# Supplementary material for: Transcriptional changes in specific subsets of Drosophila neurons following inhibition of the serotonin transporter
Source: Transl Psychiatry. 2023 Jun 24;13:226. doi: 10.1038/s41398-023-02521-3 (PMC10290657; doi:10.1038/s41398-023-02521-3)
Supplement: Supplementary file 1 — Supplementary Table Legends [file 41398_2023_2521_MOESM1_ESM.docx]

**Supplemental Table T1. DE table for bulk RNA-seq**. Differential expression table (DESeq2) for bulk RNA-seq shown in Fig. 1, with transcript per million (TPM) for each sample used in analysis, fold changes, and p-values (raw and adjusted) for each gene. **Filename: bulk_DE.csv**

**Supplemental Table T2. title: DE table for scRNA-seq, *dSERT^16^* versus *dSERT^4^*, Day 0 flies.** Differential expression table calculated using the pseudobulk method (collapsed by cell type and genotype) and DESeq2, for scRNA-seq, dSERT16 v dSERT4, Day 0 shown in Fig. 2. Fold changes, and p-values (raw and adjusted) reported here and below in Supplemental Tables T3-T4. **Filename: scRNA-seq_DEGs_dSERT16_v_dSERT4_Day0.csv**

**Supplemental Table T3. DE table for scRNA-seq, *dSERT^TMKO^* versus WT, Day 0 flies**. Differential expression table calculated using the pseudobulk method and DESeq2, for scRNA-seq, dSERT-TMKO v WT, Day0 shown in Fig. 3.  **Filename: scRNA-seq_DEGs_dSERT-TMKO_v_WT_Day0.csv**

**Supplemental Table T4. DE table for scRNA-seq, *dSERT^TMKO^* versus WT, Day 4 flies.** Differential expression table calculated using the pseudobulk method and DESeq2, for scRNA-seq, dSERT-TMKO v WT, Day4 shown in Fig 4.  **Filename: scRNA-seq_DEGs_dSERT-TMKO_v_WT_Day4.csv**

**Supplemental Table T5. DE table for scRNA-seq, citalapram versus vehicle fed Day 4 flies.** Differential expression table calculated using the pseudobulk method (collapsed by cell type and genotype) and DESeq2, for scRNA-seq, CIT v VEH Day 4 shown in Fig. 5.  **Filename: scRNA-seq_DEGs_CIT_v_VEH_Day4.csv**
