# Supplementary material for: Transcriptional changes in specific subsets of Drosophila neurons following inhibition of the serotonin transporter
Source: Transl Psychiatry. 2023 Jun 24;13:226. doi: 10.1038/s41398-023-02521-3 (PMC10290657; doi:10.1038/s41398-023-02521-3)
Supplement: Supplementary file 2 — Supplemental Table 1 [file 41398_2023_2521_MOESM2_ESM.pdf]

gene_name	FBid	TPM_Sert4_Mef2_0330	TPM_Sert4_Mef2_0331	TPM_Sert4_Mef2_0406	TPM_Sert4_Mef2_0407	TPM_Sert16_Mef2_0330	TPM_Sert16_Mef2_0331	TPM_Sert16_Mef2_0406	TPM_Sert16_Mef2_0407	chr	log2FoldChange	pvalue	padj
**a**	FBgn0000008	183.8313233	154.5827444	153.0925823	177.4596899	141.9197982	151.5251537	146.5850772	137.8673534	2R	-0.183674769	0.060570913	0.627984253
**abd-A**	FBgn0000014	0.337596841	0.216328377	0.24846226	0.15034386	0.369777938	0.189540053	0.125795712	0.197650515	3R	-0.084196305	0.886036462	0.995926915
**Abd-B**	FBgn0000015	0.394383892	0.509857254	0.424712457	0.156986302	0.257001641	0.389960418	0.201216356	0.467801566	3R	-0.139445501	0.74218393	0.995787491
**Abl**	FBgn0000017	40.6604702	43.52436174	37.88409649	39.15817363	37.15794476	40.46172522	39.93326843	47.42522478	3L	0.065003695	0.534745173	0.984760433
**abo**	FBgn0000018	10.39418948	13.74364446	21.46781078	15.60997113	22.06058532	23.39538975	21.02153566	14.0279782	2L	0.433242104	0.129628421	0.789029778
**Ace**	FBgn0000024	12.36760544	19.68800883	17.99298822	13.7131446	16.73326331	19.08138649	15.83846414	18.34995971	3R	0.169631594	0.218913126	0.863196763
**acj6**	FBgn0000028	0.435720577	0.640132676	0.400950774	0.25922101	0.390581287	0.929245938	0.137983351	0.202246075	X	-0.037213668	0.945797827	0.997604378
**Acph-1**	FBgn0000032	67.05558937	63.06365966	62.66035611	70.55234482	58.83635547	73.70216624	74.95460489	60.30740133	3R	0.051519519	0.714568646	0.995787491
**nAChRalpha1**	FBgn0000036	11.85881705	16.35232584	15.25661109	17.28622649	13.86676197	12.18860735	13.77254575	14.10548061	3R	-0.138199515	0.271910243	0.89549671
**mAChR-A**	FBgn0000037	183.8929713	185.2123073	188.9122085	195.5064767	172.3079235	162.2621084	147.1963386	167.3223208	2R	-0.184816427	0.039907886	0.529044592
**nAChRbeta1**	FBgn0000038	978.4185306	862.2053544	879.0928292	801.3448864	804.2226804	793.7083232	643.2396407	738.9217228	3L	-0.211572492	0.037340397	0.521998122
**nAChRalpha2**	FBgn0000039	409.5851053	288.4459097	255.6595445	306.0458471	304.3969977	313.515682	266.5180291	266.1526703	3R	-0.105345234	0.444485065	0.967112302
**Act5C**	FBgn0000042	2887.777487	2586.244455	2646.965035	2988.088426	3432.828404	2709.964197	2727.783291	3148.885115	X	0.144330245	0.195713347	0.844272804
**Act42A**	FBgn0000043	144.2176749	131.977835	128.8684145	98.64648594	127.4501369	126.0522866	134.6408466	128.1192524	2R	0.065078145	0.634264433	0.991585536
**Act57B**	FBgn0000044	1.545470773	0.90429433	1.569470458	0.921750609	1.297660859	3.185201815	1.833734486	1.950790762	2R	0.767252137	0.225739723	0.867702781
**Act79B**	FBgn0000045	1.35614425	7.924906677	5.624816544	1.529902871	40.98740468	8.991195624	9.820666914	16.5261535	3L	2.276014201	4.04E-05	0.004370318
**Act87E**	FBgn0000046	2.245121752	1.457758753	0.790640427	2.617208423	2.026510091	2.25324753	2.309416864	3.223371826	3R	0.480635156	0.414831345	0.958665466
**Act88F**	FBgn0000047	1.804546784	1.184428987	0.095169681	0.457307923	1.345559119	0.776687596	0.800597846	2.555111813	3R	0.631257864	0.500804856	0.976796212
**Pfas**	FBgn0000052	1.352020265	1.253271337	2.244198419	4.769752635	2.11959269	0.989551452	1.679134361	0.077246346	2L	-0.958615638	0.172348714	0.832669092
**Gart**	FBgn0000053	0.112066733	0.59525804	1.359361655	0.631425142	0.404618351	0.334576799	1.524718098	0.060826813	2L	-0.173612068	0.836935289	0.995787491
**adp**	FBgn0000057	92.5374042	97.74387939	62.79066115	68.23003159	93.51557727	87.45167012	100.5409533	69.12423375	2R	0.154914091	0.369608874	0.942282153
**al**	FBgn0000061	0.055720969	0.23366027	0.180237723	0.06495567	0.184416154	0.647911795	0.260599878	0.06048769	2L	1.157094024	0.24774659	NA
**Mps1**	FBgn0000063	4.640868069	8.409430367	7.889294859	7.187013408	13.27019357	8.623015756	8.641572373	4.70692166	3R	0.364523914	0.316059056	0.91460807
**Ald1**	FBgn0000064	1379.120921	838.0924195	738.6280642	897.0299312	1335.053745	1238.735143	1036.673098	1190.089422	3R	0.342926651	0.03409252	0.514749796
**amd**	FBgn0000075	0.24084952	0.881435525	0.106235923	0.051048326	0.237160971	0.412857194	0.167566991	0.047536964	2L	-0.517129111	0.691263613	NA
**amx**	FBgn0000077	47.60650939	47.7483528	39.33555133	38.79773388	50.83890054	49.36569202	35.45392067	41.81968227	X	0.062003662	0.747669486	0.995787491
**Amy-d**	FBgn0000078	0.161903263	0.724186695	0.523700077	1.132414087	0.876830655	0.55968516	0.826037404	0	2R	-0.124586178	0.926020811	NA
**AnxB9**	FBgn0000083	12.5135591	14.6160262	12.73543368	7.827410036	10.46862063	10.63232382	18.29696135	6.493837381	3R	-0.024284294	0.931567486	0.996479182
**AnxB10**	FBgn0000084	80.75717537	128.5770464	116.2900826	137.1366778	96.43583291	75.50585381	90.88507479	84.16629699	X	-0.380697778	0.024835172	0.44131412
**dyw**	FBgn0000092	37.52956917	1.888517622	7.283696907	5.774919328	42.68280939	0.283061189	32.93412561	0.488881012	X	0.565863525	0.706522575	0.995787491
**aop**	FBgn0000097	6.332038792	10.3536219	6.001151373	6.846435568	7.372543338	8.096643091	7.669777697	6.856832434	2L	0.055631013	0.744919116	0.995787491
**RpLP0**	FBgn0000100	1218.376521	1481.329752	1477.557941	1540.408204	1386.078023	1425.122837	1463.415768	1397.304218	3L	0.019815824	0.835821773	0.995787491
**Appl**	FBgn0000108	780.4706663	718.6589591	656.1735244	789.4423618	751.7898193	758.1822247	703.2434461	682.8219689	X	0.003969158	0.966385103	0.997604378
**Aprt**	FBgn0000109	20.30999714	22.67188035	11.67414754	21.11865924	25.03934163	21.54999869	17.33058867	11.36941256	3L	0.017188068	0.959143883	0.997604378
**bru1**	FBgn0000114	5.956044615	5.569829631	5.238414675	5.342116599	5.014072731	5.006538703	4.04311339	4.414975698	2L	-0.229875932	0.028373389	0.466846269
**Arl1**	FBgn0000115	97.77202137	154.8812242	140.0897705	77.02630786	148.9766488	102.2286902	118.3162255	153.1117956	3L	0.194916509	0.358789146	0.93823489
**Argk**	FBgn0000116	927.1055296	833.3237424	834.3001363	892.4271368	874.5318089	827.4372415	859.6955783	879.0532841	3L	0.010776636	0.90316534	0.996479182
**arm**	FBgn0000117	633.2079517	695.3330548	638.5411449	681.7928987	637.819612	663.4457641	657.8279987	707.2094028	X	0.040736947	0.625681914	0.990047511
**arr**	FBgn0000119	4.768701955	8.809022968	10.09163358	10.01238675	7.361546856	11.4067326	9.648409449	8.974125384	2R	0.181986275	0.365746113	0.940794966
**Arr1**	FBgn0000120	1.511114885	0	0.691687478	0.46531608	0.514707483	0.268805638	0.581869666	1.36182918	2L	0.037142129	0.973457063	NA
**ash2**	FBgn0000139	10.23113083	15.59710691	12.66883806	16.08868636	8.032444875	12.3838617	17.7397313	13.24665535	3R	-0.055305631	0.839090301	0.995787491
**asp**	FBgn0000140	0.12997936	0.588659785	0.063065623	0.636388169	1.36094714	0.134798122	0.198948084	0.338636668	3R	0.567267729	0.564276068	0.984934163
**aub**	FBgn0000146	0.236856018	2.622132097	1.493984427	3.975990397	0.171034327	0.803903064	0.241689916	0.77135451	2L	-2.020711583	0.014850114	0.35986804
**aurA**	FBgn0000147	0	2.731656042	0.61731685	0.7119172	1.65371704	0.671729812	1.817573489	0.220982643	3R	0.157211775	0.898821373	NA
**awd**	FBgn0000150	657.2458484	780.7127677	886.2806421	809.7970302	809.6586447	608.3679303	831.1214132	789.2127729	3R	-0.009711696	0.942358189	0.997604378
**Axs**	FBgn0000152	0.409650137	0	0.220845671	0	0.24650783	1.351754764	0.638627184	0.592925579	X	2.130743429	0.092446998	NA
**b**	FBgn0000153	8.753802929	4.309222415	0.153888433	4.214935628	8.130455859	0.089706869	18.36655301	0.068859842	2L	0.645438048	0.688751857	0.995787491
**Dll**	FBgn0000157	0.968196269	1.624013417	3.739983559	1.133500597	1.275249829	1.500455148	1.22964455	0.848034808	2R	-0.581382431	0.128888116	0.788096052
**baz**	FBgn0000163	0.627483485	0.563720176	0.562464454	0.396733321	0.307191461	0.489313131	0.309292405	0.272464946	X	-0.611126136	0.06323862	0.636110045
**ben**	FBgn0000173	296.4046325	319.4699374	261.2714709	265.0410808	306.9063568	311.2168596	272.9492583	283.2800774	X	0.070353019	0.472528748	0.973549494
**bib**	FBgn0000180	0.318069386	1.347129857	1.871207345	0.61179261	0.602906601	0.539772974	1.379383125	0.880461553	2L	-0.238247141	0.672231266	0.995787491
**bic**	FBgn0000181	1074.626882	1290.682617	1200.419723	1422.518543	1070.286739	1017.591599	1208.08505	1235.085152	2R	-0.106055551	0.363690457	0.939549067
**BicD**	FBgn0000183	25.29176401	18.43613178	19.22245749	20.6398623	19.14102207	21.55883236	20.05082894	22.20472893	2L	0.015159279	0.911550482	0.996479182
**boss**	FBgn0000206	0.437112665	1.818324032	0.763509044	0.611467034	1.073176532	3.395762919	0.490636871	0.151841929	3R	0.524570852	0.518943333	0.981083949
**brm**	FBgn0000212	58.79094033	56.57297956	52.9157419	65.49424815	58.69766486	47.20203044	55.67758254	56.04772741	3L	-0.072473601	0.544300601	0.984934163
**brn**	FBgn0000221	12.55336893	14.33848424	10.15143265	10.45275253	12.30225481	14.99130343	27.75405867	4.672204458	X	0.355176842	0.41692991	0.958846438
**Bsg25D**	FBgn0000228	25.28693945	34.51169037	23.50919976	28.36736033	25.70215214	30.79730635	32.49675962	45.2217326	2L	0.29964253	0.100090593	0.732357333
**bsk**	FBgn0000229	999.7056807	785.5540621	904.5809957	802.4690001	808.1714134	878.9068312	828.8213897	910.4956491	2L	0.00151553	0.988572096	0.998078482
**bur**	FBgn0000239	82.91795702	72.44689122	68.56784351	81.60854004	81.12609578	82.03428159	78.28772875	75.24300328	2L	0.079123103	0.47584922	0.973549494
**bw**	FBgn0000241	0.170093806	0.393997449	1.080737429	0.132189102	0.380173132	0.022909057	0.103312608	0.052755628	2R	-1.615200462	0.101588321	0.739991904
**by**	FBgn0000244	0.381098504	0.456599484	1.232719539	2.157826551	0.1310439	0.633047697	0.138884353	1.418400222	3R	-0.828139113	0.349228297	0.931946406
**c(3)G**	FBgn0000246	2.314653699	2.151750793	3.31506701	0.910257729	1.762036275	1.708984205	1.707392187	0.787099479	3R	-0.509194549	0.38129671	0.944476532
**ca**	FBgn0000247	55.84542064	81.65944696	69.39852644	76.84585464	69.16574918	63.16103498	61.89488439	78.29587309	3R	-0.023599609	0.860679333	0.995787491
**cact**	FBgn0000250	89.89988748	84.47577869	91.91297183	91.59763472	89.07676755	89.58083922	82.27259793	101.4598411	2L	0.048525403	0.630626058	0.990887368
**cad**	FBgn0000251	0.733224427	0.421673525	0.338817568	0.797759736	0.226912803	0.184341203	0.445350301	0.576115505	2L	-0.6652921	0.271053768	0.89549671
**Cam**	FBgn0000253	190.3450562	275.3405962	297.2478171	275.8554144	259.8590103	257.4759993	292.9883733	304.2459342	2R	0.136120833	0.294705964	0.906715248
**capu**	FBgn0000256	8.597607688	8.270957962	8.095476909	6.991353319	8.741172219	8.289564109	8.739431444	7.475194664	2L	0.086428987	0.471492308	0.973549494
**car**	FBgn0000257	72.99290363	72.34425945	70.64550669	68.19150749	83.5936089	81.904617	65.72761549	64.94668125	X	0.089143308	0.473213646	0.973549494
**CkIIbeta**	FBgn0000259	176.1448132	168.1890906	162.3704497	164.0514139	163.1382139	168.4854977	148.6034812	161.2830375	X	-0.034936872	0.682904703	0.995787491
**Cat**	FBgn0000261	53.87270185	55.1081888	80.00470598	78.51492218	62.16641409	62.65131574	58.48608134	72.50806984	3L	-0.033058555	0.831468841	0.995787491
**Pka-C1**	FBgn0000273	1056.847669	1331.2913	1336.64796	1485.682894	1324.967175	1350.995731	1311.203897	1283.603917	2L	0.047075502	0.653820023	0.994456599
**Cp190**	FBgn0000283	258.9541521	278.3138017	286.0785177	253.683283	213.9209711	230.3515636	229.3275822	258.8754916	3R	-0.175753276	0.068872978	0.659755633
**Cf2**	FBgn0000286	113.5401328	134.4913779	117.8795921	128.7835613	107.5911369	118.4089442	112.7575608	114.4492786	2L	-0.095455041	0.308975517	0.912131082
**salr**	FBgn0000287	0.287495362	0.127939404	0.697459739	0.068396389	0.296573036	0.359554626	0.35921968	0.547748958	2L	0.44636394	0.531572893	0.984760433
**cg**	FBgn0000289	294.4452053	266.9553993	267.5569089	285.6188969	291.1747343	309.025158	269.4589111	297.3735521	2R	0.094858755	0.286157355	0.902321374
**Col4a1**	FBgn0000299	28.82004731	67.98509142	66.87597063	30.0419467	19.71420926	26.62836316	65.68451274	22.63049541	2L	-0.481032042	0.361501189	0.939549067
**ChAT**	FBgn0000303	45.63406586	39.60386716	40.56617316	50.17269815	61.81210238	56.78666365	48.69083217	54.51900226	3R	0.361585699	0.001971122	0.101218706
**chic**	FBgn0000308	144.1464593	121.6270965	151.6236132	125.1423774	144.6708205	123.8109893	117.4442779	139.241454	2L	-0.015849148	0.891307377	0.996189576
**cin**	FBgn0000316	107.5987653	109.370328	114.0516182	121.3128134	97.68113096	146.8768049	111.8660046	128.443414	X	0.12828413	0.335808863	0.924219112
**ck**	FBgn0000317	0.342470775	1.115102381	0.635340071	0.782801686	0.569757522	0.848349678	1.062084684	0.262424384	2L	-0.032328944	0.951923513	0.997604378
**cl**	FBgn0000318	113.4303089	130.4929835	131.2709678	137.1686131	124.3922922	107.9237177	140.6946387	133.1625105	2L	0.014825847	0.918823225	0.996479182
**Chc**	FBgn0000319	274.2487871	302.4486139	311.0575368	306.55388	281.1042271	275.6022585	308.3910585	294.3140137	X	-0.010812329	0.904903815	0.996479182
**eya**	FBgn0000320	1.135453474	0.535658601	1.291216073	0.506703455	0.544474017	1.032863883	0.84860338	0.741478297	2L	-0.106323715	0.794011011	0.995787491
**clt**	FBgn0000326	24.72354286	26.35090377	21.24255192	22.21619114	20.99889206	20.03134821	16.75304552	20.22210198	2R	-0.247448313	0.22401463	0.86549481
**cm**	FBgn0000330	125.0477525	138.5654557	122.8978281	109.0458119	148.9846919	145.878872	161.3592046	141.4597818	X	0.301254098	0.008404292	0.272534513
**cni**	FBgn0000339	79.36985689	91.49011857	98.03353188	102.5226961	117.1125351	120.0459737	107.4007035	94.55267882	2L	0.273655152	0.056697644	0.621788268
**comt**	FBgn0000346	447.5817337	459.5470593	456.2716149	475.0738943	446.2377596	432.2941579	490.3676818	456.1733169	X	0.02053334	0.819664043	0.995787491
**cort**	FBgn0000351	0.487865183	4.091627018	1.075958299	0.517017867	1.601312171	0.919912629	1.357695887	0.866618569	2L	-0.329694378	0.710808644	0.995787491
**cos**	FBgn0000352	223.0783731	231.8638249	228.7272544	256.6965761	209.3667679	224.4041934	248.3004213	245.9314003	2R	0.011525422	0.911094553	0.996479182
**Cp18**	FBgn0000357	5.057999746	1.944269481	1.533829604	0.982711333	3.043661965	2.682366337	1.075255502	2.745346415	3L	0.007628665	0.993370875	NA
**crc**	FBgn0000370	41.16391861	33.61748889	38.45553971	33.92600685	30.87282164	38.15850131	34.18694232	32.15694213	2L	-0.093261606	0.442584745	0.967112302
**crm**	FBgn0000376	44.12266436	45.4011044	53.54370345	37.84557077	41.15678386	59.98857907	54.44773065	48.29200854	X	0.202536545	0.171478506	0.832652344

**crn**	FBgn0000377	81.17967853	98.70472925	87.16761987	82.33426215	120.1069589	106.4800006	80.09865836	114.2978841	X	0.302242913	0.037676471	0.521998122
**csw**	FBgn0000382	66.35069788	75.07132758	65.06568486	71.38282917	66.80263437	69.20844743	70.34400932	71.2032903	X	0.02956078	0.737060905	0.995787491
**cta**	FBgn0000384	8.338999491	14.1701744	13.85171418	11.98806334	12.21735753	14.00777926	10.47772995	11.09590864	2L	0.01892883	0.915349363	0.996479182
**cv-2**	FBgn0000395	1.204556568	1.931336072	1.675989115	0.495596169	1.279137253	1.800337004	1.283366632	1.007622645	2R	0.052640377	0.881708273	0.995787491
**CycA**	FBgn0000404	0.580906736	0.233006198	0.428908759	0.088327969	0.159582583	0.642923204	0.225507368	0.246756971	3L	-0.044167481	0.961262399	NA
**D**	FBgn0000411	1.381287638	2.751338032	3.560421078	1.408933807	11.6366983	2.387494172	1.394795716	0.687248679	3L	0.871825021	0.222469452	0.86549481
**D1**	FBgn0000412	566.9435386	523.1794672	486.0640988	535.148242	531.9314256	544.1033876	521.9449548	525.0360229	3R	0.036821996	0.683265194	0.995787491
**Dab**	FBgn0000414	75.8544553	83.52801135	96.28147454	80.96380192	74.17092362	87.59978665	93.55824117	99.00558501	3L	0.10648434	0.363124004	0.939549067
**Sap-r**	FBgn0000416	375.9491809	428.888267	374.6988449	369.6953715	355.5852696	413.0568372	353.5519033	367.7140921	3R	-0.026127147	0.761741518	0.995787491
**Ddc**	FBgn0000422	584.9620366	423.78454	437.6385376	541.7128747	580.9712183	683.6378619	523.7731835	638.0531918	2L	0.31238313	0.015802923	0.366845023
**1-Dec**	FBgn0000427	0.857464897	0.071913854	0.589389399	0.233233831	0.309588781	0.080841267	0	0.496436177	X	-0.959678389	0.405114882	NA
**Dfd**	FBgn0000439	0.400707519	0.3429238	0.495974924	0.362254192	0.265731345	0.346945042	0.062584499	0.035509107	3R	-1.120750396	0.172473455	0.832736771
**Pkg21D**	FBgn0000442	0.372952618	0.500460558	0.271433355	0.521714994	0.302973779	0.404360068	0	0.080971466	2L	-1.02244342	0.372254382	NA
**Dhod**	FBgn0000447	38.68001658	52.30438407	46.16072766	39.85973827	41.32220183	63.34918682	51.29286031	34.03509464	3R	0.132900229	0.530931313	0.984760433
**Hr3**	FBgn0000448	2.023074787	2.164995893	2.545330283	1.895165862	2.11910162	2.007729002	2.192888701	2.802265045	2R	0.113966906	0.54537151	0.984934163
**ect**	FBgn0000451	0.816658488	0.238777715	0.199937827	0.489103106	0.256984217	0.33199275	0.286694497	0.081332164	3L	-0.85875812	0.243107629	0.876959115
**Dip-B**	FBgn0000454	32.49462521	28.68919138	23.258706	45.42023328	21.5536949	34.90207952	38.74061786	23.4792754	3R	-0.107787487	0.650075542	0.993330125
**Dip-C**	FBgn0000455	144.0528054	122.7141318	150.1115772	174.5030751	143.2171725	144.5228414	132.1257418	168.3826547	3R	0.02152098	0.880733656	0.995787491
**disco**	FBgn0000459	1.309411061	0.944668113	0.990556732	0.558046496	1.347124687	0.849551656	0.790186951	0.55022954	X	-0.080855253	0.884511659	0.995787491
**Dl**	FBgn0000463	1.080607425	0.402105387	0.599464444	0.550311652	0.532634364	0.632832063	0.254026022	0.208186237	3R	-0.673600289	0.159596352	0.819277129
**Lar**	FBgn0000464	1.587101912	2.198638619	2.824507048	1.910532557	1.996449705	1.841050826	2.063105741	2.337676271	2L	-0.011588225	0.943530303	0.997604378
**DNaseII**	FBgn0000477	37.5863048	26.84528857	57.65172527	14.02096875	30.99345982	26.56567292	47.5087134	17.37361582	3R	-0.11927016	0.739344122	0.995787491
**dnc**	FBgn0000479	18.16619012	20.66552885	18.74078582	17.92007096	20.99555275	18.785839	14.87872679	17.76973856	X	-0.028424577	0.79322482	0.995787491
**Pka-C3**	FBgn0000489	19.60502649	17.05874066	25.89718285	21.76058868	19.76175638	20.1312967	20.2276878	20.68540686	3L	-0.031161598	0.810702737	0.995787491
**dpp**	FBgn0000490	0.52602731	0.360326112	0.434286752	0.218314333	0.611531602	0.524497055	0.231847801	0.077731423	2L	-0.06546914	0.905756133	0.996479182
**Dr**	FBgn0000492	0.987631837	0.442857169	1.067516314	1.162712365	0.900290676	0.590025038	0.299342974	1.061505021	3R	-0.32862476	0.554596369	0.984934163
**dsh**	FBgn0000499	11.27286349	11.08966599	15.66955576	8.097776433	11.27522594	9.220496689	9.404419864	7.408476151	X	-0.272223248	0.327262279	0.918470465
**dsx**	FBgn0000504	0.892619266	0.465359496	0.697441239	0.445282163	0.798444688	0.665284128	0.507729733	0.052377272	3R	-0.284920802	0.582168257	0.985735525
**dwg**	FBgn0000520	52.39960423	71.59125379	64.00859395	43.24977979	52.36731432	66.0551783	72.20955387	66.67010524	X	0.188132121	0.278645486	0.897654224
**dx**	FBgn0000524	0.511077935	4.921316246	2.847104165	1.191558967	1.845253323	3.337196153	0.434590264	0.534250652	X	-0.577471833	0.422142351	0.960593359
**e**	FBgn0000527	22.39231562	13.6419705	15.6641669	14.64103287	34.96867089	13.25440381	30.23287893	9.712906918	3R	0.441558246	0.327705381	0.918470465
**bsh**	FBgn0000529	1.473905122	0.044147662	0.7662157	3.865898594	0.712707992	0.645166204	0.134284415	0	2L	-2.018407572	0.0756	0.678318963
**ea**	FBgn0000533	1.805621559	0.346134498	1.877319736	0.481112993	0	1.507777094	0.789630752	0.224009803	3R	-0.817029269	0.512607393	NA
**eag**	FBgn0000535	4.307713236	7.827938803	7.22781185	5.705141172	5.642356151	6.136121851	5.172280362	5.519701481	X	-0.12211147	0.442384456	0.967112302
**eas**	FBgn0000536	69.52035052	70.17365352	65.00050052	71.91424918	58.66486214	63.01169206	72.23039894	66.34850784	X	-0.058134903	0.592414122	0.988631016
**E(bx)**	FBgn0000541	57.80330485	72.01843871	99.92156934	74.10120958	59.84518892	68.7967729	82.69597467	77.76543602	3L	-0.037663	0.805592153	0.995787491
**ec**	FBgn0000542	0.599799711	0.882692717	0.709249649	0.624447432	0.657166338	0.529922252	0.14434924	0.294842421	X	-0.754740552	0.052385891	0.605525675
**ecd**	FBgn0000543	189.5249839	209.0617773	177.8204512	240.6330181	200.0632709	213.9172749	196.6938055	175.2492367	3L	-0.027727408	0.820919333	0.995787491
**EcR**	FBgn0000546	7.313287246	11.35821323	11.73653309	11.08528731	9.842562837	11.3132235	10.03149673	10.46403454	2R	0.038052832	0.777679578	0.995787491
**ed**	FBgn0000547	5.264523345	5.770476481	5.974885498	6.292598651	4.303404043	4.712675906	5.489293269	5.79289504	2L	-0.167253433	0.189354822	0.843401316
**eEF1alpha2**	FBgn0000557	2847.731483	3390.76966	3420.05228	3579.699498	3048.726111	3563.453881	3446.560821	3739.046057	3R	0.090693645	0.371582977	0.943282319
**eEF2**	FBgn0000559	1674.232272	1802.983071	1709.302814	1802.085224	1624.44825	1774.036426	1653.365066	1822.787316	2L	0.006795326	0.934142569	0.996479182
**eg**	FBgn0000560	0.058414013	0.163302187	0.236185968	0.022698345	0.316356795	0.055072375	0.049671801	0.274781497	3L	0.598238909	0.612433214	NA
**egl**	FBgn0000562	50.7434016	63.83011137	54.41474357	56.40973191	47.28415004	53.89425689	50.93776103	48.53979658	2R	-0.136490027	0.168171297	0.830652748
**Eh**	FBgn0000564	0.078175312	1.311281189	0.189652216	0.455656992	2.469713299	1.695173752	0.398853801	1.782121308	3R	1.696038313	0.091187756	NA
**MsrA**	FBgn0000565	36.01707517	52.43953751	40.07219017	32.68726431	49.01959161	48.58008027	44.91242206	47.93247151	3L	0.274674196	0.111644665	0.758410631
**Eip55E**	FBgn0000566	54.12907294	61.18507276	43.81954907	72.73926824	39.48904115	36.82306015	52.95979145	58.69595246	2R	-0.272918662	0.224108385	0.86549481
**Eip74EF**	FBgn0000567	1.119626475	1.583378576	1.590223188	1.581536115	1.245211423	1.175406534	1.020068283	1.066581091	3L	-0.346518026	0.053381914	0.607150997
**Eip75B**	FBgn0000568	18.43654315	21.1401912	22.67931244	24.80685391	20.71335086	24.06249677	22.8962974	27.44549914	3L	0.158523366	0.184800889	0.839299945
**ems**	FBgn0000576	0.281736253	0.787621733	0.075943135	0.72984163	0.339070763	0.059026493	0	2.106882224	3R	0.464942837	0.733653601	NA
**en**	FBgn0000577	2.225207072	3.214073712	0.849735763	0.480369122	1.376218111	2.505838898	0.052560658	1.655109536	2R	-0.245085166	0.758345521	0.995787491
**ena**	FBgn0000578	15.63390072	16.86371887	13.87061992	18.27067079	14.13993541	12.29035446	13.10393659	14.85842723	2R	-0.218109053	0.094240521	0.728467429
**Eno**	FBgn0000579	2860.51952	2100.018871	2016.955997	2376.737423	2797.826786	2579.80409	2470.42326	2602.268638	2L	0.187600794	0.11943746	0.777535158
**E(Pc)**	FBgn0000581	30.80458362	47.96864268	52.76005079	51.63569662	40.71458771	48.18786637	49.28152766	52.29234232	2R	0.089946688	0.560595014	0.984934163
**Est-6**	FBgn0000592	0.137707863	0.076995207	10.4677571	4.815910467	3.148907828	8.309128436	0.585492861	53.61645546	3L	2.133207775	0.188073374	0.842945127
**exd**	FBgn0000611	201.6162382	214.9687318	211.3306709	208.0440747	195.5665792	225.9124935	210.9037432	190.1503968	X	0.006176678	0.944021057	0.997604378
**exu**	FBgn0000615	0.592302008	1.470385219	1.666823035	1.988538961	2.865608573	1.965633878	0.241756024	0.0514377	2R	-0.120559711	0.89129856	0.996189576
**e(y)1**	FBgn0000617	34.66189274	49.57239433	41.60205906	38.62005865	48.60980729	47.12658782	33.69390565	39.76589182	X	0.076985475	0.715351105	0.995787491
**e(y)2**	FBgn0000618	51.0737066	88.56320011	56.92891073	58.33135647	55.44527442	54.65900275	57.22201339	49.82366238	X	-0.196858715	0.449482072	0.967112302
**eyg**	FBgn0000625	0.03158944	0.50337512	1.072897586	0.110474387	1.397162389	0.134020492	0.362634035	1.748881784	3L	1.161556877	0.248641816	0.879503029
**E(z)**	FBgn0000629	64.95759294	74.05496478	70.99063899	88.05010615	77.50229287	78.69984863	78.19239859	77.60295038	3L	0.096679384	0.441472836	0.967112302
**CG17716**	FBgn0000633	0.727515643	0.723442146	0.703648262	0.37088848	0.628824615	0.826179789	0.519514314	0.388549004	2R	-0.068119424	0.7881669	0.995787491
**Fas2**	FBgn0000635	34.63499285	34.34079316	34.16991698	30.59855898	31.90545597	31.92633714	31.45776696	28.03143971	X	-0.086843999	0.312440827	0.914141092
**Fas3**	FBgn0000636	2.599527655	3.046891176	2.934017201	4.49664903	2.466872066	2.463067321	2.621245924	3.392443196	2L	-0.225890834	0.220975828	0.86549481
**Fbp1**	FBgn0000639	0.150031603	2.013255738	0.970598872	0	0.225704581	2.687528704	0.063788974	5.048853735	3L	1.416818472	0.215481218	0.859655565
**Fbp2**	FBgn0000640	3.440265714	5.514093642	0	0	0.966087321	4.540846102	2.535344283	0.33196157	2L	-0.078299894	0.951719746	0.997604378
**Fcp3C**	FBgn0000644	1.893294987	2.117157825	3.253450955	1.655304745	4.272353373	5.652458672	1.207459047	1.370172892	X	0.531690883	0.498777593	0.976006682
**fj**	FBgn0000658	0.195196047	1.227802443	0.118385722	0.455092234	0.308331531	0.483077806	0.124487327	0	2R	-1.063838261	0.409856978	NA
**fkh**	FBgn0000659	6.221646376	4.506428198	0.83308982	0.470958906	1.786856003	2.79955305	0.566841199	1.008697126	3R	-0.945131127	0.183952226	0.839299945
**fl(2)d**	FBgn0000662	276.5668391	319.0542668	311.0453335	294.6240949	328.8521143	333.5779916	318.2900263	330.5577545	2R	0.158164466	0.053893045	0.608597067
**Actn**	FBgn0000667	13.93743744	10.36494411	11.17217625	11.10824171	10.97765359	11.391058	11.18628618	11.96138925	X	-0.00680667	0.960275554	0.997604378
**flil**	FBgn0000709	36.05988682	45.5950151	37.68826162	37.80608306	40.2106197	40.8086674	35.20893674	48.99239342	X	0.105659944	0.450549338	0.967112302
**flw**	FBgn0000711	16.94823199	21.30903111	21.96738196	23.3631309	23.2877553	19.91517054	20.75628023	22.81237071	X	0.087275187	0.485626041	0.973549494
**FMRFa**	FBgn0000715	0.104033444	0.488604223	0.353337158	0.145530035	0.150245624	0.549259736	0.05307831	0.271039485	2R	-0.027200481	0.981973623	NA
**fog**	FBgn0000719	1.096859606	1.755130145	1.265830423	0.745602824	0.460844646	0.632104594	0.737098668	0.870940856	X	-0.813863433	0.010060788	0.304092724
**for**	FBgn0000721	0.568696874	0.983068022	0.632590925	0.897440085	0.345203311	0.920141607	0.380111251	0.479859068	2L	-0.503073692	0.165605318	0.828893393
**FER**	FBgn0000723	2.121814279	1.202526078	1.294672192	1.90381856	1.851368985	1.536696675	1.01694914	0.572342011	3R	-0.369331311	0.275521009	0.89549671
**gd**	FBgn0000808	0.497806009	2.115332689	1.610227247	0.696369583	1.497779056	2.59695084	0	0.14410439	X	-0.171135587	0.872372315	0.995787491
**fs(1)K10**	FBgn0000810	41.01217472	49.17448925	39.73337842	29.13047956	36.85848906	49.65069482	30.91132284	40.71419777	X	0.02267924	0.899321713	0.996479182
**tj**	FBgn0000964	0.297742466	1.914447999	0.902898836	0.173543838	0.403126096	0.444457148	0.253182476	0	2L	-1.516113974	0.180454142	NA
**dup**	FBgn0000996	1.506749066	9.21156277	9.426960983	2.773364783	6.012782427	3.514001545	4.248354422	6.198236906	2R	-0.144188852	0.763606338	0.995787491
**ft**	FBgn0001075	0.529514754	0.279499316	0.179663693	0.388492778	1.002702634	0.216446455	0.217262104	0.176865469	2L	0.25110295	0.688644514	0.995787491
**ftz**	FBgn0001077	2.34591082	2.194483707	0.218890266	0.315542467	1.873159932	0.085065784	0	1.763027151	3R	-0.414237663	0.740281456	NA
**ftz-f1**	FBgn0001078	5.588108586	9.25577999	11.13213848	10.02526625	5.825793935	7.304295658	8.820819125	8.812701692	3L	-0.191802698	0.307177544	0.911687199
**fu**	FBgn0001079	45.15500309	60.44972064	35.70278582	49.35966131	41.39289819	54.00801734	46.96066949	35.71378089	X	-0.070086433	0.691962408	0.995787491
**fw**	FBgn0001083	0.921386921	1.228472214	0.439412409	0.789501654	1.137326182	1.01716992	0.502238853	1.367806015	X	0.281884573	0.550641081	0.984934163
**fy**	FBgn0001084	17.76538298	19.24850301	12.69660614	21.15743726	19.84939166	32.77390394	22.29247839	11.46684326	2L	0.308325346	0.319713595	0.916263102
**fz**	FBgn0001085	17.01574173	18.80067065	17.58253196	15.96157127	19.14637385	17.63323425	16.59299657	15.74713655	3L	0.026363488	0.777625147	0.995787491
**fzy**	FBgn0001086	0.323655285	1.289355049	0.392591604	0.848913877	0.292140371	1.182418375	0	0	2L	-0.894616139	0.534644373	NA
**g**	FBgn0001087	53.37990188	55.73664712	70.83997267	72.06358446	62.07163508	56.12204337	71.42278613	86.02836769	X	0.16309795	0.295626535	0.906715248
**Gal**	FBgn0001089	0.266271889	0.893267481	0	0.186240866	0.048068901	0.502079022	0	0.462480736	2L	-0.367711193	0.815924643	NA
**Gapdh1**	FBgn0001091	2352.865462	1750.847114	1567.272068	1900.133159	2169.488911	1971.89735	2012.888114	1940.323233	2R	0.124239471	0.324874458	0.918470465
**Gapdh2**	FBgn0001092	5557.908869	3309.850865	3602.922235	3542.467895	5451.636759	4522.662702	4418.71709	4127.189189	X	0.236671542	0.124465033	0.780439453
**Gdh**	FBgn0001098	777.1874537	779.0698425	674.8549634	800.2966252	851.9797545	853.5474357	731.6005014	765.0463351	3R	0.107642679	0.256960761	0.88674759

**gdl**	FBgn0001099	3.02785378	2.720785688	2.492233745	0.756356475	2.9282422	3.619278313	2.620683587	1.995602934	3L	0.333838178	0.608267019	0.990047511
**Galphai**	FBgn0001104	3.574457354	4.163093056	6.078751353	3.211663389	3.644445118	2.160815535	3.33234859	1.701633006	3L	-0.61658629	0.064698758	0.641709151
**Gbeta13F**	FBgn0001105	1303.861879	1245.849383	1205.014756	1277.232595	1294.449469	1304.81817	1239.154249	1288.836852	X	0.056720148	0.475927082	0.973549494
**DCTN1-p150**	FBgn0001108	120.9104568	130.9787986	116.4534437	121.8045425	106.837214	118.3023052	124.67811	98.29319717	3L	-0.099874024	0.334964273	0.924122842
**Gld**	FBgn0001112	0.133356537	0.330204445	0.323521098	0.488581885	0.19488697	0.425079462	1.198783755	0.896162384	3R	1.133101435	0.078397586	0.687029953
**Glt**	FBgn0001114	1.223540732	0.247693544	0.511774612	2.508355121	0.152331353	0.019888737	1.506824198	2.244217059	2L	-0.172077813	0.86813225	0.995787491
**Galphao**	FBgn0001122	30.98955563	43.02713078	63.31485461	39.39704129	40.3952892	40.25113047	52.21968489	52.48285953	2R	0.105958634	0.543280317	0.984934163
**Galphas**	FBgn0001123	295.3843675	302.7699964	296.3768235	278.0791532	267.1555604	312.726426	279.5473604	271.2234342	2R	-0.023172217	0.781783465	0.995787491
**Got1**	FBgn0001124	89.81556344	92.47201881	105.6549528	58.22358543	83.73740928	101.1883195	96.7648766	104.6509778	2R	0.191458475	0.231030293	0.867702781
**Got2**	FBgn0001125	283.0970327	253.2950892	248.7176055	237.5336812	205.8413538	139.7531776	181.278438	151.4414614	2L	-0.560443683	3.67E-05	0.004077718
**Gpdh1**	FBgn0001128	520.9945145	423.0734373	435.7383792	427.6350028	500.2899324	445.7504606	454.6692143	405.3185625	2L	0.027726721	0.797915464	0.995787491
**grau**	FBgn0001133	124.124709	129.9324639	106.6747557	161.1270723	112.074703	134.6665901	144.3392178	130.2531325	2R	0.026398255	0.85839017	0.995787491
**Grd**	FBgn0001134	0.769518307	0.459722386	0.639329	0.135172141	0.418656314	0.710589684	0.564715239	0.240305514	3L	-0.030744307	0.957322343	0.997604378
**grk**	FBgn0001137	1.482716785	1.619474029	0.75287098	0.482357911	1.058223833	4.681327237	1.407420418	1.197810379	2L	0.956050736	0.199156075	0.847197998
**grn**	FBgn0001138	0.452460609	0.44342515	0.721497674	0.4148463	0.481824569	0.69019216	0.402037515	0.39734853	3R	-0.006378063	0.985257351	0.997950263
**gro**	FBgn0001139	50.30745325	112.6774703	89.09195314	108.0263013	93.95477328	92.35178577	91.3322023	95.8339146	3R	0.087622439	0.627536213	0.990047511
**Gs2**	FBgn0001145	307.9446224	384.2440948	346.3726709	222.2652861	355.9843169	272.953406	371.4552252	250.4607678	X	0.025162789	0.87354082	0.995787491
**gsb-n**	FBgn0001147	0.115993633	0.535048381	0.316573803	0.152119569	0.104699118	0.416927714	0.036987781	0.330530701	2R	-0.279053664	0.752270191	0.995787491
**GstD1**	FBgn0001149	1179.22593	1030.747625	1157.116402	991.0860518	1051.793923	935.2710373	1126.601731	955.1856727	3R	-0.068007472	0.526781271	0.984107922
**gt**	FBgn0001150	5.980116204	6.11252241	8.500592871	8.496159632	8.940141395	6.210628134	6.316691098	7.100289138	X	0.010698433	0.976066511	0.997757033
**h**	FBgn0001168	0.371858525	1.538559921	3.548352802	3.410098328	3.446006376	4.066796968	2.656133438	3.175534498	3L	0.635800055	0.276880728	0.896087042
**H**	FBgn0001169	57.20752127	54.51757261	46.80367709	50.88478437	40.27426341	41.94936793	44.40202634	66.73745508	3R	-0.082489054	0.616576278	0.990047511
**H2.0**	FBgn0001170	0.355048776	0.297772257	0.530057464	0.191026813	0.246520784	0.218867034	0.383196447	0	2L	-0.669186139	0.527408356	NA
**hb**	FBgn0001180	0.367133751	1.380275815	0.798523977	0.826442708	1.325541032	0.608713943	1.614769114	0.247370077	3R	0.211851899	0.748303672	0.995787491
**her**	FBgn0001185	38.43555034	57.57100002	59.87076785	52.94465146	46.98531704	72.50619124	57.13700064	68.5887445	2L	0.265673879	0.139930211	0.803344611
**Hex-A**	FBgn0001186	297.5749106	403.1462716	447.539082	412.7277168	381.7755564	380.7460631	399.1574231	434.7380113	X	0.066132788	0.592028653	0.988631016
**hfw**	FBgn0001189	0.943834276	8.05967065	1.353021558	2.800654289	2.633242465	6.127770018	3.009676896	4.703734318	X	0.367651737	0.516348561	0.980804634
**His2Av**	FBgn0001197	276.2256232	341.0604024	313.2299649	311.6914992	280.7255054	254.6493079	310.5662671	288.8563385	3R	-0.097011424	0.374904866	0.943282319
**hook**	FBgn0001202	364.2927121	333.8274869	298.0206648	340.9665821	339.6028339	328.5911115	339.7732433	325.3075167	2L	0.024813114	0.804525589	0.995787491
**Hmr**	FBgn0001206	11.0752002	16.72232925	14.56252326	11.70352445	13.24455517	21.03219197	13.79072375	22.17805989	X	0.412725504	0.061343664	0.630379895
**Hrb98DE**	FBgn0001215	403.8686836	442.0550193	432.8215247	425.2342247	412.8245018	443.0394374	436.5388071	408.7367181	3R	0.028112338	0.714714446	0.995787491
**Hsc70-1**	FBgn0001216	1.54508222	0.785351112	1.874173957	0.218321275	0.972018127	0.264853469	0.895804077	1.626432255	3L	-0.20413299	0.799943901	0.995787491
**Hsc70-3**	FBgn0001218	226.2524181	253.3796793	196.3737624	225.1556446	218.8461591	209.0311584	209.9187933	181.7425911	X	-0.106654984	0.308758084	0.912131082
**Hsc70-5**	FBgn0001220	278.6051529	305.2948653	328.0065161	312.6416538	313.3453804	323.4715206	336.7349292	313.2700739	2R	0.102757798	0.253885055	0.884040848
**Hsf**	FBgn0001222	165.0766566	159.6143176	138.4724792	128.3677173	155.140584	153.332893	154.2316351	130.3497577	2R	0.033171366	0.769294111	0.995787491
**Hsp23**	FBgn0001224	2606.885479	1287.041941	1439.529482	1247.361171	1710.309664	1815.271082	1032.754901	1010.136689	3L	-0.22123985	0.530990481	0.984760433
**Hsp26**	FBgn0001225	9329.446067	5357.585117	6020.805648	5496.005484	5150.695029	6334.60025	4096.283874	3989.438644	3L	-0.399827103	0.122608464	0.780439453
**Hsp27**	FBgn0001226	1022.424565	590.335093	684.9024096	589.0022871	583.9597185	760.6539663	431.7691433	469.8523207	3L	-0.34126594	0.212791396	0.856573136
**Hsp67Bc**	FBgn0001229	158.8293997	81.19144586	107.4116293	79.05842908	137.0203522	107.3381807	61.17996604	56.26428772	3L	-0.214755416	0.450700133	0.967112302
**Hsp68**	FBgn0001230	1666.799174	737.4605422	853.6367026	639.1673493	825.8060573	1092.455237	556.2769243	563.8313678	3R	-0.341825498	0.417304351	0.958846438
**Hsp83**	FBgn0001233	5935.404098	3816.010493	3568.9496	2955.390806	4737.533894	4170.131899	3382.385355	2383.164408	3L	-0.124591795	0.668421066	0.995787491
**lncRNA:Hsrom**	FBgn0001234	386.3646966	432.5436381	234.3143218	372.0055132	528.5644834	392.3510301	238.726484	199.0317347	3R	-0.042766471	0.897670856	0.996479182
**hth**	FBgn0001235	3.545254276	3.987557551	3.202791726	3.442098924	3.069154165	3.954885245	3.249017672	3.048628717	3R	-0.06124191	0.592517458	0.988631016
**Ide**	FBgn0001247	295.5765608	265.2642374	292.7657314	277.5817521	257.800114	309.2721253	262.5528795	276.6973168	3L	-0.003953791	0.966428396	0.997604378
**Idh**	FBgn0001248	79.21086569	78.03445424	82.70959797	88.63785553	69.08401099	55.05279189	87.02153595	69.25749188	3L	-0.196882401	0.182381292	0.838040629
**if**	FBgn0001250	2.539360314	1.953009487	1.526662406	0.614017667	1.131035307	1.756365459	1.428546363	0.710213957	X	-0.377042534	0.295507848	0.906715248
**ImpE1**	FBgn0001253	2.460168652	1.254234937	0.918205359	1.355927984	1.73316486	0.900792532	1.212799508	0.531119054	3L	-0.432904514	0.281478147	0.900509101
**ImpL2**	FBgn0001257	0.263971262	0.239337708	0.467312059	0.116434417	0.618208583	0.242144264	0.200198918	0.247829641	3L	0.310444252	0.66655651	0.995787491
**Ldh**	FBgn0001258	24.32744263	7.610619025	4.508322233	17.44319461	15.55403278	9.306277387	12.55972726	8.016885792	3L	-0.231072694	0.571181285	0.984934163
**in**	FBgn0001259	0.282973687	0.284789198	0.823788262	0	0.051084003	0.453535968	0.216561587	0.491489656	3L	-0.149519006	0.913728648	NA
**inaD**	FBgn0001263	0.362536121	0.608103485	0.811853145	1.755497048	0.100687787	0.131460323	0.071141341	0.423822797	2R	-2.25638324	0.033671521	NA
**inv**	FBgn0001269	0.290120721	0.43714832	0.23262139	0.200628761	0.403902928	0.690764109	0.100353111	0.229532156	2R	0.324311058	0.544288145	0.984934163
**ix**	FBgn0001276	81.84589051	117.4727328	139.5108872	127.6773487	147.3220431	86.0500165	122.6568833	119.672146	2R	0.065987295	0.764716483	0.995787491
**janA**	FBgn0001280	58.18920564	43.06068941	59.28057798	38.79336588	49.60525859	46.34451159	59.42527592	47.2720928	3R	0.050573751	0.824887062	0.995787491
**Jra**	FBgn0001291	79.19144038	89.30153349	98.99407194	96.98522996	79.16325061	95.39168874	108.9923727	89.77057967	2R	0.065637089	0.643877483	0.992589784
**kar**	FBgn0001296	2.255620657	4.91651013	1.785017964	2.784120696	1.480717599	3.354773724	1.815476303	2.356922525	3R	-0.347931806	0.377913183	0.943615128
**kay**	FBgn0001297	10.31668343	13.10389529	9.132390261	11.23285397	8.93675192	9.790400757	9.210149603	8.681164586	3R	-0.227375165	0.074948426	0.676944702
**kel**	FBgn0001301	43.33553404	44.47832527	34.67644704	47.14317606	46.85894669	46.72299675	47.62107145	37.36353187	2L	0.101804387	0.441997547	0.967112302
**Khc**	FBgn0001308	159.5117084	139.491115	150.0842014	145.1633883	145.1967521	164.92339	176.2075758	175.446592	2R	0.184655772	0.082885644	0.69799888
**kkv**	FBgn0001311	0.492515578	0.629429173	1.721132698	0.847541129	0.762100156	0.773901131	0.314104455	0.432810655	3R	-0.647413163	0.196369252	0.844272804
**klar**	FBgn0001316	7.822644501	7.987940325	8.211605749	8.778828563	6.574357415	6.999523606	7.021116331	8.32149389	3L	-0.150806295	0.167571799	0.830652748
**kn**	FBgn0001319	0.619223168	1.038659996	0.323650483	0.328646131	0.536207601	0.35360233	0.269696615	0.240460146	2R	-0.686930134	0.123728095	0.780439453
**knrl**	FBgn0001323	0.659268049	0.77222742	0.437817762	0.334889871	0.252657074	0.597247313	0.394613923	0.303858182	3L	-0.482167111	0.280125363	0.899310165
**kto**	FBgn0001324	25.46555625	23.12778887	22.93066052	21.16445225	23.18303059	16.88162657	21.06197052	31.76411943	3L	0.037214753	0.84014709	0.995787491
**kz**	FBgn0001330	4.168127214	8.347998415	12.425966	6.816986409	8.947087359	7.272849625	9.78657554	9.589652112	X	0.208321154	0.504881327	0.978557094
**Mybbp1A**	FBgn0001341	14.67573385	10.53916777	16.76722124	9.835769337	10.89715749	9.85881587	12.65110524	23.92114214	X	0.180672874	0.548169946	0.984934163
**egh**	FBgn0001404	91.97472841	85.90296026	87.10049788	97.68014471	93.77960644	109.3153065	91.00642056	101.1901355	X	0.152163859	0.124201501	0.780439453
**l(1)10Bb**	FBgn0001491	175.2866273	174.5198877	177.3917804	136.2247243	155.5970874	155.0311335	146.6562611	155.6007658	X	-0.083720383	0.529507647	0.984366882
**Hlc**	FBgn0001565	67.63928902	134.4590324	132.4159898	150.1295955	118.1133265	125.9590229	129.6849806	107.4275083	X	0.022899516	0.904924718	0.996479182
**Grip91**	FBgn0001612	18.86533219	17.92619816	20.66898254	16.87664086	17.01874299	15.89376537	19.4464457	16.01711507	X	-0.090647408	0.521022274	0.982083369
**dlg1**	FBgn0001624	63.42776573	67.15191567	87.34175117	62.44045058	61.43075583	63.89152763	63.10924943	65.60002898	X	-0.109865219	0.326777768	0.918470465
**ifc**	FBgn0001941	61.80104677	58.03481472	55.67813705	48.67362174	61.4615369	65.72808437	51.05296817	70.8400162	2L	0.181591316	0.225018261	0.867200583
**eIF4A**	FBgn0001942	2055.161545	2267.049087	2230.266324	2421.41732	2153.089228	2170.466723	2317.333846	2288.478714	2L	0.023997108	0.787197009	0.995787491
**Arpc1**	FBgn0001961	518.9395339	598.9174842	568.8812098	629.0654624	556.4968128	538.5684935	544.064947	486.2298288	2L	-0.093225128	0.341193006	0.927274752
**Sos**	FBgn0001965	99.68018482	131.2881441	120.8064416	168.4685854	108.1147181	122.9840559	112.1007552	137.943376	2L	-0.082349611	0.581755475	0.985637351
**CIAPIN1**	FBgn0001977	40.91430373	48.5997165	54.82485178	39.39857346	32.15045735	37.58794655	40.44141457	41.66822218	2L	-0.241637986	0.144951962	0.8037297
**stc**	FBgn0001978	71.34821716	62.49779312	57.55922038	69.35470778	83.95970383	61.94583467	80.41219422	67.00194708	2L	0.199192136	0.161763371	0.823037813
**Mtr4**	FBgn0001986	33.36600554	25.20702834	27.21292903	28.76234223	28.16088189	32.66975185	40.09813134	29.00729852	2L	0.206534143	0.234472766	0.87009833
**Gli**	FBgn0001987	0.948749286	0.473117085	0.186620476	0.538047546	0.555481824	0.410975015	0.32706484	1.419607416	2L	0.349696476	0.637455898	0.991585536
**ND-B17**	FBgn0001989	449.045925	458.1713385	507.1185979	459.4614043	591.5142468	508.2707209	555.8670549	584.8024873	2L	0.290368514	0.004031559	0.174088135
**wek**	FBgn0001990	39.47853254	36.51918111	28.82713689	41.19139209	31.33007728	34.45624909	26.82291809	29.61715888	2L	-0.229526369	0.211123873	0.856520545
**Ca-alpha1D**	FBgn0001991	41.30571	44.15323476	44.66498896	46.77419989	41.21678956	52.34783597	45.42888131	46.41089834	2L	0.09664146	0.317373968	0.91460807
**crp**	FBgn0001994	59.46539277	69.30332091	69.70587427	75.91188943	75.32501507	63.13743394	62.89650878	69.56229022	2L	0.013782197	0.900031905	0.996479182
**mRpL4**	FBgn0001995	159.3517399	191.0569688	140.4161679	146.2376123	178.5190713	158.4554374	193.7819853	178.7673383	2L	0.186818028	0.191361438	0.843483578
**l(2)37Bb**	FBgn0002021	105.7144106	86.97685875	66.04571431	96.69262892	86.53725419	56.91954073	84.06413157	121.614656	2L	0.006260813	0.977428633	0.997757033
**Catsup**	FBgn0002022	43.11136427	44.36651046	44.01793303	44.37766204	51.3194502	36.03060343	48.55680773	47.12139539	2L	0.088391553	0.6213495	0.990047511
**Lim3**	FBgn0002023	0.688883925	0.473159844	0.316956047	0.242300616	0.600364436	0.338735097	0.1287719	0.348120955	2L	-0.255685839	0.616213522	0.990047511
**l(2)37Cc**	FBgn0002031	258.7565658	256.9629011	293.8888015	248.7534783	315.7719399	262.2992	307.1098413	265.0966975	2L	0.152215078	0.169071382	0.830934498
**CG10561**	FBgn0002036	59.58249078	97.79775721	87.5803268	123.1793996	82.32937225	71.34701465	68.9687915	93.72465838	2L	-0.184351179	0.353014281	0.935162434
**swm**	FBgn0002044	248.1456066	216.5979095	220.0923801	282.1364861	205.1938929	229.3029466	208.0165591	190.0454005	2L	-0.189864683	0.098649965	0.730787365
**AspRS**	FBgn0002069	5.8598002	5.114847566	5.863588708	5.045403238	3.297835391	2.50394807	2.867813181	2.379686226	2R	-0.958458349	0.000254125	0.020710451
**l(2)gl**	FBgn0002121	1.058609052	1.032658089	1.730327841	0.437607651	0.189750704	0.835247642	0.057458179	0.472707866	2L	-1.411372662	0.034179751	0.514749796
**dre4**	FBgn0002183	52.35851742	70.74375853	52.02362837	82.17690185	62.11161401	65.90964473	56.15066836	48.9787647	3L	-0.113616392	0.484070684	0.973549494
**l(3)73Ah**	FBgn0002283	105.0710161	135.4695121	103.9893891	148.9314741	119.4701611	122.9136411	112.1494084	123.1388105	3L	-0.015895831	0.914353486	0.996479182

**Prosbeta6**	FBgn0002284	235.775205	246.6218502	253.8955075	253.9217967	220.1134924	214.8270368	240.7172801	270.2615557	3L	-0.03436664	0.784670417	0.995787491
**sas**	FBgn0002306	1.007381722	0.34768344	0.663773681	0.164310426	0.598710188	0.918485734	0.327841255	0.297016156	3R	-0.012375059	0.982437841	0.997881056
**l(3)87Df**	FBgn0002354	194.8594402	152.0772237	167.1874269	130.3075228	165.2708447	159.5113848	175.1591212	178.9965863	3R	0.1024952	0.535703448	0.984760433
**dco**	FBgn0002413	8.9961406	11.39888916	23.74152953	13.51983219	27.5866307	30.36150064	43.97591269	40.93687184	3R	1.348311762	4.28E-08	1.49E-05
**hyd**	FBgn0002431	139.1786258	175.5454787	189.0294283	170.1632798	145.9407461	156.8776507	163.4521889	189.9723773	3R	-0.004543056	0.969462946	0.997604378
**l(3)mbt**	FBgn0002441	27.75474519	28.04575078	35.78369048	29.06969612	25.68245866	30.97486799	32.22075006	31.67205822	3R	0.030280647	0.82563399	0.995787491
**sti**	FBgn0002466	0.228711812	0.85251551	0.770627313	1.27383434	0.435821408	0.73073967	0.162069096	0.358622705	3L	-0.837445446	0.251047634	0.882053535
**pho**	FBgn0002521	43.29493706	32.78898772	37.71824152	30.31743324	28.90394693	44.8928895	36.28467828	31.4351007	4	-0.001524595	0.992142049	0.998472242
**lab**	FBgn0002522	0.065608413	0.194701916	0.244869377	0.388292076	0.400873849	0.023790466	0.11587048	0.032871216	3R	-0.595536862	0.532959659	0.984760433
**lace**	FBgn0002524	54.18235193	40.00344733	48.36913822	37.12592001	46.76975915	44.31795958	45.19823998	45.52167428	2L	0.045423104	0.747379007	0.995787491
**Lam**	FBgn0002525	112.7369141	131.2217548	114.357279	114.0744396	91.22100626	87.2944179	108.4119513	122.9459395	2L	-0.170743153	0.200596582	0.848203654
**LanA**	FBgn0002526	0.08545243	0.491432448	0.177691204	0.32730499	0.26445178	0.68479425	0.140137062	0.344546682	3L	0.45133994	0.536754125	0.984934163
**lds**	FBgn0002542	0.213345433	0.572571254	0.414058435	1.144035743	0.346628867	0.100570385	1.088497564	0.046319268	3R	-0.549254912	0.610028395	NA
**robo2**	FBgn0002543	0.637293308	0.647860679	0.484121262	0.190105601	0.387366248	0.495639321	0.459807775	0.246909113	2L	-0.274987181	0.499649417	0.976796212
**lin**	FBgn0002552	59.18524332	46.71992377	51.03270212	53.67113704	33.33886619	50.73681064	55.09235314	38.72640412	2R	-0.218709063	0.200797714	0.848203654
**Lsp1alpha**	FBgn0002562	2.478485661	0.431928519	2.420726246	0.900544834	1.394587589	2.882942968	0.903238596	2.236268801	X	0.272283582	0.702464332	0.995787491
**Lsp1beta**	FBgn0002563	3.045172698	4.024366672	1.119325226	4.661422809	0.999512217	6.959927805	2.118627539	1.268845298	2L	-0.163398338	0.801311952	0.995787491
**Lsp1gamma**	FBgn0002564	0	1.41608144	1.556553006	0.787318436	2.255602705	0.318374865	0	0.219948806	3L	-0.365423631	0.787599046	NA
**lt**	FBgn0002566	21.83403215	19.35350937	24.06122013	22.78237259	18.63799017	24.5924533	19.59046342	19.26704772	2L	-0.073440704	0.549182269	0.984934163
**Rab32**	FBgn0002567	2.390737141	0.874001857	0.198287007	0.166741006	1.235747221	1.444854086	0.417013457	0.931630295	2R	0.154812827	0.833120079	0.995787491
**Mal-A2**	FBgn0002569	4.340960803	2.899057122	1.560164498	1.124531855	2.539621657	1.477894647	0.512679827	0	2R	-1.103408265	0.229998228	0.867702781
**Mal-A1**	FBgn0002570	2.165456141	0.064861587	0.093810114	0.180309981	1.116915239	0.984332647	0	3.610007976	2R	1.172990398	0.366077132	NA
**sens**	FBgn0002573	2.104874664	1.275774576	0.944039703	0.288672708	0.862148173	2.251281528	0.360980201	0.537632676	3L	-0.185294198	0.801782704	0.995787491
**lz**	FBgn0002576	0.92151923	0.474428675	0.486960808	0.382898743	1.144739588	0.756976611	0.325855594	0.574459436	X	0.326350053	0.491099357	0.973549494
**m**	FBgn0002577	0.540120337	0.295634304	0.413786654	0.530219045	0.564505897	0.578905186	0.130533971	0.394998316	X	-0.063378777	0.911088202	0.996479182
**RpL36**	FBgn0002579	1298.138948	1745.763503	1844.963925	1906.593017	1877.100432	1647.456043	1784.89549	2063.691287	X	0.151764086	0.2391918	0.873933389
**RpS5a**	FBgn0002590	221.6582394	363.7220321	333.986476	351.1185981	273.1426249	268.3567168	312.4036894	330.1842694	X	-0.066202479	0.644815285	0.992774909
**RpLP1**	FBgn0002593	1259.003799	1542.590616	1513.107768	1751.537484	1409.885045	1416.031908	1548.451042	1874.99484	2L	0.075951276	0.561066371	0.984934163
**E(spl)m3-HLH**	FBgn0002609	1.527775805	12.25605455	6.284702128	3.716819244	3.237683745	1.252502345	5.591905816	1.730578799	3R	-0.967817631	0.176752269	0.834615603
**RpS3**	FBgn0002622	638.4292485	851.4303858	914.9518991	979.7695391	831.8336333	773.7803263	955.2974542	1005.932318	3R	0.109805935	0.433411953	0.965402206
**RpL32**	FBgn0002626	276.7694587	389.7262761	418.4655806	447.4473439	381.0214926	326.4773688	373.6489794	472.7636755	3R	0.055566234	0.734674982	0.995787491
**Rcc1**	FBgn0002638	129.2892014	97.84361416	114.6493343	121.4417524	136.0047804	108.5589822	141.67808	163.2666588	3L	0.277152197	0.073705595	0.675013659
**mal**	FBgn0002641	10.26677367	9.905199246	8.738272815	15.424537	12.429829	14.53072792	8.688581548	12.44841802	X	0.146273286	0.574119292	0.984934163
**mam**	FBgn0002643	5.974708714	6.571905507	5.958874669	7.654093908	5.657499601	5.382566931	5.294622276	5.819564314	2R	-0.20930096	0.080779586	0.694684542
**Map205**	FBgn0002645	15.72184993	20.25581741	18.95075493	20.45248363	19.22227928	20.14589405	21.62307607	20.27441039	3R	0.140970154	0.255043296	0.886412683
**twe**	FBgn0002673	1.828724634	1.651692554	1.876964039	1.967816604	0.761842307	1.624642506	4.395974532	3.206805374	2L	0.471387453	0.454966833	0.967967712
**mei-9**	FBgn0002707	8.640980095	11.02944971	13.79137973	13.96090758	17.99379528	21.74230083	15.61402249	14.19369923	X	0.586461976	0.006423324	0.228769161
**mei-S332**	FBgn0002715	6.043950609	9.13587957	9.888689684	6.144437493	9.578737587	7.75220194	4.033843915	10.07035387	2R	0.052158472	0.885068535	0.995787491
**Men**	FBgn0002719	154.0189086	134.1042316	157.077849	166.2890894	127.7701281	137.2245271	140.022583	159.0393083	3R	-0.086866934	0.44157551	0.967112302
**Met**	FBgn0002723	66.55963717	72.475905	62.39646199	81.15487873	66.65965038	77.65601993	60.89822981	74.11370408	X	0.012248841	0.922603242	0.996479182
**E(spl)mbeta-H**	FBgn0002733	11.52545746	8.327776378	18.49701167	2.687117018	1.920591199	0.668684978	9.95134109	13.6663176	3R	-0.605248583	0.435742193	0.966084844
**mago**	FBgn0002736	263.0655857	227.4539307	286.3990438	239.0288592	239.000604	273.1491666	221.9174409	253.22744	2R	-0.011874243	0.922547619	0.996479182
**Mlc1**	FBgn0002772	0.475156559	0.562594214	1.559566661	0.195495817	0.30274566	4.347989749	0.285208139	2.063217748	3R	1.363494353	0.145045533	0.8037297
**mle**	FBgn0002774	130.7392774	125.0951181	126.8402736	97.4435214	119.1049285	146.5275015	123.637403	100.2373687	2R	0.055936141	0.659140384	0.995750687
**msl-3**	FBgn0002775	88.03699277	153.8069531	191.5667228	129.35065	111.9514956	122.3891305	139.0555638	152.1472167	3L	-0.060757965	0.748748796	0.995787491
**mnd**	FBgn0002778	15.30031444	16.04008191	16.70325724	17.77454994	9.310178192	6.764847752	9.690562145	13.14417684	3L	-0.725995843	0.000609976	0.041329604
**mod**	FBgn0002780	100.3214067	89.84643111	95.69275505	82.26194283	84.14043695	102.0088801	82.653348	70.34361362	3R	-0.090880339	0.491921906	0.973549494
**mod(mdg4)**	FBgn0002781	124.5176016	146.8602143	130.6941809	147.6788653	124.2053489	133.7916449	129.2838747	145.3390321	3R	-0.014274096	0.882765531	0.995787491
**mor**	FBgn0002783	162.0716369	188.7036525	188.896941	195.1437691	168.3763161	168.8798787	189.5703124	185.7345476	3R	-0.012178967	0.906064093	0.996479182
**Rpn8**	FBgn0002787	72.4096959	81.73569193	83.23181965	72.44782103	89.11489091	69.22944326	86.04816398	96.8077856	2R	0.174025628	0.18936331	0.843401316
**mr**	FBgn0002791	50.83039138	54.56304015	49.19847332	43.1435693	37.135462	46.63330868	43.42107096	46.30339811	2R	-0.158826401	0.260078663	0.890556245
**Acp26Aa**	FBgn0002855	3.163746386	2.438232229	4.148759245	0.59806667	0.154361435	1.128612182	1.963163919	0.185642872	2L	-1.578134589	0.150615269	NA
**Mst98Ca**	FBgn0002865	1.182950244	1.653527569	0.956606931	0.612889769	1.30504512	1.363117879	0.502955248	0.285366054	3R	-0.308573005	0.771385897	NA
**MtnA**	FBgn0002868	42.90436466	161.7350286	123.5059348	77.59133562	25.81779151	73.01347317	80.07973722	46.67231447	3R	-0.809461054	0.110515393	0.758410631
**mu2**	FBgn0002872	5.462741077	11.73719744	5.274419751	6.687556864	8.014650165	5.92535849	10.67306186	8.250515867	3L	0.209408816	0.4933092	0.973549494
**mus101**	FBgn0002878	5.997606078	10.08999487	7.512519027	6.638904588	4.626466246	6.090759093	4.903271132	5.52538336	X	-0.479552788	0.060374362	0.627984253
**mus201**	FBgn0002887	12.24652886	17.68686737	23.36508035	20.76092494	13.60211657	18.90405121	19.75076613	12.21261399	2L	-0.167526725	0.475405569	0.973549494
**DNApol-zeta**	FBgn0002891	2.977173829	4.837123476	6.287895078	5.906798467	4.55257	3.874569326	5.24192492	7.604376444	2R	0.128506055	0.671047411	0.995787491
**mus304**	FBgn0002901	6.340010067	5.317238736	6.235449726	5.193498172	7.166228339	6.677369576	5.391167297	4.712256326	3L	0.082501312	0.787153346	0.995787491
**DNApol-theta**	FBgn0002905	1.610669857	1.050650506	3.008125976	0.745082567	1.292298624	3.820427903	1.010908378	4.607044582	3R	0.777126167	0.152742179	0.813908772
**Blm**	FBgn0002906	4.633109459	8.005117208	9.591909685	7.02530906	6.791778972	4.335224687	5.109787983	14.29423984	3R	0.106730217	0.770037681	0.995787491
**na**	FBgn0002917	33.72537721	47.78565473	40.62337087	40.35653644	41.11226606	39.62314805	41.21720305	41.56333716	X	0.043121466	0.710918099	0.995787491
**Atpalpha**	FBgn0002921	1973.011011	1993.879648	1862.323635	1972.75064	2039.830112	2092.270799	1849.491145	2087.379738	3R	0.078481133	0.334986214	0.924122842
**nau**	FBgn0002922	0.187519204	1.048457195	0.644468546	0.145731322	0.564200373	0.17679196	0.079727586	0.237487571	3R	-0.876019194	0.375514521	NA
**ncd**	FBgn0002924	5.495694119	7.067329536	3.555327274	3.915084541	4.905450785	2.533083347	7.710798573	8.087014449	3R	0.244578995	0.573238903	0.984934163
**ndl**	FBgn0002926	0.62473915	0.739702787	0.089153439	0.042839877	0.132684033	0.051970611	0.187496823	0	3L	-1.997107995	0.084673669	NA
**net**	FBgn0002931	5.98269397	7.723831719	6.770347347	6.669218153	5.101014422	8.534671036	5.205981807	5.793919272	2L	-0.105940997	0.676225545	0.995787491
**neur**	FBgn0002932	12.65098364	11.08497858	11.46109115	11.97553213	11.34962973	13.4005634	11.48560454	10.82727128	3R	0.023911326	0.84242636	0.995787491
**ninaB**	FBgn0002937	33.1608905	51.98909536	59.91141975	35.82056541	30.44335342	10.11755877	44.38004344	28.87050233	3R	-0.622635081	0.179239212	0.836045561
**ninaC**	FBgn0002938	0.456434598	0.262493145	0.949117686	0.395259459	0.258965593	0.467208644	0.199607051	0.679516638	2L	-0.3189783	0.678076233	0.995787491
**ninaD**	FBgn0002939	2.756035089	1.027302717	2.228700895	2.447844593	0.315895219	5.196746095	4.798734969	3.704137853	2L	0.743396027	0.306298505	0.911610813
**ninaE**	FBgn0002940	0.266381073	1.638328563	0	0.207019148	0.64118148	1.130141019	0.33977192	0.578337969	3R	0.390716186	0.765599816	NA
**slou**	FBgn0002941	0.355784368	0.136406484	0.271268773	0.21329945	0.275263702	0.057502513	0.285249978	0.551743426	3R	0.277580708	0.756950685	NA
**nkd**	FBgn0002945	1.848332181	2.389683266	2.131172902	2.53232892	2.545649818	2.562987129	2.913847537	2.849610075	3L	0.321358934	0.046456938	0.565973818
**numb**	FBgn0002973	9.798253353	9.229161703	9.753583124	9.297961074	9.4236457	8.861743601	9.106586978	8.024858753	2L	-0.075197126	0.503888968	0.978429306
**odd**	FBgn0002985	0.239465924	0.803340988	0	0.07975797	0.494054134	0.19351458	0	0.594174501	2L	0.232873093	0.888086413	NA
**okr**	FBgn0002989	2.654650814	0.918103986	5.577033518	3.636963412	4.397081794	6.037654907	8.447635284	1.366601351	2L	0.690305592	0.225903262	0.867702781
**opa**	FBgn0003002	0.343122492	0.567076024	0.636549115	0.235287404	0.173074364	0.237863081	0.167339221	0.262923774	3R	-1.039748018	0.06280352	0.636110045
**or**	FBgn0003008	189.0244783	148.7630224	178.9493334	128.1278416	190.7878122	182.3593514	198.0762002	145.1629261	2R	0.17965532	0.268706213	0.89549671
**ord**	FBgn0003009	2.429116494	0.37349603	0.245541781	0.519144419	0.328888339	1.832124954	0.206557612	0.966870074	2R	-0.105173263	0.908325734	0.996479182
**ort**	FBgn0003011	0.132443801	0.074051965	0.696164762	0	0.557888346	0.166489608	0.788356289	0	3R	0.799879229	0.593510812	NA
**osp**	FBgn0003016	1.482649461	2.072715689	2.734380006	1.299358402	1.680874172	2.22312497	2.00756944	1.351841905	2L	-0.029794936	0.889027611	0.995926915
**otu**	FBgn0003023	0.133935878	0.449317292	0.83552547	0.713751903	1.865226244	0.198430458	0.146431415	0.747738487	X	0.532977225	0.574856603	0.984984955
**ovo**	FBgn0003028	0.080287039	0.391184997	0.009275011	0.08913629	0.276073682	0.61997097	0.126789595	0.041502522	X	0.929007152	0.318843686	0.915028677
**pbl**	FBgn0003041	0.5521264	1.237875925	1.167046368	0.802945552	1.075678545	0.69061928	0.725161268	0.652766969	3L	-0.214357026	0.585014403	0.987145178
**Pc**	FBgn0003042	31.04756002	25.05463048	24.01639981	23.98143995	23.25387071	27.92437218	22.79945269	23.86709258	3L	-0.063227217	0.623794826	0.990047511
**Pcl**	FBgn0003044	111.8218018	102.5688513	106.7708735	103.8054517	118.6808094	115.5880736	115.1303206	110.5098176	2R	0.143238473	0.131439948	0.790743321
**pcx**	FBgn0003048	6.107914569	8.911816251	13.4331904	15.68183443	10.339504	6.431077381	14.99213505	13.68861722	X	0.079819557	0.843462325	0.995787491
**peb**	FBgn0003053	0.673757356	1.115064999	0.61022302	0.188500789	0.810869806	0.931647669	0.939593988	0.253550195	X	0.212392674	0.732393135	0.995787491
**Fib**	FBgn0003062	2.623349951	3.200219289	6.248497474	12.45489209	2.066539209	1.753779165	3.163595895	4.142207697	2R	-1.112717437	0.060458347	0.627984253
**per**	FBgn0003068	19.26052934	26.93917929	17.83226037	18.4557283	15.9191447	18.8619199	18.41375046	18.12653101	X	-0.177901422	0.264634446	0.893759496
**Pfk**	FBgn0003071	204.9564812	158.1489735	138.8190327	169.7068103	202.9118166	183.8558531	168.0215955	172.3138005	2R	0.141692877	0.269193044	0.89549671
**Pgi**	FBgn0003074	889.2515001	641.7240488	589.4247255	701.8763739	849.8353937	865.541805	726.1374465	713.0588997	2R	0.18593719	0.157806787	0.818884898
**Pgm1**	FBgn0003076	98.02717494	75.04431458	72.41409149	58.50280393	93.83332482	113.7694781	95.75443337	77.15319242	3L	0.348596325	0.038010302	0.522230403

**Raf**	FBgn0003079	22.67814699	20.17382432	20.41263432	21.39879102	20.86566759	24.01440622	18.58417771	20.94478004	X	0.022973831	0.821424901	0.995787491
**phr**	FBgn0003082	155.0167913	109.6473952	109.5184561	141.561988	132.7973705	129.3335156	111.0267516	96.15880107	2R	-0.111970022	0.484048638	0.973549494
**pip**	FBgn0003089	0.259948052	0.592548683	0.458150252	0.352239475	0.393066154	0.450355971	0.419417494	0.395542267	3L	0.037111968	0.915015268	0.996479182
**pk**	FBgn0003090	0.638835634	0.621743051	0.390343014	0.32233743	0.314134812	0.352834262	0.212831791	0.364856255	2R	-0.635782381	0.038789617	0.525600179
**Pkc53E**	FBgn0003091	479.6943916	367.3590987	438.7873575	350.6132913	360.2402199	362.272283	351.724132	336.2523651	2R	-0.185317357	0.093957749	0.728467429
**Pkc98E**	FBgn0003093	778.2292218	670.8318763	554.1481365	699.1349894	686.5988278	765.2406259	622.9789003	635.8001945	3R	0.030635365	0.789669682	0.995787491
**pn**	FBgn0003116	44.45217885	26.41635559	51.76337562	50.93096008	38.79397094	48.80630664	42.11942921	49.0821244	X	0.070057917	0.757573654	0.995787491
**pnr**	FBgn0003117	0.975467931	0.351276854	0.467946361	0.167036542	0.338265352	0.509193166	0.224943697	0.370919585	3R	-0.427720533	0.479123088	0.973549494
**pnt**	FBgn0003118	1.130422614	1.379236386	1.18335985	0.566821137	0.91412292	0.772307778	0.691305706	0.618606018	3R	-0.473288842	0.074500174	0.676245
**polo**	FBgn0003124	1.067958433	0.038113839	0.661494194	0.423813597	0.902439385	1.156823257	0.057965636	0.147998082	3L	0.058936106	0.958100558	NA
**Poxm**	FBgn0003129	0.334342791	0.669040224	0.554970649	0.21880916	0.296492702	0.741034908	0.104744026	0.025469795	3R	-0.568954339	0.475177291	0.973549494
**Pp1alpha-96A**	FBgn0003134	289.822389	266.2874023	279.4723654	282.2088748	259.2145468	265.5208744	255.8971734	297.3427849	3R	-0.022166149	0.819525897	0.995787491
**Ppn**	FBgn0003137	0.541942327	0.916422733	1.143720849	0.328720165	1.368091915	0.585711611	0.438355726	1.807957036	3R	0.569317316	0.247798589	0.879503029
**PpV**	FBgn0003139	355.4625519	337.3868378	335.761104	395.3798282	333.6135258	309.9668431	324.997719	341.9573816	X	-0.089987476	0.400408989	0.953696242
**pr**	FBgn0003141	90.10489153	85.97088238	70.56851005	63.40607228	72.20449176	58.93459282	81.19415673	67.4796361	2L	-0.118988037	0.487359011	0.973549494
**prd**	FBgn0003145	0.122342155	3.23208588	0.519400632	0.499163225	0	1.989674277	0.546170565	0	2L	-0.746513974	0.581457765	0.985637351
**Prm**	FBgn0003149	0.848652905	0.933440127	0.303760344	0.145962465	0.301384131	0.819779482	0.177453426	0.181229903	3L	-0.566968751	0.432941863	0.965402206
**CG2841**	FBgn0003159	1.278617537	1.486359616	1.197572196	1.452789534	0.460185566	1.274138977	0.815445202	1.010252085	X	-0.575445204	0.078738012	0.689288437
**Pu**	FBgn0003162	17.70846075	17.4316072	16.0777647	20.54638787	17.10979988	23.85061233	22.20860401	18.82367304	2R	0.219128496	0.157157588	0.818884898
**pum**	FBgn0003165	24.46850809	24.77253146	26.55335582	24.3507241	29.30345337	27.62093299	24.87837516	28.4862838	3R	0.170258443	0.052735025	0.605783103
**put**	FBgn0003169	52.22017117	56.47866705	59.39067298	55.3516245	52.77102301	74.87114784	71.06112722	59.26603485	3R	0.23635607	0.055827868	0.619039537
**pwn**	FBgn0003174	0.427533295	0.501988988	0.3687786	0.420861731	0.137210164	0.738972262	0.412022057	0.556928616	2R	0.129572236	0.807731856	0.995787491
**px**	FBgn0003175	12.50550282	15.50705716	16.96754864	16.08592362	12.14386564	15.20399009	13.36523961	15.87022749	2R	-0.078497477	0.517918034	0.980804634
**qua**	FBgn0003187	0.466316262	0.83805012	0.525235633	0.582427934	0.541170237	0.235521495	0.06372765	0.289261649	2L	-1.047995434	0.121250989	0.77836519
**r**	FBgn0003189	0.914638855	0.435227906	0.566527942	0.907423855	1.264716424	0.501488695	0.595726818	0.211251662	X	-0.111510915	0.842018621	0.995787491
**ras**	FBgn0003204	117.2315277	143.108235	128.001502	149.2519303	141.4289364	142.550631	145.5628013	156.9113349	X	0.157433581	0.15154294	0.812192196
**Ras85D**	FBgn0003205	114.1314633	142.8549285	134.2480383	143.9256431	117.4944913	145.5011883	122.1215214	152.4110942	3R	0.038150059	0.766557809	0.995787491
**Ras64B**	FBgn0003206	48.68079697	57.56535714	61.71913662	59.83620117	52.7961312	46.31798983	61.37916821	56.8542089	3L	-0.034761418	0.824007677	0.995787491
**raw**	FBgn0003209	12.62971691	11.0831974	12.07011278	11.04870206	11.97907923	9.453080372	12.6132396	12.08496497	2L	0.009631207	0.941595746	0.997604378
**rdgB**	FBgn0003218	160.3648626	166.663275	143.7016692	206.0136344	188.8638534	187.6847224	168.8660625	183.8537301	X	0.136075445	0.245712184	0.877269208
**rec**	FBgn0003227	1.33355876	5.652599963	4.874651851	4.663713068	3.529791424	8.834877669	7.056720764	5.457993136	3R	0.626106291	0.08477448	0.69799888
**ref(2)P**	FBgn0003231	470.4101931	420.114398	406.2148508	443.6936883	486.7211998	458.957525	500.7583568	422.9433446	2L	0.13205401	0.189469607	0.843401316
**Rh2**	FBgn0003248	0.922625424	0.182067614	1.4482965	1.644933162	0.195950042	0.051167389	0.692246258	1.885275483	3R	-0.547726559	0.658700429	NA
**rib**	FBgn0003254	0.841845469	0.667527628	0.631258022	1.017051528	0.44210817	0.66381075	0.488083154	0.747705454	2R	-0.404410133	0.376945752	0.943615128
**rk**	FBgn0003255	16.44833911	12.53081704	9.368337802	9.548974956	10.87521144	10.31766828	9.075028599	9.003935114	2L	-0.260196945	0.148895454	0.808039863
**rl**	FBgn0003256	8.750088819	8.133463234	9.136121636	6.017814586	6.517133259	8.702339842	6.259965181	7.237048691	2R	-0.12870624	0.371689952	0.943282319
**r-l**	FBgn0003257	1.254427888	3.787428245	1.14120921	1.901023408	3.849758536	5.35156795	0.160003625	3.540522785	3R	0.710654763	0.329763842	0.921112231
**Rm62**	FBgn0003261	377.6653252	396.3441562	349.1591049	442.7827512	320.2928773	422.1483187	369.0519417	415.5090049	3R	-0.008133317	0.942996171	0.997604378
**ro**	FBgn0003267	0.098429825	0.627388445	0.620852392	0.504867947	0.426458546	0.129918652	0	0	3R	-1.664649997	0.222312519	NA
**rod**	FBgn0003268	1.793412189	0.05976551	2.247429474	1.716814081	1.715268158	5.128441524	4.241752472	1.933939011	3R	1.175423698	0.062214157	0.633840737
**RpLP2**	FBgn0003274	1065.993368	1201.111823	1160.793596	1193.263348	1371.268142	1553.225043	1627.372454	1467.649446	2R	0.412100623	1.63E-05	0.002347526
**RpII18**	FBgn0003275	387.0483069	360.0873986	321.1360188	264.3428189	295.0734213	314.1725285	365.5832982	296.4540893	3R	-0.039405352	0.791617315	0.995787491
**RpII215**	FBgn0003277	46.95753949	66.48666059	53.58070747	69.09566646	53.81257848	60.10260484	76.04296631	74.16302007	X	0.195099232	0.206645525	0.855364409
**RpI135**	FBgn0003278	9.240220016	13.44839167	19.16537577	17.98009628	11.46020522	19.15227039	12.59572507	20.41870027	2L	0.123719648	0.649100731	0.992932477
**RpL4**	FBgn0003279	618.6897817	826.6133912	884.7614462	896.0123745	793.2637869	739.2352463	776.9587133	891.1517985	3R	0.022211115	0.859903038	0.995787491
**rt**	FBgn0003292	33.78776413	28.19772167	26.38203738	29.7274219	30.8621316	31.92205484	32.40577795	30.67254839	3L	0.117421235	0.429292739	0.963666972
**run**	FBgn0003300	0.963727286	0.436896574	0.568700016	0.303634309	0.376167514	0.073669962	0.332228245	0	X	-1.530501345	0.12416746	0.780439453
**rut**	FBgn0003301	37.26862514	63.33354619	59.31356238	51.69680342	50.87484524	49.5480257	49.36860326	55.62995133	X	-0.006915496	0.959482421	0.997604378
**rux**	FBgn0003302	52.59720534	67.35719003	67.44429353	58.40387393	59.35419624	79.03014456	73.51107415	59.71497271	X	0.176162914	0.247650043	0.879384515
**ry**	FBgn0003308	0.962074469	0.370367695	0.535667367	0.514796159	0.370135786	0.817821087	0.683977621	0.273934784	3R	-0.140127914	0.848461729	0.995787491
**S**	FBgn0003310	9.409472902	11.06931755	9.408680085	11.48201863	9.96853102	9.62569483	10.46680475	10.58530498	2L	0.005824797	0.961636472	0.997604378
**sad**	FBgn0003312	0.687719298	2.249427659	0.583939099	0	0.682830277	0.259351022	0.087719335	0	3R	-1.711748927	0.228174673	NA
**sax**	FBgn0003317	143.5740332	142.3703697	129.4354411	165.311492	115.9122977	142.5629421	120.1955891	123.8662298	2R	-0.181127153	0.117275377	0.770191485
**Sb**	FBgn0003319	0.850627279	0.279374979	0.483433092	0.658757065	0.467123154	0.944974162	0.584222279	0.374523961	3R	0.073930462	0.861690965	0.995787491
**sbr**	FBgn0003321	69.98319345	55.0055209	45.61709787	51.74016286	49.3530106	62.92732494	41.52724477	44.77154584	X	-0.139256178	0.349775074	0.932509746
**sca**	FBgn0003326	69.23327101	57.86829436	52.20821901	56.40110321	61.87280701	64.26470578	60.30248114	56.13043543	2R	0.068910189	0.526333153	0.984107922
**Sce**	FBgn0003330	171.1767333	150.6542282	143.8593951	186.9085592	123.0983178	120.6550088	140.764941	123.9194053	3R	-0.332749959	0.015065689	0.361837042
**Scm**	FBgn0003334	128.0124778	164.4592091	152.3130313	163.9989673	147.28466	151.9180479	142.8581374	154.8854458	3R	0.003873255	0.971024779	0.997604378
**Scr**	FBgn0003339	0.319243748	0.384035846	0.312921568	0.097736991	0.098963489	0.41954935	0.378406994	0.15402394	3R	-0.058583212	0.923058603	0.996479182
**sd**	FBgn0003345	41.99379771	30.13993763	33.68536413	34.01881313	32.55209985	37.71035695	26.28414381	34.89753125	X	-0.063558426	0.639759471	0.991585536
**RanGAP**	FBgn0003346	12.33477444	9.111128244	7.765334424	9.008095514	6.508042882	4.648611728	5.526187149	4.668062433	2L	-0.816846784	0.000933304	0.057615996
**sei**	FBgn0003353	103.3135121	98.1430551	93.29769479	83.77360748	93.64293626	112.5046424	91.19128341	111.2822069	2R	0.139722414	0.218398142	0.862212278
**Jon99Ci**	FBgn0003358	0.989408829	0.150872228	0.218208365	0.209706312	0.649504189	10.68489851	4.589096689	1.366971554	3R	3.369197659	0.00280092	NA
**sev**	FBgn0003366	1.556540018	4.537255315	2.927572604	3.194076393	1.968019986	1.451007698	1.814357967	1.809936789	X	-0.754054135	0.014199725	0.354312909
**sgg**	FBgn0003371	58.4622347	76.99242956	78.69738934	73.78881813	75.54541235	69.043249	81.35791854	81.95525203	X	0.131258236	0.245592771	0.877269208
**Sgs7**	FBgn0003377	16.15677121	4.14038734	2.7219515	4.18543376	0	2.538750868	4.007137407	6.333499956	3L	-1.084671277	0.331318643	NA
**Sh**	FBgn0003380	26.27723517	31.31880074	28.68442408	26.06243606	28.88657609	29.86988219	22.51158524	27.07960978	X	-0.021281478	0.83559919	0.995787491
**sha**	FBgn0003382	1.026671115	0.669704298	1.003194505	0.465430983	0.630672363	0.759563444	1.145838781	0.294103906	2R	-0.140528962	0.782142055	0.995787491
**Shaw**	FBgn0003386	85.85954568	99.26379458	78.28282843	100.5497952	96.90258782	126.3096504	91.45048041	101.9896193	2L	0.222387806	0.064578109	0.641709151
**shd**	FBgn0003388	0.659671678	0.037508702	0.081373947	0.104271165	0.867926906	0.052706308	0.399317165	0.291296599	3L	0.854317355	0.429604016	0.963666972
**shf**	FBgn0003390	1.469001482	1.04588914	0.897353947	0.246397241	1.316422793	0.647645795	1.321045055	2.019149365	X	0.560419438	0.318550624	0.91460807
**shg**	FBgn0003391	1.423924494	4.507205309	1.922218116	0.348046311	1.264544099	2.901461618	4.569871519	0.498624498	2R	0.211015009	0.7416727	0.995787491
**shi**	FBgn0003392	595.4529768	624.1099222	614.3272269	619.5628794	596.0517624	681.2049361	603.6128299	624.3595623	X	0.059498707	0.434164563	0.965402206
**shn**	FBgn0003396	2.751200366	3.421196221	4.937306689	3.692043849	4.25059154	3.330503149	4.524803057	3.247798296	2R	0.087833263	0.628438547	0.990424271
**shu**	FBgn0003401	4.129198559	3.821324863	0.921138217	0.331967965	0	2.282093588	0	1.236534111	2R	-1.358513816	0.263094252	0.892691887
**skd**	FBgn0003415	19.49786217	27.45065282	30.81610767	28.45460251	24.19144202	25.01789139	25.5047996	30.08791031	3L	0.014859994	0.914216277	0.996479182
**sl**	FBgn0003416	16.98094032	15.61978931	17.24146843	14.86687518	13.43848074	15.90359115	18.02260383	12.22807654	X	-0.091302679	0.58883914	0.987588562
**slgA**	FBgn0003423	188.088217	155.1478473	159.6963114	164.4791624	168.1829884	192.9554573	161.1933477	153.5025618	X	0.044369407	0.668319393	0.995787491
**slo**	FBgn0003429	119.2659014	115.5478851	102.2472167	110.2146963	97.40208737	111.8012202	75.21648069	92.94046465	3R	-0.218154048	0.053730796	0.608413656
**slp1**	FBgn0003430	4.927020995	6.529885651	1.770797661	0.141816839	3.733508805	0	0	0.396185877	2L	-1.643760169	0.214606407	0.858753183
**sm**	FBgn0003435	9.267460216	11.72225198	11.18127014	11.73896611	11.5951063	12.59324518	10.93508845	11.84163157	2R	0.128105828	0.20042959	0.848203654
**smo**	FBgn0003444	24.61582859	18.47416449	20.89888834	25.14980135	17.90474811	16.77790404	26.02423184	16.32418306	2L	-0.185208107	0.358841572	0.93823489
**sn**	FBgn0003447	6.226753452	3.481501796	3.480992216	5.91252537	5.256828334	6.478217074	3.836571829	7.429957774	X	0.289977613	0.234255523	0.87009833
**snf**	FBgn0003449	301.4782783	267.1534929	286.4106984	246.8599749	285.9090097	253.4865779	272.2134421	273.047488	X	0.007320487	0.949175427	0.997604378
**snk**	FBgn0003450	0.631764556	0.669281946	1.290654316	1.550458188	3.061333899	0.125394477	0	1.155047858	3R	0.115829844	0.915686185	0.996479182
**stwl**	FBgn0003459	10.12132161	8.851307048	9.323994571	12.64871045	8.745371425	11.09192775	4.476677242	14.81053483	3L	-0.036728791	0.898339743	0.996479182
**so**	FBgn0003460	0.251115903	0.66457846	0.121840736	0.247197294	0.16118349	0.226228294	0.142356014	0.048461855	2R	-1.109792863	0.148033368	0.808004944
**Sod1**	FBgn0003462	2389.452468	2092.414359	2205.18966	2153.318891	2430.404498	1958.180538	2201.017427	2303.978822	3L	0.040312359	0.70091972	0.995787491
**sog**	FBgn0003463	1.689215231	1.305879673	0.850267918	1.620882137	0.98018564	1.091514398	0.59361764	0.654900471	X	-0.694753002	0.021919138	0.420318478
**sol**	FBgn0003464	278.5471178	258.9976216	207.1335188	272.5704783	243.6081115	230.2982233	251.1334458	226.6925198	X	-0.066934744	0.555399153	0.984934163
**spir**	FBgn0003475	102.747739	79.25333125	68.25828556	84.68121677	86.38175038	95.52086286	75.92606576	78.74509912	2L	0.032308051	0.799455755	0.995787491
**spn-A**	FBgn0003479	8.97832673	6.392324675	11.59579756	11.59577388	4.431013476	1.58332735	2.306868067	5.46919683	3R	-1.45588741	0.004983047	0.200621808
**spn-B**	FBgn0003480	22.64202054	34.89944987	22.1336439	32.94449804	26.31242837	13.00736154	24.12591583	14.37295524	3R	-0.500643834	0.095487686	0.730787365
**spn-E**	FBgn0003483	0.730485491	0.249210902	0.390473203	0.259794827	1.229309225	0.02334567	0.505351642	0.053761072	3R	0.174777572	0.866125477	0.995787491

spz	FBgn0003495	2.779286228	5.920279693	5.708382381	2.226189374	11.14276857	17.97232381	8.829900813	9.773000901	3R	1.557782101	2.33E-06	0.000511826
sr	FBgn0003499	11.86116754	20.33345584	25.11010592	20.934319	7.071136004	10.31275413	19.88586361	16.75203884	3R	-0.495419062	0.189372242	0.843401316
Btk29A	FBgn0003502	0.567908696	0.806296996	0.772517701	0.245896439	0.794546082	0.709432626	0.574324817	0.427140314	2L	0.102977605	0.775061243	0.995787491
srp	FBgn0003507	0.130412794	0.291665555	0.316379619	0.349097337	0.244148092	0.300550276	0.394294328	0.199245434	3R	0.107064298	0.854369136	0.995787491
Sry-delta	FBgn0003512	132.8146433	172.4671708	123.2245943	117.5084232	120.438886	124.1068499	90.53129354	115.2681225	3R	-0.245300161	0.110945349	0.758410631
ss	FBgn0003513	0.574375843	0.506548246	0.395043959	0.18637455	0.513102981	0.32658562	0.188820247	0.231406353	3R	-0.369944141	0.44573326	0.967112302
sqh	FBgn0003514	532.9167773	517.1455856	470.0992227	481.7979834	515.4356992	509.8938855	461.8051704	453.1802355	X	-0.015929595	0.863886088	0.995787491
sta	FBgn0003517	770.8809461	1073.85101	1064.066763	1244.674444	1085.384501	1043.984577	1126.292544	1273.56892	X	0.158049936	0.245547666	0.877269208
stau	FBgn0003520	161.6002206	207.5123191	173.268459	229.896484	185.3487047	149.641538	156.8030244	181.4242833	2R	-0.165305112	0.207062892	0.855489753
stg	FBgn0003525	2.375789828	0.767762725	0.475896301	0.25408553	1.534563967	0.924721158	0.556026687	0.23660824	3R	-0.247515658	0.772719186	0.995787491
Su(dx)	FBgn0003557	219.1345343	232.6646903	236.6460873	252.782786	200.6622608	223.0834222	202.3465964	246.7496576	2L	-0.078239975	0.445877639	0.967112302
su(f)	FBgn0003559	78.03077151	61.88853265	61.01119941	76.48857743	77.54651426	63.36780278	73.40210915	71.42908286	X	0.070799288	0.607866925	0.990047511
su(Hw)	FBgn0003567	25.77175669	41.15494392	36.80152172	35.31877367	36.00930408	25.40368037	34.52912279	44.3528843	3R	0.051680885	0.792295504	0.995787491
su(sable)	FBgn0003575	22.0672913	30.75642519	42.32063906	23.06868029	18.6966218	20.67001278	30.04225616	32.33037846	X	-0.177244768	0.416011794	0.958846438
Su(var)3-7	FBgn0003598	41.61461714	32.36632077	43.58979377	37.47803656	34.44839035	35.41998931	28.89602293	26.15022295	3R	-0.283967591	0.056629004	0.621788268
Su(var)205	FBgn0003607	237.2737772	186.5655113	199.3189014	198.3024103	192.2199181	248.8105956	198.3663118	188.4333476	2L	0.036468625	0.780229303	0.995787491
Su(var)2-10	FBgn0003612	198.4497982	197.2463162	210.3134025	218.5413415	189.6741735	186.8471833	191.305021	184.1530549	2R	-0.102850798	0.246511182	0.877584019
su(w[a])	FBgn0003638	30.08890924	28.34073638	23.26827026	28.77326842	26.20474357	30.16486265	23.72346096	24.12543012	X	-0.057489198	0.616434359	0.990047511
svp	FBgn0003651	0.615499248	0.59042186	0.720358087	0.568503682	0.58574151	1.088273492	0.411349674	0.619055423	3R	0.146900635	0.634429471	0.991585536
sw	FBgn0003654	54.32026544	53.84751833	49.80747808	53.57439707	45.06655503	56.42040419	45.02742543	48.61636522	X	-0.088296007	0.40764154	0.956706394
sws	FBgn0003656	42.68695252	41.47687156	41.3790037	38.3692564	39.14682248	42.66284548	45.91178987	44.42779002	X	0.100748471	0.323657632	0.918470465
CCT1	FBgn0003676	162.6597132	125.9926608	127.3952605	111.0969056	139.2842114	129.3524749	143.7124586	168.2353929	3R	0.169190303	0.250660848	0.881809839
Tbp	FBgn0003687	102.38874	74.74917723	93.47369171	64.76237118	73.76262296	80.36780635	92.50052302	85.85739579	2R	0.015002726	0.932693526	0.996479182
thr	FBgn0003701	0.166218382	0.340765355	0.537657968	0.1722364	1.266948629	0.174121861	0.094228147	0	2R	0.392717785	0.755534635	NA
tipE	FBgn0003710	7.107642014	4.230412778	2.860596562	3.39824143	3.459079526	5.095261952	2.59024964	2.808932147	3L	-0.316832579	0.392304362	0.951255325
tko	FBgn0003714	177.0387354	172.8976683	194.0870904	189.8036324	187.773399	179.4821551	212.5941124	196.0850883	X	0.111251989	0.336453707	0.924219112
tkv	FBgn0003716	4.864670103	6.024163559	5.491645231	4.788072622	4.20591593	5.032236133	5.116649201	4.031520038	2L	-0.172037106	0.174863553	0.834088743
tld	FBgn0003719	2.292094354	1.191846686	1.779388459	0.480953853	2.234421595	0.929218199	0.467774555	0.398107922	3R	-0.482144607	0.514576876	0.980804634
Tm1	FBgn0003721	22.75787474	24.68013221	26.32270239	25.81254887	26.1884259	25.83590309	23.73137695	26.71396617	3R	0.072803099	0.447654498	0.967112302
Egfr	FBgn0003731	0.728795314	0.638305581	0.794502253	0.354887606	0.607869305	0.252227967	0.15296989	0.295426087	2R	-0.906025454	0.036531444	0.521170991
tor	FBgn0003733	0.303088088	0.072626821	0.210082135	0.706638467	0.781645256	0.489856453	0.165682334	0.423020761	2R	0.560071493	0.592090603	NA
tra	FBgn0003741	131.7191687	175.3448911	138.2520641	121.4536011	134.4355698	127.9746483	124.8641807	132.6451104	3L	-0.091229321	0.536048055	0.984881942
tra2	FBgn0003742	134.2527116	99.01887254	112.8584328	145.8555493	85.99241973	81.24311098	86.94636739	86.65257036	2R	-0.502983309	0.000733069	0.048105471
trc	FBgn0003744	289.163934	275.9242096	249.2905611	327.0487598	273.5533219	267.6367401	261.882072	239.8810276	3L	-0.102239238	0.355791293	0.937787223
Treh	FBgn0003748	12.64427931	22.56194261	23.15746692	17.72297209	17.37889284	24.37804042	33.64731411	23.0298645	2R	0.40757827	0.041098916	0.533517704
trk	FBgn0003751	9.635173749	10.38963361	2.862220056	3.438374216	8.164510941	10.38169481	9.029217027	7.684477026	2L	0.43607709	0.434859852	0.96591738
trp	FBgn0003861	4.835484587	1.712849686	0.910777808	1.470489167	0.975945354	1.543218389	4.865214235	0.065206737	3R	-0.251074915	0.742086114	0.995787491
trx	FBgn0003862	14.41368811	22.66510968	31.46888071	23.26102142	20.29564731	20.75135459	26.04280698	23.18654636	3R	0.011592811	0.948529957	0.997604378
alphaTry	FBgn0003863	2.218395115	1.01483089	0.244627073	0.470191336	0	116.933269	0.257235164	1.313547586	2R	4.862050457	NA	NA
tsg	FBgn0003865	0.977632569	1.475857338	0.474344595	1.595519472	0.705950941	1.382559022	0.498792338	0	X	-0.757804475	0.592289553	NA
tsh	FBgn0003866	0.627668447	0.828453062	0.274587932	0.335858934	0	0.135814224	0.157494658	0.446795508	2L	-1.457734963	0.128802667	0.788096052
tsl	FBgn0003867	0.156704198	0.098568491	3.326419246	0.456687424	1.485179015	0.240077112	0	0.850548301	3R	-0.589077709	0.61004179	0.990047511
ttk	FBgn0003870	0.877248667	2.024947118	0.751701208	0.478481121	0.697390979	0.405944593	1.375577735	0.908610818	3R	-0.24500874	0.596898462	0.988780662
tub	FBgn0003882	118.8648257	121.016501	124.8023306	104.4352625	135.2271014	123.0664897	126.3266859	110.9631639	3R	0.109960835	0.351378394	0.933797047
alphaTub84B	FBgn0003884	6703.67218	6248.570744	6704.608861	6707.801765	8405.206017	6567.68616	7233.940515	7315.975001	3R	0.194875613	0.056390501	0.621788268
alphaTub84D	FBgn0003885	1206.090135	1022.462463	1091.234895	1084.432881	1221.947036	1270.710021	1198.726781	1217.091555	3R	0.184943809	0.040589705	0.530560761
alphaTub85E	FBgn0003886	1.835674994	0.644460129	0.82852501	0.398121595	3.545063647	0.56347173	0.653420421	0.46342097	3R	0.507910255	0.602732327	NA
betaTub60D	FBgn0003888	0.780020223	1.107086113	0.756927272	1.762631238	0.693236391	1.074812902	1.071456802	1.094259024	2R	-0.132399438	0.787557018	0.995787491
betaTub85D	FBgn0003889	12.95396617	12.08836915	9.627022145	9.039236551	15.48036287	19.3084548	10.23955792	24.46018361	3R	0.696308406	0.026151298	0.452167872
betaTub97EF	FBgn0003890	1.912603478	2.290546177	1.179648142	1.738317783	1.331352933	1.551049779	1.188761959	1.706722891	3R	-0.272943824	0.305181111	0.911292159
tud	FBgn0003891	55.35222892	63.21499169	55.56266156	78.93858553	47.83485597	50.96816112	51.44561124	47.11699712	2R	-0.329848007	0.011004305	0.318437078
ptc	FBgn0003892	0.789764407	0.736991557	1.457207065	0.920652992	0.813266907	1.347594086	0.695215441	0.905625864	2R	-0.014823027	0.968736704	0.997604378
tup	FBgn0003896	0.875977477	1.095318228	0.473319763	0.111398646	0.474409157	0.810850466	0.568816784	0.302563576	2L	-0.218003884	0.695440526	0.995787491
twi	FBgn0003900	0.155483817	0.126939168	0.136860747	0.060952112	0.203685199	0.083023968	0.017550556	0.122481063	2R	-0.134442279	0.834139477	0.995787491
RpL40	FBgn0003941	1799.087088	2195.86722	2353.898186	2380.259093	2143.077876	2002.263036	2397.290872	2460.220533	2L	0.078128519	0.509625292	0.979725655
RpS27A	FBgn0003942	385.3359217	528.4714978	574.3747174	537.2140497	519.9389077	451.8254577	585.7042947	559.5209108	2L	0.099066994	0.462431318	0.971606621
Ubi-p63E	FBgn0003943	145.7393361	110.3093167	116.4440449	105.8872087	110.8321394	131.0771626	118.8988769	110.4381169	3L	0.004603106	0.970910718	0.997604378
Ubx	FBgn0003944	0.431961227	0.513537239	0.370017195	0.246584091	0.271322417	0.531104911	0.167563402	0.101517443	3R	-0.518229986	0.220802247	0.86549481
unc	FBgn0003950	5.827045616	5.256854762	4.431930922	4.259249592	3.951849191	4.677071485	3.897019189	3.17985988	X	-0.306405683	0.255331918	0.886640087
ush	FBgn0003963	0.450996461	0.751966976	0.875288899	0.379788757	0.724241588	0.667622295	0.58040233	0.382893473	2L	-0.022942844	0.939778812	0.997188858
vap	FBgn0003969	166.8668506	157.9820658	146.8540437	137.0663501	132.3566473	148.2504602	147.4482404	137.0073537	X	-0.078187108	0.425159813	0.9620897
vg	FBgn0003975	0.258334089	0.354533885	0.427305611	0.287459551	0.370967578	0.86905138	0.194709217	0.624602131	2R	0.674404095	0.240162331	0.874750713
vir	FBgn0003977	25.57929695	19.94406986	30.55016252	32.89874536	31.99419379	22.89695412	18.57210334	28.31675822	2R	-0.067978219	0.736422984	0.995787491
vls	FBgn0003978	82.03688568	64.96038924	65.94493782	103.25226	89.21143863	81.08724916	79.31677562	103.3115675	2L	0.186134059	0.330209033	0.921112231
Vm26Ab	FBgn0003980	1.500473547	4.576061598	1.654603589	3.816324641	0.738747087	1.671844075	0.347976387	5.034576222	2L	-0.52427403	0.632049317	NA
vn	FBgn0003984	0.514468328	0.719123706	1.08558088	0.187416178	0.493396944	0.79323857	0.403295655	0.372319454	3L	-0.243565076	0.592847692	0.988631016
hid	FBgn0003997	0.614691395	0.673841435	0.317033734	0.440094992	0.882497848	0.565837671	0.77787189	0.567447273	3L	0.470381335	0.260286853	0.890556245
Yp3	FBgn0004047	14.50130549	22.14564543	23.10326051	14.02831478	13.83166398	15.22256674	20.20819391	3.665263842	X	-0.447226645	0.254248194	0.884350771
yrt	FBgn0004049	17.82775401	15.60495356	19.92448919	21.95436881	17.22938073	24.84471597	20.48179797	26.37767613	3R	0.268754157	0.135319605	0.796109055
uzip	FBgn0004055	26.40150542	25.57250911	30.20385294	24.32603883	21.49813474	22.03971243	24.75257292	23.2931655	2R	-0.186406693	0.091226533	0.720150434
Zw	FBgn0004057	1.307509295	1.566545298	0.535532352	1.741947828	0.459816115	0.352203365	0.476497758	0.479265043	X	-1.52526969	0.015256113	0.362234897
Prosalpha4	FBgn0004066	134.5641083	166.2505416	138.9728817	141.8383755	149.3630311	141.7478873	145.6322132	144.0764886	X	0.029946065	0.804651247	0.995787491
Dhfr	FBgn0004087	2.801996695	5.721686021	6.206508821	1.988228203	1.099635256	3.215989144	0.932341796	7.405871202	3R	-0.353680368	0.692106455	0.995787491
bs	FBgn0004101	3.93766153	6.479520518	3.963625246	7.005545955	6.015232553	5.617196523	5.923166429	6.115240354	2R	0.17912238	0.345358743	0.930285749
oc	FBgn0004102	1.34641947	1.486740605	1.013708748	0.541228674	0.746714603	1.539988314	0.656800767	0.815564001	X	-0.195239228	0.612632015	0.990047511
Pp1-87B	FBgn0004103	1551.621004	1559.146002	1542.78741	1506.863706	1486.995569	1603.777538	1490.551396	1515.179525	3R	0.014816935	0.840772022	0.995787491
Cdk1	FBgn0004106	0.056873438	0.858574919	0.275948333	1.32598279	0.410684526	0.428959438	0.290170723	1.111297482	2L	-0.117039672	0.93006535	NA
Cdk2	FBgn0004107	57.29031941	55.07587717	52.1007459	69.24523477	52.27211341	48.36588741	39.66444564	48.78276966	3R	-0.276312883	0.073673204	0.675013659
Nrt	FBgn0004108	29.72679193	36.82940333	35.72639588	42.1336298	32.34966125	32.20508611	37.33499831	32.86403518	3L	-0.068338184	0.595208219	0.988780662
tin	FBgn0004110	2.170520689	4.126176491	0.561669645	0.134946327	1.044892305	2.837610392	0.295309062	0.565488161	3R	-0.528071446	0.567705747	0.984934163
Tm2	FBgn0004117	0.692392052	2.322781067	0.629900047	0.565000101	0.218740395	0.293752349	1.059784199	0.601298895	3R	-0.927023747	0.228444954	0.867702781
nAChRbeta2	FBgn0004118	274.5023273	247.0275294	223.7194073	213.3878748	247.8267321	242.4192506	223.6716201	240.2371941	3R	0.023043875	0.823994153	0.995787491
boca	FBgn0004132	168.0293829	156.8795591	185.841632	151.7522154	187.4609877	189.7606212	187.5009411	173.7074304	2R	0.18683	0.148143935	0.808004944
blow	FBgn0004133	2.523244297	2.799548533	3.79396511	1.930309858	1.897959441	2.77538253	3.92245572	3.538002193	2R	0.168405909	0.629719854	0.990523437
kst	FBgn0004167	0.733982512	1.152173089	0.490513538	0.309962734	0.615012324	0.856506065	0.621779826	0.336748916	3L	-0.11465526	0.782579956	0.995787491
5-HT1A	FBgn0004168	50.45710771	42.87674861	39.1987042	44.96395229	50.8083016	51.80855976	43.00114564	48.54536208	2R	0.157089118	0.143189085	0.8037297
up	FBgn0004169	2.749634555	1.595891429	0.900181412	0.998201126	0.687030463	1.112283717	0.825220803	1.032821998	X	-0.756075854	0.10193313	0.739991904
mts	FBgn0004177	694.9045958	755.8951444	743.3788262	830.0417545	746.5087519	798.3723868	748.4695506	783.9590098	2L	0.054906261	0.532415218	0.984760433
Csp	FBgn0004179	330.7910371	329.556802	278.3301711	334.0713509	328.2919841	360.4062015	311.9832799	308.048103	3L	0.068158421	0.480221295	0.973549494
snRNA:U1:82E	FBgn0004183	4.78260252	8.913494217	7.735018004	3.716819244	10.55245072	2.004003753	6.778067655	0	3R	-0.343819994	0.80084149	NA
Ser	FBgn0004197	1.417794254	0.642035247	0.587471194	0.582793861	0.944822559	0.515526961	0.28894734	0.254393601	3R	-0.667640712	0.171729976	0.832669092
ct	FBgn0004198	2.388332312	3.191258984	2.327171721	1.020760684	1.389820556	2.000098519	1.123162698	0.96656806	X	-0.673194819	0.013267764	0.344576629
nonA	FBgn0004227	87.42557145	81.49808401	81.73307267	79.43903361	72.85582807	79.69891592	86.03417986	77.53691401	X	-0.033001005	0.731957547	0.995787491
mex1	FBgn0004228	3.555822815	2.881673476	1.163106951	0.745192542	0.577004183	3.088735488	0.407684512	0.52045082	3L	-0.834128241	0.333729225	0.923864819

**Hrb87F**	FBgn0004237	44.16707831	74.87262577	71.50850973	64.44772929	60.6801712	55.95643449	66.61930019	51.23702968	3R	-0.085384551	0.604132344	0.989286463
**Syt1**	FBgn0004242	321.8300729	293.3699074	283.1559167	291.1991851	279.5648699	330.6601662	274.7918636	286.6408925	2L	0.005749273	0.948845553	0.997604378
**Rdl**	FBgn0004244	220.4804216	195.6258772	169.2778722	195.0396744	219.9428837	216.2608334	161.3346982	188.3514349	3L	0.037441975	0.750713909	0.995787491
**T48**	FBgn0004359	0.500238647	0.839080153	0.797079821	0.821232078	0.683973656	0.633480358	0.581427244	0.186366082	3R	-0.469641289	0.240746363	0.874884741
**Wnt2**	FBgn0004360	0.028802343	0.064415853	0.489118574	0.492444905	0.242646184	0.126721807	0.048983597	0	2R	-1.309169968	0.29982923	NA
**HmgD**	FBgn0004362	16.36607759	13.24537418	17.79449276	21.82283353	13.86616365	14.5741104	14.99245175	24.16440646	2R	-0.003717861	0.985064825	0.997950263
**porin**	FBgn0004363	3411.768546	2869.767527	3111.165156	3103.996739	3429.518933	3091.652853	3380.136046	3486.90838	2L	0.130049049	0.184728659	0.839299945
**18w**	FBgn0004364	101.2703267	127.1990647	115.6302671	132.6570567	103.5819899	121.1916741	115.0681075	138.2062384	2R	0.03577106	0.773438172	0.995787491
**mei-41**	FBgn0004367	1.901946267	1.122824732	2.087942483	1.015681346	0.613810141	1.362388223	1.084224757	0.991955087	X	-0.570045368	0.20053731	0.848203654
**Ptp4E**	FBgn0004368	21.87126181	25.53046041	23.3065719	25.50202641	22.25181504	24.54077951	20.91454574	26.50351673	X	0.001197022	0.991740549	0.998449594
**Ptp99A**	FBgn0004369	10.21681531	12.92395195	13.38136567	12.36196733	13.02246555	13.04626394	12.83790839	13.9077617	3R	0.144603063	0.156336137	0.818884898
**Ptp10D**	FBgn0004370	0.557382923	0.790489971	0.672303275	0.700863264	0.333522039	0.83105318	0.347485774	0.509886203	X	-0.396063316	0.199569362	0.847197998
**fwd**	FBgn0004373	42.17640889	51.17382749	57.01811238	51.41005749	41.47947726	49.62004426	50.76202495	49.32728784	3L	-0.045819181	0.692087005	0.995787491
**neb**	FBgn0004374	1.335838377	0.823320171	0.693364476	0.14485846	0.448656864	0.199164135	0.903450636	0.822855606	2L	-0.311411217	0.622541933	0.990047511
**Klp64D**	FBgn0004380	345.6163939	366.3283932	317.4463016	337.1910769	316.2619725	311.8576259	309.4209334	295.5488131	3L	-0.118068694	0.185940349	0.839299945
**Klp68D**	FBgn0004381	268.779524	251.773548	244.8026855	278.8617439	260.9971636	253.2784816	260.0624629	247.915894	3L	-0.001624028	0.98646284	0.99797245
**Klp98A**	FBgn0004387	37.6869359	22.6815894	30.10636361	29.57273992	26.14387174	32.47852045	28.84011154	29.98645066	3R	-0.007094783	0.963237025	0.997604378
**RasGAP1**	FBgn0004390	20.19416238	38.48496913	37.80399285	39.96039781	21.90235466	33.31182837	37.36426539	32.80995851	3L	-0.088747825	0.646268308	0.99279236
**shtd**	FBgn0004391	6.304532306	14.11400179	12.43075824	17.48042132	12.85497052	18.23419682	18.87028973	11.39734724	X	0.317112557	0.22919172	0.867702781
**pdm2**	FBgn0004394	0.362630863	0.199430295	0.504767369	0.152460037	0.273661673	0.221385302	0.075826157	0.219412917	2L	-0.588038194	0.30389952	0.910208378
**unk**	FBgn0004395	74.71601869	98.19328474	86.9822829	76.68039429	71.945723	90.90484728	83.04020492	74.79639288	3R	-0.038231407	0.733470329	0.995787491
**CrebA**	FBgn0004396	3.917969919	4.835116659	2.877426119	3.422641452	2.421999261	1.283219194	1.822877592	1.466963123	3L	-1.074743376	3.49E-05	0.003975711
**Vinc**	FBgn0004397	2.876737355	6.257012692	5.981935457	7.345767214	3.880669006	3.765247561	4.542954494	3.980752302	X	-0.437531979	0.125802186	0.781248449
**Pep**	FBgn0004401	559.1518223	427.173007	435.1511976	406.690684	476.9532552	449.8835226	441.7069107	426.4832101	3L	0.002335702	0.98367935	0.997881056
**RpS14a**	FBgn0004403	1271.111175	1561.672786	1652.310149	1603.266213	1371.189654	1292.548663	1542.501167	1458.391144	X	-0.070733529	0.525988794	0.984107922
**RpS14b**	FBgn0004404	125.8580549	182.34377	158.8937666	132.5984273	189.2509177	145.5138731	181.1715314	183.6388012	X	0.258647637	0.112561819	0.758410631
**tam**	FBgn0004406	6.76133985	8.812418202	8.201458084	22.68710396	6.680284183	14.66577192	7.924905263	11.70392145	2L	-0.158609915	0.669230267	0.995787491
**me31B**	FBgn0004419	258.967443	245.6226521	253.3343625	228.0555037	212.9832295	252.6048146	230.4092887	198.4189182	2L	-0.112278523	0.266578683	0.894434069
**Cyp1**	FBgn0004432	840.4892431	889.8403741	911.9080502	800.7501415	775.2890918	842.3386173	753.5095129	795.6654187	X	-0.089890649	0.264561799	0.893759496
**Galphaq**	FBgn0004435	43.24699823	33.02612841	34.78465039	22.42140908	25.764779	27.19241641	20.79272884	24.15219625	2R	-0.418633251	0.014522096	0.358137424
**Ubc6**	FBgn0004436	411.2988099	300.5322919	310.7873901	288.7530964	339.2953566	357.3672735	326.4115094	325.1596124	3R	0.067265588	0.579896752	0.985637351
**Ten-m**	FBgn0004449	1.555551107	3.602544039	4.4948198	3.528214328	2.809530298	2.091738586	3.275437336	4.034023055	3L	-0.068242891	0.85298208	0.995787491
**mew**	FBgn0004456	0.575738889	1.364534418	0.986771483	0.989555379	0.638512375	1.022618825	0.78699453	0.588728923	X	-0.330515856	0.252508841	0.883301842
**RpIII128**	FBgn0004463	31.46017241	34.59864105	29.15603683	27.11797685	17.28692933	33.55895061	19.30822075	20.21263214	2R	-0.409249918	0.046720188	0.567588989
**Su(P)**	FBgn0004465	404.6317526	363.9338514	353.8246514	362.5063544	422.9162855	378.5089794	405.84529	367.7924696	3L	0.114296421	0.273443875	0.89549671
**GlyP**	FBgn0004507	481.5509501	403.3516309	381.3086674	376.2952216	487.8454215	441.1548575	419.945987	407.4116524	2L	0.125778139	0.248915041	0.879503029
**Fur1**	FBgn0004509	4.378935086	4.85390835	4.869191997	4.004317724	5.246417431	5.037845647	4.092954285	4.564173654	3R	0.096773166	0.381399935	0.944476532
**Ets97D**	FBgn0004510	46.24290045	43.70398953	27.37424459	27.83828947	40.14757382	61.43111645	41.65991104	44.54197761	3R	0.395276979	0.071230439	0.66775532
**dy**	FBgn0004511	0.249814131	0.709124576	0.955687286	0.940852082	0.555050501	0.371402027	0.196086527	0.250324432	X	-1.004434181	0.125525724	0.780967863
**Mdr49**	FBgn0004512	3.005181497	2.387733336	0.627892398	2.179044925	0.80468329	2.35879625	2.494292892	0.749228006	2R	-0.343757498	0.536910022	0.984934163
**Mdr65**	FBgn0004513	1.519039001	14.20772918	3.244549584	1.963268419	5.901809079	12.23546287	1.642705913	3.333821834	3L	0.17886727	0.839877215	0.995787491
**Oct-TyrR**	FBgn0004514	6.06236169	5.437096045	4.448946834	4.115517552	3.207313082	2.8402464	3.364541801	2.34790774	3L	-0.743447215	3.19E-05	0.003687992
**Gad1**	FBgn0004516	3.153619907	1.991087551	3.252237462	1.831252117	5.597141206	2.77277222	4.579897845	3.269539682	3L	0.695641696	0.015490574	0.364946783
**Akh**	FBgn0004552	9.767895031	4.577958804	11.84838855	6.028273998	8.298143069	11.24053349	10.62684106	9.356031316	3L	0.321528967	0.560272808	0.984934163
**Dbp73D**	FBgn0004556	15.40625988	23.52671118	11.13116394	7.653467591	17.37423705	12.66095394	7.517758587	3.806478374	3L	-0.446817173	0.292350168	0.905069205
**5-HT7**	FBgn0004573	0.790692676	0.997694139	0.881339456	0.900975304	0.456511749	1.015773505	0.404323393	0.394369444	3R	-0.622731216	0.04485554	0.557949353
**Rop**	FBgn0004574	2795.696672	2448.992508	2329.809227	2426.582598	2220.640967	2454.075295	2216.688759	2293.900901	3L	-0.094375804	0.301134817	0.907861127
**Syn**	FBgn0004575	103.5392603	108.8450687	101.7551245	107.0568722	104.9709676	108.2043849	101.3211502	99.90024496	3R	0.00639816	0.933053378	0.996479182
**Pxd**	FBgn0004577	1.033399396	0.097502767	0.141019454	0.090349942	0.128256615	0.066981931	0.115334803	0.014022536	3R	-2.07647401	NA	NA
**Cbp53E**	FBgn0004580	58.93798206	51.66229832	53.40249906	57.15296799	28.19289889	24.06350746	22.87531983	20.2332527	2R	-1.185059541	6.78E-24	5.65E-20
**bgcn**	FBgn0004581	0.229919251	0.539920204	0.148741572	0.750467308	0.16602527	0.101157684	0.11730579	0.465897578	2R	-0.943359876	0.345957178	NA
**ex**	FBgn0004583	0.242891956	0.291012273	0.084178928	0.208026166	0.232664042	0.462667514	0.063226796	0.096858536	2L	0.076273439	0.922163702	0.996479182
**Rrp1**	FBgn0004584	46.74534939	36.81387178	31.04000034	44.3766927	41.45041929	40.07018326	27.54993305	41.25547995	2L	-0.054536044	0.779214748	0.995787491
**B52**	FBgn0004587	482.1438548	568.5315741	491.2180018	579.5304975	518.8451597	527.7956894	499.2304346	499.8318887	3R	-0.022075451	0.81129564	0.995787491
**pros**	FBgn0004595	118.8077469	133.069717	123.4677503	138.9877915	121.2229893	126.9701223	113.2115171	124.3665478	3R	-0.052450239	0.547060447	0.984934163
**CycC**	FBgn0004597	82.00245237	80.68975893	75.41052177	84.07459573	102.4660458	82.88708027	96.22372849	95.67223663	3R	0.257008455	0.090602544	0.717758178
**Fur2**	FBgn0004598	94.5868055	86.42881107	89.02208738	115.6273004	75.07324715	87.67332056	83.52253603	83.56132367	X	-0.198434871	0.097202038	0.730787365
**zfh1**	FBgn0004606	1.220604252	1.691595549	1.471556491	0.58130525	0.382925915	0.933253079	0.512635775	1.43813408	3R	-0.565762969	0.183511207	0.839299945
**zfh2**	FBgn0004607	50.32035804	52.3600245	58.50462104	55.07744328	44.72407217	39.13200285	36.45605829	45.35802258	4	-0.352373156	0.00092907	0.057615996
**Plc21C**	FBgn0004611	113.3170009	101.1944791	95.3948929	92.15816034	98.9198341	112.7448752	100.8591654	95.06443031	2L	0.047824784	0.614942364	0.990047511
**gl**	FBgn0004618	0.828193577	1.147582274	1.747118319	0.881498806	0.747550836	1.612554464	1.19415717	0.351799224	3R	-0.197793684	0.751219344	0.995787491
**GluRIA**	FBgn0004619	190.2035186	141.8510623	155.1379553	156.1624735	141.9891285	199.0145922	166.0794383	165.0331126	3L	0.088221551	0.473262952	0.973549494
**GluRIIA**	FBgn0004620	0.06625567	0.370448638	0	1.338762532	0.398694884	0.416436272	0.619738669	0.81513409	2L	0.376386622	0.744723441	NA
**TkR99D**	FBgn0004622	1.300365062	0.366126638	1.295667011	2.089743474	0.544953551	1.077107695	0.473894173	0.352901616	3R	-1.026592862	0.036267369	0.521162798
**Rap1**	FBgn0004636	511.5578208	487.8189327	473.8119447	457.0315477	491.7787808	499.6509298	431.3910272	485.4306768	3L	0.01321604	0.882728991	0.995787491
**drk**	FBgn0004638	37.19843808	35.75517352	34.25478967	35.30563622	34.47938893	36.44240975	35.96227942	36.08586527	2R	0.033390045	0.749193799	0.995787491
**Zw10**	FBgn0004643	62.48251827	81.41104486	65.51209862	77.48867772	65.71390154	98.667243	86.97290284	65.64429479	X	0.172531118	0.277679632	0.896275
**hh**	FBgn0004644	0.680990793	0.475944384	0.535394406	0.3969261	0.751277339	0.903603352	0.659500999	0.561280149	3R	0.482545622	0.264901083	0.893759496
**ogre**	FBgn0004646	12.90610881	19.11969188	13.17942524	11.30803788	11.57431658	8.582521439	12.73501558	10.15830915	X	-0.3559005	0.044096953	0.552313482
**N**	FBgn0004647	2.353819692	2.948509717	1.84530982	1.600395303	1.595037853	1.764401606	1.72152865	1.384580415	X	-0.40611543	0.05437452	0.610724061
**svr**	FBgn0004648	182.0519867	234.1946126	259.9971165	235.5677215	181.6203609	229.4277966	238.7633115	218.0385825	X	-0.03977084	0.741412415	0.995787491
**yl**	FBgn0004649	1.348593682	0.560133281	1.526779083	0.95823098	0.741960301	0.920284641	0.524234889	1.896177877	X	-0.075783894	0.893932949	0.996479182
**fs(1)N**	FBgn0004650	2.176400685	0.818239462	0.182066128	0.58324095	0.654826841	0.188680209	0	0.135780671	X	-1.914949147	0.046372923	0.565844709
**fru**	FBgn0004652	32.647822	36.74487241	31.48667297	31.64551188	28.96156035	31.93627357	23.70300784	28.42046007	3R	-0.199954756	0.045615266	0.563196485
**Pgd**	FBgn0004654	0.984872181	5.676050749	3.553297386	0.588767286	3.236774774	4.023636397	4.50948991	2.741344453	X	0.475702957	0.422235929	0.960593359
**wapl**	FBgn0004655	47.15318446	66.10074697	70.0061736	63.39449411	64.34824371	62.11165637	66.06724219	65.76875318	X	0.100826176	0.419821011	0.960266449
**fs(1)h**	FBgn0004656	98.888064	115.2579537	115.312593	123.6058956	105.8138685	110.2874221	98.63373598	121.9484318	X	-0.021838793	0.834371002	0.995787491
**mys**	FBgn0004657	86.31580764	83.46732094	79.41192124	82.428859	69.73960515	74.57275346	76.53536506	71.96565205	X	-0.150375596	0.099178074	0.731438598
**sim**	FBgn0004666	0.450760177	0.813150128	0.433288602	0.118973251	0.560404229	0.733681427	0.401379803	0.396996311	3R	0.2391308	0.651313478	0.993444251
**Mlc-c**	FBgn0004687	45.68902466	48.58653236	45.63312096	40.17579787	38.09261905	39.69169995	42.79046097	45.86737304	X	-0.083226837	0.540163076	0.984934163
**Xpc**	FBgn0004698	93.68518232	92.97348037	88.18847792	88.20370915	73.52485871	103.2530993	84.36009798	81.88508728	2R	-0.054337537	0.623600191	0.990047511
**Ccp84Ad**	FBgn0004780	0.217907536	1.279282629	3.171843861	0.254021609	0.393378952	0.616325682	1.111773613	5.204071758	3R	0.646935793	0.622160212	NA
**inaC**	FBgn0004784	0.541364035	2.628601173	3.525285344	0.132859986	1.491669694	2.390796253	0.726858571	1.855818054	2R	-0.02359809	0.976857726	0.997757033
**retn**	FBgn0004795	0.237897928	0.418665338	0.529830739	0.354612336	0.260496215	0.264734219	0.149233457	0.110073435	2R	-0.924205497	0.084638519	0.69799888
**mdy**	FBgn0004797	0.420313692	0.578114148	0.326296049	0.039197822	0.030350958	0.840090665	0.62189297	0	2L	0.147680583	0.894300217	0.996479182
**fs(2)ltoPP43**	FBgn0004811	39.65078236	45.07859251	40.62057186	49.60106149	49.37941057	33.27017341	45.72927503	53.21539176	2L	0.087352862	0.624984315	0.990047511
**His3.3B**	FBgn0004828	71.7222473	86.97773038	149.7703589	84.8022283	79.39096019	77.48742006	107.6578175	92.93951137	X	-0.101206092	0.602008597	0.989286463
**Xpac**	FBgn0004832	56.07171457	43.04887578	42.29763603	45.69014274	34.3707733	46.6834449	56.04184924	67.83348695	X	0.157357899	0.506162934	0.978557094
**Su(H)**	FBgn0004837	22.36399322	32.60146433	29.66799742	26.51672526	22.32088021	31.93483671	24.87723542	27.57766859	2L	-0.024774442	0.884872549	0.995787491
**Hrb27C**	FBgn0004838	301.7562084	314.5624163	307.7150227	340.9426212	313.1632388	295.4346002	305.376635	298.988368	2L	-0.03007589	0.728910297	0.995787491
**otk**	FBgn0004839	6.312623465	9.932193132	8.70366696	6.704826602	5.96604047	4.422513316	4.173235559	2.743105072	2R	-0.835735768	0.000156942	0.013676217
**TkR86C**	FBgn0004841	24.52574409	16.90929596	23.43904455	22.46654961	20.17098466	15.92419504	26.36477442	25.55501563	3R	0.042454523	0.813874668	0.995787491
**RYa-R**	FBgn0004842	0.188995598	0.469561501	0.506763914	0.311427033	0.26422636	0.537231143	0.308129365	0.178939567	3R	-0.159021347	0.691334435	0.995787491
**Ac76E**	FBgn0004852	4.802045837	3.989810281	6.020458682	4.584842735	3.327669791	4.149357316	4.445240202	5.071823942	3L	-0.15994306	0.35298426	0.935162434

**B-H2**	FBgn0004854	0.821582276	0.938917788	0.416151601	0.505183687	0.146686804	0.774582751	0.115157914	0.215615856	X	-1.059796188	0.141553392	0.8037297
**RpII15**	FBgn0004855	82.48220453	93.12432425	76.79852434	119.0542598	85.86606411	86.53744579	79.94509843	68.03870928	3R	-0.184361936	0.315610652	0.91460807
**Bx42**	FBgn0004856	250.5719476	243.9744648	196.9147725	230.6807022	201.3797359	215.388806	233.4922828	266.1951748	X	0.021770829	0.863020223	0.995787491
**elB**	FBgn0004858	15.9585225	13.13069205	14.67088845	11.29222109	14.7517107	14.75210912	16.03429801	15.04767884	2L	0.167181577	0.206546565	0.855364409
**ci**	FBgn0004859	1.151073032	2.024187573	2.70240689	0.844061727	0.251368771	1.347778968	0.63938004	0.443386245	4	-1.284381679	0.019471683	0.395797586
**ph-d**	FBgn0004860	27.2493965	32.61768156	25.46412815	35.47901225	34.17385322	24.84789677	20.34550124	23.46038158	X	-0.201340054	0.248675783	0.879503029
**ph-p**	FBgn0004861	16.98746122	20.0474094	23.34445522	17.05449821	18.42574914	20.01119938	15.88055381	20.81438079	X	-0.009577988	0.946126639	0.997604378
**C15**	FBgn0004863	0.175559276	0	0.180190304	0.047228059	0.219412722	0.235542303	0.258378164	0.190577719	3R	1.179115766	0.252003872	NA
**hop**	FBgn0004864	28.37327578	31.57948536	29.55018597	34.42793703	27.33388271	43.89622324	25.77103023	29.06895957	X	0.050987516	0.754413541	0.995787491
**Eip78C**	FBgn0004865	2.321023327	4.774530806	4.371879588	4.50526368	5.138582819	3.960951544	3.506062278	4.209510525	3L	0.111617956	0.588854972	0.987588562
**RpS2**	FBgn0004867	1615.79708	1994.886409	2016.933491	2163.831855	1803.15421	1737.007623	2094.102698	2091.413218	2L	0.020990241	0.857696373	0.995787491
**Gdi**	FBgn0004868	1139.381085	1043.338952	1125.640237	1082.873522	1078.052626	1153.127861	1137.014254	1195.580185	2L	0.085549518	0.315835079	0.91460807
**bab1**	FBgn0004870	0.409536205	0.615102717	0.39286609	0.243385043	0.405899976	0.596827431	0.365316063	0.525929814	3L	0.222771015	0.473766398	0.973549494
**piwi**	FBgn0004872	1.013176028	1.61588062	0.886474099	1.974938854	1.04945056	1.252742518	0.508452554	0.829394684	2L	-0.552466951	0.316089533	0.91460807
**enc**	FBgn0004875	88.20797452	93.52576252	84.10895746	118.1434124	83.33197522	90.00317551	81.38897951	93.3561161	3L	-0.113113832	0.345721364	0.930285749
**cdi**	FBgn0004876	5.184326843	6.613181755	5.99497307	4.837278543	5.733835946	5.403644281	5.449408293	5.052859328	3R	-0.031127753	0.800893666	0.995787491
**cas**	FBgn0004878	2.713887592	1.669127429	1.207039798	3.058207932	0.694062211	2.068231288	1.326943898	1.571226982	3R	-0.591697639	0.280956471	0.899941507
**scrt**	FBgn0004880	22.16234127	21.69214833	17.9010526	19.16170422	14.31092544	21.16115539	14.66978517	21.97431552	3L	-0.137670329	0.409100051	0.957438873
**orb**	FBgn0004882	2.887845293	3.128836348	4.473379267	3.361463958	6.116939727	3.87220324	5.009529663	3.836176171	3R	0.479082601	0.025258915	0.446937992
**tok**	FBgn0004885	10.5638468	14.11526381	11.30362784	15.07672802	11.47202665	13.96665246	11.68348337	10.77418795	3R	-0.063052244	0.646793005	0.99279236
**Scsalpha1**	FBgn0004888	655.0141495	476.5454828	516.7741055	553.5153382	567.45291	593.1540083	565.5475382	512.3765964	3L	0.050178455	0.661023755	0.995750687
**tws**	FBgn0004889	39.61722882	35.23891106	35.94915243	38.10100701	35.01463371	40.25994037	33.54236799	34.34993211	3R	-0.029212019	0.777344572	0.995787491
**bowl**	FBgn0004893	1.422600605	1.005952515	1.048896921	0.390204677	0.490971516	0.532542902	0.266844464	0.529906451	2L	-1.057127976	0.035861295	0.518988565
**fd59A**	FBgn0004896	0.544127181	2.167655406	0.247508428	0.396441262	0.49114451	0.993937079	0.173510016	0.959847265	2R	-0.311054566	0.76068625	NA
**Prat**	FBgn0004901	1.985746117	3.264194329	3.468522516	1.944470774	2.509346479	0.262100903	2.634154124	0.086224773	3R	-0.923899495	0.283522786	0.901780531
**Rb97D**	FBgn0004903	266.9990041	237.3377578	253.6970298	229.670899	229.9862334	266.3036999	230.1387534	234.4326757	3R	-0.011429403	0.904886339	0.996479182
**14-3-3zeta**	FBgn0004907	4099.128433	3259.662164	3812.61098	3295.35026	3697.09879	3769.068596	3678.658966	3686.202687	2R	0.065142975	0.510391568	0.980318812
**Arl2**	FBgn0004908	73.15426964	65.11700089	71.58732444	41.8543376	60.15717052	49.71720449	70.41112872	73.32244179	3R	0.040720067	0.867168368	0.995787491
**Eip63F-1**	FBgn0004910	1.810457794	1.665701411	1.42580838	1.472629951	1.750025515	2.983870468	2.25755855	1.745335463	3L	0.478511562	0.037764666	0.521998122
**Gnf1**	FBgn0004913	62.05262004	59.5609012	59.89961567	58.49987664	56.19126623	64.97166719	45.41951979	58.22729741	3R	-0.064899367	0.62356172	0.990047511
**Hnf4**	FBgn0004914	1.021541963	0.843765658	1.117088409	0.171409265	0.356255296	0.521681075	1.13517872	0.344440715	2L	-0.391323477	0.463186118	0.971606621
**TfIIB**	FBgn0004915	194.7205443	146.0253942	205.407217	149.6998773	180.4653599	156.758347	161.436188	182.6620406	2L	0.000184059	0.998988865	0.999757791
**gol**	FBgn0004919	74.32022852	76.5623521	69.88949558	80.175787	69.91203237	98.92947484	85.85961052	91.38056542	2R	0.229651128	0.045192483	0.560365369
**Ggamma1**	FBgn0004921	1876.293902	1778.139724	1945.12599	1707.935323	1766.447453	1950.850023	1768.894349	1773.958232	2R	0.020074278	0.804440836	0.995787491
**Top1**	FBgn0004924	38.06137414	46.05096843	59.45213244	53.02589669	50.12924606	41.78670105	57.96777865	63.58719992	X	0.15476865	0.344870996	0.930285749
**eIF2beta**	FBgn0004926	242.98586	250.7537881	244.7722364	230.6734871	203.5267769	211.5863376	213.2757471	197.9297555	3L	-0.200054068	0.035059871	0.514749796
**upd1**	FBgn0004956	0.792038822	1.549956348	0.533743093	0.359062802	0.873786877	0.62227447	0.449001789	1.337458579	X	0.055157366	0.94177147	0.997604378
**por**	FBgn0004957	18.9721061	20.48881073	14.7715746	26.40115284	13.20382439	26.18261396	27.46671737	16.60505069	X	0.073504356	0.79129494	0.995787491
**gig**	FBgn0005198	18.74422713	32.51988021	22.61903203	33.59280065	23.76782902	26.80286207	20.32275562	31.24385214	3L	-0.040319863	0.831452116	0.995787491
**Sam-S**	FBgn0005278	299.4996399	286.0450278	283.7613289	309.1570175	289.8906106	338.7572841	268.8617899	320.2376036	2L	0.075390036	0.436105043	0.966084844
**nmd**	FBgn0005322	80.02666551	64.37311455	73.32206986	82.41812714	87.87994874	78.9471856	89.18317023	84.2754805	2L	0.208900678	0.155232843	0.818884898
**Su(fu)**	FBgn0005355	60.6056189	67.40725219	52.03961695	62.04019917	64.70405523	90.87905732	72.86996422	71.21376965	3R	0.335794726	0.035144305	0.514749796
**ash1**	FBgn0005386	35.85400697	45.17785895	41.35824909	43.69365798	38.00478313	42.35598259	40.99839304	41.08870761	3L	3.79E-05	0.999727612	0.999757791
**fs(1)M3**	FBgn0005390	1.218965377	0.649474968	0.591438601	1.136788756	0.750774151	1.270916618	0.256085271	0.093405412	X	-0.577738788	0.448392126	0.967112302
**Yp2**	FBgn0005391	1.233731689	2.586764791	0.124708995	0.239699916	0.928000792	1.211619479	1.180228516	0.781242496	X	-0.006728898	0.994932992	NA
**U2af50**	FBgn0005411	358.2660771	326.7318723	346.6369802	351.1450307	390.6200107	391.4463809	334.2296115	323.7809772	X	0.086730577	0.389888981	0.948624352
**ewg**	FBgn0005427	81.85033971	74.46612505	69.36374577	83.52458931	78.20499208	76.8182964	69.41009765	77.96389493	X	-0.003547761	0.973137297	0.997757033
**RpS17**	FBgn0005533	308.0604078	441.9590174	508.3374422	467.273492	454.0162472	355.9430993	507.8318549	519.2624737	3L	0.127235509	0.441229108	0.967112302
**Mbs**	FBgn0005536	25.19871602	32.3757842	32.31658125	34.03287178	25.8576632	27.32063908	29.87403957	29.7029645	3L	-0.10374653	0.370090075	0.942619319
**ey**	FBgn0005558	248.9370906	260.0358597	239.4303832	265.9951515	266.3997893	267.1265577	259.6837502	290.2203146	4	0.12582814	0.148724063	0.808039863
**sv**	FBgn0005561	0.116579065	0.242949883	0.368521971	0.247090639	0.10841626	0.156538529	0.0991319	0.207085044	4	-0.723296347	0.237974368	0.873280735
**Shal**	FBgn0005564	78.68830297	83.94129858	87.93465812	82.98106128	89.6886026	85.1477916	82.77613264	83.18545297	3L	0.062261978	0.457574074	0.968365245
**Calr**	FBgn0005585	328.3072116	389.1887426	377.1722502	351.4441878	360.8773821	338.1837513	345.7693143	353.9113763	3R	-0.015284897	0.870029782	0.995787491
**Rab3**	FBgn0005586	983.7467987	923.2227538	1036.197811	975.7015836	911.4187342	994.9557069	955.3338325	1081.108207	2R	0.039165461	0.669552488	0.995787491
**RpL7**	FBgn0005593	1263.034768	1469.998983	1696.71369	1767.229489	1483.775675	1480.229906	1662.019245	1702.358669	2L	0.062288407	0.606036195	0.989286463
**yem**	FBgn0005596	32.50326342	22.03782578	21.66479524	11.71995059	24.16504679	34.21630456	22.19850682	30.92241364	3R	0.367768779	0.138313394	0.799378522
**Sox14**	FBgn0005612	2.540403378	3.258541981	3.032055886	2.312130499	2.060056965	3.919216561	0.727770564	2.241573626	2R	-0.281807511	0.517193651	0.980804634
**Sox15**	FBgn0005613	0.141443203	0.448141023	0.133443036	0.806103227	0.312083728	0.585266178	0.140320697	0.511810477	2R	0.056960861	0.9407126	0.997188858
**trpl**	FBgn0005614	0.44697682	0.553087557	0.764384587	0.495429168	0.211647926	0.525031691	0.616855189	0.270447162	2R	-0.444755941	0.428631506	0.96338052
**msl-2**	FBgn0005616	60.14785214	76.82383638	43.60267126	63.89289277	58.50754249	62.41128482	54.36170399	51.5295318	2L	-0.078617744	0.622721889	0.990047511
**msl-1**	FBgn0005617	29.95831842	52.28150376	40.20686721	36.8911382	30.96581541	33.227901	39.66930809	40.09151506	2L	-0.109751396	0.521285732	0.982083369
**Hdc**	FBgn0005619	0.910192963	0.916032605	0.805312688	3.520157279	0.502604954	0.979271335	1.119985811	0.860190443	2R	-0.80819574	0.151801643	0.812484766
**Psc**	FBgn0005624	6.722920446	6.180902298	6.305300734	5.31900618	9.237992015	9.431258308	6.792349641	6.683601407	2R	0.418504374	0.013895584	0.35306645
**ple**	FBgn0005626	38.73881338	1.813474889	1.703150071	7.791121509	18.4782573	0.648645565	51.11315658	0.579197335	3L	0.523416597	0.727740996	0.995787491
**robo1**	FBgn0005631	25.29014844	49.59400218	60.70667299	52.22029822	45.43303333	51.35129116	74.32649548	75.48077407	2R	0.430975201	0.178638589	0.836045561
**faf**	FBgn0005632	62.42270905	94.18694444	97.983878	82.63558526	86.3853527	90.93476921	82.68597529	92.91995819	3R	0.099731323	0.432238596	0.964464916
**nvy**	FBgn0005636	3.216442998	4.5054084	3.614042288	2.328641738	3.545023482	5.554158413	5.153450944	4.924990504	2R	0.521402995	0.022039738	0.420318478
**slbo**	FBgn0005638	1.275169864	1.460724869	0.3018092	0.217537356	0.505318923	0.293224966	0	0.270098714	2R	-1.56385545	0.184090999	NA
**Eip63E**	FBgn0005640	4.406315947	5.414837224	5.388068097	3.880403217	4.268485399	4.410172171	4.866033203	4.635426316	3L	-0.036309352	0.770063513	0.995787491
**wdn**	FBgn0005642	79.38714583	67.66336043	63.65438589	69.84458186	67.97398288	66.54305215	76.68552966	57.86345522	3R	-0.033506507	0.804922119	0.995787491
**Pabp2**	FBgn0005648	107.5149342	85.9990156	98.53408936	104.2330156	116.2533048	86.7133377	78.53530269	95.52975298	2R	-0.042345474	0.776295806	0.995787491
**Rox8**	FBgn0005649	116.8157373	149.0832815	131.2282656	170.0016087	141.4187515	148.4014985	145.8394489	140.7984824	3R	0.053942965	0.655893575	0.995305364
**Ets65A**	FBgn0005658	0.385394537	0.580143191	0.391565186	0.030719072	0.208126128	0.577629542	0.201671742	0.379030277	3L	0.011289572	0.986173374	0.99797245
**Ets98B**	FBgn0005659	0.585852186	0.18425341	0.444146635	0.73416712	0.370164672	1.270377083	0.747260756	0.620070422	3R	0.643340086	0.264988543	0.893759496
**Ets21C**	FBgn0005660	0.151619835	0.472310434	1.024662613	0.075749129	0.66473075	0.275682062	0.386785456	0.6818743	2L	0.282242042	0.732046682	0.995787491
**Crys**	FBgn0005664	0.115414051	0.483977201	0.699982198	1.950855493	0.052087986	1.985812846	0	0	2L	-0.658749492	0.648314252	NA
**bt**	FBgn0005666	0.958100194	0.88445128	1.11778046	0.674786057	0.918858717	0.743333227	0.407355696	0.379189309	4	-0.537338956	0.078012298	0.684373146
**Vha55**	FBgn0005671	1605.067826	1434.251572	1444.643466	1669.155254	1510.507711	1523.348109	1461.346387	1496.635353	3R	-0.00983391	0.915083985	0.996479182
**spi**	FBgn0005672	51.0233447	49.5204958	45.66495016	47.23183091	43.89601452	42.37983698	49.87261145	52.99130927	2L	-0.001418077	0.990399342	0.998408391
**GluProRS**	FBgn0005674	47.42885553	42.20493199	46.46142749	42.35886471	48.77285826	48.55399931	45.25278531	45.93923559	3R	0.108299004	0.316381595	0.91460807
**dac**	FBgn0005677	186.4963818	255.4168492	236.4581031	264.2537806	216.4566372	221.1685945	244.1319719	250.8928357	2L	0.017684853	0.879868709	0.995787491
**pie**	FBgn0005683	0.884269793	3.775516251	0.780082249	0.374843952	0.653045569	1.288420974	3.588758791	2.181626792	2L	0.446104934	0.598572183	0.989286463
**Aef1**	FBgn0005694	156.932271	146.6824223	143.5537499	164.7474015	143.6896362	126.3063026	135.4083042	153.5954751	3L	-0.099814908	0.369810712	0.942508402
**gcl**	FBgn0005695	84.15179064	72.53771889	79.95843051	94.77588549	59.4997074	77.64975603	83.93966991	75.99994424	2R	-0.130558956	0.346708368	0.930285749
**noc**	FBgn0005771	50.32629715	71.81737686	78.73399761	66.91097986	61.80499959	78.12269344	71.60384991	55.22663953	2L	0.026767581	0.865113941	0.995787491
**Con**	FBgn0005775	16.56020313	15.72225731	14.99411018	18.95627886	13.99977005	16.1564184	13.51027646	13.79541639	3L	-0.17833637	0.089003789	0.709815866
**PpD3**	FBgn0005777	79.64896151	72.89322104	83.49245876	82.99972592	59.70066357	77.88315609	64.11348717	77.23216382	3R	-0.164769458	0.208710026	0.856520545
**betaCOP**	FBgn0008635	199.8106782	211.148441	188.1709468	214.0322931	174.0246489	194.107543	174.5342327	187.0377825	X	-0.126701475	0.178269418	0.836045561
**hbn**	FBgn0008636	0.777218676	0.372478656	0.303030268	0.032358144	0.551209572	0.32712367	0.53108077	0.271191486	2R	0.180890285	0.842401386	0.995787491
**E5**	FBgn0008646	1.72755978	0.71004205	1.66531229	0.320085345	0.185882076	1.477724468	0.350228512	0.919043784	3R	-0.569328201	0.418389195	0.959773067
**lbl**	FBgn0008651	0.114754652	0.222560585	0.208794889	0.083608172	0.369006525	0.15890404	0.018296349	0.171285793	3R	0.237556119	0.763169551	0.995787491
**CanB**	FBgn0010014	4390.701082	3645.064213	4104.845799	3545.260398	3882.259904	4164.192621	3715.49824	3911.376694	X	0.02768546	0.773120829	0.995787491
**CanA1**	FBgn0010015	0.360570111	0.604805778	0.169304141	1.05759903	0.083989866	0.208352344	0	0.17676831	3R	-2.165758144	0.027684716	0.462499435
**GstD2**	FBgn0010038	0.82718652	2.1803404	1.433387829	0	2.559914873	0	2.110170585	0	3R	0.11779621	0.947788774	NA

**GstD3**	FBgn0010039	19.88150336	48.36497856	30.46247359	21.14346303	21.61868154	26.52682283	30.54952426	43.16458421	3R	0.06523536	0.856779393	0.995787491
**GstD4**	FBgn0010040	1.076746148	6.421655502	3.482894847	2.510393108	2.375758934	4.962954045	1.220801136	1.818223213	3R	-0.318343715	0.726072554	NA
**GstD5**	FBgn0010041	0.929158773	1.385363106	1.502750816	0	1.863744141	0.486669564	0	0	3R	-0.64397427	0.731085607	NA
**GstD6**	FBgn0010042	4.114088689	9.511922648	7.822727879	2.073911979	1.405105179	1.048307483	3.120173378	3.379701171	3R	-1.368469999	0.083670649	0.69799888
**GstD7**	FBgn0010043	6.309938424	2.694118385	1.391620237	4.012195608	0.828440442	1.406120514	1.463344408	0.249081099	3R	-1.855780366	0.064112178	NA
**GstD8**	FBgn0010044	5.796016437	5.761187836	13.54027522	16.76637049	19.57023727	14.67305722	18.07148744	12.11762569	3R	0.662994917	0.161418499	0.823037813
**Itpr**	FBgn0010051	6.822536976	9.104246352	9.783788136	9.185181638	6.186269566	8.60441133	9.594246227	8.350931513	3R	-0.059786672	0.716140765	0.995787491
**Jhe**	FBgn0010052	0.901062287	1.12136543	0.987209792	1.558652723	1.04945056	0.246633683	0.222447992	0	2R	-1.547759481	0.126549493	NA
**RpL23**	FBgn0010078	441.8785865	625.1568576	623.6719646	694.2608203	596.6082407	502.0010292	666.9401427	643.6371484	2R	0.049346488	0.734721227	0.995787491
**gammaTub37C**	FBgn0010097	0.278963754	1.013832219	2.255873921	0	0.503601074	0.043834221	0	0.403770756	2L	-1.83815763	0.242286884	NA
**mAcon1**	FBgn0010100	695.7612927	726.544225	724.0652863	788.4472958	862.85393	746.9126077	773.7366175	811.7590784	2L	0.153985996	0.102352912	0.741747104
**comm**	FBgn0010105	361.7460364	312.145569	300.8822134	343.1846986	328.1945417	403.2877977	317.5393916	345.2635182	3L	0.107391626	0.306955507	0.911669416
**hdc**	FBgn0010113	1.814902151	1.861311238	2.035714241	1.648504764	1.54298677	2.308529574	1.335849848	1.043318838	3R	-0.21457737	0.274756988	0.89549671
**hig**	FBgn0010114	20.3195083	16.42946941	16.65833199	12.02511202	17.06360026	19.88714266	17.33526323	18.28968569	2R	0.177649532	0.228723203	0.867702781
**RpA-70**	FBgn0010173	23.85624784	35.46545895	31.55175012	46.69649483	39.91378782	41.68989788	24.74125545	60.42647613	3R	0.311857198	0.231891413	0.867702781
**Wnt5**	FBgn0010194	81.36144402	69.30664722	58.67396233	72.47704699	67.17112626	81.74473449	52.81306452	80.59552691	X	0.029098348	0.853875677	0.995787491
**Gyc32E**	FBgn0010197	0.192633886	0.266922177	0.040637141	0.175742082	0.166316909	0.118443946	0.042731581	0.40004226	2L	0.132887981	0.89521334	NA
**RpS15Aa**	FBgn0010198	146.6394961	221.3774906	272.82957	228.6065502	198.4846955	206.6456863	272.1459716	228.977556	X	0.095066614	0.570760352	0.984934163
**Sod2**	FBgn0010213	521.6258067	569.8452069	574.058126	576.8898173	713.3431221	690.3788494	798.2116396	868.8863486	2R	0.486512191	1.22E-05	0.001996881
**alpha-Cat**	FBgn0010215	78.55037985	58.03681742	78.92741492	59.78275951	53.94194221	83.93413912	68.17271324	61.81862791	3L	-0.014355303	0.919863918	0.996479182
**ATPsynbeta**	FBgn0010217	2761.161582	2766.349536	2954.127223	2510.192257	2951.363116	2848.695274	2976.623259	2987.481091	4	0.12998646	0.12198227	0.780439453
**Dbp45A**	FBgn0010220	44.05734954	68.65836323	70.36521248	68.03403437	46.00464902	78.84145861	51.31148757	58.57645765	2R	-0.06533949	0.742372995	0.995787491
**Nmdmc**	FBgn0010222	2.518785818	3.773530601	2.480774487	4.040449336	3.517165671	2.141285605	2.855767439	2.827737513	3R	-0.142910378	0.639759027	0.991585536
**Galphaf**	FBgn0010223	1.40148547	1.918465103	1.602291405	2.065667629	1.861179197	3.963935821	1.109550893	0.209845151	3L	0.054802376	0.930591287	0.996479182
**Gel**	FBgn0010225	29.36714493	45.452868	37.70909576	26.66117722	15.74391481	16.52839842	24.13967013	21.38173568	3R	-0.8028006	3.53E-05	0.003975711
**GstS1**	FBgn0010226	3.475808099	5.946319532	5.979851116	1.388285962	1.324941818	2.467519728	3.603266278	3.577728426	2R	-0.573635027	0.195723917	0.844272804
**HmgZ**	FBgn0010228	217.4015256	199.9855382	183.1106867	227.7235276	228.1982844	226.095063	176.3800488	265.5775287	2R	0.14314743	0.267702012	0.894917196
**Klc**	FBgn0010235	178.7004911	249.442581	266.88323	232.7089004	213.2788611	236.9418399	237.473147	217.139344	3L	-0.003189748	0.978974464	0.997757033
**Lac**	FBgn0010238	12.81170193	20.87083207	13.26010489	18.9447233	16.9063617	16.58208394	13.30408273	16.14502034	2R	-0.032996229	0.847470867	0.995787491
**Lcch3**	FBgn0010240	105.6165816	87.19943313	76.1615385	75.7301921	87.13504633	89.77222768	77.82821166	84.19690413	X	0.002792036	0.98350936	0.997881056
**Mdr50**	FBgn0010241	1.199826525	0.825657713	1.119524005	0.466225062	0.860843924	0.087014548	0.902538648	0.400759146	2R	-0.660838335	0.387651075	0.948247506
**Myo61F**	FBgn0010246	0.303323299	0.600102179	0.792462561	0.036265992	0.252727521	0.505949318	0.059521866	0.135085735	3L	-0.810268894	0.362427706	0.939549067
**Parp**	FBgn0010247	2.188488327	2.260782586	2.751578271	1.892506756	1.850996258	2.048351441	2.107642949	2.110906977	3R	-0.131328915	0.319870131	0.916396588
**Rbp9**	FBgn0010263	37.44883338	39.79746081	51.33482668	44.35688841	42.39735743	35.39612669	44.67139229	42.61250723	2L	-0.03358965	0.797116271	0.995787491
**RpS13**	FBgn0010265	1035.033214	1048.049402	1087.912332	1223.271604	1001.547792	977.1323588	1127.947104	1202.766382	2L	0.003330639	0.976416566	0.997757033
**Dsor1**	FBgn0010269	213.508351	204.3224238	179.5696149	210.7893931	196.2289526	216.3240255	201.697185	178.6359731	X	-3.22E-05	0.999757791	0.999757791
**Ssrp**	FBgn0010278	53.97663662	52.55285573	64.71797571	77.63084896	67.15014434	59.91103046	66.04705047	74.63904075	2R	0.136756835	0.405324655	0.956706394
**Taf4**	FBgn0010280	106.6125984	142.414802	151.3198606	162.8212591	119.0502734	136.3084692	156.6052492	150.8862104	3L	0.031534144	0.817944158	0.995787491
**TfIIFalpha**	FBgn0010282	112.8023293	110.9561103	114.0646704	124.2679178	110.5625876	129.6980538	102.5724312	130.1521588	3R	0.063300362	0.612225366	0.990047511
**Trf**	FBgn0010287	10.85812766	43.18685294	26.74078241	26.65779475	31.9270168	19.07800154	23.50245073	22.32386389	2L	-0.105058495	0.777180486	0.995787491
**Uch**	FBgn0010288	96.02688061	79.3028679	84.02533079	81.57404716	91.50667417	101.8987611	91.20619374	91.41090311	2L	0.168121197	0.220333663	0.865425118
**bys**	FBgn0010292	33.31093054	44.66656609	41.59784167	39.51887506	47.89494729	40.06728716	39.98175654	46.72071951	X	0.170044867	0.334007958	0.924122842
**brat**	FBgn0010300	36.67634697	37.54478156	33.60910388	32.6600396	36.67037286	33.64858919	30.08917929	32.23505304	2L	-0.05215457	0.587427764	0.987588562
**hep**	FBgn0010303	46.78418914	64.61644305	64.61795052	62.33403884	53.91908565	55.765701	58.01093921	57.8956617	X	-0.045950606	0.695696727	0.995787491
**pigeon**	FBgn0010309	109.3458059	91.27715576	94.14248378	97.9404932	111.8062264	131.7093521	103.3628344	119.5448586	2L	0.275139048	0.017908593	0.385659471
**corto**	FBgn0010313	155.2659783	156.5623175	148.2678293	168.7855094	171.1417503	170.1015443	157.312437	168.1841518	3R	0.113925132	0.202587791	0.849847813
**CycD**	FBgn0010315	0.698044727	6.516157791	5.742993396	2.052021089	0.767047606	1.230383874	2.632379183	0.329460808	X	-1.558377759	0.033618672	0.514749796
**dap**	FBgn0010316	0.044024521	0.406147271	0.427211498	0.051320757	0.397377722	0.145271174	0.280768749	2.054998187	2R	1.710566953	0.139800021	NA
**CycJ**	FBgn0010317	4.356845889	1.478999549	2.264924434	1.209264545	11.04876632	1.075802014	4.763317485	3.265647098	3L	1.131756414	0.11470993	0.762354509
**Gsc**	FBgn0010323	0.287561695	0.078218011	0.018854612	0.126839861	0.070151697	0.183183385	0.17843742	0.151862366	2L	0.174444283	0.869275522	NA
**woc**	FBgn0010328	39.36137436	44.05440749	47.63239986	47.34659731	44.05976093	47.74995617	40.49050437	46.78717987	3R	0.037273173	0.742110428	0.995787491
**Tbh**	FBgn0010329	1.298030308	0.728810391	1.485003719	0.993903521	1.090240638	0.726535922	0.404327549	0.320378471	X	-0.796523698	0.036895955	0.521170991
**Rac1**	FBgn0010333	624.3331752	542.7433546	464.1832612	520.8759554	501.348827	620.4407837	500.968333	558.3310227	3L	0.046184032	0.69205773	0.995787491
**128up**	FBgn0010339	60.12522138	59.50058715	69.8193705	70.60999238	59.30510664	40.43719549	45.10361028	42.9877408	2R	-0.437516573	0.016982918	0.375267338
**140up**	FBgn0010340	30.39456129	27.27464873	31.40640704	34.994457	52.0474437	47.28797736	42.91666895	44.1287181	3R	0.61805887	0.001254301	0.073737689
**Cdc42**	FBgn0010341	6.75421515	9.032779124	17.71419648	12.34239871	9.611669602	7.084931075	11.33153875	6.80357978	X	-0.359276218	0.283994747	0.901780531
**Arf79F**	FBgn0010348	337.1611276	322.4222181	352.4434541	310.8654575	323.6182871	313.314226	364.5836524	322.3960599	3L	0.032343026	0.737440033	0.995787491
**Cds**	FBgn0010350	656.9527969	698.3423173	681.2594539	670.3015487	670.5487553	698.2794361	686.2169046	668.890008	3L	0.039694218	0.591076364	0.988631016
**Nc73EF**	FBgn0010352	239.1939613	228.5949649	221.3351866	249.3035164	227.7359803	234.2337953	234.1097372	201.9757993	3L	-0.03501925	0.700597784	0.995787491
**Taf1**	FBgn0010355	58.97492956	61.37425906	68.23239069	69.96161139	61.46143471	70.90134207	56.42942985	60.42684608	3R	-0.023842623	0.82466375	0.995787491
**Taf5**	FBgn0010356	83.02290974	76.24491959	73.96099066	75.27044365	77.7535742	68.10450999	79.27037684	86.89934194	2R	0.045960505	0.735818713	0.995787491
**Akt1**	FBgn0010379	5.539695313	5.254976667	8.220107615	4.733626827	5.485749482	2.104328374	6.722934041	4.670753914	3R	-0.287340523	0.40052038	0.953696242
**AP-1-2beta**	FBgn0010380	900.5924444	888.5148957	744.6063075	876.72288	749.8413736	879.8251571	813.7467202	827.4053506	X	-0.031624681	0.742939525	0.995787491
**CycE**	FBgn0010382	0.960132238	0.529765505	0.674261621	0.667626436	0.965470025	0.643173259	0.773468832	0.585132903	2L	0.087660509	0.808750503	0.995787491
**Cyp18a1**	FBgn0010383	0.730793369	0.548385712	0.279930714	0.224186478	1.978903998	0.797775028	0.539656985	1.962398487	X	1.573077202	0.03099555	0.491096797
**Acbp2**	FBgn0010387	10.61073509	26.24299826	37.9555722	21.0782368	7.525216423	14.03585441	20.57736614	11.6359792	3L	-0.797137359	0.037911495	0.522230403
**htl**	FBgn0010389	6.121706599	9.969045624	6.22180366	6.707308466	7.689576079	6.538939381	4.067713156	6.124900367	3R	-0.212726023	0.389956993	0.948624352
**SrpRalpha**	FBgn0010391	98.21960343	68.30600182	75.02092462	68.89962575	78.89161485	55.02101117	69.48899496	61.01190793	X	-0.203753564	0.246713669	0.877929855
**Itgbn**	FBgn0010395	0.760567314	1.304739175	2.180603093	0.523910063	1.185789078	1.042993771	0.749631829	0.863159704	2L	-0.262665888	0.661443749	0.995750687
**Lrr47**	FBgn0010398	8.688696823	25.91780493	17.71041825	20.98830557	17.14066406	19.25460069	7.769193577	21.29488844	2L	-0.122715064	0.733404839	0.995787491
**Nmdar1**	FBgn0010399	24.77882162	19.75847113	22.25228234	26.66884946	21.5780684	24.91160243	20.05860709	22.62750296	3R	-0.041322451	0.753832133	0.995787491
**RNaseX25**	FBgn0010406	48.12641913	46.53025987	40.98811394	48.72341931	48.90180208	72.7991245	56.04270963	46.8877434	3L	0.310884858	0.077248194	0.682035833
**Ror**	FBgn0010407	79.97639635	79.10969099	73.46952087	83.32185751	76.78153863	92.46348548	61.32303531	58.60633813	2L	-0.100585079	0.503840742	0.978429306
**RpS9**	FBgn0010408	210.1663647	238.4159688	215.4545205	269.6889541	213.4991834	187.2426147	221.8549682	221.2744472	3L	-0.114395665	0.366458782	0.940794966
**RpL18A**	FBgn0010409	891.8562357	1182.819839	1358.114608	1396.801321	1104.646094	1038.807891	1190.825612	1314.968938	2R	-0.021162757	0.880633034	0.995787491
**RpS18**	FBgn0010411	555.3934001	836.127812	888.4174055	897.9561558	786.8789961	716.5495293	892.7596485	922.9687211	2R	0.0981378	0.517364336	0.980804634
**RpS19a**	FBgn0010412	405.3478197	705.1119918	666.6929042	681.4040606	555.3088253	476.9007594	620.3327536	595.8120982	X	-0.092414428	0.558903524	0.984934163
**SerT**	FBgn0010414	0.681822894	1.435184005	2.237890299	0.592220466	0.386153748	0.504171341	0.204628806	0.145127512	2R	-1.930731513	0.004530615	0.186000709
**Sdc**	FBgn0010415	29.09391093	27.22365922	30.35772817	30.24862728	27.36539214	28.17376016	26.90190574	33.4405979	2R	0.017864934	0.863901532	0.995787491
**TH1**	FBgn0010416	86.3535604	66.20737861	65.25572007	67.41857692	60.68373705	79.60441797	79.84449781	85.62376834	X	0.126397414	0.419759355	0.960266449
**Taf6**	FBgn0010417	231.3847469	266.8927086	249.7982591	307.4104398	227.1904921	312.484375	256.1229075	250.6869876	3L	0.015309644	0.902170009	0.996479182
**TfIIFbeta**	FBgn0010421	73.86733639	71.78454666	87.46721713	88.33053427	69.5851423	87.25935883	87.30174078	94.66451482	3R	0.106654848	0.525804674	0.984107922
**TfIIS**	FBgn0010422	351.2798711	349.4705627	318.5174924	339.838207	355.8693701	375.3928068	399.0113457	349.3057494	2L	0.151747693	0.112611489	0.758410631
**TpnC73F**	FBgn0010424	2.903404882	0.249746379	0.451514086	0.086784348	0.739170377	0.877343714	2.278968687	0.727333934	3L	0.301771626	NA	NA
**cora**	FBgn0010434	507.679162	479.8683506	469.1977241	476.6409894	454.860081	485.6088713	430.7222954	405.0993396	2R	-0.094015107	0.263614843	0.892790059
**emp**	FBgn0010435	0.747796379	0.211572642	0.633857397	0.084022126	1.301170239	0.688029476	0.183869362	0.46945595	2R	0.677635815	0.377365254	0.943615128
**mtSSB**	FBgn0010438	3.280994153	3.439629634	3.780831875	2.294854306	3.701897099	2.706638591	1.464730151	3.383587462	3R	-0.147710195	0.828138488	0.995787491
**trn**	FBgn0010452	221.6662408	179.951879	172.5908917	188.3637639	188.5374053	187.1000245	178.7575544	196.3571876	3L	0.005674869	0.959679094	0.997604378
**Wnt4**	FBgn0010453	0.732703071	0.881678657	0.57962851	0.306374444	0.675735774	0.292834035	0.182850777	0.285300458	2L	-0.762060323	0.132137128	0.791114096
**Fkbp14**	FBgn0010470	1.698239159	1.53903131	1.718254015	1.31353861	1.438435719	2.01084701	1.786280941	0.541697	2R	-0.091895957	0.818787054	0.995787491
**tutl**	FBgn0010473	77.4646329	79.66579416	75.9438737	101.0555949	87.64512194	94.75683148	79.45560742	84.58434399	2L	0.079356483	0.480344201	0.973549494
**l(2)01289**	FBgn0010482	0.221292335	0.445424217	0.354322397	0.392110408	0.111857027	0.250359674	0.02258086	0.3363123	2R	-0.925355916	0.187234832	0.840740887
**NAT1**	FBgn0010488	384.6902059	449.0097857	406.9119148	423.6455225	343.1209792	401.0251653	399.0216903	400.0203826	2R	-0.077874377	0.390949517	0.949207648
**dmGlut**	FBgn0010497	0.492443647	1.021637929	0.377261501	0.785551564	0.444493497	2.125881	0.099176394	0.590840936	2L	0.311131761	0.691801305	0.995787491

**wal**	FBgn0010516	49.16170699	49.97113125	42.86966822	45.68900866	41.92083787	51.47555323	59.3349797	35.08634098	2R	0.027647294	0.87769238	0.995787491
**Bka**	FBgn0010520	125.739583	134.7977802	134.2598413	117.6583319	102.7780755	130.4411439	104.6676342	113.2480598	2L	-0.152527289	0.302410755	0.910208378
**Ccs**	FBgn0010531	89.88235233	92.0080694	67.40532617	86.31014541	87.38219223	71.90560707	61.33403647	59.2861091	2R	-0.23300702	0.21707441	0.860826301
**Aldh-III**	FBgn0010548	29.95375683	30.87217284	31.34752134	34.66272205	32.75473689	35.40351412	36.8525707	31.2360245	2R	0.132657531	0.231018432	0.867702781
**l(2)03659**	FBgn0010549	0.067723413	0.851973939	0.287518098	0.078947294	0.79467777	0.207509966	0.7342465	1.727647143	2R	1.492621693	0.118542198	0.774240345
**Phb2**	FBgn0010551	360.3854353	343.6214476	361.6762491	338.949074	439.8872141	348.8363499	372.4566443	375.166434	2R	0.160934058	0.125473042	0.780967863
**dock**	FBgn0010583	317.2071644	318.6908806	289.1085301	289.7575447	351.4197477	412.0889796	329.7762934	362.4323509	2L	0.289717148	0.001429376	0.081591941
**Prosbeta1**	FBgn0010590	254.7387917	242.3471971	284.1739541	298.8077912	332.6405609	256.4655434	344.7234099	401.390893	2R	0.339284105	0.029261134	0.474440246
**Sply**	FBgn0010591	137.5498726	175.6120563	159.7668665	187.1327427	113.2857561	104.557628	116.2940646	102.6101519	2R	-0.564112097	2.96E-06	0.000574129
**lwr**	FBgn0010602	745.5465454	709.7278656	712.6121239	792.1915838	730.0600032	741.9763197	796.5225163	757.6359249	2L	0.061488714	0.506793871	0.978557094
**l(2)05714**	FBgn0010607	21.597365	22.75672048	19.87010216	10.66810243	25.1114171	21.56977098	22.70348314	15.29881634	2L	0.208129835	0.386978313	0.948247506
**Hmgs**	FBgn0010611	53.22852595	49.06920089	43.23422654	47.58615591	55.72435812	57.64924052	46.87786318	52.19728418	2R	0.165475861	0.174903665	0.834088743
**ATPsynG**	FBgn0010612	1864.020696	2003.576653	2011.494225	1817.52461	2060.778662	1594.247253	1977.347051	1951.539047	2L	0.012890316	0.90538751	0.996479182
**CG10939**	FBgn0010620	0.229278775	0.590609576	0.576092363	0.19091244	0.073911956	0.015440188	0.60578419	0.017778052	2R	-1.131637284	0.284190798	0.901780531
**CCT5**	FBgn0010621	68.01159966	65.52502057	63.79917331	77.0881641	95.81437213	68.33346362	77.99722746	55.29435192	2R	0.145520824	0.392145428	0.95114668
**DCTN3-p24**	FBgn0010622	143.994526	175.5122483	165.3489235	144.9612316	135.5723715	164.0921154	181.5944291	168.8177405	2R	0.077686142	0.570713041	0.984934163
**Sec61beta**	FBgn0010638	161.1480109	207.7167389	200.8979676	192.8484599	190.0474237	169.4286074	177.2576418	133.2926022	2R	-0.154035191	0.303289919	0.910208378
**MFS14**	FBgn0010651	0.346370354	1.979661719	1.742822974	1.824463554	1.366368465	4.644349929	3.632570441	2.590229464	2R	1.094130341	0.032585444	0.505711525
**Nup214**	FBgn0010660	23.08327752	24.90327407	24.54285902	26.23052074	22.22286135	21.42514761	28.47232113	18.23870063	2R	-0.098769481	0.538948739	0.984934163
**Pfdn2**	FBgn0010741	229.9018947	235.9636143	245.7961941	246.1691889	236.5496034	197.2284359	224.1907721	227.0058317	3L	-0.082679582	0.488796913	0.973549494
**Srp54k**	FBgn0010747	99.32830742	110.2653166	109.2219673	102.2724398	112.3407076	99.36526562	105.2715605	80.99229701	3L	-0.050307253	0.701902103	0.995787491
**atms**	FBgn0010750	10.22824084	9.223835107	8.059435168	11.54088959	9.921445583	8.26001004	7.251483155	8.08939745	3R	-0.192284677	0.205465236	0.855318319
**simj**	FBgn0010762	24.68761616	27.11296693	31.00890708	35.77483204	28.24190754	26.18435774	30.58403885	30.57948382	3L	-0.005453111	0.96691806	0.997604378
**sqz**	FBgn0010768	26.67270408	22.57529713	33.31121252	32.64786286	19.40782803	21.32351298	29.89016022	25.08401238	3R	-0.237818866	0.191618917	0.843483578
**ppan**	FBgn0010770	27.32818814	22.61047336	29.89149511	35.11057336	27.37630543	10.77258613	33.31265737	26.86517564	3R	-0.193100575	0.544070067	0.984934163
**Xe7**	FBgn0010772	491.607583	434.7962108	383.9528335	387.6711876	425.7537117	486.8836991	382.7777582	385.5713062	3R	0.012461146	0.911159109	0.996479182
**Ref1**	FBgn0010774	619.0605667	572.616797	549.7522046	663.9938658	579.1225756	637.7816598	570.2552379	535.5399108	3R	-0.023443423	0.815141313	0.995787491
**l(3)02640**	FBgn0010786	79.28842062	100.5730074	90.8602566	93.12058384	83.19365121	97.01784649	91.68631915	84.5546679	3L	0.001774951	0.986842236	0.998078482
**TrpRS**	FBgn0010803	47.20855903	42.41816502	43.7733886	41.52989402	48.73487378	41.59295375	47.55986268	39.37458613	3R	0.046787939	0.774404433	0.995787491
**Chchd3**	FBgn0010808	478.5674742	410.7041705	433.3723963	400.6296229	429.3803942	424.6609684	403.146315	362.0697689	3R	-0.061545354	0.565182784	0.984934163
**unc-45**	FBgn0010812	41.66206696	46.54095608	51.8420945	30.32653882	36.82389491	50.90509069	45.35654185	35.34650242	3R	0.014387944	0.934183031	0.996479182
**Gug**	FBgn0010825	45.93847608	65.22622887	66.74387339	68.90192358	53.96958789	52.19950953	59.032151	64.60066721	3L	-0.068730964	0.606394989	0.989286463
**l(3)04053**	FBgn0010830	73.37367542	119.6321459	103.1816975	124.459191	91.20539012	98.07785702	117.3033895	137.7009002	3L	0.11438507	0.520136429	0.981838504
**l(3)05822**	FBgn0010877	31.57461266	40.67190112	35.04692651	28.76027935	34.29386009	37.91590911	35.68365265	38.06950735	3R	0.134667379	0.263093345	0.892691887
**sinu**	FBgn0010894	23.93398893	21.84811365	29.62424659	18.03099812	23.3670425	36.22651144	30.42422304	28.98611055	3L	0.379149461	0.081298864	0.694684542
**Spn**	FBgn0010905	27.54293145	29.1190137	31.43858144	31.63415042	19.91623809	21.78649777	28.86912055	25.88088056	3L	-0.279706217	0.027567963	0.462499435
**msn**	FBgn0010909	15.43347847	25.72246053	23.10665685	29.36208628	19.90803706	18.6037284	23.08443926	27.01606073	3L	-0.043934643	0.801121744	0.995787491
**l(3)07882**	FBgn0010926	23.36541841	17.74308489	18.30970138	24.0695509	17.29579806	17.05156127	18.40543726	21.90451792	3R	-0.134982737	0.470297676	0.973549494
**SsRbeta**	FBgn0011016	150.5356626	230.2728801	221.2105674	180.2149423	199.4094352	153.5817276	250.5671988	183.5131434	3L	0.046288345	0.788844759	0.995787491
**Sas-4**	FBgn0011020	5.011223889	1.732068196	2.357752099	3.752875345	1.425515943	3.636472798	2.401793539	2.571596406	3R	-0.343517118	0.508189951	0.978557094
**dia**	FBgn0011202	9.23962919	17.30165988	10.41236411	12.55418935	8.000670468	11.677938	10.92671841	6.965045535	2L	-0.366290237	0.092754932	0.72305867
**fbl**	FBgn0011205	4.421187846	7.920671024	6.794840553	6.96183487	5.724862182	8.565683463	6.731697123	5.653748213	3L	0.066915555	0.777774976	0.995787491
**bol**	FBgn0011206	23.44929448	26.63543837	26.50681608	23.61797957	25.98765586	23.06491106	21.2817612	24.00815742	3L	-0.054364666	0.586715827	0.987588562
**pelo**	FBgn0011207	14.92456084	18.19498888	13.17083313	23.06395379	12.88056593	18.93638328	18.28373885	17.1913164	2L	-0.015936218	0.934583242	0.996479182
**blw**	FBgn0011211	4201.75804	4385.200767	4230.451897	4613.912957	4450.832912	4432.949476	4483.273102	4706.166653	2R	0.082961809	0.317554594	0.91460807
**eff**	FBgn0011217	440.9311278	376.0081213	450.5683456	402.851172	376.5653703	389.5896379	415.2554035	437.3759201	3R	-0.014649628	0.885348525	0.995787491
**heph**	FBgn0011224	6.369434581	9.222597037	8.229181989	9.237424139	7.306575276	8.673012253	6.732540829	8.54846691	3R	-0.049533461	0.700291941	0.995787491
**jar**	FBgn0011225	59.79562381	63.4059985	62.09172558	55.96417449	50.43503061	58.23385371	52.56727111	65.16122298	3R	-0.060064852	0.567800367	0.984934163
**ox**	FBgn0011227	925.2403512	1040.510895	1323.423817	788.9199137	1191.319845	1083.480317	1208.053629	1238.511733	2R	0.246737677	0.089108085	0.709968244
**poe**	FBgn0011230	51.32914534	71.51974067	79.10108734	66.0045831	69.01092747	63.29342738	67.09918733	78.83318878	2L	0.089896175	0.492966568	0.973549494
**scat**	FBgn0011232	30.80933534	53.22868805	44.84581069	44.12463767	30.82867672	57.86548107	39.74675405	43.76511844	2L	0.024750538	0.898969628	0.996479182
**ken**	FBgn0011236	0.338305325	2.249572477	1.61213643	0.140847523	0.632539527	0.353125644	1.54111456	1.180433858	2R	-0.171343806	0.819717697	0.995787491
**ms(2)35Ci**	FBgn0011239	0.519294039	0.95814719	0.251960019	0.2421429	0.093745883	2.594812413	1.324730249	0.338230629	2L	1.154658188	0.344462964	NA
**Hsp60B**	FBgn0011244	1.652119127	1.25363722	0	0	2.130356042	1.112577015	0.301042242	0.170804931	2L	0.364611331	0.7885668	NA
**Sema1a**	FBgn0011259	12.9406568	12.64159299	14.70686988	11.99612324	13.47428301	12.20100088	11.3515596	14.29814558	2L	0.00572867	0.958041903	0.997604378
**Sema2a**	FBgn0011260	19.8789147	18.57979182	22.37033399	17.08125683	18.54936423	25.07484029	19.13467582	18.75985342	2R	0.093158502	0.443901399	0.967112302
**RpL13**	FBgn0011272	681.0457107	961.9460358	887.5507308	961.6748919	870.5901485	843.6686541	982.232581	908.0913422	2L	0.079190192	0.513232928	0.980804634
**Dif**	FBgn0011274	65.86953718	84.70930478	85.37083138	81.58816123	76.80594152	79.05081784	84.55548347	79.61201297	2L	0.043938083	0.667024588	0.995787491
**HLH3B**	FBgn0011276	1.307230741	0.953346166	3.584974442	1.060090178	3.180716626	8.359220377	0.144990161	5.552841421	X	1.359064632	0.11365668	0.758410631
**HLH4C**	FBgn0011277	1.666463843	0.762343449	0.091882228	0.529813325	1.640942371	0.928396142	0.193235685	1.89125198	X	0.610178345	0.545616886	NA
**lbe**	FBgn0011278	0.08731678	0.175753979	0.381292722	0.203575785	0.031525833	0.24696518	0.133648189	1.023693185	3R	0.82064319	0.490352079	NA
**Obp84a**	FBgn0011282	0.562456141	1.533092065	0.682255376	0.491754494	0.253844373	1.060560427	1.614192411	0.763215217	3R	0.202482858	0.860871526	NA
**RpS4**	FBgn0011284	526.2110855	649.524669	656.0135985	658.5694725	585.7810262	505.7974459	655.850531	660.2458419	3L	-0.014033563	0.907987836	0.996479182
**RyR**	FBgn0011286	4.862965004	10.46728921	8.880316467	10.05621657	7.696336573	7.46166767	7.999454281	7.960841358	2R	-0.102868682	0.59332919	0.988631016
**Snap25**	FBgn0011288	5.738109934	7.24948616	7.778787613	5.528250738	5.547325006	6.041313217	5.985031481	5.234903772	3L	-0.172242392	0.135878038	0.796109055
**TfIIA-L**	FBgn0011289	54.3731428	64.29576064	50.69778371	52.64741443	65.69156495	53.73499559	58.51381291	64.10288965	3R	0.157491525	0.211551169	0.856520545
**Taf12**	FBgn0011290	445.5127098	470.2982386	506.8048255	522.3679713	474.8602826	438.0000702	429.5600377	461.9203243	3R	-0.076985062	0.451987858	0.967630712
**Taf11**	FBgn0011291	38.03760668	68.13717495	68.4567554	54.46308167	45.90281722	47.55563081	66.4477105	51.61447825	2L	-0.078100431	0.75193206	0.995787491
**Alg3**	FBgn0011297	33.62849417	37.24869989	26.16425073	27.83558635	28.9171485	35.1566158	27.91884977	33.00596354	2R	0.03039685	0.846420508	0.995787491
**babo**	FBgn0011300	51.04156351	63.89868093	74.99498282	63.71079117	48.28466128	48.88699792	75.22572629	68.03258258	2R	-0.041360412	0.798538613	0.995787491
**Rsf1**	FBgn0011305	655.0289389	607.6737944	607.3476919	676.5632322	709.4792912	626.1924994	596.0653504	708.4983699	2L	0.08235616	0.436850283	0.966363994
**Uch-L5**	FBgn0011327	93.20876433	94.45821171	99.35714703	128.0004877	117.7010135	113.7292551	138.5534648	85.21297897	3L	0.161577801	0.35660163	0.937787223
**vers**	FBgn0011335	44.32868252	55.02280821	57.74939107	53.22558708	49.93537118	47.56021905	54.28004231	39.07411932	3L	-0.107533975	0.515847798	0.980804634
**Stt3B**	FBgn0011336	112.9442697	74.65668495	93.39318035	88.58059165	99.86176865	96.23688482	94.73166392	83.32337551	3R	0.043576624	0.765042852	0.995787491
**ND-ACP**	FBgn0011361	698.7651457	697.4263393	683.6247541	666.2335885	709.327502	654.3733502	772.4579267	720.695871	3L	0.088703408	0.350857301	0.933267978
**ND-SGDH**	FBgn0011455	1196.175681	1070.712192	1192.436916	1012.891319	1314.757063	1094.57147	1127.571425	1229.797861	3L	0.123712915	0.248312348	0.879503029
**PR-Set7**	FBgn0011474	55.69148873	41.96323452	49.60988732	40.38037173	45.30324513	59.70793362	46.61290512	43.68002636	3R	0.083278082	0.57847381	0.985637351
**Ssdp**	FBgn0011481	184.4952775	162.7427557	149.4409255	168.3888532	169.426337	161.8772765	151.7286815	170.0212636	3R	0.002815402	0.978305351	0.997757033
**SrpRbeta**	FBgn0011509	83.49564431	65.81316177	76.57728443	76.12585664	73.89497216	73.15124121	56.45344186	57.47800116	3L	-0.182600189	0.28695953	0.90249226
**Smyd3**	FBgn0011566	59.55990479	66.22734321	54.25607916	49.75110374	61.66430036	57.5658291	56.7505752	56.94781083	X	0.049685111	0.72579647	0.995787491
**can**	FBgn0011569	1.406667325	0.771370783	0.590634573	0.946036129	0.683683282	0.867129044	0.828101208	0.352385189	3L	-0.431004892	0.57240615	0.984934163
**cpb**	FBgn0011570	106.9693286	115.3150862	105.5773635	109.7965245	120.5400951	124.9921947	117.0256413	129.9961508	2L	0.201112925	0.063965857	0.636907352
**Cdc37**	FBgn0011573	240.3599777	202.1161578	203.1118788	199.7326146	183.2324238	181.9617094	195.4646054	164.1932197	3L	-0.194083101	0.094587313	0.729223555
**Ms**	FBgn0011581	6.323055751	2.236213039	1.755742507	0.444035134	0.687634712	10.41438626	1.846233556	1.984762316	3R	0.464620065	0.568860822	0.984934163
**Dop1R1**	FBgn0011582	63.4089506	93.05952315	96.39301895	106.973052	87.3277638	76.28733815	88.36633348	91.0207216	3R	-0.035371698	0.800284585	0.995787491
**Trp1**	FBgn0011584	71.21030224	106.7913768	90.50930542	76.16324062	85.22042639	91.42652274	97.85676414	94.00329865	2L	0.131472603	0.324863236	0.918470465
**e(r)**	FBgn0011586	119.9646235	211.7891132	273.7543211	192.540894	158.9693144	129.3258676	197.7552783	214.6021896	X	-0.147353179	0.477421108	0.973549494
**Elk**	FBgn0011589	68.89923535	64.33616377	58.357018	67.33359007	55.49474113	62.40212835	51.17428232	52.97770267	2R	-0.194430418	0.043022997	0.544212375
**fng**	FBgn0011591	1.415462632	2.681784036	2.49090655	1.5132573	0.860584114	1.400176831	1.824146991	1.273824	3L	-0.560428581	0.056876631	0.621788268
**fra**	FBgn0011592	1.005956113	1.12076451	1.872196842	1.971702553	0.850141285	1.125073753	0.987489849	0.674477951	2R	-0.683878991	0.015150138	0.362234897
**ine**	FBgn0011603	9.053536427	16.03954732	13.7884855	9.535524077	9.913829787	4.951202022	8.523145859	12.04793844	2L	-0.406919406	0.119741093	0.777535158
**Iswi**	FBgn0011604	127.5742066	140.6268715	114.2278606	131.7224334	135.0290402	153.2551361	131.0042717	130.1233147	2R	0.124719746	0.22829378	0.867702781
**Klp3A**	FBgn0011606	0.938039849	0.326560996	0.601120797	0.041264238	0.287558889	0.250295728	0.225750925	0.038425874	X	-1.230482939	0.265830962	NA
**La**	FBgn0011638	200.4685401	195.9933683	215.3263706	198.0803707	196.353561	223.7035721	162.9599856	190.9601243	2L	-0.035684372	0.760764699	0.995787491

**lark**	FBgn0011640	863.1160321	802.3854883	766.5475471	693.5544151	774.8168989	766.3031528	863.5887751	701.6136676	3L	0.021524232	0.834486815	0.995787491
**Zyx**	FBgn0011642	98.33598369	95.17291234	106.1680856	86.40144494	76.15676904	121.7675038	81.14010943	88.51153597	4	-0.044376032	0.741806806	0.995787491
**Mad**	FBgn0011648	19.05512153	20.53114853	22.50282801	18.01427695	19.70282215	18.93272479	21.71106811	17.8824353	2L	-0.002348609	0.984482713	0.997881056
**mas**	FBgn0011653	0.537815944	0.200469066	0.398668716	0.383135386	0.755141038	1.718334711	0.038110556	0.421650739	3L	0.962075844	0.287163315	0.90249226
**Med**	FBgn0011655	108.6279639	132.3014566	109.5573846	159.1692711	123.5340763	135.8034275	120.2061881	133.6680568	3R	0.039411637	0.768616048	0.995787491
**Mef2**	FBgn0011656	135.7436723	148.6305806	113.8410821	133.7395883	134.8856987	130.1152074	109.9223208	110.9922277	2R	-0.101115662	0.342529936	0.928339671
**Mlh1**	FBgn0011659	9.732844715	10.79257781	7.804719232	15.95069396	5.807749198	8.139439783	7.168117247	13.26207542	2R	-0.335981753	0.351836902	0.934121932
**Pms2**	FBgn0011660	2.865490425	6.239963276	5.488136686	7.032403213	3.766266709	2.18021186	2.051909512	3.001477835	2R	-0.934791961	0.021618694	0.417060631
**Moe**	FBgn0011661	40.74341649	47.44876161	42.24270924	45.20681715	43.66820978	38.98351221	42.24690332	46.42731004	X	-0.002917443	0.977144224	0.997757033
**msi**	FBgn0011666	0.928069389	0.788584235	0.760359407	0.536017105	0.59460717	0.988221026	0.643947636	0.584715214	3R	-0.074723908	0.744034097	0.995787491
**Mvl**	FBgn0011672	27.37346278	32.73922545	22.89456906	22.73165654	27.36483848	31.17543111	25.64667634	22.26640075	3R	0.039199612	0.789254516	0.995787491
**insc**	FBgn0011674	0.035196361	0.541172123	0.668856076	0.287206471	0.158846133	0.077426801	0.014964446	0.02547152	2R	-2.396154377	0.012729543	0.338939333
**Nos**	FBgn0011676	1.123220022	2.670883743	1.403553076	1.020763789	1.999472176	2.476651266	1.269799202	1.663463353	2L	0.290153405	0.327349766	0.918470465
**Pdh**	FBgn0011693	10.1760157	7.919941196	4.739879454	4.175599564	11.31611492	4.835318265	5.399520813	9.72095171	3L	0.232156032	0.658709207	0.995750687
**EbpIII**	FBgn0011695	25.35482079	11.0100858	63.22773818	32.40757106	31.01804728	16.8665353	64.02403579	27.38411662	2R	0.11409205	0.855934677	0.995787491
**repo**	FBgn0011701	23.47075043	32.38133911	32.47535589	21.79371315	18.47987032	19.30219978	26.11404611	20.40492812	3R	-0.351429441	0.074972397	0.676944702
**RnrL**	FBgn0011703	2.613097572	0.14942402	1.296682826	0.900004568	3.05553089	3.079515537	1.363513918	0.064469054	2L	0.609995855	0.51052384	0.980346932
**rost**	FBgn0011705	0.552933569	1.854936987	0.524899223	0.05604973	0.954787382	1.65456846	0.061328061	0.313166077	2L	0.039835571	0.969995941	0.997604378
**rpr**	FBgn0011706	3.272244835	5.650164945	4.669659017	3.59017207	5.386013486	3.720210299	5.15585013	2.089513661	3L	-0.035977031	0.955242628	0.997604378
**Syx5**	FBgn0011708	457.6660106	413.262509	397.0542277	424.738491	364.4804937	465.8781218	340.1577472	356.5070117	2L	-0.122521989	0.277836898	0.896398793
**1-Sep**	FBgn0011710	67.44612954	64.78684975	79.49538565	84.88667427	79.47104725	74.94190576	78.00675241	59.31344333	X	0.005242232	0.970879383	0.997604378
**Snr1**	FBgn0011715	191.5741615	175.5719432	183.1206273	230.0076732	221.5500359	196.7420817	208.8772044	194.9488812	3R	0.104175723	0.409828792	0.958170848
**Tig**	FBgn0011722	7.467641151	5.414199556	7.572187203	5.091522775	3.557754851	2.209555238	10.95177681	1.480211374	2L	-0.455049398	0.479092137	0.973549494
**twin**	FBgn0011725	41.23970957	34.49129773	32.61981814	35.55340012	35.10674152	40.01729007	32.29163132	39.82093659	3R	0.060601433	0.597885934	0.989286463
**tsr**	FBgn0011726	382.5639183	336.4772062	411.6923868	353.0641156	375.6957152	359.4984206	396.7411643	373.7137825	2R	0.051649653	0.617351531	0.990047511
**Wee1**	FBgn0011737	67.15187482	81.55876453	69.07969983	106.8040854	86.70017899	110.0094836	72.64007073	95.69396715	2L	0.197018702	0.246478551	0.877584019
**wts**	FBgn0011739	17.96688925	24.99014597	20.6375627	25.53751251	18.41194746	19.64932519	18.72364411	17.26301741	3R	-0.236116205	0.069247909	0.660467387
**alpha-Man-IIa**	FBgn0011740	50.10487153	73.53840635	51.15237168	49.15932176	52.62006527	56.95294952	62.925361	53.67338525	3R	0.047720648	0.74139097	0.995787491
**Arp6**	FBgn0011741	130.1847778	120.4632917	101.4766168	130.8305647	156.251961	132.2627588	123.4514741	124.7653885	X	0.180322684	0.219685379	0.864306828
**Arp2**	FBgn0011742	80.11958896	77.27644705	75.09038686	76.61046556	76.44909096	86.26551447	83.00781206	79.02468536	X	0.099960491	0.292568492	0.905336188
**Arp1**	FBgn0011745	332.1352095	303.4803053	344.5397872	349.2899178	311.165675	322.1576932	341.0797715	304.7130468	3R	-0.026561908	0.787157561	0.995787491
**ana**	FBgn0011746	4.308489969	9.169292049	11.73305765	6.418794271	3.023042098	4.650716291	6.777090413	3.72193337	2R	-0.762590417	0.007376888	0.250934646
**Ank**	FBgn0011747	112.8059751	109.0141759	134.3439566	91.65873313	95.0485419	123.586335	107.5886464	108.557129	4	-0.012639517	0.915576002	0.996479182
**PhKgamma**	FBgn0011754	94.53431303	74.22233993	62.24362387	82.89934968	89.07088341	79.58121448	77.48216572	68.62417015	X	0.030408987	0.826598031	0.995787491
**ctp**	FBgn0011760	544.0202713	402.4411329	411.2196738	379.931397	496.6451416	476.8522283	431.2962449	448.3279535	X	0.121118578	0.312032763	0.914141092
**dhd**	FBgn0011761	0.090794807	0	0.44053387	1.270108044	1.47517107	0.599205525	0.926478011	2.956858954	X	1.809930623	0.208998613	NA
**DNApol-alpha5**	FBgn0011762	0.063534166	0.781510764	0.051377666	1.333147636	0.497013627	0.419297362	0	0.5057747	3L	-0.605456983	0.626522916	NA
**Dp**	FBgn0011763	76.45347665	82.63067166	70.73695641	87.65396707	71.58402504	79.10100511	68.33100536	91.37140079	2R	-0.002169165	0.986344768	0.99797245
**E2f1**	FBgn0011766	4.105970034	3.728322132	3.356601596	4.420565851	4.94540674	5.314435963	4.384766669	5.293963419	3R	0.380548095	0.007063576	0.24630895
**Fdh**	FBgn0011768	2.231520905	10.14567149	6.69581615	11.63763404	11.66135233	13.34292871	7.789952739	4.20735971	3R	0.306845479	0.557095916	0.984934163
**Fdx1**	FBgn0011769	65.10901686	60.03832303	71.52234209	66.99302388	67.01491434	53.24155703	51.69267628	59.13944704	3L	-0.158737748	0.384441083	0.94674182
**Gip**	FBgn0011770	6.616067004	16.06220616	4.857384003	13.39549127	6.600463925	3.857455464	5.551884562	5.859038304	X	-0.871528738	0.098103846	0.730787365
**Hem**	FBgn0011771	92.16578233	66.30911024	93.16826837	91.37833819	77.26808574	96.92848585	78.98592883	89.45033356	3L	0.02474914	0.861708337	0.995787491
**Irbp**	FBgn0011774	5.212297272	6.435743965	2.546554541	6.498091424	6.469053578	3.378458896	5.817298517	2.436165112	3R	-0.167722456	0.718578369	0.995787491
**BRWD3**	FBgn0011785	49.7161175	72.83378382	68.31178591	67.91757187	69.02371293	65.0741311	66.59502559	69.15010745	3R	0.094634852	0.438664045	0.967112302
**mRpL12**	FBgn0011787	319.1658433	406.0057275	378.4013726	343.0300757	353.5536738	324.0781113	368.3678907	336.983483	3L	-0.031536605	0.789719577	0.995787491
**Gem3**	FBgn0011802	6.981172037	11.70993673	10.92491121	4.521205499	7.196045943	8.999198194	9.454224119	10.52553523	3L	0.122382633	0.685669144	0.995787491
**nmo**	FBgn0011817	22.54235777	21.59780916	19.95553142	19.78303079	21.99118821	20.765652	17.92117081	20.71095024	3L	-0.013340916	0.89211183	0.996438268
**oaf**	FBgn0011818	47.27171274	58.91999733	55.93127487	49.46694605	38.72705937	72.26447397	46.32350736	51.4912066	2L	0.00902033	0.955647567	0.997604378
**Pp2B-14D**	FBgn0011826	151.6823871	200.747636	196.915942	214.8382217	203.229246	186.9963457	188.6136622	212.0299963	X	0.082547299	0.489048221	0.973549494
**Pxn**	FBgn0011828	155.9879281	185.4454201	164.5512494	198.2271551	181.8984055	187.8175447	180.6786108	205.1731654	3L	0.132662014	0.196419195	0.844272804
**Ret**	FBgn0011829	0.037161542	0.204809327	0.103032437	0.173281472	0.412100376	0.105107024	0.270856834	0.46103513	2L	1.326107972	0.087437635	0.706081898
**Taf2**	FBgn0011836	26.96682273	21.35816765	22.72523665	25.6215756	23.46254063	27.79452817	25.3447689	16.11155898	3L	-0.035820576	0.849051342	0.995787491
**Tis11**	FBgn0011837	2.23048064	1.826717525	1.098565278	0.732925601	0.776942551	1.386632927	0.420067412	0.47125778	X	-0.922608945	0.027614219	0.462499435
**AcCoAS**	FBgn0012034	491.6251839	408.6156018	420.181029	471.8958972	446.8834063	447.5391233	442.046052	443.1300817	3L	0.018025744	0.852599821	0.995787491
**Ance**	FBgn0012037	1.311293879	3.299267288	1.119304306	0.452923306	0.701398973	1.350750216	0.557523143	2.794219277	2L	-0.150766725	0.837377537	0.995787491
**CalpA**	FBgn0012051	59.68411863	48.64321578	38.31844576	54.49721156	42.3407384	60.03013277	37.57742802	36.67286221	2R	-0.165666749	0.346083629	0.930285749
**Cdc27**	FBgn0012058	122.1723532	159.8980156	147.7313608	169.972645	156.6517761	161.8026606	157.5213087	138.8857875	3L	0.066790696	0.571243348	0.984934163
**Dh44**	FBgn0012344	0.961349101	1.461716259	0.92282067	0.532118884	0.649244885	0.332547173	0.405796481	0.52554839	3R	-0.985935464	0.037449297	0.521998122
**Trl**	FBgn0013263	0.429130028	0.387587534	0.288294497	0.184707786	0.405222297	0.112037862	0.202102139	0.272337527	3L	-0.356085118	0.604263858	0.989286463
**Fkbp39**	FBgn0013269	101.8281635	77.19408236	84.37944215	95.30238076	101.9572723	82.92843095	97.71531879	118.734017	3R	0.190730083	0.25644276	0.88674759
**Gp150**	FBgn0013272	1.400158812	1.396781096	1.179929154	1.975831681	1.09087859	1.507648816	1.203144492	0.463980828	2R	-0.454104337	0.260282087	0.890556245
**Hsp70Aa**	FBgn0013275	9.567660061	4.820624319	4.147975146	5.004151011	5.84492393	4.630208166	7.981096379	5.212814424	3R	0.019408587	0.961666274	0.997604378
**Hsp70Ab**	FBgn0013276	231.0671229	127.0522107	180.3050943	131.8526231	133.6440366	115.5117386	124.537143	110.425604	3R	-0.442484241	0.012876083	0.34174929
**Hsp70Ba**	FBgn0013277	91.13000101	56.35223854	76.52773086	47.03117684	16.22243658	43.30994593	27.58495549	17.00034791	3R	-1.360062093	1.1E-06	0.00025391
**Hsp70Bb**	FBgn0013278	264.6545545	185.4831276	256.4431029	149.0931432	128.7071039	281.5742728	173.9652026	146.4529971	3R	-0.206574679	0.327411076	0.918470465
**Hsp70Bc**	FBgn0013279	23.64296768	11.26987078	14.27329737	7.789996001	36.51867939	45.19721671	32.05613917	23.33947334	3R	1.282029589	2.61E-05	0.003195831
**Nca**	FBgn0013303	181.8358031	157.4890948	162.3078454	137.1434987	138.6972407	175.947367	139.8291137	126.3197626	3L	-0.110632457	0.357043299	0.937787223
**Odc1**	FBgn0013307	9.046169594	3.537904766	2.501603045	0.874230212	7.446101594	9.500853829	6.217630027	3.561671012	2R	0.745369855	0.217227949	0.860826301
**Odc2**	FBgn0013308	0.190789087	0.320021929	0.694277497	0	1.033269604	1.03428002	0.973414121	2.588879811	2R	2.294373698	0.038912621	NA
**Ptth**	FBgn0013323	0.073095566	1.226075576	1.418630588	1.022517362	1.319562308	2.136335646	0	0.15869724	2L	0.013077097	0.99269253	NA
**RpL11**	FBgn0013325	213.6544454	324.8695167	384.8453979	360.5907821	281.4204641	275.4410593	324.8170682	317.1467497	2R	-0.064252716	0.68902734	0.995787491
**Sap47**	FBgn0013334	346.5659344	323.4938069	264.8844379	346.4652552	344.7690521	350.5556438	282.8538498	305.4800311	3R	0.029704969	0.792137476	0.995787491
**nSyb**	FBgn0013342	1189.281147	1499.386849	1351.940077	1556.553432	1419.773655	1402.976055	1332.77353	1576.558376	3L	0.066356248	0.531574507	0.984760433
**TfIIA-S**	FBgn0013347	1016.346528	1070.216192	1046.295474	942.720685	1044.78113	1002.074939	1027.881856	1025.634049	3R	0.040188516	0.653104354	0.99443552
**bcn92**	FBgn0013432	157.2150421	234.6236447	160.2631708	191.0311152	185.2909554	187.6045405	175.9881759	188.0562295	X	0.021171285	0.882448178	0.995787491
**beat-Ia**	FBgn0013433	0.630814803	0.64127475	0.94073335	0.42020599	0.783837234	0.756926982	0.515508383	0.486163209	2L	-0.014089105	0.966481342	0.997604378
**Cdc2rk**	FBgn0013435	44.50486291	52.53919612	60.70556723	68.53964861	55.49226016	47.23814704	59.07279947	70.4102785	2R	0.071542151	0.722500779	0.995787491
**igl**	FBgn0013467	234.9167339	153.7143138	186.6222427	148.5677682	175.3539301	193.7446881	175.3012289	153.8117053	2R	-0.025710351	0.851131168	0.995787491
**klu**	FBgn0013469	0.45137823	0.85565345	0.750025541	0.237864757	0.524631636	0.775329217	0.418001403	0.604099677	3L	0.057631042	0.88975795	0.995926915
**MED20**	FBgn0013531	58.69305834	43.61503498	73.22259724	38.98594188	37.73362294	66.3710181	46.28321063	42.29449948	2L	-0.126770142	0.619812518	0.990047511
**l(2)dtl**	FBgn0013548	15.45274239	13.05921338	14.14782201	16.63338008	9.13839741	13.00581998	10.34593498	14.13956612	2R	-0.318440402	0.176994616	0.834615603
**Pex1**	FBgn0013563	6.203102914	18.47459139	12.11837769	13.74634543	10.34816293	11.81230196	17.39100611	11.95630483	3L	0.066236361	0.821752117	0.995787491
**mtd**	FBgn0013576	74.31353115	77.95631721	78.48755807	83.75853964	69.4796447	77.38414074	68.70444532	76.85238891	3R	-0.075512844	0.38240207	0.945120535
**mt:ATPase6**	FBgn0013672	2779.494684	2042.250802	2377.576981	1265.016305	1852.057581	1829.545414	2220.140976	1717.617431	mito	-0.120139754	0.497384414	0.97594235
**mt:ATPase8**	FBgn0013673	376.601068	358.2615679	399.3863644	294.7676214	418.600407	272.3795846	468.0066267	392.4651745	mito	0.149730264	0.499486069	0.976796212
**mt:CoI**	FBgn0013674	2605.488115	1810.587338	2665.219292	1490.19915	1798.488206	2598.590687	2408.158683	2022.308912	mito	0.069848143	0.672614838	0.995787491
**mt:CoII**	FBgn0013675	5510.516022	3218.894721	5283.473407	2356.936154	3626.186159	5375.083052	4253.576357	3781.469854	mito	0.083009107	0.760963274	0.995787491
**mt:CoIII**	FBgn0013676	3205.424697	2433.791137	4017.091948	1770.62571	2296.508133	2844.945271	2878.433734	2411.081328	mito	-0.100964644	0.574316803	0.984934163
**mt:Cyt-b**	FBgn0013678	1539.761314	1207.722757	1694.210662	839.7873885	1084.941574	1351.970878	1391.923926	1192.567849	mito	-0.042188225	0.801250305	0.995787491
**mt:ND1**	FBgn0013679	134.5445457	129.8006214	183.4757815	76.42990248	108.024041	121.2758763	117.5496138	118.6879401	mito	-0.137728135	0.518638034	0.981009845
**mt:ND3**	FBgn0013681	14.96802545	15.64027852	20.83488088	17.73473809	13.2890765	14.11176297	15.64907971	15.44937862	mito	-0.208466811	0.674612379	0.995787491
**mt:ND5**	FBgn0013684	213.7189115	137.9238566	222.8700895	114.0366063	124.9304843	134.3920419	150.2716559	111.7816748	mito	-0.372578448	0.055408026	0.617340222
**mt:ND6**	FBgn0013685	12.89355565	25.50892353	21.65509811	16.9573967	22.6793476	12.46765693	24.87964757	13.27877956	mito	-0.034485963	0.938548424	0.997038394

**mt:lrRNA**	FBgn0013686	205.9720339	226.6655092	577.6717635	97.08118352	141.7123145	188.2017787	181.8827597	134.3258782	mito	-0.741970779	0.153204238	0.81584928
**mt:srRNA**	FBgn0013688	4.74469371	3.146406685	9.369061086	0.771772658	1.991950745	2.184619377	4.222261253	1.916496606	mito	-0.77716859	0.363154742	0.939549067
**mt:tRNA:Cys-C**	FBgn0013690	176.0764338	181.016994	141.2380473	76.14401829	117.9169531	113.792918	101.4265599	43.1604461	mito	-0.581954124	0.265539169	0.893759496
**mt:tRNA:Arg-T**	FBgn0013704	74.24421055	43.8176057	110.070534	16.02755388	44.67660956	28.51729149	17.5369052	32.83521467	mito	-0.944047412	0.240923984	0.874884741
**not**	FBgn0013717	54.8612424	52.5460274	56.54512573	75.20208755	64.81262854	72.67251814	59.22832916	56.75127845	3L	0.111030743	0.454732	0.967967712
**nuf**	FBgn0013718	2.303415829	2.605482925	2.528755886	1.753576259	1.926006741	2.851851494	2.695585368	2.986357773	3L	0.21809117	0.241689618	0.876519266
**pck**	FBgn0013720	118.2059724	69.88630782	99.68413146	90.44409521	73.98974368	77.38063676	88.03970385	90.45742682	X	-0.171347191	0.340055548	0.927274752
**phyl**	FBgn0013725	472.9622943	405.3650775	412.9580878	472.2599995	455.753232	532.0270316	418.777509	428.4192406	2R	0.083019391	0.431340147	0.964209983
**pnut**	FBgn0013726	264.0053138	273.3909478	271.0362067	308.6601793	255.6872189	280.3426413	272.8837494	312.7227186	2R	0.036175157	0.734915343	0.995787491
**sced**	FBgn0013732	110.5971596	145.7304953	105.5133384	131.1003366	109.6188977	120.1176334	115.5074802	93.91407214	2R	-0.136457254	0.291841679	0.905069205
**shot**	FBgn0013733	11.41109989	14.72620319	20.43519253	16.77598526	13.28686373	13.32546177	13.82432303	15.18253457	2R	-0.154235531	0.29382504	0.906604178
**alien**	FBgn0013746	65.18868248	59.36447223	55.76446215	41.56902054	54.31827607	59.64781262	63.41652224	43.67382807	2L	0.022659434	0.890048435	0.995926915
**Arf102F**	FBgn0013749	348.2681794	304.7576015	393.4127234	260.1944654	348.7900594	338.4295949	351.1015632	310.9910876	4	0.077217681	0.562616743	0.984934163
**Arf51F**	FBgn0013750	141.0118378	131.6796554	132.2393066	116.6145302	128.6937465	131.0599346	128.4834994	118.7226848	2R	-0.01176541	0.910052	0.996479182
**Awh**	FBgn0013751	0.251699668	0.412808863	0.162831969	0.299934464	0.413992863	0.427141637	0.21403042	0.036430885	3L	-0.014862554	0.984006399	0.997881056
**Bgb**	FBgn0013753	622.7998436	549.1983742	524.4053416	587.1276564	555.5415797	576.9064155	523.834848	525.3754613	3L	-0.038078838	0.70460937	0.995787491
**Mtor**	FBgn0013756	14.98288815	20.47767577	26.04347976	23.85258468	24.35226493	21.51683773	26.84525344	22.75951338	2R	0.196502041	0.252972425	0.88397157
**CASK**	FBgn0013759	94.30215785	111.8807387	91.77139115	118.9654716	98.44177063	101.9614597	90.12676132	106.2383145	3R	-0.041205106	0.70467095	0.995787491
**Chi**	FBgn0013764	357.781976	382.888873	392.484355	392.3065364	381.0050986	327.1959008	391.0299641	437.1036685	2R	0.043729796	0.702665229	0.995787491
**cnn**	FBgn0013765	17.94296066	24.57731713	23.42151347	17.80449996	21.43564593	16.92383054	23.95552878	22.42656047	2R	0.053838453	0.732472691	0.995787491
**Crz**	FBgn0013767	0.502426019	3.370996653	3.412859839	3.279884577	0	78.25262681	0.769019741	1.963464113	3R	2.928308103	NA	NA
**Cp1**	FBgn0013770	62.74072761	85.65067607	86.77474083	81.26535807	64.10108948	60.05695601	73.70540906	61.82741078	2R	-0.251243342	0.047878113	0.575780949
**Deaf1**	FBgn0013799	129.6919928	149.865103	121.7444149	158.5782475	138.6842373	148.0536254	132.557811	134.6243324	3L	0.01374225	0.895387996	0.996479182
**Dhc36C**	FBgn0013810	0.416675645	0.638663652	0.479281997	0.586228022	0.505793913	0.453797068	0.293227178	0.51470976	2L	-0.224354111	0.55767318	0.984934163
**Dhc62B**	FBgn0013811	0.812089066	1.108202427	2.036900366	1.620583591	1.466028901	0.875935614	0.175564123	0.56778458	3L	-0.813135472	0.12520369	0.780439453
**Dhc93AB**	FBgn0013812	0.338371404	0.705409127	0.117269187	0.236670057	0.261791618	0.446620291	0.061656623	0.157421923	3R	-0.553829867	0.445220192	0.967112302
**Dhc98D**	FBgn0013813	0.573984368	0.873217487	0.401551816	0.784260889	0.424114524	0.848219526	0.326901534	0.324584646	3R	-0.422818394	0.289091975	0.903033179
**Elal**	FBgn0013949	12.49772167	31.25533598	20.54771732	11.14615025	3.397829932	8.76610116	20.35835775	6.538243527	3R	-0.912169315	0.041222361	0.533583119
**Esp**	FBgn0013953	0.418939256	0.805547708	1.214651189	0.881449289	0.590277253	1.522092504	0.078199247	0.865187324	3R	-0.079893045	0.907519821	0.996479182
**Fkbp12**	FBgn0013954	4865.85357	4095.200945	4281.524405	4926.28007	5682.047673	4591.422216	5178.300658	5393.443842	2R	0.228633999	0.042747528	0.542555546
**Gycalpha99B**	FBgn0013972	1898.948697	1864.889201	1631.626203	2228.739941	1720.962216	1945.188705	1673.49595	1805.513126	3R	-0.066407014	0.542119699	0.984934163
**Gycbeta100B**	FBgn0013973	38.16366034	34.01664073	32.02822569	30.01183751	35.20296128	39.38201176	32.76259607	37.76893785	3R	0.141484506	0.174058984	0.833984823
**His4r**	FBgn0013981	3653.696755	3969.849303	3892.016733	3621.883863	3619.76756	3543.268187	4023.415194	3880.438495	3R	0.025893219	0.769132046	0.995787491
**imd**	FBgn0013983	16.60391824	18.91099871	20.49959435	17.04576504	10.89601771	20.14200301	16.38499384	16.16996767	2R	-0.16923	0.416591672	0.958846438
**MAPk-Ak2**	FBgn0013987	110.6979163	78.95384324	91.83300324	98.41104668	78.12945621	83.22226317	88.74613146	88.44553748	X	-0.139349	0.291952147	0.905069205
**Calx**	FBgn0013995	20.74626161	25.97984702	25.84156614	23.75563529	26.42341555	23.84223711	27.44499895	26.0254146	3R	0.140818367	0.180381874	0.836097072
**Nrx-IV**	FBgn0013997	65.47449282	65.46442662	78.68761201	82.89265353	57.8671108	75.44736332	67.08506854	65.60685762	3L	-0.109100916	0.36348202	0.939549067
**Hf**	FBgn0014000	10.03614328	10.16119732	5.483680996	5.059219514	3.917364513	4.603142231	6.919572199	3.337114719	2L	-0.695693514	0.201135138	0.848203654
**Ask1**	FBgn0014006	20.31294621	31.28649182	21.67606733	30.91126206	25.12395616	18.54275046	24.40383598	28.60623994	3R	-0.07238547	0.70485718	0.995787491
**Ptp69D**	FBgn0014007	26.53607435	26.47817856	22.84417215	25.73507761	21.67385573	26.8305935	24.5887376	20.89829154	3L	-0.08478149	0.527142644	0.984107922
**Rab2**	FBgn0014009	566.5404374	514.9544365	524.7208602	465.5141381	478.0397223	638.7374741	503.2821995	486.8107595	2R	0.051172559	0.637107973	0.991585536
**Rab5**	FBgn0014010	205.8013349	203.0883903	228.7455052	209.8524673	216.4312479	242.4187544	223.5238892	216.3674185	2L	0.114219995	0.184590702	0.839299945
**Rh5**	FBgn0014019	0.309535124	6.316954597	5.256495261	3.60834745	5.494770622	0.826845503	4.606179389	3.472151943	2L	-0.049626386	0.949013844	0.997604378
**Rho1**	FBgn0014020	216.4502616	219.0225291	262.1362747	201.1023977	204.2218775	214.5680464	235.6084372	208.0128791	2R	-0.027783321	0.793775098	0.995787491
**Rlb1**	FBgn0014022	45.61894271	68.2862792	48.33097083	47.25563987	49.93355729	48.39902863	44.48762112	56.29149802	3R	-0.038753298	0.835836734	0.995787491
**mRpL47**	FBgn0014023	229.227291	253.3878234	225.033706	220.7286385	256.2939941	214.9109494	267.5589156	245.7198799	3R	0.116360672	0.344943009	0.930285749
**Rnp4F**	FBgn0014024	122.9620478	156.4720383	132.4495243	167.163814	130.9707615	146.5732227	153.1742555	143.4377928	X	0.018584998	0.878323294	0.995787491
**RpL7A**	FBgn0014026	294.7352789	378.7260413	416.2398016	402.658451	348.4629722	330.8173991	369.3568453	444.7825223	X	0.035612381	0.791782745	0.995787491
**SdhB**	FBgn0014028	928.9890886	928.9723237	754.9080713	963.8863088	965.7198825	929.7239033	1023.070834	992.1000048	2R	0.15888592	0.137871959	0.798609078
**2-Sep**	FBgn0014029	195.0563547	193.9591551	169.2777604	182.0879817	188.0349625	184.924688	188.7182041	175.3896671	3R	0.022614601	0.824047839	0.995787491
**Spat**	FBgn0014031	0.146150021	3.186900042	2.245533442	2.839527263	1.846867718	2.663927373	0.248554562	0.211536816	X	-0.709610685	0.485605904	0.973549494
**Sptr**	FBgn0014032	257.27591	220.8610886	187.6859447	205.8375799	253.8585985	287.4657397	205.8728248	263.3116558	X	0.239890621	0.09197427	0.722444454
**Tie**	FBgn0014073	0.569458322	0.467431666	0.411510027	0.36722805	0.320800013	0.936687444	0.030908519	0.105220998	3L	-0.36519688	0.610198795	0.990047511
**Uggt**	FBgn0014075	37.07034049	33.64025348	32.10496742	33.49869538	38.65119923	38.60358938	39.4264588	32.01539301	3L	0.152783237	0.228706098	0.867702781
**CG4278**	FBgn0014092	24.38686954	39.2638581	57.77713699	40.69368766	46.96933576	43.5229233	49.10333457	58.78970638	2L	0.332376713	0.206875151	0.855364409
**barr**	FBgn0014127	0.108036477	2.35580845	3.276187337	2.078034582	0.633858976	2.546403271	1.51581856	4.33930807	2L	0.259809354	0.740857209	0.995787491
**bif**	FBgn0014133	35.6634286	43.937083	36.69694623	37.71302739	36.86599367	31.80564903	33.89437847	43.10864106	X	-0.045710019	0.715721407	0.995787491
**bnl**	FBgn0014135	0.686963908	0.82257555	0.575186102	0.401588766	0.618243837	0.840627627	0.222288397	0.338768708	3R	-0.269673047	0.49263541	0.973549494
**cher**	FBgn0014141	0.473309766	0.429140937	0.48412403	0.483155762	0.300210559	0.663320152	0.659187901	0.227738227	3R	0.00893493	0.982266589	0.997881056
**fax**	FBgn0014163	211.8560302	214.6211902	214.8808327	232.6238319	225.1341018	256.5174171	205.8458322	248.270868	3L	0.127381534	0.185811633	0.839299945
**gcm**	FBgn0014179	0.077096792	0.969892895	0.374071483	0.359496536	0.695897345	0.65417746	0.688364503	0.083692136	2L	0.30574132	0.804031811	NA
**Oda**	FBgn0014184	440.6563304	414.3562334	435.4051244	420.8985688	406.7847628	445.3198611	458.6777666	404.5958155	2R	0.032742028	0.698884956	0.995787491
**Hel25E**	FBgn0014189	335.8341284	287.9541894	289.7439029	315.6269497	327.3892379	312.2495054	291.8838276	298.4480054	2L	0.029273859	0.77160308	0.995787491
**prod**	FBgn0014269	91.72817107	121.5091244	123.966078	77.08799196	99.3918905	99.56386717	126.1379182	111.9201042	2R	0.112839793	0.501485771	0.976947737
**mof**	FBgn0014340	58.56002867	50.19808959	50.84240427	66.22098002	36.17278096	50.25007816	44.36901041	53.48960791	X	-0.266378686	0.109972517	0.758410631
**mia**	FBgn0014342	0	0.47589242	0.550631263	0.066147127	1.075575107	0.026748494	0	3.449442794	3R	2.146694386	0.147121294	NA
**mirr**	FBgn0014343	0.8917351	1.250840679	0.594601374	0.413377729	0.706057195	0.688310741	0.478912715	0.249081099	3L	-0.537855921	0.264931826	0.893759496
**noi**	FBgn0014366	122.2352207	121.6744732	105.9721057	105.7559769	125.060843	154.1842521	124.9915075	119.7365551	3R	0.229305039	0.065179366	0.644371095
**RhoL**	FBgn0014380	0.20548124	1.299125517	1.227063683	0.331665075	0.370946302	0.327844774	0.60483122	0.549069283	3R	-0.677429708	0.38937772	0.948297463
**sty**	FBgn0014388	26.93025432	53.92015597	42.1736066	38.28012289	23.24942057	27.64226646	39.5529404	29.97734277	3L	-0.38390572	0.03473581	0.514749796
**sun**	FBgn0014391	378.420813	438.2584798	560.7095568	379.7877254	467.42783	343.1136618	488.6817207	446.5495313	X	0.027303528	0.85662837	0.995787491
**tim**	FBgn0014396	4.069519218	3.608347447	2.676306378	4.129536172	2.655370553	2.56551616	1.672911314	2.834216152	2L	-0.546115176	0.028683007	0.469634924
**Vps26**	FBgn0014411	131.7945683	140.7444478	115.8506591	107.9465844	118.6035289	153.9479735	127.3857777	171.6972747	X	0.234602384	0.097060431	0.730787365
**CG13397**	FBgn0014417	12.26607371	8.044578086	6.910468546	11.52047665	0.734615392	9.783136104	5.709498466	5.111595498	2L	-0.845437678	0.092719559	0.72305867
**CG11899**	FBgn0014427	4.607353492	8.169799943	8.782223456	4.757114397	6.059859276	5.584866081	10.07439235	9.574291317	3R	0.288606194	0.522799497	0.983400848
**Ahcy**	FBgn0014455	10.49195543	24.24832365	13.44977494	26.1294354	25.6129496	28.77600433	26.30897864	31.06228153	X	0.623914492	0.01962483	0.396755891
**Cyp4e2**	FBgn0014469	2.062339182	1.171693191	1.533240576	0.697974121	2.281869843	3.669196963	0.084855993	3.033164991	2R	0.75190948	0.346643538	0.930285749
**Eig71Ek**	FBgn0014851	0	2.589838783	4.869430526	1.439908584	0	3.639177937	0.787754031	2.011296572	3L	-0.407517285	0.791448883	NA
**His3.3A**	FBgn0014857	543.8450804	587.6852197	538.5302452	586.2206377	565.8491209	486.0866316	545.4250779	546.242155	2L	-0.042211658	0.68285489	0.995787491
**Hr38**	FBgn0014859	18.68667736	22.72285242	40.97916085	27.54827867	18.71333603	20.87623555	35.19708596	27.43569164	2L	-0.068966885	0.824157865	0.995787491
**Ost48**	FBgn0014868	329.8156276	294.5190465	270.9491621	280.0011642	264.9187734	315.4792799	335.2092077	319.6718755	X	0.100198995	0.395205929	0.952272708
**Pglym78**	FBgn0014869	2886.174442	2137.24777	2244.105916	2668.683712	2755.219755	2182.772586	2277.824377	2625.051082	3R	0.015439375	0.904709728	0.996479182
**Psi**	FBgn0014870	47.73284521	55.54335239	59.19795324	53.70095482	40.38226003	49.44753088	51.32692527	52.18391225	2R	-0.129060706	0.29679856	0.906715248
**Roe1**	FBgn0014877	189.6650663	200.118147	177.8379624	174.7391221	211.3884447	211.7153291	200.8772778	175.0189761	2R	0.135613973	0.221315827	0.86549481
**Set**	FBgn0014879	126.1336828	104.7180986	111.3058935	102.8137901	111.1399608	103.8432685	106.2606552	108.6768216	3R	-0.0216561	0.866678716	0.995787491
**Hydr2**	FBgn0014906	119.2880808	103.9783541	95.11510206	103.7682857	99.6218502	116.5072213	83.49339561	99.63714959	2L	-0.053731799	0.656908069	0.995750687
**CG2846**	FBgn0014930	70.62823172	90.27836213	83.69070737	65.67645882	70.01558215	88.65552029	69.08411015	59.82935815	3R	-0.077031939	0.654363469	0.994669639
**betaggt-I**	FBgn0015000	44.83953627	53.66995086	43.71107047	35.9752898	34.52061526	54.05535477	35.57979325	44.73614493	2L	-0.046878437	0.794737912	0.995787491
**iotaTry**	FBgn0015001	2.650497113	2.311835281	4.629649699	4.449264652	1.148358385	0.299864699	4.868262057	1.841431867	2R	-0.772694297	0.413467284	NA
**Ag5r**	FBgn0015010	2.111520562	1.924880177	6.347469729	2.46146535	3.894701787	4.024734086	2.576168586	1.594541426	X	-0.041236484	0.945457818	0.997604378
**CCT3**	FBgn0015019	138.4139855	171.6172549	139.5584566	136.3334937	138.3129845	145.1215827	143.3060023	142.0129638	3R	-0.011039712	0.919237289	0.996479182
**CkIalpha**	FBgn0015024	470.122175	563.4819576	548.6571918	574.4552595	527.2222143	573.6021413	537.236923	613.5960148	X	0.093687562	0.339979493	0.927274752
**CkIIalpha-i1**	FBgn0015025	26.21758105	40.17152248	46.96675924	41.20116014	40.75856341	33.62015309	34.85379808	33.32787014	3L	-0.078820428	0.714175548	0.995787491
**cype**	FBgn0015031	2596.913063	2452.306477	2660.384038	2261.699538	2501.679397	2174.859252	2621.800416	2547.122784	2L	0.014431845	0.889439197	0.995926915

**Cyp4e1**	FBgn0015034	4.228964042	0.44334337	0.824416697	0.792294948	1.02245946	2.27830941	0	2.62327783	2R	-0.084925898	0.933355029	0.996479182
**Cyp9b2**	FBgn0015039	0.046518929	0.078028977	1.692814091	0.216914263	0.671828715	0.350862096	0	1.615950414	2R	0.454006889	0.763450848	NA
**Ddx1**	FBgn0015075	46.84189115	42.9066259	34.75792267	41.18258181	42.8713236	42.68195001	36.1321176	34.94096106	3L	-0.054398302	0.733561352	0.995787491
**eIF4E1**	FBgn0015218	484.8122567	468.4859797	473.6532741	486.4638941	450.3974457	483.6860848	488.9686559	456.046282	3L	0.003246133	0.969032087	0.997604378
**Fer2LCH**	FBgn0015221	603.3365858	623.7015582	791.4820074	658.870832	738.7550593	730.2389634	720.5207825	735.4486786	3R	0.159043072	0.120409551	0.77836519
**Fer1HCH**	FBgn0015222	1088.929338	1158.08086	1069.791096	1098.189534	1068.825714	1131.841895	1163.69248	1104.586705	3R	0.048201831	0.541200707	0.984934163
**glec**	FBgn0015229	197.5959297	170.4784154	146.3626344	170.7417731	148.4262487	139.6715447	116.9068263	136.8233945	3R	-0.310363561	0.010111513	0.304092724
**Hr78**	FBgn0015239	51.54720345	54.43669709	53.39536721	63.92572571	49.90280864	51.88841486	54.11258122	48.5428598	3L	-0.097565808	0.44850993	0.967112302
**Hr96**	FBgn0015240	83.69309109	68.23262499	56.00527633	79.33639172	73.43527745	55.6126865	61.09285139	59.71561999	3R	-0.173504111	0.268434863	0.89549671
**Hsp60A**	FBgn0015245	754.7288173	777.1455683	679.2852647	785.5671365	756.7056955	709.3979483	770.7208571	748.4893523	X	0.025387141	0.782623084	0.995787491
**Diap2**	FBgn0015247	383.3061273	376.3310831	349.0372249	325.7776902	410.9743259	461.7736025	397.2376541	392.3327514	2R	0.241591406	0.009761547	0.299090923
**Nap1**	FBgn0015268	16.22814701	12.61602258	15.56920291	10.57863939	13.28280511	13.14163999	13.55613531	10.82721095	2R	-0.088007846	0.734193216	0.995787491
**Nf1**	FBgn0015269	34.14626848	38.21797248	36.92715265	42.24955913	38.02287055	40.14484608	26.01427054	39.25474265	3R	-0.049568354	0.725231168	0.995787491
**Orc5**	FBgn0015271	0.747138404	1.086123878	3.262585225	2.554823425	0.359673682	1.972313066	5.209640729	1.838387522	2L	0.331263228	0.692427052	0.995787491
**Pi3K59F**	FBgn0015277	83.35147252	118.8736002	86.17117745	74.85630021	69.27200734	117.8633966	96.89374576	91.88449601	2R	0.08044445	0.626511613	0.990047511
**Pi3K68D**	FBgn0015278	9.782116526	16.28518543	14.78588576	15.57686696	13.7507875	14.59656434	15.13084592	18.21438973	3L	0.164512285	0.325555347	0.918470465
**Pi3K92E**	FBgn0015279	28.61710442	34.70815609	39.47321138	26.19002477	31.49080466	23.94601828	24.93528709	28.14313712	3R	-0.213391845	0.188571428	0.843108521
**Rpt2**	FBgn0015282	319.1252635	333.175305	366.3950259	321.5574368	329.0690809	297.4768146	309.7444173	275.555389	3R	-0.113878869	0.26703739	0.894434069
**Rpn10**	FBgn0015283	315.4283901	242.9152547	281.8620351	305.346177	250.2680872	307.6563789	280.1869792	304.9078562	3L	0.023562751	0.851087869	0.995787491
**Rala**	FBgn0015286	12.607927	10.68521804	9.845909995	6.24994597	12.10357	11.29968019	9.294501399	8.911549775	X	0.107632475	0.583150617	0.985764834
**RpL22**	FBgn0015288	261.4725096	395.3921986	321.334318	400.1225818	276.8385242	309.6209191	328.4382573	321.8176253	X	-0.123912847	0.358283862	0.93823489
**Shark**	FBgn0015295	1.402724626	1.470546111	1.336889077	3.095199677	0.04521924	1.582253107	0.894595164	0.32629768	2R	-1.332615538	0.10372002	0.745084146
**Shc**	FBgn0015296	144.6592495	169.8694124	133.8485646	137.3873042	169.7361497	176.1583846	178.7940804	169.4883657	3L	0.275546664	0.016316526	0.369626395
**Srp19**	FBgn0015298	120.715207	149.9055206	161.9546104	123.2881949	124.619355	148.6630325	140.0869303	136.1069948	3L	0.017209462	0.912524365	0.996479182
**Ssb-c31a**	FBgn0015299	172.2105561	165.4486298	187.7345148	128.1390729	161.1306147	154.0683849	169.7429992	155.9180244	2L	0.002121394	0.989695293	0.998078482
**Ssl**	FBgn0015300	5.582816614	4.11465695	1.231257749	1.183284252	0.763516277	2.392475183	1.942075244	0	2R	-1.232611352	0.244816941	NA
**Try29F**	FBgn0015316	12.39338464	10.67807337	9.036283087	14.21051298	16.01581351	13.28063785	16.23980494	19.11439392	2L	0.50631062	0.11278804	0.758410631
**Ubc2**	FBgn0015320	75.42957373	78.36734595	68.03425104	82.84148295	78.39283594	86.10610816	68.44309919	70.11308688	2L	0.021036842	0.866939392	0.995787491
**Ubc4**	FBgn0015321	123.8095763	136.5522381	134.995597	116.7433575	140.8406685	114.2903427	123.6167863	123.0036783	3L	0.003201302	0.978722437	0.997757033
**abs**	FBgn0015331	49.39064458	71.84347862	54.06172438	60.96900788	47.67894942	43.73934168	70.36749174	79.88145754	3R	0.069070682	0.746844718	0.995787491
**CG5861**	FBgn0015338	130.2456052	157.906986	158.1975665	144.883948	151.7721429	125.8496284	167.2367076	139.817357	2L	0.017283711	0.899454699	0.996479182
**CG14906**	FBgn0015351	33.2920468	36.69312658	33.95032704	35.03165349	53.98423655	38.6788913	40.02172295	35.82023418	3R	0.309825135	0.149216865	0.808039863
**CG2034**	FBgn0015359	45.96793664	52.12995683	43.71832879	34.43846844	44.69184715	48.9644689	42.80631759	38.48353945	3L	0.019281173	0.917804904	0.996479182
**oxt**	FBgn0015360	130.4595329	124.5885111	99.65601715	113.2488101	117.5724841	115.6094047	105.8898968	115.0953289	3L	-0.01426636	0.901911393	0.996479182
**chn**	FBgn0015371	193.0331885	213.8265721	169.8174201	212.1036587	183.7922262	193.0624504	163.3138319	174.3085916	2R	-0.113693685	0.253122669	0.884040848
**RabX1**	FBgn0015372	22.42343031	20.51922268	32.46118291	16.58813605	29.62459338	33.23651375	28.16220659	23.51948604	2R	0.34811251	0.090599186	0.717758178
**cutlet**	FBgn0015376	0.249233553	0.289422223	0.325573836	0.044698357	0.069220068	0.253050939	0	0.124871334	2L	-0.958878391	0.512578166	NA
**dod**	FBgn0015379	118.1472871	123.3996024	122.2983697	158.1919568	140.8355655	112.15073	130.2220433	147.5614547	X	0.055513769	0.719421963	0.995787491
**drl**	FBgn0015380	32.49309647	40.23511986	38.93681707	38.84491937	43.58529679	46.62827983	40.47217236	44.0330796	2L	0.246875792	0.012599068	0.337622622
**dsf**	FBgn0015381	0.786611207	0.861446	0.205024807	0.121253185	0.751092806	0.269677179	0.099503905	0.183483262	2L	-0.565675567	0.466822492	0.971928234
**glu**	FBgn0015391	13.97946953	13.21648308	9.64566959	13.84127594	13.60150396	16.07245873	15.97839592	17.18560387	2L	0.335521909	0.081645695	0.694684542
**jumu**	FBgn0015396	7.653930717	10.2198709	7.647945477	14.0815312	9.179616584	13.08896729	7.713476572	11.07277711	3R	0.079515221	0.72044023	0.995787491
**kek1**	FBgn0015399	372.7712818	288.2701351	281.9588046	319.6537341	288.4988009	309.9587271	265.7918444	288.2099013	2L	-0.105061227	0.351328697	0.933797047
**kek2**	FBgn0015400	1.192913586	1.231599545	1.34647757	0.7907868	0.768863914	0.941163023	1.07173878	0.761496769	2L	-0.334989083	0.151890001	0.812484766
**Rme-8**	FBgn0015477	26.3648503	22.85356853	25.59757809	23.47332971	24.59612459	33.00745884	25.7071311	29.36274882	2R	0.224805033	0.080792281	0.694684542
**Cul1**	FBgn0015509	255.0864236	266.7391925	232.4250353	249.4515863	218.5114717	280.5387796	248.8487962	243.1631152	2R	0.010296253	0.91577159	0.996479182
**mbc**	FBgn0015513	3.561684367	7.546390575	5.309154824	5.173441747	5.768676925	6.773409633	4.49292175	5.981757361	3R	0.128986981	0.539284776	0.984934163
**nAChRalpha3**	FBgn0015519	0.776973347	0.883216761	0.688367767	0.475070588	0.663501847	0.727139508	0.374068208	0.520948563	X	-0.273250332	0.274403264	0.89549671
**nonA-l**	FBgn0015520	87.30356372	87.76372971	79.44203189	78.15293302	87.51666775	89.50079298	84.98069358	99.17103534	3R	0.148584637	0.199268417	0.847197998
**RpS21**	FBgn0015521	201.2361327	290.2769084	345.7690165	330.1910079	252.569592	282.5425875	318.4292364	301.9881057	2L	0.018984799	0.907657766	0.996479182
**otp**	FBgn0015524	0.801007578	0.866350739	0.753932011	0.949067039	0.339776125	0.656144389	0.89328417	0.114036826	2R	-0.728788874	0.147817816	0.808004944
**peng**	FBgn0015527	49.74065723	52.63498397	46.30512175	73.88606367	54.02659462	53.74120063	52.33033515	49.9418789	X	-0.055269777	0.734206118	0.995787491
**spag**	FBgn0015544	73.04710003	63.83433166	54.34345982	51.94016636	72.83404801	74.64913978	63.81811392	62.30083675	2R	0.196461029	0.204761474	0.854537641
**spel1**	FBgn0015546	10.14873178	13.85599033	12.11214218	12.44444993	10.06183546	18.14356363	15.70051077	15.29361078	2L	0.317393677	0.134942571	0.795862348
**tty**	FBgn0015558	52.19174053	80.32119401	92.93942608	87.45628982	78.24767123	57.82781704	80.77216691	118.3447048	X	0.13872168	0.481505374	0.973549494
**unpg**	FBgn0015561	0.315650627	0.12457874	0.135134827	0.865797123	0.502791426	0.280087984	0.142099682	0	2R	-0.611413906	0.624646879	NA
**alpha-Est1**	FBgn0015568	0.459784259	1.756675961	1.611176954	0.178661615	0.783915738	0.313070182	0.390973174	0.221829819	3R	-1.175581265	0.206903271	0.855364409
**alpha-Est10**	FBgn0015569	0.095420816	0.64022013	0.347234353	0.639601365	0.710568494	1.293206084	0.425985965	0.440230777	3R	0.784244141	0.288969129	0.903033179
**alpha-Est2**	FBgn0015570	0.075980917	0.297377102	0	0	0.594381794	0.477562298	0.646096508	0	3R	2.210364873	0.189989439	NA
**alpha-Est8**	FBgn0015576	32.35007341	43.30912858	34.7401375	46.72841314	27.90257985	29.63467664	42.88683844	42.86029443	3R	-0.099503216	0.583493711	0.985776726
**alpha-Est9**	FBgn0015577	43.95622164	42.64975797	46.90757504	51.78318107	42.12440278	54.27892664	47.62500657	43.19051096	3R	0.042250497	0.715248897	0.995787491
**dare**	FBgn0015582	47.08377114	35.18949387	33.25525516	50.7647037	53.65291919	37.6474627	42.26715932	37.61124589	2R	0.067818913	0.74138806	0.995787491
**Apc**	FBgn0015589	91.90010058	93.75503531	106.4432397	101.8091546	88.46665027	93.84668911	84.34198935	96.46814695	3R	-0.086874645	0.363320431	0.939549067
**AstA**	FBgn0015591	0.667614754	0.767883492	2.360023661	0.489191581	2.410431478	0.899175874	0.875879784	0.331303641	3R	0.126024299	0.864756638	0.995787491
**toc**	FBgn0015600	26.17210162	33.30183167	32.71009366	32.1066452	29.33398352	29.4492023	30.01373154	32.97829379	2L	0.003617183	0.971603245	0.997604378
**BEAF-32**	FBgn0015602	130.0275747	136.5496993	124.7583357	136.0643649	140.1596828	129.2401587	172.1911473	152.2028437	2R	0.202142461	0.113100107	0.758410631
**CadN**	FBgn0015609	2.299932242	2.797634253	3.306787411	3.013356414	2.509522323	2.499276763	2.669346537	2.80035288	2L	-0.090411319	0.485759666	0.973549494
**CanB2**	FBgn0015614	129.9985304	105.89981	111.4549242	118.9915517	114.6939448	126.6677889	97.30302578	103.946953	2R	-0.048825286	0.692780821	0.995787491
**SMC3**	FBgn0015615	73.41983189	77.3375524	67.25444081	70.81559865	67.50260634	77.63789285	65.05114276	86.43052454	X	0.068882604	0.578238044	0.985637351
**Cdk8**	FBgn0015618	29.41751739	32.53032971	38.32644665	41.78655464	14.55502123	24.4475854	32.65834988	24.83761521	3L	-0.529367571	0.042332417	0.540273145
**Clp**	FBgn0015621	50.85778118	37.34360308	61.4369596	40.55164236	53.50427699	44.66876075	40.11091667	53.92455736	2L	0.04704906	0.831435428	0.995787491
**Cnx99A**	FBgn0015622	196.5583059	147.904513	173.037991	163.1678004	154.4803177	193.1082438	156.8398536	154.3003505	3R	-0.021658829	0.856313209	0.995787491
**Cpr**	FBgn0015623	14.56824873	9.231884614	15.66694438	11.28560269	9.053555283	17.19051056	17.9289177	16.24397576	2L	0.277531244	0.23194518	0.867702781
**CycB3**	FBgn0015625	0.661207982	1.058670739	0.874953459	0.700718872	0.4340545	0.311691423	0.306682867	0	3R	-1.603129075	0.130391316	NA
**Dref**	FBgn0015664	138.9489314	145.525663	120.5444748	152.0173443	120.8529831	108.2129515	137.296556	134.0556574	2L	-0.123598721	0.338354122	0.926687587
**Cyp6a17**	FBgn0015714	12.90058978	32.13299693	22.70772036	18.99615121	1.663490782	0	9.402766194	0	2R	-2.921861307	0.097694834	0.730787365
**ktub**	FBgn0015721	5.632413475	5.871379549	5.153008183	5.16552988	4.35153788	6.431498312	7.092879266	6.79648478	2R	0.205907484	0.232296627	0.867754409
**Hmu**	FBgn0015737	182.0732092	171.486409	190.4833809	193.505887	188.3839964	156.4125639	165.3910934	222.296719	3R	0.022565001	0.865531078	0.995787491
**Lis-1**	FBgn0015754	871.9505665	819.7992118	786.5738207	791.5093988	730.8223565	844.8760257	770.3677205	722.9843762	2R	-0.063103136	0.456083843	0.968365245
**RpL9**	FBgn0015756	347.4414605	460.135625	531.5775359	450.4649861	444.408944	413.5088791	530.7174872	438.2722049	2L	0.064198299	0.636596234	0.991585536
**Msr-110**	FBgn0015766	72.36597702	167.6776208	156.8605135	79.45050102	73.0487627	78.60146984	122.0615013	77.97057591	3L	-0.39574199	0.256310331	0.88674759
**MstProx**	FBgn0015770	0.4117326	0.474803777	0.187285789	0.239984764	0.743282869	0.242611862	0.131292338	0.111738698	3R	-0.066975264	0.954750457	NA
**Nak**	FBgn0015772	74.24533424	71.08811129	62.780877	91.63912578	62.97608717	72.26729251	78.47749153	67.25076567	2L	-0.066082649	0.614700439	0.990047511
**NetA**	FBgn0015773	0.676759272	0.649083663	0.395717959	0.29129331	0.441700193	0.562787976	0.203629903	0.282559043	X	-0.405789968	0.293793794	0.906604178
**NetB**	FBgn0015774	13.26939337	15.80026548	17.22866632	17.22057396	14.25725427	14.60932119	15.53153249	14.80388329	X	-0.069776123	0.515991008	0.980804634
**nrv1**	FBgn0015776	1.532974008	0.804639774	2.276922589	1.677625252	2.409718406	2.59560226	1.862214109	1.879203834	2L	0.508451759	0.182154148	0.838040629
**nrv2**	FBgn0015777	277.3460589	314.0065585	288.6462512	170.9621025	174.3404937	199.4424011	280.8481747	165.9039833	2L	-0.322331758	0.067032104	0.654151708
**rin**	FBgn0015778	183.6334441	223.2556013	206.1403376	261.5944781	220.5948425	230.2264802	200.316541	203.0047374	3R	-0.005195703	0.964036226	0.997604378
**P5cr**	FBgn0015781	273.891407	279.6949962	265.9195888	218.2279368	285.35911	255.0311005	241.2981046	253.1408365	3R	0.027262421	0.819108788	0.995787491
**Rab10**	FBgn0015789	4.184955501	6.759687771	3.384214558	4.396702673	3.217835135	6.064457225	6.458267386	4.655134818	X	0.151591708	0.678329876	0.995787491
**Rab11**	FBgn0015790	198.2274457	219.4988398	231.9109572	208.4684787	189.9981731	204.6749559	212.3838403	186.1907498	3R	-0.082292445	0.370193548	0.942619319
**Rab14**	FBgn0015791	426.9640464	360.1536869	399.2500396	375.1563647	412.9777893	410.1435389	386.307154	365.8756318	2L	0.041132899	0.66856122	0.995787491
**Rab19**	FBgn0015793	37.13873704	37.98238762	43.63683376	26.1934177	46.23788893	53.72864698	34.7485291	41.07410925	3L	0.31032955	0.147655859	0.808004944
**Rab18**	FBgn0015794	235.4081661	247.5091835	258.9882966	243.3617273	227.8053518	267.6230026	247.4359402	205.4299522	X	-0.025335439	0.828896076	0.995787491

Rab7	FBgn0015795	366.0010731	311.3271269	354.5807367	301.8465813	268.0139571	315.0914615	316.2584245	261.770578	3R	-0.171839024	0.121005295	0.77836519
Rab6	FBgn0015797	217.2541011	199.57243	189.7310797	212.2843169	203.4848639	165.6533825	208.3717493	240.3547165	2L	0.030001651	0.827529451	0.995787491
Rbf	FBgn0015799	46.65425671	34.62594981	47.68605996	50.01517054	52.69274764	53.88391224	52.15760897	45.71793047	X	0.218997646	0.137594469	0.798609078
Reg-5	FBgn0015801	0.73758595	0.753076409	1.672674972	1.981340334	1.447317044	2.328049898	0.449947253	0.800683687	2R	0.004194332	0.994568668	0.998895169
RtGEF	FBgn0015803	14.30779118	17.00931668	15.80972613	15.91594501	15.35193213	16.33263058	14.49244166	12.74011163	2L	-0.066385034	0.579578997	0.985637351
HDAC1	FBgn0015805	106.4266626	150.1276381	169.6147276	156.2079533	145.8406045	175.6967944	234.4670751	243.1725997	3L	0.492313651	0.005147594	0.20400887
ScpX	FBgn0015808	97.00810601	74.91418792	73.51649765	95.54851948	86.81329526	104.3832638	87.70497722	85.21665041	2L	0.119272054	0.412842779	0.958665466
Spx	FBgn0015818	121.9686458	146.6822496	115.8465013	148.8990474	108.241301	114.2547607	125.8030753	117.1376176	X	-0.166276132	0.21018444	0.856520545
TfIIEalpha	FBgn0015828	78.18681483	57.06351403	70.40165038	70.5157948	97.25443049	94.04896219	85.28473899	76.94660662	3L	0.382696153	0.013370422	0.344831707
TfIIEbeta	FBgn0015829	93.95205592	89.43477825	52.89430695	65.62128011	63.0959135	75.24720726	69.77842281	68.71009451	3L	-0.099497992	0.615289929	0.990047511
eIF3i	FBgn0015834	861.1379279	853.57849	791.8852866	861.4309351	790.0588789	828.9274016	755.6438223	858.6743602	2L	-0.029082947	0.748781394	0.995787491
Vang	FBgn0015838	79.70436657	72.80831678	75.47838395	100.746555	80.6012245	71.01343314	80.79692868	81.07876784	2R	-0.039373011	0.780111138	0.995787491
Drip	FBgn0015872	1.366766592	0.772064617	0.296253175	0.054751973	0.211972852	1.204445157	0.227650749	0.489464213	2R	-0.214385002	0.769872038	0.995787491
apt	FBgn0015903	0.64272814	0.566309918	0.588510513	0.2740441	0.465018558	0.52815205	0.542284552	0.396365086	2R	-0.07497987	0.830114644	0.995787491
ara	FBgn0015904	0.349949904	0.852084974	0.492952405	0.447426305	0.37701166	0.404431097	0.259179597	0.379886818	3L	-0.549091165	0.273295776	0.89549671
ast	FBgn0015905	12.80311722	24.22347423	20.16703877	14.71278922	20.10056217	17.79141195	22.21259425	24.37156038	2L	0.272022741	0.24954257	0.879541335
caup	FBgn0015919	0.861412651	0.370180704	0.155437816	0.647319798	0.886775804	0.234914963	0.363220195	0.247300047	3L	-0.217526663	0.750224172	0.995787491
crq	FBgn0015924	74.96019246	59.63893781	53.39270499	50.3809524	66.87442186	72.00738389	58.55341028	50.62142529	2L	0.082942985	0.588399536	0.987588562
csul	FBgn0015925	34.99271996	18.13762776	38.52924118	28.79957602	36.39778399	46.4067592	30.26655473	30.81285424	2R	0.280766229	0.243144996	0.876959115
dah	FBgn0015926	0.260506002	0.764683978	1.263967854	1.973919794	0.411495088	0.399105635	0.083069551	0.070697831	X	-2.066050018	0.054030519	NA
dpa	FBgn0015929	0.025157783	2.911706926	1.220648596	1.466360579	0.81749242	8.182918314	1.989519365	1.037777624	2R	1.124245687	0.203363475	0.851888994
hrg	FBgn0015949	172.8116766	163.1778448	158.2140178	177.4252654	154.8607796	172.0547199	162.1830076	151.1001431	2R	-0.041098816	0.656823794	0.995750687
lbm	FBgn0016032	101.330002	116.8821984	100.4422071	103.3442093	111.2817971	156.106636	115.1838859	96.37364363	2R	0.210031462	0.132989662	0.794759341
mael	FBgn0016034	0.740326886	0.953005076	1.044199244	3.010542322	1.398641623	1.363487347	1.317620785	0.934487326	3L	-0.164119401	0.775208925	0.995787491
mei-P22	FBgn0016036	0.174107026	0.146020056	2.534287306	2.029619889	0.628615612	0.738661685	0.444150765	4.347028419	3L	0.397373657	0.756408549	NA
Vsp37A	FBgn0016038	179.842485	188.5617177	199.7729864	200.2211347	216.304111	247.244457	218.7858635	231.5774607	X	0.280866472	0.007093752	0.246330526
Tom40	FBgn0016041	345.0971984	355.666044	413.5940453	370.5092489	335.4945463	307.3022827	357.9847967	365.4447636	X	-0.087408542	0.412682698	0.958665466
nompA	FBgn0016047	0.508847145	0.88835724	0.277122793	0.290536644	0.337444719	0.509109693	0.105965714	0	2R	-1.003290435	0.297765721	0.9074568
Sema1b	FBgn0016059	44.7635943	49.85653453	39.60230051	51.90762385	44.1673968	40.41023749	40.31678572	42.78014601	2R	-0.120147106	0.30639529	0.911610813
side	FBgn0016061	1.334104787	1.578783056	1.225903367	1.430596742	1.634003638	1.718494082	1.891132368	1.464809557	3R	0.297605546	0.061083486	0.629258058
smg	FBgn0016070	44.22362724	37.19362266	35.96282626	46.59104624	36.19783694	35.90326053	38.70919806	39.90557111	3L	-0.093918618	0.477079003	0.973549494
vkg	FBgn0016075	10.14243681	22.04655339	15.51751524	7.563423353	4.989124717	11.78611966	11.69061443	7.371307305	2L	-0.589024832	0.152348432	0.81337081
vri	FBgn0016076	0.610398338	0.991250652	0.396142356	0.235676047	0.74397514	0.205266689	0.178525522	0.270110837	2L	-0.634711649	0.271885703	0.89549671
wun	FBgn0016078	19.61167826	18.67243322	20.47399213	21.81566726	17.95597573	15.51549387	17.34298489	18.80271456	2R	-0.180161334	0.157649847	0.818884898
fry	FBgn0016081	3.331400507	4.601304528	5.081214148	6.130745346	3.473450679	4.17048914	4.941082123	5.259479476	3L	-0.06869488	0.716219789	0.995787491
ATPsynCF6	FBgn0016119	1502.374998	1725.404297	1781.16508	1680.659935	1804.347455	1461.035082	1862.232328	1700.304605	3R	0.063435945	0.564325119	0.984934163
ATPsynD	FBgn0016120	3268.254293	2825.438109	2695.377886	2949.28681	3108.53944	3040.362231	3144.100807	3146.797709	3R	0.113171819	0.235938389	0.87159155
Acer	FBgn0016122	22.68410583	34.23301558	22.07876604	31.35035008	36.43250181	49.80873157	27.86004798	46.73002182	2L	0.574448824	0.005130148	0.20400887
Alp4	FBgn0016123	17.24688968	13.11673375	17.57426365	8.780312128	9.87314717	4.048104229	12.91155253	8.957538484	3R	-0.63167284	0.061224857	0.62993575
CaMKI	FBgn0016126	109.6193243	92.89058168	109.5846132	90.29380558	87.17574372	102.0813592	85.39723003	81.49919976	4	-0.148362919	0.175515703	0.834088743
Cdk4	FBgn0016131	1.53588726	0.340257508	1.441204565	0.067563459	0.889347029	0.478121504	0.665334577	0.220206358	2R	-0.565715758	0.505510704	0.978557094
PTP-ER	FBgn0016641	116.7191423	117.3092365	118.7004543	123.2173324	119.572042	124.9087718	133.872457	100.9816085	2R	0.039212144	0.715885912	0.995787491
Lgr1	FBgn0016650	107.4975563	140.6946476	95.14037443	138.9440079	95.90438549	121.9891432	96.59412356	112.9856	3R	-0.144848089	0.291990495	0.905069205
H15	FBgn0016660	0.274353251	0.684383776	0.785039728	0.672446547	0.520676803	0.225497214	0.412750356	0.366551572	2L	-0.622083143	0.268583957	0.89549671
Ipp	FBgn0016672	595.4297563	591.0757547	548.8442849	684.942509	583.5268981	595.9229248	557.4612692	532.5841703	3R	-0.064541817	0.528058493	0.984296327
NaPi-T	FBgn0016684	7.167838336	7.324382677	6.595856348	7.491382351	0.34704405	0.466054773	0.210175972	0.775120794	2R	-3.953034204	2.66E-18	4.44E-15
Nlp	FBgn0016685	58.64274615	55.48526063	55.18535419	49.00855735	53.31178684	45.92910576	51.67890017	56.24458811	3R	-0.044602915	0.715849389	0.995787491
Nurf-38	FBgn0016687	237.3830223	240.9620209	247.1064608	254.9106844	294.5916628	270.2973321	270.0567592	257.1580232	2R	0.186149628	0.054470927	0.610983453
ATPsynO	FBgn0016691	3939.278012	3218.588956	3107.27779	2998.520533	3892.129473	3309.40113	3344.030912	3284.567174	3R	0.090257328	0.425286592	0.9620897
Past1	FBgn0016693	129.5661246	115.6664245	97.93115164	138.0716579	118.6476596	143.6916857	122.9598727	130.7903314	3R	0.127007379	0.304728244	0.911232576
Pdp1	FBgn0016694	20.04841117	15.90858881	14.6990152	13.20525274	16.18749006	16.4696341	12.45301877	14.52019714	3L	-0.071236967	0.603581841	0.989286463
Pitslre	FBgn0016696	507.5255267	456.6563073	420.8138026	460.8940706	469.856866	545.5394857	412.8524396	461.700145	3L	0.06087145	0.557329819	0.984934163
Prosalpha5	FBgn0016697	155.6880842	157.469952	183.2911069	161.0337285	188.9838419	173.8398255	175.9781182	200.8185285	2R	0.202261899	0.119793034	0.777535158
Ptpa	FBgn0016698	187.1455924	171.4567866	148.073476	157.055898	166.3134619	167.6460192	160.0187383	176.6875909	2L	0.043291216	0.721003989	0.995787491
Rab4	FBgn0016701	566.358852	429.1656504	533.4400523	478.1768403	500.233719	500.9606467	445.7171503	410.4510057	2R	-0.084748893	0.476952995	0.973549494
Reg-2	FBgn0016715	0	1.138311619	1.796022699	0	2.00471966	1.744939165	0.786912413	0.133943183	3L	0.736596109	0.618618865	NA
RpL29	FBgn0016726	664.4817932	826.5079558	917.8523172	791.9123652	814.2944309	704.9224992	840.0839363	859.5588978	2R	0.042964156	0.731904435	0.995787491
sba	FBgn0016754	34.56729391	31.26869494	31.29509839	34.75295041	30.55257082	34.32523581	32.89270387	33.77946717	3R	0.024710362	0.797826808	0.995787491
Usp47	FBgn0016756	80.35001178	77.51079766	76.66979699	74.18097878	68.806891	74.52575853	69.09339959	81.56909941	3L	-0.040168548	0.687301158	0.995787491
dos	FBgn0016794	28.83534861	30.81765577	32.05097891	28.676761	30.91410152	41.30759225	28.51507601	19.0613989	3L	0.01929916	0.916416393	0.996479182
fz2	FBgn0016797	2.628882749	3.792394446	3.499350398	2.727628267	2.637111681	4.446583677	3.017266173	3.569956601	3L	0.143513722	0.379823574	0.944208584
Stat92E	FBgn0016917	70.22563558	67.04308706	63.20862321	63.5513079	68.49159884	73.82756666	67.46794701	63.63004514	3R	0.079048568	0.36921561	0.942282153
nompB	FBgn0016919	0.116725693	0.300212827	0.453079844	0	0.323103681	0.044019308	0.079405267	0.185843008	2L	-0.401567239	0.722118408	NA
nompC	FBgn0016920	0.571255301	1.173610072	0.644584834	0.557522887	0.391719762	0.86005161	0.847258457	0.961432666	2L	0.089438816	0.826987231	0.995787491
Pino	FBgn0016926	38.82949539	40.92789298	44.33710445	35.47739796	36.33899626	49.62382934	40.31734384	43.6110537	2L	0.120353254	0.294139806	0.906715248
Dyrk2	FBgn0016930	20.50309424	21.71974806	22.59726328	24.35259749	20.1255312	20.94542759	20.87346673	22.46917405	2L	-0.048431317	0.620021213	0.990047511
l(2)k10201	FBgn0016970	29.50731445	24.12629302	35.15072997	31.80853002	40.28531888	29.41468878	46.6751477	20.43590283	2R	0.210758165	0.501165827	0.976796212
swaPsi	FBgn0016974	0.429748738	0.72084322	0	0.111327001	0.862007351	0.090036552	1.583541936	0.103669366	X	1.066413051	0.483986995	NA
spen	FBgn0016977	87.90487754	95.25964685	96.59136787	101.8316317	99.80562234	97.97553567	86.07001603	99.53225118	2L	0.037173827	0.684748831	0.995787491
snRNP-U1-70K	FBgn0016978	97.92498954	99.34068686	132.9834322	113.186522	97.61381497	102.4162807	87.69959408	116.2766706	2L	-0.102141392	0.461870974	0.971606621
smid	FBgn0016983	82.37668601	63.9954764	58.65802099	62.29591078	63.5500617	67.94098255	60.8024512	71.89422841	3L	0.009787171	0.944231904	0.997604378
cag	FBgn0017414	62.51577729	50.32507163	42.33147414	57.51104444	53.14250333	46.09825806	50.91802302	40.20042586	2R	-0.135622153	0.490721628	0.973549494
ari-1	FBgn0017418	109.3860015	158.3802034	163.3433079	145.3954121	149.4508743	137.6960955	153.5874186	159.8935879	X	0.094380415	0.453296608	0.967967712
CG5989	FBgn0017429	108.3475639	109.0092648	100.1101812	102.2517362	135.5850271	117.8430308	134.7657686	126.7053192	3L	0.324570922	0.007549638	0.252813679
Nelf-E	FBgn0017430	111.3318718	120.0133455	99.72987839	101.0438564	137.6518363	96.09769769	111.6357648	98.15043291	3L	0.068940424	0.660616193	0.995750687
CG2187	FBgn0017448	0.184799671	0.132846653	0.896642847	0.061550496	0.71488022	1.169816576	0	0.057316742	3R	0.657848227	0.639593694	NA
U2af38	FBgn0017457	144.7158494	151.1116787	126.0563876	126.5394925	154.3703989	118.6534312	119.7214577	113.4671443	2L	-0.084711275	0.545093293	0.984934163
RpS3A	FBgn0017545	967.2225089	965.2032529	1119.269203	903.1583219	979.4704223	1096.890241	1076.284644	1096.741653	4	0.134736175	0.153883747	0.817059301
Rga	FBgn0017550	94.83933663	86.42225909	74.12493683	86.94419405	80.76921747	94.91225448	74.6140185	78.06411581	3R	-0.033455771	0.775010136	0.995787491
Rca1	FBgn0017551	75.39305714	86.36836827	63.13523507	83.33087977	69.7657952	85.45409232	76.93172341	80.1451758	2L	0.047800087	0.755020748	0.995787491
Prosalpha4T2	FBgn0017556	7.579819759	1.261703022	0.421111535	3.642333889	1.357906303	4.145891531	1.623657356	0.251244128	2R	-0.809142868	0.386555421	0.948073243
Pdk	FBgn0017558	65.27797778	71.50200895	65.86730886	67.78370977	69.02015795	72.80859224	67.26779533	52.45573811	2R	-0.019105797	0.865361654	0.995787491
Ork1	FBgn0017561	2.855330165	7.20063891	4.856855866	4.560492522	2.784670458	2.258794393	2.160085308	1.339594238	X	-1.149734989	9.75E-05	0.009132299
ND-75	FBgn0017566	1015.396364	908.3493577	863.7254181	1067.406581	934.8094935	902.0873384	941.9397569	855.6682324	X	-0.056568839	0.580710541	0.985637351
ND-23	FBgn0017567	1071.181672	1250.526551	1183.555437	1253.999414	1385.664479	1138.318654	1340.879318	1208.028183	3R	0.124778904	0.237249547	0.873280735
Mo25	FBgn0017572	288.5225325	320.8110513	304.2943729	314.2993362	282.3506225	307.6316909	308.2138321	327.6383376	3L	0.028765528	0.76264327	0.995787491
Mcm5	FBgn0017577	0.098954584	1.161877026	0.800208133	0	1.548199341	0.87074192	1.178031256	1.289037369	3R	1.309458228	0.208895594	NA
Max	FBgn0017578	40.24730984	43.54232792	46.82816356	39.83077044	41.65544539	45.22431778	39.61974997	35.06786246	3L	-0.04489819	0.776708036	0.995787491
RpL14	FBgn0017579	1239.715829	1462.01949	1581.013124	1714.802561	1520.809841	1342.374901	1689.76053	1698.340421	3L	0.093061747	0.457293166	0.968365245
Lk6	FBgn0017581	126.3220105	124.9025289	119.7018722	133.4469825	118.1142407	145.2017468	119.2224317	132.0119694	3R	0.056789693	0.551807936	0.984934163
klg	FBgn0017590	17.03597119	15.18579821	16.45825422	16.67074011	14.92392069	15.17216039	16.75451665	18.1138921	3R	0.022008895	0.837886587	0.995787491
COX5A	FBgn0019624	2744.047593	2757.417883	2607.905207	2668.065974	2863.033984	2390.210985	2650.404721	2982.691835	3R	0.047310492	0.642674272	0.992589784
AANAT1	FBgn0019643	49.52741712	46.51929074	52.27098119	47.95954214	64.69013005	69.77647276	60.52588344	64.4489644	2R	0.431864927	1.34E-06	0.000302788
ATPsynB	FBgn0019644	3119.458892	2873.538814	2744.101297	2985.521151	3276.199603	2876.742888	3087.016412	3066.160874	3L	0.100618408	0.285607342	0.901952098

toy	FBgn0019650	174.3550981	181.7302128	208.0560583	181.0499685	175.4275674	202.2438671	175.7447121	182.7959876	4	0.012276096	0.890032292	0.995926915
lncRNA:roX2	FBgn0019660	0.697001245	0.106283801	0.614878291	0.147730197	0	2.329819286	3.071203442	2.751371316	X	2.38068246	0.057733333	NA
lncRNA:roX1	FBgn0019661	5.561186066	2.870187529	5.49647993	11.26648794	2.803013916	2.315307975	14.1058692	5.022171118	X	-0.026724693	0.970347831	0.997604378
qm	FBgn0019662	159.4989583	351.4161465	314.4301574	302.483324	214.0379028	120.4566115	206.9804055	163.75663	3L	-0.636784819	0.02274896	0.42700413
gcm2	FBgn0019809	0.020902086	0.631085715	0.912747057	0.097464855	0.339602606	0.098531796	0.373251699	0.045380364	2L	-0.904159531	0.486291706	NA
dj	FBgn0019828	4.58525039	5.287641069	2.780938373	0.167036542	1.16403077	4.795768285	0	1.711016149	3R	-0.709442377	0.508064332	0.978557094
colt	FBgn0019830	70.0442338	73.87291867	53.84789354	80.8334888	63.85825058	67.96980476	67.97774005	67.64429619	2L	-0.031182703	0.852015565	0.995787491
Smg5	FBgn0019890	331.9202857	412.8297148	370.8428162	403.4442784	353.8589682	415.6378679	381.9251133	354.1247427	2L	0.017055034	0.858932119	0.995787491
Surf4	FBgn0019925	169.4600319	113.9054825	119.9341674	143.9964229	116.2069957	129.7935962	116.8320719	116.5009459	3R	-0.166823864	0.247383528	0.879384515
SamDC	FBgn0019932	113.289553	91.88915089	101.1645337	83.00956866	83.45747886	96.03203613	96.65869012	62.40966754	2L	-0.17540077	0.282610034	0.901712108
RpS20	FBgn0019936	559.959314	727.0680338	780.8067295	775.9927769	695.2060674	661.2977872	772.5312705	820.2452561	3R	0.086630929	0.500515295	0.976796212
Rpl1	FBgn0019938	11.89938433	5.41759157	11.95949245	10.80894803	14.33955765	18.40152759	10.80185754	6.810957106	2R	0.347943748	0.249616861	0.879541335
Rh6	FBgn0019940	2.192155471	3.299901655	0.954537697	1.441543782	1.420606015	2.967642157	0.143390659	0.244070382	3R	-0.683291379	0.489277745	0.973549494
Cdk9	FBgn0019949	98.01812293	79.54275822	77.916516	91.9927654	119.3434672	110.9126957	95.34427524	105.8946436	2R	0.339321231	0.019275553	0.395797586
Orct	FBgn0019952	13.47851058	43.72470232	18.34113702	12.52200122	10.00459622	12.1269137	13.70122665	5.347570506	3R	-1.056369836	0.005362757	0.20691303
ND-42	FBgn0019957	1275.407375	1192.257659	1120.177438	1233.62864	1346.214489	1211.660059	1292.330368	1228.619143	3R	0.105081968	0.261636361	0.890640489
Mitofilin	FBgn0019960	281.0456759	243.3132575	226.2102313	279.4569317	272.5623131	289.3391606	269.1965316	248.4993674	3R	0.094595638	0.381915756	0.944476532
Khc-73	FBgn0019968	44.03177368	55.24738503	62.51381696	67.20953076	49.08978354	58.61750494	55.96189146	65.98684394	2R	0.035473547	0.798020773	0.995787491
Drice	FBgn0019972	38.09223991	32.06034849	55.9704457	35.2306605	49.03756626	42.951596	26.73148821	42.18083651	3R	0.029451686	0.899008435	0.996479182
Gs1l	FBgn0019982	755.8090273	645.4528034	603.8540198	741.1582393	741.8591146	677.2173681	737.9172671	740.8514572	2L	0.106598608	0.339428252	0.927274752
mGluR	FBgn0019985	104.6048105	94.895452	101.0717394	96.15770444	83.84562885	106.1582168	85.15083086	88.18767407	4	-0.099426739	0.312234802	0.914141092
Gcn2	FBgn0019990	28.45463225	37.73468078	36.18563095	41.51471993	30.88965961	46.55815203	30.95527388	29.12755073	3R	-0.037223569	0.822716812	0.995787491
Ppox	FBgn0020018	21.94259064	20.55492723	30.49112297	26.73907629	33.56147967	41.42405399	36.73843805	13.30264743	3R	0.353005187	0.245527336	0.877269208
Cbl	FBgn0020224	88.304997	87.54855534	83.31037985	111.5805244	87.42634121	106.4465535	93.99800429	93.79601022	3L	0.068914914	0.566458079	0.984934163
ATPsyngamma	FBgn0020235	3550.695021	3111.037359	2962.192917	3144.645619	3528.839728	3014.708073	3296.301128	3351.757499	3R	0.077864731	0.443407505	0.967112302
ATPCL	FBgn0020236	611.596931	609.6223481	578.1614424	635.4608034	556.2263802	625.8456066	572.6180393	584.0674078	2R	-0.029209935	0.720097496	0.995787491
14-3-3epsilon	FBgn0020238	950.2191133	879.9654035	959.2978525	941.5639264	990.6240075	941.7281959	965.2580303	1075.782942	3R	0.121845287	0.182047438	0.838040629
stet	FBgn0020248	1.933854092	2.111024235	0.863832308	1.316829007	1.462938097	1.394912457	0.877031704	1.226250841	3L	-0.300608019	0.397567975	0.952272708
stck	FBgn0020249	102.3696547	107.9699973	99.64858426	100.2972761	95.25573837	108.4648575	113.6645173	104.0418255	3R	0.068237277	0.521290025	0.982083369
sfl	FBgn0020251	6.862742085	7.416130697	9.054199761	5.495438998	6.065212585	6.168551037	7.308117926	4.816815814	3L	-0.210572445	0.183057422	0.838911159
Ran	FBgn0020255	274.223861	187.6661928	187.7833764	199.1771995	221.6833942	219.7550373	211.1862747	209.8111815	X	0.049443926	0.710367218	0.995787491
Ppa	FBgn0020257	32.67518641	35.42163355	33.66740765	35.46096297	41.96188502	39.57584345	36.0054585	29.01065841	2R	0.125157193	0.393446913	0.952091247
ppk	FBgn0020258	0.967637815	0.196736568	0.497949421	0	0	0.829348202	0.448811792	0.127323107	2L	-0.242143661	0.871825059	NA
pcm	FBgn0020261	75.64549284	87.51791	78.61563658	108.3382659	82.93613733	85.02006755	67.85873491	83.56049351	X	-0.103312305	0.444700549	0.967112302
mspo	FBgn0020269	89.23775731	80.57251204	72.73547409	77.34788341	86.22354994	87.14978117	80.52930589	82.15936439	2R	0.100102659	0.282441893	0.901712108
mre11	FBgn0020270	26.50634065	58.95730498	41.37172288	55.32183298	47.01491763	57.14601926	50.29537543	64.08152139	2L	0.29893664	0.181150566	0.837439137
mst	FBgn0020272	0.101664608	0.056842706	3.288492266	4.02946249	0	1.405781975	0.864495327	0.073574425	X	-1.655499064	0.198425986	0.847197998
loco	FBgn0020278	3.091119225	1.960243494	1.828763419	0.998348482	0.974331539	1.50971069	2.207488083	1.975563665	3R	-0.217125782	0.533150058	0.984760433
lig	FBgn0020279	191.4744352	188.2147055	195.651302	231.0709509	177.5769502	167.6211939	188.2626741	209.9365492	2R	-0.086251139	0.453766829	0.967967712
laf	FBgn0020280	0.445426105	2.18394712	0.41561443	0	0.618544832	1.098317721	0.174814087	0.148778663	3R	-0.524115367	0.677985689	NA
ko	FBgn0020294	0.757430411	0.805162229	0.809111813	0.966532614	0.869368334	0.70234602	0.80708007	1.316237958	3L	0.179891609	0.464634838	0.971928234
stumps	FBgn0020299	5.082808725	2.391526716	2.750774445	2.100480994	2.117875698	2.310023086	1.668227798	2.144896402	3R	-0.558752743	0.031710928	0.498639382
geko	FBgn0020300	0.86387878	1.328282508	1.187596126	0.637798604	0.779761304	1.628919274	0.330565999	0.593927586	3L	-0.226447534	0.712656564	0.995787491
drongo	FBgn0020304	34.67305538	37.50436668	31.66379699	33.34028167	31.94963921	36.24803528	28.45617845	27.51963694	2L	-0.115074813	0.291962388	0.905069205
dbe	FBgn0020305	71.98678523	84.05711122	74.00857387	75.21850161	73.0334272	80.49062454	78.56948255	75.28602429	2L	0.04072001	0.778595973	0.995787491
dom	FBgn0020306	22.44351785	32.56539813	32.05018451	34.45284445	25.83635935	23.70922944	31.38646113	33.26748089	2R	-0.054004267	0.727110929	0.995787491
dve	FBgn0020307	0.256761374	0.65627609	0.632786805	0.104522607	0.599633128	0.399612662	0.509447385	0.544180027	2R	0.363701353	0.430951746	0.963666972
crol	FBgn0020309	117.783847	140.0210195	145.9164147	144.1868912	147.6241011	137.5968193	131.9414496	163.9253957	2L	0.117757148	0.279225835	0.898067854
Tmtc3	FBgn0020312	208.1014689	179.7031369	187.2508817	227.1112777	211.8419802	232.2825011	184.823923	208.6538018	2R	0.088937724	0.434129385	0.965402206
Rpt6	FBgn0020369	7.312278962	9.198991611	9.13061819	4.888851632	9.997466013	7.451572612	5.760726402	7.821084651	X	0.060094114	0.875586139	0.995787491
TppII	FBgn0020370	99.89620242	125.9210582	82.09224724	98.68166976	106.8782191	110.0807814	110.8710282	125.2906593	2R	0.189085824	0.143217666	0.8037297
TM4SF	FBgn0020372	3093.796865	2763.346214	2584.585248	2930.850384	3156.134876	3160.360951	3013.272316	3393.910022	2R	0.191466511	0.046305229	0.565844709
Sr-CII	FBgn0020377	0.265537234	1.781605834	0.579770663	1.485816154	2.205068712	0.025034688	0.474173832	1.383614196	2R	0.044780484	0.964483499	0.997604378
Sp1	FBgn0020378	1.335530751	1.4789442	0.401065359	0.657512989	0.825115333	1.091042461	0.554045708	0.605248147	X	-0.308037768	0.446838169	0.967112302
Rfx	FBgn0020379	5.045826181	6.468022312	2.96363569	4.321778602	7.699510128	3.570384353	4.525528076	3.770811076	3R	0.091584455	0.739954664	0.995787491
Dredd	FBgn0020381	3.243116842	9.897547687	4.698798855	8.716388898	8.538041609	12.5700953	5.285694361	9.583728802	X	0.477321682	0.271031787	0.89549671
pug	FBgn0020385	2.375552004	2.206556391	1.921018676	0.923085011	3.066493918	1.192074925	1.889703675	0.055457455	3R	-0.228110885	0.736701381	0.995787491
Pdk1	FBgn0020386	42.90091887	48.31807649	56.93888879	45.03133507	38.56668588	43.49574682	43.49429754	51.4138527	3L	-0.093197294	0.448987446	0.967112302
Gcn5	FBgn0020388	90.19106063	111.346999	102.0445937	116.6183709	90.40625533	118.829087	119.4053531	89.2370808	3L	0.021269275	0.877844935	0.995787491
Papss	FBgn0020389	16.8670637	23.14303927	14.28947922	19.14570875	15.34472343	17.61044578	15.09663677	14.1956468	3L	-0.208323809	0.158810714	0.819015157
Nrk	FBgn0020391	36.15634457	53.25741143	37.89917644	41.49923028	25.87090428	34.30282103	23.25267524	37.93007843	2R	-0.442456516	0.019431222	0.395797586
Nmt	FBgn0020392	78.8777311	63.27143234	40.48801999	51.67523716	55.28097482	67.4237191	47.87254044	36.72588962	3L	-0.154419298	0.481582574	0.973549494
Mst89B	FBgn0020399	0	0.817958935	0.443634038	0.284232483	2.640981846	0.804563083	0.466498956	2.7791561	3R	2.19600427	0.075826573	NA
asun	FBgn0020407	34.44893586	17.02443375	21.65509811	20.04055974	17.66601813	30.10939149	22.5181895	26.27045037	3R	0.071210742	0.78038636	0.995787491
JIL-1	FBgn0020412	41.96255429	40.7166021	47.96093245	35.92564938	34.13007109	35.59791895	40.73186378	36.94449989	3L	-0.144591066	0.21210401	0.856520545
Idgf3	FBgn0020414	19.6795361	23.07965007	40.68387317	20.77413247	15.02768159	13.14382447	24.12214197	17.4589175	2L	-0.542453534	0.03256291	0.505711525
Idgf2	FBgn0020415	1.226501276	1.600109645	1.190189995	0.444817575	2.361759096	0.591017155	1.182002862	0	2L	-0.073980872	0.938112674	0.997038394
GluRIIB	FBgn0020429	0.270682018	1.327167865	0.656670799	0	1.127654809	1.001158846	0.265582939	0.180823298	2L	0.243190306	0.821565706	0.995787491
Fak	FBgn0020440	116.3178047	130.6288784	137.7018767	125.6662218	112.9167461	120.7996003	122.6690576	121.609695	2R	-0.062651367	0.496237	0.975846898
eRF3	FBgn0020443	44.77454063	47.35171401	37.98244694	41.05795875	31.47060427	34.66527276	33.85519201	31.3472959	2L	-0.353244677	0.002289124	0.113556916
E23	FBgn0020445	1.219791873	2.19165464	1.411147834	1.346044643	1.457576066	1.006772357	0.409727634	0.612591707	2L	-0.783204872	0.039536011	0.529044592
Dad	FBgn0020493	10.26680504	10.82521346	12.64574753	8.742302118	12.01291678	10.49108367	9.983236944	12.19997733	3R	0.107006655	0.469285061	0.973549494
CtBP	FBgn0020496	127.8545949	162.0778694	161.7459288	192.3096067	137.1241406	158.1393053	148.206393	165.7738935	3R	-0.049624259	0.686768342	0.995787491
emb	FBgn0020497	133.8153465	146.7483587	183.2310521	156.5855588	135.4005883	141.2092019	176.437209	148.2723642	2L	-0.012577451	0.920695231	0.996479182
CLIP-190	FBgn0020503	233.6142387	221.2710579	236.4204014	235.0610875	202.9445444	218.6068832	205.9823796	208.3152672	2L	-0.119105037	0.128944971	0.788096052
Amyrel	FBgn0020506	1.512039201	0.815217956	0.524026573	1.384924544	2.144700085	0.20364876	1.239828585	0	2R	-0.224214624	0.851983183	NA
Abi	FBgn0020510	223.0460968	196.311666	203.879856	196.3837196	188.3547751	217.0098731	193.676737	201.1570434	3R	-0.006388058	0.947940487	0.997604378
Paics	FBgn0020513	28.01003308	27.06182644	13.49650121	11.50439109	19.08206488	18.49450349	13.26653901	14.02150608	X	-0.276092941	0.327668824	0.918470465
pio	FBgn0020521	0.761043742	0.568324434	0.379372912	0.401050545	0.922192759	0.653619747	0.186165378	0.282927468	2R	-0.020200603	0.97105942	0.997604378
kraken	FBgn0020545	231.4384745	203.9473913	183.4377063	229.6564756	219.6747126	211.466527	200.6277408	197.8253842	2L	-0.004986277	0.966626924	0.997604378
lncRNA:bxd	FBgn0020556	0.234223945	0.430294744	0.331464014	0.071511041	0.18121468	0.344381697	0.092471835	0.145291962	3R	-0.441635788	0.473244601	0.973549494
SA	FBgn0020616	82.04297216	62.26712389	81.3294274	73.74829752	69.75558031	78.408391	73.84952887	59.1033864	2L	-0.064314414	0.634221513	0.991585536
Rx	FBgn0020617	0.572414308	0.95297944	0.300532871	0.657321784	0.38558005	0.306080763	0.261535737	0.667754033	2R	-0.582297725	0.231734256	0.867702781
Rack1	FBgn0020618	483.2664315	693.966252	724.2598426	821.949646	814.7351832	843.188652	905.6421417	1002.106544	2L	0.421690673	0.003329194	0.149169383
RN-tre	FBgn0020620	85.04523949	83.59350814	73.29034583	105.5321392	72.79663783	96.79827902	71.40484905	81.0058911	2R	-0.084059615	0.543331032	0.984934163
Pkn	FBgn0020621	16.79950664	15.81097881	16.23421121	15.59551826	12.45241091	16.81981492	13.47207875	12.41204381	2R	-0.197306563	0.081893524	0.694684542
Pi3K21B	FBgn0020622	29.68556766	38.23419888	38.80903883	44.13560677	35.15252613	47.04543938	34.7990584	34.19436676	2L	0.032315655	0.827129311	0.995787491
Osbp	FBgn0020626	60.42352418	81.02959059	58.72238964	68.78129387	47.8944447	29.85953638	38.02469675	32.75420161	3R	-0.822847552	2.66E-06	0.000546045
KrT95D	FBgn0020647	17.63739366	20.39372851	17.44738444	20.18941729	17.76236927	20.92723978	15.78827798	19.57399408	3R	-0.001472226	0.989589427	0.998078482
Trxr-1	FBgn0020653	285.0298855	273.1345598	233.3048167	308.6618644	281.2354023	280.1913955	275.0723312	311.138263	X	0.090394904	0.417955304	0.959305841
ArfGAP1	FBgn0020655	87.87309102	83.68109824	70.09973638	96.54561687	88.46983275	64.78055905	70.77109419	82.39425245	3L	-0.112282911	0.474409752	0.973549494
Atet	FBgn0020762	72.40943623	75.8966413	80.75561795	74.32364353	75.93667849	69.62329331	74.72912436	62.83864904	2L	-0.068671874	0.483365277	0.973549494
Alas	FBgn0020764	682.0368662	638.3974781	556.8303725	621.5089754	642.3303578	681.609631	601.670456	674.1602664	2R	0.086041583	0.383089769	0.946058474
Spred	FBgn0020767	386.7654835	398.9715385	322.3913099	429.3210713	321.3407525	360.6643084	304.6998266	348.3377637	2R	-0.175336899	0.104047086	0.745084146

**Su(z)12**	FBgn0020887	92.82422723	96.13630405	86.50945886	87.8199418	94.00061357	85.91839766	72.13110527	90.82179204	3L	-0.052418241	0.644685265	0.992758129
**Scp2**	FBgn0020907	0.123468991	4.030521799	1.105971873	1.372886523	1.954601668	2.578836408	0.605715476	0.577365617	3R	-0.172444751	0.805287682	0.995787491
**Scp1**	FBgn0020908	1.475517398	1.212900891	0.805999187	0.472730797	0.396907014	0.734708119	0.373914892	0.27579671	2R	-1.129258879	0.00379002	0.164510566
**Rtc1**	FBgn0020909	36.68609276	62.76444177	50.64556186	57.06893499	55.20819934	59.85178226	43.75698842	48.90257129	X	0.039704233	0.844220782	0.995787491
**RpL3**	FBgn0020910	367.3416663	496.8393637	472.8830817	537.4788138	467.2445781	490.7260644	447.7253727	550.3469283	3R	0.093521209	0.449339243	0.967112302
**Ptx1**	FBgn0020912	0.60393132	0.478140751	0.550888488	0.428045114	0.584374439	0.232308166	0.320453489	0.104895264	3R	-0.701896062	0.207255556	0.855506588
**Dgkepsilon**	FBgn0020930	295.0110864	300.7862307	288.9592718	308.3913302	280.4288013	356.3108091	329.9257108	371.4499078	2R	0.195060041	0.074651476	0.676245
**Crg-1**	FBgn0021738	0.420885547	0.741275284	0.867903097	0.392511524	0.341912828	1.230105802	0.375790826	0.822403336	X	0.241911851	0.759899315	0.995787491
**C901**	FBgn0021742	2.317638423	1.639312847	1.151608365	3.190010241	2.217987709	1.263646467	1.495894622	0.545617687	X	-0.565201487	0.360977696	0.939549067
**chb**	FBgn0021760	43.47114256	42.07485625	42.38975358	54.00755251	44.60804114	38.46980757	40.42016936	40.19081815	3L	-0.123025285	0.322454669	0.918365535
**Nup154**	FBgn0021761	22.73001332	23.24258266	19.25344576	21.21752855	21.01653326	23.63827701	22.20153228	18.34018301	2L	0.006686527	0.962231727	0.997604378
**sdk**	FBgn0021764	0.902647767	1.01092242	0.690813238	0.480197403	0.676259362	0.937024145	0.416103391	0.492183806	X	-0.261923374	0.376481551	0.943615128
**scu**	FBgn0021765	137.4263132	154.4959065	129.0601179	152.663959	115.7555819	106.3055472	89.41181216	134.7303727	X	-0.330407359	0.028400655	0.466846269
**org-1**	FBgn0021767	0.669184061	0.669538967	0.370256238	0.246343807	1.017304203	0.664108048	0.119796696	0.407820503	X	0.204741072	0.785154283	0.995787491
**nudC**	FBgn0021768	2.311726462	2.138674545	2.558387266	2.375358894	3.484886177	3.14564353	2.629450128	2.7035185	3L	0.382632166	0.07653006	0.681411883
**Tapdelta**	FBgn0021795	188.300534	211.8285442	177.8531	201.3618248	153.1954467	190.5473568	207.301469	165.889215	2R	-0.091530908	0.501992495	0.977207013
**Tor**	FBgn0021796	26.54093543	28.18461801	40.90839863	27.82362761	27.87611327	17.8371737	29.93686722	33.87040039	2L	-0.134615507	0.4954397	0.974969175
**Reph**	FBgn0021800	169.7722477	173.7322078	148.7397143	174.8829696	151.1895088	163.3950651	167.4137055	139.6039088	2L	-0.073259106	0.464168917	0.971928234
**Vps28**	FBgn0021814	192.0087049	203.1827965	207.2659233	155.9404411	234.0185206	205.6908084	182.6920566	234.1075416	2R	0.208587899	0.131572697	0.790743321
**DCTN2-p50**	FBgn0021825	321.0680293	400.6801965	355.7090291	366.1046402	360.1551369	333.1553179	356.300573	270.4166219	2R	-0.097582438	0.41231351	0.958665466
**Ttc7**	FBgn0021847	148.5083792	153.558762	141.961938	156.2996714	112.9885868	156.4583218	142.8635497	149.1450557	2R	-0.067840916	0.555820026	0.984934163
**l(2)k14505**	FBgn0021856	191.6332868	180.7065741	206.4154583	176.7647069	212.2228811	186.0579638	205.4992329	190.9307816	2L	0.103696053	0.384228438	0.946594947
**Xbp1**	FBgn0021872	632.6863784	827.4608908	670.1437186	770.7879628	576.3991786	627.0470939	661.8929078	613.1230084	2R	-0.195362156	0.063401225	0.636110045
**Nle**	FBgn0021874	36.62150329	51.16193313	48.73208257	41.07985349	62.81454934	38.34212992	40.54172752	60.75662798	2L	0.227360186	0.290665794	0.904896797
**Zfrp8**	FBgn0021875	57.31729955	51.71337408	42.29405148	79.15302564	67.91412576	47.98286951	59.76666464	49.80302687	2R	-0.004493379	0.98393756	0.997881056
**ytr**	FBgn0021895	74.86592058	107.8825858	104.7510799	106.0468168	96.73897954	94.78522472	94.6992881	92.0469713	2R	-0.023615274	0.852371027	0.995787491
**RFeSP**	FBgn0021906	1502.984186	1441.568452	1369.456414	1407.680336	1643.265578	1319.631261	1507.693634	1565.722922	2L	0.109503243	0.286281437	0.902371215
**Coprox**	FBgn0021944	26.88356192	33.29974429	25.80098093	27.41302088	51.30496057	45.56647959	32.40619113	36.94419787	2L	0.583908047	0.006328163	0.227322885
**ND-PDSW**	FBgn0021967	717.8937861	779.5293717	686.9992825	734.5249558	852.252826	743.9880958	764.4600702	845.6938169	2L	0.167536658	0.092229675	0.72305867
**l(2)k09913**	FBgn0021979	523.5785086	522.0067207	494.6405426	556.5956015	567.4903343	517.4850361	538.2686757	499.5406873	2R	0.047749456	0.599555698	0.989286463
**Rs1**	FBgn0021995	18.68317451	24.94633723	18.63976852	17.72140524	16.361945	17.9445311	16.07973643	12.29856435	2R	-0.320002508	0.085507652	0.701544311
**eIF3h**	FBgn0022023	410.810154	412.4064183	483.8292416	498.972067	517.4707668	428.7637713	509.8119487	573.7644034	2L	0.201326327	0.109388806	0.758410631
**Vps25**	FBgn0022027	119.1743489	128.3494259	112.4177171	138.2327055	123.9791237	147.8674374	140.6416496	116.6000216	2R	0.115690096	0.462690633	0.971606621
**l(2)k01209**	FBgn0022029	43.94988754	59.58211007	53.37069501	57.19027658	43.00716873	44.37878094	41.9967632	48.58357066	2R	-0.233258141	0.069935356	0.662558698
**Nnp-1**	FBgn0022069	88.18164334	96.52376789	75.63680599	99.46652097	83.36739084	71.57218365	83.29329956	93.60382958	2L	-0.085508906	0.547055922	0.984934163
**Vha36-1**	FBgn0022097	2167.737713	2367.146513	2375.727463	2440.844172	2133.402671	2166.493486	2206.947756	2232.084146	2R	-0.066623386	0.425206891	0.9620897
**l(2)k05819**	FBgn0022153	106.1145794	121.9641164	103.8481394	108.565468	112.1744375	119.2559825	104.5289869	81.21900738	2L	-0.049556174	0.679488815	0.995787491
**Gpo1**	FBgn0022160	203.9848431	216.7878312	192.0319101	225.439735	182.6222274	197.2614837	169.9968632	166.1796103	2R	-0.198659349	0.039880165	0.529044592
**ubl**	FBgn0022224	51.73729509	86.22217027	110.5332582	70.03948402	88.57848023	82.29254138	83.02153388	100.3691244	2R	0.194630581	0.444487805	0.967112302
**lolal**	FBgn0022238	240.5099249	174.2982994	212.0323815	188.1455655	214.6865391	213.8151431	188.712852	195.0331752	2R	0.022536455	0.851046457	0.995787491
**l(2)09851**	FBgn0022288	43.54839974	43.42644533	50.1536858	49.98471611	37.83936047	59.51033077	42.2397193	27.53309437	2R	-0.136148191	0.529067116	0.984296327
**dnk**	FBgn0022338	57.97595233	71.73380939	49.59848774	50.65949757	52.86562323	59.22208125	60.09139547	52.32123145	3R	-0.00518188	0.974914697	0.997757033
**CG3760**	FBgn0022343	57.21822398	74.0173641	80.05759871	98.93885719	90.88651586	79.55472138	100.3247916	85.26478917	2R	0.231771087	0.201798854	0.849268738
**CG10340**	FBgn0022344	511.6844199	497.1754981	518.4591205	582.6572685	533.5098048	571.2308763	571.883247	477.4317769	3R	0.058183838	0.589175409	0.987597375
**CG1910**	FBgn0022349	128.3686068	132.7808699	106.5749983	125.3203148	118.260068	125.7700922	106.3501297	132.3604419	3R	-0.000729491	0.99493986	0.998895169
**Tsf1**	FBgn0022355	175.2292423	342.2781779	420.1403946	228.9560654	52.23463109	133.6670503	217.304849	142.599693	X	-1.057306924	0.01802335	0.385711607
**Sodh-2**	FBgn0022359	2.820989314	3.335540895	3.141361645	1.617302554	1.58622239	0.741203203	3.303267569	1.30524879	3R	-0.637279641	0.343144675	0.928865024
**Pur-alpha**	FBgn0022361	179.6103044	113.3107576	135.7313884	106.5789841	110.2072993	144.9098995	106.501259	105.5565049	4	-0.172304011	0.268933878	0.89549671
**Pka-R2**	FBgn0022382	755.6439756	649.0916438	663.9842069	662.6533132	644.7364732	691.6527688	598.0941011	611.1593498	2R	-0.073726464	0.413050434	0.958665466
**D19B**	FBgn0022699	152.3395239	173.9349507	144.6590251	179.9420016	138.1897681	149.9935462	137.2978624	109.6537781	3L	-0.253958514	0.041609807	0.536003453
**Cht2**	FBgn0022702	4.227623747	7.615030958	2.505765446	4.872269708	2.818615828	7.971555692	6.311535101	2.711848012	3L	0.069126497	0.875804185	0.995787491
**Adk1**	FBgn0022709	168.0762554	167.3925767	145.0823315	163.9746642	183.4830332	171.4274644	163.4905972	173.2017879	3L	0.131474274	0.190637443	0.843401316
**Ac13E**	FBgn0022710	0.474992338	0.559052156	0.542270488	0.609550021	0.536827536	0.395135184	0.442938077	0.290310531	X	-0.360140583	0.231804785	0.867702781
**Taf8**	FBgn0022724	46.3453444	39.10913491	28.62945707	34.8599297	32.36985832	45.47643833	28.78975271	34.70085186	X	-0.051502557	0.818719501	0.995787491
**Sin3A**	FBgn0022764	68.39494669	74.57236909	81.01304968	80.99821795	73.52672543	67.0740124	73.51333358	76.17144328	2R	-0.039027006	0.699389435	0.995787491
**Pp2C1**	FBgn0022768	52.0085258	65.76392791	54.97231993	52.07112365	63.51960765	41.8412944	52.85265643	53.98288004	X	-0.047674514	0.740882385	0.995787491
**Peritrophin-A**	FBgn0022770	1.729441912	0.694580778	0.768208809	1.419763631	0.527676491	0.826736081	0.124277281	0.846147264	X	-0.960594236	0.221926238	0.86549481
**Oat**	FBgn0022774	0.741190745	10.68059185	2.370246992	3.220472321	2.736898485	1.52463296	6.016161178	1.609193718	3L	-0.468298983	0.501269157	0.976796212
**Hira**	FBgn0022786	24.12904263	42.653581	32.8646618	41.58048813	27.09063502	41.56263154	28.27514289	45.89998017	X	0.049738226	0.817121366	0.995787491
**Hel89B**	FBgn0022787	57.33030737	52.67530061	50.41741569	75.02656938	55.81385952	53.23025175	56.86807442	47.30578253	3R	-0.116124376	0.407065816	0.956706394
**Cad96Ca**	FBgn0022800	0.54176834	0.949430256	1.255472178	1.206555214	0.335741885	1.722915661	1.11390126	1.404452796	3R	0.247342715	0.62546177	0.990047511
**Df31**	FBgn0022893	392.5673733	373.7917962	415.3392962	432.7121032	458.6597169	372.6797047	393.3823588	462.4325002	2L	0.095373574	0.398064311	0.952272708
**D19A**	FBgn0022935	139.4361738	134.4362269	99.86660683	113.3308189	124.3632376	128.6572843	121.7219921	93.88534504	3L	-0.028290289	0.83921428	0.995787491
**CycH**	FBgn0022936	13.33604087	32.77883148	32.19288553	27.24439838	32.06023468	24.15038364	26.9469519	24.65371301	3L	0.072752702	0.806416839	0.995787491
**Cbp80**	FBgn0022942	31.78313841	37.60902057	40.96147402	32.58002765	37.27115263	37.59239026	36.88110719	37.13677117	X	0.092176456	0.420327491	0.960593359
**Cbp20**	FBgn0022943	220.6649908	202.4348484	200.6812972	181.9601188	232.7433143	253.0718016	211.5924539	200.1421054	3R	0.18369466	0.163025787	0.825076487
**yps**	FBgn0022959	465.2043394	529.9934686	548.4319344	499.9182616	505.3724479	499.9655879	514.7298853	547.1873467	3L	0.04928281	0.58335784	0.985764834
**vimar**	FBgn0022960	72.40477885	68.62501506	61.80905239	56.6699382	69.37364394	62.85094487	61.11662761	58.38093553	2R	-0.013868092	0.900177997	0.996479182
**qkr58E-3**	FBgn0022984	642.2796657	595.9561471	539.7682236	558.1415613	553.143072	541.69542	496.5803206	528.7622951	2R	-0.110493851	0.245800485	0.877269208
**qkr58E-2**	FBgn0022985	136.610642	138.0275691	127.2044429	160.5717761	133.8740253	136.5087055	124.1924792	146.6303004	2R	-0.025940248	0.822869074	0.995787491
**qkr58E-1**	FBgn0022986	267.2532569	277.9737269	234.7520513	254.6498019	282.7507505	227.7304669	232.0803958	262.9104815	2R	-0.009275972	0.934560299	0.996479182
**qkr54B**	FBgn0022987	148.1085461	168.0665646	173.5662634	147.1774899	149.6078036	150.6725966	148.9958403	138.8302482	2R	-0.083138815	0.364724283	0.940794966
**melt**	FBgn0023001	0.435216705	0.441404399	0.220987602	0.283169683	0.29234519	0.763385398	0.697131941	0.373563438	3L	0.636422479	0.338058537	0.926687587
**CRMP**	FBgn0023023	152.6136099	134.3765783	125.8831665	138.171755	136.4955839	137.1629717	122.4454022	127.7600173	3R	-0.044803019	0.651805989	0.993444251
**Clk**	FBgn0023076	1.498488647	2.396051008	2.344266976	0.946882488	1.56747361	0.600755612	1.339723154	1.353031885	3L	-0.523843144	0.196879495	0.84446408
**gek**	FBgn0023081	45.11229356	46.73120583	33.48950973	44.75053393	36.58208277	51.98444415	36.09173722	35.79291968	2R	-0.058571509	0.701580709	0.995787491
**dimm**	FBgn0023091	0.216566438	0.125743783	0.323315174	0.058259596	0.105257903	0.086382795	0.021248678	0.018084069	2L	-1.5849679	0.173766076	NA
**cyc**	FBgn0023094	122.4561452	121.2983487	145.8729967	117.2746763	127.8064264	125.4704623	142.0780525	122.7396516	3L	0.062838131	0.600655941	0.989286463
**caps**	FBgn0023095	25.81546732	22.09719072	23.59117235	28.39151409	22.33962009	21.02886293	22.16155404	20.00519654	3L	-0.195902345	0.077382183	0.682035833
**btv**	FBgn0023096	0.196393463	0.578319469	0.243517765	0.111927191	0.05515075	0.501985421	0.144734472	0.246357735	2L	-0.213733579	0.773518257	0.995787491
**bon**	FBgn0023097	37.94040779	42.32662388	41.30991068	41.77176584	40.37958603	40.03392853	37.33432752	45.01485127	3R	0.026780293	0.787319545	0.995787491
**a6**	FBgn0023130	113.0912803	123.8223106	97.39750536	122.9977321	129.3440893	126.6963956	117.2844159	117.3490719	X	0.131051007	0.277443035	0.896087042
**Uba1**	FBgn0023143	165.6902698	187.7034356	209.2172193	176.0575426	171.4805131	199.3851499	173.1236043	181.9357251	2R	0.006028475	0.951372023	0.997604378
**AMPKalpha**	FBgn0023169	369.4938437	327.8969448	281.7430429	318.3673235	335.5864912	311.2435524	289.6219894	290.5916498	X	-0.051947547	0.637634935	0.991585536
**RpL39**	FBgn0023170	727.113716	1028.341192	1134.65443	998.555428	842.1695493	812.5342429	1025.690502	1078.23243	2R	-0.01311924	0.929623352	0.996479182
**rnh1**	FBgn0023171	47.35914637	45.80030279	53.4052632	33.9624592	33.3726861	51.73231406	72.15523937	51.33963428	2R	0.238193379	0.32617068	0.918470465
**RhoGEF2**	FBgn0023172	1.527711141	1.921890842	1.443976332	1.815593374	0.984967914	1.22052814	0.847772741	1.507637412	2R	-0.523734644	0.068059641	0.656607807
**Prosbeta2**	FBgn0023174	174.5661573	226.7500406	190.3193264	190.580269	200.8324638	173.7729811	220.4804332	193.4174816	3L	0.04495988	0.729584087	0.995787491
**Prosalpha7**	FBgn0023175	113.3977199	184.3971688	128.8825889	168.2893158	149.4618742	140.0523072	156.5168789	151.0718377	2R	0.039318416	0.810223093	0.995787491
**Pp4-19C**	FBgn0023177	100.3464204	120.9669717	111.8795835	124.4936811	91.94338963	112.4234074	120.3747612	102.4470986	X	-0.069055618	0.579645842	0.985637351
**Pdf**	FBgn0023178	0	29.10078642	1.547383327	2.974185257	4.605836037	4.089171133	1.627135534	6.093132618	3R	-0.969521921	0.384185975	0.946594947
**amon**	FBgn0023179	527.4810173	570.6041926	535.5357398	590.9120193	510.4713802	552.3101966	555.0299466	538.1227521	3R	-0.015034271	0.855409099	0.995787491
**Orc6**	FBgn0023180	5.313485371	1.13176207	2.659932352	0.393275908	1.979343462	6.281780993	1.721246392	2.014234201	2R	0.337972262	0.711144297	0.995787491
**Orc4**	FBgn0023181	13.14279235	6.682990796	13.55707457	17.00988783	20.55017286	20.63531283	22.83569054	22.58022827	2R	0.808610004	0.008442152	0.272701148

**Jon74E**	FBgn0023197	1.567377778	2.905799115	2.001282436	0.961653233	5.361193147	3.577623842	1.473100037	4.477529273	3L	1.042372232	0.169415357	0.830934498
**EloB**	FBgn0023212	213.1404742	231.5893336	208.714827	236.0598781	241.5637934	235.2079386	211.1925975	183.0891423	3R	-0.000831953	0.994369125	0.998895169
**eIF4G1**	FBgn0023213	98.69693472	88.22185534	106.4847623	82.25634218	89.03592189	99.33264819	87.03596308	91.95459293	4	-0.002598497	0.979748033	0.997881056
**edl**	FBgn0023214	1.21083918	0.846255359	0.489579827	1.209868271	0.650558052	1.549275991	0.478040451	0.625915225	2R	-0.162229526	0.791002613	0.995787491
**Mnt**	FBgn0023215	30.88517071	38.18171981	33.45017871	41.63204204	33.19897902	36.44783083	34.11638732	36.04867496	X	-0.013673167	0.901526284	0.996479182
**Parg**	FBgn0023216	38.06002463	41.97871141	37.84789297	48.80074418	31.74302447	32.20561865	32.66799707	29.07285799	X	-0.377462678	0.016248281	0.369626395
**Dap160**	FBgn0023388	195.6906394	185.0481075	171.4452199	167.7530372	175.9833571	195.2459828	174.9725423	155.4055968	2L	-0.009045235	0.925509919	0.996479182
**Chd3**	FBgn0023395	0.254343951	0.284417532	0.411356586	0.06588815	0.204069342	0.346369001	0.072092987	0.306780146	3L	-0.079382963	0.95267453	NA
**B4**	FBgn0023407	27.16957414	25.70555906	28.05304558	23.99532532	30.11845968	26.31450234	26.79037364	29.2988393	2L	0.132770765	0.180249212	0.836097072
**Ac3**	FBgn0023416	38.85062367	29.02586617	29.12121685	31.80828948	29.76455269	38.00892147	30.21977022	33.42379366	2L	0.054131745	0.678907861	0.995787491
**fus**	FBgn0023441	0.640091423	0.848419461	0.445223549	0.48005634	0.6706917	1.042364672	0.33114491	0.758016945	2R	0.245795663	0.567391114	0.984934163
**Rbcn-3A**	FBgn0023458	47.76871097	77.01096962	90.92721267	79.17885594	70.19700904	65.85898962	73.17536	80.16180612	X	0.00850744	0.95688986	0.997604378
**Taldo**	FBgn0023477	37.31091098	46.04259161	37.36549337	39.22800248	29.73196904	44.28213221	39.42098691	23.61739032	2R	-0.194140857	0.370167494	0.942619319
**teq**	FBgn0023479	3.571120489	7.601801779	4.986612657	5.531375727	1.117681649	2.330164301	2.476506238	2.139935365	3L	-1.393918539	2.92E-07	7.73E-05
**Lip3**	FBgn0023495	2.342440537	0.523882052	2.020525536	3.155424671	0.845741106	1.325063178	3.452578212	2.712349463	3R	0.067370414	0.931542827	0.996479182
**Lip1**	FBgn0023496	0.932687102	0.408117853	0.688643535	1.323623832	0.43923633	2.829154174	0.413792145	0	2L	0.150709324	0.89724021	NA
**Es2**	FBgn0023506	66.65436122	73.81040532	77.31157389	79.59094699	60.20591235	110.6164526	86.84988187	67.47899998	X	0.155778503	0.389702789	0.948624352
**D2hgdh**	FBgn0023507	8.038816301	6.826268222	9.690084185	3.514162046	3.900133259	10.84736776	6.344412863	2.67249282	X	-0.215981153	0.61318884	0.990047511
**Ocrl**	FBgn0023508	140.8428309	153.956823	128.4490291	153.4940808	132.9628218	127.1953489	152.773736	134.7398254	X	-0.043749229	0.693965161	0.995787491
**mip130**	FBgn0023509	8.311163731	12.5816122	11.29045381	11.83695692	8.543999345	11.25665615	9.475939472	7.348500729	X	-0.23317852	0.185432729	0.839299945
**Edem1**	FBgn0023511	27.76247498	20.87320075	12.51045677	18.56668383	23.80775797	25.48885058	18.20168502	16.99590752	X	0.105857169	0.642546732	0.992589784
**eIF2Bepsilon**	FBgn0023512	77.3103027	55.52994985	65.25969184	56.90018373	47.98446045	64.6550221	54.99646003	46.38905512	X	-0.227941752	0.158320626	0.818884898
**CG14803**	FBgn0023513	32.73278531	39.46400437	40.9816033	44.35075128	30.45156697	44.29383563	41.22015367	46.13702859	X	0.072749312	0.664371538	0.995787491
**CG14805**	FBgn0023514	134.2582807	162.9558935	124.0510837	109.4645504	144.3739869	115.8595982	150.6579583	133.2421126	X	0.069553802	0.6408555	0.991992893
**CG14814**	FBgn0023515	0.022639374	1.139231226	0.713996478	0.316697092	1.88001415	0.960491866	0.577535299	0.83558701	X	1.020485637	0.273371368	0.89549671
**Pex5**	FBgn0023516	95.75027244	88.64707932	96.44134269	109.1728805	87.68962173	97.56347071	105.7737375	102.8445687	X	0.043093681	0.741498682	0.995787491
**Pgam5**	FBgn0023517	86.24649352	73.82974575	109.6668418	103.6604403	96.7737547	120.356767	99.95584372	103.9555466	X	0.203258345	0.228958861	0.867702781
**trr**	FBgn0023518	4.011496846	17.61243298	9.488539511	11.17642139	7.096944346	11.24203877	8.519317686	9.819770694	X	-0.166578327	0.588950703	0.987588562
**mRpL16**	FBgn0023519	148.2260027	161.2884419	107.466908	106.7388026	138.512066	144.475839	139.860395	146.3341296	X	0.149615443	0.346094887	0.930285749
**CG3857**	FBgn0023520	4.701075725	8.009574483	4.759460152	9.148034601	12.62973386	6.840161526	8.026506612	2.893172761	X	0.2243816	0.611998508	0.990047511
**CG3587**	FBgn0023521	42.07574666	64.42882496	66.34031444	39.92934457	57.12717	61.19752956	58.07298356	32.43931704	X	0.007776803	0.974640774	0.997757033
**CG11596**	FBgn0023522	69.04397209	54.13768287	53.60880597	75.0354197	63.72801487	57.14115463	57.38540723	32.84680084	X	-0.230226352	0.251304189	0.882557414
**Or2a**	FBgn0023523	1.815352454	0.978749702	4.246733313	0.75579034	2.809008386	1.222502289	1.157750778	1.970649111	X	-0.077231527	0.927820422	0.996479182
**CG3078**	FBgn0023524	170.3174881	146.8865487	135.0865644	139.8835587	136.4068534	193.1647655	137.8400749	137.7701315	X	0.05613624	0.6602494	0.995750687
**CG3191**	FBgn0023525	10.49918129	8.09787091	12.05317831	11.69282908	8.461479084	6.805262743	10.40257331	5.291625504	X	-0.425684568	0.215187809	0.858896167
**CG2865**	FBgn0023526	29.34207151	32.32654282	27.2027373	31.80208501	28.98605647	27.43520878	23.63377335	28.42852623	X	-0.122875427	0.277513878	0.896087042
**CG3071**	FBgn0023527	20.87029158	24.19063146	29.59643039	23.6688595	25.24857688	29.61727025	23.00792563	32.91929972	X	0.208173581	0.319580266	0.916248855
**CG2924**	FBgn0023528	90.57736926	86.3789727	86.33973078	92.47089861	83.10259256	90.03817482	82.28267655	88.69821176	X	-0.018898895	0.842615124	0.995787491
**CG2918**	FBgn0023529	116.3159299	125.7509617	112.2098359	116.343461	109.4576934	111.9951811	112.605073	78.18692495	X	-0.162107951	0.198485589	0.847197998
**CG3740**	FBgn0023530	20.51228937	25.43797205	25.70670873	19.26544356	25.79810956	26.30451727	27.27963032	20.89513661	X	0.17459907	0.526984686	0.984107922
**CG32809**	FBgn0023531	91.10822389	102.5706585	90.03785765	107.1579522	93.92587852	102.1308686	80.56039246	102.6761375	X	-0.01373243	0.898186569	0.996479182
**CG17778**	FBgn0023534	767.4228499	819.7499383	863.1685091	764.5225395	616.2953727	694.8115096	778.098994	633.8191437	X	-0.208647589	0.038194313	0.522230403
**arg**	FBgn0023535	0.214297816	0.906450539	0.395563543	0.651687772	0.260711673	0.267920848	0.475372464	0.303430693	X	-0.693563703	0.1986475	0.847197998
**CG3156**	FBgn0023536	29.4812364	29.08861074	30.40937063	33.69332982	30.04333298	38.40778425	28.48407343	32.5647071	X	0.108538406	0.500519445	0.976796212
**CG17896**	FBgn0023537	50.6019441	59.23194651	71.77790125	45.04287152	40.99755346	39.3539197	61.68742401	29.44454996	X	-0.369463905	0.084787322	0.69799888
**CG3630**	FBgn0023540	0.412263341	0.40338312	0.458399844	0.280343134	0.170555227	0.372484386	0.284833423	0.559413672	X	-0.134020582	0.838379299	0.995787491
**Nmd3**	FBgn0023542	74.16394654	111.9125335	84.84896642	78.26817692	73.28171444	85.90080842	99.61341332	73.29110348	X	-0.03943052	0.810519377	0.995787491
**PIG-K**	FBgn0023545	122.922796	175.1878888	149.5383579	190.6892839	184.5639015	187.830872	150.8584209	136.1563341	X	0.077479501	0.626845741	0.990047511
**Mct1**	FBgn0023549	5.389222945	7.118624338	6.504631606	7.873257331	5.54558595	7.57850205	7.467893608	10.23648676	X	0.230604829	0.248954392	0.879503029
**Ac78C**	FBgn0024150	22.94896506	29.1169539	25.36444863	26.28847672	19.13012055	25.66372117	20.62497811	20.19260587	3L	-0.247315298	0.025385563	0.447279658
**wit**	FBgn0024179	50.10461903	41.00384078	45.58241509	54.05724221	46.61072584	37.22069415	40.22059652	54.26658941	3L	-0.066607521	0.66281153	0.995787491
**unc-4**	FBgn0024184	0.145232289	0.730820371	0.331606124	0.059753579	0.539785449	0.805435969	0.196142129	0.092739042	X	0.412337403	0.640707562	0.991948137
**syd**	FBgn0024187	11.8471694	22.65538293	28.80582415	23.67978477	16.35159321	17.12535456	26.68149605	23.66398368	3L	-0.015450577	0.960736921	0.997604378
**san**	FBgn0024188	201.9357555	215.6277541	229.0738308	175.2520154	249.9205704	213.1653631	252.2604785	172.2369071	2R	0.142711221	0.333237192	0.922964026
**sns**	FBgn0024189	0.491721925	0.716335326	1.065228976	0.883488231	0.667799049	0.727287203	0.686655602	0.437476733	2R	-0.289826972	0.295749448	0.906715248
**sip1**	FBgn0024191	32.93469416	46.90660859	49.39217626	35.60078163	39.99737946	30.54252729	49.87561308	47.12781416	2L	0.0614411	0.756901048	0.995787491
**rasp**	FBgn0024194	8.755073872	10.16682107	8.169110422	8.092380974	8.416944983	9.475270135	14.008547	6.973376929	3L	0.169022623	0.624721741	0.990047511
**robl**	FBgn0024196	547.131993	532.0664133	589.1446514	507.1732804	585.9639948	529.5053751	621.5271389	583.3464165	2R	0.124358303	0.263480713	0.892790059
**IKKbeta**	FBgn0024222	0	0.519905502	0.410152223	0.065695244	0.915623423	0.876670152	0.071881915	0	3R	0.966286471	0.547169087	NA
**gprs**	FBgn0024232	312.2183677	341.2299719	324.2818762	338.9083197	298.8748538	286.2920734	277.2907341	261.2653283	2R	-0.198151905	0.017027364	0.375267338
**gbb**	FBgn0024234	9.355798639	14.57210832	13.96740739	17.13854397	13.92001188	25.44400907	21.7686593	31.47291199	2R	0.787250973	0.010549123	0.311527659
**foi**	FBgn0024236	56.82246723	56.89830214	45.39035658	44.82141189	47.79369454	39.00145396	39.80663455	44.92713895	3L	-0.217920777	0.091945898	0.722444454
**drm**	FBgn0024244	0.217111993	0.364174911	0.503810203	0.022008193	0.204491892	0.587376645	0.144484528	0.061483074	2L	-0.10548724	0.916053039	0.996479182
**dnt**	FBgn0024245	7.286965368	8.028278766	6.220697726	6.759531765	8.538548211	9.576915074	5.299633043	6.309892217	2L	0.098921578	0.576764379	0.985637351
**chico**	FBgn0024248	5.482407576	7.541331474	5.844744039	4.643989003	5.239668073	7.350756866	5.904014603	5.601324399	2L	0.067862229	0.796521802	0.995787491
**WASp**	FBgn0024273	91.22682482	110.4845274	90.9800884	107.2055322	90.37237828	104.0504578	91.58379321	99.48119507	3R	-0.02253038	0.829075247	0.995787491
**trio**	FBgn0024277	65.00432864	62.90058043	62.59249704	65.51224016	66.32167047	69.70963096	63.00056428	60.99630614	3L	0.051117916	0.531721477	0.984760433
**Srp54**	FBgn0024285	553.7395207	648.7302103	599.6669713	733.9402396	643.433243	582.5151766	627.569622	715.7245526	2L	0.050998752	0.664500129	0.995787491
**Sox100B**	FBgn0024288	0.29104952	0.144057464	0.659781612	0.122365262	0.404831016	0.103462186	0.048686792	0.072512593	3R	-0.904851767	0.275509489	0.89549671
**Sirt1**	FBgn0024291	91.39778265	89.35314058	97.45146189	64.12468737	76.7525551	84.10055327	85.07180425	88.29728155	2L	-0.00267246	0.984341599	0.997881056
**Spn43Aa**	FBgn0024294	1.301714399	3.912002565	1.447389614	1.770357236	0.783309372	0	1.106900466	4.710237431	2R	-0.303562397	0.777800695	0.995787491
**Plap**	FBgn0024314	33.20411296	44.91111379	41.65838932	46.54967858	47.11122593	36.57322539	54.94713887	32.15743855	2L	0.071092671	0.709228338	0.995787491
**Picot**	FBgn0024315	47.37636557	47.44977991	42.61555317	40.62813631	28.48350877	39.27130249	38.89069965	28.05923177	2R	-0.374966934	0.004477231	0.185638045
**Nach**	FBgn0024319	0.282297379	0.078919042	1.483840589	0.877554281	0.594555641	0	0.600122231	0.408595827	2R	-0.728734027	0.613956573	NA
**Npc1a**	FBgn0024320	54.90267785	71.74231202	72.0883341	47.18646942	60.76055105	77.52705319	61.38911621	68.93363138	2L	0.160425895	0.2523076	0.883301842
**NK7.1**	FBgn0024321	10.57809757	10.18144276	12.15304018	9.392784173	9.988051338	10.80388995	9.795178022	9.558740314	3R	-0.045112139	0.675681032	0.995787491
**Mkk4**	FBgn0024326	252.3243445	230.8803189	217.0876407	215.9227623	215.614883	239.9384938	226.7471781	228.5631508	3R	0.020396893	0.827439976	0.995787491
**Mekk1**	FBgn0024329	13.74263546	13.38196961	14.76632736	21.58493398	15.98270777	12.65280544	13.54695829	12.12109183	3R	-0.196192852	0.284703895	0.901780531
**MED6**	FBgn0024330	94.03931444	84.92057691	84.68006786	72.8322877	91.50156946	102.0443733	81.71140741	84.69853883	3R	0.125502846	0.312474641	0.914141092
**Stip1**	FBgn0024352	200.0743749	203.5432985	185.7528951	162.1999473	235.5937585	232.671553	199.8845452	128.980551	2L	0.112779004	0.484811531	0.973549494
**MTPAP**	FBgn0024360	21.41797869	31.64956227	24.36343835	33.23010436	42.04681582	20.06528306	26.65374984	35.05351345	X	0.199598888	0.379722221	0.944208584
**Naa30A**	FBgn0024362	238.5578334	220.9890768	223.2286321	268.6564573	233.906536	245.1427304	226.6881983	245.2354884	X	0.027773353	0.79482468	0.995787491
**CG11417**	FBgn0024364	15.9216946	24.10667017	17.51834259	27.67637222	24.73656117	36.73015336	22.10548653	19.96042126	X	0.310154937	0.21322593	0.85701028
**CG12773**	FBgn0024365	60.26308417	47.1742284	53.95609909	50.74166153	61.53541737	52.2809079	52.1229981	42.24576609	X	0.000499987	0.99727243	0.999490563
**CG11409**	FBgn0024366	0.516738229	0.650066916	0.06964443	0.033465437	0.401641572	0.378915947	0.219701721	0.077908787	X	-0.212916285	0.816828163	0.995787491
**E2f2**	FBgn0024371	67.07976515	78.26490694	81.45284703	87.32406516	68.06088545	93.93257613	84.57129692	106.8918959	2L	0.201786452	0.200408211	0.848203654
**Dlc90F**	FBgn0024432	345.1976219	320.3509822	354.2638246	321.6287586	384.8766573	329.5584171	342.9702234	340.8086514	3R	0.090295843	0.431955214	0.964264046
**Bin1**	FBgn0024491	13.24181514	24.12972856	25.0953066	15.82156596	21.1060274	20.70333454	28.07448574	18.43039338	3R	0.212120092	0.365545337	0.940794966
**Sec13**	FBgn0024509	214.3349842	208.6810017	231.8662913	248.7365386	223.829219	203.9222806	208.0189866	189.0578605	3R	-0.101846792	0.357355296	0.937922644
**Neos**	FBgn0024542	128.2936027	163.4632196	99.57406588	114.4055494	141.3625879	148.8960862	109.678196	124.5825848	3L	0.083150063	0.609822645	0.990047511
**flfl**	FBgn0024555	80.77949571	64.16099908	79.62133442	80.18014997	65.59192137	77.80247501	86.27019148	82.44412223	3R	0.062881246	0.615783868	0.990047511
**Dph5**	FBgn0024558	164.5529579	148.931308	140.4408727	169.3859379	137.9488295	159.7787002	159.8627359	181.9290841	3R	0.065099518	0.643453189	0.992589784
**fws**	FBgn0024689	75.40173529	92.77889713	80.33365851	76.81330656	67.87839733	100.0034281	105.9128747	102.13336	2L	0.239948566	0.117175478	0.7701423
**Cpsf160**	FBgn0024698	25.36062623	16.37203214	19.64131126	15.06135938	16.34723037	32.55355778	18.27881824	13.627263	2R	0.09872143	0.684957129	0.995787491

**Slip1**	FBgn0024728	176.2652947	145.3827483	181.4814551	155.2571717	129.20407	198.7401847	128.8815528	174.6944185	4	-0.034048786	0.809873396	0.995787491
**Drep1**	FBgn0024732	43.2156499	37.91649744	31.48240436	31.34539846	37.96708277	44.67973222	32.82681558	40.99355218	2R	0.147134653	0.326008117	0.918470465
**RpL10**	FBgn0024733	575.1512269	597.4022336	672.136102	586.9836898	592.3617016	553.9919457	603.8999758	594.6732061	3L	-0.020220571	0.828561188	0.995787491
**PRL-1**	FBgn0024734	15.04836567	20.56350916	22.26131304	17.13917524	16.97718754	18.76636043	18.13373506	17.98923748	2L	-0.027188393	0.844216749	0.995787491
**Lip2**	FBgn0024740	0.242111428	0	0.111877895	0.591353379	0.333008137	0.250000335	0.499987368	0.27533855	2L	0.539455368	0.612632184	NA
**Flo1**	FBgn0024754	384.1282812	427.8007794	355.7466372	475.4428317	389.2982576	399.5577629	346.8579309	379.0536511	2R	-0.088286169	0.425017969	0.9620897
**Dbp80**	FBgn0024804	4.411470895	4.0034252	4.427975567	3.397289829	4.545133399	5.175016348	3.715673455	3.993017116	3L	0.130124528	0.298800704	0.9074568
**DIP2**	FBgn0024806	120.4582282	113.598878	130.3514913	113.5871188	104.5490498	113.7681361	125.6282595	99.88281028	3L	-0.07729354	0.460875202	0.971606621
**DIP1**	FBgn0024807	403.6358457	394.1451546	290.1522994	350.5775859	349.8123434	407.0950896	345.4436102	316.5894296	X	0.007503036	0.949331164	0.997604378
**Crk**	FBgn0024811	410.5310187	253.3805451	315.0233585	231.5422942	189.9705341	294.526033	202.9331792	214.5286127	4	-0.401217392	0.019375928	0.395797586
**AP-1mu**	FBgn0024833	409.6808916	359.4984785	381.4413415	379.8344623	325.1560194	396.1991688	336.5054497	335.7628322	3R	-0.107734552	0.285853046	0.90204441
**stan**	FBgn0024836	0.686885425	1.085274291	0.980478904	0.583112813	0.742693832	0.635630592	0.522441479	0.73974371	2R	-0.298279463	0.262419984	0.89192828
**Pcd**	FBgn0024841	490.7824022	448.9910674	494.3995994	508.0025931	423.9444597	439.2755488	479.3464749	422.4631006	3R	-0.10898218	0.295102282	0.906715248
**p38b**	FBgn0024846	166.9271498	157.9895981	152.6264201	154.6650611	130.6593248	143.4841141	143.5927638	174.0645329	2L	-0.065277492	0.603498205	0.989286463
**kin17**	FBgn0024887	70.76096839	79.89040873	79.42097226	75.39891392	76.95282159	81.66315291	78.00470669	90.07942749	3L	0.129164164	0.345914062	0.930285749
**Kap-alpha1**	FBgn0024889	316.7612281	337.4802067	320.1135328	331.815927	308.2417944	305.1147934	346.4530283	281.8467494	3L	-0.04255625	0.657736964	0.995750687
**Taf7**	FBgn0024909	11.71595199	29.02148613	21.6470374	13.78450174	16.6321214	14.63644449	17.95109342	18.82137305	3R	-0.120644039	0.672136219	0.995787491
**agt**	FBgn0024912	5.689761113	3.368391555	4.709354728	1.872771366	2.29597723	3.723435875	5.976642345	3.778564116	3R	0.026627897	0.964743876	0.997604378
**Actbeta**	FBgn0024913	42.92041402	43.03229634	49.9368894	40.49764593	45.18232604	58.20892655	42.5649937	41.03732053	4	0.112962989	0.378032815	0.943615128
**Tnpo**	FBgn0024921	160.4538711	177.6093434	182.0152609	174.5431953	155.3717254	197.4817274	172.9319175	179.7223835	3L	0.0522794	0.583097655	0.985764834
**RSG7**	FBgn0024941	343.6364426	353.5930472	336.3832884	357.1819753	301.8454512	318.5384773	290.2548308	294.6714944	X	-0.176988887	0.024592593	0.440903252
**Oamb**	FBgn0024944	82.83956323	94.4023747	82.65126549	95.91286064	93.65936677	112.4286171	83.62590472	109.0709944	3R	0.193621537	0.079605866	0.692197295
**NTPase**	FBgn0024947	60.27593036	61.97268808	61.33351204	72.09035517	63.41630286	64.98457271	64.36582653	58.68991537	2L	0.00541224	0.96147061	0.997604378
**Mat1**	FBgn0024956	54.74387472	64.7331486	56.00123252	64.48313387	71.73323614	59.56289371	61.98688275	74.16739082	2R	0.188843247	0.274795685	0.89549671
**Irp-1B**	FBgn0024957	50.1656365	68.32148236	52.34829463	63.27773285	77.13572709	91.97910519	57.54134584	57.50955029	3R	0.307896226	0.057950485	0.623173342
**Irp-1A**	FBgn0024958	183.3644894	194.5204366	182.7984976	225.3129682	199.7797946	241.2899954	200.7420069	226.5654482	3R	0.172323246	0.124491078	0.780439453
**GluClalpha**	FBgn0024963	58.73914097	59.97828908	46.99659972	58.74777534	56.79567683	57.91413733	43.92893115	45.3538431	3R	-0.110590589	0.351825634	0.934121932
**CG2701**	FBgn0024973	271.9664738	260.0522711	202.7107836	264.7317983	198.3531808	303.8756073	202.9689713	256.9425761	X	-0.029506836	0.838966265	0.995787491
**CG2712**	FBgn0024975	17.5208143	32.7710402	22.50278	22.98415901	44.734391	21.96835981	16.00367345	22.27877731	X	0.171976888	0.577233794	0.985637351
**Syx4**	FBgn0024980	0.277900928	0.815744688	0.589910804	0.566926109	0.1881309	0.540381802	0.576006632	0.30167432	X	-0.446714271	0.581004535	0.985637351
**CG4293**	FBgn0024983	41.57675791	25.5339369	26.46802473	38.75667136	37.72797093	42.36058868	35.35440089	49.93493071	X	0.3450076	0.108032164	0.758410631
**Rilpl**	FBgn0024985	170.384945	227.5858774	196.6424703	226.4656317	212.6285336	244.2267863	206.6226587	212.9079282	X	0.124483187	0.26306316	0.892691887
**CG3719**	FBgn0024986	189.0938754	174.5301287	156.1188508	156.6198497	189.2103433	191.8956095	200.0460615	184.5191217	X	0.206927308	0.072058337	0.671738456
**ssx**	FBgn0024987	239.7975293	283.1243653	304.8906936	308.728315	252.4223664	279.0899799	274.2573995	299.7560809	X	-0.008366932	0.938409853	0.997038394
**CG3777**	FBgn0024989	0.250797875	0.231886061	0.284924322	0.162582297	0.225272943	0.250291722	0.149804668	0.268268516	X	-0.024304927	0.943752927	0.997604378
**CG2694**	FBgn0024991	2.592490149	2.438432824	1.116799569	0.564887214	1.530876831	5.665024314	1.112551077	3.103585325	X	0.779869944	0.214575099	0.858753183
**Spg7**	FBgn0024992	61.21567033	44.92993918	66.96357059	74.66599022	63.42347178	67.01553319	66.88545337	82.08686423	X	0.201923463	0.228097745	0.867702781
**CG2662**	FBgn0024993	13.92874876	11.96212329	21.89653574	20.52379134	22.52988544	24.51295328	18.38216599	10.3221258	X	0.17812452	0.552231882	0.984934163
**Ugalt**	FBgn0024994	24.70271082	23.32734627	33.19942692	31.90587761	27.05489699	37.68838326	41.5524369	37.77669125	X	0.379547853	0.05427479	0.610426591
**CG2680**	FBgn0024995	59.94036905	53.62975639	49.7340643	47.63801008	74.26260884	92.60703939	68.05583635	36.84487254	X	0.389225075	0.102815051	0.743157534
**eIF2Bbeta**	FBgn0024996	195.4435992	290.5799115	200.1768774	292.982906	203.0125767	231.4450841	205.6612324	210.7920676	X	-0.171748439	0.245609519	0.877269208
**CG2681**	FBgn0024997	6.124815627	0.827724279	1.971772909	0	1.04804379	0.903111276	2.665797654	1.386473542	X	-0.563465441	0.541788127	0.984934163
**CG2685**	FBgn0024998	95.97175856	82.13760656	75.03447101	101.7066101	66.33584609	79.62527932	84.21533787	89.93251626	X	-0.121760945	0.435447164	0.966084844
**Bem46**	FBgn0025109	81.62188946	74.53619475	60.49818574	91.33496709	83.33248154	67.94841207	62.37552225	61.72544948	2L	-0.13395892	0.435407638	0.966084844
**Ant2**	FBgn0025111	0.740606149	1.205725774	1.056882463	2.107584038	2.14310657	1.447819007	0.916867243	1.466048815	X	0.264832131	0.564563384	0.984934163
**az2**	FBgn0025185	61.65492426	54.57765615	52.75595025	43.57957458	71.16330663	58.89575767	50.07293497	54.10926771	2R	0.169073858	0.317735874	0.91460807
**ari-2**	FBgn0025186	113.5874398	109.4349272	81.98525292	125.299356	94.61878476	130.5246014	126.6719686	98.55012611	2R	0.090906526	0.545837655	0.984934163
**PHDP**	FBgn0025334	4.583327415	1.114186777	1.128023459	0.154867469	6.835110128	3.820132153	1.863968605	2.163223498	2R	1.062862846	0.231080691	0.867702781
**Cpes**	FBgn0025335	57.03434565	60.53748156	57.79565656	74.69392784	75.10292125	67.71739281	63.67710752	63.99778909	2R	0.143732455	0.322540907	0.918365535
**CG4882**	FBgn0025336	67.69359577	66.03462917	69.01578979	54.84157987	62.91779399	66.49160981	59.27307732	60.42754671	2R	-0.018200647	0.892680929	0.996438268
**Mtpbeta**	FBgn0025352	69.60473857	76.17504073	69.11334203	60.84239815	55.90860629	65.20520803	72.78847242	74.93391445	2R	-0.006144835	0.967489876	0.997604378
**Optix**	FBgn0025360	0.12323421	0.180869587	0.093426399	0.448931129	0.389321335	0.689845236	0.334021438	0.167220549	2R	0.934882501	0.228013176	0.867702781
**Fpps**	FBgn0025373	32.1221853	29.53340697	35.94271504	23.32855364	39.69285292	32.10653459	25.41590545	25.17361525	2R	0.049163553	0.822008088	0.995787491
**CG3795**	FBgn0025378	0.314746532	2.745304396	1.068998976	3.375570578	1.477315214	0	0.321170066	1.366687714	X	-1.201957312	0.325217507	NA
**Rab27**	FBgn0025382	1586.459421	1691.758125	1411.166421	1870.135445	1658.413097	1474.53526	1358.183932	1385.347557	X	-0.129355979	0.244191009	0.877269208
**CG14780**	FBgn0025383	0.071882362	1.326298351	4.010868949	7.709186914	5.839473864	0.203310754	1.100240692	7.178910004	X	0.175401288	0.87065152	0.995787491
**CG12179**	FBgn0025388	3.498313637	2.493870403	3.309875434	3.180912754	4.70493778	5.870775937	2.677281974	5.620420428	X	0.625399254	0.062244579	0.633840737
**Mur2B**	FBgn0025390	0.229019419	0.452174147	0.274905219	0.368481226	0.262705872	0.481879739	0.279945199	0.24472663	X	-0.029896046	0.9147235	0.996479182
**Scgdelta**	FBgn0025391	0.865226405	2.317262539	1.31044239	1.57144328	1.544696241	0.901359304	1.573095238	1.764324448	X	-0.026958064	0.937919447	0.997038394
**inc**	FBgn0025394	14.40507911	12.81544577	12.92104062	16.29846725	12.58787294	11.10021764	11.79394891	12.97376436	X	-0.190750179	0.128985243	0.788096052
**Cyp6g1**	FBgn0025454	1.034154163	0.946171926	1.140383443	0.146126758	0.226292523	1.034084787	0.479663543	1.90505587	2R	0.189558802	0.840138764	0.995787491
**CycT**	FBgn0025455	200.943094	234.34002	201.1518948	240.2587973	197.1658334	235.2269562	190.1849451	234.5435963	3L	-0.002867573	0.979080516	0.997757033
**CREG**	FBgn0025456	1.392983483	6.266160372	3.840180113	4.133421791	2.514692205	2.447506776	8.076206344	0.274936008	3R	-0.196593553	0.807275981	0.995787491
**Bub3**	FBgn0025457	190.2048463	193.8911927	218.6054304	248.22079	236.9850849	207.3920666	212.2139162	246.5744338	3R	0.117484482	0.379745045	0.944208584
**Bap60**	FBgn0025463	380.146193	363.4009557	303.3354362	334.2945303	318.4210028	345.7720023	349.3881959	334.7273265	X	-0.005863799	0.955172886	0.997604378
**slv**	FBgn0025469	46.46675722	40.91797181	34.24639136	27.3545321	36.15803515	40.24785128	42.25027805	38.24302879	2R	0.10119778	0.576102598	0.985637351
**fidipidine**	FBgn0025519	46.62917978	23.89350878	34.01751601	31.39478628	31.34059377	31.05647957	34.35130493	36.80586589	2R	-0.002053119	0.992504281	0.99849477
**bab2**	FBgn0025525	1.006122863	0.579629915	0.370688323	0.295957545	0.23764799	0.66709933	0.161914271	0.07655558	3L	-0.96432823	0.087518911	0.706081898
**CG4101**	FBgn0025558	3.17351331	2.504999237	3.170134893	4.352309891	3.370002876	1.583983569	3.333523791	1.621174392	3L	-0.392551488	0.69169383	NA
**SF1**	FBgn0025571	92.97672486	107.9454886	121.6709441	126.7563528	100.6617792	123.7645076	106.3922895	103.27776	3R	-0.020295444	0.867470976	0.995787491
**Pli**	FBgn0025574	75.99164455	57.70767329	55.27195262	58.05728031	62.46355014	64.60650977	55.28191073	49.57159692	3R	-0.065110567	0.602929226	0.989286463
**eIF3e**	FBgn0025582	553.1535993	628.9626983	583.5287263	664.0841017	581.6636866	565.9222851	708.2845323	744.6982412	3L	0.131164714	0.286940075	0.90249226
**Gk1**	FBgn0025592	60.32755016	55.07521013	52.33732884	70.13702077	71.21303676	81.11815056	69.19165818	50.90470279	3L	0.220723707	0.159010695	0.819032836
**AkhR**	FBgn0025595	0.388898246	0.622671239	0.085769247	0.08242742	0.191471184	0.449980639	0.315664294	0.038378822	2L	-0.224205753	0.848745578	NA
**Faf2**	FBgn0025608	185.4626664	148.2738794	122.0946693	126.311249	159.2292382	140.7301907	144.064563	129.3501026	2L	0.005864237	0.967951077	0.997604378
**CG3081**	FBgn0025613	0.622889245	0	0.266668345	0	0.330727863	0.604528416	0.140206222	0.079550012	X	0.366330547	0.805031917	NA
**Torsin**	FBgn0025615	130.2882778	117.3418184	105.2132547	93.26032916	144.8577014	119.8293348	137.182441	117.5351434	X	0.248052374	0.097905858	0.730787365
**CG13359**	FBgn0025616	1.875686608	4.03359122	0.350029965	0	1.562813105	1.314953601	0.490760701	0	X	-0.853524045	0.510937369	NA
**CG13360**	FBgn0025620	0.232636291	1.365751598	6.678406697	4.067870659	0.279978753	1.09664037	0	4.293136841	X	-1.074614423	0.309691351	0.912131082
**CG16989**	FBgn0025621	38.04209613	45.50983284	50.94986927	42.2056189	59.24730249	69.04020771	60.01170182	56.69554184	X	0.504268244	8.53E-05	0.008236301
**CG4025**	FBgn0025624	128.9093568	112.407004	83.26356408	101.1770285	95.98420013	108.0981023	87.70337024	106.594666	X	-0.069560593	0.667116013	0.995787491
**Sik2**	FBgn0025625	39.87980091	53.69459227	38.68641051	62.86198333	38.35221442	54.20306773	40.04468004	46.63680603	X	-0.094673746	0.574144352	0.984934163
**CG4281**	FBgn0025626	106.3362203	98.50340584	98.27081994	96.1551255	85.06559116	111.2801182	90.52613696	81.3647313	X	-0.089515104	0.466627445	0.971928234
**CG4199**	FBgn0025628	12.99027889	10.10103268	13.50610089	14.81366225	16.17371869	9.72125217	12.79139079	14.91535645	X	0.091418872	0.661320187	0.995750687
**CG4045**	FBgn0025629	74.07623723	68.89768037	69.60116201	65.06171557	86.74539893	78.26360085	80.18730066	76.07342495	X	0.238888829	0.121398848	0.77836519
**Rtca**	FBgn0025630	156.9759002	153.9709866	145.4908901	184.6630441	169.3725079	185.0196262	177.7889609	185.5508376	X	0.191565469	0.094898348	0.730787365
**CG4313**	FBgn0025632	2.864681386	2.777949102	2.528565546	2.787843087	2.989764253	2.616403984	2.903570869	2.929268006	X	0.088982759	0.671505225	0.995787491
**CG13366**	FBgn0025633	7.656861493	6.301663228	7.55824088	7.223490823	6.363560481	8.018868578	5.550240215	6.898001468	X	-0.071706527	0.604106246	0.989286463
**CG13367**	FBgn0025634	64.97701012	70.36504318	84.05386231	85.25277831	75.05670406	72.01433731	69.34901713	76.61121471	X	-0.023058534	0.879176815	0.995787491
**CG17829**	FBgn0025635	101.1479837	83.80305855	76.10373952	80.8931505	98.34632808	101.0061633	86.27817113	104.3658864	X	0.216842749	0.110837741	0.758410631
**SkpA**	FBgn0025637	791.3074127	656.0431615	702.3324125	619.9834961	692.7502518	716.8972584	684.3106727	639.6018874	X	0.009595784	0.925292101	0.996479182
**Roc1a**	FBgn0025638	353.7079172	330.1329389	342.7486381	376.4058014	331.9648309	381.4736609	315.1272005	300.7736137	X	-0.050230684	0.645446899	0.99279236
**Hmt4-20**	FBgn0025639	82.13245306	118.214066	95.85480192	108.3827774	102.4159497	101.6084316	112.7360532	107.1795967	X	0.10090068	0.397629814	0.952272708
**CG13369**	FBgn0025640	75.73252935	117.5089927	74.85135773	127.0538921	99.46315351	127.9410383	105.9677976	105.2650907	X	0.180732262	0.346968568	0.930384827

**DAAM**	FBgn0025641	16.50970451	18.97949895	16.97862789	17.52942832	16.51374695	18.45918921	15.26160334	14.80304174	X	-0.076090467	0.44745368	0.967112302
**CG32812**	FBgn0025642	0.261686543	0.702307943	2.031513289	0	0.944822559	0.394746359	0.133513599	0	X	-0.954949504	0.550988028	NA
**CycK**	FBgn0025674	77.80409601	84.29149216	88.61748332	86.06115052	86.68726812	84.75846108	79.90505067	83.25589943	2L	0.022162543	0.816265271	0.995787491
**CkIIalpha-i3**	FBgn0025676	96.85180707	65.40220839	80.03134448	72.68487696	55.89030493	77.83646479	63.88818705	66.18529312	3L	-0.231015825	0.165864159	0.828893393
**CaBP1**	FBgn0025678	48.01714537	54.21853405	58.75587725	65.42258528	33.40476621	39.48450985	47.20453066	49.32789145	2L	-0.387347673	0.038064125	0.522230403
**cry**	FBgn0025680	7.639687116	18.24962035	21.09015706	6.20336529	8.084727095	8.916384397	15.99442559	10.98138566	3R	-0.22999486	0.531590301	0.984760433
**CG3558**	FBgn0025681	123.5522893	131.9667477	104.1702366	185.6700156	128.143899	138.8245709	119.6516449	141.3633064	2L	-0.019621621	0.899338338	0.996479182
**scf**	FBgn0025682	101.5506864	149.8593545	111.8596828	116.322516	144.0736093	142.5894773	173.0913992	134.6075166	3L	0.343064595	0.018578689	0.392424388
**MFS18**	FBgn0025684	198.6709583	179.5976094	180.9403754	205.2358862	164.4053759	163.3263058	163.0530575	176.9546912	2L	-0.166223902	0.136363998	0.796109055
**ZnT41F**	FBgn0025693	0.760937323	0.790987138	1.690021359	0.649669222	0.889994326	0.353651367	0.08202116	1.116889262	2R	-0.62501076	0.366142875	0.940794966
**santa-maria**	FBgn0025697	3.147614972	10.63337934	12.06070872	5.349594144	8.576482783	4.146252797	9.680586082	3.608482582	2L	-0.215915738	0.588110445	0.987588562
**CG5885**	FBgn0025700	174.7204829	156.0327658	194.597197	171.3546397	173.0060413	167.0081432	191.6756856	181.9516088	2L	0.064901674	0.618757775	0.990047511
**Srpk79D**	FBgn0025702	47.32620088	51.53231184	40.13876468	52.85747901	49.62988584	50.02361251	39.70965317	45.53322812	3L	-0.024310028	0.843504485	0.995787491
**CG13920**	FBgn0025712	253.7233711	251.1465015	252.5396156	253.4409453	296.3472721	233.8434125	241.2727006	259.6896935	3L	0.06031302	0.581657256	0.985637351
**Bap55**	FBgn0025716	171.2996858	172.9040716	150.0961827	165.4461671	221.1492173	206.3389479	173.6153615	163.8775714	2R	0.242271218	0.057440167	0.623014047
**Ate1**	FBgn0025720	155.3696914	187.8460071	170.604374	185.5348051	209.4584692	204.9327389	200.45464	222.4245915	2R	0.292000785	0.005905371	0.22169083
**beta'COP**	FBgn0025724	190.7094822	200.6921889	150.4757363	159.7587884	168.275477	161.0265964	161.850517	125.4248588	2L	-0.156915915	0.22246783	0.86549481
**alphaCOP**	FBgn0025725	160.5989878	201.7818403	205.7621466	179.0308405	188.3379067	187.9062628	198.0825903	137.5954604	3L	-0.038315237	0.762006876	0.995787491
**unc-13**	FBgn0025726	55.96604781	72.67598995	100.9573628	65.4387448	63.34548377	83.49799132	76.72485667	71.4163253	4	0.03145368	0.830680535	0.995787491
**PlexB**	FBgn0025740	23.45591431	25.16216313	35.87165938	23.76900425	21.72548403	27.36076964	26.39847412	29.34010022	4	-0.013404048	0.931193975	0.996479182
**PlexA**	FBgn0025741	18.17845432	21.43968369	45.46851816	23.96699636	23.51974482	23.73101038	28.56132584	30.84358244	4	0.004175516	0.989435011	0.998078482
**mtm**	FBgn0025742	129.7377488	160.6204633	135.4282895	133.6883251	123.848491	106.3694746	130.411921	122.5164927	2L	-0.17859366	0.147890394	0.808004944
**mbt**	FBgn0025743	70.69012423	87.22274015	77.03090115	82.9382684	90.27438633	80.605898	73.84924904	83.77091209	X	0.080070658	0.493540337	0.973549494
**homer**	FBgn0025777	46.88089663	51.37364892	63.64986673	62.90158032	44.20899814	51.8607362	52.4966041	57.81602615	2L	-0.091498235	0.536989726	0.984934163
**Cdc16**	FBgn0025781	21.78795707	19.78966543	16.03697065	22.66895096	22.58294056	24.00706651	18.45156104	14.80250951	3R	0.017997046	0.933565453	0.996479182
**TBPH**	FBgn0025790	774.4545695	734.9792798	646.1968442	693.9858103	676.0726587	788.7233898	699.9940761	679.4237966	2R	0.025354062	0.785066547	0.995787491
**Smox**	FBgn0025800	744.3083689	693.1923032	626.071294	722.8151414	627.621924	716.7274658	597.3395339	615.329461	X	-0.096647434	0.309627551	0.912131082
**Sbf**	FBgn0025802	82.9181586	85.48477605	76.97159697	95.13017991	64.09969991	82.911303	82.20879632	89.91323315	3R	-0.064244439	0.610673657	0.990047511
**Rad9**	FBgn0025807	10.91362266	16.45229164	15.16522037	10.7898413	9.788595277	13.80589188	13.47992259	8.023132596	3L	-0.207803149	0.451156325	0.967112302
**Rad17**	FBgn0025808	38.87320574	39.18012166	38.67891734	60.3543276	30.79392237	42.1448137	34.00302659	32.84703068	3R	-0.313659622	0.124077486	0.780439453
**Paf-AHalpha**	FBgn0025809	138.5121501	101.9060427	100.3947021	77.75094691	99.84115256	128.461224	104.1652059	101.1967131	X	0.076060236	0.664517135	0.995787491
**Mgstl**	FBgn0025814	4.588756707	3.445321857	3.816774777	4.585077221	2.997976323	2.472143882	2.118231638	3.510635969	X	-0.539650883	0.252568789	0.883301842
**Mcm6**	FBgn0025815	0.248456782	0.937690643	1.280849462	1.375760631	0	2.54739846	1.426091825	0.202283502	X	0.148067177	0.892696109	0.996438268
**JTBR**	FBgn0025820	72.82878437	60.48037073	73.84448989	58.82187877	60.11322112	63.55858599	57.32582692	52.33640348	3L	-0.160160865	0.401674771	0.954265
**IntS8**	FBgn0025830	52.17881228	50.16009926	44.06696218	39.04569126	30.36108836	40.01873078	31.03271826	51.07826685	2R	-0.251672754	0.171240822	0.832652344
**Fen1**	FBgn0025832	7.825136855	8.481137097	7.447440893	14.17419393	6.845859908	12.82548475	8.599055116	13.8526479	2R	0.182222016	0.642894353	0.992589784
**CG8910**	FBgn0025833	81.81715787	90.27165971	90.35750035	90.21426681	72.71476849	83.09912579	75.49215356	75.20272287	2R	-0.172390686	0.036857955	0.521170991
**RhoGAP1A**	FBgn0025836	21.52185562	24.00648559	33.69392084	23.2479725	20.51186981	20.87348451	23.79174833	24.74155342	X	-0.154077812	0.308578018	0.912131082
**CG2652**	FBgn0025838	0.641616634	1.844951819	4.002564872	1.282204311	4.63312988	8.036691239	3.741206443	11.14407286	X	1.899873755	0.009406927	0.289289051
**ND-B14.5A**	FBgn0025839	357.8499928	351.8390319	388.2620461	347.9465191	400.0676969	329.1764177	394.1274271	425.721111	X	0.132040744	0.285793128	0.90204441
**Crag**	FBgn0025864	22.66532405	36.71094534	21.90609155	24.86745872	22.03688837	22.85425569	23.65352533	19.49168515	X	-0.236398614	0.141835822	0.8037297
**Cortactin**	FBgn0025865	56.45237866	55.38821674	60.45794647	49.7813182	47.34183905	49.09737391	54.31099743	55.72285982	3R	-0.074765657	0.582307568	0.985764834
**CalpB**	FBgn0025866	19.84407471	29.17276926	21.25786182	16.88432635	21.10300682	23.47569241	31.80887506	20.675043	3L	0.188563666	0.348885014	0.931729531
**Meics**	FBgn0025874	60.17510216	70.79686689	44.720438	38.82839595	50.72019202	66.24144204	46.809123	35.14553799	3L	-0.081177064	0.711598568	0.995787491
**wrapper**	FBgn0025878	4.134182231	16.21240783	20.88896183	11.83232319	10.8603279	15.96705491	16.51055177	14.54681169	2R	0.167862966	0.644234131	0.992589784
**Inos**	FBgn0025885	614.4372213	482.7802882	469.5595003	538.2873439	535.8932185	505.6420425	502.4829213	542.7554031	2R	0.015983355	0.886489986	0.995926915
**Eph**	FBgn0025936	98.2521064	83.19717557	96.01010702	81.08043872	74.44269446	102.186338	80.78552596	90.43803738	4	-0.016178831	0.889949442	0.995926915
**Top3beta**	FBgn0026015	63.0298397	77.54832529	69.60846853	72.08654492	66.20883325	67.24702	64.13262623	48.38102892	X	-0.167882778	0.205017608	0.854987719
**Rlip**	FBgn0026056	227.9902862	213.5085371	209.449955	211.2911018	208.2360904	251.8812009	215.7090768	221.1453021	3R	0.085046511	0.375116975	0.943338826
**OdsH**	FBgn0026058	0.613833052	0.442167597	0.401979255	0.210718329	0.319520989	1.00831857	0.134494591	0.065408019	X	-0.114392524	0.866816149	0.995787491
**Mhcl**	FBgn0026059	13.36885219	16.83629886	20.13228712	21.53177144	16.88411786	16.04743881	17.37729953	18.65138674	3R	-0.026983761	0.852654067	0.995787491
**Mipp2**	FBgn0026060	0.17794207	0.29847286	0.263807488	0.138288413	0.160615522	0.279604495	0.151311348	0.257552471	X	-0.006234974	0.994150759	NA
**Mipp1**	FBgn0026061	1.813673563	0.543771835	0.297580941	0.16342074	0.632685145	1.470366298	0.402323498	1.464730899	3L	0.495634001	0.476063173	0.973549494
**UBL3**	FBgn0026076	184.4284539	169.462762	181.0306399	159.6109817	111.1012841	76.61255722	94.82760692	79.77646667	X	-0.907151256	3.24E-11	2.46E-08
**Gasp**	FBgn0026077	0.128981504	0.444716112	1.842676498	0.70166955	1.215966624	0.695839241	0.036559399	0.093343595	3R	-0.568661092	0.512802731	0.980804634
**Nsun2**	FBgn0026079	32.2214498	31.67298492	26.5724972	23.57878233	41.97313745	48.65578079	25.45640289	38.88051602	X	0.470269341	0.023227534	0.43209435
**Tip60**	FBgn0026080	46.6455532	50.8812238	50.12888513	49.07759875	61.22042293	65.03875967	48.51855351	50.95085041	X	0.229671099	0.099056025	0.731438598
**tyf**	FBgn0026083	27.70637482	28.87688179	31.28736218	33.17614859	24.08810428	25.19769492	26.40494411	36.26261242	X	-0.080104645	0.587823432	0.987588562
**cib**	FBgn0026084	26.83243928	27.18963634	29.26690563	25.47156283	34.08105785	30.6488456	32.81793324	27.6985038	X	0.23437096	0.080416905	0.694684542
**dgt4**	FBgn0026085	0	0.473257185	1.711194265	3.453494102	0	1.995028166	0.17993893	1.071981764	X	-0.760061735	0.616791673	NA
**CG14818**	FBgn0026088	254.5773339	318.0984064	298.3633788	312.416298	328.0624151	256.4547491	318.1310742	305.3734269	X	0.063350303	0.643755949	0.992589784
**CG14817**	FBgn0026089	155.6643472	175.360213	187.743258	152.2880366	179.9194814	141.8227912	185.1940313	170.7149788	X	0.047571502	0.74444794	0.995787491
**CG14812**	FBgn0026090	211.3421151	125.282374	158.280725	122.2508338	153.9113328	126.6458139	156.8402258	140.4338571	X	-0.068228119	0.717034197	0.995787491
**TTLL4A**	FBgn0026147	74.68988554	74.97071163	68.78348373	79.24810435	59.07976794	72.89705175	62.26344437	70.21120054	2L	-0.142202479	0.181363634	0.837439137
**CG12253**	FBgn0026148	112.0906496	98.61492466	101.9189689	86.9709802	80.41789721	111.8813649	84.69042025	82.95442038	2L	-0.123522891	0.420592014	0.960593359
**BCL7-like**	FBgn0026149	87.36952635	95.10561899	73.33270641	81.19100867	70.6847787	90.9544198	94.81202535	77.81306229	X	0.016206055	0.911380979	0.996479182
**tna**	FBgn0026160	45.26484136	38.2446515	45.40915123	48.51996881	48.428014	49.44479858	42.56089775	51.85684495	3L	0.144325376	0.190196061	0.843401316
**siz**	FBgn0026179	20.95107533	20.92567453	24.13705413	25.03297916	21.79116402	23.2299521	23.01527509	24.31321708	3L	0.050653422	0.626213937	0.990047511
**Rok**	FBgn0026181	14.24463613	15.90362372	15.00513846	16.87664829	15.16777895	14.62405365	18.43298584	13.96179289	X	0.033978348	0.802437317	0.995787491
**promL**	FBgn0026189	234.1968386	250.5414409	226.3609645	252.7306416	231.5600723	245.3954982	212.5252543	240.1067483	3L	-0.022407184	0.79698697	0.995787491
**PH4alphaMP**	FBgn0026190	0.838241087	1.238647088	0.435763111	1.44247985	0.360295238	0.432777078	0.254567979	0	3R	-1.871699734	0.041232005	0.533583119
**par-6**	FBgn0026192	51.93855809	64.41808935	49.8396299	55.28735534	57.34333527	70.23380139	61.32014978	76.19692844	X	0.290776188	0.034133966	0.514749796
**myo**	FBgn0026199	12.14817265	10.6890543	12.28949188	15.91053393	5.591857259	8.837081737	8.354609076	6.514892657	4	-0.774262771	0.000600889	0.041047594
**mei-P26**	FBgn0026206	77.91453155	105.6522416	83.51670993	96.91976378	90.22946644	80.2902942	64.22691284	80.94920601	X	-0.173281637	0.162830791	0.824943352
**mbo**	FBgn0026207	36.40330168	24.63981706	27.28063952	37.31889389	33.28538046	34.79837276	35.92296582	23.97441989	3R	0.049579627	0.811694694	0.995787491
**gus**	FBgn0026238	82.40620316	61.22668243	61.94067164	57.83413606	56.9251214	66.55716328	59.96535099	59.15033111	2R	-0.092109861	0.464916088	0.971928234
**gukh**	FBgn0026239	2.146795706	3.056267325	2.516517187	2.634025062	1.717928518	1.834768711	1.495688773	2.4332599	3R	-0.433706442	0.026326206	0.453785402
**eIF1A**	FBgn0026250	97.05605582	106.7724387	107.3601641	108.2863051	99.72687073	100.7080968	108.7073642	119.4856067	3R	0.063424988	0.538452751	0.984934163
**msk**	FBgn0026252	118.4745115	114.9674039	129.6608654	129.994147	112.556099	95.5307661	118.3390363	104.8587605	3L	-0.161994741	0.15263497	0.813857865
**clumsy**	FBgn0026255	0.29279407	1.173233445	1.381166752	0.26547047	0.675392877	1.59492355	0.207478878	0.741630201	2L	0.101732541	0.897553625	0.996479182
**cav**	FBgn0026257	0.750096148	3.894369948	4.419326781	3.830750611	3.739938862	4.849275884	5.922773345	2.326468818	3R	0.429908187	0.443792108	0.967112302
**eIF5B**	FBgn0026259	17.38314741	17.54142787	18.76446105	18.78941697	13.72134121	15.62310945	15.77979618	16.74636679	3L	-0.197920066	0.058363338	0.625114381
**bonsai**	FBgn0026261	136.3961263	159.9777382	130.1380382	103.7324307	84.87742035	98.09990774	112.2936914	77.07222518	2R	-0.479940387	0.005587495	0.212630972
**bip2**	FBgn0026262	227.0843129	170.4713153	212.4657851	178.0312329	177.477043	242.001065	184.2595379	216.8837512	4	0.084852664	0.50611579	0.978557094
**bip1**	FBgn0026263	1.680947689	2.421499262	1.487254413	0.922133237	2.106328738	2.498370432	2.118842519	0.429352122	3L	0.164164161	0.776740409	0.995787491
**antdh**	FBgn0026268	0.252776981	0.847995851	2.657344869	0.982233361	2.129527109	0.079438865	1.504625524	4.756285959	X	0.908930001	0.440068857	NA
**aft**	FBgn0026309	26.94074307	29.10600105	37.14896021	31.11371241	32.22953267	63.17847394	49.46841512	66.10320168	2R	0.794658474	0.000258305	0.020710451
**X11L**	FBgn0026313	63.30482153	141.5333182	161.1485134	155.2585243	113.2491529	107.3267899	145.4156996	168.2703657	X	0.074003456	0.823450443	0.995787491
**Ugt35B1**	FBgn0026314	118.4229945	150.7914687	143.4654276	72.41364707	87.37955364	82.74075046	163.2833776	63.76433253	3R	-0.251954668	0.32236213	0.918365535
**Ubc10**	FBgn0026316	302.5563169	326.8296099	339.6264527	316.6806964	302.0819252	327.3890529	290.4870614	317.6981993	2R	-0.023450285	0.817264092	0.995787491
**Tsc1**	FBgn0026317	60.27609687	52.90867263	61.1019565	63.32045201	60.64795791	72.87126068	52.25489435	60.13300183	3R	0.077320437	0.553275069	0.984934163
**Traf4**	FBgn0026319	0.046095602	0.432985872	0.234837304	0.204193305	0.166428751	0.512812093	0.341013526	0.300233457	2L	0.572325266	0.408099606	0.956706394
**Tak1**	FBgn0026323	31.50714678	30.92646508	28.42176054	32.68768085	27.25174122	39.20193482	25.86907382	25.95676338	X	-0.037059308	0.796253625	0.995787491
**Taf10b**	FBgn0026324	437.7397924	539.8340574	604.0901464	615.956959	553.3026261	429.4985144	638.3872701	645.5812242	2L	0.080063656	0.622610322	0.990047511

**Mad1**	FBgn0026326	78.1822662	109.5802695	83.31258975	90.67490281	88.25686061	103.362756	93.87623703	99.43617347	2R	0.122077437	0.349025941	0.931729531
**5-Sep**	FBgn0026361	0.242221045	1.543908926	3.760799209	0.338837549	1.749084267	1.096149704	1.112239961	0.525884311	2R	-0.330268587	0.739039653	NA
**Sara**	FBgn0026369	96.70141208	91.7738871	73.34429684	85.4571846	80.50845791	94.89006127	92.86502261	88.69138389	2R	0.067469854	0.56712663	0.984934163
**SAK**	FBgn0026371	5.780715806	6.050516043	4.936425443	6.037929536	6.344889559	9.831842489	8.730064288	4.568370763	3L	0.395594044	0.190711203	0.843401316
**RpL23A**	FBgn0026372	499.9769149	681.315325	668.9493776	796.2659477	653.36237	685.3354033	787.2928981	831.3102721	3L	0.193006179	0.163384906	0.825738493
**RpII33**	FBgn0026373	134.6203075	147.8389023	126.8614795	150.2620392	185.2534749	153.466049	128.6112107	113.9205435	2L	0.084855984	0.615138585	0.990047511
**Rhp**	FBgn0026374	12.62229979	18.71739662	13.40246379	15.43925634	14.49748904	16.40161741	17.3598695	18.19002177	X	0.175909761	0.34200854	0.928133889
**RhoGAPp190**	FBgn0026375	80.61985949	74.1341593	84.47679453	75.90142946	72.35013078	70.26135751	79.50787043	92.67534349	X	0.030845468	0.792463425	0.995787491
**Rgl**	FBgn0026376	46.30162865	39.63599509	45.02885481	45.27875245	44.21864887	56.20487095	40.86618229	39.85918997	3L	0.064860996	0.585503942	0.98718792
**Rep**	FBgn0026378	84.91818194	103.2285848	104.7580031	110.684494	70.05108236	86.95242484	83.48530632	70.22119265	2R	-0.346412386	0.012158602	0.332228811
**Pten**	FBgn0026379	80.00742394	62.32416918	72.36984234	52.21594077	59.24137319	69.45684232	59.93617381	46.83514552	2L	-0.154294392	0.317676294	0.91460807
**Prosbeta3**	FBgn0026380	284.2368718	262.7313438	263.5314139	230.7019799	314.1064149	245.6155727	312.9329159	298.2534723	3R	0.200458726	0.137764303	0.798609078
**Or33a**	FBgn0026392	2.002734003	1.799627858	2.776345576	0	1.29123149	3.236854946	0.729859639	0.155290033	2L	-0.246871003	0.835709735	NA
**Or43b**	FBgn0026393	34.81377764	30.47064646	15.56113053	36.53086335	30.93773132	35.2227166	16.86279014	21.04869046	2R	-0.151340042	0.614302454	0.990047511
**Or22c**	FBgn0026396	0.871823668	0.086021288	0.373240952	2.032624063	0.92580304	0.024175001	0.32706484	0	2L	-1.381216612	0.286880896	NA
**Or22b**	FBgn0026397	1.332588395	2.421499262	4.175752775	0.12945332	1.00235983	1.675141598	1.274798109	0.120548865	2L	-0.937451017	0.364279358	NA
**Noa36**	FBgn0026400	102.687375	131.3402285	127.7222033	152.4873735	115.399428	115.8832699	95.46982747	113.6906253	3R	-0.190653335	0.220354224	0.865425118
**Nipped-B**	FBgn0026401	24.48597722	21.14864639	26.58685898	17.68288325	15.60540992	26.25597743	20.08181092	20.55047456	2R	-0.096946782	0.522970069	0.983400848
**NiPp1**	FBgn0026402	128.3435544	139.4270571	129.9761017	126.6944786	114.95917	149.8874137	122.8821784	141.3386037	2R	0.042925116	0.729819081	0.995787491
**Ndg**	FBgn0026403	0.284754978	0.229973143	1.049010214	0.122943613	0.475977516	1.431814686	0.21523439	0.755613731	2R	0.816458845	0.299965927	0.9074568
**Dronc**	FBgn0026404	23.27672237	25.85619902	19.32272697	28.41508067	21.32011015	27.12394853	37.57193317	30.560069	3L	0.295073574	0.180204891	0.836097072
**Mpcp2**	FBgn0026409	3128.253644	3505.289427	3421.579669	3502.010183	3583.296277	3461.581045	3523.243454	3729.781884	3L	0.108605485	0.193207121	0.844272804
**Lim1**	FBgn0026411	1.079541314	0.91423701	1.211372013	0.787046754	1.840932049	1.886383193	1.332114212	0.908502641	X	0.607865875	0.013974201	0.35398477
**Idgf4**	FBgn0026415	21.25422498	29.39984987	35.88181685	9.842803679	12.61457211	17.26592376	10.32407925	15.29734707	X	-0.758176829	0.015433264	0.364946783
**Hsc70Cb**	FBgn0026418	205.0930892	127.3983923	127.1776415	118.0120308	166.9459088	157.3978574	122.8945914	126.9034013	3L	0.016342098	0.92191287	0.996479182
**Su(var)2-HP2**	FBgn0026427	16.54310529	22.13008264	29.89876532	25.12367312	21.06491546	21.56282631	24.54718869	21.92045433	2R	-0.038565047	0.814827215	0.995787491
**HDAC6**	FBgn0026428	73.45964407	99.37420803	115.0226435	98.71063526	91.22583446	100.6831383	108.3512091	93.97476544	X	0.061235388	0.638329599	0.991585536
**Grip84**	FBgn0026430	30.30886347	39.16152763	37.42805806	43.24022969	42.69074732	49.65810862	32.79758491	38.57335261	X	0.155099136	0.304719707	0.911232576
**Grip75**	FBgn0026431	42.90341675	47.18237439	42.24018256	48.00383762	38.14601226	44.91154663	45.62470255	44.99549908	2L	-0.024601345	0.863451216	0.995787491
**Grip163**	FBgn0026432	43.14885057	43.73036497	37.4993588	47.87111376	44.52505445	58.67860186	46.06466193	43.53439851	3L	0.189449662	0.166123905	0.82902792
**Grip128**	FBgn0026433	27.33056161	28.99742212	18.17735949	30.78142253	20.00436517	28.53316521	25.8502573	14.94454142	X	-0.21315419	0.342481306	0.928339671
**Eaat2**	FBgn0026438	223.6783849	232.124117	220.1981838	217.1009489	223.8526405	251.0036322	218.7808882	211.7759766	2L	0.048904737	0.551003456	0.984934163
**Eaat1**	FBgn0026439	44.9051039	57.17380527	58.27855134	40.78569491	55.07870123	43.29378207	53.21877916	34.89772815	2L	-0.07465185	0.639429893	0.991585536
**ear**	FBgn0026441	87.93963829	95.94983369	95.56643532	108.8094793	101.4681565	125.5370113	115.1480252	119.3697623	3R	0.278970527	0.013127033	0.344576629
**Drp1**	FBgn0026479	322.3601665	267.6430783	249.2341868	270.0416844	243.0131898	247.8040721	262.4525301	219.3762715	2L	-0.161802592	0.146548381	0.808004944
**Dek**	FBgn0026533	226.6704362	203.8746153	217.9413982	205.0945978	217.9178953	218.1877244	206.1485771	205.3691263	2R	0.019168258	0.827387311	0.995787491
**SPARC**	FBgn0026562	64.06042904	129.2168921	169.8912397	78.449557	49.40030622	83.91429532	116.7740345	93.72116767	3R	-0.321525528	0.409239074	0.957495351
**CG1979**	FBgn0026563	0.296855677	1.067000899	0.308643313	0.049436274	0.842129483	1.519314473	0.378642604	0	3R	0.704344388	0.545447426	NA
**CG1307**	FBgn0026566	115.8181641	119.66871	119.9493857	159.0405545	121.59505	165.8132149	130.9827972	177.0019327	3R	0.239625191	0.147312931	0.808004944
**ADD1**	FBgn0026573	63.2984223	78.64451336	71.41890007	65.47853805	67.74841092	83.76422337	71.17366064	71.66719988	2R	0.109340284	0.300100833	0.9074568
**hang**	FBgn0026575	22.3877921	36.7805015	36.42351967	49.10691498	38.80427917	25.47693832	39.22012164	46.03002121	X	0.084246439	0.777395802	0.995787491
**Pisd**	FBgn0026576	45.57556677	61.08488349	55.80649812	46.51471156	46.70447276	60.32432976	46.96194672	39.76696344	3R	-0.077054166	0.627736525	0.990047511
**CG8677**	FBgn0026577	73.56949346	89.11802254	92.34818826	89.80232327	80.4174815	85.50049284	87.62937133	88.80481664	2L	0.021769887	0.830190942	0.995787491
**Hmg-2**	FBgn0026582	52.34084737	85.20919824	57.58138255	57.35012412	58.76325585	72.36343443	60.86367057	101.858214	2R	0.255271097	0.240841343	0.874884741
**Fie**	FBgn0026592	129.2180114	134.0989631	147.8105215	110.8519265	125.6344233	160.3258447	141.4410793	143.685282	3L	0.160436459	0.188485323	0.843108521
**CG5707**	FBgn0026593	0.707434218	1.851129786	0.274596087	1.913253247	0.408672025	0.106714346	0.866246347	0.061436207	3L	-1.714601586	0.091492401	0.720697231
**Axn**	FBgn0026597	24.25088584	24.9587846	15.92567565	20.68221569	17.19309576	17.73548946	22.55902254	14.62944443	3R	-0.222601535	0.190076755	0.843401316
**Apc2**	FBgn0026598	2.61990971	2.366285924	2.004541854	1.080685229	2.874146706	1.539021728	1.902211542	2.056454813	3R	0.077618614	0.867023988	0.995787491
**Ady43A**	FBgn0026602	85.04451298	70.322028	77.96056815	67.73155747	75.2294363	71.09916947	67.60629894	65.73767904	2R	-0.078044012	0.546020716	0.984934163
**alpha-Man-IIb**	FBgn0026616	9.782151144	11.64805735	14.65760609	12.93803517	11.39512115	13.14747993	14.57901974	11.65058976	3R	0.0825491	0.574895785	0.984984955
**Taz**	FBgn0026619	55.14626796	50.98871233	53.09187289	50.18876171	63.4149396	43.9409468	52.04547652	40.68630938	2R	-0.035380307	0.824713804	0.995787491
**tacc**	FBgn0026620	49.56282294	44.37371457	44.02704416	42.21983614	42.04132195	52.59696643	42.36525525	44.85901747	3R	0.040868563	0.697280928	0.995787491
**nes**	FBgn0026630	48.98252867	49.48498051	55.79672049	47.85619047	52.01530492	66.22961033	57.04630847	55.80237836	3L	0.223673039	0.074353239	0.676245
**Cog2**	FBgn0026634	20.75456075	18.71019896	18.1949088	26.96020273	17.82281622	17.02172772	27.20318551	14.74370313	3R	-0.114306139	0.6291608	0.990523437
**MagR**	FBgn0026666	117.594307	116.1357461	117.313586	124.7792834	155.1254728	119.4661621	132.8716722	145.4633616	X	0.248036255	0.0675084	0.656261422
**IntS4**	FBgn0026679	22.00915333	47.09002518	45.44414069	22.74899051	29.26935982	29.557358	29.44536494	43.11170478	X	-0.020494193	0.933853208	0.996479182
**l(1)G0007**	FBgn0026713	9.707025475	12.75716685	8.910716469	11.65711358	9.648388894	12.36653394	10.91115175	12.55317578	X	0.110610317	0.445605045	0.967112302
**Agpat2**	FBgn0026718	0.084783293	1.269750714	0.44074947	0.310622802	0.415436557	0.976326585	0.633403148	0.236664485	2L	0.147951779	0.832641365	0.995787491
**fat-spondin**	FBgn0026721	3.335873534	5.732603502	3.252981268	4.308104818	4.457539768	5.765908852	6.006977686	2.520670623	2R	0.203597028	0.548028699	0.984934163
**drosha**	FBgn0026722	45.12553355	53.68910176	33.20422922	34.84058109	45.97811587	50.33827086	51.99765944	57.76834979	2R	0.33609137	0.040357285	0.530560761
**CG6171**	FBgn0026737	97.47614072	107.2215225	117.8417721	118.9604023	77.37348734	106.9856574	112.8775052	91.98980498	3R	-0.151773172	0.358297227	0.93823489
**CG7857**	FBgn0026738	61.44942002	65.23451645	53.26442589	54.46643389	48.45720365	43.92392151	48.83770602	61.03834341	3L	-0.183454034	0.3223878	0.918365535
**mRpL18**	FBgn0026741	167.9620379	161.6080428	191.0832053	159.8634637	195.9362538	182.2226511	180.5992738	153.9567237	2R	0.097567406	0.493666926	0.973549494
**Yippee**	FBgn0026749	444.6172755	431.8098685	418.0323492	442.3220978	495.0096999	445.9688351	463.541359	475.0828365	X	0.144380888	0.131298561	0.790743321
**XRCC1**	FBgn0026751	3.046362684	4.258205033	4.105796322	1.420495983	5.193939564	3.957104713	7.16689936	6.834399898	X	0.886722131	0.04502942	0.559277478
**Ugt37C1**	FBgn0026754	2.685066168	2.001698587	2.050682634	0.463713384	0.359054267	1.78140058	3.932213073	1.187496282	2R	0.023886907	0.97776005	0.997757033
**Trap1**	FBgn0026761	176.846965	148.0679664	146.8933655	190.9037959	165.3974215	214.666353	179.3914749	160.6568323	2R	0.144344427	0.277264748	0.896087042
**Rad23**	FBgn0026777	224.0203656	202.1494914	224.7647711	188.7011016	196.7637121	232.7803835	217.5642983	199.2196993	4	0.040139844	0.700088852	0.995787491
**Nhe1**	FBgn0026787	108.1320915	85.18082604	80.26081798	82.23697395	86.20005047	103.9804839	87.34898234	65.58206413	2L	-0.028142452	0.856726397	0.995787491
**Thd1**	FBgn0026869	43.49547495	54.03905177	66.65063972	62.005501	46.31290583	53.76582852	57.81627131	57.96200864	4	-0.035323347	0.793084271	0.995787491
**CG14777**	FBgn0026872	19.40275792	19.82259365	17.99041815	13.02630357	20.41710578	24.96504518	15.80789524	21.31990092	X	0.261993502	0.239981546	0.874625705
**MED18**	FBgn0026873	153.8403811	154.5005664	130.6358596	139.7937016	147.2546533	143.7037691	138.9503869	135.3697194	X	-0.005724002	0.965115464	0.997604378
**CG13358**	FBgn0026874	0.347167317	1.844027494	1.965187162	0.134901255	0.522271655	2.182048174	0.590420857	0	X	-0.325899104	0.803612389	NA
**CG11403**	FBgn0026876	1.783570651	2.123134524	1.256201861	1.8108847	0.57125522	0.135608025	0.733859201	2.748081732	X	-0.704327521	0.385145472	0.947123743
**CG13364**	FBgn0026879	547.093269	745.018226	814.6251566	735.7940937	647.9110104	552.5995415	672.3083241	744.6352045	X	-0.083170599	0.577209134	0.985637351
**STUB1**	FBgn0027052	239.1216462	191.5732218	220.5668727	176.8828128	176.5148552	236.5432107	198.6334359	165.605072	2L	-0.065368205	0.640093071	0.99179631
**CSN5**	FBgn0027053	175.423486	176.5267404	168.6543641	182.1696434	198.6006898	198.6907623	189.8574608	186.9051584	3R	0.168934782	0.113520755	0.758410631
**CSN4**	FBgn0027054	144.513809	146.045101	150.8295008	145.6874659	161.7321131	146.2636304	163.311974	156.1922322	2R	0.126877034	0.238299544	0.873280735
**CSN3**	FBgn0027055	151.6168566	110.7250066	109.219182	125.6469746	150.5673801	144.8203817	142.9144633	165.2316629	3L	0.30667106	0.027110066	0.461092427
**CSN1b**	FBgn0027057	169.5830789	189.582836	166.6043587	163.7788387	178.3247908	149.3742455	198.9038972	177.7146429	3L	0.063037253	0.61634134	0.990047511
**Eb1**	FBgn0027066	178.073789	149.3982537	158.2917356	142.3594845	143.7446068	179.9521987	142.4260512	148.5731953	2R	-0.004236117	0.969038731	0.997604378
**Ugt36E1**	FBgn0027070	0.340569773	0.190419339	0.826217519	0.088225067	0.409877152	1.748140888	0.193066846	0.0821565	2L	0.795942287	0.545574708	NA
**Ugt49B1**	FBgn0027073	13.9533216	27.57175546	15.46880353	12.83889809	13.90271168	9.17344213	15.10420449	9.888233862	2R	-0.502764079	0.10742853	0.757046101
**Ugt36F1**	FBgn0027074	0.160950596	0.449953409	0.19523195	0.062541709	0.87167123	0.05058108	0.410588428	0.407678416	2L	1.041126983	0.410042891	NA
**ValRS**	FBgn0027079	71.64691373	100.1480389	111.1933058	115.7572324	90.44148067	100.5394246	113.9467119	112.0832671	2R	0.097870539	0.521068584	0.982083369
**TyrRS**	FBgn0027080	147.1578797	209.044944	174.3052444	197.811036	181.9090626	244.7803877	207.0486227	181.0269899	3L	0.191857744	0.158687198	0.818884898
**ThrRS**	FBgn0027081	232.8482877	193.9040854	193.8475298	199.1120036	190.8523606	182.0044531	190.7188451	182.8309174	2L	-0.106571154	0.313979382	0.91460807
**ProRS-m**	FBgn0027082	34.61652801	37.24721778	47.52601451	28.09803851	49.16014143	53.37840191	48.01311844	45.42743731	3L	0.443908569	0.019012862	0.393184106
**MetRS-m**	FBgn0027083	6.03669359	7.020312987	3.992328133	4.150357623	8.512550927	7.198113131	5.247616135	7.953053913	3R	0.47795039	0.040646989	0.530560761
**LysRS**	FBgn0027084	727.8887481	699.1322702	657.9940392	762.4487303	882.3231648	1196.279348	1007.97206	1017.040314	X	0.553944495	1.23E-07	3.66E-05
**LeuRS-m**	FBgn0027085	56.2134737	50.63480404	48.49261287	45.27167528	48.60422359	47.2287371	48.28623042	43.22045839	3L	-0.071203871	0.590584532	0.988141235
**IleRS**	FBgn0027086	44.57339396	57.97577486	61.03790725	44.18964808	50.36223358	80.26433896	42.06064883	52.17482844	3L	0.143350479	0.428864125	0.963643466
**GlyRS**	FBgn0027088	109.1580564	116.3347641	97.71591894	127.2489496	120.7796314	105.9309767	90.93960724	107.33398	3L	-0.05386522	0.688655996	0.995787491
**GlnRS**	FBgn0027090	107.6904105	93.89970245	103.6116273	92.99116226	111.636907	103.4266827	142.5377359	89.81332556	3R	0.197221744	0.192483734	0.844272804

**CysRS**	FBgn0027091	42.75971387	49.71138754	37.32003673	46.25973798	43.30020079	55.46819448	50.05550144	51.8705294	2R	0.21814052	0.142989503	0.8037297
**ArgRS**	FBgn0027093	39.40838533	49.28607084	45.10466096	42.83728038	41.59287052	41.62208678	50.49831567	54.53314061	X	0.124866961	0.443333837	0.967112302
**AlaRS**	FBgn0027094	32.22831927	37.38042551	46.56324754	40.88536547	38.71249906	46.45145648	24.10297891	30.82360814	2L	-0.134355829	0.49341295	0.973549494
**Manf**	FBgn0027095	101.3129659	126.8817079	119.4322954	127.0662636	129.0568775	120.887187	146.7827668	119.672146	3R	0.153830035	0.267303538	0.89466172
**Dyrk3**	FBgn0027101	119.7315508	100.700613	115.7160436	83.30680212	85.57985683	106.5802885	96.4953865	89.21301923	4	-0.122193398	0.339394853	0.927274752
**Inx7**	FBgn0027106	0.086618246	0.145289956	1.155740607	2.120445379	0.312736267	4.123989222	5.413642636	3.57306836	X	1.985995896	0.040865712	0.531318009
**Inx6**	FBgn0027107	2.756843547	1.417099158	2.049567998	0.622013898	1.043524505	0.041921536	2.155202826	0	X	-1.06760008	0.355073062	0.936745458
**Inx2**	FBgn0027108	33.83154694	73.66590565	75.05630979	31.77194164	33.8478931	43.4026467	61.30074986	42.38902051	X	-0.202936749	0.568792121	0.984934163
**miple1**	FBgn0027111	1579.150156	1324.257907	1308.619277	1251.779929	1220.258401	1341.781801	1218.399731	1160.165135	3L	-0.117255554	0.235524957	0.870926212
**Kmn1**	FBgn0027259	1.881227243	2.881103938	2.381129244	0	0	1.002474965	2.921161547	1.775790646	X	-0.2953492	0.81924653	NA
**l(1)G0196**	FBgn0027279	60.17257763	53.25009691	51.56820208	62.10051681	72.14923758	78.30291434	62.16849219	63.27788254	X	0.307010122	0.005254846	0.204644344
**Gmap**	FBgn0027287	4.359249184	5.97278572	6.883140035	4.379497276	6.376726071	7.903861391	7.665260676	6.802247878	X	0.447710806	0.007556865	0.252813679
**Idh3a**	FBgn0027291	496.6334717	457.2638347	413.8074534	459.8448329	420.0486376	351.4268266	347.91208	384.2863991	X	-0.250951124	0.019577113	0.396755891
**temp**	FBgn0027296	113.9022424	111.1707374	104.247912	123.5482741	131.0294498	156.4266894	114.5035297	105.8427392	X	0.191718265	0.158303876	0.818884898
**CCT6**	FBgn0027329	200.5680341	181.5655366	240.9809397	183.6041506	186.3897381	188.2857714	202.9872242	189.7562642	X	-0.041018427	0.729797918	0.995787491
**l(1)G0020**	FBgn0027330	0.899235435	0.896851579	0.825444776	4.193067127	1.974345119	1.672674008	1.549978995	0	X	-0.372441093	0.675515286	0.995787491
**l(1)G0004**	FBgn0027334	39.49574094	62.13658485	42.73178535	28.36574631	58.67902702	35.78112989	49.10333457	29.17434168	X	0.036065026	0.902102808	0.996479182
**Rip11**	FBgn0027335	10.66314901	10.10470707	12.51426611	10.9669799	9.833245108	13.4472808	10.39856973	8.849889192	X	-0.028146883	0.859320384	0.995787491
**Kap-alpha3**	FBgn0027338	589.371978	630.0369918	564.2396851	609.6952534	557.7081965	597.9331316	587.3357304	605.2065051	3R	0.003022621	0.971185112	0.997604378
**jim**	FBgn0027339	24.55260991	32.77488003	34.2535811	32.67290143	32.08266788	33.37882097	29.88924174	33.04824014	3L	0.079566797	0.473887543	0.973549494
**fz4**	FBgn0027342	6.995348562	5.418321392	7.006218457	8.279385454	9.036834584	4.094250443	11.51484717	9.196130756	X	0.319584494	0.331332259	0.921507984
**fz3**	FBgn0027343	5.690825413	8.823029462	9.90548598	14.28638024	14.50104425	9.798205571	13.21735062	16.04346063	X	0.504309522	0.032829827	0.508557215
**bgm**	FBgn0027348	0.903985486	0.964923805	0.398737487	0.319334565	0.346166013	0.82644557	0.628932653	0.208158382	2L	-0.344594822	0.63672385	0.991585536
**Amph**	FBgn0027356	17.61544153	20.78156267	18.25476537	16.63570511	19.08023206	22.41490787	19.02807403	21.03206856	2R	0.185552312	0.088875493	0.709815866
**Tim8**	FBgn0027359	568.7035651	489.7901253	568.9684176	541.0160104	624.9127434	549.5209061	534.4821793	519.9597657	X	0.06901287	0.538486079	0.984934163
**Stam**	FBgn0027363	119.3684296	123.1405648	122.871244	127.279101	110.3589507	117.7408513	128.7582828	135.5413919	2L	0.030138285	0.78471892	0.995787491
**RecQ5**	FBgn0027375	8.823252492	12.64842351	13.73358996	13.61094083	9.540971125	15.32356839	11.95930079	13.15481411	3L	0.069124172	0.765017349	0.995787491
**rha**	FBgn0027376	1.397677754	0.380174328	0.549850822	0.22017791	0.272774764	0.33833404	0.14454754	0.205032958	3R	-1.387741458	0.137987988	NA
**MRG15**	FBgn0027378	114.2281358	115.4310853	111.551411	115.6749569	127.98015	93.79764178	100.6191757	93.40839677	3R	-0.104992126	0.462911956	0.971606621
**Dnz1**	FBgn0027453	203.3117264	219.6778869	219.6473156	220.5968958	219.9347174	212.7160624	204.1600617	200.4854778	2L	-0.013001328	0.889690717	0.995926915
**D12**	FBgn0027490	43.21744944	30.31450543	31.51702125	26.68609531	30.35631741	30.96631199	24.45515332	26.61334	2L	-0.203713147	0.271957848	0.89549671
**Cdk5alpha**	FBgn0027491	1581.817803	1495.277937	1484.108549	1528.650787	1453.199638	1536.510289	1456.413594	1341.423391	2L	-0.044775949	0.580764639	0.985637351
**wdb**	FBgn0027492	110.2923271	119.7515336	132.4095597	115.5064021	120.0094631	127.8870546	129.8972536	128.1993745	3R	0.11382195	0.194661077	0.844272804
**AdSS**	FBgn0027493	100.4173979	90.42229615	83.97567928	102.4934017	86.71251643	92.75321072	92.22757208	105.2707796	3R	0.027522376	0.814605698	0.995787491
**Smyd4-4**	FBgn0027495	95.57196812	109.2012731	106.8575701	109.9583655	130.0206062	106.6475632	122.4887422	128.242813	2R	0.242314449	0.045952715	0.56430181
**epsilonCOP**	FBgn0027496	122.3953471	179.6383964	142.5641822	171.2160865	128.6071243	131.8889508	129.8970006	126.2225236	2L	-0.220339797	0.138262484	0.799378522
**Madm**	FBgn0027497	125.4354975	107.7431132	119.5061396	126.5442565	100.5513321	116.0717848	114.9705561	116.8240406	3R	-0.067677498	0.535696957	0.984760433
**wde**	FBgn0027499	39.83137687	35.15409706	40.67997739	35.30163764	36.56789409	48.21366471	34.50146248	38.92264998	2R	0.095281899	0.450954579	0.967112302
**spd-2**	FBgn0027500	3.094321357	2.595144994	1.324725962	2.440128771	1.314363087	1.780415469	0.580417666	1.778309498	3L	-0.765303701	0.151532431	0.812192196
**CG11970**	FBgn0027503	39.40246615	47.86656104	53.74713755	46.13697984	36.19852424	47.84681443	43.73847859	54.31467224	3R	-0.00648398	0.96488541	0.997604378
**Rab3-GAP**	FBgn0027505	20.32333837	36.67975932	46.37602956	43.41036695	35.27895798	29.58615868	23.68129056	39.49951541	2L	-0.160156708	0.481814556	0.973549494
**EDTP**	FBgn0027506	8.393793174	8.866500237	12.31341344	8.622108306	9.628308101	9.743280994	8.655111851	10.15046942	2R	0.033703746	0.836166999	0.995787491
**CG1344**	FBgn0027507	48.50286676	61.54448802	59.47718497	60.93570583	38.71396728	70.94848392	53.56727408	59.71482463	2R	-0.01807678	0.916941114	0.996479182
**Tnks**	FBgn0027508	57.01266834	84.67417067	61.92497788	64.6378829	51.40399653	60.43770866	64.33465568	67.25964717	3R	-0.10581314	0.435868384	0.966084844
**TBCD**	FBgn0027509	39.59034624	39.10843961	36.28953534	58.01232275	51.40611816	38.72401318	48.76579356	52.66118726	2L	0.177422471	0.324782654	0.918470465
**CG10254**	FBgn0027512	60.41245781	72.52341666	63.99683292	66.40331436	66.25236177	77.03065478	61.74892224	63.88121583	3R	0.060817993	0.570159402	0.984934163
**ana2**	FBgn0027513	1.519618345	26.86197562	12.19404885	7.903465842	10.23462142	12.06763191	10.43694358	9.770802745	2R	-0.138983911	0.804966868	0.995787491
**CG1024**	FBgn0027514	22.73765294	22.53237984	25.95822535	25.55692731	22.0458597	29.44149924	22.99400864	21.27390041	3R	0.015638834	0.929043448	0.996479182
**CG7115**	FBgn0027515	44.06205589	44.80840502	54.24767013	49.53693601	49.52836807	44.61425882	52.41275198	58.93831816	2L	0.126023639	0.37465051	0.943282319
**Wdr24**	FBgn0027518	42.75387766	63.8437594	39.60230051	43.7552221	43.65277059	44.41742644	51.27521283	52.13738413	3R	0.045358382	0.78958049	0.995787491
**CG3679**	FBgn0027521	0.815056204	1.03571397	0.539267722	0.345504156	0.490461782	1.979289421	0.12601369	0.21449242	X	0.06777293	0.944211477	0.997604378
**CG3909**	FBgn0027524	45.25414331	50.78623453	56.46582442	56.83543359	78.6522348	51.22284592	71.61515525	75.30694817	3R	0.437781855	0.025058103	0.444328143
**LTV1**	FBgn0027525	150.3471079	195.6733062	146.0323825	172.5492122	152.0185331	183.278314	155.3003933	185.5278889	2R	0.056184752	0.659932676	0.995750687
**Ublcp1**	FBgn0027526	123.386245	153.3744525	145.3236491	117.5082154	135.8587996	125.8703128	159.4882552	208.8350974	3R	0.26010562	0.140434754	0.8037297
**goe**	FBgn0027528	2.083963319	1.983964282	2.485133091	1.567581532	1.359943939	1.888414777	1.499681213	0.817768697	X	-0.515379666	0.055140068	0.615177141
**tapas**	FBgn0027529	84.52706468	63.82235064	76.00483575	66.35449777	117.3739309	141.3266636	107.6362731	149.2988732	2R	0.854513752	2.56E-10	1.42E-07
**CG7139**	FBgn0027532	61.87017546	49.09645293	57.46801858	55.84646757	48.29719107	48.25225615	50.58351373	48.8420395	3L	-0.166846388	0.177542339	0.834615603
**botv**	FBgn0027535	51.46179957	46.97132162	61.81031531	60.23588432	44.24796587	56.59095829	58.39444403	56.96043676	2R	0.001102252	0.99395933	0.998895169
**Nup93-1**	FBgn0027537	28.81657589	33.7313905	33.31901643	20.03756056	25.80784045	34.95310047	29.59096864	15.36646233	X	-0.103385376	0.665149054	0.995787491
**beta4GalNAcT/**	FBgn0027538	78.03813641	122.8111324	108.5365321	111.8330132	138.444805	174.9209327	110.2740452	99.08434354	2R	0.340898431	0.054663044	0.611836294
**lili**	FBgn0027539	20.98978382	21.64001208	16.50760165	16.31229693	18.65393164	15.32792692	14.70176878	13.63171688	3R	-0.246182332	0.123079771	0.780439453
**CG1927**	FBgn0027547	75.77159592	93.61108955	114.4651211	98.98663154	107.1825697	88.1640658	97.17453286	124.5858503	3L	0.159497933	0.299101904	0.9074568
**nito**	FBgn0027548	29.98959145	36.29280562	47.85414662	36.5826407	34.31068687	29.3640321	54.744667	29.14845943	2R	0.004347113	0.982654801	0.997881056
**Nulp1**	FBgn0027549	148.5739559	125.8255015	146.9835891	131.2856084	133.3092382	147.1442064	162.6325716	166.0193573	3L	0.170017358	0.158096329	0.818884898
**CG6495**	FBgn0027550	279.0657875	294.6502916	285.5759952	267.9532608	271.4031598	240.98574	276.3352249	253.8637142	2L	-0.080590003	0.389163048	0.948247506
**CG10863**	FBgn0027552	32.63789764	84.06275408	69.08339061	48.92749085	28.97693619	35.42286386	90.99451156	45.30386859	3L	-0.183928879	0.575553907	0.985548852
**NELF-B**	FBgn0027553	31.20454346	59.16034984	51.80063608	38.76702716	58.91078517	63.63855849	56.80622553	61.54567509	X	0.45108845	0.015714115	0.366465026
**CG8042**	FBgn0027554	111.2884305	95.67875134	99.19810658	109.7918967	94.66977081	121.1133306	109.6132463	99.28066873	3L	0.056365809	0.650301075	0.993330125
**CG4928**	FBgn0027556	0.60908591	0.957802657	0.722025666	0.508317243	0.668480174	1.12238302	0.194223905	0.247946605	X	-0.2936708	0.554007214	0.984934163
**Pgant3**	FBgn0027558	33.0712625	37.87229597	38.28593655	44.61854052	46.62669208	43.4548901	42.58913545	35.37006572	2R	0.158405636	0.281394812	0.900509101
**Tps1**	FBgn0027560	1.946040118	1.273041625	0	1.451878168	0.948537318	1.963144768	1.34038534	0.042250332	2L	-0.116733583	0.905579304	0.996479182
**CG18659**	FBgn0027561	56.81902797	61.37276838	60.6370689	60.40363706	50.49798305	46.99889456	59.43214165	50.73164842	2R	-0.172604786	0.119790223	0.777535158
**CG10345**	FBgn0027562	0.622832788	1.305893285	0.161891106	0.674194485	0.281093203	0.356516076	1.163272371	0.217322299	3R	-0.433454525	0.570444438	0.984934163
**CG9631**	FBgn0027563	0.468488107	0.087313675	1.136545614	1.213624873	0.939712341	0.049076414	0.13279147	0.226029122	3R	-1.067185342	0.453563159	NA
**CG3149**	FBgn0027564	31.61949233	43.13697359	34.40257639	44.32115078	28.22929135	35.19465086	30.99376406	41.27251761	X	-0.145062901	0.450999792	0.967112302
**CG5498**	FBgn0027565	22.77287209	29.92768455	34.65248725	29.74691427	14.86598452	32.20518706	31.64052897	25.60389978	3L	-0.135213885	0.607538372	0.990047511
**CG8108**	FBgn0027567	425.7426712	415.0935239	352.4760763	457.2338447	388.2386266	440.4397621	367.640368	435.2559094	3L	0.011368367	0.916766995	0.996479182
**Cand1**	FBgn0027568	166.7017669	199.7458893	188.0321477	173.1543601	169.2591867	186.2912773	186.3682542	181.1475379	2L	0.02289523	0.803442018	0.995787491
**cert**	FBgn0027569	37.78782904	35.48146154	33.11438256	37.43531879	27.66578662	39.31888389	32.35322541	22.30958538	3L	-0.216713696	0.16767543	0.830652748
**Nep2**	FBgn0027570	1.318103738	0.128678705	0.084595272	0.487795118	0.528781128	0.25643019	0.213492759	0.211979647	3R	-0.73675786	0.365761252	0.940794966
**CG5009**	FBgn0027572	70.81480566	65.7254479	76.26936826	69.09162023	38.42982453	57.57921279	48.1692667	58.02012522	2R	-0.450286456	0.002410251	0.116870433
**CG5815**	FBgn0027574	39.31656306	37.06028949	17.89427928	27.7677371	29.01361851	26.57242437	22.35544392	21.08286635	3R	-0.275866005	0.256088021	0.88674759
**GABA-B-R2**	FBgn0027575	403.4110703	485.6541684	480.0243272	531.6194394	423.4461548	491.1059194	447.0258084	484.8073887	3R	-0.011638806	0.910540191	0.996479182
**mino**	FBgn0027579	2.26266277	1.492326454	1.438913443	2.227923481	1.625943057	2.619931231	2.213572843	1.14464862	3R	0.054589326	0.886580761	0.995926915
**PCB**	FBgn0027580	83.92540904	68.30236296	72.01570537	72.19052753	77.83786422	70.50603765	74.44319191	70.59047949	2R	0.013895644	0.896912558	0.996479182
**CG6191**	FBgn0027581	0.917650691	0.379230654	0.419430451	0.217047482	0.288103424	0.288385155	0.339267627	0.288739796	2R	-0.674434212	0.269794412	0.89549671
**CG6230**	FBgn0027582	59.87828339	45.50392081	46.47726375	41.08788675	43.03128182	58.63219113	56.46778644	36.21407322	2L	0.034597351	0.840455646	0.995787491
**CG7601**	FBgn0027583	25.05825941	27.1680405	32.17508957	45.83489495	37.18510491	34.02631371	31.43811055	33.76348293	3R	0.099501473	0.675367045	0.995787491
**CG4757**	FBgn0027584	2.759987749	4.692909963	5.686751924	4.583698518	1.70633057	0.998065597	1.736084723	0.164169559	3R	-1.913394192	0.002412013	0.116870433
**CG8740**	FBgn0027585	0.488092721	0.323816975	0.724602261	0.339692479	0.433990614	0.260992415	0.232301309	0.767092273	2R	-0.109503614	0.827026496	0.995787491
**CG5867**	FBgn0027586	1.143373203	1.555012013	5.397679349	3.458246375	2.175653659	2.622077689	0.078831611	4.830556019	2L	-0.20740544	0.787705604	0.995787491
**CG7028**	FBgn0027587	74.47587983	57.49714841	52.96396543	36.62744294	44.86480965	53.98979803	49.38487721	39.08119161	3L	-0.2157743	0.246001696	0.877269208
**GCS2alpha**	FBgn0027588	690.2357345	638.3971092	611.4733148	641.1250422	618.6425104	666.3726997	636.6780431	661.1191384	X	0.030177581	0.723324591	0.995787491
**CG1688**	FBgn0027589	2.071859002	4.798646313	3.148570414	3.469557319	2.83316161	3.47125347	3.447208993	2.260704099	2R	-0.132584827	0.547954491	0.984934163

GstE12	FBgn0027590	38.06675307	96.62681376	91.91584668	57.5007786	35.27424194	40.26993166	72.64186565	42.55192537	2R	-0.534603791	0.060607209	0.627984253
MED15	FBgn0027592	86.01463418	103.7785398	74.4157571	120.6437692	94.80158614	84.04294704	85.95027774	85.36096826	2L	-0.106265024	0.487528253	0.973549494
drpr	FBgn0027594	50.90329598	54.15591311	53.68512383	53.32488686	44.8278165	53.92968917	44.9850108	53.43939269	3L	-0.074873014	0.448515095	0.967112302
Kank	FBgn0027596	5.907396493	5.978722112	5.403462577	5.882320425	6.979994974	7.154237626	6.419873817	7.019835504	2R	0.280280442	0.0192225	0.395797586
CG17712	FBgn0027597	162.6129295	128.3816473	145.9959943	119.9017397	128.690429	197.41427	142.8958364	130.787538	2L	0.131822304	0.411373769	0.958665466
cindr	FBgn0027598	16.88744891	17.88074667	10.85642392	14.58482998	15.19631578	16.62307926	12.48546013	14.78841134	3R	0.001171161	0.994081381	0.998895169
CG2790	FBgn0027599	99.53709241	117.1435251	111.832808	88.46998043	88.81228054	89.34856797	85.3700792	112.295547	2R	-0.116145369	0.427163821	0.962819343
obst-B	FBgn0027600	1.133891466	0.556666497	0.503196015	0.967179976	0.324518796	0.378070152	0.211652304	0.360260976	2L	-1.286401763	0.057826323	0.623014047
pdgy	FBgn0027601	3.074507259	3.035713001	3.776964662	3.070589469	1.637527528	1.471926732	3.048253526	3.720979668	X	-0.356714618	0.217748817	0.861225611
CG8611	FBgn0027602	13.33714145	8.52625219	12.59198433	16.99195119	13.38587311	13.59519486	11.15029539	13.87438953	X	0.041368434	0.839671161	0.995787491
Ulp1	FBgn0027603	67.38398367	99.36744014	99.12663742	87.39758746	87.71933588	94.64281578	90.39807673	89.60798967	X	0.070478052	0.569267724	0.984934163
CG8230	FBgn0027607	57.15780491	49.17726342	72.7813729	85.45959082	60.72997436	90.96621072	58.54194337	80.93949994	2R	0.164074086	0.393326759	0.952091247
CG2082	FBgn0027608	3.442143178	2.257586381	4.231591791	1.839490289	1.3126098	4.973590845	3.956374675	1.637389031	3R	0.033211693	0.926283121	0.996479182
morgue	FBgn0027609	165.101076	173.54524	165.5536017	217.5674027	185.9139355	181.8245891	179.5798471	198.8616703	2L	0.078039962	0.543941834	0.984934163
Dic1	FBgn0027610	261.5711353	208.0257569	211.9745445	230.7967759	272.5993774	203.4993085	215.6699916	200.5355368	3R	-0.002948501	0.981930593	0.997881056
LManII	FBgn0027611	3.238737893	12.3439119	13.35712602	2.642848416	3.183231633	5.292667991	4.360547472	5.820602033	2L	-0.711925541	0.110602889	0.758410631
OXA1L	FBgn0027615	72.70445505	64.88741462	68.46015935	59.94450198	73.30198474	59.01178969	57.89083178	48.16281714	3L	-0.128941168	0.417060633	0.958846438
Ythdc1	FBgn0027616	561.5862164	683.7496116	587.1791726	649.3913183	629.1725596	740.0846749	607.3418357	598.3009698	3L	0.082143057	0.39748224	0.952272708
CG5808	FBgn0027617	5.18721433	2.624058136	4.494324862	3.167422483	2.972778203	4.308274702	4.936005352	3.128311802	3R	0.006611797	0.988622908	0.998078482
eIF3j	FBgn0027619	183.6236386	159.4645856	153.9706167	162.6454668	158.751794	172.9532629	173.6955608	154.802384	2R	0.028240367	0.802015134	0.995787491
Acf	FBgn0027620	39.17026543	43.67688769	45.83377074	43.83968494	34.29455798	53.00104252	41.66356327	46.18981641	3R	0.051541234	0.711585478	0.995787491
jdp	FBgn0027654	4431.133534	3613.217628	3727.120777	3790.304463	4114.794993	4221.980095	3771.131666	4017.068024	3R	0.079480541	0.408442686	0.956706394
htt	FBgn0027655	2.937125144	5.231703959	4.091126834	5.141082846	4.30099522	4.09437802	5.264344899	3.905040696	3R	0.047854945	0.796713181	0.995787491
glob1	FBgn0027657	4.680550529	3.144152211	5.881700875	2.145868689	1.621025097	4.952488734	3.436024448	3.533517334	3R	-0.200243638	0.592952473	0.988631016
blot	FBgn0027660	0.723999777	0.895127067	0.371073245	0.285292094	0.472485433	0.634513373	0.433553753	0.642031627	3L	-0.0330193	0.937866548	0.997038394
VhaSFD	FBgn0027779	401.2862281	311.155062	285.4226338	343.7963809	351.2984265	410.9260778	318.908426	351.0027863	2L	0.119405058	0.330981001	0.921507984
Aasdh	FBgn0027780	58.28953274	59.36747048	55.54568828	58.00205651	57.37005812	60.80222906	58.52747388	36.19386381	2L	-0.091419457	0.563068716	0.984934163
SMC2	FBgn0027783	4.833607458	3.531353091	3.908754758	5.058696695	2.326906748	9.134444932	2.904549924	3.404787884	2R	0.055471935	0.89762746	0.996479182
Prp18	FBgn0027784	97.32088382	103.3643814	99.01970733	74.70340771	87.4845505	129.0440766	97.50949977	121.6664631	3R	0.24894609	0.137441584	0.798609078
Mtch	FBgn0027786	327.2873498	321.8983521	348.2694919	323.5472024	344.4927406	360.5981028	359.0025269	342.2757383	3L	0.120482485	0.177692419	0.834773745
Hey	FBgn0027788	64.81118732	73.5658297	52.82962825	74.02359622	65.24515428	72.66295332	63.25512307	76.87791634	2R	0.097620805	0.505321192	0.978557094
GV1	FBgn0027790	0.914362317	0.589890198	0.273012951	0.754329698	0.939605512	1.20687729	0.484454336	0.534467562	3L	0.337591213	0.514633342	0.980804634
O-fut2	FBgn0027791	1.697356926	16.10378566	5.40456555	9.769643072	4.30898102	9.5515384	9.607174155	9.788597369	X	0.055349034	0.920338512	0.996479182
CG14787	FBgn0027793	4.5850855	5.851724339	5.803488767	11.61951547	2.69910472	6.766107031	1.779925246	0	X	-1.273979697	0.140351166	0.8037297
Lrpprc2	FBgn0027794	56.62145633	45.37672935	36.63009688	41.48072502	65.9635473	49.01687214	53.98941594	61.62911877	X	0.38476303	0.023931293	0.438337142
CG14785	FBgn0027795	3.485454148	3.002185056	5.370488756	6.479001391	8.587918151	8.215162202	9.492243306	6.03334287	X	0.855491523	0.036460404	0.521170991
Dp1	FBgn0027835	31.51658713	25.50599751	38.39109574	39.53698205	27.27890819	32.95431839	26.28164626	36.61070489	2R	-0.103853336	0.529351589	0.984296327
Dgp-1	FBgn0027836	88.51831207	100.2895617	77.86105271	88.06900102	96.93780329	89.15965122	74.18186167	66.98998732	2R	-0.086655972	0.522604761	0.983379562
CstF64	FBgn0027841	32.97007493	48.94383124	47.93522652	59.45924897	71.44406098	93.73933534	80.88374209	79.68695664	3R	0.816858752	2.21E-05	0.002837399
CAH2	FBgn0027843	86.99510888	139.7524513	94.2058603	96.61552295	98.29476124	112.1624787	106.1370371	114.8105209	3L	0.08162957	0.552915346	0.984934163
CAH1	FBgn0027844	7.453957069	10.28985082	12.86942773	6.818254314	9.453789476	6.860833356	10.37508428	5.079408208	2L	-0.200301254	0.460954174	0.971606621
Ogg1	FBgn0027864	20.02025489	27.90272061	26.47923223	14.15263249	27.50140353	30.76125504	27.69507482	19.89543667	X	0.29171152	0.27479306	0.89549671
Tsp96F	FBgn0027865	160.9654885	122.1682869	141.0837228	119.742575	112.5036354	151.393353	122.1306522	111.5501828	3R	-0.103203023	0.415803982	0.958846438
CG9776	FBgn0027866	81.19162709	100.1030844	90.40794204	92.85632632	102.1378554	93.13540981	82.67205978	74.68750682	3R	-0.016808811	0.886554788	0.995926915
Nup107	FBgn0027868	53.47382695	74.10938347	45.46109724	51.90435053	52.34786325	54.35391279	55.83780918	69.31950286	2L	0.077156213	0.653526004	0.99443552
rdgBbeta	FBgn0027872	141.9855929	111.1867063	128.8760882	104.6439365	136.655832	151.1999908	115.0966212	125.6617032	2R	0.146422852	0.274062467	0.89549671
Cpsf100	FBgn0027873	34.76561969	36.94999678	29.04231482	23.44826626	36.75185138	21.42468415	31.33814663	21.30649124	3R	-0.132943733	0.566893917	0.984934163
ball	FBgn0027889	12.1942126	32.3306441	15.55492684	22.19401331	18.74713317	13.38801008	17.2597552	15.11623643	3R	-0.31208759	0.296632917	0.906715248
Pol31	FBgn0027903	15.24881092	15.82884799	35.32566724	15.59043878	15.34348207	22.03608888	16.42092033	25.77971399	3L	-0.003987626	0.990606829	0.998408391
Akap200	FBgn0027932	11.21092178	18.95369705	18.72860295	23.32996499	12.02292306	11.79398646	15.37566451	14.13403967	2L	-0.403654735	0.034816829	0.514749796
ppl	FBgn0027945	87.55696555	100.7725407	107.4481639	94.70626322	99.52099923	101.6967737	113.2037467	128.5822889	3L	0.215321562	0.174034314	0.833984823
msps	FBgn0027948	105.9700635	116.6263104	123.1141365	114.1040421	104.4090646	114.2986402	128.4524136	127.2055455	3R	0.077181592	0.457494893	0.968365245
MBD-like	FBgn0027950	167.2274421	187.4101121	168.6529132	193.6323769	197.2901943	180.9670208	186.6923736	233.0285032	3R	0.186870858	0.140722724	0.8037297
MTA1-like	FBgn0027951	83.31948473	95.69280929	96.90827625	102.9864519	96.615439	92.08668538	96.55576857	103.9037245	3R	0.070661711	0.493147677	0.973549494
ric8a	FBgn0028292	46.22114031	46.40275778	38.4392663	47.12544584	51.53860694	51.57106163	41.29438304	52.68549606	X	0.174587598	0.231293621	0.867702781
Pdha	FBgn0028325	555.0134552	585.205132	541.3734171	513.8441302	586.550979	514.168305	583.2112257	604.9215646	X	0.093145781	0.336432855	0.924219112
l(1)G0320	FBgn0028327	313.2195411	438.0965332	380.5772099	450.9379919	369.2324929	386.3511924	393.6732858	411.3459823	X	0.011997014	0.920607293	0.996479182
l(1)G0289	FBgn0028331	94.19051085	107.0866172	93.39185321	110.2197927	112.8787185	118.5468801	106.7585146	117.2398525	X	0.199868696	0.033349553	0.513292083
Ptpmeg2	FBgn0028341	42.43241993	42.96205991	38.91050839	48.00479499	41.39226473	38.93859022	32.43411941	40.24910503	X	-0.1419025	0.204424938	0.853973653
ATPsyndelta	FBgn0028342	343.104604	307.4641614	330.9038837	310.2489444	360.1098366	322.2768108	350.9062173	334.7851573	X	0.113424176	0.229792914	0.867702781
Ankle2	FBgn0028343	33.0030197	35.06659271	31.17116114	29.21730711	28.41129476	40.28346445	27.02514373	44.01153248	X	0.151691117	0.350955345	0.933267978
Cdc7	FBgn0028360	35.90098339	43.35083432	60.01523838	44.42994242	35.82427825	27.71617071	53.23170122	46.83211442	X	-0.129456579	0.527825956	0.984107922
kirre	FBgn0028369	0.748285229	0.654665155	0.534202698	0.347382677	0.518518806	0.726819216	0.358793153	0.399350153	X	-0.164750262	0.46740363	0.971928234
kek3	FBgn0028370	23.65616205	27.31014206	25.38692882	31.36303332	25.88541745	28.27575239	26.87211602	25.75206086	2L	0.016939823	0.875861939	0.995787491
jbug	FBgn0028371	2.335974816	1.262135064	1.235124608	0.741875279	0.95288732	0.818819754	1.203284238	0.316976098	2R	-0.740272436	0.074177892	0.676245
isopeptidase-T	FBgn0028372	2.332845371	3.404836319	3.968965167	5.650847959	9.297860332	6.969503082	6.492151265	7.038131008	2R	0.999391285	0.006369738	0.227834312
heix	FBgn0028375	25.0958563	22.40220752	26.71530691	37.87504223	29.44994791	22.05141087	36.68239651	32.65173829	2L	0.138903683	0.518289946	0.980804634
fal	FBgn0028380	17.03532773	17.7440382	18.1762044	16.00050316	10.04333534	12.90224216	10.82552418	12.2990361	3L	-0.550515501	0.001719451	0.091858384
cyp33	FBgn0028382	77.86818518	77.45508676	72.95846708	71.14437968	80.83887375	97.19163021	76.5311734	70.08207532	2R	0.14551549	0.351979885	0.934203937
chm	FBgn0028387	26.08872056	16.16366597	16.90200255	19.25729471	15.60802185	20.59978554	13.79023777	16.05558079	2L	-0.225196605	0.211248575	0.856520545
CG17834	FBgn0028394	0.331034115	0.307166925	0.341737934	0.0821057	0.483167106	0.491386674	0.359351123	0.030583222	2L	0.387938926	0.635743885	0.991585536
TotA	FBgn0028396	19.4369256	15.3167122	20.57041058	18.55237638	16.95558074	14.26645442	20.29949599	18.40911885	3R	-0.053610268	0.882305941	0.995787491
Tob	FBgn0028397	66.55118635	59.75956518	64.42559717	57.19710301	64.74486966	67.18300749	55.56391456	64.86359517	X	0.054895103	0.581372506	0.985637351
Taf10	FBgn0028398	252.1766653	304.4649627	319.8587073	209.4992203	298.6588034	241.9355735	329.3051884	284.4285354	2L	0.123490383	0.441119052	0.967112302
TMS1	FBgn0028399	1049.465884	951.9109938	946.5525035	945.3766023	1021.088607	1070.664017	954.1142864	1102.077273	3L	0.120911542	0.183183559	0.838911159
Syt4	FBgn0028400	613.2842017	655.2442249	614.1920609	690.1706602	506.053953	608.1012942	596.8011417	531.3856989	3R	-0.169574649	0.06914514	0.660467387
Sin	FBgn0028402	62.81818621	68.20928222	43.4552658	60.1421034	52.15273576	53.10927399	71.02446607	36.50630721	3L	-0.11411253	0.594672454	0.988780662
Drep4	FBgn0028406	15.99889949	20.45454671	23.56804375	22.09539722	17.10851343	22.09708844	15.33757097	19.24810819	2L	-0.117905007	0.581226996	0.985637351
Drep3	FBgn0028407	21.05033873	21.20407609	23.48039294	25.5777255	18.65881803	19.16359188	21.73566018	21.95641717	2R	-0.133292597	0.340444138	0.927274752
Drep2	FBgn0028408	130.0656101	132.675634	130.9484818	155.6825813	118.9053805	132.1803591	121.1730578	130.2758886	2R	-0.099666229	0.315495562	0.91460807
Nxt1	FBgn0028411	208.9184332	197.354044	238.7747543	199.473094	190.6110785	216.9763711	238.2226094	219.1589443	2R	0.064608107	0.647059965	0.99279236
Kap3	FBgn0028421	207.1669894	168.3510713	187.6851335	211.056548	163.7927693	223.7462373	186.5668582	192.2657296	X	0.010700361	0.928236422	0.996479182
GluRIID	FBgn0028422	0.49431943	1.469859474	3.761165045	0.314314675	3.812867586	0.889715285	0.917105081	1.512255023	3R	0.301092852	0.694377572	0.995787491
Jhl-21	FBgn0028425	32.28737031	13.38687486	16.66568299	22.04601813	24.54764493	29.03070796	20.97801662	14.47598687	2L	0.093038166	0.728434432	0.995787491
RNaseZ	FBgn0028426	18.55209892	22.49786579	20.83100423	22.43045728	22.74239857	31.02254362	24.62272855	22.31636876	2R	0.288390539	0.115346908	0.765367142
Ilk	FBgn0028427	47.95666016	53.23364347	49.68125893	48.86793642	46.66308557	44.89902294	44.96765398	59.97860076	3L	0.008621086	0.956022366	0.997604378
I-2	FBgn0028429	291.2973733	203.6919888	219.0985623	237.9795919	259.1170054	259.4262179	258.3967313	233.4890101	3L	0.111860307	0.383452344	0.946591184
Ercc1	FBgn0028434	28.29930766	23.37335931	15.44187143	12.63423264	18.63373984	12.16432268	17.3348912	25.0243642	2R	-0.100035672	0.78725631	0.995787491
ECSIT	FBgn0028436	62.30778242	52.69357311	49.64806766	30.08693862	45.88681117	64.2121857	46.88647974	47.68619212	3R	0.097791356	0.671386535	0.995787491
CG11070	FBgn0028467	142.5407194	142.5213474	166.4462504	168.4616257	122.3460566	223.1801014	146.3673202	155.2159209	2L	0.086332117	0.574367655	0.984934163
rtet	FBgn0028468	15.48827089	19.65186587	23.14297088	17.75917403	8.119618321	23.97230745	9.993392212	14.25384319	3R	-0.40195446	0.198492705	0.847197998
Nab2	FBgn0028471	197.6158751	163.5333341	180.9539454	185.9912566	160.9439501	223.0475102	182.8736718	210.3688334	3R	0.12100006	0.321239898	0.917167973
Non1	FBgn0028473	591.20724	698.6746231	617.3822572	794.129267	595.0728967	708.0450038	722.7676731	736.2298558	2R	0.062131312	0.599388601	0.989286463
CG4119	FBgn0028474	96.22157288	117.3058469	97.66693349	110.2807203	107.0101922	104.4400714	107.9366016	126.7744277	X	0.114891778	0.325265845	0.918470465

**Hrd3**	FBgn0028475	56.43176502	52.51711161	82.31253293	71.61065445	49.5258501	48.78200543	64.20005119	50.28854941	3R	-0.273858918	0.10181422	0.739991904
**Usp1**	FBgn0028476	95.22224008	118.9811411	101.902134	123.5820711	101.8264396	107.0224808	100.6054642	89.26622844	3R	-0.111048827	0.324972202	0.918470465
**Mtpalpha**	FBgn0028479	41.14771931	57.10085597	48.0438873	46.29793404	38.09217475	52.06471758	47.96994506	51.38404772	2L	0.009507165	0.950322061	0.997604378
**CG17841**	FBgn0028480	142.3071568	149.6312711	117.6707176	152.2835215	129.1290004	133.3360685	115.324802	137.11049	X	-0.096458387	0.373937268	0.943282319
**CG4341**	FBgn0028481	6.599719042	9.086537949	8.166116633	7.630577445	8.2336315	14.14446056	10.79007703	9.305600704	2L	0.460825118	0.003180322	0.143269195
**bdl**	FBgn0028482	35.3568155	60.28454871	72.25905838	31.97404482	36.25522831	29.26001095	47.5882578	29.69716982	2L	-0.444288455	0.171545965	0.832652344
**Ack**	FBgn0028484	50.74369842	39.41881231	41.45681201	48.60125542	41.63880961	44.96875679	39.81884877	34.01452484	3L	-0.142337536	0.302759376	0.910208378
**f-cup**	FBgn0028487	64.07419642	59.0802385	38.33308429	52.74172063	49.30855222	42.88963439	45.97657515	56.77105546	3R	-0.106006195	0.522865665	0.983400848
**CG31705**	FBgn0028490	185.0997155	285.5351214	295.7045359	169.1853006	189.5481001	191.9651199	287.8853487	161.320612	2L	-0.134051687	0.470555984	0.973549494
**CG2930**	FBgn0028491	0.098723233	0.369402833	0.405309699	0.354106924	0.123383588	0.365142977	0.271217787	0.280287209	X	-0.189933678	0.798127766	0.995787491
**CG30116**	FBgn0028496	47.96163336	44.38596924	42.69755209	45.72867271	42.16851147	42.98039928	40.04937384	43.65915311	2R	-0.069024381	0.435580566	0.966084844
**CG3530**	FBgn0028497	450.0738695	417.6494348	401.9705304	449.4647616	444.0113001	430.4162346	425.8278813	425.4615907	2R	0.034812433	0.691592671	0.995787491
**CG7985**	FBgn0028499	108.2728071	95.9677382	89.60972049	84.03797687	108.8608843	109.6337534	95.01611967	87.39697193	3R	0.113444235	0.305765517	0.911610813
**Rich**	FBgn0028500	42.7062116	53.06510564	44.81410002	37.05488647	30.92896375	35.9999978	38.96366687	37.63306216	3L	-0.275337346	0.049321911	0.583903663
**CG12182**	FBgn0028504	19.2150189	18.31189969	23.05464514	15.23925728	17.61599791	21.5248075	20.75427267	21.23621323	3L	0.128748946	0.479829435	0.973549494
**CG4455**	FBgn0028506	0.647613052	3.983026824	1.963875288	1.258237875	2.435640708	1.831696888	3.854841219	0.468675961	2L	0.173272233	0.834908766	0.995787491
**CG3793**	FBgn0028507	616.4409392	265.3120931	243.3948553	290.6381969	407.7640921	417.4913997	354.2780402	272.3002756	2L	0.056362808	0.873898683	0.995787491
**CenG1A**	FBgn0028509	11.19420255	9.672499112	10.50207407	10.03851578	10.93497304	12.64105466	10.11607384	10.38139	2L	0.117325092	0.259796501	0.890556245
**EndoGI**	FBgn0028515	17.77028188	29.08987046	44.37841057	37.66431203	43.7453363	53.44165739	35.75681659	36.31146065	2L	0.428099325	0.098401846	0.730787365
**ZnT35C**	FBgn0028516	0.2189864	0.352014008	0.464850708	0.765837822	0.560045985	0.563461131	0.081468189	0.148574921	2L	-0.37658142	0.564729507	0.984934163
**CG5888**	FBgn0028523	1.289570518	2.117946926	1.831836048	1.099766183	0.900118878	2.115389164	1.875919416	1.705566499	2L	0.091307573	0.727853191	0.995787491
**c(2)M**	FBgn0028525	1.845007474	1.411636341	2.512820996	0.754660609	0.87650299	2.746518596	1.734030313	0.843301477	2L	-0.042729256	0.949676536	0.997604378
**CG18507**	FBgn0028527	1.396693023	0.995671559	1.242397857	2.089483039	1.029567124	0.570610582	0.831365245	0.833896207	2L	-0.785586058	0.092605992	0.72305867
**mTTF**	FBgn0028530	50.37373151	44.49563441	54.19333482	37.62910039	52.02189353	48.70304091	64.82210028	43.89191171	2L	0.194730716	0.314943222	0.91460807
**Sec71**	FBgn0028538	2.843634913	3.61276828	3.927431402	4.529288265	2.566744918	2.893315023	3.95029264	6.357174667	2L	0.11585986	0.738891284	0.995787491
**Eato**	FBgn0028539	23.88085624	27.76612615	26.56688532	29.11680536	27.54184012	26.397444	25.30966875	27.49139384	2L	0.024295815	0.847222262	0.995787491
**CG9008**	FBgn0028540	40.4912417	26.06489335	31.27669469	30.68210358	29.60927918	32.04840645	29.39877756	19.4351674	2L	-0.194706548	0.28712912	0.90249226
**TM9SF4**	FBgn0028541	28.71742081	30.07895051	28.05901481	29.18355144	26.77977606	24.2114145	25.75042045	31.13222631	2L	-0.07277545	0.5098919	0.979810721
**NimB4**	FBgn0028542	0.051072079	0.428331237	1.115101357	0.952581033	1.382972879	0.288902664	0.390857911	0	2L	-0.244660524	0.865918252	NA
**NimB2**	FBgn0028543	0.043179584	0.579421558	1.152283756	0.100671575	0.155900432	0.569932274	0.220304093	0.656228119	2L	-0.15376375	0.912487179	NA
**ics**	FBgn0028546	7.879377654	16.30194749	25.70902603	17.34633455	21.70815926	24.02009696	16.59863909	16.51080049	2L	0.26765096	0.359721144	0.93924716
**Atf3**	FBgn0028550	1.349417728	1.924163315	1.878969544	1.327946666	1.652858835	1.47547732	0.848716375	1.097919235	X	-0.315237352	0.212427465	0.856520545
**gammaSnap1**	FBgn0028552	5.469070117	5.867796847	5.1398124	3.216451087	5.603636182	4.691682847	5.404718566	4.706754321	2R	0.077614667	0.853290187	0.995787491
**x16**	FBgn0028554	27.93947812	28.86620546	29.72966191	30.758582	24.16045115	31.09529431	28.19556849	30.8388029	2L	-0.007342684	0.955617922	0.997604378
**sut2**	FBgn0028562	1.002420518	0.993566241	2.87401423	0.424928309	0	1.503529906	0.581180972	1.286023663	2R	-0.607244928	0.57968951	NA
**sut1**	FBgn0028563	1.493775733	2.157599343	1.157628044	0.580446981	1.498137806	0.097800183	0.1058515	0.585564307	2R	-1.191972172	0.17225566	0.832669092
**robl37BC**	FBgn0028569	0.390172278	2.181530867	6.310350023	0	1.408721923	0.980938774	1.658896438	0	2L	-1.079245121	0.479153338	NA
**qtc**	FBgn0028572	37.30643917	52.04700075	34.43374128	44.06859426	46.46187519	41.98645044	36.47535069	47.32164808	2L	0.070541431	0.623132978	0.990047511
**prc**	FBgn0028573	0.08352772	1.12084826	0.521066787	0.055640496	0	1.14749161	0.273961296	0.103626521	3L	-0.184659844	0.877145593	0.995787491
**hfp**	FBgn0028577	348.3451566	332.3156352	274.0870889	316.4202879	299.0809706	357.2140182	271.8058296	275.5120461	3L	-0.052249737	0.638471576	0.991585536
**phtf**	FBgn0028579	219.6697517	190.9456632	197.6514089	206.2317725	210.4813866	212.1470875	220.7566463	211.9954949	2R	0.09947725	0.299377782	0.9074568
**lqf**	FBgn0028582	48.44514059	55.01408313	51.2614812	39.08771074	53.01369767	50.63958868	46.19175864	50.37937984	3L	0.079861949	0.489660553	0.973549494
**qsm**	FBgn0028622	0.518268435	0.395789148	0.48043629	0.648368624	0.60852511	0.715054119	0.408457956	0.356773505	2R	0.06313611	0.878961272	0.995787491
**beat-Ic**	FBgn0028644	0.447607084	0.515735137	0.527721535	0.234073809	0.434230063	0.597506407	0.533577237	0.295171808	2L	0.139462316	0.698378866	0.995787491
**beat-Ib**	FBgn0028645	0.541165633	0.481755844	0.660097346	0.18678907	0.368400168	0.316385752	0.323922104	0.252706404	2L	-0.535190729	0.144646634	0.8037297
**aralar1**	FBgn0028646	37.13782678	48.10812843	37.08983467	38.54601646	37.77775859	39.90576276	42.0032189	34.00156611	3R	-0.034496431	0.771808523	0.995787491
**CG11902**	FBgn0028647	39.8935203	24.02953315	35.53715872	29.26164878	26.73568169	29.73411012	22.73227236	27.91064304	3R	-0.239177385	0.181444917	0.837439137
**mRpL50**	FBgn0028648	300.2698152	305.5687932	286.9951629	224.4383653	292.5715604	295.1553346	287.7985008	261.6913767	3L	0.055676393	0.662097804	0.995787491
**Adat1**	FBgn0028658	13.24029702	7.231837874	12.21399252	7.91186764	10.04291159	11.09297164	10.28899352	8.51507012	2L	0.000393548	0.998872486	0.999757791
**VhaPPA1-1**	FBgn0028662	1754.532378	1717.879563	1533.582384	1984.005618	1817.700509	1733.561073	1758.529126	1930.428158	3R	0.080616773	0.447442852	0.967112302
**VhaM9.7-b**	FBgn0028663	238.2161804	235.384762	221.9382285	232.5452363	242.8155568	212.3966261	236.4799173	221.553311	3L	0.006650743	0.951694398	0.997604378
**Vha100-2**	FBgn0028670	0.264378548	0.229941146	1.187738276	0.502242588	1.237369358	0.147706358	0.949205314	0.425178	3R	0.38695586	0.608088168	0.990047511
**Vha100-1**	FBgn0028671	815.6817348	719.2141805	621.6728678	708.8235887	689.3145993	774.1575451	656.0580267	701.5187295	3R	0.005122494	0.960296913	0.997604378
**Sur**	FBgn0028675	0.601515595	0.342168374	0.177650071	0.268287315	0.500453567	0.108489696	0.120089677	0.170340788	2L	-0.60720345	0.391004302	0.949207648
**spt4**	FBgn0028683	74.73238003	67.28694348	93.35313181	71.70337228	80.19566365	72.41826768	71.25458155	87.09293645	2R	0.050851388	0.810404352	0.995787491
**Rpt5**	FBgn0028684	209.3315587	228.2058606	226.4931636	229.9215449	205.596796	203.199614	205.7871002	191.931458	3R	-0.117601694	0.228259273	0.867702781
**Rpt4**	FBgn0028685	327.9293582	354.0982245	252.990287	309.2351732	359.2502807	318.2332556	319.0610193	320.2782995	X	0.112353999	0.352785523	0.935162434
**Rpt3**	FBgn0028686	338.0374765	447.8623279	388.5744169	402.095541	372.8135724	354.5364079	397.5795528	363.7168286	X	-0.049648941	0.646966879	0.99279236
**Rpn7**	FBgn0028688	392.4578127	342.7673956	366.9808536	391.1275822	341.1566245	338.6204875	343.8291681	322.4786061	3R	-0.12136834	0.236770509	0.873280735
**Rpn6**	FBgn0028689	205.7814662	189.131094	210.8238729	197.4578284	174.3185569	225.3772074	183.6811872	235.0017532	2R	0.056201401	0.64049049	0.99179631
**Rpn5**	FBgn0028690	276.0017798	244.2494921	258.2145987	221.7916994	261.8187052	273.9019912	247.5744716	238.8748926	3R	0.059996955	0.56718437	0.984934163
**Rpn9**	FBgn0028691	214.5741878	225.3491072	198.0521648	185.8842984	226.1231561	228.0464088	211.3302114	185.8035195	3R	0.076954781	0.479239552	0.973549494
**Rpn2**	FBgn0028692	240.7713282	225.6647798	230.3539915	220.7291867	235.8723814	220.6065179	242.5817912	228.2279836	3R	0.045657925	0.623129576	0.990047511
**Rpn12**	FBgn0028693	290.9641068	289.9952437	268.9047104	323.8468845	313.837451	271.2794074	296.3242982	301.9494388	3L	0.041913002	0.727812804	0.995787491
**Rpn11**	FBgn0028694	353.8474075	318.5686692	324.4503343	348.5264445	327.0643715	304.9700429	354.2454851	322.3821076	2L	-0.01067186	0.921891777	0.996479182
**Rpn1**	FBgn0028695	228.5952736	270.1580039	243.5912832	246.6366801	224.5685802	243.0719901	234.3977447	273.4043606	3L	0.012381239	0.904014388	0.996479182
**RpL15**	FBgn0028697	3306.63333	3402.10472	3906.562176	3321.946242	4739.662667	4892.583603	5328.227592	5454.420806	3L	0.583035518	1.41E-09	6.17E-07
**Rh50**	FBgn0028699	18.48678325	27.90513087	24.19861957	19.84547048	20.20219018	17.70337527	27.35650171	20.22130565	3L	-0.044953141	0.792023895	0.995787491
**RfC38**	FBgn0028700	11.47830809	14.07947032	17.05120674	21.45615094	11.68425049	12.53789213	15.35016676	17.23574315	2L	-0.139754065	0.639523999	0.991585536
**Nhe3**	FBgn0028703	233.6530161	249.1225386	227.4179013	240.5488146	208.5307609	280.9242479	234.4686106	222.418472	2L	0.021079606	0.832372045	0.995787491
**Nckx30C**	FBgn0028704	91.9689025	87.75624631	77.7515646	96.78547872	93.13788975	95.582982	85.19498028	82.13449761	2L	0.034869383	0.727580147	0.995787491
**Mt2**	FBgn0028707	8.125119428	10.00275083	5.063485681	6.951710118	4.306179235	15.67201644	2.47206977	2.265737704	2L	-0.262602078	0.676294801	0.995787491
**Mst85C**	FBgn0028708	29.00962438	40.55639869	35.97955344	34.74662839	42.07898739	41.56030509	36.97127812	40.852148	3R	0.237489501	0.097443211	0.730787365
**Lnk**	FBgn0028717	100.0836455	99.96366879	104.4451018	80.81304199	81.6236365	107.0878909	94.96634757	77.95901221	3R	-0.062719647	0.615770905	0.990047511
**Fmr1**	FBgn0028734	97.41939454	126.0370746	125.414236	120.0635279	108.9778602	101.5603928	114.6346437	142.3443658	3R	0.030983456	0.812164831	0.995787491
**eEF1beta**	FBgn0028737	260.0184765	237.9843982	260.1856324	292.6134455	267.8062929	259.3461189	240.5970571	233.3816912	2R	-0.040970214	0.732691173	0.995787491
**CG6362**	FBgn0028740	0.201719249	0.812053782	0.97873617	1.410902508	0.436985934	0.532503093	0.205836055	0.525541396	2R	-0.94115251	0.373687437	NA
**Dhit**	FBgn0028743	43.08167642	41.91020627	42.15074561	47.3115653	44.66516429	40.52299915	45.42703137	53.11438273	2R	0.105946409	0.402533558	0.954942974
**CG5033**	FBgn0028744	25.99485812	32.68892829	21.4763841	31.06879676	32.47081139	32.79502566	33.11682099	33.34619309	2R	0.273958066	0.121235191	0.77836519
**CG18508**	FBgn0028746	194.5930541	132.1272189	157.1190316	135.2490281	153.3196721	164.1691595	134.3021249	132.4157395	X	-0.05842135	0.727194503	0.995787491
**Doc1**	FBgn0028789	0.165347074	0.396209315	0.687650905	0.110142994	0.810197583	0.957599012	0.120515411	0.256417002	3L	0.720599789	0.50169601	NA
**Dak1**	FBgn0028833	542.9326838	416.0301064	426.9180351	412.806265	455.4335811	427.8306063	374.5917996	335.5574519	3R	-0.148093989	0.268231557	0.89549671
**CSN7**	FBgn0028836	45.15768294	40.00095945	46.6285013	41.73325185	48.83395048	48.10591882	43.49589518	48.06103001	2R	0.149280633	0.172340063	0.832669092
**CSN6**	FBgn0028837	281.5092983	250.5675189	282.347597	292.8234095	317.7003347	257.6926159	282.3441738	267.6069995	3R	0.053368095	0.659996405	0.995750687
**CSN1a**	FBgn0028838	2.33202969	1.341138053	0	4.194287563	1.924530873	0.25127124	1.189811568	1.880562295	2L	-0.568273503	0.609427122	NA
**jhamt**	FBgn0028841	0.076248456	2.30212958	0	4.088719292	2.752960096	0.215659735	3.112183177	0.662169822	2L	0.097652221	0.941110455	NA
**CG15262**	FBgn0028852	1.555683693	0.838748943	2.426185891	0.129536356	0.902702502	3.771486215	0.850410541	0.12062619	2L	0.216813453	0.844936307	NA
**dao**	FBgn0028862	136.4133474	173.6458947	152.0919784	184.1954821	149.1070361	161.0034677	146.4517892	166.8531796	2L	-0.020978406	0.853454664	0.995787491
**CG4587**	FBgn0028863	4.842456687	6.269378565	5.720708887	6.541770431	4.727619741	5.356840059	5.156615619	5.695827706	2L	-0.126935249	0.303002824	0.910208378
**CG18420**	FBgn0028866	4.522401348	1.435131266	1.779126907	3.134645769	1.765209409	2.99610815	4.261319486	2.211385577	2L	0.051563715	0.930412774	0.996479182
**CG4701**	FBgn0028868	4.340777686	3.199247317	1.276444378	0.460016353	1.662227691	1.674187645	0.167779048	0.999539564	2L	-1.011894095	0.300881044	NA
**Cpr35B**	FBgn0028871	1.090160986	2.826006004	1.923429344	0.231060845	0.894554539	2.335902543	2.528203725	3.873011591	2L	0.705720511	0.484786774	NA
**CG18095**	FBgn0028872	1.17925587	2.233266777	1.015143265	0.709520172	0.068672874	2.295319186	0.485210816	0.660715782	2L	-0.510923924	0.599549015	0.989286463
**nAChRalpha5**	FBgn0028875	20.19031999	28.9770825	26.2556919	24.34641913	21.29081701	20.7760602	19.75324716	25.46323861	2L	-0.157740764	0.202621503	0.849847813

**CG15270**	FBgn0028879	17.55918002	12.7380552	11.93094821	14.6039764	16.25906514	16.30017041	13.94318229	16.29214726	2L	0.169712818	0.236414242	0.872962469
**CG15256**	FBgn0028880	0.679563786	1.139872918	2.78995922	0	0.66057751	1.034958486	0.266704895	0	2L	-1.171351724	0.401956919	NA
**CG15279**	FBgn0028886	0.025641873	0.774191594	0.155517063	0.836962849	0.23145076	1.619725058	0	0.306189835	2L	0.290461943	0.801263545	0.995787491
**CG4168**	FBgn0028888	2.353109921	2.060129243	2.233330971	1.978796527	1.609201052	3.550019634	2.010491146	1.940181438	2L	0.102613305	0.648436299	0.992932477
**GMF**	FBgn0028894	39.90578651	43.98994808	45.00572751	23.6486681	39.15226762	40.64283265	41.53733541	41.00147376	2L	0.121717593	0.592881436	0.988631016
**CG17328**	FBgn0028895	54.62369058	33.49164732	39.21283368	22.27847583	38.9633	35.90130927	40.02110419	49.23316216	2L	0.160525667	0.523542486	0.983811292
**CG4935**	FBgn0028897	40.85644523	44.06334723	57.62261798	41.23213638	53.59861505	39.6145399	44.45644672	41.47887028	2L	-0.001969847	0.992213237	0.998472242
**CG31817**	FBgn0028899	0.520300725	3.709108768	2.581859252	1.157922391	2.433579142	3.14389203	2.413269685	2.156549252	2L	0.397236945	0.415917577	0.958846438
**CG18109**	FBgn0028901	0.928920305	0.886937529	0.242689753	0.099957356	0.05159813	0.552415322	0.364568562	0.031027261	2L	-1.091020262	0.304167307	0.910208378
**CG13243**	FBgn0028903	0.114121536	0.8614029	1.38428627	0	0.309027932	2.582234084	1.310069203	0.123884209	2L	0.929131825	0.503683732	NA
**CG33090**	FBgn0028916	238.0606127	278.619116	217.0687202	251.7528336	254.3921546	283.3964739	266.6884509	210.4057839	2L	0.071300473	0.512240002	0.980804634
**CG16865**	FBgn0028919	21.87183126	42.19401725	35.13957455	29.3802752	35.99779215	38.5100487	43.2322837	34.69710811	2L	0.283047012	0.194235599	0.844272804
**NC2beta**	FBgn0028926	171.4443897	161.9231507	172.7451791	151.2514733	183.975197	174.7028278	160.009142	156.4109053	2L	0.068286231	0.61872485	0.990047511
**CG16863**	FBgn0028931	13.49065084	19.35098326	17.86828309	12.44099757	21.4370196	26.39438131	13.90018473	20.72289904	2L	0.420790873	0.095270263	0.730787365
**CG16890**	FBgn0028932	16.60833946	12.68787464	15.55814253	14.28102974	18.03391383	19.80534938	23.70103999	23.29505263	2L	0.546271042	0.016711935	0.372399106
**CG7653**	FBgn0028935	0	0	0.52239815	0.50204395	0.43732542	0.380654846	0.137330644	0	2L	-0.051899632	0.977536794	NA
**Cyp28a5**	FBgn0028940	35.15164799	54.92434586	50.5175948	24.14101698	35.79843902	27.80941952	34.01710937	18.00097124	2L	-0.473644787	0.073134573	0.671999479
**Or35a**	FBgn0028946	1.046394914	0.780080299	2.115449555	1.490885214	0.734615392	2.849989229	0	1.262122345	2L	-0.120585063	0.910443613	NA
**CG11865**	FBgn0028947	0.660487712	0	0.228904854	0	1.192349602	1.245407561	0	1.433979963	2L	2.069477035	0.292674396	NA
**Kebab**	FBgn0028952	0.676705044	1.021570001	1.559593148	2.051026036	0	1.499284448	0.604201187	0.440756788	2L	-1.022892392	0.30798241	0.912131082
**CG14478**	FBgn0028953	177.3053759	177.5031616	158.3245248	167.4780835	169.4052208	190.4502822	156.0775937	176.6091274	2R	0.054040361	0.55954363	0.984934163
**mthl3**	FBgn0028956	1.171956583	0.571866984	0.775404634	0.198718011	0.230801673	1.044645499	0.380505545	0.416360656	2R	-0.370057021	0.672898716	0.995787491
**AlaRS-m**	FBgn0028962	50.48500838	57.86054444	46.79610342	60.73478024	61.77249385	53.60096132	52.91623335	52.24897371	3L	0.061451554	0.650430574	0.993345869
**Or49b**	FBgn0028963	0.934576461	1.282598923	2.782557973	0.297126796	0.690197745	0.08010118	0.866954411	1.291214915	2R	-0.817038913	0.424819651	NA
**A16**	FBgn0028965	82.08038706	91.08852147	89.76391168	77.08173842	77.32885817	66.80675895	66.40399131	45.90434156	2L	-0.375783649	0.02599679	0.451297919
**gammaCOP**	FBgn0028968	171.2921985	144.800764	152.1176801	131.684973	123.8133762	142.1085506	153.633918	122.4687173	3R	-0.118222929	0.330022783	0.921112231
**deltaCOP**	FBgn0028969	265.3480798	214.8909198	226.079188	241.5895093	201.171234	260.4342453	236.2298587	246.1981894	X	0.020854617	0.859257155	0.995787491
**betaggt-II**	FBgn0028970	131.5012589	145.1078881	130.4783483	160.1911833	172.0441368	149.1017717	130.2807458	153.5321222	2L	0.123903336	0.357714121	0.938070952
**trbl**	FBgn0028978	0.976713914	3.495041221	1.895595467	0.341575758	0.440804736	2.271405118	2.782306967	1.555059969	3L	0.113377954	0.864288159	0.995787491
**tio**	FBgn0028979	2.933287203	0.73468718	2.414970761	2.197096185	3.905609218	1.276377811	1.320508001	2.276500479	2L	0.110791235	0.825402422	0.995787491
**tant**	FBgn0028980	47.06907882	58.78559302	58.78364969	57.9001826	61.42435253	71.02856226	62.05019449	33.86221495	3L	0.066647537	0.746897464	0.995787491
**Spt6**	FBgn0028982	115.5793761	126.8128416	100.2576506	127.4849521	97.21063313	121.2846103	109.0528558	99.85008649	X	-0.109521157	0.327836802	0.918470465
**Spn55B**	FBgn0028983	1.115855016	0.935845126	3.045429794	1.192388067	2.014404296	2.630053234	2.965177208	1.514140334	2R	0.582687238	0.397489141	0.952272708
**Spn88Ea**	FBgn0028984	5.743779463	7.827937225	7.029371256	3.826115883	2.731118968	4.448200159	5.952580079	6.736176366	3R	-0.265269918	0.476113359	0.973549494
**Spn42Dd**	FBgn0028988	3.697451791	3.28338883	7.123208671	2.915747091	2.15950969	4.306154956	5.548399386	4.367909912	2R	-0.021011925	0.96886554	0.997604378
**sds22**	FBgn0028992	91.05241951	100.1542963	88.12098468	79.30604502	80.59647833	82.57942953	68.02499119	92.70934734	3R	-0.115258549	0.447377072	0.967112302
**onecut**	FBgn0028996	133.1372155	135.4005251	139.0576598	149.6061298	133.2585259	144.3248509	133.492604	165.9502135	4	0.081056672	0.444173853	0.967112302
**nerfin-1**	FBgn0028999	3.025021363	1.585640303	0.917332454	0.503765959	1.511509865	0.28010436	1.378016873	0.293196491	3L	-0.791485858	0.338958033	0.927274752
**miple2**	FBgn0029002	15.97151517	12.86223286	14.75015394	16.02671431	12.16378618	11.76394293	16.08912298	10.04805417	3L	-0.226688234	0.293779807	0.906604178
**mab-21**	FBgn0029003	0.086652907	0.58139238	0.462481235	0.20202799	1.09501494	0.359461657	0.176842742	0.150505186	X	0.482131181	0.636341016	NA
**Smurf**	FBgn0029006	72.56783099	81.87032982	98.71106967	85.762658	97.55924419	104.8395336	105.5974153	128.0135978	2R	0.396579071	0.00125639	0.073737689
**Dd**	FBgn0029067	104.2376043	73.43721803	91.56650008	90.39266805	70.73266919	99.74598478	80.56998069	69.81888462	X	-0.140789967	0.335794011	0.924219112
**icln**	FBgn0029079	36.64981386	69.55538775	48.58074921	48.6699308	57.46571535	51.65172158	45.78337084	38.75968088	2R	-0.034117597	0.888305026	0.995926915
**hbs**	FBgn0029082	0.450872513	0.902803746	0.38283365	0.21680841	0.98181566	0.464931087	0.34505564	0.26919364	2R	0.117514458	0.812793956	0.995787491
**disp**	FBgn0029088	58.57928829	63.11128558	43.97132803	42.38020628	45.69997006	65.5626462	40.29086736	53.14138084	3R	0.004653422	0.977442552	0.997757033
**Chs2**	FBgn0029091	0.125981846	0.076842499	0.166707383	0.267019938	0.661613152	0.334729306	0.116866326	0.049730591	3L	0.912787431	0.385250978	NA
**ced-6**	FBgn0029092	6.124352	8.900212521	9.761806662	3.736848851	4.888407756	5.758095317	9.124382778	5.219714199	2R	-0.153676219	0.58826715	0.987588562
**cathD**	FBgn0029093	39.79387446	50.48958365	46.3675012	40.2422082	40.9825108	52.61964762	49.13316649	52.60090403	2R	0.175574588	0.150203882	0.809805479
**asf1**	FBgn0029094	109.3094616	126.6456995	128.2603817	122.7258787	135.2625942	133.6256777	120.1791054	135.4154897	3L	0.14012707	0.242738575	0.876959115
**aru**	FBgn0029095	3.209069516	3.662959684	4.231766319	2.20418904	2.091318618	1.506468616	1.630487359	1.300927029	2L	-0.993170427	0.004261648	0.180287197
**alpha-Catr**	FBgn0029105	179.8203009	225.2780391	154.3982269	172.5798272	162.7235551	215.9148762	143.4610487	138.5233403	2R	-0.12062879	0.404033785	0.956557569
**Uba2**	FBgn0029113	110.1244416	96.41280483	93.72123748	82.77321144	83.30008555	121.921022	112.478373	94.97179311	3L	0.133888227	0.356931442	0.937787223
**Tollo**	FBgn0029114	0.34825026	0.342427457	0.29132768	0.475960355	1.062254061	1.494459474	0.796491078	0.07821554	3L	1.257778763	0.097500312	0.730787365
**Surf1**	FBgn0029117	46.52238032	59.17050617	51.17795649	32.24640438	30.76921336	34.57512523	31.36277498	32.7581819	3L	-0.513562283	0.019858342	0.39822025
**ScsbetaG**	FBgn0029118	24.76672451	27.96284073	26.18763549	20.0932369	34.02381999	36.97412099	26.87112128	17.67161553	3L	0.252612961	0.29661367	0.906715248
**Sras**	FBgn0029121	95.61246528	100.2489762	87.27940556	89.04518198	94.59742611	86.45602419	83.63084861	82.49569734	3L	-0.070762831	0.604692277	0.989286463
**SoxN**	FBgn0029123	3.895131046	9.024220853	1.566221037	5.067494428	1.282024448	0.994160122	3.238990144	6.167309885	2L	-0.700667108	0.228748724	0.867702781
**Debcl**	FBgn0029131	3.784894688	4.891567278	2.709472639	0.723306515	2.837626109	0.623978246	5.328907727	3.502479918	2R	0.061691634	0.919172805	0.996479182
**REG**	FBgn0029133	192.6559443	183.7921428	246.8552581	225.8938679	186.6958943	228.3667142	210.356988	208.4598338	X	0.003177471	0.979663844	0.997881056
**Prosbeta5**	FBgn0029134	505.4896452	426.1838702	403.8087635	439.0668249	444.3461126	457.6539195	448.3748238	495.3714511	2R	0.085167571	0.448833871	0.967112302
**Patsas**	FBgn0029137	185.679271	206.807568	192.1231139	201.4663523	189.5653364	182.1866054	192.9059572	205.1454662	2L	0.001539833	0.987778181	0.998078482
**NtR**	FBgn0029147	0.072910981	1.161830454	0.176881023	0.594962228	0.065811504	0	1.394981096	1.741261384	2R	0.727915025	0.607114115	NA
**NHP2**	FBgn0029148	48.56808774	37.36027433	49.53174029	68.75820616	58.26574497	53.37270351	52.34766203	51.93933956	3L	0.109210388	0.646990357	0.99279236
**Mkrn1**	FBgn0029152	97.92430328	79.26314048	80.54975218	80.53141366	79.89187	102.9164562	80.3455601	61.51652897	3L	-0.034957739	0.814974378	0.995787491
**Men-b**	FBgn0029155	594.1682149	560.3726723	511.9489998	544.2138251	573.4375566	635.8894322	593.7156007	551.8552944	3R	0.119570383	0.171088945	0.832652344
**ssh**	FBgn0029157	76.64730126	85.0199789	70.31402987	77.01539869	68.37037664	67.38582935	56.10798893	76.9567279	3R	-0.169392075	0.164550629	0.827712868
**Las**	FBgn0029158	65.40762741	68.09432436	57.08099704	61.18217494	73.10833575	69.38577685	61.78463424	68.75404289	3L	0.146390226	0.300552683	0.9074568
**slmo**	FBgn0029161	8.740927985	4.90934097	4.989498676	3.043039683	3.141642864	5.034029974	7.062808397	4.121780372	2L	-0.15036426	0.740937227	0.995787491
**Hml**	FBgn0029167	0.689368701	0.260432272	0.090399938	0.086877685	0.313924696	0.626511605	0.142588732	0.417992623	3L	0.410253991	0.599591288	0.989286463
**Fad2**	FBgn0029172	2.841025601	1.994827768	0	1.540405636	2.981848464	2.803083052	1.34837532	2.868897474	3L	0.654805581	0.481055026	0.973549494
**Fkbp59**	FBgn0029174	199.6235065	159.1041852	144.132096	159.4370112	171.013777	165.9531244	182.6044772	142.6878865	2L	0.026508027	0.845329936	0.995787491
**sotv**	FBgn0029175	158.1283	161.7544303	158.1048671	176.4795951	156.7014226	170.7818761	180.3828003	159.3300559	2R	0.057103856	0.579313409	0.985637351
**eEF1gamma**	FBgn0029176	275.2823543	407.8169795	346.2828194	420.6798487	339.8605276	359.4527836	333.7860642	403.3937188	3R	0.0193047	0.887048192	0.995926915
**Coq7**	FBgn0029502	2.530742568	3.604216102	3.706895802	1.669905008	3.361828669	2.971206225	2.923462036	2.488064778	X	0.065949269	0.902560978	0.996479182
**CHORD**	FBgn0029503	112.7980941	94.24488714	95.783679	83.12972934	102.6371479	78.74252122	94.05733407	84.13735932	3R	-0.073750648	0.644454109	0.992589784
**CHES-1-like**	FBgn0029504	9.111136498	8.234905984	11.64609195	9.407825099	7.538878472	10.63399359	8.610829884	11.93124893	X	0.042217764	0.796169221	0.995787491
**Tsp42Ee**	FBgn0029506	113.0802048	108.2776487	115.8355249	99.28739004	98.22929406	116.7590561	110.0845632	99.84714344	2R	-0.009567465	0.922612983	0.996479182
**Tsp42Ed**	FBgn0029507	1.507412575	1.491150818	1.594060307	0.270344282	0.418656314	3.097442212	0.394404294	0	2R	-0.290132469	0.796398154	0.995787491
**Aos1**	FBgn0029512	144.7445315	188.3471808	161.7172836	144.606087	185.4824485	188.2243812	169.0236583	160.336537	3R	0.169623898	0.177117677	0.834615603
**CR18275**	FBgn0029523	6.14664549	12.97654988	10.28391464	7.330056658	8.545403705	10.49098416	9.101739626	9.970354742	X	0.093111812	0.761567444	0.995787491
**CG3176**	FBgn0029524	3.919810233	2.886555412	5.798417654	3.34349634	7.248853869	4.146252797	6.097268366	8.717838451	X	0.752905872	0.196067555	0.844272804
**CG18273**	FBgn0029525	4.615793273	11.31108315	5.905116264	7.146340637	8.007194178	4.742718498	4.277632289	9.120962003	X	-0.10544708	0.756708771	0.995787491
**CR18166**	FBgn0029526	121.0977818	141.3652016	130.5576684	133.1904432	123.7715144	130.319741	104.5135195	121.6326989	X	-0.100010902	0.387957227	0.948247506
**CG13365**	FBgn0029529	317.2523696	299.0405716	267.0988697	301.7042191	313.3642514	269.9147584	328.3415745	392.7278687	X	0.168544164	0.286021837	0.902235424
**CG13362**	FBgn0029531	0	0.282116419	0.91806403	1.176391334	0.455441165	0.475707687	0.107264566	0.182578864	X	-0.897569212	0.539864502	NA
**CG14778**	FBgn0029580	7.992968518	9.822550677	7.338890574	11.58235983	7.515289977	6.122774041	7.646337905	8.736992397	X	-0.261392327	0.349917217	0.932590369
**CR14798**	FBgn0029588	6.035226144	3.707103876	6.598929328	16.647265	1.381072818	4.648148056	3.252674272	6.459242425	X	-1.042241297	0.11007097	0.758410631
**Coa8**	FBgn0029594	234.6977856	219.4510027	223.1081867	202.4739977	216.7398302	221.7687187	223.8471578	244.9636798	X	0.074182265	0.517361286	0.980804634
**CG3091**	FBgn0029608	4.644409954	6.232276922	8.675800802	9.204026922	4.19217516	5.123103963	16.11326582	1.411682151	X	-0.071199163	0.901764569	0.996479182
**eIF3g1**	FBgn0029629	776.3031015	945.7855797	921.582292	922.2069062	832.5274911	774.2626757	901.7157803	955.9956715	X	-0.007804962	0.944040871	0.997604378
**CG2875**	FBgn0029672	5.15870438	8.250537653	6.014934745	6.619690538	3.898374519	11.08447228	8.26323428	10.76591526	X	0.41818504	0.280562327	0.899310165
**HIP-R**	FBgn0029676	39.92451833	21.08673999	20.02479375	21.90176741	22.69457758	35.58924487	26.78363704	29.01824593	X	0.166479356	0.508131102	0.978557094
**CG15239**	FBgn0029681	2.673402643	1.770706963	2.694034048	1.246587524	0.618740636	2.507542207	0.76942547	1.250136378	X	-0.675127312	0.185545746	0.839299945
**CG2938**	FBgn0029685	138.38762	141.4211921	131.8925284	160.0574866	122.3583269	153.8607625	144.4752173	153.3400985	X	0.034443242	0.762679409	0.995787491

**Glos**	FBgn0029686	8.480704002	3.824360459	6.565092567	6.160258349	9.155133029	7.935287922	12.06746156	9.900131145	X	0.663295363	0.024807952	0.44131412
**Vap33**	FBgn0029687	245.6304325	211.9701148	203.0511	215.2812512	228.6886974	268.982035	186.6260802	224.0855581	X	0.078729511	0.498318052	0.976006682
**Iva**	FBgn0029688	21.16764364	26.37315632	27.0829723	27.63770161	21.46385969	20.20712318	24.45882063	31.94495639	X	-0.025183064	0.878933472	0.995787491
**CG6428**	FBgn0029689	68.81039514	58.43183003	76.48379397	56.9465958	72.74517513	71.62010678	69.92650331	58.11054295	X	0.092944414	0.507098847	0.978557094
**CG6379**	FBgn0029693	93.04094196	107.0980046	76.25706613	97.00981088	92.62049662	101.871162	99.39891352	73.38512938	X	0.004454772	0.974496492	0.997757033
**CG15570**	FBgn0029697	0.100699375	0	0.122147639	0.469553603	0.181788181	0.268993141	0	0.255065609	X	0.064411373	0.96685953	NA
**CG12692**	FBgn0029703	2.020703801	0.586635084	1.602642058	0	1.823944132	0.84264357	0.1982638	1.012415771	X	-0.093421887	0.93175424	NA
**CG3626**	FBgn0029706	10.27891875	8.466780638	9.128545123	4.664598962	10.07330209	8.081519901	11.05059151	6.41598918	X	0.157887407	0.578361674	0.985637351
**mRpL30**	FBgn0029718	311.1550915	245.399422	300.4247724	267.0914302	288.1208962	275.2311683	308.4956594	322.9714083	X	0.117126185	0.385300212	0.947224769
**CG3009**	FBgn0029720	90.29916617	53.66305436	47.52752926	72.67450574	73.51608803	51.78755553	51.91720512	51.34009917	X	-0.183539065	0.333018065	0.922663747
**CG7024**	FBgn0029722	1.18384846	0.992869857	0.57439997	1.472052329	1.994673229	0.409245869	0.805339428	0.08567482	X	-0.332918155	0.736330274	NA
**Proc-R**	FBgn0029723	3.762518945	5.994532502	4.559038473	2.733696919	1.797751476	2.032966164	2.397005762	1.028725376	X	-1.198643446	1.12E-05	0.00187203
**CG6978**	FBgn0029727	1.833652378	0.805539069	0.42365858	0.101787893	1.418662501	1.111343311	2.227469814	2.938378585	X	1.266126429	0.157815195	0.818884898
**CG2861**	FBgn0029728	0.919417887	0.590222596	0.688424374	0.132320259	0.368841096	0.38525403	0.579124638	0.443586965	X	-0.375453882	0.592058689	0.988631016
**CG6927**	FBgn0029733	4.689379254	1.058854441	1.422046083	0	1.13959077	0.680172018	0.115026031	3.622113737	X	-0.348469892	0.75773659	0.995787491
**CG4041**	FBgn0029736	88.28661097	85.51325466	72.78740682	74.86137925	84.62740157	88.62527836	64.74756084	84.61639626	X	0.034249778	0.798826352	0.995787491
**CG6903**	FBgn0029737	78.81132357	100.8266485	109.6041625	112.9464688	92.22397453	120.7875833	98.66943189	114.7155889	X	0.115746268	0.413844519	0.958665466
**CG17764**	FBgn0029751	1.948161234	0.502733411	0.363554768	0.174694791	1.487932066	0.989000122	2.293754383	4.717673576	X	1.630416369	0.1230265	NA
**TrxT**	FBgn0029752	12.45924268	11.33861935	9.646579454	10.35230374	6.221226427	12.12138613	7.100635221	5.179819633	X	-0.48694806	0.21794095	0.861225611
**CG4198**	FBgn0029753	2.421006866	2.556862575	0.435061399	1.463385355	1.537781851	2.662928899	0	0.973375929	X	-0.384444038	0.69470374	0.995787491
**CG15930**	FBgn0029754	2.000421377	1.342170492	0	0.839503284	2.311214906	0.980712229	0.102062358	2.084687994	X	0.405735468	0.70749778	0.995787491
**Sas10**	FBgn0029755	41.48503221	42.83518551	53.48192503	46.659276	37.35147352	38.76788667	46.66589468	73.43471294	X	0.124132417	0.59372429	0.988631016
**CG3309**	FBgn0029756	54.62265865	49.58998489	56.78039726	49.73100439	52.49334421	53.72897561	65.33022245	61.37164374	X	0.17400536	0.222319583	0.86549481
**SK**	FBgn0029761	11.34410477	13.28836056	13.27466162	12.89123852	11.87301103	11.53314936	9.212833273	11.81907121	X	-0.161116966	0.144869051	0.8037297
**NAAT1**	FBgn0029762	1.049731011	0.854494482	0.486856269	0.071982598	1.00325304	0.62582705	0.118142045	0.167578176	X	-0.335246316	0.718884513	0.995787491
**Usp16-45**	FBgn0029763	48.41237859	35.15986266	42.61084385	38.57098775	38.69327538	48.27116724	31.82735483	37.50541052	X	-0.050541197	0.741924536	0.995787491
**CG15784**	FBgn0029766	2.286369936	5.260922255	3.057797022	1.913543489	1.269994993	0.994881051	0.149553303	0.31820005	X	-2.129344253	0.001898467	0.099353754
**SPR**	FBgn0029768	4.179463876	3.862658465	3.585370426	3.172748989	2.965169859	4.028320069	3.31755467	2.962453484	X	-0.130079736	0.375743119	0.943615128
**frma**	FBgn0029769	1.168975665	0.478242119	0.207506243	2.260106677	1.492651702	1.585953266	0.363668541	0.309506632	X	-0.11479474	0.899992662	0.996479182
**CG12730**	FBgn0029771	53.78100042	61.19239467	53.04369952	64.04797261	55.66503133	44.43713869	66.60618753	49.99183168	X	-0.068290669	0.688421665	0.995787491
**RhoGAP5A**	FBgn0029778	42.33980494	39.73686793	32.16922662	56.95018923	59.42549029	58.56059578	41.93785244	61.95585715	X	0.401598104	0.047325073	0.57243419
**Sirt4**	FBgn0029783	18.68366122	18.38272306	22.58306895	16.46977755	25.028436	18.7352203	20.04179547	22.79035098	X	0.218884566	0.396524533	0.952272708
**RpL35**	FBgn0029785	442.8414496	673.0753709	644.8896897	699.4139208	572.5311414	518.4113765	651.5189824	657.5404114	X	-0.000569177	0.996900348	0.999490563
**PGAP1**	FBgn0029789	85.50986051	84.79837938	72.1030762	79.05609943	76.10903937	85.82371639	76.02871756	90.50763081	X	0.060274374	0.603393293	0.989286463
**AdamTS-B**	FBgn0029791	1.358245615	2.581074045	0.855053681	0.681441453	0.574198188	0.470073727	0.942982854	0.485258234	X	-1.118983339	0.028843363	0.4704121
**CG4078**	FBgn0029798	46.9098813	35.83595213	34.26214018	51.25633941	25.78449129	18.48265355	31.48623751	27.96956392	X	-0.670209651	0.000897564	0.056668946
**CG15772**	FBgn0029799	518.2110556	457.355268	405.4942186	598.4037501	465.0032272	444.2256778	405.5546335	489.3449765	X	-0.105930524	0.427226533	0.962819343
**lin-52**	FBgn0029800	62.97500002	83.30081008	85.23318946	84.74569116	82.97084633	79.21534157	111.1871106	80.35565927	X	0.194937564	0.25268339	0.883331952
**CG15771**	FBgn0029801	42.34485459	35.68769322	35.01405804	39.78992776	40.20573985	31.73073909	37.87669236	40.20450393	X	0.001715716	0.990907968	0.998408391
**CG15767**	FBgn0029809	0.665363619	0.608756798	0.146742078	5.499958086	1.747129983	0	2.314577572	0	X	-0.753556968	0.604098926	NA
**CG12239**	FBgn0029810	11.46258931	13.48300463	12.06765813	5.21045572	7.54835767	14.23922641	4.689644313	14.24312682	X	-0.018841258	0.961744503	0.997604378
**Prosbeta2R1**	FBgn0029812	0.143884129	1.206727207	1.74530445	2.012762581	0.389621595	2.780888098	0	0.156192882	X	-0.56350969	0.684965642	NA
**CG15765**	FBgn0029814	376.4999148	369.2808128	393.6438236	387.5219783	306.0640729	304.699954	354.1950736	342.8073155	X	-0.191906976	0.040669814	0.530560761
**GAA1**	FBgn0029818	161.531323	175.8955506	132.5451903	168.4766012	160.7807477	196.2057867	147.7198487	154.0476506	X	0.072756958	0.564268458	0.984934163
**Usp30**	FBgn0029819	231.3860204	199.6107788	223.5555272	237.2354014	183.6357735	210.2965369	218.8243327	240.8901109	X	-0.033864212	0.777257076	0.995787491
**CG16721**	FBgn0029820	68.91093819	75.03760065	57.33657132	49.99941511	79.29400521	79.27493464	75.28620292	78.54364382	X	0.344654451	0.012367261	0.33520527
**CG4020**	FBgn0029821	0.537762944	2.641635559	0.465930501	0.44777645	3.189771237	1.122642013	0.881900244	0.083395224	X	0.414306467	0.684313233	0.995787491
**CG12236**	FBgn0029822	105.1192302	102.1496533	104.897316	120.3758771	94.12450752	105.8945132	100.5158369	117.1547708	X	-0.02084139	0.855764486	0.995787491
**Shmt**	FBgn0029823	10.63569879	20.09909307	17.52061071	20.19471789	16.55909188	28.65873114	24.76229821	17.34352357	X	0.383479358	0.1151238	0.76449542
**CG3726**	FBgn0029824	17.90798551	13.22311736	14.62581345	14.88804692	19.02568443	19.62488777	17.76289077	16.30251135	X	0.287799449	0.021970649	0.420318478
**CG12728**	FBgn0029825	24.17710487	19.50753944	23.44543247	11.99156849	20.87668348	34.34547127	19.38875994	16.92790568	X	0.2352001	0.383624011	0.946594947
**CG6041**	FBgn0029826	58.28122192	68.17560911	50.58417251	56.00878791	48.5596352	63.67971446	44.0203722	59.961288	X	-0.077850007	0.641246568	0.992099188
**Grip**	FBgn0029830	1.060458443	0.889385136	0.689472181	0.375807679	0.804047699	0.855823444	0.551871463	0.165769448	X	-0.318579449	0.520048172	0.981838504
**CG5966**	FBgn0029831	0.562602271	0	0.818919157	0.577141847	0.203128259	0.233383913	1.205576804	0	X	-0.241034225	0.857959697	NA
**MCTS1**	FBgn0029833	107.0897788	145.0311257	122.6617379	113.9250713	175.4034466	191.8405184	179.1076203	153.5494748	X	0.550507305	1.28E-05	0.002044837
**CG5937**	FBgn0029834	22.49770355	31.67365843	46.23529483	44.99825344	29.82387588	23.9102408	41.05826241	46.5972018	X	-0.00278363	0.993249059	0.998777916
**CG5921**	FBgn0029835	0.43841061	0.343173738	0.524698493	0.531511674	0.52762901	0.286576053	0.3280625	0.190366004	X	-0.431388773	0.42490623	0.9620897
**CG5928**	FBgn0029836	0.729556433	1.557472726	0	1.082412138	1.436766923	2.000934834	2.707075106	0	X	0.885473345	0.495056168	NA
**Tsp5D**	FBgn0029837	57.80096343	102.3322374	85.72730631	63.76536841	88.04241395	57.5606855	88.60210492	71.00551311	X	0.019620255	0.913323266	0.996479182
**CG4666**	FBgn0029838	7.327681224	1.949631845	2.329380665	1.531688055	1.642139091	2.66810768	4.254285405	2.413790576	X	-0.261287715	0.697164015	0.995787491
**CG4660**	FBgn0029839	645.7351196	515.2230525	504.3547256	587.6480973	629.7573964	625.3178139	550.504783	570.6631227	X	0.103793754	0.349595279	0.932328657
**Nep1**	FBgn0029843	251.8551355	316.6651832	251.9607689	329.3861946	263.1653883	265.4340331	254.0941784	229.1410504	X	-0.154752948	0.162711439	0.824840106
**Efr**	FBgn0029849	59.39716048	49.70999572	52.41954302	62.58753989	51.68866975	61.7352148	57.84131029	84.29167707	X	0.218388845	0.212042622	0.856520545
**CG14445**	FBgn0029851	1.602557736	0.761618639	1.879094979	0.498173738	0.964342482	3.34912098	3.134255398	1.62367389	X	0.955694575	0.136599859	0.796109055
**CG3566**	FBgn0029854	126.5654731	100.508805	114.302698	126.6780535	121.2924049	99.60853755	115.7796091	105.405946	X	-0.054237288	0.703214979	0.995787491
**CG11700**	FBgn0029856	13.68545754	13.03641996	12.84308039	11.42354003	6.405196624	5.362794827	5.17213277	3.668196933	X	-1.276398637	0.000232913	0.01941098
**wuho**	FBgn0029857	163.3423415	200.9413752	163.7095574	217.1415631	208.5621479	160.3658769	255.4246644	185.0977525	X	0.152149952	0.35437367	0.936745458
**mldr**	FBgn0029858	7.625057811	11.17226173	6.361069956	8.432032523	8.487623111	6.194351891	7.303992877	7.786601011	X	-0.14305669	0.626095411	0.990047511
**CG15891**	FBgn0029860	234.4341648	307.4251237	227.3920913	243.1658664	274.0219344	178.5148633	290.6170182	288.2166125	X	0.063047953	0.720807526	0.995787491
**CG3815**	FBgn0029861	66.22752208	82.1592182	89.89843414	96.77223096	74.23228899	59.40589593	53.37468021	84.86424652	X	-0.267054458	0.117061681	0.770001618
**CG3823**	FBgn0029863	3.752414974	5.350027251	4.475792307	4.884642956	4.967651893	3.921010591	8.375929814	2.376161744	X	0.118437251	0.781732586	0.995787491
**CG15894**	FBgn0029864	91.9247476	95.95085116	88.69769958	98.60304148	93.5205222	84.2537477	78.30667106	83.4832466	X	-0.113490491	0.225761445	0.867702781
**CG3842**	FBgn0029866	1.175419316	2.409734565	0.387247648	1.522469937	1.074064914	1.203946634	1.036525407	1.102691913	X	-0.284187555	0.609436426	0.990047511
**CG3847**	FBgn0029867	98.19171025	132.6886234	100.5848493	97.5178595	105.8582207	106.472212	118.0966274	77.23265682	X	-0.041876095	0.786492278	0.995787491
**ND-B16.6**	FBgn0029868	1097.456299	851.1788454	916.7029532	910.1475025	1008.320433	1042.045416	981.9780572	944.2187867	X	0.102296299	0.332505746	0.922658315
**Marf**	FBgn0029870	231.806095	211.3494291	171.697965	238.2367825	189.810484	200.095243	198.4021467	197.8842937	X	-0.089978126	0.445522671	0.967112302
**CG3918**	FBgn0029873	99.71706447	88.73673016	94.66129861	121.4048802	102.1638444	99.57860499	110.835482	84.11460742	X	-0.000798162	0.995772771	0.999129577
**CG3342**	FBgn0029874	55.50956034	50.95747606	55.1868793	63.4626376	74.58637118	73.09356279	61.00717325	75.03253599	X	0.363947943	0.017347344	0.376491575
**Pat1**	FBgn0029878	74.35036527	72.73904712	70.23762774	88.58479361	65.85786392	83.02464608	57.29782113	59.44865913	X	-0.177963521	0.184381859	0.839299945
**APC7**	FBgn0029879	0.265394855	1.168551923	0.402402635	0.464068584	0.119776433	0.562978457	0	0.0720246	X	-1.511648298	0.271772061	NA
**pigs**	FBgn0029881	8.934505273	8.110534043	7.357272521	6.967641039	9.239814356	10.26321989	8.604419796	9.425362736	X	0.286933104	0.011229378	0.319404908
**CG3226**	FBgn0029882	191.0180342	204.1739907	182.5486966	169.7423865	217.3999248	185.7027292	206.9478422	170.3275805	X	0.092663386	0.471666727	0.973549494
**CG3224**	FBgn0029885	141.920664	217.7752743	170.4168967	179.9771012	121.3141783	144.1220039	150.0619488	112.4204273	X	-0.395237584	0.015226628	0.362234897
**CG3198**	FBgn0029887	180.6007956	200.5824895	149.0355104	167.5708189	189.2014129	237.099662	176.5198913	190.1672766	X	0.212681432	0.068686849	0.659001649
**ND-ASHI**	FBgn0029888	640.1629717	622.5705916	584.5502305	616.939927	630.8645385	614.4976259	643.2510372	765.0929895	X	0.138457326	0.19343243	0.844272804
**Fum2**	FBgn0029890	0.044903186	6.251459993	1.089345027	0.41876035	1.459111665	0.211672247	3.43646976	0.097488973	X	-0.526328423	0.663258935	0.995787491
**Pink1**	FBgn0029891	214.2219511	202.2615309	186.5664709	216.0097108	193.7643937	244.4068164	198.2631214	209.8995187	X	0.074333709	0.483744198	0.973549494
**CG3184**	FBgn0029892	62.57396184	92.25542181	69.21243331	61.02987751	66.76839655	72.51244308	89.84909819	75.66948452	X	0.129667288	0.483928203	0.973549494
**CG14442**	FBgn0029893	28.58963282	49.58328204	59.2762967	58.02754695	49.06525918	42.28295275	54.98008631	71.0932235	X	0.191646056	0.360820868	0.939549067
**CG14440**	FBgn0029894	105.5068597	191.2814079	179.3263168	182.7319161	145.542202	115.1628839	155.6817496	142.2431976	X	-0.201029647	0.241045749	0.874945677
**CG14441**	FBgn0029895	4.361192289	7.147204704	7.483292404	4.469431051	6.543850261	6.551618575	7.013174313	5.647078567	X	0.171904371	0.372087657	0.943282319
**CG3168**	FBgn0029896	4.278461037	4.115409616	3.691185276	3.517046255	2.065918689	5.860522534	3.693439689	2.381054251	X	-0.134803688	0.618622386	0.990047511
**RpL17**	FBgn0029897	201.3288953	245.5029093	282.6431708	329.5006359	269.4904336	218.0203612	279.3763399	313.311008	X	0.062427909	0.69268609	0.995787491
**CG14439**	FBgn0029898	1.498512534	2.938368988	1.02404803	0.959544344	1.428802389	2.268420914	1.049906825	1.512148044	X	-0.00245183	0.995641398	0.999118051

pod1	FBgn0029903	67.56325606	81.55197102	81.74664758	80.71119757	65.50015447	75.24825711	75.70218405	78.06946959	X	-0.049334672	0.63771887	0.991585536
Nf-YC	FBgn0029905	103.2087524	128.0629453	103.0357451	165.9930661	106.2230808	129.779166	108.1043679	112.2437035	X	-0.104832073	0.505878231	0.978557094
xit	FBgn0029906	8.642829335	19.99329983	24.42354552	16.16463165	12.34485264	19.83375918	21.44232784	23.71323069	X	0.201280323	0.539682554	0.984934163
CG14435	FBgn0029911	39.66925744	23.43549486	28.59323139	26.98719913	30.28323169	26.45846367	20.57008491	29.90836849	X	-0.120334669	0.527344177	0.984107922
CG4557	FBgn0029912	50.59730949	61.37319579	53.28286052	77.12700302	63.46601652	63.91434444	76.89027575	59.8604541	X	0.153967757	0.331983051	0.921929165
Cht11	FBgn0029913	38.3972345	33.75333728	23.83234511	31.27718503	30.12946962	32.11752051	40.82463213	44.14734655	X	0.234973025	0.300967586	0.90780451
CG4558	FBgn0029914	113.0052609	115.8745815	83.99422145	140.8089601	113.022946	115.2709784	113.7227654	83.08633789	X	-0.067704363	0.687741019	0.995787491
CG14434	FBgn0029915	7.978965644	6.843884078	8.757329129	7.02884522	6.414315975	7.362239105	5.710250389	6.9636785	X	-0.18023997	0.249804937	0.879541335
CG14431	FBgn0029922	3.20283809	3.798289073	3.480728295	3.305087434	3.008470001	2.779557375	2.981378147	3.503227421	X	-0.134926863	0.304145752	0.910208378
CG4586	FBgn0029924	0.922648548	2.228563446	2.059265907	1.462761841	0.599622156	1.913708313	0.753182806	2.163407838	X	-0.249449406	0.727781992	0.995787491
CG3040	FBgn0029925	168.4558772	158.2015982	156.274136	175.4994661	174.7988021	164.3957952	168.8759678	174.1587158	X	0.080217354	0.515915021	0.980804634
CG3032	FBgn0029928	10.12832105	9.910155325	11.77368352	4.427587358	7.713653356	13.72657138	18.57074955	16.37761316	X	0.667647805	0.084616254	0.69799888
CG4593	FBgn0029929	33.19594084	52.83271118	37.2698721	30.95803456	42.36712351	52.0402445	38.796886	54.30500743	X	0.316877901	0.171868649	0.832669092
CG12541	FBgn0029930	4.453305087	3.769222369	3.295469694	4.166716837	3.884223277	5.245889244	3.868031103	3.621999199	X	0.107773656	0.490105193	0.973549494
CG4607	FBgn0029932	5.869734595	2.084440757	2.116741038	8.013776942	1.956988239	5.558877625	1.551341614	2.583189965	X	-0.6215448	0.258444618	0.887830768
CG4615	FBgn0029935	115.4056637	193.9671655	143.9665159	136.6209018	105.0296436	112.1606764	106.0893243	105.5984738	X	-0.425197888	0.001942479	0.100550432
CG4617	FBgn0029936	62.3239753	78.79878798	56.65820781	52.15577968	74.54947685	80.3977115	63.11655376	64.59576063	X	0.208529583	0.194970888	0.844272804
CG8300	FBgn0029937	86.73690078	97.52339495	106.794462	107.8895887	107.4227657	100.0093955	107.4555849	96.47542182	X	0.076596718	0.574787055	0.984984955
CG9650	FBgn0029939	0.096418894	0.076681933	0.038312991	0.019379054	0.057019922	0.087768553	0.010602012	0.010827638	X	-0.450817139	0.562796771	0.984934163
CG9657	FBgn0029950	132.5640005	169.6141479	200.3623282	60.61830484	78.62478864	103.5750248	147.1024079	87.50869076	X	-0.396131687	0.313499628	0.91460807
CG15478	FBgn0029955	1.312142777	0.536125298	0.489729242	0.15688264	1.943593036	0.459940073	1.244510616	0.474797239	X	0.731023505	0.348265953	0.931729531
CG12155	FBgn0029957	81.72521765	103.9599952	71.85842129	67.02147465	67.98385897	83.77749817	73.11014307	66.71127171	X	-0.123856602	0.401741475	0.954265
Pdp	FBgn0029958	164.1584648	146.6052548	177.3559908	152.3041746	197.637996	187.6375857	183.111669	177.841476	X	0.250644341	0.017200879	0.375267338
Rab39	FBgn0029959	126.5958975	111.7615044	121.1742123	97.28279269	119.9419507	137.5309073	124.1647331	96.02871291	X	0.091940888	0.515673432	0.980804634
CARPA	FBgn0029962	1.308575608	1.093068469	0.879704602	0.686145008	0.388975456	0.644109324	0.388786868	0.353703623	X	-1.12944293	0.005186709	0.20400887
Ir7g	FBgn0029968	1.383797534	2.007460528	0.362926867	0.087196537	2.363076283	0.105781286	0.667856234	1.461576913	X	0.293865515	0.784371156	0.995787491
CG10932	FBgn0029969	73.01054542	54.95559655	61.70721614	60.15707505	40.15495874	60.05078979	56.60976578	51.01948461	X	-0.240688646	0.180319708	0.836097072
Nek2	FBgn0029970	0.067145927	2.083615644	2.036188525	0.156548201	1.272763877	2.468886018	2.740651232	1.749360274	X	0.982015482	0.277272288	0.896087042
dpr14	FBgn0029974	284.4866824	242.6678375	229.0752086	240.9967644	206.8755183	272.3115044	191.4997899	236.8145884	X	-0.109786134	0.36703751	0.940794966
spidey	FBgn0029975	301.9029984	359.5852628	322.6000894	349.6133626	364.2943448	332.7621434	335.4868145	347.146452	X	0.080994498	0.398995355	0.952805113
snz	FBgn0029976	59.76727099	69.56999769	47.08603459	67.27335607	47.93156947	64.53380864	55.14563318	56.28370549	X	-0.094157188	0.504029873	0.978429306
hdm	FBgn0029977	9.177026026	11.16230143	8.462672011	7.507330072	11.4321188	11.28307503	6.297639564	5.476234687	X	-0.040847418	0.913801643	0.996479182
mahe	FBgn0029979	47.72267177	83.04083333	142.6109004	131.9531274	68.89419931	64.80640496	129.8145452	161.4298813	X	0.108126261	0.819730929	0.995787491
CG10778	FBgn0029980	33.54704159	30.39690232	42.91666693	35.71170537	39.53017571	35.18736103	40.86339144	33.13817742	X	0.092478141	0.635852135	0.991585536
CG15332	FBgn0029986	5.590887698	3.941448753	2.358854882	4.911718103	0.731375741	0.649332834	1.653620247	3.34244	X	-1.385614931	0.031181236	0.492167463
CG15330	FBgn0029987	0.678826377	2.04954483	3.952375971	5.064506055	0.735273355	1.919980084	0	2.947589803	X	-1.012142316	0.403728082	NA
CG2233	FBgn0029990	1.609460645	1.145303705	0.828233441	0.909671977	1.672857284	2.850853311	1.119755096	0.423550066	X	0.451147137	0.595908147	NA
Upf2	FBgn0029992	27.71717512	49.05135809	35.16999026	30.20313036	35.39393599	25.6467964	37.23653866	22.43566276	X	-0.198552218	0.327348006	0.918470465
Ldsdh1	FBgn0029994	14.15696058	18.97027072	11.23300464	8.872026601	2.498046986	9.433285867	10.7936823	4.448641987	X	-0.943379559	0.016557381	0.370938755
CG2256	FBgn0029995	0.460879554	0.545689975	0.328849227	0.758487057	0.489415128	0.051119345	0.345798131	0.176578619	X	-0.963899678	0.384898636	NA
UbcE2H	FBgn0029996	4.835742548	25.09148622	31.97140775	17.46905045	15.42096776	6.700887547	21.55425514	14.50801893	X	-0.398097531	0.51379539	0.980804634
CG2258	FBgn0029997	82.45571172	86.02572905	70.84845947	88.26983768	76.93830404	89.79303468	88.38600223	89.82112068	X	0.103509659	0.345498171	0.930285749
CG1575	FBgn0029999	53.53318392	40.47265056	33.93109173	39.47409447	28.20237346	40.13755533	39.22702648	37.9131303	X	-0.179509458	0.366992703	0.940794966
CG2260	FBgn0030000	51.34175916	47.63259082	52.20294354	59.36171313	55.20211588	40.53497905	49.43623853	50.26975238	X	-0.077011023	0.64720913	0.99279236
cyr	FBgn0030001	0.06601543	0.593924159	0.276627067	0.097944312	0.693380485	0.6393637	0.137787366	0.065148004	X	0.619444737	0.472617331	0.973549494
CG17982	FBgn0030006	15.0318045	17.86444592	17.17053608	19.80182455	32.49049933	21.73447338	31.81208251	23.70825616	X	0.684612695	0.00982015	0.299784351
alpha-PheRS	FBgn0030007	159.3102362	102.8495446	130.0656792	109.7745855	134.9112577	161.2582755	135.0775476	128.5826162	X	0.181832804	0.246491213	0.877584019
CG2129	FBgn0030008	12.71775829	8.792348512	9.90217872	3.806544047	6.980720244	12.23326993	8.439635629	19.86903615	X	0.459736331	0.274392779	0.89549671
CG10959	FBgn0030010	29.4037765	45.88505696	30.2558569	49.87660226	49.30156599	42.82702397	33.44084162	44.66706028	X	0.164949726	0.455179441	0.967967712
Gbeta5	FBgn0030011	1649.78586	1465.320719	1337.022577	1470.787582	1493.396694	1494.213558	1551.907073	1506.398605	X	0.059002699	0.539825548	0.984934163
CG18262	FBgn0030012	27.65226012	32.9996114	42.42466237	46.88742072	30.68169685	20.4544301	30.1125812	22.89896043	X	-0.493094632	0.045517872	0.562827812
GIIIspla2	FBgn0030013	47.43859732	39.27897059	34.57448115	40.85908039	47.27408686	38.22025471	42.39444874	33.34718022	X	0.017141112	0.929296581	0.996479182
Or7a	FBgn0030016	0.78088571	0.806046911	0	2.380791985	3.686904673	4.247390061	0.153235094	1.173721266	X	1.242708319	0.269910689	0.89549671
CG2278	FBgn0030017	22.33355623	33.29902888	21.47326846	31.97990517	27.65454921	20.15327979	21.1843283	26.55610188	X	-0.156841518	0.424552357	0.9620897
slpr	FBgn0030018	39.17495699	54.64038589	42.26256231	62.82543059	40.41833007	42.75692969	47.33149199	41.90442146	X	-0.175372469	0.253735992	0.884040848
CG2147	FBgn0030025	49.10896781	74.00877595	95.20493791	58.89072928	84.34876786	69.09172887	55.58694133	76.25809555	X	0.081600492	0.733816867	0.995787491
sni	FBgn0030026	21.07768045	19.58381511	17.37892978	22.32259075	20.70401131	19.51473356	15.81459284	21.90873767	X	-0.015210903	0.941177792	0.997555095
CG1632	FBgn0030027	0.708981194	0.895582791	0.679497674	0.510173754	0.497736076	0.614784245	0.323766429	0.389566765	X	-0.576761836	0.143228413	0.8037297
CG15343	FBgn0030029	3.857492721	4.313601925	7.656728348	7.903465842	2.110231221	3.967440627	6.113066956	1.77650959	X	-0.737312898	0.18948451	0.843401316
CG1636	FBgn0030030	122.3432824	135.7251134	104.0989654	144.6468273	122.1400858	149.69786	114.5555904	111.1747821	X	0.000442403	0.997376459	0.999490563
CG1387	FBgn0030033	2.070648365	0.312199744	0.451538342	0.108486263	0.672009169	0.131608627	0.356107986	0.050512014	X	-1.28449433	0.257985685	NA
CG10555	FBgn0030034	55.115053	78.03863297	64.93221161	69.75435198	71.87437699	63.73709496	60.51175816	72.24111253	X	0.037138169	0.774097543	0.995787491
PIG-T	FBgn0030035	138.5955433	161.800178	142.1181965	129.7703096	120.8299213	154.6750407	142.639067	166.8240745	X	0.063415547	0.610605429	0.990047511
Miga	FBgn0030037	90.99694577	86.34630164	75.49102338	103.0723477	80.61164489	99.60096633	68.15497477	83.77357309	X	-0.073437079	0.602618683	0.989286463
CG1440	FBgn0030038	38.48098434	32.46317052	37.6114561	34.03388397	33.67471569	36.90079717	31.35633666	35.22422363	X	-0.027280558	0.838676785	0.995787491
CG12123	FBgn0030039	49.09153977	80.36474391	90.08497505	83.4568269	100.6948289	80.03137329	84.89391222	102.6537638	X	0.320163658	0.120992475	0.77836519
CG12112	FBgn0030048	10.68709505	5.422092789	3.840999039	7.228878454	3.572767711	4.851276411	5.048705382	7.877450586	X	-0.335785466	0.509549177	0.979725655
Trf4-1	FBgn0030049	5.829142891	6.602074842	6.793008086	8.375973265	7.534664559	6.981672421	6.738792484	6.480742277	X	0.039662339	0.829073216	0.995787491
CG12065	FBgn0030052	3.538545965	4.178286591	2.290175197	2.953523641	1.649220927	3.531348548	1.926568745	1.692532511	X	-0.53208475	0.071831533	0.670374019
CG12081	FBgn0030053	141.2896702	214.4932714	187.2264457	186.6185342	182.7348437	175.515457	162.7689241	126.1691565	X	-0.14045223	0.3412469	0.927274752
Caf1-180	FBgn0030054	0.166293728	0.906536801	0	0.533097905	0.03752535	0.725110789	0	0.541558616	X	-0.261244432	0.855326117	NA
Rubicon	FBgn0030055	78.15670749	84.44105768	69.38974993	79.43570509	69.18548488	72.5611803	69.11973674	70.99134876	X	-0.114301355	0.295713995	0.906715248
CAH3	FBgn0030056	34.36810785	51.12627372	48.81124663	47.68944436	47.92262588	47.78186345	50.00260315	57.13322581	X	0.191672332	0.167357126	0.830652748
Ppt1	FBgn0030057	2.111319738	6.356435564	1.050673279	0.757301921	0	5.001868116	3.452578212	2.468238012	X	0.120455408	0.90208482	0.996479182
CG11294	FBgn0030058	2.357941131	1.93719941	0.817190328	2.019471789	0.781840667	2.359157751	0.491033346	0.522378415	X	-0.744392828	0.381262716	0.944476532
CG2004	FBgn0030060	5.828715551	7.169691943	10.11251606	6.09465385	10.13969544	8.093044926	11.80522664	6.672468174	X	0.366528222	0.260520294	0.890556245
CG1785	FBgn0030061	13.059867	11.53311532	16.48308479	11.95178231	14.175223	15.76493934	13.49246159	12.71963224	X	0.115451751	0.638804038	0.991585536
CG1789	FBgn0030063	82.47678324	96.21949256	78.66689047	80.53234399	85.58093164	82.16160553	87.66668583	76.33208093	X	0.003227362	0.983553435	0.997881056
CG12075	FBgn0030065	1.594031167	0.881414204	0.756340812	0.38688322	0.485657609	1.201800195	0.32710851	0.742377499	X	-0.374374214	0.388027862	0.948247506
Uros1	FBgn0030066	28.8220946	22.26080024	25.6831246	21.72998884	29.17042958	41.73894482	50.78318549	34.20203939	X	0.686397273	0.002443909	0.117731416
Rbm13	FBgn0030067	2.016331615	2.888888548	1.426714443	9.597877563	5.535826257	4.475235829	3.750618426	2.736026617	X	0.077960261	0.898103617	0.996479182
CG15365	FBgn0030077	0.932360491	0.929593243	1.360299616	0.760057128	1.330041331	1.26013898	0.681939423	0.792709736	X	0.067934247	0.860158734	0.995787491
CG7246	FBgn0030081	21.01481161	18.07994973	20.31740629	27.84231473	11.96909168	22.91978374	27.00243801	21.71822166	X	-0.03626238	0.892777124	0.996438268
HP1b	FBgn0030082	112.4029997	133.8083371	121.4860488	121.552283	117.5316036	139.7929552	138.6447094	118.3312816	X	0.102754381	0.393349325	0.952091247
CCT2	FBgn0030086	53.93704822	72.35992363	52.83494364	51.99545537	55.36366452	64.40863826	65.22902971	38.03129301	X	-0.021356279	0.903195255	0.996479182
CG7766	FBgn0030087	140.2296966	139.7951347	125.8691441	122.5602214	132.0490054	154.9237182	122.2104272	116.6553998	X	0.020622871	0.840847848	0.995787491
Arfrp1	FBgn0030088	36.13025655	22.27402434	11.28349514	24.51654468	30.30012859	34.44469174	26.13749482	32.05005	X	0.400682322	0.207477996	0.856000802
AP-1gamma	FBgn0030089	42.77810408	71.25675505	82.95827458	74.03763248	73.02821919	56.23418704	73.88280922	75.36374687	X	0.076568647	0.657746587	0.995750687
fend	FBgn0030090	106.3096097	129.6607147	124.0860671	127.6055083	135.9018487	142.6958765	124.4380101	147.9692776	X	0.207805817	0.034683918	0.514749796
CG7065	FBgn0030091	75.55240793	65.69118162	55.01396098	48.53313058	65.08342233	65.69642648	66.76817054	65.73785618	X	0.133955675	0.339087766	0.927274752
fh	FBgn0030092	75.34301488	69.19965601	80.36431336	66.4326744	75.89613743	48.70123448	64.88477964	85.01431967	X	-0.053257573	0.802225715	0.995787491
Bap111	FBgn0030093	161.8043119	194.3191871	145.6839065	219.135784	196.3542853	170.7975504	184.0132274	250.2480534	X	0.18556601	0.240013078	0.874625705
Zpr1	FBgn0030096	60.97642521	53.80014638	37.40952672	54.85238736	51.38037346	50.75909098	51.58847282	35.58337607	X	-0.104679166	0.594681745	0.988780662
CG12056	FBgn0030099	31.05869796	34.98569912	41.09889167	43.31989958	49.16306194	53.84100618	57.85519073	46.47025649	X	0.496477557	0.021139553	0.410927638

**CG12106**	FBgn0030100	47.26277794	44.5488366	41.58762526	46.72227415	46.20215233	47.68900498	37.57175008	40.88748682	X	-0.034420228	0.844344686	0.995787491
**CG12118**	FBgn0030101	34.30374031	38.76727184	27.02452905	21.72388523	24.80840579	40.88065287	32.0027442	28.81871305	X	0.081739915	0.732171705	0.995787491
**SmydA-9**	FBgn0030102	0.855084551	0.518783711	0.882733274	0.339335734	1.018149997	0.120067648	0.139234408	0.118497939	X	-0.858451747	0.372852962	0.943282319
**Obp8a**	FBgn0030103	0.947222613	7.944165259	0	0.490758637	0	2.182976141	0.268487249	4.798518613	X	-0.314951254	0.832377132	NA
**CG15368**	FBgn0030104	0.688356919	2.886555412	0.278324047	2.674797072	1.035550553	9.193864898	0	5.230703071	X	1.27400414	0.291217595	NA
**Gr8a**	FBgn0030108	2.152642791	0.85970388	2.984160792	8.484171423	1.387882841	1.884534298	9.675390776	2.002965757	X	0.06593233	0.932932606	0.996479182
**CG12121**	FBgn0030109	163.5396972	143.6269495	145.3108717	160.8473762	139.5613966	130.1093282	142.7451208	147.9984676	X	-0.100749112	0.359591891	0.93924716
**CG17754**	FBgn0030114	66.12605632	52.78019357	45.0044401	73.07035103	49.06110242	58.8452902	40.05564804	62.87800912	X	-0.14337652	0.419132614	0.960266449
**CG17440**	FBgn0030120	0.640753667	0.683947337	0.565257985	0.679042296	0.736097468	2.47131263	0.891587111	1.391135524	X	1.127385998	0.244259524	NA
**Cfp1**	FBgn0030121	73.82782378	93.58145605	94.75192911	97.05616596	77.99835636	110.7916172	101.9098304	115.2458603	X	0.208303942	0.164085569	0.827277154
**Aladin**	FBgn0030122	20.84251536	39.11153719	32.64591974	41.47129567	18.42910144	23.62398352	26.93011005	31.73448872	X	-0.377418099	0.12870623	0.788096052
**RpS28b**	FBgn0030136	652.7243906	743.3862764	780.818273	795.531806	730.4711745	769.1913738	865.5527097	916.1853105	X	0.175145345	0.128071791	0.788096052
**CG15317**	FBgn0030137	23.75761914	17.98680349	29.82701091	17.88859749	15.01935113	26.14615355	28.06279153	14.85182602	X	-0.06250079	0.855234684	0.995787491
**Gga**	FBgn0030141	63.00477929	38.31184371	32.65169267	38.89813354	33.19815757	45.42984014	46.02871039	42.99254723	X	-0.02216267	0.91475086	0.996479182
**CG3106**	FBgn0030148	2.43386352	3.357540994	1.655472603	0.742452864	1.437207109	3.860128026	0.580265246	1.432150907	X	-0.134114739	0.83289675	0.995787491
**CG1354**	FBgn0030151	29.97528787	34.06858747	33.22534832	26.74956666	23.32460225	34.98457267	31.83952795	26.36826606	X	-0.059939709	0.723618971	0.995787491
**CG1468**	FBgn0030157	16.17143443	38.65357049	18.38984736	9.19012833	10.94759394	39.0687196	20.11116737	16.01879201	X	0.09717364	0.836002364	0.995787491
**CG9686**	FBgn0030158	878.5564915	905.8227917	767.8604715	539.9429837	478.9802825	503.3643065	997.8314462	299.1932192	X	-0.406234266	0.280359443	0.899310165
**CG9689**	FBgn0030159	16.59720607	23.86241556	13.82947938	7.880291781	5.737444618	7.60984535	12.86924901	3.285781528	X	-1.047019563	0.015902646	0.367126451
**CG9691**	FBgn0030160	58.27307224	60.33636036	41.53824591	37.23610774	21.29931386	16.14271627	51.7542621	13.43258782	X	-0.919970072	0.026046832	0.451297919
**CG15249**	FBgn0030161	0.082046043	0.437884134	0.68760156	0.260846266	0.350087959	0.232057526	0.133191685	0.291484727	X	-0.484877817	0.527833263	0.984107922
**CG1791**	FBgn0030163	0.729416805	0.101957864	1.917017912	0	1.316784282	0.057307476	1.70569478	0.527877135	X	0.433795557	0.765490224	NA
**CG15312**	FBgn0030174	69.07746829	55.94602404	46.98003624	54.06814686	65.58862846	67.69269293	57.92737038	46.74565242	X	0.099307846	0.479021139	0.973549494
**CG2972**	FBgn0030177	28.55295545	35.70945115	32.69500182	37.59881549	39.75181452	36.69673327	56.75630681	39.97186734	X	0.395844918	0.04843115	0.580755691
**Naxe**	FBgn0030178	50.09663054	29.8208501	37.44643031	26.82989569	29.35789936	35.73168982	30.41125375	38.29943893	X	-0.084549347	0.73733355	0.995787491
**CG15309**	FBgn0030183	9.160839592	8.144607799	10.26639239	8.32654282	8.566907058	10.19348026	8.767462096	7.54136814	X	-0.003551235	0.984450083	0.997881056
**Ipod**	FBgn0030187	1.000403222	1.188608878	1.719099686	0.388733742	0.752493424	0	1.807702163	0.090498667	X	-0.672519222	0.588599412	NA
**CG2909**	FBgn0030189	67.00525046	48.23486041	39.02984541	34.90545518	43.15558881	51.82089679	33.74123171	27.88263192	X	-0.250052299	0.278840859	0.897936521
**Psf3**	FBgn0030196	1.070640197	3.359975053	0.754071731	3.301369353	2.99269155	2.083908208	1.497769176	4.049057572	X	0.36505907	0.567018544	0.984934163
**CG2889**	FBgn0030206	71.61998981	85.5799249	75.28268851	92.21614974	88.17571731	96.85455157	66.77030202	91.23291793	X	0.109661588	0.440498781	0.967112302
**CG2887**	FBgn0030207	0.561999972	0.58917257	1.704255116	0.163785222	2.536384971	1.986938027	0.716837001	0.152519245	X	0.892292467	0.45790469	NA
**PPP4R2r**	FBgn0030208	138.8402604	87.78227278	101.5820027	128.6478325	138.0370544	143.127497	116.4247179	124.7651635	X	0.217056343	0.160031149	0.820738215
**CG2124**	FBgn0030217	11.56994617	8.010735991	16.82732393	16.17168089	13.59946025	13.74899818	14.93888781	17.65148724	X	0.220908981	0.412920233	0.958665466
**CG1628**	FBgn0030218	0.113771776	0.381672388	0.230007285	0.132627307	0	0.911738353	0.193489497	0.288177234	X	0.740053259	0.564408975	NA
**CG9806**	FBgn0030222	1.713440348	0.042896355	0.062041528	0.536617836	1.431181617	0.771544403	0.065239151	0.999413088	X	0.462837861	0.688780791	0.995787491
**BTBD9**	FBgn0030228	61.01832732	68.28668088	79.24348092	81.01336927	59.61616147	80.76004921	61.5794099	71.02151215	X	-0.054972425	0.696987739	0.995787491
**Rph**	FBgn0030230	50.1516516	49.05587049	45.26127759	48.81154307	51.57654233	52.87330229	57.57593229	38.44763522	X	0.080739655	0.532582209	0.984760433
**CG15211**	FBgn0030234	0.995302823	0.74449822	0.293666444	0.031358258	0.267088427	1.153935856	0.411736035	0.438019168	X	0.146808957	0.868632873	0.995787491
**CG17333**	FBgn0030239	0.316124984	5.169989301	2.300744406	2.948133999	0	1.639223872	0.60483122	6.005445284	X	-0.325585376	0.777737028	0.995787491
**CG2202**	FBgn0030240	36.87947714	44.03465885	28.90087283	34.22354153	25.18534363	18.84886219	30.39042514	21.96623184	X	-0.549021937	0.00481323	0.19472554
**sofe**	FBgn0030242	0.825619416	1.986974115	1.045012793	0	0	1.861364861	1.282018267	0.155869233	X	-0.195558178	0.88074809	NA
**CG2186**	FBgn0030243	8.509579714	13.24200155	18.56503361	10.06092336	10.28378482	10.98854671	15.07222596	16.0234152	X	0.095861064	0.68505983	0.995787491
**CG1637**	FBgn0030245	108.287641	109.1814505	101.9724552	112.0268705	107.4589064	136.407338	115.7046515	116.3016605	X	0.169282547	0.084076114	0.69799888
**CG1582**	FBgn0030246	17.17152049	32.98129841	28.74070045	28.01989398	29.31687947	32.70111765	24.93436067	22.54197613	X	0.06925039	0.725682053	0.995787491
**CG15208**	FBgn0030247	0.422299139	0.865758133	1.593653944	0.984574545	1.270596967	1.282898935	0	0.407489121	X	-0.324098215	0.774277382	NA
**SmydA-4**	FBgn0030257	3.530635013	3.765876779	3.601806391	5.318842923	1.666967978	1.536305134	2.309416864	2.437183576	X	-1.004650073	0.005360152	0.20691303
**CG1552**	FBgn0030258	306.7599241	591.96478	447.427509	331.4266698	470.5721157	336.3224724	519.9458145	339.9948004	X	0.031038022	0.870178124	0.995787491
**CG1545**	FBgn0030259	30.6964371	69.35248611	58.25816156	33.39820751	34.30448444	31.49976969	60.72808735	34.81851114	X	-0.207297042	0.44813389	0.967112302
**CG1537**	FBgn0030260	38.57277956	72.07297017	62.139557	40.99015355	78.11566422	43.79659907	57.99606445	55.44614989	X	0.179950171	0.47462366	0.973549494
**CG15203**	FBgn0030261	1.185252403	7.077616892	8.051136237	1.215883398	3.337907611	1.743220013	4.958711127	2.367429271	X	-0.447178829	0.518001144	0.980804634
**CG11122**	FBgn0030266	27.2126416	35.17074997	32.19076837	37.97871283	28.8617202	39.88785743	35.42679556	36.96766687	X	0.12075145	0.369279314	0.942282153
**Klp10A**	FBgn0030268	86.61824564	119.2703904	105.4737255	86.18426538	83.48638998	104.542734	105.2974034	98.19852105	X	0.01133107	0.926255635	0.996479182
**CDK2AP1**	FBgn0030269	96.4160472	101.9411493	80.37937653	130.3276603	98.43502172	100.1823925	113.3804045	95.26127033	X	0.021588933	0.893839393	0.996479182
**CG15202**	FBgn0030271	3.710171972	1.316465826	2.769484755	2.328880152	1.03043251	1.412624556	0.364028016	0.464718854	X	-1.579792171	0.079386022	NA
**CG15201**	FBgn0030272	233.0772532	425.1154583	385.8060081	264.964078	55.82955128	129.6654416	267.146371	132.3763008	X	-1.123390666	0.018973166	0.393184106
**Lint-1**	FBgn0030274	57.97513658	69.77829015	65.24523574	57.76097042	59.64853546	59.03029377	66.30977989	62.58953125	X	0.013872478	0.909528395	0.996479182
**Dlic**	FBgn0030276	86.71121088	87.99051179	92.48877278	83.44680484	78.28323669	84.30850044	97.08317105	87.02807067	X	0.014904562	0.881874552	0.995787491
**Gapvd1**	FBgn0030286	20.91163494	35.24360313	45.04613505	34.15385746	29.27176894	32.41705633	42.07645858	41.98031648	X	0.144157152	0.461752553	0.971606621
**GCS1**	FBgn0030289	70.78160502	60.31923484	58.56113154	52.87029148	43.12986571	53.95142205	67.3326841	42.29247392	X	-0.20385552	0.226207847	0.867702781
**CG1738**	FBgn0030291	21.60028933	27.8563449	30.80921645	20.49839786	21.16260453	23.74166852	21.87498175	17.67445766	X	-0.218601895	0.467179902	0.971928234
**CG11752**	FBgn0030292	1130.4045	1212.682761	1355.280238	1188.7731	1511.449122	1064.194407	1425.595656	1454.275169	X	0.193693192	0.143784152	0.8037297
**CG1737**	FBgn0030293	21.39546201	33.41285942	45.04407446	45.50057104	25.14701477	17.70389586	40.33025857	25.51401693	X	-0.382942456	0.130874832	0.790743321
**CG15196**	FBgn0030296	1.410064464	0.337883618	1.954740984	1.878578409	2.090969227	3.750807605	1.027744212	2.514705394	X	0.785100979	0.312574354	0.914141092
**Gr10b**	FBgn0030297	1.415934364	0.413048915	1.045446049	0.861182	1.000222602	1.799259233	0.785234561	1.069260722	X	0.337336141	0.728698141	NA
**HP5**	FBgn0030301	70.46875062	75.70388597	90.4700432	94.26626929	71.54695951	73.14929572	88.26658858	80.07528257	X	-0.048706583	0.718747585	0.995787491
**Cyp4g15**	FBgn0030304	13.8589193	21.95231234	20.21376284	18.34694209	20.28354249	15.24819681	22.97320382	13.82632035	X	-0.004596466	0.981845998	0.997881056
**Uba5**	FBgn0030305	22.86227366	16.11192116	19.07837342	29.72892971	15.92075029	20.81298255	17.05242261	21.58622089	X	-0.193913337	0.482187108	0.973549494
**Spase25**	FBgn0030306	147.6985891	125.6382748	123.7495123	136.7286751	129.6334602	141.5453281	128.7843156	154.9034595	X	0.083456124	0.537817921	0.984934163
**CG1572**	FBgn0030309	0.937322858	4.111979879	2.6237679	4.594802628	2.690017834	3.466831963	3.67866269	2.765532786	X	0.079096727	0.871735515	0.995787491
**PGRP-SA**	FBgn0030310	0.152496911	3.708986546	0.55493307	1.599933636	0.688240024	1.078298674	0.778045794	6.787240676	X	0.692596423	0.537142372	NA
**CG11696**	FBgn0030314	98.81850247	62.65291772	59.19530576	91.58942688	68.91565403	72.52200693	81.3216935	85.36711944	X	0.005177743	0.977857667	0.997757033
**CG11695**	FBgn0030316	66.48389525	58.47864053	42.94483605	41.27157635	71.88217888	47.22279045	62.04945554	68.06403312	X	0.282658067	0.168454983	0.830712326
**rho-4**	FBgn0030318	2.403824181	8.176783556	5.277800657	7.827410036	9.721491708	7.393997101	8.564535099	5.568796872	X	0.438723138	0.185888915	0.839299945
**CG2247**	FBgn0030320	51.6769429	49.47770593	44.08830432	35.74186363	36.89028139	45.09597087	45.9897326	45.76900027	X	-0.029520497	0.837211155	0.995787491
**CG1703**	FBgn0030321	39.42604933	42.57481365	38.73147357	46.05480807	40.05681645	36.91552086	41.48702541	38.12823896	X	-0.061145251	0.660762042	0.995750687
**Vlet**	FBgn0030323	99.00221972	139.9218128	115.7850392	148.2568045	103.1150503	136.9579625	96.72765304	113.7400328	X	-0.127523875	0.446222992	0.967112302
**FucT6**	FBgn0030327	173.133863	191.5107533	173.2863986	191.4789454	175.0804338	164.5599613	195.6351901	194.4621733	X	0.032628936	0.764359526	0.995787491
**Amun**	FBgn0030328	8.494792135	11.42040423	10.72863807	9.93973259	10.05121152	5.849151448	6.493002918	13.95311601	X	-0.120068007	0.667192317	0.995787491
**prtp**	FBgn0030329	221.4037439	198.0258575	173.3711357	209.7341798	239.3359289	220.7611619	224.4153833	225.2120173	X	0.209538749	0.058288572	0.625114381
**Tango10**	FBgn0030330	92.54639934	116.7875205	117.8477538	103.4636558	93.0669855	100.4440942	102.9210791	132.6137722	X	0.029301567	0.831897275	0.995787491
**CG15221**	FBgn0030331	0.077018891	0.187910378	0.016986069	0.244863607	0.40447662	0.264047055	0.160753783	0.076006883	X	0.816605095	0.396741001	NA
**Karl**	FBgn0030334	0.67559387	0.685892648	0.172524348	0.414505704	0.032095265	0.519613658	0.09070813	0.115798171	X	-1.313506712	0.230563932	NA
**CG1578**	FBgn0030336	120.9317756	139.1677314	116.6515069	166.6785914	137.0675049	157.8448761	139.4180694	141.4220246	X	0.111378461	0.361728792	0.939549067
**Cyp28c1**	FBgn0030339	2.925365552	12.88058711	17.85312907	3.303621397	4.373356767	14.43649405	9.561546415	7.045906222	X	-0.018910816	0.972227352	0.997604378
**CG15740**	FBgn0030340	1.940530961	1.352714165	1.58961334	1.93897495	1.091892698	0.594000239	0	0.054715225	X	-1.922078978	0.036429829	0.521170991
**p24-1**	FBgn0030341	163.618179	159.989248	187.8378262	205.186307	210.3879698	207.0042537	216.7392713	211.6067628	X	0.269384758	0.027979701	0.462664349
**CG10347**	FBgn0030342	35.47518145	28.74651648	37.0715449	28.41150575	29.68131162	51.68230803	39.17959232	17.72213685	X	0.1147963	0.662619773	0.995787491
**ATP7**	FBgn0030343	19.13576121	15.86962833	16.2588775	13.28053787	13.86618364	15.58245735	9.37716269	18.37664141	X	-0.144532521	0.461482608	0.971606621
**Nrd1**	FBgn0030344	47.94428118	68.94594081	57.90839524	45.51190548	58.79471308	46.49139894	49.95413492	57.80889279	X	-0.011597478	0.939942756	0.997188858
**CG1847**	FBgn0030345	237.4415072	232.6966258	218.4432833	246.2178818	225.708557	222.5504843	237.8857492	209.6102217	X	-0.03253381	0.749995339	0.995787491
**BORCS5**	FBgn0030346	295.2043453	334.9522493	316.0538809	326.3903054	316.2228537	280.744677	335.6279274	264.1681764	X	-0.057222206	0.614378364	0.990047511
**CG15739**	FBgn0030347	0.640983564	0	0.103667812	0	0.539998488	0.92661693	0	8.164256631	X	3.628501104	0.0119711	0.331790969
**CG10352**	FBgn0030348	3.44240074	5.227941479	12.63967854	4.446742393	6.466351384	3.508620963	10.91771241	7.069783062	X	0.163764881	0.744157088	0.995787491
**CG10353**	FBgn0030349	36.42101936	42.90100232	46.64209017	43.56059573	42.1571806	51.79653349	42.15332414	37.47157548	X	0.063966711	0.599511027	0.989286463

SelG	FBgn0030350	81.90679295	121.277599	178.922186	101.1848313	101.2467308	86.53219027	142.14799	120.9775953	X	-0.060724815	0.794995131	0.995787491
CG1840	FBgn0030351	4.362069925	1.21946006	7.810760587	0.968571602	1.687425901	2.350018789	2.384514449	4.960715885	X	-0.301653336	0.732620858	0.995787491
sicily	FBgn0030352	64.50111784	83.14656185	58.45772838	79.35430628	65.48352534	71.48061373	80.78998713	80.43537683	X	0.093922455	0.555771886	0.984934163
Upf1	FBgn0030354	56.25754336	71.03665185	62.04347883	74.39170498	57.60165661	48.23823451	61.2946648	54.71998937	X	-0.216843826	0.106478374	0.753302857
CG10362	FBgn0030358	124.1149828	116.2057034	115.7972041	125.6124229	95.56615477	130.4654664	118.3206198	110.4288614	X	-0.055828751	0.622873683	0.990047511
CG1806	FBgn0030360	0.508024901	0.159776344	1.540576655	0.740275583	1.547631819	2.334943522	0	0.137871136	X	0.480708591	0.679861781	0.995787491
CG1492	FBgn0030361	103.1796542	92.63687578	84.89440094	106.3857739	88.37926903	111.6825279	110.7476608	117.7984221	X	0.174356677	0.207148827	0.855489753
regucalcin	FBgn0030362	5.025868595	11.04645522	18.9196869	13.73790333	9.886420603	16.06140042	11.22951409	7.073743725	X	-0.107190498	0.745901639	0.995787491
Lsm12a	FBgn0030364	153.7796752	186.7314871	200.8357677	211.1751645	209.7694681	186.5407952	201.727552	202.1672308	X	0.12151002	0.329402351	0.921112231
Tango4	FBgn0030365	101.484786	126.1546278	88.66229815	132.0238709	93.09401986	90.08253638	111.7993383	103.3300617	X	-0.139984568	0.385816694	0.947329421
Usp7	FBgn0030366	212.8753495	181.2665162	162.7483581	174.8252979	162.5269429	178.8548279	190.3616198	191.3954038	X	0.01213064	0.915225172	0.996479182
Cyp318a1	FBgn0030369	0.196042804	0.054805717	0.713395663	0.304710945	0.353907507	0.338851257	0.250054703	0	X	-0.374824898	0.803384509	NA
Ir11a	FBgn0030385	0.080239227	2.48991587	0	1.215985793	0.651835665	0.605192425	0.614075977	0.17420679	X	-0.842410477	0.514572007	NA
Rab40	FBgn0030391	66.84441816	60.7273458	66.66515618	73.62911319	63.84874931	62.8727471	63.97056856	72.70448919	X	0.005402569	0.966436629	0.997604378
Mks1	FBgn0030395	0	0.659809063	0.381715996	0	2.059344174	1.335092418	0.100347414	1.110232054	X	2.216429899	0.107758002	NA
CG2556	FBgn0030396	0.996845166	0.914763617	0.271113505	0.885870373	0.48418682	1.032537156	0.524559543	0.339679415	X	-0.34762307	0.471038865	0.973549494
Pits	FBgn0030400	118.5750913	153.7230721	124.4865032	166.2492998	155.36463	147.7553367	138.4024964	160.1910808	X	0.127236752	0.292283011	0.905069205
CG1824	FBgn0030403	86.75936367	100.5525793	96.8851541	102.059327	110.5633267	87.2244429	107.8545384	110.542013	X	0.141052402	0.27329301	0.89549671
CG1463	FBgn0030406	31.87961467	57.51418958	37.67503	59.03071384	47.09549108	53.87835237	36.21734115	42.61866028	X	-0.016523842	0.941906215	0.997604378
Fpgs	FBgn0030407	0.331055266	0.118992593	0.458934547	0.77184289	0.170754172	0.312116883	0.542911562	0.102678804	X	-0.554628258	0.616751991	NA
Aven	FBgn0030410	21.89327311	15.90181405	25.81181599	31.64369182	18.71492621	25.78497978	20.00864224	35.24203355	X	0.096823461	0.753535301	0.995787491
CG2540	FBgn0030411	46.15424768	72.11472256	79.4182884	73.04820909	53.39090364	51.59415744	58.78063408	55.05944755	X	-0.270962515	0.172988048	0.832736771
Tomosyn	FBgn0030412	356.7900617	332.3764725	333.6249721	343.2371455	298.8516761	311.1479438	293.537292	282.5967472	X	-0.174857533	0.027404383	0.462324143
CG4004	FBgn0030418	28.81575945	19.23990442	22.51038749	20.36503043	18.57450284	21.15944605	21.92600899	18.62677887	X	-0.155343134	0.363069983	0.939549067
CG12717	FBgn0030420	72.41040272	90.43308798	78.63240235	82.6425808	68.09834846	64.1147893	73.60538582	73.47430992	X	-0.182336514	0.144962948	0.8037297
Agpat1	FBgn0030421	5.367247917	3.256333879	4.820491774	3.035198207	4.30862754	3.983568435	4.602830703	6.148707304	X	0.234930315	0.406119817	0.956706394
CG4407	FBgn0030431	84.85624712	71.7381751	93.74939567	74.0379625	102.7995773	70.41228857	66.60618753	70.59716057	X	-0.031960016	0.851952302	0.995787491
CG4404	FBgn0030432	77.89163841	55.29072227	62.03271185	66.27918851	49.60027463	78.78895484	45.98171915	45.66961665	X	-0.226755425	0.239365169	0.874175862
mRpL49	FBgn0030433	173.8574106	235.5537275	190.3011578	234.2780296	214.4690767	174.2537528	211.1971379	199.2901739	X	-0.028265295	0.85251594	0.995787491
Brms1	FBgn0030434	271.8732832	232.4994524	248.154129	274.2827821	221.7445413	243.9412153	239.1986705	209.3217566	X	-0.140404125	0.210938286	0.856520545
CG4645	FBgn0030435	176.1414962	186.6739157	194.5507441	230.0881415	166.9305534	171.0027194	198.4196862	216.5711325	X	-0.033330387	0.806124828	0.995787491
hec	FBgn0030437	26.01259583	30.87359862	28.98099651	32.76683518	32.84932607	29.08072994	26.8579725	31.28917339	X	0.050037107	0.703720689	0.995787491
CG12716	FBgn0030439	2.238197562	3.097266498	2.579172132	7.044669033	2.525325153	6.172215046	1.855649272	10.81201836	X	0.549834825	0.396631609	0.952272708
CG2200	FBgn0030447	47.81230447	55.1554333	53.96340079	55.52490475	54.34147276	46.61581234	44.71727258	48.29357463	X	-0.098280703	0.554067887	0.984934163
MFS10	FBgn0030452	0.877937896	0.399353941	0.360994083	0.208157202	0.161176568	0.6172786	0.417559708	0.355371676	X	-0.244716995	0.769896037	0.995787491
CG4332	FBgn0030456	20.18865708	16.30932665	23.76844436	20.85230082	16.14598122	30.8248662	35.12340063	15.95335623	X	0.298244653	0.283848086	0.901780531
CG12096	FBgn0030457	54.34295473	50.06915398	38.66477666	57.78796197	54.22365736	60.22971617	43.85305495	42.87682031	X	0.027609347	0.869461582	0.995787491
CG12723	FBgn0030459	1.972111843	1.23237016	0.469050571	0.090154991	2.094216073	1.494727352	0.394580367	0.083953674	X	0.123864582	0.910560857	NA
Coq5	FBgn0030460	27.10907345	23.83273811	33.02734123	23.70141908	30.90873194	19.05660397	37.7627223	25.55602844	X	0.104307093	0.69483934	0.995787491
CG15743	FBgn0030465	76.78107025	87.35969315	76.24354767	95.11573128	79.79848699	90.49594232	82.12858614	67.95542215	X	-0.037224732	0.791223766	0.995787491
CG15744	FBgn0030466	14.86163882	18.28076378	16.00210635	17.02406491	19.11111628	22.0088723	14.43785762	19.8605294	X	0.221202786	0.173751867	0.833984823
CG1764	FBgn0030467	32.38095836	40.52093326	35.93085653	35.19200398	30.97041979	34.25081369	34.88921141	34.8550453	X	-0.061570078	0.587360058	0.987588562
CG15747	FBgn0030474	59.29052712	67.05690265	62.15800297	55.75873428	59.0956491	54.04759396	51.81305036	66.52899682	X	-0.044823218	0.74727724	0.995787491
dmrt11E	FBgn0030477	10.78456018	18.53545687	12.25248103	8.499311345	12.81930774	15.68104383	12.30274253	8.491798173	X	0.0119629	0.968702218	0.997604378
CG1640	FBgn0030478	88.38614997	96.55075665	87.77971648	91.88448679	73.22493501	82.25293286	89.57478193	73.65568459	X	-0.164076606	0.099253255	0.731438598
Rbp1-like	FBgn0030479	650.2895589	646.2642799	632.4558022	529.9238009	657.5693254	676.5508799	558.7211094	647.8778612	X	0.077868979	0.427996451	0.963202327
Tim9a	FBgn0030480	152.6916073	129.9738843	166.7540021	123.0015028	149.2708975	144.6741069	165.9663951	130.7858111	X	0.074396197	0.65364614	0.99443552
CG1662	FBgn0030481	72.07578156	85.99128528	90.63467629	69.7511172	109.5195689	126.3032145	69.94935168	86.42719872	X	0.331505815	0.062947093	0.636110045
CG1673	FBgn0030482	0.580792861	0.40311664	0.485860476	0.280157936	1.04847997	0.925201039	0.561991922	0.260887255	X	0.699305937	0.384215254	0.946594947
GstT4	FBgn0030484	4.715335191	3.017609487	1.424174536	1.766044416	1.70931497	2.481674302	1.739121163	0.616712602	X	-0.72226081	0.175390985	0.834088743
CG1998	FBgn0030485	452.8462667	370.1462597	337.3497794	410.2716372	383.8510591	419.2262263	358.3151831	419.8653522	X	0.03698578	0.749247298	0.995787491
Set2	FBgn0030486	32.92428304	51.28649556	41.61109028	45.28370949	37.25347204	42.39516034	40.43358622	46.5186638	X	-0.00438232	0.975277031	0.997757033
CG11816	FBgn0030495	0.187080444	0.806917372	0.129672728	0.249240579	0.627209421	0.4535442	0.340890166	0.348144825	X	0.407189406	0.706459905	NA
CG11178	FBgn0030499	86.48318601	91.97669755	91.90660751	79.87619707	86.55512585	105.0500538	101.352192	84.88920752	X	0.139198099	0.208990287	0.856520545
tth	FBgn0030502	58.48582398	60.37690468	59.94999906	62.7327812	51.80011259	40.38631612	70.39881256	66.01089575	X	-0.046040407	0.793646068	0.995787491
Tango2	FBgn0030503	4.647236804	6.615998886	3.78962409	7.010790252	11.77343476	18.74055222	11.35708324	7.885134959	X	1.200969923	0.000720747	0.047672253
CG2691	FBgn0030504	15.98353052	13.13611447	14.08540951	11.80517414	15.38696559	21.03637727	15.50027319	10.91986679	X	0.216823377	0.300634536	0.9074568
NFAT	FBgn0030505	19.43217253	21.34309924	22.46297943	20.75609079	17.92090403	18.60967563	18.7489831	18.36503218	X	-0.15827079	0.072939017	0.671999479
DNAlig4	FBgn0030506	13.52822925	26.51868111	16.86547129	11.26448473	19.81858277	13.84249912	18.41945662	14.45241553	X	0.003910457	0.988286688	0.998078482
CG15760	FBgn0030508	60.03481039	55.33161027	50.27406505	63.24566479	58.42562587	69.61305657	62.91059396	71.30189505	X	0.224105752	0.065648877	0.646711278
CG11162	FBgn0030509	1.139713758	2.352873777	1.48880829	0	0.158267342	0.578585092	0.223648791	0	X	-2.285272753	0.173830382	NA
CG12177	FBgn0030510	29.33897517	63.62370611	83.21364685	31.52270459	40.6400839	43.39041804	78.24251062	26.896162	X	-0.094500738	0.84087383	0.995787491
CG11158	FBgn0030511	0.520542342	3.754488039	3.662202535	2.427249747	0.563827404	0.049076414	3.983744091	1.582203854	X	-0.707437536	0.479488918	0.973549494
Nadsyn	FBgn0030512	5.824792805	6.500659574	4.989187374	6.021784336	5.728040754	6.235832559	5.487465999	4.808870401	X	-0.039336663	0.768506696	0.995787491
CG9941	FBgn0030514	181.0890242	211.5955785	189.3350183	206.2439274	197.6419917	215.3761785	185.1936124	198.0302262	X	0.045115086	0.637827445	0.991585536
Pdcd4	FBgn0030520	188.0415226	193.6411049	159.6373461	165.6012069	182.2995667	206.8705712	161.1207849	186.7934651	X	0.089403046	0.384248945	0.946594947
CtsB1	FBgn0030521	248.7804283	219.967617	238.3576487	273.3198151	235.932117	239.5054071	261.6765951	234.6857551	X	0.015674615	0.887978346	0.995926915
CG11103	FBgn0030522	92.81868405	87.45761978	87.51812128	87.61265897	94.63494411	100.8832225	112.639428	95.45587352	X	0.210861763	0.166405986	0.829442277
CG11095	FBgn0030528	48.27543033	57.20945138	70.3401841	52.27469134	59.19839117	69.81993006	34.65387102	49.78891741	X	-0.061461721	0.786819589	0.995787491
Clic	FBgn0030529	46.46360049	32.02728459	32.25395905	30.47810359	35.70874685	43.89306998	31.05249436	32.59157875	X	0.044324742	0.779255586	0.995787491
jub	FBgn0030530	53.86293619	56.87918286	52.2879499	79.72096306	57.26672053	58.32536693	40.20364227	49.1250721	X	-0.217001205	0.198418654	0.847197998
CG1368	FBgn0030539	18.38505433	9.855554985	15.40374391	14.80356782	17.79243532	15.63572403	16.4394096	6.995527135	X	-0.017392698	0.966177659	0.997604378
CG11581	FBgn0030540	0.67802932	0.758199378	2.056112092	1.975999794	0	0.372891109	0.288277894	0	X	-2.970648589	0.02931626	NA
CG11585	FBgn0030543	2.796392111	2.228013849	3.900804748	4.726770128	5.553024917	5.404663873	1.961755497	1.669587186	X	0.13550415	0.837471681	0.995787491
CG11590	FBgn0030545	53.70568866	44.04537753	34.87831735	75.34929036	77.00433459	70.23703275	73.95810886	53.39868244	X	0.424902914	0.113676598	0.758410631
CG11674	FBgn0030551	8.225921056	8.490971439	6.481426071	2.130936141	3.807665588	10.00903903	5.380641369	1.831716521	X	-0.243434651	0.693268116	0.995787491
mRpL38	FBgn0030552	154.5039912	159.9064754	162.5378904	196.5943621	135.6446459	177.3959816	159.3344046	165.8519999	X	-0.048963701	0.711052211	0.995787491
CG1434	FBgn0030554	29.01951391	31.42130281	32.23877344	42.27452498	22.97831166	37.548446	26.9569138	20.94341557	X	-0.287878454	0.19570388	0.844272804
Fbxl4	FBgn0030555	59.59631431	74.75602851	64.77655201	74.50757741	73.55093685	46.43864764	58.42037754	60.1137007	X	-0.163790557	0.306126014	0.911610813
mRNA-cap	FBgn0030556	139.9582764	170.5281171	146.1734812	169.7904881	181.1447743	194.7327531	165.205057	164.1445414	X	0.20125989	0.069924434	0.662558698
CG13404	FBgn0030559	14.0833367	28.42420813	34.1659784	27.49578246	13.74551338	12.62999192	21.90664298	20.7570326	X	-0.555269585	0.0821885	0.694684542
CG9400	FBgn0030562	1.521940145	0.887944726	0.48159203	0.15427592	2.150211155	3.119295375	2.194457657	0	X	1.283437416	0.280713117	NA
CG9411	FBgn0030569	0.823538934	1.059681886	1.067368009	1.70963358	0.509143441	1.403951053	0.460463674	1.592036326	X	-0.197088468	0.715980358	0.995787491
CG14414	FBgn0030571	158.433246	157.2897615	130.967025	146.5207613	120.8028274	139.6678575	140.3843746	123.4193679	X	-0.150605983	0.222291381	0.86549481
mRpS25	FBgn0030572	121.7365178	159.5441713	178.0928466	142.2701166	151.5624934	119.6954014	156.9739579	166.4942268	X	0.019598879	0.909572022	0.996479182
nmdyn-D6	FBgn0030573	22.50782954	10.36680195	19.24555953	26.45314484	18.65093818	24.17716348	17.41364261	22.03001623	X	0.090566611	0.79846346	0.995787491
sbm	FBgn0030574	24.8929782	26.53920859	21.82941961	26.15004934	17.8736478	11.56745955	17.03635612	15.65818104	X	-0.645108402	1.97E-05	0.002691958
CG5321	FBgn0030575	5.946225512	2.827264004	0.454345202	1.637409559	2.1976062	1.059413876	0.477762174	2.033040318	X	-0.89934048	0.277305778	0.896087042
CG15890	FBgn0030576	3.336313241	4.293937645	5.115923669	1.369796756	2.556891717	2.828931896	1.659378676	1.093350171	X	-0.757573599	0.063305774	0.636110045
CG5347	FBgn0030578	0.501511761	0.360520982	2.780938373	1.00221925	0.129336752	0.202638097	0	2.799844607	X	-0.515086453	0.720520797	NA
CG14408	FBgn0030581	284.1500985	361.0311966	364.5018681	342.6769204	259.0698518	275.1507626	305.2276964	263.955811	X	-0.261173422	0.012362257	0.33520527
CG14411	FBgn0030582	132.3354276	139.7436172	131.5448758	151.502282	151.653299	155.4649567	141.014182	117.4636144	X	0.055458013	0.627547053	0.990047511
CG14410	FBgn0030583	7.301703352	9.846096174	9.030580248	6.6759398	6.461493118	6.344079648	6.208934041	4.973387886	X	-0.419190716	0.458808695	0.970485193
CG14407	FBgn0030584	526.1432913	623.3278844	519.1222033	665.5645677	570.4103025	497.5049577	555.603893	593.3988863	X	-0.042341005	0.737317202	0.995787491

**CG9030**	FBgn0030599	1.595832571	1.18968234	1.935733779	3.720623563	2.720837532	1.170198711	0	3.464700236	X	-0.158401698	0.8813778	NA
**hiw**	FBgn0030600	12.54121413	11.28142755	12.43647648	15.58599315	13.8903915	12.05670058	12.56331094	15.49138421	X	0.089003821	0.509677845	0.979725655
**ND-B18**	FBgn0030605	659.6926906	624.5154175	679.1946628	638.3426754	756.8060374	642.9005299	764.2092313	708.4029297	X	0.174216963	0.091873076	0.722444454
**dob**	FBgn0030607	2.618497829	0.488018086	3.024971053	3.197820012	0.225098081	2.703818403	0.530146367	0.360952456	X	-1.256256267	0.167931626	0.830652748
**Lsd-2**	FBgn0030608	9.830493126	14.1951134	12.5316452	6.975477067	5.577490169	8.680955491	10.68532312	4.666276101	X	-0.524023683	0.058501943	0.625114381
**CG9065**	FBgn0030610	827.5274483	926.1692552	904.8335551	1073.334185	1005.657145	747.8882183	1012.151645	964.1390271	X	0.032328241	0.810240635	0.995787491
**CG15027**	FBgn0030611	78.4491596	66.11997986	63.54371328	66.81186608	66.71394916	60.14711263	76.08076539	57.99338812	X	-0.05130212	0.806871175	0.995787491
**CG5599**	FBgn0030612	7.679413152	9.631842114	12.83969742	8.710181839	7.306338505	8.2511093	9.089215485	4.806548996	X	-0.365133036	0.245974191	0.877269208
**Rab3-GEF**	FBgn0030613	46.6328462	62.71361296	73.59948866	70.83246982	53.26776573	59.51778794	71.26304955	72.46568742	X	0.0491386	0.737543494	0.995787491
**Cyp4s3**	FBgn0030615	4.357646309	4.035832104	12.19299855	2.617833801	2.220041401	4.007543615	4.569338078	3.192312537	X	-0.693004022	0.146259098	0.808004944
**RpL37a**	FBgn0030616	786.2761123	1126.603745	1062.885513	1151.921818	907.4992275	844.9436042	994.2687224	1153.587412	X	-0.04658045	0.735544411	0.995787491
**CG9095**	FBgn0030617	1.153025427	0.66755519	0.41507189	0.6417078	0.819173492	0.66977251	0.075906907	0.395686845	X	-0.53134181	0.335931738	0.924219112
**CG9106**	FBgn0030624	2.594118387	0	1.074465004	0.295028749	1.370648358	1.37198869	4.196559638	4.121027672	X	1.468765792	0.16586216	0.828893393
**CG5877**	FBgn0030625	20.38815158	19.31113477	19.04313579	20.6437063	22.21954144	22.37941407	21.35959454	15.36080366	X	0.062744418	0.717430021	0.995787491
**CG9123**	FBgn0030629	178.4514659	180.1128924	150.0101184	179.2512804	125.1662353	151.5263548	137.4696279	178.7828145	X	-0.184532302	0.186060149	0.839299945
**CG12608**	FBgn0030630	17.19626936	19.64044085	12.59857099	32.78214952	21.04955643	18.15249949	24.74608094	24.78342843	X	0.138514415	0.646222257	0.99279236
**CG6227**	FBgn0030631	49.95009302	42.10174727	39.15122193	39.04090145	42.99156843	51.08154763	47.7725249	46.27764591	X	0.170306826	0.192841701	0.844272804
**CG9164**	FBgn0030634	25.1654193	31.40784842	34.98099595	35.41046195	34.17275974	27.14051532	36.08659263	29.08009544	X	0.028172652	0.862280784	0.995787491
**CG11655**	FBgn0030638	4.788989543	10.12838634	7.172865654	3.301059656	1.578716732	1.884534298	11.57941616	7.232931902	X	-0.146722613	0.806618425	0.995787491
**CG15642**	FBgn0030642	50.71545088	47.10608864	45.6872551	61.11052586	55.37002565	54.71898485	56.89181644	41.48474992	X	0.054210266	0.76656887	0.995787491
**Alg14**	FBgn0030645	63.56159033	64.59654147	68.02920511	61.31058238	67.94701627	65.91802703	58.5000878	48.97579501	X	-0.06202107	0.761562421	0.995787491
**CG6324**	FBgn0030647	2.092005222	1.212684766	0.373174668	0.573815461	1.555072895	3.016504317	0.235444865	1.502846799	X	0.575720258	0.412908223	0.958665466
**CG6340**	FBgn0030648	339.8100406	410.6378221	391.3125775	446.6377792	355.5733134	339.1583658	351.5731554	354.9017752	X	-0.149356471	0.14755401	0.808004944
**CG7860**	FBgn0030653	0.125898613	0.739120414	3.665139345	1.467639382	0.568198159	3.501545074	1.927020397	0.273337543	X	0.112935725	0.918902848	NA
**CG15643**	FBgn0030654	39.66126567	60.5306373	53.69183969	65.07060034	44.55033311	52.94948355	49.4641844	58.04335855	X	-0.060744818	0.746895441	0.995787491
**CG9213**	FBgn0030655	28.14476546	36.75085501	28.37116947	20.36718193	35.03816248	23.04767309	33.15822979	28.37285934	X	0.108414112	0.621064165	0.990047511
**cerv**	FBgn0030657	17.76222252	22.43718304	28.54994057	20.62076679	26.65515862	33.28548764	33.750773	26.66113633	X	0.466059582	0.063361677	0.636110045
**CG7872**	FBgn0030658	94.2209367	102.2834253	106.2121293	96.08382727	98.91325634	131.7881148	94.03050782	96.64633099	X	0.109794674	0.427919536	0.963202327
**CG9215**	FBgn0030659	57.79005381	81.76296892	78.15193217	72.19819412	82.28631715	72.54746146	79.56558842	69.45698186	X	0.102611817	0.492226362	0.973549494
**CG8097**	FBgn0030660	10.48536658	7.090705974	6.133116748	1.062879889	4.489037324	14.02730713	6.872117398	5.668680601	X	0.342056705	0.532994495	0.984760433
**CG9220**	FBgn0030662	1.90150424	7.277713975	3.771974415	0.68581213	3.299946055	3.951922136	2.572788421	1.870298461	X	-0.176728756	0.720260081	0.995787491
**CG15646**	FBgn0030665	0.578556984	0.184847272	1.269897729	0.770790759	0.944972817	0.077922856	0.140562981	0.119628645	X	-1.085099645	0.34045204	NA
**CG12708**	FBgn0030666	0.651265005	0.899628209	0.836449315	0.357270551	0.207476515	1.155781023	0.29318665	0.415869592	X	-0.355841219	0.735925171	NA
**CG15599**	FBgn0030667	0.797508144	1.857927823	0.483685908	0.413191159	0.11997555	0.062657151	0	0.480962224	X	-2.390496866	0.029570403	NA
**CG8128**	FBgn0030668	10.5374165	12.29816555	8.107557585	3.736817878	11.69682888	7.266091559	12.87512631	6.219182391	X	0.164653093	0.670197915	0.995787491
**CG9240**	FBgn0030669	170.9256089	132.8594443	165.478625	149.7418824	71.49490555	59.35040552	84.36106468	58.30262127	X	-1.15192228	3.99E-11	2.77E-08
**Pis**	FBgn0030670	505.9641033	438.8771626	456.9681195	460.4117012	536.1963766	495.0146358	509.5820827	482.9128272	X	0.149212763	0.121245887	0.77836519
**CG8134**	FBgn0030671	137.4246646	193.2589774	160.0073524	187.4108264	165.7632444	164.6865487	165.3622707	147.3349519	X	-0.044013969	0.737372708	0.995787491
**CG9281**	FBgn0030672	180.7764696	166.9008006	171.2866051	183.2159701	177.2150154	159.0128074	162.5607753	184.9173234	X	-0.007925466	0.939175904	0.997038394
**CG15601**	FBgn0030673	20.27346423	46.59419158	36.66609517	25.15226597	37.91597941	34.6405071	27.12205844	27.72188701	X	0.026048843	0.920685145	0.996479182
**HUWE1**	FBgn0030674	21.63325118	27.99392114	30.92104196	23.06383848	27.14797656	22.94621663	26.4832974	30.66250636	X	0.086270451	0.533189223	0.984760433
**CG8191**	FBgn0030675	0	0	0.283009637	3.535775523	1.895371315	2.364656953	1.339181852	0.126637192	X	0.602982206	0.684238603	NA
**CG12379**	FBgn0030676	2.462017318	2.627983696	4.615360657	2.087309343	4.242546257	5.380905425	5.13872106	5.345267504	X	0.81238495	0.231150591	0.867702781
**CG11679**	FBgn0030678	5.098678388	5.86728774	9.49273599	12.30205265	7.17088634	1.229698401	9.981992038	1.158457266	X	-0.716188496	0.275870326	0.89549671
**CG8206**	FBgn0030679	216.0712935	195.7191569	212.6130763	272.7222644	240.6778263	243.3383082	252.4250611	264.4194161	X	0.186659675	0.170370472	0.830934498
**CG8944**	FBgn0030680	46.33934377	53.7703621	61.34659448	56.10561968	50.80044925	41.63350595	57.95358779	54.74713881	X	-0.050638087	0.729474978	0.995787491
**Mvd**	FBgn0030683	10.91571824	9.663392622	15.61093709	23.56443161	16.84713068	11.46651039	12.376103	8.923710616	X	-0.238463713	0.461156375	0.971606621
**Graf**	FBgn0030685	56.99650759	65.16316262	52.09873462	57.24697599	47.01918485	62.57927007	42.07579585	54.24339635	X	-0.140078169	0.276024388	0.89549671
**mRpL3**	FBgn0030686	65.43809512	81.73201751	56.72893181	71.78758375	72.49292972	78.54667923	37.47816349	72.16574741	X	-0.049768869	0.821751428	0.995787491
**RpIIIC160**	FBgn0030687	22.0309902	17.45783549	21.3082565	18.41482357	13.97251086	20.38465711	14.75795554	19.12679247	X	-0.187350865	0.272212745	0.89549671
**Efhc1.1**	FBgn0030691	0.732279854	1.974048312	0.190339362	0.12194878	0.70818901	0.27122424	1.067463795	1.760188138	X	0.372236265	0.699877483	0.995787491
**mRpS30**	FBgn0030692	106.4078671	126.293991	144.3093419	155.4479624	135.9935262	164.2008777	96.62905197	159.272969	X	0.092494176	0.586007127	0.98718792
**CG8974**	FBgn0030693	166.1027823	133.1982039	136.4463435	128.5038833	118.8858717	147.9281821	137.1572986	117.4638174	X	-0.087814176	0.497548003	0.97594235
**CG15602**	FBgn0030694	95.36471345	68.71694324	89.6665795	60.51837284	76.70697361	85.22760324	81.33239619	56.60063714	X	-0.041493054	0.827156275	0.995787491
**PGRP-LE**	FBgn0030695	20.16963182	24.29491009	25.58295039	20.1428796	18.97977711	23.71735134	20.17612262	22.2053927	X	-0.050123862	0.78340929	0.995787491
**CG8565**	FBgn0030697	2.080918814	0.65445926	0.315517501	1.334185567	0.328701782	1.054509182	0.862626148	0.338840053	X	-0.759804673	0.366744239	0.940794966
**CG8578**	FBgn0030699	35.8049401	63.58046555	51.7569983	51.47204856	55.7136608	45.17792995	55.469926	53.38083391	X	0.087163646	0.611903509	0.990047511
**CG16952**	FBgn0030701	316.5622204	320.3397088	360.4522193	400.2456072	330.840837	337.9445299	381.229816	339.2917149	X	0.021265375	0.845584349	0.995787491
**MSBP**	FBgn0030703	171.0216266	139.4600677	167.8101303	141.9668012	142.4044961	185.2038067	164.6371835	149.3648809	X	0.07587878	0.537906449	0.984934163
**CG15916**	FBgn0030704	638.3348792	560.6857687	590.4827697	541.5472888	571.9352189	519.9845854	542.4944604	534.5272576	X	-0.074247539	0.474596556	0.973549494
**Lrp4**	FBgn0030706	125.0896246	152.4090633	173.2165885	179.7873436	140.6126142	159.558352	159.444235	173.3906988	X	0.037040806	0.764804205	0.995787491
**CG8916**	FBgn0030707	6.355775996	5.414185827	4.278577317	3.801541209	1.441734615	5.396105379	5.432869903	7.080103913	X	-0.013534946	0.977069962	0.997757033
**CG8924**	FBgn0030710	126.9733061	190.4453157	155.2329263	149.7235906	155.9507808	158.9186268	153.6008224	143.6633592	X	0.009729049	0.935498328	0.996605275
**Rrp47**	FBgn0030711	69.83275721	86.93755488	86.10547286	82.75054521	86.51136378	93.44149228	85.47684757	90.55074362	X	0.162393247	0.244909289	0.877269208
**Or13a**	FBgn0030715	1.177187907	0.382174368	0.36849567	0.885344932	1.371048956	0.501220579	0.678103919	2.061116061	X	0.720520599	0.425471599	NA
**CG9170**	FBgn0030716	274.7871168	312.7952332	275.4085513	332.3992947	260.6449739	240.2311075	249.1128483	269.8230931	X	-0.197642898	0.056792627	0.621788268
**Slc25A46a**	FBgn0030717	113.4046596	127.762631	115.8121189	89.78178316	112.2250168	112.4681145	133.6885643	118.6147681	X	0.126167518	0.371120772	0.943251147
**ND-20**	FBgn0030718	685.2898206	744.5157957	736.3370185	772.6937753	796.1304893	657.4648446	792.4947166	917.7425729	X	0.140285405	0.243125641	0.876959115
**eIF5**	FBgn0030719	3.471009038	2.574610573	2.28499223	0.975983144	1.133558761	2.565334647	1.245875988	1.817700164	X	-0.436239717	0.374030661	0.943282319
**CG8939**	FBgn0030720	7.137446789	7.259080563	13.3194972	7.755615	5.188949992	16.9294232	9.72180114	19.35254263	X	0.563390641	0.141278187	0.8037297
**CG12698**	FBgn0030721	0	0.451561634	0.571461768	0.62765246	0.425243564	0.856606499	0.343379962	0.146119836	X	0.161640335	0.903518731	NA
**dpr18**	FBgn0030723	0.395758052	1.01787054	2.432262236	0.8304255	1.690852494	0.485053756	2.153786225	0.343690946	X	0.046625383	0.945968045	0.997604378
**Nipsnap**	FBgn0030724	83.07563241	89.13355074	85.15603597	92.17820031	82.11401826	89.61785279	82.57730617	75.97401215	X	-0.051826466	0.62815215	0.99035566
**CG9911**	FBgn0030734	87.40262821	101.007532	96.3955355	88.92990907	104.7443978	83.29007731	90.95868664	82.18032899	X	-0.016587923	0.889315331	0.995926915
**CG3632**	FBgn0030735	83.40774566	69.39417805	89.47424096	70.89602636	76.47596222	77.89805953	70.3479067	66.6427982	X	-0.075190885	0.520777054	0.982083369
**CG9915**	FBgn0030738	86.69649158	80.49789779	88.90068996	90.17996578	75.38252986	72.98290812	74.91917985	106.1178482	X	-0.040227255	0.786160915	0.995787491
**SWIP**	FBgn0030739	28.65840001	39.58531088	23.19229312	20.38961866	23.75897895	33.91011398	22.41911712	22.10509997	X	-0.099684555	0.639092029	0.991585536
**CG9917**	FBgn0030740	163.082908	147.2271552	158.0679577	156.9578352	121.1853034	131.4323078	160.3223874	153.4747784	X	-0.11333732	0.390103084	0.948624352
**CG9919**	FBgn0030742	569.5093696	552.8777127	464.7939673	563.1386665	507.0179487	591.0555331	504.7366019	602.0714943	X	0.064799733	0.551388968	0.984934163
**CG9921**	FBgn0030743	59.23955912	52.43971236	63.8831101	60.3490276	62.57422503	52.32921312	58.60003382	56.84666531	X	-0.004209536	0.976657892	0.997757033
**CG9992**	FBgn0030744	50.28784404	37.37994799	41.32980197	23.24375113	47.74680311	44.28719396	40.69226914	44.18111823	X	0.245143701	0.230403505	0.867702781
**CG4239**	FBgn0030745	79.76265436	65.00707132	62.90538031	76.85081021	67.86303923	68.59323241	74.73057139	80.46214257	X	0.063249249	0.646358851	0.99279236
**CG9981**	FBgn0030746	0.373074841	0.952274991	0.90507436	0.30254259	0.380671486	0.902270791	0.041379215	0	X	-0.885518077	0.416205006	0.958846438
**CG4301**	FBgn0030747	0.67270788	0.651595732	0.666583353	0.176720349	0.838114038	1.375642698	0.145021881	0.843393722	X	0.591933906	0.379996878	0.944214067
**AnxB11**	FBgn0030749	29.55825191	28.36261431	20.35221652	42.20075776	25.28054617	24.80516346	22.60862974	23.91896644	X	-0.292326076	0.138835109	0.801282407
**CDC50**	FBgn0030752	348.0623082	345.8967295	303.2333049	349.6249134	317.039977	295.1860757	356.4496649	322.2691424	X	-0.030494683	0.775786169	0.995787491
**rngo**	FBgn0030753	25.540713	28.17901277	19.97721435	19.76351395	20.50990261	18.95232807	25.61261885	15.44030557	X	-0.186859397	0.352946463	0.935162434
**CG9903**	FBgn0030756	0.101963797	3.164054363	1.236813748	0.118862377	0	2.018747747	2.080894696	3.099219791	X	0.698075798	0.568199996	0.984934163
**CG9902**	FBgn0030757	17.30438636	15.50908349	11.02077605	23.12886519	13.70684343	19.56828331	10.89264112	22.44407186	X	0.017640437	0.945345543	0.997604378
**CG13014**	FBgn0030759	20.48681307	18.69388267	21.27190934	31.1422802	19.8233963	10.27549606	23.41351052	24.55227972	X	-0.201084309	0.568050583	0.984934163
**CG9784**	FBgn0030761	52.32345116	73.76697679	61.92249479	53.96480143	55.47062739	57.53858825	61.15947322	51.45152049	X	-0.067513058	0.611881377	0.990047511
**CG9782**	FBgn0030763	0.514877546	0.620045536	0.288250557	0.584818839	0.834154042	0.784145501	0.235749892	1.003195859	X	0.539757895	0.420559022	0.960593359
**mthl1**	FBgn0030766	1.326026028	2.955260849	2.474554345	1.556599602	1.355938209	1.346336104	2.058019001	1.228073011	X	-0.435941403	0.239572404	0.874524157
**CG9723**	FBgn0030768	25.62355902	43.95122338	44.60239769	38.7018549	54.70706842	43.0380739	27.57446309	34.88731165	X	0.10514593	0.664690404	0.995787491

**CG13012**	FBgn0030769	1.222588974	0.897190653	1.668365887	1.399759904	0.650302256	1.204106981	0.835406448	0.54509052	X	-0.646602332	0.164867214	0.827712868
**CG4678**	FBgn0030778	60.76661658	32.93071263	37.355095	46.0144731	52.98019814	49.18608577	52.36402623	48.38558774	X	0.221199326	0.179715561	0.836097072
**CG13008**	FBgn0030780	5.588273913	1.420234171	0.616230662	3.092706878	1.069967678	2.847167599	0.719990243	2.328490296	X	-0.614736908	0.376461152	0.943615128
**mRpL22**	FBgn0030786	329.2091217	336.7116662	288.4592212	298.477582	322.1804165	317.4223502	307.7114055	270.3206888	X	-0.012022953	0.912492508	0.996479182
**CG9609**	FBgn0030787	196.1150559	179.4442243	187.8224182	186.4784304	145.5470952	158.7535144	155.8311054	197.394468	X	-0.159296271	0.211411933	0.856520545
**Sap30**	FBgn0030788	60.20353287	57.90675491	56.78352833	70.05283335	52.18894078	50.01478997	52.87811442	67.06621991	X	-0.110901567	0.485973986	0.973549494
**Rrp45**	FBgn0030789	6.935044923	10.61764319	9.597767728	12.91333385	4.621304321	12.90111067	5.580524052	8.286196032	X	-0.317438744	0.421176499	0.960593359
**CG4768**	FBgn0030790	104.3331634	97.90212383	97.94823632	91.68969373	87.70853511	108.9810243	81.02967126	88.25900578	X	-0.070765772	0.518590963	0.981009845
**CG9132**	FBgn0030791	69.581201	56.42958102	78.7453135	60.35081135	92.02231804	109.5418553	95.85981859	93.87996118	X	0.590285606	6.52E-06	0.001109181
**CG4789**	FBgn0030792	270.6213039	262.7439854	310.6850705	287.6331782	285.4083782	270.4805738	302.1231805	273.4724234	X	0.030569574	0.788507566	0.995787491
**Rai1**	FBgn0030793	51.29759	50.51777966	70.83691223	65.65067301	56.46934007	69.94752038	46.69154535	70.83672813	X	0.066405806	0.736685115	0.995787491
**CG13005**	FBgn0030794	8.192505173	12.22258658	15.87994785	11.61388244	9.661529159	22.1235728	14.91303745	10.72564046	X	0.295605909	0.363162667	0.939549067
**CG4829**	FBgn0030796	12.2360347	28.86158702	26.50295828	18.04489155	13.28521418	18.18171177	22.746178	14.71910197	X	-0.275719152	0.211846382	0.856520545
**CG13003**	FBgn0030798	0.979801184	0.425748051	0.483342152	0.534504302	0.389232837	0.674157799	0.194946597	0.33775105	X	-0.583183256	0.180325015	0.836097072
**CG4872**	FBgn0030799	5.762544409	1.71166268	7.135549642	2.099243024	4.226165769	3.056001564	1.225031216	0.781938584	X	-0.813235928	0.26980687	0.89549671
**Nprl2**	FBgn0030800	59.9700677	42.1935809	38.38127603	52.66294968	51.05554358	58.035398	38.3355236	43.87303198	X	0.008833507	0.964977516	0.997604378
**Rcp**	FBgn0030801	19.27693108	20.67395234	21.41697378	13.02564276	15.86008641	21.83313246	21.54164013	15.83339007	X	0.04260489	0.862121436	0.995787491
**CG4880**	FBgn0030803	11.05957715	8.515162241	2.968935482	3.064609204	8.346179808	9.914103337	4.393861963	2.263366446	X	-0.023135494	0.966669053	0.997604378
**CG13002**	FBgn0030804	0.104737903	2.986613359	0.508185383	0	1.890787587	3.061133906	1.068754561	7.049266158	X	1.917969256	0.125982039	NA
**wus**	FBgn0030805	2.009780519	1.878987636	1.758449002	0.614521655	3.330778043	0.621248786	1.260736053	3.075853125	X	0.437833313	0.532283417	0.984760433
**CG13001**	FBgn0030806	83.56113109	98.81344889	89.11592965	110.6466896	94.87209442	79.23842064	80.58723732	85.02037937	X	-0.137784428	0.343169596	0.928865024
**RhoGAP15B**	FBgn0030808	0.722710949	0.493878094	0.373385127	0.249625685	0.398651762	0.567806224	0.529196166	0.348682749	X	0.016505317	0.975788259	0.997757033
**Ubr1**	FBgn0030809	15.62983689	30.48906278	35.26723676	23.65541792	25.06261961	18.88874566	30.10819068	32.57405244	X	0.063973792	0.765364563	0.995787491
**wcy**	FBgn0030812	37.74215496	68.79906728	69.5184041	65.48984223	58.28886195	53.65927433	66.74683665	75.42734606	X	0.11097055	0.525329234	0.984107922
**CG4949**	FBgn0030813	6.890497675	3.509711551	5.163658763	7.233426651	5.926489023	10.88388544	5.613854785	9.242247012	X	0.496483663	0.192883182	0.844272804
**CG8945**	FBgn0030815	0.621090564	0.260448073	0.115904388	0.139235507	0.646862108	0.078825437	0.304695264	0.025931637	X	-0.100599142	0.926080199	NA
**CG4991**	FBgn0030817	0.592995544	2.19512681	0.314176775	0.06356537	0.233789309	3.405844432	0.695514654	0.38475464	X	0.596778404	0.603944807	0.989286463
**CG5162**	FBgn0030828	6.34612086	0.493464187	0.917618584	4.311342005	15.40161625	2.139645928	24.87330485	1.916147898	X	1.893132821	0.08471778	0.69799888
**CG8664**	FBgn0030836	2.730774337	1.205392332	0.11622492	0.335089346	0.259460398	1.309862519	0.366645474	2.49632163	X	0.015023985	0.989289546	NA
**CG5445**	FBgn0030838	16.68736557	17.97551435	15.85680374	16.95270065	16.04578349	16.28358791	18.97227712	17.53794768	X	0.059530914	0.586303543	0.987204104
**CG8568**	FBgn0030841	1.950312314	0.327137531	1.005428975	0	0.572132208	0.13790569	0.062191108	0.899790572	X	-0.952453169	0.426478042	NA
**ppk23**	FBgn0030844	0.485528283	0.162881116	0.706730905	0.377330304	0.64276886	0.152584366	0.743155848	0.843301477	X	0.487170164	0.64525782	NA
**CG12991**	FBgn0030847	18.61671131	22.92911963	21.61514657	21.75917787	21.46213522	20.75057651	20.2643073	20.75549913	X	0.003596799	0.972221753	0.997604378
**stas**	FBgn0030850	139.5083768	129.9962762	141.3935585	140.9828081	132.0798868	136.0703113	147.1681776	136.6767247	X	0.029925434	0.756697997	0.995787491
**CG8326**	FBgn0030851	20.80664184	35.88486859	24.20979091	32.69476165	32.49819641	28.7788822	31.94674779	27.61367827	X	0.124020671	0.610506841	0.990047511
**ND-24**	FBgn0030853	604.2537615	552.8180499	580.5339821	598.019639	703.5464822	576.5303577	647.6453191	604.5610475	X	0.147138089	0.164033969	0.827277154
**CG8289**	FBgn0030854	193.8879302	188.2656503	189.0586767	197.2267816	180.4912242	181.928707	194.2951961	214.670222	X	0.036075108	0.739559812	0.995787491
**CG5800**	FBgn0030855	6.173379741	14.41156798	17.678485	14.17043026	17.29125943	4.140159847	13.06956866	11.7448784	X	-0.135754786	0.734699075	0.995787491
**IntS2**	FBgn0030858	80.98176133	88.38442464	73.75690161	94.77629497	72.53754675	94.86774456	87.5799184	90.80654511	X	0.062005741	0.626397851	0.990047511
**CG12990**	FBgn0030859	0.161601205	0.05421267	0.470450722	0.828887675	1.925428509	3.1994892	1.401643692	0.280680939	X	2.223571017	0.022423413	0.421875556
**CG8188**	FBgn0030863	332.3635094	363.2779536	338.5076413	313.5663891	316.7339913	327.2690124	343.5260678	324.141721	X	-0.00756818	0.92889421	0.996479182
**CG8173**	FBgn0030864	5.884809762	17.09673103	12.03702069	17.66589443	10.9013308	10.91501289	15.40475394	16.03321317	X	0.054975654	0.873311759	0.995787491
**Socs16D**	FBgn0030869	129.9018925	107.6854966	89.89436966	111.219439	111.4122951	113.8360333	93.29272514	98.8677971	X	-0.045314474	0.721149099	0.995787491
**CG6398**	FBgn0030870	0.414098444	0.761379744	0.869362831	0.148531528	0.733177789	1.291354445	0.243778622	0.397654986	X	0.322079964	0.627143284	0.990047511
**CG8142**	FBgn0030871	2.863939055	9.394207278	3.242342227	3.412773781	4.251007302	3.000115831	10.55306786	5.80335168	X	0.357362807	0.5475331	0.984934163
**Ucp4A**	FBgn0030872	168.4478833	138.1452756	123.9370862	141.1286333	166.4933761	160.757184	150.7745334	159.1068282	X	0.183950274	0.12519829	0.780439453
**CG15814**	FBgn0030873	135.4380275	156.9417936	150.8224546	164.9416251	148.3115316	147.3375971	147.7059983	172.8061499	X	0.051129455	0.665202408	0.995787491
**Spt7**	FBgn0030874	40.12685697	50.3278713	42.49749162	42.82015061	38.18927822	34.74541426	44.99707251	47.6391568	X	-0.054815754	0.777421777	0.995787491
**CG6762**	FBgn0030876	7.888825551	6.92702723	7.321317469	8.147013331	8.124261213	6.089795022	4.05192553	8.391262072	X	-0.151943462	0.6968168	0.995787491
**Arp8**	FBgn0030877	20.32183541	27.66570409	19.61603974	49.0478898	29.19388531	33.52877902	22.17864368	48.08211732	X	0.218829419	0.464128604	0.971928234
**CG6769**	FBgn0030878	107.6254908	144.9621724	157.6306217	150.5776484	123.8245118	142.2311428	129.9867461	168.3278264	X	0.043262401	0.775977389	0.995787491
**CG7772**	FBgn0030883	47.39356042	42.87825523	42.53945471	56.97209032	54.66528243	60.68302582	59.64258687	76.29273048	X	0.433764012	0.027650761	0.462499435
**CG6847**	FBgn0030884	2.305091491	2.848374063	2.167360043	1.462809264	0.911049671	2.096070219	2.453040374	0.695901329	X	-0.486817281	0.220766477	0.86549481
**CG7536**	FBgn0030890	3.234921035	5.469535233	3.160076987	1.729654157	2.086777202	3.009198942	2.816803178	1.591950951	X	-0.482811588	0.161328393	0.823037813
**CG7206**	FBgn0030892	134.7826513	147.6517319	112.4880024	169.6854379	170.9180831	151.8824783	144.1722346	156.432424	X	0.17252826	0.23217869	0.867702781
**RhoGAP16F**	FBgn0030893	19.56419606	25.49722981	16.54293448	18.26420128	16.70662344	10.42793307	18.89696041	15.95041121	X	-0.332917007	0.13798861	0.798609078
**Mvb12**	FBgn0030894	61.64285692	66.99739173	56.84153628	55.59461742	54.99528869	61.18213853	52.47624733	48.7163768	X	-0.119760606	0.322166018	0.918365535
**Frq1**	FBgn0030897	30.67291785	34.30717665	29.3266637	31.48812529	22.03277442	20.87626304	23.3110953	18.06310416	X	-0.547404451	8.44E-07	0.000206985
**upd2**	FBgn0030904	2.437167368	0.994379925	0.159798217	0.460715998	2.49713369	3.260315991	0.084017112	0	X	0.521864889	0.658046163	0.995750687
**CG6023**	FBgn0030912	0.330837832	1.007102537	0.653976861	0.514223967	1.725380451	0.369673347	0.71894117	0.292632412	X	0.357502164	0.611505038	0.990047511
**CG6123**	FBgn0030913	1.013886726	0.716984706	0.569076491	0.498289913	0.630497695	0.805991459	0.445480558	0.707339954	X	-0.089620282	0.785626492	0.995787491
**CG6106**	FBgn0030914	0.095028245	0.358642239	0.518708695	0	0.643313769	1.321482727	0.181814322	0	X	1.191145106	0.399827764	NA
**CG6179**	FBgn0030915	66.03323666	108.9934672	87.69399021	106.2499474	86.0752288	101.6551345	103.6020606	109.1722277	X	0.152629211	0.38747171	0.948247506
**CG15056**	FBgn0030918	0.463199175	0.194237909	0.842786053	0.13499143	1.881434761	0.218350676	1.624742688	0	X	1.22421726	0.394540153	NA
**Hayan**	FBgn0030925	2.217463665	0.881939557	0.332754799	1.972036321	0.453958688	1.681110036	0.116634999	0.049632153	X	-1.213795137	0.208595072	0.856520545
**psh**	FBgn0030926	0.71173579	0.238767388	0	1.244538901	1.927298276	0.503265117	0.272347994	2.54965307	X	1.279769072	0.2471507	NA
**CG15046**	FBgn0030927	0.558827391	0.535631173	2.227233679	2.698833267	1.369121906	0.827921366	0.509135958	2.599855707	X	-0.134711748	0.874493392	0.995787491
**CG15043**	FBgn0030929	5.508032031	2.533260767	1.724181791	0.207125312	5.613215047	0.08375708	0	0.771512736	X	-0.594595351	0.656346661	NA
**Pgant7**	FBgn0030930	16.05932914	23.77686814	21.18861339	23.85267554	21.61624939	21.39943161	22.68496195	23.5118075	X	0.106579862	0.523837257	0.984107922
**Ggt-1**	FBgn0030932	2.17915743	1.641570756	3.039006772	3.833284807	2.708998998	5.093182481	3.095773654	4.136217159	X	0.522655064	0.139963568	0.803344611
**CG15040**	FBgn0030940	0.781055316	1.905615596	1.033543256	0.386272954	2.278796006	2.045483837	1.006307521	0.068514885	X	0.429908567	0.526223351	0.984107922
**wgn**	FBgn0030941	1.195723987	1.47081678	2.107921727	1.059358476	4.518644755	2.6003544	1.50482334	4.188257641	X	1.178188952	0.001559644	0.084403048
**Nup35**	FBgn0030943	69.98383122	52.80239401	42.28417328	50.21639874	67.35673997	53.92142385	32.96723495	48.632751	X	-0.059630091	0.804624887	0.995787491
**CG6617**	FBgn0030944	297.7915449	266.4573425	269.6861756	285.770674	281.1045453	270.8598469	302.9835048	305.4119299	X	0.080463763	0.486723731	0.973549494
**Ing3**	FBgn0030945	117.2065684	192.6790943	154.5237987	190.4584014	140.4875097	119.690032	138.2777216	153.6624648	X	-0.210824374	0.18496611	0.839299945
**CG6659**	FBgn0030946	103.3012668	88.91267708	88.44650766	88.47506093	71.39767368	86.82215025	103.7298485	81.95465367	X	-0.07436668	0.581311193	0.985637351
**CG6696**	FBgn0030947	4.843613974	3.889380136	3.875178066	0.840946016	4.092919615	3.837828126	2.497522371	1.901817906	X	-0.099130145	0.874943798	0.995787491
**CG6873**	FBgn0030951	1.658375837	0.213976371	3.404243319	0.297418526	1.381750811	2.525664523	1.301708427	0	X	-0.059943802	0.967486162	NA
**CG12609**	FBgn0030952	1.83153588	0.374651768	2.600944682	0.10415017	2.016092489	2.021573667	0.227916458	0	X	-0.175617637	0.886230526	NA
**CCKLR-17D3**	FBgn0030954	41.09465803	48.71415113	44.40922053	67.56258826	50.41332216	42.17494559	54.54668411	45.73328883	X	-0.034599652	0.828100484	0.995787491
**CG6891**	FBgn0030955	3.964869835	1.710131897	1.889391216	2.211943925	2.479599305	3.364249556	2.962094238	1.475674189	X	0.084798807	0.835366423	0.995787491
**CG18259**	FBgn0030956	37.57731877	35.19810502	32.24138729	36.42116013	25.60536954	30.77480864	28.25259156	24.12924254	X	-0.351724614	0.038057778	0.522230403
**CR6900**	FBgn0030958	8.127144036	3.587406315	0.518851002	1.994540039	5.405318194	4.839297951	5.455926063	0.464336369	X	0.173656424	0.878393651	NA
**CG6961**	FBgn0030959	25.92683465	27.54045583	27.53698826	28.2218253	20.79363477	22.98257011	24.14801318	24.46185963	X	-0.211706466	0.245983747	0.877269208
**CG7058**	FBgn0030961	145.2014223	133.4653022	112.9849864	140.0085943	152.3027977	158.6185205	117.2679768	146.7462137	X	0.140340354	0.256445567	0.88674759
**CG7101**	FBgn0030963	35.16746005	42.77139037	39.50312067	34.66847785	35.41758637	37.79319544	27.54850918	33.7568297	X	-0.142955497	0.44965082	0.967112302
**Pvf1**	FBgn0030964	0.991714917	1.222716136	0.822525319	1.778573843	0.994610889	1.198695855	0.864918311	2.281923129	X	0.181971212	0.678841526	0.995787491
**CG7322**	FBgn0030968	2.298785712	4.318597411	3.569166128	0.214381294	1.410965111	1.040294942	0.469140139	0	X	-1.778686427	0.079625873	0.692197295
**Usp39**	FBgn0030969	79.11040413	67.63095853	79.15184466	60.72461988	87.09428943	68.9920377	58.40380564	72.54603608	X	0.03298501	0.843269037	0.995787491
**CG7326**	FBgn0030970	44.50142516	49.40647196	42.04322698	35.23700292	42.92696336	53.00663745	33.15760262	42.51135392	X	0.033961559	0.844449985	0.995787491
**CG7332**	FBgn0030973	217.83084	230.284747	230.8725825	262.4337736	212.1599382	220.1354557	242.5176079	262.595195	X	0.025120435	0.822903516	0.995787491
**Flacc**	FBgn0030974	165.7052784	169.2138278	137.7288759	183.0877923	156.5695523	152.9299448	134.1539364	139.8511762	X	-0.139847936	0.221497029	0.86549481
**CG7378**	FBgn0030976	180.8884136	236.4313502	235.33317	285.5310196	210.1594279	185.7688517	223.9993365	214.75222	X	-0.136118505	0.301754113	0.908861141
**CG14190**	FBgn0030979	0.151249579	0.347168353	0.019312072	0.092798079	0.445493258	0.052535803	0	0.259244871	X	0.353736831	0.772965833	NA

tgy	FBgn0030984	0.262139092	1.193475642	1.181042164	0	0.608436317	0.17653081	0	1.463472603	X	-0.164493296	0.907191568	NA
Obp18a	FBgn0030985	4.07226822	2.399961149	1.869050306	4.875471918	4.96722152	4.980725972	2.807687491	2.389532776	X	0.219436652	0.768950659	0.995787491
Inx5	FBgn0030989	1.4395245	0.413931498	1.197348466	0.383565392	1.410728649	3.179666923	0	0.089295456	X	0.456690918	0.704269966	NA
CG7556	FBgn0030990	34.28881372	40.60778123	40.32145773	41.35548249	41.51887016	48.72136508	32.00756195	33.81070075	X	0.026278793	0.867411462	0.995787491
CG7453	FBgn0030991	28.96633931	76.92282908	94.56627135	62.51525312	33.1152395	32.95644259	64.58242464	53.44007385	X	-0.472177325	0.113278323	0.758410631
CG33253	FBgn0030992	0.426690865	0.753383229	1.688922564	1.256606765	0.60812082	1.545608028	1.374944292	0.633842619	X	0.058045218	0.933523562	0.996479182
CG7914	FBgn0030995	2.396273679	6.265555455	10.25881151	0.821591452	8.779008307	1.196042923	3.955435413	3.672375514	X	-0.108825815	0.888131371	0.995926915
CG14194	FBgn0030996	111.1170403	96.87948298	95.41569584	135.4722212	117.9376091	154.113702	109.7702868	122.8957309	X	0.226389882	0.134122441	0.795116625
CG7990	FBgn0030997	287.4157528	325.8902047	351.4547367	315.5914958	384.4369057	426.2317307	358.70894	376.652394	X	0.302346793	0.000868646	0.055686922
CG14195	FBgn0030998	0.127830941	0.51460433	0.31011608	1.192132107	0.507688745	1.012353135	0.521759163	1.221144347	X	0.654833633	0.47133	NA
CG7884	FBgn0031001	0.175638776	0.522694111	0.604783154	0.739733256	0.245475877	0.395282137	0.274616373	0.184513569	X	-0.848131765	0.150320263	0.809805479
CG14196	FBgn0031002	2.622071093	1.332013087	0.54523782	1.53704824	1.325382899	0.776937106	0.726229909	0.292770466	X	-0.936041166	0.134353102	0.795240592
CG7889	FBgn0031003	17.29102455	36.48209811	21.10580347	35.49605154	32.98160254	36.75610837	20.39036775	40.72780864	X	0.281594182	0.335108883	0.924155338
CG7992	FBgn0031004	5.00994633	5.208769924	3.013403286	2.413326708	4.783728748	2.303119513	0.316871423	2.337218913	X	-0.647209451	0.326967612	0.918470465
Hs3st-B	FBgn0031005	41.98670686	39.20184067	37.01726173	47.55186069	35.80949621	53.6828536	44.18002607	50.63052331	X	0.179973068	0.267538049	0.89472797
rictor	FBgn0031006	36.31153753	45.86763637	36.78334757	53.75524645	41.02329715	41.49801362	45.79535442	41.46754189	X	0.005715781	0.967628401	0.997604378
CG8034	FBgn0031011	5.259513085	5.964354356	6.131513287	5.093410833	5.68023639	6.241708203	5.390345381	6.220406881	X	0.100981575	0.493558977	0.973549494
CG8051	FBgn0031012	0.853705226	1.498061712	1.401959796	0.244970043	0.379361658	0.780102901	0.067009858	0.085544911	X	-1.549600395	0.063910734	0.636907352
kek5	FBgn0031016	1.341589564	1.224893212	1.015869998	0.579990298	0.945586483	1.054379266	0.725799501	0.858451116	X	-0.186529009	0.405544252	0.956706394
Naa15-16	FBgn0031020	156.0020818	158.3251997	160.7018198	152.4031511	129.2831187	155.4036303	166.1861729	166.7843615	X	0.008064305	0.940404934	0.997188858
ND-18	FBgn0031021	924.6214038	914.5653764	831.5918419	834.9060771	1047.632353	874.6694249	1048.136407	1048.47507	X	0.229307993	0.03384919	0.514749796
CG12204	FBgn0031022	30.77329917	40.74407235	53.24421846	23.30860136	62.74465229	48.52661657	43.6351093	87.32998933	X	0.752178869	0.008818261	0.281576184
CG14200	FBgn0031023	15.14315575	25.57818979	21.00704141	24.9688808	21.78513979	23.62121945	19.29113186	21.74839043	X	0.028321482	0.848989349	0.995787491
Tao	FBgn0031030	74.3632979	129.808121	120.5475325	115.3697645	84.52163817	91.60468368	129.2598147	117.6214377	X	-0.020041238	0.904953164	0.996479182
CG14218	FBgn0031031	0.584181914	0.089080292	1.546055101	0.123818013	0	1.25173441	0	3.113131941	X	0.944788388	0.541543903	NA
CG14204	FBgn0031032	0.10071889	1.647182638	0.183256967	0	2.181880932	2.919932502	1.220446252	0.492007577	X	1.862300817	0.070747729	0.6662581
CG14219	FBgn0031033	2.209962327	4.427688233	2.82959494	0.644055496	3.657086041	2.141415066	1.174512786	0.266557377	X	-0.439239747	0.552869453	0.984934163
CG14205	FBgn0031034	0.058944026	0.247175835	1.286976456	0.755841772	0.532045367	0.500148606	0.225551214	1.215742892	X	0.139194809	0.897738818	NA
CG14220	FBgn0031036	76.04472933	109.393121	66.69312806	78.19536762	70.54890397	77.91494179	82.1066256	79.42804613	X	-0.05933671	0.712727349	0.995787491
CG14207	FBgn0031037	53.76352968	58.17209686	58.16408239	64.31500919	59.31118291	49.93884472	52.05870102	58.37793154	X	-0.061659931	0.606044965	0.989286463
CG14210	FBgn0031040	28.91865117	33.26192945	27.39434304	26.64803792	21.13082885	28.69245913	36.18345878	28.40276915	X	0.00330801	0.991434708	0.998408391
CG14221	FBgn0031042	1.785207041	2.694990935	1.992208196	1.748100065	3.351666505	2.793916608	2.368132788	1.472822902	X	0.316301243	0.553404486	0.984934163
Naa20A	FBgn0031043	302.0697907	318.7785166	290.019488	292.9042879	319.9621511	273.0658847	263.1702747	255.2605505	X	-0.084246236	0.43691619	0.966363994
MKP-4	FBgn0031044	118.781273	127.3995673	143.6790687	148.969831	171.7956587	173.6317898	143.4793803	174.8239747	X	0.331927044	0.006006021	0.222463029
Rcd-1	FBgn0031047	401.7966119	474.8536805	458.1763718	479.8158845	475.8995795	416.1973523	480.785909	425.430894	X	0.019284861	0.853684749	0.995787491
CG12237	FBgn0031048	246.7432742	253.348278	253.9648879	308.8179149	315.904632	284.5070274	318.6742035	317.1627708	X	0.2486635	0.035515263	0.517454891
Sec61gamma	FBgn0031049	207.7819713	175.7874276	204.8415914	145.3137971	162.4075408	179.4395224	189.9816673	136.5815604	X	-0.106264397	0.488918783	0.973549494
Arp10	FBgn0031050	119.5279134	128.6853894	166.3193446	135.2861953	130.3226207	123.6117026	159.8470253	126.2091655	X	0.006677531	0.963687575	0.997604378
Ranbp21	FBgn0031051	41.95094641	29.87179033	24.66782811	37.05017839	22.55183545	33.30618613	36.1666687	38.349216	X	-0.00980281	0.960598749	0.997604378
Elys	FBgn0031052	38.05251005	54.56044945	45.89113646	60.42491625	39.91768882	48.22518492	41.97152809	41.70495091	X	-0.180890465	0.212273611	0.856520545
CG14223	FBgn0031053	1.090139104	2.98343252	4.500565602	1.159334655	1.519142829	3.227578797	1.024567259	0.9550217	X	-0.487020573	0.403133865	0.955279394
Ssu72	FBgn0031054	65.79980396	76.01721762	82.68639167	79.63990701	72.1125058	61.92936716	62.50289962	59.47635196	X	-0.215502598	0.116191329	0.765798592
Ubqn	FBgn0031057	354.6848422	371.7913361	351.4433892	343.7298347	368.5414138	409.1854241	368.925469	313.5898683	X	0.067447834	0.469384723	0.973549494
CG14227	FBgn0031058	0.149729033	0.879023068	2.542683821	1.221806614	0.81089784	0.63523628	0.38196198	0.162537796	X	-1.19629848	0.353606817	NA
CG14229	FBgn0031059	133.6356908	134.9039495	108.6163421	148.1686293	104.5320358	96.48183974	121.3311382	121.5791648	X	-0.213942967	0.173396337	0.832902061
CG14231	FBgn0031060	25.02304874	29.20816165	19.65600656	23.16913371	28.76850414	18.44278957	20.44069044	25.7544707	X	-0.024268239	0.91791029	0.996479182
CG14232	FBgn0031061	136.3531646	148.7166056	125.1616796	145.3119066	131.0890656	113.9502142	148.8576431	150.1546738	X	0.001851026	0.98891616	0.998078482
CG14230	FBgn0031062	12.9482875	25.86822505	18.65988242	18.92409977	14.58319495	20.69820208	14.49435779	12.00001821	X	-0.27009229	0.31216812	0.914141092
CG12531	FBgn0031064	105.6917738	180.903776	179.3673343	179.828984	129.9369821	116.0708897	161.3930282	163.39011	X	-0.140908964	0.390866995	0.949207648
CG14234	FBgn0031065	10.12259891	9.456038871	22.01984113	14.5487939	7.872812287	8.808121487	16.73292101	8.275089916	X	-0.397365112	0.196169044	0.844272804
COX6B	FBgn0031066	967.533558	937.9197301	958.1100143	952.5228445	1035.496294	900.9823846	975.1433539	987.6170491	X	0.062615662	0.48217374	0.973549494
Alr	FBgn0031068	35.99355054	36.14607102	29.03101559	24.08544336	31.22299481	43.8504248	35.77415093	22.22574973	X	0.112959105	0.628552192	0.990424271
Pmp70	FBgn0031069	36.3979843	45.75523871	41.90346779	44.4710944	42.28003233	41.98311328	38.22097394	34.3186045	X	-0.072556921	0.53550113	0.984760433
CG12702	FBgn0031070	0.546966645	0.280334944	0.258014838	0.28338492	0.411422909	0.916758777	0.077517981	0.032986534	X	0.089622524	0.932040746	NA
THADA	FBgn0031077	17.44051858	20.68390133	14.06713575	25.7106251	14.79560093	12.88302983	13.07213837	20.03851191	X	-0.326508095	0.137542119	0.798609078
Nup205	FBgn0031078	5.669777698	10.78775387	7.185344127	7.215419094	6.503517623	8.525336829	8.943454517	17.13896969	X	0.454322035	0.118427351	0.774240345
CG12655	FBgn0031080	3.258904292	3.740137475	2.496649376	2.999215529	4.644598024	1.131964496	4.813099131	8.564937183	X	0.645933853	0.365471122	0.940794966
Nep3	FBgn0031081	20.30013955	35.37211232	31.96790614	27.55197619	31.63990141	23.42244929	31.13773004	29.80407723	X	0.048623386	0.777746202	0.995787491
CG15322	FBgn0031088	6.571152057	8.883552682	5.115563426	16.46370661	9.177295623	5.008591187	4.837127882	1.277604125	X	-0.842325763	0.059862253	0.627645287
CG9572	FBgn0031089	3.234998078	2.850750146	1.336445037	3.25192345	0.571304412	0.806686084	0.717613009	0.076342177	X	-2.270752064	3.17E-05	0.003687992
Rab35	FBgn0031090	38.87906155	65.9218674	81.26980842	62.02030995	52.32581962	44.59054769	66.99986165	68.70052221	X	-0.053678534	0.790959011	0.995787491
CG9577	FBgn0031092	43.83240291	46.88105182	41.51914308	41.91085732	37.67505135	45.03952144	44.13018235	53.06206332	X	0.076342097	0.677955201	0.995787491
CG9581	FBgn0031093	51.32767625	63.01642743	47.68398584	83.63258622	79.67597517	77.87270229	80.56846773	62.16815012	X	0.318369089	0.094449312	0.728833855
CG9578	FBgn0031094	115.712411	103.0622769	111.5131762	124.9780471	125.7900092	158.9425476	107.601824	130.8029142	X	0.228240744	0.126677951	0.784349214
obst-A	FBgn0031097	1.446219963	0.389865713	0.563867592	0.060210846	1.911476885	0.170435918	0.922334549	0	X	0.300865077	0.803535226	NA
CG17068	FBgn0031098	124.9493084	143.7243312	139.8633323	127.7402953	142.1579547	143.4678452	141.0398042	155.0248012	X	0.149725111	0.141218533	0.8037297
CG17065	FBgn0031099	281.7597899	291.7969394	235.4480447	330.9130479	331.8222856	346.1644699	296.9410067	370.4865246	X	0.268135293	0.031401072	0.494700436
CG1504	FBgn0031100	3.922059012	5.349039524	5.290973806	5.277104757	4.212901716	4.409009666	4.862367792	3.72040571	X	-0.172648471	0.268247845	0.89549671
Syx16	FBgn0031106	205.328643	285.24817	275.8295411	269.072882	262.1952612	235.8837579	261.6649819	248.4412573	X	-0.004308902	0.972661687	0.997757033
HERC2	FBgn0031107	24.79381779	31.75674475	24.23634642	32.97535439	24.23653514	25.28758175	18.04654818	28.8531264	X	-0.207342623	0.178799518	0.836045561
CG15459	FBgn0031108	209.0014443	269.489434	269.2773936	287.6118694	148.4236235	80.41186267	111.1952931	118.7484094	X	-1.139052185	3.89E-10	1.91E-07
Obp19a	FBgn0031109	6.131278649	9.536406361	11.35863004	1.559437675	4.02491978	0.105100583	1.706293479	0	X	-2.254170705	0.03434137	0.514749796
cactin	FBgn0031114	59.03258061	69.00183067	51.43029938	46.7430247	55.96544854	65.63155239	80.97945982	52.55579151	X	0.203109707	0.248485134	0.879503029
CG11710	FBgn0031115	61.11526085	67.3780564	53.41619234	50.70999011	59.10466583	46.06639395	64.57023372	61.35521544	X	0.021957067	0.895016445	0.996479182
CG1695	FBgn0031116	38.43503406	40.09848122	35.87928838	39.09270225	39.12894747	37.44208943	31.4309395	33.61087387	X	-0.086481482	0.422319902	0.960593359
GstT3	FBgn0031117	4.477064384	9.433606695	10.77153297	4.83085947	2.872204075	7.011659007	3.964344397	6.988847485	X	-0.459335939	0.291262159	0.905069205
RhoGAP19D	FBgn0031118	9.261618548	9.768261096	10.52246491	8.005711824	9.645625062	10.70827862	10.34883602	11.1433206	X	0.187958459	0.096130302	0.730787365
CG1812	FBgn0031119	31.21815389	38.15387493	32.32463542	26.25690445	29.01789592	30.21384159	25.27377515	29.63070842	X	-0.132172892	0.376600323	0.943615128
Cyp6v1	FBgn0031126	0.422724415	1.215532979	0.146503362	0.187726869	0.218035975	0.227738284	0.872973529	0.65555274	X	0.026432274	0.9785209	NA
CG1835	FBgn0031127	0.39107825	0.599752139	0.487928767	0.130254888	0.363083901	1.000773933	0	0.169813417	X	-0.035687226	0.971568974	0.997604378
CG1324	FBgn0031129	0.287768258	0.386152706	0.977370492	1.341841721	0.727293644	1.465053242	0	0.124954305	X	-0.310200355	0.823375146	NA
r-cup	FBgn0031142	3.259453599	2.849963103	2.860119414	4.123020867	3.50545055	3.922969716	1.415308282	2.710177846	X	-0.145433133	0.763638212	0.995787491
CG1532	FBgn0031143	42.80552837	57.83008077	47.36487769	55.36387329	36.29475012	48.75684666	44.86498771	39.10829459	X	-0.235537227	0.15262501	0.813857865
CG1529	FBgn0031144	2.731442555	1.057295008	1.847757737	0.367399355	0.853434325	2.599948774	4.153981305	1.083404597	X	0.542352249	0.440462153	0.967112302
Ntf-2	FBgn0031145	131.2270754	108.9311322	104.226509	112.2190643	112.622597	98.05568978	111.1824586	112.9654209	X	-0.040858346	0.726992186	0.995787491
Cbs	FBgn0031148	46.15408794	39.71797173	44.96817924	56.58398723	66.33201013	49.93047753	43.48957538	45.1372961	X	0.157126982	0.358546178	0.93823489
Stt3A	FBgn0031149	135.9128346	111.4774117	84.47911014	123.9493174	115.6167147	92.20358455	90.6872666	110.271866	X	-0.128530303	0.411790706	0.958665466
bves	FBgn0031150	22.15121169	20.25411899	19.8839955	21.75187357	22.83806424	26.25584697	20.69117929	24.5026127	X	0.193861966	0.067489202	0.656261422
CG15445	FBgn0031161	7.673031806	10.92438937	9.467253898	11.06559885	7.473301965	14.46817504	8.475370077	8.186313793	X	0.013283773	0.962112607	0.997604378
CG1494	FBgn0031169	0.665642519	0.345106783	0.352328611	0.225733887	1.289048466	0.502050961	0.679227347	0.289034305	X	0.807200614	0.256946804	0.88674759
ABCA	FBgn0031170	15.99997126	23.29577754	21.99849465	24.86888712	24.59282896	23.49728249	23.85566334	28.2536245	X	0.252078662	0.084357823	0.69799888
CG1801	FBgn0031171	0.061023321	0.116980641	0.169190544	0.081299186	0.125900268	0.205472909	0.266865949	0.075707017	X	0.700452428	0.490264346	NA
CG1486	FBgn0031174	136.9978149	156.2999889	121.400894	157.3652725	123.600932	156.7797227	133.311246	124.5001889	X	-0.060141167	0.613753079	0.990047511
CG14621	FBgn0031183	2.249510429	2.028622672	3.696867722	2.988885921	2.05230446	1.550710527	2.529898137	2.258145174	X	-0.354216843	0.424170969	0.9620897

**Usp2**	FBgn0031187	141.6340874	175.5169116	139.2839833	168.2963557	153.544502	170.1813875	135.2472833	138.7837404	X	-0.03467151	0.746666093	0.995787491
**CG14613**	FBgn0031188	109.3767651	132.3278126	108.7755865	157.795982	133.5261218	134.1437379	140.4860844	140.9499877	X	0.141992577	0.319584161	0.916248855
**CG14618**	FBgn0031189	25.38580892	40.5694261	32.65169267	41.16615571	37.88920157	47.17617526	51.16189744	34.86273177	X	0.324797704	0.158312183	0.818884898
**CG12576**	FBgn0031190	76.98245626	75.20003704	79.95531267	90.07715895	68.97613774	86.89629661	83.19305965	86.46177029	X	0.043475144	0.705879189	0.995787491
**Cp110**	FBgn0031191	13.90467745	11.75616271	11.3353888	22.31336017	13.14702313	24.38046139	14.01582346	17.42038733	X	0.238952106	0.343993782	0.930285749
**CG17600**	FBgn0031195	1.507836162	2.193403365	2.420382772	2.51427136	1.865203068	2.085185065	2.339212792	1.948769217	X	-0.033095624	0.877472922	0.995787491
**CG12446**	FBgn0031201	0.491590497	0.109943213	0.954073352	1.146124739	2.189035542	3.244279628	0	1.707661561	X	1.434690271	0.159647955	0.819277129
**galectin**	FBgn0031213	88.52408042	79.26685955	93.319791	99.84007365	90.43653161	107.8452325	92.37468351	78.49046487	2L	0.059131205	0.635574366	0.991585536
**CG11374**	FBgn0031214	39.17438427	33.91786423	48.03077846	46.72227415	51.21464998	45.98176136	40.03547139	36.43179915	2L	0.080751087	0.691490199	0.995787491
**Zir**	FBgn0031216	30.00455086	33.12381596	33.40646222	31.39018352	25.71018174	40.09241766	30.61690155	27.77201701	2L	-0.014267397	0.919653353	0.996479182
**CG11377**	FBgn0031217	50.97793458	62.25534426	48.29996345	44.35502738	67.53470463	46.90301405	54.92773691	52.72405071	2L	0.141915407	0.43095594	0.963666972
**ND-15**	FBgn0031228	21.54970686	20.42304578	21.43369419	17.41075693	23.00622572	21.20070668	22.09971039	19.78269216	2L	0.122028667	0.239700897	0.874524157
**CG3436**	FBgn0031229	133.3931565	128.5154893	124.899829	117.4427088	136.3086906	143.4218477	142.2706878	139.4625917	2L	0.183811194	0.09671991	0.730787365
**mRpL10**	FBgn0031231	144.5652475	120.6645395	120.6755351	112.1481343	133.6066704	117.8131428	122.70938	142.1202611	2L	0.079796667	0.601405604	0.989286463
**CG11617**	FBgn0031232	0.203488439	0.455097747	0.658213487	3.004695693	0.122449596	0.735416021	1.211241221	3.092545668	2L	0.293333082	0.774062643	0.995787491
**Tbc1d15-17**	FBgn0031233	20.98281811	28.27131118	29.15573904	26.93768066	27.95752866	29.4088681	28.60206925	32.40337174	2L	0.203567429	0.206786719	0.855364409
**CG3645**	FBgn0031238	8.076230056	7.33465869	8.523864659	6.178076056	5.46125196	8.577721935	8.455617284	7.353386367	2L	0.015627398	0.927616255	0.996479182
**CG11601**	FBgn0031244	6.933793272	7.814219008	4.730986622	11.24033704	18.67811976	20.01998495	17.13549762	20.34629624	2L	1.339714415	2.99E-05	0.003554356
**CG3625**	FBgn0031245	571.5465286	409.835151	422.9678303	390.9514887	465.3485903	448.8560337	419.7284143	407.3508124	2L	-0.016254395	0.895936549	0.996479182
**CG11562**	FBgn0031247	35.3808027	43.966948	54.43089465	61.6152663	45.56680353	56.44215426	58.61213722	43.32553231	2L	0.095346984	0.700002213	0.995787491
**Ent1**	FBgn0031250	7.427636482	7.236182291	9.373698374	9.183392719	5.688576531	5.764873939	6.220322807	5.049582601	2L	-0.51626572	0.096878034	0.730787365
**CG11885**	FBgn0031253	69.91887216	75.65549047	64.74419124	69.49893906	77.47970577	59.29774887	68.47924497	53.19788833	2L	-0.085551042	0.704265617	0.995787491
**shv**	FBgn0031256	63.14279615	80.6331061	59.94138629	77.19973011	85.16298084	69.98944218	72.89272499	61.79337941	2L	0.076533121	0.649445036	0.992932477
**CG4133**	FBgn0031257	279.555402	265.7169757	233.4756863	294.3753993	239.7093322	275.784649	243.1027566	253.3551567	2L	-0.057108978	0.590266598	0.987807595
**CG4297**	FBgn0031258	36.67168446	38.52517364	31.02183038	32.43522688	30.81827668	33.71577636	37.99390782	35.92037706	2L	0.028284658	0.809421726	0.995787491
**Spp**	FBgn0031260	137.3272668	95.87425039	94.43116026	115.7383404	103.6033107	120.4608051	100.1266174	107.3743656	2L	-0.014813792	0.923226308	0.996479182
**Tspo**	FBgn0031263	11.40964816	6.219877359	11.99451383	12.41387708	10.12702511	8.515916056	8.489325132	10.32141764	2L	-0.138505566	0.748637638	0.995787491
**CG11835**	FBgn0031264	73.64282712	85.67236086	81.63644117	94.91703765	80.22825181	77.16678122	90.53380111	107.0144646	2L	0.112672101	0.437510188	0.967112302
**CG2794**	FBgn0031265	11.10394141	17.93314742	15.65316622	19.67869868	10.15817238	9.938218263	13.5890129	26.38814133	2L	-0.061147993	0.856716136	0.995787491
**Sf3b1**	FBgn0031266	130.6196317	106.5735388	125.9795681	116.20696	133.1542963	145.3736442	121.9734572	125.961026	2L	0.162763006	0.133914153	0.79490068
**Ipk2**	FBgn0031267	30.25657646	25.37558122	29.7022178	17.88159424	38.48866323	56.38804222	29.07906653	19.85971554	2L	0.502600404	0.106104606	0.751296334
**cold**	FBgn0031268	29.11285457	40.57647413	39.60958169	30.64894815	37.16858612	53.25365689	32.15706942	32.84142053	2L	0.185753843	0.406684046	0.956706394
**CG13689**	FBgn0031270	9.642105267	9.075006512	12.34557347	9.4916423	10.1537749	16.46393806	9.565584657	8.257261776	2L	0.165811808	0.62173125	0.990047511
**CG2839**	FBgn0031273	1.053832659	0.39914823	0.49482258	0.079257135	0.675058661	1.153796509	0.433604799	0.738054276	2L	0.565565738	0.58006332	NA
**GABA-B-R3**	FBgn0031275	1.592707424	0.723299924	2.952751306	0.97292683	2.486019787	2.498286596	3.548498564	3.171013986	2L	0.942516916	0.014727583	0.35986804
**Saf6**	FBgn0031281	51.3268967	45.19642022	38.18455998	37.23305348	41.99872477	53.31649703	34.7039121	39.02380786	2L	0.001514643	0.993142375	0.998777916
**Pex12**	FBgn0031282	7.949273138	6.563264762	13.38946451	5.281547713	10.40967127	12.93097001	15.97084206	9.299975208	2L	0.584183386	0.096222911	0.730787365
**PGAP2**	FBgn0031284	132.2004155	126.0738594	161.9022085	127.4712074	163.6995995	157.8401878	137.001396	158.8410559	2L	0.204988025	0.121415256	0.77836519
**CG3662**	FBgn0031285	3675.307647	3364.644625	3019.168957	3464.838492	3449.538482	3477.623223	3387.840105	3670.983463	2L	0.078140222	0.407632413	0.956706394
**CG3862**	FBgn0031286	66.03793141	57.37625537	55.82559067	52.99136157	73.08066812	69.50962885	76.87620624	55.2384486	2L	0.268505358	0.107461668	0.757046101
**CG4291**	FBgn0031287	51.06904003	58.54821746	31.68358907	43.69377773	44.08756704	41.1904747	54.83039692	70.06016391	2L	0.215167833	0.36742182	0.940908571
**CG15824**	FBgn0031292	1.989100132	2.096667701	1.713985773	1.368446187	2.02107137	1.311693258	0.221824576	0.755151128	2L	-0.697479562	0.191813563	0.843483578
**IA-2**	FBgn0031294	332.6926583	385.9933909	360.39881	402.4589677	358.5766642	333.0889237	331.0158817	364.3953827	2L	-0.063327296	0.50045126	0.976796212
**Atg4a**	FBgn0031298	45.10195964	44.99469652	46.78540669	56.71150359	41.5325519	40.07323659	35.84686262	32.73836351	2L	-0.336971526	0.016073234	0.36902021
**CG4629**	FBgn0031299	5.822737885	6.556122249	7.360612267	6.688921653	7.523197685	5.75886552	8.195271416	8.030629692	2L	0.193152498	0.240499896	0.874884741
**CG14339**	FBgn0031301	0.50044244	0.769470156	0.050586099	0.194460452	0.489356219	0.393178396	0.106386618	0.814880147	2L	0.275571762	0.795805769	NA
**CG14340**	FBgn0031302	23.15608906	36.77765797	38.79679108	47.40781728	23.64463839	21.08096429	29.35124134	22.93751838	2L	-0.557452871	0.039743388	0.529044592
**TBC1D23**	FBgn0031304	83.87556775	124.7756712	110.5328658	128.411001	128.3784902	125.6741833	116.0155522	139.9332783	2L	0.222067924	0.116518404	0.767033476
**CG4577**	FBgn0031306	548.8189941	928.0527995	640.2880669	834.0359121	593.148872	614.981791	571.2336861	593.9352863	2L	-0.281671837	0.038348482	0.522662821
**MFS3**	FBgn0031307	4.440372631	5.681343048	4.609534652	3.41875291	4.735038853	1.324056843	7.270665419	7.308813877	2L	0.224805961	0.61743163	0.990047511
**Tfb4**	FBgn0031309	43.16850879	64.27193659	64.98482456	70.64872899	87.57630851	49.44862993	78.55736399	54.95149562	2L	0.190677545	0.413341044	0.958665466
**Vps29**	FBgn0031310	119.6859326	134.6625341	188.4474117	183.7355095	177.363454	148.1883466	207.6805363	158.2838003	2L	0.175592167	0.337365788	0.925784156
**Tango14**	FBgn0031312	62.22734774	50.32493656	44.34528656	47.15123357	57.87326043	56.10085745	53.57586408	41.13353062	2L	0.056927544	0.760859172	0.995787491
**CG5080**	FBgn0031313	1.421155866	0.686854629	2.045248318	1.235494421	0.695742529	4.973367767	1.597633522	2.562501458	2L	0.892493473	0.159411875	0.819277129
**IntS14**	FBgn0031314	32.60202384	56.44463467	38.8038769	43.40539769	35.26566885	27.19358271	30.2126723	36.15006227	2L	-0.374361716	0.071799085	0.670374019
**CG14341**	FBgn0031315	159.4758622	265.2314511	199.1063121	189.3433834	196.7355098	150.468703	166.7423872	200.5933114	2L	-0.149095695	0.406209488	0.956706394
**Charon**	FBgn0031317	40.6075791	41.77440248	31.95029854	24.99736036	40.23507528	39.12031156	35.85835617	28.68499016	2L	0.074632246	0.718093215	0.995787491
**CG4887**	FBgn0031318	200.4890878	202.0735847	176.5498156	216.6743313	170.2295881	213.5366636	164.3246308	173.4387583	2L	-0.114439043	0.299499126	0.9074568
**CG4896**	FBgn0031319	119.0546332	150.1695512	160.247524	168.8827794	156.9589451	159.2980017	141.7905168	162.5162671	2L	0.084316573	0.483091896	0.973549494
**CG5126**	FBgn0031320	24.98506563	23.61067186	23.78192564	35.51363623	18.69520342	15.21408711	15.64581003	19.75518139	2L	-0.609156175	0.013313376	0.344576629
**Tgt**	FBgn0031321	24.1873362	36.38921733	29.33907013	28.81426374	36.2911957	33.45538843	42.08212905	45.25786783	2L	0.439000717	0.034805959	0.514749796
**CG5001**	FBgn0031322	1.450169723	0.591678553	0.741651726	0.566548647	0.778302927	1.115939918	0.359942995	1.293418411	2L	0.100068471	0.847623768	0.995787491
**CG5397**	FBgn0031327	2.033292151	0.341056234	1.761692285	1.01583088	1.363370714	0.164312179	4.742374365	1.450467229	2L	0.604095102	0.480046032	0.973549494
**CG14352**	FBgn0031351	14.56018537	19.31384489	14.7779089	10.56498964	22.52988544	26.68410057	15.35005613	12.74137404	2L	0.416403218	0.219045198	0.863196763
**CG17660**	FBgn0031356	69.89505137	55.08561107	58.90670497	63.30420842	57.66936634	61.10459694	64.49571461	57.17732729	2L	-0.013616535	0.917804565	0.996479182
**mRpL48**	FBgn0031357	257.4366802	274.3561079	249.7313157	286.5113017	303.8499423	217.7955474	249.8248418	297.9275659	2L	0.034596612	0.808753025	0.995787491
**Rim2**	FBgn0031359	44.06426715	31.6483769	33.67211761	27.40074076	24.49972229	27.80598269	26.86092283	26.07017228	2L	-0.351642796	0.020478379	0.405384344
**CG31937**	FBgn0031360	24.96772761	54.89823349	30.90737963	13.65661396	61.46338298	40.45190838	57.71613417	55.1610411	2L	0.831288352	0.010681066	0.312027572
**CG17652**	FBgn0031361	62.1320598	59.19220419	47.49074987	45.7930111	51.41355862	58.45427499	45.42893538	51.17176312	2L	-0.028653276	0.875401942	0.995787491
**CG17648**	FBgn0031364	1.924849903	1.076221894	3.113106012	0	5.212271115	1.66350867	0	0.348252277	2L	0.284698572	0.858383936	NA
**Wdr62**	FBgn0031374	3.029686069	4.692271184	4.338845525	4.498094438	5.823151701	6.986950838	5.529032016	6.35080199	2L	0.609448691	2.42E-05	0.003006757
**erm**	FBgn0031375	0.873801315	0.534286971	1.253101361	0.40142559	0.372989634	0.754825147	0.38432516	1.027987141	2L	-0.236418642	0.616302788	0.990047511
**CG15356**	FBgn0031377	32.80101366	61.85530429	40.14477267	62.7364252	40.89446272	53.56251016	47.09911683	46.80084168	2L	-0.037224751	0.843199778	0.995787491
**CG15362**	FBgn0031378	2.439950582	1.101873987	1.821318793	2.625532121	1.524716361	0.176951577	0.239398706	0	2L	-2.009716124	0.124072317	NA
**CG7289**	FBgn0031379	149.8567297	124.4800317	137.4062921	158.252034	152.2155622	156.0025751	171.0386504	137.4603436	2L	0.140833237	0.265648731	0.893759496
**Npc2a**	FBgn0031381	71.55999679	95.66784778	115.4124186	62.75896945	44.02364373	51.00806167	78.52756352	49.76257149	2L	-0.595689658	0.024473771	0.440903252
**CG4238**	FBgn0031384	21.03686476	26.85181083	20.26144275	20.39831508	18.19276626	18.77600897	19.86985238	20.10042454	2L	-0.170005381	0.147648569	0.808004944
**tho2**	FBgn0031390	55.07448233	63.86677268	59.76651113	54.0879327	46.92839733	66.0328442	47.48054988	64.3091355	2L	-0.020079078	0.879342753	0.995787491
**CG11723**	FBgn0031391	73.21825415	55.89770289	68.73464221	59.52234849	93.04615424	80.75486709	70.48984758	72.1611113	2L	0.326226014	0.037539193	0.521998122
**AIF**	FBgn0031392	80.06528346	71.8655682	65.95973652	39.61164748	73.73176912	60.09599286	62.12551188	73.30830182	2L	0.097460286	0.599915539	0.989286463
**CG15382**	FBgn0031393	24.72420512	19.33463249	14.18459586	12.65821276	12.21390435	13.46615154	18.1118857	20.31079817	2L	-0.125922914	0.723261029	0.995787491
**CG10874**	FBgn0031395	9.670969174	18.51388999	15.55608495	17.23740079	14.80183788	9.877541554	18.5031376	11.03080634	2L	-0.133572183	0.649080734	0.992932477
**CG15385**	FBgn0031397	77.31191428	111.9852512	102.2977854	114.0796043	87.34075923	129.1569385	109.7083511	95.50269644	2L	0.085785285	0.571705937	0.984934163
**daed**	FBgn0031398	37.90761161	37.0006534	27.46345389	39.21304445	29.66206565	35.80005285	42.08071428	25.70148618	2L	-0.063456301	0.785153132	0.995787491
**mio**	FBgn0031399	33.16686647	33.28381595	37.42728202	39.54168151	34.88392543	47.5286896	30.54418818	40.09234757	2L	0.123752651	0.453391384	0.967967712
**papi**	FBgn0031401	24.07467086	20.96343815	25.9820373	22.44325404	21.2251157	21.50553839	18.56732636	17.44892037	2L	-0.217104463	0.156448831	0.818884898
**CG15387**	FBgn0031403	50.07436631	41.51141593	43.20525633	42.86996188	51.56599328	45.68339262	43.66197707	62.26694914	2L	0.221075979	0.346527047	0.930285749
**CG17237**	FBgn0031410	3.510268443	6.842790906	6.444436322	0.884763106	4.966786277	1.520562541	4.598395516	3.089642324	2L	-0.27916742	0.717051051	0.995787491
**CG9967**	FBgn0031413	0.021389422	0	0.164319723	0.17454021	0.051484518	0.179811861	0.03182951	0.007739736	2L	-0.394120885	0.723781254	0.995787491
**eys**	FBgn0031414	1.94993681	1.533930432	1.77839079	0.991277622	1.220798943	1.997346919	1.467988569	1.090204106	2L	-0.089443279	0.713260765	0.995787491
**CG3597**	FBgn0031417	5.871353386	6.883270498	3.063187402	4.121370999	3.761041111	6.130688593	0.644212844	4.934420015	2L	-0.333831667	0.602745626	0.989286463
**CG3609**	FBgn0031418	9.650835841	22.40890201	16.35025637	17.20084167	12.59872162	13.34733853	18.31201704	14.89211393	2L	-0.11190683	0.697481695	0.995787491
**CG15390**	FBgn0031419	1.936586793	3.100700275	4.271029589	1.43661611	1.430196343	3.485622381	3.817484852	0.382228109	2L	-0.200660822	0.820997428	0.995787491
**Atxn7**	FBgn0031420	98.2416158	98.60407286	94.27906421	114.4150898	99.41862462	102.8097957	108.7536233	85.54240491	2L	-0.004116819	0.971911102	0.997604378

**PIG-Wa**	FBgn0031422	5.382104584	5.07809554	6.256436353	3.659884149	3.947156767	1.955687157	6.006815685	3.408138786	2L	-0.389434051	0.455071902	0.967967712
**VGlut**	FBgn0031424	0.910715614	1.976066727	2.0472268	1.149308725	1.659158356	2.150481407	0.596799475	1.66886979	2L	0.041785098	0.92321775	0.996479182
**CG18641**	FBgn0031426	0.073749038	0	0.492013881	0.601800874	0.299555811	0.191207893	0.329236918	0.520376965	2L	0.248061549	0.838912818	NA
**CG9886**	FBgn0031428	10.25712524	17.928103	15.80001211	14.33787935	17.61548268	17.15846179	14.64609547	22.71258103	2L	0.345657631	0.121866362	0.780439453
**CG3515**	FBgn0031431	0.618701755	1.470195981	0.125080152	1.923306466	0.837686429	0.826353333	0.526107156	0	2L	-0.880966284	0.482704294	NA
**insv**	FBgn0031434	3.480873005	2.200080867	2.57008024	1.646628133	3.369610331	3.519553474	4.890314625	0	2L	0.268837479	0.751051931	0.995787491
**Elba2**	FBgn0031435	4.644409954	2.337103846	0.845045533	3.383833427	1.572065685	2.736700835	0.592399479	2.520860985	2L	-0.576862727	0.455091727	0.967967712
**ND-B17.2**	FBgn0031436	872.337615	796.6258747	831.6657901	736.2440921	967.6787992	697.7952417	844.0362571	878.3058708	2L	0.098721238	0.424962738	0.9620897
**Arpc5**	FBgn0031437	165.8076939	213.6314734	169.073861	179.0919721	206.9700228	171.1965832	188.1426175	192.0468126	2L	0.09285579	0.485036357	0.973549494
**CG9962**	FBgn0031441	3.334385659	4.830287242	6.434569512	1.413453571	1.231245145	1.357480232	1.159921289	0.164528635	2L	-1.982310534	0.019388209	0.395797586
**CG31689**	FBgn0031449	0.305941811	0.718444257	1.721928516	0.299582563	0.574395517	1.240292306	0.249748523	0.783789032	2L	-0.051002382	0.933880783	0.996479182
**Hrs**	FBgn0031450	97.11665289	140.6626142	121.5199233	115.7131727	96.55997389	85.15508956	103.216918	105.6464515	2L	-0.246848072	0.065902901	0.647672366
**Cwc25**	FBgn0031452	48.14210186	64.38092297	52.24908288	54.37039433	54.46625667	66.66650674	51.82981983	49.81554507	2L	0.056103331	0.724237906	0.995787491
**Bacc**	FBgn0031453	1196.569193	1194.267672	1171.35821	1333.216566	1179.236282	1088.113519	1160.20613	1327.98984	2L	-0.010312279	0.91941625	0.996479182
**Tnpo-SR**	FBgn0031456	42.57729149	45.73060902	40.17202247	49.30261519	45.983668	39.09121418	46.14351188	36.04608686	2L	-0.058328136	0.684773187	0.995787491
**Snx21**	FBgn0031457	64.58817161	48.70992736	64.37651304	63.84368351	67.85918109	56.20923109	58.65545057	55.94024789	2L	0.011352969	0.942769232	0.997604378
**aph-1**	FBgn0031458	172.4926323	164.2830129	234.9979166	180.3650377	198.4442915	221.9242934	170.1514881	223.227051	2L	0.145484271	0.336651	0.924219112
**HINT1**	FBgn0031459	265.59925	316.2748841	340.2050019	301.2138027	383.9051183	254.9716907	356.8771611	362.1733106	2L	0.186465524	0.229442891	0.867702781
**daw**	FBgn0031461	0.024348066	0.081680931	0.236272276	0.0851499	0.351635999	0.562402599	0.559011961	0.211447623	2L	2.06781578	0.035311069	NA
**G6P**	FBgn0031463	64.41623746	36.32248894	45.89317272	31.34152145	46.22962373	27.75629636	50.12057359	67.21953707	2L	0.134868688	0.64026743	0.99179631
**CG3119**	FBgn0031466	0.676245638	0.641130151	0.213986411	0.548396956	1.433109166	0.360360145	0.600040748	1.659695168	2L	0.980262755	0.264910436	NA
**CG2975**	FBgn0031468	1.343719801	0.057792345	2.674745022	0.883619319	1.86596818	0.227383481	0.966831767	0.523625142	2L	-0.434059526	0.66795414	0.995787491
**CG18558**	FBgn0031469	1.935854762	0.22654359	2.293569528	1.154583663	1.544175954	1.188445053	0	4.398404535	2L	0.379490324	0.714888515	0.995787491
**CG3104**	FBgn0031473	29.7786103	91.16736065	110.1589689	101.16694	65.18217966	47.57368829	87.16221607	79.52709306	2L	-0.208753014	0.630160219	0.990523437
**CG2991**	FBgn0031474	75.57031059	82.06381021	74.01967408	77.2912736	78.11538783	88.01170266	70.03177618	78.0539076	2L	0.053920241	0.565740838	0.984934163
**CG8814**	FBgn0031478	158.4072043	156.0049305	149.1605524	140.5119748	160.8985535	196.5938238	128.5244216	144.5506838	2L	0.089597423	0.491452303	0.973549494
**Prx6005**	FBgn0031479	18.75083845	21.77439302	42.17426483	26.1947261	17.8158259	20.04003753	28.27422508	18.95395827	2L	-0.320573266	0.312303644	0.914141092
**CG9641**	FBgn0031483	115.4189204	121.3530184	121.9053769	116.3489987	122.4090031	144.4088306	115.8328786	111.162544	2L	0.085880268	0.456669028	0.968365245
**CG3165**	FBgn0031484	76.12096914	74.33194397	78.68612655	76.20648262	71.03516078	78.40373825	71.8376796	67.68932497	2L	-0.049528197	0.719016763	0.995787491
**CG9643**	FBgn0031485	45.92643583	50.03143288	44.80636332	30.39569854	42.07853991	38.17775678	56.18608465	37.52547338	2L	0.051649007	0.836911286	0.995787491
**Ccdc85**	FBgn0031488	1.171562996	0.943609913	0.876447948	0.421149453	0.745364397	1.065615831	0.710964089	0.44820647	2L	-0.17620587	0.676936488	0.995787491
**CG17224**	FBgn0031489	0.092788694	1.867680202	0.900416308	2.379666811	1.08879793	0.787325524	0.118352976	0.705084952	2L	-0.900826335	0.408173585	NA
**CG17264**	FBgn0031490	0.288920099	0.14538688	1.051374195	0.2020819	0.678047277	2.288093677	0.147408276	1.944544067	2L	1.639924695	0.100003241	0.732357333
**alpha4GT1**	FBgn0031491	210.8719299	208.2595108	188.2265121	255.1898031	206.9829278	234.1575012	208.0592984	239.5869211	2L	0.071550908	0.57079833	0.984934163
**CG3542**	FBgn0031492	80.71429426	64.84463011	68.08284366	71.33832807	62.53751992	57.89874334	64.23321357	71.46487898	2L	-0.125623034	0.360041571	0.939549067
**Sf3b2**	FBgn0031493	107.8866489	94.36871611	88.49292088	96.54127827	82.63487437	98.4382656	103.3594882	84.93486083	2L	-0.041761589	0.740930395	0.995787491
**GABPI**	FBgn0031495	279.1852142	274.6565944	234.119361	280.5013059	274.5543831	253.6148684	285.2092321	266.7600574	2L	0.045226653	0.683736256	0.995787491
**CG17258**	FBgn0031496	2.511873918	0.138900531	0.401787105	0.128710758	1.594576249	1.275173516	0.211247615	0.17978607	2L	0.025577182	0.981625934	0.997881056
**SerRS**	FBgn0031497	93.69282487	84.35945598	69.75150354	96.00420862	88.90879212	62.29487633	62.12471622	66.75503852	2L	-0.266922427	0.123444104	0.780439453
**CG17260**	FBgn0031498	25.82476166	30.11768756	22.45200737	34.49769467	29.71294667	29.7811882	26.57800451	27.55270412	2L	0.039659124	0.854356435	0.995787491
**CG17221**	FBgn0031500	35.93254286	27.93453694	35.79096018	19.83598205	34.8029388	33.36498724	37.05839435	18.07855079	2L	0.072460244	0.782772428	0.995787491
**CG17261**	FBgn0031501	0	2.370373558	3.83163	0	0.450196162	1.724174054	2.332644013	1.443809823	2L	-0.000542068	0.999690673	NA
**ND-B14.5B**	FBgn0031505	340.6661728	413.1429468	423.3215468	323.7924042	428.0468458	379.2090095	427.8573795	413.3034674	2L	0.169581552	0.163813509	0.826905988
**CG15404**	FBgn0031512	2.279427517	0	1.579959818	1.518399842	0	2.14903034	2.492086529	2.827913225	2L	0.480310212	0.7566415	NA
**OtopLb**	FBgn0031514	0.622099779	0.521742553	0.932156268	0.213294443	0.330308689	0.81076641	0.79349495	0.198622972	2L	-0.072202732	0.930627262	0.996479182
**CG9664**	FBgn0031515	0.175188593	0	0.060715012	2.742420517	0.722880709	0.542690701	0.446909864	1.141051956	2L	-0.039614789	NA	NA
**CG9663**	FBgn0031516	4.835587092	6.003570819	5.097122928	6.818548094	6.804415364	3.434152579	5.252093623	4.217740885	2L	-0.174560998	0.506401457	0.978557094
**CG3277**	FBgn0031518	1.320281113	0.872743389	0.347121372	0.257779099	0.352233369	0.447620526	0.282054745	0.310650026	2L	-0.988021509	0.097856439	0.730787365
**CG8837**	FBgn0031520	0.05981923	6.421655502	0.435361856	2.091994257	0.269972606	0.028198603	0.534100497	0	2L	-3.429017207	0.004631042	0.18852346
**CG3285**	FBgn0031522	0.489117404	1.790019989	1.833823998	0	1.926506778	1.341489165	0	0	2L	-0.279483345	0.850118602	NA
**CG8838**	FBgn0031526	0.980247636	0.448425789	1.945691257	2.285410461	0.321745131	0.504093537	0	0.580420461	2L	-1.942115372	0.158211829	NA
**CG15412**	FBgn0031528	1.494811142	1.558614264	1.078116238	0.941917812	0.729328981	1.942547014	1.442868506	0.877127936	2L	0.000407013	0.999562287	0.999757791
**CG9662**	FBgn0031529	115.9057409	122.6536201	169.7330756	100.1925783	166.2167341	116.9452444	127.4516386	125.5093509	2L	0.111975386	0.557061021	0.984934163
**Pgant2**	FBgn0031530	21.56214112	26.81392417	33.27753545	27.34484017	29.93326819	26.70864609	31.20054865	32.26500473	2L	0.174900344	0.191894032	0.843483578
**CG2772**	FBgn0031533	75.06553035	131.2795279	105.9226036	128.3508996	103.6905782	103.1399996	98.92922917	132.3576718	2L	0.027652017	0.879772501	0.995787491
**Snx1**	FBgn0031534	110.7126318	113.6895403	112.1354147	102.0987595	115.1948486	154.6645517	114.8142559	109.9775803	2L	0.201621214	0.108060281	0.758410631
**CG12795**	FBgn0031535	122.2331954	111.5880963	89.66180037	117.5022375	99.83783851	118.408404	88.96884243	83.30497053	2L	-0.14948521	0.356784354	0.937787223
**Cog3**	FBgn0031536	56.00472847	47.89778761	52.17480613	34.06771537	43.32980948	46.34935706	45.67616391	27.31199768	2L	-0.197537232	0.31008436	0.912401755
**CG3246**	FBgn0031538	4.038642128	2.317508496	1.160252701	0.991151798	9.689068393	4.959909287	0.542245412	3.230413757	2L	1.125591237	0.143744504	0.8037297
**Pif1**	FBgn0031540	0.45554299	0	0.868325024	0.303451809	1.409780317	0.030677368	0.830071396	0	2L	0.517633302	0.735564162	NA
**CG17593**	FBgn0031544	181.8186502	193.0922949	168.6699578	169.611991	183.1703637	167.1841387	184.1990891	210.1481193	2L	0.094518325	0.413198424	0.958665466
**CG3213**	FBgn0031545	0.14569932	0.977560678	0.176732259	0.16984624	0.986342305	0.343411072	1.022125762	0.079081678	2L	0.765374484	0.540975065	NA
**CG8851**	FBgn0031546	0.451450203	0.858210301	0.949183643	0.210507831	0.760651101	0.851248988	0.921327334	0.065342679	2L	0.11408271	0.909791211	NA
**CG8852**	FBgn0031548	1.515907433	1.005262209	0.769724014	0.739733256	0.63641894	1.462425205	0	0	2L	-0.909694417	0.458936118	NA
**Spindly**	FBgn0031549	0.798188612	1.487610468	0.358591563	0.068923952	0	0.195099683	1.131220165	0	2L	-1.019215526	0.48523891	NA
**CG2818**	FBgn0031566	146.5480289	124.4440056	129.9789436	140.2508524	133.8873815	142.188568	134.8847251	119.483842	2L	-0.001869658	0.986273877	0.99797245
**CG10019**	FBgn0031568	11.47330412	11.98968497	12.32912953	12.31701356	13.90744692	12.80858032	12.52540651	13.35416478	2L	0.160036221	0.128936682	0.788096052
**bark**	FBgn0031571	0.484336228	0.522928049	0.216090654	0.33746556	0.824100967	0.393645894	0.213026228	0.821892384	2L	0.557626166	0.325692576	0.918470465
**CG3407**	FBgn0031573	16.83229227	21.82969055	28.02869307	28.63950027	18.69944716	26.88717836	33.45388164	24.86753513	2L	0.156878676	0.507586386	0.978557094
**TTLL4B**	FBgn0031574	0.214800361	0.090074368	0	0.417332463	0.323141421	0.135008312	0	0.310900979	2L	0.113325902	0.940739576	NA
**Cep97**	FBgn0031575	108.2960416	131.0649467	96.16140632	119.6082004	120.4638625	128.2996477	101.5324717	104.6214109	2L	0.028870018	0.816549647	0.995787491
**CG2955**	FBgn0031585	1.035457198	0.45308718	0.546087983	0.629772907	0.56890693	0.084888932	0	0	2L	-1.983940693	0.204165011	NA
**Naprt**	FBgn0031589	25.30788508	19.26993114	18.48395552	17.53246611	22.01081618	27.23663971	17.1082744	20.95736013	2L	0.140243726	0.40549238	0.956706394
**CG3702**	FBgn0031590	207.4306986	221.9427277	202.1483366	213.5011655	174.6440984	251.4304021	209.6774593	207.1923149	2L	0.024619958	0.830500486	0.995787491
**Art2**	FBgn0031592	0.453623668	2.723185499	1.737607407	1.113270005	0.517204411	2.070840702	0.121810918	1.295867072	2L	-0.538668881	0.506641563	0.978557094
**CG17612**	FBgn0031597	27.36671844	25.47806974	20.66898254	24.57876743	14.05992656	26.00401442	16.79465765	28.21310546	2L	-0.178232922	0.489915444	0.973549494
**Vps53**	FBgn0031598	60.58030951	73.45548926	67.72270864	86.5855772	70.83172383	54.95406943	74.10111121	76.01380266	2L	-0.030452842	0.84891898	0.995787491
**CG3652**	FBgn0031600	286.090824	263.518672	261.4333511	254.3511937	307.7879083	265.9590206	332.2466585	301.6381113	2L	0.210847004	0.080512774	0.694684542
**Dim1**	FBgn0031601	204.3649233	171.7063113	134.3190271	163.5083693	196.7928498	124.9307041	171.0595569	221.9811458	2L	0.115620088	0.573711001	0.984934163
**CG15431**	FBgn0031602	6.89415267	8.914545957	7.765233458	9.26606659	7.698897641	7.601308763	6.630727842	6.545910052	2L	-0.174222845	0.175945286	0.834088743
**CG15432**	FBgn0031603	94.5636787	106.6187946	109.0172274	106.8953488	113.5726443	97.9957835	141.882793	114.2476943	2L	0.196475669	0.281305485	0.900509101
**Elp3**	FBgn0031604	73.2399234	76.75426093	72.7667475	57.71338463	61.30123088	67.08188164	70.21317934	55.87110801	2L	-0.110922951	0.44593885	0.967112302
**CG15439**	FBgn0031606	64.61077794	73.09136699	70.84956869	62.20814783	72.98293592	72.95794372	62.98948997	71.946706	2L	0.085178299	0.447875096	0.967112302
**Secp43**	FBgn0031607	150.6374942	153.7765186	160.2194922	181.9726527	184.6215657	168.0963498	177.9422241	176.0464079	2L	0.158961636	0.200885589	0.848203654
**CG15435**	FBgn0031608	25.98117928	30.54186477	31.46289839	26.73272027	28.06408741	30.64898699	26.4018673	21.16448393	2L	-0.07694478	0.687542437	0.995787491
**CG15443**	FBgn0031609	107.4743707	118.3423215	130.5417526	118.7990946	133.8301567	115.788314	116.4078214	110.207254	2L	0.035845139	0.773993409	0.995787491
**CG15436**	FBgn0031610	0.236661873	3.076494964	0.143534601	0.137942062	2.029367852	3.346850529	2.113053332	1.284537086	2L	1.329142494	0.216660274	NA
**FIG4**	FBgn0031611	50.0305527	54.4837334	52.16474777	44.74321275	67.58749027	53.82083498	58.65503659	33.93992343	2L	0.11803992	0.530898637	0.984760433
**CG11929**	FBgn0031620	0.608514569	0.669832946	0.461327455	0.931040781	0.755235771	1.003981682	0.048510429	0	2L	-0.531239504	0.619708627	0.990047511
**CG15631**	FBgn0031626	1.609532193	1.705114395	2.260617324	1.185020121	1.835127239	1.357724792	1.188564822	1.379385546	2L	-0.193698516	0.769034378	0.995787491
**fipi**	FBgn0031627	51.91237823	40.23504392	49.26422715	41.81473748	44.6627073	46.32871596	49.74425813	47.37782084	2L	0.067512529	0.534533066	0.984760433
**CG3294**	FBgn0031628	17.98863172	27.59937151	23.16445024	24.44832973	17.77610179	21.98314205	18.92121603	20.73060797	2L	-0.195447019	0.363469708	0.939549067
**CG15629**	FBgn0031630	2.674886836	1.562799663	2.260296436	0	2.170272498	1.700135036	0.996719317	1.95755964	2L	0.099038533	0.905066881	0.996479182
**CG3225**	FBgn0031631	5.068088841	2.760067548	3.033859012	2.762195457	8.317453908	9.897623777	2.518598088	6.144694427	2L	0.996894506	0.029052931	0.472866974

CG15628	FBgn0031632	155.8065473	163.9096	138.7515821	169.0825355	165.918997	164.4537151	139.031391	149.2701238	2L	0.008462712	0.934615368	0.996479182
Fnta	FBgn0031633	188.3463678	207.7675838	207.1509994	166.7190706	177.3017984	172.3815844	197.0607453	180.4271735	2L	-0.050553325	0.668519249	0.995787491
Ir25a	FBgn0031634	0.136131055	0.163100534	0.141536589	0	0.561717588	0.330026213	1.091430203	0.126665624	2L	2.265840629	0.054437372	NA
tank	FBgn0031635	52.83582927	64.08246922	55.76771833	46.24165884	49.65744779	50.88619889	48.93744493	63.60310163	2L	-0.005684895	0.970597724	0.997604378
CG12194	FBgn0031636	565.1576618	417.0912103	460.5035609	451.1103596	512.23352	485.5526594	450.6467192	443.1203072	2L	0.025708233	0.823758303	0.995787491
mxt	FBgn0031637	87.54209413	94.92710337	91.56564306	112.9069898	89.89889937	96.37179236	85.67953252	104.267199	2L	-0.010467151	0.93013129	0.996479182
CG11927	FBgn0031638	105.3113927	130.7861113	112.2819915	108.8393913	124.9681851	141.0845238	172.0033425	125.7687074	2L	0.333647052	0.017118753	0.375267338
mRpS2	FBgn0031639	171.0268422	203.8507032	199.8423461	195.3529853	165.4630147	222.4904268	217.8083626	165.028936	2L	0.031420127	0.824627968	0.995787491
Mon1	FBgn0031640	58.27991015	63.59615371	63.17705259	39.6014478	53.58287715	60.29755563	65.10674751	43.55679552	2L	0.016978597	0.927592106	0.996479182
CG3008	FBgn0031643	74.59909642	93.08116523	93.76738331	93.52087831	101.4974936	89.51604151	103.3530962	79.63241433	2L	0.107984899	0.433743749	0.965402206
CG15625	FBgn0031644	1.689123355	1.359968384	0.655646352	4.095652615	1.341692253	2.10209364	4.136630369	0	2L	-0.028708475	0.978211034	0.997757033
CG3036	FBgn0031645	3.530335222	1.184328416	1.338210001	1.423250051	1.540180308	1.705793973	1.35089415	1.437128356	2L	-0.296577155	0.448973903	0.967112302
snsl	FBgn0031646	1.315568798	0.100303732	1.305634727	3.067199128	0.809638247	2.11416516	0.762737334	1.687767334	2L	-0.085403189	0.918508262	0.996479182
hoe2	FBgn0031649	269.8325072	329.2185862	272.8724784	306.6891039	263.5237277	244.3415289	270.9629645	219.1134146	2L	-0.208649135	0.053833243	0.608597067
CG14044	FBgn0031650	67.99781664	78.08289368	63.45717052	69.64141359	64.62816009	46.65719485	54.85247739	53.90223339	2L	-0.312357353	0.060948573	0.62864531
mRpL24	FBgn0031651	200.1234218	168.5626455	153.2746901	135.9716752	174.3398556	178.6981934	199.5683942	141.0847187	2L	0.102998695	0.515614601	0.980804634
jet	FBgn0031652	95.51061307	95.84717613	70.17805044	70.0032363	74.61673289	105.0028753	89.19816765	79.25056564	2L	0.096537013	0.566658879	0.984934163
Marcal1	FBgn0031655	13.04097913	6.055923416	5.587482762	8.490053875	6.236745532	6.015431674	11.19513683	12.90648215	2L	0.150843492	0.691219518	0.995787491
CG3756	FBgn0031657	68.5370442	95.18170368	76.45988713	67.98221865	61.02485737	47.50208152	73.29037313	72.15028605	2L	-0.244662403	0.21975836	0.864306828
mRpL28	FBgn0031660	238.9758613	251.9804904	229.1095016	229.5265325	253.6897927	279.4220568	291.5418879	266.2258711	2L	0.229734853	0.024737539	0.44131412
Gmd	FBgn0031661	32.57610167	15.76855585	8.781018698	26.01989189	28.58732562	32.22921879	24.23818111	23.24781014	2L	0.395322956	0.242808232	0.876959115
CG3792	FBgn0031662	284.701405	327.1628804	295.6954598	311.8515002	398.7075913	297.631284	262.3409229	322.4677337	2L	0.104006404	0.446672002	0.967112302
CG8891	FBgn0031663	30.29219093	34.90205641	42.02693116	50.31756525	26.55637574	38.0486422	63.45028651	31.72792251	2L	0.0494118	0.872108355	0.995787491
CG8892	FBgn0031664	80.07708905	59.96093412	66.40618992	73.62961118	68.89524303	66.5010668	84.60228207	84.7564632	2L	0.149164868	0.358578055	0.93823489
SelT	FBgn0031670	206.0148481	215.5837684	196.3581929	189.3778524	255.379657	238.0768931	232.5175328	248.1405849	2L	0.30145727	0.005433012	0.208657706
CG31650	FBgn0031673	122.9152123	139.069372	123.3713801	124.6765433	104.8866958	108.7703363	111.1893298	125.9569529	2L	-0.146151073	0.169428503	0.830934498
CG9121	FBgn0031675	73.00070394	57.99953535	50.84947743	52.41662289	62.61473655	62.480871	68.93236302	49.15896504	2L	0.079476653	0.618564183	0.990047511
senju	FBgn0031676	38.75816419	42.4461418	37.61070629	39.75980832	28.79990449	62.61470385	47.07626273	34.6362224	2L	0.152408057	0.503160287	0.978380269
Nepl3	FBgn0031678	4.816372	5.834680482	5.749491906	4.545148334	2.69124325	7.02749992	3.900529666	1.161865495	2L	-0.472368023	0.354744244	0.936745458
Pgant5	FBgn0031681	18.72510016	23.91431164	22.76736701	24.11450302	20.79604202	18.62218756	20.4234297	23.59700984	2L	-0.067553784	0.589833966	0.987597375
CG5828	FBgn0031682	60.2386455	73.69671488	79.42524994	66.51517734	73.00952664	70.51575252	67.86619095	77.59507296	2L	0.080791696	0.566609036	0.984934163
CG4230	FBgn0031683	819.1877513	660.8395444	628.005952	761.3020159	763.6903395	793.0671697	643.5866954	684.4094011	2L	0.033829309	0.768610309	0.995787491
ND-13A	FBgn0031684	395.0446901	324.3196132	379.7933293	317.1897635	369.3159156	329.4457708	363.9657607	391.3465369	2L	0.068423427	0.580456598	0.985637351
Cyp28d2	FBgn0031688	4.710231837	3.84549931	1.921346708	0.680284048	2.85947501	1.336163233	1.914037584	1.357480001	2L	-0.559902714	0.414835021	0.958665466
Cyp28d1	FBgn0031689	56.9891329	71.51047192	66.90433373	33.87829249	37.49684208	34.02288537	98.18193194	27.00051657	2L	-0.181973137	0.700847323	0.995787491
Bub1	FBgn0031696	79.26608582	84.25370453	93.65213609	75.23880863	66.29187732	80.3507754	75.92233555	57.20859493	2L	-0.218957881	0.084457177	0.69799888
CG14024	FBgn0031697	213.2829291	149.3843434	171.0861958	172.2523789	202.7462936	185.8611435	189.8301485	159.5119803	2L	0.090474258	0.491774588	0.973549494
Ncoa6	FBgn0031698	35.23119023	56.67239211	60.14650334	64.04240828	59.48384889	41.51610477	46.14023947	67.57677551	2L	0.028029018	0.87968191	0.995787491
CG14022	FBgn0031700	1.916332868	3.535817507	5.811246012	3.350893456	7.091917558	8.762528576	5.866327581	10.60936693	2L	1.20294322	0.035931894	0.518988565
fusl	FBgn0031702	0.364564496	0.229314674	0.939705155	0.132807562	0.061699908	0.494081946	1.33689205	0.469955065	2L	0.539129408	0.541178465	0.984934163
CG12512	FBgn0031703	4.132656465	16.86150357	10.49995862	18.4239367	3.024528694	4.949281995	9.046600808	8.608634617	2L	-0.925084285	0.033381847	0.513292083
CG14020	FBgn0031707	3.43042557	4.435419441	5.201352866	9.830762011	1.935249177	7.748566489	12.94431963	5.275504787	2L	0.312218939	0.606459242	0.989286463
CG7382	FBgn0031708	208.039236	198.7415945	193.1646587	224.2962685	204.2295661	210.8749412	227.1335202	222.9300666	2L	0.098662732	0.419530005	0.960266449
Vps52	FBgn0031710	76.12608521	83.99835806	65.20035938	87.27636965	68.35504191	61.34878052	69.48956805	69.12880884	2L	-0.190451038	0.173262006	0.832736771
CG6907	FBgn0031711	48.18464084	45.58798611	38.75537364	40.38943579	38.29614766	56.43070567	47.63317485	64.07424824	2L	0.283086081	0.149906163	0.809805479
tomb	FBgn0031715	1.738158775	0	1.405582982	5.62840521	2.266204833	1.729765781	0.492675598	0.2096502	2L	-0.872703288	0.501138085	NA
CG14015	FBgn0031716	150.5665839	138.3536919	123.0711923	174.4011017	169.8320563	174.5349739	162.8510207	153.0261475	2L	0.197999684	0.127536863	0.787327567
Oscillin	FBgn0031717	27.61641851	35.74776601	35.74284098	29.55714249	30.80353262	40.05629336	31.90357082	31.46668713	2L	0.09514619	0.571670818	0.984934163
DIP-eta	FBgn0031725	0.790205286	0.814977869	0.246104818	0.087137413	0.144580041	0.251689431	0.204307162	0.23183903	2L	-1.194803339	0.085516527	0.701544311
Hsp60C	FBgn0031728	0.33610785	1.273037539	0	0.176947084	0.097864647	0.14310729	0	0.023539395	2L	-2.668405161	0.043425307	NA
CG7236	FBgn0031730	0.210210115	0.959850051	0.453303828	0.081682828	0.611389493	0.682636576	0.86395917	0.456385618	2L	0.661752674	0.381747922	0.944476532
CG11030	FBgn0031736	2.328077516	2.816233651	2.392699449	4.417126623	2.612760624	3.15070474	3.130396091	3.465923517	2L	0.07717499	0.716344736	0.995787491
obst-E	FBgn0031737	5.695710775	2.024100804	3.220230167	3.769980771	6.361033014	5.847709752	1.416047681	2.1483158	2L	0.117524489	0.818378701	0.995787491
CG9171	FBgn0031738	15.21048799	16.66960937	17.28424058	13.61660026	13.24268936	16.08163139	14.8390983	13.68103272	2L	-0.08732508	0.417817038	0.95925267
CG14005	FBgn0031739	60.35949079	42.65027507	71.7743886	53.60326354	70.89117711	59.81909967	78.50471391	73.39094277	2L	0.337815533	0.084568281	0.69799888
CG7239	FBgn0031740	113.8958147	127.4698617	129.7958813	122.6187567	119.5654678	145.6401423	134.410205	121.1456152	2L	0.107565071	0.350282495	0.932790554
CG11034	FBgn0031741	0.023614571	2.020116615	2.577987871	1.321355587	0.213151763	0.044527346	0.662654052	0.410155353	2L	-2.132491213	0.043032872	0.544212375
rau	FBgn0031745	0.812224413	1.156192562	0.479297456	0.757468257	0.451770673	0.567076484	0.604800563	0.066723788	2L	-0.896941653	0.097517218	0.730787365
CG9021	FBgn0031747	0	1.538477361	0.278139849	0.801908057	0.103486521	2.215876419	0	1.244581267	2L	0.490926171	0.755469172	NA
CG9044	FBgn0031752	46.11654219	52.11351049	49.58233451	44.68321332	48.41060104	52.08937498	50.87733577	48.40375551	2L	0.084793397	0.410238436	0.95848812
CG13999	FBgn0031753	388.0557452	311.2277042	318.6617397	265.8212867	268.4274457	304.6446042	322.2565896	314.6764117	2L	-0.056931279	0.672534079	0.995787491
CG13992	FBgn0031756	0.917177527	0.492299723	0.80102156	0.68427676	0	1.037651244	0.467947915	0.079651093	2L	-0.839209329	0.502872604	NA
lid	FBgn0031759	119.2983896	137.5227469	137.3021214	153.7245677	122.6207292	142.6780836	134.7096826	136.8578698	2L	0.000680191	0.994701572	0.998895169
Tsp26A	FBgn0031760	35.26423733	41.76568813	37.53687441	34.92362895	34.08023422	40.83157646	40.47877452	31.87847764	2L	0.008838264	0.950356729	0.997604378
CG9098	FBgn0031762	86.28737579	79.21720144	74.39628646	72.99980453	67.71551256	85.32436785	76.18717019	79.13182313	2L	0.006853837	0.951646057	0.997604378
CG9107	FBgn0031764	43.41821381	37.69487517	50.28411158	53.66606392	45.39402363	30.18656849	40.63084485	56.9616161	2L	-0.062975913	0.784145914	0.995787491
CG9109	FBgn0031765	0.577252426	0.305766305	0.663350369	0	0.987241811	0.515586374	0.800878659	0.219871658	2L	0.734165884	0.39894983	0.952805113
CG9117	FBgn0031766	0.191421537	0.481624163	1.393157939	0.223146054	3.973996762	1.443768449	0.244160227	3.324751018	2L	2.066842465	0.094271564	NA
IPIP	FBgn0031768	70.53500343	82.28395552	70.80993862	88.47443155	84.44638276	76.47661772	102.884596	77.67209153	2L	0.160580236	0.27911813	0.898067854
CG9135	FBgn0031769	99.48289896	71.58257163	63.37502781	74.55703454	71.95881605	80.57501861	60.46556213	54.14943753	2L	-0.186636636	0.27015979	0.89549671
CG13995	FBgn0031770	68.06035664	97.76798113	74.31897234	102.7462637	78.57052283	83.55614572	73.02729603	71.44662271	2L	-0.130957096	0.345445032	0.930285749
ND-51	FBgn0031771	1068.321851	1136.827518	1079.861696	1160.115361	1119.889791	1256.759676	1216.634475	1135.015091	2L	0.118574341	0.15075772	0.810890819
CG13994	FBgn0031772	68.71036788	54.53699218	67.16914217	85.57585735	68.32507885	69.68908357	81.97088916	43.56727896	2L	-0.040845996	0.870363266	0.995787491
Fbw5	FBgn0031773	41.66080405	39.92283602	25.48684658	30.65948388	37.86580898	36.61350896	42.32621186	28.55056934	2L	0.103043281	0.614199202	0.990047511
CG9147	FBgn0031774	52.72185037	48.78478367	65.68311585	82.85012469	54.12743081	52.44788575	85.12143987	44.89010117	2L	-0.051584166	0.824125678	0.995787491
CG9150	FBgn0031775	2.338505552	0.3138012	1.361563644	1.744684051	0	2.1165396	0.477246232	1.218506886	2L	-0.574379505	0.665473142	NA
Pfdn1	FBgn0031776	124.2783524	142.8014585	128.1849086	123.72258	156.1621148	134.3837655	130.7157938	111.7435566	2L	0.069943819	0.656270985	0.995515543
CG9154	FBgn0031777	49.78615682	69.4018985	71.99926264	43.72517044	43.80401953	46.75580072	63.13285873	51.00185687	2L	-0.164197974	0.482149696	0.973549494
lncRNA:CR916	FBgn0031778	2.565466356	3.397270984	2.784325135	5.823885908	1.218769552	1.145702847	1.205576804	10.26027752	2L	-0.035435288	0.965334499	0.997604378
CG9175	FBgn0031779	162.4664978	164.1238389	182.0611105	218.9541944	180.981636	158.2853705	168.8414262	184.7395411	2L	-0.039806656	0.763864535	0.995787491
WDR79	FBgn0031782	12.70035458	9.961864054	15.07294999	35.89461988	18.6388176	36.73980456	18.06412229	40.07105311	2L	0.647515275	0.090782557	0.71850126
CG13991	FBgn0031785	1.154294318	0.3614178	0.709409819	0.466473583	1.222494291	0.333733567	0.274832096	1.102675119	2L	0.145086909	0.84583229	0.995787491
CG13989	FBgn0031786	0.667027194	1.918019218	0	1.777312906	0	0.628869164	0	0	2L	-2.654890651	0.216207681	NA
Pez	FBgn0031799	0.972398267	1.25466283	1.016195227	2.964682247	1.512368821	1.636083893	3.167546712	2.66331793	2L	0.564008028	0.252522284	0.883301842
Nepl4	FBgn0031805	3.114954241	0.629505874	0.182092423	1.312481666	1.897013751	2.406020103	0.191477474	0.325920675	2L	-0.12176563	0.903859227	0.996479182
CG13982	FBgn0031811	11.67338609	14.28844929	12.10709606	13.90894477	18.53635489	29.42036767	19.31614618	19.05470404	2L	0.762262466	0.000386873	0.029046848
CG9527	FBgn0031813	5.134205713	4.09484584	4.410317283	4.406154142	4.868697361	4.881933953	4.189151589	2.602374133	2L	-0.099999783	0.624808355	0.990047511
retm	FBgn0031814	63.92229273	67.34135926	67.24871971	67.14439359	67.2183979	85.34975473	64.64669419	78.19511537	2L	0.18255767	0.094314418	0.728467429
frj	FBgn0031815	139.6558047	162.3453058	138.4784538	169.2229913	151.9246547	186.9743636	131.786639	187.1655707	2L	0.139250452	0.300142792	0.9074568
Rchy1	FBgn0031816	1.405886764	3.908461388	4.705449912	2.24588086	1.621491014	1.865464269	1.593976596	3.250145234	2L	-0.511415566	0.232140766	0.867702781
HemK1	FBgn0031817	224.8799153	174.1321212	162.1768478	210.5377747	206.9422968	169.1160635	178.1446432	212.9441572	2L	0.019681933	0.896035114	0.996479182
CG9536	FBgn0031818	123.6964541	132.4045635	123.4965596	162.3059905	144.3492978	168.0580691	151.9842076	198.6561667	2L	0.321152975	0.023087997	0.43045943
Daxx	FBgn0031820	57.25899975	61.59577182	73.78581113	74.79975245	53.8713744	74.48829489	60.40219875	75.14327447	2L	0.010466616	0.940466866	0.997188858
KFase	FBgn0031821	20.95009902	14.69339722	7.727724258	3.570494677	13.1596944	4.158236924	10.00124495	7.979755139	2L	-0.385148133	0.460932111	0.971606621

**Phf5a**	FBgn0031822	115.0713321	131.1410792	127.058164	125.0428753	119.5488927	87.9540644	117.2270742	107.6949919	2L	-0.173062462	0.320838209	0.916928013
**COX5B**	FBgn0031830	1677.970033	1425.151874	1464.950687	1324.575296	1646.531559	1506.923446	1563.605819	1430.499342	2L	0.090955984	0.373998302	0.943282319
**CG9596**	FBgn0031832	5.597057438	14.10779172	5.798336642	7.335838844	11.03261019	9.783605117	6.946158051	16.61831158	2L	0.476199485	0.190927156	0.843449603
**CG13766**	FBgn0031834	63.13209977	83.07299064	68.25059869	88.33358963	81.39607527	84.97862167	74.75858091	78.16740325	2L	0.108490101	0.456647594	0.968365245
**CG11319**	FBgn0031835	78.57103199	118.4717106	130.2374304	117.1266438	111.1684186	98.12427272	127.182717	118.177003	2L	0.068629608	0.627023068	0.990047511
**CG11050**	FBgn0031836	144.4252144	120.4721661	111.7093175	114.500677	132.1420846	128.9411165	110.0844328	107.3023098	2L	-0.01023998	0.931486981	0.996479182
**DIP-iota**	FBgn0031837	0.493226979	0	0.690324099	0.840340867	1.232863076	0.983677926	0.483935623	1.359144891	2L	1.037633645	0.248506212	0.879503029
**Tsp**	FBgn0031850	0.326348178	0.280218349	0.669188632	0.181159165	0.37873421	0.153839535	0.56492511	0.388005676	2L	0.06551764	0.907211276	0.996479182
**Aatf**	FBgn0031851	67.3206433	73.9628006	66.11379476	60.33229212	56.55491448	49.95155442	59.7507413	43.06956729	2L	-0.325354415	0.041774766	0.536003453
**TTLL3B**	FBgn0031853	2.506467663	2.257017284	3.264351948	3.506240999	1.286000488	1.368100338	0.673058195	0.973791015	2L	-1.378088848	0.006295524	0.227193262
**TTLL3A**	FBgn0031854	250.9981119	351.864613	249.8646129	254.1239082	235.5335902	209.4753836	252.803844	209.1043639	2L	-0.252804056	0.045975633	0.56430181
**meng**	FBgn0031855	93.3349565	95.12969371	100.9589803	83.94341691	80.39233213	79.10500118	98.00854109	88.88902402	2L	-0.07536326	0.483980749	0.973549494
**LUBEL**	FBgn0031857	0.288146725	0.517046106	0.357648339	0.203103305	0.20565204	0.543325944	0.08547308	1.076602273	2L	0.526013411	0.47343246	0.973549494
**CG17378**	FBgn0031858	156.4754771	142.7491139	147.2803879	150.3022724	149.8690718	163.9012678	139.6651398	147.9506652	2L	0.039402411	0.676777406	0.995787491
**CG17375**	FBgn0031861	3.009216981	1.385599033	1.574578483	0.137566198	1.171695823	3.282102195	0.150521121	0.256207397	2L	-0.311463429	0.784169112	NA
**Nha1**	FBgn0031865	0.255726579	1.240877943	0.886270164	2.406161031	1.170617617	2.092381501	1.351325424	0.277603038	2L	0.056403428	0.929044675	0.996479182
**Nlg2**	FBgn0031866	48.13973477	51.91476029	45.37389264	57.31672515	41.68208369	46.4041943	48.28999681	46.66466457	2L	-0.117885311	0.261104238	0.890640489
**Rat1**	FBgn0031868	76.01140059	63.6839806	68.76562239	104.2386037	73.12785164	74.64598978	68.67116041	74.26531935	2L	-0.078660875	0.624592975	0.990047511
**CG18304**	FBgn0031869	0.303657303	0.042445211	0.429723222	0.078662841	0.304544032	0.246524271	0.602494903	0.329634057	2L	0.819768701	0.335575779	0.924219112
**Fgop2**	FBgn0031871	53.21831577	87.20150352	94.26694411	82.74955461	79.10997941	79.5355009	69.18894774	87.30227611	2L	0.02602417	0.881666567	0.995787491
**ihog**	FBgn0031872	1.191518929	1.259990645	1.005428975	5.797526668	2.712126717	3.5654367	3.105668439	1.124738214	2L	0.204937518	0.747675737	0.995787491
**Gas41**	FBgn0031873	73.00266857	73.21188405	113.8522215	70.91790565	84.54687261	92.67902973	103.4618755	56.60555402	2L	0.058871779	0.795578708	0.995787491
**CG13775**	FBgn0031874	7.11518948	9.581404717	13.20939024	2.881621913	7.537993322	7.432512182	13.71977088	7.324982327	2L	0.174000925	0.673438726	0.995787491
**CG3430**	FBgn0031875	4.79375787	12.89309722	21.25407778	13.97058251	8.430152234	8.688398426	6.957867542	7.716047582	2L	-0.692065871	0.076721137	0.682035833
**Atac1**	FBgn0031876	30.58772873	22.69786127	24.79212782	22.3472875	18.95756866	16.54526044	26.24970774	40.39613065	2L	0.053180975	0.867542787	0.995787491
**Hmgcl**	FBgn0031877	0.950356927	2.504999237	0.164682332	0.474797443	3.186184538	0.063999336	1.212190469	4.274005215	2L	1.133592299	0.328790667	NA
**uif**	FBgn0031879	0.350802626	0.425831849	0.436719063	0.269040498	0.491631978	0.221940305	0.182513527	0.225481078	2L	-0.363979954	0.396869426	0.952272708
**MME1**	FBgn0031881	13.05620451	17.03329558	24.34218622	13.24705849	15.60407752	31.4571252	26.05937609	11.81092858	2L	0.359798646	0.299799583	0.9074568
**Rab30**	FBgn0031882	110.1892306	98.67065144	141.0179564	133.6120001	113.8904673	159.1523255	119.7373078	135.5464446	2L	0.155949403	0.304031925	0.910208378
**Caper**	FBgn0031883	249.7769887	251.3344377	223.0108534	225.7171882	254.9434295	231.6085465	209.3533095	205.6329412	2L	-0.045319088	0.654431121	0.994669639
**Mnn1**	FBgn0031885	155.1637757	152.4098616	139.0032816	151.4950354	131.2977176	154.560268	146.2281801	146.6244864	2L	-0.018622309	0.840517752	0.995787491
**Nuf2**	FBgn0031886	0.431243044	0.413342691	0	1.867221427	2.113082885	0.348491406	1.414427489	0	2L	0.541045164	0.71496672	NA
**Pvf2**	FBgn0031888	0.097523689	0.394522034	0.417513721	0.213997938	0.186411445	0.416455578	0.014634413	0.52310495	2L	0.07332032	0.93033038	0.996479182
**MICU1**	FBgn0031893	52.99617093	57.85141789	48.21298931	42.71841691	51.25268107	51.64977562	51.30143528	42.74783486	2L	-0.005302606	0.969518648	0.997604378
**CG4496**	FBgn0031894	1.77082806	0.606838681	1.385807798	2.130900107	2.543689135	0.412893497	1.21436033	1.736285258	2L	0.030150127	0.962275841	0.997604378
**CG4497**	FBgn0031895	54.25881926	45.40092675	53.5320184	48.67718867	48.56176577	60.11288139	39.91934548	47.45513223	2L	-0.014629243	0.918805934	0.996479182
**CG4502**	FBgn0031896	193.1135085	183.7865141	179.0277192	188.2192604	183.6774035	195.4207279	178.760595	159.7955349	2L	-0.024182305	0.787521918	0.995787491
**CG13784**	FBgn0031897	101.1636264	73.42792148	70.46529019	103.7300805	71.66998928	80.76720351	71.34481593	75.81718395	2L	-0.194647837	0.161527212	0.823037813
**Wnt6**	FBgn0031902	0.446248663	0.146617227	0.357144093	0.085807172	0.398644062	0.20385427	0.410759252	0.05992869	2L	0.067819049	0.926951714	0.996479182
**Wnt10**	FBgn0031903	0.379555533	0.277251311	0.341585772	0.2426392	0.143669923	0.733000242	0.046851015	0.292404908	2L	-0.002799044	0.997080357	0.999490563
**CG5149**	FBgn0031904	49.52898888	45.39587946	47.02509021	47.23001451	55.54036702	51.87680513	44.11087979	38.83932745	2L	0.03763526	0.79055063	0.995787491
**CG5160**	FBgn0031906	1.58380001	1.421843502	1.488205777	0.962148548	0.865803469	1.314433958	0.682868933	0.532737194	2L	-0.649457739	0.158652401	0.818884898
**CG5171**	FBgn0031907	0.536256827	0.085666247	0.619500754	0.059536315	0.046099096	0.312977886	0	0.332646632	2L	-0.873457952	0.562799878	NA
**CG5177**	FBgn0031908	1.977350998	1.636827251	1.059083649	2.095508824	2.457014845	1.670547747	0.851629135	1.951369117	2L	0.061424557	0.911873081	0.996479182
**CG5181**	FBgn0031909	89.87661225	57.61498245	60.93246288	59.34968033	78.30661229	69.88727506	67.36315609	71.9211912	2L	0.126350433	0.51092529	0.980373783
**CG5958**	FBgn0031913	0.348097437	0.924483978	0.985226116	0.743944732	0.994974727	0.957203735	0.22200101	0.944689699	2L	0.112798791	0.89363352	NA
**CG5973**	FBgn0031914	2.56034862	1.697069805	1.2272465	1.131289322	1.080971602	1.382141563	0.368713954	0.515529592	2L	-0.954721122	0.059494372	0.626834508
**CG6055**	FBgn0031918	0.669653664	0.634880479	0.141266995	0.984280368	0.341644661	0.38429719	0.148547903	0.031606089	2L	-1.386320277	0.138605358	0.800510781
**Cyp4d21**	FBgn0031925	2.96835492	0	1.500247423	0.576717313	0.14885115	0.272080947	0	0.895079626	2L	-1.923843453	0.162151115	NA
**CG6739**	FBgn0031926	0.350982577	0.124880856	0.567652557	0.02479705	0.28800614	0.270739824	0.244190208	0	2L	-0.381435021	0.744481622	NA
**CG13793**	FBgn0031935	0.965105801	0.69378411	0.167238087	0.72324895	0.248894761	1.462332623	0.087928773	0.673500225	2L	-0.024650311	0.982153075	NA
**CG13796**	FBgn0031939	3.535117952	1.344057272	0.762324164	0.769252813	2.581081107	1.614598888	1.242502299	1.842016396	2L	0.191982457	0.732478014	0.995787491
**CG7214**	FBgn0031940	3.219174211	2.762643929	0.181620273	0	0.94604748	1.835127032	1.336866931	4.876133866	2L	0.557207083	0.628205998	NA
**CG7203**	FBgn0031942	0.085254179	4.719063523	55.01556933	1.19260145	0	6.912432235	0.217485242	0	2L	-3.092968054	NA	NA
**CG7191**	FBgn0031945	0.643349563	3.551314246	1.021586443	0.163630395	0.823545875	0.606546038	0.029839974	0.203166757	2L	-1.633543268	0.059947882	0.627645287
**CG7154**	FBgn0031947	115.0212248	100.9665181	90.93123559	84.35528282	93.10655757	109.948857	89.67196878	87.26138557	2L	-0.015301882	0.906217852	0.996479182
**CG7149**	FBgn0031948	13.57288407	11.92851354	18.06479415	10.42574701	15.18517011	15.63802019	14.42277839	18.90399213	2L	0.282125463	0.184267745	0.839299945
**Herp**	FBgn0031950	85.99359483	114.9436028	101.9804139	117.1552544	98.02243837	97.65115801	106.5872863	124.8685327	2L	0.057786073	0.671798166	0.995787491
**r2d2**	FBgn0031951	110.6369391	105.4487839	97.68945812	103.6130836	103.9515475	116.2370967	107.1414122	98.31745466	2L	0.056086424	0.629882196	0.990523437
**cdc14**	FBgn0031952	36.35377836	22.20757376	28.95602064	23.55864157	21.14907129	29.74581475	25.7772126	21.73001699	2L	-0.151336262	0.381881195	0.944476532
**CG14535**	FBgn0031955	37.31058396	42.7347656	44.75053076	45.8154527	46.76250425	40.39892399	42.12341402	42.27262074	2L	0.040346136	0.718719948	0.995787491
**spz3**	FBgn0031959	0.958134796	0.33581961	0.971400093	0.366752347	0.929379694	0.134824423	0.474252117	0.527811772	2L	-0.32423363	0.677262901	0.995787491
**CG7102**	FBgn0031961	1.310226358	0.618109006	0.993309647	1.336450251	2.623998458	1.428284877	1.880108769	3.689120077	2L	1.201722701	0.022425111	0.421875556
**CG7231**	FBgn0031968	75.90919167	97.59349968	90.37492593	101.3648221	89.77811115	98.83571448	105.6180037	88.16584762	2L	0.097715384	0.447866789	0.967112302
**pes**	FBgn0031969	5.854166372	7.934190608	6.873828967	5.705185239	4.692312196	5.563426476	3.345239338	5.744891573	2L	-0.41051285	0.084841956	0.69799888
**CG7227**	FBgn0031970	0.345276026	0.115830419	0.39089625	0.107333074	0.498649376	0.282120911	0	0.349825566	2L	0.271519835	0.837404493	NA
**Sirup**	FBgn0031971	402.7158087	314.8849227	290.8555273	261.5053308	426.4761608	308.7413625	318.9891344	244.4497952	2L	0.060508378	0.735091652	0.995787491
**Wwox**	FBgn0031972	27.13387851	21.15139737	19.84807407	21.91844381	19.95251963	27.08541749	20.77530598	20.65522278	2L	-0.001358375	0.993671	0.998824522
**CG12560**	FBgn0031974	146.1083939	78.88935469	88.70365044	85.07562006	95.01859344	81.73265347	87.44572561	99.06962215	2L	-0.110275966	0.598930845	0.989286463
**Tg**	FBgn0031975	0.039135592	0.731466891	0.488275196	0.052138948	0.111021072	0.073793593	0.057048991	0.279177259	2L	-1.269566956	0.18546188	0.839299945
**CCDC53**	FBgn0031979	32.49584895	38.15505953	29.08720332	40.49393627	31.556829	39.19408762	55.45564176	27.73403643	2L	0.160798144	0.579432778	0.985637351
**RpL36A**	FBgn0031980	551.8986868	677.6458167	671.9846666	746.7757167	660.187467	621.7750484	656.0698428	733.7097292	2L	0.045438721	0.701467187	0.995787491
**Megf8**	FBgn0031981	17.75454597	21.08076281	20.57790319	31.77575142	16.76761929	19.43966717	26.03539771	24.77387454	2L	-0.037097883	0.848900059	0.995787491
**mon2**	FBgn0031985	82.0199172	83.95676506	84.56092962	76.42174294	73.60067148	73.78083297	80.5761674	84.85887203	2L	-0.032121728	0.752785591	0.995787491
**CG8673**	FBgn0031986	19.62168422	19.96501433	22.72885052	18.82092756	20.1044743	23.38788257	28.40978643	21.49214051	2L	0.232293721	0.323676369	0.918470465
**CG12375**	FBgn0031987	150.8376651	136.7295001	111.2578742	137.1654325	124.505887	122.0269372	131.8195491	139.2724122	2L	-0.022992006	0.874079922	0.995787491
**CG8668**	FBgn0031988	34.6448192	35.22054737	31.57432578	27.48308059	29.04491088	40.31738374	24.09492476	33.42384616	2L	0.005519331	0.971929641	0.997604378
**PAPLA1**	FBgn0031990	26.54732731	25.8524682	26.57407663	26.66011358	23.89462242	35.54892793	29.6929069	28.85538458	2L	0.186786208	0.107175059	0.756305625
**Acbp1**	FBgn0031992	525.6512743	498.9912657	483.694101	463.3086956	433.8262239	436.2007436	431.8249042	384.1392492	2L	-0.197038565	0.065160014	0.644371095
**CG8475**	FBgn0031995	73.33489112	60.73167708	55.23342998	54.10597904	47.93412667	71.47864841	54.75303084	55.52429426	2L	-0.058424448	0.683873563	0.995787491
**CG8460**	FBgn0031996	24.38143211	13.99088463	23.99685884	13.96178027	28.18487344	14.06420967	17.32256525	32.85179811	2L	0.308380437	0.355971459	0.937787223
**PGAP5**	FBgn0031997	79.76708497	57.78764341	83.10410116	48.71833074	58.66013594	93.24363392	78.27404434	51.84827117	2L	0.091099838	0.672385004	0.995787491
**SLC5A11**	FBgn0031998	2.171488052	2.179193679	2.341333212	2.423193062	2.914940564	3.097145504	2.698736944	1.732680066	2L	0.228273593	0.541115731	0.984934163
**CG8419**	FBgn0031999	0.074972515	3.814594914	0.788156528	1.281834373	1.443675785	2.214753714	1.020034648	0.596831657	2L	-0.127870619	0.88191236	0.995787491
**CG8372**	FBgn0032000	427.9724211	460.2812735	435.1223985	465.2039302	459.6824503	446.734351	412.229037	467.1987902	2L	0.028835488	0.777430061	0.995787491
**CG8360**	FBgn0032001	133.2418422	121.9397843	130.2620443	129.4797389	117.143134	128.8833434	128.1446716	128.2492313	2L	-0.00687501	0.956331856	0.997604378
**CG8353**	FBgn0032002	14.58146346	8.152786461	15.66330349	8.118479555	7.20288722	8.549325054	14.99008832	10.23756693	2L	-0.160596614	0.698626431	0.995787491
**CG8292**	FBgn0032004	1.188586561	2.367503567	2.162620814	2.078358617	0.536425844	6.303330842	1.895068658	0.967699294	2L	0.353150904	0.696603973	0.995787491
**Snx6**	FBgn0032005	146.0506898	121.5647045	142.8443447	126.1602601	111.4488981	139.6531671	135.0617562	138.4009329	2L	-0.004467629	0.970924914	0.997604378
**Pvr**	FBgn0032006	0.508764169	1.091712018	0.489254146	0.106861667	0.438539056	0.466695974	0.257235164	0.169169007	2L	-0.680955164	0.271518508	0.89549671
**CG8086**	FBgn0032010	2.129424717	0.392028062	1.858483689	1.589300988	1.101679249	0.667162619	1.159312726	0.310091219	2L	-0.86401807	0.097584873	0.730787365
**Scgalpha**	FBgn0032013	316.754961	322.8914425	300.1461108	338.2381872	318.0368816	298.4249271	342.2858022	309.7567169	2L	0.019484907	0.848015207	0.995787491
**CG7840**	FBgn0032014	22.57323977	25.66160257	24.7431283	20.80667904	30.32594104	34.78638202	19.89641609	31.58693378	2L	0.348144763	0.174153526	0.833984823
**Ostgamma**	FBgn0032015	99.00882356	104.6942328	109.4217225	81.12984929	103.2698635	96.83852491	105.9036825	73.78944297	2L	-0.023022109	0.884357673	0.995787491

**Mettl14**	FBgn0032016	10.84139711	17.8060702	13.69847821	25.802899	27.52242245	23.74300274	22.18292755	24.15063703	2L	0.553960291	0.061895837	0.633840737
**CG7810**	FBgn0032017	80.116952	59.70216529	67.38965837	43.94150141	55.4816425	60.17936667	57.62847757	54.89131792	2L	-0.112055173	0.573342975	0.984934163
**CG7806**	FBgn0032018	7.80984182	23.79213637	12.39390076	15.65342531	16.66217816	26.73114966	17.16691775	12.73367199	2L	0.329769007	0.242670214	0.876959115
**strat**	FBgn0032020	58.19804801	47.51907643	54.67453426	33.34133153	63.23350852	43.77554592	69.72981367	44.999871	2L	0.223147004	0.37582919	0.943615128
**CG7781**	FBgn0032021	1286.623938	1046.481783	1054.591993	987.5309978	1114.648407	1090.63524	1024.605473	1096.728991	2L	0.01326221	0.898105234	0.996479182
**CG14275**	FBgn0032022	0.158786885	0.239708451	1.30973021	0.037020564	0.60196715	0.119762644	0	0.034474103	2L	-1.140439699	0.397757448	NA
**CG14274**	FBgn0032023	1.034179107	3.854867491	2.39739724	0.750135448	1.244638897	0.43334122	0.644896541	0.149686572	2L	-1.652341704	0.027914233	0.462499435
**CG7778**	FBgn0032025	4.51217041	0.485162363	0.654916559	0.314699522	7.379796147	0.327234362	9.690584616	0.334917595	2L	1.591986795	0.223629602	0.86549481
**CG7627**	FBgn0032026	4.247367958	8.60058345	4.672409516	9.694416188	4.029513084	6.830315249	2.245116387	5.095324138	2L	-0.54928991	0.116209532	0.765798592
**CG17292**	FBgn0032029	0.547639066	0.459293854	1.273207872	1.383199856	1.853679137	1.269263968	2.270188413	0.89173133	2L	0.82186576	0.200655887	0.848203654
**Wdr82**	FBgn0032030	236.7671531	196.168148	206.4650248	185.9844424	194.7415633	192.3737692	171.8165393	207.9334964	2L	-0.077451897	0.557103667	0.984934163
**CG13390**	FBgn0032031	38.99493221	35.67737909	47.0571816	57.38730645	47.6952994	49.5654349	39.07412068	58.52251774	2L	0.153433401	0.488498033	0.973549494
**CG17294**	FBgn0032032	112.2116812	109.9290357	103.5420123	114.6839231	130.4625674	118.9032244	109.8220608	108.2407333	2L	0.115428369	0.395661022	0.952272708
**CG13392**	FBgn0032033	35.13924773	43.02704572	32.82022007	35.02329371	35.36520666	30.97567869	50.42304894	23.45934708	2L	-0.030377569	0.91118827	0.996479182
**Rcd4**	FBgn0032034	134.1712272	84.87415756	136.2179427	126.851243	138.491877	132.3435518	163.7805946	106.5494466	2L	0.19274245	0.337561973	0.926017606
**CG13384**	FBgn0032036	115.5573836	96.74861159	99.87315095	88.36640182	94.05818283	111.1437577	98.0598898	92.27684379	2L	0.009074549	0.937869423	0.997038394
**CG13398**	FBgn0032042	46.42648899	48.3210489	40.51439379	44.97088528	31.55500894	63.39915728	32.44890988	46.95362798	2L	-0.022130035	0.912926349	0.996479182
**CG13088**	FBgn0032047	13.78143333	5.75589601	5.236582476	5.806787893	10.39123698	2.087237135	10.58937726	3.244420572	2L	-0.199411101	0.728445443	0.995787491
**Dh31**	FBgn0032048	0.809678466	1.503635117	0.855859919	0.620255741	1.534761008	1.515814683	0.752434752	1.268189836	2L	0.45764627	0.231413701	0.867702781
**CG13096**	FBgn0032050	11.11760822	16.93404561	17.05418908	11.98542428	14.59137749	16.14577174	11.61310541	17.48109391	2L	0.106377276	0.659129025	0.995750687
**CG13097**	FBgn0032051	20.32024174	24.32285451	22.15227997	27.88580199	16.89054448	15.4899299	18.20869756	26.66573112	2L	-0.260696322	0.2569076	0.88674759
**PIG-U**	FBgn0032052	74.19199943	70.58692244	57.88519451	58.42956353	76.4403594	66.40361259	60.60221672	62.11863396	2L	0.052574211	0.742580185	0.995787491
**mRpL51**	FBgn0032053	137.3182395	151.3147386	196.7289361	180.3674963	184.7987032	187.161006	187.8378755	156.5635594	2L	0.138804583	0.399481242	0.952933016
**Uba4**	FBgn0032054	32.43890106	38.03057198	29.52600263	27.34582196	30.65346979	36.55177949	18.15293247	21.20294032	2L	-0.225938145	0.355069372	0.936745458
**CG9289**	FBgn0032058	0.198730816	0.095240876	1.51522859	0.066190488	0.820022988	0	2.969375689	0.061637571	2L	1.096536616	0.435852901	NA
**PrBP**	FBgn0032059	345.0956607	328.9559687	343.9748385	332.7133293	266.1849763	310.9981027	314.8556131	339.7305559	2L	-0.103231598	0.36160148	0.939549067
**LManIV**	FBgn0032067	0.435130904	0.319318584	0.395858064	0.253622831	0.245475877	0.076919767	0.069376768	0.826620789	2L	-0.178697023	0.884919219	NA
**LManV**	FBgn0032068	1.096764039	1.620659741	1.140314683	1.94823929	0.989970094	3.348263128	0.199847759	0.680336073	2L	-0.122094347	0.873462246	0.995787491
**LManVI**	FBgn0032069	0.313572039	0.121378409	1.111823576	0.618607343	1.175700252	0.955124595	0	0.104737527	2L	0.090748063	0.939095594	NA
**Argl**	FBgn0032076	3.239085656	3.296494794	2.560464293	2.290997408	3.285044821	1.029367474	2.413903422	0.158030445	2L	-0.698234679	0.330730088	0.921224783
**CG9515**	FBgn0032077	14.78848096	15.61424067	9.289489356	18.93254803	21.81049423	8.776299208	15.66291579	13.90354745	2L	0.065777319	0.85918997	0.995787491
**C1GalTA**	FBgn0032078	43.87273852	46.31456649	44.89154223	44.39142988	43.51954236	44.58275723	48.00244806	44.36682852	2L	0.038360675	0.698410701	0.995787491
**CG31886**	FBgn0032079	5.955976316	3.886571527	7.69613461	6.127302197	6.400706361	6.201708022	7.418394262	2.532175441	2L	-0.043070145	0.898245969	0.996479182
**CG9541**	FBgn0032083	3.970885344	7.5782862	6.593467194	10.36892606	4.141780226	8.502418733	7.923765481	9.732331353	2L	0.12134525	0.714384196	0.995787491
**CG13101**	FBgn0032084	0.99688518	0.846389341	0.238857239	0.545182779	0.111088472	0.580158802	0.219771958	0.026720124	2L	-1.45373262	0.104971298	0.747886154
**CG9555**	FBgn0032085	1.004534214	0.306357313	1.218492996	0	0.659433351	0.947068721	0.116481289	0.099133489	2L	-0.435963561	0.758181741	NA
**Or30a**	FBgn0032096	6.065043713	0.88892563	1.71421881	0.82371385	0.3189017	1.60994659	0	0.12784244	2L	-2.154841489	0.066632202	NA
**CG13108**	FBgn0032100	133.0182798	138.3365322	158.9683668	129.883744	153.7423042	144.6075479	140.2599028	141.4763909	2L	0.082654343	0.510822364	0.980373783
**CG9586**	FBgn0032101	76.31598404	72.97729707	73.24913436	79.63333405	96.71075775	67.84093582	97.6451031	95.31852801	2L	0.273051631	0.130263735	0.790690436
**borr**	FBgn0032105	1.578704356	0.794416526	2.553276498	1.104206932	0.379995464	0.893035694	0.671218121	0.114250443	2L	-1.505956214	0.133408638	NA
**CG17005**	FBgn0032109	0	0.224559437	2.273481593	1.092449963	0	0.694199909	0.597664235	0.435988167	2L	-1.014899425	0.467382982	NA
**Mco1**	FBgn0032116	0.701542076	0.476653535	0.301608077	0.351968633	0.352685968	0.552570032	0.362460538	0.15423927	2L	-0.347509762	0.600858625	0.989286463
**FucTB**	FBgn0032117	12.86733485	12.19386955	5.026301041	16.35577236	8.267891236	11.68574702	12.2397737	11.28495208	2L	-0.073446265	0.831951474	0.995787491
**CG33298**	FBgn0032120	10.14472673	9.539297705	11.83059278	11.04135912	12.13895826	11.97005298	9.242648516	8.738478349	2L	0.013175722	0.925646333	0.996479182
**Oatp30B**	FBgn0032123	20.85730594	20.57953219	23.45481781	22.35882029	23.05289352	21.92124954	18.88459544	17.49870697	2L	-0.071160209	0.547176043	0.984934163
**Cpr30B**	FBgn0032125	1.55468646	0.186269174	0.269403405	0	1.603775728	1.570445248	0.566576937	6.027443252	2L	2.214603089	0.109280017	NA
**CG13113**	FBgn0032126	2.214671053	0.825511112	2.387893816	0.286856788	3.109593563	3.595962699	1.569353733	0.801375978	2L	0.690047026	0.537614803	NA
**CG13114**	FBgn0032127	0.429600722	1.981636098	2.605513401	0.125199739	0.678596983	0.253140586	0	0	2L	-2.375496086	0.104585912	NA
**jp**	FBgn0032129	2.321350777	2.534748571	4.041475776	3.830947535	2.925226249	3.029647186	4.191716606	3.093095103	2L	0.089998161	0.705186232	0.995787491
**brwl**	FBgn0032130	38.8505587	37.26895276	50.85061192	41.79556095	53.10237766	58.30839816	46.29787921	42.57573755	2L	0.277123701	0.055857659	0.619039537
**CG4382**	FBgn0032132	1.000110266	0.948177422	0.316467855	2.331719435	1.255968943	0.94289785	0.665557246	0.472028688	2L	-0.432653035	0.638860198	NA
**GlcAT-S**	FBgn0032135	46.2181392	45.8188505	43.38774293	51.97706759	50.89029163	48.46904904	47.5540652	47.55217007	2L	0.082749811	0.459164756	0.9709919
**Apoltp**	FBgn0032136	5.29692755	5.699340508	5.290157032	5.065549231	5.160499118	5.932138219	3.711912713	5.930825381	2L	-0.010801545	0.949340022	0.997604378
**CG4364**	FBgn0032138	31.1457096	64.2985336	60.17898866	63.76374756	61.61569855	51.91691509	62.81047818	48.97464358	2L	0.075197081	0.722586737	0.995787491
**IP3K1**	FBgn0032147	0.835727963	0.593990007	0.652912258	0.660497723	1.329704487	0.921464849	1.047912537	0.369039207	2L	0.441602508	0.456996369	0.968365245
**CG4036**	FBgn0032149	69.31069656	65.35347452	72.06290705	77.8885495	85.01427331	68.98866729	72.49130759	63.26798798	2L	0.056092846	0.746545733	0.995787491
**CG13123**	FBgn0032150	25.09359298	45.51404373	27.5045361	32.77676726	35.33974395	24.86949421	46.46820798	42.00907045	2L	0.220161416	0.430606117	0.963666972
**nAChRalpha6**	FBgn0032151	21.65153225	20.45218815	19.72393663	18.20138778	18.79621253	18.81891344	14.77490821	18.34364241	2L	-0.148293878	0.157604952	0.818884898
**CG4537**	FBgn0032153	20.55022679	35.43657457	34.94478755	19.25438735	27.73714119	16.11534503	24.00728442	28.77135218	2L	-0.150241116	0.714984017	0.995787491
**mtDNA-helicas**	FBgn0032154	0	0.729433826	0.191816208	5.069418002	2.640630278	2.348141777	0.403404848	1.201637249	2L	0.160689949	0.889633934	0.995926915
**CG13124**	FBgn0032156	5.07996435	6.051486763	12.75565243	7.694662776	5.461494389	5.216435991	5.819212885	4.917421017	2L	-0.523198555	0.079232609	0.69175818
**Etl1**	FBgn0032157	54.24530683	58.72360043	55.45374585	56.0726815	50.99140827	57.0215451	51.87823185	48.18380892	2L	-0.079307098	0.47614568	0.973549494
**CG4598**	FBgn0032160	9.803662956	24.18274896	22.10471079	16.53761851	24.15273433	28.97900167	25.15646868	16.77732007	2L	0.42860131	0.166251553	0.829168429
**CG4592**	FBgn0032162	0	3.315500213	1.370071105	1.810447407	0	0.066555137	4.502139478	0.306530121	2L	-0.373251345	0.808166823	NA
**CG4619**	FBgn0032166	32.29342937	31.06676765	38.4203292	25.41790476	30.22992997	22.73720205	39.45239313	34.69738298	2L	0.033420123	0.864119038	0.995787491
**CG5853**	FBgn0032167	0.516559719	0.570593281	1.10034147	0.146870676	0.477633571	0.03563483	0.514245831	0.0820609	2L	-1.041829994	0.274624371	0.89549671
**CG13126**	FBgn0032168	20.37109997	20.32268229	12.55427531	13.50144724	19.35139063	24.20079212	16.03017821	15.60447613	2L	0.196318849	0.452338386	0.967630712
**CG4709**	FBgn0032169	32.05483429	25.27464384	26.11073519	31.42127906	27.79314744	25.45631509	23.75582718	31.29190636	2L	-0.057941898	0.778904319	0.995787491
**CG4658**	FBgn0032170	168.4148564	198.8883584	171.9283547	222.833679	189.8461594	210.0217055	198.4027026	204.5947714	2L	0.105178124	0.362027613	0.939549067
**CG5846**	FBgn0032171	24.38686954	33.24431191	41.55205058	37.0806967	18.11043354	21.06940103	22.67908249	26.03038204	2L	-0.597324885	0.026377665	0.453785402
**CG5850**	FBgn0032172	28.11730331	28.83546665	36.65399229	43.60271998	39.8820234	35.81991519	35.11186113	29.80257199	2L	0.06542212	0.704741643	0.995787491
**CG13131**	FBgn0032175	0.061344367	0.342988564	0	0.476740271	0.221484608	1.272372071	0.052163599	0.57713196	2L	1.303297893	0.306738061	NA
**CG13127**	FBgn0032176	2.708884875	0.387883177	0.160285779	0.462121691	0	0.840924916	0.33709383	0.071722437	2L	-1.569074806	0.231855854	NA
**Spn31A**	FBgn0032178	0.696512107	0.841176971	2.298030973	0.779468043	0.301771888	0.525333888	1.06609041	0.665364594	2L	-0.818452119	0.304970395	0.911292159
**Ripalpha**	FBgn0032189	47.96216908	53.47926534	60.91634014	40.31240571	47.51682632	74.23922992	59.27986799	39.81101361	2L	0.153532147	0.465580681	0.971928234
**Snx17**	FBgn0032191	184.2799246	236.4587438	166.2771505	231.9093965	186.1660536	234.4666205	174.3458007	248.8076067	2L	0.07340182	0.625099001	0.990047511
**CG5727**	FBgn0032193	109.4235325	75.80345517	98.21511085	77.80332135	86.11578365	88.07390767	91.26815447	69.72616237	2L	-0.082667136	0.670274941	0.995787491
**CG4901**	FBgn0032194	79.99924084	64.41676471	52.49290858	79.97985636	59.13845234	51.50670545	47.14148684	48.14472903	2L	-0.401188605	0.018980229	0.393184106
**CG4908**	FBgn0032195	66.14924123	95.77773619	76.54066792	59.45595715	88.14270763	72.12818143	75.05403541	64.92667587	2L	0.046220615	0.787826409	0.995787491
**CG5708**	FBgn0032196	242.2068681	167.4140749	235.4294697	149.463246	175.4568848	183.19501	162.1287259	166.5389493	2L	-0.180663662	0.23914952	0.873933389
**ova**	FBgn0032197	70.49297713	69.35637002	81.60621042	58.24214164	48.79544755	76.20094304	67.99787811	60.59695898	2L	-0.112232302	0.456099883	0.968365245
**eEF1delta**	FBgn0032198	121.5241058	137.2141373	135.2951386	126.0740752	126.1085255	109.0243019	137.8330349	110.7237913	2L	-0.072896305	0.563410168	0.984934163
**CG5676**	FBgn0032200	378.2532913	366.5707548	471.9417679	335.4750951	423.6953874	368.2810573	417.0908536	386.9759725	2L	0.072934062	0.558193731	0.984934163
**REPTOR-BP**	FBgn0032202	176.1394579	261.10079	261.3359442	173.4111645	179.1718196	158.7401505	211.3578314	177.154419	2L	-0.226559879	0.173182262	0.832736771
**CG4953**	FBgn0032204	66.88029823	58.40016803	88.88336296	76.53254947	66.5233624	75.71312368	64.07444905	77.33754962	2L	-0.004311031	0.978791557	0.997757033
**CG4957**	FBgn0032205	73.02119546	58.14413926	45.40700411	46.18171921	70.91113029	62.03867431	65.30458013	38.63170605	2L	0.112317854	0.642965167	0.992589784
**Ufd4**	FBgn0032208	111.7510009	148.7054268	161.7040817	157.3025522	131.2970194	136.841806	146.6103448	147.2944733	2L	-0.011161574	0.925988281	0.996479182
**CYLD**	FBgn0032210	41.54982546	32.82503764	55.4317096	26.48404578	31.83824583	51.60259412	35.01752345	31.64135656	2L	-0.031111585	0.88588841	0.995926915
**CG13138**	FBgn0032211	0.636586813	0	2.676874596	0.098945213	1.532269804	2.320657947	2.165262181	0	2L	0.85611589	0.516501979	0.980804634
**CG5390**	FBgn0032213	4.768291542	4.027840357	6.574510022	1.03972805	2.910614762	2.781398476	3.412924838	1.564032007	2L	-0.589708858	0.288082001	0.902924182
**CG4968**	FBgn0032214	239.3378595	227.5734926	198.1590387	189.898683	227.6597491	201.2854918	208.7656909	163.1048686	2L	-0.065705298	0.639180291	0.991585536
**Usp14**	FBgn0032216	163.3999754	132.9495137	145.743323	116.9417263	143.967282	132.8606997	159.1653137	124.1647653	2L	0.031757495	0.818838895	0.995787491
**CG4972**	FBgn0032217	60.13683025	75.02549389	78.01542316	52.86841883	59.0110925	66.81263357	50.04932967	73.67849393	2L	-0.057623351	0.725746794	0.995787491
**CG5381**	FBgn0032218	1.004493482	2.825442493	2.999245756	1.297073763	1.060122939	1.923200033	0.35480554	1.308509001	2L	-0.756388721	0.180774105	0.836519372

**CG4995**	FBgn0032219	3.342072326	1.098054558	2.256816112	1.927896696	2.674554391	3.508202273	1.142619361	0.822839508	2L	-0.061925761	0.924089439	0.996479182
**Schip1**	FBgn0032221	219.1893123	191.7767626	227.1414386	177.6387222	184.7353291	201.0213003	192.7002775	188.1207206	2L	-0.060082492	0.577497907	0.985637351
**CG5037**	FBgn0032222	356.7647765	351.3072573	342.4510494	354.4428774	401.2945765	317.9839054	362.3666165	359.2123514	2L	0.067848446	0.534965509	0.984760433
**GATAd**	FBgn0032223	68.64627063	64.07577575	63.7998267	56.79417085	55.99533967	63.14624648	59.62933832	51.78359941	2L	-0.107462908	0.334407601	0.924122842
**Sps2**	FBgn0032224	19.99291612	14.53791454	18.44644308	11.03333267	15.83839289	15.49043232	10.44463194	13.96857198	2L	-0.170948691	0.565394881	0.984934163
**CG5022**	FBgn0032225	75.97672711	69.61810379	53.11307936	62.05668519	67.02175819	70.40444617	61.53712931	76.34022795	2L	0.106206122	0.462541265	0.971606621
**ClpP**	FBgn0032229	254.7238042	273.3670718	268.6054836	241.612564	263.3761001	307.1756633	260.5768148	301.0658576	2L	0.155800392	0.141336133	0.8037297
**lft**	FBgn0032230	0.044671607	1.198885659	0.650236171	1.770552884	0.0806437	1.431948014	0.227916458	0.193972387	2L	-0.86621353	0.505728959	NA
**CG5056**	FBgn0032231	135.3841798	160.1201905	128.3597658	144.9381667	145.5196266	122.2342408	134.6230177	161.6623727	2L	0.020351108	0.887983404	0.995926915
**dpr19**	FBgn0032233	454.276424	397.7297959	392.8286608	422.7542898	385.2087334	441.26948	382.2097394	344.3084618	2L	-0.076130516	0.45015139	0.967112302
**gny**	FBgn0032234	12.36058993	5.893688896	10.35071104	7.533704973	9.28807457	14.58164954	11.92458741	3.133135297	2L	0.124405007	0.759158143	0.995787491
**CG5096**	FBgn0032235	0.035210669	0.354365746	0.256261775	1.067200539	0.572078537	1.527034567	1.437170768	0.535119352	2L	1.301887177	0.226303522	NA
**mRpS7**	FBgn0032236	113.1286784	127.5322945	115.2556748	117.9045146	132.5180444	152.1904213	136.8197459	120.4319692	2L	0.224226102	0.094207441	0.728467429
**CG5355**	FBgn0032242	138.8082549	118.4267069	123.0932331	147.0777301	143.1682261	135.4168209	122.0367221	142.3597226	2L	0.07034646	0.5646814	0.984934163
**Klp31E**	FBgn0032243	20.49651691	15.91938598	19.60242869	23.42652237	18.67363717	17.1896504	11.37254552	13.91328758	2L	-0.349425412	0.083742813	0.69799888
**RfC3**	FBgn0032244	16.7510672	28.81277603	21.13168479	32.52173275	17.37423705	30.78161038	35.58437853	24.73031991	2L	0.159878327	0.587721473	0.987588562
**Wdfy2**	FBgn0032246	150.8604406	120.4228935	137.8199454	154.7555059	151.0155045	149.0510947	138.3678629	143.0806638	2L	0.072008452	0.559462184	0.984934163
**CG5188**	FBgn0032247	29.14677809	26.45054414	35.53614541	19.16668653	29.41178006	27.69078438	41.18070854	22.55376695	2L	0.161044196	0.566114485	0.984934163
**Bug22**	FBgn0032248	118.5576388	167.5638668	160.2329389	85.79719827	171.2528756	135.0142308	162.0705251	131.3378677	2L	0.209517123	0.290218612	0.904180153
**TBC1D16**	FBgn0032249	280.8018236	280.9885234	296.1277871	319.0751823	306.8375701	304.2358149	293.2307585	290.9202633	2L	0.051905952	0.576053549	0.985637351
**holn1**	FBgn0032250	47.60397149	33.32540241	36.26850985	31.7978904	49.12397834	49.45535377	44.32681792	40.14523596	2L	0.320305118	0.122691717	0.780439453
**Nse4**	FBgn0032251	17.67939301	12.20544121	21.83726666	6.786028415	18.0013097	13.00918926	11.27512785	18.25561406	2L	0.083009105	0.820553799	0.995787491
**loh**	FBgn0032252	2.031729324	2.560769164	1.197427803	1.819826155	1.719925136	1.222891105	0.907754462	2.118311622	2L	-0.319476885	0.441867054	0.967112302
**LManI**	FBgn0032253	0.39855021	0.827681606	0.690626608	0.176991393	0.068522407	0.393643594	0.048414769	0.164817028	2L	-1.554713923	0.148227708	NA
**RluA-2**	FBgn0032256	8.321339424	11.9885138	10.57438034	11.77055736	10.88907198	13.98872375	13.32828566	8.7304993	2L	0.17004891	0.412502155	0.958665466
**Cog4**	FBgn0032258	30.69836008	26.99453458	27.39109326	24.03197622	24.43885873	27.40640832	26.08017575	40.73332078	2L	0.151050287	0.450959461	0.967112302
**CG6144**	FBgn0032259	209.5602717	180.8929839	191.2261044	167.8893072	191.854054	207.4623053	176.8220248	170.6022274	2L	0.022459901	0.846954221	0.995787491
**CG6094**	FBgn0032261	95.72636821	79.44350892	87.72182791	77.50864386	82.04805321	73.16194141	83.41370873	96.10444533	2L	0.003771609	0.982883564	0.997881056
**Lip4**	FBgn0032264	2.677216342	1.243565955	0.449646196	1.104323613	1.412741101	0.291238063	1.260855952	0.357691354	2L	-0.713708502	0.346372696	0.930285749
**CG18301**	FBgn0032265	0.42692454	1.352644714	1.26587142	0.331786165	0.342536985	0.939170981	0	0.102988077	2L	-1.201103887	0.368181313	NA
**CG18302**	FBgn0032266	0.34417846	1.090476489	1.855493649	0.980758926	0.759403739	0.144217489	0	0.166054066	2L	-1.931525779	0.10269658	NA
**CG7329**	FBgn0032271	0.750393838	0.892229139	1.336529229	0.376477908	0.188622598	0.814930027	0.12115638	1.257970172	2L	-0.452550195	0.524982306	0.984107922
**CG6431**	FBgn0032289	1.06912536	0.128093415	0.138947315	0.400600531	0.034465094	0.485983057	0.048702889	0.497393598	2L	-0.700735	0.558241826	NA
**CG6443**	FBgn0032290	184.423591	218.8128185	168.2346942	210.7938076	233.7254805	263.2464778	190.7575539	238.0530518	2L	0.272804483	0.041818568	0.536003453
**CG17118**	FBgn0032291	12.50890196	10.68890683	13.55091786	6.05291272	11.21987515	12.60920617	7.626403462	5.636562736	2L	-0.174882119	0.68785725	0.995787491
**EMC3**	FBgn0032292	200.669836	216.1596409	262.3088518	275.1851099	184.9515557	212.6594715	209.9491548	218.5717075	2L	-0.177081579	0.208552846	0.856520545
**Dpy-30L1**	FBgn0032293	335.4801471	292.8014704	330.9460175	344.9159004	322.7782572	245.9883964	335.8397791	344.1946585	2L	-0.031639741	0.834118774	0.995787491
**CG12299**	FBgn0032295	240.53864	329.9957116	280.6100188	285.7520077	226.1091447	262.1489975	273.3447698	241.1104306	2L	-0.148985692	0.178472572	0.836045561
**CG6729**	FBgn0032296	86.12375518	83.68111628	63.44693698	57.00380249	88.87145422	85.41867374	81.7843836	62.98362775	2L	0.16488958	0.307062161	0.911669416
**CG17124**	FBgn0032297	107.0035846	74.21055127	78.37593814	75.53648766	70.60180785	87.5495663	69.48222053	69.49637149	2L	-0.148857116	0.267518135	0.89472797
**CG6724**	FBgn0032298	79.40846152	71.79928546	48.31384693	59.83184754	64.07634806	52.47736951	51.96150306	72.57350415	2L	-0.076577965	0.697611187	0.995787491
**CG6700**	FBgn0032305	311.6120319	280.7545476	284.4268671	287.0431125	264.716947	350.0185128	246.626923	268.6904113	2L	-0.016639605	0.881038345	0.995787491
**YL-1**	FBgn0032321	61.8401778	72.46886883	71.06371996	86.6536986	65.86415319	90.96780307	64.14681071	75.67838567	2L	0.053249492	0.75010457	0.995787491
**CG16743**	FBgn0032322	36.09093569	136.2737351	77.12388962	50.84485711	18.57564706	36.63258455	58.96317968	18.00538367	2L	-1.143776849	0.040285158	0.530560761
**Samuel**	FBgn0032330	0.703676631	0.831895196	0.437520104	0.412964561	0.624984174	0.926058211	0.382022336	0.625781995	2L	0.128396796	0.68803857	0.995787491
**AstC**	FBgn0032336	1.191538725	1.554497043	1.043848118	0.34725346	0.985890028	0.187229239	0.675475753	0.25150814	2L	-0.949440279	0.208245921	0.856520545
**CG16854**	FBgn0032338	77.49195824	83.2376134	63.50494654	74.32910043	81.75126075	85.88843364	71.45505236	54.17894663	2L	0.001656177	0.991054604	0.998408391
**Wdr59**	FBgn0032339	23.19623336	22.27885411	26.58329983	22.39556612	25.90435946	27.54901544	25.53319615	15.5511997	2L	0.030385097	0.876264309	0.995787491
**Reps**	FBgn0032341	80.36807304	96.93668114	86.13227971	102.7506678	81.59653568	81.9218535	90.61042995	98.49618776	2L	-0.022221645	0.855404085	0.995787491
**CG6201**	FBgn0032343	56.4194723	57.44291381	51.77838763	81.37568033	76.99756732	74.7491799	59.56524711	82.01174224	2L	0.276977135	0.112526781	0.758410631
**CG14921**	FBgn0032345	0.323202409	1.219785076	0.980105671	4.709589061	3.209047515	12.11235197	1.030620362	2.982234982	2L	1.451388816	0.121244561	0.77836519
**Csl4**	FBgn0032346	30.00672357	35.26171131	22.43129256	20.74381898	34.95843467	44.08001003	30.485605	38.44452626	2L	0.475762958	0.069365669	0.660678268
**CG4751**	FBgn0032348	64.21527364	74.38316002	67.67724577	68.26167461	73.10640801	57.11458264	49.05204928	53.78136756	2L	-0.204070506	0.131524836	0.790743321
**CG6287**	FBgn0032350	16.04954919	26.05912373	20.53985247	11.01965626	11.68587474	13.85270565	18.76760901	10.97552371	2L	-0.378874815	0.143423599	0.8037297
**Ppt2**	FBgn0032358	48.48912888	78.64647419	53.50531001	62.62603638	70.47170503	61.57462146	58.98757547	74.20221137	2L	0.160336928	0.38163728	0.944476532
**spz4**	FBgn0032362	0.437213527	0.31429901	0	0.08737259	0	0.482865484	0.286801997	0.027120887	2L	-0.077182316	0.958048791	NA
**Dlg5**	FBgn0032363	22.50757454	27.57865089	23.62260125	29.51750404	21.86488298	23.46804498	27.99602697	27.31385687	2L	-0.005072186	0.972026693	0.997604378
**CG4970**	FBgn0032364	0	0.425179636	0.527093618	0.253278234	0.065371293	0.443821483	0	0.78618825	2L	0.17128978	0.919914006	NA
**CG6614**	FBgn0032369	1.046808182	0.378717894	0.846514016	0.095709563	1.148675862	0.309623329	0.209445503	0.133689263	2L	-0.367335403	0.717091346	NA
**CG14931**	FBgn0032374	0.075914326	0.891349422	4.604177383	0.530974178	0.411134444	2.862862504	1.549272607	1.812987313	2L	0.184064363	0.874455434	NA
**CG14937**	FBgn0032377	0.206973108	1.735841765	1.882927023	1.568287531	1.401148149	3.170910955	0.395994634	0	2L	-0.073127975	0.949194534	NA
**CycY**	FBgn0032378	173.0720857	187.482348	163.3557641	180.0987721	151.4498299	171.9177655	176.2687219	161.540709	2L	-0.060832568	0.533250503	0.984760433
**CG6686**	FBgn0032388	128.597293	142.5501128	141.5804887	134.1599524	125.4483571	149.7304469	164.7117916	120.8788156	2L	0.06609235	0.572691047	0.984934163
**escl**	FBgn0032391	22.00401035	21.02582389	26.03438213	29.75067758	27.02458111	28.56722476	27.14614332	32.59902364	2L	0.255348637	0.217417368	0.860826301
**Nfs1**	FBgn0032393	225.6432537	218.1176883	208.2844135	214.3942378	214.2931842	227.5782582	174.4094855	221.2913241	2L	-0.019442098	0.866746236	0.995787491
**Hacd1**	FBgn0032394	188.9318517	167.5339606	172.0579304	179.8347456	144.3980922	182.8716535	177.0717632	165.0958759	2L	-0.054346371	0.676977688	0.995787491
**Wdr81**	FBgn0032395	52.82203412	56.0990364	63.9306908	68.56980535	50.62700235	53.66505872	59.8225571	49.18958766	2L	-0.148622168	0.234604068	0.87009833
**Tom70**	FBgn0032397	317.714491	293.5696533	259.8618122	302.4013158	293.4919102	320.5771575	287.508088	317.9169815	2L	0.083738114	0.417335258	0.958846438
**CG6766**	FBgn0032398	227.3202841	248.8567026	281.0984973	242.4739938	259.1699238	245.6640329	250.27669	240.3245303	2L	0.0259747	0.798739433	0.995787491
**CG6785**	FBgn0032399	1.169952184	1.086345545	1.165725297	0.340962434	1.169178036	0.118181119	0.373071645	0.226792365	2L	-0.96251185	0.272775273	0.89549671
**CG6770**	FBgn0032400	666.7928432	906.9842656	934.6247206	641.8011635	736.4434673	677.7514833	826.836795	724.0771467	2L	-0.051315369	0.706248045	0.995787491
**Plzf**	FBgn0032401	10.16498669	9.611388101	12.94894118	13.08493275	13.9576449	12.91365671	16.21945215	15.25240828	2L	0.381475619	0.133794979	0.794759341
**PLCXD**	FBgn0032402	162.8330836	223.7608896	256.2189189	115.0584649	136.4580899	146.8446889	253.551255	109.0025655	2L	-0.193145488	0.574302442	0.984934163
**firl**	FBgn0032405	0.208844474	0.262730481	2.343273738	0.486913029	1.743707335	1.919748622	1.398512693	0.056677586	2L	0.682614178	0.487843171	0.973549494
**Pex19**	FBgn0032407	225.4146352	203.8212944	185.5535598	240.8874022	205.2217714	214.6397339	204.914747	199.3022992	2L	-0.026844829	0.808403443	0.995787491
**CG6712**	FBgn0032408	50.02446996	40.48529104	36.98999559	42.65850521	38.88705641	34.92858451	36.24819192	36.34345108	2L	-0.192251373	0.325817082	0.918470465
**Ced-12**	FBgn0032409	44.93988953	62.69593225	68.83117848	76.04142359	59.34798534	58.91393737	74.14587158	62.28853451	2L	0.045813424	0.787989322	0.995787491
**CG17211**	FBgn0032414	0.763819494	0.776201489	0.554552065	0.766920929	0.301946705	0.236537196	0.355569332	0.181568193	2L	-1.392692098	0.002690588	0.125973924
**Gr33a**	FBgn0032416	0.726663135	1.828313201	2.644311944	0.084709387	3.738667621	0.959130991	0.648806644	0	2L	0.071296064	0.949252823	0.997604378
**CG6583**	FBgn0032420	54.71615662	55.29849256	57.17122328	52.3133792	55.44166816	37.10887122	50.84433558	54.83839397	2L	-0.115626561	0.546888597	0.984934163
**crok**	FBgn0032421	6.554894265	2.486945188	6.625867525	3.274819118	3.099188231	1.765689793	6.768297468	11.01230172	2L	0.284185967	0.674147499	0.995787491
**CG6405**	FBgn0032428	32.03070542	31.78217782	24.89876781	39.70576044	32.37309013	29.13503112	31.79249104	27.05756947	2L	-0.066458268	0.759562573	0.995787491
**CG5446**	FBgn0032429	134.3466667	166.0456637	151.6556236	148.9313611	154.6274863	184.0829885	134.0548364	139.559354	2L	0.058819112	0.670997254	0.995787491
**CG6388**	FBgn0032430	46.98159736	54.16479534	37.42990607	34.45160102	37.1109519	50.6450863	52.10915836	54.16170382	2L	0.194539346	0.318302963	0.91460807
**CG5435**	FBgn0032431	9.353143896	3.819829822	0	2.6547047	4.551560692	2.377049521	1.452352149	2.648679288	2L	-0.511597531	0.589948809	0.987597375
**Oatp33Ea**	FBgn0032433	2.489580352	8.512455952	7.387002049	6.072256541	2.420024175	4.261000241	6.253265689	1.70468835	2L	-0.705266262	0.109065616	0.758410631
**Oatp33Eb**	FBgn0032435	0.170173371	0.69321618	0.058976889	1.13357945	0.0877733	1.398106182	0.744198728	0.211121223	2L	0.273870599	0.807505991	NA
**Ref2**	FBgn0032439	0.193349693	0.085133209	0.240395125	0.078887818	0.09162451	0.284826421	0.030827451	0.10494502	2L	-0.193136558	0.789774999	0.995787491
**CCT4**	FBgn0032444	257.6876138	215.8657541	213.5317518	203.8904736	232.0844968	220.8802831	260.8543772	183.5683595	2L	0.03872878	0.764998915	0.995787491
**CG6153**	FBgn0032445	24.90503469	39.93170031	30.87602236	18.35883445	44.13350067	20.37262589	22.65708831	24.84886753	2L	0.013424675	0.968443966	0.997604378
**IFT43**	FBgn0032446	14.66921902	9.894298061	15.06341619	9.77163769	17.37423705	15.6594218	19.00774241	19.88408161	2L	0.567792776	0.081475873	0.694684542
**PICK1**	FBgn0032447	206.844549	251.9381699	219.7108232	243.1789279	252.1053686	212.9675788	236.3824918	218.2207585	2L	0.02933564	0.783342696	0.995787491
**CG17036**	FBgn0032449	2.511360514	4.609856364	2.643926194	7.180835137	1.881892197	1.518898905	2.296682224	0.514376995	2L	-1.421178175	0.027165653	0.461096849
**CG5776**	FBgn0032450	7.321245206	4.688111079	5.520385018	5.824290426	8.573010173	10.33031889	15.71110405	19.65407766	2L	1.241866586	6.57E-05	0.006523136

**spict**	FBgn0032451	48.97160262	44.67035411	50.333582	44.55627237	54.78474952	32.53179738	42.09394641	52.82478355	2L	-0.01621613	0.935035088	0.996479182
**CG6180**	FBgn0032453	535.0100472	568.897864	645.2515583	664.305146	639.5901671	470.5047799	628.4166694	625.2546667	2L	0.003549487	0.978954323	0.997757033
**CG5787**	FBgn0032454	62.83987148	54.20432964	75.31508937	55.53547421	62.77278435	55.45634783	78.18761735	61.97718523	2L	0.091645988	0.550123824	0.984934163
**Pih1D1**	FBgn0032455	38.95647257	26.45644111	25.46046139	24.72774492	33.40185412	35.42200026	24.88983663	26.71465288	2L	0.08305478	0.628903372	0.990523437
**MRP**	FBgn0032456	13.56321996	12.52220119	12.20967243	13.74062193	12.27875493	16.41996534	12.9924688	13.67436931	2L	0.116080381	0.298258245	0.9074568
**Yip1d1**	FBgn0032465	31.64752127	35.00224726	34.5845348	32.9434043	42.67272267	33.05469871	34.31093718	32.64599273	2L	0.121047149	0.348911204	0.931729531
**CG9934**	FBgn0032467	73.91667438	74.57966062	78.92846035	85.69145478	57.94609947	75.3507689	76.17874421	79.12531512	2L	-0.088124779	0.479996725	0.973549494
**kmg**	FBgn0032473	0.560742641	1.770479278	2.4006244	1.307350359	0.893191928	1.399406657	1.262176346	0.644518684	2L	-0.473870262	0.509728379	0.979725655
**DnaJ-H**	FBgn0032474	82.41288897	88.50355669	89.25034629	84.43268361	87.00469319	89.56419845	82.19620361	82.36902261	2L	0.01664998	0.872462394	0.995787491
**Sfmbt**	FBgn0032475	1.61438154	3.766625814	2.738582257	3.226174103	2.059782027	4.852182853	3.468060625	4.690864468	2L	0.447134869	0.211409131	0.856520545
**CG5439**	FBgn0032476	105.6666782	96.33010195	99.20470894	84.62338023	110.005153	130.4912854	115.5593793	102.361022	2L	0.276429136	0.032002295	0.501329188
**CG5287**	FBgn0032477	31.5438856	32.86809027	27.35509996	22.62873286	36.18405725	26.27907957	24.4476348	22.48883347	2L	-0.033235065	0.859044961	0.995787491
**CG16974**	FBgn0032479	284.7215248	246.3853161	269.5048039	264.1707562	270.8265298	271.8288123	249.3126686	274.9368196	2L	0.03204676	0.726586109	0.995787491
**Edem2**	FBgn0032480	79.90021636	77.10663793	63.5781429	70.92003292	60.60804228	80.24851562	58.06855953	73.74173407	2L	-0.068722907	0.604387128	0.989286463
**CG16972**	FBgn0032481	31.27376026	42.81514116	36.00078634	41.83727282	44.74153601	49.36974722	39.42617974	48.73732412	2L	0.295227773	0.029486552	0.476301137
**Pect**	FBgn0032482	135.7501348	140.1906196	149.3484633	155.5616325	139.2397815	133.9132399	162.2369727	153.2980302	2L	0.050990741	0.63405898	0.991585536
**CG15482**	FBgn0032483	1.996962436	0.279135362	0	0	2.102933591	0	2.547147038	1.625846364	2L	1.429622634	0.394840415	NA
**kek4**	FBgn0032484	185.5900056	163.8909252	185.6712182	175.4897542	188.9902084	190.3306718	156.6176507	168.5956843	2L	0.016295488	0.878566219	0.995787491
**CG9426**	FBgn0032485	192.167096	201.9182457	169.8776449	204.8376973	186.3677451	171.2161398	174.3148103	183.6563452	2L	-0.073062574	0.483458842	0.973549494
**mRF1**	FBgn0032486	17.76314974	18.5324045	14.51247094	13.80470497	10.13803261	14.21479829	6.69590926	18.02371191	2L	-0.365105295	0.294817642	0.906715248
**Ski6**	FBgn0032487	23.24927717	34.63307085	36.4477746	20.35126609	30.45542134	52.78035174	24.40892503	36.80945576	2L	0.369041768	0.216868476	0.860826301
**CG16812**	FBgn0032488	152.7542401	114.8666249	133.9366958	141.6105601	115.4792242	123.9127821	132.375869	113.8133973	2L	-0.135319858	0.309004133	0.912131082
**Prosalpha6T**	FBgn0032492	0.680025481	2.281294693	2.268381956	0	2.301788029	0	0	0	2L	-1.118079032	0.564193095	NA
**CG5945**	FBgn0032494	1.602243333	0.570083907	1.177884842	0	1.490055083	1.922563017	0	0.105412685	2L	0.087482973	0.950433752	NA
**CG16820**	FBgn0032495	0.240788654	0.183585994	1.062090755	0.45931883	0.908887306	0	0.335049288	0.760399209	2L	0.096890157	0.934437498	NA
**Uvrag**	FBgn0032499	31.29352955	33.98289717	35.0854553	31.90169541	29.09043721	33.38227482	25.92104779	15.69954848	2L	-0.316272359	0.13471891	0.795862348
**CG9395**	FBgn0032506	63.8830185	54.60134908	57.79703675	44.19758259	44.91488481	53.63324786	51.72685559	60.85857787	2L	-0.032676381	0.82107476	0.995787491
**CG9377**	FBgn0032507	0.468488107	33.79039235	72.48635362	0.849537411	0.093971234	1.325063178	7.569113772	16.5001259	2L	-2.007539199	0.221348047	0.86549481
**CG6523**	FBgn0032509	265.4314049	203.9792525	259.231538	217.0460334	218.7205356	282.7258067	259.0545081	216.9579137	2L	0.074068871	0.601362268	0.989286463
**ND-B22**	FBgn0032511	1442.748594	1339.639761	1388.099037	1420.047641	1509.643489	1366.702201	1490.142356	1533.022431	2L	0.108444266	0.249948656	0.879675717
**Bdp1**	FBgn0032512	32.42469447	29.43552087	29.17825915	26.97548594	20.37928171	41.21287011	22.51815526	14.50697669	2L	-0.23651058	0.369406216	0.942282153
**CG6565**	FBgn0032513	88.43414178	69.99503528	79.3842033	78.06930775	80.97050098	71.97314358	87.64481982	81.13620969	2L	0.054465626	0.695468802	0.995787491
**CG9302**	FBgn0032514	9.93843529	16.11232827	8.978389037	17.16017795	12.01099441	11.64376208	8.48641404	16.88262838	2L	-0.055523327	0.864337108	0.995787491
**loqs**	FBgn0032515	428.6728083	391.1134383	386.6505508	435.9356625	377.8939721	427.22353	397.5027974	383.1513307	2L	-0.022851859	0.807101513	0.995787491
**Ing5**	FBgn0032516	114.0118575	109.0667086	102.7707743	69.38485724	101.895628	99.23411819	99.3135066	86.44934592	2L	-0.00109659	0.994761144	0.998895169
**CG7099**	FBgn0032517	7.14586967	14.260614	12.82913522	9.515670141	4.161331041	11.35204756	6.018771285	7.330307003	2L	-0.56646591	0.052047086	0.602445021
**RpL24**	FBgn0032518	988.2517985	1053.955877	1114.412184	1112.247863	998.0921649	1016.887225	1137.265874	1224.058518	2L	0.068139367	0.515963709	0.980804634
**CG7110**	FBgn0032521	0.495741452	0.530255313	0.313737874	0	0.038910451	1.090558031	1.19133422	0.015598559	2L	0.818775161	0.454055116	0.967967712
**Ance-3**	FBgn0032536	0.336336916	0.448523125	0.522004427	0.214303738	0.207419804	0.277705534	0.042633674	0.249453552	2L	-0.925754486	0.071528711	0.66904633
**CG4480**	FBgn0032553	0	0.088001185	1.400049191	1.100862877	0.94711165	1.533348117	0.267674144	0.113904439	2L	0.200010022	0.892239795	NA
**CG15258**	FBgn0032563	2.944529597	1.440551969	1.785846933	2.00230914	0.885938433	0.69402113	1.564907971	0	2L	-1.377684922	0.276463405	NA
**Tpr2**	FBgn0032586	73.93333995	81.4946459	75.20209838	79.91989595	78.35423291	86.030438	85.17891111	74.09516011	2L	0.08938853	0.310264768	0.912401755
**CG5953**	FBgn0032587	0.537893053	0.843653116	1.177818346	0.407167988	0.826010141	0.490657276	0.347499303	0.644573551	2L	-0.316447355	0.44847071	0.967112302
**Trpgamma**	FBgn0032593	0.098080598	0.405807229	0.396570272	0.198181726	0.165256576	0.184939578	0.100082285	0.35490343	2L	-0.394104124	0.60936578	0.990047511
**Prosbeta4**	FBgn0032596	204.6844588	216.6390631	200.8737371	224.1973928	193.4501882	189.8047048	206.3576652	197.5281067	2L	-0.07487242	0.527469904	0.984107922
**CG17904**	FBgn0032597	146.2186959	129.462279	172.3730277	146.1014507	127.2493004	171.8423888	162.3290404	144.8067987	2L	0.058014713	0.695689966	0.995787491
**ChLD3**	FBgn0032598	1.128801605	0.502332294	0.726529395	0.751930985	0.291110887	0.369221702	0	2.350702868	2L	-0.015854577	0.987794194	0.998078482
**BuGZ**	FBgn0032600	115.9115119	111.700735	99.87466162	114.4935992	95.69747987	120.99214	103.5418115	136.6918083	2L	0.077344232	0.52913571	0.984296327
**yellow-b**	FBgn0032601	1.843408319	0.294481795	0.212956327	0.613976728	0.475403497	0.04137986	0.111966053	0	2L	-2.232306609	0.070305508	NA
**ppk17**	FBgn0032602	1.096433489	3.371707834	1.241301764	0	0.8577155	0.99925377	1.398512693	1.03153206	2L	-0.371611391	0.698283757	0.995787491
**CG17928**	FBgn0032603	0.923435455	5.343820334	1.68018115	3.767671249	2.250500855	0.043530465	0	0.100243193	2L	-2.235131399	0.051611298	0.599821511
**CG13280**	FBgn0032609	0	0.030479877	0.011020856	0.180054652	0.172220633	0.214147881	0.069533227	0.02958875	2L	1.170692066	0.366776544	NA
**CG13284**	FBgn0032614	5.440641053	1.132247161	2.128857953	2.486569067	2.997705801	2.647433885	2.445217487	5.481065291	2L	0.294218687	0.521330734	0.982083369
**beat-IIIc**	FBgn0032629	0.547459259	0.469081215	0.526076884	0.218255566	0.314460451	0.408891117	0.181374069	0.221251703	2L	-0.614153319	0.081023118	0.694684542
**Lrch**	FBgn0032633	31.00812166	31.63232896	30.39890648	31.58617343	28.57268832	31.58520646	28.75845441	28.85854449	2L	-0.052068317	0.562112341	0.984934163
**Rpb11**	FBgn0032634	60.55936666	38.57981876	59.36007225	57.61769794	73.32516993	59.0558394	76.77597651	68.79458111	2L	0.389870516	0.097758837	0.730787365
**CG15141**	FBgn0032635	110.1670129	137.2578308	123.1544956	131.428185	125.2367591	125.752112	127.7236491	126.7306851	2L	0.042036176	0.694560766	0.995787491
**CG5050**	FBgn0032637	0.695788335	0.91877087	0.215485889	0.621269735	0.374150379	0.516413717	0.906368165	0.321408698	2L	-0.197092334	0.787710945	0.995787491
**Sgt**	FBgn0032640	142.6878482	148.2232545	151.8670336	149.8995937	148.3770806	155.1342293	154.814914	143.0888408	2L	0.051376712	0.573524943	0.984934163
**CG5110**	FBgn0032642	219.4472179	222.8019669	217.695332	208.7121589	208.7495181	186.5283679	235.2208124	200.6555302	2L	-0.033914258	0.796183629	0.995787491
**GCS2beta**	FBgn0032643	90.20399449	96.42140659	79.55939102	78.54952799	100.199675	92.93189329	79.46736293	98.69100097	2L	0.137876521	0.297755832	0.9074568
**CG5131**	FBgn0032644	33.39777414	64.30018522	65.49165585	51.251065	35.2280835	39.41356089	48.4037323	46.38608687	2L	-0.301816764	0.215063329	0.858896167
**CG15142**	FBgn0032645	1.885282251	0.175683139	0	7.325774327	1.323551311	0.098746255	0.534377281	2.501352508	2L	-1.058790224	0.462583032	NA
**mEFTs**	FBgn0032646	260.7501377	252.7159894	250.6067277	259.0813101	255.6508384	265.605184	263.2825692	228.0616624	2L	0.013862086	0.88691159	0.995926915
**CG15144**	FBgn0032648	0.391826952	0.246463029	0.891156301	0.570956118	0.751557328	0.57720634	0	0.053168287	2L	-0.552789606	0.647234918	NA
**CG15145**	FBgn0032649	1.066800152	0	1.294021144	0.138178022	0.427966154	0.111752519	0.604762246	0	2L	-1.114621101	0.448778691	NA
**Oli**	FBgn0032651	327.9683646	331.7326114	301.9361617	375.9727124	362.1314814	306.9719094	295.5393689	320.4990948	2L	-0.027666798	0.809883331	0.995787491
**elfless**	FBgn0032660	2.222192983	2.299895588	0	0.220466353	0.426768923	0.891519137	1.688597204	0.410603121	2L	-0.465875658	0.694675982	NA
**CG5758**	FBgn0032666	83.50184986	97.36061491	105.8659546	92.16257695	70.34743533	72.22806684	86.15104656	75.83619509	2L	-0.282247928	0.010519861	0.311527659
**CG5783**	FBgn0032670	0.774068326	1.687908333	5.82142478	3.428951206	0.838435032	1.532552468	2.172131422	1.512516591	2L	-0.909004737	0.295571354	0.906715248
**JMJD4**	FBgn0032671	25.36047618	32.52559601	28.18642469	21.76396517	12.72932076	18.9237926	22.58707966	26.96458424	2L	-0.376533952	0.136113211	0.796109055
**CG5790**	FBgn0032677	2.176685072	1.390889086	1.089649192	2.416599668	2.120668743	2.280183767	0.705113699	3.15052319	2L	0.248061265	0.696522157	0.995787491
**Ntf-2r**	FBgn0032680	4.387157896	3.396388576	2.456119353	2.884959699	2.639981474	3.711961496	2.869675397	0.488457739	2L	-0.412328724	0.632616958	NA
**CG10283**	FBgn0032681	36.04531029	37.2251259	41.84861965	37.50772351	44.72109662	43.8544354	45.65092244	48.32893662	2L	0.290322192	0.004466182	0.185638045
**grnd**	FBgn0032682	0.598743633	1.171693191	0.645574979	0.07755268	0.720590477	1.160344339	0.169711986	0.216654642	2L	-0.092090478	0.93381031	NA
**kon**	FBgn0032683	0.517649549	0.295395757	0.828576056	0.049768265	0.366088908	0.498099448	0.39479916	0.196966046	2L	-0.184808348	0.788627463	0.995787491
**Ugt301D1**	FBgn0032684	7.473892147	8.970331276	14.4627091	5.161099462	3.917116704	3.492174302	11.90929812	2.664764385	2L	-0.679658087	0.123535676	0.780439453
**CG10211**	FBgn0032685	0.634083842	0.401353469	0.536946289	0.306825824	0.194380276	0.518854286	0.610400568	0.493517729	2L	-0.030164822	0.955346118	0.997604378
**CG15160**	FBgn0032688	39.87340572	44.43862022	43.71245374	37.47014647	37.31747213	41.11950296	34.18189377	48.81671183	2L	-0.003099726	0.982782353	0.997881056
**CG10413**	FBgn0032689	71.60904108	56.83074534	42.03458076	49.95380709	52.54014823	64.98567611	52.76776252	46.75733699	2L	0.001814964	0.991113416	0.998408391
**CG10333**	FBgn0032690	85.05669523	63.39730961	65.63808784	82.69580948	81.17565722	85.20849951	54.22504	111.8097376	2L	0.191273025	0.330684835	0.921224783
**Atac2**	FBgn0032691	23.16948958	21.03524612	15.09384733	31.50466652	24.39413081	33.69788914	19.92239669	20.40265864	2L	0.138907858	0.581383695	0.985637351
**MESR3**	FBgn0032694	7.60925334	7.652851442	7.613369267	6.754253132	6.912230159	7.438097149	6.228910197	6.074233108	2L	-0.12291817	0.258302926	0.887830768
**CG10336**	FBgn0032698	1.697164465	3.584802972	5.032252279	0.293101856	3.517715629	3.733511345	2.886335341	1.091763306	2L	0.129297495	0.873097405	0.995787491
**CG10383**	FBgn0032699	40.69103196	33.70924899	30.50486063	38.98809947	39.64121902	30.68017558	34.94614711	42.95111413	2L	0.071739673	0.66246518	0.995787491
**CG10338**	FBgn0032700	21.21506395	16.59314252	20.09543664	19.79874303	27.48679608	19.49577851	36.02938171	17.72528616	2L	0.402317995	0.097641089	0.730787365
**CG10341**	FBgn0032701	71.45668841	77.72373085	79.87510997	74.77588448	85.43157991	64.49316841	75.40960055	68.85331653	2L	-0.014317513	0.920798888	0.996479182
**CG10376**	FBgn0032702	15.77082904	23.13941672	31.61689899	21.09172217	24.65347923	29.60656924	27.49904631	9.180816324	2L	0.020492585	0.947536448	0.997604378
**CG10343**	FBgn0032703	72.84409037	73.45954949	82.25454416	69.37431121	78.1043684	85.4091691	56.08591884	79.5548779	2L	0.038680882	0.831723561	0.995787491
**Jwa**	FBgn0032704	299.0737661	233.0025151	268.9278472	232.2481678	285.409797	315.3365349	263.090726	316.8573186	2L	0.220375926	0.087336853	0.706081898
**Grip71**	FBgn0032705	17.45794098	15.82718	8.573425368	18.20902756	6.762554936	11.52813175	13.43279716	8.05626442	2L	-0.574143363	0.072379351	0.671999479
**Irk3**	FBgn0032706	4.918182046	16.71621456	7.300158008	5.054337314	4.497701261	2.806503574	10.64792145	3.161199225	2L	-0.645401406	0.193171418	0.844272804
**CG10348**	FBgn0032707	1.078474089	1.004993883	0.5349009	0.759914126	0.562444174	1.043892885	0.207869243	0.114471724	2L	-0.785488594	0.168949934	0.830934498
**Ugt36D1**	FBgn0032713	3.636435186	1.238266668	5.240100318	0.127491906	2.813441795	3.067523736	5.789171547	1.00914012	2L	0.335521767	0.653526097	0.99443552

**CG17597**	FBgn0032715	0.323655285	0.135721584	2.355549621	3.018360451	0.219105278	0.64842298	3.302606342	1.31753547	2L	-0.056830042	0.956284243	0.997604378
**CG10600**	FBgn0032717	113.9721396	93.22508469	88.43328117	98.82521522	91.58368145	106.1388407	90.7329919	101.2901286	2L	0.009534295	0.934442162	0.996479182
**CG17321**	FBgn0032719	160.7577394	134.9852883	108.1793362	145.0414506	125.160671	144.5189345	98.57274276	120.8175071	2L	-0.141589107	0.305150358	0.911292159
**CG10602**	FBgn0032721	27.94814816	28.33894333	30.53630733	36.69539957	26.89592592	35.36359242	34.46882547	21.89883312	2L	-0.031246175	0.862644701	0.995787491
**ssp3**	FBgn0032723	2.44989135	3.45690439	5.836862383	4.607493671	3.83374593	3.643537623	6.273732096	4.27694608	2L	0.17710762	0.476148535	0.973549494
**CG10428**	FBgn0032724	24.67106566	67.46350267	56.93452138	74.63055153	42.21715044	50.7413942	62.83064304	42.04033468	2L	-0.142837287	0.591426549	0.988631016
**Nedd8**	FBgn0032725	95.26964683	86.19488349	109.7454159	88.70122781	91.26178668	85.29917777	104.2337932	79.6579937	2L	-0.046286308	0.791540636	0.995787491
**CG10621**	FBgn0032726	3.153293437	1.632471413	1.511080671	0.090762777	1.194722817	0.770753353	0.794480919	2.451069957	2L	-0.274684201	0.757017669	0.995787491
**CG10623**	FBgn0032727	13.06373541	16.83140471	8.612076057	22.40179089	7.604789005	13.03040473	9.29743466	12.434319	2L	-0.495705217	0.151657338	0.812282941
**Tango6**	FBgn0032728	33.09305958	22.01806789	22.03024617	11.80739554	17.3917514	28.12658997	20.86396726	18.51494463	2L	-0.044734006	0.870192285	0.995787491
**L2HGDH**	FBgn0032729	64.77845173	53.73657019	51.09995499	63.50436747	60.82752239	57.38042313	57.96393629	41.05830509	2L	-0.076104423	0.648373389	0.992932477
**CG10431**	FBgn0032730	1.245588807	4.763605106	5.620512795	1.33585997	3.732687684	0.845520134	1.461660951	1.081715441	2L	-0.810909585	0.204331929	0.853973653
**Swip-1**	FBgn0032731	19.15877393	30.37975614	37.02813318	38.41982494	26.19218299	25.35816171	31.10397681	27.62756204	2L	-0.146602303	0.42803713	0.963202327
**EMC5**	FBgn0032732	114.1028813	121.7734725	140.763279	116.5079878	142.8362758	153.9036343	144.8312296	115.1241661	2L	0.207020046	0.21015051	0.856520545
**CG15170**	FBgn0032733	2.159946075	4.80602907	1.283265073	1.130493114	3.501372963	6.566299318	2.923709997	4.306631209	2L	0.911976354	0.123650362	0.780439453
**CG15172**	FBgn0032740	0.916222289	0.94574422	2.564702878	1.150228033	1.526784053	0.797361949	0	0.153015646	2L	-1.104990943	0.377669872	NA
**Sidpn**	FBgn0032741	109.1051322	95.40864846	101.5080438	101.7629595	126.7244026	94.88353956	114.657086	98.19352958	2L	0.121441824	0.357697641	0.938070952
**Ttc19**	FBgn0032744	102.3523721	98.72150743	90.54159068	131.0841267	85.40627748	110.8755765	110.2770177	112.3907038	2L	0.014535647	0.928224975	0.996479182
**CG10470**	FBgn0032746	153.2971427	129.8259258	140.1764597	124.7173791	129.6616948	138.1603094	118.686649	135.6890863	2L	-0.041477439	0.778579096	0.995787491
**CG10492**	FBgn0032748	6.282242568	13.47830076	17.10140779	12.51791495	15.09943182	11.13979758	12.53206546	17.0491627	2L	0.219705699	0.422636554	0.960985399
**Phlpp**	FBgn0032749	0.086553331	1.01626749	0.419954346	0.201795832	0.078125473	0.938422442	0.883197624	1.033534111	2L	0.817186238	0.419253916	NA
**CG10495**	FBgn0032750	18.58267045	34.11420344	43.90654233	22.57969855	47.75183391	34.63978604	33.19879539	26.94027047	2L	0.299559309	0.296639293	0.906715248
**CG10700**	FBgn0032754	0.444956741	0.663424455	0	0.806866583	1.338768266	0.932227773	0	0.107338031	2L	0.34220864	0.82536502	NA
**CG17568**	FBgn0032763	61.38231028	83.76676404	52.07638167	65.76467271	67.01491434	71.1677861	48.65076927	65.38155648	2L	-0.028617363	0.872532736	0.995787491
**CG17350**	FBgn0032772	0.040045421	0.33585288	1.651543758	0	0.867506982	1.057128193	0.817253835	1.391077056	2L	1.107147822	0.36833263	NA
**fon**	FBgn0032773	1.666278609	0.757952166	0.068514723	1.646129597	1.01968134	0.612407081	0	2.146060656	2L	-0.111206752	0.917291349	0.996479182
**CG17549**	FBgn0032774	1.975755025	4.322676369	3.403834094	4.739917258	1.447374459	0.755890341	2.483573616	3.04620008	2L	-0.869818824	0.111198905	0.758410631
**CG17544**	FBgn0032775	18.28761434	18.45860327	15.80601683	25.73129757	16.97854164	9.450124799	16.94708833	15.76588603	2L	-0.372933682	0.084606864	0.69799888
**CG13085**	FBgn0032780	0.598807808	0.420453248	0.30405336	0.032467392	0.603349711	0.879651326	0.319724285	0.151170602	2L	0.538509149	0.562342211	0.984934163
**RtcB**	FBgn0032781	52.18256331	69.7811281	81.20695193	91.49855525	47.83944561	91.77469267	52.38870482	70.69076476	2L	-0.137228825	0.532404182	0.984760433
**Rab9**	FBgn0032782	28.76669621	22.23598955	20.83980057	14.58816475	15.9862126	20.69699743	15.82669461	14.50571467	2L	-0.341944719	0.133431682	0.794759341
**CG10237**	FBgn0032783	1.234344638	0.971839169	0.397229973	0.792870995	0.341067617	0.581866292	0.706882379	1.668086374	2L	-0.024043765	0.970040219	0.997604378
**CG10195**	FBgn0032787	2.416764638	1.997516577	2.634118208	2.613145866	0.821991806	1.882248167	1.608317873	1.36878776	2L	-0.735978119	0.193732896	0.844272804
**CG13084**	FBgn0032788	0.162206453	0.72554285	0.393510591	1.638772363	0.488040367	1.427321025	0.965515006	0.586942039	2L	0.296161288	0.791500572	NA
**CG13083**	FBgn0032789	2.380018407	1.81461227	2.974428933	1.17704434	2.343569028	0.203987892	2.759762767	2.035579503	2L	-0.16039655	0.860279184	0.995787491
**CG10194**	FBgn0032790	58.65919167	52.82342273	63.09950634	38.48641736	73.01578103	80.1700151	52.05575026	46.68343811	2L	0.270412483	0.212677142	0.856573136
**CG18094**	FBgn0032791	12.47009323	13.32935374	8.007954419	8.55104498	5.296873964	8.75991478	9.66819361	12.4751486	2L	-0.202817252	0.604018178	0.989286463
**CG10189**	FBgn0032793	40.504547	42.60994343	33.88846566	40.61577758	42.93794948	31.52624832	38.52440944	40.51522752	2L	-0.009224675	0.961810294	0.997604378
**cyst**	FBgn0032796	141.9251205	154.8493975	125.4140106	142.9556092	131.6080215	143.9425803	127.8565595	149.0058225	2L	-0.002516228	0.980319668	0.997881056
**Hasp**	FBgn0032797	374.8908217	538.2648609	453.377426	535.0584509	426.766937	455.001632	486.8942875	509.1489649	2L	0.01486488	0.902373026	0.996479182
**CG10132**	FBgn0032798	317.6220116	286.2402809	270.2682268	280.4838854	228.5725167	276.5016544	263.1980214	244.8787709	2L	-0.160562043	0.097684197	0.730787365
**CG10166**	FBgn0032799	69.92522186	77.4672355	63.44536716	65.29769597	73.66378878	58.49320054	81.14667763	47.92010986	2L	-0.051377739	0.806579433	0.995787491
**CG10137**	FBgn0032800	102.8445561	103.910343	99.61296748	104.6124218	98.25449631	107.7346882	100.2217846	96.01346784	2L	-0.001886507	0.982802016	0.997881056
**CG13082**	FBgn0032803	0.197608165	2.651679801	1.278385739	3.992871979	0.118911128	3.291366619	1.176239573	3.146187869	2L	-0.030354924	0.976839283	0.997757033
**CG13078**	FBgn0032809	0.141072061	0.709885777	1.026716559	0.6578084	1.78269859	3.657551637	0.359877859	0.306280504	2L	1.334537739	0.267167392	NA
**CG13077**	FBgn0032810	0.289499484	2.816449677	1.404643421	0.26998286	0.104524153	1.364691727	1.624742688	0.251412071	2L	-0.470219231	0.697040351	NA
**CG10268**	FBgn0032811	26.71532537	23.79151898	20.71279596	26.96909862	26.97288153	27.52398795	21.4290387	24.9645392	2L	0.066673346	0.779179576	0.995787491
**Hakai**	FBgn0032812	124.6707295	119.2282773	114.7727953	130.5823864	115.4705604	123.5473157	122.3495815	128.7710486	2L	0.031395365	0.787793549	0.995787491
**PCNA2**	FBgn0032813	39.41241511	35.35762419	32.591579	36.85925374	40.19746362	40.16679501	39.00496242	31.42327741	2L	0.090514202	0.660072261	0.995750687
**CG10366**	FBgn0032814	100.1707603	102.394826	72.52266351	97.8138717	67.86589738	93.84781383	77.26259378	95.94705584	2L	-0.127173844	0.428089923	0.963202327
**CG10462**	FBgn0032815	2.931694468	4.209683333	1.562539748	2.278378429	1.082548616	3.078072687	0.849865406	3.278929129	2L	-0.373875592	0.492291131	0.973549494
**Nf-YB**	FBgn0032816	57.33065876	83.77830781	98.6264189	69.25008626	45.53248331	67.19219191	77.46474333	56.9212342	2L	-0.288271621	0.193599715	0.844272804
**CG10631**	FBgn0032817	29.32366107	24.12388773	32.92164303	29.97597438	33.07851478	30.46353862	30.83816291	35.99060088	2L	0.194786579	0.130251188	0.790690436
**CG10628**	FBgn0032818	50.39032247	73.81153956	58.43890792	79.15739787	65.9713731	67.11876656	50.80582245	61.08395774	2L	-0.062191467	0.743574656	0.995787491
**CG10463**	FBgn0032819	12.04515708	20.89361964	13.06485045	28.94544283	27.08797756	14.57301637	18.1428315	13.56646743	2L	0.002788384	0.993201082	0.998777916
**fbp**	FBgn0032820	0.509718982	0	0.41219038	2.218329153	0.429414254	1.601866475	0	1.623082404	2L	0.237054186	0.854974653	NA
**CdGAPr**	FBgn0032821	13.61813199	13.71062905	21.49778346	17.14556246	13.76311711	16.50740356	16.9283515	19.86929941	2L	0.056338388	0.737375385	0.995787491
**CG10466**	FBgn0032822	33.47194743	35.35817507	49.27936618	31.87071998	28.36767023	36.85682123	35.84977966	33.83902654	2L	-0.118450904	0.665378797	0.995787491
**COX4**	FBgn0032833	2302.558986	2309.8094	2460.533828	2540.023504	2617.200068	2442.887986	2508.246997	2684.11321	2L	0.123972736	0.168233306	0.830652748
**CG13965**	FBgn0032834	1.604041586	2.690554736	1.768810234	3.059805741	2.105968127	11.27336454	4.091944548	6.331859577	2L	1.422147835	0.090271419	0.717658936
**CG16772**	FBgn0032835	2.602264891	3.491947434	1.262611666	4.853666103	0.268443148	8.832237998	0.948347663	5.972601279	2L	0.41307625	0.645004631	0.99279236
**sNPF**	FBgn0032840	468.0027216	418.761615	418.23132	401.857713	364.4633598	418.8443368	383.5557434	366.6759456	2L	-0.126090796	0.148027467	0.808004944
**CG10747**	FBgn0032845	43.20793328	53.97453963	66.89450199	62.99053362	50.96578182	63.5369858	73.82709298	84.91090267	2L	0.301757724	0.133564542	0.794759341
**Pyroxd1**	FBgn0032846	43.38479397	55.67896964	49.44344842	36.81390041	55.37089409	55.26650186	54.17953835	55.74875992	2L	0.284413774	0.088454803	0.709409025
**Taf13**	FBgn0032847	110.4069623	100.0419669	114.8699301	102.4331289	109.7248138	98.08163175	128.0493659	78.33616917	2L	-0.018231624	0.922749771	0.996479182
**mRpS18B**	FBgn0032849	57.93298248	96.85997049	77.5496944	65.78582344	80.21482171	76.09521067	70.30704723	44.16472055	2L	-0.103973599	0.650911469	0.993444251
**Kua**	FBgn0032850	3.034778023	2.883732294	2.176057878	5.019053118	1.214457371	1.071177014	2.173801401	1.460567885	2L	-1.129705304	0.006225118	0.227193262
**CG16798**	FBgn0032856	2.078837895	2.270577913	3.518533771	1.502862727	2.356431407	8.021980417	5.549818744	1.539437041	2L	0.923734309	0.051813673	0.600577406
**CG10947**	FBgn0032857	54.78845483	45.51666896	41.83892306	48.66114624	48.76468513	60.64610668	43.32882103	48.64083584	2L	0.102883568	0.394950327	0.952272708
**CG10949**	FBgn0032858	42.44045847	38.96601678	40.46145808	39.57933249	40.50337898	63.64578107	37.35511555	29.52859993	2L	0.108588568	0.623871329	0.990047511
**Arpc2**	FBgn0032859	65.42071518	58.87041925	50.68574612	51.89061516	47.72240187	63.68905192	41.97224729	53.95985277	2L	-0.103092852	0.470607273	0.973549494
**Cdc23**	FBgn0032863	17.05512168	12.41690447	5.634108911	13.45186487	13.62571083	14.43730646	13.33010507	5.436061932	2L	-0.034922584	0.925869508	0.996479182
**CG2493**	FBgn0032864	8.386919966	21.8052166	26.90266169	4.67118273	5.467417254	16.64891606	26.86287857	10.01482879	2L	-0.024691651	0.960635738	0.997604378
**CG2608**	FBgn0032870	49.67075793	68.87060763	66.45053579	72.28128662	61.88527155	61.02474224	83.48196811	63.93188044	2L	0.104672135	0.563261628	0.984934163
**CG2611**	FBgn0032871	50.45604436	30.94327689	53.34871374	22.78670566	35.94923238	33.28723722	34.28229988	39.78622626	2L	-0.108400606	0.731775821	0.995787491
**CG2614**	FBgn0032873	12.6576806	24.50987434	20.62209731	8.101032966	16.52170465	17.72488204	16.46157635	9.025621327	2L	-0.10369012	0.747893661	0.995787491
**Cen**	FBgn0032876	250.1712613	274.7346137	276.1564502	281.3452151	219.236142	266.5987854	259.8699553	243.9909028	2L	-0.099244397	0.308976481	0.912131082
**CG2617**	FBgn0032877	21.01917777	18.20445123	18.99710607	21.94033586	19.0965048	11.85119507	16.2963882	3.131784947	2L	-0.64384435	0.133704969	0.794759341
**TM9SF2**	FBgn0032880	119.703612	144.1330103	121.1256531	121.4899289	119.615798	130.9798939	144.7369032	145.7882611	2L	0.127905602	0.256244333	0.88674759
**Amacr**	FBgn0032881	36.13710746	58.3431592	58.24627985	54.54152665	64.56992576	72.26057241	48.21340207	69.50194692	2L	0.333419176	0.101893736	0.739991904
**Ns4**	FBgn0032882	106.493933	101.349579	70.01273983	80.63058544	113.6700888	128.5847993	87.8181252	90.10128662	2L	0.254849492	0.129706119	0.789029778
**Rhau**	FBgn0032883	51.37077399	31.01168462	40.28711985	29.46792364	41.45637831	42.34208105	33.2809888	45.38526735	2L	0.120956365	0.53469839	0.984760433
**Pomp**	FBgn0032884	211.4998911	250.0760542	208.8231357	228.5560305	220.0214583	215.824047	214.6823667	209.8352796	2L	-0.032163279	0.762176726	0.995787491
**CG9328**	FBgn0032886	127.180148	97.23304693	89.70180028	102.3955815	107.6771896	112.4785097	96.86501568	93.67726355	2L	0.005763398	0.962104666	0.997604378
**CG9331**	FBgn0032889	4.387295192	5.290256729	5.685091379	5.381424011	4.389504153	4.119703402	5.393775563	3.09856684	2L	-0.255802923	0.367106459	0.940794966
**Oseg5**	FBgn0032891	0.028959627	0.242878562	0.702556522	0.60766453	2.038902442	0.02730296	0.591013054	0.188622096	2L	0.913192264	0.476409038	NA
**twit**	FBgn0032895	2.10099383	0.640749529	0.37068958	0	0.586164999	0.01800731	0	0.082935493	2L	-2.152869132	0.122355109	NA
**CG9336**	FBgn0032897	2.502553885	2.631383416	1.449829449	0	1.213724192	0.598651832	0.952846078	4.297964709	2L	0.13985937	0.883683136	0.995787491
**CG9338**	FBgn0032899	1.007188903	2.15546446	1.263841153	0	0.501581823	1.211522317	0	0	2L	-1.320303846	0.354921361	NA
**CG14401**	FBgn0032900	2.531216997	2.462541623	0.122813943	2.006488628	2.193357804	0.143184955	0.774862673	2.52793295	2L	-0.311241449	0.757975787	0.995787491
**CG9272**	FBgn0032907	7.700648704	3.677033273	4.227238371	4.324631346	1.725021589	1.112865809	2.294250544	4.881407643	2L	-0.979933066	0.113752494	0.758410631
**CG9270**	FBgn0032908	0.446389928	0.935945603	0.135366967	0.867284427	0.369347105	0.070142283	0.759166865	1.85754489	2L	0.395366615	0.677734144	0.995787491
**CG9265**	FBgn0032910	17.97535788	14.8491474	15.03355358	16.98019191	19.7443671	18.93003501	16.92954558	13.64484529	2L	0.121198147	0.40298058	0.955187758
**tadr**	FBgn0032911	80.92855564	53.96925056	56.29138353	65.03929152	65.58712963	55.98437112	62.40070536	55.50455639	2L	-0.071294319	0.633830885	0.991585536

**CG9253**	FBgn0032919	38.90365649	72.30392164	59.08324437	85.01375764	45.35246855	41.06587345	51.40865544	44.53705173	2L	-0.451403653	0.038849257	0.525600179
**Mpp6**	FBgn0032921	17.1128388	20.59227719	14.59059528	10.11904261	12.87210834	15.31549468	12.4955157	15.88449182	2L	-0.112214246	0.716983096	0.995787491
**Coq3**	FBgn0032922	25.51488221	36.50749312	30.32503521	25.37197155	37.03455793	39.72239951	42.39226762	33.20517464	2L	0.405219313	0.066177792	0.648853787
**Pld3**	FBgn0032923	10.23746775	12.57860512	15.53524693	15.61751043	12.24478088	9.254627738	14.61636224	16.00673159	2L	-0.01533495	0.958056266	0.997604378
**Nbr**	FBgn0032924	103.5737584	122.2525619	102.3622843	107.6171317	89.33496142	104.6236144	88.58466304	83.46099926	2L	-0.22144343	0.058504777	0.625114381
**CG9246**	FBgn0032925	33.35279936	28.89187768	33.89268642	40.48838908	21.19812998	25.20920523	31.30863901	19.81155868	2L	-0.460368563	0.024582563	0.440903252
**Atg18b**	FBgn0032935	73.26328704	64.45590764	63.62622668	54.12184395	64.78379154	73.81359167	75.31558735	74.42467747	2L	0.203960396	0.079997299	0.694476551
**CG8671**	FBgn0032938	32.49166823	25.11112072	27.7156529	22.5935661	25.8747713	31.49346862	27.59555908	28.53933905	2L	0.100272678	0.428601884	0.96338052
**Mondo**	FBgn0032940	11.27352438	14.10352607	12.07055819	16.03143173	11.36297716	12.41707794	13.83566133	12.43405322	2L	-0.06496974	0.6368532	0.991585536
**Tsp39D**	FBgn0032943	37.59120292	37.42832583	41.55326697	37.43649036	36.19675104	40.43712622	35.26966314	34.02188226	2L	-0.048013008	0.618906505	0.990047511
**CG8665**	FBgn0032945	0.627112003	1.116293986	0.496772236	0.119354125	0.046208077	0.108594618	0.097945481	0.222288687	2L	-2.285744594	0.014854164	0.35986804
**nrv3**	FBgn0032946	1000.821509	694.4225011	758.5767989	674.1933562	811.5317109	962.9188754	671.7044182	728.9561539	2L	0.046157773	0.73629778	0.995787491
**Lamp1**	FBgn0032949	285.6956137	222.5119193	250.6452679	183.2423245	218.7638184	231.4229091	221.6789474	196.2083708	2L	-0.08950339	0.492633732	0.973549494
**CG2201**	FBgn0032955	4.547281986	5.679999285	8.919200459	6.541546515	8.113740475	5.506126476	6.641515196	9.013255514	2L	0.227945456	0.349544087	0.932328657
**Cul2**	FBgn0032956	171.0199857	151.7303729	147.428747	157.594484	130.879151	175.5519199	138.1532163	138.1150426	2L	-0.081405036	0.466459982	0.971928234
**CG2225**	FBgn0032957	20.03252052	50.2544025	45.30664868	39.49477035	33.37235958	34.01837986	47.8027617	49.85052191	2L	0.130583743	0.678083247	0.995787491
**CG1416**	FBgn0032961	112.5457807	110.8382572	88.41116956	65.44709578	92.25196904	94.30865118	71.61039812	54.40422691	2L	-0.243000668	0.201902746	0.849268738
**CG11634**	FBgn0032968	0.559660633	0.613799515	1.253288205	1.605941781	0.738318722	0.365292434	0	0.747738487	2L	-1.078050284	0.238160917	0.873280735
**CG2528**	FBgn0032969	0.019053728	0.055929921	0.161784271	0.011105762	0.18918274	0.233528594	0.048606468	0.113760368	2L	1.313297766	0.209354314	NA
**CG6675**	FBgn0032973	4.199672516	1.858928727	0.141504819	2.039870494	2.737758566	0.549920222	13.98701045	0	2L	1.080907079	0.336261522	0.924219112
**CG3651**	FBgn0032974	40.27190231	41.83006876	37.4386991	41.10903529	50.79889704	43.98913582	39.70334065	56.53092872	2L	0.28161521	0.045890899	0.56430181
**Clamp**	FBgn0032979	196.0613986	203.3550038	178.3351373	243.894139	177.3816717	161.2745877	201.4054389	169.3220272	2L	-0.18206057	0.155384404	0.818884898
**CG3635**	FBgn0032981	0.949407077	0.83013151	0.980105671	0.117739727	0.255265143	0.199968075	0.206124072	0.635917754	2L	-1.117735127	0.134942629	0.795862348
**CG3262**	FBgn0032986	42.62323174	52.79909308	59.63521693	47.51698328	48.26804799	55.19653152	53.96020427	53.7417477	2L	0.093165649	0.442694786	0.967112302
**RpL21**	FBgn0032987	1112.092109	1357.243998	1521.275892	1502.18039	1301.49864	1289.187549	1508.288618	1565.740413	2L	0.077826865	0.533723131	0.984760433
**Tif-IA**	FBgn0032988	43.69138369	34.1736394	39.47543927	29.26596164	31.38926152	40.30559718	34.34129321	29.31649474	2L	-0.088773111	0.546261698	0.984934163
**CG17486**	FBgn0032997	154.7406374	113.2181851	172.9560894	80.64446413	119.769491	160.0652149	134.4418254	115.1492387	2R	0.049507521	0.802667652	0.995787491
**CG14464**	FBgn0033000	203.078124	167.3922309	201.3594576	136.1373973	159.7496964	155.1051831	155.5488182	140.7967594	2R	-0.182566591	0.212859968	0.856573136
**CG3107**	FBgn0033005	156.8118739	140.9721793	185.9474998	113.7604423	130.9830206	192.6482496	152.2105361	120.2408759	2R	0.023551445	0.880610718	0.995787491
**Atf6**	FBgn0033010	27.89620841	23.28002284	34.02659347	19.96704124	17.53544667	28.98092736	19.4223456	18.7437068	2R	-0.285865912	0.119062892	0.776424209
**d4**	FBgn0033015	17.71923258	17.88165928	17.34587362	16.6864177	18.46244784	18.2080042	16.3746422	15.39617841	2R	0.004754003	0.960306017	0.997604378
**CG10465**	FBgn0033017	198.0325601	218.4421522	290.1781903	215.8907778	213.9902152	251.9583857	231.7646916	204.9798312	2R	0.000283746	0.998337979	0.999757791
**CG10395**	FBgn0033019	93.9185125	68.70902924	95.86075936	55.78875244	63.90697629	87.39869208	85.87335668	60.63083786	2R	-0.05105005	0.801796402	0.995787491
**CG10417**	FBgn0033021	341.9437198	259.3527879	347.8247638	303.976749	284.2031708	340.2287795	284.3141507	259.1961446	2R	-0.07551542	0.537546984	0.984934163
**hrm**	FBgn0033028	37.50060517	43.1840818	36.55283508	35.58682641	34.84775546	41.53840879	39.17950939	38.21176158	2R	0.039487902	0.702639943	0.995787491
**Not3**	FBgn0033029	159.3973966	177.1147656	197.9877875	174.6890503	157.7446135	177.6556971	170.9431447	183.779716	2R	-0.007656294	0.93804147	0.997038394
**CG8245**	FBgn0033031	889.7417547	883.8849959	886.288464	813.4578505	856.9519978	772.6668997	790.1256469	702.3659674	2R	-0.123151774	0.188710958	0.843186953
**kune**	FBgn0033032	125.3060928	133.2167583	144.2567467	124.5245589	110.7423042	120.9139035	116.8464619	101.0424878	2R	-0.198939423	0.1334828	0.794759341
**scaf**	FBgn0033033	1.180132379	1.115898278	0.308753251	0.25626103	0.563940373	1.183523955	0.280393716	0.653103849	2R	-0.073703404	0.90016975	0.996479182
**CG7791**	FBgn0033038	123.2701165	132.5103952	119.0643417	112.7006673	118.3777543	132.0139611	142.8441582	118.7596769	2R	0.100264793	0.377731668	0.943615128
**Or42b**	FBgn0033043	1.249617819	2.537334079	3.350666493	3.680130823	2.730802635	2.480277993	1.006674285	5.42607192	2R	0.156401464	0.829328786	0.995787491
**CG7881**	FBgn0033048	0.092133068	0.640238371	0.063861011	0.644414338	0.142563258	0.148907132	1.074438562	0.828693307	2R	0.647939079	0.510348883	0.980318812
**Pngl**	FBgn0033050	62.97111295	81.44298263	78.76495217	93.11705172	76.43841436	81.22113314	67.12671685	73.09763452	2R	-0.054757167	0.696760624	0.995787491
**Strica**	FBgn0033051	0.642069594	2.564242071	0.74173899	1.069257919	1.43507641	0.374733847	0.545977776	0.464664174	2R	-0.786582436	0.34632377	0.930285749
**SCAP**	FBgn0033052	42.71397027	51.92517944	43.97973962	48.67496018	49.8553861	43.64518089	40.98174498	49.52860062	2R	0.0072391	0.950470656	0.997604378
**CG14591**	FBgn0033054	156.5058277	138.8602535	137.9490344	157.2620452	134.0789839	157.6158751	143.7623178	128.1908968	2R	-0.040595624	0.705398556	0.995787491
**Tbce**	FBgn0033055	155.8202404	122.8871048	144.1219596	143.4057475	134.8289374	188.1408905	138.2848431	146.377649	2R	0.127123291	0.340447763	0.927274752
**CCHa2-R**	FBgn0033058	0.512938683	0.401511919	1.327340897	0.011389497	1.569766384	3.583212056	1.707303918	1.293940556	2R	1.882808254	0.056431953	0.621788268
**CG7849**	FBgn0033060	59.4857052	76.59173388	86.6939242	82.80177201	77.78584568	58.09314274	76.90632622	85.72682107	2R	0.002072135	0.991460024	0.998408391
**SmydA-5**	FBgn0033061	0.358853426	4.138243172	1.088216757	1.254979879	0.404889004	1.945367703	1.716455248	1.071268008	2R	-0.367133582	0.680696792	0.995787491
**Ars2**	FBgn0033062	198.6784567	208.0460092	196.7739991	176.8563891	210.6422511	212.5421022	200.6050864	157.5171836	2R	0.031330877	0.775180947	0.995787491
**Cyp6w1**	FBgn0033065	1.724775899	2.314455687	3.51479711	1.04552502	1.74365103	3.609960165	4.487939181	0	2R	0.224473684	0.791030208	0.995787491
**Fmo-2**	FBgn0033079	6.108525532	0.32527602	0.627267629	0.226060275	0.058346318	1.675922914	0.824496289	0.14034047	2R	-1.442197796	0.203592727	0.852205821
**geminin**	FBgn0033081	77.88749409	88.12458854	84.53693049	116.7758223	76.73237319	93.57687549	64.27717669	106.9143727	2R	-0.074245151	0.711929734	0.995787491
**CG15908**	FBgn0033085	104.4427486	93.6477776	126.5096397	88.26602889	135.0935575	100.690112	104.4698496	123.4518785	2R	0.200504447	0.317063479	0.91460807
**CG9410**	FBgn0033086	214.3225223	198.4616601	208.5366736	183.710504	219.2423925	261.1357856	229.5767596	193.4190497	2R	0.194202702	0.121229797	0.77836519
**Hsepi**	FBgn0033087	68.82624429	61.44798936	60.27168654	68.34073271	72.35306113	79.58223976	60.35681787	45.93293858	2R	0.021780426	0.893226357	0.996450075
**PGAP3**	FBgn0033088	137.6855572	135.6172545	111.0526321	80.11356858	121.1665221	93.15316413	99.03236008	88.28446187	2R	-0.178357398	0.330045817	0.921112231
**CG17266**	FBgn0033089	12.28501326	9.407263321	11.87811518	15.56632931	13.33869998	19.21979652	15.44256976	23.77278132	2R	0.57619285	0.108977062	0.758410631
**Rpp25**	FBgn0033092	6.25409709	6.778522157	8.616247997	6.661694715	8.061886466	7.667105706	7.238069386	8.415895851	2R	0.183031451	0.225072578	0.867200583
**CG3270**	FBgn0033093	42.00270079	25.96530797	25.47449157	41.72272393	13.2605873	22.30979704	20.24778935	19.69399329	2R	-0.819233566	0.00197968	0.101218706
**CG3420**	FBgn0033100	127.8876179	131.7635086	140.3966484	110.816579	144.2080705	122.6288967	157.5555516	121.5259314	2R	0.126633015	0.415838413	0.958846438
**CG9436**	FBgn0033101	32.59826449	53.67470715	34.69965514	14.4247985	12.61033334	16.37026337	34.62124511	16.89906209	2R	-0.714114982	0.060830379	0.628203686
**CG15237**	FBgn0033104	137.2455305	158.571309	160.948012	142.6496693	152.9029117	155.1826119	159.1437713	168.7817683	2R	0.118073733	0.39709542	0.952272708
**CG15236**	FBgn0033108	11.97451357	16.25144547	18.65249643	14.95830264	13.59858818	12.28539036	18.28858703	12.01198827	2R	-0.103773759	0.568402108	0.984934163
**Spn42Dc**	FBgn0033113	0.28729103	0	1.481048569	0.920986305	0.453804699	0	1.190939085	0.467801566	2R	-0.313141313	0.827826201	NA
**CG3358**	FBgn0033117	105.1278388	100.0204686	83.35475789	94.31953975	101.5442453	87.51489516	65.59677708	112.4968487	2R	-0.029659863	0.867920884	0.995787491
**Cyp6u1**	FBgn0033121	23.18395948	27.90717962	29.05822468	28.39257367	26.06135558	35.52212394	28.58689181	38.16982875	2R	0.275022512	0.122088327	0.780439453
**CG17002**	FBgn0033122	109.8748986	98.10478429	94.81240634	107.3918482	104.6075113	102.0009371	96.01501284	101.1153328	2R	0.005393917	0.961936736	0.997604378
**Tsp42Ec**	FBgn0033124	0.754929664	0.933054762	0	0.277908962	0.789013517	0.711743076	0	0.086264325	2R	-0.281381935	0.855718982	NA
**Tsp42Ef**	FBgn0033127	2.678624133	8.182018992	8.687207449	1.183284252	3.512174873	4.253289214	3.956079201	3.55054079	2R	-0.393561179	0.451104458	0.967112302
**Tsp42Eg**	FBgn0033128	0.180371173	0.403396453	0.097239545	0	0.217077464	0.264526641	0.051125638	0	2R	-0.310568474	0.857203244	NA
**CR12842**	FBgn0033131	151.5502248	89.34375024	143.9868505	116.7922924	140.800003	119.2066815	121.6439855	118.6709149	2R	0.022846034	0.909251636	0.996479182
**Tsp42Ej**	FBgn0033132	1055.977348	918.8234177	935.5233285	1025.808614	1160.732683	1073.873571	1051.897766	1153.688445	2R	0.203369726	0.036990146	0.521617394
**Tsp42Ek**	FBgn0033133	54.35779875	36.00418085	39.38745982	23.49823552	35.32366793	39.24125216	43.7842051	26.09348851	2R	-0.06127591	0.804836218	0.995787491
**Tsp42El**	FBgn0033134	1.470226632	2.361158544	1.821318793	0.364657239	0.677651716	0.943741745	1.356592667	1.697871338	2R	-0.331953465	0.647776401	0.992932477
**Tsp42En**	FBgn0033135	306.9914215	345.0196709	266.1027239	353.8965394	285.0026936	323.7360705	291.4544474	302.8024034	2R	-0.051397548	0.65735872	0.995750687
**Tsp42Ep**	FBgn0033137	3.169296293	1.941513885	1.069727805	1.28506	0.796019769	0.805459033	2.530938657	0.777833593	2R	-0.603277366	0.366606964	0.940794966
**Tsp42Er**	FBgn0033139	1.819286373	1.101965825	0.612994912	2.356443161	0.182459899	1.04818516	1.160259656	0	2R	-1.289363778	0.250506419	NA
**Gadd45**	FBgn0033153	13.21884873	13.03136149	6.470009482	13.24686984	11.19905641	8.199111738	10.94471915	0.503497268	2R	-0.552813688	0.350420421	0.932790554
**CG1850**	FBgn0033154	0.07212177	0.544383681	0.787348835	0.084074596	0.260396559	1.563907175	0.735936738	1.722413425	2R	1.597378091	0.146702761	NA
**Br140**	FBgn0033155	32.05440055	56.06107458	40.3245014	48.4936669	27.18862327	30.19265901	37.51019404	25.95214701	2R	-0.516855788	0.003442117	0.153404285
**CG12164**	FBgn0033158	0.041246784	0.553485546	1.601025949	0.192330647	0.595688127	1.43882698	0.210442862	0.98505644	2R	0.509469276	0.671115317	NA
**Dscam1**	FBgn0033159	20.86419954	24.51136369	24.12565919	21.34216298	21.85120927	23.37500049	19.07604104	25.06240582	2R	0.008615854	0.935062776	0.996479182
**Dhx15**	FBgn0033160	102.1083699	121.2769651	97.50773913	113.292578	103.9872546	97.0335378	82.42985479	113.9809003	2R	-0.095342849	0.461822813	0.971606621
**Eaf**	FBgn0033166	37.23265824	42.1207092	45.70893324	48.40178078	37.99079913	45.50325377	45.58700042	44.06094536	2R	0.027825406	0.813704761	0.995787491
**CG11123**	FBgn0033169	40.94391602	30.73826048	27.48434002	26.87686181	23.39422281	37.02762509	33.76840149	45.91368245	2R	0.177776443	0.445836286	0.967112302
**sPLA2**	FBgn0033170	28.06323223	13.88024499	30.76737951	36.908311	49.1999423	18.57137122	34.18877033	23.62907971	2R	0.225487609	0.547626867	0.984934163
**CG11141**	FBgn0033177	73.33366835	86.54738603	60.88807411	84.80692851	73.88499676	88.79492153	73.43661775	58.54580728	2R	-0.025212414	0.866901439	0.995787491
**CG11127**	FBgn0033178	45.32417978	55.64713072	57.95352273	58.14662536	65.16217564	62.44505422	42.91161268	52.75211831	2R	0.075117405	0.683872197	0.995787491
**p47**	FBgn0033179	217.3665222	221.8154522	195.6509345	236.3976741	268.5472252	206.385912	247.2371228	190.2165537	2R	0.096738503	0.4766784	0.973549494
**CG1620**	FBgn0033183	125.6290194	108.421264	113.9941009	115.170628	96.76013071	137.8142457	113.9714239	122.1658513	2R	0.050030079	0.696741961	0.995787491
**mEFTu2**	FBgn0033184	122.4015209	156.8020014	155.1321967	151.1845001	126.632554	146.7259272	139.5822658	196.7910816	2R	0.092641152	0.548590775	0.984934163
**CG1603**	FBgn0033185	35.47807074	44.26089454	37.00484504	36.59498801	43.39461565	44.07370635	45.33951765	38.36524492	2R	0.190028102	0.195701599	0.844272804

**CG1602**	FBgn0033186	10.88644617	22.95191551	18.47681772	20.06429994	22.06087924	28.55979118	24.80779496	27.93284282	2R	0.55288634	0.028746556	0.469752543
**PIG-G**	FBgn0033187	34.29136925	37.02421135	36.66562991	35.98016282	28.91443866	30.17605007	30.4241912	40.31012094	2R	-0.11729151	0.465246442	0.971928234
**Drat**	FBgn0033188	389.3845424	354.76746	356.27668	383.5907656	421.2803129	407.8279037	407.9304416	425.7913084	2R	0.193832466	0.028527524	0.468008631
**Gpo2**	FBgn0033190	0.43709796	0.733170508	1.855688718	0.16984624	0.065756154	0.171705536	3.066377285	2.293368652	2R	0.863324053	0.439313822	0.967112302
**CG1598**	FBgn0033191	225.0654416	177.8412573	197.7906801	188.842683	208.8112711	179.6700926	219.9084789	194.7358223	2R	0.053255024	0.684441344	0.995787491
**Corin**	FBgn0033192	7.538678919	17.15477524	22.42680342	17.60935564	17.4599511	13.36208471	22.19279348	23.6577891	2R	0.285606622	0.436164505	0.966084844
**Vps13**	FBgn0033194	11.7127658	11.64121887	12.91177894	9.991040296	11.24325898	12.39140875	12.2227145	9.208869162	2R	-0.007841506	0.956325223	0.997604378
**CG1360**	FBgn0033195	12.9037455	16.79293153	8.005986457	4.063149576	10.04076199	9.333930577	7.28352116	12.88056366	2R	-0.045235831	0.908274145	0.996479182
**CG1358**	FBgn0033196	35.92243757	34.01861025	31.76050798	28.25324047	33.06062085	34.91046834	35.9591326	31.80090998	2R	0.092454519	0.367387902	0.940908571
**CG17985**	FBgn0033199	21.50733159	26.07869752	26.05966903	34.10639057	29.47052393	28.02798449	25.59997073	26.08340414	2R	0.051365195	0.770164248	0.995787491
**CG2065**	FBgn0033204	0.058407448	0.293910902	1.416956546	3.540543933	1.792487032	0.660794225	0	0.253615953	2R	-0.926127193	0.501581955	NA
**CG2064**	FBgn0033205	6.060418507	5.945859575	10.40270573	5.598535654	4.541384734	6.737883904	5.396505086	4.468662878	2R	-0.367276808	0.390325252	0.948664524
**DCTN4-p62**	FBgn0033206	167.4003377	156.5476839	177.8062472	146.6129716	153.997589	150.12081	176.920886	139.5903062	2R	-0.033103298	0.788332833	0.995787491
**CG12826**	FBgn0033207	0.446485802	2.808439866	2.43712874	3.64337694	1.20903196	0.315708065	0.854246156	2.665745521	2R	-0.847395608	0.450682026	NA
**mRpL52**	FBgn0033208	253.2212336	196.6442847	238.9159857	188.5633453	196.2086152	220.8152412	232.0295272	234.8625664	2R	0.038565759	0.799527059	0.995787491
**CG12107**	FBgn0033209	186.9130564	156.2822879	218.8902664	185.1182474	218.7782218	206.3964556	202.0666827	233.6268728	2R	0.235320248	0.101364686	0.739731427
**U2A**	FBgn0033210	66.78266739	78.74715601	74.17753349	69.87372391	101.3265505	62.14565937	76.67485693	71.27331096	2R	0.13859832	0.479359843	0.973549494
**LRR**	FBgn0033212	16.19879721	20.33617572	21.5848003	13.58235208	14.32780187	16.85703935	14.9898897	17.50885742	2R	-0.136671961	0.343005905	0.928865024
**CG1941**	FBgn0033214	0.082180499	0.551385031	1.196212462	0.383201478	0.445070589	2.014461078	0.733754762	0.53526441	2R	0.820927815	0.451367769	NA
**CG1946**	FBgn0033216	0.046619077	0.312787849	0.565486157	0.760834366	0.58911568	0.483473995	1.308188946	0.404857329	2R	0.783514532	0.523787744	NA
**CG12825**	FBgn0033221	0.11076502	3.530062863	2.149715149	2.065955795	3.399307249	7.3100009	1.412819729	1.683367783	2R	0.869374329	0.400650028	NA
**Nop17l**	FBgn0033224	70.54746757	69.42784314	62.55258414	74.68439421	75.19631847	80.96318303	72.36603932	66.39056529	2R	0.11721242	0.284357187	0.901780531
**TTLL12**	FBgn0033225	95.36845617	86.2844431	95.63271069	83.86989982	98.28854103	101.3706383	65.61307818	90.86186188	2R	0.009533101	0.950475028	0.997604378
**puml**	FBgn0033226	88.15773827	119.1747927	89.07116194	114.247113	116.3584467	124.1378512	126.4139167	115.848187	2R	0.264772409	0.03937529	0.529044592
**CG12159**	FBgn0033232	374.8283697	391.0112179	329.0323487	392.4817675	396.7935682	351.472357	348.7318144	392.8314483	2R	0.033152848	0.76489779	0.995787491
**Kdm4A**	FBgn0033233	87.75332433	96.64112736	99.13266183	97.09525458	102.6673737	95.2429271	100.2480188	101.3787084	2R	0.101957665	0.373470919	0.943282319
**MFS12**	FBgn0033234	0	1.555566978	0.449967143	0	0.191334516	1.673732409	0.405564403	2.128504279	2R	1.193466386	0.356009866	0.937787223
**CG8728**	FBgn0033235	260.9336119	269.4456519	228.549481	207.0677257	302.2328914	261.2639661	218.4093605	230.7202317	2R	0.0987685	0.44268358	0.967112302
**CG14764**	FBgn0033236	43.48124521	38.97075769	39.403095	37.94079523	40.16827187	44.7645551	40.57172333	35.87458962	2R	0.041904766	0.683925263	0.995787491
**CG2906**	FBgn0033240	15.72631646	17.7469195	18.77629054	23.80365987	34.70718552	25.8884615	31.40139575	18.59111014	2R	0.572087248	0.020720836	0.408244558
**CG2915**	FBgn0033241	45.51290562	34.79565865	61.36675788	76.28867434	39.87041456	40.74124111	37.88669896	46.91004396	2R	-0.369268829	0.113042623	0.758410631
**CG8726**	FBgn0033244	364.8296113	384.4520744	328.6540731	382.5736567	349.3459461	381.2703786	358.0842295	339.3167382	2R	-0.003421785	0.969856761	0.997604378
**ACC**	FBgn0033246	33.75510153	30.61838251	38.29241431	44.34485582	32.53888076	32.36248123	36.49340347	46.69881125	2R	0.04175741	0.786363857	0.995787491
**Dic3**	FBgn0033248	2.813242166	0.869256145	1.975624969	0.345208853	3.875791342	0.628178099	0.188858979	0.32146364	2R	-0.232813046	0.844096903	NA
**CG11191**	FBgn0033249	3847.710008	3383.205865	2878.458033	3629.72178	3766.265595	3402.197171	3334.944848	3154.960522	2R	0.019909491	0.858294604	0.995787491
**CG14762**	FBgn0033250	1458.758876	1567.320098	1282.156818	1602.030069	1294.354427	1415.841079	1105.921274	1158.436109	2R	-0.221074121	0.034988526	0.514749796
**CG12769**	FBgn0033252	34.9391543	43.96223464	46.67752893	54.34618308	31.27594773	36.52841195	35.06183627	42.48502022	2R	-0.276427595	0.056113864	0.621053047
**sand**	FBgn0033257	0.197909168	0.110654955	0.24006244	0.038451481	0.446595964	0.186587554	0.294507814	0.035806594	2R	0.738879107	0.552592577	NA
**CG8712**	FBgn0033258	14.18041019	15.28464073	4.595143182	21.48374244	18.94531168	25.00089433	11.75345774	23.22916782	2R	0.529829752	0.179047934	0.836045561
**Tmem63**	FBgn0033259	75.59367048	77.0975414	68.92323884	67.24138537	70.24046194	93.38729414	65.58182799	71.75419909	2R	0.087304988	0.498576971	0.976006682
**Cul4**	FBgn0033260	85.25216193	83.6366034	79.22770413	81.51539652	71.74900555	77.43711494	99.64661414	104.177034	2R	0.130442797	0.359740453	0.93924716
**udd**	FBgn0033261	2.897199385	3.860825106	3.472611313	3.417403345	2.646102734	2.914998848	3.301037233	3.505543145	2R	-0.109841007	0.674953816	0.995787491
**Nup50**	FBgn0033264	83.79094386	71.42345005	71.60374769	75.80509832	68.44005842	92.97160139	80.64894862	84.0765734	2R	0.134391125	0.343331605	0.929001817
**coil**	FBgn0033265	26.0889099	22.29792775	25.74405115	20.45373904	25.72708786	21.52642156	18.47075898	16.55160976	2R	-0.171386712	0.42509918	0.9620897
**Socs44A**	FBgn0033266	143.0727466	176.6255064	156.6337755	191.7488995	195.0283243	210.4941869	179.0037394	216.9422994	2R	0.29396245	0.019805952	0.39822025
**Obp44a**	FBgn0033268	633.4857546	740.0270933	1160.236938	844.4743168	465.690249	569.1458168	1079.544315	556.4493497	2R	-0.304749587	0.37071996	0.943095283
**RagC-D**	FBgn0033272	176.899097	182.6297137	178.8625571	151.488109	187.5387169	225.7957029	179.8298398	142.5137818	2R	0.120840023	0.372881872	0.943282319
**Gasz**	FBgn0033273	35.3997968	28.56939345	42.59618898	47.06755829	27.0229843	41.27021553	24.76954756	28.54660185	2R	-0.309652693	0.162159507	0.824079516
**CG14757**	FBgn0033274	57.97666402	59.67473763	96.57109081	58.36904051	79.37341603	74.79795591	99.07157976	88.38191088	2R	0.361231837	0.075097004	0.677335968
**CG14760**	FBgn0033277	1.119512038	0.375564878	2.327931933	3.430417367	1.039374595	1.628438057	3.508676233	1.319449173	2R	0.073982576	0.919215159	0.996479182
**CG2121**	FBgn0033289	1.194734423	1.096527968	0.738274002	1.051123898	0.712151551	1.519561596	0.93446489	0.69741087	2R	-0.05552587	0.882375733	0.995787491
**Mal-A7**	FBgn0033296	0.086130175	0.19262838	1.393004016	0	0.414632107	0.21654134	0.439439852	0	2R	-0.586625345	0.708107347	NA
**Cyp6a14**	FBgn0033302	0.229832824	0.43370136	0	1.071693155	0.207453577	0.135428114	0.586308472	0.997976674	2R	0.179320824	0.886549719	NA
**Cyp6a15Psi**	FBgn0033303	3.112032778	0.994285411	0	1.727520778	1.471385345	2.09571821	1.512164281	0.643477261	2R	-0.03332894	0.976141982	NA
**Cyp6a13**	FBgn0033304	14.45389415	29.79854675	36.21252563	19.48398146	16.31997509	26.21347566	28.02286782	18.94255709	2R	-0.120977115	0.691950142	0.995787491
**CG14752**	FBgn0033307	5.133817454	3.204189238	1.882667488	0.278355863	3.232973125	0.112561203	0.152284635	0.777627413	2R	-1.263068832	0.253722132	0.884040848
**CG8736**	FBgn0033308	20.98483271	0.299566919	14.29782194	9.576876527	42.71912925	0.420944087	0.683396924	0.193872401	2R	-0.008078749	0.996205795	0.99922507
**Lnpk**	FBgn0033309	60.92246508	75.31753558	66.27323759	83.54804694	65.52818436	73.22371671	79.73954549	82.51831874	2R	0.105004291	0.46443403	0.971928234
**Cirl**	FBgn0033313	362.2743456	383.5815633	325.0761672	366.6687163	307.2609206	316.1262383	307.6770624	349.3780078	2R	-0.135874583	0.147958589	0.808004944
**beta3GalTII**	FBgn0033315	24.8346019	20.32027353	14.54778386	15.39317658	18.69856701	25.01290977	29.8225497	20.12070147	2R	0.337660521	0.232066239	0.867702781
**Gle1**	FBgn0033316	80.1768188	62.66662133	67.28721261	86.48569037	73.94311123	50.97695503	58.86600542	64.33069616	2R	-0.228862397	0.186345791	0.839751536
**CG8635**	FBgn0033317	188.7340008	171.4504264	164.7551489	164.8947621	182.563137	212.9291098	179.7937481	169.4113383	2R	0.137681075	0.223826308	0.86549481
**CG8586**	FBgn0033320	0.27625419	0	1.035746346	0.468419087	0	0.686642921	0.448464784	0	2R	-0.624580145	0.69963338	NA
**CG8738**	FBgn0033321	0.080388163	0.067419933	0	0.655976915	0.435363713	0.41684208	0	0.610855502	2R	0.909931629	0.608611119	NA
**CG12376**	FBgn0033323	1.331589078	2.669369266	1.024278412	0.227162181	0	0	0.414257603	0.916659536	2R	-1.954784935	0.128488389	NA
**CG14743**	FBgn0033326	0.79213505	0.404385563	0.167105094	0.240891267	1.119135746	0.357174824	0.175717698	0.149547697	2R	0.17345068	0.887262851	NA
**CG8272**	FBgn0033337	86.30032749	67.23962917	49.78165019	65.56376547	55.82712806	68.52947267	67.38805976	47.43760743	2R	-0.14552881	0.417409992	0.958846438
**Sec31**	FBgn0033339	156.6478861	165.8014789	152.9504708	159.8829642	141.3797126	175.8002146	159.5976752	180.2220644	2R	0.078511346	0.443887455	0.967112302
**CG13751**	FBgn0033340	135.916928	164.996838	154.2477792	178.3150623	153.0411519	156.159061	162.1977169	155.5465089	2R	0.017406971	0.92280591	0.996479182
**MrgBP**	FBgn0033341	297.328638	365.4697898	286.7597775	366.4505052	299.012982	316.4573355	349.8840199	360.0730524	2R	0.041646829	0.756321931	0.995787491
**CG8248**	FBgn0033347	918.2868493	832.7719102	717.5186146	835.7897952	832.4303576	916.0226774	731.1342096	711.1370021	2R	-0.023980829	0.831336915	0.995787491
**Spt**	FBgn0033348	19.79845615	20.05410657	18.60347053	22.34827936	18.31111744	27.07864708	28.36532667	16.95154744	2R	0.193045404	0.387692317	0.948247506
**CG8243**	FBgn0033349	207.1261758	237.4222822	232.452745	279.9345901	240.7495315	231.3736187	243.5705626	245.6259615	2R	0.037641793	0.743570036	0.995787491
**CG8237**	FBgn0033350	67.148681	103.9317813	64.47117413	74.05282115	90.17784891	102.5646326	90.27118357	88.088685	2R	0.293902061	0.094174378	0.728467429
**AIMP1**	FBgn0033351	68.11957357	71.64619266	64.33086621	62.56475388	69.36954775	67.66589389	83.44416342	91.52874229	2R	0.256218311	0.147388349	0.808004944
**PAN2**	FBgn0033352	44.97688394	53.94633136	49.99961206	43.83571719	41.67492815	58.98813883	42.18667111	54.54164255	2R	0.065505345	0.642619591	0.992589784
**FANCI**	FBgn0033354	0.332316308	0.529543288	1.289911564	0.077478296	0.599916108	1.47253719	0.339098416	0.577191573	2R	0.47321812	0.5791395	0.985637351
**CG13748**	FBgn0033355	1.681907682	5.037793195	9.034915852	0	37.51967696	1.81221976	0.612940376	0.521653896	2R	1.421442024	0.397339498	0.952272708
**Tom7**	FBgn0033357	640.064656	575.0967182	712.8988709	542.4794389	644.5653971	538.8253474	650.941732	609.220799	2R	0.016169831	0.898592022	0.996479182
**spab**	FBgn0033358	346.0933807	278.7425426	278.7842246	335.7070136	294.6386802	318.8116476	285.4938299	280.1738128	2R	-0.045563977	0.676428755	0.995787491
**CG8172**	FBgn0033362	0.922349405	0.207820979	0.166985582	0.224671051	0.049703793	0.324472159	0.351184052	0.17932889	2R	-0.760715124	0.451093536	NA
**CG8170**	FBgn0033365	0.540778192	0.580530914	0.209907256	0.15129648	0.572729366	0.428267596	0.404664124	0.375705445	2R	0.282285313	0.651907673	0.993444251
**Ance-4**	FBgn0033366	0.071763252	0.84260952	0.870483247	0.083656661	0.842081912	0.710408538	0.915347993	0	2R	0.459229111	0.729717094	NA
**PPO2**	FBgn0033367	11.28726322	5.578944379	9.528164539	16.25146824	14.62757458	10.07445759	4.513174133	11.59987363	2R	-0.036994432	0.925586051	0.996479182
**CG13743**	FBgn0033368	24.18033142	18.08978888	17.34897748	23.37553334	21.40411625	23.45004232	15.46670905	29.45315232	2R	0.140275884	0.443464948	0.967112302
**CG8080**	FBgn0033373	17.85685539	22.54579627	21.69392727	25.18674338	23.16193577	15.78803501	31.31341214	21.88663395	2R	0.11155892	0.627771882	0.990047511
**Boot**	FBgn0033374	3.662760387	1.012708799	0.390584862	1.032257885	1.307911897	0.683056112	0.718752342	0.873867237	2R	-0.769178194	0.378192811	0.943615128
**CG8078**	FBgn0033375	97.9753431	65.80887581	50.09483638	62.33250441	57.38730118	77.10354149	84.42138544	90.13485527	2R	0.185775436	0.394241513	0.952272708
**CG8777**	FBgn0033376	29.81139447	33.34678438	43.49341055	39.87296923	33.44936015	42.83156265	29.97921402	29.91618388	2R	-0.075154812	0.649241665	0.992932477
**Pgm2a**	FBgn0033377	149.3127141	107.0855459	111.820779	113.128182	101.30121	152.5736195	97.52760082	104.1012474	2R	-0.056952403	0.724617107	0.995787491
**tsu**	FBgn0033378	178.5736874	201.2424419	152.9478175	163.3205683	158.7768852	184.502117	191.4651845	134.9466396	2R	-0.027579562	0.860854023	0.995787491
**Mys45A**	FBgn0033379	19.93706962	53.69669664	47.25022922	46.48252729	33.43439648	36.49134347	41.55515231	29.29248709	2R	-0.212541323	0.397460732	0.952272708
**Phax**	FBgn0033380	43.95037407	44.59640441	30.03633178	45.42661485	50.93540566	73.01318341	39.51196569	40.05297825	2R	0.33508696	0.135607705	0.796109055
**GstE13**	FBgn0033381	13.16831437	8.835200011	9.583844098	2.899579427	13.07470035	18.07073147	12.31730605	8.735720118	2R	0.616416502	0.18153563	0.837439137

**Hydr1**	FBgn0033382	45.94583699	61.5759651	67.35900671	36.68061777	33.76668069	78.44936587	73.20905835	59.2064012	2R	0.241309411	0.298366676	0.9074568
**CG8008**	FBgn0033387	0.0245447	0.61755436	1.429082386	0.057225044	1.905307379	0	1.440123301	0	2R	0.720124537	0.627093007	0.990047511
**CG8046**	FBgn0033388	0.236500329	2.03306764	1.075774688	0.827087422	1.440935018	1.616540228	3.24283114	1.540392323	2R	0.96014003	0.180767121	0.836519372
**Rad51D**	FBgn0033389	5.185194293	5.988398568	2.680815039	1.882726398	8.823520779	5.449552893	4.120054078	3.414170996	2R	0.495822314	0.33061625	0.921224783
**CG8027**	FBgn0033392	37.85159887	25.74962001	26.47975397	22.02518974	30.1904576	42.63695198	29.14718939	26.33064377	2R	0.216520201	0.315947125	0.91460807
**CG2063**	FBgn0033400	96.431491	93.9336874	66.5003178	83.43286018	72.86943984	83.88360118	88.61965102	64.76861075	2R	-0.108344072	0.534319483	0.984760433
**Cog6**	FBgn0033401	89.13919504	96.59568681	60.39340721	95.96853205	79.85512227	85.30252944	96.06300378	95.43917784	2R	0.088920955	0.591822007	0.988631016
**Myd88**	FBgn0033402	2.290633382	1.477775444	1.531749483	2.636028611	2.730279326	2.187282278	1.835444189	0.095638021	2R	-0.193180564	0.760110447	0.995787491
**CG13954**	FBgn0033405	5.088771962	5.927872238	4.985295372	5.854052511	5.728420601	5.82544276	4.161818593	5.486566473	2R	-0.013487987	0.916154736	0.996479182
**CG8800**	FBgn0033408	0.098879276	3.151266163	0.479759488	0.230533309	5.533575499	2.889906096	1.261215785	1.288056366	2R	1.526015432	0.201269277	NA
**CG13955**	FBgn0033412	1.867642826	0.677342451	1.95929749	0	1.275730693	0.4758925	0.515069943	0.109589877	2R	-0.893646029	0.474523211	NA
**prel**	FBgn0033413	282.6241867	261.8815489	275.1515666	272.9286131	300.5829021	297.1811075	253.8909792	252.2001612	2R	0.043570072	0.670842432	0.995787491
**CG1888**	FBgn0033421	10.52632849	15.37894651	11.20627608	10.70437535	12.98856183	11.36263327	12.92652424	16.47163484	2R	0.203853954	0.30062242	0.9074568
**CG1814**	FBgn0033426	200.0842281	203.373067	185.9358911	238.0874999	218.2785935	219.4928182	188.9700726	201.4584268	2R	0.029633022	0.785625451	0.995787491
**Smyd4-1**	FBgn0033427	245.7917172	266.9389812	257.9131174	281.8120654	268.8700628	298.8155671	235.4102753	236.2861676	2R	0.010618252	0.918725308	0.996479182
**Urod**	FBgn0033428	453.3002053	472.5107596	346.4458755	511.3362488	436.2069913	463.4920823	400.3593863	420.2416348	2R	-0.024276959	0.847919376	0.995787491
**CG12929**	FBgn0033429	50.90973047	45.22270146	47.31508806	79.24086326	59.54415678	72.73969581	65.11882614	50.75027384	2R	0.183506136	0.48717727	0.973549494
**CG1902**	FBgn0033434	44.80898967	36.65186637	36.16878564	37.11405102	32.42420662	38.43013388	36.84976472	34.38250607	2R	-0.096377415	0.407656096	0.956706394
**CG12926**	FBgn0033437	8.894195436	26.74625457	22.84072345	7.098975763	15.91165095	11.86042091	15.02398511	10.26393852	2R	-0.259743167	0.496765564	0.97594235
**Mmp2**	FBgn0033438	1.610416371	1.864100561	1.840726299	2.099479741	2.455649946	2.623318874	1.741126091	1.820591393	2R	0.250926073	0.131222066	0.790743321
**CG1648**	FBgn0033446	185.2575277	196.291067	207.1026435	235.8460115	203.1976251	172.410983	202.8575087	227.5772434	2R	-0.000100667	0.999335503	0.999757791
**dila**	FBgn0033447	0.021003454	0.986449748	0.509540872	0.244843815	0.530832655	0.277226998	0.589382896	2.325622624	2R	1.148685315	0.261356605	0.890640489
**hebe**	FBgn0033448	0.913052484	1.905888386	1.058302904	0.709582498	0.805832499	0.650397564	0.181161284	0.638748027	2R	-0.963781365	0.086399272	0.705932874
**CG1663**	FBgn0033449	30.05004645	21.46328892	29.63797618	38.0675832	27.59437649	22.70847033	25.10965972	26.27256147	2R	-0.201690602	0.406080836	0.956706394
**Lsm11**	FBgn0033450	21.45142294	7.223609432	17.34695095	7.76719919	12.90843879	16.85355876	20.72842454	21.87520868	2R	0.447362109	0.287415325	0.902819904
**Marc**	FBgn0033451	6.182556827	4.923307801	9.090179774	3.93119959	2.705721127	2.296224199	5.575901366	10.44003581	2R	-0.164619873	0.777470143	0.995787491
**Sting**	FBgn0033453	0.306504762	2.005063083	2.899947479	2.14381294	0.995975372	0.433456226	2.893030858	0.66545021	2R	-0.522550144	0.518294169	0.980804634
**CG1671**	FBgn0033454	8.289309056	9.96370483	13.51505341	15.57051089	14.29789984	24.23633287	17.72171877	26.81313151	2R	0.846361392	0.002646358	0.124603093
**Sec24AB**	FBgn0033460	120.5018892	160.5187152	139.8724884	129.3451074	134.1574761	149.2335789	157.2549321	160.6998049	2R	0.161837572	0.142129087	0.8037297
**CG1513**	FBgn0033463	217.6334488	175.6802097	171.2054649	172.6862133	215.1455376	210.7369747	173.8248349	175.3087522	2R	0.099323023	0.392623503	0.951535721
**Pdrg1**	FBgn0033467	4.09776366	5.451336023	7.198740247	5.271051979	15.56028734	11.59003965	6.488368842	2.454241167	2R	0.755195949	0.264067416	0.893179858
**CG1418**	FBgn0033468	93.85986845	85.97108582	92.61616728	111.9561065	99.12622746	88.15908553	79.63349227	77.69530908	2R	-0.12822199	0.423330056	0.961840973
**CG12128**	FBgn0033473	10.38333109	17.98575059	11.85403125	21.62401237	15.23251862	16.48618888	14.42707009	16.01105531	2R	0.040279504	0.868673107	0.995787491
**CG1407**	FBgn0033474	23.74702465	31.5393477	29.79662198	35.76053416	34.54154499	32.18812814	28.84958837	29.41283948	2R	0.081878214	0.6050805	0.989286463
**CG12129**	FBgn0033475	106.8125999	76.6055362	77.02927482	70.26139296	89.40129295	78.44114903	87.02280348	88.22724108	2R	0.078842959	0.64878053	0.992932477
**oys**	FBgn0033476	15.30519201	17.1045149	15.79306383	19.64175357	14.66230002	16.496842	19.15954412	17.35202231	2R	0.026378326	0.848304368	0.995787491
**PIG-N**	FBgn0033479	3.34957102	6.918569321	8.797583863	5.163238835	2.248822629	2.818670666	8.332972406	11.35910111	2R	0.077283508	0.875932914	0.995787491
**mRpL42**	FBgn0033480	202.3530975	213.5173336	215.8139708	192.7367502	224.5150565	161.8089162	179.1608153	198.221431	2R	-0.077916703	0.61970597	0.990047511
**CG12920**	FBgn0033481	2.208522326	0.074089728	1.071568872	0	0.159477954	0	0.788758306	2.205656949	2R	-0.072782357	0.96208277	NA
**CG1371**	FBgn0033482	64.38796262	76.35199003	79.07082352	73.26214124	78.78754581	65.93984431	67.18018394	79.99647496	2R	0.028285821	0.818913641	0.995787491
**egr**	FBgn0033483	0.622513059	5.245753022	5.213813328	2.073379661	0.775954119	2.613109103	4.575079684	1.158457266	2R	-0.485805623	0.422043337	0.960593359
**CG2269**	FBgn0033484	886.6529194	703.7009913	674.8896627	783.6024572	778.6985674	818.751707	720.0272507	779.8472963	2R	0.049965919	0.64165568	0.99218509
**RpLP0-like**	FBgn0033485	31.51223489	47.16502295	30.38327579	29.95298643	44.19733623	35.04202457	51.11876994	22.80532633	2R	0.171492862	0.545409003	0.984934163
**dmpd**	FBgn0033486	133.7429979	163.9684355	140.6677294	166.0944325	151.8315014	124.6792241	167.2088131	163.9798264	2R	0.041532917	0.752101215	0.995787491
**CG12917**	FBgn0033490	1.613002712	0.270558577	1.043499222	1.00284136	0	0.05069097	0	0.70039564	2R	-2.394666504	0.12406602	NA
**CG18011**	FBgn0033491	8.554335532	6.948976684	6.301866616	10.5463625	7.761810141	4.264725152	7.197929344	3.354674207	2R	-0.494229274	0.142183489	0.8037297
**KCNQ**	FBgn0033494	5.994741908	6.890000482	6.019729477	7.674487928	7.492702784	7.106030856	6.719670471	5.08391252	2R	0.019279743	0.89302968	0.996450075
**CG12913**	FBgn0033500	56.59518839	46.58540095	34.89407352	48.7569683	37.9021404	50.35135277	42.92531343	48.10043797	2R	-0.034256633	0.827721072	0.995787491
**CG12911**	FBgn0033501	0.30193724	0.06330717	0.778276503	0.703955308	0.204402789	0.284664585	0.24070262	1.638834244	2R	0.404162361	0.690643317	0.995787491
**CG12910**	FBgn0033502	77.65989767	82.38234713	66.81957835	90.45753579	83.42398249	72.25259672	74.15219087	64.33263973	2R	-0.080146734	0.548740686	0.984934163
**CAP**	FBgn0033504	8.368296096	9.503021205	11.53285866	10.36089768	10.84884151	8.473991662	10.40061027	9.727387579	2R	0.022382191	0.868505703	0.995787491
**CG12909**	FBgn0033507	11.94154455	14.67005378	16.933181	14.70489167	10.93058797	18.15617672	7.723048738	15.5192034	2R	-0.115531913	0.757157283	0.995787491
**Prx2540-2**	FBgn0033518	1.328755446	0.835800321	2.417656634	3.485186214	1.349293577	0.469778444	1.059276153	0	2R	-1.461866306	0.208137998	NA
**CG11825**	FBgn0033519	0.107068289	0.493878094	0.649365438	0.187219264	0.096642851	0.35330165	0.682833763	0	2R	-0.304183866	0.823826033	NA
**Prx2540-1**	FBgn0033520	4.602901942	1.403767688	2.030286529	0.780472189	2.266204833	3.313867075	4.483347939	2.543755761	2R	0.508762101	0.526419905	0.984107922
**CG12896**	FBgn0033521	4.184456311	4.070926296	2.233315182	1.951180473	3.47484741	3.787276657	4.910333457	0.9084842	2R	0.087569962	0.905735269	0.996479182
**CG12895**	FBgn0033523	122.8564913	129.7231748	127.8497842	121.0652731	108.6997867	112.5993218	112.1735615	100.0248057	2R	-0.180043027	0.205754251	0.855364409
**Caf1-105**	FBgn0033526	34.21744796	17.91089254	28.30486502	35.07629221	23.89556352	23.189891	28.71935874	47.32884231	2R	0.120321743	0.661444181	0.995750687
**CG11777**	FBgn0033527	33.31470986	19.38142545	32.53301876	22.22008882	34.71463917	27.03551314	19.79433351	16.1138736	2R	-0.11042961	0.733839933	0.995787491
**trsn**	FBgn0033528	120.635593	78.85282143	74.12022404	84.08346177	106.1881779	70.4530292	92.60442506	103.9438068	2R	0.08778334	0.677512709	0.995787491
**CG17765**	FBgn0033529	233.2107032	285.3518341	250.1370743	290.9070796	257.376684	252.4780125	253.7955578	246.885195	2R	-0.036976041	0.732712434	0.995787491
**CG11883**	FBgn0033538	42.02662032	25.55586838	24.78622902	28.79702369	30.88505399	36.84227479	31.85666533	22.97642895	2R	0.038197802	0.824017527	0.995787491
**Git**	FBgn0033539	52.36935772	56.48152062	58.8656517	63.56663765	62.044657	59.87273555	52.34831803	38.35117342	2R	-0.092272823	0.557318692	0.984934163
**Elp2**	FBgn0033540	31.01405277	17.24033969	18.55621659	12.60863725	16.45128689	19.88761158	26.37250498	13.55769298	2R	-0.037686521	0.895966728	0.996479182
**CG12934**	FBgn0033541	3.861959997	3.547419302	3.123020362	1.286287764	0	1.820516148	2.345700696	4.791241515	2R	-0.374324637	0.711577066	0.995787491
**CG12338**	FBgn0033543	2.087721818	5.81159823	3.987170392	2.796191708	0.160377573	2.261441158	1.246469262	1.350147288	2R	-1.503094576	0.037768763	0.521998122
**CG7220**	FBgn0033544	73.01296328	64.80737471	59.50081511	72.22446371	72.54947941	68.70848644	66.11675007	74.34035168	2R	0.092367218	0.421823672	0.960593359
**CG12935**	FBgn0033547	44.41078572	36.95769939	33.40773542	16.69515629	40.77006977	39.7929354	46.37103455	47.08880149	2R	0.431841037	0.113607589	0.758410631
**CG7637**	FBgn0033548	61.31660021	65.76282207	74.10011546	69.08719279	64.19323374	58.23872221	67.45247538	66.31456512	2R	-0.045430145	0.856356253	0.995787491
**CG12341**	FBgn0033550	126.5843399	120.8439441	142.3375249	137.3170757	114.1907792	107.3732349	114.9861261	134.7219822	2R	-0.130250193	0.368004481	0.941359529
**CG7222**	FBgn0033551	111.6631677	126.9452644	105.774852	91.02922081	126.1606531	108.5061337	114.7604448	97.98552696	2R	0.070929765	0.59651789	0.988780662
**RpS15Ab**	FBgn0033555	97.28079594	111.5296341	134.5675596	112.0066649	102.5152078	104.3661871	128.9726589	113.1458642	2R	0.012712079	0.942022261	0.997604378
**CG12343**	FBgn0033556	158.0872096	132.4350831	146.3592201	100.7658249	145.9114167	123.1668541	145.0367012	118.4057741	2R	0.01528168	0.928873696	0.996479182
**CG12325**	FBgn0033557	10.97287047	14.84089428	14.52452327	11.43366901	13.6485514	19.82339805	8.965789327	11.53447031	2R	0.092931314	0.704334142	0.995787491
**CG12344**	FBgn0033558	0.742830709	4.12406566	2.969652703	1.756274536	1.095464638	1.085028001	0.907453822	0.249863332	2R	-1.477053053	0.006633474	0.23524839
**Pex6**	FBgn0033564	42.50412054	46.40456765	49.34388757	40.66470456	50.23350508	45.46117899	39.49715248	35.82855673	2R	-0.032917838	0.824136471	0.995787491
**CG18004**	FBgn0033566	181.3489671	163.8262112	183.635374	225.1800402	187.618697	152.9678528	185.6040865	177.1001512	2R	-0.07064862	0.625580101	0.990047511
**CG12942**	FBgn0033569	39.31208109	25.0826308	38.40680462	37.93361776	44.36938713	31.29977522	36.7875145	25.04693899	2R	-0.006547949	0.975896344	0.997757033
**ND-B14**	FBgn0033570	1335.393844	1371.137194	1427.554463	1401.366254	1455.848519	1144.606621	1567.032529	1602.665437	2R	0.09403043	0.446346973	0.967112302
**Rpb5**	FBgn0033571	58.58726819	59.16082746	67.18591035	45.92384827	58.65704521	28.96700759	43.80647341	60.67509055	2R	-0.229347251	0.376912567	0.943615128
**polyph**	FBgn0033572	0.782302269	0.801901412	0.105436355	0.101328238	0.627669377	0.573649845	0	0	2R	-0.54481755	0.747572687	NA
**Spn47C**	FBgn0033574	0.13072479	1.140216978	0.507418426	0.060955985	0.4719835	0.419038213	0.133392699	0	2R	-0.778770439	0.568989901	NA
**CG13229**	FBgn0033579	0.236935205	0.114811831	0.255467334	0.270064916	0.332677933	0.421942009	0.188043893	0.30292931	2R	0.53640715	0.258437842	0.887830768
**CG7737**	FBgn0033584	97.6606749	82.37471813	80.14942484	86.30310408	75.9848246	73.14911065	75.21363673	70.32089715	2R	-0.206175363	0.076092995	0.681158992
**Cpr47Eb**	FBgn0033598	1.910696595	0	2.575179605	0	1.533020916	0.800619146	0	0	2R	-0.905988673	0.66136881	NA
**Cpr47Ed**	FBgn0033601	4.527501148	0.999243162	3.757566059	1.944470774	2.150868411	1.909592296	2.431526884	2.586743184	2R	-0.294604482	0.743401646	NA
**Cpr47Ee**	FBgn0033602	1.001367002	1.847617934	0.364395357	2.101184521	1.446179269	2.124186058	1.021803494	1.521842319	2R	0.235938819	0.773513347	0.995787491
**CG9067**	FBgn0033605	85.08323876	84.04748073	89.63972025	64.16296445	102.1341226	72.97998223	80.83403368	99.02707779	2R	0.168774831	0.398772949	0.952805113
**CG9062**	FBgn0033607	79.91135539	100.2904857	84.72880012	99.78634623	94.93299069	98.45480208	91.50371897	71.02668603	2R	-0.005291747	0.968286467	0.997604378
**CG13220**	FBgn0033608	184.3958972	197.1664921	272.7883016	210.1241813	307.7478357	233.6668375	314.5023392	278.7385585	2R	0.427335294	0.008081353	0.265157471
**Fbl6**	FBgn0033609	175.7653544	234.556557	208.8622141	216.6652151	184.0639599	212.2818119	224.6923564	225.8382877	2R	0.051673586	0.648276352	0.992932477
**CG18335**	FBgn0033610	3.506562501	2.270155558	1.641676998	2.725139488	4.553333432	0.985989772	5.178867318	2.404127933	2R	0.3849766	0.582773394	0.985764834
**CG7741**	FBgn0033615	25.76722669	19.60292548	23.00037955	20.79109401	19.82668287	21.26206718	24.72461319	15.13213225	2R	-0.114369512	0.557112579	0.984934163
**CG7745**	FBgn0033616	97.36779203	109.8988127	95.90761213	118.579241	80.34916396	103.1887225	100.6240641	98.85006933	2R	-0.109595892	0.423045747	0.961457118

**CG12384**	FBgn0033624	258.4269637	284.0794974	263.0806719	272.8030573	272.9691749	247.0406525	287.9135865	269.2421998	2R	0.029679279	0.784212029	0.995787491
**CG13204**	FBgn0033627	230.0214468	194.8437008	213.8356575	273.1960858	260.8029869	244.6926679	222.9417683	246.5707901	2R	0.124151763	0.318567534	0.91460807
**Sod3**	FBgn0033631	135.6873553	136.6504681	154.1842815	63.31287983	91.98613851	101.4340038	163.6560567	75.33232021	2R	-0.144667884	0.685153367	0.995787491
**Prip**	FBgn0033635	0.826593002	0.024324453	0.598072853	0.405720098	1.282778928	0.054688199	0.110981921	0.314843846	2R	-0.047082236	0.965681642	0.997604378
**tou**	FBgn0033636	12.47158973	17.93140299	18.21784632	14.3028461	14.64734694	13.32532568	14.65268463	16.60610636	2R	-0.050709334	0.706150783	0.995787491
**CG9005**	FBgn0033638	27.54494113	31.89176132	27.93375142	34.35416785	30.32355659	33.89884584	34.36684319	31.72824559	2R	0.12828472	0.234801396	0.87009833
**CG9003**	FBgn0033639	47.94374145	52.77669464	45.0777075	49.87432055	49.24385542	46.76361237	47.09590463	49.83796119	2R	0.010711498	0.914716381	0.996479182
**pyr**	FBgn0033649	0.996460431	0.567458285	0.537199802	0.071704012	0.632934498	0.678496584	0.329517477	0.747844685	2R	0.157551931	0.80193082	0.995787491
**ths**	FBgn0033652	2.311042817	3.952112775	4.87336435	4.266316884	2.803969689	2.898973175	3.133792934	3.933684387	2R	-0.233558376	0.244903422	0.877269208
**CG13192**	FBgn0033653	42.27089043	44.2835824	54.93246139	35.27159632	49.44517462	48.33601003	58.01592612	58.71390269	2R	0.312712083	0.086709439	0.706081898
**Sobp**	FBgn0033654	0.841884173	0.275540019	0.780428986	0.718105338	0.531318035	0.57432008	0.366674446	0.668710417	2R	-0.263449753	0.608160659	0.990047511
**S2P**	FBgn0033656	63.91996883	74.17470366	70.89709257	58.96819825	58.68378242	59.15515242	71.34574349	98.03211961	2R	0.135249196	0.462871418	0.971606621
**Sln**	FBgn0033657	128.2073776	107.6598572	126.5010628	115.6580343	102.6891871	128.0594899	104.2954826	92.22814796	2R	-0.135755896	0.237548476	0.873280735
**CG13185**	FBgn0033661	1.022260621	1.460365471	2.040948715	1.071942787	0.759368653	1.724660861	0.449191948	1.507932948	2R	-0.295787636	0.470733197	0.973549494
**ERp60**	FBgn0033663	227.3252814	226.8584885	209.2721858	227.3459886	211.6539643	194.5694646	206.3246974	235.8007896	2R	-0.039117789	0.710056266	0.995787491
**reb**	FBgn0033667	0.553143953	0.927821382	0.049700723	0.119410584	0.351347509	0.424926529	0.130655751	0.244633222	2R	-0.487303298	0.595523907	0.988780662
**exp**	FBgn0033668	0.68044321	0.677512528	0.663313355	0.253538678	0.588946625	0.85828624	0.317045707	0.249590376	2R	-0.146667681	0.750199833	0.995787491
**PI31**	FBgn0033669	158.9876024	169.5573618	169.8815886	170.1106287	157.4963307	158.1118837	167.0788787	160.7840057	2R	-0.024243578	0.797404929	0.995787491
**rho-7**	FBgn0033672	60.22139242	62.23061429	53.9659802	53.10660202	69.93245019	60.58299867	65.0068552	74.26318999	2R	0.263382014	0.062735371	0.636110045
**CG8298**	FBgn0033673	51.77182498	39.22242048	37.40514376	53.95295865	66.45281348	53.75713608	44.96181381	51.00127006	2R	0.271572245	0.127399207	0.787284205
**CG8321**	FBgn0033677	52.21800029	47.27888361	43.49805537	42.50141579	41.01791574	44.67828504	40.86993189	45.42931386	2R	-0.081781856	0.582095641	0.985735525
**CG8888**	FBgn0033679	0.630350432	0.950621956	0.196413752	0.202243803	0.459353577	0.507055229	0.516342315	0.03766649	2R	-0.35459971	0.62993224	0.990523437
**CG18343**	FBgn0033683	0.137707863	2.078870591	7.349701792	1.605303489	2.237381878	2.077282109	4.566844312	6.577483383	2R	0.537011931	0.612114139	NA
**OSCP1**	FBgn0033685	0.387264884	0.757846267	1.722415211	0	2.679930752	0.486814622	0.987921038	1.401313185	2R	1.020024	0.393799339	NA
**Hen1**	FBgn0033686	3.271862162	4.147015209	5.072833213	3.297916157	1.110253717	2.110578505	0.941344812	2.163100623	2R	-1.279148036	0.000370437	0.028323109
**CG8407**	FBgn0033687	0.726663135	1.096987921	2.644311944	5.251982003	1.3118132	4.521617527	1.112239961	2.682009984	2R	0.029965414	0.972531852	0.997757033
**Prp8**	FBgn0033688	70.02797754	88.23788159	96.14742431	78.1909901	112.6423076	125.811711	103.175994	125.9159102	2R	0.524053607	5.59E-06	0.000991777
**EndoG**	FBgn0033690	117.0079062	126.5303444	120.3175278	114.0111411	122.2539815	120.8585576	106.6456216	133.1297439	2R	0.046721869	0.728457774	0.995787491
**CG8860**	FBgn0033691	20.25916008	15.60393319	19.55907297	14.9411994	19.03287543	11.8889077	13.18406954	8.0787879	2R	-0.403468359	0.422722879	0.960985399
**wash**	FBgn0033692	56.57830088	47.60564507	38.1148498	58.00610459	36.59679719	55.55700669	57.35687379	49.41477519	2R	0.013738563	0.945525678	0.997604378
**Cyp6t3**	FBgn0033697	1.382731401	1.739503461	2.755471541	1.381622661	0.089149449	0.13967472	0	0.321646915	2R	-3.667821574	0.000929345	NA
**CG8858**	FBgn0033698	26.05230876	19.95494762	21.92145483	16.70427283	22.82781702	15.84113938	20.01642874	19.21191727	2R	-0.090163939	0.618676587	0.990047511
**RpS11**	FBgn0033699	612.1796501	865.8989322	848.8689194	835.8651242	790.0475934	682.3045339	797.0038727	866.0456619	2R	0.02279153	0.857454855	0.995787491
**CG8854**	FBgn0033702	0.339399189	0.539331657	0.910049035	0.083294361	0.032247501	0.08420618	0.136707572	0.426607244	2R	-1.42945704	0.211387229	NA
**CG13168**	FBgn0033705	2.534179217	3.485598704	2.950984048	3.072338591	0.914968598	1.863582776	1.810128776	0	2R	-1.3609047	0.102790225	0.743157534
**Vha36-2**	FBgn0033706	1.707074914	4.242042503	5.368403615	2.063693799	1.483785208	0.178824422	1.612883252	0.823603925	2R	-1.683580566	0.047819242	0.575780949
**CG17739**	FBgn0033710	11.90889371	4.713319882	14.12078918	9.047067233	10.82982181	5.950224582	13.59697125	1.791472835	2R	-0.280855519	0.540454535	0.984934163
**mIF3**	FBgn0033712	41.33673814	37.65050809	42.05388878	35.10435246	53.05884708	49.23884781	50.88289737	52.47239244	2R	0.426434381	0.009960628	0.302963052
**CG8841**	FBgn0033713	51.01031848	63.52320112	76.9728118	49.20813691	67.4913378	71.21305603	78.40693746	77.70918572	2R	0.327911797	0.029490207	0.476301137
**CG8490**	FBgn0033715	105.4228817	106.2300014	106.3673736	99.04271093	102.1933471	133.9312452	102.6530341	91.38390452	2R	0.074004541	0.644084629	0.992589784
**Den1**	FBgn0033716	315.782259	301.474579	302.0309792	323.9015429	291.3441754	289.3970118	296.2221197	284.506362	2R	-0.068795693	0.467267384	0.971928234
**CG8839**	FBgn0033717	21.81458114	28.58210633	22.91462394	35.08968358	31.63589359	35.65321595	30.40640879	38.27227837	2R	0.358073594	0.039796941	0.529044592
**CG13155**	FBgn0033723	3.840217787	0	3.623011307	0	3.27371708	1.407985395	0	0.463192683	2R	-0.511075588	0.754670629	NA
**Cpr49Ac**	FBgn0033725	0.204348559	0.385611796	0.433778411	0.35732323	0.184450762	1.324527694	0.065162196	0.610032004	2R	0.71030502	0.519607004	NA
**Cpr49Ae**	FBgn0033728	0.389362792	1.909065808	0.072660669	0.209488775	0.162207607	0.254138443	0.458433622	2.015817121	2R	0.212160475	0.855418892	NA
**Cpr49Ah**	FBgn0033731	0.573630766	0.360819427	1.043715178	0	0	1.081631164	2.195016612	0.155675687	2R	0.817947183	0.620317906	NA
**CG8520**	FBgn0033734	76.06507331	58.73668306	70.32196155	81.6416206	47.0712617	55.85268647	55.76569365	44.33604144	2R	-0.472518159	0.002473825	0.118487709
**Dera**	FBgn0033735	0.52249612	3.992552403	1.830931987	3.519186764	8.38435032	1.642020501	1.777198436	2.772947083	2R	0.617341433	0.430813465	0.963666972
**CG13154**	FBgn0033736	0.442833567	4.456747109	3.222924168	4.749269034	1.918627404	2.672005003	0.225935589	0.384573066	2R	-1.242135045	0.230718836	NA
**Nup54**	FBgn0033737	42.19779439	46.38313304	38.14142243	60.27932775	37.7643794	39.44484367	41.85827653	59.1135242	2R	-0.038833634	0.84642134	0.995787491
**DUBAI**	FBgn0033738	87.66509384	65.30967601	75.28801117	73.77173749	66.87557209	71.76169461	60.000829	67.59759983	2R	-0.155088017	0.215126738	0.858896167
**Dyb**	FBgn0033739	235.0836209	224.5178161	219.9726986	249.4542444	234.1254039	258.7564471	226.6838995	187.005501	2R	-0.008842214	0.9329308	0.996479182
**dgt5**	FBgn0033740	0.284133986	0.214467706	0.034465254	0	0	0	0.326174356	1.29545049	2R	1.62797091	0.320307306	NA
**Nepl10**	FBgn0033742	4.441961315	0.275954332	3.891382514	2.397283701	0.371244381	2.055150572	0	0.089295456	2R	-2.090519987	0.05308868	0.606446461
**Dh44-R2**	FBgn0033744	24.50628289	23.64363164	18.19039123	22.85716853	19.55466409	17.97408284	15.85304162	10.59393595	2R	-0.452843241	0.009271512	0.287842402
**vis**	FBgn0033748	0.095569966	0.05343507	0.154567603	0.222817777	0.977660268	1.14130188	0.731403108	0.899128717	2R	2.913505715	0.005886159	NA
**achi**	FBgn0033749	1.325789474	1.317822728	2.739850954	0.629653903	4.653813496	10.27733557	6.200548626	7.462548788	2R	2.289977862	2.15E-07	5.97E-05
**CG13151**	FBgn0033750	16.96973487	30.13872314	33.24288426	46.12308539	30.96236482	29.13180049	27.08528617	27.8784248	2R	-0.101792988	0.701546313	0.995787491
**CG8818**	FBgn0033751	788.2891069	791.0720709	667.1265285	818.9399253	804.0434241	778.1289382	707.1416501	794.1039446	2R	0.037993948	0.710471864	0.995787491
**CG8569**	FBgn0033752	42.39290053	35.7609533	47.53249333	34.42468184	39.31468937	58.90511396	50.70149029	44.22152182	2R	0.298553467	0.088971397	0.709815866
**Cyp301a1**	FBgn0033753	1.596233317	0.606216783	0.511454308	0	0.543700047	0.028394699	0	0	2R	-2.212797366	0.183974792	NA
**Ak6**	FBgn0033754	39.20177504	24.41847996	39.85747373	18.4249527	21.58743739	16.17577459	31.83169348	22.57577785	2R	-0.37812052	0.28394977	0.901780531
**ClC-b**	FBgn0033755	53.13443605	44.56278123	25.16933203	34.25164161	33.296588	37.53320399	41.78570672	30.09132486	2R	-0.114386906	0.593975579	0.988780662
**CG17760**	FBgn0033756	0.12325613	1.033724338	1.121316199	1.221309869	0.055627226	0.610076916	0.628858073	0	2R	-1.396397922	0.252765341	NA
**muskelin**	FBgn0033757	10.42430938	14.52504855	17.18297511	23.70040242	23.23644906	19.70032051	14.70698896	22.58105227	2R	0.319885779	0.210112157	0.856520545
**CG8785**	FBgn0033760	2.57532347	0.559252551	0.948311428	0.696924164	0.166039961	2.026945792	0.439935636	0.898596265	2R	-0.432449647	0.543676767	0.984934163
**CG8778**	FBgn0033761	79.53395405	74.36003637	91.77888001	108.8062794	93.34623819	83.31569564	121.2885486	101.7977229	2R	0.203875948	0.275096734	0.89549671
**ZnT49B**	FBgn0033762	142.6341295	134.2621613	117.5838578	149.4326641	137.2612036	152.7918147	146.9590268	131.2169062	2R	0.090262578	0.434990153	0.965949357
**CG8646**	FBgn0033763	0.281311102	0.214481777	0.744498338	0.953987264	0.461671489	0.74743364	0.195717454	0.166568848	2R	-0.425744808	0.678063853	NA
**Nup188**	FBgn0033766	8.324387097	6.735915527	11.77187061	11.80084127	13.46699153	11.18727352	9.319515689	11.47348856	2R	0.266840356	0.245582679	0.877269208
**CG13148**	FBgn0033767	78.42416643	65.34325562	53.42406503	71.32229891	81.23500193	59.16313562	60.79368305	56.48358173	2R	-0.032065262	0.852054754	0.995787491
**CG8768**	FBgn0033769	166.4048062	150.0208154	132.8926839	167.0228839	141.6591195	140.7537122	149.1753619	174.5847629	2R	0.004586112	0.974172229	0.997757033
**wuc**	FBgn0033770	4.049684212	0.943441271	1.637412902	0.524538127	1.421528486	1.696896684	1.147870159	3.907661911	2R	0.188265311	0.859497626	NA
**Cyp9h1**	FBgn0033775	2.680450008	9.075418439	3.612630185	2.661767975	3.315542085	2.105926579	1.013020083	2.909752547	2R	-0.899121791	0.148398504	0.808039863
**CG17574**	FBgn0033777	3.323999459	4.018735382	3.67591648	3.432045254	3.413368675	3.988531765	3.138551038	5.42659142	2R	0.177799301	0.381381315	0.944476532
**CG13319**	FBgn0033781	15.50010712	15.10250856	32.62617024	20.72615784	24.89545283	11.28240928	15.11865828	28.70331923	2R	-0.028021163	0.947033668	0.997604378
**sug**	FBgn0033782	1.21154367	0.597704277	0.691573657	1.163098776	1.929835447	0.645027372	0.181804349	0.12378228	2R	-0.320072147	0.687365497	0.995787491
**CG17019**	FBgn0033783	146.1423704	160.0369094	133.8074173	170.2982594	163.0061679	176.8542906	149.5904171	139.7966672	2R	0.072398633	0.533446382	0.984760433
**SCCRO3**	FBgn0033784	160.4376197	109.2358207	117.8069126	107.7753741	119.0599608	119.5667806	122.8223564	154.6294594	2R	0.087697778	0.58439118	0.986692887
**Sans**	FBgn0033785	0.590411217	0.123791499	0.656484298	0.344130382	0.266460892	0.139159022	0.251025283	0.907968163	2R	-0.110209621	0.918796467	NA
**CG13323**	FBgn0033788	0.879373052	0.453853825	0.328207194	0	0	0.510195411	0.776526767	0.220292342	2R	-0.14371443	0.923427238	NA
**Drl-2**	FBgn0033791	19.08289839	17.89435424	21.71152126	19.9262087	19.32314398	14.32710483	19.83800589	16.65899666	2R	-0.13218637	0.326577919	0.918470465
**CG13325**	FBgn0033792	4.363582332	5.136850518	8.063546975	5.947804745	4.39000073	4.033608012	3.942438932	5.378321813	2R	-0.369562377	0.088525348	0.709409025
**CG3955**	FBgn0033793	987.6155994	1058.17601	948.4705835	995.1715423	1104.478874	1068.875147	1047.883298	1000.485604	2R	0.112110324	0.187122172	0.840740887
**CG13326**	FBgn0033794	2.677398218	2.516911597	0.571018086	0.960346639	1.327854394	0.249649584	0.825616529	3.130011971	2R	-0.25409628	0.753145791	0.995787491
**GLaz**	FBgn0033799	9.378503886	14.803666	15.89036926	15.02332316	3.685573487	5.734265095	7.052651786	4.016914304	2R	-1.396228467	5.12E-07	0.000129339
**CG17724**	FBgn0033802	2.377173122	2.502492805	3.544295223	2.150523847	1.821612761	3.548541808	2.179328957	1.827876444	2R	-0.141105902	0.61954777	0.990047511
**FLASH**	FBgn0033806	8.861855959	8.583187504	10.04403027	9.978056102	8.39785894	12.36788889	10.02768151	9.039222629	2R	0.119535448	0.599431481	0.989286463
**CG4627**	FBgn0033808	34.84453145	45.62711134	53.23145455	43.11016628	66.01880398	72.28816098	63.52219799	50.49327603	2R	0.549125347	0.00722839	0.247978871
**CG4630**	FBgn0033809	0.94150267	2.672557714	0.702791491	1.688521563	0.784455519	0.022760076	2.586546888	0.5241255	2R	-0.584179715	0.527042507	0.984107922
**CG4646**	FBgn0033810	169.1841757	144.6102366	168.5992677	117.6253744	163.500715	130.5655322	147.5684423	98.09716764	2R	-0.123303123	0.512784907	0.980804634
**Pex13**	FBgn0033812	255.9175439	264.3119624	258.3218164	251.3510087	241.9792872	259.2116932	249.9680732	218.4064687	2R	-0.057501936	0.539790281	0.984934163
**fsd**	FBgn0033813	32.72244835	65.11142461	52.9228022	41.51136456	58.20369412	53.61034761	48.28494566	46.49167895	2R	0.142802314	0.538692841	0.984934163
**Qsox1**	FBgn0033814	50.65380165	63.2820299	64.3232991	81.85196568	58.64909924	91.54213241	73.75909315	82.91908365	2R	0.267347217	0.113664646	0.758410631

**CG4676**	FBgn0033815	102.4915428	105.4196888	114.5478217	124.1740328	136.2948289	143.4232862	117.368389	123.1549633	2R	0.25149143	0.075454301	0.678318963
**CG4679**	FBgn0033816	119.8636754	122.8264997	114.5670863	128.0885588	96.13953956	118.175295	99.69397629	69.1183158	2R	-0.314429975	0.035831835	0.518988565
**CG4744**	FBgn0033834	0.072895641	0.30568053	0.088421904	0.424883608	2.434513334	1.580688001	0.046489587	0.079131592	2R	2.256147905	0.041863356	0.536003453
**cbc**	FBgn0033842	45.22219211	41.9864002	53.34445493	42.99035209	58.91891351	42.15713588	51.27234828	49.74132306	2R	0.171102836	0.329959232	0.921112231
**bbc**	FBgn0033844	110.1466583	109.7444184	110.6092847	92.13778679	114.6344897	117.2840882	102.909322	110.392755	2R	0.105719909	0.270316041	0.89549671
**mars**	FBgn0033845	1.896280702	1.698194288	2.222203224	3.25962979	1.508560842	1.499935462	3.156642937	1.360703701	2R	-0.246280664	0.578640362	0.985637351
**mip120**	FBgn0033846	30.63138498	22.93937493	25.72435625	21.88113702	20.64003198	32.43549953	28.47214454	33.12513949	2R	0.207077459	0.23501216	0.87009833
**CG6145**	FBgn0033853	22.24440739	20.22898561	19.70398448	25.72323463	20.23930083	21.58800574	19.78855723	19.93698219	2R	-0.080504204	0.540849953	0.984934163
**link**	FBgn0033855	11.25477564	8.448303241	15.53759479	11.30788224	22.1137992	47.42677007	34.10443374	39.96026125	2R	1.656776126	2.67E-08	9.66E-06
**fand**	FBgn0033859	81.47407932	63.0936471	68.96283245	83.95401088	73.80919448	95.14752523	79.41996596	54.4865972	2R	0.048888562	0.772772394	0.995787491
**CG6280**	FBgn0033866	0.397886888	1.305781508	1.17510892	0.483995636	0.718287811	0.081548984	0.088262435	0.150234655	2R	-1.64086416	0.07757244	0.682035833
**Cpr50Ca**	FBgn0033867	0.503622606	0.674180485	0.39942854	0.056450824	0.30596996	0.410895274	0.197654167	0.325920675	2R	-0.362883628	0.575705184	0.985605382
**Cpr50Cb**	FBgn0033869	0.422568445	0.097286177	0.120605312	0.367036197	0.314112378	0.484323272	0.486148377	0.017988926	2R	0.371743127	0.686791749	0.995787491
**CG13339**	FBgn0033871	72.42449951	62.26712389	91.82354706	58.83055073	55.84576195	57.30145604	56.32441318	47.67251755	2R	-0.362770594	0.09901527	0.731438598
**CG6329**	FBgn0033872	638.7913441	574.4575802	586.2674398	611.3064563	641.2803611	684.6674465	588.9405203	665.4216218	2R	0.126586689	0.154820077	0.818884898
**CG6337**	FBgn0033873	0.113896444	2.29254368	0.690777961	0	0.616836818	4.241544431	1.016932964	0.494559446	2R	1.084392873	0.389753393	NA
**CG6347**	FBgn0033874	0.38248546	0	0.356886304	0.068596188	3.611763952	4.660125574	3.377522164	7.026557486	2R	4.50173558	1.46E-10	8.7E-08
**CG6357**	FBgn0033875	35.00607206	31.29322124	49.23865307	38.35801446	33.86434133	58.0339921	56.22186532	57.75217934	2R	0.450634699	0.04186924	0.536003453
**Syngr**	FBgn0033876	34.92484671	39.11901345	125.7347773	39.67943013	632.7098662	942.2517731	727.6504489	913.9181244	2R	3.781293565	8.55E-16	8.91E-13
**Echs1**	FBgn0033879	51.44522971	49.75971028	72.57540857	64.20286036	48.58258482	44.43089172	55.72022178	49.3239966	2R	-0.232934609	0.22379698	0.86549481
**CG16935**	FBgn0033883	229.9339757	212.2657248	228.9488899	177.3909884	239.8023099	295.281709	256.1215673	237.7722441	2R	0.306687015	0.01018021	0.304092724
**CG13344**	FBgn0033884	238.3105045	261.0373962	251.110427	238.5202901	165.8559637	170.3994923	207.4553341	188.3175396	2R	-0.402474101	0.000297907	0.02320331
**DJ-1alpha**	FBgn0033885	8.219999587	11.71971173	3.739050815	2.156019704	17.62155715	15.01517046	11.79528499	10.26166851	2R	1.102938992	0.043659734	0.549637802
**Rpn13**	FBgn0033886	333.1470986	322.7015746	268.8005441	297.760741	275.6419437	276.3983637	268.8665103	224.5749486	2R	-0.197291347	0.082548288	0.696313191
**CG6701**	FBgn0033889	0.241242851	1.679301814	1.258291352	0.4499589	1.132313458	0.739187431	0.492332557	1.440341543	2R	0.125741161	0.847656027	0.995787491
**CG8067**	FBgn0033891	19.25492376	25.21721528	13.04574298	39.49968186	46.9730708	25.07680263	45.13704386	34.89962069	2R	0.67988039	0.035619922	0.518074043
**Rcd1**	FBgn0033897	130.9610842	135.753434	143.5055396	138.7867444	114.7986626	129.2663859	122.349142	110.5894452	2R	-0.173106118	0.059884486	0.627645287
**CG13016**	FBgn0033899	57.34832412	48.40101994	55.04723638	39.61643223	55.17775044	43.61554758	57.48790516	50.61321965	2R	0.074781725	0.686376303	0.995787491
**CysRS-m**	FBgn0033900	43.94896213	39.57574972	41.52245276	40.09108354	42.66554334	48.18352236	28.25822093	48.44661867	2R	0.050795314	0.789764148	0.995787491
**O-fut1**	FBgn0033901	41.60235936	32.38606156	39.20615807	55.83391831	43.23232261	37.51275374	44.35627575	36.01323113	2R	-0.042219859	0.843062608	0.995787491
**eIF3m**	FBgn0033902	977.9638195	1037.596603	897.6417303	1078.620663	842.5566623	1019.234144	897.050088	920.7164571	2R	-0.089063051	0.359074054	0.938389202
**CG8323**	FBgn0033903	39.55864208	33.92595358	29.24554488	41.22220766	42.15491433	32.24431708	42.37060931	27.33600861	2R	0.027287516	0.897920023	0.996479182
**CG18327**	FBgn0033904	7.962508592	6.039230774	11.25421418	17.43428883	15.24933036	5.026429076	15.89676299	15.784096	2R	0.317784389	0.498668713	0.976006682
**ReepB**	FBgn0033906	140.4814055	135.8942331	150.2263923	136.6488009	164.5688282	145.8086579	162.4127643	156.095569	2R	0.190120237	0.074371249	0.676245
**mRpS16**	FBgn0033907	103.2319092	140.2881704	135.0373526	105.2773818	118.9423179	101.476508	112.2936914	120.8492491	2R	-0.058041609	0.749751962	0.995787491
**VGAT**	FBgn0033911	203.3387542	233.7957101	196.1054565	277.74114	246.6564819	234.2621445	236.9134	241.4666418	2R	0.104613779	0.402208685	0.954587287
**RpS23**	FBgn0033912	278.2420196	354.731453	385.2697445	380.942817	381.881418	315.7179635	363.9933011	409.277176	2R	0.106771959	0.426476002	0.962502302
**CG8468**	FBgn0033913	0.45239897	0.880791562	0.058795371	0.376696846	0.81669609	1.317639009	0.947993868	0.29816787	2R	0.954771796	0.162401909	0.824273756
**CG8485**	FBgn0033915	252.3784941	227.7668637	214.7494618	253.8263572	214.9063571	279.3355294	229.0339524	207.1211741	2R	-0.002697079	0.981144876	0.997881056
**Usp20-33**	FBgn0033916	58.23407705	76.21576244	75.51978765	75.66916858	71.10697017	52.84086365	75.73229844	59.90724903	2R	-0.103648162	0.496394101	0.975925557
**SmydA-1**	FBgn0033917	19.15595817	16.06571626	27.00156853	21.97764316	29.6607386	28.66062578	34.3809187	47.75378903	2R	0.773184255	0.001151009	0.070018291
**CG8531**	FBgn0033918	86.50496982	100.068932	93.89190156	87.41654258	98.7014199	96.15177682	77.73267633	88.69958163	2R	0.006227257	0.959630614	0.997604378
**CG8547**	FBgn0033919	4.787035474	5.951845349	6.166621062	2.045643999	4.169506315	2.8709227	2.369950901	2.016989203	2R	-0.69220079	0.058722324	0.625114381
**CG8613**	FBgn0033924	423.8080064	314.6331426	338.5468881	392.3980909	376.5915082	467.1616206	358.2418417	338.9163241	2R	0.092073084	0.470992122	0.973549494
**CG8617**	FBgn0033925	370.991904	355.7297726	347.5838453	378.5173976	331.454939	367.6462182	353.9532034	389.7937983	2R	0.019433529	0.848378146	0.995787491
**Arc1**	FBgn0033926	23.53677223	52.53733843	67.65094614	20.27992767	9.368892385	8.57979961	33.56430464	46.26024547	2R	-0.690821055	0.326026414	0.918470465
**Arc2**	FBgn0033928	2.787934654	2.902574033	6.996714399	10.31028882	1.909044915	0.271908389	4.904883586	0.417438911	2R	-1.609709156	0.08182619	0.694684542
**Tfb1**	FBgn0033929	70.22328698	52.36429745	70.27596117	38.55733767	68.35060679	69.77569189	71.71111727	53.89652762	2R	0.216935946	0.257701178	0.88770596
**Dh44-R1**	FBgn0033932	91.36543124	73.98543877	46.29791369	66.37949823	25.55893341	20.38767505	13.60112331	12.66310881	2R	-1.918740296	1.68E-18	3.51E-15
**CG17385**	FBgn0033934	332.0556286	231.9407067	265.7829638	317.3328969	254.0896328	274.3982358	260.9463768	277.9961653	2R	-0.077798548	0.553263133	0.984934163
**Sin1**	FBgn0033935	118.5122295	148.4453151	117.3061677	109.745234	124.3381357	137.2876105	132.7316939	113.6134377	2R	0.071337618	0.569122804	0.984934163
**Achl**	FBgn0033936	0.234103367	1.701594076	1.088535379	0.363868791	0.669142913	1.618548977	0.049766893	0.296485046	2R	-0.3114071	0.743167492	0.995787491
**CG12868**	FBgn0033945	35.78213624	49.53066673	43.26301737	36.7176689	44.73633765	40.28569977	36.92597018	26.3982675	2R	-0.121351243	0.643898817	0.992589784
**CG12863**	FBgn0033948	26.52043526	26.63330989	23.47101937	15.92224801	33.39004164	34.6612331	20.76083824	10.87315913	2R	0.134017431	0.687765586	0.995787491
**Had2**	FBgn0033949	0.23347236	5.221561752	0	1.270108044	2.107387243	5.649652088	2.779434032	0	2R	0.684707126	0.600797997	0.989286463
**CG10139**	FBgn0033951	146.6219465	134.0617748	135.0027118	127.6742407	182.5750832	184.0842581	219.7305642	209.4182965	2R	0.579251864	1.37E-05	0.002119125
**CG12860**	FBgn0033954	0.073654971	0.370637642	2.680288977	1.03034275	0.1329661	4.097045937	3.194222019	0.15991176	2R	0.91620111	0.481778542	NA
**CG12866**	FBgn0033955	5.486173902	3.849033541	7.358552197	1.967816604	1.856990623	7.783363844	1.278421165	3.722184809	2R	-0.323608262	0.556023388	0.984934163
**jef**	FBgn0033958	641.1431522	664.3076892	728.0167191	762.2019111	618.9048629	627.2457132	689.2584787	690.8297197	2R	-0.059156267	0.54899126	0.984934163
**CG10151**	FBgn0033960	101.2356249	110.8260557	86.66007322	75.61898771	99.67077568	114.0406144	88.88019709	96.22667112	2R	0.121202227	0.374728768	0.943282319
**ND-B15**	FBgn0033961	580.7784528	659.553684	726.4007346	629.4504696	825.7137407	672.9866307	777.7491293	835.767883	2R	0.295884214	0.015846437	0.366845023
**Pgm2b**	FBgn0033969	0.691319331	1.50064743	0.295964304	0.094810882	2.055543538	4.025648383	0.311218318	1.059471715	2R	1.545121339	0.118195781	0.773797042
**CG10205**	FBgn0033970	0.464440995	1.947586538	0.187787896	3.609422322	0	0.583829511	0	1.68057399	2R	-1.418329072	0.346573461	NA
**CG10209**	FBgn0033971	163.490998	171.6087705	155.1819786	163.2596438	156.0284981	162.1556351	153.7732141	156.9313713	2R	-0.025577855	0.789172804	0.995787491
**Ciao1**	FBgn0033972	101.9683145	124.2014558	139.9124417	102.0813166	90.78588677	78.21745026	101.6252557	67.44632702	2R	-0.436545973	0.01297164	0.343192535
**HPS1**	FBgn0033973	287.9753734	287.8465259	280.6863781	318.7511671	298.2535454	285.7619446	331.6073962	362.4142	2R	0.152373111	0.192723775	0.844272804
**Cyp317a1**	FBgn0033982	1.159014786	1.215052944	0.746872078	0.80221543	0.163462402	0.443914275	0.692969911	0.235905786	2R	-1.340539732	0.065503195	0.646711278
**ADPS**	FBgn0033983	62.1508583	47.43471994	51.82072163	48.31384622	60.297832	50.1409859	52.5637964	53.15742197	2R	0.071981819	0.594684341	0.988780662
**Lap1**	FBgn0033984	75.11933986	90.1532499	79.82792238	74.00474455	62.64610325	71.80614256	56.48532541	63.0073815	2R	-0.297975927	0.007059624	0.24630895
**CG10257**	FBgn0033985	135.4690959	147.745363	131.3975757	165.5752341	92.03899309	81.78144816	99.22619757	116.229828	2R	-0.544114778	0.000245408	0.02024977
**ckn**	FBgn0033987	134.2847566	118.2706559	108.2068018	125.5780778	139.0365801	154.648167	125.7390993	117.1335779	2R	0.167730591	0.124492429	0.780439453
**pcs**	FBgn0033988	16.97452764	13.5871278	14.60282528	12.34979274	11.16139064	14.04155474	10.77075526	12.71381835	2R	-0.213156937	0.172226018	0.832669092
**CG7639**	FBgn0033989	12.22511344	8.138780891	8.460563821	8.351863636	6.843244353	10.59655354	7.010924684	6.50172522	2R	-0.241700062	0.344309279	0.930285749
**CG10265**	FBgn0033990	83.21806168	89.99663925	78.99273487	69.19680449	64.39911817	74.70847323	106.2925476	82.07909948	2R	0.057080871	0.749366562	0.995787491
**CG8089**	FBgn0033993	0.086661576	5.233054934	6.30719327	1.31331332	0.391115892	1.103004066	1.32645326	4.421532158	2R	-0.777385043	0.414509882	0.958665466
**CG7544**	FBgn0033994	4.731445544	12.02237059	3.295784217	12.23260359	2.198794738	2.826631577	5.377731342	5.390470612	2R	-1.002287324	0.057585505	0.623014047
**GPHR**	FBgn0033995	59.63879208	60.22557884	56.29837148	42.37580148	33.9835194	44.56092419	30.01398202	29.41956938	2R	-0.633299251	0.000579255	0.040229272
**CG11807**	FBgn0033996	65.77918437	70.54227543	57.55322038	54.24227059	64.48421315	60.36414198	53.57352609	47.1164845	2R	-0.107823873	0.449697026	0.967112302
**Ir51a**	FBgn0033997	2.526365498	7.264497787	4.878125938	2.644546391	3.071516907	10.30511215	6.444812662	1.791011709	2R	0.355052357	0.554066875	0.984934163
**row**	FBgn0033998	124.7780497	126.9319453	114.33667	138.9789858	119.2287766	121.8684958	116.4053723	123.6508738	2R	-0.040409371	0.681787175	0.995787491
**CG8093**	FBgn0033999	0.42616603	0.178708433	0.129234106	0.124198757	0	2.611613746	0.951263862	1.734836065	2R	2.587554276	0.05690728	NA
**CG11808**	FBgn0034000	36.132756	28.95697843	25.07980249	25.97466612	39.8620966	31.51081103	44.29541827	12.85668313	2R	0.169126272	0.635114616	0.991585536
**mRpL41**	FBgn0034001	150.5959718	162.1955324	155.2090712	123.6202799	208.8424312	185.3602088	213.5125602	197.2529085	2R	0.474862754	0.001907439	0.099353754
**CG8079**	FBgn0034002	19.18399736	32.56342836	25.67730208	25.40077516	30.31826029	22.74502093	26.0019606	24.53289652	2R	0.047565542	0.780224775	0.995787491
**ItgaPS4**	FBgn0034005	0.505553963	1.201327455	0.25551393	1.522461709	0.418299968	0.41705404	0.322419755	0.823203339	2R	-0.781183794	0.340410171	0.927274752
**CG8152**	FBgn0034008	48.16924882	28.50243521	53.8000615	47.67497824	70.06020277	69.78364189	45.36854229	33.60938432	2R	0.32140826	0.237863764	0.873280735
**CG8155**	FBgn0034009	89.6421256	84.45771198	81.41873319	84.7862754	84.21756384	82.18831789	64.55385555	83.70532216	2R	-0.083382411	0.486124346	0.973549494
**CG8157**	FBgn0034010	11.96763983	10.36610103	6.901364686	7.089878309	11.510678	10.17321271	16.01558364	11.92655589	2R	0.464901767	0.273820745	0.89549671
**CG8160**	FBgn0034011	4.453775042	4.250504024	3.912081358	6.803185106	10.67406585	3.113045723	13.5164764	2.334031217	2R	0.637626039	0.309580517	0.912131082
**Hr51**	FBgn0034012	201.8906969	209.632908	186.0678625	237.3048498	176.0620974	189.165869	186.5274765	223.5253284	2R	-0.076520316	0.507870289	0.978557094
**unc-5**	FBgn0034013	6.097640622	7.184094405	9.189804373	7.496458094	8.923120452	12.07868109	12.29171441	13.44803423	2R	0.674671165	2.17E-05	0.002837399
**CG8180**	FBgn0034021	1.097050677	1.008674052	1.104000443	1.307285425	1.056244076	1.838742537	1.699890276	1.429079143	2R	0.443803232	0.201208919	0.848203654
**CG12964**	FBgn0034022	0.145925401	0.20612159	0.167690362	0.429751046	0.360486919	0.383769029	0.07837028	0.050023824	2R	-0.080437308	0.928318558	0.996479182
**Pgant1**	FBgn0034025	67.49849911	59.73073045	61.5212859	77.1524468	60.22204677	55.80070341	49.28650883	56.5571732	2R	-0.232666953	0.086310987	0.705932874

**CG8187**	FBgn0034027	68.08797419	63.28827259	60.40354948	50.57052076	66.29870557	63.19978667	74.63218264	45.32477393	2R	0.069340201	0.700705046	0.995787491
**eIF2Bgamma**	FBgn0034029	58.67048218	48.62392684	47.12401892	46.21216909	64.31847137	49.33426172	59.91842828	62.39852007	2R	0.261489804	0.12947434	0.789029778
**CG8192**	FBgn0034030	0.366767448	0.99970153	0.593181809	0.249405479	0.937988098	0.273747337	1.247508853	2.62110835	2R	1.251639626	0.088968049	0.709815866
**CG12963**	FBgn0034031	0.966555531	1.600475907	0.751554563	1.155634786	1.386956262	1.250065431	0.979959252	1.022337156	2R	0.085593215	0.85043256	0.995787491
**CG8195**	FBgn0034032	158.3071223	150.0643035	147.0401006	150.9399018	139.2401807	153.0131757	132.0522182	103.8429051	2R	-0.171972508	0.158145479	0.818884898
**CG8204**	FBgn0034033	19.7370973	28.98937724	25.80165785	20.98152508	30.27671061	25.06779192	31.0446702	29.08543809	2R	0.308762454	0.265993123	0.893759496
**CG8207**	FBgn0034035	68.12838999	42.46679949	52.83592899	54.03074695	57.31099023	60.98893699	49.71076938	58.9704517	2R	0.086371442	0.638000997	0.991585536
**tun**	FBgn0034046	2.411175432	3.521180856	5.304072097	4.409455801	4.62927802	3.668185099	5.79968171	5.552150825	2R	0.366466448	0.081727049	0.694684542
**bdg**	FBgn0034049	11.14010939	16.13788379	8.669973993	10.73873138	10.12569363	10.66808136	9.315556727	11.86054466	2R	-0.120974679	0.537176707	0.984934163
**bug**	FBgn0034050	105.5295068	110.305492	108.1825317	107.5962957	112.6749713	114.3137397	105.0229002	85.4954646	2R	-0.017810729	0.901400783	0.996479182
**Mlf**	FBgn0034051	705.7936475	690.3705983	667.1021249	654.0576336	652.2571232	669.7355371	653.1683275	602.9046725	2R	-0.046240259	0.560580712	0.984934163
**Cyp4aa1**	FBgn0034053	0.098385104	0.033005442	0.286417068	1.192782065	0.852529578	0.03710275	0.35137552	0.683530139	2R	0.29792683	0.802810269	NA
**CG8314**	FBgn0034057	114.8245614	133.9604208	122.5676252	158.10587	160.5626623	168.267869	140.4637184	182.5424161	2R	0.331170833	0.020082942	0.40136988
**Pex11**	FBgn0034058	77.16585773	110.6971091	87.7645735	69.92020382	96.33393937	101.9169111	71.24367739	90.85653149	2R	0.095218211	0.610075658	0.990047511
**CG8320**	FBgn0034059	37.94132546	53.91051476	45.94167994	39.02135636	47.90955898	58.02547355	54.09304986	39.81174988	2R	0.209638455	0.289702876	0.903534148
**CG8370**	FBgn0034060	23.78973145	29.31199457	29.20577821	31.31593492	35.31951546	28.66776417	26.65486955	29.22762028	2R	0.110821734	0.453730418	0.967967712
**Ufc1**	FBgn0034061	99.26406187	88.60059317	106.9677705	74.88222268	99.194772	84.1953437	107.9131423	74.83374505	2R	0.015193865	0.93817383	0.997038394
**CG8389**	FBgn0034063	21.0376674	36.32248894	24.25221009	29.7241465	35.4084159	31.95838873	38.53571591	20.02132611	2R	0.210488009	0.311154078	0.914049378
**Rrp42**	FBgn0034065	31.09761884	27.60763607	31.83300828	22.45822393	49.15600693	36.25500747	42.18071034	37.87472222	2R	0.579300758	0.011498929	0.324854493
**EMC7**	FBgn0034066	367.7380735	356.6920676	391.7348394	353.5891703	379.1083524	392.8203282	373.5739303	400.4650642	2R	0.103478484	0.286886621	0.90249226
**CG8399**	FBgn0034067	1.885738294	0.948918434	2.104396408	0.234481485	1.134746977	1.56451904	0.96211178	0.764234314	2R	-0.198587697	0.740510158	0.995787491
**casp**	FBgn0034068	180.3709123	167.126629	155.4746647	154.2755267	150.4373863	186.3020132	158.984728	142.3329628	2R	-0.015639192	0.886475403	0.995926915
**CG8401**	FBgn0034069	4.715106114	9.178263333	1.270975741	5.292970416	13.03067779	6.750381592	7.870393343	8.341203726	2R	0.846481264	0.108798446	0.758410631
**SP2353**	FBgn0034070	10.50415217	16.90764064	13.25606201	15.27165371	16.89997132	12.56971537	13.17449553	17.96684279	2R	0.152684089	0.380272742	0.944473714
**CG8405**	FBgn0034071	36.57316992	42.08748244	35.61469293	33.01722285	32.43094298	31.65089668	43.58307268	39.47369706	2R	0.032311462	0.809861417	0.995787491
**Dg**	FBgn0034072	50.07897535	59.30918483	59.26503059	59.33679188	55.98964797	50.24340982	48.54418687	56.80189309	2R	-0.074954345	0.474550685	0.973549494
**CG8414**	FBgn0034073	5.954676487	9.724130896	5.85475116	8.990380165	6.866559465	10.09041986	6.089586768	7.802454099	2R	0.04950866	0.867889059	0.995787491
**Asph**	FBgn0034075	2.482233296	3.313439351	2.679886833	1.817979105	0.563066047	2.021668452	3.116385675	0.677171719	2R	-0.665460187	0.19184195	0.843483578
**CG10731**	FBgn0034081	37.09692921	38.28087338	36.3406831	38.82809442	46.44913018	67.37417804	66.98633943	68.67974199	2R	0.75751031	0.000258446	0.020710451
**Ibk**	FBgn0034083	19.37701422	17.18370584	17.61594918	15.88839942	20.18693577	20.51683564	21.56561949	19.53667263	2R	0.252134509	0.020259069	0.402963826
**CG8435**	FBgn0034084	86.75562589	100.700572	95.31881293	92.40564809	94.98651089	84.7716631	102.5095387	107.8228818	2R	0.088396748	0.551294126	0.984934163
**Ptp52F**	FBgn0034085	0.457469539	2.524147945	1.080609071	0.757828191	1.912494197	2.099747504	0.460663667	0.914797503	2R	0.207312429	0.733293081	0.995787491
**CG8441**	FBgn0034086	58.45479872	81.25319779	43.87927422	61.43046411	64.96508435	47.73518115	63.22409119	53.40035995	2R	-0.063643128	0.762209582	0.995787491
**clu**	FBgn0034087	46.87270982	49.06581373	46.57215496	49.3514489	58.01048393	51.29290977	54.02771032	51.72585227	2R	0.195391206	0.057421093	0.623014047
**mrj**	FBgn0034091	22.12368103	22.64647382	24.32071921	21.36063911	22.76416501	24.89169327	19.2195515	18.6895137	2R	-0.050588696	0.659275968	0.995750687
**CG15706**	FBgn0034093	2.518917087	3.173715195	2.252850772	3.518243535	4.756843513	5.165506322	5.034045959	5.77122441	2R	0.885954568	0.00047974	0.034466853
**CG15701**	FBgn0034095	3.585892916	5.305007384	3.02585259	2.284822801	3.779533182	7.916433183	1.874992333	2.707932335	2R	0.225407725	0.62648538	0.990047511
**prim**	FBgn0034103	69.10422674	92.28307079	72.5080021	67.90751292	84.88555817	57.43342623	46.13555061	100.0211154	2R	-0.028511215	0.921640737	0.996479182
**CG7747**	FBgn0034109	63.31580228	66.80534942	73.64848064	61.36337109	58.74279826	69.36553986	83.4835632	53.01049126	2R	0.026809348	0.872504147	0.995787491
**Atg9**	FBgn0034110	52.17892905	60.02445205	73.75488978	53.12522892	39.97546915	54.70162467	40.88476798	36.85423988	2R	-0.439810883	0.007460596	0.252750432
**CG8060**	FBgn0034113	28.53952606	29.91940704	37.99447612	31.80702343	36.41158844	26.38945064	22.50152806	31.70443334	2R	-0.097985239	0.583328873	0.985764834
**CG4282**	FBgn0034114	50.61287929	41.83684003	43.07157763	36.91424533	41.49884689	45.78133161	38.70056312	42.14185486	2R	-0.009489012	0.95076589	0.997604378
**CG7997**	FBgn0034117	1.743594846	9.071327841	4.516712151	4.409627926	7.682364797	1.922469311	7.68967266	2.438122388	2R	0.047437603	0.931474552	0.996479182
**Nup62**	FBgn0034118	109.4219464	92.72122606	99.63655629	85.17689422	118.1112848	111.2104688	114.2134763	81.6508784	2R	0.163357683	0.300828415	0.907713255
**CG6262**	FBgn0034121	1.87208649	0.344651977	0.553860453	1.703297239	0.865506274	2.755109007	1.456016115	1.189601873	2R	0.488750936	0.483224323	0.973549494
**CG15711**	FBgn0034122	2.475862823	1.23967539	1.434366251	0	0.533679636	0.801302268	0.094268347	0	2R	-1.806677743	0.164084617	NA
**CG7848**	FBgn0034127	0.618113075	1.105917836	0.59981348	0.960738244	1.562193531	1.087806033	0.105121316	1.073584564	2R	0.265941762	0.780944976	NA
**CG4409**	FBgn0034128	82.11240055	110.7013433	145.5767628	63.27843061	29.85731802	7.096797867	33.94260489	12.77493358	2R	-2.219173477	5.91E-05	0.005936513
**Parp16**	FBgn0034129	0.170284887	1.904193391	0.963920439	0.529350404	0.614815728	0.856232272	0.289600269	0	2R	-0.948376715	0.486510545	NA
**S-Lap8**	FBgn0034132	0.203043239	5.721686021	1.280708169	1.609518069	0.146618034	0.382855851	0.310780599	1.410642134	2R	-1.920352127	0.059738436	0.627645287
**Syn2**	FBgn0034135	116.9513211	91.19114274	115.830487	108.7233287	100.0022922	112.4507385	92.77833808	116.1310627	2R	-0.010205952	0.933129523	0.996479182
**DAT**	FBgn0034136	28.55246922	18.75200898	27.31026032	24.49037142	30.61261914	37.80180126	29.21468069	23.20046123	2R	0.309666237	0.077641718	0.682035833
**CG4945**	FBgn0034137	0.655398002	1.312113342	2.256754907	0.345040335	1.717492313	0.179391842	1.618001017	0.413108632	2R	-0.170579671	0.836826352	0.995787491
**RpS15**	FBgn0034138	805.9060744	1017.809422	1129.23883	1107.096833	914.7116215	809.6923525	1091.978225	1106.638581	2R	-0.014550079	0.916510351	0.996479182
**Lst**	FBgn0034140	4.402123097	0.79630768	0.418803499	0.503107073	0.311645508	0.569648301	1.321165953	0.562200985	2R	-1.168056878	NA	NA
**CG8311**	FBgn0034141	112.737352	108.0674706	81.04772839	122.2634861	82.46061456	95.44548027	95.03871207	75.87308369	2R	-0.256060976	0.096268396	0.730787365
**CG8306**	FBgn0034142	3.817418782	2.748269444	1.270314807	0.157525101	0.670846126	0.987347013	0.0430899	0.990155654	2R	-1.538281271	0.062183874	0.633840737
**CG8303**	FBgn0034143	2.104049413	0.621893872	1.304206531	0.108050925	0.351389064	1.249634122	0.212807389	0.623835539	2R	-0.746805003	0.313784011	0.91460807
**CG5065**	FBgn0034145	63.97892924	62.16117828	60.7732827	65.96082161	59.80809003	64.25999608	54.38251268	64.90339706	2R	-0.026084785	0.785540954	0.995787491
**CG15617**	FBgn0034151	2.23572168	0	1.549665603	1.48928598	0.691894396	1.32491841	0.488860632	2.49632163	2R	-0.060166876	0.958142652	NA
**resilin**	FBgn0034157	0.556945241	0.133456787	5.147205286	0.370999104	0.718163503	5.300853772	0.608905404	0.057579985	2R	0.126628443	0.907188453	0.996479182
**CG5522**	FBgn0034158	48.1759246	46.42146978	47.0839156	49.44849115	41.91255844	48.80674745	42.4807169	50.40616354	2R	-0.028535396	0.792065886	0.995787491
**CG15615**	FBgn0034159	1.211812439	0.132563828	0.511276535	0.491355667	0.428014964	0.894122112	0.806441626	0.171584151	2R	-0.031605411	0.974462368	NA
**CG6426**	FBgn0034162	1.131802311	0.593262375	0.514825616	0.577227543	3.384038821	0.200073057	2.34589226	0.230366961	2R	1.144054231	0.242827223	0.876959115
**CG6435**	FBgn0034165	12.93882508	13.62470124	8.719280323	6.033276715	5.96923995	6.37040363	5.317830009	5.306150872	2R	-0.820169114	0.056480767	0.621788268
**CG15614**	FBgn0034168	0.051681531	1.820458873	0	1.084441892	0.466492045	2.7773268	2.768654604	1.570875425	2R	1.399778922	0.228841972	NA
**CG6665**	FBgn0034172	62.26797276	42.43420732	77.18352268	47.24072175	54.01224856	51.77957781	61.36237501	65.98689857	2R	0.055931106	0.793200837	0.995787491
**CG9010**	FBgn0034173	1.52204585	0.696277743	0.335678364	0	1.124052078	1.695880172	0	2.253069842	2R	0.984388623	0.486509079	NA
**ste24b**	FBgn0034175	6.570645283	3.354319559	4.019726265	9.591158894	6.601293233	4.040063634	4.08114786	2.108809961	2R	-0.461036634	0.398224259	0.95231018
**ste24a**	FBgn0034176	111.2204816	111.1292649	124.4462752	138.5188664	130.9364276	105.4835776	116.1466028	137.4129196	2R	0.045834678	0.754183881	0.995787491
**AsnRS-m**	FBgn0034177	63.78988558	45.35219179	41.24138104	43.40920669	54.75320312	43.40104015	67.04670262	40.07179469	2R	0.107240109	0.629212541	0.990523437
**CG6805**	FBgn0034179	25.90096809	38.20062739	38.13175217	42.75369544	33.71449512	28.20366452	31.15067639	32.01542638	2R	-0.177512745	0.375790556	0.943615128
**Ehbp1**	FBgn0034180	105.6489697	87.81027982	79.63132029	89.91808697	81.64623222	89.95861918	82.06887615	88.76620076	2R	-0.056637173	0.616535621	0.990047511
**CG8963**	FBgn0034181	150.4256183	220.8001785	158.7485719	198.6385783	163.5318435	178.3903826	158.5504363	180.182516	2R	-0.06586705	0.610409927	0.990047511
**SmydA-7**	FBgn0034182	18.90630349	13.84738837	21.79174208	25.4303855	17.06536173	25.00303941	20.84163756	20.15219842	2R	0.082792201	0.741842265	0.995787491
**CG9646**	FBgn0034184	509.9170899	442.288299	399.7199988	464.4785116	445.7119788	468.5628376	388.2860971	427.2803687	2R	-0.043029872	0.682280796	0.995787491
**CG8950**	FBgn0034186	21.71356567	31.05501325	25.5113524	32.64904437	23.95467016	29.32258533	32.71325695	21.4075514	2R	-0.015900362	0.935129407	0.996479182
**CG6984**	FBgn0034191	14.83516914	15.5190008	11.99663781	13.94662838	24.3335309	25.56673296	34.99629922	24.76246852	2R	0.985606689	0.000457734	0.033171803
**CG15611**	FBgn0034194	5.05639505	4.398735063	2.971447272	1.354613057	2.494633021	2.872126843	3.445067164	2.931986173	2R	-0.207554559	0.604511288	0.989286463
**Cda9**	FBgn0034197	0	0.862022391	0.226682477	2.396352717	0.084340957	1.541645282	0	0.608596212	2R	-0.609049966	0.695130677	NA
**CG11400**	FBgn0034198	2.920516506	1.075336843	0.69123242	0	1.414514365	3.962274689	0	0	2R	0.191944811	0.90196156	NA
**Gbp2**	FBgn0034200	1.618345142	2.282687203	0.981520685	0.857525176	1.128772089	0.069352996	0.187656056	0.239562076	2R	-1.78667662	0.075451616	NA
**Camp**	FBgn0034210	79.47825837	55.36484889	83.14324581	91.31855477	73.0650911	75.97019642	100.6468019	88.49183854	2R	0.156633523	0.389228747	0.948247506
**CG6550**	FBgn0034214	55.89488095	43.64223005	44.51521768	30.38531698	40.25991052	50.12458598	33.24677161	41.06388063	2R	-0.056420871	0.789310595	0.995787491
**Mtap**	FBgn0034215	162.9220818	158.4807544	145.8500407	190.1226402	144.7433972	150.8082041	165.4330107	161.9510555	2R	-0.049002207	0.708627813	0.995787491
**Lhr**	FBgn0034217	27.94843141	24.72499668	28.67886625	17.35350653	20.94568932	19.95140214	22.52465516	22.33860855	2R	-0.175118351	0.507381008	0.978557094
**mthl4**	FBgn0034219	0.226157299	0.632245238	0.182884818	0	0	0.817342262	1.538485678	0.163669477	2R	1.294889448	0.403170171	NA
**CG10764**	FBgn0034221	0.260226489	0.374137225	0.541119285	0.693380872	0	0.035048563	0.474173832	0.242132484	2R	-1.326968939	0.374990709	NA
**Tes**	FBgn0034223	31.46411713	35.11100507	35.9357022	43.23065747	28.921941	40.68300796	36.24683687	36.09447945	2R	-0.008070098	0.960776874	0.997604378
**CG4853**	FBgn0034230	135.6697181	165.1840575	145.4277636	144.2658595	162.132634	175.0394532	159.6314724	121.8753507	2R	0.097082922	0.422150068	0.960593359
**APC10**	FBgn0034231	98.60433801	67.85433609	80.8734459	86.45522471	97.03276606	73.2761264	69.51439635	68.3101547	2R	-0.088823503	0.657976567	0.995750687
**CG4866**	FBgn0034232	32.42797505	47.88785855	52.53366395	36.42082207	46.87154249	53.62953326	46.7214564	52.86157694	2R	0.279906147	0.234166103	0.87009833
**eIF3b**	FBgn0034237	518.8518675	609.1364481	632.3410229	704.5053856	558.3127142	602.1716547	609.1814339	613.7348915	2R	-0.017827492	0.868201191	0.995787491
**MESR4**	FBgn0034240	58.16232199	53.73340969	62.54712289	52.07487701	57.75710282	53.00437038	57.57538877	66.98951366	2R	0.087247593	0.463884836	0.971928234

**Ns2**	FBgn0034243	93.70431057	108.4383009	94.35257985	112.1006753	96.72978297	116.3783819	107.6348029	90.26980009	2R	0.037664763	0.760385174	0.995787491
**UQCR-6.4**	FBgn0034245	829.2941629	805.4300744	881.7714408	669.2435581	883.4344982	730.3392149	854.9547163	797.0522748	2R	0.068464962	0.565268862	0.984934163
**Dcr-2**	FBgn0034246	21.26421032	17.95593862	15.98641286	18.54113268	17.15128626	20.28479784	21.16484242	24.47661282	2R	0.198589914	0.230414302	0.867702781
**CG6484**	FBgn0034247	5.011329923	0.43478291	1.781690348	0.60433071	0.935869128	4.439542976	1.212276839	0.281380892	2R	-0.19558624	0.836786796	0.995787491
**CG14483**	FBgn0034248	10.24601632	7.403309847	10.51629306	14.51667619	21.05776502	28.83103124	27.94734829	23.95620757	2R	1.284107976	5.67E-05	0.005765311
**RhoGAP54D**	FBgn0034249	0.796444959	1.59659292	1.131023041	0.090579582	0.596155701	0.421227297	0.644212844	0	2R	-1.083076453	0.285203072	0.901780531
**CG10936**	FBgn0034253	0.392717351	0.378237519	0.47943672	0.082699871	0.137217188	0.293812496	0.064634219	0.209030822	2R	-0.881636178	0.13895508	0.801419817
**eIF3c**	FBgn0034258	294.6994969	363.3662613	339.2999947	370.3768528	281.3061417	337.8225508	328.4681359	293.1418623	2R	-0.110530155	0.287762786	0.902924182
**P32**	FBgn0034259	275.9939743	236.8739324	218.817906	174.788242	242.4822145	240.0197252	235.4082479	226.486954	2R	0.08794707	0.52837645	0.984296327
**HPS4**	FBgn0034261	294.2486816	254.5180144	261.4216599	275.1976113	200.1417183	171.2920687	177.6744002	183.0227156	2R	-0.538081051	1.21E-07	3.66E-05
**swi2**	FBgn0034262	10.79742999	6.122892975	8.441801847	3.57921349	2.740599425	3.730967207	3.045992593	2.222011376	2R	-1.276679073	0.000280776	0.022075385
**CG10934**	FBgn0034263	1.413157558	2.649241036	0	2.131880008	0.900392323	1.48905941	0	0.180476228	2R	-1.219370634	0.402651658	NA
**Dlish**	FBgn0034264	0.495811366	0.083165401	0.120283146	3.583493158	1.163586568	0.701171891	0.252965089	1.076452372	2R	-0.395870383	0.748112641	NA
**Snx16**	FBgn0034265	80.4686839	67.1676185	64.64792107	69.2422083	61.61782254	91.82048509	54.84601954	77.31315542	2R	0.04654193	0.792974387	0.995787491
**Hyccin**	FBgn0034269	131.7205181	118.6789533	132.0530416	146.606566	88.99267105	68.39647228	101.0236499	68.03016581	2R	-0.667100721	1.58E-05	0.00230507
**PIG-A**	FBgn0034270	39.21543453	50.77753664	52.04983476	41.60327244	53.81549916	48.84747004	55.26802854	52.23277725	2R	0.229273045	0.144469671	0.8037297
**Vps50**	FBgn0034271	101.7146901	120.1820799	112.9189669	111.3985913	100.9781432	126.1884413	119.6622191	90.28363228	2R	-0.000331837	0.997787685	0.999596266
**Sardh**	FBgn0034276	0.453182311	1.064209651	0.659648379	0	1.145354309	0.113935337	0.077071854	0.065593383	2R	-0.579928026	0.678082767	NA
**OstDelta**	FBgn0034277	95.77979086	63.7971921	56.99565784	48.30226996	76.24199456	70.63731857	73.21236362	60.57580468	2R	0.108735215	0.576802926	0.985637351
**Mapmodulin**	FBgn0034282	298.2000422	249.4098606	279.8315366	284.7050429	313.2452603	296.3209832	303.7559028	313.0296943	2R	0.17040433	0.082091472	0.694684542
**dpr13**	FBgn0034286	1.400478041	1.307520956	1.794926895	0.899459674	0.939735274	1.908296984	1.712175455	0.613850413	2R	-0.036546058	0.911891761	0.996479182
**CG5098**	FBgn0034300	162.7416193	175.9413419	160.6972955	173.0028533	144.7643908	166.5440298	157.2257978	176.0151969	2R	-0.03045355	0.753057995	0.995787491
**CG5756**	FBgn0034301	1.276578948	0.158613489	0.458809292	0.082674882	0.256061354	0.178303827	0.180921129	0.076988085	2R	-1.508513084	0.123049879	0.780439453
**CG5742**	FBgn0034304	157.0053716	182.730397	176.5283288	190.3690365	150.2341994	161.4781657	178.2922224	188.4534514	2R	-0.026627026	0.817740636	0.995787491
**CG10914**	FBgn0034307	20.29389973	24.34473686	23.33692232	21.64071085	42.57661306	36.99305027	38.96216854	25.37339287	2R	0.715429845	0.000500033	0.035315887
**Naus**	FBgn0034308	298.753343	333.4162695	285.6599531	438.9065613	330.8355094	346.9771221	320.8547994	384.6137589	2R	0.056947648	0.678474621	0.995787491
**Nup75**	FBgn0034310	30.63155457	20.31445824	17.61144907	11.55871016	18.7309334	15.19081544	13.09890671	30.29196828	2R	-0.021754286	0.946307429	0.997604378
**CG10916**	FBgn0034312	33.40869105	25.86387344	33.62563315	24.94872736	26.61908887	16.52328386	29.12525122	36.67828211	2R	-0.081081815	0.752592651	0.995787491
**CG5726**	FBgn0034313	48.5146305	60.91476898	52.89508535	53.81716606	43.82231179	33.74264635	57.81217163	48.02245277	2R	-0.202158485	0.221090343	0.86549481
**nopo**	FBgn0034314	2.611820556	0.933647763	2.59681977	0	1.314017928	0	1.31071679	1.859181705	2R	-0.419860268	0.731807104	0.995787491
**CG5721**	FBgn0034315	56.78597977	77.7806047	63.61175985	68.08853525	48.46939726	76.82751002	46.06219149	38.74753731	2R	-0.312420153	0.128342401	0.788096052
**GstE1**	FBgn0034335	28.17701967	64.06761904	55.95151678	40.38943579	34.09955682	19.08753213	53.77702545	10.64896721	2R	-0.643254181	0.119971211	0.778085657
**CG5174**	FBgn0034345	209.6096689	194.9387489	198.2031331	221.982288	183.069098	192.9478821	177.2591569	159.6600517	2R	-0.182431569	0.064756107	0.641709151
**PIG-O**	FBgn0034346	68.55063612	76.02467776	80.51938881	77.07237683	69.98992886	89.49129257	73.76796965	80.90455884	2R	0.086948668	0.452350522	0.967630712
**rswl**	FBgn0034351	131.6573472	89.65550988	113.6058573	100.139097	94.55318263	99.54805831	105.3083911	125.6298503	2R	-0.006050651	0.971202701	0.997604378
**CG17669**	FBgn0034352	2.460460311	0.252678184	0.243634384	0.819495799	1.08777832	8.284667687	3.07429573	1.308217248	2R	1.824279119	0.047741603	0.575780949
**GstE11**	FBgn0034354	2.567372136	2.050669806	2.965909044	2.137761245	3.200194686	4.207064335	0	0.39814304	2R	-0.284627093	0.771386708	0.995787491
**Pepck2**	FBgn0034356	0.895440453	0.733523037	0.53045171	0.509783719	0.09398253	0.078531701	0.557791215	0.135633775	2R	-1.636347887	0.037184697	0.521998122
**CG10927**	FBgn0034360	14.87008509	7.981594135	20.74290594	14.90768875	14.76437312	19.06641853	18.0186056	16.46500893	2R	0.254025949	0.466609679	0.971928234
**mRpS28**	FBgn0034361	139.6826412	204.6286753	147.4583537	134.7147642	166.6249541	149.6820909	168.7319411	165.0146865	2R	0.087543083	0.589820777	0.987597375
**CG5323**	FBgn0034362	109.495056	103.8227479	113.0117158	103.446851	118.0670997	106.6230303	89.6567275	110.7508998	2R	0.015400729	0.91991851	0.996479182
**Atg7**	FBgn0034366	7.738502068	11.32995068	11.42934235	10.71934567	14.51648246	13.18240935	12.9354787	10.1463278	2R	0.337502005	0.111616596	0.758410631
**zda**	FBgn0034368	159.1711866	158.0939665	117.8845988	167.2593934	122.8743472	139.1039018	120.3542564	121.4577769	2R	-0.230720023	0.079651693	0.692197295
**Ntan1**	FBgn0034371	9.584887666	9.7501612	11.07564574	9.259790002	10.16032572	10.29727199	8.655311278	8.969502188	2R	-0.0271323	0.831400132	0.995787491
**CG15073**	FBgn0034379	64.12600222	35.53111412	48.67948229	53.60714354	47.83516044	74.50991097	50.89367403	40.13650073	2R	0.098650474	0.651065338	0.993444251
**Vps51**	FBgn0034380	55.42546054	68.60170782	67.38560654	44.06661627	54.58187335	54.66284029	51.80012663	37.91623941	2R	-0.210360501	0.22382935	0.86549481
**Cyp12b2**	FBgn0034387	66.54006993	48.42998525	50.72215829	51.26053871	36.17136421	68.52195589	53.65385911	42.87105614	2R	-0.086529807	0.670905756	0.995787491
**Mctp**	FBgn0034389	24.93274314	27.68804453	32.11686114	22.72021682	25.08400488	22.0501763	23.10104069	18.60988096	2R	-0.24163048	0.077108978	0.682035833
**CG15080**	FBgn0034391	1.157977432	0.347306667	1.265086905	0.572138975	0.609136598	1.670136195	0.25432006	0.249742669	2R	-0.24648106	0.705563722	0.995787491
**MFS15**	FBgn0034392	31.99829366	39.07312402	48.75940071	58.72153407	31.19771983	16.61012673	49.01990657	38.70671907	2R	-0.362032867	0.211530975	0.856520545
**CG15096**	FBgn0034394	0.519995471	1.177496264	0.346912984	0.242469973	0.704043825	0.073537283	0.829074762	0	2R	-0.472855188	0.649216052	0.992932477
**CG15097**	FBgn0034396	120.6958539	141.8467908	113.390462	132.0951258	131.28178	127.3275441	127.8543355	130.8206363	2R	0.057454197	0.560207763	0.984934163
**CG15082**	FBgn0034397	0	0.948628577	2.744026433	2.42891796	0.537347538	1.234769322	0.987128891	1.163234409	2R	-0.600677568	0.544923936	0.984934163
**CG15098**	FBgn0034398	31.73327032	60.47707352	86.2705358	31.0909413	48.37047037	78.9274664	70.40011225	61.92585745	2R	0.347561395	0.222114916	0.86549481
**CG15099**	FBgn0034400	66.63894016	69.29372966	66.80643527	86.38095804	69.72569658	73.98691656	81.16483517	87.6751231	2R	0.142741682	0.259530268	0.890556245
**MetRS**	FBgn0034401	108.0354809	76.27625583	91.1650752	97.00590231	89.490557	102.2917761	94.38708337	77.81637081	2R	-0.008526577	0.950805015	0.997604378
**CG15084**	FBgn0034402	15.40395968	15.06133589	14.8352438	24.54407179	21.51983249	16.85807714	27.44784251	28.90587262	2R	0.470013591	0.142580489	0.8037297
**Jheh3**	FBgn0034406	2.441383318	13.3943177	2.961380939	5.217660524	2.111842084	2.58943644	7.78502662	1.877249501	2R	-0.705045102	0.280454687	0.899310165
**sano**	FBgn0034408	1.048701343	1.126723078	0.984492093	0.921235081	1.305172377	1.565624071	1.092443098	0.827726564	2R	0.259256208	0.18493602	0.839299945
**CG15117**	FBgn0034417	1.433630604	4.686109762	4.78592157	5.199375664	6.105192366	2.056307995	4.376163641	3.059338858	2R	-0.002547297	0.995084247	0.998919792
**CG15118**	FBgn0034418	107.7317576	108.9463614	94.7498506	78.26773292	88.22397304	108.1170796	78.57674806	117.2483058	2R	0.039928468	0.791005416	0.995787491
**CG15111**	FBgn0034419	96.78914833	73.51782851	91.00861935	68.16130889	67.21011526	99.54787401	75.54565455	77.08148564	2R	-0.018910741	0.901033571	0.996479182
**CG10737**	FBgn0034420	27.35118751	30.24466337	26.22205896	28.2410508	39.15866751	53.31304699	44.33424295	44.24931024	2R	0.720646913	6.8E-11	4.36E-08
**CG7137**	FBgn0034422	10.71636294	22.32587972	17.05584677	14.00421729	10.72027393	12.99917801	18.10903119	16.59755532	2R	-0.097001459	0.774897819	0.995787491
**tbrd-2**	FBgn0034423	0.072635845	0.670100425	0.792960965	0	1.049010539	0.068480631	0	1.419292298	2R	0.800803555	0.603743535	NA
**CG11906**	FBgn0034425	54.02144616	59.70428775	58.17333766	52.25373328	46.17871342	78.03439307	56.8270207	59.45623613	2R	0.130606556	0.416411481	0.958846438
**mip40**	FBgn0034430	120.9956062	86.29504246	127.5060881	70.33907241	114.517084	72.2318447	98.43170734	73.08509742	2R	-0.146236287	0.516449688	0.980804634
**CG7461**	FBgn0034432	78.73000242	53.24528514	65.54574173	62.72534462	70.99512263	51.9152028	59.78595619	47.65525216	2R	-0.149138882	0.406313789	0.956706394
**EndoB**	FBgn0034433	191.243827	144.1612271	152.47496	160.5913085	161.9075888	187.9419859	151.8190265	164.2716162	2R	0.064144081	0.599191429	0.989286463
**CG11961**	FBgn0034436	13.27464888	8.859777344	5.373615125	6.933808719	6.235710616	9.609147822	7.468237849	3.564728241	2R	-0.340447683	0.298660994	0.9074568
**CG10051**	FBgn0034437	0.474230745	0.397727774	0.460190869	0.276412783	1.112940452	0.134130555	0.060488641	0.102959885	2R	-0.149678715	0.898457748	NA
**CG9416**	FBgn0034438	0.120787761	0.315162594	0.586058848	0.031290235	0.072684289	0.797145744	0.239658357	0.145689673	2R	0.303817516	0.783526421	NA
**CG10062**	FBgn0034439	0.285104285	1.972667574	0.086457377	1.204786708	0.418182624	1.427971199	0.454566973	0.773734744	2R	-0.16820893	0.845613342	0.995787491
**CG10073**	FBgn0034440	0.528876669	0.755691764	0.28512165	0.502356167	0.459698267	1.809812073	0.24984736	0.042527415	2R	0.328277759	0.739586733	0.995787491
**CG10081**	FBgn0034441	0.178594321	1.75460624	0.185685999	0.416385936	0.092116721	0.096215791	0.325427107	0.443136918	2R	-1.377424647	0.238162766	NA
**CG11257**	FBgn0034442	34.1682583	46.16153786	51.46638861	48.32144688	44.38666542	54.84150788	51.74970503	54.2306621	2R	0.22328565	0.211142003	0.856520545
**cer**	FBgn0034443	5.90932376	12.25965515	9.054276012	8.701494245	7.299051113	6.450949243	9.124228237	6.414844452	2R	-0.259294074	0.649686344	0.993119221
**Arl6**	FBgn0034446	4.730006588	5.123991613	1.912488335	2.756958074	0	3.158756768	7.038703249	8.771678507	2R	0.410211431	0.663804358	0.995787491
**CG7744**	FBgn0034447	12.20620994	6.440242828	10.77435384	10.6885705	10.70731182	13.91218119	14.39963814	15.3654941	2R	0.462895197	0.08572095	0.702456631
**TBCB**	FBgn0034451	262.3055883	205.5479642	215.6109871	216.6834586	231.2338891	196.3442448	213.3965839	195.6176862	2R	-0.077739086	0.550777418	0.984934163
**Oseg6**	FBgn0034452	7.921960383	9.867609494	7.573603219	4.670028513	7.753253284	8.846841566	4.373265735	8.658422231	2R	0.014375851	0.961181525	0.997604378
**CG11007**	FBgn0034455	140.4282698	116.6318452	132.1948031	85.49009017	97.2433543	86.15837234	88.90728271	100.5595565	2R	-0.317750875	0.057860916	0.623014047
**Ir56c**	FBgn0034457	1.118967354	2.388795514	1.727472214	0.94866555	0.734553768	2.109911173	0.259500887	0.110426441	2R	-0.883936016	0.383321627	NA
**Ir56d**	FBgn0034458	0.136909767	1.607528488	1.992847104	0.106399989	0.741471655	1.678009991	0.116419919	0	2R	-0.538547161	0.695699417	NA
**Obp56a**	FBgn0034468	2.427517712	2.220992954	2.944562056	7.974984136	2.589535968	4.369238755	1.407420418	0.718686227	2R	-0.733452783	0.408162687	0.956706394
**Toll-7**	FBgn0034476	124.5298124	126.1603009	147.4392808	136.9893451	136.3422537	108.4670556	140.3188253	110.0400955	2R	-0.07988779	0.514169554	0.980804634
**CG13872**	FBgn0034477	0.665015322	1.11546991	0.907491316	0.193807273	0.300130774	0.313486192	0	0	2R	-2.131580207	0.150044518	NA
**CG8654**	FBgn0034479	0.582256029	0.154208338	0	1.786194754	0.968138099	0.072229925	0.46905715	0.033266625	2R	-0.693973919	0.568480709	0.984934163
**CG16898**	FBgn0034480	0.125261382	0.945487781	0.303882366	0.146021098	1.356773392	0.472382663	0.159772238	1.903678389	2R	1.41308505	0.294333326	NA
**CG16894**	FBgn0034483	9.633557301	3.231788184	8.129000224	11.91371172	2.722075824	0.078977904	0	0	2R	-3.516564423	0.000740537	0.048215891
**CG11044**	FBgn0034484	1.397970395	1.360041548	0.271316534	1.238539832	0	0	1.783126255	0.060702463	2R	-1.204293086	0.378810912	0.943971504
**CG11099**	FBgn0034485	99.06322799	108.7347089	106.307583	109.5895484	167.073945	218.02547	206.4327371	224.1452664	2R	0.975553958	6.33E-17	7.72E-14
**Efhc1.2**	FBgn0034487	1.592572443	3.413349318	0.930115943	0.962635514	3.034723734	2.001956762	1.956108323	1.536720976	2R	0.338414568	0.573376846	0.984934163
**Hacl**	FBgn0034488	89.93707827	143.0301347	117.2640663	138.8961439	108.3780652	126.6460429	119.9548266	135.049485	2R	0.03609209	0.809223663	0.995787491

**ppk6**	FBgn0034489	4.832777654	1.403014594	2.367396873	0.86672609	1.677769672	1.0076462	1.896695327	0.807108281	2R	-0.80974189	0.287186162	0.90249226
**CG9864**	FBgn0034490	1.377415527	0.53317415	1.22096589	0.802848112	1.219384527	1.373539269	0.506800471	0.920152805	2R	0.057220418	0.91730006	0.996479182
**Hsl**	FBgn0034491	41.86406867	36.85716738	46.3471677	43.74861605	42.04599467	38.50750243	34.53255852	35.8949329	2R	-0.130731389	0.34498749	0.930285749
**CG8908**	FBgn0034493	1.169360577	0.693679407	0.207574569	0.432221489	0.514876962	0.215115319	0.29103063	0.340569398	2R	-0.870693514	0.271973961	0.89549671
**CG10444**	FBgn0034494	45.19609796	63.50692373	58.62505857	54.77976885	56.9210774	76.84255921	46.50668951	28.87581598	2R	-0.059834267	0.788568968	0.995787491
**CG11788**	FBgn0034495	9.418683022	5.07809554	9.38465453	10.8489423	20.64666617	31.23813811	15.16005863	23.00493681	2R	1.400644712	2.85E-05	0.003444005
**CG9143**	FBgn0034496	39.77608765	36.54472034	24.49904382	43.4509056	38.53395539	47.85427452	35.97628745	44.29740551	2R	0.233194664	0.226061958	0.867702781
**Mpcp1**	FBgn0034497	2.143391814	1.97385675	1.427406687	2.547611185	1.517400616	2.773615092	1.500975293	2.007393036	2R	-0.023717431	0.968613486	0.997604378
**CG16868**	FBgn0034498	85.19177865	95.57500291	87.2837454	80.48324899	66.92498887	73.36982266	75.00276035	76.46128853	2R	-0.225150776	0.027085898	0.461092427
**CG11200**	FBgn0034500	2.669026346	7.170062813	6.930295581	6.368579788	2.860851744	3.145427171	5.159750973	2.625724918	2R	-0.710237753	0.065763681	0.647077356
**CG13868**	FBgn0034501	273.8583747	246.796587	222.9905598	261.6133909	239.8156976	301.4514028	265.3208801	241.3080242	2R	0.086229193	0.413504297	0.958665466
**CG13871**	FBgn0034502	0.050476833	2.370698579	1.95929749	0.94147869	0.820112588	0.761427999	0	3.068516564	2R	-0.130294848	0.914291408	NA
**MED8**	FBgn0034503	82.74653612	71.13468727	113.8047356	94.25601179	73.47026645	75.38137192	78.55512672	87.38163676	2R	-0.169422738	0.373377132	0.943282319
**CG8929**	FBgn0034504	588.9337919	633.9963615	482.8652044	629.3659546	601.8202111	600.4643597	547.8984505	618.7709317	2R	0.050753563	0.637958898	0.991585536
**CG16739**	FBgn0034505	1.374892788	1.537459849	2.223647151	1.869881286	1.034180777	1.51228061	3.507381041	3.731274394	2R	0.513901702	0.588519304	NA
**CG18067**	FBgn0034512	4.521581376	3.015451912	1.58592193	1.524129652	1.770205285	0.154081421	0.416915105	1.89238973	2R	-1.310941782	0.144585264	0.8037297
**Mgat1**	FBgn0034521	78.37928181	72.21025202	72.20690062	68.37581649	69.63922542	73.89550813	60.06072435	61.95490268	2R	-0.104141946	0.384862315	0.94698628
**Nnf1a**	FBgn0034523	2.387117006	0.5720077	0.206825449	4.372871985	7.079659586	2.491690689	1.522396692	0.185094714	2R	0.595688297	0.591566669	0.988631016
**CG9945**	FBgn0034527	61.58698381	80.02782667	69.93620429	70.03133358	84.9774069	82.23333785	68.4837236	57.4818886	2R	0.089959301	0.548070054	0.984934163
**CG11180**	FBgn0034528	2.273083186	8.723174281	7.853939421	3.051289384	4.911762443	6.20185376	5.886542892	3.813466283	2R	-0.027359735	0.953179088	0.997604378
**FAM21**	FBgn0034529	19.0567407	29.55773009	29.48407817	21.21970148	23.54704694	34.52871566	34.89652877	31.1586454	2R	0.35663641	0.05312046	0.606446461
**Rcd6**	FBgn0034530	1.286248385	1.693228808	0.671482923	0.683279929	1.02873772	2.471384891	0.498417306	0.777676137	2R	0.166333391	0.791309645	0.995787491
**maf-S**	FBgn0034534	545.619845	429.9910829	496.4886737	420.8205665	481.758469	494.4633103	546.3455557	441.8777877	2R	0.081346738	0.52615847	0.984107922
**CG11110**	FBgn0034535	75.47251551	123.0335287	109.1876153	125.9695025	146.2118699	108.1836621	132.4165291	111.3126946	2R	0.23593501	0.234820226	0.87009833
**DMAP1**	FBgn0034537	89.60181561	123.7625757	100.1844832	137.4654116	135.6786374	104.612776	135.1885409	111.2503227	2R	0.142880201	0.386503681	0.948073243
**Lrt**	FBgn0034540	0.759602562	0.592617087	0.671402367	0.672699644	0.722843853	0.305334698	0.540770918	0.25568488	2R	-0.538709569	0.266621063	0.894434069
**CG13437**	FBgn0034541	3.43042557	9.062640804	6.033569324	6.998169567	7.12171697	12.28990024	5.250653598	1.675748579	2R	0.083539886	0.892925322	0.996450075
**Fem-1**	FBgn0034542	119.0821618	117.0257673	103.8496602	118.4433578	142.5176448	142.3528015	133.6673657	165.0940657	2R	0.378738356	0.000597484	0.041047594
**galla-1**	FBgn0034543	41.32468942	52.03809538	67.47240784	61.31170036	68.64008657	70.46624632	74.04143943	73.80171019	2R	0.403747869	0.011207789	0.319404908
**CAH14**	FBgn0034554	1.813857041	0.573222891	0.382642772	0.490311815	0.189824745	1.56138957	0.871789096	1.426830269	2R	0.3084418	0.721256104	0.995787491
**CG15649**	FBgn0034563	8.339267973	2.639236253	1.90857998	5.135804732	1.988332306	2.596013035	0.802779312	1.024829588	2R	-1.464995865	0.059204732	0.626639433
**CG9344**	FBgn0034564	131.5196156	186.1527558	135.1969293	137.7250062	173.0397359	117.8411306	118.2000306	202.9209682	2R	0.088369434	0.69083392	0.995787491
**CG15650**	FBgn0034565	7.7906844	8.672254104	6.700941599	3.632737479	3.324261218	7.678873741	2.168094903	2.306495046	2R	-0.753572004	0.19588358	0.844272804
**CG9313**	FBgn0034566	0.808571721	1.441032233	1.471187788	0.274918369	0.760249579	2.382239	2.70727253	2.084637316	2R	1.027140125	0.084008689	0.69799888
**CG15651**	FBgn0034567	229.6262057	195.6120216	190.1631802	221.2922318	205.1230361	216.8276352	187.347114	185.5130197	2R	-0.047264222	0.670562902	0.995787491
**CG3216**	FBgn0034568	0.059901968	0.234446678	0.242202231	0.791402038	1.766260613	0.847128142	1.782797417	0.173403623	2R	1.838015881	0.047797399	0.575780949
**CG10543**	FBgn0034570	74.76086948	74.71334182	78.12558426	94.25954697	68.72325171	71.0866404	68.32268662	77.65481628	2R	-0.14191023	0.210887871	0.856520545
**CG9346**	FBgn0034572	120.8582629	95.99940487	114.9479077	93.96230141	109.0900701	127.0489347	108.0662643	102.1508278	2R	0.095325582	0.438907256	0.967112302
**CG3295**	FBgn0034573	282.3511603	370.2854164	295.2323836	296.3782032	310.1188738	267.0367462	315.2211365	256.1910728	2R	-0.082092987	0.496870009	0.97594235
**ND-B14.7**	FBgn0034576	670.8617497	870.7307764	751.1063143	862.2823989	826.672404	762.8375475	865.6654831	848.4917972	2R	0.099444908	0.378198929	0.943615128
**cpa**	FBgn0034577	64.05452326	57.11780703	78.59998575	62.62674919	79.15390725	72.89683163	67.68107518	64.59946813	2R	0.147131694	0.324554398	0.918470465
**CG15653**	FBgn0034578	21.90865666	27.27738413	39.17764769	31.33209165	37.10430286	31.19596324	52.4323742	29.91246414	2R	0.367690548	0.228128547	0.867702781
**mRpL54**	FBgn0034579	234.5639459	203.1874569	191.175138	142.9826518	235.7279004	176.3228226	208.2276744	213.5074485	2R	0.141846191	0.410182947	0.95848812
**Cht8**	FBgn0034580	8.296766824	0.834999746	3.086268891	2.837061262	0.599111622	1.095099752	1.693218433	2.521826832	2R	-1.349511456	0.08898521	0.709815866
**CG10527**	FBgn0034583	5.760238558	7.741596155	7.290917425	2.836102472	1.162588353	1.922676463	5.019857623	1.786573018	2R	-1.233377609	0.0202594	0.402963826
**Rbpn-5**	FBgn0034585	109.5796904	146.7328659	119.9966808	143.6308813	117.186508	140.3682919	123.1428354	127.6455003	2R	-0.001391999	0.991084496	0.998408391
**Magi**	FBgn0034590	7.32309281	12.80567824	10.67256808	11.5348795	7.477519744	14.36985626	16.67855879	15.66400536	2R	0.390212382	0.087289178	0.706081898
**CG15657**	FBgn0034595	7.571076286	6.672575745	7.160143827	6.881163134	9.497916255	4.960280399	4.255622329	9.193860106	2R	0.011766028	0.983711631	0.997881056
**CG4266**	FBgn0034598	71.42950058	81.80665374	90.99986245	83.06407864	85.11775304	92.020947	105.7107275	98.49533008	2R	0.252961878	0.026901911	0.460370691
**hng1**	FBgn0034599	132.6542392	87.30850118	106.6238809	89.19333736	83.68590847	78.71420573	87.56761332	74.35186109	2R	-0.333323525	0.066268239	0.648977095
**Lapsyn**	FBgn0034602	3.769224	5.727300303	3.621774784	4.411132919	3.308813746	3.233080457	4.072392594	1.123202736	2R	-0.549140605	0.144287708	0.8037297
**Ugt49C1**	FBgn0034605	1.625939765	0.185951309	3.048028284	3.101577833	1.601038909	0.592266894	3.110855454	2.567320539	2R	0.013930184	0.986195237	0.99797245
**ASPP**	FBgn0034606	0.780485183	1.012412739	1.104510265	0.430655665	0.648128865	1.135641251	0.292018389	0.672153881	2R	-0.241866439	0.538836889	0.984934163
**MFS16**	FBgn0034611	35.04695653	54.10235264	46.73802459	32.1944778	34.8255559	41.68902749	41.3696683	38.4900653	2R	-0.068928372	0.689685699	0.995787491
**CG10505**	FBgn0034612	0.085003185	0.598839857	0.329946466	0.317090762	0.736572169	0.144252871	0.173475961	0.590559307	2R	0.364113894	0.713628224	NA
**CG9752**	FBgn0034614	0	1.960066857	5.315379151	2.043310411	1.7139846	2.547670032	2.04942248	7.293917837	2R	0.608977091	0.548793534	0.984934163
**Panx**	FBgn0034617	64.8982626	60.35458887	54.91434358	44.63988497	42.91069121	50.20680247	41.61173821	47.06140284	2R	-0.278059269	0.091336574	0.720150434
**CG9485**	FBgn0034618	9.724921582	12.68500869	16.32426824	14.5897683	7.218668365	9.487504376	11.83410908	10.01917429	2R	-0.43481647	0.040680145	0.530560761
**BBS9**	FBgn0034622	0.0238224	1.07888581	0.115585619	0.499869255	0.430055373	0	0.243085815	0.517206352	2R	-0.476459774	0.735809971	NA
**CG9822**	FBgn0034623	0.257792398	3.459284661	1.876202284	4.607921742	2.482033865	0	0	2.425328356	2R	-1.004258876	0.460183638	NA
**CG10795**	FBgn0034626	67.04728464	85.87585013	66.43432416	85.53261101	67.30277909	88.25794361	74.03466681	38.93604063	2R	-0.155822761	0.492440261	0.973549494
**eEFSec**	FBgn0034627	16.2441647	13.66717978	21.52967414	15.85085706	19.86221947	20.8439204	11.38585409	9.915487233	2R	-0.085465592	0.786090584	0.995787491
**Acox57D-p**	FBgn0034628	0.204357169	0.636592324	0.424943692	1.157095397	0.158107314	0.550476258	2.308700766	0	2R	0.342274457	0.774392327	NA
**Acox57D-d**	FBgn0034629	0.428237866	1.676054203	0.207779818	0.066561364	0.721540497	0	0.145659199	1.177675794	2R	-0.162130162	0.904314384	NA
**TAF1C-like**	FBgn0034631	13.16572839	14.12327247	20.64949958	10.70769772	7.019707653	16.19407168	16.09006403	12.23129948	2R	-0.155135634	0.60560353	0.989286463
**CG10494**	FBgn0034634	23.54277285	18.35436702	17.78681263	26.71440776	29.7970752	35.70809068	26.59850982	27.51652188	2R	0.49420356	0.018262743	0.388269648
**twz**	FBgn0034636	4.191293918	6.395371413	10.1280938	2.39179951	7.298962212	15.00959337	12.77731649	9.052044739	2R	0.967683188	0.035886082	0.518988565
**CG10433**	FBgn0034638	16.97703467	83.17538503	25.82708509	37.60225137	15.00470043	42.28210612	48.18101796	48.99137378	2R	-0.039459994	0.946588042	0.997604378
**CG15673**	FBgn0034639	0.77767557	2.391476398	0.539037167	0.863391103	0.467967907	1.693314629	0.802993821	0.321601076	2R	-0.43918053	0.54748523	0.984934163
**mahj**	FBgn0034641	18.48361813	17.75210504	19.06728729	16.52352544	16.43920302	16.94076045	10.64724996	14.82796302	2R	-0.256007463	0.136277457	0.796109055
**CG15674**	FBgn0034642	1.176978285	1.604050467	0.713833226	1.715050352	1.394365521	1.317706926	1.032108306	1.996350631	2R	0.169889354	0.803364151	0.995787491
**CG10321**	FBgn0034643	131.3942904	162.2404506	131.2211842	173.0665609	139.680459	144.2715701	137.3903381	112.6820794	2R	-0.133814759	0.292248427	0.905069205
**CG10082**	FBgn0034644	91.53186785	111.049569	103.6375973	110.5402316	107.5479754	120.1402636	113.3389989	108.8704502	2R	0.141003581	0.128723639	0.788096052
**ND-B12**	FBgn0034645	842.6381551	988.7615443	974.916579	965.2439554	1076.680724	976.6866357	1040.653503	1228.956876	2R	0.230516497	0.039288675	0.528969012
**Rae1**	FBgn0034646	85.98804014	93.25806375	111.2677442	86.03570194	123.8809788	96.84708595	117.3597483	82.85419972	2R	0.193683372	0.258131625	0.887830768
**pirk**	FBgn0034647	0.170844666	1.64776577	0	1.692851105	1.079464432	0.161071308	0.108957103	0.649109273	2R	-0.765719253	0.568465478	NA
**NC2alpha**	FBgn0034650	157.607939	190.5607331	131.3888445	137.6211865	136.9441946	165.2531255	147.514459	152.0371321	2R	-0.006291198	0.963997788	0.997604378
**eIF3k**	FBgn0034654	492.3464115	584.9582532	553.7208413	589.7825563	537.0683277	471.7425925	552.1718113	524.4269579	2R	-0.058170915	0.600130168	0.989286463
**CG10307**	FBgn0034655	6.964575157	4.417600006	8.944921158	2.865466728	0.845233154	1.269089539	5.37482446	1.778910279	2R	-1.311971438	0.051607198	0.599821511
**LBR**	FBgn0034657	12.23847684	11.91704131	14.638594	13.72787003	14.53872252	10.07025959	18.06202714	12.67794805	2R	0.106361088	0.651849825	0.993444251
**CG4021**	FBgn0034659	2.410627962	1.194667568	0.93038747	0.510935304	1.483568629	0.309917089	0.279525624	0.237895293	2R	-1.107771033	0.314486041	NA
**Loxl2**	FBgn0034660	0.486868013	1.113617443	1.503262739	0	3.675490107	0.292101211	0.564550346	0	2R	0.604560512	0.657431982	NA
**tpr**	FBgn0034661	0.440245213	1.015368179	0.80102156	0.128301893	0.496722152	0.051882562	1.403843746	1.911626221	2R	0.736502907	0.557492157	NA
**CG13492**	FBgn0034662	0.257912335	0.59484117	0.586585994	0.131537197	0.116399467	0.744671907	0.020560622	0.629945497	2R	-0.01192635	0.989328916	0.998078482
**CG9304**	FBgn0034674	21.81378613	31.42607499	29.03515005	43.12413717	29.46274574	17.0882149	40.6185672	24.31364241	2R	-0.135521977	0.605322484	0.989286463
**CG13500**	FBgn0034683	0	4.308433613	2.250206363	1.663485823	1.159236574	1.00901754	0	0	2R	-1.851224146	0.221195664	NA
**CG11475**	FBgn0034687	7.601985684	3.257622326	4.173077725	6.339149115	7.112195692	8.736544867	15.99554493	8.794429692	2R	0.939569353	0.033892614	0.514749796
**CG11474**	FBgn0034688	61.01312369	50.77584691	65.73247947	72.03910231	63.49579707	76.56151638	69.52045218	78.151027	2R	0.234043883	0.133056568	0.794759341
**CG2921**	FBgn0034689	247.767332	185.2652148	229.1298789	215.6385131	162.3754968	152.9915339	178.0203943	168.7193742	2R	-0.37844288	0.003588967	0.15742344
**Synj**	FBgn0034691	361.0978482	395.7208811	359.7207638	373.9508395	332.0900048	319.2764907	358.5000117	388.6585103	2R	-0.059042465	0.552321192	0.984934163
**CG13502**	FBgn0034692	2.676489084	4.341431615	7.634773572	9.60020545	4.300787372	3.41071912	1.875764054	0.574704946	2R	-1.220275282	0.029620319	0.477477245
**CG11073**	FBgn0034693	0.118433897	0.415372154	0.809717132	0.10040879	0.680283986	0.893270038	0.192262901	0.74801732	2R	0.861144693	0.283614327	0.901780531
**Plekhm1**	FBgn0034694	73.34258488	47.92586839	58.75624438	65.67270069	53.37620062	42.76616658	68.76405593	38.34025066	2R	-0.247684617	0.219890045	0.864416811
**GM130**	FBgn0034697	130.4143194	145.3394583	133.9563683	150.6863107	155.88864	159.4031356	142.7426407	138.3991331	2R	0.120067589	0.24418684	0.877269208

**CG3045**	FBgn0034703	12.93768288	17.16083693	9.794411922	14.22615191	14.74233462	9.256310765	9.596997064	11.65390829	2R	-0.226855454	0.45440631	0.967967712
**CG6758**	FBgn0034704	36.53451143	21.16154583	24.14955342	30.62247725	29.70120971	27.07984041	25.39422251	31.96805816	2R	0.044862933	0.835639965	0.995787491
**CG11170**	FBgn0034705	3.918444446	2.235230088	2.001282436	1.405493187	3.579856535	1.734968084	2.185368187	0.792178256	2R	-0.184847422	0.711686441	0.995787491
**MED16**	FBgn0034707	34.59697538	34.50240779	28.0789954	25.59170859	31.2849824	27.81410219	26.71799087	26.08477838	2R	-0.105442615	0.54322179	0.984934163
**Vps35**	FBgn0034708	199.8593195	156.6488537	144.0447832	151.7957589	136.7122516	158.235399	173.5992315	161.390106	2R	-0.022662567	0.862719037	0.995787491
**Swim**	FBgn0034709	0.552566994	0.791216501	1.078954978	0.345638551	0.547422281	1.060972968	0.498520707	0.453535329	2R	-0.071176952	0.888403106	0.995926915
**Oatp58Dc**	FBgn0034716	16.44560601	20.84478825	26.49766659	5.984537287	6.008013303	14.10287435	17.88312737	8.071082809	2R	-0.564693178	0.286478848	0.90249226
**CG5819**	FBgn0034717	0.073095566	0.204345929	0.886644117	0	0.483839513	0.298627563	0.435092838	0	2R	0.125631963	0.934104983	NA
**wdp**	FBgn0034718	2.947658727	5.071331983	5.451281604	1.030117505	4.079267783	1.267525437	4.508504085	2.507781825	2R	-0.179807167	0.768329604	0.995787491
**Liprin-gamma**	FBgn0034720	51.18281298	57.97806156	56.86670644	56.15507154	54.06107752	57.45841623	56.57315563	58.93938328	2R	0.062610961	0.450932266	0.967112302
**Rtf1**	FBgn0034722	36.08668219	55.56463245	56.96048418	49.26701949	36.99576051	46.41373659	57.87798761	42.44286145	2R	-0.073004673	0.683802201	0.995787491
**CG13506**	FBgn0034723	28.61801242	21.72472732	31.903543	33.05467564	25.9679457	26.13280934	22.46000332	16.15959696	2R	-0.320665571	0.063849857	0.636907352
**babos**	FBgn0034724	0.857396786	0.692448769	1.129894275	0.049357737	0.783464924	0.608756242	0.135014668	0.597514599	2R	-0.322131577	0.683343335	0.995787491
**CG6044**	FBgn0034725	895.2074106	798.9782049	751.5336165	766.9641886	916.5258485	1076.426295	871.0467868	830.5421694	2R	0.228298071	0.023914617	0.438337142
**Mes4**	FBgn0034726	91.42430215	113.0440135	93.01042141	118.1632926	101.5505533	72.39495145	110.621825	73.98933618	2R	-0.182270605	0.373089477	0.943282319
**mRpS29**	FBgn0034727	123.8177424	186.3830843	146.9044223	163.7750923	150.1742602	113.7268648	151.4047919	144.015264	2R	-0.115212277	0.466976017	0.971928234
**rad50**	FBgn0034728	6.299156374	7.412421103	7.617322496	8.540616876	9.447168502	5.445428411	6.922152781	12.32948651	2R	0.229601194	0.430683951	0.963666972
**CG10384**	FBgn0034731	17.16326254	23.79049401	21.97584408	19.49331266	24.56797451	25.05279152	26.44946517	16.59683278	2R	0.200836744	0.179162112	0.836045561
**CG4752**	FBgn0034733	1.667517028	2.983494278	1.752994989	1.382312241	0.100343187	0.454169407	0.51991766	1.890618777	2R	-1.357291586	0.058738046	0.625114381
**CG4554**	FBgn0034734	3.006337735	2.346607533	3.75498354	4.464582535	5.427210131	5.92128016	3.897893569	5.191472084	2R	0.622602131	0.019661649	0.396755891
**CG4610**	FBgn0034735	44.34127542	62.46498574	39.66316668	42.35307852	52.47052715	68.46781982	38.65307771	35.76474526	2R	0.078159375	0.718548091	0.995787491
**CG3927**	FBgn0034739	0.46652556	0.608635362	1.006030668	0.241708173	0	0.097741655	1.322351918	0.337623392	2R	-0.390412328	0.791435386	NA
**nsr**	FBgn0034740	1.311102106	0.860552551	0.691459874	1.79420001	1.029076232	1.18235562	1.090646622	0.556928616	2R	-0.249372214	0.720212411	0.995787491
**CG4269**	FBgn0034741	3.464729826	3.284816391	0.609085959	0.70242497	0.770509643	0.946819164	0	0.218036208	2R	-2.011983064	0.027813046	0.462499435
**CG4294**	FBgn0034742	76.28068231	97.9091948	95.36327594	97.40439759	73.42942945	83.89794843	90.49403022	83.23507381	2R	-0.116462002	0.324383559	0.918470465
**RpS16**	FBgn0034743	70.29551847	119.9772168	124.9600753	126.4638112	104.5234101	92.4426891	123.9368165	116.1769595	2R	0.021283351	0.9064279	0.996479182
**Vps20**	FBgn0034744	96.8499771	129.0447308	121.6469333	121.2775664	111.6713812	127.6861201	112.5745971	129.3840424	2R	0.071912823	0.618946568	0.990047511
**CG4329**	FBgn0034745	5.443105986	0.898626644	1.559633764	0.349735342	0.116057496	0.0404073	1.093344906	0.279153235	2R	-2.452606538	0.010578633	0.311527659
**CG17807**	FBgn0034748	47.61075697	40.49683634	29.61336023	39.90635881	47.24383024	47.09539084	44.46877647	44.20250427	2R	0.23954934	0.191699962	0.843483578
**CG3732**	FBgn0034750	317.4552489	323.7235312	326.5076502	356.95685	335.8437529	327.53134	349.6763288	276.9579907	2R	-0.00887611	0.93619546	0.996793017
**CG2852**	FBgn0034753	695.9046567	619.8353589	682.1332289	660.6693634	631.6425368	622.0442991	679.9423758	613.0101472	2R	-0.032443066	0.730766302	0.995787491
**CG3746**	FBgn0034755	1.261987685	1.347059192	0	3.477236194	1.035550553	1.406120514	0	0.249081099	2R	-1.131254527	0.477353319	NA
**CG4250**	FBgn0034761	8.63967156	11.70494528	5.911716659	21.1760469	0.3999185	4.594857709	7.629226535	0.480962224	2R	-1.844396641	0.022165257	0.420786457
**RYBP**	FBgn0034763	92.6897722	106.6566647	97.26232639	102.319904	77.43945657	105.990995	82.24107064	70.92406368	2R	-0.21633542	0.10823158	0.758410631
**CG13531**	FBgn0034786	13.42257718	21.42390673	26.07842934	16.43873617	16.19759981	26.83509779	24.17898977	11.70057941	2R	0.05946281	0.810171725	0.995787491
**CG13532**	FBgn0034788	204.811879	206.063079	176.2482516	211.3052502	194.7234927	211.4109427	198.1654035	216.8136695	2R	0.069643995	0.498981626	0.97617673
**PIP5K59B**	FBgn0034789	727.323028	732.5298593	731.1690033	753.1445413	659.4574484	694.5226389	692.4586563	726.0205924	2R	-0.056194668	0.486537765	0.973549494
**EMC8-9**	FBgn0034791	214.2028692	208.48259	184.1750382	199.5852236	211.691362	185.2941715	195.1211453	169.6355169	2R	-0.053294539	0.659592103	0.995750687
**YME1L**	FBgn0034792	134.7284941	130.5534031	109.5101749	127.1690235	116.605951	142.8638529	113.8916772	102.5712406	2R	-0.050347087	0.680763217	0.995787491
**asrij**	FBgn0034793	103.3546524	117.9557437	110.053574	129.3831079	139.2736511	108.653517	128.4593168	133.2330575	2R	0.177809039	0.238066264	0.873280735
**MED23**	FBgn0034795	14.63859382	15.38270952	19.95457346	8.302721928	18.63218618	24.03692585	21.94777025	12.04216714	2R	0.42591434	0.091266761	0.720150434
**CG3700**	FBgn0034796	18.05097791	25.39801516	25.66530147	15.10749888	31.15426171	26.24441785	33.3977642	20.95893855	2R	0.443348537	0.103842293	0.745084146
**nahoda**	FBgn0034797	0.560731403	0.848614976	0.940977772	0.629088316	0.6773796	0.437232749	0.516248249	0.338675127	2R	-0.559020255	0.14882667	0.808039863
**CG3788**	FBgn0034800	0.169673351	0.071150811	0.823250365	0	0.918911656	2.359513233	0.541050454	1.013035516	2R	2.260047218	0.062744825	NA
**CNBP**	FBgn0034802	282.9253063	331.363057	295.2609757	388.1122019	307.7484477	259.6093773	282.2854377	364.7151117	2R	-0.062796158	0.654585756	0.994669639
**CG9849**	FBgn0034803	45.24389463	45.60074212	69.80418119	59.56632139	52.57050077	49.01660037	46.19256989	52.77637596	2R	-0.100846515	0.587754773	0.987588562
**CG3831**	FBgn0034804	19.09412289	15.04836389	12.71208733	13.4199546	16.41077111	15.41944042	18.43062833	17.32323654	2R	0.186372498	0.437697955	0.967112302
**CG9890**	FBgn0034814	31.37263623	38.89123072	37.22147241	32.16739598	30.48816879	33.84190144	46.7341717	44.6214054	2R	0.188968981	0.374976379	0.943282319
**CG3085**	FBgn0034816	1.32014042	2.714364955	1.136421442	5.758572457	1.230034482	1.686259795	3.693613662	0.647194497	2R	-0.569987376	0.446023653	0.967112302
**Art7**	FBgn0034817	32.39890975	24.29742664	24.85848268	33.42870324	20.2280479	25.7152056	22.94274951	19.04569473	2R	-0.362644762	0.077011015	0.682035833
**CG13538**	FBgn0034820	0	0.882327666	1.48880829	1.226399872	0.316534683	1.239825196	0	0.380680626	2R	-0.817555255	0.633667369	NA
**Klp59C**	FBgn0034824	0.327015557	0	0.892502077	0.285909173	0.664140255	0	0.625667784	0.177495261	2R	0.000326223	0.999844363	NA
**Klp59D**	FBgn0034827	0.589475651	1.977525418	0.504726955	0.889279011	2.441295919	1.994176272	0.353827071	0.150565436	2R	0.359999912	0.703898028	0.995787491
**CG9899**	FBgn0034829	0.516149098	0.79509279	0	0.073676461	0.038031894	0.139034919	0.376202125	0.068608621	2R	-1.141274867	0.348835437	NA
**LS2**	FBgn0034834	0.184424937	0.824925228	2.833611398	0	1.109780933	2.666078803	0	0	2R	0.022919936	0.988880354	NA
**RpL22-like**	FBgn0034837	0.337561363	1.925120224	1.474054483	0	1.584400417	2.736956801	0.516675773	3.371234043	2R	1.1945626	0.283122071	NA
**CG3124**	FBgn0034840	5.56379972	4.348093566	1.227063683	0.1474067	2.396883798	4.053353575	3.225766505	8.236039246	2R	0.682516592	0.403286865	0.955370306
**CG13541**	FBgn0034841	2.119266989	3.91936518	0	0	2.158154916	7.479811998	1.940721504	2.831461422	2R	1.269759115	0.23467104	0.87009833
**CG3500**	FBgn0034849	97.29123987	96.33223386	97.93367026	149.9792125	68.28817899	89.05596139	102.7959582	96.47142701	2R	-0.280598949	0.157256138	0.818884898
**Ice1**	FBgn0034853	21.87615174	11.10315142	15.82986956	11.47577046	14.70738812	16.51754171	17.60792337	19.28466886	2R	0.199608293	0.398094326	0.952272708
**Golgin245**	FBgn0034854	74.03307926	93.66538044	92.40750034	82.5194893	71.73405314	90.3968727	92.85583084	86.30408138	2R	0.026537105	0.822351425	0.995787491
**eIF2Bdelta**	FBgn0034858	89.25341155	98.90738591	76.38241143	94.63032565	95.01943472	98.21518667	108.0816582	89.5328947	2R	0.150949406	0.238266586	0.873280735
**CG3520**	FBgn0034859	7.772028901	4.118166285	7.170148031	7.838715196	6.521220226	12.620267	6.2631352	5.635915163	2R	0.226299425	0.461997746	0.971606621
**CG9812**	FBgn0034860	3.763965047	0.276216646	3.167428951	1.892226419	1.741198662	2.905451665	1.470299467	4.673314773	2R	0.266388442	0.641154547	0.992099188
**CG9815**	FBgn0034861	0.446523535	0.331515998	0.621542546	0.102398637	0.515368647	0.055210447	0.261430751	0.508560749	2R	-0.125348299	0.874728143	0.995787491
**wmd**	FBgn0034876	332.1580682	313.849006	281.4213224	321.6105915	321.9924335	306.8502786	279.9908138	292.9902493	2R	-0.026622117	0.787581363	0.995787491
**levy**	FBgn0034877	3279.60227	3373.542822	3668.164853	3473.871997	3768.40888	3283.562171	3802.501428	4099.52456	2R	0.149483533	0.144622274	0.8037297
**pita**	FBgn0034878	35.0543814	32.80088296	33.46792168	62.35932035	32.55287861	50.74629346	37.34673984	47.33321284	2R	0.0609551	0.771176017	0.995787491
**Rrp4**	FBgn0034879	13.79823596	20.84226221	20.32995836	16.00081901	13.30237666	24.58748808	9.951718349	16.62548817	2R	-0.100403489	0.768504691	0.995787491
**ItgaPS5**	FBgn0034880	0.144725557	0.930567803	0.468136242	0.224948125	0.130633361	0.272892742	0.984528011	1.413956612	2R	0.714415122	0.486040123	NA
**Eglp2**	FBgn0034883	0.046526068	0.100338367	0.145120619	0.077481269	0.035996347	0.45117764	0.152600142	0.043291029	2R	0.931869074	0.385506666	NA
**mRpL43**	FBgn0034893	73.65886016	87.90921414	82.06578966	103.7506409	91.85982758	96.3068206	103.3114712	120.8194037	2R	0.27956642	0.121347597	0.77836519
**sigmar**	FBgn0034894	117.9381204	91.95673218	92.57595625	83.88664997	97.70181321	122.2466926	88.9740491	102.4871091	2R	0.116568334	0.402154744	0.954587287
**Sesn**	FBgn0034897	0.43007245	0.228901391	0.170547558	0.115695892	0.19409775	0.346988476	0.200436127	0.323213227	2R	0.180897139	0.765526508	0.995787491
**CG5532**	FBgn0034902	220.3586381	293.8255478	265.912843	199.0281001	259.7231547	249.6038025	292.3939036	268.6164539	2R	0.163028946	0.260920464	0.890640489
**sona**	FBgn0034903	0.630574966	0.404319971	0.407001983	0.467574394	0.483885537	0.52723439	0.280399958	0.071173158	2R	-0.466436714	0.323802719	0.918470465
**CG5543**	FBgn0034908	49.51903405	38.03613087	62.39891259	27.46182729	36.0181917	48.76370509	45.48077127	51.66762271	2R	0.067480794	0.768996631	0.995787491
**CG4797**	FBgn0034909	0.676925397	1.703171087	0.718467563	0.3287971	0.381882449	1.848124105	0.323784606	0	2R	-0.396158315	0.677022253	0.995787491
**GlyT**	FBgn0034911	99.89290246	99.20079449	110.6572759	113.6328104	97.8362791	77.20537445	88.30672211	85.81571557	2R	-0.246372184	0.023392051	0.433220779
**Snap29**	FBgn0034913	214.5831695	186.1527558	229.0139413	188.0633104	251.89929	215.6417632	187.4749969	167.3406604	2R	0.037420037	0.798002318	0.995787491
**CG5554**	FBgn0034914	122.926833	154.3391571	163.9112639	167.0082206	133.720648	134.665486	145.8172429	167.6052307	2R	-0.030318212	0.826342482	0.995787491
**CG5569**	FBgn0034919	80.82041146	114.8259328	104.5589376	103.0907776	92.18153674	78.04099694	85.89123603	87.65846824	2R	-0.195579755	0.214322368	0.858753183
**DCP1**	FBgn0034921	103.9762671	145.0977733	136.198388	104.3126632	148.6117776	114.4472361	143.2180592	147.1365869	2R	0.213624329	0.171541112	0.832652344
**Upf3**	FBgn0034923	37.46994385	21.32835373	26.58132293	37.74025884	42.73465386	31.24540208	27.14614332	29.46638473	2R	0.109270465	0.657110505	0.995750687
**CG5339**	FBgn0034925	64.28998677	72.10686692	72.69102537	106.9572096	69.03363522	99.87101145	77.7469464	84.83425462	2R	0.097310736	0.615853551	0.990047511
**CG13562**	FBgn0034928	1.311533673	4.686772766	0	0.265894903	4.426484357	2.903100894	1.163739331	0.123802653	2R	0.498902114	0.673617811	0.995787491
**CG3735**	FBgn0034933	21.67882876	18.83287809	23.07791886	27.15763333	24.35768086	16.41194481	15.76552711	22.76063442	2R	-0.162899429	0.474807076	0.973549494
**Orcokinin**	FBgn0034935	1.391129122	2.058903482	0.099260584	0	1.181809228	2.931695617	0.521882394	0.444157368	2R	0.530758077	0.649724753	NA
**Stoml2**	FBgn0034936	110.2534882	115.0546611	120.9338489	106.6762108	150.4647325	193.9061237	149.751606	164.390467	2R	0.569477005	1.58E-07	4.55E-05
**CG3803**	FBgn0034938	133.2848835	120.9645364	149.9006176	119.9842522	121.2235041	119.7016899	104.220628	129.3728849	2R	-0.112182755	0.40490573	0.956706394
**thoc5**	FBgn0034939	45.94695708	55.91937389	37.12530204	38.64475174	60.3182476	63.63729065	44.19300111	44.75981962	2R	0.290753265	0.113274329	0.758410631
**CG16787**	FBgn0034940	80.14211513	123.8359249	104.8709404	129.095206	90.60583434	89.37154398	127.2097602	122.1486724	2R	0.006024933	0.975653213	0.997757033
**CG13566**	FBgn0034942	2.204828071	2.509553781	5.539913652	5.32406199	2.843056972	1.930219978	2.008772778	2.051522503	2R	-0.775939611	0.260073351	0.890556245
**CG10904**	FBgn0034945	77.05351004	60.58026423	45.66530505	43.70764915	64.92316495	54.46614881	65.39158734	54.82204569	2R	0.101952416	0.645317981	0.99279236

**CG3065**	FBgn0034946	147.3588411	151.3185846	122.5058885	128.9403409	134.4701065	116.6071888	109.9094608	115.7794317	2R	-0.176418952	0.164825071	0.827712868
**PPP1R15**	FBgn0034948	722.7079107	568.9645912	667.2029107	639.9871951	664.8478428	717.3634574	638.0764835	576.8874672	2R	0.025746443	0.813165847	0.995787491
**Pask**	FBgn0034950	98.65219098	110.0974023	129.7871855	95.33175496	89.40044598	96.78230506	74.2111405	101.3833795	2R	-0.229083431	0.09274269	0.72305867
**CG3860**	FBgn0034951	293.7656168	289.8327914	315.1659647	287.7937685	283.1994683	294.8776104	262.7794324	281.7297644	2R	-0.04968854	0.569614723	0.984934163
**CG3907**	FBgn0034958	232.6421714	183.670719	197.5265764	199.1704052	200.5421312	202.7628034	192.8840361	190.3285756	2R	-0.020371992	0.854752883	0.995787491
**Xxylt**	FBgn0034959	436.0525358	525.6338692	483.0018187	510.4159468	508.2441652	493.6682174	477.1387208	557.9690324	2R	0.09177638	0.371680163	0.943282319
**CG3163**	FBgn0034961	109.5671831	99.1680231	107.1041743	90.53367069	89.17278047	121.6372003	116.9079795	86.58485524	2R	0.054956396	0.729901576	0.995787491
**MAN1**	FBgn0034962	77.87084872	85.96189478	74.19683236	93.84299936	76.15294023	82.91813002	81.65922269	71.08016283	2R	-0.060812498	0.630135338	0.990523437
**Not11**	FBgn0034963	152.6510672	128.2685383	176.3724349	122.0132746	152.4425736	158.7477023	149.2207192	127.469076	2R	0.050825412	0.735976421	0.995787491
**IntS1**	FBgn0034964	52.82184036	53.14289555	44.73626517	47.51880665	46.94644191	45.82662887	49.17870423	45.31823181	2R	-0.052886603	0.63445972	0.991585536
**ppk29**	FBgn0034965	0	0.413146794	0.547744363	0.526402599	0.333486541	3.212342034	1.151950266	2.406406728	2R	2.315857985	0.012439423	0.33520527
**RpL12**	FBgn0034968	387.7364653	519.5929591	601.9039229	556.2404753	481.449253	430.3019981	548.0886373	572.036088	2R	0.011674232	0.935696189	0.996688655
**yki**	FBgn0034970	75.92037467	87.98435567	84.63989522	95.6634246	73.44941409	89.30890628	86.36122329	85.48691546	2R	-0.010091625	0.936898813	0.996879532
**Gpat4**	FBgn0034971	94.94588094	107.8387805	95.49184879	93.87700977	102.5279273	119.7101777	114.3303814	119.7755422	2R	0.249645391	0.012468639	0.33520527
**CG16786**	FBgn0034974	0.807539693	1.032026323	0.69967144	0.448273427	0.13883963	1.087633557	0.147146508	0.459182802	2R	-0.662157669	0.444293572	0.967112302
**enok**	FBgn0034975	41.47089363	51.09852573	50.02182678	43.58052136	44.33962158	51.90861625	51.61123308	43.56403577	2R	0.071835171	0.525941936	0.984107922
**CG4049**	FBgn0034976	90.08057108	82.51582219	95.10744101	90.89725209	70.53385618	86.75222379	95.50104474	98.63490508	2R	0.001016294	0.993491535	0.998777916
**CG3257**	FBgn0034978	164.857103	145.0205261	113.1369403	154.6982176	158.4946048	166.2667346	144.5782466	132.6071048	2R	0.085345948	0.505260319	0.978557094
**Naa35**	FBgn0034982	104.636048	75.37865648	83.39490346	108.2211712	119.8095908	107.214899	77.14527372	89.04822851	2R	0.106552414	0.526231956	0.984107922
**CG3328**	FBgn0034985	3.419447348	8.401871937	8.013370635	5.531808858	4.451263669	4.342307119	4.173629592	2.020118838	2R	-0.721602831	0.01616569	0.369626395
**mRpS17**	FBgn0034986	71.99583562	87.62332382	94.2356446	87.70128776	80.80363598	76.71705989	96.81456429	55.25363443	2R	-0.110110626	0.598270024	0.989286463
**cN-IIIB**	FBgn0034988	42.43104381	55.49335131	36.96940641	42.92533234	64.01991599	68.36418295	67.05543661	54.8548583	2R	0.545736555	0.001892335	0.099353754
**CG3356**	FBgn0034989	137.7394885	170.2320087	140.8282681	172.277653	161.2064782	181.9312048	169.0030308	156.1603757	2R	0.135576036	0.212247244	0.856520545
**CG11406**	FBgn0034990	18.69264823	28.95339759	20.73154408	25.60684942	14.60834493	13.3210346	12.83419126	11.75351434	2R	-0.806141208	3E-06	0.000574129
**CG13575**	FBgn0034996	1.328118764	0.139233307	0.100687425	0.387057363	2.547444436	6.417229074	2.329290907	2.342818383	2R	2.733366708	0.001205646	0.072810557
**CG3376**	FBgn0034997	10.45434104	9.060443073	9.952210781	8.246176473	11.16294731	4.967841802	8.316877293	9.063265503	2R	-0.137521407	0.577772626	0.985637351
**Fatp3**	FBgn0034999	4.704229584	7.818293582	10.36538661	3.370817386	6.466644284	4.801321397	3.853400695	3.513756156	2R	-0.452074217	0.228664058	0.867702781
**CG13578**	FBgn0035000	2.668108777	16.14332842	21.49892518	21.7720831	26.3193888	52.28597909	20.17612262	38.48015256	2R	1.184024448	0.016041043	0.36902021
**Slik**	FBgn0035001	10.41783279	18.54853288	32.12465167	17.93427226	11.94509673	10.33269919	21.92686578	25.34508182	2R	-0.140025997	0.756688868	0.995787491
**CG3483**	FBgn0035005	0	0	3.452548489	0.150819402	2.569155292	1.58569229	0	3.511131991	2R	1.161642844	0.461273595	NA
**CG13579**	FBgn0035010	2.010903169	2.518457835	2.549139445	2.501212091	1.193852393	0.745877118	1.187177668	1.052910684	2R	-1.165836456	6.48E-08	2.08E-05
**CG13581**	FBgn0035014	7.667165284	3.128252636	8.546146288	15.2185075	4.489037324	0.488415986	0.264312208	0	2R	-2.683534418	0.00622178	0.227193262
**CG4612**	FBgn0035016	523.1207737	523.0438407	460.8006224	610.4410455	485.6872131	539.4224207	474.3277273	548.5977218	2R	-0.019682554	0.857379254	0.995787491
**CG13585**	FBgn0035020	321.9865781	320.2523732	333.7363982	335.1176893	343.3659737	311.565151	317.4911766	377.1589599	2R	0.073425176	0.49226725	0.973549494
**CG4622**	FBgn0035021	7.458100711	8.099126033	7.442612776	8.967208765	7.379844736	7.743858259	5.898568586	6.775470886	2R	-0.171147286	0.213864236	0.858547467
**ITP**	FBgn0035023	1.001297269	1.234357563	1.092622166	0.562526961	0.813056219	0.610388469	0.451367699	0.488911274	2R	-0.682667772	0.05976437	0.627645287
**CG11414**	FBgn0035024	92.86801185	92.41131639	72.39933519	73.49917611	77.9801674	87.01113105	77.28332707	90.73127268	2R	0.037192767	0.775109825	0.995787491
**uri**	FBgn0035025	11.99224129	17.81641071	16.54145431	9.665984435	14.47394906	17.02392468	15.20882554	15.84492756	2R	0.195303689	0.42797234	0.963202327
**Fcp1**	FBgn0035026	135.1687438	112.7759281	111.6443124	118.1975664	137.0770205	128.1817565	113.709952	143.1758304	2R	0.157027871	0.194657067	0.844272804
**CG3511**	FBgn0035027	37.6771298	60.57116027	50.04945731	54.27857069	55.83799481	45.00559714	53.78146867	37.59816827	2R	-0.041277453	0.819385227	0.995787491
**Start1**	FBgn0035028	38.12647835	38.4549931	53.05613542	25.20290427	35.96918566	37.54356693	34.38178624	36.56146136	2R	-0.066196097	0.735134308	0.995787491
**CG13587**	FBgn0035031	2.608031119	3.844351019	1.917287005	3.869425874	1.712059855	2.384325633	0	1.029504906	2R	-1.20221438	0.201973919	0.849268738
**ATPsynF**	FBgn0035032	3742.191819	4073.07408	4028.966511	4157.687418	4386.840787	3687.876791	4423.520452	4495.133965	2R	0.119946559	0.238207573	0.873280735
**CG3548**	FBgn0035033	268.6239395	274.0096521	260.4106747	249.0125545	203.6016482	251.0298325	224.7086276	248.5916851	2R	-0.15125786	0.135642001	0.796109055
**CG3565**	FBgn0035034	0	0.429005775	1.240952692	1.19260145	0.153905643	1.446788142	0.434970483	1.295662998	2R	0.284344585	0.852696311	NA
**Samtor**	FBgn0035035	40.31140576	35.46239357	43.25175975	32.37040391	43.15076907	20.89922219	40.65112124	34.76813714	2R	-0.087093379	0.748689499	0.995787491
**CG4707**	FBgn0035036	39.59895085	31.47550112	42.23464251	22.95610436	48.17271986	42.31146885	39.40603271	42.75413454	2R	0.371895114	0.062583881	0.636065933
**CG4741**	FBgn0035040	0.636167974	0.666926581	0.385833658	0.787950893	0.21533367	1.124578649	0.304289657	0.086323722	2R	-0.48291955	0.603437692	NA
**CG13594**	FBgn0035041	1.282540827	3.192427691	2.050094203	1.447150084	1.728048442	1.748539773	2.661309586	1.883403636	2R	0.049039845	0.866478683	0.995787491
**ND-19**	FBgn0035046	614.8980166	609.0733591	582.5635126	572.0866769	752.099178	592.5873748	687.7297003	651.4224432	2R	0.205918335	0.059301938	0.626639433
**Pof**	FBgn0035047	51.01735202	50.11837005	37.78568122	51.96897586	61.97342537	54.91659733	50.37596687	73.75599825	2R	0.365951095	0.040822626	0.531318009
**Mmp1**	FBgn0035049	13.41582774	3.4924643	3.160106724	1.028268643	2.351539722	1.11688504	3.833199919	3.050764172	2R	-0.996153594	0.210579438	0.856520545
**SiaT**	FBgn0035050	19.55047447	4.460656536	2.197764618	5.382532772	7.966122859	9.092051438	3.429281463	8.565314094	2R	-0.113079785	0.831135117	0.995787491
**spz6**	FBgn0035056	0.2804995	0.84689741	0.476341512	0.392384415	0.303824094	0.899140932	0.071556025	0.791687494	2R	0.10203302	0.922136266	NA
**CG3880**	FBgn0035057	3.03453926	0.748531457	3.572613931	0	2.336257532	0.925599878	1.252248872	1.065748853	2R	-0.369684573	0.706510172	0.995787491
**CG3894**	FBgn0035059	55.81957276	57.70237427	47.21138652	50.44452279	53.72290603	54.06223799	53.89992042	60.98837249	2R	0.105934248	0.407779374	0.956706394
**Eps-15**	FBgn0035060	441.9885707	402.8655743	347.4431879	422.6335221	408.5907651	399.5984492	395.8996086	374.51624	2R	-0.004235154	0.966533969	0.997604378
**CG3594**	FBgn0035063	35.67224734	35.73256452	24.86512168	35.37589793	44.98758364	39.78947126	24.09595188	27.48849758	2R	0.079246634	0.782640863	0.995787491
**TyrRS-m**	FBgn0035064	44.28565321	24.48799141	43.11669375	45.64871407	36.05105219	41.2919099	35.37433009	19.71728223	2R	-0.228051917	0.385103971	0.947123743
**CG3589**	FBgn0035065	18.39602399	27.56633785	21.25670147	8.435639583	19.35911466	22.31660394	16.56959528	14.38578101	2R	-0.023830744	0.939753119	0.997188858
**CG16896**	FBgn0035073	39.46628767	30.92334008	42.86627072	46.32397485	34.97649547	39.60837659	45.20168587	47.32256616	2R	0.095273029	0.582562092	0.985764834
**Ance-5**	FBgn0035076	1.808314105	0.060663865	0.877388959	2.108008131	0	4.80772633	1.107131552	0.235561039	2R	0.328953681	0.782515535	0.995787491
**CG2811**	FBgn0035082	139.347242	120.4205039	114.3976311	112.5394189	112.8989998	88.70489197	92.13984788	77.44915499	2R	-0.363315872	0.042771657	0.542555546
**Tina-1**	FBgn0035083	398.9865051	455.7781665	423.5600109	411.3487839	397.1295828	320.9434986	332.8069127	269.6734744	2R	-0.323981154	0.007583804	0.252813679
**CG15861**	FBgn0035084	2.96867398	6.023630006	5.343390915	3.237406406	3.889754564	0.135428114	3.664427953	4.054280237	2R	-0.537266728	0.46742121	0.971928234
**CG3770**	FBgn0035085	4.397452685	12.03470575	8.281860181	3.237630123	5.32717088	0.054551204	1.623657356	1.884330956	2R	-1.60233953	0.049947514	0.587515621
**CG12851**	FBgn0035086	33.76288557	36.61174653	42.94133964	37.62099039	31.66499176	39.63542959	31.57989627	47.78960211	2R	0.029825588	0.846106483	0.995787491
**CG2765**	FBgn0035087	63.90060452	87.00745201	80.44123213	77.11411857	82.53011514	73.73862919	73.40174683	73.13896526	2R	0.007197996	0.951222687	0.997604378
**CG3776**	FBgn0035088	152.3362519	127.0207005	124.7953478	162.8274195	138.8315204	137.8629438	128.7867407	141.241892	2R	-0.025343643	0.860821493	0.995787491
**Phk-3**	FBgn0035089	0.329764895	0.061459372	0.800005035	0	0.066145573	0.414533771	0.654295702	0.31820005	2R	0.320279112	0.834165646	NA
**CG3829**	FBgn0035091	0.643060091	0.490292314	0.567292659	1.135810905	0.211070596	0.275578694	1.193061897	0	2R	-0.748337583	0.465777008	0.971928234
**Nplp1**	FBgn0035092	9.473870617	11.62319646	16.70570513	14.66641387	9.460272075	11.0959809	8.783358971	11.9650776	2R	-0.310558347	0.12562563	0.780967863
**CG9380**	FBgn0035094	0.84817978	0.924148693	1.087459301	0.72677322	0.64126421	0.688025448	0.702748239	0.469550357	2R	-0.487779162	0.020965377	0.410927638
**p130CAS**	FBgn0035101	34.25410676	32.21054074	34.43476886	37.15387474	36.06882938	38.47413001	32.12383592	29.19987402	3L	0.00430367	0.968562881	0.997604378
**Vdup1**	FBgn0035103	0.326000172	0	0.632697496	0.836062841	0.353108318	1.567489951	1.164286819	2.123329651	3L	1.580955815	0.125080823	0.780439453
**CG13875**	FBgn0035104	2.553447517	1.112480519	0.724047436	4.948169775	1.436766923	4.595897197	0.930557068	0.43198215	3L	-0.327441594	0.67924022	0.995787491
**rno**	FBgn0035106	12.41838647	28.03963065	29.57450711	32.49170063	26.55331426	28.07963609	28.95160766	34.67544175	3L	0.241822538	0.262153543	0.891386222
**mri**	FBgn0035107	220.3992064	234.4618497	188.2500556	262.2863006	217.2394493	195.3227383	202.9794819	213.1557464	3L	-0.097714569	0.438062222	0.967112302
**CG13876**	FBgn0035109	24.18844046	17.52240672	17.34780001	19.71630749	20.99127133	33.00528004	27.91804808	25.38016593	3L	0.467034477	0.065979582	0.647672366
**thoc7**	FBgn0035110	368.5602231	344.0786868	349.0914453	340.4856338	424.4702995	321.0581806	381.3801333	338.1791376	3L	0.094102823	0.447100974	0.967112302
**Dis3l2**	FBgn0035111	122.2534974	122.6678857	111.9789187	128.1855701	103.6923962	147.9034504	103.8594138	104.0544548	3L	-0.052156016	0.683809403	0.995787491
**pyx**	FBgn0035113	0.084308201	3.026284848	0.245436623	1.375929777	0.882747882	2.559425271	0.215072032	1.317894542	3L	0.112564966	0.899564075	0.996479182
**wac**	FBgn0035120	21.53700136	29.81520264	31.71413372	28.06649175	21.5621639	30.32442812	20.39307869	24.91081432	3L	-0.154763329	0.574988218	0.984984955
**Tudor-SN**	FBgn0035121	138.7320149	125.0631547	128.9077877	135.5395906	107.0288161	119.5645263	129.092915	125.9346007	3L	-0.104211874	0.323182352	0.918470465
**mRpL17**	FBgn0035122	151.1499655	172.1301644	166.9413224	167.092462	192.1714181	163.8929192	181.4864356	189.8503479	3L	0.178712795	0.150969858	0.811129475
**ttm2**	FBgn0035124	4.124678364	2.978828458	0.833867682	0.534251796	0.930762699	0.756140305	3.215099287	0.373127439	3L	-0.685559797	0.474265308	0.973549494
**CG1231**	FBgn0035134	57.11782865	52.52430767	58.53992826	54.79565161	49.98240659	46.6834449	46.07885382	45.57562405	3L	-0.214129466	0.200697568	0.848203654
**CG1233**	FBgn0035137	41.0762695	43.35571024	52.17170713	60.40662824	44.80398407	54.69432836	47.93087434	39.80947979	3L	-0.044951443	0.774818459	0.995787491
**BORCS6**	FBgn0035140	152.1834455	156.388494	145.1161396	117.6466976	177.8446209	132.3245533	133.4996909	128.2729219	3L	0.033330144	0.828059158	0.995787491
**Cypl**	FBgn0035141	87.26065538	98.41643479	107.2751704	114.827815	110.1728505	101.4480453	98.87432882	103.2100938	3L	0.054225113	0.740902844	0.995787491
**Hipk**	FBgn0035142	20.34580364	39.8144667	43.72396226	33.29773933	30.46874537	31.12684493	36.20110715	43.43105696	3L	0.08074967	0.658086305	0.995750687
**Kah**	FBgn0035144	0.080575112	0.833446258	0.781896394	0.532263882	1.381857924	0.924249638	0.342581404	0.087468014	3L	0.348176549	0.691563561	0.995787491
**MED14**	FBgn0035145	121.551718	107.688634	104.4537193	94.25100275	100.6099654	90.96999981	100.9514743	109.4196683	3L	-0.059264644	0.612154193	0.990047511
**CG13893**	FBgn0035146	6.398539513	3.577550803	5.430830542	4.274014369	5.664128563	3.755773821	3.552347464	3.98002602	3L	-0.189610023	0.548375607	0.984934163

**Gale**	FBgn0035147	141.4303721	113.6593413	120.3928235	167.7275372	135.3753144	141.9326932	131.328258	115.4539696	3L	-0.026409842	0.853726978	0.995787491
**CG3402**	FBgn0035148	126.98403	126.6268696	111.4231558	93.96048729	95.52586495	123.8059749	106.934521	103.0868353	3L	-0.068041433	0.643636923	0.992589784
**MED30**	FBgn0035149	43.38772377	44.69446007	62.35393143	44.53100225	33.48718648	41.20209893	39.70151643	31.74088809	3L	-0.382879182	0.074484049	0.676245
**Rev1**	FBgn0035150	7.27420411	18.24178214	8.852679512	6.71664675	10.79148149	15.77583636	14.57585125	6.723739911	3L	0.255807824	0.452554177	0.967817941
**CG17129**	FBgn0035151	1.805284237	2.078115427	0	1.402984079	0.830725678	1.268165023	0.27090111	0	3L	-1.130777613	0.350756599	NA
**CG3386**	FBgn0035152	29.21342995	42.21551811	34.19595431	26.50929045	31.51963359	49.38332256	19.88033236	23.85374002	3L	-0.053899487	0.837311836	0.995787491
**ebd1**	FBgn0035153	81.09080504	108.0948545	68.70393588	73.68343015	88.27990718	82.98128939	59.47143731	81.46279609	3L	-0.054090173	0.746060591	0.995787491
**hiro**	FBgn0035154	2.964612574	3.243079369	1.563501903	2.404133083	4.731377054	0.040507516	1.424873548	0.839536739	3L	-0.496311989	0.585628337	0.98718792
**RabX6**	FBgn0035155	33.64460448	21.37808972	23.15291186	35.48862559	35.22099519	31.60603199	25.73302366	28.58874339	3L	0.115994001	0.643553501	0.992589784
**CG13894**	FBgn0035157	13.50507212	18.55377425	20.59395901	17.69245207	19.11710723	23.96943558	14.43691506	19.76436531	3L	0.169971393	0.358363473	0.93823489
**CG13895**	FBgn0035158	65.89434106	34.43957746	18.52760146	39.45888609	35.67992412	26.52152906	28.78321295	21.4135804	3L	-0.474090619	0.116146402	0.765798592
**CG13896**	FBgn0035159	2.270575371	6.488677635	2.040142289	10.19539156	3.643529323	5.867061472	3.861524369	5.112208179	3L	-0.147563649	0.822858038	0.995787491
**hng3**	FBgn0035160	1.473711518	3.091969972	2.147482023	1.326734793	2.238973218	2.36153202	1.823901163	1.267154109	3L	-0.025949401	0.932919029	0.996479182
**CG13898**	FBgn0035161	1.526924202	2.561201143	2.357279792	4.854499494	7.016518809	5.627428807	10.26920699	2.410977301	3L	1.206350346	0.124746734	0.780439453
**Sf3b3**	FBgn0035162	17.45360899	22.80169448	22.85785828	25.69410469	22.24110886	19.71433054	23.4060456	24.03959602	3L	0.043510314	0.783954038	0.995787491
**CG13901**	FBgn0035164	49.46397217	45.70212646	71.01604485	64.04910528	47.42534377	62.98111447	36.95515258	40.57712403	3L	-0.261359699	0.25348064	0.884040848
**CG13887**	FBgn0035165	179.0947301	163.047617	156.5350914	174.9233294	166.5660552	159.4995784	177.9462146	147.9012278	3L	-0.01904229	0.868215468	0.995787491
**JMJD5**	FBgn0035166	101.6822884	134.4458503	99.58555426	97.75414169	106.8515579	101.132122	101.9961656	80.12830646	3L	-0.120280119	0.438039455	0.967112302
**cep290**	FBgn0035168	2.978485942	2.701085283	1.762381793	3.528571061	1.989027138	2.385742097	1.544345859	5.888257674	3L	0.136457236	0.750052821	0.995787491
**Dci**	FBgn0035169	28.85099064	33.72216004	42.12194306	25.41460353	27.3379103	31.13907715	48.12274801	32.59452357	3L	0.130368939	0.606887997	0.989591971
**dpr20**	FBgn0035170	90.42501042	71.65028074	71.60728222	104.4523615	64.81956416	70.8240286	63.24440089	77.16618249	3L	-0.26624831	0.076467732	0.681411883
**CG12502**	FBgn0035171	40.2936264	41.08675513	38.59412076	41.25423735	43.62435317	31.39595284	38.13004378	41.27990826	3L	-0.030051492	0.827405238	0.995787491
**CG13905**	FBgn0035176	2.944260543	0.159309162	1.382464841	0.664299931	1.02873772	2.507202063	0	0	3L	-0.529214374	0.737551551	NA
**CG12038**	FBgn0035179	406.1992845	336.3631417	368.5908468	418.5707517	358.1921197	373.7513603	358.68273	352.3867602	3L	-0.056732474	0.600572193	0.989286463
**CG9205**	FBgn0035181	218.0334353	231.774233	226.0161632	216.5792044	296.4255372	268.8100037	249.1204466	281.0859195	3L	0.327365346	0.001514564	0.083591897
**galene**	FBgn0035192	1.251351064	0.853425681	0.600480045	0.28854177	0.570958417	0.700081006	0.035079379	0.328404274	3L	-0.841155615	0.306738653	0.911610813
**CG9129**	FBgn0035196	1.601076629	1.208511647	0	1.119854966	0	0.603794107	0.204219044	0.347608557	3L	-1.732254486	0.301687834	NA
**tfc**	FBgn0035199	0.139172759	0.539333317	0.291061355	0.369231365	0.277233103	0.221701728	0.220365123	0.177126483	3L	-0.533998216	0.373477556	0.943282319
**CG9149**	FBgn0035203	92.23646237	97.59748296	65.18559542	111.6233702	96.30795361	99.67234231	107.9276339	69.89794428	3L	0.054130539	0.770650923	0.995787491
**CG2277**	FBgn0035204	94.47729986	101.4992915	86.65599707	76.51659077	63.08309434	78.61032477	77.01077881	91.75796554	3L	-0.179068255	0.223555691	0.86549481
**Ctr9**	FBgn0035205	249.8523244	275.0708985	244.0791319	239.2419428	248.3935619	236.3257844	228.4414465	235.3473834	3L	-0.05663539	0.515097439	0.980804634
**sturkopf**	FBgn0035206	208.2390393	210.7493025	180.0973135	232.763003	183.7184101	194.1593177	171.521087	196.562995	3L	-0.128286414	0.284939339	0.901780531
**Herc4**	FBgn0035207	191.8723115	161.8130042	147.1033283	196.063953	162.2732745	171.9613824	161.5514542	164.9651646	3L	-0.049884126	0.672284176	0.995787491
**msd1**	FBgn0035209	1.93477181	1.248195496	2.527392767	2.77590624	2.686737688	5.051329046	1.518659832	0.323120669	3L	0.216929688	0.844762885	NA
**msd5**	FBgn0035210	18.58317547	20.08776504	24.29447153	14.20129149	27.76978771	17.32543649	44.24567454	20.84532698	3L	0.54597238	0.136305133	0.796109055
**CG2211**	FBgn0035211	27.33377657	34.47209799	44.30669393	40.38943579	31.49490235	39.63744545	33.87435227	43.46582662	3L	0.054449252	0.78557174	0.995787491
**CG2199**	FBgn0035213	45.26634788	45.36917069	38.91382514	34.37836787	39.42410976	38.81982702	43.02172891	40.3844842	3L	0.008097506	0.956743674	0.997604378
**lml1**	FBgn0035227	34.96538786	41.48014556	34.36948184	42.7093563	31.42086599	42.40793675	36.28653199	31.18333522	3L	-0.09172931	0.476571697	0.973549494
**CG12091**	FBgn0035228	699.8990562	740.3994439	559.8445866	608.278465	649.1820777	631.9254157	623.0146589	618.5333171	3L	-0.017420462	0.868486252	0.995787491
**pns**	FBgn0035229	107.0162452	109.1092408	97.68554841	87.41585536	99.89169078	98.21678188	84.75212548	93.53876726	3L	-0.061470848	0.555239062	0.984934163
**Pcyt2**	FBgn0035231	59.90065286	117.9384438	75.4270389	106.5154989	42.56783436	61.00085133	70.70395074	33.85424987	3L	-0.759288619	0.001634177	0.08786598
**CG12099**	FBgn0035232	472.9728107	407.752234	362.7027846	404.0884756	399.6130191	458.1875907	359.1654833	340.3209176	3L	-0.055455885	0.635569421	0.991585536
**Pex10**	FBgn0035233	22.30353603	23.72624965	26.4756814	21.1210811	18.1712204	27.17110409	25.54274376	25.87929763	3L	0.078700836	0.726186329	0.995787491
**CG7879**	FBgn0035235	30.85559563	37.97857968	32.66106588	26.22213512	33.9938455	33.26879783	29.47037539	26.87739402	3L	-0.014925694	0.910707458	0.996479182
**CG12004**	FBgn0035236	179.1612811	187.0336358	188.4128051	217.0882739	182.5060436	183.545047	188.263792	185.6098412	3L	-0.030683978	0.751567694	0.995787491
**CG13917**	FBgn0035237	81.38082998	76.00856129	80.108072	81.60352128	76.51327329	77.47616611	75.5981078	76.28637321	3L	-0.031566774	0.702844557	0.995787491
**CG12104**	FBgn0035238	50.82374475	36.47165727	34.41116796	40.22356541	41.98179353	42.8438102	38.79365087	21.1418451	3L	-0.139105994	0.554298945	0.984934163
**CG13926**	FBgn0035243	2.780338749	2.51118442	1.556553006	3.989080078	3.667894489	2.722105097	4.637537154	1.393009107	3L	0.21573694	0.788730226	0.995787491
**ABCB7**	FBgn0035244	43.54303887	59.26109617	49.72780512	55.62737855	49.4534945	52.97731644	49.19664957	42.24587831	3L	-0.070899963	0.585214372	0.98718792
**GC**	FBgn0035245	103.2288011	95.83156506	101.6348768	113.1788257	96.47213984	87.14560242	77.96217119	83.24606041	3L	-0.234165352	0.034797642	0.514749796
**metl**	FBgn0035247	118.9074911	116.7488507	105.798655	104.6439365	66.63483491	132.5052551	112.9604553	109.2267814	3L	-0.055755156	0.765809483	0.995787491
**CG13919**	FBgn0035248	50.19158032	38.57504639	76.15345084	53.61775875	69.09289618	69.47698475	59.52351894	66.32991039	3L	0.309756432	0.249015196	0.879503029
**CG17249**	FBgn0035249	63.90426342	73.73488948	77.58396293	60.99252697	69.05494218	55.45423495	74.0874187	55.9384479	3L	-0.085467094	0.612920744	0.990047511
**Vta1**	FBgn0035251	169.8142445	193.7859219	133.2665971	152.4233476	188.0039207	159.3210286	145.7044451	200.2260808	3L	0.126900612	0.408436237	0.956706394
**CG7970**	FBgn0035252	36.67304755	66.98179411	56.77024975	56.4583511	67.46528339	62.34498771	63.40864071	50.2999861	3L	0.203592136	0.309582565	0.912131082
**Srrm234**	FBgn0035253	453.8309209	448.5224639	417.0639664	454.936568	433.1962518	472.81131	406.8354489	417.5588905	3L	-0.007743012	0.925843411	0.996479182
**CG7974**	FBgn0035254	22.18997639	35.52419248	31.31814581	32.73319659	42.20650855	21.4814475	28.68295745	36.552138	3L	0.121375916	0.647106225	0.99279236
**CG13930**	FBgn0035256	0.037335451	4.634248588	1.630355088	0.174092396	8.896807594	2.182377362	0.190487074	0.08105872	3L	0.873402402	0.462957823	0.971606621
**CG7991**	FBgn0035260	8.459873717	6.64322951	5.944002925	6.038829974	6.586823804	6.995927432	6.01765983	6.392585386	3L	-0.033107101	0.812388143	0.995787491
**Oseg4**	FBgn0035264	1.342637239	0.669538967	0.616230662	0.846029237	0.425803464	0.975031361	0.601706132	0.393917531	3L	-0.517890046	0.448626695	0.967112302
**Gk2**	FBgn0035266	7.384903179	10.12928263	7.909259927	9.875660447	10.38887228	8.16165814	6.773235842	4.370283063	3L	-0.217580791	0.393448795	0.952091247
**CG13921**	FBgn0035267	621.1616667	605.5647198	445.678627	635.0489256	555.9724169	568.8718084	519.7493483	527.4011746	3L	-0.059183752	0.614905975	0.990047511
**CG8001**	FBgn0035268	51.8175402	49.89130327	56.85834405	64.77675992	49.49007832	50.5138427	47.90353187	64.45351255	3L	-0.041786666	0.789625289	0.995787491
**CG13933**	FBgn0035270	51.01601885	48.74917628	47.02534088	58.32147408	44.26839009	49.71226085	37.93078136	42.76053869	3L	-0.203053702	0.150292553	0.809805479
**CG2021**	FBgn0035271	187.4928609	232.9711759	222.187267	142.5213258	203.9414808	144.5906199	238.5981232	177.2699619	3L	-0.004144624	0.98471008	0.997881056
**mRpL46**	FBgn0035272	130.5951303	138.8895172	146.0297002	129.664323	108.4933085	98.03175845	144.3086435	157.6110029	3L	-0.067594698	0.711397972	0.995787491
**Cpr62Ba**	FBgn0035279	0.135800751	0.303715612	0.027454227	0	0.143007177	0.117362775	0.259822975	0.024569667	3L	0.23904681	0.867272627	NA
**Cpr62Bb**	FBgn0035280	0.99364285	0.747139804	3.491161212	0.239652507	0.865962764	11.596943	0.78666339	4.984085195	3L	1.766219073	0.036575179	0.521170991
**Cpr62Bc**	FBgn0035281	0.672576505	0.644658705	0.34964169	0	0.867266409	0	0	1.773131392	3L	0.699791173	0.686688876	NA
**CNMa**	FBgn0035282	0.24081206	0.466071287	0.494328745	0.431880194	0.066881152	0.174643184	0.094510268	0	3L	-2.198351609	0.071389708	NA
**CG12024**	FBgn0035283	353.8369142	353.3433196	339.6544224	399.1729573	372.9737254	345.0072948	365.2614943	327.7494328	3L	-0.005866614	0.954909108	0.997604378
**CG12025**	FBgn0035285	146.1696226	120.6039007	125.6875348	132.9738381	125.4102464	131.754981	113.3391053	113.6394945	3L	-0.090983919	0.406559876	0.956706394
**CG13924**	FBgn0035286	5.494231763	1.598659707	1.088075888	2.744913112	1.821764662	3.11852817	2.145291316	0.365157727	3L	-0.548223966	0.498046336	0.97594235
**CG13937**	FBgn0035287	69.64160335	67.6527344	60.74154303	59.65065362	65.31802925	62.62519446	57.57347063	58.30032876	3L	-0.050404864	0.634145654	0.991585536
**dsb**	FBgn0035290	0.061910712	0.45000165	0.375486311	0.264627907	0.316666659	0.325893814	0.447484078	0.168017359	3L	0.175653605	0.794763807	0.995787491
**CG5687**	FBgn0035293	0.617570673	1.753041514	0.96039605	1.033733274	0.171518976	3.941329501	1.252269684	0.240656966	3L	0.380736971	0.633547113	0.991585536
**Mfap1**	FBgn0035294	41.29251178	68.94024507	68.02585309	72.53978489	63.96878187	65.86468264	73.85914543	77.66180207	3L	0.202037696	0.284831651	0.901780531
**Cnb**	FBgn0035295	1.605825837	1.400644735	1.40245599	0.149756899	1.159570882	1.574521302	0.491579545	1.952382063	3L	0.213033107	0.792756339	0.995787491
**SCOT**	FBgn0035298	10.45474183	13.84658508	14.57515089	16.32342025	24.63801557	16.65821279	17.61927406	9.603089816	3L	0.344959569	0.199145015	0.847197998
**CG15822**	FBgn0035308	1.657299016	0.667717861	0.453301171	0.890219228	1.011949091	1.003364569	0.227969899	0.529139644	3L	-0.388312773	0.503103457	0.978380269
**CG15879**	FBgn0035309	3.335747586	0.186508287	0.674373093	0.648097493	0.903281901	3.983568435	1.701912751	0.241407227	3L	0.473328012	0.660746162	NA
**CG15878**	FBgn0035316	0.743960392	2.406643439	1.160252701	0.743363849	1.630833294	0.150300281	1.76229759	1.845950719	3L	0.13268878	0.890443913	NA
**Oseg2**	FBgn0035317	0.169930569	0.932840807	0.074954398	1.008475301	0.697200524	0.320419006	1.615759805	0.06707909	3L	0.337762632	0.731806309	0.995787491
**CG9018**	FBgn0035318	132.8275329	143.1008772	135.4383524	166.5613451	151.4816293	165.1035731	150.9269904	161.0861117	3L	0.151232623	0.207859118	0.856362392
**CG1275**	FBgn0035321	46.81717103	68.90327245	62.290397	67.67539572	62.66087845	74.315389	68.94825991	73.22899125	3L	0.217274778	0.112337438	0.758410631
**CG13807**	FBgn0035323	56.62543357	77.44622641	87.85802418	101.5538975	93.01207939	66.17531357	104.4504121	66.19795433	3L	0.062161628	0.800701357	0.995787491
**MsR1**	FBgn0035331	2.282978949	2.062455877	1.782497729	2.351068603	1.658019246	1.919115902	1.673542628	1.139438766	3L	-0.381767101	0.098007661	0.730787365
**CG13801**	FBgn0035332	0.244109664	3.958110584	0	0.284566246	1.542381777	1.035652949	0.311364498	1.236630912	3L	-0.061495245	0.959014378	NA
**CG1317**	FBgn0035333	254.8640372	270.3613385	222.2420236	273.4865726	236.4228151	279.1619793	231.7543125	236.1721788	3L	-0.02591698	0.802141522	0.995787491
**CG8993**	FBgn0035334	553.6146956	554.7629772	563.6699186	504.4205016	623.1628125	457.0482812	707.2838509	568.8909275	3L	0.147931391	0.297657779	0.9074568
**mRpL23**	FBgn0035335	164.9775423	165.5629728	151.7367689	139.2496245	166.5502445	143.1953891	144.9119339	146.727593	3L	-0.01784632	0.897608256	0.996479182
**CG9004**	FBgn0035336	18.64175287	17.31898385	15.98850642	10.97539016	18.97946547	20.03114294	21.21584293	13.49099037	3L	0.254412542	0.284188498	0.901780531
**CG15877**	FBgn0035337	31.51223489	39.43971746	21.17028249	39.93731524	30.48595139	42.81221263	26.38388126	31.22575452	3L	0.01571064	0.954157201	0.997604378
**CG16758**	FBgn0035348	25.94046657	4.220752139	5.608222981	6.00976489	3.656222299	26.4238355	5.845082811	6.973270672	3L	0.028866417	0.972743599	0.997757033

**CG16985**	FBgn0035355	4.399656922	10.30098099	15.56553006	8.975430175	11.41735578	8.295939344	9.586841511	8.159053341	3L	-0.019106987	0.968102796	0.997604378
**CG16986**	FBgn0035356	45.2140054	41.11524889	43.59640161	42.90330109	66.82953422	65.46606283	60.51323804	62.11317786	3L	0.589573407	0.000381261	0.028885693
**CG1246**	FBgn0035360	3.61411317	0.538859363	1.65613776	1.966106054	1.159893433	1.476524323	2.151258747	2.702708907	3L	-0.049103265	0.945117567	0.997604378
**Non2**	FBgn0035370	381.8540176	324.7903246	364.7563515	337.6417658	378.291895	364.1719353	366.2657558	352.2173027	3L	0.081154213	0.431649447	0.964209983
**AhcyL1**	FBgn0035371	80.3783202	88.36370203	95.34744293	90.06247602	88.21285995	90.57224526	85.1451058	85.91426082	3L	0.01410837	0.890253238	0.99602235
**CG12093**	FBgn0035372	98.76994147	93.27876219	99.62154379	86.38929078	117.3571479	86.12161116	103.9925147	99.94142394	3L	0.138829933	0.353706653	0.936401286
**mRpS35**	FBgn0035374	180.4905898	173.9624346	173.7182851	143.1715075	158.4426692	141.7255216	155.3717452	118.1978623	3L	-0.197069497	0.163155632	0.825084367
**Pgant6**	FBgn0035375	23.89787017	33.89454094	23.11201407	19.6837996	19.82189921	25.94517748	18.12442656	26.44305969	3L	-0.121540042	0.548966149	0.984934163
**spz5**	FBgn0035379	0.416580419	0.715798022	0.591581436	0.35533228	0.935457455	1.216565445	1.088625247	0.860315888	3L	1.016731018	0.037348045	0.521998122
**Or63a**	FBgn0035382	1.767719299	0.257834882	0.932274427	0.716760174	2.774944693	1.847746583	1.764583008	1.33491556	3L	1.072064243	0.162319439	0.824273756
**CPT2**	FBgn0035383	0.239749988	1.148991346	4.902311068	5.669533296	0.12366005	2.260348242	6.116073701	2.230797502	3L	-0.125187108	0.888854908	0.995926915
**FMRFaR**	FBgn0035385	0.222010488	0.959624116	1.470776869	0.47779301	0.693667784	0.75673663	1.851540366	1.186474634	3L	0.572080296	0.324662871	0.918470465
**CG2162**	FBgn0035388	123.2827486	112.4461046	94.72597701	113.8609829	83.24015753	104.0574103	75.00992982	93.47532285	3L	-0.293725303	0.021079264	0.410927638
**scramb2**	FBgn0035390	111.7789365	114.8215916	127.6846371	109.6904647	124.6773706	102.4193304	111.5877372	96.71065079	3L	-0.059957341	0.623689014	0.990047511
**CG1271**	FBgn0035392	30.01945052	25.99669777	35.13957455	30.73109245	32.75101792	20.75718937	25.7730922	26.41592521	3L	-0.173791516	0.396401141	0.952272708
**CG16753**	FBgn0035393	42.6847449	47.79525412	35.52735334	24.35011856	26.84695345	43.13270069	49.2320458	24.64695012	3L	-0.038001732	0.904769821	0.996479182
**PAN3**	FBgn0035397	84.50144008	92.7389112	71.50226081	94.35657896	82.05817993	88.57399204	74.04099029	77.43549198	3L	-0.062656665	0.564960062	0.984934163
**Cht7**	FBgn0035398	0.601929836	0.545758301	0.473602179	0.088501237	0.714635556	0.362992791	0.456510686	0.070640292	3L	-0.062759838	0.928948327	0.996479182
**CG11537**	FBgn0035400	227.3483241	237.9296124	216.9470352	269.550971	216.652444	232.117571	231.6529323	226.1966616	3L	-0.04093032	0.671038164	0.995787491
**Alg2**	FBgn0035401	50.58599441	42.62232441	52.10656099	50.7604359	52.33459209	86.58728612	44.9115865	55.04084764	3L	0.311020325	0.139780225	0.803344611
**Usp5**	FBgn0035402	155.9597738	144.4043414	138.4768168	128.43102	122.0550449	145.5445898	132.7436234	128.9583016	3L	-0.071709076	0.503904623	0.978429306
**CG15812**	FBgn0035405	134.7964414	132.6809655	134.494469	105.1559275	101.9322842	103.1661725	112.6616448	117.859698	3L	-0.188286951	0.144999435	0.8037297
**Asciz**	FBgn0035407	40.88353072	39.62453338	33.60107726	37.94705513	37.37858385	31.61795774	30.66965581	43.19798128	3L	-0.060222258	0.734053614	0.995787491
**CG14963**	FBgn0035409	23.72465375	25.4374466	27.76215109	37.95999604	22.84217632	17.54310482	20.88605853	38.17682854	3L	-0.174741266	0.594811209	0.988780662
**CG14964**	FBgn0035410	3.156312959	1.210119361	1.422046083	1.752101153	0.813993407	1.643749044	0.996892269	1.403161178	3L	-0.619130418	0.205631141	0.855364409
**CG14965**	FBgn0035414	12.00327749	13.0597713	16.52744484	12.96611101	14.80853912	10.92337519	11.70440639	6.942685838	3L	-0.263790049	0.388790551	0.948247506
**CG14966**	FBgn0035415	29.56254118	18.59513085	15.77802876	15.50788917	22.94822437	16.16540228	16.59122909	24.06863005	3L	0.023935833	0.952968237	0.997604378
**hob**	FBgn0035420	16.1808302	10.46424883	16.64537889	8.355029603	12.50471703	12.27831462	11.65027401	7.377079637	3L	-0.210340645	0.381303699	0.944476532
**nSMase**	FBgn0035421	199.8242286	125.6606175	170.1033505	190.5312841	171.4834251	160.9258061	156.2618553	178.5949494	3L	-0.013712893	0.929795856	0.996479182
**RpL28**	FBgn0035422	360.4232125	506.5648823	581.0751389	539.5113995	452.2389258	422.3049138	539.633425	562.333098	3L	0.027454911	0.853423483	0.995787491
**eIF1**	FBgn0035423	651.3242108	676.2809017	752.8888737	576.683388	634.3122908	593.6019098	728.559942	622.2089608	3L	-0.010310442	0.927149043	0.996479182
**Larp4B**	FBgn0035424	71.46778248	117.0617695	120.0335731	124.6422245	99.88635825	89.54767966	118.1635063	114.8060425	3L	-0.000648367	0.996731813	0.999490185
**CG17746**	FBgn0035425	106.7031832	69.99905549	92.75327035	76.03060036	91.57657726	85.99221295	81.67657076	89.17920726	3L	0.039592232	0.794427853	0.995787491
**ckd**	FBgn0035427	0.621943545	0.888149351	0.550517728	0.470282582	0.409658413	1.045947621	0.278725507	0.054741771	3L	-0.469189172	0.513528781	0.980804634
**CG14960**	FBgn0035428	0.995802786	0.055677316	0.36237055	0.116083835	0.239690567	0.234709196	0	0.360330005	3L	-0.863843567	0.486069906	NA
**CG12017**	FBgn0035429	1.706843538	0.558964829	0.394359868	0.568491636	0.939059824	1.30268706	0.228076857	0.229401425	3L	-0.251535614	0.708747322	0.995787491
**CG12009**	FBgn0035430	0.987822454	0.655066768	0.668774929	0.562377769	0.808693437	0.119121427	0.556734532	0.249378371	3L	-0.710182652	0.314875987	0.91460807
**CG14968**	FBgn0035431	1.995706401	1.731474578	1.613851964	0.802226611	1.44938683	1.362494342	1.521475144	0.896454484	3L	-0.210288389	0.702680047	0.995787491
**ZnT63C**	FBgn0035432	62.72906635	61.48130444	51.24339922	61.73494481	64.96996517	68.52696765	55.19298702	45.51249199	3L	0.00810714	0.951065044	0.997604378
**PIG-C**	FBgn0035435	36.33074257	17.01485362	28.62299298	21.63719774	23.50716957	14.58270588	22.94071902	25.61563258	3L	-0.234083847	0.464860875	0.971928234
**Strip**	FBgn0035437	101.0672723	111.5035681	100.7394012	128.702241	107.3517714	105.7946981	114.4525138	112.0755793	3L	0.022730232	0.839376396	0.995787491
**CG14961**	FBgn0035439	0.500041187	4.053953223	2.493579944	0.453377246	6.068166821	0	10.91360194	0	3L	1.245245901	0.53528288	0.984760433
**CG14969**	FBgn0035440	45.28936781	41.89102543	46.231885	40.51979437	40.6271159	55.39388694	40.17545556	51.99618178	3L	0.14343729	0.402817632	0.955073155
**CG12010**	FBgn0035443	17.63835843	13.50392491	17.04514318	12.11512438	21.33794132	14.11078561	17.17683186	22.56356957	3L	0.347937455	0.1684475	0.830712326
**CG12012**	FBgn0035444	76.16794718	73.29201816	69.76130273	68.0243129	71.41022451	92.90423469	93.03789708	63.64980074	3L	0.186914679	0.274362881	0.89549671
**Ids**	FBgn0035445	5.345037748	8.245104926	4.167960115	4.33936087	9.046090366	22.27176898	7.669859697	7.149245087	3L	1.08543036	0.01330662	0.344576629
**CG14971**	FBgn0035449	53.37855978	57.0813551	65.09953731	70.36808274	61.52890949	70.23130558	77.06205002	63.63923529	3L	0.178131962	0.201119457	0.848203654
**IntS10**	FBgn0035462	21.39980187	23.13650976	32.13824147	19.77045911	28.31708635	18.90604927	29.15252594	19.43774472	3L	0.025245497	0.915303285	0.996479182
**PIG-B**	FBgn0035464	360.6468773	307.4001216	333.6423718	329.4848778	344.0098936	403.4977901	333.9482255	349.5961817	3L	0.131634973	0.187194452	0.840740887
**CG14977**	FBgn0035469	98.49566394	171.7725731	144.7750471	85.43919109	136.7567455	99.5525727	154.0842932	148.4323416	3L	0.146878928	0.538140619	0.984934163
**Ccz1**	FBgn0035470	79.18081451	89.02404414	85.79680163	91.182926	77.91005715	84.42977842	86.99206411	67.33555412	3L	-0.094145729	0.492740829	0.973549494
**Sc2**	FBgn0035471	199.5643962	194.1593576	179.4928557	190.0371379	144.7139952	158.9864623	185.8798297	153.5650351	3L	-0.218619295	0.072253268	0.671999479
**mge**	FBgn0035473	660.9484603	576.9667506	649.9114174	641.3935654	636.2280139	585.3507959	589.2400148	596.4611722	3L	-0.04163798	0.663148002	0.995787491
**CG10866**	FBgn0035475	44.02645819	55.34855457	43.39737456	52.26376266	36.82582853	41.634682	41.0592324	35.13887103	3L	-0.304101804	0.06214573	0.633840737
**CG12766**	FBgn0035476	2.192866978	1.839113364	2.799928791	0.672708791	0.62505583	4.29806068	4.269144012	0.25057459	3L	0.351929623	0.700873448	0.995787491
**CG14982**	FBgn0035477	364.8471244	365.0378332	362.7674903	400.2250024	386.2991213	356.632714	357.0364943	399.9900012	3L	0.037669376	0.6928462	0.995787491
**CG14984**	FBgn0035480	0.765968274	2.997876758	0.929114174	0.446456549	0.806615279	0.902687763	1.302667682	0.138582336	3L	-0.669518945	0.546992957	NA
**CG12605**	FBgn0035481	50.67002697	77.63068833	107.0448308	88.76461553	63.82554801	58.91856755	94.07816159	101.9114605	3L	0.014282931	0.962135189	0.997604378
**CG14985**	FBgn0035482	1.621344065	0.230472646	1.666677156	0.832903974	0.84335605	0.077725082	0.070103111	0.357975056	3L	-1.660662462	0.109631894	NA
**Mul1**	FBgn0035483	28.64673877	41.86682188	31.66412621	33.78959648	29.98840916	21.29474584	26.70173991	40.02175138	3L	-0.168024186	0.467008221	0.971928234
**CG11594**	FBgn0035484	5.44143615	4.73914685	4.315661913	3.659568329	0.7084029	0.789254272	2.135572388	3.123857632	3L	-1.407007523	0.007225346	0.247978871
**CG11593**	FBgn0035488	71.4256571	70.75458811	78.11697241	83.3313638	70.07237072	86.43764614	79.30545316	63.172657	3L	0.006111734	0.963361579	0.997604378
**CG1136**	FBgn0035490	0.298363522	0.923076112	0.450380462	0.401915696	0.203479659	0.756371824	0.236796546	0.071974981	3L	-0.667599151	0.350235865	0.932790554
**CG14989**	FBgn0035495	7.554801132	9.582049764	11.11151058	4.846732294	12.53996153	7.744057037	8.536794361	5.650860358	3L	0.095913261	0.749063646	0.995787491
**CG14995**	FBgn0035497	50.98163544	51.6249624	51.74975535	55.2165685	56.38586121	61.76274137	49.45867678	59.2673565	3L	0.144093979	0.168088766	0.830652748
**Fit1**	FBgn0035498	108.5166647	109.5401187	116.9583937	108.5352378	104.6416254	134.3119762	86.84322854	102.6050036	3L	-0.021896137	0.863556435	0.995787491
**Chd64**	FBgn0035499	12.6200185	13.11358904	13.80314074	18.4019037	13.01297962	10.80909988	16.34251615	14.83737877	3L	-0.044117988	0.818073731	0.995787491
**CG1299**	FBgn0035501	1.146654033	2.346488562	0.389447337	0.908949442	0.496801059	1.297270102	0.702033377	0.697057774	3L	-0.552514192	0.447270854	0.967112302
**CG14997**	FBgn0035515	16.1117066	21.22548736	23.43477025	16.78737866	60.93852773	52.79539639	45.73884683	45.96930053	3L	1.442665363	4.06E-15	3.76E-12
**CG1265**	FBgn0035517	376.3179537	338.0238516	328.4485301	387.1313187	328.2873656	302.7995529	327.3811985	327.2207845	3L	-0.124467668	0.268464904	0.89549671
**CG15011**	FBgn0035518	43.62424773	31.34696293	26.12556777	36.09622148	25.92119644	38.39020729	21.87979967	32.24500992	3L	-0.18925195	0.374372351	0.943282319
**CG1309**	FBgn0035519	172.3753971	182.121255	191.9983163	199.6658055	184.8436679	179.4153861	197.3008816	213.1247282	3L	0.085889134	0.448316261	0.967112302
**CG11586**	FBgn0035520	116.3771144	144.7945083	139.3731988	165.7929525	154.2352348	102.5488698	111.8953225	146.3728367	3L	-0.102341513	0.565875576	0.984934163
**VhaM9.7-a**	FBgn0035521	1875.701562	1595.509593	1813.138156	1447.687221	1719.637431	1601.630691	1670.192671	1682.843097	3L	0.018424463	0.8631274	0.995787491
**CG1273**	FBgn0035522	0.443595824	0.475377905	0.343772465	0.588934799	0.444891197	0.365942004	0.487226344	0.187267033	3L	-0.292892958	0.58458484	0.986819942
**Ctl1**	FBgn0035523	177.9463435	204.9619	170.9845336	203.7308692	166.1222594	177.3913921	167.6601712	140.8215707	3L	-0.187406492	0.084828501	0.69799888
**CG11583**	FBgn0035524	80.87887979	75.79864408	65.93715018	87.0541533	62.52343493	67.9800656	62.9405271	84.50356083	3L	-0.127561465	0.466723848	0.971928234
**CG1316**	FBgn0035526	712.9480551	609.0223294	594.6378498	617.6340728	585.8876348	559.2869285	567.3683404	525.9210212	3L	-0.15020611	0.125081968	0.780439453
**CG15012**	FBgn0035528	507.9434116	445.6678411	416.0180324	394.55576	509.476905	466.770218	403.6123801	403.4693863	3L	0.044078313	0.721306239	0.995787491
**Fdx2**	FBgn0035529	125.6420447	102.1617794	95.99835854	93.32085427	105.8365367	81.92103656	90.71194965	104.9155806	3L	-0.094129413	0.596553148	0.988780662
**CG15014**	FBgn0035532	46.9870206	45.07262059	54.26354196	58.27418595	61.38194494	48.82805504	53.80517033	56.54723711	3L	0.140402737	0.46242675	0.971606621
**Cip4**	FBgn0035533	38.17272809	42.90856423	36.53154454	40.70877741	34.67749238	41.54495888	34.12369628	31.17925373	3L	-0.133743185	0.184589826	0.839299945
**mRpS6**	FBgn0035534	163.6122418	169.6097752	168.592884	141.6252943	140.1220936	107.7058034	138.3821247	164.7188042	3L	-0.191694999	0.274964445	0.89549671
**CG11342**	FBgn0035537	2.965147413	1.205725774	1.743856065	1.675910199	0.811038037	1.355405028	3.896685783	1.365553533	3L	-0.034701784	0.97026484	NA
**DopEcR**	FBgn0035538	268.0351224	268.9426807	232.5701348	254.2968088	207.152791	241.2903192	211.2160392	226.3377338	3L	-0.179597189	0.046294429	0.565844709
**slow**	FBgn0035539	1.525376011	0.837361397	0.695253144	0.517288254	1.310091091	0.950027607	2.146092695	0.331173252	3L	0.424107903	0.385813196	0.947329421
**Syx17**	FBgn0035540	154.3257857	112.7055933	79.75366472	93.22487261	90.41631513	110.5683141	108.0507047	113.8853311	3L	-0.032085516	0.866453995	0.995787491
**CG15019**	FBgn0035541	79.36907916	89.01348147	80.04056095	61.53755238	64.09080148	68.78280583	77.7645084	57.10291087	3L	-0.181913106	0.331314419	0.921507984
**DOR**	FBgn0035542	45.37266585	54.05570845	50.13792954	59.11799211	60.64185548	53.75884463	58.20384313	52.76002935	3L	0.142334087	0.205078048	0.854987719
**CG15020**	FBgn0035543	0.558940081	0.585964734	0.211872006	0.855190715	0.283789716	0.428159301	0.133675139	1.213510656	3L	-0.074499179	0.932554962	0.996479182
**CG15021**	FBgn0035544	7.844671304	3.563715896	2.511043057	0.889075631	1.278481594	1.643535153	2.084575524	0	3L	-1.553277588	0.087616558	0.706186065
**CG12607**	FBgn0035545	2.138008732	0.290773761	1.542015486	3.098589136	1.251779587	0.980611576	0	2.509089119	3L	-0.549523193	0.609211764	NA
**CG11349**	FBgn0035550	0.256393426	1.218514676	0.103667812	0.099628603	0.69428377	0.040287693	0	2.597718019	3L	1.045361747	0.486519797	NA
**CG7465**	FBgn0035551	3.309334966	0.965381766	2.268895785	0.167730215	1.55848638	0.135653078	4.771669555	0.624771526	3L	0.086753107	0.939540397	NA

**CG11350**	FBgn0035552	0.110977893	1.30304893	1.076923284	0.38811117	2.804809569	0.31388784	0	0.12047164	3L	0.239093685	0.868547308	NA
**CG13722**	FBgn0035553	1.178987148	1.501963705	2.968821808	0.313150346	2.774968599	0.942700077	3.769044719	0.129604569	3L	0.396814139	0.598676894	0.989286463
**CG13720**	FBgn0035555	0.316124984	0.265127656	0.639095668	1.351228083	0	0.496734507	0	1.601452076	3L	-0.253042099	0.870405145	NA
**CG11353**	FBgn0035557	0.724185332	1.047993123	0.516724563	1.191819417	0.717755367	0.18742363	0.326013943	1.479786724	3L	-0.325237794	0.652399762	0.993803622
**CG11357**	FBgn0035558	24.06568652	24.2914208	30.39822743	28.09838444	22.25017365	25.6888824	27.39299648	28.1771082	3L	-0.014639455	0.911428218	0.996479182
**CG7514**	FBgn0035567	0.291889623	0.734405842	0.177030039	2.041589004	1.580806741	1.719948461	0.186154175	0.792149239	3L	0.447895231	0.720455373	NA
**RhoGEF64C**	FBgn0035574	0.507303149	0.48444027	0.302600403	0.253727006	0.401990176	0.550892826	0.147352715	0.192654492	3L	-0.236542688	0.529230858	0.984296327
**CG7509**	FBgn0035575	0.197128461	2.909766979	6.21700165	2.114148892	5.409184408	6.616313709	2.514394692	1.198355581	3L	0.506678083	0.508300175	0.978557094
**CG13708**	FBgn0035577	0.057758332	0.710464331	0.420362726	0.359097006	0.139024791	0.018151401	0.196456995	0.62699343	3L	-0.610982006	0.616344084	NA
**CG13707**	FBgn0035578	6.467395985	9.092379621	17.41297104	4.096468455	11.81028198	12.75876623	13.16062614	5.356802393	3L	0.255950939	0.544673802	0.984934163
**Dnah3**	FBgn0035581	0.550156491	0.897527859	0.822742997	0.527124307	0.825377039	0.554210493	0.461411845	0.894466985	3L	0.008654351	0.978870235	0.997757033
**Fitm**	FBgn0035586	84.16967333	69.38127114	60.56884828	72.27690554	47.51823137	84.87856227	67.81762917	53.82528122	3L	-0.150158426	0.426631548	0.962502302
**Gdap1**	FBgn0035587	1.135856859	2.089618653	0.977783931	0.085426049	0.926038015	3.419903612	1.775945476	0.6364001	3L	0.681055523	0.440294611	0.967112302
**CG10672**	FBgn0035588	81.30336158	85.05365568	75.2721155	89.58684969	86.35255132	96.28938926	117.2616945	75.00418438	3L	0.206409155	0.228570562	0.867702781
**CHMP2B**	FBgn0035589	50.51040554	64.8190552	67.42025256	69.04985509	63.16979631	64.54640306	95.734253	70.68624081	3L	0.257016523	0.229659023	0.867702781
**Prpk**	FBgn0035590	57.24032115	54.75260782	74.9470845	52.18554287	37.4610064	47.26131326	61.85830573	32.90351256	3L	-0.385159797	0.136673488	0.796109055
**CG4611**	FBgn0035591	9.854094815	19.87248635	17.24509333	23.14982859	4.481240473	22.16216699	26.67315308	26.62020443	3L	0.221937666	0.588084673	0.987588562
**CG10674**	FBgn0035592	2.229146028	2.670771245	1.931384704	0.371226432	0.862324265	1.200928719	3.249485376	1.728457991	3L	-0.025141801	0.983420708	NA
**CG4603**	FBgn0035593	146.4662053	136.4061897	137.9483204	129.6548036	150.2837703	147.801682	122.4580937	158.9958449	3L	0.104307046	0.399002861	0.952805113
**Cyt-c1**	FBgn0035600	1069.887677	1000.46203	964.0851293	1033.232648	1066.591451	942.8037836	1035.725418	1037.035241	3L	0.036016772	0.701096733	0.995787491
**Uev1A**	FBgn0035601	174.3772284	160.684342	166.5479104	153.2284043	160.8612017	165.1106249	167.1291736	145.896961	3L	-0.006337504	0.948533777	0.997604378
**Pfdn4**	FBgn0035603	232.9732124	264.8764411	267.7877922	216.17734	287.6517238	272.1696985	283.2459966	273.7670147	3L	0.218323885	0.080138713	0.694684542
**Ir64a**	FBgn0035604	1.180670015	1.584326655	0.572857798	0.100097734	0.465035341	0.870264083	0.711907081	0.093212505	3L	-0.657646403	0.481383276	0.973549494
**CG4835**	FBgn0035607	0.246011026	0.18756772	0.108512603	0.260711566	2.059069147	1.560308111	1.026948128	0	3L	2.543456035	0.015562177	0.365338552
**blanks**	FBgn0035608	3.802752248	1.417462983	0.585741201	0.562918966	0.653804042	4.780282122	0.923895494	2.227843833	3L	0.42277768	0.654684791	0.994669639
**Lkr**	FBgn0035610	0.302037618	0.515212609	0.658223213	0.190966599	0.221798771	0.173751389	0.352603732	0.61129389	3L	-0.251907569	0.678558562	0.995787491
**frm**	FBgn0035612	1.732636699	0.205147412	0.445060849	0.760391009	0.466111474	0.928863914	0.225332999	0.309788266	3L	-0.69870011	0.292798576	0.90544836
**l(3)psg2**	FBgn0035617	25.78829185	34.17697069	37.54972796	26.86218985	28.66322577	30.63127637	31.79187031	31.64582585	3L	0.015748961	0.912262635	0.996479182
**TM9SF3**	FBgn0035622	117.8945529	104.7216538	110.424559	101.7090891	121.2219141	113.1047414	103.9294303	119.8915538	3L	0.105325965	0.354566539	0.936745458
**mthl2**	FBgn0035623	0	0.363224889	0.175112213	0.589012605	0	0.306236823	0	0.626854098	3L	-0.221849275	0.90976902	NA
**Eaf6**	FBgn0035624	31.43526841	47.87674288	36.00725648	28.57073445	33.11486833	53.67665939	40.96978574	38.99935866	3L	0.245794413	0.303986188	0.910208378
**Blimp-1**	FBgn0035625	17.01920719	21.42426376	20.09672067	20.1937613	21.36905239	21.05115239	15.57462594	19.72225342	3L	0.01332131	0.915510464	0.996479182
**lin-28**	FBgn0035626	1.185673359	0.483762339	3.576098471	0.373561189	0.404948921	0.060424087	0.572236418	0.278292607	3L	-2.082919409	0.041772819	NA
**CG10576**	FBgn0035630	64.49456964	86.65327663	74.40981125	77.99190328	71.22089631	69.32304021	65.06618645	64.68047902	3L	-0.134484239	0.261827357	0.890640489
**Txl**	FBgn0035631	185.480589	241.2892964	251.2110586	215.4864486	190.3447311	233.0500155	209.5419358	224.9687204	3L	-0.02537768	0.842059478	0.995787491
**Ppat-Dpck**	FBgn0035632	3.948271186	4.496875389	2.838059504	2.045610141	4.135791937	4.365784765	2.984333221	3.280665792	3L	0.174115673	0.73078529	0.995787491
**Cralbp**	FBgn0035636	0.469810639	0.995420764	0.779831723	0	0.178553392	0.676058074	0	0	3L	-1.3356116	0.38753103	NA
**Tektin-C**	FBgn0035638	13.32900038	34.86143466	22.507407	21.34708253	32.61935806	42.39761447	18.91328158	33.95215368	3L	0.512147261	0.076378066	0.681411883
**kri**	FBgn0035639	310.9974621	363.3985427	326.1691709	357.0683586	335.1635689	309.8644894	301.956163	342.247353	3L	-0.042490473	0.69425924	0.995787491
**mad2**	FBgn0035640	3.345381095	1.213277292	0.65804172	1.475605692	1.795458735	1.10816387	3.229133693	0	3L	-0.132997562	0.909685596	NA
**CG13288**	FBgn0035648	0.665048052	0.343238403	0.496429938	0.357815653	0.261665348	1.655931637	0.60901862	0.814496909	3L	0.857101478	0.160672644	0.822561572
**CG10483**	FBgn0035649	186.1619346	157.8127488	145.3878005	162.7536778	142.1111738	165.3134186	157.0918026	168.5945323	3L	-0.015020508	0.896891314	0.996479182
**CG10479**	FBgn0035656	1.955103394	1.631120987	1.738291882	1.240989518	1.311999229	1.302827422	1.070617493	3.022411629	3L	0.061571793	0.88336935	0.995787491
**CG10477**	FBgn0035661	0.122688733	1.543448538	0.744102889	0	1.661134562	1.850723012	1.564907971	0.133184298	3L	1.165739287	0.374303493	NA
**CG10472**	FBgn0035670	1.802200169	2.078269848	1.912799207	5.514812429	0.915028089	1.805297269	2.011384966	3.545923702	3L	-0.420995124	0.559492475	0.984934163
**CG6602**	FBgn0035673	0.185610526	0.622671239	3.152019837	2.596463729	0.893532191	1.574932235	2.840978643	1.880562295	3L	0.182353249	0.849264092	0.995787491
**CG13295**	FBgn0035674	28.72468317	36.67433071	41.30852208	43.15110661	41.87636623	20.93926998	35.50548807	37.1826277	3L	-0.108831077	0.658943747	0.995750687
**CG6610**	FBgn0035675	27.11319046	17.90970369	14.26121625	14.54467217	18.84214351	17.87088388	15.60833142	32.03728009	3L	0.212660866	0.593234196	0.988631016
**ssp6**	FBgn0035676	1.081038947	1.334783785	3.460355746	3.885619147	1.057090866	2.363970625	1.187366125	2.542621927	3L	-0.412755455	0.421417931	0.960593359
**CG13293**	FBgn0035677	47.32586361	51.69855384	48.50824725	60.22605283	55.25399493	58.26562839	45.35659975	59.07451464	3L	0.098777969	0.429479388	0.963666972
**CG10469**	FBgn0035678	11.78008141	8.265384149	12.32789981	3.051646259	11.95347509	7.912252149	5.499573472	7.187926992	3L	-0.090667985	0.857886001	0.995787491
**CG10467**	FBgn0035679	14.65156326	11.86424784	15.25280716	11.25564316	22.09219254	13.12538038	18.18700953	10.72517705	3L	0.303059412	0.340791396	0.927274752
**fmt**	FBgn0035688	118.534808	131.6102678	116.202308	129.9680138	118.1308567	109.7314454	104.6650395	113.3498224	3L	-0.123485855	0.189611719	0.843401316
**CG7376**	FBgn0035689	10.42815018	12.01203981	11.41792824	15.6634931	12.46147068	10.24922162	11.44140131	11.38034715	3L	-0.089358826	0.664762024	0.995787491
**CG10274**	FBgn0035690	24.01787649	25.4902746	18.20011454	27.67803975	17.66086787	24.48340086	29.65361591	28.57834198	3L	0.100970861	0.637940537	0.991585536
**CG7386**	FBgn0035691	5.112882555	3.783592597	9.120427768	5.171390433	2.375383972	6.876150896	4.219817815	5.223784151	3L	-0.276587211	0.53715452	0.984934163
**Sf3b6**	FBgn0035692	463.5512938	389.8728575	389.1972677	323.8811628	386.9926033	375.0312568	326.94443	392.6899275	3L	-0.050817238	0.695051091	0.995787491
**CG8219**	FBgn0035693	0.498258149	0.351898167	1.017909323	0.061140533	0.094682491	0.148343602	0.066898276	0.056934977	3L	-2.320369203	0.082538932	NA
**CG10226**	FBgn0035695	0.346774526	8.345637144	2.706695305	2.284867995	0.489926267	2.629709885	1.692334246	1.833098146	3L	-0.995453045	0.133438915	0.794759341
**Best2**	FBgn0035696	0.613397938	0.27076026	0.293702929	0.037634584	0.145702696	0.121749017	0.020589359	0.683394796	3L	-0.297906872	0.769753564	0.995787491
**CG13300**	FBgn0035699	10.27432238	13.13946274	11.49700579	9.385562118	13.50408922	11.27267315	12.45359001	15.82489991	3L	0.296374021	0.04399376	0.552174689
**CG10147**	FBgn0035702	30.08803925	30.44844885	19.73794729	18.70046983	17.94388417	32.70867977	25.55244415	26.91278782	3L	0.084511045	0.729683207	0.995787491
**CG8270**	FBgn0035703	57.02624713	51.41736275	58.07992265	50.14742126	54.03944193	49.53391719	68.76214289	65.51108665	3L	0.164938762	0.298692212	0.9074568
**Vps8**	FBgn0035704	51.26385967	49.69223714	45.67470758	34.80820566	44.44592139	36.60485069	53.02725484	47.39553361	3L	0.032328363	0.846209336	0.995787491
**axed**	FBgn0035708	36.83133821	54.58021733	53.95059154	53.27213077	45.7218633	47.73139536	63.09738359	55.90964729	3L	0.132006161	0.376867941	0.943615128
**eIF4E4**	FBgn0035709	1.956093056	4.511473573	0.148295452	0.142517416	2.758788523	3.169706006	0.623754426	0.530857387	3L	0.099732373	0.928231772	0.996479182
**SP1173**	FBgn0035710	100.2938472	86.59666236	77.63464386	92.86383239	85.04734327	88.95265656	82.19636673	78.23531315	3L	-0.068565604	0.517959698	0.980804634
**CG8519**	FBgn0035711	1.129803204	1.965274764	1.87801504	0.243897559	2.266204833	0.157803194	0.693851467	0.68136315	3L	-0.408626426	0.621387881	0.990047511
**velo**	FBgn0035713	68.61005645	73.1684824	77.5322679	83.7767903	56.06039856	78.85800498	66.08249626	78.5111042	3L	-0.087757213	0.491137779	0.973549494
**CG8549**	FBgn0035714	57.90072693	67.82801697	62.13659176	55.01070107	44.55651115	51.51510722	66.13110382	66.39261147	3L	-0.05669978	0.774808786	0.995787491
**Ist1**	FBgn0035715	123.7104137	121.0359067	130.7410379	152.1118267	115.6998084	125.5241689	119.9269748	133.686747	3L	-0.06282654	0.608764233	0.990047511
**tow**	FBgn0035719	268.1181471	276.0891679	243.7848392	301.8417568	244.7595981	240.8285206	237.0830972	279.1864667	3L	-0.090827251	0.39438399	0.952272708
**CG10077**	FBgn0035720	1841.82293	1853.653343	1736.748349	2072.753347	1572.386063	1900.371478	1723.513261	1845.871741	3L	-0.063425968	0.512855319	0.980804634
**CG9948**	FBgn0035721	23.3731774	24.66620367	22.76821322	25.50471618	31.40312411	25.64301903	27.29657419	18.81860129	3L	0.128944251	0.585708488	0.98718792
**CG10075**	FBgn0035722	46.66352993	74.27042488	65.46363631	72.12399248	67.55318675	68.3805919	84.24053138	75.25485946	3L	0.227744545	0.236956249	0.873280735
**Mis12**	FBgn0035725	77.12700649	73.72066856	55.59337389	62.79700036	15.12742061	13.14027041	14.17840608	6.683143396	3L	-2.425900126	4.06E-19	1.13E-15
**CG9953**	FBgn0035726	134.3272993	170.1802669	153.1010963	158.6009468	150.2940725	92.60308724	138.8734248	118.7526389	3L	-0.264917045	0.097259015	0.730787365
**CG8641**	FBgn0035733	42.56595959	39.96022579	39.69789714	33.48714013	39.47173151	37.45611913	39.03730996	32.05642288	3L	-0.043103496	0.698290699	0.995787491
**Cpr65Ea**	FBgn0035735	1.718047847	11.76728567	12.85121035	13.68567659	1.550758348	17.27749351	10.95694242	2.797530686	3L	-0.263838182	0.741807546	0.995787491
**BBS1**	FBgn0035741	1.330781903	0	0.094954657	2.46388334	1.201201206	0.553523404	0.099848624	0.339911847	3L	-0.811045761	0.541286869	NA
**RpL18**	FBgn0035753	823.5523965	1185.22686	1136.887062	1289.963992	1065.155752	909.0360898	1143.70563	1291.494175	3L	0.02682568	0.855134583	0.995787491
**MED4**	FBgn0035754	111.9614598	87.05750855	94.96065106	80.94075308	94.32226087	95.16457599	94.76254748	106.0878328	3L	0.084750571	0.60961078	0.990047511
**CG14830**	FBgn0035755	0.583928485	0.902422085	0.557090816	0.994286217	1.196271889	1.249504385	0.518854353	0.655248186	3L	0.288795659	0.515052432	0.980804634
**unc-13-4A**	FBgn0035756	29.98552149	33.63469941	33.01576105	34.36699594	28.2275551	30.532759	26.94406703	29.26500248	3L	-0.1579635	0.070089763	0.662558698
**CG8607**	FBgn0035760	4.243763566	10.55036424	7.169948885	5.83049598	5.745810678	0.71446328	4.059724512	2.46792952	3L	-1.061174895	0.109810494	0.758410631
**Rint1**	FBgn0035762	49.55247133	71.81821409	79.79696147	76.99427725	76.87902161	82.74745123	72.17407807	59.49882222	3L	0.099185122	0.549973466	0.984934163
**mrva**	FBgn0035763	212.1455826	204.5596613	152.7954601	160.7559287	150.8900751	226.2028011	206.8590262	180.5164118	3L	0.093101202	0.524499885	0.984107922
**eco**	FBgn0035766	0.022221202	1.379099118	1.455524809	1.657852675	2.085978179	2.597801683	0.396807347	0	3L	0.202042059	0.852248528	0.995787491
**Cln7**	FBgn0035767	3.176965057	4.139816866	4.267663515	2.32615726	2.275135013	1.361465114	2.14334046	1.938136345	3L	-0.819631153	0.062288885	0.633840737
**CG14834**	FBgn0035768	9.239279535	20.69744633	13.12419955	14.17173186	14.04569901	22.34991553	11.00948449	12.40511268	3L	0.101756781	0.770144697	0.995787491
**CTCF**	FBgn0035769	27.54653617	43.05402516	37.93818666	25.76678519	34.91473474	30.76369493	32.42229747	23.33456882	3L	-0.10956041	0.581096346	0.985637351
**pst**	FBgn0035770	1.304491651	10.4056394	7.313923762	3.919992094	6.960161228	4.591502574	11.01866996	7.363647539	3L	0.436373118	0.379069398	0.944161494
**Sec63**	FBgn0035771	28.18223078	32.87638683	36.44911244	28.8192689	33.86263915	44.3406239	39.89565278	46.30977319	3L	0.41332683	0.006915674	0.243186622
**Sh3beta**	FBgn0035772	99.67766891	83.05390327	85.47393398	69.01445508	76.980206	86.46584048	80.4282377	69.57597882	3L	-0.077831643	0.546684116	0.984934163

**CG8562**	FBgn0035779	0.212952048	1.160890857	0.645773374	0.496489684	1.057190822	2.559816102	0.407433938	4.045462834	3L	1.739601642	0.075753461	NA
**CG8560**	FBgn0035781	0.327921968	1.41439654	0	0	0	2.119973683	1.075546268	0.610241956	3L	1.151000958	0.471600487	NA
**CG8541**	FBgn0035788	3.537289613	2.28204119	0.220036289	0.634389044	0.982417592	9.919293967	0	0.787670071	3L	0.794190972	0.531050701	NA
**CG7546**	FBgn0035793	78.92796854	86.20996426	89.92966548	95.67766029	93.79617737	93.28692871	83.84801633	103.0001497	3L	0.123782539	0.263180221	0.892691887
**CG14837**	FBgn0035797	1.220835034	1.05801941	1.135329359	0.094877697	0.697908042	0.537131379	0.259531367	0.265054587	3L	-0.958606395	0.269868468	0.89549671
**frac**	FBgn0035798	1.269914764	0.92796604	0.701567293	0.234515522	0.953325825	0.580852735	0.577350864	1.47409441	3L	0.216119947	0.732234068	0.995787491
**CG14838**	FBgn0035799	0.570758076	0.421241306	1.661581358	2.182349588	0.824291689	1.22688448	0.75712838	0.396533958	3L	-0.55339112	0.459425869	0.971297614
**Pura**	FBgn0035802	0.583269026	1.209655627	0.723323973	0.477909507	0.854470675	0.65004446	0.285226546	0.635188418	3L	-0.262689334	0.463145088	0.971606621
**CG7506**	FBgn0035805	162.5365904	140.3330043	192.3350144	161.0825733	204.0144235	176.8727818	193.6693284	203.9856983	3L	0.27796731	0.060361165	0.627984253
**eIF4E5**	FBgn0035823	2.071472659	1.415579728	1.488996675	0.357745224	1.246513021	0.144664576	0.195717454	0.166568848	3L	-1.576577647	0.227990344	NA
**CG8281**	FBgn0035824	145.5852821	137.1313659	95.20716327	139.3435535	125.0945068	94.47798614	125.1001878	92.00289379	3L	-0.216487995	0.229951482	0.867702781
**CG8111**	FBgn0035825	158.0664002	131.2199806	119.3553299	199.4505792	181.4987263	124.4016986	151.9134413	153.8289624	3L	0.036829474	0.840283389	0.995787491
**Srp9**	FBgn0035827	130.7592951	165.3186276	142.7016137	125.5038965	126.3313169	133.5215619	126.586732	137.0579299	3L	-0.075606667	0.593615098	0.988631016
**HP4**	FBgn0035829	68.95586595	75.68876186	78.65375384	50.76884387	72.50589429	72.76688765	89.49699946	54.36821159	3L	0.107520973	0.63503447	0.991585536
**CG8209**	FBgn0035830	88.11890825	86.28793629	87.9565711	97.6847754	78.12988512	79.62581854	88.20224754	97.4816768	3L	-0.038394462	0.79485323	0.995787491
**Pop4**	FBgn0035831	28.59939708	36.79532607	22.38120594	33.4587042	28.12775892	25.22376676	27.71868709	38.05634939	3L	0.004555432	0.987317623	0.998078482
**Idbr**	FBgn0035838	4.26309039	11.70120449	13.63289713	8.583884563	4.722527521	10.68745961	11.36956404	15.35582187	3L	0.185774856	0.669226263	0.995787491
**CG7550**	FBgn0035839	82.7538843	115.5679626	101.9804749	73.83487717	111.958903	148.8419816	125.0291653	92.28874808	3L	0.385867682	0.042760176	0.542555546
**CG7504**	FBgn0035842	4.238856312	6.152499362	4.466530004	4.958004558	6.054788493	1.614694076	3.786515172	4.493022454	3L	-0.274953938	0.453274034	0.967967712
**CG13676**	FBgn0035844	0.479035044	0.409061716	1.035354262	0.182757628	0.11006304	0.131383655	0.088874814	0.018909626	3L	-2.530852864	0.002956507	0.135642433
**Uxs**	FBgn0035848	32.11886705	38.78754746	34.79335061	21.21848936	31.19691297	39.06209686	28.90793204	32.82911283	3L	0.088967186	0.65813178	0.995750687
**ERR**	FBgn0035849	31.97809552	25.72202842	20.69604163	29.10235781	26.97468405	25.95457307	30.30758687	26.873292	3L	0.05882934	0.747725432	0.995787491
**Atg18a**	FBgn0035850	145.0616759	176.1823988	136.80205	140.3345686	138.8634163	154.7211062	149.4015914	148.9532444	3L	0.015803459	0.879544549	0.995787491
**MED24**	FBgn0035851	53.23191826	43.94589648	48.97726467	44.32708208	50.98255756	49.32435957	64.71028732	40.53333401	3L	0.137893315	0.364941511	0.940794966
**UbcE2M**	FBgn0035853	128.4297633	111.4794894	126.4735685	114.3738228	107.1925476	95.97328485	118.1233488	100.2673635	3L	-0.160373927	0.20857899	0.856520545
**CG8005**	FBgn0035854	22.05931957	23.64922177	23.2391909	18.87356812	20.94651009	28.42946211	17.38120371	22.70149187	3L	0.059529219	0.81487752	0.995787491
**CG7366**	FBgn0035855	5.563233103	5.099796803	3.452548489	3.921304445	2.685935078	4.330159714	1.48520168	1.264007517	3L	-0.847665523	0.173034352	0.832736771
**CG13679**	FBgn0035856	4.542367312	13.13652403	4.939874367	6.573325897	4.524213626	2.215094564	2.397450332	2.040393086	3L	-1.344751347	0.082019203	0.694684542
**CG8006**	FBgn0035857	1.450305499	2.600455328	2.669146898	0	3.520991458	1.508788045	0	0.760042498	3L	-0.165480816	0.888455705	0.995926915
**CG13674**	FBgn0035858	0.346011634	0.773847967	1.678838795	1.613426197	6.662823263	2.066045147	0.882683111	0.250407762	3L	1.228497385	0.311185497	NA
**CG13678**	FBgn0035859	0.106672716	5.009998474	1.552719131	1.492220534	0.577714779	1.005703854	0	0	3L	-2.251574154	0.152854055	NA
**CG7201**	FBgn0035865	0.326472261	0.634076379	0.625276698	0.600914042	0.217134881	0.016199792	0.087667132	0	3L	-2.748918356	0.018980695	NA
**Arl5**	FBgn0035866	135.4947594	130.1963056	124.0705367	99.16240401	110.6731109	105.7104491	115.2717294	106.1154359	3L	-0.130241177	0.355039533	0.936745458
**CG13671**	FBgn0035867	41.1598339	58.44778193	42.45144561	38.22539926	43.81329343	33.74836491	35.03222091	27.08934706	3L	-0.334309867	0.088502889	0.709409025
**Gr66a**	FBgn0035870	4.952624751	3.976914847	1.533829604	2.456778333	1.141373237	2.980407041	4.704242819	1.601452076	3L	-0.301789203	0.651362094	0.993444251
**BI-1**	FBgn0035871	901.3892044	815.5167282	823.9244131	859.5123807	839.2162798	751.8830159	899.4850533	930.0197272	3L	0.040087897	0.708163449	0.995787491
**Cpsf6**	FBgn0035872	196.0280361	200.1978987	177.6223064	252.3835776	208.2515109	227.4471244	221.3179143	207.3912181	3L	0.092820478	0.436440156	0.966084844
**Pex2**	FBgn0035876	23.1924772	32.73471656	33.45000643	35.44379059	31.91186397	25.79888994	29.58225381	22.10620167	3L	-0.155007241	0.527569461	0.984107922
**CG7182**	FBgn0035878	135.0732255	139.2886878	126.0164993	132.1255638	110.5979608	107.898638	139.9482595	134.5450739	3L	-0.080511082	0.546557072	0.984934163
**GAPcenA**	FBgn0035879	216.5456141	212.3449038	222.18855	214.8734936	195.9934265	230.3078006	217.6029987	183.8136178	3L	-0.036763161	0.693146692	0.995787491
**Culd**	FBgn0035880	2.373704778	4.387085697	1.759564486	0.204970494	3.174181852	1.015350036	0	1.622408475	3L	-0.536799904	0.550600101	0.984934163
**CG7120**	FBgn0035888	115.1424114	124.7024036	112.661551	129.3167718	128.2988736	124.6417114	123.1717561	119.4956184	3L	0.07090829	0.449335251	0.967112302
**CG13667**	FBgn0035890	1.823542014	10.1702969	8.294789044	7.121295257	10.36967622	6.533052233	11.39714239	14.6485905	3L	0.699590171	0.136547713	0.796109055
**CG6983**	FBgn0035896	26.42984512	28.75984286	24.28075265	26.52250243	29.34833634	28.59177161	23.78818213	24.81689153	3L	0.037709577	0.772105508	0.995787491
**ZC3H3**	FBgn0035900	186.82959	146.1133077	160.1200429	164.5332508	180.9071605	190.8084131	160.4741496	205.8453322	3L	0.194387608	0.124099133	0.780439453
**Pus7**	FBgn0035901	41.0600472	27.03631334	25.1126303	24.53752341	26.80745297	28.29938155	30.23189657	21.09685397	3L	-0.123590711	0.559027806	0.984934163
**CG6765**	FBgn0035903	304.3461386	292.9158411	218.6160579	262.3520796	281.9130726	269.2517989	208.9379369	222.1022492	3L	-0.106524509	0.408052562	0.956706394
**GstO3**	FBgn0035904	8.846118704	2.988233132	6.706425185	2.291599194	0.665396313	6.081298621	2.507404318	1.066985699	3L	-0.994000743	0.182439557	0.838040629
**GstO2**	FBgn0035906	68.39162639	88.42212075	90.05770962	75.82765696	110.8613842	108.9761669	89.90033769	121.4378547	3L	0.453101384	0.001545153	0.084192925
**GstO1**	FBgn0035907	2.620124868	1.80966137	2.804288111	0.539004926	1.112940452	2.179621519	0.196588083	0	3L	-1.10542493	0.358199813	NA
**ergic53**	FBgn0035909	63.22333081	47.56858235	72.74424349	67.71483141	60.31195135	63.77580223	44.90626101	65.41086242	3L	-0.071304008	0.677102386	0.995787491
**CG6638**	FBgn0035911	10.26356232	15.6506236	18.86307503	4.955017556	9.544912994	15.0522149	10.71105054	9.003290458	3L	-0.128396121	0.745404383	0.995787491
**CG6282**	FBgn0035914	261.5358996	213.786968	175.2391367	231.3032761	223.3758518	241.1815369	204.8068172	199.9299577	3L	0.0050926	0.967228462	0.997604378
**S-Lap1**	FBgn0035915	7.272992488	1.752045022	6.569640869	5.321520124	9.498019719	10.06656106	6.513483862	8.147143482	3L	0.731373064	0.09098204	0.719396887
**GAPsec**	FBgn0035916	39.21545708	43.85228525	38.93472408	38.71919596	47.27994583	46.57694328	35.24522377	45.7515961	3L	0.153638935	0.358183657	0.93823489
**Zasp66**	FBgn0035917	0.875658764	1.428553782	0.712962064	0.503399698	0.552193242	1.215730361	0.336603664	0.325537026	3L	-0.500443587	0.336221549	0.924219112
**CG13305**	FBgn0035921	0.499895437	1.677008933	1.763984519	0.105953399	1.39467905	0.728370199	0.115931273	1.578646207	3L	-0.023724224	0.982530854	NA
**CG6511**	FBgn0035923	19.31891056	27.84926618	18.97537025	22.71061502	25.88703213	24.25655106	18.05963988	21.03053569	3L	0.039281767	0.825923919	0.995787491
**TrpA1**	FBgn0035934	1.155337845	1.64184648	0.934277832	0.579877968	0.420032963	2.942475591	0.429813368	0.418057568	3L	-0.016380081	0.979228926	0.997786266
**Tsp66E**	FBgn0035936	19.41672418	25.51447858	23.78862632	22.55211009	19.37219439	25.68511924	22.02196468	21.28920312	3L	-0.015464696	0.895629122	0.996479182
**CG13313**	FBgn0035941	0.335729634	2.393342294	0.203618853	0	1.136396319	2.809149155	1.605850331	0.728900114	3L	1.135672815	0.302988775	0.910208378
**ValRS-m**	FBgn0035942	14.64703381	12.88694886	14.55009634	2.311269573	15.92762676	13.76240259	15.74257121	9.577684933	3L	0.341927879	0.387134707	0.948247506
**CG5021**	FBgn0035944	114.6304056	104.6910486	103.3449127	119.0314594	85.99082153	79.71589442	88.91289345	69.92552794	3L	-0.417146832	0.004397258	0.184154493
**CG5026**	FBgn0035945	4.621072658	1.757539787	6.648180797	4.38470922	7.517698724	5.065952414	7.264978471	6.066329982	3L	0.605141859	0.133502952	0.794759341
**Srp68**	FBgn0035947	65.76366166	122.278001	76.8263009	74.51893779	68.19111383	108.8842039	88.66748631	91.75227501	3L	0.108823443	0.564055521	0.984934163
**CG5644**	FBgn0035948	215.5522057	157.626447	126.6727221	196.6648584	179.6503103	198.1276253	147.5174826	125.8501045	3L	-0.075323211	0.666323729	0.995787491
**CG5068**	FBgn0035951	106.6565285	126.8366335	110.0499022	119.8358724	106.2013673	129.6507789	124.651599	104.6910968	3L	0.035534745	0.764150531	0.995787491
**CG5280**	FBgn0035952	0.298941314	0.752148175	0.543920593	0.522727814	0.944414956	0.704600111	0.762605714	1.135801213	3L	0.791745996	0.514921309	NA
**CG5087**	FBgn0035953	87.3298515	72.18079271	65.21751069	65.57983469	74.33017389	75.55052988	57.13221162	71.54033192	3L	-0.032102706	0.817669917	0.995787491
**Doc3**	FBgn0035954	0.084382119	0.778465423	0.511774612	0.196733735	0.837822439	0.437552208	1.722084797	0	3L	0.98197175	0.465527191	NA
**Doc2**	FBgn0035956	0.277284018	0.642287914	0.288294497	1.32373913	0.715098171	1.157724576	0.235785829	0.086001324	3L	-0.174759168	0.837714693	0.995787491
**CG5144**	FBgn0035957	2.022215852	8.027694053	2.452933725	5.500506819	6.692799487	7.054171186	3.439144056	6.292932192	3L	0.426810694	0.461409708	0.971606621
**CG4911**	FBgn0035959	158.8731334	152.4809687	142.7215148	140.7720671	132.4722807	163.8721036	140.6975514	164.413322	3L	0.04513533	0.667605114	0.995787491
**CG4942**	FBgn0035960	29.50871217	29.65536542	45.82231481	25.65114681	39.3790527	58.81905507	31.96043032	32.72344081	3L	0.350277937	0.175621507	0.834088743
**Dhpr**	FBgn0035964	385.3745398	425.2624147	434.5262351	437.8500782	437.7385211	409.9418008	444.0484072	421.6009864	3L	0.057417176	0.556080111	0.984934163
**Use1**	FBgn0035965	42.42646407	33.36141647	49.82132804	35.39563326	40.36677358	41.71921942	35.72667889	47.14181295	3L	0.066108499	0.745596633	0.995787491
**Slc45-1**	FBgn0035968	1.24210684	3.012231287	0.653494092	1.221173335	0.378222852	1.551994959	0.534469606	0.3249071	3L	-1.095815112	0.099990266	0.732357333
**CG4476**	FBgn0035969	1.154567848	2.151806217	1.369360909	0.41872934	3.10327753	0.967572902	1.047227515	0.389927016	3L	0.154254149	0.837856545	0.995787491
**PGRP-LA**	FBgn0035975	0.865317139	3.04804103	3.726163976	2.269636127	3.04613247	2.488237926	0.938163111	3.569498861	3L	0.070747387	0.896083463	0.996479182
**PGRP-LC**	FBgn0035976	2.364134355	1.723468927	1.367662047	1.441571296	1.608658102	0.848693275	0.90464363	0.750171816	3L	-0.722273025	0.073689867	0.675013659
**UGP**	FBgn0035978	13.3663505	11.18307666	10.96513263	10.18746924	12.57844802	18.89750948	11.66721522	9.789739262	3L	0.235512694	0.24274642	0.876959115
**CG4452**	FBgn0035981	42.49420604	50.89870499	54.0613278	59.90017247	47.39725154	57.07759884	41.50180912	52.62743609	3L	-0.030163427	0.847357105	0.995787491
**CG4080**	FBgn0035983	51.94999136	57.2723632	48.94103919	53.93944005	46.83477804	52.15904606	42.71396116	52.65574425	3L	-0.095691425	0.363140497	0.939549067
**Cpr67B**	FBgn0035985	3.082275989	12.45041015	7.782765029	4.839692741	6.47275498	5.278155853	8.986231163	8.467310258	3L	0.098507524	0.84309803	0.995787491
**CG4022**	FBgn0035986	42.66688567	62.04408813	49.85786804	72.9579213	67.48059574	56.47797681	42.50240275	49.59058533	3L	-0.043636646	0.809211147	0.995787491
**Cpsf5**	FBgn0035987	301.1044699	288.0346101	276.8488404	267.361603	251.6872853	263.470966	255.3303175	258.5184849	3L	-0.109733783	0.216568448	0.860826301
**Tat**	FBgn0035989	137.0018719	105.4516751	109.6174249	126.0136895	119.7412564	123.5618972	114.9481381	124.618328	3L	0.041703994	0.707511633	0.995787491
**CG3529**	FBgn0035995	116.5071736	112.7192159	125.4055329	121.188909	126.8970672	126.8580118	112.4830786	125.6106261	3L	0.078163628	0.433738465	0.965402206
**CG3448**	FBgn0035996	4.132710844	5.375869833	5.115254522	1.769741588	1.979343462	5.327586412	4.733427578	1.4648976	3L	-0.249019217	0.723890874	0.995787491
**phol**	FBgn0035997	177.6446079	157.2790924	192.2508553	182.8440674	187.2101517	165.9795365	170.6475	184.1091033	3L	0.026380016	0.811130451	0.995787491
**CG3552**	FBgn0035999	15.75135201	24.75451408	26.92092084	23.02674255	25.36114878	26.54320042	26.28122445	18.48521259	3L	0.131646186	0.529050399	0.984296327
**CG3434**	FBgn0036000	45.61086641	44.70460177	36.14903869	56.48872138	47.34783342	40.14629126	64.89881282	43.66060712	3L	0.126834056	0.566509148	0.984934163
**Jarid2**	FBgn0036004	84.24564305	76.13487567	64.53250333	79.37894275	71.74285938	81.59432598	60.33936941	68.78752936	3L	-0.080899361	0.504476492	0.978557094
**pall**	FBgn0036005	135.6072534	131.8652504	119.7861488	110.9054179	144.9492981	117.0180649	132.3814191	81.41629606	3L	-0.037271075	0.831635054	0.995787491

**path**	FBgn0036007	23.89579746	19.32614003	24.51863569	27.94980449	18.8692633	26.87993504	22.91078727	28.45369479	3L	0.048442187	0.763701428	0.995787491
**CG3408**	FBgn0036008	4.76165401	4.676602554	6.23184151	4.820438991	8.53945322	9.273862573	4.475240619	15.71102676	3L	0.928072808	0.014181255	0.354312909
**Ir67a**	FBgn0036010	0.296299586	0	1.198031105	0	0.89149449	1.862329599	0.251955537	0	3L	1.050397136	0.545402607	NA
**CG3335**	FBgn0036018	16.57093545	13.14393568	8.789679182	9.494922486	10.95185371	13.47812738	19.13023881	12.31756107	3L	0.239717497	0.39153852	0.950027604
**CG8336**	FBgn0036020	22.52241014	17.0956761	21.69392727	20.97816158	16.54423983	17.38516625	20.33246444	23.99691546	3L	-0.044840659	0.827068282	0.995787491
**CG16717**	FBgn0036028	89.50707363	89.25289374	104.7847369	82.99496757	94.88071788	83.33243737	87.60151108	80.82674951	3L	-0.048785467	0.714381884	0.995787491
**Prps**	FBgn0036030	186.4658918	159.1534589	164.1391729	169.0409166	155.0929723	171.6040281	153.6027601	173.078807	3L	-0.026576508	0.782579087	0.995787491
**CG6761**	FBgn0036031	6.050332298	4.437055179	0.955778161	2.361955309	0.711226078	1.644936803	0.071788501	2.81045697	3L	-1.370449225	0.073925596	0.675782176
**CG16711**	FBgn0036032	243.348459	192.3795979	203.6883776	235.1112288	233.7179587	231.6194661	197.8296232	261.140436	3L	0.108065765	0.376992285	0.943615128
**CG18178**	FBgn0036035	111.7490179	75.21269432	69.94172637	76.4365611	81.06271426	99.58334406	81.19406316	74.91783515	3L	0.036903379	0.842038925	0.995787491
**CG14174**	FBgn0036036	11.05602607	12.91750487	15.53812596	10.84399464	12.80126445	10.71110016	9.725572428	12.25014171	3L	-0.10981476	0.746718573	0.995787491
**defl**	FBgn0036038	57.70152398	64.39137545	51.71757596	38.26108599	36.6271991	40.91863739	51.21972867	52.87112391	3L	-0.190518585	0.29707051	0.907213496
**Naa60**	FBgn0036039	151.8570332	118.0250167	113.9532398	117.7964125	138.2055916	138.1604195	103.6249681	153.3119025	3L	0.116291429	0.432805107	0.965402206
**CG6749**	FBgn0036040	2.562470482	2.318757565	2.290295976	2.358277683	4.260712083	1.176152845	1.204168968	2.488871857	3L	-0.028253	0.961247053	0.997604378
**Ae2**	FBgn0036043	55.56385498	61.13435229	59.21209937	52.88757155	77.55646581	71.55234112	60.16240292	69.53863541	3L	0.316846487	0.001497696	0.083211994
**CG10809**	FBgn0036052	234.5882016	206.9193344	194.8734797	214.6796826	216.1449379	261.5436813	213.470183	231.2806461	3L	0.143155901	0.189714998	0.843401316
**iPLA2-VIA**	FBgn0036053	156.8226272	138.1520417	156.1261842	160.3554439	124.3527799	138.0091473	136.0977588	132.3493503	3L	-0.175593233	0.083580228	0.69799888
**CG6707**	FBgn0036058	205.4424791	210.3388292	201.4348133	224.4036195	194.7131853	180.8596102	199.9123418	213.0106123	3L	-0.062820885	0.542878071	0.984934163
**nudE**	FBgn0036059	96.2628102	82.4970936	90.59631334	82.64381914	68.61219154	93.08306612	92.29224375	73.88277722	3L	-0.075549631	0.564541792	0.984934163
**CG6685**	FBgn0036062	15.5144699	17.46192	11.64690333	14.96341408	14.59679192	21.6783749	13.02069169	11.63008187	3L	0.060233918	0.834980618	0.995787491
**CG6674**	FBgn0036063	116.5461445	87.84974068	107.7434614	88.11427298	90.21904489	111.959007	106.2002352	76.4303834	3L	-0.031238242	0.864813897	0.995787491
**CG12362**	FBgn0036082	0.841383777	1.48558237	0.322292417	0.516224895	1.518913362	0.125250235	0.112967794	1.442148999	3L	0.051974904	0.963928705	NA
**Ir67b**	FBgn0036083	4.71982594	1.900043013	3.022861221	0.616229404	0.408983784	1.530739135	0.288969057	1.88548094	3L	-1.290887501	0.111952072	0.758410631
**CG8009**	FBgn0036090	55.91739226	64.77352465	75.98864184	73.42335422	62.8738709	84.27509449	85.38595515	75.49254642	3L	0.221567568	0.183306346	0.838920968
**CG18628**	FBgn0036091	18.5923002	29.71840004	12.73543368	39.77747464	14.21528486	17.52870707	14.5078034	18.0457998	3L	-0.617670336	0.208938483	0.856520545
**CG8003**	FBgn0036096	135.0770858	104.3461751	110.1895163	119.4634025	125.3176242	138.1282224	126.3783207	132.2654693	3L	0.180643898	0.169532945	0.830934498
**CG11811**	FBgn0036099	181.1609051	164.1204201	163.0078874	178.8528383	144.7448351	175.5705905	147.684362	126.5257605	3L	-0.182045791	0.149797031	0.809805479
**NijA**	FBgn0036101	1.238878364	0.923575404	0.918347721	0.080233285	0.993996875	0.55155884	2.809249177	0.224143301	3L	0.547258587	0.583309888	0.985764834
**CG12523**	FBgn0036102	4.704266789	5.510998321	4.528764133	6.441418638	2.965602531	4.364754725	4.381202697	9.727046351	3L	0.048161849	0.93033339	0.996479182
**CG6418**	FBgn0036104	256.1952593	210.7825258	223.1812233	205.8792879	209.6780629	245.4394761	206.5965978	224.2580899	3L	0.011262994	0.918573495	0.996479182
**Blos4**	FBgn0036105	48.45281737	50.18628571	47.24695796	40.6457604	35.7250858	36.64846844	39.26486046	45.35172895	3L	-0.220004627	0.271929417	0.89549671
**CG6409**	FBgn0036106	0.51344544	1.464095582	1.619294917	5.865685695	0.648830429	1.839478252	1.440791382	1.56063261	3L	-0.751817603	0.377442096	0.943615128
**galla-2**	FBgn0036107	145.0823909	170.6040183	203.3660431	129.2107133	160.031223	153.1632487	216.7592295	145.3787542	3L	0.092799824	0.622607324	0.990047511
**Aps**	FBgn0036111	84.92453714	94.42008856	77.34424873	86.36296426	82.33877559	104.3755412	79.65313175	83.32097951	3L	0.056257179	0.629422159	0.990523437
**CG7888**	FBgn0036116	137.9515694	192.3851885	178.1511449	91.56566612	98.28615786	73.41552296	152.3259827	65.80630504	3L	-0.58194865	0.106017334	0.751296334
**CG6321**	FBgn0036117	3.150635684	6.585610753	7.055444514	3.955316879	1.225044135	3.701575482	2.10207336	4.840843064	3L	-0.762124945	0.172776759	0.832736771
**Blos2**	FBgn0036118	81.50723115	87.08373124	95.60474246	49.23246443	76.24160545	66.31961021	89.89570102	58.11054295	3L	-0.077639813	0.754643314	0.995787491
**CG6310**	FBgn0036121	2.426576918	11.85679298	10.23798567	7.625289339	7.999344221	2.884569348	8.074238937	5.726438168	3L	-0.335210953	0.594841516	0.988780662
**CG7839**	FBgn0036124	22.99193664	16.7374615	26.11454129	19.70092217	18.44726152	15.99502642	22.78162019	10.00248662	3L	-0.32027698	0.190068648	0.843401316
**Elo68beta**	FBgn0036128	1.778087154	4.828796183	1.027051104	0.197406822	1.834230305	1.915851095	0	2.022109994	3L	-0.392606774	0.741366176	NA
**CG7638**	FBgn0036133	169.8459955	201.5022625	175.8481606	190.2147718	184.0724175	179.5108862	174.9875322	184.3369468	3L	0.003063197	0.975698227	0.997757033
**FoxK**	FBgn0036134	18.92400438	37.87116476	80.03162956	62.83751047	34.84829585	41.60943836	78.14290112	67.50007389	3L	0.191178439	0.704912699	0.995787491
**mRpL2**	FBgn0036135	202.1817844	171.938616	186.1997201	187.5707047	202.8594964	178.6466963	204.1242891	222.0764519	3L	0.140528779	0.283251402	0.901780531
**Ufd1-like**	FBgn0036136	266.2991344	261.1732244	265.6102049	251.3838483	290.2192557	311.9532441	282.8352071	304.1997568	3L	0.217095414	0.025822927	0.449488936
**wls**	FBgn0036141	38.07743843	36.69719681	43.49542625	63.44031179	39.59938422	42.58441285	41.2517032	21.59048225	3L	-0.300655337	0.190385354	0.843401316
**Adck5**	FBgn0036142	9.550045588	9.241651001	11.09809092	17.36091785	14.04540289	16.71670555	12.77746755	8.772090888	3L	0.175522992	0.556051282	0.984934163
**CG14142**	FBgn0036143	2.897531604	3.78493668	3.359168565	2.570671609	4.258693984	5.245975187	2.747344652	2.171164283	3L	0.229080577	0.577628327	0.985637351
**GlcAT-P**	FBgn0036144	74.06024132	51.25596971	53.31470083	50.32489946	56.90396073	63.20939117	52.36941488	51.40435074	3L	-0.006748233	0.959719768	0.997604378
**Plod**	FBgn0036147	8.70596125	10.34612551	14.69604153	7.640549318	11.13500466	11.13115588	14.69346916	9.390833369	3L	0.198475497	0.3695681	0.942282153
**Ir68a**	FBgn0036150	171.9823146	217.8454142	200.7478643	238.4046795	153.1405719	166.1188536	150.8498005	200.1066594	3L	-0.274612534	0.039107745	0.527385023
**CG6175**	FBgn0036152	1.345441871	0.837196288	0.816005803	0.910697537	0.568041572	0.521711236	0.387512223	0.871612237	3L	-0.713671078	0.170171924	0.830934498
**CG6168**	FBgn0036154	1.834955613	0.139903665	0.60703319	0	1.3551403	0.943628151	0.957479562	0.814880147	3L	0.641978965	0.578814436	NA
**CG6163**	FBgn0036155	29.84878489	24.75792968	34.7929061	33.01930418	31.500169	30.5356529	36.50991296	41.97046583	3L	0.229905509	0.214865492	0.858843649
**CG7560**	FBgn0036157	0.070022834	3.641057905	0.339748837	2.938600825	0.758454972	4.159077311	1.786297539	0	3L	-0.029050553	0.981906597	NA
**CG7551**	FBgn0036161	2.060522143	0.768052629	1.851406659	0	0	2.158497434	0	0	3L	-1.090826417	0.566374867	NA
**Fum3**	FBgn0036162	0.037758608	0.886686739	1.374027828	2.024753756	0.136327928	2.171513656	0.577938114	0	3L	-0.552581957	0.666421139	NA
**chrb**	FBgn0036165	316.2827031	318.1603777	289.6406233	303.7545355	333.4766208	310.305785	259.0697054	295.3161145	3L	-0.005404965	0.955650668	0.997604378
**CG7394**	FBgn0036173	44.16932129	41.4038734	42.00878481	35.22740082	52.12271115	52.18543451	51.80656219	53.91594834	3L	0.395917585	0.005819341	0.219449733
**CG7368**	FBgn0036179	43.64830342	49.94119991	42.04235955	61.82964289	57.4804054	45.09860611	52.91587321	59.04947195	3L	0.151663125	0.312131654	0.914141092
**Duba**	FBgn0036180	118.758796	126.7185169	164.446406	134.7818228	107.3890855	122.4035196	131.8440986	128.6847914	3L	-0.119763385	0.341688645	0.928085024
**Muc68Ca**	FBgn0036181	0.054574134	0	0.264792174	0.148443794	0	0.05145216	0.069609894	0	3L	-1.92348905	0.300425592	NA
**CG6083**	FBgn0036183	14.69852509	21.65247932	15.00021964	13.27767927	18.60359759	6.136241922	8.85520373	7.065356147	3L	-0.631334008	0.103136587	0.743546988
**PCID2**	FBgn0036184	77.09975711	49.60842288	80.98596231	62.93016486	51.30445823	60.44611711	63.47974257	68.19621508	3L	-0.125362758	0.533716008	0.984760433
**CG6071**	FBgn0036186	1.689706743	3.345339563	0.604800736	1.356217064	2.950342141	1.201312771	0.49464504	0	3L	-0.552401923	0.553736122	0.984934163
**RIOK1**	FBgn0036187	12.49004167	28.66882162	9.287201305	9.556429003	32.73957794	21.54594793	19.72901835	11.33374597	3L	0.546133354	0.143425711	0.8037297
**CG7339**	FBgn0036188	140.2887593	88.35763229	73.54110156	76.93225864	72.21821425	99.51379047	72.00695534	59.66442621	3L	-0.300742398	0.161567383	0.823037813
**Sugb**	FBgn0036191	5.512707779	5.882184443	9.460313248	7.783551188	9.622654367	11.73039946	8.301843124	8.618609382	3L	0.453818424	0.050576746	0.591173352
**Pldn**	FBgn0036192	24.30014131	22.18794573	30.30788305	28.0989853	22.64153969	44.01865652	35.36939852	25.31526166	3L	0.307391538	0.208207373	0.856520545
**CG14135**	FBgn0036193	51.56195736	38.34380788	57.05173622	32.52269071	44.82728953	61.83264023	43.41049435	36.15246232	3L	0.080600306	0.738684162	0.995787491
**Dph1**	FBgn0036194	17.66630237	19.73945897	11.71957224	15.84669822	11.86450818	20.15099201	16.33593426	5.975846461	3L	-0.235340732	0.453989088	0.967967712
**Dnai2**	FBgn0036195	1.175446478	0.611890314	1.179979349	1.512005083	0.365858993	3.362825202	0	0.264000322	3L	-0.14633174	NA	NA
**CG11658**	FBgn0036196	61.1119123	49.45665602	51.18218512	60.84949225	57.19858962	57.5888879	50.20933879	43.16759424	3L	-0.070982079	0.584077194	0.986562492
**crim**	FBgn0036198	48.67965114	31.23981984	45.89972003	22.2854111	33.97760352	31.03014002	31.92554688	44.28627929	3L	-0.04074574	0.891717571	0.996189576
**Bmcp**	FBgn0036199	28.9129389	25.1587841	19.83175064	28.92589743	29.68904984	34.03389196	26.54409797	24.00439343	3L	0.17658126	0.272602495	0.89549671
**CG6024**	FBgn0036202	30.15015484	30.41937404	35.69188029	30.34195488	33.15140681	36.96427131	34.650144	33.13039209	3L	0.153481401	0.098664864	0.730787365
**CG5964**	FBgn0036206	0.169231349	1.277378315	0.410552893	0.06575942	0.560094259	0.292508851	0	1.4696677	3L	0.334539246	0.792872956	NA
**CG10907**	FBgn0036207	32.43634934	34.94894944	30.4606959	22.69784469	20.28515177	30.66657265	35.39617999	27.06271369	3L	-0.060093724	0.790911935	0.995787491
**CG10361**	FBgn0036208	0.679181226	5.924002377	6.371045497	3.800365103	1.798274405	0.512262329	7.161437713	0.196608708	3L	-0.753783674	0.395533185	0.952272708
**CG14130**	FBgn0036210	52.95617862	67.02159096	55.60435204	59.65896123	71.51433592	55.1516554	53.58314116	88.82908784	3L	0.22898752	0.265360825	0.893759496
**CG5946**	FBgn0036211	121.3897278	126.6209776	134.9305505	139.300124	156.1439498	134.4515028	144.7756381	148.8609974	3L	0.193577934	0.06056626	0.627984253
**RpL10Ab**	FBgn0036213	812.6829518	1069.590468	1083.120542	1042.10684	893.2529624	947.7128989	1039.347898	973.1909874	3L	-0.023662728	0.829795067	0.995787491
**CG7252**	FBgn0036226	3.964725802	0.461824398	3.606888561	0.898684204	0.497037932	0.051915545	1.966630692	0	3L	-1.839764585	0.129615225	NA
**CG17826**	FBgn0036227	0.290421612	1.035175711	0.616488931	0.338553527	1.507320649	0	0	0.315266101	3L	-0.264107695	0.859532479	NA
**CG11570**	FBgn0036230	1.469489559	1.506304913	0	0.190336644	3.094939494	0.384840494	0.416522159	4.608352465	3L	1.433341243	0.265942102	NA
**CG6938**	FBgn0036235	0.86633716	1.297463438	0.788145699	4.508554629	4.049540874	5.775782917	1.973254202	1.175561356	3L	0.815395826	0.169633079	0.830934498
**CG6931**	FBgn0036236	0.137054186	0.574722926	1.329966176	2.87583008	1.237089663	0.839890022	2.797025387	0.297557325	3L	0.10974326	0.923483113	NA
**viaf**	FBgn0036237	303.5557975	298.1402485	273.3652196	395.2133705	285.2598151	259.0926729	261.4585926	295.841097	3L	-0.176231425	0.202583678	0.849847813
**Pop2**	FBgn0036239	191.0318714	198.2016177	181.7569405	221.3505608	199.3633121	211.5612609	175.4707715	193.5101515	3L	0.005810569	0.955168001	0.997604378
**CG6928**	FBgn0036240	6.422063556	7.240757598	6.051672072	3.13480098	10.02851183	3.719543117	6.318443787	5.991987703	3L	0.225657385	0.517707363	0.980804634
**ssp**	FBgn0036248	33.75397693	41.91785929	23.04348706	11.20464113	38.68376148	34.64820457	42.98144364	25.65519057	3L	0.40084806	0.217505559	0.860826301
**CG11560**	FBgn0036249	95.68121817	96.81843534	129.2165936	124.1819274	112.6494272	88.94700189	115.2201744	94.51196437	3L	-0.084209428	0.623504602	0.990047511
**Ir68b**	FBgn0036250	1.453478689	0.533313943	0	0	0.163993847	0.385405546	0	2.366727586	3L	0.55711808	0.741705219	NA
**CG5645**	FBgn0036254	10.12267743	9.157969953	11.88495838	7.706331871	13.15943065	15.38661791	13.92794754	9.611050909	3L	0.451948449	0.072688558	0.671999479
**Atg12**	FBgn0036255	134.170492	128.8466668	162.5703925	145.3200984	185.4230231	163.8780839	194.8373867	150.5720486	3L	0.314442681	0.057786667	0.623014047

RhoGAP68F	FBgn0036257	122.1332621	105.7795888	92.39963588	138.6596543	97.86870765	122.0422518	93.4889618	133.4982498	3L	-0.011971868	0.938326169	0.997038394
eIF3l	FBgn0036258	314.0932461	424.5250838	493.9075972	424.081458	405.28308	471.6809496	430.7609011	546.5051647	3L	0.196102099	0.153921659	0.817059301
CG9760	FBgn0036259	8.113575007	9.565701111	10.94222059	7.040803934	7.861692779	9.62899364	8.695764846	16.23648727	3L	0.287210574	0.25327279	0.884040848
Rh7	FBgn0036260	7.13561676	9.843178699	10.56542962	7.324323507	6.863724933	4.424597538	8.112043301	6.336912937	3L	-0.401594891	0.067861054	0.656261422
CG6910	FBgn0036262	1.867291805	9.800504664	0.876778814	3.300249447	1.304880112	9.114698222	9.603833691	8.69661979	3L	0.898608523	0.157319091	0.818884898
thoc6	FBgn0036263	24.28900672	23.59990591	31.11285178	29.4956274	31.51403225	40.3129936	35.30122749	30.6094197	3L	0.375385063	0.027754974	0.462499435
Tsen54	FBgn0036266	15.32581913	10.09914384	10.51226556	11.2724597	7.246127301	11.65387806	11.75221989	10.99222826	3L	-0.161163952	0.601870884	0.989286463
Pbgs	FBgn0036271	74.76714917	71.37858364	54.17100477	54.19396033	103.1715788	96.54629489	91.51429708	86.72640693	3L	0.597488479	0.000110947	0.010160766
Sms	FBgn0036272	103.2142705	79.82508024	84.99280179	98.68160629	108.1301713	105.6731416	90.59762695	92.09545464	3L	0.138958636	0.261779016	0.890640489
INPP5E	FBgn0036273	77.369294	84.41342626	84.47182185	88.47411084	72.02510204	79.46181694	77.43316997	54.13814509	3L	-0.213038457	0.108993064	0.758410631
CG4328	FBgn0036274	0.620258388	0.168712935	0.569360399	0.273588204	0	0.047414256	0.042764661	0.145582527	3L	-2.796854217	0.028045796	NA
CG10418	FBgn0036277	126.8338597	135.4309945	157.2257514	119.7258275	152.1797013	138.3898788	132.9735837	178.653418	3L	0.193415194	0.277474674	0.896087042
CrzR	FBgn0036278	1.268468032	0.794956308	0.152173471	0.422483638	0.880734579	0.854217081	0.391151442	0.211843194	3L	-0.161902905	0.805589369	0.995787491
Ncc69	FBgn0036279	2.773546773	5.875837319	4.193844564	0.885810944	2.15367988	1.923550887	2.490920538	1.229071769	3L	-0.772983351	0.157094287	0.818884898
toe	FBgn0036285	1.159685116	0.72945341	1.261883639	0.994030217	0.431020647	0.50647555	0.304539287	0.684985282	3L	-1.073096119	0.024564413	0.440903252
CG10616	FBgn0036286	61.37063108	79.96328675	67.08743455	80.25530481	63.76344998	65.48175039	67.10789058	71.94892038	3L	-0.07325235	0.591681127	0.988631016
CG10663	FBgn0036287	1.199291901	0.396854923	0.732314426	0.627696798	0.574395517	1.023000785	0.291373277	0.194840211	3L	-0.485085338	0.434795532	0.96591738
CG10660	FBgn0036288	1.728907099	1.848749437	2.254438672	5.290532382	1.287462177	1.202127251	2.094977598	0.422281952	3L	-1.129974771	0.052771791	0.605783103
CG10657	FBgn0036289	0.627202299	0.21580387	0.858329452	0	0.116129323	0.28808019	0	0.2094945	3L	-1.425354844	0.282372735	NA
CG10638	FBgn0036290	1.486428552	2.58393993	2.163632961	2.394381529	2.976117361	4.866665275	3.99874269	3.461877547	3L	0.866420757	0.036744272	0.521170991
CG10681	FBgn0036291	35.74721249	51.12053998	44.10233516	23.68516296	35.43792795	36.29473463	39.75031846	27.03101005	3L	-0.124987225	0.632889985	0.991585536
CG10646	FBgn0036292	94.50918603	78.90659825	79.66562633	80.80717531	85.25186253	69.24485986	82.99736719	65.89216169	3L	-0.111199717	0.48831075	0.973549494
CG10654	FBgn0036294	40.95574794	67.87268005	52.66148308	57.57050559	36.20689546	51.32923237	53.04593808	52.00906265	3L	-0.152116346	0.409872699	0.958170848
nst	FBgn0036298	94.96946351	101.5456863	103.5034028	102.1349907	97.33334424	106.0847453	97.62526971	75.19704439	3L	-0.066700726	0.587996465	0.987588562
Tsf2	FBgn0036299	76.5812774	62.1282475	56.49605709	41.15103707	56.83087576	73.76507426	45.36234147	56.73715925	3L	0.003633892	0.984595099	0.997881056
Pmm2	FBgn0036300	114.3961446	90.35366695	95.16390695	105.8964622	126.0432192	105.7285674	133.1056805	114.0969539	3L	0.265553789	0.109618698	0.758410631
CG4069	FBgn0036301	5.364920455	7.537541378	6.45199049	11.01141314	8.857273838	14.61204113	19.30080779	10.65220569	3L	0.846265737	0.019327448	0.395797586
sowah	FBgn0036302	7.944971973	6.918371811	6.496783585	8.278744724	6.370074123	8.031332947	7.027177944	8.246149432	3L	0.02874449	0.835437907	0.995787491
CG10984	FBgn0036305	139.1485311	129.4908348	118.860815	136.866425	129.79079	163.5750513	132.9289936	116.8549217	3L	0.076783313	0.523437318	0.983811292
CG10973	FBgn0036306	67.43244531	61.42878259	40.38418466	40.78412083	55.21272204	64.00050355	41.88978281	42.02168515	3L	-0.023108698	0.917496984	0.996479182
Hip1	FBgn0036309	13.86633265	14.10225402	17.28981898	18.3445502	14.29547223	18.96631095	15.85996636	10.75443133	3L	-0.058149683	0.773406379	0.995787491
CG10969	FBgn0036310	0.073811884	0.495236321	0.537199802	0.172089628	0.199874052	0.313152269	0.188295701	2.724291351	3L	1.496623206	0.233667834	NA
CG17666	FBgn0036311	1.050090134	1.856588556	0.75736606	0	0.051234644	0.481630653	0.868800808	1.910138635	3L	-0.105760869	0.925032819	0.996479182
Sf3a2	FBgn0036314	267.3715574	258.1792251	255.0877809	234.3483913	244.7295409	263.6129427	206.9177852	236.8256048	3L	-0.062650497	0.593119049	0.988631016
CG10960	FBgn0036316	47.80969775	55.16331031	61.86670487	52.07679913	53.86146943	50.84568525	59.6360618	56.42925046	3L	0.05898618	0.585465262	0.98718792
CG10948	FBgn0036317	186.1634851	225.2081047	203.575788	199.5016306	202.0544054	228.6512567	196.5431527	211.0540661	3L	0.072739341	0.418802279	0.960266449
Wbp2	FBgn0036318	124.6795254	144.8546663	135.9859424	123.6287823	110.6132768	114.7742449	124.164091	117.7542502	3L	-0.147465544	0.155427359	0.818884898
Ent3	FBgn0036319	389.3240502	320.3524434	313.5351302	401.3313054	350.4432309	340.2991248	362.9972447	397.4440619	3L	0.055533263	0.644456842	0.992589784
CG14120	FBgn0036321	0.102994347	0.276413709	0.649643407	0.62433135	0.632166057	0	0.052548158	0.313054365	3L	-0.675580599	0.610735279	NA
CG10748	FBgn0036327	0	2.791597403	2.385808319	1.058238492	2.321622942	5.563079413	0	0	3L	0.375805476	0.802450979	NA
CG11263	FBgn0036330	0.162816251	0.409652131	2.17244475	1.518399842	0.146962531	5.833082351	1.246043264	2.120934919	3L	1.187422378	0.312061079	NA
CR14117	FBgn0036331	0.371752127	0.623562042	1.442984761	0.346690436	1.207994164	2.663690825	0	1.452794906	3L	0.996132441	0.435305008	NA
Cul6	FBgn0036332	2.803915213	0.5720077	1.011146638	0.176681696	1.162842637	0.142892656	0.096660107	2.385665203	3L	-0.251563939	0.82194526	0.995787491
MICAL-like	FBgn0036333	43.36074275	49.00721987	54.41210247	41.40037518	39.97731183	56.35633556	49.0521243	45.78126921	3L	0.05410315	0.712520578	0.995787491
CG11267	FBgn0036334	808.3015654	700.0489075	779.9370694	629.9899685	880.6477294	741.8613973	815.9903488	677.0465189	3L	0.124710029	0.307444099	0.912131082
mRpL20	FBgn0036335	79.62122859	105.6286308	125.5539935	79.87882844	155.7147847	110.4386093	147.4125873	157.9358027	3L	0.58752214	0.004110538	0.176583611
ste14	FBgn0036336	4.63264368	5.418977941	2.3660482	7.674277032	6.602454615	3.103314748	5.442487822	4.7642676	3L	0.012957035	0.980511776	0.997881056
AdenoK	FBgn0036337	29.68852752	36.79896962	36.18867937	31.11059803	38.13215498	37.38430915	43.62813934	46.99824369	3L	0.346341488	0.027382962	0.462324143
Srrm1	FBgn0036340	190.5392937	207.5321028	205.1784112	189.1644814	207.5500015	209.9067324	214.9024278	166.0145005	3L	0.041083256	0.690186809	0.995787491
Syx13	FBgn0036341	123.272552	98.1002506	115.3177989	125.9296636	118.3566929	106.9256161	115.3329541	99.83798198	3L	-0.043102345	0.742438963	0.995787491
CG11279	FBgn0036342	19.08991202	25.13770604	20.12618227	24.54146974	17.15057283	18.16605667	27.76285343	15.97800096	3L	-0.140289598	0.572263305	0.984934163
CG17687	FBgn0036348	0.630537424	1.672543929	2.134430226	2.871772986	1.69418665	0.09677367	1.084812115	0.222853158	3L	-1.201172263	0.110867002	0.758410631
CG10171	FBgn0036353	19.83253049	18.96697025	14.78765043	25.84841505	16.90466308	20.15551556	17.24000298	23.20734703	3L	-0.008037807	0.974379521	0.997757033
CG10222	FBgn0036356	19.32803828	18.33862702	13.103814	15.62776817	25.61100909	24.66460366	25.06815615	20.769546	3L	0.556063239	0.030641394	0.487338512
CG10713	FBgn0036360	45.85751565	63.64109329	60.40960292	57.73982722	48.42762694	44.46104992	55.74265868	60.30159846	3L	-0.088183984	0.536305362	0.984934163
CG10140	FBgn0036363	0.375621187	1.008082954	0.91125176	3.327837294	0.813711015	0.424960033	0	0.652406694	3L	-1.519127383	0.24272723	NA
cmb	FBgn0036365	25.43458634	33.77210518	20.41001167	22.77257318	25.60290142	29.63988139	24.88389553	24.40122177	3L	0.060402471	0.706860127	0.995787491
JMJD7	FBgn0036366	91.91333913	64.16089286	71.8982627	80.70516822	117.5614434	67.65523982	67.53948619	105.1810845	3L	0.243351844	0.280975258	0.899941507
CG10738	FBgn0036368	6.727004384	11.09669376	4.894462834	10.80223924	7.665415981	7.956752457	5.296337732	9.639011828	3L	-0.101183957	0.684227887	0.995787491
CG10089	FBgn0036369	98.33304681	98.2496777	84.62271628	107.1293156	91.85675409	83.06915631	86.04116513	68.66546895	3L	-0.207895347	0.081574694	0.694684542
Abp1	FBgn0036372	336.1935694	298.9894866	246.0878004	276.7859952	270.2715317	315.3856532	241.3159871	279.9963518	3L	-0.037968217	0.754052627	0.995787491
Tgi	FBgn0036373	17.9693538	19.6898207	15.53081878	19.66073841	16.61816162	18.01324214	15.83852381	19.41070989	3L	-0.030093285	0.813225026	0.995787491
Spt20	FBgn0036374	25.29715573	60.40864925	45.90897506	43.32011593	44.37569737	42.89303028	53.05261227	51.4951346	3L	0.172381939	0.366992504	0.940794966
Liprin-beta	FBgn0036376	65.14999085	65.33721041	68.70054266	70.70156033	61.47099357	58.15324456	67.63488754	61.73123085	3L	-0.086223044	0.481099099	0.973549494
CG10710	FBgn0036377	4.055356039	7.23833513	2.585690542	4.000154205	3.848195361	5.048802371	3.581065516	4.232958346	3L	-0.065560664	0.86568205	0.995787491
CG8757	FBgn0036380	2.077481173	1.635667637	1.954262584	0.09884835	1.91346223	1.798747862	1.622356869	0.828441539	3L	0.120861073	0.885622376	0.995787491
CG8745	FBgn0036381	65.09591622	91.90994438	83.71168317	82.09425884	67.77164959	86.69734553	79.8982217	67.06919611	3L	-0.06774278	0.587984904	0.987588562
CG8833	FBgn0036386	15.4179366	19.99018313	25.13798159	26.36064844	30.94263578	30.45998936	30.3313093	20.08426055	3L	0.396759135	0.069172225	0.660467387
CG17364	FBgn0036391	33.52928283	24.21030434	26.34989585	27.91680266	27.00491288	34.24634835	24.99849337	27.8534396	3L	0.05123639	0.707053274	0.995787491
CG17361	FBgn0036395	26.26618097	24.04683295	32.83353997	32.48918735	32.57669447	24.00745468	30.94533064	28.94847051	3L	0.042351182	0.876193902	0.995787491
CG17359	FBgn0036396	43.46940825	28.49877	32.663418	38.71526228	34.14404099	33.48733288	33.69266554	14.75496693	3L	-0.282096448	0.320875079	0.916928013
Nprl3	FBgn0036397	72.51679525	55.09395122	75.56442222	69.95450278	73.61811723	69.80566311	98.99232249	74.9968243	3L	0.245615162	0.134208094	0.795116625
upSET	FBgn0036398	48.82940777	89.97480196	99.66266594	92.15990231	70.10144555	68.39022828	90.53912664	102.2877288	3L	0.040767781	0.878507832	0.995787491
CG6650	FBgn0036402	28.171589	24.69803237	27.88430385	24.34575704	27.89220536	30.07772675	27.40502671	25.38949383	3L	0.104267549	0.486176219	0.973549494
CG6833	FBgn0036405	64.9465389	61.54404301	64.88718587	74.99067848	77.99835636	49.98069454	78.20376682	60.89865229	3L	0.03403694	0.863334776	0.995787491
CG13484	FBgn0036406	75.0425354	81.23739461	73.27276016	68.55656919	65.33353656	74.88925083	76.37039363	74.24032407	3L	-0.00695022	0.970537394	0.997604378
Sox21a	FBgn0036411	1.139467972	1.848632174	3.670672227	3.39699805	3.675679652	7.519984105	8.196243467	3.041668581	3L	1.194571155	0.007230485	0.247978871
nan	FBgn0036414	0.050966899	0.726663503	0.185467481	1.010032963	1.058095637	0.6486923	0.195026483	0.774576997	3L	0.492718425	0.637355836	NA
CG7906	FBgn0036417	0.052912795	1.065045491	0.898559921	0.24672838	0.955211567	0.29931516	0	0	3L	-0.775732874	0.65424782	NA
CG13481	FBgn0036421	0.44306008	0.185792782	1.612286361	2.065955795	1.9995925	5.952429305	0	0.240481112	3L	0.967037576	0.499177129	NA
CG3919	FBgn0036423	0.329034171	2.55257757	2.694034048	2.589066397	0.965235392	3.48987833	1.363981516	0.089295456	3L	-0.427272162	0.641337028	0.992099188
Gbs-70E	FBgn0036428	0.621290465	0.607907765	0.711753168	0.100591342	0.311552368	0.42304084	0.044025703	0.936721605	3L	-0.211271991	0.795403864	0.995787491
CG9628	FBgn0036433	1.352906775	1.837061545	2.461599724	0.675910974	0.639661386	1.746237	0.944997235	0.594450707	3L	-0.648109596	0.260514615	0.890556245
CG9384	FBgn0036446	50.64305534	53.55887003	60.13368828	64.8366097	51.16751081	53.80414603	46.49025844	53.35163514	3L	-0.131317447	0.282867815	0.901780531
CG17173	FBgn0036447	2.960370421	2.482803041	0.265993235	0.12781467	0.395868692	2.067421593	0.559405077	2.142412741	3L	-0.163418071	0.880325922	NA
mop	FBgn0036448	82.90494136	85.01645613	99.49510661	73.96003047	79.86569221	101.3210185	91.96024466	80.8132029	3L	0.08213975	0.481406918	0.973549494
bmm	FBgn0036449	15.06822043	18.13926636	18.54253971	16.94433772	17.88235814	12.88854723	21.70430773	14.62195975	3L	-5.95E-05	0.999728513	0.999757791
Tdrd3	FBgn0036450	118.1431882	143.0150608	139.5779603	82.91498648	144.4528164	106.6962906	120.0161356	125.305386	3L	0.074524905	0.64787826	0.992932477
Helz	FBgn0036451	142.9503914	166.1703394	153.6352984	177.9575777	163.7982133	167.0529102	166.4864539	178.8843997	3L	0.108981996	0.26884291	0.89549671
CG17839	FBgn0036454	1.228125551	1.421085339	2.022622387	1.493227117	1.338768266	1.194946509	1.699774911	1.468579417	3L	-0.077675062	0.682249631	0.995787491
CG5114	FBgn0036460	26.704232	42.06456623	47.85622688	33.39284061	38.35802184	21.12063373	38.27740254	52.96556432	3L	0.049003063	0.860122706	0.995787491
Zip71B	FBgn0036461	2.646922154	4.108128359	7.602342657	1.986133126	3.40527723	2.811025285	3.570210838	2.212814783	3L	-0.40381046	0.321977765	0.918330833
mRpL39	FBgn0036462	219.2444022	190.3858886	183.5716399	204.2243281	203.4177736	183.9723018	167.4613128	174.3286827	3L	-0.100862788	0.400389312	0.953696242
Reck	FBgn0036463	1.148245614	1.393532794	0.507967431	0.299204336	1.560754919	0.566751451	0.430764593	0.234630355	3L	-0.228333449	0.714460964	0.995787491

**CG13458**	FBgn0036479	3.137450659	2.528127781	3.805705769	0.071714197	3.16512202	2.435974732	2.903304406	2.404127933	3L	0.226456613	0.746264141	0.995787491
**Cep135**	FBgn0036480	0.053695335	0.360265565	0.037218324	0.858436467	0.512364589	0.028927791	0.078273116	0.416348365	3L	-0.296393722	0.780130941	0.995787491
**CG12316**	FBgn0036483	41.63294962	42.73549011	47.40228874	47.15574562	42.10354644	47.34837432	57.99007887	46.54616958	3L	0.146859405	0.270936474	0.89549671
**Pex3**	FBgn0036484	102.1102441	109.613519	97.90364644	88.00267382	103.5243571	108.5345264	106.9889849	92.54150422	3L	0.079456781	0.509673718	0.979725655
**FucTA**	FBgn0036485	53.11901507	59.77346484	48.95807425	65.47006228	66.78716089	72.61697285	58.05735266	54.72234177	3L	0.177577114	0.167899355	0.830652748
**Msh6**	FBgn0036486	9.464178838	7.719263068	9.368442727	13.29520582	11.08917001	13.75200704	14.44515975	12.55445838	3L	0.407091519	0.075499291	0.678318963
**Prp31**	FBgn0036487	105.1914416	124.9721749	95.1689339	96.21722804	99.82087421	98.53272474	122.5173976	117.650487	3L	0.088829387	0.528999971	0.984296327
**CG7011**	FBgn0036489	26.81640454	28.56172458	24.89240472	22.45488254	28.5235476	27.18148549	32.27759166	18.51861853	3L	0.080388856	0.688850093	0.995787491
**Best4**	FBgn0036491	2.051944327	1.904001436	0.741402515	1.221454711	0.551702084	1.852238851	0.779614435	0	3L	-0.86796041	0.39316912	NA
**CG7255**	FBgn0036493	0.087367107	1.38172004	0.121115075	1.0184656	0.540754352	0.482448022	0	0.027097439	3L	-1.264972215	0.271861758	0.89549671
**Toll-6**	FBgn0036494	54.64346626	75.01787561	75.8888505	60.18942549	61.84571985	53.12443596	71.53897858	67.94003095	3L	-0.026018713	0.852238827	0.995787491
**CG7275**	FBgn0036500	42.38995292	41.08386389	54.4356318	51.05406209	37.38389208	44.70573352	51.03436458	41.5554964	3L	-0.082936802	0.655763108	0.995305364
**CG7272**	FBgn0036501	18.48019088	18.21898067	23.97509495	16.05019967	13.64285728	21.08645877	16.00064505	25.77386684	3L	0.028849909	0.904757217	0.996479182
**CG7841**	FBgn0036502	24.93384225	21.10118483	27.63846811	20.62972161	38.32652357	31.02346932	30.86586892	27.92610457	3L	0.472892752	0.008401393	0.272534513
**CG7945**	FBgn0036505	182.5909762	140.0197512	141.6200174	165.0705919	142.7036469	189.4140814	133.0708463	150.538004	3L	-0.007176191	0.959029776	0.997604378
**CG7739**	FBgn0036509	494.0461801	446.4917494	422.5152998	434.2857247	443.7004283	466.6216497	464.0359587	400.4737404	3L	0.010083352	0.917579502	0.996479182
**SCCRO**	FBgn0036510	140.3280584	155.5801918	153.0671297	140.9462487	154.1678314	158.117024	146.4463194	148.8043429	3L	0.073962216	0.451004047	0.967112302
**CG16979**	FBgn0036512	24.70102062	44.2637175	43.44514034	49.71893474	40.13250574	45.76086373	41.69349202	48.44414276	3L	0.154728664	0.465631839	0.971928234
**CG12301**	FBgn0036514	14.30732779	21.00577673	8.656927133	18.05202144	15.31471809	13.36190726	16.48864402	14.91001586	3L	-0.016273427	0.953321781	0.997604378
**AIMP2**	FBgn0036515	103.4958792	128.2870352	135.3763932	112.8493598	131.9384719	133.4611972	143.2587631	153.5040609	3L	0.262212528	0.043752116	0.549970032
**CG7656**	FBgn0036516	190.5348873	188.9722493	167.1734525	148.7373355	154.4306274	167.6118357	155.7384551	139.6029663	3L	-0.142521166	0.204765019	0.854537641
**RhoGAP71E**	FBgn0036518	47.18375592	44.36487768	45.24142626	49.96610684	45.79545384	46.61227189	39.53708908	55.40072489	3L	0.034670874	0.770133397	0.995787491
**CG7650**	FBgn0036519	36.11243118	43.26681231	52.94041655	63.98802811	44.70321862	59.28968636	53.95810142	57.57051932	3L	0.166720539	0.414403135	0.958665466
**CG7304**	FBgn0036527	2.227326317	1.577433805	0.840538623	0.807788716	2.412536916	1.306610447	0.50506287	0.107460703	3L	-0.308385584	0.754424871	NA
**Pgant8**	FBgn0036529	1.33454377	0.479680705	2.77507249	0.666736834	0.860426266	0.029957136	0	0.551889155	3L	-1.823496496	0.111040389	NA
**DCP2**	FBgn0036534	85.86347617	96.70880394	81.7964657	73.1464291	85.80012421	103.5525739	71.72956872	88.17046523	3L	0.079197761	0.515752148	0.980804634
**CG12713**	FBgn0036536	15.82841846	9.601089456	16.72008724	7.353437399	11.49300509	12.33482552	6.853938136	6.467206812	3L	-0.384600376	0.330247582	0.921112231
**CG18081**	FBgn0036537	32.24395459	27.1791631	33.90119682	26.24172382	24.24540425	31.93731592	28.57425684	24.31863356	3L	-0.104061033	0.618117557	0.990047511
**CG15715**	FBgn0036538	59.21415004	80.31960102	84.57068315	67.84251232	55.15276256	67.51246683	87.69432865	65.90903067	3L	-0.046158852	0.795670899	0.995787491
**sff**	FBgn0036544	29.84744486	36.34454266	33.61474032	37.68372778	31.49515613	29.20106427	29.09529096	34.05640844	3L	-0.118183556	0.288031258	0.902924182
**GXIVsPLA2**	FBgn0036545	79.84822937	116.7413815	122.3355155	99.86723166	107.0247926	129.503802	89.41900152	94.78363647	3L	0.040665165	0.809471482	0.995787491
**CG17032**	FBgn0036547	0.049047704	0.740435788	0.95191237	0.228705752	2.125048246	1.24853065	2.001947955	0.638922637	3L	1.687030191	0.110262879	NA
**CG10516**	FBgn0036549	39.22816896	51.68146836	42.07643284	47.45401908	42.5453932	38.04214014	32.272863	35.88352081	3L	-0.244871528	0.17148016	0.832652344
**hzg**	FBgn0036556	266.8462154	252.725298	211.5103612	240.304926	229.2457652	227.7391582	178.3358075	183.1783311	3L	-0.219425047	0.053381866	0.607150997
**mRpS31**	FBgn0036557	85.75575956	107.2624282	83.0973461	78.13603509	96.75695314	80.09494159	94.13800735	97.23817839	3L	0.088364536	0.569916062	0.984934163
**CG5895**	FBgn0036560	41.02184423	66.72328622	67.79315531	49.03769041	29.2629228	52.45755919	73.31674697	37.56806421	3L	-0.188361173	0.465831157	0.971928234
**CG13075**	FBgn0036563	2.589687911	2.514853679	0.661320711	3.813322026	0.984221139	0	0	0.443877804	3L	-2.723170512	0.031349254	NA
**CG5235**	FBgn0036565	0.187960027	0.157638288	0.683982605	0.438221726	0.113105341	1.772075651	0.079915011	0.068013103	3L	0.514457444	0.701531146	NA
**ClC-c**	FBgn0036566	103.1603996	117.3642928	114.0119749	120.9485298	111.2445225	116.1660859	126.8308879	145.8038205	3L	0.167279193	0.155111354	0.818884898
**CG13074**	FBgn0036567	0	0.336904245	1.339989109	3.160912366	2.719445799	0.80479629	0.896669588	2.071343977	3L	0.472768347	0.67591998	0.995787491
**IleRS-m**	FBgn0036569	24.07565229	12.25118303	14.76586432	10.34332834	19.72914613	22.18830201	15.87193981	21.55422742	3L	0.389909542	0.14295074	0.8037297
**IntS9**	FBgn0036570	35.47599218	23.76268524	28.24133187	33.94920685	34.9067815	51.56497947	28.99221941	30.41456103	3L	0.286781854	0.200335803	0.848203654
**Strump**	FBgn0036571	34.58313659	42.94125359	53.2341128	39.29243877	47.37375303	71.70226463	36.82750093	62.45324163	3L	0.391832238	0.042461739	0.541094998
**elg1**	FBgn0036574	0.408664251	0.623160865	0.856220986	0.259850541	0.335338052	0.245182102	0.14216063	0	3L	-1.51416109	0.160725949	NA
**CG5151**	FBgn0036576	3.004380207	3.527058296	3.812249973	3.063104539	3.272807879	4.37475755	3.162627873	3.222942183	3L	0.098868814	0.553936918	0.984934163
**CG13073**	FBgn0036577	0.081484709	0.410037504	0.247100959	0.807408759	0.110325588	0.326498965	0	0.442277115	3L	-0.763188253	0.544511417	NA
**CG5027**	FBgn0036579	179.2687575	153.2705713	187.0497304	200.1644182	202.5346937	208.5033713	188.779323	208.9001274	3L	0.196883968	0.099526202	0.731438598
**PDCD-5**	FBgn0036580	178.3955718	167.4554035	155.2405451	179.3679228	166.6619604	159.9983404	191.179754	187.2702055	3L	0.079757516	0.562512123	0.984934163
**MED10**	FBgn0036581	219.6078753	258.7125619	220.141068	193.787697	245.0444343	218.7111282	238.1830579	229.8465027	3L	0.094234998	0.484933655	0.973549494
**CG13055**	FBgn0036583	97.43390494	150.7480757	317.1191335	192.1930218	106.2967634	89.23050769	254.5643356	170.9663252	3L	-0.245022449	0.605885203	0.989286463
**CG13054**	FBgn0036584	167.9320132	127.6034264	168.2332718	161.9800375	153.331859	153.0802941	186.6403445	114.6033482	3L	-0.015534833	0.925046828	0.996479182
**CG13070**	FBgn0036586	0.964807192	0.719257207	2.860744076	0.99973851	1.161149506	0.606409551	0.546943083	0.232742858	3L	-1.067729144	0.411622496	NA
**CG4950**	FBgn0036587	0	1.539740947	0.712622826	0.513642676	0.397714625	0.034617699	1.686040738	0.079718622	3L	-0.28761651	0.841237468	NA
**CG13068**	FBgn0036588	7.176463512	1.959594779	4.048837298	3.501974201	3.314160825	0	0.425751456	0	3L	-2.135113047	0.132858087	NA
**CG13067**	FBgn0036589	74.90571557	0.456886653	8.920810859	22.22689076	0.245861845	9.630088787	0	26.61173058	3L	-1.531388333	0.392648349	0.951535721
**CG4998**	FBgn0036612	0.430452968	0.512404461	0.303176002	0.258989649	0.476273571	0.183277355	0.035422412	0.391909423	3L	-0.433313637	0.64319475	NA
**Golgin104**	FBgn0036614	37.49672494	51.28681022	38.36728266	33.15362551	35.82216291	35.60656199	30.55165339	31.1666223	3L	-0.234483578	0.148488904	0.808039863
**roq**	FBgn0036621	55.17541368	56.22652718	45.26104345	66.50384288	56.49588042	62.68192977	56.02337713	53.71978552	3L	0.063913413	0.620722481	0.990047511
**Agpat4**	FBgn0036622	0	1.511105087	0.890401084	1.867000113	1.325153673	0.283115707	0	0.289763065	3L	-1.115571222	0.399598794	NA
**Agpat3**	FBgn0036623	136.788527	123.5572168	108.9587104	108.8150271	128.0836228	145.2569917	122.2831429	128.7859387	3L	0.16106628	0.150173914	0.809805479
**RAF2**	FBgn0036624	43.16440533	37.35010223	33.56349926	41.25318848	32.98744094	36.25632964	41.64321557	46.37519403	3L	0.045809517	0.77160862	0.995787491
**GluRS-m**	FBgn0036629	14.15300778	7.013997863	7.357499884	8.463568772	10.2862857	9.097751784	10.55005332	7.582107925	3L	0.035273874	0.920029749	0.996479182
**CG13033**	FBgn0036638	2.938979117	4.51891474	2.772747579	0.190336644	1.32640264	2.232074868	0.624783239	3.544886512	3L	-0.391958879	0.682777	0.995787491
**nxf2**	FBgn0036640	0.301543066	0.758694286	2.011732997	0.234345435	0.453635432	0	0.576932129	0.054556492	3L	-1.574849418	0.205537099	NA
**Smn**	FBgn0036641	46.66378943	60.69554287	73.33681198	99.12811916	63.41823342	59.98581467	72.30882689	75.28795957	3L	-0.013078424	0.954781319	0.997604378
**Syx8**	FBgn0036643	146.7672698	177.7467214	134.4064658	139.1711855	170.7032521	156.7003177	178.5881552	194.001105	3L	0.258797225	0.076998657	0.682035833
**CR18217**	FBgn0036646	16.98098354	17.24320354	15.24053547	12.96012665	12.64691717	14.71731547	12.72369922	10.00211154	3L	-0.28893916	0.226785555	0.867702781
**CG4098**	FBgn0036648	21.56500574	18.6269174	26.07129724	27.72806592	29.87678977	25.73503826	23.028611	26.75407069	3L	0.198559279	0.433689159	0.965402206
**CG9701**	FBgn0036659	2.471404052	0.399559865	0.830714521	0.79834739	0.564408869	0.898322755	0.113938642	0.161615873	3L	-1.351391753	0.092567508	0.72305867
**CG13025**	FBgn0036660	40.48490857	39.12175719	34.81482129	37.17592374	37.86011593	28.81779281	32.95515528	32.5651193	3L	-0.168531447	0.275148687	0.89549671
**CG9705**	FBgn0036661	96.59110963	55.76026762	64.24556746	58.34610172	73.43031364	61.37942024	51.93011127	49.22385997	3L	-0.19621613	0.327867727	0.918470465
**CG9706**	FBgn0036662	33.23723379	20.53292756	20.58116709	19.14704917	26.64412292	31.70108083	29.84411077	21.86700342	3L	0.255004752	0.277454803	0.896087042
**CG9674**	FBgn0036663	122.1498596	108.6755607	111.1655903	110.6010941	107.4827293	113.0150301	101.8995283	112.4157935	3L	-0.028718674	0.74624538	0.995787491
**CG13024**	FBgn0036665	0.147218897	0.20952404	0.194809631	0.312032106	0.265767841	0.134591105	0.068283376	0.096856319	3L	-0.566357115	0.423606671	0.961944958
**TSG101**	FBgn0036666	276.7171278	372.1350765	321.3932055	302.7885463	337.1026675	308.2953337	312.8810467	329.4883042	3L	0.050724694	0.636923498	0.991585536
**kud**	FBgn0036667	117.5387069	106.8207476	147.5413541	113.6251267	135.2972502	134.5607981	117.0122015	138.2635635	3L	0.146230746	0.453610587	0.967967712
**Zcchc7**	FBgn0036668	42.78672674	62.75591425	44.29604749	52.66169973	41.74157957	47.21077737	50.10105454	42.0453115	3L	-0.129083514	0.346640502	0.930285749
**CG9951**	FBgn0036671	53.58465717	51.26944192	61.37083372	64.51244127	57.40638324	61.84026155	61.02266455	63.99064027	3L	0.113054348	0.454295839	0.967967712
**CG13023**	FBgn0036677	0.664401316	0.495306667	0.656670799	0	0.799609773	0.069599278	0	0.213700261	3L	-0.703606808	0.624430137	NA
**CG11905**	FBgn0036678	0.447921047	1.360506923	0.352427096	0.08467387	0.415233338	0.833181015	0.339708604	0.341681479	3L	-0.17757999	0.827218591	0.995787491
**Cpr73D**	FBgn0036680	0.209684279	0.203213667	0.203476454	0.0108638	0.235532182	0.092254893	0.023773738	0.040466131	3L	-0.636918557	0.531519337	0.984760433
**CG3764**	FBgn0036684	16.59277128	13.94451447	18.59046512	17.73034678	14.53466682	19.03856899	15.26500739	15.05672075	3L	-0.03813401	0.776894463	0.995787491
**CG6664**	FBgn0036685	104.1838158	107.0046851	130.5207971	127.1519275	132.158131	140.4437648	134.900008	118.0632309	3L	0.194754605	0.097645132	0.730787365
**CG7728**	FBgn0036686	13.15279734	15.13599164	14.68804102	19.52408452	7.119063388	11.54744101	10.17835591	21.25327499	3L	-0.286374946	0.339974456	0.927274752
**CG6652**	FBgn0036687	0.345367806	0.115861209	1.424357715	0	0.99756385	0.162805329	0.088104069	0.524877833	3L	-0.027439193	0.984504648	NA
**Fit2**	FBgn0036688	0.217399684	0.312563548	0.37672043	0.072408454	0.616726235	1.815387408	0.079227324	0	3L	1.388869767	0.320887816	NA
**beg**	FBgn0036691	40.16134197	55.46993298	43.71222163	71.70221474	51.86144443	44.34507051	62.39446272	39.90499773	3L	-0.057705075	0.808451153	0.995787491
**Papst2**	FBgn0036695	103.8588077	109.5771968	135.5544902	116.4707351	121.5884107	120.781174	147.7074228	113.194517	3L	0.144451986	0.305717476	0.911610813
**Pop5**	FBgn0036696	27.60361674	43.56417825	57.72930104	33.9220536	31.6663881	55.76683127	33.99461624	54.32079469	3L	0.150263868	0.638134071	0.991585536
**rogdi**	FBgn0036697	80.99141245	56.14031275	62.3472565	55.02397891	52.49990344	63.61809742	53.2889352	58.39174408	3L	-0.133995788	0.348556525	0.931729531
**CG7724**	FBgn0036698	1.156887527	1.293677688	0.575711386	0.968239899	0.053550731	1.006805995	0.454037683	0.322014092	3L	-1.085956688	0.252232353	NA
**CG6512**	FBgn0036702	155.6664176	169.0158262	174.085951	178.2191036	151.4746784	174.5012636	158.9816498	157.881631	3L	-0.044537925	0.639665839	0.991585536
**CG6479**	FBgn0036710	43.10190214	33.92416191	47.65733342	28.60113156	36.21156775	39.54215825	48.52772132	34.28202078	3L	0.077341219	0.705378615	0.995787491
**Mip**	FBgn0036713	0.312038605	0.523400985	0.757001303	0.128383458	0.397630346	0.069220727	0.234122701	0.159403458	3L	-0.962760921	0.358798122	NA

**CG7692**	FBgn0036714	2.167540314	2.750114658	7.516847648	6.44650122	5.819282477	2.515130792	6.699131545	6.184075559	3L	0.209412208	0.611968867	0.990047511
**Cad74A**	FBgn0036715	0.375098117	0.567490699	0.5709696	0.514427729	0.424877326	0.201089533	0.431535631	0.4471066	3L	-0.399013269	0.474004975	0.973549494
**CG13728**	FBgn0036716	0.577027543	0.430170113	1.710940943	0.597919109	0.98381135	1.057812862	1.063118087	0	3L	-0.055335586	0.95749257	NA
**CG13731**	FBgn0036717	0.797799631	0.464272415	0.355490959	0.417559956	0.999345214	0.122801734	0.124604326	0.035348915	3L	-0.638805665	0.537309309	NA
**CG18265**	FBgn0036725	7.529936083	14.8186101	16.8247349	18.58450783	10.39856746	11.91938378	16.27261669	16.16075682	3L	-0.041308319	0.855858915	0.995787491
**QIL1**	FBgn0036726	612.3267961	518.8422599	603.5945641	526.0932584	609.8927198	612.6224861	640.8790623	564.3971892	3L	0.131672437	0.223073646	0.86549481
**SecCl**	FBgn0036727	0.295962115	0	0.957333284	0.138004906	0.356191648	0.14881668	0.805339428	0.299861869	3L	0.250713306	0.836093406	NA
**UQCR-Q**	FBgn0036728	962.5238067	1008.288842	1008.893021	930.1719658	1088.381865	880.440229	1087.478545	988.7094206	3L	0.082112342	0.42830999	0.963309722
**CG6333**	FBgn0036731	0.454316319	0.45246831	0.447754553	0.132402674	0	0.214163325	0.253524838	0	3L	-1.646413416	0.202507242	NA
**Oatp74D**	FBgn0036732	1.339366565	1.661301223	1.2398586	0.788888268	0.668103927	1.821013542	0.791248036	0.979499384	3L	-0.21060604	0.543357287	0.984934163
**U4-U6-60K**	FBgn0036733	46.06726336	70.56133485	39.53432756	55.3710301	41.88567892	45.4963409	45.11682175	28.06672943	3L	-0.367268688	0.083343997	0.69799888
**CG7564**	FBgn0036734	60.23128535	77.98795268	61.63976735	61.82964289	68.19579389	74.4916234	66.91164026	69.64311811	3L	0.125669293	0.224815666	0.867012385
**Edc3**	FBgn0036735	89.92764416	156.0070152	167.8107145	168.8542446	109.7132421	140.656124	155.2806201	166.750389	3L	0.008888051	0.960410956	0.997604378
**CG7542**	FBgn0036738	3.15248381	0.503604699	1.092554536	1.574977999	0.135500982	19.24816083	0.765909898	2.281444725	3L	1.799727551	0.092963883	0.72339963
**Vps60**	FBgn0036740	381.2471518	437.7077561	424.0466989	369.2056946	352.8173131	370.9638431	322.0322218	368.600226	3L	-0.156716022	0.112098904	0.758410631
**anchor**	FBgn0036741	49.03348376	64.67920627	63.1299917	52.94296874	50.99443994	63.48602606	65.41743031	62.72652849	3L	0.111559239	0.360408613	0.939549067
**CG7497**	FBgn0036742	2.302462154	1.053288343	0.923262987	0.931654384	0.75573351	1.722245756	0.194169601	1.941705357	3L	-0.157479564	0.821095356	0.995787491
**CG7484**	FBgn0036745	64.54238926	58.22904841	74.40565633	45.7720746	97.95824703	57.99037123	72.65191817	74.63710274	3L	0.353418026	0.11067797	0.758410631
**Crtc**	FBgn0036746	48.98493607	38.74501687	37.91618311	42.72289512	39.92236543	42.98243265	36.53807494	39.56450622	3L	-0.0560871	0.645793411	0.99279236
**Adgf-B**	FBgn0036751	0.197420503	3.063093084	1.796022699	2.646601205	1.871071682	0.232658555	1.259059861	1.6073182	3L	-0.584424059	0.535451715	0.984760433
**CG5589**	FBgn0036754	6.656952811	22.98519749	12.27753045	18.24331819	14.47722944	21.47098627	15.59168803	18.17172554	3L	0.250797249	0.451295865	0.967112302
**Cln3**	FBgn0036756	2.427720878	2.407628154	1.289697151	0.123944668	1.215627879	0.601447116	0.13561682	0.615568673	3L	-1.244125605	0.12299125	0.780439453
**Ir75a**	FBgn0036757	0.70001985	0.308996078	0.357523872	0	1.197203914	0.451561458	0.751901338	1.679787673	3L	1.570255491	0.153793113	NA
**CG5577**	FBgn0036759	1.216460778	3.243780071	4.918528133	3.127017895	1.351399065	3.087731745	3.182787261	2.573329751	3L	-0.251267704	0.64041102	0.99179631
**CG5567**	FBgn0036760	16.16528137	11.23749658	12.26834068	13.1003659	12.01631365	10.55426976	12.01855569	13.04382031	3L	-0.125325004	0.640402274	0.99179631
**MED19**	FBgn0036761	66.33659591	75.83758857	97.42193876	69.98313622	65.0031794	81.25130704	79.75754163	84.54401093	3L	0.037896513	0.800317595	0.995787491
**CG7430**	FBgn0036762	1088.905871	1075.849125	919.5836892	1012.669206	1034.533901	1048.208157	958.6671694	1041.698436	3L	0.024948274	0.783665425	0.995787491
**TrpRS-m**	FBgn0036763	82.32864067	71.89880789	82.55840658	71.04451379	73.19359439	74.62322594	71.57450474	67.67540759	3L	-0.071824673	0.550611901	0.984934163
**CG5535**	FBgn0036764	41.14885962	34.73719086	52.15572392	57.00043875	40.19823479	32.29317486	38.31119881	46.67582673	3L	-0.201318879	0.277470525	0.896087042
**CG7408**	FBgn0036765	0.551224297	0.255062463	1.014474968	0.443158172	0.480393651	0.017920376	0.193956555	0.90788612	3L	-0.461926175	0.658284112	NA
**CG7402**	FBgn0036768	0.234166655	0	0.340851023	0.327570444	0.211365414	0.55192723	0.418154917	0.101679497	3L	0.536886158	0.685363212	NA
**Tsp74F**	FBgn0036769	1.050048005	1.006461841	1.091743635	0.249810959	0.618973314	1.192015344	0.382670717	0.325678774	3L	-0.394128318	0.618795622	0.990047511
**Prestin**	FBgn0036770	21.43372032	13.889088	17.6387826	17.81154655	18.29522933	24.15208568	18.22596034	20.63286435	3L	0.224270855	0.173081832	0.832736771
**Wdr92**	FBgn0036771	67.10101757	61.606715	57.99170695	83.40156221	78.18406673	60.82314052	72.02885246	57.14955544	3L	0.01773186	0.922788137	0.996479182
**CG5290**	FBgn0036772	7.588230475	6.269813145	2.181800449	5.635125694	6.240519278	11.26114211	5.377149714	1.342387102	3L	0.172292328	0.739411062	0.995787491
**CG13698**	FBgn0036773	0.879273867	0.966851724	1.422070759	0.95666383	1.225759675	1.805321224	0.722759436	1.368105888	3L	0.315478673	0.35442472	0.936745458
**mRpS26**	FBgn0036774	120.8889428	112.9155634	98.21511085	128.1925571	124.5846107	139.5374743	141.9459981	116.1042808	3L	0.208543341	0.177491798	0.834615603
**RpIIIC53**	FBgn0036775	63.08176986	83.75637657	72.43923534	116.7689945	90.41448273	80.95344996	66.18708452	65.37296505	3L	-0.119774549	0.546843097	0.984934163
**CG7341**	FBgn0036777	0.603011158	1.679757072	0.67920202	0.175737227	0.058317305	0.832468739	0.219756346	0.187027578	3L	-1.227585846	0.149024355	0.808039863
**Cyp312a1**	FBgn0036778	0.67002644	0.116262968	0.448406841	0.377068619	0.166837166	0.544566237	0.47151775	0.100323409	3L	-0.318300126	0.770458942	NA
**CG13699**	FBgn0036781	1.113257452	0.247707466	0.440938294	0.13242438	0.26659485	0.203488512	0.173874103	0.12331556	3L	-1.324899268	0.13195721	0.791114096
**skl**	FBgn0036786	2.885184142	4.418666286	1.825934735	1.608558268	2.943932998	3.784534241	1.920043495	1.906435346	3L	0.006497081	0.992242273	0.998472242
**AstC-R2**	FBgn0036789	0.472372752	0.600509576	0.313633815	0.104335512	0.565510471	0.553171759	0.139530141	0.086363366	3L	-0.118078105	0.868478932	0.995787491
**AstC-R1**	FBgn0036790	0.43125838	0.43402526	0.510035572	0.075409701	0.350339358	0.381176031	0.178774276	0.643707532	3L	0.135676163	0.833832807	0.995787491
**MYPT-75D**	FBgn0036801	6.031208817	6.841860452	5.867945771	5.134665523	5.189761914	6.835311698	5.527073627	5.858222102	3L	0.001780979	0.988963379	0.998078482
**Sgf11**	FBgn0036804	71.00744793	49.64225021	52.07341908	71.27547492	67.71386005	60.81298621	76.88144124	41.89487965	3L	0.042266735	0.864853974	0.995787491
**Chmp1**	FBgn0036805	293.5808806	297.0593949	357.1928711	325.9559097	293.8272559	337.018089	303.5899991	308.5347915	3L	-0.004751912	0.965470291	0.997604378
**Cyp12c1**	FBgn0036806	0.378429229	0.070529105	0.612042687	0.784260889	1.518137218	0.158569229	0.429058263	1.460630908	3L	0.998672327	0.389536186	NA
**CG12477**	FBgn0036809	2.512494435	0.632153542	1.219055291	0.29288931	0.45356964	0	2.083063867	1.772828849	3L	-0.108028824	0.932762247	NA
**MED11**	FBgn0036811	99.15361105	140.5542988	137.8066908	145.0754832	132.5153673	115.6273586	124.4548662	95.62181159	3L	-0.124471932	0.491135141	0.973549494
**Nufip**	FBgn0036812	20.22407786	29.70335656	21.42033222	24.15085853	17.45339075	28.64721082	21.51766284	12.1015683	3L	-0.230357006	0.405630751	0.956706394
**Atg3**	FBgn0036813	160.7935585	153.784079	140.4017745	171.4751937	116.4054535	132.7708277	172.6135316	126.1735838	3L	-0.16517344	0.260393279	0.890556245
**CG14073**	FBgn0036814	10.92020359	10.01482639	9.690620465	8.501307098	7.703742371	9.661181263	9.721980147	8.406859144	3L	-0.113179697	0.414159573	0.958665466
**HipHop**	FBgn0036815	4.396865261	0.491674977	0.888894483	1.195964681	2.381240611	6.563451376	3.738832581	1.431900224	3L	0.994045721	0.255242661	0.886640087
**Indy**	FBgn0036816	1.920483927	2.150307822	1.048588019	0.618380928	0.478813678	0.889102657	0.77685457	0.138629486	3L	-1.303135761	0.010106097	0.304092724
**CG14074**	FBgn0036818	35.76773328	50.59849205	40.42391388	48.5610961	49.14056599	29.32018674	53.31742672	44.90895032	3L	0.045088367	0.851723154	0.995787491
**Dysb**	FBgn0036819	11.78438839	14.14770978	15.38278558	11.71516784	14.90248786	12.68937114	9.919009228	6.623527418	3L	-0.228789956	0.501284216	0.976796212
**Grx1**	FBgn0036820	203.6104831	210.4684002	214.2001388	155.7948007	235.5921334	169.3498274	201.7274235	166.908431	3L	0.013075582	0.936272587	0.996793017
**CG3961**	FBgn0036821	4.120969424	4.548847629	4.741041854	1.848940083	2.224152311	4.65292386	3.88355074	4.273655832	3L	0.010306736	0.973082616	0.997757033
**NijB**	FBgn0036822	3.580866021	0.324670292	3.052235224	1.12819653	1.572416985	1.368656194	1.728217921	0	3L	-0.778980167	0.522055357	NA
**CG3902**	FBgn0036824	1.908849798	7.049638293	5.890107305	3.708676203	3.143685652	2.446895168	4.314221064	1.2360162	3L	-0.69519765	0.121384866	0.77836519
**RpL26**	FBgn0036825	589.6594987	824.5418232	785.4348191	876.6852419	683.5113257	674.2403956	791.7552352	792.9520334	3L	-0.03058366	0.813565026	0.995787491
**arx**	FBgn0036826	3.432214113	0.454504554	3.725014753	1.474067	5.706866185	3.661642936	8.294828155	1.960961725	3L	1.128482122	0.163483143	0.825738493
**CG6843**	FBgn0036827	83.04635923	85.37657193	80.15445633	104.8400303	71.62075038	82.92598518	64.92270782	87.10410039	3L	-0.175148023	0.288761186	0.903033179
**CG6841**	FBgn0036828	34.02687884	27.06539709	32.14441847	40.98372787	22.74761297	31.94430385	38.52471502	39.46988047	3L	0.010695489	0.958161003	0.997604378
**CG3819**	FBgn0036833	13.05984276	4.329259706	0.876604643	0.601749639	5.684419631	5.548033035	4.213873765	3.586292814	3L	0.016417797	0.982560394	0.997881056
**CG6836**	FBgn0036834	3.336352773	6.326211177	4.750793976	3.424266589	3.142420099	3.760915562	2.821617195	2.440754914	3L	-0.512392764	0.12616348	0.78246933
**CG11619**	FBgn0036836	1.217957135	2.643820535	1.998799455	2.254993313	3.556760687	2.093926998	1.005217147	1.088828458	3L	-0.020797283	0.973818605	0.997757033
**CG18135**	FBgn0036837	114.5119014	107.7929356	100.0919948	130.9954088	90.82494466	113.6479702	87.24063439	105.2677598	3L	-0.165047201	0.175676428	0.834088743
**CG3808**	FBgn0036838	13.18448232	5.422457185	11.07185892	6.945859795	10.12702511	8.276872798	6.144654381	11.00951215	3L	-0.017534444	0.959898098	0.997604378
**Ugt316A1**	FBgn0036842	75.63882516	70.81609377	84.89046399	86.07584676	90.72647556	77.6230262	80.59789137	84.33903499	3L	0.101344257	0.418188475	0.959576749
**Sfxn2**	FBgn0036843	70.22865797	72.30437851	75.29942454	75.88236261	103.3202704	85.6834086	78.88435899	104.1048048	3L	0.373287864	0.00518957	0.20400887
**Mkp3**	FBgn0036844	98.24084089	129.8155828	117.8230229	137.450803	97.11008002	109.6496323	104.7978671	113.2734725	3L	-0.154889558	0.170297984	0.830934498
**MESR6**	FBgn0036846	25.48814405	22.38438553	23.42649512	16.61957645	23.2681768	29.07051951	18.18467988	30.50290037	3L	0.229654436	0.326986796	0.918470465
**CNPYb**	FBgn0036847	103.5894166	94.15927802	85.19400513	78.83092591	106.5235815	127.6948322	115.061657	140.439882	3L	0.46457686	0.002322257	0.114518881
**Naxd**	FBgn0036848	44.76378038	31.16525005	34.32734072	50.56387381	52.28186447	39.77182562	46.89181034	52.82800949	3L	0.280277498	0.216890013	0.860826301
**CG14079**	FBgn0036849	1.626633721	4.229097303	0.98654768	3.982057049	1.615069923	1.763617387	0	0.706314477	3L	-1.347651535	0.19782648	NA
**Gem2**	FBgn0036850	39.7465982	50.26563791	43.21317518	56.18691672	50.06302338	53.67376583	70.20984854	50.04722235	3L	0.27254308	0.179108754	0.836045561
**CG14082**	FBgn0036851	0.541460725	0.679005866	0.650532958	0.414787026	0.605103809	0.413250461	0.414383382	0.235112271	3L	-0.420034368	0.252149769	0.883301842
**mRpL21**	FBgn0036853	84.0749657	85.31990625	85.43023838	70.81264716	89.08833804	68.29086926	86.71410439	76.56044124	3L	0.008881452	0.952608899	0.997604378
**Aldh7A1**	FBgn0036857	4.934400059	8.991304638	4.650521071	6.37292327	3.332435631	7.128791627	6.70139771	5.395055763	3L	-0.114122657	0.76801925	0.995787491
**CG14085**	FBgn0036859	0.143854259	0.301619173	0	0.167695395	0.422002436	0.101718687	0.045871913	0	3L	-0.069806681	0.967039376	NA
**CG14086**	FBgn0036860	0.377695839	0.563139363	1.119903689	0.831662316	0	1.088050729	1.070566887	0.592231343	3L	-0.039164518	0.967650411	0.997604378
**CG14089**	FBgn0036861	1.910696595	7.264497787	4.635323289	2.078868018	6.668640986	5.163993493	3.574433914	0.276553279	3L	0.029254827	0.974671929	0.997757033
**Gbs-76A**	FBgn0036862	14.06160083	12.423935	15.73890511	15.33820882	18.95288199	20.79180257	18.97252591	19.31361557	3L	0.468748193	0.000268638	0.021322174
**CG9449**	FBgn0036875	0.488850769	0.358740631	0.852398075	0.142467146	0.910079084	0.475288192	0.623534407	0.66333767	3L	0.57455342	0.438909861	0.967112302
**CG9451**	FBgn0036876	0.23311331	2.178511447	1.696588916	2.639832404	0.240473869	1.758222439	0.084953866	0.216904532	3L	-1.493447616	0.1409111	0.8037297
**CG9452**	FBgn0036877	0.666294197	1.828824618	0.808210215	0.564887214	1.640225176	2.569819497	0.386302457	1.052062822	3L	0.588370066	0.448101044	0.967112302
**Cpr76Bc**	FBgn0036880	0.684512768	0.275561794	0.298911317	1.819343954	1.903010317	1.742457032	0.104772407	3.120895226	3L	1.182210263	0.129606621	0.789029778
**Cpr76Bd**	FBgn0036881	0.234272211	1.178876309	1.970249207	0.218478737	0.394726626	0.161971634	0.438264526	0.033908444	3L	-1.764658195	0.0628729	0.636110045
**CG9300**	FBgn0036886	39.23101624	36.70939231	37.06678632	40.26897305	34.17846292	38.65690792	37.56499242	22.51546676	3L	-0.17868266	0.349035229	0.931729531
**CG9231**	FBgn0036887	209.0260082	190.4735354	244.5413192	178.2017414	225.4450452	165.8390182	222.9075551	227.5273721	3L	0.067131108	0.674806383	0.995787491
**CG9330**	FBgn0036888	139.5347806	141.0705483	136.7566339	110.1594304	145.7539215	129.5238181	127.1051327	150.5502635	3L	0.100220637	0.410763871	0.958665466
**CG14100**	FBgn0036889	75.52251996	80.14962434	91.31884835	77.05147185	93.0455009	81.38194371	79.59001234	90.53120137	3L	0.121764001	0.397340306	0.952272708

**CG9368**	FBgn0036890	671.3523535	530.6851459	676.790749	522.7727937	588.0623853	778.4432514	613.3178261	626.0979412	3L	0.144460315	0.235549627	0.870926212
**CG9372**	FBgn0036891	3.44868941	1.076221894	1.459268443	0	1.520245742	0.037807015	3.88734732	1.915387522	3L	0.318704103	0.774528812	0.995787491
**Lon**	FBgn0036892	39.75821363	40.05344934	52.79984096	42.97540151	46.38465129	46.13535562	33.07859365	44.87016437	3L	-0.010284947	0.946644529	0.997604378
**PIG-F**	FBgn0036893	163.602934	180.5586007	206.2733807	172.0652463	177.8422872	211.1869158	176.7292646	262.0783623	3L	0.22966102	0.140327635	0.8037297
**wnd**	FBgn0036896	11.63335496	23.68927379	24.86079341	17.73272671	13.30441709	10.55153685	20.39318413	17.68657515	3L	-0.28930896	0.213275657	0.85701028
**Rnf146**	FBgn0036897	41.12397721	40.17556095	29.89614108	32.79976467	29.77378615	33.28257578	33.36733102	34.90221695	3L	-0.104795621	0.4648728	0.971928234
**tey**	FBgn0036899	1.095627169	0.8547725	0.757214159	0.103958689	0.885449792	1.135044558	0.861240278	0.152126675	3L	0.130989178	0.840487588	0.995787491
**CG8765**	FBgn0036900	64.5828889	84.56795196	73.3500513	69.95942717	70.30149963	67.56596737	72.79152	74.46188921	3L	-0.003245034	0.978687161	0.997757033
**CG14102**	FBgn0036906	0.710598734	0.627331415	0.5443903	1.04635844	0.945230513	0.987292004	1.717344603	1.055583326	3L	0.706441601	0.372390529	NA
**TORIP**	FBgn0036908	89.26455568	95.27210213	108.8253247	108.3472197	80.63096003	106.9775133	94.51111266	73.84920486	3L	-0.144510612	0.358902427	0.93823489
**Ccdc58**	FBgn0036909	15.38450851	17.17591139	17.52122201	15.33922033	24.82600863	22.94590019	18.67665381	12.78052045	3L	0.308639609	0.243016222	0.876959115
**Cyp305a1**	FBgn0036910	2.065148908	3.463997582	6.58460093	8.32275499	4.367229886	2.837071389	2.859901298	5.316303489	3L	-0.369783736	0.440456017	0.967112302
**Fibp**	FBgn0036911	183.9141921	171.383476	186.4638019	171.9019026	184.1189074	198.1264379	191.0255388	203.8441594	3L	0.152639733	0.13725445	0.798609078
**Usp32**	FBgn0036913	14.49333855	20.77520788	24.06559051	17.0376331	8.359025549	5.938301424	12.53878837	10.9717135	3L	-0.976204009	1.34E-05	0.002110205
**Prp3**	FBgn0036915	209.746632	203.2345434	186.6804247	203.871358	237.6738871	228.4641565	211.9419337	177.4759334	3L	0.118758498	0.303517871	0.910208378
**Mtr3**	FBgn0036916	65.92243581	37.26521002	53.57631682	65.36305639	57.77265389	53.79793967	51.44605091	51.24891138	3L	-0.027695422	0.897341115	0.996479182
**Pfdn6**	FBgn0036918	290.0902111	325.009488	335.9675028	350.6772055	362.0914005	335.405103	348.720584	371.8594665	3L	0.155736914	0.165395166	0.828893393
**Grasp65**	FBgn0036919	166.0959191	167.9426908	177.0892865	164.7606688	169.7490983	141.9226569	156.3188816	163.3696366	3L	-0.066476271	0.548398651	0.984934163
**CG8004**	FBgn0036920	278.9054774	196.2630681	207.2560989	217.623444	204.4447894	233.864874	220.6286814	260.7598554	3L	0.057677363	0.692447831	0.995787491
**RhoGDI**	FBgn0036921	230.3544698	195.7788226	239.193162	188.9245522	211.7150582	223.4960758	205.8188492	190.2570308	3L	-0.010434943	0.922983794	0.996479182
**CG14182**	FBgn0036922	90.24375911	99.68027901	113.002349	94.81778374	90.0646301	101.8002907	93.6939878	84.53118958	3L	-0.071318625	0.623515469	0.990047511
**Gabat**	FBgn0036927	697.7839964	681.1811047	770.6115792	628.3329622	547.6041498	581.4515407	647.2073935	520.5082582	3L	-0.243682828	0.016517437	0.370938755
**Tom20**	FBgn0036928	780.8620424	713.1463144	802.0566939	641.1430692	723.7015722	662.2328821	747.5042586	729.161418	3L	-0.00516131	0.960588273	0.997604378
**CG7668**	FBgn0036929	8.917798408	9.76448225	18.42935017	6.256704781	9.689159843	15.491866	11.4800694	10.21849865	3L	0.149410339	0.670263778	0.995787491
**CG14183**	FBgn0036931	3.329354423	2.676879744	2.202809289	2.437735959	1.589510887	2.049361207	1.403843746	1.493457985	3L	-0.676992407	0.149179265	0.808039863
**CG14184**	FBgn0036932	138.54592	154.7259395	181.9650641	172.3529212	155.5869438	134.9039928	166.505687	221.5649997	3L	0.102488062	0.551609158	0.984934163
**sNPF-R**	FBgn0036934	0.613508036	0.524065292	0.172264113	0.185418449	0.364052252	0.393641247	0.38402234	0.172664429	3L	-0.169021318	0.743543033	0.995787491
**CG14186**	FBgn0036935	401.5690041	438.0455483	480.2398323	490.8902347	314.2882404	406.3108012	453.972854	386.8201704	3L	-0.183832506	0.124701221	0.780439453
**CG14185**	FBgn0036936	19.8931856	9.136483888	9.048860775	4.831272223	8.12301992	7.814655781	5.135208605	6.298534001	3L	-0.630320875	0.170807048	0.832459611
**CG7365**	FBgn0036939	2.163348983	4.288480692	0.954228083	1.703090059	4.666191462	1.456851576	7.883928191	0.060997804	3L	0.664119672	0.411604193	0.958665466
**CG7328**	FBgn0036942	0.310015196	3.640048999	1.805022097	2.891155031	2.014764783	3.098172587	2.056224103	0.134614337	3L	-0.199317561	0.835761273	0.995787491
**obst-F**	FBgn0036947	1.449677751	4.98484367	0.879224501	1.858928007	1.962779918	2.391807826	0.369815909	2.675276904	3L	-0.263734722	0.768752823	0.995787491
**CG7298**	FBgn0036948	1.178479533	5.391092977	4.418415768	0.874230212	4.448320433	0.151508632	0.136651209	0.232598923	3L	-1.192224426	0.272763478	0.89549671
**CG7290**	FBgn0036949	0	2.091104717	1.451707467	2.906551222	0.630153675	0.987292004	0	0	3L	-1.935925921	0.188883514	NA
**CG6933**	FBgn0036952	1.624092106	2.875530374	1.203896465	0	0.325766945	0.935723627	0.11508594	2.154810962	3L	-0.648237372	0.576580818	NA
**CG17145**	FBgn0036953	0.501045528	0.735379386	0.303882366	0	0	0.354286997	1.118405668	0	3L	-0.057770075	0.977260297	NA
**CG17233**	FBgn0036958	152.2715024	149.6336761	150.343764	182.6546306	137.5430581	149.7906148	144.1714401	152.3406248	3L	-0.0921272	0.37488398	0.943282319
**CG6951**	FBgn0036959	1.563197017	1.760001936	1.350680111	3.994010955	0.773142811	1.675659256	0.983280618	2.464024762	3L	-0.52577459	0.358226623	0.93823489
**FRG1**	FBgn0036964	47.27325981	61.76998118	102.1013554	56.31717736	53.50492163	71.60370551	75.04115245	77.53900992	3L	0.091109074	0.700626935	0.995787491
**SCCRO4**	FBgn0036967	47.5581418	31.8599398	44.6023861	36.80672769	39.52161615	41.65216947	39.70413673	33.41856567	3L	-0.033515106	0.814004313	0.995787491
**Rbbp5**	FBgn0036973	30.27319465	35.6968069	24.19501476	27.37180712	29.13295687	40.46138991	40.96767329	27.36448307	3L	0.25760997	0.207667379	0.856358208
**eRF1**	FBgn0036974	249.3688746	228.1773752	245.2459556	254.721342	226.2775545	276.6601995	251.1184396	249.4693004	3L	0.065768887	0.487264486	0.973549494
**CG5618**	FBgn0036975	24.05608833	21.13391439	6.53576215	7.423129103	15.42557144	16.31723696	12.21798334	9.630275024	3L	-0.118618066	0.748938678	0.995787491
**Toll-9**	FBgn0036978	0.195206044	0.506029348	0.817979607	0	0.768866053	0.117115769	0.407433938	0.15411287	3L	-0.014047578	0.990369894	NA
**RhoBTB**	FBgn0036980	232.6704532	224.2836987	189.0734013	244.5279844	182.0992769	224.1226395	185.2861207	189.9807815	3L	-0.161806032	0.142484781	0.8037297
**CG13248**	FBgn0036984	540.7699887	440.9464149	473.9103845	469.149597	531.5071611	586.3334202	540.8314719	533.323774	3L	0.215190434	0.026408198	0.453785402
**zye**	FBgn0036985	0.631127806	0.488597703	0.628146566	0.282971292	1.110133409	1.41890205	0.55731482	0.614849886	3L	0.891940076	0.083170346	0.69799888
**CG5282**	FBgn0036986	520.0776146	597.0385067	574.1384038	659.056519	523.0309941	548.0901593	539.7341939	533.7965226	3L	-0.101676888	0.307802848	0.912131082
**CG5274**	FBgn0036987	97.01007334	102.3071058	61.02069759	83.14662309	70.3496715	96.86018137	84.65053383	115.2437289	3L	0.124548208	0.529320525	0.984296327
**CG5262**	FBgn0036988	70.79740412	59.95451019	44.48533103	32.38548074	54.50795261	54.8537215	42.11788208	43.32851447	3L	-0.064594455	0.764361134	0.995787491
**mRpL15**	FBgn0036990	162.832844	164.988048	160.0388598	146.0855389	160.0674706	154.0379581	169.4936303	143.9083612	3L	0.014630235	0.905319519	0.996479182
**CtIP**	FBgn0036991	29.82609402	34.67769698	20.6169851	36.48385355	27.73321612	31.85633371	33.04052206	28.20843442	3L	0.017898647	0.932807779	0.996479182
**Hpd**	FBgn0036992	6.498907059	10.47518438	5.296477256	8.759725229	4.399572337	4.308138196	5.958024769	6.283238616	3L	-0.539024589	0.184203962	0.839299945
**CG5910**	FBgn0036993	2.526055932	2.973094303	2.291629012	1.26201521	1.781918443	1.541003008	1.353786335	2.719107349	3L	-0.258639269	0.495289081	0.97491773
**CG5199**	FBgn0036994	30.90325059	13.89350777	20.00424253	21.82276548	22.99925805	28.01480374	25.39392002	12.338166	3L	0.052287965	0.858802994	0.995787491
**atk**	FBgn0036995	0.732320991	0.485632963	0.764351383	0.27794539	1.214420226	0.216762203	0.152060071	0.794968331	3L	0.111385189	0.874214463	0.995787491
**CG5955**	FBgn0036997	7.261801561	9.32752737	7.062682917	9.685533129	0.826719739	4.039982263	6.759125926	6.107566364	3L	-0.883668865	0.068715195	0.659001649
**CG5969**	FBgn0036998	86.53231485	73.36566217	80.05129744	88.75257963	85.31991409	85.79491958	77.38159421	80.59552691	3L	0.030007503	0.850533927	0.995787491
**isoQC**	FBgn0036999	16.93443037	29.09235219	27.83021062	36.13875413	30.44771696	25.46139297	24.73553866	32.54106611	3L	0.077991124	0.738722595	0.995787491
**ZnT77C**	FBgn0037000	0.179300094	0.683524444	0.533838512	0.380028564	0.750355176	0.983523106	1.143495983	1.114748067	3L	1.218831116	0.018474479	0.391771766
**ND-39**	FBgn0037001	450.4945343	420.7076607	418.2108153	418.1865013	461.7892978	404.2832178	441.7974851	456.9067086	3L	0.07888947	0.401789763	0.954265
**CG5059**	FBgn0037007	30.02284135	39.45099528	43.16634743	29.18652659	29.67570416	41.96413113	30.36828752	32.15977229	3L	-0.04712086	0.774900088	0.995787491
**mTerf3**	FBgn0037008	100.9080577	103.4975827	106.4895591	110.0287506	122.6338915	109.3678266	94.40552268	101.6649159	3L	0.055818113	0.694344933	0.995787491
**CG5104**	FBgn0037009	10.63732841	14.07018519	8.552849148	16.29749163	7.022849503	9.283811068	10.544295	9.237849868	3L	-0.430148718	0.223294673	0.86549481
**CG4858**	FBgn0037011	99.27983328	75.14997711	115.3062729	93.40435487	88.85085845	99.90574629	85.13943993	114.4690092	3L	0.04959917	0.775108432	0.995787491
**Rcd2**	FBgn0037012	1.726100837	0.817219518	1.148184539	0.308316304	0.502589421	0.885859835	0.55041504	0.105776907	3L	-0.948425466	0.126788614	0.784451606
**CG13251**	FBgn0037014	1.05034251	2.662278898	0.226499225	0.163255601	0.210681937	0.308079801	0.535889262	0.810805624	3L	-1.1047583	0.253946939	0.884040848
**cmpy**	FBgn0037015	168.8524807	190.4390036	181.3327979	184.6156543	174.2120921	163.5188471	169.9784246	165.2068951	3L	-0.076380152	0.379451585	0.944208584
**CG13252**	FBgn0037016	3.99575438	4.269856427	2.627249415	2.53939472	3.089835216	2.540787901	3.127836897	4.553779502	3L	0.017486116	0.947739576	0.997604378
**CG4074**	FBgn0037017	64.24354027	56.85747688	61.59399423	46.23075403	53.93672662	70.48420623	53.66048859	35.6332376	3L	-0.072452669	0.737093963	0.995787491
**CG4042**	FBgn0037018	67.8213243	52.93922874	49.76062597	39.82682634	51.47308792	66.57582554	58.48120748	64.52369163	3L	0.221206671	0.26575693	0.893759496
**Pex16**	FBgn0037019	31.74460404	60.03637023	41.66221146	47.58835391	46.24237859	55.98668761	43.95699043	51.84727821	3L	0.165586278	0.430228194	0.963666972
**Pex14**	FBgn0037020	143.2300439	125.8750218	143.9934753	147.0799593	131.7097008	174.7043259	143.8581544	178.7665614	3L	0.195739254	0.15608877	0.818884898
**CG11399**	FBgn0037021	124.7646425	126.8274216	131.859766	194.0378401	117.8577671	127.3900492	125.6934511	149.3321487	3L	-0.121926511	0.419536621	0.960266449
**CG3698**	FBgn0037023	3.513614903	0.64060827	5.744421807	0.356167864	4.412504648	2.088387508	0.389709005	0.331668835	3L	-0.467548328	0.666329175	0.995787491
**tzn**	FBgn0037024	179.9319128	170.8876986	196.9646565	222.3387813	191.1045085	192.2723677	166.954757	202.5926093	3L	-0.002593965	0.984206342	0.997881056
**Spc105R**	FBgn0037025	0.147881531	0.293150617	0.554445002	0.689560443	0.097077842	1.064675593	0.03429536	0.204313768	3L	-0.227541533	0.825681746	0.995787491
**CG3634**	FBgn0037026	10.55704907	16.19704997	28.75802901	19.82509743	6.569543045	11.61763656	37.40963281	22.13806694	3L	0.084732185	0.839394422	0.995787491
**HIPP1**	FBgn0037027	160.8219188	158.0221632	180.6481111	169.0335929	167.3817776	166.0238875	157.7459704	145.9579198	3L	-0.039422726	0.687366084	0.995787491
**CG3618**	FBgn0037028	525.5964597	495.9180672	508.3529421	549.8275324	499.493358	551.481292	486.6587834	507.2405719	3L	0.004008861	0.96384228	0.997604378
**CG11456**	FBgn0037031	70.71553331	56.80695252	51.08245776	69.00362595	52.36637874	69.20370884	50.52238546	50.83032806	3L	-0.127268937	0.446895505	0.967112302
**CG12983**	FBgn0037040	0.06327118	0.28300941	0.051165	0.344200208	0.076147131	0.715820186	0	1.327886694	3L	1.561794384	0.245010678	NA
**Pdss2**	FBgn0037044	94.55630589	79.49827767	71.08328652	74.93637457	82.82031867	65.30117349	70.57777132	68.00775593	3L	-0.130973809	0.383942192	0.946594947
**CG10584**	FBgn0037045	4.651720599	1.479805105	7.004488526	2.056869415	3.619632719	5.141754072	2.864361183	2.089513661	3L	-0.114617615	0.872037434	0.995787491
**CG10581**	FBgn0037046	5.472030927	14.77524974	6.151862034	12.44666742	19.75683659	10.44382387	13.61879849	17.67554699	3L	0.704200482	0.164847951	0.827712868
**ICA69**	FBgn0037050	495.9638067	544.9932245	507.7502627	525.2360104	492.5092873	530.2409548	476.5447008	503.7364558	3L	-0.019758113	0.81351456	0.995787491
**CG10565**	FBgn0037051	116.2074627	96.73511072	101.3088769	117.7320163	116.1044729	98.84846998	113.0104621	110.179254	3L	0.049159601	0.697485602	0.995787491
**CG10512**	FBgn0037057	6.029112226	13.3997117	11.41682875	10.19106993	6.772324419	8.889473142	10.33904318	7.381170645	3L	-0.260952377	0.319598683	0.916248855
**CG10508**	FBgn0037060	454.3450541	263.1202094	279.6994527	356.625707	388.0266122	346.0609038	338.1870167	346.7246451	3L	0.093260865	0.546139228	0.984934163
**CG12975**	FBgn0037061	180.1125842	193.490415	235.2811016	154.3316711	192.3105717	194.7724251	229.0810188	196.5232161	3L	0.123626398	0.416729829	0.958846438
**CG9391**	FBgn0037063	305.8068938	294.0914512	306.631537	362.7727873	331.7272004	318.2642515	294.7980256	313.0482853	3L	0.016100613	0.884577591	0.995787491
**CG9389**	FBgn0037064	2.414859682	0.796837052	2.208910915	2.122845117	1.143459843	2.314038154	2.726716248	1.633029415	3L	0.069273161	0.914652904	0.996479182
**CG12974**	FBgn0037065	0.534934534	0.759234923	0.648871859	0.623589864	0	0.213371658	0.05248575	0.9380477	3L	-1.055204968	0.353752205	NA
**Cpr78Cb**	FBgn0037068	0	0.526890139	0.666791745	0.640811538	0.354415534	0.22211193	0	0	3L	-1.591856922	0.395122694	NA

**CG11309**	FBgn0037070	0.271341528	0.252854082	0.511988371	0.281165582	0.435414225	0.17054609	0.461465379	0.458194781	3L	0.246476712	0.825702888	NA
**CG7632**	FBgn0037071	89.73650249	105.7710878	102.1254427	107.1666363	108.3109938	82.80643706	117.848003	102.5533305	3L	0.056593142	0.715111779	0.995787491
**Tsr1**	FBgn0037073	50.01292142	58.270085	75.29186705	54.35237298	57.5820339	55.16390641	60.05677977	63.57883793	3L	0.025504758	0.866005671	0.995787491
**CG7324**	FBgn0037074	128.4992797	123.6469284	114.9386079	100.0533662	110.7143387	121.3919047	107.0224874	105.9471715	3L	-0.040477571	0.69726658	0.995787491
**CG12971**	FBgn0037078	1.30393058	3.645267707	1.355707457	5.211540102	1.120918519	0	1.900774241	4.853063986	3L	-0.508699142	0.624766249	0.990047511
**barc**	FBgn0037081	17.03007403	19.26408483	19.81655863	16.27175214	19.13810515	16.51715112	19.22565441	14.90167831	3L	-0.020516315	0.877207574	0.995787491
**CG5664**	FBgn0037082	7.790891564	16.89294549	17.90178238	18.9025513	12.97826921	18.69145657	16.40069328	20.49022171	3L	0.195395876	0.495234982	0.97491773
**Syx6**	FBgn0037084	301.8237951	262.8162409	265.9371604	309.6843852	267.8621749	274.1727208	264.2413888	294.0736482	2R	-0.022780637	0.830430524	0.995787491
**Neu2**	FBgn0037085	0.167304776	0.408189516	0.221388575	0.106381306	0.27457091	1.132816344	0.232798952	0.165106435	3L	1.034783581	0.293860511	NA
**CG7202**	FBgn0037086	1.702105264	1.691877214	0.152936431	0.293955137	0	0.6537807	0.964912688	0.273735414	3L	-1.020296191	0.473991926	NA
**CG7519**	FBgn0037087	93.13563791	80.05053387	115.0130216	96.07195891	111.2035512	86.12291248	96.26994781	95.85390336	3L	0.05122067	0.779940436	0.995787491
**M6**	FBgn0037092	98.09777218	80.16611403	70.97462133	73.0341952	79.98819161	92.86953208	62.58494236	68.97059181	3L	-0.056870027	0.696532642	0.995787491
**Cdk12**	FBgn0037093	40.04973446	50.32071374	48.72830917	42.65018031	46.90063332	44.80077956	45.14227066	51.51054137	3L	0.085590548	0.473122347	0.973549494
**CG7611**	FBgn0037094	141.1266534	143.7135729	130.6744544	175.3698427	131.2650315	157.6326924	129.0183456	160.1708573	3L	-0.003514137	0.977658798	0.997757033
**Wnk**	FBgn0037098	95.68040007	123.1646483	116.2588288	93.93697536	100.895416	104.935456	100.5093576	123.5317911	3L	0.037524298	0.746521161	0.995787491
**CG7173**	FBgn0037099	0.42504572	2.138863702	1.178462843	1.557251292	0.8769331	0.658342978	0.697050491	0.395491545	3L	-0.965479208	0.217405608	0.860826301
**CG7634**	FBgn0037101	2.166374096	1.416561671	1.692479211	1.112892466	0.198857313	1.869355768	1.779709668	0.318874488	3L	-0.599738325	0.476311087	0.973549494
**CRIF**	FBgn0037102	183.9734371	166.2435727	195.1071476	192.5598846	174.6328261	160.892958	206.2164474	128.2099681	3L	-0.110844005	0.499827183	0.976796212
**S1P**	FBgn0037105	14.71469499	20.58172885	16.96812392	18.45116723	12.62654109	19.00681933	17.47222592	14.87005111	3L	-0.112617539	0.574490722	0.984942538
**CG11307**	FBgn0037106	22.79022285	24.15041931	30.35278361	20.01520929	35.23495274	18.6554936	37.31853427	38.94853463	3L	0.457227509	0.070910839	0.6662581
**CG7166**	FBgn0037107	175.015793	146.5249332	158.1126532	138.2553203	138.1754889	153.8252094	127.4843259	146.262256	3L	-0.098738191	0.361922418	0.939549067
**Alg11**	FBgn0037108	152.9054817	107.1940361	110.5011897	116.0557206	131.4284058	121.5053205	109.7233102	127.9326465	3L	0.038460655	0.787158085	0.995787491
**MED1**	FBgn0037109	81.50973614	88.70259183	73.84408505	94.29260536	72.37231206	99.10045742	73.71516768	65.83965034	3L	-0.095590748	0.473802311	0.973549494
**ORMDL**	FBgn0037110	82.77261529	131.774611	128.1688122	109.9395319	110.2511047	89.60502402	105.1102035	88.85955028	3L	-0.164425548	0.36817086	0.941496148
**CG11249**	FBgn0037115	4.25991372	1.404652788	1.943237674	4.668808256	2.892054591	5.286307503	1.393221977	1.976210902	3L	-0.071970961	0.904595124	0.996479182
**Als2**	FBgn0037116	65.96946409	53.30821543	54.95407338	67.16504757	48.41841103	54.56396451	54.11808474	59.10739538	3L	-0.131809346	0.320304625	0.916560752
**CG11248**	FBgn0037117	58.73484937	81.52862968	78.01604165	72.62734131	75.9965842	63.30755583	66.68544651	64.73485421	3L	-0.068979878	0.614440069	0.990047511
**CG11247**	FBgn0037120	220.0644803	216.1836708	210.275371	215.2901175	193.5055652	232.1566776	199.2630854	191.2464691	3L	-0.050365579	0.579577771	0.985637351
**Rpb8**	FBgn0037121	111.5124658	103.9147321	111.409975	109.7193334	100.5516869	103.4185598	118.3119715	90.32622044	3L	-0.053554108	0.74062345	0.995787491
**CG14570**	FBgn0037122	0.88953269	3.170640883	0	3.110867968	4.616774159	0.62898449	5.95669463	0.72422168	3L	0.767411593	0.516566666	NA
**Syn1**	FBgn0037130	19.11622533	20.86288739	21.25135387	27.04126873	19.43499065	18.87578498	22.89961071	23.50796243	3L	-0.027978363	0.839832779	0.995787491
**CG7407**	FBgn0037134	265.0830455	230.893227	218.2750073	222.5057656	254.2743071	246.5344373	265.5561418	241.5929578	3L	0.133347935	0.261583879	0.890640489
**CG7414**	FBgn0037135	492.4397374	509.9136572	410.760651	493.6558607	476.6819319	517.4969889	434.0586647	407.4953983	3L	-0.026975579	0.798569628	0.995787491
**Nopp140**	FBgn0037137	31.11701231	41.4954949	41.58635223	34.90572564	30.38603463	47.64901942	32.54304352	43.94870716	3L	0.084237035	0.625803398	0.990047511
**P5CDh1**	FBgn0037138	526.5968338	469.0409486	460.8090394	436.6983976	474.2711155	492.6424141	474.6202081	434.8825362	3L	0.016086404	0.861701755	0.995787491
**CG14563**	FBgn0037139	5.844483703	20.10933643	30.90215526	23.40910206	19.47838341	16.10657341	29.43651458	11.06213114	3L	-0.034764463	0.946596769	0.997604378
**SLC22A**	FBgn0037140	19.40759777	21.83783414	23.67488619	15.20253432	9.305733273	14.14359286	20.84893618	6.600142336	3L	-0.625928607	0.032553803	0.505711525
**DNApol-eta**	FBgn0037141	9.212387359	17.82979318	14.01779327	18.23752056	17.71319322	7.734615805	21.69322899	27.10187322	3L	0.367128521	0.272566705	0.89549671
**CG14562**	FBgn0037142	81.23647106	119.3487145	104.6962595	107.7756037	90.22103816	113.3884066	94.51348178	85.33251275	3L	-0.076765302	0.539322059	0.984934163
**CG7470**	FBgn0037146	0.63912225	0.126800184	0.900291289	1.40997713	1.104154544	0.233249247	0.175313397	0.14920361	3L	-0.858597914	0.257200246	0.88684603
**CG7140**	FBgn0037147	3.307617972	0.970911399	2.40727052	5.01253141	2.761632906	1.403280909	0.527362782	1.16693603	3L	-0.968286674	0.176314405	0.834204539
**CG14561**	FBgn0037149	9.330154802	10.98831598	6.581757744	14.0391087	18.396251	19.27724542	16.88044351	11.92411539	3L	0.727296868	0.035066186	0.514749796
**CG7133**	FBgn0037150	70.306631	43.63691454	65.71221195	77.03139815	72.21950906	71.72887246	51.93816626	54.67201722	3L	-0.009646742	0.963357946	0.997604378
**CG7130**	FBgn0037151	0	2.075570796	1.801154192	0.57699194	0	2.916541176	2.525314349	7.790900934	3L	1.641986237	0.241638633	NA
**olf413**	FBgn0037153	25.17324332	39.92310551	40.25641696	37.78684089	34.73773312	33.99704691	33.81826529	35.44714686	3L	-0.01878272	0.884320096	0.995787491
**CG11523**	FBgn0037156	97.3339049	144.2913993	172.8255245	164.2411192	146.1585302	145.0853954	156.1688527	145.8351287	3L	0.071143665	0.694478881	0.995787491
**CG11449**	FBgn0037162	0.375202797	1.888050167	1.430372038	0.124967314	1.161149506	0.656943681	0.957150395	0	3L	-0.409381075	0.748671162	NA
**laza**	FBgn0037163	0.336744799	1.569006002	0.635396367	0.34893681	0.06754563	0.98771848	0.286347739	1.543442056	3L	0.046894862	0.966350303	NA
**CG11438**	FBgn0037164	0.669819563	1.06439528	1.368398247	0.164385168	0.254567576	0.664738729	4.226843818	1.148084429	3L	0.982849283	0.303520253	0.910208378
**CG11426**	FBgn0037166	13.93283667	17.22027921	15.29933999	12.58323093	8.631224434	4.286416763	7.782038801	3.629937378	3L	-1.248581616	7.31E-05	0.007165591
**CG11425**	FBgn0037167	9.46979817	10.78380815	8.852212179	12.76095514	5.803030028	10.15670106	8.643565915	11.50594784	3L	-0.183430062	0.659654401	0.995750687
**CG14459**	FBgn0037171	0.179112449	0.372181918	0.402097116	0.115305787	0.330582918	0.480134275	0.088656062	0.174120764	3L	0.044260889	0.930128187	0.996479182
**CG14457**	FBgn0037174	0.137516564	0.129748919	0.333613266	0.060115255	0.093094741	0.421361773	0.021925482	0.018660074	3L	-0.202709496	0.865075399	NA
**ArfGAP3**	FBgn0037182	139.5339263	139.8148747	122.7716877	181.2822409	126.282598	157.8720879	138.2385237	145.0752407	3L	-0.013094487	0.923143187	0.996479182
**CG14450**	FBgn0037184	112.2416495	118.567816	110.6754232	133.6357859	117.6531937	148.2015547	108.8483564	93.12109111	3L	0.0050491	0.973630902	0.997757033
**CG11367**	FBgn0037185	9.968810277	12.93584423	10.2408642	12.56728461	13.68770728	6.995325376	7.538075166	10.39861063	3L	-0.207900561	0.402269085	0.954587287
**CG11241**	FBgn0037186	3.920601098	2.653180088	2.131848389	1.481429282	1.635190408	3.071768355	2.103771709	2.817761515	3L	-0.062217144	0.87428254	0.995787491
**CG7369**	FBgn0037188	8.051014182	8.17934951	7.30126278	5.50684288	8.023581104	7.758059388	5.928250406	6.313572283	3L	-0.021346686	0.920948742	0.996479182
**CG14448**	FBgn0037191	1.822413584	4.31554324	0.520135287	0	0.193524918	0.404273034	2.187772332	3.491142873	3L	-0.053689159	0.968891803	NA
**CG11226**	FBgn0037195	1.493418028	2.636841302	0.762739222	0.366510307	1.702738295	2.223134834	1.203076255	0	3L	-0.006924944	0.995418096	NA
**CG13239**	FBgn0037197	10.96589454	3.100070179	7.173871606	11.20332856	4.671025324	4.530388284	20.11630207	4.547590186	3L	0.072280639	0.916272999	0.996479182
**EMC4**	FBgn0037199	263.828795	236.1641977	296.7069859	256.7830234	302.4874192	231.9141786	285.1027881	264.476083	3L	0.072821485	0.588355207	0.987588562
**CG11109**	FBgn0037200	0.980366296	0.580385975	0.139903233	0.134452183	1.561599202	0.761174528	0.147113852	0.876427168	3L	0.85727537	0.508855652	NA
**Ssl1**	FBgn0037202	16.17736712	17.67668618	13.90432087	17.78083486	21.19397327	24.83884587	18.62992043	16.25672848	3L	0.333435789	0.158302127	0.818884898
**slif**	FBgn0037203	0.908488174	0.682326029	0.361847404	0.30032846	0.660917283	0.57527268	1.158786426	0.426865267	3L	0.335409926	0.531922275	0.984760433
**BoYb**	FBgn0037205	0.559926827	0.040834726	0	1.078413828	1.01081143	0.527895592	0	0.052854477	3L	-0.06683543	0.964797412	NA
**CG12768**	FBgn0037206	5.745324935	5.269699338	6.187539899	6.612333825	5.659516831	8.059234553	5.811747328	6.80642131	3L	0.174877911	0.326152925	0.918470465
**Mes2**	FBgn0037207	0.091993282	2.402762127	1.721631226	2.359267604	0.521938846	1.06554865	1.005757877	0.19972593	3L	-1.199093884	0.11321249	0.758410631
**CG12581**	FBgn0037213	0.235861218	0.179829139	0.710910214	0.816520486	0.812871723	1.70482529	0.455823512	0.729321131	3R	0.966214018	0.108890958	0.758410631
**beta-Man**	FBgn0037215	0.623237445	1.972440344	0.770246498	0.411241879	0.700536031	1.286033904	3.719747582	0.842500078	3R	0.832497448	0.183743038	0.839299945
**aux**	FBgn0037218	39.09518783	50.59063664	52.77738542	53.00551948	50.55257038	53.30551278	47.7177686	58.22751509	3R	0.134668751	0.261443687	0.890640489
**CG14641**	FBgn0037220	370.7460418	371.2655683	326.5624305	409.8377809	343.2495623	377.0193931	366.7199133	308.3737147	3R	-0.055701022	0.617921731	0.990047511
**CG14642**	FBgn0037222	1.761375809	0.695167252	0.125678622	0.483127222	0.187043222	0.048841599	1.585873246	1.5746335	3R	0.13749088	0.914902316	NA
**TwdlG**	FBgn0037225	1.455768834	1.109930907	0.160530677	0.771379599	0.298640438	0.436701352	0	0.143664041	3R	-1.933549962	0.104752666	NA
**CG1092**	FBgn0037228	0.840409887	0.187955958	0.815529065	0.914379206	1.11258051	1.479022174	2.143903676	1.94624817	3R	1.30003649	0.155799113	NA
**CG9766**	FBgn0037229	3.780284064	3.237194374	2.775408914	3.710985257	4.741149332	6.349598544	5.760773661	5.874732332	3R	0.774646001	0.000553046	0.038731776
**Vps24**	FBgn0037231	7.226408307	6.329545338	4.726833088	4.140147176	3.3392967	5.510867665	3.775028569	3.694726604	3R	-0.433437193	0.202590627	0.849847813
**Suv3**	FBgn0037232	15.89015435	31.71956099	34.44830423	33.52648976	29.86578453	23.86357552	19.77932915	20.50365864	3R	-0.261360464	0.263744795	0.892790059
**CG9795**	FBgn0037234	58.2888015	65.11576421	48.67366355	55.37261358	49.1335951	60.86929453	45.3743851	48.27787611	3R	-0.130879355	0.299735284	0.9074568
**CG1103**	FBgn0037235	50.10939732	46.63545129	62.58294751	43.21426432	53.30567218	41.56915473	58.74660176	64.71782236	3R	0.14238715	0.450573968	0.967112302
**Skp2**	FBgn0037236	1.611502245	2.339194278	1.879558638	0.361265079	0.167836995	0.262958274	0.237171741	2.153058799	3R	-1.094389443	0.251449406	0.882557414
**CG1090**	FBgn0037238	211.5264272	198.7421555	240.5155699	263.8753299	240.4059126	217.1124788	219.0951066	227.1132318	3R	0.012277625	0.913936968	0.996479182
**Sfxn1-3**	FBgn0037239	181.1682004	147.1269552	175.5952813	156.2189097	165.2948966	139.108957	143.2462795	154.9842719	3R	-0.101381077	0.404570145	0.956706394
**Cont**	FBgn0037240	17.95448229	16.63508947	25.16318357	16.58144729	21.21591447	23.68974867	20.03849315	14.51904713	3R	0.088387913	0.645478547	0.99279236
**CG14646**	FBgn0037241	98.03497716	86.42462697	90.43617564	74.76096137	81.90285042	98.18297697	82.56113675	83.95174942	3R	0.015272845	0.91203579	0.996479182
**CG9855**	FBgn0037242	9.365369387	7.279823732	6.982322423	9.373075816	6.762756827	10.03560898	6.409849845	6.943004252	3R	-0.103351615	0.760260535	0.995787491
**srl**	FBgn0037248	5.3725561	5.216189304	5.244166103	7.10292909	2.578750596	4.886440849	5.095223421	4.885290257	3R	-0.368079347	0.183081882	0.838911159
**eIF3a**	FBgn0037249	304.4852267	340.8303062	312.406685	347.110577	286.7448833	353.9401041	315.5641317	390.5253601	3R	0.076260151	0.497344483	0.97594235
**CG1074**	FBgn0037250	2.508401684	3.352845132	3.423007967	4.294804258	2.547173215	2.660519009	1.999683308	1.957146279	3R	-0.526941024	0.279233183	0.898067854
**CG9804**	FBgn0037251	0.860705241	1.181219152	1.138941224	0.72970977	0.988777718	1.106546787	0.199607051	1.189154116	3R	-0.118462515	0.917437965	NA
**CG14650**	FBgn0037252	120.9490499	134.0826108	133.9401815	146.553903	120.5792331	153.6079555	132.6014306	122.444122	3R	0.011672996	0.915735435	0.996479182
**CG14651**	FBgn0037254	0.738151373	0.341557345	1.975994412	0	0.41355075	0.143984401	0.454526899	0.055261895	3R	-1.486428743	0.22886275	NA
**Fip1**	FBgn0037255	155.1274688	181.9254523	143.7639121	157.3810911	151.0367359	161.7046696	175.0996578	187.7039323	3R	0.113953585	0.336684572	0.924219112
**CG9775**	FBgn0037261	383.1069794	373.3967044	352.8044495	410.4887668	371.5581353	407.3344414	373.2392179	359.1638175	3R	0.020024465	0.826396777	0.995787491

**slx1**	FBgn0037263	5.379210167	7.392231194	7.232468512	6.950669832	6.493401726	12.25100049	6.117250435	2.743805817	3R	0.064215932	0.874525377	0.995787491
**spartin**	FBgn0037265	0.271956815	0.547403287	1.649408601	2.409416892	0.294571052	0.666637983	5.550141427	0.354266052	3R	0.526411984	0.580539494	0.985637351
**eIF3f1**	FBgn0037270	606.1278313	778.1953553	717.0441023	735.5537633	685.4938405	675.4799102	676.6628363	782.0245972	3R	0.024568393	0.820017421	0.995787491
**CG14655**	FBgn0037275	2.068956313	1.19132584	1.423372	0.647958863	0.668954582	0.349361082	1.02408024	0.201129657	3R	-1.245995846	0.130972118	0.790743321
**CG17387**	FBgn0037276	0.384756227	0.36302321	1.108428223	1.738024027	0.08682295	0.090686455	0.061345088	0.052208837	3R	-3.562634664	0.001211537	NA
**CG1129**	FBgn0037279	41.03069047	49.82547926	48.89839403	49.81451914	33.14417324	47.24019979	36.56220786	38.83457609	3R	-0.252319407	0.068486365	0.659001649
**BBS5**	FBgn0037280	1.485223691	3.188805613	6.053261689	7.618034871	4.504431828	5.769070473	3.48573054	6.449116236	3R	0.185638701	0.74899289	0.995787491
**CG14657**	FBgn0037282	129.9273685	144.7007149	135.0778682	123.7306731	122.032373	140.9470061	111.2506314	135.7578574	3R	-0.033799144	0.753033507	0.995787491
**CG14661**	FBgn0037288	0	0.166426066	0.842464256	1.50361588	1.253810921	1.028974435	0	3.554327361	3R	1.276053596	0.35264756	NA
**CG1124**	FBgn0037290	0.398899815	10.3232337	0	0.664299931	0.617242632	0.295491672	0.726858571	3.897217913	3R	-0.959716412	NA	NA
**plh**	FBgn0037292	0.095336213	0.242831038	0.334077354	0.115252558	0.318715367	0.386161418	0.090075825	0.114990968	3R	0.259266085	0.703092491	0.995787491
**CG12007**	FBgn0037293	62.34455802	89.90404389	81.46689139	82.32727867	76.66429006	76.63637136	90.55747258	93.82503024	3R	0.130367654	0.405196099	0.956706394
**dpr16**	FBgn0037295	8.169065794	3.954960019	5.391994238	3.720881765	7.365490074	7.178944739	6.75097373	5.931508616	3R	0.380855949	0.095169338	0.730787365
**CG1116**	FBgn0037297	117.9950295	82.36426893	112.5681683	115.230569	105.1825837	137.0265384	101.3097091	115.0613736	3R	0.124385673	0.425524625	0.962105868
**Sccpdh1**	FBgn0037298	96.16474589	113.5156377	77.72435912	94.04003081	69.65735663	83.59517211	75.86507693	103.0891044	3R	-0.168381477	0.316122619	0.91460807
**Vps37B**	FBgn0037299	217.6853279	215.5983992	187.5543967	227.3381985	217.8637883	167.5049759	196.8979107	173.622089	3R	-0.136292046	0.309415299	0.912131082
**Mms19**	FBgn0037301	27.33852239	21.80319229	36.86474469	29.01137043	26.17965813	23.6158063	37.34869119	27.96991183	3R	0.032375252	0.874648853	0.995787491
**CG1113**	FBgn0037304	4.388657779	12.39807622	16.25041338	21.98980393	3.127362669	1.270315712	4.713920119	4.346188414	3R	-2.002977194	4.85E-05	0.005048487
**CG12173**	FBgn0037305	72.61367885	107.1175745	86.97570231	66.77154097	100.8745113	82.78544362	100.0326622	109.7628114	3R	0.275137716	0.12792072	0.788096052
**CG11999**	FBgn0037312	94.35502098	90.41831226	101.8031002	90.97426313	86.38200771	108.3027834	121.2089594	115.7549995	3R	0.222628772	0.16610735	0.82902792
**CG1161**	FBgn0037313	497.2703269	488.6866265	509.7288118	459.6249501	493.8438032	394.0641636	490.6330468	502.2782022	3R	-0.022866692	0.840807285	0.995787491
**Cerk**	FBgn0037315	52.61555683	43.15726238	42.6818616	41.04025676	59.01309818	58.9986629	47.78634701	57.59508607	3R	0.343610548	0.005045481	0.202158844
**CG14667**	FBgn0037317	69.69214896	96.79261307	90.45434766	98.44573883	95.58246823	100.2900751	90.04554469	92.72035235	3R	0.125496528	0.401400527	0.954265
**CG2663**	FBgn0037323	0.440307377	0.902676935	0.534089777	1.026560074	1.059823554	1.106984281	2.371270887	0.106216453	3R	0.707609729	0.399513794	0.952933016
**Orco**	FBgn0037324	1.293552204	0.723250785	0.40232559	0.115994933	0.119753501	1.532259044	0.676898351	0.252037834	3R	0.02876322	0.975150979	0.997757033
**CG14669**	FBgn0037326	22.90100561	24.8536674	28.84219943	25.89998926	19.93322093	22.42679014	25.5686135	23.09387257	3R	-0.139695032	0.209726162	0.856520545
**PEK**	FBgn0037327	40.88828979	47.57717937	43.69035573	53.89608071	52.99844007	69.35431258	57.79359354	52.1028164	3R	0.348284539	0.00783238	0.258004155
**RpL35A**	FBgn0037328	356.6010167	533.1578389	521.4082213	568.7744135	460.0688522	409.5371034	541.6722026	509.1964391	3R	-0.009024038	0.951444134	0.997604378
**POLDIP2**	FBgn0037329	236.213963	232.723956	197.0893835	195.170428	220.4720718	275.5903046	234.3118851	186.5837534	3R	0.117417068	0.365067041	0.940794966
**mRpL44**	FBgn0037330	123.4660756	145.042232	147.6137735	148.9209802	140.1047811	157.8264004	167.6546078	145.8918745	3R	0.145586085	0.261617553	0.890640489
**Hcs**	FBgn0037332	30.54832544	19.71535134	22.56734868	21.72654517	23.00670841	21.78150991	21.20371266	25.41106664	3R	-0.022126359	0.888315652	0.995926915
**CG2519**	FBgn0037336	109.0561965	115.212386	105.1563141	106.8499714	118.7376171	132.1036122	120.1204803	142.4585666	3R	0.265725603	0.005959695	0.222031582
**Snm1**	FBgn0037338	2.40428143	6.560094227	1.944251071	3.736994429	2.372721492	1.722723803	3.107575948	8.143071371	3R	0.110805947	0.847018757	0.995787491
**Pi4KIIalpha**	FBgn0037339	49.59324834	54.70510105	52.31437164	57.72809657	46.60496214	52.79674872	44.08969472	47.54001192	3R	-0.135670276	0.214636775	0.858753183
**CG14671**	FBgn0037340	86.30815646	91.02294363	73.47190232	78.0798162	82.47579355	78.34985077	85.43277065	78.76818203	3R	0.010431378	0.950561706	0.997604378
**CG12746**	FBgn0037341	72.32792029	65.46527967	59.4427606	60.89846836	59.80928996	66.28378871	40.32396405	67.90512877	3R	-0.11078037	0.519087809	0.981083949
**CG2931**	FBgn0037342	255.5744103	271.6517501	270.7395296	204.668839	260.0515858	281.3820228	309.3113016	282.1688259	3R	0.207739805	0.094243158	0.728467429
**CG2926**	FBgn0037344	106.9204405	144.6658442	119.7297692	132.2143443	115.2394736	119.926734	125.924985	114.5032312	3R	-0.05011864	0.636125691	0.991585536
**Rev7**	FBgn0037345	9.601058553	5.601540462	11.1396685	7.299295624	5.840255587	4.919467043	9.31780144	11.32868852	3R	-0.074659668	0.886595003	0.995926915
**SecS**	FBgn0037347	143.6715878	171.826934	189.1451782	152.2672508	182.2509866	171.7163558	207.2177535	191.5983845	3R	0.230625983	0.06784512	0.656261422
**RpL13A**	FBgn0037351	1086.520927	1542.460508	1437.703368	1761.176823	1244.469537	1199.223123	1441.147867	1467.170529	3R	-0.089521745	0.519011099	0.981083949
**CG12171**	FBgn0037354	28.2171975	31.2667427	57.87519147	80.93835602	36.42929862	34.82574734	46.46154609	68.87350555	3R	-0.054002004	0.873970996	0.995787491
**CG12170**	FBgn0037356	83.27038654	100.5696461	83.13830724	73.2289138	93.84285733	77.93293159	105.435867	74.27097748	3R	0.078625558	0.639195867	0.991585536
**elm**	FBgn0037358	224.4040457	173.680699	198.497302	175.6694956	200.089255	212.1182932	204.5493253	209.2241976	3R	0.124970139	0.285160705	0.901780531
**MED27**	FBgn0037359	23.51790295	26.82462842	43.71222163	20.07662013	54.9966451	50.41693401	33.22782039	26.39385676	3R	0.567833688	0.060026781	0.627682799
**CG2182**	FBgn0037360	192.2755398	137.7408857	145.7108916	136.9571048	140.9695305	145.9984333	142.3408959	148.9204799	3R	-0.05606107	0.664366109	0.995787491
**Atg17**	FBgn0037363	18.83779405	32.47942242	35.12870628	22.61207524	26.23225808	31.18817879	36.00927222	33.94104797	3R	0.261603116	0.144747118	0.8037297
**Rab23**	FBgn0037364	5.216414793	3.159651454	7.225819948	7.31964682	6.016394726	5.251942548	4.189299362	3.740151775	3R	-0.225560413	0.482482227	0.973549494
**CG1239**	FBgn0037368	61.41226331	30.67096627	31.45834208	39.57417384	28.14363378	57.76174231	35.30979904	35.90308165	3R	-0.038053035	0.895782969	0.996479182
**CG2100**	FBgn0037369	18.40049636	11.57409424	26.37779626	18.15940233	24.44136787	20.10776487	24.80355524	19.63411448	3R	0.28767131	0.309994182	0.912401755
**CG1236**	FBgn0037370	19.30936385	35.94921435	23.58823804	21.23239114	35.10557305	16.33263058	29.52022604	36.57061853	3R	0.272588008	0.384623639	0.94674182
**Sym**	FBgn0037371	41.85809591	25.11643488	20.97435257	32.13863899	22.37468638	27.15138332	21.43843819	25.37752747	3R	-0.296341839	0.159499271	0.819277129
**CG2091**	FBgn0037372	156.4006647	121.440849	125.705553	142.3061852	175.6993588	134.1048067	127.7833343	119.7308549	3R	0.057412342	0.717815205	0.995787491
**jagn**	FBgn0037374	5.389193407	3.790806673	4.744635981	4.053129532	3.216805555	5.736498449	2.439152094	3.396901285	3R	-0.250478538	0.586105811	0.98718792
**kat-60L1**	FBgn0037375	173.1909267	228.9533285	187.0688493	213.5618861	217.1551143	184.0750185	194.1919355	191.6505007	3R	0.004566924	0.96744398	0.997604378
**Hat1**	FBgn0037376	66.30717681	69.34454654	72.47438543	98.55653725	79.90281759	73.32529314	84.84538074	83.87656936	3R	0.100584871	0.5363329	0.984934163
**CG1218**	FBgn0037377	64.8485004	85.39648994	77.403324	64.31695393	54.4793231	98.49531874	73.60602911	69.52078695	3R	0.05097856	0.784510488	0.995787491
**PSMG1**	FBgn0037378	86.53240001	113.4672796	86.42802988	62.24536437	91.58926351	80.20632759	69.85910483	93.00303121	3R	-0.024616696	0.901700981	0.996479182
**CG10979**	FBgn0037379	39.53421157	45.57308311	40.7237705	48.41539476	31.33866921	41.04001899	47.91961834	35.37832261	3R	-0.133541341	0.376775981	0.943615128
**Hpr1**	FBgn0037382	49.23425603	56.77619586	47.33191793	37.47394741	56.02305008	60.55295012	49.93842529	52.3799853	3R	0.228938207	0.131720596	0.790743321
**Sec20**	FBgn0037383	27.85886113	21.04559215	28.7614473	27.07671673	27.26770723	29.39352112	22.83716538	20.63665726	3R	-0.036442262	0.853568516	0.995787491
**dgrn**	FBgn0037384	70.09849864	64.01352461	63.97266165	50.66701277	64.34935426	82.8708181	64.00210258	55.23732186	3R	0.126344254	0.440807024	0.967112302
**glob3**	FBgn0037385	0.301630571	0.168647656	0.731751558	0	0.453767073	0.56875092	0	0.654867891	3R	0.533914742	0.753428008	NA
**CG1208**	FBgn0037386	0.776083959	0.223742057	1.117894011	0.141360198	1.247792749	1.45194182	0.773362052	0.789820367	3R	0.94390337	0.161287203	0.823037813
**CG1213**	FBgn0037387	3.610430765	3.041453316	4.476747946	2.65621521	5.301105673	4.916732993	4.093460644	7.525036228	3R	0.697522117	0.02367345	0.435528771
**CG14676**	FBgn0037388	0.905101835	1.214545085	0	2.532253027	0.653576315	5.973271373	2.308934227	0.589517961	3R	1.048132446	0.380289129	NA
**CG2017**	FBgn0037391	156.4820134	161.257732	162.2441801	166.6695019	172.0700701	176.3636903	172.804655	175.0463237	3R	0.137059578	0.110297217	0.758410631
**CG10280**	FBgn0037395	2.294523063	0.213818919	1.700869179	0.445799512	3.566896348	2.103171709	0.162593823	0.276756776	3R	0.40812561	0.72655085	NA
**Or83c**	FBgn0037399	2.284051102	1.571764227	0.142078875	1.365430554	1.057255804	0	0.896409759	0	3R	-1.45261376	0.315624596	NA
**Osi1**	FBgn0037406	0.304564858	1.736940399	0.886644117	0.852097801	0.879708205	1.89513646	0	0.1322477	3R	-0.318292225	0.810384925	NA
**NPFR**	FBgn0037408	0.428379059	0.502982043	0.233829365	0.124843706	0.096666749	1.7063641	1.50260575	0.162758853	3R	1.434895792	0.0967964	0.730787365
**Osi24**	FBgn0037409	0.879174705	1.146982847	0.545065646	1.047656505	0.564314906	1.086754488	0.024919928	0.042417104	3R	-1.046375814	0.214825017	0.858843649
**Osi2**	FBgn0037410	0.925049225	0.211587314	0.408028458	0.44114675	1.745857801	0.059463461	0.750851961	0	3R	0.381376753	0.745810752	0.995787491
**Osi4**	FBgn0037412	0.225112391	0.377594456	0.121359894	0.349894044	0.180615805	0.330142692	0.063807394	0	3R	-0.843498194	0.570412445	NA
**Osi7**	FBgn0037414	0.032212066	0.540312219	0.703314207	0.375506097	0.523359316	0.060738678	0.32869469	0.76928915	3R	0.08865316	0.94439715	NA
**Osi12**	FBgn0037419	1.158114788	0.329871242	0.371075326	0.764179537	0.276129398	0	0	0.094882053	3R	-2.809116389	0.033367353	NA
**Osi17**	FBgn0037427	0.406998708	0.205740154	0.256987542	0.311967836	0.100648902	0.346921221	0.241787146	0	3R	-0.761954382	0.390194898	0.948624352
**CG17917**	FBgn0037431	8.422547643	14.44872488	14.16377238	7.586965838	3.455649358	7.399313303	14.16129317	6.02611122	3R	-0.49375903	0.345282402	0.930285749
**CG10298**	FBgn0037432	9.455191181	14.03679623	11.86456651	8.361677421	3.923918029	9.631538738	15.24852611	4.71910237	3R	-0.356765292	0.507602036	0.978557094
**CG17919**	FBgn0037433	8.432589132	13.73250999	14.30027903	10.87995198	11.67588142	10.96046098	12.53109672	12.62003052	3R	0.052317643	0.884025734	0.995787491
**CG18048**	FBgn0037435	33.03550988	37.25118894	45.66846923	35.42681335	42.86796841	51.50346576	46.76674123	33.65779107	3R	0.24061631	0.234949879	0.87009833
**CG10286**	FBgn0037439	25.01857235	19.79998001	25.41163279	37.75942601	21.60062123	33.91869561	30.42122867	32.18822904	3R	0.155674441	0.519148094	0.981083949
**CRAT**	FBgn0037440	14.13717481	13.57931232	11.26469159	19.51860936	13.08780711	16.7459913	24.21896235	8.956843132	3R	0.130897739	0.631319854	0.991585536
**gzl**	FBgn0037442	236.9547369	212.0047313	174.4624461	228.1366183	180.5631285	220.369306	210.8801463	236.2739862	3R	0.022258221	0.859163733	0.995787491
**Dmtn**	FBgn0037443	14.06970607	20.42203884	22.27806606	20.37212921	21.09142875	18.99926219	21.97369087	20.88140049	3R	0.139839019	0.332573263	0.922658315
**CG9727**	FBgn0037445	71.98887171	92.13351471	72.8229578	104.2433819	72.6559004	75.10050203	84.91331988	84.80967211	3R	-0.072557627	0.600530235	0.989286463
**Zif**	FBgn0037446	162.2468638	125.5995638	140.8168801	178.0722241	155.4663565	152.0234473	155.3381441	155.8459064	3R	0.055095055	0.691956673	0.995787491
**Neurochondrin**	FBgn0037447	3.157082238	1.295722754	0.733312099	1.487784568	0.424419129	0.601628502	0	0	3R	-2.628538185	0.015215414	0.362234897
**CG15186**	FBgn0037448	0.647797099	0.408557372	0.716625307	0.13290768	0.318087623	0.488591318	0.052881418	0.112514197	3R	-0.935580412	0.195763872	0.844272804
**CG1138**	FBgn0037456	1.240060782	0.6240084	1.203348065	0.722788758	0.335794131	0.233824346	0	1.0769147	3R	-1.176056596	0.373134077	NA
**CG15177**	FBgn0037461	4.187453236	0.650357904	0.376248265	0.180794252	1.819861893	1.681515235	0.791280235	2.69373292	3R	0.351992709	0.752941291	NA
**Mst84B**	FBgn0037464	0.197308077	1.787165743	1.14879994	0.644022894	0.498668307	0	0.100667429	0	3R	-2.609957098	0.077237878	NA
**CG1105**	FBgn0037465	54.23183041	66.93188131	63.32532508	70.44191428	51.89301785	63.2820229	62.02000834	63.36538714	3R	-0.052089955	0.703105983	0.995787491
**CG1965**	FBgn0037466	108.8932669	113.5690608	101.0119231	120.2692766	95.56858927	104.3849632	109.5668297	111.1059182	3R	-0.047443135	0.665317675	0.995787491

**Ufl1**	FBgn0037467	68.05719301	98.17860871	92.13825077	90.6905648	80.72753708	109.500239	95.63690481	104.1358046	3R	0.192823766	0.178069813	0.835604629
**CG1943**	FBgn0037468	237.5759249	202.251809	245.7404319	189.0101399	200.4937983	231.9369561	195.9808137	166.4315701	3R	-0.110365366	0.372452366	0.943282319
**Dpck**	FBgn0037469	90.02139468	89.15828902	105.1656135	94.45611737	80.01250609	79.29499787	91.5692212	90.94955157	3R	-0.117410943	0.448175332	0.967112302
**Tailor**	FBgn0037470	126.1389567	108.128669	128.3747757	147.2560955	145.3709344	120.0374057	114.9667471	140.9434722	3R	0.061828165	0.655860853	0.995305364
**CG10098**	FBgn0037472	207.6622595	219.0125317	177.244014	145.2366924	186.362021	221.806313	191.6913808	197.9640513	3R	0.120871904	0.363398105	0.939549067
**CG10068**	FBgn0037473	58.4525177	58.00014748	67.13299652	47.15167618	66.0735507	79.80294442	51.08871997	60.07946738	3R	0.186678754	0.284572933	0.901780531
**Fer1**	FBgn0037475	0.61591191	0.604744873	0.692431228	0.455309283	1.301711829	0.552352231	0.459864736	1.043669674	3R	0.538783327	0.395043711	0.952272708
**CG2656**	FBgn0037478	10.30791527	10.43175668	12.6799861	12.03168344	10.2477741	11.77666	9.856673026	9.968761323	3R	-0.086238148	0.610237959	0.990047511
**MAGE**	FBgn0037481	0.275854285	2.776241193	3.346093245	4.930769762	0.663983582	0.693529961	1.641990487	6.787592146	3R	-0.161286997	0.876757026	0.995787491
**CG10055**	FBgn0037482	75.2689654	82.58705882	91.09647791	80.48050482	69.24700778	81.31389371	67.52663078	69.68024365	3R	-0.163113684	0.177072512	0.834615603
**CG14609**	FBgn0037483	3.667304755	3.046402298	1.779366037	0.732872827	1.197981668	1.580577153	1.336481889	3.033164991	3R	-0.347478386	0.595526237	0.988780662
**CG14606**	FBgn0037485	0.347230577	2.276774088	0.382898425	0.662363196	0.455883771	0.20832437	0	0.137067223	3R	-2.120255534	0.075034971	NA
**thw**	FBgn0037487	0.383266574	0.22587532	0.188472844	0.108677626	0.112199091	0.214851634	0	0.640947446	3R	0.12282732	0.896807533	0.996479182
**CG1234**	FBgn0037489	35.41315925	43.52859471	31.40119628	30.17771143	35.70700892	45.26233211	37.62137106	24.46036452	3R	0.054236905	0.784598431	0.995787491
**CG10053**	FBgn0037490	22.85071814	35.83818908	46.830009	49.23664553	50.33564677	57.70862806	62.29108728	50.86473254	3R	0.552444574	0.030261025	0.483132909
**CG1227**	FBgn0037491	30.40098477	26.54951932	22.24487849	27.10444179	46.01381117	21.7150917	35.50477405	34.00890854	3R	0.397815752	0.123525357	0.780439453
**CG10050**	FBgn0037492	12.49429426	8.620785098	7.52400302	8.057227599	7.598451498	10.98588707	9.042046264	7.983972913	3R	-0.024192216	0.940621802	0.997188858
**CR10032**	FBgn0037493	0.094458283	1.584405188	0	0.660677794	2.387299748	1.246765693	3.855441755	0	3R	1.721080257	0.256556702	NA
**Ir84a**	FBgn0037501	0.551050469	1.848619715	1.503945847	0.160594178	0.310871041	0.357174824	0.175717698	1.046833882	3R	-1.053180776	0.310353439	NA
**wa-cup**	FBgn0037502	4.950693796	1.394580727	2.567090036	1.321643841	2.387820537	0.49881507	0.578442423	1.72302828	3R	-0.961001609	0.199578007	0.847197998
**CG14598**	FBgn0037503	1.368067959	1.901357724	0.853434974	0	1.058448557	0.405367636	0.09971345	0	3R	-1.351277553	0.325082453	NA
**CG1142**	FBgn0037504	51.59121509	57.7645629	75.12750732	61.82056498	64.41468839	79.25584909	68.14340308	85.14412807	3R	0.30437469	0.087425768	0.706081898
**CG2616**	FBgn0037512	0.075385766	3.224450715	2.011732997	0.175759077	0	0.24875634	1.730796388	0.245504216	3R	-1.26220387	0.332936281	NA
**pyd3**	FBgn0037513	2.959256776	4.494195307	4.365678444	3.076757574	0.360960604	4.448869826	2.856426665	0.694575178	3R	-0.803720491	0.248468015	0.879503029
**Sp7**	FBgn0037515	3.913537655	2.514029515	3.989578901	5.581332751	2.104449765	2.727992852	3.876579045	0.04519496	3R	-0.852159946	0.195247442	0.844272804
**CG2641**	FBgn0037518	3.28670023	1.607952079	1.7442007	0.798210193	2.843056972	7.746702253	3.580855821	2.30424629	3R	1.14974576	0.048777552	0.582395583
**CG3014**	FBgn0037519	19.58667045	11.69236255	14.35036896	8.469306456	20.69252432	16.54552404	8.765967917	19.02410709	3R	0.292539617	0.331495193	0.921507984
**CG18268**	FBgn0037520	0.162307768	0.998244543	2.362538291	0.756828903	0.879021362	2.040303633	0.552067472	1.174617298	3R	0.187320009	0.854172938	NA
**CG2993**	FBgn0037521	336.7893574	343.0298843	293.0649714	320.4953453	287.0695589	286.2256106	262.2332876	312.3020165	3R	-0.141477592	0.148059852	0.808004944
**CG17816**	FBgn0037525	228.2115911	223.3289124	225.9735108	215.4315015	229.1736103	272.8484143	225.1926433	249.4879713	3R	0.158153207	0.061953123	0.633840737
**ArgRS-m**	FBgn0037526	4.184456311	13.89730011	14.48271058	28.68235295	12.59002685	12.33494967	15.51380715	24.9530327	3R	0.130998063	0.747465113	0.995787491
**mRpS9**	FBgn0037529	12.16795519	14.29028168	15.09705294	13.86030302	13.96686241	16.04598621	17.29434846	16.26356338	3R	0.230403262	0.072723266	0.671999479
**EMC1**	FBgn0037530	94.19459346	119.4040001	118.3795741	118.7074694	125.3687735	109.9898824	131.0593271	118.8751471	3R	0.140250822	0.23330848	0.869196636
**CD98hc**	FBgn0037533	442.7035043	420.8672965	457.9670192	419.4712739	395.8031903	438.7337143	444.733996	430.5781098	3R	0.003959759	0.963501942	0.997604378
**ELOVL**	FBgn0037534	0.38881008	0.734990157	0.389276883	0.359720661	0.501358461	0.779683825	0.236157826	0.509165424	3R	0.152755071	0.77513582	0.995787491
**PIG-H**	FBgn0037535	31.98969299	35.96869846	25.07288506	29.01351516	37.69588932	45.07381817	37.8438443	36.78697346	3R	0.394227369	0.060470651	0.627984253
**CG2698**	FBgn0037536	150.7440325	186.040035	157.0252483	185.9124555	172.4211358	177.4634743	159.187717	187.1729414	3R	0.066128211	0.543792133	0.984934163
**CG2767**	FBgn0037537	2.377444986	7.643341679	9.708874764	14.04207283	11.80271822	8.069186198	8.89520608	7.226314398	3R	0.131486549	0.769009463	0.995787491
**CG3223**	FBgn0037538	94.22009415	105.1511361	89.48255738	98.35251978	105.6306365	117.0437896	84.94452006	125.0608794	3R	0.191254073	0.196993539	0.8445186
**CG10435**	FBgn0037539	93.4525536	130.6596245	133.5555049	118.6681766	121.8771677	104.9090286	117.7067292	132.8854176	3R	0.039092443	0.783586905	0.995787491
**Pbp95**	FBgn0037540	9.43306187	16.95650778	7.976022763	18.62584836	11.98138234	14.8320448	13.32531073	16.21059374	3R	0.12084174	0.68315516	0.995787491
**CG2747**	FBgn0037541	47.6400351	49.89484283	57.7942366	59.43452296	52.78765109	58.13318684	46.98158555	52.31523091	3R	-0.00162066	0.98832912	0.998078482
**CG10903**	FBgn0037543	3.596605355	2.010933643	2.181328607	1.746947915	0.631243907	2.54314317	2.166323584	1.843688524	3R	-0.393821748	0.569491101	0.984934163
**CG11035**	FBgn0037544	53.42764593	48.24956487	62.29111403	58.6818306	46.18643644	55.54311305	51.15475116	64.45354538	3R	-0.004048189	0.980900142	0.997881056
**mAChR-B**	FBgn0037546	61.09292651	52.43834491	56.80030944	68.42856328	59.00482385	59.48924656	63.62741651	66.08552605	3R	0.084845019	0.451601362	0.967518189
**CG7910**	FBgn0037547	1.104630221	0	0.462037502	0	1.787850251	1.5442021	1.651893181	0	3R	1.651248673	0.250788532	NA
**CG7878**	FBgn0037549	149.8821998	159.4312073	139.0451451	174.8412389	126.9110283	145.7950089	144.8240029	160.1577778	3R	-0.079670718	0.515458801	0.980804634
**CG9667**	FBgn0037550	73.38603005	77.34273183	87.04734569	62.64715673	73.1281149	78.52520608	78.07292137	78.9589417	3R	0.070986669	0.662957127	0.995787491
**Arl8**	FBgn0037551	202.4936139	181.3200655	188.4055613	187.692084	172.1985916	172.5039295	155.2227856	179.7305202	3R	-0.131632957	0.191144792	0.843449603
**CG7800**	FBgn0037552	2.193483992	4.415114018	1.77378719	2.272900157	2.287884902	2.06800331	6.466055312	1.164107217	3R	0.194667342	0.771377998	0.995787491
**DNApol-iota**	FBgn0037554	51.74335694	62.76436264	65.33553053	62.52975504	61.7899837	70.17729988	80.08700743	96.15961243	3R	0.380900489	0.013205797	0.344576629
**Ada2b**	FBgn0037555	40.28651858	37.82852054	39.1262828	33.18112288	40.18938893	52.2829251	31.86721687	40.6333187	3R	0.161645929	0.308079875	0.912131082
**CG9636**	FBgn0037556	401.0894133	374.7956001	326.2090371	351.1692323	335.1259241	404.8025058	325.1705057	319.3559154	3R	-0.043009377	0.685620071	0.995787491
**CR18228**	FBgn0037560	1.235327329	6.581927525	5.112336424	2.37186284	6.427884681	8.563669559	6.117319785	4.732958795	3R	0.808087205	0.180673024	0.836519372
**CG9630**	FBgn0037561	19.11544489	24.17779601	15.70899116	5.894003686	10.08826668	13.08784786	19.91287301	14.44364281	3R	-0.141119393	0.705204459	0.995787491
**CG9626**	FBgn0037565	0.327777524	0.162903945	0.530122467	0.028303739	0.810878898	0.343362941	1.207797508	0.052713729	3R	1.234160734	0.206896071	0.855364409
**mRpL1**	FBgn0037566	222.6023787	221.3213912	207.4487241	234.5927755	191.8742811	248.3051056	210.4982421	183.248777	3R	-0.059963128	0.623338146	0.990047511
**tex**	FBgn0037569	15.29861506	19.21496855	19.90183173	11.54476194	22.81480446	18.53446986	7.352931073	22.62451225	3R	0.151494294	0.689217748	0.995787491
**CG7483**	FBgn0037573	226.7452264	231.5339331	247.8218495	210.6055423	220.1051444	191.0898053	278.3411845	223.9413554	3R	0.027673454	0.837005083	0.995787491
**Coq2**	FBgn0037574	62.63695762	60.95735494	46.11255096	64.06283448	63.56066824	50.83635727	59.16686619	81.03844339	3R	0.153393114	0.425817434	0.962175354
**CG9601**	FBgn0037578	67.75367894	78.55839655	79.97577735	60.74745329	84.21119425	94.47392246	83.14478377	75.42524782	3R	0.264710327	0.057748403	0.623014047
**DppIII**	FBgn0037580	141.4717578	130.4054383	140.2040362	176.448465	140.8078461	155.8127629	138.7069072	151.7948162	3R	0.024453837	0.843358426	0.995787491
**CG7352**	FBgn0037581	2.471924308	5.246346684	6.608777759	8.703605848	9.289196047	3.519553474	5.533777076	4.271515986	3R	0.016277849	0.974612703	0.997757033
**CG9684**	FBgn0037583	0.200196253	2.014806782	3.15687657	3.967375241	3.373118441	1.793066146	4.681461982	2.680308355	3R	0.470645757	0.501290784	0.976796212
**CG11737**	FBgn0037592	25.7140205	31.29322124	32.44814343	32.73730621	27.486934	23.89403363	34.75005216	32.41425038	3R	-0.010460019	0.952862285	0.997604378
**SLIRP2**	FBgn0037602	23.50764124	22.7348586	29.28526988	24.68791919	25.23299953	21.76352975	30.25437729	31.72586756	3R	0.151264745	0.690765981	0.995787491
**CG11753**	FBgn0037603	136.0058418	175.027666	153.8120141	141.9141482	173.8947761	140.562503	168.415823	177.9752425	3R	0.157444017	0.278069062	0.896398793
**CG8036**	FBgn0037607	7.720515553	14.19528193	14.94787147	18.83086482	6.83474015	12.13609077	10.49148209	15.18246213	3R	-0.28530296	0.306823654	0.911610813
**mRpL19**	FBgn0037608	174.5114609	211.5860165	179.590704	197.8599315	181.8301018	163.8521955	182.7484269	155.3583202	3R	-0.127856034	0.316742369	0.91460807
**CG9773**	FBgn0037609	92.02374518	90.34194992	119.4449622	108.2619087	85.70854834	122.187725	89.21669774	83.14064881	3R	-0.079426288	0.634214697	0.991585536
**CG11755**	FBgn0037611	8.449524943	6.053748156	7.10749571	4.553710898	4.752364841	7.806036676	6.82391929	4.424852458	3R	-0.113629255	0.768185562	0.995787491
**Cks85A**	FBgn0037613	41.45190278	41.72832242	41.26968915	40.97162585	43.78307737	41.15528625	44.94945789	40.45750234	3R	0.072094313	0.513446645	0.980804634
**TMEM216**	FBgn0037614	0.955411021	1.459481533	0.620842985	1.392190519	0.585187027	0.643396911	0.696364012	0.888979615	3R	-0.627046852	0.284942565	0.901780531
**CG8136**	FBgn0037616	3.557535089	2.335017146	1.375881675	0.120206654	1.954601668	2.38184196	0.394580367	0.447752927	3R	-0.490127255	0.620829821	0.990047511
**nom**	FBgn0037617	15.673022	11.18127007	15.69031099	15.35649643	13.25153673	13.01822135	9.615975964	6.460933215	3R	-0.422718448	0.15649239	0.818884898
**CG8159**	FBgn0037619	34.56730779	30.76390224	34.93003513	33.83547721	33.41904699	23.32463404	27.9850139	26.91833891	3R	-0.235159096	0.263636071	0.892790059
**ranshi**	FBgn0037620	7.120202696	8.32732778	10.93460879	10.35626559	3.066041833	17.36727686	11.49817902	8.934798232	3R	0.182229151	0.702621424	0.995787491
**M1BP**	FBgn0037621	245.9177341	242.226074	220.0466678	231.1596639	227.8319764	190.461756	199.5402016	217.624819	3R	-0.138240552	0.216254243	0.860826301
**CG8202**	FBgn0037622	36.30289526	27.22732219	33.20868025	30.78265862	39.65555974	32.87454207	41.17287076	30.07092404	3R	0.200265037	0.232002135	0.867702781
**CG9801**	FBgn0037623	54.80828972	50.88104503	52.56929207	70.79108044	49.6040654	53.46596558	54.19224831	60.45347763	3R	-0.04466002	0.735149771	0.995787491
**CG8223**	FBgn0037624	1.716980765	1.474557115	2.565868775	1.088836677	1.413413195	1.579908762	1.436657981	0.984119184	3R	-0.304148614	0.482761036	0.973549494
**CG8236**	FBgn0037626	1.874426728	2.316696333	5.504666382	6.095216676	1.424766592	1.023114672	3.020022855	0.749654673	3R	-1.323706443	0.054194623	0.610348628
**CCT7**	FBgn0037632	56.90466331	48.16035364	35.50240758	29.88126352	49.45518911	42.92998724	52.04743172	52.55104485	3R	0.239064916	0.199231036	0.847197998
**hng2**	FBgn0037634	10.11348857	9.809091531	17.02441214	15.55907574	12.79262728	11.80515793	11.93456742	10.75460566	3R	-0.114115452	0.73827032	0.995787491
**CG9837**	FBgn0037635	14.07913518	9.653163161	12.10945474	9.275878897	12.90699678	9.301294705	8.50153496	10.26340778	3R	-0.110971492	0.61302242	0.990047511
**IscU**	FBgn0037637	520.2964951	463.3305441	511.4120538	511.1027624	485.5889928	468.4562492	525.9566134	542.3609629	3R	0.041945861	0.693351003	0.995787491
**CG8379**	FBgn0037638	72.30267785	81.6116174	75.46676968	98.50910158	62.1546974	112.6997068	76.50566615	76.84864049	3R	0.026720172	0.876634008	0.995787491
**ScsbetaA**	FBgn0037643	396.5356312	335.7909853	344.1363977	349.1250439	370.6248183	374.0411529	364.1326643	356.6206908	3R	0.068310623	0.465877629	0.971928234
**CG11964**	FBgn0037644	78.77816242	105.4864151	95.81163879	100.0938583	98.24096672	78.10570676	95.9368877	96.66781979	3R	-0.008778665	0.951740187	0.997604378
**CAHbeta**	FBgn0037646	7.438504282	6.689501325	5.870290436	8.566822904	8.170295644	12.29383213	19.09009619	8.074840685	3R	0.759807516	0.037477965	0.521998122
**RagA-B**	FBgn0037647	58.05556452	38.18106228	46.32785525	35.59271189	35.95578053	43.95262557	48.43642257	45.38059359	3R	-0.012283541	0.953298111	0.997604378
**CG11975**	FBgn0037648	322.0660037	305.0421582	283.1829971	239.4707997	314.0932135	327.3612761	297.1014929	282.4203303	3R	0.116035585	0.299910958	0.9074568
**CG11980**	FBgn0037652	129.6636904	105.3739177	100.7494925	113.4223882	101.1513801	106.2739661	119.5632564	98.0354012	3R	-0.054426382	0.712690196	0.995787491
**Iru**	FBgn0037653	394.2946188	369.132486	347.910364	426.9886985	377.3101467	394.04806	385.0743394	370.2581036	3R	0.017159058	0.86963825	0.995787491

**CG11983**	FBgn0037654	3.548110421	5.802671043	13.6646704	4.239546511	11.92977567	3.010623149	9.843295179	5.007129509	3R	0.176667055	0.738094642	0.995787491
**Kcmf1**	FBgn0037655	259.2931317	264.6431266	215.3119522	285.3098571	267.8568891	256.6379742	253.9641019	247.4359898	3R	0.03102343	0.773266448	0.995787491
**CG11986**	FBgn0037656	10.70138209	19.00595351	12.52255943	12.91522656	9.886647973	7.715268807	26.81770053	13.25687083	3R	0.098534697	0.810612246	0.995787491
**hyx**	FBgn0037657	63.37684956	62.74712494	66.21964215	69.04128965	67.44437743	62.07218756	66.0617027	65.23544321	3R	0.027261566	0.811391829	0.995787491
**Kdm2**	FBgn0037659	47.62794884	47.27667583	54.08768303	44.63502299	40.6557147	52.83416079	51.46943013	50.80142236	3R	0.046043646	0.688608363	0.995787491
**beag**	FBgn0037660	182.5392731	179.3352897	200.5241808	215.2304466	155.002122	183.805041	187.0140618	183.9974619	3R	-0.102204498	0.389177466	0.948247506
**CG16734**	FBgn0037667	18.62369665	4.845882979	4.630722015	15.03478104	15.08733628	9.289852416	14.4765638	11.31249504	3R	0.230224134	0.614281472	0.990047511
**CG16736**	FBgn0037668	1.265115564	0	3.069152713	3.539483175	4.71997287	1.113227011	3.657648583	0.1831122	3R	0.330760476	0.777625193	NA
**Ibf2**	FBgn0037669	52.82146246	62.50846933	61.62863936	58.4446616	63.63819385	35.66168693	49.67430358	52.96674163	3R	-0.187575417	0.434101841	0.965402206
**Ibf1**	FBgn0037670	28.71189758	36.32248894	44.19266438	34.87667157	26.3517328	28.20677148	38.00721196	28.15605107	3R	-0.222188263	0.321053287	0.916949312
**ATP6AP2**	FBgn0037671	1333.219872	1313.936265	1161.876711	1290.930788	1245.65803	1185.328951	1151.010336	1255.024847	3R	-0.04591961	0.614311029	0.990047511
**sage**	FBgn0037672	7.101764764	6.541953652	3.530488168	2.171475042	5.779736116	1.42690993	2.672963777	7.835676227	3R	-0.096767431	0.879209595	0.995787491
**CG8861**	FBgn0037676	0.93650746	0.750012677	1.108858203	0.64865881	1.372240352	1.271705298	0.614689424	0.787410293	3R	0.263515495	0.391570053	0.950027604
**CG16749**	FBgn0037678	17.58350387	7.409787743	9.245924854	8.279834336	11.10213748	5.063115481	4.640265117	7.146136719	3R	-0.585500246	0.244657235	0.877269208
**Aduk**	FBgn0037679	141.6616893	138.1625239	132.5830734	119.4917647	127.5727942	151.9859133	128.6600014	118.4399422	3R	0.014127348	0.899385158	0.996479182
**pasi2**	FBgn0037680	44.00918291	40.74973857	53.70413876	46.93573578	43.31035239	46.55762977	39.19256979	37.70983369	3R	-0.121240948	0.441006458	0.967112302
**Or85f**	FBgn0037685	16.1017018	5.124113061	13.58695791	9.199651061	16.65935294	13.56052356	10.22835808	16.30465472	3R	0.389556532	0.329815845	0.921112231
**RpL34b**	FBgn0037686	560.8728464	713.9627754	724.741467	798.8042103	654.5642796	579.3212142	834.4836156	764.5845284	3R	0.052037392	0.719013946	0.995787491
**CG8132**	FBgn0037687	97.80225777	97.80873245	135.8484061	93.51071509	107.6336306	122.3129325	135.5698031	122.7472985	3R	0.23291518	0.153488383	0.816319199
**CG9356**	FBgn0037688	86.56744315	91.94596026	99.9526134	95.11060685	103.6340122	112.7957332	92.66274427	97.06127972	3R	0.152502098	0.221378048	0.86549481
**Task7**	FBgn0037690	532.0583073	501.4130957	501.0491818	458.6997786	544.1833973	549.4383415	499.4214153	512.2712971	3R	0.108578649	0.2256895	0.867702781
**GstZ1**	FBgn0037696	0.673416876	1.835538799	3.471612389	1.373790333	0.607845028	1.745956622	1.073688074	7.310251874	3R	0.606319854	0.537696817	NA
**GstZ2**	FBgn0037697	35.96113248	34.15468858	31.32578137	39.36838411	33.33571362	32.71208393	23.38071208	28.96565848	3R	-0.221061966	0.169806646	0.830934498
**CG16779**	FBgn0037698	10.99437555	15.96749675	19.7478738	16.30175276	13.58679943	15.76487461	15.85730519	19.29432796	3R	0.068678232	0.670932598	0.995787491
**CG8149**	FBgn0037700	138.6321798	141.0838776	179.7181423	191.5909133	168.0084412	172.2423821	173.4456495	168.3017703	3R	0.098060906	0.49603752	0.975846898
**CG8176**	FBgn0037702	75.34938929	81.72721731	74.21140203	80.09552404	63.64726074	76.31832501	59.95372858	60.43587997	3R	-0.229718971	0.021038868	0.410927638
**mura**	FBgn0037705	218.2023935	241.8147085	226.1340313	254.6523848	236.0549037	227.012563	226.0995981	281.4160769	3R	0.077170679	0.469273208	0.973549494
**RnpS1**	FBgn0037707	261.9784768	225.8652377	215.0414779	223.8748936	226.5649196	241.557032	244.1837452	209.2441175	3R	0.019078336	0.865927879	0.995787491
**CG9386**	FBgn0037708	49.83584811	26.56294167	32.28120004	33.73650302	34.70788009	43.12119757	47.23901404	28.12055767	3R	0.124506139	0.610756059	0.990047511
**CG8199**	FBgn0037709	5.351102066	5.473001849	7.282416499	5.882941426	5.65469895	3.445356527	7.324806814	7.556252313	3R	0.030755263	0.939137506	0.997038394
**CG9393**	FBgn0037710	56.90279965	50.74720942	38.00521526	57.87864124	51.92319838	50.24823667	42.60712787	54.07741335	3R	-0.006752247	0.970028962	0.997604378
**CG16789**	FBgn0037712	7.145860613	6.259988073	4.000559579	9.240735977	7.938529154	9.02822432	6.494629422	4.01990604	3R	0.067389002	0.852213525	0.995787491
**CG16790**	FBgn0037713	22.56633242	31.73438507	32.81049889	31.00066343	40.60084869	35.38736626	30.23731655	28.70331923	3R	0.229855099	0.334839659	0.924122842
**CG9396**	FBgn0037714	2.865781494	0.961389285	3.302364317	0.501109625	0.258673504	1.013190483	2.74150131	6.688517673	3R	0.517780643	0.603608166	0.989286463
**CG9399**	FBgn0037715	60.55745174	77.06684609	72.08532322	74.80825933	72.20128597	74.06492912	58.60376235	54.61807012	3R	-0.100576255	0.495026375	0.97491773
**Son**	FBgn0037716	36.60117512	28.16333732	46.47653084	33.37235819	42.24838039	36.99599953	37.76467579	32.79020023	3R	0.081414957	0.649994292	0.993330125
**CG8301**	FBgn0037717	97.19014683	109.1714493	110.377732	90.61538523	101.2792478	100.1757097	84.34063017	93.95770805	3R	-0.069122899	0.513387212	0.980804634
**P58IPK**	FBgn0037718	51.53112757	45.91581019	44.90941488	37.0376789	41.12144169	50.53275055	36.17161137	37.98180004	3R	-0.085897717	0.599924882	0.989286463
**bocks**	FBgn0037719	127.9510721	163.6643048	176.9843385	179.435466	159.4261804	125.5823844	148.2222886	205.832079	3R	0.015973329	0.925799531	0.996479182
**CG8312**	FBgn0037720	1.236523943	1.716937932	1.282067856	0.962963387	1.051284395	1.175461803	0.700250432	0.930144246	3R	-0.395162799	0.120901986	0.77836519
**CG8319**	FBgn0037722	31.13142851	47.07180116	41.7167693	40.5384743	49.27615719	53.81933251	55.93431506	34.14157376	3R	0.301257553	0.161713908	0.823037813
**SpdS**	FBgn0037723	11.59456831	11.29715207	9.100319739	7.652525973	9.542149877	9.283557246	10.87426208	7.033599133	3R	-0.086301288	0.777492867	0.995787491
**CG9492**	FBgn0037726	1.125919064	0.829732956	0.510470311	0.645501126	0.919655746	0.473328878	0.169509466	0.512938914	3R	-0.555690645	0.236887329	0.873280735
**Nepl12**	FBgn0037727	3.610694053	5.669851931	1.304605402	2.32843754	2.912399825	1.231284789	0.195977832	0	3R	-1.53058825	0.09873609	0.730787365
**p23**	FBgn0037728	507.85013	518.0413673	579.0539041	500.3763434	534.6868758	516.0677237	540.2217814	478.6688313	3R	0.006411336	0.945484242	0.997604378
**CG9444**	FBgn0037730	0.304030346	1.223923811	0.516301366	0.070883531	0	0.143318977	0	0.462054618	3R	-1.752800635	0.260125072	NA
**trbd**	FBgn0037734	190.5395211	182.3257806	163.822495	203.8300429	182.8896082	192.5669309	178.8847823	175.9692986	3R	0.008002256	0.936889411	0.996879532
**side-VII**	FBgn0037736	141.1411434	138.0339801	161.7829074	176.259579	131.5515385	147.9024122	153.383116	156.1935098	3R	-0.037854238	0.743874792	0.995787491
**Pnn**	FBgn0037737	116.0105552	90.46073851	97.88277539	82.23247595	93.25811559	96.14839084	89.74928247	68.74446228	3R	-0.125750019	0.420878532	0.960593359
**CG12948**	FBgn0037739	84.97046672	56.01540462	61.69457554	60.18717444	65.14512599	68.14756451	64.59408216	63.44065572	3R	0.015382294	0.929805018	0.996479182
**CG16908**	FBgn0037741	9.846265278	9.731761905	13.89995778	11.95518319	8.040051332	13.84505928	11.72993776	9.251237524	3R	-0.052571278	0.832518113	0.995787491
**Rpt3R**	FBgn0037742	0.536789128	0.982241954	2.959643039	0.910184468	0.088094723	0.23003705	0	0	3R	-3.834499752	0.005940133	NA
**CG8412**	FBgn0037743	86.28061251	82.32411029	84.29214399	106.6269655	84.05756856	82.53129416	89.49787433	113.2202459	3R	0.06933079	0.647622213	0.992932477
**CG8478**	FBgn0037746	2.021673339	1.227802443	0.169122459	0.975197645	1.636044857	0.953010638	0.622436635	4.918975821	3R	0.901833016	0.290916683	0.905001729
**Naa80**	FBgn0037747	173.4734769	151.0538528	145.1682643	139.2358115	132.4815285	174.6094501	118.4928621	154.955589	3R	-0.041749283	0.761410424	0.995787491
**CG9471**	FBgn0037749	60.54701131	115.8004103	100.4901374	82.57840089	81.59010528	68.40299739	81.603908	92.53856043	3R	-0.109766427	0.602099161	0.989286463
**CG12947**	FBgn0037753	0.782171449	0.437327626	0.070279149	0.5403269	0.313782208	0.300433004	0.517309378	0.44026542	3R	-0.225227357	0.835568289	NA
**CG8500**	FBgn0037754	427.4897914	574.1597716	431.7434737	478.7921125	448.076737	475.290042	390.0718428	398.1396018	3R	-0.129050937	0.237113005	0.873280735
**CG12945**	FBgn0037755	39.93851561	38.23195311	31.96762164	50.76851253	31.77878072	29.25963188	39.8450847	40.81455318	3R	-0.154636942	0.412739127	0.958665466
**CG8507**	FBgn0037756	89.62172661	83.21560933	95.11358104	84.53021236	87.43060342	102.6478021	126.0116224	94.55211041	3R	0.249130006	0.097495436	0.730787365
**CG8516**	FBgn0037757	46.36863699	40.01257359	41.14096311	37.90755523	42.46732667	31.02430613	39.89164151	51.15770494	3R	0.024416905	0.885401431	0.995787491
**CG9467**	FBgn0037758	120.9251345	152.3827107	166.1979961	132.0686187	114.8395994	141.8887879	137.3822954	134.8207847	3R	-0.079111364	0.517143067	0.980804634
**CG8526**	FBgn0037759	0.119144767	0.066616211	0.192695695	4.259316933	1.003738592	0	1.924958783	0.517348637	3R	-0.410112953	0.768342366	NA
**FBXO11**	FBgn0037760	131.6355828	196.4452598	203.6684244	169.090421	160.5950177	137.100244	178.9283104	193.0118084	3R	-0.028052681	0.848017694	0.995787491
**Teh1**	FBgn0037766	1.339276854	1.458463839	1.169664108	0.951153487	1.870957233	1.476343305	1.193155314	2.21879421	3R	0.491615639	0.036816258	0.521170991
**Spn85F**	FBgn0037772	1.346967324	0.78385568	1.467141361	0.06408987	0.843623697	1.062579901	0.420752153	0.656495882	3R	-0.264435343	0.768045626	0.995787491
**CG5359**	FBgn0037773	19.9711171	27.03068944	31.83435317	28.71541282	21.29931386	29.02975868	26.28088771	34.98720548	3R	0.089688561	0.714728702	0.995787491
**CG11722**	FBgn0037777	75.26447983	126.3991898	98.84769749	63.40202129	108.4328706	103.8485972	124.4971966	101.7809614	3R	0.306132425	0.137964355	0.798609078
**mtTFB2**	FBgn0037778	67.86637159	32.40155597	40.13247874	27.30958292	43.21918754	42.91432591	42.85620215	31.45424478	3R	-0.043118073	0.871377214	0.995787491
**CG12811**	FBgn0037779	2.466797934	7.429065698	5.168360466	5.75124652	3.441111028	4.65136405	6.292854527	1.217192424	3R	-0.380170377	0.516555514	0.980804634
**ohgt**	FBgn0037780	199.7651894	232.9756893	204.7188141	196.0483782	193.6466189	205.0348491	212.1377953	207.4434696	3R	0.004962799	0.95981592	0.997604378
**CAH7**	FBgn0037788	44.94342934	77.16445125	77.39406177	36.55851531	30.12139848	50.64906157	74.50949002	36.273467	3R	-0.264259079	0.473155111	0.973549494
**TAF1B**	FBgn0037792	12.67118173	6.123467872	11.58546865	10.83715564	10.46030395	14.26753934	18.24680394	15.89791697	3R	0.538128109	0.045862844	0.56430181
**CG6254**	FBgn0037794	78.06335719	46.10481449	51.21251556	41.27589803	54.19617977	72.29074716	43.24326584	48.58334231	3R	0.032478386	0.881545826	0.995787491
**CG12814**	FBgn0037796	1.108142569	1.646099677	1.013822538	0.755372437	0.771372047	0.934077371	0.407264606	0.203888144	3R	-0.929505717	0.040671344	0.530560761
**CG12420**	FBgn0037797	3.474657418	0	0.802806708	2.12169911	0.746743713	0.857970088	0.422091701	8.26226032	3R	0.6964805	0.573687901	NA
**CG12817**	FBgn0037798	69.36200371	77.11108935	120.3572814	85.33643437	75.8111651	95.07243328	100.5949636	99.0770952	3R	0.107520747	0.573184397	0.984934163
**CG3999**	FBgn0037801	0.161687392	0.697391788	0.056035908	0.053852581	0.208490845	1.807477785	0.058924001	2.557564721	3R	2.287066627	0.047236794	0.572196862
**Sirt6**	FBgn0037802	2.865065048	2.615219203	2.424630644	1.646646228	1.130661888	3.078072687	1.393779266	3.066763126	3R	-0.111444525	0.789999448	0.995787491
**CG6293**	FBgn0037807	1.751931945	1.183080806	0.441575321	0	1.478658472	0.900933129	2.553837732	1.185539666	3R	0.867993787	0.315046322	0.91460807
**CG12818**	FBgn0037809	34.75615422	31.3012472	50.55304107	32.81188455	40.84661296	29.68902777	36.29856016	33.76233922	3R	-0.053902158	0.824676093	0.995787491
**sle**	FBgn0037810	4.457055358	10.04334451	11.5880082	9.450343713	8.197940978	7.310041455	4.54801759	10.26093213	3R	-0.187201436	0.554119582	0.984934163
**CG6325**	FBgn0037814	21.88524919	24.49538855	12.21486767	19.82992216	18.32382481	28.51969305	22.31142715	26.34877694	3R	0.310447682	0.159170049	0.819277129
**Rrp46**	FBgn0037815	11.69024144	24.39512541	18.31141528	7.940537323	37.5549396	17.70380786	22.30783797	14.18915228	3R	0.600231708	0.155097044	0.818884898
**CG6345**	FBgn0037816	23.02870207	29.8051058	28.7383282	25.74053211	33.87405955	37.61508466	33.96672132	22.42683699	3R	0.285812997	0.177992249	0.835604629
**Cyp12e1**	FBgn0037817	3.25666511	7.283465144	7.790893243	1.160009905	1.224815667	6.737743165	5.538548442	4.026269142	3R	-0.113917095	0.856251408	0.995787491
**CG6465**	FBgn0037818	7.376124999	12.28649537	18.14290937	10.15110006	16.0899203	12.99076767	10.06168269	16.57030053	3R	0.261354856	0.444472002	0.967112302
**CG14688**	FBgn0037819	25.83875521	44.07007127	41.85555408	25.65514663	26.8788385	35.24733325	36.66577414	20.64607287	3R	-0.167850276	0.45831816	0.969693716
**CG14684**	FBgn0037824	16.04041586	17.04017999	13.40365088	20.77645874	23.48739453	24.86861447	22.73302526	31.34270491	3R	0.636572842	0.102272338	0.741747104
**CG4073**	FBgn0037827	1.884712507	3.556508436	1.714607345	0.183089011	0.850597281	0	0.600992762	0	3R	-2.323544771	0.115486981	NA
**Cap-H2**	FBgn0037831	26.35446806	32.04062373	26.66232963	35.30182048	29.1718736	36.84288775	28.11765495	26.03642986	3R	0.026732265	0.862977256	0.995787491
**Art1**	FBgn0037834	96.99257994	99.33003851	85.01723036	93.9719824	82.88855212	100.3825003	73.69721103	70.58844143	3R	-0.168001861	0.244586495	0.877269208
**CG14687**	FBgn0037835	24.61411581	56.81839947	19.90450055	16.94279444	6.982609478	39.67127456	16.44529811	7.379743781	3R	-0.718450478	0.181776746	0.837439137
**CG14692**	FBgn0037836	13.23297842	12.65433382	16.43368936	10.9370201	12.24465024	19.97640631	13.61914251	14.06098917	3R	0.198472849	0.265870448	0.893759496

**CG14693**	FBgn0037837	0.049981677	0.838372509	0.060627425	0.349591193	0.541378419	1.107376857	0.828778151	0.054257423	3R	1.00386471	0.399815766	NA
**TTLL15**	FBgn0037838	0.786723394	4.090816193	3.721730961	3.851853549	2.059344174	2.373497632	7.526056048	3.245293696	3R	0.331086343	0.594651476	0.988780662
**CG4565**	FBgn0037841	23.25511595	12.31806147	7.765845975	15.36554622	12.91736755	14.11352317	17.53309283	21.46293923	3R	0.182826811	0.658912586	0.995750687
**CG6567**	FBgn0037842	83.64250276	53.01666909	44.87555323	46.31471977	49.50931617	47.61788304	70.98782909	47.66884925	3R	-0.057144375	0.820309769	0.995787491
**CG4511**	FBgn0037843	80.72584798	60.44244627	76.20267737	85.28067682	77.32490116	72.64644686	79.78328301	96.04546308	3R	0.135019695	0.41640615	0.958846438
**CG14694**	FBgn0037845	3.11847191	7.124709189	3.000060263	7.019889522	1.455941652	6.285680299	4.251964725	1.050593461	3R	-0.606249759	0.34693679	0.930384827
**CG6574**	FBgn0037846	18.29665018	25.07242215	30.44686099	42.57575852	22.59287236	31.67480046	33.63488485	25.25785744	3R	-0.010623893	0.966428437	0.997604378
**Tsp86D**	FBgn0037848	69.21956176	88.41684367	82.14251298	106.6044935	97.10571147	97.4475306	114.1313564	96.11536578	3R	0.256513563	0.101839358	0.739991904
**CG4596**	FBgn0037849	308.1348754	302.972962	308.6765757	369.4426772	272.107597	354.5156685	342.9126737	279.3492496	3R	-0.019093356	0.873602157	0.995787491
**Tpc1**	FBgn0037852	26.05831778	19.60525779	32.05035461	19.22203653	29.3489119	45.85748151	26.94489173	46.43896186	3R	0.645768408	0.006297293	0.227193262
**CG14696**	FBgn0037853	2.968226486	2.998585535	0.900109507	0.393199336	0.365346106	4.48384134	1.290683444	1.318151141	3R	0.052526949	0.949343589	0.997604378
**Leash**	FBgn0037856	13.64010432	12.47479488	10.64388632	12.34554826	10.55103971	7.85251498	11.51748099	11.35805688	3R	-0.221371383	0.20649119	0.855364409
**mAcon2**	FBgn0037862	0.540308745	0.396502803	0.983086109	0.472391062	0.060962235	1.273499461	0	0	3R	-0.798165778	NA	NA
**CG18577**	FBgn0037870	1.379502655	1.948566045	3.522793894	0.507830291	1.966070412	0.273807721	1.666961484	1.261064405	3R	-0.471684116	0.63445965	NA
**SdhC**	FBgn0037873	1025.102626	1238.891379	1074.482715	1072.083412	1267.037007	969.6862742	1122.741109	1278.078616	3R	0.10745712	0.364535828	0.940794966
**Tctp**	FBgn0037874	343.0628154	477.4064263	488.6171675	450.0082806	544.0209319	515.9469098	468.9650996	542.5268449	3R	0.269663287	0.025499927	0.447402929
**ZnT86D**	FBgn0037875	33.93292097	39.52980867	37.11028883	38.74199578	31.68014179	31.51590079	31.59472738	25.0348275	3R	-0.286004689	0.083739614	0.69799888
**CG4820**	FBgn0037876	37.49432497	48.28785763	33.30086988	43.4119761	33.26981563	60.87290049	45.7168977	34.90698903	3R	0.132577227	0.567631986	0.984934163
**CG6689**	FBgn0037877	86.25833604	90.75591418	93.81357901	83.53862988	92.83906724	94.36882352	85.48876947	98.97696287	3R	0.100464133	0.389243427	0.948247506
**CG6693**	FBgn0037878	186.3145196	200.290311	221.9365292	242.1598557	174.1605688	212.5267174	232.0857827	231.5382314	3R	0.029971637	0.829289225	0.995787491
**CG17726**	FBgn0037880	39.61380518	24.69433377	32.84698715	31.29147414	37.78451826	33.83585926	27.75260138	21.9505906	3R	-0.057412402	0.815702624	0.995787491
**GCC88**	FBgn0037881	106.9426743	98.16888901	78.99410893	84.35140152	73.82983533	110.1215244	70.57850301	84.86403147	3R	-0.092768514	0.56196507	0.984934163
**CG17187**	FBgn0037882	16.47256653	30.96909516	29.13911629	22.56303663	40.6408461	35.13715692	25.38821379	17.5837045	3R	0.298469317	0.330022616	0.921112231
**CG14701**	FBgn0037883	17.54468452	38.4659213	27.14658233	30.92014223	24.44031432	16.93175419	33.12713002	16.49616048	3R	-0.291472044	0.447986076	0.967112302
**Arfip**	FBgn0037884	117.5581221	123.8113323	110.2719014	133.9541272	120.1807713	108.9684796	107.3185302	117.0257032	3R	-0.068300221	0.559859869	0.984934163
**CG17721**	FBgn0037885	16.17736712	16.74633428	27.36011527	7.758909756	19.85891983	18.65092286	16.74334621	8.429414767	3R	-0.059798386	0.888945076	0.995926915
**CG17734**	FBgn0037890	389.5644716	322.0553458	332.8598651	255.732106	321.1178084	309.3259795	303.5030227	287.1768775	3R	-0.061083443	0.632596408	0.991585536
**CG5214**	FBgn0037891	828.016292	767.6531021	721.352873	737.6594024	814.1526762	818.4498103	770.7880858	767.2106486	3R	0.082958049	0.340587133	0.927274752
**mRpL40**	FBgn0037892	111.4253804	113.9087123	129.3907904	134.7117817	121.6611253	147.9291959	143.706358	138.0126266	3R	0.202640833	0.1758786	0.834088743
**Ranbp9**	FBgn0037894	0.789613842	8.950182356	13.17695217	2.175674027	8.207167226	8.730287342	8.789768177	14.02629336	3R	0.717665624	0.369531905	0.942282153
**CG6723**	FBgn0037895	272.4408983	160.9456897	188.692344	58.93560528	210.9374209	184.0587471	251.2687778	88.50156431	3R	0.138943742	0.788127653	0.995787491
**ninaG**	FBgn0037896	1.345816505	1.191415261	1.632466035	0.52295342	1.214771861	0.810639843	0.762934849	1.704437125	3R	-0.031591216	0.966995147	0.997604378
**CG5270**	FBgn0037897	12.83273996	14.49763936	18.11367669	15.71583353	13.3995798	13.01095048	16.41037556	18.3830515	3R	0.035358435	0.841823059	0.995787491
**Dtd**	FBgn0037898	29.40819182	31.91819284	39.26910115	41.19607217	48.18190703	42.52075722	30.89097082	30.31434227	3R	0.135579583	0.630629541	0.990887368
**RpL24-like**	FBgn0037899	616.306306	783.2198445	727.235614	797.715341	669.8573128	732.3497241	757.6317946	773.4426634	3R	0.036418419	0.745188733	0.995787491
**CG5276**	FBgn0037900	98.66322052	126.1326098	137.9554388	100.8057663	112.6197083	133.3303666	106.3814902	122.5230614	3R	0.068669536	0.625630664	0.990047511
**Exd2**	FBgn0037901	15.5639105	17.05435146	19.73273517	25.34596648	13.69544826	12.5352018	18.55507496	16.72554275	3R	-0.30664377	0.23112762	0.867702781
**CG5281**	FBgn0037902	526.6472174	586.4573049	538.2109982	622.7408823	507.7992045	510.3825026	554.7238633	552.2535895	3R	-0.06661158	0.508515072	0.978744714
**dpr5**	FBgn0037908	72.95848879	101.6567061	108.1135233	120.4846585	104.5598881	98.59248426	103.6502515	117.6809051	3R	0.107547265	0.456959429	0.968365245
**CG10898**	FBgn0037911	39.45438414	56.98187698	30.40501445	27.00959414	45.54845201	73.81167415	45.22368034	33.56548598	3R	0.392408186	0.123006795	0.780439453
**wkd**	FBgn0037917	111.1473064	89.4181948	96.81183659	117.4098911	117.9575112	111.1398501	96.05370717	92.43954642	3R	0.036208735	0.785888102	0.995787491
**CG6791**	FBgn0037918	72.41082889	122.429224	88.09693728	105.1457036	109.9014761	100.4770346	94.57412905	92.18365672	3R	0.067124948	0.645610522	0.99279236
**CG14710**	FBgn0037920	42.51591544	49.05088249	51.18664692	41.30177347	45.34484945	40.43273322	58.6811828	43.85682518	3R	0.06463149	0.726770906	0.995787491
**CG6808**	FBgn0037921	4.561037348	4.833725191	4.47630071	3.528517009	2.881844489	4.925589884	3.860805507	3.735918393	3R	-0.146137569	0.643867321	0.992589784
**CG6813**	FBgn0037923	35.53149207	38.54257805	43.29806707	32.5762305	34.38719507	37.51256363	35.22630524	29.62448479	3R	-0.100475	0.58635233	0.987204104
**CG14712**	FBgn0037924	38.04034939	31.62596743	34.96731398	28.60956212	28.93882929	35.22148129	46.80661912	30.99735017	3R	0.11886988	0.497244758	0.97594235
**Elp1**	FBgn0037926	48.88792508	44.01793151	45.36593522	50.31565306	40.41718704	37.47508246	38.48161749	38.08935495	3R	-0.259355793	0.033332198	0.513292083
**CG14715**	FBgn0037930	177.5550648	167.4352909	204.1479135	210.0020522	152.8041874	180.7756975	181.30462	197.4322523	3R	-0.061618166	0.687925799	0.995787491
**CG18476**	FBgn0037931	48.37721166	65.3713881	48.91745429	37.09104945	56.99902237	74.4575806	43.69520761	47.07289849	3R	0.184213922	0.331093479	0.921507984
**Ho**	FBgn0037933	121.6027439	95.68162535	117.7640285	101.2192673	106.654988	123.0576951	117.7404774	98.06987473	3R	0.057220412	0.699392589	0.995787491
**CG6830**	FBgn0037934	0.873893279	0.172450986	1.434153437	0.239699916	0.278400238	1.18738709	0.065568251	1.116060709	3R	-0.004387709	0.996647574	NA
**CG6834**	FBgn0037935	2.853071368	5.667189837	2.853605554	3.734359853	5.150525055	2.548286771	4.022188775	1.195387763	3R	-0.191233466	0.690509803	0.995787491
**CG6908**	FBgn0037936	6.794604898	4.523998622	7.549748112	3.869646542	1.872672257	3.765307051	7.674233726	4.053906743	3R	-0.368812793	0.481127875	0.973549494
**CG14718**	FBgn0037939	0.8637147	1.517748825	0.099779039	0.191782696	1.856221907	1.51228061	0.944294896	1.250136378	3R	1.070348879	0.263447065	NA
**CG12594**	FBgn0037941	1.571945826	0.865384567	1.896981071	0.469863143	1.469816797	2.150830365	1.028222455	2.275226601	3R	0.556680813	0.21753062	0.860826301
**CG14721**	FBgn0037942	108.4848098	131.1673656	138.571315	115.660657	133.6236777	67.44348503	116.9723841	126.3790465	3R	-0.114200377	0.569839211	0.984934163
**CG14722**	FBgn0037943	88.15848586	96.54269403	80.23859575	89.96430246	69.29369321	89.11486785	66.58936059	74.80009126	3R	-0.21395013	0.111446493	0.758410631
**CG6923**	FBgn0037944	65.09809186	89.85432263	90.28544176	73.01435073	87.18871442	76.50992637	70.28973022	76.74639571	3R	0.000352508	0.997797136	0.999596266
**prd1**	FBgn0037949	222.3834748	189.2004676	232.1375366	206.8903021	229.7014124	260.4253632	237.3411304	227.0548016	3R	0.194378484	0.048899399	0.582455792
**HisCl1**	FBgn0037950	25.39551631	19.39820789	26.25498406	48.18676484	37.09952107	40.11329433	34.08661791	44.95705981	3R	0.416753192	0.105715175	0.749812996
**Kyat**	FBgn0037955	46.83619154	44.57760006	42.58410638	48.38317829	39.46222308	57.3034056	46.55749407	59.21278058	3R	0.179488864	0.296133247	0.906715248
**CG6959**	FBgn0037956	1.689461483	3.020271119	2.305468121	1.576430136	2.217205023	2.390967457	2.603869243	2.111500074	3R	0.154655708	0.442093728	0.967112302
**CG6962**	FBgn0037958	5.780666817	13.23616122	10.01701219	10.48242772	10.60122939	4.556065735	11.62563059	1.992121075	3R	-0.420294059	0.355789367	0.937787223
**Cad87A**	FBgn0037963	0.775588153	1.418495172	1.583084682	1.270861654	1.23706742	1.911743264	1.211757677	1.781930577	3R	0.319835448	0.177314446	0.834615603
**CG10005**	FBgn0037972	2.376590767	0.498299701	0.20591343	1.187342551	1.149202353	0.20005672	0	1.658506678	3R	-0.493848338	0.680885733	NA
**CG18547**	FBgn0037973	0	0.080005482	1.272842078	0.778430756	0.688846403	0.224843483	2.190181773	4.452873274	3R	1.910721189	0.12559555	NA
**Tk**	FBgn0037976	0.904669232	0.54011136	0.911364678	0.160871359	0.581294176	0.368633457	2.112251779	0.099870541	3R	0.35596109	0.637837568	0.991585536
**KLHL18**	FBgn0037978	175.6669559	154.9661293	130.7125648	140.5710755	146.8528312	175.4736308	185.5568201	168.8564386	3R	0.196823293	0.118481704	0.774240345
**GCC185**	FBgn0037979	33.82293987	27.3498937	34.70383398	24.58978946	45.70311902	69.05270103	62.67633468	52.94701865	3R	0.961957707	4.74E-09	1.98E-06
**DCAF12**	FBgn0037980	71.47664619	56.84956602	57.96818091	55.7095666	71.21939412	50.4919207	58.83934056	56.38084274	3R	-0.002375046	0.988727206	0.998078482
**Spt3**	FBgn0037981	48.83926906	42.21121383	62.40727941	68.66783559	53.05748001	77.38667504	70.06207494	63.6747061	3R	0.278835228	0.170227607	0.830934498
**ssp5**	FBgn0037985	2.508039845	1.219386955	1.23453008	0	0	0.034269053	1.483608934	0.157831498	3R	-1.568364827	0.269584914	NA
**CG14736**	FBgn0037986	2.457885261	1.291466273	1.005772711	1.795086035	1.336479773	1.200518145	1.133153875	0.64292728	3R	-0.587777309	0.357432484	0.937922644
**ATP8B**	FBgn0037989	10.81705572	10.66300345	10.60264801	12.12391437	13.18479337	11.55704197	11.96392799	10.34279663	3R	0.119398451	0.338272494	0.926687587
**CG4702**	FBgn0037992	1.570593756	0.219537558	0.190511927	0.549267032	0.070883107	0.345507447	0.400661841	0.625149146	3R	-0.815873698	0.363684356	0.939549067
**dpr15**	FBgn0037993	1.17027991	2.03650024	0.96762745	1.338684366	2.175955708	3.334792248	0.737966318	2.064983392	3R	0.621703248	0.09600202	0.730787365
**eIF3d2**	FBgn0037994	1.463653198	2.769825846	1.36569317	3.149955997	1.016257367	1.592219186	0.095738737	0.814801687	3R	-1.260950333	0.108618398	0.758410631
**CG4830**	FBgn0037996	0.629664666	0.528087072	0.286417068	0.091752467	0.710441315	0.667849501	0	0	3R	-0.127575265	0.937142629	NA
**Cog1**	FBgn0037998	45.92501148	50.16373599	48.1306049	52.5254539	43.53581727	41.10868251	46.71231709	47.28526163	3R	-0.108054079	0.354922973	0.936745458
**Rsbp15**	FBgn0038000	0.834372023	1.661956146	0.759065584	0.364745055	0.188281919	1.966602117	0.26606262	1.811498899	3R	0.26299295	0.808742639	NA
**CG3916**	FBgn0038003	0.256773455	0.71783575	1.245857643	0	1.081597761	1.129727412	0.218344867	1.858263137	3R	1.019074765	0.478234402	NA
**CG10038**	FBgn0038013	21.22329978	18.85174441	15.34477571	25.59378852	24.34699966	20.60060224	23.06997342	24.74125409	3R	0.221557341	0.367489489	0.940908571
**CG10041**	FBgn0038014	1.143253242	0.338408282	1.142036173	0	1.578249794	1.458270588	1.715566813	0.146006389	3R	0.911800033	0.478893529	NA
**MBD-R2**	FBgn0038016	138.6060039	105.6013361	128.4666018	120.2276509	151.3607114	128.1013942	128.3333875	139.4681935	3R	0.180190977	0.144146702	0.8037297
**CG4115**	FBgn0038017	7.015891162	1.073901736	0.258866222	0.684145047	1.155785403	3.998904008	0	3.475015019	3R	-0.066222706	0.963216466	0.997604378
**GstD9**	FBgn0038020	53.41767611	38.26694766	33.89752477	27.3875546	32.36742234	42.31795076	47.631286	37.04747989	3R	0.080879589	0.738412183	0.995787491
**CG10035**	FBgn0038028	40.17146958	43.53914151	32.17758999	35.46822023	27.80640793	26.13041611	26.92328827	40.25352881	3R	-0.291338066	0.172024311	0.832669092
**GstD11**	FBgn0038029	1488.826812	1511.320344	1407.847088	1648.852819	1221.694385	1148.138244	1279.530314	1346.22945	3R	-0.246612788	0.013878738	0.35306645
**Cyp9f2**	FBgn0038037	37.89548247	53.94322017	47.45228276	30.50243849	28.27191699	33.6921267	37.32072639	23.85683566	3R	-0.430206194	0.03418618	0.514749796
**Sccpdh2**	FBgn0038038	3.464729826	3.966646411	5.870428479	0.897543017	0.496406773	3.525774221	4.489447732	7.402848397	3R	0.203359962	0.784472923	0.995787491
**CG5196**	FBgn0038039	172.5590021	190.6194118	186.576686	245.0042831	193.6482533	214.0937236	191.2000207	179.6122215	3R	-0.001773156	0.988595509	0.998078482
**Scgbeta**	FBgn0038042	966.668334	904.2564303	830.3225208	1032.418981	913.0430995	858.3872067	1003.140377	1083.56959	3R	0.078103274	0.511528709	0.980804634
**CG17202**	FBgn0038043	534.6971178	541.2831048	587.2737039	491.6136576	578.4501357	510.2485306	521.2981098	510.5345131	3R	0.008522488	0.935260398	0.996479182
**NANS**	FBgn0038045	3.734119905	6.487157377	4.85299436	3.575662137	5.898413041	9.241326773	4.25259826	5.211719985	3R	0.439498721	0.372680565	0.943282319

CG5641	FBgn0038046	395.1120109	421.1161461	390.7345819	453.1928286	422.8354167	452.7444739	384.7198382	452.1190452	3R	0.074559127	0.470418342	0.973549494
CG5245	FBgn0038047	0	0	1.379988784	0	0.09335411	2.193935451	1.187274657	1.684085637	3R	1.985154046	0.224658275	NA
Srlp	FBgn0038049	170.545439	200.7728515	176.9483412	188.6147438	199.6856488	182.6188954	208.5223159	155.192034	3R	0.048727155	0.699595528	0.995787491
CG18549	FBgn0038053	39.48682738	35.52492755	54.83326759	43.19878739	43.15131378	34.93734549	37.46920409	41.61266899	3R	-0.106082607	0.485252581	0.973549494
trus	FBgn0038055	135.9225918	125.1776636	166.7824499	154.8873339	125.5290948	118.2391669	137.4683413	110.160143	3R	-0.215917227	0.115657005	0.765596089
CG5961	FBgn0038056	113.9957157	117.67478	104.1137002	130.7858666	107.7796371	133.8961786	97.91828864	88.9577252	3R	-0.095575765	0.512558867	0.980804634
CG12267	FBgn0038057	12.79391253	15.73249521	8.703181851	13.60422613	14.74729402	16.87053159	15.65720099	20.08185285	3R	0.433777973	0.106638375	0.753794926
CG5608	FBgn0038058	76.7407264	70.78637098	61.82689982	49.89630591	57.15209556	54.32271137	79.06606011	59.31744372	3R	-0.023315678	0.893465752	0.996479182
Octbeta2R	FBgn0038063	0.468621994	1.008331622	1.425747332	0.743308907	1.029741367	1.292849028	0.832905786	1.009082207	3R	0.232979836	0.440659017	0.967112302
Snx3	FBgn0038065	129.4352193	139.9241335	139.8623521	111.3823929	125.2721978	153.6486919	124.0237836	143.9423899	3R	0.102118153	0.390153722	0.948624352
Dtg	FBgn0038071	0.01785207	0.538998187	0.519706486	0	0	0.387108928	0.500951167	0	3R	-0.231115667	0.893303054	NA
CG6225	FBgn0038072	1.240751569	0.161870076	0.468229378	0.03214184	0.199099963	0.376926856	0.10550613	0.059861922	3R	-1.342554948	0.223608695	NA
CG14395	FBgn0038073	0.381406725	0.169731257	0.188834162	0.163328955	0.2107766	0.102739409	0	0.337987472	3R	-0.447200448	0.66050968	NA
CG12279	FBgn0038080	59.5799535	71.84227093	69.36765571	72.24353501	78.6160229	75.68504918	54.02045026	77.66410581	3R	0.10188921	0.619670222	0.990047511
beat-Vc	FBgn0038084	1.185817295	1.156692493	1.090801199	0.434528626	1.333208669	1.320052579	1.045988192	0.703061213	3R	0.215712316	0.499851525	0.976796212
beat-Va	FBgn0038087	1.642208754	2.18147535	1.871666358	1.207725844	2.666157257	5.517640183	4.751633279	2.345019784	3R	1.173827945	0.000875627	0.055705891
CG10126	FBgn0038088	1.403861356	2.433273165	2.611073519	1.036465802	1.140448299	0.44118397	4.178160559	2.336733267	3R	0.152743805	0.81641907	0.995787491
beat-Vb	FBgn0038092	2.184274148	1.471722109	2.274208835	1.065883175	2.059120806	2.459864658	3.274951834	1.574069842	3R	0.446711476	0.141922054	0.8037297
CG7381	FBgn0038098	49.56250277	52.65562134	57.43160456	69.5026543	51.17121364	48.27693817	50.36434223	53.91876216	3R	-0.139069328	0.275659536	0.89549671
CG7091	FBgn0038099	0.888392263	1.834034944	1.740760462	2.78822535	0.431785773	1.127499153	0.522994096	0.074183917	3R	-1.692744986	0.045251487	0.560365369
Paip2	FBgn0038100	91.15574318	92.19848128	87.52627485	78.6974739	101.6925638	112.1129359	81.49731466	82.41476154	3R	0.140256796	0.250900706	0.881909103
CG7488	FBgn0038106	92.52554362	72.81197782	98.38488481	106.4671717	127.1314863	69.27288144	81.27448481	71.47577763	3R	-0.053929385	0.802359413	0.995787491
CG7518	FBgn0038108	92.76507607	88.70927206	70.66961909	85.55590615	80.07139903	101.0121346	81.7238484	85.50496521	3R	0.071556861	0.533861811	0.984760433
CG8031	FBgn0038110	60.89327795	72.89325189	77.48984743	77.09330105	71.93147903	71.89483319	78.08537791	52.70201919	3R	-0.039523261	0.790684348	0.995787491
CG11668	FBgn0038113	0.861873091	5.059849205	2.555534786	4.130484036	1.210145864	1.263995733	1.58791878	0	3R	-1.595949301	0.070119603	0.662558698
CG7966	FBgn0038115	4.214999788	3.393634003	7.3623821	0.2948134	1.445739433	0.238432563	1.505357702	2.836857963	3R	-1.302106244	0.119443407	0.777535158
timeout	FBgn0038118	0.287863894	0.451948815	0.332416405	0.169128246	0.342980951	0.30613505	0.217366705	0.272490914	3R	-0.086890929	0.795413099	0.995787491
CG8483	FBgn0038126	8.235634282	0.737770744	1.324609982	0.353610889	0.574983506	0.900854252	0.077382247	0.592717936	3R	-2.297530341	0.005214761	0.204036711
Ravus	FBgn0038128	85.70510149	107.982821	78.64226199	100.6694521	75.12264243	106.7524348	106.8219379	79.86725407	3R	0.011321669	0.947658126	0.997604378
TBC1D5	FBgn0038129	24.98487322	27.04597297	27.05282625	34.50106641	30.57720634	28.67018834	27.83205707	31.66987302	3R	0.095929409	0.578614927	0.985637351
CG8630	FBgn0038130	0.144183513	5.985728514	3.497871923	2.016950601	1.952161466	0.900571599	0.55172286	2.543415504	3R	-0.90973874	0.216926992	0.860826301
apn	FBgn0038132	0	1.881994245	1.6331709	0	2.295559818	1.763021436	0.572448201	0	3R	0.459981481	0.781751	NA
CG8774	FBgn0038136	0.145740178	0.407431171	2.062454558	0.169893869	0.482347019	0.022900492	0.557679487	0	3R	-1.348477937	0.310887499	NA
PK2-R2	FBgn0038139	1.087827261	0.79829646	0.49482258	0.079257135	0.889850053	1.538395345	0.08672096	0.885665131	3R	0.483408834	0.58336839	0.985764834
PK2-R1	FBgn0038140	0.524106858	1.458045601	0.558208944	0.208623119	0.738456356	0.060259115	0.293489529	0.777091857	3R	-0.508111374	0.551536889	0.984934163
CG9813	FBgn0038143	461.6826503	508.5910728	529.2268553	499.1616	473.9330371	468.5562646	510.1969691	476.0464018	3R	-0.019557219	0.818199556	0.995787491
CG8870	FBgn0038144	0.155881666	0.784408879	2.773222812	0.84800855	1.407031795	1.42065473	0.397657479	1.466545281	3R	0.110294277	0.913539965	NA
Droj2	FBgn0038145	914.303704	857.7915328	887.3674983	866.0781596	893.4689099	912.6634899	980.814765	832.0171108	3R	0.067372863	0.44215503	0.967112302
CG9799	FBgn0038146	41.03118464	40.29767149	40.84616798	39.51857569	34.94145811	32.06766083	33.49485873	41.46943969	3R	-0.157172065	0.293424919	0.906604178
CCHa2	FBgn0038147	0.282236056	0.899481642	0.034235037	0.32901137	0.636885522	0.332613037	0	0	3R	-0.627676605	0.641375755	NA
GILT1	FBgn0038149	27.47065464	42.15847637	43.21114325	43.28304345	37.82646394	36.9638372	39.27825189	52.76397805	3R	0.132449307	0.481782799	0.973549494
yellow-e3	FBgn0038150	0.059860571	1.204892515	0.871325456	0.139562667	1.620955081	0.05643618	0.458116805	1.16966556	3R	0.608762061	0.664609513	NA
Ir87a	FBgn0038153	0.242204112	0.253914638	2.864471016	0	0.765170873	0.542327824	0	0.723040379	3R	-0.665175799	0.622386994	NA
side-IV	FBgn0038156	8.926891434	6.925694521	11.26880713	9.37389068	8.546529584	9.215188802	10.17537836	12.03537734	3R	0.162392541	0.378283914	0.943615128
CG9759	FBgn0038160	2.077559397	0.201046987	0.775404634	1.210937881	1.370384933	1.431365226	0.305763384	0	3R	-0.445548322	0.697426591	NA
CG10841	FBgn0038163	4.124678364	1.84163356	1.511760114	0.691836858	1.392795982	0	1.438278556	2.512570383	3R	-0.585280205	0.535870005	0.984771911
Task6	FBgn0038165	69.64356543	74.79641665	73.22194783	83.7854491	74.57525042	61.6068743	67.03234027	78.17278695	3R	-0.067078714	0.579997652	0.985637351
CG9588	FBgn0038166	40.33826418	49.28337706	68.10531638	54.52192559	50.77782056	31.65796236	41.99586638	50.17988753	3R	-0.244436093	0.272666563	0.89549671
Lkb1	FBgn0038167	73.29929985	69.59393923	54.33888183	84.04535493	59.26900442	81.29466246	52.84445985	72.19374862	3R	-0.057017078	0.740100797	0.995787491
omd	FBgn0038168	24.05690786	16.5671687	13.87375527	21.07839924	20.90945826	23.19569865	20.10582483	16.36113399	3R	0.114134818	0.543423047	0.984934163
CG14367	FBgn0038170	2.729068013	5.075197084	0.287855693	0.829919913	1.285217535	0.61527033	2.87557199	2.704918369	3R	-0.22906066	0.804272042	0.995787491
Adgf-D	FBgn0038172	65.16920639	110.1897076	84.94226595	67.25764544	120.1430741	70.53047115	94.65843422	49.48848143	3R	0.069063446	0.76520242	0.995787491
Adgf-C	FBgn0038173	7.449461261	11.88290532	4.429482626	7.662414041	3.362046714	4.64777641	16.39537984	3.567677203	3R	-0.139478441	0.783303352	0.995787491
CG9312	FBgn0038179	4.501319373	1.72579012	0.468005915	0.749618333	1.044775947	0.788137333	0.328084641	0	3R	-1.760708958	0.140249253	NA
Cht5	FBgn0038180	68.33126447	1.186785689	1.650444987	5.519762666	32.37404962	31.42864038	23.46408165	20.38309744	3R	0.492544115	NA	NA
CG9297	FBgn0038181	0.188252388	0.612959414	0.18805198	0.103271378	0.399816245	0.135722448	0.225993358	0.72125887	3R	0.484017736	0.588859037	0.987588562
CG9286	FBgn0038183	27.11662431	33.26854085	42.47802523	34.50081501	34.54644809	31.26281704	52.37542619	27.75427358	3R	0.12051975	0.649399183	0.992932477
CG14362	FBgn0038186	1.722234124	3.738455002	0.245771527	0.944782124	1.645980352	2.674348867	3.618140442	1.319692838	3R	0.492711249	0.629305923	NA
CG9925	FBgn0038191	0.600473107	0.083934117	0.485579794	0	0.316168958	0.070765297	0.638258248	0.543201125	3R	0.428682637	0.746194228	NA
CG3061	FBgn0038195	382.0610783	334.9831339	294.9755393	310.938147	311.0964513	335.9200032	340.3854187	292.790167	3R	-0.019465057	0.859689951	0.995787491
CG9922	FBgn0038196	54.48162117	67.27556646	58.16770906	50.63313327	63.45373532	66.0406367	80.7002629	76.17640019	3R	0.344329797	0.040526162	0.530560761
foxo	FBgn0038197	1.669117098	1.231310488	0.643276264	0.344897383	0.851552043	1.142068391	0.954123377	0.690826222	3R	-0.075092373	0.85036455	0.995787491
Npc2b	FBgn0038198	4.199853038	1.898562008	4.696957574	3.333378469	3.226302616	2.246579172	3.41933468	1.61671449	3R	-0.403293307	0.398707676	0.952805113
CCHa1	FBgn0038199	0.11999757	1.006395445	0.194074953	0.373026421	0.722087894	0.075421984	0	0	3R	-1.024547688	0.482295387	NA
PK1-R	FBgn0038201	1.132092572	0.485771888	1.085802173	0	0.332698156	0.099286508	0.201487847	0	3R	-2.071048164	0.131911174	NA
CG12402	FBgn0038202	49.87385028	45.82717362	53.01336085	40.77931555	36.71571096	44.80501189	34.41589051	42.24273769	3R	-0.230336295	0.105084556	0.747886154
Kif19A	FBgn0038205	1.039592213	1.186732427	1.856498876	1.548519792	0.703773896	1.52463296	2.025850193	1.41065683	3R	0.041632788	0.930735368	0.996479182
twf	FBgn0038206	75.19605941	76.1574823	89.1340408	67.46367296	90.72788403	85.83605029	80.98003226	65.09638712	3R	0.098526223	0.514462505	0.980804634
CG14356	FBgn0038207	1.991224038	2.504999237	2.566299675	2.031077949	1.572668009	3.695961662	1.904870738	3.512544516	3R	0.271152542	0.680846165	0.995787491
CG14355	FBgn0038208	0.605721998	2.497700288	0.18368414	0.588424181	0.683427157	0.404508625	0.064383743	0.273974693	3R	-1.385083704	0.172511464	NA
CG9722	FBgn0038209	0.734052929	1.07736196	6.9006084	4.920323216	0.165644209	1.038090927	2.57480409	3.18739372	3R	-0.933869914	0.365453856	0.940794966
CG9649	FBgn0038211	10.97474264	26.5635371	28.87942236	5.500831122	2.710467027	6.808071353	15.13833715	5.122456106	3R	-1.231491117	0.013046871	0.344090585
Afti	FBgn0038223	41.08301525	43.19793698	59.72894879	59.63937156	45.43290797	40.47968845	62.90939678	54.639914	3R	0.031934901	0.854743251	0.995787491
ATPsynE	FBgn0038224	1645.087137	1437.043039	1519.742898	1521.267074	1622.02173	1463.096945	1575.917933	1419.013122	3R	0.019312895	0.844332014	0.995787491
soti	FBgn0038225	4.096808916	6.170615881	1.216996076	5.652961573	4.226165769	1.576508744	3.412586959	2.722821855	3R	-0.498604229	0.485979729	0.973549494
HtrA2	FBgn0038233	10.63618713	19.4229156	10.02144246	15.09105843	10.33450676	7.574464427	18.58661707	11.86385526	3R	-0.156432648	0.629971492	0.990523437
mRpL11	FBgn0038234	51.17889309	47.86222458	57.15268481	75.52303644	54.81133869	45.76200128	62.4297378	54.45473467	3R	-0.062003383	0.787989441	0.995787491
CG8461	FBgn0038235	37.84123758	36.97760397	30.74114203	18.41402133	24.75567644	35.02187319	25.0190838	21.29293981	3R	-0.197403918	0.505791979	0.978557094
Pde6	FBgn0038237	6.057015353	7.273823201	6.298881311	6.612868459	8.064388119	9.60948526	4.916636838	6.255411817	3R	0.163979781	0.331469409	0.921507984
CG8066	FBgn0038243	3.854371772	4.819488823	2.040142289	4.738242871	5.566503133	11.09984603	3.039162698	15.2149053	3R	1.204006191	0.036779405	0.521170991
CG7987	FBgn0038244	78.6682423	91.14496392	85.6837156	98.3816543	98.38342211	115.0824598	99.6892906	80.50126313	3R	0.181947364	0.136696911	0.796109055
CG14853	FBgn0038246	183.664049	130.4733343	132.1970724	149.8666331	118.6189083	119.0210961	113.3790478	120.2996404	3R	-0.312087816	0.011635763	0.327609616
CG7886	FBgn0038248	0.010904292	0.182904206	0.01322683	0.025422947	0.009842521	0.0308415	0.125176877	0.426136102	3R	1.407635519	0.283609001	NA
Hexim	FBgn0038251	74.61422195	93.40665355	80.23542923	68.51571913	84.1636417	107.4441023	85.76847542	89.64842681	3R	0.244099945	0.086901375	0.706081898
CG7530	FBgn0038256	0.551872064	7.2117551	3.5494667	2.812718154	0.949932356	1.960206071	3.306781088	2.089823264	3R	-0.719232081	0.196056428	0.844272804
CG14855	FBgn0038260	0.638954325	2.947329669	0.968808925	3.072502031	3.099967607	0.414151536	0.407496552	1.127123551	3R	-0.547331115	0.530729741	0.984760433
CG14856	FBgn0038261	0.238727365	0	1.544398021	4.638198873	0.071827347	0.112535351	0.202999546	2.677879243	3R	-1.034173167	0.445055127	0.967112302
CG14857	FBgn0038262	4.754057079	13.23388464	6.543702788	11.16251437	5.538925709	5.594672991	6.536917253	7.905828252	3R	-0.443140915	0.234149464	0.87009833
CG3631	FBgn0038268	8.705488456	9.429656863	10.3279793	6.108043219	12.90751448	14.07364356	10.99951824	9.539084103	3R	0.491087436	0.054018219	0.609185163
Rrp6	FBgn0038269	24.17360858	33.06625727	34.25620989	38.95619273	25.40773567	44.01269181	32.36653897	24.28474254	3R	-0.021260096	0.917712802	0.996479182
UQCR-C1	FBgn0038271	3041.682457	3115.175688	2682.530673	2994.831703	3217.87632	2846.165416	2949.411378	3068.561295	3R	0.061347946	0.507763304	0.978557094
CG7265	FBgn0038272	82.68105266	72.55196253	90.26911269	87.91554525	75.68137574	79.51962585	80.77529973	83.55379209	3R	-0.031676776	0.824208045	0.995787491
Nup93-2	FBgn0038274	28.07223023	21.1212075	21.33626516	20.12662031	20.32961495	17.50143255	20.61456965	13.95096794	3R	-0.300350726	0.164994274	0.827852065
CG3817	FBgn0038275	51.79337012	54.77332044	41.1653312	36.80130823	44.88014765	34.32084673	48.82370282	46.69277679	3R	-0.051740213	0.829645136	0.995787491

**RpS5b**	FBgn0038277	0.689269859	1.669999491	0.928977258	0.357112606	1.520821811	1.660700722	1.562970862	5.320777491	3R	1.50425061	0.111781595	NA
**dpr9**	FBgn0038282	141.7325846	141.5526984	120.5566933	158.5588148	135.2672801	144.0286385	123.0305694	134.8456704	3R	-0.03823386	0.714763947	0.995787491
**CG6966**	FBgn0038286	9.202956269	16.19380415	12.45786354	11.06865171	10.21938755	14.88971962	11.60977315	12.9359996	3R	0.055634851	0.745862705	0.995787491
**CG6912**	FBgn0038290	0.974803408	0.389308563	0.112612356	0.10822464	1.005582852	0.262582485	0	24.3888572	3R	4.040917269	NA	NA
**CG3987**	FBgn0038292	0.098597889	1.571149208	1.19598552	0	0.444986151	1.812671009	0.503050667	5.993824046	3R	1.682185734	0.152304944	0.81337081
**Gyc88E**	FBgn0038295	7.331404597	6.146923764	4.432331056	7.110920559	7.380872818	6.965956666	6.4126189	5.448370951	3R	0.091815567	0.582963551	0.985764834
**CG6752**	FBgn0038296	38.21492088	58.46003072	49.18912944	55.15133555	62.64437645	58.25156967	56.61370484	78.66094225	3R	0.385552997	0.014509024	0.358137424
**Spn88Eb**	FBgn0038299	0.252408681	0	0	0	0.455662361	0.832892769	0.429266645	0.822003917	3R	3.131703538	0.071906273	NA
**Mau2**	FBgn0038300	46.68412213	56.00070156	41.37975956	32.22862118	54.36036371	70.80267761	37.53208681	53.00324899	3R	0.320847266	0.12495981	0.780439453
**CG6654**	FBgn0038301	48.67501582	63.64444512	63.8964682	65.22359538	67.05244194	59.16377101	52.1555368	52.0491622	3R	-0.03343252	0.828066958	0.995787491
**SIDL**	FBgn0038303	73.87407283	73.31630792	82.58210208	66.64453352	64.1139769	67.48331557	68.72914448	70.15521789	3R	-0.100989127	0.366255414	0.940794966
**CG12241**	FBgn0038304	99.2147353	93.56393297	69.01051456	77.21319105	95.72842071	77.38992854	70.86486053	79.3277321	3R	-0.038857352	0.797498766	0.995787491
**Art3**	FBgn0038306	4.467829642	8.138816156	0.11654723	1.680093003	6.07086486	3.306383895	1.838311194	4.589281251	3R	0.170021271	0.828659539	0.995787491
**mRpS10**	FBgn0038307	251.2030908	274.3607037	273.3479012	265.3078097	252.8349608	257.7523252	296.0048253	311.1435618	3R	0.102555287	0.412191731	0.958665466
**Amt**	FBgn0038309	0.394916014	2.208054039	1.383865514	0.306910606	1.346635395	1.365189486	5.037196176	0.285799741	3R	0.949505318	0.317860064	0.91460807
**CG14864**	FBgn0038311	4.952283732	0.783656719	1.246753621	6.971207903	2.44588774	2.995196547	0.357548553	1.420057828	3R	-0.934565682	0.24756609	0.879384515
**Zip88E**	FBgn0038312	42.24376472	15.46133754	25.84543895	36.39368947	31.35724602	25.63436939	20.08772778	45.05304321	3R	0.049876588	0.875397618	0.995787491
**CG4338**	FBgn0038313	13.92078948	22.28186296	11.69867306	11.03072826	15.6039629	8.578062281	9.980562226	7.70398419	3R	-0.45979428	0.271081174	0.89549671
**CG6276**	FBgn0038316	163.1171035	239.1911812	187.3869599	215.5834836	202.9601389	165.0768557	214.2152707	232.601167	3R	0.05298716	0.723454957	0.995787491
**CG6236**	FBgn0038318	41.33785161	62.6760339	69.16456571	45.41067679	43.55362413	65.57462415	40.38015387	33.15257578	3R	-0.228037023	0.275651206	0.89549671
**mRpL9**	FBgn0038319	126.258831	177.5956047	167.3660729	170.3483851	169.9813592	159.5794082	213.1662407	173.675459	3R	0.193301529	0.230388757	0.867702781
**Sra-1**	FBgn0038320	72.19602745	65.33631467	70.0998332	70.02208518	68.75413909	86.62796511	65.86915247	70.1476659	3R	0.097251062	0.396129036	0.952272708
**CG6218**	FBgn0038321	25.02340866	24.26027122	38.7813959	25.03571048	15.49646798	24.93566356	23.50955276	18.53134497	3R	-0.425583652	0.031080677	0.491511116
**CG5038**	FBgn0038324	23.21792293	32.65124144	20.61669152	15.25258219	20.66768739	29.99253895	20.69757364	12.32356631	3R	-0.103302981	0.707816823	0.995787491
**Atg4b**	FBgn0038325	91.02485427	109.0581597	96.86919057	95.67909414	99.02339039	119.4616722	92.34461375	103.7713526	3R	0.109548059	0.332971262	0.922663747
**CG5044**	FBgn0038326	49.56914353	60.78748886	46.07672393	55.89786713	50.27765943	37.94084538	48.3428847	46.78291647	3R	-0.18040694	0.28932206	0.903248331
**Trax**	FBgn0038327	78.53986004	96.98543678	96.35708309	61.56075489	91.41729392	83.77877864	95.40803349	62.52301238	3R	0.031675752	0.870307172	0.995787491
**CG14868**	FBgn0038330	219.0884231	234.0744707	207.3710248	238.5613692	235.3233911	260.1023203	242.0340033	187.5262448	3R	0.069029679	0.555238712	0.984934163
**Ccm3**	FBgn0038331	93.60849003	80.41926498	81.19676831	62.17847351	54.31735162	77.86653265	76.65935062	66.97441154	3R	-0.17538228	0.285581279	0.901952098
**CG6136**	FBgn0038332	33.11874098	32.58340919	32.44726303	32.38950618	26.3008901	31.59943693	26.40206868	16.33392802	3R	-0.34702616	0.099399601	0.731438598
**CG6125**	FBgn0038337	0.193690174	1.429507615	0.516878626	2.393381146	0.62938873	0.639134694	1.235269485	0.04205193	3R	-0.80495431	0.37772987	0.943615128
**CG6118**	FBgn0038339	1.232864245	0.980680552	0.662933984	1.081595309	0.975149768	0.491297031	0.567408305	0.303539035	3R	-0.735702767	0.10904864	0.758410631
**obe**	FBgn0038344	22.34655291	24.41511653	25.4419232	14.49235265	24.10136933	25.66877616	26.78363704	20.90377096	3R	0.200896944	0.24416978	0.877269208
**AOX3**	FBgn0038349	0.700588752	0.961477648	1.622363151	3.044056741	0.172464853	1.381068027	0	0.691383197	3R	-1.463898516	0.09030657	0.717658936
**AOX4**	FBgn0038350	0.556169473	0.760137699	0.349807988	0.048025488	0.371862386	1.573059545	0	0.178888209	3R	0.330229395	0.784298579	NA
**CG5399**	FBgn0038353	1.77701819	2.369273345	0.994851079	1.487249082	0.082255725	1.460571909	0	0	3R	-2.029974739	0.108075666	NA
**CG5404**	FBgn0038354	27.63578407	41.67710811	45.34837835	24.93440421	19.22354382	18.99244163	35.27913873	24.87066153	3R	-0.466986707	0.04998183	0.587515621
**CG4520**	FBgn0038355	1.084576781	1.15768889	1.674379727	0.229877267	0.266991691	0.790138645	0.251525334	1.391423439	3R	-0.57773486	0.589903618	NA
**CG5614**	FBgn0038359	0.538419553	1.354684902	0.326549582	0.156913115	0.607490806	1.332498999	0.515069943	1.461198364	3R	0.749056308	0.536677679	NA
**CG9590**	FBgn0038360	79.36476175	83.47955892	83.74559631	91.96249897	83.91883935	85.85612654	105.8903205	70.34866285	3R	0.060588407	0.696715105	0.995787491
**Uros2**	FBgn0038361	0.267065506	0.14932164	1.295794383	7.056735955	0.482121686	2.601806516	2.270966141	0.193274645	3R	-0.641982898	0.615319801	NA
**Acyp2**	FBgn0038363	65.04499047	63.28023104	87.578023	63.69297759	61.25560172	68.88731548	84.9014477	78.14101149	3R	0.100372151	0.626242487	0.990047511
**Arpc3A**	FBgn0038369	104.597998	78.94122868	80.96229636	73.54230843	109.4463046	86.00503941	91.73346917	94.5075006	3R	0.202298015	0.227942062	0.867702781
**Hmt-1**	FBgn0038376	78.44217582	87.12557035	87.74084461	114.7656511	89.21058142	97.78267033	90.48400422	91.4176729	3R	0.032551311	0.808352052	0.995787491
**CG14877**	FBgn0038380	0.51588438	0.61205821	0.39682605	0.058671464	0.363435522	0.462647148	0.224688414	0.464403821	3R	-0.034158604	0.967289272	0.997604378
**EndoU**	FBgn0038381	3.519278645	3.320491624	1.814265692	1.641013135	0.079414999	1.285707334	0	0	3R	-2.81291786	0.011899683	0.331790969
**Fbxl7**	FBgn0038385	0.079472909	0.278727916	0.315491184	0.084221861	0.208682137	0.694773676	0.552919357	0.031371462	3R	1.015050333	0.259674538	0.890556245
**blp**	FBgn0038387	95.81103292	82.01637941	86.70698056	62.81979949	91.56048071	70.38123517	65.29889917	70.71464804	3R	-0.106010839	0.577885036	0.985637351
**CG4287**	FBgn0038388	112.3999117	101.446575	89.36903302	116.8917789	114.2407187	117.7870888	112.1722352	113.2762229	3R	0.150042556	0.261512119	0.890640489
**CG5516**	FBgn0038389	69.53163618	54.33877478	87.69587687	75.06816458	112.3283057	107.2701849	95.49114836	112.7908514	3R	0.610005502	0.001331888	0.077083046
**Rbf2**	FBgn0038390	0.257409348	0.215884035	0.234176808	0.150035051	1.916845618	0.242683961	0.492492583	0.279429761	3R	1.799948112	0.11827524	NA
**GATAe**	FBgn0038391	0.354143206	0.188349564	0.628643765	0.181244975	0.218304135	0.293166484	0.572904881	0.393815399	3R	0.157773819	0.825608047	0.995787491
**CG5013**	FBgn0038396	57.66082628	46.15761057	50.12009385	42.24505991	59.45963237	33.28791277	32.61555801	47.06001441	3R	-0.155579154	0.538834406	0.984934163
**CG10185**	FBgn0038397	0.630625343	0.850376615	0.149989048	0.720725121	1.071473291	1.340651333	0.662421858	0.590611998	3R	0.661962937	0.296530831	0.906715248
**CG5903**	FBgn0038400	852.20494	794.2991223	794.8981294	907.6924954	872.7049535	843.087196	843.6108953	817.8223638	3R	0.041079221	0.655267301	0.995179752
**CG5916**	FBgn0038401	117.1190318	98.53189173	116.5533994	122.0823759	87.27105262	102.2747197	75.77396048	88.50977888	3R	-0.332760883	0.011199991	0.319404908
**Fer2**	FBgn0038402	3.298893555	0.822537063	2.920038964	2.494458206	1.609553613	1.176823614	0.568618131	1.742157555	3R	-0.876622364	0.226400148	0.867702781
**CG8925**	FBgn0038404	0.684043194	0.702132035	0.297220164	0.309442872	0.387049835	1.068435876	0.026044909	0.044331973	3R	-0.36402565	0.701173056	0.995787491
**CG8927**	FBgn0038405	0.963347698	0.250740096	0.644708436	0.968107282	0.029984302	1.315379644	0.466081507	0.108181915	3R	-0.547205432	0.576280185	0.985637351
**CG6126**	FBgn0038407	3.374072917	1.972261843	1.364242306	1.430277089	1.907303615	4.787648941	2.651753952	0.44396513	3R	0.274242973	0.636574943	0.991585536
**Zip89B**	FBgn0038412	0.500513316	0.987695144	0.214277351	0.068642821	0.265751416	0.277576998	0.450642431	0.06392122	3R	-0.723896907	0.565813651	NA
**pad**	FBgn0038418	29.13590091	31.86364737	30.22443782	44.51925847	45.07107692	48.28615934	38.20158313	26.5667587	3R	0.246587119	0.223617367	0.86549481
**CG14879**	FBgn0038419	0.305621567	0.81167573	0	0.712545177	0.045977105	0.048023024	0.714676584	1.327065424	3R	0.258520206	0.844311427	NA
**CG10311**	FBgn0038420	1.487903722	4.711602353	4.891559655	3.1447867	2.03336311	2.569590299	7.094718432	3.894566319	3R	0.174685169	0.691063301	0.995787491
**CG17931**	FBgn0038421	3.139967475	5.567818086	9.032199083	7.634460933	4.615734649	7.992923822	7.89569414	7.596264059	3R	0.188943167	0.654758536	0.994669639
**CG14880**	FBgn0038422	0.108584989	0.200349695	0.342453369	0.556955991	0.431252217	0.102373264	0.110801056	0.023574806	3R	-0.808736807	0.440631724	NA
**CG10317**	FBgn0038423	1.496978024	1.818289077	2.379355459	0.962799423	0.838684864	0.146000869	0.26336711	0.11207165	3R	-2.257270287	0.02980404	NA
**CG17565**	FBgn0038424	224.749983	171.9792702	157.9025628	188.6154191	200.9806262	201.6732973	165.0261035	180.2835265	3R	0.034912654	0.801207597	0.995787491
**CG14881**	FBgn0038425	77.71148517	67.85177882	60.32143075	37.72449444	72.35543451	46.25479355	56.80334333	80.57250619	3R	0.10424673	0.676623833	0.995787491
**mRpS33**	FBgn0038426	219.213205	258.7319321	222.6970537	192.4351741	292.9069746	303.5738945	219.7641088	267.775718	3R	0.311404342	0.039676797	0.529044592
**ema**	FBgn0038427	180.9181763	166.2887759	163.8971201	189.8666742	159.9003983	207.9412725	168.6469996	175.7446629	3R	0.049539475	0.648175298	0.992932477
**CG14894**	FBgn0038428	198.8350106	195.3425951	188.4712892	173.8411283	192.1017447	209.7003128	160.4084448	160.1062916	3R	-0.037592677	0.768106387	0.995787491
**CG14882**	FBgn0038429	57.02487941	49.15522629	65.61609052	72.58001772	48.48019224	70.83814263	53.06591648	81.77264775	3R	0.085753597	0.670856763	0.995787491
**CG14883**	FBgn0038432	129.907511	88.59956036	159.2629922	104.1680772	98.36638199	112.1791867	102.3267564	93.12275461	3R	-0.219296472	0.195811405	0.844272804
**Gyc89Db**	FBgn0038436	0.809311891	0.366893828	0.106128614	0.509967624	0.434355926	0.288708116	0.72539017	0.284933681	3R	-0.057063092	0.95288044	NA
**Sdhaf3**	FBgn0038437	81.34200733	94.40159561	77.86715671	83.03411926	85.724662	54.44210194	98.70518015	111.6882175	3R	0.091339418	0.713632826	0.995787491
**Der-2**	FBgn0038438	166.2118592	159.2583164	156.5358881	139.6063886	173.9582701	172.3525144	178.7663256	160.9977792	3R	0.171388733	0.127946137	0.788096052
**Cad89D**	FBgn0038439	0.207449741	0.267667568	0.425843997	0.111614137	0.60496146	0.105313572	0.08141673	0	3R	-0.306475892	0.777868016	0.995787491
**CG14903**	FBgn0038446	112.618836	172.67445	190.6981169	170.2277267	160.1886463	183.9915016	214.4093248	211.6863316	3R	0.289673081	0.131391625	0.790743321
**CG14892**	FBgn0038447	0.398315244	1.182054822	0	0.21430546	0.110624785	0.519963478	1.016110744	0.598693337	3R	0.345782098	0.780477238	NA
**CG12783**	FBgn0038448	1.005663629	0.366708621	1.803275693	0	1.657612722	0	2.342385218	0.759439594	3R	0.62315326	0.639174782	NA
**Arl6IP1**	FBgn0038453	857.6091307	817.835862	928.6985649	826.7454433	756.1787195	872.7423545	742.7584861	878.3162594	3R	-0.048296731	0.620629609	0.990047511
**CG10324**	FBgn0038454	22.99956523	25.56862575	20.23035506	20.90550507	35.12617491	13.18783836	24.24667763	31.53738009	3R	0.250492166	0.387621194	0.948247506
**CG18622**	FBgn0038460	1.907981252	2.443293903	3.931936951	0.19132845	1.99997139	1.392644957	0.994394862	0.757213619	3R	-0.676674806	0.324428612	0.918470465
**EMC2B**	FBgn0038461	67.60037953	59.47101815	66.14342144	71.15237393	66.13237769	57.96814482	67.97822386	61.99522072	3R	-0.028872068	0.852166927	0.995787491
**EMC2A**	FBgn0038462	60.38917584	47.51143092	51.94957481	53.491568	46.73658404	44.81058554	49.13544924	43.04753454	3R	-0.189930108	0.261746505	0.890640489
**CG3534**	FBgn0038463	16.70062079	11.85349289	11.12601075	19.36756162	8.539585044	13.84167327	11.25795733	5.448182472	3R	-0.5750366	0.063702155	0.636563265
**Irc**	FBgn0038465	20.61006491	4.560220166	10.28122479	4.520857687	9.671511601	8.763758621	14.94178321	5.945927402	3R	-0.006351889	0.987947341	0.998078482
**CG8907**	FBgn0038466	0.16251078	0.272589035	0.640654438	0.284165824	0.476732114	0.57455314	0	0	3R	-0.317548224	0.818758804	NA
**CG5225**	FBgn0038468	0	0.651200001	1.77902846	0.905141738	0.155745153	2.602809655	0.110042333	0.187307001	3R	-0.083514711	0.952228373	NA
**CG18213**	FBgn0038470	1.751012054	0.495870156	1.930877646	2.438847527	0.533679636	2.679940697	1.740338715	0.83931633	3R	-0.172618542	0.796765684	0.995787491
**CG5220**	FBgn0038471	90.47429285	67.19510731	83.67274422	61.59971658	93.58884163	81.66315291	69.76883523	58.29278011	3R	0.029235949	0.882569051	0.995787491
**CG3995**	FBgn0038472	55.53347201	52.96053225	59.48166466	45.43811526	57.50312005	76.3945624	59.69619102	61.87656582	3R	0.289384693	0.097192107	0.730787365
**Ns1**	FBgn0038473	41.98086906	74.73790939	77.14051025	51.61289424	77.89341964	60.90583663	66.02253651	57.32569076	3R	0.133493773	0.497722219	0.97594235

mRpS11	FBgn0038474	224.8163121	220.0534607	256.3919554	219.6151824	229.0359208	264.4257501	277.9211782	218.8387167	3R	0.134430157	0.277072504	0.896087042
Keap1	FBgn0038475	15.82878818	17.66811056	19.70778131	13.22092976	8.67409436	12.96434861	12.25742909	13.37664667	3R	-0.458633805	0.01762002	0.38141622
kuk	FBgn0038476	104.2779056	97.22290184	95.64206837	105.2244062	85.40450402	100.418499	99.98703029	94.46333412	3R	-0.053341837	0.612358247	0.990047511
cal1	FBgn0038478	26.40222632	36.85868751	30.84808916	31.3575718	25.43440115	44.64907212	26.75984075	34.75287346	3R	0.099659259	0.600476587	0.989286463
TwdlW	FBgn0038487	0.869079388	3.279957195	1.9326766	0.337704313	0.130742587	0	0.554260047	0.9434259	3R	-1.969012777	0.151099872	NA
m-cup	FBgn0038488	75.00364597	87.52123321	67.46271611	68.92464989	72.53048929	91.4554532	74.43078879	59.00326097	3R	0.021016707	0.877552195	0.995787491
CG5285	FBgn0038490	48.91480986	29.81021144	46.33841882	35.62634796	49.47409784	32.88449111	44.27774321	36.24094163	3R	0.046048389	0.856073254	0.995787491
CG5292	FBgn0038491	57.77573034	51.80233501	57.86954395	40.91809332	51.00384667	62.59416538	34.12810295	48.44170937	3R	-0.057667865	0.770949044	0.995787491
Mur89F	FBgn0038492	0.419561259	0.431715346	0.342134374	0.180842086	0.381890221	0.558437354	0.112427499	0.172230189	3R	-0.138545783	0.773067997	0.995787491
beat-IIb	FBgn0038494	1.175299406	0.68223726	1.037986774	0.89902086	1.325476921	0.859061707	0.660280844	0.527539124	3R	-0.143387479	0.660724318	0.995750687
beat-IIa	FBgn0038498	0.888440425	1.112162081	1.089063921	0.451058429	0.97316826	1.407832735	0.680408344	0.607619607	3R	0.082302431	0.796857653	0.995787491
Brf	FBgn0038499	5.972586254	8.371955157	7.639408232	9.201664964	5.106787684	3.716009927	5.890972034	5.617530363	3R	-0.583410983	0.003481097	0.154316288
Sur-8	FBgn0038504	109.8119503	121.7766551	97.39838952	126.1755816	118.5585069	130.8552681	119.1507582	99.65746331	3R	0.068702515	0.552491009	0.984934163
cysu	FBgn0038511	1.123935559	0.462550628	0.557493901	0.44810044	0.346965131	0.571181186	0.564909811	0.208638912	3R	-0.603320131	0.211832288	0.856520545
CG5823	FBgn0038515	179.3250417	189.2237885	184.1232926	197.4832714	170.6776841	175.9981744	182.2814767	193.0444583	3R	-0.02444536	0.812431877	0.995787491
Prx3	FBgn0038519	409.7989269	405.3467673	450.4490788	397.5690452	432.9091793	430.1508322	400.4041201	398.5240493	3R	0.029582222	0.752946557	0.995787491
sll	FBgn0038524	55.70602574	75.30017419	65.49987833	66.51401951	71.47340146	72.87639888	75.77239411	50.04535387	3R	0.070278446	0.658622212	0.995750687
CG14322	FBgn0038532	2.481352953	1.849831769	1.940632076	1.738270347	1.990879131	2.255200493	1.555254455	1.829471971	3R	-0.044155788	0.84356559	0.995787491
CG7523	FBgn0038533	24.1897899	29.78817482	34.92243566	22.6051668	34.02413412	24.34504045	27.38401268	33.61599076	3R	0.135326932	0.548223803	0.984934163
alt	FBgn0038535	70.98692157	90.96544081	84.04907728	80.38339264	75.82510309	79.93374993	83.57574274	81.2562212	3R	0.00698899	0.944678813	0.997604378
CG7655	FBgn0038536	48.17355328	42.63944353	48.91721607	46.27956184	61.18753049	45.49170203	52.23900947	62.00404667	3R	0.278457226	0.161229264	0.823037813
CG14321	FBgn0038540	196.6886855	295.7754655	222.2113601	203.2367522	217.2778151	188.7743382	204.3150243	182.2920538	3R	-0.177024431	0.249704039	0.879541335
TyrRII	FBgn0038541	1.044938777	0.976716711	0.967559885	0.948458065	0.403196219	1.549185883	0.406971186	0.519540186	3R	-0.421313601	0.415212057	0.958818865
TyrR	FBgn0038542	5.206778684	8.789269089	5.680184814	6.37125643	5.579872134	4.58374233	5.346882744	8.381095454	3R	-0.086658354	0.708951644	0.995787491
pasi1	FBgn0038545	40.24052153	40.59228232	22.50234237	21.62558357	21.27714537	29.12783957	25.08539918	26.34604181	3R	-0.270126905	0.335591574	0.924219112
CG7379	FBgn0038546	374.5071036	377.3168093	320.5425429	429.1377552	354.423622	325.0854246	299.7009801	353.4832861	3R	-0.142448167	0.234446208	0.87009833
CG17803	FBgn0038547	30.07806253	28.83743714	36.28511134	37.55374485	20.35456159	29.13220635	27.75689444	30.33164991	3R	-0.273415787	0.152300462	0.81337081
CG17806	FBgn0038548	27.37701245	23.51024895	24.82964792	13.40045308	24.6657533	29.09071571	31.63992513	30.97783058	3R	0.411941908	0.100007511	0.732357333
CG17802	FBgn0038549	15.32081618	18.87908022	20.45282964	16.86219034	9.73437985	17.22834303	18.99600344	22.48498396	3R	-0.030418933	0.913715887	0.996479182
CG17801	FBgn0038550	2.09460907	1.756706435	5.978226338	2.010853844	1.557008441	4.065733075	0.47147583	1.07002122	3R	-0.6720057	0.413000432	0.958665466
Odj	FBgn0038551	84.04117387	77.56802596	67.15046851	89.90265597	70.12873864	66.32037873	74.50720849	66.10461399	3R	-0.174802793	0.242606117	0.876959115
Alg1	FBgn0038552	43.33411366	62.12340289	41.22664914	44.39808146	42.9493546	59.091514	49.72668908	48.83168105	3R	0.101459884	0.579634532	0.985637351
CG7785	FBgn0038564	89.64511588	63.56870251	71.62081135	69.89363828	75.00280983	71.61651323	63.57107149	70.84741627	3R	-0.042317721	0.782381472	0.995787491
CG7794	FBgn0038565	5.59830672	2.608437266	1.509046002	0.483416347	0.655043049	0.977416552	1.45458711	1.463034699	3R	-1.162863149	0.167134974	0.830652748
CG7218	FBgn0038569	60.27246311	60.24335602	49.01976496	49.93236404	40.327888	59.54208587	57.7229715	37.36092146	3R	-0.144845125	0.404247151	0.956557569
Arp5	FBgn0038576	47.00779844	49.20421303	41.90316653	55.56308583	61.31051762	35.76413218	37.83456343	42.48472664	3R	-0.094908505	0.632627424	0.991585536
CG12321	FBgn0038577	60.8824476	82.47057355	71.2873666	88.65973709	76.45049541	59.48191958	72.26682555	74.63872521	3R	-0.067156807	0.7335426	0.995787491
MED17	FBgn0038578	104.5106603	116.0067088	97.25794602	85.73316169	89.34303294	80.95562902	83.93244397	107.9629698	3R	-0.122919596	0.400795174	0.953805533
CG14314	FBgn0038581	208.6796409	183.5527486	175.6734702	164.7293495	214.1708837	201.671344	189.3311266	238.9881249	3R	0.234446562	0.062209621	0.633840737
CG7988	FBgn0038582	47.83668688	39.27584865	43.49143879	30.28141437	53.4987067	41.82326205	40.36636321	51.03532309	3R	0.243205772	0.301204225	0.907861127
CG7183	FBgn0038583	34.41649729	32.84964908	34.83031324	32.2862261	35.29312103	36.19162459	27.10111549	33.97097248	3R	0.011318224	0.943748974	0.997604378
mTerf5	FBgn0038584	61.51499482	40.82916812	39.20567883	34.5928038	49.66136091	67.33429414	42.96541775	28.46962363	3R	0.11711096	0.63335214	0.991585536
Non3	FBgn0038585	83.92030029	88.84123523	88.18765882	82.93076774	98.43502172	89.29397212	99.4342525	76.91808073	3R	0.112545373	0.446826403	0.967112302
CG7168	FBgn0038586	56.37526496	68.70491127	52.17750351	45.69482778	44.39264806	57.71785553	52.24511572	41.59548804	3R	-0.156620983	0.426739382	0.962502302
CG7156	FBgn0038588	19.0376721	23.4988866	22.01849077	22.04534739	22.60744098	16.69637299	16.29606832	9.818196718	3R	-0.372161173	0.13138893	0.790743321
CG12320	FBgn0038590	19.82075171	23.29874966	23.09588109	13.94830365	20.28559569	16.82309997	32.38165847	24.84827056	3R	0.267391361	0.340159498	0.927274752
CG18599	FBgn0038592	0.332851668	0.858941506	3.851122867	0.537252324	1.432873821	0.096557083	0.718479179	0.44470879	3R	-0.997452273	0.294559403	0.906715248
Vps39	FBgn0038593	39.91832567	32.87648062	39.09782051	44.08487695	33.22282725	35.20266094	30.86861453	25.98259234	3R	-0.288647407	0.075598602	0.678318963
CG14312	FBgn0038596	315.6053896	369.0364876	382.0630105	412.7065984	408.5472869	356.6452605	316.1808352	283.7939463	3R	-0.08440828	0.569858852	0.984934163
CG8064	FBgn0038597	51.40401285	45.34183841	32.54109548	33.58972983	33.60543219	66.48218218	37.4984473	34.32470315	3R	0.099126892	0.660085724	0.995750687
CG7131	FBgn0038598	2.208624715	4.849734267	6.527131172	2.246978347	6.379413883	2.801610253	2.561022318	1.482130691	3R	-0.211037496	0.717732442	0.995787491
CG18600	FBgn0038601	12.09152288	26.72652806	30.5675331	22.92159761	21.01228669	21.62768433	28.82778938	31.52671296	3R	0.200095207	0.504797097	0.978557094
PKD	FBgn0038603	21.05520799	16.58143816	24.49179085	28.50997647	30.14105376	30.69706764	25.63495886	32.83548291	3R	0.42455746	0.010265131	0.305534303
CG15803	FBgn0038606	4.61638388	4.149609086	5.613746342	5.069690222	4.570970743	4.27007626	3.611557179	4.922937944	3R	-0.130746167	0.414689043	0.958665466
Nup43	FBgn0038609	46.53607707	36.23702426	55.04703924	89.64870845	49.91516104	50.7272291	54.93796609	62.24292457	3R	-0.034753075	0.89326596	0.996450075
CG14309	FBgn0038611	0.520900103	0.665704265	0.722112219	0.23132552	0.806021307	0.140314696	0	0	3R	-1.124147203	0.411404216	NA
Wdr37	FBgn0038617	223.0706335	218.8960921	228.0718863	279.778294	260.3466021	236.24108	213.6089362	255.2363678	3R	0.053274858	0.665551135	0.995787491
CG7685	FBgn0038619	47.30563321	33.79154338	41.74991749	40.50353024	24.00000542	26.91346847	27.46457696	52.7690738	3R	-0.287887687	0.321733184	0.918261766
CG7694	FBgn0038627	12.18598625	9.869995292	10.21181741	10.62699076	9.895482673	9.386516074	8.758408783	8.818968061	3R	-0.191534611	0.149361387	0.808297271
CG7702	FBgn0038638	5.600952923	1.867974474	3.25790164	3.550971382	3.074737357	4.771018009	1.08623372	2.560039425	3R	-0.295940407	0.51768374	0.980804634
CG7706	FBgn0038640	55.81910916	38.07839032	44.66305908	46.06945233	34.85953595	52.82167182	52.70319949	46.35664945	3R	0.040139357	0.828004258	0.995787491
ChT	FBgn0038641	67.26150322	21.83493711	25.99709922	24.35874693	26.04861164	33.47723384	23.59121086	22.71388199	3R	-0.38104533	0.36182296	0.939549067
Muc91C	FBgn0038642	1.37569168	12.91653877	5.494514532	5.671574849	5.542391715	4.903268914	7.575209372	1.784767624	3R	-0.318048793	0.525260448	0.984107922
CG7718	FBgn0038649	55.74571623	43.6310585	40.47588668	39.20511462	43.7166723	54.82743532	38.20527397	48.20248146	3R	0.071923286	0.695495706	0.995787491
Epg5	FBgn0038651	19.85536175	18.0988199	22.78928648	16.49183434	21.59166475	10.91810318	22.52339751	12.17004875	3R	-0.166524607	0.463129825	0.971606621
CG7720	FBgn0038652	0.643616497	0.699500803	0.397661108	0.522669625	0.4786824	0.552254088	0.399708495	0.146538681	3R	-0.4948811	0.224110607	0.86549481
Octalpha2R	FBgn0038653	11.99967247	14.26019868	13.78766753	13.51328432	14.68690762	13.39305794	12.58700626	13.37072863	3R	0.045151463	0.64899748	0.992932477
EndoA	FBgn0038659	689.2062966	656.503143	655.3910357	684.068061	673.7859875	657.7567121	657.2456461	616.2855739	3R	-0.014193362	0.863177394	0.995787491
Sgsh	FBgn0038660	3.753920758	7.229516257	14.16638129	11.66949991	5.019843771	2.097288036	4.610826761	6.03711812	3R	-1.012435998	0.040203065	0.530560761
Mpc1	FBgn0038662	96.77492508	97.15119109	106.2677363	94.59822753	116.1242603	114.3185929	129.6824086	98.94720431	3R	0.247743003	0.057857066	0.623014047
euc	FBgn0038665	43.95552764	104.0883265	64.29493199	59.90597286	18.67082191	27.88126303	51.11876994	22.80532633	3R	-1.144001116	0.001848852	0.098142261
Smu1	FBgn0038666	59.53156729	77.16979305	72.70443935	80.12409403	58.63805005	61.59575698	70.13565023	89.13415995	3R	-0.016733579	0.921532279	0.996479182
CG6005	FBgn0038672	97.96898267	81.24037602	74.45610895	89.28326295	74.30470464	83.01381575	96.70733068	81.34747839	3R	-0.006879003	0.964882089	0.997604378
CG14286	FBgn0038673	26.40661365	16.61002122	20.43359333	20.96429321	19.31485486	25.53985597	20.90601892	17.05109045	3R	-0.004852234	0.988289841	0.998078482
CG14285	FBgn0038674	1.569171117	5.13252561	3.045429794	3.658463387	1.982929229	2.293077663	6.604932232	1.533067088	3R	-0.079946972	0.914328972	0.996479182
CG6013	FBgn0038675	212.721409	264.2801844	256.0581236	233.1531448	236.2781178	220.3823498	241.695718	205.0767712	3R	-0.064177535	0.604683535	0.989286463
CG6026	FBgn0038676	0.792026988	1.276415568	0.640482142	0.832771872	0.799012426	0.871171241	0.138660246	0.286593985	3R	-0.717167015	0.220818011	0.86549481
mRpL55	FBgn0038678	372.7466187	484.2050775	454.8119947	417.7263564	442.7880413	324.2554545	433.7140912	407.0258117	3R	-0.070317819	0.632502154	0.991585536
CG6040	FBgn0038679	3.282120095	3.353036899	3.509455473	2.06752145	1.363883571	2.218599727	2.919407246	1.34986598	3R	-0.608594226	0.016321396	0.369626395
Cyp12a5	FBgn0038680	0.884719435	1.695991701	2.350728153	1.375126705	0.532381777	0.079438865	0.214946503	1.829340753	3R	-1.20832745	0.22660632	NA
Cyp12a4	FBgn0038681	1.488887943	9.842696698	10.51735638	2.144029706	4.031736505	9.082858262	11.50626672	4.658622469	3R	0.334658982	0.571029944	0.984934163
CG5835	FBgn0038682	1.371084221	3.133479457	2.577829177	1.318610389	0.928184409	1.1310686	2.492086529	1.265119074	3R	-0.496790885	0.325642887	0.918470465
CG11779	FBgn0038683	47.60182778	33.32545949	42.78299427	36.88915775	48.79572959	38.43719756	41.28043695	46.61556423	3R	0.154191769	0.312216584	0.914141092
CG14282	FBgn0038685	2.108354864	2.357646341	1.704946497	0.614443749	1.427295336	0	0	3.433075183	3R	-0.439180731	0.754588714	NA
CG5555	FBgn0038686	238.1030699	235.8518564	197.7133247	229.1972875	223.5560718	220.6203483	200.4268803	206.1488793	3R	-0.053630114	0.60759499	0.990047511
CG11703	FBgn0038690	0.13565896	2.730586482	1.151873603	0.316283757	0	3.645105494	0.69213784	1.030848556	3R	0.357304293	0.788423086	NA
Gdn1	FBgn0038692	28.40875989	50.62330817	37.75977014	37.76367992	39.40614028	31.31713315	33.41107097	32.96822138	3R	-0.135862081	0.54378198	0.984934163
unc79	FBgn0038693	308.6881264	326.0466969	320.7131295	353.4667042	321.3758963	343.4784311	305.6783238	364.0942878	3R	0.058157088	0.533861929	0.984760433
CG14280	FBgn0038695	0.025817659	1.039332023	0.626332804	0.240771599	2.190552093	0.486814622	0.987921038	0	3R	0.985693652	0.41413347	0.958665466
CG5316	FBgn0038704	92.38465932	107.2658497	102.8098052	107.7668109	84.60072314	97.57006505	91.76391038	88.16584762	3R	-0.149032839	0.172578762	0.832736771
CG11626	FBgn0038705	3.838433962	3.279957195	1.9326766	2.954912735	4.379876648	4.779615973	1.478026793	0.15723765	3R	-0.132802487	0.854181124	0.995787491
CG7342	FBgn0038716	0.02794137	0.79675137	0.406712237	1.758894784	0.907944001	2.186465062	0.498953238	0.1213266	3R	0.345230978	0.751021266	0.995787491
CG6231	FBgn0038720	11.64837435	12.75878011	10.58603364	15.13333658	6.996944752	7.733883117	8.44469112	7.232065371	3R	-0.692940425	4.44E-06	0.000804869
subdued	FBgn0038721	28.14348201	29.81067557	32.92952796	32.86199795	24.90730587	31.13276047	25.17443099	34.17917928	3R	-0.069812914	0.609996257	0.990047511

**Nup58**	FBgn0038722	55.38038897	69.16729276	62.03443296	67.88862611	62.38090431	59.29266156	65.46675963	59.2177063	3R	-0.014672623	0.91840319	0.996479182
**CG6195**	FBgn0038723	146.6072333	107.5931024	108.1909512	129.6282567	122.5588073	122.1636625	146.0160645	93.01159457	3R	0.000201798	0.999052003	0.999757791
**CG6184**	FBgn0038725	130.3764534	118.674168	98.62605274	118.0749924	111.9069014	133.6886871	119.9087887	85.25306964	3R	-0.021914068	0.873402429	0.995787491
**CG7432**	FBgn0038727	0.506017243	1.173303766	0.505478553	0.208192968	0.564214915	0.3227238	0.379664958	0.387744803	3R	-0.49586492	0.517929061	0.980804634
**CG11391**	FBgn0038732	0.744820524	5.879209135	4.676744995	0.817186359	1.265498298	2.437089541	2.235356657	3.043904574	3R	-0.378152483	0.607987296	0.990047511
**CG11407**	FBgn0038733	52.77372839	142.0000831	111.6513258	56.57498641	20.66793128	46.14247661	90.87607654	36.04658637	3R	-0.863929908	0.110128692	0.758410631
**CG11453**	FBgn0038734	1.648218996	1.964360944	1.052251656	7.078769417	3.680171394	2.412682034	0.442593902	0.188338738	3R	-0.77711458	0.37873108	0.943971504
**MICU3**	FBgn0038735	80.30385859	74.9394611	63.37817664	72.21348643	81.57953946	80.01653266	71.97252719	55.81759207	3R	0.019821153	0.881401685	0.995787491
**CG11447**	FBgn0038737	55.20819495	81.3036722	57.5217088	68.74363566	77.39432868	56.99923097	91.73395686	58.69633828	3R	0.149093183	0.498159198	0.97594235
**CG4572**	FBgn0038738	30.19633937	30.66888192	25.98680178	17.54658324	25.00556505	25.726505	26.73723681	18.74547703	3R	-0.090066999	0.702402232	0.995787491
**CG4686**	FBgn0038739	53.54781558	50.75658378	52.51152587	75.6557278	55.21976226	53.82265957	52.89006454	61.49668523	3R	-0.028053763	0.869008884	0.995787491
**CG4562**	FBgn0038740	36.34066343	53.45186463	47.18353444	58.37397259	56.11026873	51.41252976	54.84824765	71.09152266	3R	0.29182574	0.059082347	0.626639433
**CG17186**	FBgn0038741	24.97305264	20.56701971	23.33313386	17.83429632	39.19357112	70.68635249	28.5532702	47.74674397	3R	1.126337832	2.18E-05	0.002837399
**Arc42**	FBgn0038742	9.312012413	14.69535812	15.77346653	5.524000442	4.078303735	14.13001119	18.55431344	12.20212176	3R	0.147216005	0.74531163	0.995787491
**CG4733**	FBgn0038744	0.473500163	0.812280781	0.600459322	0.451615011	0.854789151	1.045012393	0.109809917	0.327094945	3R	0.040580351	0.954108497	0.997604378
**CG4538**	FBgn0038745	68.97099503	89.49235678	104.9509006	102.6199849	82.57782068	93.06522682	93.36007596	86.48145426	3R	-0.010623247	0.936249909	0.996793017
**Surf6**	FBgn0038746	36.28894082	32.00485309	44.71693423	39.95297472	19.62725532	33.0589821	42.24601104	34.85996606	3R	-0.208830042	0.42641211	0.962502302
**RhoGAP92B**	FBgn0038747	21.05542335	24.83711694	28.17022559	27.44956165	25.27161753	28.11766003	28.70021976	25.54079445	3R	0.116445706	0.367851266	0.94125651
**CG4465**	FBgn0038750	0.263678069	0.810852112	0.213226439	5.02050318	0.079334416	0.9115116	0.336324209	1.049527409	3R	-1.381182587	0.235244102	NA
**CG4462**	FBgn0038752	0.09552605	4.125962349	0.463489729	1.559007723	0.043112251	2.27404975	1.644900786	1.348073329	3R	-0.191771172	0.844816537	0.995787491
**CG4459**	FBgn0038753	2.723140367	0.750852484	0.723978142	2.348223011	1.279498077	1.019910179	3.42581404	1.133844622	3R	0.066851828	0.929034413	0.996479182
**Hs6st**	FBgn0038755	1.738158775	2.176553006	2.365622679	1.842718626	1.533388524	2.182906012	1.79873072	1.310115967	3R	-0.220348496	0.196138505	0.844272804
**MED25**	FBgn0038760	94.91981349	78.08349882	73.87012131	84.5767932	66.61904565	55.14108734	49.30756182	77.39891107	3R	-0.385845232	0.021057446	0.410927638
**PIG-L**	FBgn0038763	27.6026863	58.08757761	49.02457267	53.76045787	44.88769162	42.86716825	46.2233736	51.28806258	3R	0.012751495	0.949626744	0.997604378
**CG4424**	FBgn0038765	38.37305409	34.61512146	31.25634609	34.58979369	36.75104231	27.60666792	38.27404153	38.6284206	3R	0.051723359	0.801996949	0.995787491
**CG4854**	FBgn0038766	8.932686677	14.69332886	12.44310337	12.76445908	17.79038644	9.671351443	17.2008687	19.01832793	3R	0.421186366	0.202082573	0.849297107
**trem**	FBgn0038767	59.67695026	54.49411719	48.41867712	36.14034706	47.8313044	39.4074906	41.81784839	33.11682028	3R	-0.264937475	0.187920662	0.84291216
**CG4936**	FBgn0038768	56.69746354	60.27186038	58.42196054	70.40360367	58.13127259	52.86768934	50.68004715	54.38403125	3R	-0.155080369	0.265797553	0.893759496
**Regnase-1**	FBgn0038769	38.70939529	38.27638834	47.72713561	35.65412263	24.4437542	21.62665567	31.79864218	22.13443436	3R	-0.651857875	0.000920758	0.057615996
**CG4390**	FBgn0038771	77.31172345	68.58976602	79.8410787	75.09380393	76.96390723	80.09459295	92.24559506	87.59401804	3R	0.19291664	0.11547858	0.765469429
**CG10887**	FBgn0038773	0.527012359	0.947131393	0.547939129	0.175529925	0	1.206669795	0.192059979	0	3R	-0.610982574	0.691446369	NA
**CG5023**	FBgn0038774	0.238968859	0.267224491	0.386490079	0.074286253	0.086280007	0.30039784	0.162563918	0.726352919	3R	0.438662135	0.679780864	NA
**CG4360**	FBgn0038787	35.37666616	42.66358314	34.99426689	39.58792915	40.17180795	52.95293745	38.5162922	38.98296202	3R	0.190725032	0.191769997	0.843483578
**Sirt2**	FBgn0038788	148.841965	138.173713	127.4531014	116.1402347	106.0750358	141.9605585	116.7055989	132.5604524	3R	-0.065301291	0.612558018	0.990047511
**MFS9**	FBgn0038799	14.02683174	33.43157374	21.89937607	31.86755129	5.97624466	15.25151083	23.33279019	21.8954357	3R	-0.570460566	0.240765175	0.874884741
**CG5191**	FBgn0038803	13.44777707	16.41471193	13.039258	20.15311361	15.15359909	19.49552155	13.83876425	15.48151407	3R	0.048713599	0.770521672	0.995787491
**TFAM**	FBgn0038805	160.6240895	158.8723029	172.1556443	163.8460965	158.8487611	202.145221	160.1213836	216.2913007	3R	0.200086703	0.125108622	0.780439453
**CG5412**	FBgn0038806	74.88701757	102.2959892	59.84354297	70.43797707	81.81041491	82.11767881	65.38760369	81.64222206	3R	0.048102763	0.803112753	0.995787491
**Srp14**	FBgn0038808	89.15406485	111.2621079	96.81088148	120.4215073	104.5666717	78.64325204	84.43655066	91.85489173	3R	-0.183338768	0.346448799	0.930285749
**CG16953**	FBgn0038809	200.9082813	197.5342642	177.5516301	226.9482695	176.6117067	185.8776368	191.3253482	206.7091117	3R	-0.048827643	0.664397793	0.995787491
**Srp72**	FBgn0038810	160.8518301	125.0778709	136.0597084	132.4926103	129.5794322	174.3713437	115.7480922	143.00025	3R	0.046652012	0.742500013	0.995787491
**Pus1**	FBgn0038811	29.97717062	17.99584151	22.83794984	35.80362857	22.40611991	27.46896583	21.19761438	21.00931768	3R	-0.188085265	0.473636636	0.973549494
**CG5466**	FBgn0038815	460.8213458	419.2640749	390.2944035	426.0811123	440.6570377	438.4390008	391.8115456	376.6678524	3R	-0.014108519	0.885351281	0.995787491
**Lrrk**	FBgn0038816	95.45824844	109.0954356	107.1808541	94.37513351	101.8996337	102.4524997	112.9309728	114.7914545	3R	0.122469864	0.212961308	0.856573136
**Nep4**	FBgn0038818	13.09249481	20.11920336	12.34401481	16.927561	12.14799456	14.10211459	12.43812829	5.972073254	3R	-0.455654514	0.058718856	0.625114381
**Cpr92F**	FBgn0038819	0.906160108	2.548162301	0.517254537	0.434963155	0.769812349	0.603050975	1.223806185	0	3R	-0.73046284	0.464405572	0.971928234
**CG4000**	FBgn0038820	1.383432495	9.024220853	33.37542448	9.317882258	1.109977877	6.159155278	1.568518229	0.33372889	3R	-2.484193803	0.002626252	0.124359022
**CG17267**	FBgn0038821	0.487990116	2.864872367	1.657400102	1.137730586	0.969041954	1.472237123	1.369360598	1.271366058	3R	-0.22506362	0.782392024	0.995787491
**Syp**	FBgn0038826	10.5327222	12.45257913	10.40613496	11.29097048	11.23596418	11.85542633	8.433966266	9.779467235	3R	-0.083556862	0.483862107	0.973549494
**Fancd2**	FBgn0038827	1.527800471	1.969210317	0.858329452	0.712401857	2.206457138	1.228131338	0.533335513	1.29188275	3R	0.085782464	0.881457095	0.995787491
**CG17270**	FBgn0038828	66.42824394	54.045056	67.93000346	80.24229694	70.63005062	67.29648712	57.55408961	49.05814681	3R	-0.108590288	0.51950147	0.981306721
**CG17271**	FBgn0038829	111.4982934	130.7556167	125.5861771	113.9341569	108.3477615	99.32329779	104.5906444	136.1142551	3R	-0.069426732	0.601585275	0.989286463
**CG17272**	FBgn0038830	316.9142724	281.9503785	320.1943837	275.6600801	313.2428592	345.9660355	313.7893978	326.1536766	3R	0.150047078	0.173823529	0.833984823
**CG15695**	FBgn0038832	0.213420528	2.659302252	0	0.071083132	0.110079643	0.54614569	1.322212283	0	3R	-0.528595301	0.725799848	NA
**RpS30**	FBgn0038834	1719.529059	2379.980252	2659.783442	2727.949488	2299.006344	2082.724144	2500.227527	2524.618755	3R	0.021628935	0.875627816	0.995787491
**KaiR1D**	FBgn0038837	68.54650379	71.53919727	78.50497233	59.57508604	72.59579459	92.6074206	75.75107405	92.72130439	3R	0.293702164	0.017311127	0.376491575
**Ktl**	FBgn0038839	1988.323641	1626.305247	1943.965372	1881.460468	1639.084796	1815.068927	1724.420765	1716.832725	3R	-0.081641201	0.394708863	0.952272708
**Grik**	FBgn0038840	25.7368943	27.55141701	30.58563723	31.60327661	20.13174637	23.51360166	20.63554595	26.74590738	3R	-0.311593599	0.034052967	0.514749796
**CG5697**	FBgn0038846	0.073969467	1.737027652	0.897244474	0	0.801203075	1.255283307	1.698279291	1.605945597	3R	1.045048538	0.424853792	NA
**CG7079**	FBgn0038849	2.281094984	0.106283801	1.537195726	0.147730197	1.372653695	1.194779121	0.646569146	1.375685658	3R	0.179660438	0.876387336	NA
**HHEX**	FBgn0038852	0.085254179	0.393255294	1.602897227	0.298150363	0.153905643	0.582734113	0.05437131	0.647831499	3R	-0.660931303	0.552801595	NA
**RhoGAP93B**	FBgn0038853	58.08531435	66.36519691	59.12879925	61.60591024	61.52552979	58.15958719	63.56629929	72.98881525	3R	0.096779682	0.367795853	0.94125651
**CG7044**	FBgn0038854	27.38318284	29.14960419	28.72864168	24.25218343	33.0304713	23.68676146	28.24602823	24.52438647	3R	0.031270084	0.853711925	0.995787491
**CG5745**	FBgn0038855	22.59089454	30.11500798	26.60936444	32.71073042	33.5381206	31.41537214	30.17984633	25.49149301	3R	0.139664524	0.43307777	0.965402206
**Bdbt**	FBgn0038857	119.5661269	103.4045631	99.49318537	102.2875601	96.4191894	103.7342753	124.9701983	138.5479328	3R	0.154377449	0.368850292	0.942282153
**CG5793**	FBgn0038858	12.85606111	12.82391621	15.64612082	16.13187524	5.243112956	10.02668889	12.31215268	11.22031744	3R	-0.538653348	0.088277028	0.709409025
**Ice2**	FBgn0038860	23.52252677	26.05239222	25.51155565	31.21148492	28.60088064	38.71172624	32.91531433	27.78786138	3R	0.298387584	0.102935994	0.743386979
**CG7009**	FBgn0038861	57.16653178	54.59456925	47.63296469	39.08655076	50.85031716	68.13569079	46.23504912	44.26781338	3R	0.104919357	0.61825512	0.990047511
**Usp8**	FBgn0038862	79.21010621	101.1000451	102.9404589	107.0544888	94.08993456	117.4114461	98.51729246	104.7823424	3R	0.118932378	0.334855561	0.924122842
**CG5862**	FBgn0038868	82.46479882	70.17492195	74.01429898	73.24326629	72.52645772	74.04505583	73.59078651	61.97493874	3R	-0.061218863	0.699630565	0.995787491
**Smyd5**	FBgn0038869	53.17666427	30.05012217	36.14923166	16.30958832	35.42154041	32.17195781	29.30724302	19.38596719	3R	-0.198969391	0.5287136	0.984296327
**Oga**	FBgn0038870	14.90404227	16.43481709	15.34440529	21.34226692	18.68445567	16.06808187	16.07658299	14.88071067	3R	-0.018876185	0.923410604	0.996479182
**CG3337**	FBgn0038871	29.92540084	25.17350275	35.2413329	21.24652612	37.95184906	24.61236692	31.76371955	25.53124504	3R	0.135454263	0.525710254	0.984107922
**Nelf-A**	FBgn0038872	155.7129998	201.9250211	177.8619091	181.0500965	162.1834773	175.5870271	157.0495081	165.261535	3R	-0.08658521	0.370947494	0.943098356
**ETHR**	FBgn0038874	0.32022416	0.721519396	0.336252553	0.178290287	0.319241899	0.175735969	0.70716605	0.134896595	3R	-0.186943294	0.774219872	0.995787491
**Idi**	FBgn0038876	104.6580831	87.9446222	86.05358369	108.062219	93.9184577	76.43976872	75.14878504	79.06576262	3R	-0.224940643	0.16589803	0.828893393
**CG3308**	FBgn0038877	58.0617375	46.94472831	52.85972174	48.29331953	56.29024447	64.87733506	46.87136482	44.07889715	3R	0.067692578	0.667952047	0.995787491
**SIFaR**	FBgn0038880	10.86850607	12.4721042	12.41363145	12.25616893	12.48491385	12.68242804	9.445890684	15.76356879	3R	0.102517159	0.511680908	0.980804634
**CG16791**	FBgn0038881	31.53854975	36.53484686	30.84785608	35.07187836	29.59909239	36.24110594	26.4020038	34.17347155	3R	-0.054576439	0.632294617	0.991585536
**CG7907**	FBgn0038887	0	1.08168495	0.142223117	0.478385872	0.317498748	0.552711695	0.074776652	0.06364001	3R	-0.691810234	0.645227452	NA
**Fancm**	FBgn0038889	0.947572134	6.102234906	3.160845574	5.365269845	3.726687299	3.397978428	2.676270823	6.575906441	3R	0.118151073	0.805441055	0.995787491
**CG7956**	FBgn0038890	52.97603524	37.29803427	38.6023116	48.44625935	39.36725759	45.52694157	31.34418853	36.48339888	3R	-0.191674553	0.214170843	0.858753183
**Archease**	FBgn0038893	21.77580756	22.37107381	29.76711529	28.49422682	31.5187727	24.59953878	28.4557398	16.21537477	3R	0.009042263	0.971286718	0.997604378
**CG5849**	FBgn0038897	0.40045421	2.015117278	1.651543758	0.933643916	0.506045739	0.604073253	0.664018741	0.478182738	3R	-1.10357825	0.110800303	0.758410631
**CG6800**	FBgn0038902	2.014377806	2.162455155	2.541163279	2.630009772	1.890963474	3.266526117	4.933172217	4.72327274	3R	0.695030258	0.30629264	0.911610813
**Rpl12**	FBgn0038903	1.748504959	7.444982204	3.262960493	2.195078032	6.312999177	6.593919179	6.519153891	4.088178901	3R	0.726902732	0.329165186	0.921112231
**CG6569**	FBgn0038909	1.338690688	1.164315616	2.646229208	1.502755195	4.833359592	2.711197979	0.505930179	4.413454727	3R	0.953952561	0.194816911	0.844272804
**CG6656**	FBgn0038912	45.16646797	39.53983164	29.64625204	43.86988817	30.9602637	39.76782797	38.08214525	35.72691493	3R	-0.106082103	0.560351579	0.984934163
**dnd**	FBgn0038916	4.797794428	2.942143237	5.006185963	1.804173725	8.940643724	0.826845503	14.4765638	0.672029408	3R	0.809625901	0.303343165	0.910208378
**CG6678**	FBgn0038917	0.829520241	0.64163182	0.271101129	0.150310509	0.263807698	0.871214106	0.241216202	0.755845511	3R	0.189685328	0.751627113	0.995787491
**Qsox2**	FBgn0038919	1.107403919	2.422845288	0.116806368	2.357360065	0.173838948	0.45393637	0.122826573	1.358938845	3R	-1.476911468	0.19162631	NA
**Idh3b**	FBgn0038922	586.4614151	542.8335859	576.939416	617.0495715	595.3072336	660.5132047	595.9627883	615.7253945	3R	0.115198809	0.19492482	0.844272804
**mRpL35**	FBgn0038923	203.6699289	198.4796447	196.1945189	206.4128414	225.3314749	212.9863313	270.9128692	207.4625234	3R	0.216791509	0.112042134	0.758410631
**CG6028**	FBgn0038924	85.99309391	63.96486424	66.39378859	97.65368797	83.88955997	72.03646207	71.23330572	95.91320123	3R	0.068347785	0.729091915	0.995787491

**Cchl**	FBgn0038925	513.4361258	521.7888444	484.2172666	461.8619202	538.4050948	505.5518908	485.9761916	510.1884285	3R	0.073009417	0.427513765	0.963202327
**CG13409**	FBgn0038926	114.4698848	129.4025581	118.1366784	118.6234445	132.5315278	114.7267709	117.3090639	111.8399446	3R	0.018886312	0.865137745	0.995787491
**CG13408**	FBgn0038929	3.670977088	0.094008383	0.951758389	0.392003583	0.050588202	1.479500667	0.428919454	0	3R	-1.385941393	0.265504593	0.893759496
**CG7084**	FBgn0038938	63.67945112	84.66406682	111.5977816	38.44729017	36.23062184	35.71944854	70.79537756	39.82170202	3R	-0.667718725	0.095178342	0.730787365
**CG7080**	FBgn0038941	0.278068204	0.699630605	2.023768756	0.324152775	0	2.162828287	3.901468798	0	3R	0.910626094	0.534226945	NA
**CG5388**	FBgn0038944	0.071585327	0.360223031	0.138932004	0	0.180921807	0.188972585	0.292185131	0.155418371	3R	0.559772072	0.66251923	NA
**rdhB**	FBgn0038946	0.132733739	0.286254291	0.092002914	1.237854862	0.034231203	0.196649449	0.725585614	0.123504529	3R	-0.686577833	0.569453378	NA
**Sar1**	FBgn0038947	145.2243314	138.2528118	155.1739522	137.9922229	141.4537362	148.072781	142.205745	159.7497031	3R	0.067404175	0.497501324	0.97594235
**PSR**	FBgn0038948	112.2384945	105.332932	87.80957296	82.3547967	80.89024903	106.8970158	97.89764878	103.2001461	3R	0.031605722	0.837072734	0.995787491
**CG5382**	FBgn0038950	125.5043091	127.2575144	142.1762139	141.7927001	104.7999192	119.7726243	139.3176524	142.4425908	3R	-0.053314692	0.712593664	0.995787491
**CG5380**	FBgn0038951	41.53388422	31.35026887	32.82846131	28.58185131	58.65316684	41.68420798	43.23599877	46.25048799	3R	0.525243143	0.016242222	0.369626395
**CG7069**	FBgn0038952	30.58785715	49.50659935	32.92926457	44.52552597	28.72067758	18.27302328	27.21799267	12.47311726	3R	-0.829090492	0.00122775	0.073611993
**CG18596**	FBgn0038953	12.61034479	8.936485373	8.703493927	13.32290417	12.95311423	15.55488627	14.90636942	11.10054132	3R	0.345327975	0.136003492	0.796109055
**CAH8**	FBgn0038956	10.85078178	13.15493553	8.861492461	10.70789693	11.00637715	17.54808216	4.864655936	9.096673425	3R	-0.007912017	0.98085526	0.997881056
**CG7059**	FBgn0038957	28.05230844	23.58346109	30.34642168	28.69237848	35.54651224	28.2912441	28.55600695	26.05995239	3R	0.128250321	0.48366247	0.973549494
**CG13856**	FBgn0038959	0.576494147	0.604367536	0.34964169	0.25201395	0.065044981	0.441606068	0.735324478	0.547584695	3R	0.013142182	0.991033014	NA
**CG13855**	FBgn0038960	2.789999027	2.474393915	2.333965077	1.196280956	1.042068272	1.541955127	1.227128872	2.22798939	3R	-0.516928171	0.371736908	0.943282319
**CG13850**	FBgn0038961	136.7307173	118.137001	108.7797963	83.73629301	120.2883002	129.0131803	109.212589	93.66785375	3R	0.042838139	0.783886729	0.995787491
**Nop56**	FBgn0038964	96.75956077	124.597367	102.0319312	95.80464241	86.86237926	87.99587587	110.8744833	90.83506673	3R	-0.122480523	0.384649364	0.94674182
**mats**	FBgn0038965	37.94858216	56.52803976	58.69244473	55.55669705	55.21453008	48.86057865	56.7635465	54.28246137	3R	0.079572418	0.642903468	0.992589784
**pinta**	FBgn0038966	3.973806682	0.540446209	2.084410721	1.627596606	2.908272786	0	0.821940504	0.349763601	3R	-0.992287496	0.386995942	0.948247506
**CG13847**	FBgn0038967	0.031681875	0.225853077	0.096074733	0.203129021	0.328864948	0.462976909	0.040410572	0.034392141	3R	0.686182623	0.528331569	NA
**CG12499**	FBgn0038968	6.770625883	10.79232305	9.95976224	6.182376729	7.071192982	4.720853595	8.515823058	7.364439755	3R	-0.246800953	0.3444391	0.930285749
**Pebp1**	FBgn0038973	0.402875561	2.477813199	2.606321312	0.939289204	1.212156073	223.0860084	1.027744212	2.624040411	3R	5.148095011	NA	NA
**CG5377**	FBgn0038974	0.069183902	3.945573878	7.049245641	3.548592601	4.121524285	1.36974937	7.942032948	13.66862371	3R	0.955373177	0.272291905	0.89549671
**Nrx-1**	FBgn0038975	306.6155431	290.926271	239.2918782	340.1054402	272.9431736	274.6956086	261.7532038	267.4683903	3R	-0.100162675	0.374364036	0.943282319
**Pfdn5**	FBgn0038976	190.7865933	207.0144467	225.6347845	224.0086355	164.252241	162.3350631	179.2890661	193.6750897	3R	-0.244800414	0.059263473	0.626639433
**Octbeta1R**	FBgn0038980	165.2463815	162.9309004	143.7018477	168.0552432	141.0206248	144.2419238	147.1263736	146.8965666	3R	-0.113812815	0.206658978	0.855364409
**CG5346**	FBgn0038981	0.717105345	1.321433833	0.637068686	0.211931508	0.401130939	0.457069886	0.901792817	1.359548301	3R	0.14759768	0.818311651	0.995787491
**CG5326**	FBgn0038983	1.409154158	0.975478076	1.383714325	0.46934141	0.343222501	0.991134458	0.798840562	0.971239403	3R	-0.423634499	0.443143908	0.967112302
**AdipoR**	FBgn0038984	293.0587454	273.1577112	259.8727648	267.3502504	268.0435209	314.3431646	260.9227997	269.9714038	3R	0.053765826	0.56531906	0.984934163
**sit**	FBgn0038986	0.696222305	1.442595305	1.490316708	1.766441045	1.256859702	2.490436578	0.5223759	0.622408324	3R	-0.097574861	0.879809106	0.995787491
**CG6937**	FBgn0038989	25.33402728	27.72245674	18.87699902	28.82950674	33.10300318	18.42539105	27.23593732	39.81134662	3R	0.266811945	0.346151846	0.930285749
**wfs1**	FBgn0039003	63.3002965	95.1705524	69.60062595	96.27714345	73.77622647	74.55575377	60.96391803	74.81165717	3R	-0.158581031	0.320935807	0.916928013
**Nup133**	FBgn0039004	15.72230341	13.49993106	15.488917	8.582149021	17.57029687	22.88136842	13.31625844	12.01031381	3R	0.330246468	0.187211024	0.840740887
**Cyp6d4**	FBgn0039006	3.267790957	4.750426118	6.130689153	5.58706984	0.943872033	4.313194696	1.222643391	2.081105155	3R	-1.158290037	0.044144146	0.552313482
**CG4813**	FBgn0039013	65.55812317	72.17017409	54.93716491	72.19941626	66.63530916	53.42798436	71.63309984	58.38346993	3R	-0.053873273	0.755998781	0.995787491
**Dcr-1**	FBgn0039016	14.22035851	5.927272333	14.41635273	11.06091268	12.42733754	13.47603413	8.390681324	10.85756604	3R	0.008749457	0.973233721	0.997757033
**CG6985**	FBgn0039017	0.313996748	0.225722355	2.28525519	0.732071596	1.295644814	1.184136863	0.34329105	1.071268008	3R	0.196969193	0.848173343	NA
**HP1c**	FBgn0039019	247.3676396	286.7448467	273.7083892	224.0896107	285.590124	273.923916	318.099876	296.4988064	3R	0.218938765	0.070209278	0.662654724
**CG17141**	FBgn0039020	28.39696171	41.58342185	35.72106898	33.97897026	39.87183976	33.57184257	43.50308436	35.06685978	3R	0.156053143	0.478419184	0.973549494
**CG7029**	FBgn0039026	47.82490397	58.80455071	58.71531306	59.14337956	49.34067399	52.15732361	57.22270739	65.81030562	3R	0.033849028	0.773039109	0.995787491
**CG7031**	FBgn0039027	0.808706528	1.744060823	0.280272965	0.134676345	0.469260821	1.225355746	0	0	3R	-0.768814575	0.572897678	NA
**CG13840**	FBgn0039028	1.430861875	0.514300728	0.495892993	0.834000149	0.553515517	1.011755877	1.173265516	1.997057304	3R	0.530184176	0.574751547	NA
**CG4704**	FBgn0039029	2.038998992	1.973154673	0.761012787	0.548521084	0.991015785	0.369683807	0.700205993	0.085131838	3R	-1.293445449	0.182252292	NA
**Imd**	FBgn0039039	2.602806507	1.561119539	2.104790761	0.698779166	1.452335605	1.100541489	0.362171745	0.547969345	3R	-0.97516518	0.10174055	0.739991904
**CG17121**	FBgn0039043	0.707585669	1.800776916	0.947085773	0.284432646	1.7178471	1.564251943	0.124487327	0.052973586	3R	-0.069579036	0.944198955	0.997604378
**p53**	FBgn0039044	1.742152285	1.93719941	2.421890948	2.966455736	1.731534133	2.93433702	3.195878047	2.082430563	3R	0.165721166	0.688441295	0.995787491
**CG17111**	FBgn0039048	5.963816737	9.076627239	9.89195256	11.68326178	9.183962084	12.23330557	10.69342306	11.21056713	3R	0.27856136	0.253347794	0.884040848
**Cow**	FBgn0039054	230.828781	209.5818109	212.5588652	250.001365	227.4327717	212.7258006	229.9472378	237.4023908	3R	0.037190185	0.710409449	0.995787491
**Rassf**	FBgn0039055	36.00063924	40.13370746	38.14290025	32.73843723	44.63098627	53.12170376	37.96513882	28.23003904	3R	0.185725614	0.290338404	0.904215344
**CenB1A**	FBgn0039056	43.85262737	54.42441354	58.26838573	62.38595162	51.98830499	71.1341234	66.5516056	46.10077071	3R	0.135215189	0.381161493	0.944476532
**VhaAC39-2**	FBgn0039058	0.933529976	2.013255738	3.073563095	0.310927142	1.805636645	4.086300954	1.360831443	0.434309999	3R	0.329661068	0.733875095	NA
**CG4467**	FBgn0039064	130.602284	147.6433017	160.5283494	146.3392265	152.2920469	164.587349	162.5203042	146.8323431	3R	0.128672113	0.147513111	0.808004944
**Rad60**	FBgn0039065	62.25952563	50.6443356	62.08993516	64.53572582	58.27288356	76.52407545	69.20093832	82.10124472	3R	0.28443296	0.077500778	0.682035833
**EloA**	FBgn0039066	80.39595434	93.93318689	80.32798558	110.9327861	83.35473477	92.02236131	89.88513767	81.21584596	3R	-0.048284574	0.715481111	0.995787491
**wda**	FBgn0039067	18.28830708	13.91740047	19.64135662	27.04231365	20.93892142	25.74135181	19.18887354	8.414846197	3R	-0.065138143	0.82892199	0.995787491
**CG13827**	FBgn0039068	8.983206522	16.43198874	17.65995319	18.23573095	14.39956884	14.27371157	12.34717286	10.92861414	3R	-0.200161251	0.476020273	0.973549494
**CG6763**	FBgn0039069	1.401864692	2.280175863	1.958096351	3.070310224	3.374300085	4.806070794	1.625539521	1.106755642	3R	0.364913332	0.566890756	0.984934163
**CG4393**	FBgn0039075	26.3701609	40.65294727	40.72561133	26.86086898	36.93178878	39.63648342	41.70093336	45.88240472	3R	0.323540444	0.039736771	0.529044592
**CG4374**	FBgn0039078	0.569337149	0.463783706	0.489038731	0.323908108	0.452428477	0.600974236	0.288393488	0.286842379	3R	-0.153418133	0.626567602	0.990047511
**Ir94h**	FBgn0039080	0.051589187	1.038403495	1.126391842	0	0	0.535017678	0	1.008044113	3R	-0.455280746	0.807820438	NA
**Irk2**	FBgn0039081	73.80683045	80.99640806	89.63533876	36.59166454	66.86159722	70.14511728	88.34050497	43.17419955	3R	-0.031735913	0.923725879	0.996479182
**CG10177**	FBgn0039083	0	1.314383733	1.462314932	4.075482386	1.958682256	0.284144582	2.614060609	6.41249495	3R	0.770170369	0.486112533	0.973549494
**CG10175**	FBgn0039084	0.496550365	0.816235706	0.433664733	0.463075393	0.376488283	0.149806288	0	0.043122272	3R	-1.898329355	0.041662116	0.536003453
**Ugt303B3**	FBgn0039085	1.37866582	0.797420174	1.153318638	1.662572823	0.171644493	0.582667941	0.606380367	0.103214176	3R	-1.742692835	0.094441548	NA
**beat-IV**	FBgn0039089	52.7391039	44.97482151	43.7792122	57.65718927	41.42220487	47.15673264	47.75144925	44.32368752	3R	-0.113835037	0.33437162	0.924122842
**CG10184**	FBgn0039094	1.014604618	5.154195217	2.672395221	0.878618918	4.029567027	4.263538104	2.144573347	0.692308743	3R	0.241125173	0.719968142	0.995787491
**GILT3**	FBgn0039098	46.2833147	26.92579613	22.53560749	26.40701587	23.83032702	26.35038706	32.42760195	26.18280523	3R	-0.15025869	0.608677445	0.990047511
**GILT2**	FBgn0039099	5.142958335	22.70155558	22.98347798	11.7802521	9.284357924	13.18519656	10.8180784	5.680865265	3R	-0.638803259	0.198749349	0.847197998
**CG16710**	FBgn0039101	3.987840693	1.770629115	3.414510317	0	5.293437152	1.879855924	0.149603931	3.565047004	3R	0.279931866	0.785906353	0.995787491
**SPE**	FBgn0039102	1.327658099	0.614333851	0.111064829	0.10673741	0.16529401	0	6.77376867	1.590327522	3R	1.978426371	0.139943726	0.803344611
**CG10252**	FBgn0039104	17.93268367	28.23505976	19.49491435	17.35483569	14.04869949	15.232092	32.79949301	18.1812175	3R	-0.0184877	0.958400944	0.997604378
**CG10365**	FBgn0039109	10.67881652	13.85662041	12.78610724	13.20271713	7.523589184	9.548030244	11.03742927	7.390554561	3R	-0.478742197	0.008657627	0.277510248
**RanBP3**	FBgn0039110	23.46957209	21.21524393	35.2255248	29.38187738	17.80470049	17.6068685	20.03478174	18.33969554	3R	-0.53473976	0.02131165	0.41304951
**PTPMT1**	FBgn0039111	70.2106853	47.13036449	52.40927484	41.76019012	57.63529465	42.92632979	55.11045127	58.62842197	3R	0.044944153	0.837311573	0.995787491
**SdhD**	FBgn0039112	1307.415955	1417.520087	1203.806323	1326.668218	1427.331682	1155.566513	1389.092452	1388.61932	3R	0.061625122	0.567113429	0.984934163
**udt**	FBgn0039113	19.99146366	26.22811553	37.76766972	16.78895787	26.24706595	30.550942	35.25292844	29.109258	3R	0.303022063	0.206194659	0.855364409
**CG10214**	FBgn0039115	85.7243525	108.4051992	85.00803412	116.70838	108.9251704	92.45044246	94.40610145	100.9176876	3R	0.035182945	0.837410814	0.995787491
**CG10375**	FBgn0039116	93.15547173	83.26759256	89.04290839	73.98379354	81.99492533	87.76220915	92.06875232	77.91323343	3R	0.028839692	0.8457364	0.995787491
**tst**	FBgn0039117	31.98660327	45.83474613	32.792314	34.77476086	31.65928801	49.51057522	30.5189335	42.29156909	3R	0.113872699	0.520965073	0.982083369
**CG10208**	FBgn0039118	13.34949047	6.820520509	16.37896343	6.618286644	3.878040511	12.94747951	13.11306942	5.663340834	3R	-0.255572776	0.616644822	0.990047511
**Nup98-96**	FBgn0039120	30.25080158	27.38428858	36.87868891	44.3158831	28.58433159	34.43853082	27.45630613	35.52116719	3R	-0.111708196	0.503627447	0.978429306
**tbrd-1**	FBgn0039124	1.897394072	3.774726308	0.535238553	0.205753621	1.115208288	1.788851541	0.112564954	2.011808873	3R	-0.31233115	0.755226619	0.995787491
**Ndc1**	FBgn0039125	17.77438718	19.18654791	23.10759101	19.62962269	27.94207196	23.49268906	12.36623006	17.72547671	3R	0.067541522	0.799971271	0.995787491
**CG13601**	FBgn0039126	139.6050013	120.1923247	128.3786209	109.1346878	124.5245437	142.8781629	123.0890641	129.3445382	3R	0.091799454	0.491229432	0.973549494
**CG5854**	FBgn0039130	10.72175165	15.57689584	13.31262439	18.50198326	9.677754849	13.05336947	14.53717142	8.889597591	3R	-0.301136628	0.30051673	0.9074568
**CG12268**	FBgn0039131	0.487633993	0.363527829	0.744847612	0.842148369	1.630193218	1.174886896	1.059673713	1.333175939	3R	1.137174834	0.0535899	0.608413656
**AP-1sigma**	FBgn0039132	215.0147076	178.1886035	201.4149288	156.2711136	157.2493063	203.6558547	156.7810106	159.154145	3R	-0.122715925	0.327307832	0.918470465
**CG13603**	FBgn0039135	254.8083937	313.0805683	353.9436237	325.4089867	293.5938553	305.6948114	324.6034595	314.2591563	3R	0.022351828	0.852150177	0.995787491
**CG5902**	FBgn0039136	191.0477813	176.8295215	160.3078791	177.2818746	183.449065	166.9107572	171.7328216	148.3993337	3R	-0.044969667	0.697960025	0.995787491
**CG13604**	FBgn0039137	64.88992174	97.99118183	66.41482186	85.93810607	72.66742942	67.21811346	54.5692167	49.78228166	3R	-0.336603762	0.037252309	0.521998122
**Mettl3**	FBgn0039139	30.89384095	17.15805192	30.61962127	17.11742755	23.23183697	33.2485694	26.93668639	24.98461334	3R	0.202980511	0.42547253	0.962105868
**Miro**	FBgn0039140	123.0230156	131.4574449	93.89294319	136.567624	120.2134502	151.5092743	123.0210967	119.7102642	3R	0.111817954	0.399336121	0.952933016

**spas**	FBgn0039141	112.5292207	119.9320277	110.1146151	115.1865701	110.7402598	125.3984223	130.8546698	116.8102571	3R	0.109594835	0.257769189	0.88770596
**CG18428**	FBgn0039149	125.3008746	167.4930202	174.9043648	226.5164203	175.8561501	141.5171552	193.7541814	172.9894099	3R	0.012326376	0.949967507	0.997604378
**CG13605**	FBgn0039150	124.8082124	133.048757	107.8605288	135.3748165	112.7513371	101.1019893	117.2153833	121.8327474	3R	-0.115304665	0.368637469	0.942282153
**Root**	FBgn0039152	0.284718189	0.682249742	1.226384588	0.460602676	1.027978606	0.651902811	0.355749657	0.504611872	3R	-0.011756221	0.98245228	0.997881056
**GatB**	FBgn0039153	29.56642746	53.15004274	42.29364785	34.97000107	33.74035881	38.61045716	34.75741475	47.65341444	3R	-0.010013892	0.961279495	0.997604378
**Kal1**	FBgn0039155	0.227381777	0.023837565	0.034476564	0.132533013	0.179586043	0.133983844	0	0.246833443	3R	0.420000041	0.782010987	NA
**CG6178**	FBgn0039156	34.72885329	39.10118071	30.08989775	38.66230809	37.23635034	54.33531084	55.21957966	45.00364848	3R	0.455292053	0.008956083	0.281896641
**Myo95E**	FBgn0039157	72.89590067	72.73208216	53.95093905	60.08170716	65.05964306	70.76983665	71.07177787	63.1081981	3R	0.084935401	0.489590833	0.973549494
**TBC1d7**	FBgn0039158	76.95518867	64.05256113	51.54512726	40.98659142	54.22443338	64.81463951	55.38974938	46.50852986	3R	-0.05516056	0.79187424	0.995787491
**mRpS24**	FBgn0039159	90.61042736	103.4886556	84.22995281	90.25572243	115.3105789	78.35556711	106.161958	107.160533	3R	0.174161332	0.366273384	0.940794966
**CG5510**	FBgn0039160	84.50672508	77.14919748	77.50594617	121.6820045	100.1849885	76.12373396	77.28505759	89.19466103	3R	-0.045017021	0.805125674	0.995787491
**CG13606**	FBgn0039161	1.985690939	0.454188816	0.675146081	0.438405651	1.167737016	0.255285994	2.628708385	0.277609925	3R	0.300436748	0.668387728	0.995787491
**CG5515**	FBgn0039163	74.42369741	79.7111276	86.95845397	58.75766896	96.19575446	87.65550031	87.10395271	79.26602163	3R	0.256307148	0.122485645	0.780439453
**CG6204**	FBgn0039165	28.70224019	30.9360831	22.40609027	23.6532621	16.5192057	31.80254676	29.79547811	30.47892958	3R	0.066470014	0.762714041	0.995787491
**Spps**	FBgn0039169	62.31349859	82.41009527	69.92442974	69.45732723	54.72212409	71.13050207	76.56826934	75.45965068	3R	0.000453251	0.997451765	0.999490563
**CG13609**	FBgn0039170	19.32473071	17.6457768	9.847894759	6.265027993	9.082769469	12.50551253	15.16855484	17.12987437	3R	0.045564628	0.907415219	0.996479182
**Spase22-23**	FBgn0039172	93.11589313	90.36640484	107.115303	88.38916692	85.52681133	89.76676171	100.4979647	94.75012624	3R	-0.001844527	0.988981357	0.998078482
**beta-PheRS**	FBgn0039175	50.00359291	68.29086248	64.48927471	65.34388214	104.3628687	62.26861412	73.58861609	60.76445089	3R	0.313853879	0.097976021	0.730787365
**CG6356**	FBgn0039178	14.71506876	22.87490938	26.5034701	37.98963448	14.79158019	9.248851295	24.26728504	12.70650199	3R	-0.709411577	0.016773407	0.3727722
**CG5715**	FBgn0039180	2.934790511	1.125188428	0.203421738	0	0.60549132	2.213522054	4.278122083	0.182048625	3R	0.747058553	0.578607063	NA
**CG5728**	FBgn0039182	25.28518629	16.61822987	24.03515092	15.24678352	19.7832549	21.70516222	27.72858535	26.4230415	3R	0.264593753	0.198509512	0.847197998
**Dis3**	FBgn0039183	12.73730952	23.23115937	13.10931798	17.5744334	13.60792144	10.27478726	11.46817067	11.58406656	3R	-0.470672608	0.046519326	0.565973818
**CG6432**	FBgn0039184	0.418782815	1.443923815	0.507980634	0.108486263	0.084001146	1.623173064	0.474810649	1.868944531	3R	0.749742936	0.444609339	0.967112302
**CG5746**	FBgn0039186	275.6887504	365.5098825	300.0513437	396.4285819	317.7824608	312.741854	283.5627308	312.8893066	3R	-0.093800705	0.4367332	0.966363994
**CG6454**	FBgn0039187	172.3407367	176.4720329	167.565941	178.0877162	157.5643198	167.6529837	178.8630177	151.5319214	3R	-0.053472153	0.555662278	0.984934163
**Golgin84**	FBgn0039188	73.72752172	85.08951369	77.05687324	59.13549619	81.36500307	114.2759538	83.6307596	73.18890729	3R	0.285969113	0.081739299	0.694684542
**CG18528**	FBgn0039189	27.39317871	31.62485127	32.50985158	31.44028798	33.27306305	30.13047516	41.41071847	27.80919352	3R	0.140099179	0.464247905	0.971928234
**CG17784**	FBgn0039192	2.023340555	1.454515686	2.103683984	0.673906048	0.521806897	1.998430883	1.966318181	0.418367585	3R	-0.328862077	0.764629731	NA
**CG13614**	FBgn0039194	1.340609133	0.4997075	2.168199372	2.604649771	1.21007154	1.966094825	1.139974233	1.940391018	3R	-0.039360099	0.965305276	NA
**CG17782**	FBgn0039195	0.172288902	0.288990464	1.462896659	2.410110539	1.55512813	2.842576183	1.758050765	3.179468822	3R	1.161619499	0.176324068	0.834204539
**CG17781**	FBgn0039196	0.274978558	1.515496137	0.095299164	0.366344089	0.141830507	0.481460765	1.503163303	1.194007806	3R	0.593147327	0.608306041	NA
**CG5768**	FBgn0039198	0.061358852	0.274455642	0.099237146	0	0.553842267	0.925580009	0.052175916	0.621673486	3R	2.342706849	0.064279424	NA
**CG13615**	FBgn0039199	1.355528101	2.273708228	1.47982152	0.158018137	0.734122692	0	1.728990654	0	3R	-1.078259651	0.471080389	NA
**CG13617**	FBgn0039201	0.911771007	1.427410091	0.81104604	3.401215645	1.920310411	0.143268689	0	0.197953926	3R	-1.493979367	0.160682774	0.822561572
**CG13618**	FBgn0039203	1.233337561	0.053044891	0.30687792	0.884763106	0.399626482	0.357779421	0.564715239	0.274634873	3R	-0.640718203	0.569606606	NA
**CG6607**	FBgn0039204	158.1958796	148.9057691	124.678311	151.3461607	157.1641251	133.0259504	139.602891	124.874442	3R	-0.043780289	0.740820788	0.995787491
**CG13623**	FBgn0039205	76.68749137	96.24302801	92.61986102	89.01111326	96.0120259	91.96207284	103.8676268	77.85767461	3R	0.092818869	0.59467116	0.988780662
**CG5789**	FBgn0039207	4.639861926	3.636959982	8.067426474	2.514517403	8.071921805	1.864165358	6.1904593	5.683151941	3R	0.252677809	0.53295305	0.984760433
**REPTOR**	FBgn0039209	23.98173031	28.8915938	29.75314196	26.39982536	27.05286463	27.47835695	27.72462554	23.56603597	3R	-0.01120178	0.912983542	0.996479182
**CG13625**	FBgn0039210	75.03052047	63.16954597	58.43209253	53.64674223	41.76291763	50.29326522	56.79845819	42.58894064	3R	-0.361154381	0.033155369	0.51264721
**Syx18**	FBgn0039212	76.52918862	53.24546673	63.75676488	51.63422189	57.0388305	87.92708794	76.33336556	39.85509863	3R	0.112951202	0.616413055	0.990047511
**atl**	FBgn0039213	30.65115405	39.10602148	35.18123066	41.89460441	30.19117119	43.95162725	36.21255537	42.13224824	3R	0.084583834	0.556561779	0.984934163
**puf**	FBgn0039214	11.45098719	13.53272721	18.07900545	15.21473026	17.43304108	18.45201411	13.98512693	14.1299949	3R	0.166975795	0.288620571	0.903033179
**CG6695**	FBgn0039215	45.14660398	46.11122321	38.69984061	49.5893049	41.99220081	51.60321538	38.44235935	46.80233253	3R	0.022628944	0.871757145	0.995787491
**Rpb10**	FBgn0039218	212.1880227	199.8855654	217.9014398	151.3567152	219.9428571	184.7901325	220.5112787	177.2439616	3R	0.068519953	0.694087236	0.995787491
**CG13630**	FBgn0039219	268.1705741	295.5688448	259.4654965	305.680383	323.8036681	270.1694078	265.5606058	267.2586078	3R	0.028270209	0.809800135	0.995787491
**CG5805**	FBgn0039223	143.3775004	150.1008814	142.4579664	188.2212466	176.3161073	173.9433696	151.340619	177.6341519	3R	0.151369424	0.228069023	0.867702781
**Ets96B**	FBgn0039225	1.889661757	1.129918856	0.488142317	0.387536249	0.379035976	0.783557169	0.290126762	2.032321639	3R	-0.136874145	0.837419246	0.995787491
**polybromo**	FBgn0039227	69.92311893	58.83529387	58.9266495	57.58471145	63.55779574	55.69204816	54.11387891	73.48833759	3R	0.039738398	0.774432775	0.995787491
**Saf-B**	FBgn0039229	404.8685006	403.8144902	300.7514629	374.7141065	409.0342026	406.376975	331.6284048	337.0078479	3R	0.027988618	0.81396341	0.995787491
**CG7006**	FBgn0039233	22.5116021	54.81916536	26.6133601	34.90116153	19.8038797	41.6934567	29.73407727	21.58429019	3R	-0.265098035	0.457057937	0.968365245
**Nct**	FBgn0039234	56.90617301	56.59550602	43.40296593	54.07092459	55.93462302	54.10058937	40.09554722	47.8196838	3R	-0.06347595	0.678486327	0.995787491
**CG7016**	FBgn0039238	1.617772681	2.713586722	0	0.157157338	1.338559897	0.127102184	4.814801678	0	3R	0.510695566	0.731119996	NA
**CG3744**	FBgn0039240	14.2669084	43.4111038	40.50834982	35.74450251	25.69806449	18.42363868	40.93445628	38.49426854	3R	-0.071384542	0.866941802	0.995787491
**CG11089**	FBgn0039241	11.05931131	10.71730556	14.97914533	9.70330456	8.994783224	17.61109276	12.51121507	9.163127805	3R	0.084332422	0.73773832	0.995787491
**CG11069**	FBgn0039244	0.874931774	1.012120904	0.073192148	1.688168685	0.108929386	1.592872366	0	0.327509978	3R	-0.821521779	0.502997475	NA
**CG11168**	FBgn0039249	5.040416725	4.821494208	2.946501366	2.218162517	4.969868229	3.874180893	10.48279882	7.691011955	3R	0.872454485	0.025708038	0.449488936
**CG11771**	FBgn0039252	90.48911946	107.0820437	121.2843201	89.41490769	97.34709168	114.7888045	106.5706094	118.1735261	3R	0.131081721	0.308490849	0.912131082
**Nmnat**	FBgn0039254	185.6505718	162.1497703	151.5534272	175.7117849	150.7293381	181.1842214	142.2021672	157.3655919	3R	-0.069596935	0.562715189	0.984934163
**tnc**	FBgn0039257	1.467533616	0.970286065	1.283206717	0.960328814	2.222627368	2.798994327	1.102442145	1.32430523	3R	0.691590036	0.014634041	0.359764307
**beta4GalT7**	FBgn0039258	74.32495466	63.75693001	83.29970857	73.46528695	63.26149608	48.49166013	65.78485076	51.53906452	3R	-0.334855831	0.069474916	0.660963415
**EMC6**	FBgn0039259	203.3299627	254.2574225	282.3684437	203.4320847	274.2191164	275.463025	267.2701722	256.8488575	3R	0.221484027	0.131680966	0.790743321
**Smg6**	FBgn0039260	97.99279227	97.30395202	81.1173919	102.7749023	75.78197945	98.13337256	90.44454638	89.50842834	3R	-0.072184331	0.558062711	0.984934163
**Ythdf**	FBgn0039261	115.6683254	105.2426033	86.43171213	100.6541123	109.1637574	108.8842039	99.85167197	105.8056917	3R	0.082760435	0.467584319	0.972024872
**CG11790**	FBgn0039265	24.91079334	28.99153491	33.52062262	47.22492635	25.21201466	25.77361944	36.51165615	29.89115597	3R	-0.167195289	0.497223457	0.97594235
**CG11791**	FBgn0039266	9.643731803	7.226683744	7.352889922	4.613599519	2.532277465	4.605065014	9.073750952	3.480508584	3R	-0.52957174	0.19239563	0.844272804
**veli**	FBgn0039269	626.1915798	589.8301528	621.088535	538.5258105	597.4782389	533.4212086	605.0635958	549.5795009	3R	-0.024745174	0.809354345	0.995787491
**PQBP1**	FBgn0039270	125.9561975	110.4955974	109.7143187	99.77541311	145.572491	108.6199218	134.9149872	137.5427439	3R	0.269035843	0.100875784	0.737033314
**CG11839**	FBgn0039271	18.69269131	21.63892958	19.3741172	11.86721011	27.09113558	37.97708935	22.61141394	15.05211968	3R	0.550220257	0.108806642	0.758410631
**CG11836**	FBgn0039272	1.583814075	0.916077905	1.722415211	4.456589699	4.042303576	4.99453079	5.015599118	4.387188203	3R	1.123142218	0.066991644	0.654151708
**shams**	FBgn0039273	13.65229005	17.71640035	16.56013262	11.39850957	13.3220987	14.08885066	18.70789983	8.711870054	3R	-0.083018898	0.780254157	0.995787491
**CG11920**	FBgn0039274	13.35244717	19.30763531	14.93980609	13.82096972	11.94839558	21.43318631	14.82888077	11.24588748	3R	-0.013924692	0.963577303	0.997604378
**Nepl16**	FBgn0039277	210.3585966	203.4942602	226.2939469	199.4918941	199.9040636	216.7176256	222.9127214	204.9755741	3R	0.038458312	0.680375747	0.995787491
**Bili**	FBgn0039282	189.9573557	240.2406931	255.8330124	258.6852536	211.384943	228.8472697	256.0543957	258.7121883	3R	0.048348358	0.689539921	0.995787491
**danr**	FBgn0039283	67.22257076	89.0307648	109.830074	69.48482945	105.3840943	101.0642843	125.2657978	95.10653658	3R	0.382829875	0.025468761	0.447402929
**CG13654**	FBgn0039290	19.64314859	25.93421181	26.79628048	23.51411012	16.76649377	25.43736224	20.26443634	25.57001998	3R	-0.091111639	0.546494652	0.984934163
**CG13663**	FBgn0039291	43.96151981	59.59750212	67.69111739	53.35336825	44.93545434	58.00638788	39.43060354	44.23570635	3R	-0.229868393	0.303669129	0.910208378
**Alg9**	FBgn0039293	46.92249764	51.99185842	64.7271241	49.90786352	49.5258501	57.92949637	44.29784809	59.85973298	3R	0.020644628	0.896759518	0.996479182
**Cad96Cb**	FBgn0039294	3.89331245	6.370337312	3.731723832	3.611057945	3.045013254	5.760927872	3.734619812	3.201447139	3R	-0.129683411	0.653902347	0.994456599
**Sil1**	FBgn0039296	39.16810263	51.73028535	38.51913659	55.58081364	43.69716989	47.15151935	40.85459691	40.03521419	3R	-0.075949621	0.672219269	0.995787491
**CG11852**	FBgn0039297	4.25991372	43.09928429	2.296553615	10.24712461	2.319277546	4.844964576	0	5.431756823	3R	-2.199306832	0.087247536	0.706081898
**RpS27**	FBgn0039300	305.802966	396.4486669	444.1268062	437.4442741	395.383696	342.5766646	390.0549681	435.2192918	3R	0.01555291	0.909104459	0.996479182
**Nup37**	FBgn0039301	10.81226367	31.33507367	24.33598302	26.18871183	23.96488037	21.29358217	22.9852641	42.64520598	3R	0.303077726	0.370858773	0.943098356
**Nup358**	FBgn0039302	38.4040999	46.49777219	45.59227892	37.94631676	39.46098435	50.95626594	42.75213106	42.31466853	3R	0.090357785	0.430542994	0.963666972
**CG11857**	FBgn0039303	176.7272857	173.8639073	157.4033907	150.2938435	162.8379595	207.3392786	186.1757812	171.7869909	3R	0.173485344	0.131790554	0.790743321
**CG10425**	FBgn0039304	28.94798698	19.49819601	25.87325729	20.65518377	35.24649784	27.98359507	30.22974018	24.99248913	3R	0.34417126	0.145379449	0.805044735
**CG11858**	FBgn0039305	76.71452609	105.209968	111.3599265	98.59127247	122.0295778	104.0427102	100.2563546	116.0419565	3R	0.212101694	0.280391463	0.899310165
**RIOK2**	FBgn0039306	19.78681304	24.75929199	25.5940276	15.20146041	20.10797743	18.05724709	25.87358141	16.51513149	3R	-0.050795069	0.840111341	0.995787491
**CG10513**	FBgn0039311	44.2228174	99.61864054	124.8690465	29.39737415	41.67617622	82.4671502	139.2297081	43.24499791	3R	0.081059383	0.892572252	0.996438268
**CG10514**	FBgn0039312	22.52440637	67.5767236	63.38099554	14.23165461	18.29253711	22.67461751	28.18510642	17.22854815	3R	-0.913085477	0.018918571	0.393184106
**CG11892**	FBgn0039313	1.076958649	2.752681222	2.23944571	4.423946489	1.110963648	1.208750043	1.177433422	1.558784614	3R	-1.014596934	0.155631374	0.818884898
**CG13658**	FBgn0039315	0	1.097657633	2.453495294	0	1.503539745	0.448698642	1.365855117	1.549913979	3R	0.527978445	0.701663673	NA
**CG11893**	FBgn0039316	2.404433359	6.393938472	3.129967791	3.008014852	2.117374861	5.19724873	2.24405896	3.565047004	3R	-0.143482156	0.810934219	0.995787491
**CG10550**	FBgn0039321	6.417783321	10.91139285	4.342500447	8.651970872	6.935683339	3.128225616	10.24636115	1.516582496	3R	-0.445255998	0.406338801	0.956706394

**CG10553**	FBgn0039324	0.104991813	0.440272593	15.15516608	3.182197971	0.284305697	0	1.473100037	9.801718626	3R	-0.645484794	0.729949021	0.995787491
**CG10562**	FBgn0039326	5.794882631	6.053748156	7.399107598	4.740544106	3.211786779	5.12790925	9.855246727	0.331084911	3R	-0.34826889	0.595878045	0.988780662
**CHKov2**	FBgn0039328	5.488065773	3.126382789	5.777761008	3.138449882	1.775848497	5.466989316	7.396318178	2.58535163	3R	-0.006336455	0.991079064	0.998408391
**CG10669**	FBgn0039329	19.69002388	26.5491182	27.72910922	38.98413325	28.81526171	36.36956913	27.67757544	28.20843442	3R	0.130225839	0.519494709	0.981306721
**Vps33B**	FBgn0039335	22.24705162	26.03165138	21.88516274	19.78476334	29.46566328	23.96557665	26.91365469	28.29972385	3R	0.3031997	0.10885645	0.758410631
**CG4553**	FBgn0039336	12.25793781	28.3991313	24.15152025	14.36840757	26.77452793	28.47675231	11.747944	30.28896268	3R	0.34017739	0.340665993	0.927274752
**MED28**	FBgn0039337	110.6658987	146.07319	117.5281431	134.7221746	129.3935767	103.7848426	117.0340259	128.2431969	3R	-0.056101139	0.740619372	0.995787491
**XNP**	FBgn0039338	312.6175628	378.7603236	325.9922248	352.2892491	295.3235449	347.981067	294.6941667	278.5246814	3R	-0.141688083	0.141349979	0.8037297
**CG5116**	FBgn0039339	1.036779872	0.895875679	0.381092956	0.512742203	0.340300617	0.592405897	0.561028306	0.477473198	3R	-0.508368346	0.57827069	NA
**CG5111**	FBgn0039343	1.685684421	0.217499934	1.258291352	1.31003659	1.638588225	0.978001831	0.551309894	0.844563905	3R	-0.134060707	0.882235604	NA
**Npl4**	FBgn0039348	20.30759661	16.97834855	21.63082947	17.74010312	22.91276042	24.66826723	19.88021297	22.5684009	3R	0.261118428	0.037701113	0.521998122
**Ssadh**	FBgn0039349	224.0874104	287.3573405	289.1706731	266.4673489	237.8360048	281.8708355	277.9007739	211.3968563	3R	-0.049932484	0.681267263	0.995787491
**jigr1**	FBgn0039350	67.10429265	67.90759958	58.28836721	70.35263421	63.30061992	64.48193793	64.44222099	56.1984014	3R	-0.057157785	0.571568084	0.984934163
**Lgr3**	FBgn0039354	57.18854798	46.90553416	53.78194303	62.84178326	50.13926033	52.37755051	46.05294329	47.5705587	3R	-0.143378029	0.223500577	0.86549481
**CG4730**	FBgn0039355	11.29803204	4.963321469	9.78888148	7.776847884	6.701491434	5.224412888	12.62657175	10.39565492	3R	0.067853135	0.873875123	0.995787491
**CG5039**	FBgn0039356	6.552680522	13.73900291	12.91607813	11.21928772	5.729801581	7.722283964	3.395443348	6.224083244	3R	-0.895668884	0.073121486	0.671999479
**CG4743**	FBgn0039357	18.14480069	30.10212386	12.2096589	15.7481745	29.52823315	23.16286677	15.37294763	21.42230748	3R	0.265457873	0.420783671	0.960593359
**CG5028**	FBgn0039358	778.7968941	747.2903482	746.3181097	824.5775102	863.4611036	820.6938189	764.8325355	829.7540618	3R	0.111867113	0.215832519	0.860397245
**RpL27**	FBgn0039359	181.3645339	254.6643972	293.1290156	285.667578	259.9565468	221.3140068	269.3978114	277.4751229	3R	0.053996704	0.72796072	0.995787491
**CLS**	FBgn0039360	80.00213785	80.40648441	75.42790362	69.93391061	68.95145904	93.18171385	72.89567194	86.36134248	3R	0.100991534	0.464486521	0.971928234
**CG5886**	FBgn0039379	65.37711455	85.1274624	63.47724859	88.84990435	81.79970664	70.29891271	69.46757281	70.69063186	3R	-0.019521563	0.898740493	0.996479182
**CG5890**	FBgn0039380	91.9571518	91.91903291	87.45856284	85.73829127	82.80429252	80.88192391	78.50706301	71.73372763	3R	-0.155927778	0.069575216	0.661162882
**SppL**	FBgn0039381	52.96110793	60.3164994	52.07948241	63.79151201	43.88622126	45.10692884	64.59847905	58.19927818	3R	-0.081557132	0.590022478	0.987597375
**CG5913**	FBgn0039385	23.36599311	33.30945426	41.99948052	23.61106795	21.75467827	27.09659156	23.23668576	27.14593515	3R	-0.263175031	0.285228399	0.901780531
**CCAP-R**	FBgn0039396	1.599097941	2.092608071	0.865648852	1.201663052	1.154712651	1.248458686	0.438275468	0.367263699	3R	-0.815489457	0.034321768	0.514749796
**CG14540**	FBgn0039398	0.179929883	1.509035685	0	1.761898944	0.584675198	0.678547178	0.275402998	0.468773318	3R	-0.738517593	0.537468444	NA
**Vps2**	FBgn0039402	163.3618787	250.8947438	241.00371	224.3514184	208.2914463	227.1822678	198.6487234	239.566658	3R	0.025062681	0.860750248	0.995787491
**Sld5**	FBgn0039403	0.778590972	7.67261564	7.555403354	3.857418025	0.175694532	6.973482721	4.468955169	4.225982684	3R	-0.281299374	0.755914775	0.995787491
**CG14543**	FBgn0039404	55.70034407	64.16973045	65.37522624	59.46222491	56.03191449	54.55552299	59.23089733	27.94724521	3R	-0.276765039	0.285525153	0.901952098
**RpL34a**	FBgn0039406	197.6777672	217.6776314	282.3296792	209.5768244	194.767864	198.9032874	235.052503	217.3636938	3R	-0.067266736	0.650192266	0.993330125
**CG14544**	FBgn0039407	1.542450674	0.646811155	1.520172802	2.022843195	0	1.726878025	1.229632752	1.569751498	3R	-0.319140852	0.751771797	NA
**CG14551**	FBgn0039408	0.546644121	0.750206312	1.446710195	0.115861835	0.089712067	1.265005811	0.126772809	0.755245901	3R	-0.297711819	0.817818174	NA
**dysf**	FBgn0039411	0.419013169	1.050969434	0.598511837	0.35607125	0.699870935	0.553798677	0.429562604	0.663157733	3R	-0.004218182	0.992063487	0.998472242
**CG6142**	FBgn0039415	0.086748368	3.019295525	0.683963577	0.050562639	0.704713671	1.226787475	0.719215091	0.376677475	3R	-0.287293594	0.779333278	0.995787491
**CG6073**	FBgn0039417	2.227187982	3.329730679	1.996807797	1.354592592	1.223674605	3.78873208	4.817012419	1.892125282	3R	0.419081844	0.518191966	0.980804634
**CG12290**	FBgn0039419	1.848322852	3.720362748	5.333606492	4.037129139	4.706498177	3.283401701	4.665478554	3.294785691	3R	0.137566205	0.716786795	0.995787491
**CG6154**	FBgn0039420	89.48986805	91.77058089	90.36023876	108.8897841	77.879764	84.72239947	86.95467483	74.77548573	3R	-0.201987802	0.052719976	0.605783103
**CG5447**	FBgn0039427	143.8006467	166.3290181	160.5465654	167.6821286	121.9839169	161.9725529	145.4425926	146.7040308	3R	-0.117736349	0.320071719	0.916451164
**CG14237**	FBgn0039428	0.237310262	7.164984119	0	3.319679654	4.712464296	5.593366638	1.816150731	1.030445093	3R	0.344413981	0.807856074	NA
**CG5455**	FBgn0039430	41.17966825	39.62453338	45.63943355	40.52754375	32.52368676	42.42516823	44.86519745	28.44902044	3R	-0.143739228	0.323462365	0.918470465
**plum**	FBgn0039431	0.662212904	1.307049756	1.929113498	1.593528454	1.005822133	1.287524494	0.868405125	1.157686609	3R	-0.31077187	0.244457257	0.877269208
**CG6425**	FBgn0039449	2.843134862	1.110579698	0.472425035	0.544821526	0.773403538	0.51406661	1.291611993	0.169115314	3R	-0.865056025	0.373132353	NA
**Yif1**	FBgn0039450	135.4109525	160.3820907	129.9902754	148.0945979	154.9943566	105.634559	134.4061042	182.9053353	3R	0.04581097	0.784204388	0.995787491
**CG6420**	FBgn0039451	58.59517008	79.80258511	65.32217182	81.09482544	57.37478542	71.76136254	67.24842971	76.14718622	3R	-0.031975318	0.817176167	0.995787491
**CG14247**	FBgn0039454	3.172183971	2.808249399	0.855074896	1.232637918	2.067940727	2.741489365	4.495727478	0	3R	0.213146407	0.840667922	NA
**IntS12**	FBgn0039459	30.60560159	21.86104152	14.38791811	18.94855187	18.63728387	31.20181329	27.27040644	17.16827792	3R	0.152455295	0.635041498	0.991585536
**CG5500**	FBgn0039461	1.606136182	1.100394024	1.756152777	1.107571365	0.980108436	1.343635057	0.807915925	0.834932332	3R	-0.455641613	0.424381286	0.9620897
**CG14252**	FBgn0039462	35.42554854	34.24570719	33.10172524	22.85749867	33.11647167	43.54732892	35.71021385	41.0344398	3R	0.316499692	0.118984988	0.776424209
**Spag1**	FBgn0039463	0.608011444	0.3138012	1.13463637	1.199470285	0.844320375	0.220472875	0.238623116	0.101542241	3R	-1.169778973	0.331902584	NA
**CG6330**	FBgn0039464	62.87427377	42.22143617	50.19923246	40.43992832	52.9224118	57.25423234	52.46004053	45.97215019	3R	0.117796719	0.419222448	0.960266449
**Tsp97E**	FBgn0039465	534.8036206	432.5018347	425.5884675	422.566266	420.4815356	514.2189261	430.2723849	442.0047962	3R	0.019940342	0.855586882	0.995787491
**CG5521**	FBgn0039466	28.5536398	36.21632163	27.71597031	37.59301705	25.43767315	31.69407533	25.16837431	27.14001119	3R	-0.219876832	0.104207575	0.745392907
**TwdlC**	FBgn0039469	6.025617088	4.151141593	6.264884148	2.508660608	5.827383856	8.572094933	1.646944141	8.293162914	3R	0.391108247	0.479392243	0.973549494
**Nep5**	FBgn0039478	0.413057919	1.247124084	0.367426675	0.353110629	0.621396175	0.817799815	0.245867913	0.269036094	3R	-0.239370665	0.754130953	0.995787491
**CG14257**	FBgn0039479	0.772931956	0.209110472	0.483902489	0.174393073	0.472615257	0.364357763	0.413434812	0.216529913	3R	-0.152768443	0.849078352	0.995787491
**Cpr97Ea**	FBgn0039480	0.11181357	0.93775789	0.497306629	1.129652894	2.758646072	0.368960028	0.095079607	0.080919204	3R	0.354183723	0.737033358	0.995787491
**Cpr97Eb**	FBgn0039481	2.794136956	3.769795486	3.536628148	0.141617938	4.386202902	1.374414916	0	0	3R	-0.78386524	0.533372935	0.984760433
**gb**	FBgn0039487	4.783021716	10.20047809	6.409576669	9.027352201	6.003089005	7.279465905	6.798029359	8.703101863	3R	-0.042607619	0.887050798	0.995926915
**CG6066**	FBgn0039488	6.795772327	9.156537404	13.24321303	8.831130654	11.5642028	10.6083324	8.952279968	10.27960097	3R	0.165416896	0.62708901	0.990047511
**CG5880**	FBgn0039489	45.80539571	67.31957658	38.21699112	31.19049882	47.77672125	43.73342884	39.32113697	36.51679441	3R	-0.091715925	0.684275586	0.995787491
**CG5882**	FBgn0039490	0.411907418	0.838970532	2.141317824	0.068596188	0.106228352	3.49509418	0.37528024	0.894289135	3R	0.531504932	0.619944292	0.990047511
**CG6051**	FBgn0039492	7.710373672	9.382274223	11.70077046	9.164763651	8.279012159	10.36076034	8.81844378	8.877454845	3R	-0.030434247	0.828715945	0.995787491
**grass**	FBgn0039494	14.79078608	5.786233917	8.139422019	6.390036213	8.360107517	3.56413106	6.509607365	5.129738938	3R	-0.558038994	0.209127982	0.856520545
**CG5909**	FBgn0039495	0.19764574	0.497284503	0	0.115200901	0.089200304	0.978280782	1.638645221	3.754687981	3R	2.995890741	0.01917571	NA
**TTLL6B**	FBgn0039501	2.18654064	1.257468063	1.74291276	0.218478737	1.691685541	3.82842044	0.079684459	0.339084438	3R	0.151288893	0.875187424	0.995787491
**CG14262**	FBgn0039503	0.656198831	2.338948151	1.392938059	0.956189294	0.148075884	0.773325312	0.836988657	1.424668405	3R	-0.709622722	0.540045312	NA
**CG5934**	FBgn0039505	389.9835432	378.9364776	413.6720607	389.1005529	377.4654012	405.1821785	386.6887597	401.9155229	3R	0.029772676	0.765216511	0.995787491
**mrt**	FBgn0039507	142.6192141	162.6860923	136.7117744	174.3342979	144.4064913	158.9051484	135.0205605	135.7952226	3R	-0.073623093	0.497968562	0.97594235
**CG3368**	FBgn0039508	76.53482392	57.39507794	49.21092402	63.64529412	52.77637656	66.12310909	56.38583566	51.01307206	3R	-0.101522137	0.51819891	0.980804634
**bigmax**	FBgn0039509	98.86427348	104.0827183	107.0599841	81.47524116	115.6584987	110.4564542	92.76720492	52.36393286	3R	-0.047419363	0.826395962	0.995787491
**CG3339**	FBgn0039510	0.540611041	0.66844054	0.314763563	0.237678124	0.390376595	0.436872675	0.543763522	1.059698429	3R	0.495267812	0.27570197	0.89549671
**Cyp6a18**	FBgn0039519	0.286747562	0.309200662	0.745334556	0.47752939	0.36975203	0.289654125	0.261249711	0.533619001	3R	-0.275667846	0.759487126	NA
**CG12885**	FBgn0039523	0.285015654	0.144533932	0.289441675	0.061814257	0.41880048	0.193721896	0.050726585	0.07195295	3R	-0.053630103	0.953681721	0.997604378
**CG5646**	FBgn0039525	42.014314	51.22594316	57.19128776	88.79430084	48.60308478	67.68780922	43.05400624	55.35057063	3R	-0.128482673	0.570907579	0.984934163
**CG5639**	FBgn0039527	0.825277403	0.496537753	0.457003705	0.167313321	0.825888029	0.16914489	0.297488098	0.019475583	3R	-0.542926175	0.538906299	0.984934163
**dsd**	FBgn0039528	79.49181823	105.7943744	86.20799364	108.2952786	72.35584331	80.38418154	72.71867772	84.93417217	3R	-0.259705967	0.032347122	0.505711525
**Tusp**	FBgn0039530	39.37571378	43.02361422	42.16208596	45.58050769	41.08471122	48.50645785	39.18120474	38.97650089	3R	0.007925549	0.935217743	0.996479182
**CG5611**	FBgn0039531	27.76612752	41.20767386	44.66276656	45.31497674	46.63802169	47.97354558	34.18413327	41.93588392	3R	0.140240859	0.51630066	0.980804634
**Mtl**	FBgn0039532	27.06976685	39.73526977	36.38932027	34.91310003	38.8320979	26.86691761	37.05108506	35.93673538	3R	0.042399853	0.81278083	0.995787491
**unc80**	FBgn0039536	130.6718363	182.0187808	162.7181864	185.5957941	142.2886017	140.2534014	155.4291362	151.3557607	3R	-0.132685588	0.253855012	0.884040848
**CG5590**	FBgn0039537	52.40084344	65.0930184	69.34020835	43.47756166	37.85975599	41.95437635	48.31293884	32.16253421	3R	-0.490358682	0.015742078	0.366465026
**CG12883**	FBgn0039538	58.93356235	31.97769361	25.53789597	29.37413512	23.34299409	20.78698438	23.6823738	21.4150156	3R	-0.690661233	0.024721544	0.44131412
**CROT**	FBgn0039543	21.34631143	31.79833551	18.44598402	13.8943492	20.17204569	20.07673599	17.69292749	17.23291071	3R	-0.151659999	0.51575852	0.980804634
**CG12877**	FBgn0039544	30.68642133	36.62219539	29.97928547	30.15500862	32.96341807	54.79712763	44.99287444	33.73699725	3R	0.413127325	0.017903611	0.385659471
**CG5003**	FBgn0039554	108.6285775	121.0484407	100.0422019	101.8214874	86.09096213	112.8197775	94.38811848	87.40254841	3R	-0.151883125	0.212515672	0.856520545
**mRpS22**	FBgn0039555	71.81557531	126.6407972	134.2352726	100.0693484	122.2938835	114.511242	134.2279959	150.3046431	3R	0.307847418	0.103105689	0.743546988
**CG12259**	FBgn0039557	30.8903623	40.02767455	46.58406827	34.22558615	53.2530832	42.56978815	48.80753135	34.74131146	3R	0.277338785	0.209438611	0.856520545
**BCAS2**	FBgn0039558	58.84874285	81.78483995	77.9437027	57.02702257	73.73930618	68.11605019	67.5456867	71.85745953	3R	0.06495086	0.717720098	0.995787491
**NSD**	FBgn0039559	23.68620472	24.93582488	20.4051924	26.86548387	14.44583454	21.89531466	13.79046637	26.75486902	3R	-0.289113212	0.18172456	0.837439137
**BOD1**	FBgn0039560	103.739346	127.2128468	96.88538067	120.9099148	117.2502285	136.3574684	117.2825509	121.4296456	3R	0.163519083	0.150703039	0.810890819
**mfrn**	FBgn0039561	177.712458	163.753598	134.4459625	169.790231	173.7257445	169.8593581	143.701261	162.3222191	3R	0.036799514	0.752030045	0.995787491
**Gp93**	FBgn0039562	147.6790729	146.6414902	141.4132931	142.1261977	140.776776	132.0141841	153.7119937	118.7378286	3R	-0.054058625	0.622401421	0.990047511
**CG4951**	FBgn0039563	46.81840158	51.03829595	39.15172626	52.42199938	38.6937157	42.71367241	34.62964425	41.60778294	3R	-0.235418115	0.156159927	0.818884898
**Nep7**	FBgn0039564	1.510156213	0.542801827	1.22120597	0.083830294	0.064909977	0	0.550348706	0	3R	-2.501153671	0.103822311	NA

**CG4849**	FBgn0039566	86.17818682	76.27415119	67.43525363	88.40532727	69.59992844	87.90947367	87.50039594	100.6894347	3R	0.146894697	0.317604544	0.91460807
**Gfat2**	FBgn0039580	122.0730556	140.7397241	120.219062	124.2865012	127.9530939	146.3156272	129.3924723	110.3272344	3R	0.048445895	0.667261432	0.995787491
**Moca-cyp**	FBgn0039581	161.9143132	185.3160614	175.0615085	192.215288	169.4338472	191.0934668	169.1856154	158.1850435	3R	-0.025307714	0.793709405	0.995787491
**beat-VI**	FBgn0039584	15.45803479	11.13942706	11.5524688	13.69955777	14.70422878	14.86804321	13.35408409	15.69737632	3R	0.203931582	0.112157482	0.758410631
**mIF2**	FBgn0039588	12.90928632	17.36852007	29.08666883	31.20929663	14.75751161	29.38332085	16.5093791	21.0759046	3R	-0.118562367	0.6927325	0.995787491
**CG9986**	FBgn0039589	51.71407688	44.73384595	46.27911085	50.20056345	36.96098052	46.40518458	33.43866417	44.45114538	3R	-0.230289553	0.158559233	0.818884898
**CG10011**	FBgn0039590	12.78225068	15.64702211	20.87420108	20.23189521	17.62308014	15.34641872	14.97778259	16.38914194	3R	-0.07953815	0.602071465	0.989286463
**snu**	FBgn0039594	0.489087615	0.507520661	0.422320583	0.086971223	0.314262686	0.515816642	0.042294	0.557923544	3R	-0.040718576	0.953138317	0.997604378
**AstA-R2**	FBgn0039595	1.986812359	1.211854186	0.684656118	0.500064694	1.181982502	0.915291431	1.094313855	0.171561789	3R	-0.367410789	0.564125187	0.984934163
**CG1646**	FBgn0039600	82.04984236	73.94517359	60.25567243	72.9809112	57.34292175	63.54861119	69.41969901	50.59471672	3R	-0.237098113	0.083463655	0.69799888
**CG1523**	FBgn0039601	57.01312729	74.518881	50.22995211	75.53484337	69.44466116	84.71762166	63.26856995	75.1093023	3R	0.214947041	0.19387194	0.844272804
**CG1647**	FBgn0039602	46.30954491	36.19539835	58.91809103	41.12430306	44.5723954	44.32707181	47.36805228	49.04288886	3R	0.052754056	0.741176209	0.995787491
**DIP-gamma**	FBgn0039617	10.37670087	10.33118932	11.10237987	11.77817483	11.98059496	10.44369629	10.36443158	15.32039817	3R	0.175231522	0.215873607	0.860397245
**wat**	FBgn0039620	0.571581263	0.979935171	1.233427379	0.301864244	0.934936523	0.506133508	0.330291488	0.32223715	3R	-0.515512318	0.270671512	0.89549671
**CG1951**	FBgn0039623	151.3830495	162.5583459	156.0967864	164.3071051	143.1051421	155.7600007	159.2476053	154.5291869	3R	-0.019865627	0.830822704	0.995787491
**Slu7**	FBgn0039626	105.4651366	92.24071706	91.2358118	92.99803024	89.87477914	105.1113906	85.51005431	108.2747988	3R	0.053663884	0.687950357	0.995787491
**CG11837**	FBgn0039627	29.27245272	28.63205968	21.21880889	14.80068901	26.74047885	27.53138869	17.09419831	21.89905604	3R	0.017738858	0.952197254	0.997604378
**Sirt7**	FBgn0039631	19.05759997	31.70043282	20.64795824	28.10128581	28.7732635	24.8453352	30.14078654	19.4540927	3R	0.085808862	0.687573068	0.995787491
**Cul5**	FBgn0039632	50.05753326	53.43908175	67.47234829	54.20888357	53.7555047	69.18101828	64.62465596	66.07106522	3R	0.203530785	0.13794056	0.798609078
**CG11873**	FBgn0039633	14.14140784	25.07792997	32.2104551	29.17386274	23.43069596	21.15756602	28.12360651	29.74785088	3R	0.063054457	0.820334192	0.995787491
**alpha-Man-Ib**	FBgn0039634	240.2742792	223.2353764	226.0801351	227.8268519	235.942047	279.6350893	242.6569801	209.0123495	3R	0.103421888	0.308783201	0.912131082
**Pdhb**	FBgn0039635	446.6963333	407.0535612	407.5644331	478.3809533	460.5006515	424.9566566	456.2793061	414.9148479	3R	0.042994333	0.673096062	0.995787491
**Atg14**	FBgn0039636	121.4561994	146.7435821	89.56214444	105.8732584	127.7384502	117.9397311	133.3085526	109.973853	3R	0.107592727	0.485515277	0.973549494
**Ctl2**	FBgn0039637	74.04376996	60.68395322	67.51712233	75.94240848	64.99040528	84.2920467	66.10374064	73.95772977	3R	0.082969052	0.517892855	0.980804634
**dgt6**	FBgn0039638	4.679126335	4.137234921	2.023910001	6.173427162	3.797885989	4.035281425	4.626570468	3.150020594	3R	-0.104131761	0.817578523	0.995787491
**CG14512**	FBgn0039639	27.57993891	29.87720483	28.22704331	13.73106855	32.67393834	19.23079223	15.02415461	20.27140118	3R	-0.154724399	0.699012913	0.995787491
**CG14516**	FBgn0039640	0.133726414	1.284669082	0.678329415	0.170060782	1.492355519	1.065919049	1.922783206	0.184756997	3R	1.093557295	0.164750197	0.827712868
**CG11882**	FBgn0039642	9.216301223	14.45358556	13.99685856	9.084129156	24.61851236	23.80664579	34.40631575	19.5214079	3R	1.16714088	0.000223904	0.018848622
**rdog**	FBgn0039644	11.11204262	11.56969987	16.05792299	15.84535872	12.52041747	8.567233167	11.46151037	11.12840049	3R	-0.288189246	0.157783673	0.818884898
**CG11898**	FBgn0039645	2.850353181	4.08744121	2.436343835	0.757517125	3.039380599	2.575905079	1.393982132	2.533067711	3R	-0.050093754	0.914873547	0.996479182
**jus**	FBgn0039647	139.4123266	130.9642184	120.617981	143.2711007	123.8305808	123.0282176	106.4304411	110.362453	3R	-0.176495875	0.071012193	0.666459028
**CG14515**	FBgn0039648	0	0.702307943	0.888787064	1.098202182	0.094482256	0	1.335135985	0	3R	-0.892296812	0.634662124	NA
**Mesh1**	FBgn0039650	2.703300254	5.479075054	1.366285148	5.514812429	5.693508111	0.530969785	1.149362838	0.733639387	3R	-0.855688742	0.391331518	NA
**Brd8**	FBgn0039654	50.77300246	58.35544263	53.6065478	39.8684685	68.79973769	57.816693	43.8604848	59.96776933	3R	0.219344852	0.196259193	0.844272804
**CG14507**	FBgn0039655	2.164032436	0.28656796	2.210489476	0.531090543	1.850510455	2.738212228	1.452761378	1.236398616	3R	0.505465465	0.572417493	0.984934163
**CG11951**	FBgn0039656	0.611243133	0.555357079	0.432502967	0.415651353	1.195404725	0.240115122	0.194911796	0.055294393	3R	-0.223510581	0.836980385	NA
**CG14506**	FBgn0039659	0.831656126	1.183624894	1.58961334	0.293784083	0.409459762	1.71072069	0.321450401	0.930158831	3R	-0.163778759	0.841706653	0.995787491
**CG2321**	FBgn0039663	0.869661101	1.166987596	0.98456666	0	0.418656314	2.077108307	1.035311271	0.461539162	3R	0.427751443	0.638827244	0.991585536
**CG2006**	FBgn0039664	12.57434584	19.49844315	14.81367025	19.93107928	21.14848385	19.31770667	22.7311259	22.48818618	3R	0.393671302	0.103507025	0.745084146
**CG2310**	FBgn0039665	0.130318323	6.266266898	2.52920348	3.949819448	1.097870546	2.989674104	1.329779372	1.414665718	3R	-0.867887061	0.298580402	0.9074568
**FipoQ**	FBgn0039667	0.606599643	1.017485805	0.608938872	0.146303197	0.981784948	0.256368266	0.106720602	0.499545647	3R	-0.323896034	0.735004715	NA
**Trc8**	FBgn0039668	74.56574877	110.0635352	96.08420346	131.231811	99.94191263	127.4746073	91.82179506	122.0079812	3R	0.12875061	0.426100818	0.962364286
**CG7567**	FBgn0039670	1.698396973	3.183978269	0.242369845	1.63048472	0	1.412857317	0	0	3R	-2.158761804	0.213023923	NA
**CG11470**	FBgn0039671	0.675825064	2.267203471	0	2.888711272	0	3.50440058	0	0.244546462	3R	-0.60750637	0.735468786	NA
**CG1907**	FBgn0039674	336.8032552	243.9807113	250.8459181	334.4712875	333.5176196	294.5758268	281.1332676	298.2462942	3R	0.076719427	0.586024288	0.98718792
**ppk21**	FBgn0039675	1.901797809	2.754999161	0.314572838	4.433969997	2.340840321	5.745760759	1.323143746	0.469202169	3R	0.08761567	0.916140186	0.996479182
**ppk30**	FBgn0039677	2.677764571	0.249531912	1.263153792	0.086709824	0.738535624	1.437608102	0.094875485	0.645963862	3R	-0.541384677	0.629119031	NA
**Obp99a**	FBgn0039678	14.32975408	21.46085018	36.62612907	59.95772964	8.545968889	8.202502804	52.54854638	51.66685181	3R	-0.092049088	0.874070648	0.995787491
**Cap-D2**	FBgn0039680	0.33849944	1.198657907	0.63870716	0.306910606	1.901132322	1.081513749	0.479732969	2.653854736	3R	1.352609469	0.055656348	0.61863954
**CG7582**	FBgn0039681	198.494831	280.0237439	296.7881221	306.1428024	309.8192374	299.6320103	330.0915621	297.917257	3R	0.22750907	0.090379551	0.717658936
**Obp99c**	FBgn0039682	15.02190554	11.0542288	8.405386231	10.78934578	9.010303635	6.281780993	6.366264496	4.629076417	3R	-0.760336452	0.008943099	0.281896641
**Obp99b**	FBgn0039685	2.833307101	3.847241819	4.909688219	0.314559469	3.897025133	5.97845512	0	3.222147234	3R	0.18124115	0.871517695	NA
**Naa40**	FBgn0039687	11.05997827	14.32694886	7.704051855	20.67979961	15.22167671	11.56292431	9.777212636	9.985286519	3R	-0.176694879	0.692599313	0.995787491
**Kul**	FBgn0039688	33.79293151	50.70665192	55.21271543	59.96962397	47.81287942	54.54207432	51.17206372	68.03965154	3R	0.183567775	0.27225825	0.89549671
**CIA30**	FBgn0039689	96.10313951	96.90379853	88.23753872	105.9994242	104.4341018	95.11355457	90.58565387	94.28339467	3R	0.01904903	0.896471439	0.996479182
**Gnpnat**	FBgn0039690	38.9157851	38.0116773	35.94634172	31.79967816	40.99507225	44.99579761	42.90699319	27.97572954	3R	0.144392031	0.382402981	0.945120535
**IntS11**	FBgn0039691	49.08101879	55.68558046	59.87292582	52.71415333	62.5903696	52.95345072	60.9278508	47.44617186	3R	0.075360315	0.620609887	0.990047511
**Rnb**	FBgn0039696	47.98702917	61.8982092	59.81683803	42.68667468	57.21098238	53.83917465	49.90896762	47.8239796	3R	0.009446141	0.947616615	0.997604378
**CG7834**	FBgn0039697	122.012031	118.3044513	120.9913508	112.8263585	115.9401168	86.88085808	139.0470726	83.82892843	3R	-0.125120472	0.479181919	0.973549494
**CG7789**	FBgn0039698	151.019238	146.020668	147.2527848	175.0321735	137.4938575	154.7013626	167.0665843	165.6723363	3R	0.041921907	0.757247866	0.995787491
**Vps16B**	FBgn0039702	53.5561179	51.75361169	52.19030666	40.78541065	48.03465538	60.84705511	38.84940294	19.66601092	3R	-0.218756001	0.415828145	0.958846438
**neo**	FBgn0039704	0.168846483	1.459653177	0	0.121125913	0.046894027	1.44493208	0.033133154	0.394779909	3R	0.167925638	0.894716859	0.996479182
**Atg16**	FBgn0039705	15.56248531	15.32790454	17.70827854	15.64961515	17.1964955	22.12841701	16.81255477	12.02405559	3R	0.113229441	0.517644226	0.980804634
**Cad99C**	FBgn0039709	0.551243244	0.980334006	1.031177578	0.727765482	0.839270767	0.41326239	0.135540565	0.418159075	3R	-0.817886101	0.135929539	0.796109055
**dgt1**	FBgn0039710	75.80645461	82.88178806	81.54367007	72.6605372	81.70738304	59.99145783	80.21440653	77.0115818	3R	-0.032712976	0.816624255	0.995787491
**CG15514**	FBgn0039712	112.8023574	99.85529272	139.1649249	88.4132889	109.2077652	136.0863382	123.6114173	102.718691	3R	0.12903333	0.425748311	0.962175354
**RpS8**	FBgn0039713	615.3255631	794.0847581	845.4296884	928.7829307	764.7884528	705.1894851	888.431712	895.9420276	3R	0.06536878	0.628542337	0.990424271
**Zip99C**	FBgn0039714	25.36510428	23.58094584	23.5460858	14.73584536	22.78988776	23.28516162	19.4843998	23.57038994	3R	0.061485357	0.735268201	0.995787491
**CG15522**	FBgn0039723	23.78992187	19.23096701	27.25770518	32.20997361	20.80079075	17.24059946	27.78514299	16.05509834	3R	-0.296973232	0.282512278	0.901712108
**eIF2Balpha**	FBgn0039726	82.24090704	86.32848825	78.03622362	85.97444984	97.22122846	86.41537315	101.0077399	96.04403143	3R	0.224442852	0.113112117	0.758410631
**Vps13B**	FBgn0039727	1.629094488	4.19391237	5.32682503	4.062393165	4.513692952	6.544270983	4.228130779	3.613804393	3R	0.349553599	0.256886078	0.88674759
**CG7896**	FBgn0039728	0.753304487	0.078972662	0.713869601	0.192095428	0.786196622	0.34400839	0.210185492	0.127772951	3R	-0.219814842	0.809366372	0.995787491
**CG7903**	FBgn0039730	334.4371899	313.4986573	283.370652	286.0151674	278.9871083	273.4041435	264.6295027	287.760301	3R	-0.110366341	0.287507082	0.902819904
**Sas-6**	FBgn0039731	0.361554991	6.497761885	6.014587643	4.094337557	5.221595393	11.39485855	2.635241569	4.93409308	3R	0.555365514	0.419871723	0.960266449
**CG15525**	FBgn0039732	42.27939543	47.7526428	50.84376986	48.01541317	54.01808247	48.65524915	57.79084761	45.50171706	3R	0.15684	0.4764539	0.973549494
**CG11504**	FBgn0039733	331.9510018	438.0629232	311.4070991	307.3298578	348.4775546	368.2545952	296.8435546	311.0277692	3R	-0.036443759	0.770295988	0.995787491
**Tace**	FBgn0039734	409.9508392	352.4250642	307.3252717	355.3320915	352.1263336	396.4865404	389.0051019	341.8226636	3R	0.081218652	0.463789863	0.971928234
**Nph**	FBgn0039735	138.075356	116.1088375	125.0123122	138.7959001	150.4049419	95.71198346	126.586732	129.0894456	3R	-0.015469712	0.92771222	0.996479182
**CG7920**	FBgn0039737	536.8241439	498.1801602	443.3456631	428.2137041	491.5774412	507.3204481	454.2007384	434.0782102	3R	0.014196301	0.887105987	0.995926915
**Mgat2**	FBgn0039738	142.9433481	181.6774223	150.9912106	188.7308981	152.1722086	189.1229995	154.9851448	152.7606432	3R	-0.005032752	0.965346362	0.997604378
**ZIPIC**	FBgn0039740	40.91782156	53.30269542	40.73776149	51.91157544	50.93955759	46.04198621	46.09086006	58.72430723	3R	0.143721848	0.430650807	0.963666972
**CG7943**	FBgn0039741	242.2548565	210.951749	178.5426574	181.3384463	205.7362959	191.6758833	181.8053759	178.4606496	3R	-0.073431038	0.559546079	0.984934163
**CG15528**	FBgn0039742	3.960972407	3.217522747	4.532671609	3.078286018	3.103077713	2.794913775	3.241074284	3.731918272	3R	-0.172232134	0.635288607	0.991585536
**Jasper**	FBgn0039743	84.43592613	70.54848973	69.49941433	73.72968466	77.00217292	65.83928041	76.21926211	58.06225316	3R	-0.078815898	0.599302015	0.989286463
**AdoR**	FBgn0039747	0.164427856	0.699361821	0.954509962	0.232752681	0.890503269	0.758498437	0.344555602	0.828722367	3R	0.516372617	0.379448521	0.944208584
**CG11498**	FBgn0039749	0.44175189	0.857973165	0.366628711	0.027103366	0.335779108	0.197280466	0.35586902	0.050478118	3R	-0.817417951	0.408215519	0.956706394
**CG1983**	FBgn0039751	130.198899	154.1713167	123.8411138	128.6173517	162.3320173	141.3958666	121.3344937	130.2834693	3R	0.080612875	0.535040197	0.984760433
**CG15530**	FBgn0039752	0.1882187	1.010273486	1.095878257	2.808478803	0.339782993	0.070980576	0	0.735552397	3R	-2.12168999	0.077629164	NA
**CG9747**	FBgn0039754	0.50523595	1.196417546	0.324448774	0.069290506	0.885254487	0.08405883	0.151631502	0.451670477	3R	-0.367152856	0.712311378	0.995787491
**CG15531**	FBgn0039755	0.361411317	1.010361306	1.461298024	0.42130844	0.108740009	5.167835129	0	1.176986137	3R	1.024750361	0.431233704	NA
**CG9743**	FBgn0039756	0.861223895	4.902824659	3.640476863	2.312140042	1.766738477	6.569467258	1.59781866	1.643154762	3R	0.015499744	0.976759055	0.997757033
**RpS7**	FBgn0039757	366.6042344	449.8737184	490.423089	503.2589628	367.8570975	404.7610529	434.4585943	384.29835	3R	-0.154766909	0.181743762	0.837439137
**CG15535**	FBgn0039764	56.1039027	51.02155636	58.80022141	86.45230403	83.67525537	81.93627383	94.84005256	114.8883096	3R	0.603411053	0.006670841	0.235571127
**mRpS18C**	FBgn0039765	192.5842094	197.4645275	179.4448865	205.0700735	233.2088314	171.6048729	218.3073509	199.0423322	3R	0.116202486	0.462795241	0.971606621

**CG15536**	FBgn0039766	20.64205552	12.27185147	15.21336874	15.53439838	35.8491045	19.46120386	39.32710506	27.51350567	3R	0.960660747	0.010149113	0.304092724
**CG2218**	FBgn0039767	104.0391845	100.208762	83.19827719	127.4485882	94.95151664	124.6015183	108.5823123	126.4709497	3R	0.158766416	0.296117275	0.906715248
**CG15534**	FBgn0039769	0.845056055	1.559209281	0.068336473	0	0	0.106228492	1.149736614	0.489251979	3R	-0.476018237	0.745744533	NA
**CG15537**	FBgn0039770	0.514237982	0.914315798	0.87327392	0	0.334199288	1.34780079	0.367312999	2.478537339	3R	1.021292251	0.23002994	0.867702781
**CG2224**	FBgn0039773	70.89426191	52.69873526	44.02611361	57.99182718	70.44496534	72.9798376	50.39145982	53.34508642	3R	0.155101462	0.388732434	0.948247506
**CDase**	FBgn0039774	16.61286385	29.64242379	29.21260541	12.44501521	10.59227421	9.132082265	21.4387608	16.55136887	3R	-0.563466717	0.051363104	0.598685469
**PH4alphaEFB**	FBgn0039776	28.12275007	25.56497019	23.64267584	21.61711156	21.80555165	25.50500416	25.30876716	23.56773345	3R	-0.012318536	0.913891849	0.996479182
**PH4alphaNE1**	FBgn0039780	48.04586884	73.60003073	56.01631523	69.82005963	23.96915724	27.20582241	35.97248482	22.09331926	3R	-1.149082905	5.79E-10	2.68E-07
**CG15539**	FBgn0039782	10.66405537	10.73247412	9.397982093	10.45254243	5.578963556	8.699793175	8.438864534	6.8985451	3R	-0.451243298	0.139482415	0.803344611
**CG9698**	FBgn0039784	0.747047965	0	0.095385681	0.366676675	0	0.926726656	1.604829818	1.19509179	3R	1.598957053	0.26378594	NA
**CG9717**	FBgn0039789	0.158351455	0	1.280528067	0.061531743	1.619901444	0.298585568	0.134652654	0.458394222	3R	0.813437451	0.542971586	NA
**CG2246**	FBgn0039790	86.93347993	85.45464643	82.34673409	82.22033535	82.22268957	89.1602867	84.87878589	79.69981079	3R	0.024855915	0.773610076	0.995787491
**CG2267**	FBgn0039792	1.841910092	2.195203584	2.586996322	0.791063376	1.137540982	4.295655497	0.123651374	0.210471438	3R	-0.328381332	0.740830803	0.995787491
**Spn100A**	FBgn0039795	0.977816508	1.708489602	1.976807674	0.284967797	1.250356665	1.075526003	0.831476973	0.353821692	3R	-0.448438786	0.613424838	NA
**dj-1beta**	FBgn0039802	400.2950051	344.0722645	354.3734265	369.8846704	499.6152557	385.837057	456.5583564	383.9754371	3R	0.262322954	0.044694041	0.557604994
**CG15544**	FBgn0039804	0.597157093	0.214638696	0.266086796	0.227306005	0.462009258	0.384905469	0.031088991	0.07937653	3R	-0.426719519	0.605229589	0.989286463
**Cpr100A**	FBgn0039805	10.78170586	0	10.86685532	0	15.13846632	0	2.258812444	0.565412596	3R	-0.231796334	0.92905097	0.996479182
**CG12071**	FBgn0039808	522.279683	475.2967637	497.704172	571.2703104	557.2225826	541.631949	491.5252508	589.5894264	3R	0.106300025	0.308426598	0.912131082
**CG15547**	FBgn0039809	6.682412873	2.802202536	7.123208671	0.944229942	7.493972498	7.82744423	6.198901384	6.045057054	3R	0.675179269	0.221846676	0.86549481
**CG11317**	FBgn0039816	118.728202	105.3016928	115.8936027	113.9447769	118.1574306	139.1819874	134.1704596	118.1149052	3R	0.194962735	0.049534591	0.583905634
**CG15556**	FBgn0039821	3.752067657	1.936482194	0.155597672	0.747675598	0.173678045	2.479221969	0.490851549	1.113994695	3R	-0.623933616	0.514267048	0.980804634
**CG1544**	FBgn0039827	2.227180868	1.280840045	1.173246682	0.563766735	1.81502829	1.535833613	1.038918627	0.414464214	3R	-0.108113819	0.848887806	0.995787491
**CG1542**	FBgn0039828	27.76225822	28.92650703	30.42673749	40.66357223	16.98115477	24.20333265	24.6627608	36.4483039	3R	-0.290500693	0.295161427	0.906715248
**CG15561**	FBgn0039829	16.1944919	29.03307006	26.67232338	25.80628616	26.41897281	20.80099178	34.86926331	18.5475698	3R	0.078514112	0.79391126	0.995787491
**ATPsynC**	FBgn0039830	4195.678459	3629.580728	3790.081509	3718.725469	4194.345227	3991.113782	3904.264605	4093.096447	3R	0.107570957	0.227351776	0.867702781
**CG12054**	FBgn0039831	161.4461703	144.2440112	127.4900512	161.7375578	149.1696005	141.8217529	126.630509	144.5736817	3R	-0.053206149	0.632909267	0.991585536
**CG15564**	FBgn0039833	0.029392007	0.345106783	0.641741399	1.50757989	1.061202127	2.133716584	1.199674275	1.978195831	3R	1.391541545	0.124857167	0.780439453
**mRpL32**	FBgn0039835	89.3849218	101.9171557	131.6568482	91.79417224	131.7795326	112.6630476	119.9834551	85.4801043	3R	0.151425985	0.431661561	0.964209983
**CG1750**	FBgn0039836	49.27677794	37.81519396	25.39903172	38.07867838	44.22660342	34.61306884	51.99176602	22.57887042	3R	0.049145735	0.864500537	0.995787491
**CAH6**	FBgn0039838	0.459607018	0.770926296	0.428846236	0	0.191471184	0.199991395	0.090189798	1.611910538	3R	0.375316393	0.785721922	NA
**ppk24**	FBgn0039839	0.249171509	0.574681594	0.377804128	2.396352717	0.89963687	2.143621058	0.556186638	0.067621801	3R	0.054766308	0.958140247	0.997604378
**CG1607**	FBgn0039844	106.4667848	109.5763066	129.2723317	113.1515162	81.93984263	111.6441469	100.2066363	114.7289078	3R	-0.136189169	0.255655594	0.88674759
**PNPase**	FBgn0039846	20.31923388	10.86314931	12.63399523	8.042603478	21.29370779	15.33806906	13.05805531	18.40940992	3R	0.417600166	0.134833387	0.795862348
**Loxl1**	FBgn0039848	20.18571608	4.976351741	10.16097731	4.88253757	13.86205078	5.868339397	10.98147106	10.7352448	3R	0.061206031	0.901137842	0.996479182
**CG11334**	FBgn0039849	29.851638	15.27465165	30.32736556	28.89446417	18.91981459	30.60780692	25.84225259	25.9125691	3R	-0.01961423	0.93410839	0.996479182
**mey**	FBgn0039851	0.200996316	0.655556072	0.108358724	0.130170929	0.020158326	0.484272919	0.199401191	0.242434226	3R	-0.173671817	0.868891741	NA
**nyo**	FBgn0039852	0.475533532	0.35799626	0.281596608	0.18332645	0.371773974	0.557765018	0.238798476	0.317044609	3R	0.21477367	0.664463611	0.995787491
**CG1638**	FBgn0039855	0.099848122	3.852067991	0.726690452	1.978733165	2.343268859	3.341835076	1.018858815	0.108389758	3R	0.077003855	0.942189259	0.997604378
**CG1774**	FBgn0039856	0.226039263	3.260682723	3.290208598	0.210800813	0.652894085	0.809812059	0	1.472256494	3R	-1.185122784	0.305294901	0.911292159
**RpL6**	FBgn0039857	1273.754428	1562.381507	1547.436367	1806.225866	1475.419718	1317.946125	1489.166426	1589.288234	3R	-0.043457801	0.71687068	0.995787491
**CycG**	FBgn0039858	753.1062346	608.5365784	499.97149	594.7875064	642.0735946	701.6853661	531.201418	625.5082125	3R	0.052178045	0.680896953	0.995787491
**CG11539**	FBgn0039859	11.66775455	9.45379849	14.63266439	3.457999639	9.639131515	8.949386621	10.5942126	8.157690319	3R	-0.040814334	0.934937261	0.996479182
**CG1792**	FBgn0039860	75.15572683	74.34981438	61.23219761	72.53774159	57.69515224	41.47040164	61.93934315	47.56572166	3R	-0.412903364	0.024276923	0.44079275
**pasha**	FBgn0039861	25.49518174	24.19214829	16.60262021	8.954261708	19.50913741	15.52370312	15.84277398	10.64469223	3R	-0.260449676	0.361274289	0.939549067
**kek6**	FBgn0039862	11.73044555	16.39168731	14.76034068	14.62228692	12.24534266	14.35588666	12.91171663	13.66515391	3R	-0.08032579	0.48066324	0.973549494
**CG1815**	FBgn0039863	36.19107042	47.41651977	49.21093749	35.56511569	33.8252252	27.91869841	40.00542071	41.56427361	3R	-0.19507473	0.22194092	0.86549481
**CstF50**	FBgn0039867	48.52994859	53.93640823	47.06440578	44.1240754	82.1468208	91.45154453	69.77045724	84.36769198	3R	0.78939	2.97E-07	7.73E-05
**CG11563**	FBgn0039868	53.8219182	26.52596702	57.76523214	38.33644579	57.90811582	37.5277767	51.1153488	53.64674595	3R	0.211033901	0.45025939	0.967112302
**CG1890**	FBgn0039869	18.39780679	34.69324801	54.6128327	46.62368907	38.16372651	24.56358689	59.17664397	71.69953867	3R	0.371100018	0.330641924	0.921224783
**CG1896**	FBgn0039870	165.2127029	136.5336414	119.8424209	122.8361747	136.2985628	127.1429669	158.8185978	119.2142169	3R	0.017772422	0.915581393	0.996479182
**salt**	FBgn0039872	0.842121833	0.908062223	0.656670799	0.420723289	1.031595325	1.899802516	0.07672396	0.326486509	3R	0.269200004	0.782048546	NA
**sip3**	FBgn0039875	143.2555156	172.8772809	155.6024285	134.1055485	185.6871585	142.494435	145.9840135	182.4988613	3R	0.151397326	0.249592673	0.879541335
**CG2126**	FBgn0039876	60.57333335	81.39137882	76.5556076	79.36788692	68.86831081	75.44799093	84.2230932	69.21595469	3R	0.032091874	0.834782071	0.995787491
**Mccc1**	FBgn0039877	2.194851473	2.980306784	2.345394348	2.680445211	2.264153969	0.88683967	1.666402757	0.510559899	3R	-0.907329405	0.109211053	0.758410631
**CG1971**	FBgn0039881	3.595223109	4.802050805	1.292142388	3.104491605	1.923051603	7.406804031	1.868266765	2.746401506	3R	0.148869678	0.832401018	0.995787491
**Rift**	FBgn0039882	63.46959126	73.84879643	56.53818849	64.55738381	58.60860441	67.24477159	57.05544133	63.17646756	3R	-0.040403411	0.747841205	0.995787491
**RhoGAP100F**	FBgn0039883	18.01433023	27.06779983	36.35173001	28.8699848	30.96881911	29.8845636	29.20738229	35.41178373	3R	0.220854633	0.174422312	0.833984823
**Arl4**	FBgn0039889	441.0273438	290.7934685	412.4446012	264.5794985	281.9376439	459.4502621	329.4446408	282.8675279	4	-0.034488496	0.844882391	0.995787491
**ABCD**	FBgn0039890	16.14600334	19.72356212	32.25382751	13.31406091	15.31134702	18.70557609	23.83422392	21.82709441	4	0.006007543	0.978806199	0.997757033
**CG1674**	FBgn0039897	0.725006076	1.465060517	1.343377158	0.758649522	0.566812584	0.871906068	0.961160638	0.69407163	4	-0.434340418	0.261615481	0.890640489
**Syt7**	FBgn0039900	10.20248008	12.70686007	23.06521927	10.28044532	10.09788457	14.62803419	16.87684224	16.09532918	4	0.072375604	0.818284151	0.995787491
**Zip102B**	FBgn0039902	122.5605649	86.75057541	106.4318386	69.02154136	90.34383131	107.1424705	101.4185281	115.9193609	4	0.137360021	0.408081857	0.956706394
**Hcf**	FBgn0039904	43.29248253	49.84107921	51.99540919	40.47737385	39.41245987	45.75772208	46.63810568	47.96541764	4	-0.013255172	0.905159872	0.996479182
**Igs**	FBgn0039907	93.5561753	90.27867397	97.69418207	98.56323343	90.37151688	88.95576407	98.02660946	88.37615155	4	-0.025557349	0.792116164	0.995787491
**Asator**	FBgn0039908	91.5849186	91.83274762	106.3387579	92.80186931	80.18407783	98.99233665	96.90418345	100.5431084	4	0.007919604	0.938535686	0.997038394
**ND-49**	FBgn0039909	564.7732941	594.0923236	561.2620548	594.8701891	629.4081345	594.9278497	600.2272251	601.052645	4	0.098177761	0.23272443	0.868185048
**CG1909**	FBgn0039911	368.8216004	306.4360749	310.9252046	317.9697168	282.1893511	340.4594428	292.7432213	296.0378753	4	-0.079480226	0.430189304	0.963666972
**mav**	FBgn0039914	8.902088546	11.67693101	9.340978653	4.452604941	8.007471984	10.54186505	7.936509802	8.22578813	4	0.046707767	0.883333661	0.995787491
**Gat**	FBgn0039915	80.98553414	108.6016103	103.6377141	58.56075643	96.65589872	81.77859826	104.0202818	57.92224915	4	-0.01201878	0.949297803	0.997604378
**Ekar**	FBgn0039916	0.75609212	0.473847338	0.624860847	0.639257257	0.419981558	0.587504697	0.74184894	0.487052105	4	-0.138628262	0.780996189	0.995787491
**CG11360**	FBgn0039920	19.76750203	22.53677497	27.41401984	20.66262913	22.73221378	25.05402759	22.32530377	25.0446014	4	0.107147592	0.372402048	0.943282319
**MED26**	FBgn0039923	65.13341029	64.97913429	88.45660716	63.56452497	57.7524821	72.42901892	69.04364397	71.34345008	4	-0.02920147	0.819294935	0.995787491
**PIP4K**	FBgn0039924	264.1768485	223.6920768	271.5287051	199.5305611	223.4133097	262.4320739	218.3706998	239.4330724	4	0.005866828	0.9597141	0.997604378
**Kif3C**	FBgn0039925	0.278476884	0.447087082	0.386046306	0.259703349	0.193907114	0.093766538	0.476983202	0.285025287	4	-0.357697788	0.540838656	0.984934163
**CG11155**	FBgn0039927	173.1905684	147.7292145	186.6646368	146.1828479	125.2796559	177.6500099	154.2088552	132.2724738	4	-0.122741551	0.317889966	0.91460807
**Cals**	FBgn0039928	580.1018002	416.7436496	582.2351628	397.922439	369.9852316	618.514835	438.1780536	443.4740101	4	-0.055902871	0.717202135	0.995787491
**CG11076**	FBgn0039929	220.6521487	232.9894158	249.5734182	270.8421598	200.2368082	218.6864198	241.592726	266.9185192	4	-0.039362376	0.755875868	0.995787491
**fuss**	FBgn0039932	0.6490468	1.007708388	1.132134943	0.487734703	0.329236843	0.849604266	0.424195886	0.687101656	4	-0.476073127	0.294762593	0.906715248
**Gyf**	FBgn0039936	101.9314356	111.8585945	113.3834677	116.5829608	94.55465392	121.033427	109.334036	116.8718919	4	0.023314456	0.816519857	0.995787491
**fd102C**	FBgn0039937	0.310048075	0.115569367	0.278582336	1.713458763	0.041460462	0.119089843	0.205058337	0.797799197	4	-1.034009091	0.305770604	0.911610813
**Sox102F**	FBgn0039938	1.145318853	1.069661305	0.853979518	0.112995742	0.594030368	0.531482663	0.475025633	0.481812055	4	-0.578720592	0.227865577	0.867702781
**CG17167**	FBgn0039941	0.930726302	1.244405134	0.965346468	0.880560774	0.693995453	0.623140153	0.584973152	0.497851888	X	-0.711195129	0.063138974	0.636110045
**CG17163**	FBgn0039942	145.82959	120.2553788	130.4990093	134.0100767	137.4435799	141.0888011	123.8537185	127.3960406	X	0.025381307	0.830741571	0.995787491
**CG17168**	FBgn0039943	262.1639285	237.8549928	234.6008235	270.9476296	247.3134733	272.0920285	249.5774492	231.6427114	X	0.020317759	0.84815487	0.995787491
**CG17162**	FBgn0039944	1.32410057	1.826149762	2.177191338	1.646448338	1.248289133	2.115266381	2.007919796	2.251883512	X	0.165102657	0.595776236	0.988780662
**ATbp**	FBgn0039946	2.864950565	16.66340987	20.7607263	9.281936489	9.000571256	5.998453242	12.14927866	6.462413383	X	-0.513522059	0.271156892	0.89549671
**CG41099**	FBgn0039955	18.42915885	17.22267886	21.20544907	16.63324633	14.04802306	22.7444994	17.73062683	17.12915268	3R	-0.009136688	0.94861715	0.997604378
**CG12567**	FBgn0039958	31.62778181	33.2181421	32.83648864	26.79631404	25.79219923	29.56790404	24.22644313	30.38567575	2L	-0.147394681	0.209711561	0.856520545
**Coa7**	FBgn0039965	119.0965849	97.48669305	107.548698	95.32220449	100.1140525	130.8301305	98.50850135	98.68236951	X	0.055762874	0.667576917	0.995787491
**Rab21**	FBgn0039966	213.3716595	228.6399067	185.958407	213.2460361	217.8556816	207.9224957	185.2693191	225.5489214	X	0.022515793	0.85388989	0.995787491
**Fis1**	FBgn0039969	379.9533309	373.0701189	432.5706988	283.4062255	303.3316077	365.8797396	349.7874266	291.2081628	2R	-0.134724153	0.314550146	0.91460807
**CG17508**	FBgn0039970	110.9805445	101.274632	102.2546139	94.23289237	87.96038684	92.01287138	94.94531584	81.63335477	2R	-0.168774475	0.132076328	0.791114096
**Marf1**	FBgn0039972	0.333252523	0.401694386	0.234490131	0.134539517	0.104174237	0.231220932	0.172971057	0.178531422	2L	-0.655865287	0.126503859	0.783853651
**CG17454**	FBgn0039977	201.8839423	174.7665494	213.7924619	132.4899559	170.2591801	230.4312616	180.9101246	154.7569571	3L	0.054116023	0.734863292	0.995787491

**CG17691**	FBgn0039993	11.19525961	10.97032815	12.70025293	7.708681868	6.96365704	12.15445219	8.508613976	7.524767929	2R	-0.246885843	0.284540056	0.901780531
**conu**	FBgn0039994	44.64949275	35.15592725	40.27836541	31.32392374	34.19554218	49.62973804	33.03184901	29.37520664	2R	-0.026278881	0.866262829	0.995787491
**CG17883**	FBgn0040005	188.0770194	150.1709173	183.308955	129.2416716	128.9643116	183.3534396	116.6685332	124.6688554	2R	-0.207210321	0.203870446	0.852939908
**RpL38**	FBgn0040007	606.7324777	557.397447	814.2226943	418.2382696	476.2651841	641.0939107	541.4675183	517.7697682	2R	-0.108654238	0.503624352	0.978429306
**CG17493**	FBgn0040010	78.77798075	61.18453601	68.52730229	62.16252435	62.16078745	74.66457746	58.40625328	66.18637637	2L	-0.02328819	0.851482604	0.995787491
**Slmap**	FBgn0040011	95.53464163	75.29500143	92.6069728	77.75458507	74.61607432	100.3693337	86.12120066	82.54365883	2L	0.037051631	0.764478001	0.995787491
**MED21**	FBgn0040020	337.6586014	277.9274594	319.9310415	246.0119743	295.7530352	303.9595242	280.854738	268.8357644	3L	-0.011193581	0.929902483	0.996479182
**Set1**	FBgn0040022	74.16102102	71.11278746	84.15358348	59.60449956	58.08665965	77.31410022	67.60608726	61.57812733	3L	-0.098224732	0.434382771	0.965629772
**CG12061**	FBgn0040031	0.350590557	0.650626739	0.597179094	0.260868729	0.161592973	0.351632591	0.133203155	0.356289791	X	-0.842948572	0.201325435	0.848253881
**CR12460**	FBgn0040045	0.942869147	0.738047671	0.609969973	1.025856497	0.794322884	0.711145018	0.801759817	0.818822479	3L	-0.065525995	0.950078388	NA
**CG17698**	FBgn0040056	51.88665499	47.06410082	53.88747796	44.38179577	38.91591139	60.40483171	38.54642735	41.53233181	3L	-0.110916568	0.429844289	0.963666972
**yip3**	FBgn0040063	0.191774714	1.072250596	0.155080927	0	0.577004183	2.59152441	0.978442829	3.19209836	2R	2.395595085	0.051097463	NA
**yip2**	FBgn0040064	6.693428235	16.76706658	30.66021668	17.45349284	3.100334115	21.46408239	18.31077575	14.91447473	2L	-0.275663633	0.561727918	0.984934163
**wds**	FBgn0040066	76.78318333	101.839361	75.33660882	98.12989875	83.04217369	96.30192105	77.34269082	111.0515329	X	0.094814638	0.551589317	0.984934163
**Vav**	FBgn0040068	39.88520788	35.76465664	36.70938751	39.50833989	36.88159378	38.55840492	32.06499165	38.84198038	X	-0.024663907	0.823404234	0.995787491
**vanin-like**	FBgn0040069	0.767179738	1.348114309	0.443135082	0.170347684	0.527602306	2.479859557	0	0	X	0.161300572	0.907424558	NA
**Trx-2**	FBgn0040070	167.1641189	161.6676067	158.5419901	158.4144023	188.8683663	145.6216939	156.95476	158.6688137	2L	0.040937035	0.719845283	0.995787491
**tara**	FBgn0040071	27.03604276	34.66546784	37.12108257	40.72952793	32.96635976	33.38538897	34.36369672	35.7696202	3R	-0.000263469	0.998296522	0.999757791
**rept**	FBgn0040075	38.49156371	44.05460544	74.75145858	68.30398655	70.19348735	77.05202062	73.98771784	106.7173973	3L	0.574893795	0.010699081	0.312027572
**pont**	FBgn0040078	84.71615579	67.24568897	69.33497088	85.16803886	77.39166065	77.34089144	79.75144627	56.22627131	3R	-0.050790043	0.760601699	0.995787491
**pkaap**	FBgn0040079	23.10813782	22.30613434	20.32627824	19.88189094	15.77138145	21.67376623	25.93771666	24.85837938	2L	0.070450598	0.731413706	0.995787491
**pins**	FBgn0040080	138.8929448	126.1425804	120.102415	129.9292512	103.4789119	140.3239019	132.1040786	116.7905769	3R	-0.037253959	0.754273593	0.995787491
**p115**	FBgn0040087	44.09922168	45.9921245	52.17870676	46.73439783	47.96698148	41.87995477	58.52586634	34.62521792	X	-0.015904818	0.926629516	0.996479182
**meso18E**	FBgn0040089	0.326762659	0.591946402	0.158544331	0.304733935	0.165169297	0.616139678	0.666862851	0.141886396	X	0.222855382	0.797800069	0.995787491
**Ugt317A1**	FBgn0040091	1.788457442	1.159047043	2.465211823	1.611028645	4.769558271	5.479976944	6.428827004	8.383548474	2R	1.872077913	5.38E-05	0.005530503
**lectin-30A**	FBgn0040097	3.896707709	0	0.540191917	0.259572209	2.411847817	3.043999273	1.7041003	1.450305111	2L	0.836315342	0.525042768	NA
**krz**	FBgn0040206	48.43826134	42.24481121	40.74328181	39.57910724	34.69644764	35.10329571	34.80389068	40.99563858	3R	-0.203040596	0.108888352	0.758410631
**kat80**	FBgn0040207	27.62945457	24.10676034	27.48954398	23.55646805	24.01860897	28.91550427	25.75074577	34.76979888	X	0.172637593	0.230911804	0.867702781
**Kat60**	FBgn0040208	46.055555	49.78838727	48.13450347	40.71780518	52.59944327	41.34612071	42.8905651	39.59883206	3R	-0.033887484	0.800760741	0.995787491
**hgo**	FBgn0040211	1.635058965	2.809474282	1.838194503	3.533145856	2.447751629	3.19584162	2.848535983	0.692656462	2L	-0.062730809	0.919916865	0.996479182
**Dhap-at**	FBgn0040212	98.41057544	87.31603959	80.05506066	94.93568021	75.97879971	107.5687074	74.79071268	100.9237456	3R	0.020493904	0.882856622	0.995787491
**eIF3d1**	FBgn0040227	625.9099831	560.3038899	518.9717179	621.4441283	573.5099891	659.2919426	587.3323568	549.9695349	3R	0.053400524	0.602656672	0.989286463
**DCTN5-p25**	FBgn0040228	48.56171667	91.42960602	56.2603083	73.24628801	69.23852134	66.52650443	69.01996169	54.22216227	2L	-0.022543474	0.921094114	0.996479182
**dbo**	FBgn0040230	203.9151975	240.1146552	264.9684645	252.7892365	227.9502618	262.8512782	232.4969947	237.6433783	3L	0.029181625	0.774300423	0.995787491
**cmet**	FBgn0040232	0.173757765	0.655772117	0.052691739	0.784900018	0.117628987	0.348112738	0.193926158	0.330088668	2L	-0.716088149	0.436442217	0.966084844
**cana**	FBgn0040233	0.139602925	0.198139042	0.442882364	0	0.232632978	0.151865521	0.438313921	0.442978968	2L	0.747847795	0.471345185	NA
**c12.2**	FBgn0040234	59.45555202	63.96861162	41.29337617	60.87327202	61.97685776	59.54571534	64.17908869	62.40265547	X	0.166558452	0.248879175	0.879503029
**c12.1**	FBgn0040235	201.7119084	209.2708537	168.2041167	204.8806624	154.6944684	184.4288637	187.009374	141.9048153	X	-0.203626277	0.130842912	0.790743321
**c11.1**	FBgn0040236	58.91906567	71.53653418	67.95225053	57.51053781	60.77084402	66.83624889	56.72162343	68.62521208	X	0.015990584	0.884823038	0.995787491
**Best1**	FBgn0040238	41.30758185	39.11219134	37.47164592	41.51745727	33.44680235	31.84890115	32.57048181	34.34740842	3R	-0.240578062	0.025834557	0.449488936
**bc10**	FBgn0040239	75.83331415	90.16690865	76.57449321	79.51505611	73.80086612	76.60578686	65.54046775	76.62004133	3L	-0.10673743	0.441902512	0.967112302
**Ugt304A1**	FBgn0040250	0.088071424	5.982962082	1.816110396	2.053352099	0.079495747	2.200379819	0.44934419	1.912112145	3R	-1.046690068	0.318423614	0.91460807
**Ugt302K1**	FBgn0040251	3.06830484	2.573325465	2.530846014	4.07757322	0.720079963	2.574573365	5.713937445	0.39969443	3R	-0.365176707	0.595441011	0.988780662
**Ugt303A1**	FBgn0040252	13.75123765	19.34351784	17.65095807	11.53499216	8.931569152	3.944251401	11.32202044	17.61299419	3R	-0.534027447	0.162165876	0.824079516
**Ugt35E1**	FBgn0040253	0.57710521	1.584021322	1.272771991	1.345499072	0.284133495	0.049462842	0.401511215	0.569522197	3R	-1.871846623	0.101818128	NA
**Ugt35E2**	FBgn0040255	25.35168165	14.57731818	27.14045166	7.724143762	7.280999563	18.69561095	20.33270007	14.28143206	3R	-0.278450868	0.48195171	0.973549494
**Ugt35C1**	FBgn0040256	2.25177415	0.419670583	2.185109072	1.166650369	0.903339881	3.349557399	0.893561548	0.108640225	3R	-0.177217596	0.858638062	0.995787491
**Ugt302E1**	FBgn0040257	1.239370764	1.000937692	1.781745889	1.391263024	1.905917896	0.562597238	0.117098572	1.295564909	3R	-0.436717615	0.635170299	NA
**Ugt302C1**	FBgn0040259	0.916595192	2.416008334	4.764958181	4.655622795	2.009265509	0.617257392	1.419654231	1.99001301	3R	-1.038854141	0.110159518	0.758410631
**Ugt37D1**	FBgn0040260	0.270787794	9.311275006	0.492695559	0.552415105	1.588734455	2.170025165	0	3.674408547	2L	-0.450841843	0.674223358	0.995787491
**Ugt37E1**	FBgn0040261	2.529376661	2.424385435	0.876604643	2.768048341	1.58418252	3.650021733	0.790101331	2.241433009	2L	-0.030212255	0.966924415	0.997604378
**Top3alpha**	FBgn0040268	11.17586633	7.370807511	9.891704528	16.79609464	9.191395162	9.998756772	16.27595895	12.9345992	2L	0.122725877	0.67146128	0.995787491
**Sulf1**	FBgn0040271	2.055590922	1.476269657	2.63455429	1.718077815	1.588833375	1.724228042	1.986424636	1.561035763	3R	-0.17145667	0.450630393	0.967112302
**Spt5**	FBgn0040273	135.2931873	130.8432308	134.5528136	136.4458516	110.5733438	110.555077	115.1120074	125.8251646	2R	-0.186686492	0.063567248	0.636110045
**Osi14**	FBgn0040279	1.829205188	0.451211043	0	0.250866061	1.068353707	2.028898209	0.686226725	2.803322675	3R	1.343948114	0.236094804	NA
**Aplip1**	FBgn0040281	1098.291839	854.7203286	924.9540672	933.2205009	804.1937559	1019.005901	834.5779529	835.4874046	3L	-0.100005857	0.362723688	0.939549067
**SMC1**	FBgn0040283	108.4832312	111.2135571	125.6259967	84.82851907	108.1937637	111.529359	88.08344414	121.5637331	3R	0.030142306	0.825813905	0.995787491
**Scamp**	FBgn0040285	223.2103549	240.4893949	218.0203786	227.1360549	207.7450283	244.2618983	232.4785709	219.3312395	X	0.021578682	0.809408517	0.995787491
**RecQ4**	FBgn0040290	0.064906891	0.108872204	0.708584452	0.378319931	0.205053753	0.749624296	0.165578873	0.105689151	3L	0.019610482	0.985919979	NA
**POSH**	FBgn0040294	34.86444706	37.51637576	26.57413196	46.18346348	33.45145585	32.66526117	31.32902168	32.21576076	2R	-0.134516966	0.447487895	0.967112302
**Nhe2**	FBgn0040297	5.97548311	2.882601271	2.534045433	2.857069261	2.907508663	4.324265718	2.054311366	2.210786677	2L	-0.293946521	0.298420514	0.9074568
**Myt1**	FBgn0040298	45.62303813	53.03255122	40.53661445	36.5701038	40.88490676	22.24017782	47.01383097	46.41387791	3L	-0.131276812	0.581883313	0.985653564
**Myo28B1**	FBgn0040299	0.547561095	0.23582002	0.735991875	0.207019148	0.21372716	0.840629585	0.113257307	0.273104041	2L	-0.2376983	0.705609908	0.995787491
**MTF-1**	FBgn0040305	25.8967603	31.97708767	25.89478053	29.17650461	28.63966358	24.40640972	28.81838218	23.99366362	3L	-0.061717077	0.634013767	0.991585536
**Jafrac2**	FBgn0040308	28.15689528	13.44832648	14.24053039	24.2559146	22.14138309	18.08737602	14.85274418	21.86218425	3L	-0.037278923	0.902067859	0.996479182
**Jafrac1**	FBgn0040309	167.3688037	196.0410094	197.4491074	210.8812477	186.7049925	166.3745618	205.6909268	178.3501015	X	-0.033971802	0.778957091	0.995787491
**HGTX**	FBgn0040318	0.976697953	0.739003942	0.656751284	0.23513889	0.613283524	0.395354154	0.148953	0.39183183	3L	-0.719374939	0.199576108	0.847197998
**Gclc**	FBgn0040319	28.24427113	29.82574131	28.45228656	26.5194849	23.05327129	28.84464652	29.90289707	25.7725636	X	-0.041918164	0.708575386	0.995787491
**GNBP3**	FBgn0040321	0.339679395	1.804254352	1.510772035	1.979874303	0	0.05337461	2.310745156	0	3L	-1.242490407	0.397266252	NA
**GNBP1**	FBgn0040323	1.543774128	3.219334279	1.214651189	1.459155917	4.218022868	1.180103366	0.851502912	0.181171705	3L	-0.168125878	0.84468846	0.995787491
**Ephrin**	FBgn0040324	36.32645201	46.91926143	52.16322219	39.67983294	33.07895258	34.47284046	42.62430011	38.92833625	4	-0.197067377	0.160297584	0.821599055
**Tsp3A**	FBgn0040334	61.5951969	53.19960501	57.62783742	59.66621196	57.09806086	65.82545053	56.69203173	75.79235914	X	0.166955135	0.284538215	0.901780531
**Seipin**	FBgn0040336	3.404048953	0.248252808	0	4.054471341	0.73474988	0	1.22705898	0	X	-1.977998586	0.167945437	0.830652748
**MED22**	FBgn0040339	142.962153	126.1463416	181.8641126	202.5073653	162.4406823	139.5409215	211.1579595	208.9223853	X	0.175850448	0.38778472	0.948247506
**CG3708**	FBgn0040345	8.728453984	0.782330531	2.262988601	0.310687968	0.601415898	2.763983637	0.849865406	1.44658638	X	-1.090566852	0.277483812	0.896087042
**CG3704**	FBgn0040346	46.83212792	46.47501337	29.43170408	60.52239613	40.35923689	22.92561908	51.22063432	41.40687621	X	-0.2035339	0.455914111	0.968365245
**mus81**	FBgn0040347	8.605526726	10.4549411	8.097110692	7.125341504	5.589761503	10.42589	6.770515491	9.079780809	X	-0.07421313	0.820526171	0.995787491
**CG3703**	FBgn0040348	194.7138253	202.2784986	166.4388771	170.4828413	214.0996844	192.3378897	172.6728658	173.8622309	X	0.067226268	0.550892088	0.984934163
**CG3690**	FBgn0040350	0.287421026	0.043828041	0.380334219	0.365515256	0.094339749	1.059280717	0	0.113457755	X	0.264629215	0.857878296	NA
**CG14625**	FBgn0040358	0.215885963	0.201176898	0.989279742	0.950734434	0.606245879	0.316611504	0.244768768	0.937416818	X	-0.11074663	0.908017267	NA
**CG11380**	FBgn0040359	1.132899326	0.29861538	0.510416765	0.150932122	0.175300598	0.305168733	0.041286436	0.035137562	X	-1.859961482	0.087697052	NA
**CG11378**	FBgn0040364	1.721278711	2.544653991	4.458903103	1.156315945	1.106005659	2.750527212	0.297696202	1.963537593	X	-0.643934262	0.345870026	0.930285749
**CG11398**	FBgn0040366	11.97941004	16.92832134	17.58310192	15.24009339	16.83660673	19.1071665	19.60567305	16.92327925	X	0.267286071	0.177434543	0.834615603
**CG11382**	FBgn0040367	0.874002479	0.154317531	3.682657271	0.750733007	0.498252151	3.122542225	0.352041963	0.299611624	X	-0.312388924	0.777217258	NA
**G9a**	FBgn0040372	39.37374589	38.91934585	37.87367009	30.01728709	36.53157731	45.1821734	32.38543843	42.69865265	X	0.130983235	0.332904887	0.922663747
**CG13760**	FBgn0040375	94.28710772	98.29750332	103.3466756	99.83673227	85.0661852	97.08470022	73.7303908	76.5121302	X	-0.220803529	0.083772852	0.69799888
**AANATL7**	FBgn0040376	6.651313802	9.077452732	5.35421636	12.05339183	5.403296758	6.18530687	3.779157163	8.533092436	X	-0.438965663	0.229918773	0.867702781
**Vha36-3**	FBgn0040377	0.943425412	4.252871407	3.147013225	0.824835312	1.91601525	4.391687597	1.955442527	0.512065975	X	-0.014478724	0.986329567	0.99797245
**CG5273**	FBgn0040382	546.747664	643.6128189	613.1989124	691.5291458	607.8019346	632.4454243	653.8491146	586.7835526	X	0.022238408	0.821453898	0.995787491
**CG5254**	FBgn0040383	1.489850379	3.765876779	4.015951377	2.99109534	2.428076607	2.643420263	3.721460318	0.808650772	X	-0.316927072	0.456906642	0.968365245
**CG12496**	FBgn0040385	1.973723045	1.891796299	0.820838499	1.183284252	0.814417362	1.43548511	0.287714851	0	X	-1.164311535	0.299243627	NA
**boi**	FBgn0040388	1.563252462	1.880976008	1.210004008	1.225293983	1.19650991	2.319617305	0.896634935	1.104674095	X	-0.064638932	0.832593854	0.995787491
**mRpL14**	FBgn0040389	100.1689528	148.4096787	140.8417862	155.1772034	154.6893345	160.4455504	143.3561309	208.2463154	X	0.32797041	0.0871666	0.706081898
**CG16903**	FBgn0040394	120.5598278	145.3604164	116.8886761	116.2518435	119.2856809	106.8512059	126.4494212	117.8315463	X	-0.053235714	0.670382168	0.995787491

**Unc-76**	FBgn0040395	399.7570361	374.204967	347.7036927	357.9270738	354.3664395	367.44133	353.1165912	367.1594145	X	-0.007215676	0.932133894	0.996479182
**CG3939**	FBgn0040396	0.646941254	1.085153196	5.057182587	4.692548556	3.244152147	2.575269552	0.916867243	3.901581524	X	-0.057272932	0.945853807	0.997604378
**CG3655**	FBgn0040397	0.718376203	0.892574298	0.6182929	0.192626038	0.667386424	0.580903521	0.371519854	0.465162123	X	-0.181158232	0.62394607	0.990047511
**Dlip3**	FBgn0040465	233.4045306	234.9351853	220.4494848	241.9447081	220.0935029	223.9677225	232.5681908	237.5390615	2R	0.003885777	0.969267129	0.997604378
**Dlip2**	FBgn0040466	77.92198043	64.55726662	96.50319182	59.21910317	86.73302035	72.08437355	77.53543436	69.53967928	3R	0.068291347	0.729633194	0.995787491
**Dlip1**	FBgn0040467	21.28071108	18.13712595	33.48759455	12.33674665	28.44944792	23.42528689	29.34462225	24.97426686	X	0.351915868	0.323293492	0.918470465
**SH3PX1**	FBgn0040475	37.52185384	44.50438292	45.79145803	37.30536727	41.00932252	44.53699705	56.84738469	43.4655661	3L	0.202832113	0.182837332	0.838911159
**Buffy**	FBgn0040491	0.18154955	0.192330885	0.370894042	0.089110724	0.086248281	0.180172428	0.316883075	0.062235928	2R	-0.340816689	0.728954031	NA
**grsm**	FBgn0040493	5.053956495	6.150361454	5.589320216	8.560183193	4.406979653	7.995317178	6.014950067	6.502680034	3R	0.002366664	0.991078215	0.998408391
**CG17104**	FBgn0040496	2.934456309	1.505595396	1.423790015	1.04635844	0.685551801	1.041538818	0.088068954	0.224857987	2L	-1.708377825	0.049427657	0.583905634
**Alk**	FBgn0040505	147.4299274	169.2680304	146.5859839	159.7685115	141.5366404	159.5866017	143.5717046	148.5999421	2R	-0.040438527	0.636329985	0.991585536
**ACXE**	FBgn0040506	0.411292714	0.724380121	0.798232311	0.431511066	1.633475278	0.096941065	0.052460828	0.669715917	2L	0.105096913	0.917051012	0.996479182
**ACXD**	FBgn0040507	0.255134744	0.118875762	0.515794442	1.189674103	0.434995626	0.160359652	0.361585674	0.153866985	3L	-0.877472383	0.341767811	0.928085024
**ACXC**	FBgn0040508	3.041027003	4.142338512	2.772747579	1.189604023	1.32640264	1.05831136	1.457827558	6.02630707	2L	-0.132500049	0.822916138	0.995787491
**ACXB**	FBgn0040509	0.041305792	0.831416056	0.450932738	0.963028989	0.671107869	0.038942848	0.210743925	0	2L	-1.266436456	0.328909415	NA
**ACXA**	FBgn0040510	1.767314045	0.666162108	1.25252202	0.416672309	0.215086841	1.572605421	0.455911291	0.431123864	2L	-0.595767981	0.456984131	0.968365245
**zetaCOP**	FBgn0040512	168.5643279	172.0276102	187.6876963	180.0544388	168.0275236	173.129339	160.7868047	176.1224656	3L	-0.031901719	0.756465599	0.995787491
**CG17169**	FBgn0040514	1.604041586	0.336319342	1.945691257	1.636146126	1.990797995	0.756140305	3.580451479	0.870630692	X	0.399924871	0.711425161	NA
**CG15864**	FBgn0040528	3.031179328	3.158477299	1.671272448	1.49907768	0.414549665	3.593871523	1.523088586	1.196540166	3R	-0.450657865	0.549072712	0.984934163
**COX7A**	FBgn0040529	2733.085445	2282.599068	2412.628832	2276.178076	2614.29307	2376.925328	2397.239091	2459.104707	3R	0.051066865	0.605067983	0.989286463
**CG8369**	FBgn0040532	439.4571475	1092.650486	1086.370853	509.4908346	314.997011	402.6091828	843.1279188	530.1826228	3R	-0.535896051	0.238105961	0.873280735
**Sf3b5**	FBgn0040534	445.5323313	561.8665768	526.1908454	391.3068643	444.5262114	406.7665991	600.6176168	464.2797426	3R	0.027801612	0.866528802	0.995787491
**CG11686**	FBgn0040551	1.435149981	1.009497287	0.449245656	0	1.170044825	0.698348715	1.299099445	0.502555397	3R	0.343489351	0.761786305	NA
**CG17193**	FBgn0040571	3.579011675	3.204708314	2.798093099	2.617225586	2.431828057	2.780763999	2.863695691	2.905524179	3R	-0.126530904	0.510695877	0.980373783
**CG15922**	FBgn0040575	78.59242918	78.23305309	90.37259672	63.19675704	82.28462967	82.51975941	90.394775	106.1925911	3R	0.250777275	0.232580557	0.868036885
**CG13631**	FBgn0040600	18.61580501	31.13356194	18.8616442	21.44571812	15.41936082	18.89299239	26.53814858	14.26469503	3R	-0.231231794	0.41920322	0.960266449
**CG13643**	FBgn0040601	0.843379961	0.454085689	0.505192104	0.509783719	0.413523132	0.942380416	0.132807432	0.113028146	3R	-0.5032139	0.509730075	0.979725655
**CG14545**	FBgn0040602	3.637769681	4.2877853	1.431110028	0.229224948	4.792212944	0	0.752434752	1.494203866	3R	-0.40769768	0.749699474	NA
**CG6503**	FBgn0040606	0	4.997688895	4.130410924	0.496184715	3.073575105	3.00969851	0.542911562	0	3R	-0.468099823	0.783637179	NA
**CG3348**	FBgn0040609	10.02406997	20.26299086	14.34153437	18.87636836	16.00801366	14.9029196	13.76933269	11.43962227	3R	-0.138831804	0.736427055	0.995787491
**CG4186**	FBgn0040634	14.90712561	12.37070275	15.98850642	8.414465789	16.42998504	19.38020477	11.60866877	14.30862615	3L	0.28725641	0.527357658	0.984107922
**CG13255**	FBgn0040636	21.67058995	7.420434453	8.399164628	5.530751755	6.828808197	9.792324776	5.888038638	8.073472471	3L	-0.479566437	0.316236733	0.91460807
**CG15456**	FBgn0040650	158.2094307	161.9352862	190.1873398	157.3270985	205.2491703	224.6739117	209.3923703	233.3005269	X	0.418644603	0.00297847	0.135642433
**CG15458**	FBgn0040651	27.55315309	38.51379083	33.42178255	19.56709412	6.003164173	2.687270114	13.73457073	0.687591333	X	-2.352523697	0.000200601	0.017235151
**CG13551**	FBgn0040660	100.5039702	107.9610502	122.7234783	112.6006311	118.4717902	109.7412899	132.666467	131.1453662	2R	0.181854627	0.109983033	0.758410631
**CG12848**	FBgn0040666	85.46989769	126.3032002	109.4451332	102.8859681	127.3452602	114.7614763	119.689378	105.0692949	2R	0.173810745	0.362406269	0.939549067
**e(y)2b**	FBgn0040670	3.937192984	0	2.653215351	2.80482193	2.764083167	3.815071538	8.648882794	0.237444734	3R	0.723929529	0.521043102	0.982083369
**CG12589**	FBgn0040684	0.717335368	1.278431288	0.2175307	0.104527525	1.699653625	1.09898653	0.686226725	0.097337593	3R	0.649848676	0.582479281	NA
**CG18675**	FBgn0040696	1.52503304	0.631894064	0.594596071	0.582010838	0.33594076	0.924294751	0.544191745	0.660226754	3L	-0.422952136	0.356361779	0.937787223
**dpr1**	FBgn0040726	1.010232238	1.526748318	1.751155834	0.99109934	1.173686408	2.211360037	1.692566682	1.947193969	2R	0.447414122	0.058748272	0.625114381
**CG13018**	FBgn0040751	83.05859171	91.88428708	72.4436827	57.1722607	76.39904238	49.59451752	94.33894073	88.87588926	2R	0.051757815	0.854926385	0.995787491
**Prosap**	FBgn0040752	11.14841403	9.163926552	9.764106895	10.54846787	10.24947378	10.96035927	8.663247738	9.06548564	2R	-0.034706669	0.757140142	0.995787491
**CG17059**	FBgn0040754	140.5771528	181.8109282	163.0553067	123.0443194	160.4268692	114.5738498	157.8752704	144.8957833	2R	-0.039877137	0.824421934	0.995787491
**CG18336**	FBgn0040763	15.18492701	27.44365831	14.78725355	25.92902051	25.48221434	40.22666421	22.36929686	25.5385003	2R	0.480706156	0.161666079	0.823037813
**luna**	FBgn0040765	0.677066809	0.535304196	0.552578454	0.379320242	0.440562714	0.572849124	0.191557705	0.259487219	2R	-0.525131313	0.076774969	0.682035833
**COX7C**	FBgn0040773	524.917693	604.0601663	638.6228943	529.6839901	590.7849152	501.3249948	587.6920989	605.5564283	2R	0.026999656	0.806008758	0.995787491
**CG14767**	FBgn0040777	628.4955632	648.0900293	626.5780873	596.7535669	563.6182207	610.6501824	591.9979658	607.4196831	2R	-0.044026127	0.567076445	0.984934163
**CG17977**	FBgn0040778	28.78912002	34.19206055	31.80169664	25.88316486	32.27003478	49.84941195	28.16063793	24.91983345	2R	0.194670633	0.432032871	0.964264046
**Atg10**	FBgn0040780	8.59195027	6.164079389	4.269243081	5.913003745	5.60626711	5.953334123	4.753355363	6.124328954	2R	-0.132371866	0.677966652	0.995787491
**CG14104**	FBgn0040786	12.94808827	9.36146622	13.53960411	5.204824199	16.12042613	9.260769918	8.542461555	16.47915413	3L	0.324593164	0.599335611	0.989286463
**CG7630**	FBgn0040793	2571.432092	2706.002865	2880.280487	2539.517133	2944.577062	2328.870809	2916.463583	2816.404848	3L	0.07510019	0.483880683	0.973549494
**CG13053**	FBgn0040801	3.287699835	2.969429752	1.840595525	1.474067	4.108943653	0.71529769	2.580613204	0.274534642	3L	-0.301031231	0.782028624	NA
**CG12355**	FBgn0040805	62.25791118	67.72703933	64.46297177	37.3426505	48.99736484	63.49823496	58.12619102	50.92431745	3L	-0.034257903	0.850571454	0.995787491
**dpr6**	FBgn0040823	2.912885529	4.344346891	3.462503298	3.61449759	2.458842356	2.711048699	2.106230132	2.328024891	3L	-0.545307557	1.86E-05	0.002579947
**CG13315**	FBgn0040827	9.546257218	5.18051706	5.449196908	9.491844715	3.548061375	21.17683058	14.68324996	2.438330366	3L	0.505291924	0.478658708	0.973549494
**CG8012**	FBgn0040832	74.35204528	92.11193272	95.22441918	74.13529336	86.01950697	82.99751816	74.85851496	95.05238613	3L	0.048313019	0.780040663	0.995787491
**CG8620**	FBgn0040837	3.464729826	10.12342917	4.338263861	0	3.026480003	6.216936803	6.842787269	0.485306399	3L	-0.076371194	0.940641468	0.997188858
**CG15213**	FBgn0040843	5.924361141	9.937284709	2.478003016	5.23919568	2.212756606	0.963008879	0	0.887057686	3L	-2.475095987	0.029559164	NA
**Ir41a**	FBgn0040849	0.077962724	0.137727466	0.144870497	0.106353124	0.121278283	0.123547227	0.095210706	0.192673183	2R	0.233368913	0.551674413	0.984934163
**ksh**	FBgn0040890	105.2441971	154.9420857	143.2736289	112.9774428	148.9871202	131.7746331	139.8414895	97.31581104	X	0.039048736	0.844873355	0.995787491
**CG17776**	FBgn0040899	166.8985709	238.5154058	221.1736246	239.4570677	241.7398536	194.888425	237.5477452	264.9722858	X	0.152563843	0.37922115	0.944208584
**CG17777**	FBgn0040900	5.670268902	0.163984149	1.660206084	0	2.294340559	0.184341203	5.486715714	1.485771565	X	0.327414616	0.804312418	NA
**CG15579**	FBgn0040906	8.495251015	12.01436172	16.16420429	11.26243883	13.23114975	14.76218533	17.84717353	11.21104445	X	0.290847504	0.542071205	0.984934163
**mRpL33**	FBgn0040907	172.123623	192.2680785	220.4839001	124.5413009	186.8381552	151.9599141	182.6391695	159.4652395	X	-0.025139999	0.898775442	0.996479182
**schlank**	FBgn0040918	33.87715695	37.00638517	41.76316381	34.58028577	37.70173092	32.71651419	34.69335495	48.05662851	X	0.092034781	0.52181689	0.982562577
**CR15345**	FBgn0040928	12.67769239	9.295501112	7.735018004	6.017707348	9.319047393	7.443442509	6.390749504	7.251949251	X	-0.211763148	0.621272437	0.990047511
**CG12659**	FBgn0040929	37.86143442	38.98766108	59.47207239	38.10324131	42.77996104	43.82760408	46.09228104	58.54580728	X	0.168755059	0.479907757	0.973549494
**CG9034**	FBgn0040931	649.153841	680.2702575	729.2219374	692.1758326	838.8074966	617.1791445	717.9309103	734.7475731	X	0.11353157	0.363545857	0.939549067
**CG12643**	FBgn0040942	8.15725226	2.68010598	3.67225612	0	7.135244925	1.664976904	2.145291316	4.564471589	X	0.116190445	0.901682875	0.996479182
**CG18661**	FBgn0040964	75.82555689	49.97726912	76.98223811	71.40198446	62.28081141	70.9660626	96.71631234	119.5629063	2L	0.379751003	0.118067648	0.773565864
**lncRNA:CR131**	FBgn0040965	0	1.355316751	1.764190207	1.130301375	0.583463185	1.0664964	0.412248145	1.22797911	2L	-0.30419888	0.822568572	NA
**CG6115**	FBgn0040985	523.7523325	544.8797173	598.283034	554.4710759	617.6267582	524.8548964	564.1440282	571.6294956	2L	0.06864874	0.517449237	0.980804634
**CG17715**	FBgn0041004	0.344089533	0.30969664	0.08143965	0.254366178	0.10605338	0.110772613	0.149864842	0.145765898	2L	-0.94171944	0.283937472	NA
**wun2**	FBgn0041087	17.84770929	35.82492059	40.47970681	27.03003051	38.15489558	34.86531871	38.34095987	43.52557137	2R	0.394892262	0.068071652	0.656607807
**tai**	FBgn0041092	8.121660922	9.412249479	8.527991855	8.156988158	9.642635967	8.240409483	7.603592475	7.3073999	2L	-0.029579267	0.789342694	0.995787491
**scyl**	FBgn0041094	833.9107168	797.8730428	711.0326076	720.2173129	697.6082084	779.3812992	656.1488665	730.4755945	3L	-0.068047556	0.465060495	0.971928234
**rols**	FBgn0041096	1.114960996	1.241662934	0.927599392	0.837969895	1.118216867	0.938706652	0.577441481	0.577776317	3L	-0.328683837	0.199297249	0.847197998
**robo3**	FBgn0041097	1.358159605	1.374105458	2.311643342	0.412147357	1.525580734	4.227565596	3.536188023	2.368313101	2L	1.125323861	0.038450813	0.52275543
**park**	FBgn0041100	4.209176301	2.837964833	2.402683058	2.212856177	2.532880675	3.229176604	1.263258726	1.523085231	3L	-0.421299555	0.462614608	0.971606621
**nerfin-2**	FBgn0041105	0	1.153967122	1.192140634	0.53465592	3.252741052	5.837494668	0.250716723	2.987277472	3R	2.15251209	0.02537113	0.447279658
**lilli**	FBgn0041111	7.636350146	9.465053851	10.0346149	7.859416035	8.266317788	8.669934747	8.925467024	10.22985024	2L	0.079003709	0.512762353	0.980804634
**ida**	FBgn0041147	22.38251953	18.65407694	26.1285295	23.22924213	17.35312619	34.39841192	19.3310819	21.40291635	3L	0.060308889	0.801408025	0.995787491
**hoe1**	FBgn0041150	76.3636468	69.90129703	81.20945673	74.49893619	57.8489175	60.1486534	66.59677141	59.07223575	2L	-0.279483949	0.003522623	0.155330886
**exex**	FBgn0041156	0.230845191	0.731397467	0.746702952	0.717609164	0.416734735	2.708401873	0	0	3L	0.386135968	0.764049394	NA
**comm2**	FBgn0041160	0.729497636	2.640464621	0.931447942	1.79031187	0.415872695	2.425279807	1.763018661	0.75022432	3L	-0.14956954	0.8518896	0.995787491
**ago**	FBgn0041171	73.6412813	83.32779453	74.31108762	86.45459412	77.42804851	69.13114577	85.16157253	77.55577766	3L	-0.007077634	0.95010143	0.997604378
**Vhl**	FBgn0041174	146.8139531	124.8516133	113.421305	100.8191956	132.2195379	118.7487132	118.0845099	118.1821258	2R	0.031607176	0.833227924	0.995787491
**Tep4**	FBgn0041180	0.850481264	0.128024885	0.211615968	0.228792121	0.255889443	0.041119412	0.139076665	0.189381903	2L	-1.18817585	0.231399368	NA
**Tep3**	FBgn0041181	0.142807507	0.773897038	0.586298342	1.63914007	0.456115037	1.688153954	0.672563111	1.430992234	2L	0.470953663	0.490433429	0.973549494
**Tep2**	FBgn0041182	0.40701995	3.708824118	3.923011735	0.820714977	0.615622573	1.929050856	2.581760289	1.265809012	2L	-0.423872112	0.489327529	0.973549494
**Tep1**	FBgn0041183	1.088800226	1.320562352	0.894017101	0.351484164	1.179338079	0.789626309	0.683705297	0.545512172	2L	-0.162601637	0.797917997	0.995787491
**Socs36E**	FBgn0041184	46.00688218	45.99121725	49.00899227	52.68378959	44.52543343	40.27297244	53.72173509	47.78724736	2L	-0.024479744	0.84009745	0.995787491
**Slbp**	FBgn0041186	132.3253568	114.8180691	106.3013401	149.0885214	137.8715673	137.3612597	103.809063	107.5959299	3R	-0.019903989	0.904243821	0.996479182
**Atx2**	FBgn0041188	165.2927353	231.5548414	210.229589	240.949853	181.7642681	178.4894212	211.5064854	207.4905473	3R	-0.088483438	0.481591155	0.973549494

**Rheb**	FBgn0041191	242.2552335	216.4542716	230.0773715	212.6233674	213.7363149	231.3095803	231.9428848	189.2540398	3R	-0.029354543	0.793349619	0.995787491
**Prat2**	FBgn0041194	0.632249968	1.161511638	0.43823703	1.614454333	0.271755533	0.738005553	0.076803964	0.980480863	3L	-0.853725025	0.372942263	NA
**key**	FBgn0041205	84.06124946	135.9460577	118.0830485	113.0563543	83.08860228	98.7103899	102.6200158	95.56468381	2R	-0.213257848	0.164672987	0.827712868
**HDAC4**	FBgn0041210	35.87041055	65.34723658	68.21935284	64.91882292	48.68572436	48.47470659	63.24154245	58.60109133	X	-0.061498669	0.715608901	0.995787491
**Gr59e**	FBgn0041233	1.242072956	1.535139155	0.634368772	0.30482593	5.66465691	0.431427978	0.83383021	0.28385846	2R	0.985723074	0.386685265	NA
**Gr59f**	FBgn0041234	1.322896843	1.162319646	0	2.496801485	0.796055952	0.059391384	0	0	2R	-2.503736124	0.140112727	NA
**Gr47b**	FBgn0041241	0.230214606	2.992683474	0.279248712	2.952051785	1.55848638	1.356530779	1.321385415	0.249908611	2R	-0.483757143	0.65075322	NA
**Gr43a**	FBgn0041243	1.965160896	6.846123569	1.83363574	7.401205511	4.434523855	7.339707461	3.085026256	0.492293794	2R	-0.206871759	0.75913071	0.995787491
**Gr39b**	FBgn0041245	2.505088306	0.888870697	0.46748533	0.336953024	1.652388507	2.316363069	0.122894886	1.359694651	2L	0.37518962	0.712812268	NA
**Gr32a**	FBgn0041246	0.143645515	0.401575334	2.090892097	0.111634704	1.555901826	1.083424914	4.030870748	5.093839272	2L	2.188535967	0.022848729	0.427913049
**Gr28a**	FBgn0041247	2.508448012	3.091275654	3.849984828	0	1.201410009	1.689249617	0.26118795	0.222288687	2L	-1.43455913	0.184407495	0.839299945
**Cyp6t2Psi**	FBgn0041336	1.421913745	2.484438368	3.449542913	5.93962291	1.81823411	0.670285797	0.453416564	2.829847502	2L	-1.164724094	0.14676596	0.808004944
**Pcyt1**	FBgn0041342	149.6186453	168.126275	161.9669844	167.0178369	160.4608774	164.9348893	160.5066905	159.7158614	3L	0.028465533	0.731672969	0.995787491
**AttB**	FBgn0041581	2.574639998	4.010126167	2.676874596	4.073244586	0.995975372	0.433456226	1.172850348	0.998175316	2R	-1.884519086	0.030437656	NA
**tamo**	FBgn0041582	67.92384054	83.60530073	81.19046226	70.95757235	73.55839289	89.28252708	70.2561959	74.04731037	2R	0.047578909	0.661389201	0.995750687
**olf186-F**	FBgn0041585	7.100270428	8.747913321	8.434809987	7.411013743	6.55943337	5.326686404	5.504787478	6.374601477	2R	-0.381408457	0.011980519	0.331790969
**eIF2D**	FBgn0041588	142.3405206	120.4880876	87.68527259	140.0223854	115.3400352	146.2880505	147.1547772	162.7905084	3R	0.246778341	0.148416651	0.808039863
**dlp**	FBgn0041604	18.96550544	19.66971535	20.71826	21.83688476	16.69588997	18.69702673	19.24833595	23.10206756	3L	-0.031153691	0.795597438	0.995787491
**cpx**	FBgn0041605	687.5498648	714.7112226	778.0920121	756.8608888	734.711544	747.2029453	697.6940825	722.8399269	3R	0.012950482	0.872007745	0.995787491
**Or65b**	FBgn0041624	0.361662821	0.404425764	1.316083439	2.108008131	0	1.534380744	0.615073084	0.130867244	3L	-0.834572554	0.540857605	NA
**Ku80**	FBgn0041627	20.51574897	16.48802582	15.03929328	36.65233497	17.12691695	17.50155353	11.88265748	19.18485852	2L	-0.407741638	0.168243561	0.830652748
**Hexo2**	FBgn0041629	25.22534188	58.71236003	38.88903927	24.15362579	25.0653802	35.13885136	33.77809002	40.92518794	X	-0.082950607	0.744253778	0.995787491
**Hexo1**	FBgn0041630	44.48874874	80.84227109	45.29750946	87.06516508	90.5961676	70.06507867	79.10031328	94.17446199	3L	0.408906642	0.057118036	0.622250606
**CG15107**	FBgn0041702	36.89034403	32.06317427	27.20798882	44.89542333	26.10380078	21.87880888	32.92882412	29.40292838	2R	-0.330102642	0.196530986	0.844272804
**CG3253**	FBgn0041706	121.9546029	135.2137528	147.9836612	123.166153	128.7784475	132.4267345	141.1877369	171.274057	2R	0.152442895	0.257065296	0.88674759
**7B2**	FBgn0041707	8018.602546	8939.040515	8292.142116	9469.717254	8814.190352	8410.621377	8656.096072	9353.240165	3R	0.052737454	0.570919831	0.984934163
**yellow-e**	FBgn0041711	0.301648078	0.07589585	0.622024926	0.035164057	0.626234907	0.056878393	0.153902146	0.458434131	3R	0.370026373	0.748438543	NA
**yellow-c**	FBgn0041713	1.097329634	1.781241795	1.030492795	0.990341703	2.300471109	1.702010952	1.625406661	1.767590224	2L	0.626030296	0.309734936	0.912131082
**rho-5**	FBgn0041723	410.0176019	429.7367763	392.26328	497.288483	376.5344654	384.5604746	371.2212094	410.5227477	2L	-0.13487642	0.192254098	0.84417579
**tral**	FBgn0041775	276.7522743	278.6018083	242.6440376	308.0422448	258.6506649	298.2817657	277.3026482	295.5944058	3L	0.059311945	0.56647654	0.984934163
**SCAR**	FBgn0041781	89.77612229	116.9967846	107.5898407	112.4482691	98.33144583	104.9726604	124.806128	126.2180704	2L	0.123802672	0.32565053	0.918470465
**Pax**	FBgn0041789	11.68741333	13.27359771	12.29437757	10.49472048	12.06508757	12.45344912	9.661821626	13.65141379	2L	0.035080407	0.789166392	0.995787491
**Mccc2**	FBgn0042083	0.269314405	5.025575616	0.326676496	1.491253945	2.734771087	2.189956297	0.171756709	0.219264939	2R	-0.370232048	0.718012827	0.995787491
**Bap170**	FBgn0042085	44.58335634	46.25121581	39.00035948	42.04066315	37.34004692	45.72074914	41.16163036	51.11552319	2R	0.058562861	0.658013023	0.995750687
**CG13773**	FBgn0042092	8.014761269	21.3371497	17.12472577	9.08590873	17.38898598	21.35165628	10.5042787	9.099494973	2L	0.112732134	0.787734339	0.995787491
**CG18766**	FBgn0042111	237.0854932	277.3173417	223.8359545	243.5286159	247.3235165	258.1329106	223.8464984	209.9982741	3R	-0.033822577	0.744595597	0.995787491
**mRpL36**	FBgn0042112	212.2779268	313.1157623	292.577997	260.9098589	333.8516718	214.2912662	316.1251175	278.9958295	3L	0.12076849	0.500828721	0.976796212
**CG18787**	FBgn0042125	1.864698344	1.412541236	3.356317419	1.121928772	0.977300834	2.325131437	1.534479205	0.914162226	2L	-0.390236558	0.627631063	0.990047511
**CG18788**	FBgn0042126	0.981762702	2.021034933	3.247830847	3.641499878	1.530651487	0	0.227681613	3.584791597	2L	-0.840559754	0.425274752	0.9620897
**CG18789**	FBgn0042127	1.020450572	1.81234872	2.329975393	1.259545815	1.950538047	2.54666797	1.071902314	0.781938584	2L	0.037535764	0.963312943	NA
**CG18808**	FBgn0042131	0.192058194	2.040287627	0.155310167	1.7911058	2.773714119	0.120714195	0	1.250928356	3L	0.043331817	0.975242548	NA
**CG18810**	FBgn0042133	0.519561028	0	0.540191917	1.384385117	2.009873181	0.629792953	0	4.189770322	2L	1.527222996	0.280316659	NA
**Capr**	FBgn0042134	148.3113415	184.00772	160.313521	172.4225092	176.4471202	163.2789283	169.7298399	176.7095763	3L	0.077801729	0.436337925	0.966084844
**Gdap2**	FBgn0042135	240.2338513	255.2747997	241.6408936	257.9106438	251.9592188	249.7724933	243.0908663	251.6745074	2R	0.03272033	0.676680618	0.995787491
**CG18815**	FBgn0042138	813.642997	718.1459649	826.8240825	808.3844301	845.7832411	763.2433396	800.9981452	798.1294989	3L	0.048778274	0.609092472	0.990047511
**CG18858**	FBgn0042175	19.28305648	14.34640497	13.39278327	10.42366498	13.05407218	15.21104509	11.3061089	15.36186434	2L	-0.040576972	0.880070164	0.995787491
**Arts**	FBgn0042177	26.10622562	36.54357614	34.69924318	32.75247787	30.67185653	29.75982953	25.12050623	35.3366997	3L	-0.070571035	0.652037453	0.993444251
**Apl**	FBgn0042178	2.330388572	1.761757705	2.149918797	2.716606613	2.19235352	3.218252332	2.302591002	2.565374862	3L	0.227329092	0.5268015	0.984107922
**Ugt305A1**	FBgn0042179	0.040231419	0.944755866	1.171210343	0	0.581024184	0.113789809	0.821049719	0.611422948	3L	0.042961457	0.975544353	NA
**CG18870**	FBgn0042180	95.55729386	81.51800913	74.52848993	95.63142046	71.08570927	102.945712	79.3799824	90.4045623	2R	0.011066641	0.939734998	0.997188858
**MCU**	FBgn0042185	2.285653443	3.080542555	2.329857417	2.846762464	2.537245071	2.171912474	2.874457968	1.884828187	3L	-0.123751022	0.493945514	0.973868444
**CG18764**	FBgn0042205	12.46903728	28.4444827	9.948793684	19.38072735	25.21098505	29.51994971	12.7235128	19.85238507	3R	0.347538844	0.327405978	0.918470465
**GstD10**	FBgn0042206	4.064464081	2.121021252	3.505896238	4.843362998	0	1.447626277	3.917002184	2.745346415	3R	-0.830774676	0.386024804	0.947329421
**CG18731**	FBgn0042213	80.47788736	71.87798715	86.84297992	81.93633655	81.83973199	67.74469699	92.31871273	78.00220679	3R	0.023187311	0.90725053	0.996479182
**FASN2**	FBgn0042627	1.278393261	0.595645932	1.791899328	0.5629882	1.07698616	0.850378847	0.380474345	0.75555553	2L	-0.430639134	0.389080892	0.948247506
**Sox21b**	FBgn0042630	0.54482643	0.407017689	0.333212098	0.074720136	0.752127499	0.444595631	0.338706336	0.159041158	3L	0.340223962	0.595465625	0.988780662
**wrd**	FBgn0042693	52.0329585	72.16036282	90.87828101	69.51175676	65.56470549	55.10203305	73.1456726	66.29089182	3R	-0.093556415	0.535294364	0.984760433
**Nfl**	FBgn0042696	92.23141241	92.20255917	115.0688553	86.6790613	92.36291994	106.1900042	99.29132908	102.8181484	4	0.08425146	0.439556652	0.967112302
**HBS1**	FBgn0042712	100.9016274	96.20301436	80.88269074	85.69271984	74.57149222	129.6086666	90.10951054	83.22624144	3L	0.07842196	0.633608284	0.991585536
**Chrac-16**	FBgn0043001	14.82866705	27.2418667	18.55808781	3.841386556	18.05881976	17.97477007	21.01569346	17.63027151	X	0.250245437	0.60573825	0.989286463
**Chrac-14**	FBgn0043002	20.37214238	30.72968607	33.06687477	63.89868437	37.04152062	36.20261601	46.36152401	32.77460513	2L	0.073977331	0.839955424	0.995787491
**prt**	FBgn0043005	2877.911602	3672.908192	3639.48475	4309.11924	3451.701393	3371.19644	3679.920409	4042.677701	3R	0.036876097	0.76521791	0.995787491
**Fsn**	FBgn0043010	181.7273588	187.251989	187.6004789	191.1855201	189.4934881	183.5809168	190.8730607	226.4098026	2R	0.111430765	0.347675283	0.931680323
**AP-2sigma**	FBgn0043012	1047.320612	1195.706137	1224.167827	1073.631422	1144.593069	965.43575	1179.585705	1271.442521	3R	0.041224123	0.719926253	0.995787491
**Desat2**	FBgn0043043	4.252853577	0.361193178	0	0.753065925	0.097183427	0.050753979	0.823983862	0.818143944	3R	-1.612900934	0.25759738	NA
**MESK2**	FBgn0043070	146.6687581	127.9633802	123.5637289	128.8440071	130.162222	137.9926098	118.7993314	130.5987431	2R	0.002071208	0.982712358	0.997881056
**bchs**	FBgn0043362	20.14635943	23.64557491	26.88786491	34.16034807	25.1780047	21.97637228	27.56576317	31.15995447	2L	0.046984732	0.779721009	0.995787491
**CG5986**	FBgn0043455	71.21804958	83.82112831	87.98816864	86.75814944	77.84364264	94.70081158	111.9256705	79.01636927	3R	0.171245466	0.296793487	0.906715248
**Ndf**	FBgn0043456	121.2689696	165.1139758	144.3143483	178.5457459	134.1049228	127.7510782	153.0631628	155.2198342	2L	-0.062397378	0.641400339	0.992099188
**CG5180**	FBgn0043457	191.5067879	200.547775	180.1646311	151.2948537	181.2332301	174.660973	184.8619801	185.1788664	3R	0.036055427	0.757692882	0.995787491
**CG12084**	FBgn0043458	103.8445025	125.2781277	108.6483296	132.8338436	103.3480179	108.8507275	115.8008805	109.6554156	3L	-0.073834142	0.505486213	0.978557094
**PGRP-SC2**	FBgn0043575	0.116266102	1.950200748	1.410299703	0	1.259340672	2.521144318	1.482986615	7.825158541	2R	1.986026864	0.112250174	NA
**CG32444**	FBgn0043783	0.040993017	1.65024086	4.07738802	1.624752504	0.518020319	0.270535765	1.568611019	3.737984684	3L	-0.219940161	0.831735992	0.995787491
**phu**	FBgn0043791	0.135200175	1.058303528	0.655987479	2.626784325	0.244070968	2.464340723	0.114966184	0.587064192	3R	-0.352163739	0.762071319	NA
**CG30427**	FBgn0043792	0.863205884	0.732274691	0.914675478	1.873736712	1.450838352	0.654801913	0.734018919	0.581617204	2R	-0.325934508	0.528579242	0.984296327
**CG12219**	FBgn0043796	70.81653073	107.4533514	107.3749474	141.2996622	112.5626277	134.3726734	109.541594	94.91318438	X	0.109453307	0.547482909	0.984934163
**CG32032**	FBgn0043806	24.54904244	66.88334069	44.63758911	19.26506643	25.75781233	17.30189929	32.59938518	19.71304901	3L	-0.658876515	0.048671928	0.582395583
**vir-1**	FBgn0043841	5.634545009	8.428243751	7.491041574	3.574552178	3.38801499	4.186608167	5.97084278	3.263408216	2L	-0.544467732	0.029825138	0.47985078
**slam**	FBgn0043854	0.212299622	0.356102833	0.557955581	0.123742144	0.22356555	0.416989139	0	0.768203552	2L	0.225573799	0.843909863	NA
**mask**	FBgn0043884	52.0211073	74.19485548	92.08735525	86.48870596	73.42614171	65.82914628	70.29888397	90.96166905	3R	0.01463934	0.926862904	0.996479182
**pygo**	FBgn0043900	60.52948539	88.62567818	65.19185764	83.16204758	62.9137412	47.48013907	65.32641406	76.12720308	3R	-0.204069174	0.225944461	0.867702781
**dome**	FBgn0043903	26.18177896	33.84318803	39.47950285	39.05160682	44.95819742	49.03950634	40.86237665	37.92900216	X	0.349899491	0.016549182	0.370938755
**Roc2**	FBgn0044020	7.744123173	9.023949428	8.990647352	7.76719919	8.195246938	6.969522412	9.254643815	8.689416723	2R	0.015431259	0.910032929	0.996479182
**Notum**	FBgn0044028	0.635250292	0.893175957	0.294623655	1.001894977	0.42161382	0.44037507	0.357471428	0.141975152	3L	-1.022169963	0.109215083	0.758410631
**mRpS14**	FBgn0044030	156.9449755	168.5431379	173.802963	166.7951583	187.6052255	159.224068	196.4420201	195.0863689	X	0.180538041	0.192198452	0.84417579
**Cka**	FBgn0044323	0.274298254	0.373828684	0.623853506	0.159879014	0.386858321	0.339421318	0.328003472	0.167491941	2L	-0.184667565	0.794885059	0.995787491
**Chro**	FBgn0044324	168.5792437	172.0814016	180.7746831	165.2816461	187.3930869	169.7888575	183.7331133	173.4589076	3L	0.088485145	0.329257201	0.921112231
**CG32052**	FBgn0044328	8.147320101	12.61991493	9.174661207	12.12596482	10.85418714	9.852193738	9.831091769	8.583911521	3L	-0.072061708	0.648256671	0.992932477
**Atg2**	FBgn0044452	21.80828523	25.3266132	28.0444737	26.66505581	16.55198258	27.02781114	13.87349301	16.99893035	3L	-0.423252607	0.025730586	0.449488936
**mRpS21**	FBgn0044511	18.36371812	23.17006799	22.07793757	14.39773508	20.82952622	8.733207723	21.55756152	17.28847138	3R	-0.159144805	0.722811725	0.995787491
**Spec2**	FBgn0044823	8.000696129	13.01220029	13.2018832	14.68510431	8.44442266	16.03571336	10.60372103	12.89571524	3R	0.004261122	0.985166568	0.997950263
**Pak3**	FBgn0044826	40.77366703	24.13257264	22.57743579	25.66154305	31.52554722	33.44724098	27.62689314	22.38155034	3R	0.044921434	0.81340767	0.995787491
**Gos28**	FBgn0044871	95.17517877	98.52677687	112.0986022	92.41432408	96.57152411	70.16056732	96.75140779	81.98244802	3R	-0.172548904	0.271814552	0.89549671
**FucTC**	FBgn0044872	0.136567987	0.515415688	1.325248125	0.23880234	0.184905164	1.319743504	2.438715079	0.074125435	X	0.908407642	0.432006757	NA

tefu	FBgn0045035	1.071494659	2.898841894	3.018694177	0.342488232	2.698691848	2.574377127	1.829618462	1.350763021	3R	0.250464478	0.621059241	0.990047511
Proc	FBgn0045038	24.12299006	22.6814953	19.68271592	20.55162012	17.61732284	18.20883851	18.77714407	14.76199857	2L	-0.300509067	0.037757882	0.521998122
fdl	FBgn0045063	43.72300137	51.18982698	46.36659968	45.7281058	38.71845494	47.68270507	45.44441745	35.05363577	2R	-0.13480523	0.216815065	0.860826301
bwa	FBgn0045064	22.58835527	14.92949463	14.19441247	15.04682856	16.92138781	20.17062047	16.73520443	13.13929987	2L	0.026324212	0.891073656	0.996189576
Stim	FBgn0045073	48.10832045	43.9821676	56.34403044	53.43666118	43.71271696	53.30865321	44.11138207	46.69831487	X	-0.074867097	0.549317103	0.984934163
Gr93b	FBgn0045470	3.202925482	1.401510827	0.337837067	0	1.634072134	2.035014261	0	0.907023615	3R	-0.106141098	0.938656473	NA
Gr85a	FBgn0045473	1.948040866	3.851059068	2.531742841	0.324413139	2.637534781	4.657095467	1.419855457	4.380426228	3R	0.63653675	0.424227226	0.9620897
Gr64a	FBgn0045479	0.236532621	0.396750289	2.065769418	1.433813941	0	1.427209663	1.448159512	0	3L	-0.493558346	0.721299829	NA
Gr36a	FBgn0045487	8.508929733	4.014150734	0.806349408	0.309972646	1.080056179	6.894049747	0	0.577302316	2L	-0.673263494	0.558081665	0.984934163
Gr28b	FBgn0045495	0.326817259	0.646081194	0.594640584	0.136064667	0.105355163	0.28611265	0.357307649	0.582845072	2L	-0.317561546	0.70097684	0.995787491
Gr22d	FBgn0045498	9.58480587	1.309991404	0.688966085	1.324243796	3.460606232	5.154149815	2.354545141	0.924866702	2L	-0.130004292	0.884936596	0.995787491
bin	FBgn0045759	0.090581172	3.34261859	0	0.422373185	0.286163904	1.046142351	0.057768629	0.5408153	3L	-0.941189642	0.449973654	0.967112302
CHKov1	FBgn0045761	2.067026317	2.382189209	1.944427821	0.327836329	0.761533118	2.532088021	2.726191627	1.312730173	3R	0.152281184	0.791251707	0.995787491
S-Lap3	FBgn0045770	0.434577942	3.683083114	1.553675828	1.706445663	1.527758368	0.388153354	0.583482983	0.248291827	3L	-1.374396185	0.076488551	0.681411883
Uhg1	FBgn0045800	6.13894324	6.055173573	7.273321027	6.13402074	4.528674456	7.902885766	3.850108409	5.357853488	2R	-0.208589464	0.47592242	0.973549494
vsg	FBgn0045823	379.4400094	482.6008052	443.8722903	480.2199131	450.7108611	443.4415134	460.4306189	481.5060002	3L	0.072373889	0.476706069	0.973549494
yuri	FBgn0045842	0.513293308	0.391353416	0.084902891	0.271982733	0.65285012	0.483929795	0.17855758	0.785150588	2L	0.746128964	0.348582623	0.931729531
ham	FBgn0045852	0.830649132	0.599891932	0.510782827	0.336219997	0.718526985	0.467703193	0.286948429	0.194117712	2L	-0.423542682	0.350699834	0.93318404
btz	FBgn0045862	134.1403182	149.2943806	126.598635	121.60049	151.5006429	138.197145	124.5256743	140.79826	3R	0.09376649	0.397698409	0.952272708
bai	FBgn0045866	211.4003769	231.4581802	250.8249025	228.0170673	229.944533	216.0368535	222.4119233	213.7402421	3R	-0.031155465	0.78099824	0.995787491
niki	FBgn0045980	4.61501586	3.713910171	4.206576109	0.248780017	0.963154502	0.679059171	3.266498388	0.57916917	3R	-1.196376354	0.113381722	0.758410631
GluRIIC	FBgn0046113	0.861757217	0.820405773	0.734538996	0	0.042045747	0.087833453	0	0.758495144	2L	-1.406830276	0.32298583	NA
Gclm	FBgn0046114	88.40359512	152.7283378	153.8286277	144.0753477	90.86818394	127.870163	118.8568414	137.6661626	3R	-0.146856275	0.4294854	0.963666972
vig2	FBgn0046214	161.8210858	189.7955165	190.2064805	215.4343013	215.6153648	197.7222732	198.1404814	220.7944243	3R	0.168457635	0.157068446	0.818884898
Wdr33	FBgn0046222	38.47577122	42.97173151	39.85791883	39.73023459	40.55776907	35.12200004	30.36197584	36.33375761	3R	-0.145399418	0.27546884	0.89549671
CG17230	FBgn0046225	3.551151924	4.753461897	5.035504477	5.965519802	3.345422939	4.548648934	5.160604956	5.332719658	3R	-0.038040235	0.844821995	0.995787491
CG5938	FBgn0046247	245.891506	273.4153207	256.3418571	218.3399949	244.3252085	253.8902445	256.4170892	234.2102736	3R	0.023953747	0.804037827	0.995787491
CG3502	FBgn0046253	0.061344367	0.411586277	0	0.047674027	0.110742304	0.385567294	0.312981594	0.843500557	2R	1.698874163	0.206377239	NA
CG12880	FBgn0046258	0.685030062	0.893699201	0.923262987	0.650679253	1.992388344	0.03588012	0.161808001	0.523296479	3R	-0.169063945	0.84772804	0.995787491
DEF8	FBgn0046296	16.31789225	12.65262055	9.087576938	11.84405848	12.50574527	17.22279765	9.686854509	13.81455714	3L	0.115458917	0.712498899	0.995787491
CG7148	FBgn0046301	167.053094	165.8634896	125.4168221	137.6644023	132.1214656	173.6954715	161.6186407	154.8245259	3L	0.089680023	0.513874486	0.980804634
CG10650	FBgn0046302	0.100953666	1.072458881	0.163274791	0.078456557	2.126217821	2.760176497	0.858449905	0.8036591	2L	2.248836858	0.023517709	0.434002865
ORY	FBgn0046323	2.112117521	2.631413306	2.970416027	2.01968589	1.099666345	1.806591996	0.89020075	1.548471621	Y	-0.831502442	0.000121861	0.010920275
Tre1	FBgn0046687	1.028653316	0.779985121	0.44440386	1.544089379	1.373658566	2.271742175	1.222191344	1.68262447	X	0.808285058	0.112537478	0.758410631
Stlk	FBgn0046692	10.62809136	7.932722167	12.15141232	7.376123426	7.654384156	11.0392417	7.702276687	7.253163879	2R	-0.152108813	0.415030598	0.958665466
Ppr-Y	FBgn0046697	0.040819483	0.043890357	0.050783332	0.030909618	0.02141408	0.030261206	0.011570153	0.026511117	Y	-0.848518834	0.072749489	0.671999479
Liprin-alpha	FBgn0046704	85.24025031	91.73446828	90.9010798	103.742018	83.67979883	86.58339704	88.95443302	103.4171344	2L	-0.004009614	0.97173315	0.997604378
Haspin	FBgn0046706	6.566868216	4.536316066	5.58871938	4.109906823	3.30214559	5.780928823	4.020282381	4.248893266	2R	-0.237407981	0.212313106	0.856520545
CG17278	FBgn0046763	1.333015643	3.353920107	0.26948978	0.84171636	0.852279022	1.309123965	1.416896477	0.060293757	3R	-0.642061941	0.479801405	0.973549494
Gr98d	FBgn0046885	0.18429414	2.163892958	1.341285037	0.859349698	0.665396313	1.042508335	0	0	3R	-1.332623886	0.36118557	NA
Gr98c	FBgn0046886	1.492345941	2.39890092	2.111906088	0	2.469561333	3.576060537	0.317250546	0.945006178	3R	0.322327623	0.764188118	NA
CG6429	FBgn0046999	15.09966715	0.589013334	0.851897253	9.278654167	7.184481808	2.979601525	11.34685164	1.016520159	2R	-0.199859803	0.835716277	0.995787491
ND-13B	FBgn0047038	607.7338599	620.9415966	674.5231483	636.0024791	717.8718854	625.3771321	705.9401477	683.4337863	3L	0.137790121	0.174327263	0.833984823
CG33939	FBgn0047095	1.653971209	0.988108514	0.384761337	0.475418418	1.431568054	0.961245588	1.791764366	0.049190748	X	0.280276944	0.749007263	0.995787491
CG31142	FBgn0047114	16.97485082	5.26554202	8.461798219	3.252840466	13.64285728	20.49799946	14.53326882	10.6018277	3R	0.807221732	0.142004198	0.8037297
CG32276	FBgn0047135	368.2738586	327.0251115	362.4112898	272.2193392	346.8097284	344.0336206	341.8985559	254.4477023	3L	-0.018157953	0.894173279	0.996479182
CG32147	FBgn0047178	25.56803636	25.67372712	18.77709274	19.66754654	20.56649145	22.71153952	29.28451881	15.2937295	3L	-0.007025015	0.981564499	0.997881056
CG31468	FBgn0047351	1.350771862	1.274473296	1.433667242	0.393659218	1.67646147	3.104154935	1.076827513	0.549872016	3R	0.556271035	0.596112834	NA
GstT1	FBgn0050000	27.30357743	24.41745912	33.55829369	22.45733679	21.83401195	35.8471198	33.0708495	22.64222161	2R	0.102913157	0.697279999	0.995787491
CG30001	FBgn0050001	0.339299227	0	3.292540773	3.277262557	0.70002522	2.83330469	1.48381649	0	2R	-0.440821996	0.72940981	0.995787491
GstT2	FBgn0050005	11.13429245	11.6726335	9.732134341	12.02521009	14.9273927	12.34981519	12.32224511	5.510154171	2R	0.046124037	0.90949389	0.996479182
CG30010	FBgn0050010	27.73153319	24.26284834	28.23943991	19.75570229	33.83855853	37.60378385	43.01393883	36.97943642	2R	0.625335232	0.009296929	0.287842402
CG30015	FBgn0050015	6.602792618	4.476538769	7.711633755	7.620648262	6.549496521	5.020810629	6.201070133	7.186089903	2R	-0.051243858	0.782599306	0.995787491
CG30020	FBgn0050020	29.51074534	49.52290452	47.19055975	40.54134716	46.952099	57.76954588	39.99601652	47.12457466	2R	0.235627615	0.141430036	0.8037297
metro	FBgn0050021	646.4626511	532.1862195	514.6876894	541.3037787	628.6215785	576.0366211	523.3420105	551.0066148	2R	0.056963697	0.593627022	0.988631016
CG30036	FBgn0050036	7.130516848	2.156800962	1.98507772	4.224266226	8.757459566	4.959300784	2.385587105	0.761361253	2R	0.132771028	0.854592967	0.995787491
CG30037	FBgn0050037	7.85802644	1.911709944	2.764929681	1.678231404	0.758017267	10.40583112	1.836274284	1.302328459	2R	0.004280834	NA	NA
CG30043	FBgn0050043	0.092913109	2.298768407	1.183381006	0.7040261	0	0.087597911	0.118511668	0.40344592	2R	-2.782245683	0.016131886	NA
Cpr49Aa	FBgn0050045	4.837721166	1.352433099	0.279434383	0	3.742854258	0.108594618	0	2.000598186	2R	-0.136543856	0.932550371	NA
CG30048	FBgn0050048	0.245435407	1.578119944	0.942752883	0.572223411	0.221536907	0.906297093	0.521759163	0.310836743	2R	-0.715085594	0.397765478	0.952272708
Tmem18	FBgn0050051	15.8302311	15.69799522	14.00897705	12.99889887	14.55841243	12.20254009	21.84251779	12.75323855	2R	0.090180079	0.804910254	0.995787491
Obp49a	FBgn0050052	2.803492326	0.972923811	7.035758564	6.085461865	3.315842116	2.551973529	5.918705506	6.29652554	2R	0.13499918	0.856538821	0.995787491
CG30053	FBgn0050053	4.395326551	1.906692333	2.022293275	0.353363393	0.684025081	2.357728824	6.186246875	0.987171808	2R	0.233662151	0.806950331	0.995787491
CG30054	FBgn0050054	17.6326195	16.35182159	17.05890195	15.8974409	12.50175729	12.00337856	11.82285085	16.07614754	2R	-0.324806061	0.216817223	0.860826301
CG30069	FBgn0050069	1.822641602	3.035151765	2.777531913	1.79488149	1.187846653	0.41563592	2.450690581	1.585706584	2R	-0.702923872	0.10395135	0.745084146
Blos1	FBgn0050077	195.4827074	191.1396549	208.4122406	208.2925138	212.3359627	224.4621088	190.1747999	226.0785272	2R	0.117611343	0.362530724	0.939549067
Rif1	FBgn0050085	4.619162335	3.964091393	8.25249785	7.388311424	4.686519089	6.194786507	3.745169586	5.09205582	2R	-0.261874038	0.408387246	0.956706394
CG30089	FBgn0050089	22.61725013	25.55557396	26.09784765	27.07349452	22.38857128	20.04885415	23.04441321	21.94522829	2R	-0.180944642	0.081276895	0.694684542
CG30094	FBgn0050094	71.13804076	100.2956584	103.2039237	94.22364965	85.75714823	58.48997992	91.03878811	79.27609127	2R	-0.194468985	0.366645611	0.940794966
CG30096	FBgn0050096	23.49081528	11.9872538	22.47429474	16.04469565	15.76821514	11.97809424	17.55566125	19.82563763	2R	-0.15932817	0.627528683	0.990047511
CG30103	FBgn0050103	3.384994402	2.52349033	1.094926561	2.104529872	2.715903317	1.702054425	0	0	2R	-1.016850576	0.340083325	0.927274752
NT5E-2	FBgn0050104	0.190905892	1.24529165	0.849080891	0.593453201	0.229756043	0.279976525	0.189390846	0.046052707	2R	-1.890039058	0.017112546	0.375267338
CG30105	FBgn0050105	0.12286276	0	2.086443391	0	3.326981563	0.579171297	2.507404318	1.867224973	2R	2.014723301	0.202689289	NA
CCHa1-R	FBgn0050106	0.513434167	0.996775218	0.311396032	0.798034955	0.377617885	0.380975192	0.230424539	0.21674977	2R	-1.08156032	0.030518875	0.486317979
CG30108	FBgn0050108	1.718047847	2.881784246	0.34733001	2.00278194	0.775379174	1.889725853	1.826157071	0.932510229	2R	-0.336960388	0.782384691	NA
CG30109	FBgn0050109	131.618115	105.1497878	82.03455031	97.33490156	93.21043391	90.98207127	102.8515792	122.2647858	2R	0.000985384	0.996227634	0.99922507
GEFmeso	FBgn0050115	0.703065298	1.597589787	0.656010233	0.514867587	0.542398742	0.409321364	0.643832962	0.300065439	2R	-0.834387616	0.017193387	0.375267338
CG30122	FBgn0050122	66.10536811	96.27395512	104.6738173	89.19188283	69.24166555	72.47575412	90.77963064	96.65580909	2R	-0.077898869	0.612252205	0.990047511
Obp57e	FBgn0050145	2.188250416	3.05873591	5.308664988	4.251519557	0	0	3.256326398	1.979539257	2R	-1.499809835	0.291334361	NA
Hil	FBgn0050147	0.20630669	1.307302001	0.519034083	0.267220156	0.468994049	0.100854282	0.058476977	0.232250107	2R	-1.359839926	0.094075647	0.728467429
CG30158	FBgn0050158	273.7684577	226.3948372	212.2732576	227.873759	238.1886095	260.0870708	217.5273106	224.4386763	2R	0.026928201	0.795867254	0.995787491
Brca2	FBgn0050169	21.69902663	20.24427368	17.5677257	17.8903045	20.73288247	33.4893188	15.55635123	14.28763961	2R	0.142448213	0.566443479	0.984934163
CG30172	FBgn0050172	393.9606246	489.1373538	494.9255514	476.1002164	393.8489082	410.5404135	407.8196357	422.0058634	2R	-0.149382646	0.171813231	0.832669092
ppk3	FBgn0050181	0.808760464	1.220923998	3.237368646	0.094279729	3.285044821	3.164351864	1.856848786	0.087794692	2R	0.691681275	0.481936142	0.973549494
CG30183	FBgn0050183	3.886789431	4.08430257	4.714648473	5.623710539	5.241851115	4.634440511	6.269958585	5.956644459	2R	0.303830964	0.201973707	0.849268738
AIMP3	FBgn0050185	417.1898171	331.3339926	370.6708238	371.4262253	397.0716456	370.6035902	407.1601471	474.7508461	2R	0.175767281	0.182424348	0.838040629
CG30203	FBgn0050203	0.137707863	2.263659088	0.400892825	0.06421214	0.497195973	0.233694237	0	0.239181214	2R	-1.490233845	0.261820626	NA
stum	FBgn0050263	1.277960056	0.848807544	1.394100598	0.679889502	1.672220855	0.816712392	0.546997241	0.018621272	2R	-0.43011536	0.550043645	0.984934163
CG30271	FBgn0050271	0.184392221	0.412389443	0.372777463	0.21495176	0.471573225	0.376661695	0.313592344	0.066722097	2R	0.099678165	0.917166016	NA
MFS1	FBgn0050272	1.202492438	1.171168474	0.376416759	0.180875216	1.820676879	0.621707837	0.098954324	0.084216852	2R	-0.130715788	0.913241508	NA
CG30275	FBgn0050275	1.019038184	1.090411433	0.639354936	0.163851666	0.222023719	1.971179553	0	0.267016959	2R	-0.225175641	0.831537636	0.995787491
CG30285	FBgn0050285	20.91250479	48.2036769	35.34975518	13.84061662	16.56235682	15.77294542	32.35313166	16.69658112	2R	-0.501703009	0.25087625	0.881909103
CG30288	FBgn0050288	0.82852235	1.010712736	0	0.175606243	1.90361206	0.994160122	1.152860899	0.81763578	2R	1.261835765	0.334411275	NA
CG30291	FBgn0050291	126.3836786	89.91860523	64.04433496	109.12467	62.16942813	57.17766061	75.96051262	83.95920623	2R	-0.453416088	0.031941893	0.501325306

Ipk1	FBgn0050295	0.767267199	0.713058198	0.37730666	0.048347421	0.299484096	0.312810737	0.396753062	0.067532761	2R	-0.804162147	0.362212249	0.939549067
RIC-3	FBgn0050296	83.84995857	69.16241796	77.379274	86.42342827	64.3095183	85.95579131	70.74349211	75.70752607	2R	-0.068462335	0.575475094	0.985548852
CG30324	FBgn0050324	0	2.021034933	2.273481593	0	2.175136323	0	1.024567259	2.906587781	2R	0.584979801	0.748376399	NA
Prp38	FBgn0050342	63.93078884	85.35025015	69.28289069	61.17563077	82.43675655	76.05948887	62.4988886	120.5448299	2R	0.324275929	0.129267006	0.788661222
CG30344	FBgn0050344	15.04898794	28.19125525	13.24649583	21.54848435	28.32128863	31.18869493	18.00606595	21.16499999	2R	0.370666171	0.143748953	0.8037297
CG30345	FBgn0050345	0.243385642	1.723702254	0.852872471	0	1.07402402	0.586404158	0.068986889	0	2R	-0.644771376	0.641919882	NA
CG30349	FBgn0050349	29.45586316	32.32021884	23.96546119	28.06987815	26.33226364	26.46109291	30.58202928	31.10998509	2R	0.036975875	0.82188536	0.995787491
CG30356	FBgn0050356	5.645509493	6.76396442	4.695746498	5.264917142	3.785448296	4.410114403	6.172206859	4.202373842	2R	-0.247277194	0.723541127	0.995787491
Mal-A5	FBgn0050359	12.18358393	45.40699344	42.58393623	14.41863142	21.66022883	17.63142036	43.83401564	28.86369661	2R	0.017973468	0.973645633	0.997757033
mtt	FBgn0050361	9.166941764	8.358070065	8.987040457	9.228824763	6.776006058	7.190915296	8.957836027	6.87908385	2R	-0.23310326	0.054693789	0.611836294
Asap	FBgn0050372	58.62012769	83.73969566	69.51442486	56.95767824	71.98496288	69.49098589	61.18055748	58.95233722	2R	-0.005502669	0.965694122	0.997604378
CG30377	FBgn0050377	0.890186313	0.517087458	0.428554702	0.815638224	0.362672521	0.751090701	0.265083783	0.421128038	2R	-0.536870554	0.21193195	0.856520545
PIG-X	FBgn0050381	18.30591789	22.36053074	26.15077822	27.80863215	17.61732284	33.37485263	28.3121583	32.40438711	2R	0.272707991	0.31634506	0.91460807
CG30389	FBgn0050389	226.357176	237.030861	195.3328254	248.7995823	188.3930403	191.9948606	192.4266913	221.1038958	2R	-0.162220008	0.133004605	0.794759341
Sgf29	FBgn0050390	87.11074455	91.07728569	75.72016381	84.25230564	79.90667232	106.272376	74.2831895	66.69513752	2R	-0.020096089	0.903603548	0.996479182
CG30392	FBgn0050392	220.6816866	196.5485273	215.1102198	199.3969383	186.3582333	224.4286491	172.8305608	159.4099372	2R	-0.135120099	0.292653671	0.905336188
CG30394	FBgn0050394	65.09572424	54.43943768	61.75908786	57.56725985	69.74590831	62.11877028	69.05156424	41.33796995	2R	0.047817553	0.775630831	0.995787491
Tango11	FBgn0050404	229.4036477	200.3385535	217.1985603	227.6426258	242.2895489	274.0834001	248.4554681	266.5163842	2R	0.266049649	0.007333182	0.250470256
Rpi	FBgn0050410	2.891865117	7.969003932	8.518972532	3.371137327	8.576630532	2.986093032	13.70053294	10.31468985	2R	0.694803405	0.218664869	0.862856541
CG30414	FBgn0050414	3.620478722	6.874919041	8.451788206	2.548229387	2.466374345	9.531661381	6.621979662	3.262805243	2R	0.064305487	0.914714548	0.996479182
nord	FBgn0050418	0.278021229	1.411296977	2.254168535	2.55866854	0.792473014	0.986386592	1.679774517	2.620937398	2R	-0.053897737	0.921246168	0.996479182
CG30419	FBgn0050419	18.53101857	21.39382843	21.0207518	16.89895487	14.27771907	10.48161481	12.66743444	13.76948929	2R	-0.569953231	4.53E-05	0.004835041
Usp15-31	FBgn0050421	20.03869598	34.76545777	21.28830716	25.60409912	24.67228355	20.15802482	25.6932168	19.67716198	2R	-0.137455272	0.415891131	0.958846438
CG30423	FBgn0050423	396.0665563	470.0268984	551.219367	499.0753117	494.9605484	420.5330867	521.2189164	516.1498311	2R	0.061473118	0.641873047	0.992277865
CG30424	FBgn0050424	1.798302678	2.082752702	1.558091862	2.196163093	2.937216546	3.269755505	3.932150371	6.228258711	2R	1.1297853	0.030162745	0.482946307
Ugt50B3	FBgn0050438	0.122876703	0.098932047	0.091416465	0.103133667	0.09168723	0.091133292	0.066871703	0.192079222	2R	0.111675795	0.840041793	0.995787491
CG30440	FBgn0050440	0.496902288	0.622475302	0.36622117	0.168643712	0.499614979	0.723469948	0.168479599	0.341399008	2R	0.09691259	0.850345133	0.995787491
Opbp	FBgn0050443	76.7733426	72.78672417	86.00145181	72.01140868	59.80127641	83.81427987	81.37979473	67.88371209	2R	-0.041785214	0.777642164	0.995787491
CG30456	FBgn0050456	1.055886024	1.17410045	1.669334343	1.465991026	0.899529052	0.570445254	1.059276153	1.777274323	2R	-0.283004691	0.558983392	0.984934163
CG30460	FBgn0050460	0.355877752	0.798149051	0.455928761	0.540713525	0.66410619	0.716277308	0.173409893	0.451432182	2R	-0.059904252	0.901429832	0.996479182
Pgant9	FBgn0050463	26.08808441	25.08936318	23.35455458	24.48839218	25.17621009	28.09176336	22.28865692	24.09272516	2R	0.037193325	0.69289221	0.995787491
CG30466	FBgn0050466	2.263184566	1.173363063	2.046442016	0.767495217	1.671393351	2.83202383	1.469600987	1.116723453	2R	0.204063747	0.708280158	0.995787491
CG30467	FBgn0050467	118.5996425	109.5007497	123.1784442	118.3790424	95.74253305	120.9183064	100.5697171	121.4849378	2R	-0.068874595	0.595923642	0.988780662
ave	FBgn0050476	38.79293087	42.34386411	39.61094447	30.77804599	33.44772908	25.98373047	31.90403824	22.12426229	2R	-0.388364243	0.154734403	0.818884898
antr	FBgn0050488	0.149858556	1.885250291	1.090664303	0	2.975864132	6.28721506	2.293754383	3.253567984	2R	2.301715867	0.021997119	0.420318478
CG30491	FBgn0050491	44.73745667	49.11450801	58.90700598	34.49038981	67.59376323	60.30953696	52.57353489	55.59730694	2R	0.368628084	0.048255143	0.579478905
Coq9	FBgn0050493	27.69010772	18.40065824	18.35215567	16.21091997	25.87730646	26.18296004	23.19924493	23.68410611	2R	0.318114347	0.098613807	0.730787365
CG30495	FBgn0050495	17.55559088	21.46481341	25.80601036	13.33261615	30.46507494	15.34483067	14.89422475	13.37057218	2R	-0.039315937	0.897893733	0.996479182
Rpe	FBgn0050499	2.565004996	12.2453949	2.074222115	8.433633137	9.379713397	8.370938226	4.865592378	12.85122297	2R	0.529673245	0.361156989	0.939549067
Ugt35D1	FBgn0051002	0.452708601	2.699929491	2.440588337	0.703648707	3.359826329	0.37938747	0.641594093	0.218416062	3R	-0.392577711	0.705808864	NA
mesh	FBgn0051004	0.653139364	0.710852831	0.229813991	0.511464679	0.261018585	0.615775803	0.34340961	0.519582053	3R	-0.252049451	0.621735546	0.990047511
qless	FBgn0051005	4.703820737	5.470399589	5.439435668	5.544317047	4.943999393	6.041092468	6.426424466	6.10476386	3R	0.183924645	0.246194194	0.87758016
CG31013	FBgn0051013	1.762699746	1.031400538	1.19338186	0.28672103	1.406055152	1.120791024	0.627444408	0.266998906	3R	-0.297792177	0.749600879	NA
PH4alphaSG1	FBgn0051014	3.384881343	3.129994037	1.684446139	1.51763912	0.548385238	2.454804196	2.214078212	3.109146078	3R	-0.204460913	0.762439749	0.995787491
PH4alphaPV	FBgn0051015	0.070853371	1.010196011	0.257833933	0	0.511633975	0.701401313	0	0.692231519	3R	0.56887403	0.712435937	NA
CG31016	FBgn0051016	0.913480444	0.204298039	0.640204013	0.047327673	0.21987551	0.344489513	0.362492393	0	3R	-0.950389896	0.445828495	NA
Ppi1	FBgn0051025	1.366610301	0.583493798	1.205591829	1.448272941	0.941976708	0.983893409	1.140955278	1.02497658	3R	-0.148580761	0.813616204	0.995787491
CG31028	FBgn0051028	1.987525304	1.619271797	0.321447244	0.308922695	0.956798019	1.570445248	0.096575614	0.328769632	3R	-0.501159127	0.570918322	0.984934163
CG31029	FBgn0051029	1.278703241	0.606151933	0.404623856	1.296194987	1.60583449	2.253861088	2.69469519	2.957238525	3R	1.4144895	0.019877565	0.39822025
CG31030	FBgn0051030	3064.139248	2748.478781	2427.438554	2828.079052	2747.986596	2832.533981	2633.539496	2489.376642	3R	-0.020406421	0.834915909	0.995787491
CR31032	FBgn0051032	39.52483054	32.97235646	36.21252563	26.46880501	45.35434939	49.85020135	50.95066876	50.66563463	3R	0.5671017	0.014524892	0.358137424
CG31036	FBgn0051036	1.176632386	0.819817932	0.30740702	0.126612651	1.013043289	0.375462772	0.184714738	0.668120355	3R	-0.099287245	0.911409471	0.996479182
CG31038	FBgn0051038	3.55448555	5.112794178	4.167044412	6.358110784	2.688585513	2.839426454	2.279556454	2.24902949	3R	-0.900883388	6.42E-06	0.001109181
Cog7	FBgn0051040	112.2995531	113.3447181	96.01124536	117.654781	97.83927904	94.63279971	110.4825028	94.70349624	3R	-0.114982706	0.354773943	0.936745458
CG31041	FBgn0051041	2.901089567	2.25929357	1.256786219	2.657199723	0	2.14903034	1.585873246	0	3R	-1.260261736	0.343760937	NA
lncRNA:CR310	FBgn0051044	2.646904274	5.407461624	2.963701316	2.966902727	4.686449446	4.25512217	2.467190072	5.52564827	3R	0.315730607	0.381240699	0.944476532
CG31051	FBgn0051051	309.747563	382.8322722	360.9448431	357.9498084	325.9960259	310.7760111	321.7360164	313.2973998	3R	-0.117697666	0.275634149	0.89549671
CG31064	FBgn0051064	129.1769181	137.130666	117.7236636	128.1668944	121.5199117	132.4937772	131.9562418	106.5913701	3R	-0.027370866	0.781688707	0.995787491
ppk31	FBgn0051065	0.474738765	0.434349643	2.198718371	0	0.545379638	0.935850781	0.440388651	0.655901149	3R	-0.21279107	0.860088696	NA
Lerp	FBgn0051072	26.49928233	32.91308171	27.90882669	34.5209688	26.13196889	37.57184638	26.70653106	35.12178468	3R	0.071804421	0.604542726	0.989286463
CG31075	FBgn0051075	0.119762524	1.707521084	2.251702153	1.116887267	0	2.738100875	1.909479827	0.260015526	3R	-0.048305499	0.964606359	0.997604378
CG31091	FBgn0051091	0.419920979	0.896456316	2.037444878	0.356010893	0.068914999	1.043733554	0.486921564	0.248641996	3R	-0.945255811	0.395903487	NA
LpR2	FBgn0051092	1.168385459	1.507272769	1.590937544	1.790226082	0.561963199	1.242419428	0.74117353	1.184982231	3R	-0.667437984	0.013193549	0.344576629
CG31098	FBgn0051098	61.01546613	57.5011897	61.32741354	76.53132046	79.30306706	71.44890666	60.36318447	77.70338292	3R	0.202443073	0.226694831	0.867702781
CG31100	FBgn0051100	2.345203658	5.365773214	4.516609987	3.189523192	1.16983641	0.601248721	2.991332459	1.027263574	3R	-1.380020362	0.0021061	0.105103203
CG31102	FBgn0051102	0.040551613	3.741080321	1.475664718	0	1.31770899	0.038231813	0.620688218	0.968454365	3R	-0.766525634	0.568697867	NA
CG31103	FBgn0051103	0.040742354	0.34169792	0	2.754688706	1.250356665	2.611991721	2.390496297	1.857563885	3R	1.401777235	0.209210484	0.856520545
CG31104	FBgn0051104	3.924077751	2.129499684	2.239943033	1.479959339	3.646159008	3.971625691	0.294423725	1.754022127	3R	0.00754472	0.992379334	0.998489602
ppk22	FBgn0051105	1.368067959	0.721204654	1.706869948	0.637919789	1.340701505	1.105548099	0.997134502	0.848629194	3R	-0.017019355	0.982517863	0.997881056
TTLL5	FBgn0051108	10.93390567	9.899892538	11.30835062	7.030887559	10.03257198	14.14259915	9.693182876	11.65413617	3R	0.245676328	0.190333568	0.843401316
CG31109	FBgn0051109	131.4114817	142.9137181	160.3831485	151.4603842	145.2859682	138.7128789	120.6358792	131.9615753	3R	-0.094804489	0.4597905	0.971606621
CG31111	FBgn0051111	15.97048489	28.50375154	31.87328566	26.22928845	20.16737053	26.99853557	21.27365176	16.90968821	3R	-0.225834015	0.447662115	0.967112302
ClC-a	FBgn0051116	3.423924889	7.490674283	7.450743292	2.327775539	3.536233551	3.918685406	3.972746764	2.226563138	3R	-0.559978412	0.077437317	0.682035833
RabX4	FBgn0051118	410.0956733	505.1825067	483.0446302	431.3378679	489.3677021	541.2525141	445.7310147	508.5281984	3R	0.149412292	0.134236752	0.795116625
HDAC11	FBgn0051119	23.55103989	37.4244305	26.94342467	16.99269336	20.1389983	10.89153671	23.90520095	10.33394277	3R	-0.649098579	0.048746324	0.582395583
CG31121	FBgn0051121	0.533988728	1.161081159	0.831646404	0.476471767	0.809272629	0.19267507	0.437254749	0.357820666	3R	-0.699666072	0.209759733	0.856520545
CG31122	FBgn0051122	129.5585096	129.3101941	97.66170126	142.2526604	105.6001536	133.2392499	115.9011747	118.9918198	3R	-0.047489557	0.709037779	0.995787491
CG31125	FBgn0051125	76.15710487	92.24759095	71.93015132	81.94957986	91.07985138	96.65445635	94.08930879	97.85672671	3R	0.26711928	0.052991419	0.606446461
CG31126	FBgn0051126	141.4944013	163.1127919	148.4647025	147.3838107	185.0194374	140.8816194	145.4800658	200.0287534	3R	0.194794528	0.258206827	0.887830768
Slimp	FBgn0051133	3.847185306	10.7235565	4.254849333	3.561446003	7.864367261	6.080731177	7.216362037	5.895949715	3R	0.312045187	0.514029405	0.980804634
CG31139	FBgn0051139	1.521843262	0.212723215	2.615145789	0.147838345	0.915772378	0.119565378	0.970563714	0.27533855	3R	-0.961676395	0.461845401	NA
CG31140	FBgn0051140	485.3161729	538.2655212	482.4336233	580.9355238	485.6442374	503.0396462	486.3169572	519.3960752	3R	-0.035199887	0.705566909	0.995787491
CG31145	FBgn0051145	1.47191924	1.577755982	1.374417696	1.156547967	1.054557575	1.47971977	1.365731633	0.885725184	3R	-0.193847855	0.320109112	0.916451164
Nlg1	FBgn0051146	0.262501031	0.301472592	0.091794427	0.038595305	0.051230448	0.189515057	0.072394137	0.128359018	3R	-0.62656207	0.386695167	0.94813696
Gba1a	FBgn0051148	21.87285336	29.51202226	25.73725875	23.71657898	14.10781043	21.97990104	16.8173971	15.54497058	3R	-0.524894552	0.018673443	0.392991093
wge	FBgn0051151	75.12763976	98.66188482	94.2300531	108.6152062	75.11518791	89.23530852	79.80308178	83.3587124	3R	-0.171247987	0.149985395	0.809805479
Rpb7	FBgn0051155	136.0344676	103.6891719	117.1687316	98.25632387	105.7041849	110.9353057	100.8494751	80.76903719	3R	-0.166533174	0.335721155	0.924219112
CG31156	FBgn0051156	13.32454762	18.82416996	14.32927857	16.44415456	17.65649	16.06729904	11.96561402	11.39048963	3R	-0.107193633	0.59346492	0.988631016
CG31157	FBgn0051157	0	0.929558258	1.209988421	1.162843641	1.200523098	0.156743096	0.141372364	0.240634971	3R	-0.863598396	0.556247197	NA
Efa6	FBgn0051158	10.74564094	12.22979425	11.17090858	14.37486203	12.5839011	15.15378752	14.13580844	9.738578392	3R	0.116431023	0.445291931	0.967112302
mRRF2	FBgn0051159	1.622616999	12.2229629	9.089612154	15.54498039	11.55718153	6.953606345	9.633351741	17.422103	3R	0.288860836	0.544402378	0.984934163
CG31161	FBgn0051161	0.677839821	6.984313132	2.819022772	3.38648148	3.583618486	3.560495208	3.952420446	3.889368636	3R	0.161165194	0.799512129	0.995787491
SKIP	FBgn0051163	0.580671435	0.767200005	0.539915657	0.437998515	0.661907303	0.766837415	0.530982601	0.385855008	3R	0.042189491	0.849183868	0.995787491
CG31174	FBgn0051174	3.847389062	3.139518852	4.288462363	2.181902293	8.44725811	14.21507464	9.549508044	10.15910003	3R	1.679954725	0.001417834	0.081491242

**CG31176**	FBgn0051176	0.334715859	0.371224627	0.581279218	0.277183239	0.277359698	0.439725996	0.18197173	0.111188936	3R	-0.594537655	0.17729403	0.834615603
**CG31183**	FBgn0051183	1.577230619	0.900624034	0.616401193	0.268249518	0.588500834	1.609037106	0.330200086	0.520412413	3R	-0.12936963	0.815753671	0.995787491
**CG31189**	FBgn0051189	0	1.231270811	2.22600271	1.711416771	1.656442092	0	2.57480409	0	3R	-0.24343489	0.886849493	NA
**CG31191**	FBgn0051191	46.70664164	43.03050263	52.67687051	50.51320796	53.53639815	46.87024697	54.45485034	60.23785327	3R	0.189135435	0.100906529	0.737033314
**GluRIIE**	FBgn0051201	1.177187907	0.891740193	1.013363092	1.150948411	1.816639867	1.396257327	1.307771844	0.742001782	3R	0.341102892	0.526612589	0.984107922
**alpha-Man-Ic**	FBgn0051202	1.490759124	3.966372679	2.244761902	2.516849114	2.969602535	2.568633296	4.196368058	3.683000339	3R	0.431093912	0.452216971	0.967630712
**CG31205**	FBgn0051205	0	0.810167039	0.390584862	1.313782763	2.615823794	0	1.232146871	0	3R	0.66674286	0.718646888	NA
**CG31211**	FBgn0051211	78.21082988	94.84853696	92.62704767	101.6686807	91.99814002	92.79392685	115.4514267	103.9962168	3R	0.170469794	0.179584048	0.836097072
**Naam**	FBgn0051216	0.758528533	0.69738327	0.785348633	0.606759075	0.756287323	0.8019099	0.777247565	0.330745237	3R	-0.067138771	0.845459051	0.995787491
**CG31220**	FBgn0051220	0.453930778	1.938122325	1.201341529	1.154533658	2.383887695	0	0	0	3R	-0.936774086	0.535165781	NA
**CG31221**	FBgn0051221	99.23393066	136.2043758	103.284656	127.9015762	87.86062146	98.93996333	78.30425925	80.66491535	3R	-0.403035827	0.000490443	0.034934638
**CG31223**	FBgn0051223	39.59132162	35.11903298	21.04511236	35.73613483	39.68076881	26.196162	28.28618294	21.94933401	3R	-0.15423611	0.574161078	0.984934163
**CG31224**	FBgn0051224	43.27141353	58.66627512	60.53835806	67.12523775	47.95879409	54.54579554	52.86037029	58.18936225	3R	-0.072466397	0.596214539	0.988780662
**CG31229**	FBgn0051229	27.16055469	37.06015118	25.94676497	26.41226305	32.51847455	25.93849942	28.5405653	24.44272682	3R	-0.033572048	0.887207824	0.995926915
**CG31233**	FBgn0051233	1.94532703	0.901622066	1.924992414	1.312895371	0.924161545	0.313717786	0	0.500149546	3R	-1.767588534	0.039932562	0.529044592
**CG31248**	FBgn0051248	6.818186842	23.05608869	18.52572532	8.011758358	5.218835689	14.03906848	16.00187383	9.592288782	3R	-0.290108408	0.516095137	0.980804634
**CG31249**	FBgn0051249	104.6689365	66.96794631	94.42374909	85.48415317	85.99115878	99.98122629	89.53719473	69.39846383	3R	-0.003053741	0.986476727	0.99797245
**CG31251**	FBgn0051251	18.28197866	30.17085038	45.96136726	31.62406887	25.68429767	25.50670393	34.6021796	25.92775249	3R	-0.135286589	0.644375223	0.992589784
**CG31259**	FBgn0051259	3.276415461	1.173844666	0.848873013	0.964125349	0.057424948	0.209930984	0	0	3R	-4.303895656	0.000213873	NA
**CG31262**	FBgn0051262	0.290762825	1.511912933	0.246885295	0.033895129	0.419921137	0.17818412	0	0.126254602	3R	-1.458992794	0.22527063	NA
**CG31267**	FBgn0051267	2.711805112	0.802706938	0	1.859550451	0.43195617	1.052747828	0.203466856	0.173164116	3R	-1.499238148	0.29237854	NA
**CG31268**	FBgn0051268	0.91017421	0.084816086	1.472046625	1.53258221	0	0.238362968	0.773957988	0.329345411	3R	-1.580175414	0.24053172	NA
**CG31278**	FBgn0051278	33.83812569	34.08607242	36.58395281	56.38228032	45.98086303	42.65209261	42.69175267	50.70730603	3R	0.209700535	0.395837923	0.952272708
**Tpl94D**	FBgn0051281	0.389170552	0.466270718	2.292868516	2.203531477	0.702552589	2.725599455	0.425478188	1.448443359	3R	0.040354466	0.967601439	NA
**CG31286**	FBgn0051286	1.229208784	0.893457628	2.087430355	0.095528467	0.887614759	1.699706116	0.313573802	0.800617817	3R	-0.175362526	0.858885596	NA
**CG31300**	FBgn0051300	3.456089602	9.692035701	2.358119579	4.910187019	3.412022862	5.04030682	4.683796876	2.462082805	3R	-0.353964531	0.524991868	0.984107922
**CG31301**	FBgn0051301	153.1997305	126.9578529	140.1285336	118.0167244	136.3786266	107.3777816	141.6034734	110.7315042	3R	-0.088203107	0.549352073	0.984934163
**CG31321**	FBgn0051321	0.106936106	0.502236884	0.363195701	0.199454004	0.88801656	0.14114619	0.054559261	0.464336369	3R	0.46373917	0.687350419	NA
**CG31323**	FBgn0051323	0.052623479	0.29128606	0.370225092	0.552103466	0.256496938	0.124032735	0.523550135	0.091400355	3R	-0.311682426	0.692469158	0.995787491
**CG31324**	FBgn0051324	6.301890341	6.048153665	7.891303968	7.677148489	7.258119276	8.318623281	8.595043063	8.665785252	3R	0.265091577	0.065633149	0.646711278
**CG31326**	FBgn0051326	1.548997143	3.089781562	0	0.097606176	0.302306686	1.10515625	1.1747777	0.545353976	3R	-0.57258626	0.635274869	NA
**CG31337**	FBgn0051337	4.349033254	1.823723294	3.956510255	0.563311517	1.199476617	0.113895611	2.157259469	0.393423074	3R	-1.465753937	0.141060481	0.8037297
**CG31342**	FBgn0051342	100.0628079	132.4562737	125.4620764	126.0793456	111.8336251	122.0346135	129.5990682	114.125727	3R	0.012822624	0.907713626	0.996479182
**CG31344**	FBgn0051344	264.6495963	264.2177155	288.4370109	418.9032461	236.6761138	230.3394826	251.3179638	249.1672748	3R	-0.325126411	0.032438365	0.505711525
**CG31345**	FBgn0051345	8.97979773	6.790404118	14.21373097	9.507030687	8.264869053	7.022698058	9.050402165	8.469561659	3R	-0.234845819	0.340862977	0.927274752
**Unc-115a**	FBgn0051352	20.76921945	19.33511513	21.41159941	22.5545213	21.00784823	24.06734749	23.4566242	18.5481985	3R	0.078792735	0.533324068	0.984760433
**Hsp70Bbb**	FBgn0051354	8.455063194	4.459026775	6.897002769	4.131911589	4.465756582	8.110620047	4.521023132	4.088178901	3R	-0.153807251	0.70699629	0.995787491
**CG31357**	FBgn0051357	116.3159299	167.4293776	130.5836789	97.4875965	108.8992358	142.0355552	128.3701461	138.4675928	3R	0.050771423	0.752304679	0.995787491
**CG31360**	FBgn0051360	17.68964172	19.44020535	28.70853748	23.8923423	25.98794331	19.20809371	23.96381046	18.80562295	3R	0.007822647	0.981397604	0.997881056
**dpr17**	FBgn0051361	17.32993188	10.82824682	11.38060031	11.64516494	13.85612208	12.87339838	12.28770582	10.34400198	3R	-0.028184279	0.87015986	0.995787491
**Jupiter**	FBgn0051363	67.13794304	63.50037293	60.8408165	66.35798224	59.7769101	62.26262136	55.52569144	60.48759819	3R	-0.086203392	0.324483009	0.918470465
**CG31365**	FBgn0051365	37.8866798	34.60972116	36.64543387	50.81516413	33.62550512	40.89796926	53.71425745	43.87978687	3R	0.133260051	0.497836588	0.97594235
**CG31368**	FBgn0051368	26.72956705	15.44993274	18.92789226	20.02207748	21.76307946	26.55414657	18.15267143	20.05645566	3R	0.114893225	0.521444553	0.982083369
**CG31370**	FBgn0051370	3.187005096	1.813737949	1.242583378	2.255651802	0.513692948	2.146206384	0	0	3R	-1.630541039	0.168824468	NA
**CG31371**	FBgn0051371	24.98183659	39.21974876	34.93422489	41.45767023	19.10290799	28.86665607	37.10579439	35.44804661	3R	-0.188965337	0.424545846	0.9620897
**sals**	FBgn0051374	0.285808589	0.102000811	0.088515019	0.170132417	0.098800421	0.183461169	0.139615631	0.026404975	3R	-0.519297863	0.578932452	NA
**lncRNA:CR313**	FBgn0051386	0.595914767	0.895191339	0.685101798	0.775582512	0.626457584	0.798738876	0.604413438	0.815760728	3R	-0.01738142	0.947913751	0.997604378
**CG31388**	FBgn0051388	97.99654499	118.0520389	107.672303	120.5182816	111.3417288	139.8112962	110.0499919	118.1179623	3R	0.140149458	0.255053709	0.886412683
**MED7**	FBgn0051390	139.1660471	141.3591141	163.7782073	159.0780587	162.7139786	127.635854	167.3907638	161.0512766	3R	0.068618429	0.651591614	0.993444251
**CG31391**	FBgn0051391	0.20344861	0.938455381	0	2.371663875	1.469107537	0	1.038003549	0	3R	-0.456709819	0.784510703	NA
**Gba1b**	FBgn0051414	1.705267511	1.528130602	0.793388668	0.490163056	0.801239087	0.836893153	0.238365702	0.50716351	3R	-0.889230304	0.239775342	0.874524157
**Ir94c**	FBgn0051423	0.859497476	0.560655838	0.926723951	0.111327001	0	0.81032897	0.243621836	0	3R	-1.175013465	0.472501278	NA
**CG31431**	FBgn0051431	1.580731756	2.111344406	0.923200583	0.682484552	1.004053577	0.027598227	0.896106815	2.097281369	3R	-0.360970171	0.696331127	0.995787491
**CG31436**	FBgn0051436	4.920190968	4.318393665	4.302625052	4.401754891	3.718132632	10.78773486	4.232486897	0.248423024	3R	0.098456209	0.890641188	0.996189576
**CG31441**	FBgn0051441	5.373179663	8.904167691	9.737181359	5.13169214	16.82885741	16.35704408	13.8722425	19.53648423	3R	1.235983713	0.000412972	0.030457608
**CG31445**	FBgn0051445	2.936903078	0.547360323	1.682264727	0.665707677	2.025017033	1.480589304	0.208113968	0.221398904	3R	-0.549719951	0.502108105	0.977207013
**mRpS18A**	FBgn0051450	273.0524874	312.7172864	343.4893412	259.3049576	289.6048862	235.5667873	366.4227385	327.7074531	3R	0.072000052	0.659733692	0.995750687
**lncRNA:CR314**	FBgn0051451	1169.56813	1161.947585	904.9781238	1355.062985	1193.483282	1221.758144	943.9817427	1111.482145	3R	-0.011488106	0.929427687	0.996479182
**pch2**	FBgn0051453	1.908405592	4.768959878	4.629765348	8.807948002	7.312178903	2.97424253	9.637412563	4.396998171	3R	0.312169123	0.547785201	0.984934163
**CG31457**	FBgn0051457	14.53949123	23.03883584	23.71519687	9.520367007	25.01890135	20.5907807	23.20132558	29.28304144	3R	0.510798089	0.10278969	0.743157534
**CG31467**	FBgn0051467	3.683705662	6.093081428	2.482394043	7.157017564	6.372948145	8.778909528	9.005661774	4.887462549	3R	0.609659719	0.185213685	0.839299945
**CG31469**	FBgn0051469	8.772873597	8.009574483	11.85374981	4.660319514	4.811327184	2.722105097	3.966038561	4.580856871	3R	-1.02216444	0.081286269	0.694684542
**sgll**	FBgn0051472	74.62977831	48.6405504	67.9130844	46.97466988	69.91241909	81.24891953	66.92997995	51.10223626	3R	0.201332769	0.333784134	0.923864819
**CG31473**	FBgn0051473	8.915518171	9.604942681	3.692742905	9.12564658	8.767083634	12.09571386	12.01901704	13.06164606	3R	0.572248475	0.170394415	0.830934498
**CG31475**	FBgn0051475	18.61463693	13.53584556	14.09671738	18.13245943	16.40423145	19.90731652	14.4989026	12.67081411	3R	0.002124233	0.989356581	0.998078482
**pb**	FBgn0051481	0.723019533	0.364677238	0.54583616	0.20629108	0.502013679	0.769844975	0.412741452	0.417134327	3R	0.21157194	0.61132482	0.990047511
**asRNA:CR315**	FBgn0051514	0.436638919	1.235925899	1.257894915	0.636254502	0.147795967	1.029151265	0.487320428	0.651738665	3R	-0.571162971	0.512677497	NA
**CG31522**	FBgn0051522	44.90688811	42.37285322	48.23661187	46.87454782	44.17611	42.10589899	42.85905921	52.98826774	3R	0.029577406	0.791916758	0.995787491
**CG31523**	FBgn0051523	31.02574329	41.60777203	38.77720617	44.72252555	36.84054095	44.31467046	39.65380994	41.40074397	3R	0.086030779	0.48763436	0.973549494
**CG31533**	FBgn0051533	0.288407033	0.322508226	1.554825422	0.896546854	0.057849846	0.574028822	0.245244179	0	3R	-1.735110873	0.174786212	NA
**NKCC**	FBgn0051547	1.608052602	3.996382052	1.576368184	1.698578227	1.101935016	1.862590667	0.920896818	1.239743272	3R	-0.753699527	0.043542433	0.548990375
**CG31548**	FBgn0051548	10.01893483	7.791574914	10.60616559	6.582925917	2.795224258	2.490253874	14.17335998	4.745898535	3R	-0.514268086	0.406299009	0.956706394
**CG31549**	FBgn0051549	52.32386495	54.0494078	52.4871328	84.60674547	28.11579371	8.752206823	27.98759592	4.830496594	3R	-1.777254474	2.32E-05	0.0029321
**CG31550**	FBgn0051550	232.6795245	270.770992	231.8617058	280.0443394	263.5591626	259.4346895	263.522888	252.0601616	3R	0.063270042	0.525102572	0.984107922
**CG31551**	FBgn0051551	0.517927802	0.590750837	0	0.193204668	0.673194547	0.390639335	0.105699596	0.134936304	3R	0.022689527	0.985428084	NA
**CG31559**	FBgn0051559	0.559690869	0.591854013	0.472279041	0.340408224	0.483227832	0.435903893	1.427783433	0.079248306	3R	0.323574294	0.681367268	0.995787491
**CG31601**	FBgn0051601	2.474807018	2.075570796	0.400256487	0.897543017	0.744610159	2.048060025	0.070147621	1.373108977	2L	-0.443914261	0.606581933	0.989286463
**CG31612**	FBgn0051612	1.301901015	0.925246149	0.837164596	0.463223541	0.665436478	1.032712434	0.813621787	0.726502199	2L	-0.101781107	0.753849125	0.995787491
**nolo**	FBgn0051619	4.295740784	5.139860488	5.38135474	3.167710783	3.148585177	2.863524953	4.621361781	2.267804569	2L	-0.445176306	0.035140114	0.514749796
**CG31626**	FBgn0051626	0.735785003	0.445674711	0.991668975	0.571818345	0.84862366	1.503002814	0.26069491	0.044373815	2L	-0.015225306	0.986801817	0.998078482
**CG31627**	FBgn0051627	3.258366607	5.769089152	6.806869728	1.688168685	1.797334867	3.157300583	1.616253959	3.340601777	2L	-0.773998481	0.289008614	0.903033179
**sens-2**	FBgn0051632	0.467081566	1.030425218	0.49266668	0.497144453	0.247461439	0.258473141	0.569864618	0.308632227	2L	-0.818723543	0.10453304	0.746511016
**CG31633**	FBgn0051633	38.17639526	52.77348168	36.34616083	38.42300586	56.09619138	49.59427864	40.48429955	39.03281124	2L	0.19305558	0.332089195	0.921929165
**Oatp26F**	FBgn0051634	2.764538862	3.817634	2.977558061	1.472444694	3.054653316	5.257718471	2.583852727	5.562262943	2L	0.610835465	0.08638156	0.705932874
**CG31635**	FBgn0051635	33.12155152	29.08292446	37.67123487	32.98536601	30.9650306	29.37991589	29.3744237	28.91842852	2L	-0.133253641	0.229192478	0.867702781
**CG31637**	FBgn0051637	26.79302254	36.54670974	28.67864768	29.96902119	28.95256738	33.94541585	30.74912508	27.72185907	2L	0.023823669	0.828722664	0.995787491
**CG31638**	FBgn0051638	130.3292584	145.9272999	130.4699736	158.5389275	139.5889648	163.1196334	124.5561133	123.6151628	2L	-0.009648298	0.932704066	0.996479182
**CG31643**	FBgn0051643	9.46617593	15.41927515	14.20366909	9.950650636	13.28585847	17.51398636	13.40027829	15.50305619	2L	0.321463468	0.169596801	0.830934498
**DIP-theta**	FBgn0051646	0.437533671	0.643852108	0.527469309	0.331686773	0.489422712	0.690881054	0.417703224	0.390460422	2L	0.071095128	0.840585322	0.995787491
**lncRNA:CR316**	FBgn0051647	0.328188211	0.668451758	0.398090011	0.054654176	0.677101525	1.989088975	0.179403252	0.050894785	2L	1.025695449	0.366964398	NA
**smog**	FBgn0051660	78.08601541	70.58051009	72.44235939	75.53607704	71.38119031	79.53010918	69.16699648	77.79875524	2L	0.034521716	0.70543109	0.995787491
**CG31663**	FBgn0051663	5.877815454	6.924316897	6.716297205	3.487785216	2.741621334	4.141893987	5.420023966	3.807006	2L	-0.48117114	0.038538855	0.523099051
**wry**	FBgn0051665	200.5299745	208.5094358	199.5190944	194.0663582	194.7995164	209.9565152	199.3322786	195.3607941	2L	0.024470265	0.736922827	0.995787491
**CG31673**	FBgn0051673	3.460264968	1.591449257	8.529950589	1.04096484	9.470750352	17.73226967	6.406846167	14.41925986	2L	1.754826135	0.002369469	0.116159757

**CG31674**	FBgn0051674	0.1084762	1.910512881	3.552683598	3.540714471	3.13323749	1.789737227	4.981052098	2.001850908	2L	0.435189041	0.604088542	0.989286463
**CG31675**	FBgn0051675	0.13712651	3.066806454	2.883114011	3.410189829	1.815355639	4.137025161	0.816229572	2.480952895	2L	0.008774667	0.991581243	0.99840982
**CG31676**	FBgn0051676	14.19668115	18.76432185	23.30122191	6.155173741	11.77582599	12.17393065	20.09963938	13.84996661	2L	-0.067233628	0.871654753	0.995787491
**CG31678**	FBgn0051678	17.90182553	17.48596469	23.10243583	20.89274461	17.34943	20.40485706	22.82082513	20.41778921	2L	0.059521008	0.658827836	0.995750687
**CG31687**	FBgn0051687	2.103567642	2.796933844	2.178206116	1.644764526	1.968205441	2.491131275	2.421355014	2.812628339	2L	0.182745636	0.579873993	0.985637351
**CG31690**	FBgn0051690	0.600347099	0.620227166	0.861765262	0.779756237	1.77004962	2.976904632	1.997836945	1.249288331	2L	1.510619373	5.34E-09	2.12E-06
**CG31694**	FBgn0051694	116.7321973	117.0350002	112.5593346	109.2416418	115.2621468	124.2360965	106.4491941	111.5761798	2L	0.035693491	0.68886791	0.995787491
**CG31698**	FBgn0051698	4.774234012	0	0.992762153	0	0	3.729498322	2.08785832	3.257666967	2L	0.630882177	0.702206847	NA
**CG31706**	FBgn0051706	72.51159988	140.1680159	148.8888549	86.9355532	64.4416836	53.60411527	168.8506175	59.10605424	2L	-0.331238395	0.478985218	0.973549494
**DIP-zeta**	FBgn0051708	0.674037441	0.6099307	0.290464123	0.310163076	0.328218577	0.271750624	0.429870998	0.356221804	2L	-0.428343258	0.379881375	0.944208584
**CG31712**	FBgn0051712	308.8363644	262.2295994	205.2666715	249.2101362	278.3696442	297.8388826	215.5179918	259.8270209	2L	0.062615673	0.646574449	0.99279236
**CG31715**	FBgn0051715	177.8204778	170.9199369	195.5387536	183.4668968	167.574229	201.0516212	154.2126224	168.5663887	2L	-0.043489216	0.741052726	0.995787491
**Cnot4**	FBgn0051716	182.5255974	190.3320807	171.4750596	169.5236103	164.439524	199.4770359	145.7892379	148.7643578	2L	-0.088759802	0.40420655	0.956557569
**CG31717**	FBgn0051717	148.3237717	162.0942188	184.3248721	122.9872789	146.2850297	197.5128253	185.9091449	148.7896268	2L	0.166261627	0.295820484	0.906715248
**Ir31a**	FBgn0051718	1.744212393	1.324832423	0.239515185	0.844004167	0.415872695	2.016757196	0	0.714499352	2L	-0.382132326	0.720456302	0.995787491
**RluA-1**	FBgn0051719	1.723080734	1.121777295	0.839851472	0.623690079	1.193107749	0.630517576	0.401426116	0.444133161	2L	-0.665621647	0.193884159	0.844272804
**mthl15**	FBgn0051720	1.299737877	0.155723425	0.506755601	0.162336961	0.041899285	0	0.769706611	0.554292209	2L	-0.641097396	0.631887547	NA
**Trim9**	FBgn0051721	0.541552798	0.564243014	0.540679039	0.410348237	0.629826799	1.130132967	0.794369872	0.434127233	2L	0.563821603	0.034673394	0.514749796
**CG31729**	FBgn0051729	70.38706552	81.34046533	81.92208755	79.02431379	77.50746741	67.55000242	86.10649005	82.12857523	2L	0.03662996	0.742955257	0.995787491
**CG31730**	FBgn0051730	2.041077953	2.139763708	1.856859992	0.297418526	1.381750811	1.924315827	0.325427107	1.384802868	2L	-0.29731048	0.809364842	NA
**AspRS-m**	FBgn0051739	37.96349031	38.58947578	50.86809015	48.76531081	63.79445684	45.02464961	51.68334028	49.53584828	2L	0.286562176	0.067878251	0.656261422
**Prosbeta5R2**	FBgn0051742	0.16950733	2.274598133	1.370741395	0	2.244030617	1.331753961	0	1.717408488	2L	0.538011041	0.686748377	NA
**CG31760**	FBgn0051760	110.6375651	93.22702296	94.74550875	102.4229363	83.95313051	103.7782934	89.92242941	95.1403919	2L	-0.078321679	0.439400842	0.967112302
**fred**	FBgn0051774	0.39804296	0.396014747	0.499390802	0.200161646	0.295881584	0.47093016	0.194123674	0.237228109	2L	-0.286174553	0.400038715	0.953639202
**CG31776**	FBgn0051776	0.555754097	0.066585681	0.963036919	0.740411288	0.788290315	0.261980784	0.405068754	0.172370513	2L	-0.48018375	0.693107951	NA
**lncRNA:CR317**	FBgn0051781	0.764221734	1.070933335	0.727515561	0.202984656	0.820784261	1.340683882	0.271455781	0.525062066	2L	0.130450109	0.838320224	0.995787491
**CG31792**	FBgn0051792	0.559358092	0.60709953	0.199558078	0.038356539	0	0.465317111	0.503623944	0.357181822	2L	-0.068014716	0.954293438	NA
**CG31793**	FBgn0051793	35.23816034	41.04657677	38.0375245	29.12819388	27.63028452	42.28295569	32.32526895	24.53579324	2L	-0.149434937	0.385932162	0.947329421
**CG31797**	FBgn0051797	1.017949468	0.93134587	1.197348466	1.078777665	0.111373314	1.51228061	0	1.20548865	2L	-0.542871705	0.601445783	0.989286463
**CG31800**	FBgn0051800	62.11253959	73.67259997	85.51725926	59.55134439	73.33478294	44.16385963	89.68438202	73.66729336	2L	0.035941336	0.880938518	0.995787491
**CG31810**	FBgn0051810	0.672261011	0.086740272	0.815447918	0.18084822	0.513447602	0.365655909	0.263838813	0.336817127	2L	-0.219219879	0.840399322	NA
**Tsen2**	FBgn0051812	6.822252821	9.399912748	8.866441172	11.21928772	13.74544071	8.441971504	14.45129783	7.141375802	2L	0.303590068	0.427131484	0.962819343
**DIP-kappa**	FBgn0051814	45.13953805	45.78824087	43.57982938	45.3906981	44.89303604	49.86377145	45.18150003	41.39468066	2L	0.040098206	0.632135175	0.991585536
**Tap42**	FBgn0051852	80.75732446	89.71965216	54.92836088	73.7883923	93.66495744	89.92253163	70.19258724	74.33846677	2L	0.16140876	0.37498132	0.943282319
**CG31855**	FBgn0051855	197.87457	170.0861082	172.2170246	142.170814	181.9430104	185.5386834	167.3441642	127.5439138	2L	-0.015596103	0.919650162	0.996479182
**Qtzl**	FBgn0051864	23.26268245	19.36214059	27.36239668	18.69500843	24.497144	32.06711803	26.7495785	25.06141003	2L	0.319894482	0.235653781	0.870926212
**Ada1-1**	FBgn0051865	6.219130094	6.409850989	5.998653668	5.066148953	4.463796198	4.167929085	5.35209356	5.368387173	2L	-0.267588348	0.57320668	0.984934163
**Ada1-2**	FBgn0051866	8.872355023	13.11645434	10.03247055	12.09579457	14.93098497	8.790131043	10.35773464	6.20324368	2L	-0.096533408	0.809624222	0.995787491
**CG31869**	FBgn0051869	2.398530761	1.459346671	1.207130388	0.375121174	1.360845439	1.002846328	1.406161674	1.028548742	2L	-0.154709794	0.695129225	0.995787491
**CG31871**	FBgn0051871	0.570947398	0.169911707	0.647874231	0.558220992	0.299237338	0.564331803	0.493330333	0.399864405	2L	-0.123269125	0.847614557	0.995787491
**CG31875**	FBgn0051875	0.241444587	0.303742068	0.08786118	0.591064914	0.239728149	0	0.061593033	0.026209927	2L	-1.879944751	0.151063437	NA
**CG31897**	FBgn0051897	0.514360129	0.949043654	1.372613547	1.25917184	0.510703524	1.382066424	0.459250308	1.730921352	2L	0.044755152	0.951569622	0.997604378
**CG31898**	FBgn0051898	0	1.861101056	0.897244474	0	2.804210762	5.300085074	0.754790796	2.248323836	2L	2.06868575	0.098217702	NA
**CG31908**	FBgn0051908	87.56563271	113.6780396	79.53925057	129.5301827	87.46842466	94.80581659	140.0020465	102.9901694	2L	0.082593866	0.653649841	0.99443552
**CG31922**	FBgn0051922	12.39438712	14.57678832	10.3684863	6.642999307	6.943979611	10.34220851	13.08345428	8.660484251	2L	-0.14998148	0.769451073	0.995787491
**CG31935**	FBgn0051935	13.9405796	16.02770127	14.690773	16.98655793	15.41185766	21.12693506	17.80164967	20.69942458	2L	0.313634239	0.018599428	0.392424388
**Sbat**	FBgn0051950	20.69872841	36.93062802	25.90701236	26.74186388	34.98647735	21.37909026	21.86107361	20.89513661	2L	-0.11583417	0.735910448	0.995787491
**CG31955**	FBgn0051955	19.74386482	16.97423503	18.88465044	14.02410965	19.03500971	18.08064905	25.27377515	12.29125683	2L	0.125799912	0.683772411	0.995787491
**Pgant4**	FBgn0051956	1.583514546	0.21248988	2.458613888	0.073838091	1.715189764	1.015190932	1.454248666	0.825109599	2L	0.240553781	0.796155627	0.995787491
**CG31957**	FBgn0051957	35.17269958	25.5818035	28.50183621	26.0302598	28.45452134	21.25856297	18.05695496	25.34877566	2L	-0.280833036	0.283840489	0.901780531
**TBCC**	FBgn0051961	203.5500705	172.3140758	201.6911375	173.1536732	174.0013514	217.6272747	179.2924089	224.74121	2L	0.112762621	0.389152256	0.948247506
**Cda5**	FBgn0051973	3.066132589	5.055837668	5.053485797	4.432518952	3.834736736	4.807677744	3.474500987	3.798545908	2L	-0.111584106	0.513930813	0.980804634
**CG31988**	FBgn0051988	0.463199175	0.221986181	2.086898797	0	1.552930279	1.809191317	0.844022176	0	2L	0.653275633	0.660274428	NA
**Cap-D3**	FBgn0051989	5.298937737	4.13338579	8.71641813	6.267534052	5.661792558	7.692729117	6.308261872	5.293933446	2L	0.06209896	0.797029808	0.995787491
**gw**	FBgn0051992	333.7726675	308.1774932	286.743789	354.4332739	268.9364267	303.7711144	260.0689536	307.3899136	4	-0.14243804	0.17440978	0.833984823
**CG31997**	FBgn0051997	794.6577173	498.0779422	683.4607261	467.3798864	605.0089943	545.3309692	536.6966529	506.5212951	4	-0.127421709	0.413496476	0.958665466
**CG31998**	FBgn0051998	74.69538896	85.68502568	100.9190386	84.61303592	64.21720433	74.87110427	80.04320266	75.1819187	4	-0.201014055	0.083119634	0.69799888
**CG31999**	FBgn0051999	26.51107149	36.393771	26.59055382	26.10934953	22.85559812	39.1744664	23.19100498	21.62668782	4	-0.086863503	0.617382105	0.990047511
**anne**	FBgn0052000	123.4963431	109.4838371	124.1573377	84.85004372	94.08189998	132.7597345	99.26502772	80.99716165	4	-0.092542156	0.527085484	0.984107922
**CG32006**	FBgn0052006	0.093675103	1.047512298	1.060520974	0	0.05636919	0.264948946	0.477934403	0.271169761	4	-0.993057837	0.47701847	NA
**4E-T**	FBgn0052016	63.35144681	59.53706654	68.1940642	51.94612106	57.11431678	67.84598587	57.22509779	58.33861133	4	0.014673617	0.897710992	0.996479182
**CG32017**	FBgn0052017	19.56102604	14.70338174	21.71483577	15.71382809	14.24975961	16.92811905	14.24174321	14.74890192	4	-0.224748266	0.104287033	0.745392907
**CG32022**	FBgn0052022	44.53155774	50.52926888	42.15402873	32.36854985	43.6632792	39.76139401	37.1987459	31.27951397	3L	-0.128475962	0.57376943	0.984934163
**Cpr66D**	FBgn0052029	0.275415725	0.369576994	0.400892825	0.06421214	0.049719597	0.57125258	0.843109719	0	3L	0.421736776	0.768069746	NA
**CG32036**	FBgn0052036	2.85554656	0.98251872	0	0	1.321793182	3.106375217	1.681052451	4.927931626	3L	1.496187914	0.208492687	NA
**CG32037**	FBgn0052037	1.181303018	0.535532458	0	1.116552777	0.230546456	0.993322538	0.16289348	0.069316708	3L	-0.940703038	0.465256666	NA
**CG32039**	FBgn0052039	81.49239159	109.5868071	100.3250714	59.34856914	87.67280502	84.03851042	72.69460564	70.921908	3L	-0.118632457	0.553570737	0.984934163
**CG32055**	FBgn0052055	7.93130924	8.927455112	11.39284278	12.40880256	11.20952282	10.97041151	12.51247622	8.949663932	3L	0.136977474	0.665292286	0.995787491
**scramb1**	FBgn0052056	91.17039225	93.0014537	83.40009042	88.04676248	83.36127527	89.81745135	73.39178238	96.30123501	3L	-0.022456926	0.844400935	0.995787491
**dpr10**	FBgn0052057	39.71478159	33.76843179	32.09590231	34.35620243	32.59725823	36.14144867	33.13274622	29.71613415	3L	-0.061734632	0.550380493	0.984934163
**Ir67c**	FBgn0052058	3.774763756	0	2.808307428	0.58671464	0.726871045	2.420000464	0.385180138	0	3L	-1.010661346	0.440062759	NA
**Rbfox1**	FBgn0052062	28.38170669	31.31088869	26.11380318	29.06353494	30.19043714	29.79037006	25.12446684	27.72409777	3L	0.004232828	0.964195929	0.997604378
**CG32066**	FBgn0052066	64.45697982	71.22551456	62.63385456	71.69042035	68.29742424	67.98761841	60.5103185	61.94142319	3L	-0.031674347	0.731484857	0.995787491
**Adi1**	FBgn0052068	33.28954294	55.97573366	60.32194085	36.23226062	63.04928516	81.66315291	77.82808978	45.28266148	3L	0.563044648	0.024501716	0.440903252
**CG32069**	FBgn0052069	32.45942539	40.9098012	54.81773629	30.5220374	46.2953273	29.08087433	48.95083974	54.50905201	3L	0.210446764	0.531017432	0.984760433
**CG32071**	FBgn0052071	2.491232729	0	1.394699043	2.680714765	4.151366375	0.722682769	0.488860632	4.16053605	3L	0.557271661	0.697903196	NA
**Elo68alpha**	FBgn0052072	2.399812866	9.182828372	1.273543368	1.573614381	5.009195617	2.403936969	0.382623386	1.953830955	3L	-0.514567866	0.561666046	0.984934163
**CG32075**	FBgn0052075	20.25654684	18.30063099	18.68917706	22.42844515	20.04900673	25.91837314	26.93478623	24.19685119	3L	0.312331818	0.122640385	0.780439453
**Alg10**	FBgn0052076	43.91319185	45.28746953	40.39580708	27.18755229	55.85253524	42.44106856	46.22979195	49.2663985	3L	0.336750531	0.09816201	0.730787365
**CG32081**	FBgn0052081	1.486211185	1.635972692	0.788709164	0	2.179931083	0.13136164	0.118479896	1.109178833	3L	-0.105891754	0.933154214	NA
**IRSp53**	FBgn0052082	1.593763317	1.066205152	0.767271698	0.79520994	0.459000786	0.991008013	0.878006645	1.151158591	3L	-0.259000289	0.459875156	0.971606621
**CG32085**	FBgn0052085	55.39711181	94.25556186	95.03156106	105.4794348	79.95341145	73.20579953	91.09148451	95.05266085	3L	-0.010185153	0.949969675	0.997604378
**CG32087**	FBgn0052087	0.294820441	2.175887697	1.716552668	0.274945104	1.2773435	1.334183574	0.300837312	2.560329877	3L	0.358175457	0.734146176	NA
**sunn**	FBgn0052088	0.212531137	0.911032983	0.515597574	0.110112966	0.042630353	0.5788555	0.180723832	1.076657802	3L	0.154842416	0.892128231	NA
**CG32091**	FBgn0052091	1.69323243	0.6942613	1.606589897	0.438634185	1.304199897	0.624357729	0.268767166	0.310431656	3L	-0.790957638	0.185147727	0.839299945
**CG32095**	FBgn0052095	73.78170563	80.7907426	77.85316755	59.45854645	75.26177702	63.67048447	79.40250692	67.33029592	3L	0.000156612	0.999218812	0.999757791
**CG32100**	FBgn0052100	171.7135331	132.6449398	144.9236373	152.3258721	154.0312031	153.4549357	141.2233486	173.5903656	3L	0.076872671	0.548869657	0.984934163
**SCaMC**	FBgn0052103	62.65618853	62.4838289	50.66209721	59.16616161	56.09412359	56.9045147	52.65683961	47.90210483	3L	-0.109389087	0.307578752	0.912131082
**CG32104**	FBgn0052104	10.50155968	12.74380909	14.81376968	17.89637015	11.47682023	16.69373622	10.13123349	9.269897501	3L	-0.204629978	0.374540413	0.943282319
**Lmx1a**	FBgn0052105	0.051952764	1.089293415	0.630183403	0.454222175	0.257917149	1.506158016	0.132532617	0.479375604	3L	0.139797326	0.879087072	0.995787491
**CG32106**	FBgn0052106	1.054324921	0.579330359	0.396896517	0.296669518	0.131263911	0.891182361	0.417352581	1.1445185	3L	0.16129271	0.84884651	0.995787491
**CG32107**	FBgn0052107	0.45214595	1.841856262	1.723699637	0.853368641	0.583028089	2.212598476	0.329552581	0.327216904	3L	-0.447066341	0.573551101	0.984934163
**CG32109**	FBgn0052109	81.94110335	55.83372773	77.51110024	56.36393914	69.19235078	84.35471546	54.23404765	65.0152322	3L	0.032503096	0.867790473	0.995787491
**lncRNA:CR321**	FBgn0052111	0.225634868	0.164242061	0.413122296	0.119107743	0.122967175	0.180617413	0.152045123	0.258801456	3L	-0.330902176	0.612183428	0.990047511

**CG32112**	FBgn0052112	73.71121952	53.53232944	54.85762114	43.1400551	56.61290979	62.62584631	56.01295688	47.72556231	3L	0.010879148	0.948108943	0.997604378
**Vps13D**	FBgn0052113	5.642070736	5.187395882	5.611444777	4.126539434	4.452500702	6.837391455	4.401043929	6.020684832	3L	0.107202211	0.596627261	0.988780662
**CG32121**	FBgn0052121	0.406023026	0.479255062	1.02148791	0.560964386	1.00444808	1.899802516	0.306895841	0.195891906	3L	0.504701035	0.514343813	0.980804634
**Ptip**	FBgn0052133	14.6560511	23.21405493	23.05133744	22.22198687	15.73270114	16.79445491	19.49691226	26.24586714	3L	-0.050029511	0.796681653	0.995787491
**CG32137**	FBgn0052137	4.05010731	6.122831831	4.43674488	5.903828285	6.268893841	5.860257031	6.289810391	5.513808326	3L	0.255480527	0.126186559	0.78246933
**meru**	FBgn0052150	0.674875821	0.947191634	0.233891333	0.1284447	0.621593789	0.220746319	0.105405473	0.089707217	3L	-0.89832172	0.354531847	0.936745458
**CG32176**	FBgn0052176	22.06094728	19.93620402	19.92258862	13.97830827	22.75207791	27.128169	17.23343938	20.25855555	3L	0.231173513	0.240867921	0.874884741
**Ndfip**	FBgn0052177	150.7395617	130.331762	133.1242072	143.0286908	133.637814	160.1257026	133.1024946	148.6463762	3L	0.073914023	0.472446625	0.973549494
**Ccn**	FBgn0052183	17.18258378	19.51475487	17.03611066	18.94370552	16.6591713	16.3342965	15.75048056	17.08119873	3L	-0.111595716	0.226561484	0.867702781
**CG32187**	FBgn0052187	29.53030597	36.39769078	44.08622178	61.88029499	30.97146605	56.13285045	65.57277598	50.74533176	3L	0.272095943	0.31158582	0.914141092
**NUCB1**	FBgn0052190	172.5879512	162.1513974	155.2062619	174.1966747	148.620318	154.1367284	183.2535149	189.0501404	3L	0.054213381	0.646161398	0.99279236
**CG32191**	FBgn0052191	2.15340351	0.485198368	0.701748387	0.074934018	1.218452988	0.51512935	0.409953628	0.069779677	3L	-0.618082042	0.583015672	NA
**CG32196**	FBgn0052196	0.888392263	1.955826327	4.579857882	0.12945332	2.00471966	9.003886091	0.99150964	2.652075031	3L	0.99868846	0.268783612	0.89549671
**CG32202**	FBgn0052202	76.62383269	114.770468	72.73891931	66.02119311	96.68917128	78.40145894	89.56273894	65.93095618	3L	0.034850929	0.878350826	0.995787491
**CG32204**	FBgn0052204	458.2746607	365.2763818	417.0575494	435.0769381	342.2236994	291.247612	308.5314754	319.5817324	3L	-0.379813026	0.000396468	0.029501497
**CG32206**	FBgn0052206	9.505323591	10.13352958	11.77305238	10.12086673	8.687477542	8.689316763	10.71173912	9.932465233	3L	-0.094693882	0.41408186	0.958665466
**hpRNA:CR322(**	FBgn0052207	0.249081943	2.402350095	1.812807958	0	0	1.056748205	2.382800204	1.622339866	3L	0.235018756	0.862893899	NA
**Csas**	FBgn0052220	20.75603189	40.39181342	61.47092628	11.73137139	15.73413791	23.63487517	34.38252161	30.82249404	3L	-0.317555436	0.453997495	0.967967712
**CG32221**	FBgn0052221	28.07762983	50.51389167	71.12991791	40.08819502	38.66590105	44.22835132	36.00347767	41.43063231	3L	-0.204466161	0.40074368	0.953805533
**Pex23**	FBgn0052226	83.14452909	89.3712956	69.26226084	78.77229346	75.25138426	85.03591282	77.68163204	68.70130809	3L	-0.035327306	0.758026416	0.995787491
**gogo**	FBgn0052227	3.270354711	4.844104485	3.766972021	3.681689073	5.338441643	4.16490161	5.895427758	5.897782335	3L	0.488611389	0.006256941	0.227193262
**CG32228**	FBgn0052228	1.566438468	1.946282059	2.814931759	1.961308838	0.314202545	1.258039194	0.814003704	2.330234591	3L	-0.776146775	0.245645902	0.877269208
**ND-MLRQ**	FBgn0052230	101.7666105	103.3514935	132.1527631	86.49205198	108.1092844	97.53866751	107.4070443	102.8476958	3L	0.006531397	0.958872798	0.997604378
**CG32240**	FBgn0052240	0	7.976703452	0.549371649	0	0.204402789	5.977956292	4.910333457	1.474950819	3L	0.609197643	0.678988765	NA
**CG32243**	FBgn0052243	180.1944966	172.7469144	205.1572129	168.9825776	160.7966851	244.442795	190.5851619	155.6160457	3L	0.074224729	0.601618289	0.989286463
**PMP34**	FBgn0052250	243.4804297	211.3925652	241.8244616	238.6648478	265.9055388	284.1558562	262.7362152	324.6646204	3L	0.311933971	0.005967732	0.222031582
**Claspin**	FBgn0052251	0.60844036	0.595334453	2.214101739	0.788086552	0.610217106	1.465953184	0.388036107	1.100816953	3L	-0.189344335	0.781075664	0.995787491
**CG32260**	FBgn0052260	0.336661699	0.36302321	0.46670662	0.504587621	0	1.677699421	0.797486139	0.991967895	3L	1.082234007	0.297015092	NA
**CG32262**	FBgn0052262	144.8587041	96.30648495	119.8768179	123.4762751	198.3641465	158.8931632	122.6881888	155.4598163	3L	0.417850357	0.02214929	0.420786457
**CG32264**	FBgn0052264	21.17318089	22.21057588	24.04212163	23.23980446	23.69932925	25.99624775	21.93421246	23.13120559	3L	0.093121338	0.284201553	0.901780531
**CG32267**	FBgn0052267	209.2498121	180.7011277	272.6478329	152.3651655	206.8094668	218.207457	211.857129	161.7687995	3L	0.001559044	0.993480441	0.998777916
**CG32278**	FBgn0052278	214.0144481	217.9642141	232.6642078	232.4712386	199.856248	199.3357735	217.2243646	225.0459271	3L	-0.061215734	0.551287803	0.984934163
**Drsl2**	FBgn0052279	1.043593321	5.689064532	5.696421392	5.474471718	6.593836953	5.411413747	7.986686948	6.230778687	3L	0.607676149	0.505562358	NA
**CG32280**	FBgn0052280	52.20643172	29.13997061	20.42601553	34.18444168	44.47608618	39.52253643	35.64682957	24.26065232	3L	0.101717532	0.68467865	0.995787491
**Mrtf**	FBgn0052296	11.26616171	10.81336704	11.83808817	13.84127248	11.50682285	10.46308625	11.1022257	10.91334264	3L	-0.089003689	0.440910028	0.967112302
**CG32305**	FBgn0052305	0.359675177	0	1.591151522	0.295965577	0.267361244	0.19947033	0.539728885	0.229672972	3L	-0.82615802	0.487147941	NA
**zormin**	FBgn0052311	0.549049463	0.814852822	0.669062262	0.401133615	0.48873606	0.5725056	0.587363893	0.521859607	3L	-0.131812109	0.671987001	0.995787491
**CG32333**	FBgn0052333	25.06896903	28.67826356	29.1631949	34.72677265	27.11077956	22.6132025	23.90625801	27.71836234	3L	-0.182807195	0.161733644	0.823037813
**Atac3**	FBgn0052343	47.06824522	50.49152103	41.30016015	34.3182602	40.3530667	39.10706358	52.39376421	40.58812535	3L	0.023803452	0.887829321	0.995926915
**CG32344**	FBgn0052344	23.89640158	21.541413	23.14072493	20.27000649	14.34981437	21.99073719	16.66584355	18.39035682	3L	-0.286826155	0.120125259	0.77836519
**Vps11**	FBgn0052350	7.373062063	17.11999127	23.57824664	9.944637725	9.90019839	10.59926255	17.33211264	12.63409772	3L	-0.158398674	0.641693981	0.99218509
**S-Lap2**	FBgn0052351	1.612050872	1.516871985	0.476928406	0.183338338	0.567837071	0.667243192	0.100301864	0.256091098	3L	-1.211402436	0.277245839	NA
**CG32354**	FBgn0052354	0.873277839	2.815164549	1.158587422	0.413565426	1.945980237	1.775285933	0.626554836	1.895967027	3L	0.293740516	0.61763065	0.990047511
**tut**	FBgn0052364	70.42375375	83.11183575	81.81915414	39.48881372	65.04163356	45.59409309	50.21931945	60.64248909	3L	-0.27476118	0.249720434	0.879541335
**CG32365**	FBgn0052365	0.961229949	0.518651091	0.60336662	0.321272479	0.455052383	0.500651023	0.342953602	0.350252173	3L	-0.522899405	0.209333883	0.856520545
**CG32368**	FBgn0052368	1.058902758	10.3017328	5.65154331	0.493758384	12.61649977	18.76854859	25.93232339	38.16300011	3L	2.508985353	0.00111592	0.068382914
**CG32369**	FBgn0052369	8.815365464	8.618820419	10.55168825	10.33189418	8.047797578	7.964154063	9.366701183	7.230279237	3L	-0.202778692	0.121176236	0.77836519
**CG32373**	FBgn0052373	0.069406285	0.175298035	0.129670937	0.137638418	0.13249706	0.069948637	0.059018975	0.071013635	3L	-0.578812769	0.193346043	0.844272804
**SMSr**	FBgn0052380	31.88680837	34.24803644	28.27647106	29.37809176	34.94271732	37.92760658	27.91826175	34.27263988	3L	0.155477746	0.22569323	0.867702781
**Rsph3**	FBgn0052392	116.3200439	149.3300573	150.0825726	171.275135	125.5765847	132.9664213	129.1995778	142.7829072	3L	-0.114394117	0.34809416	0.931729531
**PVRAP**	FBgn0052406	98.88685765	96.63037353	93.71562408	106.0900842	109.482941	107.1700884	101.0735185	105.3370717	3L	0.127217939	0.144965556	0.8037297
**CG32407**	FBgn0052407	9.853398314	17.39251454	24.46011866	8.414465789	6.825593127	15.3928562	17.24462345	9.825689237	3L	-0.249160806	0.541026215	0.984934163
**CG32409**	FBgn0052409	19.1938158	10.42207779	20.4463408	18.78911963	21.21187038	29.23169678	12.86870061	14.82545559	3L	0.208081486	0.538156958	0.984934163
**QC**	FBgn0052412	1.977097526	3.585193232	2.333389145	3.239128101	5.980767597	2.518912798	2.589966136	2.204237113	3L	0.295913897	0.621317387	0.990047511
**vito**	FBgn0052418	34.33825397	61.62943613	62.26983358	41.43019429	48.50666578	53.01225487	45.9887524	36.53024873	3L	-0.079291982	0.744063097	0.995787491
**shep**	FBgn0052423	34.39820245	33.36756703	31.10474008	34.26667738	37.11539255	36.55158816	30.07706401	34.13940541	3L	0.079380316	0.402733399	0.955073155
**CG32425**	FBgn0052425	50.6838531	45.30789015	43.48174807	49.42796777	58.86164605	57.42791908	47.56592976	51.83198984	3L	0.219165255	0.041768144	0.536003453
**CG32428**	FBgn0052428	116.9100302	108.3305449	109.1932121	102.6444293	103.6532651	116.2323414	103.4153336	108.0313138	3L	0.009719974	0.921272863	0.996479182
**CG32432**	FBgn0052432	56.54694164	74.3448163	67.20749822	78.90353479	63.06240785	68.51101991	67.2154173	79.61182065	3L	0.039465066	0.741618194	0.995787491
**CG32436**	FBgn0052436	1.50182325	0.672638684	1.335278314	1.833216947	0.496812334	0.948881951	0.862517723	1.451051153	3L	-0.483895807	0.288976458	0.903033179
**CG32437**	FBgn0052437	0.35694332	0.598722345	1.058370519	0.092466657	0.572777046	0.112174661	0	1.20548865	3L	-0.099292671	0.942761019	NA
**SMC5**	FBgn0052438	5.678904994	9.425297336	12.71346089	6.689761621	6.726671488	7.476865622	9.759668983	3.763720258	3L	-0.279533021	0.396933737	0.952272708
**EMC10**	FBgn0052441	202.1589348	227.479225	241.7211617	239.8339991	218.8974444	241.1944413	264.5748518	210.8214988	3L	0.068628293	0.551923447	0.984934163
**Arv1**	FBgn0052442	15.53690505	8.860732727	12.6269255	11.22934986	7.152264401	17.870681	13.47589272	12.14354127	3L	0.090575453	0.818169056	0.995787491
**Atox1**	FBgn0052446	555.4584007	524.094566	535.0311311	494.9216711	514.3380749	481.9347915	495.7936752	470.8440259	3L	-0.073716159	0.420502818	0.960593359
**CG32447**	FBgn0052447	0.780790382	0.295136075	0.186750789	0.730717273	0.804025824	0.316222455	0.2945639	0.131315846	3L	-0.350577805	0.595573015	0.988780662
**CG32448**	FBgn0052448	56.80488347	61.32635526	92.56639936	80.66445391	73.31140535	65.23798618	65.7261348	72.98499557	3L	-0.035688596	0.872392045	0.995787491
**SPoCk**	FBgn0052451	35.37813333	45.77811159	43.51048062	39.62050605	35.98100895	37.99057039	39.24910657	43.33167735	3L	-0.035693753	0.736082585	0.995787491
**CG32452**	FBgn0052452	42.39014821	32.79682974	53.79067712	42.66874932	40.45098893	49.03475999	42.29759023	46.69567774	3L	0.086512275	0.633188633	0.991585536
**CG32459**	FBgn0052459	0.117368897	0.984349294	2.562617753	1.641846983	1.906928457	0.165982018	0.89823173	0.12740937	3L	-0.724728954	0.550399951	NA
**CG32473**	FBgn0052473	20.21690532	25.78441653	30.20284043	19.62296254	19.26642978	29.03363945	22.93135897	27.60458414	3R	0.077795899	0.639177353	0.991585536
**mthl14**	FBgn0052476	0.184687091	0.206524457	0.67207246	1.650598833	1.055790524	2.727909159	2.120133316	1.002431927	3L	1.394340069	0.099411685	0.731438598
**Usp10**	FBgn0052479	15.47467846	19.44728459	16.97124089	18.09537804	18.3455737	16.75883872	17.88368863	21.14769164	3L	0.116804425	0.336543378	0.924219112
**CG32483**	FBgn0052483	1.445490621	0.839287292	1.348746186	0	0.602187934	0.157246122	2.269217002	0	3L	-0.249798877	0.863397612	NA
**Sk2**	FBgn0052484	71.1668138	61.76714915	58.62154948	67.44720234	46.11638311	47.74317143	55.24125139	50.44216571	3L	-0.349430114	0.01070793	0.312027572
**CG32485**	FBgn0052485	797.5245821	726.248267	766.219154	678.9458554	734.5615368	752.0145683	669.4841257	718.8390685	3L	-0.016816533	0.856148192	0.995787491
**Cda4**	FBgn0052499	0.638923403	0.432234362	0.441027581	0.234554348	0.404653047	0.45760386	0.238632841	0.379361943	X	-0.213666648	0.557971278	0.984934163
**tbc**	FBgn0052506	48.79419612	74.75051067	70.71634294	81.76275768	59.12080382	58.08150151	61.17842434	68.31274558	X	-0.128511051	0.363801877	0.939549067
**CG32521**	FBgn0052521	2.911595239	4.669288727	4.722842938	3.026771891	2.448759475	2.982937171	3.996308876	2.312867772	X	-0.349625819	0.07291409	0.671999479
**parvin**	FBgn0052528	54.54405042	69.48911412	57.81528593	50.21524805	67.2182419	78.04794507	77.25090182	62.32231683	X	0.326865387	0.022407268	0.421875556
**Hers**	FBgn0052529	27.48697772	36.02298984	33.72210028	36.54222701	32.49812907	31.04258501	34.04889486	38.84188887	X	0.062229519	0.60115259	0.989286463
**CG32532**	FBgn0052532	0.775801573	0.390389491	1.922414163	0.581384624	1.990740104	2.701033207	1.639814513	2.466350652	X	1.299612907	0.002037956	0.102315209
**CG32533**	FBgn0052533	13.66441972	21.28891835	15.31523752	29.59073792	25.20338897	15.02726334	23.37898061	30.45401546	X	0.272227042	0.289961391	0.903716616
**CG32536**	FBgn0052536	4.664762965	13.7312049	9.319594026	9.063099371	13.7049156	10.3480236	5.949955271	11.7162804	X	0.227028605	0.561898807	0.984934163
**CG32537**	FBgn0052537	14.39111918	28.42035498	24.30711222	31.21001856	29.26387952	33.4332532	20.15699581	30.36017497	X	0.23826458	0.318199291	0.91460807
**CG32544**	FBgn0052544	316.2081551	331.8318811	274.9261746	379.853027	370.350478	314.5975748	324.3450617	343.8742481	X	0.084904188	0.497784304	0.97594235
**CG32547**	FBgn0052547	4.765666505	4.662349101	4.045927821	4.08214676	3.499626904	2.893823192	3.775677413	3.883882084	X	-0.291436924	0.058806056	0.625114381
**CG32548**	FBgn0052548	1.959688815	2.002144598	0.691516761	1.121467263	1.73670901	1.46126991	0.318131435	0.116036341	X	-0.640541695	0.393991473	0.952272708
**CG32549**	FBgn0052549	41.15314593	45.25982537	43.63989594	49.07339063	44.15015964	51.78456475	44.46068752	51.5560853	X	0.129557592	0.207873904	0.856362392
**CG32554**	FBgn0052554	22.18531047	20.83201571	30.38711934	26.23333452	12.83905017	34.82693286	32.49485376	14.74950819	X	-0.043459789	0.908736608	0.996479182
**CG32572**	FBgn0052572	0.06009939	0.756062215	0.583201126	0.140119465	0.623845112	0.665770718	0.038328709	0.065240669	X	-0.083665438	0.94065278	NA
**CG32576**	FBgn0052576	18.29726507	17.62626207	26.2261082	15.10690137	19.33388937	19.8460453	22.26779126	18.29539287	X	0.078698948	0.721078631	0.995787491
**CG32579**	FBgn0052579	1.256348446	1.697586107	4.148508515	1.545929251	1.134015472	1.678009991	1.602485939	1.742664495	X	-0.444054154	0.47609095	0.973549494
**Muc14A**	FBgn0052580	0.227214832	0.229224953	0.335525226	0.095967904	0.107003741	0.127288217	0.130206721	0.117964104	X	-0.846346638	0.072433773	0.671999479

CG32581	FBgn0052581	35.20067126	37.19268166	39.06229848	34.31489332	5.272394917	0.217381951	8.136688315	0.166864445	X	-3.362187609	0.002488839	0.11852564
lncRNA:CR325	FBgn0052582	4.324360221	2.307934244	2.066377099	0.916553006	0.82797045	1.544310759	3.175745163	0.711256541	X	-0.619872476	0.465724902	0.971928234
CG32590	FBgn0052590	89.14556846	88.44574229	90.29658949	87.31403227	86.27207364	84.37081714	92.60602355	67.34109012	X	-0.074849431	0.671029575	0.995787491
dpr8	FBgn0052600	23.00444611	20.78592502	19.50109591	20.14211435	21.04399829	22.84936476	17.81123809	20.7120199	X	0.010333484	0.917892275	0.996479182
CG32603	FBgn0052603	3.267301401	2.332102018	2.867005737	3.889833302	2.133433603	2.228368539	0.709357963	3.320414966	X	-0.523891457	0.462655739	0.971606621
AMPdeam	FBgn0052626	26.45793685	23.23678697	22.45141182	20.45507955	19.91173719	20.86797263	19.95624447	19.11024295	X	-0.185660505	0.105697825	0.749812996
lncRNA:CR326	FBgn0052636	1.109741515	0.293910902	6.518000112	1.6340972	0.738082896	1.046257522	0.595994621	1.648503697	X	-1.19632968	0.219165389	0.863196763
CG32638	FBgn0052638	7.903658778	9.525600343	7.705174097	11.4998647	5.97147654	7.078945158	7.579570147	5.942297213	X	-0.434231582	0.049283205	0.583903663
Mur11Da	FBgn0052644	2.517043116	1.139266258	0.775404634	0.186298136	0.793380751	0.226005036	0	0.086741803	X	-2.024177142	0.104742459	NA
CG32645	FBgn0052645	1.900828079	0.957530382	0.854505366	0.580511417	1.852780941	1.482910791	0.402796757	0.672429903	X	0.057567475	0.908845215	0.996479182
CG32647	FBgn0052647	124.6169316	119.0548928	123.8951623	138.347831	132.222528	125.8449487	116.2955977	123.3536439	X	0.005629486	0.952928108	0.997604378
Coq8	FBgn0052649	46.41054338	50.53563678	52.84716407	46.48400538	52.05605807	65.65076999	46.99980297	52.50504275	X	0.177950285	0.223500795	0.86549481
CG32650	FBgn0052650	3.788619664	0.872238156	0.720873605	3.290734477	5.096045516	0.210111028	4.358657914	0.967699294	X	0.302411656	0.762356067	0.995787491
CG32651	FBgn0052651	0.246775629	0.451561634	0.326549582	0.679956831	0.404993871	0.042301556	0	0.048706612	X	-1.729484243	0.200136463	NA
Sec16	FBgn0052654	40.10069253	49.0692904	46.06500894	46.68835811	42.77198024	38.23671107	45.63390398	49.30999019	X	-0.014050727	0.905301305	0.996479182
Drak	FBgn0052666	3.762178364	4.555125248	4.531251259	3.66316721	4.010864092	3.946634688	2.855306161	3.064912543	X	-0.218271196	0.12773368	0.787958909
Atg8a	FBgn0052672	1396.030771	1117.094998	1348.627216	1084.386311	1112.834948	1215.176038	1250.687477	1149.697521	X	-0.035605287	0.739396734	0.995787491
Tango5	FBgn0052675	102.433765	109.6414924	99.12193729	109.3244407	118.8612753	121.6247783	105.5974803	98.01326964	X	0.107953692	0.304020712	0.910208378
stx	FBgn0052676	9.546469169	17.43221405	21.03212763	17.3430659	14.36846318	14.50258302	16.41865494	19.44583805	X	0.023582855	0.89955183	0.996479182
X11Lbeta	FBgn0052677	14.08730583	14.91487564	14.34653262	13.6346503	14.5919788	13.01055664	13.91487356	13.77481935	X	-0.011510618	0.896872861	0.996479182
CG32683	FBgn0052683	6.956085069	7.165905376	8.248814571	6.520719399	6.65119712	8.528903801	7.16458078	8.282181595	X	0.115175402	0.347830839	0.931729531
ZAP3	FBgn0052685	24.68872508	47.56012187	55.90285323	39.17782868	44.11363555	40.55511666	50.28543478	40.58576703	X	0.106731498	0.565929242	0.984934163
CG32687	FBgn0052687	1.70659862	1.094515796	0.202950218	0.702153606	0.422861477	0.063096892	1.195097577	0.108975988	X	-1.048614645	0.266818269	0.894434069
CG32694	FBgn0052694	0.906891823	0.405649016	0.708923338	0.481609722	0.391102242	0.641259053	0.385583421	0.546929471	X	-0.326753162	0.466841007	0.971928234
CARPB	FBgn0052698	3.036543599	3.48811762	3.657121567	2.329105863	3.777897058	3.315099332	3.460130996	3.426494586	X	0.194394037	0.182510648	0.838040629
LPCAT	FBgn0052699	0.953313236	2.475947175	1.914836508	1.792430582	1.757984932	0.879449339	3.373311919	2.915427936	X	0.366636541	0.416653404	0.958846438
Cubn	FBgn0052702	3.377584047	2.125970245	2.32688854	1.852872954	1.471787383	1.724595573	2.499248264	2.037784026	X	-0.304778924	0.310491561	0.912746622
Erk7	FBgn0052703	0.09590758	0.436651243	0.166193179	0.159717794	0.074201898	0.103338378	0.454372837	0.594926382	X	0.555120743	0.601071106	NA
APC4	FBgn0052707	12.89242961	14.49930417	17.24879157	21.32276072	21.83614943	17.46745914	19.19144292	15.56459937	X	0.200336034	0.380441355	0.944473714
CG32708	FBgn0052708	25.46502072	58.45894142	39.23114818	31.41882076	38.58870246	39.42963392	36.51138504	28.4505669	X	-0.073530605	0.795637222	0.995787491
CG32712	FBgn0052712	2.639337125	0	1.746271378	0	1.949187259	0.79174802	0	0.520931384	X	-0.409188642	0.827446246	NA
CG32718	FBgn0052718	2.230512763	0.249424817	1.082238571	0	1.744880459	0.210291381	0.948347663	0.968529937	X	0.118698792	0.930833582	NA
CG32719	FBgn0052719	0.686939647	0.476408945	0.576867462	0.22586299	0.254379923	0.67255183	0.224666384	0.320270643	X	-0.392857628	0.373668267	0.943282319
CR32745	FBgn0052745	70.42947912	86.22217027	103.6502348	78.5998654	97.31581331	112.1884933	113.6756387	103.2678717	X	0.367019033	0.067752133	0.656261422
CG32750	FBgn0052750	0.40713629	0.768278262	0	0.474611466	1.561843871	2.207112241	0	2.762283041	X	2.004445718	0.108618272	NA
CG32751	FBgn0052751	0.4176386	0.910689211	0	0.389483469	0.150788943	2.519981575	0	0.816058855	X	1.035556779	0.47354411	NA
CG32755	FBgn0052755	4.924187937	5.373738089	2.590701236	10.5123189	0.428405845	1.062739661	0.756729471	1.416862003	X	-2.648853162	9.66E-05	0.009132299
CG32756	FBgn0052756	50.45974924	43.18990073	47.36093702	33.79647659	33.48654899	47.42027337	44.59009285	39.00529806	X	-0.060460139	0.728907747	0.995787491
Snx27	FBgn0052758	23.74260107	63.27281398	73.19853824	58.12597689	42.95367337	31.05537171	60.25995328	55.52885073	X	-0.159211684	0.685686328	0.995787491
CG32767	FBgn0052767	35.11124578	62.10852194	62.04179452	55.4946909	51.72062926	45.66028701	49.0139973	52.5807462	X	-0.073622755	0.632139443	0.991585536
CG32772	FBgn0052772	77.78344191	70.81056929	48.41524665	58.56101558	51.51193174	73.26636396	55.25036861	60.67831797	X	-0.060587199	0.698561282	0.995787491
lncRNA:CR327	FBgn0052773	0.392225091	0.36629521	0.325944533	0.237416054	0.299163472	0.38548422	0.132356368	0.236216694	X	-0.300254775	0.275683149	0.89549671
Muc4B	FBgn0052774	2.71211728	2.274598133	0.205611209	2.173599773	2.983540707	2.7567307	0.972937891	0.092004026	X	-0.098396885	0.916246627	0.996479182
DIP-alpha	FBgn0052791	0.597144604	0.742196066	0.469837129	0.307291838	0.373900574	0.379126805	0.277904563	0.414632392	X	-0.517379471	0.090417792	0.717658936
CG32803	FBgn0052803	23.58417579	19.65969698	29.99446819	29.49228603	26.19037219	26.14298955	30.81112701	35.37691445	X	0.238139705	0.35265053	0.935162434
CG32813	FBgn0052813	15.05236174	13.62892619	13.95143326	15.0499658	11.70565372	15.80687569	11.87346538	11.17712018	X	-0.162915887	0.256642544	0.88674759
CG32815	FBgn0052815	202.5158983	197.7847745	183.2260645	201.3376804	179.5828908	197.7849171	186.4134209	185.5156595	X	-0.038000678	0.658561335	0.995750687
CG32816	FBgn0052816	0.243868431	0.291239678	0.219472459	0.086464572	0.153172807	0.318918054	0.149077915	0.198852841	X	-0.00707984	0.984260224	0.997881056
CG32817	FBgn0052817	0.708694737	1.981226669	1.146189032	1.6522951	2.843056972	1.039349219	1.205263667	2.051522503	X	0.426606219	0.641482697	NA
CG33695	FBgn0052831	103.0938222	93.42714645	68.25893714	79.50189808	85.63966106	75.74975785	64.11478234	57.25118255	2L	-0.256943831	0.141127546	0.8037297
CG32834	FBgn0052834	2.176586413	3.73389378	5.520385018	3.229309543	2.053950354	3.031470554	3.281033778	0.859194652	2R	-0.628467316	0.303568553	0.910208378
lncRNA:CR328	FBgn0052835	1.339120691	0.383495358	0.554654069	0.152298023	0.471698743	0.030793044	0	0.141822194	2R	-1.895120026	0.182612782	NA
Dh31-R	FBgn0052843	1.296394519	0.655971573	0.500516262	0.595883816	0.44471722	1.152556853	0.628433305	0.414500559	2R	-0.195802703	0.639344391	0.991585536
CG32850	FBgn0052850	15.39388889	12.53990689	13.72052691	10.10717173	14.14189448	17.40500508	14.06531377	14.30068023	4	0.238150168	0.103637343	0.745084146
CG32856	FBgn0052856	42.47254244	26.98550441	42.4640612	30.00701024	32.99297913	47.56605638	44.32433247	44.98819159	3R	0.284578112	0.313987115	0.91460807
CG32944	FBgn0052944	41.46118276	41.83070866	44.6266053	38.16247394	42.37423226	43.52591172	42.1856921	41.59741064	3R	0.061745056	0.504667366	0.978557094
CG32982	FBgn0052982	3.589330209	3.31247635	2.970785335	3.652530327	3.643265536	3.973080944	3.810343267	3.054725505	2L	0.125107771	0.395158385	0.952272708
mRpL27	FBgn0053002	169.665461	148.9117571	161.7815231	139.2138271	190.8500804	142.3428686	184.1021954	168.9440911	2L	0.177747074	0.233071133	0.868700724
CG33051	FBgn0053051	14.26532719	16.69801624	11.95078611	21.05610396	6.669730811	8.127612375	11.78130954	16.26552695	3L	-0.552511252	0.201211738	0.848203654
Gorab	FBgn0053052	30.26829616	27.71042918	21.54875307	24.27059499	16.9543502	31.95050855	39.6899912	11.42340257	3L	-0.032122449	0.924030237	0.996479182
CG33054	FBgn0053054	0.823277056	1.380931767	1.421126351	1.181193516	1.429063548	0.641841633	0.484660952	0.481225957	3L	-0.617433198	0.294739438	0.906715248
CG33080	FBgn0053080	1.371760914	3.640522809	5.90355525	2.606759333	2.730802635	1.408443575	3.499391562	2.284661861	X	-0.401310505	0.327659226	0.918470465
LRP1	FBgn0053087	1.925475895	2.398661485	2.986820141	2.08232796	2.180712209	1.743463713	3.128079584	2.021839209	2R	-0.014322433	0.944591631	0.997604378
Synd	FBgn0053094	80.71272514	90.18339513	90.3243103	100.601095	97.14522515	83.42606836	99.53841008	97.4839916	3R	0.093868447	0.41212578	0.958665466
eIF4EHP	FBgn0053100	11.87053001	12.17214633	10.53810437	10.10550145	10.79305797	9.433008096	7.560862601	9.057913979	3R	-0.247480646	0.049324236	0.583903663
CG33107	FBgn0053107	81.03815368	74.24040198	64.4556521	69.63186038	67.4237413	72.33705346	77.64509712	84.94194258	3R	0.090163335	0.573268735	0.984934163
CG33108	FBgn0053108	9.282167438	26.20614586	20.40034059	17.56994024	19.32826265	13.86324081	19.69343286	17.85785945	3R	-0.011791176	0.969680798	0.997604378
CG33110	FBgn0053110	0.509319359	0.256293532	1.297381744	0.197909819	1.517095768	0.58422287	1.60245104	0.423882132	3R	0.911034	0.165513778	0.828893393
CG33111	FBgn0053111	0.840329953	0.22386742	0.491669285	0.610808679	0.490797657	1.169745556	0.315249965	0.600990062	3R	0.263117251	0.618857032	0.990047511
Rtnl1	FBgn0053113	255.0761878	254.9618795	251.2247283	274.811517	260.7553276	279.5147392	256.464173	260.5121419	2L	0.058153054	0.471258179	0.973549494
CG33116	FBgn0053116	448.5460285	306.3283851	252.4970233	319.8710208	326.6660749	382.8117984	298.7920209	309.1985276	2L	0.013178893	0.931217222	0.996479182
CG33125	FBgn0053125	0.106830594	0.358386669	0.673840781	0.697400223	0.347141885	0.080575385	0	0.603041683	2L	-0.780876763	0.523471287	NA
NLaz	FBgn0053126	7.11631756	11.49723944	7.731769089	3.460788349	4.807689587	13.45960232	5.680048026	3.791456239	2L	-0.073080841	0.878152763	0.995787491
CG33128	FBgn0053128	0.249980507	1.362748503	1.970959975	0.728525177	1.241016932	1.88544076	0	0.135682705	2L	-0.337565646	0.796214169	NA
CG33129	FBgn0053129	165.0908276	137.1461426	128.4677979	142.641648	134.9075535	164.2843652	137.3110286	142.8106711	2L	0.041256132	0.714339291	0.995787491
AGBE	FBgn0053138	16.81337964	8.944212297	21.84905257	14.45454035	19.62086152	21.21558199	21.21784737	15.89753692	2R	0.356467142	0.164312182	0.827712868
CG33143	FBgn0053143	59.76816788	48.83937012	43.65218806	57.85690589	22.36770324	0.851519007	10.54717555	0.66632598	2R	-2.570136517	0.013488269	0.345880718
CG33144	FBgn0053144	6.742799584	9.397836367	9.035199077	8.507569332	7.955033846	8.541530017	6.965549319	6.088462701	2R	-0.157593995	0.23243011	0.867864038
GalT1	FBgn0053145	62.58796312	81.16450239	77.02287509	88.96201914	75.65351896	105.4858633	90.65230998	86.0837469	2R	0.238977937	0.106808368	0.754356729
Hs3st-A	FBgn0053147	10.20863818	14.18684232	17.5873758	13.40017658	15.70053932	15.25781564	15.01868217	16.15099588	2R	0.200682691	0.155335155	0.818884898
CG33156	FBgn0053156	77.04793508	88.84323755	74.9098923	75.18449816	66.0088858	81.41796731	69.04050714	67.18982577	2R	-0.12579224	0.245721411	0.877269208
CG33158	FBgn0053158	1.454085384	1.329003922	1.903042437	1.424805662	1.751352352	1.723178155	1.248908923	1.273044132	3L	0.004100253	0.981626726	0.997881056
CG33169	FBgn0053169	39.06044305	31.49481636	43.55639226	55.59938154	36.37043611	40.83157646	50.34645696	43.44336946	3L	0.039177057	0.889188659	0.995926915
CG33170	FBgn0053170	31.69608035	32.42416389	28.06568486	27.93545303	28.07700386	26.59965049	28.60875711	31.716468	3L	-0.03429147	0.829143573	0.995787491
CG33172	FBgn0053172	4.61650993	6.518579767	5.378333557	6.202533048	6.686020762	4.180287864	4.125212389	4.756616463	X	-0.165943253	0.608040312	0.990047511
CG33177	FBgn0053177	0.34578142	2.174999337	1.468006577	1.813896817	3.901400536	2.771005189	3.087335407	6.005787767	X	1.506226067	0.06472073	0.641709151
CG33178	FBgn0053178	0.946249742	0.488369599	0.529751233	1.187924582	0.657008964	1.509738961	1.114109272	0.474091335	X	0.273870929	0.809346238	NA
beat-IIIb	FBgn0053179	12.93490291	10.3266924	9.363679918	9.839765862	8.15033252	9.345551075	7.815686551	9.29795075	2L	-0.268575571	0.063513885	0.636110045
Ranbp16	FBgn0053180	91.60897066	79.88555783	79.53165886	87.04098703	71.49126725	90.80473304	86.07780747	83.0520702	X	-0.001449672	0.989625669	0.998078482
CG33181	FBgn0053181	49.75988376	47.18603743	43.74863702	47.80751648	42.46303455	53.43315002	40.62320412	45.32072926	X	-0.024805949	0.80972312	0.995787491
Kdm4B	FBgn0053182	13.5549761	11.85182939	11.61412622	13.64608187	11.81518306	15.16376485	9.514560008	10.20393239	2R	-0.092719843	0.536896762	0.984934163
sav	FBgn0053193	0.482105263	5.930202275	3.664686019	3.821634926	11.08212003	1.030258701	3.033656848	1.744491924	3R	0.336668353	0.644337828	0.992589784
dpy	FBgn0053196	1.114710319	1.022480798	0.932136805	1.164415763	1.2272861	1.300995874	0.728271515	1.014232138	2L	0.039609562	0.82670761	0.995787491
pen-2	FBgn0053198	33.84855102	46.78270684	52.57337193	29.00224036	38.53062094	26.97414801	37.57908258	38.66310991	2R	-0.156777179	0.52213457	0.982938673

**CG33199**	FBgn0053199	13.20260484	9.600657868	8.578184186	8.243952385	7.255848777	11.34410318	7.722418697	7.842579907	2R	-0.191359461	0.479373487	0.973549494
**VepD**	FBgn0053200	332.7964175	343.9299564	299.8984194	233.5863942	355.2255272	424.3085485	310.7573946	297.8516045	2R	0.226440019	0.128237945	0.788096052
**dpr11**	FBgn0053202	0.777608041	0.724626889	0.835154334	1.123659901	0.962662875	0.577844351	0.520031107	0.776716398	3R	-0.254424286	0.321916936	0.918330833
**CG33203**	FBgn0053203	49.44983631	45.56717082	39.94315418	44.34974005	40.67601156	42.78685104	39.06906255	41.83792043	3R	-0.0969243	0.339391836	0.927274752
**pxb**	FBgn0053207	14.34737073	23.62380045	23.18131591	19.8295031	16.22166744	15.80230678	19.79875512	20.68386642	3R	-0.122191395	0.437879624	0.967112302
**Mical**	FBgn0053208	39.2454551	50.69210521	62.77455792	52.14644385	51.99663121	53.45113216	50.98000104	62.23355205	3R	0.127652516	0.329495797	0.921112231
**CG33213**	FBgn0053213	17.4185361	8.981147499	30.08970423	18.0140676	33.89199763	27.09325261	27.66385046	21.483732	3R	0.596940022	0.08070313	0.694684542
**CG33217**	FBgn0053217	8.418105697	5.521206274	9.480525382	4.702521631	4.652609405	8.913749712	6.789028308	4.713569787	3L	-0.140644985	0.634306884	0.991585536
**CG33223**	FBgn0053223	0.671237848	0.351346966	0.219431627	0.177584769	0.275008534	0.332128239	0.255029685	0.320403579	X	-0.254062157	0.661139817	0.995750687
**CG33229**	FBgn0053229	0.839994396	1.574734261	0.958971618	0.691205404	1.784006086	1.63046797	1.008397059	1.394598393	3L	0.555593304	0.487093077	NA
**CG33258**	FBgn0053258	2.071825563	2.940551082	1.739845356	0.743135893	0	2.328939964	0	0	3L	-1.626629167	0.289738055	NA
**Tsen34**	FBgn0053260	11.97215933	12.83906709	13.014382	10.06685333	14.64474966	10.9788831	15.18717183	15.48197055	3L	0.26238284	0.396895407	0.952272708
**Muc68E**	FBgn0053265	0.015368745	0.335125874	0.223706163	0.358316499	0	0.202853822	0.117617994	0.13346787	3L	-0.987285119	0.485188469	NA
**CG33267**	FBgn0053267	1.354549733	0.891349422	1.466114524	2.186364261	1.44837559	1.905768274	1.541678133	1.357316705	3L	0.113846438	0.773276191	0.995787491
**Urm1**	FBgn0053276	32.32838459	33.40524967	28.79010245	15.58332562	21.66991456	19.80492212	17.05084295	16.58448638	3L	-0.521938067	0.148958074	0.808039863
**CG33281**	FBgn0053281	0.469182164	0.181612445	2.101346558	0.589012605	0.521227112	1.020789411	0	0	2L	-1.059166391	0.44430368	NA
**CG33286**	FBgn0053286	0.757464082	0.672638684	0.594516773	1.714057846	0.844580968	1.974366351	0.340995379	0.096736744	3L	-0.172648471	0.84282717	0.995787491
**CR33294**	FBgn0053294	1.611153699	0.659908959	0.95443447	0.567819471	1.79247563	1.289371723	1.170897077	1.56594892	3R	0.631521724	0.15892397	0.819032836
**CG33296**	FBgn0053296	3.534102958	12.02058946	2.381579023	14.19047045	1.358695531	9.409619055	3.255623429	3.836432335	2L	-0.824226938	0.162704913	0.824840106
**Muc30E**	FBgn0053300	0.94646915	2.189750652	1.34600194	0.114137441	0.029458955	0.415392828	0.333029398	0.03542883	2L	-2.450232556	0.012102766	0.331790969
**CG33301**	FBgn0053301	0.053435068	0.627408816	0.388898191	1.868727751	1.446959903	2.87156059	0	0.232024959	2L	0.667084822	0.623552681	NA
**Cpr31A**	FBgn0053302	0.100601911	1.603083135	3.050735421	0.469099138	1.180479521	1.470126446	0	0.109208031	2L	-0.853764172	0.511886391	NA
**CG33303**	FBgn0053303	116.4340739	83.59648207	107.7952066	100.6353195	93.70301465	95.5472568	100.7667048	84.73555397	2L	-0.097559127	0.483505549	0.973549494
**CR33318**	FBgn0053318	0.8975984	1.756527962	2.903414933	1.743930733	1.620395165	0.56416686	1.526528536	0.974384609	2L	-0.602207365	0.620974089	NA
**CG33322**	FBgn0053322	1.183433729	1.908696213	2.429302379	1.697926885	4.190633109	0.085825699	1.625596468	0.691746509	2L	-0.085522786	0.92654429	0.996479182
**CG33339**	FBgn0053339	3.076787104	2.468246352	0.973596861	0.935662605	1.086728341	2.207112241	3.412586959	1.016520159	3R	0.052160895	0.950736575	0.997604378
**CG33340**	FBgn0053340	1.881369367	1.136063946	3.651340674	0.350907348	2.309520651	2.483240966	0.191976547	1.960620985	3R	0.030653063	0.975919687	NA
**CG33458**	FBgn0053458	3.074294433	1.67592673	1.678093968	0.716760174	7.076108968	3.912875117	3.333101237	2.836695564	2R	1.267825111	0.080678582	0.694684542
**CG33459**	FBgn0053459	2.071472659	0.128689066	0	1.252108284	0.831008681	0.65099059	0.391434908	0	2R	-0.883415821	0.599701988	NA
**CG33462**	FBgn0053462	7.051978881	2.150673687	1.03684863	0	0.642961075	1.07451517	1.272002499	0	2R	-1.773540705	0.167682648	NA
**CG33468**	FBgn0053468	1.801865437	2.158840353	4.995771906	1.20028041	0	5.581731106	0	1.117719046	2R	-0.555961375	0.691259201	NA
**dpr7**	FBgn0053481	133.2985701	118.2924829	142.9118852	101.2176836	117.8872084	141.6372041	125.0651248	104.2567976	4	0.008213513	0.947832352	0.997604378
**CG33493**	FBgn0053493	39.56971661	29.87909608	43.54438707	34.55611068	65.78753496	25.12712397	33.30084856	40.74059602	3L	0.194266022	0.567893295	0.984934163
**CG33494**	FBgn0053494	0.360909357	1.297231748	0.625400761	0.120206654	0.55845762	1.361052549	0.657633945	1.567135245	3R	0.83014787	0.444600418	NA
**U3-55K**	FBgn0053505	18.70012308	7.442977346	16.36699619	17.62947145	19.12710049	20.02091935	13.28331189	9.338913642	2R	0.059637791	0.863767952	0.995787491
**CG33506**	FBgn0053506	46.46745785	71.85337291	56.18856261	51.62941289	58.06461285	70.43703634	53.52798542	28.37394912	2R	-0.072930176	0.772708104	0.995787491
**CG33511**	FBgn0053511	0.633637496	0.850270407	0.922317436	0.147730197	2.058980543	0	0	0	2L	-0.258076409	0.89101486	NA
**dpr4**	FBgn0053512	23.00425413	41.2417189	46.48446748	38.82779058	32.83962068	22.59410495	37.25545625	36.25635102	3R	-0.174081971	0.385414042	0.947225192
**CG33514**	FBgn0053514	2.45079919	2.701430058	0	0	0.63204581	1.518393304	1.250408277	0	3L	-0.584715758	0.710901395	NA
**dpr3**	FBgn0053516	0.537464451	0.528968285	0.510035572	0.632468459	0.606030583	0.589839168	0.358990281	0.629455356	2L	0.016267215	0.952175991	0.997604378
**Dop2R**	FBgn0053517	13.98108587	24.70825635	29.57587519	28.05808011	19.65043996	15.98895461	21.48873826	22.32977322	X	-0.240839085	0.188380916	0.843108521
**Unc-89**	FBgn0053519	1.217497654	0.48919177	0.909673835	2.924893672	0.376065737	0.305511234	0.318852822	0.221112557	2R	-2.162992791	1.51E-05	0.002247684
**CG33523**	FBgn0053523	117.6779042	99.73883827	98.6538221	117.0764406	94.12572048	108.1273386	109.1054438	112.4490909	3L	-0.003672505	0.974541399	0.997757033
**PNUTS**	FBgn0053526	252.0270726	266.7517398	237.084833	267.1122431	256.9737681	333.3367401	282.6523303	259.639477	2L	0.174268484	0.080873768	0.694684542
**SIFa**	FBgn0053527	0.600473107	5.539651692	14.20320897	7.699892438	11.11108054	3.962856641	6.893189082	6.192492824	2R	0.062531874	0.939254672	0.997038394
**Acp53C14c**	FBgn0053530	1.084233951	0.808289044	2.045817233	0.280872293	0	5.111045732	2.151258747	1.0462099	2R	1.007935993	0.459842441	NA
**Ddr**	FBgn0053531	0.657551472	0.627896828	0.743834202	0.933040006	0.746907195	0.848638312	0.735052689	1.136202572	2L	0.258618881	0.255573669	0.88674759
**upd3**	FBgn0053542	1.152870851	1.00635444	0.513706886	0.411409437	0.552162362	1.896563785	0.240081495	0.280947783	X	-0.037122945	0.959152491	0.997604378
**CG33543**	FBgn0053543	110.0430692	100.2407631	79.64174176	103.703198	89.21013368	100.8931365	66.06640162	84.44257573	2L	-0.182722947	0.184998953	0.839299945
**Vkor**	FBgn0053544	0	2.771827455	10.35639798	4.815910467	4.723361742	11.4250516	3.86425288	1.19590607	2R	0.284493139	0.791926257	0.995787491
**Rim**	FBgn0053547	144.4882454	153.5903447	130.3764962	148.9562023	140.5136962	147.5395868	118.3471026	146.853766	3R	-0.031967524	0.746845327	0.995787491
**SmydA-8**	FBgn0053548	0.271530551	0.595594224	0.354700408	0.900897712	0.452475911	0.649839413	0.293057037	0.430801587	X	-0.173555339	0.791634811	0.995787491
**Nipped-A**	FBgn0053554	3.550948516	3.772602845	5.709477418	2.894160674	2.94513259	3.949189253	3.375404769	3.675818822	2R	-0.15872072	0.374247254	0.943282319
**form3**	FBgn0053556	4.364520612	4.974110989	4.050841884	5.055947842	3.958756425	3.234524965	2.405251298	2.885655124	3L	-0.531517756	0.002977634	0.135642433
**mim**	FBgn0053558	52.25875242	59.92011529	50.36518171	62.22725959	52.65892563	52.07308779	48.04282805	57.95496451	2R	-0.061984484	0.558076285	0.984934163
**CG33635**	FBgn0053635	94.0456556	67.38084904	74.61302995	53.90136056	87.24888606	120.3249355	78.72545488	64.95662032	2L	0.299590916	0.199651652	0.847197998
**CG33639**	FBgn0053639	0.919297418	0.904254835	0.468067236	0.68797519	0.583921882	0.454754193	0.810668259	0.455849089	X	-0.353274614	0.414143978	0.958665466
**Cadps**	FBgn0053653	66.61997614	59.3770886	68.999056	53.37619587	47.59595011	62.65724244	55.78931718	51.64400339	4	-0.161916316	0.142309367	0.8037297
**CG33658**	FBgn0053658	1.191994206	1.555091269	5.14090901	2.470301883	0.956380021	1.373539269	2.027201886	1.725286509	3R	-0.725286173	0.483720631	NA
**CG33689**	FBgn0053689	0.124272949	2.709855702	2.713359974	2.317901623	0.897378097	1.40596533	0	1.888656537	3L	-0.841522701	0.537244684	NA
**CG33700**	FBgn0053700	1.12963839	1.017347611	1.342654693	1.361044444	0.780129856	1.150789288	0.328788192	0.181060702	3L	-0.955950113	0.070871617	0.6662581
**CG33774**	FBgn0053774	46.76783749	42.12399758	56.18183505	29.45063026	49.67945907	29.7730245	47.95247517	36.24000255	2R	-0.061999977	0.842070227	0.995787491
**CG33798**	FBgn0053798	1.672628192	6.212398108	0.579681809	3.064026163	1.294081105	0	0	0	2R	-3.102517054	0.053097014	NA
**CG33914**	FBgn0053914	0.227742977	0.254671263	0.460417738	0	0.479656851	1.39564547	0.968295379	0.494451085	2R	1.860571818	0.11775832	NA
**CG33958**	FBgn0053958	1.431875592	0.823464149	1.06692528	0.143072745	0.830861496	1.967089762	0.73054908	0.2886682	2R	0.154986541	0.820069514	0.995787491
**CG33965**	FBgn0053965	2.064159428	0.384704208	0.556402442	0	0.966087321	1.657769212	1.560211866	0.829903925	3L	0.710125343	0.576292458	NA
**CG33966**	FBgn0053966	4.565923026	0.788395109	1.303160656	0.313096401	1.090940466	3.101933715	0.513872106	0.145780023	3L	-0.518733868	0.647904943	NA
**CG33969**	FBgn0053969	54.02047975	56.622638	52.75864752	40.54078806	68.47384003	80.04562794	61.15573227	63.82636481	3L	0.452914157	0.004236509	0.18013811
**CG33970**	FBgn0053970	3.61880311	3.586838597	3.273184677	4.077020116	3.079073186	3.313936642	2.467766436	3.124846351	3R	-0.250835813	0.086840335	0.706081898
**CG33977**	FBgn0053977	165.3140908	214.9818857	212.6972735	174.8567037	176.7087037	196.8546735	238.0314084	211.3721441	3R	0.133317559	0.425901713	0.962175354
**CG33978**	FBgn0053978	6.041795256	4.262825318	3.58289825	1.632212584	1.315763745	5.158196273	1.223234088	1.030644747	4	-0.821534479	0.20425862	0.853973653
**obst-H**	FBgn0053983	0.708533707	0	1.71889289	0	4.31691166	4.676008756	0.225935589	0	3L	1.957432627	0.226034567	NA
**Mid1**	FBgn0053988	43.51371302	52.08461361	43.50931231	60.08171963	47.12749699	48.12268211	46.11043286	49.30217581	2R	-0.033164903	0.776192421	0.995787491
**CG33993**	FBgn0053993	5.691971879	6.356744744	4.054351547	5.775813085	5.847610938	6.307090632	6.344812894	6.712482597	3L	0.232641098	0.175807049	0.834088743
**CG34001**	FBgn0054001	0.392270572	0.534607819	1.130075986	2.114929414	0.973704765	0.670317417	0.750517992	1.543625999	3L	-0.040314418	0.957227837	0.997604378
**CG34008**	FBgn0054008	3.923114821	11.94875679	7.012837142	10.10939394	10.80971602	6.813373902	6.847544869	10.75887844	3R	0.13928822	0.789494834	0.995787491
**CG34015**	FBgn0054015	80.95383721	67.49846718	99.34158795	80.88892453	102.6831612	89.33993432	63.82095529	60.46494571	X	-0.024127189	0.915458967	0.996479182
**CG34025**	FBgn0054025	7.702300668	5.802839227	4.592241912	2.28274882	3.063731326	5.169332965	2.164691087	3.826313636	3L	-0.493220735	0.394288892	0.952272708
**CG34027**	FBgn0054027	1.32463561	0.54192449	0.901360888	0.52727723	0.7873815	1.264088342	0.288466065	0.455936401	3R	-0.212878098	0.759419159	0.995787491
**CG34039**	FBgn0054039	12.43086189	17.9040818	17.04881249	13.29311702	15.73854539	15.09502597	15.89797973	12.81056596	3L	0.00814539	0.970850556	0.997604378
**CG34041**	FBgn0054041	1.95839651	2.874318484	2.573481418	1.902469891	1.399432188	1.846364268	2.289792804	3.543216758	3R	-0.003395419	0.995286191	0.999002182
**CG34043**	FBgn0054043	5.681363327	2.420238721	2.297151365	1.892269246	0.895392748	2.125537556	0.460104124	0.489474829	2L	-1.591477651	0.033836059	0.514749796
**lncRNA:CR340**	FBgn0054044	0.349468426	0.502443651	1.816726131	0.581980343	0.721005803	0	0.509429408	0	3R	-1.367788725	0.373529991	NA
**CG34051**	FBgn0054051	10.00481297	13.15320099	12.90108709	22.69541657	9.274696782	19.62974852	18.85445417	12.32834803	2L	0.062477791	0.877979712	0.995787491
**CG34056**	FBgn0054056	0.122601905	3.290359932	1.933298319	0.285841725	2.102614675	3.698826459	3.90950116	0.399270126	3L	0.896931771	0.383718349	0.946594947
**CG34057**	FBgn0054057	0.062225751	4.905623506	2.113423262	1.59584696	6.852339182	1.818647802	5.0796553	3.242348784	3L	1.031018953	0.238614998	0.873349755
**ND-AGGG**	FBgn0058002	63.76065304	58.68772518	90.71785487	42.77353443	63.70553586	71.36074126	64.70387316	55.06739126	3L	0.026479677	0.910427079	0.996479182
**CG40006**	FBgn0058006	0.587233574	0.546741165	0.475396475	0.22617505	0.217158504	0.493276369	0.268922258	0.296268736	2L	-0.498390657	0.104065028	0.745084146
**CG40045**	FBgn0058045	65.82467714	63.97980894	68.44255128	54.63903112	58.14833503	67.79687409	61.4694317	58.63953778	3L	-0.009796028	0.926371556	0.996479182
**CG40160**	FBgn0058160	0.279865091	0.277393072	0.370335713	0.252100311	0.241131649	0.359802407	0.210937368	0.469519151	3L	0.156284481	0.794745803	0.995787491
**CG40191**	FBgn0058191	13.92807942	29.72663913	42.99400498	26.87403546	22.02245961	10.30128488	30.38498552	27.31950769	2R	-0.288748764	0.528237455	0.984296327
**CG40198**	FBgn0058198	1.01276718	1.072910442	2.069018149	0.124275187	0.384906175	2.361949654	1.223806185	0.231453821	3R	0.011389394	0.991745031	NA
**MFS17**	FBgn0058263	7.480683389	7.619994706	8.302108125	6.635043845	7.109755315	7.674669425	6.152600625	6.591271947	2R	-0.095332166	0.374877903	0.943282319
**CR40354**	FBgn0058354	17.45986081	14.02011724	15.32075403	10.24892759	14.97736232	16.34430509	15.16271661	15.189674	3L	0.137173507	0.635928872	0.991585536

lncRNA:CR404	FBgn0058469	577.8616294	488.3925273	513.0047935	367.8662227	307.5974081	476.1766945	500.0240971	505.897744	X	-0.093826164	0.643474517	0.992589784
CG40470	FBgn0058470	0.41373437	0.512554808	0.464576107	0.209457326	0.362778338	0.539405882	0.343773601	0.314389466	3L	-0.005087571	0.984882827	0.99793477
pain	FBgn0060296	3.019483895	1.588102914	5.86638847	4.295479138	0.923888529	1.978250676	0.97916546	0.666669056	2R	-1.665416811	0.000303617	0.023429144
salto	FBgn0061197	0.209278983	0.741076284	0.507707893	0.758996109	0.377802202	0	0	0	2R	-2.498988724	0.10765106	NA
HSPC300	FBgn0061198	40.15789933	65.19073574	56.06204436	52.93578105	45.94772578	36.94648243	53.79838289	51.39969704	2R	-0.152805539	0.494100562	0.973943729
Nup153	FBgn0061200	25.64264519	36.76931577	41.84710474	38.43441139	32.38038635	27.40789541	46.7615371	37.34973072	X	0.04876899	0.784280938	0.995787491
CG18003	FBgn0061356	3.429808143	3.242612403	3.479551536	2.762416415	0.78803235	1.704990234	0	0	2R	-2.291738091	0.027393947	0.462324143
Ube3a	FBgn0061469	70.23551089	82.92805693	57.97514656	86.68052429	89.25121773	77.36509224	80.18677203	73.57739523	3L	0.135438753	0.344732586	0.930285749
Zwilch	FBgn0061476	1.150117784	0.120572577	1.046313639	0	0.973245644	0.271081006	1.100240692	0	3R	0.031356383	0.982462037	NA
loj	FBgn0061492	64.70892469	79.29223075	62.628368	62.0360615	50.79409303	63.35566177	56.46883476	65.02074861	3L	-0.158169618	0.323446104	0.918470465
endos	FBgn0061515	278.1394536	232.9284199	256.7653833	266.1297332	272.2290542	286.5313165	260.8571363	246.1441198	3L	0.071117053	0.498540982	0.976006682
Ctr1B	FBgn0062412	1.196234403	1.915310151	3.561604335	2.155117415	2.453988284	0	1.109679877	0.708309715	3R	-1.005923947	0.334660932	0.924122842
Ctr1A	FBgn0062413	10.31138395	19.9770942	20.21564681	11.8828396	16.98265313	12.82201675	20.08772778	15.81595274	X	0.115976011	0.614809721	0.990047511
EMRE	FBgn0062440	590.1177335	579.7069234	492.3154793	443.9952977	547.5118502	554.4928083	530.6316777	518.229238	2R	0.059840539	0.611698969	0.990047511
Cisd2	FBgn0062442	499.2529765	512.1740884	580.429747	529.9654291	610.3651254	506.5677991	552.5037464	561.6840478	3R	0.104845211	0.326982691	0.918470465
CG13197	FBgn0062449	33.91437464	24.47576947	25.08684506	29.57749451	25.42786416	27.75290843	25.97188681	27.08009704	2R	-0.064656646	0.661660556	0.995787491
CG16984	FBgn0062517	1.067603685	0.835685859	1.726661099	0.33187704	1.541838621	1.006530233	1.089391975	0.618097714	3L	0.139426278	0.895031259	NA
hpRNA:CR339	FBgn0062928	0.102264753	1.372278212	1.116417888	1.072918896	1.107684535	0.578487506	0	0.777091857	3L	-0.508642215	0.684032227	NA
lncRNA:CR339	FBgn0062961	6.187017546	11.41563938	4.502885481	3.245579661	2.513059288	2.479059999	1.972901835	4.365591041	2R	-1.130111298	0.155690079	0.818884898
CG31808	FBgn0062978	151.7146684	121.8659306	149.2593042	104.6618514	120.7982894	164.9994782	128.7493638	118.9859509	2L	0.042819241	0.749683019	0.995787491
Gpb5	FBgn0063368	94.78122167	122.4695438	117.1693238	107.0531511	84.11950635	103.3616085	102.0950877	104.6124313	2L	-0.130363763	0.434123801	0.965402206
Uhg2	FBgn0063449	6.218004278	9.459613145	15.78640987	6.911380915	8.353556379	10.36126815	14.94003794	7.37783204	2L	0.136107529	0.755260832	0.995787491
GstE9	FBgn0063491	0.550306516	7.753720637	3.471093425	6.158490844	0	5.603316718	3.08845624	2.389532776	2R	-0.655471077	0.533647132	0.984760433
GstE7	FBgn0063493	0	1.901700991	0	0	1.228021991	1.49644521	5.205981807	1.72302828	2R	2.38656809	0.136609808	NA
GstE6	FBgn0063494	1.732364913	11.42947652	4.482872657	8.077887157	2.084908446	8.819620515	8.249360208	0	2R	-0.394257815	0.67715238	0.995787491
GstE3	FBgn0063497	3.216592676	2.119614739	8.918181678	4.017516822	9.747085242	19.60348896	17.87647923	0.49882289	2R	1.417003085	0.073096044	0.671999479
GstE2	FBgn0063498	51.34737396	70.765524	70.91158238	42.93365221	34.8266484	42.71469753	74.81492314	44.42273137	2R	-0.228657545	0.404626232	0.956706394
CG6006	FBgn0063649	2.740365136	7.167545481	3.79354151	3.946536862	2.720417956	4.685510978	2.817356482	4.985999887	3R	-0.176262727	0.53862826	0.984934163
CG40228	FBgn0063670	104.0850654	101.7429938	132.7958403	78.10849565	82.96557164	125.5317888	94.35144866	81.43922664	3L	-0.087871179	0.62084628	0.990047511
Kaz1-ORFB	FBgn0063923	14.32555772	12.26802836	11.29125506	7.75093848	11.13018117	15.7285626	11.1022194	8.530115083	3L	0.051548989	0.881483861	0.995787491
RpL5	FBgn0064225	459.4999181	568.0348237	542.5170299	529.5796162	548.7316041	473.3270824	566.9755713	591.7747711	2L	0.089376761	0.40804255	0.956706394
Idgf5	FBgn0064237	0.160801199	1.438514413	1.040270573	2.749280901	0.870862129	0.656943681	0.410207312	0.349114287	2R	-1.183676854	0.27270463	NA
CG7600	FBgn0064766	112.2557814	120.1461488	94.88109646	104.724647	135.0215927	127.0452735	96.63465058	91.4438325	3L	0.087974716	0.535376073	0.984760433
CG31178	FBgn0064912	2.416231704	2.82474132	0.888143093	1.536369071	3.70102091	0.06903056	2.61497048	5.086897162	3R	0.61216722	0.531515003	0.984760433
AlkB	FBgn0065035	0.256077592	0.322150678	2.48496267	0	2.311428433	3.13856907	1.14320402	3.474800988	X	1.79764784	0.098169257	NA
snRNA:7SK	FBgn0065099	490.9670763	803.1943603	1074.864853	651.8836702	1252.709945	666.3934487	930.2477097	954.2898506	3R	0.373544832	0.077664027	0.682035833
ppk10	FBgn0065110	0	1.669999491	0.650284081	0.53566891	1.382565283	1.119167878	0	1.496468669	2L	0.546446909	0.673060188	NA
LpR1	FBgn0066101	1.170463033	0.634395633	1.487371282	1.058141214	2.220791159	1.643997839	1.63512276	0.458104525	3R	0.479671291	0.238580207	0.873349755
GlcAT-I	FBgn0066114	133.5587129	171.804029	133.9576042	130.2322745	153.5724023	159.1978357	186.635054	184.114237	X	0.29619304	0.027853954	0.462499435
dyl	FBgn0066365	0.536863853	0.487336879	0.475001774	0.191433085	0.319258257	0.881299886	0.660607061	0.137127191	3L	0.261725078	0.679758444	0.995787491
GlcT	FBgn0067102	57.71996858	43.03460228	55.61477185	44.99618841	52.72182278	64.93580506	53.07588551	55.86816366	2R	0.19728361	0.177409956	0.834615603
CG33332	FBgn0067629	9.402426665	10.80222719	10.93637614	11.18592181	11.62588353	13.11467735	12.32146871	13.07305387	3R	0.27761611	0.217515732	0.860826301
dbr	FBgn0067779	19.60734941	22.29081839	35.36496041	20.0795076	24.34092379	23.81010021	22.96091134	20.61415066	2L	-0.05057451	0.794531707	0.995787491
DPCoAC	FBgn0067783	4.345671165	5.638856669	4.654629823	5.237933267	6.512871897	4.359903279	5.563867047	3.916354646	3R	0.066358199	0.809405985	0.995787491
Patj	FBgn0067864	195.6019833	211.2956854	217.9036093	224.6445844	180.6951486	219.1451601	209.2664738	206.0775895	3L	-0.029484985	0.766907307	0.995787491
eca	FBgn0069242	83.69369713	78.3440377	72.82720444	75.80539587	87.42329607	91.39461106	105.3259212	80.4888132	3R	0.25731926	0.112105483	0.758410631
Porin2	FBgn0069354	1.172380713	1.619474029	0.501913987	0	0	2.470700486	0	3.892883731	2L	0.976945794	0.529284913	NA
CG4681	FBgn0069913	0.603038419	0.735645346	0.53198647	1.150332032	1.28657325	0.155056619	0.139851269	0.11902293	2R	-0.787078228	0.548332147	NA
CG41128	FBgn0069923	99.37158533	110.4641724	138.8578253	65.15079843	98.92838193	93.35862959	102.1546759	87.88557457	3R	-0.079337328	0.668373078	0.995787491
CG40498	FBgn0069969	43.25705294	38.7953004	39.31734993	33.68062546	34.06306123	44.98898449	34.7021988	32.24709315	2R	-0.060134557	0.609852508	0.990047511
CG40485	FBgn0069973	0.296807238	0.331901669	0.96006696	1.499322288	0.982324082	0	0.126193607	0.107399331	X	-1.302140246	0.384121944	NA
tmod	FBgn0082582	35.61230087	39.14839081	34.05417777	34.25398929	33.07514646	32.56966	28.77919097	32.27964179	3R	-0.144104704	0.108186584	0.758410631
sprt	FBgn0082585	0.505142263	1.351108423	1.092985049	1.114059621	1.219989378	0.984668799	0.574658774	0.563175721	2R	-0.236833548	0.596767509	0.988780662
akirin	FBgn0082598	210.745685	203.6613599	202.2029961	201.5295012	221.1307368	227.2127331	227.4202914	205.4130221	3L	0.136373621	0.116228159	0.765798592
pps	FBgn0082831	52.18332137	71.63697131	55.28667422	65.23903207	55.65466908	59.33037635	63.34548455	53.68330193	3R	-0.042988711	0.726193619	0.995787491
snoRNA:Psi28	FBgn0083006	5.303157897	3.953468183	2.858974909	1.373790333	7.446101594	20.55467114	10.52214312	20.46870525	2R	2.114565688	0.049403289	NA
CG33947	FBgn0083068	17.91231682	13.75317542	15.40307428	10.48948939	12.44872519	9.395226816	14.90977465	8.581206587	3L	-0.320703833	0.282112537	0.901329548
Uhg5	FBgn0083123	38.74948096	34.95585059	39.89439444	31.22966385	23.53348505	20.77519631	24.40759468	19.14966782	2R	-0.692530201	4.58E-05	0.004835041
Uhg4	FBgn0083124	12.40437848	16.67378599	21.33294446	19.31324173	12.50024804	8.61087684	16.58050312	12.17431206	2R	-0.45013321	0.12493031	0.780439453
Neb-cGP	FBgn0083167	1052.237235	1075.425566	1081.770264	990.8143989	1117.991077	850.7412699	1145.070843	1029.863099	X	0.014166833	0.902464797	0.996479182
Frq2	FBgn0083228	1.228398333	2.08757727	2.148792524	1.326467755	1.126294076	1.32575001	1.129770695	1.256281585	X	-0.450158701	0.072229793	0.671999479
BG642163	FBgn0083938	0.989294684	2.396915513	1.733344242	0.128139073	0.595310153	0.155450164	0	1.312575206	2L	-1.297835522	0.309971557	NA
RhoU	FBgn0083940	0.501518656	0.335558768	0.380843576	0.123081249	0.19060415	0.369356511	0.092144062	0.171922646	X	-0.676523328	0.153841572	0.817059301
lobo	FBgn0083946	0.307677053	0.312051452	0.173586102	0.183504933	0.232508067	0.151783978	0.109519643	0.116510819	3R	-0.650387014	0.266884669	0.894434069
side-III	FBgn0083949	7.246405926	8.846023058	9.488550079	9.097961645	8.729705083	7.771892989	8.190491053	9.205400083	3R	0.000765917	0.99466291	0.998895169
side-VI	FBgn0083950	23.212808	23.30421456	22.94101899	25.2323647	21.53745212	22.35412553	24.26257909	23.22842776	3R	-0.021069368	0.817663614	0.995787491
CG34115	FBgn0083951	1.916862974	1.205725774	1.598534726	1.256932649	0	1.016553771	0	0	2R	-2.446883324	0.097128582	NA
CG34117	FBgn0083953	159.7864531	174.7690772	224.9267434	171.8432516	169.6416189	154.5344136	168.8117744	186.8881783	3R	-0.070897157	0.683251312	0.995787491
CG34120	FBgn0083956	0.550211181	0.427890959	0.582460211	0.268221119	1.038420936	0.778103611	0.548680212	0.065157786	X	0.436925462	0.484156155	0.973549494
CG34124	FBgn0083960	0.308513484	0.393290947	0.209565834	0.632973064	0.891113455	1.431053969	0.283328829	0.053584907	2L	0.807109459	0.351670548	0.934121932
CG34125	FBgn0083961	292.7839873	337.0086577	350.513868	319.3324537	345.9052649	267.5986788	354.2744717	416.5212355	2L	0.126316103	0.407990563	0.956706394
CG34126	FBgn0083962	50.21483433	55.84648191	65.4017584	56.78853751	58.1303613	47.65156921	53.8462703	56.71537079	2L	-0.043521819	0.713552988	0.995787491
Nlg3	FBgn0083963	27.48240753	30.66609104	32.81483302	31.51874687	27.36945451	28.91446935	29.56297242	27.00658561	3R	-0.087299071	0.325568965	0.918470465
CG34133	FBgn0083969	35.84924975	38.51663307	31.19016292	28.441301	30.9011931	34.55955315	30.08998781	37.97476501	3R	0.025527925	0.857012339	0.995787491
CG34134	FBgn0083970	95.92433815	84.35222637	60.34858916	55.91099582	64.93800584	81.49442739	67.56781162	69.54972122	2L	-0.04128999	0.870905503	0.995787491
Nlg4	FBgn0083975	9.598215003	12.4659811	12.44871104	12.66887796	8.617474599	9.37246544	9.308308443	10.15570234	3R	-0.300353599	0.012669674	0.338426487
CG17672	FBgn0083978	0.241180477	0.327489956	1.14233902	0.53552686	0.476858535	0.368144683	0.38087312	0	3L	-0.831883852	0.370520349	0.942875294
Ir10a	FBgn0083979	2.26664568	0.706082028	1.57110023	0.075494272	1.110652163	2.411734236	0.826037404	0.281205577	X	0.015161151	0.987170504	0.998078482
RunxA	FBgn0083981	0.416023912	0.394420989	0.244481119	0.192783918	0.443154222	0.163223986	0.309816392	0.25245459	X	-0.070180822	0.88180191	0.995787491
mRpL34	FBgn0083983	135.6054283	149.3984121	187.9867872	141.2072819	149.5345646	132.2565201	137.4640909	171.6194382	2R	-0.020919857	0.915952413	0.996479182
CG34149	FBgn0083985	22.79022285	22.47151315	17.93149063	18.66889476	15.84530419	16.04227271	28.87276073	20.22649223	3R	0.007299848	0.980953275	0.997881056
CG34150	FBgn0083986	41.88277447	55.89748297	61.02713057	46.25436883	46.58272239	44.13233264	87.32748723	28.71517276	3R	0.043897923	0.891614827	0.996189576
CG34159	FBgn0085188	310.5146012	311.1161657	290.1191324	276.0057159	281.8455116	293.7055967	257.9769514	254.2997427	2L	-0.097310097	0.314551601	0.91460807
CG34163	FBgn0085192	5.383054814	8.208472076	15.03788497	4.944092893	6.773007664	8.612309912	11.23550876	8.145561728	2L	0.094144586	0.878293734	0.995787491
CG34172	FBgn0085201	0.611006745	0.72820286	0.351069594	0.374878743	0.725673536	2.622559023	0.123054579	0.24436488	2L	0.860995892	0.323379935	0.918470465
CG34174	FBgn0085203	21.43825614	19.35049355	17.25856218	10.53442554	16.31365654	12.96096656	15.4503833	9.3923755	2L	-0.317103031	0.400952021	0.953906407
CG34180	FBgn0085209	2.023790786	6.789250268	12.27422055	4.718392031	1.461384425	1.526414073	4.13018702	1.318151141	2L	-1.587782305	0.104190349	NA
CG34183	FBgn0085212	40.68986504	61.91681658	64.72502056	38.62843366	62.01942846	51.45582631	51.27879876	49.46063981	2L	0.095212106	0.702940201	0.995787491
CG34190	FBgn0085219	1.261048162	0	0.764821313	0.918776974	0.995975372	3.120884825	1.809540537	0.171115768	2R	1.050657479	0.419928368	NA
CG34195	FBgn0085224	35.41260003	41.54475843	42.70206258	35.68544527	26.12415402	35.61694139	34.07653098	25.82940983	2R	-0.321000979	0.131256253	0.790743321
CG34196	FBgn0085225	0.680693482	5.137955213	4.541220459	0	0.614413098	1.443945729	0	2.216772842	2R	-1.207558841	0.439388162	NA
CG34200	FBgn0085229	287.8719966	413.2692074	438.9479476	317.6305012	383.7775918	252.4500431	421.1974921	289.7458943	2R	-0.076570033	0.69855106	0.995787491
CG34205	FBgn0085234	2.40808939	4.651236999	3.186540694	1.70132417	9.616574341	1.169564953	6.701550295	2.376447718	2R	0.774514002	0.291769943	0.905069205
CG34213	FBgn0085242	9.066322008	11.23014151	11.16657591	11.05668935	16.11523437	18.5418753	24.90748855	19.68382434	2R	0.93117657	0.020317393	0.403155126

CG34214	FBgn0085243	1.601345718	3.418587194	2.119004932	1.357628094	1.576821514	5.07821287	2.599588301	0.31606089	2R	0.211574153	0.8481996	NA	
CG34216	FBgn0085245	0.381578175	2.743045105	1.983649992	3.050177655	0.98406629	1.850140658	0.13905916	0	2R	-1.395736928	0.215837895	NA	
CG34219	FBgn0085248	389.3745773	411.385589	263.1626485	331.1657331	418.8097741	379.6742824	338.724799	331.22992	2R	0.103257507	0.477109628	0.973549494	
CG34228	FBgn0085257	97.58030578	145.8928186	149.0996935	135.3297485	142.4182792	118.9367912	121.0264761	151.3813631	2R	0.053581928	0.791591287	0.995787491	
CG34231	FBgn0085260	1.948252334	1.678121069	2.938052938	0.613821213	2.851698483	3.226811513	2.283528933	1.486158652	2R	0.491278222	0.484501529	0.973549494	
CG34232	FBgn0085261	9.975966276	8.849332852	7.91204684	13.86569778	5.887615213	0.994789238	3.42581404	3.540371983	2R	-1.532073763	0.012058789	0.331790969	
CG34242	FBgn0085271	143.6543386	144.5760002	156.9556151	136.9469813	149.8367864	129.4170359	182.4182847	143.6989861	3L	0.085774791	0.651272251	0.993444251	
CG34250	FBgn0085279	73.83689271	89.38383202	97.07696357	58.22313672	82.71004562	86.20514439	104.0868074	90.93297815	3L	0.226082537	0.247406511	0.879384515	
CG34251	FBgn0085280	0.34041789	0.911175406	0.105427539	0.084433138	0.039226056	0.355086876	0.129338159	0.204426038	3L	-0.944552334	0.307780328	0.912131082	
CG34256	FBgn0085285	0.080276409	1.61582898	2.336993391	1.310129984	4.057745817	2.270523251	0.40957369	0.174287516	3L	0.434973815	0.719704036	NA	
CG34259	FBgn0085288	2.854833564	0.979482848	1.888850838	2.117798131	2.225464072	3.242057756	2.813786588	1.831259163	3L	0.381217987	0.59237778	0.988631016	
CG34261	FBgn0085290	2.769491867	0.800936911	0.926723951	1.781232008	0.17240147	0.090036552	2.436218363	1.244032389	3L	-0.691374184	0.588518365	NA	
CG34279	FBgn0085308	5.238252436	2.196608402	4.084690933	2.835111583	4.221601875	2.38110705	0.715869348	2.640098254	3R	-0.497900243	0.526825284	0.984107922	
CG34282	FBgn0085311	0	2.737630391	0.282819187	2.717997025	4.209101843	2.527930709	0.892187102	3.543455198	3R	1.016977356	0.415396125	NA	
CG34293	FBgn0085322	45.85671828	53.12216375	56.22980408	33.77432635	70.51896215	73.56277096	66.26401551	57.91351011	3R	0.53816059	0.012085525	0.331790969	
CG34295	FBgn0085324	0	2.684340984	0	1.39917214	3.972400619	0.848693275	1.020623582	0	3R	0.570376821	0.738750392	NA	
CG34299	FBgn0085328	4.33894737	0.673886622	1.169580644	0	1.595593199	1.969612222	0.204976814	0.174449192	3R	-0.641704242	0.63808722	NA	
CG34325	FBgn0085354	0.311039377	0.772152882	0.482929401	0.087021192	0.291983013	0.727250141	0.222171077	0.081035434	X	-0.276448364	0.764251077	0.995787491	
Diedel3	FBgn0085358	0.796709397	0.16036419	0.695810118	0	0.632836449	2.013035778	0.081296912	0	X	0.724910669	0.62110145	NA	
CG34330	FBgn0085359	4.591511589	4.995643177	2.408420124	6.075774724	1.568161797	9.125681844	3.165687759	6.735538304	X	0.215169692	0.775754841	0.995787491	
Vml	FBgn0085362	1.54754395	1.112480519	2.359858309	1.030868703	1.277126154	0.416861424	1.804716738	1.055956368	X	-0.387993838	0.626563496	0.990047511	
Drgx	FBgn0085369	0.575115186	1.142768898	1.123332117	0.653239198	0.654883735	0.906472398	0.925421216	0.633918987	2L	-0.124491811	0.695958268	0.995787491	
Pde11	FBgn0085370	0.730767647	0.737449233	0.749490001	0.360143811	0.388795366	0.691763937	0.484996154	0.141887856	2L	-0.568493616	0.143981275	0.8037297	
CG34342	FBgn0085371	0.389852988	0.416133261	0.25793943	0.055086519	0.405209293	0.24503401	0.060274142	0.025648695	3L	-0.566201079	0.60649053	NA	
CG34345	FBgn0085374	0.385483959	0.775914316	0.654625096	0	0.069589735	0.363431922	0.78670229	0.083692136	2L	-0.454225918	0.757135098	NA	
CG34347	FBgn0085376	1.465295205	1.34281288	1.321339867	0.966557441	1.483911418	1.14291485	0.831476973	1.474257051	3R	-0.014893142	0.946610464	0.997604378	
CG34348	FBgn0085377	21.61446862	21.19123197	23.30026321	20.60600715	22.35668643	23.04913512	15.1370802	21.81931977	X	-0.042422719	0.800974246	0.995787491	
CG34351	FBgn0085380	2.172949069	1.356332848	1.098238361	1.856132159	1.645743043	2.387140175	1.457489764	1.321761993	2L	0.089518061	0.744887051	0.995787491	
CG34353	FBgn0085382	10.01647344	11.97498536	12.66962143	12.1042051	11.02017851	9.913155859	11.10939039	11.41619444	3R	-0.072401749	0.489711704	0.973549494	
CG34354	FBgn0085383	15.55957679	17.54724601	15.50103797	17.79755748	16.91987161	17.38239356	16.64917967	16.78621641	3R	0.059014571	0.505168089	0.978557094	
CG34355	FBgn0085384	5.418730201	5.256476485	6.341121366	4.429050554	5.080522453	5.796802241	6.028715437	4.399811255	3R	0.020602871	0.882980203	0.995787491	
bma	FBgn0085385	33.88295935	40.90745281	37.80989244	36.92024957	34.2156031	30.25557855	33.00771654	31.26008194	3L	-0.182912102	0.048992262	0.582455792	
CG34357	FBgn0085386	15.66160161	12.94171313	13.62678307	15.38333949	13.9781628	14.29018726	13.91139856	14.51005509	3R	0.004707563	0.964053279	0.997604378	
shakB	FBgn0085387	6.354199983	7.639191969	6.797323558	6.835438527	6.242327357	6.490582021	5.38637064	7.090390254	X	-0.099866959	0.350440032	0.932790554	
Dgk	FBgn0085390	13.63513716	10.36081783	9.790131315	12.78696228	12.51678473	13.89566294	12.16883693	13.99488219	2R	0.200949934	0.125663295	0.780967863	
trv	FBgn0085391	84.33003811	76.77880281	65.29002545	77.21704297	86.19816218	85.37795084	69.76463942	86.64223381	3R	0.140174639	0.205203108	0.855081349	
Shawl	FBgn0085395	29.40581956	25.45417045	29.22871057	28.96796223	29.61604847	30.60433498	27.57689781	29.87715198	2L	0.086459519	0.363062779	0.939549067	
CG34367	FBgn0085396	1.502298611	1.655931111	2.152023234	0.967371686	2.272942806	1.173553734	1.970946531	1.801661927	2L	0.23622569	0.584972596	0.987145178	
Fili	FBgn0085397	0.939359115	1.009977464	1.259749179	1.13448886	0.876330592	0.899924007	0.815643637	0.76257157	2R	-0.339088248	0.050926633	0.594429359	
ppk9	FBgn0085398	1.347780436	3.536560315	1.16543639	0.062223749	0.313169887	0.490658291	0.170208755	0	2R	-2.590860782	0.006173522	0.227193262	
CG34370	FBgn0085399	12.11213204	14.09415813	16.32125491	11.98425373	15.32074661	14.89635726	14.21995749	11.6579027	2R	0.073210236	0.553712732	0.984934163	
side-V	FBgn0085400	3.222729093	3.472543802	3.452567646	3.130696483	3.878567994	3.674353429	4.105231055	3.613199204	2R	0.23243523	0.067005721	0.654151708	
CG34375	FBgn0085404	1.371199322	0.250908491	0.342731378	0.155001193	0.27003995	0.564112732	0.508794026	0.090212141	3R	-0.567613355	0.482395691	0.973549494	
CG34376	FBgn0085405	14.7353727	12.2204358	14.30244574	11.25476373	11.27333895	16.20997446	13.62471971	13.69168279	3R	0.088772981	0.557426243	0.984934163	
Pvf3	FBgn0085407	2.637542829	1.811638079	2.334162493	1.771496251	2.288749966	2.573115965	1.789791666	2.082596253	2L	0.056132228	0.752760708	0.995787491	
Shrm	FBgn0085408	0.795554661	0.702157138	0.582370439	0.656814004	0.927607571	1.365416895	1.022330076	0.719317231	2R	0.582574355	0.02152457	0.416208265	
smal	FBgn0085409	1.603389557	3.516288601	3.390434395	2.570041644	3.31501812	3.577439018	3.012436835	4.233778969	2L	0.391966838	0.08216429	0.694684542	
TrissinR	FBgn0085410	0.674164534	0.208424989	0.282206295	0.073966552	0.400907219	0.810076144	0.236052107	0.206636274	2L	0.420447543	0.487950816	0.973549494	
CG34382	FBgn0085411	0.345969754	1.87283712	0.457809708	0.659958101	0.45422842	1.764327378	1.083161792	0.1707119	3L	0.093446821	0.906100913	0.996479182	
kmr	FBgn0085412	13.18187495	19.29659764	15.57528605	17.49174514	16.65798009	16.54066854	16.96775356	19.65731311	3R	0.125510369	0.317694049	0.91460807	
CG34384	FBgn0085413	7.299990091	9.20654834	8.450249373	9.484346319	8.963414246	8.277571908	8.207225475	10.28486215	3R	0.086551051	0.504954342	0.978557094	
dpr12	FBgn0085414	97.19876336	122.0617891	120.3615242	129.9005553	113.5082352	110.158455	126.0845954	111.5355502	2R	0.006489748	0.951320128	0.997604378	
CG34386	FBgn0085415	0.502287587	0.600467891	0.41740136	0.187629135	0.465903195	0.633149105	0.375200887	0.361495972	2R	0.133353417	0.748259154	0.995787491	
Rgk2	FBgn0085419	90.39348674	170.8209806	110.4192229	140.6786774	105.1619022	143.2063986	131.9809097	127.174428	2R	0.019031901	0.904788969	0.996479182	
DIP-delta	FBgn0085420	0.786147008	0.611663564	0.356160434	0.165620978	0.589907119	0.486674886	0.169136691	0.4729681	3L	-0.134766263	0.813929426	0.995787491	
Epac	FBgn0085421	11.01442434	13.43041298	13.00685627	11.34170283	13.59341598	20.14410512	17.63731791	15.686534	2R	0.48865833	8.6E-05	0.008236301	
CG34393	FBgn0085422	0.907676227	0.898618147	0.720206234	0.421651453	0.99793719	0.627336701	0.356900616	0.400056586	2L	-0.275360936	0.502834087	0.978202447	
GramD1B	FBgn0085423	57.37312252	65.52446823	62.78935343	71.66575153	58.83441242	70.98685421	58.88600406	59.91962673	2L	-0.021008944	0.839744327	0.995787491	
nub	FBgn0085424	0.186022892	0.261816015	0.295672066	0.179464291	0.223879332	0.316492595	0.261819804	0.143908741	2L	0.073368617	0.865508219	0.995787491	
CG34396	FBgn0085425	1.050941774	0.645313421	0.933324763	0.065631192	0.186334034	0.65464991	0.119686386	0.407444978	2R	-0.946344178	0.237796656	0.873280735	
Rgk3	FBgn0085426	23.44695213	40.21769971	39.9036193	44.83024096	37.81772086	38.49518195	40.00125304	41.9941282	2R	0.126802571	0.420954197	0.960593359	
CG34398	FBgn0085427	0.646999077	0.517580858	0.458809292	0.812244457	0.458215054	0.316723904	0.184095184	0.194496215	2L	-1.047279751	0.008617141	0.277278958	
Nox	FBgn0085428	1.757553378	2.569689972	0.912042777	0.854594182	1.018021702	1.329152879	0.551453464	1.305946038	2R	-0.501915743	0.288755962	0.903033179	
CG34401	FBgn0085430	22.30339125	31.086636	34.78054849	29.27105197	26.82927527	32.06876509	25.00061206	30.87392706	X	-0.000494133	0.997094664	0.999490563	
CG34402	FBgn0085431	315.1764003	286.2961567	266.1730877	293.1769049	278.1536791	274.2308796	284.8939911	252.6952649	3R	-0.061787405	0.504035432	0.978429306	
pan	FBgn0085432	30.48906742	32.51761447	45.82902937	31.12962922	32.51638874	37.08674382	35.19687778	37.1811066	4	0.052745593	0.679995088	0.995787491	
CG34404	FBgn0085433	167.9442706	202.9339985	174.8684374	209.0275647	166.8491117	174.7368314	167.3614814	180.6386984	3R	-0.099196033	0.348719052	0.931729531	
NaCP60E	FBgn0085434	33.35017109	53.53830721	54.19199635	53.77331495	46.46884557	49.88669804	41.81452006	56.89290513	2R	0.035169761	0.812135619	0.995787491	
Not1	FBgn0085436	99.38098381	105.163222	94.16110888	119.4473361	94.10538473	109.8684033	99.20316788	107.4293692	2R	0.002439255	0.981605808	0.997881056	
CG34408	FBgn0085437	40.54858944	44.891655	37.48643889	39.95226811	42.92366382	38.90826107	41.19010928	51.49024041	X	0.132311053	0.288952848	0.903033179	
CG34409	FBgn0085438	20.45084081	14.77877773	14.51073124	17.35935636	12.36607588	15.88804608	12.15624099	18.05124265	3R	-0.173808743	0.433973792	0.965402206	
Lgr4	FBgn0085440	0.258750315	0.566339718	0.352138221	0.182922749	0.19368002	0.428396868	0.136845359	0.219227663	X	-0.433338786	0.419180481	0.960266449	
NKAIN	FBgn0085442	95.3108077	122.4189038	129.4214722	123.9775087	97.4165703	118.1652492	127.6860948	138.3794881	2R	0.065013311	0.623375995	0.990047511	
spri	FBgn0085443	4.75454257	5.927217055	6.734675449	4.358617917	4.936059502	3.700633265	4.458555795	4.896999756	X	-0.238021222	0.113254593	0.758410631	
mute	FBgn0085444	17.3982129	21.61631049	11.90863544	18.83295069	16.62392925	21.12462908	13.82220121	16.8899856	2R	0.001112758	0.995430809	0.999027018	
CG34417	FBgn0085446	1.165003056	0.750433638	0.542680715	0.421401287	0.772116522	0.889146762	0.716737033	0.668271383	X	0.100407209	0.7455543	0.995787491	
sif	FBgn0085447	2.471406001	2.819087224	2.972587402	2.202595586	2.389369751	2.245411606	2.446169324	2.282863069	3L	-0.12751789	0.315875572	0.91460807	
Snoo	FBgn0085450	7.162542628	9.497607016	9.037175146	8.498330897	8.574196913	9.342063102	7.063068752	8.570894824	2L	0.004371002	0.969654934	0.997604378	
htk	FBgn0085451	44.94631065	63.38192411	58.2242724	67.31572631	56.82850082	51.34164768	57.05771238	59.92509288	X	-0.021121223	0.87166979	0.995787491	
CG34426	FBgn0085455	3.049938227	4.476363073	0.92488845	0.711081616	0.963536034	1.293958409	0	0	3L	-1.951550587	0.128292418	NA	
CG34435	FBgn0085464	1.516957017	2.671706717	1.840058282	0.176836409	0.13692481	3.718462304	0.580468491	1.152708937	X	-0.11253785	0.922607926	NA	
CG34436	FBgn0085465	1.700151516	1.200743436	2.387893816	0	0.484611984	3.880687019	0	0.582818893	3R	-0.064899023	0.964488731	NA	
CG34437	FBgn0085466	2.841353982	3.755008796	4.595390087	0	0.466306561	2.922339468	0	0.934673109	3R	-1.320047669	0.300019037	NA	
ND-MWFE	FBgn0085468	13.07230741	9.438127046	11.03069059	5.167939618	7.797885921	8.895067837	9.424360474	9.007942749	2R	-0.117344619	0.755757359	0.995787491	
CG34443	FBgn0085472	0.875735939	2.136616996	2.124523174	0.185613216	0	0.90069654	0.812371344	0	2R	-1.589699497	0.30095455	NA	
Zdhhc8	FBgn0085478	14.83916728	11.46965696	11.47429691	11.88673612	11.83150542	12.01299762	9.731962318	12.20151077	X	-0.09033229	0.492249598	0.973549494	
CG34451	FBgn0085480	0.658601136	0.286406559	0.473409531	0	0.176139829	0.275966723	0.062226135	0.211834671	3L	-0.935691191	0.493221842	NA	
CG34452	FBgn0085481	0.391179174	0.562412732	0.406712237	0	0.201765334	1.159089912	0	0.970612797	3L	0.80924972	0.622431505	NA	
Pdxk	FBgn0085484	54.50024031	44.27724028	47.23768624	42.9401012	42.03639277	70.73372747	59.40194819	44.12675141	3L	0.219044617	0.266068792	0.893759496	
CG34459	FBgn0085488	0.04365839	1.464616489	0.529573225	0.610727356	0.4728875	1.111343311	0.445493963	1.042650466	2R	0.272957035	0.810443998	NA	
CG41284	FBgn0085599	0.048682448	0.204144943	0.088577205	0.056750647	0.483363627	0.17211568	0.062094985	0.237811728	3L	1.316097002	0.254337623	NA	
CR41320	FBgn0085612	0.964696496	0.975172295	0.821443927	0.551117098	0.703529735	1.060091084	0.603017053	0.693524965	3L	-0.084585177	0.833371493	0.995787491	
CG41378	FBgn0085638	0.964593711	1.16684235	1.470127688	0.783151202	0.880912606	1.207914522	0.631096992	0.586382227	2R	-0.375219555	0.134469399	0.795364069	
CR40190	FBgn0085732	1.199791013	0.670827037	2.021302961	1.087826281	0.18049419	0.596998809	1.105250124	1.013000082	2R	-0.760672684	0.362852281	0.939549067	

**CG40472**	FBgn0085736	46.74398429	20.54099374	47.46144812	37.95214225	32.08242738	33.08765678	38.09741475	36.31430638	3L	-0.105311886	0.752309203	0.995787491
**CR41379**	FBgn0085784	2.718145784	6.990939293	4.835734756	8.449672759	12.75806947	18.28024553	12.01901704	13.7698076	2R	1.355878759	0.023311979	0.432699404
**CR41501**	FBgn0085786	6.363087536	4.519277247	3.893957883	1.871118799	3.725514762	6.053125828	5.410791003	3.484827548	2R	0.18067983	0.73531183	0.995787491
**CR41504**	FBgn0085788	0.329764895	0.184378116	0	0.512556292	6.349974963	8.394308866	3.925774211	7.159501121	Y	4.63952569	2.69E-06	0.000546045
**CR41506**	FBgn0085789	2.503808663	2.497171114	1.313341599	2.051026036	1.832439064	1.722582132	1.035773464	1.616108222	Y	-0.403632857	0.599481804	NA
**18SrRNA-Psi:C**	FBgn0085813	0.877591141	1.840045032	3.087084609	1.636856567	1.980346168	1.075603838	1.902940777	2.095864766	X	-0.029186937	0.965893948	0.997604378
**snama**	FBgn0086129	60.55271812	58.54567533	79.27630914	79.36038862	65.40870352	70.02502151	65.30925026	73.86681841	2R	0.014050552	0.917454463	0.996479182
**Dbp21E2**	FBgn0086130	13.35927285	14.39956132	20.3582573	8.882977247	11.66666475	6.865888692	15.62503102	14.34504646	2L	-0.197331095	0.577007806	0.985637351
**Prosalpha2**	FBgn0086134	291.7667222	338.9314676	339.6184396	329.9833256	340.591319	308.9975009	326.885073	320.0762707	3R	0.028322272	0.785517913	0.995787491
**del**	FBgn0086251	1.016347852	7.245319001	8.568118848	8.352758061	3.853013556	6.683490661	3.305724636	4.964816853	2L	-0.381720162	0.448642536	0.967112302
**rumi**	FBgn0086253	5.307624654	18.81518694	1.194701138	11.8642373	18.47178835	9.646879087	15.49407177	19.48276793	3R	0.805629605	0.139905321	0.803344611
**Akr1B**	FBgn0086254	9.301439275	11.86194646	11.14598798	16.48762719	13.53889455	23.46276234	15.51236389	8.312417838	3L	0.342655285	0.236525052	0.872984846
**psd**	FBgn0086265	4.600432574	0	2.02919732	0.108340761	4.613866599	1.620996061	2.015238785	0	2L	0.299834566	0.813806742	0.995787491
**ALiX**	FBgn0086346	172.1192966	148.3600234	128.0183688	168.6957225	145.7261749	178.6101341	139.5887604	130.5659332	3R	-0.029442576	0.822595241	0.995787491
**Myo31DF**	FBgn0086347	6.442105406	4.902868537	5.012901578	3.805486432	5.161767987	5.631565239	4.296746851	4.297710022	2L	-0.030959796	0.877618934	0.995787491
**Tpi**	FBgn0086355	1923.749479	1147.952283	1040.283432	1304.16939	1700.334326	1517.228702	1403.086815	1431.807596	3R	0.185714987	0.251800534	0.882837883
**tum**	FBgn0086356	1.637524928	2.346155252	5.710630661	12.32840202	2.832979181	1.029232333	0.870283598	2.370145468	2R	-1.599530879	0.014122761	0.354312909
**Sec61alpha**	FBgn0086357	144.92537	216.5432674	162.1994293	196.7095274	175.0788503	157.838773	212.3686658	190.5503439	2L	0.065585295	0.646106246	0.99279236
**Tab2**	FBgn0086358	1.894480371	0.933911561	1.789275807	0.87663856	2.036349346	1.697481015	0.866963458	1.240207206	2R	0.115013245	0.774379267	0.995787491
**Invadolysin**	FBgn0086359	17.62782491	22.0455864	26.87329692	15.64930095	11.17687336	14.93714245	20.52930786	9.034914936	3R	-0.529770061	0.015027393	0.361837042
**alph**	FBgn0086361	37.15630659	38.95280737	36.05959149	40.93006671	37.90349725	42.04398053	37.80468172	39.43641454	3R	0.067317228	0.454979755	0.967967712
**spn-F**	FBgn0086362	90.91523433	93.0348868	95.87736579	92.53715379	95.63768998	87.20686565	87.54945064	83.59418555	3R	-0.042260371	0.73910929	0.995787491
**Orct2**	FBgn0086365	8.440384811	5.329272182	6.695657798	7.482296367	4.866588815	10.71091372	5.239629624	4.877338296	3R	-0.097761663	0.788315644	0.995787491
**tw**	FBgn0086368	0.249692262	0.335058993	2.241274795	0.407503676	1.262123879	2.071593386	2.102002071	0.542105014	X	0.942606495	0.254110755	0.884241768
**sra**	FBgn0086370	141.8215225	126.7460984	125.2867088	146.7145778	121.1945363	135.9458118	125.564904	121.9478469	3R	-0.071820638	0.462063325	0.971606621
**poly**	FBgn0086371	45.74069116	48.17328921	45.54155084	36.63117262	33.3057371	47.90135471	43.29074903	44.48522899	3R	-0.029532016	0.836218127	0.995787491
**lap**	FBgn0086372	57.27970743	60.83697867	88.44127263	60.82885916	52.8850997	50.38441463	80.38835866	69.31935725	3R	-0.043963104	0.793952916	0.995787491
**Alg-2**	FBgn0086378	73.05497516	58.88924311	73.80361673	48.63254034	50.13959503	60.68612133	51.60871694	47.89292142	3R	-0.246136654	0.098667199	0.730787365
**Mer**	FBgn0086384	22.36632059	27.96535567	27.62748564	29.41854776	26.06820842	18.70328851	27.4245287	18.93913644	X	-0.204071477	0.282153737	0.901329548
**stl**	FBgn0086408	1.672084663	1.714968124	0.361722019	0.198644711	0.442206697	1.134628831	0.923744029	0.439329689	2R	-0.406683098	0.555664261	0.984934163
**mib2**	FBgn0086442	181.133068	215.5499428	188.3128661	206.0731957	156.7426943	183.0718279	209.7564101	183.6840612	2L	-0.078087076	0.477097762	0.973549494
**AsnRS**	FBgn0086443	185.5059571	207.3232125	162.6112447	254.1202529	183.2999488	150.0827442	195.356184	183.2129302	2L	-0.154315412	0.306406393	0.911610813
**l(2)37Cb**	FBgn0086444	29.49325087	40.44505484	30.48293193	29.90554262	43.476432	40.25571138	21.44354822	35.36315933	2L	0.142220965	0.50181784	0.977207013
**l(2)37Cd**	FBgn0086445	5.324548498	12.44739698	5.695808676	6.158118235	2.421965281	4.901370262	6.844995332	9.011406929	2L	-0.317433369	0.495184008	0.97491773
**DCTN6-p27**	FBgn0086446	62.44571198	96.67225737	109.1397049	86.62333773	97.78058992	94.21859891	80.50662993	67.78771062	2L	-0.023606378	0.911606542	0.996479182
**l(2)37Cg**	FBgn0086447	5.447934907	7.823305309	3.993509295	5.48272884	5.872649356	6.097022729	7.598796572	4.339759141	2L	0.098154885	0.794885196	0.995787491
**PIG-Q**	FBgn0086448	33.19282678	25.89595834	25.9218642	12.5980182	21.70964705	22.40757245	11.91884411	20.99314381	X	-0.31239984	0.294065005	0.906715248
**su(r)**	FBgn0086450	1.192469862	4.406239679	8.427187911	2.699613126	1.591136234	2.232213078	4.144196034	1.801017361	X	-0.733301963	0.15649855	0.818884898
**l(2)k09022**	FBgn0086451	6.676850007	10.87454268	10.90306549	9.243742945	10.14166364	7.305485575	11.43206455	6.505001526	2L	-0.055246288	0.81857173	0.995787491
**RpS25**	FBgn0086472	2201.025349	2560.980697	2505.592893	2454.484299	2330.36957	2181.489403	2536.271085	2673.116984	3R	0.033610073	0.740779729	0.995787491
**Cpr47Eg**	FBgn0086519	2.307453265	1.786351915	3.444830423	2.482957118	4.165544539	0.167342526	3.62237714	0.385361126	2R	-0.239870521	0.84900593	NA
**Ubi-p5E**	FBgn0086558	227.7206494	255.1880962	234.6288795	259.2299226	201.9637494	247.4355221	225.1913301	203.5296399	X	-0.124536394	0.218087183	0.861392692
**side-VIII**	FBgn0086604	5.132416145	5.129164021	6.06013372	3.627527924	4.349206136	4.210782023	4.592226783	2.975126476	2R	-0.275528093	0.105295277	0.748746451
**CG9853**	FBgn0086605	99.99347118	116.1602361	98.90045785	91.1697906	124.9315339	104.0611652	101.3311819	117.5480381	3R	0.174004091	0.149067952	0.808039863
**CG34112**	FBgn0086608	1.461767882	1.482550569	1.484467741	1.268114153	0.368213815	1.314046024	1.647698236	0.885665131	3R	-0.412373142	0.563887111	0.984934163
**Ino80**	FBgn0086613	1.262834075	1.807723848	1.445683123	2.695939856	1.854004744	2.094294805	1.753074434	2.604092644	3R	0.235797458	0.348667558	0.931729531
**jing**	FBgn0086655	4.381312928	5.088168603	6.274954199	5.568526639	5.357344095	6.484730357	5.965645635	6.852766112	2R	0.242263752	0.056763192	0.621788268
**shrb**	FBgn0086656	177.1558689	174.7095282	192.9007615	168.2512413	159.9943176	183.7882836	154.2255752	137.6384394	2R	-0.136271008	0.238775453	0.87355339
**IKKepsilon**	FBgn0086657	27.60329232	32.48473448	35.23730424	26.22422467	25.58169873	32.8990616	30.50130445	21.60018723	2L	-0.104731418	0.564839515	0.984934163
**Tpst**	FBgn0086674	0.4271843	0.711600069	0.843368912	0.576972315	0.29251547	0.508294173	0.496027018	0.29422763	X	-0.648433264	0.081995466	0.694684542
**fne**	FBgn0086675	78.86888408	65.24374697	75.7862693	57.9050123	70.00488355	79.52116858	60.68823331	64.39812593	X	0.011005865	0.925435577	0.996479182
**spin**	FBgn0086676	15.85093641	13.86375455	18.89420757	17.79243456	12.05463019	15.43696622	15.71773405	15.19259423	2R	-0.156142399	0.28979443	0.903534148
**jeb**	FBgn0086677	35.47832202	48.57039902	43.90267189	45.59277228	38.38909573	38.0860338	40.95611551	41.21750194	2R	-0.096044191	0.374576611	0.943282319
**p**	FBgn0086679	54.7729117	49.42027224	52.46384106	67.14366894	53.20449322	64.43048267	51.01104003	49.45103999	3R	-0.011749954	0.927470721	0.996479182
**vvl**	FBgn0086680	3.78490766	8.535746929	7.556588082	3.377749177	2.026933781	1.843951603	4.712195394	3.145410486	3L	-0.949960628	0.020669337	0.408194924
**Mst36Fa**	FBgn0086681	2.195283361	1.440892123	2.083980057	1.446453623	2.584597247	0.269960836	0.730462828	0.103612248	2L	-0.926904207	0.338473364	0.926687587
**Spf45**	FBgn0086683	234.4765076	211.7850942	199.4333539	205.0191268	180.4645456	228.0303119	171.5693892	209.6976209	2L	-0.079557935	0.523431735	0.983811292
**l(3)L1231**	FBgn0086686	125.4782559	141.7256514	136.2395572	191.2181545	139.1657207	142.0966407	138.2640582	134.2084821	3R	-0.074513801	0.558479659	0.984934163
**Desat1**	FBgn0086687	494.1432601	468.5492323	460.9540653	484.6379592	482.8178233	514.8059015	474.2367709	470.2813343	3R	0.054140191	0.496197314	0.975846898
**Plp**	FBgn0086690	3.671212869	7.476779484	6.45597518	5.576937421	3.772313662	5.995796899	5.045250625	5.482210369	3L	-0.156346955	0.440552846	0.967112302
**Bre1**	FBgn0086694	100.0251055	131.763764	95.59898121	143.9701397	101.113996	106.8118836	111.3692002	97.32696856	3L	-0.148112886	0.291134906	0.905069205
**hd**	FBgn0086695	4.42228174	2.390847373	2.925925587	9.799124716	4.090643154	5.582038301	1.398512693	2.856550321	3R	-0.465279749	0.408959931	0.957379793
**frtz**	FBgn0086698	1.523292955	0.702655315	2.078711697	2.9743814	0.790606083	0.107711347	0.777190611	0.702782128	2L	-1.598005485	0.024227937	0.44079275
**pix**	FBgn0086706	237.6965811	313.6418092	262.261082	290.1359987	234.8696696	299.1301094	259.546197	275.1771479	3L	-0.01567139	0.889597266	0.995926915
**ncm**	FBgn0086707	46.83224697	42.83604987	40.27974413	32.62921835	45.22377253	51.45696197	46.92402927	38.49870225	2L	0.191032916	0.189829279	0.843401316
**stv**	FBgn0086708	295.5227792	213.1194982	253.5052669	269.048811	410.0279228	326.7209605	322.3855019	365.3466018	3L	0.494971222	0.000117195	0.010616299
**RpL30**	FBgn0086710	93.34389766	198.2780572	181.9518902	170.2691901	168.8775841	117.4668411	175.0389456	191.7436065	2L	0.061555036	0.772748681	0.995787491
**mol**	FBgn0086711	0.861734793	0.686084057	0.346820448	0.536995066	0.086026848	0.453018591	0.435608621	0.41384096	2L	-0.79043218	0.135031121	0.795862348
**Egm**	FBgn0086712	104.2716393	104.0772524	90.43399532	93.19196651	105.9876856	101.7945987	74.27050326	107.6477859	2R	0.021907166	0.879960818	0.995787491
**cbs**	FBgn0086757	59.55582974	80.48659821	49.93224631	75.46939462	64.56382853	74.30270486	55.65452759	65.11957785	2R	-0.001846373	0.991080608	0.998408391
**chinmo**	FBgn0086758	13.94510952	11.81650303	11.98488125	10.03612159	13.78289796	13.3032798	10.67517744	11.11990171	2L	0.061218788	0.628870731	0.990523437
**Pcmt**	FBgn0086768	335.1598159	289.6067705	254.469831	328.4177833	275.9745691	325.6133619	348.8375875	318.5493666	3R	0.09846808	0.443556057	0.967112302
**nAChRalpha7**	FBgn0086778	55.64610297	54.40555473	51.55043984	50.6001845	44.77695007	45.38624978	37.19970476	37.21176279	X	-0.337839333	0.000695368	0.046361561
**step**	FBgn0086779	9.902068598	12.14015355	13.65121551	10.36102026	10.1403725	12.59276281	11.38746485	11.93630931	2L	0.033456253	0.807895711	0.995787491
**stmA**	FBgn0086784	720.2734625	861.8762347	822.7283764	941.4809444	835.1139423	775.0686916	872.1261455	918.7811743	2R	0.055978306	0.605899736	0.989286463
**Vps36**	FBgn0086785	62.6889213	58.96829718	56.90549682	51.79545653	47.46508826	50.63561119	54.26152933	40.15115949	3L	-0.231928503	0.169730946	0.830934498
**CG17078**	FBgn0086855	93.65876704	105.7448075	103.082656	98.14147391	109.4666441	109.8736353	103.336239	114.5345324	2L	0.158105901	0.112531214	0.758410631
**CG11555**	FBgn0086856	44.5199155	51.24204891	58.06946939	33.22356815	47.07857782	66.20862075	59.39919504	48.53063986	2L	0.274303878	0.231338468	0.867702781
**pea**	FBgn0086895	29.40594122	27.60251879	34.76551097	32.38920097	32.51571103	31.07042297	38.56965693	17.31658677	2R	-0.027401297	0.897045522	0.996479182
**Nacalpha**	FBgn0086904	1369.974335	1595.964552	1629.750328	1748.250234	1510.999881	1291.791023	1515.96206	1539.44039	2R	-0.081952338	0.457462378	0.968365245
**sls**	FBgn0086906	1.432800165	1.681942198	1.087619119	0.630336908	0.508664941	1.142187125	0.439427313	0.718244522	3L	-0.754361297	0.013862271	0.35306645
**Cyt-c-d**	FBgn0086907	2.568008885	1.629855273	0.785759931	0.3236333	1.085889816	1.48319829	0.472147448	0.853887794	2L	-0.42860005	0.559788863	0.984934163
**egg**	FBgn0086908	1.36859605	1.896388248	1.15485041	1.43356138	1.145816412	1.196803707	1.517964316	1.464142844	2R	-0.110542132	0.821149628	0.995787491
**CG31751**	FBgn0086909	23.64862173	28.63737963	30.80508043	19.96459638	18.68519735	28.70911274	28.51380885	18.12799502	2L	-0.101595157	0.58945289	0.987597375
**mbm**	FBgn0086912	9.992565221	7.897069142	5.244625352	9.632533216	6.677951346	6.83923244	5.392379504	4.693583098	2L	-0.449826824	0.235389142	0.870926212
**Rab26**	FBgn0086913	142.0170436	226.5926166	196.6238753	199.2881808	200.3066842	192.7095654	206.8530462	190.1852116	3L	0.081524476	0.505053333	0.978557094
**snky**	FBgn0086916	0.865054125	0	0.54746328	0	1.15425891	1.13470295	0.095946594	0	3L	0.754438965	0.659261928	NA
**spok**	FBgn0086917	0.317202819	0.146776063	0.218917908	0.325145414	0.133285124	0.211402277	0.097661008	0.32059087	3R	-0.377310145	0.493498754	0.973549494
**apolpp**	FBgn0087002	5.594272121	8.037999269	11.52234926	7.394655657	2.129153927	3.571260306	7.697995778	4.452263528	4	-0.829613117	0.060826496	0.628203686
**bbg**	FBgn0087007	0.510105183	0.483719232	0.321718051	0.265013957	0.326801394	0.538476172	0.144985463	0.193467218	3L	-0.37009234	0.296442703	0.906715248
**e(y)3**	FBgn0087008	66.70945845	58.07419346	70.76779161	90.88562659	71.78833096	76.28848639	68.95693305	76.28200831	X	0.061290017	0.65109836	0.993444251
**CG41520**	FBgn0087011	0.628248386	0.652191661	0.524220244	0.211469527	0.40574133	0.552066958	0.255203046	0.36778375	2R	-0.320860416	0.301730019	0.908861141
**5-HT2A**	FBgn0087012	4.022742249	2.026408798	2.961670787	2.450959204	2.487013966	1.618556177	2.141091862	0.855888683	3R	-0.667147023	0.035299373	0.515210107
**Karybeta3**	FBgn0087013	176.4163094	189.1062915	177.3493295	196.1274823	171.2070331	182.123221	187.9500433	217.5004225	3R	0.069611176	0.516571763	0.980804634

AGO2	FBgn0087035	136.1715327	154.2434571	132.8315475	135.8749817	122.6753029	183.6802191	156.2285412	143.6815816	3L	0.145002221	0.214286467	0.858753183
Sbp2	FBgn0087039	49.61267666	43.66162197	55.59213068	38.90249594	57.96866592	52.22806098	77.37558842	63.59778308	3L	0.449695075	0.029107362	0.472866974
alphaTub67C	FBgn0087040	3.049357839	4.008952157	3.898787619	1.921476488	5.430482275	5.283629304	6.517521572	2.862892171	3L	0.673175638	0.146676107	0.808004944
rdo	FBgn0243486	0.332306545	0.783369616	0.388559917	0.5165068	0.635027674	0.900374858	0.389493495	0.243739281	2L	0.132857448	0.728735981	0.995787491
psidin	FBgn0243511	49.58160357	46.64604272	48.46554849	63.35680645	49.61366478	73.35668738	50.59349437	65.6110788	3R	0.22736026	0.150813627	0.810890819
puc	FBgn0243512	24.11849865	27.52988549	29.34848482	24.90705685	21.05558406	21.00074714	22.83643761	22.46208707	3R	-0.24513886	0.02194048	0.420318478
cnir	FBgn0243513	115.5202769	73.38174235	99.52625177	70.86584575	86.90642712	68.24588033	94.34712712	73.62039003	2L	-0.12657714	0.548707614	0.984934163
Vrp1	FBgn0243516	70.90536426	62.63670289	55.3718546	59.80483761	70.6341134	75.48940863	54.15690474	66.86824664	2R	0.13047632	0.279396925	0.898067854
Prosbeta7	FBgn0250746	229.3589641	196.3348736	219.1493082	222.5601306	242.0698989	185.0105306	224.56059	212.536922	3R	0.024744492	0.850739807	0.995787491
kra	FBgn0250753	519.664454	453.0620086	488.9671798	443.6050384	459.4600734	469.6621663	497.5158026	449.7450089	3R	0.007661028	0.933747043	0.996479182
CG42232	FBgn0250754	22.02171685	28.82058509	27.11831761	26.45201123	26.74591548	20.51084549	21.9547941	28.63054077	3R	-0.058157795	0.690233108	0.995787491
CG42233	FBgn0250755	21.37831612	15.18800033	20.44010029	23.72271078	18.3685458	23.19384021	27.87510347	23.59032786	3R	0.230661003	0.219102428	0.863196763
CG42235	FBgn0250757	20.67250914	16.03742523	13.5904259	13.20490324	6.846123356	11.78595419	15.5513734	7.428918362	3R	-0.585292016	0.011373998	0.322418032
vari	FBgn0250785	1.540109618	5.704104468	5.767418953	3.181029549	3.881213814	1.646906067	4.693528758	1.52599327	2L	-0.417702772	0.339669573	0.927274752
Chd1	FBgn0250786	38.46397773	50.70282979	57.21347937	53.57378292	43.70490288	52.36408274	56.53411734	53.34519926	2L	0.075369913	0.585977939	0.98718792
beta-Spec	FBgn0250788	344.7910048	380.2676106	365.5772097	368.5506562	336.9101887	367.7802417	390.9565021	366.8499177	X	0.034422678	0.683288701	0.995787491
alpha-Spec	FBgn0250789	109.559764	129.8405518	125.7963388	143.2802225	102.0389571	130.1917106	125.9302433	139.4985769	3L	-0.000538545	0.996380788	0.999258422
alphaSnap	FBgn0250791	320.5316268	307.6002968	289.1224534	309.3323342	342.8417785	312.9820058	296.3707205	310.5960951	3L	0.071775945	0.450831837	0.967112302
UQCR-C2	FBgn0250814	2294.707826	2138.840712	2100.394765	2387.286002	2275.64032	2182.300834	2236.784158	2337.405485	3L	0.047720984	0.60248642	0.989286463
Jon65Aiv	FBgn0250815	1.944491229	4.447651706	1.072115591	4.945645198	0	103.4955469	0	2.110835229	3L	3.06643759	NA	NA
AGO3	FBgn0250816	0.14816186	0.135556765	0.110476801	0.061310761	0.064841244	0.134243781	0.044177621	0.072411264	3L	-0.49927486	0.260255695	0.890556245
lncRNA:CR404	FBgn0250817	1.368376708	2.524785961	2.655730247	0	1.482162403	0.12900972	0	0	2L	-1.95902132	0.307069739	NA
CG33521	FBgn0250819	2.736450049	2.536585066	3.756044145	2.462429229	2.166663897	3.586977896	2.235062409	1.797960348	4	-0.197556352	0.548422336	0.984934163
meigo	FBgn0250820	42.42020049	60.12715591	57.97509835	50.41449861	40.97666123	58.15819768	63.0725887	52.69164997	3R	0.059934053	0.7354482	0.995787491
gish	FBgn0250823	28.38760489	51.28489062	69.69375048	47.98088996	39.79848675	38.71140734	41.23093773	46.80456286	3R	-0.207863954	0.256941082	0.88674759
CG12547	FBgn0250830	124.607727	121.4642717	110.6754232	119.5449378	102.2174969	138.512261	95.34888665	72.80376214	2R	-0.195681093	0.212537136	0.856520545
CG34461	FBgn0250833	0.955348298	1.12765897	0.686714561	0.494968576	0.063875872	0.767260015	0.270790448	2.458251365	3L	0.156998853	0.880540535	NA
Acbp3	FBgn0250836	2.745427754	9.440393321	5.328295551	0.320043073	2.973720446	6.082675415	1.750911296	2.980288898	3L	-0.319905228	0.735601196	0.995787491
roh	FBgn0250838	1532.770117	1598.334949	1791.298027	1568.072339	1693.440276	1470.181987	1749.803963	1706.812645	2R	0.061717063	0.545438477	0.984934163
CG2016	FBgn0250839	3.065075788	4.607080398	4.587038672	3.062618063	3.988249728	2.364328416	2.03092836	7.475580812	3R	0.093007885	0.830745529	0.995787491
CG17575	FBgn0250842	0.988040825	0.828649938	0.998738858	0	0.14863891	0.62101257	1.680342248	0	2R	-0.192466118	0.909587819	NA
Prosalpha6	FBgn0250843	194.5467542	214.834604	278.8900118	249.4343398	242.4687459	256.1967868	249.6257385	268.1571674	2L	0.148931574	0.264423615	0.893759496
CG4218	FBgn0250844	1.613002712	0.090186192	0.913061819	0.12535517	0.485313884	0.709673582	0.960121067	0	2L	-0.351651138	0.807995934	NA
26-29-p	FBgn0250848	185.3778522	211.1671536	200.97494	212.90756	230.5427609	188.8024176	209.6334668	223.8994253	3L	0.106517405	0.346264258	0.930285749
rig	FBgn0250850	45.0593078	41.63341862	43.81793924	48.52074027	31.0208641	29.50913931	35.55572095	29.0167584	2R	-0.488619585	9.93E-05	0.009197541
CG42237	FBgn0250862	2.532505733	1.20846088	1.4829919	0.432653061	0.512359354	1.51285236	0.835406448	0.853185162	X	-0.5890493	0.292036644	0.905069205
CG42238	FBgn0250867	5.048498921	5.127841335	5.37196185	4.489061754	6.074686502	5.689012407	4.539468514	4.855290445	2L	0.109181838	0.356800636	0.937787223
CG42239	FBgn0250868	17.28907099	20.87999364	16.77721803	24.58837907	9.675473328	20.37503815	31.31440199	37.53617354	2R	0.343979151	0.462249605	0.971606621
CG42240	FBgn0250869	31.65964239	34.76058868	37.29745949	28.26890977	35.05049652	31.41752589	41.84790133	34.92718626	X	0.149523303	0.401574516	0.954265
pot	FBgn0250871	0.281370811	1.061910142	0.661270361	0.225501875	0.492073103	0.256984848	0.201876464	0.209990714	X	-0.889645295	0.211522822	0.856520545
ttm50	FBgn0250874	144.9513093	116.3419807	148.1782476	169.9299972	151.4839419	152.0797476	142.0115843	165.1833232	X	0.104409445	0.452280631	0.967630712
CG34434	FBgn0250904	0.429275446	1.920131574	3.124248737	0	2.841491412	3.057871365	1.703474713	3.106655773	X	1.029717329	0.257676073	0.88770596
Pgk	FBgn0250906	488.2424706	343.0796434	323.3180442	389.3063829	442.1164937	370.5986846	373.9407787	391.7331606	2L	0.059802848	0.657879368	0.995750687
Cht10	FBgn0250907	1.095018253	0.722561186	0.70999523	0.452332241	0.534267188	0.576642741	0.327156722	0.564004485	2L	-0.548484733	0.142505652	0.8037297
beat-VII	FBgn0250908	23.20586954	19.89795926	18.34754396	20.54483894	24.31796427	25.4913522	18.67878318	15.65084692	3R	0.061929939	0.668941438	0.995787491
Octbeta3R	FBgn0250910	14.85262261	13.02730801	11.28659094	13.4194421	10.45468494	16.61375909	9.963587501	11.92696837	3R	-0.079527785	0.602953007	0.989286463
CG42246	FBgn0259098	3.388820252	2.046337405	3.617341492	0	0	0.766790168	0.345798131	0.294297699	X	-2.619157657	0.10596114	NA
DCX-EMAP	FBgn0259099	0.671201689	0.598222159	0.386928935	0.16526801	0.219943925	1.023347781	0.327757412	0.163518792	3L	-0.056595062	0.917316759	0.996479182
CG42249	FBgn0259101	0.145883361	0.354813366	0.389302099	0.255091173	1.02709174	0.87336593	0.186075794	0.649288876	X	1.309085521	0.038381287	0.522662821
futsch	FBgn0259108	57.25691819	78.79139721	92.04724012	82.51332613	83.97203931	72.22971073	82.31029411	91.48020719	X	0.122390335	0.365388061	0.940794966
mmd	FBgn0259110	73.22401211	79.68425212	75.28563059	78.88284917	68.44348228	75.14205913	65.63308654	74.46088664	X	-0.084076068	0.325961535	0.918470465
Ndae1	FBgn0259111	50.00651283	49.51530838	47.66498573	60.55698416	51.34332485	55.51775985	46.21451452	49.43749964	2L	-0.009329906	0.931288505	0.996479182
asRNA:CR4225	FBgn0259112	5.873289062	3.597982395	2.97360362	2.976815727	9.312017382	10.20789411	14.85260061	13.30586529	2R	1.63415035	3.75E-05	0.004116601
DNApol-alpha1	FBgn0259113	0.934969498	0.504090055	0	0.038925825	0.452105243	0.094444664	0.042591559	0.616221261	3R	-0.27926317	0.831909068	NA
glo	FBgn0259139	271.9682975	298.5949393	290.4159579	291.6091856	263.968175	279.8835134	279.4007063	233.2843669	3R	-0.09560024	0.292106297	0.905069205
CG42255	FBgn0259140	0.791497559	0.658139443	0.24617462	0.8674707	0.818233751	0.17858234	0.138059985	0.264371457	3L	-0.844114502	0.2090435	0.856520545
CG42258	FBgn0259143	59.03385365	52.9372918	52.07188377	54.63671915	58.27475094	57.9417687	55.85286023	58.31093312	X	0.104208723	0.265961948	0.893759496
CG42260	FBgn0259145	22.73180875	30.06226454	30.87647988	33.12088834	25.8721162	24.69993216	28.7536065	28.29213897	2R	-0.084879123	0.491187355	0.973549494
fid	FBgn0259146	35.00729508	41.18997797	41.4590894	42.25317081	24.27808014	25.57780801	33.85359875	31.64979209	3R	-0.43838869	0.001272265	0.074147226
OtopLc	FBgn0259150	25.07518596	51.06538952	51.44829298	45.12354812	52.68234983	60.77906696	57.11947191	53.82868647	X	0.41237211	0.016473474	0.370938755
CG42266	FBgn0259151	1.383561721	0.855006045	3.444830423	3.480384336	4.00943932	1.956620309	3.43661421	1.264774977	2L	0.257436209	0.68780758	0.995787491
Clbn	FBgn0259152	72.29647713	55.48302443	72.59510966	59.48403081	59.75634118	69.72553352	74.58249435	56.11313718	3R	0.028908784	0.841844229	0.995787491
RunxB	FBgn0259162	0.957040756	0.513086907	0.448189301	0.218893795	0.606883315	0.328365825	0.09271259	0.243289528	X	-0.72296289	0.197151324	0.84476048
CG42268	FBgn0259163	73.7057808	89.09149467	85.59743569	77.04962677	68.24769954	72.6156073	72.59692485	70.41684004	3L	-0.16491045	0.057000988	0.621788268
CG42269	FBgn0259164	1.88121166	0.691988739	0.360299467	0.038473458	0.744752017	0.248925595	0	0.107481175	3L	-1.407660163	0.224253904	0.865647077
CG42271	FBgn0259166	57.50032391	63.25894676	68.26052505	84.02697305	58.08479949	66.1354605	49.3363424	60.90066039	X	-0.190988192	0.175627317	0.834088743
mnb	FBgn0259168	16.56783651	13.19309812	14.18034069	11.808043	13.80375164	15.17386291	12.6317202	13.21186069	X	0.003206646	0.98005922	0.997881056
alpha-Man-Ia	FBgn0259170	17.48585887	14.37036171	15.29323983	15.22641999	15.54536999	15.10394284	15.27476714	16.82354093	X	0.037986516	0.719340685	0.995787491
Pde9	FBgn0259171	5.332845534	5.870668911	5.589727745	5.100899092	5.361321064	5.849052817	4.608545041	4.729841997	X	-0.061357736	0.54794669	0.984934163
corn	FBgn0259173	124.6190638	114.6884781	95.83047574	106.9680449	102.2133694	123.1210339	97.23903845	112.0698677	3L	0.003089421	0.977738072	0.997757033
Nedd4	FBgn0259174	17.86282537	22.9603525	21.06543205	20.52482035	22.01358487	21.89965486	21.77796122	20.66701551	3L	0.100232183	0.35227929	0.934700923
ome	FBgn0259175	11.90251919	15.361863	12.73483959	12.60201644	8.963725253	8.934849489	7.942830372	8.584581773	3L	-0.579084205	1.15E-08	4.34E-06
bun	FBgn0259176	72.41917251	74.88404907	73.12522659	78.83769864	69.09517219	66.71340947	67.66448238	74.94289879	2L	-0.072932166	0.409361488	0.957512951
5PtaseI	FBgn0259178	168.1024716	153.9938206	162.1826746	141.7563178	152.495341	188.2021054	156.3687582	145.9784491	3R	0.066386179	0.527103828	0.984107922
Ir7f	FBgn0259188	0.166014846	0.417699921	0.4027497	0	1.048947709	1.056495031	1.482276032	1.982384786	X	2.545140167	0.014354298	NA
Ir7e	FBgn0259189	2.673687199	1.005199268	0.670999433	0	0.249656413	0.043460965	0.940777033	0	X	-1.841509502	0.219878321	NA
CG42296	FBgn0259192	0.245934826	3.052656651	0.656297903	0.057338779	2.797044977	0.950649991	0.125477005	0.106789455	2L	0.049466387	0.964922315	0.997604378
Mlp60A	FBgn0259209	0.574106019	0.401242628	1.508837628	0.111542214	0.129551063	1.172737904	0	0	2R	-0.954062071	0.491622792	NA
prom	FBgn0259210	0.493931585	1.388298876	18.02264559	0.497981793	110.5795146	194.5076977	158.7637907	223.8357494	2R	5.118045916	3.03E-06	0.000574129
grh	FBgn0259211	0.405165945	0.529746528	0.332682961	0.242212602	0.592644528	0.395700367	0.185515621	0.451103985	2R	0.141149021	0.729469927	0.995787491
cno	FBgn0259212	26.07564779	27.90551835	29.33347298	34.43384103	28.87333696	29.37168151	28.11655365	38.4062404	3R	0.114990859	0.38088639	0.944476532
side-II	FBgn0259213	20.27298095	24.00688365	24.24244065	24.33549039	21.59830169	24.64851024	22.45755337	24.86679555	2L	0.042162654	0.663683062	0.995787491
PMCA	FBgn0259214	108.2728071	99.73670279	116.2566358	94.42036276	89.23325361	117.8991171	99.55076185	104.7456794	4	0.003373717	0.974173905	0.997757033
Ir93a	FBgn0259215	2.38736544	1.005527863	1.071594893	1.520243455	1.632790126	1.090692912	1.180483274	0.936171128	3R	-0.291501483	0.506916458	0.978557094
RhoGAP102A	FBgn0259216	9.376750616	7.555022753	8.364062597	5.937938229	7.288407847	12.19118072	5.724157278	6.418483391	4	0.041869119	0.859303181	0.995787491
CG42319	FBgn0259219	0.56958269	1.794278523	1.078477784	0.129557132	0.225711821	0.890632622	1.630214957	0.361936608	2R	-0.162470136	0.838463803	0.995787491
ATP8A	FBgn0259221	57.48043344	63.58887441	63.40789015	73.99053372	55.32936322	67.01853732	59.47963428	70.83844509	2R	-0.003117513	0.978246154	0.997757033
CG42322	FBgn0259222	185.8433208	185.9739558	161.3604332	194.922823	196.1530448	207.8702929	178.445516	197.4527841	3R	0.127850351	0.178806905	0.836045561
CG42323	FBgn0259223	0.830689365	1.646703945	0.595412059	0.088032772	0.34081982	1.548538427	1.781975851	0.450875877	X	0.409084015	0.624861488	0.990047511
CG42324	FBgn0259224	51.63797359	45.10080165	55.16253331	50.63911652	43.78942189	50.01783156	47.86195813	46.69123924	3L	-0.076066415	0.42996004	0.963666972
CG42326	FBgn0259226	0.774258264	0.827609072	0.902339684	0.230068647	0.835900109	0.472330917	0.193642105	0.824012924	2R	-0.193836033	0.746839559	0.995787491
CG42327	FBgn0259227	3.41613614	2.184443654	4.432573945	4.894315921	2.114149799	3.623873995	3.532960818	3.386594651	3R	-0.212169664	0.429890659	0.963666972
C3G	FBgn0259228	66.44036901	68.20720853	54.2407834	64.40204733	57.21086425	67.16985029	58.86779848	56.72283235	X	-0.049813887	0.624554829	0.990047511
CCKLR-17D1	FBgn0259231	30.2832424	33.54661154	32.66901085	32.05241943	28.68688894	28.41816082	25.955686	28.89154044	X	-0.167912933	0.069264278	0.660467387

**CG42331**	FBgn0259233	1.343243935	0.809708985	1.222009144	0.668753278	0.606224893	1.160868663	0.517566042	1.048047802	3R	-0.251463136	0.565765237	0.984934163
**comm3**	FBgn0259236	0.345691419	0.273117217	0.19446784	0.029201686	0.244198022	0.283405008	0.115026031	0.34807099	3L	0.258936212	0.682424967	0.995787491
**CG42336**	FBgn0259238	101.1029992	93.72760215	90.27903107	114.4412465	123.9835804	103.6388606	107.1502557	107.0105488	2R	0.174101157	0.175757573	0.834088743
**CG42337**	FBgn0259239	86.60962691	78.35537912	75.12575306	89.53996312	72.55170212	81.66315291	72.09395555	91.74524308	3L	-0.022782612	0.846391911	0.995787491
**CG42339**	FBgn0259241	47.52069036	43.07631102	35.61392156	42.30933152	43.86947315	45.22196514	37.63314556	40.80340001	X	0.019353504	0.858114582	0.995787491
**CG42340**	FBgn0259242	4.66215004	4.206517493	4.350119603	3.152930264	5.913249474	5.466266777	3.934574506	4.342760951	X	0.293127453	0.052687156	0.605783103
**Pka-R1**	FBgn0259243	591.450938	527.760479	528.299401	570.97449	503.4614529	540.3576664	514.4836989	513.7530118	3L	-0.06999455	0.41214688	0.958665466
**CG42342**	FBgn0259244	21.24131486	18.62485357	18.61382602	18.81892274	20.89756505	17.53674927	17.60169099	19.04880501	3R	-0.011537443	0.912588238	0.996479182
**DIP-beta**	FBgn0259245	0.627333671	0.530210657	0.454211282	0.470527913	0.425784155	0.298015604	0.434201229	0.580697449	X	-0.236809703	0.48722169	0.973549494
**brp**	FBgn0259246	135.8966947	199.4162297	189.8271982	188.1741676	173.1342722	164.2963258	161.4389037	189.4193884	2R	-0.017361969	0.884452416	0.995787491
**Mob2**	FBgn0259481	28.80765394	26.51405515	23.51212824	21.17386193	24.46371447	32.05003618	23.70520819	26.52013956	3L	0.121502522	0.320477326	0.916560752
**Mob3**	FBgn0259482	127.6811903	95.48634855	111.5382492	88.37234735	98.71213612	121.6344206	102.4246217	84.88437585	2L	-0.028045813	0.86772931	0.995787491
**Mob4**	FBgn0259483	894.6819309	857.6521499	838.9086193	936.7633846	943.2627159	890.7459873	879.4321521	945.62987	2R	0.082605425	0.372902704	0.943282319
**DNApol-alpha6**	FBgn0259676	0.148318914	4.395187017	1.439278464	0	1.428019484	2.004289712	0	4.937549403	3L	0.551843183	0.66137539	0.995750687
**CG42346**	FBgn0259677	1.775004131	2.837886682	2.688863158	2.736102684	2.504032245	3.200939888	2.25779982	3.298114596	X	0.200541708	0.274353601	0.89549671
**sqa**	FBgn0259678	0.054523177	0.128036974	0.046295364	0.127118735	0.10827114	0.128510296	0.215589185	0.01775623	2R	0.429770271	0.598826221	0.989286463
**Pkcdelta**	FBgn0259680	0.658955196	0.413641711	0.264801442	0.556094631	0.54735415	0.266798315	0.350639886	0.403742488	X	-0.253440954	0.589995283	0.987597375
**Jabba**	FBgn0259682	0.237724726	1.123749658	1.101004933	0.352702159	0.390140054	0.32600061	1.157750778	0.422281952	2R	-0.253885312	0.769827724	0.995787491
**Ir40a**	FBgn0259683	0.170796541	0.116933566	0.380525534	0.040633235	0.169896966	0.305620653	0.133379279	0.068108896	2L	-0.027522585	0.971121242	0.997604378
**crb**	FBgn0259685	25.15369287	24.19384199	19.55268261	26.53131257	21.67606351	27.31895333	18.13086954	28.38766902	3R	0.028659491	0.850902568	0.995787491
**Nsun5**	FBgn0259704	30.85356864	32.08128964	34.17910348	39.27005569	32.75134463	28.38360381	34.53519396	29.90444357	3R	-0.088679285	0.616832013	0.990047511
**CG42365**	FBgn0259711	21.4874369	19.06229849	39.3857679	27.83175013	21.55018377	22.32906496	27.04202383	22.18524427	2R	-0.176330628	0.573463147	0.984934163
**CG42366**	FBgn0259712	0.016603076	1.503863832	0.523624789	0.309675567	0.779292979	0.92354342	0.804741251	0.468608584	2L	0.393206149	0.67193536	0.995787491
**DIP-epsilon**	FBgn0259714	0.278789168	0.090928001	0.482204396	0.102312682	0.237662787	0.569488237	0.309502466	0.08967068	2L	0.361409133	0.571363355	0.984934163
**CG42376**	FBgn0259722	192.8860007	216.1412305	251.9670178	162.0563782	249.2720399	272.96665	226.761927	199.6646387	X	0.23731705	0.141655857	0.8037297
**Nost**	FBgn0259734	0.646882261	0.840618948	0.724304928	2.287132597	1.456527136	1.112522231	0.643763455	0.733087476	X	-0.162342504	0.700572508	0.995787491
**mtgo**	FBgn0259735	6.750539575	8.461583182	7.864805309	7.28501016	6.277108622	6.766458433	6.440030153	7.008237515	2L	-0.163981017	0.092833307	0.72305867
**CG42390**	FBgn0259736	0.963437574	1.048236566	1.579247375	0.546377457	0.434812937	0.466436166	0.199277023	1.059989269	3R	-0.8959643	0.092339227	0.72305867
**CG42394**	FBgn0259740	18.13555729	11.54622165	25.79849824	16.66604329	12.5859221	12.35555599	15.53396371	13.41205916	3R	-0.391208694	0.175438265	0.834088743
**CG42360**	FBgn0259742	15.24287563	12.49980625	11.26979159	10.83068669	13.19083137	26.39225361	18.51662058	14.13048205	2R	0.556256614	0.024104069	0.43956961
**wech**	FBgn0259745	41.06668146	48.47413291	38.71304825	37.13103554	46.5683402	53.12199263	34.92737645	43.31978959	2R	0.135465811	0.306129074	0.911610813
**mmy**	FBgn0259749	107.0540822	97.39246567	106.085498	113.957521	100.301736	118.1574207	110.0707156	114.3547292	2L	0.089591287	0.390699776	0.949207648
**pzg**	FBgn0259785	186.2775665	176.7907227	120.661505	153.9901191	151.4665439	175.3886381	149.9686502	170.8181139	3L	0.050438913	0.709505243	0.995787491
**zld**	FBgn0259789	1.333524475	1.190692139	0.701145898	1.430405002	0.768888557	1.013439584	0.776087358	0.308234761	X	-0.676512248	0.098670215	0.730787365
**loopin-1**	FBgn0259795	1.010478834	0.338987298	0	0	0.583735449	1.028887134	2.268422829	0.26326117	2R	1.571291046	0.244042076	NA
**CG42402**	FBgn0259821	115.2360448	132.411144	145.8925567	129.4629355	178.3949024	198.2668825	202.2490929	221.5453045	3R	0.64652371	3.27E-10	1.7E-07
**Ca-beta**	FBgn0259822	54.90120322	76.45598249	87.48152161	72.51209274	65.74411886	67.92405839	69.70559289	76.8307996	2L	-0.022042794	0.861100233	0.995787491
**CG42404**	FBgn0259823	132.4849271	111.2923713	115.216577	131.8145646	140.0199073	123.7349532	122.623912	157.8044517	3R	0.179112513	0.14629326	0.808004944
**Hip14**	FBgn0259824	97.02554668	133.5031878	97.41341659	106.7052975	99.93062834	116.91274	99.61194433	120.4040239	3L	0.039823154	0.753283042	0.995787491
**CG34309**	FBgn0259831	1.368966526	2.087499364	1.207670436	1.160615971	0.224666858	3.989291953	1.587392281	2.161565856	3R	0.482754046	0.673381561	NA
**CG34229**	FBgn0259832	17.91564433	33.70936023	28.72344216	21.42964669	25.31138851	21.7376738	34.17804043	26.72021965	2R	0.121492703	0.735892774	0.995787491
**out**	FBgn0259834	2.510457219	7.644860951	6.507232073	8.500838723	6.514776888	7.065310306	7.090397832	9.781584263	X	0.314221818	0.281584679	0.900509101
**Cap-G**	FBgn0259876	10.01506684	11.67964306	13.87435886	14.4188366	9.581143123	14.31931913	12.60308773	9.754518205	2R	-0.08211017	0.652044643	0.993444251
**Fs**	FBgn0259878	0.509264742	1.192684755	0.4195933	0.358439721	0.459676692	0.163063592	0.220609579	0.219046027	2R	-1.183239562	0.035112291	0.514749796
**CG42445**	FBgn0259916	0.862281658	1.136422977	1.892656298	0.382928995	0.037062843	0.251628582	0.314242719	0.66860475	3L	-1.719097346	0.05340073	0.607150997
**4-Sep**	FBgn0259923	64.06445767	64.61604037	74.92891304	79.49239598	59.06796609	73.67347509	62.4961716	63.45202887	X	-0.101971163	0.365477375	0.940794966
**CG42449**	FBgn0259926	2.208195407	3.22601053	2.419313471	0.332149965	1.928883225	2.283344736	0	0	X	-0.912413559	0.475765561	NA
**CG42450**	FBgn0259927	22.74178265	30.11269993	34.46360653	24.51600273	25.99280914	26.79624411	29.79521117	29.35871114	X	0.036637885	0.785610555	0.995787491
**CG42458**	FBgn0259935	0.269192346	0.319835448	0.244896329	0.091526724	0.339160406	0.229999816	0.135912435	0.225253496	3L	0.041218072	0.93896745	0.997038394
**Uhg3**	FBgn0259936	52.30029433	44.19466334	54.17159567	45.61088376	32.93838249	39.80690572	40.49624817	24.74047733	2L	-0.481125744	0.023538432	0.434002865
**Nop60B**	FBgn0259937	3.271036138	7.450766961	5.149820188	6.99224054	6.614211122	3.169379299	5.877753942	7.446147582	2R	0.057166206	0.846337719	0.995787491
**cwo**	FBgn0259938	36.02276328	33.86300233	36.48007591	58.38268051	34.81800443	34.30192304	36.43252053	37.97549759	3R	-0.171414731	0.299043771	0.9074568
**Sfp77F**	FBgn0259972	0.678471898	0.885143333	0.182884818	0	0.13609063	0.497512681	0	1.145686341	3L	0.048588988	0.979414446	NA
**vlc**	FBgn0259978	164.4887514	183.98154	180.3229463	190.2321788	159.9401137	191.0873596	168.1645308	152.7850881	2R	-0.068307825	0.500768508	0.976796212
**Cndp2**	FBgn0259979	61.5951969	64.01450614	52.53366395	62.82801122	67.7595245	96.21594555	60.52930281	67.66809624	2R	0.3046829	0.056940272	0.621788268
**Uxt**	FBgn0259982	41.7171758	35.46663387	39.0165195	33.87992257	27.5596993	28.09335609	30.8226398	30.30877968	2L	-0.336044389	0.124866465	0.780439453
**kuz**	FBgn0259984	25.83740997	24.32457745	22.80664351	24.09422218	22.22069788	23.42811866	21.4895844	20.47148532	2L	-0.119309316	0.167539699	0.830652748
**Mppe**	FBgn0259985	32.90086254	22.35230088	29.31644505	28.17418837	23.16011532	27.78029855	33.04445616	36.11973447	2R	0.117108068	0.592314639	0.988631016
**nab**	FBgn0259986	1.255336893	0.734991573	0.632070335	0.2484994	0.598620145	0.792737744	0.151056197	0.617083608	3L	-0.387400302	0.581153223	0.985637351
**CG42489**	FBgn0259992	0.808676567	6.330060312	4.141658153	3.561309172	0	2.20253317	0.458433622	0.975395381	3R	-1.957299262	0.069711962	NA
**OtopLa**	FBgn0259994	155.8376573	189.9004879	164.5076798	202.5934524	173.025648	206.049907	177.5682573	198.2212082	X	0.112105675	0.293756649	0.906604178
**Dys**	FBgn0260003	7.186230669	9.038286932	10.02823185	9.966659595	8.232681256	8.377183854	8.038601225	9.376370962	3R	-0.057738801	0.629585633	0.990523437
**drd**	FBgn0260006	1.116639436	2.727800692	2.781675232	0.646482577	0.811739748	2.204438805	0.401476477	1.171487154	X	-0.623834878	0.234769322	0.87009833
**UQCR-11**	FBgn0260008	169.6366484	137.8153169	197.5158042	102.5264605	118.5500624	141.7219016	135.9691292	123.3968375	3L	-0.194939945	0.221725411	0.86549481
**pds5**	FBgn0260012	70.96961834	72.23998845	52.05774922	56.11598439	58.02510876	81.64892588	61.90099807	60.77426927	2R	0.088320921	0.537391114	0.984934163
**CG42494**	FBgn0260026	1.009536663	1.286950657	2.938946235	0.376591476	1.093483451	0.685285199	2.163293761	0.526031411	3L	-0.292870547	0.734496816	0.995787491
**CG42495**	FBgn0260027	20.47950769	13.3276316	26.60619673	8.61015104	19.7985492	22.68420914	25.9790898	22.83887024	3L	0.43288874	0.26906236	0.89549671
**flr**	FBgn0260049	44.19689986	49.70445854	62.22547009	59.57721484	66.31568209	62.61595769	48.90610511	64.17874645	3L	0.198976094	0.195983964	0.844272804
**E(var)3-9**	FBgn0260243	64.70052116	60.03300255	48.79428856	56.00878791	78.86274787	54.37121379	47.95247517	55.82919312	3R	0.075894775	0.674786804	0.995787491
**mtg**	FBgn0260386	0.703386595	1.236691728	0.84292348	0.632258069	0.16828583	1.182481815	0.108093633	0.165591044	3R	-1.033882058	NA	NA
**CG42521**	FBgn0260396	1.698396973	3.70346946	1.23608621	3.563773746	3.066041833	2.561981268	1.29979415	8.480967211	3L	0.635054969	0.527065063	NA
**Su(var)3-3**	FBgn0260397	62.34991319	44.34929897	53.55202135	63.2096236	49.79207558	55.06827758	48.54490947	47.14772359	3L	-0.130676617	0.377626075	0.943615128
**gwl**	FBgn0260399	0.325632502	0.291308182	1.579959818	0	0.862180185	0.040933911	0	0.047131887	3R	-1.147547122	0.446967922	NA
**mRpS23**	FBgn0260407	105.7964947	94.56219747	125.262751	96.7970663	111.4741195	88.1207015	106.4502947	89.9100951	2L	-0.062387691	0.724348711	0.995787491
**CG42524**	FBgn0260429	0.515394543	0.804187947	0.426472549	0.479717699	0.638310877	0.259905791	0.101921128	0.472742828	2R	-0.554835169	0.225540355	0.867702781
**CR42530**	FBgn0260435	0.094048041	0.907076271	0.912636942	0.1644521	0.169780818	0.620675429	0.239918573	0.612561008	2L	-0.276085444	0.795251415	NA
**Pp2A-29B**	FBgn0260439	909.6412159	855.998794	825.5512304	844.4701382	851.303313	886.3837928	828.3461092	861.2436945	2L	0.025941514	0.74848026	0.995787491
**rhea**	FBgn0260442	14.15263433	21.91107704	18.31389835	20.8072617	21.33791212	20.22418195	19.52077953	22.50305602	3L	0.187005736	0.163055073	0.825076487
**Not10**	FBgn0260444	92.48142481	95.77358358	81.12636652	93.30420625	108.6761604	93.68283776	78.00299488	64.46161978	3R	-0.044217257	0.780084301	0.995787491
**GABA-B-R1**	FBgn0260446	41.16641381	51.25834067	47.54420966	52.62739306	43.14102588	51.85230185	45.13588776	50.0514321	2L	0.012424059	0.908446815	0.996479182
**CG4806**	FBgn0260456	60.2958131	69.2870178	55.6832795	68.67128327	63.26289702	72.35691718	68.66601317	63.25918931	2R	0.104784299	0.441938498	0.967112302
**CG12163**	FBgn0260462	30.15721097	33.56492805	31.08633158	22.02304626	24.97407893	32.08816098	30.60335539	32.07656986	3R	0.06765173	0.624619838	0.990047511
**HIP**	FBgn0260484	18.75535567	13.50836365	15.36935292	11.5994454	12.98760117	23.35161232	13.97010159	14.45390854	X	0.152388664	0.568292495	0.984934163
**Ziz**	FBgn0260486	32.83586516	39.94649451	43.83030582	38.31174102	41.03398442	42.50300436	40.96312682	43.21641854	2L	0.14715312	0.151052669	0.811129475
**CG42534**	FBgn0260487	0.252112445	0.386506204	0.309098924	0.985971454	0.606836772	0.406373351	0.179803773	0.565016213	3R	-0.10402543	0.836641222	0.995787491
**qvr**	FBgn0260499	199.1581841	184.1393307	186.1166272	192.7586822	158.2585054	184.7226083	154.0853896	155.6698011	2R	-0.19604028	0.029975869	0.481346608
**dl**	FBgn0260632	6.720050091	12.14149106	11.0858685	9.504976576	8.561311282	11.6092734	9.436809016	6.600170607	2L	-0.090792679	0.653185843	0.99443552
**Diap1**	FBgn0260635	125.9065912	124.5046531	124.6426095	116.4587661	129.8113149	127.6310208	120.8621169	112.2766002	3L	0.027138296	0.749813247	0.995787491
**gammaTub23C**	FBgn0260639	124.8412437	111.8296863	124.3501471	123.5091288	98.57472572	135.0119844	113.1223999	118.1685306	2L	-0.031830849	0.803188004	0.995787491
**Antp**	FBgn0260642	0.185895919	0.234213476	0.244876511	0.103939796	0.151850579	0.152263417	0.109436419	0.127836233	3R	-0.468923498	0.1859387	0.839299945
**serp**	FBgn0260653	0.529210651	0.392229207	2.418429542	0.143469152	3.599266499	0.777412794	0.784899849	0.347361606	3L	0.713425623	0.377080002	0.943615128
**l(3)76BDm**	FBgn0260655	46.7099177	53.87764554	43.97399746	49.67409015	55.59141926	53.67260227	58.38612043	57.11091224	3L	0.241442764	0.036270029	0.521162798
**CG42540**	FBgn0260657	80.64003652	97.15824399	93.75622415	100.114667	92.45706389	90.56517635	85.49212227	99.33095968	3L	0.01699224	0.863399744	0.995787491
**CG42541**	FBgn0260658	16.13966032	15.16062638	13.24330639	13.28538669	15.12426869	20.38601161	11.14408603	11.93366211	X	0.042686474	0.788235204	0.995787491

**CG42542**	FBgn0260659	48.01721771	50.72584773	48.54253321	51.07928955	42.68975602	52.21851702	50.94205579	49.65660166	3R	0.008664264	0.936470105	0.996875956
**Mp**	FBgn0260660	33.31385652	34.20651256	30.87659947	38.50061484	34.57745281	31.29155832	34.46109681	38.8629814	3L	0.055462994	0.621278163	0.990047511
**asRNA:CR4254**	FBgn0260720	2.513549702	2.440915637	4.252438624	3.624099813	3.164379944	3.398733741	3.121689655	4.164666402	2R	0.149468921	0.72838029	0.995787491
**CG3281**	FBgn0260741	62.32728081	52.23483841	55.75893572	50.11904493	41.32935057	60.97118857	60.24228039	45.39963084	3R	-0.058661532	0.738271343	0.995787491
**CG12213**	FBgn0260742	130.9752856	157.5218579	138.046698	107.7676361	101.9994984	132.3067588	121.2853991	131.5832616	3R	-0.10091899	0.445306725	0.967112302
**GC1**	FBgn0260743	35.20907572	40.72355471	36.29022336	38.17603785	48.19188829	49.69997315	38.38281017	47.46316098	3R	0.320679919	0.015501699	0.364946783
**Tango9**	FBgn0260744	112.7834805	96.37604289	80.54354216	90.03025656	92.58692197	86.05701971	102.4716014	94.34130086	3R	0.012263027	0.926463038	0.996479182
**mfas**	FBgn0260745	31.21248979	59.19538715	61.07900736	23.41278071	40.60428877	35.04955859	54.16295889	43.51567311	3R	0.030733607	0.929603953	0.996479182
**Chchd2**	FBgn0260747	1304.95702	1451.398993	1469.027633	1454.976668	1572.136234	1425.284968	1504.065566	1546.7225	X	0.122774189	0.16729924	0.830652748
**CG5004**	FBgn0260748	5.006940764	7.728406785	8.144696755	6.995565165	5.268052655	4.697514323	7.436546692	6.143637997	X	-0.20691875	0.264074295	0.893179858
**Utx**	FBgn0260749	48.23698786	42.40423778	46.10262337	44.58654953	37.77811673	51.60919886	41.1541361	46.68062433	2L	-0.005362377	0.965926411	0.997604378
**Mulk**	FBgn0260750	122.9440021	76.85777008	89.31465921	81.40727864	73.53946871	76.40760347	91.73016851	87.77730653	2L	-0.144510847	0.434698381	0.96591738
**Pdfr**	FBgn0260753	1.10427286	1.170718036	0.842637267	0.236829937	0.638865038	1.291336836	0.359442205	0.40550825	X	-0.290081796	0.549942232	0.984934163
**CG42553**	FBgn0260755	23.99971082	35.76053763	26.15600146	21.44446134	32.87909417	26.1299118	18.33605531	18.91142111	3L	-0.121609974	0.65454745	0.994669639
**CG42554**	FBgn0260756	40.08185608	64.82578522	54.90178679	43.84501769	68.33014112	58.52400747	51.83631685	47.576323	3L	0.188851222	0.378139985	0.943615128
**CG42564**	FBgn0260766	1.688347287	2.983678172	2.047953485	4.076900932	4.245288691	5.542747935	1.692039722	4.451035017	3R	0.599782731	0.223781907	0.86549481
**CG42566**	FBgn0260768	0.132545135	4.780006195	3.215526485	1.081583973	7.417616125	7.872652844	5.071882301	1.007187151	2R	1.267540535	0.143521121	0.8037297
**Larp7**	FBgn0260771	50.21228471	59.20496773	52.53366395	61.40803686	50.07044122	60.24013415	51.04836704	46.56800369	X	-0.07239075	0.625240617	0.990047511
**CG42570**	FBgn0260776	1.460289856	0.06445872	1.305184197	0.179190043	0.554988939	1.304291706	0.686226725	1.00118667	3L	0.249758605	0.814472005	NA
**CG42571**	FBgn0260777	0.467845463	1.008958026	0.972845629	1.714057846	0.361963272	0.630116921	0.852488447	0.725525577	3L	-0.661012249	0.549653726	NA
**GatA**	FBgn0260779	18.69249505	30.75836558	48.24560118	31.46251241	33.74474442	32.11130314	43.1552974	32.31230778	3R	0.168072662	0.488683452	0.973549494
**wisp**	FBgn0260780	0.536201286	0.534779827	0.457043755	1.317707877	0.052323284	0.696807897	0.665445893	0.125853257	X	-0.86600956	0.320415033	0.916560752
**mxc**	FBgn0260789	8.641034106	16.52112008	18.34659011	24.56933125	17.75737729	13.13274826	13.42496658	16.19326068	X	-0.134738393	0.593662251	0.988631016
**ctrip**	FBgn0260794	10.26183927	21.89593708	30.33324529	23.77644029	19.93069732	18.30871943	28.17278139	29.46301689	3R	0.191959575	0.59208101	0.988631016
**NaPi-III**	FBgn0260795	373.4421456	271.3834039	313.7069912	337.0902668	316.3569873	333.5595173	310.3469614	308.9340869	3L	-0.003144783	0.977396417	0.997757033
**Gprk1**	FBgn0260798	7.24084632	6.414576206	6.362023928	5.173398978	5.2124463	7.707675395	4.733744313	5.109557217	2R	-0.121140227	0.40364625	0.955949943
**p120ctn**	FBgn0260799	175.7672271	138.0070461	177.6025445	120.3095627	120.5677132	177.837556	127.1279939	133.8133139	2R	-0.103022478	0.467803198	0.97223737
**gkt**	FBgn0260817	31.37214532	39.9858841	37.58227	41.06561662	21.83662787	38.14726294	21.47393684	46.53412619	2L	-0.196734335	0.420975787	0.960593359
**Sec22**	FBgn0260855	219.1963608	231.2737007	225.8650749	228.5692176	253.7669285	314.2339683	184.0334722	232.856819	X	0.149448306	0.27395403	0.89549671
**Membrin**	FBgn0260856	54.44040925	45.65807464	65.5819822	47.10646938	42.5537469	38.36228026	62.75787955	57.8790771	3L	-0.048064284	0.843047401	0.995787491
**Bet1**	FBgn0260857	114.0238267	103.6392398	93.88566596	106.1045581	105.5447421	120.4198746	122.2404681	106.1985802	3L	0.147776429	0.308904987	0.912131082
**Ykt6**	FBgn0260858	213.9587494	242.2432403	289.7617893	298.4460236	262.9234254	244.7272136	319.8812605	306.0593998	X	0.1512855	0.293078716	0.905978493
**Bet5**	FBgn0260860	122.1220561	140.423785	137.7437696	128.6728835	133.0500785	131.5467057	124.5791539	111.723996	3R	-0.046678654	0.707290073	0.995787491
**Trs23**	FBgn0260861	111.8335449	133.2561957	145.6353461	101.096578	170.081295	86.53708043	119.4987363	139.7250913	2L	0.106761428	0.620407698	0.990047511
**Vti1a**	FBgn0260862	151.0530327	128.4594375	124.937004	131.0613234	151.4577594	151.5868714	148.646382	135.3007485	3L	0.159228551	0.202885497	0.850527029
**dnr1**	FBgn0260866	1.46145772	3.976808127	2.671442189	2.757027433	1.877760358	2.1968955	3.083370533	2.075355544	2R	-0.196857703	0.457136695	0.968365245
**Ir76a**	FBgn0260874	13.00630683	13.09675668	12.9656476	4.331959249	4.070320177	1.673021932	2.716128051	3.127471639	3L	-1.871303724	9.23E-07	0.000219837
**cuff**	FBgn0260932	1.071449653	0.662128705	3.14654758	0.262952056	1.221626043	0	0.143857425	0	2R	-1.863739642	0.220760843	NA
**rempA**	FBgn0260933	0.685771738	1.880275006	1.567691555	1.383621049	0.547574158	0.484906054	0.201855973	0.114528763	2L	-1.970594108	0.002010101	0.101732313
**par-1**	FBgn0260934	87.02909245	85.15775289	78.22026911	77.64936081	79.03700309	87.58121377	71.70357851	76.06095905	2R	-0.03271148	0.715454024	0.995787491
**Vps15**	FBgn0260935	28.88969265	57.86587439	42.01048869	45.50922342	36.58378083	44.44188017	47.50401822	34.46961796	3R	-0.062304429	0.735097739	0.995787491
**scny**	FBgn0260936	82.16161834	90.72377344	83.19817572	80.17878091	79.63610264	83.30338133	90.43484545	82.68368542	3L	0.030206601	0.748584109	0.995787491
**tay**	FBgn0260938	102.1086876	120.8735956	129.5987199	107.7740588	113.3000941	111.8045652	114.6118406	139.2162583	X	0.09139748	0.421878009	0.960593359
**lsn**	FBgn0260940	98.51813547	107.6775093	110.9589538	80.21266453	107.7040321	104.2540812	122.6665545	112.1307237	3R	0.200666535	0.196509673	0.844272804
**app**	FBgn0260941	2.000982524	1.699760385	1.581132997	1.996045743	1.800982743	2.119633344	1.462088795	1.908396962	3L	0.029446299	0.860884446	0.995787491
**bond**	FBgn0260942	0.446946572	1.274473296	1.048143278	0.347346369	1.290964982	0.603975847	0.076011354	0.711599079	3R	-0.162580704	0.836772565	0.995787491
**Rbp6**	FBgn0260943	20.31960026	21.86013122	19.61075662	21.69965981	25.32121877	23.91713949	20.41707884	24.55427167	3L	0.204827775	0.033487409	0.51396698
**Rbp1**	FBgn0260944	379.2980526	382.1363101	364.0517307	367.7145799	376.2749033	345.5975218	333.676262	314.5109467	3R	-0.094103816	0.318042003	0.91460807
**Atg1**	FBgn0260945	79.92309532	84.5739666	76.63053334	85.36897954	81.4532318	83.02900833	74.22467364	88.13289237	3L	0.032012851	0.731730177	0.995787491
**MCPH1**	FBgn0260959	0.37179906	1.027173024	1.432555411	0.509903242	0.651451635	1.154686672	1.506389383	0.427346564	2R	0.20568835	0.731403395	0.995787491
**Baldspot**	FBgn0260960	114.7611206	112.7532997	111.2008415	124.8200054	98.98685683	116.1745605	101.6844958	113.6217665	3L	-0.078101663	0.413705259	0.958665466
**pic**	FBgn0260962	99.01161517	87.49828138	90.66747536	97.72800981	78.25387393	98.05046864	90.70416188	86.55623775	3R	-0.057287967	0.606538975	0.989286463
**Vmat**	FBgn0260964	0.80388163	4.347897724	1.203953471	0.994483953	2.60625896	1.233512277	1.621742311	1.15759789	2R	-0.099890229	0.82264186	0.995787491
**CG42588**	FBgn0260965	17.83022756	18.99279048	20.51622505	19.48374572	15.38712972	16.81022963	17.0888673	12.04746526	3L	-0.295036882	0.035288346	0.515210107
**Ubr3**	FBgn0260970	39.6853818	44.44239376	49.47574733	50.444692	45.67510831	53.01953076	47.46810371	50.29957858	X	0.124348549	0.242913407	0.876959115
**CG42594**	FBgn0260971	1.110693028	1.074061003	1.345664721	1.087422064	1.110765087	1.279441567	0.853717736	1.327280741	X	0.018256918	0.923487965	0.996479182
**alc**	FBgn0260972	307.5320199	345.4470332	276.3459664	351.7101963	318.2990113	321.3212018	290.847996	263.1169841	2R	-0.073116405	0.512972491	0.980804634
**RfC4**	FBgn0260985	64.19162007	59.6946847	49.5903517	71.14437968	41.28224916	44.43640154	43.51772605	40.2293462	3L	-0.503182971	0.007812161	0.258004155
**mei-38**	FBgn0260986	1.848863173	0.100038987	0	0.18540021	0.7177789	0.018742977	0.405719542	1.942283756	X	0.532931755	0.676589039	0.995787491
**vtd**	FBgn0260987	32.52722049	26.87397337	30.54066595	23.83981087	23.00302382	34.68231138	25.45717723	22.05111231	3L	-0.088273619	0.542131873	0.984934163
**yata**	FBgn0260990	51.40969901	69.51148758	74.05574326	78.93019331	64.09061187	63.00476066	62.51449206	76.19305887	3R	-0.009599037	0.947372752	0.997604378
**dpr21**	FBgn0260995	11.12850228	9.450521649	11.83222558	8.469610707	10.37707118	12.38930351	8.82887515	8.862711662	2R	0.011950139	0.92718331	0.996479182
**CG42598**	FBgn0260997	0.241500685	0.13502784	0.170880877	0.023460406	0.054496329	0.028460671	0.141183496	0.087386724	3L	-0.873676241	0.378238422	NA
**asl**	FBgn0261004	1.571543616	5.49175823	5.401102432	10.25918538	6.998029559	2.864507329	3.674954734	5.629745	3R	-0.2047954	0.677474363	0.995787491
**clos**	FBgn0261016	0.147072325	0.407043768	0.01783977	0.120012756	0.159301944	0.0623965	0.075036932	0.031930763	2R	-1.038207326	0.357096415	NA
**wol**	FBgn0261020	60.84403596	48.28233285	58.171423	77.33099291	56.92181444	52.13495189	65.21162359	46.55538363	2L	-0.121127277	0.531879751	0.984760433
**stj**	FBgn0261041	133.3444416	132.3502174	133.0786725	152.2849127	131.0123659	167.2147451	131.886132	137.9333816	2R	0.070504394	0.493086978	0.973549494
**Dscam3**	FBgn0261046	26.66352652	28.04427641	24.54837921	28.63867091	24.28899943	27.12754616	28.40401669	24.91454647	3R	-0.01365086	0.891088477	0.996189576
**Vps45**	FBgn0261049	129.0883541	144.8878618	161.6718662	123.7951111	174.0388866	146.4135957	161.2392245	143.2107172	3R	0.193041368	0.123102779	0.780439453
**Cad86C**	FBgn0261053	1.507365033	1.111963581	1.206184986	1.076389228	0.512892607	1.555063456	0.694573613	0.591129326	3R	-0.524099065	0.15548658	0.818884898
**Sfp78E**	FBgn0261059	3.119724949	10.7736196	7.123208671	6.631739986	1.325153673	1.989674277	3.979242685	4.382666364	3L	-1.212994363	0.067689335	0.656261422
**Rbsn-5**	FBgn0261064	102.77114	109.4842502	119.6956042	100.0500505	106.0728954	97.38994913	82.18820329	92.49920229	2L	-0.159512626	0.213656642	0.858127449
**Cpsf73**	FBgn0261065	37.09156467	32.45267214	44.86214988	35.97002406	48.17759439	47.70122466	43.72034869	33.88261148	3R	0.236433289	0.176370098	0.834204539
**LSm1**	FBgn0261067	49.16170699	39.88756964	49.61927861	33.03545601	31.58502838	43.2129344	33.94625975	40.66080637	2R	-0.17207817	0.51154443	0.980804634
**LSm7**	FBgn0261068	78.56330051	87.91430443	55.94667906	84.89499242	81.27159039	56.80010381	81.91186831	80.9719816	2L	-0.001080451	0.996137361	0.99922507
**Syt12**	FBgn0261085	78.29684213	100.6178901	91.57362897	97.23131446	105.4184897	105.4918593	88.22765758	90.62747862	X	0.115048397	0.288468165	0.903033179
**Syt14**	FBgn0261086	158.8939816	164.4037406	123.5577202	193.8062654	136.5764148	142.1229124	133.2303089	137.4351118	3R	-0.193815288	0.140916058	0.8037297
**Sytalpha**	FBgn0261089	276.0898546	222.2779376	217.3083795	201.8805682	209.8533332	251.002916	199.2092361	180.2560906	2L	-0.101067571	0.405389133	0.956706394
**Sytbeta**	FBgn0261090	11.03012265	10.10458962	12.80483245	10.82792241	10.06338809	10.47059279	9.597620861	9.477238086	3L	-0.146720312	0.167267038	0.830652748
**Atg13**	FBgn0261108	36.5285473	30.63575682	36.88906182	18.76857526	36.87829397	41.5932581	38.12787238	31.57239828	3R	0.29938136	0.146912817	0.808004944
**mrn**	FBgn0261109	31.59998215	34.6789374	31.13986415	37.00841967	32.63565681	27.02089619	39.74370575	20.5287626	3L	-0.136202587	0.551161618	0.984934163
**BHD**	FBgn0261111	69.68114887	58.61080422	63.54629931	59.29159687	56.65245939	59.17341791	49.04566771	63.49520372	3L	-0.108430192	0.488162953	0.973549494
**APP-BP1**	FBgn0261112	45.50857043	33.9412362	39.48513615	27.50434801	44.25377	51.68983502	49.47739044	26.13339107	3L	0.252752782	0.269321773	0.89549671
**Xrp1**	FBgn0261113	103.5225302	110.0582595	108.5896749	127.1724602	87.79041118	108.4018743	95.97024325	85.52561404	3R	-0.22320844	0.036116823	0.520757094
**Prp19**	FBgn0261119	65.71688637	67.43349033	66.46650525	91.31524613	65.01175114	72.98644292	53.13968205	71.23651735	2R	-0.120593637	0.430697261	0.963666972
**Alh**	FBgn0261238	23.23461474	28.4059074	30.61578631	29.40242816	26.14326798	25.87697191	28.6614516	34.95587083	3R	0.0848169	0.516263228	0.980804634
**Hr39**	FBgn0261239	14.66619749	16.72859156	14.95540293	15.51638748	12.73955763	15.62251621	14.39598096	11.95844741	2L	-0.147734268	0.194246347	0.844272804
**Psa**	FBgn0261243	53.80478939	48.49601435	40.99963801	48.92083631	50.63291072	56.65562656	48.63231364	52.07556006	3L	0.141265538	0.190630604	0.843401316
**inaE**	FBgn0261244	28.9174416	31.76305487	27.60291439	33.68088412	33.57701559	33.27790385	27.91990963	34.89793955	X	0.118655358	0.284631199	0.901780531
**sing**	FBgn0261245	2.089704358	0.584197651	0.844932271	0.081201117	0.125748398	0.19701605	0.266544036	2.192855108	X	-0.357185625	0.760835524	NA
**rgn**	FBgn0261258	18.10479423	23.04396101	23.46750146	20.62593041	20.74960177	24.47069547	24.2658218	21.98438564	3L	0.133698548	0.205985899	0.855364409
**mgl**	FBgn0261260	9.331308325	10.24999285	10.99982587	8.717636794	7.560448327	8.792311247	9.806937825	10.37918808	X	-0.071590688	0.54961711	0.984934163
**plx**	FBgn0261261	147.1694779	122.5019084	114.1480858	136.0235724	114.5204464	140.2365351	126.4681114	120.972944	3R	-0.023541655	0.82764675	0.995787491
**CG42613**	FBgn0261262	13.26769291	16.0498865	15.67027233	11.71402682	11.34414157	11.76375657	13.62162576	11.97371845	3R	-0.185586848	0.119771114	0.777535158

**Cul3**	FBgn0261268	117.8718566	91.58046628	102.9749707	108.6743338	82.693577	96.74535788	94.59218855	93.90328023	2L	-0.167635924	0.143624878	0.8037297
**conv**	FBgn0261269	0.545226053	1.036478912	0.92589756	1.313546485	0.951463674	1.096609691	0.231813896	0.039457874	2R	-0.677640493	0.423728703	0.961959959
**Sps1**	FBgn0261270	122.3494201	149.7580456	95.59678402	124.9588324	134.3140913	141.2001812	116.3405358	108.5860201	2R	0.051824114	0.728780071	0.995787491
**Ero1L**	FBgn0261274	60.97822138	63.05197784	74.2455091	61.38955602	66.42758521	87.09127977	54.04993341	50.27795795	3L	0.017619055	0.914044541	0.996479182
**Opa1**	FBgn0261276	172.0011339	145.0797388	142.9419496	152.5665438	179.9946538	173.8928899	172.1407826	185.8017315	2R	0.245500183	0.018933359	0.393184106
**rtv**	FBgn0261277	1.955524802	1.15768889	1.395316439	0.268190145	4.049373974	1.084504023	0.586892445	3.121783357	X	0.910755993	0.31681835	0.91460807
**grp**	FBgn0261278	46.72207	42.60288708	48.24045389	43.31214154	46.04015443	45.77525465	44.70004642	43.17192627	2L	0.02020601	0.824172687	0.995787491
**lqfR**	FBgn0261279	85.94765277	79.86260793	75.72981853	92.5569522	78.18406673	77.11109071	67.34443137	80.12408641	3R	-0.113301764	0.312611245	0.914141092
**SREBP**	FBgn0261283	152.492074	160.1818882	146.1027434	158.3868041	145.0528344	152.1550974	168.3960114	180.2640502	3L	0.097031315	0.364463846	0.940794966
**Ppcs**	FBgn0261285	543.5353599	479.3452944	548.1866478	540.144854	505.8657434	524.327974	496.7720484	593.8126017	3R	0.036545194	0.714680532	0.995787491
**Mat89Ba**	FBgn0261286	38.87857624	28.94420557	29.00537099	34.69577083	36.12616691	40.71165993	36.93565805	31.11258226	3R	0.163689608	0.299713376	0.9074568
**ymp**	FBgn0261287	0.108119229	0.148381231	0.178838005	0.034373988	0.035487803	0.092667408	0.025074043	0.021339714	3R	-1.358347045	0.206068368	NA
**CheB53a**	FBgn0261292	0.639249045	0.357416865	2.584682113	3.974360225	9.23206692	4.218760667	13.31769406	9.483770489	2R	2.335980619	0.002874087	0.133070218
**CheB53b**	FBgn0261293	3.942623595	2.204399328	2.318727236	1.949834831	3.66656313	1.914860137	6.705144996	1.037551611	2R	0.358679612	0.68630112	NA
**verm**	FBgn0261341	0.679423775	0.444736807	1.246254894	1.159061674	3.619771217	1.437346483	0.465011491	1.00737984	3L	0.929221696	0.159266279	0.819277129
**CG42633**	FBgn0261356	0.06652707	2.678155866	1.936724938	0	1.681377779	0.501770525	1.357695887	4.188656416	3R	0.796303645	0.516895929	NA
**PPO3**	FBgn0261363	0.179408131	4.393606781	1.30572487	1.422163232	0.453428722	1.995909703	3.478322374	1.402241976	2R	0.05141693	0.952409094	0.997604378
**CG33228**	FBgn0261373	14.32641277	27.3505575	49.84695861	24.39187907	51.04509253	59.00362735	59.86973491	58.31994058	2R	1.022301562	0.001253337	0.073737689
**mRpL37**	FBgn0261380	169.5542245	138.7333783	139.7690941	125.3419704	159.8452535	137.2090516	166.7714954	153.003237	3R	0.133959844	0.338458727	0.926687587
**IntS6**	FBgn0261383	48.57719794	50.76946189	50.46069082	41.79514424	42.48204208	38.03215863	42.77322829	43.93305232	X	-0.164052913	0.147392371	0.808004944
**scra**	FBgn0261385	2.823589112	0.794089159	1.476644077	1.30085046	2.441821331	2.773646029	1.207694741	1.541745391	2R	0.328928298	0.522308924	0.983044846
**CG17528**	FBgn0261387	58.12860565	57.99835184	56.49746564	50.91258014	47.70344609	49.44968834	46.06647029	45.33819363	2R	-0.215553064	0.023023423	0.430217959
**alpha-Est5**	FBgn0261393	1.556374835	1.004077096	1.161767275	0.837376001	1.404827737	2.172792942	1.985172821	2.01442402	3R	0.747927298	0.19685945	0.84446408
**Prosalpha3**	FBgn0261394	329.4764631	367.3998881	319.1700437	295.625468	375.2578441	353.4043342	440.8415136	423.8213053	2R	0.313480285	0.008963596	0.281896641
**Rpn3**	FBgn0261396	229.0276194	242.4828664	256.4731811	269.9748643	167.6967608	241.966497	251.5799349	190.2440862	2L	-0.200953437	0.14270604	0.8037297
**didum**	FBgn0261397	50.89000726	45.32813552	50.9845467	51.35622998	38.30816386	58.57438463	46.88795058	39.017564	2R	-0.094184613	0.491997319	0.973549494
**sxc**	FBgn0261403	67.85296891	53.14531322	59.9346656	45.01117569	45.21031316	63.14985539	50.2224769	46.63612067	2R	-0.112688018	0.406311283	0.956706394
**lncRNA:CR426**	FBgn0261429	0.253269724	0.743442756	1.484869644	0.393659218	0	0.079593716	0.323048254	0.045822668	2R	-2.696131534	0.023003774	NA
**hkb**	FBgn0261434	3.292661698	14.16376638	5.797706628	5.69562859	10.5459808	4.556313253	7.451303069	5.765059149	3R	0.014554184	0.976749987	0.997757033
**DhpD**	FBgn0261436	11.18409547	9.262136563	10.55781901	18.32907945	14.78358906	15.66203095	14.08636988	8.026170787	3R	0.117911648	0.723302466	0.995787491
**CSN8**	FBgn0261437	160.8011986	185.2087307	225.998782	136.4643066	180.7522731	126.0321184	179.9442743	131.4997149	2L	-0.161221863	0.399156637	0.952899287
**SdhA**	FBgn0261439	524.1795489	572.9899155	453.7230761	557.0261647	502.2584034	581.6213554	498.4621973	499.3011915	2R	0.010383516	0.916952227	0.996479182
**CG3638**	FBgn0261444	38.09259515	62.12257607	57.04426645	57.95539512	52.21285092	52.95222811	54.76875085	59.98110352	X	0.065926446	0.627435904	0.990047511
**sgl**	FBgn0261445	21.85429603	23.95441622	31.94014464	25.68966804	22.68599938	20.68255871	23.39579517	32.12567025	3L	-0.02949073	0.873537416	0.995787491
**hpo**	FBgn0261456	83.46849126	105.2590169	92.57680639	93.34761208	103.7169053	82.77674136	86.29996896	81.46911479	2R	-0.047572708	0.712121301	0.995787491
**capt**	FBgn0261458	110.2330316	126.7535701	94.79038999	136.9216596	116.2548623	105.4526315	81.62012356	100.8260161	2L	-0.184376835	0.185622472	0.839299945
**RhoGAP18B**	FBgn0261461	1.750541462	1.951271076	1.580673924	0.808440684	1.145942415	1.687309697	1.005845546	0.619265066	X	-0.42157222	0.190002964	0.843401316
**slim**	FBgn0261477	171.0525189	141.2762451	144.0212528	129.9085777	118.998858	164.2394435	140.5920733	138.990912	2R	-0.032100425	0.794273901	0.995787491
**nero**	FBgn0261479	120.2382655	111.7414215	156.7041907	142.0706569	139.7900205	121.9444386	169.148414	137.5361822	3R	0.130610131	0.431924678	0.964264046
**lncRNA:CR426**	FBgn0261503	0.296989897	0.21075953	0.249401543	0.106526271	0.433038481	0.26923102	0.029139523	0.19839771	3R	0.139247439	0.889526197	NA
**haf**	FBgn0261509	0.522008226	0.570342067	0.650619649	0.483839537	0.527375017	1.01589805	0.431667722	0.689701247	2L	0.290823003	0.313789238	0.91460807
**NimA**	FBgn0261514	0.207575392	0.4725281	0	0.449386054	0.91005076	0.629038323	0.226941122	0.836950011	2L	1.237126363	0.189693526	0.843401316
**lncRNA:CR426**	FBgn0261522	1.253066845	0.788191442	1.519961344	0.182592386	0.989671731	0.590691884	0	0.510098272	X	-0.793294566	0.576981791	NA
**lic**	FBgn0261524	99.51494325	114.4011373	92.67818504	136.1952815	119.6565724	109.0566709	126.5434932	133.5788353	X	0.173875555	0.231118821	0.867702781
**NT1**	FBgn0261526	0.153284021	0.299964516	0.21692119	0.029781327	0.691792362	0.590103892	0	0.027732817	3L	0.94132145	0.464650201	NA
**ms(2)34Fe**	FBgn0261529	0.637954304	0.240767741	0.425608767	0.074368322	0.518251961	1.263064785	0.040685878	0.207758677	2L	0.567955836	0.585801574	NA
**nbs**	FBgn0261530	74.74718392	59.16123378	59.03423243	40.46207866	69.07196687	71.67557306	67.40227508	45.11996584	3L	0.144234476	0.455077867	0.967967712
**cdm**	FBgn0261532	98.44480916	169.2399889	198.0884017	149.6349815	146.3626558	133.0037127	197.2075882	167.7028554	3R	0.104358587	0.559233551	0.984934163
**l(2)34Fc**	FBgn0261534	1.563506239	1.107304115	1.011478488	0.567039812	0.815397407	0.131027923	0.354536712	0	2L	-1.686894087	0.127323495	NA
**l(2)34Fd**	FBgn0261535	7.128358232	25.37179075	15.52178911	13.13345764	26.67862574	19.17821937	29.62758405	33.14198504	2L	0.876111951	0.005710382	0.216319634
**CG42663**	FBgn0261545	0.872290357	0.491892331	0.516579707	0.544355495	0.532770472	0.526540934	0.74809708	0.519077855	2R	-0.042687865	0.885559693	0.995787491
**Exn**	FBgn0261547	42.64638219	30.57198699	33.43188493	45.96524918	39.08722204	40.18329666	38.8082539	31.97148779	3L	-0.000196499	0.998897207	0.999757791
**rdgA**	FBgn0261549	1.289989721	1.102659703	0.952754182	0.706109017	1.129251601	1.184838896	0.572233079	0.788125333	X	-0.114146678	0.622528192	0.990047511
**CG42668**	FBgn0261550	62.7299926	59.55596893	59.65039784	67.80976174	64.80244152	62.94089088	59.0797224	63.04416688	3R	0.029940617	0.755989389	0.995787491
**ps**	FBgn0261552	116.7685903	128.4632436	116.4971762	121.173684	116.5766899	114.9371916	94.34546843	118.8819785	3R	-0.087860111	0.362305355	0.939549067
**CG42671**	FBgn0261553	19.30776165	21.91874332	19.05962734	19.64757036	16.49375693	22.27131184	17.96020981	17.72869996	3L	-0.073174272	0.533653242	0.984760433
**CG42672**	FBgn0261554	28.76906566	35.63585878	33.90003492	34.13904814	26.62464391	47.33574586	32.75773644	35.48591361	2R	0.13010757	0.378066363	0.943615128
**CG42673**	FBgn0261555	6.230395298	6.117719026	6.364371709	5.10855814	5.127704536	5.887178583	4.585114273	3.697062438	3L	-0.275421888	0.055673105	0.61863954
**CG42674**	FBgn0261556	3.014556113	3.460492139	4.426794178	3.474770259	2.928109926	5.055425868	3.593295781	4.019599098	3L	0.147731736	0.394634443	0.952272708
**Thor**	FBgn0261560	2.205897597	5.796797895	7.670450084	5.485831686	7.698940357	2.079706781	1.313034838	0	2L	-0.884808417	0.326262102	0.918470465
**CG42675**	FBgn0261561	2.410217927	2.230511698	1.411382259	1.705176717	2.55062202	1.175347624	1.696142818	4.619304811	3R	0.402228664	0.405122641	0.956706394
**CG42676**	FBgn0261562	35.91488234	26.67269054	32.51493494	34.74360068	40.88618648	29.44842257	36.91442658	25.49831383	3L	0.059351234	0.749975961	0.995787491
**wb**	FBgn0261563	0.434186653	0.601338055	0.44452491	0.321951508	0.409886577	0.67974388	0.226943441	0.276744049	2L	-0.146245207	0.675865386	0.995787491
**ReepA**	FBgn0261564	97.16926447	96.05012787	89.9062503	104.1321982	90.81839552	107.4793983	89.25240675	96.86102174	2R	0.017504612	0.862679165	0.995787491
**Lmpt**	FBgn0261565	14.23039999	10.31144094	8.840117181	11.46528343	11.15782544	11.01178898	10.16117577	8.121745817	3L	-0.124240822	0.414001803	0.958665466
**raskol**	FBgn0261570	26.41398189	24.96753557	19.5211446	23.08284765	21.27589729	23.16484583	19.94665351	21.76013127	X	-0.097290174	0.386000478	0.947329421
**CG42685**	FBgn0261571	2.14987732	0.235181583	0.415734031	1.234928792	0.815589185	0.57281708	0.31793526	0.135292251	2L	-1.12268107	0.174553311	0.834088743
**CoRest**	FBgn0261573	101.5327735	118.263788	108.1723621	121.9520435	109.8082008	101.2797952	108.5234621	105.3634991	X	-0.050653179	0.607673985	0.990047511
**kug**	FBgn0261574	7.999602817	6.359163607	9.13203026	6.265739158	9.476365697	9.936120698	9.064761442	9.565483131	3L	0.384364724	0.01173354	0.329250239
**CG42694**	FBgn0261584	1.086039166	0.355449433	0.449829538	0.123515094	1.291113029	1.323592387	0.878454151	0.690114603	2R	1.043785316	0.219753115	0.864306828
**CG42697**	FBgn0261587	0.957989814	1.472985726	0.774689975	1.489011458	0.432354286	3.010623149	0.814617532	1.559913424	2R	0.347814188	0.737286484	NA
**pdm3**	FBgn0261588	0.562848609	1.087720994	0.870597135	0.901261578	1.077460318	1.105224529	0.886558243	0.669774331	2R	0.163460329	0.490267621	0.973549494
**RpS24**	FBgn0261596	927.8724948	1272.466678	1373.52294	1460.894486	1152.011142	1270.671344	1388.14522	1333.451338	2R	0.063197727	0.638006759	0.991585536
**RpS26**	FBgn0261597	875.995028	1138.378974	1246.741248	1397.407925	1220.911435	1069.694791	1281.414981	1462.326015	2L	0.145827634	0.309234546	0.912131082
**RpS29**	FBgn0261599	413.5642348	604.5274538	679.4730963	572.1383737	597.4820066	518.2257954	618.2856616	619.5984518	3R	0.088288837	0.527369107	0.984107922
**RpL8**	FBgn0261602	1175.143122	1779.521967	1749.10963	2019.188752	1628.442068	1390.448232	1743.099557	1838.127742	3L	0.00824328	0.955956714	0.997604378
**RpL37A**	FBgn0261608	344.2641384	606.962684	701.022991	603.0826956	560.3903508	457.774778	654.6440465	760.95977	2L	0.149189928	0.436330616	0.966084844
**eIF2alpha**	FBgn0261609	103.7798133	99.15219874	114.2614419	154.9336089	128.2270329	109.8879521	135.9597558	146.2429566	X	0.170898005	0.314481434	0.91460807
**CG42699**	FBgn0261610	28.23928578	26.08751034	24.89066538	23.66259556	25.66454558	26.69888718	27.31266284	25.28158717	X	0.057955885	0.552934667	0.984934163
**CG42700**	FBgn0261611	6.847389853	6.724083283	8.365806073	5.753727178	12.24972963	16.34506029	13.37939745	13.10418456	2R	1.02218929	6.94E-13	5.79E-10
**CngA**	FBgn0261612	2.298035089	3.026147337	3.178507398	5.042618634	3.238115609	3.568473909	2.281271366	2.61879023	2R	-0.179278689	0.495296577	0.97491773
**nej**	FBgn0261617	9.569505588	18.09405525	24.06092341	24.05734059	14.19490594	14.31295549	17.25905912	19.74138184	X	-0.174852759	0.581385805	0.985637351
**larp**	FBgn0261618	19.85613575	41.90658971	57.31049672	41.75283889	34.53650043	25.81517111	53.62735336	50.91860595	3R	0.078678384	0.840165444	0.995787491
**GLS**	FBgn0261625	17.33315717	22.64673295	19.67561596	19.33018003	24.32202471	30.68419017	28.08753858	33.15239347	2R	0.59038216	1.68E-05	0.002375277
**lncRNA:CR427**	FBgn0261638	0.054657356	0.17190009	0.298345465	0.047786838	0.037001451	0.012882655	0	0.133499453	3R	-1.593927851	0.231239203	NA
**CG42724**	FBgn0261641	120.6711563	104.443626	94.20486533	111.5654382	110.3747254	109.8922386	102.7340322	107.9561003	3R	0.028180763	0.795219236	0.995787491
**Axud1**	FBgn0261647	102.2897855	117.3355861	92.30664658	91.95508022	89.60494459	91.47909116	88.71800307	93.83189579	2L	-0.120064257	0.267128198	0.894434069
**salm**	FBgn0261648	0.551757382	1.002620238	0.985325733	0.857601043	0.401192238	0.368470388	0.664673997	0.532407267	2L	-0.757962063	0.110192219	0.758410631
**tinc**	FBgn0261649	6.804034681	7.637934392	7.334687326	8.638884668	7.756081022	9.629570684	7.774332201	6.243247194	3R	0.073049212	0.581731286	0.985637351
**tweek**	FBgn0261671	10.57784612	16.35414914	19.62885168	14.76317168	11.97862461	15.63099422	15.81305481	16.03896698	2L	-0.00990848	0.951764651	0.997604378
**nemy**	FBgn0261673	4.499754041	10.56949874	10.42637912	10.28382389	5.585617899	6.489181709	6.38865433	3.314925141	2R	-0.682482322	0.012402149	0.33520527
**Npc1b**	FBgn0261675	1.121513755	5.33001564	2.720776726	4.357945165	0.742360582	3.119201136	2.098070834	1.339200598	X	-0.854517415	0.114058864	0.759845383
**CG42729**	FBgn0261682	0	2.15777162	4.161082293	4.332200208	8.515096377	3.368941952	0.364628722	1.241295244	3L	0.39843284	0.750917276	NA
**SLO2**	FBgn0261698	15.41569224	17.23231044	15.70673485	18.22830994	17.7050405	16.37036063	15.31821412	16.87528975	2R	0.024014892	0.806595362	0.995787491

**lncRNA:CR427**	FBgn0261700	5.375462353	2.663990726	5.038489631	2.848338208	2.426021817	1.842892026	1.246629086	0.530483017	2R	-1.360684122	0.129709275	NA
**gce**	FBgn0261703	6.987833878	6.87311223	8.569880602	6.027708175	7.87417905	9.542953386	6.968672974	10.34362216	X	0.319475257	0.050151251	0.587848846
**nocte**	FBgn0261710	62.01893435	118.7612121	113.9254633	117.4320481	88.79170982	78.7115495	110.6549969	147.2886924	X	0.085646498	0.751470984	0.995787491
**Cpn**	FBgn0261714	0.02625591	0.484446654	1.146536467	0.061214665	1.801148552	1.782281603	0	0.11400802	3R	1.162704488	0.380701612	NA
**fwe**	FBgn0261722	343.0651862	297.9947726	394.4050523	317.7102324	316.1210725	321.3236684	344.3478985	337.1878799	3L	-0.006177151	0.953930242	0.997604378
**Dbx**	FBgn0261723	0.260664912	0.66175173	0.256365989	0.082125734	0.190770395	0.09298773	0.143775522	0.030590684	3L	-1.422956452	0.111427626	0.758410631
**mi**	FBgn0261786	32.74736126	37.21323117	31.44385517	32.14463498	36.57111205	28.59322929	32.51256213	32.96535176	2R	-0.000227639	0.998721497	0.999757791
**brun**	FBgn0261787	50.79218535	41.63389106	52.46585357	60.10377039	55.41942698	47.0583354	56.51456071	48.58321546	2L	0.046858078	0.746679654	0.995787491
**SmD2**	FBgn0261789	191.0165263	199.8334957	188.4522654	185.0997743	219.0183735	214.8700083	222.4203184	218.4011973	3R	0.224422493	0.047040082	0.570643441
**SNRPG**	FBgn0261791	286.02511	370.9784584	416.4737759	370.402297	409.7923498	270.0650082	394.3989508	379.2004101	X	0.046035829	0.784249385	0.995787491
**snRNP-U1-C**	FBgn0261792	155.0542356	171.975866	186.5481128	163.971689	144.0592716	168.0880057	156.5437211	171.0598938	3R	-0.050405173	0.722198382	0.995787491
**Trf2**	FBgn0261793	13.95291172	13.07909497	14.34003717	9.366184086	9.913393187	15.09490353	9.308100828	13.05516939	X	-0.070211711	0.679728279	0.995787491
**kcc**	FBgn0261794	210.9491561	206.3280919	215.0102818	208.8828792	226.9277719	220.9951623	214.670574	201.0490875	2R	0.067837776	0.39716468	0.952272708
**Dhc64C**	FBgn0261797	33.65624065	45.31041635	57.11340418	48.15247981	40.69037023	53.748165	48.05527497	68.41021415	3L	0.228773544	0.170063128	0.830934498
**LanB1**	FBgn0261800	0.454762031	0.747232174	0.495335093	0.086551882	0.234560577	0.533746097	0.236756676	0.604488041	2L	-0.110794456	0.881347612	0.995787491
**CG42747**	FBgn0261801	1.230678333	1.170842289	0.927926128	1.073910315	1.217991528	1.000534799	1.115514295	0.914134692	3L	-0.024741627	0.885522166	0.995787491
**CG42748**	FBgn0261802	12.22554667	14.52484443	17.34511487	17.41361674	13.2734207	15.30600809	14.06600093	16.95729886	2L	-0.013738609	0.919578871	0.996479182
**CG42749**	FBgn0261803	0.466053953	1.321851485	0.349470666	0.908782061	1.315560505	0.910741482	0.670114965	1.195818325	X	0.465640269	0.365126827	0.940794966
**CG42750**	FBgn0261804	6.58606121	7.858455164	8.560578428	7.143795031	7.097038491	6.659308669	6.450267283	7.492473892	2L	-0.088550734	0.41464924	0.958665466
**cu**	FBgn0261808	6.644837792	6.013722468	7.5938527	2.725308365	2.1526996	4.704081628	5.523624811	5.024598106	3R	-0.369211059	0.249238182	0.879541335
**pico**	FBgn0261811	11.06321669	15.78342176	17.4406762	17.63016253	15.15433705	15.4772685	15.9320584	14.54930543	X	0.014119491	0.922634167	0.996479182
**Bsg**	FBgn0261822	70.78100548	84.57366455	80.30061912	77.46462262	86.49810433	81.64342215	68.42300752	81.37687341	2L	0.053855319	0.58422368	0.98660996
**Asx**	FBgn0261823	54.52839295	60.55024869	62.53567963	49.92484447	55.40213657	52.3945431	56.49040916	58.80750749	2R	0.004708479	0.965379826	0.997604378
**CG42764**	FBgn0261832	0.261094938	0.766412728	1.266825355	1.978382311	0.824850742	1.107714207	0.333029398	0.42514596	3L	-0.601633077	0.600063338	NA
**CG42766**	FBgn0261834	6.671354778	1.467575311	1.59192921	4.3347248	2.171779631	2.165310873	3.068958411	3.561671012	X	-0.361507774	0.664697409	0.995787491
**Msp300**	FBgn0261836	11.70825662	13.61378864	15.31384149	15.51449093	15.26035108	13.26694388	13.00700485	17.20459771	2L	0.098236718	0.441908374	0.967112302
**Xpd**	FBgn0261850	23.9356157	14.35064935	22.85506819	13.37474893	19.99797996	14.47696773	21.69910417	12.83055633	2R	-0.083697234	0.745335315	0.995787491
**aPKC**	FBgn0261854	103.4683511	120.2442938	128.5600441	141.8026812	106.2586976	120.7587642	131.8914346	142.7971672	2R	0.053739124	0.666550363	0.995787491
**CG42788**	FBgn0261859	74.90266375	89.70500534	96.55830273	90.93209458	76.83857652	70.58825898	84.21107888	77.21133188	3R	-0.155865145	0.139702468	0.803344611
**CG42789**	FBgn0261860	4.916561522	1.697879282	4.560518406	1.685702662	0.783145911	4.771636648	0	3.767403595	3R	-0.433349026	0.706275535	NA
**whd**	FBgn0261862	25.31683494	33.18311393	29.96972163	29.35717732	27.70057873	31.21411035	30.56674478	35.8576536	2R	0.122638867	0.341051076	0.927274752
**dpr2**	FBgn0261871	0.896722472	0.49623834	0.583979802	0.312744369	0.534075894	0.556109128	0.468761945	0.267295332	2L	-0.30548515	0.400455681	0.953696242
**scaf6**	FBgn0261872	147.4707839	114.0456241	114.7927201	119.154136	102.1659861	127.774613	104.5475722	101.1522046	3L	-0.160305387	0.188353788	0.843108521
**sdt**	FBgn0261873	36.9776333	48.04833272	41.41951729	46.98023512	43.91438368	38.76180951	33.75224849	37.85115614	X	-0.137074281	0.223578786	0.86549481
**l(2)35Be**	FBgn0261881	135.1367205	122.3314014	106.5541297	108.5942377	118.198082	119.7020036	142.2722795	105.7816291	2L	0.065878033	0.688139489	0.995787491
**osa**	FBgn0261885	35.46207489	51.95928949	55.89229463	56.25444638	48.20821565	38.04189126	53.74315902	55.97586183	3R	0.010338011	0.947237521	0.997604378
**CG42795**	FBgn0261928	54.61414613	78.76899465	76.86130627	78.27061295	66.71633693	68.7308398	69.2248206	81.24334859	3R	0.020828512	0.868733373	0.995787491
**5-HT2B**	FBgn0261929	1.866462554	1.761736445	1.513394733	0.900731755	1.43110763	1.95521055	1.378071418	1.405220227	3R	0.057568988	0.799411593	0.995787491
**vnd**	FBgn0261930	0.010807018	0.054381768	0.039326511	0.60470771	0.019509436	0.697932124	0.165413604	0.152509731	X	0.552293857	0.625955115	0.990047511
**CG42797**	FBgn0261931	9.214964792	17.55023806	14.21278675	13.19288418	9.198125498	15.21081179	17.60363302	19.91446677	X	0.2303258	0.294777298	0.906715248
**SmD1**	FBgn0261933	31.11866403	27.6132474	23.59170153	24.93974784	32.97900489	28.42165867	31.54111547	25.33556259	3L	0.165194148	0.472080756	0.973549494
**dikar**	FBgn0261934	130.6992381	141.375502	141.3625159	144.1636294	134.7156538	145.0993448	124.0505294	141.2254628	3L	-0.002582739	0.97618562	0.997757033
**mtRNApol**	FBgn0261938	28.24103379	28.86499023	29.67910844	35.82106632	33.75401201	44.84374347	37.42462587	15.02539516	2L	0.118711654	0.617282684	0.990047511
**TfAP-2**	FBgn0261953	0.342932612	0.613569437	0.309009901	0.060916912	0.188672383	0.172434545	0.116643795	0.127635162	3L	-1.090725425	0.086833609	0.706081898
**east**	FBgn0261954	28.51673439	55.53623462	47.58398458	48.54231892	41.43126072	43.26304989	51.52698142	56.47006527	X	0.13421695	0.434025113	0.965402206
**kdn**	FBgn0261955	25.4168744	48.25789441	64.21545307	49.66533389	57.8155601	35.44794799	73.28996397	60.87999498	X	0.319406252	0.357203387	0.937912106
**mid**	FBgn0261963	0.638285515	0.681312805	0.527888099	1.420495983	0.628510334	0.574418426	0	0.251959443	2L	-1.126278446	0.19604727	0.844272804
**l(2)gd1**	FBgn0261983	32.11613049	34.53796543	37.63926792	30.74681972	29.3290281	33.30887046	31.6068163	26.56270181	2L	-0.129369985	0.35367868	0.936401286
**Ire1**	FBgn0261984	37.1438935	34.28079411	33.72742342	28.69329415	36.30163629	37.07637662	33.6101451	33.24033312	3R	0.096043428	0.421147412	0.960593359
**Ptpmeg**	FBgn0261985	35.76531289	37.66738129	32.30990414	40.91971601	36.3328096	40.45909222	37.95921571	35.53511265	3L	0.063583618	0.532922915	0.984760433
**RASSF8**	FBgn0261986	122.0540065	91.97288746	85.36549605	94.0144214	99.63639193	98.35684295	71.83517736	79.28196305	3R	-0.14674373	0.327236518	0.918470465
**Gprk2**	FBgn0261988	19.0837303	18.67887513	18.41493183	18.32674102	17.09032271	19.81682994	15.14862317	16.61589775	3R	-0.089336169	0.356856252	0.937787223
**CG42808**	FBgn0261990	4.879901163	2.046337405	0	1.264145095	0	1.277983614	0.691596262	1.765786192	2R	-1.131377008	0.459135636	NA
**CG42813**	FBgn0261995	308.5375604	257.7410684	298.7830116	300.0087199	330.7747102	325.6667386	342.4883487	321.6253421	3R	0.209345538	0.05284429	0.605783103
**CG42816**	FBgn0261998	0.393290254	0.63330166	0.686964004	0.990296737	0.085198765	0.044494998	0	0.239083474	3L	-2.852064888	0.004957629	NA
**Ca-Ma2d**	FBgn0261999	34.97646827	26.81186509	36.62514096	38.7339993	40.39873086	28.31238439	37.61153248	44.41359816	2L	0.168165361	0.312324352	0.914141092
**CG42827**	FBgn0262009	0.896934496	3.22388955	0.310850083	1.792430582	0.693941421	3.7449079	2.28809917	0.834569066	3R	0.314469766	0.792137822	NA
**CadN2**	FBgn0262018	0.810488365	0.6287186	0.616530833	0.150986835	0.540263273	0.512499283	0.29787072	0.321678493	2L	-0.371003249	0.329608968	0.921112231
**lncRNA:CR428**	FBgn0262028	0.069257659	0.464680029	0.896096613	1.453244625	0.083351884	0.74001791	0.706711105	0.100243193	3R	-0.788992687	0.46871393	NA
**d**	FBgn0262029	0.356714657	0.551801333	0.500000096	0.535963045	0.22180892	0.15694392	0.101109639	0.163497225	2L	-1.552316564	0.001463159	0.082296708
**CG42841**	FBgn0262030	0.545197455	0.571557654	0.082665089	0.71499788	0.615138212	1.542026491	1.303884769	0.36989817	3L	1.011331576	0.300860153	NA
**Spn77Ba**	FBgn0262057	1.598559557	2.780669009	1.436327107	1.449381667	3.687423516	3.572414071	0.679659962	1.928122653	3L	0.478531116	0.42836883	0.963309722
**Csk**	FBgn0262081	46.93229823	46.07629019	48.14480609	45.17971372	35.61977794	43.56336543	38.9744335	38.14429488	3R	-0.224527352	0.01476496	0.35986804
**lncRNA:CR428**	FBgn0262097	1.181754741	0.880990131	0.955640173	0.204090125	0.395068553	2.021978016	0.446619516	0.570155319	2L	0.118266297	0.908426614	NA
**Sik3**	FBgn0262103	19.97223737	19.16318038	19.17881513	17.48312023	15.11213557	16.35758283	16.55393354	14.32262122	2R	-0.252971624	0.018096141	0.385711607
**lncRNA:CR428**	FBgn0262108	305.2186722	374.6253786	362.7507014	313.9762709	370.0902721	337.382077	382.6565516	301.3017691	X	0.069238414	0.604715698	0.989286463
**lncRNA:CR428**	FBgn0262109	2087.464355	1920.992525	1857.323169	1966.290599	2000.682158	2208.060675	1733.405706	1882.180159	3L	0.025953495	0.780914006	0.995787491
**f**	FBgn0262111	0.593423906	0.414491773	0.433204176	0.281755466	0.530756856	0.382620661	0.188654476	0.505169581	X	-0.071506511	0.851106014	0.995787491
**sro**	FBgn0262112	5.162676893	4.209559976	0.521857589	6.519817863	3.494983116	5.002544136	5.487541529	6.071351779	3R	0.30421596	0.649418924	0.992932477
**RanBPM**	FBgn0262114	157.2814936	166.0486836	154.1843546	155.7869842	151.0443821	187.7907947	137.4721903	158.2594546	2R	0.031312158	0.757775857	0.995787491
**CG17683**	FBgn0262115	119.6781179	124.6830935	116.955424	94.61748304	95.62752385	146.5381836	112.5072817	111.0355905	2R	0.058962661	0.676380332	0.995787491
**RNASEK**	FBgn0262116	792.905566	645.4832399	940.5640204	509.8697351	614.4589889	719.040269	663.7501444	670.2778147	2R	-0.084327275	0.578886535	0.985637351
**IntS3**	FBgn0262117	36.68110193	36.53062619	42.7905232	25.20458851	34.1146969	40.44922627	32.36496518	28.01788129	2R	-0.035319749	0.826471286	0.995787491
**l(2)41Ab**	FBgn0262123	1.630797386	1.933244813	1.964810041	1.317933738	1.157755661	1.985801049	1.086793603	1.565427928	2R	-0.209716761	0.282165681	0.901329548
**uex**	FBgn0262124	6.779279055	6.699776958	10.84027986	6.361706003	5.454110313	8.605042853	7.212614345	5.80930721	2R	-0.151318342	0.396664832	0.952272708
**Sec23**	FBgn0262125	255.0183085	276.1487543	248.8715999	268.8096007	263.9918796	285.8897752	239.378756	251.5858872	3R	0.018450577	0.838338507	0.995787491
**Sec24CD**	FBgn0262126	95.70576462	88.2171847	105.6532733	93.58270282	79.21961728	103.6800399	83.48764937	92.65161392	2L	-0.065177292	0.565070361	0.984934163
**kibra**	FBgn0262127	2.151439189	3.083830161	1.803052142	0.37999996	1.447635136	0.915836955	1.480196108	1.691458635	3R	-0.379218552	0.360197517	0.939549067
**trh**	FBgn0262139	0.318486023	0.322755273	0.289740994	0.015469545	0.185660582	0.181411118	0.22004252	0.605029809	3L	0.370539849	0.606378257	0.989286463
**CG42866**	FBgn0262140	1.691028657	0.945487781	4.102411935	2.190316474	0.678386696	3.011439478	1.437950145	6.118966251	2L	0.381591165	0.743247649	NA
**CG9932**	FBgn0262160	5.739545123	7.770917104	6.484349615	7.556133994	6.116301625	7.376398402	6.33357575	4.279812287	2L	-0.163834799	0.310189556	0.912401755
**calypso**	FBgn0262166	87.80061712	96.09225606	84.01685048	83.32210773	93.02217011	110.4304459	80.66049741	70.05529287	2R	0.040533559	0.780394779	0.995787491
**ana1**	FBgn0262167	1.399229265	0.1498092	2.491715286	2.429335371	2.633455418	2.35769199	3.265679752	1.034163833	3R	0.547069863	0.381869175	0.944476532
**magu**	FBgn0262169	0.178338524	0.130059937	0.319782396	0.433867361	0.447925904	0.453237443	0.712088461	0.606035651	2R	1.106988526	0.076019639	0.681158992
**CG43052**	FBgn0262354	1.278498091	2.948689139	1.938511585	0	2.30801673	2.260050357	0	1.734836065	2L	0.082609109	0.956695722	NA
**CG43060**	FBgn0262362	0.193842541	0.197056769	0.142502818	0.136950481	0.243894417	0.271361352	0.164832162	0.0382591	3R	0.127735072	0.87525306	0.995787491
**mir-317**	FBgn0262378	124.6921998	183.6081858	196.2796235	179.7551813	166.6781203	145.3783601	208.8240712	169.4572617	3R	0.048099786	0.884606336	0.995787491
**Scox**	FBgn0262467	156.5881975	116.7061657	176.2235327	146.1050306	181.8860519	170.285813	167.6769807	158.3900609	2L	0.216516452	0.15405905	0.817268058
**Tl**	FBgn0262473	14.78594747	21.64506052	21.99624562	21.79587651	17.80697023	22.61452747	20.07244031	18.05013669	3R	9.58E-05	0.999420005	0.999757791
**bru2**	FBgn0262475	9.688881779	16.06095986	13.57563928	17.39937708	14.62220536	14.82250028	14.81003142	14.5586685	2L	0.084575666	0.569879817	0.984934163
**CG43066**	FBgn0262476	190.2535272	191.2219089	207.0707455	190.1179931	171.7743153	184.5318831	207.4072707	161.9201382	2R	-0.071584198	0.466334634	0.971928234
**FoxP**	FBgn0262477	143.744871	123.011364	108.5320378	102.0973681	115.0523983	142.8431943	102.5128251	85.4624868	3R	-0.073749498	0.624414793	0.990047511
**Rbp**	FBgn0262483	303.3749118	374.6365127	321.2482116	358.357107	340.717579	337.0697259	330.6003228	380.1353043	3R	0.064973145	0.507422878	0.978557094
**CG43078**	FBgn0262508	5.897590227	9.866900848	5.850125232	7.348685103	9.00133102	8.80754773	6.403519167	7.544015518	3L	0.166272162	0.356750541	0.937787223

**nrm**	FBgn0262509	10.13107891	13.03964185	13.63208986	12.38328079	9.625695298	9.058751293	9.776046683	11.20407511	3L	-0.275935316	0.027081681	0.461092427
**Vha44**	FBgn0262511	226.0689327	185.3479104	176.990108	195.0129304	176.6547299	184.3567778	160.0239634	202.3862923	2R	-0.086243047	0.448721428	0.967112302
**Vha14-1**	FBgn0262512	2480.467481	2060.776834	2450.080667	2092.516514	2489.295134	2070.620126	2270.09641	2310.405105	2R	0.040140354	0.716161809	0.995787491
**Vha16-4**	FBgn0262513	3.978930012	0	2.598838499	5.351957039	2.76268787	9.378277278	1.171192609	2.990293401	2R	0.463368846	0.679907433	NA
**VhaAC45**	FBgn0262515	316.6869523	318.2197991	320.0921485	305.2477693	308.3554155	279.9352363	305.5367903	346.88004	2R	0.009667218	0.926742806	0.996479182
**Ltn1**	FBgn0262517	35.60669481	34.01375931	37.61000384	39.57605508	32.76940745	38.68849686	35.51570952	25.98939458	3L	-0.115530729	0.392941568	0.951969484
**Rab8**	FBgn0262518	124.3297548	111.5373588	117.1837273	102.3799069	107.3669774	119.0598725	89.67855518	88.84216299	3L	-0.141615309	0.249055333	0.879503029
**Mi-2**	FBgn0262519	49.81071959	43.75619247	42.72230646	43.83266244	37.59853003	41.91311161	41.8879479	41.50538	3L	-0.116187197	0.229781983	0.867702781
**ver**	FBgn0262524	3.426001117	4.997075004	5.239815148	4.167439634	4.974738554	10.46243318	4.749892639	7.923263322	3L	0.698185605	0.165564566	0.828893393
**nsl1**	FBgn0262527	143.6176321	152.85132	140.0981894	168.3783149	119.5877925	126.5386259	147.0267152	140.6353197	3R	-0.149794059	0.175723146	0.834088743
**CG43085**	FBgn0262531	2.14641536	2.206642014	3.023520227	1.614286003	0.874961578	1.762514092	1.413045599	2.405195581	3L	-0.443972528	0.565563717	0.984934163
**Mdh2**	FBgn0262559	1863.85902	1706.625126	1711.75582	1754.731388	1955.948508	1883.914777	1883.531591	1976.77711	3R	0.160092725	0.060658426	0.627984253
**wcd**	FBgn0262560	28.45581355	35.75426709	35.14887744	67.80176044	36.22246172	36.94987424	63.28480304	44.92077202	2R	0.147630771	0.596949476	0.988780662
**CG43102**	FBgn0262562	40.06151677	46.277993	43.85753438	45.77664522	41.94588003	47.13276489	45.03074318	35.83526289	3R	-0.021028547	0.837429748	0.995787491
**lncRNA:CR431**	FBgn0262564	7.272054205	13.55316751	6.076655158	15.070685	10.93993471	11.57910377	8.657211039	5.262767615	2R	-0.170453149	0.712080029	0.995787491
**CG43110**	FBgn0262570	0.260205013	0.184655056	0.509858784	0.256663081	0.216801572	0.141530594	0.02553033	0.065184135	2R	-1.370871168	0.159344802	NA
**CG43117**	FBgn0262577	54.47688405	74.47252448	54.18566596	36.19807924	26.06135558	39.29076225	38.91207645	16.55841014	3R	-0.829491016	0.039959211	0.529044592
**Sarm**	FBgn0262579	2.150882852	2.739736855	2.510993155	2.493894931	2.177616322	2.377904482	2.115272581	2.130215157	3L	-0.135756572	0.342441052	0.928339671
**cic**	FBgn0262582	12.01977961	16.02187173	17.80962249	19.36912284	15.83482505	14.40898553	13.38174299	18.5990889	3R	-0.034508888	0.819143118	0.995787491
**Shab**	FBgn0262593	45.51073586	62.77249815	52.69802998	58.06739924	52.83073782	53.99897248	43.49343717	52.9692781	3L	-0.076041965	0.497293347	0.97594235
**mtsh**	FBgn0262598	3.643761907	2.598760388	1.252873072	0.602028688	1.13948292	5.004201156	1.976169311	2.304763329	2L	0.374076962	0.563145001	0.984934163
**SmydA-3**	FBgn0262599	14.12907973	19.95580167	18.12723336	28.50699591	14.61024236	12.38762145	14.40753229	5.393501539	2L	-0.758391177	0.008922524	0.281896641
**SmB**	FBgn0262601	164.3778071	164.2132105	138.6815255	157.1092563	202.8411801	156.5448377	194.8895897	196.1110854	2L	0.295641907	0.036669772	0.521170991
**CG43133**	FBgn0262607	4.966307507	7.105242355	0.35435861	0.68110347	2.636899384	4.95762817	2.235733614	0.317126862	X	-0.347008928	0.765533254	NA
**pyd**	FBgn0262614	17.68258916	19.87639996	18.4881132	19.04885818	21.8304998	20.66896987	17.59870294	19.18091736	3R	0.108822029	0.243224306	0.876959115
**Nuak1**	FBgn0262617	3.371680086	4.023677359	3.016810574	3.736121129	2.595453949	3.988755255	2.251630455	4.258423257	3R	-0.081814285	0.682502994	0.995787491
**DNAlig1**	FBgn0262619	5.752881606	4.524977984	5.992583054	8.335530838	2.581687757	7.231708851	3.399461624	2.469781625	2R	-0.621574236	0.155363446	0.818884898
**lncRNA:CR431**	FBgn0262622	4.003006436	2.285781015	2.479464965	2.184286105	1.230034482	5.942058324	1.08635696	0	3L	-0.387726569	0.709667655	0.995787491
**Tmhs**	FBgn0262624	0.208898439	0.901022981	0.217193443	0.069576978	0.16162081	0.126609539	0	0.064791121	3L	-1.904935762	0.135971851	NA
**lncRNA:CR431**	FBgn0262631	8.484116714	4.423122412	8.344195465	6.949869824	4.139460846	8.323048014	6.141978977	4.356034435	2L	-0.271335054	0.541574253	0.984934163
**dati**	FBgn0262636	94.35813797	94.69135508	85.29583023	98.4296518	98.64546161	106.1927762	82.30987961	89.36085393	4	0.042123331	0.675127823	0.995787491
**Nup160**	FBgn0262647	26.22859076	13.75000301	16.44630717	15.44216464	17.86501705	18.46897863	22.18396421	12.48520127	2L	0.003729497	0.987214801	0.998078482
**CG43155**	FBgn0262685	1.005140546	0.365006469	1.546031981	0.410707267	0.664082873	3.17996845	1.401022287	1.282354956	X	0.991305145	0.049514368	0.583905634
**fzr**	FBgn0262699	77.60066883	88.96296057	98.21268635	83.65134635	72.25339	78.03575394	105.4216157	82.51932531	X	-0.009646834	0.940388893	0.997188858
**Sap130**	FBgn0262714	75.33868997	65.33363863	54.40408035	70.20311239	48.02391091	57.23517866	57.0222281	59.73302718	3L	-0.229534107	0.081370749	0.694684542
**Arp3**	FBgn0262716	198.5449887	210.568529	153.5372655	200.3283639	201.6177496	204.5015549	163.5760983	192.653997	3L	0.027778928	0.826400089	0.995787491
**Skeletor**	FBgn0262717	1.448745183	2.352180049	1.194074918	1.429024746	1.324443288	0.639156231	0.402745266	1.350891752	3R	-0.750567603	0.110443387	0.758410631
**CG43163**	FBgn0262719	0.332962766	0.668505577	0.446717431	0.229358455	0.277773265	0.458982164	0.321740595	0.394305266	3L	-0.168833723	0.679655102	0.995787491
**Pal2**	FBgn0262728	971.7807958	1118.801068	934.1516715	929.8639934	919.85919	1043.963291	965.0112413	948.1820971	2R	0.002177105	0.980092566	0.997881056
**dtn**	FBgn0262730	23.33636965	40.93023254	68.67990121	39.94622563	37.46859162	25.16099603	58.63275382	61.39268261	X	0.123646904	0.772750979	0.995787491
**CG33941**	FBgn0262731	5.159216943	3.4688479	4.11925186	2.436155966	2.63298943	4.330466264	3.387500714	1.937749537	4	-0.282567959	0.478347966	0.973549494
**mbf1**	FBgn0262732	570.1152456	482.8280583	503.2013051	465.5981366	440.1425818	498.832746	473.1464226	391.4813253	3L	-0.137305741	0.198623045	0.847197998
**Src64B**	FBgn0262733	17.65781297	20.69851276	17.04787364	18.78912388	18.09290827	18.37051933	17.37654819	19.33553931	3L	0.011559563	0.908083035	0.996479182
**eIF4H1**	FBgn0262734	37.21824095	59.66402178	54.8742315	55.2016706	53.92432064	42.5342409	52.93770785	56.53315539	X	0.029694556	0.850177778	0.995787491
**Vha16-1**	FBgn0262736	270.7482395	356.5416995	440.2974362	365.5789464	337.7416196	315.0503671	382.5780448	387.0632358	2R	0.024131339	0.858434192	0.995787491
**mub**	FBgn0262737	138.1917387	220.1296359	235.6097242	218.6608727	204.1141827	190.4278386	203.9189757	205.3165716	3L	0.018556165	0.891560354	0.996189576
**norpA**	FBgn0262738	13.30189972	17.84965657	11.99450305	11.20145518	16.97381169	19.1855535	12.11686932	13.41563303	X	0.213044233	0.176104419	0.834204539
**AGO1**	FBgn0262739	151.6437916	186.687019	167.1415226	164.6126372	173.2578489	199.5809242	190.7254741	219.7272418	2R	0.257740055	0.010973297	0.318437078
**Evi5**	FBgn0262740	35.89159841	49.88919655	44.90997546	52.22377912	44.79455896	49.87807291	41.76139526	51.62621461	X	0.071514058	0.567650932	0.984934163
**lncRNA:CR431**	FBgn0262741	0.746429243	1.279248393	0.27556062	0.264823951	0.497987686	0.780221206	0.289763027	0.070459434	3R	-0.611932461	0.494887511	0.97491773
**Fs(2)Ket**	FBgn0262743	274.8108631	421.7162841	481.4106221	436.5753764	334.6902303	358.4538395	423.7733226	482.1272583	2L	0.02172883	0.889531178	0.995926915
**Mdh1**	FBgn0262782	226.6702644	272.2908709	249.2371171	221.289266	268.9067315	243.9678712	252.7535599	239.7386099	2L	0.085307009	0.397360433	0.952272708
**CR43170**	FBgn0262789	1.141860112	11.76547664	1.58293526	1.90157419	1.766871565	4.459946204	2.912909678	4.60401315	3L	-0.204596766	0.839191167	NA
**CG43175**	FBgn0262794	1.435200908	0.859053709	0.837938125	0.319338956	0.37627258	0.387401688	0.212685464	0.129292698	3R	-1.611388717	0.004375633	0.184154493
**twr**	FBgn0262801	79.12243593	95.92862462	98.19754107	84.66247117	103.4435344	73.18274857	81.58707898	99.81446025	3R	0.035865719	0.825882294	0.995787491
**CG43183**	FBgn0262812	2.797746952	2.815696817	1.085967213	3.130964015	3.63646822	3.798286182	1.71290702	0.728900114	2R	0.034723579	0.969907726	NA
**CG43184**	FBgn0262813	13.09967295	4.932683683	9.728456286	1.869881286	4.826176959	7.057309511	4.091944548	2.321681845	3L	-0.6730452	0.472734996	NA
**CG43191**	FBgn0262820	3.411811927	2.335851378	1.858103869	1.136358724	1.885467847	1.903723018	2.486744436	0.907023615	2R	-0.273461011	0.728583726	0.995787491
**CG43219**	FBgn0262855	10.48469566	12.72222571	18.01375085	16.56888186	15.01548301	19.40927034	10.97508306	17.08973093	3L	0.149360388	0.567193184	0.984934163
**CG43222**	FBgn0262858	282.9529358	191.5674972	227.6458771	212.7924904	324.8982329	226.6908634	267.6131734	277.2088123	3R	0.288881232	0.105012788	0.747886154
**Ptr**	FBgn0262867	2.680814493	3.549493841	2.696622333	1.316564782	1.470109407	1.423445021	1.39505768	1.445395121	2R	-0.803254705	0.008215335	0.268496474
**Gfrl**	FBgn0262869	28.16051962	30.50363511	28.88980272	29.53760538	31.83055833	31.82885443	25.81707405	29.83596336	3R	0.057132697	0.53035312	0.984760433
**axo**	FBgn0262870	3.03320698	5.631549755	7.165543336	2.431918101	5.868580339	6.690059206	8.143616981	7.647635204	3L	0.676153538	0.059400666	0.626639433
**milt**	FBgn0262872	65.99122514	73.91917086	59.75787319	71.68697351	61.87834478	66.93531756	63.04434013	73.17721097	2L	-0.003148113	0.976080065	0.997757033
**lncRNA:CR432**	FBgn0262887	5.593150242	2.789161454	10.63508729	4.934137007	4.548229595	6.460842814	10.54050637	2.625566903	2L	0.041806309	0.93776144	0.997038394
**CG43245**	FBgn0262890	1.46910186	2.661349257	2.851216496	1.5070685	1.909515877	3.324144623	1.798900995	1.403404697	3L	0.038741576	0.955797619	0.997604378
**CG43248**	FBgn0262893	9.79759695	8.269385089	3.93623995	4.073862399	2.70377176	11.11983855	1.910360279	4.606564698	3L	-0.337513554	0.577745422	0.985637351
**lncRNA:CR432**	FBgn0262898	0.268808115	0.589622642	0.702288179	0.337462462	0.410610997	0.409387971	0.263744337	0.359142954	3L	-0.342177513	0.699056206	NA
**lncRNA:CR432**	FBgn0262901	0.296203087	0.212931054	1.437169116	0.493275962	0.458333562	0.757987252	0	0.643084322	2R	-0.326805142	0.800498426	NA
**asRNA:CR4325**	FBgn0262904	4.353008773	3.298675016	5.628606851	3.142545386	1.316364389	2.770714117	4.396752662	1.583126421	3R	-0.682902727	0.129203364	0.788661222
**Rabex-5**	FBgn0262937	54.37105048	63.76114931	47.81701919	50.8775594	67.83617555	55.65356512	64.22009256	48.90626401	3L	0.156539857	0.317337659	0.91460807
**mt:ND4**	FBgn0262952	30.55591326	36.70254487	55.59616453	15.54600854	23.02281188	26.00037604	27.57933926	24.73684334	mito	-0.412251872	0.190762117	0.843401316
**Rpb12**	FBgn0262954	142.2677174	145.817324	160.1752367	114.717726	123.7323161	125.6812226	176.4511841	127.6461521	2R	0.005499743	0.977064671	0.997757033
**RpII140**	FBgn0262955	74.14682231	103.6872531	92.51761928	95.08346342	74.56443401	107.3152128	94.06357529	93.81045705	3R	0.048142413	0.720748013	0.995787491
**CG43273**	FBgn0262962	4.511366961	5.405132282	4.690505709	2.103616447	2.559597423	3.159586273	1.644084863	2.518610216	3R	-0.718823259	0.368424571	NA
**lncRNA:CR432**	FBgn0262967	0.320585688	0.369694544	1.020779036	0.280287549	0.470225708	0.79339635	0.153341433	0	X	-0.439636683	0.69543969	NA
**lncRNA:CR432**	FBgn0262969	4.478945217	1.400691948	2.025838049	0.353982785	0	0.28628625	0	0.988902171	3L	-2.681639562	0.051789829	NA
**CR43281**	FBgn0262970	0.181807412	0.435652041	0.378053354	1.089969781	0.562643958	0.759087778	0.331281867	0.281943372	2R	-0.056710024	0.9537041	NA
**lncRNA:cherub**	FBgn0262972	1.132197982	0.684361544	0.470155259	0.546960094	1.381018607	0.557755872	0.650509319	1.528012227	3R	0.556717292	0.312339708	0.914141092
**cnc**	FBgn0262975	3.316810602	3.52883873	3.749941342	3.029379529	2.85888591	4.463270228	2.922490653	3.269072088	3R	0.017902746	0.912293251	0.996479182
**lawc**	FBgn0262976	34.9558016	26.23180297	28.15228676	25.22956537	32.25896014	32.82187543	23.186191	21.89695822	X	-0.030810061	0.870353937	0.995787491
**asRNA:CR4329**	FBgn0262991	0.481494051	0.425073013	0.676267178	0.236333738	0.04574843	0.621194259	0.064647456	0	X	-1.246270524	0.365291346	NA
**lncRNA:CR433**	FBgn0262993	0.293787153	0.903442348	2.256957863	1.712384219	1.237509253	1.431067123	5.121291819	0.106306517	3R	0.654435159	0.511008365	0.980373783
**CG43313**	FBgn0263005	1.835849384	1.961169256	1.567519189	1.661870693	0.870203336	1.107149489	0.784909143	1.347155513	X	-0.74310411	0.009300326	0.287842402
**SERCA**	FBgn0263006	167.6500272	237.8926406	235.6261459	223.2126448	194.7547956	219.5618184	228.4484137	216.4204058	2R	0.02413628	0.833696738	0.995787491
**lncRNA:CR433**	FBgn0263019	1.178198853	0.899699631	0.700671288	0.491667737	0.571049074	0.328485213	0.45610433	0.806211209	2L	-0.573251909	0.187141247	0.840740887
**CG43316**	FBgn0263021	3.455198244	1.398940427	2.601391887	0	1.720694729	0.112328959	0	0	2R	-1.982961127	0.268728632	NA
**HSPBAP1**	FBgn0263025	46.18303066	40.13410814	32.10390574	58.12214332	40.66054088	50.47236534	26.42714187	26.5542361	3R	-0.268707038	0.314981109	0.91460807
**CG43322**	FBgn0263027	10.39935686	8.450869909	3.290699308	6.776750971	11.89377302	8.769197628	4.448959844	14.16381743	2L	0.463703104	0.339772113	0.927274752
**CG43324**	FBgn0263029	2611.452907	2544.621936	3425.617979	2374.30247	2672.950577	2442.550384	2833.98742	2641.369644	2R	-0.015714244	0.896216745	0.996479182
**CG43333**	FBgn0263038	0.44373068	0.32985844	0.207957221	0.105805368	0.436935057	0.361299314	0.038589767	0.602112738	2L	0.428963099	0.560370807	0.984934163
**CG43342**	FBgn0263047	6.762019177	9.541967354	8.072087149	4.754642379	4.650353412	8.095479843	10.67860622	10.48640685	3R	0.248740075	0.633280918	0.991585536
**Gpdh3**	FBgn0263048	6.909498877	10.75268008	6.053070076	6.622686125	8.246314151	6.966610652	10.18407294	6.667182963	3R	0.110761378	0.679697292	0.995787491
**lncRNA:let7C**	FBgn0263049	4.476395124	6.173675983	6.286543535	4.673253696	5.459191797	5.055003092	3.146673637	3.714690952	2L	-0.2790614	0.180345887	0.836097072

**CG43347**	FBgn0263072	26.54963493	41.66871886	36.9163567	39.32152889	38.93832943	30.05189136	34.38688416	50.26906219	X	0.126920729	0.451262485	0.967112302
**Klp54D**	FBgn0263076	6.005531698	3.031147312	5.142118636	3.86471464	2.305663458	7.327266426	3.91671304	3.598879999	2R	-0.054658026	0.879436544	0.995787491
**CG43340**	FBgn0263077	0.576544637	0.834736477	0.507945043	0.665021363	0.651876261	0.884004703	0.371564907	0.520456897	2R	-0.055698149	0.87431868	0.995787491
**lncRNA:CR433**	FBgn0263089	0.456419327	0.170128754	0.492118632	0.709416319	0.732403435	1.09967946	0.646853061	1.321238146	2L	1.094119667	0.30615621	NA
**lncRNA:CR433**	FBgn0263095	7.98511952	4.313295561	6.895043393	5.36422194	1.954601668	4.338354999	5.86938296	5.289081453	2L	-0.477778898	0.486226035	0.973549494
**psq**	FBgn0263102	13.59681277	14.28632377	14.57342146	15.05627203	15.35720842	14.12660347	14.0930671	14.32776196	2R	0.04080153	0.656227468	0.995515543
**DnaJ-1**	FBgn0263106	950.5951449	513.0887881	613.8655917	541.3306324	707.7829819	699.2785538	568.1665004	478.4115652	3L	-0.070786924	0.689581764	0.995787491
**BtbVII**	FBgn0263108	115.6684542	104.2122793	106.8754933	112.7031583	107.4169948	108.8925693	87.77937836	108.9963272	3L	-0.060486518	0.563116759	0.984934163
**CG43366**	FBgn0263109	0.672731651	0.942282995	0.6000963	0.538985745	0.66356713	1.07451517	0.595640695	0.376433682	2R	0.006161537	0.985471859	0.99797245
**CG43367**	FBgn0263110	11.40093536	16.79476363	15.92990143	15.51110455	14.4279694	14.04410304	14.30639806	15.71626203	3L	0.006803113	0.957961179	0.997604378
**cac**	FBgn0263111	49.75876694	50.34533099	51.81215229	48.57736609	47.97959157	52.75542579	40.29006764	47.12327656	X	-0.062612846	0.500495467	0.976796212
**Mitf**	FBgn0263112	3.303768339	3.77759557	2.387241258	2.980130144	3.00954479	3.022317742	2.915720159	3.810313609	4	0.064925619	0.735451074	0.995787491
**Cc2d2a**	FBgn0263113	0.413205704	2.252557453	0.835359395	1.043654672	1.398641623	0.941455549	0.702731085	0.934487326	2R	-0.143406238	0.820659817	0.995787491
**5-HT1B**	FBgn0263116	136.4046367	97.35286072	103.0814668	134.2203941	107.034192	116.8945859	106.8736433	112.8665477	2R	-0.060941342	0.637786972	0.991585536
**CG34377**	FBgn0263117	1.992708004	1.628874496	0.285971601	0.719790995	1.134938173	1.624689777	0.615740732	0.255925139	3R	-0.33458997	0.562726522	0.984934163
**tx**	FBgn0263118	0.411312746	0.619158462	0.511712299	0.639306788	0.533094818	0.208806308	0.322851585	0.43505033	3R	-0.507040201	0.429037875	0.963666972
**Acsl**	FBgn0263120	41.82177948	44.77149371	48.04926902	45.69599133	39.41687274	50.71381923	45.99318089	47.63420909	2R	0.057046964	0.578844309	0.985637351
**CG43373**	FBgn0263131	3.526451125	4.308062027	3.759686265	4.028597763	3.853674018	4.498984924	4.437995435	3.864619013	3L	0.123159315	0.31280141	0.914376341
**Cht6**	FBgn0263132	1.038153778	0.868539402	0.680688266	0.808787807	0.271680307	1.337084428	0.344871468	0.393191363	X	-0.516161273	0.237432322	0.873280735
**mEFG1**	FBgn0263133	30.21418156	37.44752178	27.19663133	46.54018689	37.53527098	33.06273669	43.0197356	44.24036666	2L	0.190097818	0.345353038	0.930285749
**bin3**	FBgn0263144	17.25111791	24.37125439	22.17253973	23.18583844	20.98602821	19.17518836	22.12705575	21.43208238	2R	-0.020925768	0.865474391	0.995787491
**Patronin**	FBgn0263197	46.8835012	51.66497714	51.4294651	53.7542574	48.34395386	63.93116442	50.27537711	51.09519689	2R	0.096751055	0.35636937	0.937787223
**Acn**	FBgn0263198	402.0667844	426.7630156	396.8400865	430.0856587	376.5120626	386.6851721	377.2744506	422.6647953	2L	-0.052091187	0.565470755	0.984934163
**Galk**	FBgn0263199	16.23394292	11.27411103	17.01012681	20.77260232	26.68618313	18.25263047	16.12093235	16.04707707	3L	0.268484189	0.265944396	0.893759496
**CG43386**	FBgn0263216	0.738542966	0.511679177	0.447923733	0.112296856	0.318822886	0.105957752	0.204786845	0.087143758	X	-1.31815859	0.095789929	0.730787365
**Dscam4**	FBgn0263219	24.66435291	25.9490232	26.86780335	23.90076781	23.32780524	19.38032312	23.45458357	23.60212923	3L	-0.142086315	0.179468708	0.836097072
**Hk**	FBgn0263220	13.99376993	15.15666669	14.24464469	16.36993026	16.78448841	17.03280175	11.95079166	15.26888292	X	0.059777055	0.638150011	0.991585536
**bel**	FBgn0263231	50.91404311	68.6272812	106.193179	72.31845053	60.18524598	65.84144631	97.0397648	83.52998785	3R	0.077180264	0.693967483	0.995787491
**SP1029**	FBgn0263236	3.502595726	7.277730023	5.01414206	1.839227495	2.962165006	4.626091176	3.823620315	2.363548239	3R	-0.318698867	0.421635743	0.960593359
**Cdk7**	FBgn0263237	94.32144931	113.7051828	99.80704006	76.89424863	98.47690092	97.96350557	110.3369395	71.48118866	X	0.006865194	0.967429654	0.997604378
**dar1**	FBgn0263239	0.482811188	0.874206821	0.667094145	0.298187049	0.363646822	0.437104362	0.187604102	0.701132491	3L	-0.416289369	0.371691255	0.943282319
**Mocs1**	FBgn0263241	2.179268406	11.42961381	12.88210328	11.02050516	8.754842341	7.263448399	6.890475014	5.997540982	3L	-0.332853019	0.440107947	0.967112302
**Cngl**	FBgn0263257	60.84330132	56.59608737	54.91801143	52.73069292	47.16475703	44.69036528	39.97572066	42.39728953	X	-0.339693819	0.00021914	0.01863584
**chas**	FBgn0263258	0.840729775	0.72998912	0.559889841	0.630316256	0.535670372	0.500447915	0.15139198	0.529695531	X	-0.6533492	0.117733792	0.771985382
**sel**	FBgn0263260	7.234780671	11.13332994	19.16170424	8.201686194	15.45707986	21.90199503	14.39235668	18.0130488	2R	0.653743884	0.074029118	0.675782176
**Fic**	FBgn0263278	33.75344904	39.55313783	49.46167082	61.82461547	42.26165769	56.06065092	52.11950991	33.7299871	2L	0.025022306	0.909878759	0.996479182
**scrib**	FBgn0263289	58.55025443	67.31096235	61.56513046	65.92269914	56.56654934	59.6397582	58.64135323	60.08266116	3R	-0.077798233	0.347142599	0.930552081
**Mrp4**	FBgn0263316	3.87301562	3.454628827	2.92246493	1.993197818	1.543337342	0	0.892187102	2.081076862	3R	-1.389788447	0.245325601	0.877269208
**asRNA:CR4342**	FBgn0263345	0.340622294	0.571346113	0.388868204	0.186858366	0.325540875	1.208984915	0.715593356	0.217506627	3L	0.759656555	0.397315485	NA
**smash**	FBgn0263346	12.85099072	15.56342781	15.48032702	14.07112856	13.07674359	13.87748984	14.048123	13.38203754	3R	-0.0597718	0.527708968	0.984107922
**AP-2mu**	FBgn0263351	740.7714956	807.4820725	790.1571763	803.5579788	800.1855321	696.1137476	757.7412621	781.5539852	3R	-0.017324054	0.85201115	0.995787491
**Unr**	FBgn0263352	88.24572834	63.53817629	75.4345603	68.35969224	66.85946337	82.85656016	67.35050274	73.73434426	3L	0.002617176	0.983324628	0.997881056
**CG11000**	FBgn0263353	72.98756106	85.96433154	84.51527418	82.70041838	94.42174573	84.98048502	87.09215407	110.7002544	3R	0.24369231	0.034524621	0.514749796
**CG42784**	FBgn0263354	0.407634456	0.526487204	0.645267367	0.810202692	0.515119269	0.503453011	0.48614589	0.911562503	2L	0.051973851	0.853797459	0.995787491
**CG31688**	FBgn0263355	23.05722949	30.87729503	27.00984473	28.25548615	29.97548159	24.13099893	27.86239843	31.86542025	2L	0.095211237	0.456130292	0.968365245
**CG43428**	FBgn0263376	2.706820176	2.270155558	1.89424269	4.490412032	2.631194553	11.63111012	2.523037924	4.633597	2R	0.935597179	0.128586396	0.788096052
**lncRNA:CR434**	FBgn0263379	0.12424324	0.521001515	0.854001709	0.048278073	0.448581784	0.449020444	0.316947175	0.719316202	3L	0.386039126	0.705990547	NA
**Nha2**	FBgn0263390	0.390743741	0.42559598	0.492435774	0.851848198	0.403081485	0.354037923	0.32363496	0.308487575	3R	-0.602318571	0.206528435	0.855364409
**hts**	FBgn0263391	65.48813004	66.61099546	64.12382934	68.95810347	66.64622932	67.76597182	66.09684803	71.60319712	2R	0.067665506	0.426525876	0.962502302
**Tet**	FBgn0263392	8.128701754	11.98570624	12.46551938	10.07226519	12.03659987	10.68945554	10.95660844	13.17694508	3L	0.172060639	0.198868937	0.847197998
**hppy**	FBgn0263395	13.00659076	14.62044834	14.25905992	15.57062554	10.94948048	11.81423387	12.5405792	13.61083885	2R	-0.200672592	0.070891437	0.6662581
**sqd**	FBgn0263396	163.6223354	199.2308517	183.045981	194.4107827	185.0807229	183.7385255	178.3826041	197.5231398	3R	0.040719883	0.663902791	0.995787491
**Ih**	FBgn0263397	94.4758741	86.78596772	72.42696669	87.72251753	86.68921432	87.91269276	69.88081691	83.022529	2R	-0.031946009	0.776883291	0.995787491
**Uck**	FBgn0263398	143.4250851	122.8538075	130.4555881	145.13178	121.8586298	138.1049257	133.6565445	108.6364214	3R	-0.082892887	0.481664888	0.973549494
**CG43450**	FBgn0263404	2.784157896	2.075570796	14.25913736	5.048679473	0.55845762	2.624887058	1.578321468	0	3R	-2.261932768	0.101128694	NA
**lncRNA:CR434**	FBgn0263406	3.673541668	8.107698423	7.973859706	6.310849342	3.664878128	7.655920586	5.425480047	7.136062278	3R	-0.081533395	0.882129291	0.995787491
**lncRNA:CR434**	FBgn0263413	11.65554273	15.36113686	14.19342705	13.16179771	17.97358532	12.39731604	12.7138522	16.98572395	3R	0.179949155	0.31858738	0.91460807
**nwk**	FBgn0263456	11.12446765	19.92102802	23.87588965	20.88308629	27.10939937	27.81530601	20.25442027	22.97584726	3L	0.407631402	0.04311608	0.544438502
**snoRNA:2R:94**	FBgn0263459	2.309819884	3.87439882	8.405386231	4.038943579	15.63681335	14.69936752	33.88130085	40.11866228	2R	2.567562391	0.003743848	NA
**snoRNA:2R:94**	FBgn0263472	0.577454971	3.87439882	1.400897705	4.038943579	14.59435912	10.34399937	10.31170026	31.34270491	2R	2.834753646	0.012008713	NA
**mld**	FBgn0263490	25.67565369	25.5623101	22.92218229	25.74633804	19.24449104	22.1591853	21.55572964	20.81864968	3R	-0.225167201	0.016860382	0.373708572
**asRNA:CR4348**	FBgn0263492	2.898008219	1.129323752	0.497108006	1.023726828	1.532502829	3.035804941	0.224026704	0.762647771	3L	0.006719671	0.99350479	0.998777916
**lncRNA:CR434**	FBgn0263495	0.278068204	1.865681615	2.023768756	0	3.011906263	5.112139589	1.418715927	0	3L	1.251783506	0.406366307	NA
**lncRNA:CR434**	FBgn0263497	8.969344963	0.21492597	4.662751238	1.493692152	3.238393297	7.127405358	0.32687131	2.781896885	3R	-0.180761583	0.868043885	0.995787491
**lncRNA:CR434**	FBgn0263503	13.65367928	24.65261297	33.96754978	35.27992885	19.46751862	15.0863656	25.06932292	47.76930326	X	0.040032119	0.920428101	0.996479182
**nclb**	FBgn0263510	79.34410357	63.44703494	50.49747542	57.40100693	46.46598281	38.40489362	55.09850914	50.17262452	2R	-0.373328421	0.063567337	0.636110045
**Vsx1**	FBgn0263511	17.7146181	23.86627591	19.61332064	23.04867319	21.72853268	20.76025666	15.61359389	24.63627552	X	0.007146577	0.96209882	0.997604378
**Vsx2**	FBgn0263512	0.867401807	1.492131936	0.47733429	0.652567989	1.170659817	0.668856128	0.735230374	0.457264955	X	-0.168369647	0.66246933	0.995787491
**Lpin**	FBgn0263593	94.05663373	84.58995745	82.70553032	90.27453718	86.72277811	99.95722713	84.27183785	85.75738875	2R	0.047923612	0.609336139	0.990047511
**lost**	FBgn0263594	184.9023193	198.9333003	202.4580366	219.4564813	161.890145	201.1745718	204.7666454	181.7815818	3R	-0.074932868	0.486660547	0.973549494
**Vha68-2**	FBgn0263598	5.963216445	13.15577662	10.59255208	9.001612877	3.64919798	8.880986991	7.219393414	12.02506578	2L	-0.246479197	0.470733911	0.973549494
**l(3)72Ab**	FBgn0263599	50.91714951	50.46740361	67.61581306	49.16650334	48.35944548	57.02260805	71.47759418	48.96518083	3L	0.080825638	0.592946172	0.988631016
**DNApol-delta**	FBgn0263600	7.860859968	10.64821891	9.505088925	14.80059485	9.803785064	12.85849817	18.09218196	18.84330956	3L	0.510240889	0.070089432	0.662558698
**mib1**	FBgn0263601	118.564899	121.4800809	104.5879332	131.4960243	80.8892949	124.404343	115.6915638	107.6496781	3L	-0.124548493	0.356971768	0.937787223
**Tasp1**	FBgn0263602	46.1663219	49.50200315	35.20485119	40.23166455	43.97853754	56.4978584	37.69064548	45.95297621	3L	0.133712788	0.44401832	0.967112302
**Zn72D**	FBgn0263603	340.1833951	390.3704099	346.2414363	386.7194735	384.206251	342.4112174	364.8193784	377.3595161	3L	0.037394496	0.693496426	0.995787491
**l(3)72Dn**	FBgn0263605	17.68193218	22.92587582	25.75376861	16.31974521	22.398193	27.96126339	16.50255691	18.94246968	3L	0.088906438	0.696514326	0.995787491
**Hsc20**	FBgn0263606	19.02597303	26.73447162	19.6368609	28.40490185	21.99397638	24.62482357	26.60946599	27.53809911	3L	0.135459223	0.61860874	0.990047511
**l(3)72Dp**	FBgn0263607	16.48140106	13.51770837	13.00938582	13.2794927	11.1574324	13.93900616	11.20668968	9.998092647	3L	-0.255843565	0.256808669	0.88674759
**l(3)72Dr**	FBgn0263608	11.90440524	14.62089836	10.27110163	6.735443576	6.564044706	4.320304812	5.336705137	5.515162566	3L	-0.973150604	0.009060974	0.283887822
**lncRNA:CR436**	FBgn0263617	0	1.041000845	0.406922261	0.899454902	0.121121715	0.838138479	0.042789505	0.182083878	3L	-0.94162937	0.44373105	NA
**lncRNA:CR436**	FBgn0263618	0.42298655	0.551833798	0.684106313	0.219150493	0.593910436	0.177239616	0	0.204076212	3L	-0.889571741	0.554119643	NA
**CG43646**	FBgn0263655	1.649871346	3.228665683	6.670941453	2.884959699	1.489220319	2.203608888	1.402952416	2.686517564	2R	-0.836254937	0.364636528	NA
**lncRNA:CR436**	FBgn0263659	0.608051922	0.489003601	0.929531125	0.388397305	0.811989538	0.801004096	0.276232818	0.289345098	2R	-0.108843757	0.854183178	0.995787491
**lncRNA:CR436**	FBgn0263660	7.982155849	10.3399786	21.09015706	13.81937812	8.560299277	4.396100385	15.72561171	14.06990038	2R	-0.2779151	0.563353416	0.984934163
**Lpt**	FBgn0263667	32.3204944	42.24804007	34.77522539	23.04573689	36.79250199	32.50513734	39.08047746	25.18478524	2R	0.046582398	0.80323911	0.995787491
**Uba3**	FBgn0263697	103.3566364	103.7645157	101.0918074	118.5757422	112.6273178	96.78187177	84.50418456	109.7841772	2R	-0.049256473	0.738757787	0.995787491
**Myo10A**	FBgn0263705	0.852983386	0.643185674	0.696103479	0.486531781	0.786408429	0.683682362	0.578930178	0.611638643	X	0.015622818	0.957647525	0.997604378
**CG43658**	FBgn0263706	8.947139923	8.089727469	8.382493958	6.786637538	8.08710833	9.195863131	7.848696525	8.146458826	X	0.076305845	0.472966195	0.973549494
**eIF2gamma**	FBgn0263740	36.53580436	39.51866092	41.77240396	48.81409366	33.50745717	43.11535184	38.88732196	40.59924298	3R	-0.064482805	0.638904303	0.991585536
**CR43670**	FBgn0263745	66.27811707	45.7048598	50.08969642	44.2011105	55.91009469	78.37057564	48.16782576	30.07899815	2L	0.063273724	0.789589152	0.995787491
**CG43672**	FBgn0263747	3.234582568	3.150324286	1.410299703	0.625546243	1.991265165	3.681938748	1.102733636	0.970863343	X	-0.099373224	0.860207589	0.995787491
**lncRNA:CR436**	FBgn0263768	72.86123016	102.7712775	82.8177761	92.36113625	87.72712194	96.91494765	105.4458005	61.25655121	3R	0.033013171	0.858340542	0.995787491
**CG43689**	FBgn0263772	1.948306604	1.189168426	0.433162259	1.212123846	1.924501703	1.138748949	0.401900701	0.490264209	X	-0.254454596	0.622438414	0.990047511

**CG43691**	FBgn0263774	0.0672154	0.751629362	0	0	0.44491709	1.542009871	0	0.486436186	2R	1.628153407	0.291467212	NA
**CG43693**	FBgn0263776	3.796363527	9.522768031	6.970429533	6.09352375	7.749143495	5.749547234	11.08283815	7.127422538	3L	0.307076198	0.227296574	0.867702781
**CG17684**	FBgn0263780	1.903245859	1.821833727	2.06083554	1.5848393	1.648827314	2.039748781	1.25146643	1.395576801	2R	-0.191187142	0.168984801	0.830934498
**Hmgcr**	FBgn0263782	4.304438515	5.756337391	4.912022461	5.052108506	4.80574465	4.354012341	4.202187111	3.576346677	3R	-0.208185518	0.221160562	0.86549481
**Gen**	FBgn0263831	1.096867377	0.402465251	0.665246239	0.958989374	0	0.90485488	0	2.38140061	3L	0.09799793	0.938947432	NA
**Rcd5**	FBgn0263832	99.35129529	173.3222054	131.4865786	150.874464	144.9505376	120.7572155	135.9127114	129.2204555	3L	-0.028626185	0.85680395	0.995787491
**lncRNA:CR437**	FBgn0263838	3.604400339	7.368411279	4.918626533	2.363491041	3.253433206	10.72558965	9.194902702	6.847300942	2L	0.751370752	0.240257305	0.874750713
**CG43707**	FBgn0263846	26.62298511	33.27612484	36.65238922	37.01843715	31.00853115	33.21372019	33.30053756	38.48799349	2L	0.059115184	0.655333151	0.995179752
**Dad1**	FBgn0263852	194.8498059	241.5964407	307.3969822	223.1035501	306.4815416	225.2347532	242.0092918	325.2477264	2L	0.222254165	0.22889776	0.867702781
**BubR1**	FBgn0263855	1.474771492	3.820526645	3.538022014	3.744007479	1.952383051	0.695202777	2.46631391	1.672085279	2R	-0.854390654	0.072957267	0.671999479
**mRpL45**	FBgn0263863	191.5470949	144.9632167	153.1116083	162.8899284	171.0203199	168.8358139	150.2363269	129.2710363	3R	-0.049504124	0.732332465	0.995787491
**Dark**	FBgn0263864	33.03326145	26.70323183	23.10348073	26.83150805	17.66051118	25.60230363	18.79989862	19.4366085	2R	-0.403715654	0.013405984	0.344831707
**sick**	FBgn0263873	5.449617933	5.547697111	5.786972453	5.207592672	5.68509643	5.922958301	5.16351746	5.650076708	2L	0.05847018	0.50220514	0.977207013
**COX8**	FBgn0263911	3212.240586	3560.839374	3861.045614	3227.033492	3855.2191	3066.812488	3780.079917	4013.145522	3L	0.121410105	0.289377615	0.903248331
**Ent2**	FBgn0263916	14.96926057	16.63765013	20.09677077	24.3963035	12.7901787	15.48311456	17.05434607	20.43060546	2L	-0.178522756	0.47597778	0.973549494
**jvl**	FBgn0263929	14.22788373	15.82275759	14.44999945	16.15250066	13.01963606	13.35679797	13.5218242	15.21465866	3R	-0.106769798	0.295911484	0.906715248
**dally**	FBgn0263930	2.116255911	4.717754463	4.229946908	1.728158219	1.983849951	2.538870457	3.018505422	3.499091367	3L	-0.168462196	0.655892932	0.995305364
**ebi**	FBgn0263933	158.4547094	139.0913644	135.7485626	130.7504652	121.7048026	126.1317882	144.2100815	135.5262536	2L	-0.06720382	0.556464697	0.984934163
**esn**	FBgn0263934	0.49821334	0.989532573	0.586629285	0.821358135	0.835428514	1.804167654	0.680677722	0.724128723	2R	0.51028547	0.130400707	0.790743321
**nonC**	FBgn0263968	3.261056804	7.815759398	7.001404207	7.321437428	6.414926362	4.207216597	6.535219231	7.728716314	X	0.012182012	0.963276973	0.997604378
**qin**	FBgn0263974	0.502623215	0.238422022	0.433707122	0.095660988	0.328026536	0.096708183	0.037382001	0.206795042	3R	-0.889676238	0.135253817	0.796109055
**CR43727**	FBgn0263975	0.229959944	0.771451092	0.185959872	1.251000223	0	0.578146215	1.759898274	0.499264326	3L	0.24103894	0.874953841	NA
**Tim17b**	FBgn0263977	70.6223396	68.37010835	83.82464644	51.28387837	59.81817985	64.88669236	76.24148982	64.76072814	3R	-0.012088746	0.930999128	0.996479182
**Caf1-55**	FBgn0263979	98.07729927	113.425559	92.17174036	139.4735798	121.8381483	147.839883	108.3706642	133.6637084	3R	0.235860186	0.131002642	0.790743321
**Stacl**	FBgn0263980	50.56891334	62.42575018	55.67434409	62.6170909	56.05466682	59.80648095	53.27158254	58.25152488	2R	0.006146425	0.948153563	0.997604378
**spoon**	FBgn0263987	29.78172436	34.3138523	34.16763239	29.74083557	31.56493863	34.54868443	27.87791015	32.17422343	X	0.011389841	0.920267538	0.996479182
**Dic61B**	FBgn0263988	0.05953144	1.897257154	0.866534663	0.69397656	0.322408523	1.487335775	0.759329916	1.163234409	3L	0.139014242	0.884826302	0.995787491
**CG43736**	FBgn0263993	133.8000246	126.1145066	110.1270929	145.148108	127.6346493	125.0195849	104.6249068	124.9598142	X	-0.067164875	0.554087699	0.984934163
**CG43737**	FBgn0263994	105.8428335	137.0979686	127.5309981	140.275939	105.5403965	112.1698918	98.78614949	103.0350867	X	-0.253093971	0.014262094	0.354806828
**cpo**	FBgn0263995	46.57151606	52.21110281	44.57459643	52.05858229	50.29320715	46.01363359	39.89906173	47.50764327	3R	-0.058278047	0.568236715	0.984934163
**CG43740**	FBgn0263997	46.00407602	49.42652928	48.27658723	45.88150798	51.43717698	57.61230919	44.5773696	46.02337789	X	0.103997464	0.297577444	0.9074568
**Ack-like**	FBgn0263998	100.2318214	92.40222726	113.1904851	106.5849431	86.6593689	88.75672152	86.78763705	91.45841414	2R	-0.19169244	0.055135803	0.615177141
**GluRIB**	FBgn0264000	29.09713177	28.51013224	31.2119886	27.33780358	28.56165967	31.35217916	29.79738254	32.57431952	3L	0.104935008	0.251983815	0.883108964
**bru3**	FBgn0264001	19.36850598	17.37896893	16.58947986	17.77179367	17.74697462	18.53669121	14.0227958	15.60938922	3L	-0.082199974	0.431529463	0.964209983
**MsR2**	FBgn0264002	1.098254706	0.587241391	1.082028846	0.413711623	0.614702258	0.66014176	0.660656126	1.270298433	3L	0.038884921	0.928748782	0.996479182
**Hmx**	FBgn0264005	0.102234577	0.300097282	0.289356268	0.218493063	0.153799679	0.168675736	0	0.110980366	3R	-1.004774049	0.339395134	NA
**dysc**	FBgn0264006	175.6931977	182.8271826	162.7191451	189.6630267	175.8803203	165.5558453	141.6268362	154.2531458	3L	-0.128317347	0.18670409	0.840622305
**tgo**	FBgn0264075	83.68564884	106.3402631	92.29367615	102.061332	100.2507916	96.21623557	105.7557968	81.90198974	3R	0.03050709	0.807657964	0.995787491
**Flo2**	FBgn0264078	1.771382966	1.900430107	2.05090713	1.437141286	1.623702094	1.361772149	1.754728975	1.648499674	X	-0.131294105	0.394041081	0.952272708
**lncRNA:CR437**	FBgn0264084	0.103178375	0.449974848	0.600742316	0.120278248	1.043075101	0.661476402	0.447457426	0.358415684	2L	1.039954659	0.169006503	0.830934498
**Slob**	FBgn0264087	162.7211992	155.5036995	161.40207	167.209553	141.1298879	154.5014605	148.1072852	160.2739679	2L	-0.069112159	0.419851574	0.960266449
**sli**	FBgn0264089	1.193360852	1.046403535	1.257860946	0.468189876	0.677497186	0.965252477	0.713833323	0.607520647	2R	-0.390606097	0.180218833	0.836097072
**CG43759**	FBgn0264090	4.631671304	4.811268297	5.857620575	3.790592967	5.369019985	4.954921399	4.678089981	3.378713871	X	-0.023626291	0.87802842	0.995787491
**Send2**	FBgn0264253	1.344655819	3.195251291	1.087372087	3.135014421	2.22516102	1.05644441	0.285853824	0.243281021	2L	-1.145511279	0.328058448	NA
**lncRNA:CR437**	FBgn0264261	1.783700689	1.167573728	2.955183507	1.927170467	0.549762398	0.861339132	0.443927686	0.094453154	2L	-1.945675937	0.024600433	0.440903252
**lncRNA:CR437**	FBgn0264263	1.622824274	9.527210213	4.921186318	0.472944213	2.563412024	3.824972033	0	0.440412715	2L	-1.199502819	0.377554429	NA
**lncRNA:CR437**	FBgn0264264	0.587811652	0.492985793	0.713011435	0	0.530575344	1.029201152	0.321325747	0.364626717	2L	0.356676907	0.77891009	NA
**lncRNA:CR437**	FBgn0264268	0	1.099293486	0	0.509324537	1.971855403	1.544700244	0	4.505760857	X	2.38040669	0.159734971	NA
**Sxl**	FBgn0264270	52.05644895	52.57588706	51.23054834	44.97262199	38.41507904	39.11339456	34.43793624	34.75795689	X	-0.42261482	4.2E-06	0.000777239
**mwh**	FBgn0264272	0.236127949	0.464359701	0.329220179	0.27842564	0.122491801	0.143295625	0.110780225	0.377125985	3L	-0.756029165	0.156294523	0.818884898
**Sema2b**	FBgn0264273	24.41679379	25.92054523	28.27252764	28.787094	26.45198208	26.39239582	27.14556608	29.25568569	2R	0.056013471	0.570962075	0.984934163
**Cyt-b5**	FBgn0264294	175.624731	194.0188915	174.8527877	136.1273577	181.2068575	164.3344929	190.4118196	163.9881277	2R	0.073034856	0.558954747	0.984934163
**CG43783**	FBgn0264305	6.41077999	7.957751807	8.486629017	6.918823801	7.613695476	10.45015616	6.597766196	5.70048379	3L	0.057682789	0.744265322	0.995787491
**orb2**	FBgn0264307	0.094784695	0.050206746	0.072614637	0.151201597	0.054034948	0.216351151	0.063630999	0.119139465	3L	0.32946547	0.725426323	NA
**CG43778**	FBgn0264308	27.34768577	30.11691682	32.49497594	32.49677196	30.15636846	29.40843197	35.66262246	41.50365756	2L	0.192913223	0.14007893	0.803453409
**spg**	FBgn0264324	10.26589164	13.779138	23.07649047	14.64315633	10.86606005	14.83427769	13.42347704	18.76153968	3R	-0.05850263	0.777796767	0.995787491
**Atg6**	FBgn0264325	111.9765211	92.32532768	83.81775596	81.68649934	75.93908105	60.04943454	77.51706188	72.31125926	3R	-0.344126082	0.038224208	0.522230403
**DNApol-epsilo**	FBgn0264326	1.058872655	2.313073922	1.13505571	0.373178867	2.178262556	0.266986854	1.507650358	0.668287951	3R	-0.033397887	0.96011764	0.997604378
**CG43795**	FBgn0264339	1.49672564	1.866972896	1.721119621	1.244764266	1.385292281	2.22329896	1.560513412	0.954819552	2R	-0.018866543	0.936219883	0.996793017
**lncRNA:CR438**	FBgn0264352	0.670419858	2.811338153	0.813214612	0.625223464	0.605139835	4.614094553	1.026153277	1.0188805	2L	0.601865257	0.552154901	NA
**SNF4Agamma**	FBgn0264357	4.853354713	5.641273271	6.215733392	5.182569382	4.937941057	5.257627715	4.886292657	6.667517886	3R	0.024462117	0.854985497	0.995787491
**lncRNA:CR438**	FBgn0264366	1.242729493	0	2.713359974	1.158950812	2.019100719	3.163421992	1.585114818	0	2L	0.43837429	0.766531878	NA
**lncRNA:CR438**	FBgn0264371	0.667761806	0.160010964	0.115712916	0.667226362	0.774952203	0.854405234	0	0	2L	0.031724365	0.984935499	NA
**lncRNA:CR438**	FBgn0264385	0	1.06106384	1.205780497	1.15879967	4.16002859	6.773313153	4.4953757	4.610663946	X	2.622860088	0.000677983	0.045567039
**Ca-alpha1T**	FBgn0264386	6.692488719	8.229448725	8.859610648	8.61721712	7.493104067	6.632047951	6.776580127	7.681972947	X	-0.147301234	0.21038868	0.856520545
**opm**	FBgn0264389	53.02382581	31.58873098	38.97234217	29.60879691	56.5315871	44.56679586	46.24205004	32.04968321	X	0.251475238	0.298243176	0.9074568
**lncRNA:CR438**	FBgn0264390	1.281124422	0.716302165	0.86333055	0	2.055784828	2.013060466	0.363130658	0.463573285	X	0.788049542	0.53520586	NA
**lncRNA:CR438**	FBgn0264438	14.13138045	13.64224937	9.372202957	10.66622424	14.86598452	5.942623804	10.11459533	12.58122662	2L	-0.111221589	0.803579475	0.995787491
**lncRNA:CR438**	FBgn0264439	0.287086988	0.687925927	1.492433635	0.478094647	0.74037942	0	0	0.445208877	2L	-1.256423728	0.395647852	NA
**ab**	FBgn0264442	72.156666	59.32726041	59.23144031	52.33990712	62.66266338	60.15127479	52.78399006	55.20947635	2L	-0.045583793	0.683596559	0.995787491
**CG43861**	FBgn0264443	20.93182182	18.59249835	17.12278771	20.15814982	17.16580913	18.82362729	17.43694795	15.88869662	2L	-0.120981717	0.244889285	0.877269208
**CG43867**	FBgn0264449	7.081608269	9.18510876	10.04543249	8.638883396	8.739056408	9.901890842	9.278987734	10.86806029	X	0.183554074	0.122587223	0.780439453
**lncRNA:CR438**	FBgn0264462	1.383407443	0.528815028	0.559354527	0.274265508	0.399245017	0.816276626	0.228071225	0.326912176	3L	-0.618252549	0.27108876	0.89549671
**asRNA:CR4387**	FBgn0264467	0.644900852	0.94651437	0.391130118	0.187945257	0.65486887	0.380005365	0.411288982	0.262526146	3L	-0.321259025	0.778952759	NA
**lncRNA:CR438**	FBgn0264479	9.386387836	4.69568298	3.195964346	1.151789994	3.270056023	3.726075418	4.200855619	2.86017079	3L	-0.389702852	0.660778905	NA
**CG43896**	FBgn0264488	0.520637567	0.151877649	0.906107885	0	0.143025864	0.480183181	0.086618975	0.024572877	3L	-1.067488829	0.375488683	NA
**CG43897**	FBgn0264489	0.904298203	1.134081028	0.768860337	0.620678714	0.770474265	0.398395711	0.48509246	0.155964284	3L	-0.889047436	0.053669122	0.608413656
**Eip93F**	FBgn0264490	12.97056099	15.29403019	16.36432665	12.74806036	16.81151625	15.24295641	14.83682491	14.46962261	3R	0.129981583	0.222037434	0.86549481
**how**	FBgn0264491	0.905140838	0.868687589	0.871552302	0.674426345	0.871753397	0.961131102	0.565356195	0.415314054	3R	-0.20864769	0.490870261	0.973549494
**CkIIalpha**	FBgn0264492	162.6600618	110.5823051	137.6335942	120.9217219	124.4482765	153.9429692	125.4602497	143.7505586	3L	0.068403828	0.606398725	0.989286463
**rdx**	FBgn0264493	26.74136435	19.94618554	21.42535485	22.20934265	26.15357836	27.77471934	23.1959541	22.6115366	3R	0.1688389	0.147031967	0.808004944
**CG17646**	FBgn0264494	3.61547852	5.285315727	4.763114157	3.126117321	2.818222662	3.972997267	3.774778125	3.514098051	2L	-0.219669422	0.289690759	0.903534148
**gpp**	FBgn0264495	16.9156639	21.05418228	21.79665917	20.20471551	18.69679089	17.11847888	18.19540217	19.16516372	3R	-0.094476697	0.37733945	0.943615128
**CG43901**	FBgn0264502	28.3547418	45.41493155	59.07175478	50.39250167	49.38220526	47.08722632	54.57531747	66.30767499	X	0.282878013	0.11422168	0.760322269
**lncRNA:CR439**	FBgn0264513	0.194842342	0.363134106	0.997890142	0.151422529	0.429904891	0.326570969	0.055227445	0.376018454	3L	-0.456913787	0.676996607	NA
**hwt**	FBgn0264542	14.37731157	21.37936456	19.29485299	17.28815635	13.23839209	14.03078178	12.91641904	14.29529291	X	-0.374879172	0.001461493	0.082296708
**garz**	FBgn0264560	51.60137621	61.64168522	73.0147884	53.26020266	49.07874482	58.39459854	63.99146561	63.06152228	2R	0.003308346	0.980512296	0.997881056
**Glg1**	FBgn0264561	23.61212162	20.13027318	20.27928803	30.95216047	22.6826935	24.80388487	30.15069602	25.30259009	3L	0.142918015	0.388650295	0.948247506
**Hr4**	FBgn0264562	1.024734153	1.016783197	0.719377609	0.535336226	0.677431019	0.900551008	0.518807196	0.558334661	X	-0.284505645	0.278148222	0.896398793
**lncRNA:CR439**	FBgn0264571	8.10712644	12.40571051	4.830681742	8.290114078	4.62171823	6.57059851	5.0796553	3.087951223	3L	-0.76461307	0.224376842	0.865720648
**Glut1**	FBgn0264574	16.68602479	17.00341216	17.50111624	18.43552727	18.87661576	21.52885514	17.53826919	18.46968187	3L	0.162572176	0.075612949	0.678318963
**PsGEF**	FBgn0264598	23.36327212	39.17446648	38.13376804	37.05101092	35.12060399	36.43695953	32.91402994	38.41132935	X	0.087393812	0.545520907	0.984934163
**Fife**	FBgn0264606	77.72477211	82.70748525	77.73581989	81.69395875	72.02798322	73.49901434	72.25977783	77.06044693	3L	-0.086495225	0.27942044	0.898067854
**CaMKII**	FBgn0264607	233.9327629	197.9462232	250.0391299	181.385085	186.4699544	226.4173553	198.5865241	189.8944114	4	-0.078904218	0.484120346	0.973549494

asRNA:CR4395	FBgn0264617	3.058498488	1.753915265	1.934240833	1.55414307	0.967420171	3.240894706	1.133666846	1.049959331	4	-0.357512665	0.456734486	0.968365245
Eogt	FBgn0264672	42.52009022	49.04271279	48.70529977	51.40659464	39.88337007	65.38011534	56.24767095	59.95719866	2L	0.238861132	0.178491449	0.836045561
Lst8	FBgn0264691	53.87988944	84.21213964	71.31842863	74.10293177	76.50407852	104.9247783	71.09838196	63.09985881	X	0.186114891	0.306621309	0.911610813
ens	FBgn0264693	143.2403763	138.1396384	112.2595172	120.1831146	125.3711694	125.128459	124.3272139	118.2801484	3L	-0.029622393	0.771354276	0.995787491
mgr	FBgn0264694	243.3788749	181.1093631	243.313812	244.4623745	252.0948855	252.1528932	262.5872227	255.9509531	3R	0.192909348	0.154971802	0.818884898
Mhc	FBgn0264695	0.085837901	0.346863408	0.283965751	0.081870478	0.302875213	0.952736784	0.358321631	0.118594019	2L	1.155601549	0.093974144	0.728467429
CG43968	FBgn0264699	9.496896498	3.845102798	1.91994487	2.099639857	1.280898384	3.447656739	0.765789244	0.414742787	3L	-1.537827329	0.022394274	0.421875556
RhoGEF3	FBgn0264707	29.55064399	39.75066425	39.80148784	40.57793432	37.03884202	36.98368961	37.30742851	41.33793907	3L	0.061744193	0.596367189	0.988780662
CG43980	FBgn0264711	1.201002469	0.687384557	2.234427902	1.759517298	1.076733915	0.237170483	0.424375362	0.17618159	3L	-1.586438143	0.002014139	0.101732313
CG1172	FBgn0264712	46.23876642	49.06266043	43.77178585	49.65558766	48.83006315	44.11973133	47.68858209	55.36014629	3R	0.085832028	0.491128364	0.973549494
lncRNA:CR439	FBgn0264717	2.229776621	6.617166301	2.080541146	1.199686211	4.025318287	1.617092137	1.312663399	1.861942866	3L	-0.417217974	0.697908679	NA
CG44002	FBgn0264744	25.87651993	26.27098256	31.55323841	16.03511405	23.23394436	27.22105097	14.59202867	16.1148813	2L	-0.263336166	0.383123032	0.946058474
CR44003	FBgn0264745	0.63834308	1.317822728	0.714743727	0.228965055	0	0.092588609	1.002108869	0	2L	-1.415370518	0.376675295	NA
CG44004	FBgn0264746	2.596816159	0.967954402	0.839978637	0.807250549	0.62505583	0.054405831	0.58884745	0.877011063	3L	-1.292936491	0.252720224	NA
CG44006	FBgn0264748	0.182738915	0.408691859	0.886644117	0.1420163	0.879708205	3.56055941	2.020073891	1.1902293	3L	2.314710088	0.027646995	NA
Vti1b	FBgn0264751	52.63405357	48.48791585	57.24661227	45.353568	59.24594052	55.80152644	53.25890983	68.15913579	3R	0.246740153	0.079292092	0.69175818
Rgk1	FBgn0264753	108.283783	96.49069146	99.61911795	114.4294657	113.5932114	109.1367561	102.3528098	112.1490981	2R	0.090880585	0.348771681	0.931729531
Hph	FBgn0264785	35.19604256	32.23875762	28.1583809	39.12625375	32.11377625	33.37907702	31.48795629	35.7110787	3R	0.006113362	0.961896633	0.997604378
asRNA:CR4401	FBgn0264788	8.843224032	6.846123569	4.238934387	6.717060464	5.441822939	6.186983559	7.213517274	4.778145651	3R	-0.15390687	0.563495688	0.984934163
lncRNA:CR440	FBgn0264793	2.937990161	4.928066336	0.254554398	4.403451509	0.852400485	4.896821404	3.881275083	4.100559818	4	0.154992387	0.83989901	0.995787491
lncRNA:CR440	FBgn0264794	60.66009632	62.59450099	62.87941029	51.82916557	55.14108357	75.1676385	64.82569564	61.31531411	4	0.137218065	0.26903855	0.89549671
Pde1c	FBgn0264815	48.55091359	48.89009042	45.87053526	52.9031149	44.15825374	44.9505638	41.42514482	44.29058187	2L	-0.137161741	0.112499048	0.758410631
koko	FBgn0264816	18.2917354	58.03051762	40.79084495	22.57056706	30.90570167	58.46121006	39.773701	39.71305081	3R	0.31269878	0.311015218	0.913963619
asRNA:CR4402	FBgn0264819	93.9140477	154.6635013	166.8273342	154.898553	157.7692816	116.6407399	174.2376388	161.4317812	4	0.135382335	0.534038837	0.984760433
asRNA:CR4403	FBgn0264822	14.21147882	16.75866907	13.44248875	10.97757287	10.88409282	11.99097671	14.06207526	8.913503749	4	-0.244486757	0.305212615	0.911292159
asRNA:CR4403	FBgn0264823	0.578998968	0.097118954	0.842786053	1.214922868	2.195007221	1.200928719	0.590815523	0	4	0.580818319	0.65695485	NA
vih	FBgn0264848	231.0764055	231.1906228	275.4152061	237.7403022	247.3868639	284.512502	249.868211	264.557848	3L	0.131957468	0.217941542	0.861225611
AP-2alpha	FBgn0264855	381.7647504	353.6960906	366.5443551	351.9035101	321.5663038	348.160752	367.4562565	330.9867682	2L	-0.058057083	0.50579148	0.978557094
lncRNA:iab8	FBgn0264857	0.15797498	0.305989811	0.271465278	0.15565597	0.229166781	0.172874286	0.095951802	0.095952264	3R	-0.542135247	0.175131887	0.834088743
lncRNA:CR440	FBgn0264868	0.099934521	0.921943763	0	0.698980717	0.090203711	0.42397945	0.44613644	0.379692416	2L	-0.324563769	0.799893274	NA
lncRNA:CR440	FBgn0264875	0.26116054	0.438060168	1.478334262	2.029619889	1.100077321	0.082073521	2.66490459	0	2L	-0.09940489	0.946543646	NA
asRNA:CR4406	FBgn0264878	0.03361857	0.22556174	0.040779091	0	0.333795744	0.110933839	0.30016593	0	2R	1.350629224	0.43538173	NA
lncRNA:CR440	FBgn0264886	0.855488846	1.614332842	2.59425501	1.246587524	0.772188313	3.024561219	0	1.160840923	2L	-0.284827307	0.820559678	NA
CG44085	FBgn0264894	0.793420874	0.416285525	0.784527041	0.341911872	0.597369036	0.740942	0.306962456	0.400032787	2L	-0.164135317	0.710970781	0.995787491
RapGAP1	FBgn0264895	5.266986886	6.237579252	6.663216557	5.891110807	7.47215263	6.498847771	7.409507178	6.859679648	2L	0.26471485	0.016696728	0.372399106
asRNA:CR4409	FBgn0264902	1.981204155	1.063421451	0.961274729	1.478112929	1.001442758	1.49429374	1.010818873	1.032330628	3R	-0.255413275	0.792436524	NA
asRNA:CR4409	FBgn0264904	0.381158397	1.598349348	1.618198669	2.221641132	1.548199341	0.718707616	1.215429074	0.206882541	3R	-0.61167284	0.606112217	NA
lncRNA:CR440	FBgn0264905	0.038208313	0.51271268	1.205006848	0.445406217	0.62078218	0.468293334	0	0	3R	-0.941932144	0.554017184	NA
pHCl-1	FBgn0264908	33.97531195	31.32095615	29.17252639	32.57909832	30.37417906	32.70346161	24.57064639	28.42966169	3L	-0.102865532	0.331635197	0.921589773
CG44102	FBgn0264911	0.695554294	0.656265798	0.228502762	0.050676833	0.235435082	0.423514469	0.147864496	0.12584273	2R	-0.779992598	0.343017653	0.928865024
lncRNA:CR441	FBgn0264914	0.324818421	1.452899557	1.050673279	1.767037816	2.345522002	0.204157882	1.381031285	1.410421721	2R	0.266179482	0.830334135	NA
smt3	FBgn0264922	1943.697392	1736.248603	1780.550712	1796.955916	1725.297936	1672.676872	1681.533462	1778.263188	2L	-0.051831346	0.56849068	0.984934163
lncRNA:CR441	FBgn0264941	2.334723602	1.174851394	1.888002298	1.905162065	1.545417312	0.513604735	0.397062005	0.422408422	X	-1.31208807	0.095935355	0.730787365
Piezo	FBgn0264953	3.302912707	3.714922013	3.493962278	3.842846481	4.125289953	3.433811263	4.384355897	3.90509906	2L	0.173964088	0.266613475	0.894434069
fd3F	FBgn0264954	0.446792876	0.349735107	0.289043543	0.115742308	0.501869296	0.702055991	0.30394086	0.452680057	X	0.722885293	0.300560014	0.9074568
lncRNA:CR441	FBgn0264956	5.572566709	1.361242558	1.050017018	1.387519655	0.879021362	1.887280861	0.966118076	0.587308649	2R	-1.109573218	0.19668865	0.84446408
lncRNA:CR441	FBgn0264957	3.803812544	3.463625043	0.922799618	2.533841643	1.863861064	0.204926356	0.415868894	0	2R	-2.081307345	0.044791617	0.557949353
Src42A	FBgn0264959	25.99023125	35.72750002	36.68241883	34.28097568	28.73645256	30.55079473	29.79897041	30.44650466	2R	-0.117630504	0.299225915	0.9074568
Pcf11	FBgn0264962	91.02821257	90.57147907	99.76990268	66.61168418	82.31119803	105.0300146	93.98916024	86.72667158	2R	0.111122764	0.374479699	0.943282319
Nrg	FBgn0264975	43.00776824	34.73781861	31.92298842	40.38729861	35.04496279	36.24576166	33.17076598	31.4496766	X	-0.116530755	0.315311223	0.91460807
Slh	FBgn0264978	15.31404388	23.15416595	26.67780163	16.92673047	25.75845203	24.422284	30.42343394	20.25996929	2L	0.334072196	0.098603702	0.730787365
CG4267	FBgn0264979	3.499424979	0.100338367	0.798163402	0.069733142	0.107989042	0.281986025	1.144501065	0.129873087	2L	-1.445726084	0.238089225	NA
lncRNA:CR441	FBgn0264999	1.574877194	2.946439662	3.37978817	0.988552624	0.765438416	3.483533096	2.472335727	2.630157055	2L	0.115029092	0.878790303	0.995787491
ppk18	FBgn0265001	0.061442274	0.180356391	0.335380724	0.322313284	0.083189289	0.304118853	0.391851402	0.233444513	2L	0.221045021	0.836329055	NA
CG44153	FBgn0265002	1.4185896	1.083403812	0.841370947	0.95639513	1.030022148	1.582142283	0.97796541	1.269523261	2L	0.19938751	0.315284144	0.91460807
koi	FBgn0265003	36.7584672	46.17711407	43.95853106	45.48030478	44.73261088	51.38636837	44.9316028	39.28751312	2R	0.096070896	0.42356435	0.961944958
Np	FBgn0265011	0.615889886	1.192821803	0.338877175	0.932610488	0.458490349	0.736297303	0.453527353	0.137850923	2R	-0.75448113	0.188130302	0.842945127
Irk1	FBgn0265042	0.329974269	0.050316868	0.351740549	0.198158848	0.045127888	0.103699242	0.114787016	0.108546164	3R	-1.304809392	0.115546021	0.765469429
Strn-Mlck	FBgn0265045	0.500601161	0.450564411	0.468995473	0.493421981	0.922081558	0.744398058	0.643713396	0.53458954	2R	0.60135975	0.028109953	0.46389772
cv-d	FBgn0265048	0.133039544	0.06375862	0.069161159	0.398798598	0.257325508	0.349408992	0.654531558	0.144420582	3R	1.104275654	0.230891918	0.867702781
St3	FBgn0265052	145.3485399	160.5670226	140.8810662	159.9168949	121.7857394	108.8428075	124.5238376	126.1198306	2R	-0.302444552	0.004518786	0.186000709
lncRNA:CR441	FBgn0265066	0.274782285	0.944862905	0.63328709	0.384386731	0.620065562	0.576415307	0.280390205	0.23863111	3R	-0.333297566	0.57356321	0.984934163
lncRNA:CR441	FBgn0265079	1.109601225	0.581625123	0.729050047	0.161687093	0.417315542	0.043588552	0.176913535	0	2L	-2.002159649	0.100964437	NA
Cdep	FBgn0265082	32.31367056	41.02250949	46.04795235	46.39872513	39.27508195	41.92044958	44.73052448	42.05277493	3R	0.050673663	0.672945603	0.995787491
lncRNA:CR441	FBgn0265085	1.792752361	1.582679256	0.915619415	1.099930168	0	2.846645853	1.925620964	0.409708561	2L	-0.042527106	0.973321772	NA
eIF4E3	FBgn0265089	0.128896199	1.945847622	0	1.803099812	2.559597423	0.789896568	2.794944267	0	3L	0.70522278	0.610443741	NA
Sgt1	FBgn0265101	124.2540332	129.6082908	118.2492067	94.45315472	158.8204012	170.1817919	126.7898833	86.56831965	3R	0.244992108	0.210141943	0.856520545
Oseg1	FBgn0265102	13.48295528	13.62175039	7.941025322	15.7720129	7.738210672	11.46252641	14.15575706	10.7953819	3L	-0.178386999	0.4734983	0.973549494
lncRNA:CR442	FBgn0265104	1.546754387	2.538062115	2.433624701	1.907627337	1.618717738	1.949644373	2.273126028	2.129259844	2R	-0.043885732	0.865490132	0.995787491
Meltrin	FBgn0265140	1.463028574	1.628539569	1.557273966	0.573695861	1.042937064	1.399507484	1.31002875	1.109116561	3R	-0.071700527	0.80993527	0.995787491
lncRNA:CR442	FBgn0265161	4.205238488	2.10800159	0.469050571	0.225387476	2.094216073	3.645676469	1.849595471	3.043320677	3R	0.597997744	0.473187413	0.973549494
PIG-V	FBgn0265174	27.67101793	29.60773693	35.55251203	53.27039098	36.85216551	28.29517893	44.67076329	29.07247655	2R	-0.042937059	0.864528853	0.995787491
CG44242	FBgn0265177	618.1103485	643.9229472	670.5767692	520.088231	689.2845295	596.8615735	701.0764699	721.4583656	2R	0.1763963	0.153349732	0.816102596
CG44245	FBgn0265180	0.659036788	0.430540474	0.538547892	0.291130003	0.200375632	0.431664912	0.47781957	0.572331949	2R	-0.171283091	0.763144993	0.995787491
CG44247	FBgn0265182	1320.153845	1942.459355	1736.788443	1713.608744	1379.49141	1393.663728	1532.999602	1586.189949	2R	-0.153485135	0.183203541	0.838911159
Fatp2	FBgn0265187	0.584584045	0.741397305	0.345900668	0.432150342	1.13254286	1.236709476	0.03637284	1.176318802	2R	0.797029236	0.316063965	0.91460807
PIG-S	FBgn0265190	15.69117101	23.56898163	18.67160345	23.89650353	23.01114529	17.28841275	15.92583409	17.51900761	3R	-0.115324079	0.55578376	0.984934163
Snp	FBgn0265192	8.153346842	5.543025449	4.461812267	6.007739398	3.923851197	3.5606507	4.139800831	4.377373344	2R	-0.573386072	0.018924948	0.393184106
Atf-2	FBgn0265193	85.69085543	111.0674822	94.64308908	140.3972536	101.3435155	110.2831444	120.8131294	123.4915996	2R	0.109714783	0.525668369	0.984107922
Trpm	FBgn0265194	26.96030103	31.55750263	29.6562462	27.34267646	26.1553914	22.38896364	24.17991201	23.23237611	2R	-0.234554448	0.015028139	0.361837042
lncRNA:CR442	FBgn0265200	2.417253367	0.675767236	1.759266886	0	0	0.531760065	1.438841897	0.174936027	3R	-1.183831219	0.452654589	NA
CG44261	FBgn0265201	4.741209235	4.588103866	3.539109992	8.928191069	9.217489973	7.564586796	3.256326398	1.583631406	3R	0.023978866	0.977001742	0.997757033
lncRNA:CR442	FBgn0265255	0	3.125976597	0.982856201	3.211507991	3.803153854	2.902899729	2.067025309	0	2L	0.302877473	0.813404084	NA
Vha68-1	FBgn0265262	1285.931827	1118.258083	962.0729011	1193.285728	1138.85859	1240.976471	1102.772311	1186.608321	2L	0.061912553	0.561770912	0.984934163
CG32727	FBgn0265265	2.949847375	1.124535261	0.325285845	1.56305866	1.936447473	1.896203241	2.394357645	0	X	0.055712938	0.967124339	NA
CG13639	FBgn0265266	10.2231835	3.42959123	0	1.300089135	3.691093279	1.577185357	30.82129906	0.40355414	3R	1.281382099	0.300473138	0.9074568
l(3)neo38	FBgn0265276	214.6460373	196.528124	165.6469568	209.6438173	191.6702649	217.6679468	173.0866406	193.3549039	3R	0.007132048	0.948145867	0.997604378
Dscam2	FBgn0265296	44.25613996	53.63039366	47.80884789	52.8787241	51.71057336	47.88130783	44.55681828	47.90746519	3L	-0.016541938	0.865874337	0.995787491
pAbp	FBgn0265297	1349.663998	1328.832189	1088.339761	1338.137837	1145.116838	1378.916722	1082.085483	1176.164414	2R	-0.067114767	0.519979624	0.981838504
SC35	FBgn0265298	170.2446633	195.5546145	191.5795901	212.1289169	212.9045784	222.5605259	195.8525481	259.2975866	2L	0.242529516	0.039992572	0.529044592
lncRNA:CR442	FBgn0265310	1.011107926	1.130661134	0.817644575	1.178680033	2.433745268	0.953266376	4.298930069	1.829340753	2R	1.218234478	0.3185991	NA
lncRNA:CR442	FBgn0265340	10.34247709	6.144102606	7.318122341	3.516493166	3.500779107	9.344539886	4.397313544	7.017023487	2R	-0.149741619	0.847398132	0.995787491
CG44296	FBgn0265342	1.811408179	2.209733167	3.994955433	2.303579988	1.189111281	1.863037709	0.840171124	0	2R	-1.339386144	0.303928532	NA
nac	FBgn0265351	26.69163591	20.21236482	26.56152343	40.78215281	30.64207253	31.8223655	22.64188913	26.5838649	3R	-0.004217856	0.987484338	0.998078482

**tn**	FBgn0265356	1.030596719	0.571742881	0.797733415	0.402024477	0.839754791	0.491491198	0.347815287	0.252482901	2R	-0.50988304	0.275971937	0.89549671
**lncRNA:CR443**	FBgn0265376	3.223818144	1.550989141	1.940077607	0.233060795	0.225574341	1.013132018	0.70127382	0.16277227	3R	-1.700379122	0.046191138	0.565844709
**lncRNA:CR443**	FBgn0265379	0.346294888	0.36096368	0.366046433	0.132639931	0.223268224	0.398777747	0.170371099	0.118146012	3R	-0.372513388	0.468489942	0.973421884
**CG44325**	FBgn0265413	4.023338632	3.321014757	7.243365472	2.814078768	2.006925072	2.655225361	2.376839077	1.563112529	X	-0.982732729	0.00365288	0.159387967
**Neto**	FBgn0265416	0.708877617	0.682003503	0.544838501	0.277935143	0.464998197	0.52984363	0.325830997	0.314278219	X	-0.4081853	0.180328672	0.836097072
**lncRNA:CR443**	FBgn0265425	2.933287203	6.946133342	3.767354387	1.609140868	4.049373974	1.626756034	0	0.374614003	3R	-1.271119696	0.305276687	NA
**zip**	FBgn0265434	21.86609697	22.04305662	25.88992534	22.88954938	24.20618173	25.12301344	22.68002581	25.97364609	2R	0.111481857	0.283489224	0.901780531
**lncRNA:CR443**	FBgn0265447	14.13080311	23.77744758	19.09328829	14.07479048	17.43702469	14.88234021	17.68176348	10.7765831	3L	-0.190316138	0.490639018	0.973549494
**lncRNA:CR443**	FBgn0265450	1.236018614	0.243911509	1.705065758	0.113009054	1.05003783	0.822572329	0.123651374	0	3R	-0.691574068	0.555730552	0.984934163
**lncRNA:CR443**	FBgn0265457	0.124130164	0.130511228	0.057066977	0.04809351	0.07513113	0.143301013	0.017540862	0.02357126	X	-0.451640734	0.414646407	0.958665466
**Traf6**	FBgn0265464	50.87695304	59.79461042	44.63570729	39.3460058	50.15899222	63.96507457	45.74697101	47.03092642	X	0.117543458	0.475350033	0.973549494
**lncRNA:CR443**	FBgn0265470	2.324282083	1.559462852	0.751823455	4.335180943	0.559456649	0.14608793	0.790572471	2.018492624	3L	-1.357290728	0.312979229	NA
**mbl**	FBgn0265487	0.672935574	0.542516248	0.518796831	0.365875548	0.389895178	0.638953348	0.194058699	0.449273605	2R	-0.303738796	0.287926843	0.902924182
**lncRNA:CR443**	FBgn0265509	0.866182456	3.632248894	2.626683197	2.524339737	6.450185505	1.020789411	1.104825028	2.115632581	2R	0.204577467	0.831486558	NA
**Smr**	FBgn0265523	9.249758047	13.0752255	10.34935213	12.98836859	11.13033808	10.13117982	8.794721184	12.49737709	X	-0.06821065	0.634466493	0.991585536
**lncRNA:CR443**	FBgn0265530	3.858449124	2.641635559	2.101346558	6.058415368	2.985209821	2.895329967	1.406140945	0	2R	-0.980506083	0.286663436	0.90249226
**CR44383**	FBgn0265533	4.5371462	3.805213127	10.00641218	3.365786316	1.116915239	0	0	0.447752927	X	-3.78605461	0.007241897	NA
**lncRNA:CR443**	FBgn0265537	2.43017122	5.240920247	2.526669208	2.428222592	0.626725986	0	0	0	2R	-4.221732845	0.014674008	NA
**CR44391**	FBgn0265541	1.423861572	0.199027337	2.302845543	1.383199856	1.285217535	3.579754648	0.605383577	0.515222546	2R	0.217194963	0.863464684	NA
**CR44394**	FBgn0265544	3.759956001	1.167925689	1.689185336	0.974021763	0	2.49453361	4.262990461	1.814047229	2L	0.168695371	0.892643458	NA
**lncRNA:CR444**	FBgn0265551	0.096135678	2.902574033	1.632566693	4.034460844	0.173549538	0.725089038	0	0	3R	-3.101754975	0.046616811	NA
**Cdc5**	FBgn0265574	98.34064695	103.4141984	86.68352024	105.0331166	91.02289989	78.03316565	108.5431157	85.775364	3L	-0.084161316	0.535745597	0.984760433
**yin**	FBgn0265575	1.196997934	3.027886072	4.42610609	3.623481089	0.958458413	0.018201973	2.117796777	1.970053	X	-1.226885151	0.243645557	0.877269208
**IBIN**	FBgn0265577	21.74995595	10.24070173	5.554219977	15.12379754	13.77692806	6.835241874	0.973414121	3.313766158	2R	-1.052482213	0.269919018	NA
**CG44405**	FBgn0265578	3.72117722	2.331257054	4.024317942	0.313582576	3.399307249	1.183523955	0.343113363	1.849414265	2L	-0.578684298	0.488981399	0.973549494
**lncRNA:CR444**	FBgn0265580	1.918758606	1.379335023	1.329966176	0.319536676	0.742253798	1.938207743	2.09776904	0	2L	-0.039663713	0.978322373	NA
**lncRNA:CR444**	FBgn0265584	24.29331841	11.52202744	16.66445046	16.40576695	9.37603895	21.00812251	27.78090012	21.46096236	2L	0.223759578	0.636760932	0.991585536
**CG44422**	FBgn0265595	1.814326353	1.318695704	1.053142369	0.796611936	1.23176183	1.33063432	0.818644031	0.728288357	X	-0.25435452	0.317262161	0.91460807
**rad**	FBgn0265597	53.73098647	45.61001142	41.52282171	41.86963013	47.44546781	49.83298562	37.28715765	43.81083327	X	-0.007237701	0.950277624	0.997604378
**Bx**	FBgn0265598	53.90676653	58.59605367	59.46509368	59.04863575	63.79425253	61.46404294	56.53417066	64.9992018	X	0.126642575	0.147206238	0.808004944
**Ric**	FBgn0265605	1901.231838	1729.553734	1805.429342	1775.05066	1684.491949	1873.847714	1680.517324	1736.947758	2R	-0.019256897	0.814531264	0.995787491
**CG44426**	FBgn0265608	2.827743415	0.249639099	1.805280548	2.77590624	2.149390151	5.331958438	0.379664958	2.584965353	X	0.468576562	0.689874059	NA
**lncRNA:CR444**	FBgn0265612	0.233158131	1.759905251	2.262553494	0.271799702	2.104550921	1.209010339	0	1.265519714	X	0.090492378	0.950737316	NA
**Su(z)2**	FBgn0265623	43.72665161	49.59722854	59.44913838	52.37175306	48.21236578	47.99588987	55.83191157	48.37379785	2R	-0.001272488	0.991433455	0.998408391
**sno**	FBgn0265630	9.890812965	13.18613014	18.91536685	13.13436984	10.5276598	11.60197375	17.55425237	16.74194562	X	0.070822076	0.731195023	0.995787491
**lncRNA:CR444**	FBgn0265633	0.266517679	0.894092035	1.1314943	0	0.721699078	0.25127124	0	2.169879571	4	0.521349477	0.742266833	NA
**lncRNA:CR444**	FBgn0265634	0	2.114665452	3.958015777	0.518699946	1.204891439	3.635688315	1.135094207	1.610070458	4	0.26339772	0.820492686	NA
**lncRNA:CR444**	FBgn0265637	3.966429565	8.810900781	1.820473503	2.249411647	13.74026915	8.38866546	8.477617788	7.91325718	2R	1.220432602	0.050124654	0.587848846
**lncRNA:CR444**	FBgn0265638	10.43498793	3.816493403	5.139162777	2.195078032	4.107496259	5.177917304	5.003736539	6.472949927	2R	-0.04731333	0.936951929	0.996879532
**lncRNA:CR444**	FBgn0265641	1.039418948	1.162319646	0.840538623	1.346314526	2.71038098	2.395452485	1.473100037	1.002966557	2R	0.823324978	0.442737087	NA
**lncRNA:CR444**	FBgn0265644	0.824935673	2.075570796	0.200128244	0	0	1.088842039	0	1.611910538	2R	-0.159326581	0.931563961	NA
**lncRNA:CR444**	FBgn0265653	1.616873919	1.356039587	1.681077246	2.961891958	1.042454223	1.851031466	0	0.250741639	2R	-1.206511462	0.356565186	NA
**lncRNA:CR444**	FBgn0265657	3.103585349	16.56400147	4.106866237	5.920275604	11.20553399	9.576135195	0	6.738171088	2R	-0.057503652	0.956370873	NA
**lncRNA:CR444**	FBgn0265659	5.197094739	2.03405938	0.420269312	1.211683074	0.312736267	2.123241976	3.535440089	0.752224918	2R	-0.421098776	0.749975513	NA
**lncRNA:CR444**	FBgn0265664	2.0570915	0.184847272	1.069387561	0.085643418	2.188358102	1.350662834	0.281125961	0.957029158	X	0.497185794	0.631998736	0.991585536
**lncRNA:CR444**	FBgn0265665	14.75157895	10.99720189	10.09380443	11.1702952	6.828302771	17.35490586	16.72515326	17.5190665	2R	0.332670471	0.468864785	0.973549494
**lncRNA:CR444**	FBgn0265677	5.284402254	4.624613976	1.11477271	2.678344548	4.562465433	5.198715305	2.930570366	1.995291559	2L	0.11222352	0.913909611	NA
**lncRNA:CR444**	FBgn0265687	0.807195121	0.729053541	0.451902486	1.01335502	0.336275556	0.64393884	0	1.482880662	2L	-0.252980259	0.84798319	NA
**lncRNA:CR444**	FBgn0265691	2.020130431	0.242034672	0.450074227	0	1.339660353	0.855111549	0.262928374	0.492293794	X	0.119356145	0.910321799	0.996479182
**lncRNA:CR445**	FBgn0265694	0.796585996	0.984539874	1.017108692	0	0.075686415	0.474326154	0.213906104	0	X	-1.811956814	0.24399206	NA
**lncRNA:CR445**	FBgn0265699	6.69504609	7.613065338	5.568716034	7.541091203	5.179831765	6.853081293	4.70232696	5.096886363	2R	-0.297717665	0.22991906	0.867702781
**lncRNA:CR445**	FBgn0265707	4.187650553	4.80602907	16.04081342	8.221768099	9.947082281	5.402651338	17.99206152	37.80265172	3L	1.154647385	0.114618865	0.762354509
**lncRNA:CR445**	FBgn0265710	1.256794046	1.096209765	0.487834372	0.11720672	0.272260244	0.4739591	0.128244344	1.418880431	X	-0.349426473	0.77898214	NA
**lncRNA:CR445**	FBgn0265730	0	0.489603895	0	1.361059336	0.658669475	3.439896921	1.489233399	2.376447718	3L	2.162971253	0.107576833	NA
**lncRNA:CR445**	FBgn0265740	2.905598449	0.658612673	0.381023855	2.19706813	1.842960775	5.700872869	3.205294732	0.852476108	3L	0.907283498	0.334746835	0.924122842
**lncRNA:CR445**	FBgn0265746	1.525992583	3.900401496	0	0	1.57417584	2.466336833	0	1.104357052	3L	-0.040478689	0.978658208	NA
**lncRNA:CR445**	FBgn0265751	0.126449994	1.272612751	0	6.485894798	0.228274647	2.861190759	1.935459903	0	3L	-0.633189474	0.700915935	NA
**lncRNA:CR445**	FBgn0265757	1.178479533	6.177294037	0.714743727	1.03034275	1.728559298	1.111063305	0	2.558588156	3L	-0.6986288	0.528571649	NA
**lncRNA:CR445**	FBgn0265759	4.215488201	6.397471979	6.087330701	6.55216803	6.341697186	3.406574166	5.120857603	4.576107553	3L	-0.222627015	0.694944153	0.995787491
**lncRNA:CR445**	FBgn0265764	1.732364913	1.452899557	4.202693116	1.615577431	4.378307737	2.613220893	2.209650056	1.880562295	2R	0.35194461	0.73511023	NA
**lncRNA:CR445**	FBgn0265765	1.443637427	2.421499262	0.700448853	3.197497	0.390920334	2.10963145	1.104825028	0	2R	-1.071330487	0.346031918	NA
**zyd**	FBgn0265767	19.27208042	31.02856943	25.74365448	16.98322782	9.294090766	11.68921672	23.69977178	10.16680848	X	-0.723819421	0.048945175	0.582455792
**PDZ-GEF**	FBgn0265778	113.1740057	150.2016645	144.0104829	176.2952761	111.3919301	149.8756912	130.8596791	121.6675029	2L	-0.155792246	0.241510104	0.876249547
**CrebB**	FBgn0265784	4.207838647	6.363616728	3.652292618	6.405728714	3.20699396	3.221958062	4.493422378	4.216434101	X	-0.415743501	0.096329385	0.730787365
**lncRNA:CR445**	FBgn0265787	1.434670735	3.30888098	2.175306996	0.418110101	2.266204833	1.014449105	0.914968967	0.389350372	2L	-0.631664855	0.648880167	NA
**lncRNA:CR445**	FBgn0265798	0.302332446	0.507120264	1.283545018	0.176219179	0.955128215	1.567704506	0.771256564	2.789664835	2L	1.494833082	0.193449346	NA
**lncRNA:CR445**	FBgn0265800	2.558344808	6.130377879	5.319864703	6.816782411	7.257592692	10.68167823	7.458734365	4.760917201	2L	0.586280955	0.536193723	NA
**lncRNA:CR445**	FBgn0265806	15.0902867	6.70763733	8.236985636	5.101453126	3.269020212	7.540325966	5.196912152	4.259113211	2L	-0.777128445	0.135959655	0.796109055
**lncRNA:CR445**	FBgn0265807	10.48246285	11.72190726	8.030623788	6.145597094	4.979876862	6.183975486	5.942441763	5.057421599	2L	-0.688764091	0.08552544	0.701544311
**lncRNA:CR445**	FBgn0265808	31.26802141	31.59893656	16.01923385	17.20626188	15.42645263	13.00018802	8.422432948	9.276316251	2L	-1.029175804	0.033857297	0.514749796
**lncRNA:CR445**	FBgn0265809	16.60927185	15.87704671	9.965148625	7.078560911	5.480944884	13.47021079	8.656361043	6.203916848	2L	-0.525906084	0.376846685	0.943615128
**lncRNA:CR446**	FBgn0265814	0.035191595	0.550936994	0.113832425	0.437588687	0.232942777	0.608271047	0.179549029	0.254680701	2L	0.213526415	0.828101443	NA
**lncRNA:CR446**	FBgn0265819	2.580804339	10.01066734	4.695746498	2.632458571	1.164753322	6.995353881	13.57885509	5.252967303	2L	0.474542834	0.577949895	0.985637351
**lncRNA:CR446**	FBgn0265820	0.45554299	1.964852895	0.473631831	1.213807236	1.057335238	0	0.664057117	0.141289429	2L	-1.111581233	0.434145127	NA
**lncRNA:CR446**	FBgn0265821	3.899873619	6.197201904	0	0.957095635	0.741081201	1.161087482	1.047227515	7.575724883	2L	-0.046492261	0.973395296	NA
**lncRNA:CR446**	FBgn0265830	0.032033375	2.632843133	0.310850083	1.120269114	0.636112969	1.238235677	0.898896103	0.904116488	X	-0.106525981	0.916742056	0.996479182
**lncRNA:CR446**	FBgn0265839	3.622217545	2.377472003	3.438567095	1.835883445	3.695974064	9.79957835	2.410527333	8.889930847	2R	1.161672333	0.157600834	0.818884898
**lncRNA:CR446**	FBgn0265847	11.35167892	1.241794494	4.789393864	1.726044264	0.890986515	11.86558632	3.777179582	0	2R	-0.21246148	0.870155268	NA
**lncRNA:CR446**	FBgn0265854	1.795517601	0.934674483	0.901220825	0.433053279	0.894170885	1.517685472	0	0.403265681	2R	-0.498000268	0.687451854	NA
**lncRNA:CR446**	FBgn0265868	1.954269007	0.740196281	0.152936431	1.322798115	0.284512615	1.069822964	0.241228172	0.342169268	X	-1.084825574	0.274969706	NA
**lncRNA:CR446**	FBgn0265873	0.207496463	1.121482224	0.167794508	0.430017948	1.290234798	0.804241857	0.705770579	1.301427193	X	1.134381165	0.170274817	0.830934498
**lncRNA:CR446**	FBgn0265874	49.24360904	36.85197128	30.63187402	25.90576056	41.48542316	63.09252339	39.94119634	22.47902451	X	0.246854769	0.549657189	0.984934163
**lncRNA:CR446**	FBgn0265888	1.453152114	0.609364704	1.510854326	3.024972722	2.71700172	2.201822577	0.132393652	0.676055948	3L	-0.169273104	0.857998413	0.995787491
**lncRNA:CR446**	FBgn0265892	1.866772535	0.626249809	0	0	3.707003164	0.703992698	3.333523791	4.863523175	3L	2.25273659	0.138420935	NA
**asRNA:CR446**	FBgn0265901	0.207925375	0.52314847	0.294247368	0.242385092	0.187679296	0.326717955	0.176807366	0.300950157	X	-0.307506643	0.75227871	NA
**lncRNA:CR446**	FBgn0265902	1.213464415	0.630009815	0.70091613	0.134721267	1.147464622	0.680980261	0.442224828	0.31363614	X	-0.035721494	0.969885323	NA
**lncRNA:CR447**	FBgn0265916	3.248184212	7.264497787	0.875561066	0.841446579	11.07607612	4.083157646	2.76206257	0	X	0.580700978	0.693060591	NA
**lncRNA:CR447**	FBgn0265918	0.448025409	2.880749122	1.449204523	1.566831561	0	0	0.380974148	0.648469757	X	-2.647808536	0.073831931	NA
**lncRNA:CR447**	FBgn0265919	18.4063772	18.16124447	22.5894755	13.12656663	17.98233535	13.67857811	17.12478793	19.27576352	X	-0.056850476	0.902238696	0.996479182
**lncRNA:CR447**	FBgn0265921	2.056897654	1.533403227	2.032965008	0.177614054	0.962688597	0.933703595	0.194340374	1.323174877	X	-0.728770574	0.521363299	NA
**lncRNA:CR447**	FBgn0265928	0.904786688	0.556472672	0.365833314	0.562526961	0.272228645	0.255908209	0.153875352	0.851229451	3L	-0.627121287	0.545281509	NA
**lncRNA:CR447**	FBgn0265929	57.30725002	96.38431885	86.49292528	104.0388591	46.77082563	64.09099233	56.42499249	73.5434183	3L	-0.480958555	0.02789855	0.462499435
**coro**	FBgn0265935	94.16713446	91.29257833	82.52839098	86.30382379	69.96222823	82.56230865	79.62740053	78.40209323	2R	-0.161155724	0.092650179	0.72305867
**lncRNA:CR447**	FBgn0265940	4.093897182	2.891342403	6.795399316	0	2.722828195	3.250274743	3.847649351	0.935603132	2L	-0.325742306	0.791646607	NA

**lncRNA:CR447**	FBgn0265941	1.050979725	1.76287105	0.424943692	0.408386611	1.106751197	2.229428846	2.234226548	0.760591423	2L	0.80613578	0.462249241	NA
**lncRNA:CR447**	FBgn0265945	1.410975947	0.657420614	1.806587538	0.639651698	3.25472133	0.960742975	0.099984165	2.042239596	2L	0.532350595	0.557430856	0.984934163
**lncRNA:CR447**	FBgn0265950	0.163071689	0.592648078	0.131869881	0.063365917	0.539708023	0.97370562	0.06933323	0.177021866	2L	0.926522285	0.47271701	NA
**lncRNA:CR447**	FBgn0265951	1.807443328	3.264942826	0	0.972458326	2.760914074	3.539173561	1.064036945	0	2L	0.306834513	0.83531951	NA
**lncRNA:CR447**	FBgn0265952	3.911791739	3.593192454	0.225951243	0.434295008	1.345102223	0.878098418	0	2.628742992	2L	-0.726830109	0.569629353	NA
**lncRNA:CR447**	FBgn0265958	2.403836604	2.150452629	2.721447901	0	1.735816468	2.039690221	1.022039804	1.043790358	2L	-0.282614213	0.791428152	NA
**rdgC**	FBgn0265959	201.4020442	186.5970692	168.5983675	195.8566351	191.3572259	197.7506382	170.7755752	161.6999455	3L	-0.033080273	0.740151582	0.995787491
**lncRNA:CR447**	FBgn0265967	2.015848557	0.848640793	0.728768542	0.645080886	0.727824661	1.207395343	0.766329307	0.377588487	X	-0.449684714	0.387266599	0.948247506
**ttv**	FBgn0265974	9.517469521	15.20536568	13.29659841	14.21000588	11.11697533	15.59746351	11.87082074	13.00684124	2R	0.012870409	0.927813221	0.996479182
**lncRNA:CR447**	FBgn0265982	10.56742597	13.07609602	13.02834866	15.3479856	5.629252805	11.43284141	7.512810189	5.265574425	2R	-0.76176262	0.168640606	0.830934498
**mv**	FBgn0265988	3.403038053	4.336075669	6.589430166	6.318128291	4.339801859	6.010530502	5.145018512	5.341274106	3L	0.04684892	0.83862	0.995787491
**Zasp52**	FBgn0265991	1.018308147	0.9447757	0.621460342	1.461584315	0.715216393	0.556531709	0.515486245	0.31435399	2R	-0.920019858	0.002761056	0.128551053
**lncRNA:CR447**	FBgn0265997	86.98527211	43.0944784	35.61604335	26.52695994	45.0552249	33.91097028	22.47101752	23.10860447	2L	-0.608002187	0.227947921	0.867702781
**Doa**	FBgn0265998	17.22180111	18.06336981	18.64620321	18.54824806	16.48897664	17.26272304	17.97700829	20.92524574	3R	0.035330825	0.747745105	0.995787491
**CG44774**	FBgn0266000	55.87297027	40.52270333	42.57426205	37.77353054	41.58974129	39.05784221	38.00883935	38.98867024	X	-0.1370359	0.306667908	0.911610813
**lncRNA:CR447**	FBgn0266005	0.056186158	0.160564529	0.302904078	0.160106114	0.289264518	0.035314644	0.127406307	0.171683085	X	-0.071014589	0.9169069	0.996479182
**lncRNA:CR447**	FBgn0266010	2.351066667	1.660456637	3.302116019	1.44247985	1.563681335	0	0.315664294	0	2R	-2.190955536	0.153222043	NA
**rudhira**	FBgn0266019	32.45762001	36.50973647	39.24312218	42.41853253	37.2630996	45.86353905	39.69675764	49.049001	X	0.220646677	0.071444009	0.669004915
**lncRNA:CR448**	FBgn0266045	0.329974269	0.083022832	0.160102595	0	0.208490845	0.699969882	0.126265717	0.394022576	2R	1.302367993	0.28820252	NA
**lncRNA:CR448**	FBgn0266048	0.072181871	2.300424299	0.700448853	0.673157263	5.212271115	0.476368392	0.368275009	2.977556966	2R	1.338374937	0.253104632	NA
**lncRNA:CR448**	FBgn0266049	8.343959443	0.666467687	3.373721538	0	5.379637619	0	3.040802829	1.725286509	2R	-0.282263787	0.852870589	NA
**Patr-1**	FBgn0266053	68.10687258	69.36944768	68.53063512	83.65610954	56.62992521	65.03460479	68.10471759	65.12468148	3R	-0.155449025	0.205361973	0.855315685
**GlyS**	FBgn0266064	38.99953008	38.82572657	33.50775893	33.72887131	45.19500904	44.3059933	45.91783344	39.74043386	3R	0.30039091	0.012044144	0.331790969
**ocm**	FBgn0266083	31.32056047	43.39917742	46.49538209	44.47212244	36.32868595	37.23200695	33.14346421	40.25239894	2R	-0.139177199	0.313378938	0.91460807
**Fhos**	FBgn0266084	0.528160034	0.985781074	1.006409881	0.483598566	0.579012822	0.490703203	0.421352339	0.788084864	3L	-0.355396261	0.275081544	0.89549671
**lncRNA:CR448**	FBgn0266086	3.320366083	1.452899557	1.751122132	6.731572631	1.563681335	0.680526274	3.314475083	15.67135245	2R	0.711019152	0.488269686	0.973549494
**lncRNA:CR448**	FBgn0266095	45.2372687	110.708956	174.5125138	61.20659362	59.97681832	266.5239203	85.1320655	198.6826945	X	0.670344955	0.273620064	0.89549671
**lncRNA:CR448**	FBgn0266096	1.025957098	0.768306923	0.57030446	0.389873963	0.580426751	0.877394118	0.230811593	0.261330494	X	-0.479536679	0.188791319	0.843186953
**CG44836**	FBgn0266099	0.891918007	0.280512625	0.067618145	0.281595337	0.167722979	0.13138982	0.260711687	0.544622782	3L	-0.463908368	0.599322964	0.989286463
**CG44837**	FBgn0266100	8.965484707	7.091010115	5.959138458	9.279402716	6.935161643	8.618287109	7.881678766	9.267780675	3L	0.089392586	0.555385832	0.984934163
**CG44838**	FBgn0266101	28.23424548	33.54007297	35.70468224	36.25949348	36.85626716	33.49746106	31.70866978	35.83823742	3L	0.076839484	0.507839	0.978557094
**ana3**	FBgn0266111	0.245841757	1.649460235	0	0.127371289	1.232798277	0.296160914	1.28913674	0.444787676	2R	0.742607575	0.462531382	0.971606621
**asRNA:CR4484**	FBgn0266115	1.023721879	0.858574919	0.275948333	0	0.718697921	0.750679016	0.580341446	0.246954996	X	0.093604181	0.946382235	NA
**ghi**	FBgn0266124	345.5252994	322.848906	418.8898718	365.2283225	401.3562813	336.927516	400.3479751	333.1105421	3L	0.050270983	0.699664978	0.995787491
**lov**	FBgn0266129	0.868643613	0.955162671	0.425365897	0.262527207	0.467533372	0.746156169	0.18466071	0.499415767	2R	-0.376306295	0.37746618	0.943615128
**Dop1R2**	FBgn0266137	198.6236921	190.3112402	156.7938709	176.0505831	167.5109435	162.9466052	144.3345203	149.7543458	3R	-0.179524711	0.074032773	0.675782176
**lncRNA:CR448**	FBgn0266153	0	4.600848598	2.451570984	0.673157263	3.387976225	2.858210352	1.473100037	2.820843442	2L	0.514208651	0.682488472	NA
**lncRNA:CR448**	FBgn0266158	0.669235893	0	2.319367062	1.560298292	0.172591759	0.630951513	0	3.113513733	2L	-0.168444252	0.916644218	NA
**lncRNA:CR448**	FBgn0266165	1.755077683	2.078044659	0.751375408	3.610497836	0.186374414	1.168006955	2.106936883	0.224143301	2L	-1.145704996	0.334615045	NA
**lncRNA:CR448**	FBgn0266180	0.096333065	0.128439519	0.143817029	0.097901219	0.115937248	0.086164675	0.006301224	0.128706462	X	-0.422929346	0.587989923	0.987588562
**Vamp7**	FBgn0266186	187.1855618	236.7347591	226.5375719	190.8699075	247.6378597	230.0006522	216.9248578	170.9962761	2R	0.07327907	0.589457509	0.987597375
**asRNA:CR4489**	FBgn0266199	0.095594982	0.195808566	0.194003386	0.143583643	0.154320483	0.214916717	0.105518446	0.043903867	X	-0.240416993	0.609179356	0.990047511
**lncRNA:CR449**	FBgn0266214	0.427493104	0.317555015	0.607439781	0.208829045	0.437316284	0.458695501	0.264846423	0.212143338	2L	-0.152111154	0.714796902	0.995787491
**lncRNA:CR449**	FBgn0266219	1.717789727	1.833587182	3.662202535	1.941799797	1.033683575	5.349329127	5.046075848	1.695218415	2L	0.55339556	0.421798939	0.960593359
**lncRNA:CR449**	FBgn0266220	1.298191858	2.903379632	0.524899223	1.17704434	1.301982793	0.883947534	0.735936738	0	2L	-0.976113785	0.413112822	NA
**lncRNA:CR449**	FBgn0266222	1.039537077	0.485455699	0.60181763	0.39935003	0.479820389	0.929952031	0.301350161	0.384704186	2L	-0.249956886	0.634113065	0.991585536
**lncRNA:CR449**	FBgn0266227	29.57276352	24.19574382	20.29519927	17.17674733	21.81447331	46.99850772	25.02981979	29.22495458	3R	0.453041568	0.074573928	0.676245
**lncRNA:CR449**	FBgn0266236	0.28492844	0.152936796	0.248843671	0.186003981	0.205747544	0.096706365	0.029074343	0.222698166	3R	-0.624707963	0.55131784	NA
**lncRNA:CR449**	FBgn0266245	0	1.362093335	1.313341599	0.420723289	2.443252085	0	0	1.763027151	3R	0.517538795	0.776907138	NA
**lncRNA:CR449**	FBgn0266246	0.361878675	0	0.501664353	0.060264751	1.166578137	0.48739572	0.395640117	0	3R	1.17581803	0.422857691	NA
**lncRNA:CR449**	FBgn0266251	0.353363572	0.148179455	0.535784436	0.720872133	1.116345675	0.666298035	0.901438065	0.095898128	3R	0.700215978	0.555143676	NA
**lncRNA:CR449**	FBgn0266252	7.120313413	7.018243625	6.05472737	5.134250312	6.493253	1.107296989	5.805012858	1.59369686	3R	-0.731677596	0.258181194	0.887830768
**lncRNA:CR449**	FBgn0266255	0	1.210749631	0.175112213	1.514603842	0.781840667	1.633263058	1.288962532	0	3R	0.391579337	0.803734608	NA
**lncRNA:CR449**	FBgn0266256	0.621810809	2.81610108	3.469560002	1.159782794	2.469561333	3.165692934	2.062128552	0.810005296	3R	0.132953642	0.871125667	0.995787491
**lncRNA:CR449**	FBgn0266258	0.742442106	1.094391874	0	0.104907625	0.081230199	0.212112085	0.172180524	0.928069704	3R	-0.45084045	0.726668235	NA
**lncRNA:CR449**	FBgn0266262	1.918580034	2.318960206	2.943254136	2.499671922	2.954186235	2.92604131	4.462485454	4.532951459	X	0.656226405	0.117706387	0.771985382
**FeCH**	FBgn0266268	110.6788694	70.58749484	96.14003859	113.0728213	93.08843675	95.09576303	102.6596676	119.0203369	3R	0.096665111	0.58117057	0.985637351
**asRNA:CR4496**	FBgn0266275	2.742530205	1.788970431	1.293705005	1.95375459	2.062904135	2.298347312	2.332084491	1.653968597	X	0.113501424	0.882711703	NA
**jnj**	FBgn0266282	21.96652971	12.42589605	32.86807788	20.45794655	17.48548302	14.3606139	20.81810709	14.06228837	3R	-0.364759871	0.158126258	0.818884898
**Ns3**	FBgn0266284	84.02990581	73.4399145	70.04488526	58.13630909	75.74904108	90.5649865	99.0436578	75.03253599	X	0.279864203	0.067328445	0.656261422
**lncRNA:CR449**	FBgn0266315	0.060032447	0.251740022	0.384900112	0.269929398	0.294157875	0.291076585	0.13126634	0.158265144	2L	-0.088976325	0.896082673	0.996479182
**asRNA:CR4498**	FBgn0266322	0.247746144	0.363614476	0.60102869	0.505409457	0.391339626	0.875900818	0.553005019	0.605114789	2L	0.551623375	0.565893295	NA
**CngB**	FBgn0266346	187.857325	190.5281867	171.6029032	212.7665862	228.7159988	271.551493	237.855628	240.7929125	2R	0.387781348	0.000155379	0.013676217
**nAChRalpha4**	FBgn0266347	4.454278865	4.967762138	6.100171676	4.234993267	4.279948292	5.281178411	4.491570076	4.711940088	3L	-0.042380061	0.741950907	0.995787491
**lncRNA:CR449**	FBgn0266348	1.41321323	1.29495814	1.196209621	1.035936454	1.684468415	1.374841693	1.042183799	1.101342429	X	0.103941709	0.543695326	0.984934163
**lncRNA:CR449**	FBgn0266350	0.219978435	0.235954896	0.212061466	0.12011989	0.118090191	0.182834471	0.088605744	0.106830135	X	-0.633652114	0.051676487	0.599821511
**CG45002**	FBgn0266354	1.693997207	1.731643596	2.766996379	1.863084364	1.943734209	1.404770454	1.733094358	1.338266073	X	-0.293492035	0.116013683	0.765798592
**asRNA:CR4500**	FBgn0266361	0.437957422	1.632471413	1.180531774	0.567267357	0.702778128	0.87168534	0.248275287	0.633897403	X	-0.573710624	0.5767478	NA
**Mtp**	FBgn0266369	4.740871721	9.757707234	4.000448233	5.019311163	2.563412024	3.497998618	10.69185511	0.596688195	2L	-0.406502127	0.481946566	0.973549494
**Pde8**	FBgn0266377	61.63042421	78.57154972	71.37193748	80.72855179	70.66359049	75.81127599	74.05370144	77.32466263	2R	0.058612228	0.569165347	0.984934163
**asRNA:CR4502**	FBgn0266382	3.340035179	4.668700377	5.942111089	7.787166283	1.406911227	3.253930258	3.976234033	0.483435037	3R	-1.222939759	0.127435372	0.787284205
**asRNA:CR4502**	FBgn0266388	1.237403509	2.554548672	3.001923654	0.221919977	1.718331137	1.435835656	0.97127475	0	3R	-0.714421715	0.572208025	NA
**lncRNA:CR450**	FBgn0266396	0.570795688	0.478714846	0.692371189	0.831742912	0.772824383	1.278088884	1.274098055	2.788313122	3L	1.286760676	0.211026182	NA
**CG45049**	FBgn0266409	148.9448498	155.7389831	172.7817114	130.9007015	152.5712427	165.68765	154.6550252	182.5836015	3R	0.140155541	0.203414819	0.851888994
**CG45050**	FBgn0266410	26.32239426	30.62328025	35.60056356	26.01251308	29.01096374	28.15080677	29.35464513	31.71973104	3R	0.030311943	0.797171786	0.995787491
**sima**	FBgn0266411	8.808218909	8.9640518	9.393453039	8.477156455	9.278100379	10.88721984	8.502803859	10.21043399	3R	0.155362844	0.129822233	0.789160095
**lncRNA:CR450**	FBgn0266412	1.208064793	0.810543686	2.637673504	3.379869104	1.962779918	0.569478054	0	0	3R	-1.606438367	0.279566744	NA
**lncRNA:CR450**	FBgn0266414	44.29121659	77.66830081	94.83120524	65.97770376	69.0129794	63.85363552	81.87211222	76.23332034	3R	0.079365486	0.656715804	0.995750687
**ringer**	FBgn0266417	300.4073201	305.630238	283.4259614	276.1785106	278.3032158	277.9198374	283.9225702	252.5223226	3L	-0.063077454	0.463379747	0.971768196
**wake**	FBgn0266418	22.22766766	18.25572469	19.24289051	19.42443844	16.87691683	18.68907977	19.70257495	16.9134831	3R	-0.105266403	0.326245497	0.918470465
**Ote**	FBgn0266420	0.165512571	3.053865312	1.204593568	0.771772658	1.394365521	0.052014747	0.422226125	0.718686227	2R	-0.950383757	0.431716883	NA
**RPA3**	FBgn0266421	22.38318057	26.78966882	33.93830241	36.42116013	29.67416799	45.83248286	51.15206051	59.98563443	X	0.678521315	0.024102887	0.43956961
**AstA-R1**	FBgn0266429	0.899180599	0.663503616	0.79551563	0.435929715	0.496982938	0.860143636	0.486569794	0.628295642	X	-0.150814631	0.557256768	0.984934163
**CG45062**	FBgn0266432	0.234879244	0.175100881	0.56981377	0.304228953	1.319164729	0.738142389	0.199727333	0.283302545	2R	1.042036047	0.311565856	NA
**CG45067**	FBgn0266437	0.241052632	0.673886622	0.25990681	0.874230212	0.580215709	1.994863661	0.273302419	0	3L	0.503780511	0.663303514	NA
**CG45076**	FBgn0266446	0.240272526	0.542531575	0.560478651	0.861825153	0.333656531	0.59245646	0.094298519	0.501590284	3R	-0.487315048	0.488900102	0.973549494
**Kr-h2**	FBgn0266449	147.6096189	177.5657453	210.2053844	169.1842909	175.8944133	174.4309554	202.2971441	218.3756283	2L	0.164751029	0.209972511	0.856520545
**Kr-h1**	FBgn0266450	29.16157993	42.66160629	56.04599024	38.74711616	33.43430907	40.18802732	44.73368555	43.68805388	2L	-0.004911609	0.976262923	0.997757033
**CTPsyn**	FBgn0266452	33.54167999	24.52472114	23.49374805	25.48263163	29.05800558	35.0758098	28.62923891	21.46151681	3L	0.11677042	0.454310033	0.967967712
**lncRNA:CR450**	FBgn0266457	0.261797941	0.19791744	0.250469131	0.206322945	0.173069323	0.264203296	0.216346479	0.344234723	X	0.149190705	0.774120363	0.995787491
**Nsf2**	FBgn0266464	50.1707373	37.27621695	57.65376737	45.52124883	52.33148036	56.93161061	52.16819557	49.08543773	3R	0.17159245	0.243284074	0.876959115
**GckIII**	FBgn0266465	340.9434138	424.1879678	329.7628295	431.1470277	352.8549598	385.1861201	390.7732644	387.8137354	3R	0.021836735	0.848411745	0.995787491
**Dpit47**	FBgn0266518	39.0458351	39.45416313	37.51895076	63.88824534	57.00589794	40.6929764	35.70242452	41.49237921	2R	-0.010647599	0.964261886	0.997604378
**stai**	FBgn0266521	140.0321168	119.2435771	135.0432841	116.7793087	137.8172921	135.849595	140.2922076	123.8524879	2L	0.103015104	0.287671116	0.902924182

CG45095	FBgn0266534	0	0.409652131	0	2.277599762	1.02873772	2.839790092	1.038369387	1.237212036	3R	1.234855208	0.399799974	NA
lncRNA:CR451	FBgn0266549	5.337819059	7.22380032	8.534880977	6.363881689	3.175543327	3.545599076	5.725283758	2.633840749	X	-0.836330751	0.0765211	0.681411883
kis	FBgn0266557	44.71357951	75.26170477	100.1557291	89.58358319	72.9221277	60.99680173	79.07513395	97.18052408	2L	0.039491848	0.836718962	0.995787491
CG45105	FBgn0266566	0.82817707	0.418648203	0.577973513	0.238051684	0.798737028	0.053479471	0.086823185	0.098523237	3R	-0.959138457	0.314227091	0.91460807
CG42259	FBgn0266569	13.02032228	18.69335736	10.43981092	17.23625604	19.72031237	16.43666008	9.288974756	16.7693453	X	0.101866656	0.799955315	0.995787491
NO66	FBgn0266570	15.69415981	18.45251714	17.12410719	16.19429047	20.13061796	23.68125241	18.48558564	18.83007759	X	0.299106456	0.133714485	0.794759341
ebo	FBgn0266572	64.24705846	87.4614176	72.14875142	87.46201563	70.90001159	89.27133874	74.31736698	78.63862113	X	0.038872726	0.759851329	0.995787491
tau	FBgn0266579	172.988432	155.5901729	182.4278303	155.3494313	135.6774582	149.9098755	154.8563052	143.3997443	3R	-0.160835703	0.087394778	0.706081898
Gp210	FBgn0266580	4.516862906	3.324120218	4.448698328	5.670482368	4.797209859	2.681259366	3.676731633	5.182651993	2R	-0.105721936	0.683996729	0.995787491
pit	FBgn0266581	13.57212624	13.97320733	10.71791199	13.57371356	7.299657841	9.77769326	7.163334955	9.998234805	3R	-0.569213227	0.00754197	0.252813679
ND-30	FBgn0266582	1286.51473	1204.989432	1299.451179	1342.311304	1385.138397	1150.008463	1362.786004	1398.119781	3L	0.076818398	0.472254025	0.973549494
Hsc70-4	FBgn0266599	3053.695701	2915.455514	2889.766221	2709.75368	3014.27749	2676.085093	2881.09783	2483.520274	3R	-0.034668711	0.709068721	0.995787491
asRNA:CR4512	FBgn0266619	0.842487496	0.176644323	1.149672889	0.613821213	0.190113232	0.297859524	0.402975694	0.914559171	4	-0.604095199	0.625829334	NA
asRNA:CR4513	FBgn0266627	7.602133261	9.467031691	10.33907216	9.130590537	7.485708517	7.38443404	12.63496707	14.75441162	2R	0.241941517	0.612649587	0.990047511
Sem1	FBgn0266666	183.8611825	198.4521849	220.6195451	143.1683743	160.2692096	158.4027478	180.9983621	161.0793483	2L	-0.143388935	0.413182565	0.958665466
Exo70	FBgn0266667	21.97999335	24.0897118	24.3836962	22.93610483	18.56847508	26.97962258	25.69487131	19.45888553	3L	-0.012690856	0.940272507	0.997188858
Exo84	FBgn0266668	56.23670739	70.91944395	45.00508661	44.6906069	79.85546008	65.20767198	58.25918199	59.55858415	3R	0.309727937	0.078910485	0.69007343
Sec3	FBgn0266669	54.84980453	52.69408307	46.43792102	44.09866969	55.16193629	37.11745055	52.37450423	28.45515542	3L	-0.164202443	0.406389224	0.956706394
Sec5	FBgn0266670	125.3350471	117.4644406	107.5011877	118.7304458	104.2284165	124.1333212	119.1697077	109.7659362	2L	-0.009011658	0.934498906	0.996479182
Sec6	FBgn0266671	89.28450189	76.20514082	63.66169402	45.31338077	91.00891364	99.98692234	84.2155003	88.42984301	2R	0.433588124	0.015631543	0.365936171
Sec8	FBgn0266672	144.0654265	126.0966701	102.4051053	133.0417085	114.8238323	118.3462814	103.7149399	116.1646229	3R	-0.130965795	0.296051379	0.906715248
Sec10	FBgn0266673	55.16433524	66.71422792	68.23360267	70.05112709	61.86563389	78.22406927	72.89314738	68.42829184	3R	0.144913368	0.27804454	0.896398793
Sec15	FBgn0266674	33.62895661	32.67286086	48.47603987	33.12872382	35.96573952	43.45506804	32.6236782	33.67787568	3R	0.010914041	0.950684849	0.997604378
lncRNA:CR451	FBgn0266687	3.331470986	5.293965999	1.276121391	6.949599275	3.481881514	0.330620052	4.025678239	1.142041879	3L	-0.896738981	0.408599313	NA
lncRNA:CR451	FBgn0266690	0.080650136	3.517261498	0	0.37606551	2.475100809	2.585239478	5.349245945	0	3L	1.4371056	0.309476017	NA
Svil	FBgn0266696	7.107810403	8.913083629	9.691661786	8.759222796	6.967501945	8.091685846	8.05914926	8.3593609	3L	-0.098704079	0.380404376	0.944473714
lncRNA:CR451	FBgn0266698	1.732364913	2.300424299	0.175112213	1.009735895	0.781840667	1.905473568	0	0	3R	-0.917830587	0.53083961	NA
Zmynd10	FBgn0266709	3.622029388	0.867920883	0.878699277	0.120637502	1.120918519	1.951329819	2.639964224	0	3L	0.049865481	0.965554232	0.997604378
EloC	FBgn0266711	524.1810236	542.9319911	553.9436471	512.6868847	612.8433008	523.2969399	552.8724744	552.8947096	2R	0.103211122	0.29167824	0.905069205
Bruce	FBgn0266717	25.23114062	35.09255688	43.05181904	34.80802313	30.19846421	30.43447109	37.42241551	40.11554651	3R	0.035841574	0.81705693	0.995787491
stac	FBgn0266719	0.687616934	0.991186748	1.563889773	0.776527232	0.581871	0.577375306	0.274082121	0.97970251	3R	-0.690045253	0.216367425	0.860826301
Snap24	FBgn0266720	28.68495831	32.44415272	58.88326127	31.19509268	46.3595496	36.11768714	52.21720457	49.0271254	3R	0.319750317	0.186108267	0.839299945
gammaSnap2	FBgn0266721	151.0843725	158.2078439	169.8291074	171.0801276	145.1208751	172.7897681	180.3480615	150.2946889	3R	0.026284221	0.842562143	0.995787491
Trs33	FBgn0266722	40.77720487	37.55186548	73.06220339	40.07874782	43.30194465	58.7974701	46.23267809	33.27148675	3R	-0.042034118	0.888006541	0.995926915
Trs31	FBgn0266723	170.0243057	159.596	199.9235088	123.6991032	145.1704155	221.806313	240.7444818	165.2105238	2R	0.271817474	0.156695622	0.818884898
Trs20	FBgn0266724	227.8705036	230.2534956	279.0694795	216.9892034	207.6648112	217.9409659	268.5897928	220.2433495	3L	-0.030218208	0.838542695	0.995787491
btsz	FBgn0266756	38.58003546	35.92566641	37.00519675	43.16367794	35.32059063	35.777099	33.49323898	40.60029583	3R	-0.061730102	0.55993406	0.984934163
mfr	FBgn0266757	0.500238647	0.086059503	0.622344605	0.059809619	0.208398223	0.157207313	0.818025343	0.612651717	3L	0.523305465	0.588402146	0.987588562
Esyt2	FBgn0266758	31.90158041	36.25494077	46.64527524	42.96712167	24.71753251	32.83093451	29.43709626	23.06589932	3R	-0.490982599	0.000774616	0.050043811
lncRNA:CR452	FBgn0266766	0.222832679	0.106791588	0.386134979	0.371090002	0.086200735	0.120048736	0.162414558	0	2R	-1.531056547	0.235321537	NA
lncRNA:CR452	FBgn0266775	4.61161956	4.932683683	3.567100638	3.428115692	1.447853088	4.977923673	2.898460721	5.659099497	3L	-0.114804533	0.848390408	0.995787491
lncRNA:CR452	FBgn0266780	1.993441437	1.186480708	0.156001972	1.349313	0.464344866	0	0.820211602	0.977278104	3L	-1.056772609	0.431030189	NA
lncRNA:CR452	FBgn0266783	0.447639512	2.627983696	2.986409837	0.521827336	1.414182086	2.11015899	1.427422517	0	3L	-0.352203539	0.788892183	NA
lncRNA:CR452	FBgn0266784	1.621558421	2.946598166	1.966939056	1.260200805	3.171272598	3.312389978	0	0.293379453	3L	-0.151918094	0.906525847	NA
lncRNA:CR452	FBgn0266786	3.235739061	2.504999237	2.415340871	0	7.18933947	4.927948883	1.904870738	7.565480495	3L	1.415191211	0.225215759	NA
CG45263	FBgn0266801	63.79483604	56.38002837	55.15411019	62.95909058	62.99500932	64.24119012	52.52768199	57.14111332	3R	0.018918792	0.849712986	0.995787491
lncRNA:CR452	FBgn0266804	1.5550852	4.347395444	4.275630339	4.229893036	4.491723762	3.420957932	7.272935551	3.376233922	2R	0.407810069	0.45200418	0.967630712
lncRNA:CR452	FBgn0266805	0.105076723	0.979174793	0.141619259	0.326643233	0.147536991	0.055036496	0.238269315	0	2R	-1.790603409	0.124274291	NA
asRNA:CR4528	FBgn0266819	2.130794248	1.958415579	1.628676355	1.769377136	1.422726067	2.118976504	2.345542603	2.376447718	2R	0.164357599	0.65994725	0.995750687
lncRNA:CR452	FBgn0266822	0.109991423	0.161433284	0.200128244	0.096165323	0.049640677	0.27221051	0.140295242	0.149250976	2L	0.15395579	0.897313921	NA
lncRNA:CR452	FBgn0266828	7.503236565	11.29004752	12.5813106	16.97899848	6.374242383	9.050565992	11.82233151	11.49897964	2L	-0.285654828	0.538671458	0.984934163
lncRNA:CR453	FBgn0266846	1.021040224	7.706932819	5.77973513	2.777269651	1.843239294	1.764822558	3.038811471	3.325159263	2R	-0.748554743	0.442578151	NA
wap	FBgn0266848	121.4005786	149.2354713	150.8510038	124.1033204	123.0263741	106.6733919	152.5697524	150.8421856	X	0.002839794	0.983929725	0.997881056
lncRNA:CR453	FBgn0266851	23.32689388	24.0231907	16.02016683	25.39335814	18.57839209	10.83451732	16.40829249	8.937325756	2R	-0.671891319	0.056389139	0.621788268
lncRNA:CR453	FBgn0266872	7.927771635	2.955049947	19.23266341	10.26850062	17.49202849	13.28756386	47.9381707	14.66201111	2L	1.250577861	0.07935215	0.69175818
lncRNA:CR453	FBgn0266877	1.408426758	1.181219152	1.138941224	0	4.237618793	2.877021647	4.790569226	2.038549913	2L	1.895336043	0.16264794	NA
lncRNA:CR453	FBgn0266886	0.520750976	0.097054079	1.060577198	0.164879312	0.290150919	0.345490961	0.50841796	0.069790035	2L	-0.575005177	0.470184829	0.973549494
lncRNA:CR453	FBgn0266898	4.612805981	15.90452178	13.05570347	7.169722329	13.4162008	5.798567071	15.68982288	11.68396692	2L	0.238547666	0.732646381	0.995787491
lncRNA:CR453	FBgn0266901	0.577638348	0.599799754	0.734036587	0.128261149	1.539349678	0.907656511	0.280679588	0	2L	0.455236361	0.692150196	NA
lncRNA:CR453	FBgn0266902	2.008351245	0.760680397	1.728856555	0	0.584772376	0.366476378	0.661077055	0.421966109	2L	-1.133495229	0.37948178	NA
lncRNA:CR453	FBgn0266915	6.992618789	2.270155558	4.925030995	0.525904112	4.886504171	2.764637989	0.575429702	3.428108349	2L	-0.314320331	0.788412453	NA
Cenp-C	FBgn0266916	0.656836784	1.15825164	1.389184969	0.824594742	0.273636633	1.556093522	1.417819402	0.87757136	3R	0.070638994	0.911992333	0.996479182
Sf3a1	FBgn0266917	136.6183437	117.7577292	90.60333021	132.4789807	122.6839849	120.3919925	104.7928956	102.1618157	3R	-0.059365179	0.682500325	0.995787491
CG32486	FBgn0266918	200.9431915	210.486038	194.2735777	197.9166211	200.0184168	218.7857105	191.6480358	188.3836999	3L	0.020628898	0.795125314	0.995787491
lncRNA:CR453	FBgn0266937	2.177773867	3.65291015	2.79701419	1.585250893	1.939036012	2.415519881	1.885366942	2.225011133	3L	-0.233599184	0.470073948	0.973549494
lncRNA:CR454	FBgn0266951	6.307153809	3.526455236	4.080284578	0.980326111	3.415808741	2.180326898	0	5.477365906	3L	-0.401114498	0.738392474	NA
lncRNA:CR454	FBgn0266954	28.97549854	24.81821308	17.19963375	25.15356321	7.790583162	15.40265873	10.22258033	12.04630651	3L	-1.05264233	0.059385103	0.626639433
lncRNA:CR454	FBgn0266955	0.384969981	1.506710652	0.933931803	2.393448047	3.706503904	4.355368155	0.982066691	1.95021275	3L	1.13700965	0.292852452	NA
lncRNA:CR454	FBgn0266958	49.46416612	25.90441071	30.95006558	36.00608617	30.30390183	37.91252319	25.12263629	27.05029313	3L	-0.220709119	0.411251167	0.958665466
lncRNA:CR454	FBgn0266967	7.033105416	6.829869714	8.082102145	9.493243454	5.011799149	4.536841829	3.305032135	4.821954601	3L	-0.794083582	0.248441435	0.879503029
lncRNA:CR454	FBgn0266978	0.64882581	1.813857125	1.049361577	0	1.561729173	2.446836042	0	0.469553632	3L	0.399180304	0.80244754	NA
lncRNA:CR454	FBgn0266993	25.21165075	13.8172004	8.478055276	5.237823085	12.16697292	6.354193454	10.82537491	31.97497849	3L	0.232919892	0.744957434	0.995787491
Ten-a	FBgn0267001	9.648416116	11.8747461	10.35478097	10.06978757	11.37214011	10.08841753	7.413101697	8.713976411	X	-0.127262376	0.302632561	0.910208378
unc-104	FBgn0267002	321.1315807	333.1346037	339.6819563	335.6436909	377.5350796	430.965253	384.0686626	453.1543911	2R	0.338110939	0.000157537	0.013676217
lncRNA:CR454	FBgn0267005	8.833588791	1.852138246	2.083488088	0.858132488	5.537115208	0.347010565	1.877889566	0	2L	-0.803793507	0.517392939	NA
lncRNA:CR454	FBgn0267006	0	2.512218255	0.605575377	1.745941028	0	2.118064485	2.865540418	0	2L	0.069886285	0.969281341	NA
lncRNA:CR454	FBgn0267020	0.385827375	1.132549766	1.404017745	2.248854999	1.741293246	1.364083846	0.246063481	0.209416737	X	-0.472386504	0.710864109	NA
asRNA:CR4546	FBgn0267025	1.604041586	5.136513587	12.73543368	7.13954673	3.422198207	4.399361773	7.067904218	5.065487662	2R	-0.368521432	0.627837904	0.990047511
mamo	FBgn0267033	28.48587519	26.8786066	29.16051064	22.88653586	26.71049377	27.87080039	19.60226497	25.91826893	X	-0.071999167	0.544012293	0.984934163
asRNA:CR4547	FBgn0267035	1.167066889	1.03997021	0.1769555	0.76527352	0.592552927	0.687689709	1.395568456	1.346086695	X	0.355213255	0.688866307	NA
lncRNA:CR454	FBgn0267038	0.915740159	1.716734552	1.437488317	1.5070685	0.388975456	0.3554988	0.274832096	0.935603132	X	-1.479575743	0.131454768	NA
lncRNA:CR454	FBgn0267039	4.508894979	1.990273366	0.287855693	1.936479798	1.285217535	1.23054066	0	0.77283382	X	-1.378776171	0.293277336	NA
lncRNA:CR454	FBgn0267040	0.092763851	0.311196692	0.393826664	0	0.251193789	0.153050086	0.177481932	0.050349727	X	-0.279362682	0.856034997	NA
lncRNA:CR455	FBgn0267075	1.08313835	0.605604211	0.557386355	0.15304826	0.296263989	0	0.586114073	0	X	-1.453419312	0.331282122	NA
asRNA:CR4552	FBgn0267076	0.973629417	0.291629779	1.60279344	0.243212179	1.255464741	1.737513892	0.798348073	0.075494271	X	0.344433396	0.737062008	NA
lncRNA:CR455	FBgn0267078	4.432987656	3.326504037	5.801697567	1.495905029	1.052984064	3.189537286	1.041585885	2.152832256	X	-0.976276892	0.158615448	0.818884898
lncRNA:CR455	FBgn0267083	34.93443056	26.89068242	48.18004539	12.27303297	15.98237828	12.63297393	24.41602271	9.350862238	X	-0.945060479	0.077117298	0.682035833
lncRNA:CR455	FBgn0267086	8.348746568	12.25336976	5.696421392	7.90757026	5.180871892	11.8067209	4.65890072	9.629385243	X	-0.09472063	0.899106595	0.996479182
lncRNA:CR455	FBgn0267089	0.054914357	0.27633339	0.621700165	0.128030756	0.793075909	1.225292447	0	0.079482768	X	1.010722405	0.420691834	NA
lncRNA:CR455	FBgn0267097	0.244339198	2.663990726	1.0373361	0.14241691	0.882189752	0.287951879	0	0.928345279	X	-0.89689764	0.520027425	NA
lncRNA:CR455	FBgn0267118	1.866591734	0.844299985	0.356160434	0.048897622	0.227169201	0.553648494	0	0.45534196	3R	-1.310688247	0.252672055	NA
lncRNA:CR455	FBgn0267123	4.504148774	1.549759528	0.560359082	0	0.833963378	5.008673379	3.830060096	0	3R	0.529337943	0.707322075	NA
lncRNA:CR455	FBgn0267131	0.577454971	0.505356368	0.487268767	0	0.40791687	2.816787014	0	0.27254526	3R	1.160254952	0.368369516	NA
lncRNA:CR455	FBgn0267133	0.458567183	0.384591059	0.370825863	0.178188687	0.229953137	0.048037149	0.129979415	0.553106557	3R	-0.518154316	0.672307949	NA

lncRNA:CR455	FBgn0267143	3.836910106	0.643587844	1.39624356	0	1.55848638	4.160027723	0.244701003	0	3R	0.017038268	0.990883689	NA
lncRNA:CR455	FBgn0267145	0.681100735	0.273194789	0.035920454	0.276167082	0.026729595	0.2093927	0.339946162	0	3R	-1.146998442	0.388422701	NA
lncRNA:CR455	FBgn0267155	3.153126715	6.533381307	0.899934286	2.162175363	2.009012421	1.748675651	6.151060112	2.013449994	3R	-0.08694481	0.934694428	NA
lncRNA:CR455	FBgn0267157	22.33126646	12.86421483	20.24734965	20.51026036	32.16948579	31.04901126	36.25207123	36.24000255	3R	0.861242823	0.030811039	0.489103237
lncRNA:CR456	FBgn0267162	3.030375941	3.388687014	0.61263748	3.532603713	7.294140337	2.618934933	3.221064221	0	X	0.335478112	0.802743589	NA
lncRNA:CR456	FBgn0267173	0.371752127	0.727489049	0.751554563	1.444543483	0.111851311	0	1.896695327	0	X	-0.708997217	0.665930865	NA
lncRNA:CR456	FBgn0267177	4.624382724	4.475049971	3.020415997	0.414675932	3.853013556	4.024467495	0.453726911	0	X	-0.55201112	0.676809318	NA
lncRNA:CR456	FBgn0267182	4.005982584	1.19457312	2.591588766	1.66040846	0.160707229	2.433948031	0	0.773098579	X	-1.440275674	0.230613542	NA
lncRNA:CR456	FBgn0267185	3.665360061	2.912270828	2.340029574	2.248854999	3.482586491	2.910045538	3.937015689	1.256500419	X	0.066988277	0.947316401	NA
lncRNA:CR456	FBgn0267186	0.789232307	1.158348167	2.63266653	1.150040882	1.78095824	2.418271043	2.516685713	7.068172633	X	1.328218385	0.137630159	NA
lncRNA:CR456	FBgn0267201	8.802148674	7.703149347	3.404243319	11.00448545	7.829921263	10.46346731	5.857687924	12.46322581	3R	0.267294203	0.638197185	0.991585536
lncRNA:CR456	FBgn0267203	23.02271453	26.90554736	12.81867181	13.19916202	20.7809502	12.09824488	17.81199391	24.17280509	3R	0.003197775	0.994616753	0.998895169
lncRNA:CR456	FBgn0267211	0.866182456	1.981226669	0.191031505	0	2.558751275	2.078698438	0.602631833	0	3R	0.811901811	0.589376514	NA
lncRNA:CR456	FBgn0267219	2.489523413	1.439940097	8.538672832	0.600437598	3.874334328	2.428042208	3.503904945	1.30465174	3L	-0.188730738	0.829783874	0.995787491
lncRNA:CR456	FBgn0267220	1.310869841	0.507415445	0.978508292	1.880765345	0.546105239	1.426015476	1.543410982	0.218924598	3L	-0.305231241	0.807350061	NA
lncRNA:CR456	FBgn0267228	4.496886475	3.66772291	1.980251197	2.053035459	3.652668147	3.176229775	2.22113433	2.524028438	X	-0.051085116	0.859202093	0.995787491
lncRNA:CR456	FBgn0267229	4.901439885	4.012081168	2.473291557	1.919823793	5.309013133	10.05539954	2.50073569	5.618701287	3R	0.835465493	0.099490254	0.731438598
lncRNA:CR456	FBgn0267230	45.82555504	38.21081405	10.92443165	11.11635847	30.60416068	29.7183951	7.433073582	18.9781516	3L	-0.26816705	0.667020354	0.995787491
lncRNA:CR456	FBgn0267233	0.382139319	2.350278696	1.545107763	0.593962291	2.069578237	2.161671695	1.949691226	0.829659836	3R	0.581743731	0.624025802	NA
CG45676	FBgn0267236	2.112640138	2.48056022	4.100188405	0.98510819	0.381385691	1.19507053	4.850451342	1.376021191	3R	-0.29713632	0.818079836	NA
Ggamma30A	FBgn0267252	419.5283094	313.3108257	396.5341742	373.4820299	398.3334449	351.5297281	398.1372689	392.0557425	2L	0.065237344	0.563820418	0.984934163
CG32700	FBgn0267253	104.1324073	88.38684136	83.96282672	82.43274208	93.7550916	90.96702375	77.42427494	81.0985635	X	-0.035966329	0.735437204	0.995787491
lncRNA:CR456	FBgn0267257	0.518911149	0.870400657	0.484181235	0.186126432	1.080885715	1.317147628	0.305481575	0	2L	0.427183275	0.733077943	NA
lncRNA:CR457	FBgn0267264	0.082024854	0.733787655	0.397982303	0.127491906	0.296151768	0.077332531	0	0.534250652	2L	-0.502168001	0.729283156	NA
lncRNA:CR457	FBgn0267270	0.504245932	0.657845854	0.135921511	0	0.505718414	0.739511087	0.428780735	1.216405106	2L	1.152919999	0.412315184	NA
lncRNA:CR457	FBgn0267275	2.723844203	0	0.991201207	0.952581033	2.704480296	0.898808287	0	5.322346117	X	0.938787364	0.53096549	NA
lncRNA:CR457	FBgn0267284	3.22400418	1.982865401	2.085703779	0.250554813	1.358035899	3.242209545	0.822450393	2.099883455	2L	0.016530227	0.987493103	NA
lncRNA:CR457	FBgn0267304	16.76482174	8.095334777	2.464922648	9.475527457	4.127018185	4.071183576	11.01585072	5.514845438	3L	-0.581007317	0.489018685	0.973549494
lncRNA:CR457	FBgn0267313	0	2.648859722	0	0	1.140332787	1.488845085	0	2.742843821	3L	1.084255196	0.561005702	NA
lncRNA:CR457	FBgn0267322	59.62339944	76.18863533	115.0330636	86.19696662	51.06330646	65.72887918	79.34380281	68.80105956	3L	-0.312611282	0.19403371	0.844272804
Ntl	FBgn0267326	3.953645414	1.307656304	0.675456946	2.207072994	3.116304813	0.026249808	0.852324033	0.181346412	2L	-0.942241903	0.338018919	0.926687587
KdelR	FBgn0267330	178.1506496	148.786906	135.2327899	162.7150336	161.296642	175.166293	156.1317203	174.3696156	2L	0.120769103	0.341910605	0.928133889
Glut4EF	FBgn0267336	2.293158894	1.615630375	1.784742028	1.477584739	1.328085454	1.637553177	1.364060631	1.82472962	3R	-0.193397399	0.251620334	0.882577383
rn	FBgn0267337	14.40205025	13.38589509	13.70542146	15.68129775	11.77450524	10.6767534	11.54615586	9.216848596	3R	-0.375342074	0.001545658	0.084192925
LanB2	FBgn0267348	0.821659952	0.304490311	0.208604887	1.358788652	0.810644416	0.153129671	0.755560906	0.082972084	3L	-0.566107526	0.489947519	0.973549494
Pi4KIIIalpha	FBgn0267350	149.0228432	182.1694878	156.8427251	175.3260409	161.3405978	193.577034	160.8150819	158.4312235	X	0.052529296	0.597017553	0.988780662
Topors	FBgn0267351	59.77590328	58.55341442	59.83188909	45.25353988	50.34045071	48.34809892	62.01275963	34.8207341	2R	-0.162325216	0.332840838	0.922663747
JYalpha	FBgn0267363	0.666294197	0.419105642	0.353591969	0.533994944	0.338296443	1.177833936	0.159349764	0.226029122	4	-0.024972795	0.978717115	NA
l(2)SH0834	FBgn0267365	131.1246655	115.0962376	135.2153409	108.5778922	140.7164137	123.3121394	139.8672676	124.9525185	2L	0.139496992	0.334400545	0.924122842
SelR	FBgn0267376	228.7429441	221.7135167	219.7928213	200.8394679	216.9397123	217.9589088	198.0200863	215.9071899	3R	-0.007009197	0.936646231	0.996879532
sau	FBgn0267378	84.32396375	72.29374085	77.87836833	70.38646682	65.11777851	89.13077358	69.72219326	61.43354647	2L	-0.069236395	0.596160414	0.988780662
Ubc7	FBgn0267384	129.9669202	129.4341113	118.4046417	136.4757191	134.5908363	126.6338207	114.7201878	139.0242171	X	0.031988905	0.787902404	0.995787491
PyK	FBgn0267385	1970.174392	1662.6079	1638.929316	1856.828725	2223.548855	1956.467322	2055.016963	2176.795975	3R	0.268746521	0.009325348	0.287842402
dop	FBgn0267390	40.59284192	65.16889277	47.35170253	58.05491231	60.17895933	51.97899325	55.82048005	53.95205418	3L	0.106058269	0.449166442	0.967112302
Yeti	FBgn0267398	234.5153667	227.5870635	273.9342803	207.2429966	208.7642155	226.4772405	226.37324	212.1660025	2R	-0.07951135	0.479787887	0.973549494
AOX1	FBgn0267408	0.067211054	0.676422684	0.285342889	0.117525031	0.424666124	0.269306227	1.071605265	3.173790875	3R	2.173845164	0.018796067	0.393184106
DIP-lambda	FBgn0267428	0.42454548	0.437310962	0.437364787	0.209895506	0.281733236	0.502154715	0.19468796	0.243082074	2R	-0.279186041	0.290797731	0.904969488
lovit	FBgn0267429	0.433450441	0.457252971	0.470096652	0.271254275	0.359901772	0.522294979	0.22705371	0.275992577	3L	-0.209431324	0.354366497	0.936745458
Pzl	FBgn0267430	0.430148684	0.411650096	0.382402301	0.230863469	0.284778592	0.390001356	0.142654524	0.257397027	3R	-0.410146959	0.08228469	0.694792913
Myo81F	FBgn0267431	0.498377794	0.478619875	0.410893584	0.252709283	0.334712769	0.450052597	0.207830225	0.28698373	3R	-0.332234744	0.243258532	0.876959115
kl-3	FBgn0267432	0.013749898	0.017297644	0.010192435	0.015138202	0.012411045	0.00972249	0.006820389	0.010779998	Y	-0.472231096	0.506904806	0.978557094
kl-5	FBgn0267433	0.211216348	0.234509457	0.187822118	0.112157828	0.124838317	0.128267474	0.082452491	0.096283432	Y	-0.754408657	0.030191388	0.482946307
chp	FBgn0267435	1.772037392	1.086901959	0.70579579	1.479918258	1.694982821	0.511173934	0.404821384	0.057421749	3R	-0.892995136	0.230313338	0.867702781
asRNA:CR457	FBgn0267439	1.833190384	2.81867639	4.817902161	3.561678641	1.654689243	2.304427595	3.507381041	1.99001301	2L	-0.42467878	0.646090161	NA
WDY	FBgn0267449	0.009698108	0.013556013	0.014704671	0.007536924	0.00364741	0.005714573	0.00618502	0.003509248	Y	-1.220616702	0.1714734	NA
asRNA:CR458	FBgn0267465	1.860149066	1.085265776	0.19620416	1.697035117	0.438005976	0.381247212	1.237899191	0.52676815	3L	-0.921014009	0.452588003	NA
Ptp61F	FBgn0267487	3.10031261	3.84882027	2.934452501	3.594356782	2.99983155	3.319695371	3.591654982	3.111984695	3L	-0.018878374	0.8746316	0.995787491
Mcr	FBgn0267488	36.31810706	44.4638071	40.79433081	33.25920826	30.53279628	43.76555437	43.96867116	43.38127346	2L	0.094235406	0.504124723	0.978429306
28SrRNA-Psi:C	FBgn0267513	69.22639317	146.4339711	146.4324176	2283.434244	136.6682111	238.2377807	43.49704834	558.2456575	X	-1.424284483	NA	NA
28SrRNA-Psi:C	FBgn0267519	30.80619153	51.07850007	67.46510712	44.32620371	48.10879701	38.73531249	66.99645816	49.88247455	X	0.112347649	0.619319783	0.990047511
asRNA:CR459	FBgn0267569	0.280393566	0.431127465	0	0.708204296	0.210909271	0.13217667	0.417252506	0.202920129	3R	-0.54782248	0.66677726	NA
lncRNA:CR459	FBgn0267571	3.270541428	3.900113856	6.074689164	5.30185809	8.856248267	4.071112933	6.061871834	8.099176844	3R	0.588481656	0.104911991	0.747886154
asRNA:CR459	FBgn0267572	0.350350191	0.078355106	0.254983403	0.081682828	0.316235945	0	0.029791696	0.152128539	3L	-0.59512872	0.667129394	NA
lncRNA:CR459	FBgn0267576	1.533656389	0.820215301	0.05392216	0.414569523	0.802505176	0.670572464	0.340207861	0.048256667	3L	-0.590824289	0.576506436	NA
lncRNA:CR459	FBgn0267579	3.321020223	6.178241408	6.07918314	4.540116083	3.515421613	3.87110101	3.966433526	4.588336027	2L	-0.296429004	0.303160654	0.910208378
CCY	FBgn0267592	0.455293165	0.634978545	0.608114702	0.489005099	0.249346776	0.356902511	0.221852415	0.344303279	Y	-0.858931693	0.063580474	0.636110045
asRNA:CR459	FBgn0267613	1.311533673	2.104265323	1.106699965	0.398842355	1.029414967	0.215044511	1.163739331	0.619013263	3L	-0.685164138	0.508232417	NA
lncRNA:CR459	FBgn0267618	1.007188903	0.938565606	0.542983607	0.260913668	1.919247116	1.160587445	0	0.242966705	3L	0.301454852	0.816693844	NA
lncRNA:CR459	FBgn0267635	11.74931191	8.086393522	9.586435027	5.957687456	2.615647001	0.124183627	1.478476315	0	3L	-3.031834855	0.011185926	0.319404908
lncRNA:CR459	FBgn0267658	2.223796481	0.828912584	0.865848027	1.280172291	2.874596431	0.69886058	2.451275815	1.132509781	3R	0.47010017	0.484733275	0.973549494
lncRNA:CR460	FBgn0267665	1.017680953	1.009006019	0.669696139	0.384240458	0.86280281	0.866236175	0.767276098	0.438317806	3L	-0.045222324	0.910029929	0.996479182
lncRNA:CR460	FBgn0267666	4.124974635	3.610855099	5.599767081	4.148606032	4.470228209	5.590072168	3.444146402	6.389211071	3L	0.219183239	0.342413171	0.928339671
lncRNA:CR460	FBgn0267668	7.60957124	8.664701677	8.33385754	7.526666798	6.916645463	8.580865097	6.923224935	6.401331526	3L	-0.126822911	0.266958403	0.894434069
CG46025	FBgn0267689	0.265586989	0.284615363	0.286360148	0.017200169	0.332953184	0.118241513	0.056459843	0.176187593	2L	-0.280911626	0.77178816	NA
asRNA:CR460	FBgn0267693	0.227314645	0	0.248157972	0.238488992	0	0.117870973	0.20295959	0.148056341	3R	-0.595214868	0.678355046	NA
Pak	FBgn0267698	62.25231817	76.44057291	63.0521602	62.10280852	51.47105569	50.48516992	55.1690945	56.77831298	3R	-0.269681315	0.01119073	0.319404908
lncRNA:flam	FBgn0267704	0.119969869	0.091469375	0.107157562	0.052126885	0.064972909	0.178400366	0.050080208	0.050909203	X	-0.083399791	0.860298154	0.995787491
Pen	FBgn0267727	0.460879554	0.227370823	0.131539691	0	0.538356641	0.79234984	0.207478878	0.353157238	2L	1.186592706	0.351585498	NA
otk2	FBgn0267728	13.97462759	5.125468806	6.226944174	3.419613566	24.78648212	26.54244528	8.210835108	11.94148211	2R	1.322176938	0.003114794	0.141079859
lncRNA:CR460	FBgn0267730	0.255815799	0.363080482	0.322237502	0.435848969	0.577266353	0.514829896	0.200797415	0.138849945	3R	0.095101433	0.871662263	0.995787491
lncRNA:CR460	FBgn0267733	26.36775716	26.36154987	25.60297768	28.03340696	18.40319036	28.063045	25.58893124	23.40948914	2R	-0.127831199	0.489188239	0.973549494
lncRNA:CR460	FBgn0267744	0.35115505	0.589013334	4.827417768	0.818704779	2.113082885	1.324267345	2.388810871	3.303690517	2R	0.543674139	0.628218302	NA
lncRNA:CR460	FBgn0267767	5.197094739	8.233097492	7.004488526	5.385258105	1.042454223	5.444210194	5.892400148	2.507416393	3R	-0.763314831	0.438883842	NA
rump	FBgn0267790	245.8865519	230.1697062	262.2100748	170.6004238	209.5284025	221.9206683	235.9892421	190.2013575	3R	-0.052523723	0.684600901	0.995787491
HnRNP-K	FBgn0267791	75.07243691	74.33474604	70.36783997	88.01645425	68.20803418	78.53674407	71.54490415	75.17183166	2R	-0.040680589	0.693604036	0.995787491
rgr	FBgn0267792	1.08637362	0.40358321	0.619083582	0.42497302	0.605465837	0.57054223	1.190383868	0.364081926	2R	0.11617408	0.832724798	0.995787491
lncRNA:CR452	FBgn0267793	0.17799553	0.455700705	0.386360496	0.283940442	0.236767669	0.547600636	0.597461079	0.14237439	2R	0.264179792	0.722406626	0.995787491
lncRNA:CR431	FBgn0267794	0.652396983	1.190507004	0.191316108	0.300864859	0.737707632	0.439339922	0.073155109	0.498079734	3L	-0.378510372	0.5998164	0.989286463
Frl	FBgn0267795	5.555111791	5.323396135	3.855366888	4.892557416	4.946095685	7.358042143	5.305181155	4.341019234	3L	0.185458252	0.340951606	0.927274752
Tmc	FBgn0267796	4.283700884	5.664133891	7.713263216	6.280921683	3.515080909	5.421745325	2.395137093	6.150018959	3L	-0.419865816	0.116238927	0.765798592
asRNA:CR461	FBgn0267810	0.60072099	0.431839366	0.763368961	0.400159536	0.413125848	1.375436195	0.656765208	0.372634537	3R	0.393889132	0.659267989	NA
asRNA:CR461	FBgn0267814	2.046100291	0.889789755	0.55153453	0	2.052075242	0.35723164	1.739881934	0.329057269	3R	0.352963224	0.790533524	NA
da	FBgn0267821	241.1040763	317.9192668	310.0542383	358.60111	266.0669567	293.8151932	301.4061017	292.6379585	2L	-0.058304411	0.625665157	0.990047511
Gmer	FBgn0267823	28.82774037	22.53523086	29.6448735	26.75839471	29.85985401	31.31587781	42.02296287	27.11644774	2R	0.300220074	0.206913205	0.855364409

**PRAS40**	FBgn0267824	8.884749042	10.19056992	9.278503871	9.079795642	11.04516614	11.3239572	8.45490905	6.344346594	2R	0.019016155	0.913745087	0.996479182
**Fatp1**	FBgn0267828	122.689333	85.80674746	88.27838426	85.54835429	93.18547031	106.4140994	83.71420926	99.37160062	2L	0.027357436	0.84295907	0.995787491
**Syx7**	FBgn0267849	309.5450644	290.0098986	263.9965466	310.9317507	246.0719101	323.9471192	260.6500894	251.3428071	3L	-0.092136924	0.417696362	0.959239869
**Maf1**	FBgn0267861	26.74038945	27.63407839	28.30235056	22.08200865	23.79729242	27.26869019	23.23342236	23.10386298	2R	-0.074598759	0.506946439	0.978557094
**CanA-14F**	FBgn0267912	65.48035906	77.14277928	87.12242486	77.33887735	77.65276456	63.52880281	67.84613373	80.78721817	X	-0.04909213	0.678469889	0.995787491
**lncRNA:CR462**	FBgn0267919	4.092200582	1.1440154	3.309207178	2.782736718	3.078112863	4.661872903	0.434970483	0	3L	-0.437672989	0.732859826	NA
**lncRNA:CR462**	FBgn0267923	1.19157375	1.460586857	0.667094145	0	2.812971713	1.598696644	1.052214312	0.199001301	2L	0.79320157	0.469351802	NA
**lncRNA:CR462**	FBgn0267927	6.93747143	7.788299078	8.03962736	7.047137209	4.404736154	7.725124829	5.806311897	6.56239943	2L	-0.250822242	0.313216638	0.91460807
**lncRNA:CR462**	FBgn0267936	0	0.183957908	0	0.051138815	0.673147194	0.516986281	0.055954673	0.142863684	3L	2.610949443	0.057174237	NA
**lncRNA:CR462**	FBgn0267953	0.405231559	0.254894659	0.614428818	0.236195531	0.914433529	0.334293608	1.292193015	0.109974403	3R	0.83653866	0.515235546	NA
**lncRNA:CR462**	FBgn0267963	3.473993809	2.330854905	0	3.779760034	4.599062749	0.436701352	2.363262092	0	X	-0.362646584	0.823103765	NA
**lncRNA:CR462**	FBgn0267965	4.677205372	3.620933476	3.927750575	1.467944187	1.948512567	0.763207037	1.606183841	1.366971554	X	-1.255015195	0.127342773	NA
**Der-1**	FBgn0267972	209.5257288	231.9051217	202.0525536	222.7521768	245.6139569	268.9948361	251.9243882	274.4639338	2L	0.29555666	0.004637306	0.18852346
**vib**	FBgn0267975	62.77255227	69.58167444	78.90587235	67.8198291	74.51080265	77.49153959	74.6990551	72.52663356	3R	0.132205887	0.167328147	0.830652748
**Tim23**	FBgn0267976	455.1154542	405.1931919	516.746229	403.8943579	440.5844264	441.7734445	492.5554167	471.4077989	2L	0.083063557	0.475459764	0.973549494
**mh**	FBgn0267977	4.671245913	4.365414695	5.26146095	8.868250539	0.84327961	5.00171083	1.106527378	4.56376828	X	-0.979513206	0.083642095	0.69799888
**ap**	FBgn0267978	1.417707064	2.057757662	1.202289922	0.918673562	1.825993774	0.945964394	1.202079475	1.340549964	2R	-0.039297272	0.901715474	0.996479182
**lncRNA:CR462**	FBgn0267986	2.094396268	1.505595396	2.7219515	0	3.510856192	1.904063151	0.572448201	0	2L	-0.042670814	0.973860419	NA
**ltl**	FBgn0268063	0.97032418	2.691151576	0.582671517	0.403177598	1.04060426	2.545180343	0.441145752	2.482108619	3L	0.524418811	0.394302168	0.952272708
**zf30C**	FBgn0270924	141.1707733	140.9669221	115.5662451	118.6245641	131.9028986	125.7446515	116.7558708	130.5155929	2L	-0.002009491	0.985569131	0.99797245
**CG4836**	FBgn0270925	0.138557523	0.564426245	0.816336643	0.138446421	0.21439872	0.690479127	0.454452708	0.171897833	3R	-0.057455585	0.954922832	NA
**betaGlu**	FBgn0270927	14.06910839	13.59072384	15.37971845	7.429759225	12.17896617	14.67060164	8.734820181	7.801941418	3R	-0.18691605	0.54101744	0.984934163
**PheRS-m**	FBgn0275436	172.2040279	164.3398935	137.8926286	134.0402229	170.9847281	167.7322225	177.8532636	146.7640837	2R	0.152719412	0.239193912	0.873933389
**lncRNA:CR462**	FBgn0278598	1.728636481	1.43555914	1.788467526	0.908782061	0.841346834	1.302200541	0.648498353	0.84627143	2L	-0.65389926	0.103823591	0.745084146
**dmt**	FBgn0278604	1.103260983	0.328989427	1.546419771	0.800243562	0.35407446	0.369830299	1.000690656	0.159685448	3R	-0.993176565	0.256609363	0.88674759
**Dsp1**	FBgn0278608	179.8823293	204.6740135	224.3394229	208.8016325	190.53185	244.3139792	222.6036508	229.3846164	X	0.147357342	0.15745461	0.818884898
**FASN1**	FBgn0283427	144.323846	129.7182453	130.5742161	152.5024728	147.8468197	145.5887784	146.0632311	161.6968495	2L	0.139276891	0.173121443	0.832736771
**CG46280**	FBgn0283438	113.3004657	99.10660829	108.5727735	107.0190832	93.44210901	117.1761739	110.7854713	103.1386999	3R	0.015921136	0.883758069	0.995787491
**Glo1**	FBgn0283450	7.5509619	10.04729367	10.0399626	14.55780167	12.58285064	9.206784633	9.446115074	12.21656143	2R	0.078459532	0.792517305	0.995787491
**Drs**	FBgn0283461	1.570797201	1.505595396	5.443903	2.6158961	0.810197583	3.173438585	4.007137407	5.846307651	3L	0.362918261	0.746483783	NA
**Pol32**	FBgn0283467	53.31250944	27.68825419	40.32887333	41.06939262	36.64384542	43.38870549	54.75765795	40.01735253	2L	0.127735818	0.589486882	0.987597375
**slmb**	FBgn0283468	88.43835453	100.1482447	90.83799829	97.55179529	92.34323635	102.0367854	97.18380726	102.6965635	3R	0.095482695	0.297935106	0.9074568
**Vps4**	FBgn0283469	164.3584391	174.7746428	169.3419957	186.9419994	151.9595165	187.0937609	169.11448	138.1840488	X	-0.07762856	0.484824074	0.973549494
**wupA**	FBgn0283471	1.068042486	1.100487599	2.146875116	0.284582954	0.798780319	1.632687662	1.128762579	0.712208725	X	-0.071677397	0.890852272	0.996189576
**S6k**	FBgn0283472	34.84277509	35.35077686	35.04861394	33.72828762	32.47250358	39.82467015	30.4813481	40.03678058	3L	0.0692465	0.547198492	0.984934163
**S6KL**	FBgn0283473	21.86011542	20.01009281	18.04771585	22.83140675	18.28279717	22.46630251	19.48028896	16.62180622	X	-0.080825363	0.5264848	0.984107922
**Dhc16F**	FBgn0283476	0.390088604	1.422883976	0.585835412	0.346467388	0.503007077	0.881488033	0.505460017	0.053772601	X	-0.459608136	0.500035476	0.976796212
**SF2**	FBgn0283477	139.1012221	171.4373763	172.4553382	170.9753127	171.2089842	191.1641885	162.3312701	169.9608353	3R	0.118556581	0.263743158	0.892790059
**und**	FBgn0283478	255.9395394	236.7879472	249.835058	293.8439145	282.3997372	265.8925715	270.9037702	267.232773	2L	0.097082682	0.378561222	0.943971504
**Adk2**	FBgn0283494	3.267634417	4.436998648	4.907336273	2.055749726	2.715374773	3.569706684	2.381658743	4.729557866	2R	-0.091719887	0.865116019	0.995787491
**InR**	FBgn0283499	1.394420844	1.566205176	1.832731495	1.037040513	1.051524954	1.775611521	1.166220456	1.64400225	3R	-0.015670578	0.945264222	0.997604378
**Sac1**	FBgn0283500	162.6147398	175.5724864	162.5871027	187.303325	152.5687559	154.2422877	152.2594856	177.0612942	3L	-0.082264972	0.460136687	0.971606621
**lncRNA:CR461**	FBgn0283501	0.681324877	0.603757608	0.249492022	0.569456278	0.30168978	0.460552065	0.327938566	0.167458797	3R	-0.718744772	0.335267761	0.924219112
**Neurl4**	FBgn0283503	75.22846342	87.85846307	72.14690989	79.4690496	74.24837315	69.21209136	76.43578121	89.08246042	3L	0.006488053	0.9570759	0.997604378
**nw**	FBgn0283508	0.302909141	0.484992699	1.369499426	0.930928022	0.621396175	0.259618989	0.210743925	0.926679878	2R	-0.564195096	0.453801673	0.967967712
**Phm**	FBgn0283509	787.4866113	727.822946	711.6389501	713.7108836	733.5000309	698.6183143	708.0985285	663.5862194	2R	-0.039012727	0.653086463	0.99443552
**Pal1**	FBgn0283510	59.71104434	58.39954076	50.87367828	69.95893211	47.48446804	77.67865893	63.35288496	65.05941581	2R	0.111410472	0.47866696	0.973549494
**lola**	FBgn0283521	119.3711357	129.4468974	121.2885309	135.5283086	119.3243935	121.0057006	114.9865943	127.0146053	2R	-0.037454019	0.66504832	0.995787491
**l(2)10685**	FBgn0283525	34.87089791	57.59804638	42.75019733	60.9441087	48.72688609	14.15427727	44.54611053	19.99614486	2L	-0.585264906	0.081810011	0.694684542
**Duox**	FBgn0283531	0.303327657	1.210919228	0.691716544	1.089083399	0.591390896	0.566231415	0.123807259	0.355618308	2L	-0.961943965	0.09629825	0.730787365
**Vha26**	FBgn0283535	1008.730101	914.0367825	895.2800753	923.1228686	912.3577929	1076.280808	924.1417029	926.515829	3R	0.064949247	0.466615417	0.971928234
**Vha13**	FBgn0283536	2486.982056	2240.948879	2329.760471	2174.327484	2735.985119	2255.347646	2426.415434	2497.656164	3R	0.134610659	0.177558611	0.834615603
**CR46300**	FBgn0283627	0.541364035	0.990613335	2.98486727	1.72114073	1.510374016	0.092799037	0.125548299	1.816452216	3R	-0.762341812	0.479861841	NA
**elgi**	FBgn0283649	112.4243188	134.6420461	119.4073721	191.2224217	141.4205689	152.9612004	142.6943424	129.8317038	3L	0.051735412	0.753153606	0.995787491
**CG46301**	FBgn0283651	4.703697146	4.274065994	3.503670031	4.532435756	4.20417358	3.350535222	3.559094378	3.438449564	2L	-0.19709484	0.171289806	0.832652344
**Rpp30**	FBgn0283652	34.54340831	20.47564594	29.77170161	24.82708946	28.13219792	23.56845118	24.01793539	14.94299874	2L	-0.249510279	0.374334994	0.943282319
**Tlk**	FBgn0283657	7.915246133	8.499180216	13.40093006	9.242970376	8.573428759	7.927165855	11.25682138	13.09543974	X	0.101720681	0.575970627	0.985637351
**muc**	FBgn0283658	500.900083	431.842193	428.3397445	421.5640071	492.2683774	539.4295764	414.8118687	425.1493562	2L	0.097105127	0.368883317	0.942282153
**THG**	FBgn0283659	10.45029846	18.35241546	11.40001776	7.848959181	20.00499197	12.89418204	17.35514619	19.79539257	2L	0.579921997	0.099641527	0.73164096
**Rap2l**	FBgn0283666	131.9879554	116.8368251	147.4691807	135.2891679	119.3685369	118.5399293	124.0933464	116.636404	2R	-0.121567801	0.298466888	0.9074568
**IP3K2**	FBgn0283680	1.177630219	1.035764001	0.573020098	0.519225314	0.76143423	0.843036055	0.568121947	1.120864944	X	0.019325363	0.95756753	0.997604378
**Tcs3**	FBgn0283681	48.82727361	52.05755943	44.62318945	41.94887346	51.43847479	39.91720139	39.58600872	37.90168022	3L	-0.120740282	0.53060995	0.984760433
**Ge-1**	FBgn0283682	99.17774031	87.81205411	80.12802838	86.33259497	94.18059764	81.62045695	90.0726992	84.03209695	2L	0.01465845	0.8975813	0.996479182
**LIMK1**	FBgn0283712	61.69109427	63.67600263	63.78318829	71.17810405	65.12668673	81.35984459	61.89170942	69.91895928	X	0.124600822	0.26317801	0.892691887
**Girdin**	FBgn0283724	27.86445058	34.57978654	40.62537234	36.40576798	30.08309379	42.70069534	39.66038561	31.41666127	3L	0.075255415	0.625203536	0.990047511
**prage**	FBgn0283741	23.58198748	24.30464637	25.26410056	21.52136344	29.00950812	25.96432286	18.31056639	26.32382628	X	0.104504159	0.414986444	0.958665466
**CG46302**	FBgn0284080	0.343269137	0.575785822	0.832766139	2.13418419	1.445940468	0.647264092	1.751373888	0.745269073	2R	0.283881302	0.826659736	NA
**wg**	FBgn0284084	0.84658729	0.19146491	0.369224082	0.155241627	0.103031935	0.412530753	0.315456301	0.227170934	2L	-0.558748848	0.500468985	0.976796212
**Top2**	FBgn0284220	52.90430318	45.18814134	60.00416538	56.34738429	49.29744746	44.25142931	54.51071403	61.68244326	2L	-0.000263376	0.998562446	0.999757791
**Sema5c**	FBgn0284221	1.155393877	1.511851624	0.880513957	0.84620649	0.971908924	1.146329614	1.064780039	0.669800105	3L	-0.161703762	0.65167301	0.993444251
**betaTub56D**	FBgn0284243	2456.389043	1747.619944	1930.872916	1927.484637	2589.004828	1851.01239	2263.484791	2136.838875	2R	0.16354053	0.222943448	0.86549481
**l(2)k05911**	FBgn0284244	0.058555515	0.245546655	0.74578803	0.443689932	0.184988497	0.441646255	0.224064565	0.127129444	2L	-0.550334551	0.585390221	NA
**eEF1alpha1**	FBgn0284245	101.8751876	179.4536626	148.8699809	142.6331058	94.89555193	122.3270613	132.1874077	112.0127444	2R	-0.277871719	0.068691509	0.659001649
**l(2)k09848**	FBgn0284246	29.78644312	33.2573	28.37261175	22.58059174	28.45306226	28.55626075	41.9553808	30.64840448	2R	0.213991649	0.316456453	0.91460807
**ds**	FBgn0284247	0.554578604	0.428292387	0.689517478	0.167009805	0.256546357	0.626334706	0.21221129	0.258367235	2L	-0.416572976	0.314993644	0.91460807
**Cyt-c-p**	FBgn0284248	2229.749109	2058.970822	2013.558001	1986.037319	2312.138097	1826.821593	2164.267494	2056.344943	2L	0.044532313	0.667808581	0.995787491
**Adf1**	FBgn0284249	191.2596348	168.1568972	169.1186817	181.8218587	169.022668	168.8967993	158.661986	189.0579035	2R	-0.021593638	0.835101729	0.995787491
**Oaz**	FBgn0284250	0.280233801	0.45551517	0.161199956	0.262688946	0.312924021	0.642802484	0.147398443	0.633502741	2R	0.612829057	0.23838657	0.873280735
**l(2)05287**	FBgn0284251	8.902076164	9.16944515	9.601489699	10.01212062	9.281935297	7.243870726	5.684748904	4.233344037	2L	-0.479928635	0.042160281	0.538901512
**Letm1**	FBgn0284252	46.27488863	37.52240598	32.86517586	37.42207703	44.90337487	35.5625327	36.00376123	28.02076647	2R	-0.065840049	0.68964132	0.995787491
**LeuRS**	FBgn0284253	61.02103303	40.88797738	37.96795305	52.53986217	42.30755182	51.26589336	38.24001598	42.06063542	2L	-0.123496922	0.472133538	0.973549494
**Impbeta11**	FBgn0284254	14.23962641	10.5502289	11.14214849	13.63229466	8.09145154	15.56399529	9.316667611	6.487459363	2R	-0.307389654	0.247583035	0.879384515
**Arpc4**	FBgn0284255	236.8380786	240.9687071	278.3812128	289.829199	312.7362669	206.7262356	302.0895134	308.5184355	2L	0.145278646	0.378879851	0.943971504
**bsf**	FBgn0284256	95.33132361	70.66765895	87.99129669	82.65096574	77.56722991	80.55176089	83.85338673	64.83674341	2L	-0.107215293	0.413039627	0.958665466
**Ttd14**	FBgn0284257	145.5715103	157.4737197	142.8957024	169.349406	124.0787295	136.5044612	131.9700447	127.1230496	2R	-0.214323807	0.021152863	0.410927638
**trol**	FBgn0284408	2.473750636	4.182316546	5.01742145	1.901832918	2.25060648	2.891058837	3.354251555	2.919917445	X	-0.210704712	0.363367471	0.939549067
**Psn**	FBgn0284421	112.6552778	123.7775293	84.35092767	132.8283528	105.525221	113.4716801	94.94590083	121.8027885	3L	-0.028963245	0.848999747	0.995787491
**tyn**	FBgn0284435	1.475574721	1.820187365	1.337173052	1.16125066	0.754049662	1.242302997	0.922747227	0.841414897	X	-0.593381307	0.004208298	0.179856182
**CCT8**	FBgn0284436	262.720485	309.8074732	236.5728464	248.0581378	292.0426276	283.8802986	267.4026652	162.9061186	2R	-0.042284527	0.790331464	0.995787491
**Mcm3**	FBgn0284442	0.360533801	0.25197703	0.145774995	2.171475042	0.216951972	0.283257554	0	2.80486225	X	0.208439838	0.869632457	NA
**disco-r**	FBgn0285879	2.297863521	2.011779394	0.917767018	0.705606449	1.123058092	1.955054453	0.757757703	0.778751128	X	-0.341571858	0.370430386	0.942875294
**tea**	FBgn0285892	26.48209553	21.0690375	19.44979202	17.55259103	19.45660215	19.73712982	17.65563631	19.55693024	2R	-0.119678937	0.455897814	0.968365245
**btl**	FBgn0285896	0.151004049	0.448125115	0.986283083	0.081244674	0.775864414	0.536609158	0.444478354	0.252187514	3L	0.328528605	0.694275915	0.995787491
**VhaAC39-1**	FBgn0285910	1213.084822	1048.523367	1026.620011	1122.586053	1111.912023	1195.505869	1022.174755	1104.946877	X	0.03541809	0.711542721	0.995787491

**Vps16A**	FBgn0285911	62.9434927	122.3971546	101.8218784	71.83144586	77.42784019	88.49177225	98.86546583	84.02387537	3R	-0.003879734	0.982662654	0.997881056
**red**	FBgn0285913	32.43674553	25.82321288	29.83737066	26.71728297	33.21564668	27.28495499	26.80696243	28.75316375	3R	0.045089757	0.722974245	0.995787491
**sbb**	FBgn0285917	21.97815609	23.8384415	23.60156113	24.43086494	25.65016223	26.00175107	22.74237162	24.92580735	2R	0.112013415	0.191069923	0.843449603
**Fas1**	FBgn0285925	1714.287906	1414.900031	1676.537077	1544.069816	1792.25662	1732.323109	1581.32976	1774.338281	3R	0.145063406	0.136617672	0.796109055
**Imp**	FBgn0285926	31.97033578	30.50019588	26.02442219	23.78459414	25.13795554	26.49207589	17.7825606	21.9264638	X	-0.269236919	0.044203635	0.552313482
**Rab1**	FBgn0285937	74.44425269	52.58432248	61.07840044	53.76818654	55.71678791	54.82827984	66.1218278	52.36537699	3R	-0.052466027	0.738122973	0.995787491
**para**	FBgn0285944	19.40974765	18.21591268	18.33284838	17.72719027	16.30800116	19.57869619	13.37071932	16.02364624	X	-0.147487552	0.181281728	0.837439137
**RpS10b**	FBgn0285947	265.6618193	407.957825	397.3024816	417.8884441	343.9217989	361.5262285	386.1596886	421.5637955	X	0.057648493	0.684791225	0.995787491
**RpL27A**	FBgn0285948	1260.817537	1275.048865	1254.386163	1335.114287	1168.247829	1197.287194	1404.572124	1271.853973	2L	0.007164031	0.941546385	0.997604378
**RpL31**	FBgn0285949	692.7283314	1190.245982	1117.726297	1340.563937	1036.822875	919.2234298	1195.320746	1173.697673	2R	0.029837403	0.859024707	0.995787491
**RpL19**	FBgn0285950	575.4667278	769.5147656	894.8968095	972.2486938	838.6762703	664.1121436	851.0460574	951.0915652	2R	0.075853	0.629679882	0.990523437
**mRpS34**	FBgn0285951	139.3477144	184.8958335	140.9801716	178.8430525	165.4233502	129.0693052	192.5641362	136.7923138	3L	-0.014069655	0.938870643	0.997038394
**eEF5**	FBgn0285952	309.8208703	356.8010901	421.2669835	371.7745692	349.6706985	350.3518887	360.887653	381.1347321	2R	0.015314988	0.892353494	0.996438268
**caz**	FBgn0285954	30.3439714	31.37152548	37.20855688	25.98301283	32.30280124	30.54999478	32.93363777	37.17204875	X	0.123401975	0.41096905	0.958665466
**cv-c**	FBgn0285955	11.00647262	14.72910614	19.04819422	14.68115391	15.7670767	15.84009374	17.40378601	16.85257869	3R	0.181565012	0.184708624	0.839299945
**CG46338**	FBgn0285962	20.92057798	23.31627296	22.93933517	21.0321811	21.73342359	27.90402565	23.57768995	24.94478105	2R	0.185015408	0.109960078	0.758410631
**CG46339**	FBgn0285963	71.70893207	93.05578761	71.64263308	83.55233053	68.90189176	73.30835018	67.9541426	62.14231164	3R	-0.202053109	0.050269675	0.588409366
**prg**	FBgn0285971	43.07353376	50.97662092	49.91412495	35.6108126	64.2200947	39.53592353	51.18455025	38.19092964	2L	0.139575148	0.489426939	0.973549494
**hpRNA:CR188**	FBgn0285991	210.7350885	164.6142356	206.3594171	218.6834563	209.5632041	224.8753817	200.9878738	228.8716788	2L	0.138454093	0.243978023	0.877269208
**hpRNA:CR463**	FBgn0285992	0.550084893	0.36007424	0.65097477	0.594330297	0.993044218	0.392124805	0.68452604	0.582578158	X	0.329888161	0.604651952	0.989286463
**Rnmt**	FBgn0286027	42.76330922	40.60157667	32.35497988	28.43858903	27.16093058	45.91035336	31.60102271	28.33724005	2L	-0.09068417	0.683455184	0.995787491
**lncRNA:CR463**	FBgn0286028	7.421455439	5.22312603	10.95369386	6.049945444	5.340313725	5.871526872	6.619682611	9.126755294	3L	-0.104807814	0.797201144	0.995787491
**lncRNA:CR463**	FBgn0286033	17.49645361	2.536233641	5.764292303	4.532480325	2.72961829	8.349599176	1.65310478	6.096585992	3L	-0.678257372	0.460167158	0.971606621
**Rexo5**	FBgn0286051	27.50193556	30.38011537	27.47754846	23.53054172	28.45869096	22.3422632	26.48957337	32.55177068	3L	0.045814452	0.77766064	0.995787491
**cnk**	FBgn0286070	21.72271392	27.4599701	32.6497113	26.69435958	20.25507377	30.21955998	27.41474063	27.06987964	2R	-0.015696603	0.923938069	0.996479182
**AdamTS-A**	FBgn0286071	1.189032977	0.70190452	0.61900803	0.362882668	0.824517509	0.488338293	0.397056156	0.238207142	3R	-0.536536417	0.214422992	0.858753183
**LKRSDH**	FBgn0286198	2.118381008	2.459967896	1.625205323	3.419256165	3.072450783	2.458088215	1.847533491	3.655775363	2L	0.229688275	0.571944481	0.984934163
**stw**	FBgn0286203	0.302696521	0.384228827	0.332436588	0.233652376	0.313836541	0.169685662	0.06782709	0.084367981	2R	-0.93760571	0.068694274	0.659001649
**ich**	FBgn0286204	0.629778504	0.306686639	0.443564671	0.126305796	0.305621401	0.504369407	0.414600902	0.588089217	3R	0.282227176	0.646802225	0.99279236
**RpS12**	FBgn0286213	496.4884123	707.9021248	669.8973601	726.1504945	665.3963126	582.7621593	645.0297609	743.0221661	3L	0.05444153	0.682376288	0.995787491
**Fum1**	FBgn0286222	326.5211622	358.7443017	309.0287791	357.6135662	341.4867948	313.1654204	301.0575536	308.1681445	X	-0.066494686	0.490112455	0.973549494
**Mpi**	FBgn0286506	87.87437405	91.33296713	104.0073801	101.7375763	94.84624489	103.8038564	124.4147226	83.35657585	3R	0.108098249	0.481794572	0.973549494
**vrs**	FBgn0286507	3.544321609	1.66880064	1.961055653	2.029619889	4.265605938	0.234495773	0.158625273	0.810005296	3R	-0.71986491	0.478698312	0.973549494
**Pld**	FBgn0286511	0.124094908	0.810696481	0.776398592	0.426370156	0.978627287	0.394091524	0.199938174	0.794084516	2R	0.204403409	0.755177786	0.995787491
**aqz**	FBgn0286516	737.158365	763.9641624	657.5181165	730.6545527	669.1956034	755.4409381	614.1671943	718.3632914	3R	-0.038402068	0.679884089	0.995787491
**gry**	FBgn0286567	17.05795766	21.46517457	21.11975003	20.69225459	20.89500756	26.64811647	20.38761799	11.35271739	3L	0.007539369	0.968836864	0.997604378
**hll**	FBgn0286723	1.50035829	1.043485107	1.420428598	0.426588887	0	1.794036312	0.560114083	0	2L	-0.876230454	0.488786926	NA
**CG46385**	FBgn0286778	72.80277028	63.91191069	59.6067878	54.08366621	60.38707105	53.60252067	54.10537753	53.48418433	2R	-0.145724293	0.179267269	0.836045561
**cass**	FBgn0286781	490.3710358	465.5039409	470.6140728	522.6468992	498.003081	560.6383996	467.694412	475.764567	2L	0.066202616	0.480228189	0.973549494
**flz**	FBgn0286782	0.15174152	1.193635012	0.812408963	0.463573324	0.566756555	0.12332843	0.800888023	0.295364381	2R	-0.503956322	0.443953861	0.967112302
**Etf-QO**	FBgn0286783	36.85030566	39.97573349	39.51665203	32.91855749	30.18899224	33.48346617	45.55540577	33.69793707	2R	-0.032883438	0.851322821	0.995787491
**TER94**	FBgn0286784	182.6070106	216.3499523	175.4624376	210.0709632	192.5460334	227.1534838	191.1347298	201.1774255	2R	0.079203352	0.445323753	0.967112302
**scb**	FBgn0286785	1.150273182	2.452908456	1.773835884	0.810782374	0.853151233	3.085276623	1.751524133	0.17423369	2R	-0.051035808	0.932474131	0.996479182
**hoip**	FBgn0286786	226.5300526	245.5574252	291.294448	253.9455543	243.6814774	209.2923919	248.4202383	208.8888058	2L	-0.127959397	0.366289154	0.940794966
**Orc1**	FBgn0286788	0.105664222	0.31016459	2.499314296	0.184764116	0.190751001	0.149429374	1.347758497	2.752881675	2R	0.588211976	0.597961735	0.989286463
**xmas**	FBgn0286809	9.804220002	10.90349389	7.702768806	10.97892403	11.83169406	11.03337459	10.01525598	9.827830195	X	0.145552151	0.408776761	0.957219873
**SRPK**	FBgn0286813	277.0398358	244.8136774	265.4767	289.3622269	281.976042	279.4788779	268.0909784	307.6132346	2R	0.108252433	0.280198405	0.899310165
**Pdi**	FBgn0286818	153.0334901	132.3613709	138.6345063	156.8237403	147.0203717	148.4566333	149.9807554	148.7641988	3L	0.061188929	0.549812119	0.984934163
**lncRNA:CR433**	FBgn0286827	1.36102384	1.597109116	1.440302199	0.940200251	1.900886459	1.193396221	1.328790528	0.608005915	3L	-0.05326857	0.885012378	0.995787491
**dind**	FBgn0286828	57.98337762	55.21317577	61.17205222	59.55119351	54.80668186	47.20932012	50.89856462	67.78545861	3R	-0.051971043	0.733846173	0.995787491
**CG46387**	FBgn0286833	1.024394495	1.718278057	0.552259279	0	1.232863076	1.39503415	0.290361374	1.976938023	3L	0.595845604	0.680052052	NA
**naz**	FBgn0286852	0.552008716	0.813000452	1.43715316	0.69057868	1.458317869	1.180488571	1.425359094	0.833077258	3R	0.531666755	0.193011454	0.844272804
**CG46395**	FBgn0286873	17.90841366	8.582529031	6.206508821	2.556293404	1.319562308	12.40452956	10.25575975	6.347889602	2R	-0.215976501	0.828364781	NA
**CR46407**	FBgn0286885	13.38894018	7.333246977	2.320098723	4.777930101	3.946198952	3.735380812	2.091151472	5.042517193	3R	-0.896807994	0.234457703	0.87009833
**Tango1**	FBgn0286898	90.02551948	77.74385407	61.06304016	72.35207452	71.18880893	85.00148466	83.46904086	87.64595191	2L	0.147434623	0.2797109	0.898654833
**lncRNA:CR464**	FBgn0286937	1.994499077	0.955854972	1.382464841	5.978699376	3.600582021	8.596121359	10.17601999	12.37212036	3R	1.79435917	0.074462884	NA
**l(3)80Fj**	FBgn0287182	26.21751243	20.48891097	27.52066708	16.20742435	16.80384263	24.2873833	19.25861905	17.65501984	3L	-0.186325587	0.23349111	0.869488344
**l(3)80Fg**	FBgn0287183	7.383421174	6.423393632	7.791714427	5.605092076	5.400029944	8.377183743	5.378870035	5.481133107	3L	-0.117975126	0.412583968	0.958665466
**FASN3**	FBgn0287184	3.641109721	3.192863947	1.71581725	0.936448612	0.772382917	2.519044838	1.269657794	0.872034935	3L	-0.782255184	0.097231593	0.730787365
**nvd**	FBgn0287185	0.283128829	0.23840048	0.249023345	0.102565992	0.177161151	0.305217217	0.12949023	0.171429971	3L	-0.128690174	0.75233957	0.995787491
**scro**	FBgn0287186	1.24273683	1.046547476	1.414379805	0.429243576	0.480081652	0.754577755	0.619698752	0.377511472	3L	-0.859261633	0.018053284	0.385711607
**mRpS5**	FBgn0287187	56.4435186	44.26983567	55.65331944	37.58051663	39.79963765	49.45431331	44.10178025	36.33922376	3L	-0.165380772	0.276041642	0.89549671
**Cog8**	FBgn0287204	47.59155474	35.98365975	39.98831136	20.91057543	34.86192528	37.5939981	33.9074099	32.01516966	2L	-0.034941585	0.878242312	0.995787491
**Gfat1**	FBgn0287209	8.775397439	8.051908386	9.129101742	13.69851493	1.817916237	0.563932612	1.873566918	0.493154448	3R	-3.040783244	6.48E-17	7.72E-14
**CG46448**	FBgn0287218	31.92222986	22.5281055	16.99965755	6.807208279	18.27223133	15.96560518	6.95170804	14.79093939	2L	-0.458116533	0.417384933	0.958846438
**bor**	FBgn0287225	126.7262594	129.2870041	105.6002781	133.6070829	122.8849571	82.78750067	115.4462094	116.1042808	3R	-0.147338096	0.33197019	0.921929165
**slf**	FBgn0287234	2.310687748	0.682368757	2.28965058	1.251973869	0.470015055	1.6875722	0.083022734	1.095198781	2L	-0.942857186	0.191177862	0.843449603
**Nplp2**	FBgn0287423	1.43170654	0.51460433	0	1.907411371	0	4.724314631	1.043518326	1.554183714	3L	0.922589925	0.52823577	NA
**Pa1**	FBgn0287454	214.4735297	139.7976007	138.5175876	127.275629	140.5743055	157.2003635	134.0407919	107.4130974	X	-0.177946336	0.313077362	0.91460807
**lncRNA:CR464**	FBgn0287461	46.00945182	56.00805848	70.29684528	55.32965274	49.77906407	52.3858355	59.74533424	52.6512345	2L	-0.05107749	0.76800472	0.995787491
**lncRNA:CR464**	FBgn0287462	0.139867986	0.335155607	1.260323195	0.60560861	0.793563768	0.904228683	0.152916959	1.084522661	3L	0.388545862	0.661789259	0.995787491
**lncRNA:CR464**	FBgn0287465	0.749741173	0.458009585	0.325598687	0.118690904	0.568125298	0.7286678	0.354186267	0.200957715	2L	0.178777709	0.765338477	0.995787491
**lncRNA:CR464**	FBgn0287466	0	0.966666372	0.699050751	1.477989999	2.913045733	0.978001831	0	0.125120578	2R	0.406515171	0.786583305	NA
**Camta**	FBgn0287478	8.654188516	12.4412263	13.06008807	13.5956799	12.6316779	12.95379663	10.8956591	12.03051459	2R	0.055231682	0.69536842	0.995787491
**sov**	FBgn0287582	15.66231238	21.34419503	23.10166959	23.96982499	21.70028927	23.23342238	22.92569516	24.43816185	X	0.167779761	0.233010706	0.868700724
**l(3)77CDf**	FBgn0287585	23.24356779	27.85956302	41.81308228	39.64308574	21.85971228	23.42293433	45.09248373	42.15400751	3L	0.035573611	0.889955529	0.995926915
**CG46466**	FBgn0287597	53.07671222	42.79699406	64.08361843	33.89657141	34.45274241	59.8090893	38.44686622	33.61004952	4	-0.194169403	0.396220961	0.952272708
**asRNA:CR4647**	FBgn0287604	0.010872128	0.05470941	0.131878157	0.088717852	0.117761868	0.179378082	0.083205098	0.023604397	X	0.564052849	0.617943102	NA
**lncRNA:CR464**	FBgn0287605	0.872161425	0.726863815	0.372602771	0.396451304	0.485215537	0.558521975	0.125937879	0.500181219	3R	-0.47673732	0.388868691	0.948247506
**lncRNA:CR464**	FBgn0287608	0.388453187	0.422952664	0.396792426	0.142999576	0.31987187	0.494733499	0.226006693	0.192347048	2R	-0.097808235	0.880887647	0.995787491
**lncRNA:CR464**	FBgn0287612	0.093565483	0.588536143	1.815908449	0.708969302	0.337819354	1.014449105	0.775734559	0.761772466	2R	-0.09449167	0.915842423	0.996479182
**lncRNA:CR464**	FBgn0287613	0.304206958	0.157190485	0.209858342	0.080672653	0.122327307	0.180781959	0.147116301	0.165897904	3L	-0.268316084	0.567222147	0.984934163
**lncRNA:CR464**	FBgn0287614	0.787611129	0.827681606	1.151044346	1.039824431	0.745181179	0.635197618	0.496251382	0.700472371	3L	-0.524723638	0.124213478	0.780439453
**dbf**	FBgn0287630	1.440636102	2.36612194	0.72811731	1.259545815	0.379271287	0.339555729	0.995337863	0.325807743	2L	-1.502495933	0.05768389	0.623014047
**NA**	FBti0018862	0.069359332	0.116340549	0.1963089	0.026951445	0.125211371	0.098087332	0	0.075292765	2R	-0.387046284	0.798476692	NA
**NA**	FBti0018897	1.922713555	1.290032903	0	3.362050704	1.041297226	0.996997427	0	0.626158367	2R	-1.268066694	0.379480041	NA
**NA**	FBti0018900	0	0.788666829	0.126739841	2.679636874	2.169159873	0.738810189	0	1.361082481	2R	0.289902898	0.840864991	NA
**NA**	FBti0018905	0.972620131	1.323616122	0.801360975	1.15520632	0.89447873	1.297613659	0.280887719	0.916375694	2R	-0.287333928	0.647336712	0.99280533
**NA**	FBti0018908	0.600993899	3.024248862	6.743263022	1.751493313	0.54247401	7.436800569	1.341504804	3.262033468	2R	0.103249925	0.910181446	0.996479182
**NA**	FBti0018920	12.17006748	7.184668141	1.979290321	5.072456614	5.400469288	11.73026451	3.642280312	6.937710192	2R	0.07941163	0.891629593	0.996189576
**NA**	FBti0018929	3.18595846	0.834999746	0.241534087	1.624862359	3.954136708	0.563194158	2.285844885	0.216156586	2R	0.247833899	0.838056693	NA
**NA**	FBti0018968	0.117733538	0.141058209	0.163211383	0.235278267	0.182176466	0.126855383	0.128717479	0.073031545	2R	-0.322204652	0.798491483	NA
**NA**	FBti0018990	0.157233604	0.29304146	0.169531792	0.122194743	0.252308404	0.148238882	0	0	2R	-0.829357774	0.605669978	NA
**NA**	FBti0019006	0.057977407	2.236726226	2.953699981	2.703442824	1.465297101	0.32796447	0.29580322	2.517486338	2R	-0.732241214	0.502081442	0.977207013

**NA**	FBti0019027	0.066600145	0.063835657	0.138489448	0	0.068703046	0.08073027	0.0970848	0.16525152	2R	0.654807367	0.638475846	NA
**NA**	FBti0019039	0.685468131	0.836201184	0.302352023	0	1.349940721	2.232517849	0.31793526	0	2R	1.105485422	0.469182692	NA
**NA**	FBti0019051	0.095268638	0.303619573	0.138672232	0.088846097	0.137587447	0.026945607	0.048606468	0.103418516	X	-0.94660117	0.42048949	NA
**NA**	FBti0019058	1.780078353	3.355212961	1.157521409	0.727352128	1.722699775	0.484442433	0.468146198	0.199212107	X	-1.245283996	0.084758678	0.69799888
**NA**	FBti0019082	27.31246885	12.65379468	16.3168568	18.11803127	26.49890195	27.03538798	35.35903814	20.91706225	X	0.579123559	0.038181733	0.522230403
**NA**	FBti0019100	0.068590402	0.153401035	0.221865811	0.07995797	0.082548836	0.096999918	0.14581299	0.074458056	2L	-0.345900315	0.773922383	NA
**NA**	FBti0019110	1.329739877	1.185809977	1.020864049	1.098818696	0.850817671	1.586924853	0.772905189	0.694338974	2L	-0.216093638	0.688088238	0.995787491
**NA**	FBti0019149	1.062126479	1.535834627	2.13244471	0.512339566	1.520704892	2.278971709	1.962057132	1.749360274	2L	0.560515999	0.396987259	0.952272708
**NA**	FBti0019150	0.294860586	0.296752361	0.057226213	0.137491271	0.1490437	0.333591311	0.12035131	0.230461066	2L	0.099060654	0.921998599	NA
**NA**	FBti0019154	0.477741703	0.472587892	0.505202821	0.65687811	0.464394117	0.404215861	0.624989409	0.63829013	2L	0.040122885	0.947716659	0.997604378
**NA**	FBti0019209	0.883859649	0.776574107	0.61263748	0.392511524	0.379903143	0	0	0.639647039	2L	-1.360417993	0.364030297	NA
**NA**	FBti0019232	0.29253038	1.030425218	0.070967462	0.88663064	0.739329236	2.151207676	0.447750771	0.063511054	2L	0.604983425	0.583530486	NA
**NA**	FBti0019237	0.70998562	0.480202128	0.55561781	0.533969273	0.08269071	0.086370336	0.175276313	0.174034057	2L	-2.144394119	0.00552256	0.211123931
**NA**	FBti0019242	3.070342207	1.160578778	2.098199259	1.512335339	0.078066966	1.263883046	0.882536218	2.347182095	2L	-0.760170827	0.383046253	0.946058474
**NA**	FBti0019243	4.444741016	3.000355782	2.103976528	0.252749911	0.782819191	2.759580887	1.659311681	0.706093477	2L	-0.712274789	0.416157085	0.958846438
**NA**	FBti0019277	1.493418028	3.220713305	0.776359566	1.989627379	0.192571593	0.905133468	0	0	2R	-2.613430069	0.087999368	NA
**NA**	FBti0019285	0.74922077	0.383995558	0.605866379	0.557999173	0.20663737	0.019621132	0.026545532	0.022592051	3R	-3.033042763	0.001471347	0.082296708
**NA**	FBti0019287	2.041208658	1.339763936	2.583622817	1.448391652	1.441919264	3.012165476	2.263985713	0.963402815	3R	0.083448972	0.924432019	NA
**NA**	FBti0019326	0.350307933	0.185555499	0.089457069	0.300900437	0.199703874	0.139060286	0.047033845	0.240173984	3R	-0.548024865	0.654916804	NA
**NA**	FBti0019355	0.318387825	0.449727083	0.162611457	0.23441344	0.121004553	0.04739591	0	0.218289297	3R	-1.562038998	0.227307931	NA
**NA**	FBti0019405	3.02919841	2.201790664	2.122984671	2.746513022	1.367119877	1.22169473	1.846025887	1.169185806	3R	-0.833384095	0.039092845	0.527385023
**NA**	FBti0019406	0.142604948	0.047839959	0.115319205	0.066495614	0.137300523	0.179262766	0.218272972	0.123843417	3R	0.831068152	0.43389508	NA
**NA**	FBti0019407	0.903800081	0.983349954	0.237038529	0.455605593	0.837843919	0.529682041	0.747766516	1.060666833	3R	0.310995723	0.695579869	0.995787491
**NA**	FBti0019417	0.523660732	0.558961083	0.288725825	0.277476201	0.343760667	0.201969875	0.303606768	0.361745976	3R	-0.432049112	0.665922222	NA
**NA**	FBti0019476	0.124272949	0.451642617	0.954700732	0.67605464	0.224344524	0.566291591	0.317022964	0.179872051	4	-0.717359951	0.459394995	NA
**NA**	FBti0019486	22.30203999	20.27301708	30.01923654	23.98542075	6.23394552	5.2226435	11.92917389	1.561928816	4	-1.932717602	2.18E-05	0.002837399
**NA**	FBti0019508	9.003330192	9.199961355	16.31870087	6.273150122	5.978232104	11.41527944	12.14383543	11.4586233	4	0.042645652	0.928987792	0.996479182
**NA**	FBti0019517	1.523730979	4.718483001	1.990450055	4.918875806	3.808696079	3.97817795	2.392043362	0.254473923	4	-0.291273621	0.759209649	NA
**NA**	FBti0019573	3.97103705	9.158679158	6.62315534	5.207806907	10.30506324	5.147820528	5.065100414	7.004959836	X	0.185789004	0.814427047	NA
**NA**	FBti0019598	0.483900814	1.623351461	0.146742078	0.141024566	0.218391248	0.855410121	0.462915514	0.919269278	X	0.068581894	0.959463084	NA
**NA**	FBti0019601	0.060487602	0.608756798	0.440226234	0.282049133	1.091956239	2.110011633	0.462915514	1.838538556	X	2.061054455	0.078205274	NA
**NA**	FBti0019622	1.117578886	1.6772574	2.311680988	1.097091832	0.48845132	1.663652443	0.690234297	0.459732735	X	-0.867141365	0.105390098	0.748781821
**NA**	FBti0019715	7.679386996	25.92697887	16.08781391	15.34645859	21.10526414	9.416734079	14.20190962	15.53507982	X	-0.064039572	0.833390549	0.995787491
**NA**	FBti0019731	0.320511547	5.107324994	1.749502222	0.934075758	1.735816468	3.097307372	2.044079607	0	X	-0.188434567	0.870336337	NA
**NA**	FBti0019780	0.381242278	0.127896088	0.30829615	0.474054411	0.045882668	0.599054819	0.389022897	0.220723274	2L	-0.018643053	0.987533581	NA
**NA**	FBti0019783	0.155844271	0.941064845	0.680536849	0.29067604	0.675213243	1.557447681	0	0.067670468	2L	0.204616863	0.873888231	NA
**NA**	FBti0019784	1.211443995	0.580579244	1.679397848	0	1.249695372	2.44744714	0.220744261	0	2L	0.205692558	0.896236803	NA
**NA**	FBti0019789	2.191133487	0.80397604	0.332228705	2.075346503	0.618055863	0.322779261	0.524027681	1.337949459	2L	-0.947786553	0.395297965	NA
**NA**	FBti0019805	0.05471778	0.367125599	1.592934851	0.127572444	0.592677701	0.515875887	0	0.831581558	2L	-0.063734861	0.967170922	NA
**NA**	FBti0019819	5.224711069	5.633819912	6.29226996	7.221721608	7.478183031	8.162796845	6.806978844	6.279344154	2L	0.271488719	0.259630395	0.890556245
**NA**	FBti0019834	0	0.470956096	0.875763789	0.32730499	0.108614124	1.304644027	0.05116115	1.175623569	2L	0.7112595	0.564284343	NA
**NA**	FBti0019907	1.067626771	0.98964939	2.590047655	0.589531174	0.507194724	1.350898735	1.93514601	0.487982431	2R	-0.257329443	0.73493814	0.995787491
**NA**	FBti0019969	0.083810591	0.210870763	0.237210545	0.032566873	0	0.171201578	0.249436388	0	2R	-0.391034311	0.813380202	NA
**NA**	FBti0020018	11.32601132	11.0100858	16.54849444	11.40266389	7.899727396	11.52748815	14.11812071	13.97148807	3L	-0.050177901	0.9100659	0.996479182
**NA**	FBti0020027	65.20164645	70.25008849	72.73891931	34.39759641	58.42325866	74.48397464	61.9187653	41.33103944	3L	-0.007971723	0.984539226	0.997881056
**NA**	FBti0020046	1.879736234	2.080975929	0.638429944	1.31476028	1.153757929	0.921545996	1.150859404	0.571351392	3L	-0.622023127	0.406776281	0.956706394
**NA**	FBti0020054	0.275036766	0.215289943	0.222411786	0.085498382	0.198604742	0.190155521	0.2338749	0.039808685	3L	-0.25176817	0.839768364	NA
**NA**	FBti0020060	0.147309942	0.277978232	0.134014449	0.128792844	0.19944915	0.364567647	0.187895413	0.15991176	3L	0.443229785	0.701770727	NA
**NA**	FBti0020075	0.574582061	2.409452002	0.348482016	0.669808222	1.815218796	0.9479968	2.565099567	1.559338553	3L	0.809432715	0.532398126	NA
**NA**	FBti0020092	0.609012913	1.62516729	0.604414159	0.70994534	0.699633707	0.182691617	0.353092051	0.240404256	3L	-1.232092871	0.178123818	NA
**NA**	FBti0020097	0.588439169	0.658016104	0.380678724	0.731692677	0.566551208	0.554776854	1.000747308	0.511022363	3L	0.17539621	0.856186376	NA
**NA**	FBti0020099	6.971760219	4.106091899	3.088119517	3.789648621	1.060602307	0.03692659	0.849288966	0	3L	-3.178339872	0.007668279	0.254611293
**NA**	FBti0020120	1.735836586	5.240920247	0.842223069	2.428222592	2.506903943	3.109418648	5.313787709	6.406725252	3L	0.786952135	0.41365686	NA
**NA**	FBti0020144	2.396427619	2.274287622	1.759409204	3.161168072	1.593850651	1.099940538	0.241315987	3.628314584	3L	-0.513077836	0.460593249	0.971606621
**NA**	FBti0020150	0.380655881	0.191549052	0.346299696	0.088748486	0.12025675	0.215328024	0.121382666	0.18594881	3L	-0.621433663	0.489733162	NA
**NA**	FBti0020152	1.173566642	1.135669274	0.547510828	0.105235633	0.814841759	0.765991012	0	0.097996993	3L	-0.777935527	0.568387223	NA
**NA**	FBti0020235	1.04654856	1.398864969	1.269456104	0.762496428	1.505526677	0.801677167	0.041715123	0.923062482	3L	-0.409171698	0.578391233	0.985637351
**NA**	FBti0020264	0.09669913	0.608247094	0.087971528	0.028181298	0.305491748	0.091167349	0.123340779	0.078728536	3L	-0.406203402	0.739575585	NA
**NA**	FBti0020269	6.129565254	3.117011538	2.01858459	0.581980343	2.052863744	3.530107475	0.353770422	3.673208452	3L	-0.28268835	0.69034622	0.995787491
**NA**	FBti0020273	3.179657119	3.678226728	0.265993235	2.300664064	1.187606077	0.516855398	0.559405077	2.380458601	3L	-1.005509255	0.374204508	NA
**NA**	FBti0020288	0.45225245	0.219591854	0.606324233	0.11099048	0.451185876	0.538586334	0.333967445	0.723491952	3L	0.59044679	0.435874035	0.966084844
**NA**	FBti0020415	0.903057296	0.883605291	0.730268135	2.631805112	1.901958183	0.851396903	0.959882735	2.287391149	4	0.257316201	0.791736101	NA
**NA**	FBti0020420	1.114061037	1.167925689	3.040533605	1.298695684	0.754187139	1.444203669	3.907741256	0.60468241	4	0.04917809	0.967042948	NA
**NA**	FBti0020422	4.254649693	3.231657959	4.673986783	5.053358509	2.173792402	2.043470926	2.047868448	3.311462798	4	-0.818548804	0.194793312	0.844272804
**NA**	FBti0020428	7.457199956	6.350421907	6.679777137	3.209756486	4.763532543	5.191829589	6.146046512	2.241729887	4	-0.347061406	0.58878029	0.987588562
**NA**	FBti0020434	0.121329518	0.148009633	0.107034079	0.154295565	0.139383285	0.072792827	0.028137655	0.191576447	4	-0.261295183	0.826042552	NA
**NA**	FBti0059634	0.727884417	0.813949332	0.672699978	0.161622392	0.876011952	0.91499331	1.14947782	0.225757778	3L	0.434596488	0.653370355	NA
**NA**	FBti0059646	0.850124548	0.633762075	2.3679296	1.027721012	1.079968933	0	0.080321703	1.23046606	2L	-0.981961527	0.388205035	0.948247506
**NA**	FBti0059648	0.553618614	0.270846788	0.503651638	0.430246986	0.249855872	0	0.235382163	0	2L	-1.862254696	0.195565118	NA
**NA**	FBti0059670	4.067901415	3.411666977	4.764186528	4.087999574	3.165346831	3.107828491	2.50486646	6.699979025	2L	-0.044831698	0.938459699	0.997038394
**NA**	FBti0059673	4.243451825	3.531730413	5.893829164	5.286575365	5.379905863	5.863622049	5.701789598	4.571299519	2L	0.217192884	0.534557141	0.984760433
**NA**	FBti0059684	1.0648168	1.587627381	0.861076902	1.195316433	0.759414216	1.251780004	0.637173335	0.428114045	2R	-0.57642353	0.277062139	0.896087042
**NA**	FBti0059697	0.105246957	0.057374527	0.057448721	0.073613795	0.052249376	0.096745534	0.05369745	0.028562611	X	-0.321809642	0.697317257	NA
**NA**	FBti0059700	0.4722485	0.33005442	0.572834137	0.091752467	0.071044132	0.333924751	0.100393006	0.683530139	2L	-0.271161419	0.847906122	NA
**NA**	FBti0059702	0.11231619	0.056518396	0.081743253	0.052372194	0.081103804	0.031767305	0.028652101	0.060962211	2L	-0.562653259	0.626336949	NA
**NA**	FBti0059704	0.21007744	0.370921511	0.080470254	0.283561267	0.279442669	0.156362943	0.028205898	0.024005135	2R	-0.905494575	0.427615302	NA
**NA**	FBti0059711	0.198135522	0	0.480674012	0.46194551	0.059614233	0.37360192	0	0.286780373	2L	-0.612163105	0.727930873	NA
**NA**	FBti0059727	0.074806667	0.050631348	0.057309452	0.012239223	0.04027664	0.032170333	0.046871365	0.039890715	2R	-0.276669892	0.716426529	0.995787491
**NA**	FBti0059733	0.286146556	0.508204276	0.367511058	0.510165823	0.151931727	0.047607746	0	0	2R	-2.978567054	0.023699661	NA
**NA**	FBti0059738	0.483900814	1.623351461	0.483385669	0.862738523	0.616634112	0.563564315	0.798756182	1.359591537	2L	-0.012212524	0.987857211	0.998078482
**NA**	FBti0059745	0.012635776	0.063584226	0.1839253	0.029459836	0.114054073	0.500343169	0.193404819	0.384068156	2L	2.140136834	0.075336243	NA
**NA**	FBti0059761	0.037305588	0.16090686	0.025857953	0.02485045	0.038483513	0.045220475	0	0.208269989	3L	0.278658926	0.840079803	NA
**NA**	FBti0059765	0.188617381	0.316379197	0.187193101	0.039977666	0.030954792	0.169744255	0.065613681	0.148911198	X	-0.776677601	0.470923906	NA
**NA**	FBti0059796	23.07590328	14.024127	15.14484006	17.93353326	22.94204275	13.03247228	17.34731704	13.79563073	3R	-0.04374504	0.908421882	0.996479182
**NA**	FBti0059798	26.68240211	24.38199257	19.80579514	17.64136276	28.03842393	27.03331959	17.27082802	9.943202936	3R	-0.077625351	0.866263533	0.995787491
**NA**	FBti0059859	0.329346942	0.138108323	0.066582591	0.191964999	0.123865758	0.064688809	0	0.268141012	3L	-0.656988235	0.627692031	NA
**NA**	FBti0059860	0.874801827	0.77683624	0.84266088	0.239949121	0.627051775	0.679212322	0.656364193	0.307235783	3L	-0.242349894	0.709955567	0.995787491
**NA**	FBti0059889	1.789923228	0.058869512	0.085143702	0.081826248	0.887015344	0.231621584	0	1.066769535	3L	0.10400928	0.943691312	NA
**NA**	FBti0059906	0.169728764	0.759189841	1.372532043	0.52762163	0	1.120134691	2.02058137	0	3L	0.189807688	0.89956075	NA
**NA**	FBti0059915	0.123037281	0.584737038	0.348234515	0.239047324	0.666341478	0.48332832	0.104623582	0.178083551	3L	0.206003741	0.846941816	NA
**NA**	FBti0059924	3.072696176	2.488146031	2.827499955	1.111635847	1.434570032	0.899043885	0.946027547	1.380229207	3L	-1.003260574	0.168177283	0.830652748
**NA**	FBti0060009	24.25310878	5.81159823	8.405386231	4.038943579	7.818406673	7.349683762	15.46755039	22.56674753	4	0.306287496	0.786791623	NA
**NA**	FBti0060088	6.420489906	6.853299799	7.929609652	10.34230836	8.640287698	11.11587391	8.6360986	12.16536255	X	0.39975974	0.399962754	0.953639202
**NA**	FBti0060252	0.228142877	0.255118447	0.245987306	0.177302176	0.320332729	0.621376054	0.258665503	0.110070957	2L	0.569487182	0.629952854	NA

NA	FBti0060363	0.517070631	0.328980962	0	0	0.418440868	0.537921139	0.090969537	0.077421255	2R	0.406377995	0.782340024	NA
NA	FBti0060378	0.616743838	0.996186405	0.591096078	0.2840326	0.371126899	0.186642227	0.194237874	0.628176575	2L	-0.813759867	0.19963917	0.847197998
NA	FBti0060396	1.310361358	2.42337785	2.282300373	0.548343775	0.606548229	0.665215753	0.771406148	0.583572473	2L	-1.284262966	0.088527164	0.709409025
NA	FBti0060415	4.182772425	3.702896256	2.395901508	0.406332352	6.39735489	0.931102347	3.556780774	0.630637926	2L	0.135746261	0.875126561	0.995787491
NA	FBti0060449	1.09991423	1.0147235	0.667094145	1.15398388	1.389938964	1.399939764	0	0	3L	-0.460285283	0.731311664	NA
NA	FBti0060472	34.52924954	26.11259	24.58133963	19.55252357	19.5349173	23.44163759	17.88047319	20.34339109	3L	-0.342131055	0.086781747	0.706081898
NA	FBti0060502	16.24092106	30.26874078	39.40024796	63.10849342	32.57669447	79.96183723	55.24125139	47.01405736	2R	0.577717846	0.492358824	NA
NA	FBti0060577	10.77596658	13.59941529	14.44053322	12.68151716	8.151893214	21.673633	9.948661388	13.36892626	2R	0.081774345	0.846895799	0.995787491
NA	FBti0060579	1.364562765	1.16203794	1.171375929	0.783120422	1.099048926	1.147955131	0.589097203	0.820410114	X	-0.261544013	0.660321832	0.995750687
NA	FBti0060586	0	1.25340005	0	0.290362587	2.58552629	0.939331737	0	1.081559911	2L	1.635771721	0.32942299	NA
NA	FBti0060608	2.184645596	0.990388246	0.716205371	0.68829986	1.705446582	1.22466896	0.753118628	1.922865332	X	0.293928984	0.744548149	NA
NA	FBti0060623	1.543997249	1.294919392	3.183858421	0.899942865	0.557462151	0.800619146	0	0	2L	-2.243321934	0.096117216	NA
NA	FBti0060672	2.750579394	3.690968853	2.669146898	2.331953567	3.61127329	2.168882814	1.275779478	3.908789989	X	-0.025326986	0.965922137	0.997604378
NA	FBti0060690	0.114773659	0.445194895	0.243634384	0.08362202	0.246045096	0.473409582	0.091496897	0.467220446	2L	0.57666028	0.477287728	0.973549494
NA	FBti0060692	2.071305874	1.473956073	1.979529366	0.585354142	2.719445799	3.787276657	0.480358708	0.81763578	X	0.382681709	0.672321536	0.995787491
NA	FBti0060736	15.18051728	7.489172976	17.33069326	4.163859359	4.030106533	12.20737853	0	3.87744803	3L	-1.081546383	0.344182264	NA
NA	FBti0060797	0.436463904	0.565719021	0.673817036	0.046254507	0.358149641	0.523721549	0	0.086145776	2R	-0.776537544	0.532878309	NA
NA	FBti0060873	3.666000438	14.22756413	10.98629321	7.374004874	5.96923995	2.507499301	10.08553967	4.369771307	2R	-0.621042261	0.270248623	0.89549671
NA	FBti0060941	1.824161423	2.46756039	1.855808781	0.548769508	0.106228352	1.664330562	1.200896769	1.91633386	2R	-0.423534779	0.660966765	NA
NA	FBti0060949	1.085744026	1.112945343	0.804833292	0.632842831	0.272228645	1.421712272	0.307750704	0.720271074	3L	-0.38107897	0.658252715	NA
NA	FBti0060960	5.895482688	3.042721586	8.067997255	1.762191788	3.274725308	5.273187885	6.555680793	4.922937944	4	0.118927387	0.877785693	0.995787491
NA	FBti0060971	2.559175439	0.990613335	3.820630105	7.802504641	2.487674851	3.711961496	4.51973875	4.274005215	2R	0.01633556	0.986625875	NA
NA	FBti0060981	0.143608876	0.148236149	0.616387844	0.051510567	0.039884743	0.354106262	0.056361435	0.143902229	2R	-0.629222914	0.585307635	NA
NA	FBti0061033	0.29816952	0.250068771	0.482236732	0.173792753	0.134568101	0.187408268	0.190159213	0.161838407	3L	-0.803141208	0.512207121	NA
NA	FBti0061069	7.504055935	4.675171843	3.640947006	0.99973851	1.161149506	2.223501688	0.546943083	0.930971433	2R	-1.739635935	0.147646442	NA
NA	FBti0061100	4.230711227	1.867480151	0.540191917	0.778716628	2.009873181	0.419861969	3.124183883	0	2R	-0.428145595	0.74519857	NA
NA	FBti0061122	14.3170654	16.8104081	10.41990029	10.0139097	16.79988211	13.4980418	7.304628283	3.108367429	2R	-0.305000129	0.763392351	NA
NA	FBti0061131	1.094454367	5.507380994	5.310262059	0.182262797	1.834644165	0.22110962	0.1994269	0.339451678	2R	-2.157850237	0.065388052	NA
NA	FBti0061146	5.470626041	15.54857421	11.05971873	13.81743856	6.035261292	8.023046602	1.550631618	4.618924934	X	-1.125926577	0.087870426	0.707547954
NA	FBti0061160	0.426224281	0.052957884	1.034014162	0.22082797	0.541460641	0.491139793	0.845683455	0.754007116	2R	0.642849822	0.456995216	0.968365245
NA	FBti0061184	36.58410375	26.17024048	39.15552592	32.6125879	15.53969028	33.47682045	17.84189486	39.7137379	2R	-0.304162029	0.602703049	0.989286463
NA	FBti0061207	3.440265714	5.385858907	2.782012212	3.03009889	3.036274436	3.675923035	3.120423733	2.821673346	2R	-0.179261101	0.76778297	0.995787491
NA	FBti0061267	15.46754387	19.02606563	17.51122132	4.808266165	11.16915239	13.61052549	5.261071561	4.477529273	2R	-0.670241937	0.57403506	NA
NA	FBti0061279	1.134117783	1.664533044	2.063515441	0.661038229	1.023686635	2.138478636	0.723289707	0	2R	-0.450064476	0.758487827	NA
NA	FBti0061312	0.303315518	1.017538459	0.946078991	0.404096406	1.799133101	0.817040049	1.436991032	1.599277589	2R	1.137064487	0.187999155	NA
NA	FBti0061329	0.467448708	1.72497519	0.907219237	0.980855159	0.506318835	0.396637008	1.550213207	0.304462325	X	-0.543090748	0.590351289	NA
NA	FBti0061341	0.158255016	0.530901178	0.383924462	0.122988538	0.476151442	1.193614903	1.076565192	0.68717258	2R	1.568798379	0.196337209	NA
NA	FBti0061370	2.694789864	4.197265388	7.004488526	1.346314526	5.907240598	0.544421019	2.946200074	1.253708196	4	-0.474010115	0.668283644	NA
NA	FBti0061381	8.842697931	12.13558795	12.18878514	12.65098337	15.96333613	6.44210487	9.740916719	12.21711003	4	-0.009718035	0.986453559	0.99797245
NA	FBti0061436	2.794136956	4.686772766	2.711414913	1.302885025	1.008826668	2.37086573	1.425580681	5.459696984	2R	-0.132823831	0.916674192	NA
NA	FBti0061437	8.604143158	8.062705646	8.862504905	5.603417839	1.735495377	4.350534229	3.5560406	5.6354253	2R	-0.998128206	0.026735368	0.458462048
NA	FBti0061511	7.398404687	3.436556115	2.485166757	2.653708002	2.671203331	5.902067556	5.807227479	4.942345058	2R	0.273733152	0.700388791	0.995787491
NA	FBti0061531	14.88751097	16.64780743	15.32231865	10.51808224	35.01994656	84.64045536	62.14640782	70.52108604	2R	2.160675376	1.42E-05	0.00214926
NA	FBti0061658	3.429254776	3.471091093	3.729352253	0.413543711	0.640415564	5.964476015	4.977368726	1.540392323	2R	0.271082372	0.740220665	0.995787491
NA	FBti0061663	5.923740204	9.273826962	12.77414321	9.514228795	2.614056942	1.613405757	1.007439235	1.428998704	2R	-2.439036262	0.000438567	0.032061559
NA	FBti0061704	10.06529532	7.371503483	4.814869363	5.288305831	9.980944689	14.16742096	13.38085958	17.23592283	3R	1.000261289	0.052769911	0.605783103
NA	FBti0061710	7.064012266	8.181376149	3.67225612	6.274087113	3.33990188	5.074215327	3.003407843	5.477365906	3R	-0.548583545	0.453199825	0.967967712
NA	FBti0061762	10.35965729	3.861527063	4.18873068	2.012762581	1.55848638	3.798286182	4.404618051	4.998172211	2R	-0.473140506	0.655856971	NA
NA	FBti0061781	0.522246098	0.312855202	0.180994536	0.347884891	0.538736033	0.562709064	0.380646004	0.809889016	X	0.753410541	0.494626858	NA
NA	FBti0061801	1.150090515	1.377939641	0.717455198	0.229833663	1.364365353	1.951736963	0.838258746	0.642073621	2R	0.487236308	0.549906738	0.984934163
NA	FBti0061802	5.725725692	7.147238407	3.714909364	1.397021222	2.043242328	1.820316245	3.22700623	2.023664268	2R	-0.961016182	0.148033606	0.808004944
NA	FBti0061827	0.113944116	0.121625172	0.025129712	0.048301167	0.074799394	0.195319667	0.184974293	0	2R	0.560487643	0.700491889	NA
NA	FBti0061829	1.30253001	3.433275001	0.225708545	0.433828526	1.175700252	1.315732861	0.237341574	0.807975207	3R	-0.565161831	0.658435615	NA
NA	FBti0061874	13.24199799	5.487570271	6.424980483	3.632143506	5.624753002	3.08439966	0	5.411690056	2R	-1.00061017	0.275489766	0.89549671
NA	FBti0061876	1.078683009	2.533075193	2.093495948	0.754472649	1.168379578	0.20339515	1.375871766	1.639344466	2R	-0.530337139	0.650633663	NA
NA	FBti0061898	0.1882187	0.8839893	0.730585505	2.457418953	0.883435782	1.951965845	0.384119958	0.572096309	2R	-0.122110717	0.899219967	NA
NA	FBti0061935	14.91182373	4.784998542	5.347738246	1.511580681	8.661109189	5.868010988	0.661571873	0	2R	-0.788022473	0.453427524	0.967967712
NA	FBti0061958	7.114434961	4.773386636	7.766783992	5.805463049	6.903315954	7.042818116	4.083542197	7.529972226	3L	0.038356965	0.938947531	0.997038394
NA	FBti0061997	13.56671317	11.37812906	5.063485681	7.299295624	10.36174378	19.18592147	6.655572457	12.46155737	4	0.399403781	0.657214437	NA
NA	FBti0061998	8.033732733	2.450083571	2.126151661	1.702758676	2.900589322	6.885594681	6.334578574	5.391156662	4	0.574354035	0.486405825	0.973549494
NA	FBti0062035	49.24865967	56.45552566	67.24308985	43.85138743	37.97511813	56.93088375	39.14237241	52.65574425	4	-0.179221172	0.571439178	0.984934163
NA	FBti0062045	0	5.681730113	3.521809873	9.025572243	5.241389949	4.562187314	3.703324116	3.151780382	2L	-0.073443977	0.9597835	NA
NA	FBti0062048	0.231955874	0.281457381	0.323265666	0.069037837	0.133640362	0.195422051	0.075539287	0.203582039	3L	-0.532543483	0.469598419	0.973549494
NA	FBti0062174	2.398375279	2.320925811	0.447571152	0.430132436	0.666104935	1.739364279	3.765111916	0.600818624	2L	0.268428822	0.811094605	NA
NA	FBti0062208	0.132762803	0.254503973	0.253063413	0.198984521	0.359506328	0.178811371	0.435446694	0.267651739	2L	0.604285593	0.438387049	0.967112302
NA	FBti0062240	4.024686161	2.348120497	2.547086737	1.223922297	2.211266534	7.588899059	1.339181852	3.419204172	X	0.538956962	0.595347137	NA
NA	FBti0062241	22.74590535	18.11307141	24.52499961	6.428026916	12.44308754	25.77696339	17.58342219	8.978812016	X	-0.121476176	0.834539076	0.995787491
NA	FBti0062301	2.52474707	5.700839071	0.942307873	0.226398183	0.175300598	1.556360538	0	2.108253693	2R	-1.242471111	0.348181463	NA
NA	FBti0062352	0.331047938	0.090046455	0.06511765	0.125160941	0.290737155	0.312109974	0.114123027	0.174827607	X	0.537641842	0.57163494	NA
NA	FBti0062353	0.288457057	0.075600976	0.174948199	0.252197538	0.13018473	0.101983332	0.114978148	0.078283372	X	-0.881159265	0.369829347	NA
NA	FBti0062359	2.054812397	1.590765939	1.917287005	4.422200999	2.853433092	4.172569857	2.822545692	0	2R	0.010340755	0.992953047	NA
NA	FBti0062388	2.235774867	2.419483026	2.274563302	0.336298383	1.562379352	1.087935426	0	0.469749116	3R	-1.166525575	0.313240436	NA
NA	FBti0062414	0.93753964	1.135760328	1.137229045	1.092919188	0.84624967	0.491059248	1.860198483	2.374732903	X	0.399940423	0.646567098	NA
NA	FBti0062417	0.117501577	0.394184822	0.095019062	0.365267337	0.141413641	0.258486127	0.149874523	0.1275534	X	-0.473326113	0.696903684	NA
NA	FBti0062433	2.201061549	5.646545365	2.198718371	0	0.701202392	0.488269973	1.321165953	1.686602955	2R	-1.232767517	0.338927085	NA
NA	FBti0062445	2.12995686	5.359055745	1.722415211	2.482957118	1.922559018	5.354960847	0.905594285	2.697527881	3L	-0.056228316	0.957326094	NA
NA	FBti0062476	2.651578948	1.779060683	2.144231181	0.4121371	2.872067758	1.999913949	1.803795964	0	X	-0.044106952	0.974975381	NA
NA	FBti0062477	1.001367002	0.923808967	1.457581427	0.116732473	0	0.802470289	0	0.652218137	X	-1.214562047	0.412802451	NA
NA	FBti0062484	0.486923305	0.998244543	1.181269146	0	0.09766904	0.765113862	0	0.11746173	3L	-1.358921322	0.424913518	NA
NA	FBti0062516	0	5.429650408	1.895544575	2.34217089	4.231611859	7.576993569	1.138994874	0.484681004	3L	0.520834917	0.662224052	NA
NA	FBti0062558	0.242628139	0.610461999	2.354449925	0.707097965	0.876011952	1.944360784	0	0.131692037	3R	-0.341829905	0.800914374	NA
NA	FBti0062576	0.072696807	0.091454022	0.2204518	0.296607271	0.196854553	0.633977478	0.370902233	0.039457874	2R	0.918052652	0.44752038	NA
NA	FBti0062662	2.249824562	7.075809533	6.140298383	1.967017977	1.01537749	3.446821389	1.434837698	0.610572174	4	-1.344314704	0.290075106	NA
NA	FBti0062664	0	2.125795638	0.118252479	0.227290016	0.527973439	0.597423178	0.870430523	0	3L	-0.257202919	0.875725615	NA
NA	FBti0062666	0.212828932	0.120746847	0.129080005	0.043782586	0.045201267	0.008852374	0.095811385	0.054361331	3L	-1.315302773	0.151154835	0.811155438
NA	FBti0062678	0.221247115	0.556666497	0.805113624	0.773743981	6.789931721	8.865093228	13.54574747	9.366785375	4	4.161690087	5.47E-08	1.82E-05
NA	FBti0062695	4.441961315	1.241794494	0.552622369	0	3.084184092	2.684521792	0.435828413	0	3L	-0.008333502	0.994899285	0.998895169
NA	FBti0062731	0.789370623	0.712954567	0.184134819	0.07078415	0.246637434	0.286236078	0	0	3L	-1.672586404	0.206919849	NA
NA	FBti0062808	1.393856826	1.335999593	0.724602261	0	1.07840092	1.595716781	0	0	4	-0.336907282	0.855550644	NA
NA	FBti0062856	1.976004193	2.767753155	3.014631703	1.852290682	1.066480684	1.862964683	1.662953946	1.680653038	X	-0.57907691	0.187899837	0.84291216
NA	FBti0062909	66.76823102	33.29561486	39.40024796	33.65786316	39.09203337	32.32499803	41.43093854	19.58919057	4	-0.376332348	0.664446887	NA
NA	FBti0062928	0.175536013	0.132496666	0.191631564	0.122776682	0.015844375	0.057922998	0.044779614	0.11433148	2L	-1.39569859	0.22911341	NA
NA	FBti0063002	58.90040704	142.3841566	79.8511692	44.42837937	21.89153869	9.79957835	17.67720045	0	X	-2.689635784	0.005157027	NA
NA	FBti0063031	0.558827391	0.843619098	1.355707457	0.912019518	0.907944001	0.421487241	0.427674204	0	2R	-1.013738472	0.41821732	NA

NA	FBti0063036	0.435267566	3.650501401	5.279765221	1.014809944	3.14307806	1.436286609	0.555188456	0	X	-0.939812539	0.540109895	NA
NA	FBti0063040	0	0	0.544037944	0.914971037	1.92297381	1.902830748	0	0.973753939	2R	1.780389121	0.268943492	NA
NA	FBti0063052	3.036985403	0.57398501	0	0.199454004	0.772188313	1.774409249	0.109118521	0.092867274	2R	-0.481145256	0.72637807	NA
NA	FBti0063065	3.600002653	5.672529758	5.690044206	4.323806098	3.840275318	3.908312104	3.89610841	3.552699549	2R	-0.305139199	0.50697972	0.978557094
NA	FBti0063079	3.41400743	3.2722963	0.709914378	2.729015932	0.17609024	1.287482141	0.497668931	0.635325099	3R	-1.915523964	0.082336281	NA
NA	FBti0063083	1.024764811	2.062679052	0.870122798	0.179190043	0.601238017	1.835669808	0.392129557	0	2R	-0.513739658	0.624412411	0.990047511
NA	FBti0063095	1.292225184	0.788191442	1.139971008	0.730369544	0.141381676	0.664528369	0.099893764	0.255049136	2R	-1.696343697	0.111618557	NA
NA	FBti0063138	0.339946019	1.026381163	2.144231181	1.585142692	1.595593199	1.089696703	3.121954553	1.476108551	2R	0.568902215	0.535583223	NA
NA	FBti0063229	0.702357556	0.795221732	0.511172891	0	0.158491925	0.115881222	0.313552613	0.991174126	2R	-0.313895092	0.777509428	NA
NA	FBti0063232	0.353146229	0.817038975	0.295423388	0.539434331	0.571569165	0.229616626	0.062129905	0.528767691	2R	-0.482566134	0.556269653	0.984934163
NA	FBti0063240	0.602112929	0.144279996	0.695579794	0.668477918	0.362322719	0.405477423	0.512001006	0.746995946	2R	-0.033973218	0.972052965	NA
NA	FBti0063242	0.096528506	0.151118825	0.218565021	0.210049072	0.209110431	0.206281367	0.098498517	0.321344226	2R	0.356407711	0.646067828	0.99279236
NA	FBti0063252	1.277063878	5.774344395	7.273891931	8.543919109	5.913922996	5.444210194	6.798923248	5.183601196	2R	0.073949875	0.892469257	0.996438268
NA	FBti0063255	51.97094739	60.24217677	55.35254347	35.46389484	36.61302637	46.60775069	30.18058613	27.52042382	2R	-0.490941358	0.251508742	0.882557414
NA	FBti0063288	14.91439991	0	12.52458213	8.024391216	3.106651658	8.112233733	0	3.736216479	3L	-1.207602698	0.424457872	NA
NA	FBti0063347	5.470626041	30.5873591	4.42388749	8.503039113	14.81382317	1.719224272	6.977842281	11.87723554	3L	-0.429707643	0.735587452	NA
NA	FBti0063352	0.07089203	0.257641444	0.114655384	0.055094033	0.255956568	0.122533717	0.090423546	0.102608773	3L	0.246256059	0.835897133	NA
NA	FBti0063378	10.53219758	15.22955511	11.01334674	16.08803522	11.47355277	17.63376258	8.338302097	16.55841014	4	0.069239838	0.891117497	0.996189576
NA	FBti0063419	54.07911633	46.12379547	44.47294302	42.74014369	40.53988645	28.94937167	23.38254027	30.84520166	4	-0.570456805	0.208152634	0.856520545
NA	FBti0063420	63.29794874	59.14046275	54.55418948	58.25399392	45.60737226	30.62368234	31.86995273	59.06894387	4	-0.464238629	0.186409928	0.839751536
NA	FBti0063425	1.05448299	0.800147582	0.852720342	0.819495799	0.317268677	0.426068624	0.576430449	0.490581468	4	-0.947274557	0.216948134	NA
NA	FBti0063446	2.466392885	4.322268127	6.519264714	2.832238379	3.056920586	3.574715151	6.291821238	6.233908159	4	0.290174741	0.538602936	0.984934163
NA	FBti0063514	1.174846735	0.89574572	1.295528087	3.237131105	1.54247234	0.604166359	2.724599329	1.159409553	4	-0.111177122	0.917519091	NA
NA	FBti0063548	74.43755485	52.21357784	52.53366395	53.6422194	63.52455422	43.38354999	34.52578212	52.89081453	4	-0.236944083	0.712709803	0.995787491
NA	FBti0063553	20.12343081	22.30714472	17.82960716	28.15021282	7.581485259	15.50775025	8.035091111	6.838408344	4	-1.17955902	0.014104964	0.354312909
NA	FBti0063565	2.715995838	3.324431191	4.184885094	3.166121026	2.186503561	0.276824247	1.310809355	0.318739372	X	-1.682525946	0.018075409	0.385711607
NA	FBti0063603	22.98607166	17.8673732	27.20189719	20.91362371	6.072548872	12.42125627	7.150971054	1.217192424	4	-1.682668242	0.014080814	0.354312909
NA	FBti0063605	79.40005851	72.64497787	171.6099689	134.6314526	44.30430448	54.44210194	33.14475083	21.93989344	4	-1.525024279	0.014850579	0.35986804
NA	FBti0063660	0.233199069	0.170069011	0.073791873	0.118194533	0.237947528	0.296331235	0.155190218	0.088051612	3L	0.395750893	0.689633601	NA
NA	FBti0063812	5.636762189	3.781951126	6.381529677	4.380632949	1.017580044	5.314304962	1.437950145	0.815862167	2R	-1.175206496	0.240934096	NA
NA	FBti0063843	5.84540308	11.58754248	18.04837534	6.19469874	4.796568511	10.5210197	4.066840593	2.307438398	2R	-0.873675704	0.416299246	NA
NA	FBti0063924	10.98888191	8.674027208	4.704507219	8.540054831	8.946435497	2.8439904	10.44361967	13.56624541	3L	0.135823188	0.85151438	0.995787491
NA	FBti0063939	0.663843084	0.979883209	0.450932738	0.557181058	1.390152014	1.326838475	0.677391188	0.40355414	3L	0.548894595	0.481370717	0.973549494
NA	FBti0063941	2.024842106	5.283271118	0	0	2.843056972	0.848448342	0	2.198059825	3L	-0.263206652	0.86912639	NA
NA	FBti0063974	9.061601083	10.28205841	13.5779316	1.24275187	4.330194465	8.040679672	10.8782772	2.893172761	4	-0.357069398	0.68903615	0.995787491
NA	FBti0063991	12.48551289	19.30654817	15.14484006	19.55794751	15.84812163	12.13911733	10.94871649	9.741651526	4	-0.412014904	0.398019369	0.952272708
NA	FBti0063994	3.634331985	2.032027353	1.469473117	0	4.00943932	2.474640997	0	0	X	-0.117361627	0.948348922	NA
NA	FBti0064131	11.97911908	9.582954528	4.470950123	0.429674849	0.665396313	3.301276394	0	1.200358911	X	-2.286647826	0.056705836	NA
NA	FBti0064138	5.470626041	1.529367955	7.741803108	1.594319834	2.880465616	3.008642476	0	4.948848143	2R	-0.552249762	0.650633959	NA
NA	FBti0064154	13.86400701	11.17943272	12.46613817	9.963913674	5.832232878	9.785188367	8.371361444	8.284415394	4	-0.530510246	0.034343785	0.514749796
NA	FBti0064179	38.75026779	61.17471821	118.8919763	50.48679473	84.35649305	42.9806068	93.03789708	106.4002351	4	0.326513419	0.579328311	0.985637351
NA	FBti0064264	17.83316822	6.409850989	37.08258631	11.87924582	13.79718825	36.02786158	16.24742688	13.82766393	2L	0.173143924	0.871672905	NA
NA	FBti0102105	0.896359907	0.626465832	1.848373136	0.801101262	8.468367352	11.2677686	8.49865406	8.692492151	4	3.197455879	1.63E-22	6.79E-19
NA	FBti0102111	0.175696239	0.221029344	0.674874655	0.238950347	1.559452311	3.865057947	1.307264232	2.034414923	4	2.811565131	2.66E-06	0.000546045
NA	FBti0102113	1.975580869	1.767338539	2.343113242	2.047107744	1.743587905	1.655613845	1.567921986	2.478186501	4	-0.095649416	0.873398318	0.995787491
NA	FBti0102121	8.849630222	7.207221852	11.114218	11.80900061	9.195103775	10.86516982	10.0174674	11.86162814	4	0.137470804	0.598672731	0.989286463
NA	RR41919_trans	0.202939447	0.216619742	0.179028461	0.215066218	0.466273454	0.260904642	0	0.08010915	2L	0.03288475	0.980243955	NA
NA	RR47158_trans	0.267807454	0.136715915	0.197733915	0.027147087	0.231220523	0.15368788	0.20792513	0.176958409	2R	0.298611786	0.778739399	NA
NA	RR48307_trans	0.512209267	0.889843446	0.355032153	0.469148673	0.396286716	0.120726942	0.046666316	0.119148614	3L	-1.658424329	0.096564462	NA
NA	RR48323_trans	0.771998624	2.136616996	0.842786053	0.149990478	0.301958665	0.303264828	0.065646169	1.201191008	3R	-1.007084757	0.243416345	0.877056557
NA	RR48332_trans	0.23836081	0.5215002	0.075425217	0.217459274	0.505137545	0.586239432	0.079312637	0.292501912	3R	0.507978008	0.565552018	0.984934163
NA	RR48342_trans	0.131493335	0.120306339	0.087000272	0	0.021579925	0.123971113	0.030494756	0.155718655	2R	-0.007997204	0.996021462	NA
NA	RR48345_trans	0.634041468	0.686139106	0.396948582	0.715279264	0.295382543	0.30852667	0.052175916	0.399647241	2L	-1.163141739	0.206195917	NA
NA	RR48349_trans	0.174525293	0.180148736	0.13027567	0.093899804	0.121178033	0.101256234	0.273980168	0.0874409	3R	0.01729652	0.988169618	NA
NA	RR48350_trans	0.131211365	0.157206185	0.227369245	0.262212308	0.304547328	0.300427094	0.239087866	0.162784012	3R	0.421050278	0.701298525	NA
NA	RR48355_trans	0.171402485	0.143751811	0.33265603	0.179828298	0.21659779	0.145437494	0.240488282	0.130245731	2R	-0.140093796	0.867523359	NA
NA	RR48360_trans	1.347353917	0.897807099	1.208957108	1.506105106	1.599333136	0.957058046	1.742106372	0.801432898	3L	0.062436217	0.906916809	0.996479182
NA	RR48361_trans	63.27670565	55.46555837	61.40336213	43.72629519	61.86230287	47.56703196	50.04946203	42.59549195	X	-0.11719444	0.468611369	0.973431492
NA	RR48366_trans	0.403250678	0.324670292	0.195656104	0.112819653	0.29118833	0.243316657	0.246888274	0.070039564	2L	-0.269998275	0.799858299	NA
NA	RR48378_trans	0.295350497	0.382816064	0.227982423	0.093899804	0.07270682	0.316425732	0.205485126	0.204028767	2L	-0.296812889	0.769537391	NA
NA	RR48383_trans	1.851476572	1.207730305	1.147867731	0.383702038	1.002716923	1.173404762	0.65599396	0.446636336	2R	-0.462403078	0.40138886	0.954265
NA	RR48389_trans	0.165283441	0.652328907	0.353801755	0.181342174	0.105310226	0.091663658	0.198419581	0.189977109	3L	-1.178298073	0.180101944	NA
NA	RR48390_trans	0.042951196	0.408252768	0.069466002	0.300417291	0.155075835	0.134980418	0.036523141	0.031083674	3L	-1.130986412	0.402988771	NA
NA	RR48393_trans	1.016712371	0.568464124	0.205544169	0	0.50984067	0.585780686	0.216137925	1.165004356	3L	0.469954136	0.685457781	NA
NA	RR48395_trans	0.985044492	1.184129662	1.314337309	0.842084522	1.3040557	0.758433376	0.293168127	0.85544911	3L	-0.386948552	0.521372996	0.982083369
NA	RR48396_trans	0.834569958	0.572675738	0.674885026	0.38325742	0.388066901	0.393413729	0.161288325	0.411801962	3L	-0.831731564	0.228872011	0.867702781
NA	RR48403_trans	0.417514718	0.134677378	0.623313773	0.449270698	0.028989272	0.378490698	0	0.38350362	3L	-0.997175195	0.40241576	NA
NA	RR48408_trans	0.455107031	0.362604645	0.414031241	0.212212982	0.308094313	0.246716474	0.464395125	0.321125835	2R	-0.092069344	0.899989051	0.996479182
NA	RR50207_trans	0.2004779	0.494519931	0.772448701	0.219956083	0.170312467	0.489200644	0.090251194	0.179223091	2R	-0.795369109	0.369051266	0.942282153
