# Supplementary material for: Transcriptional changes in specific subsets of Drosophila neurons following inhibition of the serotonin transporter
Source: Transl Psychiatry. 2023 Jun 24;13:226. doi: 10.1038/s41398-023-02521-3 (PMC10290657; doi:10.1038/s41398-023-02521-3)
Supplement: Supplementary file 7 — Supplementary Figure 1 [file 41398_2023_2521_MOESM7_ESM.pdf]

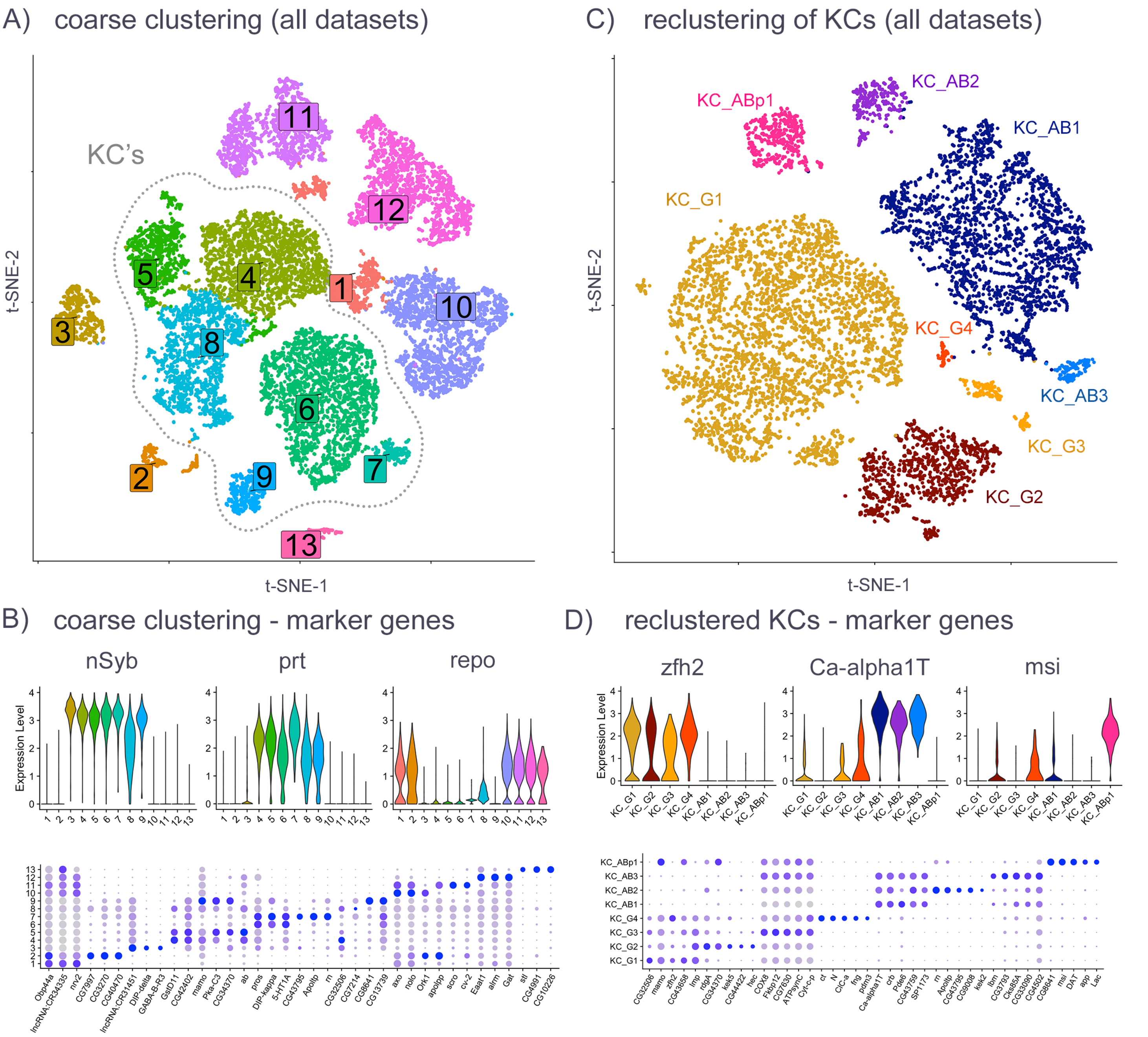

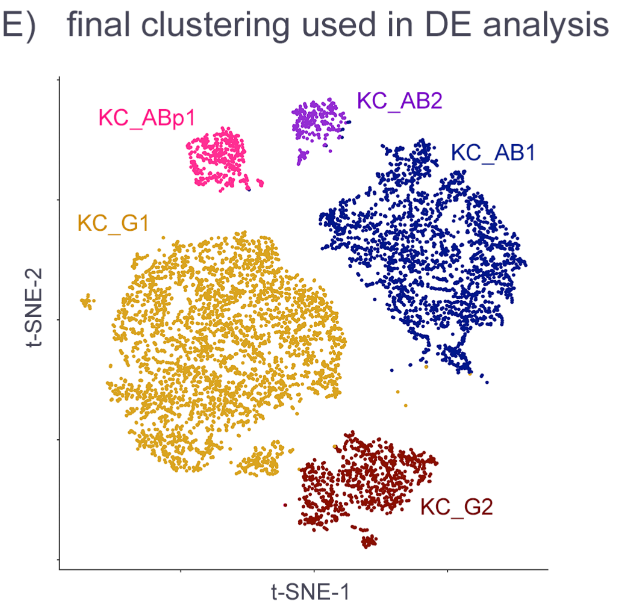

F) cell count per cluster, by experiment

	Fig. 2: day0		Fig. 3: day0		Fig. 4: day4-6		Fig. 5: day4-6	
	dSERT[4]	*dSERT*[16]	*w*[1118]	*dSERT*[TMKO]	*w*[1118]	*dSERT*[TMKO]	VEH	CIT
KC_G1	1015	395	588	492	251	251	383	270
KC_G2	220	67	173	155	72	46	99	97
KC_AB1	496	184	296	265	182	193	415	378
KC_AB2	92	39	19	25	17	27	32	31
KC_ABp1	129	51	62	53	16	17	35	49

Supplemental Figure S1. Clustering and integration for all scRNA-seq experiments

A) Coarse clustering performed on all scRNA-seq datasets, integrated using Seurat. Clusters 4-9 are KCs. B) Expression of marker genes in each cluster, including *prt*, a marker for KCs. Clusters 1,2, and 10-13 are non-neuronal. C) Reclustering of KC's (clusters 4-6 from A), defining transcriptionally-defined subclusters for each KC type. D) Expression of marker genes in each of the clusters from C). E) Final clustering used in DE analysis for all experiments. KC_AB3, KC_G3, and KC_G4 were removed because the number of cells in these clusters was too low to proceed with DE analysis. F) Table showing the number of cells in each cluster, by experiment and genotype.
