# Supplementary material for: Transcriptional changes in specific subsets of Drosophila neurons following inhibition of the serotonin transporter
Source: Transl Psychiatry. 2023 Jun 24;13:226. doi: 10.1038/s41398-023-02521-3 (PMC10290657; doi:10.1038/s41398-023-02521-3)
Supplement: Supplementary file 8 — Supplementary Figure 2 [file 41398_2023_2521_MOESM8_ESM.pdf]

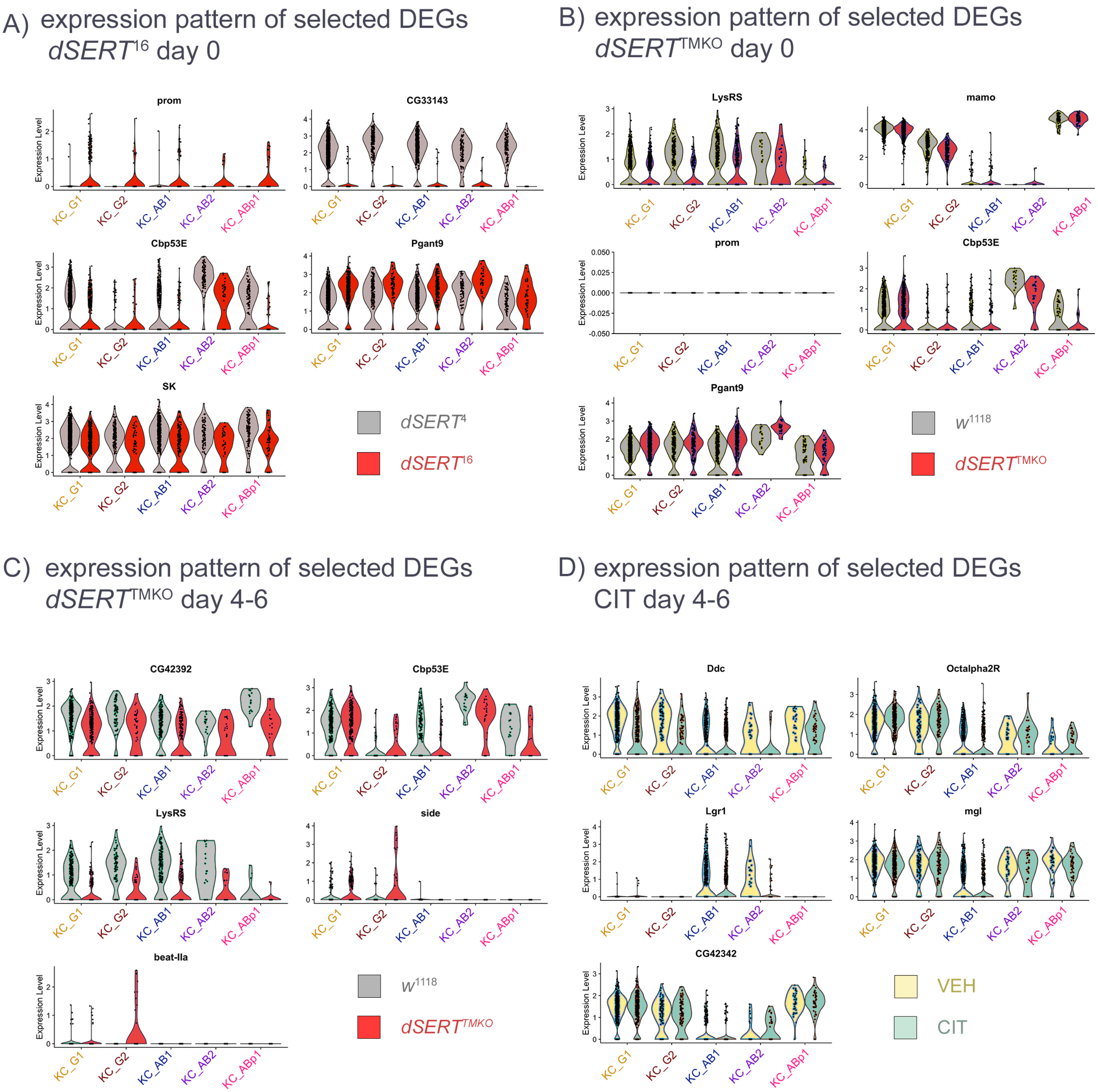

A) expression pattern of selected DEGs
dSERT16 day 0
prom
CG33143
Cbp53E
Pgant9
SK
dSERT4
dSERT16
B) expression pattern of selected DEGs
dSERTTMKO day 0
LysRS
mamo
prom
Cbp53E
Pgant9
w1118
dSERTTMKO
C) expression pattern of selected DEGs
dSERTTMKO day 4-6
CG42392
Cbp53E
LysRS
side
beat-IIa
w1118
dSERTTMKO
D) expression pattern of selected DEGs
CIT day 4-6
Ddc
Octalpha2R
Lgr1
mgl
CG42342
VEH
CIT
Expression Level
KC_G1
KC_G2
KC_AB1
KC_AB2
KC_ABp1

Supplemental Figure S2. Violin plots for selected DEGs from each experiment

A) Violin plots derived from the normalized single-cell dataset examining d*SERT*^{16} vs d*SERT*^{4} at day 0, reflecting the same observations as in Fig. 2C for downregulation and upregulation of genes in different cell types. *Cbp53E* is enriched in KC_AB2, and downregulated in *dSERT*^{16} compared to controls. *SK* is highly expressed in all KC types, but undergoes downregulation in response to *dSERT* LOF in KC_ABp1 alone. B) Violin plots derived from the normalized single-cell dataset examining *dSERT*^{TMKO} vs controls at day 0, reflecting the same observations as in Fig. 3C for downregulation and upregulation of genes in different cell types. *LysRS*, identified as downregulated in KC_G1 and KC_G2, is de-enriched in KC_ABp1. Notably, *prom*, is no longer expressed in any cell-type, consistent with the idea that it was an artifact of the *dSERT*^{16} deletion. *mamo*, identified as DE only in KC_G2, appears to be highly expressed in KC_G1 and KC_ABp1, suggesting that the cell-type specific DE may be a true observation. C) Violin plots derived from the normalized single-cell dataset examining *dSERT*^{TMKO} vs controls at day 4-6, reflecting the same observations as in Fig. 4C for downregulation and upregulation of genes in different cell types. *LysRS*, identified as downregulated in KC_AB1 and KC_G1, is robustly expressed in the other KC clusters, suggesting that it may be a true cell-type specific change. D) Violin plots derived from the normalized single-cell dataset examining CIT- vs VEH-fed WT flies at day 4-6, reflecting the same observations as in Fig. 5C for downregulation and upregulation of genes in different cell types. *LysRS*, identified as downregulated in KC_AB1 and KC_G1, is robustly expressed in the other KC clusters, again suggesting that it may be a true cell-type specific change.
