# Supplementary material for: Transcriptional changes in specific subsets of Drosophila neurons following inhibition of the serotonin transporter
Source: Transl Psychiatry. 2023 Jun 24;13:226. doi: 10.1038/s41398-023-02521-3 (PMC10290657; doi:10.1038/s41398-023-02521-3)
Supplement: Supplementary file 3 — Supplemental Table 2 [file 41398_2023_2521_MOESM3_ESM.pdf]

scRNA-seq_DEGs_dSERT16_v_dSERT4_Day0

	cell_type	gene	avg_logFC	p_val	p_val_adj	de_family	de_method	de_type	minuslogP
1	KC_G1	CG33143	-6.78141921732661	2.04969882979795E-109	1.87957382692472E-105	pseudobulk	DESeq2	LRT	18
2	KC_AB1	CG33143	-6.43186537586646	5.7200480358085E-74	4.96557369988536E-70	pseudobulk	DESeq2	LRT	18
3	KC_G2	CG33143	-7.35123500851136	6.51015999777772E-40	4.98678255829773E-36	pseudobulk	DESeq2	LRT	18
4	KC_G3	CG33143	-5.97152161981526	4.31815305716457E-32	3.31072794892807E-28	pseudobulk	DESeq2	LRT	18
5	KC_ABp1	CG33143	-7.98054349459212	1.59688457467421E-30	1.18201396217385E-26	pseudobulk	DESeq2	LRT	18
6	KC_AB3	CG33143	-7.97417581373557	2.26561338845858E-29	1.63078851701249E-25	pseudobulk	DESeq2	LRT	18
7	KC_AB2	CG33143	-6.13949708247361	3.5024020470289E-22	2.46674176172246E-18	pseudobulk	DESeq2	LRT	17.6078763135541
8	KC_G1	prom	6.62766505828738	2.09980004229425E-16	9.62758319391915E-13	pseudobulk	DESeq2	LRT	12.0164827198614
9	KC_G1	CG42392	-1.55956819960516	2.80094698469896E-13	8.56156128322983E-10	pseudobulk	DESeq2	LRT	9.06744703031554
10	KC_G1	Pgant9	1.02670295887919	7.28806292483069E-13	1.67078842551744E-09	pseudobulk	DESeq2	LRT	8.77707854199641
11	KC_AB2	Cbp53E	-1.67344845282431	5.1529423644502E-09	1.81460865364114E-05	pseudobulk	DESeq2	LRT	4.74121702235787
12	KC_AB3	Cbp53E	-3.99428021266821	1.50954777350952E-08	5.43286243686075E-05	pseudobulk	DESeq2	LRT	4.26497129140814
13	KC_G2	mt:Cyt-b	-2.114946535196	2.79599550157712E-08	0.000107086627710404	pseudobulk	DESeq2	LRT	3.97026475768513
14	KC_G1	Cbp53E	-1.09051572991314	6.09569905915973E-08	0.000111795120744989	pseudobulk	DESeq2	LRT	3.95157715065146
15	KC_AB1	Pgant9	1.09243986639643	6.46469292260591E-08	0.00028059999630571	pseudobulk	DESeq2	LRT	3.55191233902544
16	KC_G2	mt:ATPase6	-1.62365311477441	6.70513224149142E-07	0.00171204376566081	pseudobulk	DESeq2	LRT	2.76648513747309
17	KC_AB1	CG31690	2.45549848709916	8.69134534249802E-07	0.00251498563060751	pseudobulk	DESeq2	LRT	2.5994644919463
18	KC_AB1	CG42392	-1.35289017134148	1.56017225604786E-06	0.00338596383868786	pseudobulk	DESeq2	LRT	2.47031768436713
19	KC_G2	CG42392	-1.64970132400198	3.01564374801425E-06	0.00577495777744729	pseudobulk	DESeq2	LRT	2.23845118668927
20	KC_ABp1	CG42392	-1.61367912839201	3.38871037046349E-06	0.0125416170810854	pseudobulk	DESeq2	LRT	1.90164646317718
21	KC_AB1	lncRNA:CR45312	-2.42180456420242	8.36991783090179E-06	0.0145318513380117	pseudobulk	DESeq2	LRT	1.83767905365349
22	KC_G1	SK	-0.806477869514589	1.17699763815059E-05	0.0179884472364015	pseudobulk	DESeq2	LRT	1.74500632335633
23	KC_AB1	Cbp53E	-1.85087448125114	1.66223443365615E-05	0.0240497618642817	pseudobulk	DESeq2	LRT	1.6188892195621
24	KC_AB1	prom	5.01038017493503	2.59804768586682E-05	0.0322195028014426	pseudobulk	DESeq2	LRT	1.49188116572487
25	KC_AB1	sr	-1.956429757962	3.90806903524272E-05	0.0424074341186775	pseudobulk	DESeq2	LRT	1.37255800393291
26	KC_AB1	SK	-0.881719953006432	4.7930444741234E-05	0.0462315767554058	pseudobulk	DESeq2	LRT	1.33506129443787
27	KC_G2	mt:ND1	-1.50281591729015	3.88455946070061E-05	0.0595114509379333	pseudobulk	DESeq2	LRT	1.22539946115083
28	KC_G1	dpr1	3.09830905097847	6.1178153694323E-05	0.0801433813395631	pseudobulk	DESeq2	LRT	1.09613233814322
29	KC_ABp1	Cbp53E	-2.16796372071001	3.38987168030897E-05	0.0836394339254899	pseudobulk	DESeq2	LRT	1.07758891516412
30	KC_AB1	dpr21	-0.878527869087709	0.000101190782987938	0.0878437187118287	pseudobulk	DESeq2	LRT	1.05628928739942
31	KC_AB1	CG17684	-0.651715041693591	0.000114720758141696	0.0905355364934603	pseudobulk	DESeq2	LRT	1.04318092053266
32	KC_AB1	MFS17	-0.868797586633713	0.000130013517751863	0.0940539456336599	pseudobulk	DESeq2	LRT	1.02662298075174
33	KC_G1	Snp	-2.37530715830634	8.46276293004588E-05	0.0970044200856509	pseudobulk	DESeq2	LRT	1.01320847630002
34	KC_AB1	brp	-0.450771519966115	0.000210415926702827	0.140509281515941	pseudobulk	DESeq2	LRT	0.852294986947366
35	KC_G1	hwt	-0.804196436643486	0.000147237363158604	0.1500185133516	pseudobulk	DESeq2	LRT	0.823855142608924
36	KC_G1	lncRNA:CR31451	-0.485089851540546	0.000169974533288351	0.155866647025418	pseudobulk	DESeq2	LRT	0.807246806956676
37	KC_G3	CG42392	-1.38802835869246	4.77232114163906E-05	0.182946930964733	pseudobulk	DESeq2	LRT	0.737674871644774
38	KC_AB2	lncRNA:CR44832	5.55980441868709	8.97231316991406E-05	0.189687279235234	pseudobulk	DESeq2	LRT	0.721961792691635
39	KC_AB2	Pgant9	0.949529568245631	0.00010773095512437	0.189687279235234	pseudobulk	DESeq2	LRT	0.721961792691635
40	KC_G2	mt:ND5	-1.72140013646837	0.000202355923982146	0.221435196814748	pseudobulk	DESeq2	LRT	0.654753347470239
41	KC_G2	prom	5.33607882392887	0.000191168305610674	0.221435196814748	pseudobulk	DESeq2	LRT	0.654753347470239
42	KC_G2	CG9650	2.41479756316242	0.000257245367654616	0.246312439529295	pseudobulk	DESeq2	LRT	0.608513654411369
43	KC_G2	mt:ND3	-1.70908256926641	0.00030076390319113	0.255983499827117	pseudobulk	DESeq2	LRT	0.591788027520101
44	KC_AB1	slo	-0.425049743644355	0.000463708769218378	0.287532558970339	pseudobulk	DESeq2	LRT	0.541312970520309
45	KC_G1	CG8910	-0.566031935957746	0.000350041094992896	0.291806985553169	pseudobulk	DESeq2	LRT	0.534904315763742
46	KC_AB1	mtd	-0.529495249123994	0.000537839281694458	0.311265520292639	pseudobulk	DESeq2	LRT	0.50686898460495
47	KC_G2	Sulf1	5.03187512277609	0.000416760863105967	0.319238821139171	pseudobulk	DESeq2	LRT	0.495884301590229
48	KC_G2	GstD11	-1.63707785004952	0.000499091709162578	0.347549317471395	pseudobulk	DESeq2	LRT	0.458983560035073
49	KC_AB1	CG42340	1.47769838156506	0.000773457156410508	0.419648848424976	pseudobulk	DESeq2	LRT	0.377113964313927
50	KC_G1	mt:ND1	-0.595242853664893	0.000556199456868507	0.425029084957017	pseudobulk	DESeq2	LRT	0.371581349939944
51	KC_G1	CG34370	-1.26103778517949	0.000691224667810954	0.448764303366191	pseudobulk	DESeq2	LRT	0.34798169602028

52	KC_G1	CG6044	0.731699257893253	0.000686165540246236	0.448764303366191	pseudobulk	DESeq2	LRT	0.34798169602028
53	KC_G1	otk	-2.65663388547953	0.000734074651089734	0.448764303366191	pseudobulk	DESeq2	LRT	0.34798169602028
54	KC_G1	tyn	-1.55337690676233	0.000799890789896804	0.458437408959606	pseudobulk	DESeq2	LRT	0.338719950722501
55	KC_G2	mt:ND4	-1.58469293811762	0.000805571834394145	0.514223354288263	pseudobulk	DESeq2	LRT	0.288848203056449
56	KC_AB2	sr	-1.51903680791065	0.000374832094098127	0.527988487746621	pseudobulk	DESeq2	LRT	0.27737554671358
57	KC_G3	beta4GalNAcTA	4.76106046162155	0.000208991573967785	0.534112799203669	pseudobulk	DESeq2	LRT	0.272367014697173
58	KC_AB1	CG41378	-1.74063398701002	0.00111420520040077	0.566112835538046	pseudobulk	DESeq2	LRT	0.247096998197634
59	KC_AB1	CG42540	-0.537722636471385	0.00117754671540092	0.566112835538046	pseudobulk	DESeq2	LRT	0.247096998197634
60	KC_AB1	EcR	-0.724867632223414	0.00123904433535571	0.566112835538046	pseudobulk	DESeq2	LRT	0.247096998197634
61	KC_AB1	CG10947	0.998955195794154	0.00144179475699988	0.596010489786475	pseudobulk	DESeq2	LRT	0.224746096608266
62	KC_AB1	mei-P26	-0.394553247834229	0.00141110819624778	0.596010489786475	pseudobulk	DESeq2	LRT	0.224746096608266
63	KC_AB1	shakB	-0.559077511090078	0.00169405607177874	0.668459125414149	pseudobulk	DESeq2	LRT	0.174925143599913
64	KC_G1	cwo	-1.11704838865959	0.00127812134937334	0.689433692573735	pseudobulk	DESeq2	LRT	0.161507496470131
65	KC_G2	lncRNA:CR44330	4.61896977469622	0.00142656865491829	0.780536849762435	pseudobulk	DESeq2	LRT	0.107606588731344
66	KC_G2	Rgk1	-0.785431134987682	0.00135196325316988	0.780536849762435	pseudobulk	DESeq2	LRT	0.107606588731344
67	KC_AB1	CG34370	-2.30434911337349	0.00216855894272843	0.809886712163272	pseudobulk	DESeq2	LRT	0.0915757264587476
68	KC_AB1	jim	-0.609375148412871	0.00223906014190975	0.809886712163272	pseudobulk	DESeq2	LRT	0.0915757264587476
69	KC_AB2	Prosap	0.843159594355185	0.000698555668574077	0.819987928961204	pseudobulk	DESeq2	LRT	0.0861925408164376
70	KC_AB1	CG42806	-4.08778190945727	0.00253035422897052	0.844846348526657	pseudobulk	DESeq2	LRT	0.0732222686298386
71	KC_AB1	dpr10	0.675484217963457	0.00251525411959746	0.844846348526657	pseudobulk	DESeq2	LRT	0.0732222686298386
72	KC_AB1	Tet	-0.510814984751828	0.00279215741728382	0.897730316275586	pseudobulk	DESeq2	LRT	0.0468541084816039
73	KC_G1	Hsp70Ab	-1.08237282922769	0.00189158840423959	0.963659203715391	pseudobulk	DESeq2	LRT	0.0160765263811816
74	KC_AB1	18w	2.76986267366739	0.00324518470542482	0.971429256130788	pseudobulk	DESeq2	LRT	0.0125888211997812
75	KC_AB1	CG46339	-0.689963071959244	0.00322986536410907	0.971429256130788	pseudobulk	DESeq2	LRT	0.0125888211997812
76	KC_AB1	128up	0.49605003100763	0.828150015021173	1	pseudobulk	DESeq2	LRT	0
77	KC_AB1	14-3-3epsilon	-0.145158189119524	0.333295313431955	1	pseudobulk	DESeq2	LRT	0
78	KC_AB1	14-3-3zeta	-0.184066883642442	0.251706265770561	1	pseudobulk	DESeq2	LRT	0
79	KC_AB1	140up	0.248091066769659	0.723904998099219	1	pseudobulk	DESeq2	LRT	0
80	KC_AB1	18SrRNA-Psi:CR4	-1.13584521858219	0.54202769046723	1	pseudobulk	DESeq2	LRT	0
81	KC_AB1	26-29-p	0.0990763065684494	0.67346206318803	1	pseudobulk	DESeq2	LRT	0
82	KC_AB1	28SrRNA-Psi:CR4	0.180096355886811	0.786464909800271	1	pseudobulk	DESeq2	LRT	0
83	KC_AB1	28SrRNA-Psi:CR4	2.19691424616663	0.593804541109954	1	pseudobulk	DESeq2	LRT	0
84	KC_AB1	28SrRNA-Psi:CR4	2.31997030691033	0.813169507846918	1	pseudobulk	DESeq2	LRT	0
85	KC_AB1	28SrRNA-Psi:CR4	0.849606887838128	1	1	pseudobulk	DESeq2	LRT	0
86	KC_AB1	28SrRNA-Psi:CR4	0.500697155367556	0.315418818568568	1	pseudobulk	DESeq2	LRT	0
87	KC_AB1	28SrRNA-Psi:CR4	-0.504039151378979	0.826589305333094	1	pseudobulk	DESeq2	LRT	0
88	KC_AB1	2mit	-0.060905867055275	0.75163296609107	1	pseudobulk	DESeq2	LRT	0
89	KC_AB1	312	0.278161194607222	0.538486378403988	1	pseudobulk	DESeq2	LRT	0
90	KC_AB1	4E-T	-0.463905455857595	0.291750671899864	1	pseudobulk	DESeq2	LRT	0
91	KC_AB1	5-HT1A	0.0952973700549798	0.671255485400968	1	pseudobulk	DESeq2	LRT	0
92	KC_AB1	5-HT1B	0.400042763196106	0.366447209881136	1	pseudobulk	DESeq2	LRT	0
93	KC_AB1	5-HT2A	-0.10506771737906	1	1	pseudobulk	DESeq2	LRT	0
94	KC_AB1	5-HT2B	1.55381337748976	0.0750545155903152	1	pseudobulk	DESeq2	LRT	0
95	KC_AB1	5.8SrRNA-Psi:CR4	-0.0065790798335215	1	1	pseudobulk	DESeq2	LRT	0
96	KC_AB1	5.8SrRNA-Psi:CR4	-0.564356698759404	0.803702590456371	1	pseudobulk	DESeq2	LRT	0
97	KC_AB1	5.8SrRNA-Psi:CR4	0.849606887838128	1	1	pseudobulk	DESeq2	LRT	0
98	KC_AB1	5PtaseI	-0.0523678684756901	0.547950624290497	1	pseudobulk	DESeq2	LRT	0
99	KC_AB1	7B2	0.0007949717647494	0.600819066839301	1	pseudobulk	DESeq2	LRT	0
100	KC_AB1	a	0.0161225092262836	0.932077286253461	1	pseudobulk	DESeq2	LRT	0
101	KC_AB1	A16	-0.476104292941614	0.550858396809943	1	pseudobulk	DESeq2	LRT	0
102	KC_AB1	a6	-0.106046795365338	0.716643551183048	1	pseudobulk	DESeq2	LRT	0
103	KC_AB1	AANAT1	-0.0078374109612325	0.514355023786346	1	pseudobulk	DESeq2	LRT	0
104	KC_AB1	AANATL2	0.849606887838128	1	1	pseudobulk	DESeq2	LRT	0

105	KC_AB1	AANATL7	-1.198186108106	0.334231603007075	1	pseudobulk	DESeq2	LRT	0
106	KC_AB1	Aasdh	-0.86895624028877	0.460839891209034	1	pseudobulk	DESeq2	LRT	0
107	KC_AB1	Aatf	-0.112781044462648	0.7654255860441	1	pseudobulk	DESeq2	LRT	0
108	KC_AB1	aay	1.18918094830324	0.207449319737647	1	pseudobulk	DESeq2	LRT	0
109	KC_AB1	ab	-1.03937392314279	0.164827412474705	1	pseudobulk	DESeq2	LRT	0
110	KC_AB1	ABCA	-0.266390656481678	0.632316064710968	1	pseudobulk	DESeq2	LRT	0
111	KC_AB1	ABCB7	0.939187554901897	0.155537960634725	1	pseudobulk	DESeq2	LRT	0
112	KC_AB1	ABCD	0.233546897033327	0.454057548244758	1	pseudobulk	DESeq2	LRT	0
113	KC_AB1	Abd-B	0.849606887838128	1	1	pseudobulk	DESeq2	LRT	0
114	KC_AB1	Abi	-0.215842067485953	0.561442488752896	1	pseudobulk	DESeq2	LRT	0
115	KC_AB1	Abl	0.280855886783895	0.226371222158122	1	pseudobulk	DESeq2	LRT	0
116	KC_AB1	Abp1	-0.438449163952333	0.419217265069106	1	pseudobulk	DESeq2	LRT	0
117	KC_AB1	abs	0.718343865880961	0.599099962372323	1	pseudobulk	DESeq2	LRT	0
118	KC_AB1	Ac13E	3.08753393561965	0.382167145512329	1	pseudobulk	DESeq2	LRT	0
119	KC_AB1	Ac3	-0.276747740269355	0.472756617105766	1	pseudobulk	DESeq2	LRT	0
120	KC_AB1	Ac76E	0.691076284329732	0.573216197570888	1	pseudobulk	DESeq2	LRT	0
121	KC_AB1	Ac78C	0.0904589351007534	0.829728786182411	1	pseudobulk	DESeq2	LRT	0
122	KC_AB1	Acbp1	-0.21528832862625	0.442614116631857	1	pseudobulk	DESeq2	LRT	0
123	KC_AB1	Acbp2	1.41455081392864	0.169241503921145	1	pseudobulk	DESeq2	LRT	0
124	KC_AB1	ACC	-0.262169878360119	0.612359175348349	1	pseudobulk	DESeq2	LRT	0
125	KC_AB1	AcCoAS	-0.0785183662228638	0.582099019484912	1	pseudobulk	DESeq2	LRT	0
126	KC_AB1	Ace	0.444475596867021	0.373961374651706	1	pseudobulk	DESeq2	LRT	0
127	KC_AB1	Acer	1.63711542526446	0.293838269137575	1	pseudobulk	DESeq2	LRT	0
128	KC_AB1	Acf	0.587175363078602	0.392670239018917	1	pseudobulk	DESeq2	LRT	0
129	KC_AB1	achi	-1.61736334507379	0.343055968929855	1	pseudobulk	DESeq2	LRT	0
130	KC_AB1	Achl	2.68993930934622	0.478412374021253	1	pseudobulk	DESeq2	LRT	0
131	KC_AB1	acj6	0.849606887838128	1	1	pseudobulk	DESeq2	LRT	0
132	KC_AB1	Ack	-0.0909974101819327	0.70965652696223	1	pseudobulk	DESeq2	LRT	0
133	KC_AB1	Ack-like	0.0288595320428082	0.879804192776861	1	pseudobulk	DESeq2	LRT	0
134	KC_AB1	Acn	-0.367378234664277	0.393151452779345	1	pseudobulk	DESeq2	LRT	0
135	KC_AB1	Acp1	0.769893673767581	1	1	pseudobulk	DESeq2	LRT	0
136	KC_AB1	Acp36DE	2.78674512494791	0.54962276837928	1	pseudobulk	DESeq2	LRT	0
137	KC_AB1	Acp53C14b	0.849606887838128	1	1	pseudobulk	DESeq2	LRT	0
138	KC_AB1	Acp53Ea	2.78674512494791	0.54962276837928	1	pseudobulk	DESeq2	LRT	0
139	KC_AB1	Acp63F	0.849606887838128	1	1	pseudobulk	DESeq2	LRT	0
140	KC_AB1	Acph-1	-0.732045466934019	0.576416548263396	1	pseudobulk	DESeq2	LRT	0
141	KC_AB1	Acsl	0.16373799269102	0.546375872668487	1	pseudobulk	DESeq2	LRT	0
142	KC_AB1	Act57B	0.849606887838128	1	1	pseudobulk	DESeq2	LRT	0
143	KC_AB1	Act5C	0.326470779822806	0.148173388953326	1	pseudobulk	DESeq2	LRT	0
144	KC_AB1	Act79B	2.78674512494791	0.54962276837928	1	pseudobulk	DESeq2	LRT	0
145	KC_AB1	Act87E	2.31997030691033	0.813169507846918	1	pseudobulk	DESeq2	LRT	0
146	KC_AB1	Actbeta	0.0476841464187501	0.484459220821554	1	pseudobulk	DESeq2	LRT	0
147	KC_AB1	Actn	0.582673624301417	0.476326241865138	1	pseudobulk	DESeq2	LRT	0
148	KC_AB1	ACXD	1.34102046816198	0.79729618590623	1	pseudobulk	DESeq2	LRT	0
149	KC_AB1	Acyp2	0.302359884597569	0.569360369923163	1	pseudobulk	DESeq2	LRT	0
150	KC_AB1	Ada	0.237022465848507	1	1	pseudobulk	DESeq2	LRT	0
151	KC_AB1	Ada1-1	-1.00017018989402	0.509074585551041	1	pseudobulk	DESeq2	LRT	0
152	KC_AB1	Ada1-2	1.26241255734151	0.486661641911772	1	pseudobulk	DESeq2	LRT	0
153	KC_AB1	Ada2a	1.4229055769363	0.771821430526778	1	pseudobulk	DESeq2	LRT	0
154	KC_AB1	Ada2b	0.0759160309333391	0.724178374215126	1	pseudobulk	DESeq2	LRT	0
155	KC_AB1	Ada3	-0.0551155883516888	0.766871377131997	1	pseudobulk	DESeq2	LRT	0
156	KC_AB1	AdamTS-A	-1.5805998677877	0.524230026009756	1	pseudobulk	DESeq2	LRT	0
157	KC_AB1	AdamTS-B	-1.25393399638069	0.612054104673377	1	pseudobulk	DESeq2	LRT	0

158	KC_AB1	Adar	0.0791455437463415	0.677492923396148	1	pseudobulk	DESeq2	LRT	0
159	KC_AB1	Adat1	1.09242128868511	0.901015655054996	1	pseudobulk	DESeq2	LRT	0
160	KC_AB1	Adck5	-0.797252183417281	0.490620184909438	1	pseudobulk	DESeq2	LRT	0
161	KC_AB1	ADD1	-0.211856638396092	0.759687322820635	1	pseudobulk	DESeq2	LRT	0
162	KC_AB1	AdenoK	-0.668606200682696	0.499718218988108	1	pseudobulk	DESeq2	LRT	0
163	KC_AB1	Adf1	-0.292941628736463	0.512553286508322	1	pseudobulk	DESeq2	LRT	0
164	KC_AB1	Adgf-D	0.751302237845922	1	1	pseudobulk	DESeq2	LRT	0
165	KC_AB1	Adi1	-0.146751556634271	0.765246854072479	1	pseudobulk	DESeq2	LRT	0
166	KC_AB1	AdipoR	-0.388889450002095	0.336204346183988	1	pseudobulk	DESeq2	LRT	0
167	KC_AB1	Adk1	-0.175716095024412	0.704719889325829	1	pseudobulk	DESeq2	LRT	0
168	KC_AB1	Adk2	-0.0512532535626096	0.477250931753472	1	pseudobulk	DESeq2	LRT	0
169	KC_AB1	Adk3	-1.22642399786237	0.413750197695165	1	pseudobulk	DESeq2	LRT	0
170	KC_AB1	adp	-0.394478168625197	0.633154649900118	1	pseudobulk	DESeq2	LRT	0
171	KC_AB1	ADPS	-0.192208953455337	0.774123219555679	1	pseudobulk	DESeq2	LRT	0
172	KC_AB1	AdSL	0.0047555593347729	0.80148752657842	1	pseudobulk	DESeq2	LRT	0
173	KC_AB1	AdSS	-0.154818528816661	0.526444377449873	1	pseudobulk	DESeq2	LRT	0
174	KC_AB1	Aduk	0.672302588966555	0.381608210094449	1	pseudobulk	DESeq2	LRT	0
175	KC_AB1	Ady43A	-0.218186128802392	0.691806672948089	1	pseudobulk	DESeq2	LRT	0
176	KC_AB1	Aef1	-0.367710894456933	0.36462549911621	1	pseudobulk	DESeq2	LRT	0
177	KC_AB1	aft	-0.517122225038665	0.714347075545985	1	pseudobulk	DESeq2	LRT	0
178	KC_AB1	Afti	-0.57984151972585	0.384491352287029	1	pseudobulk	DESeq2	LRT	0
179	KC_AB1	Ag5r	0.672748670242853	0.431534282383807	1	pseudobulk	DESeq2	LRT	0
180	KC_AB1	Ag5r2	2.19691424616663	0.593804541109954	1	pseudobulk	DESeq2	LRT	0
181	KC_AB1	AGBE	3.20547843809887	0.383970068137593	1	pseudobulk	DESeq2	LRT	0
182	KC_AB1	ago	-1.15099599333651	0.17836242919644	1	pseudobulk	DESeq2	LRT	0
183	KC_AB1	AGO1	0.0926119291197732	0.464983695827867	1	pseudobulk	DESeq2	LRT	0
184	KC_AB1	AGO2	-0.338439662944973	0.465003380656583	1	pseudobulk	DESeq2	LRT	0
185	KC_AB1	AGO3	-0.641578582194754	0.511042913288835	1	pseudobulk	DESeq2	LRT	0
186	KC_AB1	Agpat1	-1.42050143506042	0.295983893672374	1	pseudobulk	DESeq2	LRT	0
187	KC_AB1	Agpat2	2.13009086641515	0.809121577229065	1	pseudobulk	DESeq2	LRT	0
188	KC_AB1	Agpat3	-0.255746825712294	0.478435071740088	1	pseudobulk	DESeq2	LRT	0
189	KC_AB1	agt	0.806106592302826	1	1	pseudobulk	DESeq2	LRT	0
190	KC_AB1	Ahcy	-0.888177784936213	0.552510090899109	1	pseudobulk	DESeq2	LRT	0
191	KC_AB1	AhcyL1	-0.371768247461379	0.497531767566392	1	pseudobulk	DESeq2	LRT	0
192	KC_AB1	AIF	-0.104782725303815	0.668082541082161	1	pseudobulk	DESeq2	LRT	0
193	KC_AB1	AIMP1	-0.267842275927345	0.693514206096691	1	pseudobulk	DESeq2	LRT	0
194	KC_AB1	AIMP2	-0.176613354179858	0.662831175897079	1	pseudobulk	DESeq2	LRT	0
195	KC_AB1	AIMP3	0.417337832797945	0.385484495896783	1	pseudobulk	DESeq2	LRT	0
196	KC_AB1	Ak6	-0.632746841153161	0.544417006382791	1	pseudobulk	DESeq2	LRT	0
197	KC_AB1	Akap200	-0.0969931207179119	0.741583307475671	1	pseudobulk	DESeq2	LRT	0
198	KC_AB1	akirin	0.383811865905514	0.178998389719173	1	pseudobulk	DESeq2	LRT	0
199	KC_AB1	Akt1	-0.81947160462718	0.210149959326533	1	pseudobulk	DESeq2	LRT	0
200	KC_AB1	Aladin	1.02686918315853	0.237321350358104	1	pseudobulk	DESeq2	LRT	0
201	KC_AB1	AlaRS	-1.56643461440907	0.219073745893137	1	pseudobulk	DESeq2	LRT	0
202	KC_AB1	AlaRS-m	-0.995303284311191	0.502580488424685	1	pseudobulk	DESeq2	LRT	0
203	KC_AB1	Alas	0.11326575548193	0.756455900055514	1	pseudobulk	DESeq2	LRT	0
204	KC_AB1	alc	0.291619051869946	0.504268326148356	1	pseudobulk	DESeq2	LRT	0
205	KC_AB1	Ald1	0.14812892754747	0.555559261326494	1	pseudobulk	DESeq2	LRT	0
206	KC_AB1	Aldh	1.37129945894492	1	1	pseudobulk	DESeq2	LRT	0
207	KC_AB1	Aldh-III	-0.243072255480263	0.590202486580924	1	pseudobulk	DESeq2	LRT	0
208	KC_AB1	Aldh7A1	-0.601783288718601	0.7767390045737	1	pseudobulk	DESeq2	LRT	0
209	KC_AB1	Alg-2	0.177981837305102	0.688420469424937	1	pseudobulk	DESeq2	LRT	0
210	KC_AB1	Alg1	-0.345256895221066	0.683862191781536	1	pseudobulk	DESeq2	LRT	0

211	KC_AB1	Alg10	-1.01270333661881	0.359186296020102	1	pseudobulk	DESeq2	LRT		0
212	KC_AB1	Alg11	0.414096292528924	0.403518524445212	1	pseudobulk	DESeq2	LRT		0
213	KC_AB1	Alg14	-0.45385500396076	0.652491609447022	1	pseudobulk	DESeq2	LRT		0
214	KC_AB1	Alg2	0.355449478668943	0.740960901490723	1	pseudobulk	DESeq2	LRT		0
215	KC_AB1	Alg3	0.619268771877414	0.572503754093251	1	pseudobulk	DESeq2	LRT		0
216	KC_AB1	Alg9	0.552797829073086	0.890778049133657	1	pseudobulk	DESeq2	LRT		0
217	KC_AB1	Alh	-0.0405880672696755	0.772451282424027	1	pseudobulk	DESeq2	LRT		0
218	KC_AB1	alien	0.277068574670503	0.632421439221381	1	pseudobulk	DESeq2	LRT		0
219	KC_AB1	ALiX	-0.149674372875759	0.725064779107627	1	pseudobulk	DESeq2	LRT		0
220	KC_AB1	Alk	-0.34931917441828	0.0849126227661847	1	pseudobulk	DESeq2	LRT		0
221	KC_AB1	Alp12	0.183159795463042	1	1	pseudobulk	DESeq2	LRT		0
222	KC_AB1	Alp4	0.849606887838128	1	1	pseudobulk	DESeq2	LRT		0
223	KC_AB1	Alp6	2.06541384650887	0.768363369045814	1	pseudobulk	DESeq2	LRT		0
224	KC_AB1	alph	-0.193789491840816	0.442067449760715	1	pseudobulk	DESeq2	LRT		0
225	KC_AB1	alpha-Cat	-0.080461758301697	0.747687695273731	1	pseudobulk	DESeq2	LRT		0
226	KC_AB1	alpha-Catr	-0.0686917699586224	0.878394481567865	1	pseudobulk	DESeq2	LRT		0
227	KC_AB1	alpha-Est4	2.78674512494791	0.54962276837928	1	pseudobulk	DESeq2	LRT		0
228	KC_AB1	alpha-Est8	0.211574452274434	0.488571863672787	1	pseudobulk	DESeq2	LRT		0
229	KC_AB1	alpha-Est9	-0.452267910696792	0.34298545126177	1	pseudobulk	DESeq2	LRT		0
230	KC_AB1	alpha-Man-Ia	0.270488787898664	0.197936349170398	1	pseudobulk	DESeq2	LRT		0
231	KC_AB1	alpha-Man-Ib	-0.303361940971253	0.453944859977209	1	pseudobulk	DESeq2	LRT		0
232	KC_AB1	alpha-Man-IIa	-0.151742465556529	0.674722222208337	1	pseudobulk	DESeq2	LRT		0
233	KC_AB1	alpha-Man-IIb	0.0155253686904648	0.905685914726747	1	pseudobulk	DESeq2	LRT		0
234	KC_AB1	alpha-PheRS	0.538810562613336	0.608264468276246	1	pseudobulk	DESeq2	LRT		0
235	KC_AB1	alpha-Spec	-0.0506196840780242	0.801727166314574	1	pseudobulk	DESeq2	LRT		0
236	KC_AB1	alpha4GT1	-0.619972731854131	0.295866353308012	1	pseudobulk	DESeq2	LRT		0
237	KC_AB1	alpha4GT2	1.33016929913258	1	1	pseudobulk	DESeq2	LRT		0
238	KC_AB1	alphaCOP	1.51572339922816	0.0586278262069125	1	pseudobulk	DESeq2	LRT		0
239	KC_AB1	alphaKap4	-0.790596054643963	0.667058293819746	1	pseudobulk	DESeq2	LRT		0
240	KC_AB1	alphaSnap	-0.24329548820738	0.403753795954488	1	pseudobulk	DESeq2	LRT		0
241	KC_AB1	alphaTub67C	1.26095156218009	0.653320799429466	1	pseudobulk	DESeq2	LRT		0
242	KC_AB1	alphaTub84B	-0.0219140225653545	0.668215744630982	1	pseudobulk	DESeq2	LRT		0
243	KC_AB1	alphaTub84D	0.0050260575763776	0.916673641396607	1	pseudobulk	DESeq2	LRT		0
244	KC_AB1	Alr	-0.407252691649509	0.5933797316151	1	pseudobulk	DESeq2	LRT		0
245	KC_AB1	alrm	-0.711192148130106	0.51733961928359	1	pseudobulk	DESeq2	LRT		0
246	KC_AB1	Als2	-0.384730196798372	0.544903726739813	1	pseudobulk	DESeq2	LRT		0
247	KC_AB1	alt	-0.105227405061371	0.780576260434926	1	pseudobulk	DESeq2	LRT		0
248	KC_AB1	Amacr	-0.104449242834156	0.433000412519945	1	pseudobulk	DESeq2	LRT		0
249	KC_AB1	amn	-0.915307867034278	0.378512450947941	1	pseudobulk	DESeq2	LRT		0
250	KC_AB1	Amnionless	0.0870821993996777	0.860697787850464	1	pseudobulk	DESeq2	LRT		0
251	KC_AB1	amon	-0.283794769952875	0.0999961016161937	1	pseudobulk	DESeq2	LRT		0
252	KC_AB1	AMPdeam	-0.729658983707845	0.229539109160258	1	pseudobulk	DESeq2	LRT		0
253	KC_AB1	Amph	-0.37212581298142	0.399500794957958	1	pseudobulk	DESeq2	LRT		0
254	KC_AB1	AMPKalpha	-0.093537741924011	0.830797753484026	1	pseudobulk	DESeq2	LRT		0
255	KC_AB1	Amun	-0.619352157830924	0.237196297330606	1	pseudobulk	DESeq2	LRT		0
256	KC_AB1	amx	-0.556573541794494	0.479977782421208	1	pseudobulk	DESeq2	LRT		0
257	KC_AB1	Amyrel	1.60090069278089	0.173809202492446	1	pseudobulk	DESeq2	LRT		0
258	KC_AB1	ana	1.43060365239813	1	1	pseudobulk	DESeq2	LRT		0
259	KC_AB1	ana2	0.237022465848507	1	1	pseudobulk	DESeq2	LRT		0
260	KC_AB1	ana3	0.849606887838128	1	1	pseudobulk	DESeq2	LRT		0
261	KC_AB1	Ance-3	0.849606887838128	1	1	pseudobulk	DESeq2	LRT		0
262	KC_AB1	anchor	0.18305301669646	0.635297854992975	1	pseudobulk	DESeq2	LRT		0
263	KC_AB1	angel	0.367598322052773	0.751069555033464	1	pseudobulk	DESeq2	LRT		0

264	KC_AB1	Ank	-0.287465196202177	0.297218946880473	1	pseudobulk	DESeq2	LRT		0
265	KC_AB1	Ank2	-0.161042835822007	0.237132300372313	1	pseudobulk	DESeq2	LRT		0
266	KC_AB1	Ankle2	-0.622343721911063	0.501313708507272	1	pseudobulk	DESeq2	LRT		0
267	KC_AB1	anne	-0.0332182284551435	0.506357837803483	1	pseudobulk	DESeq2	LRT		0
268	KC_AB1	Ant2	0.138525318549997	1	1	pseudobulk	DESeq2	LRT		0
269	KC_AB1	antr	2.31997030691033	0.813169507846918	1	pseudobulk	DESeq2	LRT		0
270	KC_AB1	AnxB10	0.193852133210078	0.727803349804936	1	pseudobulk	DESeq2	LRT		0
271	KC_AB1	AnxB11	0.281544684297209	0.578133069033619	1	pseudobulk	DESeq2	LRT		0
272	KC_AB1	aop	-0.375766302974801	0.539447714153295	1	pseudobulk	DESeq2	LRT		0
273	KC_AB1	aos	0.849606887838128	1	1	pseudobulk	DESeq2	LRT		0
274	KC_AB1	Aos1	0.761515569269224	0.476042524214743	1	pseudobulk	DESeq2	LRT		0
275	KC_AB1	ap	0.116783351990007	1	1	pseudobulk	DESeq2	LRT		0
276	KC_AB1	AP-1-2beta	-0.113034793973805	0.734815449847684	1	pseudobulk	DESeq2	LRT		0
277	KC_AB1	AP-1gamma	-0.0959782932027446	0.706028441193053	1	pseudobulk	DESeq2	LRT		0
278	KC_AB1	AP-1mu	-0.0666607483580713	0.884569890844191	1	pseudobulk	DESeq2	LRT		0
279	KC_AB1	AP-1sigma	-0.0362380308687093	0.902408014350305	1	pseudobulk	DESeq2	LRT		0
280	KC_AB1	AP-2alpha	-0.242744592135089	0.344624091691372	1	pseudobulk	DESeq2	LRT		0
281	KC_AB1	AP-2mu	0.212005546793164	0.638670768910299	1	pseudobulk	DESeq2	LRT		0
282	KC_AB1	AP-2sigma	0.274357081021247	0.275078631472301	1	pseudobulk	DESeq2	LRT		0
283	KC_AB1	Apc	-0.303420638607935	0.372546477428896	1	pseudobulk	DESeq2	LRT		0
284	KC_AB1	APC10	-0.592022180789313	0.477136707273572	1	pseudobulk	DESeq2	LRT		0
285	KC_AB1	Apc2	-0.216210029276812	1	1	pseudobulk	DESeq2	LRT		0
286	KC_AB1	APC4	0.376431477381175	1	1	pseudobulk	DESeq2	LRT		0
287	KC_AB1	ApepP	-1.7019327049077	0.373659531810403	1	pseudobulk	DESeq2	LRT		0
288	KC_AB1	Apf	1.18950564134102	0.255703474332739	1	pseudobulk	DESeq2	LRT		0
289	KC_AB1	aph-1	-0.499910209269756	0.360250489016492	1	pseudobulk	DESeq2	LRT		0
290	KC_AB1	aPKC	-0.0506100692800903	0.834352719132809	1	pseudobulk	DESeq2	LRT		0
291	KC_AB1	Apl	-0.453264059247586	0.663077417474921	1	pseudobulk	DESeq2	LRT		0
292	KC_AB1	Aplip1	-0.173362930379389	0.420331871516466	1	pseudobulk	DESeq2	LRT		0
293	KC_AB1	apolpp	-0.179346935229247	0.77486714081092	1	pseudobulk	DESeq2	LRT		0
294	KC_AB1	Apoltp	-0.217196733655969	0.690476388014154	1	pseudobulk	DESeq2	LRT		0
295	KC_AB1	app	-3.15285430999518	0.0673817458757195	1	pseudobulk	DESeq2	LRT		0
296	KC_AB1	APP-BP1	0.0093515250017868	0.822169271874785	1	pseudobulk	DESeq2	LRT		0
297	KC_AB1	Appl	-0.0520503298541005	0.711227767406242	1	pseudobulk	DESeq2	LRT		0
298	KC_AB1	Aprt	-0.146916748239051	0.811702674040931	1	pseudobulk	DESeq2	LRT		0
299	KC_AB1	Aps	-0.204597900210905	0.615827978780326	1	pseudobulk	DESeq2	LRT		0
300	KC_AB1	apt	-1.25393399638069	0.612054104673377	1	pseudobulk	DESeq2	LRT		0
301	KC_AB1	aqz	-0.0975918491972279	0.527525273965493	1	pseudobulk	DESeq2	LRT		0
302	KC_AB1	aralar1	0.63039452256801	0.149447089429076	1	pseudobulk	DESeq2	LRT		0
303	KC_AB1	Arc1	0.222381330747391	0.909737123604422	1	pseudobulk	DESeq2	LRT		0
304	KC_AB1	Arc2	0.849606887838128	1	1	pseudobulk	DESeq2	LRT		0
305	KC_AB1	Arc42	0.0160940546605626	1	1	pseudobulk	DESeq2	LRT		0
306	KC_AB1	Archease	-0.494817221611313	0.57726248349831	1	pseudobulk	DESeq2	LRT		0
307	KC_AB1	Arf102F	0.374147463694957	0.241773322506527	1	pseudobulk	DESeq2	LRT		0
308	KC_AB1	Arf51F	-0.379832216113123	0.390562994092613	1	pseudobulk	DESeq2	LRT		0
309	KC_AB1	Arf79F	0.236044281591309	0.265511342744633	1	pseudobulk	DESeq2	LRT		0
310	KC_AB1	ArfGAP1	-0.129095386599299	0.647292428354372	1	pseudobulk	DESeq2	LRT		0
311	KC_AB1	ArfGAP3	0.182066670886197	0.718985162689858	1	pseudobulk	DESeq2	LRT		0
312	KC_AB1	Arfip	-0.250416313461835	0.664022951759731	1	pseudobulk	DESeq2	LRT		0
313	KC_AB1	Arfrp1	-0.0214996347178565	0.853996660197828	1	pseudobulk	DESeq2	LRT		0
314	KC_AB1	arg	-0.714388304116082	0.408552208933876	1	pseudobulk	DESeq2	LRT		0
315	KC_AB1	Argk	-0.0221130787577225	0.726364771296326	1	pseudobulk	DESeq2	LRT		0
316	KC_AB1	Argl	0.781801500057156	1	1	pseudobulk	DESeq2	LRT		0

317	KC_AB1	ArgRS	0.269810596488339	1	1	pseudobulk	DESeq2	LRT	0
318	KC_AB1	ArgRS-m	1.19440279227984	0.675542440826627	1	pseudobulk	DESeq2	LRT	0
319	KC_AB1	ari-1	-0.560843539370843	0.0814921272284992	1	pseudobulk	DESeq2	LRT	0
320	KC_AB1	ari-2	0.0012900387564592	0.801561846803474	1	pseudobulk	DESeq2	LRT	0
321	KC_AB1	Arl1	0.133225548001225	0.640207966365744	1	pseudobulk	DESeq2	LRT	0
322	KC_AB1	Arl2	-0.195735304196804	0.697681250767964	1	pseudobulk	DESeq2	LRT	0
323	KC_AB1	Arl4	0.174390484479537	0.544014892447891	1	pseudobulk	DESeq2	LRT	0
324	KC_AB1	Arl5	-0.117798741777494	0.695720503187336	1	pseudobulk	DESeq2	LRT	0
325	KC_AB1	Arl6IP1	-0.179399831412582	0.504613750868475	1	pseudobulk	DESeq2	LRT	0
326	KC_AB1	Arl8	-0.156562412606658	0.631357489652302	1	pseudobulk	DESeq2	LRT	0
327	KC_AB1	arm	0.0685642881323905	0.652851560958246	1	pseudobulk	DESeq2	LRT	0
328	KC_AB1	armi	-1.66345780610205	0.37434052101801	1	pseudobulk	DESeq2	LRT	0
329	KC_AB1	Arp1	-0.0509049762343968	0.761948938312861	1	pseudobulk	DESeq2	LRT	0
330	KC_AB1	Arp10	0.626856848091389	0.729577093067151	1	pseudobulk	DESeq2	LRT	0
331	KC_AB1	Arp2	-0.370713789509394	0.468024491575807	1	pseudobulk	DESeq2	LRT	0
332	KC_AB1	Arp3	-0.262003438367336	0.555972888963742	1	pseudobulk	DESeq2	LRT	0
333	KC_AB1	Arp5	-0.470417067746284	0.636862672037165	1	pseudobulk	DESeq2	LRT	0
334	KC_AB1	Arp6	0.531031608628823	0.583391654713958	1	pseudobulk	DESeq2	LRT	0
335	KC_AB1	Arp8	0.35171002352277	0.801476304668637	1	pseudobulk	DESeq2	LRT	0
336	KC_AB1	Arpc1	-0.282373245069189	0.499694456923693	1	pseudobulk	DESeq2	LRT	0
337	KC_AB1	Arpc2	0.0461987565123012	0.856362184217274	1	pseudobulk	DESeq2	LRT	0
338	KC_AB1	Arpc3A	-0.186739597030954	0.657041104833387	1	pseudobulk	DESeq2	LRT	0
339	KC_AB1	Arpc4	0.616734936494706	0.243321552227403	1	pseudobulk	DESeq2	LRT	0
340	KC_AB1	Arpc5	0.312334678003687	0.350829307553853	1	pseudobulk	DESeq2	LRT	0
341	KC_AB1	arr	0.280086783651792	0.465438940878828	1	pseudobulk	DESeq2	LRT	0
342	KC_AB1	Ars2	-0.220020437887023	0.612058714565866	1	pseudobulk	DESeq2	LRT	0
343	KC_AB1	Art1	0.214165175742086	1	1	pseudobulk	DESeq2	LRT	0
344	KC_AB1	Art3	-0.0806766248263104	1	1	pseudobulk	DESeq2	LRT	0
345	KC_AB1	Art4	0.796323406385266	0.362607235022707	1	pseudobulk	DESeq2	LRT	0
346	KC_AB1	Art7	0.849606887838128	1	1	pseudobulk	DESeq2	LRT	0
347	KC_AB1	Art8	0.849606887838128	1	1	pseudobulk	DESeq2	LRT	0
348	KC_AB1	Arts	-0.748850715961542	0.63180991411194	1	pseudobulk	DESeq2	LRT	0
349	KC_AB1	aru	-1.10012950436128	0.546091009501692	1	pseudobulk	DESeq2	LRT	0
350	KC_AB1	Arv1	0.519983821193156	0.640233927277579	1	pseudobulk	DESeq2	LRT	0
351	KC_AB1	Asap	-0.238341671736237	0.428477554140613	1	pseudobulk	DESeq2	LRT	0
352	KC_AB1	Asator	-0.180499919627307	0.379231828036634	1	pseudobulk	DESeq2	LRT	0
353	KC_AB1	Asciz	1.05813547642144	0.715224126413024	1	pseudobulk	DESeq2	LRT	0
354	KC_AB1	asf1	-0.301571749817412	0.585581327841257	1	pseudobulk	DESeq2	LRT	0
355	KC_AB1	ash1	-0.265937545388062	0.525426909915437	1	pseudobulk	DESeq2	LRT	0
356	KC_AB1	ash2	-1.00424456266051	0.491231383578524	1	pseudobulk	DESeq2	LRT	0
357	KC_AB1	Ask1	0.328765227685698	0.675719972977511	1	pseudobulk	DESeq2	LRT	0
358	KC_AB1	asl	0.183159795463042	1	1	pseudobulk	DESeq2	LRT	0
359	KC_AB1	AsnRS	-1.28144093141294	0.241926009067995	1	pseudobulk	DESeq2	LRT	0
360	KC_AB1	AsnRS-m	0.475419692279278	1	1	pseudobulk	DESeq2	LRT	0
361	KC_AB1	AsnS	0.183159795455075	1	1	pseudobulk	DESeq2	LRT	0
362	KC_AB1	Asph	3.41844045086015	0.351942490385471	1	pseudobulk	DESeq2	LRT	0
363	KC_AB1	ASPP	1.84981579152528	0.66160499764878	1	pseudobulk	DESeq2	LRT	0
364	KC_AB1	AspRS	-1.34285870030088	0.385182738666667	1	pseudobulk	DESeq2	LRT	0
365	KC_AB1	AspRS-m	0.455084055243365	0.293123054674257	1	pseudobulk	DESeq2	LRT	0
366	KC_AB1	asrij	0.447625650545051	0.351609777924426	1	pseudobulk	DESeq2	LRT	0
367	KC_AB1	asRNA:ASTR	0.849606887838128	1	1	pseudobulk	DESeq2	LRT	0
368	KC_AB1	asRNA:CR30267	0.348844643305223	1	1	pseudobulk	DESeq2	LRT	0
369	KC_AB1	asRNA:CR31144	0.237022465848507	1	1	pseudobulk	DESeq2	LRT	0

370	KC_AB1	asRNA:CR31429	0.849606887838128	1	1	pseudobulk	DESeq2	LRT	0
371	KC_AB1	asRNA:CR31912	0.35583936471955	0.832576412259691	1	pseudobulk	DESeq2	LRT	0
372	KC_AB1	asRNA:CR42254	0.769502227940538	1	1	pseudobulk	DESeq2	LRT	0
373	KC_AB1	asRNA:CR42547	1.25184190956606	0.64126412515282	1	pseudobulk	DESeq2	LRT	0
374	KC_AB1	asRNA:CR42860	0.849606887838128	1	1	pseudobulk	DESeq2	LRT	0
375	KC_AB1	asRNA:CR43159	0.183159795455075	1	1	pseudobulk	DESeq2	LRT	0
376	KC_AB1	asRNA:CR43259	1.21267130850476	1	1	pseudobulk	DESeq2	LRT	0
377	KC_AB1	asRNA:CR43425	-0.4747256355529	0.404228263954485	1	pseudobulk	DESeq2	LRT	0
378	KC_AB1	asRNA:CR43426	2.71397898638463	0.463880806276523	1	pseudobulk	DESeq2	LRT	0
379	KC_AB1	asRNA:CR43460	-0.195251010403479	0.890027049148088	1	pseudobulk	DESeq2	LRT	0
380	KC_AB1	asRNA:CR43466	2.31997030691033	0.813169507846918	1	pseudobulk	DESeq2	LRT	0
381	KC_AB1	asRNA:CR43609	1.59863986092676	1	1	pseudobulk	DESeq2	LRT	0
382	KC_AB1	asRNA:CR43629	-1.2303566332608	0.340015739445654	1	pseudobulk	DESeq2	LRT	0
383	KC_AB1	asRNA:CR43786	0.849606887838128	1	1	pseudobulk	DESeq2	LRT	0
384	KC_AB1	asRNA:CR43809	1.59863986092676	1	1	pseudobulk	DESeq2	LRT	0
385	KC_AB1	asRNA:CR43820	0.0160940546605626	1	1	pseudobulk	DESeq2	LRT	0
386	KC_AB1	asRNA:CR43833	0.849606887838128	1	1	pseudobulk	DESeq2	LRT	0
387	KC_AB1	asRNA:CR43842	0.0075578628771858	0.902546991606208	1	pseudobulk	DESeq2	LRT	0
388	KC_AB1	asRNA:CR43865	0.742456727585392	1	1	pseudobulk	DESeq2	LRT	0
389	KC_AB1	asRNA:CR43875	0.849606887838128	1	1	pseudobulk	DESeq2	LRT	0
390	KC_AB1	asRNA:CR43889	0.769893673767581	1	1	pseudobulk	DESeq2	LRT	0
391	KC_AB1	asRNA:CR43906	0.480041951352051	0.7585269425535	1	pseudobulk	DESeq2	LRT	0
392	KC_AB1	asRNA:CR43933	0.914725505927293	0.715675365336446	1	pseudobulk	DESeq2	LRT	0
393	KC_AB1	asRNA:CR43944	1.24634228756186	0.713440436700707	1	pseudobulk	DESeq2	LRT	0
394	KC_AB1	asRNA:CR43957	-0.55178066149661	0.729427371202381	1	pseudobulk	DESeq2	LRT	0
395	KC_AB1	asRNA:CR43959	-0.0021789030708211	0.915999085813942	1	pseudobulk	DESeq2	LRT	0
396	KC_AB1	asRNA:CR43964	0.0160940546605174	1	1	pseudobulk	DESeq2	LRT	0
397	KC_AB1	asRNA:CR43979	2.78674512494791	0.54962276837928	1	pseudobulk	DESeq2	LRT	0
398	KC_AB1	asRNA:CR43981	2.78674512494791	0.54962276837928	1	pseudobulk	DESeq2	LRT	0
399	KC_AB1	asRNA:CR44018	0.494670098792379	0.628064812089702	1	pseudobulk	DESeq2	LRT	0
400	KC_AB1	asRNA:CR44027	-0.381578022779931	0.676900412598846	1	pseudobulk	DESeq2	LRT	0
401	KC_AB1	asRNA:CR44029	-0.374964549248688	0.594637091772331	1	pseudobulk	DESeq2	LRT	0
402	KC_AB1	asRNA:CR44030	-0.143538693223024	0.754194137058593	1	pseudobulk	DESeq2	LRT	0
403	KC_AB1	asRNA:CR44031	-0.452992883833848	0.722580117488299	1	pseudobulk	DESeq2	LRT	0
404	KC_AB1	asRNA:CR44045	0.849606887838128	1	1	pseudobulk	DESeq2	LRT	0
405	KC_AB1	asRNA:CR44046	0.566834853631585	0.803143554484192	1	pseudobulk	DESeq2	LRT	0
406	KC_AB1	asRNA:CR44047	0.849606887838128	1	1	pseudobulk	DESeq2	LRT	0
407	KC_AB1	asRNA:CR44049	2.31997030691033	0.813169507846918	1	pseudobulk	DESeq2	LRT	0
408	KC_AB1	asRNA:CR44054	0.116783351990007	1	1	pseudobulk	DESeq2	LRT	0
409	KC_AB1	asRNA:CR44065	0.237022465848507	1	1	pseudobulk	DESeq2	LRT	0
410	KC_AB1	asRNA:CR44069	1.59863986092676	1	1	pseudobulk	DESeq2	LRT	0
411	KC_AB1	asRNA:CR44093	1.33016929913258	1	1	pseudobulk	DESeq2	LRT	0
412	KC_AB1	asRNA:CR44095	0.849606887838128	1	1	pseudobulk	DESeq2	LRT	0
413	KC_AB1	asRNA:CR44106	1.40624762719955	0.571260018222558	1	pseudobulk	DESeq2	LRT	0
414	KC_AB1	asRNA:CR44107	-1.32956250134239	0.337210353535117	1	pseudobulk	DESeq2	LRT	0
415	KC_AB1	asRNA:CR44114	0.849606887838128	1	1	pseudobulk	DESeq2	LRT	0
416	KC_AB1	asRNA:CR44116	0.634960112221505	1	1	pseudobulk	DESeq2	LRT	0
417	KC_AB1	asRNA:CR44119	0.769522064442666	1	1	pseudobulk	DESeq2	LRT	0
418	KC_AB1	asRNA:CR44137	0.849606887838128	1	1	pseudobulk	DESeq2	LRT	0
419	KC_AB1	asRNA:CR44169	2.78674512494791	0.54962276837928	1	pseudobulk	DESeq2	LRT	0
420	KC_AB1	asRNA:CR44179	-1.20054795286106	0.522310314157169	1	pseudobulk	DESeq2	LRT	0
421	KC_AB1	asRNA:CR44228	0.695961368907295	0.897060839873424	1	pseudobulk	DESeq2	LRT	0
422	KC_AB1	asRNA:CR44266	2.31997030691033	0.813169507846918	1	pseudobulk	DESeq2	LRT	0

423	KC_AB1	asRNA:CR44340	-2.09645572734659	0.269393890185116	1	pseudobulk	DESeq2	LRT	0
424	KC_AB1	asRNA:CR44351	0.849606887838128	1	1	pseudobulk	DESeq2	LRT	0
425	KC_AB1	asRNA:CR44368	2.06541384650887	0.768363369045814	1	pseudobulk	DESeq2	LRT	0
426	KC_AB1	asRNA:CR44382	0.849606887838128	1	1	pseudobulk	DESeq2	LRT	0
427	KC_AB1	asRNA:CR44390	0.849606887838128	1	1	pseudobulk	DESeq2	LRT	0
428	KC_AB1	asRNA:CR44431	2.13009086641515	0.687046658320206	1	pseudobulk	DESeq2	LRT	0
429	KC_AB1	asRNA:CR44512	2.06541384650887	0.768363369045814	1	pseudobulk	DESeq2	LRT	0
430	KC_AB1	asRNA:CR44690	0.0160940546605626	1	1	pseudobulk	DESeq2	LRT	0
431	KC_AB1	asRNA:CR44724	-0.267798911930929	1	1	pseudobulk	DESeq2	LRT	0
432	KC_AB1	asRNA:CR44840	-0.404469744007938	0.734792506510936	1	pseudobulk	DESeq2	LRT	0
433	KC_AB1	asRNA:CR44841	0.849606887838128	1	1	pseudobulk	DESeq2	LRT	0
434	KC_AB1	asRNA:CR44843	1.59863986092676	1	1	pseudobulk	DESeq2	LRT	0
435	KC_AB1	asRNA:CR44892	1.71267232945624	0.415638712901798	1	pseudobulk	DESeq2	LRT	0
436	KC_AB1	asRNA:CR44894	0.128534093952184	0.74651211604111	1	pseudobulk	DESeq2	LRT	0
437	KC_AB1	asRNA:CR44960	-0.307631767383303	0.797088330828503	1	pseudobulk	DESeq2	LRT	0
438	KC_AB1	asRNA:CR44961	0.43253641533918	1	1	pseudobulk	DESeq2	LRT	0
439	KC_AB1	asRNA:CR44969	1.59863986092676	1	1	pseudobulk	DESeq2	LRT	0
440	KC_AB1	asRNA:CR44981	2.48009176776177	0.312384287758656	1	pseudobulk	DESeq2	LRT	0
441	KC_AB1	asRNA:CR44987	2.48417587387807	0.622507045344447	1	pseudobulk	DESeq2	LRT	0
442	KC_AB1	asRNA:CR44989	-0.930708748038168	0.483237865549271	1	pseudobulk	DESeq2	LRT	0
443	KC_AB1	asRNA:CR45000	1.59863986092676	1	1	pseudobulk	DESeq2	LRT	0
444	KC_AB1	asRNA:CR45007	1.59863986092676	1	1	pseudobulk	DESeq2	LRT	0
445	KC_AB1	asRNA:CR45009	1.82975745499112	0.336261307302743	1	pseudobulk	DESeq2	LRT	0
446	KC_AB1	asRNA:CR45016	0.930993226399931	1	1	pseudobulk	DESeq2	LRT	0
447	KC_AB1	asRNA:CR45026	2.78674512494791	0.54962276837928	1	pseudobulk	DESeq2	LRT	0
448	KC_AB1	asRNA:CR45041	2.04017343731623	0.530151172476187	1	pseudobulk	DESeq2	LRT	0
449	KC_AB1	asRNA:CR45044	2.31997030691033	0.813169507846918	1	pseudobulk	DESeq2	LRT	0
450	KC_AB1	asRNA:CR45053	2.31997030691033	0.813169507846918	1	pseudobulk	DESeq2	LRT	0
451	KC_AB1	asRNA:CR45124	0.0160940546605174	1	1	pseudobulk	DESeq2	LRT	0
452	KC_AB1	asRNA:CR45126	0.354104679756174	0.713683490682513	1	pseudobulk	DESeq2	LRT	0
453	KC_AB1	asRNA:CR45129	-0.714733468959387	0.513049020769814	1	pseudobulk	DESeq2	LRT	0
454	KC_AB1	asRNA:CR45131	-2.55883185549639	0.12699187137121	1	pseudobulk	DESeq2	LRT	0
455	KC_AB1	asRNA:CR45133	0.849606887838128	1	1	pseudobulk	DESeq2	LRT	0
456	KC_AB1	asRNA:CR45134	1.51741202869167	0.132558030258097	1	pseudobulk	DESeq2	LRT	0
457	KC_AB1	asRNA:CR45137	-0.145489161020015	0.848762515713808	1	pseudobulk	DESeq2	LRT	0
458	KC_AB1	asRNA:CR45139	-0.524684756236559	0.687843985405584	1	pseudobulk	DESeq2	LRT	0
459	KC_AB1	asRNA:CR45145	-0.764164334613662	0.540001670321965	1	pseudobulk	DESeq2	LRT	0
460	KC_AB1	asRNA:CR45149	0.849606887838128	1	1	pseudobulk	DESeq2	LRT	0
461	KC_AB1	asRNA:CR45151	-0.216210032712384	1	1	pseudobulk	DESeq2	LRT	0
462	KC_AB1	asRNA:CR45152	1.59863986092676	1	1	pseudobulk	DESeq2	LRT	0
463	KC_AB1	asRNA:CR45161	0.985243145755336	1	1	pseudobulk	DESeq2	LRT	0
464	KC_AB1	asRNA:CR45162	2.85142250261825	0.484249113053674	1	pseudobulk	DESeq2	LRT	0
465	KC_AB1	asRNA:CR45164	0.593877445539172	0.979245044647934	1	pseudobulk	DESeq2	LRT	0
466	KC_AB1	asRNA:CR45167	0.849606887838128	1	1	pseudobulk	DESeq2	LRT	0
467	KC_AB1	asRNA:CR45175	0.849606887838128	1	1	pseudobulk	DESeq2	LRT	0
468	KC_AB1	asRNA:CR45182	0.467638171232937	0.685347483598577	1	pseudobulk	DESeq2	LRT	0
469	KC_AB1	asRNA:CR45185	-4.1366530727217	0.263878877880707	1	pseudobulk	DESeq2	LRT	0
470	KC_AB1	asRNA:CR45198	-0.10506771737906	1	1	pseudobulk	DESeq2	LRT	0
471	KC_AB1	asRNA:CR45202	0.849606887838128	1	1	pseudobulk	DESeq2	LRT	0
472	KC_AB1	asRNA:CR45208	0.849606887838128	1	1	pseudobulk	DESeq2	LRT	0
473	KC_AB1	asRNA:CR45214	3.41844045086015	0.351942490385471	1	pseudobulk	DESeq2	LRT	0
474	KC_AB1	asRNA:CR45271	0.849606887838128	1	1	pseudobulk	DESeq2	LRT	0
475	KC_AB1	asRNA:CR45281	0.465082759499822	1	1	pseudobulk	DESeq2	LRT	0

476	KC_AB1	asRNA:CR45350	0.985243145755336	1	1	pseudobulk	DESeq2	LRT	0
477	KC_AB1	asRNA:CR45367	0.849606887838128	1	1	pseudobulk	DESeq2	LRT	0
478	KC_AB1	asRNA:CR45384	0.930993226399931	1	1	pseudobulk	DESeq2	LRT	0
479	KC_AB1	asRNA:CR45396	0.849606887838128	1	1	pseudobulk	DESeq2	LRT	0
480	KC_AB1	asRNA:CR45469	-0.667276867089511	0.61499952067632	1	pseudobulk	DESeq2	LRT	0
481	KC_AB1	asRNA:CR45520	0.769522064442666	1	1	pseudobulk	DESeq2	LRT	0
482	KC_AB1	asRNA:CR45528	0.849606887838128	1	1	pseudobulk	DESeq2	LRT	0
483	KC_AB1	asRNA:CR45538	2.78674512494791	0.54962276837928	1	pseudobulk	DESeq2	LRT	0
484	KC_AB1	asRNA:CR45600	0.2543249331732	0.967943066013212	1	pseudobulk	DESeq2	LRT	0
485	KC_AB1	asRNA:CR45604	0.237022465848507	1	1	pseudobulk	DESeq2	LRT	0
486	KC_AB1	asRNA:CR45620	-1.32075372376881	0.313504963957792	1	pseudobulk	DESeq2	LRT	0
487	KC_AB1	asRNA:CR45623	2.78674512494791	0.54962276837928	1	pseudobulk	DESeq2	LRT	0
488	KC_AB1	asRNA:CR45629	0.849606887838128	1	1	pseudobulk	DESeq2	LRT	0
489	KC_AB1	asRNA:CR45683	0.516467005704292	1	1	pseudobulk	DESeq2	LRT	0
490	KC_AB1	asRNA:CR45789	-0.186428723038723	0.837466856081456	1	pseudobulk	DESeq2	LRT	0
491	KC_AB1	asRNA:CR45798	0.183159795455075	1	1	pseudobulk	DESeq2	LRT	0
492	KC_AB1	asRNA:CR45804	0.340532871387421	1	1	pseudobulk	DESeq2	LRT	0
493	KC_AB1	asRNA:CR45813	0.849606887838128	1	1	pseudobulk	DESeq2	LRT	0
494	KC_AB1	asRNA:CR45814	0.849606887838128	1	1	pseudobulk	DESeq2	LRT	0
495	KC_AB1	asRNA:CR45815	-0.619292669500288	0.675665203383677	1	pseudobulk	DESeq2	LRT	0
496	KC_AB1	asRNA:CR45818	0.849606887838128	1	1	pseudobulk	DESeq2	LRT	0
497	KC_AB1	asRNA:CR45822	-0.518297648476751	0.639162081001915	1	pseudobulk	DESeq2	LRT	0
498	KC_AB1	asRNA:CR45832	-1.00752466718157	0.580536516624078	1	pseudobulk	DESeq2	LRT	0
499	KC_AB1	asRNA:CR45834	0.849606887838128	1	1	pseudobulk	DESeq2	LRT	0
500	KC_AB1	asRNA:CR45835	0.389892024658762	0.736635924043329	1	pseudobulk	DESeq2	LRT	0
501	KC_AB1	asRNA:CR45870	0.849606887838128	1	1	pseudobulk	DESeq2	LRT	0
502	KC_AB1	asRNA:CR45873	2.85142250261825	0.484249113053674	1	pseudobulk	DESeq2	LRT	0
503	KC_AB1	asRNA:CR45874	0.372662209609587	0.62956502843684	1	pseudobulk	DESeq2	LRT	0
504	KC_AB1	asRNA:CR45882	0.849606887838128	1	1	pseudobulk	DESeq2	LRT	0
505	KC_AB1	asRNA:CR45889	1.99875784706794	0.63487605805892	1	pseudobulk	DESeq2	LRT	0
506	KC_AB1	asRNA:CR45891	1.59863986092676	1	1	pseudobulk	DESeq2	LRT	0
507	KC_AB1	asRNA:CR45896	2.06531092668974	0.944672961903726	1	pseudobulk	DESeq2	LRT	0
508	KC_AB1	asRNA:CR45907	-0.572809606158377	0.787374717193114	1	pseudobulk	DESeq2	LRT	0
509	KC_AB1	asRNA:CR45912	0.849606887838128	1	1	pseudobulk	DESeq2	LRT	0
510	KC_AB1	asRNA:CR45925	0.849606887838128	1	1	pseudobulk	DESeq2	LRT	0
511	KC_AB1	asRNA:CR45944	3.64217331632531	0.246682905127758	1	pseudobulk	DESeq2	LRT	0
512	KC_AB1	asRNA:CR45951	-0.115606844850552	0.817218022247486	1	pseudobulk	DESeq2	LRT	0
513	KC_AB1	asRNA:CR45960	0.849606887838128	1	1	pseudobulk	DESeq2	LRT	0
514	KC_AB1	asRNA:CR45999	0.849606887838128	1	1	pseudobulk	DESeq2	LRT	0
515	KC_AB1	asRNA:CR46029	2.78674512494791	0.54962276837928	1	pseudobulk	DESeq2	LRT	0
516	KC_AB1	asRNA:CR46033	-1.03343975534598	0.627153393210199	1	pseudobulk	DESeq2	LRT	0
517	KC_AB1	asRNA:CR46050	0.849606887838128	1	1	pseudobulk	DESeq2	LRT	0
518	KC_AB1	asRNA:CR46058	0.849606887838128	1	1	pseudobulk	DESeq2	LRT	0
519	KC_AB1	asRNA:CR46066	0.849606887838128	1	1	pseudobulk	DESeq2	LRT	0
520	KC_AB1	asRNA:CR46067	0.680825752226896	1	1	pseudobulk	DESeq2	LRT	0
521	KC_AB1	asRNA:CR46091	0.237022465848507	1	1	pseudobulk	DESeq2	LRT	0
522	KC_AB1	asRNA:CR46107	0.849606887838128	1	1	pseudobulk	DESeq2	LRT	0
523	KC_AB1	asRNA:CR46135	-0.960022699105854	0.610500321913265	1	pseudobulk	DESeq2	LRT	0
524	KC_AB1	asRNA:CR46137	-0.10506771737906	1	1	pseudobulk	DESeq2	LRT	0
525	KC_AB1	asRNA:CR46144	0.849606887838128	1	1	pseudobulk	DESeq2	LRT	0
526	KC_AB1	asRNA:CR46246	0.849606887838128	1	1	pseudobulk	DESeq2	LRT	0
527	KC_AB1	asRNA:CR46256	0.849606887838128	1	1	pseudobulk	DESeq2	LRT	0
528	KC_AB1	asRNA:CR46262	2.19984397754212	0.254545187295706	1	pseudobulk	DESeq2	LRT	0

529	KC_AB1	asRNA:CR46263	0.0160940546605626	1	1	pseudobulk	DESeq2	LRT		0
530	KC_AB1	asRNA:CR46354	-0.747916810024492	0.594565015610323	1	pseudobulk	DESeq2	LRT		0
531	KC_AB1	ast	2.27201025045383	0.0939438369307866	1	pseudobulk	DESeq2	LRT		0
532	KC_AB1	AstA	0.849606887838128	1	1	pseudobulk	DESeq2	LRT		0
533	KC_AB1	AstA-R1	-0.395388837421284	0.734517171574983	1	pseudobulk	DESeq2	LRT		0
534	KC_AB1	AstC	0.183159795455075	1	1	pseudobulk	DESeq2	LRT		0
535	KC_AB1	asun	1.60172288900978	0.749416984916788	1	pseudobulk	DESeq2	LRT		0
536	KC_AB1	Asx	0.279424983583722	0.587818007459657	1	pseudobulk	DESeq2	LRT		0
537	KC_AB1	Atac1	0.253161562067212	1	1	pseudobulk	DESeq2	LRT		0
538	KC_AB1	Atac2	0.131876065984052	0.958115530694076	1	pseudobulk	DESeq2	LRT		0
539	KC_AB1	Atac3	0.286338585980019	0.546308079007876	1	pseudobulk	DESeq2	LRT		0
540	KC_AB1	ATbp	-0.0898518858188937	0.822763793824986	1	pseudobulk	DESeq2	LRT		0
541	KC_AB1	Ate1	0.082203947493002	0.81932465644374	1	pseudobulk	DESeq2	LRT		0
542	KC_AB1	Atet	0.051130975605355	0.732728676961554	1	pseudobulk	DESeq2	LRT		0
543	KC_AB1	Atf-2	-0.282564714214468	0.68940973667715	1	pseudobulk	DESeq2	LRT		0
544	KC_AB1	Atf3	-1.08340404578512	0.42193797162881	1	pseudobulk	DESeq2	LRT		0
545	KC_AB1	Atf6	-0.0342071974261139	0.597201264720699	1	pseudobulk	DESeq2	LRT		0
546	KC_AB1	Atg1	-0.166181311230429	0.555523565203013	1	pseudobulk	DESeq2	LRT		0
547	KC_AB1	Atg10	-0.89488794912958	0.528193713586017	1	pseudobulk	DESeq2	LRT		0
548	KC_AB1	Atg101	-0.223938071549091	0.551204307108375	1	pseudobulk	DESeq2	LRT		0
549	KC_AB1	Atg12	-0.407794618646382	0.354548744085352	1	pseudobulk	DESeq2	LRT		0
550	KC_AB1	Atg13	0.18763929151404	0.60977569146287	1	pseudobulk	DESeq2	LRT		0
551	KC_AB1	Atg14	0.495649661616764	0.481890684632964	1	pseudobulk	DESeq2	LRT		0
552	KC_AB1	Atg16	0.0525376540552532	0.818759343368414	1	pseudobulk	DESeq2	LRT		0
553	KC_AB1	Atg17	-0.33506080657431	0.296022435763971	1	pseudobulk	DESeq2	LRT		0
554	KC_AB1	Atg18a	-0.368126717041569	0.484906882524219	1	pseudobulk	DESeq2	LRT		0
555	KC_AB1	Atg18b	0.158703262067518	0.741524401453456	1	pseudobulk	DESeq2	LRT		0
556	KC_AB1	Atg2	0.678354066460313	0.224279033838754	1	pseudobulk	DESeq2	LRT		0
557	KC_AB1	Atg3	-0.34450846503546	0.564595310872637	1	pseudobulk	DESeq2	LRT		0
558	KC_AB1	Atg4a	0.00156812359134208	0.753816823241267	1	pseudobulk	DESeq2	LRT		0
559	KC_AB1	Atg4b	-0.0979071701229214	0.769914745058192	1	pseudobulk	DESeq2	LRT		0
560	KC_AB1	Atg5	-0.0158776574304678	0.665378887014574	1	pseudobulk	DESeq2	LRT		0
561	KC_AB1	Atg6	-0.800584654946562	0.37401985430244	1	pseudobulk	DESeq2	LRT		0
562	KC_AB1	Atg7	0.0461365922897516	0.89972127301891	1	pseudobulk	DESeq2	LRT		0
563	KC_AB1	Atg8a	-0.165872651386703	0.390880860654475	1	pseudobulk	DESeq2	LRT		0
564	KC_AB1	Atg9	-0.124957684019366	0.805503306133961	1	pseudobulk	DESeq2	LRT		0
565	KC_AB1	atilla	3.64217331632531	0.246682905127758	1	pseudobulk	DESeq2	LRT		0
566	KC_AB1	atl	0.0691809300878431	0.917059502934501	1	pseudobulk	DESeq2	LRT		0
567	KC_AB1	atms	0.87930660729347	0.362363400356887	1	pseudobulk	DESeq2	LRT		0
568	KC_AB1	Atox1	0.253557942622422	0.266295388266622	1	pseudobulk	DESeq2	LRT		0
569	KC_AB1	ATP6AP2	-0.106815739799779	0.529475547257748	1	pseudobulk	DESeq2	LRT		0
570	KC_AB1	ATP7	0.428245279540673	0.859996228136203	1	pseudobulk	DESeq2	LRT		0
571	KC_AB1	ATP8A	-0.237655897187422	0.375046080939851	1	pseudobulk	DESeq2	LRT		0
572	KC_AB1	ATP8B	0.931109140710098	1	1	pseudobulk	DESeq2	LRT		0
573	KC_AB1	Atpalpha	0.0192243138391509	0.8776705529919	1	pseudobulk	DESeq2	LRT		0
574	KC_AB1	ATPCL	-0.377487857497106	0.223291341247103	1	pseudobulk	DESeq2	LRT		0
575	KC_AB1	ATPsynB	0.0171264797153256	0.697231463253845	1	pseudobulk	DESeq2	LRT		0
576	KC_AB1	ATPsynbeta	-0.163644740339348	0.439804929181954	1	pseudobulk	DESeq2	LRT		0
577	KC_AB1	ATPsynC	0.0356191123887153	0.686970225487179	1	pseudobulk	DESeq2	LRT		0
578	KC_AB1	ATPsynCF6	-0.0717418609834534	0.575982888245765	1	pseudobulk	DESeq2	LRT		0
579	KC_AB1	ATPsynCF6L	0.237022465848507	1	1	pseudobulk	DESeq2	LRT		0
580	KC_AB1	ATPsynD	0.216628555953551	0.366222784164653	1	pseudobulk	DESeq2	LRT		0
581	KC_AB1	ATPsyndelta	0.116860039171748	0.657061439356945	1	pseudobulk	DESeq2	LRT		0

582	KC_AB1	ATPsynE	-0.231769078775325	0.754587101453725	1	pseudobulk	DESeq2	LRT	0
583	KC_AB1	ATPsynF	0.144682069427428	0.416214046825253	1	pseudobulk	DESeq2	LRT	0
584	KC_AB1	ATPsynG	0.0643194506973736	0.524552373244094	1	pseudobulk	DESeq2	LRT	0
585	KC_AB1	ATPsyngamma	0.177254317845823	0.547869841724619	1	pseudobulk	DESeq2	LRT	0
586	KC_AB1	ATPsynO	0.288320399382739	0.35505243680683	1	pseudobulk	DESeq2	LRT	0
587	KC_AB1	AttA	0.849606887838128	1	1	pseudobulk	DESeq2	LRT	0
588	KC_AB1	Atu	-0.692369339009322	0.606368888176565	1	pseudobulk	DESeq2	LRT	0
589	KC_AB1	Atx2	-0.307085948842577	0.279541235080519	1	pseudobulk	DESeq2	LRT	0
590	KC_AB1	Atxn7	-0.364141209275531	0.439265593973424	1	pseudobulk	DESeq2	LRT	0
591	KC_AB1	aub	0.849606887838128	1	1	pseudobulk	DESeq2	LRT	0
592	KC_AB1	aux	-0.246248700528437	0.4837553442867	1	pseudobulk	DESeq2	LRT	0
593	KC_AB1	ave	-0.515859553134107	0.435467608720735	1	pseudobulk	DESeq2	LRT	0
594	KC_AB1	Aven	-0.310423448325964	0.698764856268262	1	pseudobulk	DESeq2	LRT	0
595	KC_AB1	awd	-0.089840850879052	0.582762543014409	1	pseudobulk	DESeq2	LRT	0
596	KC_AB1	axed	-0.457818194162715	0.305953392035242	1	pseudobulk	DESeq2	LRT	0
597	KC_AB1	Axn	0.33901521782723	0.656981549910273	1	pseudobulk	DESeq2	LRT	0
598	KC_AB1	axo	0.184824350087733	0.671153265528625	1	pseudobulk	DESeq2	LRT	0
599	KC_AB1	Axud1	-0.0374241819863371	0.945185705719526	1	pseudobulk	DESeq2	LRT	0
600	KC_AB1	az2	-0.550481023171346	0.609545420940377	1	pseudobulk	DESeq2	LRT	0
601	KC_AB1	azot	1.39302308053166	0.675623807317343	1	pseudobulk	DESeq2	LRT	0
602	KC_AB1	b	-1.04911819251966	0.645964455482897	1	pseudobulk	DESeq2	LRT	0
603	KC_AB1	B4	-0.208335851888947	0.343879396989671	1	pseudobulk	DESeq2	LRT	0
604	KC_AB1	B52	-0.389003766602372	0.0800776942958402	1	pseudobulk	DESeq2	LRT	0
605	KC_AB1	bab1	0.849606887838128	1	1	pseudobulk	DESeq2	LRT	0
606	KC_AB1	bab2	0.0160940546605174	1	1	pseudobulk	DESeq2	LRT	0
607	KC_AB1	babo	-0.251356412770676	0.470775827271893	1	pseudobulk	DESeq2	LRT	0
608	KC_AB1	Bacc	-0.384372079624985	0.0152330937160746	1	pseudobulk	DESeq2	LRT	0
609	KC_AB1	baf	-2.49893396807395	0.128102498054845	1	pseudobulk	DESeq2	LRT	0
610	KC_AB1	bai	-0.0022515244833825	0.919905588104478	1	pseudobulk	DESeq2	LRT	0
611	KC_AB1	Baldspot	-0.469464174037016	0.116678057219417	1	pseudobulk	DESeq2	LRT	0
612	KC_AB1	ball	0.516467005704292	1	1	pseudobulk	DESeq2	LRT	0
613	KC_AB1	Bap111	-1.66492055129527	0.155937280218957	1	pseudobulk	DESeq2	LRT	0
614	KC_AB1	Bap170	0.922080640288379	0.627489530272423	1	pseudobulk	DESeq2	LRT	0
615	KC_AB1	Bap55	0.0617353269256108	0.655364728381316	1	pseudobulk	DESeq2	LRT	0
616	KC_AB1	Bap60	-0.38939286887368	0.429597679664651	1	pseudobulk	DESeq2	LRT	0
617	KC_AB1	barc	0.0719827473121861	0.583521223788676	1	pseudobulk	DESeq2	LRT	0
618	KC_AB1	bark	2.78674512494791	0.54962276837928	1	pseudobulk	DESeq2	LRT	0
619	KC_AB1	barr	0.849606887838128	1	1	pseudobulk	DESeq2	LRT	0
620	KC_AB1	baz	-1.8219618299953	0.568901687397251	1	pseudobulk	DESeq2	LRT	0
621	KC_AB1	bbc	-0.0030244717981957	0.691011091458316	1	pseudobulk	DESeq2	LRT	0
622	KC_AB1	bbg	-2.59610313770632	0.291483550812208	1	pseudobulk	DESeq2	LRT	0
623	KC_AB1	BBS5	-1.05548521056871	0.62314630615428	1	pseudobulk	DESeq2	LRT	0
624	KC_AB1	BBS9	0.849606887838128	1	1	pseudobulk	DESeq2	LRT	0
625	KC_AB1	bbx	-1.29195350762757	0.486995519272942	1	pseudobulk	DESeq2	LRT	0
626	KC_AB1	bc10	-0.641782419217104	0.332200489946963	1	pseudobulk	DESeq2	LRT	0
627	KC_AB1	BCAS2	0.255393320230862	0.803384424046742	1	pseudobulk	DESeq2	LRT	0
628	KC_AB1	bcd	0.237022465848507	1	1	pseudobulk	DESeq2	LRT	0
629	KC_AB1	bchs	-0.30279930466505	0.370297616333876	1	pseudobulk	DESeq2	LRT	0
630	KC_AB1	BCL7-like	-0.4251571979752	0.673059808929959	1	pseudobulk	DESeq2	LRT	0
631	KC_AB1	bcn92	0.0316984733987027	0.884379662203608	1	pseudobulk	DESeq2	LRT	0
632	KC_AB1	Bdbt	-0.13797662419298	0.709094903189028	1	pseudobulk	DESeq2	LRT	0
633	KC_AB1	bdg	0.344453897616826	0.768336231972874	1	pseudobulk	DESeq2	LRT	0
634	KC_AB1	bdl	0.183159795455075	1	1	pseudobulk	DESeq2	LRT	0

635	KC_AB1	Bdp1	-1.62295259850125	0.385695124829572	1	pseudobulk	DESeq2	LRT	0
636	KC_AB1	be	0.40265452544986	0.737870148793645	1	pseudobulk	DESeq2	LRT	0
637	KC_AB1	BEAF-32	0.547548148257963	0.367784674005077	1	pseudobulk	DESeq2	LRT	0
638	KC_AB1	beag	-0.259649784508783	0.701385267932203	1	pseudobulk	DESeq2	LRT	0
639	KC_AB1	beat-Ia	0.237022465848507	1	1	pseudobulk	DESeq2	LRT	0
640	KC_AB1	beat-Ib	-0.563757576445448	0.700109513678433	1	pseudobulk	DESeq2	LRT	0
641	KC_AB1	beat-Ic	1.63062725578732	0.558856661580835	1	pseudobulk	DESeq2	LRT	0
642	KC_AB1	beat-IIa	2.78674512494791	0.54962276837928	1	pseudobulk	DESeq2	LRT	0
643	KC_AB1	beat-IIIb	0.0890739047309585	0.618308111550498	1	pseudobulk	DESeq2	LRT	0
644	KC_AB1	beat-IIIc	0.183159795455075	1	1	pseudobulk	DESeq2	LRT	0
645	KC_AB1	beat-IV	0.825505232840244	0.0986095624665313	1	pseudobulk	DESeq2	LRT	0
646	KC_AB1	beat-Va	3.41844045086015	0.351942490385471	1	pseudobulk	DESeq2	LRT	0
647	KC_AB1	beat-Vb	0.0160940546605174	1	1	pseudobulk	DESeq2	LRT	0
648	KC_AB1	beat-Vc	-0.283054125455946	1	1	pseudobulk	DESeq2	LRT	0
649	KC_AB1	beat-VI	-0.154688477638524	0.786730464531634	1	pseudobulk	DESeq2	LRT	0
650	KC_AB1	beat-VII	-0.107770501715141	0.560259992521317	1	pseudobulk	DESeq2	LRT	0
651	KC_AB1	beg	0.16027806085858	0.859198191694061	1	pseudobulk	DESeq2	LRT	0
652	KC_AB1	bel	-0.131129833373264	0.494346037271203	1	pseudobulk	DESeq2	LRT	0
653	KC_AB1	Bem46	0.3893842140542	0.647715379785338	1	pseudobulk	DESeq2	LRT	0
654	KC_AB1	ben	0.0300459695852129	0.515356150787097	1	pseudobulk	DESeq2	LRT	0
655	KC_AB1	Best1	-0.289452307729187	0.713758597428107	1	pseudobulk	DESeq2	LRT	0
656	KC_AB1	Best2	2.06541384650887	0.904905526327343	1	pseudobulk	DESeq2	LRT	0
657	KC_AB1	Bet1	-0.3911860710532	0.441895662677394	1	pseudobulk	DESeq2	LRT	0
658	KC_AB1	Bet3	-0.0311360978536442	0.963588530632782	1	pseudobulk	DESeq2	LRT	0
659	KC_AB1	Bet5	-0.284328583301465	0.671611980146709	1	pseudobulk	DESeq2	LRT	0
660	KC_AB1	beta-Man	0.849606887838128	1	1	pseudobulk	DESeq2	LRT	0
661	KC_AB1	beta-PheRS	1.17697543643323	0.524764955824479	1	pseudobulk	DESeq2	LRT	0
662	KC_AB1	beta-Spec	-0.0895458679560314	0.519558143180156	1	pseudobulk	DESeq2	LRT	0
663	KC_AB1	beta'COP	0.0355538785120557	0.68122568602587	1	pseudobulk	DESeq2	LRT	0
664	KC_AB1	beta3GalTII	-1.13386816727682	0.585241461181947	1	pseudobulk	DESeq2	LRT	0
665	KC_AB1	beta4GalNAcTA	1.3298310238887	0.235359664589361	1	pseudobulk	DESeq2	LRT	0
666	KC_AB1	beta4GalNAcTB	-0.49387752714312	0.620224310836351	1	pseudobulk	DESeq2	LRT	0
667	KC_AB1	beta4GalT7	-0.566034287696167	0.651704569949213	1	pseudobulk	DESeq2	LRT	0
668	KC_AB1	betaCOP	-0.426485826253674	0.526891144452289	1	pseudobulk	DESeq2	LRT	0
669	KC_AB1	betaggt-I	-0.70625498361251	0.518862777821797	1	pseudobulk	DESeq2	LRT	0
670	KC_AB1	betaggt-II	0.252093452253549	0.761990319558735	1	pseudobulk	DESeq2	LRT	0
671	KC_AB1	betaGlu	1.74488096961208	0.717340193332887	1	pseudobulk	DESeq2	LRT	0
672	KC_AB1	betaNACtes3	0.849606887838128	1	1	pseudobulk	DESeq2	LRT	0
673	KC_AB1	betaNACtes6	0.849606887838128	1	1	pseudobulk	DESeq2	LRT	0
674	KC_AB1	betaTub56D	-0.157134208043875	0.413906441543356	1	pseudobulk	DESeq2	LRT	0
675	KC_AB1	betaTub85D	2.06531092668974	0.944672961903726	1	pseudobulk	DESeq2	LRT	0
676	KC_AB1	Bgb	0.0413791965804725	0.566302546105866	1	pseudobulk	DESeq2	LRT	0
677	KC_AB1	bgm	3.41844045086015	0.351942490385471	1	pseudobulk	DESeq2	LRT	0
678	KC_AB1	BHD	-1.18031397173339	0.450617504841961	1	pseudobulk	DESeq2	LRT	0
679	KC_AB1	BI-1	0.130278834584095	0.499523976269894	1	pseudobulk	DESeq2	LRT	0
680	KC_AB1	bib	0.930993226399931	1	1	pseudobulk	DESeq2	LRT	0
681	KC_AB1	bic	-0.281074724792182	0.246659538915632	1	pseudobulk	DESeq2	LRT	0
682	KC_AB1	BicD	-0.283110902538817	0.355280301015319	1	pseudobulk	DESeq2	LRT	0
683	KC_AB1	bif	-0.27703009175481	0.525903641467668	1	pseudobulk	DESeq2	LRT	0
684	KC_AB1	bigmax	-0.311874983290879	0.700164689496387	1	pseudobulk	DESeq2	LRT	0
685	KC_AB1	Bili	-0.207363947806644	0.474729261120154	1	pseudobulk	DESeq2	LRT	0
686	KC_AB1	Bin1	-0.783426208534381	0.373210530295346	1	pseudobulk	DESeq2	LRT	0
687	KC_AB1	bin3	-0.506399933767625	0.249050198678134	1	pseudobulk	DESeq2	LRT	0

688	KC_AB1	bip1	-0.267798911930929	1	1	pseudobulk	DESeq2	LRT	0
689	KC_AB1	bip2	-0.0280201067097056	0.687186124699211	1	pseudobulk	DESeq2	LRT	0
690	KC_AB1	Bka	0.366384536568578	0.716077682222192	1	pseudobulk	DESeq2	LRT	0
691	KC_AB1	Blimp-1	-0.294127878218529	0.304309550531099	1	pseudobulk	DESeq2	LRT	0
692	KC_AB1	Blm	-0.0127846790391957	1	1	pseudobulk	DESeq2	LRT	0
693	KC_AB1	Blos1	0.287445532339671	0.54948890293561	1	pseudobulk	DESeq2	LRT	0
694	KC_AB1	Blos2	0.573821630503186	0.545464473285486	1	pseudobulk	DESeq2	LRT	0
695	KC_AB1	Blos3	-0.0994984315474619	0.823681985415318	1	pseudobulk	DESeq2	LRT	0
696	KC_AB1	Blos4	0.701667145627921	0.58501433752847	1	pseudobulk	DESeq2	LRT	0
697	KC_AB1	blot	1.00693893053477	1	1	pseudobulk	DESeq2	LRT	0
698	KC_AB1	blow	0.849606887838128	1	1	pseudobulk	DESeq2	LRT	0
699	KC_AB1	blp	-0.205603957041449	0.683459363648209	1	pseudobulk	DESeq2	LRT	0
700	KC_AB1	blw	-0.275972675950057	0.239178863358774	1	pseudobulk	DESeq2	LRT	0
701	KC_AB1	bma	0.0966160264649667	0.589963825771374	1	pseudobulk	DESeq2	LRT	0
702	KC_AB1	Bmcp	-0.212772310390799	0.64964641193516	1	pseudobulk	DESeq2	LRT	0
703	KC_AB1	bmm	-0.626284704114558	0.410553143386678	1	pseudobulk	DESeq2	LRT	0
704	KC_AB1	bnb	-0.928294685435351	0.453870718445653	1	pseudobulk	DESeq2	LRT	0
705	KC_AB1	boca	0.288453505779691	0.641666123277267	1	pseudobulk	DESeq2	LRT	0
706	KC_AB1	bocks	-0.529353176613549	0.614386845799638	1	pseudobulk	DESeq2	LRT	0
707	KC_AB1	BOD1	-0.911083946585506	0.378710359818574	1	pseudobulk	DESeq2	LRT	0
708	KC_AB1	boi	1.59863986092676	1	1	pseudobulk	DESeq2	LRT	0
709	KC_AB1	bol	-0.110749149251783	0.447819636041821	1	pseudobulk	DESeq2	LRT	0
710	KC_AB1	Bom065	0.849606887838128	1	1	pseudobulk	DESeq2	LRT	0
711	KC_AB1	Bom067	0.183159795463042	1	1	pseudobulk	DESeq2	LRT	0
712	KC_AB1	Bom068	0.849606887838128	1	1	pseudobulk	DESeq2	LRT	0
713	KC_AB1	Bom1	0.00330394891908091	1	1	pseudobulk	DESeq2	LRT	0
714	KC_AB1	Bom2	2.31997030691033	0.813169507846918	1	pseudobulk	DESeq2	LRT	0
715	KC_AB1	Bom23	0.849606887838128	1	1	pseudobulk	DESeq2	LRT	0
716	KC_AB1	Bom3	-1.08327842095589	0.442677750533466	1	pseudobulk	DESeq2	LRT	0
717	KC_AB1	Bom836	1.59863986092676	1	1	pseudobulk	DESeq2	LRT	0
718	KC_AB1	bon	-0.526033874966123	0.22604801758814	1	pseudobulk	DESeq2	LRT	0
719	KC_AB1	bonsai	0.308690732719984	0.641054749867203	1	pseudobulk	DESeq2	LRT	0
720	KC_AB1	bor	0.532109827636516	0.541195381284911	1	pseudobulk	DESeq2	LRT	0
721	KC_AB1	BORCS5	-0.071425824110621	0.584869941754307	1	pseudobulk	DESeq2	LRT	0
722	KC_AB1	BORCS6	-0.288228281883687	0.645213042553611	1	pseudobulk	DESeq2	LRT	0
723	KC_AB1	botv	-0.207225497076221	0.7325747495395	1	pseudobulk	DESeq2	LRT	0
724	KC_AB1	bou	0.849606887838128	1	1	pseudobulk	DESeq2	LRT	0
725	KC_AB1	bowl	1.00693893053477	1	1	pseudobulk	DESeq2	LRT	0
726	KC_AB1	br	-2.47151232288594	0.0460541809822082	1	pseudobulk	DESeq2	LRT	0
727	KC_AB1	Br140	-0.755423677626275	0.45522934336673	1	pseudobulk	DESeq2	LRT	0
728	KC_AB1	brat	-0.152633880111794	0.389859861508539	1	pseudobulk	DESeq2	LRT	0
729	KC_AB1	Brca2	-0.0785708402630631	0.754390093288141	1	pseudobulk	DESeq2	LRT	0
730	KC_AB1	Brd8	-0.772286997526068	0.48360082435319	1	pseudobulk	DESeq2	LRT	0
731	KC_AB1	Bre1	-0.0917828699441453	0.845426856283201	1	pseudobulk	DESeq2	LRT	0
732	KC_AB1	Brf	-1.21572343205896	0.298365579647571	1	pseudobulk	DESeq2	LRT	0
733	KC_AB1	brk	2.06541384650887	0.904905526327343	1	pseudobulk	DESeq2	LRT	0
734	KC_AB1	brm	0.0941473343504414	0.837619342957886	1	pseudobulk	DESeq2	LRT	0
735	KC_AB1	Brms1	-0.194779335094288	0.521628519419057	1	pseudobulk	DESeq2	LRT	0
736	KC_AB1	brn	3.28337310078207	0.18326727042555	1	pseudobulk	DESeq2	LRT	0
737	KC_AB1	bru1	-0.509290198677104	0.0850392211773771	1	pseudobulk	DESeq2	LRT	0
738	KC_AB1	bru2	0.0384652891564378	0.81211547287035	1	pseudobulk	DESeq2	LRT	0
739	KC_AB1	bru3	-0.301809223433103	0.0330796885830282	1	pseudobulk	DESeq2	LRT	0
740	KC_AB1	Bruce	0.0368989854303352	0.520694739968564	1	pseudobulk	DESeq2	LRT	0

741	KC_AB1	brun	-0.860885491254983	0.417655988951665	1	pseudobulk	DESeq2	LRT	0
742	KC_AB1	BRWD3	-0.363236189357561	0.546585340047954	1	pseudobulk	DESeq2	LRT	0
743	KC_AB1	brwl	-0.940457278568912	0.20015495119163	1	pseudobulk	DESeq2	LRT	0
744	KC_AB1	bs	-1.63363591227014	0.19903531383766	1	pseudobulk	DESeq2	LRT	0
745	KC_AB1	bsf	-0.0060309999904620	0.758298030587748	1	pseudobulk	DESeq2	LRT	0
746	KC_AB1	Bsg	-0.0567634158107131	0.656898030792592	1	pseudobulk	DESeq2	LRT	0
747	KC_AB1	Bsg25D	-0.515328715587142	0.398199358004437	1	pseudobulk	DESeq2	LRT	0
748	KC_AB1	bsk	0.0120733367074014	0.586845842051652	1	pseudobulk	DESeq2	LRT	0
749	KC_AB1	bt	-1.96439762405016	0.0418229665663814	1	pseudobulk	DESeq2	LRT	0
750	KC_AB1	BTBD9	-1.26585520439075	0.285509413759593	1	pseudobulk	DESeq2	LRT	0
751	KC_AB1	BtbVII	-0.0228856720291948	0.818997036696259	1	pseudobulk	DESeq2	LRT	0
752	KC_AB1	Btk29A	-1.455604640701	0.427099582025952	1	pseudobulk	DESeq2	LRT	0
753	KC_AB1	btsz	-0.0976866620182992	0.656490219051827	1	pseudobulk	DESeq2	LRT	0
754	KC_AB1	btv	2.31997030691033	0.813169507846918	1	pseudobulk	DESeq2	LRT	0
755	KC_AB1	btz	0.960089640193856	0.284265302947093	1	pseudobulk	DESeq2	LRT	0
756	KC_AB1	Bub1	-0.385765357321296	0.605757203188231	1	pseudobulk	DESeq2	LRT	0
757	KC_AB1	Bub3	0.192716365885362	0.778980489105931	1	pseudobulk	DESeq2	LRT	0
758	KC_AB1	Buffy	2.78674512494791	0.54962276837928	1	pseudobulk	DESeq2	LRT	0
759	KC_AB1	bug	-0.138418953081378	0.760624873443475	1	pseudobulk	DESeq2	LRT	0
760	KC_AB1	Bug22	-0.801199269582757	0.375374015548867	1	pseudobulk	DESeq2	LRT	0
761	KC_AB1	BuGZ	-0.0280109024461131	0.804713713623293	1	pseudobulk	DESeq2	LRT	0
762	KC_AB1	bun	-0.03414152634825	0.648432111809404	1	pseudobulk	DESeq2	LRT	0
763	KC_AB1	bur	0.100494468624866	0.710205063880827	1	pseudobulk	DESeq2	LRT	0
764	KC_AB1	bves	0.135536901452445	0.595923204815159	1	pseudobulk	DESeq2	LRT	0
765	KC_AB1	bw	0.0160940546605174	1	1	pseudobulk	DESeq2	LRT	0
766	KC_AB1	bwa	0.0369823105966479	0.709905052802797	1	pseudobulk	DESeq2	LRT	0
767	KC_AB1	Bx	-0.038504598109907	0.758869260293807	1	pseudobulk	DESeq2	LRT	0
768	KC_AB1	Bx42	-0.22528519139388	0.535522508249027	1	pseudobulk	DESeq2	LRT	0
769	KC_AB1	by	2.31997030691033	0.813169507846918	1	pseudobulk	DESeq2	LRT	0
770	KC_AB1	bys	0.425011620766152	0.794112396144258	1	pseudobulk	DESeq2	LRT	0
771	KC_AB1	c(2)M	0.849606887838128	1	1	pseudobulk	DESeq2	LRT	0
772	KC_AB1	c(3)G	2.31997030691033	0.813169507846918	1	pseudobulk	DESeq2	LRT	0
773	KC_AB1	c11.1	0.0405330794761641	0.786529522645146	1	pseudobulk	DESeq2	LRT	0
774	KC_AB1	c12.1	-0.711243304931433	0.325528791936333	1	pseudobulk	DESeq2	LRT	0
775	KC_AB1	c12.2	0.600121177806781	0.443446904404439	1	pseudobulk	DESeq2	LRT	0
776	KC_AB1	C1GalTA	-0.571615804202721	0.203191070548241	1	pseudobulk	DESeq2	LRT	0
777	KC_AB1	C3G	-0.130190143654523	0.474124236114471	1	pseudobulk	DESeq2	LRT	0
778	KC_AB1	C901	-1.42340527600048	0.513120142384162	1	pseudobulk	DESeq2	LRT	0
779	KC_AB1	ca	-0.339488504252087	0.619629085265621	1	pseudobulk	DESeq2	LRT	0
780	KC_AB1	Ca-alpha1D	-0.121561032276219	0.482733972672001	1	pseudobulk	DESeq2	LRT	0
781	KC_AB1	Ca-alpha1T	-0.102763420695232	0.540200544883112	1	pseudobulk	DESeq2	LRT	0
782	KC_AB1	Ca-beta	-0.391277030016135	0.0596997677035638	1	pseudobulk	DESeq2	LRT	0
783	KC_AB1	Ca-Ma2d	-0.142624821930229	0.754476726540079	1	pseudobulk	DESeq2	LRT	0
784	KC_AB1	CaBP1	-0.4299202915027	0.560767741299369	1	pseudobulk	DESeq2	LRT	0
785	KC_AB1	cac	-0.142688129186716	0.292924611303294	1	pseudobulk	DESeq2	LRT	0
786	KC_AB1	cact	-0.0820924571405174	0.445481356902925	1	pseudobulk	DESeq2	LRT	0
787	KC_AB1	cactin	-0.210051026889788	0.734238445214958	1	pseudobulk	DESeq2	LRT	0
788	KC_AB1	Cad86C	0.0571155708876178	1	1	pseudobulk	DESeq2	LRT	0
789	KC_AB1	Cad87A	0.0610601824346463	0.828855660092931	1	pseudobulk	DESeq2	LRT	0
790	KC_AB1	Cad96Ca	0.553663151357419	1	1	pseudobulk	DESeq2	LRT	0
791	KC_AB1	Cad99C	3.81892523345342	0.259683132508622	1	pseudobulk	DESeq2	LRT	0
792	KC_AB1	CadN	-0.557249659603727	0.137662771932747	1	pseudobulk	DESeq2	LRT	0
793	KC_AB1	CadN2	-1.07357325230907	0.573798217974042	1	pseudobulk	DESeq2	LRT	0

794	KC_AB1	Cadps	-0.36400219339594	0.0644024591121815	1	pseudobulk	DESeq2	LRT		0
795	KC_AB1	Caf1-105	0.729165426791457	1	1	pseudobulk	DESeq2	LRT		0
796	KC_AB1	Caf1-180	0.849606887838128	1	1	pseudobulk	DESeq2	LRT		0
797	KC_AB1	Caf1-55	0.682092149508777	0.457577543683333	1	pseudobulk	DESeq2	LRT		0
798	KC_AB1	cag	-0.581481673170329	0.571720799911659	1	pseudobulk	DESeq2	LRT		0
799	KC_AB1	CAH1	-2.86261772566742	0.0434228289433301	1	pseudobulk	DESeq2	LRT		0
800	KC_AB1	CAH2	0.211788993319012	1	1	pseudobulk	DESeq2	LRT		0
801	KC_AB1	CAH3	-0.305550924941481	0.569091312540763	1	pseudobulk	DESeq2	LRT		0
802	KC_AB1	CAH7	-0.489375207759863	0.599182593162369	1	pseudobulk	DESeq2	LRT		0
803	KC_AB1	CAH8	-0.147271671984344	0.667320678385577	1	pseudobulk	DESeq2	LRT		0
804	KC_AB1	CAHbeta	-1.05479360218703	0.201966898488721	1	pseudobulk	DESeq2	LRT		0
805	KC_AB1	cal1	0.161608764965907	0.712631915229639	1	pseudobulk	DESeq2	LRT		0
806	KC_AB1	CalpA	0.198938790866276	0.63462939333618	1	pseudobulk	DESeq2	LRT		0
807	KC_AB1	CalpB	0.385709132434428	0.763200116979291	1	pseudobulk	DESeq2	LRT		0
808	KC_AB1	CalpC	0.849606887838128	1	1	pseudobulk	DESeq2	LRT		0
809	KC_AB1	Calr	-0.151715858045016	0.523678156014948	1	pseudobulk	DESeq2	LRT		0
810	KC_AB1	Cals	-0.0384280374036429	0.662194899325781	1	pseudobulk	DESeq2	LRT		0
811	KC_AB1	Calx	-0.665951265905033	0.170492541061585	1	pseudobulk	DESeq2	LRT		0
812	KC_AB1	calypso	0.198383194125719	0.745833446523837	1	pseudobulk	DESeq2	LRT		0
813	KC_AB1	Cam	0.311452806461415	0.0146069624478815	1	pseudobulk	DESeq2	LRT		0
814	KC_AB1	CaMKI	-0.192448444561008	0.271941425517969	1	pseudobulk	DESeq2	LRT		0
815	KC_AB1	CaMKII	-0.14507226170929	0.317922132729177	1	pseudobulk	DESeq2	LRT		0
816	KC_AB1	Camp	-0.190675160363677	0.71878295317961	1	pseudobulk	DESeq2	LRT		0
817	KC_AB1	Camta	-0.701800150053325	0.150406789814515	1	pseudobulk	DESeq2	LRT		0
818	KC_AB1	CanA-14F	0.20566112524012	0.212314000319813	1	pseudobulk	DESeq2	LRT		0
819	KC_AB1	CanB	-0.158923891538311	0.341040763507467	1	pseudobulk	DESeq2	LRT		0
820	KC_AB1	CanB2	0.25113539497544	0.4661094367423	1	pseudobulk	DESeq2	LRT		0
821	KC_AB1	Cand1	-1.46681791111685	0.121630962582859	1	pseudobulk	DESeq2	LRT		0
822	KC_AB1	CAP	0.828205135347231	0.0170083213460023	1	pseudobulk	DESeq2	LRT		0
823	KC_AB1	Cap-D3	1.57973095756595	0.604201189652865	1	pseudobulk	DESeq2	LRT		0
824	KC_AB1	Cap-G	1.46091799385084	1	1	pseudobulk	DESeq2	LRT		0
825	KC_AB1	Cap-H2	0.0791655390147026	0.692359152820911	1	pseudobulk	DESeq2	LRT		0
826	KC_AB1	Capa	0.849606887838128	1	1	pseudobulk	DESeq2	LRT		0
827	KC_AB1	Caper	-0.0910776200411172	0.458843390926733	1	pseudobulk	DESeq2	LRT		0
828	KC_AB1	Capr	-0.36503268847147	0.43512840537858	1	pseudobulk	DESeq2	LRT		0
829	KC_AB1	caps	-0.122288135729178	0.611621900854924	1	pseudobulk	DESeq2	LRT		0
830	KC_AB1	capt	0.12066167562805	0.456614417207776	1	pseudobulk	DESeq2	LRT		0
831	KC_AB1	capu	-0.438853743265092	0.179384704100642	1	pseudobulk	DESeq2	LRT		0
832	KC_AB1	car	0.147292508437647	0.687434473015414	1	pseudobulk	DESeq2	LRT		0
833	KC_AB1	CARPB	-1.59623373582255	0.336594258933996	1	pseudobulk	DESeq2	LRT		0
834	KC_AB1	CarT	1.59863986092676	1	1	pseudobulk	DESeq2	LRT		0
835	KC_AB1	cas	2.31997030691033	0.813169507846918	1	pseudobulk	DESeq2	LRT		0
836	KC_AB1	CASK	0.0538363747668072	0.722666985606099	1	pseudobulk	DESeq2	LRT		0
837	KC_AB1	casp	-0.0272606437707978	0.730853432536041	1	pseudobulk	DESeq2	LRT		0
838	KC_AB1	cass	-0.200291935868076	0.477577097297486	1	pseudobulk	DESeq2	LRT		0
839	KC_AB1	Cat	-0.0827798323241159	0.714543930409325	1	pseudobulk	DESeq2	LRT		0
840	KC_AB1	cathD	-0.339679827572775	0.551333077141456	1	pseudobulk	DESeq2	LRT		0
841	KC_AB1	Catsup	-0.0761078153738956	0.847874517314026	1	pseudobulk	DESeq2	LRT		0
842	KC_AB1	cav	0.272635854031061	1	1	pseudobulk	DESeq2	LRT		0
843	KC_AB1	caz	-0.525051157782579	0.250442623643274	1	pseudobulk	DESeq2	LRT		0
844	KC_AB1	cbc	-0.119943655182149	0.796592667534239	1	pseudobulk	DESeq2	LRT		0
845	KC_AB1	Cbl	0.155194622954485	0.568645185905069	1	pseudobulk	DESeq2	LRT		0
846	KC_AB1	Cbp20	-0.0982372184208003	0.708130197814021	1	pseudobulk	DESeq2	LRT		0

847	KC_AB1	Cbp80	-0.0763393120918949	0.673176932695277	1	pseudobulk	DESeq2	LRT	0
848	KC_AB1	cbs	0.540155676651739	0.428256094438421	1	pseudobulk	DESeq2	LRT	0
849	KC_AB1	Cbs	-0.485738487007362	0.565751794905869	1	pseudobulk	DESeq2	LRT	0
850	KC_AB1	cbt	0.232378231960548	0.6554368897732	1	pseudobulk	DESeq2	LRT	0
851	KC_AB1	CCAP-R	-0.486107420439249	0.612399807608933	1	pseudobulk	DESeq2	LRT	0
852	KC_AB1	CCDC53	-0.93633086889391	0.466502824089423	1	pseudobulk	DESeq2	LRT	0
853	KC_AB1	Ccdc58	0.128104381720756	0.762545265382452	1	pseudobulk	DESeq2	LRT	0
854	KC_AB1	Ccdc85	-0.389346928562833	0.820127740800904	1	pseudobulk	DESeq2	LRT	0
855	KC_AB1	CchI	0.969980693299675	0.0272250450949896	1	pseudobulk	DESeq2	LRT	0
856	KC_AB1	CCKLR-17D1	0.291805497136617	0.665141115211689	1	pseudobulk	DESeq2	LRT	0
857	KC_AB1	CCKLR-17D3	-0.386029499617288	0.615823355857063	1	pseudobulk	DESeq2	LRT	0
858	KC_AB1	Ccm3	-0.477445568915142	0.320122012184035	1	pseudobulk	DESeq2	LRT	0
859	KC_AB1	Ccn	0.16305154409917	0.51753168157003	1	pseudobulk	DESeq2	LRT	0
860	KC_AB1	Ccs	0.647552430574112	0.661192923609423	1	pseudobulk	DESeq2	LRT	0
861	KC_AB1	CCT1	0.188961938005151	0.758053727539513	1	pseudobulk	DESeq2	LRT	0
862	KC_AB1	CCT2	0.808702052782283	0.417723971679897	1	pseudobulk	DESeq2	LRT	0
863	KC_AB1	CCT3	-0.52875978003167	0.572740849012911	1	pseudobulk	DESeq2	LRT	0
864	KC_AB1	CCT4	-0.0194823255053251	0.716551781416957	1	pseudobulk	DESeq2	LRT	0
865	KC_AB1	CCT5	0.345632640090459	0.778060742802859	1	pseudobulk	DESeq2	LRT	0
866	KC_AB1	CCT6	-1.02976848957274	0.32848017509352	1	pseudobulk	DESeq2	LRT	0
867	KC_AB1	CCT7	0.544095643413044	0.43473996722166	1	pseudobulk	DESeq2	LRT	0
868	KC_AB1	CCT8	0.735502204393345	0.385594838755112	1	pseudobulk	DESeq2	LRT	0
869	KC_AB1	Ccz1	-0.287877388567286	0.693728423617919	1	pseudobulk	DESeq2	LRT	0
870	KC_AB1	CD98hc	0.194429935605947	0.573745002632874	1	pseudobulk	DESeq2	LRT	0
871	KC_AB1	Cda5	0.126650801583393	0.417221105228997	1	pseudobulk	DESeq2	LRT	0
872	KC_AB1	cdc14	0.174980985906862	0.582728229461287	1	pseudobulk	DESeq2	LRT	0
873	KC_AB1	Cdc16	-0.137492980608447	0.848789029973766	1	pseudobulk	DESeq2	LRT	0
874	KC_AB1	Cdc23	0.799796899886614	1	1	pseudobulk	DESeq2	LRT	0
875	KC_AB1	Cdc27	0.401848575528805	0.39320808525224	1	pseudobulk	DESeq2	LRT	0
876	KC_AB1	Cdc2rk	0.428351707396322	0.724333267141454	1	pseudobulk	DESeq2	LRT	0
877	KC_AB1	Cdc37	0.527618545783351	0.296161062133762	1	pseudobulk	DESeq2	LRT	0
878	KC_AB1	Cdc42	0.262959308969923	0.563291041731776	1	pseudobulk	DESeq2	LRT	0
879	KC_AB1	CDC45L	-0.155934067093443	0.914618786789969	1	pseudobulk	DESeq2	LRT	0
880	KC_AB1	Cdc5	-0.109701050341389	0.732374691129089	1	pseudobulk	DESeq2	LRT	0
881	KC_AB1	CDC50	0.684332320690048	0.195212549873261	1	pseudobulk	DESeq2	LRT	0
882	KC_AB1	Cdc7	-0.929367747612911	0.278008242667961	1	pseudobulk	DESeq2	LRT	0
883	KC_AB1	Cdep	-0.442768130878481	0.0876752773950031	1	pseudobulk	DESeq2	LRT	0
884	KC_AB1	CdGAPr	-0.753296754539203	0.311853839744763	1	pseudobulk	DESeq2	LRT	0
885	KC_AB1	cdi	-0.23682806111435	0.412671603402547	1	pseudobulk	DESeq2	LRT	0
886	KC_AB1	cDIP	0.849606887838128	1	1	pseudobulk	DESeq2	LRT	0
887	KC_AB1	Cdk12	-1.04365511336313	0.0529197685757204	1	pseudobulk	DESeq2	LRT	0
888	KC_AB1	Cdk2	-0.51506715067314	0.574958291265875	1	pseudobulk	DESeq2	LRT	0
889	KC_AB1	CDK2AP1	-0.533280533067402	0.445622514120167	1	pseudobulk	DESeq2	LRT	0
890	KC_AB1	Cdk5	0.389048938864051	0.526006791516213	1	pseudobulk	DESeq2	LRT	0
891	KC_AB1	Cdk5alpha	-0.0926078133626076	0.478003150183593	1	pseudobulk	DESeq2	LRT	0
892	KC_AB1	Cdk7	0.129537403634361	0.816139579365126	1	pseudobulk	DESeq2	LRT	0
893	KC_AB1	Cdk8	-0.0806766248263104	1	1	pseudobulk	DESeq2	LRT	0
894	KC_AB1	Cdk9	-0.133262092273432	0.768694560302247	1	pseudobulk	DESeq2	LRT	0
895	KC_AB1	cdm	-0.252007755767526	0.478218470411848	1	pseudobulk	DESeq2	LRT	0
896	KC_AB1	Cds	-0.100102837846583	0.719703604399935	1	pseudobulk	DESeq2	LRT	0
897	KC_AB1	Ced-12	-0.102628265165026	0.788913869082859	1	pseudobulk	DESeq2	LRT	0
898	KC_AB1	ced-6	-0.142364677196449	0.930943914900445	1	pseudobulk	DESeq2	LRT	0
899	KC_AB1	Cen	-0.223772821034657	0.560255259896992	1	pseudobulk	DESeq2	LRT	0

900	KC_AB1	CenB1A	-0.467558183226146	0.554659679380581	1	pseudobulk	DESeq2	LRT		0
901	KC_AB1	CenG1A	-0.690762237401711	0.173963020851409	1	pseudobulk	DESeq2	LRT		0
902	KC_AB1	Cep97	-0.830286346832503	0.276423604177326	1	pseudobulk	DESeq2	LRT		0
903	KC_AB1	cer	-0.579356275199074	0.54631486948535	1	pseudobulk	DESeq2	LRT		0
904	KC_AB1	Cerk	-0.148237430793558	0.60394618600045	1	pseudobulk	DESeq2	LRT		0
905	KC_AB1	cert	0.0643270979184859	0.704113322402075	1	pseudobulk	DESeq2	LRT		0
906	KC_AB1	cerv	0.603061041589212	0.88797127252248	1	pseudobulk	DESeq2	LRT		0
907	KC_AB1	Cf2	-0.270087144673098	0.471329931783852	1	pseudobulk	DESeq2	LRT		0
908	KC_AB1	Cfp1	-1.55475071969949	0.249445567673804	1	pseudobulk	DESeq2	LRT		0
909	KC_AB1	cg	-0.518030355712204	0.0399026213289305	1	pseudobulk	DESeq2	LRT		0
910	KC_AB1	CG10011	0.58972449057805	0.11003792131392	1	pseudobulk	DESeq2	LRT		0
911	KC_AB1	CG10019	2.04017343731623	0.530151172476187	1	pseudobulk	DESeq2	LRT		0
912	KC_AB1	CG10026	3.41844045086015	0.351942490385471	1	pseudobulk	DESeq2	LRT		0
913	KC_AB1	CG10035	3.150585340203	0.0855758437014555	1	pseudobulk	DESeq2	LRT		0
914	KC_AB1	CG10038	1.23458570765338	0.666562252120211	1	pseudobulk	DESeq2	LRT		0
915	KC_AB1	CG10050	3.10917861892161	0.295975473292483	1	pseudobulk	DESeq2	LRT		0
916	KC_AB1	CG10053	0.811967575378733	0.824576705147507	1	pseudobulk	DESeq2	LRT		0
917	KC_AB1	CG10055	-0.210617017148462	0.591939243107672	1	pseudobulk	DESeq2	LRT		0
918	KC_AB1	CG10068	0.0715787292539758	0.797407737092682	1	pseudobulk	DESeq2	LRT		0
919	KC_AB1	CG10075	0.44709359722457	0.558499510184499	1	pseudobulk	DESeq2	LRT		0
920	KC_AB1	CG10077	-0.399925396593794	0.0133375316019156	1	pseudobulk	DESeq2	LRT		0
921	KC_AB1	CG10082	0.0536333352285686	0.841746420981543	1	pseudobulk	DESeq2	LRT		0
922	KC_AB1	CG10089	-0.163936666037269	0.592361878267179	1	pseudobulk	DESeq2	LRT		0
923	KC_AB1	CG10098	0.260035447421855	0.640290237059377	1	pseudobulk	DESeq2	LRT		0
924	KC_AB1	CG10103	-0.623700878595126	0.411703231902445	1	pseudobulk	DESeq2	LRT		0
925	KC_AB1	CG10116	-0.287567257126996	0.920650037231581	1	pseudobulk	DESeq2	LRT		0
926	KC_AB1	CG10126	0.931109140710098	1	1	pseudobulk	DESeq2	LRT		0
927	KC_AB1	CG10132	-0.227583918906297	0.475983530197218	1	pseudobulk	DESeq2	LRT		0
928	KC_AB1	CG10137	-0.517597324848768	0.224512763723349	1	pseudobulk	DESeq2	LRT		0
929	KC_AB1	CG10139	0.537821991295403	0.361992963050052	1	pseudobulk	DESeq2	LRT		0
930	KC_AB1	CG10147	-0.398882617605776	0.685978051541032	1	pseudobulk	DESeq2	LRT		0
931	KC_AB1	CG10151	0.454995958355617	0.621187717192779	1	pseudobulk	DESeq2	LRT		0
932	KC_AB1	CG10165	0.757610437163003	1	1	pseudobulk	DESeq2	LRT		0
933	KC_AB1	CG10166	0.944079396186517	0.773436119617132	1	pseudobulk	DESeq2	LRT		0
934	KC_AB1	CG10171	-0.226251395958154	0.740602201005774	1	pseudobulk	DESeq2	LRT		0
935	KC_AB1	CG10188	-0.497963840293346	0.175043442505502	1	pseudobulk	DESeq2	LRT		0
936	KC_AB1	CG10189	-0.0127846790391957	1	1	pseudobulk	DESeq2	LRT		0
937	KC_AB1	CG10194	-0.0461489237270862	0.761536383627201	1	pseudobulk	DESeq2	LRT		0
938	KC_AB1	CG10205	-2.44173222908943	0.322526248395761	1	pseudobulk	DESeq2	LRT		0
939	KC_AB1	CG10208	-2.49490202879426	0.249225272330293	1	pseudobulk	DESeq2	LRT		0
940	KC_AB1	CG10209	1.10628801461199	0.286923185565829	1	pseudobulk	DESeq2	LRT		0
941	KC_AB1	CG10214	-0.686717508550228	0.458515789217738	1	pseudobulk	DESeq2	LRT		0
942	KC_AB1	CG10222	0.492502386827429	1	1	pseudobulk	DESeq2	LRT		0
943	KC_AB1	CG10237	2.06541384650887	0.795866856626082	1	pseudobulk	DESeq2	LRT		0
944	KC_AB1	CG1024	-0.561798184976518	0.621679575608405	1	pseudobulk	DESeq2	LRT		0
945	KC_AB1	CG10252	0.634960112221505	1	1	pseudobulk	DESeq2	LRT		0
946	KC_AB1	CG10254	0.193553091648977	0.645908184693433	1	pseudobulk	DESeq2	LRT		0
947	KC_AB1	CG10257	-0.18514273433041	0.810963970822343	1	pseudobulk	DESeq2	LRT		0
948	KC_AB1	CG10265	-0.568175753141124	0.564916031795706	1	pseudobulk	DESeq2	LRT		0
949	KC_AB1	CG10268	0.162118002617676	1	1	pseudobulk	DESeq2	LRT		0
950	KC_AB1	CG10274	-0.157923675920927	0.821138458042109	1	pseudobulk	DESeq2	LRT		0
951	KC_AB1	CG10283	-0.570796619646087	0.478468691177378	1	pseudobulk	DESeq2	LRT		0
952	KC_AB1	CG10286	-1.627192898534	0.304970217103771	1	pseudobulk	DESeq2	LRT		0

953	KC_AB1	CG10311	1.64178394674445	0.488899249824213	1	pseudobulk	DESeq2	LRT	0
954	KC_AB1	CG10321	0.242817372885887	0.532365833478647	1	pseudobulk	DESeq2	LRT	0
955	KC_AB1	CG10324	-0.760300983525441	0.530235377688841	1	pseudobulk	DESeq2	LRT	0
956	KC_AB1	CG10333	0.495669370175915	0.489210506288562	1	pseudobulk	DESeq2	LRT	0
957	KC_AB1	CG10336	0.769893673767581	1	1	pseudobulk	DESeq2	LRT	0
958	KC_AB1	CG10338	0.849374877002964	0.690605773320115	1	pseudobulk	DESeq2	LRT	0
959	KC_AB1	CG10340	-0.381240509409431	0.507203714074043	1	pseudobulk	DESeq2	LRT	0
960	KC_AB1	CG10341	-0.0530517156364519	0.830245990512008	1	pseudobulk	DESeq2	LRT	0
961	KC_AB1	CG10343	0.183764970291085	0.699344428044431	1	pseudobulk	DESeq2	LRT	0
962	KC_AB1	CG10347	-0.641351637293636	0.513414599395929	1	pseudobulk	DESeq2	LRT	0
963	KC_AB1	CG10352	0.0160940546605626	1	1	pseudobulk	DESeq2	LRT	0
964	KC_AB1	CG10353	0.419530637258656	0.378613944839545	1	pseudobulk	DESeq2	LRT	0
965	KC_AB1	CG10359	-0.830062897938551	0.717724257445671	1	pseudobulk	DESeq2	LRT	0
966	KC_AB1	CG10362	-0.0827086611575242	0.873103660422865	1	pseudobulk	DESeq2	LRT	0
967	KC_AB1	CG10365	-1.86869955983474	0.182659808680287	1	pseudobulk	DESeq2	LRT	0
968	KC_AB1	CG10366	0.0505764833529874	0.694051322549982	1	pseudobulk	DESeq2	LRT	0
969	KC_AB1	CG10375	-0.0474309482391059	0.843905135690161	1	pseudobulk	DESeq2	LRT	0
970	KC_AB1	CG10376	0.156052446553484	0.766503759715252	1	pseudobulk	DESeq2	LRT	0
971	KC_AB1	CG10383	-2.53791486313253	0.132351107369381	1	pseudobulk	DESeq2	LRT	0
972	KC_AB1	CG10384	1.99749445572676	0.662793464935471	1	pseudobulk	DESeq2	LRT	0
973	KC_AB1	CG10395	0.292191165764077	0.564668714870593	1	pseudobulk	DESeq2	LRT	0
974	KC_AB1	CG10413	-0.150999205706447	0.686272020112664	1	pseudobulk	DESeq2	LRT	0
975	KC_AB1	CG10417	0.203654558104813	0.458483208921993	1	pseudobulk	DESeq2	LRT	0
976	KC_AB1	CG10418	-0.25356083300775	0.706886661864525	1	pseudobulk	DESeq2	LRT	0
977	KC_AB1	CG10425	-1.87153086029172	0.188545383190298	1	pseudobulk	DESeq2	LRT	0
978	KC_AB1	CG10428	-1.20613899009612	0.363100250252327	1	pseudobulk	DESeq2	LRT	0
979	KC_AB1	CG10431	-0.932844210943508	0.607958962231227	1	pseudobulk	DESeq2	LRT	0
980	KC_AB1	CG10433	2.00276415192897	0.50522933963966	1	pseudobulk	DESeq2	LRT	0
981	KC_AB1	CG10435	0.730737886840971	0.561092293658729	1	pseudobulk	DESeq2	LRT	0
982	KC_AB1	CG10444	1.18192749288364	0.227888342777116	1	pseudobulk	DESeq2	LRT	0
983	KC_AB1	CG10463	-0.665790677704216	0.548882228764948	1	pseudobulk	DESeq2	LRT	0
984	KC_AB1	CG10465	0.0332513887459855	0.571407920972715	1	pseudobulk	DESeq2	LRT	0
985	KC_AB1	CG10466	0.0834230340082815	0.887285967406931	1	pseudobulk	DESeq2	LRT	0
986	KC_AB1	CG10467	2.94557542850455	0.360649399262036	1	pseudobulk	DESeq2	LRT	0
987	KC_AB1	CG10470	-0.409956903204788	0.498185042332706	1	pseudobulk	DESeq2	LRT	0
988	KC_AB1	CG10479	2.06531092668974	0.944672961903726	1	pseudobulk	DESeq2	LRT	0
989	KC_AB1	CG10483	0.641848987256819	0.226643529664916	1	pseudobulk	DESeq2	LRT	0
990	KC_AB1	CG10492	0.416914315923449	0.560718766601746	1	pseudobulk	DESeq2	LRT	0
991	KC_AB1	CG10494	0.250523013159441	0.316602169713957	1	pseudobulk	DESeq2	LRT	0
992	KC_AB1	CG10495	0.667434521010749	0.814898364568048	1	pseudobulk	DESeq2	LRT	0
993	KC_AB1	CG10508	0.425622275366017	0.0900572786564119	1	pseudobulk	DESeq2	LRT	0
994	KC_AB1	CG10512	1.33016929913258	1	1	pseudobulk	DESeq2	LRT	0
995	KC_AB1	CG10513	-0.521394547461525	0.668933627786741	1	pseudobulk	DESeq2	LRT	0
996	KC_AB1	CG10514	0.237022465848507	1	1	pseudobulk	DESeq2	LRT	0
997	KC_AB1	CG10516	1.3171144649733	0.553338063991534	1	pseudobulk	DESeq2	LRT	0
998	KC_AB1	CG10527	-0.19840274673302	0.851090966472495	1	pseudobulk	DESeq2	LRT	0
999	KC_AB1	CG10543	-0.0923217660247545	0.553155598716138	1	pseudobulk	DESeq2	LRT	0
100	KC_AB1	CG10550	-2.59610313770632	0.291483550812208	1	pseudobulk	DESeq2	LRT	0
100	KC_AB1	CG10555	-0.647043728920833	0.315839593135864	1	pseudobulk	DESeq2	LRT	0
100	KC_AB1	CG10559	0.849606887838128	1	1	pseudobulk	DESeq2	LRT	0
100	KC_AB1	CG10561	-0.643631591515388	0.435525877364073	1	pseudobulk	DESeq2	LRT	0
100	KC_AB1	CG10562	0.366247714696288	1	1	pseudobulk	DESeq2	LRT	0
100	KC_AB1	CG10565	0.280016527420781	0.668693079031815	1	pseudobulk	DESeq2	LRT	0

100	KC_AB1	CG10576	0.601189526907326	0.44492129585771	1	pseudobulk	DESeq2	LRT	0
100	KC_AB1	CG10581	-0.582303408297223	0.779363959297745	1	pseudobulk	DESeq2	LRT	0
100	KC_AB1	CG10584	0.237022465848507	1	1	pseudobulk	DESeq2	LRT	0
100	KC_AB1	CG10600	-0.387826852430439	0.378950736925737	1	pseudobulk	DESeq2	LRT	0
101	KC_AB1	CG10602	-0.319630084779878	0.705872055106584	1	pseudobulk	DESeq2	LRT	0
101	KC_AB1	CG10616	-0.254155720784554	0.701501409784063	1	pseudobulk	DESeq2	LRT	0
101	KC_AB1	CG10623	1.59863986092676	1	1	pseudobulk	DESeq2	LRT	0
101	KC_AB1	CG10628	0.661620405393269	0.870922194329637	1	pseudobulk	DESeq2	LRT	0
101	KC_AB1	CG10631	-0.412277690511642	0.532285262166559	1	pseudobulk	DESeq2	LRT	0
101	KC_AB1	CG10638	-0.546774565518499	0.509621030571564	1	pseudobulk	DESeq2	LRT	0
101	KC_AB1	CG10646	1.28181171881137	0.295715467294824	1	pseudobulk	DESeq2	LRT	0
101	KC_AB1	CG10654	-0.27640204588722	0.706674709536585	1	pseudobulk	DESeq2	LRT	0
101	KC_AB1	CG10657	3.16697464674408	0.406679230261689	1	pseudobulk	DESeq2	LRT	0
101	KC_AB1	CG10660	0.849606887838128	1	1	pseudobulk	DESeq2	LRT	0
102	KC_AB1	CG10669	0.146431221448733	0.739826778349288	1	pseudobulk	DESeq2	LRT	0
102	KC_AB1	CG10672	-0.433406574444181	0.640884484167899	1	pseudobulk	DESeq2	LRT	0
102	KC_AB1	CG10674	0.550618848155738	0.323933977789861	1	pseudobulk	DESeq2	LRT	0
102	KC_AB1	CG10681	0.24660551183679	0.708756072977024	1	pseudobulk	DESeq2	LRT	0
102	KC_AB1	CG10713	-0.171042685236645	0.721981255791192	1	pseudobulk	DESeq2	LRT	0
102	KC_AB1	CG10730	0.849606887838128	1	1	pseudobulk	DESeq2	LRT	0
102	KC_AB1	CG10731	-0.0324556458618587	0.831649896247166	1	pseudobulk	DESeq2	LRT	0
102	KC_AB1	CG10734	0.849606887838128	1	1	pseudobulk	DESeq2	LRT	0
102	KC_AB1	CG10737	1.09190320489132	0.0389956979967688	1	pseudobulk	DESeq2	LRT	0
102	KC_AB1	CG10738	0.380333149837687	0.554215669516322	1	pseudobulk	DESeq2	LRT	0
103	KC_AB1	CG1074	1.16545121576438	0.257441084692782	1	pseudobulk	DESeq2	LRT	0
103	KC_AB1	CG10747	0.375403994261234	1	1	pseudobulk	DESeq2	LRT	0
103	KC_AB1	CG10752	0.849606887838128	1	1	pseudobulk	DESeq2	LRT	0
103	KC_AB1	CG10778	-0.299520144004726	0.71199514089121	1	pseudobulk	DESeq2	LRT	0
103	KC_AB1	CG10793	0.849606887838128	1	1	pseudobulk	DESeq2	LRT	0
103	KC_AB1	CG10795	0.148883153367057	0.802925958209471	1	pseudobulk	DESeq2	LRT	0
103	KC_AB1	CG10802	0.861079740098373	0.523036038207122	1	pseudobulk	DESeq2	LRT	0
103	KC_AB1	CG10803	-0.78878277272578	0.192458978478481	1	pseudobulk	DESeq2	LRT	0
103	KC_AB1	CG10804	-0.0905161473498433	0.776561017893507	1	pseudobulk	DESeq2	LRT	0
103	KC_AB1	CG10809	-0.545525103613364	0.353908177710779	1	pseudobulk	DESeq2	LRT	0
104	KC_AB1	CG10841	0.849606887838128	1	1	pseudobulk	DESeq2	LRT	0
104	KC_AB1	CG10853	2.31997030691033	0.813169507846918	1	pseudobulk	DESeq2	LRT	0
104	KC_AB1	CG10863	-0.86762028319303	0.50365464940089	1	pseudobulk	DESeq2	LRT	0
104	KC_AB1	CG10864	1.59863986092676	1	1	pseudobulk	DESeq2	LRT	0
104	KC_AB1	CG10866	-0.0828852622748296	0.613234315205138	1	pseudobulk	DESeq2	LRT	0
104	KC_AB1	CG10874	-1.1408326826432	0.263968673518829	1	pseudobulk	DESeq2	LRT	0
104	KC_AB1	CG10889	-0.216693811357252	0.792972130381734	1	pseudobulk	DESeq2	LRT	0
104	KC_AB1	CG10898	-0.432516097481131	0.694576006651378	1	pseudobulk	DESeq2	LRT	0
104	KC_AB1	CG1090	0.0163266017762952	0.746196527924937	1	pseudobulk	DESeq2	LRT	0
104	KC_AB1	CG10903	1.03084564893558	0.459294569170696	1	pseudobulk	DESeq2	LRT	0
105	KC_AB1	CG10904	-0.861166666178722	0.425297775217952	1	pseudobulk	DESeq2	LRT	0
105	KC_AB1	CG10907	0.0685808820662935	0.838906273652398	1	pseudobulk	DESeq2	LRT	0
105	KC_AB1	CG10914	-0.693759677532312	0.58662232916293	1	pseudobulk	DESeq2	LRT	0
105	KC_AB1	CG10916	-2.01466652963376	0.158598378984463	1	pseudobulk	DESeq2	LRT	0
105	KC_AB1	CG10932	-0.394989550495416	0.626019849993487	1	pseudobulk	DESeq2	LRT	0
105	KC_AB1	CG10936	-0.216210029276812	1	1	pseudobulk	DESeq2	LRT	0
105	KC_AB1	CG10948	-0.428355470902696	0.265169758182506	1	pseudobulk	DESeq2	LRT	0
105	KC_AB1	CG10949	1.02167340221035	0.138967086233358	1	pseudobulk	DESeq2	LRT	0
105	KC_AB1	CG10958	0.0662018739088962	0.901509670753111	1	pseudobulk	DESeq2	LRT	0

105	KC_AB1	CG10959	-0.645214632668674	0.547665760157391	1	pseudobulk	DESeq2	LRT	0
106	KC_AB1	CG10960	-0.134411338305934	0.742375457877691	1	pseudobulk	DESeq2	LRT	0
106	KC_AB1	CG10973	0.0194440017531989	0.757953570340267	1	pseudobulk	DESeq2	LRT	0
106	KC_AB1	CG10979	0.693771467485011	0.402363506181607	1	pseudobulk	DESeq2	LRT	0
106	KC_AB1	CG10984	-0.381987795264345	0.479670953148352	1	pseudobulk	DESeq2	LRT	0
106	KC_AB1	CG11000	-0.235498080275164	0.313479225715065	1	pseudobulk	DESeq2	LRT	0
106	KC_AB1	CG11007	-0.325281266118045	0.649826579925418	1	pseudobulk	DESeq2	LRT	0
106	KC_AB1	CG1103	-0.726110080268322	0.422567070134674	1	pseudobulk	DESeq2	LRT	0
106	KC_AB1	CG11030	-0.43614826865473	0.394346570566717	1	pseudobulk	DESeq2	LRT	0
106	KC_AB1	CG11035	0.425788307953799	0.471260560858037	1	pseudobulk	DESeq2	LRT	0
106	KC_AB1	CG11041	0.849606887838128	1	1	pseudobulk	DESeq2	LRT	0
107	KC_AB1	CG1105	0.0734373504778922	0.783583823151703	1	pseudobulk	DESeq2	LRT	0
107	KC_AB1	CG11050	0.1854171057685	0.571228556347103	1	pseudobulk	DESeq2	LRT	0
107	KC_AB1	CG11069	-0.830062897938551	0.717724257445671	1	pseudobulk	DESeq2	LRT	0
107	KC_AB1	CG11070	-1.22141097842353	0.272440819825247	1	pseudobulk	DESeq2	LRT	0
107	KC_AB1	CG11076	0.181738999312553	0.763315739194043	1	pseudobulk	DESeq2	LRT	0
107	KC_AB1	CG11077	0.300060883941439	0.536948234424551	1	pseudobulk	DESeq2	LRT	0
107	KC_AB1	CG11089	0.0160940546605174	1	1	pseudobulk	DESeq2	LRT	0
107	KC_AB1	CG11095	-1.30534605254628	0.402197043097855	1	pseudobulk	DESeq2	LRT	0
107	KC_AB1	CG11099	1.30156770856991	0.0749201092033278	1	pseudobulk	DESeq2	LRT	0
107	KC_AB1	CG11103	0.227882794835851	0.793616241696888	1	pseudobulk	DESeq2	LRT	0
108	KC_AB1	CG11109	0.985223513365244	0.281319807461732	1	pseudobulk	DESeq2	LRT	0
108	KC_AB1	CG11110	0.131491014629656	0.732141371065829	1	pseudobulk	DESeq2	LRT	0
108	KC_AB1	CG11122	-0.0321857867135121	0.554432173734583	1	pseudobulk	DESeq2	LRT	0
108	KC_AB1	CG11123	0.120336175449406	1	1	pseudobulk	DESeq2	LRT	0
108	KC_AB1	CG11127	1.69394476264167	0.298903002235196	1	pseudobulk	DESeq2	LRT	0
108	KC_AB1	CG1113	0.236697511454165	1	1	pseudobulk	DESeq2	LRT	0
108	KC_AB1	CG11134	0.220458610892702	1	1	pseudobulk	DESeq2	LRT	0
108	KC_AB1	CG11137	0.0730129419213387	0.551087681118789	1	pseudobulk	DESeq2	LRT	0
108	KC_AB1	CG11141	-0.117900824014375	0.733218119909652	1	pseudobulk	DESeq2	LRT	0
108	KC_AB1	CG11147	2.68453167034132	0.550970543274	1	pseudobulk	DESeq2	LRT	0
109	KC_AB1	CG11151	0.494253768969903	0.108755125997938	1	pseudobulk	DESeq2	LRT	0
109	KC_AB1	CG11155	0.125716369882381	0.492060112078115	1	pseudobulk	DESeq2	LRT	0
109	KC_AB1	CG11158	0.849606887838128	1	1	pseudobulk	DESeq2	LRT	0
109	KC_AB1	CG11159	1.16179233607502	0.611294215080484	1	pseudobulk	DESeq2	LRT	0
109	KC_AB1	CG1116	0.243589995987951	0.754314802227182	1	pseudobulk	DESeq2	LRT	0
109	KC_AB1	CG11170	-1.90368758369071	0.180924259324415	1	pseudobulk	DESeq2	LRT	0
109	KC_AB1	CG11178	0.660558061207439	0.437908707068858	1	pseudobulk	DESeq2	LRT	0
109	KC_AB1	CG11180	-1.05491653522407	0.470583751611065	1	pseudobulk	DESeq2	LRT	0
109	KC_AB1	CG11191	-0.0710698680181678	0.550073376794385	1	pseudobulk	DESeq2	LRT	0
109	KC_AB1	CG11200	-0.770029509264707	0.610834024620634	1	pseudobulk	DESeq2	LRT	0
110	KC_AB1	CG11241	1.66720351338878	0.732248328474997	1	pseudobulk	DESeq2	LRT	0
110	KC_AB1	CG11247	-0.702397010484026	0.180049180085906	1	pseudobulk	DESeq2	LRT	0
110	KC_AB1	CG11248	-0.399106425754497	0.572888468355537	1	pseudobulk	DESeq2	LRT	0
110	KC_AB1	CG11249	2.31997030691033	0.813169507846918	1	pseudobulk	DESeq2	LRT	0
110	KC_AB1	CG11257	-0.357349766270791	0.728680495896593	1	pseudobulk	DESeq2	LRT	0
110	KC_AB1	CG11267	0.187741977197469	0.587535012998451	1	pseudobulk	DESeq2	LRT	0
110	KC_AB1	CG11269	0.849606887838128	1	1	pseudobulk	DESeq2	LRT	0
110	KC_AB1	CG11275	2.78674512494791	0.54962276837928	1	pseudobulk	DESeq2	LRT	0
110	KC_AB1	CG11279	0.142309988831414	0.746749105076853	1	pseudobulk	DESeq2	LRT	0
110	KC_AB1	CG1129	-0.91149125194315	0.190313182460646	1	pseudobulk	DESeq2	LRT	0
111	KC_AB1	CG11291	0.849606887838128	1	1	pseudobulk	DESeq2	LRT	0
111	KC_AB1	CG11307	-0.137492980608447	0.848789029973766	1	pseudobulk	DESeq2	LRT	0

111	KC_AB1	CG11309	-0.360297879864743	0.723489150842172	1	pseudobulk	DESeq2	LRT	0
111	KC_AB1	CG11317	-0.162996967115056	0.557052898474019	1	pseudobulk	DESeq2	LRT	0
111	KC_AB1	CG11319	-0.118824608723801	0.483682772128136	1	pseudobulk	DESeq2	LRT	0
111	KC_AB1	CG11334	0.346509705931319	0.757087198148734	1	pseudobulk	DESeq2	LRT	0
111	KC_AB1	CG11342	-0.216209988775614	1	1	pseudobulk	DESeq2	LRT	0
111	KC_AB1	CG11357	-0.356173118236351	0.327058168887932	1	pseudobulk	DESeq2	LRT	0
111	KC_AB1	CG1136	3.81892523345342	0.259683132508622	1	pseudobulk	DESeq2	LRT	0
111	KC_AB1	CG11360	-0.368286054954638	0.292316474125459	1	pseudobulk	DESeq2	LRT	0
112	KC_AB1	CG11362	0.849606887838128	1	1	pseudobulk	DESeq2	LRT	0
112	KC_AB1	CG11367	-0.545580689235114	0.542037846088523	1	pseudobulk	DESeq2	LRT	0
112	KC_AB1	CG11374	-0.302458995746278	0.779143818314263	1	pseudobulk	DESeq2	LRT	0
112	KC_AB1	CG11377	-0.81083887460086	0.442157568433867	1	pseudobulk	DESeq2	LRT	0
112	KC_AB1	CG11396	-0.284017014510159	0.68847202351527	1	pseudobulk	DESeq2	LRT	0
112	KC_AB1	CG11398	-0.074123206896811	0.846920466238955	1	pseudobulk	DESeq2	LRT	0
112	KC_AB1	CG11399	0.299065054576811	0.67472542230082	1	pseudobulk	DESeq2	LRT	0
112	KC_AB1	CG11403	0.849606887838128	1	1	pseudobulk	DESeq2	LRT	0
112	KC_AB1	CG11406	-1.52884912538657	0.00489038283397832	1	pseudobulk	DESeq2	LRT	0
112	KC_AB1	CG11407	-1.17984099755339	0.468358879369449	1	pseudobulk	DESeq2	LRT	0
113	KC_AB1	CG11413	0.500352090722316	0.803700652248629	1	pseudobulk	DESeq2	LRT	0
113	KC_AB1	CG11414	0.0189555934325481	0.750409679775361	1	pseudobulk	DESeq2	LRT	0
113	KC_AB1	CG11417	0.666044499293101	0.908857518545142	1	pseudobulk	DESeq2	LRT	0
113	KC_AB1	CG1142	0.345092948043745	0.284341475038645	1	pseudobulk	DESeq2	LRT	0
113	KC_AB1	CG11425	-0.10506771737906	1	1	pseudobulk	DESeq2	LRT	0
113	KC_AB1	CG1143	0.849606887838128	1	1	pseudobulk	DESeq2	LRT	0
113	KC_AB1	CG11436	-0.512773483757323	0.635523863107354	1	pseudobulk	DESeq2	LRT	0
113	KC_AB1	CG11444	-0.154006741110401	0.615193911217661	1	pseudobulk	DESeq2	LRT	0
113	KC_AB1	CG11447	0.644087439640281	0.608143714842084	1	pseudobulk	DESeq2	LRT	0
113	KC_AB1	CG11454	-0.465362276197251	0.536880519607505	1	pseudobulk	DESeq2	LRT	0
114	KC_AB1	CG11456	-0.139966091467046	0.676016107438731	1	pseudobulk	DESeq2	LRT	0
114	KC_AB1	CG1146	-0.914566795469385	0.499668143203699	1	pseudobulk	DESeq2	LRT	0
114	KC_AB1	CG11474	1.10705531101828	0.304609479211666	1	pseudobulk	DESeq2	LRT	0
114	KC_AB1	CG11475	3.16697464674408	0.406679230261689	1	pseudobulk	DESeq2	LRT	0
114	KC_AB1	CG11486	0.0538371340589657	0.740979171999	1	pseudobulk	DESeq2	LRT	0
114	KC_AB1	CG11504	-0.239589077734694	0.643645846334237	1	pseudobulk	DESeq2	LRT	0
114	KC_AB1	CG11523	0.383105799585239	0.485394663956782	1	pseudobulk	DESeq2	LRT	0
114	KC_AB1	CG11537	-0.214450605732209	0.359325373829457	1	pseudobulk	DESeq2	LRT	0
114	KC_AB1	CG11539	0.56299079442748	0.904997173168881	1	pseudobulk	DESeq2	LRT	0
114	KC_AB1	CG11555	0.180515464683207	0.765205179922496	1	pseudobulk	DESeq2	LRT	0
115	KC_AB1	CG11560	-0.77737763458979	0.478248870354127	1	pseudobulk	DESeq2	LRT	0
115	KC_AB1	CG11562	1.29664765505568	0.38217358810603	1	pseudobulk	DESeq2	LRT	0
115	KC_AB1	CG11563	-0.086766966507715	0.748187240160193	1	pseudobulk	DESeq2	LRT	0
115	KC_AB1	CG11583	-0.227261527508685	0.723272840969628	1	pseudobulk	DESeq2	LRT	0
115	KC_AB1	CG11586	-0.349373021807148	0.635667499982058	1	pseudobulk	DESeq2	LRT	0
115	KC_AB1	CG11590	1.79590159336616	0.652741537999668	1	pseudobulk	DESeq2	LRT	0
115	KC_AB1	CG11593	0.259308622795117	0.752603298454331	1	pseudobulk	DESeq2	LRT	0
115	KC_AB1	CG11594	1.90244890691395	0.564198562139563	1	pseudobulk	DESeq2	LRT	0
115	KC_AB1	CG11596	-0.344928382982608	0.66899093027169	1	pseudobulk	DESeq2	LRT	0
115	KC_AB1	CG11597	2.29743640924755	0.34282403959746	1	pseudobulk	DESeq2	LRT	0
116	KC_AB1	CG1161	-0.039740328243014	0.63904507258649	1	pseudobulk	DESeq2	LRT	0
116	KC_AB1	CG11619	-0.851943617129873	0.694524594037258	1	pseudobulk	DESeq2	LRT	0
116	KC_AB1	CG11626	0.769522064442666	1	1	pseudobulk	DESeq2	LRT	0
116	KC_AB1	CG11638	1.55791057386243	0.611068930051323	1	pseudobulk	DESeq2	LRT	0
116	KC_AB1	CG11655	2.31997030691033	0.813169507846918	1	pseudobulk	DESeq2	LRT	0

116	KC_AB1	CG11658	0.548594743405325	0.235059786990442	1	pseudobulk	DESeq2	LRT	0
116	KC_AB1	CG11679	2.06541384650887	0.768363369045814	1	pseudobulk	DESeq2	LRT	0
116	KC_AB1	CG11686	-0.457622207737357	0.755249650709674	1	pseudobulk	DESeq2	LRT	0
116	KC_AB1	CG11695	-0.594870044829891	0.630106645199967	1	pseudobulk	DESeq2	LRT	0
116	KC_AB1	CG11696	1.38777243606053	0.170043730638998	1	pseudobulk	DESeq2	LRT	0
117	KC_AB1	CG11699	0.224640069825962	0.483962309697434	1	pseudobulk	DESeq2	LRT	0
117	KC_AB1	CG11700	2.31997030691033	0.813169507846918	1	pseudobulk	DESeq2	LRT	0
117	KC_AB1	CG11710	0.414683340160396	0.673622436895578	1	pseudobulk	DESeq2	LRT	0
117	KC_AB1	CG1172	0.0499362881078592	0.743656874439158	1	pseudobulk	DESeq2	LRT	0
117	KC_AB1	CG11722	-0.426187448580843	0.563140261347003	1	pseudobulk	DESeq2	LRT	0
117	KC_AB1	CG11723	-0.113778398639621	0.488911971053648	1	pseudobulk	DESeq2	LRT	0
117	KC_AB1	CG11737	1.13163834359318	0.723494571733966	1	pseudobulk	DESeq2	LRT	0
117	KC_AB1	CG11752	0.347296516651609	0.373183629484398	1	pseudobulk	DESeq2	LRT	0
117	KC_AB1	CG11753	0.0100973222069115	0.809185139619246	1	pseudobulk	DESeq2	LRT	0
117	KC_AB1	CG11755	0.0455209597628663	0.825326880385729	1	pseudobulk	DESeq2	LRT	0
118	KC_AB1	CG11760	-0.908416068420516	0.317741021409083	1	pseudobulk	DESeq2	LRT	0
118	KC_AB1	CG11768	-0.714331156564779	0.0216239812175867	1	pseudobulk	DESeq2	LRT	0
118	KC_AB1	CG11771	-0.023399943683862	0.702900250527753	1	pseudobulk	DESeq2	LRT	0
118	KC_AB1	CG11777	1.4762816727683	0.377988500024854	1	pseudobulk	DESeq2	LRT	0
118	KC_AB1	CG11779	-0.327338651855054	0.622625962544583	1	pseudobulk	DESeq2	LRT	0
118	KC_AB1	CG11781	0.234448799855027	0.717704580078385	1	pseudobulk	DESeq2	LRT	0
118	KC_AB1	CG11788	0.880742696173337	1	1	pseudobulk	DESeq2	LRT	0
118	KC_AB1	CG11790	0.33027973469198	1	1	pseudobulk	DESeq2	LRT	0
118	KC_AB1	CG11791	-0.600179851358006	0.684167225149329	1	pseudobulk	DESeq2	LRT	0
118	KC_AB1	CG11807	-1.13369278361357	0.218450506209127	1	pseudobulk	DESeq2	LRT	0
119	KC_AB1	CG11808	1.21435546155796	0.537124137671421	1	pseudobulk	DESeq2	LRT	0
119	KC_AB1	CG11811	-0.220591611917207	0.562542742758874	1	pseudobulk	DESeq2	LRT	0
119	KC_AB1	CG11828	0.849606887838128	1	1	pseudobulk	DESeq2	LRT	0
119	KC_AB1	CG11835	-0.593346002816939	0.584658927408779	1	pseudobulk	DESeq2	LRT	0
119	KC_AB1	CG11837	2.7141870817367	0.45608503377963	1	pseudobulk	DESeq2	LRT	0
119	KC_AB1	CG11839	-0.459309024830653	0.676849161473474	1	pseudobulk	DESeq2	LRT	0
119	KC_AB1	CG11857	0.406157188621464	0.370332215415415	1	pseudobulk	DESeq2	LRT	0
119	KC_AB1	CG11858	0.742004509914067	0.537453971553856	1	pseudobulk	DESeq2	LRT	0
119	KC_AB1	CG11873	-0.026572941791131	0.56192605966566	1	pseudobulk	DESeq2	LRT	0
119	KC_AB1	CG11882	-1.61782813191161	0.326805557133356	1	pseudobulk	DESeq2	LRT	0
120	KC_AB1	CG11883	0.266164531427993	0.399599380690106	1	pseudobulk	DESeq2	LRT	0
120	KC_AB1	CG11885	0.375812411882478	0.673368601083751	1	pseudobulk	DESeq2	LRT	0
120	KC_AB1	CG11898	0.503796073322249	0.8261134283531	1	pseudobulk	DESeq2	LRT	0
120	KC_AB1	CG11899	0.713780324542466	1	1	pseudobulk	DESeq2	LRT	0
120	KC_AB1	CG11902	-0.222840700629708	0.760257493763136	1	pseudobulk	DESeq2	LRT	0
120	KC_AB1	CG11905	0.849606887838128	1	1	pseudobulk	DESeq2	LRT	0
120	KC_AB1	CG11906	-0.513169884355657	0.659712181461903	1	pseudobulk	DESeq2	LRT	0
120	KC_AB1	CG11911	0.183159795455075	1	1	pseudobulk	DESeq2	LRT	0
120	KC_AB1	CG11920	-0.209177628788863	0.836714394059029	1	pseudobulk	DESeq2	LRT	0
120	KC_AB1	CG11927	0.548449050099305	0.606989812896767	1	pseudobulk	DESeq2	LRT	0
121	KC_AB1	CG11961	-0.155934067093443	0.914618786789969	1	pseudobulk	DESeq2	LRT	0
121	KC_AB1	CG11964	0.753597064622224	0.596313585589627	1	pseudobulk	DESeq2	LRT	0
121	KC_AB1	CG11970	-0.100381679682868	0.780160814484399	1	pseudobulk	DESeq2	LRT	0
121	KC_AB1	CG11975	-0.105855800640468	0.707134466941401	1	pseudobulk	DESeq2	LRT	0
121	KC_AB1	CG11980	0.301671447975681	0.613542362712343	1	pseudobulk	DESeq2	LRT	0
121	KC_AB1	CG11983	1.33605976138209	0.809986558087114	1	pseudobulk	DESeq2	LRT	0
121	KC_AB1	CG11986	0.849606887838128	1	1	pseudobulk	DESeq2	LRT	0
121	KC_AB1	CG11999	-0.645080728818547	0.538547453647153	1	pseudobulk	DESeq2	LRT	0

121	KC_AB1	CG12004	0.514212591636613	0.0827783643073503	1	pseudobulk	DESeq2	LRT	0
121	KC_AB1	CG12007	0.319726825598464	0.565362870494678	1	pseudobulk	DESeq2	LRT	0
122	KC_AB1	CG12009	0.849606887838128	1	1	pseudobulk	DESeq2	LRT	0
122	KC_AB1	CG12010	1.68846603811467	0.491557163805824	1	pseudobulk	DESeq2	LRT	0
122	KC_AB1	CG12012	-0.550331646211207	0.490247487543568	1	pseudobulk	DESeq2	LRT	0
122	KC_AB1	CG12017	0.849606887838128	1	1	pseudobulk	DESeq2	LRT	0
122	KC_AB1	CG12020	0.849606887838128	1	1	pseudobulk	DESeq2	LRT	0
122	KC_AB1	CG12024	0.404693677418469	0.424923883134238	1	pseudobulk	DESeq2	LRT	0
122	KC_AB1	CG12025	-0.210882741541152	0.497728876182715	1	pseudobulk	DESeq2	LRT	0
122	KC_AB1	CG12038	0.204340977267644	0.593648151576469	1	pseudobulk	DESeq2	LRT	0
122	KC_AB1	CG12054	-0.374216072173401	0.16898698507065	1	pseudobulk	DESeq2	LRT	0
122	KC_AB1	CG12056	1.17254933875124	0.593648329065046	1	pseudobulk	DESeq2	LRT	0
123	KC_AB1	CG12065	-1.05992744849371	0.613288832576002	1	pseudobulk	DESeq2	LRT	0
123	KC_AB1	CG12071	0.553428063684887	0.106324702120752	1	pseudobulk	DESeq2	LRT	0
123	KC_AB1	CG12081	0.588419798613297	0.318928311465099	1	pseudobulk	DESeq2	LRT	0
123	KC_AB1	CG12084	-0.805552060164244	0.237440423663588	1	pseudobulk	DESeq2	LRT	0
123	KC_AB1	CG12091	-0.230167803421539	0.394752836236258	1	pseudobulk	DESeq2	LRT	0
123	KC_AB1	CG12093	-1.10118520547517	0.394896339712453	1	pseudobulk	DESeq2	LRT	0
123	KC_AB1	CG12096	0.0173048317624363	0.807089757042769	1	pseudobulk	DESeq2	LRT	0
123	KC_AB1	CG12099	-0.644737232477432	0.189150850756772	1	pseudobulk	DESeq2	LRT	0
123	KC_AB1	CG12104	-0.0965361153400467	0.712881350252806	1	pseudobulk	DESeq2	LRT	0
123	KC_AB1	CG12106	-0.308886495770974	0.657413398609662	1	pseudobulk	DESeq2	LRT	0
124	KC_AB1	CG12107	0.367362690197305	0.577393756940614	1	pseudobulk	DESeq2	LRT	0
124	KC_AB1	CG12112	1.06759111434625	1	1	pseudobulk	DESeq2	LRT	0
124	KC_AB1	CG12116	0.849606887838128	1	1	pseudobulk	DESeq2	LRT	0
124	KC_AB1	CG12118	0.227578312922571	0.752363020053317	1	pseudobulk	DESeq2	LRT	0
124	KC_AB1	CG12121	-0.0837130144322547	0.565469122119106	1	pseudobulk	DESeq2	LRT	0
124	KC_AB1	CG12123	-1.07691884810122	0.243368307070624	1	pseudobulk	DESeq2	LRT	0
124	KC_AB1	CG12128	-0.0027972489394451	0.862430626642598	1	pseudobulk	DESeq2	LRT	0
124	KC_AB1	CG12129	0.494367536359908	0.61451624188419	1	pseudobulk	DESeq2	LRT	0
124	KC_AB1	CG12134	0.779570991707761	1	1	pseudobulk	DESeq2	LRT	0
124	KC_AB1	CG12155	-0.0767199567597192	0.726172397296882	1	pseudobulk	DESeq2	LRT	0
125	KC_AB1	CG12158	0.849606887838128	1	1	pseudobulk	DESeq2	LRT	0
125	KC_AB1	CG12159	-0.0427004698596331	0.547060780486604	1	pseudobulk	DESeq2	LRT	0
125	KC_AB1	CG12163	0.10410708579072	0.654642811666973	1	pseudobulk	DESeq2	LRT	0
125	KC_AB1	CG12170	-0.115937483478741	0.835996750066795	1	pseudobulk	DESeq2	LRT	0
125	KC_AB1	CG12171	-0.301152936934865	0.775796239422475	1	pseudobulk	DESeq2	LRT	0
125	KC_AB1	CG12173	-0.498898292699859	0.594650880706262	1	pseudobulk	DESeq2	LRT	0
125	KC_AB1	CG12177	0.0523646012774308	0.391249102326872	1	pseudobulk	DESeq2	LRT	0
125	KC_AB1	CG12179	0.849606887838128	1	1	pseudobulk	DESeq2	LRT	0
125	KC_AB1	CG1218	1.29999645182016	0.37869509479009	1	pseudobulk	DESeq2	LRT	0
125	KC_AB1	CG12182	0.183159795455075	1	1	pseudobulk	DESeq2	LRT	0
126	KC_AB1	CG12194	-0.285850201676604	0.304176882853317	1	pseudobulk	DESeq2	LRT	0
126	KC_AB1	CG12204	-2.14687097891971	0.136909560002204	1	pseudobulk	DESeq2	LRT	0
126	KC_AB1	CG12206	-1.69444915626913	0.253328387832523	1	pseudobulk	DESeq2	LRT	0
126	KC_AB1	CG12213	-0.448891543335677	0.350455163706457	1	pseudobulk	DESeq2	LRT	0
126	KC_AB1	CG12219	-0.383282621079617	0.661428606441716	1	pseudobulk	DESeq2	LRT	0
126	KC_AB1	CG12236	-0.166938284523606	0.800272203056282	1	pseudobulk	DESeq2	LRT	0
126	KC_AB1	CG12237	1.03335564574598	0.0404794915356885	1	pseudobulk	DESeq2	LRT	0
126	KC_AB1	CG12241	0.402718237498368	0.512069044266399	1	pseudobulk	DESeq2	LRT	0
126	KC_AB1	CG12253	0.459272210247551	0.640985261016553	1	pseudobulk	DESeq2	LRT	0
126	KC_AB1	CG12259	0.128267304499652	0.822186569063059	1	pseudobulk	DESeq2	LRT	0
127	KC_AB1	CG12268	2.78674512494791	0.54962276837928	1	pseudobulk	DESeq2	LRT	0

127	KC_AB1	CG1227	0.54719258217882	0.84334192471431	1	pseudobulk	DESeq2	LRT		0
127	KC_AB1	CG12279	0.456097334433305	0.579759244402312	1	pseudobulk	DESeq2	LRT		0
127	KC_AB1	CG12288	-1.27467172141861	0.491626102067384	1	pseudobulk	DESeq2	LRT		0
127	KC_AB1	CG12290	-0.128402507746718	1	1	pseudobulk	DESeq2	LRT		0
127	KC_AB1	CG12299	-0.275706684507393	0.439883596420646	1	pseudobulk	DESeq2	LRT		0
127	KC_AB1	CG12301	0.490820782134795	1	1	pseudobulk	DESeq2	LRT		0
127	KC_AB1	CG1231	1.28558727260066	0.37347191960083	1	pseudobulk	DESeq2	LRT		0
127	KC_AB1	CG12316	-0.692006943973182	0.444393525509227	1	pseudobulk	DESeq2	LRT		0
127	KC_AB1	CG12320	2.31997030691033	0.813169507846918	1	pseudobulk	DESeq2	LRT		0
128	KC_AB1	CG12321	0.367634104528991	0.833631834135566	1	pseudobulk	DESeq2	LRT		0
128	KC_AB1	CG12325	0.181522037750249	1	1	pseudobulk	DESeq2	LRT		0
128	KC_AB1	CG1233	0.400255662724248	0.754330923465052	1	pseudobulk	DESeq2	LRT		0
128	KC_AB1	CG12333	-0.480770557898057	0.377468073120892	1	pseudobulk	DESeq2	LRT		0
128	KC_AB1	CG12338	0.183159795455075	1	1	pseudobulk	DESeq2	LRT		0
128	KC_AB1	CG1234	-1.04821843629993	0.37503779833192	1	pseudobulk	DESeq2	LRT		0
128	KC_AB1	CG12341	0.69346335201564	0.190778175041031	1	pseudobulk	DESeq2	LRT		0
128	KC_AB1	CG12343	-0.0565077706908431	0.679483113919588	1	pseudobulk	DESeq2	LRT		0
128	KC_AB1	CG12344	0.849606887838128	1	1	pseudobulk	DESeq2	LRT		0
128	KC_AB1	CG12355	-1.02338367993598	0.214519837333779	1	pseudobulk	DESeq2	LRT		0
129	KC_AB1	CG1236	-0.0810476480571921	1	1	pseudobulk	DESeq2	LRT		0
129	KC_AB1	CG12375	0.230071085925996	0.724947243650318	1	pseudobulk	DESeq2	LRT		0
129	KC_AB1	CG12379	-0.442784575045254	0.685466773274669	1	pseudobulk	DESeq2	LRT		0
129	KC_AB1	CG12384	-0.183018923353769	0.707972945393066	1	pseudobulk	DESeq2	LRT		0
129	KC_AB1	CG1239	1.53568827361621	0.423803999113646	1	pseudobulk	DESeq2	LRT		0
129	KC_AB1	CG12391	-0.0140488335818363	0.58109378722315	1	pseudobulk	DESeq2	LRT		0
129	KC_AB1	CG12402	0.459458518155192	0.484625059627388	1	pseudobulk	DESeq2	LRT		0
129	KC_AB1	CG12420	1.2250355339054	0.716644027560673	1	pseudobulk	DESeq2	LRT		0
129	KC_AB1	CG12470	0.849606887838128	1	1	pseudobulk	DESeq2	LRT		0
129	KC_AB1	CG12499	0.351889883624226	1	1	pseudobulk	DESeq2	LRT		0
130	KC_AB1	CG12502	-0.160746597020143	0.782558532634225	1	pseudobulk	DESeq2	LRT		0
130	KC_AB1	CG12512	0.849606887838128	1	1	pseudobulk	DESeq2	LRT		0
130	KC_AB1	CG12520	0.366247714696288	1	1	pseudobulk	DESeq2	LRT		0
130	KC_AB1	CG12531	0.275385665017571	0.0931460615175686	1	pseudobulk	DESeq2	LRT		0
130	KC_AB1	CG12535	-0.524127444717734	0.565225296059882	1	pseudobulk	DESeq2	LRT		0
130	KC_AB1	CG12539	-1.25393399638069	0.612054104673377	1	pseudobulk	DESeq2	LRT		0
130	KC_AB1	CG12541	1.1169458212053	0.0285792093858061	1	pseudobulk	DESeq2	LRT		0
130	KC_AB1	CG12547	-0.121971096341043	0.76041316454877	1	pseudobulk	DESeq2	LRT		0
130	KC_AB1	CG12560	1.61359809683368	0.126228463694363	1	pseudobulk	DESeq2	LRT		0
130	KC_AB1	CG12567	-0.50146392525662	0.261311872056418	1	pseudobulk	DESeq2	LRT		0
131	KC_AB1	CG12576	0.0460687749204419	0.727225224971802	1	pseudobulk	DESeq2	LRT		0
131	KC_AB1	CG12605	-0.502956509306084	0.0484279209085165	1	pseudobulk	DESeq2	LRT		0
131	KC_AB1	CG12608	-1.15109932185075	0.435965043808501	1	pseudobulk	DESeq2	LRT		0
131	KC_AB1	CG12617	0.849606887838128	1	1	pseudobulk	DESeq2	LRT		0
131	KC_AB1	CG12640	0.0863909509812212	1	1	pseudobulk	DESeq2	LRT		0
131	KC_AB1	CG12643	0.536600542756646	1	1	pseudobulk	DESeq2	LRT		0
131	KC_AB1	CG1265	-0.597292800634726	0.239202544368734	1	pseudobulk	DESeq2	LRT		0
131	KC_AB1	CG12655	1.66680428118609	0.723089691568339	1	pseudobulk	DESeq2	LRT		0
131	KC_AB1	CG12659	-0.300431451384834	0.693667000236455	1	pseudobulk	DESeq2	LRT		0
131	KC_AB1	CG12672	-0.305066726257421	0.701058960524192	1	pseudobulk	DESeq2	LRT		0
132	KC_AB1	CG12674	2.13009086641515	0.687046658320206	1	pseudobulk	DESeq2	LRT		0
132	KC_AB1	CG12693	0.849606887838128	1	1	pseudobulk	DESeq2	LRT		0
132	KC_AB1	CG1271	1.06082415621725	0.750737044738196	1	pseudobulk	DESeq2	LRT		0
132	KC_AB1	CG12713	-0.392211694638649	0.690217745602098	1	pseudobulk	DESeq2	LRT		0

132	KC_AB1	CG12715	1.60172288900978	0.749416984916788	1	pseudobulk	DESeq2	LRT	0
132	KC_AB1	CG12717	0.744905681550488	0.570911078129405	1	pseudobulk	DESeq2	LRT	0
132	KC_AB1	CG12728	0.0907064876949162	0.725299175163974	1	pseudobulk	DESeq2	LRT	0
132	KC_AB1	CG12730	-0.360964433596376	0.405458297936021	1	pseudobulk	DESeq2	LRT	0
132	KC_AB1	CG12746	0.359280136142761	0.674803004096696	1	pseudobulk	DESeq2	LRT	0
132	KC_AB1	CG1275	0.106520123335792	0.610781144542834	1	pseudobulk	DESeq2	LRT	0
133	KC_AB1	CG12768	-1.7805202744015	0.305685268637521	1	pseudobulk	DESeq2	LRT	0
133	KC_AB1	CG12769	-1.14540155217786	0.0743280642505512	1	pseudobulk	DESeq2	LRT	0
133	KC_AB1	CG12773	-0.507606808399074	0.592004850219072	1	pseudobulk	DESeq2	LRT	0
133	KC_AB1	CG12795	0.176630366970365	0.586413989159903	1	pseudobulk	DESeq2	LRT	0
133	KC_AB1	CG12811	0.849606887838128	1	1	pseudobulk	DESeq2	LRT	0
133	KC_AB1	CG12814	-0.999263960990402	0.56854704845248	1	pseudobulk	DESeq2	LRT	0
133	KC_AB1	CG12817	-0.076022613742960	0.823352306329908	1	pseudobulk	DESeq2	LRT	0
133	KC_AB1	CG12818	-0.434351273456229	0.662118745374778	1	pseudobulk	DESeq2	LRT	0
133	KC_AB1	CG12822	0.695197988398985	0.104246645483962	1	pseudobulk	DESeq2	LRT	0
133	KC_AB1	CG12824	0.849606887838128	1	1	pseudobulk	DESeq2	LRT	0
134	KC_AB1	CG12825	0.237022465848507	1	1	pseudobulk	DESeq2	LRT	0
134	KC_AB1	CG12836	0.849606887838128	1	1	pseudobulk	DESeq2	LRT	0
134	KC_AB1	CG12848	0.25240215174687	0.720272615414607	1	pseudobulk	DESeq2	LRT	0
134	KC_AB1	CG12851	0.0450369526707364	0.817304936951972	1	pseudobulk	DESeq2	LRT	0
134	KC_AB1	CG12853	2.78674512494791	0.54962276837928	1	pseudobulk	DESeq2	LRT	0
134	KC_AB1	CG12863	-0.805104657895581	0.548533538596495	1	pseudobulk	DESeq2	LRT	0
134	KC_AB1	CG12866	1.59863986092676	1	1	pseudobulk	DESeq2	LRT	0
134	KC_AB1	CG12868	0.233410332173921	0.831463018475116	1	pseudobulk	DESeq2	LRT	0
134	KC_AB1	CG12877	-0.170570386307133	0.70593476916659	1	pseudobulk	DESeq2	LRT	0
134	KC_AB1	CG12883	0.958967915779258	0.667033284144861	1	pseudobulk	DESeq2	LRT	0
135	KC_AB1	CG12885	-0.683829746723457	0.723342236517982	1	pseudobulk	DESeq2	LRT	0
135	KC_AB1	CG12895	-0.047672622444654	0.637129164435338	1	pseudobulk	DESeq2	LRT	0
135	KC_AB1	CG12896	1.59863986092676	1	1	pseudobulk	DESeq2	LRT	0
135	KC_AB1	CG12909	1.06572926637885	1	1	pseudobulk	DESeq2	LRT	0
135	KC_AB1	CG12910	0.843353859179659	0.0730200183115373	1	pseudobulk	DESeq2	LRT	0
135	KC_AB1	CG12913	-0.311269009804189	0.651452809656903	1	pseudobulk	DESeq2	LRT	0
135	KC_AB1	CG12923	0.849606887838128	1	1	pseudobulk	DESeq2	LRT	0
135	KC_AB1	CG12926	1.64673267098683	0.852532516449	1	pseudobulk	DESeq2	LRT	0
135	KC_AB1	CG12929	-1.71643890484814	0.235164705443928	1	pseudobulk	DESeq2	LRT	0
135	KC_AB1	CG12935	-0.559085271814125	0.585509444714878	1	pseudobulk	DESeq2	LRT	0
136	KC_AB1	CG12942	-0.203744207145655	0.76119049659941	1	pseudobulk	DESeq2	LRT	0
136	KC_AB1	CG12945	-0.510713210720646	0.409088848638046	1	pseudobulk	DESeq2	LRT	0
136	KC_AB1	CG12947	-3.06096481305749	0.127181751569372	1	pseudobulk	DESeq2	LRT	0
136	KC_AB1	CG12948	-0.090808375527080	0.619638536029768	1	pseudobulk	DESeq2	LRT	0
136	KC_AB1	CG12963	0.849606887838128	1	1	pseudobulk	DESeq2	LRT	0
136	KC_AB1	CG12975	0.504846465262498	0.316561114444091	1	pseudobulk	DESeq2	LRT	0
136	KC_AB1	CG1299	3.41844045086015	0.351942490385471	1	pseudobulk	DESeq2	LRT	0
136	KC_AB1	CG12990	2.31997030691033	0.813169507846918	1	pseudobulk	DESeq2	LRT	0
136	KC_AB1	CG12991	0.345793333560515	0.386771127415763	1	pseudobulk	DESeq2	LRT	0
136	KC_AB1	CG12992	0.0470630602785356	0.859332623876854	1	pseudobulk	DESeq2	LRT	0
137	KC_AB1	CG12994	-2.36275568366809	0.0907737907314271	1	pseudobulk	DESeq2	LRT	0
137	KC_AB1	CG13001	-0.347349559173927	0.624317536845595	1	pseudobulk	DESeq2	LRT	0
137	KC_AB1	CG13005	-0.68112953579674	0.619242938323651	1	pseudobulk	DESeq2	LRT	0
137	KC_AB1	CG13008	-0.40200137056069	0.861588439126694	1	pseudobulk	DESeq2	LRT	0
137	KC_AB1	CG13010	2.31997030691033	0.813169507846918	1	pseudobulk	DESeq2	LRT	0
137	KC_AB1	CG13012	0.849606887838128	1	1	pseudobulk	DESeq2	LRT	0
137	KC_AB1	CG13014	0.444559027168278	1	1	pseudobulk	DESeq2	LRT	0

137	KC_AB1	CG13016	0.411097579523941	0.636718889333328	1	pseudobulk	DESeq2	LRT	0
137	KC_AB1	CG13018	0.603705299118008	0.500950549260724	1	pseudobulk	DESeq2	LRT	0
137	KC_AB1	CG13024	-0.389346928562833	0.820127740800904	1	pseudobulk	DESeq2	LRT	0
138	KC_AB1	CG13025	0.19105858823047	1	1	pseudobulk	DESeq2	LRT	0
138	KC_AB1	CG13028	0.849606887838128	1	1	pseudobulk	DESeq2	LRT	0
138	KC_AB1	CG13054	0.0577751952669666	0.779653955839589	1	pseudobulk	DESeq2	LRT	0
138	KC_AB1	CG13055	-0.526027567432448	0.124319242399388	1	pseudobulk	DESeq2	LRT	0
138	KC_AB1	CG13067	-0.0810476480571921	1	1	pseudobulk	DESeq2	LRT	0
138	KC_AB1	CG1307	-0.693773483803095	0.413312358789728	1	pseudobulk	DESeq2	LRT	0
138	KC_AB1	CG13082	-0.216210029276812	1	1	pseudobulk	DESeq2	LRT	0
138	KC_AB1	CG13085	1.37008314861419	1	1	pseudobulk	DESeq2	LRT	0
138	KC_AB1	CG13088	-0.40200137056069	0.861588439126694	1	pseudobulk	DESeq2	LRT	0
138	KC_AB1	CG1309	0.0969002007797844	0.760812164349132	1	pseudobulk	DESeq2	LRT	0
139	KC_AB1	CG13096	0.751302237845922	1	1	pseudobulk	DESeq2	LRT	0
139	KC_AB1	CG13097	-0.691810385964798	0.60559331091582	1	pseudobulk	DESeq2	LRT	0
139	KC_AB1	CG13101	1.37008314861419	1	1	pseudobulk	DESeq2	LRT	0
139	KC_AB1	CG13108	0.478023937875139	0.350349261729706	1	pseudobulk	DESeq2	LRT	0
139	KC_AB1	CG13116	1.37129945894492	1	1	pseudobulk	DESeq2	LRT	0
139	KC_AB1	CG13117	-1.05548521056871	0.62314630615428	1	pseudobulk	DESeq2	LRT	0
139	KC_AB1	CG13123	-0.166450952015876	0.754896852581948	1	pseudobulk	DESeq2	LRT	0
139	KC_AB1	CG13124	-1.4963552996872	0.750624930179005	1	pseudobulk	DESeq2	LRT	0
139	KC_AB1	CG13126	1.26147353437088	0.631969672974718	1	pseudobulk	DESeq2	LRT	0
139	KC_AB1	CG13133	2.31997030691033	0.813169507846918	1	pseudobulk	DESeq2	LRT	0
140	KC_AB1	CG13135	2.31997030691033	0.813169507846918	1	pseudobulk	DESeq2	LRT	0
140	KC_AB1	CG1314	3.16697464674408	0.406679230261689	1	pseudobulk	DESeq2	LRT	0
140	KC_AB1	CG13148	0.513911163797922	0.705334892663822	1	pseudobulk	DESeq2	LRT	0
140	KC_AB1	CG13151	-0.267798911930929	1	1	pseudobulk	DESeq2	LRT	0
140	KC_AB1	CG1316	-0.183033660894376	0.606011749692406	1	pseudobulk	DESeq2	LRT	0
140	KC_AB1	CG13168	-0.572809606158377	0.787374717193114	1	pseudobulk	DESeq2	LRT	0
140	KC_AB1	CG1317	-0.414489615665544	0.357079323102406	1	pseudobulk	DESeq2	LRT	0
140	KC_AB1	CG13185	-0.19698351532854	1	1	pseudobulk	DESeq2	LRT	0
140	KC_AB1	CG13192	-1.1183102425874	0.40638229181724	1	pseudobulk	DESeq2	LRT	0
140	KC_AB1	CG13197	0.379461116564979	0.578253422298721	1	pseudobulk	DESeq2	LRT	0
141	KC_AB1	CG13202	0.849606887838128	1	1	pseudobulk	DESeq2	LRT	0
141	KC_AB1	CG13204	-0.11067162718817	0.60973656197023	1	pseudobulk	DESeq2	LRT	0
141	KC_AB1	CG13220	0.45720020993832	0.435922753544396	1	pseudobulk	DESeq2	LRT	0
141	KC_AB1	CG13229	0.237022465848507	1	1	pseudobulk	DESeq2	LRT	0
141	KC_AB1	CG13248	0.32726990205378	0.405341542262289	1	pseudobulk	DESeq2	LRT	0
141	KC_AB1	CG13252	2.06541384650887	0.904905526327343	1	pseudobulk	DESeq2	LRT	0
141	KC_AB1	CG13255	0.235883549019826	1	1	pseudobulk	DESeq2	LRT	0
141	KC_AB1	CG13282	2.31997030691033	0.813169507846918	1	pseudobulk	DESeq2	LRT	0
141	KC_AB1	CG13284	1.37129945894492	1	1	pseudobulk	DESeq2	LRT	0
141	KC_AB1	CG13293	-0.448986514215074	0.227796638596611	1	pseudobulk	DESeq2	LRT	0
142	KC_AB1	CG13295	-0.168404738724522	0.865252793946466	1	pseudobulk	DESeq2	LRT	0
142	KC_AB1	CG13300	2.06541384650887	0.768363369045814	1	pseudobulk	DESeq2	LRT	0
142	KC_AB1	CG13314	0.849606887838128	1	1	pseudobulk	DESeq2	LRT	0
142	KC_AB1	CG13315	0.37765466845954	1	1	pseudobulk	DESeq2	LRT	0
142	KC_AB1	CG13319	0.662839024766543	1	1	pseudobulk	DESeq2	LRT	0
142	KC_AB1	CG13325	-0.766487484571281	0.674970337807573	1	pseudobulk	DESeq2	LRT	0
142	KC_AB1	CG13339	-0.271020131270515	0.603951957626421	1	pseudobulk	DESeq2	LRT	0
142	KC_AB1	CG13344	-0.315062923008025	0.515034142443826	1	pseudobulk	DESeq2	LRT	0
142	KC_AB1	CG13358	2.78674512494791	0.54962276837928	1	pseudobulk	DESeq2	LRT	0
142	KC_AB1	CG13360	2.31997030691033	0.813169507846918	1	pseudobulk	DESeq2	LRT	0

143	KC_AB1	CG13364	-0.0479179358306014	0.909103178094427	1	pseudobulk	DESeq2	LRT		0
143	KC_AB1	CG13365	0.183069104070322	0.685202645825209	1	pseudobulk	DESeq2	LRT		0
143	KC_AB1	CG13366	-0.454225535613193	0.479471230361282	1	pseudobulk	DESeq2	LRT		0
143	KC_AB1	CG13367	-0.444468478108535	0.642193415749354	1	pseudobulk	DESeq2	LRT		0
143	KC_AB1	CG13369	0.142043693387408	0.576337112634333	1	pseudobulk	DESeq2	LRT		0
143	KC_AB1	CG13375	-0.0240894642289829	0.843049392337123	1	pseudobulk	DESeq2	LRT		0
143	KC_AB1	CG13377	0.869279691014102	0.659539235841269	1	pseudobulk	DESeq2	LRT		0
143	KC_AB1	CG13384	-0.0852443342707003	0.796675521112916	1	pseudobulk	DESeq2	LRT		0
143	KC_AB1	CG13390	0.916877772500438	0.912034834646281	1	pseudobulk	DESeq2	LRT		0
143	KC_AB1	CG13392	1.44832487001358	0.528791451952054	1	pseudobulk	DESeq2	LRT		0
144	KC_AB1	CG13398	0.618364150326862	0.576805097915522	1	pseudobulk	DESeq2	LRT		0
144	KC_AB1	CG13404	0.0407453462446358	0.582948049330173	1	pseudobulk	DESeq2	LRT		0
144	KC_AB1	CG13409	-0.992769285558554	0.227316531058678	1	pseudobulk	DESeq2	LRT		0
144	KC_AB1	CG13436	0.849606887838128	1	1	pseudobulk	DESeq2	LRT		0
144	KC_AB1	CG13437	0.849606887838128	1	1	pseudobulk	DESeq2	LRT		0
144	KC_AB1	CG1344	0.527308461511222	0.593000543235995	1	pseudobulk	DESeq2	LRT		0
144	KC_AB1	CG13481	0.849606887838128	1	1	pseudobulk	DESeq2	LRT		0
144	KC_AB1	CG13482	-1.81047400710126	0.215881315435735	1	pseudobulk	DESeq2	LRT		0
144	KC_AB1	CG13484	-0.774359121422117	0.424244175735762	1	pseudobulk	DESeq2	LRT		0
144	KC_AB1	CG13506	-0.769525022610133	0.174255385538298	1	pseudobulk	DESeq2	LRT		0
145	KC_AB1	CG13516	-0.216210029276812	1	1	pseudobulk	DESeq2	LRT		0
145	KC_AB1	CG13526	0.849606887838128	1	1	pseudobulk	DESeq2	LRT		0
145	KC_AB1	CG13527	0.849606887838128	1	1	pseudobulk	DESeq2	LRT		0
145	KC_AB1	CG13531	-1.36642492898125	0.345391555576809	1	pseudobulk	DESeq2	LRT		0
145	KC_AB1	CG13532	0.213479172895293	0.739762758125506	1	pseudobulk	DESeq2	LRT		0
145	KC_AB1	CG1354	-1.75238640183631	0.213782863266687	1	pseudobulk	DESeq2	LRT		0
145	KC_AB1	CG13551	0.492369382419056	0.228683340273457	1	pseudobulk	DESeq2	LRT		0
145	KC_AB1	CG13561	2.78674512494791	0.54962276837928	1	pseudobulk	DESeq2	LRT		0
145	KC_AB1	CG13578	0.941769298407471	0.259121681436165	1	pseudobulk	DESeq2	LRT		0
145	KC_AB1	CG1358	0.270014278912411	0.461636584892812	1	pseudobulk	DESeq2	LRT		0
146	KC_AB1	CG13585	-0.291076428536292	0.337437834756837	1	pseudobulk	DESeq2	LRT		0
146	KC_AB1	CG13594	-0.216210029276812	1	1	pseudobulk	DESeq2	LRT		0
146	KC_AB1	CG1360	0.880742696173337	1	1	pseudobulk	DESeq2	LRT		0
146	KC_AB1	CG13601	0.36201495942229	0.482094898777101	1	pseudobulk	DESeq2	LRT		0
146	KC_AB1	CG13603	0.168843701670026	0.791860384161709	1	pseudobulk	DESeq2	LRT		0
146	KC_AB1	CG13604	0.485394159773125	0.7373555536719	1	pseudobulk	DESeq2	LRT		0
146	KC_AB1	CG13605	-0.694221182763842	0.405959414462227	1	pseudobulk	DESeq2	LRT		0
146	KC_AB1	CG13606	2.78674512494791	0.54962276837928	1	pseudobulk	DESeq2	LRT		0
146	KC_AB1	CG13609	0.849606887838128	1	1	pseudobulk	DESeq2	LRT		0
146	KC_AB1	CG13623	-0.35839370019074	0.637113599128335	1	pseudobulk	DESeq2	LRT		0
147	KC_AB1	CG13625	-0.38761874638843	0.598019970663562	1	pseudobulk	DESeq2	LRT		0
147	KC_AB1	CG13630	0.711303630117963	0.757872099572056	1	pseudobulk	DESeq2	LRT		0
147	KC_AB1	CG13631	1.36979324890833	0.168102284746199	1	pseudobulk	DESeq2	LRT		0
147	KC_AB1	CG13639	-1.84530939222163	0.393119215817718	1	pseudobulk	DESeq2	LRT		0
147	KC_AB1	CG13640	-3.52447474120153	0.313140678524834	1	pseudobulk	DESeq2	LRT		0
147	KC_AB1	CG13641	0.849606887838128	1	1	pseudobulk	DESeq2	LRT		0
147	KC_AB1	CG13643	2.78674512494791	0.54962276837928	1	pseudobulk	DESeq2	LRT		0
147	KC_AB1	CG13654	-0.445780633926877	0.52548411056033	1	pseudobulk	DESeq2	LRT		0
147	KC_AB1	CG13663	-0.957050263712849	0.516516714477728	1	pseudobulk	DESeq2	LRT		0
147	KC_AB1	CG13667	2.85142250261825	0.484249113053674	1	pseudobulk	DESeq2	LRT		0
148	KC_AB1	CG13671	0.731192521170083	0.691088343115929	1	pseudobulk	DESeq2	LRT		0
148	KC_AB1	CG13674	-1.08474447522085	0.580213796993363	1	pseudobulk	DESeq2	LRT		0
148	KC_AB1	CG13676	0.849606887838128	1	1	pseudobulk	DESeq2	LRT		0

148	KC_AB1	CG13685	0.849606887838128	1	1	pseudobulk	DESeq2	LRT	0
148	KC_AB1	CG13689	-1.68377775440576	0.28788704354562	1	pseudobulk	DESeq2	LRT	0
148	KC_AB1	CG13693	0.849606887838128	1	1	pseudobulk	DESeq2	LRT	0
148	KC_AB1	CG13698	1.99934590185188	0.626688701321165	1	pseudobulk	DESeq2	LRT	0
148	KC_AB1	CG13707	-0.283054125455946	1	1	pseudobulk	DESeq2	LRT	0
148	KC_AB1	CG1371	0.0577167187170093	0.648373712786265	1	pseudobulk	DESeq2	LRT	0
148	KC_AB1	CG13737	-0.922741054215178	0.366494641817667	1	pseudobulk	DESeq2	LRT	0
149	KC_AB1	CG13739	-0.127835426746998	0.402054456622321	1	pseudobulk	DESeq2	LRT	0
149	KC_AB1	CG13741	-0.10506771737906	1	1	pseudobulk	DESeq2	LRT	0
149	KC_AB1	CG13743	0.185369276237389	1	1	pseudobulk	DESeq2	LRT	0
149	KC_AB1	CG13748	-2.73573142459503	0.264943650326734	1	pseudobulk	DESeq2	LRT	0
149	KC_AB1	CG13751	-0.418928104893064	0.440884311254976	1	pseudobulk	DESeq2	LRT	0
149	KC_AB1	CG13760	-0.769151030785249	0.361151149247387	1	pseudobulk	DESeq2	LRT	0
149	KC_AB1	CG13766	0.582063463807245	0.571031265838975	1	pseudobulk	DESeq2	LRT	0
149	KC_AB1	CG13773	-1.0124032166989	0.592614381977149	1	pseudobulk	DESeq2	LRT	0
149	KC_AB1	CG13775	0.183159795455075	1	1	pseudobulk	DESeq2	LRT	0
149	KC_AB1	CG13784	0.451413248157462	0.071255245234992	1	pseudobulk	DESeq2	LRT	0
150	KC_AB1	CG13795	0.849606887838128	1	1	pseudobulk	DESeq2	LRT	0
150	KC_AB1	CG13796	-0.830062897938551	0.717724257445671	1	pseudobulk	DESeq2	LRT	0
150	KC_AB1	CG13807	0.390892513907977	0.703325188002498	1	pseudobulk	DESeq2	LRT	0
150	KC_AB1	CG13827	-0.896587419711557	0.544347577356121	1	pseudobulk	DESeq2	LRT	0
150	KC_AB1	CG13841	0.0160940546605626	1	1	pseudobulk	DESeq2	LRT	0
150	KC_AB1	CG13850	-0.194431276273717	0.795253971562443	1	pseudobulk	DESeq2	LRT	0
150	KC_AB1	CG13856	-3.978644847653	0.275743075334038	1	pseudobulk	DESeq2	LRT	0
150	KC_AB1	CG13868	0.224886852383783	0.384224789594084	1	pseudobulk	DESeq2	LRT	0
150	KC_AB1	CG13870	0.849606887838128	1	1	pseudobulk	DESeq2	LRT	0
150	KC_AB1	CG13871	0.849606887838128	1	1	pseudobulk	DESeq2	LRT	0
151	KC_AB1	CG13876	0.403792209238783	0.864193642398759	1	pseudobulk	DESeq2	LRT	0
151	KC_AB1	CG13877	0.849606887838128	1	1	pseudobulk	DESeq2	LRT	0
151	KC_AB1	CG13887	-0.239282654482528	0.519400962621505	1	pseudobulk	DESeq2	LRT	0
151	KC_AB1	CG13893	-0.921848355101375	0.439213774015132	1	pseudobulk	DESeq2	LRT	0
151	KC_AB1	CG13894	0.849606887838128	1	1	pseudobulk	DESeq2	LRT	0
151	KC_AB1	CG13895	0.387449332529058	0.716984551227535	1	pseudobulk	DESeq2	LRT	0
151	KC_AB1	CG13896	0.849606887838128	1	1	pseudobulk	DESeq2	LRT	0
151	KC_AB1	CG13898	-0.564356698759404	0.803702590456371	1	pseudobulk	DESeq2	LRT	0
151	KC_AB1	CG13901	1.4669126015118	0.269344142402521	1	pseudobulk	DESeq2	LRT	0
151	KC_AB1	CG13907	-0.448000525621288	0.462023578935207	1	pseudobulk	DESeq2	LRT	0
152	KC_AB1	CG13912	0.849606887838128	1	1	pseudobulk	DESeq2	LRT	0
152	KC_AB1	CG13917	-0.063521794631131	0.710069925800332	1	pseudobulk	DESeq2	LRT	0
152	KC_AB1	CG13919	0.881827669936858	0.437888615405362	1	pseudobulk	DESeq2	LRT	0
152	KC_AB1	CG13920	0.0881035248336755	0.766447565885233	1	pseudobulk	DESeq2	LRT	0
152	KC_AB1	CG13921	-0.274447077049239	0.376277045821103	1	pseudobulk	DESeq2	LRT	0
152	KC_AB1	CG13926	0.582580455143757	0.501215630215993	1	pseudobulk	DESeq2	LRT	0
152	KC_AB1	CG13928	-0.141983179754563	0.416685934582778	1	pseudobulk	DESeq2	LRT	0
152	KC_AB1	CG13933	-0.411227080139563	0.496112217252783	1	pseudobulk	DESeq2	LRT	0
152	KC_AB1	CG13937	0.417417433006449	0.619513304264368	1	pseudobulk	DESeq2	LRT	0
152	KC_AB1	CG13954	-0.210413644041582	0.363616794499232	1	pseudobulk	DESeq2	LRT	0
153	KC_AB1	CG13955	0.849606887838128	1	1	pseudobulk	DESeq2	LRT	0
153	KC_AB1	CG13982	0.679295535891305	0.808482696395939	1	pseudobulk	DESeq2	LRT	0
153	KC_AB1	CG13994	0.107980845384072	0.772468194672351	1	pseudobulk	DESeq2	LRT	0
153	KC_AB1	CG13995	0.450350504952217	0.898564801962159	1	pseudobulk	DESeq2	LRT	0
153	KC_AB1	CG13999	0.203554521832136	0.592914387160037	1	pseudobulk	DESeq2	LRT	0
153	KC_AB1	CG14005	0.278252914907684	0.620636683440078	1	pseudobulk	DESeq2	LRT	0

153	KC_AB1	CG14015	0.142872146716767	0.830042253039884	1	pseudobulk	DESeq2	LRT	0
153	KC_AB1	CG14024	0.290804950686519	0.551011544271083	1	pseudobulk	DESeq2	LRT	0
153	KC_AB1	CG14043	0.325856740704634	0.609860599663907	1	pseudobulk	DESeq2	LRT	0
153	KC_AB1	CG14050	1.59863986092676	1	1	pseudobulk	DESeq2	LRT	0
154	KC_AB1	CG14052	2.31997030691033	0.813169507846918	1	pseudobulk	DESeq2	LRT	0
154	KC_AB1	CG14053	0.849606887838128	1	1	pseudobulk	DESeq2	LRT	0
154	KC_AB1	CG14057	0.0863909509812212	1	1	pseudobulk	DESeq2	LRT	0
154	KC_AB1	CG1407	-0.715263002027267	0.400835680784401	1	pseudobulk	DESeq2	LRT	0
154	KC_AB1	CG14073	-0.105722574463614	0.662281348339241	1	pseudobulk	DESeq2	LRT	0
154	KC_AB1	CG14074	-0.446764375687507	0.625084306032884	1	pseudobulk	DESeq2	LRT	0
154	KC_AB1	CG14082	2.15837425806378	0.0176016754731204	1	pseudobulk	DESeq2	LRT	0
154	KC_AB1	CG14086	0.849606887838128	1	1	pseudobulk	DESeq2	LRT	0
154	KC_AB1	CG14089	0.849606887838128	1	1	pseudobulk	DESeq2	LRT	0
154	KC_AB1	CG14100	-0.190456992445745	0.729858180313591	1	pseudobulk	DESeq2	LRT	0
155	KC_AB1	CG14102	0.38791462042315	0.872661205144507	1	pseudobulk	DESeq2	LRT	0
155	KC_AB1	CG14104	0.855536372532076	0.726720341825399	1	pseudobulk	DESeq2	LRT	0
155	KC_AB1	CG14117	0.849606887838128	1	1	pseudobulk	DESeq2	LRT	0
155	KC_AB1	CG14130	-1.67728108686538	0.203269726682845	1	pseudobulk	DESeq2	LRT	0
155	KC_AB1	CG14132	-2.44173222908943	0.322526248395761	1	pseudobulk	DESeq2	LRT	0
155	KC_AB1	CG14135	-1.26543797905561	0.299587545434848	1	pseudobulk	DESeq2	LRT	0
155	KC_AB1	CG14137	0.849606887838128	1	1	pseudobulk	DESeq2	LRT	0
155	KC_AB1	CG14141	-1.40556673452188	0.332474275705785	1	pseudobulk	DESeq2	LRT	0
155	KC_AB1	CG14142	-1.38372700554157	0.457202799405001	1	pseudobulk	DESeq2	LRT	0
155	KC_AB1	CG14153	1.53445747936661	0.741101785179312	1	pseudobulk	DESeq2	LRT	0
156	KC_AB1	CG14154	3.27119979990127	0.0371946238556393	1	pseudobulk	DESeq2	LRT	0
156	KC_AB1	CG1416	0.143694795679023	0.535030518241679	1	pseudobulk	DESeq2	LRT	0
156	KC_AB1	CG14174	0.985243145755336	1	1	pseudobulk	DESeq2	LRT	0
156	KC_AB1	CG1418	0.330303735457967	0.390620361499367	1	pseudobulk	DESeq2	LRT	0
156	KC_AB1	CG14182	-0.719940425313989	0.440073488291552	1	pseudobulk	DESeq2	LRT	0
156	KC_AB1	CG14183	-1.30745874410672	0.5410399285464	1	pseudobulk	DESeq2	LRT	0
156	KC_AB1	CG14184	-0.13303798306241	0.837223607211772	1	pseudobulk	DESeq2	LRT	0
156	KC_AB1	CG14185	2.31997030691033	0.813169507846918	1	pseudobulk	DESeq2	LRT	0
156	KC_AB1	CG14186	0.0572494504340166	0.516251452690934	1	pseudobulk	DESeq2	LRT	0
156	KC_AB1	CG14190	0.849606887838128	1	1	pseudobulk	DESeq2	LRT	0
157	KC_AB1	CG14194	-0.0570772004658343	0.692486171392384	1	pseudobulk	DESeq2	LRT	0
157	KC_AB1	CG14200	-0.0960458511466958	0.710988061153559	1	pseudobulk	DESeq2	LRT	0
157	KC_AB1	CG14207	0.227633039649931	0.618473740238721	1	pseudobulk	DESeq2	LRT	0
157	KC_AB1	CG14210	0.0917064358749574	1	1	pseudobulk	DESeq2	LRT	0
157	KC_AB1	CG14220	-1.29798302837625	0.312325714809261	1	pseudobulk	DESeq2	LRT	0
157	KC_AB1	CG14223	2.78674512494791	0.54962276837928	1	pseudobulk	DESeq2	LRT	0
157	KC_AB1	CG14229	-0.733522054107837	0.275654242776556	1	pseudobulk	DESeq2	LRT	0
157	KC_AB1	CG14230	0.750850669314555	0.263613869313835	1	pseudobulk	DESeq2	LRT	0
157	KC_AB1	CG14231	0.353314845633942	1	1	pseudobulk	DESeq2	LRT	0
157	KC_AB1	CG14232	-0.0767614525973085	0.685095388177802	1	pseudobulk	DESeq2	LRT	0
158	KC_AB1	CG14234	0.197242958776691	0.623480574583412	1	pseudobulk	DESeq2	LRT	0
158	KC_AB1	CG14237	0.183159795463042	1	1	pseudobulk	DESeq2	LRT	0
158	KC_AB1	CG14252	-1.37872537761554	0.314428477992686	1	pseudobulk	DESeq2	LRT	0
158	KC_AB1	CG14253	3.41844045086015	0.351942490385471	1	pseudobulk	DESeq2	LRT	0
158	KC_AB1	CG14259	0.849606887838128	1	1	pseudobulk	DESeq2	LRT	0
158	KC_AB1	CG14265	-0.683829746723457	0.723342236517982	1	pseudobulk	DESeq2	LRT	0
158	KC_AB1	CG14270	0.395207989461165	0.661608343243171	1	pseudobulk	DESeq2	LRT	0
158	KC_AB1	CG14274	0.849606887838128	1	1	pseudobulk	DESeq2	LRT	0
158	KC_AB1	CG14286	-0.722313492736721	0.562859374670592	1	pseudobulk	DESeq2	LRT	0

158	KC_AB1	CG14301	-0.24753590046261	0.852822912687924	1	pseudobulk	DESeq2	LRT	0
159	KC_AB1	CG14302	0.849606887838128	1	1	pseudobulk	DESeq2	LRT	0
159	KC_AB1	CG14304	-1.38372700554157	0.457202799405001	1	pseudobulk	DESeq2	LRT	0
159	KC_AB1	CG14305	0.849606887838128	1	1	pseudobulk	DESeq2	LRT	0
159	KC_AB1	CG14312	0.0397301748611494	0.750593041273139	1	pseudobulk	DESeq2	LRT	0
159	KC_AB1	CG14314	0.595639705925952	0.583644480034074	1	pseudobulk	DESeq2	LRT	0
159	KC_AB1	CG14321	0.34802063738034	0.491837976301815	1	pseudobulk	DESeq2	LRT	0
159	KC_AB1	CG14322	-1.15398036752679	0.453041197563316	1	pseudobulk	DESeq2	LRT	0
159	KC_AB1	CG1434	-1.31451666162458	0.49342546530929	1	pseudobulk	DESeq2	LRT	0
159	KC_AB1	CG14340	-0.602885138026742	0.624554020788244	1	pseudobulk	DESeq2	LRT	0
159	KC_AB1	CG14341	0.739771057907444	0.348929307106989	1	pseudobulk	DESeq2	LRT	0
160	KC_AB1	CG14352	-0.738368321166691	0.608486364661884	1	pseudobulk	DESeq2	LRT	0
160	KC_AB1	CG14355	0.0160940546605626	1	1	pseudobulk	DESeq2	LRT	0
160	KC_AB1	CG14356	-0.100548307932341	1	1	pseudobulk	DESeq2	LRT	0
160	KC_AB1	CG14367	-0.622166910409198	0.780289788827665	1	pseudobulk	DESeq2	LRT	0
160	KC_AB1	CG14397	0.849606887838128	1	1	pseudobulk	DESeq2	LRT	0
160	KC_AB1	CG1440	0.269753587694192	0.64210711403468	1	pseudobulk	DESeq2	LRT	0
160	KC_AB1	CG14400	2.78674512494791	0.54962276837928	1	pseudobulk	DESeq2	LRT	0
160	KC_AB1	CG14401	-4.08890327346962	0.267407065217271	1	pseudobulk	DESeq2	LRT	0
160	KC_AB1	CG14406	0.849606887838128	1	1	pseudobulk	DESeq2	LRT	0
160	KC_AB1	CG14407	-0.59018495529637	0.255138608026433	1	pseudobulk	DESeq2	LRT	0
161	KC_AB1	CG14408	-0.393101792301521	0.177504049174786	1	pseudobulk	DESeq2	LRT	0
161	KC_AB1	CG14410	-0.395388837421284	0.734517171574983	1	pseudobulk	DESeq2	LRT	0
161	KC_AB1	CG14411	-0.584205424841348	0.345701347739415	1	pseudobulk	DESeq2	LRT	0
161	KC_AB1	CG14414	0.0309245414072114	0.924165034157968	1	pseudobulk	DESeq2	LRT	0
161	KC_AB1	CG14427	0.849606887838128	1	1	pseudobulk	DESeq2	LRT	0
161	KC_AB1	CG14431	-0.554659624698267	0.311939310141318	1	pseudobulk	DESeq2	LRT	0
161	KC_AB1	CG14434	-0.524746831409561	0.430693463746958	1	pseudobulk	DESeq2	LRT	0
161	KC_AB1	CG14435	-0.193891961592067	0.679177029825664	1	pseudobulk	DESeq2	LRT	0
161	KC_AB1	CG14439	-1.25393399638069	0.612054104673377	1	pseudobulk	DESeq2	LRT	0
161	KC_AB1	CG14440	-0.0241544722333833	0.601179341492612	1	pseudobulk	DESeq2	LRT	0
162	KC_AB1	CG14441	1.56129728754526	0.486655305057187	1	pseudobulk	DESeq2	LRT	0
162	KC_AB1	CG14442	-0.119008516532498	0.756424984447038	1	pseudobulk	DESeq2	LRT	0
162	KC_AB1	CG14450	0.0206258933808748	0.727640348181399	1	pseudobulk	DESeq2	LRT	0
162	KC_AB1	CG14459	0.849606887838128	1	1	pseudobulk	DESeq2	LRT	0
162	KC_AB1	CG14464	0.171096893221258	0.480802032864491	1	pseudobulk	DESeq2	LRT	0
162	KC_AB1	CG14478	-0.289901449958682	0.274625189852092	1	pseudobulk	DESeq2	LRT	0
162	KC_AB1	CG14483	0.207199517575018	0.652580446559664	1	pseudobulk	DESeq2	LRT	0
162	KC_AB1	CG14491	2.31997030691033	0.813169507846918	1	pseudobulk	DESeq2	LRT	0
162	KC_AB1	CG14492	0.849606887838128	1	1	pseudobulk	DESeq2	LRT	0
162	KC_AB1	CG14502	-1.66849992718111	0.245437353584758	1	pseudobulk	DESeq2	LRT	0
163	KC_AB1	CG14512	0.582386458356713	0.823275527850096	1	pseudobulk	DESeq2	LRT	0
163	KC_AB1	CG14535	-0.219089533748682	0.684116517625098	1	pseudobulk	DESeq2	LRT	0
163	KC_AB1	CG14543	0.632295691947619	0.7705043117907	1	pseudobulk	DESeq2	LRT	0
163	KC_AB1	CG14561	-0.830062897938551	0.717724257445671	1	pseudobulk	DESeq2	LRT	0
163	KC_AB1	CG14562	0.754424922194984	0.144669228822349	1	pseudobulk	DESeq2	LRT	0
163	KC_AB1	CG14563	-1.21743681886707	0.501157600450438	1	pseudobulk	DESeq2	LRT	0
163	KC_AB1	CG14566	0.849606887838128	1	1	pseudobulk	DESeq2	LRT	0
163	KC_AB1	CG14589	-1.00752466718157	0.580536516624078	1	pseudobulk	DESeq2	LRT	0
163	KC_AB1	CG14591	0.295608142868179	0.415860355609788	1	pseudobulk	DESeq2	LRT	0
163	KC_AB1	CG14609	0.849606887838128	1	1	pseudobulk	DESeq2	LRT	0
164	KC_AB1	CG1461	-0.10506771737906	1	1	pseudobulk	DESeq2	LRT	0
164	KC_AB1	CG14613	-0.353668384822544	0.615223869919826	1	pseudobulk	DESeq2	LRT	0

164	KC_AB1	CG14615	0.849606887838128	1	1	pseudobulk	DESeq2	LRT	0
164	KC_AB1	CG14618	-0.853698737059242	0.572215810208669	1	pseudobulk	DESeq2	LRT	0
164	KC_AB1	CG14621	0.462847362661402	0.406993351401349	1	pseudobulk	DESeq2	LRT	0
164	KC_AB1	CG1463	-0.115639388181918	0.718671072231016	1	pseudobulk	DESeq2	LRT	0
164	KC_AB1	CG14641	0.361148278447055	0.450599777897569	1	pseudobulk	DESeq2	LRT	0
164	KC_AB1	CG14644	0.849606887838128	1	1	pseudobulk	DESeq2	LRT	0
164	KC_AB1	CG14646	0.294310030664294	0.597943784190583	1	pseudobulk	DESeq2	LRT	0
164	KC_AB1	CG14647	0.466595259577662	0.625189965452268	1	pseudobulk	DESeq2	LRT	0
165	KC_AB1	CG14650	0.0739761485811165	0.747057572198138	1	pseudobulk	DESeq2	LRT	0
165	KC_AB1	CG14657	-0.335456960260792	0.596863097474164	1	pseudobulk	DESeq2	LRT	0
165	KC_AB1	CG14661	-1.26843202587724	0.560029244163373	1	pseudobulk	DESeq2	LRT	0
165	KC_AB1	CG14667	0.995139258950311	0.0847220232602944	1	pseudobulk	DESeq2	LRT	0
165	KC_AB1	CG14669	-0.0962520868269984	0.738704880688824	1	pseudobulk	DESeq2	LRT	0
165	KC_AB1	CG14671	-1.93144238241005	0.204642447661588	1	pseudobulk	DESeq2	LRT	0
165	KC_AB1	CG1468	-1.30745874410672	0.5410399285464	1	pseudobulk	DESeq2	LRT	0
165	KC_AB1	CG14683	0.43253641533918	1	1	pseudobulk	DESeq2	LRT	0
165	KC_AB1	CG14687	-1.77314390790237	0.348473966080666	1	pseudobulk	DESeq2	LRT	0
165	KC_AB1	CG14688	1.39302308053166	0.675623807317343	1	pseudobulk	DESeq2	LRT	0
166	KC_AB1	CG14694	-0.0806766248263104	1	1	pseudobulk	DESeq2	LRT	0
166	KC_AB1	CG14696	0.849606887838128	1	1	pseudobulk	DESeq2	LRT	0
166	KC_AB1	CG14701	-0.682090141825051	0.582487343308912	1	pseudobulk	DESeq2	LRT	0
166	KC_AB1	CG14710	0.0396116186141901	0.844831796636546	1	pseudobulk	DESeq2	LRT	0
166	KC_AB1	CG14711	-0.212841780129023	0.740415205376792	1	pseudobulk	DESeq2	LRT	0
166	KC_AB1	CG14712	-0.789359387491958	0.499566042770873	1	pseudobulk	DESeq2	LRT	0
166	KC_AB1	CG14715	0.289442816470534	0.663882011896233	1	pseudobulk	DESeq2	LRT	0
166	KC_AB1	CG14721	0.248016754297845	0.706211934473442	1	pseudobulk	DESeq2	LRT	0
166	KC_AB1	CG14722	1.2364323511888	0.309042089658657	1	pseudobulk	DESeq2	LRT	0
166	KC_AB1	CG14742	2.78674512494791	0.54962276837928	1	pseudobulk	DESeq2	LRT	0
167	KC_AB1	CG14752	0.366247714696288	1	1	pseudobulk	DESeq2	LRT	0
167	KC_AB1	CG14757	-0.811334823348136	0.437675926933485	1	pseudobulk	DESeq2	LRT	0
167	KC_AB1	CG14760	2.31997030691033	0.813169507846918	1	pseudobulk	DESeq2	LRT	0
167	KC_AB1	CG14762	-0.0307970142778832	0.860586909494804	1	pseudobulk	DESeq2	LRT	0
167	KC_AB1	CG14763	1.59863986092676	1	1	pseudobulk	DESeq2	LRT	0
167	KC_AB1	CG14764	-0.10777027137092	0.486354518230984	1	pseudobulk	DESeq2	LRT	0
167	KC_AB1	CG14767	0.245314230775955	0.244494599261758	1	pseudobulk	DESeq2	LRT	0
167	KC_AB1	CG14777	-0.116912230338746	0.571936766972353	1	pseudobulk	DESeq2	LRT	0
167	KC_AB1	CG14785	2.06541384650887	0.768363369045814	1	pseudobulk	DESeq2	LRT	0
167	KC_AB1	CG14787	1.59863986092676	1	1	pseudobulk	DESeq2	LRT	0
168	KC_AB1	CG14797	0.0530698057625935	1	1	pseudobulk	DESeq2	LRT	0
168	KC_AB1	CG14803	1.19415811754596	0.765010256606659	1	pseudobulk	DESeq2	LRT	0
168	KC_AB1	CG14805	0.192650452920462	0.664499161036403	1	pseudobulk	DESeq2	LRT	0
168	KC_AB1	CG14806	0.117140811724264	0.734694338472024	1	pseudobulk	DESeq2	LRT	0
168	KC_AB1	CG14811	-0.590637494330849	0.764308822189376	1	pseudobulk	DESeq2	LRT	0
168	KC_AB1	CG14812	-0.239144114845824	0.728337957869773	1	pseudobulk	DESeq2	LRT	0
168	KC_AB1	CG14817	0.101902614842344	0.873918209512119	1	pseudobulk	DESeq2	LRT	0
168	KC_AB1	CG14818	0.0485317702022362	0.707437509588923	1	pseudobulk	DESeq2	LRT	0
168	KC_AB1	CG14829	2.31997030691033	0.813169507846918	1	pseudobulk	DESeq2	LRT	0
168	KC_AB1	CG14830	2.78674512494791	0.54962276837928	1	pseudobulk	DESeq2	LRT	0
169	KC_AB1	CG14834	2.78674512494791	0.54962276837928	1	pseudobulk	DESeq2	LRT	0
169	KC_AB1	CG14853	-0.279682106623375	0.370174103091709	1	pseudobulk	DESeq2	LRT	0
169	KC_AB1	CG14857	2.68993930934622	0.478412374021253	1	pseudobulk	DESeq2	LRT	0
169	KC_AB1	CG1486	-0.435267528039608	0.56842957842887	1	pseudobulk	DESeq2	LRT	0
169	KC_AB1	CG14868	0.572456432618173	0.239363743801567	1	pseudobulk	DESeq2	LRT	0

169	KC_AB1	CG14880	3.41844045086015	0.351942490385471	1	pseudobulk	DESeq2	LRT	0
169	KC_AB1	CG14881	0.889598245658659	0.822134554047309	1	pseudobulk	DESeq2	LRT	0
169	KC_AB1	CG14882	-0.680394925896106	0.553286080704912	1	pseudobulk	DESeq2	LRT	0
169	KC_AB1	CG14883	-0.184755355972321	0.796160521968792	1	pseudobulk	DESeq2	LRT	0
169	KC_AB1	CG14894	0.469871685019625	0.443377775539564	1	pseudobulk	DESeq2	LRT	0
170	KC_AB1	CG14903	0.0182811706441658	0.963747497900923	1	pseudobulk	DESeq2	LRT	0
170	KC_AB1	CG14906	0.186688522330959	1	1	pseudobulk	DESeq2	LRT	0
170	KC_AB1	CG1492	0.384692158117039	0.705209358492783	1	pseudobulk	DESeq2	LRT	0
170	KC_AB1	CG14937	3.412819005277	0.165326531479949	1	pseudobulk	DESeq2	LRT	0
170	KC_AB1	CG14961	2.78674512494791	0.54962276837928	1	pseudobulk	DESeq2	LRT	0
170	KC_AB1	CG14965	0.109984153126441	0.922429741903205	1	pseudobulk	DESeq2	LRT	0
170	KC_AB1	CG14966	0.442295766124506	0.730383060803311	1	pseudobulk	DESeq2	LRT	0
170	KC_AB1	CG14968	0.849606887838128	1	1	pseudobulk	DESeq2	LRT	0
170	KC_AB1	CG14969	-0.67600837691768	0.443277898627752	1	pseudobulk	DESeq2	LRT	0
170	KC_AB1	CG14971	-0.650942313554214	0.536195138824173	1	pseudobulk	DESeq2	LRT	0
171	KC_AB1	CG14982	-0.076524736793396	0.545469082837963	1	pseudobulk	DESeq2	LRT	0
171	KC_AB1	CG14985	0.849606887838128	1	1	pseudobulk	DESeq2	LRT	0
171	KC_AB1	CG14989	0.4621496925327	0.95215603782892	1	pseudobulk	DESeq2	LRT	0
171	KC_AB1	CG14995	-0.192629462036724	0.536760960864275	1	pseudobulk	DESeq2	LRT	0
171	KC_AB1	CG14997	1.73668516450085	0.149690693563211	1	pseudobulk	DESeq2	LRT	0
171	KC_AB1	CG15011	3.47261803475683	0.32867999329132	1	pseudobulk	DESeq2	LRT	0
171	KC_AB1	CG15012	-0.355407205972002	0.293734544689215	1	pseudobulk	DESeq2	LRT	0
171	KC_AB1	CG15014	0.402613968574384	0.73301433819782	1	pseudobulk	DESeq2	LRT	0
171	KC_AB1	CG15019	-0.503150497087114	0.534795616368765	1	pseudobulk	DESeq2	LRT	0
171	KC_AB1	CG15027	0.514912663003135	0.837133088035014	1	pseudobulk	DESeq2	LRT	0
172	KC_AB1	CG1503	-0.40200137056069	0.861588439126694	1	pseudobulk	DESeq2	LRT	0
172	KC_AB1	CG1504	2.68993930934622	0.478412374021253	1	pseudobulk	DESeq2	LRT	0
172	KC_AB1	CG15040	-1.59387782197811	0.406125972401365	1	pseudobulk	DESeq2	LRT	0
172	KC_AB1	CG15043	0.849606887838128	1	1	pseudobulk	DESeq2	LRT	0
172	KC_AB1	CG15046	3.81892523345342	0.259683132508622	1	pseudobulk	DESeq2	LRT	0
172	KC_AB1	CG15047	-0.19698351532854	1	1	pseudobulk	DESeq2	LRT	0
172	KC_AB1	CG15073	-0.681895256814707	0.580309397605961	1	pseudobulk	DESeq2	LRT	0
172	KC_AB1	CG15082	1.51280379024899	0.85518877994987	1	pseudobulk	DESeq2	LRT	0
172	KC_AB1	CG15083	2.78674512494791	0.54962276837928	1	pseudobulk	DESeq2	LRT	0
172	KC_AB1	CG15084	0.572207669466247	1	1	pseudobulk	DESeq2	LRT	0
173	KC_AB1	CG15093	0.849606887838128	1	1	pseudobulk	DESeq2	LRT	0
173	KC_AB1	CG15097	-0.181852907578203	0.586514728706208	1	pseudobulk	DESeq2	LRT	0
173	KC_AB1	CG15098	-0.980909183526803	0.308778606783244	1	pseudobulk	DESeq2	LRT	0
173	KC_AB1	CG15099	0.392827450136601	0.442644291926527	1	pseudobulk	DESeq2	LRT	0
173	KC_AB1	CG15107	0.179656589031598	0.862766495746633	1	pseudobulk	DESeq2	LRT	0
173	KC_AB1	CG15109	1.70415992268272	0.492690881845078	1	pseudobulk	DESeq2	LRT	0
173	KC_AB1	CG15111	-0.131926491232681	0.688203439403094	1	pseudobulk	DESeq2	LRT	0
173	KC_AB1	CG15117	2.71656517801154	0.391234425153119	1	pseudobulk	DESeq2	LRT	0
173	KC_AB1	CG15118	-0.165782876704724	0.71711924844984	1	pseudobulk	DESeq2	LRT	0
173	KC_AB1	CG1513	-0.049523339057001	0.583750273326765	1	pseudobulk	DESeq2	LRT	0
174	KC_AB1	CG15141	1.03483814891003	0.226284683864193	1	pseudobulk	DESeq2	LRT	0
174	KC_AB1	CG15160	-0.172473714435605	0.69692646385809	1	pseudobulk	DESeq2	LRT	0
174	KC_AB1	CG15168	0.104826975486212	0.586674803168463	1	pseudobulk	DESeq2	LRT	0
174	KC_AB1	CG15172	0.849606887838128	1	1	pseudobulk	DESeq2	LRT	0
174	KC_AB1	CG15201	0.494158385889054	0.520905476241723	1	pseudobulk	DESeq2	LRT	0
174	KC_AB1	CG15202	0.849606887838128	1	1	pseudobulk	DESeq2	LRT	0
174	KC_AB1	CG15203	2.31997030691033	0.813169507846918	1	pseudobulk	DESeq2	LRT	0
174	KC_AB1	CG15209	-0.395003056856662	0.597156494935841	1	pseudobulk	DESeq2	LRT	0

174	KC_AB1	CG15210	-1.8476447304053	0.456955818574401	1	pseudobulk	DESeq2	LRT	0
174	KC_AB1	CG15213	3.8636724609959	0.018541250422827	1	pseudobulk	DESeq2	LRT	0
175	KC_AB1	CG1523	-0.853777418295258	0.456792190484576	1	pseudobulk	DESeq2	LRT	0
175	KC_AB1	CG15233	2.85142250261825	0.484249113053674	1	pseudobulk	DESeq2	LRT	0
175	KC_AB1	CG15236	-0.860601783997805	0.644483780366323	1	pseudobulk	DESeq2	LRT	0
175	KC_AB1	CG15237	0.30760347727836	0.529612997103706	1	pseudobulk	DESeq2	LRT	0
175	KC_AB1	CG15239	0.183159795455075	1	1	pseudobulk	DESeq2	LRT	0
175	KC_AB1	CG15270	-0.245804097588933	0.609478304936554	1	pseudobulk	DESeq2	LRT	0
175	KC_AB1	CG15286	1.02362625135634	0.890529674363423	1	pseudobulk	DESeq2	LRT	0
175	KC_AB1	CG15309	-0.253211545093157	0.524732069837612	1	pseudobulk	DESeq2	LRT	0
175	KC_AB1	CG15312	0.369741842147874	0.325594820468121	1	pseudobulk	DESeq2	LRT	0
175	KC_AB1	CG15317	3.20547843809887	0.383970068137593	1	pseudobulk	DESeq2	LRT	0
176	KC_AB1	CG1532	-0.0175483656632571	0.747570929446806	1	pseudobulk	DESeq2	LRT	0
176	KC_AB1	CG15330	2.31997030691033	0.813169507846918	1	pseudobulk	DESeq2	LRT	0
176	KC_AB1	CG15332	-0.564356698759404	0.803702590456371	1	pseudobulk	DESeq2	LRT	0
176	KC_AB1	CG15343	0.849606887838128	1	1	pseudobulk	DESeq2	LRT	0
176	KC_AB1	CG15356	0.200210137956425	0.660829598321709	1	pseudobulk	DESeq2	LRT	0
176	KC_AB1	CG15365	3.08753393561965	0.382167145512329	1	pseudobulk	DESeq2	LRT	0
176	KC_AB1	CG1537	1.71056345536611	0.156143963690853	1	pseudobulk	DESeq2	LRT	0
176	KC_AB1	CG15382	1.50875020934616	0.732232096088675	1	pseudobulk	DESeq2	LRT	0
176	KC_AB1	CG15385	0.200073840065049	0.729729144030179	1	pseudobulk	DESeq2	LRT	0
176	KC_AB1	CG15387	1.31255798272826	0.560650621130882	1	pseudobulk	DESeq2	LRT	0
177	KC_AB1	CG15390	-0.485027100453874	0.813470998077292	1	pseudobulk	DESeq2	LRT	0
177	KC_AB1	CG15399	0.0160940546605174	1	1	pseudobulk	DESeq2	LRT	0
177	KC_AB1	CG15403	0.849606887838128	1	1	pseudobulk	DESeq2	LRT	0
177	KC_AB1	CG15412	0.849606887838128	1	1	pseudobulk	DESeq2	LRT	0
177	KC_AB1	CG1542	1.01265988621066	0.962519979213338	1	pseudobulk	DESeq2	LRT	0
177	KC_AB1	CG15431	-0.334027622965191	0.0879436856899085	1	pseudobulk	DESeq2	LRT	0
177	KC_AB1	CG15432	-0.767550724568639	0.252029450955997	1	pseudobulk	DESeq2	LRT	0
177	KC_AB1	CG15435	-0.558566403247552	0.635216785885753	1	pseudobulk	DESeq2	LRT	0
177	KC_AB1	CG15439	1.70085350812671	0.348499695592741	1	pseudobulk	DESeq2	LRT	0
177	KC_AB1	CG15443	-0.156334273939777	0.749303225569592	1	pseudobulk	DESeq2	LRT	0
178	KC_AB1	CG15445	-2.15403455142108	0.24772159333887	1	pseudobulk	DESeq2	LRT	0
178	KC_AB1	CG15446	0.769522064442666	1	1	pseudobulk	DESeq2	LRT	0
178	KC_AB1	CG15449	0.109048745868637	0.628634508705252	1	pseudobulk	DESeq2	LRT	0
178	KC_AB1	CG1545	0.500710785727311	1	1	pseudobulk	DESeq2	LRT	0
178	KC_AB1	CG15450	2.06541384650887	0.904905526327343	1	pseudobulk	DESeq2	LRT	0
178	KC_AB1	CG15456	0.332458624541505	0.563388934475115	1	pseudobulk	DESeq2	LRT	0
178	KC_AB1	CG15458	0.849606887838128	1	1	pseudobulk	DESeq2	LRT	0
178	KC_AB1	CG15459	-0.453443561764756	0.343511905947045	1	pseudobulk	DESeq2	LRT	0
178	KC_AB1	CG15461	0.849606887838128	1	1	pseudobulk	DESeq2	LRT	0
178	KC_AB1	CG15483	2.31997030691033	0.813169507846918	1	pseudobulk	DESeq2	LRT	0
179	KC_AB1	CG15514	0.434086419703617	0.54665457449619	1	pseudobulk	DESeq2	LRT	0
179	KC_AB1	CG1552	0.902085911831396	0.0649365632975811	1	pseudobulk	DESeq2	LRT	0
179	KC_AB1	CG15522	-0.331236259846864	0.789946846393125	1	pseudobulk	DESeq2	LRT	0
179	KC_AB1	CG15525	0.873196706287336	0.493027808676027	1	pseudobulk	DESeq2	LRT	0
179	KC_AB1	CG15529	0.849606887838128	1	1	pseudobulk	DESeq2	LRT	0
179	KC_AB1	CG15535	-0.25333651533385	0.533119992486288	1	pseudobulk	DESeq2	LRT	0
179	KC_AB1	CG15536	3.16697464674408	0.406679230261689	1	pseudobulk	DESeq2	LRT	0
179	KC_AB1	CG15537	2.31997030691033	0.813169507846918	1	pseudobulk	DESeq2	LRT	0
179	KC_AB1	CG15539	2.19691424616663	0.593804541109954	1	pseudobulk	DESeq2	LRT	0
179	KC_AB1	CG15547	-1.59387782197811	0.406125972401365	1	pseudobulk	DESeq2	LRT	0
180	KC_AB1	CG15561	-0.794346985539992	0.59546676641635	1	pseudobulk	DESeq2	LRT	0

180	KC_AB1	CG15570	0.849606887838128	1	1	pseudobulk	DESeq2	LRT	0
180	KC_AB1	CG15579	2.31997030691033	0.813169507846918	1	pseudobulk	DESeq2	LRT	0
180	KC_AB1	CG15601	-0.0453615587205798	0.754304623798004	1	pseudobulk	DESeq2	LRT	0
180	KC_AB1	CG15602	-1.28148813652929	0.281940273361682	1	pseudobulk	DESeq2	LRT	0
180	KC_AB1	CG15611	0.849606887838128	1	1	pseudobulk	DESeq2	LRT	0
180	KC_AB1	CG15614	2.13009086641515	0.687046658320206	1	pseudobulk	DESeq2	LRT	0
180	KC_AB1	CG15628	0.218686495725257	0.27935099212932	1	pseudobulk	DESeq2	LRT	0
180	KC_AB1	CG15631	1.59863986092676	1	1	pseudobulk	DESeq2	LRT	0
180	KC_AB1	CG15638	0.849606887838128	1	1	pseudobulk	DESeq2	LRT	0
181	KC_AB1	CG15642	-0.0675435987690132	0.696355862200282	1	pseudobulk	DESeq2	LRT	0
181	KC_AB1	CG15643	-0.602984945619093	0.594301498474692	1	pseudobulk	DESeq2	LRT	0
181	KC_AB1	CG15651	0.565124764208246	0.313546080103739	1	pseudobulk	DESeq2	LRT	0
181	KC_AB1	CG15653	1.80222118524055	0.164991806179666	1	pseudobulk	DESeq2	LRT	0
181	KC_AB1	CG15657	1.59863986092676	1	1	pseudobulk	DESeq2	LRT	0
181	KC_AB1	CG15673	-0.216210029276812	1	1	pseudobulk	DESeq2	LRT	0
181	KC_AB1	CG15706	2.06541384650887	0.768363369045814	1	pseudobulk	DESeq2	LRT	0
181	KC_AB1	CG15715	0.114446767258402	0.566419075297747	1	pseudobulk	DESeq2	LRT	0
181	KC_AB1	CG15717	0.849606887838128	1	1	pseudobulk	DESeq2	LRT	0
181	KC_AB1	CG15719	1.13500620204015	0.225433134399722	1	pseudobulk	DESeq2	LRT	0
182	KC_AB1	CG1572	1.39876208311159	0.189935027187886	1	pseudobulk	DESeq2	LRT	0
182	KC_AB1	CG15725	0.980149469708189	0.952825560416467	1	pseudobulk	DESeq2	LRT	0
182	KC_AB1	CG15739	0.849606887838128	1	1	pseudobulk	DESeq2	LRT	0
182	KC_AB1	CG15743	-0.256606698804154	0.681223060881817	1	pseudobulk	DESeq2	LRT	0
182	KC_AB1	CG15744	-1.6186157998159	0.26241971884862	1	pseudobulk	DESeq2	LRT	0
182	KC_AB1	CG15747	0.350672585386214	0.75374373664193	1	pseudobulk	DESeq2	LRT	0
182	KC_AB1	CG1575	1.20541473026147	0.234671025886793	1	pseudobulk	DESeq2	LRT	0
182	KC_AB1	CG15760	0.751746789665312	0.178147090604564	1	pseudobulk	DESeq2	LRT	0
182	KC_AB1	CG15765	-0.261447383061233	0.226433908808144	1	pseudobulk	DESeq2	LRT	0
182	KC_AB1	CG15767	2.31997030691033	0.813169507846918	1	pseudobulk	DESeq2	LRT	0
183	KC_AB1	CG15771	-0.258472406837803	0.46687343064096	1	pseudobulk	DESeq2	LRT	0
183	KC_AB1	CG15772	0.3554943461343	0.234802504792508	1	pseudobulk	DESeq2	LRT	0
183	KC_AB1	CG1578	-0.732694319593833	0.0923728241870044	1	pseudobulk	DESeq2	LRT	0
183	KC_AB1	CG15803	-1.03269237224961	0.0214697403105645	1	pseudobulk	DESeq2	LRT	0
183	KC_AB1	CG15812	0.781942441589468	0.617519677355798	1	pseudobulk	DESeq2	LRT	0
183	KC_AB1	CG15814	0.0131157348471842	0.772398195958143	1	pseudobulk	DESeq2	LRT	0
183	KC_AB1	CG1582	0.422876418997045	0.924355037840588	1	pseudobulk	DESeq2	LRT	0
183	KC_AB1	CG15864	0.849606887838128	1	1	pseudobulk	DESeq2	LRT	0
183	KC_AB1	CG15870	0.849606887838128	1	1	pseudobulk	DESeq2	LRT	0
183	KC_AB1	CG15877	0.503908089666771	0.753870801605894	1	pseudobulk	DESeq2	LRT	0
184	KC_AB1	CG15878	-0.216210032712384	1	1	pseudobulk	DESeq2	LRT	0
184	KC_AB1	CG15879	0.849606887838128	1	1	pseudobulk	DESeq2	LRT	0
184	KC_AB1	CG15891	0.0650065884687969	0.875732414881808	1	pseudobulk	DESeq2	LRT	0
184	KC_AB1	CG15894	-0.430387370831342	0.0676994847909048	1	pseudobulk	DESeq2	LRT	0
184	KC_AB1	CG15908	0.471944185738102	0.826766565261483	1	pseudobulk	DESeq2	LRT	0
184	KC_AB1	CG15912	-0.48504100977615	0.636044492931173	1	pseudobulk	DESeq2	LRT	0
184	KC_AB1	CG15916	0.363590228399364	0.358016694487552	1	pseudobulk	DESeq2	LRT	0
184	KC_AB1	CG15922	0.0029732462875603	0.686278813769905	1	pseudobulk	DESeq2	LRT	0
184	KC_AB1	CG15923	0.116783351990007	1	1	pseudobulk	DESeq2	LRT	0
184	KC_AB1	CG1598	-0.489935040837216	0.523424256579554	1	pseudobulk	DESeq2	LRT	0
185	KC_AB1	CG1602	2.71656517801154	0.391234425153119	1	pseudobulk	DESeq2	LRT	0
185	KC_AB1	CG1603	-0.556628421020755	0.675401935723456	1	pseudobulk	DESeq2	LRT	0
185	KC_AB1	CG1607	-0.555224875146231	0.21455237716203	1	pseudobulk	DESeq2	LRT	0
185	KC_AB1	CG1620	-1.08551105920426	0.27667669806053	1	pseudobulk	DESeq2	LRT	0

185	KC_AB1	CG1622	-0.467874528134579	0.481814714680058	1	pseudobulk	DESeq2	LRT	0
185	KC_AB1	CG1628	0.366247714696288	1	1	pseudobulk	DESeq2	LRT	0
185	KC_AB1	CG1635	0.968264371716599	0.26211753252481	1	pseudobulk	DESeq2	LRT	0
185	KC_AB1	CG1636	-0.576532378134817	0.195620729258449	1	pseudobulk	DESeq2	LRT	0
185	KC_AB1	CG1637	-0.278642008005091	0.493317409454646	1	pseudobulk	DESeq2	LRT	0
185	KC_AB1	CG1640	0.381137527113433	0.17639854799567	1	pseudobulk	DESeq2	LRT	0
186	KC_AB1	CG1646	-0.0693369634937592	0.747576081575207	1	pseudobulk	DESeq2	LRT	0
186	KC_AB1	CG1647	-0.313963448743768	0.603545264464399	1	pseudobulk	DESeq2	LRT	0
186	KC_AB1	CG1648	-0.584348095698639	0.183126159661997	1	pseudobulk	DESeq2	LRT	0
186	KC_AB1	CG1662	-0.292894710611161	0.748634425746599	1	pseudobulk	DESeq2	LRT	0
186	KC_AB1	CG1663	0.367634104528991	0.833631834135566	1	pseudobulk	DESeq2	LRT	0
186	KC_AB1	CG16700	0.366247714696288	1	1	pseudobulk	DESeq2	LRT	0
186	KC_AB1	CG1671	0.849606887838128	1	1	pseudobulk	DESeq2	LRT	0
186	KC_AB1	CG16711	-0.226067870327013	0.260239545432493	1	pseudobulk	DESeq2	LRT	0
186	KC_AB1	CG16717	-0.199972201042594	0.656051563898635	1	pseudobulk	DESeq2	LRT	0
186	KC_AB1	CG1673	2.31997030691033	0.813169507846918	1	pseudobulk	DESeq2	LRT	0
187	KC_AB1	CG16734	0.183159795455075	1	1	pseudobulk	DESeq2	LRT	0
187	KC_AB1	CG16739	2.06531092668974	0.944672961903726	1	pseudobulk	DESeq2	LRT	0
187	KC_AB1	CG1674	0.849606887838128	1	1	pseudobulk	DESeq2	LRT	0
187	KC_AB1	CG16743	-0.954459377968857	0.536823324487888	1	pseudobulk	DESeq2	LRT	0
187	KC_AB1	CG16749	-0.874332157180961	0.546189162320603	1	pseudobulk	DESeq2	LRT	0
187	KC_AB1	CG16753	1.27391167016016	0.893087792489736	1	pseudobulk	DESeq2	LRT	0
187	KC_AB1	CG16758	1.22837664205667	0.689763751191378	1	pseudobulk	DESeq2	LRT	0
187	KC_AB1	CG1677	-0.14008010379697	0.558938222060771	1	pseudobulk	DESeq2	LRT	0
187	KC_AB1	CG16779	-0.170673706466037	0.237649589062919	1	pseudobulk	DESeq2	LRT	0
187	KC_AB1	CG16781	0.849606887838128	1	1	pseudobulk	DESeq2	LRT	0
188	KC_AB1	CG16787	0.485397873298996	0.594666187267901	1	pseudobulk	DESeq2	LRT	0
188	KC_AB1	CG16789	0.366247714696288	1	1	pseudobulk	DESeq2	LRT	0
188	KC_AB1	CG16790	-0.482669143513822	0.703620610536866	1	pseudobulk	DESeq2	LRT	0
188	KC_AB1	CG16791	-0.352498250148614	0.0896547093725324	1	pseudobulk	DESeq2	LRT	0
188	KC_AB1	CG16798	2.67648348568903	0.142827971353346	1	pseudobulk	DESeq2	LRT	0
188	KC_AB1	CG16799	0.849606887838128	1	1	pseudobulk	DESeq2	LRT	0
188	KC_AB1	CG16812	0.272986042106717	0.366811827444887	1	pseudobulk	DESeq2	LRT	0
188	KC_AB1	CG16820	0.366247714696288	1	1	pseudobulk	DESeq2	LRT	0
188	KC_AB1	CG16825	0.769522064442666	1	1	pseudobulk	DESeq2	LRT	0
188	KC_AB1	CG16852	0.183159795455075	1	1	pseudobulk	DESeq2	LRT	0
189	KC_AB1	CG16854	-0.264189512405487	0.699603136233016	1	pseudobulk	DESeq2	LRT	0
189	KC_AB1	CG16863	0.375949898457391	1	1	pseudobulk	DESeq2	LRT	0
189	KC_AB1	CG16865	0.813047889876332	0.585933383046552	1	pseudobulk	DESeq2	LRT	0
189	KC_AB1	CG16868	0.222471547743086	0.44686082874521	1	pseudobulk	DESeq2	LRT	0
189	KC_AB1	CG1688	-1.47477375861756	0.015951021096635	1	pseudobulk	DESeq2	LRT	0
189	KC_AB1	CG16890	0.325394210509413	1	1	pseudobulk	DESeq2	LRT	0
189	KC_AB1	CG16896	1.20660435421263	0.820893338469406	1	pseudobulk	DESeq2	LRT	0
189	KC_AB1	CG16903	-0.438113947056702	0.535181090776427	1	pseudobulk	DESeq2	LRT	0
189	KC_AB1	CG16908	-0.564356698759404	0.803702590456371	1	pseudobulk	DESeq2	LRT	0
189	KC_AB1	CG16935	0.541143015810333	0.495879945938039	1	pseudobulk	DESeq2	LRT	0
190	KC_AB1	CG1695	0.352693528919901	0.344308741065071	1	pseudobulk	DESeq2	LRT	0
190	KC_AB1	CG16952	-0.308470532758196	0.352656275262827	1	pseudobulk	DESeq2	LRT	0
190	KC_AB1	CG16953	0.667328719757402	0.186340931479793	1	pseudobulk	DESeq2	LRT	0
190	KC_AB1	CG16959	0.849606887838128	1	1	pseudobulk	DESeq2	LRT	0
190	KC_AB1	CG16972	-0.372949734327595	0.580866089575226	1	pseudobulk	DESeq2	LRT	0
190	KC_AB1	CG16974	-0.362154152386968	0.430287263531975	1	pseudobulk	DESeq2	LRT	0
190	KC_AB1	CG16978	0.366247714696288	1	1	pseudobulk	DESeq2	LRT	0

190	KC_AB1	CG16979	-0.677119842443523	0.607244928904796	1	pseudobulk	DESeq2	LRT	0
190	KC_AB1	CG1698	2.06541384650887	0.904905526327343	1	pseudobulk	DESeq2	LRT	0
190	KC_AB1	CG16985	-0.102964149517689	0.840930765812077	1	pseudobulk	DESeq2	LRT	0
191	KC_AB1	CG16986	1.40927271939444	0.261003389460208	1	pseudobulk	DESeq2	LRT	0
191	KC_AB1	CG16989	1.4017523968542	0.345107003428343	1	pseudobulk	DESeq2	LRT	0
191	KC_AB1	CG17002	0.176714067743619	0.787095836321193	1	pseudobulk	DESeq2	LRT	0
191	KC_AB1	CG17005	0.849606887838128	1	1	pseudobulk	DESeq2	LRT	0
191	KC_AB1	CG17010	-0.360297879864743	0.723489150842172	1	pseudobulk	DESeq2	LRT	0
191	KC_AB1	CG17019	1.31382138355412	0.0056308470274238	1	pseudobulk	DESeq2	LRT	0
191	KC_AB1	CG17026	0.695649380909695	0.600562028435839	1	pseudobulk	DESeq2	LRT	0
191	KC_AB1	CG17029	-0.4511661235214	0.575978458795498	1	pseudobulk	DESeq2	LRT	0
191	KC_AB1	CG1703	0.632956631962984	0.189984755381434	1	pseudobulk	DESeq2	LRT	0
191	KC_AB1	CG17030	1.37008314861419	1	1	pseudobulk	DESeq2	LRT	0
192	KC_AB1	CG17036	0.849606887838128	1	1	pseudobulk	DESeq2	LRT	0
192	KC_AB1	CG17059	-0.541687383801157	0.15608652927556	1	pseudobulk	DESeq2	LRT	0
192	KC_AB1	CG17065	-0.409718099114817	0.402224683332597	1	pseudobulk	DESeq2	LRT	0
192	KC_AB1	CG17068	0.335918766912742	1	1	pseudobulk	DESeq2	LRT	0
192	KC_AB1	CG17075	0.369057439973934	0.59708577053932	1	pseudobulk	DESeq2	LRT	0
192	KC_AB1	CG17078	0.599613345536009	0.44623274470088	1	pseudobulk	DESeq2	LRT	0
192	KC_AB1	CG17107	2.78674512494791	0.54962276837928	1	pseudobulk	DESeq2	LRT	0
192	KC_AB1	CG17109	2.78674512494791	0.54962276837928	1	pseudobulk	DESeq2	LRT	0
192	KC_AB1	CG17111	-0.0811469691453732	0.853080383108617	1	pseudobulk	DESeq2	LRT	0
192	KC_AB1	CG17121	0.849606887838128	1	1	pseudobulk	DESeq2	LRT	0
193	KC_AB1	CG17124	-0.134536509611698	0.510898749613089	1	pseudobulk	DESeq2	LRT	0
193	KC_AB1	CG17141	0.501541304409255	1	1	pseudobulk	DESeq2	LRT	0
193	KC_AB1	CG17162	-0.188834487991199	0.762489414198217	1	pseudobulk	DESeq2	LRT	0
193	KC_AB1	CG17163	-0.552935286363526	0.318629677113047	1	pseudobulk	DESeq2	LRT	0
193	KC_AB1	CG17167	2.06541384650887	0.768363369045814	1	pseudobulk	DESeq2	LRT	0
193	KC_AB1	CG17168	-0.156130886388447	0.818656718550059	1	pseudobulk	DESeq2	LRT	0
193	KC_AB1	CG17169	0.849606887838128	1	1	pseudobulk	DESeq2	LRT	0
193	KC_AB1	CG17186	0.399505027584516	1	1	pseudobulk	DESeq2	LRT	0
193	KC_AB1	CG17187	1.23740510256999	0.802272659328411	1	pseudobulk	DESeq2	LRT	0
193	KC_AB1	CG17189	-1.8220115466871	0.455944165919265	1	pseudobulk	DESeq2	LRT	0
194	KC_AB1	CG17193	1.59863986092676	1	1	pseudobulk	DESeq2	LRT	0
194	KC_AB1	CG17202	0.0566581174219192	0.649546292810878	1	pseudobulk	DESeq2	LRT	0
194	KC_AB1	CG17211	2.06541384650887	0.768363369045814	1	pseudobulk	DESeq2	LRT	0
194	KC_AB1	CG1722	0.849606887838128	1	1	pseudobulk	DESeq2	LRT	0
194	KC_AB1	CG17221	1.59811977941717	0.393780924951314	1	pseudobulk	DESeq2	LRT	0
194	KC_AB1	CG17224	3.16697464674408	0.406679230261689	1	pseudobulk	DESeq2	LRT	0
194	KC_AB1	CG17230	0.371484475875394	0.568885091267288	1	pseudobulk	DESeq2	LRT	0
194	KC_AB1	CG17233	0.117088538271973	0.834034413797335	1	pseudobulk	DESeq2	LRT	0
194	KC_AB1	CG17239	3.41844045086015	0.351942490385471	1	pseudobulk	DESeq2	LRT	0
194	KC_AB1	CG17249	0.339569641361069	0.739899841718594	1	pseudobulk	DESeq2	LRT	0
195	KC_AB1	CG17260	-0.420145690518528	0.726273546772655	1	pseudobulk	DESeq2	LRT	0
195	KC_AB1	CG17264	0.366247714696288	1	1	pseudobulk	DESeq2	LRT	0
195	KC_AB1	CG17266	1.25212272806003	0.618535322179697	1	pseudobulk	DESeq2	LRT	0
195	KC_AB1	CG17270	-0.0443297851662103	0.89690378306589	1	pseudobulk	DESeq2	LRT	0
195	KC_AB1	CG17271	-0.14440933110628	0.619423334120514	1	pseudobulk	DESeq2	LRT	0
195	KC_AB1	CG17272	-0.3242062204723	0.326840410734304	1	pseudobulk	DESeq2	LRT	0
195	KC_AB1	CG17279	2.85142250261825	0.484249113053674	1	pseudobulk	DESeq2	LRT	0
195	KC_AB1	CG17292	2.85142250261825	0.484249113053674	1	pseudobulk	DESeq2	LRT	0
195	KC_AB1	CG17294	0.343084899413927	0.540804359989042	1	pseudobulk	DESeq2	LRT	0
195	KC_AB1	CG17321	-0.165691753718291	0.579348076888438	1	pseudobulk	DESeq2	LRT	0

196	KC_AB1	CG17328	-0.590637494330849	0.764308822189376	1	pseudobulk	DESeq2	LRT		0
196	KC_AB1	CG17343	0.276511028914071	0.702624799178901	1	pseudobulk	DESeq2	LRT		0
196	KC_AB1	CG17350	-2.92303448934338	0.184467852201505	1	pseudobulk	DESeq2	LRT		0
196	KC_AB1	CG17359	-1.77899149233245	0.346071786945344	1	pseudobulk	DESeq2	LRT		0
196	KC_AB1	CG17361	-0.289450712162219	0.712668959538654	1	pseudobulk	DESeq2	LRT		0
196	KC_AB1	CG17364	-0.349864850570547	0.420300052432946	1	pseudobulk	DESeq2	LRT		0
196	KC_AB1	CG1737	-0.335168723439075	0.481392664091149	1	pseudobulk	DESeq2	LRT		0
196	KC_AB1	CG17378	-0.132633055114665	0.455621846911561	1	pseudobulk	DESeq2	LRT		0
196	KC_AB1	CG1738	0.193868295755685	0.824147656618461	1	pseudobulk	DESeq2	LRT		0
196	KC_AB1	CG17385	-0.256010063542652	0.495760159674203	1	pseudobulk	DESeq2	LRT		0
197	KC_AB1	CG17454	-0.303393135789015	0.364913209041611	1	pseudobulk	DESeq2	LRT		0
197	KC_AB1	CG17486	-0.050555683162106	0.911034856638391	1	pseudobulk	DESeq2	LRT		0
197	KC_AB1	CG17490	2.78674512494791	0.54962276837928	1	pseudobulk	DESeq2	LRT		0
197	KC_AB1	CG17493	-0.220983670480082	0.526135408047018	1	pseudobulk	DESeq2	LRT		0
197	KC_AB1	CG1750	0.0394354842550928	0.905798526983969	1	pseudobulk	DESeq2	LRT		0
197	KC_AB1	CG17508	-0.122923493171237	0.668534806575161	1	pseudobulk	DESeq2	LRT		0
197	KC_AB1	CG17528	-0.038630436121921	0.5939993277419	1	pseudobulk	DESeq2	LRT		0
197	KC_AB1	CG17544	1.10451565279745	0.757254849515156	1	pseudobulk	DESeq2	LRT		0
197	KC_AB1	CG17556	0.198402639157198	0.727253917820056	1	pseudobulk	DESeq2	LRT		0
197	KC_AB1	CG17565	-0.590006618770172	0.37949633412729	1	pseudobulk	DESeq2	LRT		0
198	KC_AB1	CG17568	0.224114432675152	0.732715880132291	1	pseudobulk	DESeq2	LRT		0
198	KC_AB1	CG17574	0.002219812972515	0.686704956014511	1	pseudobulk	DESeq2	LRT		0
198	KC_AB1	CG17593	0.0497918791314134	0.506700847313804	1	pseudobulk	DESeq2	LRT		0
198	KC_AB1	CG17598	-1.00435978471628	0.232408060893193	1	pseudobulk	DESeq2	LRT		0
198	KC_AB1	CG17600	-0.272604995716208	0.587687331333385	1	pseudobulk	DESeq2	LRT		0
198	KC_AB1	CG17612	-0.971634996989608	0.499428427999027	1	pseudobulk	DESeq2	LRT		0
198	KC_AB1	CG1764	0.132347835953584	0.831140896121556	1	pseudobulk	DESeq2	LRT		0
198	KC_AB1	CG17646	1.97466512143323	0.330792509832706	1	pseudobulk	DESeq2	LRT		0
198	KC_AB1	CG17652	0.929794981961883	0.50532925737138	1	pseudobulk	DESeq2	LRT		0
198	KC_AB1	CG17660	0.155788639780071	0.680318589561857	1	pseudobulk	DESeq2	LRT		0
199	KC_AB1	CG17669	-0.830062897938551	0.717724257445671	1	pseudobulk	DESeq2	LRT		0
199	KC_AB1	CG17683	0.518362389814626	0.315087404100804	1	pseudobulk	DESeq2	LRT		0
199	KC_AB1	CG17687	0.366247714696288	1	1	pseudobulk	DESeq2	LRT		0
199	KC_AB1	CG17691	-0.96690118846624	0.329307065215355	1	pseudobulk	DESeq2	LRT		0
199	KC_AB1	CG17698	-0.278679143779022	0.346024887588625	1	pseudobulk	DESeq2	LRT		0
199	KC_AB1	CG17707	0.849606887838128	1	1	pseudobulk	DESeq2	LRT		0
199	KC_AB1	CG17712	-0.371829134087186	0.661361590168952	1	pseudobulk	DESeq2	LRT		0
199	KC_AB1	CG17715	-0.33551357857043	0.469680994067142	1	pseudobulk	DESeq2	LRT		0
199	KC_AB1	CG17716	-1.5051987887174	0.435059343705501	1	pseudobulk	DESeq2	LRT		0
199	KC_AB1	CG17721	0.653919700887554	0.653143430075654	1	pseudobulk	DESeq2	LRT		0
200	KC_AB1	CG17724	-0.122442322509209	0.755274905792324	1	pseudobulk	DESeq2	LRT		0
200	KC_AB1	CG17726	2.68993930934622	0.478412374021253	1	pseudobulk	DESeq2	LRT		0
200	KC_AB1	CG17734	-0.205813272509414	0.433244207536196	1	pseudobulk	DESeq2	LRT		0
200	KC_AB1	CG17739	0.116783351990007	1	1	pseudobulk	DESeq2	LRT		0
200	KC_AB1	CG1774	0.849606887838128	1	1	pseudobulk	DESeq2	LRT		0
200	KC_AB1	CG17746	0.53263135030874	0.489334134762071	1	pseudobulk	DESeq2	LRT		0
200	KC_AB1	CG17754	-1.45144640400911	0.166634240412333	1	pseudobulk	DESeq2	LRT		0
200	KC_AB1	CG17760	0.979458518756415	1	1	pseudobulk	DESeq2	LRT		0
200	KC_AB1	CG17765	-0.197531854534786	0.537663049172467	1	pseudobulk	DESeq2	LRT		0
200	KC_AB1	CG17776	0.0346847533780686	0.846222141298033	1	pseudobulk	DESeq2	LRT		0
201	KC_AB1	CG17777	-0.216210029276812	1	1	pseudobulk	DESeq2	LRT		0
201	KC_AB1	CG17778	-0.543654613738034	0.060012303358789	1	pseudobulk	DESeq2	LRT		0
201	KC_AB1	CG17782	0.849606887838128	1	1	pseudobulk	DESeq2	LRT		0

201	KC_AB1	CG17786	-2.65260656000192	0.103185876081134	1	pseudobulk	DESeq2	LRT	0
201	KC_AB1	CG17802	-0.330369787381405	0.532898171949087	1	pseudobulk	DESeq2	LRT	0
201	KC_AB1	CG17803	0.484142268667207	0.721118418027724	1	pseudobulk	DESeq2	LRT	0
201	KC_AB1	CG17806	-0.406768652671335	0.669630032429296	1	pseudobulk	DESeq2	LRT	0
201	KC_AB1	CG17807	0.468786380899664	0.780899399076416	1	pseudobulk	DESeq2	LRT	0
201	KC_AB1	CG17816	-0.086281007004501	0.614860451985089	1	pseudobulk	DESeq2	LRT	0
201	KC_AB1	CG17829	0.600740722208826	0.224815787902723	1	pseudobulk	DESeq2	LRT	0
202	KC_AB1	CG17834	-2.73573142459503	0.264943650326734	1	pseudobulk	DESeq2	LRT	0
202	KC_AB1	CG17839	-0.394467016562885	0.488211359560487	1	pseudobulk	DESeq2	LRT	0
202	KC_AB1	CG17841	0.161736471731706	0.381744292749926	1	pseudobulk	DESeq2	LRT	0
202	KC_AB1	CG1785	-0.683829746723457	0.723342236517982	1	pseudobulk	DESeq2	LRT	0
202	KC_AB1	CG17883	0.315064283040325	0.363555607888322	1	pseudobulk	DESeq2	LRT	0
202	KC_AB1	CG1789	-0.905214641954151	0.423773108052529	1	pseudobulk	DESeq2	LRT	0
202	KC_AB1	CG17896	-0.939948041397983	0.504540689136537	1	pseudobulk	DESeq2	LRT	0
202	KC_AB1	CG17904	0.707423988877813	0.359625900708227	1	pseudobulk	DESeq2	LRT	0
202	KC_AB1	CG17919	-2.13115735256597	0.520462507736437	1	pseudobulk	DESeq2	LRT	0
202	KC_AB1	CG1792	-0.174782377332377	0.719338528505741	1	pseudobulk	DESeq2	LRT	0
203	KC_AB1	CG17928	0.849606887838128	1	1	pseudobulk	DESeq2	LRT	0
203	KC_AB1	CG17931	0.530324326659355	0.362520145654329	1	pseudobulk	DESeq2	LRT	0
203	KC_AB1	CG17977	0.859864455363861	0.501437360704411	1	pseudobulk	DESeq2	LRT	0
203	KC_AB1	CG17982	0.444559027168278	1	1	pseudobulk	DESeq2	LRT	0
203	KC_AB1	CG17985	-0.526689717821872	0.550243021254633	1	pseudobulk	DESeq2	LRT	0
203	KC_AB1	CG17996	0.131852466720189	0.834332939777208	1	pseudobulk	DESeq2	LRT	0
203	KC_AB1	CG18004	1.2683007047092	0.231619771885196	1	pseudobulk	DESeq2	LRT	0
203	KC_AB1	CG18011	1.59863986092676	1	1	pseudobulk	DESeq2	LRT	0
203	KC_AB1	CG18048	-0.151410296776845	0.799801854692475	1	pseudobulk	DESeq2	LRT	0
203	KC_AB1	CG18081	-1.07151703731329	0.293087028352698	1	pseudobulk	DESeq2	LRT	0
204	KC_AB1	CG18094	0.849606887838128	1	1	pseudobulk	DESeq2	LRT	0
204	KC_AB1	CG1812	-0.305627774427871	0.704407144850965	1	pseudobulk	DESeq2	LRT	0
204	KC_AB1	CG18135	0.545451956753308	0.282800841098735	1	pseudobulk	DESeq2	LRT	0
204	KC_AB1	CG1814	0.154058579188567	0.482929535892512	1	pseudobulk	DESeq2	LRT	0
204	KC_AB1	CG1815	0.0339967184060647	0.895029638424304	1	pseudobulk	DESeq2	LRT	0
204	KC_AB1	CG18171	0.366247714696288	1	1	pseudobulk	DESeq2	LRT	0
204	KC_AB1	CG18178	-0.817920104878204	0.51944044367678	1	pseudobulk	DESeq2	LRT	0
204	KC_AB1	CG18179	0.849606887838128	1	1	pseudobulk	DESeq2	LRT	0
204	KC_AB1	CG1824	-0.033197385296347	0.703444954403884	1	pseudobulk	DESeq2	LRT	0
204	KC_AB1	CG18249	2.78674512494791	0.54962276837928	1	pseudobulk	DESeq2	LRT	0
205	KC_AB1	CG18259	0.265369701213173	0.791270629880576	1	pseudobulk	DESeq2	LRT	0
205	KC_AB1	CG18262	-0.060906620375367	0.563901090030539	1	pseudobulk	DESeq2	LRT	0
205	KC_AB1	CG18265	-0.046861943453129	0.578807726147684	1	pseudobulk	DESeq2	LRT	0
205	KC_AB1	CG18268	0.849606887838128	1	1	pseudobulk	DESeq2	LRT	0
205	KC_AB1	CG1827	2.06541384650887	0.768363369045814	1	pseudobulk	DESeq2	LRT	0
205	KC_AB1	CG18273	0.239252786875915	1	1	pseudobulk	DESeq2	LRT	0
205	KC_AB1	CG18281	-0.408374627804089	0.713495395395173	1	pseudobulk	DESeq2	LRT	0
205	KC_AB1	CG18324	0.0899830660338098	0.687862939876684	1	pseudobulk	DESeq2	LRT	0
205	KC_AB1	CG18327	-0.170954946521023	0.884275839978581	1	pseudobulk	DESeq2	LRT	0
205	KC_AB1	CG18335	2.31997030691033	0.813169507846918	1	pseudobulk	DESeq2	LRT	0
206	KC_AB1	CG18336	0.849606887838128	1	1	pseudobulk	DESeq2	LRT	0
206	KC_AB1	CG1840	-0.319773024027304	0.640048920905981	1	pseudobulk	DESeq2	LRT	0
206	KC_AB1	CG18428	0.0738985648921821	0.795515075239366	1	pseudobulk	DESeq2	LRT	0
206	KC_AB1	CG18467	-0.089175203410485	0.558998288252076	1	pseudobulk	DESeq2	LRT	0
206	KC_AB1	CG1847	0.37684422623215	0.563417126428414	1	pseudobulk	DESeq2	LRT	0
206	KC_AB1	CG18476	-1.07277979878073	0.378784669453718	1	pseudobulk	DESeq2	LRT	0

206	KC_AB1	CG18507	-0.830062897938551	0.717724257445671	1	pseudobulk	DESeq2	LRT		0
206	KC_AB1	CG18508	0.348874343862889	0.404005009751706	1	pseudobulk	DESeq2	LRT		0
206	KC_AB1	CG18528	-1.20041180824337	0.444839340799142	1	pseudobulk	DESeq2	LRT		0
206	KC_AB1	CG18538	0.849606887838128	1	1	pseudobulk	DESeq2	LRT		0
207	KC_AB1	CG18540	0.849606887838128	1	1	pseudobulk	DESeq2	LRT		0
207	KC_AB1	CG18549	0.0079511105023399	0.901419072800875	1	pseudobulk	DESeq2	LRT		0
207	KC_AB1	CG18596	1.15778133810046	0.447234175656778	1	pseudobulk	DESeq2	LRT		0
207	KC_AB1	CG18600	1.21044688550022	0.691466013624207	1	pseudobulk	DESeq2	LRT		0
207	KC_AB1	CG18609	2.78674512494791	0.54962276837928	1	pseudobulk	DESeq2	LRT		0
207	KC_AB1	CG18622	2.19691424616663	0.593804541109954	1	pseudobulk	DESeq2	LRT		0
207	KC_AB1	CG18628	0.249732363955365	0.396042432562645	1	pseudobulk	DESeq2	LRT		0
207	KC_AB1	CG18641	2.78674512494791	0.54962276837928	1	pseudobulk	DESeq2	LRT		0
207	KC_AB1	CG18659	-0.443357309086629	0.518976537324612	1	pseudobulk	DESeq2	LRT		0
207	KC_AB1	CG18661	0.480041951352051	0.7585269425535	1	pseudobulk	DESeq2	LRT		0
208	KC_AB1	CG18675	-0.524210161138662	0.632668851783341	1	pseudobulk	DESeq2	LRT		0
208	KC_AB1	CG18731	-0.159431117191572	0.682727894720489	1	pseudobulk	DESeq2	LRT		0
208	KC_AB1	CG18764	0.690830055946358	1	1	pseudobulk	DESeq2	LRT		0
208	KC_AB1	CG18766	-0.190910511059173	0.685096987572033	1	pseudobulk	DESeq2	LRT		0
208	KC_AB1	CG18787	-1.7880194738789	0.285474511189094	1	pseudobulk	DESeq2	LRT		0
208	KC_AB1	CG18789	0.573427873845082	1	1	pseudobulk	DESeq2	LRT		0
208	KC_AB1	CG18809	-1.07777536343512	0.274538363680658	1	pseudobulk	DESeq2	LRT		0
208	KC_AB1	CG18815	-0.0440538432672548	0.682110200910431	1	pseudobulk	DESeq2	LRT		0
208	KC_AB1	CG1882	0.84706298624354	0.155551838495134	1	pseudobulk	DESeq2	LRT		0
208	KC_AB1	CG18858	2.31997030691033	0.813169507846918	1	pseudobulk	DESeq2	LRT		0
209	KC_AB1	CG18870	-0.275822784190169	0.644477231051807	1	pseudobulk	DESeq2	LRT		0
209	KC_AB1	CG1888	-1.05142589618342	0.240012826457541	1	pseudobulk	DESeq2	LRT		0
209	KC_AB1	CG1890	-2.55698711677011	0.0739545856485268	1	pseudobulk	DESeq2	LRT		0
209	KC_AB1	CG1896	-0.178168153562706	0.59532864146999	1	pseudobulk	DESeq2	LRT		0
209	KC_AB1	CG1902	-0.206652129339166	0.729419920956916	1	pseudobulk	DESeq2	LRT		0
209	KC_AB1	CG1907	0.270914226196718	0.592063068492506	1	pseudobulk	DESeq2	LRT		0
209	KC_AB1	CG1909	-0.224910062132436	0.269329417420061	1	pseudobulk	DESeq2	LRT		0
209	KC_AB1	CG1910	-0.110700706747885	0.613822113735277	1	pseudobulk	DESeq2	LRT		0
209	KC_AB1	CG1924	0.930993226399931	1	1	pseudobulk	DESeq2	LRT		0
209	KC_AB1	CG1927	-0.062150709850367	0.722204260920369	1	pseudobulk	DESeq2	LRT		0
210	KC_AB1	CG1943	-0.0888591016108129	0.522916458284513	1	pseudobulk	DESeq2	LRT		0
210	KC_AB1	CG1951	0.201928227890788	0.757688909891064	1	pseudobulk	DESeq2	LRT		0
210	KC_AB1	CG1965	-0.0108050002347799	0.735958786416931	1	pseudobulk	DESeq2	LRT		0
210	KC_AB1	CG1983	-0.51788830828742	0.54016152956001	1	pseudobulk	DESeq2	LRT		0
210	KC_AB1	CG1998	-0.020257783783665	0.817643049219985	1	pseudobulk	DESeq2	LRT		0
210	KC_AB1	CG2003	-0.770692022699588	0.491707199605552	1	pseudobulk	DESeq2	LRT		0
210	KC_AB1	CG2004	0.374420150816607	1	1	pseudobulk	DESeq2	LRT		0
210	KC_AB1	CG2006	-0.40200137056069	0.861588439126694	1	pseudobulk	DESeq2	LRT		0
210	KC_AB1	CG2016	0.849606887838128	1	1	pseudobulk	DESeq2	LRT		0
210	KC_AB1	CG2017	0.0426280558166414	0.818121259177501	1	pseudobulk	DESeq2	LRT		0
211	KC_AB1	CG2021	-0.926956272000854	0.341049468111203	1	pseudobulk	DESeq2	LRT		0
211	KC_AB1	CG2023	0.482431622225684	0.8203640554397	1	pseudobulk	DESeq2	LRT		0
211	KC_AB1	CG2034	-0.379189065536346	0.65733024882403	1	pseudobulk	DESeq2	LRT		0
211	KC_AB1	CG2053	2.31997030691033	0.813169507846918	1	pseudobulk	DESeq2	LRT		0
211	KC_AB1	CG2059	0.637934884914969	0.290214631838338	1	pseudobulk	DESeq2	LRT		0
211	KC_AB1	CG2061	-0.513995900480139	0.484213377288034	1	pseudobulk	DESeq2	LRT		0
211	KC_AB1	CG2063	0.481780618516364	0.553747365457669	1	pseudobulk	DESeq2	LRT		0
211	KC_AB1	CG2064	-0.216210029276812	1	1	pseudobulk	DESeq2	LRT		0
211	KC_AB1	CG2076	-0.211054644557048	0.726831286504806	1	pseudobulk	DESeq2	LRT		0

211	KC_AB1	CG2082	0.43499411719518	1	1	pseudobulk	DESeq2	LRT	0
212	KC_AB1	CG2091	0.270781438271274	0.66241347987892	1	pseudobulk	DESeq2	LRT	0
212	KC_AB1	CG2100	-0.521394547461525	0.668933627786741	1	pseudobulk	DESeq2	LRT	0
212	KC_AB1	CG2104	0.0160940546605174	1	1	pseudobulk	DESeq2	LRT	0
212	KC_AB1	CG2111	1.81827378179382	0.686482515515535	1	pseudobulk	DESeq2	LRT	0
212	KC_AB1	CG2116	-1.3762776182315	0.281253239796804	1	pseudobulk	DESeq2	LRT	0
212	KC_AB1	CG2124	0.906189876260672	1	1	pseudobulk	DESeq2	LRT	0
212	KC_AB1	CG2126	0.974034884932129	0.506005588861804	1	pseudobulk	DESeq2	LRT	0
212	KC_AB1	CG2129	-0.190088533275455	0.842561350568363	1	pseudobulk	DESeq2	LRT	0
212	KC_AB1	CG2147	-0.437870571349238	0.64421662692832	1	pseudobulk	DESeq2	LRT	0
212	KC_AB1	CG2162	-0.18481592703427	0.526628254298946	1	pseudobulk	DESeq2	LRT	0
213	KC_AB1	CG2182	0.0427678106961386	0.581982782219635	1	pseudobulk	DESeq2	LRT	0
213	KC_AB1	CG2186	0.0587249969152273	0.726730462144638	1	pseudobulk	DESeq2	LRT	0
213	KC_AB1	CG2199	0.126766728931126	0.687261870792949	1	pseudobulk	DESeq2	LRT	0
213	KC_AB1	CG2200	1.39536314073085	0.274377357470197	1	pseudobulk	DESeq2	LRT	0
213	KC_AB1	CG2201	-1.12277761482222	0.288071548621753	1	pseudobulk	DESeq2	LRT	0
213	KC_AB1	CG2202	-0.458452596666515	0.66250089043062	1	pseudobulk	DESeq2	LRT	0
213	KC_AB1	CG2209	0.134879185374237	0.651720092296611	1	pseudobulk	DESeq2	LRT	0
213	KC_AB1	CG2211	-2.24099711987565	0.178616498547539	1	pseudobulk	DESeq2	LRT	0
213	KC_AB1	CG2218	-1.01529186750234	0.271316803715402	1	pseudobulk	DESeq2	LRT	0
213	KC_AB1	CG2224	0.899569305464615	0.351380420079665	1	pseudobulk	DESeq2	LRT	0
214	KC_AB1	CG2225	-0.589932792147192	0.0825703358048861	1	pseudobulk	DESeq2	LRT	0
214	KC_AB1	CG2233	0.849606887838128	1	1	pseudobulk	DESeq2	LRT	0
214	KC_AB1	CG2246	-0.48480007703484	0.316678636170526	1	pseudobulk	DESeq2	LRT	0
214	KC_AB1	CG2247	0.739349193292739	0.338950512161202	1	pseudobulk	DESeq2	LRT	0
214	KC_AB1	CG2256	2.78674512494791	0.54962276837928	1	pseudobulk	DESeq2	LRT	0
214	KC_AB1	CG2258	0.271975942371916	0.478729819248246	1	pseudobulk	DESeq2	LRT	0
214	KC_AB1	CG2260	0.801934514347863	0.658344022395336	1	pseudobulk	DESeq2	LRT	0
214	KC_AB1	CG2269	0.363992486112408	0.0232787874900503	1	pseudobulk	DESeq2	LRT	0
214	KC_AB1	CG2277	0.643869696806171	0.399505753829619	1	pseudobulk	DESeq2	LRT	0
214	KC_AB1	CG2278	0.458897816740046	0.45056679040355	1	pseudobulk	DESeq2	LRT	0
215	KC_AB1	CG2310	-0.216210029276812	1	1	pseudobulk	DESeq2	LRT	0
215	KC_AB1	CG2321	1.36676643962438	0.444370608241979	1	pseudobulk	DESeq2	LRT	0
215	KC_AB1	CG2444	0.237022465848507	1	1	pseudobulk	DESeq2	LRT	0
215	KC_AB1	CG2493	0.849606887838128	1	1	pseudobulk	DESeq2	LRT	0
215	KC_AB1	CG2519	-0.300194322415838	0.412851670509315	1	pseudobulk	DESeq2	LRT	0
215	KC_AB1	CG2528	1.15040783121559	0.87372258286756	1	pseudobulk	DESeq2	LRT	0
215	KC_AB1	CG2540	1.10476752039546	0.536341520323436	1	pseudobulk	DESeq2	LRT	0
215	KC_AB1	CG2608	-0.134347297930435	0.724364916470848	1	pseudobulk	DESeq2	LRT	0
215	KC_AB1	CG2611	-0.0725975973699454	0.752846537901386	1	pseudobulk	DESeq2	LRT	0
215	KC_AB1	CG2614	-0.579829924209202	0.64894638993951	1	pseudobulk	DESeq2	LRT	0
216	KC_AB1	CG2617	-0.328106360513151	0.707359375086824	1	pseudobulk	DESeq2	LRT	0
216	KC_AB1	CG2656	0.314363137266825	0.780420905754515	1	pseudobulk	DESeq2	LRT	0
216	KC_AB1	CG2662	-0.018316044529512	0.874991406673796	1	pseudobulk	DESeq2	LRT	0
216	KC_AB1	CG2680	-0.0920220101329262	0.729605412619713	1	pseudobulk	DESeq2	LRT	0
216	KC_AB1	CG2681	2.31997030691033	0.813169507846918	1	pseudobulk	DESeq2	LRT	0
216	KC_AB1	CG2685	1.66570670513728	0.586338927578276	1	pseudobulk	DESeq2	LRT	0
216	KC_AB1	CG2691	-1.16020557067325	0.492962443274044	1	pseudobulk	DESeq2	LRT	0
216	KC_AB1	CG2694	0.183159795455075	1	1	pseudobulk	DESeq2	LRT	0
216	KC_AB1	CG2698	-0.521869529872319	0.32539149696932	1	pseudobulk	DESeq2	LRT	0
216	KC_AB1	CG2701	-0.440823382224252	0.504914545050556	1	pseudobulk	DESeq2	LRT	0
217	KC_AB1	CG2712	0.563036248278008	0.867142305120976	1	pseudobulk	DESeq2	LRT	0
217	KC_AB1	CG2747	-0.242701105036225	0.562999204891895	1	pseudobulk	DESeq2	LRT	0

217	KC_AB1	CG2765	0.321673095524563	0.502286414215916	1	pseudobulk	DESeq2	LRT	0
217	KC_AB1	CG2767	0.0160940546605626	1	1	pseudobulk	DESeq2	LRT	0
217	KC_AB1	CG2772	-1.56610117064475	0.0321092290065757	1	pseudobulk	DESeq2	LRT	0
217	KC_AB1	CG2790	-0.915501861183412	0.381237501947327	1	pseudobulk	DESeq2	LRT	0
217	KC_AB1	CG2811	-0.920264295712031	0.296484293548126	1	pseudobulk	DESeq2	LRT	0
217	KC_AB1	CG2812	0.849606887838128	1	1	pseudobulk	DESeq2	LRT	0
217	KC_AB1	CG2818	-0.399641144022823	0.480792087458919	1	pseudobulk	DESeq2	LRT	0
217	KC_AB1	CG2841	-0.821352715712547	0.587907872721277	1	pseudobulk	DESeq2	LRT	0
218	KC_AB1	CG2846	-0.376537628760473	0.586237393821705	1	pseudobulk	DESeq2	LRT	0
218	KC_AB1	CG2852	0.189566986511408	0.556372802264987	1	pseudobulk	DESeq2	LRT	0
218	KC_AB1	CG2865	-0.180244671466985	0.393291243212867	1	pseudobulk	DESeq2	LRT	0
218	KC_AB1	CG2875	-0.216210029276812	1	1	pseudobulk	DESeq2	LRT	0
218	KC_AB1	CG2889	0.0298825582558319	0.693785026909537	1	pseudobulk	DESeq2	LRT	0
218	KC_AB1	CG2898	0.849606887838128	1	1	pseudobulk	DESeq2	LRT	0
218	KC_AB1	CG2906	-0.960022699105854	0.610500321913265	1	pseudobulk	DESeq2	LRT	0
218	KC_AB1	CG2909	-0.446181751829801	0.564539135021094	1	pseudobulk	DESeq2	LRT	0
218	KC_AB1	CG2911	0.26793883179505	0.8945526941538	1	pseudobulk	DESeq2	LRT	0
218	KC_AB1	CG2915	-1.45848502649866	0.288784711442751	1	pseudobulk	DESeq2	LRT	0
219	KC_AB1	CG2918	-0.452857664768718	0.389882033471482	1	pseudobulk	DESeq2	LRT	0
219	KC_AB1	CG2921	-0.276421910667449	0.57477621182205	1	pseudobulk	DESeq2	LRT	0
219	KC_AB1	CG2924	-0.26889082964218	0.494862617594685	1	pseudobulk	DESeq2	LRT	0
219	KC_AB1	CG2926	-0.527903600201906	0.158816509310306	1	pseudobulk	DESeq2	LRT	0
219	KC_AB1	CG2930	-0.216210029276812	1	1	pseudobulk	DESeq2	LRT	0
219	KC_AB1	CG2931	0.163946995273208	0.567145149780999	1	pseudobulk	DESeq2	LRT	0
219	KC_AB1	CG2938	-1.02296355784785	0.231000600948294	1	pseudobulk	DESeq2	LRT	0
219	KC_AB1	CG2962	0.849606887838128	1	1	pseudobulk	DESeq2	LRT	0
219	KC_AB1	CG2972	-0.475865273071454	0.626145004958177	1	pseudobulk	DESeq2	LRT	0
219	KC_AB1	CG2975	0.849606887838128	1	1	pseudobulk	DESeq2	LRT	0
220	KC_AB1	CG2991	0.0357534854463073	0.910345794003989	1	pseudobulk	DESeq2	LRT	0
220	KC_AB1	CG2993	-0.350513533575233	0.206467048176284	1	pseudobulk	DESeq2	LRT	0
220	KC_AB1	CG30001	2.31997030691033	0.813169507846918	1	pseudobulk	DESeq2	LRT	0
220	KC_AB1	CG30008	0.931109140710098	1	1	pseudobulk	DESeq2	LRT	0
220	KC_AB1	CG30010	0.956927511440727	0.765229656706532	1	pseudobulk	DESeq2	LRT	0
220	KC_AB1	CG30015	0.45055086900421	1	1	pseudobulk	DESeq2	LRT	0
220	KC_AB1	CG30020	-0.525288870199929	0.45481866695654	1	pseudobulk	DESeq2	LRT	0
220	KC_AB1	CG30022	-0.770029509264707	0.610834024620634	1	pseudobulk	DESeq2	LRT	0
220	KC_AB1	CG30054	2.42874708135467	0.60279296430569	1	pseudobulk	DESeq2	LRT	0
220	KC_AB1	CG30060	-1.54683965356528	0.221584747277808	1	pseudobulk	DESeq2	LRT	0
221	KC_AB1	CG30069	0.613608288119773	0.748307232777068	1	pseudobulk	DESeq2	LRT	0
221	KC_AB1	CG3008	0.0733061613049356	0.783091770115805	1	pseudobulk	DESeq2	LRT	0
221	KC_AB1	CG30089	-0.770308021631666	0.295277284541716	1	pseudobulk	DESeq2	LRT	0
221	KC_AB1	CG3009	0.889336357980238	0.0743022852155359	1	pseudobulk	DESeq2	LRT	0
221	KC_AB1	CG30094	-0.782340809723584	0.317164867115667	1	pseudobulk	DESeq2	LRT	0
221	KC_AB1	CG30096	-0.824577097460447	0.49283171226726	1	pseudobulk	DESeq2	LRT	0
221	KC_AB1	CG30099	0.849606887838128	1	1	pseudobulk	DESeq2	LRT	0
221	KC_AB1	CG30105	2.31997030691033	0.813169507846918	1	pseudobulk	DESeq2	LRT	0
221	KC_AB1	CG30109	-0.0178898408791787	0.615514570580584	1	pseudobulk	DESeq2	LRT	0
221	KC_AB1	CG30110	0.290331930089864	1	1	pseudobulk	DESeq2	LRT	0
222	KC_AB1	CG30116	-0.0593737750419605	0.524749129022485	1	pseudobulk	DESeq2	LRT	0
222	KC_AB1	CG30122	-1.00534509631305	0.0644730313505645	1	pseudobulk	DESeq2	LRT	0
222	KC_AB1	CG3014	0.849606887838128	1	1	pseudobulk	DESeq2	LRT	0
222	KC_AB1	CG30148	0.849606887838128	1	1	pseudobulk	DESeq2	LRT	0
222	KC_AB1	CG30156	-0.475595807395326	0.581231452581282	1	pseudobulk	DESeq2	LRT	0

222	KC_AB1	CG30158	0.0252984508113382	0.853660513783153	1	pseudobulk	DESeq2	LRT	0
222	KC_AB1	CG30172	0.169197078935509	0.393031525682544	1	pseudobulk	DESeq2	LRT	0
222	KC_AB1	CG30183	1.1568069755842	0.42136635847895	1	pseudobulk	DESeq2	LRT	0
222	KC_AB1	CG30197	-0.47224577375091	0.650943038825067	1	pseudobulk	DESeq2	LRT	0
222	KC_AB1	CG3021	0.0802720078689544	0.557535973589782	1	pseudobulk	DESeq2	LRT	0
223	KC_AB1	CG30281	1.59863986092676	1	1	pseudobulk	DESeq2	LRT	0
223	KC_AB1	CG30285	0.352768710515767	1	1	pseudobulk	DESeq2	LRT	0
223	KC_AB1	CG30289	-0.216210029276812	1	1	pseudobulk	DESeq2	LRT	0
223	KC_AB1	CG30291	0.121245841377609	0.462768013635557	1	pseudobulk	DESeq2	LRT	0
223	KC_AB1	CG3032	-0.260657448875346	0.807136242124327	1	pseudobulk	DESeq2	LRT	0
223	KC_AB1	CG30323	2.06541384650887	0.768363369045814	1	pseudobulk	DESeq2	LRT	0
223	KC_AB1	CG30325	0.849606887838128	1	1	pseudobulk	DESeq2	LRT	0
223	KC_AB1	CG30334	2.32944802598004	0.0750207817870391	1	pseudobulk	DESeq2	LRT	0
223	KC_AB1	CG30338	0.123955503162957	1	1	pseudobulk	DESeq2	LRT	0
223	KC_AB1	CG30344	-0.585825495997831	0.558479429659539	1	pseudobulk	DESeq2	LRT	0
224	KC_AB1	CG30345	-1.8476447304053	0.456955818574401	1	pseudobulk	DESeq2	LRT	0
224	KC_AB1	CG30349	0.501338251227562	0.759485072429182	1	pseudobulk	DESeq2	LRT	0
224	KC_AB1	CG30356	0.931109140710098	1	1	pseudobulk	DESeq2	LRT	0
224	KC_AB1	CG30357	1.59863986092676	1	1	pseudobulk	DESeq2	LRT	0
224	KC_AB1	CG3036	0.769522064442666	1	1	pseudobulk	DESeq2	LRT	0
224	KC_AB1	CG30373	-0.808658264600773	0.32943221931234	1	pseudobulk	DESeq2	LRT	0
224	KC_AB1	CG30377	2.31997030691033	0.813169507846918	1	pseudobulk	DESeq2	LRT	0
224	KC_AB1	CG30389	-0.23333666413831	0.284993818040149	1	pseudobulk	DESeq2	LRT	0
224	KC_AB1	CG30392	-0.595555047849252	0.375720798347786	1	pseudobulk	DESeq2	LRT	0
224	KC_AB1	CG30394	-0.116310923667134	0.783040965594552	1	pseudobulk	DESeq2	LRT	0
225	KC_AB1	CG30398	0.849606887838128	1	1	pseudobulk	DESeq2	LRT	0
225	KC_AB1	CG3040	-0.767136924667957	0.363080763817736	1	pseudobulk	DESeq2	LRT	0
225	KC_AB1	CG30403	0.0160940546605174	1	1	pseudobulk	DESeq2	LRT	0
225	KC_AB1	CG30419	-0.531612034377185	0.0492169352721044	1	pseudobulk	DESeq2	LRT	0
225	KC_AB1	CG30423	0.106067665634382	0.605589557176584	1	pseudobulk	DESeq2	LRT	0
225	KC_AB1	CG30424	0.849606887838128	1	1	pseudobulk	DESeq2	LRT	0
225	KC_AB1	CG30428	-1.17146448134731	0.00903033668472294	1	pseudobulk	DESeq2	LRT	0
225	KC_AB1	CG3045	-0.155934067093443	0.914618786789969	1	pseudobulk	DESeq2	LRT	0
225	KC_AB1	CG30456	2.06541384650887	0.904905526327343	1	pseudobulk	DESeq2	LRT	0
225	KC_AB1	CG30460	1.65792752010766	0.762646744542806	1	pseudobulk	DESeq2	LRT	0
226	KC_AB1	CG30466	0.183159795455075	1	1	pseudobulk	DESeq2	LRT	0
226	KC_AB1	CG30467	0.383795919638795	0.664709921764632	1	pseudobulk	DESeq2	LRT	0
226	KC_AB1	CG30479	0.849606887838128	1	1	pseudobulk	DESeq2	LRT	0
226	KC_AB1	CG30480	1.62694375280936	0.614508164331177	1	pseudobulk	DESeq2	LRT	0
226	KC_AB1	CG30491	0.215004295222834	0.766419535141288	1	pseudobulk	DESeq2	LRT	0
226	KC_AB1	CG30495	1.81831001177022	0.695681163491059	1	pseudobulk	DESeq2	LRT	0
226	KC_AB1	CG30496	0.849606887838128	1	1	pseudobulk	DESeq2	LRT	0
226	KC_AB1	CG3061	-0.28211474893129	0.594287969299138	1	pseudobulk	DESeq2	LRT	0
226	KC_AB1	CG3065	0.13908897473846	0.677985717750982	1	pseudobulk	DESeq2	LRT	0
226	KC_AB1	CG3071	-0.33219086652516	0.723607056447924	1	pseudobulk	DESeq2	LRT	0
227	KC_AB1	CG3078	-0.0114504067987068	0.564372156083347	1	pseudobulk	DESeq2	LRT	0
227	KC_AB1	CG3091	-0.0718207395094281	1	1	pseudobulk	DESeq2	LRT	0
227	KC_AB1	CG3097	1.90244890691395	0.564198562139563	1	pseudobulk	DESeq2	LRT	0
227	KC_AB1	CG31019	1.59863986092676	1	1	pseudobulk	DESeq2	LRT	0
227	KC_AB1	CG31030	-0.113886141445063	0.471542454739265	1	pseudobulk	DESeq2	LRT	0
227	KC_AB1	CG31038	-0.782130839262277	0.523214133380365	1	pseudobulk	DESeq2	LRT	0
227	KC_AB1	CG3104	0.23279663340399	0.42184562019658	1	pseudobulk	DESeq2	LRT	0
227	KC_AB1	CG31051	0.249385412711314	0.533277596394232	1	pseudobulk	DESeq2	LRT	0

227	KC_AB1	CG31064	-0.0883249398822673	0.667443308799003	1	pseudobulk	DESeq2	LRT		0
227	KC_AB1	CG31068	0.849606887838128	1	1	pseudobulk	DESeq2	LRT		0
228	KC_AB1	CG3107	-0.0213481099460939	0.704262820541218	1	pseudobulk	DESeq2	LRT		0
228	KC_AB1	CG31097	2.78674512494791	0.54962276837928	1	pseudobulk	DESeq2	LRT		0
228	KC_AB1	CG31098	-0.308753478281285	0.412028668314231	1	pseudobulk	DESeq2	LRT		0
228	KC_AB1	CG31104	0.849606887838128	1	1	pseudobulk	DESeq2	LRT		0
228	KC_AB1	CG31109	0.0911964680577632	0.540661850988837	1	pseudobulk	DESeq2	LRT		0
228	KC_AB1	CG31111	1.59863986092676	1	1	pseudobulk	DESeq2	LRT		0
228	KC_AB1	CG31115	2.85142250261825	0.484249113053674	1	pseudobulk	DESeq2	LRT		0
228	KC_AB1	CG31121	-0.894314452976069	0.643015595841411	1	pseudobulk	DESeq2	LRT		0
228	KC_AB1	CG31122	-0.0170492884315879	0.592303878520453	1	pseudobulk	DESeq2	LRT		0
228	KC_AB1	CG31125	-1.34029795272554	0.333855464202924	1	pseudobulk	DESeq2	LRT		0
229	KC_AB1	CG31126	0.274534479721005	0.672882883273201	1	pseudobulk	DESeq2	LRT		0
229	KC_AB1	CG31140	-0.356553058494855	0.0648492265099293	1	pseudobulk	DESeq2	LRT		0
229	KC_AB1	CG31142	0.7909921588382	0.783492222796219	1	pseudobulk	DESeq2	LRT		0
229	KC_AB1	CG31145	-0.514145979421497	0.738501280881092	1	pseudobulk	DESeq2	LRT		0
229	KC_AB1	CG31156	-0.146802630607922	0.811468174402697	1	pseudobulk	DESeq2	LRT		0
229	KC_AB1	CG31157	2.31997030691033	0.813169507846918	1	pseudobulk	DESeq2	LRT		0
229	KC_AB1	CG31174	1.12044150062859	0.614020105094219	1	pseudobulk	DESeq2	LRT		0
229	KC_AB1	CG31176	2.78674512494791	0.54962276837928	1	pseudobulk	DESeq2	LRT		0
229	KC_AB1	CG31183	2.78674512494791	0.54962276837928	1	pseudobulk	DESeq2	LRT		0
229	KC_AB1	CG31191	-0.209674989657228	0.383324667419456	1	pseudobulk	DESeq2	LRT		0
230	KC_AB1	CG31195	-1.58973249459572	0.26097910861178	1	pseudobulk	DESeq2	LRT		0
230	KC_AB1	CG31199	0.849606887838128	1	1	pseudobulk	DESeq2	LRT		0
230	KC_AB1	CG31200	0.849606887838128	1	1	pseudobulk	DESeq2	LRT		0
230	KC_AB1	CG31205	0.849606887838128	1	1	pseudobulk	DESeq2	LRT		0
230	KC_AB1	CG31211	0.0947940860618506	0.569867618478348	1	pseudobulk	DESeq2	LRT		0
230	KC_AB1	CG31213	2.13009086641515	0.687046658320206	1	pseudobulk	DESeq2	LRT		0
230	KC_AB1	CG31221	0.138647381371404	0.485888460642705	1	pseudobulk	DESeq2	LRT		0
230	KC_AB1	CG31223	1.9163124448524	0.436714599119659	1	pseudobulk	DESeq2	LRT		0
230	KC_AB1	CG31224	0.589691133108937	0.112337201152899	1	pseudobulk	DESeq2	LRT		0
230	KC_AB1	CG31229	0.119943782553177	0.659160107923962	1	pseudobulk	DESeq2	LRT		0
231	KC_AB1	CG31248	-1.68376590330768	0.235327654320754	1	pseudobulk	DESeq2	LRT		0
231	KC_AB1	CG31249	-0.265030861118907	0.666839560567901	1	pseudobulk	DESeq2	LRT		0
231	KC_AB1	CG31251	-1.13584521858219	0.54202769046723	1	pseudobulk	DESeq2	LRT		0
231	KC_AB1	CG31278	0.621375926231566	1	1	pseudobulk	DESeq2	LRT		0
231	KC_AB1	CG31300	-0.267798911929862	1	1	pseudobulk	DESeq2	LRT		0
231	KC_AB1	CG31301	0.315309585134816	0.688082473694197	1	pseudobulk	DESeq2	LRT		0
231	KC_AB1	CG31313	1.59863986092676	1	1	pseudobulk	DESeq2	LRT		0
231	KC_AB1	CG31324	0.652666925641943	0.260043271188088	1	pseudobulk	DESeq2	LRT		0
231	KC_AB1	CG31337	0.849606887838128	1	1	pseudobulk	DESeq2	LRT		0
231	KC_AB1	CG31342	-0.0023277506429854	0.602496560709909	1	pseudobulk	DESeq2	LRT		0
232	KC_AB1	CG31344	0.653074672193802	0.195350546477606	1	pseudobulk	DESeq2	LRT		0
232	KC_AB1	CG31345	-0.202310336013133	0.774757478881628	1	pseudobulk	DESeq2	LRT		0
232	KC_AB1	CG31357	0.191888202973328	0.638427260939057	1	pseudobulk	DESeq2	LRT		0
232	KC_AB1	CG31360	0.228538105011024	0.681884148475947	1	pseudobulk	DESeq2	LRT		0
232	KC_AB1	CG31365	0.373737903362398	0.716681430830604	1	pseudobulk	DESeq2	LRT		0
232	KC_AB1	CG31368	1.11356971457167	0.360590716435862	1	pseudobulk	DESeq2	LRT		0
232	KC_AB1	CG31370	0.849606887838128	1	1	pseudobulk	DESeq2	LRT		0
232	KC_AB1	CG31381	-0.267798911929862	1	1	pseudobulk	DESeq2	LRT		0
232	KC_AB1	CG31388	-0.285012828320208	0.654539967474299	1	pseudobulk	DESeq2	LRT		0
232	KC_AB1	CG31415	1.43060365239813	1	1	pseudobulk	DESeq2	LRT		0
233	KC_AB1	CG31431	2.78674512494791	0.54962276837928	1	pseudobulk	DESeq2	LRT		0

233	KC_AB1	CG31441	-0.370862531082229	0.881125640939123	1	pseudobulk	DESeq2	LRT	0
233	KC_AB1	CG31457	-0.61108112023135	0.620049237751923	1	pseudobulk	DESeq2	LRT	0
233	KC_AB1	CG31460	1.03522715385609	0.204323807838705	1	pseudobulk	DESeq2	LRT	0
233	KC_AB1	CG31467	-1.20054795286106	0.522310314157169	1	pseudobulk	DESeq2	LRT	0
233	KC_AB1	CG31473	0.849606887838128	1	1	pseudobulk	DESeq2	LRT	0
233	KC_AB1	CG31475	-0.177605076076936	0.566956192483759	1	pseudobulk	DESeq2	LRT	0
233	KC_AB1	CG3149	0.920377725503859	0.616569726877031	1	pseudobulk	DESeq2	LRT	0
233	KC_AB1	CG31510	0.25665505982643	0.615536233874617	1	pseudobulk	DESeq2	LRT	0
233	KC_AB1	CG31522	0.005999291896892	0.963798392569869	1	pseudobulk	DESeq2	LRT	0
234	KC_AB1	CG31523	-0.921894405913602	0.0316866675994105	1	pseudobulk	DESeq2	LRT	0
234	KC_AB1	CG31533	0.849606887838128	1	1	pseudobulk	DESeq2	LRT	0
234	KC_AB1	CG31538	-0.0065790798335215	1	1	pseudobulk	DESeq2	LRT	0
234	KC_AB1	CG31550	0.285944256331902	0.595441809982576	1	pseudobulk	DESeq2	LRT	0
234	KC_AB1	CG3156	-2.50181846928574	0.139419462802994	1	pseudobulk	DESeq2	LRT	0
234	KC_AB1	CG31612	0.183159795455075	1	1	pseudobulk	DESeq2	LRT	0
234	KC_AB1	CG31626	0.0160940546605174	1	1	pseudobulk	DESeq2	LRT	0
234	KC_AB1	CG31627	2.13009086641515	0.718583316467961	1	pseudobulk	DESeq2	LRT	0
234	KC_AB1	CG3163	-0.0824496038089324	0.712345867498531	1	pseudobulk	DESeq2	LRT	0
234	KC_AB1	CG31633	0.763870745020894	0.528183366501534	1	pseudobulk	DESeq2	LRT	0
235	KC_AB1	CG31635	-0.225705270830261	0.394804036770735	1	pseudobulk	DESeq2	LRT	0
235	KC_AB1	CG31637	-0.110007325397478	0.662344004150719	1	pseudobulk	DESeq2	LRT	0
235	KC_AB1	CG31638	0.173491260940963	0.674705092971774	1	pseudobulk	DESeq2	LRT	0
235	KC_AB1	CG3164	-0.699198163334299	0.41291964176278	1	pseudobulk	DESeq2	LRT	0
235	KC_AB1	CG31642	-0.216210032712384	1	1	pseudobulk	DESeq2	LRT	0
235	KC_AB1	CG31643	0.781801500057156	1	1	pseudobulk	DESeq2	LRT	0
235	KC_AB1	CG31648	-0.489831805407219	0.571999643507969	1	pseudobulk	DESeq2	LRT	0
235	KC_AB1	CG3165	0.344555108089194	0.706310670844997	1	pseudobulk	DESeq2	LRT	0
235	KC_AB1	CG31650	-0.371803745612745	0.299299635308455	1	pseudobulk	DESeq2	LRT	0
235	KC_AB1	CG31663	0.849606887838128	1	1	pseudobulk	DESeq2	LRT	0
236	KC_AB1	CG31664	2.31997030691033	0.813169507846918	1	pseudobulk	DESeq2	LRT	0
236	KC_AB1	CG31673	0.849606887838128	1	1	pseudobulk	DESeq2	LRT	0
236	KC_AB1	CG31674	2.78674512494791	0.54962276837928	1	pseudobulk	DESeq2	LRT	0
236	KC_AB1	CG31676	0.741060761967125	0.377190811597492	1	pseudobulk	DESeq2	LRT	0
236	KC_AB1	CG31678	-0.2446447600804	0.483729621754195	1	pseudobulk	DESeq2	LRT	0
236	KC_AB1	CG3168	0.713780324542466	1	1	pseudobulk	DESeq2	LRT	0
236	KC_AB1	CG31683	2.13009086641515	0.687046658320206	1	pseudobulk	DESeq2	LRT	0
236	KC_AB1	CG31687	-0.268866293892939	0.566990266362536	1	pseudobulk	DESeq2	LRT	0
236	KC_AB1	CG31688	-0.0008116659601887	0.875850148861861	1	pseudobulk	DESeq2	LRT	0
236	KC_AB1	CG31689	0.849606887838128	1	1	pseudobulk	DESeq2	LRT	0
237	KC_AB1	CG31694	0.166073293505334	0.519712501814968	1	pseudobulk	DESeq2	LRT	0
237	KC_AB1	CG31697	2.78674512494791	0.54962276837928	1	pseudobulk	DESeq2	LRT	0
237	KC_AB1	CG31704	0.849606887838128	1	1	pseudobulk	DESeq2	LRT	0
237	KC_AB1	CG31705	0.740912000628272	0.531862821112736	1	pseudobulk	DESeq2	LRT	0
237	KC_AB1	CG31706	-0.859913115111515	0.403946753586125	1	pseudobulk	DESeq2	LRT	0
237	KC_AB1	CG31710	2.78674512494791	0.54962276837928	1	pseudobulk	DESeq2	LRT	0
237	KC_AB1	CG31712	0.157233133282632	0.573967530105854	1	pseudobulk	DESeq2	LRT	0
237	KC_AB1	CG31715	-0.0697503193753636	0.795526309643729	1	pseudobulk	DESeq2	LRT	0
237	KC_AB1	CG31717	-0.13101542530028	0.669911770382857	1	pseudobulk	DESeq2	LRT	0
237	KC_AB1	CG31729	0.338097635255482	0.415721236822323	1	pseudobulk	DESeq2	LRT	0
238	KC_AB1	CG31751	-0.872613805992935	0.521783290210312	1	pseudobulk	DESeq2	LRT	0
238	KC_AB1	CG31752	2.31997030691033	0.813169507846918	1	pseudobulk	DESeq2	LRT	0
238	KC_AB1	CG3176	1.02362625135634	0.890529674363423	1	pseudobulk	DESeq2	LRT	0
238	KC_AB1	CG31760	0.401668452451304	0.148465816289131	1	pseudobulk	DESeq2	LRT	0

238	KC_AB1	CG31776	0.849606887838128	1	1	pseudobulk	DESeq2	LRT	0
238	KC_AB1	CG31788	0.183159795455075	1	1	pseudobulk	DESeq2	LRT	0
238	KC_AB1	CG31793	0.592664483892331	0.879657374752307	1	pseudobulk	DESeq2	LRT	0
238	KC_AB1	CG31800	0.425567403208401	0.58681917082039	1	pseudobulk	DESeq2	LRT	0
238	KC_AB1	CG31806	2.06541384650887	0.768363369045814	1	pseudobulk	DESeq2	LRT	0
238	KC_AB1	CG31808	0.248735003173789	0.231401923265075	1	pseudobulk	DESeq2	LRT	0
239	KC_AB1	CG31815	0.191567758394413	0.927292788092387	1	pseudobulk	DESeq2	LRT	0
239	KC_AB1	CG31817	-0.556204207671607	0.658836255417628	1	pseudobulk	DESeq2	LRT	0
239	KC_AB1	CG31832	0.849606887838128	1	1	pseudobulk	DESeq2	LRT	0
239	KC_AB1	CG3184	-0.241259245531147	0.737496581844562	1	pseudobulk	DESeq2	LRT	0
239	KC_AB1	CG31849	1.143100321871	0.74789604418055	1	pseudobulk	DESeq2	LRT	0
239	KC_AB1	CG31855	0.68441455519856	0.222666875296257	1	pseudobulk	DESeq2	LRT	0
239	KC_AB1	CG31861	1.59863986092676	1	1	pseudobulk	DESeq2	LRT	0
239	KC_AB1	CG31869	2.31997030691033	0.813169507846918	1	pseudobulk	DESeq2	LRT	0
239	KC_AB1	CG31886	-1.74181238087108	0.368878660842145	1	pseudobulk	DESeq2	LRT	0
239	KC_AB1	CG31897	0.849606887838128	1	1	pseudobulk	DESeq2	LRT	0
240	KC_AB1	CG31898	2.32443062293958	0.126089769327006	1	pseudobulk	DESeq2	LRT	0
240	KC_AB1	CG31908	0.187812793647189	0.598047933113058	1	pseudobulk	DESeq2	LRT	0
240	KC_AB1	CG3191	1.95817321197357	0.13979477301244	1	pseudobulk	DESeq2	LRT	0
240	KC_AB1	CG31915	-0.419706562660969	0.714763166690707	1	pseudobulk	DESeq2	LRT	0
240	KC_AB1	CG31921	0.411645734263316	0.727803978060135	1	pseudobulk	DESeq2	LRT	0
240	KC_AB1	CG31922	1.34593154681262	0.375219272580539	1	pseudobulk	DESeq2	LRT	0
240	KC_AB1	CG31935	-1.38828522205426	0.293764956389612	1	pseudobulk	DESeq2	LRT	0
240	KC_AB1	CG31937	0.536600542756646	1	1	pseudobulk	DESeq2	LRT	0
240	KC_AB1	CG31955	-1.0868008871937	0.596161533311927	1	pseudobulk	DESeq2	LRT	0
240	KC_AB1	CG31957	-0.836856735258715	0.429752135803664	1	pseudobulk	DESeq2	LRT	0
241	KC_AB1	CG31974	2.78674512494791	0.54962276837928	1	pseudobulk	DESeq2	LRT	0
241	KC_AB1	CG3198	-0.223928169133536	0.478869738962156	1	pseudobulk	DESeq2	LRT	0
241	KC_AB1	CG31997	0.0274232439411308	0.750152055146715	1	pseudobulk	DESeq2	LRT	0
241	KC_AB1	CG31998	-0.381554758002279	0.22087025212194	1	pseudobulk	DESeq2	LRT	0
241	KC_AB1	CG31999	-0.310075340690795	0.636952348744021	1	pseudobulk	DESeq2	LRT	0
241	KC_AB1	CG32017	-0.303510202413058	0.265027338944536	1	pseudobulk	DESeq2	LRT	0
241	KC_AB1	CG32022	1.41697436193229	0.164142553727322	1	pseudobulk	DESeq2	LRT	0
241	KC_AB1	CG32032	1.2099849464845	0.215158219209193	1	pseudobulk	DESeq2	LRT	0
241	KC_AB1	CG32036	2.06541384650887	0.795866856626082	1	pseudobulk	DESeq2	LRT	0
241	KC_AB1	CG32039	-0.650652946689664	0.362244478744	1	pseudobulk	DESeq2	LRT	0
242	KC_AB1	CG32052	-1.80500692422852	0.210817593253523	1	pseudobulk	DESeq2	LRT	0
242	KC_AB1	CG32055	-0.0182703573405389	1	1	pseudobulk	DESeq2	LRT	0
242	KC_AB1	CG32066	-0.408512380973732	0.0758142220671508	1	pseudobulk	DESeq2	LRT	0
242	KC_AB1	CG32069	-0.282726514695765	0.639179047047182	1	pseudobulk	DESeq2	LRT	0
242	KC_AB1	CG32075	2.7141870817367	0.45608503377963	1	pseudobulk	DESeq2	LRT	0
242	KC_AB1	CG32085	-0.671074251716728	0.117752971639662	1	pseudobulk	DESeq2	LRT	0
242	KC_AB1	CG32091	2.91158659104676	0.319439684358899	1	pseudobulk	DESeq2	LRT	0
242	KC_AB1	CG32095	0.0706929954712526	1	1	pseudobulk	DESeq2	LRT	0
242	KC_AB1	CG32099	-0.814305327028655	0.57893569598182	1	pseudobulk	DESeq2	LRT	0
242	KC_AB1	CG32100	0.434920700511285	0.568596362907553	1	pseudobulk	DESeq2	LRT	0
243	KC_AB1	CG32104	2.40453966086703	0.330523371073339	1	pseudobulk	DESeq2	LRT	0
243	KC_AB1	CG32109	-0.640320995685178	0.45545837413238	1	pseudobulk	DESeq2	LRT	0
243	KC_AB1	CG32110	0.781801500057156	1	1	pseudobulk	DESeq2	LRT	0
243	KC_AB1	CG32112	-0.722486236519038	0.471468987415734	1	pseudobulk	DESeq2	LRT	0
243	KC_AB1	CG32115	0.849606887838128	1	1	pseudobulk	DESeq2	LRT	0
243	KC_AB1	CG32119	2.31997030691033	0.813169507846918	1	pseudobulk	DESeq2	LRT	0
243	KC_AB1	CG32137	-0.44860183736145	0.766877376616102	1	pseudobulk	DESeq2	LRT	0

243	KC_AB1	CG32147	0.419531704146103	0.687627775275577	1	pseudobulk	DESeq2	LRT		0
243	KC_AB1	CG32163	-1.1695169228714	0.387011779202057	1	pseudobulk	DESeq2	LRT		0
243	KC_AB1	CG32176	-0.733837944794847	0.523408523344641	1	pseudobulk	DESeq2	LRT		0
244	KC_AB1	CG32187	1.48698305419881	0.613405091313477	1	pseudobulk	DESeq2	LRT		0
244	KC_AB1	CG32192	-0.40200137056069	0.861588439126694	1	pseudobulk	DESeq2	LRT		0
244	KC_AB1	CG32195	0.849606887838128	1	1	pseudobulk	DESeq2	LRT		0
244	KC_AB1	CG32196	-1.48424094283886	0.434809854972627	1	pseudobulk	DESeq2	LRT		0
244	KC_AB1	CG32202	-0.272615277594458	0.575798580310867	1	pseudobulk	DESeq2	LRT		0
244	KC_AB1	CG32204	0.0347667801101942	0.787403788029247	1	pseudobulk	DESeq2	LRT		0
244	KC_AB1	CG32206	-0.230120397843623	0.397982829575616	1	pseudobulk	DESeq2	LRT		0
244	KC_AB1	CG3222	0.93038672702804	1	1	pseudobulk	DESeq2	LRT		0
244	KC_AB1	CG32221	-1.50973222868949	0.341588934562225	1	pseudobulk	DESeq2	LRT		0
244	KC_AB1	CG32225	0.655543955405497	0.0744159780206359	1	pseudobulk	DESeq2	LRT		0
245	KC_AB1	CG3223	-0.184215727601096	0.69728206745768	1	pseudobulk	DESeq2	LRT		0
245	KC_AB1	CG32232	2.78674512494791	0.54962276837928	1	pseudobulk	DESeq2	LRT		0
245	KC_AB1	CG3224	-0.421505227567335	0.534059712413294	1	pseudobulk	DESeq2	LRT		0
245	KC_AB1	CG32240	0.849606887838128	1	1	pseudobulk	DESeq2	LRT		0
245	KC_AB1	CG32243	0.10809474205499	0.555010784340095	1	pseudobulk	DESeq2	LRT		0
245	KC_AB1	CG3225	0.305556281478426	1	1	pseudobulk	DESeq2	LRT		0
245	KC_AB1	CG3226	-1.42054153731149	0.104623115773564	1	pseudobulk	DESeq2	LRT		0
245	KC_AB1	CG32262	0.726812929807508	0.321505398206852	1	pseudobulk	DESeq2	LRT		0
245	KC_AB1	CG32264	-0.503374709588429	0.0106108212277604	1	pseudobulk	DESeq2	LRT		0
245	KC_AB1	CG32266	2.31997030691033	0.813169507846918	1	pseudobulk	DESeq2	LRT		0
246	KC_AB1	CG32267	-0.161161079515547	0.477927911850927	1	pseudobulk	DESeq2	LRT		0
246	KC_AB1	CG32276	0.252275964001521	0.467243494376447	1	pseudobulk	DESeq2	LRT		0
246	KC_AB1	CG32278	-0.268500844930658	0.559832811285656	1	pseudobulk	DESeq2	LRT		0
246	KC_AB1	CG32280	6.7313433563258E-0	0.707959877263468	1	pseudobulk	DESeq2	LRT		0
246	KC_AB1	CG32281	0.402593091507332	0.918875596748027	1	pseudobulk	DESeq2	LRT		0
246	KC_AB1	CG32284	1.59863986092676	1	1	pseudobulk	DESeq2	LRT		0
246	KC_AB1	CG32333	1.01497337145931	0.485631284630197	1	pseudobulk	DESeq2	LRT		0
246	KC_AB1	CG32344	-0.190088533275455	0.842561350568363	1	pseudobulk	DESeq2	LRT		0
246	KC_AB1	CG32354	0.849606887838128	1	1	pseudobulk	DESeq2	LRT		0
246	KC_AB1	CG32365	0.82825245407851	0.309212248215568	1	pseudobulk	DESeq2	LRT		0
247	KC_AB1	CG32368	-0.679381631425979	0.707507904409577	1	pseudobulk	DESeq2	LRT		0
247	KC_AB1	CG32369	-0.732023849616103	0.0470927632964986	1	pseudobulk	DESeq2	LRT		0
247	KC_AB1	CG32373	-0.331062249151192	0.536602834363546	1	pseudobulk	DESeq2	LRT		0
247	KC_AB1	CG32392	-0.738374706094816	0.470804177611922	1	pseudobulk	DESeq2	LRT		0
247	KC_AB1	CG32396	2.06541384650887	0.768363369045814	1	pseudobulk	DESeq2	LRT		0
247	KC_AB1	CG32409	0.120336175449406	1	1	pseudobulk	DESeq2	LRT		0
247	KC_AB1	CG32425	0.347638054468355	0.364514540911657	1	pseudobulk	DESeq2	LRT		0
247	KC_AB1	CG32428	-0.0361402749403168	0.748287990133576	1	pseudobulk	DESeq2	LRT		0
247	KC_AB1	CG32432	-0.094458618336614	0.663169495372451	1	pseudobulk	DESeq2	LRT		0
247	KC_AB1	CG32436	2.31997030691033	0.813169507846918	1	pseudobulk	DESeq2	LRT		0
248	KC_AB1	CG32441	0.243952465065426	0.45210445546295	1	pseudobulk	DESeq2	LRT		0
248	KC_AB1	CG32444	2.78674512494791	0.54962276837928	1	pseudobulk	DESeq2	LRT		0
248	KC_AB1	CG32448	-0.429621270773782	0.58976918209505	1	pseudobulk	DESeq2	LRT		0
248	KC_AB1	CG32452	-1.13584521858219	0.54202769046723	1	pseudobulk	DESeq2	LRT		0
248	KC_AB1	CG32457	0.0017198393240381	1	1	pseudobulk	DESeq2	LRT		0
248	KC_AB1	CG32459	1.36004237795471	0.790165338156147	1	pseudobulk	DESeq2	LRT		0
248	KC_AB1	CG32473	-0.316012408801007	0.732692647387188	1	pseudobulk	DESeq2	LRT		0
248	KC_AB1	CG32485	-0.06526376192745	0.742887467937539	1	pseudobulk	DESeq2	LRT		0
248	KC_AB1	CG32486	-0.496221446652533	0.0289282157393965	1	pseudobulk	DESeq2	LRT		0
248	KC_AB1	CG32506	-1.44606698569928	0.0917233148947631	1	pseudobulk	DESeq2	LRT		0

249	KC_AB1	CG32512	0.0160940546605174	1	1	pseudobulk	DESeq2	LRT		0
249	KC_AB1	CG32521	0.20171308630228	0.705844766541328	1	pseudobulk	DESeq2	LRT		0
249	KC_AB1	CG3253	0.801043145233386	0.3494285792198	1	pseudobulk	DESeq2	LRT		0
249	KC_AB1	CG32533	-1.13646098538957	0.387785199519501	1	pseudobulk	DESeq2	LRT		0
249	KC_AB1	CG32537	-0.767122540393086	0.488244224615378	1	pseudobulk	DESeq2	LRT		0
249	KC_AB1	CG32544	-0.434911706669705	0.320738889712567	1	pseudobulk	DESeq2	LRT		0
249	KC_AB1	CG32547	-0.0650637562681289	0.652157110396802	1	pseudobulk	DESeq2	LRT		0
249	KC_AB1	CG32548	0.849606887838128	1	1	pseudobulk	DESeq2	LRT		0
249	KC_AB1	CG32549	-0.034686375005048	0.81590106923322	1	pseudobulk	DESeq2	LRT		0
249	KC_AB1	CG32554	2.48413880439228	0.570388686515026	1	pseudobulk	DESeq2	LRT		0
250	KC_AB1	CG3257	0.173044071856041	0.421874595092668	1	pseudobulk	DESeq2	LRT		0
250	KC_AB1	CG32576	-0.740243848775682	0.462921938019886	1	pseudobulk	DESeq2	LRT		0
250	KC_AB1	CG32581	6.05671531228365	0.106335868798984	1	pseudobulk	DESeq2	LRT		0
250	KC_AB1	CG32590	-0.53841176301274	0.589114959800547	1	pseudobulk	DESeq2	LRT		0
250	KC_AB1	CG3262	0.0624517173350178	0.814744559899763	1	pseudobulk	DESeq2	LRT		0
250	KC_AB1	CG32638	-0.321136280772472	0.713417631699094	1	pseudobulk	DESeq2	LRT		0
250	KC_AB1	CG32647	-0.109106036284153	0.582601303420867	1	pseudobulk	DESeq2	LRT		0
250	KC_AB1	CG32655	-0.589969456535054	0.659107663519984	1	pseudobulk	DESeq2	LRT		0
250	KC_AB1	CG32668	2.78674512494791	0.54962276837928	1	pseudobulk	DESeq2	LRT		0
250	KC_AB1	CG32669	0.849606887838128	1	1	pseudobulk	DESeq2	LRT		0
251	KC_AB1	CG32681	0.849606887838128	1	1	pseudobulk	DESeq2	LRT		0
251	KC_AB1	CG32683	0.426871753481764	0.124686276940517	1	pseudobulk	DESeq2	LRT		0
251	KC_AB1	CG32686	0.116783351990007	1	1	pseudobulk	DESeq2	LRT		0
251	KC_AB1	CG32694	0.849606887838128	1	1	pseudobulk	DESeq2	LRT		0
251	KC_AB1	CG3270	0.687527733529118	0.355253084809673	1	pseudobulk	DESeq2	LRT		0
251	KC_AB1	CG32700	-0.186640701086715	0.221579156468407	1	pseudobulk	DESeq2	LRT		0
251	KC_AB1	CG32708	0.291249510304839	1	1	pseudobulk	DESeq2	LRT		0
251	KC_AB1	CG32755	-0.564356698759404	0.803702590456371	1	pseudobulk	DESeq2	LRT		0
251	KC_AB1	CG32756	0.752039686686063	0.405309033552583	1	pseudobulk	DESeq2	LRT		0
251	KC_AB1	CG32762	0.849606887838128	1	1	pseudobulk	DESeq2	LRT		0
252	KC_AB1	CG32767	-0.408124327016327	0.0267672537898766	1	pseudobulk	DESeq2	LRT		0
252	KC_AB1	CG32772	-0.438962744413243	0.321375664938699	1	pseudobulk	DESeq2	LRT		0
252	KC_AB1	CG32795	0.182852045666246	0.407377242564494	1	pseudobulk	DESeq2	LRT		0
252	KC_AB1	CG32803	0.361104422935429	0.523278227331699	1	pseudobulk	DESeq2	LRT		0
252	KC_AB1	CG32808	0.863188969783109	0.736717989269941	1	pseudobulk	DESeq2	LRT		0
252	KC_AB1	CG32809	-0.218466838171053	0.442405403470532	1	pseudobulk	DESeq2	LRT		0
252	KC_AB1	CG3281	-0.80258314479175	0.499954783088498	1	pseudobulk	DESeq2	LRT		0
252	KC_AB1	CG32813	-0.848043123724095	0.244864681948859	1	pseudobulk	DESeq2	LRT		0
252	KC_AB1	CG32815	-0.235473957928933	0.411974489772232	1	pseudobulk	DESeq2	LRT		0
252	KC_AB1	CG32816	1.35182037181477	0.240592817261217	1	pseudobulk	DESeq2	LRT		0
253	KC_AB1	CG32829	1.64673267098683	0.852532516449	1	pseudobulk	DESeq2	LRT		0
253	KC_AB1	CG32850	-0.159587236186295	0.428078144317241	1	pseudobulk	DESeq2	LRT		0
253	KC_AB1	CG32856	-1.11520601615477	0.362522096996359	1	pseudobulk	DESeq2	LRT		0
253	KC_AB1	CG3294	0.256760650594231	0.986608338301519	1	pseudobulk	DESeq2	LRT		0
253	KC_AB1	CG32944	0.973081458516875	0.252978523498521	1	pseudobulk	DESeq2	LRT		0
253	KC_AB1	CG32945	0.849606887838128	1	1	pseudobulk	DESeq2	LRT		0
253	KC_AB1	CG3295	-0.504327076337349	0.603661705232301	1	pseudobulk	DESeq2	LRT		0
253	KC_AB1	CG32971	2.13009086641515	0.687046658320206	1	pseudobulk	DESeq2	LRT		0
253	KC_AB1	CG32982	-0.0213842108439974	0.901481853811563	1	pseudobulk	DESeq2	LRT		0
253	KC_AB1	CG33051	0.635848183543954	1	1	pseudobulk	DESeq2	LRT		0
254	KC_AB1	CG33054	1.15040783121559	0.87372258286756	1	pseudobulk	DESeq2	LRT		0
254	KC_AB1	CG33056	-0.475777139510637	0.718410711256783	1	pseudobulk	DESeq2	LRT		0
254	KC_AB1	CG3308	0.952142190586462	0.595240358301534	1	pseudobulk	DESeq2	LRT		0

254	KC_AB1	CG33080	2.13009086641515	0.718583316467961	1	pseudobulk	DESeq2	LRT		0
254	KC_AB1	CG33082	0.849606887838128	1	1	pseudobulk	DESeq2	LRT		0
254	KC_AB1	CG3309	0.137651546404081	0.832796521896123	1	pseudobulk	DESeq2	LRT		0
254	KC_AB1	CG33090	0.020046307951636	0.655091001400152	1	pseudobulk	DESeq2	LRT		0
254	KC_AB1	CG33096	0.580097764538252	0.817207589502997	1	pseudobulk	DESeq2	LRT		0
254	KC_AB1	CG33098	1.42209826321324	0.334078187976002	1	pseudobulk	DESeq2	LRT		0
254	KC_AB1	CG33107	0.0002812445978657	0.772509434570885	1	pseudobulk	DESeq2	LRT		0
255	KC_AB1	CG33108	-2.30114390700363	0.230265648210238	1	pseudobulk	DESeq2	LRT		0
255	KC_AB1	CG33111	-1.38482772358242	0.229520141901161	1	pseudobulk	DESeq2	LRT		0
255	KC_AB1	CG33116	0.381447214583476	0.184494949054924	1	pseudobulk	DESeq2	LRT		0
255	KC_AB1	CG33127	-0.216210029276812	1	1	pseudobulk	DESeq2	LRT		0
255	KC_AB1	CG33129	0.326110274836847	0.340441317592353	1	pseudobulk	DESeq2	LRT		0
255	KC_AB1	CG33144	-1.11136298548854	0.0336810982454197	1	pseudobulk	DESeq2	LRT		0
255	KC_AB1	CG33155	0.12021435320541	0.931836737089629	1	pseudobulk	DESeq2	LRT		0
255	KC_AB1	CG33156	0.446654403602798	0.522063612446094	1	pseudobulk	DESeq2	LRT		0
255	KC_AB1	CG33158	0.577367997777857	0.679982912112294	1	pseudobulk	DESeq2	LRT		0
255	KC_AB1	CG33169	-0.449503931091095	0.564111470152702	1	pseudobulk	DESeq2	LRT		0
256	KC_AB1	CG33170	0.0843959480232315	0.838288778509946	1	pseudobulk	DESeq2	LRT		0
256	KC_AB1	CG33178	2.78674512494791	0.54962276837928	1	pseudobulk	DESeq2	LRT		0
256	KC_AB1	CG33181	-0.190487974046654	0.304362616447267	1	pseudobulk	DESeq2	LRT		0
256	KC_AB1	CG33199	-0.479514020830351	0.255223789573154	1	pseudobulk	DESeq2	LRT		0
256	KC_AB1	CG33203	-0.429674884163547	0.411621417714455	1	pseudobulk	DESeq2	LRT		0
256	KC_AB1	CG33213	2.91158659104676	0.319439684358899	1	pseudobulk	DESeq2	LRT		0
256	KC_AB1	CG33217	0.537345686230171	0.567674093907038	1	pseudobulk	DESeq2	LRT		0
256	KC_AB1	CG33223	0.849606887838128	1	1	pseudobulk	DESeq2	LRT		0
256	KC_AB1	CG33228	2.34429204191651	0.107332373622806	1	pseudobulk	DESeq2	LRT		0
256	KC_AB1	CG33229	0.0340565153582787	0.528539831150925	1	pseudobulk	DESeq2	LRT		0
257	KC_AB1	CG33230	0.235883549019826	1	1	pseudobulk	DESeq2	LRT		0
257	KC_AB1	CG33233	2.2835980085604	0.118361152541389	1	pseudobulk	DESeq2	LRT		0
257	KC_AB1	CG33267	0.0861051903727775	0.947571577851206	1	pseudobulk	DESeq2	LRT		0
257	KC_AB1	CG3328	2.06541384650887	0.904905526327343	1	pseudobulk	DESeq2	LRT		0
257	KC_AB1	CG33296	0.849606887838128	1	1	pseudobulk	DESeq2	LRT		0
257	KC_AB1	CG33298	-0.615398411714004	0.212748486236938	1	pseudobulk	DESeq2	LRT		0
257	KC_AB1	CG33303	-0.31608567981526	0.378482613369982	1	pseudobulk	DESeq2	LRT		0
257	KC_AB1	CG33307	0.849606887838128	1	1	pseudobulk	DESeq2	LRT		0
257	KC_AB1	CG33310	-0.830652688528626	0.434393349596663	1	pseudobulk	DESeq2	LRT		0
257	KC_AB1	CG33331	-0.131495121339242	0.73110009511337	1	pseudobulk	DESeq2	LRT		0
258	KC_AB1	CG33332	-0.418233008488479	0.758535203974294	1	pseudobulk	DESeq2	LRT		0
258	KC_AB1	CG3335	0.341114110130812	0.861509272445218	1	pseudobulk	DESeq2	LRT		0
258	KC_AB1	CG3337	0.0941245278760401	0.803000063953083	1	pseudobulk	DESeq2	LRT		0
258	KC_AB1	CG3342	-1.44966531455004	0.252457665555911	1	pseudobulk	DESeq2	LRT		0
258	KC_AB1	CG33468	0.849606887838128	1	1	pseudobulk	DESeq2	LRT		0
258	KC_AB1	CG33475	0.849606887838128	1	1	pseudobulk	DESeq2	LRT		0
258	KC_AB1	CG33477	0.849606887838128	1	1	pseudobulk	DESeq2	LRT		0
258	KC_AB1	CG3348	0.237022465848507	1	1	pseudobulk	DESeq2	LRT		0
258	KC_AB1	CG33493	-0.624857218212682	0.503729259948832	1	pseudobulk	DESeq2	LRT		0
258	KC_AB1	CG33494	2.31997030691033	0.813169507846918	1	pseudobulk	DESeq2	LRT		0
259	KC_AB1	CG33500	0.849606887838128	1	1	pseudobulk	DESeq2	LRT		0
259	KC_AB1	CG33506	-0.211986337667571	0.748669616111508	1	pseudobulk	DESeq2	LRT		0
259	KC_AB1	CG33521	-0.948932455545349	0.362032404082597	1	pseudobulk	DESeq2	LRT		0
259	KC_AB1	CG33523	0.416647972238865	0.45662924803307	1	pseudobulk	DESeq2	LRT		0
259	KC_AB1	CG33543	-1.47437682203446	0.294717388930426	1	pseudobulk	DESeq2	LRT		0
259	KC_AB1	CG3356	0.372171390328476	0.303908399636685	1	pseudobulk	DESeq2	LRT		0

259	KC_AB1	CG3358	-1.23380970217206	0.227564002097252	1	pseudobulk	DESeq2	LRT		0
259	KC_AB1	CG33635	0.60296721788872	0.462709192155491	1	pseudobulk	DESeq2	LRT		0
259	KC_AB1	CG33639	0.237022465848507	1	1	pseudobulk	DESeq2	LRT		0
259	KC_AB1	CG33641	0.0160940546605174	1	1	pseudobulk	DESeq2	LRT		0
260	KC_AB1	CG33642	-0.874332157180961	0.546189162320603	1	pseudobulk	DESeq2	LRT		0
260	KC_AB1	CG33655	0.849606887838128	1	1	pseudobulk	DESeq2	LRT		0
260	KC_AB1	CG33667	0.849606887838128	1	1	pseudobulk	DESeq2	LRT		0
260	KC_AB1	CG3368	-0.997398500061394	0.430249162257005	1	pseudobulk	DESeq2	LRT		0
260	KC_AB1	CG33695	0.812333015363572	0.403103258889192	1	pseudobulk	DESeq2	LRT		0
260	KC_AB1	CG33700	0.428399365957321	0.788521472725227	1	pseudobulk	DESeq2	LRT		0
260	KC_AB1	CG3376	1.59863986092676	1	1	pseudobulk	DESeq2	LRT		0
260	KC_AB1	CG33774	0.042116503070464	0.634753941980172	1	pseudobulk	DESeq2	LRT		0
260	KC_AB1	CG33777	1.00693893053477	1	1	pseudobulk	DESeq2	LRT		0
260	KC_AB1	CG33798	0.849606887838128	1	1	pseudobulk	DESeq2	LRT		0
261	KC_AB1	CG3386	-0.183476077869041	0.77974162108544	1	pseudobulk	DESeq2	LRT		0
261	KC_AB1	CG33914	2.31997030691033	0.813169507846918	1	pseudobulk	DESeq2	LRT		0
261	KC_AB1	CG33927	0.849606887838128	1	1	pseudobulk	DESeq2	LRT		0
261	KC_AB1	CG33928	2.78674512494791	0.54962276837928	1	pseudobulk	DESeq2	LRT		0
261	KC_AB1	CG33939	-0.830062897938551	0.717724257445671	1	pseudobulk	DESeq2	LRT		0
261	KC_AB1	CG33941	-1.30324646252305	0.242452213343055	1	pseudobulk	DESeq2	LRT		0
261	KC_AB1	CG33947	-0.532757593284641	0.541896014527119	1	pseudobulk	DESeq2	LRT		0
261	KC_AB1	CG33969	-0.641075503407477	0.514248668399604	1	pseudobulk	DESeq2	LRT		0
261	KC_AB1	CG33970	-0.819907355577798	0.363929857840282	1	pseudobulk	DESeq2	LRT		0
261	KC_AB1	CG33977	0.119518592817846	0.550709834145446	1	pseudobulk	DESeq2	LRT		0
262	KC_AB1	CG33978	-0.647808412501558	0.496637582988826	1	pseudobulk	DESeq2	LRT		0
262	KC_AB1	CG33993	0.896439592643686	0.397367065736907	1	pseudobulk	DESeq2	LRT		0
262	KC_AB1	CG33998	-1.07721701221392	0.401513800488041	1	pseudobulk	DESeq2	LRT		0
262	KC_AB1	CG34001	0.931109140710098	1	1	pseudobulk	DESeq2	LRT		0
262	KC_AB1	CG34008	0.849606887838128	1	1	pseudobulk	DESeq2	LRT		0
262	KC_AB1	CG3402	-1.02916225500035	0.199417278252163	1	pseudobulk	DESeq2	LRT		0
262	KC_AB1	CG34025	0.849606887838128	1	1	pseudobulk	DESeq2	LRT		0
262	KC_AB1	CG34026	-1.05992744849371	0.613288832576002	1	pseudobulk	DESeq2	LRT		0
262	KC_AB1	CG34027	0.849606887838128	1	1	pseudobulk	DESeq2	LRT		0
262	KC_AB1	CG34028	-0.0162073643721956	0.768036035899192	1	pseudobulk	DESeq2	LRT		0
263	KC_AB1	CG34031	1.3802998342441	0.309190019307469	1	pseudobulk	DESeq2	LRT		0
263	KC_AB1	CG34038	0.849606887838128	1	1	pseudobulk	DESeq2	LRT		0
263	KC_AB1	CG34039	0.540736801436624	0.627625697468892	1	pseudobulk	DESeq2	LRT		0
263	KC_AB1	CG34041	0.366247714696288	1	1	pseudobulk	DESeq2	LRT		0
263	KC_AB1	CG34045	-0.13014218306452	0.754161048903269	1	pseudobulk	DESeq2	LRT		0
263	KC_AB1	CG34051	0.849606887838128	1	1	pseudobulk	DESeq2	LRT		0
263	KC_AB1	CG34056	-0.830062897938551	0.717724257445671	1	pseudobulk	DESeq2	LRT		0
263	KC_AB1	CG34057	-0.19698351532854	1	1	pseudobulk	DESeq2	LRT		0
263	KC_AB1	CG3407	0.276499057106296	0.828444646188944	1	pseudobulk	DESeq2	LRT		0
263	KC_AB1	CG3408	-0.10506771737906	1	1	pseudobulk	DESeq2	LRT		0
264	KC_AB1	CG34109	0.959106482335399	0.796808479971156	1	pseudobulk	DESeq2	LRT		0
264	KC_AB1	CG34116	-0.656304882785011	0.592678497647616	1	pseudobulk	DESeq2	LRT		0
264	KC_AB1	CG34117	-0.0120767280314141	0.6966014529249	1	pseudobulk	DESeq2	LRT		0
264	KC_AB1	CG34120	1.20092489142827	1	1	pseudobulk	DESeq2	LRT		0
264	KC_AB1	CG34125	-0.0166463162286918	0.673911752734761	1	pseudobulk	DESeq2	LRT		0
264	KC_AB1	CG34126	-0.265251556582716	0.518357091903233	1	pseudobulk	DESeq2	LRT		0
264	KC_AB1	CG34132	-0.0108852402712452	0.931299203890657	1	pseudobulk	DESeq2	LRT		0
264	KC_AB1	CG34133	-0.801878859981651	0.353255089387748	1	pseudobulk	DESeq2	LRT		0
264	KC_AB1	CG34134	-0.0751066525556263	0.539166826265269	1	pseudobulk	DESeq2	LRT		0

264	KC_AB1	CG34136	0.237022465848507	1	1	pseudobulk	DESeq2	LRT	0
265	KC_AB1	CG34141	2.31997030691033	0.813169507846918	1	pseudobulk	DESeq2	LRT	0
265	KC_AB1	CG34148	1.39781688034856	0.564996570126306	1	pseudobulk	DESeq2	LRT	0
265	KC_AB1	CG34149	2.35647596842778	0.47460136078314	1	pseudobulk	DESeq2	LRT	0
265	KC_AB1	CG34150	0.377206733735503	0.70404028707476	1	pseudobulk	DESeq2	LRT	0
265	KC_AB1	CG34155	0.475419692279278	1	1	pseudobulk	DESeq2	LRT	0
265	KC_AB1	CG34159	-0.204416216101819	0.588267780078838	1	pseudobulk	DESeq2	LRT	0
265	KC_AB1	CG34163	-1.13584521858219	0.54202769046723	1	pseudobulk	DESeq2	LRT	0
265	KC_AB1	CG34174	-0.121469889925361	0.861025083872889	1	pseudobulk	DESeq2	LRT	0
265	KC_AB1	CG34183	0.146113255480853	0.802993706566316	1	pseudobulk	DESeq2	LRT	0
265	KC_AB1	CG34190	0.849606887838128	1	1	pseudobulk	DESeq2	LRT	0
266	KC_AB1	CG34195	-0.853777418295258	0.456792190484576	1	pseudobulk	DESeq2	LRT	0
266	KC_AB1	CG3420	-0.519532799982725	0.523909685448327	1	pseudobulk	DESeq2	LRT	0
266	KC_AB1	CG34200	0.245583827548943	0.422647296285238	1	pseudobulk	DESeq2	LRT	0
266	KC_AB1	CG34205	0.366247714696288	1	1	pseudobulk	DESeq2	LRT	0
266	KC_AB1	CG34206	0.849606887838128	1	1	pseudobulk	DESeq2	LRT	0
266	KC_AB1	CG34213	1.06759111434625	1	1	pseudobulk	DESeq2	LRT	0
266	KC_AB1	CG34215	4.33051932034915	0.0669245779236724	1	pseudobulk	DESeq2	LRT	0
266	KC_AB1	CG34219	0.167962657133593	0.402477332018778	1	pseudobulk	DESeq2	LRT	0
266	KC_AB1	CG34228	-0.331515255010618	0.631620494445568	1	pseudobulk	DESeq2	LRT	0
266	KC_AB1	CG34229	-0.132749431341547	0.762158825859008	1	pseudobulk	DESeq2	LRT	0
267	KC_AB1	CG34231	1.26993512808297	1	1	pseudobulk	DESeq2	LRT	0
267	KC_AB1	CG34232	1.17261808986456	0.859088728563237	1	pseudobulk	DESeq2	LRT	0
267	KC_AB1	CG34234	-0.173314816631053	0.840339458725541	1	pseudobulk	DESeq2	LRT	0
267	KC_AB1	CG34242	-0.268052284934843	0.733981564803231	1	pseudobulk	DESeq2	LRT	0
267	KC_AB1	CG34250	0.932919485780541	0.0879783849478829	1	pseudobulk	DESeq2	LRT	0
267	KC_AB1	CG34253	0.849606887838128	1	1	pseudobulk	DESeq2	LRT	0
267	KC_AB1	CG34279	0.849606887838128	1	1	pseudobulk	DESeq2	LRT	0
267	KC_AB1	CG34286	-0.216210029276812	1	1	pseudobulk	DESeq2	LRT	0
267	KC_AB1	CG34293	-0.621133199509796	0.470391762059859	1	pseudobulk	DESeq2	LRT	0
267	KC_AB1	CG34309	0.849606887838128	1	1	pseudobulk	DESeq2	LRT	0
268	KC_AB1	CG34310	-0.679381631425979	0.707507904409577	1	pseudobulk	DESeq2	LRT	0
268	KC_AB1	CG34316	0.849606887838128	1	1	pseudobulk	DESeq2	LRT	0
268	KC_AB1	CG34330	-0.645870411351165	0.615397558375281	1	pseudobulk	DESeq2	LRT	0
268	KC_AB1	CG34334	2.78674512494791	0.54962276837928	1	pseudobulk	DESeq2	LRT	0
268	KC_AB1	CG3434	0.903923071279047	0.698728668713201	1	pseudobulk	DESeq2	LRT	0
268	KC_AB1	CG34347	-0.318965163823604	0.460831638961552	1	pseudobulk	DESeq2	LRT	0
268	KC_AB1	CG34348	0.339298430115018	0.474772131194302	1	pseudobulk	DESeq2	LRT	0
268	KC_AB1	CG34351	1.24596994024205	0.06734080368339	1	pseudobulk	DESeq2	LRT	0
268	KC_AB1	CG34353	0.240040049445061	0.535985460638767	1	pseudobulk	DESeq2	LRT	0
268	KC_AB1	CG34354	-0.178507981925635	0.311420151792477	1	pseudobulk	DESeq2	LRT	0
269	KC_AB1	CG34355	-0.0645021461752805	0.763501062826205	1	pseudobulk	DESeq2	LRT	0
269	KC_AB1	CG34357	-0.0027484171037299	0.845370887301963	1	pseudobulk	DESeq2	LRT	0
269	KC_AB1	CG3436	0.545258830002039	0.49218381799879	1	pseudobulk	DESeq2	LRT	0
269	KC_AB1	CG3437	-0.934441020220233	0.439769608754167	1	pseudobulk	DESeq2	LRT	0
269	KC_AB1	CG34376	0.5139813165914	0.758214401129524	1	pseudobulk	DESeq2	LRT	0
269	KC_AB1	CG34377	-0.763228806811091	0.58424853834871	1	pseudobulk	DESeq2	LRT	0
269	KC_AB1	CG34384	-0.224806400652745	0.357870693969213	1	pseudobulk	DESeq2	LRT	0
269	KC_AB1	CG34396	3.16697464674408	0.406679230261689	1	pseudobulk	DESeq2	LRT	0
269	KC_AB1	CG34398	-2.81746082419635	0.416900528353106	1	pseudobulk	DESeq2	LRT	0
269	KC_AB1	CG34401	0.0846439067875271	0.594336648972177	1	pseudobulk	DESeq2	LRT	0
270	KC_AB1	CG34402	-0.15128001940458	0.617014176027615	1	pseudobulk	DESeq2	LRT	0
270	KC_AB1	CG34404	-0.0562280709715232	0.610011088313097	1	pseudobulk	DESeq2	LRT	0

270	KC_AB1	CG34408	-0.460445720323118	0.556656894961949	1	pseudobulk	DESeq2	LRT	0
270	KC_AB1	CG34409	2.01572994627287	0.135266073320957	1	pseudobulk	DESeq2	LRT	0
270	KC_AB1	CG34417	1.73776272004912	0.225766815945447	1	pseudobulk	DESeq2	LRT	0
270	KC_AB1	CG34428	0.849606887838128	1	1	pseudobulk	DESeq2	LRT	0
270	KC_AB1	CG34430	2.31997030691033	0.813169507846918	1	pseudobulk	DESeq2	LRT	0
270	KC_AB1	CG34446	2.31997030691033	0.813169507846918	1	pseudobulk	DESeq2	LRT	0
270	KC_AB1	CG34457	1.59863986092676	1	1	pseudobulk	DESeq2	LRT	0
270	KC_AB1	CG3448	0.349540486374305	1	1	pseudobulk	DESeq2	LRT	0
271	KC_AB1	CG3491	-0.216209988775614	1	1	pseudobulk	DESeq2	LRT	0
271	KC_AB1	CG3500	0.0742850347906132	0.662392959888558	1	pseudobulk	DESeq2	LRT	0
271	KC_AB1	CG3511	-0.0223576445796841	0.813753489222033	1	pseudobulk	DESeq2	LRT	0
271	KC_AB1	CG3520	-0.283054125455946	1	1	pseudobulk	DESeq2	LRT	0
271	KC_AB1	CG3527	-0.502530982936478	0.634271956793322	1	pseudobulk	DESeq2	LRT	0
271	KC_AB1	CG3529	-0.171578341449781	0.591270250274418	1	pseudobulk	DESeq2	LRT	0
271	KC_AB1	CG3530	-0.301881966843093	0.392500553194928	1	pseudobulk	DESeq2	LRT	0
271	KC_AB1	CG3534	-1.07674319282786	0.562491167513556	1	pseudobulk	DESeq2	LRT	0
271	KC_AB1	CG3542	-0.264507634750129	0.703316917740164	1	pseudobulk	DESeq2	LRT	0
271	KC_AB1	CG3548	0.19603242813567	0.594892268839926	1	pseudobulk	DESeq2	LRT	0
272	KC_AB1	CG3552	1.55175399343324	0.348870222942045	1	pseudobulk	DESeq2	LRT	0
272	KC_AB1	CG3556	0.11068360152305	0.61243212597497	1	pseudobulk	DESeq2	LRT	0
272	KC_AB1	CG3558	0.0863003228659957	0.821233519399755	1	pseudobulk	DESeq2	LRT	0
272	KC_AB1	CG3566	-0.04887984511088	0.850746036083453	1	pseudobulk	DESeq2	LRT	0
272	KC_AB1	CG3568	-1.28486350245085	0.378381015782117	1	pseudobulk	DESeq2	LRT	0
272	KC_AB1	CG3587	0.591893401900672	1	1	pseudobulk	DESeq2	LRT	0
272	KC_AB1	CG3589	-0.775489018376136	0.539448477173966	1	pseudobulk	DESeq2	LRT	0
272	KC_AB1	CG3594	-1.69160126474558	0.362318418586504	1	pseudobulk	DESeq2	LRT	0
272	KC_AB1	CG3609	-0.0602154351905578	0.839517166726746	1	pseudobulk	DESeq2	LRT	0
272	KC_AB1	CG3610	2.31997030691033	0.813169507846918	1	pseudobulk	DESeq2	LRT	0
273	KC_AB1	CG3618	-0.239487801210566	0.463295859592526	1	pseudobulk	DESeq2	LRT	0
273	KC_AB1	CG3625	0.239875940849014	0.190780366893111	1	pseudobulk	DESeq2	LRT	0
273	KC_AB1	CG3626	-0.743134514596773	0.623789677514114	1	pseudobulk	DESeq2	LRT	0
273	KC_AB1	CG3631	1.00335676338205	1	1	pseudobulk	DESeq2	LRT	0
273	KC_AB1	CG3632	-0.98037818736625	0.214228041773932	1	pseudobulk	DESeq2	LRT	0
273	KC_AB1	CG3634	0.487986963647385	0.488166741198448	1	pseudobulk	DESeq2	LRT	0
273	KC_AB1	CG3635	0.849606887838128	1	1	pseudobulk	DESeq2	LRT	0
273	KC_AB1	CG3638	-0.404705118683466	0.210694813842195	1	pseudobulk	DESeq2	LRT	0
273	KC_AB1	CG3645	0.10378941610932	0.868799444319281	1	pseudobulk	DESeq2	LRT	0
273	KC_AB1	CG3651	-0.56944758466283	0.458345811732991	1	pseudobulk	DESeq2	LRT	0
274	KC_AB1	CG3652	-0.09114700126755	0.615215013964301	1	pseudobulk	DESeq2	LRT	0
274	KC_AB1	CG3655	1.8672486729626	0.410958940841775	1	pseudobulk	DESeq2	LRT	0
274	KC_AB1	CG3662	0.123643918160171	0.533587696689952	1	pseudobulk	DESeq2	LRT	0
274	KC_AB1	CG3678	0.0778291113320838	0.673289373812713	1	pseudobulk	DESeq2	LRT	0
274	KC_AB1	CG3700	0.791346497497451	0.832717228459211	1	pseudobulk	DESeq2	LRT	0
274	KC_AB1	CG3702	-0.254356932554183	0.53076851844882	1	pseudobulk	DESeq2	LRT	0
274	KC_AB1	CG3703	-0.49019105573999	0.255669685016666	1	pseudobulk	DESeq2	LRT	0
274	KC_AB1	CG3704	-2.44973952918366	0.138019378972288	1	pseudobulk	DESeq2	LRT	0
274	KC_AB1	CG3709	0.849606887838128	1	1	pseudobulk	DESeq2	LRT	0
274	KC_AB1	CG3713	4.19351050123574	0.00944420026651257	1	pseudobulk	DESeq2	LRT	0
275	KC_AB1	CG3719	0.136443087678243	0.540919966817733	1	pseudobulk	DESeq2	LRT	0
275	KC_AB1	CG3726	1.27210737936317	0.33057107257816	1	pseudobulk	DESeq2	LRT	0
275	KC_AB1	CG3732	-0.0418882833332061	0.939269716161945	1	pseudobulk	DESeq2	LRT	0
275	KC_AB1	CG3735	0.962412707187032	0.669655572429527	1	pseudobulk	DESeq2	LRT	0
275	KC_AB1	CG3740	0.0006407912231934	0.802146258173072	1	pseudobulk	DESeq2	LRT	0

275	KC_AB1	CG3744	-0.288295614609161	0.496443004335547	1	pseudobulk	DESeq2	LRT		0
275	KC_AB1	CG3756	-1.13220862963476	0.427243912627255	1	pseudobulk	DESeq2	LRT		0
275	KC_AB1	CG3760	0.605638035868284	0.436368427861764	1	pseudobulk	DESeq2	LRT		0
275	KC_AB1	CG3764	-0.352412889712269	0.629543018886096	1	pseudobulk	DESeq2	LRT		0
275	KC_AB1	CG3776	0.176788832978896	0.663913915109081	1	pseudobulk	DESeq2	LRT		0
276	KC_AB1	CG3777	0.177748886857028	0.787760822816653	1	pseudobulk	DESeq2	LRT		0
276	KC_AB1	CG3781	0.486213376390747	0.546891914765854	1	pseudobulk	DESeq2	LRT		0
276	KC_AB1	CG3792	-0.032964277524488	0.899034259833023	1	pseudobulk	DESeq2	LRT		0
276	KC_AB1	CG3793	0.363976876690013	0.362932262328626	1	pseudobulk	DESeq2	LRT		0
276	KC_AB1	CG3803	0.362637019124869	0.659001870475371	1	pseudobulk	DESeq2	LRT		0
276	KC_AB1	CG3808	2.13004336645676	0.828063380524205	1	pseudobulk	DESeq2	LRT		0
276	KC_AB1	CG3815	-0.181759595512661	0.744781869530651	1	pseudobulk	DESeq2	LRT		0
276	KC_AB1	CG3817	-1.74906290820352	0.247065690238076	1	pseudobulk	DESeq2	LRT		0
276	KC_AB1	CG3819	0.341924565583324	0.818875241572659	1	pseudobulk	DESeq2	LRT		0
276	KC_AB1	CG3823	-0.763813596978388	0.704153856296894	1	pseudobulk	DESeq2	LRT		0
277	KC_AB1	CG3829	-0.830062897938551	0.717724257445671	1	pseudobulk	DESeq2	LRT		0
277	KC_AB1	CG3842	-0.320759537211391	0.820543175055409	1	pseudobulk	DESeq2	LRT		0
277	KC_AB1	CG3847	-1.21199109290281	0.331267594823454	1	pseudobulk	DESeq2	LRT		0
277	KC_AB1	CG3857	0.855185708823415	0.440135219058393	1	pseudobulk	DESeq2	LRT		0
277	KC_AB1	CG3860	-0.468356934843405	0.354700241484489	1	pseudobulk	DESeq2	LRT		0
277	KC_AB1	CG3862	-0.875661295010559	0.431144155913909	1	pseudobulk	DESeq2	LRT		0
277	KC_AB1	CG3894	0.452860684838667	0.517748592736562	1	pseudobulk	DESeq2	LRT		0
277	KC_AB1	CG3902	0.539227591542996	1	1	pseudobulk	DESeq2	LRT		0
277	KC_AB1	CG3907	0.0189045295755942	0.537382464676334	1	pseudobulk	DESeq2	LRT		0
277	KC_AB1	CG3909	1.15689353799939	0.231844117431862	1	pseudobulk	DESeq2	LRT		0
278	KC_AB1	CG3918	-0.164783340662578	0.436545571255803	1	pseudobulk	DESeq2	LRT		0
278	KC_AB1	CG3919	0.237022465848507	1	1	pseudobulk	DESeq2	LRT		0
278	KC_AB1	CG3939	-0.283054125455946	1	1	pseudobulk	DESeq2	LRT		0
278	KC_AB1	CG3955	-0.200738686286548	0.436357805255347	1	pseudobulk	DESeq2	LRT		0
278	KC_AB1	CG3961	-0.712738672799771	0.597758110805039	1	pseudobulk	DESeq2	LRT		0
278	KC_AB1	CG3995	-0.0439590111083317	0.770084997246122	1	pseudobulk	DESeq2	LRT		0
278	KC_AB1	CG3999	0.237022465848507	1	1	pseudobulk	DESeq2	LRT		0
278	KC_AB1	CG4000	-1.88509129173436	0.142264902138403	1	pseudobulk	DESeq2	LRT		0
278	KC_AB1	CG4004	0.602386745236829	0.452656770905067	1	pseudobulk	DESeq2	LRT		0
278	KC_AB1	CG40045	-0.194144226109541	0.363335316191951	1	pseudobulk	DESeq2	LRT		0
279	KC_AB1	CG40160	-1.25393399638069	0.612054104673377	1	pseudobulk	DESeq2	LRT		0
279	KC_AB1	CG40191	-0.0390849763650142	0.842562455334207	1	pseudobulk	DESeq2	LRT		0
279	KC_AB1	CG4020	0.849606887838128	1	1	pseudobulk	DESeq2	LRT		0
279	KC_AB1	CG4022	-0.276620483766084	0.590157205124587	1	pseudobulk	DESeq2	LRT		0
279	KC_AB1	CG40228	-0.344708619541532	0.282088049504015	1	pseudobulk	DESeq2	LRT		0
279	KC_AB1	CG4025	-0.0728996827381454	0.721214258994105	1	pseudobulk	DESeq2	LRT		0
279	KC_AB1	CG4036	-1.79169854155779	0.229134224490733	1	pseudobulk	DESeq2	LRT		0
279	KC_AB1	CG4038	0.39549644962617	0.482810018958394	1	pseudobulk	DESeq2	LRT		0
279	KC_AB1	CG4041	-0.197508440777108	0.687840972710133	1	pseudobulk	DESeq2	LRT		0
279	KC_AB1	CG4042	0.926934732302313	0.553126940648418	1	pseudobulk	DESeq2	LRT		0
280	KC_AB1	CG40439	-0.731520070204393	0.540619210860351	1	pseudobulk	DESeq2	LRT		0
280	KC_AB1	CG4045	-0.0920902432382107	0.829838205197318	1	pseudobulk	DESeq2	LRT		0
280	KC_AB1	CG40470	1.43060365239813	1	1	pseudobulk	DESeq2	LRT		0
280	KC_AB1	CG40472	0.0507585837174574	0.611961723604489	1	pseudobulk	DESeq2	LRT		0
280	KC_AB1	CG4049	0.66220953357978	0.160284820218537	1	pseudobulk	DESeq2	LRT		0
280	KC_AB1	CG40498	-0.313164526834879	0.121936008277895	1	pseudobulk	DESeq2	LRT		0
280	KC_AB1	CG4069	0.849606887838128	1	1	pseudobulk	DESeq2	LRT		0
280	KC_AB1	CG4074	-1.00731060628475	0.374666351231335	1	pseudobulk	DESeq2	LRT		0

280	KC_AB1	CG4078	0.316289694689672	0.767386727366117	1	pseudobulk	DESeq2	LRT	0
280	KC_AB1	CG4080	0.305442203750377	0.395392997656928	1	pseudobulk	DESeq2	LRT	0
281	KC_AB1	CG4098	1.59863986092676	1	1	pseudobulk	DESeq2	LRT	0
281	KC_AB1	CG4101	-0.354055039070008	0.479135561222252	1	pseudobulk	DESeq2	LRT	0
281	KC_AB1	CG41099	-0.42320767654883	0.336031088056615	1	pseudobulk	DESeq2	LRT	0
281	KC_AB1	CG41128	0.0536684570386326	0.694640560858986	1	pseudobulk	DESeq2	LRT	0
281	KC_AB1	CG4115	5.98503584706672	0.160241992970958	1	pseudobulk	DESeq2	LRT	0
281	KC_AB1	CG4119	-0.589389296018961	0.443942904738859	1	pseudobulk	DESeq2	LRT	0
281	KC_AB1	CG4133	-0.300668489340341	0.370706045624716	1	pseudobulk	DESeq2	LRT	0
281	KC_AB1	CG41520	1.01265988621066	0.962519979213338	1	pseudobulk	DESeq2	LRT	0
281	KC_AB1	CG4168	2.31997030691033	0.813169507846918	1	pseudobulk	DESeq2	LRT	0
281	KC_AB1	CG4186	0.355542466162835	0.822350886578439	1	pseudobulk	DESeq2	LRT	0
282	KC_AB1	CG4199	-0.171849727889796	0.679674370900892	1	pseudobulk	DESeq2	LRT	0
282	KC_AB1	CG42231	1.33739971103221	0.620955655860259	1	pseudobulk	DESeq2	LRT	0
282	KC_AB1	CG42232	0.093669387453297	0.826815284500567	1	pseudobulk	DESeq2	LRT	0
282	KC_AB1	CG42233	-0.645776819068304	0.634258639138015	1	pseudobulk	DESeq2	LRT	0
282	KC_AB1	CG42235	1.1259344621399	0.648472562805826	1	pseudobulk	DESeq2	LRT	0
282	KC_AB1	CG42237	-0.564356698759404	0.803702590456371	1	pseudobulk	DESeq2	LRT	0
282	KC_AB1	CG42238	-0.340579805716348	0.0511221969858824	1	pseudobulk	DESeq2	LRT	0
282	KC_AB1	CG42239	-1.26635257088884	0.428990046215644	1	pseudobulk	DESeq2	LRT	0
282	KC_AB1	CG42240	0.688694930793186	0.416327826579121	1	pseudobulk	DESeq2	LRT	0
282	KC_AB1	CG42258	0.0204362511461466	0.700096245536702	1	pseudobulk	DESeq2	LRT	0
283	KC_AB1	CG42259	0.503739008031488	1	1	pseudobulk	DESeq2	LRT	0
283	KC_AB1	CG42260	-0.257223255794398	0.108196391441698	1	pseudobulk	DESeq2	LRT	0
283	KC_AB1	CG42268	-0.182333210860512	0.417015085867996	1	pseudobulk	DESeq2	LRT	0
283	KC_AB1	CG42271	0.311798820053699	0.642992051416212	1	pseudobulk	DESeq2	LRT	0
283	KC_AB1	CG42287	2.31997030691033	0.813169507846918	1	pseudobulk	DESeq2	LRT	0
283	KC_AB1	CG42288	2.85142250261825	0.484249113053674	1	pseudobulk	DESeq2	LRT	0
283	KC_AB1	CG4230	0.131595974594889	0.50688123277104	1	pseudobulk	DESeq2	LRT	0
283	KC_AB1	CG42302	0.849606887838128	1	1	pseudobulk	DESeq2	LRT	0
283	KC_AB1	CG42307	-0.5771888292326	0.648802557833071	1	pseudobulk	DESeq2	LRT	0
283	KC_AB1	CG42322	-0.111574102693333	0.616952120802696	1	pseudobulk	DESeq2	LRT	0
284	KC_AB1	CG42324	0.286506358398054	0.267419546902957	1	pseudobulk	DESeq2	LRT	0
284	KC_AB1	CG42327	-0.824031178598066	0.642231983836704	1	pseudobulk	DESeq2	LRT	0
284	KC_AB1	CG42336	-0.518524483201658	0.467845861066741	1	pseudobulk	DESeq2	LRT	0
284	KC_AB1	CG42337	-0.354907227984558	0.154114158803374	1	pseudobulk	DESeq2	LRT	0
284	KC_AB1	CG42339	0.176057990872732	0.409354834960186	1	pseudobulk	DESeq2	LRT	0
284	KC_AB1	CG42342	0.874209486432333	0.0148482232467642	1	pseudobulk	DESeq2	LRT	0
284	KC_AB1	CG42346	-0.278613296493212	0.495574933862035	1	pseudobulk	DESeq2	LRT	0
284	KC_AB1	CG42354	2.06541384650887	0.768363369045814	1	pseudobulk	DESeq2	LRT	0
284	KC_AB1	CG42359	-0.224676316979344	0.7805842003479	1	pseudobulk	DESeq2	LRT	0
284	KC_AB1	CG42360	0.657806795450563	0.193119359074154	1	pseudobulk	DESeq2	LRT	0
285	KC_AB1	CG42361	0.208644296779458	0.528632346834342	1	pseudobulk	DESeq2	LRT	0
285	KC_AB1	CG42365	-0.0283638099376234	0.784025389290044	1	pseudobulk	DESeq2	LRT	0
285	KC_AB1	CG42376	-0.467148362385375	0.40413469460808	1	pseudobulk	DESeq2	LRT	0
285	KC_AB1	CG4238	0.262916716505908	0.544141314018605	1	pseudobulk	DESeq2	LRT	0
285	KC_AB1	CG42382	-0.165522834448479	0.806076562335353	1	pseudobulk	DESeq2	LRT	0
285	KC_AB1	CG4239	-0.1498911891125	0.722193538737723	1	pseudobulk	DESeq2	LRT	0
285	KC_AB1	CG42390	-4.411070721625	0.244552085569936	1	pseudobulk	DESeq2	LRT	0
285	KC_AB1	CG42391	2.06541384650887	0.768363369045814	1	pseudobulk	DESeq2	LRT	0
285	KC_AB1	CG42393	0.849606887838128	1	1	pseudobulk	DESeq2	LRT	0
285	KC_AB1	CG42394	-0.123515236593407	0.835728971132741	1	pseudobulk	DESeq2	LRT	0
286	KC_AB1	CG42399	-0.925681209973711	0.347079854509474	1	pseudobulk	DESeq2	LRT	0

286	KC_AB1	CG42402	-0.814795050872369	0.0210940064187856	1	pseudobulk	DESeq2	LRT	0
286	KC_AB1	CG42404	-0.254017707536918	0.414255447591299	1	pseudobulk	DESeq2	LRT	0
286	KC_AB1	CG42450	-0.144545616474319	0.471173156475041	1	pseudobulk	DESeq2	LRT	0
286	KC_AB1	CG42458	-0.216210029276812	1	1	pseudobulk	DESeq2	LRT	0
286	KC_AB1	CG42486	2.78674512494791	0.54962276837928	1	pseudobulk	DESeq2	LRT	0
286	KC_AB1	CG42494	0.366247714696288	1	1	pseudobulk	DESeq2	LRT	0
286	KC_AB1	CG42495	0.811967575378733	0.824576705147507	1	pseudobulk	DESeq2	LRT	0
286	KC_AB1	CG4250	-0.830062897938551	0.717724257445671	1	pseudobulk	DESeq2	LRT	0
286	KC_AB1	CG42514	0.337398599182116	1	1	pseudobulk	DESeq2	LRT	0
287	KC_AB1	CG42518	0.849606887838128	1	1	pseudobulk	DESeq2	LRT	0
287	KC_AB1	CG42524	3.81892523345342	0.259683132508622	1	pseudobulk	DESeq2	LRT	0
287	KC_AB1	CG42526	0.0459493376759376	1	1	pseudobulk	DESeq2	LRT	0
287	KC_AB1	CG42534	0.849606887838128	1	1	pseudobulk	DESeq2	LRT	0
287	KC_AB1	CG42541	-0.113594087618032	0.396154150535462	1	pseudobulk	DESeq2	LRT	0
287	KC_AB1	CG42542	-0.690194224074858	0.288743175277415	1	pseudobulk	DESeq2	LRT	0
287	KC_AB1	CG42553	1.1662315253072	0.456063767897227	1	pseudobulk	DESeq2	LRT	0
287	KC_AB1	CG42554	-0.798444455528828	0.49278605906188	1	pseudobulk	DESeq2	LRT	0
287	KC_AB1	CG42561	-0.0065790798335215	1	1	pseudobulk	DESeq2	LRT	0
287	KC_AB1	CG42562	-0.130537289152049	0.825353098550245	1	pseudobulk	DESeq2	LRT	0
288	KC_AB1	CG42568	0.849606887838128	1	1	pseudobulk	DESeq2	LRT	0
288	KC_AB1	CG42581	0.849606887838128	1	1	pseudobulk	DESeq2	LRT	0
288	KC_AB1	CG42588	0.364271114071169	0.499722774883299	1	pseudobulk	DESeq2	LRT	0
288	KC_AB1	CG42594	-2.0466951202295	0.086007146234134	1	pseudobulk	DESeq2	LRT	0
288	KC_AB1	CG42613	-0.208070824522822	0.300005781218922	1	pseudobulk	DESeq2	LRT	0
288	KC_AB1	CG42615	0.849606887838128	1	1	pseudobulk	DESeq2	LRT	0
288	KC_AB1	CG4266	0.186354318293067	0.620101296338063	1	pseudobulk	DESeq2	LRT	0
288	KC_AB1	CG42663	1.55810288896539	0.527984947737291	1	pseudobulk	DESeq2	LRT	0
288	KC_AB1	CG42668	0.0371296640318636	0.707679714627081	1	pseudobulk	DESeq2	LRT	0
288	KC_AB1	CG42671	-0.844700568978063	0.0920313398865921	1	pseudobulk	DESeq2	LRT	0
289	KC_AB1	CG42672	-0.544542084031963	0.419719645306227	1	pseudobulk	DESeq2	LRT	0
289	KC_AB1	CG42673	-0.0618458826164134	0.537391966925609	1	pseudobulk	DESeq2	LRT	0
289	KC_AB1	CG42674	-1.19501628018881	0.0555242158251103	1	pseudobulk	DESeq2	LRT	0
289	KC_AB1	CG42675	-0.830062897938551	0.717724257445671	1	pseudobulk	DESeq2	LRT	0
289	KC_AB1	CG42676	0.693267817455519	0.590082758737282	1	pseudobulk	DESeq2	LRT	0
289	KC_AB1	CG4269	0.0160940546605626	1	1	pseudobulk	DESeq2	LRT	0
289	KC_AB1	CG42692	0.0160940546605174	1	1	pseudobulk	DESeq2	LRT	0
289	KC_AB1	CG42697	0.849606887838128	1	1	pseudobulk	DESeq2	LRT	0
289	KC_AB1	CG42699	0.0703825077030637	0.592303598183636	1	pseudobulk	DESeq2	LRT	0
289	KC_AB1	CG42700	0.0121974882856203	0.592981435892331	1	pseudobulk	DESeq2	LRT	0
290	KC_AB1	CG42709	0.30726543352942	0.594019709211674	1	pseudobulk	DESeq2	LRT	0
290	KC_AB1	CG42724	-0.34008329332729	0.295092961509042	1	pseudobulk	DESeq2	LRT	0
290	KC_AB1	CG42748	-0.505322204102041	0.0551883323577729	1	pseudobulk	DESeq2	LRT	0
290	KC_AB1	CG42750	-0.385822860191894	0.160215883968104	1	pseudobulk	DESeq2	LRT	0
290	KC_AB1	CG42751	0.849606887838128	1	1	pseudobulk	DESeq2	LRT	0
290	KC_AB1	CG42762	0.183159795463042	1	1	pseudobulk	DESeq2	LRT	0
290	KC_AB1	CG4278	0.558914423444395	0.818418794089849	1	pseudobulk	DESeq2	LRT	0
290	KC_AB1	CG42784	-0.196397697293059	0.388117519853555	1	pseudobulk	DESeq2	LRT	0
290	KC_AB1	CG42788	-0.13036211830807	0.435224368357658	1	pseudobulk	DESeq2	LRT	0
290	KC_AB1	CG42789	0.849606887838128	1	1	pseudobulk	DESeq2	LRT	0
291	KC_AB1	CG42795	-0.531534308340644	0.0193727882865126	1	pseudobulk	DESeq2	LRT	0
291	KC_AB1	CG42797	-0.385325548721829	0.652193180690378	1	pseudobulk	DESeq2	LRT	0
291	KC_AB1	CG42808	2.72139278170804	0.311778555417195	1	pseudobulk	DESeq2	LRT	0
291	KC_AB1	CG42809	-0.279392942036039	0.562547134104577	1	pseudobulk	DESeq2	LRT	0

291	KC_AB1	CG4281	-0.478962094294579	0.550647529784199	1	pseudobulk	DESeq2	LRT		0
291	KC_AB1	CG42810	2.31997030691033	0.813169507846918	1	pseudobulk	DESeq2	LRT		0
291	KC_AB1	CG42813	-0.0613785487970059	0.542607902969826	1	pseudobulk	DESeq2	LRT		0
291	KC_AB1	CG4282	1.21066938057947	0.226645635472411	1	pseudobulk	DESeq2	LRT		0
291	KC_AB1	CG42846	0.849606887838128	1	1	pseudobulk	DESeq2	LRT		0
291	KC_AB1	CG42855	0.0776509426685793	0.74931334725892	1	pseudobulk	DESeq2	LRT		0
292	KC_AB1	CG4287	-0.286201451075247	0.542793902484273	1	pseudobulk	DESeq2	LRT		0
292	KC_AB1	CG4291	2.00211528911164	0.107226286966002	1	pseudobulk	DESeq2	LRT		0
292	KC_AB1	CG4293	-0.20703539344243	0.715938258003669	1	pseudobulk	DESeq2	LRT		0
292	KC_AB1	CG4294	-0.0892203630442988	0.63985971007669	1	pseudobulk	DESeq2	LRT		0
292	KC_AB1	CG4297	-0.258553299054555	0.38573175673074	1	pseudobulk	DESeq2	LRT		0
292	KC_AB1	CG43049	1.11528148199936	1	1	pseudobulk	DESeq2	LRT		0
292	KC_AB1	CG43051	-1.8583574571615	0.3993047817149	1	pseudobulk	DESeq2	LRT		0
292	KC_AB1	CG43060	0.849606887838128	1	1	pseudobulk	DESeq2	LRT		0
292	KC_AB1	CG43066	-1.10990999842872	0.00603092087978577	1	pseudobulk	DESeq2	LRT		0
292	KC_AB1	CG43068	2.31997030691033	0.813169507846918	1	pseudobulk	DESeq2	LRT		0
293	KC_AB1	CG43078	0.184744427668609	0.442129713995917	1	pseudobulk	DESeq2	LRT		0
293	KC_AB1	CG43093	1.59863986092676	1	1	pseudobulk	DESeq2	LRT		0
293	KC_AB1	CG43094	0.849606887838128	1	1	pseudobulk	DESeq2	LRT		0
293	KC_AB1	CG43102	-0.233244699857406	0.375578860100231	1	pseudobulk	DESeq2	LRT		0
293	KC_AB1	CG43103	-0.216210029276812	1	1	pseudobulk	DESeq2	LRT		0
293	KC_AB1	CG43107	0.849606887838128	1	1	pseudobulk	DESeq2	LRT		0
293	KC_AB1	CG43110	3.47261803475683	0.32867999329132	1	pseudobulk	DESeq2	LRT		0
293	KC_AB1	CG43117	0.465416665836636	0.777420633043633	1	pseudobulk	DESeq2	LRT		0
293	KC_AB1	CG4313	1.82008206908452	0.260726556141237	1	pseudobulk	DESeq2	LRT		0
293	KC_AB1	CG43163	-0.473681291894004	0.729181397106467	1	pseudobulk	DESeq2	LRT		0
294	KC_AB1	CG43175	0.849606887838128	1	1	pseudobulk	DESeq2	LRT		0
294	KC_AB1	CG43219	-2.45361645356134	0.134797625260058	1	pseudobulk	DESeq2	LRT		0
294	KC_AB1	CG43222	0.763352350879454	0.126219621811203	1	pseudobulk	DESeq2	LRT		0
294	KC_AB1	CG43232	2.31997030691033	0.813169507846918	1	pseudobulk	DESeq2	LRT		0
294	KC_AB1	CG43236	0.849606887838128	1	1	pseudobulk	DESeq2	LRT		0
294	KC_AB1	CG4324	2.78674512494791	0.54962276837928	1	pseudobulk	DESeq2	LRT		0
294	KC_AB1	CG4329	0.849606887838128	1	1	pseudobulk	DESeq2	LRT		0
294	KC_AB1	CG43290	0.849606887838128	1	1	pseudobulk	DESeq2	LRT		0
294	KC_AB1	CG43293	0.849606887838128	1	1	pseudobulk	DESeq2	LRT		0
294	KC_AB1	CG43295	2.78674512494791	0.54962276837928	1	pseudobulk	DESeq2	LRT		0
295	KC_AB1	CG43313	2.06531092668974	0.944672961903726	1	pseudobulk	DESeq2	LRT		0
295	KC_AB1	CG43315	0.849606887838128	1	1	pseudobulk	DESeq2	LRT		0
295	KC_AB1	CG43317	0.849606887838128	1	1	pseudobulk	DESeq2	LRT		0
295	KC_AB1	CG4332	1.61630010745505	0.199323647934436	1	pseudobulk	DESeq2	LRT		0
295	KC_AB1	CG43322	1.59863986092676	1	1	pseudobulk	DESeq2	LRT		0
295	KC_AB1	CG43324	0.0057696580735737	0.588192529194265	1	pseudobulk	DESeq2	LRT		0
295	KC_AB1	CG43333	2.78674512494791	0.54962276837928	1	pseudobulk	DESeq2	LRT		0
295	KC_AB1	CG43340	3.25571491907373	0.255296478506207	1	pseudobulk	DESeq2	LRT		0
295	KC_AB1	CG43341	1.59863986092676	1	1	pseudobulk	DESeq2	LRT		0
295	KC_AB1	CG43342	1.51280379024899	0.85518877994987	1	pseudobulk	DESeq2	LRT		0
296	KC_AB1	CG43347	-0.272768317147945	0.296491072718281	1	pseudobulk	DESeq2	LRT		0
296	KC_AB1	CG4335	1.59863986092676	1	1	pseudobulk	DESeq2	LRT		0
296	KC_AB1	CG43366	0.849606887838128	1	1	pseudobulk	DESeq2	LRT		0
296	KC_AB1	CG43367	-0.319100817966482	0.516651477060212	1	pseudobulk	DESeq2	LRT		0
296	KC_AB1	CG43373	0.37575529587925	0.160018555399967	1	pseudobulk	DESeq2	LRT		0
296	KC_AB1	CG4338	0.190683149238066	0.827453831945451	1	pseudobulk	DESeq2	LRT		0
296	KC_AB1	CG43394	0.713780324542466	1	1	pseudobulk	DESeq2	LRT		0

296	KC_AB1	CG43396	0.849606887838128	1	1	pseudobulk	DESeq2	LRT	0
296	KC_AB1	CG4341	0.574402412779335	0.147447967777185	1	pseudobulk	DESeq2	LRT	0
296	KC_AB1	CG43445	0.183159795455075	1	1	pseudobulk	DESeq2	LRT	0
297	KC_AB1	CG4360	-0.229878252395467	0.714311828859759	1	pseudobulk	DESeq2	LRT	0
297	KC_AB1	CG4364	-0.356127675189468	0.66467186499974	1	pseudobulk	DESeq2	LRT	0
297	KC_AB1	CG43647	2.78674512494791	0.54962276837928	1	pseudobulk	DESeq2	LRT	0
297	KC_AB1	CG43658	-1.16933883800846	0.284326817728798	1	pseudobulk	DESeq2	LRT	0
297	KC_AB1	CG43691	0.849606887838128	1	1	pseudobulk	DESeq2	LRT	0
297	KC_AB1	CG43693	0.584365021815201	1	1	pseudobulk	DESeq2	LRT	0
297	KC_AB1	CG43707	0.0552642242710716	0.87722236434733	1	pseudobulk	DESeq2	LRT	0
297	KC_AB1	CG43736	0.0445903326169762	0.824020249793602	1	pseudobulk	DESeq2	LRT	0
297	KC_AB1	CG43737	0.0196776458485523	0.616291961867136	1	pseudobulk	DESeq2	LRT	0
297	KC_AB1	CG43740	-0.17885352588832	0.691644229674253	1	pseudobulk	DESeq2	LRT	0
298	KC_AB1	CG4375	0.479929632770381	1	1	pseudobulk	DESeq2	LRT	0
298	KC_AB1	CG43755	0.510591489885733	0.358115746792867	1	pseudobulk	DESeq2	LRT	0
298	KC_AB1	CG43759	0.159837367240547	0.362745883273087	1	pseudobulk	DESeq2	LRT	0
298	KC_AB1	CG43778	0.398244680275871	0.206519723150225	1	pseudobulk	DESeq2	LRT	0
298	KC_AB1	CG43783	-0.430433600362722	0.574130602326644	1	pseudobulk	DESeq2	LRT	0
298	KC_AB1	CG43789	0.800242620847685	0.745281970798207	1	pseudobulk	DESeq2	LRT	0
298	KC_AB1	CG43800	-0.0892950451142593	0.513339585892041	1	pseudobulk	DESeq2	LRT	0
298	KC_AB1	CG43843	0.634960112221505	1	1	pseudobulk	DESeq2	LRT	0
298	KC_AB1	CG43845	-0.582303408297223	0.779363959297745	1	pseudobulk	DESeq2	LRT	0
298	KC_AB1	CG43850	0.481421037665488	0.273212918187006	1	pseudobulk	DESeq2	LRT	0
299	KC_AB1	CG43861	-0.367927184324976	0.38195329632433	1	pseudobulk	DESeq2	LRT	0
299	KC_AB1	CG43867	-9.41212646566189E-	0.905055007503137	1	pseudobulk	DESeq2	LRT	0
299	KC_AB1	CG43897	-1.05548521056871	0.62314630615428	1	pseudobulk	DESeq2	LRT	0
299	KC_AB1	CG4390	-0.780625823996778	0.377707863336175	1	pseudobulk	DESeq2	LRT	0
299	KC_AB1	CG43901	-0.154692909267494	0.561526718807665	1	pseudobulk	DESeq2	LRT	0
299	KC_AB1	CG43902	0.423199651628215	0.758188678120158	1	pseudobulk	DESeq2	LRT	0
299	KC_AB1	CG4393	-0.185098247094906	0.575305044265083	1	pseudobulk	DESeq2	LRT	0
299	KC_AB1	CG43980	2.78674512494791	0.54962276837928	1	pseudobulk	DESeq2	LRT	0
299	KC_AB1	CG44002	0.247400601739167	1	1	pseudobulk	DESeq2	LRT	0
299	KC_AB1	CG44008	1.21267130850476	1	1	pseudobulk	DESeq2	LRT	0
300	KC_AB1	CG4404	-0.0132930611460254	0.751736677055493	1	pseudobulk	DESeq2	LRT	0
300	KC_AB1	CG4407	-0.0158964301805524	0.728085374499682	1	pseudobulk	DESeq2	LRT	0
300	KC_AB1	CG44085	0.849606887838128	1	1	pseudobulk	DESeq2	LRT	0
300	KC_AB1	CG4409	-0.749378108562714	0.510618341226511	1	pseudobulk	DESeq2	LRT	0
300	KC_AB1	CG44098	-1.03116098321432	0.441637083435329	1	pseudobulk	DESeq2	LRT	0
300	KC_AB1	CG44102	1.59863986092676	1	1	pseudobulk	DESeq2	LRT	0
300	KC_AB1	CG44153	1.38160466972972	0.0634949297885282	1	pseudobulk	DESeq2	LRT	0
300	KC_AB1	CG44158	-0.0211670629492539	0.743916923168221	1	pseudobulk	DESeq2	LRT	0
300	KC_AB1	CG44195	0.849606887838128	1	1	pseudobulk	DESeq2	LRT	0
300	KC_AB1	CG4424	0.801659832640106	0.63035292480987	1	pseudobulk	DESeq2	LRT	0
301	KC_AB1	CG44242	-0.208637195208881	0.513738259857848	1	pseudobulk	DESeq2	LRT	0
301	KC_AB1	CG44243	-0.926087014993963	0.373361228245231	1	pseudobulk	DESeq2	LRT	0
301	KC_AB1	CG44245	1.27781582596797	0.856769467717085	1	pseudobulk	DESeq2	LRT	0
301	KC_AB1	CG44247	-0.521920677762114	0.0809867592144183	1	pseudobulk	DESeq2	LRT	0
301	KC_AB1	CG44249	1.59863986092676	1	1	pseudobulk	DESeq2	LRT	0
301	KC_AB1	CG44251	2.31997030691033	0.813169507846918	1	pseudobulk	DESeq2	LRT	0
301	KC_AB1	CG44296	0.849606887838128	1	1	pseudobulk	DESeq2	LRT	0
301	KC_AB1	CG44325	-0.39108419863745	0.748908135248462	1	pseudobulk	DESeq2	LRT	0
301	KC_AB1	CG44362	2.78674512494791	0.54962276837928	1	pseudobulk	DESeq2	LRT	0
301	KC_AB1	CG4438	1.82422053292417	0.606839105763612	1	pseudobulk	DESeq2	LRT	0

302	KC_AB1	CG44385	1.59863986092676	1	1	pseudobulk	DESeq2	LRT		0
302	KC_AB1	CG44422	1.15186207494354	0.0160220003990932	1	pseudobulk	DESeq2	LRT		0
302	KC_AB1	CG4452	0.415292253064842	0.487709019853845	1	pseudobulk	DESeq2	LRT		0
302	KC_AB1	CG44521	0.849606887838128	1	1	pseudobulk	DESeq2	LRT		0
302	KC_AB1	CG4455	0.34400370901222	1	1	pseudobulk	DESeq2	LRT		0
302	KC_AB1	CG4459	0.366247714696288	1	1	pseudobulk	DESeq2	LRT		0
302	KC_AB1	CG4462	0.849606887838128	1	1	pseudobulk	DESeq2	LRT		0
302	KC_AB1	CG44622	0.116783351990007	1	1	pseudobulk	DESeq2	LRT		0
302	KC_AB1	CG4467	-0.382472996518314	0.063273761067317	1	pseudobulk	DESeq2	LRT		0
302	KC_AB1	CG4476	2.06541384650887	0.768363369045814	1	pseudobulk	DESeq2	LRT		0
303	KC_AB1	CG44774	-0.269479861562897	0.28160226007015	1	pseudobulk	DESeq2	LRT		0
303	KC_AB1	CG44815	0.849606887838128	1	1	pseudobulk	DESeq2	LRT		0
303	KC_AB1	CG44837	0.437254220526138	0.802054389609419	1	pseudobulk	DESeq2	LRT		0
303	KC_AB1	CG44838	-0.293501962484199	0.264943203855738	1	pseudobulk	DESeq2	LRT		0
303	KC_AB1	CG4496	1.21267130850476	1	1	pseudobulk	DESeq2	LRT		0
303	KC_AB1	CG4497	-0.297741339327722	0.656789535230804	1	pseudobulk	DESeq2	LRT		0
303	KC_AB1	CG45002	0.868162777079863	0.355310727059471	1	pseudobulk	DESeq2	LRT		0
303	KC_AB1	CG4502	-0.207837378158048	0.371820317324731	1	pseudobulk	DESeq2	LRT		0
303	KC_AB1	CG45049	-0.012538113009191	0.548477758950941	1	pseudobulk	DESeq2	LRT		0
303	KC_AB1	CG45050	-0.306803350066626	0.160686847750485	1	pseudobulk	DESeq2	LRT		0
304	KC_AB1	CG45071	-0.167267799620524	0.621873689935248	1	pseudobulk	DESeq2	LRT		0
304	KC_AB1	CG45081	0.849606887838128	1	1	pseudobulk	DESeq2	LRT		0
304	KC_AB1	CG4511	-0.060356158943970	0.729487668737535	1	pseudobulk	DESeq2	LRT		0
304	KC_AB1	CG45218	2.06541384650887	0.768363369045814	1	pseudobulk	DESeq2	LRT		0
304	KC_AB1	CG45263	-0.134010454761066	0.352470235639387	1	pseudobulk	DESeq2	LRT		0
304	KC_AB1	CG4537	0.483791181192489	1	1	pseudobulk	DESeq2	LRT		0
304	KC_AB1	CG4538	-1.05489102470797	0.231082728479111	1	pseudobulk	DESeq2	LRT		0
304	KC_AB1	CG4552	0.190676105827672	0.703151726273635	1	pseudobulk	DESeq2	LRT		0
304	KC_AB1	CG4553	0.846221160477797	1	1	pseudobulk	DESeq2	LRT		0
304	KC_AB1	CG4554	-1.34691172843453	0.438925392949886	1	pseudobulk	DESeq2	LRT		0
305	KC_AB1	CG4557	0.119057398220571	0.575565498539613	1	pseudobulk	DESeq2	LRT		0
305	KC_AB1	CG4558	0.114088742319102	0.751806329387926	1	pseudobulk	DESeq2	LRT		0
305	KC_AB1	CG4562	-1.3330464352482	0.133710225432074	1	pseudobulk	DESeq2	LRT		0
305	KC_AB1	CG4565	3.1098503736322	0.25718827200394	1	pseudobulk	DESeq2	LRT		0
305	KC_AB1	CG45676	0.849606887838128	1	1	pseudobulk	DESeq2	LRT		0
305	KC_AB1	CG4572	-1.54870265514401	0.418573736618684	1	pseudobulk	DESeq2	LRT		0
305	KC_AB1	CG4576	1.01752621803122	1	1	pseudobulk	DESeq2	LRT		0
305	KC_AB1	CG4577	-0.12803369605091	0.480097779010074	1	pseudobulk	DESeq2	LRT		0
305	KC_AB1	CG4587	-0.115561204252047	0.563015084345865	1	pseudobulk	DESeq2	LRT		0
305	KC_AB1	CG4593	-1.45656132047757	0.199018272116693	1	pseudobulk	DESeq2	LRT		0
306	KC_AB1	CG4596	0.0132110432787379	0.752954549104598	1	pseudobulk	DESeq2	LRT		0
306	KC_AB1	CG4598	-1.63458565181836	0.298117035476654	1	pseudobulk	DESeq2	LRT		0
306	KC_AB1	CG4603	0.2155641347026	0.650507323895239	1	pseudobulk	DESeq2	LRT		0
306	KC_AB1	CG4607	2.78674512494791	0.54962276837928	1	pseudobulk	DESeq2	LRT		0
306	KC_AB1	CG4610	2.78452262432147	0.205497859676046	1	pseudobulk	DESeq2	LRT		0
306	KC_AB1	CG4612	-0.364636898165223	0.193014601856734	1	pseudobulk	DESeq2	LRT		0
306	KC_AB1	CG4615	-0.172345033109685	0.627895559856035	1	pseudobulk	DESeq2	LRT		0
306	KC_AB1	CG4617	0.327712188712253	0.607811458244627	1	pseudobulk	DESeq2	LRT		0
306	KC_AB1	CG4619	-0.743804462416192	0.576006624096938	1	pseudobulk	DESeq2	LRT		0
306	KC_AB1	CG4622	-0.79485699249031	0.359062753695039	1	pseudobulk	DESeq2	LRT		0
307	KC_AB1	CG4627	-0.366563325728877	0.733758723068592	1	pseudobulk	DESeq2	LRT		0
307	KC_AB1	CG46270	0.849606887838128	1	1	pseudobulk	DESeq2	LRT		0
307	KC_AB1	CG46280	-0.413060948252396	0.378004380986731	1	pseudobulk	DESeq2	LRT		0

307	KC_AB1	CG46281	-0.246735869871477	0.774680178118884	1	pseudobulk	DESeq2	LRT	0
307	KC_AB1	CG4629	-1.8906674396992	0.00947486531853093	1	pseudobulk	DESeq2	LRT	0
307	KC_AB1	CG46298	0.183159795455075	1	1	pseudobulk	DESeq2	LRT	0
307	KC_AB1	CG46301	-0.721256261188348	0.0589443720560045	1	pseudobulk	DESeq2	LRT	0
307	KC_AB1	CG46304	-0.473533893544948	0.839148280595286	1	pseudobulk	DESeq2	LRT	0
307	KC_AB1	CG4631	1.93706461079025	0.658817357590498	1	pseudobulk	DESeq2	LRT	0
307	KC_AB1	CG46338	0.198266076589651	0.574523993556546	1	pseudobulk	DESeq2	LRT	0
308	KC_AB1	CG46385	-0.181486565166822	0.489978590068233	1	pseudobulk	DESeq2	LRT	0
308	KC_AB1	CG46394	0.849606887838128	1	1	pseudobulk	DESeq2	LRT	0
308	KC_AB1	CG46397	0.849606887838128	1	1	pseudobulk	DESeq2	LRT	0
308	KC_AB1	CG46399	0.849606887838128	1	1	pseudobulk	DESeq2	LRT	0
308	KC_AB1	CG46448	1.09659342715769	0.556574680378168	1	pseudobulk	DESeq2	LRT	0
308	KC_AB1	CG4645	0.170090055408433	0.796149036329678	1	pseudobulk	DESeq2	LRT	0
308	KC_AB1	CG4646	-0.330760546641015	0.660342767164628	1	pseudobulk	DESeq2	LRT	0
308	KC_AB1	CG4653	0.849606887838128	1	1	pseudobulk	DESeq2	LRT	0
308	KC_AB1	CG4658	0.491193901506528	0.323767862226955	1	pseudobulk	DESeq2	LRT	0
308	KC_AB1	CG4660	-0.0690887448674269	0.696341307889556	1	pseudobulk	DESeq2	LRT	0
309	KC_AB1	CG4661	0.162118002617676	1	1	pseudobulk	DESeq2	LRT	0
309	KC_AB1	CG4676	0.236531471019087	0.681998207758941	1	pseudobulk	DESeq2	LRT	0
309	KC_AB1	CG4678	0.525506434330952	0.285706166212207	1	pseudobulk	DESeq2	LRT	0
309	KC_AB1	CG4679	-0.195812563435689	0.726771735979089	1	pseudobulk	DESeq2	LRT	0
309	KC_AB1	CG4686	0.59371129274207	0.288406823557681	1	pseudobulk	DESeq2	LRT	0
309	KC_AB1	CG4707	-0.377691909679564	0.721759495952915	1	pseudobulk	DESeq2	LRT	0
309	KC_AB1	CG4709	0.115737035665194	1	1	pseudobulk	DESeq2	LRT	0
309	KC_AB1	CG4730	0.0160940546605174	1	1	pseudobulk	DESeq2	LRT	0
309	KC_AB1	CG4733	2.78674512494791	0.54962276837928	1	pseudobulk	DESeq2	LRT	0
309	KC_AB1	CG4734	0.849606887838128	1	1	pseudobulk	DESeq2	LRT	0
310	KC_AB1	CG4743	1.74488096961208	0.717340193332887	1	pseudobulk	DESeq2	LRT	0
310	KC_AB1	CG4751	-1.05585599245649	0.306859575310634	1	pseudobulk	DESeq2	LRT	0
310	KC_AB1	CG4763	0.849606887838128	1	1	pseudobulk	DESeq2	LRT	0
310	KC_AB1	CG4768	-0.317983303664446	0.492621737487524	1	pseudobulk	DESeq2	LRT	0
310	KC_AB1	CG4788	-0.572523455136493	0.640705390645634	1	pseudobulk	DESeq2	LRT	0
310	KC_AB1	CG4789	0.0578364780663049	0.659855713263844	1	pseudobulk	DESeq2	LRT	0
310	KC_AB1	CG4797	2.55649584056861	0.281351496292498	1	pseudobulk	DESeq2	LRT	0
310	KC_AB1	CG4806	0.0063834584771983	0.517812998888161	1	pseudobulk	DESeq2	LRT	0
310	KC_AB1	CG4813	0.158388086681724	0.775720860674929	1	pseudobulk	DESeq2	LRT	0
310	KC_AB1	CG4820	-0.0985820710080572	0.623290676133215	1	pseudobulk	DESeq2	LRT	0
311	KC_AB1	CG4825	0.482713809150727	0.360146171587963	1	pseudobulk	DESeq2	LRT	0
311	KC_AB1	CG4829	-0.341110876298625	0.671936139729051	1	pseudobulk	DESeq2	LRT	0
311	KC_AB1	CG4849	-1.16050862155789	0.365418763059499	1	pseudobulk	DESeq2	LRT	0
311	KC_AB1	CG4853	-0.821484353519767	0.353297200394391	1	pseudobulk	DESeq2	LRT	0
311	KC_AB1	CG4854	0.664514874013562	1	1	pseudobulk	DESeq2	LRT	0
311	KC_AB1	CG4858	1.10759960582688	0.25691087052135	1	pseudobulk	DESeq2	LRT	0
311	KC_AB1	CG4866	-0.0942017424745486	0.912966158116196	1	pseudobulk	DESeq2	LRT	0
311	KC_AB1	CG4872	0.849606887838128	1	1	pseudobulk	DESeq2	LRT	0
311	KC_AB1	CG4880	1.43060365239813	1	1	pseudobulk	DESeq2	LRT	0
311	KC_AB1	CG4882	-0.98016942376919	0.442608595044464	1	pseudobulk	DESeq2	LRT	0
312	KC_AB1	CG4887	-0.257230677098523	0.606212284445489	1	pseudobulk	DESeq2	LRT	0
312	KC_AB1	CG4896	-0.426359028555803	0.399853842458362	1	pseudobulk	DESeq2	LRT	0
312	KC_AB1	CG4901	0.462471038503802	0.88970382374659	1	pseudobulk	DESeq2	LRT	0
312	KC_AB1	CG4908	0.0893228691262288	0.746483526381658	1	pseudobulk	DESeq2	LRT	0
312	KC_AB1	CG4911	-0.258204428919107	0.513130351118843	1	pseudobulk	DESeq2	LRT	0
312	KC_AB1	CG4928	1.93554247728556	0.377492694673431	1	pseudobulk	DESeq2	LRT	0

312	KC_AB1	CG4935	0.141743934570787	1	1	pseudobulk	DESeq2	LRT	0
312	KC_AB1	CG4936	0.75134012678546	0.50523410147402	1	pseudobulk	DESeq2	LRT	0
312	KC_AB1	CG4942	1.89745682871468	0.503732169685088	1	pseudobulk	DESeq2	LRT	0
312	KC_AB1	CG4949	-1.46887697150435	0.438721421029884	1	pseudobulk	DESeq2	LRT	0
313	KC_AB1	CG4951	0.454577307260253	0.72584106225236	1	pseudobulk	DESeq2	LRT	0
313	KC_AB1	CG4953	-0.077079122246418	0.795704373796982	1	pseudobulk	DESeq2	LRT	0
313	KC_AB1	CG4957	-0.323364991394261	0.671742236242862	1	pseudobulk	DESeq2	LRT	0
313	KC_AB1	CG4968	-0.503382834623515	0.499348001850963	1	pseudobulk	DESeq2	LRT	0
313	KC_AB1	CG4972	-1.24978086409783	0.298498965504743	1	pseudobulk	DESeq2	LRT	0
313	KC_AB1	CG4975	2.31997030691033	0.813169507846918	1	pseudobulk	DESeq2	LRT	0
313	KC_AB1	CG4984	-0.216209988775614	1	1	pseudobulk	DESeq2	LRT	0
313	KC_AB1	CG5001	1.01949336055865	1	1	pseudobulk	DESeq2	LRT	0
313	KC_AB1	CG5002	0.849606887838128	1	1	pseudobulk	DESeq2	LRT	0
313	KC_AB1	CG5003	-0.951865611262934	0.304429221367226	1	pseudobulk	DESeq2	LRT	0
314	KC_AB1	CG5004	-0.511867629702288	0.527037310031036	1	pseudobulk	DESeq2	LRT	0
314	KC_AB1	CG5009	0.0911193407013137	0.551084386734619	1	pseudobulk	DESeq2	LRT	0
314	KC_AB1	CG5013	0.209349798144191	0.841645966976527	1	pseudobulk	DESeq2	LRT	0
314	KC_AB1	CG5021	-0.0629862758310272	0.844049988277729	1	pseudobulk	DESeq2	LRT	0
314	KC_AB1	CG5022	-0.872327971554936	0.339600231278434	1	pseudobulk	DESeq2	LRT	0
314	KC_AB1	CG5027	-0.158947521815109	0.736264410732913	1	pseudobulk	DESeq2	LRT	0
314	KC_AB1	CG5028	-0.178166346052946	0.471349959935031	1	pseudobulk	DESeq2	LRT	0
314	KC_AB1	CG5033	0.377267202023289	1	1	pseudobulk	DESeq2	LRT	0
314	KC_AB1	CG5037	-0.290654922990794	0.661722576126824	1	pseudobulk	DESeq2	LRT	0
314	KC_AB1	CG5038	1.02173759533639	1	1	pseudobulk	DESeq2	LRT	0
315	KC_AB1	CG5039	0.849606887838128	1	1	pseudobulk	DESeq2	LRT	0
315	KC_AB1	CG5044	-0.231957752394674	0.639163502511879	1	pseudobulk	DESeq2	LRT	0
315	KC_AB1	CG5050	0.539227591542996	1	1	pseudobulk	DESeq2	LRT	0
315	KC_AB1	CG5056	-0.494644724339827	0.447799632963494	1	pseudobulk	DESeq2	LRT	0
315	KC_AB1	CG5059	-0.787404602723196	0.308118625932399	1	pseudobulk	DESeq2	LRT	0
315	KC_AB1	CG5062	2.06531092668974	0.944672961903726	1	pseudobulk	DESeq2	LRT	0
315	KC_AB1	CG5065	0.602412900735555	0.0562283649737049	1	pseudobulk	DESeq2	LRT	0
315	KC_AB1	CG5068	0.124338353695469	0.605403513949688	1	pseudobulk	DESeq2	LRT	0
315	KC_AB1	CG5071	0.634960112221505	1	1	pseudobulk	DESeq2	LRT	0
315	KC_AB1	CG5078	0.849606887838128	1	1	pseudobulk	DESeq2	LRT	0
316	KC_AB1	CG5079	0.500710785727311	1	1	pseudobulk	DESeq2	LRT	0
316	KC_AB1	CG5087	-0.178593434391907	0.443152304993092	1	pseudobulk	DESeq2	LRT	0
316	KC_AB1	CG5098	-0.19316691626849	0.590368626714169	1	pseudobulk	DESeq2	LRT	0
316	KC_AB1	CG5104	0.183159795463042	1	1	pseudobulk	DESeq2	LRT	0
316	KC_AB1	CG5114	1.43891125305538	0.54124168123036	1	pseudobulk	DESeq2	LRT	0
316	KC_AB1	CG5116	0.849606887838128	1	1	pseudobulk	DESeq2	LRT	0
316	KC_AB1	CG5126	-0.830062897938551	0.717724257445671	1	pseudobulk	DESeq2	LRT	0
316	KC_AB1	CG5131	0.542982564383309	1	1	pseudobulk	DESeq2	LRT	0
316	KC_AB1	CG5149	0.609480836259794	0.336707425677093	1	pseudobulk	DESeq2	LRT	0
316	KC_AB1	CG5151	-0.235230328887278	0.477192137486086	1	pseudobulk	DESeq2	LRT	0
317	KC_AB1	CG5162	0.849606887838128	1	1	pseudobulk	DESeq2	LRT	0
317	KC_AB1	CG5174	0.166681234231555	0.596895554116696	1	pseudobulk	DESeq2	LRT	0
317	KC_AB1	CG5177	2.06541384650887	0.795866856626082	1	pseudobulk	DESeq2	LRT	0
317	KC_AB1	CG5180	-0.131909277831941	0.780140714440158	1	pseudobulk	DESeq2	LRT	0
317	KC_AB1	CG5181	0.142488192234117	0.741982105553346	1	pseudobulk	DESeq2	LRT	0
317	KC_AB1	CG5188	0.0265099561146486	0.897346031714047	1	pseudobulk	DESeq2	LRT	0
317	KC_AB1	CG5196	-0.932796500803761	0.134552556837766	1	pseudobulk	DESeq2	LRT	0
317	KC_AB1	CG5199	0.971223487312944	0.308738841554941	1	pseudobulk	DESeq2	LRT	0
317	KC_AB1	CG5214	-0.398494087866567	0.361908484348439	1	pseudobulk	DESeq2	LRT	0

317	KC_AB1	CG5220	0.907553846723968	0.337854892592005	1	pseudobulk	DESeq2	LRT	0
318	KC_AB1	CG5245	0.849606887838128	1	1	pseudobulk	DESeq2	LRT	0
318	KC_AB1	CG5254	0.536990687379332	1	1	pseudobulk	DESeq2	LRT	0
318	KC_AB1	CG5262	0.900871501446576	0.381693913597108	1	pseudobulk	DESeq2	LRT	0
318	KC_AB1	CG5270	-0.489759504996966	0.631038363467046	1	pseudobulk	DESeq2	LRT	0
318	KC_AB1	CG5273	-0.207555982292358	0.529163691157787	1	pseudobulk	DESeq2	LRT	0
318	KC_AB1	CG5274	2.0009799258733	0.139411113928626	1	pseudobulk	DESeq2	LRT	0
318	KC_AB1	CG5276	-0.342536639191045	0.714066476701779	1	pseudobulk	DESeq2	LRT	0
318	KC_AB1	CG5280	0.849606887838128	1	1	pseudobulk	DESeq2	LRT	0
318	KC_AB1	CG5281	-0.232698497643894	0.651720196178848	1	pseudobulk	DESeq2	LRT	0
318	KC_AB1	CG5282	0.0659154346313209	0.860462538555677	1	pseudobulk	DESeq2	LRT	0
319	KC_AB1	CG5285	-0.289450712162219	0.712668959538654	1	pseudobulk	DESeq2	LRT	0
319	KC_AB1	CG5287	-0.054956074979213	0.792498991316846	1	pseudobulk	DESeq2	LRT	0
319	KC_AB1	CG5290	1.59863986092676	1	1	pseudobulk	DESeq2	LRT	0
319	KC_AB1	CG5292	0.354738118743056	0.492707181521593	1	pseudobulk	DESeq2	LRT	0
319	KC_AB1	CG5316	-0.560101483443804	0.456621198795414	1	pseudobulk	DESeq2	LRT	0
319	KC_AB1	CG5321	0.56665731550197	1	1	pseudobulk	DESeq2	LRT	0
319	KC_AB1	CG5323	-0.197829362461612	0.804590811593751	1	pseudobulk	DESeq2	LRT	0
319	KC_AB1	CG5326	2.78674512494791	0.54962276837928	1	pseudobulk	DESeq2	LRT	0
319	KC_AB1	CG5339	-0.579738248564335	0.49434323417926	1	pseudobulk	DESeq2	LRT	0
319	KC_AB1	CG5346	-0.267798911930929	1	1	pseudobulk	DESeq2	LRT	0
320	KC_AB1	CG5348	0.662603566462587	0.386509416297883	1	pseudobulk	DESeq2	LRT	0
320	KC_AB1	CG5355	0.131800063029808	0.708566066600996	1	pseudobulk	DESeq2	LRT	0
320	KC_AB1	CG5359	0.931114208482955	0.613911454174465	1	pseudobulk	DESeq2	LRT	0
320	KC_AB1	CG5380	-0.069903954806580	0.769098855091062	1	pseudobulk	DESeq2	LRT	0
320	KC_AB1	CG5382	1.10803019972862	0.447402923207893	1	pseudobulk	DESeq2	LRT	0
320	KC_AB1	CG5390	1.4378296602924	0.707172127558917	1	pseudobulk	DESeq2	LRT	0
320	KC_AB1	CG5397	-0.830062897938551	0.717724257445671	1	pseudobulk	DESeq2	LRT	0
320	KC_AB1	CG5399	0.849606887838128	1	1	pseudobulk	DESeq2	LRT	0
320	KC_AB1	CG5404	-0.370862531082229	0.881125640939123	1	pseudobulk	DESeq2	LRT	0
320	KC_AB1	CG5412	1.17794114082294	0.155992446034968	1	pseudobulk	DESeq2	LRT	0
321	KC_AB1	CG5421	0.849606887838128	1	1	pseudobulk	DESeq2	LRT	0
321	KC_AB1	CG5435	0.0160940546605174	1	1	pseudobulk	DESeq2	LRT	0
321	KC_AB1	CG5439	0.0108650450374866	0.854512300538081	1	pseudobulk	DESeq2	LRT	0
321	KC_AB1	CG5445	0.174868609087936	0.510532174359074	1	pseudobulk	DESeq2	LRT	0
321	KC_AB1	CG5446	-0.456290443999905	0.406033313828194	1	pseudobulk	DESeq2	LRT	0
321	KC_AB1	CG5447	0.140308170346855	0.587408141460109	1	pseudobulk	DESeq2	LRT	0
321	KC_AB1	CG5455	0.289047718098098	0.434904733825763	1	pseudobulk	DESeq2	LRT	0
321	KC_AB1	CG5458	2.31997030691033	0.813169507846918	1	pseudobulk	DESeq2	LRT	0
321	KC_AB1	CG5466	-0.090409499775692	0.848957190560541	1	pseudobulk	DESeq2	LRT	0
321	KC_AB1	CG5498	0.210645577999987	1	1	pseudobulk	DESeq2	LRT	0
322	KC_AB1	CG5500	0.28513597980298	0.667245008117404	1	pseudobulk	DESeq2	LRT	0
322	KC_AB1	CG5504	0.262056506571905	0.636192581335235	1	pseudobulk	DESeq2	LRT	0
322	KC_AB1	CG5509	2.31997030691033	0.813169507846918	1	pseudobulk	DESeq2	LRT	0
322	KC_AB1	CG5510	-0.000300110155942	0.752910757325569	1	pseudobulk	DESeq2	LRT	0
322	KC_AB1	CG5515	0.311468474735546	0.615391403219387	1	pseudobulk	DESeq2	LRT	0
322	KC_AB1	CG5516	0.13311065565531	0.671497616077116	1	pseudobulk	DESeq2	LRT	0
322	KC_AB1	CG5521	-0.74526313346166	0.269860658646484	1	pseudobulk	DESeq2	LRT	0
322	KC_AB1	CG5522	0.190141337168962	0.394076094036945	1	pseudobulk	DESeq2	LRT	0
322	KC_AB1	CG5532	0.675669120790123	0.105057165061698	1	pseudobulk	DESeq2	LRT	0
322	KC_AB1	CG5535	-0.853808049437734	0.237956712318939	1	pseudobulk	DESeq2	LRT	0
323	KC_AB1	CG5541	1.30317068079209	0.698273434779912	1	pseudobulk	DESeq2	LRT	0
323	KC_AB1	CG5543	0.05524634179482	0.950759493315034	1	pseudobulk	DESeq2	LRT	0

323	KC_AB1	CG5554	-0.259724537835751	0.582507118700457	1	pseudobulk	DESeq2	LRT	0
323	KC_AB1	CG5555	-0.315307943608519	0.640765871965562	1	pseudobulk	DESeq2	LRT	0
323	KC_AB1	CG5567	1.13636105692968	0.109760082524016	1	pseudobulk	DESeq2	LRT	0
323	KC_AB1	CG5569	-0.33071807427343	0.608636344157923	1	pseudobulk	DESeq2	LRT	0
323	KC_AB1	CG5577	0.849606887838128	1	1	pseudobulk	DESeq2	LRT	0
323	KC_AB1	CG5589	0.663546949070083	1	1	pseudobulk	DESeq2	LRT	0
323	KC_AB1	CG5590	0.0905099787911974	1	1	pseudobulk	DESeq2	LRT	0
323	KC_AB1	CG5599	0.621375926231566	1	1	pseudobulk	DESeq2	LRT	0
324	KC_AB1	CG5608	0.151446858253503	0.824930065706905	1	pseudobulk	DESeq2	LRT	0
324	KC_AB1	CG5611	0.461976580885506	0.340573623523855	1	pseudobulk	DESeq2	LRT	0
324	KC_AB1	CG5612	0.849606887838128	1	1	pseudobulk	DESeq2	LRT	0
324	KC_AB1	CG5618	-1.05915213103846	0.379972437950841	1	pseudobulk	DESeq2	LRT	0
324	KC_AB1	CG5641	-0.868280729363264	0.32721598604596	1	pseudobulk	DESeq2	LRT	0
324	KC_AB1	CG5644	0.471794339854754	0.360697386344124	1	pseudobulk	DESeq2	LRT	0
324	KC_AB1	CG5645	-0.10506771737906	1	1	pseudobulk	DESeq2	LRT	0
324	KC_AB1	CG5646	0.150704031971811	0.736421640278214	1	pseudobulk	DESeq2	LRT	0
324	KC_AB1	CG5662	0.849606887838128	1	1	pseudobulk	DESeq2	LRT	0
324	KC_AB1	CG5664	1.79590159336616	0.652741537999668	1	pseudobulk	DESeq2	LRT	0
325	KC_AB1	CG5674	0.461662601297517	0.262218823697622	1	pseudobulk	DESeq2	LRT	0
325	KC_AB1	CG5676	0.182920131165683	0.659488504068877	1	pseudobulk	DESeq2	LRT	0
325	KC_AB1	CG5704	-0.830062897938551	0.717724257445671	1	pseudobulk	DESeq2	LRT	0
325	KC_AB1	CG5708	-0.31954980894674	0.29781671410733	1	pseudobulk	DESeq2	LRT	0
325	KC_AB1	CG5721	-0.789384044587011	0.503594092373308	1	pseudobulk	DESeq2	LRT	0
325	KC_AB1	CG5726	-0.404749107621518	0.510149882161403	1	pseudobulk	DESeq2	LRT	0
325	KC_AB1	CG5727	-0.677224403386601	0.416940052192791	1	pseudobulk	DESeq2	LRT	0
325	KC_AB1	CG5728	0.784722815526686	0.721033624642002	1	pseudobulk	DESeq2	LRT	0
325	KC_AB1	CG5731	-0.216210029276812	1	1	pseudobulk	DESeq2	LRT	0
325	KC_AB1	CG5742	0.0011163984682659	0.74155459389216	1	pseudobulk	DESeq2	LRT	0
326	KC_AB1	CG5745	-1.0875569477506	0.278698769008849	1	pseudobulk	DESeq2	LRT	0
326	KC_AB1	CG5746	-0.0208219829645087	0.7694633533277	1	pseudobulk	DESeq2	LRT	0
326	KC_AB1	CG5758	-0.233022201675496	0.591909823753889	1	pseudobulk	DESeq2	LRT	0
326	KC_AB1	CG5776	1.17261808986456	0.859088728563237	1	pseudobulk	DESeq2	LRT	0
326	KC_AB1	CG5783	-0.830062897938551	0.717724257445671	1	pseudobulk	DESeq2	LRT	0
326	KC_AB1	CG5787	0.723961679810498	0.310588081644449	1	pseudobulk	DESeq2	LRT	0
326	KC_AB1	CG5789	0.103720425499334	0.916485611139835	1	pseudobulk	DESeq2	LRT	0
326	KC_AB1	CG5793	-0.151014623067717	0.889262136934981	1	pseudobulk	DESeq2	LRT	0
326	KC_AB1	CG5800	1.00335676338205	1	1	pseudobulk	DESeq2	LRT	0
326	KC_AB1	CG5805	-0.135059032301676	0.547840997986547	1	pseudobulk	DESeq2	LRT	0
327	KC_AB1	CG5808	0.355539821138042	0.956139963784331	1	pseudobulk	DESeq2	LRT	0
327	KC_AB1	CG5810	-0.216209988775614	1	1	pseudobulk	DESeq2	LRT	0
327	KC_AB1	CG5815	0.516467005704292	1	1	pseudobulk	DESeq2	LRT	0
327	KC_AB1	CG5823	-0.13009336424914	0.788252476880344	1	pseudobulk	DESeq2	LRT	0
327	KC_AB1	CG5828	0.369857515942518	0.565255048592717	1	pseudobulk	DESeq2	LRT	0
327	KC_AB1	CG5830	-0.207255398319265	0.144416275737349	1	pseudobulk	DESeq2	LRT	0
327	KC_AB1	CG5835	3.16697464674408	0.406679230261689	1	pseudobulk	DESeq2	LRT	0
327	KC_AB1	CG5846	0.501338251227562	0.759485072429182	1	pseudobulk	DESeq2	LRT	0
327	KC_AB1	CG5850	-0.620703826401051	0.542516212331537	1	pseudobulk	DESeq2	LRT	0
327	KC_AB1	CG5853	0.849606887838128	1	1	pseudobulk	DESeq2	LRT	0
328	KC_AB1	CG5854	-1.99642785165477	0.26261355435939	1	pseudobulk	DESeq2	LRT	0
328	KC_AB1	CG5861	0.318301947538487	0.387935621322638	1	pseudobulk	DESeq2	LRT	0
328	KC_AB1	CG5862	0.319150258133953	0.576608177671333	1	pseudobulk	DESeq2	LRT	0
328	KC_AB1	CG5867	4.58586740073107	0.0393089112468113	1	pseudobulk	DESeq2	LRT	0
328	KC_AB1	CG5877	0.892444107652462	0.585729295510941	1	pseudobulk	DESeq2	LRT	0

328	KC_AB1	CG5880	0.169315334809772	0.819589849438902	1	pseudobulk	DESeq2	LRT	0
328	KC_AB1	CG5885	0.23238539626459	0.722202556033417	1	pseudobulk	DESeq2	LRT	0
328	KC_AB1	CG5886	0.44088597771691	0.776504745815546	1	pseudobulk	DESeq2	LRT	0
328	KC_AB1	CG5888	-0.413177752081446	0.547526205419892	1	pseudobulk	DESeq2	LRT	0
328	KC_AB1	CG5890	-0.539598702140949	0.0838837219926904	1	pseudobulk	DESeq2	LRT	0
329	KC_AB1	CG5895	0.183159795455075	1	1	pseudobulk	DESeq2	LRT	0
329	KC_AB1	CG5902	0.247903634913126	0.569001458977557	1	pseudobulk	DESeq2	LRT	0
329	KC_AB1	CG5903	0.0117416724025477	0.940368352389745	1	pseudobulk	DESeq2	LRT	0
329	KC_AB1	CG5910	-2.38607821948916	0.279830879164901	1	pseudobulk	DESeq2	LRT	0
329	KC_AB1	CG5913	1.72376504323795	0.486313498520827	1	pseudobulk	DESeq2	LRT	0
329	KC_AB1	CG5916	0.366794636557981	0.483789114679051	1	pseudobulk	DESeq2	LRT	0
329	KC_AB1	CG5934	0.133809156783702	0.706772170847379	1	pseudobulk	DESeq2	LRT	0
329	KC_AB1	CG5937	0.065615240440178	0.694641841998663	1	pseudobulk	DESeq2	LRT	0
329	KC_AB1	CG5938	0.0330944602182526	0.677224207413813	1	pseudobulk	DESeq2	LRT	0
329	KC_AB1	CG5945	0.183159795455075	1	1	pseudobulk	DESeq2	LRT	0
330	KC_AB1	CG5946	-0.281769950382232	0.420235561519698	1	pseudobulk	DESeq2	LRT	0
330	KC_AB1	CG5953	-0.263426399461851	0.8458556303128	1	pseudobulk	DESeq2	LRT	0
330	KC_AB1	CG5955	2.31997030691033	0.813169507846918	1	pseudobulk	DESeq2	LRT	0
330	KC_AB1	CG5961	0.126232666621216	0.750778297712974	1	pseudobulk	DESeq2	LRT	0
330	KC_AB1	CG5968	0.279103127511873	0.827829212393648	1	pseudobulk	DESeq2	LRT	0
330	KC_AB1	CG5969	-0.0199595628199974	0.783719157709019	1	pseudobulk	DESeq2	LRT	0
330	KC_AB1	CG5973	-2.2782245289447	0.302721542053929	1	pseudobulk	DESeq2	LRT	0
330	KC_AB1	CG5986	-0.256740826002351	0.55607078652099	1	pseudobulk	DESeq2	LRT	0
330	KC_AB1	CG5989	-0.421956831073274	0.66875091632021	1	pseudobulk	DESeq2	LRT	0
330	KC_AB1	CG6000	2.31997030691033	0.813169507846918	1	pseudobulk	DESeq2	LRT	0
331	KC_AB1	CG6005	0.868662485418998	0.352109297352868	1	pseudobulk	DESeq2	LRT	0
331	KC_AB1	CG6006	-1.44344995550072	0.0641118920033433	1	pseudobulk	DESeq2	LRT	0
331	KC_AB1	CG6013	0.103266974134596	0.875141991317638	1	pseudobulk	DESeq2	LRT	0
331	KC_AB1	CG6024	-0.294531525729269	0.183463081848398	1	pseudobulk	DESeq2	LRT	0
331	KC_AB1	CG6028	0.0892830175955321	0.794359180488452	1	pseudobulk	DESeq2	LRT	0
331	KC_AB1	CG6034	0.116783351990007	1	1	pseudobulk	DESeq2	LRT	0
331	KC_AB1	CG6036	-0.216210032712384	1	1	pseudobulk	DESeq2	LRT	0
331	KC_AB1	CG6040	0.947040974337908	0.538760201210719	1	pseudobulk	DESeq2	LRT	0
331	KC_AB1	CG6041	-0.143780996856688	0.605186660168673	1	pseudobulk	DESeq2	LRT	0
331	KC_AB1	CG6044	0.0485316879459058	0.544110566053816	1	pseudobulk	DESeq2	LRT	0
332	KC_AB1	CG6051	-0.263684338856863	0.505172231659336	1	pseudobulk	DESeq2	LRT	0
332	KC_AB1	CG6055	2.78674512494791	0.54962276837928	1	pseudobulk	DESeq2	LRT	0
332	KC_AB1	CG6066	0.986161561286372	0.285737223248401	1	pseudobulk	DESeq2	LRT	0
332	KC_AB1	CG6083	-0.660222021119874	0.76330493518027	1	pseudobulk	DESeq2	LRT	0
332	KC_AB1	CG6084	0.120055169989382	1	1	pseudobulk	DESeq2	LRT	0
332	KC_AB1	CG6094	-0.481946358472159	0.670640315860944	1	pseudobulk	DESeq2	LRT	0
332	KC_AB1	CG6106	2.78674512494791	0.54962276837928	1	pseudobulk	DESeq2	LRT	0
332	KC_AB1	CG6115	0.157362709511082	0.494726033220743	1	pseudobulk	DESeq2	LRT	0
332	KC_AB1	CG6126	0.849606887838128	1	1	pseudobulk	DESeq2	LRT	0
332	KC_AB1	CG6136	-1.72076692992746	0.235281275964182	1	pseudobulk	DESeq2	LRT	0
333	KC_AB1	CG6144	0.241368384366625	0.659884659890155	1	pseudobulk	DESeq2	LRT	0
333	KC_AB1	CG6145	-0.98080144671863	0.225258401105259	1	pseudobulk	DESeq2	LRT	0
333	KC_AB1	CG6153	-1.16972781619816	0.42337935707319	1	pseudobulk	DESeq2	LRT	0
333	KC_AB1	CG6154	-0.419624007992571	0.175235004687616	1	pseudobulk	DESeq2	LRT	0
333	KC_AB1	CG6163	-1.39957980045653	0.360131653767529	1	pseudobulk	DESeq2	LRT	0
333	KC_AB1	CG6171	-0.177218370768558	0.750092236206269	1	pseudobulk	DESeq2	LRT	0
333	KC_AB1	CG6175	0.34400370901222	1	1	pseudobulk	DESeq2	LRT	0
333	KC_AB1	CG6178	0.671143894959203	0.522676668006706	1	pseudobulk	DESeq2	LRT	0

333	KC_AB1	CG6179	-0.496436264844756	0.540006522858396	1	pseudobulk	DESeq2	LRT	0
333	KC_AB1	CG6180	0.335454649593038	0.599189168313538	1	pseudobulk	DESeq2	LRT	0
334	KC_AB1	CG6184	-0.116439363317139	0.810551904305108	1	pseudobulk	DESeq2	LRT	0
334	KC_AB1	CG6191	-0.157370030122856	0.592401034710182	1	pseudobulk	DESeq2	LRT	0
334	KC_AB1	CG6195	0.059657553511817	0.774539753129579	1	pseudobulk	DESeq2	LRT	0
334	KC_AB1	CG6201	-0.191247834940026	0.607343641044783	1	pseudobulk	DESeq2	LRT	0
334	KC_AB1	CG6204	0.217539260035621	0.820468157179961	1	pseudobulk	DESeq2	LRT	0
334	KC_AB1	CG6218	0.631984369835012	0.44526804136037	1	pseudobulk	DESeq2	LRT	0
334	KC_AB1	CG6220	-0.790596054643963	0.667058293819746	1	pseudobulk	DESeq2	LRT	0
334	KC_AB1	CG6227	-0.666849538061765	0.556660160725727	1	pseudobulk	DESeq2	LRT	0
334	KC_AB1	CG6230	0.842165294701663	0.35183808438153	1	pseudobulk	DESeq2	LRT	0
334	KC_AB1	CG6231	-2.13305419425166	0.213741366541797	1	pseudobulk	DESeq2	LRT	0
335	KC_AB1	CG6236	0.865929810762137	0.311539983732877	1	pseudobulk	DESeq2	LRT	0
335	KC_AB1	CG6254	-0.139272420603192	0.775439086606276	1	pseudobulk	DESeq2	LRT	0
335	KC_AB1	CG6276	0.211694735834364	0.637505593228245	1	pseudobulk	DESeq2	LRT	0
335	KC_AB1	CG6282	-0.253386542580322	0.286004798715525	1	pseudobulk	DESeq2	LRT	0
335	KC_AB1	CG6287	-1.17107835973552	0.481260770148885	1	pseudobulk	DESeq2	LRT	0
335	KC_AB1	CG6293	0.194265322970028	1	1	pseudobulk	DESeq2	LRT	0
335	KC_AB1	CG6294	0.849606887838128	1	1	pseudobulk	DESeq2	LRT	0
335	KC_AB1	CG6310	0.849606887838128	1	1	pseudobulk	DESeq2	LRT	0
335	KC_AB1	CG6324	2.78674512494791	0.54962276837928	1	pseudobulk	DESeq2	LRT	0
335	KC_AB1	CG6325	0.550271586200908	0.813213385153984	1	pseudobulk	DESeq2	LRT	0
336	KC_AB1	CG6329	-0.163152104355914	0.34639656148441	1	pseudobulk	DESeq2	LRT	0
336	KC_AB1	CG6330	0.677680454555987	0.166530713315579	1	pseudobulk	DESeq2	LRT	0
336	KC_AB1	CG6340	-0.0344265383412025	0.800608236915278	1	pseudobulk	DESeq2	LRT	0
336	KC_AB1	CG6345	0.366247714696288	1	1	pseudobulk	DESeq2	LRT	0
336	KC_AB1	CG6356	0.99846836156641	0.257629039864688	1	pseudobulk	DESeq2	LRT	0
336	KC_AB1	CG6357	2.12815897200743	0.092413595075169	1	pseudobulk	DESeq2	LRT	0
336	KC_AB1	CG6362	1.11477144852983	1	1	pseudobulk	DESeq2	LRT	0
336	KC_AB1	CG6379	-0.0541327652206273	0.716557228351842	1	pseudobulk	DESeq2	LRT	0
336	KC_AB1	CG6388	2.43834171605157	0.344901026596326	1	pseudobulk	DESeq2	LRT	0
336	KC_AB1	CG6398	1.65792752010766	0.762646744542806	1	pseudobulk	DESeq2	LRT	0
337	KC_AB1	CG6418	-1.51360252890022	0.172145551966528	1	pseudobulk	DESeq2	LRT	0
337	KC_AB1	CG6420	-0.380796726797656	0.536881567144831	1	pseudobulk	DESeq2	LRT	0
337	KC_AB1	CG6421	0.849606887838128	1	1	pseudobulk	DESeq2	LRT	0
337	KC_AB1	CG6424	0.849606887838128	1	1	pseudobulk	DESeq2	LRT	0
337	KC_AB1	CG6425	-0.0938530176988777	1	1	pseudobulk	DESeq2	LRT	0
337	KC_AB1	CG6426	-0.617233250411185	0.726931347358628	1	pseudobulk	DESeq2	LRT	0
337	KC_AB1	CG6428	0.78628050381877	0.388302471759486	1	pseudobulk	DESeq2	LRT	0
337	KC_AB1	CG6429	-0.287567257126996	0.920650037231581	1	pseudobulk	DESeq2	LRT	0
337	KC_AB1	CG6435	-0.704858387530061	0.698842745464243	1	pseudobulk	DESeq2	LRT	0
337	KC_AB1	CG6443	-0.179993592297172	0.601349583512527	1	pseudobulk	DESeq2	LRT	0
338	KC_AB1	CG6454	-0.310910920023929	0.51223482274654	1	pseudobulk	DESeq2	LRT	0
338	KC_AB1	CG6465	2.6840296908559	0.560387737355481	1	pseudobulk	DESeq2	LRT	0
338	KC_AB1	CG6472	-1.2252782056983	0.286158363588489	1	pseudobulk	DESeq2	LRT	0
338	KC_AB1	CG6479	-0.376823193002063	0.746583230189694	1	pseudobulk	DESeq2	LRT	0
338	KC_AB1	CG6481	2.13004336645676	0.828063380524205	1	pseudobulk	DESeq2	LRT	0
338	KC_AB1	CG6495	0.360651492100724	0.474135699006015	1	pseudobulk	DESeq2	LRT	0
338	KC_AB1	CG6503	0.849606887838128	1	1	pseudobulk	DESeq2	LRT	0
338	KC_AB1	CG6511	-0.509594232313236	0.640192906989619	1	pseudobulk	DESeq2	LRT	0
338	KC_AB1	CG6512	0.0241123043374371	0.59464296452777	1	pseudobulk	DESeq2	LRT	0
338	KC_AB1	CG6523	0.182602874826908	0.617135709357386	1	pseudobulk	DESeq2	LRT	0
339	KC_AB1	CG6550	-0.0182703573405389	1	1	pseudobulk	DESeq2	LRT	0

339	KC_AB1	CG6565	0.361605585504236	0.803728142315178	1	pseudobulk	DESeq2	LRT		0
339	KC_AB1	CG6567	-1.28509776297998	0.260493138982531	1	pseudobulk	DESeq2	LRT		0
339	KC_AB1	CG6574	0.725979373215586	0.619584448243789	1	pseudobulk	DESeq2	LRT		0
339	KC_AB1	CG6583	-0.48662383767307	0.567781103885782	1	pseudobulk	DESeq2	LRT		0
339	KC_AB1	CG6607	-0.0815512614261973	0.690031116950787	1	pseudobulk	DESeq2	LRT		0
339	KC_AB1	CG6610	-0.246735869871477	0.774680178118884	1	pseudobulk	DESeq2	LRT		0
339	KC_AB1	CG6617	-0.299602931678785	0.566319630948307	1	pseudobulk	DESeq2	LRT		0
339	KC_AB1	CG6621	-0.0752114657333862	0.685532079178268	1	pseudobulk	DESeq2	LRT		0
339	KC_AB1	CG6628	1.59863986092676	1	1	pseudobulk	DESeq2	LRT		0
340	KC_AB1	CG6638	0.849606887838128	1	1	pseudobulk	DESeq2	LRT		0
340	KC_AB1	CG6650	0.19732597505578	1	1	pseudobulk	DESeq2	LRT		0
340	KC_AB1	CG6654	0.713780324542466	1	1	pseudobulk	DESeq2	LRT		0
340	KC_AB1	CG6656	0.849606887838128	1	1	pseudobulk	DESeq2	LRT		0
340	KC_AB1	CG6659	-0.989774321347864	0.289826198824118	1	pseudobulk	DESeq2	LRT		0
340	KC_AB1	CG6660	2.06541384650887	0.768363369045814	1	pseudobulk	DESeq2	LRT		0
340	KC_AB1	CG6664	-0.171298066152838	0.76860383750317	1	pseudobulk	DESeq2	LRT		0
340	KC_AB1	CG6665	-0.565398977015703	0.540915443973664	1	pseudobulk	DESeq2	LRT		0
340	KC_AB1	CG6674	0.147223882242023	0.779865329562851	1	pseudobulk	DESeq2	LRT		0
340	KC_AB1	CG6675	0.183159795455075	1	1	pseudobulk	DESeq2	LRT		0
341	KC_AB1	CG6683	0.849606887838128	1	1	pseudobulk	DESeq2	LRT		0
341	KC_AB1	CG6685	-0.571429007446914	0.614695562031428	1	pseudobulk	DESeq2	LRT		0
341	KC_AB1	CG6686	0.584281479648394	0.242928531660284	1	pseudobulk	DESeq2	LRT		0
341	KC_AB1	CG6688	2.78674512494791	0.54962276837928	1	pseudobulk	DESeq2	LRT		0
341	KC_AB1	CG6689	0.256044423111992	0.834847376799848	1	pseudobulk	DESeq2	LRT		0
341	KC_AB1	CG6693	0.934605642402758	0.273431907723351	1	pseudobulk	DESeq2	LRT		0
341	KC_AB1	CG6695	-1.03503607729309	0.43158503998701	1	pseudobulk	DESeq2	LRT		0
341	KC_AB1	CG6700	-0.0528829782563996	0.792297219914321	1	pseudobulk	DESeq2	LRT		0
341	KC_AB1	CG6701	0.849606887838128	1	1	pseudobulk	DESeq2	LRT		0
341	KC_AB1	CG6707	0.261055165979634	0.475450233953154	1	pseudobulk	DESeq2	LRT		0
342	KC_AB1	CG6712	1.31276386626902	0.49826796805415	1	pseudobulk	DESeq2	LRT		0
342	KC_AB1	CG6723	1.40964799290255	0.148737417705842	1	pseudobulk	DESeq2	LRT		0
342	KC_AB1	CG6724	-0.0811946788699932	0.829707952505221	1	pseudobulk	DESeq2	LRT		0
342	KC_AB1	CG6729	0.0120199596703158	0.887878864316488	1	pseudobulk	DESeq2	LRT		0
342	KC_AB1	CG6733	0.849606887838128	1	1	pseudobulk	DESeq2	LRT		0
342	KC_AB1	CG6749	3.41844045086015	0.351942490385471	1	pseudobulk	DESeq2	LRT		0
342	KC_AB1	CG6752	-0.29646208184655	0.696768563617141	1	pseudobulk	DESeq2	LRT		0
342	KC_AB1	CG6758	-0.303537808102992	0.691989319934164	1	pseudobulk	DESeq2	LRT		0
342	KC_AB1	CG6762	-0.289302406251966	0.691327746045313	1	pseudobulk	DESeq2	LRT		0
342	KC_AB1	CG6763	1.59863986092676	1	1	pseudobulk	DESeq2	LRT		0
343	KC_AB1	CG6765	0.249260049985002	0.481675077496959	1	pseudobulk	DESeq2	LRT		0
343	KC_AB1	CG6766	-0.305521931157336	0.613366983117947	1	pseudobulk	DESeq2	LRT		0
343	KC_AB1	CG6767	0.209898831263243	0.231365487907582	1	pseudobulk	DESeq2	LRT		0
343	KC_AB1	CG6769	0.0958592682341114	0.738994821489641	1	pseudobulk	DESeq2	LRT		0
343	KC_AB1	CG6770	-0.805586296218808	0.236942521972861	1	pseudobulk	DESeq2	LRT		0
343	KC_AB1	CG6791	-0.0927420408330473	0.594382362242769	1	pseudobulk	DESeq2	LRT		0
343	KC_AB1	CG6805	-0.81540684809366	0.479914160511942	1	pseudobulk	DESeq2	LRT		0
343	KC_AB1	CG6808	1.64453392304166	0.483247631368695	1	pseudobulk	DESeq2	LRT		0
343	KC_AB1	CG6813	-0.701290191993194	0.529539285459567	1	pseudobulk	DESeq2	LRT		0
343	KC_AB1	CG6833	0.664006856592019	0.673578116109923	1	pseudobulk	DESeq2	LRT		0
344	KC_AB1	CG6834	0.849606887838128	1	1	pseudobulk	DESeq2	LRT		0
344	KC_AB1	CG6836	0.249609997857266	1	1	pseudobulk	DESeq2	LRT		0
344	KC_AB1	CG6841	0.639775652996716	0.404193620029005	1	pseudobulk	DESeq2	LRT		0
344	KC_AB1	CG6843	-0.188688718073871	0.714784341278584	1	pseudobulk	DESeq2	LRT		0

344	KC_AB1	CG6847	0.915380531920192	1	1	pseudobulk	DESeq2	LRT	0
344	KC_AB1	CG6867	-0.379041322293851	0.366827977889495	1	pseudobulk	DESeq2	LRT	0
344	KC_AB1	CG6878	-0.155837625974543	0.815120669980052	1	pseudobulk	DESeq2	LRT	0
344	KC_AB1	CG6903	0.463723805331401	0.534718011641052	1	pseudobulk	DESeq2	LRT	0
344	KC_AB1	CG6907	0.535626103983383	1	1	pseudobulk	DESeq2	LRT	0
344	KC_AB1	CG6908	-1.42552888527949	0.502705832947673	1	pseudobulk	DESeq2	LRT	0
345	KC_AB1	CG6910	2.31997030691033	0.813169507846918	1	pseudobulk	DESeq2	LRT	0
345	KC_AB1	CG6912	-0.119679228631457	0.952170065394095	1	pseudobulk	DESeq2	LRT	0
345	KC_AB1	CG6923	0.219566752586292	0.715480973871307	1	pseudobulk	DESeq2	LRT	0
345	KC_AB1	CG6928	-0.485027100453874	0.813470998077292	1	pseudobulk	DESeq2	LRT	0
345	KC_AB1	CG6937	0.109984153126441	0.922429741903205	1	pseudobulk	DESeq2	LRT	0
345	KC_AB1	CG6938	0.784722815526686	0.721033624642002	1	pseudobulk	DESeq2	LRT	0
345	KC_AB1	CG6951	0.930993226399931	1	1	pseudobulk	DESeq2	LRT	0
345	KC_AB1	CG6959	-1.55348476958828	0.370893969093652	1	pseudobulk	DESeq2	LRT	0
345	KC_AB1	CG6961	-1.11733789562429	0.366773871181774	1	pseudobulk	DESeq2	LRT	0
345	KC_AB1	CG6962	0.183159795463042	1	1	pseudobulk	DESeq2	LRT	0
346	KC_AB1	CG6966	0.180930293248982	0.558096080581847	1	pseudobulk	DESeq2	LRT	0
346	KC_AB1	CG6983	0.0290204980641629	0.691587521198445	1	pseudobulk	DESeq2	LRT	0
346	KC_AB1	CG6984	1.36467083488334	0.483716252213573	1	pseudobulk	DESeq2	LRT	0
346	KC_AB1	CG7006	1.9163124448524	0.436714599119659	1	pseudobulk	DESeq2	LRT	0
346	KC_AB1	CG7009	-0.52276710845623	0.633918433950265	1	pseudobulk	DESeq2	LRT	0
346	KC_AB1	CG7011	1.58900263029462	0.213408601226318	1	pseudobulk	DESeq2	LRT	0
346	KC_AB1	CG7016	2.78674512494791	0.54962276837928	1	pseudobulk	DESeq2	LRT	0
346	KC_AB1	CG7028	-0.0263251351604122	0.601015630781568	1	pseudobulk	DESeq2	LRT	0
346	KC_AB1	CG7029	0.0662301134191828	0.840938920090556	1	pseudobulk	DESeq2	LRT	0
346	KC_AB1	CG7044	-0.404469744007938	0.734792506510936	1	pseudobulk	DESeq2	LRT	0
347	KC_AB1	CG7049	-0.30025623092474	0.671781597820943	1	pseudobulk	DESeq2	LRT	0
347	KC_AB1	CG7058	-0.271596358119598	0.631247912919425	1	pseudobulk	DESeq2	LRT	0
347	KC_AB1	CG7059	-1.25373709335668	0.231667122595412	1	pseudobulk	DESeq2	LRT	0
347	KC_AB1	CG7065	-0.46749075755577	0.391539554594352	1	pseudobulk	DESeq2	LRT	0
347	KC_AB1	CG7069	-1.55822740538383	0.329749923663552	1	pseudobulk	DESeq2	LRT	0
347	KC_AB1	CG7083	-0.802982227882136	0.443992713001294	1	pseudobulk	DESeq2	LRT	0
347	KC_AB1	CG7084	-0.204776607228549	0.847140528761976	1	pseudobulk	DESeq2	LRT	0
347	KC_AB1	CG7099	0.642247207535423	0.825888865965155	1	pseudobulk	DESeq2	LRT	0
347	KC_AB1	CG7101	-0.731520070204393	0.540619210860351	1	pseudobulk	DESeq2	LRT	0
347	KC_AB1	CG7102	0.849606887838128	1	1	pseudobulk	DESeq2	LRT	0
348	KC_AB1	CG7110	-0.651372869174996	0.602712888982485	1	pseudobulk	DESeq2	LRT	0
348	KC_AB1	CG7115	-0.151483352978127	0.761906211821007	1	pseudobulk	DESeq2	LRT	0
348	KC_AB1	CG7120	0.0241133655614756	0.825640100595173	1	pseudobulk	DESeq2	LRT	0
348	KC_AB1	CG7133	0.157584630065768	0.740584622610382	1	pseudobulk	DESeq2	LRT	0
348	KC_AB1	CG7137	-0.216210032712384	1	1	pseudobulk	DESeq2	LRT	0
348	KC_AB1	CG7139	0.0894839530968835	0.709958221783539	1	pseudobulk	DESeq2	LRT	0
348	KC_AB1	CG7148	0.670440146922972	0.414205761129692	1	pseudobulk	DESeq2	LRT	0
348	KC_AB1	CG7149	0.0392991766086434	0.781940989768464	1	pseudobulk	DESeq2	LRT	0
348	KC_AB1	CG7154	0.0408351730719896	0.845221121281835	1	pseudobulk	DESeq2	LRT	0
348	KC_AB1	CG7156	-0.839225435699114	0.522367976923168	1	pseudobulk	DESeq2	LRT	0
349	KC_AB1	CG7166	-0.179553454832092	0.509324271432015	1	pseudobulk	DESeq2	LRT	0
349	KC_AB1	CG7168	-0.32329366788109	0.664654511361055	1	pseudobulk	DESeq2	LRT	0
349	KC_AB1	CG7182	0.175694336818655	0.553084267981781	1	pseudobulk	DESeq2	LRT	0
349	KC_AB1	CG7183	-0.0615519787821832	0.797556787115986	1	pseudobulk	DESeq2	LRT	0
349	KC_AB1	CG7203	-0.689854829609766	0.509913759587427	1	pseudobulk	DESeq2	LRT	0
349	KC_AB1	CG7206	-0.447517436568423	0.55587120776732	1	pseudobulk	DESeq2	LRT	0
349	KC_AB1	CG7218	-0.20141395660648	0.736627243024765	1	pseudobulk	DESeq2	LRT	0

349	KC_AB1	CG7220	-0.308170232840266	0.473906932630278	1	pseudobulk	DESeq2	LRT		0
349	KC_AB1	CG7222	0.0002738265238723	0.648532412325048	1	pseudobulk	DESeq2	LRT		0
349	KC_AB1	CG7231	0.374716147517451	0.498461861819191	1	pseudobulk	DESeq2	LRT		0
350	KC_AB1	CG7239	0.0034774453540518	0.807944574842781	1	pseudobulk	DESeq2	LRT		0
350	KC_AB1	CG7246	1.59863986092676	1	1	pseudobulk	DESeq2	LRT		0
350	KC_AB1	CG7265	2.13009086641515	0.718583316467961	1	pseudobulk	DESeq2	LRT		0
350	KC_AB1	CG7272	0.24096706419244	0.80163454659661	1	pseudobulk	DESeq2	LRT		0
350	KC_AB1	CG7275	0.951993753379764	0.858943944621224	1	pseudobulk	DESeq2	LRT		0
350	KC_AB1	CG7289	-0.138825576555217	0.725383685112146	1	pseudobulk	DESeq2	LRT		0
350	KC_AB1	CG7324	-0.303784745950172	0.494628042564798	1	pseudobulk	DESeq2	LRT		0
350	KC_AB1	CG7326	-0.756268456112326	0.42062922162757	1	pseudobulk	DESeq2	LRT		0
350	KC_AB1	CG7328	0.849606887838128	1	1	pseudobulk	DESeq2	LRT		0
350	KC_AB1	CG7330	2.78674512494791	0.54962276837928	1	pseudobulk	DESeq2	LRT		0
351	KC_AB1	CG7332	0.44947688459546	0.272589435365519	1	pseudobulk	DESeq2	LRT		0
351	KC_AB1	CG7339	-0.563948576817788	0.573655027344847	1	pseudobulk	DESeq2	LRT		0
351	KC_AB1	CG7341	0.849606887838128	1	1	pseudobulk	DESeq2	LRT		0
351	KC_AB1	CG7367	-0.473533893544948	0.839148280595286	1	pseudobulk	DESeq2	LRT		0
351	KC_AB1	CG7368	0.474974006264443	0.49312715149469	1	pseudobulk	DESeq2	LRT		0
351	KC_AB1	CG7369	-0.413945375555986	0.550775326990549	1	pseudobulk	DESeq2	LRT		0
351	KC_AB1	CG7370	0.849606887838128	1	1	pseudobulk	DESeq2	LRT		0
351	KC_AB1	CG7376	-0.960022699105854	0.610500321913265	1	pseudobulk	DESeq2	LRT		0
351	KC_AB1	CG7378	-0.196536579656225	0.252028085051059	1	pseudobulk	DESeq2	LRT		0
351	KC_AB1	CG7379	-0.206334306722364	0.619271562271623	1	pseudobulk	DESeq2	LRT		0
352	KC_AB1	CG7381	1.91040834498725	0.517599141821231	1	pseudobulk	DESeq2	LRT		0
352	KC_AB1	CG7382	0.187730937159676	0.773753069143406	1	pseudobulk	DESeq2	LRT		0
352	KC_AB1	CG7394	-0.164881137384125	0.549414406545241	1	pseudobulk	DESeq2	LRT		0
352	KC_AB1	CG7406	0.849606887838128	1	1	pseudobulk	DESeq2	LRT		0
352	KC_AB1	CG7407	-0.319936340975922	0.327611700675874	1	pseudobulk	DESeq2	LRT		0
352	KC_AB1	CG7409	-0.0065790798335215	1	1	pseudobulk	DESeq2	LRT		0
352	KC_AB1	CG7414	0.248786500702852	0.53627360745752	1	pseudobulk	DESeq2	LRT		0
352	KC_AB1	CG7420	1.51280379024899	0.85518877994987	1	pseudobulk	DESeq2	LRT		0
352	KC_AB1	CG7430	0.0520380892894326	0.886385123162268	1	pseudobulk	DESeq2	LRT		0
352	KC_AB1	CG7453	0.0775148353914581	0.710748851267012	1	pseudobulk	DESeq2	LRT		0
353	KC_AB1	CG7460	0.849606887838128	1	1	pseudobulk	DESeq2	LRT		0
353	KC_AB1	CG7461	1.13126142658608	0.205933423729251	1	pseudobulk	DESeq2	LRT		0
353	KC_AB1	CG7483	0.189403622612212	0.719515959495432	1	pseudobulk	DESeq2	LRT		0
353	KC_AB1	CG7484	-0.0895392822825802	0.725092610839541	1	pseudobulk	DESeq2	LRT		0
353	KC_AB1	CG7488	-0.489521898985821	0.655114415024516	1	pseudobulk	DESeq2	LRT		0
353	KC_AB1	CG7492	-0.36538446448863	0.403804828774142	1	pseudobulk	DESeq2	LRT		0
353	KC_AB1	CG7497	-1.97244306741341	0.361136320499796	1	pseudobulk	DESeq2	LRT		0
353	KC_AB1	CG7504	-0.865573757760995	0.508332417439105	1	pseudobulk	DESeq2	LRT		0
353	KC_AB1	CG7506	0.611080929256583	0.389816803844155	1	pseudobulk	DESeq2	LRT		0
353	KC_AB1	CG7509	0.366247714696288	1	1	pseudobulk	DESeq2	LRT		0
354	KC_AB1	CG7518	0.0699715504716659	0.627556324059999	1	pseudobulk	DESeq2	LRT		0
354	KC_AB1	CG7519	0.485816130152614	0.509993838792444	1	pseudobulk	DESeq2	LRT		0
354	KC_AB1	CG7523	-0.679547270234905	0.441247744987069	1	pseudobulk	DESeq2	LRT		0
354	KC_AB1	CG7530	0.116783351990007	1	1	pseudobulk	DESeq2	LRT		0
354	KC_AB1	CG7536	-0.660222021119874	0.76330493518027	1	pseudobulk	DESeq2	LRT		0
354	KC_AB1	CG7546	-0.322750205072392	0.500883154633796	1	pseudobulk	DESeq2	LRT		0
354	KC_AB1	CG7550	2.59089838995353	0.105014195913749	1	pseudobulk	DESeq2	LRT		0
354	KC_AB1	CG7556	-0.583894416762585	0.50137149296218	1	pseudobulk	DESeq2	LRT		0
354	KC_AB1	CG7560	2.31997030691033	0.813169507846918	1	pseudobulk	DESeq2	LRT		0
354	KC_AB1	CG7564	0.191611090426094	0.673369760998238	1	pseudobulk	DESeq2	LRT		0

355	KC_AB1	CG7565	-0.31356279699028	0.573788426315593	1	pseudobulk	DESeq2	LRT		0
355	KC_AB1	CG7568	-0.40200137056069	0.861588439126694	1	pseudobulk	DESeq2	LRT		0
355	KC_AB1	CG7582	0.475503089195862	0.228483758850192	1	pseudobulk	DESeq2	LRT		0
355	KC_AB1	CG7600	-0.412939201124699	0.570684847628813	1	pseudobulk	DESeq2	LRT		0
355	KC_AB1	CG7601	0.594040568208585	0.523609308979755	1	pseudobulk	DESeq2	LRT		0
355	KC_AB1	CG7607	-0.533669627141449	0.665097697502647	1	pseudobulk	DESeq2	LRT		0
355	KC_AB1	CG7611	-0.815155487501464	0.20716656888686	1	pseudobulk	DESeq2	LRT		0
355	KC_AB1	CG7627	2.31997030691033	0.813169507846918	1	pseudobulk	DESeq2	LRT		0
355	KC_AB1	CG7630	0.0541055263943913	0.516140050195039	1	pseudobulk	DESeq2	LRT		0
355	KC_AB1	CG7632	0.372009473000098	0.868292850410423	1	pseudobulk	DESeq2	LRT		0
356	KC_AB1	CG7637	-0.448728261972547	0.518954992108642	1	pseudobulk	DESeq2	LRT		0
356	KC_AB1	CG7638	-0.709309549611561	0.374566914994772	1	pseudobulk	DESeq2	LRT		0
356	KC_AB1	CG7639	-0.329347762048144	0.494693591270565	1	pseudobulk	DESeq2	LRT		0
356	KC_AB1	CG7646	-0.0357495395640013	0.687590504063692	1	pseudobulk	DESeq2	LRT		0
356	KC_AB1	CG7650	0.189218952035872	0.879649129164866	1	pseudobulk	DESeq2	LRT		0
356	KC_AB1	CG7655	-0.290722332707448	0.74235465815119	1	pseudobulk	DESeq2	LRT		0
356	KC_AB1	CG7656	0.235415623446426	0.642202646892077	1	pseudobulk	DESeq2	LRT		0
356	KC_AB1	CG7668	-1.6551278205788	0.211928715953546	1	pseudobulk	DESeq2	LRT		0
356	KC_AB1	CG7685	-0.704733842259958	0.538550033927944	1	pseudobulk	DESeq2	LRT		0
356	KC_AB1	CG7692	-0.521394547461525	0.668933627786741	1	pseudobulk	DESeq2	LRT		0
357	KC_AB1	CG7694	-0.0953281895039817	0.678428325307741	1	pseudobulk	DESeq2	LRT		0
357	KC_AB1	CG7695	0.849606887838128	1	1	pseudobulk	DESeq2	LRT		0
357	KC_AB1	CG7702	2.13004336645676	0.828063380524205	1	pseudobulk	DESeq2	LRT		0
357	KC_AB1	CG7706	-0.239780172088155	0.705087702370901	1	pseudobulk	DESeq2	LRT		0
357	KC_AB1	CG7718	0.85879998658552	1	1	pseudobulk	DESeq2	LRT		0
357	KC_AB1	CG7720	0.237022465848507	1	1	pseudobulk	DESeq2	LRT		0
357	KC_AB1	CG7728	0.0508343163501531	1	1	pseudobulk	DESeq2	LRT		0
357	KC_AB1	CG7737	0.582325939327861	0.558636477357695	1	pseudobulk	DESeq2	LRT		0
357	KC_AB1	CG7739	0.925455034537101	0.0114040454599478	1	pseudobulk	DESeq2	LRT		0
357	KC_AB1	CG7741	-0.212798871652652	0.744828897640517	1	pseudobulk	DESeq2	LRT		0
358	KC_AB1	CG7744	0.285480299679657	1	1	pseudobulk	DESeq2	LRT		0
358	KC_AB1	CG7745	0.0684837128612693	0.800985713105111	1	pseudobulk	DESeq2	LRT		0
358	KC_AB1	CG7747	0.72189878846513	0.521051952343793	1	pseudobulk	DESeq2	LRT		0
358	KC_AB1	CG7766	-0.198759749289324	0.496960841798979	1	pseudobulk	DESeq2	LRT		0
358	KC_AB1	CG7772	0.13114963848637	0.449059254252833	1	pseudobulk	DESeq2	LRT		0
358	KC_AB1	CG7778	1.58205617221276	1	1	pseudobulk	DESeq2	LRT		0
358	KC_AB1	CG7781	0.0928644433132214	0.502868290647908	1	pseudobulk	DESeq2	LRT		0
358	KC_AB1	CG7785	0.429293269034772	0.58922730965342	1	pseudobulk	DESeq2	LRT		0
358	KC_AB1	CG7789	0.0475433354220633	0.669287308640914	1	pseudobulk	DESeq2	LRT		0
358	KC_AB1	CG7791	0.524362380888343	0.441721394150638	1	pseudobulk	DESeq2	LRT		0
359	KC_AB1	CG7806	-0.154018274646341	0.78387295351941	1	pseudobulk	DESeq2	LRT		0
359	KC_AB1	CG7810	-0.0549679299711188	0.767843327093635	1	pseudobulk	DESeq2	LRT		0
359	KC_AB1	CG7834	-0.308845655738107	0.627664079186866	1	pseudobulk	DESeq2	LRT		0
359	KC_AB1	CG7839	0.695027241997098	0.633434027710684	1	pseudobulk	DESeq2	LRT		0
359	KC_AB1	CG7840	-1.31054743783693	0.404491325240096	1	pseudobulk	DESeq2	LRT		0
359	KC_AB1	CG7841	0.184083593520607	0.745668334172138	1	pseudobulk	DESeq2	LRT		0
359	KC_AB1	CG7849	1.82422053292417	0.606839105763612	1	pseudobulk	DESeq2	LRT		0
359	KC_AB1	CG7857	-0.427844997302807	0.582436496662761	1	pseudobulk	DESeq2	LRT		0
359	KC_AB1	CG7860	-0.216210029276812	1	1	pseudobulk	DESeq2	LRT		0
359	KC_AB1	CG7872	-0.603166950342568	0.489538508202515	1	pseudobulk	DESeq2	LRT		0
360	KC_AB1	CG7878	-0.981111779163717	0.303272354646458	1	pseudobulk	DESeq2	LRT		0
360	KC_AB1	CG7879	-0.0282812644753762	0.742898634517157	1	pseudobulk	DESeq2	LRT		0
360	KC_AB1	CG7884	-0.216210029276812	1	1	pseudobulk	DESeq2	LRT		0

360	KC_AB1	CG7888	0.202708685287469	0.733917844727674	1	pseudobulk	DESeq2	LRT		0
360	KC_AB1	CG7889	-1.0425838022098	0.492607663805008	1	pseudobulk	DESeq2	LRT		0
360	KC_AB1	CG7900	0.849606887838128	1	1	pseudobulk	DESeq2	LRT		0
360	KC_AB1	CG7903	-0.0619030984163493	0.892149470220513	1	pseudobulk	DESeq2	LRT		0
360	KC_AB1	CG7920	-0.0796900762620367	0.437227473492653	1	pseudobulk	DESeq2	LRT		0
360	KC_AB1	CG7943	0.314703846208993	0.558780175951058	1	pseudobulk	DESeq2	LRT		0
360	KC_AB1	CG7945	0.437582067894605	0.279308966521599	1	pseudobulk	DESeq2	LRT		0
361	KC_AB1	CG7946	0.40318615652177	0.617765256868686	1	pseudobulk	DESeq2	LRT		0
361	KC_AB1	CG7956	-0.0768465466164586	0.625933255728761	1	pseudobulk	DESeq2	LRT		0
361	KC_AB1	CG7966	2.31997030691033	0.813169507846918	1	pseudobulk	DESeq2	LRT		0
361	KC_AB1	CG7970	0.441166876623851	0.754818494814619	1	pseudobulk	DESeq2	LRT		0
361	KC_AB1	CG7971	-0.0581565213517264	0.622493511607361	1	pseudobulk	DESeq2	LRT		0
361	KC_AB1	CG7974	0.933465239592238	0.286485732354642	1	pseudobulk	DESeq2	LRT		0
361	KC_AB1	CG7985	-0.48216365439984	0.0410126020229071	1	pseudobulk	DESeq2	LRT		0
361	KC_AB1	CG7987	0.176742560457042	0.602134333184611	1	pseudobulk	DESeq2	LRT		0
361	KC_AB1	CG7988	1.32705300980944	0.474472565715158	1	pseudobulk	DESeq2	LRT		0
361	KC_AB1	CG7990	-0.113159023485666	0.55172307477192	1	pseudobulk	DESeq2	LRT		0
362	KC_AB1	CG7991	2.68453167034132	0.550970543274	1	pseudobulk	DESeq2	LRT		0
362	KC_AB1	CG7997	2.31997030691033	0.813169507846918	1	pseudobulk	DESeq2	LRT		0
362	KC_AB1	CG8001	0.405403724917295	0.654025215380071	1	pseudobulk	DESeq2	LRT		0
362	KC_AB1	CG8003	0.384363550905579	0.680815161766565	1	pseudobulk	DESeq2	LRT		0
362	KC_AB1	CG8004	-0.602552646810865	0.30380803054181	1	pseudobulk	DESeq2	LRT		0
362	KC_AB1	CG8005	-0.267798911929862	1	1	pseudobulk	DESeq2	LRT		0
362	KC_AB1	CG8008	-1.5805998677877	0.524230026009756	1	pseudobulk	DESeq2	LRT		0
362	KC_AB1	CG8009	0.154215052195836	0.646060471877168	1	pseudobulk	DESeq2	LRT		0
362	KC_AB1	CG8010	0.20700602091169	0.780416329116034	1	pseudobulk	DESeq2	LRT		0
362	KC_AB1	CG8012	0.069173631314144	0.912285568367581	1	pseudobulk	DESeq2	LRT		0
363	KC_AB1	CG8027	0.304204963197816	0.697073339297826	1	pseudobulk	DESeq2	LRT		0
363	KC_AB1	CG8031	-1.26987746117219	0.328084771459587	1	pseudobulk	DESeq2	LRT		0
363	KC_AB1	CG8032	0.48111578202157	0.51316856737437	1	pseudobulk	DESeq2	LRT		0
363	KC_AB1	CG8034	0.70001595141736	0.912466360179237	1	pseudobulk	DESeq2	LRT		0
363	KC_AB1	CG8036	0.444559027168278	1	1	pseudobulk	DESeq2	LRT		0
363	KC_AB1	CG8038	1.06572926637885	1	1	pseudobulk	DESeq2	LRT		0
363	KC_AB1	CG8042	0.474206638292796	0.516711471296796	1	pseudobulk	DESeq2	LRT		0
363	KC_AB1	CG8043	1.41457185224298	0.484927033144171	1	pseudobulk	DESeq2	LRT		0
363	KC_AB1	CG8060	0.844106062059195	0.28071882812087	1	pseudobulk	DESeq2	LRT		0
363	KC_AB1	CG8064	0.880742696173337	1	1	pseudobulk	DESeq2	LRT		0
364	KC_AB1	CG8065	2.78674512494791	0.54962276837928	1	pseudobulk	DESeq2	LRT		0
364	KC_AB1	CG8066	-2.26572866289156	0.188327959155355	1	pseudobulk	DESeq2	LRT		0
364	KC_AB1	CG8067	-0.331236259846864	0.789946846393125	1	pseudobulk	DESeq2	LRT		0
364	KC_AB1	CG8072	2.85142250261825	0.484249113053674	1	pseudobulk	DESeq2	LRT		0
364	KC_AB1	CG8078	0.400842505890726	0.691481841954691	1	pseudobulk	DESeq2	LRT		0
364	KC_AB1	CG8079	-0.0590325784010749	0.727405337904214	1	pseudobulk	DESeq2	LRT		0
364	KC_AB1	CG8080	0.582386458356713	0.823275527850096	1	pseudobulk	DESeq2	LRT		0
364	KC_AB1	CG8086	4.35696122833019	0.122203345306448	1	pseudobulk	DESeq2	LRT		0
364	KC_AB1	CG8097	0.1772226318482	1	1	pseudobulk	DESeq2	LRT		0
364	KC_AB1	CG8108	-0.0101611672706914	0.946602023246863	1	pseudobulk	DESeq2	LRT		0
365	KC_AB1	CG8111	0.0959619971773431	0.661109642092294	1	pseudobulk	DESeq2	LRT		0
365	KC_AB1	CG8112	-0.515281586991448	0.755410408991726	1	pseudobulk	DESeq2	LRT		0
365	KC_AB1	CG8128	-0.816090144415082	0.633737824480411	1	pseudobulk	DESeq2	LRT		0
365	KC_AB1	CG8132	-0.624652056600654	0.509292338465616	1	pseudobulk	DESeq2	LRT		0
365	KC_AB1	CG8134	0.87030862967684	0.139842211221063	1	pseudobulk	DESeq2	LRT		0
365	KC_AB1	CG8149	-0.113829107697537	0.717488514164694	1	pseudobulk	DESeq2	LRT		0

365	KC_AB1	CG8152	-0.700244950924106	0.510897790978192	1	pseudobulk	DESeq2	LRT	0
365	KC_AB1	CG8155	0.703337099442771	0.377530028277202	1	pseudobulk	DESeq2	LRT	0
365	KC_AB1	CG8157	0.849606887838128	1	1	pseudobulk	DESeq2	LRT	0
365	KC_AB1	CG8159	-0.744821221219041	0.601875371239769	1	pseudobulk	DESeq2	LRT	0
366	KC_AB1	CG8173	0.748048075449835	0.755414637845287	1	pseudobulk	DESeq2	LRT	0
366	KC_AB1	CG8176	0.167264152283327	0.645403888659211	1	pseudobulk	DESeq2	LRT	0
366	KC_AB1	CG8177	0.450571259840146	0.0644077641730758	1	pseudobulk	DESeq2	LRT	0
366	KC_AB1	CG8180	-0.660222021119874	0.76330493518027	1	pseudobulk	DESeq2	LRT	0
366	KC_AB1	CG8187	0.742490720550818	0.361036690345624	1	pseudobulk	DESeq2	LRT	0
366	KC_AB1	CG8188	-0.12571709960817	0.708903296201397	1	pseudobulk	DESeq2	LRT	0
366	KC_AB1	CG8195	0.197615214880851	0.546817646310256	1	pseudobulk	DESeq2	LRT	0
366	KC_AB1	CG8202	0.0992475112383186	0.879921950689094	1	pseudobulk	DESeq2	LRT	0
366	KC_AB1	CG8204	-1.52933761066118	0.423015923609909	1	pseudobulk	DESeq2	LRT	0
366	KC_AB1	CG8206	-0.129645873881905	0.717607153482453	1	pseudobulk	DESeq2	LRT	0
367	KC_AB1	CG8207	0.290477944078669	0.860590641798726	1	pseudobulk	DESeq2	LRT	0
367	KC_AB1	CG8209	-0.381472839612204	0.679317353992132	1	pseudobulk	DESeq2	LRT	0
367	KC_AB1	CG8223	0.628981257860916	0.185961719644252	1	pseudobulk	DESeq2	LRT	0
367	KC_AB1	CG8229	-1.08547593077328	0.378303861802969	1	pseudobulk	DESeq2	LRT	0
367	KC_AB1	CG8230	1.21471335846935	0.191441147679025	1	pseudobulk	DESeq2	LRT	0
367	KC_AB1	CG8236	2.31997030691033	0.813169507846918	1	pseudobulk	DESeq2	LRT	0
367	KC_AB1	CG8237	-0.200499858572623	0.673088665995545	1	pseudobulk	DESeq2	LRT	0
367	KC_AB1	CG8243	-0.0727618512602816	0.8718845361708	1	pseudobulk	DESeq2	LRT	0
367	KC_AB1	CG8245	-0.18245247927017	0.376476416700478	1	pseudobulk	DESeq2	LRT	0
367	KC_AB1	CG8248	0.148826147790571	0.48465892249169	1	pseudobulk	DESeq2	LRT	0
368	KC_AB1	CG8270	0.453177474902423	0.842902326449178	1	pseudobulk	DESeq2	LRT	0
368	KC_AB1	CG8272	-0.372340956310102	0.660501916340158	1	pseudobulk	DESeq2	LRT	0
368	KC_AB1	CG8281	0.229589593076526	0.630082211866335	1	pseudobulk	DESeq2	LRT	0
368	KC_AB1	CG8289	-1.23418399508825	0.14576793847722	1	pseudobulk	DESeq2	LRT	0
368	KC_AB1	CG8292	0.849606887838128	1	1	pseudobulk	DESeq2	LRT	0
368	KC_AB1	CG8298	1.03457138526647	0.700583425066724	1	pseudobulk	DESeq2	LRT	0
368	KC_AB1	CG8300	-0.402919689413622	0.520492007891724	1	pseudobulk	DESeq2	LRT	0
368	KC_AB1	CG8301	0.30792818409744	0.50138317279975	1	pseudobulk	DESeq2	LRT	0
368	KC_AB1	CG8303	3.41844045086015	0.351942490385471	1	pseudobulk	DESeq2	LRT	0
368	KC_AB1	CG8306	0.544524589037743	0.877254787760959	1	pseudobulk	DESeq2	LRT	0
369	KC_AB1	CG8311	-0.669271317304209	0.347985330155917	1	pseudobulk	DESeq2	LRT	0
369	KC_AB1	CG8312	-1.37297653161864	0.460471036916857	1	pseudobulk	DESeq2	LRT	0
369	KC_AB1	CG8314	0.246188605843144	0.703480134209176	1	pseudobulk	DESeq2	LRT	0
369	KC_AB1	CG8319	0.0556878207923385	0.899179551055565	1	pseudobulk	DESeq2	LRT	0
369	KC_AB1	CG8320	-1.25544588152895	0.213888159118767	1	pseudobulk	DESeq2	LRT	0
369	KC_AB1	CG8321	0.0043616475726152	0.669908273292223	1	pseudobulk	DESeq2	LRT	0
369	KC_AB1	CG8323	0.610996756001472	0.341218480577165	1	pseudobulk	DESeq2	LRT	0
369	KC_AB1	CG8326	-1.80414037570206	0.300484747084585	1	pseudobulk	DESeq2	LRT	0
369	KC_AB1	CG8336	-0.291784925009456	0.720642052660468	1	pseudobulk	DESeq2	LRT	0
369	KC_AB1	CG8353	-0.763902867717893	0.535826889653047	1	pseudobulk	DESeq2	LRT	0
370	KC_AB1	CG8360	-0.707666238493846	0.398893768988975	1	pseudobulk	DESeq2	LRT	0
370	KC_AB1	CG8366	0.293759090373141	0.78739119753095	1	pseudobulk	DESeq2	LRT	0
370	KC_AB1	CG8369	-0.352545312412402	0.265012350704249	1	pseudobulk	DESeq2	LRT	0
370	KC_AB1	CG8370	0.407098562815397	0.495176105750721	1	pseudobulk	DESeq2	LRT	0
370	KC_AB1	CG8372	0.0378276996245229	0.863482092659071	1	pseudobulk	DESeq2	LRT	0
370	KC_AB1	CG8379	0.830541943574205	0.381516753607032	1	pseudobulk	DESeq2	LRT	0
370	KC_AB1	CG8388	-0.73478285930218	0.511743268335329	1	pseudobulk	DESeq2	LRT	0
370	KC_AB1	CG8389	-0.693969481055523	0.41515207980499	1	pseudobulk	DESeq2	LRT	0
370	KC_AB1	CG8397	0.0585218359066858	0.780916095589473	1	pseudobulk	DESeq2	LRT	0

370	KC_AB1	CG8399	-0.802742798496205	0.638576459732922	1	pseudobulk	DESeq2	LRT	0
371	KC_AB1	CG8401	-0.314046915178736	0.835033458921175	1	pseudobulk	DESeq2	LRT	0
371	KC_AB1	CG8405	-0.538913882739328	0.417731550249049	1	pseudobulk	DESeq2	LRT	0
371	KC_AB1	CG8407	2.85142250261825	0.484249113053674	1	pseudobulk	DESeq2	LRT	0
371	KC_AB1	CG8412	-0.358718183942628	0.504284461075704	1	pseudobulk	DESeq2	LRT	0
371	KC_AB1	CG8414	-1.40698406551946	0.406855345918477	1	pseudobulk	DESeq2	LRT	0
371	KC_AB1	CG8435	-0.188388972835883	0.748210383528105	1	pseudobulk	DESeq2	LRT	0
371	KC_AB1	CG8441	-1.97960609844546	0.208644197126792	1	pseudobulk	DESeq2	LRT	0
371	KC_AB1	CG8460	-0.660222021119874	0.76330493518027	1	pseudobulk	DESeq2	LRT	0
371	KC_AB1	CG8461	0.874292972088697	0.533860612117805	1	pseudobulk	DESeq2	LRT	0
371	KC_AB1	CG8475	0.102164185917843	0.650142740658027	1	pseudobulk	DESeq2	LRT	0
372	KC_AB1	CG8485	0.0551543475284224	0.760847802090939	1	pseudobulk	DESeq2	LRT	0
372	KC_AB1	CG8490	-0.136078417910449	0.7446290610851	1	pseudobulk	DESeq2	LRT	0
372	KC_AB1	CG8500	-0.429106336806045	0.124409372597724	1	pseudobulk	DESeq2	LRT	0
372	KC_AB1	CG8507	-0.305445316459303	0.646942036002876	1	pseudobulk	DESeq2	LRT	0
372	KC_AB1	CG8509	0.849606887838128	1	1	pseudobulk	DESeq2	LRT	0
372	KC_AB1	CG8516	-0.463342143110233	0.637065122297158	1	pseudobulk	DESeq2	LRT	0
372	KC_AB1	CG8519	0.406550567727916	0.908095439982153	1	pseudobulk	DESeq2	LRT	0
372	KC_AB1	CG8520	-0.989136067265479	0.320907081127648	1	pseudobulk	DESeq2	LRT	0
372	KC_AB1	CG8531	-0.597910759684586	0.528103411882423	1	pseudobulk	DESeq2	LRT	0
372	KC_AB1	CG8545	1.33452863118797	0.627020104177396	1	pseudobulk	DESeq2	LRT	0
373	KC_AB1	CG8547	-1.0868008871937	0.596161533311927	1	pseudobulk	DESeq2	LRT	0
373	KC_AB1	CG8549	-0.670948170432554	0.476404748370011	1	pseudobulk	DESeq2	LRT	0
373	KC_AB1	CG8569	-0.0210754013395289	0.779846497954989	1	pseudobulk	DESeq2	LRT	0
373	KC_AB1	CG8578	0.429285049590345	0.474971982797552	1	pseudobulk	DESeq2	LRT	0
373	KC_AB1	CG8586	0.536600542756646	1	1	pseudobulk	DESeq2	LRT	0
373	KC_AB1	CG8611	-1.38841560390631	0.309670636856713	1	pseudobulk	DESeq2	LRT	0
373	KC_AB1	CG8613	-0.0449891424458327	0.831207016144422	1	pseudobulk	DESeq2	LRT	0
373	KC_AB1	CG8617	0.234655550877854	0.429558424499507	1	pseudobulk	DESeq2	LRT	0
373	KC_AB1	CG8620	1.44105098463091	0.618980069329691	1	pseudobulk	DESeq2	LRT	0
373	KC_AB1	CG8635	-0.239642347184809	0.515154922919158	1	pseudobulk	DESeq2	LRT	0
374	KC_AB1	CG8641	-0.891145964526884	0.459263301870175	1	pseudobulk	DESeq2	LRT	0
374	KC_AB1	CG8654	0.849606887838128	1	1	pseudobulk	DESeq2	LRT	0
374	KC_AB1	CG8668	-0.171970489524938	0.542666479781061	1	pseudobulk	DESeq2	LRT	0
374	KC_AB1	CG8671	-0.147261531804151	0.518206385431325	1	pseudobulk	DESeq2	LRT	0
374	KC_AB1	CG8673	0.849606887838128	1	1	pseudobulk	DESeq2	LRT	0
374	KC_AB1	CG8675	1.72130518181245	0.335698284667933	1	pseudobulk	DESeq2	LRT	0
374	KC_AB1	CG8677	-0.0698740469360464	0.63602281838587	1	pseudobulk	DESeq2	LRT	0
374	KC_AB1	CG8679	-0.566377030942222	0.765841855334795	1	pseudobulk	DESeq2	LRT	0
374	KC_AB1	CG8712	2.85142250261825	0.484249113053674	1	pseudobulk	DESeq2	LRT	0
374	KC_AB1	CG8726	-0.0192529009615184	0.492988618551183	1	pseudobulk	DESeq2	LRT	0
375	KC_AB1	CG8728	-0.0868402154995881	0.650596050120778	1	pseudobulk	DESeq2	LRT	0
375	KC_AB1	CG8736	0.183159795455075	1	1	pseudobulk	DESeq2	LRT	0
375	KC_AB1	CG8738	-0.216210029276812	1	1	pseudobulk	DESeq2	LRT	0
375	KC_AB1	CG8740	-1.23330714229684	0.425695006391347	1	pseudobulk	DESeq2	LRT	0
375	KC_AB1	CG8745	-0.0574283946912483	0.826053074442521	1	pseudobulk	DESeq2	LRT	0
375	KC_AB1	CG8757	0.475419692279278	1	1	pseudobulk	DESeq2	LRT	0
375	KC_AB1	CG8765	0.209566550403984	0.742261676638106	1	pseudobulk	DESeq2	LRT	0
375	KC_AB1	CG8768	-0.0037037073363521	0.882741134852029	1	pseudobulk	DESeq2	LRT	0
375	KC_AB1	CG8777	0.229423914325719	0.993245617489675	1	pseudobulk	DESeq2	LRT	0
375	KC_AB1	CG8778	0.425994121331493	0.633903609107533	1	pseudobulk	DESeq2	LRT	0
376	KC_AB1	CG8785	-1.5805998677877	0.524230026009756	1	pseudobulk	DESeq2	LRT	0
376	KC_AB1	CG8814	-0.0592099700976997	0.814046022873805	1	pseudobulk	DESeq2	LRT	0

376	KC_AB1	CG8818	-0.387701055741911	0.318094954520922	1	pseudobulk	DESeq2	LRT		0
376	KC_AB1	CG8833	-0.845658284187517	0.489965893558397	1	pseudobulk	DESeq2	LRT		0
376	KC_AB1	CG8839	0.0890703346653177	0.90476813495268	1	pseudobulk	DESeq2	LRT		0
376	KC_AB1	CG8841	1.23840911626265	0.348445329380744	1	pseudobulk	DESeq2	LRT		0
376	KC_AB1	CG8850	0.162118002617676	1	1	pseudobulk	DESeq2	LRT		0
376	KC_AB1	CG8854	2.78674512494791	0.54962276837928	1	pseudobulk	DESeq2	LRT		0
376	KC_AB1	CG8858	0.392115212657322	0.754376136184171	1	pseudobulk	DESeq2	LRT		0
376	KC_AB1	CG8860	-0.0995541800017993	0.609284214484201	1	pseudobulk	DESeq2	LRT		0
377	KC_AB1	CG8861	-2.5201045593367	0.253361243959694	1	pseudobulk	DESeq2	LRT		0
377	KC_AB1	CG8878	-0.139824625395357	0.54866491745995	1	pseudobulk	DESeq2	LRT		0
377	KC_AB1	CG8891	-0.967118397768889	0.461710976662423	1	pseudobulk	DESeq2	LRT		0
377	KC_AB1	CG8892	-0.281362773732212	0.627120971432868	1	pseudobulk	DESeq2	LRT		0
377	KC_AB1	CG8910	-0.649668125309594	0.0283233240741239	1	pseudobulk	DESeq2	LRT		0
377	KC_AB1	CG8915	-0.678137758944623	0.656755656276817	1	pseudobulk	DESeq2	LRT		0
377	KC_AB1	CG8916	0.849606887838128	1	1	pseudobulk	DESeq2	LRT		0
377	KC_AB1	CG8924	0.173453486033386	0.59963785982221	1	pseudobulk	DESeq2	LRT		0
377	KC_AB1	CG8929	-0.374424376208225	0.294389952270181	1	pseudobulk	DESeq2	LRT		0
377	KC_AB1	CG8939	0.930993226399931	1	1	pseudobulk	DESeq2	LRT		0
378	KC_AB1	CG8944	1.08808066690221	0.124709473543863	1	pseudobulk	DESeq2	LRT		0
378	KC_AB1	CG8950	1.93706461079025	0.658817357590498	1	pseudobulk	DESeq2	LRT		0
378	KC_AB1	CG8958	0.849606887838128	1	1	pseudobulk	DESeq2	LRT		0
378	KC_AB1	CG8963	-0.445151154842785	0.279404013760683	1	pseudobulk	DESeq2	LRT		0
378	KC_AB1	CG8974	0.286298601941064	0.423590109884589	1	pseudobulk	DESeq2	LRT		0
378	KC_AB1	CG8993	0.292855309318381	0.408329775047946	1	pseudobulk	DESeq2	LRT		0
378	KC_AB1	CG9003	0.132850155020551	0.648109241585533	1	pseudobulk	DESeq2	LRT		0
378	KC_AB1	CG9004	-0.433919513776447	0.672545915314399	1	pseudobulk	DESeq2	LRT		0
378	KC_AB1	CG9005	-0.0472653799041352	0.642701861536704	1	pseudobulk	DESeq2	LRT		0
378	KC_AB1	CG9008	-0.216210029276812	1	1	pseudobulk	DESeq2	LRT		0
379	KC_AB1	CG9018	-0.302749955675939	0.684891939830803	1	pseudobulk	DESeq2	LRT		0
379	KC_AB1	CG9034	0.188569578079062	0.400767911784168	1	pseudobulk	DESeq2	LRT		0
379	KC_AB1	CG9044	-0.396262491289322	0.547237717717879	1	pseudobulk	DESeq2	LRT		0
379	KC_AB1	CG9062	-1.15506706160016	0.382402506471314	1	pseudobulk	DESeq2	LRT		0
379	KC_AB1	CG9065	-0.216237261644205	0.443659476207238	1	pseudobulk	DESeq2	LRT		0
379	KC_AB1	CG9067	-0.398229867999874	0.667127398216782	1	pseudobulk	DESeq2	LRT		0
379	KC_AB1	CG9072	0.849606887838128	1	1	pseudobulk	DESeq2	LRT		0
379	KC_AB1	CG9095	2.78674512494791	0.54962276837928	1	pseudobulk	DESeq2	LRT		0
379	KC_AB1	CG9098	-0.905951370532408	0.556710309688352	1	pseudobulk	DESeq2	LRT		0
379	KC_AB1	CG9107	-0.384323068419032	0.736644834060995	1	pseudobulk	DESeq2	LRT		0
380	KC_AB1	CG9117	0.849606887838128	1	1	pseudobulk	DESeq2	LRT		0
380	KC_AB1	CG9121	-0.631012187106785	0.448995628170021	1	pseudobulk	DESeq2	LRT		0
380	KC_AB1	CG9123	-0.275519552744193	0.46400334073591	1	pseudobulk	DESeq2	LRT		0
380	KC_AB1	CG9125	-0.223752418352032	0.77100990703309	1	pseudobulk	DESeq2	LRT		0
380	KC_AB1	CG9132	0.332269314730816	0.326590226129561	1	pseudobulk	DESeq2	LRT		0
380	KC_AB1	CG9135	-0.51451659911529	0.539962656410793	1	pseudobulk	DESeq2	LRT		0
380	KC_AB1	CG9143	0.129953675885512	0.866517250168784	1	pseudobulk	DESeq2	LRT		0
380	KC_AB1	CG9147	0.415028344355792	0.884564451227325	1	pseudobulk	DESeq2	LRT		0
380	KC_AB1	CG9149	0.0984731088219555	0.756747280857705	1	pseudobulk	DESeq2	LRT		0
380	KC_AB1	CG9154	0.485360050841702	0.656038519318001	1	pseudobulk	DESeq2	LRT		0
381	KC_AB1	CG9164	0.623203980573811	0.456454290949068	1	pseudobulk	DESeq2	LRT		0
381	KC_AB1	CG9170	-0.410052618564833	0.181188981380254	1	pseudobulk	DESeq2	LRT		0
381	KC_AB1	CG9171	-0.149955322222597	0.456251593565365	1	pseudobulk	DESeq2	LRT		0
381	KC_AB1	CG9175	0.303268631905725	0.618443587231284	1	pseudobulk	DESeq2	LRT		0
381	KC_AB1	CG9205	-0.105188238241644	0.597574215365296	1	pseudobulk	DESeq2	LRT		0

381	KC_AB1	CG9213	2.23045641149244	0.449115898480754	1	pseudobulk	DESeq2	LRT	0
381	KC_AB1	CG9215	-0.0663565364246291	0.823585025844698	1	pseudobulk	DESeq2	LRT	0
381	KC_AB1	CG9220	2.31997030691033	0.813169507846918	1	pseudobulk	DESeq2	LRT	0
381	KC_AB1	CG9231	-0.111903123249598	0.874344497771119	1	pseudobulk	DESeq2	LRT	0
381	KC_AB1	CG9240	0.396303906107267	0.712822621989624	1	pseudobulk	DESeq2	LRT	0
382	KC_AB1	CG9246	0.183982119200679	0.924284231986297	1	pseudobulk	DESeq2	LRT	0
382	KC_AB1	CG9248	-0.216209988775614	1	1	pseudobulk	DESeq2	LRT	0
382	KC_AB1	CG9253	0.0433353441604222	0.804004012529773	1	pseudobulk	DESeq2	LRT	0
382	KC_AB1	CG9257	0.0013877959379096	0.679582700246381	1	pseudobulk	DESeq2	LRT	0
382	KC_AB1	CG9265	0.263256096921618	0.552487411705079	1	pseudobulk	DESeq2	LRT	0
382	KC_AB1	CG9270	2.31997030691033	0.813169507846918	1	pseudobulk	DESeq2	LRT	0
382	KC_AB1	CG9272	-0.412504720963328	0.753961068789293	1	pseudobulk	DESeq2	LRT	0
382	KC_AB1	CG9281	-0.166349377006755	0.443118251716378	1	pseudobulk	DESeq2	LRT	0
382	KC_AB1	CG9286	0.448303681384459	1	1	pseudobulk	DESeq2	LRT	0
382	KC_AB1	CG9300	2.13009086641515	0.718583316467961	1	pseudobulk	DESeq2	LRT	0
383	KC_AB1	CG9302	-0.412504720963328	0.753961068789293	1	pseudobulk	DESeq2	LRT	0
383	KC_AB1	CG9304	0.0427818236215402	0.553820389091867	1	pseudobulk	DESeq2	LRT	0
383	KC_AB1	CG9313	0.849606887838128	1	1	pseudobulk	DESeq2	LRT	0
383	KC_AB1	CG9328	0.0882445335282325	0.538112105768881	1	pseudobulk	DESeq2	LRT	0
383	KC_AB1	CG9330	0.125081769293806	0.728408534017772	1	pseudobulk	DESeq2	LRT	0
383	KC_AB1	CG9331	0.708819175480693	1	1	pseudobulk	DESeq2	LRT	0
383	KC_AB1	CG9336	1.00693893053477	1	1	pseudobulk	DESeq2	LRT	0
383	KC_AB1	CG9344	-0.111734960347351	0.67529832425704	1	pseudobulk	DESeq2	LRT	0
383	KC_AB1	CG9346	-0.645175061204422	0.365112308102678	1	pseudobulk	DESeq2	LRT	0
383	KC_AB1	CG9356	0.417089388435304	0.801133523406137	1	pseudobulk	DESeq2	LRT	0
384	KC_AB1	CG9360	2.43484285911389	0.528783108084678	1	pseudobulk	DESeq2	LRT	0
384	KC_AB1	CG9368	-0.133699343542487	0.460185870781584	1	pseudobulk	DESeq2	LRT	0
384	KC_AB1	CG9372	-0.830062897938551	0.717724257445671	1	pseudobulk	DESeq2	LRT	0
384	KC_AB1	CG9380	1.59788849941864	0.321700648157905	1	pseudobulk	DESeq2	LRT	0
384	KC_AB1	CG9384	-0.0607593744530367	0.831289154425384	1	pseudobulk	DESeq2	LRT	0
384	KC_AB1	CG9386	-2.0416759217972	0.229592750346425	1	pseudobulk	DESeq2	LRT	0
384	KC_AB1	CG9391	0.0770447440361017	0.483528812705136	1	pseudobulk	DESeq2	LRT	0
384	KC_AB1	CG9393	-0.105067208064077	0.803585989754427	1	pseudobulk	DESeq2	LRT	0
384	KC_AB1	CG9394	-0.396508936075362	0.609996027212845	1	pseudobulk	DESeq2	LRT	0
384	KC_AB1	CG9395	0.56233211089092	0.435702150671049	1	pseudobulk	DESeq2	LRT	0
385	KC_AB1	CG9396	2.78674512494791	0.54962276837928	1	pseudobulk	DESeq2	LRT	0
385	KC_AB1	CG9399	0.803443053023606	0.2723120325373	1	pseudobulk	DESeq2	LRT	0
385	KC_AB1	CG9410	0.201457867472508	0.643127012426868	1	pseudobulk	DESeq2	LRT	0
385	KC_AB1	CG9422	-0.387543776074321	0.444349620882291	1	pseudobulk	DESeq2	LRT	0
385	KC_AB1	CG9425	-0.157212570352863	0.556968581849653	1	pseudobulk	DESeq2	LRT	0
385	KC_AB1	CG9426	-0.702076224415803	0.22855886714649	1	pseudobulk	DESeq2	LRT	0
385	KC_AB1	CG9436	1.46081863254101	1	1	pseudobulk	DESeq2	LRT	0
385	KC_AB1	CG9449	2.31997030691033	0.813169507846918	1	pseudobulk	DESeq2	LRT	0
385	KC_AB1	CG9467	0.394979155629894	0.546281921875426	1	pseudobulk	DESeq2	LRT	0
385	KC_AB1	CG9471	-0.173614356318889	0.722095996728316	1	pseudobulk	DESeq2	LRT	0
386	KC_AB1	CG9485	0.931109140710098	1	1	pseudobulk	DESeq2	LRT	0
386	KC_AB1	CG9503	0.849606887838128	1	1	pseudobulk	DESeq2	LRT	0
386	KC_AB1	CG9512	1.21191750555156	1	1	pseudobulk	DESeq2	LRT	0
386	KC_AB1	CG9514	1.42067046402716	0.763128348850882	1	pseudobulk	DESeq2	LRT	0
386	KC_AB1	CG9515	1.6129998745446	0.521654626937693	1	pseudobulk	DESeq2	LRT	0
386	KC_AB1	CG9518	-1.06254413415076	0.388609453965464	1	pseudobulk	DESeq2	LRT	0
386	KC_AB1	CG9521	0.849606887838128	1	1	pseudobulk	DESeq2	LRT	0
386	KC_AB1	CG9522	2.31997030691033	0.813169507846918	1	pseudobulk	DESeq2	LRT	0

386	KC_AB1	CG9527	0.546325034219889	0.794541380156047	1	pseudobulk	DESeq2	LRT	0
386	KC_AB1	CG9536	0.440263571912829	0.592894739567167	1	pseudobulk	DESeq2	LRT	0
387	KC_AB1	CG9541	-2.71406895760489	0.0999071286233991	1	pseudobulk	DESeq2	LRT	0
387	KC_AB1	CG9547	-0.216210029276812	1	1	pseudobulk	DESeq2	LRT	0
387	KC_AB1	CG9572	-2.26914832756227	0.359196159304271	1	pseudobulk	DESeq2	LRT	0
387	KC_AB1	CG9577	0.330343729824852	0.952889422506907	1	pseudobulk	DESeq2	LRT	0
387	KC_AB1	CG9578	0.0906934261221832	0.800379563603048	1	pseudobulk	DESeq2	LRT	0
387	KC_AB1	CG9581	0.134121923362633	0.480806999098434	1	pseudobulk	DESeq2	LRT	0
387	KC_AB1	CG9586	0.354350149435062	0.561471833675801	1	pseudobulk	DESeq2	LRT	0
387	KC_AB1	CG9588	0.0700354688021866	0.833203394173725	1	pseudobulk	DESeq2	LRT	0
387	KC_AB1	CG9590	0.410520989148544	0.34526769664264	1	pseudobulk	DESeq2	LRT	0
387	KC_AB1	CG9596	-0.590637494330849	0.764308822189376	1	pseudobulk	DESeq2	LRT	0
388	KC_AB1	CG9601	-0.128266149366436	0.708915419884407	1	pseudobulk	DESeq2	LRT	0
388	KC_AB1	CG9609	-0.0263508357366952	0.66687208272623	1	pseudobulk	DESeq2	LRT	0
388	KC_AB1	CG9628	-1.0634716716243	0.470193184448581	1	pseudobulk	DESeq2	LRT	0
388	KC_AB1	CG9630	0.47572993756592	1	1	pseudobulk	DESeq2	LRT	0
388	KC_AB1	CG9636	-0.109313789396947	0.712139671796779	1	pseudobulk	DESeq2	LRT	0
388	KC_AB1	CG9641	-0.201296816387754	0.723409352219565	1	pseudobulk	DESeq2	LRT	0
388	KC_AB1	CG9643	0.428245279540673	0.859996228136203	1	pseudobulk	DESeq2	LRT	0
388	KC_AB1	CG9646	-0.177122542225445	0.424287932163242	1	pseudobulk	DESeq2	LRT	0
388	KC_AB1	CG9650	-0.549744749555481	0.689271847920307	1	pseudobulk	DESeq2	LRT	0
388	KC_AB1	CG9657	-0.334114789628426	0.765666448636952	1	pseudobulk	DESeq2	LRT	0
389	KC_AB1	CG9662	0.538006682876977	0.405362015432555	1	pseudobulk	DESeq2	LRT	0
389	KC_AB1	CG9663	0.56299079442748	0.904997173168881	1	pseudobulk	DESeq2	LRT	0
389	KC_AB1	CG9667	0.0878609280955265	0.927517937490124	1	pseudobulk	DESeq2	LRT	0
389	KC_AB1	CG9673	0.849606887838128	1	1	pseudobulk	DESeq2	LRT	0
389	KC_AB1	CG9674	0.134741580812105	0.402166920948034	1	pseudobulk	DESeq2	LRT	0
389	KC_AB1	CG9676	0.849606887838128	1	1	pseudobulk	DESeq2	LRT	0
389	KC_AB1	CG9684	0.374420150816607	1	1	pseudobulk	DESeq2	LRT	0
389	KC_AB1	CG9686	-0.370712957040074	0.392773478051994	1	pseudobulk	DESeq2	LRT	0
389	KC_AB1	CG9689	0.849606887838128	1	1	pseudobulk	DESeq2	LRT	0
389	KC_AB1	CG9691	0.4598779706348	1	1	pseudobulk	DESeq2	LRT	0
390	KC_AB1	CG9705	1.2107582670317	0.039763077965635	1	pseudobulk	DESeq2	LRT	0
390	KC_AB1	CG9706	-0.360937196953018	0.724339930712613	1	pseudobulk	DESeq2	LRT	0
390	KC_AB1	CG9723	-0.446623723516908	0.670728036978905	1	pseudobulk	DESeq2	LRT	0
390	KC_AB1	CG9727	-0.13295551407323	0.657294498126312	1	pseudobulk	DESeq2	LRT	0
390	KC_AB1	CG9743	-1.52366673500008	0.330722247015658	1	pseudobulk	DESeq2	LRT	0
390	KC_AB1	CG9747	-0.830062897938551	0.717724257445671	1	pseudobulk	DESeq2	LRT	0
390	KC_AB1	CG9752	0.237022465848507	1	1	pseudobulk	DESeq2	LRT	0
390	KC_AB1	CG9760	0.843887699130038	0.813035377317032	1	pseudobulk	DESeq2	LRT	0
390	KC_AB1	CG9766	0.218474789904676	0.837675769437618	1	pseudobulk	DESeq2	LRT	0
390	KC_AB1	CG9773	0.258937644122459	0.734227989978591	1	pseudobulk	DESeq2	LRT	0
391	KC_AB1	CG9775	-0.115977649248009	0.505171633043676	1	pseudobulk	DESeq2	LRT	0
391	KC_AB1	CG9776	-0.46349408442609	0.332312384795909	1	pseudobulk	DESeq2	LRT	0
391	KC_AB1	CG9782	0.475419692279278	1	1	pseudobulk	DESeq2	LRT	0
391	KC_AB1	CG9784	0.235912263236016	0.543333818124812	1	pseudobulk	DESeq2	LRT	0
391	KC_AB1	CG9795	0.243128655014723	0.673176103325759	1	pseudobulk	DESeq2	LRT	0
391	KC_AB1	CG9799	-1.79399142443853	0.25414147810037	1	pseudobulk	DESeq2	LRT	0
391	KC_AB1	CG9801	-0.783039139384661	0.451708691233605	1	pseudobulk	DESeq2	LRT	0
391	KC_AB1	CG9804	0.064097098759816	0.872463833717926	1	pseudobulk	DESeq2	LRT	0
391	KC_AB1	CG9812	-1.8476447304053	0.456955818574401	1	pseudobulk	DESeq2	LRT	0
391	KC_AB1	CG9813	-0.0726067139492813	0.650467680445365	1	pseudobulk	DESeq2	LRT	0
392	KC_AB1	CG9837	-0.0421347980721914	0.605305388938497	1	pseudobulk	DESeq2	LRT	0

392	KC_AB1	CG9839	0.253551333521533	0.749240777696411	1	pseudobulk	DESeq2	LRT	0
392	KC_AB1	CG9849	0.841577071637704	0.442135268596079	1	pseudobulk	DESeq2	LRT	0
392	KC_AB1	CG9853	0.520763546046037	0.417357594846725	1	pseudobulk	DESeq2	LRT	0
392	KC_AB1	CG9855	0.264977885917644	0.581150356955014	1	pseudobulk	DESeq2	LRT	0
392	KC_AB1	CG9864	0.849606887838128	1	1	pseudobulk	DESeq2	LRT	0
392	KC_AB1	CG9886	0.371454830748247	1	1	pseudobulk	DESeq2	LRT	0
392	KC_AB1	CG9890	-1.86920913220687	0.325720263604925	1	pseudobulk	DESeq2	LRT	0
392	KC_AB1	CG9896	0.849606887838128	1	1	pseudobulk	DESeq2	LRT	0
392	KC_AB1	CG9902	-0.697731717448614	0.532914211516654	1	pseudobulk	DESeq2	LRT	0
393	KC_AB1	CG9911	-0.357644642986163	0.471751202558884	1	pseudobulk	DESeq2	LRT	0
393	KC_AB1	CG9915	-0.654170696441406	0.434394540599007	1	pseudobulk	DESeq2	LRT	0
393	KC_AB1	CG9917	-0.191830565911175	0.586736765977289	1	pseudobulk	DESeq2	LRT	0
393	KC_AB1	CG9919	0.118047485180741	0.574719592666963	1	pseudobulk	DESeq2	LRT	0
393	KC_AB1	CG9921	0.508980116721947	0.623091856237524	1	pseudobulk	DESeq2	LRT	0
393	KC_AB1	CG9922	-0.202774067006403	0.656747129770669	1	pseudobulk	DESeq2	LRT	0
393	KC_AB1	CG9932	-0.346409870175266	0.604645106977758	1	pseudobulk	DESeq2	LRT	0
393	KC_AB1	CG9934	-0.0354927659361702	0.837630000275037	1	pseudobulk	DESeq2	LRT	0
393	KC_AB1	CG9941	0.51709965319652	0.312854623330657	1	pseudobulk	DESeq2	LRT	0
393	KC_AB1	CG9945	0.0833729067166114	0.766640308836218	1	pseudobulk	DESeq2	LRT	0
394	KC_AB1	CG9948	1.26616506462613	0.390937448146934	1	pseudobulk	DESeq2	LRT	0
394	KC_AB1	CG9951	0.26441213532692	0.758850697377599	1	pseudobulk	DESeq2	LRT	0
394	KC_AB1	CG9953	-0.534119108225142	0.431890621845365	1	pseudobulk	DESeq2	LRT	0
394	KC_AB1	CG9967	0.63526686404269	0.374649485776702	1	pseudobulk	DESeq2	LRT	0
394	KC_AB1	CG9986	-0.0321638698884442	0.780223222730929	1	pseudobulk	DESeq2	LRT	0
394	KC_AB1	CG9992	0.502226542234293	0.482174270600537	1	pseudobulk	DESeq2	LRT	0
394	KC_AB1	Charon	0.811617551156565	0.402062292874032	1	pseudobulk	DESeq2	LRT	0
394	KC_AB1	chas	0.308526058365601	1	1	pseudobulk	DESeq2	LRT	0
394	KC_AB1	ChAT	0.0582567277406105	0.780103758493212	1	pseudobulk	DESeq2	LRT	0
394	KC_AB1	chb	-0.690847903870685	0.244322896450261	1	pseudobulk	DESeq2	LRT	0
395	KC_AB1	Chc	0.290463409394311	0.382306603403363	1	pseudobulk	DESeq2	LRT	0
395	KC_AB1	Chchd2	0.0118260627456741	0.566886267264912	1	pseudobulk	DESeq2	LRT	0
395	KC_AB1	Chchd3	0.20873322019174	0.43181415102087	1	pseudobulk	DESeq2	LRT	0
395	KC_AB1	Chd1	0.0320510764277042	0.551561014835779	1	pseudobulk	DESeq2	LRT	0
395	KC_AB1	Chd64	0.794134127142895	0.345778893620122	1	pseudobulk	DESeq2	LRT	0
395	KC_AB1	CheB42c	-1.57763372212448	0.277542551875405	1	pseudobulk	DESeq2	LRT	0
395	KC_AB1	CheB53a	2.06531092668974	0.944672961903726	1	pseudobulk	DESeq2	LRT	0
395	KC_AB1	CheB74a	0.849606887838128	1	1	pseudobulk	DESeq2	LRT	0
395	KC_AB1	cher	1.41364343305001	0.621353306629252	1	pseudobulk	DESeq2	LRT	0
395	KC_AB1	CHES-1-like	0.194402261825302	0.507293443232661	1	pseudobulk	DESeq2	LRT	0
396	KC_AB1	Chi	-1.12429108711699	0.208539366222379	1	pseudobulk	DESeq2	LRT	0
396	KC_AB1	chic	0.119997835937443	0.591735614058216	1	pseudobulk	DESeq2	LRT	0
396	KC_AB1	chico	0.27055317272242	0.583495513385882	1	pseudobulk	DESeq2	LRT	0
396	KC_AB1	chif	0.147370312337383	0.771272878319506	1	pseudobulk	DESeq2	LRT	0
396	KC_AB1	chinmo	-0.261348038006049	0.176526189597353	1	pseudobulk	DESeq2	LRT	0
396	KC_AB1	CHKov1	-1.71765652649625	0.433593857784211	1	pseudobulk	DESeq2	LRT	0
396	KC_AB1	chm	-0.402421110391024	0.608683148272735	1	pseudobulk	DESeq2	LRT	0
396	KC_AB1	Chmp1	0.23417735378837	0.592109937613494	1	pseudobulk	DESeq2	LRT	0
396	KC_AB1	CHMP2B	-0.678776324833114	0.425277955750911	1	pseudobulk	DESeq2	LRT	0
396	KC_AB1	chn	-0.127328334599558	0.46019542121853	1	pseudobulk	DESeq2	LRT	0
397	KC_AB1	CHOp24	0.0808998162954757	0.74351815442381	1	pseudobulk	DESeq2	LRT	0
397	KC_AB1	CHORD	-0.596075722376207	0.528070015597828	1	pseudobulk	DESeq2	LRT	0
397	KC_AB1	Chrac-14	0.769893673767581	1	1	pseudobulk	DESeq2	LRT	0
397	KC_AB1	Chrac-16	1.61899258724414	0.293410925406139	1	pseudobulk	DESeq2	LRT	0

397	KC_AB1	chrb	-0.257588197345837	0.133323075829874	1	pseudobulk	DESeq2	LRT		0
397	KC_AB1	Chro	-0.0522223388532367	0.535793273152949	1	pseudobulk	DESeq2	LRT		0
397	KC_AB1	ChT	0.161120803421358	0.488980328220784	1	pseudobulk	DESeq2	LRT		0
397	KC_AB1	Cht10	0.849606887838128	1	1	pseudobulk	DESeq2	LRT		0
397	KC_AB1	Cht11	1.72376504323795	0.486313498520827	1	pseudobulk	DESeq2	LRT		0
397	KC_AB1	Cht2	2.7141870817367	0.45608503377963	1	pseudobulk	DESeq2	LRT		0
398	KC_AB1	Cht5	-3.95513701671172	0.129151976663025	1	pseudobulk	DESeq2	LRT		0
398	KC_AB1	Cht6	-0.216210029276812	1	1	pseudobulk	DESeq2	LRT		0
398	KC_AB1	ci	-0.830062897938551	0.717724257445671	1	pseudobulk	DESeq2	LRT		0
398	KC_AB1	CIA30	0.0075658647981053	0.778879864633932	1	pseudobulk	DESeq2	LRT		0
398	KC_AB1	Ciao1	-1.07929747314516	0.284110725745236	1	pseudobulk	DESeq2	LRT		0
398	KC_AB1	CIAPIN1	0.0219827767188123	0.736141525416805	1	pseudobulk	DESeq2	LRT		0
398	KC_AB1	cib	-2.01571396806062	0.0613926187725207	1	pseudobulk	DESeq2	LRT		0
398	KC_AB1	cic	-0.358062249918941	0.13029238739523	1	pseudobulk	DESeq2	LRT		0
398	KC_AB1	cid	0.849606887838128	1	1	pseudobulk	DESeq2	LRT		0
398	KC_AB1	cin	-0.701195443444902	0.500606392744851	1	pseudobulk	DESeq2	LRT		0
399	KC_AB1	cindr	-0.532312212739728	0.319700092459412	1	pseudobulk	DESeq2	LRT		0
399	KC_AB1	Cip4	-0.356585142815044	0.12646763895773	1	pseudobulk	DESeq2	LRT		0
399	KC_AB1	Cirl	-0.190004337526262	0.276895309036742	1	pseudobulk	DESeq2	LRT		0
399	KC_AB1	Cisd2	-0.029128050382581	0.632503891119756	1	pseudobulk	DESeq2	LRT		0
399	KC_AB1	ck	0.849606887838128	1	1	pseudobulk	DESeq2	LRT		0
399	KC_AB1	Cka	-0.0736743742631597	0.763805670345278	1	pseudobulk	DESeq2	LRT		0
399	KC_AB1	CkIalpha	0.0003206492348127	0.970267549877341	1	pseudobulk	DESeq2	LRT		0
399	KC_AB1	CkIIalpha	0.0658036426558099	0.447451451663014	1	pseudobulk	DESeq2	LRT		0
399	KC_AB1	CkIIalpha-i1	-0.371217781874023	0.354826097638621	1	pseudobulk	DESeq2	LRT		0
399	KC_AB1	CkIIalpha-i3	-1.08244290362935	0.419856847263001	1	pseudobulk	DESeq2	LRT		0
400	KC_AB1	CkIIbeta	-0.227670722692015	0.345987197978198	1	pseudobulk	DESeq2	LRT		0
400	KC_AB1	ckn	0.136899732854904	0.650240793374169	1	pseudobulk	DESeq2	LRT		0
400	KC_AB1	Cks85A	-0.280582924571999	0.219220327711569	1	pseudobulk	DESeq2	LRT		0
400	KC_AB1	cl	0.217447762669522	0.775475634978491	1	pseudobulk	DESeq2	LRT		0
400	KC_AB1	Clamp	-0.247599873811024	0.490598073166034	1	pseudobulk	DESeq2	LRT		0
400	KC_AB1	Clbn	-0.62204704284758	0.462183202991999	1	pseudobulk	DESeq2	LRT		0
400	KC_AB1	Clc	0.155442704489126	0.658672235101666	1	pseudobulk	DESeq2	LRT		0
400	KC_AB1	ClC-a	0.183159795463042	1	1	pseudobulk	DESeq2	LRT		0
400	KC_AB1	ClC-b	-0.533408142761625	0.492620706388895	1	pseudobulk	DESeq2	LRT		0
400	KC_AB1	ClC-c	0.14757476486981	0.747590600998306	1	pseudobulk	DESeq2	LRT		0
401	KC_AB1	Clic	-0.397258596620803	0.439149220281491	1	pseudobulk	DESeq2	LRT		0
401	KC_AB1	CLIP-190	-0.338894790261674	0.080119593102264	1	pseudobulk	DESeq2	LRT		0
401	KC_AB1	Clk	2.06541384650887	0.904905526327343	1	pseudobulk	DESeq2	LRT		0
401	KC_AB1	Cln7	0.849606887838128	1	1	pseudobulk	DESeq2	LRT		0
401	KC_AB1	clos	2.06541384650887	0.795866856626082	1	pseudobulk	DESeq2	LRT		0
401	KC_AB1	Clp	-0.485807922504866	0.604775551320146	1	pseudobulk	DESeq2	LRT		0
401	KC_AB1	ClpP	0.0474844385514836	0.686846904180078	1	pseudobulk	DESeq2	LRT		0
401	KC_AB1	CLS	1.06656273564959	0.279872460274433	1	pseudobulk	DESeq2	LRT		0
401	KC_AB1	clt	0.183159795463042	1	1	pseudobulk	DESeq2	LRT		0
401	KC_AB1	clu	-0.0526865676013913	0.838727540579229	1	pseudobulk	DESeq2	LRT		0
402	KC_AB1	cm	0.75590858506677	0.1966584896935	1	pseudobulk	DESeq2	LRT		0
402	KC_AB1	cmb	-0.549759716738224	0.609024235410469	1	pseudobulk	DESeq2	LRT		0
402	KC_AB1	cmpy	-0.190524144465681	0.595333311769652	1	pseudobulk	DESeq2	LRT		0
402	KC_AB1	cN-IIIB	1.90550287265121	0.107790990639552	1	pseudobulk	DESeq2	LRT		0
402	KC_AB1	Cnb	0.183159795455075	1	1	pseudobulk	DESeq2	LRT		0
402	KC_AB1	CNBP	-0.255908865649424	0.419030046958105	1	pseudobulk	DESeq2	LRT		0
402	KC_AB1	cnc	-0.619990712356317	0.321798241233318	1	pseudobulk	DESeq2	LRT		0

402	KC_AB1	Cndp2	-0.836840666402475	0.50976435083342	1	pseudobulk	DESeq2	LRT	0
402	KC_AB1	CngA	-2.73573142459503	0.264943650326734	1	pseudobulk	DESeq2	LRT	0
402	KC_AB1	CngB	0.717296340372419	0.113172077459829	1	pseudobulk	DESeq2	LRT	0
403	KC_AB1	CngI	0.1764615516917	0.402560981144657	1	pseudobulk	DESeq2	LRT	0
403	KC_AB1	cni	-0.3896039678808	0.365431082788942	1	pseudobulk	DESeq2	LRT	0
403	KC_AB1	cnir	0.103249748920497	0.75042296996961	1	pseudobulk	DESeq2	LRT	0
403	KC_AB1	cnk	0.550490961283812	0.490053147309111	1	pseudobulk	DESeq2	LRT	0
403	KC_AB1	CNMaR	1.36004237795471	0.790165338156147	1	pseudobulk	DESeq2	LRT	0
403	KC_AB1	cnn	0.194115940681274	0.330534141875329	1	pseudobulk	DESeq2	LRT	0
403	KC_AB1	cno	0.586884785617825	0.582589949330515	1	pseudobulk	DESeq2	LRT	0
403	KC_AB1	Cnot4	-0.439209771303059	0.30580715441429	1	pseudobulk	DESeq2	LRT	0
403	KC_AB1	CNPYb	-0.159718714375724	0.506231892339444	1	pseudobulk	DESeq2	LRT	0
403	KC_AB1	Cnx14D	0.422531845287059	1	1	pseudobulk	DESeq2	LRT	0
404	KC_AB1	Cnx99A	0.0454092840420412	0.865789724819224	1	pseudobulk	DESeq2	LRT	0
404	KC_AB1	Coa7	0.156352115185747	0.624366122056354	1	pseudobulk	DESeq2	LRT	0
404	KC_AB1	Cog1	-0.0355527778410938	0.794209701587686	1	pseudobulk	DESeq2	LRT	0
404	KC_AB1	Cog2	0.210414547667586	1	1	pseudobulk	DESeq2	LRT	0
404	KC_AB1	Cog3	-0.287567257126996	0.920650037231581	1	pseudobulk	DESeq2	LRT	0
404	KC_AB1	Cog4	0.286231917340975	0.876374960076666	1	pseudobulk	DESeq2	LRT	0
404	KC_AB1	Cog6	-0.1971025153341	0.710091347524887	1	pseudobulk	DESeq2	LRT	0
404	KC_AB1	Cog7	-0.501425755823596	0.586811055363964	1	pseudobulk	DESeq2	LRT	0
404	KC_AB1	Cog8	0.352175941940346	0.940495928719139	1	pseudobulk	DESeq2	LRT	0
404	KC_AB1	coil	-0.0352876233234891	0.836524763524013	1	pseudobulk	DESeq2	LRT	0
405	KC_AB1	Col4a1	3.79402714561127	0.0314262819050138	1	pseudobulk	DESeq2	LRT	0
405	KC_AB1	cold	-0.633552239129675	0.507645865143503	1	pseudobulk	DESeq2	LRT	0
405	KC_AB1	colt	-0.435410803619278	0.695357321042872	1	pseudobulk	DESeq2	LRT	0
405	KC_AB1	comm	-0.439421098136028	0.187329623855928	1	pseudobulk	DESeq2	LRT	0
405	KC_AB1	comt	0.0077096574521585	0.529286762481791	1	pseudobulk	DESeq2	LRT	0
405	KC_AB1	Con	0.292832858629378	0.11280518858388	1	pseudobulk	DESeq2	LRT	0
405	KC_AB1	Cont	0.942357804054851	0.325984277434574	1	pseudobulk	DESeq2	LRT	0
405	KC_AB1	conu	0.0075136591562040	0.755608359309648	1	pseudobulk	DESeq2	LRT	0
405	KC_AB1	Coop	-0.216210029276812	1	1	pseudobulk	DESeq2	LRT	0
405	KC_AB1	Coprox	-0.10186371051557	0.752232074705779	1	pseudobulk	DESeq2	LRT	0
406	KC_AB1	Coq2	-0.446658903485539	0.599497946578654	1	pseudobulk	DESeq2	LRT	0
406	KC_AB1	Coq3	0.304378705804448	1	1	pseudobulk	DESeq2	LRT	0
406	KC_AB1	Coq4	0.849606887838128	1	1	pseudobulk	DESeq2	LRT	0
406	KC_AB1	Coq5	-1.30099855104554	0.406591950890794	1	pseudobulk	DESeq2	LRT	0
406	KC_AB1	Coq6	-0.105695632775556	0.740369056762697	1	pseudobulk	DESeq2	LRT	0
406	KC_AB1	Coq7	0.354314555419191	0.411661999650346	1	pseudobulk	DESeq2	LRT	0
406	KC_AB1	Coq8	-0.321122323685933	0.720836373917598	1	pseudobulk	DESeq2	LRT	0
406	KC_AB1	Coq9	0.156960437451696	0.758229049161568	1	pseudobulk	DESeq2	LRT	0
406	KC_AB1	cora	-0.228574852177479	0.234425540684029	1	pseudobulk	DESeq2	LRT	0
406	KC_AB1	CoRest	-0.823491403359114	0.103540023858755	1	pseudobulk	DESeq2	LRT	0
407	KC_AB1	Corin	-1.47492617139073	0.00722250593106871	1	pseudobulk	DESeq2	LRT	0
407	KC_AB1	corn	0.203181700639299	0.430187333509611	1	pseudobulk	DESeq2	LRT	0
407	KC_AB1	coro	-0.511215859088784	0.19821014774602	1	pseudobulk	DESeq2	LRT	0
407	KC_AB1	Corp	0.849606887838128	1	1	pseudobulk	DESeq2	LRT	0
407	KC_AB1	cort	0.849606887838128	1	1	pseudobulk	DESeq2	LRT	0
407	KC_AB1	Cortactin	-0.100244182562574	0.695415755934799	1	pseudobulk	DESeq2	LRT	0
407	KC_AB1	corto	-0.229206344945789	0.301927507866416	1	pseudobulk	DESeq2	LRT	0
407	KC_AB1	cos	-0.0263134017955405	0.870753525994042	1	pseudobulk	DESeq2	LRT	0
407	KC_AB1	Cow	0.11758784845552	0.477666348513872	1	pseudobulk	DESeq2	LRT	0
407	KC_AB1	COX4	0.0931795215075184	0.577229267886219	1	pseudobulk	DESeq2	LRT	0

408	KC_AB1	COX5A	0.301327942460006	0.167445921027142	1	pseudobulk	DESeq2	LRT	0
408	KC_AB1	COX5B	0.310728262916857	0.253237731537557	1	pseudobulk	DESeq2	LRT	0
408	KC_AB1	COX6B	0.0338393370648912	0.598114453783687	1	pseudobulk	DESeq2	LRT	0
408	KC_AB1	COX7A	0.189285841785476	0.373418103353838	1	pseudobulk	DESeq2	LRT	0
408	KC_AB1	COX7C	0.39224915097341	0.203724790852396	1	pseudobulk	DESeq2	LRT	0
408	KC_AB1	COX8	-0.078852550839859	0.74826373997331	1	pseudobulk	DESeq2	LRT	0
408	KC_AB1	Cp1	-0.880510250359998	0.0427471595358778	1	pseudobulk	DESeq2	LRT	0
408	KC_AB1	Cp110	-0.0883613516516318	0.597111646506607	1	pseudobulk	DESeq2	LRT	0
408	KC_AB1	Cp18	0.0160940546605626	1	1	pseudobulk	DESeq2	LRT	0
408	KC_AB1	Cp190	-0.491877074087174	0.286074445349039	1	pseudobulk	DESeq2	LRT	0
409	KC_AB1	Cp7Fa	0.865817449292603	1	1	pseudobulk	DESeq2	LRT	0
409	KC_AB1	Cp7Fb	2.43275481406755	0.550258497427138	1	pseudobulk	DESeq2	LRT	0
409	KC_AB1	cpa	-0.798699478742673	0.402613158671747	1	pseudobulk	DESeq2	LRT	0
409	KC_AB1	cpb	0.200209209107191	0.631891199432752	1	pseudobulk	DESeq2	LRT	0
409	KC_AB1	Cpes	0.0882822044420654	0.789876831396798	1	pseudobulk	DESeq2	LRT	0
409	KC_AB1	cpo	-0.118391530710333	0.381035469765097	1	pseudobulk	DESeq2	LRT	0
409	KC_AB1	Cpr	0.919645223364894	0.27955286011425	1	pseudobulk	DESeq2	LRT	0
409	KC_AB1	Cpr11A	2.78674512494791	0.54962276837928	1	pseudobulk	DESeq2	LRT	0
409	KC_AB1	Cpr11B	0.0160940546605174	1	1	pseudobulk	DESeq2	LRT	0
409	KC_AB1	Cpr50Cb	5.33300544757014	0.0974888510042745	1	pseudobulk	DESeq2	LRT	0
410	KC_AB1	Cpr51A	4.18723362042445	0.0870352217807931	1	pseudobulk	DESeq2	LRT	0
410	KC_AB1	Cpr64Ad	0.849606887838128	1	1	pseudobulk	DESeq2	LRT	0
410	KC_AB1	Cpr65Av	2.78674512494791	0.54962276837928	1	pseudobulk	DESeq2	LRT	0
410	KC_AB1	Cpr67B	1.43365546465148	0.217318002200089	1	pseudobulk	DESeq2	LRT	0
410	KC_AB1	Cpr73D	-0.622166910409198	0.780289788827665	1	pseudobulk	DESeq2	LRT	0
410	KC_AB1	Cpr76Bd	0.849606887838128	1	1	pseudobulk	DESeq2	LRT	0
410	KC_AB1	Cpr97Ea	2.06541384650887	0.768363369045814	1	pseudobulk	DESeq2	LRT	0
410	KC_AB1	Cpsf100	2.09184378415274	0.557642343718699	1	pseudobulk	DESeq2	LRT	0
410	KC_AB1	Cpsf160	0.459966163948786	0.964879936083596	1	pseudobulk	DESeq2	LRT	0
410	KC_AB1	Cpsf5	0.0291914817564957	0.687156650488464	1	pseudobulk	DESeq2	LRT	0
411	KC_AB1	Cpsf6	-0.0587889595088453	0.600754950317229	1	pseudobulk	DESeq2	LRT	0
411	KC_AB1	Cpsf73	0.0711780900876238	0.858856071949416	1	pseudobulk	DESeq2	LRT	0
411	KC_AB1	cpx	-0.239134275823289	0.118969982047895	1	pseudobulk	DESeq2	LRT	0
411	KC_AB1	CR12842	0.933714680939821	0.443419766740748	1	pseudobulk	DESeq2	LRT	0
411	KC_AB1	CR14578	-0.939569060349697	0.579584221631933	1	pseudobulk	DESeq2	LRT	0
411	KC_AB1	CR14798	-0.960979635715674	0.488014336378815	1	pseudobulk	DESeq2	LRT	0
411	KC_AB1	CR15345	0.0531311896183023	0.813515441811836	1	pseudobulk	DESeq2	LRT	0
411	KC_AB1	CR18166	-0.31992491433207	0.724621943485843	1	pseudobulk	DESeq2	LRT	0
411	KC_AB1	CR18228	2.31997030691033	0.813169507846918	1	pseudobulk	DESeq2	LRT	0
411	KC_AB1	CR18275	2.4005608883654	0.129282339224583	1	pseudobulk	DESeq2	LRT	0
412	KC_AB1	CR31032	-0.0352138241105627	0.81188613796887	1	pseudobulk	DESeq2	LRT	0
412	KC_AB1	CR32009	0.0160940546605174	1	1	pseudobulk	DESeq2	LRT	0
412	KC_AB1	CR32010	0.849606887838128	1	1	pseudobulk	DESeq2	LRT	0
412	KC_AB1	CR32745	-0.994995308781593	0.347006934716099	1	pseudobulk	DESeq2	LRT	0
412	KC_AB1	CR33294	2.31997030691033	0.813169507846918	1	pseudobulk	DESeq2	LRT	0
412	KC_AB1	CR40100	0.849606887838128	1	1	pseudobulk	DESeq2	LRT	0
412	KC_AB1	CR40190	1.24327838959006	0.781410300801615	1	pseudobulk	DESeq2	LRT	0
412	KC_AB1	CR40354	0.849606887838128	1	1	pseudobulk	DESeq2	LRT	0
412	KC_AB1	CR41320	-1.09700044704809	0.405757381217753	1	pseudobulk	DESeq2	LRT	0
412	KC_AB1	CR41440	2.31997030691033	0.813169507846918	1	pseudobulk	DESeq2	LRT	0
413	KC_AB1	CR41597	-0.301452671158207	0.558751789101214	1	pseudobulk	DESeq2	LRT	0
413	KC_AB1	CR42875	-0.564356698759404	0.803702590456371	1	pseudobulk	DESeq2	LRT	0
413	KC_AB1	CR43087	-0.216210029276812	1	1	pseudobulk	DESeq2	LRT	0

413	KC_AB1	CR43170	0.849606887838128	1	1	pseudobulk	DESeq2	LRT	0
413	KC_AB1	CR43212	0.849606887838128	1	1	pseudobulk	DESeq2	LRT	0
413	KC_AB1	CR43281	-0.23653290774987	0.734176862569001	1	pseudobulk	DESeq2	LRT	0
413	KC_AB1	CR43361	1.59863986092676	1	1	pseudobulk	DESeq2	LRT	0
413	KC_AB1	CR43670	0.16987958244178	0.753971461866233	1	pseudobulk	DESeq2	LRT	0
413	KC_AB1	CR43972	0.0160940546605626	1	1	pseudobulk	DESeq2	LRT	0
413	KC_AB1	CR43975	0.849606887838128	1	1	pseudobulk	DESeq2	LRT	0
414	KC_AB1	CR45470	0.849606887838128	1	1	pseudobulk	DESeq2	LRT	0
414	KC_AB1	CR4575	2.31997030691033	0.813169507846918	1	pseudobulk	DESeq2	LRT	0
414	KC_AB1	CR46027	2.78674512494791	0.54962276837928	1	pseudobulk	DESeq2	LRT	0
414	KC_AB1	CR46418	0.849606887838128	1	1	pseudobulk	DESeq2	LRT	0
414	KC_AB1	CR46425	0.849606887838128	1	1	pseudobulk	DESeq2	LRT	0
414	KC_AB1	CR6900	0.492581815826022	1	1	pseudobulk	DESeq2	LRT	0
414	KC_AB1	Crag	0.0909854713396982	0.874081327571047	1	pseudobulk	DESeq2	LRT	0
414	KC_AB1	CRAT	0.721021277409401	0.665751071260094	1	pseudobulk	DESeq2	LRT	0
414	KC_AB1	crb	-0.274809082017287	0.119020842666793	1	pseudobulk	DESeq2	LRT	0
414	KC_AB1	crc	0.133058953196613	0.745597169627901	1	pseudobulk	DESeq2	LRT	0
415	KC_AB1	CrebA	-1.60704036694905	0.17614037190336	1	pseudobulk	DESeq2	LRT	0
415	KC_AB1	CrebB	-0.449221522317385	0.356586991816171	1	pseudobulk	DESeq2	LRT	0
415	KC_AB1	CREG	-2.46820091056286	0.215083669970982	1	pseudobulk	DESeq2	LRT	0
415	KC_AB1	Crg-1	0.795637095636752	0.506470053889274	1	pseudobulk	DESeq2	LRT	0
415	KC_AB1	CRIF	0.406567302759291	0.356921108401652	1	pseudobulk	DESeq2	LRT	0
415	KC_AB1	crim	1.2643649495819	0.312374123270393	1	pseudobulk	DESeq2	LRT	0
415	KC_AB1	Crk	-0.163766753219506	0.419338950921252	1	pseudobulk	DESeq2	LRT	0
415	KC_AB1	crm	-0.271921508211117	0.758937686909386	1	pseudobulk	DESeq2	LRT	0
415	KC_AB1	CRMP	-0.110714091084278	0.497086892437937	1	pseudobulk	DESeq2	LRT	0
415	KC_AB1	crn	-0.586314964914339	0.46017275273912	1	pseudobulk	DESeq2	LRT	0
416	KC_AB1	crok	-0.216210029276812	1	1	pseudobulk	DESeq2	LRT	0
416	KC_AB1	crol	-0.104880999446181	0.454910556521621	1	pseudobulk	DESeq2	LRT	0
416	KC_AB1	CROT	-0.36747841843669	0.657257143859108	1	pseudobulk	DESeq2	LRT	0
416	KC_AB1	crp	-0.226487400222686	0.41356223192085	1	pseudobulk	DESeq2	LRT	0
416	KC_AB1	crq	0.0326156103454881	0.635248499946866	1	pseudobulk	DESeq2	LRT	0
416	KC_AB1	Crtc	-0.018968475185051	0.637981209401663	1	pseudobulk	DESeq2	LRT	0
416	KC_AB1	cry	-0.080676624826310	1	1	pseudobulk	DESeq2	LRT	0
416	KC_AB1	Crz	2.78674512494791	0.54962276837928	1	pseudobulk	DESeq2	LRT	0
416	KC_AB1	Csas	0.849606887838128	1	1	pseudobulk	DESeq2	LRT	0
416	KC_AB1	Cse1	0.584365021815201	1	1	pseudobulk	DESeq2	LRT	0
417	KC_AB1	Csk	-0.227661245386993	0.424803090819063	1	pseudobulk	DESeq2	LRT	0
417	KC_AB1	Csl4	-0.985726180656546	0.489641775880387	1	pseudobulk	DESeq2	LRT	0
417	KC_AB1	CSN1b	-0.630057748567808	0.587920915190088	1	pseudobulk	DESeq2	LRT	0
417	KC_AB1	CSN3	0.303153072387998	0.777175659909502	1	pseudobulk	DESeq2	LRT	0
417	KC_AB1	CSN4	0.747154010244268	0.399319150976668	1	pseudobulk	DESeq2	LRT	0
417	KC_AB1	CSN5	-0.409563392955413	0.56866940809202	1	pseudobulk	DESeq2	LRT	0
417	KC_AB1	CSN6	0.566855003971728	0.48171493626695	1	pseudobulk	DESeq2	LRT	0
417	KC_AB1	CSN7	0.0795955501188347	0.627560312659974	1	pseudobulk	DESeq2	LRT	0
417	KC_AB1	CSN8	-0.986491315046721	0.229762806381437	1	pseudobulk	DESeq2	LRT	0
417	KC_AB1	Csp	0.121989383222284	0.674551713004211	1	pseudobulk	DESeq2	LRT	0
418	KC_AB1	CstF50	1.36004237795471	0.790165338156147	1	pseudobulk	DESeq2	LRT	0
418	KC_AB1	CstF64	0.0757733623244993	0.89714005530086	1	pseudobulk	DESeq2	LRT	0
418	KC_AB1	csul	-0.595304404980016	0.683619870592495	1	pseudobulk	DESeq2	LRT	0
418	KC_AB1	csw	0.0499026139064412	0.809446421055145	1	pseudobulk	DESeq2	LRT	0
418	KC_AB1	ct	-0.094476903026557	1	1	pseudobulk	DESeq2	LRT	0
418	KC_AB1	cta	-0.244799685884351	0.46037595166079	1	pseudobulk	DESeq2	LRT	0

418	KC_AB1	CtBP	0.14405278172263	0.418988659997087	1	pseudobulk	DESeq2	LRT	0
418	KC_AB1	CTCF	-0.491815190042991	0.557093931988961	1	pseudobulk	DESeq2	LRT	0
418	KC_AB1	CtIP	0.519924988261737	0.427141634087149	1	pseudobulk	DESeq2	LRT	0
418	KC_AB1	Ctl1	-0.39876027020344	0.404683352558098	1	pseudobulk	DESeq2	LRT	0
419	KC_AB1	Ctl2	-0.278923133633473	0.716999025426006	1	pseudobulk	DESeq2	LRT	0
419	KC_AB1	ctp	0.177822378040779	0.193968441460912	1	pseudobulk	DESeq2	LRT	0
419	KC_AB1	CTPsyn	-0.134175323130036	0.752606611379192	1	pseudobulk	DESeq2	LRT	0
419	KC_AB1	Ctr1A	-0.824597687236738	0.269724617474286	1	pseudobulk	DESeq2	LRT	0
419	KC_AB1	Ctr9	0.831910032659263	0.0836101865175814	1	pseudobulk	DESeq2	LRT	0
419	KC_AB1	ctrip	-0.315187373685739	0.342066169984309	1	pseudobulk	DESeq2	LRT	0
419	KC_AB1	CtsB1	-0.211402577244319	0.630130509921742	1	pseudobulk	DESeq2	LRT	0
419	KC_AB1	cu	0.0554393740136241	1	1	pseudobulk	DESeq2	LRT	0
419	KC_AB1	Cubn	0.183159795455075	1	1	pseudobulk	DESeq2	LRT	0
419	KC_AB1	cue	0.205391738439212	0.549530362010531	1	pseudobulk	DESeq2	LRT	0
420	KC_AB1	Cul1	0.0082921777957040	0.984429480853016	1	pseudobulk	DESeq2	LRT	0
420	KC_AB1	Cul2	-0.145523953653444	0.694543160684843	1	pseudobulk	DESeq2	LRT	0
420	KC_AB1	Cul3	-0.638633891596899	0.222216331479164	1	pseudobulk	DESeq2	LRT	0
420	KC_AB1	Cul4	0.654972262805659	0.482286730704547	1	pseudobulk	DESeq2	LRT	0
420	KC_AB1	Cul5	0.136566734946455	0.777854284787914	1	pseudobulk	DESeq2	LRT	0
420	KC_AB1	cv	0.849606887838128	1	1	pseudobulk	DESeq2	LRT	0
420	KC_AB1	cv-2	3.63862896487685	0.199781996084938	1	pseudobulk	DESeq2	LRT	0
420	KC_AB1	cv-c	-0.0377595084339432	0.85375405104558	1	pseudobulk	DESeq2	LRT	0
420	KC_AB1	Cwc25	0.468300619399486	0.517567861229663	1	pseudobulk	DESeq2	LRT	0
420	KC_AB1	cwo	-1.18140194600112	0.0293119483438484	1	pseudobulk	DESeq2	LRT	0
421	KC_AB1	cyc	-0.881768777033746	0.368425541858718	1	pseudobulk	DESeq2	LRT	0
421	KC_AB1	CycC	-0.464099400003524	0.519678810097514	1	pseudobulk	DESeq2	LRT	0
421	KC_AB1	CycD	0.0160940546605626	1	1	pseudobulk	DESeq2	LRT	0
421	KC_AB1	CycE	2.06541384650887	0.768363369045814	1	pseudobulk	DESeq2	LRT	0
421	KC_AB1	CycG	-0.0080227090741417	0.884546307360647	1	pseudobulk	DESeq2	LRT	0
421	KC_AB1	CycH	0.542982564383309	1	1	pseudobulk	DESeq2	LRT	0
421	KC_AB1	CycK	-0.394133125050079	0.384674898699011	1	pseudobulk	DESeq2	LRT	0
421	KC_AB1	CycT	-0.0935640544410595	0.760172930286544	1	pseudobulk	DESeq2	LRT	0
421	KC_AB1	CycY	0.0783768157043623	0.752173515699021	1	pseudobulk	DESeq2	LRT	0
421	KC_AB1	CYLD	0.574671433262471	0.423101957574719	1	pseudobulk	DESeq2	LRT	0
422	KC_AB1	Cyp1	0.0812048638308318	0.48534355105161	1	pseudobulk	DESeq2	LRT	0
422	KC_AB1	Cyp12a4	2.69386945292999	0.0744469813566584	1	pseudobulk	DESeq2	LRT	0
422	KC_AB1	Cyp12b2	1.15364441734406	0.349999254464626	1	pseudobulk	DESeq2	LRT	0
422	KC_AB1	Cyp18a1	3.150585340203	0.0855575843701455	1	pseudobulk	DESeq2	LRT	0
422	KC_AB1	Cyp28a5	-0.302448632011014	0.82885033068175	1	pseudobulk	DESeq2	LRT	0
422	KC_AB1	Cyp28d1	2.39585391709709	0.27260984245649	1	pseudobulk	DESeq2	LRT	0
422	KC_AB1	Cyp301a1	0.849606887838128	1	1	pseudobulk	DESeq2	LRT	0
422	KC_AB1	Cyp303a1	0.931109140710098	1	1	pseudobulk	DESeq2	LRT	0
422	KC_AB1	Cyp305a1	2.06531092668974	0.944672961903726	1	pseudobulk	DESeq2	LRT	0
422	KC_AB1	Cyp313a1	0.849606887838128	1	1	pseudobulk	DESeq2	LRT	0
423	KC_AB1	cyp33	-0.741243748172912	0.483520004478381	1	pseudobulk	DESeq2	LRT	0
423	KC_AB1	Cyp49a1	-0.224369133505456	0.658919747112613	1	pseudobulk	DESeq2	LRT	0
423	KC_AB1	Cyp4ac2	-0.10506771737906	1	1	pseudobulk	DESeq2	LRT	0
423	KC_AB1	Cyp4ae1	0.812815273246077	0.738558176739467	1	pseudobulk	DESeq2	LRT	0
423	KC_AB1	Cyp4d2	2.03230813134526	0.13228346197626	1	pseudobulk	DESeq2	LRT	0
423	KC_AB1	Cyp4e2	2.78674512494791	0.54962276837928	1	pseudobulk	DESeq2	LRT	0
423	KC_AB1	Cyp4g1	0.849606887838128	1	1	pseudobulk	DESeq2	LRT	0
423	KC_AB1	Cyp4g15	-0.216210029276812	1	1	pseudobulk	DESeq2	LRT	0
423	KC_AB1	Cyp4p1	2.31997030691033	0.813169507846918	1	pseudobulk	DESeq2	LRT	0

423	KC_AB1	Cyp4p2	-0.0395282947650686	1	1	pseudobulk	DESeq2	LRT	0
424	KC_AB1	Cyp4s3	1.75766265097088	0.573192547160778	1	pseudobulk	DESeq2	LRT	0
424	KC_AB1	Cyp6a13	0.849606887838128	1	1	pseudobulk	DESeq2	LRT	0
424	KC_AB1	Cyp6a17	0.849606887838128	1	1	pseudobulk	DESeq2	LRT	0
424	KC_AB1	Cyp6a2	0.183159795455075	1	1	pseudobulk	DESeq2	LRT	0
424	KC_AB1	Cyp6a20	3.41844045086015	0.351942490385471	1	pseudobulk	DESeq2	LRT	0
424	KC_AB1	Cyp6a21	-0.216210029276812	1	1	pseudobulk	DESeq2	LRT	0
424	KC_AB1	Cyp6d4	0.849606887838128	1	1	pseudobulk	DESeq2	LRT	0
424	KC_AB1	Cyp6d5	-3.26283664165193	0.129606541725589	1	pseudobulk	DESeq2	LRT	0
424	KC_AB1	Cyp6g2	0.849606887838128	1	1	pseudobulk	DESeq2	LRT	0
424	KC_AB1	Cyp6t1	0.849606887838128	1	1	pseudobulk	DESeq2	LRT	0
425	KC_AB1	Cyp6t2Psi	1.59863986092676	1	1	pseudobulk	DESeq2	LRT	0
425	KC_AB1	Cyp6u1	-1.24867676713803	0.20656654664183	1	pseudobulk	DESeq2	LRT	0
425	KC_AB1	Cyp6v1	-0.830062897938551	0.717724257445671	1	pseudobulk	DESeq2	LRT	0
425	KC_AB1	Cyp9b2	3.41844045086015	0.351942490385471	1	pseudobulk	DESeq2	LRT	0
425	KC_AB1	Cyp9c1	0.849606887838128	1	1	pseudobulk	DESeq2	LRT	0
425	KC_AB1	Cyp9f2	-1.14792238939349	0.534097302778179	1	pseudobulk	DESeq2	LRT	0
425	KC_AB1	Cyp9f3	0.849606887838128	1	1	pseudobulk	DESeq2	LRT	0
425	KC_AB1	cype	0.0910473191514412	0.540106678175375	1	pseudobulk	DESeq2	LRT	0
425	KC_AB1	Cypl	-2.65579324640552	0.0598260959911427	1	pseudobulk	DESeq2	LRT	0
425	KC_AB1	cyr	0.237022465848507	1	1	pseudobulk	DESeq2	LRT	0
426	KC_AB1	CysRS	0.12345369826182	0.858047342701161	1	pseudobulk	DESeq2	LRT	0
426	KC_AB1	CysRS-m	-0.467668324874733	0.703838456841189	1	pseudobulk	DESeq2	LRT	0
426	KC_AB1	Cyt-b5	-0.158697983265794	0.594932132908479	1	pseudobulk	DESeq2	LRT	0
426	KC_AB1	Cyt-c-d	0.849606887838128	1	1	pseudobulk	DESeq2	LRT	0
426	KC_AB1	Cyt-c-p	0.341305964083948	0.103778478595873	1	pseudobulk	DESeq2	LRT	0
426	KC_AB1	Cyt-c1	0.0863169465494895	0.711569259884829	1	pseudobulk	DESeq2	LRT	0
426	KC_AB1	d	0.849606887838128	1	1	pseudobulk	DESeq2	LRT	0
426	KC_AB1	D1	0.111064917649512	0.664867664196291	1	pseudobulk	DESeq2	LRT	0
426	KC_AB1	D12	1.64766624272741	0.0426582353101107	1	pseudobulk	DESeq2	LRT	0
426	KC_AB1	D19A	-0.633669296601804	0.479802239218677	1	pseudobulk	DESeq2	LRT	0
427	KC_AB1	D19B	-0.324052555700954	0.619410147910755	1	pseudobulk	DESeq2	LRT	0
427	KC_AB1	D2hgdh	0.366247714696288	1	1	pseudobulk	DESeq2	LRT	0
427	KC_AB1	d4	0.0562912509405921	0.59682531887484	1	pseudobulk	DESeq2	LRT	0
427	KC_AB1	da	-0.156172207778148	0.462139358819931	1	pseudobulk	DESeq2	LRT	0
427	KC_AB1	DAAM	0.0211149679499398	0.804434982561464	1	pseudobulk	DESeq2	LRT	0
427	KC_AB1	Dab	-0.146564243848284	0.648576312632594	1	pseudobulk	DESeq2	LRT	0
427	KC_AB1	dac	-0.439076451356082	0.0117685935572192	1	pseudobulk	DESeq2	LRT	0
427	KC_AB1	Dad	0.243062999400567	0.694095178153271	1	pseudobulk	DESeq2	LRT	0
427	KC_AB1	Dad1	-0.240260693207167	0.56055575999329	1	pseudobulk	DESeq2	LRT	0
427	KC_AB1	daed	0.922442135604408	0.658029847491992	1	pseudobulk	DESeq2	LRT	0
428	KC_AB1	Dak1	0.194870462062549	0.51765734030646	1	pseudobulk	DESeq2	LRT	0
428	KC_AB1	dally	0.492494586665129	0.908299348410506	1	pseudobulk	DESeq2	LRT	0
428	KC_AB1	Damm	0.849606887838128	1	1	pseudobulk	DESeq2	LRT	0
428	KC_AB1	danr	-1.16020557067325	0.492962443274044	1	pseudobulk	DESeq2	LRT	0
428	KC_AB1	dao	-0.620609300130642	0.15596797934876	1	pseudobulk	DESeq2	LRT	0
428	KC_AB1	dap	0.849606887838128	1	1	pseudobulk	DESeq2	LRT	0
428	KC_AB1	Dap160	-0.0598822246892011	0.683836588128789	1	pseudobulk	DESeq2	LRT	0
428	KC_AB1	dar1	0.849606887838128	1	1	pseudobulk	DESeq2	LRT	0
428	KC_AB1	dare	0.0835216216068552	0.854543440312867	1	pseudobulk	DESeq2	LRT	0
428	KC_AB1	Dark	-0.254306421118736	0.427579949353449	1	pseudobulk	DESeq2	LRT	0
429	KC_AB1	DAT	0.75551953162779	1	1	pseudobulk	DESeq2	LRT	0
429	KC_AB1	dati	-0.0694617453503176	0.634230666828852	1	pseudobulk	DESeq2	LRT	0

429	KC_AB1	Daxx	-0.755875845298597	0.290675360014183	1	pseudobulk	DESeq2	LRT	0
429	KC_AB1	dbe	2.21255633841496	0.261989490376044	1	pseudobulk	DESeq2	LRT	0
429	KC_AB1	dbo	-0.378424313238374	0.282901795098412	1	pseudobulk	DESeq2	LRT	0
429	KC_AB1	Dbp21E2	0.849606887838128	1	1	pseudobulk	DESeq2	LRT	0
429	KC_AB1	Dbp45A	-0.564648006886177	0.575660552765266	1	pseudobulk	DESeq2	LRT	0
429	KC_AB1	Dbp73D	-1.21354781253754	0.509253089247373	1	pseudobulk	DESeq2	LRT	0
429	KC_AB1	Dbp80	-0.360751928809209	0.105250283760949	1	pseudobulk	DESeq2	LRT	0
429	KC_AB1	dbr	0.190242424135167	0.714886578711871	1	pseudobulk	DESeq2	LRT	0
430	KC_AB1	DCAF12	0.30163096260939	0.772214153904056	1	pseudobulk	DESeq2	LRT	0
430	KC_AB1	Dci	1.4304643047634	0.429388905674059	1	pseudobulk	DESeq2	LRT	0
430	KC_AB1	dco	0.104289533223615	0.78979440920822	1	pseudobulk	DESeq2	LRT	0
430	KC_AB1	Dcp-1	-0.602207686486536	0.516783458318043	1	pseudobulk	DESeq2	LRT	0
430	KC_AB1	DCP1	-0.238156428633822	0.65916759488233	1	pseudobulk	DESeq2	LRT	0
430	KC_AB1	DCP2	0.0040895069173923	0.816257475213394	1	pseudobulk	DESeq2	LRT	0
430	KC_AB1	Dcr-1	2.06541384650887	0.768363369045814	1	pseudobulk	DESeq2	LRT	0
430	KC_AB1	Dcr-2	-1.25440990967483	0.276452587157847	1	pseudobulk	DESeq2	LRT	0
430	KC_AB1	DCTN1-p150	-0.199383906504867	0.646527067943572	1	pseudobulk	DESeq2	LRT	0
430	KC_AB1	DCTN2-p50	-0.45958744714206	0.543076679985169	1	pseudobulk	DESeq2	LRT	0
431	KC_AB1	DCTN3-p24	-0.337173774914383	0.609732935437177	1	pseudobulk	DESeq2	LRT	0
431	KC_AB1	DCTN4-p62	-0.115490383726686	0.768614518158912	1	pseudobulk	DESeq2	LRT	0
431	KC_AB1	DCTN5-p25	1.09931401152618	0.298216282074528	1	pseudobulk	DESeq2	LRT	0
431	KC_AB1	DCTN6-p27	0.580311900757064	0.268159430808426	1	pseudobulk	DESeq2	LRT	0
431	KC_AB1	Dd	0.0309935803779976	0.927924380846969	1	pseudobulk	DESeq2	LRT	0
431	KC_AB1	Ddc	0.64797910067378	0.020341403840361	1	pseudobulk	DESeq2	LRT	0
431	KC_AB1	Ddx1	-0.161735846764317	0.774686794101728	1	pseudobulk	DESeq2	LRT	0
431	KC_AB1	Deaf1	-0.376703134795277	0.349774274234208	1	pseudobulk	DESeq2	LRT	0
431	KC_AB1	DEF8	-0.624308167201518	0.573552006465027	1	pseudobulk	DESeq2	LRT	0
431	KC_AB1	defl	2.08600308111381	0.486909638608969	1	pseudobulk	DESeq2	LRT	0
432	KC_AB1	Dek	0.157767948092137	0.54752556000256	1	pseudobulk	DESeq2	LRT	0
432	KC_AB1	del	0.849606887838128	1	1	pseudobulk	DESeq2	LRT	0
432	KC_AB1	deltaCOP	-0.0257425970279971	0.637283832918213	1	pseudobulk	DESeq2	LRT	0
432	KC_AB1	Den1	0.497568994001732	0.303302401714531	1	pseudobulk	DESeq2	LRT	0
432	KC_AB1	DENR	-0.233382507211518	0.584848075649266	1	pseudobulk	DESeq2	LRT	0
432	KC_AB1	Der-1	0.309446462232483	0.5098441609019	1	pseudobulk	DESeq2	LRT	0
432	KC_AB1	Der-2	-0.389703947427742	0.548716927141442	1	pseudobulk	DESeq2	LRT	0
432	KC_AB1	Desat1	-0.0595394293822088	0.634217447962673	1	pseudobulk	DESeq2	LRT	0
432	KC_AB1	Det	2.31997030691033	0.813169507846918	1	pseudobulk	DESeq2	LRT	0
432	KC_AB1	Df31	-0.288866386600127	0.251057060560874	1	pseudobulk	DESeq2	LRT	0
433	KC_AB1	Dg	-0.715610696044305	0.0151261611735005	1	pseudobulk	DESeq2	LRT	0
433	KC_AB1	Dgk	-0.248681998530238	0.518260004362482	1	pseudobulk	DESeq2	LRT	0
433	KC_AB1	Dgkepsilon	-0.0003965999083059	0.870330350498542	1	pseudobulk	DESeq2	LRT	0
433	KC_AB1	Dgp-1	0.0749171718683366	0.644322845559055	1	pseudobulk	DESeq2	LRT	0
433	KC_AB1	dgrn	1.01528740487861	0.446733969187623	1	pseudobulk	DESeq2	LRT	0
433	KC_AB1	dgt1	-0.0824417386723228	0.798768293545217	1	pseudobulk	DESeq2	LRT	0
433	KC_AB1	dgt2	0.849606887838128	1	1	pseudobulk	DESeq2	LRT	0
433	KC_AB1	Dh31	2.06541384650887	0.768363369045814	1	pseudobulk	DESeq2	LRT	0
433	KC_AB1	Dh44	2.13009086641515	0.687046658320206	1	pseudobulk	DESeq2	LRT	0
433	KC_AB1	Dh44-R1	-1.63667579461385	0.00555215906400447	1	pseudobulk	DESeq2	LRT	0
434	KC_AB1	Dh44-R2	-0.4964670925182	0.176840303967925	1	pseudobulk	DESeq2	LRT	0
434	KC_AB1	Dhap-at	0.253367880642923	0.621135085532501	1	pseudobulk	DESeq2	LRT	0
434	KC_AB1	Dhc64C	-0.0853410155757703	0.777066960927506	1	pseudobulk	DESeq2	LRT	0
434	KC_AB1	Dhit	0.0207892322349552	0.612500832116692	1	pseudobulk	DESeq2	LRT	0
434	KC_AB1	Dhod	0.0917064358749574	1	1	pseudobulk	DESeq2	LRT	0

434	KC_AB1	DhpD	-0.62526376855221	0.653326062158568	1	pseudobulk	DESeq2	LRT	0
434	KC_AB1	Dhpr	-0.267850577264665	0.445969220447156	1	pseudobulk	DESeq2	LRT	0
434	KC_AB1	Dhx15	-0.634628265858944	0.476809329517014	1	pseudobulk	DESeq2	LRT	0
434	KC_AB1	dia	2.06541384650887	0.904905526327343	1	pseudobulk	DESeq2	LRT	0
434	KC_AB1	Diap1	-0.228991832038023	0.350100170071963	1	pseudobulk	DESeq2	LRT	0
435	KC_AB1	Diap2	0.0212467309032569	0.844902678374743	1	pseudobulk	DESeq2	LRT	0
435	KC_AB1	dib	0.849606887838128	1	1	pseudobulk	DESeq2	LRT	0
435	KC_AB1	Dic1	0.104162915146045	0.723449964036418	1	pseudobulk	DESeq2	LRT	0
435	KC_AB1	Dic3	0.849606887838128	1	1	pseudobulk	DESeq2	LRT	0
435	KC_AB1	didum	-0.693761534186147	0.327434702980506	1	pseudobulk	DESeq2	LRT	0
435	KC_AB1	Dif	-0.179201395404301	0.476757907817122	1	pseudobulk	DESeq2	LRT	0
435	KC_AB1	dikar	-0.130024917178574	0.6562327292601	1	pseudobulk	DESeq2	LRT	0
435	KC_AB1	Dim1	-0.447133780035529	0.59158789155744	1	pseudobulk	DESeq2	LRT	0
435	KC_AB1	dind	0.122016170577254	0.712337466085917	1	pseudobulk	DESeq2	LRT	0
435	KC_AB1	DIP-alpha	-0.121681951010078	0.806259033794954	1	pseudobulk	DESeq2	LRT	0
436	KC_AB1	Dip-B	0.931109140710098	1	1	pseudobulk	DESeq2	LRT	0
436	KC_AB1	Dip-C	-0.203765597954176	0.782567207712753	1	pseudobulk	DESeq2	LRT	0
436	KC_AB1	DIP-gamma	0.959106482335399	0.796808479971156	1	pseudobulk	DESeq2	LRT	0
436	KC_AB1	DIP-kappa	-0.0817510803719724	0.495046835777108	1	pseudobulk	DESeq2	LRT	0
436	KC_AB1	DIP-lambda	-0.73226530067167	0.57065335221453	1	pseudobulk	DESeq2	LRT	0
436	KC_AB1	DIP-theta	0.849606887838128	1	1	pseudobulk	DESeq2	LRT	0
436	KC_AB1	DIP1	-0.0327115024058891	0.698470430497332	1	pseudobulk	DESeq2	LRT	0
436	KC_AB1	DIP2	0.223168859201951	0.62305171643494	1	pseudobulk	DESeq2	LRT	0
436	KC_AB1	Dis3	0.769893673767581	1	1	pseudobulk	DESeq2	LRT	0
436	KC_AB1	Dis3l2	-0.89692984952467	0.321030595310477	1	pseudobulk	DESeq2	LRT	0
437	KC_AB1	disco-r	0.849606887838128	1	1	pseudobulk	DESeq2	LRT	0
437	KC_AB1	disp	-0.247426448219422	0.663425652804236	1	pseudobulk	DESeq2	LRT	0
437	KC_AB1	DJ-1alpha	0.74563012801613	0.436291170865169	1	pseudobulk	DESeq2	LRT	0
437	KC_AB1	dj-1beta	-0.179204116711776	0.780183816489081	1	pseudobulk	DESeq2	LRT	0
437	KC_AB1	dl	-0.847326435092832	0.479243311511568	1	pseudobulk	DESeq2	LRT	0
437	KC_AB1	Dl	1.59863986092676	1	1	pseudobulk	DESeq2	LRT	0
437	KC_AB1	Dlc90F	0.0419425052205275	0.65735620501595	1	pseudobulk	DESeq2	LRT	0
437	KC_AB1	dlg1	-0.240335707969288	0.0742872629864573	1	pseudobulk	DESeq2	LRT	0
437	KC_AB1	Dlg5	-0.450157076382631	0.406544772472987	1	pseudobulk	DESeq2	LRT	0
437	KC_AB1	Dlic	-0.105062880615138	0.477091311062931	1	pseudobulk	DESeq2	LRT	0
438	KC_AB1	Dlip1	1.86730827006053	0.636504446122226	1	pseudobulk	DESeq2	LRT	0
438	KC_AB1	Dlip2	-0.75715232463087	0.368861606706881	1	pseudobulk	DESeq2	LRT	0
438	KC_AB1	Dlip3	-0.566401670375258	0.413279862678873	1	pseudobulk	DESeq2	LRT	0
438	KC_AB1	Dlish	-0.216210029276812	1	1	pseudobulk	DESeq2	LRT	0
438	KC_AB1	Dll	0.849606887838128	1	1	pseudobulk	DESeq2	LRT	0
438	KC_AB1	dlp	2.44122474001094	0.404182156854988	1	pseudobulk	DESeq2	LRT	0
438	KC_AB1	dlt	0.0418184222819095	1	1	pseudobulk	DESeq2	LRT	0
438	KC_AB1	DMAP1	0.431146540899307	0.60428912360221	1	pseudobulk	DESeq2	LRT	0
438	KC_AB1	dmpd	0.424930099044665	0.534426295340314	1	pseudobulk	DESeq2	LRT	0
438	KC_AB1	dmrt11E	0.849606887838128	1	1	pseudobulk	DESeq2	LRT	0
439	KC_AB1	dmt	0.162970982752759	0.801479754956558	1	pseudobulk	DESeq2	LRT	0
439	KC_AB1	Dmtn	0.0414990072639405	0.616261275071217	1	pseudobulk	DESeq2	LRT	0
439	KC_AB1	Dnah3	0.849606887838128	1	1	pseudobulk	DESeq2	LRT	0
439	KC_AB1	DnaJ-1	-0.369122806204079	0.675083528520609	1	pseudobulk	DESeq2	LRT	0
439	KC_AB1	DnaJ-H	-0.628148993786482	0.305637375468196	1	pseudobulk	DESeq2	LRT	0
439	KC_AB1	DNAlig3	-0.0015555042744908	0.797698460521648	1	pseudobulk	DESeq2	LRT	0
439	KC_AB1	DNAlig4	-1.37846396121506	0.508668417798892	1	pseudobulk	DESeq2	LRT	0
439	KC_AB1	DNApol-alpha50	2.06531092668974	0.944672961903726	1	pseudobulk	DESeq2	LRT	0

439	KC_AB1	DNApol-delta	1.59863986092676	1	1	pseudobulk	DESeq2	LRT	0
439	KC_AB1	DNApol-epsilon25	0.849606887838128	1	1	pseudobulk	DESeq2	LRT	0
440	KC_AB1	DNApol-eta	-1.02645081659277	0.538168704635352	1	pseudobulk	DESeq2	LRT	0
440	KC_AB1	DNApol-iota	0.0139619037307553	0.881030923556158	1	pseudobulk	DESeq2	LRT	0
440	KC_AB1	DNApol-zeta	1.28605039148513	0.191696309668761	1	pseudobulk	DESeq2	LRT	0
440	KC_AB1	DNaseII	-1.00752466718157	0.580536516624078	1	pseudobulk	DESeq2	LRT	0
440	KC_AB1	dnc	-0.0616360409516506	0.557907460464184	1	pseudobulk	DESeq2	LRT	0
440	KC_AB1	dnd	1.11528148199936	1	1	pseudobulk	DESeq2	LRT	0
440	KC_AB1	dnk	0.939212571786077	0.384749872656166	1	pseudobulk	DESeq2	LRT	0
440	KC_AB1	dnr1	-1.26201389899237	0.37348226830611	1	pseudobulk	DESeq2	LRT	0
440	KC_AB1	dnt	0.478950777462741	0.325715775866643	1	pseudobulk	DESeq2	LRT	0
440	KC_AB1	Dnz1	-0.468112309981463	0.407741161145127	1	pseudobulk	DESeq2	LRT	0
441	KC_AB1	Doa	-0.802923399078155	0.0645893262277048	1	pseudobulk	DESeq2	LRT	0
441	KC_AB1	dock	0.206302195249755	0.576953928344105	1	pseudobulk	DESeq2	LRT	0
441	KC_AB1	dod	0.202139301404354	0.638985394746032	1	pseudobulk	DESeq2	LRT	0
441	KC_AB1	Dok	0.849606887838128	1	1	pseudobulk	DESeq2	LRT	0
441	KC_AB1	dom	-0.329595918886843	0.487265064870936	1	pseudobulk	DESeq2	LRT	0
441	KC_AB1	dome	1.0671043855741	0.390274219177512	1	pseudobulk	DESeq2	LRT	0
441	KC_AB1	dop	-0.0906850061530906	0.878118521762395	1	pseudobulk	DESeq2	LRT	0
441	KC_AB1	Dop1R1	-0.35788665261792	0.0719271049485674	1	pseudobulk	DESeq2	LRT	0
441	KC_AB1	Dop1R2	-0.101084014700325	0.510105979025093	1	pseudobulk	DESeq2	LRT	0
441	KC_AB1	Dop2R	-0.090890900888599	0.499351397805361	1	pseudobulk	DESeq2	LRT	0
442	KC_AB1	DopEcR	-0.320229533087468	0.296593354680635	1	pseudobulk	DESeq2	LRT	0
442	KC_AB1	dor	-2.11652384306353	0.206380143099425	1	pseudobulk	DESeq2	LRT	0
442	KC_AB1	DOR	0.0482720853709264	0.782003412672534	1	pseudobulk	DESeq2	LRT	0
442	KC_AB1	dos	-0.161786199813253	0.723094152269818	1	pseudobulk	DESeq2	LRT	0
442	KC_AB1	Dp	-0.0981857643017765	0.778925561784316	1	pseudobulk	DESeq2	LRT	0
442	KC_AB1	Dp1	-0.386816186279973	0.589926587970604	1	pseudobulk	DESeq2	LRT	0
442	KC_AB1	Dpck	0.233024927780487	0.60174435927809	1	pseudobulk	DESeq2	LRT	0
442	KC_AB1	DPCoAC	1.20936298497022	0.263892524531553	1	pseudobulk	DESeq2	LRT	0
442	KC_AB1	Dph1	-0.673973387349693	0.737308747629933	1	pseudobulk	DESeq2	LRT	0
442	KC_AB1	Dph5	0.134032735584527	0.684704037837025	1	pseudobulk	DESeq2	LRT	0
443	KC_AB1	Dpit47	-1.63602097691	0.387761117184358	1	pseudobulk	DESeq2	LRT	0
443	KC_AB1	dpn	2.06541384650887	0.768363369045814	1	pseudobulk	DESeq2	LRT	0
443	KC_AB1	dpp	-0.216210029276812	1	1	pseudobulk	DESeq2	LRT	0
443	KC_AB1	DppIII	0.026287857373267	0.691157157465737	1	pseudobulk	DESeq2	LRT	0
443	KC_AB1	dpr1	-0.758421438230061	0.371256519096403	1	pseudobulk	DESeq2	LRT	0
443	KC_AB1	dpr12	-0.257255785168647	0.198758478927664	1	pseudobulk	DESeq2	LRT	0
443	KC_AB1	dpr14	-0.232902902643874	0.336576194213335	1	pseudobulk	DESeq2	LRT	0
443	KC_AB1	dpr16	0.849606887838128	1	1	pseudobulk	DESeq2	LRT	0
443	KC_AB1	dpr17	0.210869981205978	0.326989176282083	1	pseudobulk	DESeq2	LRT	0
443	KC_AB1	dpr19	0.326376650758376	0.305509681901412	1	pseudobulk	DESeq2	LRT	0
444	KC_AB1	dpr2	-0.425433072499985	0.639887744012494	1	pseudobulk	DESeq2	LRT	0
444	KC_AB1	dpr20	-1.37328433054683	0.0454567463339101	1	pseudobulk	DESeq2	LRT	0
444	KC_AB1	dpr3	-0.344697397454955	0.652693255719208	1	pseudobulk	DESeq2	LRT	0
444	KC_AB1	dpr4	0.19495641101401	0.485889402270579	1	pseudobulk	DESeq2	LRT	0
444	KC_AB1	dpr5	0.584031169630489	0.145921957716584	1	pseudobulk	DESeq2	LRT	0
444	KC_AB1	dpr6	-0.306370374516126	0.317778611061949	1	pseudobulk	DESeq2	LRT	0
444	KC_AB1	dpr7	-0.0416984319774342	0.685396786825288	1	pseudobulk	DESeq2	LRT	0
444	KC_AB1	dpr8	-0.373253811630246	0.0155616406654292	1	pseudobulk	DESeq2	LRT	0
444	KC_AB1	dpr9	-0.047517298639459	0.815648182555749	1	pseudobulk	DESeq2	LRT	0
444	KC_AB1	dpy	1.94653483709093	0.146191975260949	1	pseudobulk	DESeq2	LRT	0
445	KC_AB1	Dpy-30L1	-0.351658315187922	0.458580712668889	1	pseudobulk	DESeq2	LRT	0

445	KC_AB1	Drak	-0.623112528668527	0.0775074292260222	1	1	pseudobulk	DESeq2	LRT	0
445	KC_AB1	Drat	-0.110874141168714	0.473343982605067	1	1	pseudobulk	DESeq2	LRT	0
445	KC_AB1	drd	-1.25393399638069	0.612054104673377	1	1	pseudobulk	DESeq2	LRT	0
445	KC_AB1	dre4	-0.252330990450303	0.678335872452378	1	1	pseudobulk	DESeq2	LRT	0
445	KC_AB1	Dredd	3.150585340203	0.085575843701455	1	1	pseudobulk	DESeq2	LRT	0
445	KC_AB1	Dref	-0.0604634363016463	0.792929905352482	1	1	pseudobulk	DESeq2	LRT	0
445	KC_AB1	Drep1	0.341566979329183	0.69023433184503	1	1	pseudobulk	DESeq2	LRT	0
445	KC_AB1	Drep2	-0.177249595323057	0.38146301727549	1	1	pseudobulk	DESeq2	LRT	0
445	KC_AB1	Drep3	2.06531092668974	0.944672961903726	1	1	pseudobulk	DESeq2	LRT	0
446	KC_AB1	Drep4	0.162361251464331	0.666880357316474	1	1	pseudobulk	DESeq2	LRT	0
446	KC_AB1	Drice	0.337335040743366	1	1	1	pseudobulk	DESeq2	LRT	0
446	KC_AB1	Drip	2.06541384650887	0.795866856626082	1	1	pseudobulk	DESeq2	LRT	0
446	KC_AB1	drk	0.0125463367599251	0.899635097652433	1	1	pseudobulk	DESeq2	LRT	0
446	KC_AB1	drl	0.0403354211201317	0.948162044273888	1	1	pseudobulk	DESeq2	LRT	0
446	KC_AB1	Drl-2	-2.77514004697929	0.107349805077204	1	1	pseudobulk	DESeq2	LRT	0
446	KC_AB1	Dro	0.931109140710098	1	1	1	pseudobulk	DESeq2	LRT	0
446	KC_AB1	Droj2	-0.261839073260626	0.32220664871074	1	1	pseudobulk	DESeq2	LRT	0
446	KC_AB1	Dronc	-0.592288094814511	0.613650626645975	1	1	pseudobulk	DESeq2	LRT	0
446	KC_AB1	drongo	-0.337187818279289	0.616321291050704	1	1	pseudobulk	DESeq2	LRT	0
447	KC_AB1	drosha	-0.694549668826606	0.5355507092204	1	1	pseudobulk	DESeq2	LRT	0
447	KC_AB1	Drp1	-0.131366377285673	0.525260741301086	1	1	pseudobulk	DESeq2	LRT	0
447	KC_AB1	drpr	-0.59332394269418	0.265972201919848	1	1	pseudobulk	DESeq2	LRT	0
447	KC_AB1	Drs	0.849606887838128	1	1	1	pseudobulk	DESeq2	LRT	0
447	KC_AB1	Drsl2	0.849606887838128	1	1	1	pseudobulk	DESeq2	LRT	0
447	KC_AB1	ds	-1.46245891300785	0.380550779422469	1	1	pseudobulk	DESeq2	LRT	0
447	KC_AB1	dsb	-0.216209988775614	1	1	1	pseudobulk	DESeq2	LRT	0
447	KC_AB1	Dscam1	-0.307262450966815	0.251390880292602	1	1	pseudobulk	DESeq2	LRT	0
447	KC_AB1	Dscam2	0.0585451175235473	0.684421499872894	1	1	pseudobulk	DESeq2	LRT	0
447	KC_AB1	Dscam3	1.06759111434625	1	1	1	pseudobulk	DESeq2	LRT	0
448	KC_AB1	Dscam4	-0.683432360596655	0.0141758452804868	1	1	pseudobulk	DESeq2	LRT	0
448	KC_AB1	dsd	-0.210947190725035	0.390158815360888	1	1	pseudobulk	DESeq2	LRT	0
448	KC_AB1	dsh	0.320616463483683	0.408806052884059	1	1	pseudobulk	DESeq2	LRT	0
448	KC_AB1	Dsk	0.849606887838128	1	1	1	pseudobulk	DESeq2	LRT	0
448	KC_AB1	Dsor1	0.0521077477559108	0.840802614756986	1	1	pseudobulk	DESeq2	LRT	0
448	KC_AB1	Dsp1	0.0579348051842537	0.787097469453244	1	1	pseudobulk	DESeq2	LRT	0
448	KC_AB1	dsx	1.59863986092676	1	1	1	pseudobulk	DESeq2	LRT	0
448	KC_AB1	dsx-c73A	1.59863986092676	1	1	1	pseudobulk	DESeq2	LRT	0
448	KC_AB1	Dtd	2.10726702383923	0.118735137694877	1	1	pseudobulk	DESeq2	LRT	0
448	KC_AB1	dtn	-0.0208238768251284	0.799125766019643	1	1	pseudobulk	DESeq2	LRT	0
449	KC_AB1	dtr	0.849606887838128	1	1	1	pseudobulk	DESeq2	LRT	0
449	KC_AB1	Duba	-0.384265816868533	0.413820999507247	1	1	pseudobulk	DESeq2	LRT	0
449	KC_AB1	DUBAI	0.0014026146558534	0.720484274287912	1	1	pseudobulk	DESeq2	LRT	0
449	KC_AB1	dunk	0.86496253043377	0.50733079267364	1	1	pseudobulk	DESeq2	LRT	0
449	KC_AB1	Duox	2.78674512494791	0.54962276837928	1	1	pseudobulk	DESeq2	LRT	0
449	KC_AB1	dup	2.44682123549748	0.230307111765361	1	1	pseudobulk	DESeq2	LRT	0
449	KC_AB1	dUTPase	0.183159795463042	1	1	1	pseudobulk	DESeq2	LRT	0
449	KC_AB1	dve	1.59863986092676	1	1	1	pseudobulk	DESeq2	LRT	0
449	KC_AB1	dwg	0.0090323591528656	1	1	1	pseudobulk	DESeq2	LRT	0
449	KC_AB1	Dyb	-0.305116168073631	0.123435543990764	1	1	pseudobulk	DESeq2	LRT	0
450	KC_AB1	Dyrk2	-0.278172434442966	0.372217534610448	1	1	pseudobulk	DESeq2	LRT	0
450	KC_AB1	Dyrk3	0.0011529996542204	0.51179825714113	1	1	pseudobulk	DESeq2	LRT	0
450	KC_AB1	Dys	-0.0867815827922641	0.736614693012799	1	1	pseudobulk	DESeq2	LRT	0
450	KC_AB1	Dysb	1.59863986092676	1	1	1	pseudobulk	DESeq2	LRT	0

450	KC_AB1	dysc	-0.110930021715816	0.338353511406132	1	pseudobulk	DESeq2	LRT		0
450	KC_AB1	dyw	-0.405319165074576	0.757016930501376	1	pseudobulk	DESeq2	LRT		0
450	KC_AB1	e	-1.67838920401787	0.425843067852459	1	pseudobulk	DESeq2	LRT		0
450	KC_AB1	E(bx)	-0.131586008595949	0.517158012908063	1	pseudobulk	DESeq2	LRT		0
450	KC_AB1	E(Pc)	-0.211627023187312	0.427808881905018	1	pseudobulk	DESeq2	LRT		0
450	KC_AB1	e(r)	-0.307391855467011	0.322501740733171	1	pseudobulk	DESeq2	LRT		0
451	KC_AB1	E(spl)mbeta-HLH	-0.216209988775614	1	1	pseudobulk	DESeq2	LRT		0
451	KC_AB1	E(var)3-9	0.19604492882729	0.602230904859312	1	pseudobulk	DESeq2	LRT		0
451	KC_AB1	e(y)1	-0.0747054678111253	0.894145296027091	1	pseudobulk	DESeq2	LRT		0
451	KC_AB1	e(y)2	0.551121729332217	0.416426417676886	1	pseudobulk	DESeq2	LRT		0
451	KC_AB1	e(y)2b	0.237022465848507	1	1	pseudobulk	DESeq2	LRT		0
451	KC_AB1	e(y)3	-0.313071572297869	0.485768587829736	1	pseudobulk	DESeq2	LRT		0
451	KC_AB1	E(z)	0.893227189591427	0.270201819437068	1	pseudobulk	DESeq2	LRT		0
451	KC_AB1	E23	0.366247714696288	1	1	pseudobulk	DESeq2	LRT		0
451	KC_AB1	E2f1	-0.0749316834179626	0.781456653300105	1	pseudobulk	DESeq2	LRT		0
451	KC_AB1	E2f2	-0.906082625467925	0.371472622147135	1	pseudobulk	DESeq2	LRT		0
452	KC_AB1	Eaat1	-0.301948171172976	0.808165217781496	1	pseudobulk	DESeq2	LRT		0
452	KC_AB1	Eaat2	0.0288795792238045	0.782484937116327	1	pseudobulk	DESeq2	LRT		0
452	KC_AB1	Eaf	-0.189552627690421	0.637584234335615	1	pseudobulk	DESeq2	LRT		0
452	KC_AB1	Eaf6	0.122303030163598	0.823687646642134	1	pseudobulk	DESeq2	LRT		0
452	KC_AB1	eag	-0.689191443053297	0.0152312371833494	1	pseudobulk	DESeq2	LRT		0
452	KC_AB1	ear	-0.0425193068891583	0.948137074516058	1	pseudobulk	DESeq2	LRT		0
452	KC_AB1	eas	-0.0324113161240999	0.770913172771345	1	pseudobulk	DESeq2	LRT		0
452	KC_AB1	east	-0.505816656053464	0.101599838031648	1	pseudobulk	DESeq2	LRT		0
452	KC_AB1	eater	-0.0065790798335215	1	1	pseudobulk	DESeq2	LRT		0
452	KC_AB1	Eato	-0.0373503477706651	0.766619159240951	1	pseudobulk	DESeq2	LRT		0
453	KC_AB1	Eb1	0.327113532533665	0.30919305982747	1	pseudobulk	DESeq2	LRT		0
453	KC_AB1	ebd1	0.144905902879444	0.861300583376949	1	pseudobulk	DESeq2	LRT		0
453	KC_AB1	ebd2	0.472044356714715	0.48233679997353	1	pseudobulk	DESeq2	LRT		0
453	KC_AB1	ebi	-1.25589147756086	0.244901733360751	1	pseudobulk	DESeq2	LRT		0
453	KC_AB1	ebo	-0.961672462757892	0.426601885911053	1	pseudobulk	DESeq2	LRT		0
453	KC_AB1	ec	3.64217331632531	0.246682905127758	1	pseudobulk	DESeq2	LRT		0
453	KC_AB1	eca	0.0967600751524335	0.850614724266351	1	pseudobulk	DESeq2	LRT		0
453	KC_AB1	ecd	0.84568947843537	0.343914590199979	1	pseudobulk	DESeq2	LRT		0
453	KC_AB1	Echs1	-0.176127043233816	0.767398020165225	1	pseudobulk	DESeq2	LRT		0
453	KC_AB1	ECSIT	-0.530708105627659	0.611679733536277	1	pseudobulk	DESeq2	LRT		0
454	KC_AB1	ect	3.16697464674408	0.406679230261689	1	pseudobulk	DESeq2	LRT		0
454	KC_AB1	Ect3	1.06759111434625	1	1	pseudobulk	DESeq2	LRT		0
454	KC_AB1	Ect4	0.791157241655684	0.719970122933164	1	pseudobulk	DESeq2	LRT		0
454	KC_AB1	ed	-1.88346432495891	0.053609272384822	1	pseudobulk	DESeq2	LRT		0
454	KC_AB1	Edc3	-0.362283006297148	0.483992261055932	1	pseudobulk	DESeq2	LRT		0
454	KC_AB1	Edem1	-0.332956819336416	0.715578288990054	1	pseudobulk	DESeq2	LRT		0
454	KC_AB1	Edem2	-0.31542927970265	0.555382290219587	1	pseudobulk	DESeq2	LRT		0
454	KC_AB1	Edg78E	2.31997030691033	0.813169507846918	1	pseudobulk	DESeq2	LRT		0
454	KC_AB1	EDTP	-0.0018437909470013	0.75044210482574	1	pseudobulk	DESeq2	LRT		0
454	KC_AB1	eEF1alpha1	-0.632615530143398	0.372377306164982	1	pseudobulk	DESeq2	LRT		0
455	KC_AB1	eEF1alpha2	-0.221885999720974	0.261071429219354	1	pseudobulk	DESeq2	LRT		0
455	KC_AB1	eEF1beta	-1.28391472895074	0.119858845399146	1	pseudobulk	DESeq2	LRT		0
455	KC_AB1	eEF1delta	-0.641851014773723	0.21607216012354	1	pseudobulk	DESeq2	LRT		0
455	KC_AB1	eEF1gamma	-0.602004745411371	0.361661230944801	1	pseudobulk	DESeq2	LRT		0
455	KC_AB1	eEF2	-0.2921654072562	0.246203383923976	1	pseudobulk	DESeq2	LRT		0
455	KC_AB1	eEF5	0.0940938579616599	0.732806034599011	1	pseudobulk	DESeq2	LRT		0
455	KC_AB1	eEFSec	0.849606887838128	1	1	pseudobulk	DESeq2	LRT		0

455	KC_AB1	Efa6	-0.737952446254476	0.246486471215685	1	pseudobulk	DESeq2	LRT	0
455	KC_AB1	eff	0.0589377073234146	0.516030005758638	1	pseudobulk	DESeq2	LRT	0
455	KC_AB1	Efhc1.1	0.849606887838128	1	1	pseudobulk	DESeq2	LRT	0
456	KC_AB1	Efr	0.267517244832809	0.636906608640448	1	pseudobulk	DESeq2	LRT	0
456	KC_AB1	eg	0.849606887838128	1	1	pseudobulk	DESeq2	LRT	0
456	KC_AB1	Egfr	3.63836937689872	0.172338795213305	1	pseudobulk	DESeq2	LRT	0
456	KC_AB1	egg	0.199244326523609	0.802194531655882	1	pseudobulk	DESeq2	LRT	0
456	KC_AB1	egh	-0.0885099508325047	0.51812123335349	1	pseudobulk	DESeq2	LRT	0
456	KC_AB1	egl	0.0432311257971187	0.940641479783732	1	pseudobulk	DESeq2	LRT	0
456	KC_AB1	Egm	-0.938658506702721	0.475664283912025	1	pseudobulk	DESeq2	LRT	0
456	KC_AB1	Ehbp1	-0.278566885015605	0.456869929654841	1	pseudobulk	DESeq2	LRT	0
456	KC_AB1	eIF1	0.0809438991669981	0.672869206659336	1	pseudobulk	DESeq2	LRT	0
456	KC_AB1	eIF1A	-0.0612570382278323	0.64968892932013	1	pseudobulk	DESeq2	LRT	0
457	KC_AB1	eIF2alpha	-0.230591159286297	0.577317376524437	1	pseudobulk	DESeq2	LRT	0
457	KC_AB1	eIF2Balpha	-0.239826031717758	0.736971459415984	1	pseudobulk	DESeq2	LRT	0
457	KC_AB1	eIF2Bbeta	0.311437138184587	0.80252389704943	1	pseudobulk	DESeq2	LRT	0
457	KC_AB1	eIF2Bdelta	-0.201301738099584	0.746934438765046	1	pseudobulk	DESeq2	LRT	0
457	KC_AB1	eIF2Bepsilon	-0.742167283396107	0.553113914988146	1	pseudobulk	DESeq2	LRT	0
457	KC_AB1	eIF2beta	0.256262479770969	0.518501181147126	1	pseudobulk	DESeq2	LRT	0
457	KC_AB1	eIF2Bgamma	0.022103439969825	0.871616471867028	1	pseudobulk	DESeq2	LRT	0
457	KC_AB1	eIF2D	0.240086918805004	0.675621659932549	1	pseudobulk	DESeq2	LRT	0
457	KC_AB1	eIF2gamma	-0.0830959115850061	0.716178134202284	1	pseudobulk	DESeq2	LRT	0
457	KC_AB1	eIF3a	0.186066470273378	0.542086268359501	1	pseudobulk	DESeq2	LRT	0
458	KC_AB1	eIF3b	-0.17692196640418	0.688538601047823	1	pseudobulk	DESeq2	LRT	0
458	KC_AB1	eIF3c	-0.309022734107685	0.347154044240683	1	pseudobulk	DESeq2	LRT	0
458	KC_AB1	eIF3d1	-0.182639572714595	0.714472244239046	1	pseudobulk	DESeq2	LRT	0
458	KC_AB1	eIF3e	-0.0983078152494151	0.529963714024548	1	pseudobulk	DESeq2	LRT	0
458	KC_AB1	eIF3f1	-0.454258315354068	0.285953910136882	1	pseudobulk	DESeq2	LRT	0
458	KC_AB1	eIF3g1	-0.0351621548691572	0.955398808149834	1	pseudobulk	DESeq2	LRT	0
458	KC_AB1	eIF3g2	-0.84087894522729	0.689871361737426	1	pseudobulk	DESeq2	LRT	0
458	KC_AB1	eIF3h	-0.300798964831245	0.534037108716314	1	pseudobulk	DESeq2	LRT	0
458	KC_AB1	eIF3i	0.0056765993199566	0.509130810824082	1	pseudobulk	DESeq2	LRT	0
458	KC_AB1	eIF3j	-0.140599466698044	0.745271132108632	1	pseudobulk	DESeq2	LRT	0
459	KC_AB1	eIF3k	0.307297766784732	0.649787294589346	1	pseudobulk	DESeq2	LRT	0
459	KC_AB1	eIF3l	-0.257612132891475	0.426311789098402	1	pseudobulk	DESeq2	LRT	0
459	KC_AB1	eIF3m	-0.43394589356799	0.313763802942159	1	pseudobulk	DESeq2	LRT	0
459	KC_AB1	eIF4A	-0.149555960088706	0.388715319886239	1	pseudobulk	DESeq2	LRT	0
459	KC_AB1	eIF4B	-0.161369475805165	0.647462462836986	1	pseudobulk	DESeq2	LRT	0
459	KC_AB1	eIF4E1	-0.25215173444813	0.448376527457079	1	pseudobulk	DESeq2	LRT	0
459	KC_AB1	eIF4EHP	-0.112342462105274	0.547414602078948	1	pseudobulk	DESeq2	LRT	0
459	KC_AB1	eIF4G1	-0.296828773362374	0.37567574233229	1	pseudobulk	DESeq2	LRT	0
459	KC_AB1	eIF4G2	0.0593520251902966	0.612778773034585	1	pseudobulk	DESeq2	LRT	0
459	KC_AB1	eIF4H1	-0.111687565886132	0.767478626270718	1	pseudobulk	DESeq2	LRT	0
460	KC_AB1	eIF5	0.161068844152668	0.514968988356326	1	pseudobulk	DESeq2	LRT	0
460	KC_AB1	eIF5B	-0.303846055906583	0.325150750266203	1	pseudobulk	DESeq2	LRT	0
460	KC_AB1	eIF6	0.114393070116907	0.902138257903094	1	pseudobulk	DESeq2	LRT	0
460	KC_AB1	Eip55E	-0.0070516781099352	0.796946897829901	1	pseudobulk	DESeq2	LRT	0
460	KC_AB1	Eip63E	-0.180628779050482	0.548074438374101	1	pseudobulk	DESeq2	LRT	0
460	KC_AB1	Eip63F-1	2.7043030917924	0.0147673015751486	1	pseudobulk	DESeq2	LRT	0
460	KC_AB1	Eip71CD	0.268852683558029	0.534932653894004	1	pseudobulk	DESeq2	LRT	0
460	KC_AB1	Eip74EF	-0.986037514986749	0.214661169462571	1	pseudobulk	DESeq2	LRT	0
460	KC_AB1	Eip75B	-0.0856731837176231	0.61301910335033	1	pseudobulk	DESeq2	LRT	0
460	KC_AB1	Eip78C	-0.681914961199471	0.41915802312007	1	pseudobulk	DESeq2	LRT	0

461	KC_AB1	Eip93F	-0.20633216608752	0.0792664551821166	1	pseudobulk	DESeq2	LRT		0
461	KC_AB1	Ekar	0.849606887838128	1	1	pseudobulk	DESeq2	LRT		0
461	KC_AB1	elav	-0.310379003671986	0.129686288679228	1	pseudobulk	DESeq2	LRT		0
461	KC_AB1	elB	-1.31334671690253	0.014937687126822	1	pseudobulk	DESeq2	LRT		0
461	KC_AB1	elgi	0.73580586305359	0.454529930214921	1	pseudobulk	DESeq2	LRT		0
461	KC_AB1	Elk	-0.40851249945732	0.0112479799310868	1	pseudobulk	DESeq2	LRT		0
461	KC_AB1	elm	-0.256852717045216	0.556959869114796	1	pseudobulk	DESeq2	LRT		0
461	KC_AB1	EloA	-0.609307941685688	0.340045158198146	1	pseudobulk	DESeq2	LRT		0
461	KC_AB1	EloB	-0.198261213657082	0.579518558152286	1	pseudobulk	DESeq2	LRT		0
461	KC_AB1	EloC	-0.10208982578582	0.616605101263708	1	pseudobulk	DESeq2	LRT		0
462	KC_AB1	ELOVL	4.10705356122222	0.288827886762194	1	pseudobulk	DESeq2	LRT		0
462	KC_AB1	Elp1	-0.10639339909891	0.794108960627831	1	pseudobulk	DESeq2	LRT		0
462	KC_AB1	Elp2	-0.515281586991448	0.755410408991726	1	pseudobulk	DESeq2	LRT		0
462	KC_AB1	Elp3	-0.276994764059515	0.740372092000983	1	pseudobulk	DESeq2	LRT		0
462	KC_AB1	Elys	-0.553854691963132	0.545340368867584	1	pseudobulk	DESeq2	LRT		0
462	KC_AB1	ema	-0.237143135489499	0.72822709350625	1	pseudobulk	DESeq2	LRT		0
462	KC_AB1	emb	0.0297471054373133	0.568186892749851	1	pseudobulk	DESeq2	LRT		0
462	KC_AB1	emc	-0.0461760056845001	0.808126193759016	1	pseudobulk	DESeq2	LRT		0
462	KC_AB1	EMC1	-0.87206176085139	0.304392769293796	1	pseudobulk	DESeq2	LRT		0
462	KC_AB1	EMC3	0.181450126227566	0.778096553019341	1	pseudobulk	DESeq2	LRT		0
463	KC_AB1	EMC8-9	-0.0401076775305463	0.594301192308689	1	pseudobulk	DESeq2	LRT		0
463	KC_AB1	emp	2.06541384650887	0.904905526327343	1	pseudobulk	DESeq2	LRT		0
463	KC_AB1	EMRE	0.289749170877317	0.490716051867684	1	pseudobulk	DESeq2	LRT		0
463	KC_AB1	ena	-0.287983284379331	0.487478315055022	1	pseudobulk	DESeq2	LRT		0
463	KC_AB1	enc	0.381663488306409	0.0339681982299063	1	pseudobulk	DESeq2	LRT		0
463	KC_AB1	EndoA	-0.100574368390172	0.598922774859087	1	pseudobulk	DESeq2	LRT		0
463	KC_AB1	EndoB	-0.79111650491874	0.224329899617871	1	pseudobulk	DESeq2	LRT		0
463	KC_AB1	EndoG	0.178326611092034	0.743050875867408	1	pseudobulk	DESeq2	LRT		0
463	KC_AB1	EndoGI	0.542982564383309	1	1	pseudobulk	DESeq2	LRT		0
463	KC_AB1	endos	0.270245534528091	0.406247090693211	1	pseudobulk	DESeq2	LRT		0
464	KC_AB1	Eno	0.225516496548056	0.392768173175887	1	pseudobulk	DESeq2	LRT		0
464	KC_AB1	enok	-0.421849997823218	0.444139510408255	1	pseudobulk	DESeq2	LRT		0
464	KC_AB1	ens	-0.0967399393829617	0.512753927082121	1	pseudobulk	DESeq2	LRT		0
464	KC_AB1	Ent1	-0.632242873273803	0.51673362582636	1	pseudobulk	DESeq2	LRT		0
464	KC_AB1	Ent2	-0.604146903514765	0.579875172163428	1	pseudobulk	DESeq2	LRT		0
464	KC_AB1	Ent3	-0.171214725800697	0.554241890752818	1	pseudobulk	DESeq2	LRT		0
464	KC_AB1	Eogt	-1.79388115252064	0.231210300982156	1	pseudobulk	DESeq2	LRT		0
464	KC_AB1	Epac	-0.19698351532854	1	1	pseudobulk	DESeq2	LRT		0
464	KC_AB1	Epg5	0.415899868295729	0.56742883917436	1	pseudobulk	DESeq2	LRT		0
464	KC_AB1	Eph	-0.171137462275918	0.493860766218247	1	pseudobulk	DESeq2	LRT		0
465	KC_AB1	Ephrin	-0.17518263634466	0.438585935807585	1	pseudobulk	DESeq2	LRT		0
465	KC_AB1	Eps-15	-0.10466233748845	0.755814396570326	1	pseudobulk	DESeq2	LRT		0
465	KC_AB1	epsilonCOP	-0.876579238768588	0.363089509006089	1	pseudobulk	DESeq2	LRT		0
465	KC_AB1	Ercc1	1.59863986092676	1	1	pseudobulk	DESeq2	LRT		0
465	KC_AB1	eRF1	-0.123918115137655	0.758920412601001	1	pseudobulk	DESeq2	LRT		0
465	KC_AB1	eRF3	-0.65640456187884	0.463182757398184	1	pseudobulk	DESeq2	LRT		0
465	KC_AB1	ergic53	0.149799725768501	0.73273858115416	1	pseudobulk	DESeq2	LRT		0
465	KC_AB1	Ero1L	-0.288019933457771	0.670182109000268	1	pseudobulk	DESeq2	LRT		0
465	KC_AB1	ERp60	0.318387519942604	0.665758087053324	1	pseudobulk	DESeq2	LRT		0
465	KC_AB1	ERR	0.144086743441525	0.641976483159808	1	pseudobulk	DESeq2	LRT		0
466	KC_AB1	Es2	-1.14122489788792	0.384238221432274	1	pseudobulk	DESeq2	LRT		0
466	KC_AB1	esc	2.06541384650887	0.768363369045814	1	pseudobulk	DESeq2	LRT		0
466	KC_AB1	escl	2.49437977965853	0.298423236653912	1	pseudobulk	DESeq2	LRT		0

466	KC_AB1	esn	-0.556225867709297	0.589049502550166	1	pseudobulk	DESeq2	LRT	0
466	KC_AB1	Est-Q	0.849606887838128	1	1	pseudobulk	DESeq2	LRT	0
466	KC_AB1	Esyt2	-1.1500919287557	0.0965339317387351	1	pseudobulk	DESeq2	LRT	0
466	KC_AB1	Etf-QO	-0.190088533275455	0.842561350568363	1	pseudobulk	DESeq2	LRT	0
466	KC_AB1	ETHR	0.849606887838128	1	1	pseudobulk	DESeq2	LRT	0
466	KC_AB1	Etl1	-0.029839847111397	0.831168278323276	1	pseudobulk	DESeq2	LRT	0
466	KC_AB1	Ets97D	-1.01185411356949	0.402903721318971	1	pseudobulk	DESeq2	LRT	0
467	KC_AB1	Ets98B	-1.5805998677877	0.524230026009756	1	pseudobulk	DESeq2	LRT	0
467	KC_AB1	euc	1.07614194220411	0.854049384657625	1	pseudobulk	DESeq2	LRT	0
467	KC_AB1	Evi5	0.160318933623415	0.447955054128448	1	pseudobulk	DESeq2	LRT	0
467	KC_AB1	ewg	-0.122185465287758	0.511305764265678	1	pseudobulk	DESeq2	LRT	0
467	KC_AB1	ex	3.64217331632531	0.246682905127758	1	pseudobulk	DESeq2	LRT	0
467	KC_AB1	exd	-0.007695059755141	0.520116095253494	1	pseudobulk	DESeq2	LRT	0
467	KC_AB1	Exd2	-1.22288537547408	0.426956038395241	1	pseudobulk	DESeq2	LRT	0
467	KC_AB1	Exn	-0.952003464772405	0.122163362306406	1	pseudobulk	DESeq2	LRT	0
467	KC_AB1	Exo70	0.899351654882833	0.317212132693741	1	pseudobulk	DESeq2	LRT	0
467	KC_AB1	Exo84	-0.636040241931026	0.521404657877091	1	pseudobulk	DESeq2	LRT	0
468	KC_AB1	ey	-0.139325517828469	0.384691894666919	1	pseudobulk	DESeq2	LRT	0
468	KC_AB1	eys	-0.080431215520881	0.773729912012923	1	pseudobulk	DESeq2	LRT	0
468	KC_AB1	f-cup	-0.861789219881105	0.389166665521259	1	pseudobulk	DESeq2	LRT	0
468	KC_AB1	fabp	-0.043455672031846	0.914414213161547	1	pseudobulk	DESeq2	LRT	0
468	KC_AB1	Fadd	0.1772226318482	1	1	pseudobulk	DESeq2	LRT	0
468	KC_AB1	faf	0.146992583726582	0.675755909206254	1	pseudobulk	DESeq2	LRT	0
468	KC_AB1	Faf2	0.158673625474146	0.81930832208099	1	pseudobulk	DESeq2	LRT	0
468	KC_AB1	Fak	-0.055200399510078	0.686088034048799	1	pseudobulk	DESeq2	LRT	0
468	KC_AB1	fal	-0.885366864674278	0.444629837473484	1	pseudobulk	DESeq2	LRT	0
468	KC_AB1	FAM21	0.167697904157137	0.848959549727716	1	pseudobulk	DESeq2	LRT	0
469	KC_AB1	fand	-0.728859603151019	0.381899903022922	1	pseudobulk	DESeq2	LRT	0
469	KC_AB1	FarO	2.31997030691033	0.813169507846918	1	pseudobulk	DESeq2	LRT	0
469	KC_AB1	Fas1	-0.007906710249247	0.943915468936431	1	pseudobulk	DESeq2	LRT	0
469	KC_AB1	Fas2	0.012004184251432	0.894330648731384	1	pseudobulk	DESeq2	LRT	0
469	KC_AB1	Fas3	-1.16813476907824	0.470505835400246	1	pseudobulk	DESeq2	LRT	0
469	KC_AB1	FASN1	-0.413587468281785	0.313551458601964	1	pseudobulk	DESeq2	LRT	0
469	KC_AB1	FASN3	0.849606887838128	1	1	pseudobulk	DESeq2	LRT	0
469	KC_AB1	fat-spondin	2.85142250261825	0.484249113053674	1	pseudobulk	DESeq2	LRT	0
469	KC_AB1	Fatp1	-0.728213944459186	0.319957356568772	1	pseudobulk	DESeq2	LRT	0
469	KC_AB1	fax	-0.19968473801144	0.214275515404948	1	pseudobulk	DESeq2	LRT	0
470	KC_AB1	fbl	-0.459822574227332	0.633168280191042	1	pseudobulk	DESeq2	LRT	0
470	KC_AB1	Fbl6	-0.208958770789127	0.524984828068021	1	pseudobulk	DESeq2	LRT	0
470	KC_AB1	fbp	0.366247714696288	1	1	pseudobulk	DESeq2	LRT	0
470	KC_AB1	Fbw5	0.742798980668769	1	1	pseudobulk	DESeq2	LRT	0
470	KC_AB1	Fbxl4	0.157942359511419	0.829597715807388	1	pseudobulk	DESeq2	LRT	0
470	KC_AB1	FBXO11	-0.343106197733612	0.457452154251825	1	pseudobulk	DESeq2	LRT	0
470	KC_AB1	Fcp1	-0.465168924068426	0.521188983410716	1	pseudobulk	DESeq2	LRT	0
470	KC_AB1	fd102C	2.78674512494791	0.54962276837928	1	pseudobulk	DESeq2	LRT	0
470	KC_AB1	Fdh	2.31997030691033	0.813169507846918	1	pseudobulk	DESeq2	LRT	0
470	KC_AB1	fdl	-0.852174730266618	0.052740885097452	1	pseudobulk	DESeq2	LRT	0
471	KC_AB1	Fdx1	-0.683884660502142	0.473192533849592	1	pseudobulk	DESeq2	LRT	0
471	KC_AB1	Fdx2	-0.779532708993135	0.395546161944329	1	pseudobulk	DESeq2	LRT	0
471	KC_AB1	FeCH	-0.107819248362577	0.76400184274342	1	pseudobulk	DESeq2	LRT	0
471	KC_AB1	Fem-1	0.217519714337618	0.654951095034189	1	pseudobulk	DESeq2	LRT	0
471	KC_AB1	Fen1	3.16697464674408	0.406679230261689	1	pseudobulk	DESeq2	LRT	0
471	KC_AB1	fend	-0.109346439486636	0.39710226157923	1	pseudobulk	DESeq2	LRT	0

471	KC_AB1	FER	1.30447632591393	1	1	pseudobulk	DESeq2	LRT	0
471	KC_AB1	Fer1HCH	-0.530691002290693	0.150170296084813	1	pseudobulk	DESeq2	LRT	0
471	KC_AB1	Fer2	1.59863986092676	1	1	pseudobulk	DESeq2	LRT	0
471	KC_AB1	Fer2LCH	-0.046914652515072	0.660736754018117	1	pseudobulk	DESeq2	LRT	0
472	KC_AB1	fest	0.318832812977104	0.512283075007807	1	pseudobulk	DESeq2	LRT	0
472	KC_AB1	Fgop2	-0.262225388847221	0.680327706361582	1	pseudobulk	DESeq2	LRT	0
472	KC_AB1	fh	0.720501237090148	0.745626069432631	1	pseudobulk	DESeq2	LRT	0
472	KC_AB1	Fhos	-1.94072364244559	0.164191189987444	1	pseudobulk	DESeq2	LRT	0
472	KC_AB1	Fib	0.0702218262325044	0.888868588631627	1	pseudobulk	DESeq2	LRT	0
472	KC_AB1	Fibp	0.0361220003367051	0.597789258735174	1	pseudobulk	DESeq2	LRT	0
472	KC_AB1	Fic	-0.743777774370501	0.50143343034711	1	pseudobulk	DESeq2	LRT	0
472	KC_AB1	fid	-0.347866270358258	0.544395408993032	1	pseudobulk	DESeq2	LRT	0
472	KC_AB1	fidipidine	0.0911506254984636	0.896849126214569	1	pseudobulk	DESeq2	LRT	0
472	KC_AB1	Fie	-0.170692446838319	0.771849995175861	1	pseudobulk	DESeq2	LRT	0
473	KC_AB1	Fife	-0.022775004888598	0.754223523357957	1	pseudobulk	DESeq2	LRT	0
473	KC_AB1	FIG4	1.2479602775867	0.726568014190941	1	pseudobulk	DESeq2	LRT	0
473	KC_AB1	Fili	0.905477808468391	1	1	pseudobulk	DESeq2	LRT	0
473	KC_AB1	Fim	0.156579865208029	0.469105502016366	1	pseudobulk	DESeq2	LRT	0
473	KC_AB1	Fip1	0.170017062389164	0.725133585332796	1	pseudobulk	DESeq2	LRT	0
473	KC_AB1	fipi	0.482855930484289	0.283680524655715	1	pseudobulk	DESeq2	LRT	0
473	KC_AB1	Fis1	0.181136283951602	0.399374037580091	1	pseudobulk	DESeq2	LRT	0
473	KC_AB1	Fit1	0.221370933642923	0.676482972195118	1	pseudobulk	DESeq2	LRT	0
473	KC_AB1	Fitm	-0.405034199097342	0.699537166886544	1	pseudobulk	DESeq2	LRT	0
473	KC_AB1	fiz	-0.216209988775614	1	1	pseudobulk	DESeq2	LRT	0
474	KC_AB1	Fkbp12	0.455531334445088	0.0444921990299356	1	pseudobulk	DESeq2	LRT	0
474	KC_AB1	Fkbp14	2.43275481406755	0.550258497427138	1	pseudobulk	DESeq2	LRT	0
474	KC_AB1	Fkbp39	0.870198354555823	0.341632983305903	1	pseudobulk	DESeq2	LRT	0
474	KC_AB1	Fkbp59	-0.285609694395629	0.573151459406684	1	pseudobulk	DESeq2	LRT	0
474	KC_AB1	fkh	3.81892523345342	0.259683132508622	1	pseudobulk	DESeq2	LRT	0
474	KC_AB1	fl(2)d	0.0463929976069402	0.6881594709517	1	pseudobulk	DESeq2	LRT	0
474	KC_AB1	Flacc	0.141442862996388	0.806605539318032	1	pseudobulk	DESeq2	LRT	0
474	KC_AB1	FLASH	1.53711870859642	0.563188001421	1	pseudobulk	DESeq2	LRT	0
474	KC_AB1	flfl	-0.133081144359029	0.675790970125228	1	pseudobulk	DESeq2	LRT	0
474	KC_AB1	fliI	0.0489144064541268	0.763339858251511	1	pseudobulk	DESeq2	LRT	0
475	KC_AB1	fln	0.849606887838128	1	1	pseudobulk	DESeq2	LRT	0
475	KC_AB1	Flo1	0.115578555327185	0.62767481081125	1	pseudobulk	DESeq2	LRT	0
475	KC_AB1	Flo2	-0.784763145031305	0.0444271199286618	1	pseudobulk	DESeq2	LRT	0
475	KC_AB1	flr	1.15641485435057	0.591142640503974	1	pseudobulk	DESeq2	LRT	0
475	KC_AB1	flw	-0.527716193056269	0.0462503194115127	1	pseudobulk	DESeq2	LRT	0
475	KC_AB1	Fmr1	0.0014428828156520	0.77313198380087	1	pseudobulk	DESeq2	LRT	0
475	KC_AB1	FMRFaR	-1.72288719393595	0.161123322407825	1	pseudobulk	DESeq2	LRT	0
475	KC_AB1	fmt	-0.563914563780048	0.114429012158681	1	pseudobulk	DESeq2	LRT	0
475	KC_AB1	fne	-0.071456567876848	0.567363584598831	1	pseudobulk	DESeq2	LRT	0
475	KC_AB1	fng	-1.25232636752206	0.556603619318279	1	pseudobulk	DESeq2	LRT	0
476	KC_AB1	Fnta	-0.396663726325962	0.576259588974844	1	pseudobulk	DESeq2	LRT	0
476	KC_AB1	fog	0.0180122005395675	0.707340258138916	1	pseudobulk	DESeq2	LRT	0
476	KC_AB1	foi	0.524759439793952	0.445373690955626	1	pseudobulk	DESeq2	LRT	0
476	KC_AB1	fok	0.096823048061638	0.452792909243102	1	pseudobulk	DESeq2	LRT	0
476	KC_AB1	for	-1.05548521056871	0.62314630615428	1	pseudobulk	DESeq2	LRT	0
476	KC_AB1	form3	-2.03591551091146	0.335053449683047	1	pseudobulk	DESeq2	LRT	0
476	KC_AB1	FoxK	-0.120561544117243	0.651391021853745	1	pseudobulk	DESeq2	LRT	0
476	KC_AB1	foxo	-1.62811888019599	0.403667960093648	1	pseudobulk	DESeq2	LRT	0
476	KC_AB1	FoxP	-0.184782608319075	0.425007268782479	1	pseudobulk	DESeq2	LRT	0

476	KC_AB1	Fpgs	0.116783351990007	1	1	pseudobulk	DESeq2	LRT	0
477	KC_AB1	Fpps	-1.26430997000707	0.541406370841364	1	pseudobulk	DESeq2	LRT	0
477	KC_AB1	fra	-1.13409122162485	0.59392837149035	1	pseudobulk	DESeq2	LRT	0
477	KC_AB1	fray	-1.5850666209215	0.276102568308198	1	pseudobulk	DESeq2	LRT	0
477	KC_AB1	fred	0.849606887838128	1	1	pseudobulk	DESeq2	LRT	0
477	KC_AB1	FRG1	-0.370711467575244	0.654302698461919	1	pseudobulk	DESeq2	LRT	0
477	KC_AB1	frj	0.275260956224992	0.542443057908121	1	pseudobulk	DESeq2	LRT	0
477	KC_AB1	Frl	0.363454074877344	0.162863495097848	1	pseudobulk	DESeq2	LRT	0
477	KC_AB1	frm	2.78674512494791	0.54962276837928	1	pseudobulk	DESeq2	LRT	0
477	KC_AB1	Frq1	0.153491266392874	0.450548223179083	1	pseudobulk	DESeq2	LRT	0
477	KC_AB1	Frq2	-1.61796373924629	0.121366146722254	1	pseudobulk	DESeq2	LRT	0
478	KC_AB1	frtz	-0.216209988775614	1	1	pseudobulk	DESeq2	LRT	0
478	KC_AB1	fru	-0.48118918811951	0.17459084006527	1	pseudobulk	DESeq2	LRT	0
478	KC_AB1	fry	-0.975190008972759	0.136311291729641	1	pseudobulk	DESeq2	LRT	0
478	KC_AB1	Fs	0.849606887838128	1	1	pseudobulk	DESeq2	LRT	0
478	KC_AB1	fs(1)h	-0.244668848331314	0.0802586192561926	1	pseudobulk	DESeq2	LRT	0
478	KC_AB1	fs(1)K10	1.32835012406946	0.0862595582031	1	pseudobulk	DESeq2	LRT	0
478	KC_AB1	Fs(2)Ket	0.0214469324247142	0.845651934811945	1	pseudobulk	DESeq2	LRT	0
478	KC_AB1	fs(2)ltoPP43	-0.598614666793333	0.532234271121864	1	pseudobulk	DESeq2	LRT	0
478	KC_AB1	fsd	0.25193247750809	0.772536144593526	1	pseudobulk	DESeq2	LRT	0
478	KC_AB1	Fsn	-0.233170030037969	0.545856895412402	1	pseudobulk	DESeq2	LRT	0
479	KC_AB1	ftz-f1	-1.33708487756577	0.0106847631157593	1	pseudobulk	DESeq2	LRT	0
479	KC_AB1	fu	-0.0910223628115864	0.812343148294813	1	pseudobulk	DESeq2	LRT	0
479	KC_AB1	FucT6	0.214864036166636	0.632090064266462	1	pseudobulk	DESeq2	LRT	0
479	KC_AB1	FucTA	0.0049439628182386	0.913117148928411	1	pseudobulk	DESeq2	LRT	0
479	KC_AB1	FucTB	0.120336175449406	1	1	pseudobulk	DESeq2	LRT	0
479	KC_AB1	Fum1	0.372101381180328	0.432728583682836	1	pseudobulk	DESeq2	LRT	0
479	KC_AB1	Fum2	2.06541384650887	0.904905526327343	1	pseudobulk	DESeq2	LRT	0
479	KC_AB1	Fur1	-0.11605632753531	0.680073698271453	1	pseudobulk	DESeq2	LRT	0
479	KC_AB1	Fur2	0.0764101146396033	0.751295153071295	1	pseudobulk	DESeq2	LRT	0
479	KC_AB1	fus	0.589289816377549	1	1	pseudobulk	DESeq2	LRT	0
480	KC_AB1	fuss	0.985243145755336	1	1	pseudobulk	DESeq2	LRT	0
480	KC_AB1	futsch	-0.0714609163301116	0.554486705003888	1	pseudobulk	DESeq2	LRT	0
480	KC_AB1	fw	2.68453167034132	0.550970543274	1	pseudobulk	DESeq2	LRT	0
480	KC_AB1	fwd	0.0560898018992268	0.64088011782501	1	pseudobulk	DESeq2	LRT	0
480	KC_AB1	fwe	0.0068632852757407	0.538642819376959	1	pseudobulk	DESeq2	LRT	0
480	KC_AB1	fws	0.774118947163253	0.731107609987475	1	pseudobulk	DESeq2	LRT	0
480	KC_AB1	fy	-0.234514680509696	0.789323893135924	1	pseudobulk	DESeq2	LRT	0
480	KC_AB1	fz	-0.330815325859476	0.0509451338612022	1	pseudobulk	DESeq2	LRT	0
480	KC_AB1	fz2	-0.697906926199294	0.206846444464521	1	pseudobulk	DESeq2	LRT	0
480	KC_AB1	fz3	0.26091452086035	0.720737573172793	1	pseudobulk	DESeq2	LRT	0
481	KC_AB1	fz4	-0.74324646566374	0.563217991542495	1	pseudobulk	DESeq2	LRT	0
481	KC_AB1	fzr	-0.0777802062976619	0.746354749269487	1	pseudobulk	DESeq2	LRT	0
481	KC_AB1	fzy	2.13009086641515	0.718583316467961	1	pseudobulk	DESeq2	LRT	0
481	KC_AB1	g	-0.607590820439245	0.042499647653031	1	pseudobulk	DESeq2	LRT	0
481	KC_AB1	G6P	0.611183902525028	0.620766032379883	1	pseudobulk	DESeq2	LRT	0
481	KC_AB1	G9a	0.127948674844518	0.682246909439812	1	pseudobulk	DESeq2	LRT	0
481	KC_AB1	GAA1	0.101141131171932	0.42609832656437	1	pseudobulk	DESeq2	LRT	0
481	KC_AB1	GABA-B-R1	-0.1033847144146	0.400997831393417	1	pseudobulk	DESeq2	LRT	0
481	KC_AB1	GABA-B-R2	0.146526024832512	0.594933378397906	1	pseudobulk	DESeq2	LRT	0
481	KC_AB1	Gabat	-0.163311103415595	0.536425556742734	1	pseudobulk	DESeq2	LRT	0
482	KC_AB1	GABPI	0.188374225318448	0.67357947906173	1	pseudobulk	DESeq2	LRT	0
482	KC_AB1	Gad1	0.849606887838128	1	1	pseudobulk	DESeq2	LRT	0

482	KC_AB1	Gadd45	-0.830062897938551	0.717724257445671	1	pseudobulk	DESeq2	LRT		0
482	KC_AB1	Gagr	0.849606887838128	1	1	pseudobulk	DESeq2	LRT		0
482	KC_AB1	Gale	0.365832255734681	0.740998054265613	1	pseudobulk	DESeq2	LRT		0
482	KC_AB1	galectin	0.23834928455897	0.55005771549815	1	pseudobulk	DESeq2	LRT		0
482	KC_AB1	Galk	0.258130182948211	1	1	pseudobulk	DESeq2	LRT		0
482	KC_AB1	galla-1	0.718835258687976	0.265716115185209	1	pseudobulk	DESeq2	LRT		0
482	KC_AB1	galla-2	0.162876927576019	0.707636632401473	1	pseudobulk	DESeq2	LRT		0
482	KC_AB1	Galphaf	0.237022465848507	1	1	pseudobulk	DESeq2	LRT		0
483	KC_AB1	Galphai	-1.31170390114703	0.65473030797909	1	pseudobulk	DESeq2	LRT		0
483	KC_AB1	Galphao	-0.187619423102166	0.210629125217438	1	pseudobulk	DESeq2	LRT		0
483	KC_AB1	Galphaq	-0.45657950964385	0.14432699069849	1	pseudobulk	DESeq2	LRT		0
483	KC_AB1	Galphas	-0.0518695510409293	0.700123307599003	1	pseudobulk	DESeq2	LRT		0
483	KC_AB1	Galt	-0.0764777951430399	0.90713010045115	1	pseudobulk	DESeq2	LRT		0
483	KC_AB1	GalT1	-1.51066569914349	0.263132524032933	1	pseudobulk	DESeq2	LRT		0
483	KC_AB1	gammaCOP	-0.461691847673844	0.644889578708162	1	pseudobulk	DESeq2	LRT		0
483	KC_AB1	gammaSnap1	0.126303057656048	0.503477628347585	1	pseudobulk	DESeq2	LRT		0
483	KC_AB1	gammaSnap2	-0.180857693937338	0.789820919834869	1	pseudobulk	DESeq2	LRT		0
483	KC_AB1	gammaTub23C	-0.281516833599345	0.692402258996063	1	pseudobulk	DESeq2	LRT		0
484	KC_AB1	GAPcenA	-0.480063206803014	0.343878290298185	1	pseudobulk	DESeq2	LRT		0
484	KC_AB1	Gapdh1	0.298173691631698	0.39288533679927	1	pseudobulk	DESeq2	LRT		0
484	KC_AB1	Gapdh2	0.320803455783743	0.248155074795157	1	pseudobulk	DESeq2	LRT		0
484	KC_AB1	GAPsec	-0.801911425322951	0.454965052666593	1	pseudobulk	DESeq2	LRT		0
484	KC_AB1	Gapvd1	-0.475669925434946	0.411383214409469	1	pseudobulk	DESeq2	LRT		0
484	KC_AB1	Gart	2.78674512494791	0.54962276837928	1	pseudobulk	DESeq2	LRT		0
484	KC_AB1	garz	0.345781897934742	0.561474798171739	1	pseudobulk	DESeq2	LRT		0
484	KC_AB1	gas	0.366247714696288	1	1	pseudobulk	DESeq2	LRT		0
484	KC_AB1	Gas41	0.686309479096506	0.660769275565165	1	pseudobulk	DESeq2	LRT		0
484	KC_AB1	Gas8	0.376431477381175	1	1	pseudobulk	DESeq2	LRT		0
485	KC_AB1	Gasp	-2.54638402068839	0.248646166714586	1	pseudobulk	DESeq2	LRT		0
485	KC_AB1	Gasz	0.316239333531959	0.592246600659016	1	pseudobulk	DESeq2	LRT		0
485	KC_AB1	Gat	0.428388606224242	1	1	pseudobulk	DESeq2	LRT		0
485	KC_AB1	GatA	0.39184935565729	1	1	pseudobulk	DESeq2	LRT		0
485	KC_AB1	GATAd	-0.0724093381803757	0.873338760175713	1	pseudobulk	DESeq2	LRT		0
485	KC_AB1	GatB	-0.0920705004275841	0.842044344985412	1	pseudobulk	DESeq2	LRT		0
485	KC_AB1	gb	-1.04341300690344	0.428979034755974	1	pseudobulk	DESeq2	LRT		0
485	KC_AB1	Gba1a	0.849606887838128	1	1	pseudobulk	DESeq2	LRT		0
485	KC_AB1	gbb	2.98830558580523	0.0262698597101136	1	pseudobulk	DESeq2	LRT		0
485	KC_AB1	Gbeta13F	-0.187382793435902	0.243823503923707	1	pseudobulk	DESeq2	LRT		0
486	KC_AB1	Gbeta5	0.236760256568038	0.519481137420863	1	pseudobulk	DESeq2	LRT		0
486	KC_AB1	Gbp1	-2.59610313770632	0.291483550812208	1	pseudobulk	DESeq2	LRT		0
486	KC_AB1	Gbs-70E	1.00693893053477	1	1	pseudobulk	DESeq2	LRT		0
486	KC_AB1	Gbs-76A	0.335390218036738	0.628408195839024	1	pseudobulk	DESeq2	LRT		0
486	KC_AB1	GC	-0.699259507374599	0.0536493965142153	1	pseudobulk	DESeq2	LRT		0
486	KC_AB1	GC1	0.558305674252424	0.321955213157181	1	pseudobulk	DESeq2	LRT		0
486	KC_AB1	GCC185	-0.166538581853933	0.688099922121702	1	pseudobulk	DESeq2	LRT		0
486	KC_AB1	GCC88	-1.08219423905856	0.288885400242823	1	pseudobulk	DESeq2	LRT		0
486	KC_AB1	gce	-0.309552927558896	0.256396937659176	1	pseudobulk	DESeq2	LRT		0
486	KC_AB1	GckIII	0.0523181730702619	0.922708361553248	1	pseudobulk	DESeq2	LRT		0
487	KC_AB1	gcl	-0.354968133252381	0.62003340903971	1	pseudobulk	DESeq2	LRT		0
487	KC_AB1	Gclc	-0.861223441831928	0.206341124243586	1	pseudobulk	DESeq2	LRT		0
487	KC_AB1	Gclm	-0.423952627644019	0.541504823009631	1	pseudobulk	DESeq2	LRT		0
487	KC_AB1	Gcn2	-0.199525527344097	0.534599902454405	1	pseudobulk	DESeq2	LRT		0
487	KC_AB1	Gcn5	0.0261954544250205	0.818052590078494	1	pseudobulk	DESeq2	LRT		0

487	KC_AB1	GCS1	0.187571925780821	0.708377738972021	1	pseudobulk	DESeq2	LRT	0
487	KC_AB1	GCS2alpha	-0.195447307461185	0.506574719307368	1	pseudobulk	DESeq2	LRT	0
487	KC_AB1	GCS2beta	0.0211972751448305	0.69348786604804	1	pseudobulk	DESeq2	LRT	0
487	KC_AB1	Gdap1	0.849606887838128	1	1	pseudobulk	DESeq2	LRT	0
487	KC_AB1	Gdap2	-0.169100098451965	0.28480850511203	1	pseudobulk	DESeq2	LRT	0
488	KC_AB1	Gdh	-0.148162100787169	0.417374715959168	1	pseudobulk	DESeq2	LRT	0
488	KC_AB1	Gdi	0.0406722377061628	0.867571294825208	1	pseudobulk	DESeq2	LRT	0
488	KC_AB1	gdl	-0.356000799145971	0.68248600851713	1	pseudobulk	DESeq2	LRT	0
488	KC_AB1	Gdn1	-0.437395786589452	0.732969626343787	1	pseudobulk	DESeq2	LRT	0
488	KC_AB1	Ge-1	0.0086698482529446	0.649813918350936	1	pseudobulk	DESeq2	LRT	0
488	KC_AB1	GEFmeso	-2.66707380020106	0.22649589872017	1	pseudobulk	DESeq2	LRT	0
488	KC_AB1	gek	0.476707728409068	0.398105246712686	1	pseudobulk	DESeq2	LRT	0
488	KC_AB1	geko	0.849606887838128	1	1	pseudobulk	DESeq2	LRT	0
488	KC_AB1	Gel	-0.511908815565556	0.655579183324897	1	pseudobulk	DESeq2	LRT	0
488	KC_AB1	Gem2	-0.216209988775614	1	1	pseudobulk	DESeq2	LRT	0
489	KC_AB1	Gem3	0.592664483892331	0.879657374752307	1	pseudobulk	DESeq2	LRT	0
489	KC_AB1	geminin	-1.3642730092591	0.181157336478224	1	pseudobulk	DESeq2	LRT	0
489	KC_AB1	Gen	-0.216210029276812	1	1	pseudobulk	DESeq2	LRT	0
489	KC_AB1	Gfat1	-0.833404182616462	0.691823612159792	1	pseudobulk	DESeq2	LRT	0
489	KC_AB1	Gfat2	0.0045956880517478	0.659754467717894	1	pseudobulk	DESeq2	LRT	0
489	KC_AB1	Gfrl	-0.0176109178255402	0.894277770232808	1	pseudobulk	DESeq2	LRT	0
489	KC_AB1	gfzf	1.59863986092676	1	1	pseudobulk	DESeq2	LRT	0
489	KC_AB1	Gga	1.18406059566835	0.327476690197567	1	pseudobulk	DESeq2	LRT	0
489	KC_AB1	Ggamma1	0.0424228334318658	0.618328240152603	1	pseudobulk	DESeq2	LRT	0
489	KC_AB1	Ggamma30A	0.0858432118993159	0.576432242486827	1	pseudobulk	DESeq2	LRT	0
490	KC_AB1	Ggt-1	0.931109140710098	1	1	pseudobulk	DESeq2	LRT	0
490	KC_AB1	ghi	0.259975453523258	0.384639397158193	1	pseudobulk	DESeq2	LRT	0
490	KC_AB1	gig	-0.013833526076196	0.645537053147424	1	pseudobulk	DESeq2	LRT	0
490	KC_AB1	GIIIspla2	-0.55630637739799	0.533385950710938	1	pseudobulk	DESeq2	LRT	0
490	KC_AB1	GILT1	-1.44484484240587	0.214297640079498	1	pseudobulk	DESeq2	LRT	0
490	KC_AB1	GILT2	0.742456727585392	1	1	pseudobulk	DESeq2	LRT	0
490	KC_AB1	GILT3	0.592664483892331	0.879657374752307	1	pseudobulk	DESeq2	LRT	0
490	KC_AB1	Gip	2.38637069871024	0.104465763020034	1	pseudobulk	DESeq2	LRT	0
490	KC_AB1	Girdin	-0.141697576630774	0.69999672235769	1	pseudobulk	DESeq2	LRT	0
490	KC_AB1	gish	-0.158005579569506	0.303017167374565	1	pseudobulk	DESeq2	LRT	0
491	KC_AB1	Git	-0.384513465756373	0.643202941733747	1	pseudobulk	DESeq2	LRT	0
491	KC_AB1	Gk1	0.161062069268461	0.679756069338254	1	pseudobulk	DESeq2	LRT	0
491	KC_AB1	Gk2	1.37129945894492	1	1	pseudobulk	DESeq2	LRT	0
491	KC_AB1	gkt	0.100639215495319	1	1	pseudobulk	DESeq2	LRT	0
491	KC_AB1	GLaz	-0.267798911930929	1	1	pseudobulk	DESeq2	LRT	0
491	KC_AB1	GlcAT-I	0.459639652224057	0.754802870147247	1	pseudobulk	DESeq2	LRT	0
491	KC_AB1	GlcAT-P	-0.0626038709836392	0.628813852924846	1	pseudobulk	DESeq2	LRT	0
491	KC_AB1	GlcAT-S	-0.158530678258147	0.642657916657125	1	pseudobulk	DESeq2	LRT	0
491	KC_AB1	GlcT	-0.256918252133983	0.734391772422151	1	pseudobulk	DESeq2	LRT	0
491	KC_AB1	Gld	2.78674512494791	0.54962276837928	1	pseudobulk	DESeq2	LRT	0
492	KC_AB1	Gle1	-0.964434567775807	0.359874287972708	1	pseudobulk	DESeq2	LRT	0
492	KC_AB1	glec	-0.368364268793846	0.281842620308262	1	pseudobulk	DESeq2	LRT	0
492	KC_AB1	Glg1	-0.110280025999573	0.689081012377653	1	pseudobulk	DESeq2	LRT	0
492	KC_AB1	Gli	-0.216209988775614	1	1	pseudobulk	DESeq2	LRT	0
492	KC_AB1	GlnRS	0.289453119444341	0.423028750580662	1	pseudobulk	DESeq2	LRT	0
492	KC_AB1	glo	-0.164368734073707	0.509158115748432	1	pseudobulk	DESeq2	LRT	0
492	KC_AB1	Glo1	-1.46920884245505	0.198273436974338	1	pseudobulk	DESeq2	LRT	0
492	KC_AB1	Glos	-0.847199302416132	0.677009019822614	1	pseudobulk	DESeq2	LRT	0

492	KC_AB1	GLS	-0.0963890306446986	0.789992518770079	1	pseudobulk	DESeq2	LRT	0
492	KC_AB1	glu	-1.93180818643489	0.365426685858663	1	pseudobulk	DESeq2	LRT	0
493	KC_AB1	GluClalpha	0.0323423871727168	0.837252667810303	1	pseudobulk	DESeq2	LRT	0
493	KC_AB1	GluProRS	-0.536943139709038	0.571403849249855	1	pseudobulk	DESeq2	LRT	0
493	KC_AB1	GluRIA	0.103196910825691	0.588702673646759	1	pseudobulk	DESeq2	LRT	0
493	KC_AB1	GluRIB	-0.239980665480725	0.0811234945031175	1	pseudobulk	DESeq2	LRT	0
493	KC_AB1	GluRIIE	-0.440797203820518	0.739710782180082	1	pseudobulk	DESeq2	LRT	0
493	KC_AB1	GluRS-m	0.849606887838128	1	1	pseudobulk	DESeq2	LRT	0
493	KC_AB1	Glut1	-0.007371110547660	0.974793857733941	1	pseudobulk	DESeq2	LRT	0
493	KC_AB1	Glut4EF	-0.441947886661794	0.00441818316224511	1	pseudobulk	DESeq2	LRT	0
493	KC_AB1	GlyP	0.0313315117066558	0.919019537939281	1	pseudobulk	DESeq2	LRT	0
493	KC_AB1	GlyRS	-0.392197239739788	0.606657137938749	1	pseudobulk	DESeq2	LRT	0
494	KC_AB1	GlyS	0.613832357913101	0.591061342112906	1	pseudobulk	DESeq2	LRT	0
494	KC_AB1	GlyT	-0.232199336878925	0.271981254995033	1	pseudobulk	DESeq2	LRT	0
494	KC_AB1	GM130	0.375352591285336	0.588283725311021	1	pseudobulk	DESeq2	LRT	0
494	KC_AB1	Gmap	0.32192611906056	0.626376215520613	1	pseudobulk	DESeq2	LRT	0
494	KC_AB1	Gmd	0.539227591542996	1	1	pseudobulk	DESeq2	LRT	0
494	KC_AB1	Gmer	1.51872195779367	0.623907569846402	1	pseudobulk	DESeq2	LRT	0
494	KC_AB1	GMF	0.624427434028843	0.456100146738871	1	pseudobulk	DESeq2	LRT	0
494	KC_AB1	Gnf1	0.917483130743281	0.339186097219731	1	pseudobulk	DESeq2	LRT	0
494	KC_AB1	Gnpnat	-0.899226002017773	0.382315734667215	1	pseudobulk	DESeq2	LRT	0
494	KC_AB1	gny	0.286809738130804	1	1	pseudobulk	DESeq2	LRT	0
495	KC_AB1	goe	0.849606887838128	1	1	pseudobulk	DESeq2	LRT	0
495	KC_AB1	gogo	-1.50643016592806	0.265830842316493	1	pseudobulk	DESeq2	LRT	0
495	KC_AB1	gol	0.431503210525138	0.190105722141487	1	pseudobulk	DESeq2	LRT	0
495	KC_AB1	Golgin104	0.271708831055059	0.768058148103735	1	pseudobulk	DESeq2	LRT	0
495	KC_AB1	Golgin245	0.358450864522032	0.502284322443321	1	pseudobulk	DESeq2	LRT	0
495	KC_AB1	Golgin84	0.207367226894526	0.725084676080667	1	pseudobulk	DESeq2	LRT	0
495	KC_AB1	gom	-1.76860416527997	0.0990330011786271	1	pseudobulk	DESeq2	LRT	0
495	KC_AB1	Gorab	2.3742045836616	0.0518222284975893	1	pseudobulk	DESeq2	LRT	0
495	KC_AB1	Gos28	-0.885757162824556	0.299472679501445	1	pseudobulk	DESeq2	LRT	0
495	KC_AB1	Got1	-0.333050473159593	0.569852611067721	1	pseudobulk	DESeq2	LRT	0
496	KC_AB1	Got2	-0.34646339201539	0.578375265742935	1	pseudobulk	DESeq2	LRT	0
496	KC_AB1	Gp150	0.741390795038255	0.810185849551454	1	pseudobulk	DESeq2	LRT	0
496	KC_AB1	Gp210	0.466750716186451	0.481939298243281	1	pseudobulk	DESeq2	LRT	0
496	KC_AB1	Gp93	-0.042467319088135	0.673135531313891	1	pseudobulk	DESeq2	LRT	0
496	KC_AB1	Gpat4	0.0403379658955046	0.926100078173652	1	pseudobulk	DESeq2	LRT	0
496	KC_AB1	Gpb5	0.136826684411147	0.813291513154314	1	pseudobulk	DESeq2	LRT	0
496	KC_AB1	Gpdh1	-0.0255255364793457	0.666708132755973	1	pseudobulk	DESeq2	LRT	0
496	KC_AB1	Gpdh3	-0.805492004106347	0.578859573251408	1	pseudobulk	DESeq2	LRT	0
496	KC_AB1	GPHR	-0.259645106179589	0.684564202487846	1	pseudobulk	DESeq2	LRT	0
496	KC_AB1	Gpo1	-0.978177451070807	0.194488709900165	1	pseudobulk	DESeq2	LRT	0
497	KC_AB1	gpp	-0.0685623092904075	0.526955782474798	1	pseudobulk	DESeq2	LRT	0
497	KC_AB1	Gprk1	-0.473953565233289	0.00359997109775224	1	pseudobulk	DESeq2	LRT	0
497	KC_AB1	Gprk2	-0.145652714180779	0.356267817952755	1	pseudobulk	DESeq2	LRT	0
497	KC_AB1	gprs	-0.271961416168488	0.276340946987248	1	pseudobulk	DESeq2	LRT	0
497	KC_AB1	Gr2a	3.41844045086015	0.351942490385471	1	pseudobulk	DESeq2	LRT	0
497	KC_AB1	Gr39a	0.985243145755336	1	1	pseudobulk	DESeq2	LRT	0
497	KC_AB1	Gr43a	0.849606887838128	1	1	pseudobulk	DESeq2	LRT	0
497	KC_AB1	Gr47a	2.78674512494791	0.54962276837928	1	pseudobulk	DESeq2	LRT	0
497	KC_AB1	Gr47b	0.849606887838128	1	1	pseudobulk	DESeq2	LRT	0
497	KC_AB1	Gr77a	2.68810840688657	0.500242849464732	1	pseudobulk	DESeq2	LRT	0
498	KC_AB1	Graf	-0.484536116515466	0.135636073065971	1	pseudobulk	DESeq2	LRT	0

498	KC_AB1	GramD1B	0.149841033790816	0.446340067344797	1	pseudobulk	DESeq2	LRT		0
498	KC_AB1	Grasp65	-0.195392238795121	0.745970335780066	1	pseudobulk	DESeq2	LRT		0
498	KC_AB1	grass	-0.370862531082229	0.881125640939123	1	pseudobulk	DESeq2	LRT		0
498	KC_AB1	grau	0.388203835684853	0.617197708162447	1	pseudobulk	DESeq2	LRT		0
498	KC_AB1	grh	-0.216210029276812	1	1	pseudobulk	DESeq2	LRT		0
498	KC_AB1	Grik	0.85879998658552	1	1	pseudobulk	DESeq2	LRT		0
498	KC_AB1	grim	0.849606887838128	1	1	pseudobulk	DESeq2	LRT		0
498	KC_AB1	Grip	2.31997030691033	0.813169507846918	1	pseudobulk	DESeq2	LRT		0
498	KC_AB1	Grip128	1.15040783121559	0.87372258286756	1	pseudobulk	DESeq2	LRT		0
499	KC_AB1	Grip163	0.174335752682643	0.709955992195015	1	pseudobulk	DESeq2	LRT		0
499	KC_AB1	Grip71	-0.0065790798335215	1	1	pseudobulk	DESeq2	LRT		0
499	KC_AB1	Grip75	0.784916633286949	0.477005699766278	1	pseudobulk	DESeq2	LRT		0
499	KC_AB1	Grip84	-0.541678577562469	0.597566166427492	1	pseudobulk	DESeq2	LRT		0
499	KC_AB1	Grip91	0.393887204513028	0.704629815339566	1	pseudobulk	DESeq2	LRT		0
499	KC_AB1	grk	0.849606887838128	1	1	pseudobulk	DESeq2	LRT		0
499	KC_AB1	grn	-1.5805998677877	0.524230026009756	1	pseudobulk	DESeq2	LRT		0
499	KC_AB1	grnd	-1.96283072402402	0.372690411015166	1	pseudobulk	DESeq2	LRT		0
499	KC_AB1	gro	0.0239818034930018	0.533008623722345	1	pseudobulk	DESeq2	LRT		0
499	KC_AB1	grp	0.309907230268205	0.196901411292597	1	pseudobulk	DESeq2	LRT		0
500	KC_AB1	grsm	-0.15301154010748	0.800528793094557	1	pseudobulk	DESeq2	LRT		0
500	KC_AB1	Grx1	0.0315994043149084	0.552438039633584	1	pseudobulk	DESeq2	LRT		0
500	KC_AB1	gry	-1.2124621392535	0.325285160058661	1	pseudobulk	DESeq2	LRT		0
500	KC_AB1	Gs1	-0.0438371820799014	0.617452630547818	1	pseudobulk	DESeq2	LRT		0
500	KC_AB1	Gs1l	0.375169361126541	0.321229318480452	1	pseudobulk	DESeq2	LRT		0
500	KC_AB1	Gs2	-1.22232243759079	0.246680899707583	1	pseudobulk	DESeq2	LRT		0
500	KC_AB1	Gss1	0.985243145755336	1	1	pseudobulk	DESeq2	LRT		0
500	KC_AB1	GstD1	0.0562317860655877	0.797439880201944	1	pseudobulk	DESeq2	LRT		0
500	KC_AB1	GstD11	0.114232821959008	0.529087823116557	1	pseudobulk	DESeq2	LRT		0
500	KC_AB1	GstD3	1.59863986092676	1	1	pseudobulk	DESeq2	LRT		0
501	KC_AB1	GstD7	2.78674512494791	0.54962276837928	1	pseudobulk	DESeq2	LRT		0
501	KC_AB1	GstD9	0.849606887838128	1	1	pseudobulk	DESeq2	LRT		0
501	KC_AB1	GstE1	-3.27542082902335	0.0417844622061842	1	pseudobulk	DESeq2	LRT		0
501	KC_AB1	GstE11	0.849606887838128	1	1	pseudobulk	DESeq2	LRT		0
501	KC_AB1	GstE12	-0.705034709037549	0.624651761683295	1	pseudobulk	DESeq2	LRT		0
501	KC_AB1	GstE2	2.75591047303857	0.0510378197016062	1	pseudobulk	DESeq2	LRT		0
501	KC_AB1	GstE3	2.06541384650887	0.904905526327343	1	pseudobulk	DESeq2	LRT		0
501	KC_AB1	GstE4	2.31997030691033	0.813169507846918	1	pseudobulk	DESeq2	LRT		0
501	KC_AB1	GstO2	-0.0751335027730247	0.76677918525206	1	pseudobulk	DESeq2	LRT		0
501	KC_AB1	GstO3	-0.724804015939394	0.596212412615739	1	pseudobulk	DESeq2	LRT		0
502	KC_AB1	GstS1	0.561623199255563	1	1	pseudobulk	DESeq2	LRT		0
502	KC_AB1	GstT1	-0.960022699105854	0.610500321913265	1	pseudobulk	DESeq2	LRT		0
502	KC_AB1	GstT2	0.634960112221505	1	1	pseudobulk	DESeq2	LRT		0
502	KC_AB1	GstT3	0.194265322970028	1	1	pseudobulk	DESeq2	LRT		0
502	KC_AB1	GstT4	-0.0616466674399467	0.943863910951334	1	pseudobulk	DESeq2	LRT		0
502	KC_AB1	GstZ2	-0.0013308035823898	0.731678388484371	1	pseudobulk	DESeq2	LRT		0
502	KC_AB1	gt	-0.683829746723457	0.723342236517982	1	pseudobulk	DESeq2	LRT		0
502	KC_AB1	Gtp-bp	-0.2269166940378	0.591513328100128	1	pseudobulk	DESeq2	LRT		0
502	KC_AB1	Gug	-0.0820270451654386	0.541100807712834	1	pseudobulk	DESeq2	LRT		0
502	KC_AB1	gukh	-0.23840962158986	0.816943768041783	1	pseudobulk	DESeq2	LRT		0
503	KC_AB1	gus	-0.0208314951949589	0.853210987954446	1	pseudobulk	DESeq2	LRT		0
503	KC_AB1	GV1	-1.25393399638069	0.612054104673377	1	pseudobulk	DESeq2	LRT		0
503	KC_AB1	gw	0.0425641010661489	0.756081313240631	1	pseudobulk	DESeq2	LRT		0
503	KC_AB1	GXIVsPLA2	0.457729660532988	0.504002835956225	1	pseudobulk	DESeq2	LRT		0

503	KC_AB1	Gyc88E	0.185773738078409	0.457021270568359	1	pseudobulk	DESeq2	LRT	0
503	KC_AB1	Gyc89Da	2.31997030691033	0.813169507846918	1	pseudobulk	DESeq2	LRT	0
503	KC_AB1	Gyc89Db	-0.239225661985278	0.726497274228714	1	pseudobulk	DESeq2	LRT	0
503	KC_AB1	Gycalpha99B	-0.259159586794314	0.284515797408304	1	pseudobulk	DESeq2	LRT	0
503	KC_AB1	Gycbeta100B	-0.0551677280173847	0.867660837141855	1	pseudobulk	DESeq2	LRT	0
503	KC_AB1	Gyf	-0.155100364100482	0.420801153002474	1	pseudobulk	DESeq2	LRT	0
504	KC_AB1	Gyg	0.23042114998891	0.903370613671843	1	pseudobulk	DESeq2	LRT	0
504	KC_AB1	gzl	0.0358926977849489	0.863023663380797	1	pseudobulk	DESeq2	LRT	0
504	KC_AB1	H	-0.310069991835335	0.551100946297896	1	pseudobulk	DESeq2	LRT	0
504	KC_AB1	h-cup	0.237022465848507	1	1	pseudobulk	DESeq2	LRT	0
504	KC_AB1	Hacd1	0.309573370858986	0.650294839997189	1	pseudobulk	DESeq2	LRT	0
504	KC_AB1	Hacd2	-0.117779764382237	0.513985683523195	1	pseudobulk	DESeq2	LRT	0
504	KC_AB1	Hacl	-0.399823031516359	0.623359412328799	1	pseudobulk	DESeq2	LRT	0
504	KC_AB1	Had1	0.849606887838128	1	1	pseudobulk	DESeq2	LRT	0
504	KC_AB1	haf	0.341607200160629	0.610988761660295	1	pseudobulk	DESeq2	LRT	0
504	KC_AB1	Hakai	0.674845445897497	0.40817614719614	1	pseudobulk	DESeq2	LRT	0
505	KC_AB1	Hand	0.849606887838128	1	1	pseudobulk	DESeq2	LRT	0
505	KC_AB1	hang	-0.121781461955044	0.66628286572493	1	pseudobulk	DESeq2	LRT	0
505	KC_AB1	Hasp	-0.238964427053435	0.237735717715387	1	pseudobulk	DESeq2	LRT	0
505	KC_AB1	Haspin	-0.377419338331734	0.273736315942626	1	pseudobulk	DESeq2	LRT	0
505	KC_AB1	Hat1	-0.266123028226715	0.683642734605794	1	pseudobulk	DESeq2	LRT	0
505	KC_AB1	hay	0.362838644651637	0.871020849133208	1	pseudobulk	DESeq2	LRT	0
505	KC_AB1	hbs	1.59863986092676	1	1	pseudobulk	DESeq2	LRT	0
505	KC_AB1	HBS1	-0.674794159403976	0.546473198573713	1	pseudobulk	DESeq2	LRT	0
505	KC_AB1	Hcf	-0.157480832173269	0.520093731435499	1	pseudobulk	DESeq2	LRT	0
505	KC_AB1	Hcs	-0.288418864693456	0.581831726673943	1	pseudobulk	DESeq2	LRT	0
506	KC_AB1	hd	1.59863986092676	1	1	pseudobulk	DESeq2	LRT	0
506	KC_AB1	HDAC1	-0.44453898392616	0.619348780211014	1	pseudobulk	DESeq2	LRT	0
506	KC_AB1	HDAC11	0.202106862107108	1	1	pseudobulk	DESeq2	LRT	0
506	KC_AB1	HDAC4	-0.391188520419977	0.0839360514719992	1	pseudobulk	DESeq2	LRT	0
506	KC_AB1	HDAC6	0.301044037064875	0.525656871527768	1	pseudobulk	DESeq2	LRT	0
506	KC_AB1	hdc	-1.67345566113897	0.0390048395046805	1	pseudobulk	DESeq2	LRT	0
506	KC_AB1	hdly	-1.02846249615172	0.360553024589707	1	pseudobulk	DESeq2	LRT	0
506	KC_AB1	hdm	0.90409604875497	1	1	pseudobulk	DESeq2	LRT	0
506	KC_AB1	HEATR2	-0.505244560657932	0.719569300523196	1	pseudobulk	DESeq2	LRT	0
506	KC_AB1	hebe	0.849606887838128	1	1	pseudobulk	DESeq2	LRT	0
507	KC_AB1	hec	-0.945174809315091	0.43327767577196	1	pseudobulk	DESeq2	LRT	0
507	KC_AB1	heix	-0.0878551097929883	0.774989936550536	1	pseudobulk	DESeq2	LRT	0
507	KC_AB1	Hel25E	0.0240898304665838	0.825697233006814	1	pseudobulk	DESeq2	LRT	0
507	KC_AB1	Hel89B	0.128155109703866	0.589787950108418	1	pseudobulk	DESeq2	LRT	0
507	KC_AB1	Hem	-1.46543948149147	0.358336759994262	1	pseudobulk	DESeq2	LRT	0
507	KC_AB1	HemK1	-0.0268008253678101	0.784434264955171	1	pseudobulk	DESeq2	LRT	0
507	KC_AB1	Hen1	0.849606887838128	1	1	pseudobulk	DESeq2	LRT	0
507	KC_AB1	hep	-0.0934698567651397	0.792662285781235	1	pseudobulk	DESeq2	LRT	0
507	KC_AB1	heph	-0.199111084606757	0.298006374094129	1	pseudobulk	DESeq2	LRT	0
507	KC_AB1	her	-0.5826939831489	0.544581335131837	1	pseudobulk	DESeq2	LRT	0
508	KC_AB1	HERC2	-0.115160036032259	0.599709346658123	1	pseudobulk	DESeq2	LRT	0
508	KC_AB1	Herc4	0.350478530436071	0.475912612308911	1	pseudobulk	DESeq2	LRT	0
508	KC_AB1	Herp	0.0265554149184804	0.711693263046492	1	pseudobulk	DESeq2	LRT	0
508	KC_AB1	Hers	-0.482162225814695	0.0776874089677731	1	pseudobulk	DESeq2	LRT	0
508	KC_AB1	Hesr	0.849606887838128	1	1	pseudobulk	DESeq2	LRT	0
508	KC_AB1	Hex-A	-0.102414817149836	0.47040522052708	1	pseudobulk	DESeq2	LRT	0
508	KC_AB1	Hexim	0.187799331650938	0.575105655981535	1	pseudobulk	DESeq2	LRT	0

508	KC_AB1	Hexo1	0.0588965639316058	0.733631416935773	1	pseudobulk	DESeq2	LRT	0
508	KC_AB1	Hey	-0.393178788370145	0.418245451402082	1	pseudobulk	DESeq2	LRT	0
508	KC_AB1	Hf	0.237022465848507	1	1	pseudobulk	DESeq2	LRT	0
509	KC_AB1	hfp	-0.259999058269168	0.33328314152013	1	pseudobulk	DESeq2	LRT	0
509	KC_AB1	hfw	0.849606887838128	1	1	pseudobulk	DESeq2	LRT	0
509	KC_AB1	hh	2.78674512494791	0.54962276837928	1	pseudobulk	DESeq2	LRT	0
509	KC_AB1	hid	-3.03597874216142	0.167721488208701	1	pseudobulk	DESeq2	LRT	0
509	KC_AB1	hig	-0.370002666238906	0.699546033927651	1	pseudobulk	DESeq2	LRT	0
509	KC_AB1	HINT1	0.755001892653136	0.253804171393762	1	pseudobulk	DESeq2	LRT	0
509	KC_AB1	HIP	-0.351419685639588	0.673436604828948	1	pseudobulk	DESeq2	LRT	0
509	KC_AB1	HIP-R	0.187515891011434	0.539955372608359	1	pseudobulk	DESeq2	LRT	0
509	KC_AB1	Hip1	-0.452333173867879	0.579225163039769	1	pseudobulk	DESeq2	LRT	0
509	KC_AB1	Hip14	0.182597108978342	0.718087192521478	1	pseudobulk	DESeq2	LRT	0
510	KC_AB1	Hipk	-0.601547050867102	0.0407459100580055	1	pseudobulk	DESeq2	LRT	0
510	KC_AB1	HIPP1	0.097834208256318	0.869468448078007	1	pseudobulk	DESeq2	LRT	0
510	KC_AB1	Hira	1.0890563004518	1	1	pseudobulk	DESeq2	LRT	0
510	KC_AB1	His2Av	-0.313661872028031	0.290392499985323	1	pseudobulk	DESeq2	LRT	0
510	KC_AB1	His3.3A	0.0983889690538377	0.735647176346234	1	pseudobulk	DESeq2	LRT	0
510	KC_AB1	His3.3B	-0.092841602658753	0.403358612382927	1	pseudobulk	DESeq2	LRT	0
510	KC_AB1	His4r	-0.0800671134781358	0.515472497946969	1	pseudobulk	DESeq2	LRT	0
510	KC_AB1	HisCl1	0.237022465848507	1	1	pseudobulk	DESeq2	LRT	0
510	KC_AB1	HisRS	0.0227054127463378	0.838907335845119	1	pseudobulk	DESeq2	LRT	0
510	KC_AB1	hiw	-0.240198825626432	0.320398546873554	1	pseudobulk	DESeq2	LRT	0
511	KC_AB1	Hk	-0.322150224364316	0.198042438780181	1	pseudobulk	DESeq2	LRT	0
511	KC_AB1	Hlc	-0.593345070207138	0.394237713882068	1	pseudobulk	DESeq2	LRT	0
511	KC_AB1	Hmg-2	-0.160311429644358	0.754520999434519	1	pseudobulk	DESeq2	LRT	0
511	KC_AB1	Hmgcl	2.31997030691033	0.813169507846918	1	pseudobulk	DESeq2	LRT	0
511	KC_AB1	Hmgcr	1.0260012885517	0.114711021076884	1	pseudobulk	DESeq2	LRT	0
511	KC_AB1	HmgD	0.273566057578676	0.687152573745539	1	pseudobulk	DESeq2	LRT	0
511	KC_AB1	Hmgs	-0.13074903240941	0.730981943782099	1	pseudobulk	DESeq2	LRT	0
511	KC_AB1	HmgZ	0.373334992391654	0.0384287967153289	1	pseudobulk	DESeq2	LRT	0
511	KC_AB1	Hmr	1.33605976138209	0.809986558087114	1	pseudobulk	DESeq2	LRT	0
511	KC_AB1	Hmt-1	-0.207921525366194	0.667498665157297	1	pseudobulk	DESeq2	LRT	0
512	KC_AB1	Hmt4-20	-0.435657665512821	0.269331578962398	1	pseudobulk	DESeq2	LRT	0
512	KC_AB1	Hmu	-0.402237219053533	0.600657078463787	1	pseudobulk	DESeq2	LRT	0
512	KC_AB1	Hnf4	0.624433261972519	1	1	pseudobulk	DESeq2	LRT	0
512	KC_AB1	hng1	-1.93718863625975	0.11405761579142	1	pseudobulk	DESeq2	LRT	0
512	KC_AB1	hng2	-0.36107699501945	0.716281717438519	1	pseudobulk	DESeq2	LRT	0
512	KC_AB1	hng3	-1.7034018237942	0.343851880292217	1	pseudobulk	DESeq2	LRT	0
512	KC_AB1	HnRNP-K	-0.339703260562607	0.221093672031235	1	pseudobulk	DESeq2	LRT	0
512	KC_AB1	Ho	-1.82856985036944	0.0875415813622191	1	pseudobulk	DESeq2	LRT	0
512	KC_AB1	hob	-0.137969801323837	0.74601965572852	1	pseudobulk	DESeq2	LRT	0
512	KC_AB1	hoe1	-0.149896412325356	0.720089440357995	1	pseudobulk	DESeq2	LRT	0
513	KC_AB1	hoe2	-0.557526926667295	0.252730494038368	1	pseudobulk	DESeq2	LRT	0
513	KC_AB1	hog	0.849606887838128	1	1	pseudobulk	DESeq2	LRT	0
513	KC_AB1	hoip	-0.208451247389889	0.512331495205718	1	pseudobulk	DESeq2	LRT	0
513	KC_AB1	holn1	-0.855684726589259	0.414788176681657	1	pseudobulk	DESeq2	LRT	0
513	KC_AB1	homer	-1.17534064468515	0.152931504041741	1	pseudobulk	DESeq2	LRT	0
513	KC_AB1	hook	0.0610991797053963	0.61488662842182	1	pseudobulk	DESeq2	LRT	0
513	KC_AB1	hop	0.107491454783114	0.647847512031943	1	pseudobulk	DESeq2	LRT	0
513	KC_AB1	how	-2.08869599479716	0.424904614113793	1	pseudobulk	DESeq2	LRT	0
513	KC_AB1	HP1b	-0.817547284838116	0.274860464544637	1	pseudobulk	DESeq2	LRT	0
513	KC_AB1	HP1c	-0.249488959119277	0.712016171987973	1	pseudobulk	DESeq2	LRT	0

514	KC_AB1	HP4	0.393977631385596	0.668445107950685	1	pseudobulk	DESeq2	LRT	0
514	KC_AB1	HP5	-0.47277575175255	0.42293310307303	1	pseudobulk	DESeq2	LRT	0
514	KC_AB1	Hpd	-0.916453283982564	0.624449962842147	1	pseudobulk	DESeq2	LRT	0
514	KC_AB1	Hph	0.45503175721443	0.516484573498498	1	pseudobulk	DESeq2	LRT	0
514	KC_AB1	hpo	-0.684252108311117	0.483922615873738	1	pseudobulk	DESeq2	LRT	0
514	KC_AB1	hppy	-0.526404901512396	0.121161279484112	1	pseudobulk	DESeq2	LRT	0
514	KC_AB1	Hpr1	-0.186958561692184	0.777551626584	1	pseudobulk	DESeq2	LRT	0
514	KC_AB1	hpRNA:CR18854	0.199690343504756	0.458449512738607	1	pseudobulk	DESeq2	LRT	0
514	KC_AB1	hpRNA:CR33940	-0.216210029276812	1	1	pseudobulk	DESeq2	LRT	0
514	KC_AB1	hpRNA:CR46342	0.105124965767337	0.529166513858934	1	pseudobulk	DESeq2	LRT	0
515	KC_AB1	HPS1	0.661045422610423	0.405436186338401	1	pseudobulk	DESeq2	LRT	0
515	KC_AB1	HPS4	-0.360605640570921	0.458282438389206	1	pseudobulk	DESeq2	LRT	0
515	KC_AB1	Hr3	0.0300084468747264	0.628679913009466	1	pseudobulk	DESeq2	LRT	0
515	KC_AB1	Hr38	-0.903087049258015	0.0747086090419992	1	pseudobulk	DESeq2	LRT	0
515	KC_AB1	Hr39	0.0630069692522542	0.749808720747323	1	pseudobulk	DESeq2	LRT	0
515	KC_AB1	Hr4	-0.457484615439545	0.638659919987704	1	pseudobulk	DESeq2	LRT	0
515	KC_AB1	Hr51	-0.529064638046601	0.111589939797394	1	pseudobulk	DESeq2	LRT	0
515	KC_AB1	Hr78	-0.283903460050036	0.491378897012698	1	pseudobulk	DESeq2	LRT	0
515	KC_AB1	Hr96	0.434471570599729	0.570931187861228	1	pseudobulk	DESeq2	LRT	0
515	KC_AB1	Hrb27C	-0.0952220684833261	0.541842430882274	1	pseudobulk	DESeq2	LRT	0
516	KC_AB1	Hrb87F	-0.481440769049685	0.324247290848798	1	pseudobulk	DESeq2	LRT	0
516	KC_AB1	Hrb98DE	0.0769215767414704	0.692716437150316	1	pseudobulk	DESeq2	LRT	0
516	KC_AB1	Hrd3	-0.0210544538182547	0.604698505438677	1	pseudobulk	DESeq2	LRT	0
516	KC_AB1	hrg	0.155311487151159	0.564138772384758	1	pseudobulk	DESeq2	LRT	0
516	KC_AB1	hrm	-0.0518252091132082	0.816482818086015	1	pseudobulk	DESeq2	LRT	0
516	KC_AB1	Hrs	-2.00265787777588	0.0722047082099868	1	pseudobulk	DESeq2	LRT	0
516	KC_AB1	Hs2st	0.0400535116314188	0.672920618743877	1	pseudobulk	DESeq2	LRT	0
516	KC_AB1	Hs3st-A	-0.119558376442646	0.406159168209432	1	pseudobulk	DESeq2	LRT	0
516	KC_AB1	Hs3st-B	0.0324306869782502	0.742973019635603	1	pseudobulk	DESeq2	LRT	0
516	KC_AB1	Hs6st	0.310425468147516	0.506656069607983	1	pseudobulk	DESeq2	LRT	0
517	KC_AB1	Hsc20	0.995186982460451	0.424618415380927	1	pseudobulk	DESeq2	LRT	0
517	KC_AB1	Hsc70-2	2.48418530981904	0.632843361752602	1	pseudobulk	DESeq2	LRT	0
517	KC_AB1	Hsc70-3	-0.507823656466182	0.286642733400081	1	pseudobulk	DESeq2	LRT	0
517	KC_AB1	Hsc70-4	-0.212440707847162	0.374766254048155	1	pseudobulk	DESeq2	LRT	0
517	KC_AB1	Hsc70-5	0.3429263918538	0.435079069061732	1	pseudobulk	DESeq2	LRT	0
517	KC_AB1	Hsc70Cb	0.0696707637908407	0.75319360240565	1	pseudobulk	DESeq2	LRT	0
517	KC_AB1	Hsepi	-0.468464407318903	0.610137357386512	1	pseudobulk	DESeq2	LRT	0
517	KC_AB1	Hsf	-0.0910964184636517	0.857956245346735	1	pseudobulk	DESeq2	LRT	0
517	KC_AB1	Hsl	0.170531876339407	1	1	pseudobulk	DESeq2	LRT	0
517	KC_AB1	Hsp23	-0.664888382043009	0.620754418863351	1	pseudobulk	DESeq2	LRT	0
518	KC_AB1	Hsp26	-0.944311710423046	0.341512225982598	1	pseudobulk	DESeq2	LRT	0
518	KC_AB1	Hsp27	-1.39247692113038	0.275457412204452	1	pseudobulk	DESeq2	LRT	0
518	KC_AB1	Hsp60A	-0.657122842317348	0.122634162523904	1	pseudobulk	DESeq2	LRT	0
518	KC_AB1	Hsp60D	-0.559019799096384	0.650688555919123	1	pseudobulk	DESeq2	LRT	0
518	KC_AB1	Hsp67Ba	-0.590637494330849	0.764308822189376	1	pseudobulk	DESeq2	LRT	0
518	KC_AB1	Hsp67Bc	-1.33009018572286	0.279330424017948	1	pseudobulk	DESeq2	LRT	0
518	KC_AB1	Hsp68	-1.55336639942678	0.0288419063228636	1	pseudobulk	DESeq2	LRT	0
518	KC_AB1	Hsp70Aa	0.34947255889994	0.670589913885868	1	pseudobulk	DESeq2	LRT	0
518	KC_AB1	Hsp70Ab	-0.66195999489343	0.28899816899093	1	pseudobulk	DESeq2	LRT	0
518	KC_AB1	Hsp70Ba	-0.0231152081004256	0.83155275963134	1	pseudobulk	DESeq2	LRT	0
519	KC_AB1	Hsp70Bb	-0.116869949401143	0.870326044418798	1	pseudobulk	DESeq2	LRT	0
519	KC_AB1	Hsp70Bbb	0.183159795455075	1	1	pseudobulk	DESeq2	LRT	0
519	KC_AB1	Hsp70Bc	-0.075779909299627	0.928927957502234	1	pseudobulk	DESeq2	LRT	0

519	KC_AB1	Hsp83	-0.0410974332394569	0.950166692625806	1	pseudobulk	DESeq2	LRT	0
519	KC_AB1	HSPBAP1	1.1189393800705	0.55512662174914	1	pseudobulk	DESeq2	LRT	0
519	KC_AB1	HSPC300	0.078151907523448	0.710059249278128	1	pseudobulk	DESeq2	LRT	0
519	KC_AB1	hth	0.059859228978445	0.566881069718815	1	pseudobulk	DESeq2	LRT	0
519	KC_AB1	htk	-0.274293178601661	0.545749599185329	1	pseudobulk	DESeq2	LRT	0
519	KC_AB1	htl	0.183159795455075	1	1	pseudobulk	DESeq2	LRT	0
519	KC_AB1	HtrA2	0.849606887838128	1	1	pseudobulk	DESeq2	LRT	0
520	KC_AB1	hts	-0.0896340507092411	0.555892796531719	1	pseudobulk	DESeq2	LRT	0
520	KC_AB1	htt	0.316107920190279	0.648796328260346	1	pseudobulk	DESeq2	LRT	0
520	KC_AB1	Hug	-0.383237810938154	0.642055882606621	1	pseudobulk	DESeq2	LRT	0
520	KC_AB1	hui	2.78674512494791	0.54962276837928	1	pseudobulk	DESeq2	LRT	0
520	KC_AB1	Hus1-like	1.46091799385084	1	1	pseudobulk	DESeq2	LRT	0
520	KC_AB1	HUWE1	0.10245484352545	0.584759883825303	1	pseudobulk	DESeq2	LRT	0
520	KC_AB1	hwt	0.0163748971285638	0.864608017955553	1	pseudobulk	DESeq2	LRT	0
520	KC_AB1	Hyccin	-0.116284984012829	0.675122186850872	1	pseudobulk	DESeq2	LRT	0
520	KC_AB1	hyd	-0.194097473194226	0.681800398520058	1	pseudobulk	DESeq2	LRT	0
520	KC_AB1	Hydr1	-0.175801884892714	0.794668508970486	1	pseudobulk	DESeq2	LRT	0
521	KC_AB1	Hydr2	-1.30602425508506	0.0947318002369854	1	pseudobulk	DESeq2	LRT	0
521	KC_AB1	hyx	-1.03636125932971	0.329336485092214	1	pseudobulk	DESeq2	LRT	0
521	KC_AB1	I-2	0.339911656144115	0.446358297603262	1	pseudobulk	DESeq2	LRT	0
521	KC_AB1	I-3	-0.241788759271823	0.791703124576277	1	pseudobulk	DESeq2	LRT	0
521	KC_AB1	IA-2	0.395343407551801	0.0145454572928286	1	pseudobulk	DESeq2	LRT	0
521	KC_AB1	Ibf1	-0.269889586956917	0.669268143224896	1	pseudobulk	DESeq2	LRT	0
521	KC_AB1	Ibf2	-0.203291336364388	0.78045957908614	1	pseudobulk	DESeq2	LRT	0
521	KC_AB1	ICA69	-0.33151197947156	0.384893724190605	1	pseudobulk	DESeq2	LRT	0
521	KC_AB1	Ice1	1.19950513707505	0.524568410096412	1	pseudobulk	DESeq2	LRT	0
521	KC_AB1	Ice2	-0.314990971515788	0.359210786528659	1	pseudobulk	DESeq2	LRT	0
522	KC_AB1	icln	-0.100548307932341	1	1	pseudobulk	DESeq2	LRT	0
522	KC_AB1	ics	0.254450008036219	0.658689458277054	1	pseudobulk	DESeq2	LRT	0
522	KC_AB1	ida	-0.70631177060131	0.63135365345511	1	pseudobulk	DESeq2	LRT	0
522	KC_AB1	Ide	-0.209146152228894	0.760199262542222	1	pseudobulk	DESeq2	LRT	0
522	KC_AB1	Idgf2	-0.216210029276812	1	1	pseudobulk	DESeq2	LRT	0
522	KC_AB1	Idgf3	-0.916453283982564	0.624449962842147	1	pseudobulk	DESeq2	LRT	0
522	KC_AB1	Idgf4	0.0160940546605626	1	1	pseudobulk	DESeq2	LRT	0
522	KC_AB1	Idgf6	0.258589040337223	0.373366782579965	1	pseudobulk	DESeq2	LRT	0
522	KC_AB1	Idh	0.0539376694938803	0.685090691060325	1	pseudobulk	DESeq2	LRT	0
522	KC_AB1	Idh3a	-0.211618275812107	0.434611692705807	1	pseudobulk	DESeq2	LRT	0
523	KC_AB1	Idh3b	-0.442737614250305	0.301306650675416	1	pseudobulk	DESeq2	LRT	0
523	KC_AB1	Idi	0.212561656246669	0.698048835251414	1	pseudobulk	DESeq2	LRT	0
523	KC_AB1	Ids	0.183159795455075	1	1	pseudobulk	DESeq2	LRT	0
523	KC_AB1	if	1.65792752010766	0.762646744542806	1	pseudobulk	DESeq2	LRT	0
523	KC_AB1	ifc	-0.852593962284434	0.324483572422984	1	pseudobulk	DESeq2	LRT	0
523	KC_AB1	IFT43	1.08564364618872	1	1	pseudobulk	DESeq2	LRT	0
523	KC_AB1	igl	0.134166399467655	0.510176210650574	1	pseudobulk	DESeq2	LRT	0
523	KC_AB1	Ih	-0.239610223347216	0.159975377384507	1	pseudobulk	DESeq2	LRT	0
523	KC_AB1	IKKbeta	0.849606887838128	1	1	pseudobulk	DESeq2	LRT	0
523	KC_AB1	IKKepsilon	-0.54861606769073	0.63974745596045	1	pseudobulk	DESeq2	LRT	0
524	KC_AB1	IleRS	-0.835760925837883	0.388863274792115	1	pseudobulk	DESeq2	LRT	0
524	KC_AB1	IleRS-m	0.849606887838128	1	1	pseudobulk	DESeq2	LRT	0
524	KC_AB1	Ilk	-0.706859169109606	0.454181576426928	1	pseudobulk	DESeq2	LRT	0
524	KC_AB1	Ilp2	-1.13584521858219	0.54202769046723	1	pseudobulk	DESeq2	LRT	0
524	KC_AB1	Ilp3	0.849606887838128	1	1	pseudobulk	DESeq2	LRT	0
524	KC_AB1	Ilp5	3.86768574026245	0.0384140894002853	1	pseudobulk	DESeq2	LRT	0

524	KC_AB1	Ilp6	2.06531092668974	0.944672961903726	1	pseudobulk	DESeq2	LRT	0
524	KC_AB1	Ilp8	0.849606887838128	1	1	pseudobulk	DESeq2	LRT	0
524	KC_AB1	IM14	0.325488636334516	1	1	pseudobulk	DESeq2	LRT	0
524	KC_AB1	IM33	2.85868541466546	0.0256292481517104	1	pseudobulk	DESeq2	LRT	0
525	KC_AB1	IM4	0.237022465848507	1	1	pseudobulk	DESeq2	LRT	0
525	KC_AB1	imd	0.128177075263526	0.77827762300695	1	pseudobulk	DESeq2	LRT	0
525	KC_AB1	Iml1	0.0951432663306894	0.513809114256333	1	pseudobulk	DESeq2	LRT	0
525	KC_AB1	Imp	-0.38731500847829	0.611743178167549	1	pseudobulk	DESeq2	LRT	0
525	KC_AB1	Impbeta11	-0.185087285659563	0.755466452520621	1	pseudobulk	DESeq2	LRT	0
525	KC_AB1	ImpE1	5.81402651936298	0.114674316982137	1	pseudobulk	DESeq2	LRT	0
525	KC_AB1	ImpE3	0.0508343163501531	1	1	pseudobulk	DESeq2	LRT	0
525	KC_AB1	ImpL2	0.359225134651982	0.92784226113361	1	pseudobulk	DESeq2	LRT	0
525	KC_AB1	inaE	-0.0052789402182282	0.957820135925055	1	pseudobulk	DESeq2	LRT	0
525	KC_AB1	inc	-0.731016795357149	0.265291146045805	1	pseudobulk	DESeq2	LRT	0
526	KC_AB1	ind	0.849606887838128	1	1	pseudobulk	DESeq2	LRT	0
526	KC_AB1	Indy	1.0890563004518	1	1	pseudobulk	DESeq2	LRT	0
526	KC_AB1	ine	0.849606887838128	1	1	pseudobulk	DESeq2	LRT	0
526	KC_AB1	Ing3	0.107837998822046	0.689058587153762	1	pseudobulk	DESeq2	LRT	0
526	KC_AB1	Ing5	0.32345111977734	0.623924639506398	1	pseudobulk	DESeq2	LRT	0
526	KC_AB1	Ino80	-0.132569596466481	0.778894655846885	1	pseudobulk	DESeq2	LRT	0
526	KC_AB1	Inos	-0.254446007405129	0.496296769475096	1	pseudobulk	DESeq2	LRT	0
526	KC_AB1	INPP5E	-0.76548031623702	0.386744940973923	1	pseudobulk	DESeq2	LRT	0
526	KC_AB1	InR	-0.733238287116829	0.481656704267483	1	pseudobulk	DESeq2	LRT	0
526	KC_AB1	insc	2.78674512494791	0.54962276837928	1	pseudobulk	DESeq2	LRT	0
527	KC_AB1	insv	2.06541384650887	0.904905526327343	1	pseudobulk	DESeq2	LRT	0
527	KC_AB1	IntS1	1.17064997130226	0.30973285333993	1	pseudobulk	DESeq2	LRT	0
527	KC_AB1	IntS10	-2.30617242917746	0.160147708627331	1	pseudobulk	DESeq2	LRT	0
527	KC_AB1	IntS11	0.686942917512098	0.685271927650469	1	pseudobulk	DESeq2	LRT	0
527	KC_AB1	IntS12	0.490034462753877	0.812952344511667	1	pseudobulk	DESeq2	LRT	0
527	KC_AB1	IntS14	1.4576192915312	0.632234789693062	1	pseudobulk	DESeq2	LRT	0
527	KC_AB1	IntS2	-0.217924050817263	0.719054464244209	1	pseudobulk	DESeq2	LRT	0
527	KC_AB1	IntS3	0.00436067212630263	0.62598519158315	1	pseudobulk	DESeq2	LRT	0
527	KC_AB1	IntS4	-0.686023663615068	0.64597210976136	1	pseudobulk	DESeq2	LRT	0
527	KC_AB1	IntS6	0.0164566809772209	0.900285196906257	1	pseudobulk	DESeq2	LRT	0
528	KC_AB1	IntS8	0.517525758057102	0.723397915304756	1	pseudobulk	DESeq2	LRT	0
528	KC_AB1	IntS9	1.59863986092676	1	1	pseudobulk	DESeq2	LRT	0
528	KC_AB1	Invadolysin	-0.924344306192199	0.514013274837772	1	pseudobulk	DESeq2	LRT	0
528	KC_AB1	Inx2	0.131128740021146	1	1	pseudobulk	DESeq2	LRT	0
528	KC_AB1	Inx3	1.07128099293315	0.636000873088564	1	pseudobulk	DESeq2	LRT	0
528	KC_AB1	Inx7	1.5986549622256	0.238662755015134	1	pseudobulk	DESeq2	LRT	0
528	KC_AB1	Ip259	0.0351781032667407	0.81125155529801	1	pseudobulk	DESeq2	LRT	0
528	KC_AB1	IP3K1	2.31997030691033	0.813169507846918	1	pseudobulk	DESeq2	LRT	0
528	KC_AB1	IP3K2	-1.25232636752206	0.556603619318279	1	pseudobulk	DESeq2	LRT	0
528	KC_AB1	IPIP	0.884415281262129	0.153636055606364	1	pseudobulk	DESeq2	LRT	0
529	KC_AB1	Ipk2	1.39307486506261	0.551800093719379	1	pseudobulk	DESeq2	LRT	0
529	KC_AB1	iPLA2-VIA	-0.106138571464764	0.657461012307056	1	pseudobulk	DESeq2	LRT	0
529	KC_AB1	Ipp	0.332979815924192	0.297664397586257	1	pseudobulk	DESeq2	LRT	0
529	KC_AB1	Ir40a	1.59863986092676	1	1	pseudobulk	DESeq2	LRT	0
529	KC_AB1	Ir41a	-0.791656470982457	0.572074790174465	1	pseudobulk	DESeq2	LRT	0
529	KC_AB1	Ir47a	-2.02001322054791	0.273212954531656	1	pseudobulk	DESeq2	LRT	0
529	KC_AB1	Ir47b	0.183159795455075	1	1	pseudobulk	DESeq2	LRT	0
529	KC_AB1	Ir56a	0.228837926575705	0.614724359049187	1	pseudobulk	DESeq2	LRT	0
529	KC_AB1	Ir68a	-0.0278835855788963	0.756402943455364	1	pseudobulk	DESeq2	LRT	0

529	KC_AB1	Ir76a	0.237022465848507	1	1	pseudobulk	DESeq2	LRT	0
530	KC_AB1	Ir93a	2.78674512494791	0.54962276837928	1	pseudobulk	DESeq2	LRT	0
530	KC_AB1	Irbp18	0.0741482152868582	0.755903808187335	1	pseudobulk	DESeq2	LRT	0
530	KC_AB1	Irc	-0.118820410179067	0.83874594457554	1	pseudobulk	DESeq2	LRT	0
530	KC_AB1	Ire1	-0.262152646211668	0.628845515972313	1	pseudobulk	DESeq2	LRT	0
530	KC_AB1	Irk2	-0.85684764757434	0.475951781670273	1	pseudobulk	DESeq2	LRT	0
530	KC_AB1	Irk3	1.59863986092676	1	1	pseudobulk	DESeq2	LRT	0
530	KC_AB1	Irp-1A	-0.128233779945678	0.742240628028614	1	pseudobulk	DESeq2	LRT	0
530	KC_AB1	Irp-1B	0.871049198821558	0.786428943251427	1	pseudobulk	DESeq2	LRT	0
530	KC_AB1	IRSp53	0.849606887838128	1	1	pseudobulk	DESeq2	LRT	0
530	KC_AB1	Iru	-0.209797189080178	0.581883153848674	1	pseudobulk	DESeq2	LRT	0
531	KC_AB1	IscU	-0.011646417558945	0.802634443187063	1	pseudobulk	DESeq2	LRT	0
531	KC_AB1	isopeptidase-T-3	-0.60087698907792	0.572472453375869	1	pseudobulk	DESeq2	LRT	0
531	KC_AB1	isoQC	-2.18043139254377	0.119312165323099	1	pseudobulk	DESeq2	LRT	0
531	KC_AB1	Iswi	-1.00149798289164	0.296283350103339	1	pseudobulk	DESeq2	LRT	0
531	KC_AB1	Itgbn	0.849606887838128	1	1	pseudobulk	DESeq2	LRT	0
531	KC_AB1	ITP	-0.456915458888921	0.708770679258358	1	pseudobulk	DESeq2	LRT	0
531	KC_AB1	Itpr	-1.33662955787955	0.0878185624143093	1	pseudobulk	DESeq2	LRT	0
531	KC_AB1	ix	-0.23325540921332	0.625036948894676	1	pseudobulk	DESeq2	LRT	0
531	KC_AB1	Jafrac1	-0.334527124454721	0.615132523068011	1	pseudobulk	DESeq2	LRT	0
531	KC_AB1	Jafrac2	1.69254084318166	0.439983398691188	1	pseudobulk	DESeq2	LRT	0
532	KC_AB1	jagn	-0.328858474373134	0.464857125980649	1	pseudobulk	DESeq2	LRT	0
532	KC_AB1	janA	1.01150626833962	0.264705374835826	1	pseudobulk	DESeq2	LRT	0
532	KC_AB1	jar	0.184794970590311	0.369375442689126	1	pseudobulk	DESeq2	LRT	0
532	KC_AB1	Jarid2	-0.22263307538055	0.43995205722461	1	pseudobulk	DESeq2	LRT	0
532	KC_AB1	jbug	1.37129945894492	1	1	pseudobulk	DESeq2	LRT	0
532	KC_AB1	jdp	-0.099116221793586	0.606796901137017	1	pseudobulk	DESeq2	LRT	0
532	KC_AB1	jeb	-0.944402696245138	0.0522048833788218	1	pseudobulk	DESeq2	LRT	0
532	KC_AB1	jef	-0.211701440827012	0.382067014033021	1	pseudobulk	DESeq2	LRT	0
532	KC_AB1	jet	-0.006047735701502	0.777989011442607	1	pseudobulk	DESeq2	LRT	0
532	KC_AB1	JHDM2	-0.272342033250727	0.747325418875611	1	pseudobulk	DESeq2	LRT	0
533	KC_AB1	Jheh1	0.849606887838128	1	1	pseudobulk	DESeq2	LRT	0
533	KC_AB1	Jheh2	0.849606887838128	1	1	pseudobulk	DESeq2	LRT	0
533	KC_AB1	Jheh3	0.849606887838128	1	1	pseudobulk	DESeq2	LRT	0
533	KC_AB1	JhI-21	1.87918346471565	0.416153938342468	1	pseudobulk	DESeq2	LRT	0
533	KC_AB1	JhI-26	0.849606887838128	1	1	pseudobulk	DESeq2	LRT	0
533	KC_AB1	jigr1	-0.904111656937975	0.0420737023612525	1	pseudobulk	DESeq2	LRT	0
533	KC_AB1	JIL-1	-0.413613702818406	0.127607556594537	1	pseudobulk	DESeq2	LRT	0
533	KC_AB1	jing	-0.471570541967051	0.275197447200951	1	pseudobulk	DESeq2	LRT	0
533	KC_AB1	JMJD4	0.179197026544259	0.940781833796052	1	pseudobulk	DESeq2	LRT	0
533	KC_AB1	JMJD5	0.256966398428518	1	1	pseudobulk	DESeq2	LRT	0
534	KC_AB1	JMJD7	0.518999521654433	0.624752404541754	1	pseudobulk	DESeq2	LRT	0
534	KC_AB1	jnj	-0.137969801323837	0.74601965572852	1	pseudobulk	DESeq2	LRT	0
534	KC_AB1	jp	-0.299152867645822	0.433571746007675	1	pseudobulk	DESeq2	LRT	0
534	KC_AB1	Jra	-0.909879427077698	0.364929220204114	1	pseudobulk	DESeq2	LRT	0
534	KC_AB1	JTBR	0.275874106414765	0.760420727870095	1	pseudobulk	DESeq2	LRT	0
534	KC_AB1	jub	-0.309381970921449	0.698968666663817	1	pseudobulk	DESeq2	LRT	0
534	KC_AB1	jumu	0.259928158939877	0.711248134976605	1	pseudobulk	DESeq2	LRT	0
534	KC_AB1	Jupiter	-0.434803740913081	0.212644855430949	1	pseudobulk	DESeq2	LRT	0
534	KC_AB1	jus	-0.241182818197071	0.137884132692881	1	pseudobulk	DESeq2	LRT	0
534	KC_AB1	jv	-0.071841840962736	0.844751225944333	1	pseudobulk	DESeq2	LRT	0
535	KC_AB1	jvl	-0.450295208283748	0.10853176917205	1	pseudobulk	DESeq2	LRT	0
535	KC_AB1	Jwa	0.424641441190161	0.296295011878823	1	pseudobulk	DESeq2	LRT	0

535	KC_AB1	KaiR1D	0.118460166691258	0.854841526031402	1	pseudobulk	DESeq2	LRT	0
535	KC_AB1	Kank	-0.0025043492297962	0.807604821336984	1	pseudobulk	DESeq2	LRT	0
535	KC_AB1	Kap-alpha1	0.120807535833327	0.671858245178679	1	pseudobulk	DESeq2	LRT	0
535	KC_AB1	Kap-alpha3	-0.451468370278762	0.28447043509347	1	pseudobulk	DESeq2	LRT	0
535	KC_AB1	Kap3	-0.288981666305357	0.519009803228214	1	pseudobulk	DESeq2	LRT	0
535	KC_AB1	kar	3.16697464674408	0.406679230261689	1	pseudobulk	DESeq2	LRT	0
535	KC_AB1	Karybeta3	-0.131605017313073	0.821395036414894	1	pseudobulk	DESeq2	LRT	0
535	KC_AB1	kat-60L1	0.360561092089497	0.517879588966066	1	pseudobulk	DESeq2	LRT	0
536	KC_AB1	Kat60	-0.421017752645348	0.600405023349766	1	pseudobulk	DESeq2	LRT	0
536	KC_AB1	kat80	-0.32272441764722	0.645931509133749	1	pseudobulk	DESeq2	LRT	0
536	KC_AB1	kay	-0.428446939367276	0.455244771724369	1	pseudobulk	DESeq2	LRT	0
536	KC_AB1	Kaz1-ORFB	-0.278129493418767	0.794244422965159	1	pseudobulk	DESeq2	LRT	0
536	KC_AB1	kcc	-0.0469575540802918	0.648855305464081	1	pseudobulk	DESeq2	LRT	0
536	KC_AB1	Kcmf1	0.262828861189903	0.550027837724502	1	pseudobulk	DESeq2	LRT	0
536	KC_AB1	KCNQ	-0.08639378848332	0.566492986060154	1	pseudobulk	DESeq2	LRT	0
536	KC_AB1	KdelR	-0.474677352369093	0.381822647870542	1	pseudobulk	DESeq2	LRT	0
536	KC_AB1	Kdm2	-0.56403655752337	0.321193916622468	1	pseudobulk	DESeq2	LRT	0
536	KC_AB1	Kdm4A	-0.443856289025472	0.639155603256284	1	pseudobulk	DESeq2	LRT	0
537	KC_AB1	Kdm4B	-0.364997383155231	0.19999007023671	1	pseudobulk	DESeq2	LRT	0
537	KC_AB1	kdn	0.331982053526013	0.382229346063936	1	pseudobulk	DESeq2	LRT	0
537	KC_AB1	Keap1	0.0723747220455526	0.760233419328873	1	pseudobulk	DESeq2	LRT	0
537	KC_AB1	Kebab	0.849606887838128	1	1	pseudobulk	DESeq2	LRT	0
537	KC_AB1	kek1	-0.267280249857229	0.186936560674104	1	pseudobulk	DESeq2	LRT	0
537	KC_AB1	kek2	0.539227591542996	1	1	pseudobulk	DESeq2	LRT	0
537	KC_AB1	kek3	-0.372063563923449	0.220210454978195	1	pseudobulk	DESeq2	LRT	0
537	KC_AB1	kek4	-0.547255495611545	0.368179063867104	1	pseudobulk	DESeq2	LRT	0
537	KC_AB1	kek5	-0.2431166961764	0.770415239589528	1	pseudobulk	DESeq2	LRT	0
537	KC_AB1	kek6	0.0697425791714352	0.651046443349412	1	pseudobulk	DESeq2	LRT	0
538	KC_AB1	kel	-0.55495093517301	0.466771370811456	1	pseudobulk	DESeq2	LRT	0
538	KC_AB1	ken	2.06541384650887	0.904905526327343	1	pseudobulk	DESeq2	LRT	0
538	KC_AB1	kermit	0.849606887838128	1	1	pseudobulk	DESeq2	LRT	0
538	KC_AB1	key	-0.194986639657359	0.661872274952282	1	pseudobulk	DESeq2	LRT	0
538	KC_AB1	KFase	2.31997030691033	0.813169507846918	1	pseudobulk	DESeq2	LRT	0
538	KC_AB1	Khc	0.183607263168608	0.748797923467091	1	pseudobulk	DESeq2	LRT	0
538	KC_AB1	Khc-73	-0.265347476039135	0.352390301086783	1	pseudobulk	DESeq2	LRT	0
538	KC_AB1	kibra	1.21267130850476	1	1	pseudobulk	DESeq2	LRT	0
538	KC_AB1	Kif19A	1.39706235106669	1	1	pseudobulk	DESeq2	LRT	0
538	KC_AB1	Kif3C	0.40737517849924	0.646937651993385	1	pseudobulk	DESeq2	LRT	0
539	KC_AB1	kin17	-0.0756196570291044	0.478645019099297	1	pseudobulk	DESeq2	LRT	0
539	KC_AB1	kirre	-0.0630048829410672	0.720232562123976	1	pseudobulk	DESeq2	LRT	0
539	KC_AB1	kis	-0.130510695270578	0.524032929305357	1	pseudobulk	DESeq2	LRT	0
539	KC_AB1	kl-2	0.849606887838128	1	1	pseudobulk	DESeq2	LRT	0
539	KC_AB1	kl-3	0.366247714696288	1	1	pseudobulk	DESeq2	LRT	0
539	KC_AB1	kl-5	0.183159795463042	1	1	pseudobulk	DESeq2	LRT	0
539	KC_AB1	klar	-0.467103124303419	0.444329299426308	1	pseudobulk	DESeq2	LRT	0
539	KC_AB1	Klc	-0.0462905172429622	0.837122305814876	1	pseudobulk	DESeq2	LRT	0
539	KC_AB1	klg	-0.0425417604960005	0.580329803871308	1	pseudobulk	DESeq2	LRT	0
539	KC_AB1	KLHL18	-0.277541093513739	0.580276202315529	1	pseudobulk	DESeq2	LRT	0
540	KC_AB1	Klp10A	0.472415128592653	0.368057808629796	1	pseudobulk	DESeq2	LRT	0
540	KC_AB1	Klp31E	0.432766872695096	0.73498094272889	1	pseudobulk	DESeq2	LRT	0
540	KC_AB1	Klp54D	-0.847199302416132	0.677009019822614	1	pseudobulk	DESeq2	LRT	0
540	KC_AB1	Klp64D	-0.541595062956668	0.393851298415955	1	pseudobulk	DESeq2	LRT	0
540	KC_AB1	Klp68D	-0.34775552260251	0.485510578686391	1	pseudobulk	DESeq2	LRT	0

540	KC_AB1	Klp98A	0.344442089410614	0.426452924045089	1	pseudobulk	DESeq2	LRT	0
540	KC_AB1	klu	0.849606887838128	1	1	pseudobulk	DESeq2	LRT	0
540	KC_AB1	Kmn1	-0.580682610791802	0.768938018276591	1	pseudobulk	DESeq2	LRT	0
540	KC_AB1	kmr	0.17299403779209	0.473240379213992	1	pseudobulk	DESeq2	LRT	0
540	KC_AB1	kni	-3.59878431419227	0.306666859703826	1	pseudobulk	DESeq2	LRT	0
541	KC_AB1	knk	0.376431477381175	1	1	pseudobulk	DESeq2	LRT	0
541	KC_AB1	knrl	-0.300289249643225	0.901958007500605	1	pseudobulk	DESeq2	LRT	0
541	KC_AB1	ko	-0.989592528332913	0.640503837706122	1	pseudobulk	DESeq2	LRT	0
541	KC_AB1	koi	0.391943169528158	0.29312118834185	1	pseudobulk	DESeq2	LRT	0
541	KC_AB1	koko	-1.88712392008431	0.26719760928779	1	pseudobulk	DESeq2	LRT	0
541	KC_AB1	Kr-h1	-0.286657760647653	0.537864668902916	1	pseudobulk	DESeq2	LRT	0
541	KC_AB1	Kr-h2	-0.0080109729450591	0.945160802933615	1	pseudobulk	DESeq2	LRT	0
541	KC_AB1	kra	-0.268247398156018	0.236083476486424	1	pseudobulk	DESeq2	LRT	0
541	KC_AB1	kraken	0.0390209942642912	0.901247865149814	1	pseudobulk	DESeq2	LRT	0
541	KC_AB1	kri	0.504905687605675	0.234660576487769	1	pseudobulk	DESeq2	LRT	0
542	KC_AB1	Krn	-0.0381738336470014	0.74164063965834	1	pseudobulk	DESeq2	LRT	0
542	KC_AB1	KrT95D	-0.512481706580749	0.0718854800446462	1	pseudobulk	DESeq2	LRT	0
542	KC_AB1	krz	-0.365053555245551	0.425226350843725	1	pseudobulk	DESeq2	LRT	0
542	KC_AB1	ksh	0.141087713031438	0.575020129059953	1	pseudobulk	DESeq2	LRT	0
542	KC_AB1	ksr	-0.657848455973433	0.299411088778612	1	pseudobulk	DESeq2	LRT	0
542	KC_AB1	kst	4.73786001624398	0.0628075777075593	1	pseudobulk	DESeq2	LRT	0
542	KC_AB1	Ktl	0.174121112555408	0.589303903398762	1	pseudobulk	DESeq2	LRT	0
542	KC_AB1	kto	-0.531569622369456	0.567437376497131	1	pseudobulk	DESeq2	LRT	0
542	KC_AB1	ktub	-1.41567692939154	0.448661989255937	1	pseudobulk	DESeq2	LRT	0
542	KC_AB1	Ku80	0.237022465848507	1	1	pseudobulk	DESeq2	LRT	0
543	KC_AB1	kud	0.0348325765521928	0.596172126542774	1	pseudobulk	DESeq2	LRT	0
543	KC_AB1	kug	-0.242160456024088	0.429903640433751	1	pseudobulk	DESeq2	LRT	0
543	KC_AB1	kuk	0.552457374621858	0.348920580934671	1	pseudobulk	DESeq2	LRT	0
543	KC_AB1	Kul	-0.091742977205749	0.8702652277282	1	pseudobulk	DESeq2	LRT	0
543	KC_AB1	kune	-0.853343338059512	0.23202607958402	1	pseudobulk	DESeq2	LRT	0
543	KC_AB1	kuz	-0.298890194501713	0.220698342602908	1	pseudobulk	DESeq2	LRT	0
543	KC_AB1	Kyat	0.268151595085632	1	1	pseudobulk	DESeq2	LRT	0
543	KC_AB1	kz	-0.650934862190717	0.584265663183686	1	pseudobulk	DESeq2	LRT	0
543	KC_AB1	l(1)10Bb	0.245044733229078	0.563056757029409	1	pseudobulk	DESeq2	LRT	0
543	KC_AB1	l(1)G0004	-0.152019868250592	0.769835405185865	1	pseudobulk	DESeq2	LRT	0
544	KC_AB1	l(1)G0007	0.540801799415879	0.362363734413646	1	pseudobulk	DESeq2	LRT	0
544	KC_AB1	l(1)G0193	-0.380286249710391	0.402266129538065	1	pseudobulk	DESeq2	LRT	0
544	KC_AB1	l(1)G0196	-0.0585393591454602	0.706430755075908	1	pseudobulk	DESeq2	LRT	0
544	KC_AB1	l(1)G0289	-0.0390939437778761	0.86404745623278	1	pseudobulk	DESeq2	LRT	0
544	KC_AB1	l(1)G0320	0.718065563143415	0.080884316456633	1	pseudobulk	DESeq2	LRT	0
544	KC_AB1	l(1)G0469	0.125494772583795	0.581412059442483	1	pseudobulk	DESeq2	LRT	0
544	KC_AB1	l(2)05287	0.0989845864158605	0.393337829159854	1	pseudobulk	DESeq2	LRT	0
544	KC_AB1	l(2)05714	-1.1442824565274	0.307167565406451	1	pseudobulk	DESeq2	LRT	0
544	KC_AB1	l(2)09851	-0.0873127613564943	0.845018057100727	1	pseudobulk	DESeq2	LRT	0
544	KC_AB1	l(2)10685	0.437195883853447	1	1	pseudobulk	DESeq2	LRT	0
545	KC_AB1	l(2)34Fc	2.78674512494791	0.54962276837928	1	pseudobulk	DESeq2	LRT	0
545	KC_AB1	l(2)34Fd	0.842158677354496	0.788767743533491	1	pseudobulk	DESeq2	LRT	0
545	KC_AB1	l(2)35Be	-0.119261740160671	0.822439636659705	1	pseudobulk	DESeq2	LRT	0
545	KC_AB1	l(2)37Bb	-1.0124032166989	0.592614381977149	1	pseudobulk	DESeq2	LRT	0
545	KC_AB1	l(2)37Cb	-1.00752466718157	0.580536516624078	1	pseudobulk	DESeq2	LRT	0
545	KC_AB1	l(2)37Cc	0.368989802335291	0.49792175649299	1	pseudobulk	DESeq2	LRT	0
545	KC_AB1	l(2)37Cd	0.849606887838128	1	1	pseudobulk	DESeq2	LRT	0
545	KC_AB1	l(2)37Cg	1.87223267308124	0.417804035158437	1	pseudobulk	DESeq2	LRT	0

545	KC_AB1	l(2)41Ab	0.438102080115011	0.975726964765433	1	pseudobulk	DESeq2	LRT	0
545	KC_AB1	l(2)dtl	2.78674512494791	0.54962276837928	1	pseudobulk	DESeq2	LRT	0
546	KC_AB1	l(2)efl	-1.86748544403173	0.194856046879364	1	pseudobulk	DESeq2	LRT	0
546	KC_AB1	l(2)gd1	-0.463646647894343	0.626509776298229	1	pseudobulk	DESeq2	LRT	0
546	KC_AB1	l(2)gl	1.37129945894492	1	1	pseudobulk	DESeq2	LRT	0
546	KC_AB1	l(2)k01209	0.0075304815638714	0.584977773669381	1	pseudobulk	DESeq2	LRT	0
546	KC_AB1	l(2)k05819	-0.316250521621562	0.526539341290253	1	pseudobulk	DESeq2	LRT	0
546	KC_AB1	l(2)k09022	0.566580361464048	1	1	pseudobulk	DESeq2	LRT	0
546	KC_AB1	l(2)k09848	1.69065819005582	0.417021113459439	1	pseudobulk	DESeq2	LRT	0
546	KC_AB1	l(2)k09913	-0.290555615341756	0.234383637927827	1	pseudobulk	DESeq2	LRT	0
546	KC_AB1	l(2)k10201	-0.989592528332913	0.640503837706122	1	pseudobulk	DESeq2	LRT	0
546	KC_AB1	l(2)k14505	-0.247686672847845	0.615117129839549	1	pseudobulk	DESeq2	LRT	0
547	KC_AB1	l(2)SH0834	-0.162032084797715	0.733748202546283	1	pseudobulk	DESeq2	LRT	0
547	KC_AB1	l(3)02640	0.346926132943339	0.494180384792703	1	pseudobulk	DESeq2	LRT	0
547	KC_AB1	l(3)04053	-0.33816101913279	0.655348666657866	1	pseudobulk	DESeq2	LRT	0
547	KC_AB1	l(3)05822	-0.321990133659583	0.533142195258492	1	pseudobulk	DESeq2	LRT	0
547	KC_AB1	l(3)07882	-0.404963105319387	0.631960880351504	1	pseudobulk	DESeq2	LRT	0
547	KC_AB1	l(3)72Ab	-0.0064127888917290	0.832564781935559	1	pseudobulk	DESeq2	LRT	0
547	KC_AB1	l(3)72Dn	-0.405319165074576	0.757016930501376	1	pseudobulk	DESeq2	LRT	0
547	KC_AB1	l(3)72Dp	0.194265322970028	1	1	pseudobulk	DESeq2	LRT	0
547	KC_AB1	l(3)72Dr	-1.20054795286106	0.522310314157169	1	pseudobulk	DESeq2	LRT	0
547	KC_AB1	l(3)73Ah	-0.262061565680891	0.482956745076596	1	pseudobulk	DESeq2	LRT	0
548	KC_AB1	l(3)76BDm	-0.796206659706647	0.426956523307482	1	pseudobulk	DESeq2	LRT	0
548	KC_AB1	l(3)80Fg	-0.37991024419734	0.0267161539943504	1	pseudobulk	DESeq2	LRT	0
548	KC_AB1	l(3)80Fj	-0.130481982003914	0.442255659161604	1	pseudobulk	DESeq2	LRT	0
548	KC_AB1	l(3)87Df	-0.182200157218673	0.674429285657118	1	pseudobulk	DESeq2	LRT	0
548	KC_AB1	l(3)L1231	-0.20789593746801	0.393917325878353	1	pseudobulk	DESeq2	LRT	0
548	KC_AB1	l(3)mbt	0.240099602818837	0.400170726695626	1	pseudobulk	DESeq2	LRT	0
548	KC_AB1	l(3)neo38	-0.280119461576889	0.15585334728127	1	pseudobulk	DESeq2	LRT	0
548	KC_AB1	l(3)neo43	-0.796581424289674	0.368281733072921	1	pseudobulk	DESeq2	LRT	0
548	KC_AB1	l(3)psg2	-0.285647264109788	0.647937608255396	1	pseudobulk	DESeq2	LRT	0
548	KC_AB1	L2HGDH	0.739834314379904	0.351659281716249	1	pseudobulk	DESeq2	LRT	0
549	KC_AB1	La	-0.0962728386647723	0.672001381102858	1	pseudobulk	DESeq2	LRT	0
549	KC_AB1	Lac	-2.10820399710285	0.197365451321858	1	pseudobulk	DESeq2	LRT	0
549	KC_AB1	lace	-1.09212815074561	0.197900494801817	1	pseudobulk	DESeq2	LRT	0
549	KC_AB1	Lam	0.321135741635351	0.547369327565286	1	pseudobulk	DESeq2	LRT	0
549	KC_AB1	lama	2.06541384650887	0.904905526327343	1	pseudobulk	DESeq2	LRT	0
549	KC_AB1	LamC	0.724703920326507	0.874558524268367	1	pseudobulk	DESeq2	LRT	0
549	KC_AB1	Lamp1	0.10461983506033	0.690644296892545	1	pseudobulk	DESeq2	LRT	0
549	KC_AB1	LanA	2.78674512494791	0.54962276837928	1	pseudobulk	DESeq2	LRT	0
549	KC_AB1	LanB1	2.78674512494791	0.54962276837928	1	pseudobulk	DESeq2	LRT	0
549	KC_AB1	LanB2	3.16697464674408	0.406679230261689	1	pseudobulk	DESeq2	LRT	0
550	KC_AB1	lap	-0.224247553654297	0.280275600329724	1	pseudobulk	DESeq2	LRT	0
550	KC_AB1	Lap1	-0.733073002496204	0.421142979123502	1	pseudobulk	DESeq2	LRT	0
550	KC_AB1	Lar	-0.346319743928398	0.0189947457252159	1	pseudobulk	DESeq2	LRT	0
550	KC_AB1	lark	0.0439240864377561	0.91026408410168	1	pseudobulk	DESeq2	LRT	0
550	KC_AB1	larp	-0.0780744326914855	0.593610817050235	1	pseudobulk	DESeq2	LRT	0
550	KC_AB1	Larp4B	-0.0500337996054788	0.524835312474713	1	pseudobulk	DESeq2	LRT	0
550	KC_AB1	Larp7	0.590613862662558	0.785276424632034	1	pseudobulk	DESeq2	LRT	0
550	KC_AB1	Las	-0.469462173115722	0.551546774319952	1	pseudobulk	DESeq2	LRT	0
550	KC_AB1	lawc	0.296494433404078	0.319606146905665	1	pseudobulk	DESeq2	LRT	0
550	KC_AB1	lbk	-0.351016274834088	0.277753839748661	1	pseudobulk	DESeq2	LRT	0
551	KC_AB1	lbm	-0.383747448531127	0.128058874514866	1	pseudobulk	DESeq2	LRT	0

551	KC_AB1	LBR	0.319144022961168	0.840802535083676	1	pseudobulk	DESeq2	LRT	0
551	KC_AB1	Lcch3	-0.0756273335310687	0.764494505362358	1	pseudobulk	DESeq2	LRT	0
551	KC_AB1	ldbr	0.849606887838128	1	1	pseudobulk	DESeq2	LRT	0
551	KC_AB1	Ldh	1.7454737383338	0.70756651762728	1	pseudobulk	DESeq2	LRT	0
551	KC_AB1	Ldsdh1	0.849606887838128	1	1	pseudobulk	DESeq2	LRT	0
551	KC_AB1	Leash	-0.842908502933556	0.52748823271097	1	pseudobulk	DESeq2	LRT	0
551	KC_AB1	lectin-30A	3.41844045086015	0.351942490385471	1	pseudobulk	DESeq2	LRT	0
551	KC_AB1	lectin-46Ca	0.849606887838128	1	1	pseudobulk	DESeq2	LRT	0
551	KC_AB1	Lerp	-1.00538173552738	0.029877400674749	1	pseudobulk	DESeq2	LRT	0
552	KC_AB1	Letm1	0.0462653573124129	0.697510581453568	1	pseudobulk	DESeq2	LRT	0
552	KC_AB1	LeuRS	1.07022652702202	0.256652200459881	1	pseudobulk	DESeq2	LRT	0
552	KC_AB1	LeuRS-m	-0.185720913579601	0.733350479630558	1	pseudobulk	DESeq2	LRT	0
552	KC_AB1	levy	-0.0015963409359754	0.729334910846397	1	pseudobulk	DESeq2	LRT	0
552	KC_AB1	lft	-0.389346928562833	0.820127740800904	1	pseudobulk	DESeq2	LRT	0
552	KC_AB1	Lgr1	0.496001197900266	0.0344562873455599	1	pseudobulk	DESeq2	LRT	0
552	KC_AB1	Lgr3	0.849606887838128	1	1	pseudobulk	DESeq2	LRT	0
552	KC_AB1	lgs	-0.0735181894423825	0.489378440215584	1	pseudobulk	DESeq2	LRT	0
552	KC_AB1	Lhr	-1.13743771763179	0.397191242539682	1	pseudobulk	DESeq2	LRT	0
552	KC_AB1	lic	-1.29894109409001	0.201644826778906	1	pseudobulk	DESeq2	LRT	0
553	KC_AB1	lid	-0.110636790493724	0.569273459057937	1	pseudobulk	DESeq2	LRT	0
553	KC_AB1	lig	0.276448504273543	0.303768690571041	1	pseudobulk	DESeq2	LRT	0
553	KC_AB1	lili	0.196955796990222	0.564144662463347	1	pseudobulk	DESeq2	LRT	0
553	KC_AB1	lilli	0.0497000236379099	0.804831500050902	1	pseudobulk	DESeq2	LRT	0
553	KC_AB1	LIMK1	0.359935450368875	0.462441121681447	1	pseudobulk	DESeq2	LRT	0
553	KC_AB1	lin	-1.13895862469189	0.253050909472374	1	pseudobulk	DESeq2	LRT	0
553	KC_AB1	lin-52	-0.348553118667644	0.652805609394537	1	pseudobulk	DESeq2	LRT	0
553	KC_AB1	link	3.11011620171721	0.0764187156066316	1	pseudobulk	DESeq2	LRT	0
553	KC_AB1	Lint-1	0.426813470395105	0.345395501658529	1	pseudobulk	DESeq2	LRT	0
553	KC_AB1	Liprin-alpha	0.130633090133637	0.738855501779855	1	pseudobulk	DESeq2	LRT	0
554	KC_AB1	Liprin-beta	0.0285227365962909	0.805975479600742	1	pseudobulk	DESeq2	LRT	0
554	KC_AB1	Liprin-gamma	-0.301713707013842	0.193899107816527	1	pseudobulk	DESeq2	LRT	0
554	KC_AB1	Lis-1	-0.233824716071041	0.174168922265088	1	pseudobulk	DESeq2	LRT	0
554	KC_AB1	List	0.713780324542466	1	1	pseudobulk	DESeq2	LRT	0
554	KC_AB1	Lk6	0.21375895591627	0.331634233725958	1	pseudobulk	DESeq2	LRT	0
554	KC_AB1	Lkb1	0.212951857671543	0.753930482168808	1	pseudobulk	DESeq2	LRT	0
554	KC_AB1	LKRSDH	2.31997030691033	0.813169507846918	1	pseudobulk	DESeq2	LRT	0
554	KC_AB1	LManII	0.849606887838128	1	1	pseudobulk	DESeq2	LRT	0
554	KC_AB1	Lmpt	0.584189058118167	0.0124477014077105	1	pseudobulk	DESeq2	LRT	0
554	KC_AB1	lms	0.865817449292603	1	1	pseudobulk	DESeq2	LRT	0
555	KC_AB1	lncRNA:alphagamm	-0.79925658509401	0.0986232112116597	1	pseudobulk	DESeq2	LRT	0
555	KC_AB1	lncRNA:cherub	2.35164802177858	0.0283399641817236	1	pseudobulk	DESeq2	LRT	0
555	KC_AB1	lncRNA:CR30055	-0.146014313761031	0.692164259414067	1	pseudobulk	DESeq2	LRT	0
555	KC_AB1	lncRNA:CR30121	1.58205617221276	1	1	pseudobulk	DESeq2	LRT	0
555	KC_AB1	lncRNA:CR31044	0.276313778440695	0.561746239407362	1	pseudobulk	DESeq2	LRT	0
555	KC_AB1	lncRNA:CR31386	2.43275481406755	0.550258497427138	1	pseudobulk	DESeq2	LRT	0
555	KC_AB1	lncRNA:CR31451	-0.173292423902543	0.314285417000664	1	pseudobulk	DESeq2	LRT	0
555	KC_AB1	lncRNA:CR32194	0.849606887838128	1	1	pseudobulk	DESeq2	LRT	0
555	KC_AB1	lncRNA:CR32218	0.0125488695985611	0.562113498695296	1	pseudobulk	DESeq2	LRT	0
555	KC_AB1	lncRNA:CR32553	0.849606887838128	1	1	pseudobulk	DESeq2	LRT	0
556	KC_AB1	lncRNA:CR32582	1.46081863254101	1	1	pseudobulk	DESeq2	LRT	0
556	KC_AB1	lncRNA:CR32773	3.4445176613275	0.313892688617837	1	pseudobulk	DESeq2	LRT	0
556	KC_AB1	lncRNA:CR33963	2.78674512494791	0.54962276837928	1	pseudobulk	DESeq2	LRT	0
556	KC_AB1	lncRNA:CR34024	0.849606887838128	1	1	pseudobulk	DESeq2	LRT	0

556	KC_AB1	lncRNA:CR34052	0.849606887838128	1	1	pseudobulk	DESeq2	LRT	0
556	KC_AB1	lncRNA:CR34335	-0.737649474343643	0.186951152942635	1	pseudobulk	DESeq2	LRT	0
556	KC_AB1	lncRNA:CR40341	-1.07674319282786	0.562491167513556	1	pseudobulk	DESeq2	LRT	0
556	KC_AB1	lncRNA:CR40469	-0.696938067807957	0.528149026630469	1	pseudobulk	DESeq2	LRT	0
556	KC_AB1	lncRNA:CR42491	-0.704858387530061	0.698842745464243	1	pseudobulk	DESeq2	LRT	0
556	KC_AB1	lncRNA:CR42549	-0.422623708838822	0.609540734437255	1	pseudobulk	DESeq2	LRT	0
557	KC_AB1	lncRNA:CR42746	0.849606887838128	1	1	pseudobulk	DESeq2	LRT	0
557	KC_AB1	lncRNA:CR42844	0.849606887838128	1	1	pseudobulk	DESeq2	LRT	0
557	KC_AB1	lncRNA:CR42861	0.711302391545227	0.0856115316958362	1	pseudobulk	DESeq2	LRT	0
557	KC_AB1	lncRNA:CR42862	-0.256688983993734	0.0905402478425279	1	pseudobulk	DESeq2	LRT	0
557	KC_AB1	lncRNA:CR43126	2.78674512494791	0.54962276837928	1	pseudobulk	DESeq2	LRT	0
557	KC_AB1	lncRNA:CR43144	0.849606887838128	1	1	pseudobulk	DESeq2	LRT	0
557	KC_AB1	lncRNA:CR43146	0.930993226399931	1	1	pseudobulk	DESeq2	LRT	0
557	KC_AB1	lncRNA:CR43148	-0.485027100453874	0.813470998077292	1	pseudobulk	DESeq2	LRT	0
557	KC_AB1	lncRNA:CR43174	0.849606887838128	1	1	pseudobulk	DESeq2	LRT	0
557	KC_AB1	lncRNA:CR43241	0.635848183543954	1	1	pseudobulk	DESeq2	LRT	0
558	KC_AB1	lncRNA:CR43242	2.31997030691033	0.813169507846918	1	pseudobulk	DESeq2	LRT	0
558	KC_AB1	lncRNA:CR43256	1.93706461079025	0.658817357590498	1	pseudobulk	DESeq2	LRT	0
558	KC_AB1	lncRNA:CR43264	0.521825140548128	0.395660502775771	1	pseudobulk	DESeq2	LRT	0
558	KC_AB1	lncRNA:CR43285	2.31997030691033	0.813169507846918	1	pseudobulk	DESeq2	LRT	0
558	KC_AB1	lncRNA:CR43314	1.57310851682448	0.566847312691645	1	pseudobulk	DESeq2	LRT	0
558	KC_AB1	lncRNA:CR43334	-2.19091245841659	0.141838228125169	1	pseudobulk	DESeq2	LRT	0
558	KC_AB1	lncRNA:CR43363	2.31997030691033	0.813169507846918	1	pseudobulk	DESeq2	LRT	0
558	KC_AB1	lncRNA:CR43406	0.183159795463042	1	1	pseudobulk	DESeq2	LRT	0
558	KC_AB1	lncRNA:CR43408	1.35647311563626	0.383409832941808	1	pseudobulk	DESeq2	LRT	0
558	KC_AB1	lncRNA:CR43459	-0.284629469777731	0.577606181736069	1	pseudobulk	DESeq2	LRT	0
559	KC_AB1	lncRNA:CR43461	0.203254739366544	1	1	pseudobulk	DESeq2	LRT	0
559	KC_AB1	lncRNA:CR43492	1.20092489142827	1	1	pseudobulk	DESeq2	LRT	0
559	KC_AB1	lncRNA:CR43496	-0.0705425057432341	0.805657819091573	1	pseudobulk	DESeq2	LRT	0
559	KC_AB1	lncRNA:CR43604	0.849606887838128	1	1	pseudobulk	DESeq2	LRT	0
559	KC_AB1	lncRNA:CR43605	2.06531092668974	0.944672961903726	1	pseudobulk	DESeq2	LRT	0
559	KC_AB1	lncRNA:CR43626	1.00693893053477	1	1	pseudobulk	DESeq2	LRT	0
559	KC_AB1	lncRNA:CR43635	2.42818428071509	0.612687220587	1	pseudobulk	DESeq2	LRT	0
559	KC_AB1	lncRNA:CR43651	-0.447482086186883	0.442946823877191	1	pseudobulk	DESeq2	LRT	0
559	KC_AB1	lncRNA:CR43654	0.849606887838128	1	1	pseudobulk	DESeq2	LRT	0
559	KC_AB1	lncRNA:CR43656	2.31997030691033	0.813169507846918	1	pseudobulk	DESeq2	LRT	0
560	KC_AB1	lncRNA:CR43685	0.573057534216947	0.393632479401547	1	pseudobulk	DESeq2	LRT	0
560	KC_AB1	lncRNA:CR43700	-0.260657448875346	0.807136242124327	1	pseudobulk	DESeq2	LRT	0
560	KC_AB1	lncRNA:CR43801	0.849606887838128	1	1	pseudobulk	DESeq2	LRT	0
560	KC_AB1	lncRNA:CR43807	0.849606887838128	1	1	pseudobulk	DESeq2	LRT	0
560	KC_AB1	lncRNA:CR43818	0.0160940546605626	1	1	pseudobulk	DESeq2	LRT	0
560	KC_AB1	lncRNA:CR43821	0.849606887838128	1	1	pseudobulk	DESeq2	LRT	0
560	KC_AB1	lncRNA:CR43827	1.59863986092676	1	1	pseudobulk	DESeq2	LRT	0
560	KC_AB1	lncRNA:CR43828	0.366247714696288	1	1	pseudobulk	DESeq2	LRT	0
560	KC_AB1	lncRNA:CR43836	-0.0789324499915628	0.916666342452182	1	pseudobulk	DESeq2	LRT	0
560	KC_AB1	lncRNA:CR43837	0.849606887838128	1	1	pseudobulk	DESeq2	LRT	0
561	KC_AB1	lncRNA:CR43838	0.849606887838128	1	1	pseudobulk	DESeq2	LRT	0
561	KC_AB1	lncRNA:CR43849	0.849606887838128	1	1	pseudobulk	DESeq2	LRT	0
561	KC_AB1	lncRNA:CR43855	2.31997030691033	0.813169507846918	1	pseudobulk	DESeq2	LRT	0
561	KC_AB1	lncRNA:CR43856	-0.403170783264911	0.637575652223243	1	pseudobulk	DESeq2	LRT	0
561	KC_AB1	lncRNA:CR43857	-1.25393399638069	0.612054104673377	1	pseudobulk	DESeq2	LRT	0
561	KC_AB1	lncRNA:CR43878	-0.124421197091681	0.625208437515512	1	pseudobulk	DESeq2	LRT	0
561	KC_AB1	lncRNA:CR43887	0.849606887838128	1	1	pseudobulk	DESeq2	LRT	0

561	KC_AB1	lncRNA:CR43890	3.150585340203	0.085575843701455	1	pseudobulk	DESeq2	LRT	0
561	KC_AB1	lncRNA:CR43893	0.849606887838128	1	1	pseudobulk	DESeq2	LRT	0
561	KC_AB1	lncRNA:CR43900	0.0571155708876178	1	1	pseudobulk	DESeq2	LRT	0
562	KC_AB1	lncRNA:CR43905	2.31997030691033	0.813169507846918	1	pseudobulk	DESeq2	LRT	0
562	KC_AB1	lncRNA:CR43910	0.237022465848507	1	1	pseudobulk	DESeq2	LRT	0
562	KC_AB1	lncRNA:CR43913	2.31997030691033	0.813169507846918	1	pseudobulk	DESeq2	LRT	0
562	KC_AB1	lncRNA:CR43943	0.116783351990007	1	1	pseudobulk	DESeq2	LRT	0
562	KC_AB1	lncRNA:CR43951	0.849606887838128	1	1	pseudobulk	DESeq2	LRT	0
562	KC_AB1	lncRNA:CR43956	0.237022465848507	1	1	pseudobulk	DESeq2	LRT	0
562	KC_AB1	lncRNA:CR43958	1.38689474342661	0.477504151481303	1	pseudobulk	DESeq2	LRT	0
562	KC_AB1	lncRNA:CR43963	-0.258328162989287	0.818312428923155	1	pseudobulk	DESeq2	LRT	0
562	KC_AB1	lncRNA:CR43966	3.16697464674408	0.406679230261689	1	pseudobulk	DESeq2	LRT	0
562	KC_AB1	lncRNA:CR43973	2.78674512494791	0.54962276837928	1	pseudobulk	DESeq2	LRT	0
563	KC_AB1	lncRNA:CR43974	2.78674512494791	0.54962276837928	1	pseudobulk	DESeq2	LRT	0
563	KC_AB1	lncRNA:CR43995	-0.361254580414261	0.695666205948683	1	pseudobulk	DESeq2	LRT	0
563	KC_AB1	lncRNA:CR44017	0.0160940546605174	1	1	pseudobulk	DESeq2	LRT	0
563	KC_AB1	lncRNA:CR44023	0.44130730256556	0.877620929682955	1	pseudobulk	DESeq2	LRT	0
563	KC_AB1	lncRNA:CR44024	-0.305045115158105	0.179959537155169	1	pseudobulk	DESeq2	LRT	0
563	KC_AB1	lncRNA:CR44043	-1.4837896443873	0.328769260941889	1	pseudobulk	DESeq2	LRT	0
563	KC_AB1	lncRNA:CR44048	2.31032285938233	0.167873101705742	1	pseudobulk	DESeq2	LRT	0
563	KC_AB1	lncRNA:CR44050	-0.364248393414505	0.655020206441853	1	pseudobulk	DESeq2	LRT	0
563	KC_AB1	lncRNA:CR44051	3.150585340203	0.085575843701455	1	pseudobulk	DESeq2	LRT	0
563	KC_AB1	lncRNA:CR44070	0.849606887838128	1	1	pseudobulk	DESeq2	LRT	0
564	KC_AB1	lncRNA:CR44074	0.688676318369418	0.343963364070558	1	pseudobulk	DESeq2	LRT	0
564	KC_AB1	lncRNA:CR44075	2.78674512494791	0.54962276837928	1	pseudobulk	DESeq2	LRT	0
564	KC_AB1	lncRNA:CR44092	-0.325466691417655	0.711142158185114	1	pseudobulk	DESeq2	LRT	0
564	KC_AB1	lncRNA:CR44109	-0.351896215003217	0.827141835548642	1	pseudobulk	DESeq2	LRT	0
564	KC_AB1	lncRNA:CR44110	0.849606887838128	1	1	pseudobulk	DESeq2	LRT	0
564	KC_AB1	lncRNA:CR44126	0.849606887838128	1	1	pseudobulk	DESeq2	LRT	0
564	KC_AB1	lncRNA:CR44127	2.31997030691033	0.813169507846918	1	pseudobulk	DESeq2	LRT	0
564	KC_AB1	lncRNA:CR44129	0.237022465848507	1	1	pseudobulk	DESeq2	LRT	0
564	KC_AB1	lncRNA:CR44157	1.51280379024899	0.85518877994987	1	pseudobulk	DESeq2	LRT	0
564	KC_AB1	lncRNA:CR44171	0.849606887838128	1	1	pseudobulk	DESeq2	LRT	0
565	KC_AB1	lncRNA:CR44178	-1.15786623345146	0.28614543185844	1	pseudobulk	DESeq2	LRT	0
565	KC_AB1	lncRNA:CR44187	0.275716154190184	0.880095358094991	1	pseudobulk	DESeq2	LRT	0
565	KC_AB1	lncRNA:CR44188	0.849606887838128	1	1	pseudobulk	DESeq2	LRT	0
565	KC_AB1	lncRNA:CR44206	-1.01577466119714	0.323894283273717	1	pseudobulk	DESeq2	LRT	0
565	KC_AB1	lncRNA:CR44218	0.758154272134397	1	1	pseudobulk	DESeq2	LRT	0
565	KC_AB1	lncRNA:CR44219	0.849606887838128	1	1	pseudobulk	DESeq2	LRT	0
565	KC_AB1	lncRNA:CR44221	2.06541384650887	0.795866856626082	1	pseudobulk	DESeq2	LRT	0
565	KC_AB1	lncRNA:CR44222	0.366247714696288	1	1	pseudobulk	DESeq2	LRT	0
565	KC_AB1	lncRNA:CR44260	0.849606887838128	1	1	pseudobulk	DESeq2	LRT	0
565	KC_AB1	lncRNA:CR44268	1.60154856568448	0.759528993631368	1	pseudobulk	DESeq2	LRT	0
566	KC_AB1	lncRNA:CR44294	1.59863986092676	1	1	pseudobulk	DESeq2	LRT	0
566	KC_AB1	lncRNA:CR44299	1.902583705109	0.571350176208571	1	pseudobulk	DESeq2	LRT	0
566	KC_AB1	lncRNA:CR44317	-0.283054125455946	1	1	pseudobulk	DESeq2	LRT	0
566	KC_AB1	lncRNA:CR44331	2.6840296908559	0.560387737355481	1	pseudobulk	DESeq2	LRT	0
566	KC_AB1	lncRNA:CR44332	-1.12676148946619	0.455170585867974	1	pseudobulk	DESeq2	LRT	0
566	KC_AB1	lncRNA:CR44347	0.446726681575979	0.620227240799238	1	pseudobulk	DESeq2	LRT	0
566	KC_AB1	lncRNA:CR44348	0.194265322970028	1	1	pseudobulk	DESeq2	LRT	0
566	KC_AB1	lncRNA:CR44357	0.509869034586251	0.214193367838212	1	pseudobulk	DESeq2	LRT	0
566	KC_AB1	lncRNA:CR44360	0.849606887838128	1	1	pseudobulk	DESeq2	LRT	0
566	KC_AB1	lncRNA:CR44363	0.116783351990007	1	1	pseudobulk	DESeq2	LRT	0

567	KC_AB1	lncRNA:CR44369	2.78674512494791	0.54962276837928	1	pseudobulk	DESeq2	LRT	0
567	KC_AB1	lncRNA:CR44384	-1.38168903504741	0.385538474372507	1	pseudobulk	DESeq2	LRT	0
567	KC_AB1	lncRNA:CR44400	2.31997030691033	0.813169507846918	1	pseudobulk	DESeq2	LRT	0
567	KC_AB1	lncRNA:CR44411	2.85142250261825	0.484249113053674	1	pseudobulk	DESeq2	LRT	0
567	KC_AB1	lncRNA:CR44417	-0.190101045536633	0.629081287527333	1	pseudobulk	DESeq2	LRT	0
567	KC_AB1	lncRNA:CR44440	0.849606887838128	1	1	pseudobulk	DESeq2	LRT	0
567	KC_AB1	lncRNA:CR44444	1.11528148199936	1	1	pseudobulk	DESeq2	LRT	0
567	KC_AB1	lncRNA:CR44464	1.4229055769363	0.771821430526778	1	pseudobulk	DESeq2	LRT	0
567	KC_AB1	lncRNA:CR44472	0.849606887838128	1	1	pseudobulk	DESeq2	LRT	0
567	KC_AB1	lncRNA:CR44473	1.59863986092676	1	1	pseudobulk	DESeq2	LRT	0
568	KC_AB1	lncRNA:CR44506	-1.82682724733575	0.3306311643005	1	pseudobulk	DESeq2	LRT	0
568	KC_AB1	lncRNA:CR44517	1.59863986092676	1	1	pseudobulk	DESeq2	LRT	0
568	KC_AB1	lncRNA:CR44538	0.849606887838128	1	1	pseudobulk	DESeq2	LRT	0
568	KC_AB1	lncRNA:CR44564	0.849606887838128	1	1	pseudobulk	DESeq2	LRT	0
568	KC_AB1	lncRNA:CR44565	0.849606887838128	1	1	pseudobulk	DESeq2	LRT	0
568	KC_AB1	lncRNA:CR44567	0.849606887838128	1	1	pseudobulk	DESeq2	LRT	0
568	KC_AB1	lncRNA:CR44589	0.931109140710098	1	1	pseudobulk	DESeq2	LRT	0
568	KC_AB1	lncRNA:CR44595	0.486914680447219	0.802732224560645	1	pseudobulk	DESeq2	LRT	0
568	KC_AB1	lncRNA:CR44596	1.68466407915539	0.680527244683373	1	pseudobulk	DESeq2	LRT	0
568	KC_AB1	lncRNA:CR44597	-0.0273012833936009	0.833071926609927	1	pseudobulk	DESeq2	LRT	0
569	KC_AB1	lncRNA:CR44598	-1.05812073967145	0.294019738369885	1	pseudobulk	DESeq2	LRT	0
569	KC_AB1	lncRNA:CR44603	-0.3819411685274	0.856470199429309	1	pseudobulk	DESeq2	LRT	0
569	KC_AB1	lncRNA:CR44612	0.849606887838128	1	1	pseudobulk	DESeq2	LRT	0
569	KC_AB1	lncRNA:CR44619	2.78674512494791	0.54962276837928	1	pseudobulk	DESeq2	LRT	0
569	KC_AB1	lncRNA:CR44628	0.47572993756592	1	1	pseudobulk	DESeq2	LRT	0
569	KC_AB1	lncRNA:CR44642	0.183159795463042	1	1	pseudobulk	DESeq2	LRT	0
569	KC_AB1	lncRNA:CR44661	1.59863986092676	1	1	pseudobulk	DESeq2	LRT	0
569	KC_AB1	lncRNA:CR44662	0.758154272134397	1	1	pseudobulk	DESeq2	LRT	0
569	KC_AB1	lncRNA:CR44663	0.364382449400306	0.489407383518778	1	pseudobulk	DESeq2	LRT	0
569	KC_AB1	lncRNA:CR44708	0.0311534257790393	0.6191952190824	1	pseudobulk	DESeq2	LRT	0
570	KC_AB1	lncRNA:CR44716	0.849606887838128	1	1	pseudobulk	DESeq2	LRT	0
570	KC_AB1	lncRNA:CR44718	-0.34918661580976	0.760306269292669	1	pseudobulk	DESeq2	LRT	0
570	KC_AB1	lncRNA:CR44719	0.849606887838128	1	1	pseudobulk	DESeq2	LRT	0
570	KC_AB1	lncRNA:CR44727	-1.33105432892297	0.443282250729798	1	pseudobulk	DESeq2	LRT	0
570	KC_AB1	lncRNA:CR44728	-0.370862531082229	0.881125640939123	1	pseudobulk	DESeq2	LRT	0
570	KC_AB1	lncRNA:CR44749	1.59863986092676	1	1	pseudobulk	DESeq2	LRT	0
570	KC_AB1	lncRNA:CR44761	0.849606887838128	1	1	pseudobulk	DESeq2	LRT	0
570	KC_AB1	lncRNA:CR44766	2.43275481406755	0.550258497427138	1	pseudobulk	DESeq2	LRT	0
570	KC_AB1	lncRNA:CR44772	0.865817449292603	1	1	pseudobulk	DESeq2	LRT	0
570	KC_AB1	lncRNA:CR44773	0.849606887838128	1	1	pseudobulk	DESeq2	LRT	0
571	KC_AB1	lncRNA:CR44779	-0.302458995746278	0.779143818314263	1	pseudobulk	DESeq2	LRT	0
571	KC_AB1	lncRNA:CR44785	0.849606887838128	1	1	pseudobulk	DESeq2	LRT	0
571	KC_AB1	lncRNA:CR44805	-0.267798911929862	1	1	pseudobulk	DESeq2	LRT	0
571	KC_AB1	lncRNA:CR44811	-1.77234859294432	0.265042689185988	1	pseudobulk	DESeq2	LRT	0
571	KC_AB1	lncRNA:CR44813	0.587194502832704	0.288348213269163	1	pseudobulk	DESeq2	LRT	0
571	KC_AB1	lncRNA:CR44814	5.63849700412075	0.124344102548845	1	pseudobulk	DESeq2	LRT	0
571	KC_AB1	lncRNA:CR44816	0.345187706064789	0.918022087452894	1	pseudobulk	DESeq2	LRT	0
571	KC_AB1	lncRNA:CR44831	2.31997030691033	0.813169507846918	1	pseudobulk	DESeq2	LRT	0
571	KC_AB1	lncRNA:CR44832	-2.19761372238053	0.261375484389507	1	pseudobulk	DESeq2	LRT	0
571	KC_AB1	lncRNA:CR44833	-2.64659265198904	0.116171041832348	1	pseudobulk	DESeq2	LRT	0
572	KC_AB1	lncRNA:CR44834	0.237022465848507	1	1	pseudobulk	DESeq2	LRT	0
572	KC_AB1	lncRNA:CR44845	-2.73573142459503	0.264943650326734	1	pseudobulk	DESeq2	LRT	0
572	KC_AB1	lncRNA:CR44851	2.78674512494791	0.54962276837928	1	pseudobulk	DESeq2	LRT	0

572	KC_AB1	lncRNA:CR44886	0.372595365991275	0.355796627101757	1	pseudobulk	DESeq2	LRT	0
572	KC_AB1	lncRNA:CR44899	0.849606887838128	1	1	pseudobulk	DESeq2	LRT	0
572	KC_AB1	lncRNA:CR44900	1.59863986092676	1	1	pseudobulk	DESeq2	LRT	0
572	KC_AB1	lncRNA:CR44901	1.59863986092676	1	1	pseudobulk	DESeq2	LRT	0
572	KC_AB1	lncRNA:CR44914	1.20092489142827	1	1	pseudobulk	DESeq2	LRT	0
572	KC_AB1	lncRNA:CR44922	-0.846552024493242	0.145780921485573	1	pseudobulk	DESeq2	LRT	0
572	KC_AB1	lncRNA:CR44938	2.85142250261825	0.484249113053674	1	pseudobulk	DESeq2	LRT	0
573	KC_AB1	lncRNA:CR44947	2.42874708135467	0.60279296430569	1	pseudobulk	DESeq2	LRT	0
573	KC_AB1	lncRNA:CR44951	0.376431477381175	1	1	pseudobulk	DESeq2	LRT	0
573	KC_AB1	lncRNA:CR44973	0.849606887838128	1	1	pseudobulk	DESeq2	LRT	0
573	KC_AB1	lncRNA:CR44977	-0.267798911929862	1	1	pseudobulk	DESeq2	LRT	0
573	KC_AB1	lncRNA:CR44978	1.52701240178692	0.0409096280064111	1	pseudobulk	DESeq2	LRT	0
573	KC_AB1	lncRNA:CR44979	-0.216210029276812	1	1	pseudobulk	DESeq2	LRT	0
573	KC_AB1	lncRNA:CR44985	0.849606887838128	1	1	pseudobulk	DESeq2	LRT	0
573	KC_AB1	lncRNA:CR44986	-1.06107868158694	0.416628350081987	1	pseudobulk	DESeq2	LRT	0
573	KC_AB1	lncRNA:CR44997	-0.569389321063983	0.0949665260229088	1	pseudobulk	DESeq2	LRT	0
573	KC_AB1	lncRNA:CR44999	-0.60651716331841	0.355789871946534	1	pseudobulk	DESeq2	LRT	0
574	KC_AB1	lncRNA:CR45004	2.78674512494791	0.54962276837928	1	pseudobulk	DESeq2	LRT	0
574	KC_AB1	lncRNA:CR45013	2.31997030691033	0.813169507846918	1	pseudobulk	DESeq2	LRT	0
574	KC_AB1	lncRNA:CR45025	0.511195751388297	1	1	pseudobulk	DESeq2	LRT	0
574	KC_AB1	lncRNA:CR45030	0.376431477381175	1	1	pseudobulk	DESeq2	LRT	0
574	KC_AB1	lncRNA:CR45035	-0.161142344209633	0.727135360522127	1	pseudobulk	DESeq2	LRT	0
574	KC_AB1	lncRNA:CR45036	0.849606887838128	1	1	pseudobulk	DESeq2	LRT	0
574	KC_AB1	lncRNA:CR45054	-0.0141586514300254	0.787663032932165	1	pseudobulk	DESeq2	LRT	0
574	KC_AB1	lncRNA:CR45102	-0.123465086144878	0.732883276504358	1	pseudobulk	DESeq2	LRT	0
574	KC_AB1	lncRNA:CR45125	2.78674512494791	0.54962276837928	1	pseudobulk	DESeq2	LRT	0
574	KC_AB1	lncRNA:CR45128	0.849606887838128	1	1	pseudobulk	DESeq2	LRT	0
575	KC_AB1	lncRNA:CR45155	2.78674512494791	0.54962276837928	1	pseudobulk	DESeq2	LRT	0
575	KC_AB1	lncRNA:CR45156	-0.116922919641532	0.907762700221791	1	pseudobulk	DESeq2	LRT	0
575	KC_AB1	lncRNA:CR45168	0.849606887838128	1	1	pseudobulk	DESeq2	LRT	0
575	KC_AB1	lncRNA:CR45169	2.31997030691033	0.813169507846918	1	pseudobulk	DESeq2	LRT	0
575	KC_AB1	lncRNA:CR45170	-0.12667568312555	0.79427956649788	1	pseudobulk	DESeq2	LRT	0
575	KC_AB1	lncRNA:CR45177	0.849606887838128	1	1	pseudobulk	DESeq2	LRT	0
575	KC_AB1	lncRNA:CR45180	-1.6221705253141	0.455350281911715	1	pseudobulk	DESeq2	LRT	0
575	KC_AB1	lncRNA:CR45187	0.536600542756646	1	1	pseudobulk	DESeq2	LRT	0
575	KC_AB1	lncRNA:CR45188	0.0251319372616546	0.916098078775446	1	pseudobulk	DESeq2	LRT	0
575	KC_AB1	lncRNA:CR45196	0.0160940546605626	1	1	pseudobulk	DESeq2	LRT	0
576	KC_AB1	lncRNA:CR45200	0.0659634603015876	1	1	pseudobulk	DESeq2	LRT	0
576	KC_AB1	lncRNA:CR45203	0.985243145755336	1	1	pseudobulk	DESeq2	LRT	0
576	KC_AB1	lncRNA:CR45211	2.31997030691033	0.813169507846918	1	pseudobulk	DESeq2	LRT	0
576	KC_AB1	lncRNA:CR45224	0.849606887838128	1	1	pseudobulk	DESeq2	LRT	0
576	KC_AB1	lncRNA:CR45240	0.249609997857266	1	1	pseudobulk	DESeq2	LRT	0
576	KC_AB1	lncRNA:CR45245	0.849606887838128	1	1	pseudobulk	DESeq2	LRT	0
576	KC_AB1	lncRNA:CR45246	2.78674512494791	0.54962276837928	1	pseudobulk	DESeq2	LRT	0
576	KC_AB1	lncRNA:CR45251	-0.580682610791802	0.768938018276591	1	pseudobulk	DESeq2	LRT	0
576	KC_AB1	lncRNA:CR45254	0.366247714696288	1	1	pseudobulk	DESeq2	LRT	0
576	KC_AB1	lncRNA:CR45262	0.29312497632079	1	1	pseudobulk	DESeq2	LRT	0
577	KC_AB1	lncRNA:CR45290	1.11528148199936	1	1	pseudobulk	DESeq2	LRT	0
577	KC_AB1	lncRNA:CR45306	-0.19698351532854	1	1	pseudobulk	DESeq2	LRT	0
577	KC_AB1	lncRNA:CR45318	-0.504039151378979	0.826589305333094	1	pseudobulk	DESeq2	LRT	0
577	KC_AB1	lncRNA:CR45327	0.849606887838128	1	1	pseudobulk	DESeq2	LRT	0
577	KC_AB1	lncRNA:CR45339	0.116783351990007	1	1	pseudobulk	DESeq2	LRT	0
577	KC_AB1	lncRNA:CR45358	0.849606887838128	1	1	pseudobulk	DESeq2	LRT	0

577	KC_AB1	lncRNA:CR45380	2.31997030691033	0.813169507846918	1	pseudobulk	DESeq2	LRT	0
577	KC_AB1	lncRNA:CR45387	2.31997030691033	0.813169507846918	1	pseudobulk	DESeq2	LRT	0
577	KC_AB1	lncRNA:CR45388	-0.439574452401261	0.46648270935843	1	pseudobulk	DESeq2	LRT	0
577	KC_AB1	lncRNA:CR45405	1.21267130850476	1	1	pseudobulk	DESeq2	LRT	0
578	KC_AB1	lncRNA:CR45409	-0.728186254323099	0.423510424156791	1	pseudobulk	DESeq2	LRT	0
578	KC_AB1	lncRNA:CR45418	0.849606887838128	1	1	pseudobulk	DESeq2	LRT	0
578	KC_AB1	lncRNA:CR45425	2.31997030691033	0.813169507846918	1	pseudobulk	DESeq2	LRT	0
578	KC_AB1	lncRNA:CR45444	1.59863986092676	1	1	pseudobulk	DESeq2	LRT	0
578	KC_AB1	lncRNA:CR45457	0.237022465848507	1	1	pseudobulk	DESeq2	LRT	0
578	KC_AB1	lncRNA:CR45471	0.849606887838128	1	1	pseudobulk	DESeq2	LRT	0
578	KC_AB1	lncRNA:CR45473	-0.731520070204393	0.540619210860351	1	pseudobulk	DESeq2	LRT	0
578	KC_AB1	lncRNA:CR45480	0.849606887838128	1	1	pseudobulk	DESeq2	LRT	0
578	KC_AB1	lncRNA:CR45522	0.106645811795416	0.553333835198145	1	pseudobulk	DESeq2	LRT	0
578	KC_AB1	lncRNA:CR45530	-0.0065790798335215	1	1	pseudobulk	DESeq2	LRT	0
579	KC_AB1	lncRNA:CR45531	0.769893673767581	1	1	pseudobulk	DESeq2	LRT	0
579	KC_AB1	lncRNA:CR45556	1.34196946250982	0.145362011651871	1	pseudobulk	DESeq2	LRT	0
579	KC_AB1	lncRNA:CR45585	2.31997030691033	0.813169507846918	1	pseudobulk	DESeq2	LRT	0
579	KC_AB1	lncRNA:CR45625	2.06541384650887	0.795866856626082	1	pseudobulk	DESeq2	LRT	0
579	KC_AB1	lncRNA:CR45641	1.29219678305741	0.728275381236252	1	pseudobulk	DESeq2	LRT	0
579	KC_AB1	lncRNA:CR45643	-0.366842083154775	0.683033243892977	1	pseudobulk	DESeq2	LRT	0
579	KC_AB1	lncRNA:CR45659	2.78674512494791	0.54962276837928	1	pseudobulk	DESeq2	LRT	0
579	KC_AB1	lncRNA:CR45660	0.409442668345093	0.529542447889913	1	pseudobulk	DESeq2	LRT	0
579	KC_AB1	lncRNA:CR45668	0.444276413577542	0.495895298089926	1	pseudobulk	DESeq2	LRT	0
579	KC_AB1	lncRNA:CR45669	2.06541384650887	0.904905526327343	1	pseudobulk	DESeq2	LRT	0
580	KC_AB1	lncRNA:CR45670	0.849606887838128	1	1	pseudobulk	DESeq2	LRT	0
580	KC_AB1	lncRNA:CR45674	1.59863986092676	1	1	pseudobulk	DESeq2	LRT	0
580	KC_AB1	lncRNA:CR45684	-0.336845706754219	0.74781472164682	1	pseudobulk	DESeq2	LRT	0
580	KC_AB1	lncRNA:CR45687	0.849606887838128	1	1	pseudobulk	DESeq2	LRT	0
580	KC_AB1	lncRNA:CR45736	0.539227591542996	1	1	pseudobulk	DESeq2	LRT	0
580	KC_AB1	lncRNA:CR45753	2.78674512494791	0.54962276837928	1	pseudobulk	DESeq2	LRT	0
580	KC_AB1	lncRNA:CR45758	-1.02510869253878	0.081093402339394	1	pseudobulk	DESeq2	LRT	0
580	KC_AB1	lncRNA:CR45762	0.849606887838128	1	1	pseudobulk	DESeq2	LRT	0
580	KC_AB1	lncRNA:CR45805	0.849606887838128	1	1	pseudobulk	DESeq2	LRT	0
580	KC_AB1	lncRNA:CR45808	0.849606887838128	1	1	pseudobulk	DESeq2	LRT	0
581	KC_AB1	lncRNA:CR45811	0.849606887838128	1	1	pseudobulk	DESeq2	LRT	0
581	KC_AB1	lncRNA:CR45819	0.366247714696288	1	1	pseudobulk	DESeq2	LRT	0
581	KC_AB1	lncRNA:CR45825	0.505459084862637	0.42614691803458	1	pseudobulk	DESeq2	LRT	0
581	KC_AB1	lncRNA:CR45876	0.849606887838128	1	1	pseudobulk	DESeq2	LRT	0
581	KC_AB1	lncRNA:CR45880	0.0863909509812212	1	1	pseudobulk	DESeq2	LRT	0
581	KC_AB1	lncRNA:CR45883	-0.370862531082229	0.881125640939123	1	pseudobulk	DESeq2	LRT	0
581	KC_AB1	lncRNA:CR45910	0.849606887838128	1	1	pseudobulk	DESeq2	LRT	0
581	KC_AB1	lncRNA:CR45911	-1.74247561962399	0.160263129527378	1	pseudobulk	DESeq2	LRT	0
581	KC_AB1	lncRNA:CR45918	0.326723824121506	0.601529951219551	1	pseudobulk	DESeq2	LRT	0
581	KC_AB1	lncRNA:CR45919	-0.790181448644479	0.369112204835745	1	pseudobulk	DESeq2	LRT	0
582	KC_AB1	lncRNA:CR45938	3.150585340203	0.085575843701455	1	pseudobulk	DESeq2	LRT	0
582	KC_AB1	lncRNA:CR45939	0.742456727585392	1	1	pseudobulk	DESeq2	LRT	0
582	KC_AB1	lncRNA:CR45950	0.849606887838128	1	1	pseudobulk	DESeq2	LRT	0
582	KC_AB1	lncRNA:CR45953	1.59863986092676	1	1	pseudobulk	DESeq2	LRT	0
582	KC_AB1	lncRNA:CR45954	-0.216210032712384	1	1	pseudobulk	DESeq2	LRT	0
582	KC_AB1	lncRNA:CR45959	0.492502386827429	1	1	pseudobulk	DESeq2	LRT	0
582	KC_AB1	lncRNA:CR45961	1.17261808986456	0.859088728563237	1	pseudobulk	DESeq2	LRT	0
582	KC_AB1	lncRNA:CR45971	1.59863986092676	1	1	pseudobulk	DESeq2	LRT	0
582	KC_AB1	lncRNA:CR45972	1.59863986092676	1	1	pseudobulk	DESeq2	LRT	0

582	KC_AB1	lncRNA:CR45973	0.849606887838128	1	1	pseudobulk	DESeq2	LRT	0
583	KC_AB1	lncRNA:CR45976	2.78674512494791	0.54962276837928	1	pseudobulk	DESeq2	LRT	0
583	KC_AB1	lncRNA:CR45979	0.181837713712423	0.7722570257888	1	pseudobulk	DESeq2	LRT	0
583	KC_AB1	lncRNA:CR45981	0.237022465848507	1	1	pseudobulk	DESeq2	LRT	0
583	KC_AB1	lncRNA:CR45982	-0.216209988775614	1	1	pseudobulk	DESeq2	LRT	0
583	KC_AB1	lncRNA:CR45984	0.849606887838128	1	1	pseudobulk	DESeq2	LRT	0
583	KC_AB1	lncRNA:CR45993	1.33605976138209	0.809986558087114	1	pseudobulk	DESeq2	LRT	0
583	KC_AB1	lncRNA:CR45994	0.781801500057156	1	1	pseudobulk	DESeq2	LRT	0
583	KC_AB1	lncRNA:CR45996	-0.116922919641532	0.907762700221791	1	pseudobulk	DESeq2	LRT	0
583	KC_AB1	lncRNA:CR46002	0.849606887838128	1	1	pseudobulk	DESeq2	LRT	0
583	KC_AB1	lncRNA:CR46003	-0.136746289666646	0.801926513183763	1	pseudobulk	DESeq2	LRT	0
584	KC_AB1	lncRNA:CR46004	-0.724641124401844	0.157775145126049	1	pseudobulk	DESeq2	LRT	0
584	KC_AB1	lncRNA:CR46006	-0.676664072472548	0.0128499717547144	1	pseudobulk	DESeq2	LRT	0
584	KC_AB1	lncRNA:CR46007	0.849606887838128	1	1	pseudobulk	DESeq2	LRT	0
584	KC_AB1	lncRNA:CR46008	-0.881829211786643	0.676206792569243	1	pseudobulk	DESeq2	LRT	0
584	KC_AB1	lncRNA:CR46009	0.657738703487695	0.962188133534062	1	pseudobulk	DESeq2	LRT	0
584	KC_AB1	lncRNA:CR46010	1.69632125922203	0.729252927817565	1	pseudobulk	DESeq2	LRT	0
584	KC_AB1	lncRNA:CR46016	0.621375926231566	1	1	pseudobulk	DESeq2	LRT	0
584	KC_AB1	lncRNA:CR46019	-0.216210029276812	1	1	pseudobulk	DESeq2	LRT	0
584	KC_AB1	lncRNA:CR46021	0.849606887838128	1	1	pseudobulk	DESeq2	LRT	0
584	KC_AB1	lncRNA:CR46024	0.849606887838128	1	1	pseudobulk	DESeq2	LRT	0
585	KC_AB1	lncRNA:CR46035	-0.830062897938551	0.717724257445671	1	pseudobulk	DESeq2	LRT	0
585	KC_AB1	lncRNA:CR46036	2.31997030691033	0.813169507846918	1	pseudobulk	DESeq2	LRT	0
585	KC_AB1	lncRNA:CR46041	0.849606887838128	1	1	pseudobulk	DESeq2	LRT	0
585	KC_AB1	lncRNA:CR46064	-0.0789859988112128	0.623688771872263	1	pseudobulk	DESeq2	LRT	0
585	KC_AB1	lncRNA:CR46079	-1.60193975250819	0.398497879394378	1	pseudobulk	DESeq2	LRT	0
585	KC_AB1	lncRNA:CR46080	0.865817449292603	1	1	pseudobulk	DESeq2	LRT	0
585	KC_AB1	lncRNA:CR46084	0.0418184222819095	1	1	pseudobulk	DESeq2	LRT	0
585	KC_AB1	lncRNA:CR46088	-0.590637494330849	0.764308822189376	1	pseudobulk	DESeq2	LRT	0
585	KC_AB1	lncRNA:CR46094	-0.216210029276812	1	1	pseudobulk	DESeq2	LRT	0
585	KC_AB1	lncRNA:CR46095	0.366247714696288	1	1	pseudobulk	DESeq2	LRT	0
586	KC_AB1	lncRNA:CR46124	2.78674512494791	0.54962276837928	1	pseudobulk	DESeq2	LRT	0
586	KC_AB1	lncRNA:CR46125	0.849606887838128	1	1	pseudobulk	DESeq2	LRT	0
586	KC_AB1	lncRNA:CR46126	0.849606887838128	1	1	pseudobulk	DESeq2	LRT	0
586	KC_AB1	lncRNA:CR46127	0.849606887838128	1	1	pseudobulk	DESeq2	LRT	0
586	KC_AB1	lncRNA:CR46194	-1.30533495487828	0.549530974560874	1	pseudobulk	DESeq2	LRT	0
586	KC_AB1	lncRNA:CR46200	-0.216209988775614	1	1	pseudobulk	DESeq2	LRT	0
586	KC_AB1	lncRNA:CR46204	2.42874708135467	0.60279296430569	1	pseudobulk	DESeq2	LRT	0
586	KC_AB1	lncRNA:CR46208	0.458492136589768	0.388004739291675	1	pseudobulk	DESeq2	LRT	0
586	KC_AB1	lncRNA:CR46230	0.849606887838128	1	1	pseudobulk	DESeq2	LRT	0
586	KC_AB1	lncRNA:CR46248	2.78674512494791	0.54962276837928	1	pseudobulk	DESeq2	LRT	0
587	KC_AB1	lncRNA:CR46268	-2.76045852156223	0.0365544833529723	1	pseudobulk	DESeq2	LRT	0
587	KC_AB1	lncRNA:CR46271	0.931404298831974	1	1	pseudobulk	DESeq2	LRT	0
587	KC_AB1	lncRNA:CR46322	0.200280139999782	1	1	pseudobulk	DESeq2	LRT	0
587	KC_AB1	lncRNA:CR46348	0.366247714696288	1	1	pseudobulk	DESeq2	LRT	0
587	KC_AB1	lncRNA:CR46349	0.849606887838128	1	1	pseudobulk	DESeq2	LRT	0
587	KC_AB1	lncRNA:CR9162	1.59863986092676	1	1	pseudobulk	DESeq2	LRT	0
587	KC_AB1	lncRNA:flam	-1.4984266423025	0.299345548902306	1	pseudobulk	DESeq2	LRT	0
587	KC_AB1	lncRNA:Hsromega	-0.476836472141091	0.392342206225722	1	pseudobulk	DESeq2	LRT	0
587	KC_AB1	lncRNA:let7C	-1.55897129933071	0.00726854468234856	1	pseudobulk	DESeq2	LRT	0
587	KC_AB1	lncRNA:noe	0.0939903200882682	0.423411148032242	1	pseudobulk	DESeq2	LRT	0
588	KC_AB1	Lnk	-0.332995361048634	0.516331539775093	1	pseudobulk	DESeq2	LRT	0
588	KC_AB1	Lnpk	0.151091630788808	0.731483164301552	1	pseudobulk	DESeq2	LRT	0

588	KC_AB1	loco	-1.8476447304053	0.456955818574401	1	pseudobulk	DESeq2	LRT		0
588	KC_AB1	loj	-0.37270454031033	0.603905471758473	1	pseudobulk	DESeq2	LRT		0
588	KC_AB1	lola	-0.239853834981721	0.102807459954817	1	pseudobulk	DESeq2	LRT		0
588	KC_AB1	lolal	0.176426680388923	0.366095140442405	1	pseudobulk	DESeq2	LRT		0
588	KC_AB1	Lon	-0.500620322033034	0.525842136729041	1	pseudobulk	DESeq2	LRT		0
588	KC_AB1	loqs	-0.400014773006865	0.225315775270613	1	pseudobulk	DESeq2	LRT		0
588	KC_AB1	lost	-0.106923129077226	0.589901962254116	1	pseudobulk	DESeq2	LRT		0
588	KC_AB1	lov	2.78674512494791	0.54962276837928	1	pseudobulk	DESeq2	LRT		0
589	KC_AB1	lovit	-2.14228146238098	0.126963580246929	1	pseudobulk	DESeq2	LRT		0
589	KC_AB1	Loxl1	1.54583996749589	0.772563523328761	1	pseudobulk	DESeq2	LRT		0
589	KC_AB1	LPCAT	-0.408935677123805	0.464246604514891	1	pseudobulk	DESeq2	LRT		0
589	KC_AB1	Lpin	-0.206676055327601	0.509930932477417	1	pseudobulk	DESeq2	LRT		0
589	KC_AB1	LpR2	-1.3268430421649	0.361116726823422	1	pseudobulk	DESeq2	LRT		0
589	KC_AB1	Lpt	-1.30889914456843	0.297322440764741	1	pseudobulk	DESeq2	LRT		0
589	KC_AB1	lqf	-0.0515027865530085	0.8422456631774	1	pseudobulk	DESeq2	LRT		0
589	KC_AB1	lqfR	0.470405217278306	0.508957118672612	1	pseudobulk	DESeq2	LRT		0
589	KC_AB1	Lrch	0.0518057609415343	0.51850767473603	1	pseudobulk	DESeq2	LRT		0
589	KC_AB1	LRP1	-0.495318636007828	0.383523643969613	1	pseudobulk	DESeq2	LRT		0
590	KC_AB1	Lrp4	-0.191655695063505	0.491200602206573	1	pseudobulk	DESeq2	LRT		0
590	KC_AB1	Lrpprc2	0.655500119151095	1	1	pseudobulk	DESeq2	LRT		0
590	KC_AB1	LRR	0.10416650024641	0.768368083196788	1	pseudobulk	DESeq2	LRT		0
590	KC_AB1	Lrr47	2.37267802278275	0.347940025187477	1	pseudobulk	DESeq2	LRT		0
590	KC_AB1	Lrrk	0.220764417817162	0.630646494669959	1	pseudobulk	DESeq2	LRT		0
590	KC_AB1	Lrt	0.849606887838128	1	1	pseudobulk	DESeq2	LRT		0
590	KC_AB1	Lsd-1	0.849606887838128	1	1	pseudobulk	DESeq2	LRT		0
590	KC_AB1	Lsd-2	0.644903176327931	0.65247758160071	1	pseudobulk	DESeq2	LRT		0
590	KC_AB1	LSm1	0.535822306701024	0.576765374481645	1	pseudobulk	DESeq2	LRT		0
590	KC_AB1	Lsm11	-0.026294104586324	1	1	pseudobulk	DESeq2	LRT		0
591	KC_AB1	Lsm12a	0.53973572722782	0.257584185120585	1	pseudobulk	DESeq2	LRT		0
591	KC_AB1	LSm3	-0.1562722767228	0.762249600973458	1	pseudobulk	DESeq2	LRT		0
591	KC_AB1	LSm7	-0.412038613856102	0.61281089027587	1	pseudobulk	DESeq2	LRT		0
591	KC_AB1	lsn	-0.663610044207964	0.505464263634954	1	pseudobulk	DESeq2	LRT		0
591	KC_AB1	Lst8	0.735926427398844	0.506444896497222	1	pseudobulk	DESeq2	LRT		0
591	KC_AB1	lt	0.0506354241015677	0.652933544134475	1	pseudobulk	DESeq2	LRT		0
591	KC_AB1	Ltn1	-0.170969882987099	0.708983189083384	1	pseudobulk	DESeq2	LRT		0
591	KC_AB1	LTV1	-0.510873628564715	0.458354797672052	1	pseudobulk	DESeq2	LRT		0
591	KC_AB1	luna	-0.701631988382402	0.592663114714166	1	pseudobulk	DESeq2	LRT		0
591	KC_AB1	lute	-0.0598419653689355	0.629606634862285	1	pseudobulk	DESeq2	LRT		0
592	KC_AB1	lva	0.00489591310862073	0.782310135913657	1	pseudobulk	DESeq2	LRT		0
592	KC_AB1	lwr	0.256459664884334	0.524409846584543	1	pseudobulk	DESeq2	LRT		0
592	KC_AB1	LysRS	-0.180734770887519	0.703110054785983	1	pseudobulk	DESeq2	LRT		0
592	KC_AB1	Lztr1	0.181189340745794	0.783771017086296	1	pseudobulk	DESeq2	LRT		0
592	KC_AB1	m-cup	-0.53551758334065	0.475879084336674	1	pseudobulk	DESeq2	LRT		0
592	KC_AB1	M1BP	-0.420020378910467	0.523308954563679	1	pseudobulk	DESeq2	LRT		0
592	KC_AB1	M6	-0.946569288525839	0.200141564684632	1	pseudobulk	DESeq2	LRT		0
592	KC_AB1	mAChR-A	-0.38854635105834	0.121421985763866	1	pseudobulk	DESeq2	LRT		0
592	KC_AB1	mAChR-B	0.200892973622906	0.342337269579507	1	pseudobulk	DESeq2	LRT		0
592	KC_AB1	mAcon1	-0.167614410548521	0.682028599320137	1	pseudobulk	DESeq2	LRT		0
593	KC_AB1	Mad	-0.28946115933012	0.458099849872592	1	pseudobulk	DESeq2	LRT		0
593	KC_AB1	Mad1	0.0728574931067903	0.847328976612745	1	pseudobulk	DESeq2	LRT		0
593	KC_AB1	Madm	0.247770570086661	0.707937447781286	1	pseudobulk	DESeq2	LRT		0
593	KC_AB1	maf-S	-0.151985656579222	0.6701520631578	1	pseudobulk	DESeq2	LRT		0
593	KC_AB1	Maf1	-0.148767933798734	0.602704789614122	1	pseudobulk	DESeq2	LRT		0

593	KC_AB1	Magi	-0.95495554056351	0.214888727641371	1	pseudobulk	DESeq2	LRT	0
593	KC_AB1	mago	-0.573413282937616	0.423094187706389	1	pseudobulk	DESeq2	LRT	0
593	KC_AB1	MagR	-0.300555631635602	0.504229606808466	1	pseudobulk	DESeq2	LRT	0
593	KC_AB1	magu	0.849606887838128	1	1	pseudobulk	DESeq2	LRT	0
593	KC_AB1	mahe	-0.607503208243916	0.0362438804798317	1	pseudobulk	DESeq2	LRT	0
594	KC_AB1	mahj	-0.943135116827356	0.391184747607833	1	pseudobulk	DESeq2	LRT	0
594	KC_AB1	mal	1.72667320451856	0.129451952417779	1	pseudobulk	DESeq2	LRT	0
594	KC_AB1	Mal-A5	2.31997030691033	0.813169507846918	1	pseudobulk	DESeq2	LRT	0
594	KC_AB1	Mal-B2	2.78674512494791	0.54962276837928	1	pseudobulk	DESeq2	LRT	0
594	KC_AB1	mam	-1.25940031896351	0.00841908940735615	1	pseudobulk	DESeq2	LRT	0
594	KC_AB1	mamo	-0.828589152560841	0.150241141799663	1	pseudobulk	DESeq2	LRT	0
594	KC_AB1	MAN1	0.978128223074769	0.396932299669646	1	pseudobulk	DESeq2	LRT	0
594	KC_AB1	Manf	0.506015933756854	0.342406347386138	1	pseudobulk	DESeq2	LRT	0
594	KC_AB1	Map205	-0.708998305883876	0.272084649674305	1	pseudobulk	DESeq2	LRT	0
594	KC_AB1	Map60	0.283489384080504	0.43820747487194	1	pseudobulk	DESeq2	LRT	0
595	KC_AB1	MAPk-Ak2	-0.85279800410094	0.22839714721479	1	pseudobulk	DESeq2	LRT	0
595	KC_AB1	Mapmodulin	-0.120998275386193	0.439913153465413	1	pseudobulk	DESeq2	LRT	0
595	KC_AB1	Marc	0.456943103792357	1	1	pseudobulk	DESeq2	LRT	0
595	KC_AB1	Marcal1	0.181522037750249	1	1	pseudobulk	DESeq2	LRT	0
595	KC_AB1	Marf	-0.0847053867191785	0.539796145938892	1	pseudobulk	DESeq2	LRT	0
595	KC_AB1	Marf1	-1.56330878796277	0.414990018128068	1	pseudobulk	DESeq2	LRT	0
595	KC_AB1	mars	0.849606887838128	1	1	pseudobulk	DESeq2	LRT	0
595	KC_AB1	mask	-0.0532703794188929	0.7893833133791	1	pseudobulk	DESeq2	LRT	0
595	KC_AB1	Mat1	-0.369048075351678	0.654566233567585	1	pseudobulk	DESeq2	LRT	0
595	KC_AB1	Mat89Ba	-0.66610684231133	0.661091705192795	1	pseudobulk	DESeq2	LRT	0
596	KC_AB1	mats	-0.603921827133034	0.395699399638272	1	pseudobulk	DESeq2	LRT	0
596	KC_AB1	Mau2	0.421043227099207	0.75040150512905	1	pseudobulk	DESeq2	LRT	0
596	KC_AB1	mav	0.905477808468391	1	1	pseudobulk	DESeq2	LRT	0
596	KC_AB1	Max	0.237194342155231	0.650153632579365	1	pseudobulk	DESeq2	LRT	0
596	KC_AB1	mbc	-1.12699139412813	0.188105306985707	1	pseudobulk	DESeq2	LRT	0
596	KC_AB1	MBD-like	-0.0225870827518763	0.658888311548574	1	pseudobulk	DESeq2	LRT	0
596	KC_AB1	MBD-R2	-0.206315746339558	0.682313153529986	1	pseudobulk	DESeq2	LRT	0
596	KC_AB1	mbf1	0.0479167872175068	0.732873593232447	1	pseudobulk	DESeq2	LRT	0
596	KC_AB1	mbl	-0.197007755309848	0.684837189524504	1	pseudobulk	DESeq2	LRT	0
596	KC_AB1	mbm	0.141743934570787	1	1	pseudobulk	DESeq2	LRT	0
597	KC_AB1	mbo	-0.113597661246092	0.913271748330675	1	pseudobulk	DESeq2	LRT	0
597	KC_AB1	Mbs	-0.225747910990262	0.494561377873	1	pseudobulk	DESeq2	LRT	0
597	KC_AB1	mbt	-0.608288170826557	0.306766960188125	1	pseudobulk	DESeq2	LRT	0
597	KC_AB1	Mcad	1.00693893053477	1	1	pseudobulk	DESeq2	LRT	0
597	KC_AB1	Mccc1	2.31997030691033	0.813169507846918	1	pseudobulk	DESeq2	LRT	0
597	KC_AB1	Mcr	0.899339231666093	0.422371749742711	1	pseudobulk	DESeq2	LRT	0
597	KC_AB1	Mct1	-0.328752386921656	0.623215755870044	1	pseudobulk	DESeq2	LRT	0
597	KC_AB1	Mctp	-0.0587965989301386	0.929671161323327	1	pseudobulk	DESeq2	LRT	0
597	KC_AB1	MCTS1	-0.709020342008202	0.178030210926718	1	pseudobulk	DESeq2	LRT	0
597	KC_AB1	MCU	-0.511089549186458	0.347958192256911	1	pseudobulk	DESeq2	LRT	0
598	KC_AB1	Mdh1	-0.169524509236791	0.496530832043685	1	pseudobulk	DESeq2	LRT	0
598	KC_AB1	Mdh2	0.206174833551575	0.529137841868626	1	pseudobulk	DESeq2	LRT	0
598	KC_AB1	mdlc	0.702053237098659	1	1	pseudobulk	DESeq2	LRT	0
598	KC_AB1	mdy	2.13009086641515	0.809121577229065	1	pseudobulk	DESeq2	LRT	0
598	KC_AB1	me31B	-0.0619262935211695	0.606010529218943	1	pseudobulk	DESeq2	LRT	0
598	KC_AB1	Med	-0.0979409592060008	0.872905368465617	1	pseudobulk	DESeq2	LRT	0
598	KC_AB1	MED1	-0.608429583133965	0.431214998490586	1	pseudobulk	DESeq2	LRT	0
598	KC_AB1	MED10	0.317205861133834	0.541553882951194	1	pseudobulk	DESeq2	LRT	0

598	KC_AB1	MED11	-0.0079131568734032	0.759998085370834	1	pseudobulk	DESeq2	LRT	0
598	KC_AB1	MED14	0.383299756420015	0.561597925829619	1	pseudobulk	DESeq2	LRT	0
599	KC_AB1	MED15	-0.366605156298873	0.662302842722838	1	pseudobulk	DESeq2	LRT	0
599	KC_AB1	MED16	0.64551664670976	0.284867555780678	1	pseudobulk	DESeq2	LRT	0
599	KC_AB1	MED17	0.0887592454252838	0.877539700465358	1	pseudobulk	DESeq2	LRT	0
599	KC_AB1	MED18	-0.181099786700594	0.691865437795247	1	pseudobulk	DESeq2	LRT	0
599	KC_AB1	MED19	-0.138211652617994	0.588492937049664	1	pseudobulk	DESeq2	LRT	0
599	KC_AB1	MED20	0.418775020968066	0.731026463562842	1	pseudobulk	DESeq2	LRT	0
599	KC_AB1	MED21	-0.194547958020642	0.651278443662308	1	pseudobulk	DESeq2	LRT	0
599	KC_AB1	MED22	0.300073088169797	0.489458397319583	1	pseudobulk	DESeq2	LRT	0
599	KC_AB1	MED23	0.849606887838128	1	1	pseudobulk	DESeq2	LRT	0
599	KC_AB1	MED24	-0.184807728831189	0.781411723209673	1	pseudobulk	DESeq2	LRT	0
600	KC_AB1	MED25	-0.510072773866228	0.701005459429574	1	pseudobulk	DESeq2	LRT	0
600	KC_AB1	MED26	0.0366289353877142	0.682125392071604	1	pseudobulk	DESeq2	LRT	0
600	KC_AB1	MED27	0.200280139999782	1	1	pseudobulk	DESeq2	LRT	0
600	KC_AB1	MED28	-0.66811697409516	0.420876145681552	1	pseudobulk	DESeq2	LRT	0
600	KC_AB1	MED30	-0.300388745572977	0.791353828360273	1	pseudobulk	DESeq2	LRT	0
600	KC_AB1	MED31	-0.144637767413523	0.562262542299079	1	pseudobulk	DESeq2	LRT	0
600	KC_AB1	MED4	-0.394019744673933	0.619985040867031	1	pseudobulk	DESeq2	LRT	0
600	KC_AB1	MED6	0.616116865645197	0.270676728905836	1	pseudobulk	DESeq2	LRT	0
600	KC_AB1	MED7	0.375917214356099	0.653487227536624	1	pseudobulk	DESeq2	LRT	0
600	KC_AB1	MED8	-0.841527117396467	0.39409358172309	1	pseudobulk	DESeq2	LRT	0
601	KC_AB1	Mef2	-0.130312278720795	0.380778095783199	1	pseudobulk	DESeq2	LRT	0
601	KC_AB1	mEFG1	0.852584958542025	0.51014529355204	1	pseudobulk	DESeq2	LRT	0
601	KC_AB1	mEFTs	0.0587517051359988	0.817365133417048	1	pseudobulk	DESeq2	LRT	0
601	KC_AB1	mEFTu1	0.701584802674639	0.429650795046087	1	pseudobulk	DESeq2	LRT	0
601	KC_AB1	mEFTu2	-0.835187585788606	0.433627980903669	1	pseudobulk	DESeq2	LRT	0
601	KC_AB1	Megf8	-0.548855164301044	0.413355747295237	1	pseudobulk	DESeq2	LRT	0
601	KC_AB1	mei-41	1.15768992830972	1	1	pseudobulk	DESeq2	LRT	0
601	KC_AB1	mei-9	0.865287014183669	0.716803761889828	1	pseudobulk	DESeq2	LRT	0
601	KC_AB1	mei-S332	-0.10506771737906	1	1	pseudobulk	DESeq2	LRT	0
601	KC_AB1	mei-W68	0.806581675788948	0.364283766513649	1	pseudobulk	DESeq2	LRT	0
602	KC_AB1	Meics	0.102274765403483	0.895747226600197	1	pseudobulk	DESeq2	LRT	0
602	KC_AB1	meigo	-0.857838349612069	0.487871818871235	1	pseudobulk	DESeq2	LRT	0
602	KC_AB1	Mekk1	0.511134275303083	0.560359077010871	1	pseudobulk	DESeq2	LRT	0
602	KC_AB1	Meltrin	-2.47225658244856	0.191636490714256	1	pseudobulk	DESeq2	LRT	0
602	KC_AB1	Membrin	-0.807419233152187	0.475066559435171	1	pseudobulk	DESeq2	LRT	0
602	KC_AB1	Men	-0.496797969521821	0.325304830401983	1	pseudobulk	DESeq2	LRT	0
602	KC_AB1	Men-b	0.247874600400693	0.357529838084475	1	pseudobulk	DESeq2	LRT	0
602	KC_AB1	meng	-0.403418066026874	0.247373705001251	1	pseudobulk	DESeq2	LRT	0
602	KC_AB1	MEP-1	-0.232954520285661	0.722955043737799	1	pseudobulk	DESeq2	LRT	0
602	KC_AB1	Mer	0.283569445551349	0.792241608153827	1	pseudobulk	DESeq2	LRT	0
603	KC_AB1	Mes4	-0.494512990139797	0.483418244626488	1	pseudobulk	DESeq2	LRT	0
603	KC_AB1	MESK2	-0.0059589693105997	0.947039112472136	1	pseudobulk	DESeq2	LRT	0
603	KC_AB1	MESK4	0.849606887838128	1	1	pseudobulk	DESeq2	LRT	0
603	KC_AB1	MESR3	0.406306622076877	0.290031860487036	1	pseudobulk	DESeq2	LRT	0
603	KC_AB1	MESR4	-0.520255889416114	0.294642828953266	1	pseudobulk	DESeq2	LRT	0
603	KC_AB1	MESR6	-0.799212229470597	0.482843491548818	1	pseudobulk	DESeq2	LRT	0
603	KC_AB1	Met	0.251475716811267	0.458185977649374	1	pseudobulk	DESeq2	LRT	0
603	KC_AB1	metl	0.803022745738245	0.395855683481349	1	pseudobulk	DESeq2	LRT	0
603	KC_AB1	metro	0.0747921879982713	0.613699880361756	1	pseudobulk	DESeq2	LRT	0
603	KC_AB1	MetRS	-0.261967761970597	0.676531193573142	1	pseudobulk	DESeq2	LRT	0
604	KC_AB1	MetRS-m	-1.64701305382841	0.286160900062893	1	pseudobulk	DESeq2	LRT	0

604	KC_AB1	Mettl14	0.287521062231708	0.878712500770759	1	pseudobulk	DESeq2	LRT	0
604	KC_AB1	Mettl3	-0.670094167901268	0.564712619413071	1	pseudobulk	DESeq2	LRT	0
604	KC_AB1	mew	-0.446564331220973	0.736621612576868	1	pseudobulk	DESeq2	LRT	0
604	KC_AB1	Mf	1.12259013952689	0.440686708185004	1	pseudobulk	DESeq2	LRT	0
604	KC_AB1	Mfap1	-0.661505150332154	0.49945149800235	1	pseudobulk	DESeq2	LRT	0
604	KC_AB1	mfas	-2.75068307682146	0.204134188833398	1	pseudobulk	DESeq2	LRT	0
604	KC_AB1	Mfe2	0.0562466004206807	0.762959454754587	1	pseudobulk	DESeq2	LRT	0
604	KC_AB1	mfrn	-0.695481048424367	0.40888063670209	1	pseudobulk	DESeq2	LRT	0
604	KC_AB1	MFS10	-0.509146728821761	0.541172564595095	1	pseudobulk	DESeq2	LRT	0
605	KC_AB1	MFS15	-0.843553998278746	0.434431205758269	1	pseudobulk	DESeq2	LRT	0
605	KC_AB1	MFS16	0.752316437946966	0.516292972456437	1	pseudobulk	DESeq2	LRT	0
605	KC_AB1	MFS18	-0.235708735686308	0.626687279841468	1	pseudobulk	DESeq2	LRT	0
605	KC_AB1	MFS3	-0.267798911930929	1	1	pseudobulk	DESeq2	LRT	0
605	KC_AB1	MFS9	0.427578985863165	0.779587689976707	1	pseudobulk	DESeq2	LRT	0
605	KC_AB1	Mgat1	0.0985632022333415	0.737898012244447	1	pseudobulk	DESeq2	LRT	0
605	KC_AB1	Mgat2	-0.444165349678811	0.3736199693102	1	pseudobulk	DESeq2	LRT	0
605	KC_AB1	mge	0.0391824087782012	0.654553945594652	1	pseudobulk	DESeq2	LRT	0
605	KC_AB1	mgl	-1.25502035022949	0.110503643159042	1	pseudobulk	DESeq2	LRT	0
605	KC_AB1	mGluR	-0.18686542494069	0.755694556947802	1	pseudobulk	DESeq2	LRT	0
606	KC_AB1	mgr	-0.568147532459398	0.336726956513462	1	pseudobulk	DESeq2	LRT	0
606	KC_AB1	Mgstl	0.233936532205972	0.988970925999217	1	pseudobulk	DESeq2	LRT	0
606	KC_AB1	mh	0.865817449292603	1	1	pseudobulk	DESeq2	LRT	0
606	KC_AB1	Mhcl	0.39931194321386	0.485631972197333	1	pseudobulk	DESeq2	LRT	0
606	KC_AB1	mi	0.246042296928824	0.687558007872467	1	pseudobulk	DESeq2	LRT	0
606	KC_AB1	Mi-2	-0.194372943910344	0.423476910739629	1	pseudobulk	DESeq2	LRT	0
606	KC_AB1	mia	0.849606887838128	1	1	pseudobulk	DESeq2	LRT	0
606	KC_AB1	mib1	-0.182900401253232	0.627150166492186	1	pseudobulk	DESeq2	LRT	0
606	KC_AB1	mib2	0.0083563003254351	0.864037613390459	1	pseudobulk	DESeq2	LRT	0
606	KC_AB1	Mical	-0.502739760799851	0.366006757673633	1	pseudobulk	DESeq2	LRT	0
607	KC_AB1	MICAL-like	-1.66467501404813	0.213550560133992	1	pseudobulk	DESeq2	LRT	0
607	KC_AB1	Mics1	0.849606887838128	1	1	pseudobulk	DESeq2	LRT	0
607	KC_AB1	MICU1	0.667388723652439	0.161121182270584	1	pseudobulk	DESeq2	LRT	0
607	KC_AB1	MICU3	-0.212123516780805	0.510933526856614	1	pseudobulk	DESeq2	LRT	0
607	KC_AB1	Mid1	-0.356350170610654	0.113657467055459	1	pseudobulk	DESeq2	LRT	0
607	KC_AB1	mIF2	0.194492339049739	1	1	pseudobulk	DESeq2	LRT	0
607	KC_AB1	mIF3	-0.017270064017230	0.910956748482241	1	pseudobulk	DESeq2	LRT	0
607	KC_AB1	Miga	0.63937119057219	0.245972719062964	1	pseudobulk	DESeq2	LRT	0
607	KC_AB1	milt	-0.157323832311444	0.544667599122758	1	pseudobulk	DESeq2	LRT	0
607	KC_AB1	mim	-0.201006046400029	0.520285001700116	1	pseudobulk	DESeq2	LRT	0
608	KC_AB1	mino	2.31997030691033	0.813169507846918	1	pseudobulk	DESeq2	LRT	0
608	KC_AB1	mio	0.0312427275180073	0.862909324801589	1	pseudobulk	DESeq2	LRT	0
608	KC_AB1	mip120	-0.38472371563436	0.593487883764311	1	pseudobulk	DESeq2	LRT	0
608	KC_AB1	mip130	1.33933619648017	0.351358208150301	1	pseudobulk	DESeq2	LRT	0
608	KC_AB1	mip40	0.498848588868923	0.654369537216381	1	pseudobulk	DESeq2	LRT	0
608	KC_AB1	miple1	-0.001565866741155	0.58117837208963	1	pseudobulk	DESeq2	LRT	0
608	KC_AB1	miple2	-0.529307209499195	0.620597987793058	1	pseudobulk	DESeq2	LRT	0
608	KC_AB1	Mipp1	-0.389346928562833	0.820127740800904	1	pseudobulk	DESeq2	LRT	0
608	KC_AB1	Mipp2	0.761719603433624	1	1	pseudobulk	DESeq2	LRT	0
608	KC_AB1	mira	1.59863986092676	1	1	pseudobulk	DESeq2	LRT	0
609	KC_AB1	Miro	-0.290742389815373	0.551112825294742	1	pseudobulk	DESeq2	LRT	0
609	KC_AB1	mirr	-2.07348984597132	0.403224287902523	1	pseudobulk	DESeq2	LRT	0
609	KC_AB1	Mis12	0.0565737839339299	0.775160832615338	1	pseudobulk	DESeq2	LRT	0
609	KC_AB1	Mitf	-0.375010554165156	0.44810321780049	1	pseudobulk	DESeq2	LRT	0

609	KC_AB1	Mitofilin	-0.709678142327743	0.327594880180611	1	pseudobulk	DESeq2	LRT		0
609	KC_AB1	mkg-p	0.849606887838128	1	1	pseudobulk	DESeq2	LRT		0
609	KC_AB1	Mkk4	-0.0961664520336408	0.78590241976001	1	pseudobulk	DESeq2	LRT		0
609	KC_AB1	Mkp	2.31997030691033	0.813169507846918	1	pseudobulk	DESeq2	LRT		0
609	KC_AB1	MKP-4	0.585197607852081	0.358514436827023	1	pseudobulk	DESeq2	LRT		0
609	KC_AB1	Mkp3	-0.375861709495559	0.0241052101492505	1	pseudobulk	DESeq2	LRT		0
610	KC_AB1	Mkrn1	-0.0940321180442628	0.596874989289542	1	pseudobulk	DESeq2	LRT		0
610	KC_AB1	Mlc-c	-0.557895262743817	0.35499482131232	1	pseudobulk	DESeq2	LRT		0
610	KC_AB1	Mlc2	0.849606887838128	1	1	pseudobulk	DESeq2	LRT		0
610	KC_AB1	mld	-1.21021725737607	0.0127462805955299	1	pseudobulk	DESeq2	LRT		0
610	KC_AB1	mldr	0.146542282856618	1	1	pseudobulk	DESeq2	LRT		0
610	KC_AB1	mle	-0.297496559150897	0.577882569196499	1	pseudobulk	DESeq2	LRT		0
610	KC_AB1	Mlf	-0.263241174995893	0.333631471905693	1	pseudobulk	DESeq2	LRT		0
610	KC_AB1	Mlh1	-0.216210029276812	1	1	pseudobulk	DESeq2	LRT		0
610	KC_AB1	Mlp84B	-2.27471852236244	0.214384394574612	1	pseudobulk	DESeq2	LRT		0
610	KC_AB1	mlt	-0.46173796905856	0.372294562654502	1	pseudobulk	DESeq2	LRT		0
611	KC_AB1	mmd	-0.185458004025481	0.148603040679347	1	pseudobulk	DESeq2	LRT		0
611	KC_AB1	MME1	3.57402442309135	0.0441288592559412	1	pseudobulk	DESeq2	LRT		0
611	KC_AB1	Mmp2	0.194304552048173	0.431709508737521	1	pseudobulk	DESeq2	LRT		0
611	KC_AB1	Mms19	-0.815746214459807	0.546419520309935	1	pseudobulk	DESeq2	LRT		0
611	KC_AB1	mmy	-0.415912901940146	0.5042916425973	1	pseudobulk	DESeq2	LRT		0
611	KC_AB1	mnb	0.118518128280136	0.636435198480968	1	pseudobulk	DESeq2	LRT		0
611	KC_AB1	mnd	0.203847710048007	0.912094260389099	1	pseudobulk	DESeq2	LRT		0
611	KC_AB1	Mnn1	0.0571015139497774	0.758893864263281	1	pseudobulk	DESeq2	LRT		0
611	KC_AB1	Mnt	-0.166984706428158	0.468625328247556	1	pseudobulk	DESeq2	LRT		0
611	KC_AB1	Mo25	0.402839589492144	0.397005005448604	1	pseudobulk	DESeq2	LRT		0
612	KC_AB1	Mob2	-0.188802045383843	0.454439275866711	1	pseudobulk	DESeq2	LRT		0
612	KC_AB1	Mob3	0.251366397877737	0.726860366196804	1	pseudobulk	DESeq2	LRT		0
612	KC_AB1	Mob4	-0.440671848459092	0.286602633730348	1	pseudobulk	DESeq2	LRT		0
612	KC_AB1	Moca-cyp	0.34826599829974	0.413972147745987	1	pseudobulk	DESeq2	LRT		0
612	KC_AB1	mod	0.0630111909107112	0.915264312981232	1	pseudobulk	DESeq2	LRT		0
612	KC_AB1	modSP	0.405038903029178	1	1	pseudobulk	DESeq2	LRT		0
612	KC_AB1	Moe	-0.153961104763836	0.507990238217135	1	pseudobulk	DESeq2	LRT		0
612	KC_AB1	mof	-2.52824700516371	0.121360864481648	1	pseudobulk	DESeq2	LRT		0
612	KC_AB1	mol	-0.493403067484676	0.791396581717061	1	pseudobulk	DESeq2	LRT		0
612	KC_AB1	Mon1	1.17455245950194	0.61249782287032	1	pseudobulk	DESeq2	LRT		0
613	KC_AB1	mon2	-0.47873364143275	0.404721548302217	1	pseudobulk	DESeq2	LRT		0
613	KC_AB1	Mondo	-0.198821153424978	0.808932177712601	1	pseudobulk	DESeq2	LRT		0
613	KC_AB1	moody	0.849606887838128	1	1	pseudobulk	DESeq2	LRT		0
613	KC_AB1	mop	-0.0322913329679064	0.364848569548492	1	pseudobulk	DESeq2	LRT		0
613	KC_AB1	mor	-0.0829313358679554	0.897443845955255	1	pseudobulk	DESeq2	LRT		0
613	KC_AB1	morgue	-0.443781212638001	0.535207606622319	1	pseudobulk	DESeq2	LRT		0
613	KC_AB1	Mp	-0.498872928833975	0.195829727430405	1	pseudobulk	DESeq2	LRT		0
613	KC_AB1	MP1	1.66254982011319	0.324835478829394	1	pseudobulk	DESeq2	LRT		0
613	KC_AB1	Mpc1	-0.126584769886789	0.835386099854132	1	pseudobulk	DESeq2	LRT		0
613	KC_AB1	Mpcp2	0.120343307356445	0.623584745706241	1	pseudobulk	DESeq2	LRT		0
614	KC_AB1	Mpi	0.0948622989704584	0.754200164323121	1	pseudobulk	DESeq2	LRT		0
614	KC_AB1	Mpp6	-0.184368523887911	0.689164414823844	1	pseudobulk	DESeq2	LRT		0
614	KC_AB1	Mppe	1.0608268074276	0.259633308884626	1	pseudobulk	DESeq2	LRT		0
614	KC_AB1	Mps1	0.849606887838128	1	1	pseudobulk	DESeq2	LRT		0
614	KC_AB1	mr	0.481337012440576	0.624382180073871	1	pseudobulk	DESeq2	LRT		0
614	KC_AB1	mre11	-1.77204424177703	0.21612482240886	1	pseudobulk	DESeq2	LRT		0
614	KC_AB1	mRF1	-1.14792238939349	0.534097302778179	1	pseudobulk	DESeq2	LRT		0

614	KC_AB1	MRG15	0.337139580551436	0.63291574472364	1	pseudobulk	DESeq2	LRT	0
614	KC_AB1	MrgBP	0.116625687614423	0.788961200410965	1	pseudobulk	DESeq2	LRT	0
614	KC_AB1	mri	0.142284501324187	0.687510697159992	1	pseudobulk	DESeq2	LRT	0
615	KC_AB1	mrj	-0.17200845639721	0.54858890331421	1	pseudobulk	DESeq2	LRT	0
615	KC_AB1	mrn	-2.26863936507334	0.202092726802476	1	pseudobulk	DESeq2	LRT	0
615	KC_AB1	mRNA-cap	-0.685277180205524	0.363139522760524	1	pseudobulk	DESeq2	LRT	0
615	KC_AB1	MRP	-0.592727430079731	0.359270347584798	1	pseudobulk	DESeq2	LRT	0
615	KC_AB1	Mrp4	0.849606887838128	1	1	pseudobulk	DESeq2	LRT	0
615	KC_AB1	mRpL1	0.359512673021585	0.767888142398564	1	pseudobulk	DESeq2	LRT	0
615	KC_AB1	mRpL10	-0.598532351340308	0.430662175822901	1	pseudobulk	DESeq2	LRT	0
615	KC_AB1	mRpL11	0.26277196458462	0.743985482886808	1	pseudobulk	DESeq2	LRT	0
615	KC_AB1	mRpL12	-0.0898332865310377	0.76360802384407	1	pseudobulk	DESeq2	LRT	0
615	KC_AB1	mRpL13	0.676952270121035	0.350456891289063	1	pseudobulk	DESeq2	LRT	0
616	KC_AB1	mRpL14	-1.10228175399905	0.378846742701133	1	pseudobulk	DESeq2	LRT	0
616	KC_AB1	mRpL15	0.292741749859244	0.646304282172986	1	pseudobulk	DESeq2	LRT	0
616	KC_AB1	mRpL16	0.0399231637812933	0.685282641039977	1	pseudobulk	DESeq2	LRT	0
616	KC_AB1	mRpL17	0.521031897465637	0.483443264432303	1	pseudobulk	DESeq2	LRT	0
616	KC_AB1	mRpL18	0.912339730684144	0.261551595012547	1	pseudobulk	DESeq2	LRT	0
616	KC_AB1	mRpL19	-0.486709780236214	0.623563941496046	1	pseudobulk	DESeq2	LRT	0
616	KC_AB1	mRpL2	-0.228021741234083	0.676024131740062	1	pseudobulk	DESeq2	LRT	0
616	KC_AB1	mRpL20	-0.600106870355889	0.544112125534274	1	pseudobulk	DESeq2	LRT	0
616	KC_AB1	mRpL21	0.0251566881680917	0.663315938342542	1	pseudobulk	DESeq2	LRT	0
616	KC_AB1	mRpL22	0.41346785094474	0.427430296620117	1	pseudobulk	DESeq2	LRT	0
617	KC_AB1	mRpL23	0.205713388805522	0.588940062826131	1	pseudobulk	DESeq2	LRT	0
617	KC_AB1	mRpL24	-0.50560618068932	0.523392484341488	1	pseudobulk	DESeq2	LRT	0
617	KC_AB1	mRpL27	-0.58407220397894	0.436755511170541	1	pseudobulk	DESeq2	LRT	0
617	KC_AB1	mRpL28	-0.167887236392459	0.792424831670897	1	pseudobulk	DESeq2	LRT	0
617	KC_AB1	mRpL3	-0.73381914453944	0.544472176153359	1	pseudobulk	DESeq2	LRT	0
617	KC_AB1	mRpL30	0.178054107260999	0.635406860785267	1	pseudobulk	DESeq2	LRT	0
617	KC_AB1	mRpL32	-0.714262062480379	0.511475868461695	1	pseudobulk	DESeq2	LRT	0
617	KC_AB1	mRpL33	0.421810106158385	0.366462802758293	1	pseudobulk	DESeq2	LRT	0
617	KC_AB1	mRpL34	0.624844573214907	0.339803490178686	1	pseudobulk	DESeq2	LRT	0
617	KC_AB1	mRpL35	-0.0043270650860173	0.643115723392907	1	pseudobulk	DESeq2	LRT	0
618	KC_AB1	mRpL36	0.400231244071134	0.483842942976936	1	pseudobulk	DESeq2	LRT	0
618	KC_AB1	mRpL37	0.849078360305442	0.415744277158834	1	pseudobulk	DESeq2	LRT	0
618	KC_AB1	mRpL38	0.374666933266338	0.654333867748202	1	pseudobulk	DESeq2	LRT	0
618	KC_AB1	mRpL39	0.0022153842783461	0.735275146268334	1	pseudobulk	DESeq2	LRT	0
618	KC_AB1	mRpL4	-0.0499196177438385	0.749998419843916	1	pseudobulk	DESeq2	LRT	0
618	KC_AB1	mRpL40	0.553248897334096	0.562256212928831	1	pseudobulk	DESeq2	LRT	0
618	KC_AB1	mRpL41	-0.0056829262745693	0.7202384616237	1	pseudobulk	DESeq2	LRT	0
618	KC_AB1	mRpL42	-0.0494475322967969	0.935576844746253	1	pseudobulk	DESeq2	LRT	0
618	KC_AB1	mRpL43	-0.375039118666416	0.550265942093609	1	pseudobulk	DESeq2	LRT	0
618	KC_AB1	mRpL44	-0.340764867855513	0.642955641585859	1	pseudobulk	DESeq2	LRT	0
619	KC_AB1	mRpL45	0.120051505710496	0.834548457934674	1	pseudobulk	DESeq2	LRT	0
619	KC_AB1	mRpL46	1.16063705865299	0.230622429603766	1	pseudobulk	DESeq2	LRT	0
619	KC_AB1	mRpL47	0.53418820835818	0.416027447214817	1	pseudobulk	DESeq2	LRT	0
619	KC_AB1	mRpL48	0.360510535256387	0.553233425751754	1	pseudobulk	DESeq2	LRT	0
619	KC_AB1	mRpL49	0.471513165332343	0.535435402105906	1	pseudobulk	DESeq2	LRT	0
619	KC_AB1	mRpL50	1.09145735018475	0.211950497627109	1	pseudobulk	DESeq2	LRT	0
619	KC_AB1	mRpL51	-0.0202540387472609	0.827671419654595	1	pseudobulk	DESeq2	LRT	0
619	KC_AB1	mRpL52	0.659306691581196	0.391464225363868	1	pseudobulk	DESeq2	LRT	0
619	KC_AB1	mRpL54	0.415097611789364	0.55792374557279	1	pseudobulk	DESeq2	LRT	0
619	KC_AB1	mRpL55	0.498783179306311	0.272051751049718	1	pseudobulk	DESeq2	LRT	0

620	KC_AB1	mRpL9	-0.919401233095308	0.464898553264601	1	pseudobulk	DESeq2	LRT	0
620	KC_AB1	mRpS10	-0.755173526502167	0.385380048883412	1	pseudobulk	DESeq2	LRT	0
620	KC_AB1	mRpS11	-1.56956148198346	0.0730556061282292	1	pseudobulk	DESeq2	LRT	0
620	KC_AB1	mRpS14	-0.455532895358851	0.581688563244277	1	pseudobulk	DESeq2	LRT	0
620	KC_AB1	mRpS16	0.261928858209221	0.64469032340063	1	pseudobulk	DESeq2	LRT	0
620	KC_AB1	mRpS17	0.222552618622775	0.720907506296204	1	pseudobulk	DESeq2	LRT	0
620	KC_AB1	mRpS18A	-0.454323631306342	0.495129672268305	1	pseudobulk	DESeq2	LRT	0
620	KC_AB1	mRpS18B	0.0037280916318399	0.885489839413176	1	pseudobulk	DESeq2	LRT	0
620	KC_AB1	mRpS18C	-0.223839272419452	0.672146798220725	1	pseudobulk	DESeq2	LRT	0
620	KC_AB1	mRpS2	-0.424375043205591	0.602015750174226	1	pseudobulk	DESeq2	LRT	0
621	KC_AB1	mRpS21	-0.493464276622807	0.518468281583361	1	pseudobulk	DESeq2	LRT	0
621	KC_AB1	mRpS22	1.68854534765648	0.567014088138706	1	pseudobulk	DESeq2	LRT	0
621	KC_AB1	mRpS23	0.694147732058656	0.463727836691343	1	pseudobulk	DESeq2	LRT	0
621	KC_AB1	mRpS24	-0.517923510563236	0.343197692375266	1	pseudobulk	DESeq2	LRT	0
621	KC_AB1	mRpS25	-0.532578785868128	0.500651583683259	1	pseudobulk	DESeq2	LRT	0
621	KC_AB1	mRpS26	0.076404466963552	0.512153201889743	1	pseudobulk	DESeq2	LRT	0
621	KC_AB1	mRpS28	-0.482083573837531	0.480919164592306	1	pseudobulk	DESeq2	LRT	0
621	KC_AB1	mRpS29	-0.665295472875645	0.501099167675059	1	pseudobulk	DESeq2	LRT	0
621	KC_AB1	mRpS30	0.201853698909167	0.785759025087485	1	pseudobulk	DESeq2	LRT	0
621	KC_AB1	mRpS31	0.472663202934898	0.661923563365291	1	pseudobulk	DESeq2	LRT	0
622	KC_AB1	mRpS33	-0.177536304651622	0.601744418416274	1	pseudobulk	DESeq2	LRT	0
622	KC_AB1	mRpS34	0.24999012722865	0.664823819331356	1	pseudobulk	DESeq2	LRT	0
622	KC_AB1	mRpS35	-0.228956492929466	0.670183576739317	1	pseudobulk	DESeq2	LRT	0
622	KC_AB1	mRpS5	0.103551122763219	0.809562664490129	1	pseudobulk	DESeq2	LRT	0
622	KC_AB1	mRpS6	0.502931077331063	0.525517305049059	1	pseudobulk	DESeq2	LRT	0
622	KC_AB1	mRpS7	-0.660080169071945	0.395550773232101	1	pseudobulk	DESeq2	LRT	0
622	KC_AB1	mRpS9	-0.129634042776204	0.62675477487634	1	pseudobulk	DESeq2	LRT	0
622	KC_AB1	mRRF1	0.0438849181154018	0.86606178847258	1	pseudobulk	DESeq2	LRT	0
622	KC_AB1	mRRF2	1.51280379024899	0.85518877994987	1	pseudobulk	DESeq2	LRT	0
622	KC_AB1	mrt	0.0122487849585444	0.71142301477014	1	pseudobulk	DESeq2	LRT	0
623	KC_AB1	Mrtf	-0.304005010920699	0.477920925850532	1	pseudobulk	DESeq2	LRT	0
623	KC_AB1	mrva	-0.236670582260593	0.404540727225867	1	pseudobulk	DESeq2	LRT	0
623	KC_AB1	Ms	0.849606887838128	1	1	pseudobulk	DESeq2	LRT	0
623	KC_AB1	ms(3)76Ba	0.237022465848507	1	1	pseudobulk	DESeq2	LRT	0
623	KC_AB1	MSBP	0.0127178154582169	0.963084049647697	1	pseudobulk	DESeq2	LRT	0
623	KC_AB1	msd5	0.849606887838128	1	1	pseudobulk	DESeq2	LRT	0
623	KC_AB1	Msh6	-0.851943617129873	0.694524594037258	1	pseudobulk	DESeq2	LRT	0
623	KC_AB1	msi	-1.64494121892571	0.111537474726297	1	pseudobulk	DESeq2	LRT	0
623	KC_AB1	msk	0.249023355573369	0.680488447437548	1	pseudobulk	DESeq2	LRT	0
623	KC_AB1	msl-1	-0.504394314469237	0.464207575094019	1	pseudobulk	DESeq2	LRT	0
624	KC_AB1	msl-2	-0.835241878716224	0.385000147461147	1	pseudobulk	DESeq2	LRT	0
624	KC_AB1	msl-3	2.36824519058358	0.0906900900849061	1	pseudobulk	DESeq2	LRT	0
624	KC_AB1	msn	0.0392649879917658	0.875622391374371	1	pseudobulk	DESeq2	LRT	0
624	KC_AB1	Msp300	-0.311781993887873	0.342096884284924	1	pseudobulk	DESeq2	LRT	0
624	KC_AB1	mspo	-0.314839271275074	0.0845101683795346	1	pseudobulk	DESeq2	LRT	0
624	KC_AB1	msps	-0.542683234948221	0.240063034647558	1	pseudobulk	DESeq2	LRT	0
624	KC_AB1	Msr-110	-1.44001149356605	0.230212715146793	1	pseudobulk	DESeq2	LRT	0
624	KC_AB1	Mst27D	2.31997030691033	0.813169507846918	1	pseudobulk	DESeq2	LRT	0
624	KC_AB1	Mst36Fa	0.849606887838128	1	1	pseudobulk	DESeq2	LRT	0
624	KC_AB1	Mst77F	1.84355258516387	0.268010021459732	1	pseudobulk	DESeq2	LRT	0
625	KC_AB1	Mst85C	-0.195463289711239	0.661408410742991	1	pseudobulk	DESeq2	LRT	0
625	KC_AB1	mt:ATPase6	-0.326850104919323	0.103826359591911	1	pseudobulk	DESeq2	LRT	0
625	KC_AB1	mt:ATPase8	0.155948807342214	0.846136248387499	1	pseudobulk	DESeq2	LRT	0

625	KC_AB1	mt:CoI	0.0584584932875673	0.71421732363181	1	pseudobulk	DESeq2	LRT		0
625	KC_AB1	mt:CoII	-0.0190135268167013	0.687578154767664	1	pseudobulk	DESeq2	LRT		0
625	KC_AB1	mt:CoIII	-0.056138622981354	0.673156933933294	1	pseudobulk	DESeq2	LRT		0
625	KC_AB1	mt:Cyt-b	0.0348671776427689	0.623955405052681	1	pseudobulk	DESeq2	LRT		0
625	KC_AB1	mt:lrRNA	-0.33594341608559	0.0230209386978075	1	pseudobulk	DESeq2	LRT		0
625	KC_AB1	mt:ND1	0.232311569467873	0.364161954039608	1	pseudobulk	DESeq2	LRT		0
625	KC_AB1	mt:ND2	-0.167814407033925	0.669065707605456	1	pseudobulk	DESeq2	LRT		0
626	KC_AB1	mt:ND3	-0.274013047216806	0.347215146832055	1	pseudobulk	DESeq2	LRT		0
626	KC_AB1	mt:ND4	-0.0117541873517988	0.793478803951971	1	pseudobulk	DESeq2	LRT		0
626	KC_AB1	mt:ND4L	-1.67682081858299	0.377739521376721	1	pseudobulk	DESeq2	LRT		0
626	KC_AB1	mt:ND5	-0.145465551036269	0.481447745705592	1	pseudobulk	DESeq2	LRT		0
626	KC_AB1	mt:ND6	0.0085329041932069	0.502943556185703	1	pseudobulk	DESeq2	LRT		0
626	KC_AB1	mt:srRNA	0.144147522122077	0.370058170604739	1	pseudobulk	DESeq2	LRT		0
626	KC_AB1	mt:tRNA:Ala-TGC	0.116783351990007	1	1	pseudobulk	DESeq2	LRT		0
626	KC_AB1	mt:tRNA:Asp-GTC	0.849606887838128	1	1	pseudobulk	DESeq2	LRT		0
626	KC_AB1	mt:tRNA:Lys-CTT	2.78674512494791	0.54962276837928	1	pseudobulk	DESeq2	LRT		0
626	KC_AB1	Mt2	0.849606887838128	1	1	pseudobulk	DESeq2	LRT		0
627	KC_AB1	MTA1-like	0.10987589422248	0.770307881959925	1	pseudobulk	DESeq2	LRT		0
627	KC_AB1	Mtap	0.673237840916328	0.625434149229803	1	pseudobulk	DESeq2	LRT		0
627	KC_AB1	Mtch	0.0160146537270936	0.907848554322038	1	pseudobulk	DESeq2	LRT		0
627	KC_AB1	mTerf3	-0.0616327993167622	0.802955105725567	1	pseudobulk	DESeq2	LRT		0
627	KC_AB1	mTerf5	-0.170370067468414	0.753538392022669	1	pseudobulk	DESeq2	LRT		0
627	KC_AB1	MTF-1	-0.0771151134196236	0.681001327115406	1	pseudobulk	DESeq2	LRT		0
627	KC_AB1	mtg	2.13523672791691	0.322056359518768	1	pseudobulk	DESeq2	LRT		0
627	KC_AB1	mtgo	-0.099686888563608	0.677910495839057	1	pseudobulk	DESeq2	LRT		0
627	KC_AB1	mth	-0.216210029276812	1	1	pseudobulk	DESeq2	LRT		0
627	KC_AB1	mthl1	0.0690420684361077	1	1	pseudobulk	DESeq2	LRT		0
628	KC_AB1	mthl10	2.06541384650887	0.904905526327343	1	pseudobulk	DESeq2	LRT		0
628	KC_AB1	mthl13	0.849606887838128	1	1	pseudobulk	DESeq2	LRT		0
628	KC_AB1	mthl14	0.849606887838128	1	1	pseudobulk	DESeq2	LRT		0
628	KC_AB1	mthl5	-0.216210029276812	1	1	pseudobulk	DESeq2	LRT		0
628	KC_AB1	mthl8	2.76023102824727	0.0172909900467463	1	pseudobulk	DESeq2	LRT		0
628	KC_AB1	Mtl	-0.460560411158332	0.5332846021203	1	pseudobulk	DESeq2	LRT		0
628	KC_AB1	mtm	0.975381797901727	0.276440220827761	1	pseudobulk	DESeq2	LRT		0
628	KC_AB1	MtnA	0.325846801348632	0.611331679979753	1	pseudobulk	DESeq2	LRT		0
628	KC_AB1	MtnE	-0.216210029276812	1	1	pseudobulk	DESeq2	LRT		0
628	KC_AB1	Mtor	0.384737233595516	0.547269592856894	1	pseudobulk	DESeq2	LRT		0
629	KC_AB1	Mtp	2.13004336645676	0.828063380524205	1	pseudobulk	DESeq2	LRT		0
629	KC_AB1	Mtpalpha	-0.131271264700582	0.719347176852014	1	pseudobulk	DESeq2	LRT		0
629	KC_AB1	MTPAP	0.144184940013552	1	1	pseudobulk	DESeq2	LRT		0
629	KC_AB1	Mtpbeta	-0.482581958555461	0.636941871179559	1	pseudobulk	DESeq2	LRT		0
629	KC_AB1	Mtr3	0.7320558342228	0.639172554187921	1	pseudobulk	DESeq2	LRT		0
629	KC_AB1	Mtr4	-0.604985779190547	0.655552504247949	1	pseudobulk	DESeq2	LRT		0
629	KC_AB1	mtrm	2.06531092668974	0.944672961903726	1	pseudobulk	DESeq2	LRT		0
629	KC_AB1	mtRNApol	0.0605751455452726	0.820992273145563	1	pseudobulk	DESeq2	LRT		0
629	KC_AB1	mts	0.0888624267170815	0.674070501748329	1	pseudobulk	DESeq2	LRT		0
629	KC_AB1	mtSSB	-0.607717839818027	0.640973213990844	1	pseudobulk	DESeq2	LRT		0
630	KC_AB1	mtt	1.17353381161674	0.598751301432125	1	pseudobulk	DESeq2	LRT		0
630	KC_AB1	mTTF	-0.720018920723874	0.538006527828305	1	pseudobulk	DESeq2	LRT		0
630	KC_AB1	mtTFB2	0.407753327808333	0.923068229374996	1	pseudobulk	DESeq2	LRT		0
630	KC_AB1	mu2	-0.370862531082229	0.881125640939123	1	pseudobulk	DESeq2	LRT		0
630	KC_AB1	mub	-0.26088154740535	0.0258991437542407	1	pseudobulk	DESeq2	LRT		0
630	KC_AB1	muc	0.146429034462721	0.644799376895457	1	pseudobulk	DESeq2	LRT		0

630	KC_AB1	Muc12Ea	0.183159795463042	1	1	pseudobulk	DESeq2	LRT	0
630	KC_AB1	Muc14A	-1.41567692939154	0.448661989255937	1	pseudobulk	DESeq2	LRT	0
630	KC_AB1	Muc91C	2.31946552793885	0.139237400047265	1	pseudobulk	DESeq2	LRT	0
630	KC_AB1	Muc96D	0.849606887838128	1	1	pseudobulk	DESeq2	LRT	0
631	KC_AB1	Mul1	0.241917848710181	1	1	pseudobulk	DESeq2	LRT	0
631	KC_AB1	Mulk	1.22474742804222	0.260812964426841	1	pseudobulk	DESeq2	LRT	0
631	KC_AB1	Mur2B	-1.98143973573117	0.220685463695425	1	pseudobulk	DESeq2	LRT	0
631	KC_AB1	mura	-0.211533343698418	0.298289281166753	1	pseudobulk	DESeq2	LRT	0
631	KC_AB1	mus101	1.7454737383338	0.70756651762728	1	pseudobulk	DESeq2	LRT	0
631	KC_AB1	mus201	-1.27467172141861	0.491626102067384	1	pseudobulk	DESeq2	LRT	0
631	KC_AB1	mus304	0.917643762774595	0.229711840873052	1	pseudobulk	DESeq2	LRT	0
631	KC_AB1	mus81	-1.62295259850125	0.385695124829572	1	pseudobulk	DESeq2	LRT	0
631	KC_AB1	muskelin	-0.0012997446430116	1	1	pseudobulk	DESeq2	LRT	0
631	KC_AB1	mute	-1.25992910135218	0.340464884073266	1	pseudobulk	DESeq2	LRT	0
632	KC_AB1	mv	-2.22260211965662	0.116608544758786	1	pseudobulk	DESeq2	LRT	0
632	KC_AB1	Mvb12	-0.855284200528346	0.408719986682585	1	pseudobulk	DESeq2	LRT	0
632	KC_AB1	Mvd	-0.216210032712384	1	1	pseudobulk	DESeq2	LRT	0
632	KC_AB1	Mvl	0.972409982242986	0.110461717759871	1	pseudobulk	DESeq2	LRT	0
632	KC_AB1	mwh	2.78674512494791	0.54962276837928	1	pseudobulk	DESeq2	LRT	0
632	KC_AB1	mxc	-1.18757069110428	0.316015765699093	1	pseudobulk	DESeq2	LRT	0
632	KC_AB1	mxt	-0.67216187016783	0.281312003681072	1	pseudobulk	DESeq2	LRT	0
632	KC_AB1	Myb	1.59863986092676	1	1	pseudobulk	DESeq2	LRT	0
632	KC_AB1	Mybbp1A	2.03995107795309	0.53727460709934	1	pseudobulk	DESeq2	LRT	0
632	KC_AB1	Myc	-0.133128409144287	0.754410966956828	1	pseudobulk	DESeq2	LRT	0
633	KC_AB1	Myd88	-1.61613514129859	0.38485325864237	1	pseudobulk	DESeq2	LRT	0
633	KC_AB1	myo	-1.20106544347512	0.339097337811662	1	pseudobulk	DESeq2	LRT	0
633	KC_AB1	Myo10A	2.06541384650887	0.904905526327343	1	pseudobulk	DESeq2	LRT	0
633	KC_AB1	Myo28B1	-0.0058816974662282	0.693130129492068	1	pseudobulk	DESeq2	LRT	0
633	KC_AB1	Myo31DF	1.51872195779367	0.623907569846402	1	pseudobulk	DESeq2	LRT	0
633	KC_AB1	Myo81F	-1.10042851940886	0.0242957481684974	1	pseudobulk	DESeq2	LRT	0
633	KC_AB1	Myo95E	-0.191672214022833	0.780035347928933	1	pseudobulk	DESeq2	LRT	0
633	KC_AB1	MYPT-75D	-0.345383453385328	0.168027351356934	1	pseudobulk	DESeq2	LRT	0
633	KC_AB1	mys	-0.624100314066847	0.173778344068831	1	pseudobulk	DESeq2	LRT	0
633	KC_AB1	Mys45A	0.12393427938949	0.756518116507937	1	pseudobulk	DESeq2	LRT	0
634	KC_AB1	Myt1	0.407363085727121	1	1	pseudobulk	DESeq2	LRT	0
634	KC_AB1	N	0.264044685843466	1	1	pseudobulk	DESeq2	LRT	0
634	KC_AB1	na	-0.14086038927413	0.609978104621262	1	pseudobulk	DESeq2	LRT	0
634	KC_AB1	Naa15-16	-0.671996132396472	0.376834024557364	1	pseudobulk	DESeq2	LRT	0
634	KC_AB1	Naa20A	0.448946929065833	0.513940147706559	1	pseudobulk	DESeq2	LRT	0
634	KC_AB1	Naa30A	0.180187156017276	0.693011296715833	1	pseudobulk	DESeq2	LRT	0
634	KC_AB1	Naa35	-0.256841837273593	0.695892924399518	1	pseudobulk	DESeq2	LRT	0
634	KC_AB1	Naa40	-0.155934067093443	0.914618786789969	1	pseudobulk	DESeq2	LRT	0
634	KC_AB1	Naa60	-0.197524801381872	0.762487030100563	1	pseudobulk	DESeq2	LRT	0
634	KC_AB1	Naa80	0.263965472250279	0.56661729978132	1	pseudobulk	DESeq2	LRT	0
635	KC_AB1	Naam	0.849606887838128	1	1	pseudobulk	DESeq2	LRT	0
635	KC_AB1	Nab2	0.111637265129279	0.587716113206638	1	pseudobulk	DESeq2	LRT	0
635	KC_AB1	nac	2.71397898638463	0.463880806276523	1	pseudobulk	DESeq2	LRT	0
635	KC_AB1	Nacalpha	-0.0327218065745986	0.598042081445334	1	pseudobulk	DESeq2	LRT	0
635	KC_AB1	nAChRalpha1	-0.241045011477918	0.247332694399243	1	pseudobulk	DESeq2	LRT	0
635	KC_AB1	nAChRalpha2	0.502146225911389	0.177920331582004	1	pseudobulk	DESeq2	LRT	0
635	KC_AB1	nAChRalpha3	-0.0868497715783982	0.706398189738876	1	pseudobulk	DESeq2	LRT	0
635	KC_AB1	nAChRalpha4	-0.0283520801586413	0.504063297764555	1	pseudobulk	DESeq2	LRT	0
635	KC_AB1	nAChRalpha5	-0.0549103375133405	0.713730427728801	1	pseudobulk	DESeq2	LRT	0

635	KC_AB1	nAChRalpha6	-0.0355180287200261	0.882859392681374	1	pseudobulk	DESeq2	LRT		0
636	KC_AB1	nAChRalpha7	-1.50883877203359	0.0578310606045457	1	pseudobulk	DESeq2	LRT		0
636	KC_AB1	nAChRbeta1	-0.0135604827974465	0.807352340704849	1	pseudobulk	DESeq2	LRT		0
636	KC_AB1	nAChRbeta2	-0.0110124938119146	0.914709171248891	1	pseudobulk	DESeq2	LRT		0
636	KC_AB1	NaCP60E	-0.413671007979544	0.0515404213864847	1	pseudobulk	DESeq2	LRT		0
636	KC_AB1	Nadsyn	-0.0020862978175281	0.84452897610973	1	pseudobulk	DESeq2	LRT		0
636	KC_AB1	nahoda	2.68453167034132	0.550970543274	1	pseudobulk	DESeq2	LRT		0
636	KC_AB1	Nak	-0.172672738718667	0.505744478610573	1	pseudobulk	DESeq2	LRT		0
636	KC_AB1	Nap1	0.0373652978672667	0.55854170003047	1	pseudobulk	DESeq2	LRT		0
636	KC_AB1	NaPi-III	-0.109835612313455	0.44105156337822	1	pseudobulk	DESeq2	LRT		0
636	KC_AB1	NaPi-T	-1.97396849215676	0.197820492728281	1	pseudobulk	DESeq2	LRT		0
637	KC_AB1	Naprt	0.374863041030204	0.579700578337016	1	pseudobulk	DESeq2	LRT		0
637	KC_AB1	NAT1	-0.307721772737909	0.212622741293884	1	pseudobulk	DESeq2	LRT		0
637	KC_AB1	natalisin	0.849606887838128	1	1	pseudobulk	DESeq2	LRT		0
637	KC_AB1	Naus	-0.863405080180625	0.277077350246126	1	pseudobulk	DESeq2	LRT		0
637	KC_AB1	Naxd	-0.503170689153208	0.660017308463675	1	pseudobulk	DESeq2	LRT		0
637	KC_AB1	Naxe	1.45597301642256	0.555635827445551	1	pseudobulk	DESeq2	LRT		0
637	KC_AB1	naz	1.4229055769363	0.771821430526778	1	pseudobulk	DESeq2	LRT		0
637	KC_AB1	Nbr	-0.338875467311146	0.425279502625683	1	pseudobulk	DESeq2	LRT		0
637	KC_AB1	nbs	0.265044037421444	0.823256874133877	1	pseudobulk	DESeq2	LRT		0
637	KC_AB1	NC2alpha	0.435710292898793	0.526097173363067	1	pseudobulk	DESeq2	LRT		0
638	KC_AB1	NC2beta	-0.314220551726901	0.503431536463368	1	pseudobulk	DESeq2	LRT		0
638	KC_AB1	Nc73EF	-0.0955409784827684	0.578560825776658	1	pseudobulk	DESeq2	LRT		0
638	KC_AB1	Nca	-0.0295090031896412	0.657543993663919	1	pseudobulk	DESeq2	LRT		0
638	KC_AB1	ncd	1.53445747936661	0.741101785179312	1	pseudobulk	DESeq2	LRT		0
638	KC_AB1	Nckx30C	-0.208199914507571	0.213121823905869	1	pseudobulk	DESeq2	LRT		0
638	KC_AB1	nclb	0.767093879837038	0.313761429548021	1	pseudobulk	DESeq2	LRT		0
638	KC_AB1	ncm	-0.550136295072134	0.36500435984444	1	pseudobulk	DESeq2	LRT		0
638	KC_AB1	Ncoa6	0.0986799047520076	0.685539197932181	1	pseudobulk	DESeq2	LRT		0
638	KC_AB1	Nct	-0.776355771293107	0.456825710147232	1	pseudobulk	DESeq2	LRT		0
638	KC_AB1	ND-13A	0.138334682884795	0.594558012616975	1	pseudobulk	DESeq2	LRT		0
639	KC_AB1	ND-13B	0.265043689836519	0.442314815445554	1	pseudobulk	DESeq2	LRT		0
639	KC_AB1	ND-15	0.204570370392128	0.483902625000399	1	pseudobulk	DESeq2	LRT		0
639	KC_AB1	ND-18	0.165362998286591	0.609613862483437	1	pseudobulk	DESeq2	LRT		0
639	KC_AB1	ND-19	0.554038439145127	0.239806358710586	1	pseudobulk	DESeq2	LRT		0
639	KC_AB1	ND-20	0.156304691417104	0.701378667802734	1	pseudobulk	DESeq2	LRT		0
639	KC_AB1	ND-23	0.0700687924374416	0.586447669873374	1	pseudobulk	DESeq2	LRT		0
639	KC_AB1	ND-24	0.0384505408318293	0.82882188074135	1	pseudobulk	DESeq2	LRT		0
639	KC_AB1	ND-30	0.465018311056106	0.281522626241475	1	pseudobulk	DESeq2	LRT		0
639	KC_AB1	ND-39	0.282360790261511	0.488860430414719	1	pseudobulk	DESeq2	LRT		0
639	KC_AB1	ND-42	0.0543548014343358	0.860189116802565	1	pseudobulk	DESeq2	LRT		0
640	KC_AB1	ND-49	0.130979901486494	0.642764399378314	1	pseudobulk	DESeq2	LRT		0
640	KC_AB1	ND-51	0.106919440856498	0.509372862496401	1	pseudobulk	DESeq2	LRT		0
640	KC_AB1	ND-75	0.389163544897163	0.174298836043188	1	pseudobulk	DESeq2	LRT		0
640	KC_AB1	ND-ACP	0.174778193861057	0.505279042359382	1	pseudobulk	DESeq2	LRT		0
640	KC_AB1	ND-AGGG	-0.0623891496257293	0.595180648361369	1	pseudobulk	DESeq2	LRT		0
640	KC_AB1	ND-ASHI	0.106120918399317	0.830241537433721	1	pseudobulk	DESeq2	LRT		0
640	KC_AB1	ND-B12	0.0189654111010017	0.47934784405569	1	pseudobulk	DESeq2	LRT		0
640	KC_AB1	ND-B14	-0.0133991998783106	0.857523968216912	1	pseudobulk	DESeq2	LRT		0
640	KC_AB1	ND-B14.5A	-0.213962157464134	0.59341600588987	1	pseudobulk	DESeq2	LRT		0
640	KC_AB1	ND-B14.5B	-0.0531879048594282	0.474726893778344	1	pseudobulk	DESeq2	LRT		0
641	KC_AB1	ND-B14.7	-0.0843466194584117	0.695200563818597	1	pseudobulk	DESeq2	LRT		0
641	KC_AB1	ND-B15	-0.259984713904052	0.521998036057401	1	pseudobulk	DESeq2	LRT		0

641	KC_AB1	ND-B16.6	0.198057107336031	0.439245966105707	1	pseudobulk	DESeq2	LRT	0
641	KC_AB1	ND-B17	0.27738496865089	0.446877743118549	1	pseudobulk	DESeq2	LRT	0
641	KC_AB1	ND-B17.2	-0.18168583058283	0.567158632577655	1	pseudobulk	DESeq2	LRT	0
641	KC_AB1	ND-B18	0.245453483635128	0.489271584371414	1	pseudobulk	DESeq2	LRT	0
641	KC_AB1	ND-B22	0.0337134392287737	0.57152358667228	1	pseudobulk	DESeq2	LRT	0
641	KC_AB1	ND-MLRQ	0.234676620423256	0.274676824409972	1	pseudobulk	DESeq2	LRT	0
641	KC_AB1	ND-MNLL	-1.09854487964116	0.379670067809041	1	pseudobulk	DESeq2	LRT	0
641	KC_AB1	ND-MWFE	-0.417044162828685	0.189298859477285	1	pseudobulk	DESeq2	LRT	0
642	KC_AB1	ND-PDSW	0.154760976931386	0.6358376672449	1	pseudobulk	DESeq2	LRT	0
642	KC_AB1	ND-SGDH	-0.135525009970484	0.473397979089501	1	pseudobulk	DESeq2	LRT	0
642	KC_AB1	Ndae1	-0.175213297867622	0.416715417503194	1	pseudobulk	DESeq2	LRT	0
642	KC_AB1	Ndc1	0.683921476677681	0.879942985604416	1	pseudobulk	DESeq2	LRT	0
642	KC_AB1	Ndf	-0.174539152058398	0.696425642197617	1	pseudobulk	DESeq2	LRT	0
642	KC_AB1	Ndfip	0.395187204111678	0.382530047764992	1	pseudobulk	DESeq2	LRT	0
642	KC_AB1	neb	0.239252786875915	1	1	pseudobulk	DESeq2	LRT	0
642	KC_AB1	Neb-cGP	0.274681330258724	0.274579793996095	1	pseudobulk	DESeq2	LRT	0
642	KC_AB1	nec	-3.28436990320244	0.17540672087871	1	pseudobulk	DESeq2	LRT	0
642	KC_AB1	Nedd4	0.177149370420585	0.601395492874882	1	pseudobulk	DESeq2	LRT	0
643	KC_AB1	Nedd8	-0.114420655879141	0.827762771000831	1	pseudobulk	DESeq2	LRT	0
643	KC_AB1	nej	-0.188106998541332	0.331018471437081	1	pseudobulk	DESeq2	LRT	0
643	KC_AB1	Nelf-A	0.29372879415192	0.59299901568551	1	pseudobulk	DESeq2	LRT	0
643	KC_AB1	NELF-B	0.0826665774422919	0.865887024222829	1	pseudobulk	DESeq2	LRT	0
643	KC_AB1	Nelf-E	-0.0822401264152414	0.656552126962712	1	pseudobulk	DESeq2	LRT	0
643	KC_AB1	nemy	-1.0760652632624	0.418188875409692	1	pseudobulk	DESeq2	LRT	0
643	KC_AB1	Neos	-1.467499765688	0.231757476218388	1	pseudobulk	DESeq2	LRT	0
643	KC_AB1	Nep1	-0.138479881952909	0.375135560716175	1	pseudobulk	DESeq2	LRT	0
643	KC_AB1	Nep2	0.849606887838128	1	1	pseudobulk	DESeq2	LRT	0
643	KC_AB1	Nep3	-0.145759681996554	0.772826560117241	1	pseudobulk	DESeq2	LRT	0
644	KC_AB1	Nep4	0.992726656740002	0.735615530786155	1	pseudobulk	DESeq2	LRT	0
644	KC_AB1	Nep6	0.849606887838128	1	1	pseudobulk	DESeq2	LRT	0
644	KC_AB1	Nepl11	3.67779196297239	0.0204052806578904	1	pseudobulk	DESeq2	LRT	0
644	KC_AB1	Nepl16	-0.557586786932784	0.305087594880916	1	pseudobulk	DESeq2	LRT	0
644	KC_AB1	Nepl21	2.31997030691033	0.813169507846918	1	pseudobulk	DESeq2	LRT	0
644	KC_AB1	Nepl3	0.930993226399931	1	1	pseudobulk	DESeq2	LRT	0
644	KC_AB1	Nepl5	-0.28750455936872	0.707053146260815	1	pseudobulk	DESeq2	LRT	0
644	KC_AB1	nero	0.0719695936892521	0.753232261452283	1	pseudobulk	DESeq2	LRT	0
644	KC_AB1	nes	-0.216871786815857	0.639721077755164	1	pseudobulk	DESeq2	LRT	0
644	KC_AB1	net	-0.0386660862138616	1	1	pseudobulk	DESeq2	LRT	0
645	KC_AB1	NetA	2.31997030691033	0.813169507846918	1	pseudobulk	DESeq2	LRT	0
645	KC_AB1	NetB	-0.607481655865285	0.0467023572231219	1	pseudobulk	DESeq2	LRT	0
645	KC_AB1	neur	-0.580115009837595	0.154725241563277	1	pseudobulk	DESeq2	LRT	0
645	KC_AB1	Neurl4	-0.638819605480454	0.399684173326203	1	pseudobulk	DESeq2	LRT	0
645	KC_AB1	Nf-YA	0.0788406313328618	0.697919547276851	1	pseudobulk	DESeq2	LRT	0
645	KC_AB1	Nf-YB	0.930782078926583	0.574169770273964	1	pseudobulk	DESeq2	LRT	0
645	KC_AB1	Nf-YC	0.754817121462795	0.310535700016976	1	pseudobulk	DESeq2	LRT	0
645	KC_AB1	Nf1	0.0032349182662982	0.934627733556872	1	pseudobulk	DESeq2	LRT	0
645	KC_AB1	NFAT	-0.045855266771355	0.685253865929776	1	pseudobulk	DESeq2	LRT	0
645	KC_AB1	NfI	-0.438510960223123	0.164035037428775	1	pseudobulk	DESeq2	LRT	0
646	KC_AB1	Nfs1	0.40181490644092	0.555443941984984	1	pseudobulk	DESeq2	LRT	0
646	KC_AB1	Nhe1	-1.87020047863275	0.115644016710511	1	pseudobulk	DESeq2	LRT	0
646	KC_AB1	Nhe2	-0.406051094868132	0.405781723377048	1	pseudobulk	DESeq2	LRT	0
646	KC_AB1	Nhe3	-0.187924300927351	0.562921138799588	1	pseudobulk	DESeq2	LRT	0
646	KC_AB1	NHP2	0.250638712658593	1	1	pseudobulk	DESeq2	LRT	0

646	KC_AB1	NijA	0.183159795455075	1	1	pseudobulk	DESeq2	LRT	0
646	KC_AB1	NijC	-3.36328693465652	0.32769601941714	1	pseudobulk	DESeq2	LRT	0
646	KC_AB1	NimC2	0.849606887838128	1	1	pseudobulk	DESeq2	LRT	0
646	KC_AB1	NimC4	1.59863986092676	1	1	pseudobulk	DESeq2	LRT	0
646	KC_AB1	ninaB	-1.65357397100891	0.344324481860042	1	pseudobulk	DESeq2	LRT	0
647	KC_AB1	NiPp1	-0.304769008493854	0.690712380942619	1	pseudobulk	DESeq2	LRT	0
647	KC_AB1	Nipped-A	-0.366857880399591	0.308980301678632	1	pseudobulk	DESeq2	LRT	0
647	KC_AB1	Nipped-B	-0.188640585917034	0.367623768737988	1	pseudobulk	DESeq2	LRT	0
647	KC_AB1	Nipsnap	-0.438281011740775	0.430414638277642	1	pseudobulk	DESeq2	LRT	0
647	KC_AB1	NitFhit	1.30479668573778	1	1	pseudobulk	DESeq2	LRT	0
647	KC_AB1	nito	-0.506752641644782	0.466960702623564	1	pseudobulk	DESeq2	LRT	0
647	KC_AB1	NK7.1	-0.188744459472447	0.556374536432797	1	pseudobulk	DESeq2	LRT	0
647	KC_AB1	NKAIN	-0.420891839447508	0.418138535456312	1	pseudobulk	DESeq2	LRT	0
647	KC_AB1	nkd	0.169817676833983	0.70180463876731	1	pseudobulk	DESeq2	LRT	0
647	KC_AB1	NLaz	0.0160940546605626	1	1	pseudobulk	DESeq2	LRT	0
648	KC_AB1	Nle	0.673100822969769	0.775882015108641	1	pseudobulk	DESeq2	LRT	0
648	KC_AB1	Nlg2	-0.033181675100915	0.73613008190201	1	pseudobulk	DESeq2	LRT	0
648	KC_AB1	Nlg3	-0.406825234199524	0.0521958308780598	1	pseudobulk	DESeq2	LRT	0
648	KC_AB1	Nlg4	0.100639215495319	1	1	pseudobulk	DESeq2	LRT	0
648	KC_AB1	Nlp	-0.017303687938314	0.827854869512312	1	pseudobulk	DESeq2	LRT	0
648	KC_AB1	nmd	0.242383437180937	0.600501728942655	1	pseudobulk	DESeq2	LRT	0
648	KC_AB1	Nmd3	-1.05992744849371	0.613288832576002	1	pseudobulk	DESeq2	LRT	0
648	KC_AB1	Nmda1	-0.296930366605683	0.459294962388923	1	pseudobulk	DESeq2	LRT	0
648	KC_AB1	Nmdar1	-0.126458209746807	0.52312838798027	1	pseudobulk	DESeq2	LRT	0
648	KC_AB1	Nmdar2	-0.263393574867051	0.458976063210152	1	pseudobulk	DESeq2	LRT	0
649	KC_AB1	Nmdmc	-0.676463064817226	0.615155968820918	1	pseudobulk	DESeq2	LRT	0
649	KC_AB1	nmdyn-D6	0.54719258217882	0.84334192471431	1	pseudobulk	DESeq2	LRT	0
649	KC_AB1	Nmnat	-0.007329245857881	0.720887758812982	1	pseudobulk	DESeq2	LRT	0
649	KC_AB1	nmo	-0.198164415201913	0.287475282699854	1	pseudobulk	DESeq2	LRT	0
649	KC_AB1	Nmt	0.421020629302786	0.760404673501275	1	pseudobulk	DESeq2	LRT	0
649	KC_AB1	Nna1	0.243550181812117	0.468916476431655	1	pseudobulk	DESeq2	LRT	0
649	KC_AB1	Nnp-1	0.637318363614905	0.438900382882973	1	pseudobulk	DESeq2	LRT	0
649	KC_AB1	NO66	2.31997030691033	0.813169507846918	1	pseudobulk	DESeq2	LRT	0
649	KC_AB1	Noa36	1.00560681787083	0.110751237264405	1	pseudobulk	DESeq2	LRT	0
649	KC_AB1	noc	-1.43772419667425	0.201758140077354	1	pseudobulk	DESeq2	LRT	0
650	KC_AB1	nocte	-0.153699891366043	0.52820201651752	1	pseudobulk	DESeq2	LRT	0
650	KC_AB1	noi	0.0808692881737732	0.775976864479039	1	pseudobulk	DESeq2	LRT	0
650	KC_AB1	nolo	-0.216209988775614	1	1	pseudobulk	DESeq2	LRT	0
650	KC_AB1	nom	0.325488636334516	1	1	pseudobulk	DESeq2	LRT	0
650	KC_AB1	nompC	2.31997030691033	0.813169507846918	1	pseudobulk	DESeq2	LRT	0
650	KC_AB1	Non1	-0.637921813278289	0.342158151064662	1	pseudobulk	DESeq2	LRT	0
650	KC_AB1	Non2	0.259984308142156	0.368608594676655	1	pseudobulk	DESeq2	LRT	0
650	KC_AB1	Non3	0.497909276872025	0.556605070878948	1	pseudobulk	DESeq2	LRT	0
650	KC_AB1	nonA	-0.216146681459975	0.408556545689172	1	pseudobulk	DESeq2	LRT	0
650	KC_AB1	nonA-l	0.0146487887252999	0.725640628092121	1	pseudobulk	DESeq2	LRT	0
651	KC_AB1	nonC	-0.869217467124677	0.378485131897374	1	pseudobulk	DESeq2	LRT	0
651	KC_AB1	Nop17l	0.452106827120403	0.349254065329666	1	pseudobulk	DESeq2	LRT	0
651	KC_AB1	nop5	-1.63750658444466	0.456514745645447	1	pseudobulk	DESeq2	LRT	0
651	KC_AB1	Nop56	-0.751923774779173	0.443441726409709	1	pseudobulk	DESeq2	LRT	0
651	KC_AB1	Nop60B	-1.66033054620543	0.270916120329408	1	pseudobulk	DESeq2	LRT	0
651	KC_AB1	Nopp140	-0.368367823718252	0.719790831267103	1	pseudobulk	DESeq2	LRT	0
651	KC_AB1	nord	3.81892523345342	0.259683132508622	1	pseudobulk	DESeq2	LRT	0
651	KC_AB1	norpA	-0.136691242076672	0.489988064255351	1	pseudobulk	DESeq2	LRT	0

651	KC_AB1	Nos	0.237022465848507	1	1	pseudobulk	DESeq2	LRT	0
651	KC_AB1	Nost	-0.568977408916664	0.619217777426034	1	pseudobulk	DESeq2	LRT	0
652	KC_AB1	not	-0.182711884299716	0.669210608499363	1	pseudobulk	DESeq2	LRT	0
652	KC_AB1	Not1	0.129035635743833	0.469058124490739	1	pseudobulk	DESeq2	LRT	0
652	KC_AB1	Not10	0.620670509738396	0.668300164382393	1	pseudobulk	DESeq2	LRT	0
652	KC_AB1	Not11	0.3493895355571	0.61226794728737	1	pseudobulk	DESeq2	LRT	0
652	KC_AB1	Not3	-0.674804713295829	0.124207027613266	1	pseudobulk	DESeq2	LRT	0
652	KC_AB1	NP15.6	0.0382675921394007	0.550207576673404	1	pseudobulk	DESeq2	LRT	0
652	KC_AB1	Npc1a	-0.290113543655877	0.568451475917467	1	pseudobulk	DESeq2	LRT	0
652	KC_AB1	Npc2a	0.431318235698724	0.361216260663778	1	pseudobulk	DESeq2	LRT	0
652	KC_AB1	Npc2b	-0.449646612484476	0.745210259090743	1	pseudobulk	DESeq2	LRT	0
652	KC_AB1	Nph	0.277454087337661	0.600702690511218	1	pseudobulk	DESeq2	LRT	0
653	KC_AB1	Npl4	-0.376131847213502	0.61648377489276	1	pseudobulk	DESeq2	LRT	0
653	KC_AB1	Nplp1	-1.42950221496985	0.0428820464190171	1	pseudobulk	DESeq2	LRT	0
653	KC_AB1	Nplp2	0.849606887838128	1	1	pseudobulk	DESeq2	LRT	0
653	KC_AB1	Nplp3	-0.183288084058187	0.772475101569762	1	pseudobulk	DESeq2	LRT	0
653	KC_AB1	Nprl2	-0.76837225306773	0.565184494209276	1	pseudobulk	DESeq2	LRT	0
653	KC_AB1	Nprl3	-1.4304063477577	0.246651880377283	1	pseudobulk	DESeq2	LRT	0
653	KC_AB1	Nrd1	0.854810620248206	0.429451057167406	1	pseudobulk	DESeq2	LRT	0
653	KC_AB1	Nrg	-0.189358979456833	0.301844770956824	1	pseudobulk	DESeq2	LRT	0
653	KC_AB1	Nrk	-0.930186395988565	0.477943105075617	1	pseudobulk	DESeq2	LRT	0
653	KC_AB1	nrm	-1.17015918157527	0.00353314428770992	1	pseudobulk	DESeq2	LRT	0
654	KC_AB1	Nrt	0.103638330536466	0.874930687723908	1	pseudobulk	DESeq2	LRT	0
654	KC_AB1	nrv1	-0.820071584052029	0.678106535840569	1	pseudobulk	DESeq2	LRT	0
654	KC_AB1	nrv2	0.338908092934676	0.278149150174227	1	pseudobulk	DESeq2	LRT	0
654	KC_AB1	nrv3	0.0045195512980349	0.903814257101003	1	pseudobulk	DESeq2	LRT	0
654	KC_AB1	Nrx-1	-0.018000940119725	0.632827940728219	1	pseudobulk	DESeq2	LRT	0
654	KC_AB1	Nrx-IV	-0.456351698763722	0.334108880133918	1	pseudobulk	DESeq2	LRT	0
654	KC_AB1	Ns1	-0.892984804124552	0.428828574478291	1	pseudobulk	DESeq2	LRT	0
654	KC_AB1	Ns2	-0.768043072346133	0.4002724928752	1	pseudobulk	DESeq2	LRT	0
654	KC_AB1	Ns3	-0.764419997924579	0.515028618316528	1	pseudobulk	DESeq2	LRT	0
654	KC_AB1	Ns4	1.08263028829517	0.165154214489665	1	pseudobulk	DESeq2	LRT	0
655	KC_AB1	NSD	-0.328106360513151	0.707359375086824	1	pseudobulk	DESeq2	LRT	0
655	KC_AB1	Nse4	1.23274792104101	0.762341915340625	1	pseudobulk	DESeq2	LRT	0
655	KC_AB1	Nsf2	-0.538328410297516	0.493965664836399	1	pseudobulk	DESeq2	LRT	0
655	KC_AB1	nsl1	0.0682905401138175	0.576572689769833	1	pseudobulk	DESeq2	LRT	0
655	KC_AB1	nSMase	0.506866777401329	0.358188874207016	1	pseudobulk	DESeq2	LRT	0
655	KC_AB1	nst	-0.105956266651268	0.678398463452511	1	pseudobulk	DESeq2	LRT	0
655	KC_AB1	Nsun2	-0.571155570398758	0.670765255013795	1	pseudobulk	DESeq2	LRT	0
655	KC_AB1	Nsun5	0.899469884611849	0.55874305907911	1	pseudobulk	DESeq2	LRT	0
655	KC_AB1	nSyb	-0.170981089512981	0.230139303901301	1	pseudobulk	DESeq2	LRT	0
655	KC_AB1	NT5E-2	1.59863986092676	1	1	pseudobulk	DESeq2	LRT	0
656	KC_AB1	Ntan1	-0.616923741545765	0.435288912351011	1	pseudobulk	DESeq2	LRT	0
656	KC_AB1	ntc	0.827189359208984	0.335971760776835	1	pseudobulk	DESeq2	LRT	0
656	KC_AB1	Ntf-2	0.317292027932528	0.362750060584031	1	pseudobulk	DESeq2	LRT	0
656	KC_AB1	Ntl	0.849606887838128	1	1	pseudobulk	DESeq2	LRT	0
656	KC_AB1	Ntmt	0.15556275753995	0.911209744539248	1	pseudobulk	DESeq2	LRT	0
656	KC_AB1	NTPase	-1.2144199861638	0.138415200567467	1	pseudobulk	DESeq2	LRT	0
656	KC_AB1	Nuak1	0.0168391211286965	0.627829717357651	1	pseudobulk	DESeq2	LRT	0
656	KC_AB1	NUCB1	-0.288859620127609	0.535116800352409	1	pseudobulk	DESeq2	LRT	0
656	KC_AB1	nudC	0.19521939022247	0.747563093259109	1	pseudobulk	DESeq2	LRT	0
656	KC_AB1	nudE	0.0730188913043884	0.734919381995267	1	pseudobulk	DESeq2	LRT	0
657	KC_AB1	nuf	0.161184900932306	0.64458664945302	1	pseudobulk	DESeq2	LRT	0

657	KC_AB1	Nufip	2.23092733464796	0.133415061709484	1	pseudobulk	DESeq2	LRT		0
657	KC_AB1	Nulp1	-0.137865359473254	0.616275270810796	1	pseudobulk	DESeq2	LRT		0
657	KC_AB1	numb	0.0199979168730169	0.508137833454356	1	pseudobulk	DESeq2	LRT		0
657	KC_AB1	Nup107	-0.410518364110504	0.659969580722974	1	pseudobulk	DESeq2	LRT		0
657	KC_AB1	Nup133	-0.366518181431787	0.748541561119953	1	pseudobulk	DESeq2	LRT		0
657	KC_AB1	Nup153	-0.302972222228595	0.437149562292961	1	pseudobulk	DESeq2	LRT		0
657	KC_AB1	Nup154	-0.729654599369619	0.531661617500114	1	pseudobulk	DESeq2	LRT		0
657	KC_AB1	Nup160	0.386232461385949	0.928711973133828	1	pseudobulk	DESeq2	LRT		0
657	KC_AB1	Nup188	0.301800204634993	0.329983929857931	1	pseudobulk	DESeq2	LRT		0
658	KC_AB1	Nup205	0.0690902400571678	1	1	pseudobulk	DESeq2	LRT		0
658	KC_AB1	Nup214	-0.0966695397768699	0.803435042522314	1	pseudobulk	DESeq2	LRT		0
658	KC_AB1	Nup35	-1.0124473963607	0.441484213402043	1	pseudobulk	DESeq2	LRT		0
658	KC_AB1	Nup358	-0.507956234776892	0.342848317025405	1	pseudobulk	DESeq2	LRT		0
658	KC_AB1	Nup37	0.0709812202678292	0.843832165628304	1	pseudobulk	DESeq2	LRT		0
658	KC_AB1	Nup43	0.601811007030181	0.796306111667652	1	pseudobulk	DESeq2	LRT		0
658	KC_AB1	Nup44A	-0.494798565445363	0.582158062649561	1	pseudobulk	DESeq2	LRT		0
658	KC_AB1	Nup50	-0.682786194307581	0.528713130948692	1	pseudobulk	DESeq2	LRT		0
658	KC_AB1	Nup54	0.158507836517252	1	1	pseudobulk	DESeq2	LRT		0
658	KC_AB1	Nup58	0.959274269319765	0.282683040166024	1	pseudobulk	DESeq2	LRT		0
659	KC_AB1	Nup62	-1.15133897048371	0.291575986500174	1	pseudobulk	DESeq2	LRT		0
659	KC_AB1	Nup75	0.880742696173337	1	1	pseudobulk	DESeq2	LRT		0
659	KC_AB1	Nup93-1	0.41607477741567	1	1	pseudobulk	DESeq2	LRT		0
659	KC_AB1	Nup93-2	1.37899233131595	0.0993330429285471	1	pseudobulk	DESeq2	LRT		0
659	KC_AB1	Nup98-96	-0.177398899106126	0.681256674295199	1	pseudobulk	DESeq2	LRT		0
659	KC_AB1	Nurf-38	0.642812582985664	0.294637314224007	1	pseudobulk	DESeq2	LRT		0
659	KC_AB1	nvd	0.849606887838128	1	1	pseudobulk	DESeq2	LRT		0
659	KC_AB1	nvy	-0.467363835151776	0.687633421272069	1	pseudobulk	DESeq2	LRT		0
659	KC_AB1	nwk	-0.231559782186855	0.510321741374842	1	pseudobulk	DESeq2	LRT		0
659	KC_AB1	Nxf3	-0.558890828368712	0.596370505247327	1	pseudobulk	DESeq2	LRT		0
660	KC_AB1	Nxt1	-0.0988416856358299	0.685560587560423	1	pseudobulk	DESeq2	LRT		0
660	KC_AB1	O-fut1	1.04000370444717	0.485963571573433	1	pseudobulk	DESeq2	LRT		0
660	KC_AB1	O-fut2	-0.939569060349697	0.579584221631933	1	pseudobulk	DESeq2	LRT		0
660	KC_AB1	oaf	-0.133727131400205	0.751341031018038	1	pseudobulk	DESeq2	LRT		0
660	KC_AB1	Oamb	-0.290277716578631	0.143346216961621	1	pseudobulk	DESeq2	LRT		0
660	KC_AB1	Oatp30B	-0.0867464819499176	0.647976293653016	1	pseudobulk	DESeq2	LRT		0
660	KC_AB1	Oatp33Ea	0.849606887838128	1	1	pseudobulk	DESeq2	LRT		0
660	KC_AB1	Oatp58Dc	1.59863986092676	1	1	pseudobulk	DESeq2	LRT		0
660	KC_AB1	Oatp74D	0.849606887838128	1	1	pseudobulk	DESeq2	LRT		0
660	KC_AB1	obe	0.330126010913164	0.751114926194683	1	pseudobulk	DESeq2	LRT		0
661	KC_AB1	Obp19a	-0.564356698759404	0.803702590456371	1	pseudobulk	DESeq2	LRT		0
661	KC_AB1	Obp44a	-0.145897935581881	0.440109565527226	1	pseudobulk	DESeq2	LRT		0
661	KC_AB1	Obp49a	-0.216210029276812	1	1	pseudobulk	DESeq2	LRT		0
661	KC_AB1	Obp73a	-0.10506771737906	1	1	pseudobulk	DESeq2	LRT		0
661	KC_AB1	Obp85a	0.826817161601989	0.81549788882273	1	pseudobulk	DESeq2	LRT		0
661	KC_AB1	Obp99a	0.116783351990007	1	1	pseudobulk	DESeq2	LRT		0
661	KC_AB1	obst-E	0.99874174340391	0.759166820836522	1	pseudobulk	DESeq2	LRT		0
661	KC_AB1	oc	0.849606887838128	1	1	pseudobulk	DESeq2	LRT		0
661	KC_AB1	ocm	-0.386687805515769	0.598447769751204	1	pseudobulk	DESeq2	LRT		0
661	KC_AB1	Ocrl	0.00392062418303203	0.620360228395626	1	pseudobulk	DESeq2	LRT		0
662	KC_AB1	Octalpha2R	0.0194961489539972	0.844076101802649	1	pseudobulk	DESeq2	LRT		0
662	KC_AB1	Octbeta1R	-0.0288848953627579	0.841381252387812	1	pseudobulk	DESeq2	LRT		0
662	KC_AB1	Octbeta2R	-0.669677180911625	0.58928000959429	1	pseudobulk	DESeq2	LRT		0
662	KC_AB1	Octbeta3R	0.197118790029996	0.272201028751728	1	pseudobulk	DESeq2	LRT		0

662	KC_AB1	Oda	0.250634597070068	0.211008073705865	1	pseudobulk	DESeq2	LRT	0
662	KC_AB1	Odc1	1.59863986092676	1	1	pseudobulk	DESeq2	LRT	0
662	KC_AB1	Odj	-0.496572846596139	0.588179730572379	1	pseudobulk	DESeq2	LRT	0
662	KC_AB1	Oga	0.623741115984691	0.422891288530311	1	pseudobulk	DESeq2	LRT	0
662	KC_AB1	Ogg1	-1.08804194635895	0.394097225456835	1	pseudobulk	DESeq2	LRT	0
662	KC_AB1	ogre	-1.52567617281408	0.279541378340586	1	pseudobulk	DESeq2	LRT	0
663	KC_AB1	ohgt	-0.386805721498859	0.585023491063179	1	pseudobulk	DESeq2	LRT	0
663	KC_AB1	okr	0.376431477381175	1	1	pseudobulk	DESeq2	LRT	0
663	KC_AB1	olf186-F	-0.624864224646947	0.357076722061362	1	pseudobulk	DESeq2	LRT	0
663	KC_AB1	olf186-M	3.63822266309189	0.158295863680395	1	pseudobulk	DESeq2	LRT	0
663	KC_AB1	olf413	0.186175937615853	0.348063337440599	1	pseudobulk	DESeq2	LRT	0
663	KC_AB1	Oli	-0.801546114717991	0.172382943545125	1	pseudobulk	DESeq2	LRT	0
663	KC_AB1	omd	-0.511630891101041	0.515930236140857	1	pseudobulk	DESeq2	LRT	0
663	KC_AB1	ome	0.224343998899851	0.248587097579806	1	pseudobulk	DESeq2	LRT	0
663	KC_AB1	onecut	-0.188086099588662	0.512359527669553	1	pseudobulk	DESeq2	LRT	0
663	KC_AB1	Opa1	-0.184140114244275	0.616167441157349	1	pseudobulk	DESeq2	LRT	0
664	KC_AB1	Opbp	-0.0941383445999442	0.808264682286233	1	pseudobulk	DESeq2	LRT	0
664	KC_AB1	opm	-0.187554088806198	0.796546021416664	1	pseudobulk	DESeq2	LRT	0
664	KC_AB1	or	-0.148288099344954	0.733227965447787	1	pseudobulk	DESeq2	LRT	0
664	KC_AB1	Or13a	0.849606887838128	1	1	pseudobulk	DESeq2	LRT	0
664	KC_AB1	Or19b	2.31997030691033	0.813169507846918	1	pseudobulk	DESeq2	LRT	0
664	KC_AB1	Or43b	-1.03811891812446	0.421555289690508	1	pseudobulk	DESeq2	LRT	0
664	KC_AB1	Or45a	-0.529308127244193	0.574418133828154	1	pseudobulk	DESeq2	LRT	0
664	KC_AB1	Or45b	0.849606887838128	1	1	pseudobulk	DESeq2	LRT	0
664	KC_AB1	Or63a	0.849606887838128	1	1	pseudobulk	DESeq2	LRT	0
664	KC_AB1	Or98P	0.849606887838128	1	1	pseudobulk	DESeq2	LRT	0
665	KC_AB1	orb	-0.299390737043112	0.473305481260179	1	pseudobulk	DESeq2	LRT	0
665	KC_AB1	orb2	-0.322711440878618	0.204085503084207	1	pseudobulk	DESeq2	LRT	0
665	KC_AB1	Orc1	0.849606887838128	1	1	pseudobulk	DESeq2	LRT	0
665	KC_AB1	Orc4	0.733884498510037	0.94081897942149	1	pseudobulk	DESeq2	LRT	0
665	KC_AB1	Orc5	0.849606887838128	1	1	pseudobulk	DESeq2	LRT	0
665	KC_AB1	Orc6	0.849606887838128	1	1	pseudobulk	DESeq2	LRT	0
665	KC_AB1	Orct	2.31997030691033	0.813169507846918	1	pseudobulk	DESeq2	LRT	0
665	KC_AB1	Orct2	0.849606887838128	1	1	pseudobulk	DESeq2	LRT	0
665	KC_AB1	Ork1	0.366247714696288	1	1	pseudobulk	DESeq2	LRT	0
665	KC_AB1	ORMDL	0.420105721351687	0.52873677366886	1	pseudobulk	DESeq2	LRT	0
666	KC_AB1	ORY	0.849606887838128	1	1	pseudobulk	DESeq2	LRT	0
666	KC_AB1	osa	-0.282731677429141	0.238462743453389	1	pseudobulk	DESeq2	LRT	0
666	KC_AB1	Osbp	-0.354966385755459	0.606475974340554	1	pseudobulk	DESeq2	LRT	0
666	KC_AB1	Oscillin	-0.205506355181047	0.759013066348923	1	pseudobulk	DESeq2	LRT	0
666	KC_AB1	Oseg1	2.06541384650887	0.795866856626082	1	pseudobulk	DESeq2	LRT	0
666	KC_AB1	Oseg4	-0.216210029276812	1	1	pseudobulk	DESeq2	LRT	0
666	KC_AB1	Osi2	2.78674512494791	0.54962276837928	1	pseudobulk	DESeq2	LRT	0
666	KC_AB1	osp	1.40127974217904	0.365820456291311	1	pseudobulk	DESeq2	LRT	0
666	KC_AB1	Ost48	-0.556046846126967	0.373589607799763	1	pseudobulk	DESeq2	LRT	0
666	KC_AB1	OstDelta	0.689442908976637	0.571038569431151	1	pseudobulk	DESeq2	LRT	0
667	KC_AB1	Ostgamma	0.0833090217695317	0.869871550442799	1	pseudobulk	DESeq2	LRT	0
667	KC_AB1	otk	-0.940113164442304	0.188499116278723	1	pseudobulk	DESeq2	LRT	0
667	KC_AB1	otk2	2.63188966752769	0.0769711439566967	1	pseudobulk	DESeq2	LRT	0
667	KC_AB1	OtopLa	-0.086280131649279	0.720169585822252	1	pseudobulk	DESeq2	LRT	0
667	KC_AB1	OtopLc	-0.0708039786970358	0.760444135258829	1	pseudobulk	DESeq2	LRT	0
667	KC_AB1	out	0.387697955805104	0.943239053034252	1	pseudobulk	DESeq2	LRT	0
667	KC_AB1	ova	-0.169368935801019	0.686321158938059	1	pseudobulk	DESeq2	LRT	0

667	KC_AB1	ovo	-2.26914832756227	0.359196159304271	1	pseudobulk	DESeq2	LRT	0
667	KC_AB1	ox	-0.0083423514865329	0.621067479226028	1	pseudobulk	DESeq2	LRT	0
667	KC_AB1	OXA1L	-0.0637394187311516	0.753485962148069	1	pseudobulk	DESeq2	LRT	0
668	KC_AB1	oxt	0.0989749007114858	0.803851278349336	1	pseudobulk	DESeq2	LRT	0
668	KC_AB1	oys	0.157286287429242	0.647745552014884	1	pseudobulk	DESeq2	LRT	0
668	KC_AB1	p	-0.0674690712135567	0.586508935043877	1	pseudobulk	DESeq2	LRT	0
668	KC_AB1	p115	0.524902332299426	0.795691861252983	1	pseudobulk	DESeq2	LRT	0
668	KC_AB1	p120ctn	0.0009679805289758	0.5378391411399	1	pseudobulk	DESeq2	LRT	0
668	KC_AB1	p130CAS	0.208350180516362	0.541769247431213	1	pseudobulk	DESeq2	LRT	0
668	KC_AB1	p23	-0.108055575602408	0.501086082375373	1	pseudobulk	DESeq2	LRT	0
668	KC_AB1	p24-1	0.320050082734071	0.533126076981721	1	pseudobulk	DESeq2	LRT	0
668	KC_AB1	P32	0.440000375618464	0.636347715945812	1	pseudobulk	DESeq2	LRT	0
668	KC_AB1	p38b	-0.0568550972617971	0.60949337619616	1	pseudobulk	DESeq2	LRT	0
669	KC_AB1	p47	0.233649141948612	0.653553132159443	1	pseudobulk	DESeq2	LRT	0
669	KC_AB1	P58IPK	0.121513967497085	0.618573512755434	1	pseudobulk	DESeq2	LRT	0
669	KC_AB1	P5CDh1	-0.110071472975805	0.548853797514399	1	pseudobulk	DESeq2	LRT	0
669	KC_AB1	P5cr	0.142004429496016	0.666581658543242	1	pseudobulk	DESeq2	LRT	0
669	KC_AB1	Pa1	-0.179417713482174	0.718438080760074	1	pseudobulk	DESeq2	LRT	0
669	KC_AB1	pAbp	-0.241832945560866	0.0973912709908001	1	pseudobulk	DESeq2	LRT	0
669	KC_AB1	Pabp2	-0.366776683338476	0.527320545573307	1	pseudobulk	DESeq2	LRT	0
669	KC_AB1	pad	-1.39864591891361	0.301696338767563	1	pseudobulk	DESeq2	LRT	0
669	KC_AB1	Paf-AHalpha	-0.146797181444968	0.577685223313149	1	pseudobulk	DESeq2	LRT	0
669	KC_AB1	Paics	1.13002034545874	0.210705511364584	1	pseudobulk	DESeq2	LRT	0
670	KC_AB1	pain	3.53095723328851	0.146030482494715	1	pseudobulk	DESeq2	LRT	0
670	KC_AB1	Paip2	0.314171503700283	0.364969116709707	1	pseudobulk	DESeq2	LRT	0
670	KC_AB1	Pak	0.152692231897664	0.618461374675422	1	pseudobulk	DESeq2	LRT	0
670	KC_AB1	Pak3	-0.671183938424417	0.433163617270857	1	pseudobulk	DESeq2	LRT	0
670	KC_AB1	Pal1	-0.0291012744355836	0.791519628339491	1	pseudobulk	DESeq2	LRT	0
670	KC_AB1	Pal2	-0.596825281622776	0.117144857714474	1	pseudobulk	DESeq2	LRT	0
670	KC_AB1	pall	-0.143808692628664	0.798250813939206	1	pseudobulk	DESeq2	LRT	0
670	KC_AB1	pan	-0.252263290149923	0.0983774794316378	1	pseudobulk	DESeq2	LRT	0
670	KC_AB1	PAN2	-0.392870109583702	0.643543761937019	1	pseudobulk	DESeq2	LRT	0
670	KC_AB1	Panx	-1.20108564225256	0.258214756833222	1	pseudobulk	DESeq2	LRT	0
671	KC_AB1	papi	-0.627701653736421	0.554069002439166	1	pseudobulk	DESeq2	LRT	0
671	KC_AB1	PAPLA1	-0.25965485302258	0.581142615298285	1	pseudobulk	DESeq2	LRT	0
671	KC_AB1	Papss	-0.818330903423793	0.444195092507954	1	pseudobulk	DESeq2	LRT	0
671	KC_AB1	Papst2	-0.236630014743168	0.621079133769832	1	pseudobulk	DESeq2	LRT	0
671	KC_AB1	par-1	-0.200185917406157	0.346623925979097	1	pseudobulk	DESeq2	LRT	0
671	KC_AB1	par-6	0.712522257271513	0.187718119893271	1	pseudobulk	DESeq2	LRT	0
671	KC_AB1	para	-0.391361559575691	0.0051130868929976	1	pseudobulk	DESeq2	LRT	0
671	KC_AB1	Parg	-0.429068474506832	0.66273262048826	1	pseudobulk	DESeq2	LRT	0
671	KC_AB1	park	0.499931828801246	0.808644499454173	1	pseudobulk	DESeq2	LRT	0
671	KC_AB1	Parp	-0.54417952707278	0.0613241244887195	1	pseudobulk	DESeq2	LRT	0
672	KC_AB1	parvin	-0.0400949652111557	0.733841388199519	1	pseudobulk	DESeq2	LRT	0
672	KC_AB1	pasha	-0.602205943720152	0.483808704281121	1	pseudobulk	DESeq2	LRT	0
672	KC_AB1	pasi1	-1.58901953738079	0.223692327701165	1	pseudobulk	DESeq2	LRT	0
672	KC_AB1	pasi2	-1.28617527674986	0.232454611224652	1	pseudobulk	DESeq2	LRT	0
672	KC_AB1	Pask	-0.236310204169906	0.666272357189078	1	pseudobulk	DESeq2	LRT	0
672	KC_AB1	Past1	0.322727690928648	0.257043527333738	1	pseudobulk	DESeq2	LRT	0
672	KC_AB1	Pat1	0.077771313963776	0.816022179886033	1	pseudobulk	DESeq2	LRT	0
672	KC_AB1	path	-0.163010011606276	0.648975131631059	1	pseudobulk	DESeq2	LRT	0
672	KC_AB1	Patj	-1.02374190643664	0.241195241145063	1	pseudobulk	DESeq2	LRT	0
672	KC_AB1	Patr-1	-0.0595001940762936	0.634804322144552	1	pseudobulk	DESeq2	LRT	0

673	KC_AB1	Patronin	-0.0033619911500343	0.899698956286071	1	pseudobulk	DESeq2	LRT	0
673	KC_AB1	Patsas	0.362460931915201	0.387477120919855	1	pseudobulk	DESeq2	LRT	0
673	KC_AB1	Pax	0.763397683596025	0.404658621642956	1	pseudobulk	DESeq2	LRT	0
673	KC_AB1	pb	-0.338544850340201	0.803042014543195	1	pseudobulk	DESeq2	LRT	0
673	KC_AB1	Pbgs	1.07971397891278	0.745725138174763	1	pseudobulk	DESeq2	LRT	0
673	KC_AB1	Pbp45	-1.03343975534598	0.627153393210199	1	pseudobulk	DESeq2	LRT	0
673	KC_AB1	Pbp95	-0.692369339009322	0.606368888176565	1	pseudobulk	DESeq2	LRT	0
673	KC_AB1	Pc	0.184212242405368	0.317541786761548	1	pseudobulk	DESeq2	LRT	0
673	KC_AB1	PCB	-0.152023023458834	0.66949402217133	1	pseudobulk	DESeq2	LRT	0
673	KC_AB1	Pcd	0.239394452876459	0.462388024478857	1	pseudobulk	DESeq2	LRT	0
674	KC_AB1	Pcf11	-0.372662682479223	0.241107702687126	1	pseudobulk	DESeq2	LRT	0
674	KC_AB1	PCID2	0.925710360058984	0.528909502017092	1	pseudobulk	DESeq2	LRT	0
674	KC_AB1	pck	-0.559149124481645	0.431690819951012	1	pseudobulk	DESeq2	LRT	0
674	KC_AB1	Pcl	-0.0706814736548216	0.638117409301159	1	pseudobulk	DESeq2	LRT	0
674	KC_AB1	pcm	-0.332429461620049	0.611190333165525	1	pseudobulk	DESeq2	LRT	0
674	KC_AB1	Pcmt	-0.400670756383005	0.377511764118515	1	pseudobulk	DESeq2	LRT	0
674	KC_AB1	PCNA2	-1.29370472899562	0.345289275590837	1	pseudobulk	DESeq2	LRT	0
674	KC_AB1	pcs	0.619323684503567	0.0606232885916405	1	pseudobulk	DESeq2	LRT	0
674	KC_AB1	pcx	-0.468399359817376	0.434002387654021	1	pseudobulk	DESeq2	LRT	0
674	KC_AB1	Pcyt1	-0.634084529633337	0.0903190580618243	1	pseudobulk	DESeq2	LRT	0
675	KC_AB1	Pcyt2	-0.25479942242785	0.701319816041526	1	pseudobulk	DESeq2	LRT	0
675	KC_AB1	PDCD-5	-0.0652294114800391	0.585219604118796	1	pseudobulk	DESeq2	LRT	0
675	KC_AB1	Pdcd4	-0.128177668752615	0.629172331706168	1	pseudobulk	DESeq2	LRT	0
675	KC_AB1	Pde11	-0.216210029276812	1	1	pseudobulk	DESeq2	LRT	0
675	KC_AB1	Pde1c	-0.148818082490443	0.294861583744376	1	pseudobulk	DESeq2	LRT	0
675	KC_AB1	Pde6	-0.539668245397561	0.0386994864177174	1	pseudobulk	DESeq2	LRT	0
675	KC_AB1	Pde8	-0.14127800110821	0.413836829309928	1	pseudobulk	DESeq2	LRT	0
675	KC_AB1	Pde9	-0.331303654418802	0.0774327205984439	1	pseudobulk	DESeq2	LRT	0
675	KC_AB1	Pdf	0.849606887838128	1	1	pseudobulk	DESeq2	LRT	0
675	KC_AB1	pdgy	1.59619117898907	0.710470202745399	1	pseudobulk	DESeq2	LRT	0
676	KC_AB1	Pdha	0.0854802340151211	0.683162669608492	1	pseudobulk	DESeq2	LRT	0
676	KC_AB1	Pdhb	-0.597019173704255	0.190668268698266	1	pseudobulk	DESeq2	LRT	0
676	KC_AB1	Pdi	-0.158095996757629	0.638832000697754	1	pseudobulk	DESeq2	LRT	0
676	KC_AB1	Pdk	0.105057557268128	0.722275929777196	1	pseudobulk	DESeq2	LRT	0
676	KC_AB1	Pdk1	-0.77013953823439	0.0585405203716226	1	pseudobulk	DESeq2	LRT	0
676	KC_AB1	Pdp	-1.18020533935049	0.282853228094694	1	pseudobulk	DESeq2	LRT	0
676	KC_AB1	Pdp1	-0.0850548764676954	0.554957579330861	1	pseudobulk	DESeq2	LRT	0
676	KC_AB1	pds5	-0.648929708513594	0.341185846801153	1	pseudobulk	DESeq2	LRT	0
676	KC_AB1	Pdss2	-0.155256148529265	0.784196993084446	1	pseudobulk	DESeq2	LRT	0
676	KC_AB1	Pdxk	-0.0007315644914293	0.846701373309227	1	pseudobulk	DESeq2	LRT	0
677	KC_AB1	PDZ-GEF	-0.268548916328256	0.293014155456159	1	pseudobulk	DESeq2	LRT	0
677	KC_AB1	pea	0.41771940960791	0.768819251518223	1	pseudobulk	DESeq2	LRT	0
677	KC_AB1	peb	-0.773547157744851	0.674522890084326	1	pseudobulk	DESeq2	LRT	0
677	KC_AB1	Pect	-0.0524724323138323	0.854067086757248	1	pseudobulk	DESeq2	LRT	0
677	KC_AB1	PEK	-0.311581608394615	0.662520795206614	1	pseudobulk	DESeq2	LRT	0
677	KC_AB1	pelo	-0.185673067696453	0.668686444668925	1	pseudobulk	DESeq2	LRT	0
677	KC_AB1	pen-2	-0.0299751295619151	0.704173407987506	1	pseudobulk	DESeq2	LRT	0
677	KC_AB1	peng	-0.13403288676787	0.698090332152339	1	pseudobulk	DESeq2	LRT	0
677	KC_AB1	Pep	0.0083265378147497	0.621402982635984	1	pseudobulk	DESeq2	LRT	0
677	KC_AB1	per	-0.241322173607667	0.668132061612031	1	pseudobulk	DESeq2	LRT	0
678	KC_AB1	pes	-0.965931837376196	0.462129724721911	1	pseudobulk	DESeq2	LRT	0
678	KC_AB1	Pex1	1.81165311764527	0.504901054226294	1	pseudobulk	DESeq2	LRT	0
678	KC_AB1	Pex10	0.322645121864862	0.842034847591044	1	pseudobulk	DESeq2	LRT	0

678	KC_AB1	Pex11	-0.23844317086391	0.690326003271504	1	pseudobulk	DESeq2	LRT		0
678	KC_AB1	Pex12	0.0403172072004106	0.853986096642709	1	pseudobulk	DESeq2	LRT		0
678	KC_AB1	Pex13	0.313283069050831	0.466341315012658	1	pseudobulk	DESeq2	LRT		0
678	KC_AB1	Pex14	1.3716907301445	0.0430582368271885	1	pseudobulk	DESeq2	LRT		0
678	KC_AB1	Pex16	-0.752504437047696	0.464832051704929	1	pseudobulk	DESeq2	LRT		0
678	KC_AB1	Pex19	-0.143373089895631	0.754781133777645	1	pseudobulk	DESeq2	LRT		0
678	KC_AB1	Pex2	-0.141004526294857	0.770437249446977	1	pseudobulk	DESeq2	LRT		0
679	KC_AB1	Pex23	-0.0488285390368373	0.936472197298667	1	pseudobulk	DESeq2	LRT		0
679	KC_AB1	Pex3	0.831104446593754	0.312386747826895	1	pseudobulk	DESeq2	LRT		0
679	KC_AB1	Pex5	0.280506775291822	0.740074429369223	1	pseudobulk	DESeq2	LRT		0
679	KC_AB1	Pex6	-0.101300570030256	0.814551712750365	1	pseudobulk	DESeq2	LRT		0
679	KC_AB1	Pfas	0.849606887838128	1	1	pseudobulk	DESeq2	LRT		0
679	KC_AB1	Pfdn1	0.782212614212081	0.287504919627982	1	pseudobulk	DESeq2	LRT		0
679	KC_AB1	Pfdn2	0.0552306072364801	0.660322885028261	1	pseudobulk	DESeq2	LRT		0
679	KC_AB1	Pfdn4	-0.296009955901375	0.541240122525341	1	pseudobulk	DESeq2	LRT		0
679	KC_AB1	Pfdn5	0.840690070841938	0.247155561233508	1	pseudobulk	DESeq2	LRT		0
679	KC_AB1	Pfdn6	0.0181479176644496	0.957394900563275	1	pseudobulk	DESeq2	LRT		0
680	KC_AB1	Pfk	0.256817541077017	0.395866630061522	1	pseudobulk	DESeq2	LRT		0
680	KC_AB1	Pfrx	-0.802283831415956	0.0715308936030217	1	pseudobulk	DESeq2	LRT		0
680	KC_AB1	Pgam5	-0.55994947749533	0.562015575850049	1	pseudobulk	DESeq2	LRT		0
680	KC_AB1	Pgant1	-0.413040513509046	0.5361233366972	1	pseudobulk	DESeq2	LRT		0
680	KC_AB1	Pgant2	-0.0741185690055906	0.537027758575912	1	pseudobulk	DESeq2	LRT		0
680	KC_AB1	Pgant3	0.743937493968051	0.379806709814874	1	pseudobulk	DESeq2	LRT		0
680	KC_AB1	Pgant35A	-0.21718169448226	0.416845091997146	1	pseudobulk	DESeq2	LRT		0
680	KC_AB1	Pgant5	0.179862301365095	0.481418487033969	1	pseudobulk	DESeq2	LRT		0
680	KC_AB1	Pgant6	-0.209177628788863	0.836714394059029	1	pseudobulk	DESeq2	LRT		0
680	KC_AB1	Pgant7	-0.671746157779338	0.541812432442221	1	pseudobulk	DESeq2	LRT		0
681	KC_AB1	Pgant8	2.78674512494791	0.54962276837928	1	pseudobulk	DESeq2	LRT		0
681	KC_AB1	PGAP1	0.820283751829016	0.550097981936311	1	pseudobulk	DESeq2	LRT		0
681	KC_AB1	PGAP2	0.637028879914269	0.352326198747181	1	pseudobulk	DESeq2	LRT		0
681	KC_AB1	PGAP3	0.0027961296337988	0.809043295525462	1	pseudobulk	DESeq2	LRT		0
681	KC_AB1	PGAP5	-0.0602328169531986	0.616913549792337	1	pseudobulk	DESeq2	LRT		0
681	KC_AB1	Pgd	-0.830062897938551	0.717724257445671	1	pseudobulk	DESeq2	LRT		0
681	KC_AB1	Pgi	0.457275179262052	0.26442964230925	1	pseudobulk	DESeq2	LRT		0
681	KC_AB1	Pgk	0.0733246817982833	0.533350410523772	1	pseudobulk	DESeq2	LRT		0
681	KC_AB1	Pglym78	0.657119717377909	0.0356606637406584	1	pseudobulk	DESeq2	LRT		0
681	KC_AB1	Pgm1	0.598913305121131	0.553879901310301	1	pseudobulk	DESeq2	LRT		0
682	KC_AB1	Pgm2a	0.661112180763826	0.252699991161766	1	pseudobulk	DESeq2	LRT		0
682	KC_AB1	PGRP-LA	0.337335040743366	1	1	pseudobulk	DESeq2	LRT		0
682	KC_AB1	PGRP-LC	-0.663002251527988	0.718344636482651	1	pseudobulk	DESeq2	LRT		0
682	KC_AB1	PGRP-LD	0.237022465848507	1	1	pseudobulk	DESeq2	LRT		0
682	KC_AB1	PGRP-LE	-0.456476400249943	0.645075078102842	1	pseudobulk	DESeq2	LRT		0
682	KC_AB1	ph-d	-0.565464597895493	0.547093511770099	1	pseudobulk	DESeq2	LRT		0
682	KC_AB1	ph-p	0.202987929946054	0.75832154297288	1	pseudobulk	DESeq2	LRT		0
682	KC_AB1	PH4alphaEFB	-0.771150098038705	0.213297929334835	1	pseudobulk	DESeq2	LRT		0
682	KC_AB1	PH4alphaNE1	0.101879950370915	1	1	pseudobulk	DESeq2	LRT		0
682	KC_AB1	Phax	0.811654262148948	0.395622702871023	1	pseudobulk	DESeq2	LRT		0
683	KC_AB1	Phb2	-0.287494598798696	0.488029959511689	1	pseudobulk	DESeq2	LRT		0
683	KC_AB1	pHCl-1	-0.0445756090904134	0.751364724652523	1	pseudobulk	DESeq2	LRT		0
683	KC_AB1	PheRS-m	0.0680221012043135	0.809880939223087	1	pseudobulk	DESeq2	LRT		0
683	KC_AB1	Phf5a	-0.815843682601044	0.433288114841232	1	pseudobulk	DESeq2	LRT		0
683	KC_AB1	PHGPx	-0.24068758121247	0.549770391448787	1	pseudobulk	DESeq2	LRT		0
683	KC_AB1	Phk-3	2.78674512494791	0.54962276837928	1	pseudobulk	DESeq2	LRT		0

683	KC_AB1	PhKgamma	0.0991420638407298	0.819893502662332	1	pseudobulk	DESeq2	LRT		0
683	KC_AB1	Phm	-0.633887444756733	0.0605377013334122	1	pseudobulk	DESeq2	LRT		0
683	KC_AB1	pho	-0.0361032812362433	0.765213832830206	1	pseudobulk	DESeq2	LRT		0
683	KC_AB1	phol	0.254192509338126	0.636815772196909	1	pseudobulk	DESeq2	LRT		0
684	KC_AB1	phr	-0.40200137056069	0.861588439126694	1	pseudobulk	DESeq2	LRT		0
684	KC_AB1	Phs	0.849606887838128	1	1	pseudobulk	DESeq2	LRT		0
684	KC_AB1	phtf	0.371306505219214	0.29109499587181	1	pseudobulk	DESeq2	LRT		0
684	KC_AB1	phu	2.31997030691033	0.813169507846918	1	pseudobulk	DESeq2	LRT		0
684	KC_AB1	phyl	0.0051615749653767	0.985401973094752	1	pseudobulk	DESeq2	LRT		0
684	KC_AB1	PI31	0.649332110679883	0.158906084349966	1	pseudobulk	DESeq2	LRT		0
684	KC_AB1	Pi3K21B	-0.575371696585866	0.273838981160685	1	pseudobulk	DESeq2	LRT		0
684	KC_AB1	Pi3K59F	0.183809088135214	0.788609760421082	1	pseudobulk	DESeq2	LRT		0
684	KC_AB1	Pi3K68D	0.0529352536000392	0.844915960079591	1	pseudobulk	DESeq2	LRT		0
684	KC_AB1	Pi3K92E	0.358026603502715	0.619060378861416	1	pseudobulk	DESeq2	LRT		0
685	KC_AB1	Pi4KIIalpha	-0.158984671963582	0.830248869311213	1	pseudobulk	DESeq2	LRT		0
685	KC_AB1	PI4KIIIalpha	-0.450777373021722	0.278706784649141	1	pseudobulk	DESeq2	LRT		0
685	KC_AB1	pic	-0.31727480069425	0.60331420832266	1	pseudobulk	DESeq2	LRT		0
685	KC_AB1	PICK1	-0.0288427451152025	0.801316706164876	1	pseudobulk	DESeq2	LRT		0
685	KC_AB1	pico	0.400930910358017	0.286491508840455	1	pseudobulk	DESeq2	LRT		0
685	KC_AB1	Picot	0.624709582830846	0.376408472991616	1	pseudobulk	DESeq2	LRT		0
685	KC_AB1	pie	2.31997030691033	0.813169507846918	1	pseudobulk	DESeq2	LRT		0
685	KC_AB1	Piezo	1.93546526816425	0.306034851040899	1	pseudobulk	DESeq2	LRT		0
685	KC_AB1	Pif1A	-0.216209988775614	1	1	pseudobulk	DESeq2	LRT		0
685	KC_AB1	PIG-A	0.355121999510257	0.71101322059583	1	pseudobulk	DESeq2	LRT		0
686	KC_AB1	PIG-B	-0.0846732650864976	0.613794676890457	1	pseudobulk	DESeq2	LRT		0
686	KC_AB1	PIG-C	0.463361041201507	1	1	pseudobulk	DESeq2	LRT		0
686	KC_AB1	PIG-F	0.272615893459757	0.644982415662265	1	pseudobulk	DESeq2	LRT		0
686	KC_AB1	PIG-G	1.66832495676424	0.164160007041841	1	pseudobulk	DESeq2	LRT		0
686	KC_AB1	PIG-H	-0.0669874497224087	0.700998867939746	1	pseudobulk	DESeq2	LRT		0
686	KC_AB1	PIG-K	0.0843874708672333	0.855853661251685	1	pseudobulk	DESeq2	LRT		0
686	KC_AB1	PIG-L	-0.250089544535388	0.657082953538333	1	pseudobulk	DESeq2	LRT		0
686	KC_AB1	PIG-N	2.31997030691033	0.813169507846918	1	pseudobulk	DESeq2	LRT		0
686	KC_AB1	PIG-O	-2.15433367392458	0.122106923836338	1	pseudobulk	DESeq2	LRT		0
686	KC_AB1	PIG-Q	0.598584328159757	0.721913906678635	1	pseudobulk	DESeq2	LRT		0
687	KC_AB1	PIG-S	-0.443314119003181	0.706123327634846	1	pseudobulk	DESeq2	LRT		0
687	KC_AB1	PIG-T	-0.400627326103607	0.365256867770213	1	pseudobulk	DESeq2	LRT		0
687	KC_AB1	PIG-U	-1.59169164088812	0.211099096260961	1	pseudobulk	DESeq2	LRT		0
687	KC_AB1	PIG-V	-0.512413235480837	0.652724950919993	1	pseudobulk	DESeq2	LRT		0
687	KC_AB1	PIG-X	-0.312046737312187	0.516058830515509	1	pseudobulk	DESeq2	LRT		0
687	KC_AB1	Pig1	-0.704858387530061	0.698842745464243	1	pseudobulk	DESeq2	LRT		0
687	KC_AB1	pigeon	-0.301618026078432	0.608502603642807	1	pseudobulk	DESeq2	LRT		0
687	KC_AB1	pigs	0.259925926253285	0.4489270520241	1	pseudobulk	DESeq2	LRT		0
687	KC_AB1	Pih1D1	0.15541361497745	0.633661874001118	1	pseudobulk	DESeq2	LRT		0
687	KC_AB1	Pink1	-0.417672178882282	0.436835310395984	1	pseudobulk	DESeq2	LRT		0
688	KC_AB1	Pino	0.0099842742751093	0.956407250886431	1	pseudobulk	DESeq2	LRT		0
688	KC_AB1	pins	0.154408167328223	0.61643965292476	1	pseudobulk	DESeq2	LRT		0
688	KC_AB1	pinta	0.849606887838128	1	1	pseudobulk	DESeq2	LRT		0
688	KC_AB1	pio	-0.621924174207416	0.832538680499164	1	pseudobulk	DESeq2	LRT		0
688	KC_AB1	PIP4K	-0.0983353647300599	0.493821352121094	1	pseudobulk	DESeq2	LRT		0
688	KC_AB1	PIP5K59B	0.239772158133614	0.424738788200166	1	pseudobulk	DESeq2	LRT		0
688	KC_AB1	pirk	-2.07348984597132	0.403224287902523	1	pseudobulk	DESeq2	LRT		0
688	KC_AB1	Pis	0.0988724860426616	0.727104201321074	1	pseudobulk	DESeq2	LRT		0
688	KC_AB1	Pisd	0.72564860066039	0.214030467044219	1	pseudobulk	DESeq2	LRT		0

688	KC_AB1	pit	0.114393070116907	0.902138257903094	1	pseudobulk	DESeq2	LRT	0
689	KC_AB1	pita	0.534155182257251	0.707486935960621	1	pseudobulk	DESeq2	LRT	0
689	KC_AB1	Pits	-0.0744923734027043	0.499958423340471	1	pseudobulk	DESeq2	LRT	0
689	KC_AB1	Pitslre	-0.1883972746117	0.220643581457418	1	pseudobulk	DESeq2	LRT	0
689	KC_AB1	piwi	0.849606887838128	1	1	pseudobulk	DESeq2	LRT	0
689	KC_AB1	pix	-0.0750319778247373	0.883463567888423	1	pseudobulk	DESeq2	LRT	0
689	KC_AB1	pk	0.33027973469198	1	1	pseudobulk	DESeq2	LRT	0
689	KC_AB1	Pka-C1	-0.189672285944031	0.136404672045687	1	pseudobulk	DESeq2	LRT	0
689	KC_AB1	Pka-C3	-0.901737053405303	0.267609698340833	1	pseudobulk	DESeq2	LRT	0
689	KC_AB1	Pka-R1	0.0995541987695331	0.595883597728595	1	pseudobulk	DESeq2	LRT	0
689	KC_AB1	Pka-R2	-0.173413825219188	0.212607047133598	1	pseudobulk	DESeq2	LRT	0
690	KC_AB1	pkaap	-0.0004578884086618	0.772524558886128	1	pseudobulk	DESeq2	LRT	0
690	KC_AB1	Pkc53E	-0.327053749278538	0.0363435949946206	1	pseudobulk	DESeq2	LRT	0
690	KC_AB1	Pkc98E	-0.0400413533028193	0.700987157279302	1	pseudobulk	DESeq2	LRT	0
690	KC_AB1	PKD	0.717375709369023	0.406668005929236	1	pseudobulk	DESeq2	LRT	0
690	KC_AB1	Pkd2	0.849606887838128	1	1	pseudobulk	DESeq2	LRT	0
690	KC_AB1	Pkn	-0.0352947931159494	0.537006963576315	1	pseudobulk	DESeq2	LRT	0
690	KC_AB1	Plap	-0.298258978595433	0.710768897812957	1	pseudobulk	DESeq2	LRT	0
690	KC_AB1	Plc21C	-0.122889123211408	0.502497541729757	1	pseudobulk	DESeq2	LRT	0
690	KC_AB1	PLCXD	-0.852972622175161	0.506498188283187	1	pseudobulk	DESeq2	LRT	0
690	KC_AB1	Pld	-0.830062897938551	0.717724257445671	1	pseudobulk	DESeq2	LRT	0
691	KC_AB1	Pldn	0.971128942203519	0.0987555834299908	1	pseudobulk	DESeq2	LRT	0
691	KC_AB1	ple	-2.58004409830365	0.233393372861252	1	pseudobulk	DESeq2	LRT	0
691	KC_AB1	Plekhm1	-1.72076692992746	0.235281275964182	1	pseudobulk	DESeq2	LRT	0
691	KC_AB1	PlexA	0.0258912627716001	0.846761718159279	1	pseudobulk	DESeq2	LRT	0
691	KC_AB1	PlexB	-0.0262555374930134	0.80850487353008	1	pseudobulk	DESeq2	LRT	0
691	KC_AB1	plh	-0.216209988775614	1	1	pseudobulk	DESeq2	LRT	0
691	KC_AB1	Pli	0.178182109944453	0.436696149074526	1	pseudobulk	DESeq2	LRT	0
691	KC_AB1	pll	-1.34285870030088	0.385182738666667	1	pseudobulk	DESeq2	LRT	0
691	KC_AB1	Plod	0.779272624643143	0.616062521238259	1	pseudobulk	DESeq2	LRT	0
691	KC_AB1	Plp	0.554950023205165	0.544482394939479	1	pseudobulk	DESeq2	LRT	0
692	KC_AB1	plum	-1.14992312341278	0.455962334342801	1	pseudobulk	DESeq2	LRT	0
692	KC_AB1	plx	-0.107245030452733	0.714763890335354	1	pseudobulk	DESeq2	LRT	0
692	KC_AB1	Plzf	-1.25320958561915	0.388175167394963	1	pseudobulk	DESeq2	LRT	0
692	KC_AB1	PMCA	-0.106263367887192	0.528774158289283	1	pseudobulk	DESeq2	LRT	0
692	KC_AB1	Pmm2	0.289582378311683	0.754734335530845	1	pseudobulk	DESeq2	LRT	0
692	KC_AB1	PMP34	-0.49716200200796	0.375498660514417	1	pseudobulk	DESeq2	LRT	0
692	KC_AB1	Pmp70	0.520957492830942	0.495432583277495	1	pseudobulk	DESeq2	LRT	0
692	KC_AB1	Pms2	0.849606887838128	1	1	pseudobulk	DESeq2	LRT	0
692	KC_AB1	pn	0.116783351990007	1	1	pseudobulk	DESeq2	LRT	0
692	KC_AB1	Pngl	-0.684214063841987	0.507855186891741	1	pseudobulk	DESeq2	LRT	0
693	KC_AB1	Pnn	0.141103621695812	0.798749280836759	1	pseudobulk	DESeq2	LRT	0
693	KC_AB1	PNPase	-0.0158630671089883	1	1	pseudobulk	DESeq2	LRT	0
693	KC_AB1	pns	-0.314869919749912	0.445519289238236	1	pseudobulk	DESeq2	LRT	0
693	KC_AB1	pnt	1.40320639851534	0.562150267238202	1	pseudobulk	DESeq2	LRT	0
693	KC_AB1	pnut	-0.256921185413593	0.644488918149337	1	pseudobulk	DESeq2	LRT	0
693	KC_AB1	PNUTS	0.0197293068701233	0.857403339968584	1	pseudobulk	DESeq2	LRT	0
693	KC_AB1	pod1	-0.667113442149025	0.279092511911525	1	pseudobulk	DESeq2	LRT	0
693	KC_AB1	poe	-0.503064746026913	0.282515287145949	1	pseudobulk	DESeq2	LRT	0
693	KC_AB1	Pof	1.45443576081883	0.161771696786471	1	pseudobulk	DESeq2	LRT	0
693	KC_AB1	Pol31	2.06541384650887	0.795866856626082	1	pseudobulk	DESeq2	LRT	0
694	KC_AB1	Pol32	0.512199452327089	1	1	pseudobulk	DESeq2	LRT	0
694	KC_AB1	POLDIP2	-0.217795042951842	0.72938080525357	1	pseudobulk	DESeq2	LRT	0

694	KC_AB1	poly	0.146172749257014	0.619903137845835	1	pseudobulk	DESeq2	LRT	0
694	KC_AB1	polybromo	0.328983017966667	0.55118908482692	1	pseudobulk	DESeq2	LRT	0
694	KC_AB1	Pomp	0.548656551707637	0.21958531590422	1	pseudobulk	DESeq2	LRT	0
694	KC_AB1	pont	0.634741412090048	0.334748341158875	1	pseudobulk	DESeq2	LRT	0
694	KC_AB1	Pop2	0.0412485290178251	0.930871311325051	1	pseudobulk	DESeq2	LRT	0
694	KC_AB1	por	0.145610255744606	0.956736683412958	1	pseudobulk	DESeq2	LRT	0
694	KC_AB1	porin	-0.146143148610178	0.380044484238642	1	pseudobulk	DESeq2	LRT	0
694	KC_AB1	Porin2	0.849606887838128	1	1	pseudobulk	DESeq2	LRT	0
695	KC_AB1	POSH	-1.39088941056278	0.377431096387751	1	pseudobulk	DESeq2	LRT	0
695	KC_AB1	pot	-0.663002251527988	0.718344636482651	1	pseudobulk	DESeq2	LRT	0
695	KC_AB1	Poxn	3.16697464674408	0.406679230261689	1	pseudobulk	DESeq2	LRT	0
695	KC_AB1	Pp1-87B	-0.15611403871658	0.512989490104832	1	pseudobulk	DESeq2	LRT	0
695	KC_AB1	Pp1alpha-96A	-0.304503424760877	0.258199019660709	1	pseudobulk	DESeq2	LRT	0
695	KC_AB1	Pp2A-29B	0.312802158291503	0.397994882369694	1	pseudobulk	DESeq2	LRT	0
695	KC_AB1	Pp2B-14D	-0.380405255735748	0.218095823347794	1	pseudobulk	DESeq2	LRT	0
695	KC_AB1	Pp2C1	-0.425299074024417	0.245173755900919	1	pseudobulk	DESeq2	LRT	0
695	KC_AB1	Pp4-19C	-0.158689660678027	0.836165163880434	1	pseudobulk	DESeq2	LRT	0
695	KC_AB1	Ppa	-0.0239144792939481	0.813147006359237	1	pseudobulk	DESeq2	LRT	0
696	KC_AB1	ppan	1.67681420345114	0.157415036072084	1	pseudobulk	DESeq2	LRT	0
696	KC_AB1	Ppat-Dpck	0.6353594047639	0.681427176239029	1	pseudobulk	DESeq2	LRT	0
696	KC_AB1	Ppcs	0.177769279449422	0.504835236428976	1	pseudobulk	DESeq2	LRT	0
696	KC_AB1	PpD3	-0.598934916544544	0.538465920433534	1	pseudobulk	DESeq2	LRT	0
696	KC_AB1	PpD6	0.849606887838128	1	1	pseudobulk	DESeq2	LRT	0
696	KC_AB1	ppk13	-0.216210029276812	1	1	pseudobulk	DESeq2	LRT	0
696	KC_AB1	ppk19	2.78674512494791	0.54962276837928	1	pseudobulk	DESeq2	LRT	0
696	KC_AB1	ppk21	1.59863986092676	1	1	pseudobulk	DESeq2	LRT	0
696	KC_AB1	ppk22	-0.267798911930929	1	1	pseudobulk	DESeq2	LRT	0
696	KC_AB1	ppk25	1.21191750555156	1	1	pseudobulk	DESeq2	LRT	0
697	KC_AB1	ppk30	0.849606887838128	1	1	pseudobulk	DESeq2	LRT	0
697	KC_AB1	ppk9	-1.0124032166989	0.592614381977149	1	pseudobulk	DESeq2	LRT	0
697	KC_AB1	ppl	0.166814679923802	0.719660739318313	1	pseudobulk	DESeq2	LRT	0
697	KC_AB1	Ppm1	0.849606887838128	1	1	pseudobulk	DESeq2	LRT	0
697	KC_AB1	PPO1	0.849606887838128	1	1	pseudobulk	DESeq2	LRT	0
697	KC_AB1	PPO2	2.31997030691033	0.813169507846918	1	pseudobulk	DESeq2	LRT	0
697	KC_AB1	PPP1R15	-0.22395859012962	0.335621445340577	1	pseudobulk	DESeq2	LRT	0
697	KC_AB1	PPP4R2r	0.0297326204371809	0.863735313023371	1	pseudobulk	DESeq2	LRT	0
697	KC_AB1	Ppr-Y	2.06541384650887	0.768363369045814	1	pseudobulk	DESeq2	LRT	0
697	KC_AB1	pps	-0.853710766651016	0.254908793352819	1	pseudobulk	DESeq2	LRT	0
698	KC_AB1	Ppt2	-0.388728328415968	0.708199665591652	1	pseudobulk	DESeq2	LRT	0
698	KC_AB1	PpV	-1.53448784663544	0.175116534859102	1	pseudobulk	DESeq2	LRT	0
698	KC_AB1	PQBP1	0.723089682712032	0.362346998904999	1	pseudobulk	DESeq2	LRT	0
698	KC_AB1	pr	1.16638296700886	0.167041236866278	1	pseudobulk	DESeq2	LRT	0
698	KC_AB1	PR-Set7	-0.122781985177196	0.822877346042864	1	pseudobulk	DESeq2	LRT	0
698	KC_AB1	prage	-0.134787270699961	0.723128593641826	1	pseudobulk	DESeq2	LRT	0
698	KC_AB1	PRAS40	0.637482620342034	0.402791988908903	1	pseudobulk	DESeq2	LRT	0
698	KC_AB1	PrBP	0.140241046114751	0.619009205369424	1	pseudobulk	DESeq2	LRT	0
698	KC_AB1	prd1	0.101894981804184	0.808782445701104	1	pseudobulk	DESeq2	LRT	0
698	KC_AB1	pre-lola-G	1.47734119370697	0.566150893513107	1	pseudobulk	DESeq2	LRT	0
699	KC_AB1	pre-mod(mdg4)-AE	0.0160940546605174	1	1	pseudobulk	DESeq2	LRT	0
699	KC_AB1	pre-mod(mdg4)-B	1.51872195779367	0.623907569846402	1	pseudobulk	DESeq2	LRT	0
699	KC_AB1	pre-rRNA:CR4584	-0.745141523368405	0.135761565181708	1	pseudobulk	DESeq2	LRT	0
699	KC_AB1	pre-rRNA:CR4584	0.849606887838128	1	1	pseudobulk	DESeq2	LRT	0
699	KC_AB1	pre-rRNA:CR4585	0.767072704705723	0.838310513697889	1	pseudobulk	DESeq2	LRT	0

699	KC_AB1	prel	-0.348076269798111	0.304610019647564	1	pseudobulk	DESeq2	LRT	0
699	KC_AB1	Prestin	-1.1341274676294	0.301552504811729	1	pseudobulk	DESeq2	LRT	0
699	KC_AB1	prg	-0.223583624427108	0.706878563356622	1	pseudobulk	DESeq2	LRT	0
699	KC_AB1	prim	0.0431116680264898	1	1	pseudobulk	DESeq2	LRT	0
699	KC_AB1	PRL-1	0.258349420875912	0.598756353290972	1	pseudobulk	DESeq2	LRT	0
700	KC_AB1	Proc	0.849606887838128	1	1	pseudobulk	DESeq2	LRT	0
700	KC_AB1	Proc-R	0.849606887838128	1	1	pseudobulk	DESeq2	LRT	0
700	KC_AB1	prod	0.50823807359332	0.517648939506062	1	pseudobulk	DESeq2	LRT	0
700	KC_AB1	prominin-like	-0.171147524340216	0.451758565620243	1	pseudobulk	DESeq2	LRT	0
700	KC_AB1	ProRS-m	-1.34356802107991	0.397036566329918	1	pseudobulk	DESeq2	LRT	0
700	KC_AB1	pros	-0.182078570469194	0.109733272002161	1	pseudobulk	DESeq2	LRT	0
700	KC_AB1	Prosalpha1	3.16697464674408	0.406679230261689	1	pseudobulk	DESeq2	LRT	0
700	KC_AB1	Prosalpha2	-0.23690413674034	0.718899130453029	1	pseudobulk	DESeq2	LRT	0
700	KC_AB1	Prosalpha3	-0.210128498373684	0.575257373998751	1	pseudobulk	DESeq2	LRT	0
700	KC_AB1	Prosalpha4	0.159638990127864	0.410482711745921	1	pseudobulk	DESeq2	LRT	0
701	KC_AB1	Prosalpha5	-0.325756458700849	0.626914495550864	1	pseudobulk	DESeq2	LRT	0
701	KC_AB1	Prosalpha6	-0.0227955490493941	0.716488054127066	1	pseudobulk	DESeq2	LRT	0
701	KC_AB1	Prosalpha7	-0.376650150761951	0.557422535386238	1	pseudobulk	DESeq2	LRT	0
701	KC_AB1	Prosap	0.597262811665037	0.00480369798161405	1	pseudobulk	DESeq2	LRT	0
701	KC_AB1	Prosbeta1	-0.729269912822167	0.343006109455343	1	pseudobulk	DESeq2	LRT	0
701	KC_AB1	Prosbeta2	0.342872207148614	0.632078510751937	1	pseudobulk	DESeq2	LRT	0
701	KC_AB1	Prosbeta3	-0.115108243420125	0.711970655540704	1	pseudobulk	DESeq2	LRT	0
701	KC_AB1	Prosbeta4	-1.0754482938588	0.134423338568103	1	pseudobulk	DESeq2	LRT	0
701	KC_AB1	Prosbeta5	-0.324411672103434	0.513664703299732	1	pseudobulk	DESeq2	LRT	0
701	KC_AB1	Prosbeta6	-0.847783427610009	0.392494010078528	1	pseudobulk	DESeq2	LRT	0
702	KC_AB1	Prosbeta7	0.334811650957251	0.600400461529605	1	pseudobulk	DESeq2	LRT	0
702	KC_AB1	Prp18	-0.214248408928537	0.789189911630375	1	pseudobulk	DESeq2	LRT	0
702	KC_AB1	Prp19	-0.612153025738031	0.486784126002262	1	pseudobulk	DESeq2	LRT	0
702	KC_AB1	Prp3	-0.0408772846165774	0.672145398785013	1	pseudobulk	DESeq2	LRT	0
702	KC_AB1	Prp31	0.605588102876756	0.590307962106747	1	pseudobulk	DESeq2	LRT	0
702	KC_AB1	Prp38	-0.244538614247051	0.743870759193885	1	pseudobulk	DESeq2	LRT	0
702	KC_AB1	Prp8	-1.18653481105616	0.15608311852668	1	pseudobulk	DESeq2	LRT	0
702	KC_AB1	Prpk	1.78459775991824	0.460382591080016	1	pseudobulk	DESeq2	LRT	0
702	KC_AB1	prt	0.0016540908440421	0.751505011888077	1	pseudobulk	DESeq2	LRT	0
702	KC_AB1	prtp	0.134384790978207	0.743737528129261	1	pseudobulk	DESeq2	LRT	0
703	KC_AB1	Prx2540-1	0.183159795463042	1	1	pseudobulk	DESeq2	LRT	0
703	KC_AB1	Prx3	0.361309477516182	0.478634100159488	1	pseudobulk	DESeq2	LRT	0
703	KC_AB1	Prx6005	0.542982564383309	1	1	pseudobulk	DESeq2	LRT	0
703	KC_AB1	PRY	0.849606887838128	1	1	pseudobulk	DESeq2	LRT	0
703	KC_AB1	ps	0.152441735963134	0.227090734880215	1	pseudobulk	DESeq2	LRT	0
703	KC_AB1	Psa	-0.132223180624778	0.630040123780461	1	pseudobulk	DESeq2	LRT	0
703	KC_AB1	Psc	0.836949295300604	0.0950519346149728	1	pseudobulk	DESeq2	LRT	0
703	KC_AB1	Psf3	-0.565682355907419	0.423637202557863	1	pseudobulk	DESeq2	LRT	0
703	KC_AB1	PsGEF	-0.692707240390329	0.0820946640857786	1	pseudobulk	DESeq2	LRT	0
703	KC_AB1	psh	2.06541384650887	0.795866856626082	1	pseudobulk	DESeq2	LRT	0
704	KC_AB1	Psi	-0.257306560604277	0.344375064864918	1	pseudobulk	DESeq2	LRT	0
704	KC_AB1	psidin	0.582334128505502	0.48834518461246	1	pseudobulk	DESeq2	LRT	0
704	KC_AB1	PSMG1	-0.657432611377816	0.47907278836477	1	pseudobulk	DESeq2	LRT	0
704	KC_AB1	Psn	0.0641974905009382	0.653900639886944	1	pseudobulk	DESeq2	LRT	0
704	KC_AB1	psq	-0.0846913279391742	0.559429819629503	1	pseudobulk	DESeq2	LRT	0
704	KC_AB1	PSR	-0.0720672141636919	0.742584927973948	1	pseudobulk	DESeq2	LRT	0
704	KC_AB1	pst	1.65792752010766	0.762646744542806	1	pseudobulk	DESeq2	LRT	0
704	KC_AB1	Pstk	0.849606887838128	1	1	pseudobulk	DESeq2	LRT	0

704	KC_AB1	ptc	0.849606887838128	1	1	pseudobulk	DESeq2	LRT	0
704	KC_AB1	Pten	-0.0591293006105575	0.931490847231547	1	pseudobulk	DESeq2	LRT	0
705	KC_AB1	Ptip	0.464522363848461	0.347740920746395	1	pseudobulk	DESeq2	LRT	0
705	KC_AB1	PTP-ER	-0.431203585842617	0.299022136768439	1	pseudobulk	DESeq2	LRT	0
705	KC_AB1	Ptp10D	-1.05548521056871	0.62314630615428	1	pseudobulk	DESeq2	LRT	0
705	KC_AB1	Ptp36E	-0.633735026138048	0.106069992018659	1	pseudobulk	DESeq2	LRT	0
705	KC_AB1	Ptp4E	-1.87209452280324	0.0363708324249744	1	pseudobulk	DESeq2	LRT	0
705	KC_AB1	Ptp61F	0.186355951253862	0.766409319054276	1	pseudobulk	DESeq2	LRT	0
705	KC_AB1	Ptp69D	-1.09373832468931	0.315755783917328	1	pseudobulk	DESeq2	LRT	0
705	KC_AB1	Ptp99A	-0.334050568319432	0.0291578105162809	1	pseudobulk	DESeq2	LRT	0
705	KC_AB1	Ptpa	0.894371001607383	0.425944278725807	1	pseudobulk	DESeq2	LRT	0
705	KC_AB1	Ptpmeg	-0.183086285859791	0.471310310602041	1	pseudobulk	DESeq2	LRT	0
706	KC_AB1	Ptpmeg2	-0.389168792733194	0.0808316888975908	1	pseudobulk	DESeq2	LRT	0
706	KC_AB1	PTPMT1	0.92991223033755	0.399420053913338	1	pseudobulk	DESeq2	LRT	0
706	KC_AB1	Ptr	0.849606887838128	1	1	pseudobulk	DESeq2	LRT	0
706	KC_AB1	Pu	0.254819465507032	0.734715792417897	1	pseudobulk	DESeq2	LRT	0
706	KC_AB1	puc	-0.673660512880576	0.383056799051166	1	pseudobulk	DESeq2	LRT	0
706	KC_AB1	puf	0.526092011379953	0.315187689408266	1	pseudobulk	DESeq2	LRT	0
706	KC_AB1	pug	2.78674512494791	0.54962276837928	1	pseudobulk	DESeq2	LRT	0
706	KC_AB1	pum	-0.0163398713735684	0.856804641972036	1	pseudobulk	DESeq2	LRT	0
706	KC_AB1	Pur-alpha	-0.0493942803608638	0.69737513352408	1	pseudobulk	DESeq2	LRT	0
706	KC_AB1	Pura	-0.802742798496205	0.638576459732922	1	pseudobulk	DESeq2	LRT	0
707	KC_AB1	Pus1	0.7379233744128	0.6641704780724	1	pseudobulk	DESeq2	LRT	0
707	KC_AB1	Pus7	-2.24335095517823	0.185518392119573	1	pseudobulk	DESeq2	LRT	0
707	KC_AB1	put	-0.0111455992049675	0.50854223766879	1	pseudobulk	DESeq2	LRT	0
707	KC_AB1	Pvf1	-2.07348984597132	0.403224287902523	1	pseudobulk	DESeq2	LRT	0
707	KC_AB1	Pvf3	0.136910309152538	0.686549361940454	1	pseudobulk	DESeq2	LRT	0
707	KC_AB1	Pvr	1.00693893053477	1	1	pseudobulk	DESeq2	LRT	0
707	KC_AB1	PVRAP	-0.102172639632795	0.526219928423481	1	pseudobulk	DESeq2	LRT	0
707	KC_AB1	pwn	-0.216210029276812	1	1	pseudobulk	DESeq2	LRT	0
707	KC_AB1	px	-0.419061399399565	0.139656130652074	1	pseudobulk	DESeq2	LRT	0
707	KC_AB1	pxb	-1.56421291392065	0.00486662471535507	1	pseudobulk	DESeq2	LRT	0
708	KC_AB1	Pxn	0.4377509867174	0.0445054754886408	1	pseudobulk	DESeq2	LRT	0
708	KC_AB1	pyd	0.256091532050311	0.438125512267573	1	pseudobulk	DESeq2	LRT	0
708	KC_AB1	pyd3	0.183159795463042	1	1	pseudobulk	DESeq2	LRT	0
708	KC_AB1	pygo	0.535308236430704	0.310975169243833	1	pseudobulk	DESeq2	LRT	0
708	KC_AB1	PyK	-0.104310299893216	0.487443138865174	1	pseudobulk	DESeq2	LRT	0
708	KC_AB1	Pym	-1.74961725376103	0.0946980375560994	1	pseudobulk	DESeq2	LRT	0
708	KC_AB1	pyr	3.41844045086015	0.351942490385471	1	pseudobulk	DESeq2	LRT	0
708	KC_AB1	Pyroxd1	1.17271416152056	0.20659585973501	1	pseudobulk	DESeq2	LRT	0
708	KC_AB1	pzg	-0.138708404490241	0.652888048227929	1	pseudobulk	DESeq2	LRT	0
708	KC_AB1	Pzl	-1.43638698438186	0.241727179932665	1	pseudobulk	DESeq2	LRT	0
709	KC_AB1	QC	-3.44613056289704	0.320125970675908	1	pseudobulk	DESeq2	LRT	0
709	KC_AB1	QIL1	-0.0297685608134804	0.632135270489769	1	pseudobulk	DESeq2	LRT	0
709	KC_AB1	qin	-1.5585037442691	0.52226429478726	1	pseudobulk	DESeq2	LRT	0
709	KC_AB1	qkr54B	-0.266739399210497	0.379642443749234	1	pseudobulk	DESeq2	LRT	0
709	KC_AB1	qkr58E-1	-0.331458694328128	0.2919808925532	1	pseudobulk	DESeq2	LRT	0
709	KC_AB1	qkr58E-2	0.0921657349832417	0.598703682082677	1	pseudobulk	DESeq2	LRT	0
709	KC_AB1	qkr58E-3	-0.148100913701943	0.394036728003915	1	pseudobulk	DESeq2	LRT	0
709	KC_AB1	qless	-0.31805678250188	0.672260224438401	1	pseudobulk	DESeq2	LRT	0
709	KC_AB1	qm	-0.393130880751361	0.0297462051869349	1	pseudobulk	DESeq2	LRT	0
709	KC_AB1	qsm	-1.52328167018149	0.418644940957251	1	pseudobulk	DESeq2	LRT	0
710	KC_AB1	Qsox1	-0.0647148625227483	0.731737806035068	1	pseudobulk	DESeq2	LRT	0

710	KC_AB1	qtc	0.157408721782592	0.544058466998316	1	pseudobulk	DESeq2	LRT	0
710	KC_AB1	Qtzl	-0.287824199420804	0.605176122051499	1	pseudobulk	DESeq2	LRT	0
710	KC_AB1	qvr	-0.193363321263589	0.270805537419972	1	pseudobulk	DESeq2	LRT	0
710	KC_AB1	r	0.849606887838128	1	1	pseudobulk	DESeq2	LRT	0
710	KC_AB1	r-cup	2.31997030691033	0.813169507846918	1	pseudobulk	DESeq2	LRT	0
710	KC_AB1	r-l	-0.216209988775614	1	1	pseudobulk	DESeq2	LRT	0
710	KC_AB1	r2d2	-0.353850736726277	0.615374051897567	1	pseudobulk	DESeq2	LRT	0
710	KC_AB1	Rab1	-0.37781332896609	0.21904386101554	1	pseudobulk	DESeq2	LRT	0
710	KC_AB1	Rab10	-0.0334149015885338	0.648733380906148	1	pseudobulk	DESeq2	LRT	0
711	KC_AB1	Rab11	-0.0726309701817262	0.527934894412664	1	pseudobulk	DESeq2	LRT	0
711	KC_AB1	Rab14	0.0077280788198415	0.499650741001831	1	pseudobulk	DESeq2	LRT	0
711	KC_AB1	Rab18	-0.226589292046001	0.561676302976399	1	pseudobulk	DESeq2	LRT	0
711	KC_AB1	Rab19	-0.236640475195339	0.697984743240616	1	pseudobulk	DESeq2	LRT	0
711	KC_AB1	Rab2	0.169536252828466	0.419379168064939	1	pseudobulk	DESeq2	LRT	0
711	KC_AB1	Rab21	-0.121478422591209	0.795319464497469	1	pseudobulk	DESeq2	LRT	0
711	KC_AB1	Rab23	0.75551953162779	1	1	pseudobulk	DESeq2	LRT	0
711	KC_AB1	Rab26	-0.090511949308065	0.486141602285479	1	pseudobulk	DESeq2	LRT	0
711	KC_AB1	Rab27	-0.140624198358197	0.44472746402857	1	pseudobulk	DESeq2	LRT	0
711	KC_AB1	Rab3	-0.176890622503297	0.347191985991843	1	pseudobulk	DESeq2	LRT	0
712	KC_AB1	Rab3-GAP	0.120055169989382	1	1	pseudobulk	DESeq2	LRT	0
712	KC_AB1	Rab3-GEF	-0.166693717425667	0.387903096753543	1	pseudobulk	DESeq2	LRT	0
712	KC_AB1	Rab30	-0.152478761293484	0.694529225224489	1	pseudobulk	DESeq2	LRT	0
712	KC_AB1	Rab35	-0.117568774571609	0.577678043231447	1	pseudobulk	DESeq2	LRT	0
712	KC_AB1	Rab39	-0.118878892419994	0.715386269881582	1	pseudobulk	DESeq2	LRT	0
712	KC_AB1	Rab4	0.0613653427110029	0.744965578439292	1	pseudobulk	DESeq2	LRT	0
712	KC_AB1	Rab40	-0.80972386218254	0.325753097241478	1	pseudobulk	DESeq2	LRT	0
712	KC_AB1	Rab5	-0.335688308118107	0.194857769788756	1	pseudobulk	DESeq2	LRT	0
712	KC_AB1	Rab6	-0.385287827915648	0.593273285281553	1	pseudobulk	DESeq2	LRT	0
712	KC_AB1	Rab7	-0.461085368586046	0.373563603286737	1	pseudobulk	DESeq2	LRT	0
713	KC_AB1	Rab8	-0.413029356142424	0.326317411826624	1	pseudobulk	DESeq2	LRT	0
713	KC_AB1	Rab9	2.48413894093893	0.550425935634389	1	pseudobulk	DESeq2	LRT	0
713	KC_AB1	Rab9E	0.849606887838128	1	1	pseudobulk	DESeq2	LRT	0
713	KC_AB1	Rabex-5	-0.818419573769359	0.388635910101963	1	pseudobulk	DESeq2	LRT	0
713	KC_AB1	RabX1	-0.258888108339888	0.722569022605331	1	pseudobulk	DESeq2	LRT	0
713	KC_AB1	RabX4	-0.160775362628236	0.524944491259745	1	pseudobulk	DESeq2	LRT	0
713	KC_AB1	RabX6	0.671991174662694	0.608859476042514	1	pseudobulk	DESeq2	LRT	0
713	KC_AB1	Rac1	-0.0923664041587701	0.523515486016121	1	pseudobulk	DESeq2	LRT	0
713	KC_AB1	Rac2	-0.441535490434487	0.758560476543402	1	pseudobulk	DESeq2	LRT	0
713	KC_AB1	Rack1	0.377454532042021	0.197834164068224	1	pseudobulk	DESeq2	LRT	0
714	KC_AB1	rad	0.143934629757881	0.245810394129138	1	pseudobulk	DESeq2	LRT	0
714	KC_AB1	Rad17	-0.741993850594816	0.595811787875558	1	pseudobulk	DESeq2	LRT	0
714	KC_AB1	Rad23	-0.015757978205729	0.779392770073625	1	pseudobulk	DESeq2	LRT	0
714	KC_AB1	rad50	1.59863986092676	1	1	pseudobulk	DESeq2	LRT	0
714	KC_AB1	Rad51D	0.849606887838128	1	1	pseudobulk	DESeq2	LRT	0
714	KC_AB1	Rad60	-0.766945597483069	0.417906052936886	1	pseudobulk	DESeq2	LRT	0
714	KC_AB1	Rad9	1.59863986092676	1	1	pseudobulk	DESeq2	LRT	0
714	KC_AB1	Rae1	0.950997565265736	0.599528221544687	1	pseudobulk	DESeq2	LRT	0
714	KC_AB1	Raf	-0.742762439170123	0.417527396989406	1	pseudobulk	DESeq2	LRT	0
714	KC_AB1	RAF2	1.32020132756379	0.269194147466131	1	pseudobulk	DESeq2	LRT	0
715	KC_AB1	RagA-B	2.37262253842278	0.189192960286764	1	pseudobulk	DESeq2	LRT	0
715	KC_AB1	RagC-D	0.174528369384528	0.776756605683118	1	pseudobulk	DESeq2	LRT	0
715	KC_AB1	Rala	-0.297634660274566	0.550237659642798	1	pseudobulk	DESeq2	LRT	0
715	KC_AB1	Ran	0.140583103842013	0.518960182286397	1	pseudobulk	DESeq2	LRT	0

715	KC_AB1	Ranbp16	-0.148233250349072	0.56250731563189	1	pseudobulk	DESeq2	LRT		0
715	KC_AB1	Ranbp21	0.729034232407747	0.59644752182463	1	pseudobulk	DESeq2	LRT		0
715	KC_AB1	RanBP3	-0.415175678645826	0.514536779723435	1	pseudobulk	DESeq2	LRT		0
715	KC_AB1	Ranbp9	0.366247714696288	1	1	pseudobulk	DESeq2	LRT		0
715	KC_AB1	RanBPM	-0.110519109118622	0.817276185086696	1	pseudobulk	DESeq2	LRT		0
715	KC_AB1	RanGAP	0.849606887838128	1	1	pseudobulk	DESeq2	LRT		0
716	KC_AB1	ranshi	1.11528148199936	1	1	pseudobulk	DESeq2	LRT		0
716	KC_AB1	Rap1	-0.279976058740354	0.122477313956215	1	pseudobulk	DESeq2	LRT		0
716	KC_AB1	Rap2l	-0.0976548282265042	0.624903767881145	1	pseudobulk	DESeq2	LRT		0
716	KC_AB1	RapGAP1	0.590140214674122	0.460022737984308	1	pseudobulk	DESeq2	LRT		0
716	KC_AB1	raptor	-0.535734181807533	0.28214033977639	1	pseudobulk	DESeq2	LRT		0
716	KC_AB1	ras	-0.831972490470811	0.265973636319231	1	pseudobulk	DESeq2	LRT		0
716	KC_AB1	Ras64B	-0.42638856183834	0.594406802650265	1	pseudobulk	DESeq2	LRT		0
716	KC_AB1	Ras85D	-0.212820796834812	0.528307659565767	1	pseudobulk	DESeq2	LRT		0
716	KC_AB1	RasGAP1	-0.74005688588577	0.188982651837808	1	pseudobulk	DESeq2	LRT		0
716	KC_AB1	raskol	-0.090990505773043	0.681788808458267	1	pseudobulk	DESeq2	LRT		0
717	KC_AB1	rasp	0.849606887838128	1	1	pseudobulk	DESeq2	LRT		0
717	KC_AB1	Rassf	0.111567204326936	0.896370033002226	1	pseudobulk	DESeq2	LRT		0
717	KC_AB1	RASSF8	-0.519029144313021	0.239991025177519	1	pseudobulk	DESeq2	LRT		0
717	KC_AB1	Rat1	0.297718510215775	0.72775664511409	1	pseudobulk	DESeq2	LRT		0
717	KC_AB1	rau	0.25655505129873	1	1	pseudobulk	DESeq2	LRT		0
717	KC_AB1	Ravus	-0.330146162967311	0.653577158535797	1	pseudobulk	DESeq2	LRT		0
717	KC_AB1	raw	-0.386047635209658	0.310059207610682	1	pseudobulk	DESeq2	LRT		0
717	KC_AB1	rb	-0.438689923996792	0.433946426927557	1	pseudobulk	DESeq2	LRT		0
717	KC_AB1	Rb97D	-0.079194495802531	0.55383730286325	1	pseudobulk	DESeq2	LRT		0
717	KC_AB1	Rbbp5	0.17965685793534	0.857273346240851	1	pseudobulk	DESeq2	LRT		0
718	KC_AB1	Rbcn-3A	-0.110312117902904	0.610398605945342	1	pseudobulk	DESeq2	LRT		0
718	KC_AB1	Rbcn-3B	-0.309166210518841	0.481257770775901	1	pseudobulk	DESeq2	LRT		0
718	KC_AB1	Rbf	-0.368655130997252	0.575722516867115	1	pseudobulk	DESeq2	LRT		0
718	KC_AB1	Rbf2	0.116783351990007	1	1	pseudobulk	DESeq2	LRT		0
718	KC_AB1	Rbfox1	-0.166259383622563	0.237562158557394	1	pseudobulk	DESeq2	LRT		0
718	KC_AB1	Rbm13	0.366247714696288	1	1	pseudobulk	DESeq2	LRT		0
718	KC_AB1	Rbp	-0.190422784284542	0.209469966067116	1	pseudobulk	DESeq2	LRT		0
718	KC_AB1	Rbp1	0.0712856988589752	0.530254043469803	1	pseudobulk	DESeq2	LRT		0
718	KC_AB1	Rbp1-like	-0.1206633623824	0.339001317190396	1	pseudobulk	DESeq2	LRT		0
718	KC_AB1	Rbp4	0.849606887838128	1	1	pseudobulk	DESeq2	LRT		0
719	KC_AB1	Rbp6	-0.167575517856582	0.673434421971205	1	pseudobulk	DESeq2	LRT		0
719	KC_AB1	Rbp9	-0.707909469835012	0.0553290609669283	1	pseudobulk	DESeq2	LRT		0
719	KC_AB1	Rbpn-5	-0.077202042234653	0.766164445330033	1	pseudobulk	DESeq2	LRT		0
719	KC_AB1	Rbsn-5	0.139137721203631	0.735450396072849	1	pseudobulk	DESeq2	LRT		0
719	KC_AB1	Rca1	-0.021895394044706	0.631079107256769	1	pseudobulk	DESeq2	LRT		0
719	KC_AB1	Rcc1	-0.005104890235929	0.912011569114181	1	pseudobulk	DESeq2	LRT		0
719	KC_AB1	Rcd-1	0.299553517822897	0.428406512272663	1	pseudobulk	DESeq2	LRT		0
719	KC_AB1	Rcd1	-0.145156148132892	0.796864643138523	1	pseudobulk	DESeq2	LRT		0
719	KC_AB1	Rcd4	-0.657045599389527	0.551487751013772	1	pseudobulk	DESeq2	LRT		0
719	KC_AB1	Rcd5	0.105095081223215	0.678800116349874	1	pseudobulk	DESeq2	LRT		0
720	KC_AB1	Rcd7	0.849606887838128	1	1	pseudobulk	DESeq2	LRT		0
720	KC_AB1	Rchy1	0.849606887838128	1	1	pseudobulk	DESeq2	LRT		0
720	KC_AB1	Rcp	-1.27945444963978	0.340680489877414	1	pseudobulk	DESeq2	LRT		0
720	KC_AB1	rdgA	-1.02679823286643	0.389666650863642	1	pseudobulk	DESeq2	LRT		0
720	KC_AB1	rdgB	0.115891781151351	0.551435599674774	1	pseudobulk	DESeq2	LRT		0
720	KC_AB1	rdgBbeta	-0.308044425871942	0.335997022239118	1	pseudobulk	DESeq2	LRT		0
720	KC_AB1	rdgC	-0.870454738828866	0.0897001339687301	1	pseudobulk	DESeq2	LRT		0

720	KC_AB1	Rdh	0.157758626750089	0.503445899320195	1	pseudobulk	DESeq2	LRT	0
720	KC_AB1	Rdl	-0.14151500516405	0.26058357843179	1	pseudobulk	DESeq2	LRT	0
720	KC_AB1	rdo	-0.386999196237009	0.787275937133681	1	pseudobulk	DESeq2	LRT	0
721	KC_AB1	rdog	0.731815619342312	0.547063214252526	1	pseudobulk	DESeq2	LRT	0
721	KC_AB1	rdx	-0.229813473981327	0.316785173982243	1	pseudobulk	DESeq2	LRT	0
721	KC_AB1	rec	-0.563283233299884	0.572138612169023	1	pseudobulk	DESeq2	LRT	0
721	KC_AB1	Reck	-0.216210029276812	1	1	pseudobulk	DESeq2	LRT	0
721	KC_AB1	RecQ5	-0.402888138702669	0.695248156450173	1	pseudobulk	DESeq2	LRT	0
721	KC_AB1	red	-0.83925561170226	0.261013767218169	1	pseudobulk	DESeq2	LRT	0
721	KC_AB1	ReepA	-0.50689099714615	0.293946163362789	1	pseudobulk	DESeq2	LRT	0
721	KC_AB1	ReepB	-0.191320760263862	0.450868695119799	1	pseudobulk	DESeq2	LRT	0
721	KC_AB1	ref(2)P	-0.101503139482799	0.700184773169723	1	pseudobulk	DESeq2	LRT	0
721	KC_AB1	Ref1	0.132182741191506	0.660841539270903	1	pseudobulk	DESeq2	LRT	0
722	KC_AB1	REG	-0.301518898502706	0.473993312795162	1	pseudobulk	DESeq2	LRT	0
722	KC_AB1	Reg-5	4.11599205977059	0.175712481619698	1	pseudobulk	DESeq2	LRT	0
722	KC_AB1	regucalcin	0.780994196176025	0.837010123424364	1	pseudobulk	DESeq2	LRT	0
722	KC_AB1	Rel	-1.11516056362467	0.439781280573908	1	pseudobulk	DESeq2	LRT	0
722	KC_AB1	rempA	2.31997030691033	0.813169507846918	1	pseudobulk	DESeq2	LRT	0
722	KC_AB1	Rep	0.319778093921225	0.796101526359694	1	pseudobulk	DESeq2	LRT	0
722	KC_AB1	Reph	-0.15535358724973	0.360140885946417	1	pseudobulk	DESeq2	LRT	0
722	KC_AB1	repo	0.292882946602088	1	1	pseudobulk	DESeq2	LRT	0
722	KC_AB1	Reps	-0.113361398539933	0.839142838911922	1	pseudobulk	DESeq2	LRT	0
722	KC_AB1	rept	1.81827378179382	0.686482515515535	1	pseudobulk	DESeq2	LRT	0
723	KC_AB1	REPTOR	-0.526675435149103	0.117988913988902	1	pseudobulk	DESeq2	LRT	0
723	KC_AB1	REPTOR-BP	-0.842687483655458	0.121031852716051	1	pseudobulk	DESeq2	LRT	0
723	KC_AB1	retm	0.427887461451812	0.260867085068812	1	pseudobulk	DESeq2	LRT	0
723	KC_AB1	retn	0.930460990050852	0.504395459799573	1	pseudobulk	DESeq2	LRT	0
723	KC_AB1	Rev1	0.849606887838128	1	1	pseudobulk	DESeq2	LRT	0
723	KC_AB1	Rev7	0.849606887838128	1	1	pseudobulk	DESeq2	LRT	0
723	KC_AB1	Rexo5	0.875756382658116	0.596220893260083	1	pseudobulk	DESeq2	LRT	0
723	KC_AB1	RfC3	0.798346791900698	0.822165887287426	1	pseudobulk	DESeq2	LRT	0
723	KC_AB1	RfC38	-0.0715439913912816	0.829681542650881	1	pseudobulk	DESeq2	LRT	0
723	KC_AB1	RfC4	0.358207943231009	0.776648286178879	1	pseudobulk	DESeq2	LRT	0
724	KC_AB1	RFeSP	0.0642923170044407	0.537946110227654	1	pseudobulk	DESeq2	LRT	0
724	KC_AB1	Rfx	0.43727432428607	0.835785598476836	1	pseudobulk	DESeq2	LRT	0
724	KC_AB1	rg	0.129181880773239	0.512771951954021	1	pseudobulk	DESeq2	LRT	0
724	KC_AB1	Rga	-0.235791253914083	0.511081023950981	1	pseudobulk	DESeq2	LRT	0
724	KC_AB1	Rgk1	-0.190768896255946	0.143830100742124	1	pseudobulk	DESeq2	LRT	0
724	KC_AB1	Rgk2	0.449448880079957	0.153527700296387	1	pseudobulk	DESeq2	LRT	0
724	KC_AB1	Rgk3	-0.47808525191744	0.101605864074557	1	pseudobulk	DESeq2	LRT	0
724	KC_AB1	Rgl	-0.114363962969248	0.525031952225082	1	pseudobulk	DESeq2	LRT	0
724	KC_AB1	rgn	-0.201577958629491	0.440919148533232	1	pseudobulk	DESeq2	LRT	0
724	KC_AB1	Rh4	2.43275481406755	0.550258497427138	1	pseudobulk	DESeq2	LRT	0
725	KC_AB1	Rh5	0.849606887838128	1	1	pseudobulk	DESeq2	LRT	0
725	KC_AB1	Rh50	0.51792430742098	0.352464608799499	1	pseudobulk	DESeq2	LRT	0
725	KC_AB1	Rh7	1.93706461079025	0.658817357590498	1	pseudobulk	DESeq2	LRT	0
725	KC_AB1	Rhau	1.75080248448828	0.603717156600772	1	pseudobulk	DESeq2	LRT	0
725	KC_AB1	rhea	-0.285185742318569	0.42868735854998	1	pseudobulk	DESeq2	LRT	0
725	KC_AB1	Rheb	-0.246639845949427	0.53239343297257	1	pseudobulk	DESeq2	LRT	0
725	KC_AB1	rho	1.42479196492156	0.909837944709441	1	pseudobulk	DESeq2	LRT	0
725	KC_AB1	rho-4	-1.28587202470122	0.364515457674196	1	pseudobulk	DESeq2	LRT	0
725	KC_AB1	rho-5	0.235452346252857	0.52319499917514	1	pseudobulk	DESeq2	LRT	0
725	KC_AB1	rho-7	0.788699787867468	0.285214525989055	1	pseudobulk	DESeq2	LRT	0

726	KC_AB1	Rho1	-0.179026090584369	0.558910241662727	1	pseudobulk	DESeq2	LRT		0
726	KC_AB1	RhoBTB	-0.16742812275816	0.639571351940996	1	pseudobulk	DESeq2	LRT		0
726	KC_AB1	RhoGAP100F	-0.361238939523587	0.155162207473074	1	pseudobulk	DESeq2	LRT		0
726	KC_AB1	RhoGAP102A	-0.371596612934922	0.477218437232998	1	pseudobulk	DESeq2	LRT		0
726	KC_AB1	RhoGAP15B	0.849606887838128	1	1	pseudobulk	DESeq2	LRT		0
726	KC_AB1	RhoGAP16F	0.492222556853792	0.98869368368255	1	pseudobulk	DESeq2	LRT		0
726	KC_AB1	RhoGAP18B	-1.0485618136326	0.711820015438189	1	pseudobulk	DESeq2	LRT		0
726	KC_AB1	RhoGAP19D	0.0735898168739448	0.757379091635265	1	pseudobulk	DESeq2	LRT		0
726	KC_AB1	RhoGAP1A	-0.58925581801865	0.334619010398064	1	pseudobulk	DESeq2	LRT		0
726	KC_AB1	RhoGAP5A	-0.727830925545998	0.513681631628161	1	pseudobulk	DESeq2	LRT		0
727	KC_AB1	RhoGAP68F	-0.39754680182178	0.49046635846457	1	pseudobulk	DESeq2	LRT		0
727	KC_AB1	RhoGAP71E	-0.184401785897397	0.390000094870926	1	pseudobulk	DESeq2	LRT		0
727	KC_AB1	RhoGAP92B	-0.623086794568854	0.422910221834668	1	pseudobulk	DESeq2	LRT		0
727	KC_AB1	RhoGAP93B	-0.219074390236962	0.330752917348902	1	pseudobulk	DESeq2	LRT		0
727	KC_AB1	RhoGAPp190	-0.306122406082304	0.263414578532958	1	pseudobulk	DESeq2	LRT		0
727	KC_AB1	RhoGDI	0.0050884366272620	0.7725706506568	1	pseudobulk	DESeq2	LRT		0
727	KC_AB1	RhoGEF2	-1.40307130648885	0.334300496344265	1	pseudobulk	DESeq2	LRT		0
727	KC_AB1	RhoGEF3	-0.0708675657561872	0.62589787074073	1	pseudobulk	DESeq2	LRT		0
727	KC_AB1	RhoGEF64C	0.366247714696288	1	1	pseudobulk	DESeq2	LRT		0
727	KC_AB1	RhoL	-1.12499981461738	0.601851881127761	1	pseudobulk	DESeq2	LRT		0
728	KC_AB1	RhoU	-0.040670535102592	0.798452037634844	1	pseudobulk	DESeq2	LRT		0
728	KC_AB1	Rhp	-1.94227467810108	0.215984733869739	1	pseudobulk	DESeq2	LRT		0
728	KC_AB1	Ric	0.0111953348145854	0.893478181959382	1	pseudobulk	DESeq2	LRT		0
728	KC_AB1	RIC-3	-0.720158753787283	0.129184107631788	1	pseudobulk	DESeq2	LRT		0
728	KC_AB1	ric8a	-0.451944941184641	0.612406080694431	1	pseudobulk	DESeq2	LRT		0
728	KC_AB1	Rich	-1.05719555676598	0.34453609247126	1	pseudobulk	DESeq2	LRT		0
728	KC_AB1	rictor	0.0221576704521799	0.737098657734204	1	pseudobulk	DESeq2	LRT		0
728	KC_AB1	Rif1	0.0640469555519605	1	1	pseudobulk	DESeq2	LRT		0
728	KC_AB1	Rift	0.275866436402766	0.751357050155192	1	pseudobulk	DESeq2	LRT		0
728	KC_AB1	rig	-0.105026257456743	0.73732738624999	1	pseudobulk	DESeq2	LRT		0
729	KC_AB1	Rilpl	0.48189877337899	0.497125773783096	1	pseudobulk	DESeq2	LRT		0
729	KC_AB1	Rim	-0.176677624651675	0.137734408063233	1	pseudobulk	DESeq2	LRT		0
729	KC_AB1	Rim2	0.369351900629761	0.316699569201275	1	pseudobulk	DESeq2	LRT		0
729	KC_AB1	rin	-0.0181563766277961	0.933801564249066	1	pseudobulk	DESeq2	LRT		0
729	KC_AB1	ringer	-0.349884432714647	0.264725308102422	1	pseudobulk	DESeq2	LRT		0
729	KC_AB1	Rint1	1.68354035716633	0.485954601043398	1	pseudobulk	DESeq2	LRT		0
729	KC_AB1	RIOK1	1.02230943207598	0.656086514238543	1	pseudobulk	DESeq2	LRT		0
729	KC_AB1	RIOK2	-0.347486708516507	0.687214598147674	1	pseudobulk	DESeq2	LRT		0
729	KC_AB1	Rip11	-0.819200008649513	0.230325036299473	1	pseudobulk	DESeq2	LRT		0
729	KC_AB1	Ripalpha	-0.589217779974244	0.464120770042604	1	pseudobulk	DESeq2	LRT		0
730	KC_AB1	rk	0.0817903838308293	0.749874047259748	1	pseudobulk	DESeq2	LRT		0
730	KC_AB1	rl	-0.0238482112352378	0.851394318873166	1	pseudobulk	DESeq2	LRT		0
730	KC_AB1	Rlb1	0.415682685046798	0.598046900490151	1	pseudobulk	DESeq2	LRT		0
730	KC_AB1	Rlip	-0.143524689246425	0.779811593128457	1	pseudobulk	DESeq2	LRT		0
730	KC_AB1	RluA-2	-1.12762045096436	0.331846442416899	1	pseudobulk	DESeq2	LRT		0
730	KC_AB1	Rm62	-0.300011404582076	0.202570377560206	1	pseudobulk	DESeq2	LRT		0
730	KC_AB1	Rme-8	-0.182085915413681	0.70412461372529	1	pseudobulk	DESeq2	LRT		0
730	KC_AB1	rn	-1.57593973285425	0.187135088831406	1	pseudobulk	DESeq2	LRT		0
730	KC_AB1	RN-tre	-0.194227493692983	0.743638707598017	1	pseudobulk	DESeq2	LRT		0
730	KC_AB1	RNASEK	-0.089803427340651	0.560653652088813	1	pseudobulk	DESeq2	LRT		0
731	KC_AB1	RNaseMRP:RNA	1.86149361285156	0.488727711818157	1	pseudobulk	DESeq2	LRT		0
731	KC_AB1	RNaseP:RNA	0.849606887838128	1	1	pseudobulk	DESeq2	LRT		0
731	KC_AB1	RNaseX25	-0.240995035236427	0.635658325371749	1	pseudobulk	DESeq2	LRT		0

731	KC_AB1	RNaseZ	1.32260900172455	0.734821849617029	1	pseudobulk	DESeq2	LRT		0
731	KC_AB1	Rnb	0.256518445573614	0.785583894135635	1	pseudobulk	DESeq2	LRT		0
731	KC_AB1	Rnf146	0.15802413936286	0.653659370925933	1	pseudobulk	DESeq2	LRT		0
731	KC_AB1	rngo	0.875332955154018	0.289474740635687	1	pseudobulk	DESeq2	LRT		0
731	KC_AB1	rnh1	-1.33470454049406	0.475328403325108	1	pseudobulk	DESeq2	LRT		0
731	KC_AB1	Rnmt	0.770509610991622	1	1	pseudobulk	DESeq2	LRT		0
731	KC_AB1	rno	0.0304403650437352	0.858567077460784	1	pseudobulk	DESeq2	LRT		0
732	KC_AB1	Rnp4F	-0.0463889582327253	0.674356431591066	1	pseudobulk	DESeq2	LRT		0
732	KC_AB1	RnpS1	-0.803924813607377	0.319734061372018	1	pseudobulk	DESeq2	LRT		0
732	KC_AB1	RnrL	0.849606887838128	1	1	pseudobulk	DESeq2	LRT		0
732	KC_AB1	ro	-0.216209988775614	1	1	pseudobulk	DESeq2	LRT		0
732	KC_AB1	robl	-0.069430178019769	0.746005383192744	1	pseudobulk	DESeq2	LRT		0
732	KC_AB1	robo1	-0.725995587648524	0.117464849792974	1	pseudobulk	DESeq2	LRT		0
732	KC_AB1	robo2	-0.216210029276812	1	1	pseudobulk	DESeq2	LRT		0
732	KC_AB1	robo3	0.849606887838128	1	1	pseudobulk	DESeq2	LRT		0
732	KC_AB1	Roc1a	-0.0694805668724652	0.534938460748931	1	pseudobulk	DESeq2	LRT		0
732	KC_AB1	Roc2	-0.0351438448589127	0.531589370728024	1	pseudobulk	DESeq2	LRT		0
733	KC_AB1	Roe1	-0.120027454467546	0.670204290951667	1	pseudobulk	DESeq2	LRT		0
733	KC_AB1	rogdi	-0.309349013511306	0.612089982526178	1	pseudobulk	DESeq2	LRT		0
733	KC_AB1	roh	0.165280141055779	0.373475963536602	1	pseudobulk	DESeq2	LRT		0
733	KC_AB1	Rok	0.0770724780885894	0.894084196919227	1	pseudobulk	DESeq2	LRT		0
733	KC_AB1	Rop	-0.105767260634384	0.758092917293572	1	pseudobulk	DESeq2	LRT		0
733	KC_AB1	roq	-0.372419755297015	0.46785012300624	1	pseudobulk	DESeq2	LRT		0
733	KC_AB1	Ror	-0.258773325872832	0.692955666894801	1	pseudobulk	DESeq2	LRT		0
733	KC_AB1	rost	2.78674512494791	0.54962276837928	1	pseudobulk	DESeq2	LRT		0
733	KC_AB1	row	0.0291387528714784	0.60825454901402	1	pseudobulk	DESeq2	LRT		0
733	KC_AB1	Rox8	0.415779305759137	0.315141106491531	1	pseudobulk	DESeq2	LRT		0
734	KC_AB1	RpA-70	-1.01388386037552	0.410248828376099	1	pseudobulk	DESeq2	LRT		0
734	KC_AB1	RPA2	0.849606887838128	1	1	pseudobulk	DESeq2	LRT		0
734	KC_AB1	RPA3	0.0017374500558627	1	1	pseudobulk	DESeq2	LRT		0
734	KC_AB1	Rpb10	-0.118201994887184	0.602438423206688	1	pseudobulk	DESeq2	LRT		0
734	KC_AB1	Rpb11	1.11981634634947	0.219730743120825	1	pseudobulk	DESeq2	LRT		0
734	KC_AB1	Rpb12	-0.116489365071067	0.656150199121829	1	pseudobulk	DESeq2	LRT		0
734	KC_AB1	Rpb4	0.366247714696288	1	1	pseudobulk	DESeq2	LRT		0
734	KC_AB1	Rpb5	1.59134387529418	0.136076392364227	1	pseudobulk	DESeq2	LRT		0
734	KC_AB1	Rpb7	0.362640122270604	0.521967933616589	1	pseudobulk	DESeq2	LRT		0
734	KC_AB1	Rpb8	0.214937657039429	0.725645692988221	1	pseudobulk	DESeq2	LRT		0
735	KC_AB1	Rpe	-0.572809606158377	0.787374717193114	1	pseudobulk	DESeq2	LRT		0
735	KC_AB1	Rph	0.134683573172532	0.59978009219182	1	pseudobulk	DESeq2	LRT		0
735	KC_AB1	Rpi	0.849606887838128	1	1	pseudobulk	DESeq2	LRT		0
735	KC_AB1	RpI1	0.656251763380176	1	1	pseudobulk	DESeq2	LRT		0
735	KC_AB1	RpI12	0.237022465848507	1	1	pseudobulk	DESeq2	LRT		0
735	KC_AB1	RpI135	-0.283054125455946	1	1	pseudobulk	DESeq2	LRT		0
735	KC_AB1	RpII140	-0.460598334516251	0.585477704517409	1	pseudobulk	DESeq2	LRT		0
735	KC_AB1	RpII15	-0.122752190812722	0.711293526079174	1	pseudobulk	DESeq2	LRT		0
735	KC_AB1	RpII18	-0.200661311006819	0.572387881064878	1	pseudobulk	DESeq2	LRT		0
735	KC_AB1	RpII215	-0.0406714268854195	0.712292375275357	1	pseudobulk	DESeq2	LRT		0
736	KC_AB1	RpII33	0.181544514765763	0.621285423768789	1	pseudobulk	DESeq2	LRT		0
736	KC_AB1	RpIII128	-0.673973387349693	0.737308747629933	1	pseudobulk	DESeq2	LRT		0
736	KC_AB1	RpIIIC160	-0.645776819068304	0.634258639138015	1	pseudobulk	DESeq2	LRT		0
736	KC_AB1	RpIIIC53	-0.89573457214911	0.467837256518422	1	pseudobulk	DESeq2	LRT		0
736	KC_AB1	RpL10	-0.208548027261411	0.336702677568002	1	pseudobulk	DESeq2	LRT		0
736	KC_AB1	RpL10Ab	-0.336848333071806	0.23390424402468	1	pseudobulk	DESeq2	LRT		0

736	KC_AB1	RpL11	-0.25681688218007	0.466196282638555	1	pseudobulk	DESeq2	LRT		0
736	KC_AB1	RpL12	-0.085654193906202	0.748850745263139	1	pseudobulk	DESeq2	LRT		0
736	KC_AB1	RpL13	-0.453392998513595	0.0730892256663207	1	pseudobulk	DESeq2	LRT		0
736	KC_AB1	RpL13A	-0.129563085315092	0.470897848298668	1	pseudobulk	DESeq2	LRT		0
737	KC_AB1	RpL14	-0.205353050266062	0.368260089415146	1	pseudobulk	DESeq2	LRT		0
737	KC_AB1	RpL15	-0.275591418365151	0.185761336581102	1	pseudobulk	DESeq2	LRT		0
737	KC_AB1	RpL17	-0.114232487862585	0.582658516666945	1	pseudobulk	DESeq2	LRT		0
737	KC_AB1	RpL18	-0.034797714399289	0.580876829345893	1	pseudobulk	DESeq2	LRT		0
737	KC_AB1	RpL18A	-0.165132902271553	0.531372052917102	1	pseudobulk	DESeq2	LRT		0
737	KC_AB1	RpL19	-0.255707536437989	0.376553069216003	1	pseudobulk	DESeq2	LRT		0
737	KC_AB1	RpL21	-0.11828398470976	0.539629231822697	1	pseudobulk	DESeq2	LRT		0
737	KC_AB1	RpL22	-0.402630724819791	0.28438022315029	1	pseudobulk	DESeq2	LRT		0
737	KC_AB1	RpL23	0.0116605543788536	0.89127518270864	1	pseudobulk	DESeq2	LRT		0
737	KC_AB1	RpL23A	-0.329788476777854	0.389342409264886	1	pseudobulk	DESeq2	LRT		0
738	KC_AB1	RpL24	-0.232976890408168	0.29076351115267	1	pseudobulk	DESeq2	LRT		0
738	KC_AB1	RpL24-like	-0.160733210564238	0.4896955513412	1	pseudobulk	DESeq2	LRT		0
738	KC_AB1	RpL26	-0.365486703003448	0.179380881139572	1	pseudobulk	DESeq2	LRT		0
738	KC_AB1	RpL27	-0.204438764736328	0.484752798020583	1	pseudobulk	DESeq2	LRT		0
738	KC_AB1	RpL27A	-0.068559558931037	0.744533505938609	1	pseudobulk	DESeq2	LRT		0
738	KC_AB1	RpL28	-0.036116783270081	0.629757391101459	1	pseudobulk	DESeq2	LRT		0
738	KC_AB1	RpL29	0.126941412479457	0.603497000610535	1	pseudobulk	DESeq2	LRT		0
738	KC_AB1	RpL3	-0.054247389077999	0.483834878885337	1	pseudobulk	DESeq2	LRT		0
738	KC_AB1	RpL30	-0.158484875732295	0.747900062476957	1	pseudobulk	DESeq2	LRT		0
738	KC_AB1	RpL31	-0.072489256514272	0.660692701124514	1	pseudobulk	DESeq2	LRT		0
739	KC_AB1	RpL32	-0.307698011543767	0.242785479325247	1	pseudobulk	DESeq2	LRT		0
739	KC_AB1	RpL34a	-0.654881746015355	0.307456715132417	1	pseudobulk	DESeq2	LRT		0
739	KC_AB1	RpL34b	-0.296639686987247	0.246017385158804	1	pseudobulk	DESeq2	LRT		0
739	KC_AB1	RpL35	-0.118579826398	0.549482059707899	1	pseudobulk	DESeq2	LRT		0
739	KC_AB1	RpL35A	-0.327583849806271	0.257347420624639	1	pseudobulk	DESeq2	LRT		0
739	KC_AB1	RpL36	-0.118719920076495	0.37452711700415	1	pseudobulk	DESeq2	LRT		0
739	KC_AB1	RpL36A	-0.3673673525998	0.20844236044773	1	pseudobulk	DESeq2	LRT		0
739	KC_AB1	RpL37a	0.0058576633165831	0.52044145684189	1	pseudobulk	DESeq2	LRT		0
739	KC_AB1	RpL37A	-0.116088611935118	0.565533017033136	1	pseudobulk	DESeq2	LRT		0
739	KC_AB1	RpL37b	-0.216210032712384	1	1	pseudobulk	DESeq2	LRT		0
740	KC_AB1	RpL38	-0.070452161629898	0.72036850387381	1	pseudobulk	DESeq2	LRT		0
740	KC_AB1	RpL39	0.0185070686376227	0.574597030164957	1	pseudobulk	DESeq2	LRT		0
740	KC_AB1	RpL4	0.0871758317944919	0.684785424264377	1	pseudobulk	DESeq2	LRT		0
740	KC_AB1	RpL40	-0.21208723472656	0.297465147462791	1	pseudobulk	DESeq2	LRT		0
740	KC_AB1	RpL41	0.0975778957666786	0.555490458040954	1	pseudobulk	DESeq2	LRT		0
740	KC_AB1	RpL5	-0.056641285674099	0.733096731853469	1	pseudobulk	DESeq2	LRT		0
740	KC_AB1	RpL6	-0.074003111454248	0.706572447522117	1	pseudobulk	DESeq2	LRT		0
740	KC_AB1	RpL7	-0.121019948125262	0.454516892205884	1	pseudobulk	DESeq2	LRT		0
740	KC_AB1	RpL7A	-0.294431983968536	0.329015404298193	1	pseudobulk	DESeq2	LRT		0
740	KC_AB1	RpL8	-0.22463065187546	0.342539823377851	1	pseudobulk	DESeq2	LRT		0
741	KC_AB1	RpL9	-0.114202548111541	0.668448091477625	1	pseudobulk	DESeq2	LRT		0
741	KC_AB1	RpLP0	-0.087454309775941	0.539207404541319	1	pseudobulk	DESeq2	LRT		0
741	KC_AB1	RpLP0-like	-0.023676338005484	0.844188641027155	1	pseudobulk	DESeq2	LRT		0
741	KC_AB1	RpLP1	0.0371167853144663	0.657831269864395	1	pseudobulk	DESeq2	LRT		0
741	KC_AB1	RpLP2	0.6700239147914	0.0321400474241272	1	pseudobulk	DESeq2	LRT		0
741	KC_AB1	Rpn1	-1.26210955747876	0.152107943458692	1	pseudobulk	DESeq2	LRT		0
741	KC_AB1	Rpn10	0.104909792625032	0.797739597610327	1	pseudobulk	DESeq2	LRT		0
741	KC_AB1	Rpn11	-1.04922040700903	0.188538060029867	1	pseudobulk	DESeq2	LRT		0
741	KC_AB1	Rpn12	0.418147130619095	0.542616159039098	1	pseudobulk	DESeq2	LRT		0

741	KC_AB1	Rpn13	-0.37230286598031	0.499438953364563	1	pseudobulk	DESeq2	LRT	0
742	KC_AB1	Rpn13R	0.486164817500061	1	1	pseudobulk	DESeq2	LRT	0
742	KC_AB1	Rpn2	-0.185124107139369	0.579920974826314	1	pseudobulk	DESeq2	LRT	0
742	KC_AB1	Rpn3	-0.994846383244859	0.32286799365088	1	pseudobulk	DESeq2	LRT	0
742	KC_AB1	Rpn5	-0.392197912601751	0.477060742558421	1	pseudobulk	DESeq2	LRT	0
742	KC_AB1	Rpn6	0.296191661066464	0.323434416888197	1	pseudobulk	DESeq2	LRT	0
742	KC_AB1	Rpn7	-1.12192187043604	0.229663291982436	1	pseudobulk	DESeq2	LRT	0
742	KC_AB1	Rpn8	0.143608499929801	0.747115092749609	1	pseudobulk	DESeq2	LRT	0
742	KC_AB1	Rpn9	0.21819089121536	0.552864966314878	1	pseudobulk	DESeq2	LRT	0
742	KC_AB1	Rpp30	0.542245173624972	1	1	pseudobulk	DESeq2	LRT	0
742	KC_AB1	rpr	0.849606887838128	1	1	pseudobulk	DESeq2	LRT	0
743	KC_AB1	RpS10b	-0.0943030623641481	0.692679881215829	1	pseudobulk	DESeq2	LRT	0
743	KC_AB1	RpS11	-0.0224214792108721	0.829925583082552	1	pseudobulk	DESeq2	LRT	0
743	KC_AB1	RpS12	-0.341237476228318	0.27537836434991	1	pseudobulk	DESeq2	LRT	0
743	KC_AB1	RpS13	0.0494273767591244	0.685189411367936	1	pseudobulk	DESeq2	LRT	0
743	KC_AB1	RpS14a	-0.0129031186757309	0.881038183277307	1	pseudobulk	DESeq2	LRT	0
743	KC_AB1	RpS14b	-0.0412310301355811	0.920336629127221	1	pseudobulk	DESeq2	LRT	0
743	KC_AB1	RpS15	0.0815665971623755	0.696053793953103	1	pseudobulk	DESeq2	LRT	0
743	KC_AB1	RpS15Aa	-0.124809637238236	0.44227433460961	1	pseudobulk	DESeq2	LRT	0
743	KC_AB1	RpS15Ab	0.293918637510868	0.61769506231194	1	pseudobulk	DESeq2	LRT	0
743	KC_AB1	RpS16	0.448739527602142	0.26619673031706	1	pseudobulk	DESeq2	LRT	0
744	KC_AB1	RpS17	-0.270862041243399	0.32512861394098	1	pseudobulk	DESeq2	LRT	0
744	KC_AB1	RpS18	-0.0277503165740938	0.562990099049561	1	pseudobulk	DESeq2	LRT	0
744	KC_AB1	RpS19a	-0.251386369347466	0.373941412935457	1	pseudobulk	DESeq2	LRT	0
744	KC_AB1	RpS2	0.0943489323689513	0.621238487689601	1	pseudobulk	DESeq2	LRT	0
744	KC_AB1	RpS20	-0.0743116876028237	0.628546362608451	1	pseudobulk	DESeq2	LRT	0
744	KC_AB1	RpS21	-0.364924763154941	0.235799670643216	1	pseudobulk	DESeq2	LRT	0
744	KC_AB1	RpS23	0.141978598127473	0.518655148322822	1	pseudobulk	DESeq2	LRT	0
744	KC_AB1	RpS24	0.0288023719617855	0.898386535482371	1	pseudobulk	DESeq2	LRT	0
744	KC_AB1	RpS25	0.117673960984466	0.474751736170017	1	pseudobulk	DESeq2	LRT	0
744	KC_AB1	RpS26	-0.128612546542269	0.504327991852435	1	pseudobulk	DESeq2	LRT	0
745	KC_AB1	RpS27	-0.0053074421066339	0.904968242808051	1	pseudobulk	DESeq2	LRT	0
745	KC_AB1	RpS27A	-0.126091367201732	0.57101972053165	1	pseudobulk	DESeq2	LRT	0
745	KC_AB1	RpS28b	-0.257161803092	0.224633581605428	1	pseudobulk	DESeq2	LRT	0
745	KC_AB1	RpS29	-0.163243850880508	0.375810353827734	1	pseudobulk	DESeq2	LRT	0
745	KC_AB1	RpS3	-0.24182466291108	0.344066435201304	1	pseudobulk	DESeq2	LRT	0
745	KC_AB1	RpS30	-0.181310605154163	0.441286032057584	1	pseudobulk	DESeq2	LRT	0
745	KC_AB1	RpS3A	-0.18002260006626	0.429353606440801	1	pseudobulk	DESeq2	LRT	0
745	KC_AB1	RpS4	-0.144942147794624	0.410456385449059	1	pseudobulk	DESeq2	LRT	0
745	KC_AB1	RpS5a	-0.310713503791858	0.373504840499941	1	pseudobulk	DESeq2	LRT	0
745	KC_AB1	RpS6	0.0717036725431138	0.754030919388817	1	pseudobulk	DESeq2	LRT	0
746	KC_AB1	RpS7	0.112645974940533	0.707758360299783	1	pseudobulk	DESeq2	LRT	0
746	KC_AB1	RpS8	0.215128235624807	0.378023794621715	1	pseudobulk	DESeq2	LRT	0
746	KC_AB1	RpS9	0.0564166120914465	0.497508968967747	1	pseudobulk	DESeq2	LRT	0
746	KC_AB1	Rpt1	0.349848353167452	0.469718304913792	1	pseudobulk	DESeq2	LRT	0
746	KC_AB1	Rpt2	-0.953057678078586	0.321149829235597	1	pseudobulk	DESeq2	LRT	0
746	KC_AB1	Rpt3	-0.222651279687997	0.650786884217589	1	pseudobulk	DESeq2	LRT	0
746	KC_AB1	Rpt4	0.0854769678630748	0.653761022973609	1	pseudobulk	DESeq2	LRT	0
746	KC_AB1	Rpt5	-0.619664601089613	0.377537462322698	1	pseudobulk	DESeq2	LRT	0
746	KC_AB1	Rpt6	0.133324574845037	0.604428204322143	1	pseudobulk	DESeq2	LRT	0
746	KC_AB1	Rrp1	-0.45560956407913	0.710292978199623	1	pseudobulk	DESeq2	LRT	0
747	KC_AB1	Rrp4	2.48417587387807	0.622507045344447	1	pseudobulk	DESeq2	LRT	0
747	KC_AB1	Rrp40	-0.216210029276812	1	1	pseudobulk	DESeq2	LRT	0

747	KC_AB1	Rrp42	1.63265388209318	0.641062427469355	1	pseudobulk	DESeq2	LRT	0
747	KC_AB1	Rrp46	-0.517122225038665	0.714347075545985	1	pseudobulk	DESeq2	LRT	0
747	KC_AB1	Rrp47	-0.0258969186795672	0.967701640596786	1	pseudobulk	DESeq2	LRT	0
747	KC_AB1	Rrp6	-1.87616864159618	0.269403214098234	1	pseudobulk	DESeq2	LRT	0
747	KC_AB1	Rs1	0.382681250973463	1	1	pseudobulk	DESeq2	LRT	0
747	KC_AB1	Rsf1	-0.21917460185257	0.393122682904071	1	pseudobulk	DESeq2	LRT	0
747	KC_AB1	RSG7	-0.0457619162705474	0.670926919289062	1	pseudobulk	DESeq2	LRT	0
747	KC_AB1	rst	-0.964145141546908	0.587253772095583	1	pseudobulk	DESeq2	LRT	0
748	KC_AB1	rswl	0.239768019893098	0.727756872002239	1	pseudobulk	DESeq2	LRT	0
748	KC_AB1	rt	0.84098351489873	0.425711898176477	1	pseudobulk	DESeq2	LRT	0
748	KC_AB1	Rtc1	0.860799096402194	0.587698301860433	1	pseudobulk	DESeq2	LRT	0
748	KC_AB1	Rtca	-0.319262073526839	0.593849752982816	1	pseudobulk	DESeq2	LRT	0
748	KC_AB1	RtcB	-0.573505995193938	0.633939057885262	1	pseudobulk	DESeq2	LRT	0
748	KC_AB1	rtet	0.0160940546605626	1	1	pseudobulk	DESeq2	LRT	0
748	KC_AB1	Rtf1	-0.117898668540474	0.71548695024306	1	pseudobulk	DESeq2	LRT	0
748	KC_AB1	RtGEF	0.825118387024964	0.385558796246124	1	pseudobulk	DESeq2	LRT	0
748	KC_AB1	Rtnl1	0.0341851830047826	0.852894628343497	1	pseudobulk	DESeq2	LRT	0
748	KC_AB1	rtv	-1.5805998677877	0.524230026009756	1	pseudobulk	DESeq2	LRT	0
749	KC_AB1	Rubicon	-0.700946196752172	0.449835743781622	1	pseudobulk	DESeq2	LRT	0
749	KC_AB1	rudhira	-0.188833396715522	0.373927104774084	1	pseudobulk	DESeq2	LRT	0
749	KC_AB1	rumi	0.963579652842034	0.657127222388853	1	pseudobulk	DESeq2	LRT	0
749	KC_AB1	rump	-0.104721728517022	0.528063068115342	1	pseudobulk	DESeq2	LRT	0
749	KC_AB1	rush	-0.645048010351892	0.433960551532011	1	pseudobulk	DESeq2	LRT	0
749	KC_AB1	rut	-0.0534473817125742	0.640489108611912	1	pseudobulk	DESeq2	LRT	0
749	KC_AB1	rux	-0.20866271369863	0.767550289513843	1	pseudobulk	DESeq2	LRT	0
749	KC_AB1	RYa-R	2.31997030691033	0.813169507846918	1	pseudobulk	DESeq2	LRT	0
749	KC_AB1	RYBP	0.593998293083043	0.434491963845852	1	pseudobulk	DESeq2	LRT	0
749	KC_AB1	RyR	-0.44416571436678	0.370108407114682	1	pseudobulk	DESeq2	LRT	0
750	KC_AB1	S	0.411539108064754	0.35690865698977	1	pseudobulk	DESeq2	LRT	0
750	KC_AB1	s-cup	0.114800176438394	0.623239655508264	1	pseudobulk	DESeq2	LRT	0
750	KC_AB1	S-Lap1	-1.04206097169077	0.465082236160534	1	pseudobulk	DESeq2	LRT	0
750	KC_AB1	S-Lap4	1.59863986092676	1	1	pseudobulk	DESeq2	LRT	0
750	KC_AB1	S1P	-0.469036981332096	0.651809389400378	1	pseudobulk	DESeq2	LRT	0
750	KC_AB1	S2P	-0.0323258764968137	0.558483768684517	1	pseudobulk	DESeq2	LRT	0
750	KC_AB1	S6k	0.0754523393618803	0.836799345444456	1	pseudobulk	DESeq2	LRT	0
750	KC_AB1	S6kII	-0.378466047949224	0.49802179882244	1	pseudobulk	DESeq2	LRT	0
750	KC_AB1	S6KL	-0.933906249197389	0.208694716636484	1	pseudobulk	DESeq2	LRT	0
750	KC_AB1	SA	0.119219013132199	0.71051867222398	1	pseudobulk	DESeq2	LRT	0
751	KC_AB1	Sac1	-0.384551268587328	0.582737969940857	1	pseudobulk	DESeq2	LRT	0
751	KC_AB1	Saf-B	-0.377053950004795	0.256611489818443	1	pseudobulk	DESeq2	LRT	0
751	KC_AB1	Saf6	-1.42358156167198	0.262096499848807	1	pseudobulk	DESeq2	LRT	0
751	KC_AB1	sage	-1.09348562801015	0.567616642317403	1	pseudobulk	DESeq2	LRT	0
751	KC_AB1	SAK	-0.429068474506832	0.66273262048826	1	pseudobulk	DESeq2	LRT	0
751	KC_AB1	sals	0.262328543990178	0.265730984744824	1	pseudobulk	DESeq2	LRT	0
751	KC_AB1	Sam-S	-0.156622365054121	0.502762255313318	1	pseudobulk	DESeq2	LRT	0
751	KC_AB1	SamDC	-0.115088558056369	0.607647084808177	1	pseudobulk	DESeq2	LRT	0
751	KC_AB1	Samtor	-1.05992744849371	0.613288832576002	1	pseudobulk	DESeq2	LRT	0
751	KC_AB1	Samuel	-0.457622207737357	0.755249650709674	1	pseudobulk	DESeq2	LRT	0
752	KC_AB1	san	-0.0344922224001349	0.935119227128976	1	pseudobulk	DESeq2	LRT	0
752	KC_AB1	santa-maria	-2.42327466222736	0.171313845981994	1	pseudobulk	DESeq2	LRT	0
752	KC_AB1	Sap-r	-0.421817382198931	0.358484651324587	1	pseudobulk	DESeq2	LRT	0
752	KC_AB1	Sap130	0.0357324446422338	0.704411403423131	1	pseudobulk	DESeq2	LRT	0
752	KC_AB1	Sap30	0.0650848660217826	0.706282147470002	1	pseudobulk	DESeq2	LRT	0

752	KC_AB1	Sap47	-0.151684511540136	0.381940198967549	1	pseudobulk	DESeq2	LRT	0
752	KC_AB1	Sar1	-0.206794190184619	0.612491550544025	1	pseudobulk	DESeq2	LRT	0
752	KC_AB1	Sara	-0.431589753597687	0.527282656143486	1	pseudobulk	DESeq2	LRT	0
752	KC_AB1	sas	-0.830062897938551	0.717724257445671	1	pseudobulk	DESeq2	LRT	0
752	KC_AB1	Sas-6	0.849606887838128	1	1	pseudobulk	DESeq2	LRT	0
753	KC_AB1	Sas10	-0.579712491063223	0.610167200226413	1	pseudobulk	DESeq2	LRT	0
753	KC_AB1	saturn	0.849606887838128	1	1	pseudobulk	DESeq2	LRT	0
753	KC_AB1	sau	-0.479326244354787	0.314565756293599	1	pseudobulk	DESeq2	LRT	0
753	KC_AB1	sav	2.85142250261825	0.484249113053674	1	pseudobulk	DESeq2	LRT	0
753	KC_AB1	sax	-0.302634085617808	0.548871178686482	1	pseudobulk	DESeq2	LRT	0
753	KC_AB1	sba	-0.339066354592828	0.265257695843715	1	pseudobulk	DESeq2	LRT	0
753	KC_AB1	Sbat	0.585042129706728	1	1	pseudobulk	DESeq2	LRT	0
753	KC_AB1	sbb	-0.125198168136398	0.406343738693461	1	pseudobulk	DESeq2	LRT	0
753	KC_AB1	Sbf	-0.492040133471866	0.38386067821346	1	pseudobulk	DESeq2	LRT	0
753	KC_AB1	sbm	-0.361947826780604	0.250297920930218	1	pseudobulk	DESeq2	LRT	0
754	KC_AB1	Sbp2	-0.645119008720676	0.513462314519051	1	pseudobulk	DESeq2	LRT	0
754	KC_AB1	sbr	-0.0569519826077023	0.831509532886809	1	pseudobulk	DESeq2	LRT	0
754	KC_AB1	Sc2	-0.823974020407549	0.327486693114296	1	pseudobulk	DESeq2	LRT	0
754	KC_AB1	SC35	-0.541686985352401	0.195075980381937	1	pseudobulk	DESeq2	LRT	0
754	KC_AB1	sca	-0.008297538593298	0.961403792286419	1	pseudobulk	DESeq2	LRT	0
754	KC_AB1	scaf	-0.361723760442942	0.819493995411841	1	pseudobulk	DESeq2	LRT	0
754	KC_AB1	scaf6	-0.40102885960024	0.255078569857081	1	pseudobulk	DESeq2	LRT	0
754	KC_AB1	SCaMC	-0.447909701031711	0.240963879187548	1	pseudobulk	DESeq2	LRT	0
754	KC_AB1	Scamp	0.0679878026226884	0.543418895054283	1	pseudobulk	DESeq2	LRT	0
754	KC_AB1	SCAP	-0.106310880219331	0.716652507399276	1	pseudobulk	DESeq2	LRT	0
755	KC_AB1	SCAR	0.00536555141260085	0.756823931754637	1	pseudobulk	DESeq2	LRT	0
755	KC_AB1	scat	-0.0207301249614513	0.765962378100522	1	pseudobulk	DESeq2	LRT	0
755	KC_AB1	scb	-0.0718207395094281	1	1	pseudobulk	DESeq2	LRT	0
755	KC_AB1	Sccpdh1	-0.175205757250305	0.644936725442904	1	pseudobulk	DESeq2	LRT	0
755	KC_AB1	SCCRO	-0.127165847065726	0.603576518740858	1	pseudobulk	DESeq2	LRT	0
755	KC_AB1	SCCRO3	-0.0436618197751423	0.936900336249807	1	pseudobulk	DESeq2	LRT	0
755	KC_AB1	SCCRO4	0.356992707669304	0.410699707343225	1	pseudobulk	DESeq2	LRT	0
755	KC_AB1	Sce	-0.142099931636617	0.408982194403648	1	pseudobulk	DESeq2	LRT	0
755	KC_AB1	sced	-0.120946169230353	0.653835898225812	1	pseudobulk	DESeq2	LRT	0
755	KC_AB1	scf	0.568001884307491	0.369413078059206	1	pseudobulk	DESeq2	LRT	0
756	KC_AB1	Scgalpha	-0.131446803346054	0.564474155589778	1	pseudobulk	DESeq2	LRT	0
756	KC_AB1	Scgbeta	0.41918964578296	0.218412999013832	1	pseudobulk	DESeq2	LRT	0
756	KC_AB1	Scgdelta	-1.54063829811534	0.215186797663147	1	pseudobulk	DESeq2	LRT	0
756	KC_AB1	Schip1	-0.481395993685209	0.21546934033128	1	pseudobulk	DESeq2	LRT	0
756	KC_AB1	schlank	0.147709645787183	0.598110870462746	1	pseudobulk	DESeq2	LRT	0
756	KC_AB1	Sclp	0.849606887838128	1	1	pseudobulk	DESeq2	LRT	0
756	KC_AB1	Scm	-0.498843334528998	0.374644461028786	1	pseudobulk	DESeq2	LRT	0
756	KC_AB1	scny	-0.107771165299369	0.590154269654399	1	pseudobulk	DESeq2	LRT	0
756	KC_AB1	SCOT	1.18250193032345	0.646464536809229	1	pseudobulk	DESeq2	LRT	0
756	KC_AB1	Scox	0.0332073858274988	0.700964888687296	1	pseudobulk	DESeq2	LRT	0
757	KC_AB1	Scp1	0.237022465848507	1	1	pseudobulk	DESeq2	LRT	0
757	KC_AB1	Scp2	-0.0065790798335215	1	1	pseudobulk	DESeq2	LRT	0
757	KC_AB1	scpr-C	0.849606887838128	1	1	pseudobulk	DESeq2	LRT	0
757	KC_AB1	ScpX	-0.350757847822397	0.726259974811167	1	pseudobulk	DESeq2	LRT	0
757	KC_AB1	scramb1	0.443503911884915	0.486381737418311	1	pseudobulk	DESeq2	LRT	0
757	KC_AB1	scramb2	0.631321991840965	0.326443745564363	1	pseudobulk	DESeq2	LRT	0
757	KC_AB1	scrib	-0.161719560919166	0.247194738311565	1	pseudobulk	DESeq2	LRT	0
757	KC_AB1	scro	2.43484285911389	0.528783108084678	1	pseudobulk	DESeq2	LRT	0

757	KC_AB1	scrt	-0.471372016910414	0.0678130995618523	1	pseudobulk	DESeq2	LRT		0
757	KC_AB1	Scsalpha1	0.159049907380913	0.590044846793185	1	pseudobulk	DESeq2	LRT		0
758	KC_AB1	ScsbetaA	0.199726230696657	0.532144377841378	1	pseudobulk	DESeq2	LRT		0
758	KC_AB1	ScsbetaG	0.247093001687432	1	1	pseudobulk	DESeq2	LRT		0
758	KC_AB1	scu	1.00588933998108	0.222454306283683	1	pseudobulk	DESeq2	LRT		0
758	KC_AB1	scyl	-0.383649117828722	0.0550895214154081	1	pseudobulk	DESeq2	LRT		0
758	KC_AB1	sd	-0.445847414810902	0.101511281543621	1	pseudobulk	DESeq2	LRT		0
758	KC_AB1	Sdc	0.127914417241593	0.364766044951859	1	pseudobulk	DESeq2	LRT		0
758	KC_AB1	SdhA	0.0151248258188302	0.413815110405965	1	pseudobulk	DESeq2	LRT		0
758	KC_AB1	Sdhaf3	-0.264809558323218	0.74490148388155	1	pseudobulk	DESeq2	LRT		0
758	KC_AB1	SdhB	-0.421767763067452	0.386509276520945	1	pseudobulk	DESeq2	LRT		0
758	KC_AB1	SdhC	-0.117540239333999	0.648757307239959	1	pseudobulk	DESeq2	LRT		0
759	KC_AB1	SdhD	-0.0539959854749832	0.727809583693918	1	pseudobulk	DESeq2	LRT		0
759	KC_AB1	SdicC	4.56074667486801	0.0868375374371263	1	pseudobulk	DESeq2	LRT		0
759	KC_AB1	sdk	-0.980741402264348	0.496824932793845	1	pseudobulk	DESeq2	LRT		0
759	KC_AB1	sds22	0.48771637511794	0.667535071415399	1	pseudobulk	DESeq2	LRT		0
759	KC_AB1	sdt	-0.338663651161805	0.0531833112383449	1	pseudobulk	DESeq2	LRT		0
759	KC_AB1	Sec10	0.555094465970481	0.370558754109079	1	pseudobulk	DESeq2	LRT		0
759	KC_AB1	Sec13	-0.107542210256923	0.607646535614178	1	pseudobulk	DESeq2	LRT		0
759	KC_AB1	Sec15	-1.45974796019564	0.327665000647492	1	pseudobulk	DESeq2	LRT		0
759	KC_AB1	Sec16	-0.205789545961641	0.46609575082471	1	pseudobulk	DESeq2	LRT		0
759	KC_AB1	Sec22	-0.0450285093167243	0.938869422714195	1	pseudobulk	DESeq2	LRT		0
760	KC_AB1	Sec23	-0.282418632083718	0.662248195583989	1	pseudobulk	DESeq2	LRT		0
760	KC_AB1	Sec24AB	0.154334522505679	0.634434457656701	1	pseudobulk	DESeq2	LRT		0
760	KC_AB1	Sec24CD	-0.4576071109593	0.593495151180824	1	pseudobulk	DESeq2	LRT		0
760	KC_AB1	Sec3	-1.14645364286717	0.361260277722296	1	pseudobulk	DESeq2	LRT		0
760	KC_AB1	Sec31	0.124160584331177	0.864657132333579	1	pseudobulk	DESeq2	LRT		0
760	KC_AB1	Sec5	-1.14398858683184	0.367965653853423	1	pseudobulk	DESeq2	LRT		0
760	KC_AB1	Sec6	-0.569676801057051	0.553140049297454	1	pseudobulk	DESeq2	LRT		0
760	KC_AB1	Sec61alpha	0.0514147165342068	0.744866391258548	1	pseudobulk	DESeq2	LRT		0
760	KC_AB1	Sec61beta	-0.0540910599957322	0.508295341587924	1	pseudobulk	DESeq2	LRT		0
760	KC_AB1	Sec61gamma	-0.228791814325093	0.697808705461139	1	pseudobulk	DESeq2	LRT		0
761	KC_AB1	Sec63	0.16172205088414	0.690259232350975	1	pseudobulk	DESeq2	LRT		0
761	KC_AB1	Sec71	-0.646881130358836	0.467201937224706	1	pseudobulk	DESeq2	LRT		0
761	KC_AB1	Sec8	-0.735359512862362	0.386651584654755	1	pseudobulk	DESeq2	LRT		0
761	KC_AB1	Secp43	-0.302333363417429	0.687194116531169	1	pseudobulk	DESeq2	LRT		0
761	KC_AB1	SecS	0.4703555700637	0.528313086048174	1	pseudobulk	DESeq2	LRT		0
761	KC_AB1	sei	-0.350115236984555	0.454842899589395	1	pseudobulk	DESeq2	LRT		0
761	KC_AB1	sel	-1.07674319282786	0.562491167513556	1	pseudobulk	DESeq2	LRT		0
761	KC_AB1	SelG	-0.0974099122873679	0.689534872578768	1	pseudobulk	DESeq2	LRT		0
761	KC_AB1	SelR	0.119944854132026	0.638696387917358	1	pseudobulk	DESeq2	LRT		0
761	KC_AB1	SelT	-0.393517168027883	0.380695758935429	1	pseudobulk	DESeq2	LRT		0
762	KC_AB1	Sem1	-0.256710220237475	0.706000548832684	1	pseudobulk	DESeq2	LRT		0
762	KC_AB1	Sema1a	0.0194835616901358	0.833825286654863	1	pseudobulk	DESeq2	LRT		0
762	KC_AB1	Sema1b	-1.04136881879332	0.12730453963938	1	pseudobulk	DESeq2	LRT		0
762	KC_AB1	Sema2a	-0.657231028989627	0.0598225120128566	1	pseudobulk	DESeq2	LRT		0
762	KC_AB1	Sema2b	-0.508440701386892	0.190295631938284	1	pseudobulk	DESeq2	LRT		0
762	KC_AB1	Sema5c	-0.216210029276812	1	1	pseudobulk	DESeq2	LRT		0
762	KC_AB1	senju	-0.849867248309013	0.455419448908674	1	pseudobulk	DESeq2	LRT		0
762	KC_AB1	Sep1	-0.37054074919485	0.519667053604104	1	pseudobulk	DESeq2	LRT		0
762	KC_AB1	Sep2	0.205980099682083	0.392085046298555	1	pseudobulk	DESeq2	LRT		0
762	KC_AB1	Sep4	-0.404545659112817	0.275007435029666	1	pseudobulk	DESeq2	LRT		0
763	KC_AB1	Sep5	0.849606887838128	1	1	pseudobulk	DESeq2	LRT		0

763	KC_AB1	SERCA	0.211462598673258	0.451711433042019	1	pseudobulk	DESeq2	LRT	0
763	KC_AB1	SerRS	-0.101775731914311	0.80923320701004	1	pseudobulk	DESeq2	LRT	0
763	KC_AB1	SerRS-m	-0.824349280752776	0.479475707585529	1	pseudobulk	DESeq2	LRT	0
763	KC_AB1	sesB	-0.06245106499417	0.529168990213575	1	pseudobulk	DESeq2	LRT	0
763	KC_AB1	Sesn	1.15833265070908	0.543474376660166	1	pseudobulk	DESeq2	LRT	0
763	KC_AB1	Set	0.0894606547460905	0.689927508425444	1	pseudobulk	DESeq2	LRT	0
763	KC_AB1	Set1	-0.169175748459944	0.48525721349822	1	pseudobulk	DESeq2	LRT	0
763	KC_AB1	Set2	0.195955901499936	0.651036078829808	1	pseudobulk	DESeq2	LRT	0
763	KC_AB1	Setd3	-0.461683291351368	0.597237443527085	1	pseudobulk	DESeq2	LRT	0
764	KC_AB1	SF1	-0.08186158698857	0.886205784787395	1	pseudobulk	DESeq2	LRT	0
764	KC_AB1	SF2	-0.123519118168613	0.710046322331843	1	pseudobulk	DESeq2	LRT	0
764	KC_AB1	Sf3a1	-0.459689881413936	0.545300407090097	1	pseudobulk	DESeq2	LRT	0
764	KC_AB1	Sf3a2	-0.073351721623788	0.828430334380357	1	pseudobulk	DESeq2	LRT	0
764	KC_AB1	Sf3b1	0.141099734149254	0.673666674469857	1	pseudobulk	DESeq2	LRT	0
764	KC_AB1	Sf3b2	0.279792354735535	0.649860292697632	1	pseudobulk	DESeq2	LRT	0
764	KC_AB1	Sf3b3	0.558750721830129	0.687982108624037	1	pseudobulk	DESeq2	LRT	0
764	KC_AB1	Sf3b5	0.0455828362601714	0.56192929321297	1	pseudobulk	DESeq2	LRT	0
764	KC_AB1	Sf3b6	-0.272351804908762	0.605209732286757	1	pseudobulk	DESeq2	LRT	0
764	KC_AB1	sff	-0.358000012854301	0.22200900782095	1	pseudobulk	DESeq2	LRT	0
765	KC_AB1	sfl	-0.715617893943095	0.0120666288553407	1	pseudobulk	DESeq2	LRT	0
765	KC_AB1	Sfmbt	0.222318044546553	0.735154829249801	1	pseudobulk	DESeq2	LRT	0
765	KC_AB1	Sfp33A4	0.849606887838128	1	1	pseudobulk	DESeq2	LRT	0
765	KC_AB1	Sfp78E	0.849606887838128	1	1	pseudobulk	DESeq2	LRT	0
765	KC_AB1	Sfxn1-3	-0.759172035765399	0.234108022132595	1	pseudobulk	DESeq2	LRT	0
765	KC_AB1	Sfxn2	-0.02792858271888	0.95866211997966	1	pseudobulk	DESeq2	LRT	0
765	KC_AB1	Sgf11	-0.596461209455093	0.507937193796327	1	pseudobulk	DESeq2	LRT	0
765	KC_AB1	Sgf29	-0.212068330934162	0.660769775598723	1	pseudobulk	DESeq2	LRT	0
765	KC_AB1	sgg	0.0540114977751526	0.593242077642748	1	pseudobulk	DESeq2	LRT	0
765	KC_AB1	sgl	-0.584837927172949	0.524685206626995	1	pseudobulk	DESeq2	LRT	0
766	KC_AB1	sgll	0.275364136893637	0.760355759632337	1	pseudobulk	DESeq2	LRT	0
766	KC_AB1	Sgs4	0.849606887838128	1	1	pseudobulk	DESeq2	LRT	0
766	KC_AB1	Sgs7	2.78674512494791	0.54962276837928	1	pseudobulk	DESeq2	LRT	0
766	KC_AB1	Sgsh	0.849606887838128	1	1	pseudobulk	DESeq2	LRT	0
766	KC_AB1	Sgt	0.691897077576647	0.225279731587231	1	pseudobulk	DESeq2	LRT	0
766	KC_AB1	Sgt1	-0.0508455837007998	0.606329179285398	1	pseudobulk	DESeq2	LRT	0
766	KC_AB1	Sh	-0.157418278381997	0.208429482333933	1	pseudobulk	DESeq2	LRT	0
766	KC_AB1	Sh3beta	0.197872642965637	0.606379747781665	1	pseudobulk	DESeq2	LRT	0
766	KC_AB1	SH3PX1	0.401366458847782	0.588536589075103	1	pseudobulk	DESeq2	LRT	0
766	KC_AB1	Shab	-0.281178301569902	0.0710792707110865	1	pseudobulk	DESeq2	LRT	0
767	KC_AB1	Shal	-0.351712994727455	0.134596693331085	1	pseudobulk	DESeq2	LRT	0
767	KC_AB1	shams	-0.133405888188449	0.818857506725242	1	pseudobulk	DESeq2	LRT	0
767	KC_AB1	Shark	0.849606887838128	1	1	pseudobulk	DESeq2	LRT	0
767	KC_AB1	Shaw	0.0874060045502578	0.750526593174252	1	pseudobulk	DESeq2	LRT	0
767	KC_AB1	Shawl	0.00777592767351765	0.593072028754773	1	pseudobulk	DESeq2	LRT	0
767	KC_AB1	Shc	0.122885340202896	0.775416331443529	1	pseudobulk	DESeq2	LRT	0
767	KC_AB1	shep	0.139667399090121	0.32280754172503	1	pseudobulk	DESeq2	LRT	0
767	KC_AB1	shf	0.849606887838128	1	1	pseudobulk	DESeq2	LRT	0
767	KC_AB1	shg	2.06541384650887	0.904905526327343	1	pseudobulk	DESeq2	LRT	0
767	KC_AB1	shi	-0.189047891621931	0.305911428761079	1	pseudobulk	DESeq2	LRT	0
768	KC_AB1	Shmt	-0.691810385964798	0.60559331091582	1	pseudobulk	DESeq2	LRT	0
768	KC_AB1	shn	-0.358948900842569	0.608806543224896	1	pseudobulk	DESeq2	LRT	0
768	KC_AB1	shop	0.256681053088514	0.766016545971763	1	pseudobulk	DESeq2	LRT	0
768	KC_AB1	shot	-0.0862656638314328	0.463818772860596	1	pseudobulk	DESeq2	LRT	0

768	KC_AB1	shrb	-0.344339798861947	0.485954867610258	1	pseudobulk	DESeq2	LRT		0
768	KC_AB1	Shrm	4.73786001624398	0.0628075777075593	1	pseudobulk	DESeq2	LRT		0
768	KC_AB1	shtd	1.59863986092676	1	1	pseudobulk	DESeq2	LRT		0
768	KC_AB1	shv	-1.87403163101145	0.196945796695454	1	pseudobulk	DESeq2	LRT		0
768	KC_AB1	SiaT	2.09975560790004	0.086872560090648	1	pseudobulk	DESeq2	LRT		0
768	KC_AB1	sicily	0.567764515121969	0.615159092254378	1	pseudobulk	DESeq2	LRT		0
769	KC_AB1	sick	0.539161342028913	0.188635667908171	1	pseudobulk	DESeq2	LRT		0
769	KC_AB1	side-II	-0.180068291121485	0.378378446184154	1	pseudobulk	DESeq2	LRT		0
769	KC_AB1	side-III	-0.166178566084359	0.289482756926978	1	pseudobulk	DESeq2	LRT		0
769	KC_AB1	side-IV	-0.393928018390381	0.405401474577691	1	pseudobulk	DESeq2	LRT		0
769	KC_AB1	side-V	-0.591494642095766	0.599791285541845	1	pseudobulk	DESeq2	LRT		0
769	KC_AB1	side-VI	-0.364351998773037	0.0207709727378157	1	pseudobulk	DESeq2	LRT		0
769	KC_AB1	side-VII	-0.0275863145047648	0.83241303632768	1	pseudobulk	DESeq2	LRT		0
769	KC_AB1	side-VIII	-1.29195350762757	0.486995519272942	1	pseudobulk	DESeq2	LRT		0
769	KC_AB1	SIDL	0.359485467403885	0.722547987513012	1	pseudobulk	DESeq2	LRT		0
769	KC_AB1	Sidpn	0.237215227651024	0.640375465762186	1	pseudobulk	DESeq2	LRT		0
770	KC_AB1	sif	-0.6866899370806	0.00639157385210755	1	pseudobulk	DESeq2	LRT		0
770	KC_AB1	SIFaR	0.453373536773301	0.543806159474784	1	pseudobulk	DESeq2	LRT		0
770	KC_AB1	sigmar	0.336156021912503	0.517780672334653	1	pseudobulk	DESeq2	LRT		0
770	KC_AB1	Sik2	-0.0759837166978183	0.686425885832556	1	pseudobulk	DESeq2	LRT		0
770	KC_AB1	Sik3	0.191669957824297	0.64832701257546	1	pseudobulk	DESeq2	LRT		0
770	KC_AB1	Sil1	0.106423585666807	0.819205962210525	1	pseudobulk	DESeq2	LRT		0
770	KC_AB1	sima	0.173671939376079	0.467071594698336	1	pseudobulk	DESeq2	LRT		0
770	KC_AB1	simj	-0.207737311426735	0.470883325111755	1	pseudobulk	DESeq2	LRT		0
770	KC_AB1	Sin	1.23655099691048	0.524421500981735	1	pseudobulk	DESeq2	LRT		0
770	KC_AB1	Sin1	0.091490856235909	0.76380774277539	1	pseudobulk	DESeq2	LRT		0
771	KC_AB1	Sin3A	-0.12474520686636	0.591466658240984	1	pseudobulk	DESeq2	LRT		0
771	KC_AB1	sina	-0.277674183442897	0.342302268855613	1	pseudobulk	DESeq2	LRT		0
771	KC_AB1	sing	0.116783351990007	1	1	pseudobulk	DESeq2	LRT		0
771	KC_AB1	sinu	-0.839902291711688	0.463844430631184	1	pseudobulk	DESeq2	LRT		0
771	KC_AB1	sip1	0.0366098818926194	0.946039577746226	1	pseudobulk	DESeq2	LRT		0
771	KC_AB1	sip3	0.248463296839385	0.561884370924984	1	pseudobulk	DESeq2	LRT		0
771	KC_AB1	Sirt1	-0.410495307302607	0.351566494552946	1	pseudobulk	DESeq2	LRT		0
771	KC_AB1	Sirt2	-1.24462048228618	0.188086358412088	1	pseudobulk	DESeq2	LRT		0
771	KC_AB1	Sirt4	-0.273204404105848	0.65420750896212	1	pseudobulk	DESeq2	LRT		0
771	KC_AB1	Sirt6	1.84981579152528	0.66160499764878	1	pseudobulk	DESeq2	LRT		0
772	KC_AB1	Sirt7	-1.28587461230697	0.340597787555948	1	pseudobulk	DESeq2	LRT		0
772	KC_AB1	Sirup	-0.440982095943521	0.522742808818882	1	pseudobulk	DESeq2	LRT		0
772	KC_AB1	sisRNA:1	-0.673973387349693	0.737308747629933	1	pseudobulk	DESeq2	LRT		0
772	KC_AB1	siz	-0.480805057319918	0.0849080413231001	1	pseudobulk	DESeq2	LRT		0
772	KC_AB1	Sk1	-0.704858387530061	0.698842745464243	1	pseudobulk	DESeq2	LRT		0
772	KC_AB1	Sk2	-1.65222913695873	0.145274113258276	1	pseudobulk	DESeq2	LRT		0
772	KC_AB1	Skadu	1.59863986092676	1	1	pseudobulk	DESeq2	LRT		0
772	KC_AB1	skd	-0.10133239941482	0.532054967200949	1	pseudobulk	DESeq2	LRT		0
772	KC_AB1	Skeletor	-4.40864753874082	0.244715118468961	1	pseudobulk	DESeq2	LRT		0
772	KC_AB1	Ski6	0.892609344370087	0.654771043023561	1	pseudobulk	DESeq2	LRT		0
773	KC_AB1	SKIP	-0.433439417951897	0.744883260817895	1	pseudobulk	DESeq2	LRT		0
773	KC_AB1	skl	0.849606887838128	1	1	pseudobulk	DESeq2	LRT		0
773	KC_AB1	SkpA	-0.219889289157596	0.328404284153078	1	pseudobulk	DESeq2	LRT		0
773	KC_AB1	sktl	-0.216210029276812	1	1	pseudobulk	DESeq2	LRT		0
773	KC_AB1	sky	-0.474246454014467	0.0666566911726739	1	pseudobulk	DESeq2	LRT		0
773	KC_AB1	sl	-1.03776216208369	0.394141016067975	1	pseudobulk	DESeq2	LRT		0
773	KC_AB1	Slbp	-1.00098886868143	0.299948301790561	1	pseudobulk	DESeq2	LRT		0

773	KC_AB1	SLC22A	-0.484506573106544	0.642028208342465	1	pseudobulk	DESeq2	LRT	0
773	KC_AB1	Slc25A46a	0.0582307521246916	0.766353162721661	1	pseudobulk	DESeq2	LRT	0
773	KC_AB1	SLC5A11	0.721569306918665	0.32854459536435	1	pseudobulk	DESeq2	LRT	0
774	KC_AB1	Sld5	0.849606887838128	1	1	pseudobulk	DESeq2	LRT	0
774	KC_AB1	sle	0.849606887838128	1	1	pseudobulk	DESeq2	LRT	0
774	KC_AB1	slgA	-0.0821205866404724	0.716006285858388	1	pseudobulk	DESeq2	LRT	0
774	KC_AB1	Slh	-0.398900281995945	0.737216060312039	1	pseudobulk	DESeq2	LRT	0
774	KC_AB1	sli	2.78674512494791	0.54962276837928	1	pseudobulk	DESeq2	LRT	0
774	KC_AB1	slif	1.58205617221276	1	1	pseudobulk	DESeq2	LRT	0
774	KC_AB1	Slik	-0.551311794956103	0.184107488378506	1	pseudobulk	DESeq2	LRT	0
774	KC_AB1	slim	0.0503647608381037	0.739524673234019	1	pseudobulk	DESeq2	LRT	0
774	KC_AB1	Slimp	1.59863986092676	1	1	pseudobulk	DESeq2	LRT	0
774	KC_AB1	Slip1	-0.0616242722122641	0.626668249414041	1	pseudobulk	DESeq2	LRT	0
775	KC_AB1	SLIRP2	-0.627459769143426	0.638064069136767	1	pseudobulk	DESeq2	LRT	0
775	KC_AB1	sll	-1.46833861422601	0.319175673301862	1	pseudobulk	DESeq2	LRT	0
775	KC_AB1	Slmap	0.0191203812465011	0.791697628161422	1	pseudobulk	DESeq2	LRT	0
775	KC_AB1	slmb	0.278246485236102	0.425031343765687	1	pseudobulk	DESeq2	LRT	0
775	KC_AB1	slmo	-0.467057517398218	0.590093226838308	1	pseudobulk	DESeq2	LRT	0
775	KC_AB1	Sln	-0.01161147646379	0.659934816603481	1	pseudobulk	DESeq2	LRT	0
775	KC_AB1	SLO2	0.147047713370011	0.328180113883246	1	pseudobulk	DESeq2	LRT	0
775	KC_AB1	Slob	-0.413014586131442	0.0395383475365429	1	pseudobulk	DESeq2	LRT	0
775	KC_AB1	slow	2.31997030691033	0.813169507846918	1	pseudobulk	DESeq2	LRT	0
775	KC_AB1	slpr	0.322665444431841	0.435321950433505	1	pseudobulk	DESeq2	LRT	0
776	KC_AB1	sls	-0.683829746723457	0.723342236517982	1	pseudobulk	DESeq2	LRT	0
776	KC_AB1	Slu7	0.41898458260941	0.532765161393154	1	pseudobulk	DESeq2	LRT	0
776	KC_AB1	slv	-0.703390452321825	0.496020095928025	1	pseudobulk	DESeq2	LRT	0
776	KC_AB1	slx1	1.46091799385084	1	1	pseudobulk	DESeq2	LRT	0
776	KC_AB1	sm	-0.393056837060013	0.180202692538155	1	pseudobulk	DESeq2	LRT	0
776	KC_AB1	smal	1.59863986092676	1	1	pseudobulk	DESeq2	LRT	0
776	KC_AB1	smash	-0.0704599077878179	0.74032454141762	1	pseudobulk	DESeq2	LRT	0
776	KC_AB1	SmB	0.575405917683796	0.482336704444755	1	pseudobulk	DESeq2	LRT	0
776	KC_AB1	SMC1	0.674963777677139	0.259371505724131	1	pseudobulk	DESeq2	LRT	0
776	KC_AB1	SMC2	1.74488096961208	0.717340193332887	1	pseudobulk	DESeq2	LRT	0
777	KC_AB1	SMC3	0.0297111253491337	0.856273455002805	1	pseudobulk	DESeq2	LRT	0
777	KC_AB1	SMC5	0.407363085727121	1	1	pseudobulk	DESeq2	LRT	0
777	KC_AB1	SmD1	-0.315740867458215	0.656760136871712	1	pseudobulk	DESeq2	LRT	0
777	KC_AB1	SmD2	0.00894498822089035	0.7764789139801	1	pseudobulk	DESeq2	LRT	0
777	KC_AB1	SmD3	0.170068399663398	0.724168636218067	1	pseudobulk	DESeq2	LRT	0
777	KC_AB1	SmE	-0.111197102935088	0.850347847537639	1	pseudobulk	DESeq2	LRT	0
777	KC_AB1	SmF	-0.481741129943024	0.600724259718334	1	pseudobulk	DESeq2	LRT	0
777	KC_AB1	smg	0.238946664243373	0.600762852910536	1	pseudobulk	DESeq2	LRT	0
777	KC_AB1	Smg5	-0.415053548483046	0.305166323325192	1	pseudobulk	DESeq2	LRT	0
777	KC_AB1	Smg6	-0.596000637334392	0.484483805871134	1	pseudobulk	DESeq2	LRT	0
778	KC_AB1	smid	-0.343039008095567	0.654597446945778	1	pseudobulk	DESeq2	LRT	0
778	KC_AB1	Smn	0.586863319979459	0.577579549640056	1	pseudobulk	DESeq2	LRT	0
778	KC_AB1	smo	-0.214482803882399	0.724509943890629	1	pseudobulk	DESeq2	LRT	0
778	KC_AB1	smog	0.10884363541117	0.611274697815329	1	pseudobulk	DESeq2	LRT	0
778	KC_AB1	Smox	-0.332996839872657	0.194207297228093	1	pseudobulk	DESeq2	LRT	0
778	KC_AB1	smp-30	1.22772839314209	0.443517226883431	1	pseudobulk	DESeq2	LRT	0
778	KC_AB1	Smr	-0.283742188977872	0.0890909024607343	1	pseudobulk	DESeq2	LRT	0
778	KC_AB1	Sms	-0.499825042712312	0.275415201870694	1	pseudobulk	DESeq2	LRT	0
778	KC_AB1	SMSr	-1.06728084463614	0.333971396816548	1	pseudobulk	DESeq2	LRT	0
778	KC_AB1	smt3	-0.034625374452998	0.814434490665738	1	pseudobulk	DESeq2	LRT	0

779	KC_AB1	Smu1	-1.15826959624471	0.355157655163391	1	pseudobulk	DESeq2	LRT	0
779	KC_AB1	Smurf	-0.023460012063062	0.580417001445711	1	pseudobulk	DESeq2	LRT	0
779	KC_AB1	Smyd3	0.545615856131233	0.669746421920512	1	pseudobulk	DESeq2	LRT	0
779	KC_AB1	Smyd4-1	-0.521223725908939	0.714202258510727	1	pseudobulk	DESeq2	LRT	0
779	KC_AB1	Smyd4-4	-0.824643702502294	0.502179848256061	1	pseudobulk	DESeq2	LRT	0
779	KC_AB1	Smyd5	-0.0097165467464642	0.360442466719095	1	pseudobulk	DESeq2	LRT	0
779	KC_AB1	SmydA-1	1.26737066893878	0.771338963979384	1	pseudobulk	DESeq2	LRT	0
779	KC_AB1	SmydA-3	0.260318855097993	0.695243282500257	1	pseudobulk	DESeq2	LRT	0
779	KC_AB1	SmydA-4	-0.19698351532854	1	1	pseudobulk	DESeq2	LRT	0
779	KC_AB1	SmydA-7	-0.0672927064560813	0.777430941378593	1	pseudobulk	DESeq2	LRT	0
780	KC_AB1	sn	-0.198991811494155	0.807644856856482	1	pseudobulk	DESeq2	LRT	0
780	KC_AB1	snama	0.383985275253024	0.534792190540941	1	pseudobulk	DESeq2	LRT	0
780	KC_AB1	Snap24	0.135937596364542	0.681146999684013	1	pseudobulk	DESeq2	LRT	0
780	KC_AB1	Snap25	-0.259555478190635	0.108398466842278	1	pseudobulk	DESeq2	LRT	0
780	KC_AB1	Snap29	0.313855549195561	0.582407279190076	1	pseudobulk	DESeq2	LRT	0
780	KC_AB1	snf	0.121390364587638	0.839486480797463	1	pseudobulk	DESeq2	LRT	0
780	KC_AB1	SNF4Agamma	-0.0537045067218642	0.664523979249245	1	pseudobulk	DESeq2	LRT	0
780	KC_AB1	sni	0.807598379880877	0.71018833315747	1	pseudobulk	DESeq2	LRT	0
780	KC_AB1	Snm1	0.237022465848507	1	1	pseudobulk	DESeq2	LRT	0
780	KC_AB1	sno	0.699281409403104	0.0797871397636658	1	pseudobulk	DESeq2	LRT	0
781	KC_AB1	Snoo	-0.353902856619137	0.141502418586321	1	pseudobulk	DESeq2	LRT	0
781	KC_AB1	snoRNA:Dek-a	0.237022465848507	1	1	pseudobulk	DESeq2	LRT	0
781	KC_AB1	snoRNA:Irbp-a	2.31997030691033	0.813169507846918	1	pseudobulk	DESeq2	LRT	0
781	KC_AB1	snoRNA:lola-d	0.849606887838128	1	1	pseudobulk	DESeq2	LRT	0
781	KC_AB1	snoRNA:Me28S-A	0.849606887838128	1	1	pseudobulk	DESeq2	LRT	0
781	KC_AB1	snoRNA:nop5-x16	0.849606887838128	1	1	pseudobulk	DESeq2	LRT	0
781	KC_AB1	snoRNA:Or-CD2	0.116783351990007	1	1	pseudobulk	DESeq2	LRT	0
781	KC_AB1	snoRNA:Psi18S-92	0.849606887838128	1	1	pseudobulk	DESeq2	LRT	0
781	KC_AB1	snoRNA:Psi28S-1	3.16697464674408	0.406679230261689	1	pseudobulk	DESeq2	LRT	0
781	KC_AB1	snoRNA:Psi28S-2	1.44445815960193	0.840903376662985	1	pseudobulk	DESeq2	LRT	0
782	KC_AB1	snoRNA:Psi28S-2	2.31997030691033	0.813169507846918	1	pseudobulk	DESeq2	LRT	0
782	KC_AB1	snoRNA:Psi28S-3	0.849606887838128	1	1	pseudobulk	DESeq2	LRT	0
782	KC_AB1	snoRNA:Psi28S-3	0.849606887838128	1	1	pseudobulk	DESeq2	LRT	0
782	KC_AB1	snoRNA:Psi28S-3	0.183159795463042	1	1	pseudobulk	DESeq2	LRT	0
782	KC_AB1	snoRNA:Tudor-SN	0.849606887838128	1	1	pseudobulk	DESeq2	LRT	0
782	KC_AB1	Snp	-2.10971956209718	0.023366310939848	1	pseudobulk	DESeq2	LRT	0
782	KC_AB1	sNPF	-0.176865728775941	0.353900287136798	1	pseudobulk	DESeq2	LRT	0
782	KC_AB1	sNPF-R	2.06541384650887	0.904905526327343	1	pseudobulk	DESeq2	LRT	0
782	KC_AB1	Snr1	0.465903582026074	0.439141560602269	1	pseudobulk	DESeq2	LRT	0
782	KC_AB1	snRNA:7SK	-0.160796206486047	0.597299453320857	1	pseudobulk	DESeq2	LRT	0
783	KC_AB1	snRNA:U12	-0.35586282077459	0.778037081131139	1	pseudobulk	DESeq2	LRT	0
783	KC_AB1	snRNA:U2:14B	2.78674512494791	0.54962276837928	1	pseudobulk	DESeq2	LRT	0
783	KC_AB1	snRNA:U2:34ABa	0.849606887838128	1	1	pseudobulk	DESeq2	LRT	0
783	KC_AB1	snRNA:U4:38AB	0.849606887838128	1	1	pseudobulk	DESeq2	LRT	0
783	KC_AB1	snRNP-U1-70K	-0.170081000172619	0.624704778468387	1	pseudobulk	DESeq2	LRT	0
783	KC_AB1	snRNP-U1-C	-0.367019088800449	0.530038312707495	1	pseudobulk	DESeq2	LRT	0
783	KC_AB1	SNRPG	0.169473888326596	0.645893854119566	1	pseudobulk	DESeq2	LRT	0
783	KC_AB1	sns	1.37981166648799	0.219751097792525	1	pseudobulk	DESeq2	LRT	0
783	KC_AB1	snu	-0.759959182091963	0.802752140923966	1	pseudobulk	DESeq2	LRT	0
783	KC_AB1	Snx1	-0.835106511955608	0.295388789763263	1	pseudobulk	DESeq2	LRT	0
784	KC_AB1	Snx16	-0.432871025222947	0.580223270831186	1	pseudobulk	DESeq2	LRT	0
784	KC_AB1	Snx17	0.0194361033220329	0.809418264747766	1	pseudobulk	DESeq2	LRT	0
784	KC_AB1	Snx21	-0.685006510487393	0.507012745908278	1	pseudobulk	DESeq2	LRT	0

784	KC_AB1	Snx27	-0.315648672787825	0.452881354309358	1	pseudobulk	DESeq2	LRT		0
784	KC_AB1	Snx3	-0.319142523882651	0.414767952983633	1	pseudobulk	DESeq2	LRT		0
784	KC_AB1	Snx6	0.022681464055097	0.900479060700358	1	pseudobulk	DESeq2	LRT		0
784	KC_AB1	snz	0.086223378760575	0.698472454157361	1	pseudobulk	DESeq2	LRT		0
784	KC_AB1	Socs16D	-0.162765177904457	0.516950831857663	1	pseudobulk	DESeq2	LRT		0
784	KC_AB1	Socs36E	-0.10430907782672	0.755692653115301	1	pseudobulk	DESeq2	LRT		0
784	KC_AB1	Socs44A	-0.77331123012131	0.277033540258744	1	pseudobulk	DESeq2	LRT		0
785	KC_AB1	Sod1	0.441654348104813	0.108926875261844	1	pseudobulk	DESeq2	LRT		0
785	KC_AB1	Sod2	-0.0138016440467149	0.568322053979095	1	pseudobulk	DESeq2	LRT		0
785	KC_AB1	Sod3	-0.311263343349099	0.571515021401813	1	pseudobulk	DESeq2	LRT		0
785	KC_AB1	Sodh-2	0.849606887838128	1	1	pseudobulk	DESeq2	LRT		0
785	KC_AB1	sog	1.77338707100774	0.460389313744518	1	pseudobulk	DESeq2	LRT		0
785	KC_AB1	sol	-0.3086046479337	0.451598109943917	1	pseudobulk	DESeq2	LRT		0
785	KC_AB1	Son	-0.275855885943777	0.717679831139158	1	pseudobulk	DESeq2	LRT		0
785	KC_AB1	sona	1.00693893053477	1	1	pseudobulk	DESeq2	LRT		0
785	KC_AB1	Sos	-0.132500139085722	0.850410106291259	1	pseudobulk	DESeq2	LRT		0
785	KC_AB1	soti	0.849606887838128	1	1	pseudobulk	DESeq2	LRT		0
786	KC_AB1	sotv	0.613959400351794	0.149967945816995	1	pseudobulk	DESeq2	LRT		0
786	KC_AB1	sov	0.273366418399391	0.413518597713551	1	pseudobulk	DESeq2	LRT		0
786	KC_AB1	sowah	0.320601816785458	0.592152959799653	1	pseudobulk	DESeq2	LRT		0
786	KC_AB1	Sox102F	0.849606887838128	1	1	pseudobulk	DESeq2	LRT		0
786	KC_AB1	Sox14	-1.25393399638069	0.612054104673377	1	pseudobulk	DESeq2	LRT		0
786	KC_AB1	SP1029	4.11599205977059	0.175712481619698	1	pseudobulk	DESeq2	LRT		0
786	KC_AB1	SP1173	-0.150027154297685	0.463435592160288	1	pseudobulk	DESeq2	LRT		0
786	KC_AB1	SP2353	-0.882054092940087	0.269005320000205	1	pseudobulk	DESeq2	LRT		0
786	KC_AB1	Sp7	-0.0718207395094281	1	1	pseudobulk	DESeq2	LRT		0
786	KC_AB1	spab	0.439730463200944	0.240155326449375	1	pseudobulk	DESeq2	LRT		0
787	KC_AB1	spag	0.0096845433268193	0.867945073788503	1	pseudobulk	DESeq2	LRT		0
787	KC_AB1	Spag1	3.41844045086015	0.351942490385471	1	pseudobulk	DESeq2	LRT		0
787	KC_AB1	SPARC	-1.77900922033605	0.163488556741411	1	pseudobulk	DESeq2	LRT		0
787	KC_AB1	spartin	0.234803646391757	0.613832320279541	1	pseudobulk	DESeq2	LRT		0
787	KC_AB1	spas	-0.497968635270688	0.466740620966201	1	pseudobulk	DESeq2	LRT		0
787	KC_AB1	Spase12	-0.0741523648939893	0.834481555134685	1	pseudobulk	DESeq2	LRT		0
787	KC_AB1	Spase22-23	-0.0317731819925747	0.684444344600453	1	pseudobulk	DESeq2	LRT		0
787	KC_AB1	Spase25	0.402844463901853	0.481649149611854	1	pseudobulk	DESeq2	LRT		0
787	KC_AB1	Spc105R	2.78674512494791	0.54962276837928	1	pseudobulk	DESeq2	LRT		0
787	KC_AB1	spdo	1.59863986092676	1	1	pseudobulk	DESeq2	LRT		0
788	KC_AB1	SpdS	0.962439817833451	0.217537883806615	1	pseudobulk	DESeq2	LRT		0
788	KC_AB1	SPE	0.849606887838128	1	1	pseudobulk	DESeq2	LRT		0
788	KC_AB1	Spec2	0.314197113040988	0.654587766479721	1	pseudobulk	DESeq2	LRT		0
788	KC_AB1	spel1	0.930993226399931	1	1	pseudobulk	DESeq2	LRT		0
788	KC_AB1	spen	-0.333192560129474	0.0166790838332033	1	pseudobulk	DESeq2	LRT		0
788	KC_AB1	Spf45	0.140904472988321	0.768128002880639	1	pseudobulk	DESeq2	LRT		0
788	KC_AB1	spg	-0.0783513359453197	0.849230888578968	1	pseudobulk	DESeq2	LRT		0
788	KC_AB1	Spg7	0.858316770068222	0.189381662487235	1	pseudobulk	DESeq2	LRT		0
788	KC_AB1	sphe	2.06531092668974	0.944672961903726	1	pseudobulk	DESeq2	LRT		0
788	KC_AB1	spi	0.0462757027170832	0.814050028279554	1	pseudobulk	DESeq2	LRT		0
789	KC_AB1	spict	0.0843587384244169	0.735534678766619	1	pseudobulk	DESeq2	LRT		0
789	KC_AB1	spidey	-0.608417235180774	0.175883184223849	1	pseudobulk	DESeq2	LRT		0
789	KC_AB1	spin	0.713980465041143	0.430684936470557	1	pseudobulk	DESeq2	LRT		0
789	KC_AB1	spir	0.424142579764265	0.0172640655450484	1	pseudobulk	DESeq2	LRT		0
789	KC_AB1	sPLA2	1.177541179001	0.647884013797716	1	pseudobulk	DESeq2	LRT		0
789	KC_AB1	Sply	0.261549449411198	0.600959280869949	1	pseudobulk	DESeq2	LRT		0

789	KC_AB1	Spn	-0.467219012498671	0.0494186996099792	1	pseudobulk	DESeq2	LRT	0
789	KC_AB1	spn-A	0.366247714696288	1	1	pseudobulk	DESeq2	LRT	0
789	KC_AB1	spn-B	1.30479668573778	1	1	pseudobulk	DESeq2	LRT	0
789	KC_AB1	spn-F	0.139371570469137	0.712231882187487	1	pseudobulk	DESeq2	LRT	0
790	KC_AB1	Spn27A	1.78483343304283	0.480292680194443	1	pseudobulk	DESeq2	LRT	0
790	KC_AB1	Spn28Dc	2.06541384650887	0.904905526327343	1	pseudobulk	DESeq2	LRT	0
790	KC_AB1	Spn42Da	0.69620356948217	0.550553807072166	1	pseudobulk	DESeq2	LRT	0
790	KC_AB1	Spn42Dd	0.183159795455075	1	1	pseudobulk	DESeq2	LRT	0
790	KC_AB1	Spn43Aa	-1.25393399638069	0.612054104673377	1	pseudobulk	DESeq2	LRT	0
790	KC_AB1	Spn43Ab	-0.212116125262889	0.735240852009139	1	pseudobulk	DESeq2	LRT	0
790	KC_AB1	Spn75F	2.78674512494791	0.54962276837928	1	pseudobulk	DESeq2	LRT	0
790	KC_AB1	Spn77Ba	-0.493403067484676	0.791396581717061	1	pseudobulk	DESeq2	LRT	0
790	KC_AB1	spo	0.849606887838128	1	1	pseudobulk	DESeq2	LRT	0
790	KC_AB1	SPoCk	0.195390393060742	0.356773720484311	1	pseudobulk	DESeq2	LRT	0
791	KC_AB1	spoon	0.434261447085749	0.501723247973801	1	pseudobulk	DESeq2	LRT	0
791	KC_AB1	Spp	0.250340586460465	0.699453360152639	1	pseudobulk	DESeq2	LRT	0
791	KC_AB1	SppL	-0.142110452416991	0.561454750803407	1	pseudobulk	DESeq2	LRT	0
791	KC_AB1	Spps	0.841140062946712	0.198728827999069	1	pseudobulk	DESeq2	LRT	0
791	KC_AB1	Spred	-0.415448761385841	0.034849408546006	1	pseudobulk	DESeq2	LRT	0
791	KC_AB1	spri	-1.35575641541948	0.36716346665365	1	pseudobulk	DESeq2	LRT	0
791	KC_AB1	sprt	-1.5817641761383	0.61071285705648	1	pseudobulk	DESeq2	LRT	0
791	KC_AB1	Sps1	-0.484506573106544	0.642028208342465	1	pseudobulk	DESeq2	LRT	0
791	KC_AB1	Sps2	1.27407311614374	0.744359737766178	1	pseudobulk	DESeq2	LRT	0
791	KC_AB1	Spt	-0.725951880349337	0.509286548839072	1	pseudobulk	DESeq2	LRT	0
792	KC_AB1	Spt-I	0.136692826810455	0.83713635852482	1	pseudobulk	DESeq2	LRT	0
792	KC_AB1	Spt20	0.050949974371014	0.905882642235327	1	pseudobulk	DESeq2	LRT	0
792	KC_AB1	Spt3	-0.430850862004475	0.637046139731612	1	pseudobulk	DESeq2	LRT	0
792	KC_AB1	spt4	-0.536521103244134	0.384613113230413	1	pseudobulk	DESeq2	LRT	0
792	KC_AB1	Spt5	-0.541988653307818	0.353277633396493	1	pseudobulk	DESeq2	LRT	0
792	KC_AB1	Spt6	0.149977716580264	0.812847252265943	1	pseudobulk	DESeq2	LRT	0
792	KC_AB1	Spt7	0.663546949070083	1	1	pseudobulk	DESeq2	LRT	0
792	KC_AB1	Sptr	-0.234855169828895	0.497235847298866	1	pseudobulk	DESeq2	LRT	0
792	KC_AB1	Spx	-0.485877238581862	0.633108966568847	1	pseudobulk	DESeq2	LRT	0
792	KC_AB1	spz	2.06541384650887	0.904905526327343	1	pseudobulk	DESeq2	LRT	0
793	KC_AB1	spz4	0.542982564383309	1	1	pseudobulk	DESeq2	LRT	0
793	KC_AB1	spz5	0.849606887838128	1	1	pseudobulk	DESeq2	LRT	0
793	KC_AB1	sqa	0.849606887838128	1	1	pseudobulk	DESeq2	LRT	0
793	KC_AB1	sqd	-0.262246899190813	0.119304554198019	1	pseudobulk	DESeq2	LRT	0
793	KC_AB1	sqh	0.309363885671284	0.47173277910862	1	pseudobulk	DESeq2	LRT	0
793	KC_AB1	squ	1.58205617221276	1	1	pseudobulk	DESeq2	LRT	0
793	KC_AB1	sqz	-0.968227765533368	0.330369177475557	1	pseudobulk	DESeq2	LRT	0
793	KC_AB1	sra	0.406942749887643	0.211852847093926	1	pseudobulk	DESeq2	LRT	0
793	KC_AB1	Sra-1	-0.736118079005185	0.454211964133415	1	pseudobulk	DESeq2	LRT	0
793	KC_AB1	Sras	0.439963920745984	0.504660976670862	1	pseudobulk	DESeq2	LRT	0
794	KC_AB1	Src42A	-0.191925161262864	0.638339741342174	1	pseudobulk	DESeq2	LRT	0
794	KC_AB1	Src64B	-0.161201262663491	0.497571664979931	1	pseudobulk	DESeq2	LRT	0
794	KC_AB1	SREBP	0.39196104303381	0.593027892474861	1	pseudobulk	DESeq2	LRT	0
794	KC_AB1	srl	2.78674512494791	0.54962276837928	1	pseudobulk	DESeq2	LRT	0
794	KC_AB1	Srlp	-0.445691793544731	0.509862233685504	1	pseudobulk	DESeq2	LRT	0
794	KC_AB1	SRm160	0.134671723317183	0.727942741911029	1	pseudobulk	DESeq2	LRT	0
794	KC_AB1	Srp14	-0.394885910731698	0.467202569715094	1	pseudobulk	DESeq2	LRT	0
794	KC_AB1	Srp19	-0.26757842875452	0.741957769269243	1	pseudobulk	DESeq2	LRT	0
794	KC_AB1	Srp54	0.260500055197915	0.568352756208556	1	pseudobulk	DESeq2	LRT	0

794	KC_AB1	Srp54k	-0.700747761729294	0.422073723204369	1	pseudobulk	DESeq2	LRT	0
795	KC_AB1	Srp68	0.811967575378733	0.824576705147507	1	pseudobulk	DESeq2	LRT	0
795	KC_AB1	Srp72	0.467828765571383	0.642737153793531	1	pseudobulk	DESeq2	LRT	0
795	KC_AB1	Srp9	0.608630522641199	0.307408630537151	1	pseudobulk	DESeq2	LRT	0
795	KC_AB1	SRPK	-0.0495514297417588	0.75486092481912	1	pseudobulk	DESeq2	LRT	0
795	KC_AB1	Srpk79D	-0.266689899333656	0.555356255391019	1	pseudobulk	DESeq2	LRT	0
795	KC_AB1	SrpRbeta	0.412658329012117	0.249397063743365	1	pseudobulk	DESeq2	LRT	0
795	KC_AB1	Sry-delta	-0.402095558938465	0.690189911274928	1	pseudobulk	DESeq2	LRT	0
795	KC_AB1	Ssadh	-0.793363785084203	0.288862114841387	1	pseudobulk	DESeq2	LRT	0
795	KC_AB1	Ssb-c31a	-0.0322176939679417	0.961524202451833	1	pseudobulk	DESeq2	LRT	0
795	KC_AB1	Ssdp	-0.0859389246370405	0.569580357131435	1	pseudobulk	DESeq2	LRT	0
796	KC_AB1	ssh	0.598659574643131	0.285432054441679	1	pseudobulk	DESeq2	LRT	0
796	KC_AB1	Ssk	2.9455837830248	0.354126936037652	1	pseudobulk	DESeq2	LRT	0
796	KC_AB1	Ssl1	1.34104631165686	0.212403111107005	1	pseudobulk	DESeq2	LRT	0
796	KC_AB1	ssp	-1.35533948076358	0.322702873685284	1	pseudobulk	DESeq2	LRT	0
796	KC_AB1	ssp3	-0.573371528424052	0.288964229343341	1	pseudobulk	DESeq2	LRT	0
796	KC_AB1	ssp7	2.31997030691033	0.813169507846918	1	pseudobulk	DESeq2	LRT	0
796	KC_AB1	SsRbeta	-0.242126844900293	0.641251496221173	1	pseudobulk	DESeq2	LRT	0
796	KC_AB1	Ssrp	0.478706463443362	0.741991092340705	1	pseudobulk	DESeq2	LRT	0
796	KC_AB1	sstn	0.603061041589212	0.88797127252248	1	pseudobulk	DESeq2	LRT	0
796	KC_AB1	Ssu72	-0.896617143228746	0.412280099500312	1	pseudobulk	DESeq2	LRT	0
797	KC_AB1	ssx	-0.1271698365977	0.714914797493664	1	pseudobulk	DESeq2	LRT	0
797	KC_AB1	St1	1.26344600836598	0.615715273419676	1	pseudobulk	DESeq2	LRT	0
797	KC_AB1	St3	-0.270536785818373	0.333214677956655	1	pseudobulk	DESeq2	LRT	0
797	KC_AB1	sta	-0.0272187901929082	0.645143686913299	1	pseudobulk	DESeq2	LRT	0
797	KC_AB1	stac	0.849606887838128	1	1	pseudobulk	DESeq2	LRT	0
797	KC_AB1	Stacl	-0.0809897944889774	0.549173650108911	1	pseudobulk	DESeq2	LRT	0
797	KC_AB1	stai	0.0294098694580003	0.713765940130676	1	pseudobulk	DESeq2	LRT	0
797	KC_AB1	Stam	-1.06192971575129	0.290353485873818	1	pseudobulk	DESeq2	LRT	0
797	KC_AB1	stan	0.116783351990007	1	1	pseudobulk	DESeq2	LRT	0
797	KC_AB1	Start1	-0.679220926102518	0.441869489923016	1	pseudobulk	DESeq2	LRT	0
798	KC_AB1	stas	-0.373076431954003	0.326425891402118	1	pseudobulk	DESeq2	LRT	0
798	KC_AB1	Stat92E	-0.371023127786859	0.210216096655962	1	pseudobulk	DESeq2	LRT	0
798	KC_AB1	stau	-0.765928028192781	0.0847156785380781	1	pseudobulk	DESeq2	LRT	0
798	KC_AB1	stc	-0.79056154792089	0.444975343598754	1	pseudobulk	DESeq2	LRT	0
798	KC_AB1	stck	-0.618709397749543	0.204761230043195	1	pseudobulk	DESeq2	LRT	0
798	KC_AB1	ste14	3.20547843809887	0.383970068137593	1	pseudobulk	DESeq2	LRT	0
798	KC_AB1	ste24a	0.113341573344023	0.739042888459775	1	pseudobulk	DESeq2	LRT	0
798	KC_AB1	step	0.133080104557421	0.600718719181318	1	pseudobulk	DESeq2	LRT	0
798	KC_AB1	stet	0.849606887838128	1	1	pseudobulk	DESeq2	LRT	0
798	KC_AB1	Stim	-0.185473067058436	0.520244378220811	1	pseudobulk	DESeq2	LRT	0
799	KC_AB1	Stip1	1.02929510921384	0.28912217525889	1	pseudobulk	DESeq2	LRT	0
799	KC_AB1	stj	0.342799808330099	0.23272107233352	1	pseudobulk	DESeq2	LRT	0
799	KC_AB1	Stlk	-0.374743350782986	0.307375322982158	1	pseudobulk	DESeq2	LRT	0
799	KC_AB1	stmA	0.0231222686892154	0.82503964017612	1	pseudobulk	DESeq2	LRT	0
799	KC_AB1	Stoml2	0.539393441017276	0.34636716292473	1	pseudobulk	DESeq2	LRT	0
799	KC_AB1	stops	-0.784747297719904	0.470094184429032	1	pseudobulk	DESeq2	LRT	0
799	KC_AB1	strat	0.426023595345519	0.598997828260197	1	pseudobulk	DESeq2	LRT	0
799	KC_AB1	Strica	0.849606887838128	1	1	pseudobulk	DESeq2	LRT	0
799	KC_AB1	Strip	-0.48627828358323	0.429328384010743	1	pseudobulk	DESeq2	LRT	0
799	KC_AB1	Strump	-1.04398830605922	0.494390000440799	1	pseudobulk	DESeq2	LRT	0
800	KC_AB1	Stt3A	0.206672921391197	0.642683328272238	1	pseudobulk	DESeq2	LRT	0
800	KC_AB1	Stt3B	-0.679223285928799	0.438321489115804	1	pseudobulk	DESeq2	LRT	0

800	KC_AB1	STUB1	0.195234777320486	0.7187523992873	1	pseudobulk	DESeq2	LRT	0
800	KC_AB1	sturkopf	0.374221203203771	0.490738493372276	1	pseudobulk	DESeq2	LRT	0
800	KC_AB1	stv	-0.235471685108431	0.532240157215436	1	pseudobulk	DESeq2	LRT	0
800	KC_AB1	stw	1.58205617221276	1	1	pseudobulk	DESeq2	LRT	0
800	KC_AB1	stwl	0.141729152325207	1	1	pseudobulk	DESeq2	LRT	0
800	KC_AB1	stx	0.109406644641082	0.642028855605736	1	pseudobulk	DESeq2	LRT	0
800	KC_AB1	sty	-2.22124325223156	0.165824697859277	1	pseudobulk	DESeq2	LRT	0
800	KC_AB1	Su(dx)	-0.622967129300956	0.2334240424975	1	pseudobulk	DESeq2	LRT	0
801	KC_AB1	su(f)	0.242479129387537	0.655042874682491	1	pseudobulk	DESeq2	LRT	0
801	KC_AB1	Su(fu)	-0.472891157005362	0.593182926684919	1	pseudobulk	DESeq2	LRT	0
801	KC_AB1	Su(H)	-1.0720278864858	0.279181105576738	1	pseudobulk	DESeq2	LRT	0
801	KC_AB1	su(Hw)	-0.174556821850764	0.705353615462578	1	pseudobulk	DESeq2	LRT	0
801	KC_AB1	Su(P)	-0.283425049348718	0.681034140052373	1	pseudobulk	DESeq2	LRT	0
801	KC_AB1	su(sable)	1.03236261761415	0.0892728052944177	1	pseudobulk	DESeq2	LRT	0
801	KC_AB1	Su(Tpl)	-0.286585495012592	0.244905735729547	1	pseudobulk	DESeq2	LRT	0
801	KC_AB1	Su(var)2-10	-0.0091030146436719	0.846996614767637	1	pseudobulk	DESeq2	LRT	0
801	KC_AB1	Su(var)2-HP2	0.0415460940056676	0.958594850714823	1	pseudobulk	DESeq2	LRT	0
801	KC_AB1	Su(var)205	0.376206057004283	0.441881774414105	1	pseudobulk	DESeq2	LRT	0
802	KC_AB1	Su(var)3-3	-0.164205236283318	0.596740312582451	1	pseudobulk	DESeq2	LRT	0
802	KC_AB1	Su(var)3-7	0.577236966831769	0.414937798921741	1	pseudobulk	DESeq2	LRT	0
802	KC_AB1	su(w[a])	-0.0937777201751334	0.716452768525011	1	pseudobulk	DESeq2	LRT	0
802	KC_AB1	Su(z)12	0.475738186657671	0.412776900901986	1	pseudobulk	DESeq2	LRT	0
802	KC_AB1	Su(z)2	-0.271470684042004	0.581921799775029	1	pseudobulk	DESeq2	LRT	0
802	KC_AB1	subdued	-0.240167302180701	0.743177885720993	1	pseudobulk	DESeq2	LRT	0
802	KC_AB1	sud1	-1.15873060950853	0.418156582328336	1	pseudobulk	DESeq2	LRT	0
802	KC_AB1	sug	-0.216209988775614	1	1	pseudobulk	DESeq2	LRT	0
802	KC_AB1	Sugb	1.68208627965226	0.400974457037314	1	pseudobulk	DESeq2	LRT	0
802	KC_AB1	Sulf1	3.64217331632531	0.246682905127758	1	pseudobulk	DESeq2	LRT	0
803	KC_AB1	sun	-0.142153789119722	0.393333840901692	1	pseudobulk	DESeq2	LRT	0
803	KC_AB1	Sur	0.849606887838128	1	1	pseudobulk	DESeq2	LRT	0
803	KC_AB1	Sur-8	0.143593383899951	0.686676604333463	1	pseudobulk	DESeq2	LRT	0
803	KC_AB1	Surf1	-0.333551203013868	0.73601988456838	1	pseudobulk	DESeq2	LRT	0
803	KC_AB1	Surf4	0.0221750347590482	0.846131080157642	1	pseudobulk	DESeq2	LRT	0
803	KC_AB1	Surf6	-0.0247672191748029	0.814425053078568	1	pseudobulk	DESeq2	LRT	0
803	KC_AB1	sut1	2.78674512494791	0.54962276837928	1	pseudobulk	DESeq2	LRT	0
803	KC_AB1	Suv3	0.56211334887708	0.446344162504995	1	pseudobulk	DESeq2	LRT	0
803	KC_AB1	sv	-0.820071584052029	0.678106535840569	1	pseudobulk	DESeq2	LRT	0
803	KC_AB1	Svil	0.229243174378637	0.595578203599493	1	pseudobulk	DESeq2	LRT	0
804	KC_AB1	svr	-0.241878378119735	0.24339625685008	1	pseudobulk	DESeq2	LRT	0
804	KC_AB1	sw	0.424711025603006	0.417779799674537	1	pseudobulk	DESeq2	LRT	0
804	KC_AB1	Swim	2.06541384650887	0.795866856626082	1	pseudobulk	DESeq2	LRT	0
804	KC_AB1	SWIP	0.906189876260672	1	1	pseudobulk	DESeq2	LRT	0
804	KC_AB1	Swip-1	-0.0523193959965906	0.77551690368371	1	pseudobulk	DESeq2	LRT	0
804	KC_AB1	swm	0.0382299594041405	0.8437184148492	1	pseudobulk	DESeq2	LRT	0
804	KC_AB1	sws	0.0025993685467536	0.726150219094327	1	pseudobulk	DESeq2	LRT	0
804	KC_AB1	sxc	-0.31460483976799	0.314714470438326	1	pseudobulk	DESeq2	LRT	0
804	KC_AB1	Sxl	-0.106752705021775	0.526012477652082	1	pseudobulk	DESeq2	LRT	0
804	KC_AB1	Syb	-2.96279137506692	0.129708054178091	1	pseudobulk	DESeq2	LRT	0
805	KC_AB1	syd	-0.270899144617145	0.481458266993618	1	pseudobulk	DESeq2	LRT	0
805	KC_AB1	Sym	-0.116612002330998	0.827687199391974	1	pseudobulk	DESeq2	LRT	0
805	KC_AB1	Syn	0.129728924805203	0.524759731619063	1	pseudobulk	DESeq2	LRT	0
805	KC_AB1	Syn1	-0.537198511249469	0.245994014155615	1	pseudobulk	DESeq2	LRT	0
805	KC_AB1	Syn2	0.369074365876411	0.373343353963202	1	pseudobulk	DESeq2	LRT	0

805	KC_AB1	Synd	-0.0628083678214336	0.668724010983308	1	pseudobulk	DESeq2	LRT	0
805	KC_AB1	Syngr	0.0289345293919878	0.641343877385734	1	pseudobulk	DESeq2	LRT	0
805	KC_AB1	Synj	0.167899953291644	0.517594627530021	1	pseudobulk	DESeq2	LRT	0
805	KC_AB1	Syp	-0.466817551826522	0.0527781207735863	1	pseudobulk	DESeq2	LRT	0
805	KC_AB1	Syt1	-0.247072640618169	0.0869215326200447	1	pseudobulk	DESeq2	LRT	0
806	KC_AB1	Syt12	0.0449130815826133	0.657462609190867	1	pseudobulk	DESeq2	LRT	0
806	KC_AB1	Syt14	0.0026267148190137	0.847789609138845	1	pseudobulk	DESeq2	LRT	0
806	KC_AB1	Syt4	-0.378304908134159	0.070115446549752	1	pseudobulk	DESeq2	LRT	0
806	KC_AB1	Syt7	0.0051333016980907	0.692658149430015	1	pseudobulk	DESeq2	LRT	0
806	KC_AB1	Sytalpha	-0.486853103474005	0.0509254626253089	1	pseudobulk	DESeq2	LRT	0
806	KC_AB1	Sytbeta	0.25760471000562	0.558884836412261	1	pseudobulk	DESeq2	LRT	0
806	KC_AB1	Syx13	0.082538249023372	0.649081332332536	1	pseudobulk	DESeq2	LRT	0
806	KC_AB1	Syx16	-0.0519415823113747	0.894340050000954	1	pseudobulk	DESeq2	LRT	0
806	KC_AB1	Syx17	-0.539852078428446	0.460040815516363	1	pseudobulk	DESeq2	LRT	0
806	KC_AB1	Syx18	0.289299549909332	0.517236490700505	1	pseudobulk	DESeq2	LRT	0
807	KC_AB1	Syx1A	0.10093178664945	0.490230122126208	1	pseudobulk	DESeq2	LRT	0
807	KC_AB1	Syx4	2.06541384650887	0.904905526327343	1	pseudobulk	DESeq2	LRT	0
807	KC_AB1	Syx5	-0.307552266032665	0.626762936652407	1	pseudobulk	DESeq2	LRT	0
807	KC_AB1	Syx6	0.163860755050438	0.707955277798986	1	pseudobulk	DESeq2	LRT	0
807	KC_AB1	Syx7	-0.538425647841721	0.269316149090287	1	pseudobulk	DESeq2	LRT	0
807	KC_AB1	Syx8	-0.535308575205816	0.510539230759539	1	pseudobulk	DESeq2	LRT	0
807	KC_AB1	t-cup	0.849606887838128	1	1	pseudobulk	DESeq2	LRT	0
807	KC_AB1	T48	0.849606887838128	1	1	pseudobulk	DESeq2	LRT	0
807	KC_AB1	tacc	-0.0313619432066754	0.825694834970056	1	pseudobulk	DESeq2	LRT	0
807	KC_AB1	Tace	-0.0185277304690274	0.686782107566599	1	pseudobulk	DESeq2	LRT	0
808	KC_AB1	tadr	0.767093879837038	0.313761429548021	1	pseudobulk	DESeq2	LRT	0
808	KC_AB1	Taf1	0.0267788737500972	0.843847498503494	1	pseudobulk	DESeq2	LRT	0
808	KC_AB1	Taf10	0.0708485484086288	0.644334510165458	1	pseudobulk	DESeq2	LRT	0
808	KC_AB1	Taf10b	0.196111897546937	0.510298776661344	1	pseudobulk	DESeq2	LRT	0
808	KC_AB1	Taf11	-0.459082458089285	0.557249519076669	1	pseudobulk	DESeq2	LRT	0
808	KC_AB1	Taf12	-0.77676663947168	0.102397746720983	1	pseudobulk	DESeq2	LRT	0
808	KC_AB1	Taf13	-0.535153451157865	0.523250354752793	1	pseudobulk	DESeq2	LRT	0
808	KC_AB1	TAF1B	1.86730827006053	0.636504446122226	1	pseudobulk	DESeq2	LRT	0
808	KC_AB1	TAF1C-like	-0.320759537211391	0.820543175055409	1	pseudobulk	DESeq2	LRT	0
808	KC_AB1	Taf2	0.90445057126493	0.587576816505139	1	pseudobulk	DESeq2	LRT	0
809	KC_AB1	Taf4	0.0380021469163653	0.602454417018853	1	pseudobulk	DESeq2	LRT	0
809	KC_AB1	Taf5	-0.360265306619606	0.612946887544507	1	pseudobulk	DESeq2	LRT	0
809	KC_AB1	Taf6	0.196541638582857	0.671998128793159	1	pseudobulk	DESeq2	LRT	0
809	KC_AB1	Taf7	-0.704905712263576	0.509486251831276	1	pseudobulk	DESeq2	LRT	0
809	KC_AB1	Taf8	-0.777755326292596	0.563035162383272	1	pseudobulk	DESeq2	LRT	0
809	KC_AB1	tai	-0.195486672041191	0.233429118322212	1	pseudobulk	DESeq2	LRT	0
809	KC_AB1	Tailor	-0.593819562070421	0.49284101923183	1	pseudobulk	DESeq2	LRT	0
809	KC_AB1	Tak1	-1.00457770937778	0.298706395799729	1	pseudobulk	DESeq2	LRT	0
809	KC_AB1	Taldo	0.183159795455075	1	1	pseudobulk	DESeq2	LRT	0
809	KC_AB1	tam	0.172618327828008	1	1	pseudobulk	DESeq2	LRT	0
810	KC_AB1	tamo	-0.418953793193161	0.47225865963674	1	pseudobulk	DESeq2	LRT	0
810	KC_AB1	Tango1	-0.199302456901942	0.617358209977329	1	pseudobulk	DESeq2	LRT	0
810	KC_AB1	Tango10	0.283536185226646	0.645272051726855	1	pseudobulk	DESeq2	LRT	0
810	KC_AB1	Tango11	0.326065908882685	0.430723202082143	1	pseudobulk	DESeq2	LRT	0
810	KC_AB1	Tango14	-0.333721990236932	0.567149166425991	1	pseudobulk	DESeq2	LRT	0
810	KC_AB1	Tango2	-1.08554625838624	0.504617021804754	1	pseudobulk	DESeq2	LRT	0
810	KC_AB1	Tango4	0.973410861239336	0.415749389177165	1	pseudobulk	DESeq2	LRT	0
810	KC_AB1	Tango5	0.0685323141482505	0.567970126939653	1	pseudobulk	DESeq2	LRT	0

810	KC_AB1	Tango6	0.85879998658552	1	1	pseudobulk	DESeq2	LRT		0
810	KC_AB1	Tango9	0.0851280583493339	0.881430487772189	1	pseudobulk	DESeq2	LRT		0
811	KC_AB1	tank	-1.84632984716614	0.205405581389766	1	pseudobulk	DESeq2	LRT		0
811	KC_AB1	tant	-0.396421728006151	0.612062397118104	1	pseudobulk	DESeq2	LRT		0
811	KC_AB1	Tao	-0.332012185918222	0.408032405761542	1	pseudobulk	DESeq2	LRT		0
811	KC_AB1	Tap42	1.62427550163868	0.27637062162393	1	pseudobulk	DESeq2	LRT		0
811	KC_AB1	tapas	0.649278818539376	0.180287860505036	1	pseudobulk	DESeq2	LRT		0
811	KC_AB1	Tapdelta	-1.23668578306335	0.143814445149629	1	pseudobulk	DESeq2	LRT		0
811	KC_AB1	tara	-0.241440432434731	0.560055249372662	1	pseudobulk	DESeq2	LRT		0
811	KC_AB1	Task6	-0.285445655062808	0.118556204268688	1	pseudobulk	DESeq2	LRT		0
811	KC_AB1	Task7	-0.0978124738052538	0.686275762210164	1	pseudobulk	DESeq2	LRT		0
811	KC_AB1	Tasp1	-0.276631907837659	0.708066758460226	1	pseudobulk	DESeq2	LRT		0
812	KC_AB1	Tat	0.316058703496199	0.25345738299013	1	pseudobulk	DESeq2	LRT		0
812	KC_AB1	tau	-0.398035768443223	0.271937351062674	1	pseudobulk	DESeq2	LRT		0
812	KC_AB1	tay	-0.0204285106420837	0.92354224224686	1	pseudobulk	DESeq2	LRT		0
812	KC_AB1	Taz	-0.200450503250375	0.730122947985572	1	pseudobulk	DESeq2	LRT		0
812	KC_AB1	Tbc1d15-17	0.75980798914669	0.582919032404186	1	pseudobulk	DESeq2	LRT		0
812	KC_AB1	TBC1D16	0.211704336880743	0.523035705215117	1	pseudobulk	DESeq2	LRT		0
812	KC_AB1	TBC1D5	-2.44595091341338	0.0753734865365363	1	pseudobulk	DESeq2	LRT		0
812	KC_AB1	TBC1d7	-0.105161659850439	0.807438493536816	1	pseudobulk	DESeq2	LRT		0
812	KC_AB1	TBCB	0.348014393992601	0.60375421940268	1	pseudobulk	DESeq2	LRT		0
812	KC_AB1	TBCC	0.0650252152162156	0.70593525806871	1	pseudobulk	DESeq2	LRT		0
813	KC_AB1	TBCD	-1.04450449908661	0.448642098516774	1	pseudobulk	DESeq2	LRT		0
813	KC_AB1	Tbce	0.92316032844904	0.277643603994407	1	pseudobulk	DESeq2	LRT		0
813	KC_AB1	Tbp	0.185872045236121	0.797295915036935	1	pseudobulk	DESeq2	LRT		0
813	KC_AB1	TBPH	-0.0839914614508255	0.694772223044291	1	pseudobulk	DESeq2	LRT		0
813	KC_AB1	tbrd-1	0.849606887838128	1	1	pseudobulk	DESeq2	LRT		0
813	KC_AB1	Tcs3	0.325893323582329	1	1	pseudobulk	DESeq2	LRT		0
813	KC_AB1	Tctp	0.195035429015269	0.408136986814047	1	pseudobulk	DESeq2	LRT		0
813	KC_AB1	Tdc1	3.41844045086015	0.351942490385471	1	pseudobulk	DESeq2	LRT		0
813	KC_AB1	Tdc2	2.78674512494791	0.54962276837928	1	pseudobulk	DESeq2	LRT		0
813	KC_AB1	Tdrd3	-0.373288264094198	0.582655587732424	1	pseudobulk	DESeq2	LRT		0
814	KC_AB1	tea	-0.0887801722811587	0.507186962131195	1	pseudobulk	DESeq2	LRT		0
814	KC_AB1	tef	-0.392379561141797	0.766630922187207	1	pseudobulk	DESeq2	LRT		0
814	KC_AB1	Teh1	0.200496668467607	0.79092517621108	1	pseudobulk	DESeq2	LRT		0
814	KC_AB1	Teh2	-0.538441037409414	0.0970363537982418	1	pseudobulk	DESeq2	LRT		0
814	KC_AB1	Teh3	-0.556628421020755	0.675401935723456	1	pseudobulk	DESeq2	LRT		0
814	KC_AB1	Teh4	-0.814025080750268	0.298575904721876	1	pseudobulk	DESeq2	LRT		0
814	KC_AB1	Tehao	1.18547808669509	0.63008522780602	1	pseudobulk	DESeq2	LRT		0
814	KC_AB1	tej	0.849606887838128	1	1	pseudobulk	DESeq2	LRT		0
814	KC_AB1	Tektin-C	2.06531092668974	0.944672961903726	1	pseudobulk	DESeq2	LRT		0
814	KC_AB1	temp	-1.09635862575369	0.322706254045488	1	pseudobulk	DESeq2	LRT		0
815	KC_AB1	Ten-a	-0.322228822710273	0.00635692869327339	1	pseudobulk	DESeq2	LRT		0
815	KC_AB1	Ten-m	-0.881326080147915	0.482366556163584	1	pseudobulk	DESeq2	LRT		0
815	KC_AB1	Tep3	0.849606887838128	1	1	pseudobulk	DESeq2	LRT		0
815	KC_AB1	Tep4	2.78674512494791	0.54962276837928	1	pseudobulk	DESeq2	LRT		0
815	KC_AB1	TER94	0.278260982659192	0.575103379927123	1	pseudobulk	DESeq2	LRT		0
815	KC_AB1	Tes	0.724322479577756	0.842077641943204	1	pseudobulk	DESeq2	LRT		0
815	KC_AB1	tex	2.43275481406755	0.550258497427138	1	pseudobulk	DESeq2	LRT		0
815	KC_AB1	TFAM	-0.149326520416707	0.796711081820627	1	pseudobulk	DESeq2	LRT		0
815	KC_AB1	Tfb1	0.24546018564654	0.756016901042568	1	pseudobulk	DESeq2	LRT		0
815	KC_AB1	Tfb4	-2.23462431491213	0.186903899880871	1	pseudobulk	DESeq2	LRT		0
816	KC_AB1	TfIIA-L	0.523037843127061	0.265496221922605	1	pseudobulk	DESeq2	LRT		0

816	KC_AB1	TfIIA-S	0.119102575403142	0.644727509841243	1	pseudobulk	DESeq2	LRT	0
816	KC_AB1	TfIIB	-0.234177659216139	0.686568885196406	1	pseudobulk	DESeq2	LRT	0
816	KC_AB1	TfIIEalpha	-0.127783698208498	0.700481662042891	1	pseudobulk	DESeq2	LRT	0
816	KC_AB1	TfIIEbeta	-0.0979265776501384	0.704788839944155	1	pseudobulk	DESeq2	LRT	0
816	KC_AB1	TfIIFalpha	0.377775044906034	0.460575063694677	1	pseudobulk	DESeq2	LRT	0
816	KC_AB1	TfIIFbeta	0.553736439494898	0.25511144541481	1	pseudobulk	DESeq2	LRT	0
816	KC_AB1	TfIIS	0.0499333595422123	0.519292175436632	1	pseudobulk	DESeq2	LRT	0
816	KC_AB1	Tg	-0.571155570398758	0.670765255013795	1	pseudobulk	DESeq2	LRT	0
816	KC_AB1	Tgi	-0.988883707698776	0.13525487483373	1	pseudobulk	DESeq2	LRT	0
817	KC_AB1	tgo	-1.38733554004595	0.194158823406853	1	pseudobulk	DESeq2	LRT	0
817	KC_AB1	Tgt	0.705885161925424	1	1	pseudobulk	DESeq2	LRT	0
817	KC_AB1	TH1	-0.33748995556885	0.686625842838997	1	pseudobulk	DESeq2	LRT	0
817	KC_AB1	THADA	0.194265322970028	1	1	pseudobulk	DESeq2	LRT	0
817	KC_AB1	Thd1	0.190512179190291	0.403723641807535	1	pseudobulk	DESeq2	LRT	0
817	KC_AB1	Theg	0.314419026731899	1	1	pseudobulk	DESeq2	LRT	0
817	KC_AB1	THG	-1.79237465788111	0.29736730774098	1	pseudobulk	DESeq2	LRT	0
817	KC_AB1	tho2	-0.851983240603211	0.193140527215089	1	pseudobulk	DESeq2	LRT	0
817	KC_AB1	thoc5	0.321394458808109	0.786325527862494	1	pseudobulk	DESeq2	LRT	0
817	KC_AB1	thoc6	0.727094345503981	0.499656394382769	1	pseudobulk	DESeq2	LRT	0
818	KC_AB1	thoc7	0.0013584980013438	0.851050258953868	1	pseudobulk	DESeq2	LRT	0
818	KC_AB1	ThrRS	-0.403286491820582	0.470676362092847	1	pseudobulk	DESeq2	LRT	0
818	KC_AB1	ths	-0.265540904329132	0.686623882170528	1	pseudobulk	DESeq2	LRT	0
818	KC_AB1	Tie	3.08753393561965	0.382167145512329	1	pseudobulk	DESeq2	LRT	0
818	KC_AB1	Tif-IA	-0.218454622969017	0.533843469833612	1	pseudobulk	DESeq2	LRT	0
818	KC_AB1	tim	-0.499050902629637	0.763605461396524	1	pseudobulk	DESeq2	LRT	0
818	KC_AB1	Tim17b	-0.321555069068675	0.222916398401739	1	pseudobulk	DESeq2	LRT	0
818	KC_AB1	Tim17b1	1.75080248448828	0.603717156600772	1	pseudobulk	DESeq2	LRT	0
818	KC_AB1	Tim23	-0.199875600710672	0.680952181604785	1	pseudobulk	DESeq2	LRT	0
818	KC_AB1	Tim8	0.143701329249452	0.529997919614996	1	pseudobulk	DESeq2	LRT	0
819	KC_AB1	Tim9a	0.589244373089334	0.384146677984175	1	pseudobulk	DESeq2	LRT	0
819	KC_AB1	timeout	-0.35461399000119	0.640314447582811	1	pseudobulk	DESeq2	LRT	0
819	KC_AB1	Timp	0.28600602903418	0.719264613807072	1	pseudobulk	DESeq2	LRT	0
819	KC_AB1	Tina-1	-0.315473695792267	0.333353336734286	1	pseudobulk	DESeq2	LRT	0
819	KC_AB1	tinc	-0.150833247664716	0.436405050747913	1	pseudobulk	DESeq2	LRT	0
819	KC_AB1	Tip60	0.214087241898656	0.754930660888871	1	pseudobulk	DESeq2	LRT	0
819	KC_AB1	tipE	-0.143619136703595	0.592601117024581	1	pseudobulk	DESeq2	LRT	0
819	KC_AB1	Tis11	2.06541384650887	0.904905526327343	1	pseudobulk	DESeq2	LRT	0
819	KC_AB1	tj	2.78674512494791	0.54962276837928	1	pseudobulk	DESeq2	LRT	0
819	KC_AB1	Tk	0.849606887838128	1	1	pseudobulk	DESeq2	LRT	0
820	KC_AB1	tko	0.514410806292008	0.419480508443978	1	pseudobulk	DESeq2	LRT	0
820	KC_AB1	TkR86C	-0.164494799102923	0.77284225122866	1	pseudobulk	DESeq2	LRT	0
820	KC_AB1	tkv	-0.712847325673036	0.232320457987229	1	pseudobulk	DESeq2	LRT	0
820	KC_AB1	Tl	-0.184846709805737	0.364072028628564	1	pseudobulk	DESeq2	LRT	0
820	KC_AB1	tld	0.849606887838128	1	1	pseudobulk	DESeq2	LRT	0
820	KC_AB1	Tlk	-0.285142692660782	0.130101619533796	1	pseudobulk	DESeq2	LRT	0
820	KC_AB1	Tm1	-0.121405589681481	0.529842644764572	1	pseudobulk	DESeq2	LRT	0
820	KC_AB1	Tm2	-0.876229688618519	0.686418568776057	1	pseudobulk	DESeq2	LRT	0
820	KC_AB1	TM4SF	0.0639908815578163	0.674253920618687	1	pseudobulk	DESeq2	LRT	0
820	KC_AB1	TM9SF2	-0.519698749077919	0.418465982377906	1	pseudobulk	DESeq2	LRT	0
821	KC_AB1	TM9SF3	0.0565607105026822	0.441761242438851	1	pseudobulk	DESeq2	LRT	0
821	KC_AB1	TM9SF4	-0.0339739402876142	0.570792890036789	1	pseudobulk	DESeq2	LRT	0
821	KC_AB1	Tmc	-2.01951118655521	0.0665229939145247	1	pseudobulk	DESeq2	LRT	0
821	KC_AB1	Tmem18	-0.457654172212919	0.613420349433126	1	pseudobulk	DESeq2	LRT	0

821	KC_AB1	TMEM216	0.749750705007676	0.298450360377438	1	pseudobulk	DESeq2	LRT	0
821	KC_AB1	Tmem63	-0.153243121379032	0.675573069795509	1	pseudobulk	DESeq2	LRT	0
821	KC_AB1	tmod	0.0815326764768029	0.801791543712466	1	pseudobulk	DESeq2	LRT	0
821	KC_AB1	TMS1	0.224542094802212	0.353938939988216	1	pseudobulk	DESeq2	LRT	0
821	KC_AB1	Tmtc3	0.253217096239709	0.536208539577358	1	pseudobulk	DESeq2	LRT	0
821	KC_AB1	tna	-0.177254232566946	0.345919964962779	1	pseudobulk	DESeq2	LRT	0
822	KC_AB1	tnc	-0.697581683371428	0.608317657917838	1	pseudobulk	DESeq2	LRT	0
822	KC_AB1	Tnks	-0.710186697808388	0.257400859775747	1	pseudobulk	DESeq2	LRT	0
822	KC_AB1	Tnpo	-0.24861598159696	0.418768622047648	1	pseudobulk	DESeq2	LRT	0
822	KC_AB1	Tnpo-SR	-0.130342345464615	0.723257695661074	1	pseudobulk	DESeq2	LRT	0
822	KC_AB1	Tob	0.0552108899878761	0.677479205036859	1	pseudobulk	DESeq2	LRT	0
822	KC_AB1	toc	-0.108658097426141	0.448701997688682	1	pseudobulk	DESeq2	LRT	0
822	KC_AB1	tok	-0.567626927107421	0.475604915311543	1	pseudobulk	DESeq2	LRT	0
822	KC_AB1	Toll-6	-0.0060684632269341	0.955209866248046	1	pseudobulk	DESeq2	LRT	0
822	KC_AB1	Toll-7	0.375790891221989	1	1	pseudobulk	DESeq2	LRT	0
822	KC_AB1	Tollo	-0.014838026305582	1	1	pseudobulk	DESeq2	LRT	0
823	KC_AB1	Tom20	-0.0517256618483142	0.670766654711411	1	pseudobulk	DESeq2	LRT	0
823	KC_AB1	Tom40	-0.147733117189491	0.508808287368982	1	pseudobulk	DESeq2	LRT	0
823	KC_AB1	Tom7	0.0356159713917376	0.577389944241438	1	pseudobulk	DESeq2	LRT	0
823	KC_AB1	Tom70	-0.638466669969444	0.363652918545324	1	pseudobulk	DESeq2	LRT	0
823	KC_AB1	tomboy40	0.116783351990007	1	1	pseudobulk	DESeq2	LRT	0
823	KC_AB1	Tomosyn	-0.231763091623296	0.0683407673457151	1	pseudobulk	DESeq2	LRT	0
823	KC_AB1	Top1	-0.257491630003145	0.278554395682883	1	pseudobulk	DESeq2	LRT	0
823	KC_AB1	Top2	0.0693873960217605	0.714326281461894	1	pseudobulk	DESeq2	LRT	0
823	KC_AB1	Top3alpha	0.0160940546605626	1	1	pseudobulk	DESeq2	LRT	0
823	KC_AB1	Top3beta	-0.534426764268472	0.512745893804295	1	pseudobulk	DESeq2	LRT	0
824	KC_AB1	Topors	-0.804740043199234	0.439801754724399	1	pseudobulk	DESeq2	LRT	0
824	KC_AB1	Tor	0.188170615053683	0.754204496458437	1	pseudobulk	DESeq2	LRT	0
824	KC_AB1	TORIP	-0.675212667786328	0.522881610929314	1	pseudobulk	DESeq2	LRT	0
824	KC_AB1	Torsin	0.161265256999325	0.635424967841592	1	pseudobulk	DESeq2	LRT	0
824	KC_AB1	TotA	-0.177837381280383	0.758165449133929	1	pseudobulk	DESeq2	LRT	0
824	KC_AB1	TotB	0.849606887838128	1	1	pseudobulk	DESeq2	LRT	0
824	KC_AB1	tou	0.141241675088401	0.439166350267637	1	pseudobulk	DESeq2	LRT	0
824	KC_AB1	tow	0.0212353846687645	0.829956634964306	1	pseudobulk	DESeq2	LRT	0
824	KC_AB1	toy	-0.156377036289612	0.256347936678902	1	pseudobulk	DESeq2	LRT	0
824	KC_AB1	Tpc1	-0.0077612150583608	0.798628638650761	1	pseudobulk	DESeq2	LRT	0
825	KC_AB1	Tpc2	2.42818428071509	0.612687220587	1	pseudobulk	DESeq2	LRT	0
825	KC_AB1	Tpi	0.297006219304246	0.304148102915117	1	pseudobulk	DESeq2	LRT	0
825	KC_AB1	TpnC41C	2.31997030691033	0.813169507846918	1	pseudobulk	DESeq2	LRT	0
825	KC_AB1	TppII	0.341098296587589	0.637061892263391	1	pseudobulk	DESeq2	LRT	0
825	KC_AB1	Tpr2	0.16536047383847	0.561651847889098	1	pseudobulk	DESeq2	LRT	0
825	KC_AB1	Tps1	1.59863986092676	1	1	pseudobulk	DESeq2	LRT	0
825	KC_AB1	Tpst	0.345757289788815	0.628735939367727	1	pseudobulk	DESeq2	LRT	0
825	KC_AB1	tra	0.247327060966797	0.675870078349326	1	pseudobulk	DESeq2	LRT	0
825	KC_AB1	tra2	-0.554894103595379	0.439481185115845	1	pseudobulk	DESeq2	LRT	0
825	KC_AB1	Traf4	-1.8476447304053	0.456955818574401	1	pseudobulk	DESeq2	LRT	0
826	KC_AB1	Traf6	-0.957721155376195	0.461484360466818	1	pseudobulk	DESeq2	LRT	0
826	KC_AB1	tral	-0.0418824870984486	0.821344089080243	1	pseudobulk	DESeq2	LRT	0
826	KC_AB1	TRAM	-0.0870708600351776	0.73668060822731	1	pseudobulk	DESeq2	LRT	0
826	KC_AB1	Trap1	-0.38515059456775	0.541809913758584	1	pseudobulk	DESeq2	LRT	0
826	KC_AB1	Trax	-0.168844864826502	0.849712971931995	1	pseudobulk	DESeq2	LRT	0
826	KC_AB1	trbd	0.148671860238454	0.584071370168204	1	pseudobulk	DESeq2	LRT	0
826	KC_AB1	trbl	0.849606887838128	1	1	pseudobulk	DESeq2	LRT	0

826	KC_AB1	trc	-0.142945510340201	0.54537587690491	1	pseudobulk	DESeq2	LRT		0
826	KC_AB1	Trc8	-0.262778971659626	0.700717761943612	1	pseudobulk	DESeq2	LRT		0
826	KC_AB1	Tre1	-1.30133167140708	0.516301705836476	1	pseudobulk	DESeq2	LRT		0
827	KC_AB1	Treh	1.29988169273607	0.167322923856131	1	pseudobulk	DESeq2	LRT		0
827	KC_AB1	trem	0.323256308716571	1	1	pseudobulk	DESeq2	LRT		0
827	KC_AB1	Tret1-1	1.43060365239813	1	1	pseudobulk	DESeq2	LRT		0
827	KC_AB1	Trf	0.260325438673914	1	1	pseudobulk	DESeq2	LRT		0
827	KC_AB1	Trf2	0.0524314358092028	0.456205133188662	1	pseudobulk	DESeq2	LRT		0
827	KC_AB1	Trf4-1	-0.0241340991563432	0.608904330315826	1	pseudobulk	DESeq2	LRT		0
827	KC_AB1	trh	2.31997030691033	0.813169507846918	1	pseudobulk	DESeq2	LRT		0
827	KC_AB1	Trh	0.849606887838128	1	1	pseudobulk	DESeq2	LRT		0
827	KC_AB1	Trim9	0.305556281478426	1	1	pseudobulk	DESeq2	LRT		0
827	KC_AB1	trio	-0.157750920035847	0.636490608734199	1	pseudobulk	DESeq2	LRT		0
828	KC_AB1	Trissin	0.366247714696288	1	1	pseudobulk	DESeq2	LRT		0
828	KC_AB1	Trl	0.216549154430144	0.6811109111382	1	pseudobulk	DESeq2	LRT		0
828	KC_AB1	trn	0.410136007540014	0.122035017787621	1	pseudobulk	DESeq2	LRT		0
828	KC_AB1	tRNA:Ala-AGC-2-1	0.849606887838128	1	1	pseudobulk	DESeq2	LRT		0
828	KC_AB1	tRNA:Arg-ACG-1-	2.31997030691033	0.813169507846918	1	pseudobulk	DESeq2	LRT		0
828	KC_AB1	tRNA:Arg-TCG-2-	0.849606887838128	1	1	pseudobulk	DESeq2	LRT		0
828	KC_AB1	tRNA:Cys-GCA-4-	0.769522064442666	1	1	pseudobulk	DESeq2	LRT		0
828	KC_AB1	tRNA:Gln-CTG-1-1	0.849606887838128	1	1	pseudobulk	DESeq2	LRT		0
828	KC_AB1	tRNA:Gln-CTG-2-	2.31997030691033	0.813169507846918	1	pseudobulk	DESeq2	LRT		0
828	KC_AB1	tRNA:Gln-CTG-3-1	0.849606887838128	1	1	pseudobulk	DESeq2	LRT		0
829	KC_AB1	tRNA:Glu-CTC-2-2	0.849606887838128	1	1	pseudobulk	DESeq2	LRT		0
829	KC_AB1	tRNA:Gly-GCC-1-	0.849606887838128	1	1	pseudobulk	DESeq2	LRT		0
829	KC_AB1	tRNA:Ile-AAT-1-1	0.183159795455075	1	1	pseudobulk	DESeq2	LRT		0
829	KC_AB1	tRNA:Ile-AAT-1-5	0.849606887838128	1	1	pseudobulk	DESeq2	LRT		0
829	KC_AB1	tRNA:Ile-TAT-1-2	0.849606887838128	1	1	pseudobulk	DESeq2	LRT		0
829	KC_AB1	tRNA:Phe-GAA-1-	2.31997030691033	0.813169507846918	1	pseudobulk	DESeq2	LRT		0
829	KC_AB1	tRNA:Phe-GAA-1-	0.849606887838128	1	1	pseudobulk	DESeq2	LRT		0
829	KC_AB1	trol	0.624433261972519	1	1	pseudobulk	DESeq2	LRT		0
829	KC_AB1	trp	0.183159795455075	1	1	pseudobulk	DESeq2	LRT		0
829	KC_AB1	Trp1	-0.031854943495707	0.57659313661775	1	pseudobulk	DESeq2	LRT		0
830	KC_AB1	Trpm	0.11282290521248	0.844371311314437	1	pseudobulk	DESeq2	LRT		0
830	KC_AB1	TrpRS	-0.805104657895581	0.548533538596495	1	pseudobulk	DESeq2	LRT		0
830	KC_AB1	TrpRS-m	2.35960105871759	0.412293848936182	1	pseudobulk	DESeq2	LRT		0
830	KC_AB1	trr	-0.112020321174444	0.582911225641725	1	pseudobulk	DESeq2	LRT		0
830	KC_AB1	Trs20	-0.23394769035251	0.544491991729718	1	pseudobulk	DESeq2	LRT		0
830	KC_AB1	Trs23	-0.255179304218508	0.719784934329277	1	pseudobulk	DESeq2	LRT		0
830	KC_AB1	Trs31	-0.91702204775697	0.265278949382756	1	pseudobulk	DESeq2	LRT		0
830	KC_AB1	Trs33	0.358468068236569	0.897731174871691	1	pseudobulk	DESeq2	LRT		0
830	KC_AB1	trsn	0.532639872717061	0.483272510004573	1	pseudobulk	DESeq2	LRT		0
830	KC_AB1	trus	1.01711836439449	0.318119393884635	1	pseudobulk	DESeq2	LRT		0
831	KC_AB1	trv	0.0087194447564189	0.925438907903233	1	pseudobulk	DESeq2	LRT		0
831	KC_AB1	trx	-0.37926144391037	0.171358501914973	1	pseudobulk	DESeq2	LRT		0
831	KC_AB1	Trx-2	0.427232598230122	0.232922775991562	1	pseudobulk	DESeq2	LRT		0
831	KC_AB1	Trxr-1	-0.553622003114325	0.368053709759586	1	pseudobulk	DESeq2	LRT		0
831	KC_AB1	Try29F	0.0571155708876178	1	1	pseudobulk	DESeq2	LRT		0
831	KC_AB1	Tsc1	-0.0953748225898782	0.71962911114452	1	pseudobulk	DESeq2	LRT		0
831	KC_AB1	Tsen15	0.849606887838128	1	1	pseudobulk	DESeq2	LRT		0
831	KC_AB1	Tsen2	-1.99750092438004	0.257470239770557	1	pseudobulk	DESeq2	LRT		0
831	KC_AB1	Tsen34	-0.305122110428823	0.715648875247252	1	pseudobulk	DESeq2	LRT		0
831	KC_AB1	Tsen54	1.21267130850476	1	1	pseudobulk	DESeq2	LRT		0

832	KC_AB1	Tsf1	-3.04237815814362	0.0492846273121642	1	pseudobulk	DESeq2	LRT		0
832	KC_AB1	Tsf2	0.343033648324813	0.705918541829006	1	pseudobulk	DESeq2	LRT		0
832	KC_AB1	Tsf3	2.31997030691033	0.813169507846918	1	pseudobulk	DESeq2	LRT		0
832	KC_AB1	TSG101	-0.611711054790228	0.445281567612453	1	pseudobulk	DESeq2	LRT		0
832	KC_AB1	tsh	0.849606887838128	1	1	pseudobulk	DESeq2	LRT		0
832	KC_AB1	tsl	-0.833404182616462	0.691823612159792	1	pseudobulk	DESeq2	LRT		0
832	KC_AB1	Tsp26A	-0.473890838755881	0.484737215338886	1	pseudobulk	DESeq2	LRT		0
832	KC_AB1	Tsp39D	-0.0711147776954516	0.719495647714812	1	pseudobulk	DESeq2	LRT		0
832	KC_AB1	Tsp3A	0.511116253607799	0.478278695677301	1	pseudobulk	DESeq2	LRT		0
832	KC_AB1	Tsp42A	0.849606887838128	1	1	pseudobulk	DESeq2	LRT		0
833	KC_AB1	Tsp42Ea	0.501214492091809	0.788383036999197	1	pseudobulk	DESeq2	LRT		0
833	KC_AB1	Tsp42Ed	3.41844045086015	0.351942490385471	1	pseudobulk	DESeq2	LRT		0
833	KC_AB1	Tsp42Ee	0.0281472196618564	0.731462906889874	1	pseudobulk	DESeq2	LRT		0
833	KC_AB1	Tsp42Ef	-0.3056891428917	0.836825835077291	1	pseudobulk	DESeq2	LRT		0
833	KC_AB1	Tsp42Eg	-0.830062897938551	0.717724257445671	1	pseudobulk	DESeq2	LRT		0
833	KC_AB1	Tsp42Ei	-1.25393399638069	0.612054104673377	1	pseudobulk	DESeq2	LRT		0
833	KC_AB1	Tsp42Ej	0.316904361200216	0.228436882955487	1	pseudobulk	DESeq2	LRT		0
833	KC_AB1	Tsp42Ek	-0.337372597830673	0.653492813511745	1	pseudobulk	DESeq2	LRT		0
833	KC_AB1	Tsp42El	-3.28422984766061	0.357802653325262	1	pseudobulk	DESeq2	LRT		0
833	KC_AB1	Tsp42En	0.0721169730576095	0.626289123615637	1	pseudobulk	DESeq2	LRT		0
834	KC_AB1	Tsp42Eo	0.814717522193354	1	1	pseudobulk	DESeq2	LRT		0
834	KC_AB1	Tsp42Ep	2.31997030691033	0.813169507846918	1	pseudobulk	DESeq2	LRT		0
834	KC_AB1	Tsp5D	-0.805104657895581	0.548533538596495	1	pseudobulk	DESeq2	LRT		0
834	KC_AB1	Tsp66E	-0.523108177032275	0.0609594443573201	1	pseudobulk	DESeq2	LRT		0
834	KC_AB1	Tsp74F	0.849606887838128	1	1	pseudobulk	DESeq2	LRT		0
834	KC_AB1	Tsp86D	-0.0118099675128135	0.695586051577044	1	pseudobulk	DESeq2	LRT		0
834	KC_AB1	Tsp96F	0.0368139300027952	0.918505815050661	1	pseudobulk	DESeq2	LRT		0
834	KC_AB1	Tsp97E	-0.0922533809490309	0.54981966611567	1	pseudobulk	DESeq2	LRT		0
834	KC_AB1	Tspo	-0.906681239498541	0.379793242098493	1	pseudobulk	DESeq2	LRT		0
834	KC_AB1	tsr	0.066809723183287	0.560943679306345	1	pseudobulk	DESeq2	LRT		0
835	KC_AB1	Tsr1	0.409954645590393	1	1	pseudobulk	DESeq2	LRT		0
835	KC_AB1	tst	-0.209177628788863	0.836714394059029	1	pseudobulk	DESeq2	LRT		0
835	KC_AB1	tsu	0.395831976857732	0.613218945437997	1	pseudobulk	DESeq2	LRT		0
835	KC_AB1	Ttc19	-1.12560606060517	0.360900713033912	1	pseudobulk	DESeq2	LRT		0
835	KC_AB1	Ttc7	-0.247847622023124	0.689598600528074	1	pseudobulk	DESeq2	LRT		0
835	KC_AB1	Ttd14	0.128795265173572	0.758864098043643	1	pseudobulk	DESeq2	LRT		0
835	KC_AB1	tth	-0.517795253800928	0.341389899237587	1	pseudobulk	DESeq2	LRT		0
835	KC_AB1	ttk	-0.663002251527988	0.718344636482651	1	pseudobulk	DESeq2	LRT		0
835	KC_AB1	TTLL12	-0.872919007832744	0.566129956589624	1	pseudobulk	DESeq2	LRT		0
835	KC_AB1	TTLL15	-0.564356698759404	0.803702590456371	1	pseudobulk	DESeq2	LRT		0
836	KC_AB1	TTLL3A	2.31997030691033	0.813169507846918	1	pseudobulk	DESeq2	LRT		0
836	KC_AB1	TTLL4A	-0.187922851523242	0.533462099366284	1	pseudobulk	DESeq2	LRT		0
836	KC_AB1	TTLL5	-0.158146255733797	0.737517788506454	1	pseudobulk	DESeq2	LRT		0
836	KC_AB1	ttm50	-0.312081982826977	0.539143847035275	1	pseudobulk	DESeq2	LRT		0
836	KC_AB1	ttv	-0.487627711950542	0.102162652007954	1	pseudobulk	DESeq2	LRT		0
836	KC_AB1	tty	-0.2751674188512	0.368728503719342	1	pseudobulk	DESeq2	LRT		0
836	KC_AB1	tub	0.0761042192954759	0.674518992455403	1	pseudobulk	DESeq2	LRT		0
836	KC_AB1	tud	-0.383051453708091	0.484274178439321	1	pseudobulk	DESeq2	LRT		0
836	KC_AB1	Tudor-SN	0.0467210244457203	0.889626379971893	1	pseudobulk	DESeq2	LRT		0
836	KC_AB1	tun	0.548099896786603	0.0942977314489072	1	pseudobulk	DESeq2	LRT		0
837	KC_AB1	Tusp	-0.470589497743722	0.350637816858774	1	pseudobulk	DESeq2	LRT		0
837	KC_AB1	tut	1.50875020934616	0.732232096088675	1	pseudobulk	DESeq2	LRT		0
837	KC_AB1	tutl	0.0068284499658275	0.890268729212224	1	pseudobulk	DESeq2	LRT		0

837	KC_AB1	tw	2.31997030691033	0.813169507846918	1	pseudobulk	DESeq2	LRT	0
837	KC_AB1	tweek	0.110576122214209	0.621114945097739	1	pseudobulk	DESeq2	LRT	0
837	KC_AB1	twf	-0.83022829518673	0.446318570302875	1	pseudobulk	DESeq2	LRT	0
837	KC_AB1	twin	0.229247228246712	0.497601186403994	1	pseudobulk	DESeq2	LRT	0
837	KC_AB1	twit	0.849606887838128	1	1	pseudobulk	DESeq2	LRT	0
837	KC_AB1	twr	0.466587407311188	0.384406965676997	1	pseudobulk	DESeq2	LRT	0
837	KC_AB1	tws	-0.0212584582127761	0.787501130993782	1	pseudobulk	DESeq2	LRT	0
838	KC_AB1	twz	-0.284411192716741	0.730958159258234	1	pseudobulk	DESeq2	LRT	0
838	KC_AB1	tx	2.06541384650887	0.768363369045814	1	pseudobulk	DESeq2	LRT	0
838	KC_AB1	Txl	-0.313251241366523	0.60803655493543	1	pseudobulk	DESeq2	LRT	0
838	KC_AB1	tyf	0.0615620919502033	0.803161125093892	1	pseudobulk	DESeq2	LRT	0
838	KC_AB1	tyn	-0.788996319851018	0.290845904757405	1	pseudobulk	DESeq2	LRT	0
838	KC_AB1	TyrR	1.05474038785733	0.467004455077801	1	pseudobulk	DESeq2	LRT	0
838	KC_AB1	TyrRS	0.0352517095321935	0.547533228946423	1	pseudobulk	DESeq2	LRT	0
838	KC_AB1	TyrRS-m	0.0917064358749574	1	1	pseudobulk	DESeq2	LRT	0
838	KC_AB1	tzn	0.0863016123718256	0.642310134952002	1	pseudobulk	DESeq2	LRT	0
838	KC_AB1	U2A	0.595141163004889	0.63362927283475	1	pseudobulk	DESeq2	LRT	0
839	KC_AB1	U2af38	0.245163516607458	0.672652309733885	1	pseudobulk	DESeq2	LRT	0
839	KC_AB1	U2af50	-0.0001296847533698	0.786907218912331	1	pseudobulk	DESeq2	LRT	0
839	KC_AB1	U3-55K	2.7695050463561	0.270878570003605	1	pseudobulk	DESeq2	LRT	0
839	KC_AB1	U4-U6-60K	0.171595606284684	0.788545997427407	1	pseudobulk	DESeq2	LRT	0
839	KC_AB1	Uba1	-0.439926118377449	0.36160018205497	1	pseudobulk	DESeq2	LRT	0
839	KC_AB1	Uba2	0.0241093652015925	0.688152622750189	1	pseudobulk	DESeq2	LRT	0
839	KC_AB1	Uba3	-0.816507379449825	0.489261460535512	1	pseudobulk	DESeq2	LRT	0
839	KC_AB1	Uba4	2.94565678162588	0.316359210641813	1	pseudobulk	DESeq2	LRT	0
839	KC_AB1	Uba5	-0.482669143513822	0.703620610536866	1	pseudobulk	DESeq2	LRT	0
839	KC_AB1	Ubc10	-0.303905327409592	0.511650240360746	1	pseudobulk	DESeq2	LRT	0
840	KC_AB1	Ubc2	0.286231607595593	0.658545767206594	1	pseudobulk	DESeq2	LRT	0
840	KC_AB1	Ubc4	-0.0368354833048032	0.912621475841528	1	pseudobulk	DESeq2	LRT	0
840	KC_AB1	Ubc6	0.214020997083721	0.549728984941849	1	pseudobulk	DESeq2	LRT	0
840	KC_AB1	Ubc7	-0.0519972166145669	0.70013563102028	1	pseudobulk	DESeq2	LRT	0
840	KC_AB1	UbcE2H	-0.224940160058061	0.483877530454019	1	pseudobulk	DESeq2	LRT	0
840	KC_AB1	UbcE2M	-0.247555016148736	0.632668023091135	1	pseudobulk	DESeq2	LRT	0
840	KC_AB1	Ube3a	0.0773668544688638	0.78272315337199	1	pseudobulk	DESeq2	LRT	0
840	KC_AB1	Ubi-p5E	-0.381768910117492	0.223285965537217	1	pseudobulk	DESeq2	LRT	0
840	KC_AB1	Ubi-p63E	-0.586423393986229	0.137024042536099	1	pseudobulk	DESeq2	LRT	0
840	KC_AB1	ubl	-0.123154719851858	0.691541721727463	1	pseudobulk	DESeq2	LRT	0
841	KC_AB1	UBL3	0.231382595496181	0.311134285376606	1	pseudobulk	DESeq2	LRT	0
841	KC_AB1	Ublcp1	-0.0839770064941475	0.729181694153038	1	pseudobulk	DESeq2	LRT	0
841	KC_AB1	Ubqn	-0.610409253746152	0.238233098041561	1	pseudobulk	DESeq2	LRT	0
841	KC_AB1	Ubr1	0.119961671787442	0.339807223238965	1	pseudobulk	DESeq2	LRT	0
841	KC_AB1	Ubr3	-0.104745483301293	0.424213472905642	1	pseudobulk	DESeq2	LRT	0
841	KC_AB1	Uch	-0.298357583229827	0.677494728043746	1	pseudobulk	DESeq2	LRT	0
841	KC_AB1	Uch-L5	-0.183045138925573	0.786132511957335	1	pseudobulk	DESeq2	LRT	0
841	KC_AB1	Uck	0.162867182796515	0.686982599932009	1	pseudobulk	DESeq2	LRT	0
841	KC_AB1	Ucp4A	-0.127202533233993	0.720709244116425	1	pseudobulk	DESeq2	LRT	0
841	KC_AB1	udd	0.696862234906232	0.403864108200734	1	pseudobulk	DESeq2	LRT	0
842	KC_AB1	Ude	2.31997030691033	0.813169507846918	1	pseudobulk	DESeq2	LRT	0
842	KC_AB1	udt	-0.707953580573672	0.552377476820983	1	pseudobulk	DESeq2	LRT	0
842	KC_AB1	Uev1A	-0.0222149925654434	0.51534359570888	1	pseudobulk	DESeq2	LRT	0
842	KC_AB1	uex	-0.156605037797412	0.416999527715361	1	pseudobulk	DESeq2	LRT	0
842	KC_AB1	Ufc1	-0.260787255553401	0.606764116623025	1	pseudobulk	DESeq2	LRT	0
842	KC_AB1	Ufd1-like	0.171326930499888	0.787401272523839	1	pseudobulk	DESeq2	LRT	0

842	KC_AB1	Ufd4	0.160389947498533	0.668947431003258	1	pseudobulk	DESeq2	LRT	0
842	KC_AB1	Ufl1	0.0779338305128068	0.758313883016737	1	pseudobulk	DESeq2	LRT	0
842	KC_AB1	Ufm1	0.0660815448921778	0.598322494885552	1	pseudobulk	DESeq2	LRT	0
842	KC_AB1	Ugalt	-0.95386123499568	0.354558999345573	1	pseudobulk	DESeq2	LRT	0
843	KC_AB1	Uggt	-1.81608320436247	0.212794475442387	1	pseudobulk	DESeq2	LRT	0
843	KC_AB1	UGP	0.141701272271015	0.819254079325746	1	pseudobulk	DESeq2	LRT	0
843	KC_AB1	Ugt301D1	0.183159795455075	1	1	pseudobulk	DESeq2	LRT	0
843	KC_AB1	Ugt302C1	0.849606887838128	1	1	pseudobulk	DESeq2	LRT	0
843	KC_AB1	Ugt302K1	0.366247714696288	1	1	pseudobulk	DESeq2	LRT	0
843	KC_AB1	Ugt316A1	0.448307910323414	0.46215833463058	1	pseudobulk	DESeq2	LRT	0
843	KC_AB1	Ugt35B1	-0.251137557101777	0.734811947840221	1	pseudobulk	DESeq2	LRT	0
843	KC_AB1	Ugt35C1	0.849606887838128	1	1	pseudobulk	DESeq2	LRT	0
843	KC_AB1	Ugt35E2	2.31997030691033	0.813169507846918	1	pseudobulk	DESeq2	LRT	0
843	KC_AB1	Ugt36D1	0.849606887838128	1	1	pseudobulk	DESeq2	LRT	0
844	KC_AB1	Ugt37D1	0.849606887838128	1	1	pseudobulk	DESeq2	LRT	0
844	KC_AB1	Ugt49B1	0.56665731550197	1	1	pseudobulk	DESeq2	LRT	0
844	KC_AB1	Uhg1	0.140985356493145	0.811339774319344	1	pseudobulk	DESeq2	LRT	0
844	KC_AB1	Uhg2	0.273131489366144	1	1	pseudobulk	DESeq2	LRT	0
844	KC_AB1	Uhg3	0.80565420850995	0.164282456558659	1	pseudobulk	DESeq2	LRT	0
844	KC_AB1	Uhg4	0.768735879104643	0.596349539559397	1	pseudobulk	DESeq2	LRT	0
844	KC_AB1	Uhg5	-0.529088480862243	0.342352335878456	1	pseudobulk	DESeq2	LRT	0
844	KC_AB1	Uhg8	0.85879998658552	1	1	pseudobulk	DESeq2	LRT	0
844	KC_AB1	uif	-0.389346928562833	0.820127740800904	1	pseudobulk	DESeq2	LRT	0
844	KC_AB1	UK114	0.849606887838128	1	1	pseudobulk	DESeq2	LRT	0
845	KC_AB1	Ulp1	-0.0054023221446361	0.970985471206738	1	pseudobulk	DESeq2	LRT	0
845	KC_AB1	unc-104	0.00496224185427589	0.667649510622232	1	pseudobulk	DESeq2	LRT	0
845	KC_AB1	Unc-115a	0.0439924260456139	0.924459419503134	1	pseudobulk	DESeq2	LRT	0
845	KC_AB1	Unc-115b	1.43060365239813	1	1	pseudobulk	DESeq2	LRT	0
845	KC_AB1	unc-119	-0.167740466041401	0.543435964683053	1	pseudobulk	DESeq2	LRT	0
845	KC_AB1	unc-13	-0.306544675490259	0.0219041732135246	1	pseudobulk	DESeq2	LRT	0
845	KC_AB1	unc-13-4A	-0.735912240340109	0.00444302776086829	1	pseudobulk	DESeq2	LRT	0
845	KC_AB1	unc-45	-0.151248483157865	0.793150935639499	1	pseudobulk	DESeq2	LRT	0
845	KC_AB1	unc-5	-0.476147910234439	0.296501958418684	1	pseudobulk	DESeq2	LRT	0
845	KC_AB1	Unc-76	-0.225995906393781	0.345096320564518	1	pseudobulk	DESeq2	LRT	0
846	KC_AB1	Unc-89	-1.49098669779345	0.444448378968039	1	pseudobulk	DESeq2	LRT	0
846	KC_AB1	unc79	-0.177423818571985	0.35712006376504	1	pseudobulk	DESeq2	LRT	0
846	KC_AB1	unc80	-0.645965717854651	0.0656750022003456	1	pseudobulk	DESeq2	LRT	0
846	KC_AB1	und	-0.342950046507833	0.538810556908558	1	pseudobulk	DESeq2	LRT	0
846	KC_AB1	unk	-0.30427223103808	0.387089167853051	1	pseudobulk	DESeq2	LRT	0
846	KC_AB1	Unr	-0.0755595087648694	0.476108540603888	1	pseudobulk	DESeq2	LRT	0
846	KC_AB1	Updo	-0.532018727255156	0.172462135004369	1	pseudobulk	DESeq2	LRT	0
846	KC_AB1	Upf1	0.729735816217761	0.188901088561655	1	pseudobulk	DESeq2	LRT	0
846	KC_AB1	Upf2	0.629966525147039	0.583055817035871	1	pseudobulk	DESeq2	LRT	0
846	KC_AB1	Upf3	2.01864121824969	0.326253449244482	1	pseudobulk	DESeq2	LRT	0
847	KC_AB1	upSET	-0.305617821412038	0.210306446207713	1	pseudobulk	DESeq2	LRT	0
847	KC_AB1	UQCR-11	0.14213192754984	0.495523042469561	1	pseudobulk	DESeq2	LRT	0
847	KC_AB1	UQCR-11L	0.849606887838128	1	1	pseudobulk	DESeq2	LRT	0
847	KC_AB1	UQCR-14	0.013855740264999	0.697051657197491	1	pseudobulk	DESeq2	LRT	0
847	KC_AB1	UQCR-6.4	-0.0191876590200483	0.612888869395913	1	pseudobulk	DESeq2	LRT	0
847	KC_AB1	UQCR-C1	-0.0891476053612141	0.744547375216339	1	pseudobulk	DESeq2	LRT	0
847	KC_AB1	UQCR-C2	0.0249523741073886	0.575993520709302	1	pseudobulk	DESeq2	LRT	0
847	KC_AB1	UQCR-Q	0.125084072717529	0.506436697268023	1	pseudobulk	DESeq2	LRT	0
847	KC_AB1	uri	0.237022465848507	1	1	pseudobulk	DESeq2	LRT	0

847	KC_AB1	Urm1	-0.531146052610669	0.620586233797598	1	pseudobulk	DESeq2	LRT		0
848	KC_AB1	Uros1	0.162118002617676	1	1	pseudobulk	DESeq2	LRT		0
848	KC_AB1	Uros2	0.849606887838128	1	1	pseudobulk	DESeq2	LRT		0
848	KC_AB1	Use1	-0.478811769428506	0.523550048398174	1	pseudobulk	DESeq2	LRT		0
848	KC_AB1	Usf	-0.169012069803608	0.630279724618223	1	pseudobulk	DESeq2	LRT		0
848	KC_AB1	ush	0.340532871387421	1	1	pseudobulk	DESeq2	LRT		0
848	KC_AB1	usp	-0.214991636499574	0.646062935464614	1	pseudobulk	DESeq2	LRT		0
848	KC_AB1	Usp1	0.282354875543434	0.399942741815929	1	pseudobulk	DESeq2	LRT		0
848	KC_AB1	Usp10	0.0008901855267678	0.941365401100568	1	pseudobulk	DESeq2	LRT		0
848	KC_AB1	Usp12-46	-0.0333211380992981	0.732013278298093	1	pseudobulk	DESeq2	LRT		0
848	KC_AB1	Usp14	0.162532571205068	0.76899880822725	1	pseudobulk	DESeq2	LRT		0
849	KC_AB1	Usp15-31	0.422076590066007	0.5330315464803	1	pseudobulk	DESeq2	LRT		0
849	KC_AB1	Usp16-45	-0.204925540128823	0.688783821068079	1	pseudobulk	DESeq2	LRT		0
849	KC_AB1	Usp2	0.0229111082068762	0.855997983380329	1	pseudobulk	DESeq2	LRT		0
849	KC_AB1	Usp20-33	0.482290300549752	0.588378421640333	1	pseudobulk	DESeq2	LRT		0
849	KC_AB1	Usp30	0.0458801275150917	0.595624873662301	1	pseudobulk	DESeq2	LRT		0
849	KC_AB1	Usp32	-0.52326865275699	0.466600803415306	1	pseudobulk	DESeq2	LRT		0
849	KC_AB1	Usp39	0.0809627571577082	0.860736394940859	1	pseudobulk	DESeq2	LRT		0
849	KC_AB1	Usp47	-0.140538609068307	0.55539582250395	1	pseudobulk	DESeq2	LRT		0
849	KC_AB1	Usp5	-0.383981664180002	0.619034048184287	1	pseudobulk	DESeq2	LRT		0
849	KC_AB1	Usp7	-0.190379769779102	0.37899473072108	1	pseudobulk	DESeq2	LRT		0
850	KC_AB1	Usp8	0.0856598367893428	0.876976710097748	1	pseudobulk	DESeq2	LRT		0
850	KC_AB1	Utx	-1.34193665567418	0.0693197617228307	1	pseudobulk	DESeq2	LRT		0
850	KC_AB1	Uvrag	-0.275455178247629	0.698450482422816	1	pseudobulk	DESeq2	LRT		0
850	KC_AB1	Uxs	-0.132145053906491	0.789676944630615	1	pseudobulk	DESeq2	LRT		0
850	KC_AB1	Uxt	-0.309305245328974	0.665171368376879	1	pseudobulk	DESeq2	LRT		0
850	KC_AB1	uzip	-0.433998241217183	0.273660897823	1	pseudobulk	DESeq2	LRT		0
850	KC_AB1	v	0.849606887838128	1	1	pseudobulk	DESeq2	LRT		0
850	KC_AB1	VAChT	-0.108024824145564	0.505361500130753	1	pseudobulk	DESeq2	LRT		0
850	KC_AB1	ValRS	-0.665770341292554	0.508385460885514	1	pseudobulk	DESeq2	LRT		0
850	KC_AB1	Vamp7	0.130199203539337	0.594486977645393	1	pseudobulk	DESeq2	LRT		0
851	KC_AB1	Vang	0.363168225128316	0.563800441330553	1	pseudobulk	DESeq2	LRT		0
851	KC_AB1	vap	0.0819874737373664	0.799507550709573	1	pseudobulk	DESeq2	LRT		0
851	KC_AB1	Vap33	-0.118374234434264	0.550689939784207	1	pseudobulk	DESeq2	LRT		0
851	KC_AB1	vari	0.203041112050382	0.861726901816197	1	pseudobulk	DESeq2	LRT		0
851	KC_AB1	Vav	-0.896404063508088	0.226272880880218	1	pseudobulk	DESeq2	LRT		0
851	KC_AB1	Vdup1	0.773968368885371	1	1	pseudobulk	DESeq2	LRT		0
851	KC_AB1	veli	0.380592170491893	0.374737990100915	1	pseudobulk	DESeq2	LRT		0
851	KC_AB1	velo	-0.26724928935185	0.649028331871226	1	pseudobulk	DESeq2	LRT		0
851	KC_AB1	VepD	-0.345926701848752	0.210348060553953	1	pseudobulk	DESeq2	LRT		0
851	KC_AB1	ver	0.116783351990007	1	1	pseudobulk	DESeq2	LRT		0
852	KC_AB1	verm	3.81892523345342	0.259683132508622	1	pseudobulk	DESeq2	LRT		0
852	KC_AB1	vers	-0.976958734051115	0.311427111076436	1	pseudobulk	DESeq2	LRT		0
852	KC_AB1	VGAT	-0.255682586511966	0.601991727788226	1	pseudobulk	DESeq2	LRT		0
852	KC_AB1	VGlut	1.59863986092676	1	1	pseudobulk	DESeq2	LRT		0
852	KC_AB1	Vha100-1	-0.381840104937125	0.138455168056226	1	pseudobulk	DESeq2	LRT		0
852	KC_AB1	Vha13	0.141799111273562	0.475999235105088	1	pseudobulk	DESeq2	LRT		0
852	KC_AB1	Vha14-1	0.0543694717949051	0.612289663869039	1	pseudobulk	DESeq2	LRT		0
852	KC_AB1	Vha14-2	0.849606887838128	1	1	pseudobulk	DESeq2	LRT		0
852	KC_AB1	Vha16-1	0.0684488707110073	0.581619863307621	1	pseudobulk	DESeq2	LRT		0
852	KC_AB1	Vha26	0.199103349724495	0.346105257831364	1	pseudobulk	DESeq2	LRT		0
853	KC_AB1	Vha36-1	0.0445321427955956	0.622193181099484	1	pseudobulk	DESeq2	LRT		0
853	KC_AB1	Vha44	-0.103810606030246	0.434880154169426	1	pseudobulk	DESeq2	LRT		0

853	KC_AB1	Vha55	0.0141552908139592	0.52075661211763	1	pseudobulk	DESeq2	LRT		0
853	KC_AB1	Vha68-1	0.176215617119668	0.43110818907368	1	pseudobulk	DESeq2	LRT		0
853	KC_AB1	Vha68-2	0.0554393740136241	1	1	pseudobulk	DESeq2	LRT		0
853	KC_AB1	VhaAC39-1	0.121923727847408	0.532424930956103	1	pseudobulk	DESeq2	LRT		0
853	KC_AB1	VhaAC45	-0.803809654027172	0.34816657573138	1	pseudobulk	DESeq2	LRT		0
853	KC_AB1	VhaM9.7-a	0.356732884568248	0.0804637902715523	1	pseudobulk	DESeq2	LRT		0
853	KC_AB1	VhaM9.7-b	0.30545388559521	0.250091758862949	1	pseudobulk	DESeq2	LRT		0
853	KC_AB1	VhaM9.7-c	0.0221500568615403	0.847165357669084	1	pseudobulk	DESeq2	LRT		0
854	KC_AB1	VhaPPA1-1	0.0340310521408513	0.495892718176587	1	pseudobulk	DESeq2	LRT		0
854	KC_AB1	VhaSFD	0.1637070189063	0.511186209318485	1	pseudobulk	DESeq2	LRT		0
854	KC_AB1	Vhl	0.0766368099585016	0.889399800761211	1	pseudobulk	DESeq2	LRT		0
854	KC_AB1	viaf	0.115817635559037	0.852696607720448	1	pseudobulk	DESeq2	LRT		0
854	KC_AB1	vib	0.0241575284042285	0.5394118571432	1	pseudobulk	DESeq2	LRT		0
854	KC_AB1	vig	-0.120483876140885	0.441709552379139	1	pseudobulk	DESeq2	LRT		0
854	KC_AB1	vig2	-0.223428495081333	0.50090014091157	1	pseudobulk	DESeq2	LRT		0
854	KC_AB1	vih	0.0036066216367021	0.704129240070909	1	pseudobulk	DESeq2	LRT		0
854	KC_AB1	vimar	0.367448698668977	0.509408435670171	1	pseudobulk	DESeq2	LRT		0
854	KC_AB1	Vinc	-1.75126583973683	0.247731196700987	1	pseudobulk	DESeq2	LRT		0
855	KC_AB1	vir	-1.18753819119296	0.33441994056501	1	pseudobulk	DESeq2	LRT		0
855	KC_AB1	vir-1	-0.591003422229726	0.620414017612915	1	pseudobulk	DESeq2	LRT		0
855	KC_AB1	vis	0.849606887838128	1	1	pseudobulk	DESeq2	LRT		0
855	KC_AB1	vito	-0.483096944047981	0.63388594537714	1	pseudobulk	DESeq2	LRT		0
855	KC_AB1	Vkor	2.31997030691033	0.813169507846918	1	pseudobulk	DESeq2	LRT		0
855	KC_AB1	vlc	-0.454927958162794	0.372385950791795	1	pseudobulk	DESeq2	LRT		0
855	KC_AB1	Vlet	0.0338204246157983	0.702167111446675	1	pseudobulk	DESeq2	LRT		0
855	KC_AB1	vls	0.409346762770009	0.680605086438025	1	pseudobulk	DESeq2	LRT		0
855	KC_AB1	Vm26Ab	2.78674512494791	0.54962276837928	1	pseudobulk	DESeq2	LRT		0
855	KC_AB1	Vm32E	-0.766487484571281	0.674970337807573	1	pseudobulk	DESeq2	LRT		0
856	KC_AB1	Vmat	0.183159795455075	1	1	pseudobulk	DESeq2	LRT		0
856	KC_AB1	vnc	0.849606887838128	1	1	pseudobulk	DESeq2	LRT		0
856	KC_AB1	vnd	-0.40200137056069	0.861588439126694	1	pseudobulk	DESeq2	LRT		0
856	KC_AB1	Vps11	-0.590121984872928	0.314382094736545	1	pseudobulk	DESeq2	LRT		0
856	KC_AB1	Vps13	-0.0578291475227762	0.845332818151897	1	pseudobulk	DESeq2	LRT		0
856	KC_AB1	Vps13B	-1.17454542974403	0.44967995890894	1	pseudobulk	DESeq2	LRT		0
856	KC_AB1	Vps13D	0.69172171947036	0.64932892932268	1	pseudobulk	DESeq2	LRT		0
856	KC_AB1	Vps15	-0.535460910078942	0.643933169339705	1	pseudobulk	DESeq2	LRT		0
856	KC_AB1	Vps16A	-0.525822606705479	0.560486638845297	1	pseudobulk	DESeq2	LRT		0
856	KC_AB1	Vps16B	-1.1498219243931	0.399171626307316	1	pseudobulk	DESeq2	LRT		0
857	KC_AB1	Vps2	-0.462731373891271	0.380448596653989	1	pseudobulk	DESeq2	LRT		0
857	KC_AB1	Vps20	0.38965144094411	0.239312761009616	1	pseudobulk	DESeq2	LRT		0
857	KC_AB1	Vps24	-0.366940239287379	0.35071520066231	1	pseudobulk	DESeq2	LRT		0
857	KC_AB1	Vps25	-0.106820982317622	0.623747265389006	1	pseudobulk	DESeq2	LRT		0
857	KC_AB1	Vps26	0.857823733024828	0.187256074270713	1	pseudobulk	DESeq2	LRT		0
857	KC_AB1	Vps28	0.20875230089743	0.770307872879733	1	pseudobulk	DESeq2	LRT		0
857	KC_AB1	Vps29	0.0455314818387494	0.665147511815392	1	pseudobulk	DESeq2	LRT		0
857	KC_AB1	Vps33B	-0.984137767810984	0.444831971240951	1	pseudobulk	DESeq2	LRT		0
857	KC_AB1	Vps35	0.709560984186348	0.310644486775912	1	pseudobulk	DESeq2	LRT		0
857	KC_AB1	Vps36	-0.773103495425828	0.472203130262023	1	pseudobulk	DESeq2	LRT		0
858	KC_AB1	Vps37B	-0.32652402856902	0.539881118543071	1	pseudobulk	DESeq2	LRT		0
858	KC_AB1	Vps39	0.432141054722593	0.858953376317962	1	pseudobulk	DESeq2	LRT		0
858	KC_AB1	Vps4	-0.0817270156117655	0.653020048472927	1	pseudobulk	DESeq2	LRT		0
858	KC_AB1	Vps45	-0.826106784547727	0.416210757395427	1	pseudobulk	DESeq2	LRT		0
858	KC_AB1	Vps50	0.0441740222579112	0.739591330762325	1	pseudobulk	DESeq2	LRT		0

858	KC_AB1	Vps51	0.13969269499592	0.517937832609371	1	pseudobulk	DESeq2	LRT	0
858	KC_AB1	Vps52	-0.644634083550138	0.538961448907796	1	pseudobulk	DESeq2	LRT	0
858	KC_AB1	Vps53	-0.465022094781669	0.60620685677478	1	pseudobulk	DESeq2	LRT	0
858	KC_AB1	Vps60	0.0869876656204298	0.628211433108164	1	pseudobulk	DESeq2	LRT	0
858	KC_AB1	Vps8	-0.98195075920546	0.316546229938969	1	pseudobulk	DESeq2	LRT	0
859	KC_AB1	vri	0.183159795455075	1	1	pseudobulk	DESeq2	LRT	0
859	KC_AB1	Vrp1	0.410863430490149	0.163071598871674	1	pseudobulk	DESeq2	LRT	0
859	KC_AB1	vsg	0.317353845694722	0.318015974318799	1	pseudobulk	DESeq2	LRT	0
859	KC_AB1	Vsp37A	0.285069929258868	0.586415201278204	1	pseudobulk	DESeq2	LRT	0
859	KC_AB1	Vsx1	-1.08390588230547	0.0138792292509087	1	pseudobulk	DESeq2	LRT	0
859	KC_AB1	Vsx2	0.849606887838128	1	1	pseudobulk	DESeq2	LRT	0
859	KC_AB1	Vta1	0.417458260490054	0.353840352851205	1	pseudobulk	DESeq2	LRT	0
859	KC_AB1	vtd	-0.617633130807426	0.161381336784131	1	pseudobulk	DESeq2	LRT	0
859	KC_AB1	Vti1a	0.0089554499530342	0.959318053536072	1	pseudobulk	DESeq2	LRT	0
859	KC_AB1	Vti1b	0.639181037316062	0.227362297666814	1	pseudobulk	DESeq2	LRT	0
860	KC_AB1	vvl	0.222423402322448	0.929052676333832	1	pseudobulk	DESeq2	LRT	0
860	KC_AB1	w	0.225718024854992	0.601080027144682	1	pseudobulk	DESeq2	LRT	0
860	KC_AB1	wac	0.598114192711129	1	1	pseudobulk	DESeq2	LRT	0
860	KC_AB1	wake	-0.194758904512976	0.620925962540782	1	pseudobulk	DESeq2	LRT	0
860	KC_AB1	wal	0.540568592249868	0.456717588256034	1	pseudobulk	DESeq2	LRT	0
860	KC_AB1	wap	0.0715883636490355	0.625936754678458	1	pseudobulk	DESeq2	LRT	0
860	KC_AB1	wapl	-0.533760494920702	0.318906185383041	1	pseudobulk	DESeq2	LRT	0
860	KC_AB1	wash	0.152423126446881	0.597805456436426	1	pseudobulk	DESeq2	LRT	0
860	KC_AB1	WASp	0.145341296452202	0.660868820275927	1	pseudobulk	DESeq2	LRT	0
860	KC_AB1	wat	2.78674512494791	0.54962276837928	1	pseudobulk	DESeq2	LRT	0
861	KC_AB1	wb	1.93706461079025	0.658817357590498	1	pseudobulk	DESeq2	LRT	0
861	KC_AB1	Wbp2	0.355836197476874	0.606629174426921	1	pseudobulk	DESeq2	LRT	0
861	KC_AB1	wcd	-0.711717172786096	0.61271156555813	1	pseudobulk	DESeq2	LRT	0
861	KC_AB1	wcy	-0.0147297404772046	0.770853608444638	1	pseudobulk	DESeq2	LRT	0
861	KC_AB1	wda	-1.55270134360179	0.369168660716194	1	pseudobulk	DESeq2	LRT	0
861	KC_AB1	wdb	-0.0816481263597523	0.474023519204867	1	pseudobulk	DESeq2	LRT	0
861	KC_AB1	wde	0.29128653863361	0.61105741312243	1	pseudobulk	DESeq2	LRT	0
861	KC_AB1	Wdfy2	0.8605946470494	0.24802428536701	1	pseudobulk	DESeq2	LRT	0
861	KC_AB1	wdn	-0.0574497727611406	0.74256206831732	1	pseudobulk	DESeq2	LRT	0
861	KC_AB1	wdp	-1.8476447304053	0.456955818574401	1	pseudobulk	DESeq2	LRT	0
862	KC_AB1	Wdr24	1.81744475646881	0.446738835676213	1	pseudobulk	DESeq2	LRT	0
862	KC_AB1	Wdr33	-0.0319928457572775	0.663718447651722	1	pseudobulk	DESeq2	LRT	0
862	KC_AB1	Wdr59	0.0160940546605174	1	1	pseudobulk	DESeq2	LRT	0
862	KC_AB1	Wdr62	0.227676512379117	0.333296640556046	1	pseudobulk	DESeq2	LRT	0
862	KC_AB1	Wdr81	-0.209233368404682	0.339591077532519	1	pseudobulk	DESeq2	LRT	0
862	KC_AB1	Wdr82	-0.430025777565068	0.537883296457124	1	pseudobulk	DESeq2	LRT	0
862	KC_AB1	Wdr92	0.0489434638589907	0.326341493859534	1	pseudobulk	DESeq2	LRT	0
862	KC_AB1	wds	0.620266891455678	0.205355091393296	1	pseudobulk	DESeq2	LRT	0
862	KC_AB1	WDY	-1.02430668501573	0.472234197425808	1	pseudobulk	DESeq2	LRT	0
862	KC_AB1	wech	-0.112451837390239	0.718643998152846	1	pseudobulk	DESeq2	LRT	0
863	KC_AB1	Wee1	-0.531055020427431	0.55339942464439	1	pseudobulk	DESeq2	LRT	0
863	KC_AB1	wek	1.68854534765648	0.567014088138706	1	pseudobulk	DESeq2	LRT	0
863	KC_AB1	wfs1	0.275036119762393	0.870579349713188	1	pseudobulk	DESeq2	LRT	0
863	KC_AB1	wge	-0.270108782647636	0.370899056313158	1	pseudobulk	DESeq2	LRT	0
863	KC_AB1	wgn	2.31997030691033	0.813169507846918	1	pseudobulk	DESeq2	LRT	0
863	KC_AB1	whd	-0.710104224139132	0.559728928256334	1	pseudobulk	DESeq2	LRT	0
863	KC_AB1	wisp	-0.216209988775614	1	1	pseudobulk	DESeq2	LRT	0
863	KC_AB1	wit	-0.0357916798679616	0.568013252610786	1	pseudobulk	DESeq2	LRT	0

863	KC_AB1	wkd	-0.0377179926417212	0.671678558124143	1	pseudobulk	DESeq2	LRT	0
863	KC_AB1	wls	-0.123764884426649	0.48950411055619	1	pseudobulk	DESeq2	LRT	0
864	KC_AB1	wmd	-0.184418112756739	0.588198391288283	1	pseudobulk	DESeq2	LRT	0
864	KC_AB1	wnd	0.174903205394402	0.568659073240165	1	pseudobulk	DESeq2	LRT	0
864	KC_AB1	Wnk	-0.16691351587064	0.542719054659024	1	pseudobulk	DESeq2	LRT	0
864	KC_AB1	Wnt5	1.0982828623148	0.129296133689329	1	pseudobulk	DESeq2	LRT	0
864	KC_AB1	Wnt6	0.0160940546605174	1	1	pseudobulk	DESeq2	LRT	0
864	KC_AB1	woc	-0.390972983451803	0.591980778117361	1	pseudobulk	DESeq2	LRT	0
864	KC_AB1	wol	0.256844725430131	0.688016535336484	1	pseudobulk	DESeq2	LRT	0
864	KC_AB1	wrapper	-1.23673751520861	0.511971583553302	1	pseudobulk	DESeq2	LRT	0
864	KC_AB1	wrd	0.0201401449441355	0.930536823567975	1	pseudobulk	DESeq2	LRT	0
864	KC_AB1	wry	-0.279618926797115	0.0826292804274474	1	pseudobulk	DESeq2	LRT	0
865	KC_AB1	Wsck	-0.851943617129873	0.694524594037258	1	pseudobulk	DESeq2	LRT	0
865	KC_AB1	wtrw	-0.697820930581184	0.61609999620834	1	pseudobulk	DESeq2	LRT	0
865	KC_AB1	wts	-0.136111859103768	0.418621883136688	1	pseudobulk	DESeq2	LRT	0
865	KC_AB1	wuho	0.868238864715238	0.330432656072584	1	pseudobulk	DESeq2	LRT	0
865	KC_AB1	wun	-0.788244974821805	0.301480626347602	1	pseudobulk	DESeq2	LRT	0
865	KC_AB1	wun2	0.536600542756646	1	1	pseudobulk	DESeq2	LRT	0
865	KC_AB1	Wwox	-0.621410672079686	0.483746405640081	1	pseudobulk	DESeq2	LRT	0
865	KC_AB1	X11L	0.0254022385640038	0.718035613511238	1	pseudobulk	DESeq2	LRT	0
865	KC_AB1	X11Lbeta	-0.296447498110767	0.26601050973638	1	pseudobulk	DESeq2	LRT	0
865	KC_AB1	x16	0.044798767723025	0.766077524281197	1	pseudobulk	DESeq2	LRT	0
866	KC_AB1	Xbp1	-0.237804360084525	0.531006999453319	1	pseudobulk	DESeq2	LRT	0
866	KC_AB1	Xe7	-0.566620185960786	0.0509717743494236	1	pseudobulk	DESeq2	LRT	0
866	KC_AB1	xit	0.374420150816607	1	1	pseudobulk	DESeq2	LRT	0
866	KC_AB1	xmas	-0.45560956407913	0.710292978199623	1	pseudobulk	DESeq2	LRT	0
866	KC_AB1	XNP	0.0850590072800648	0.806896221179417	1	pseudobulk	DESeq2	LRT	0
866	KC_AB1	Xpac	-1.40307130648885	0.334300496344265	1	pseudobulk	DESeq2	LRT	0
866	KC_AB1	Xpc	0.0076274159918316	0.531529442190677	1	pseudobulk	DESeq2	LRT	0
866	KC_AB1	Xpd	0.985243145755336	1	1	pseudobulk	DESeq2	LRT	0
866	KC_AB1	XRCC1	-1.02807122549234	0.574547736228934	1	pseudobulk	DESeq2	LRT	0
866	KC_AB1	Xrp1	0.0832891251142354	0.840938688476737	1	pseudobulk	DESeq2	LRT	0
867	KC_AB1	Xxylt	0.162516394858254	0.563626520248687	1	pseudobulk	DESeq2	LRT	0
867	KC_AB1	yata	-0.676315458051712	0.38206680888135	1	pseudobulk	DESeq2	LRT	0
867	KC_AB1	yellow-b	-0.564356698759404	0.803702590456371	1	pseudobulk	DESeq2	LRT	0
867	KC_AB1	yellow-h	0.703797494175466	1	1	pseudobulk	DESeq2	LRT	0
867	KC_AB1	yem	-1.13253335832992	0.303192996364633	1	pseudobulk	DESeq2	LRT	0
867	KC_AB1	Yeti	0.187334166690938	0.496671206809625	1	pseudobulk	DESeq2	LRT	0
867	KC_AB1	Yif1	-0.185311623185967	0.71390679424673	1	pseudobulk	DESeq2	LRT	0
867	KC_AB1	Yip1d1	-0.443304223187281	0.552382233242013	1	pseudobulk	DESeq2	LRT	0
867	KC_AB1	yip2	0.349540486374305	1	1	pseudobulk	DESeq2	LRT	0
867	KC_AB1	Yippee	0.298617345466459	0.551091100450101	1	pseudobulk	DESeq2	LRT	0
868	KC_AB1	yki	-0.0657392479395881	0.707890775005847	1	pseudobulk	DESeq2	LRT	0
868	KC_AB1	Ykt6	-0.344933770285112	0.548240595362421	1	pseudobulk	DESeq2	LRT	0
868	KC_AB1	yl	0.366247714696288	1	1	pseudobulk	DESeq2	LRT	0
868	KC_AB1	YL-1	-1.42819579050462	0.141911156262138	1	pseudobulk	DESeq2	LRT	0
868	KC_AB1	YME1L	0.433136080573176	0.425571986043036	1	pseudobulk	DESeq2	LRT	0
868	KC_AB1	Yp1	0.849606887838128	1	1	pseudobulk	DESeq2	LRT	0
868	KC_AB1	Yp3	2.78674512494791	0.54962276837928	1	pseudobulk	DESeq2	LRT	0
868	KC_AB1	yps	0.564681040054388	0.271304171655718	1	pseudobulk	DESeq2	LRT	0
868	KC_AB1	yrt	0.394114867714174	0.317198038613662	1	pseudobulk	DESeq2	LRT	0
868	KC_AB1	Ythdc1	0.262420773691106	0.437830277866333	1	pseudobulk	DESeq2	LRT	0
869	KC_AB1	Ythdf	0.337841879249306	0.479133044918539	1	pseudobulk	DESeq2	LRT	0

869	KC_AB1	ytr	-0.498008994219774	0.566469181149055	1	pseudobulk	DESeq2	LRT		0
869	KC_AB1	yuri	-0.830062897938551	0.717724257445671	1	pseudobulk	DESeq2	LRT		0
869	KC_AB1	z	0.265439734073927	0.60339124514445	1	pseudobulk	DESeq2	LRT		0
869	KC_AB1	ZAP3	-0.604392553721041	0.171404192178627	1	pseudobulk	DESeq2	LRT		0
869	KC_AB1	Zasp52	0.862116781147037	0.582029645938739	1	pseudobulk	DESeq2	LRT		0
869	KC_AB1	Zasp67	0.849606887838128	1	1	pseudobulk	DESeq2	LRT		0
869	KC_AB1	ZC3H3	-0.273561201947378	0.50395523956847	1	pseudobulk	DESeq2	LRT		0
869	KC_AB1	Zcchc7	-0.278016263201847	0.711255279899125	1	pseudobulk	DESeq2	LRT		0
869	KC_AB1	zda	0.0517105343350926	0.597894557204498	1	pseudobulk	DESeq2	LRT		0
870	KC_AB1	Zdhhc8	-0.119572577649139	0.542747111716488	1	pseudobulk	DESeq2	LRT		0
870	KC_AB1	zetaCOP	-0.081668108637947	0.567595816759811	1	pseudobulk	DESeq2	LRT		0
870	KC_AB1	zf30C	-0.430110719733073	0.500983103658239	1	pseudobulk	DESeq2	LRT		0
870	KC_AB1	zfh2	0.111829237781382	0.734960217595136	1	pseudobulk	DESeq2	LRT		0
870	KC_AB1	Zfrp8	-0.898415405690777	0.430168182570776	1	pseudobulk	DESeq2	LRT		0
870	KC_AB1	Zif	0.0514462000965655	0.658455954569212	1	pseudobulk	DESeq2	LRT		0
870	KC_AB1	zip	-0.587177968582403	0.170804157522892	1	pseudobulk	DESeq2	LRT		0
870	KC_AB1	Zip102B	-0.116330789954631	0.701445286694763	1	pseudobulk	DESeq2	LRT		0
870	KC_AB1	Zip48C	-0.114854824432736	0.702251393878983	1	pseudobulk	DESeq2	LRT		0
870	KC_AB1	Zip71B	-3.48385457305913	0.31672377740814	1	pseudobulk	DESeq2	LRT		0
871	KC_AB1	Zip88E	-0.152507969833924	0.753938526199874	1	pseudobulk	DESeq2	LRT		0
871	KC_AB1	Zip99C	1.03189270376543	0.284405222368742	1	pseudobulk	DESeq2	LRT		0
871	KC_AB1	ZIPIC	1.0732807014031	0.708442304123541	1	pseudobulk	DESeq2	LRT		0
871	KC_AB1	Zir	-0.12338251289312	0.686619581765986	1	pseudobulk	DESeq2	LRT		0
871	KC_AB1	Ziz	-0.373675980469473	0.297482427871228	1	pseudobulk	DESeq2	LRT		0
871	KC_AB1	Zmynd10	2.78674512494791	0.54962276837928	1	pseudobulk	DESeq2	LRT		0
871	KC_AB1	Zn72D	-0.312001604828382	0.567079081909813	1	pseudobulk	DESeq2	LRT		0
871	KC_AB1	ZnT33D	0.211788993319012	1	1	pseudobulk	DESeq2	LRT		0
871	KC_AB1	ZnT49B	-1.13044569169246	0.143195931871463	1	pseudobulk	DESeq2	LRT		0
871	KC_AB1	ZnT63C	0.590394204517086	0.119468089227132	1	pseudobulk	DESeq2	LRT		0
872	KC_AB1	ZnT77C	1.59863986092676	1	1	pseudobulk	DESeq2	LRT		0
872	KC_AB1	ZnT86D	-0.604985779190547	0.655552504247949	1	pseudobulk	DESeq2	LRT		0
872	KC_AB1	zormin	3.81892523345342	0.259683132508622	1	pseudobulk	DESeq2	LRT		0
872	KC_AB1	Zpr1	-1.16020557067325	0.492962443274044	1	pseudobulk	DESeq2	LRT		0
872	KC_AB1	Zw	0.849606887838128	1	1	pseudobulk	DESeq2	LRT		0
872	KC_AB1	Zw10	0.442808397454391	0.716521263454741	1	pseudobulk	DESeq2	LRT		0
872	KC_AB1	zyd	2.5153355929419	0.0656230040131003	1	pseudobulk	DESeq2	LRT		0
872	KC_AB1	Zyx	-0.407860872727394	0.238287925433109	1	pseudobulk	DESeq2	LRT		0
872	KC_AB2	128up	0.222396124134733	1	1	pseudobulk	DESeq2	LRT		0
872	KC_AB2	14-3-3epsilon	-0.17515954909035	0.431739613373405	1	pseudobulk	DESeq2	LRT		0
873	KC_AB2	14-3-3zeta	0.0276621111742113	0.881700277641046	1	pseudobulk	DESeq2	LRT		0
873	KC_AB2	140up	-0.174965807374141	0.91631653210935	1	pseudobulk	DESeq2	LRT		0
873	KC_AB2	18SrRNA-Psi:CR4	2.12684015898615	0.445939223035924	1	pseudobulk	DESeq2	LRT		0
873	KC_AB2	18w	1.53811683574911	0.54844951579854	1	pseudobulk	DESeq2	LRT		0
873	KC_AB2	26-29-p	-0.476958205248542	0.710403082690575	1	pseudobulk	DESeq2	LRT		0
873	KC_AB2	28SrRNA-Psi:CR4	-1.16267027545273	0.479506304188474	1	pseudobulk	DESeq2	LRT		0
873	KC_AB2	28SrRNA-Psi:CR4	0.443892390635762	1	1	pseudobulk	DESeq2	LRT		0
873	KC_AB2	28SrRNA-Psi:CR4	-0.314805936676809	0.847697048432822	1	pseudobulk	DESeq2	LRT		0
873	KC_AB2	28SrRNA-Psi:CR4	0.0163320842711025	1	1	pseudobulk	DESeq2	LRT		0
873	KC_AB2	2mit	-0.464694004714349	0.247083957473369	1	pseudobulk	DESeq2	LRT		0
874	KC_AB2	312	-0.678378597058692	0.647673827281084	1	pseudobulk	DESeq2	LRT		0
874	KC_AB2	4E-T	-0.058859278123189	0.839327686754743	1	pseudobulk	DESeq2	LRT		0
874	KC_AB2	5-HT1A	0.316620251666231	0.153245286219862	1	pseudobulk	DESeq2	LRT		0
874	KC_AB2	5-HT1B	-0.138059458486345	0.750746423131643	1	pseudobulk	DESeq2	LRT		0

874	KC_AB2	5-HT2B	-1.7824418856176	0.467224294431682	1	pseudobulk	DESeq2	LRT		0
874	KC_AB2	5PtaseI	0.208484739465905	0.735690816210197	1	pseudobulk	DESeq2	LRT		0
874	KC_AB2	7B2	0.103730636097024	0.612652790838233	1	pseudobulk	DESeq2	LRT		0
874	KC_AB2	a	0.123638472008214	0.742868538810714	1	pseudobulk	DESeq2	LRT		0
874	KC_AB2	A16	-1.63713385259394	0.397503634782758	1	pseudobulk	DESeq2	LRT		0
874	KC_AB2	a6	-0.519667034314251	0.780427814827476	1	pseudobulk	DESeq2	LRT		0
875	KC_AB2	AANAT1	-0.0715850075945668	0.919574208531439	1	pseudobulk	DESeq2	LRT		0
875	KC_AB2	AANATL2	-1.29244479833796	0.693295472370086	1	pseudobulk	DESeq2	LRT		0
875	KC_AB2	AANATL7	-2.02285942584217	0.357240028002837	1	pseudobulk	DESeq2	LRT		0
875	KC_AB2	Aasdh	1.07173252967008	0.608626529368362	1	pseudobulk	DESeq2	LRT		0
875	KC_AB2	Aatf	-3.45708264399634	0.0742910185488935	1	pseudobulk	DESeq2	LRT		0
875	KC_AB2	aay	-0.841882716738579	1	1	pseudobulk	DESeq2	LRT		0
875	KC_AB2	ab	-2.26236596825427	0.0929880385802446	1	pseudobulk	DESeq2	LRT		0
875	KC_AB2	ABCA	0.0650327709337841	0.904904749609656	1	pseudobulk	DESeq2	LRT		0
875	KC_AB2	ABCB7	-0.11617890383455	0.882805354715668	1	pseudobulk	DESeq2	LRT		0
875	KC_AB2	ABCD	-0.16969254474715	0.761595872889682	1	pseudobulk	DESeq2	LRT		0
876	KC_AB2	Abi	0.653511765645453	0.499976381320909	1	pseudobulk	DESeq2	LRT		0
876	KC_AB2	Abl	0.0323745785923106	0.857131909170774	1	pseudobulk	DESeq2	LRT		0
876	KC_AB2	Abp1	0.07643939664351	0.868169463940269	1	pseudobulk	DESeq2	LRT		0
876	KC_AB2	abs	0.920212920242661	0.67286879185691	1	pseudobulk	DESeq2	LRT		0
876	KC_AB2	Ac3	0.389843965698705	0.540936115060945	1	pseudobulk	DESeq2	LRT		0
876	KC_AB2	Ac76E	-1.3000336711682	0.247955107675309	1	pseudobulk	DESeq2	LRT		0
876	KC_AB2	Ac78C	-0.0923338887672531	0.869895799161087	1	pseudobulk	DESeq2	LRT		0
876	KC_AB2	Acbp1	0.550367544118721	0.291837230019853	1	pseudobulk	DESeq2	LRT		0
876	KC_AB2	Acbp2	-1.93220872672389	0.398686710033904	1	pseudobulk	DESeq2	LRT		0
876	KC_AB2	ACC	0.153543200419069	0.771235850638368	1	pseudobulk	DESeq2	LRT		0
877	KC_AB2	AcCoAS	-0.328061353319867	0.527914127144723	1	pseudobulk	DESeq2	LRT		0
877	KC_AB2	Ace	-0.946363052032143	0.381651622616693	1	pseudobulk	DESeq2	LRT		0
877	KC_AB2	Acer	-0.525980220958878	0.813340603474339	1	pseudobulk	DESeq2	LRT		0
877	KC_AB2	Acf	0.872263915466572	0.537517495282304	1	pseudobulk	DESeq2	LRT		0
877	KC_AB2	achi	2.12684015898615	0.445939223035924	1	pseudobulk	DESeq2	LRT		0
877	KC_AB2	Ack	0.786405264766822	0.634618360629617	1	pseudobulk	DESeq2	LRT		0
877	KC_AB2	Ack-like	0.187839703439702	0.751416607927466	1	pseudobulk	DESeq2	LRT		0
877	KC_AB2	Acn	0.678397105124988	0.347404483159261	1	pseudobulk	DESeq2	LRT		0
877	KC_AB2	Acph-1	-3.04547355004204	0.142660039361559	1	pseudobulk	DESeq2	LRT		0
877	KC_AB2	Acsl	-0.124684932195773	0.824668981429144	1	pseudobulk	DESeq2	LRT		0
878	KC_AB2	Act42A	-0.0852743682753834	0.917718671153184	1	pseudobulk	DESeq2	LRT		0
878	KC_AB2	Act5C	0.356938566941998	0.351531544252297	1	pseudobulk	DESeq2	LRT		0
878	KC_AB2	Act87E	-0.427431139129811	1	1	pseudobulk	DESeq2	LRT		0
878	KC_AB2	Actbeta	-0.550853091720432	0.459233942302459	1	pseudobulk	DESeq2	LRT		0
878	KC_AB2	Actn	-1.699061583586	0.254865900926878	1	pseudobulk	DESeq2	LRT		0
878	KC_AB2	ACXC	-0.600547221779134	1	1	pseudobulk	DESeq2	LRT		0
878	KC_AB2	Acyp2	0.995105944635339	0.374491152405468	1	pseudobulk	DESeq2	LRT		0
878	KC_AB2	Ada1-1	0.189813678325555	1	1	pseudobulk	DESeq2	LRT		0
878	KC_AB2	Ada1-2	-0.600547221779133	0.841940696994101	1	pseudobulk	DESeq2	LRT		0
878	KC_AB2	Ada2a	-0.600547221779134	1	1	pseudobulk	DESeq2	LRT		0
879	KC_AB2	Ada2b	0.979295435729602	0.417409715565363	1	pseudobulk	DESeq2	LRT		0
879	KC_AB2	Ada3	1.04332896635476	0.529312245057685	1	pseudobulk	DESeq2	LRT		0
879	KC_AB2	Adar	0.756222340310176	0.302623314356707	1	pseudobulk	DESeq2	LRT		0
879	KC_AB2	Adck5	-1.70380348762697	0.473446675363872	1	pseudobulk	DESeq2	LRT		0
879	KC_AB2	ADD1	0.314323242605539	0.780789868843449	1	pseudobulk	DESeq2	LRT		0
879	KC_AB2	AdenoK	-1.6150095599849	0.519549602003096	1	pseudobulk	DESeq2	LRT		0
879	KC_AB2	Adf1	-0.0775583938986776	0.890706156338606	1	pseudobulk	DESeq2	LRT		0

879	KC_AB2	Adgf-D	-1.12475160732157	0.574429473125171	1	pseudobulk	DESeq2	LRT	0
879	KC_AB2	Adi1	1.53811683574911	0.54844951579854	1	pseudobulk	DESeq2	LRT	0
879	KC_AB2	AdipoR	0.0662746062022291	0.906072678614656	1	pseudobulk	DESeq2	LRT	0
880	KC_AB2	Adk1	0.0345937513907985	0.913250068166204	1	pseudobulk	DESeq2	LRT	0
880	KC_AB2	Adk2	-0.336102021618619	0.587349617610215	1	pseudobulk	DESeq2	LRT	0
880	KC_AB2	Adk3	1.5243995994652	0.741477491078038	1	pseudobulk	DESeq2	LRT	0
880	KC_AB2	adp	1.02681148972642	0.601157884532627	1	pseudobulk	DESeq2	LRT	0
880	KC_AB2	ADPS	0.3950609931031	0.676505330379909	1	pseudobulk	DESeq2	LRT	0
880	KC_AB2	AdSL	1.11471276021981	0.644913768495318	1	pseudobulk	DESeq2	LRT	0
880	KC_AB2	AdSS	0.0761287327599584	0.839682953237123	1	pseudobulk	DESeq2	LRT	0
880	KC_AB2	Aduk	-0.554395503918041	0.748596607568247	1	pseudobulk	DESeq2	LRT	0
880	KC_AB2	Ady43A	-0.183748591876916	0.853724345129629	1	pseudobulk	DESeq2	LRT	0
880	KC_AB2	Aef1	-0.71430962603479	0.354622822966869	1	pseudobulk	DESeq2	LRT	0
881	KC_AB2	aft	-1.54470851095603	0.55301747381519	1	pseudobulk	DESeq2	LRT	0
881	KC_AB2	Afti	0.776903978643653	0.554755147580385	1	pseudobulk	DESeq2	LRT	0
881	KC_AB2	Ag5r	2.71751852760692	0.213382210335911	1	pseudobulk	DESeq2	LRT	0
881	KC_AB2	ago	-1.70917090055421	0.225085450667554	1	pseudobulk	DESeq2	LRT	0
881	KC_AB2	AGO1	0.0989950353863229	0.673061668163366	1	pseudobulk	DESeq2	LRT	0
881	KC_AB2	AGO2	-0.212026176338979	0.862960929601621	1	pseudobulk	DESeq2	LRT	0
881	KC_AB2	AGO3	2.68767245534939	0.209891813239836	1	pseudobulk	DESeq2	LRT	0
881	KC_AB2	Agpat1	1.48722631396354	0.366836405323111	1	pseudobulk	DESeq2	LRT	0
881	KC_AB2	Agpat3	0.586236117164697	0.468534792327668	1	pseudobulk	DESeq2	LRT	0
881	KC_AB2	Ahcy	-0.284824451364236	0.921837079470819	1	pseudobulk	DESeq2	LRT	0
882	KC_AB2	AhcyL1	0.275974329299634	0.794513396172319	1	pseudobulk	DESeq2	LRT	0
882	KC_AB2	AIF	0.379378645244074	0.822219735623116	1	pseudobulk	DESeq2	LRT	0
882	KC_AB2	AIMP1	1.29699351328743	0.46055405427585	1	pseudobulk	DESeq2	LRT	0
882	KC_AB2	AIMP2	0.529499457275229	0.682303541369736	1	pseudobulk	DESeq2	LRT	0
882	KC_AB2	AIMP3	-0.205203030372485	0.779342393342721	1	pseudobulk	DESeq2	LRT	0
882	KC_AB2	Ak6	0.355234727600781	0.802879983356527	1	pseudobulk	DESeq2	LRT	0
882	KC_AB2	Akap200	-1.70380348762697	0.473446675363872	1	pseudobulk	DESeq2	LRT	0
882	KC_AB2	Akh	-0.841882716738579	1	1	pseudobulk	DESeq2	LRT	0
882	KC_AB2	akirin	0.558228801559918	0.310982090716914	1	pseudobulk	DESeq2	LRT	0
882	KC_AB2	Akt1	0.500636629895666	0.660464775874788	1	pseudobulk	DESeq2	LRT	0
883	KC_AB2	AlaRS	0.222396124134733	1	1	pseudobulk	DESeq2	LRT	0
883	KC_AB2	AlaRS-m	1.23085716405798	0.614623732585597	1	pseudobulk	DESeq2	LRT	0
883	KC_AB2	Alas	-0.963195885656634	0.187521737995996	1	pseudobulk	DESeq2	LRT	0
883	KC_AB2	alc	-0.220306433961154	0.723015438513716	1	pseudobulk	DESeq2	LRT	0
883	KC_AB2	Ald1	0.0492220879193366	0.83268213630119	1	pseudobulk	DESeq2	LRT	0
883	KC_AB2	Aldh	1.5243995994652	0.741477491078038	1	pseudobulk	DESeq2	LRT	0
883	KC_AB2	Aldh-III	-0.584264594669257	0.632378753478571	1	pseudobulk	DESeq2	LRT	0
883	KC_AB2	Alg-2	0.110469696605421	0.867197494244356	1	pseudobulk	DESeq2	LRT	0
883	KC_AB2	Alg10	-1.02988290140464	0.610579185122146	1	pseudobulk	DESeq2	LRT	0
883	KC_AB2	Alg11	-0.124821244088904	0.875378614383082	1	pseudobulk	DESeq2	LRT	0
884	KC_AB2	Alg14	0.222396124134733	1	1	pseudobulk	DESeq2	LRT	0
884	KC_AB2	Alg2	-0.621805355124546	0.74877258313458	1	pseudobulk	DESeq2	LRT	0
884	KC_AB2	Alg3	-1.50331603016663	0.431246352444201	1	pseudobulk	DESeq2	LRT	0
884	KC_AB2	Alh	0.169005384805635	0.815098971825753	1	pseudobulk	DESeq2	LRT	0
884	KC_AB2	alien	-1.06301868568402	0.548325471714763	1	pseudobulk	DESeq2	LRT	0
884	KC_AB2	ALiX	1.5513871945706	0.2257748254797	1	pseudobulk	DESeq2	LRT	0
884	KC_AB2	Alk	0.0137165379184377	0.96162912416638	1	pseudobulk	DESeq2	LRT	0
884	KC_AB2	Alp6	-0.600547221779134	1	1	pseudobulk	DESeq2	LRT	0
884	KC_AB2	alph	0.265594609369961	0.586333267122881	1	pseudobulk	DESeq2	LRT	0
884	KC_AB2	alpha-Cat	-0.29073141119732	0.571673445199272	1	pseudobulk	DESeq2	LRT	0

885	KC_AB2	alpha-Catr	0.576779477568473	0.575060009090993	1	pseudobulk	DESeq2	LRT		0
885	KC_AB2	alpha-Est10	2.12684015898615	0.445939223035924	1	pseudobulk	DESeq2	LRT		0
885	KC_AB2	alpha-Est8	0.12045821616144	0.745233311423879	1	pseudobulk	DESeq2	LRT		0
885	KC_AB2	alpha-Est9	-0.180914872340651	0.799742014187354	1	pseudobulk	DESeq2	LRT		0
885	KC_AB2	alpha-Man-Ia	0.377263007111213	0.0977015345464247	1	pseudobulk	DESeq2	LRT		0
885	KC_AB2	alpha-Man-Ib	0.164458538374668	0.809194830508533	1	pseudobulk	DESeq2	LRT		0
885	KC_AB2	alpha-Man-Ic	-0.427431139129811	1	1	pseudobulk	DESeq2	LRT		0
885	KC_AB2	alpha-Man-IIa	-1.01337037550694	0.608094630643134	1	pseudobulk	DESeq2	LRT		0
885	KC_AB2	alpha-Man-IIb	0.55483752013202	0.688375908112313	1	pseudobulk	DESeq2	LRT		0
885	KC_AB2	alpha-PheRS	0.300368105702188	0.875838324087404	1	pseudobulk	DESeq2	LRT		0
886	KC_AB2	alpha-Spec	0.4250370990194	0.302393606731837	1	pseudobulk	DESeq2	LRT		0
886	KC_AB2	alpha4GT1	0.235849193784524	0.827764315775177	1	pseudobulk	DESeq2	LRT		0
886	KC_AB2	alphaCOP	0.853641878115967	0.565450095973286	1	pseudobulk	DESeq2	LRT		0
886	KC_AB2	alphaSnap	-0.214136805921997	0.595337847271835	1	pseudobulk	DESeq2	LRT		0
886	KC_AB2	alphaTub67C	0.360869486893906	0.827089164634664	1	pseudobulk	DESeq2	LRT		0
886	KC_AB2	alphaTub84B	-0.153954335105097	0.529729673541183	1	pseudobulk	DESeq2	LRT		0
886	KC_AB2	alphaTub84D	-0.211026270860156	0.659705755348407	1	pseudobulk	DESeq2	LRT		0
886	KC_AB2	Alr	0.949681456329294	0.563171829148646	1	pseudobulk	DESeq2	LRT		0
886	KC_AB2	alrm	-1.12785316439435	0.661319045116135	1	pseudobulk	DESeq2	LRT		0
886	KC_AB2	Als2	0.6546639689275	0.714019207783944	1	pseudobulk	DESeq2	LRT		0
887	KC_AB2	alt	-0.692237798209826	0.349160421117273	1	pseudobulk	DESeq2	LRT		0
887	KC_AB2	Amacr	-1.04977914081511	0.661390933829843	1	pseudobulk	DESeq2	LRT		0
887	KC_AB2	amn	-0.239387199396269	0.85912161403618	1	pseudobulk	DESeq2	LRT		0
887	KC_AB2	Amnionless	-0.326043386873417	0.744743413530652	1	pseudobulk	DESeq2	LRT		0
887	KC_AB2	amon	-0.633001870946766	0.0472329636238099	1	pseudobulk	DESeq2	LRT		0
887	KC_AB2	AMPdeam	-1.92388523021778	0.182744775188565	1	pseudobulk	DESeq2	LRT		0
887	KC_AB2	Amph	0.661932647779233	0.629669454293477	1	pseudobulk	DESeq2	LRT		0
887	KC_AB2	AMPKalpha	0.147190210988506	0.843823991637561	1	pseudobulk	DESeq2	LRT		0
887	KC_AB2	Amun	-1.28118745223113	0.154511610730798	1	pseudobulk	DESeq2	LRT		0
887	KC_AB2	amx	1.9758607418779	0.322720287118553	1	pseudobulk	DESeq2	LRT		0
888	KC_AB2	Amyrel	-0.427431139129811	1	1	pseudobulk	DESeq2	LRT		0
888	KC_AB2	ana2	-0.600547221779133	0.841940696994101	1	pseudobulk	DESeq2	LRT		0
888	KC_AB2	anchor	-1.06259161081327	0.41810235608371	1	pseudobulk	DESeq2	LRT		0
888	KC_AB2	angel	0.864341462967788	0.925947094335722	1	pseudobulk	DESeq2	LRT		0
888	KC_AB2	Ank	0.0429307365214572	0.91116527279221	1	pseudobulk	DESeq2	LRT		0
888	KC_AB2	Ank2	-0.106174879689984	0.64419786222779	1	pseudobulk	DESeq2	LRT		0
888	KC_AB2	Ankle2	1.94559924579109	0.42325307792885	1	pseudobulk	DESeq2	LRT		0
888	KC_AB2	anne	0.234521028283081	0.569968062655077	1	pseudobulk	DESeq2	LRT		0
888	KC_AB2	Ant2	-0.600547221779133	0.841940696994101	1	pseudobulk	DESeq2	LRT		0
888	KC_AB2	AnxB10	-1.04445428854771	0.603034233836386	1	pseudobulk	DESeq2	LRT		0
889	KC_AB2	AnxB11	1.09558592040113	0.183718452359425	1	pseudobulk	DESeq2	LRT		0
889	KC_AB2	AnxB9	2.51068564084663	0.263718309739592	1	pseudobulk	DESeq2	LRT		0
889	KC_AB2	aop	-0.764265644338521	0.672554234005952	1	pseudobulk	DESeq2	LRT		0
889	KC_AB2	Aos1	0.0957839571421125	0.935712208577892	1	pseudobulk	DESeq2	LRT		0
889	KC_AB2	AP-1-2beta	0.645688454742908	0.391798400011742	1	pseudobulk	DESeq2	LRT		0
889	KC_AB2	AP-1gamma	-0.158864821970646	0.701354586108289	1	pseudobulk	DESeq2	LRT		0
889	KC_AB2	AP-1mu	-0.325610568585254	0.640584848658812	1	pseudobulk	DESeq2	LRT		0
889	KC_AB2	AP-1sigma	-0.108061151554568	0.811760283145897	1	pseudobulk	DESeq2	LRT		0
889	KC_AB2	AP-2alpha	-0.131570471890031	0.782800857017951	1	pseudobulk	DESeq2	LRT		0
889	KC_AB2	AP-2mu	-0.778562153672425	0.225390837501851	1	pseudobulk	DESeq2	LRT		0
890	KC_AB2	AP-2sigma	0.12346065413665	0.735534810508742	1	pseudobulk	DESeq2	LRT		0
890	KC_AB2	Apc	-0.358794228606121	0.587386476114148	1	pseudobulk	DESeq2	LRT		0
890	KC_AB2	APC10	-0.498101586388589	0.71526928953407	1	pseudobulk	DESeq2	LRT		0

890	KC_AB2	APC4	0.189813678325555	1	1	pseudobulk	DESeq2	LRT	0
890	KC_AB2	ApepP	-1.12475160732157	0.574429473125171	1	pseudobulk	DESeq2	LRT	0
890	KC_AB2	Apf	-2.71678297683606	0.227234918915191	1	pseudobulk	DESeq2	LRT	0
890	KC_AB2	aph-1	0.066595207219245	0.935705969432816	1	pseudobulk	DESeq2	LRT	0
890	KC_AB2	aPKC	-0.369367134153965	0.484156681913329	1	pseudobulk	DESeq2	LRT	0
890	KC_AB2	Apl	-0.592481036256484	0.706847545932384	1	pseudobulk	DESeq2	LRT	0
890	KC_AB2	Aplip1	-0.0094433144998418	0.978400659014157	1	pseudobulk	DESeq2	LRT	0
891	KC_AB2	apolpp	-1.12785316439435	0.661319045116135	1	pseudobulk	DESeq2	LRT	0
891	KC_AB2	Apoltp	0.235547095082686	0.495019131595388	1	pseudobulk	DESeq2	LRT	0
891	KC_AB2	app	-2.19123523702538	0.122922243045303	1	pseudobulk	DESeq2	LRT	0
891	KC_AB2	APP-BP1	-0.841882716738579	1	1	pseudobulk	DESeq2	LRT	0
891	KC_AB2	Appl	-0.0332885410659147	0.858139334759784	1	pseudobulk	DESeq2	LRT	0
891	KC_AB2	Aprt	-2.17936304678827	0.330362909067606	1	pseudobulk	DESeq2	LRT	0
891	KC_AB2	Aps	-0.548401334737253	0.577743242173795	1	pseudobulk	DESeq2	LRT	0
891	KC_AB2	aqz	0.0734248343590282	0.676147667359963	1	pseudobulk	DESeq2	LRT	0
891	KC_AB2	aralar1	-1.1208501072248	0.384117235032495	1	pseudobulk	DESeq2	LRT	0
891	KC_AB2	Arc42	0.864341462967788	0.925947094335722	1	pseudobulk	DESeq2	LRT	0
892	KC_AB2	Archease	0.70216704605812	0.690489897393318	1	pseudobulk	DESeq2	LRT	0
892	KC_AB2	Arf102F	0.05292969247656	0.874628698124721	1	pseudobulk	DESeq2	LRT	0
892	KC_AB2	Arf51F	0.680627943257433	0.60218080721442	1	pseudobulk	DESeq2	LRT	0
892	KC_AB2	Arf79F	0.166869045637421	0.59110442173803	1	pseudobulk	DESeq2	LRT	0
892	KC_AB2	ArfGAP1	-0.351910095803846	0.747084827956258	1	pseudobulk	DESeq2	LRT	0
892	KC_AB2	ArfGAP3	0.683217878354314	0.635777627204594	1	pseudobulk	DESeq2	LRT	0
892	KC_AB2	Arfip	-0.500724552634949	0.616781333645935	1	pseudobulk	DESeq2	LRT	0
892	KC_AB2	Arfrp1	-1.78094334699383	0.470930349923561	1	pseudobulk	DESeq2	LRT	0
892	KC_AB2	arg	-1.14257094086606	0.633194643355871	1	pseudobulk	DESeq2	LRT	0
892	KC_AB2	Argk	-0.132291387735039	0.611934670790351	1	pseudobulk	DESeq2	LRT	0
893	KC_AB2	ArgRS	-0.167620063280405	0.874493033115399	1	pseudobulk	DESeq2	LRT	0
893	KC_AB2	ArgRS-m	-1.07776915880798	0.775491075088671	1	pseudobulk	DESeq2	LRT	0
893	KC_AB2	ari-1	0.214545926572762	0.62325599496745	1	pseudobulk	DESeq2	LRT	0
893	KC_AB2	ari-2	0.0907375054369488	0.831301640003983	1	pseudobulk	DESeq2	LRT	0
893	KC_AB2	Arl1	-0.862048091466641	0.52569836261071	1	pseudobulk	DESeq2	LRT	0
893	KC_AB2	Arl2	-1.05090242456006	0.467922509431907	1	pseudobulk	DESeq2	LRT	0
893	KC_AB2	Arl4	0.364676022145486	0.427212262463418	1	pseudobulk	DESeq2	LRT	0
893	KC_AB2	Arl5	1.77778305961943	0.104549666970421	1	pseudobulk	DESeq2	LRT	0
893	KC_AB2	Arl6	-0.427431139129811	1	1	pseudobulk	DESeq2	LRT	0
893	KC_AB2	Arl6IP1	0.0757907860685769	0.809238005612089	1	pseudobulk	DESeq2	LRT	0
894	KC_AB2	Arl8	0.0865960419386298	0.797828633491157	1	pseudobulk	DESeq2	LRT	0
894	KC_AB2	arm	-0.302944495942191	0.444358279598434	1	pseudobulk	DESeq2	LRT	0
894	KC_AB2	armi	-0.599026426864273	0.757820721998031	1	pseudobulk	DESeq2	LRT	0
894	KC_AB2	Arp1	-0.148581281945318	0.828053303790441	1	pseudobulk	DESeq2	LRT	0
894	KC_AB2	Arp10	-0.152452621351761	0.934552222330741	1	pseudobulk	DESeq2	LRT	0
894	KC_AB2	Arp2	-0.916669297163211	0.288685441987151	1	pseudobulk	DESeq2	LRT	0
894	KC_AB2	Arp3	0.778319003925421	0.435731491931567	1	pseudobulk	DESeq2	LRT	0
894	KC_AB2	Arp5	-2.90660662728959	0.448848165232629	1	pseudobulk	DESeq2	LRT	0
894	KC_AB2	Arp6	-0.470154205142474	0.703083080196972	1	pseudobulk	DESeq2	LRT	0
894	KC_AB2	Arp8	-0.259134671513485	1	1	pseudobulk	DESeq2	LRT	0
895	KC_AB2	Arpc1	0.339266084743829	0.579455314715735	1	pseudobulk	DESeq2	LRT	0
895	KC_AB2	Arpc2	0.25886144602744	0.816692140941214	1	pseudobulk	DESeq2	LRT	0
895	KC_AB2	Arpc3A	0.130470654632234	0.924188420429145	1	pseudobulk	DESeq2	LRT	0
895	KC_AB2	Arpc4	-0.0279493328411267	0.791598616480272	1	pseudobulk	DESeq2	LRT	0
895	KC_AB2	Arpc5	-0.10628813780033	0.840655116651446	1	pseudobulk	DESeq2	LRT	0
895	KC_AB2	arr	-0.365159813524311	0.577519788851915	1	pseudobulk	DESeq2	LRT	0

895	KC_AB2	Ars2	0.869567581283344	0.298814451510235	1	pseudobulk	DESeq2	LRT	0
895	KC_AB2	Art1	-2.97706175481125	0.157153445908316	1	pseudobulk	DESeq2	LRT	0
895	KC_AB2	Art4	-1.92942763539083	0.427765257981976	1	pseudobulk	DESeq2	LRT	0
895	KC_AB2	Arts	-0.713896150588864	0.790597169673125	1	pseudobulk	DESeq2	LRT	0
896	KC_AB2	Arv1	2.07946313289983	0.357635956314506	1	pseudobulk	DESeq2	LRT	0
896	KC_AB2	Asap	-1.28963497138975	0.134701382194059	1	pseudobulk	DESeq2	LRT	0
896	KC_AB2	Asator	0.116928485606017	0.743280081682608	1	pseudobulk	DESeq2	LRT	0
896	KC_AB2	Asciz	1.35968315642719	0.485168225381963	1	pseudobulk	DESeq2	LRT	0
896	KC_AB2	asf1	-0.830292433523662	0.59076467168751	1	pseudobulk	DESeq2	LRT	0
896	KC_AB2	ash1	-0.568652618648196	0.644483735441664	1	pseudobulk	DESeq2	LRT	0
896	KC_AB2	ash2	-1.12785316439435	0.661319045116135	1	pseudobulk	DESeq2	LRT	0
896	KC_AB2	Ask1	-1.01337037550694	0.608094630643134	1	pseudobulk	DESeq2	LRT	0
896	KC_AB2	AsnRS	-1.21120681873304	0.542862412873055	1	pseudobulk	DESeq2	LRT	0
896	KC_AB2	AsnRS-m	-1.9379626606486	0.399953775626921	1	pseudobulk	DESeq2	LRT	0
897	KC_AB2	AspRS	-1.18742575401949	0.563135617327774	1	pseudobulk	DESeq2	LRT	0
897	KC_AB2	AspRS-m	-1.58287442524021	0.399167833255611	1	pseudobulk	DESeq2	LRT	0
897	KC_AB2	asrij	-0.0050917041419925	0.926690814727381	1	pseudobulk	DESeq2	LRT	0
897	KC_AB2	asRNA:CR30267	-0.427431139129811	1	1	pseudobulk	DESeq2	LRT	0
897	KC_AB2	asRNA:CR31429	2.1817927492653	0.458599861292202	1	pseudobulk	DESeq2	LRT	0
897	KC_AB2	asRNA:CR31845	-0.427431139129811	1	1	pseudobulk	DESeq2	LRT	0
897	KC_AB2	asRNA:CR31912	-0.427431139129811	1	1	pseudobulk	DESeq2	LRT	0
897	KC_AB2	asRNA:CR42254	-2.18471391069127	0.356826310757868	1	pseudobulk	DESeq2	LRT	0
897	KC_AB2	asRNA:CR42547	-0.600547221779134	1	1	pseudobulk	DESeq2	LRT	0
897	KC_AB2	asRNA:CR43259	1.41392113638259	0.617674323943697	1	pseudobulk	DESeq2	LRT	0
898	KC_AB2	asRNA:CR43425	0.763390655415291	0.38364171808948	1	pseudobulk	DESeq2	LRT	0
898	KC_AB2	asRNA:CR43426	2.1817927492653	0.458599861292202	1	pseudobulk	DESeq2	LRT	0
898	KC_AB2	asRNA:CR43430	-0.427431139129811	1	1	pseudobulk	DESeq2	LRT	0
898	KC_AB2	asRNA:CR43480	-0.600547221779133	0.841940696994101	1	pseudobulk	DESeq2	LRT	0
898	KC_AB2	asRNA:CR43629	-1.68804044802334	0.37693629092235	1	pseudobulk	DESeq2	LRT	0
898	KC_AB2	asRNA:CR43683	2.12684015898615	0.445939223035924	1	pseudobulk	DESeq2	LRT	0
898	KC_AB2	asRNA:CR43730	1.5243995994652	0.741477491078038	1	pseudobulk	DESeq2	LRT	0
898	KC_AB2	asRNA:CR43820	-0.427431139129811	1	1	pseudobulk	DESeq2	LRT	0
898	KC_AB2	asRNA:CR43833	-0.427431139129811	1	1	pseudobulk	DESeq2	LRT	0
898	KC_AB2	asRNA:CR43906	-0.734008597994575	0.694445711446769	1	pseudobulk	DESeq2	LRT	0
899	KC_AB2	asRNA:CR43933	-1.3723417613553	0.619532036522	1	pseudobulk	DESeq2	LRT	0
899	KC_AB2	asRNA:CR43944	-1.40673172715063	0.587364787535786	1	pseudobulk	DESeq2	LRT	0
899	KC_AB2	asRNA:CR43957	1.5243995994652	0.741477491078038	1	pseudobulk	DESeq2	LRT	0
899	KC_AB2	asRNA:CR43959	1.40241945196173	0.57265124481703	1	pseudobulk	DESeq2	LRT	0
899	KC_AB2	asRNA:CR44018	-0.94307066344797	0.629943854378022	1	pseudobulk	DESeq2	LRT	0
899	KC_AB2	asRNA:CR44027	-2.18471391069127	0.356826310757868	1	pseudobulk	DESeq2	LRT	0
899	KC_AB2	asRNA:CR44029	0.366326605070335	0.803155912068754	1	pseudobulk	DESeq2	LRT	0
899	KC_AB2	asRNA:CR44030	-0.165850012437211	0.746344121045989	1	pseudobulk	DESeq2	LRT	0
899	KC_AB2	asRNA:CR44046	2.76205395729416	0.251829971474322	1	pseudobulk	DESeq2	LRT	0
899	KC_AB2	asRNA:CR44047	1.5243995994652	0.741477491078038	1	pseudobulk	DESeq2	LRT	0
900	KC_AB2	asRNA:CR44053	-0.600547221779134	1	1	pseudobulk	DESeq2	LRT	0
900	KC_AB2	asRNA:CR44065	2.12684015898615	0.445939223035924	1	pseudobulk	DESeq2	LRT	0
900	KC_AB2	asRNA:CR44093	-0.427431139129811	1	1	pseudobulk	DESeq2	LRT	0
900	KC_AB2	asRNA:CR44106	-0.841882716738579	1	1	pseudobulk	DESeq2	LRT	0
900	KC_AB2	asRNA:CR44107	-2.88584229053393	0.174566486388664	1	pseudobulk	DESeq2	LRT	0
900	KC_AB2	asRNA:CR44116	1.5243995994652	0.741477491078038	1	pseudobulk	DESeq2	LRT	0
900	KC_AB2	asRNA:CR44119	-1.51928970377954	0.486908946651791	1	pseudobulk	DESeq2	LRT	0
900	KC_AB2	asRNA:CR44228	-0.841882716738579	1	1	pseudobulk	DESeq2	LRT	0
900	KC_AB2	asRNA:CR44291	-0.600547221779134	1	1	pseudobulk	DESeq2	LRT	0

900	KC_AB2	asRNA:CR44321	-0.427431139129811	1	1	pseudobulk	DESeq2	LRT	0
901	KC_AB2	asRNA:CR44340	0.443892390635762	1	1	pseudobulk	DESeq2	LRT	0
901	KC_AB2	asRNA:CR44351	-0.427431139129811	1	1	pseudobulk	DESeq2	LRT	0
901	KC_AB2	asRNA:CR44368	-0.600547221779134	1	1	pseudobulk	DESeq2	LRT	0
901	KC_AB2	asRNA:CR44429	-0.427431139129811	1	1	pseudobulk	DESeq2	LRT	0
901	KC_AB2	asRNA:CR44724	1.5243995994652	0.741477491078038	1	pseudobulk	DESeq2	LRT	0
901	KC_AB2	asRNA:CR44840	-0.600547221779133	0.841940696994101	1	pseudobulk	DESeq2	LRT	0
901	KC_AB2	asRNA:CR44847	1.5243995994652	0.741477491078038	1	pseudobulk	DESeq2	LRT	0
901	KC_AB2	asRNA:CR44892	-0.913409445928892	0.707710875350411	1	pseudobulk	DESeq2	LRT	0
901	KC_AB2	asRNA:CR44894	0.771570297722526	0.665615306522169	1	pseudobulk	DESeq2	LRT	0
901	KC_AB2	asRNA:CR44960	-0.592481036256484	0.706847545932384	1	pseudobulk	DESeq2	LRT	0
902	KC_AB2	asRNA:CR44961	2.1817927492653	0.458599861292202	1	pseudobulk	DESeq2	LRT	0
902	KC_AB2	asRNA:CR44981	-1.85733156562156	0.448702818121367	1	pseudobulk	DESeq2	LRT	0
902	KC_AB2	asRNA:CR44987	-0.427431139129811	1	1	pseudobulk	DESeq2	LRT	0
902	KC_AB2	asRNA:CR44990	2.12684015898615	0.445939223035924	1	pseudobulk	DESeq2	LRT	0
902	KC_AB2	asRNA:CR45000	1.5243995994652	0.741477491078038	1	pseudobulk	DESeq2	LRT	0
902	KC_AB2	asRNA:CR45009	0.222396124134733	1	1	pseudobulk	DESeq2	LRT	0
902	KC_AB2	asRNA:CR45041	-1.07776915880798	0.775491075088671	1	pseudobulk	DESeq2	LRT	0
902	KC_AB2	asRNA:CR45053	1.5243995994652	0.741477491078038	1	pseudobulk	DESeq2	LRT	0
902	KC_AB2	asRNA:CR45104	-0.600547221779133	0.841940696994101	1	pseudobulk	DESeq2	LRT	0
902	KC_AB2	asRNA:CR45121	-0.592481036256484	0.706847545932384	1	pseudobulk	DESeq2	LRT	0
903	KC_AB2	asRNA:CR45126	1.14814388261213	0.568837005180859	1	pseudobulk	DESeq2	LRT	0
903	KC_AB2	asRNA:CR45129	-1.12569474978191	0.563105123273263	1	pseudobulk	DESeq2	LRT	0
903	KC_AB2	asRNA:CR45131	-1.72976330140494	0.41918502381035	1	pseudobulk	DESeq2	LRT	0
903	KC_AB2	asRNA:CR45133	1.5243995994652	0.741477491078038	1	pseudobulk	DESeq2	LRT	0
903	KC_AB2	asRNA:CR45134	-0.892276647940849	0.722011423451975	1	pseudobulk	DESeq2	LRT	0
903	KC_AB2	asRNA:CR45137	-0.592481036256484	0.706847545932384	1	pseudobulk	DESeq2	LRT	0
903	KC_AB2	asRNA:CR45141	-0.427431139129811	1	1	pseudobulk	DESeq2	LRT	0
903	KC_AB2	asRNA:CR45145	-1.40673172715063	0.587364787535786	1	pseudobulk	DESeq2	LRT	0
903	KC_AB2	asRNA:CR45151	2.12684015898615	0.445939223035924	1	pseudobulk	DESeq2	LRT	0
903	KC_AB2	asRNA:CR45152	-0.600547221779134	1	1	pseudobulk	DESeq2	LRT	0
904	KC_AB2	asRNA:CR45160	1.5243995994652	0.741477491078038	1	pseudobulk	DESeq2	LRT	0
904	KC_AB2	asRNA:CR45164	-0.600547221779134	1	1	pseudobulk	DESeq2	LRT	0
904	KC_AB2	asRNA:CR45182	-0.734008597994575	0.694445711446769	1	pseudobulk	DESeq2	LRT	0
904	KC_AB2	asRNA:CR45198	2.72066226053615	0.290765320578625	1	pseudobulk	DESeq2	LRT	0
904	KC_AB2	asRNA:CR45281	0.674459942220112	0.74558757773504	1	pseudobulk	DESeq2	LRT	0
904	KC_AB2	asRNA:CR45350	1.40241945196173	0.57265124481703	1	pseudobulk	DESeq2	LRT	0
904	KC_AB2	asRNA:CR45469	-1.92942763539083	0.427765257981976	1	pseudobulk	DESeq2	LRT	0
904	KC_AB2	asRNA:CR45520	0.864341462967788	0.925947094335722	1	pseudobulk	DESeq2	LRT	0
904	KC_AB2	asRNA:CR45600	3.75006552120102	0.0630070317191532	1	pseudobulk	DESeq2	LRT	0
904	KC_AB2	asRNA:CR45604	-0.600547221779134	1	1	pseudobulk	DESeq2	LRT	0
905	KC_AB2	asRNA:CR45620	-1.78094334699383	0.470930349923561	1	pseudobulk	DESeq2	LRT	0
905	KC_AB2	asRNA:CR45789	1.21092382796727	0.637125262974216	1	pseudobulk	DESeq2	LRT	0
905	KC_AB2	asRNA:CR45813	1.5243995994652	0.741477491078038	1	pseudobulk	DESeq2	LRT	0
905	KC_AB2	asRNA:CR45815	1.5243995994652	0.741477491078038	1	pseudobulk	DESeq2	LRT	0
905	KC_AB2	asRNA:CR45822	0.181386468203001	0.814284240620835	1	pseudobulk	DESeq2	LRT	0
905	KC_AB2	asRNA:CR45832	1.40241945196173	0.57265124481703	1	pseudobulk	DESeq2	LRT	0
905	KC_AB2	asRNA:CR45835	-0.493357239699781	0.783807521297462	1	pseudobulk	DESeq2	LRT	0
905	KC_AB2	asRNA:CR45867	1.5243995994652	0.741477491078038	1	pseudobulk	DESeq2	LRT	0
905	KC_AB2	asRNA:CR45869	-0.427431139129811	1	1	pseudobulk	DESeq2	LRT	0
905	KC_AB2	asRNA:CR45874	-0.668740092924914	0.59199934760861	1	pseudobulk	DESeq2	LRT	0
906	KC_AB2	asRNA:CR45889	-0.841882716738579	1	1	pseudobulk	DESeq2	LRT	0
906	KC_AB2	asRNA:CR45944	-0.841882716738579	1	1	pseudobulk	DESeq2	LRT	0

906	KC_AB2	asRNA:CR45951	2.1817927492653	0.458599861292202	1	pseudobulk	DESeq2	LRT		0
906	KC_AB2	asRNA:CR46067	1.5243995994652	0.741477491078038	1	pseudobulk	DESeq2	LRT		0
906	KC_AB2	asRNA:CR46068	1.5243995994652	0.741477491078038	1	pseudobulk	DESeq2	LRT		0
906	KC_AB2	asRNA:CR46092	-0.427431139129811	1	1	pseudobulk	DESeq2	LRT		0
906	KC_AB2	asRNA:CR46135	-1.59432579116312	0.459120231439715	1	pseudobulk	DESeq2	LRT		0
906	KC_AB2	asRNA:CR46246	-0.427431139129811	1	1	pseudobulk	DESeq2	LRT		0
906	KC_AB2	asRNA:CR46251	2.12684015898615	0.445939223035924	1	pseudobulk	DESeq2	LRT		0
906	KC_AB2	asRNA:CR46262	-2.28539133851304	0.280298638885039	1	pseudobulk	DESeq2	LRT		0
907	KC_AB2	asRNA:CR46263	-0.592481036256484	0.706847545932384	1	pseudobulk	DESeq2	LRT		0
907	KC_AB2	ast	-1.07776915880798	0.775491075088671	1	pseudobulk	DESeq2	LRT		0
907	KC_AB2	AstA-R1	1.5243995994652	0.741477491078038	1	pseudobulk	DESeq2	LRT		0
907	KC_AB2	AstC-R2	-0.427431139129811	1	1	pseudobulk	DESeq2	LRT		0
907	KC_AB2	asun	-0.427431139129811	1	1	pseudobulk	DESeq2	LRT		0
907	KC_AB2	Asx	-0.0413311300007399	0.872335040240091	1	pseudobulk	DESeq2	LRT		0
907	KC_AB2	Atac1	-1.43450874381432	0.6168250034656	1	pseudobulk	DESeq2	LRT		0
907	KC_AB2	Atac2	-1.12785316439435	0.661319045116135	1	pseudobulk	DESeq2	LRT		0
907	KC_AB2	Atac3	0.316121837207955	0.850585050377942	1	pseudobulk	DESeq2	LRT		0
907	KC_AB2	ATbp	0.108468324554008	1	1	pseudobulk	DESeq2	LRT		0
908	KC_AB2	Ate1	0.876734422586968	0.272781477443533	1	pseudobulk	DESeq2	LRT		0
908	KC_AB2	Atet	0.324272916388376	0.603295745926646	1	pseudobulk	DESeq2	LRT		0
908	KC_AB2	Atf-2	-0.855909128841314	0.703076219237071	1	pseudobulk	DESeq2	LRT		0
908	KC_AB2	Atf3	0.613659617664411	0.76686169970757	1	pseudobulk	DESeq2	LRT		0
908	KC_AB2	Atf6	-0.0746942698538024	0.889002281911636	1	pseudobulk	DESeq2	LRT		0
908	KC_AB2	Atg1	-0.0067303130700659	0.858665162695651	1	pseudobulk	DESeq2	LRT		0
908	KC_AB2	Atg101	0.408036735764249	0.671636559676703	1	pseudobulk	DESeq2	LRT		0
908	KC_AB2	Atg12	0.0686561774289558	0.915821121891929	1	pseudobulk	DESeq2	LRT		0
908	KC_AB2	Atg13	0.177744917511271	0.871941332318327	1	pseudobulk	DESeq2	LRT		0
908	KC_AB2	Atg14	1.24049317184181	0.33716073070869	1	pseudobulk	DESeq2	LRT		0
909	KC_AB2	Atg16	-1.32661248755526	0.55265479009941	1	pseudobulk	DESeq2	LRT		0
909	KC_AB2	Atg17	-0.0052707405486641	0.855795179913882	1	pseudobulk	DESeq2	LRT		0
909	KC_AB2	Atg18a	0.300118631433799	0.706016142010511	1	pseudobulk	DESeq2	LRT		0
909	KC_AB2	Atg18b	-0.662489294546068	0.373391722463481	1	pseudobulk	DESeq2	LRT		0
909	KC_AB2	Atg2	0.864341462967788	0.925947094335722	1	pseudobulk	DESeq2	LRT		0
909	KC_AB2	Atg3	0.972700190833546	0.524902259511665	1	pseudobulk	DESeq2	LRT		0
909	KC_AB2	Atg4a	-1.93210948743547	0.291583645102348	1	pseudobulk	DESeq2	LRT		0
909	KC_AB2	Atg4b	-0.864501303525073	0.738774175713262	1	pseudobulk	DESeq2	LRT		0
909	KC_AB2	Atg5	-0.979848106426026	0.39862670531261	1	pseudobulk	DESeq2	LRT		0
909	KC_AB2	Atg6	0.380657795674128	0.821883499538588	1	pseudobulk	DESeq2	LRT		0
910	KC_AB2	Atg7	-1.63925793886161	0.546290859679341	1	pseudobulk	DESeq2	LRT		0
910	KC_AB2	Atg8a	-0.0412598772428814	0.783987365791574	1	pseudobulk	DESeq2	LRT		0
910	KC_AB2	Atg9	0.674459942220112	0.74558757773504	1	pseudobulk	DESeq2	LRT		0
910	KC_AB2	atl	1.01858524426521	0.530147347193427	1	pseudobulk	DESeq2	LRT		0
910	KC_AB2	atms	0.356775117037679	0.757731381969355	1	pseudobulk	DESeq2	LRT		0
910	KC_AB2	Atox1	-0.254536305138094	0.51577404762213	1	pseudobulk	DESeq2	LRT		0
910	KC_AB2	ATP6AP2	-0.142411141998235	0.683923393256428	1	pseudobulk	DESeq2	LRT		0
910	KC_AB2	ATP7	-0.841882716738579	1	1	pseudobulk	DESeq2	LRT		0
910	KC_AB2	ATP8A	0.463739170780097	0.393708806328269	1	pseudobulk	DESeq2	LRT		0
910	KC_AB2	ATP8B	0.112236198515868	0.822246389328101	1	pseudobulk	DESeq2	LRT		0
911	KC_AB2	Atpalpha	0.059387543029414	0.743843988626391	1	pseudobulk	DESeq2	LRT		0
911	KC_AB2	ATPCL	-0.320244262374578	0.586889290133435	1	pseudobulk	DESeq2	LRT		0
911	KC_AB2	ATPsynB	0.146823141805821	0.612754338453218	1	pseudobulk	DESeq2	LRT		0
911	KC_AB2	ATPsynbeta	0.0117295996437143	0.849033565392496	1	pseudobulk	DESeq2	LRT		0
911	KC_AB2	ATPsynC	-0.349561751020561	0.169841976039659	1	pseudobulk	DESeq2	LRT		0

911	KC_AB2	ATPsynCF6	-0.144562932576156	0.621518533282624	1	pseudobulk	DESeq2	LRT	0
911	KC_AB2	ATPsynD	-0.335434594933666	0.2787286902629	1	pseudobulk	DESeq2	LRT	0
911	KC_AB2	ATPsyndelta	-0.627093956639965	0.120483454385211	1	pseudobulk	DESeq2	LRT	0
911	KC_AB2	ATPsynE	0.0195602451012691	0.899596628116434	1	pseudobulk	DESeq2	LRT	0
911	KC_AB2	ATPsynF	-0.17261093480683	0.518351501264433	1	pseudobulk	DESeq2	LRT	0
912	KC_AB2	ATPsynG	0.0624594796608492	0.781282301944128	1	pseudobulk	DESeq2	LRT	0
912	KC_AB2	ATPsyngamma	-0.235891325691103	0.533197375805629	1	pseudobulk	DESeq2	LRT	0
912	KC_AB2	ATPsynO	-0.3925193658013	0.244142260852184	1	pseudobulk	DESeq2	LRT	0
912	KC_AB2	AttA	-0.427431139129811	1	1	pseudobulk	DESeq2	LRT	0
912	KC_AB2	Atu	1.21092382796727	0.637125262974216	1	pseudobulk	DESeq2	LRT	0
912	KC_AB2	Atx2	-0.823817767292311	0.150457917368319	1	pseudobulk	DESeq2	LRT	0
912	KC_AB2	Atxn7	-0.16830032012324	0.813040552825648	1	pseudobulk	DESeq2	LRT	0
912	KC_AB2	aub	-0.427431139129811	1	1	pseudobulk	DESeq2	LRT	0
912	KC_AB2	aux	0.29817623848159	0.773273038920726	1	pseudobulk	DESeq2	LRT	0
912	KC_AB2	ave	-2.81423787116358	0.186938591355401	1	pseudobulk	DESeq2	LRT	0
913	KC_AB2	Aven	-1.3723417613553	0.619532036522	1	pseudobulk	DESeq2	LRT	0
913	KC_AB2	awd	-0.173131222827986	0.669308699980519	1	pseudobulk	DESeq2	LRT	0
913	KC_AB2	axed	-0.824251592318981	0.324093590665999	1	pseudobulk	DESeq2	LRT	0
913	KC_AB2	Axn	-2.60085768680526	0.11992048857523	1	pseudobulk	DESeq2	LRT	0
913	KC_AB2	axo	1.0547002356457	0.651955074600822	1	pseudobulk	DESeq2	LRT	0
913	KC_AB2	Axud1	0.895284993895749	0.378311767428078	1	pseudobulk	DESeq2	LRT	0
913	KC_AB2	az2	-1.3723417613553	0.619532036522	1	pseudobulk	DESeq2	LRT	0
913	KC_AB2	B4	0.659386901947538	0.0919421883529243	1	pseudobulk	DESeq2	LRT	0
913	KC_AB2	B52	0.286956822367348	0.421559487300188	1	pseudobulk	DESeq2	LRT	0
913	KC_AB2	bab1	-0.427431139129811	1	1	pseudobulk	DESeq2	LRT	0
914	KC_AB2	babo	0.232616301329473	0.751284435045634	1	pseudobulk	DESeq2	LRT	0
914	KC_AB2	babos	-0.427431139129811	1	1	pseudobulk	DESeq2	LRT	0
914	KC_AB2	Bacc	-0.16898313883421	0.411175457651666	1	pseudobulk	DESeq2	LRT	0
914	KC_AB2	baf	-1.40673172715063	0.587364787535786	1	pseudobulk	DESeq2	LRT	0
914	KC_AB2	bai	-1.13332186378172	0.265239591646207	1	pseudobulk	DESeq2	LRT	0
914	KC_AB2	Baldspot	-0.308228345600521	0.534451532645203	1	pseudobulk	DESeq2	LRT	0
914	KC_AB2	ball	1.5243995994652	0.741477491078038	1	pseudobulk	DESeq2	LRT	0
914	KC_AB2	Bap111	-0.245919581482988	0.832204672574379	1	pseudobulk	DESeq2	LRT	0
914	KC_AB2	Bap170	-1.40673172715063	0.587364787535786	1	pseudobulk	DESeq2	LRT	0
914	KC_AB2	Bap55	-1.19962071437726	0.428266670016381	1	pseudobulk	DESeq2	LRT	0
915	KC_AB2	Bap60	-0.862476470718677	0.337839503587933	1	pseudobulk	DESeq2	LRT	0
915	KC_AB2	barc	-0.491442287863105	0.551084379380212	1	pseudobulk	DESeq2	LRT	0
915	KC_AB2	bbc	0.121194571830637	0.865275691156927	1	pseudobulk	DESeq2	LRT	0
915	KC_AB2	BBS5	-0.592481036256484	0.706847545932384	1	pseudobulk	DESeq2	LRT	0
915	KC_AB2	bbx	0.108468324554008	1	1	pseudobulk	DESeq2	LRT	0
915	KC_AB2	bc10	-0.97489058536879	0.435627974338177	1	pseudobulk	DESeq2	LRT	0
915	KC_AB2	BCAS2	-0.533946064220369	0.73906067333976	1	pseudobulk	DESeq2	LRT	0
915	KC_AB2	bchs	0.849345590737178	0.338883624780653	1	pseudobulk	DESeq2	LRT	0
915	KC_AB2	BCL7-like	1.43840889475515	0.422185533641736	1	pseudobulk	DESeq2	LRT	0
915	KC_AB2	bcn92	-0.451523144747172	0.613138281391617	1	pseudobulk	DESeq2	LRT	0
916	KC_AB2	Bdbt	0.41931709209241	0.684915702795864	1	pseudobulk	DESeq2	LRT	0
916	KC_AB2	bdg	-1.70380348762697	0.473446675363872	1	pseudobulk	DESeq2	LRT	0
916	KC_AB2	bdl	2.1817927492653	0.458599861292202	1	pseudobulk	DESeq2	LRT	0
916	KC_AB2	Bdp1	0.791468033138705	0.699865413482108	1	pseudobulk	DESeq2	LRT	0
916	KC_AB2	be	0.674459942220112	0.74558757773504	1	pseudobulk	DESeq2	LRT	0
916	KC_AB2	BEAF-32	0.320220476301991	0.728183991043835	1	pseudobulk	DESeq2	LRT	0
916	KC_AB2	beag	0.0951652990582188	0.829122231837765	1	pseudobulk	DESeq2	LRT	0
916	KC_AB2	beat-Ib	-1.36278536056344	0.518581808073597	1	pseudobulk	DESeq2	LRT	0

916	KC_AB2	beat-Ic	-2.72954001372517	0.206956892822938	1	pseudobulk	DESeq2	LRT		0
916	KC_AB2	beat-IIb	-0.592481036256484	0.706847545932384	1	pseudobulk	DESeq2	LRT		0
917	KC_AB2	beat-IIIb	-1.66828165461268	0.531269883399465	1	pseudobulk	DESeq2	LRT		0
917	KC_AB2	beat-IV	-0.237224957982124	0.575867664289178	1	pseudobulk	DESeq2	LRT		0
917	KC_AB2	beat-Vb	1.5243995994652	0.741477491078038	1	pseudobulk	DESeq2	LRT		0
917	KC_AB2	beat-Vc	-0.600547221779133	0.841940696994101	1	pseudobulk	DESeq2	LRT		0
917	KC_AB2	beat-VI	-1.40673172715063	0.587364787535786	1	pseudobulk	DESeq2	LRT		0
917	KC_AB2	beat-VII	-0.151884172301593	0.683146823763849	1	pseudobulk	DESeq2	LRT		0
917	KC_AB2	bel	-0.0070016119136897	0.906662583133322	1	pseudobulk	DESeq2	LRT		0
917	KC_AB2	Bem46	-1.62718723652348	0.411060170992501	1	pseudobulk	DESeq2	LRT		0
917	KC_AB2	ben	0.0848052210426022	0.773209701191594	1	pseudobulk	DESeq2	LRT		0
917	KC_AB2	Best1	1.62847150712622	0.350526012135992	1	pseudobulk	DESeq2	LRT		0
918	KC_AB2	Bet1	-0.356653285945803	0.715810562044033	1	pseudobulk	DESeq2	LRT		0
918	KC_AB2	Bet3	-1.15335789244447	0.345863038319351	1	pseudobulk	DESeq2	LRT		0
918	KC_AB2	Bet5	-0.233788446286757	0.761255272552235	1	pseudobulk	DESeq2	LRT		0
918	KC_AB2	beta-PheRS	-0.463052496301942	0.794041648114126	1	pseudobulk	DESeq2	LRT		0
918	KC_AB2	beta-Spec	0.159906435154716	0.611539600835487	1	pseudobulk	DESeq2	LRT		0
918	KC_AB2	beta'COP	0.541626970998422	0.56400364344127	1	pseudobulk	DESeq2	LRT		0
918	KC_AB2	beta3GalTII	2.72066226053615	0.290765320578625	1	pseudobulk	DESeq2	LRT		0
918	KC_AB2	beta4GalNAcTA	0.910858885337268	0.593576541362697	1	pseudobulk	DESeq2	LRT		0
918	KC_AB2	beta4GalNAcTB	-1.84905028965745	0.445198155344163	1	pseudobulk	DESeq2	LRT		0
918	KC_AB2	beta4GalT7	1.5243995994652	0.741477491078038	1	pseudobulk	DESeq2	LRT		0
919	KC_AB2	betaCOP	-2.11674245501194	0.261651386987452	1	pseudobulk	DESeq2	LRT		0
919	KC_AB2	betaggt-I	-1.51812168653838	0.552401469179882	1	pseudobulk	DESeq2	LRT		0
919	KC_AB2	betaggt-II	-0.752313144181686	0.678208821989755	1	pseudobulk	DESeq2	LRT		0
919	KC_AB2	betaTub56D	-0.654638265226242	0.0830412961502782	1	pseudobulk	DESeq2	LRT		0
919	KC_AB2	betaTub85D	-1.07776915880798	0.775491075088671	1	pseudobulk	DESeq2	LRT		0
919	KC_AB2	betaTub97EF	-0.600547221779134	1	1	pseudobulk	DESeq2	LRT		0
919	KC_AB2	Bgb	-0.931617406030653	0.217417340207847	1	pseudobulk	DESeq2	LRT		0
919	KC_AB2	BHD	0.183555857030446	1	1	pseudobulk	DESeq2	LRT		0
919	KC_AB2	BI-1	-0.588517412038815	0.167017878032854	1	pseudobulk	DESeq2	LRT		0
919	KC_AB2	bic	-0.534954716545358	0.17183086528036	1	pseudobulk	DESeq2	LRT		0
920	KC_AB2	BicD	-0.293255142405436	0.553098304927869	1	pseudobulk	DESeq2	LRT		0
920	KC_AB2	bif	0.165326497736857	0.825113238482007	1	pseudobulk	DESeq2	LRT		0
920	KC_AB2	bigmax	-1.3929484167291	0.512855059074155	1	pseudobulk	DESeq2	LRT		0
920	KC_AB2	Bili	-0.434935520499499	0.51139473597126	1	pseudobulk	DESeq2	LRT		0
920	KC_AB2	Bin1	-1.0726365359201	0.440547981716032	1	pseudobulk	DESeq2	LRT		0
920	KC_AB2	bin3	0.356296904228739	0.639167785316914	1	pseudobulk	DESeq2	LRT		0
920	KC_AB2	bip1	2.1817927492653	0.458599861292202	1	pseudobulk	DESeq2	LRT		0
920	KC_AB2	bip2	0.174628004933399	0.625488934906891	1	pseudobulk	DESeq2	LRT		0
920	KC_AB2	Bka	0.52880282091781	0.716312240888837	1	pseudobulk	DESeq2	LRT		0
920	KC_AB2	Blimp-1	0.374962933654893	0.431488899759787	1	pseudobulk	DESeq2	LRT		0
921	KC_AB2	Blm	1.7512149445917	0.356426896003883	1	pseudobulk	DESeq2	LRT		0
921	KC_AB2	Blos1	0.412147049037067	0.671337704425309	1	pseudobulk	DESeq2	LRT		0
921	KC_AB2	Blos2	-1.92909806167416	0.301388940011989	1	pseudobulk	DESeq2	LRT		0
921	KC_AB2	Blos3	0.835414796448957	0.467053182778283	1	pseudobulk	DESeq2	LRT		0
921	KC_AB2	Blos4	0.629312849717786	0.606510833949087	1	pseudobulk	DESeq2	LRT		0
921	KC_AB2	blot	-0.427431139129811	1	1	pseudobulk	DESeq2	LRT		0
921	KC_AB2	blp	0.930679613580921	0.466189134637888	1	pseudobulk	DESeq2	LRT		0
921	KC_AB2	blw	-0.071933198593947	0.76182203569193	1	pseudobulk	DESeq2	LRT		0
921	KC_AB2	bma	-0.322798814055842	0.289253714766042	1	pseudobulk	DESeq2	LRT		0
921	KC_AB2	Bmcp	0.269463852832676	0.799940963525289	1	pseudobulk	DESeq2	LRT		0
922	KC_AB2	bmm	2.79253611440997	0.0665058894494272	1	pseudobulk	DESeq2	LRT		0

922	KC_AB2	bnb	1.13264315058701	0.469836404777228	1	pseudobulk	DESeq2	LRT	0
922	KC_AB2	boca	-0.280802054374547	0.749027521006649	1	pseudobulk	DESeq2	LRT	0
922	KC_AB2	bocks	-0.39917336535616	0.810147819664692	1	pseudobulk	DESeq2	LRT	0
922	KC_AB2	BOD1	0.773551146205319	0.702323084538382	1	pseudobulk	DESeq2	LRT	0
922	KC_AB2	boi	-0.600547221779134	1	1	pseudobulk	DESeq2	LRT	0
922	KC_AB2	bol	-0.226417134333345	0.434998601924063	1	pseudobulk	DESeq2	LRT	0
922	KC_AB2	Bom065	-0.600547221779133	0.841940696994101	1	pseudobulk	DESeq2	LRT	0
922	KC_AB2	Bom067	-0.427431139129811	1	1	pseudobulk	DESeq2	LRT	0
922	KC_AB2	bon	0.0133277886599415	0.880286689666584	1	pseudobulk	DESeq2	LRT	0
923	KC_AB2	bonsai	-0.531539015928775	0.64478530625098	1	pseudobulk	DESeq2	LRT	0
923	KC_AB2	bor	-0.674228641611906	0.587006023618918	1	pseudobulk	DESeq2	LRT	0
923	KC_AB2	BORCS5	-1.70031435243266	0.0841308440166533	1	pseudobulk	DESeq2	LRT	0
923	KC_AB2	BORCS6	-2.10488109534752	0.250434931643258	1	pseudobulk	DESeq2	LRT	0
923	KC_AB2	botv	1.85296564410137	0.274352822955943	1	pseudobulk	DESeq2	LRT	0
923	KC_AB2	br	-1.02247440244601	0.0952546984971293	1	pseudobulk	DESeq2	LRT	0
923	KC_AB2	Br140	-0.720855549198881	0.659484835319849	1	pseudobulk	DESeq2	LRT	0
923	KC_AB2	brat	0.200353150238393	0.608941009118111	1	pseudobulk	DESeq2	LRT	0
923	KC_AB2	Brca2	-0.841882716738579	1	1	pseudobulk	DESeq2	LRT	0
923	KC_AB2	Brd8	-1.16116875819275	0.736255625566845	1	pseudobulk	DESeq2	LRT	0
924	KC_AB2	Bre1	0.271543141472767	0.791421003235011	1	pseudobulk	DESeq2	LRT	0
924	KC_AB2	Brf	0.773551146205319	0.702323084538382	1	pseudobulk	DESeq2	LRT	0
924	KC_AB2	brm	0.777916063165026	0.395791542363798	1	pseudobulk	DESeq2	LRT	0
924	KC_AB2	Brms1	-0.155688685887525	0.871812765464523	1	pseudobulk	DESeq2	LRT	0
924	KC_AB2	brp	0.0221898270957411	0.902330410961562	1	pseudobulk	DESeq2	LRT	0
924	KC_AB2	bru1	0.665123816898181	0.156245939935389	1	pseudobulk	DESeq2	LRT	0
924	KC_AB2	bru2	0.475614181274588	0.504024843740109	1	pseudobulk	DESeq2	LRT	0
924	KC_AB2	bru3	-0.0246010807513416	0.888399954914313	1	pseudobulk	DESeq2	LRT	0
924	KC_AB2	Bruce	-0.0960667466002158	0.896058936380949	1	pseudobulk	DESeq2	LRT	0
924	KC_AB2	brun	-0.245919581482988	0.832204672574379	1	pseudobulk	DESeq2	LRT	0
925	KC_AB2	BRWD3	-0.34683484955706	0.789885247857429	1	pseudobulk	DESeq2	LRT	0
925	KC_AB2	brwl	-0.321648857277193	0.815018278876993	1	pseudobulk	DESeq2	LRT	0
925	KC_AB2	bs	-0.94307066344797	0.629943854378022	1	pseudobulk	DESeq2	LRT	0
925	KC_AB2	bsf	-0.432428277403744	0.739770885427475	1	pseudobulk	DESeq2	LRT	0
925	KC_AB2	Bsg	-0.0051578333036028	0.945043830840042	1	pseudobulk	DESeq2	LRT	0
925	KC_AB2	Bsg25D	-0.348994603728114	0.729611587265814	1	pseudobulk	DESeq2	LRT	0
925	KC_AB2	bsk	0.169097487830914	0.617817346028933	1	pseudobulk	DESeq2	LRT	0
925	KC_AB2	bt	-0.600547221779133	0.841940696994101	1	pseudobulk	DESeq2	LRT	0
925	KC_AB2	BTBD9	-0.2488904916109	0.790972801778357	1	pseudobulk	DESeq2	LRT	0
925	KC_AB2	BtbVII	0.0572896233555058	0.813906234880374	1	pseudobulk	DESeq2	LRT	0
926	KC_AB2	btsz	-0.294022342820692	0.522264409874599	1	pseudobulk	DESeq2	LRT	0
926	KC_AB2	btz	2.29782712851375	0.103095982716972	1	pseudobulk	DESeq2	LRT	0
926	KC_AB2	Bub1	0.997249947847885	0.601888101051434	1	pseudobulk	DESeq2	LRT	0
926	KC_AB2	Bub3	-0.823350754707956	0.431962335480673	1	pseudobulk	DESeq2	LRT	0
926	KC_AB2	BubR1	-0.600547221779134	1	1	pseudobulk	DESeq2	LRT	0
926	KC_AB2	bug	0.458061275320087	0.802259721126042	1	pseudobulk	DESeq2	LRT	0
926	KC_AB2	Bug22	1.26642586668089	0.271490733353906	1	pseudobulk	DESeq2	LRT	0
926	KC_AB2	BuGZ	-0.102304495840616	0.835505892362273	1	pseudobulk	DESeq2	LRT	0
926	KC_AB2	bun	0.110074798051157	0.586339184651434	1	pseudobulk	DESeq2	LRT	0
926	KC_AB2	bur	0.0744052852432164	0.854125178399904	1	pseudobulk	DESeq2	LRT	0
927	KC_AB2	bves	0.557940381459108	0.298203838889155	1	pseudobulk	DESeq2	LRT	0
927	KC_AB2	bwa	0.65628999924013	0.671243274897585	1	pseudobulk	DESeq2	LRT	0
927	KC_AB2	Bx	0.414914893318161	0.081836308851367	1	pseudobulk	DESeq2	LRT	0
927	KC_AB2	Bx42	0.449905875814008	0.645420378171048	1	pseudobulk	DESeq2	LRT	0

927	KC_AB2	bys	-1.59432579116312	0.459120231439715	1	pseudobulk	DESeq2	LRT		0
927	KC_AB2	c(3)G	-0.427431139129811	1	1	pseudobulk	DESeq2	LRT		0
927	KC_AB2	c11.1	0.325694839125882	0.685217606339927	1	pseudobulk	DESeq2	LRT		0
927	KC_AB2	c12.1	-0.179329326646023	0.860207090528527	1	pseudobulk	DESeq2	LRT		0
927	KC_AB2	c12.2	-0.597149594069719	0.720919359858713	1	pseudobulk	DESeq2	LRT		0
927	KC_AB2	C1GalTA	-0.218449853307312	0.732078559703742	1	pseudobulk	DESeq2	LRT		0
928	KC_AB2	C3G	0.913910906827043	0.263885858023394	1	pseudobulk	DESeq2	LRT		0
928	KC_AB2	C901	-1.12785316439435	0.661319045116135	1	pseudobulk	DESeq2	LRT		0
928	KC_AB2	ca	-0.38409858233708	0.760561531756852	1	pseudobulk	DESeq2	LRT		0
928	KC_AB2	Ca-alpha1D	0.160743159657578	0.639744614221864	1	pseudobulk	DESeq2	LRT		0
928	KC_AB2	Ca-alpha1T	0.287306921556587	0.280505177909786	1	pseudobulk	DESeq2	LRT		0
928	KC_AB2	Ca-beta	0.0928902830595651	0.72668790536117	1	pseudobulk	DESeq2	LRT		0
928	KC_AB2	Ca-Ma2d	0.514442040410779	0.429657160819016	1	pseudobulk	DESeq2	LRT		0
928	KC_AB2	CaBP1	-0.0738748688508091	0.942862902944051	1	pseudobulk	DESeq2	LRT		0
928	KC_AB2	cac	0.0296863069150403	0.884043031661829	1	pseudobulk	DESeq2	LRT		0
928	KC_AB2	cact	0.639740875092845	0.189956807061738	1	pseudobulk	DESeq2	LRT		0
929	KC_AB2	cactin	1.69515175799364	0.368369504470473	1	pseudobulk	DESeq2	LRT		0
929	KC_AB2	Cad86C	-0.427431139129811	1	1	pseudobulk	DESeq2	LRT		0
929	KC_AB2	Cad87A	1.11471276021981	0.644913768495318	1	pseudobulk	DESeq2	LRT		0
929	KC_AB2	Cad96Ca	-1.07776915880798	0.775491075088671	1	pseudobulk	DESeq2	LRT		0
929	KC_AB2	CadN	-0.992280894369818	0.515831635501147	1	pseudobulk	DESeq2	LRT		0
929	KC_AB2	CadN2	-0.592481036256484	0.706847545932384	1	pseudobulk	DESeq2	LRT		0
929	KC_AB2	Cadps	0.347190268051234	0.318462298438826	1	pseudobulk	DESeq2	LRT		0
929	KC_AB2	Caf1-105	-1.07776915880798	0.775491075088671	1	pseudobulk	DESeq2	LRT		0
929	KC_AB2	Caf1-55	-1.69726920070713	0.250525319820446	1	pseudobulk	DESeq2	LRT		0
929	KC_AB2	cag	-0.0852743682753834	0.917718671153184	1	pseudobulk	DESeq2	LRT		0
930	KC_AB2	CAH1	-0.0529809127348567	0.863884257519641	1	pseudobulk	DESeq2	LRT		0
930	KC_AB2	CAH13	-0.600547221779134	1	1	pseudobulk	DESeq2	LRT		0
930	KC_AB2	CAH2	0.222396124134733	1	1	pseudobulk	DESeq2	LRT		0
930	KC_AB2	CAH3	-0.558882839747815	0.442124323168044	1	pseudobulk	DESeq2	LRT		0
930	KC_AB2	CAH8	-0.0852743682753834	0.917718671153184	1	pseudobulk	DESeq2	LRT		0
930	KC_AB2	CAHbeta	-0.533123160922757	0.744971957102297	1	pseudobulk	DESeq2	LRT		0
930	KC_AB2	cal1	0.779110570428844	0.722427818657622	1	pseudobulk	DESeq2	LRT		0
930	KC_AB2	CalpA	-2.30239837462272	0.196646503505144	1	pseudobulk	DESeq2	LRT		0
930	KC_AB2	CalpB	-1.22182103121519	0.534749509500509	1	pseudobulk	DESeq2	LRT		0
930	KC_AB2	Calr	0.28985725550014	0.641397289151942	1	pseudobulk	DESeq2	LRT		0
931	KC_AB2	Cals	0.0929594252012103	0.737206142739071	1	pseudobulk	DESeq2	LRT		0
931	KC_AB2	Calx	-0.161934935903857	0.822868505745758	1	pseudobulk	DESeq2	LRT		0
931	KC_AB2	calypso	-1.54132252871259	0.432696240183291	1	pseudobulk	DESeq2	LRT		0
931	KC_AB2	Cam	0.230812996024867	0.285166632591931	1	pseudobulk	DESeq2	LRT		0
931	KC_AB2	CaMKI	-0.0634171878421633	0.822006227781684	1	pseudobulk	DESeq2	LRT		0
931	KC_AB2	CaMKII	0.175136782050223	0.449756113004765	1	pseudobulk	DESeq2	LRT		0
931	KC_AB2	Camp	-1.03398376725774	0.539004535377113	1	pseudobulk	DESeq2	LRT		0
931	KC_AB2	Camta	-0.157299724398507	0.864886677139766	1	pseudobulk	DESeq2	LRT		0
931	KC_AB2	CanA-14F	-0.142377188686975	0.59253815846844	1	pseudobulk	DESeq2	LRT		0
931	KC_AB2	CanB	-0.384173915355987	0.137613119904609	1	pseudobulk	DESeq2	LRT		0
932	KC_AB2	CanB2	-0.0238234572553691	0.9288779081666	1	pseudobulk	DESeq2	LRT		0
932	KC_AB2	Cand1	0.73049788259523	0.526192545247875	1	pseudobulk	DESeq2	LRT		0
932	KC_AB2	CAP	0.0636352008331748	0.929924185853014	1	pseudobulk	DESeq2	LRT		0
932	KC_AB2	Cap-D3	1.5243995994652	0.741477491078038	1	pseudobulk	DESeq2	LRT		0
932	KC_AB2	Cap-G	1.84998908200093	0.351217765903382	1	pseudobulk	DESeq2	LRT		0
932	KC_AB2	Cap-H2	0.329740041077442	0.855811003303737	1	pseudobulk	DESeq2	LRT		0
932	KC_AB2	Caper	0.34297990738693	0.282057710008895	1	pseudobulk	DESeq2	LRT		0

932	KC_AB2	Capr	0.20442558756581	0.799535304221631	1	pseudobulk	DESeq2	LRT	0
932	KC_AB2	caps	-0.159407192588595	0.63285086365276	1	pseudobulk	DESeq2	LRT	0
932	KC_AB2	capt	-0.0917315186681407	0.862273682241098	1	pseudobulk	DESeq2	LRT	0
933	KC_AB2	capu	0.764296328913256	0.274046851806292	1	pseudobulk	DESeq2	LRT	0
933	KC_AB2	car	0.0046631200022412	0.945761246794169	1	pseudobulk	DESeq2	LRT	0
933	KC_AB2	CARPB	-1.29244479833796	0.693295472370086	1	pseudobulk	DESeq2	LRT	0
933	KC_AB2	CarT	2.12684015898615	0.445939223035924	1	pseudobulk	DESeq2	LRT	0
933	KC_AB2	CASK	0.283937331281818	0.323370167878576	1	pseudobulk	DESeq2	LRT	0
933	KC_AB2	casp	1.23049953843431	0.287136417274066	1	pseudobulk	DESeq2	LRT	0
933	KC_AB2	cass	0.140987107788322	0.74782716559229	1	pseudobulk	DESeq2	LRT	0
933	KC_AB2	Cat	0.164054477342436	0.842806774525055	1	pseudobulk	DESeq2	LRT	0
933	KC_AB2	cathD	-0.685923464590775	0.589689566338648	1	pseudobulk	DESeq2	LRT	0
933	KC_AB2	Catsup	0.864341462967788	0.925947094335722	1	pseudobulk	DESeq2	LRT	0
934	KC_AB2	cav	-1.29244479833796	0.693295472370086	1	pseudobulk	DESeq2	LRT	0
934	KC_AB2	caz	0.712608737171298	0.334925857587829	1	pseudobulk	DESeq2	LRT	0
934	KC_AB2	cbc	1.40241945196173	0.57265124481703	1	pseudobulk	DESeq2	LRT	0
934	KC_AB2	Cbl	0.357674183845841	0.682779191249231	1	pseudobulk	DESeq2	LRT	0
934	KC_AB2	Cbp20	-0.750602743517619	0.525483487789058	1	pseudobulk	DESeq2	LRT	0
934	KC_AB2	Cbp80	-1.05776592699768	0.596524436181984	1	pseudobulk	DESeq2	LRT	0
934	KC_AB2	cbs	-2.05560996500493	0.122328657909258	1	pseudobulk	DESeq2	LRT	0
934	KC_AB2	Cbs	-2.53564472550049	0.241603852619635	1	pseudobulk	DESeq2	LRT	0
934	KC_AB2	cbt	-0.405206940929088	0.721853127669005	1	pseudobulk	DESeq2	LRT	0
934	KC_AB2	CCAP-R	-0.427431139129811	1	1	pseudobulk	DESeq2	LRT	0
935	KC_AB2	CCDC53	-1.84423614433138	0.419369668145802	1	pseudobulk	DESeq2	LRT	0
935	KC_AB2	Ccdc58	-0.611149939622605	0.724818174113419	1	pseudobulk	DESeq2	LRT	0
935	KC_AB2	Cchl	-0.424430335790383	0.543859237647578	1	pseudobulk	DESeq2	LRT	0
935	KC_AB2	CCKLR-17D1	-1.70380348762697	0.473446675363872	1	pseudobulk	DESeq2	LRT	0
935	KC_AB2	Ccm3	-0.614042930498202	0.391832422108167	1	pseudobulk	DESeq2	LRT	0
935	KC_AB2	Ccn	0.44993726248549	0.311649928095043	1	pseudobulk	DESeq2	LRT	0
935	KC_AB2	CCT1	-1.77049677143504	0.34188273241158	1	pseudobulk	DESeq2	LRT	0
935	KC_AB2	CCT2	-1.36192722140614	0.504761005035368	1	pseudobulk	DESeq2	LRT	0
935	KC_AB2	CCT3	0.613559242226206	0.691831166136014	1	pseudobulk	DESeq2	LRT	0
935	KC_AB2	CCT4	-0.398008277315517	0.690928878421296	1	pseudobulk	DESeq2	LRT	0
936	KC_AB2	CCT5	-0.600547221779134	1	1	pseudobulk	DESeq2	LRT	0
936	KC_AB2	CCT6	-0.569072434057771	0.664325773518098	1	pseudobulk	DESeq2	LRT	0
936	KC_AB2	CCT7	1.07173252967008	0.608626529368362	1	pseudobulk	DESeq2	LRT	0
936	KC_AB2	CCT8	0.0828162449678417	0.929932733850168	1	pseudobulk	DESeq2	LRT	0
936	KC_AB2	Ccz1	-1.87282361414576	0.298350536076648	1	pseudobulk	DESeq2	LRT	0
936	KC_AB2	CD98hc	0.354606537809127	0.549880330115999	1	pseudobulk	DESeq2	LRT	0
936	KC_AB2	Cda5	2.70714859398546	0.251391037333528	1	pseudobulk	DESeq2	LRT	0
936	KC_AB2	cdc14	-0.153051219182842	0.781985568508623	1	pseudobulk	DESeq2	LRT	0
936	KC_AB2	Cdc16	-1.07776915880798	0.775491075088671	1	pseudobulk	DESeq2	LRT	0
936	KC_AB2	Cdc23	-0.0852743682753834	0.917718671153184	1	pseudobulk	DESeq2	LRT	0
937	KC_AB2	Cdc27	-0.117731627259495	0.890289771756269	1	pseudobulk	DESeq2	LRT	0
937	KC_AB2	Cdc2rk	-0.740207704288144	0.726967462633097	1	pseudobulk	DESeq2	LRT	0
937	KC_AB2	Cdc37	0.511995197579496	0.448583037201796	1	pseudobulk	DESeq2	LRT	0
937	KC_AB2	Cdc42	-1.12432007107868	0.231792694270171	1	pseudobulk	DESeq2	LRT	0
937	KC_AB2	Cdc5	1.3560255041741	0.33814593088297	1	pseudobulk	DESeq2	LRT	0
937	KC_AB2	CDC50	0.835414796448957	0.467053182778283	1	pseudobulk	DESeq2	LRT	0
937	KC_AB2	Cdc7	-1.39655208244106	0.286039806916015	1	pseudobulk	DESeq2	LRT	0
937	KC_AB2	Cdep	-0.0936131569169561	0.815743831077747	1	pseudobulk	DESeq2	LRT	0
937	KC_AB2	CdGAPr	0.740875519797777	0.575630124146364	1	pseudobulk	DESeq2	LRT	0
937	KC_AB2	cdi	-0.145769368233844	0.810236462444498	1	pseudobulk	DESeq2	LRT	0

938	KC_AB2	Cdk12	-0.179141172372805	0.836951602759328	1	pseudobulk	DESeq2	LRT		0
938	KC_AB2	Cdk2	0.83721407599818	0.620952546821801	1	pseudobulk	DESeq2	LRT		0
938	KC_AB2	CDK2AP1	0.0468064013040354	0.796429399653503	1	pseudobulk	DESeq2	LRT		0
938	KC_AB2	Cdk5	-0.557418729674801	0.605624283896387	1	pseudobulk	DESeq2	LRT		0
938	KC_AB2	Cdk5alpha	-0.252012789347943	0.516480380798702	1	pseudobulk	DESeq2	LRT		0
938	KC_AB2	Cdk7	-0.843996296624293	0.67626831722335	1	pseudobulk	DESeq2	LRT		0
938	KC_AB2	Cdk9	1.12684041175347	0.411310930109686	1	pseudobulk	DESeq2	LRT		0
938	KC_AB2	cdm	0.0991092191605412	0.859220317499135	1	pseudobulk	DESeq2	LRT		0
938	KC_AB2	Cds	-0.087592605001003	0.77950411262565	1	pseudobulk	DESeq2	LRT		0
938	KC_AB2	Ced-12	-0.669774949668414	0.69553201717938	1	pseudobulk	DESeq2	LRT		0
939	KC_AB2	Cen	-0.994443854230012	0.356712091627614	1	pseudobulk	DESeq2	LRT		0
939	KC_AB2	CenB1A	1.70053690678249	0.319223901856326	1	pseudobulk	DESeq2	LRT		0
939	KC_AB2	CenG1A	-0.822828830119574	0.38995386260655	1	pseudobulk	DESeq2	LRT		0
939	KC_AB2	Cep97	-0.449589580186246	0.691942078676082	1	pseudobulk	DESeq2	LRT		0
939	KC_AB2	cer	0.497049603063354	0.707379335399985	1	pseudobulk	DESeq2	LRT		0
939	KC_AB2	Cerk	-0.380588256102009	0.653191181873088	1	pseudobulk	DESeq2	LRT		0
939	KC_AB2	cert	0.576016423747365	0.710743166083386	1	pseudobulk	DESeq2	LRT		0
939	KC_AB2	Cf2	0.215466609081379	0.729597951994203	1	pseudobulk	DESeq2	LRT		0
939	KC_AB2	Cfp1	-0.421548404092889	0.761931442609479	1	pseudobulk	DESeq2	LRT		0
939	KC_AB2	cg	-0.691445588018782	0.141444718273918	1	pseudobulk	DESeq2	LRT		0
940	KC_AB2	CG10011	-0.256282425388363	0.716073269531836	1	pseudobulk	DESeq2	LRT		0
940	KC_AB2	CG10019	-1.12785316439435	0.661319045116135	1	pseudobulk	DESeq2	LRT		0
940	KC_AB2	CG10035	-0.600547221779134	1	1	pseudobulk	DESeq2	LRT		0
940	KC_AB2	CG10038	-1.78094334699383	0.470930349923561	1	pseudobulk	DESeq2	LRT		0
940	KC_AB2	CG10053	-0.772768427838894	0.67287259907463	1	pseudobulk	DESeq2	LRT		0
940	KC_AB2	CG10055	-0.046714363877816	0.914977784990712	1	pseudobulk	DESeq2	LRT		0
940	KC_AB2	CG10068	-1.93220872672389	0.398686710033904	1	pseudobulk	DESeq2	LRT		0
940	KC_AB2	CG10075	0.364166285909227	0.779637045418912	1	pseudobulk	DESeq2	LRT		0
940	KC_AB2	CG10077	-0.193379612233846	0.389631253498143	1	pseudobulk	DESeq2	LRT		0
940	KC_AB2	CG10082	0.104649276777865	0.803385213441416	1	pseudobulk	DESeq2	LRT		0
941	KC_AB2	CG10089	0.340270457836025	0.464896826206241	1	pseudobulk	DESeq2	LRT		0
941	KC_AB2	CG10098	-0.071313713332491	0.882555399066333	1	pseudobulk	DESeq2	LRT		0
941	KC_AB2	CG10103	-0.453627949817996	0.63397130164795	1	pseudobulk	DESeq2	LRT		0
941	KC_AB2	CG10132	0.365749351659915	0.439015495409808	1	pseudobulk	DESeq2	LRT		0
941	KC_AB2	CG10137	-0.312875775876714	0.642885817595841	1	pseudobulk	DESeq2	LRT		0
941	KC_AB2	CG10139	0.261883095029506	0.750349567710368	1	pseudobulk	DESeq2	LRT		0
941	KC_AB2	CG10147	0.953439861695601	0.666910728299079	1	pseudobulk	DESeq2	LRT		0
941	KC_AB2	CG10151	0.504868159666234	0.687839192211512	1	pseudobulk	DESeq2	LRT		0
941	KC_AB2	CG10165	-1.63925793886161	0.546290859679341	1	pseudobulk	DESeq2	LRT		0
941	KC_AB2	CG10166	2.76205395729416	0.251829971474322	1	pseudobulk	DESeq2	LRT		0
942	KC_AB2	CG10171	0.329740041077442	0.855811003303737	1	pseudobulk	DESeq2	LRT		0
942	KC_AB2	CG10188	0.175579073345082	0.796510053928138	1	pseudobulk	DESeq2	LRT		0
942	KC_AB2	CG10189	0.864341462967788	0.925947094335722	1	pseudobulk	DESeq2	LRT		0
942	KC_AB2	CG10194	-0.572620596212965	0.68527735518322	1	pseudobulk	DESeq2	LRT		0
942	KC_AB2	CG10209	0.284766838279805	0.811581706168461	1	pseudobulk	DESeq2	LRT		0
942	KC_AB2	CG10214	0.535286876966951	0.766915728963733	1	pseudobulk	DESeq2	LRT		0
942	KC_AB2	CG10222	-0.600547221779133	0.841940696994101	1	pseudobulk	DESeq2	LRT		0
942	KC_AB2	CG1024	-1.12181309299261	0.580924140803933	1	pseudobulk	DESeq2	LRT		0
942	KC_AB2	CG10252	2.03955752684245	0.330463272096619	1	pseudobulk	DESeq2	LRT		0
942	KC_AB2	CG10254	-0.273000392465228	0.770022467237668	1	pseudobulk	DESeq2	LRT		0
943	KC_AB2	CG10257	-2.16775308664436	0.227864624886289	1	pseudobulk	DESeq2	LRT		0
943	KC_AB2	CG10265	-0.868944949717054	0.637727733429232	1	pseudobulk	DESeq2	LRT		0
943	KC_AB2	CG10274	-2.31584321869877	0.294173754941528	1	pseudobulk	DESeq2	LRT		0

943	KC_AB2	CG10283	-1.27891660184938	0.431439081376688	1	pseudobulk	DESeq2	LRT		0
943	KC_AB2	CG10286	-0.94307066344797	0.629943854378022	1	pseudobulk	DESeq2	LRT		0
943	KC_AB2	CG10321	-0.465507348622065	0.684014955200003	1	pseudobulk	DESeq2	LRT		0
943	KC_AB2	CG10324	1.37785179899409	0.533959925164643	1	pseudobulk	DESeq2	LRT		0
943	KC_AB2	CG10333	0.68356454334838	0.550768831110349	1	pseudobulk	DESeq2	LRT		0
943	KC_AB2	CG10338	0.70378358684898	0.932301451973468	1	pseudobulk	DESeq2	LRT		0
943	KC_AB2	CG10340	0.0095033580845650	0.917984069683634	1	pseudobulk	DESeq2	LRT		0
944	KC_AB2	CG10341	-2.36899097638582	0.27901845799825	1	pseudobulk	DESeq2	LRT		0
944	KC_AB2	CG10343	1.55246637584339	0.354652172354936	1	pseudobulk	DESeq2	LRT		0
944	KC_AB2	CG10347	0.70378358684898	0.932301451973468	1	pseudobulk	DESeq2	LRT		0
944	KC_AB2	CG10353	-0.377321114579447	0.734763823888909	1	pseudobulk	DESeq2	LRT		0
944	KC_AB2	CG10362	-1.51823417326523	0.327066103605636	1	pseudobulk	DESeq2	LRT		0
944	KC_AB2	CG10365	0.409311045158768	0.784287302555254	1	pseudobulk	DESeq2	LRT		0
944	KC_AB2	CG10366	0.758978991638335	0.632424526822082	1	pseudobulk	DESeq2	LRT		0
944	KC_AB2	CG10375	-0.332097361739802	0.679027882273829	1	pseudobulk	DESeq2	LRT		0
944	KC_AB2	CG10376	-0.427431139129811	1	1	pseudobulk	DESeq2	LRT		0
944	KC_AB2	CG10383	-0.493357239699781	0.783807521297462	1	pseudobulk	DESeq2	LRT		0
945	KC_AB2	CG10384	-0.427431139129811	1	1	pseudobulk	DESeq2	LRT		0
945	KC_AB2	CG10395	-0.173089822794233	0.843016531098925	1	pseudobulk	DESeq2	LRT		0
945	KC_AB2	CG10413	0.649399836698127	0.608510306406511	1	pseudobulk	DESeq2	LRT		0
945	KC_AB2	CG10417	0.372774103772641	0.555786018115191	1	pseudobulk	DESeq2	LRT		0
945	KC_AB2	CG10418	-0.426012504797552	0.767550494040207	1	pseudobulk	DESeq2	LRT		0
945	KC_AB2	CG10425	-0.174965807374141	0.91631653210935	1	pseudobulk	DESeq2	LRT		0
945	KC_AB2	CG10428	1.5243995994652	0.741477491078038	1	pseudobulk	DESeq2	LRT		0
945	KC_AB2	CG10431	1.5243995994652	0.741477491078038	1	pseudobulk	DESeq2	LRT		0
945	KC_AB2	CG10433	-0.427431139129811	1	1	pseudobulk	DESeq2	LRT		0
945	KC_AB2	CG10435	-0.621805355124546	0.74877258313458	1	pseudobulk	DESeq2	LRT		0
946	KC_AB2	CG10444	1.57510921701807	0.391975105979649	1	pseudobulk	DESeq2	LRT		0
946	KC_AB2	CG10463	1.46667553000072	0.486649107648129	1	pseudobulk	DESeq2	LRT		0
946	KC_AB2	CG10465	-0.0289788164595053	0.838633003404507	1	pseudobulk	DESeq2	LRT		0
946	KC_AB2	CG10466	-1.45439488874766	0.456946233886225	1	pseudobulk	DESeq2	LRT		0
946	KC_AB2	CG10467	-0.600547221779133	0.841940696994101	1	pseudobulk	DESeq2	LRT		0
946	KC_AB2	CG10470	-0.092518516583034	0.92835759696914	1	pseudobulk	DESeq2	LRT		0
946	KC_AB2	CG10479	1.53811683574911	0.54844951579854	1	pseudobulk	DESeq2	LRT		0
946	KC_AB2	CG10483	-0.500716931505733	0.623609620850261	1	pseudobulk	DESeq2	LRT		0
946	KC_AB2	CG10492	-1.61845427191002	0.397357186117697	1	pseudobulk	DESeq2	LRT		0
946	KC_AB2	CG10494	-2.62548793044389	0.206246834544296	1	pseudobulk	DESeq2	LRT		0
947	KC_AB2	CG10495	-0.427431139129811	1	1	pseudobulk	DESeq2	LRT		0
947	KC_AB2	CG10508	0.22761218588032	0.59126210030588	1	pseudobulk	DESeq2	LRT		0
947	KC_AB2	CG10512	-1.51928970377954	0.486908946651791	1	pseudobulk	DESeq2	LRT		0
947	KC_AB2	CG10516	-0.493357239699781	0.783807521297462	1	pseudobulk	DESeq2	LRT		0
947	KC_AB2	CG10543	-0.35004481543984	0.410183559746464	1	pseudobulk	DESeq2	LRT		0
947	KC_AB2	CG10555	0.886114694120956	0.447014373291109	1	pseudobulk	DESeq2	LRT		0
947	KC_AB2	CG10561	-0.34320096042909	0.812623012266645	1	pseudobulk	DESeq2	LRT		0
947	KC_AB2	CG10565	-0.132869816240665	0.913896465864267	1	pseudobulk	DESeq2	LRT		0
947	KC_AB2	CG10576	0.480664848071903	0.780499999966716	1	pseudobulk	DESeq2	LRT		0
947	KC_AB2	CG10581	-0.600547221779134	1	1	pseudobulk	DESeq2	LRT		0
948	KC_AB2	CG10600	0.115182296292129	0.78266781248171	1	pseudobulk	DESeq2	LRT		0
948	KC_AB2	CG10602	-1.54470851095603	0.55301747381519	1	pseudobulk	DESeq2	LRT		0
948	KC_AB2	CG10616	-0.0662839178509967	0.911172387002726	1	pseudobulk	DESeq2	LRT		0
948	KC_AB2	CG10628	0.183555857030446	1	1	pseudobulk	DESeq2	LRT		0
948	KC_AB2	CG10631	0.429381082106657	0.743385385662229	1	pseudobulk	DESeq2	LRT		0
948	KC_AB2	CG10638	0.302708283566489	0.766610381895137	1	pseudobulk	DESeq2	LRT		0

948	KC_AB2	CG10646	-2.30493359034205	0.318713340463956	1	pseudobulk	DESeq2	LRT		0
948	KC_AB2	CG10654	1.73369784822028	0.346131467007105	1	pseudobulk	DESeq2	LRT		0
948	KC_AB2	CG10669	-0.427431139129811	1	1	pseudobulk	DESeq2	LRT		0
948	KC_AB2	CG10672	-2.17489218640147	0.354960305801642	1	pseudobulk	DESeq2	LRT		0
949	KC_AB2	CG10674	0.20388576127656	0.806979618523452	1	pseudobulk	DESeq2	LRT		0
949	KC_AB2	CG10681	-0.167679005593927	0.896089390589033	1	pseudobulk	DESeq2	LRT		0
949	KC_AB2	CG10713	0.520806735676611	0.706772908600337	1	pseudobulk	DESeq2	LRT		0
949	KC_AB2	CG10731	0.323327068789264	0.801664003269	1	pseudobulk	DESeq2	LRT		0
949	KC_AB2	CG10737	2.07637094691477	0.0223708609574814	1	pseudobulk	DESeq2	LRT		0
949	KC_AB2	CG10738	-0.598044388314997	0.845391648036006	1	pseudobulk	DESeq2	LRT		0
949	KC_AB2	CG1074	0.976909465230314	0.598676537442	1	pseudobulk	DESeq2	LRT		0
949	KC_AB2	CG10747	-2.63181173635671	0.221727059081464	1	pseudobulk	DESeq2	LRT		0
949	KC_AB2	CG10778	2.178309445114	0.246730071368082	1	pseudobulk	DESeq2	LRT		0
949	KC_AB2	CG10793	-0.427431139129811	1	1	pseudobulk	DESeq2	LRT		0
950	KC_AB2	CG10795	-2.01096988624633	0.270277822042983	1	pseudobulk	DESeq2	LRT		0
950	KC_AB2	CG10802	-0.465605661365784	0.791706020110304	1	pseudobulk	DESeq2	LRT		0
950	KC_AB2	CG10803	-0.164077937153952	0.81967351707669	1	pseudobulk	DESeq2	LRT		0
950	KC_AB2	CG10804	-0.061931861570003	0.866614666740729	1	pseudobulk	DESeq2	LRT		0
950	KC_AB2	CG10809	0.151082843566906	0.839226178739045	1	pseudobulk	DESeq2	LRT		0
950	KC_AB2	CG10863	-1.51812168653838	0.552401469179882	1	pseudobulk	DESeq2	LRT		0
950	KC_AB2	CG10866	-0.172799150721876	0.902830203088964	1	pseudobulk	DESeq2	LRT		0
950	KC_AB2	CG10874	-0.461811710275548	0.731947869632253	1	pseudobulk	DESeq2	LRT		0
950	KC_AB2	CG10889	0.892659559600706	0.712043317464255	1	pseudobulk	DESeq2	LRT		0
950	KC_AB2	CG10898	-1.00810919569491	0.56505667967343	1	pseudobulk	DESeq2	LRT		0
951	KC_AB2	CG1090	0.262281333857203	0.605600832894129	1	pseudobulk	DESeq2	LRT		0
951	KC_AB2	CG10903	-0.523092312393629	0.773910803298929	1	pseudobulk	DESeq2	LRT		0
951	KC_AB2	CG10904	0.83721407599818	0.620952546821801	1	pseudobulk	DESeq2	LRT		0
951	KC_AB2	CG10907	-2.53693580180331	0.244367126599884	1	pseudobulk	DESeq2	LRT		0
951	KC_AB2	CG10914	-1.12785316439435	0.661319045116135	1	pseudobulk	DESeq2	LRT		0
951	KC_AB2	CG10916	-1.02988290140464	0.610579185122146	1	pseudobulk	DESeq2	LRT		0
951	KC_AB2	CG1092	-0.600547221779133	0.841940696994101	1	pseudobulk	DESeq2	LRT		0
951	KC_AB2	CG10927	-0.427431139129811	1	1	pseudobulk	DESeq2	LRT		0
951	KC_AB2	CG10932	-2.31658951096177	0.321432784490917	1	pseudobulk	DESeq2	LRT		0
951	KC_AB2	CG10947	-0.493430223231315	0.321224946305955	1	pseudobulk	DESeq2	LRT		0
952	KC_AB2	CG10948	-0.260688059230057	0.771985424394843	1	pseudobulk	DESeq2	LRT		0
952	KC_AB2	CG10949	1.26307719581879	0.569064211306759	1	pseudobulk	DESeq2	LRT		0
952	KC_AB2	CG10958	-1.59432579116312	0.459120231439715	1	pseudobulk	DESeq2	LRT		0
952	KC_AB2	CG10959	0.713982351041985	0.921569637923553	1	pseudobulk	DESeq2	LRT		0
952	KC_AB2	CG10960	-1.17758805213335	0.11450737178473	1	pseudobulk	DESeq2	LRT		0
952	KC_AB2	CG10973	1.69613111137042	0.377852418948745	1	pseudobulk	DESeq2	LRT		0
952	KC_AB2	CG10979	-0.760748794683506	0.606495958413642	1	pseudobulk	DESeq2	LRT		0
952	KC_AB2	CG10984	0.103355255408507	0.901770772892483	1	pseudobulk	DESeq2	LRT		0
952	KC_AB2	CG11000	-0.166457703929998	0.760919099408663	1	pseudobulk	DESeq2	LRT		0
952	KC_AB2	CG11007	0.195713384286393	0.886583498634163	1	pseudobulk	DESeq2	LRT		0
953	KC_AB2	CG1103	-1.97714314412646	0.266803939749778	1	pseudobulk	DESeq2	LRT		0
953	KC_AB2	CG11030	-0.267316637615104	0.652549176058899	1	pseudobulk	DESeq2	LRT		0
953	KC_AB2	CG11035	-0.923473724045651	0.600525917567165	1	pseudobulk	DESeq2	LRT		0
953	KC_AB2	CG1105	0.137461227792054	0.884678758594551	1	pseudobulk	DESeq2	LRT		0
953	KC_AB2	CG11050	0.477093780735071	0.371070718313694	1	pseudobulk	DESeq2	LRT		0
953	KC_AB2	CG11070	0.397247314929831	0.714324297426645	1	pseudobulk	DESeq2	LRT		0
953	KC_AB2	CG11076	-1.16307534840584	0.25538548083437	1	pseudobulk	DESeq2	LRT		0
953	KC_AB2	CG11077	-0.452090275040053	0.543376679494513	1	pseudobulk	DESeq2	LRT		0
953	KC_AB2	CG11089	-1.72976330140494	0.41918502381035	1	pseudobulk	DESeq2	LRT		0

953	KC_AB2	CG11095	0.443892390635762	1	1	pseudobulk	DESeq2	LRT		0
954	KC_AB2	CG11099	2.15951262603702	0.0228016930658599	1	pseudobulk	DESeq2	LRT		0
954	KC_AB2	CG11103	0.260985262118427	0.878783347119633	1	pseudobulk	DESeq2	LRT		0
954	KC_AB2	CG11109	1.94559924579109	0.42325307792885	1	pseudobulk	DESeq2	LRT		0
954	KC_AB2	CG11110	0.630057297425705	0.692457702154453	1	pseudobulk	DESeq2	LRT		0
954	KC_AB2	CG11122	0.0244266974870157	0.952738272587392	1	pseudobulk	DESeq2	LRT		0
954	KC_AB2	CG11123	-0.482173055655404	0.804949101714764	1	pseudobulk	DESeq2	LRT		0
954	KC_AB2	CG11127	-1.43450874381432	0.6168250034656	1	pseudobulk	DESeq2	LRT		0
954	KC_AB2	CG1113	-1.63925793886161	0.546290859679341	1	pseudobulk	DESeq2	LRT		0
954	KC_AB2	CG11134	-1.3723417613553	0.619532036522	1	pseudobulk	DESeq2	LRT		0
954	KC_AB2	CG11137	-0.21082984444053	0.71619813502437	1	pseudobulk	DESeq2	LRT		0
955	KC_AB2	CG11141	0.164054477342436	0.842806774525055	1	pseudobulk	DESeq2	LRT		0
955	KC_AB2	CG11151	-0.0947832649593568	0.777961465053921	1	pseudobulk	DESeq2	LRT		0
955	KC_AB2	CG11155	0.2552159476511	0.433972670226809	1	pseudobulk	DESeq2	LRT		0
955	KC_AB2	CG11159	0.183555857030446	1	1	pseudobulk	DESeq2	LRT		0
955	KC_AB2	CG1116	-0.382929749762112	0.851961810578875	1	pseudobulk	DESeq2	LRT		0
955	KC_AB2	CG11170	-0.427431139129811	1	1	pseudobulk	DESeq2	LRT		0
955	KC_AB2	CG11178	1.14814388261213	0.568837005180859	1	pseudobulk	DESeq2	LRT		0
955	KC_AB2	CG11180	-0.600547221779134	1	1	pseudobulk	DESeq2	LRT		0
955	KC_AB2	CG11191	-0.112938880191609	0.688757899340226	1	pseudobulk	DESeq2	LRT		0
955	KC_AB2	CG11200	-0.427431139129811	1	1	pseudobulk	DESeq2	LRT		0
956	KC_AB2	CG11241	-0.600547221779133	0.841940696994101	1	pseudobulk	DESeq2	LRT		0
956	KC_AB2	CG11247	-1.43718915913708	0.173422672607253	1	pseudobulk	DESeq2	LRT		0
956	KC_AB2	CG11248	0.48048026792585	0.787708333227872	1	pseudobulk	DESeq2	LRT		0
956	KC_AB2	CG11257	0.943586647867311	0.690632779168748	1	pseudobulk	DESeq2	LRT		0
956	KC_AB2	CG11267	0.064993965959585	0.84826152712632	1	pseudobulk	DESeq2	LRT		0
956	KC_AB2	CG11279	-1.37866851291071	0.486618970380267	1	pseudobulk	DESeq2	LRT		0
956	KC_AB2	CG1129	-0.24148666990399	0.791587085693882	1	pseudobulk	DESeq2	LRT		0
956	KC_AB2	CG11307	-0.841882716738579	1	1	pseudobulk	DESeq2	LRT		0
956	KC_AB2	CG11317	0.621558761276874	0.437356366520944	1	pseudobulk	DESeq2	LRT		0
956	KC_AB2	CG11319	0.00356270461588418	0.984906101803487	1	pseudobulk	DESeq2	LRT		0
957	KC_AB2	CG11334	-1.80180327134037	0.333651825469671	1	pseudobulk	DESeq2	LRT		0
957	KC_AB2	CG11357	1.24563111035253	0.0856490396230441	1	pseudobulk	DESeq2	LRT		0
957	KC_AB2	CG11360	-0.255306151142871	0.66328371387978	1	pseudobulk	DESeq2	LRT		0
957	KC_AB2	CG11367	0.818049755224763	0.631984103515073	1	pseudobulk	DESeq2	LRT		0
957	KC_AB2	CG11374	0.674459942220112	0.74558757773504	1	pseudobulk	DESeq2	LRT		0
957	KC_AB2	CG11377	0.568724199816521	0.713152060086387	1	pseudobulk	DESeq2	LRT		0
957	KC_AB2	CG11396	-1.60676268448416	0.413552313870337	1	pseudobulk	DESeq2	LRT		0
957	KC_AB2	CG11398	-0.600547221779134	1	1	pseudobulk	DESeq2	LRT		0
957	KC_AB2	CG11399	-1.17521051578874	0.426686828179601	1	pseudobulk	DESeq2	LRT		0
957	KC_AB2	CG11406	1.00634871790136	0.392306544374783	1	pseudobulk	DESeq2	LRT		0
958	KC_AB2	CG11414	-3.08149556295888	0.139893777771122	1	pseudobulk	DESeq2	LRT		0
958	KC_AB2	CG11417	0.222396124134733	1	1	pseudobulk	DESeq2	LRT		0
958	KC_AB2	CG1142	0.738997480046779	0.59701044701952	1	pseudobulk	DESeq2	LRT		0
958	KC_AB2	CG11425	1.5243995994652	0.741477491078038	1	pseudobulk	DESeq2	LRT		0
958	KC_AB2	CG11426	-0.600547221779133	0.841940696994101	1	pseudobulk	DESeq2	LRT		0
958	KC_AB2	CG1143	2.1817927492653	0.458599861292202	1	pseudobulk	DESeq2	LRT		0
958	KC_AB2	CG11436	-0.0852743682753834	0.917718671153184	1	pseudobulk	DESeq2	LRT		0
958	KC_AB2	CG11444	0.0216292275692997	0.821551682189263	1	pseudobulk	DESeq2	LRT		0
958	KC_AB2	CG11447	-1.11043983282862	0.592034775657771	1	pseudobulk	DESeq2	LRT		0
958	KC_AB2	CG11454	0.160340217751021	0.858410773711483	1	pseudobulk	DESeq2	LRT		0
959	KC_AB2	CG11456	-0.527175403416086	0.716104466890697	1	pseudobulk	DESeq2	LRT		0
959	KC_AB2	CG1146	2.48629812612306	0.259054692310381	1	pseudobulk	DESeq2	LRT		0

959	KC_AB2	CG11474	1.86554897087304	0.283513176743011	1	pseudobulk	DESeq2	LRT	0
959	KC_AB2	CG11486	-0.128345944429599	0.618104151477555	1	pseudobulk	DESeq2	LRT	0
959	KC_AB2	CG11504	1.14814388261213	0.568837005180859	1	pseudobulk	DESeq2	LRT	0
959	KC_AB2	CG11523	0.341801163214575	0.70118359760514	1	pseudobulk	DESeq2	LRT	0
959	KC_AB2	CG11537	-0.322529725964483	0.458720422498495	1	pseudobulk	DESeq2	LRT	0
959	KC_AB2	CG11555	0.488527246554852	0.735314515612831	1	pseudobulk	DESeq2	LRT	0
959	KC_AB2	CG11560	-1.70380348762697	0.473446675363872	1	pseudobulk	DESeq2	LRT	0
959	KC_AB2	CG11562	1.21340176778324	0.579318232741745	1	pseudobulk	DESeq2	LRT	0
960	KC_AB2	CG11563	1.55612481604137	0.486620099396909	1	pseudobulk	DESeq2	LRT	0
960	KC_AB2	CG11583	-0.401210989251639	0.822383044849055	1	pseudobulk	DESeq2	LRT	0
960	KC_AB2	CG11586	0.0700803771965153	0.934903890620354	1	pseudobulk	DESeq2	LRT	0
960	KC_AB2	CG11590	-1.7824418856176	0.467224294431682	1	pseudobulk	DESeq2	LRT	0
960	KC_AB2	CG11593	1.12256220424622	0.478579626845228	1	pseudobulk	DESeq2	LRT	0
960	KC_AB2	CG11596	1.50255200571414	0.44025635980316	1	pseudobulk	DESeq2	LRT	0
960	KC_AB2	CG11597	1.69707806671825	0.422682165079481	1	pseudobulk	DESeq2	LRT	0
960	KC_AB2	CG1161	0.056371043949147	0.88019552306694	1	pseudobulk	DESeq2	LRT	0
960	KC_AB2	CG11626	1.5243995994652	0.741477491078038	1	pseudobulk	DESeq2	LRT	0
960	KC_AB2	CG11638	-1.35233349590671	0.521987811674718	1	pseudobulk	DESeq2	LRT	0
961	KC_AB2	CG11658	0.880163064200857	0.178705211446166	1	pseudobulk	DESeq2	LRT	0
961	KC_AB2	CG11695	2.1542884907318	0.280930633341219	1	pseudobulk	DESeq2	LRT	0
961	KC_AB2	CG11696	-0.427431139129811	1	1	pseudobulk	DESeq2	LRT	0
961	KC_AB2	CG11699	-0.535763657504906	0.497063125813031	1	pseudobulk	DESeq2	LRT	0
961	KC_AB2	CG11700	-0.600547221779133	0.841940696994101	1	pseudobulk	DESeq2	LRT	0
961	KC_AB2	CG11710	-1.68564117043918	0.405908616473656	1	pseudobulk	DESeq2	LRT	0
961	KC_AB2	CG1172	-0.246940748764947	0.815540980572445	1	pseudobulk	DESeq2	LRT	0
961	KC_AB2	CG11722	-1.66363594822232	0.289006588553597	1	pseudobulk	DESeq2	LRT	0
961	KC_AB2	CG11723	-1.66828165461268	0.531269883399465	1	pseudobulk	DESeq2	LRT	0
961	KC_AB2	CG11737	-0.427431139129811	1	1	pseudobulk	DESeq2	LRT	0
962	KC_AB2	CG11752	0.118517633926651	0.796020286450113	1	pseudobulk	DESeq2	LRT	0
962	KC_AB2	CG11753	0.0726728274229612	0.934965892048865	1	pseudobulk	DESeq2	LRT	0
962	KC_AB2	CG11755	0.694278586043927	0.756746500572413	1	pseudobulk	DESeq2	LRT	0
962	KC_AB2	CG11768	-0.760293344023607	0.0721571240639542	1	pseudobulk	DESeq2	LRT	0
962	KC_AB2	CG11771	0.443830477529118	0.761875479059546	1	pseudobulk	DESeq2	LRT	0
962	KC_AB2	CG11777	0.864341462967788	0.925947094335722	1	pseudobulk	DESeq2	LRT	0
962	KC_AB2	CG11779	-1.8717316292751	0.19597038233834	1	pseudobulk	DESeq2	LRT	0
962	KC_AB2	CG11781	-0.272602709907525	0.785900231310163	1	pseudobulk	DESeq2	LRT	0
962	KC_AB2	CG11790	1.21092382796727	0.637125262974216	1	pseudobulk	DESeq2	LRT	0
962	KC_AB2	CG11791	2.12684015898615	0.445939223035924	1	pseudobulk	DESeq2	LRT	0
963	KC_AB2	CG11807	-0.316694328129489	0.729345546645342	1	pseudobulk	DESeq2	LRT	0
963	KC_AB2	CG11808	-0.841882716738579	1	1	pseudobulk	DESeq2	LRT	0
963	KC_AB2	CG11811	-1.02738481196322	0.389879673899853	1	pseudobulk	DESeq2	LRT	0
963	KC_AB2	CG11835	-0.578903707615405	0.307497668235339	1	pseudobulk	DESeq2	LRT	0
963	KC_AB2	CG11839	1.94691684546882	0.398076844044942	1	pseudobulk	DESeq2	LRT	0
963	KC_AB2	CG11852	-0.427431139129811	1	1	pseudobulk	DESeq2	LRT	0
963	KC_AB2	CG11857	-0.0666878962061944	0.937130563789835	1	pseudobulk	DESeq2	LRT	0
963	KC_AB2	CG11858	1.50325750081456	0.280654141389462	1	pseudobulk	DESeq2	LRT	0
963	KC_AB2	CG11873	0.0635377639483385	0.836084857956922	1	pseudobulk	DESeq2	LRT	0
963	KC_AB2	CG11882	-1.59432579116312	0.459120231439715	1	pseudobulk	DESeq2	LRT	0
964	KC_AB2	CG11883	0.207030982521161	0.723947519912807	1	pseudobulk	DESeq2	LRT	0
964	KC_AB2	CG11885	0.443892390635762	1	1	pseudobulk	DESeq2	LRT	0
964	KC_AB2	CG11898	-1.3723417613553	0.619532036522	1	pseudobulk	DESeq2	LRT	0
964	KC_AB2	CG11902	-0.825973974745624	0.678387419789199	1	pseudobulk	DESeq2	LRT	0
964	KC_AB2	CG11906	-1.3723417613553	0.619532036522	1	pseudobulk	DESeq2	LRT	0

964	KC_AB2	CG11927	-0.740207704288144	0.726967462633097	1	pseudobulk	DESeq2	LRT	0
964	KC_AB2	CG11961	-0.055716669860236	0.908649369064948	1	pseudobulk	DESeq2	LRT	0
964	KC_AB2	CG11964	-1.61670796634688	0.303363802197906	1	pseudobulk	DESeq2	LRT	0
964	KC_AB2	CG11970	0.222396124134733	1	1	pseudobulk	DESeq2	LRT	0
964	KC_AB2	CG11975	1.54053875912195	0.39238818226048	1	pseudobulk	DESeq2	LRT	0
965	KC_AB2	CG11980	-1.49601144928094	0.349406870569553	1	pseudobulk	DESeq2	LRT	0
965	KC_AB2	CG11983	-0.600547221779134	1	1	pseudobulk	DESeq2	LRT	0
965	KC_AB2	CG11999	0.0229837019841527	0.911199773266245	1	pseudobulk	DESeq2	LRT	0
965	KC_AB2	CG12004	0.505133968646424	0.297885281170556	1	pseudobulk	DESeq2	LRT	0
965	KC_AB2	CG12007	-0.598123821701242	0.71217014349458	1	pseudobulk	DESeq2	LRT	0
965	KC_AB2	CG12010	-0.427431139129811	1	1	pseudobulk	DESeq2	LRT	0
965	KC_AB2	CG12012	-0.664230824189655	0.577077515130721	1	pseudobulk	DESeq2	LRT	0
965	KC_AB2	CG12024	-0.612742894961157	0.593807627650563	1	pseudobulk	DESeq2	LRT	0
965	KC_AB2	CG12025	-0.181388239592739	0.787873683847163	1	pseudobulk	DESeq2	LRT	0
965	KC_AB2	CG12038	-0.259506039972831	0.691802017197198	1	pseudobulk	DESeq2	LRT	0
966	KC_AB2	CG12054	0.153818083047942	0.680845183026071	1	pseudobulk	DESeq2	LRT	0
966	KC_AB2	CG12056	-0.085274368275383	0.917718671153184	1	pseudobulk	DESeq2	LRT	0
966	KC_AB2	CG12071	-0.158294439684869	0.739362600738425	1	pseudobulk	DESeq2	LRT	0
966	KC_AB2	CG12081	0.255982601553642	0.833834283885821	1	pseudobulk	DESeq2	LRT	0
966	KC_AB2	CG12084	0.713662468638379	0.489859599520009	1	pseudobulk	DESeq2	LRT	0
966	KC_AB2	CG12091	-0.407876141283376	0.493961800366085	1	pseudobulk	DESeq2	LRT	0
966	KC_AB2	CG12093	-1.85852543389552	0.304856081690745	1	pseudobulk	DESeq2	LRT	0
966	KC_AB2	CG12096	-0.112094292991318	0.858629129827245	1	pseudobulk	DESeq2	LRT	0
966	KC_AB2	CG12099	0.51397252654045	0.487606376427192	1	pseudobulk	DESeq2	LRT	0
966	KC_AB2	CG12104	1.70271200804115	0.279469868828566	1	pseudobulk	DESeq2	LRT	0
967	KC_AB2	CG12106	0.818097236913285	0.642607168556225	1	pseudobulk	DESeq2	LRT	0
967	KC_AB2	CG12107	0.0025734666473445	0.986081958383881	1	pseudobulk	DESeq2	LRT	0
967	KC_AB2	CG12118	0.83721407599818	0.620952546821801	1	pseudobulk	DESeq2	LRT	0
967	KC_AB2	CG12121	0.340266581012575	0.65355807831577	1	pseudobulk	DESeq2	LRT	0
967	KC_AB2	CG12123	-0.540435842582463	0.684384239193462	1	pseudobulk	DESeq2	LRT	0
967	KC_AB2	CG12128	-1.51812168653838	0.552401469179882	1	pseudobulk	DESeq2	LRT	0
967	KC_AB2	CG12129	-0.018189855879368	0.865014560126369	1	pseudobulk	DESeq2	LRT	0
967	KC_AB2	CG12155	-0.598478207158216	0.716299384490056	1	pseudobulk	DESeq2	LRT	0
967	KC_AB2	CG12158	0.713982351041985	0.921569637923553	1	pseudobulk	DESeq2	LRT	0
967	KC_AB2	CG12159	0.457159101106935	0.480461165587382	1	pseudobulk	DESeq2	LRT	0
968	KC_AB2	CG12163	-0.013043920792984	0.914160278921935	1	pseudobulk	DESeq2	LRT	0
968	KC_AB2	CG12170	-0.187579055727378	0.895795928577846	1	pseudobulk	DESeq2	LRT	0
968	KC_AB2	CG12171	0.189813678325555	1	1	pseudobulk	DESeq2	LRT	0
968	KC_AB2	CG12173	0.37274599773354	0.732547790864351	1	pseudobulk	DESeq2	LRT	0
968	KC_AB2	CG12177	-0.600547221779133	0.841940696994101	1	pseudobulk	DESeq2	LRT	0
968	KC_AB2	CG12179	-0.600547221779134	1	1	pseudobulk	DESeq2	LRT	0
968	KC_AB2	CG1218	-1.99153573919778	0.406760593108112	1	pseudobulk	DESeq2	LRT	0
968	KC_AB2	CG12182	0.864341462967788	0.925947094335722	1	pseudobulk	DESeq2	LRT	0
968	KC_AB2	CG12194	0.346589616049749	0.84654468695694	1	pseudobulk	DESeq2	LRT	0
968	KC_AB2	CG12204	-1.93220872672389	0.398686710033904	1	pseudobulk	DESeq2	LRT	0
969	KC_AB2	CG12213	-0.777448691763072	0.538524952984983	1	pseudobulk	DESeq2	LRT	0
969	KC_AB2	CG12219	-0.617874106857047	0.716176596915688	1	pseudobulk	DESeq2	LRT	0
969	KC_AB2	CG12236	-0.746024530408185	0.591131743003275	1	pseudobulk	DESeq2	LRT	0
969	KC_AB2	CG12237	-0.442391438215517	0.606100253746484	1	pseudobulk	DESeq2	LRT	0
969	KC_AB2	CG12239	-0.427431139129811	1	1	pseudobulk	DESeq2	LRT	0
969	KC_AB2	CG12241	-0.938615631390307	0.527527434761187	1	pseudobulk	DESeq2	LRT	0
969	KC_AB2	CG12253	-2.77125686148606	0.191776729603111	1	pseudobulk	DESeq2	LRT	0
969	KC_AB2	CG12259	1.53811683574911	0.54844951579854	1	pseudobulk	DESeq2	LRT	0

969	KC_AB2	CG12279	-1.7463816821023	0.261305681365524	1	pseudobulk	DESeq2	LRT		0
969	KC_AB2	CG12288	-0.600547221779134	1	1	pseudobulk	DESeq2	LRT		0
970	KC_AB2	CG12299	-0.820074316723004	0.49548854516793	1	pseudobulk	DESeq2	LRT		0
970	KC_AB2	CG12301	-0.841882716738579	1	1	pseudobulk	DESeq2	LRT		0
970	KC_AB2	CG1231	1.31428215495784	0.441196858753469	1	pseudobulk	DESeq2	LRT		0
970	KC_AB2	CG12316	-2.09102007051366	0.283409601898511	1	pseudobulk	DESeq2	LRT		0
970	KC_AB2	CG12321	-0.539576916214097	0.747924312632912	1	pseudobulk	DESeq2	LRT		0
970	KC_AB2	CG12325	0.222396124134733	1	1	pseudobulk	DESeq2	LRT		0
970	KC_AB2	CG1233	0.864341462967788	0.925947094335722	1	pseudobulk	DESeq2	LRT		0
970	KC_AB2	CG12333	0.0078442458967902	0.955361917868957	1	pseudobulk	DESeq2	LRT		0
970	KC_AB2	CG12338	1.5243995994652	0.741477491078038	1	pseudobulk	DESeq2	LRT		0
970	KC_AB2	CG1234	0.183555857030446	1	1	pseudobulk	DESeq2	LRT		0
971	KC_AB2	CG12341	0.993196808685059	0.40994622194069	1	pseudobulk	DESeq2	LRT		0
971	KC_AB2	CG12343	1.28856754329153	0.24761613046713	1	pseudobulk	DESeq2	LRT		0
971	KC_AB2	CG12347	-0.600547221779134	1	1	pseudobulk	DESeq2	LRT		0
971	KC_AB2	CG12355	-1.50055174886969	0.452037089275982	1	pseudobulk	DESeq2	LRT		0
971	KC_AB2	CG1236	-0.427431139129811	1	1	pseudobulk	DESeq2	LRT		0
971	KC_AB2	CG12375	1.24332632358774	0.294168864068285	1	pseudobulk	DESeq2	LRT		0
971	KC_AB2	CG12379	-1.3723417613553	0.619532036522	1	pseudobulk	DESeq2	LRT		0
971	KC_AB2	CG12384	-0.116680119111535	0.814362274770048	1	pseudobulk	DESeq2	LRT		0
971	KC_AB2	CG1239	1.37785179899409	0.533959925164643	1	pseudobulk	DESeq2	LRT		0
971	KC_AB2	CG12391	-0.0426844898251506	0.888523197109286	1	pseudobulk	DESeq2	LRT		0
972	KC_AB2	CG12402	0.568522373668797	0.671921965921597	1	pseudobulk	DESeq2	LRT		0
972	KC_AB2	CG12420	-1.12475160732157	0.574429473125171	1	pseudobulk	DESeq2	LRT		0
972	KC_AB2	CG12470	0.864341462967788	0.925947094335722	1	pseudobulk	DESeq2	LRT		0
972	KC_AB2	CG12499	-2.02285942584217	0.357240028002837	1	pseudobulk	DESeq2	LRT		0
972	KC_AB2	CG12502	-2.10923289300043	0.365665725771537	1	pseudobulk	DESeq2	LRT		0
972	KC_AB2	CG12531	0.232623175559983	0.558057838218346	1	pseudobulk	DESeq2	LRT		0
972	KC_AB2	CG12535	-1.12785316439435	0.661319045116135	1	pseudobulk	DESeq2	LRT		0
972	KC_AB2	CG12541	1.22666409774829	0.506946508491293	1	pseudobulk	DESeq2	LRT		0
972	KC_AB2	CG12547	-0.115040903677135	0.878851373271212	1	pseudobulk	DESeq2	LRT		0
972	KC_AB2	CG12560	-0.519974981067827	0.679585413198816	1	pseudobulk	DESeq2	LRT		0
973	KC_AB2	CG12567	-0.569111678813456	0.645393086739726	1	pseudobulk	DESeq2	LRT		0
973	KC_AB2	CG12576	0.693605370515191	0.475074893253263	1	pseudobulk	DESeq2	LRT		0
973	KC_AB2	CG12605	-0.29689265048813	0.476441384243031	1	pseudobulk	DESeq2	LRT		0
973	KC_AB2	CG12608	-0.427431139129811	1	1	pseudobulk	DESeq2	LRT		0
973	KC_AB2	CG12640	-0.600547221779133	0.841940696994101	1	pseudobulk	DESeq2	LRT		0
973	KC_AB2	CG1265	-0.363326368877488	0.589540337060012	1	pseudobulk	DESeq2	LRT		0
973	KC_AB2	CG12659	-0.211959943697087	0.913646816097592	1	pseudobulk	DESeq2	LRT		0
973	KC_AB2	CG12672	0.190393219485123	0.893175556614257	1	pseudobulk	DESeq2	LRT		0
973	KC_AB2	CG1271	-0.290063816371847	0.881698537689548	1	pseudobulk	DESeq2	LRT		0
973	KC_AB2	CG12713	-1.3929484167291	0.512855059074155	1	pseudobulk	DESeq2	LRT		0
974	KC_AB2	CG12715	1.43191469273003	0.651609831456347	1	pseudobulk	DESeq2	LRT		0
974	KC_AB2	CG12717	0.184766766395136	0.923010422932084	1	pseudobulk	DESeq2	LRT		0
974	KC_AB2	CG12728	-1.5349257242429	0.440351429378741	1	pseudobulk	DESeq2	LRT		0
974	KC_AB2	CG12730	0.340082706435182	0.803835787783267	1	pseudobulk	DESeq2	LRT		0
974	KC_AB2	CG12746	1.09075308028859	0.406500092388886	1	pseudobulk	DESeq2	LRT		0
974	KC_AB2	CG1275	0.179794820336081	0.734512626552634	1	pseudobulk	DESeq2	LRT		0
974	KC_AB2	CG12768	-2.17489218640147	0.354960305801642	1	pseudobulk	DESeq2	LRT		0
974	KC_AB2	CG12769	-0.593106221071813	0.60193915099925	1	pseudobulk	DESeq2	LRT		0
974	KC_AB2	CG12773	-0.817575110288588	0.748631920773504	1	pseudobulk	DESeq2	LRT		0
974	KC_AB2	CG12795	-0.017128473349243	0.975090044325049	1	pseudobulk	DESeq2	LRT		0
975	KC_AB2	CG12817	0.49682719904822	0.618207815411341	1	pseudobulk	DESeq2	LRT		0

975	KC_AB2	CG12818	-2.28539133851304	0.280298638885039	1	pseudobulk	DESeq2	LRT	0
975	KC_AB2	CG12822	0.234860442844689	0.737403633093488	1	pseudobulk	DESeq2	LRT	0
975	KC_AB2	CG12848	0.993288056286251	0.457571243380878	1	pseudobulk	DESeq2	LRT	0
975	KC_AB2	CG12851	0.384349711901467	0.667038315168685	1	pseudobulk	DESeq2	LRT	0
975	KC_AB2	CG12860	1.5243995994652	0.741477491078038	1	pseudobulk	DESeq2	LRT	0
975	KC_AB2	CG12868	-0.559968532137786	0.845570507403904	1	pseudobulk	DESeq2	LRT	0
975	KC_AB2	CG12877	1.25230092358671	0.476991461296781	1	pseudobulk	DESeq2	LRT	0
975	KC_AB2	CG12883	0.443892390635762	1	1	pseudobulk	DESeq2	LRT	0
975	KC_AB2	CG12895	0.690299077759694	0.535496004342984	1	pseudobulk	DESeq2	LRT	0
976	KC_AB2	CG12896	-0.427431139129811	1	1	pseudobulk	DESeq2	LRT	0
976	KC_AB2	CG12909	-0.841882716738579	1	1	pseudobulk	DESeq2	LRT	0
976	KC_AB2	CG12910	-0.622031754654327	0.643439228403025	1	pseudobulk	DESeq2	LRT	0
976	KC_AB2	CG12913	-0.247023689372284	0.856036941832863	1	pseudobulk	DESeq2	LRT	0
976	KC_AB2	CG12926	1.5243995994652	0.741477491078038	1	pseudobulk	DESeq2	LRT	0
976	KC_AB2	CG12929	-0.828178378785339	0.673584255563891	1	pseudobulk	DESeq2	LRT	0
976	KC_AB2	CG12935	-0.112094292991318	0.858629129827245	1	pseudobulk	DESeq2	LRT	0
976	KC_AB2	CG12942	1.94691684546882	0.398076844044942	1	pseudobulk	DESeq2	LRT	0
976	KC_AB2	CG12945	0.10575379089628	0.902964145651533	1	pseudobulk	DESeq2	LRT	0
976	KC_AB2	CG12947	-3.49855286901308	0.394124953750094	1	pseudobulk	DESeq2	LRT	0
977	KC_AB2	CG12948	-2.65693476663437	0.201732236875042	1	pseudobulk	DESeq2	LRT	0
977	KC_AB2	CG12975	-0.771287099059976	0.639734106236813	1	pseudobulk	DESeq2	LRT	0
977	KC_AB2	CG12991	0.545340092880895	0.470434479487169	1	pseudobulk	DESeq2	LRT	0
977	KC_AB2	CG12992	-0.600547221779133	0.841940696994101	1	pseudobulk	DESeq2	LRT	0
977	KC_AB2	CG12994	-1.29244479833796	0.693295472370086	1	pseudobulk	DESeq2	LRT	0
977	KC_AB2	CG13001	-2.81345759926264	0.191558610371653	1	pseudobulk	DESeq2	LRT	0
977	KC_AB2	CG13005	0.183555857030446	1	1	pseudobulk	DESeq2	LRT	0
977	KC_AB2	CG13014	-1.7824418856176	0.467224294431682	1	pseudobulk	DESeq2	LRT	0
977	KC_AB2	CG13016	-1.51812168653838	0.552401469179882	1	pseudobulk	DESeq2	LRT	0
977	KC_AB2	CG13018	0.550897539145824	0.747701414668312	1	pseudobulk	DESeq2	LRT	0
978	KC_AB2	CG13024	-0.427431139129811	1	1	pseudobulk	DESeq2	LRT	0
978	KC_AB2	CG13025	0.920707161609556	0.66023874320038	1	pseudobulk	DESeq2	LRT	0
978	KC_AB2	CG13054	-3.1890852737721	0.121402890065498	1	pseudobulk	DESeq2	LRT	0
978	KC_AB2	CG13055	-1.06957108822585	0.0509498525952363	1	pseudobulk	DESeq2	LRT	0
978	KC_AB2	CG1307	1.14548642295141	0.315254862049543	1	pseudobulk	DESeq2	LRT	0
978	KC_AB2	CG1309	1.09901490132735	0.502734648474513	1	pseudobulk	DESeq2	LRT	0
978	KC_AB2	CG13096	-1.29244479833796	0.693295472370086	1	pseudobulk	DESeq2	LRT	0
978	KC_AB2	CG13097	-0.539665365830432	0.805145917072468	1	pseudobulk	DESeq2	LRT	0
978	KC_AB2	CG13108	-0.0608305834638444	0.933547274069067	1	pseudobulk	DESeq2	LRT	0
978	KC_AB2	CG13123	0.195204845255013	0.85782625149847	1	pseudobulk	DESeq2	LRT	0
979	KC_AB2	CG13124	1.5243995994652	0.741477491078038	1	pseudobulk	DESeq2	LRT	0
979	KC_AB2	CG13126	1.41392113638259	0.617674323943697	1	pseudobulk	DESeq2	LRT	0
979	KC_AB2	CG13133	1.5243995994652	0.741477491078038	1	pseudobulk	DESeq2	LRT	0
979	KC_AB2	CG13148	-0.642855674003565	0.691860437530049	1	pseudobulk	DESeq2	LRT	0
979	KC_AB2	CG1316	-0.0040283122086145	0.849580016740561	1	pseudobulk	DESeq2	LRT	0
979	KC_AB2	CG1317	0.399623641756951	0.680449107689638	1	pseudobulk	DESeq2	LRT	0
979	KC_AB2	CG13185	-1.29244479833796	0.693295472370086	1	pseudobulk	DESeq2	LRT	0
979	KC_AB2	CG13192	-0.772768427838894	0.67287259907463	1	pseudobulk	DESeq2	LRT	0
979	KC_AB2	CG13197	-2.37404667811699	0.160875025576593	1	pseudobulk	DESeq2	LRT	0
979	KC_AB2	CG13204	0.922175542365384	0.290654959672066	1	pseudobulk	DESeq2	LRT	0
980	KC_AB2	CG13220	0.0704907710346216	0.929397629949365	1	pseudobulk	DESeq2	LRT	0
980	KC_AB2	CG13229	1.5243995994652	0.741477491078038	1	pseudobulk	DESeq2	LRT	0
980	KC_AB2	CG13248	0.912309666827502	0.142845701342042	1	pseudobulk	DESeq2	LRT	0
980	KC_AB2	CG13255	-0.782183163725771	0.672768983012932	1	pseudobulk	DESeq2	LRT	0

980	KC_AB2	CG13284	-0.600547221779134	1	1	pseudobulk	DESeq2	LRT		0
980	KC_AB2	CG13293	0.0361917256810598	0.831494257834269	1	pseudobulk	DESeq2	LRT		0
980	KC_AB2	CG13295	-1.29244479833796	0.693295472370086	1	pseudobulk	DESeq2	LRT		0
980	KC_AB2	CG13300	-1.00127798029295	0.353775924133852	1	pseudobulk	DESeq2	LRT		0
980	KC_AB2	CG13315	-1.63925793886161	0.546290859679341	1	pseudobulk	DESeq2	LRT		0
980	KC_AB2	CG13318	-0.427431139129811	1	1	pseudobulk	DESeq2	LRT		0
981	KC_AB2	CG13319	0.613659617664411	0.76686169970757	1	pseudobulk	DESeq2	LRT		0
981	KC_AB2	CG13325	-0.185739403830919	0.883696114334917	1	pseudobulk	DESeq2	LRT		0
981	KC_AB2	CG13339	-0.381986294860539	0.769178789256189	1	pseudobulk	DESeq2	LRT		0
981	KC_AB2	CG13344	-1.46626498821536	0.169562147695773	1	pseudobulk	DESeq2	LRT		0
981	KC_AB2	CG13364	0.0135642999266361	0.940185691073695	1	pseudobulk	DESeq2	LRT		0
981	KC_AB2	CG13365	-0.080167278307174	0.810172938841568	1	pseudobulk	DESeq2	LRT		0
981	KC_AB2	CG13366	-0.110716453763003	0.876486518291423	1	pseudobulk	DESeq2	LRT		0
981	KC_AB2	CG13367	1.05494029059943	0.515522233135476	1	pseudobulk	DESeq2	LRT		0
981	KC_AB2	CG13369	0.719274947052845	0.545922160664551	1	pseudobulk	DESeq2	LRT		0
981	KC_AB2	CG13375	0.108296958768984	0.875661334888693	1	pseudobulk	DESeq2	LRT		0
982	KC_AB2	CG13377	-0.592481036256484	0.706847545932384	1	pseudobulk	DESeq2	LRT		0
982	KC_AB2	CG13384	0.492332403399651	0.58121365507272	1	pseudobulk	DESeq2	LRT		0
982	KC_AB2	CG13390	-0.869983840300699	0.681381135624691	1	pseudobulk	DESeq2	LRT		0
982	KC_AB2	CG13392	-0.109785922225664	0.939774912519229	1	pseudobulk	DESeq2	LRT		0
982	KC_AB2	CG13398	1.36390873566401	0.513938015567603	1	pseudobulk	DESeq2	LRT		0
982	KC_AB2	CG13404	-0.420735167019196	0.566539961338901	1	pseudobulk	DESeq2	LRT		0
982	KC_AB2	CG13408	-0.600547221779134	1	1	pseudobulk	DESeq2	LRT		0
982	KC_AB2	CG13409	-0.549553222057563	0.63815613863236	1	pseudobulk	DESeq2	LRT		0
982	KC_AB2	CG13436	-0.592481036256484	0.706847545932384	1	pseudobulk	DESeq2	LRT		0
982	KC_AB2	CG1344	-3.37128700288313	0.0855303599770896	1	pseudobulk	DESeq2	LRT		0
983	KC_AB2	CG13482	-0.427431139129811	1	1	pseudobulk	DESeq2	LRT		0
983	KC_AB2	CG13484	0.195187461475637	0.890945022865778	1	pseudobulk	DESeq2	LRT		0
983	KC_AB2	CG13506	-2.31898344098742	0.0424970848100665	1	pseudobulk	DESeq2	LRT		0
983	KC_AB2	CG13531	1.5243995994652	0.741477491078038	1	pseudobulk	DESeq2	LRT		0
983	KC_AB2	CG13532	0.607064224562108	0.622457922961346	1	pseudobulk	DESeq2	LRT		0
983	KC_AB2	CG1354	-0.713896150588864	0.790597169673125	1	pseudobulk	DESeq2	LRT		0
983	KC_AB2	CG13551	0.458838057391041	0.495752832468605	1	pseudobulk	DESeq2	LRT		0
983	KC_AB2	CG13578	-1.12475160732157	0.574429473125171	1	pseudobulk	DESeq2	LRT		0
983	KC_AB2	CG1358	-0.422988962337433	0.536855811772867	1	pseudobulk	DESeq2	LRT		0
983	KC_AB2	CG13585	0.355620451498794	0.494017754942262	1	pseudobulk	DESeq2	LRT		0
984	KC_AB2	CG13601	0.458802951075224	0.650562795354477	1	pseudobulk	DESeq2	LRT		0
984	KC_AB2	CG13603	-0.2031587127385	0.816048760214919	1	pseudobulk	DESeq2	LRT		0
984	KC_AB2	CG13604	-1.07776915880798	0.775491075088671	1	pseudobulk	DESeq2	LRT		0
984	KC_AB2	CG13605	-0.390316128605982	0.80692093990243	1	pseudobulk	DESeq2	LRT		0
984	KC_AB2	CG13623	-0.546330988306464	0.681052831083189	1	pseudobulk	DESeq2	LRT		0
984	KC_AB2	CG13625	2.15255017993272	0.262460181179107	1	pseudobulk	DESeq2	LRT		0
984	KC_AB2	CG13630	-2.66938810389172	0.212278219588769	1	pseudobulk	DESeq2	LRT		0
984	KC_AB2	CG13631	-1.63925793886161	0.546290859679341	1	pseudobulk	DESeq2	LRT		0
984	KC_AB2	CG13643	-0.600547221779133	0.841940696994101	1	pseudobulk	DESeq2	LRT		0
984	KC_AB2	CG13654	0.829231146772707	0.562086097794089	1	pseudobulk	DESeq2	LRT		0
985	KC_AB2	CG13663	0.355234727600781	0.802879983356527	1	pseudobulk	DESeq2	LRT		0
985	KC_AB2	CG13667	-0.600547221779134	1	1	pseudobulk	DESeq2	LRT		0
985	KC_AB2	CG13671	-1.3775796060334	0.471521132477908	1	pseudobulk	DESeq2	LRT		0
985	KC_AB2	CG13674	-0.427431139129811	1	1	pseudobulk	DESeq2	LRT		0
985	KC_AB2	CG13685	-1.07776915880798	0.775491075088671	1	pseudobulk	DESeq2	LRT		0
985	KC_AB2	CG13689	0.427408177268532	0.783010727256973	1	pseudobulk	DESeq2	LRT		0
985	KC_AB2	CG13707	-0.427431139129811	1	1	pseudobulk	DESeq2	LRT		0

985	KC_AB2	CG1371	-0.488583110729315	0.75900105988985	1	pseudobulk	DESeq2	LRT		0
985	KC_AB2	CG13737	-0.938817101015206	0.629705053774855	1	pseudobulk	DESeq2	LRT		0
985	KC_AB2	CG13739	0.324259461185271	0.189709354605052	1	pseudobulk	DESeq2	LRT		0
986	KC_AB2	CG13743	-0.427431139129811	1	1	pseudobulk	DESeq2	LRT		0
986	KC_AB2	CG13751	0.733222691338993	0.578480261404914	1	pseudobulk	DESeq2	LRT		0
986	KC_AB2	CG13760	-0.882463046358329	0.542305652345026	1	pseudobulk	DESeq2	LRT		0
986	KC_AB2	CG13766	-0.640130310563385	0.734563302816648	1	pseudobulk	DESeq2	LRT		0
986	KC_AB2	CG13775	-0.600547221779133	0.841940696994101	1	pseudobulk	DESeq2	LRT		0
986	KC_AB2	CG13784	0.217737224149554	0.578252492831773	1	pseudobulk	DESeq2	LRT		0
986	KC_AB2	CG13793	2.12684015898615	0.445939223035924	1	pseudobulk	DESeq2	LRT		0
986	KC_AB2	CG13796	-0.600547221779134	1	1	pseudobulk	DESeq2	LRT		0
986	KC_AB2	CG13807	0.355234727600781	0.802879983356527	1	pseudobulk	DESeq2	LRT		0
986	KC_AB2	CG13827	-1.07776915880798	0.775491075088671	1	pseudobulk	DESeq2	LRT		0
987	KC_AB2	CG13850	-0.080724554680448	0.93328310207127	1	pseudobulk	DESeq2	LRT		0
987	KC_AB2	CG13868	0.330170138032069	0.311891621672865	1	pseudobulk	DESeq2	LRT		0
987	KC_AB2	CG13876	1.39827770914961	0.587246212652858	1	pseudobulk	DESeq2	LRT		0
987	KC_AB2	CG13887	0.502691376702925	0.403383700756895	1	pseudobulk	DESeq2	LRT		0
987	KC_AB2	CG13893	0.383113085611061	0.835927103043888	1	pseudobulk	DESeq2	LRT		0
987	KC_AB2	CG13895	2.76205395729416	0.251829971474322	1	pseudobulk	DESeq2	LRT		0
987	KC_AB2	CG13898	-0.600547221779134	1	1	pseudobulk	DESeq2	LRT		0
987	KC_AB2	CG13901	0.0456926662205872	0.981608052892825	1	pseudobulk	DESeq2	LRT		0
987	KC_AB2	CG13907	0.410028470123208	0.619475450202378	1	pseudobulk	DESeq2	LRT		0
987	KC_AB2	CG13917	0.120860062998657	0.689970724184826	1	pseudobulk	DESeq2	LRT		0
988	KC_AB2	CG13919	-2.6623837226904	0.103362034180175	1	pseudobulk	DESeq2	LRT		0
988	KC_AB2	CG13920	0.291959432590208	0.482690292259643	1	pseudobulk	DESeq2	LRT		0
988	KC_AB2	CG13921	-0.167793636552364	0.745399953884711	1	pseudobulk	DESeq2	LRT		0
988	KC_AB2	CG13926	0.646691048334021	0.599471454456185	1	pseudobulk	DESeq2	LRT		0
988	KC_AB2	CG13928	-0.147817696282997	0.608901995534608	1	pseudobulk	DESeq2	LRT		0
988	KC_AB2	CG13933	-0.916632851900887	0.599005613580599	1	pseudobulk	DESeq2	LRT		0
988	KC_AB2	CG13937	1.01663977246644	0.539305554779201	1	pseudobulk	DESeq2	LRT		0
988	KC_AB2	CG13954	0.270441606813978	0.585907115989278	1	pseudobulk	DESeq2	LRT		0
988	KC_AB2	CG13982	1.15869638324589	0.607216082893751	1	pseudobulk	DESeq2	LRT		0
988	KC_AB2	CG13994	-2.92394755656208	0.149070712987843	1	pseudobulk	DESeq2	LRT		0
989	KC_AB2	CG13995	-0.0949529622946533	0.868618193758953	1	pseudobulk	DESeq2	LRT		0
989	KC_AB2	CG13999	-0.223165486336986	0.677123892742965	1	pseudobulk	DESeq2	LRT		0
989	KC_AB2	CG14005	-3.64011843104531	0.0545612907874712	1	pseudobulk	DESeq2	LRT		0
989	KC_AB2	CG14015	1.56546102124329	0.277091153635623	1	pseudobulk	DESeq2	LRT		0
989	KC_AB2	CG14024	-0.860416704128076	0.327602069531676	1	pseudobulk	DESeq2	LRT		0
989	KC_AB2	CG14043	-1.42101048879286	0.456613161512226	1	pseudobulk	DESeq2	LRT		0
989	KC_AB2	CG14057	0.70378358684898	0.932301451973468	1	pseudobulk	DESeq2	LRT		0
989	KC_AB2	CG1407	0.0563146874970796	0.877574419167179	1	pseudobulk	DESeq2	LRT		0
989	KC_AB2	CG14073	0.104218469903463	0.77866684831998	1	pseudobulk	DESeq2	LRT		0
989	KC_AB2	CG14074	-0.450031319336008	0.818760642519443	1	pseudobulk	DESeq2	LRT		0
990	KC_AB2	CG14082	0.554561984706809	0.625412559717209	1	pseudobulk	DESeq2	LRT		0
990	KC_AB2	CG14086	1.98295763317476	0.470185203902984	1	pseudobulk	DESeq2	LRT		0
990	KC_AB2	CG14100	-1.15947420603738	0.496969277976602	1	pseudobulk	DESeq2	LRT		0
990	KC_AB2	CG14102	1.39827770914961	0.587246212652858	1	pseudobulk	DESeq2	LRT		0
990	KC_AB2	CG14104	-0.0852743682753834	0.917718671153184	1	pseudobulk	DESeq2	LRT		0
990	KC_AB2	CG14130	1.44249618489118	0.5203038246131	1	pseudobulk	DESeq2	LRT		0
990	KC_AB2	CG14135	1.99945984800115	0.325387796783553	1	pseudobulk	DESeq2	LRT		0
990	KC_AB2	CG14142	-0.592481036256484	0.706847545932384	1	pseudobulk	DESeq2	LRT		0
990	KC_AB2	CG1416	0.337403968844324	0.671547199304062	1	pseudobulk	DESeq2	LRT		0
990	KC_AB2	CG14174	-0.600547221779134	1	1	pseudobulk	DESeq2	LRT		0

991	KC_AB2	CG1418	0.313651020975298	0.83985313330738	1	pseudobulk	DESeq2	LRT	0
991	KC_AB2	CG14182	0.0953012929358155	0.870468164973558	1	pseudobulk	DESeq2	LRT	0
991	KC_AB2	CG14184	0.82513458454287	0.38616340725344	1	pseudobulk	DESeq2	LRT	0
991	KC_AB2	CG14185	0.658649697766144	0.774289187460886	1	pseudobulk	DESeq2	LRT	0
991	KC_AB2	CG14186	-0.674539453588177	0.0800726997531954	1	pseudobulk	DESeq2	LRT	0
991	KC_AB2	CG14190	-0.600547221779133	0.841940696994101	1	pseudobulk	DESeq2	LRT	0
991	KC_AB2	CG14194	-1.72005644564701	0.367926060612262	1	pseudobulk	DESeq2	LRT	0
991	KC_AB2	CG14200	-0.990261878901888	0.362824542546984	1	pseudobulk	DESeq2	LRT	0
991	KC_AB2	CG14207	0.431695017393863	0.587722826552057	1	pseudobulk	DESeq2	LRT	0
991	KC_AB2	CG14210	-0.938817101015206	0.629705053774855	1	pseudobulk	DESeq2	LRT	0
992	KC_AB2	CG14220	-0.0949529622946533	0.868618193758953	1	pseudobulk	DESeq2	LRT	0
992	KC_AB2	CG14229	-1.10951415344068	0.319453263055065	1	pseudobulk	DESeq2	LRT	0
992	KC_AB2	CG14230	0.222396124134733	1	1	pseudobulk	DESeq2	LRT	0
992	KC_AB2	CG14231	-1.09272849286634	0.644096748745442	1	pseudobulk	DESeq2	LRT	0
992	KC_AB2	CG14232	0.0495353073457683	0.923194051641149	1	pseudobulk	DESeq2	LRT	0
992	KC_AB2	CG14234	0.771688942768856	0.304601022500942	1	pseudobulk	DESeq2	LRT	0
992	KC_AB2	CG14260	2.12684015898615	0.445939223035924	1	pseudobulk	DESeq2	LRT	0
992	KC_AB2	CG14270	1.27496040324747	0.501655537161972	1	pseudobulk	DESeq2	LRT	0
992	KC_AB2	CG14286	-0.427431139129811	1	1	pseudobulk	DESeq2	LRT	0
992	KC_AB2	CG14304	0.70378358684898	0.932301451973468	1	pseudobulk	DESeq2	LRT	0
993	KC_AB2	CG14312	-0.353673044583826	0.640159314703156	1	pseudobulk	DESeq2	LRT	0
993	KC_AB2	CG14314	-2.0190056050487	0.253746295339958	1	pseudobulk	DESeq2	LRT	0
993	KC_AB2	CG14321	0.712168223931594	0.424357354903758	1	pseudobulk	DESeq2	LRT	0
993	KC_AB2	CG14322	-0.427431139129811	1	1	pseudobulk	DESeq2	LRT	0
993	KC_AB2	CG1434	2.76205395729416	0.251829971474322	1	pseudobulk	DESeq2	LRT	0
993	KC_AB2	CG14341	0.61935477401623	0.606747002791581	1	pseudobulk	DESeq2	LRT	0
993	KC_AB2	CG14352	0.864341462967788	0.925947094335722	1	pseudobulk	DESeq2	LRT	0
993	KC_AB2	CG14355	2.12684015898615	0.445939223035924	1	pseudobulk	DESeq2	LRT	0
993	KC_AB2	CG14356	2.12684015898615	0.445939223035924	1	pseudobulk	DESeq2	LRT	0
993	KC_AB2	CG14367	1.5243995994652	0.741477491078038	1	pseudobulk	DESeq2	LRT	0
994	KC_AB2	CG1440	0.987557173191977	0.386892028937997	1	pseudobulk	DESeq2	LRT	0
994	KC_AB2	CG14401	1.5243995994652	0.741477491078038	1	pseudobulk	DESeq2	LRT	0
994	KC_AB2	CG14407	-0.185289781708421	0.783004411135932	1	pseudobulk	DESeq2	LRT	0
994	KC_AB2	CG14408	0.355277607363776	0.475863724988251	1	pseudobulk	DESeq2	LRT	0
994	KC_AB2	CG14411	-0.899484590672455	0.47321307221886	1	pseudobulk	DESeq2	LRT	0
994	KC_AB2	CG14414	-0.0162875123322863	0.905335105023269	1	pseudobulk	DESeq2	LRT	0
994	KC_AB2	CG14431	1.20490324641321	0.247249775673943	1	pseudobulk	DESeq2	LRT	0
994	KC_AB2	CG14434	-0.0583169041972162	0.914331626090543	1	pseudobulk	DESeq2	LRT	0
994	KC_AB2	CG14435	0.802237805275299	0.347238998927557	1	pseudobulk	DESeq2	LRT	0
994	KC_AB2	CG14440	0.684689300625766	0.377145630181858	1	pseudobulk	DESeq2	LRT	0
995	KC_AB2	CG14442	0.220891373723873	0.727700060318961	1	pseudobulk	DESeq2	LRT	0
995	KC_AB2	CG14443	2.12684015898615	0.445939223035924	1	pseudobulk	DESeq2	LRT	0
995	KC_AB2	CG14450	0.933722893549181	0.381780042525005	1	pseudobulk	DESeq2	LRT	0
995	KC_AB2	CG14464	-0.114136802288166	0.817486782498252	1	pseudobulk	DESeq2	LRT	0
995	KC_AB2	CG14478	0.172232952709343	0.662351593619887	1	pseudobulk	DESeq2	LRT	0
995	KC_AB2	CG14483	-0.218302940615186	0.800569684931733	1	pseudobulk	DESeq2	LRT	0
995	KC_AB2	CG14502	-3.21335635562541	0.101317179615526	1	pseudobulk	DESeq2	LRT	0
995	KC_AB2	CG14512	-1.70380348762697	0.473446675363872	1	pseudobulk	DESeq2	LRT	0
995	KC_AB2	CG14535	1.88054640636039	0.158064986286148	1	pseudobulk	DESeq2	LRT	0
995	KC_AB2	CG14543	0.573760393110584	0.792134620722927	1	pseudobulk	DESeq2	LRT	0
996	KC_AB2	CG14561	2.12684015898615	0.445939223035924	1	pseudobulk	DESeq2	LRT	0
996	KC_AB2	CG14562	-1.16719563004499	0.37630527163117	1	pseudobulk	DESeq2	LRT	0
996	KC_AB2	CG14563	0.122418697168221	0.886972372045921	1	pseudobulk	DESeq2	LRT	0

996	KC_AB2	CG14589	-0.600547221779133	0.841940696994101	1	pseudobulk	DESeq2	LRT	0
996	KC_AB2	CG14591	0.465101419234274	0.566388653052087	1	pseudobulk	DESeq2	LRT	0
996	KC_AB2	CG14613	-0.0745492687879989	0.920659452974254	1	pseudobulk	DESeq2	LRT	0
996	KC_AB2	CG14618	0.65360470678452	0.777547207962487	1	pseudobulk	DESeq2	LRT	0
996	KC_AB2	CG14621	-0.763696756123279	0.599409791913358	1	pseudobulk	DESeq2	LRT	0
996	KC_AB2	CG1463	-0.865499373982915	0.638875415729182	1	pseudobulk	DESeq2	LRT	0
996	KC_AB2	CG14641	0.561749901372434	0.49563637801283	1	pseudobulk	DESeq2	LRT	0
997	KC_AB2	CG14646	0.910858885337268	0.593576541362697	1	pseudobulk	DESeq2	LRT	0
997	KC_AB2	CG14647	0.0471936707755822	0.953916869426286	1	pseudobulk	DESeq2	LRT	0
997	KC_AB2	CG14650	0.556781325987863	0.654918050159717	1	pseudobulk	DESeq2	LRT	0
997	KC_AB2	CG14657	0.472090993941785	0.636464537266379	1	pseudobulk	DESeq2	LRT	0
997	KC_AB2	CG14661	-0.592481036256484	0.706847545932384	1	pseudobulk	DESeq2	LRT	0
997	KC_AB2	CG14667	0.628916363195527	0.676797978493874	1	pseudobulk	DESeq2	LRT	0
997	KC_AB2	CG14669	-1.10109102980809	0.439284674587735	1	pseudobulk	DESeq2	LRT	0
997	KC_AB2	CG14671	-1.70380348762697	0.473446675363872	1	pseudobulk	DESeq2	LRT	0
997	KC_AB2	CG14683	-2.06407076736304	0.362444518317458	1	pseudobulk	DESeq2	LRT	0
997	KC_AB2	CG14687	0.222396124134733	1	1	pseudobulk	DESeq2	LRT	0
998	KC_AB2	CG14688	0.864341462967788	0.925947094335722	1	pseudobulk	DESeq2	LRT	0
998	KC_AB2	CG14694	-0.427431139129811	1	1	pseudobulk	DESeq2	LRT	0
998	KC_AB2	CG14701	-0.600547221779133	0.841940696994101	1	pseudobulk	DESeq2	LRT	0
998	KC_AB2	CG14710	-0.780640540820601	0.628347559796334	1	pseudobulk	DESeq2	LRT	0
998	KC_AB2	CG14711	-0.319604581482846	0.837100547724082	1	pseudobulk	DESeq2	LRT	0
998	KC_AB2	CG14715	-0.565165753738175	0.52377305092925	1	pseudobulk	DESeq2	LRT	0
998	KC_AB2	CG14721	-2.24432354911821	0.231065186915512	1	pseudobulk	DESeq2	LRT	0
998	KC_AB2	CG14722	-1.24330161354718	0.542016636259656	1	pseudobulk	DESeq2	LRT	0
998	KC_AB2	CG14739	1.5243995994652	0.741477491078038	1	pseudobulk	DESeq2	LRT	0
998	KC_AB2	CG14757	1.32778982316151	0.726339004545413	1	pseudobulk	DESeq2	LRT	0
999	KC_AB2	CG14760	0.864341462967788	0.925947094335722	1	pseudobulk	DESeq2	LRT	0
999	KC_AB2	CG14762	-0.14213677114514	0.666191792183406	1	pseudobulk	DESeq2	LRT	0
999	KC_AB2	CG14764	-0.209688942584084	0.689071267298033	1	pseudobulk	DESeq2	LRT	0
999	KC_AB2	CG14767	-0.335167825357158	0.467509975984996	1	pseudobulk	DESeq2	LRT	0
999	KC_AB2	CG14777	-0.350749981478291	0.765306200357044	1	pseudobulk	DESeq2	LRT	0
999	KC_AB2	CG14778	1.5243995994652	0.741477491078038	1	pseudobulk	DESeq2	LRT	0
999	KC_AB2	CG14803	-1.63925793886161	0.546290859679341	1	pseudobulk	DESeq2	LRT	0
999	KC_AB2	CG14805	-0.131023558643448	0.853530169706317	1	pseudobulk	DESeq2	LRT	0
999	KC_AB2	CG14806	0.435569048022507	0.539355586470607	1	pseudobulk	DESeq2	LRT	0
999	KC_AB2	CG14812	0.376567274176268	0.673116287249755	1	pseudobulk	DESeq2	LRT	0
100	KC_AB2	CG14817	-1.27154126452803	0.158518784123403	1	pseudobulk	DESeq2	LRT	0
100	KC_AB2	CG14818	-0.0158699373645832	0.790500888038432	1	pseudobulk	DESeq2	LRT	0
100	KC_AB2	CG14853	-0.0299279597454284	0.948635377747426	1	pseudobulk	DESeq2	LRT	0
100	KC_AB2	CG1486	0.872263915466572	0.537517495282304	1	pseudobulk	DESeq2	LRT	0
100	KC_AB2	CG14868	1.16007010204435	0.0942429246146212	1	pseudobulk	DESeq2	LRT	0
100	KC_AB2	CG14881	-0.209808076863057	0.877444409505505	1	pseudobulk	DESeq2	LRT	0
100	KC_AB2	CG14882	1.400777848138	0.492920641857113	1	pseudobulk	DESeq2	LRT	0
100	KC_AB2	CG14883	0.351856207049651	0.77483256566977	1	pseudobulk	DESeq2	LRT	0
100	KC_AB2	CG14894	0.891916865776145	0.371598146587893	1	pseudobulk	DESeq2	LRT	0
100	KC_AB2	CG14903	-0.145669929375454	0.867360803637671	1	pseudobulk	DESeq2	LRT	0
100	KC_AB2	CG14906	-1.63925793886161	0.546290859679341	1	pseudobulk	DESeq2	LRT	0
100	KC_AB2	CG1492	-0.381022595176465	0.906491838131301	1	pseudobulk	DESeq2	LRT	0
100	KC_AB2	CG14937	1.5243995994652	0.741477491078038	1	pseudobulk	DESeq2	LRT	0
100	KC_AB2	CG14965	-0.841882716738579	1	1	pseudobulk	DESeq2	LRT	0
100	KC_AB2	CG14966	-0.0189308032219217	0.876009482337566	1	pseudobulk	DESeq2	LRT	0
100	KC_AB2	CG14969	-1.93220872672389	0.398686710033904	1	pseudobulk	DESeq2	LRT	0

10(	KC_AB2	CG14971	0.717340694333406	0.615866173404391	1	pseudobulk	DESeq2	LRT	0
10(	KC_AB2	CG14977	-0.231723605003279	0.809639891302342	1	pseudobulk	DESeq2	LRT	0
10(	KC_AB2	CG14982	-0.492882154806174	0.534504955166944	1	pseudobulk	DESeq2	LRT	0
10(	KC_AB2	CG14984	1.5243995994652	0.741477491078038	1	pseudobulk	DESeq2	LRT	0
10(	KC_AB2	CG14995	-0.125100693521519	0.870708303754601	1	pseudobulk	DESeq2	LRT	0
10(	KC_AB2	CG15011	1.94691684546882	0.398076844044942	1	pseudobulk	DESeq2	LRT	0
10(	KC_AB2	CG15012	0.601282872606675	0.306471594641158	1	pseudobulk	DESeq2	LRT	0
10(	KC_AB2	CG15014	-1.14928602554562	0.572019929472691	1	pseudobulk	DESeq2	LRT	0
10(	KC_AB2	CG15019	-2.47242271668572	0.279151826981215	1	pseudobulk	DESeq2	LRT	0
10(	KC_AB2	CG15021	-0.600547221779133	0.841940696994101	1	pseudobulk	DESeq2	LRT	0
10(	KC_AB2	CG15027	1.96602891120698	0.44112716469798	1	pseudobulk	DESeq2	LRT	0
10(	KC_AB2	CG1504	-0.427431139129811	1	1	pseudobulk	DESeq2	LRT	0
10(	KC_AB2	CG15040	-0.841882716738579	1	1	pseudobulk	DESeq2	LRT	0
10(	KC_AB2	CG15073	-0.463052496301942	0.794041648114126	1	pseudobulk	DESeq2	LRT	0
10(	KC_AB2	CG15084	-0.600547221779133	0.841940696994101	1	pseudobulk	DESeq2	LRT	0
10(	KC_AB2	CG15097	-0.414144738047206	0.564852780598581	1	pseudobulk	DESeq2	LRT	0
10(	KC_AB2	CG15098	-0.764771705114834	0.650480942350562	1	pseudobulk	DESeq2	LRT	0
10(	KC_AB2	CG15099	-0.154276666309413	0.851902843357533	1	pseudobulk	DESeq2	LRT	0
10(	KC_AB2	CG15107	2.1817927492653	0.458599861292202	1	pseudobulk	DESeq2	LRT	0
10(	KC_AB2	CG15109	-0.427431139129811	1	1	pseudobulk	DESeq2	LRT	0
10(	KC_AB2	CG15111	-0.006608265340708	0.84518248965419	1	pseudobulk	DESeq2	LRT	0
10(	KC_AB2	CG15117	-0.427431139129811	1	1	pseudobulk	DESeq2	LRT	0
10(	KC_AB2	CG15118	-0.268406529346261	0.780983213867695	1	pseudobulk	DESeq2	LRT	0
10(	KC_AB2	CG1513	0.427767362880268	0.402374890284049	1	pseudobulk	DESeq2	LRT	0
10(	KC_AB2	CG15141	-1.82264455627709	0.313971529081416	1	pseudobulk	DESeq2	LRT	0
10(	KC_AB2	CG15160	-0.207166741761006	0.860740676387662	1	pseudobulk	DESeq2	LRT	0
10(	KC_AB2	CG15168	0.151520361399871	0.847536928882064	1	pseudobulk	DESeq2	LRT	0
10(	KC_AB2	CG15196	1.5243995994652	0.741477491078038	1	pseudobulk	DESeq2	LRT	0
10(	KC_AB2	CG15201	0.651869231857958	0.64198904947059	1	pseudobulk	DESeq2	LRT	0
10(	KC_AB2	CG15209	1.19434746569918	0.491673587701676	1	pseudobulk	DESeq2	LRT	0
10(	KC_AB2	CG15213	2.1817927492653	0.458599861292202	1	pseudobulk	DESeq2	LRT	0
10(	KC_AB2	CG15221	-1.63925793886161	0.546290859679341	1	pseudobulk	DESeq2	LRT	0
10(	KC_AB2	CG1523	0.920212920242661	0.67286879185691	1	pseudobulk	DESeq2	LRT	0
10(	KC_AB2	CG15237	-0.477172548602799	0.617247266561302	1	pseudobulk	DESeq2	LRT	0
10(	KC_AB2	CG15239	2.12684015898615	0.445939223035924	1	pseudobulk	DESeq2	LRT	0
10(	KC_AB2	CG15270	0.493500704523333	0.524192295419432	1	pseudobulk	DESeq2	LRT	0
10(	KC_AB2	CG15309	0.694367319097508	0.401147577434952	1	pseudobulk	DESeq2	LRT	0
10(	KC_AB2	CG15312	3.17195708651769	0.15530090920866	1	pseudobulk	DESeq2	LRT	0
10(	KC_AB2	CG15317	2.81573400598773	0.162704827723198	1	pseudobulk	DESeq2	LRT	0
10(	KC_AB2	CG1532	1.07419424641734	0.36937640973791	1	pseudobulk	DESeq2	LRT	0
10(	KC_AB2	CG15356	1.72763303534666	0.167354731208905	1	pseudobulk	DESeq2	LRT	0
10(	KC_AB2	CG15382	-0.841882716738579	1	1	pseudobulk	DESeq2	LRT	0
10(	KC_AB2	CG15385	0.0751604116153772	0.900920279002769	1	pseudobulk	DESeq2	LRT	0
10(	KC_AB2	CG15387	0.0981463281699375	0.947527364080578	1	pseudobulk	DESeq2	LRT	0
10(	KC_AB2	CG15390	-0.841882716738579	1	1	pseudobulk	DESeq2	LRT	0
10(	KC_AB2	CG1542	-1.54470851095603	0.55301747381519	1	pseudobulk	DESeq2	LRT	0
10(	KC_AB2	CG15431	0.233529307865598	0.548204482754664	1	pseudobulk	DESeq2	LRT	0
10(	KC_AB2	CG15432	0.717659674158859	0.41458380166829	1	pseudobulk	DESeq2	LRT	0
10(	KC_AB2	CG15435	2.12684015898615	0.445939223035924	1	pseudobulk	DESeq2	LRT	0
10(	KC_AB2	CG15439	-0.206071851008905	0.969636392453816	1	pseudobulk	DESeq2	LRT	0
10(	KC_AB2	CG15443	-2.23889186715597	0.192259214810764	1	pseudobulk	DESeq2	LRT	0
10(	KC_AB2	CG15445	0.864341462967788	0.925947094335722	1	pseudobulk	DESeq2	LRT	0
10(	KC_AB2	CG15449	1.9374194786065	0.174541356356973	1	pseudobulk	DESeq2	LRT	0

100	KC_AB2	CG15456	-1.48579216665348	0.197694426430052	1	pseudobulk	DESeq2	LRT	0
100	KC_AB2	CG15459	0.470513442773017	0.810304627149072	1	pseudobulk	DESeq2	LRT	0
100	KC_AB2	CG15514	-0.182328666103502	0.886848451451546	1	pseudobulk	DESeq2	LRT	0
100	KC_AB2	CG1552	2.02633506802478	0.157268648197423	1	pseudobulk	DESeq2	LRT	0
100	KC_AB2	CG15525	-2.35804099162616	0.305169355581043	1	pseudobulk	DESeq2	LRT	0
100	KC_AB2	CG15535	-1.50265668554613	0.163078285676571	1	pseudobulk	DESeq2	LRT	0
100	KC_AB2	CG15536	0.943586647867311	0.690632779168748	1	pseudobulk	DESeq2	LRT	0
100	KC_AB2	CG15554	-0.427431139129811	1	1	pseudobulk	DESeq2	LRT	0
100	KC_AB2	CG15561	-0.427431139129811	1	1	pseudobulk	DESeq2	LRT	0
100	KC_AB2	CG15579	-0.427431139129811	1	1	pseudobulk	DESeq2	LRT	0
100	KC_AB2	CG15601	3.50499421812744	0.0978647741102649	1	pseudobulk	DESeq2	LRT	0
100	KC_AB2	CG15602	-0.0529809127348567	0.863884257519641	1	pseudobulk	DESeq2	LRT	0
100	KC_AB2	CG15628	0.0907148391726456	0.789071164971709	1	pseudobulk	DESeq2	LRT	0
100	KC_AB2	CG15638	0.864341462967788	0.925947094335722	1	pseudobulk	DESeq2	LRT	0
100	KC_AB2	CG15642	-0.385593519773769	0.739898234377577	1	pseudobulk	DESeq2	LRT	0
100	KC_AB2	CG15643	-1.40673172715063	0.587364787535786	1	pseudobulk	DESeq2	LRT	0
100	KC_AB2	CG15651	-0.0176016391791131	0.95131192074042	1	pseudobulk	DESeq2	LRT	0
100	KC_AB2	CG15653	0.222396124134733	1	1	pseudobulk	DESeq2	LRT	0
100	KC_AB2	CG15657	-0.600547221779133	0.841940696994101	1	pseudobulk	DESeq2	LRT	0
100	KC_AB2	CG15674	1.5243995994652	0.741477491078038	1	pseudobulk	DESeq2	LRT	0
100	KC_AB2	CG15706	-0.427431139129811	1	1	pseudobulk	DESeq2	LRT	0
100	KC_AB2	CG15708	-1.63925793886161	0.546290859679341	1	pseudobulk	DESeq2	LRT	0
100	KC_AB2	CG15715	-0.0120017332355433	0.898293715546112	1	pseudobulk	DESeq2	LRT	0
100	KC_AB2	CG15719	1.5243995994652	0.741477491078038	1	pseudobulk	DESeq2	LRT	0
100	KC_AB2	CG1572	0.713982351041985	0.921569637923553	1	pseudobulk	DESeq2	LRT	0
100	KC_AB2	CG15725	-1.12785316439435	0.661319045116135	1	pseudobulk	DESeq2	LRT	0
100	KC_AB2	CG15743	-3.25162815993712	0.10891581569568	1	pseudobulk	DESeq2	LRT	0
100	KC_AB2	CG15744	-1.51812168653838	0.552401469179882	1	pseudobulk	DESeq2	LRT	0
100	KC_AB2	CG15747	0.181448001997556	0.88844185789269	1	pseudobulk	DESeq2	LRT	0
100	KC_AB2	CG1575	0.613659617664411	0.76686169970757	1	pseudobulk	DESeq2	LRT	0
100	KC_AB2	CG15760	-0.0518492696181388	0.962280426154907	1	pseudobulk	DESeq2	LRT	0
101	KC_AB2	CG15765	0.0699292081178546	0.827030442889585	1	pseudobulk	DESeq2	LRT	0
101	KC_AB2	CG15771	-0.608140332901926	0.502520137906323	1	pseudobulk	DESeq2	LRT	0
101	KC_AB2	CG15772	0.197008927741015	0.634438901412333	1	pseudobulk	DESeq2	LRT	0
101	KC_AB2	CG1578	0.744089115004934	0.224455227108494	1	pseudobulk	DESeq2	LRT	0
101	KC_AB2	CG15803	-0.295781934016409	0.729222263941854	1	pseudobulk	DESeq2	LRT	0
101	KC_AB2	CG15812	-0.463052496301942	0.794041648114126	1	pseudobulk	DESeq2	LRT	0
101	KC_AB2	CG15814	0.154415993437786	0.821052134659264	1	pseudobulk	DESeq2	LRT	0
101	KC_AB2	CG1582	0.864341462967788	0.925947094335722	1	pseudobulk	DESeq2	LRT	0
101	KC_AB2	CG15877	2.60449690258161	0.170888565624672	1	pseudobulk	DESeq2	LRT	0
101	KC_AB2	CG15880	-0.427431139129811	1	1	pseudobulk	DESeq2	LRT	0
101	KC_AB2	CG15890	-0.600547221779133	0.841940696994101	1	pseudobulk	DESeq2	LRT	0
101	KC_AB2	CG15891	0.328323323969981	0.674423602069876	1	pseudobulk	DESeq2	LRT	0
101	KC_AB2	CG15894	-0.362342910671677	0.527159202808079	1	pseudobulk	DESeq2	LRT	0
101	KC_AB2	CG15908	2.12684015898615	0.445939223035924	1	pseudobulk	DESeq2	LRT	0
101	KC_AB2	CG15912	0.341286121848233	0.813452069665491	1	pseudobulk	DESeq2	LRT	0
101	KC_AB2	CG15916	-0.118278002163321	0.769445535412156	1	pseudobulk	DESeq2	LRT	0
101	KC_AB2	CG15922	-0.790816491013546	0.518236280362606	1	pseudobulk	DESeq2	LRT	0
101	KC_AB2	CG1598	-0.215857665558152	0.795200188549047	1	pseudobulk	DESeq2	LRT	0
101	KC_AB2	CG1602	-0.600547221779134	1	1	pseudobulk	DESeq2	LRT	0
101	KC_AB2	CG1603	1.62571710983431	0.470438722277963	1	pseudobulk	DESeq2	LRT	0
101	KC_AB2	CG1607	0.399508807970897	0.563967386942966	1	pseudobulk	DESeq2	LRT	0
101	KC_AB2	CG1620	-1.25976123586122	0.346585117299897	1	pseudobulk	DESeq2	LRT	0

101	KC_AB2	CG1622	-2.23888222951327	0.216847118912908	1	pseudobulk	DESeq2	LRT		0
101	KC_AB2	CG1635	-1.57560465757076	0.408904235984595	1	pseudobulk	DESeq2	LRT		0
101	KC_AB2	CG1636	0.602401981054255	0.461174570361339	1	pseudobulk	DESeq2	LRT		0
101	KC_AB2	CG1637	0.51070101428773	0.675386602317034	1	pseudobulk	DESeq2	LRT		0
101	KC_AB2	CG1640	0.636033392211084	0.21293804480084	1	pseudobulk	DESeq2	LRT		0
101	KC_AB2	CG1646	-0.0074084658482360	0.898382710338604	1	pseudobulk	DESeq2	LRT		0
101	KC_AB2	CG1647	-0.682838133336604	0.671027848057055	1	pseudobulk	DESeq2	LRT		0
101	KC_AB2	CG1648	-0.412314500261633	0.58682615553022	1	pseudobulk	DESeq2	LRT		0
101	KC_AB2	CG1662	-2.62548793044389	0.206246834544296	1	pseudobulk	DESeq2	LRT		0
101	KC_AB2	CG1663	0.864341462967788	0.925947094335722	1	pseudobulk	DESeq2	LRT		0
101	KC_AB2	CG16710	-0.592481036256484	0.706847545932384	1	pseudobulk	DESeq2	LRT		0
101	KC_AB2	CG16711	-0.0131971646060435	0.913548205722206	1	pseudobulk	DESeq2	LRT		0
101	KC_AB2	CG16717	-0.233433110438695	0.776001472622519	1	pseudobulk	DESeq2	LRT		0
101	KC_AB2	CG16721	-1.04077127086163	0.605194231525991	1	pseudobulk	DESeq2	LRT		0
101	KC_AB2	CG16734	-0.427431139129811	1	1	pseudobulk	DESeq2	LRT		0
101	KC_AB2	CG1677	0.485929464781396	0.290153192203147	1	pseudobulk	DESeq2	LRT		0
101	KC_AB2	CG16779	0.0116460630991764	0.969419307309589	1	pseudobulk	DESeq2	LRT		0
101	KC_AB2	CG16787	0.158885872213844	0.913023095756665	1	pseudobulk	DESeq2	LRT		0
101	KC_AB2	CG16790	-1.07776915880798	0.775491075088671	1	pseudobulk	DESeq2	LRT		0
101	KC_AB2	CG16791	0.239616526733171	0.451537712881934	1	pseudobulk	DESeq2	LRT		0
101	KC_AB2	CG16798	1.23085716405798	0.614623732585597	1	pseudobulk	DESeq2	LRT		0
101	KC_AB2	CG16812	0.609449960179138	0.564550521164391	1	pseudobulk	DESeq2	LRT		0
101	KC_AB2	CG16854	-1.84423614433138	0.419369668145802	1	pseudobulk	DESeq2	LRT		0
101	KC_AB2	CG16863	-0.427431139129811	1	1	pseudobulk	DESeq2	LRT		0
101	KC_AB2	CG16868	0.0203015795782856	0.967047078004919	1	pseudobulk	DESeq2	LRT		0
101	KC_AB2	CG1688	-0.0210949685507608	0.792536305623943	1	pseudobulk	DESeq2	LRT		0
101	KC_AB2	CG16896	-0.427431139129811	1	1	pseudobulk	DESeq2	LRT		0
101	KC_AB2	CG16903	0.176575964931395	0.88062168618173	1	pseudobulk	DESeq2	LRT		0
101	KC_AB2	CG16935	-0.681631453907657	0.610204907598957	1	pseudobulk	DESeq2	LRT		0
101	KC_AB2	CG1695	0.472421796511505	0.509835420764336	1	pseudobulk	DESeq2	LRT		0
101	KC_AB2	CG16952	-0.339963192344522	0.603899058000812	1	pseudobulk	DESeq2	LRT		0
101	KC_AB2	CG16953	-2.46666071156584	0.0523254875991512	1	pseudobulk	DESeq2	LRT		0
101	KC_AB2	CG16972	-1.3723417613553	0.619532036522	1	pseudobulk	DESeq2	LRT		0
101	KC_AB2	CG16974	-0.478607673353634	0.680240766726692	1	pseudobulk	DESeq2	LRT		0
101	KC_AB2	CG16979	-0.39917336535616	0.810147819664692	1	pseudobulk	DESeq2	LRT		0
101	KC_AB2	CG16984	-0.427431139129811	1	1	pseudobulk	DESeq2	LRT		0
101	KC_AB2	CG16986	-0.427431139129811	1	1	pseudobulk	DESeq2	LRT		0
101	KC_AB2	CG16989	-1.41358380305188	0.473113093157609	1	pseudobulk	DESeq2	LRT		0
101	KC_AB2	CG17002	-0.882630140105986	0.598840614274749	1	pseudobulk	DESeq2	LRT		0
101	KC_AB2	CG17003	2.12684015898615	0.445939223035924	1	pseudobulk	DESeq2	LRT		0
101	KC_AB2	CG17005	-0.427431139129811	1	1	pseudobulk	DESeq2	LRT		0
101	KC_AB2	CG17019	1.67158739086685	0.154174499209636	1	pseudobulk	DESeq2	LRT		0
101	KC_AB2	CG17026	-0.529556196993598	0.843734517510881	1	pseudobulk	DESeq2	LRT		0
101	KC_AB2	CG17027	1.5243995994652	0.741477491078038	1	pseudobulk	DESeq2	LRT		0
101	KC_AB2	CG17029	0.0920390296490068	0.921929622402554	1	pseudobulk	DESeq2	LRT		0
101	KC_AB2	CG1703	-0.27476783516425	0.80532273619366	1	pseudobulk	DESeq2	LRT		0
101	KC_AB2	CG17059	0.212303011730959	0.671029678299759	1	pseudobulk	DESeq2	LRT		0
101	KC_AB2	CG17065	-0.210106009611177	0.769524056601833	1	pseudobulk	DESeq2	LRT		0
101	KC_AB2	CG17068	1.40241945196173	0.57265124481703	1	pseudobulk	DESeq2	LRT		0
101	KC_AB2	CG17075	0.590815037719792	0.597728898591738	1	pseudobulk	DESeq2	LRT		0
101	KC_AB2	CG17078	0.386572106423214	0.755721866428068	1	pseudobulk	DESeq2	LRT		0
101	KC_AB2	CG17118	1.5243995994652	0.741477491078038	1	pseudobulk	DESeq2	LRT		0
101	KC_AB2	CG17124	0.108359318804624	0.748966108315514	1	pseudobulk	DESeq2	LRT		0

101	KC_AB2	CG17141	0.0806715714550146	0.88136911310611	1	pseudobulk	DESeq2	LRT			0
101	KC_AB2	CG17162	-0.631578663457669	0.750664130745045	1	pseudobulk	DESeq2	LRT			0
101	KC_AB2	CG17163	0.390796062685279	0.665725098786183	1	pseudobulk	DESeq2	LRT			0
101	KC_AB2	CG17168	0.87836319242015	0.309647995624072	1	pseudobulk	DESeq2	LRT			0
101	KC_AB2	CG17186	-1.29244479833796	0.693295472370086	1	pseudobulk	DESeq2	LRT			0
101	KC_AB2	CG17189	-0.427431139129811	1	1	pseudobulk	DESeq2	LRT			0
101	KC_AB2	CG17202	0.05807189154129	0.913009926455307	1	pseudobulk	DESeq2	LRT			0
101	KC_AB2	CG17221	-1.14995976321342	0.586703842885709	1	pseudobulk	DESeq2	LRT			0
101	KC_AB2	CG17230	-2.29972426504075	0.180265036844968	1	pseudobulk	DESeq2	LRT			0
101	KC_AB2	CG17233	0.836119506854296	0.344755658762319	1	pseudobulk	DESeq2	LRT			0
101	KC_AB2	CG17249	-1.54470851095603	0.55301747381519	1	pseudobulk	DESeq2	LRT			0
101	KC_AB2	CG17260	-1.12785316439435	0.661319045116135	1	pseudobulk	DESeq2	LRT			0
101	KC_AB2	CG17266	0.725498497157359	0.93879047033739	1	pseudobulk	DESeq2	LRT			0
101	KC_AB2	CG17270	0.626150764043901	0.480397536324343	1	pseudobulk	DESeq2	LRT			0
101	KC_AB2	CG17271	-0.578326585659894	0.646265172651463	1	pseudobulk	DESeq2	LRT			0
101	KC_AB2	CG17272	0.207074217464007	0.645620859021781	1	pseudobulk	DESeq2	LRT			0
101	KC_AB2	CG17294	-0.413386166840315	0.743021142717934	1	pseudobulk	DESeq2	LRT			0
101	KC_AB2	CG17298	1.5243995994652	0.741477491078038	1	pseudobulk	DESeq2	LRT			0
101	KC_AB2	CG17321	-0.549456311301258	0.442029450524272	1	pseudobulk	DESeq2	LRT			0
101	KC_AB2	CG17328	-2.12214992510756	0.367174323489033	1	pseudobulk	DESeq2	LRT			0
101	KC_AB2	CG17329	-0.427431139129811	1	1	pseudobulk	DESeq2	LRT			0
101	KC_AB2	CG17343	0.756080480905839	0.527350547464786	1	pseudobulk	DESeq2	LRT			0
101	KC_AB2	CG17359	1.26307719581879	0.569064211306759	1	pseudobulk	DESeq2	LRT			0
101	KC_AB2	CG17361	-0.482173055655404	0.804949101714764	1	pseudobulk	DESeq2	LRT			0
101	KC_AB2	CG17364	-0.819651323710324	0.334449629052182	1	pseudobulk	DESeq2	LRT			0
102	KC_AB2	CG1737	-0.527938416249376	0.441087112546326	1	pseudobulk	DESeq2	LRT			0
102	KC_AB2	CG17378	0.556398692403767	0.411210805767069	1	pseudobulk	DESeq2	LRT			0
102	KC_AB2	CG1738	-0.0738748688508091	0.942862902944051	1	pseudobulk	DESeq2	LRT			0
102	KC_AB2	CG17385	0.407451611701522	0.549822219809071	1	pseudobulk	DESeq2	LRT			0
102	KC_AB2	CG17454	-1.19805438262475	0.119284081780769	1	pseudobulk	DESeq2	LRT			0
102	KC_AB2	CG17486	-0.618799344905012	0.551369259145027	1	pseudobulk	DESeq2	LRT			0
102	KC_AB2	CG17493	-0.141605694914151	0.780243065074309	1	pseudobulk	DESeq2	LRT			0
102	KC_AB2	CG17508	0.102082948329972	0.814808082192878	1	pseudobulk	DESeq2	LRT			0
102	KC_AB2	CG17528	-1.27238120872759	0.166181066781076	1	pseudobulk	DESeq2	LRT			0
102	KC_AB2	CG17544	-1.40673172715063	0.587364787535786	1	pseudobulk	DESeq2	LRT			0
102	KC_AB2	CG17556	-0.864310991081923	0.557536737369029	1	pseudobulk	DESeq2	LRT			0
102	KC_AB2	CG17565	-0.621487474067558	0.656783926783925	1	pseudobulk	DESeq2	LRT			0
102	KC_AB2	CG17568	-0.290063816371847	0.881698537689548	1	pseudobulk	DESeq2	LRT			0
102	KC_AB2	CG17574	0.414501402739449	0.691569883295192	1	pseudobulk	DESeq2	LRT			0
102	KC_AB2	CG17593	0.311466913551275	0.75937131386354	1	pseudobulk	DESeq2	LRT			0
102	KC_AB2	CG17598	1.15914538950102	0.526907502356858	1	pseudobulk	DESeq2	LRT			0
102	KC_AB2	CG17600	1.01373711007917	0.59169164640204	1	pseudobulk	DESeq2	LRT			0
102	KC_AB2	CG17612	-0.631578663457669	0.750664130745045	1	pseudobulk	DESeq2	LRT			0
102	KC_AB2	CG17637	-0.427431139129811	1	1	pseudobulk	DESeq2	LRT			0
102	KC_AB2	CG1764	0.511475075550361	0.592581015388211	1	pseudobulk	DESeq2	LRT			0
102	KC_AB2	CG17652	0.164054477342436	0.842806774525055	1	pseudobulk	DESeq2	LRT			0
102	KC_AB2	CG17660	0.978084434320466	0.589675648799423	1	pseudobulk	DESeq2	LRT			0
102	KC_AB2	CG17669	-0.600547221779133	0.841940696994101	1	pseudobulk	DESeq2	LRT			0
102	KC_AB2	CG17683	-1.29786825733546	0.252266103025902	1	pseudobulk	DESeq2	LRT			0
102	KC_AB2	CG17684	-0.204814329754479	0.470550988269049	1	pseudobulk	DESeq2	LRT			0
102	KC_AB2	CG17691	-2.24984370964259	0.323653956973513	1	pseudobulk	DESeq2	LRT			0
102	KC_AB2	CG17698	0.0770924786995257	0.888511427168402	1	pseudobulk	DESeq2	LRT			0
102	KC_AB2	CG17707	-0.600547221779133	0.841940696994101	1	pseudobulk	DESeq2	LRT			0

102	KC_AB2	CG17712	-0.125471416539298	0.872719208024247	1	pseudobulk	DESeq2	LRT	0
102	KC_AB2	CG17715	0.0706397009552882	0.921327371222487	1	pseudobulk	DESeq2	LRT	0
102	KC_AB2	CG17716	1.43191469273003	0.651609831456347	1	pseudobulk	DESeq2	LRT	0
102	KC_AB2	CG17721	-0.592481036256484	0.706847545932384	1	pseudobulk	DESeq2	LRT	0
102	KC_AB2	CG17724	0.380012323533951	0.555633373350145	1	pseudobulk	DESeq2	LRT	0
102	KC_AB2	CG17726	-2.06407076736304	0.362444518317458	1	pseudobulk	DESeq2	LRT	0
102	KC_AB2	CG17734	0.0539380033473886	0.860951133848614	1	pseudobulk	DESeq2	LRT	0
102	KC_AB2	CG17746	0.0184670635050711	0.959065624404202	1	pseudobulk	DESeq2	LRT	0
102	KC_AB2	CG17754	0.631166171275459	0.671522600747857	1	pseudobulk	DESeq2	LRT	0
102	KC_AB2	CG17765	-0.114685432513886	0.864817909322271	1	pseudobulk	DESeq2	LRT	0
102	KC_AB2	CG17776	0.115204085769276	0.887140968486966	1	pseudobulk	DESeq2	LRT	0
102	KC_AB2	CG17778	-0.493832926266234	0.515089023468849	1	pseudobulk	DESeq2	LRT	0
102	KC_AB2	CG17786	3.17195708651769	0.15530090920866	1	pseudobulk	DESeq2	LRT	0
102	KC_AB2	CG17802	-0.600547221779134	1	1	pseudobulk	DESeq2	LRT	0
102	KC_AB2	CG17803	0.189813678325555	1	1	pseudobulk	DESeq2	LRT	0
102	KC_AB2	CG17806	-0.427431139129811	1	1	pseudobulk	DESeq2	LRT	0
102	KC_AB2	CG17807	-1.84905028965745	0.445198155344163	1	pseudobulk	DESeq2	LRT	0
102	KC_AB2	CG17816	0.39275542528466	0.143239562078653	1	pseudobulk	DESeq2	LRT	0
102	KC_AB2	CG17829	-0.946346179677933	0.645187518702097	1	pseudobulk	DESeq2	LRT	0
102	KC_AB2	CG17839	1.00588749571511	0.0384871802319856	1	pseudobulk	DESeq2	LRT	0
102	KC_AB2	CG17841	-0.209342807201392	0.46930187359327	1	pseudobulk	DESeq2	LRT	0
102	KC_AB2	CG17883	0.445730967025093	0.486269647948988	1	pseudobulk	DESeq2	LRT	0
102	KC_AB2	CG1789	-0.638893724814917	0.818738110341823	1	pseudobulk	DESeq2	LRT	0
102	KC_AB2	CG17896	-1.07776915880798	0.775491075088671	1	pseudobulk	DESeq2	LRT	0
102	KC_AB2	CG17904	-2.4614989296155	0.141504452461259	1	pseudobulk	DESeq2	LRT	0
102	KC_AB2	CG17919	-1.63925793886161	0.546290859679341	1	pseudobulk	DESeq2	LRT	0
102	KC_AB2	CG1792	0.945107916771697	0.500247078932519	1	pseudobulk	DESeq2	LRT	0
102	KC_AB2	CG17931	0.631375901422047	0.571708622985527	1	pseudobulk	DESeq2	LRT	0
102	KC_AB2	CG17977	-1.16116875819275	0.736255625566845	1	pseudobulk	DESeq2	LRT	0
102	KC_AB2	CG17985	-2.3627362995981	0.281646618575938	1	pseudobulk	DESeq2	LRT	0
102	KC_AB2	CG17996	-0.466723165886526	0.651753332306742	1	pseudobulk	DESeq2	LRT	0
102	KC_AB2	CG18004	-1.79042605033057	0.33570609509706	1	pseudobulk	DESeq2	LRT	0
102	KC_AB2	CG18011	-0.841882716738579	1	1	pseudobulk	DESeq2	LRT	0
102	KC_AB2	CG18048	-1.54470851095603	0.55301747381519	1	pseudobulk	DESeq2	LRT	0
102	KC_AB2	CG18081	-0.145160341844869	0.905482154071934	1	pseudobulk	DESeq2	LRT	0
102	KC_AB2	CG1812	-2.02285942584217	0.357240028002837	1	pseudobulk	DESeq2	LRT	0
102	KC_AB2	CG18135	0.412587604557133	0.615136006774484	1	pseudobulk	DESeq2	LRT	0
102	KC_AB2	CG1814	-0.191758086360972	0.766627724115106	1	pseudobulk	DESeq2	LRT	0
102	KC_AB2	CG1815	-0.302639641990549	0.817634780205163	1	pseudobulk	DESeq2	LRT	0
102	KC_AB2	CG18178	1.27496040324747	0.501655537161972	1	pseudobulk	DESeq2	LRT	0
102	KC_AB2	CG1824	-0.689786510989523	0.709532577240393	1	pseudobulk	DESeq2	LRT	0
102	KC_AB2	CG18259	-0.338771435603146	0.816656863441963	1	pseudobulk	DESeq2	LRT	0
102	KC_AB2	CG18262	-0.173873523005693	0.922926085715966	1	pseudobulk	DESeq2	LRT	0
102	KC_AB2	CG18265	-0.216377501480409	0.714889318034441	1	pseudobulk	DESeq2	LRT	0
102	KC_AB2	CG1827	-0.600547221779133	0.841940696994101	1	pseudobulk	DESeq2	LRT	0
102	KC_AB2	CG18273	0.713982351041985	0.921569637923553	1	pseudobulk	DESeq2	LRT	0
102	KC_AB2	CG18281	0.70378358684898	0.932301451973468	1	pseudobulk	DESeq2	LRT	0
102	KC_AB2	CG18324	-1.45304901990099	0.338075685843113	1	pseudobulk	DESeq2	LRT	0
102	KC_AB2	CG18327	2.12684015898615	0.445939223035924	1	pseudobulk	DESeq2	LRT	0
102	KC_AB2	CG18336	-0.600547221779133	0.841940696994101	1	pseudobulk	DESeq2	LRT	0
102	KC_AB2	CG1840	-0.0842035680160362	0.95034678905991	1	pseudobulk	DESeq2	LRT	0
102	KC_AB2	CG18420	-0.600547221779134	1	1	pseudobulk	DESeq2	LRT	0
102	KC_AB2	CG18428	-0.063074530309406	0.858813714906678	1	pseudobulk	DESeq2	LRT	0

102	KC_AB2	CG18467	0.230210909968943	0.541148970513946	1	pseudobulk	DESeq2	LRT	0
102	KC_AB2	CG1847	0.37690128527012	0.664849921614294	1	pseudobulk	DESeq2	LRT	0
102	KC_AB2	CG18476	-1.3723417613553	0.619532036522	1	pseudobulk	DESeq2	LRT	0
102	KC_AB2	CG18508	0.103544724721986	0.875370627901637	1	pseudobulk	DESeq2	LRT	0
102	KC_AB2	CG18528	0.212754629509105	0.843360808043461	1	pseudobulk	DESeq2	LRT	0
102	KC_AB2	CG18549	1.26307719581879	0.569064211306759	1	pseudobulk	DESeq2	LRT	0
102	KC_AB2	CG18596	-1.12785316439435	0.661319045116135	1	pseudobulk	DESeq2	LRT	0
102	KC_AB2	CG18598	-0.427431139129811	1	1	pseudobulk	DESeq2	LRT	0
102	KC_AB2	CG18600	0.0163320842711025	1	1	pseudobulk	DESeq2	LRT	0
102	KC_AB2	CG18628	-3.92575021003544	0.359779808874255	1	pseudobulk	DESeq2	LRT	0
102	KC_AB2	CG18649	-0.600547221779133	0.841940696994101	1	pseudobulk	DESeq2	LRT	0
102	KC_AB2	CG18659	-1.22062910604874	0.332629907572524	1	pseudobulk	DESeq2	LRT	0
102	KC_AB2	CG18661	0.855953920316062	0.639291761619508	1	pseudobulk	DESeq2	LRT	0
102	KC_AB2	CG18675	4.4900996670122	0.0102920185460301	1	pseudobulk	DESeq2	LRT	0
102	KC_AB2	CG18731	-2.12967468770992	0.340688355153583	1	pseudobulk	DESeq2	LRT	0
102	KC_AB2	CG18764	-0.427431139129811	1	1	pseudobulk	DESeq2	LRT	0
102	KC_AB2	CG18766	0.563305363206599	0.569937792326304	1	pseudobulk	DESeq2	LRT	0
102	KC_AB2	CG18787	0.183555857030446	1	1	pseudobulk	DESeq2	LRT	0
102	KC_AB2	CG18789	0.864341462967788	0.925947094335722	1	pseudobulk	DESeq2	LRT	0
103	KC_AB2	CG18809	-1.81817250360743	0.31760599657489	1	pseudobulk	DESeq2	LRT	0
103	KC_AB2	CG18815	-0.0442399576933378	0.804206969143439	1	pseudobulk	DESeq2	LRT	0
103	KC_AB2	CG1882	-0.0661253606101283	0.868398449146627	1	pseudobulk	DESeq2	LRT	0
103	KC_AB2	CG18858	-0.592481036256484	0.706847545932384	1	pseudobulk	DESeq2	LRT	0
103	KC_AB2	CG18870	1.12485409509294	0.408096023194889	1	pseudobulk	DESeq2	LRT	0
103	KC_AB2	CG1888	-2.32695906920448	0.186898397890132	1	pseudobulk	DESeq2	LRT	0
103	KC_AB2	CG1896	-1.21718423682306	0.341622946714914	1	pseudobulk	DESeq2	LRT	0
103	KC_AB2	CG1902	-0.620672294547888	0.582064333672046	1	pseudobulk	DESeq2	LRT	0
103	KC_AB2	CG1907	0.0410507445446318	0.906377147919955	1	pseudobulk	DESeq2	LRT	0
103	KC_AB2	CG1909	-0.142980775567277	0.721368403860051	1	pseudobulk	DESeq2	LRT	0
103	KC_AB2	CG1910	-0.0889129066163655	0.879604077398431	1	pseudobulk	DESeq2	LRT	0
103	KC_AB2	CG1927	-0.0812448209993101	0.8887642318531	1	pseudobulk	DESeq2	LRT	0
103	KC_AB2	CG1943	-0.208038715474437	0.583962800953529	1	pseudobulk	DESeq2	LRT	0
103	KC_AB2	CG1946	-0.427431139129811	1	1	pseudobulk	DESeq2	LRT	0
103	KC_AB2	CG1951	0.931539725855514	0.485038944080291	1	pseudobulk	DESeq2	LRT	0
103	KC_AB2	CG1965	0.925998566940998	0.259281369756225	1	pseudobulk	DESeq2	LRT	0
103	KC_AB2	CG1983	0.233337576596852	0.843214394467505	1	pseudobulk	DESeq2	LRT	0
103	KC_AB2	CG1998	0.326302185030443	0.552819588961403	1	pseudobulk	DESeq2	LRT	0
103	KC_AB2	CG2006	-1.85733156562156	0.448702818121367	1	pseudobulk	DESeq2	LRT	0
103	KC_AB2	CG2017	0.376936260701673	0.697539291483562	1	pseudobulk	DESeq2	LRT	0
103	KC_AB2	CG2021	-0.270271373462586	0.829468381294659	1	pseudobulk	DESeq2	LRT	0
103	KC_AB2	CG2023	-1.93210948743547	0.291583645102348	1	pseudobulk	DESeq2	LRT	0
103	KC_AB2	CG2034	1.64759826860276	0.469402419161652	1	pseudobulk	DESeq2	LRT	0
103	KC_AB2	CG2059	-0.223714255437344	0.853048761794787	1	pseudobulk	DESeq2	LRT	0
103	KC_AB2	CG2061	-1.12445031492436	0.215471221574185	1	pseudobulk	DESeq2	LRT	0
103	KC_AB2	CG2063	0.0413362970451304	0.902872569883266	1	pseudobulk	DESeq2	LRT	0
103	KC_AB2	CG2064	1.5243995994652	0.741477491078038	1	pseudobulk	DESeq2	LRT	0
103	KC_AB2	CG2076	-1.58008548963187	0.419276379693269	1	pseudobulk	DESeq2	LRT	0
103	KC_AB2	CG2082	2.12684015898615	0.445939223035924	1	pseudobulk	DESeq2	LRT	0
103	KC_AB2	CG2091	-1.17911912095232	0.551525988478076	1	pseudobulk	DESeq2	LRT	0
103	KC_AB2	CG2100	1.5243995994652	0.741477491078038	1	pseudobulk	DESeq2	LRT	0
103	KC_AB2	CG2111	0.0163320842711025	1	1	pseudobulk	DESeq2	LRT	0
103	KC_AB2	CG2116	0.0471936707755822	0.953916869426286	1	pseudobulk	DESeq2	LRT	0
103	KC_AB2	CG2121	0.713982351041985	0.921569637923553	1	pseudobulk	DESeq2	LRT	0

103	KC_AB2	CG2124	-1.40673172715063	0.587364787535786	1	pseudobulk	DESeq2	LRT		0
103	KC_AB2	CG2126	-1.11096679211408	0.579551054175852	1	pseudobulk	DESeq2	LRT		0
103	KC_AB2	CG2129	-0.427431139129811	1	1	pseudobulk	DESeq2	LRT		0
103	KC_AB2	CG2147	-0.588612193849309	0.728877075024375	1	pseudobulk	DESeq2	LRT		0
103	KC_AB2	CG2162	-2.72158281988503	0.0861952952604964	1	pseudobulk	DESeq2	LRT		0
103	KC_AB2	CG2182	-0.508619613835208	0.52820044105289	1	pseudobulk	DESeq2	LRT		0
103	KC_AB2	CG2186	-0.0898078525866969	0.848474308379083	1	pseudobulk	DESeq2	LRT		0
103	KC_AB2	CG2199	-1.93950607598024	0.29674388380508	1	pseudobulk	DESeq2	LRT		0
103	KC_AB2	CG2200	1.47129758695777	0.269798101882687	1	pseudobulk	DESeq2	LRT		0
103	KC_AB2	CG2201	-2.30955060491846	0.323436524908491	1	pseudobulk	DESeq2	LRT		0
103	KC_AB2	CG2202	-0.382929749762112	0.851961810578875	1	pseudobulk	DESeq2	LRT		0
103	KC_AB2	CG2209	-2.30493359034205	0.318713340463956	1	pseudobulk	DESeq2	LRT		0
103	KC_AB2	CG2211	1.5243995994652	0.741477491078038	1	pseudobulk	DESeq2	LRT		0
103	KC_AB2	CG2218	-1.83732545811997	0.320183338854187	1	pseudobulk	DESeq2	LRT		0
103	KC_AB2	CG2224	0.376879668789492	0.794741453238472	1	pseudobulk	DESeq2	LRT		0
103	KC_AB2	CG2225	-0.0362249881750668	0.881776107510486	1	pseudobulk	DESeq2	LRT		0
103	KC_AB2	CG2246	0.201383987974341	0.77968709626295	1	pseudobulk	DESeq2	LRT		0
103	KC_AB2	CG2247	0.859393490148729	0.631990595266451	1	pseudobulk	DESeq2	LRT		0
103	KC_AB2	CG2258	0.243557613127211	0.767220180022342	1	pseudobulk	DESeq2	LRT		0
103	KC_AB2	CG2260	-0.493357239699781	0.783807521297462	1	pseudobulk	DESeq2	LRT		0
103	KC_AB2	CG2269	-0.0441607719039792	0.838531231018714	1	pseudobulk	DESeq2	LRT		0
103	KC_AB2	CG2277	-0.382438560856989	0.820463296322043	1	pseudobulk	DESeq2	LRT		0
103	KC_AB2	CG2278	-0.117326976251101	0.842055699943014	1	pseudobulk	DESeq2	LRT		0
103	KC_AB2	CG2321	-0.841882716738579	1	1	pseudobulk	DESeq2	LRT		0
103	KC_AB2	CG2519	-0.706396543903827	0.435072298084237	1	pseudobulk	DESeq2	LRT		0
103	KC_AB2	CG2528	2.12684015898615	0.445939223035924	1	pseudobulk	DESeq2	LRT		0
103	KC_AB2	CG2540	0.864341462967788	0.925947094335722	1	pseudobulk	DESeq2	LRT		0
103	KC_AB2	CG2574	1.5243995994652	0.741477491078038	1	pseudobulk	DESeq2	LRT		0
103	KC_AB2	CG2608	0.76984282854887	0.689091656722747	1	pseudobulk	DESeq2	LRT		0
103	KC_AB2	CG2611	-0.946346179677933	0.645187518702097	1	pseudobulk	DESeq2	LRT		0
103	KC_AB2	CG2614	-2.22988039125053	0.338836482281886	1	pseudobulk	DESeq2	LRT		0
103	KC_AB2	CG2617	2.12684015898615	0.445939223035924	1	pseudobulk	DESeq2	LRT		0
103	KC_AB2	CG2656	-0.45725565689515	0.807848404541048	1	pseudobulk	DESeq2	LRT		0
103	KC_AB2	CG2680	0.613352576708142	0.592848971245876	1	pseudobulk	DESeq2	LRT		0
103	KC_AB2	CG2685	-0.0852743682753834	0.917718671153184	1	pseudobulk	DESeq2	LRT		0
103	KC_AB2	CG2691	-1.07776915880798	0.775491075088671	1	pseudobulk	DESeq2	LRT		0
103	KC_AB2	CG2698	-0.212997795401169	0.855522667989675	1	pseudobulk	DESeq2	LRT		0
103	KC_AB2	CG2701	0.624501317525968	0.612400371696688	1	pseudobulk	DESeq2	LRT		0
103	KC_AB2	CG2747	-0.732633544020219	0.437033530148327	1	pseudobulk	DESeq2	LRT		0
103	KC_AB2	CG2765	0.213289382378275	0.786314677233963	1	pseudobulk	DESeq2	LRT		0
103	KC_AB2	CG2767	-0.841882716738579	1	1	pseudobulk	DESeq2	LRT		0
103	KC_AB2	CG2772	-0.14644173313968	0.812267360217892	1	pseudobulk	DESeq2	LRT		0
103	KC_AB2	CG2790	-1.53064326013361	0.439237031116924	1	pseudobulk	DESeq2	LRT		0
103	KC_AB2	CG2794	-0.600547221779134	1	1	pseudobulk	DESeq2	LRT		0
103	KC_AB2	CG2811	-1.77341819080315	0.230601796347209	1	pseudobulk	DESeq2	LRT		0
103	KC_AB2	CG2812	1.5243995994652	0.741477491078038	1	pseudobulk	DESeq2	LRT		0
103	KC_AB2	CG2818	0.488977541964243	0.595446790208887	1	pseudobulk	DESeq2	LRT		0
103	KC_AB2	CG2841	-1.66828165461268	0.531269883399465	1	pseudobulk	DESeq2	LRT		0
103	KC_AB2	CG2846	0.953874023609845	0.491570089011346	1	pseudobulk	DESeq2	LRT		0
103	KC_AB2	CG2852	-0.0775945606973409	0.868321985177407	1	pseudobulk	DESeq2	LRT		0
103	KC_AB2	CG2865	-0.428179314397154	0.331927004358186	1	pseudobulk	DESeq2	LRT		0
103	KC_AB2	CG2875	0.713982351041985	0.921569637923553	1	pseudobulk	DESeq2	LRT		0
103	KC_AB2	CG2889	0.508489170660969	0.863841523886068	1	pseudobulk	DESeq2	LRT		0

103	KC_AB2	CG2906	0.613659617664411	0.76686169970757	1	pseudobulk	DESeq2	LRT	0
103	KC_AB2	CG2909	0.715179995500779	0.755051078020394	1	pseudobulk	DESeq2	LRT	0
103	KC_AB2	CG2911	0.864341462967788	0.925947094335722	1	pseudobulk	DESeq2	LRT	0
103	KC_AB2	CG2915	0.892659559600706	0.712043317464255	1	pseudobulk	DESeq2	LRT	0
103	KC_AB2	CG2918	0.67804189239458	0.608390601481178	1	pseudobulk	DESeq2	LRT	0
103	KC_AB2	CG2921	-0.261028581146792	0.777786707819808	1	pseudobulk	DESeq2	LRT	0
103	KC_AB2	CG2924	-0.135665150027052	0.785802411106099	1	pseudobulk	DESeq2	LRT	0
103	KC_AB2	CG2926	0.619299643764501	0.347850917937018	1	pseudobulk	DESeq2	LRT	0
103	KC_AB2	CG2931	0.776909560310074	0.499968263076488	1	pseudobulk	DESeq2	LRT	0
103	KC_AB2	CG2938	-0.672618927747464	0.707148843902201	1	pseudobulk	DESeq2	LRT	0
103	KC_AB2	CG2972	2.51068564084663	0.263718309739592	1	pseudobulk	DESeq2	LRT	0
103	KC_AB2	CG2991	0.942330641587687	0.253114926893822	1	pseudobulk	DESeq2	LRT	0
103	KC_AB2	CG2993	-0.383801146091059	0.49517943873121	1	pseudobulk	DESeq2	LRT	0
104	KC_AB2	CG30008	2.12684015898615	0.445939223035924	1	pseudobulk	DESeq2	LRT	0
104	KC_AB2	CG30010	0.623447689168001	0.707182629626653	1	pseudobulk	DESeq2	LRT	0
104	KC_AB2	CG30015	-1.99153573919778	0.406760593108112	1	pseudobulk	DESeq2	LRT	0
104	KC_AB2	CG30020	-1.12785316439435	0.661319045116135	1	pseudobulk	DESeq2	LRT	0
104	KC_AB2	CG30054	0.864341462967788	0.925947094335722	1	pseudobulk	DESeq2	LRT	0
104	KC_AB2	CG30060	-1.11096679211408	0.579551054175852	1	pseudobulk	DESeq2	LRT	0
104	KC_AB2	CG30069	-0.209808076863057	0.877444409505505	1	pseudobulk	DESeq2	LRT	0
104	KC_AB2	CG3008	-0.134866061918005	0.930289006564755	1	pseudobulk	DESeq2	LRT	0
104	KC_AB2	CG30089	0.388806698653097	0.528446568420418	1	pseudobulk	DESeq2	LRT	0
104	KC_AB2	CG3009	0.487664652337637	0.570451010888906	1	pseudobulk	DESeq2	LRT	0
104	KC_AB2	CG30094	-0.285256418039142	0.845250603657592	1	pseudobulk	DESeq2	LRT	0
104	KC_AB2	CG30096	-1.07776915880798	0.775491075088671	1	pseudobulk	DESeq2	LRT	0
104	KC_AB2	CG30109	-1.37866851291071	0.486618970380267	1	pseudobulk	DESeq2	LRT	0
104	KC_AB2	CG30110	0.864341462967788	0.925947094335722	1	pseudobulk	DESeq2	LRT	0
104	KC_AB2	CG30116	0.578355261113754	0.0966608549043187	1	pseudobulk	DESeq2	LRT	0
104	KC_AB2	CG30122	0.090354248321592	0.897760866276897	1	pseudobulk	DESeq2	LRT	0
104	KC_AB2	CG30156	1.16188189401431	0.540741154759098	1	pseudobulk	DESeq2	LRT	0
104	KC_AB2	CG30158	-0.0728826723912896	0.78737803967456	1	pseudobulk	DESeq2	LRT	0
104	KC_AB2	CG30172	0.0448921420446055	0.896259020128752	1	pseudobulk	DESeq2	LRT	0
104	KC_AB2	CG30183	-2.02285942584217	0.357240028002837	1	pseudobulk	DESeq2	LRT	0
104	KC_AB2	CG30197	-1.12785316439435	0.661319045116135	1	pseudobulk	DESeq2	LRT	0
104	KC_AB2	CG3021	-1.54470851095603	0.55301747381519	1	pseudobulk	DESeq2	LRT	0
104	KC_AB2	CG30270	-0.427431139129811	1	1	pseudobulk	DESeq2	LRT	0
104	KC_AB2	CG30285	1.5243995994652	0.741477491078038	1	pseudobulk	DESeq2	LRT	0
104	KC_AB2	CG30291	-0.347254644246228	0.805764202656959	1	pseudobulk	DESeq2	LRT	0
104	KC_AB2	CG30334	1.5243995994652	0.741477491078038	1	pseudobulk	DESeq2	LRT	0
104	KC_AB2	CG30338	1.5243995994652	0.741477491078038	1	pseudobulk	DESeq2	LRT	0
104	KC_AB2	CG30344	0.864341462967788	0.925947094335722	1	pseudobulk	DESeq2	LRT	0
104	KC_AB2	CG30349	-1.54470851095603	0.55301747381519	1	pseudobulk	DESeq2	LRT	0
104	KC_AB2	CG30356	0.864341462967788	0.925947094335722	1	pseudobulk	DESeq2	LRT	0
104	KC_AB2	CG30373	0.167243719964387	0.843320955812055	1	pseudobulk	DESeq2	LRT	0
104	KC_AB2	CG3038	1.5243995994652	0.741477491078038	1	pseudobulk	DESeq2	LRT	0
104	KC_AB2	CG30389	-0.0887204186859187	0.837482244401782	1	pseudobulk	DESeq2	LRT	0
104	KC_AB2	CG30392	-0.385330686915802	0.749666376971994	1	pseudobulk	DESeq2	LRT	0
104	KC_AB2	CG30394	0.590705330521892	0.748095145085763	1	pseudobulk	DESeq2	LRT	0
104	KC_AB2	CG3040	-1.04028567568019	0.462854713694476	1	pseudobulk	DESeq2	LRT	0
104	KC_AB2	CG30403	-0.427431139129811	1	1	pseudobulk	DESeq2	LRT	0
104	KC_AB2	CG30419	0.244694246541121	0.51546541779804	1	pseudobulk	DESeq2	LRT	0
104	KC_AB2	CG30423	-0.333432790375655	0.514182080008138	1	pseudobulk	DESeq2	LRT	0
104	KC_AB2	CG30428	-0.737825428894362	0.308249079648519	1	pseudobulk	DESeq2	LRT	0

104	KC_AB2	CG3045	-1.51928970377954	0.486908946651791	1	pseudobulk	DESeq2	LRT		0
104	KC_AB2	CG30460	2.12684015898615	0.445939223035924	1	pseudobulk	DESeq2	LRT		0
104	KC_AB2	CG30466	-0.427431139129811	1	1	pseudobulk	DESeq2	LRT		0
104	KC_AB2	CG30467	0.374717669693449	0.792923075639426	1	pseudobulk	DESeq2	LRT		0
104	KC_AB2	CG30472	1.41392113638259	0.617674323943697	1	pseudobulk	DESeq2	LRT		0
104	KC_AB2	CG30480	0.222396124134733	1	1	pseudobulk	DESeq2	LRT		0
104	KC_AB2	CG30491	-2.25375902476868	0.307909557410438	1	pseudobulk	DESeq2	LRT		0
104	KC_AB2	CG30495	1.5243995994652	0.741477491078038	1	pseudobulk	DESeq2	LRT		0
104	KC_AB2	CG30496	2.12684015898615	0.445939223035924	1	pseudobulk	DESeq2	LRT		0
104	KC_AB2	CG3061	-0.241559331196383	0.752023759460365	1	pseudobulk	DESeq2	LRT		0
104	KC_AB2	CG3065	-0.831304672821689	0.614141931635772	1	pseudobulk	DESeq2	LRT		0
104	KC_AB2	CG3078	0.559565011356637	0.511525275167158	1	pseudobulk	DESeq2	LRT		0
104	KC_AB2	CG3097	-0.427431139129811	1	1	pseudobulk	DESeq2	LRT		0
104	KC_AB2	CG31030	-0.187870892247831	0.584693415227452	1	pseudobulk	DESeq2	LRT		0
104	KC_AB2	CG31038	-0.609256093348873	0.729816784107073	1	pseudobulk	DESeq2	LRT		0
104	KC_AB2	CG3104	0.543154539811717	0.444363737852558	1	pseudobulk	DESeq2	LRT		0
104	KC_AB2	CG31041	-1.07776915880798	0.775491075088671	1	pseudobulk	DESeq2	LRT		0
104	KC_AB2	CG31051	-0.413608693999222	0.689022522455316	1	pseudobulk	DESeq2	LRT		0
104	KC_AB2	CG31064	-0.19800640787978	0.74088687582651	1	pseudobulk	DESeq2	LRT		0
104	KC_AB2	CG3107	0.644008720425144	0.35978311478939	1	pseudobulk	DESeq2	LRT		0
104	KC_AB2	CG31098	-0.39917336535616	0.810147819664692	1	pseudobulk	DESeq2	LRT		0
104	KC_AB2	CG31109	-0.656025717506732	0.594706701270014	1	pseudobulk	DESeq2	LRT		0
104	KC_AB2	CG31111	-1.12785316439435	0.661319045116135	1	pseudobulk	DESeq2	LRT		0
104	KC_AB2	CG31122	0.47469005480611	0.632534879923667	1	pseudobulk	DESeq2	LRT		0
104	KC_AB2	CG31125	-0.705781503434747	0.669589432045155	1	pseudobulk	DESeq2	LRT		0
104	KC_AB2	CG31126	-1.34001049951678	0.189088664378885	1	pseudobulk	DESeq2	LRT		0
104	KC_AB2	CG31128	-0.600547221779134	1	1	pseudobulk	DESeq2	LRT		0
104	KC_AB2	CG31140	-0.208880090908878	0.404874051468554	1	pseudobulk	DESeq2	LRT		0
104	KC_AB2	CG31142	-1.84905028965745	0.445198155344163	1	pseudobulk	DESeq2	LRT		0
104	KC_AB2	CG31145	-1.40673172715063	0.587364787535786	1	pseudobulk	DESeq2	LRT		0
104	KC_AB2	CG31156	-1.07776915880798	0.775491075088671	1	pseudobulk	DESeq2	LRT		0
104	KC_AB2	CG31183	-0.600547221779134	1	1	pseudobulk	DESeq2	LRT		0
104	KC_AB2	CG31191	0.131441203062051	0.771925238641129	1	pseudobulk	DESeq2	LRT		0
104	KC_AB2	CG31195	-0.94307066344797	0.629943854378022	1	pseudobulk	DESeq2	LRT		0
104	KC_AB2	CG31200	-0.427431139129811	1	1	pseudobulk	DESeq2	LRT		0
104	KC_AB2	CG31211	0.321068051871346	0.620112045094567	1	pseudobulk	DESeq2	LRT		0
104	KC_AB2	CG31221	-0.256249205023958	0.218326000467717	1	pseudobulk	DESeq2	LRT		0
104	KC_AB2	CG31224	0.0587451345182143	0.916292019862862	1	pseudobulk	DESeq2	LRT		0
104	KC_AB2	CG31226	2.12684015898615	0.445939223035924	1	pseudobulk	DESeq2	LRT		0
104	KC_AB2	CG31229	0.101926453465527	0.912214896590882	1	pseudobulk	DESeq2	LRT		0
104	KC_AB2	CG31249	-0.78124650078617	0.591293978914265	1	pseudobulk	DESeq2	LRT		0
104	KC_AB2	CG31300	-0.427431139129811	1	1	pseudobulk	DESeq2	LRT		0
104	KC_AB2	CG31301	-0.250034425709092	0.810340337833663	1	pseudobulk	DESeq2	LRT		0
104	KC_AB2	CG31324	1.6188540710898	0.0878885345607802	1	pseudobulk	DESeq2	LRT		0
104	KC_AB2	CG31342	0.210115559385765	0.820586438502803	1	pseudobulk	DESeq2	LRT		0
104	KC_AB2	CG31344	0.652906972996107	0.140105644375594	1	pseudobulk	DESeq2	LRT		0
104	KC_AB2	CG31345	3.71762459443429	0.0858728353048306	1	pseudobulk	DESeq2	LRT		0
104	KC_AB2	CG31357	-1.61396488220263	0.285705728500956	1	pseudobulk	DESeq2	LRT		0
104	KC_AB2	CG31360	0.952060844262399	0.475645113848659	1	pseudobulk	DESeq2	LRT		0
104	KC_AB2	CG31365	2.00472098532514	0.307389476608588	1	pseudobulk	DESeq2	LRT		0
104	KC_AB2	CG31368	1.21340176778324	0.579318232741745	1	pseudobulk	DESeq2	LRT		0
104	KC_AB2	CG31371	0.70378358684898	0.932301451973468	1	pseudobulk	DESeq2	LRT		0
104	KC_AB2	CG31388	-0.207341539597698	0.773670070054791	1	pseudobulk	DESeq2	LRT		0

104	KC_AB2	CG31457	0.458061275320087	0.802259721126042	1	pseudobulk	DESeq2	LRT	0
104	KC_AB2	CG31460	-0.70727250916738	0.705390475165233	1	pseudobulk	DESeq2	LRT	0
104	KC_AB2	CG31475	-0.0774393009494329	0.852604506316885	1	pseudobulk	DESeq2	LRT	0
104	KC_AB2	CG3149	1.41392113638259	0.617674323943697	1	pseudobulk	DESeq2	LRT	0
104	KC_AB2	CG31510	-0.81981628137141	0.452483871780317	1	pseudobulk	DESeq2	LRT	0
104	KC_AB2	CG31522	1.07104651598766	0.12586717813245	1	pseudobulk	DESeq2	LRT	0
104	KC_AB2	CG31523	0.170709948831413	0.807138367949229	1	pseudobulk	DESeq2	LRT	0
105	KC_AB2	CG31538	0.943586647867311	0.690632779168748	1	pseudobulk	DESeq2	LRT	0
105	KC_AB2	CG31550	-0.0750040698136519	0.914603670508815	1	pseudobulk	DESeq2	LRT	0
105	KC_AB2	CG3156	-0.889913991337288	0.621714276066693	1	pseudobulk	DESeq2	LRT	0
105	KC_AB2	CG3163	-0.56682065025685	0.654402412688627	1	pseudobulk	DESeq2	LRT	0
105	KC_AB2	CG31633	0.536038856924057	0.762696974399166	1	pseudobulk	DESeq2	LRT	0
105	KC_AB2	CG31635	-0.160482942444805	0.722837984204707	1	pseudobulk	DESeq2	LRT	0
105	KC_AB2	CG31637	-0.143530796511214	0.669368908271442	1	pseudobulk	DESeq2	LRT	0
105	KC_AB2	CG31638	-0.565183735334984	0.526900398465814	1	pseudobulk	DESeq2	LRT	0
105	KC_AB2	CG3164	-1.36771957505847	0.50359885895216	1	pseudobulk	DESeq2	LRT	0
105	KC_AB2	CG31648	-0.534087734036669	0.773480946157286	1	pseudobulk	DESeq2	LRT	0
105	KC_AB2	CG3165	-0.620339094770264	0.664453663999398	1	pseudobulk	DESeq2	LRT	0
105	KC_AB2	CG31650	0.328309581771588	0.550749362350259	1	pseudobulk	DESeq2	LRT	0
105	KC_AB2	CG31663	2.1817927492653	0.458599861292202	1	pseudobulk	DESeq2	LRT	0
105	KC_AB2	CG31676	-0.600547221779133	0.841940696994101	1	pseudobulk	DESeq2	LRT	0
105	KC_AB2	CG31678	0.954012380353803	0.395981930574604	1	pseudobulk	DESeq2	LRT	0
105	KC_AB2	CG3168	0.864341462967788	0.925947094335722	1	pseudobulk	DESeq2	LRT	0
105	KC_AB2	CG31687	0.0111143962153818	0.960271571286893	1	pseudobulk	DESeq2	LRT	0
105	KC_AB2	CG31688	-0.158114341720364	0.744684683418883	1	pseudobulk	DESeq2	LRT	0
105	KC_AB2	CG31690	-0.592481036256484	0.706847545932384	1	pseudobulk	DESeq2	LRT	0
105	KC_AB2	CG31694	0.559601626041981	0.550831558873664	1	pseudobulk	DESeq2	LRT	0
105	KC_AB2	CG31704	1.5243995994652	0.741477491078038	1	pseudobulk	DESeq2	LRT	0
105	KC_AB2	CG31706	0.183555857030446	1	1	pseudobulk	DESeq2	LRT	0
105	KC_AB2	CG31712	0.693616764968872	0.138961267483963	1	pseudobulk	DESeq2	LRT	0
105	KC_AB2	CG31715	0.294628189567238	0.664301752525106	1	pseudobulk	DESeq2	LRT	0
105	KC_AB2	CG31717	-0.703913045759954	0.567406936726755	1	pseudobulk	DESeq2	LRT	0
105	KC_AB2	CG31729	0.0999114223590401	0.840556026422174	1	pseudobulk	DESeq2	LRT	0
105	KC_AB2	CG31760	-0.0860237328264441	0.819797495360739	1	pseudobulk	DESeq2	LRT	0
105	KC_AB2	CG31769	1.5243995994652	0.741477491078038	1	pseudobulk	DESeq2	LRT	0
105	KC_AB2	CG31788	-1.9379626606486	0.399953775626921	1	pseudobulk	DESeq2	LRT	0
105	KC_AB2	CG31793	0.222396124134733	1	1	pseudobulk	DESeq2	LRT	0
105	KC_AB2	CG31800	-0.626925960306407	0.67354463090145	1	pseudobulk	DESeq2	LRT	0
105	KC_AB2	CG31808	-0.481829145090256	0.411178460462574	1	pseudobulk	DESeq2	LRT	0
105	KC_AB2	CG31815	0.943586647867311	0.690632779168748	1	pseudobulk	DESeq2	LRT	0
105	KC_AB2	CG31817	2.34817442587308	0.244503819929596	1	pseudobulk	DESeq2	LRT	0
105	KC_AB2	CG3184	1.14814388261213	0.568837005180859	1	pseudobulk	DESeq2	LRT	0
105	KC_AB2	CG31849	2.12684015898615	0.445939223035924	1	pseudobulk	DESeq2	LRT	0
105	KC_AB2	CG31855	-0.483839590792444	0.536072921965867	1	pseudobulk	DESeq2	LRT	0
105	KC_AB2	CG31861	-0.600547221779134	1	1	pseudobulk	DESeq2	LRT	0
105	KC_AB2	CG31886	0.183555857030446	1	1	pseudobulk	DESeq2	LRT	0
105	KC_AB2	CG31897	-1.12475160732157	0.574429473125171	1	pseudobulk	DESeq2	LRT	0
105	KC_AB2	CG31904	-0.600547221779134	1	1	pseudobulk	DESeq2	LRT	0
105	KC_AB2	CG31908	0.986396011852576	0.489306989333767	1	pseudobulk	DESeq2	LRT	0
105	KC_AB2	CG3191	0.190393219485123	0.893175556614257	1	pseudobulk	DESeq2	LRT	0
105	KC_AB2	CG31915	-0.0852743682753834	0.917718671153184	1	pseudobulk	DESeq2	LRT	0
105	KC_AB2	CG31922	0.892659559600706	0.712043317464255	1	pseudobulk	DESeq2	LRT	0
105	KC_AB2	CG31935	1.11471276021981	0.644913768495318	1	pseudobulk	DESeq2	LRT	0

105	KC_AB2	CG31957	-0.0842035680160362	0.95034678905991	1	pseudobulk	DESeq2	LRT		0
105	KC_AB2	CG31974	1.5243995994652	0.741477491078038	1	pseudobulk	DESeq2	LRT		0
105	KC_AB2	CG3198	0.0910717859629327	0.857224030725798	1	pseudobulk	DESeq2	LRT		0
105	KC_AB2	CG31988	-0.600547221779133	0.841940696994101	1	pseudobulk	DESeq2	LRT		0
105	KC_AB2	CG31997	0.279770773112518	0.514532395568306	1	pseudobulk	DESeq2	LRT		0
105	KC_AB2	CG31998	0.626450289830924	0.344798965587291	1	pseudobulk	DESeq2	LRT		0
105	KC_AB2	CG31999	-3.27171621189432	0.0969850763500578	1	pseudobulk	DESeq2	LRT		0
105	KC_AB2	CG32017	-0.586225210410222	0.303748729039573	1	pseudobulk	DESeq2	LRT		0
105	KC_AB2	CG32022	0.713982351041985	0.921569637923553	1	pseudobulk	DESeq2	LRT		0
105	KC_AB2	CG32037	-0.600547221779133	0.841940696994101	1	pseudobulk	DESeq2	LRT		0
105	KC_AB2	CG32039	-1.49468362653554	0.081642664076066	1	pseudobulk	DESeq2	LRT		0
105	KC_AB2	CG32052	-0.427431139129811	1	1	pseudobulk	DESeq2	LRT		0
105	KC_AB2	CG32066	-0.407805285345925	0.111188119019762	1	pseudobulk	DESeq2	LRT		0
105	KC_AB2	CG32069	1.14062395543429	0.508434599770014	1	pseudobulk	DESeq2	LRT		0
105	KC_AB2	CG32075	-0.592481036256484	0.706847545932384	1	pseudobulk	DESeq2	LRT		0
105	KC_AB2	CG32085	-0.0982494894773803	0.842677395909942	1	pseudobulk	DESeq2	LRT		0
105	KC_AB2	CG32095	-2.13580979111316	0.396045572646122	1	pseudobulk	DESeq2	LRT		0
105	KC_AB2	CG32099	-1.28022120945988	0.506214723005637	1	pseudobulk	DESeq2	LRT		0
105	KC_AB2	CG32100	0.249913788134016	0.81017477837707	1	pseudobulk	DESeq2	LRT		0
105	KC_AB2	CG32104	1.5243995994652	0.741477491078038	1	pseudobulk	DESeq2	LRT		0
105	KC_AB2	CG32109	-0.379415893290377	0.694883398730027	1	pseudobulk	DESeq2	LRT		0
105	KC_AB2	CG32112	1.11042604883343	0.559537088780678	1	pseudobulk	DESeq2	LRT		0
105	KC_AB2	CG32137	1.2224385214167	0.489015734827038	1	pseudobulk	DESeq2	LRT		0
105	KC_AB2	CG32147	0.772278413675747	0.668572171405162	1	pseudobulk	DESeq2	LRT		0
105	KC_AB2	CG32163	-0.427431139129811	1	1	pseudobulk	DESeq2	LRT		0
105	KC_AB2	CG32176	-0.0112358251547259	0.94854210423839	1	pseudobulk	DESeq2	LRT		0
105	KC_AB2	CG32187	1.41524016620164	0.505408490038387	1	pseudobulk	DESeq2	LRT		0
105	KC_AB2	CG32202	0.360527522870149	0.753318004781853	1	pseudobulk	DESeq2	LRT		0
105	KC_AB2	CG32204	-0.351732658259481	0.189312779656337	1	pseudobulk	DESeq2	LRT		0
105	KC_AB2	CG32206	0.390118913775669	0.332758650884051	1	pseudobulk	DESeq2	LRT		0
105	KC_AB2	CG3222	-0.427431139129811	1	1	pseudobulk	DESeq2	LRT		0
105	KC_AB2	CG32225	0.15341598651851	0.782267541787164	1	pseudobulk	DESeq2	LRT		0
105	KC_AB2	CG3223	0.624967361818474	0.706215091814379	1	pseudobulk	DESeq2	LRT		0
105	KC_AB2	CG3224	-0.466668803774774	0.698184415995564	1	pseudobulk	DESeq2	LRT		0
105	KC_AB2	CG32243	-1.39477386000456	0.293906830869227	1	pseudobulk	DESeq2	LRT		0
105	KC_AB2	CG3226	0.268917236607019	0.759246222059888	1	pseudobulk	DESeq2	LRT		0
105	KC_AB2	CG32262	0.196479234259133	0.856768340350604	1	pseudobulk	DESeq2	LRT		0
105	KC_AB2	CG32264	-0.365567439567452	0.147322308808054	1	pseudobulk	DESeq2	LRT		0
105	KC_AB2	CG32267	-0.455000269187202	0.39302838619598	1	pseudobulk	DESeq2	LRT		0
105	KC_AB2	CG32276	-0.0708192219841819	0.87643317430768	1	pseudobulk	DESeq2	LRT		0
105	KC_AB2	CG32278	-0.397657868167203	0.461309445259774	1	pseudobulk	DESeq2	LRT		0
105	KC_AB2	CG32280	0.330413740745341	0.724773467336738	1	pseudobulk	DESeq2	LRT		0
105	KC_AB2	CG32281	1.11042604883343	0.559537088780678	1	pseudobulk	DESeq2	LRT		0
105	KC_AB2	CG32333	0.963929122181584	0.475000466683992	1	pseudobulk	DESeq2	LRT		0
105	KC_AB2	CG32365	-0.841882716738579	1	1	pseudobulk	DESeq2	LRT		0
105	KC_AB2	CG32369	0.115129350774409	0.870370466724176	1	pseudobulk	DESeq2	LRT		0
105	KC_AB2	CG32373	-0.389472303413376	0.689763271593774	1	pseudobulk	DESeq2	LRT		0
105	KC_AB2	CG32392	2.49750470281195	0.235076905519563	1	pseudobulk	DESeq2	LRT		0
105	KC_AB2	CG32409	-1.78094334699383	0.470930349923561	1	pseudobulk	DESeq2	LRT		0
105	KC_AB2	CG32425	-0.0650900629830213	0.900357214256412	1	pseudobulk	DESeq2	LRT		0
105	KC_AB2	CG32428	-0.238590038737545	0.768539424290801	1	pseudobulk	DESeq2	LRT		0
105	KC_AB2	CG32432	-0.0349181096154404	0.927417880390263	1	pseudobulk	DESeq2	LRT		0
105	KC_AB2	CG32441	-0.463257063164435	0.48640397664742	1	pseudobulk	DESeq2	LRT		0

105	KC_AB2	CG32448	0.109775929079565	0.898136290451027	1	pseudobulk	DESeq2	LRT	0
106	KC_AB2	CG32452	-1.3723417613553	0.619532036522	1	pseudobulk	DESeq2	LRT	0
106	KC_AB2	CG32459	-0.427431139129811	1	1	pseudobulk	DESeq2	LRT	0
106	KC_AB2	CG32473	2.12684015898615	0.445939223035924	1	pseudobulk	DESeq2	LRT	0
106	KC_AB2	CG32485	0.181439198653134	0.674713058901365	1	pseudobulk	DESeq2	LRT	0
106	KC_AB2	CG32486	-0.354528693053578	0.293787186969154	1	pseudobulk	DESeq2	LRT	0
106	KC_AB2	CG32506	-0.257673172265791	0.799422316878108	1	pseudobulk	DESeq2	LRT	0
106	KC_AB2	CG32521	-3.11558114315342	0.128937242833061	1	pseudobulk	DESeq2	LRT	0
106	KC_AB2	CG3253	-1.64244448697354	0.27363924487546	1	pseudobulk	DESeq2	LRT	0
106	KC_AB2	CG32533	-1.70614408517477	0.52816093424714	1	pseudobulk	DESeq2	LRT	0
106	KC_AB2	CG32536	-0.427431139129811	1	1	pseudobulk	DESeq2	LRT	0
106	KC_AB2	CG32537	0.222396124134733	1	1	pseudobulk	DESeq2	LRT	0
106	KC_AB2	CG32544	-0.792889336172726	0.253357348334239	1	pseudobulk	DESeq2	LRT	0
106	KC_AB2	CG32547	0.297543026715574	0.857938585075858	1	pseudobulk	DESeq2	LRT	0
106	KC_AB2	CG32549	-2.63157369712608	0.0368101766748763	1	pseudobulk	DESeq2	LRT	0
106	KC_AB2	CG32554	0.725498497157359	0.93879047033739	1	pseudobulk	DESeq2	LRT	0
106	KC_AB2	CG3257	0.113608530644814	0.822111621638344	1	pseudobulk	DESeq2	LRT	0
106	KC_AB2	CG32576	0.290677432798505	0.842113648321692	1	pseudobulk	DESeq2	LRT	0
106	KC_AB2	CG32581	3.20650492957429	0.177944556633249	1	pseudobulk	DESeq2	LRT	0
106	KC_AB2	CG32590	1.43423382283507	0.524724861492854	1	pseudobulk	DESeq2	LRT	0
106	KC_AB2	CG3262	-1.57102881340945	0.0566486395575658	1	pseudobulk	DESeq2	LRT	0
106	KC_AB2	CG32638	-1.59432579116312	0.459120231439715	1	pseudobulk	DESeq2	LRT	0
106	KC_AB2	CG32647	0.0714523963482425	0.814585186019123	1	pseudobulk	DESeq2	LRT	0
106	KC_AB2	CG32655	1.18889395892318	0.651704638100559	1	pseudobulk	DESeq2	LRT	0
106	KC_AB2	CG32683	-0.0146256063830009	0.916816567894676	1	pseudobulk	DESeq2	LRT	0
106	KC_AB2	CG3270	0.864341462967788	0.925947094335722	1	pseudobulk	DESeq2	LRT	0
106	KC_AB2	CG32700	-0.271321366484322	0.189533006359756	1	pseudobulk	DESeq2	LRT	0
106	KC_AB2	CG32756	-0.155092008916832	0.855432308034536	1	pseudobulk	DESeq2	LRT	0
106	KC_AB2	CG32767	-0.221475128739689	0.4302173506913	1	pseudobulk	DESeq2	LRT	0
106	KC_AB2	CG32772	0.497634270055804	0.550278919092042	1	pseudobulk	DESeq2	LRT	0
106	KC_AB2	CG32795	-0.67519998837058	0.452150730942392	1	pseudobulk	DESeq2	LRT	0
106	KC_AB2	CG32803	-0.137724360522742	0.915187716550757	1	pseudobulk	DESeq2	LRT	0
106	KC_AB2	CG32808	-1.40673172715063	0.587364787535786	1	pseudobulk	DESeq2	LRT	0
106	KC_AB2	CG32809	0.190243554234318	0.644257748005249	1	pseudobulk	DESeq2	LRT	0
106	KC_AB2	CG3281	-1.07776915880798	0.775491075088671	1	pseudobulk	DESeq2	LRT	0
106	KC_AB2	CG32813	0.426685192676755	0.770147623767993	1	pseudobulk	DESeq2	LRT	0
106	KC_AB2	CG32815	0.323499758381088	0.610799400157319	1	pseudobulk	DESeq2	LRT	0
106	KC_AB2	CG32816	-0.841882716738579	1	1	pseudobulk	DESeq2	LRT	0
106	KC_AB2	CG32850	0.380686394570625	0.414284926925615	1	pseudobulk	DESeq2	LRT	0
106	KC_AB2	CG32856	-0.454932278062884	0.762190045481453	1	pseudobulk	DESeq2	LRT	0
106	KC_AB2	CG3294	0.27224451317105	0.879463751979412	1	pseudobulk	DESeq2	LRT	0
106	KC_AB2	CG32944	-0.841882716738579	1	1	pseudobulk	DESeq2	LRT	0
106	KC_AB2	CG3295	-1.22182103121519	0.534749509500509	1	pseudobulk	DESeq2	LRT	0
106	KC_AB2	CG32982	1.06136351914996	0.230801118595997	1	pseudobulk	DESeq2	LRT	0
106	KC_AB2	CG33054	2.12684015898615	0.445939223035924	1	pseudobulk	DESeq2	LRT	0
106	KC_AB2	CG3308	-0.0715309630154252	1	1	pseudobulk	DESeq2	LRT	0
106	KC_AB2	CG3309	0.243578547264208	0.798563062722021	1	pseudobulk	DESeq2	LRT	0
106	KC_AB2	CG33090	-0.0242595844794031	0.86547387546609	1	pseudobulk	DESeq2	LRT	0
106	KC_AB2	CG33096	-0.073870656886408	0.963551931599223	1	pseudobulk	DESeq2	LRT	0
106	KC_AB2	CG33098	-1.51491138922076	0.440189277482886	1	pseudobulk	DESeq2	LRT	0
106	KC_AB2	CG33107	-2.06407076736304	0.362444518317458	1	pseudobulk	DESeq2	LRT	0
106	KC_AB2	CG33108	0.864341462967788	0.925947094335722	1	pseudobulk	DESeq2	LRT	0
106	KC_AB2	CG33111	0.899269135956093	0.642144625480232	1	pseudobulk	DESeq2	LRT	0

106	KC_AB2	CG33116	-0.3385052867089	0.556173251681845	1	pseudobulk	DESeq2	LRT		0
106	KC_AB2	CG33129	-0.164402158474891	0.745769189512895	1	pseudobulk	DESeq2	LRT		0
106	KC_AB2	CG33144	0.405563861725644	0.500870228947608	1	pseudobulk	DESeq2	LRT		0
106	KC_AB2	CG33155	0.674829464434859	0.816518190918181	1	pseudobulk	DESeq2	LRT		0
106	KC_AB2	CG33156	-0.777879774372922	0.639887304060927	1	pseudobulk	DESeq2	LRT		0
106	KC_AB2	CG33158	1.44249618489118	0.5203038246131	1	pseudobulk	DESeq2	LRT		0
106	KC_AB2	CG33169	0.322022189526554	0.738326601290059	1	pseudobulk	DESeq2	LRT		0
106	KC_AB2	CG33170	-0.517694921364895	0.346403683145849	1	pseudobulk	DESeq2	LRT		0
106	KC_AB2	CG33181	0.160269722455607	0.619284863291007	1	pseudobulk	DESeq2	LRT		0
106	KC_AB2	CG33199	0.368864506622439	0.613802763045444	1	pseudobulk	DESeq2	LRT		0
106	KC_AB2	CG33203	-0.339667053143544	0.763927696266179	1	pseudobulk	DESeq2	LRT		0
106	KC_AB2	CG33213	-0.600547221779134	1	1	pseudobulk	DESeq2	LRT		0
106	KC_AB2	CG33217	0.551325277045612	0.712321324254121	1	pseudobulk	DESeq2	LRT		0
106	KC_AB2	CG33228	2.76205395729416	0.251829971474322	1	pseudobulk	DESeq2	LRT		0
106	KC_AB2	CG33229	0.119178539367068	0.713587977620784	1	pseudobulk	DESeq2	LRT		0
106	KC_AB2	CG33230	0.864341462967788	0.925947094335722	1	pseudobulk	DESeq2	LRT		0
106	KC_AB2	CG33233	-0.600547221779134	1	1	pseudobulk	DESeq2	LRT		0
106	KC_AB2	CG33267	-1.45972214055569	0.452659000139653	1	pseudobulk	DESeq2	LRT		0
106	KC_AB2	CG3328	-0.600547221779134	1	1	pseudobulk	DESeq2	LRT		0
106	KC_AB2	CG33288	2.12684015898615	0.445939223035924	1	pseudobulk	DESeq2	LRT		0
106	KC_AB2	CG33298	-0.344523446935168	0.725326498933815	1	pseudobulk	DESeq2	LRT		0
106	KC_AB2	CG33303	-0.113319884418091	0.859781334007398	1	pseudobulk	DESeq2	LRT		0
106	KC_AB2	CG33310	-0.39917336535616	0.810147819664692	1	pseudobulk	DESeq2	LRT		0
106	KC_AB2	CG33331	-3.12352868074192	0.121845467339742	1	pseudobulk	DESeq2	LRT		0
106	KC_AB2	CG33332	-1.54470851095603	0.55301747381519	1	pseudobulk	DESeq2	LRT		0
106	KC_AB2	CG3335	0.183555857030446	1	1	pseudobulk	DESeq2	LRT		0
106	KC_AB2	CG3337	-0.0967647750159098	0.814617163482459	1	pseudobulk	DESeq2	LRT		0
106	KC_AB2	CG3342	-1.12475160732157	0.574429473125171	1	pseudobulk	DESeq2	LRT		0
106	KC_AB2	CG3345	-0.600547221779134	1	1	pseudobulk	DESeq2	LRT		0
106	KC_AB2	CG33493	0.713982351041985	0.921569637923553	1	pseudobulk	DESeq2	LRT		0
106	KC_AB2	CG33506	-0.740207704288144	0.726967462633097	1	pseudobulk	DESeq2	LRT		0
106	KC_AB2	CG33521	0.773551146205319	0.702323084538382	1	pseudobulk	DESeq2	LRT		0
106	KC_AB2	CG33523	-0.02716387615138	0.791817397540164	1	pseudobulk	DESeq2	LRT		0
106	KC_AB2	CG33543	-0.17001799707889	0.763332856122759	1	pseudobulk	DESeq2	LRT		0
106	KC_AB2	CG3356	-1.50796803119171	0.327125440871517	1	pseudobulk	DESeq2	LRT		0
106	KC_AB2	CG3358	0.207731536122681	0.802102997622261	1	pseudobulk	DESeq2	LRT		0
106	KC_AB2	CG33635	-0.461964843723736	0.710267124636019	1	pseudobulk	DESeq2	LRT		0
106	KC_AB2	CG33640	-0.427431139129811	1	1	pseudobulk	DESeq2	LRT		0
106	KC_AB2	CG33642	-0.427431139129811	1	1	pseudobulk	DESeq2	LRT		0
106	KC_AB2	CG3368	1.11471276021981	0.644913768495318	1	pseudobulk	DESeq2	LRT		0
106	KC_AB2	CG33695	-1.13898311207644	0.50341302473168	1	pseudobulk	DESeq2	LRT		0
106	KC_AB2	CG33700	0.70378358684898	0.932301451973468	1	pseudobulk	DESeq2	LRT		0
106	KC_AB2	CG33774	0.5287710239273	0.512658479219241	1	pseudobulk	DESeq2	LRT		0
106	KC_AB2	CG33792	-0.600547221779133	0.841940696994101	1	pseudobulk	DESeq2	LRT		0
106	KC_AB2	CG3386	0.107479031250535	0.88712690359169	1	pseudobulk	DESeq2	LRT		0
106	KC_AB2	CG33941	0.239648604166778	0.811833887266772	1	pseudobulk	DESeq2	LRT		0
106	KC_AB2	CG33947	-0.116411347810869	0.916848826069363	1	pseudobulk	DESeq2	LRT		0
106	KC_AB2	CG33969	0.222396124134733	1	1	pseudobulk	DESeq2	LRT		0
107	KC_AB2	CG33970	-0.0536607318989657	0.966212996789475	1	pseudobulk	DESeq2	LRT		0
107	KC_AB2	CG33977	-1.18599140876239	0.149148241766063	1	pseudobulk	DESeq2	LRT		0
107	KC_AB2	CG33978	-2.24514585595614	0.341664214848398	1	pseudobulk	DESeq2	LRT		0
107	KC_AB2	CG33993	1.84032404200623	0.19189455857822	1	pseudobulk	DESeq2	LRT		0
107	KC_AB2	CG33998	-1.75385665017519	0.338637401272894	1	pseudobulk	DESeq2	LRT		0

107	KC_AB2	CG34001	-1.70614408517477	0.52816093424714	1	pseudobulk	DESeq2	LRT	0
107	KC_AB2	CG34008	-0.427431139129811	1	1	pseudobulk	DESeq2	LRT	0
107	KC_AB2	CG34015	-0.198369966735485	0.820414674442933	1	pseudobulk	DESeq2	LRT	0
107	KC_AB2	CG3402	-2.37930527043192	0.158461009459792	1	pseudobulk	DESeq2	LRT	0
107	KC_AB2	CG34028	-0.362794625744925	0.741362465373358	1	pseudobulk	DESeq2	LRT	0
107	KC_AB2	CG34039	-1.3929484167291	0.512855059074155	1	pseudobulk	DESeq2	LRT	0
107	KC_AB2	CG34045	0.498593948417392	0.805619674491474	1	pseudobulk	DESeq2	LRT	0
107	KC_AB2	CG3407	-2.02285942584217	0.357240028002837	1	pseudobulk	DESeq2	LRT	0
107	KC_AB2	CG34109	-0.380396221655848	0.800275859940725	1	pseudobulk	DESeq2	LRT	0
107	KC_AB2	CG34116	0.108468324554008	1	1	pseudobulk	DESeq2	LRT	0
107	KC_AB2	CG34117	-0.817061359754372	0.420224289025578	1	pseudobulk	DESeq2	LRT	0
107	KC_AB2	CG34125	-0.26619839014017	0.765226873815151	1	pseudobulk	DESeq2	LRT	0
107	KC_AB2	CG34126	0.698368998295314	0.447734922546575	1	pseudobulk	DESeq2	LRT	0
107	KC_AB2	CG34132	-0.0096312093660293	0.964046034170718	1	pseudobulk	DESeq2	LRT	0
107	KC_AB2	CG34133	-0.541248839257114	0.70929582698467	1	pseudobulk	DESeq2	LRT	0
107	KC_AB2	CG34134	-1.04077127086163	0.605194231525991	1	pseudobulk	DESeq2	LRT	0
107	KC_AB2	CG34148	-0.073870656886408	0.963551931599223	1	pseudobulk	DESeq2	LRT	0
107	KC_AB2	CG34149	0.222396124134733	1	1	pseudobulk	DESeq2	LRT	0
107	KC_AB2	CG34150	0.107479031250535	0.88712690359169	1	pseudobulk	DESeq2	LRT	0
107	KC_AB2	CG34159	-0.399885528630487	0.553470517036215	1	pseudobulk	DESeq2	LRT	0
107	KC_AB2	CG34163	-0.841882716738579	1	1	pseudobulk	DESeq2	LRT	0
107	KC_AB2	CG34174	-1.6150095599849	0.519549602003096	1	pseudobulk	DESeq2	LRT	0
107	KC_AB2	CG34183	-1.62799124195291	0.299218347343067	1	pseudobulk	DESeq2	LRT	0
107	KC_AB2	CG34195	-1.16116875819275	0.736255625566845	1	pseudobulk	DESeq2	LRT	0
107	KC_AB2	CG3420	-0.77458164533582	0.566331788258707	1	pseudobulk	DESeq2	LRT	0
107	KC_AB2	CG34200	-0.204728397925253	0.665507573846935	1	pseudobulk	DESeq2	LRT	0
107	KC_AB2	CG34206	2.12684015898615	0.445939223035924	1	pseudobulk	DESeq2	LRT	0
107	KC_AB2	CG34219	-0.209025479753393	0.66989192643825	1	pseudobulk	DESeq2	LRT	0
107	KC_AB2	CG34228	1.35968315642719	0.485168225381963	1	pseudobulk	DESeq2	LRT	0
107	KC_AB2	CG34229	-0.248014251067743	0.888879863269151	1	pseudobulk	DESeq2	LRT	0
107	KC_AB2	CG34231	0.713982351041985	0.921569637923553	1	pseudobulk	DESeq2	LRT	0
107	KC_AB2	CG34232	-0.427431139129811	1	1	pseudobulk	DESeq2	LRT	0
107	KC_AB2	CG34234	1.11471276021981	0.644913768495318	1	pseudobulk	DESeq2	LRT	0
107	KC_AB2	CG34242	-1.70955857380389	0.354817565104641	1	pseudobulk	DESeq2	LRT	0
107	KC_AB2	CG34250	1.06703342016055	0.170949226015637	1	pseudobulk	DESeq2	LRT	0
107	KC_AB2	CG34293	0.107470577047687	0.898906881908683	1	pseudobulk	DESeq2	LRT	0
107	KC_AB2	CG34309	1.5243995994652	0.741477491078038	1	pseudobulk	DESeq2	LRT	0
107	KC_AB2	CG34310	0.613659617664411	0.76686169970757	1	pseudobulk	DESeq2	LRT	0
107	KC_AB2	CG34325	1.5243995994652	0.741477491078038	1	pseudobulk	DESeq2	LRT	0
107	KC_AB2	CG34330	1.5243995994652	0.741477491078038	1	pseudobulk	DESeq2	LRT	0
107	KC_AB2	CG3434	2.76205395729416	0.251829971474322	1	pseudobulk	DESeq2	LRT	0
107	KC_AB2	CG34347	-0.539665365830432	0.805145917072468	1	pseudobulk	DESeq2	LRT	0
107	KC_AB2	CG34348	-3.38866527417271	0.0814481425502979	1	pseudobulk	DESeq2	LRT	0
107	KC_AB2	CG34351	-0.630394080400611	0.640646125313854	1	pseudobulk	DESeq2	LRT	0
107	KC_AB2	CG34353	0.492234830258253	0.62047473795772	1	pseudobulk	DESeq2	LRT	0
107	KC_AB2	CG34354	-0.266864852905348	0.446903531858582	1	pseudobulk	DESeq2	LRT	0
107	KC_AB2	CG34355	-0.20868547813941	0.690598293481918	1	pseudobulk	DESeq2	LRT	0
107	KC_AB2	CG34357	0.325393731080688	0.519783011951283	1	pseudobulk	DESeq2	LRT	0
107	KC_AB2	CG3436	0.737851663616983	0.496392179930659	1	pseudobulk	DESeq2	LRT	0
107	KC_AB2	CG3437	-0.747852219079764	0.708491663089672	1	pseudobulk	DESeq2	LRT	0
107	KC_AB2	CG34370	-0.267187390779156	0.835895271211131	1	pseudobulk	DESeq2	LRT	0
107	KC_AB2	CG34376	-1.16116875819275	0.736255625566845	1	pseudobulk	DESeq2	LRT	0
107	KC_AB2	CG34384	0.362757974837031	0.631596204423858	1	pseudobulk	DESeq2	LRT	0

107	KC_AB2	CG34401	-0.656158133102401	0.170185010177272	1	pseudobulk	DESeq2	LRT	0
107	KC_AB2	CG34402	-0.30497889896546	0.419941018353918	1	pseudobulk	DESeq2	LRT	0
107	KC_AB2	CG34404	-0.716043006628606	0.495740184880173	1	pseudobulk	DESeq2	LRT	0
107	KC_AB2	CG34408	-1.98854952664738	0.278883695537438	1	pseudobulk	DESeq2	LRT	0
107	KC_AB2	CG34409	0.222396124134733	1	1	pseudobulk	DESeq2	LRT	0
107	KC_AB2	CG34425	-0.427431139129811	1	1	pseudobulk	DESeq2	LRT	0
107	KC_AB2	CG34454	-0.427431139129811	1	1	pseudobulk	DESeq2	LRT	0
107	KC_AB2	CG3448	-1.12785316439435	0.661319045116135	1	pseudobulk	DESeq2	LRT	0
107	KC_AB2	CG3500	-0.875806341768744	0.62420730782344	1	pseudobulk	DESeq2	LRT	0
107	KC_AB2	CG3511	-1.54470851095603	0.55301747381519	1	pseudobulk	DESeq2	LRT	0
107	KC_AB2	CG3520	-0.427431139129811	1	1	pseudobulk	DESeq2	LRT	0
107	KC_AB2	CG3527	-1.99338034826303	0.401583286802675	1	pseudobulk	DESeq2	LRT	0
107	KC_AB2	CG3529	-0.412465294738416	0.708858743633788	1	pseudobulk	DESeq2	LRT	0
107	KC_AB2	CG3530	-0.352188586361324	0.583232347806511	1	pseudobulk	DESeq2	LRT	0
107	KC_AB2	CG3534	1.18889395892318	0.651704638100559	1	pseudobulk	DESeq2	LRT	0
107	KC_AB2	CG3542	-3.74482900982104	0.0530412027452156	1	pseudobulk	DESeq2	LRT	0
107	KC_AB2	CG3548	0.51001099967901	0.571764503291925	1	pseudobulk	DESeq2	LRT	0
107	KC_AB2	CG3552	0.443892390635762	1	1	pseudobulk	DESeq2	LRT	0
107	KC_AB2	CG3556	-0.621192069069662	0.343615652829453	1	pseudobulk	DESeq2	LRT	0
107	KC_AB2	CG3558	-0.196738802868023	0.754939913670935	1	pseudobulk	DESeq2	LRT	0
107	KC_AB2	CG3566	0.118849955980394	0.858689786502747	1	pseudobulk	DESeq2	LRT	0
107	KC_AB2	CG3568	-0.0949529622946533	0.868618193758953	1	pseudobulk	DESeq2	LRT	0
107	KC_AB2	CG3587	0.674459942220112	0.74558757773504	1	pseudobulk	DESeq2	LRT	0
107	KC_AB2	CG3589	0.864341462967788	0.925947094335722	1	pseudobulk	DESeq2	LRT	0
107	KC_AB2	CG3594	1.82013411219004	0.375922530320745	1	pseudobulk	DESeq2	LRT	0
107	KC_AB2	CG3618	-0.141716443384802	0.789999037281938	1	pseudobulk	DESeq2	LRT	0
107	KC_AB2	CG3625	0.164564797165314	0.590040384361548	1	pseudobulk	DESeq2	LRT	0
107	KC_AB2	CG3626	-1.12785316439435	0.661319045116135	1	pseudobulk	DESeq2	LRT	0
107	KC_AB2	CG3632	0.2420598045139	0.807197023645815	1	pseudobulk	DESeq2	LRT	0
107	KC_AB2	CG3634	0.5587771922818	0.669181750731575	1	pseudobulk	DESeq2	LRT	0
107	KC_AB2	CG3638	-0.113150776669499	0.84765489924115	1	pseudobulk	DESeq2	LRT	0
107	KC_AB2	CG3645	0.674459942220112	0.74558757773504	1	pseudobulk	DESeq2	LRT	0
107	KC_AB2	CG3651	-1.25152427095392	0.410368496968226	1	pseudobulk	DESeq2	LRT	0
107	KC_AB2	CG3652	0.0438000453686511	0.9394938932692	1	pseudobulk	DESeq2	LRT	0
107	KC_AB2	CG3662	0.222580122961213	0.497715800319052	1	pseudobulk	DESeq2	LRT	0
107	KC_AB2	CG3678	-0.202975661271382	0.838728130121489	1	pseudobulk	DESeq2	LRT	0
107	KC_AB2	CG3700	-0.427431139129811	1	1	pseudobulk	DESeq2	LRT	0
107	KC_AB2	CG3702	-0.0620821647746945	0.920444068437502	1	pseudobulk	DESeq2	LRT	0
107	KC_AB2	CG3703	-0.130736572244267	0.858581973888284	1	pseudobulk	DESeq2	LRT	0
107	KC_AB2	CG3704	-0.638893724814917	0.818738110341823	1	pseudobulk	DESeq2	LRT	0
107	KC_AB2	CG3713	1.21092382796727	0.637125262974216	1	pseudobulk	DESeq2	LRT	0
107	KC_AB2	CG3719	-1.41210458553491	0.25178470165051	1	pseudobulk	DESeq2	LRT	0
108	KC_AB2	CG3726	0.0234981640340532	0.878560945168135	1	pseudobulk	DESeq2	LRT	0
108	KC_AB2	CG3732	-0.168561826024952	0.777805829188241	1	pseudobulk	DESeq2	LRT	0
108	KC_AB2	CG3735	1.5243995994652	0.741477491078038	1	pseudobulk	DESeq2	LRT	0
108	KC_AB2	CG3740	-1.3723417613553	0.619532036522	1	pseudobulk	DESeq2	LRT	0
108	KC_AB2	CG3744	-0.375809429758283	0.701792508312053	1	pseudobulk	DESeq2	LRT	0
108	KC_AB2	CG3756	-2.25375902476868	0.307909557410438	1	pseudobulk	DESeq2	LRT	0
108	KC_AB2	CG3760	1.36747920939214	0.31903902852787	1	pseudobulk	DESeq2	LRT	0
108	KC_AB2	CG3764	-1.50331603016663	0.431246352444201	1	pseudobulk	DESeq2	LRT	0
108	KC_AB2	CG3776	-0.164188178441572	0.828077406809117	1	pseudobulk	DESeq2	LRT	0
108	KC_AB2	CG3777	-1.21120681873304	0.542862412873055	1	pseudobulk	DESeq2	LRT	0
108	KC_AB2	CG3781	-0.638893724814917	0.818738110341823	1	pseudobulk	DESeq2	LRT	0

108	KC_AB2	CG3792	-0.409432477000056	0.723450531237882	1	pseudobulk	DESeq2	LRT		0
108	KC_AB2	CG3793	0.58421085473091	0.261780766633497	1	pseudobulk	DESeq2	LRT		0
108	KC_AB2	CG3803	-0.476791627912098	0.764225599255553	1	pseudobulk	DESeq2	LRT		0
108	KC_AB2	CG3808	1.23085716405798	0.614623732585597	1	pseudobulk	DESeq2	LRT		0
108	KC_AB2	CG3815	1.64759826860276	0.469402419161652	1	pseudobulk	DESeq2	LRT		0
108	KC_AB2	CG3817	0.725498497157359	0.93879047033739	1	pseudobulk	DESeq2	LRT		0
108	KC_AB2	CG3819	0.899269135956093	0.642144625480232	1	pseudobulk	DESeq2	LRT		0
108	KC_AB2	CG3823	-1.3723417613553	0.619532036522	1	pseudobulk	DESeq2	LRT		0
108	KC_AB2	CG3847	-0.246880080867625	0.883966189422952	1	pseudobulk	DESeq2	LRT		0
108	KC_AB2	CG3857	1.23322816775853	0.613440801106173	1	pseudobulk	DESeq2	LRT		0
108	KC_AB2	CG3860	-0.283463442075296	0.688730068092478	1	pseudobulk	DESeq2	LRT		0
108	KC_AB2	CG3862	1.14814388261213	0.568837005180859	1	pseudobulk	DESeq2	LRT		0
108	KC_AB2	CG3894	0.702593589496404	0.570018904695911	1	pseudobulk	DESeq2	LRT		0
108	KC_AB2	CG3907	0.457843665836626	0.549981849024499	1	pseudobulk	DESeq2	LRT		0
108	KC_AB2	CG3909	-1.12785316439435	0.661319045116135	1	pseudobulk	DESeq2	LRT		0
108	KC_AB2	CG3918	1.73619993266463	0.3112906377858	1	pseudobulk	DESeq2	LRT		0
108	KC_AB2	CG3919	-0.600547221779134	1	1	pseudobulk	DESeq2	LRT		0
108	KC_AB2	CG3955	0.1205713978626	0.75466846302028	1	pseudobulk	DESeq2	LRT		0
108	KC_AB2	CG3961	-0.682270860669584	0.575036598256691	1	pseudobulk	DESeq2	LRT		0
108	KC_AB2	CG3995	-1.85437434640594	0.329186813557381	1	pseudobulk	DESeq2	LRT		0
108	KC_AB2	CG4000	-1.70388801125551	0.376012626446968	1	pseudobulk	DESeq2	LRT		0
108	KC_AB2	CG4004	0.506981234019831	0.677039596305403	1	pseudobulk	DESeq2	LRT		0
108	KC_AB2	CG40045	0.343535580047989	0.445525748668033	1	pseudobulk	DESeq2	LRT		0
108	KC_AB2	CG40191	-0.157214556025032	0.829506745394319	1	pseudobulk	DESeq2	LRT		0
108	KC_AB2	CG4022	0.200889550194162	0.736725199779269	1	pseudobulk	DESeq2	LRT		0
108	KC_AB2	CG40228	-0.351510886668371	0.529819919045496	1	pseudobulk	DESeq2	LRT		0
108	KC_AB2	CG4025	0.691576011972506	0.601466494683019	1	pseudobulk	DESeq2	LRT		0
108	KC_AB2	CG4036	-0.0852743682753834	0.917718671153184	1	pseudobulk	DESeq2	LRT		0
108	KC_AB2	CG4041	0.222396124134733	1	1	pseudobulk	DESeq2	LRT		0
108	KC_AB2	CG4042	-2.89992575416664	0.169147414761037	1	pseudobulk	DESeq2	LRT		0
108	KC_AB2	CG40439	-1.72976330140494	0.41918502381035	1	pseudobulk	DESeq2	LRT		0
108	KC_AB2	CG4045	1.15572398393975	0.592367151364093	1	pseudobulk	DESeq2	LRT		0
108	KC_AB2	CG40472	-0.688614229260297	0.498082544991116	1	pseudobulk	DESeq2	LRT		0
108	KC_AB2	CG4049	0.0173669518264436	0.954562511931616	1	pseudobulk	DESeq2	LRT		0
108	KC_AB2	CG40498	-0.201400636967419	0.549653383589428	1	pseudobulk	DESeq2	LRT		0
108	KC_AB2	CG4074	-1.28960240277274	0.566679180342473	1	pseudobulk	DESeq2	LRT		0
108	KC_AB2	CG4078	0.864341462967788	0.925947094335722	1	pseudobulk	DESeq2	LRT		0
108	KC_AB2	CG4080	0.0921161265570636	0.824312168253946	1	pseudobulk	DESeq2	LRT		0
108	KC_AB2	CG4098	2.12684015898615	0.445939223035924	1	pseudobulk	DESeq2	LRT		0
108	KC_AB2	CG4101	0.737194067405082	0.511410708730012	1	pseudobulk	DESeq2	LRT		0
108	KC_AB2	CG41099	-0.667816917799522	0.409747985041531	1	pseudobulk	DESeq2	LRT		0
108	KC_AB2	CG41128	-0.162302708588635	0.622339007754186	1	pseudobulk	DESeq2	LRT		0
108	KC_AB2	CG4115	-0.600547221779133	0.841940696994101	1	pseudobulk	DESeq2	LRT		0
108	KC_AB2	CG4119	-0.289469814412896	0.828762874114481	1	pseudobulk	DESeq2	LRT		0
108	KC_AB2	CG4133	0.203271161687687	0.774671024271054	1	pseudobulk	DESeq2	LRT		0
108	KC_AB2	CG41378	-1.05160017276764	0.197519042214195	1	pseudobulk	DESeq2	LRT		0
108	KC_AB2	CG4168	0.214987427462135	0.75499037469271	1	pseudobulk	DESeq2	LRT		0
108	KC_AB2	CG4186	0.222396124134733	1	1	pseudobulk	DESeq2	LRT		0
108	KC_AB2	CG4199	0.765807229836984	0.578854226018603	1	pseudobulk	DESeq2	LRT		0
108	KC_AB2	CG42231	0.713982351041985	0.921569637923553	1	pseudobulk	DESeq2	LRT		0
108	KC_AB2	CG42232	-0.0747446750633757	0.848708472311525	1	pseudobulk	DESeq2	LRT		0
108	KC_AB2	CG42233	1.5243995994652	0.741477491078038	1	pseudobulk	DESeq2	LRT		0
108	KC_AB2	CG42235	-0.427431139129811	1	1	pseudobulk	DESeq2	LRT		0

108	KC_AB2	CG42237	-0.427431139129811	1	1	pseudobulk	DESeq2	LRT		0
108	KC_AB2	CG42238	0.0489414506055687	0.833016439054187	1	pseudobulk	DESeq2	LRT		0
108	KC_AB2	CG42239	-0.246880080867625	0.883966189422952	1	pseudobulk	DESeq2	LRT		0
108	KC_AB2	CG42240	-0.5530991855172	0.734046349074196	1	pseudobulk	DESeq2	LRT		0
108	KC_AB2	CG42258	-0.506020442258302	0.220999766815924	1	pseudobulk	DESeq2	LRT		0
108	KC_AB2	CG42259	-0.600547221779134	1	1	pseudobulk	DESeq2	LRT		0
108	KC_AB2	CG42260	0.471392278202225	0.115808968212488	1	pseudobulk	DESeq2	LRT		0
108	KC_AB2	CG42266	1.5243995994652	0.741477491078038	1	pseudobulk	DESeq2	LRT		0
108	KC_AB2	CG42268	-0.339589784407315	0.450627900178208	1	pseudobulk	DESeq2	LRT		0
108	KC_AB2	CG42271	0.279141948228911	0.827912278911508	1	pseudobulk	DESeq2	LRT		0
108	KC_AB2	CG4230	-0.292442828543352	0.51775292370461	1	pseudobulk	DESeq2	LRT		0
108	KC_AB2	CG42307	-2.24514585595614	0.341664214848398	1	pseudobulk	DESeq2	LRT		0
108	KC_AB2	CG42322	0.224796107766855	0.556034913232506	1	pseudobulk	DESeq2	LRT		0
108	KC_AB2	CG42324	-0.718745116919672	0.141817768581327	1	pseudobulk	DESeq2	LRT		0
108	KC_AB2	CG42327	0.222396124134733	1	1	pseudobulk	DESeq2	LRT		0
108	KC_AB2	CG42336	-3.00196951853976	0.064713892986397	1	pseudobulk	DESeq2	LRT		0
108	KC_AB2	CG42337	-0.455280261779184	0.264938591708378	1	pseudobulk	DESeq2	LRT		0
108	KC_AB2	CG42339	0.252293584922708	0.556027001777648	1	pseudobulk	DESeq2	LRT		0
108	KC_AB2	CG42340	-0.343030228262703	0.479066796218602	1	pseudobulk	DESeq2	LRT		0
108	KC_AB2	CG42342	-0.334820890657685	0.60476363522133	1	pseudobulk	DESeq2	LRT		0
108	KC_AB2	CG42346	0.769327310278838	0.63845663087918	1	pseudobulk	DESeq2	LRT		0
108	KC_AB2	CG42359	-0.65240740284843	0.676590917368097	1	pseudobulk	DESeq2	LRT		0
108	KC_AB2	CG42360	0.741184268602222	0.552727325146142	1	pseudobulk	DESeq2	LRT		0
108	KC_AB2	CG42361	0.0024842387977239	0.872341620543209	1	pseudobulk	DESeq2	LRT		0
108	KC_AB2	CG42364	-0.600547221779134	1	1	pseudobulk	DESeq2	LRT		0
108	KC_AB2	CG42365	2.51068564084663	0.263718309739592	1	pseudobulk	DESeq2	LRT		0
108	KC_AB2	CG42376	0.255903333334801	0.769646803291297	1	pseudobulk	DESeq2	LRT		0
108	KC_AB2	CG4238	-0.342368207055256	0.648412402040585	1	pseudobulk	DESeq2	LRT		0
108	KC_AB2	CG42382	0.401733800039527	0.836927022098515	1	pseudobulk	DESeq2	LRT		0
108	KC_AB2	CG4239	0.0068519607898035	0.88078345434118	1	pseudobulk	DESeq2	LRT		0
108	KC_AB2	CG42392	-0.984422648038282	0.009254489665825	1	pseudobulk	DESeq2	LRT		0
108	KC_AB2	CG42399	0.522867664761254	0.789441383158121	1	pseudobulk	DESeq2	LRT		0
108	KC_AB2	CG42402	-0.468442278826723	0.0721817123570173	1	pseudobulk	DESeq2	LRT		0
108	KC_AB2	CG42404	0.680186119241929	0.300395071025288	1	pseudobulk	DESeq2	LRT		0
108	KC_AB2	CG42450	0.307285273611217	0.386952575881583	1	pseudobulk	DESeq2	LRT		0
108	KC_AB2	CG42495	-1.3723417613553	0.619532036522	1	pseudobulk	DESeq2	LRT		0
109	KC_AB2	CG42518	-0.600547221779134	1	1	pseudobulk	DESeq2	LRT		0
109	KC_AB2	CG42526	-0.600547221779134	1	1	pseudobulk	DESeq2	LRT		0
109	KC_AB2	CG42540	0.214440983163785	0.40763084386782	1	pseudobulk	DESeq2	LRT		0
109	KC_AB2	CG42541	0.062810352949413	0.789126434341221	1	pseudobulk	DESeq2	LRT		0
109	KC_AB2	CG42542	-0.22040788489923	0.807989632833785	1	pseudobulk	DESeq2	LRT		0
109	KC_AB2	CG42553	0.222396124134733	1	1	pseudobulk	DESeq2	LRT		0
109	KC_AB2	CG42554	0.229920203951763	0.871897429832218	1	pseudobulk	DESeq2	LRT		0
109	KC_AB2	CG42561	-0.592481036256484	0.706847545932384	1	pseudobulk	DESeq2	LRT		0
109	KC_AB2	CG42588	-0.614691417743657	0.653868648121902	1	pseudobulk	DESeq2	LRT		0
109	KC_AB2	CG42594	-1.35377814890605	0.117864074049362	1	pseudobulk	DESeq2	LRT		0
109	KC_AB2	CG42613	0.35302985491607	0.278856919116475	1	pseudobulk	DESeq2	LRT		0
109	KC_AB2	CG4266	-0.066362408527940	0.912411655128331	1	pseudobulk	DESeq2	LRT		0
109	KC_AB2	CG42663	1.53811683574911	0.54844951579854	1	pseudobulk	DESeq2	LRT		0
109	KC_AB2	CG42668	-0.289415460174277	0.482510344855045	1	pseudobulk	DESeq2	LRT		0
109	KC_AB2	CG4267	1.5243995994652	0.741477491078038	1	pseudobulk	DESeq2	LRT		0
109	KC_AB2	CG42671	-0.359013634254925	0.661765448251498	1	pseudobulk	DESeq2	LRT		0
109	KC_AB2	CG42672	0.175458733123396	0.844721414588962	1	pseudobulk	DESeq2	LRT		0

109	KC_AB2	CG42673	0.344006868967012	0.686243813380046	1	pseudobulk	DESeq2	LRT	0
109	KC_AB2	CG42674	-2.12967468770992	0.340688355153583	1	pseudobulk	DESeq2	LRT	0
109	KC_AB2	CG42676	0.717340694333406	0.615866173404391	1	pseudobulk	DESeq2	LRT	0
109	KC_AB2	CG4269	-0.600547221779134	1	1	pseudobulk	DESeq2	LRT	0
109	KC_AB2	CG42697	2.12684015898615	0.445939223035924	1	pseudobulk	DESeq2	LRT	0
109	KC_AB2	CG42699	-1.69720890033463	0.260636340875763	1	pseudobulk	DESeq2	LRT	0
109	KC_AB2	CG42700	1.76481463767611	0.00263427043025482	1	pseudobulk	DESeq2	LRT	0
109	KC_AB2	CG42709	-1.06252717759971	0.373494831946854	1	pseudobulk	DESeq2	LRT	0
109	KC_AB2	CG42724	0.447089403602203	0.476140656175363	1	pseudobulk	DESeq2	LRT	0
109	KC_AB2	CG42747	-0.0771730160570212	0.907594347051564	1	pseudobulk	DESeq2	LRT	0
109	KC_AB2	CG42748	-0.187163011207096	0.685711461060753	1	pseudobulk	DESeq2	LRT	0
109	KC_AB2	CG42750	0.376685001461547	0.259363120702661	1	pseudobulk	DESeq2	LRT	0
109	KC_AB2	CG42751	-0.600547221779134	1	1	pseudobulk	DESeq2	LRT	0
109	KC_AB2	CG4278	-2.42100284528813	0.295221564390726	1	pseudobulk	DESeq2	LRT	0
109	KC_AB2	CG42784	-0.266620086398723	0.57527784655772	1	pseudobulk	DESeq2	LRT	0
109	KC_AB2	CG42788	0.921269184592486	0.141572183243119	1	pseudobulk	DESeq2	LRT	0
109	KC_AB2	CG42789	-0.600547221779134	1	1	pseudobulk	DESeq2	LRT	0
109	KC_AB2	CG42795	-0.206352225523494	0.531937000652313	1	pseudobulk	DESeq2	LRT	0
109	KC_AB2	CG42797	1.41524016620164	0.505408490038387	1	pseudobulk	DESeq2	LRT	0
109	KC_AB2	CG42806	-1.79505857988765	0.330719697656799	1	pseudobulk	DESeq2	LRT	0
109	KC_AB2	CG42808	-1.29244479833796	0.693295472370086	1	pseudobulk	DESeq2	LRT	0
109	KC_AB2	CG42809	-0.74419767317593	0.558983774098232	1	pseudobulk	DESeq2	LRT	0
109	KC_AB2	CG4281	0.884872796438086	0.389440413300901	1	pseudobulk	DESeq2	LRT	0
109	KC_AB2	CG42813	0.385010982252448	0.607249846308732	1	pseudobulk	DESeq2	LRT	0
109	KC_AB2	CG4282	0.70378358684898	0.932301451973468	1	pseudobulk	DESeq2	LRT	0
109	KC_AB2	CG42846	1.5243995994652	0.741477491078038	1	pseudobulk	DESeq2	LRT	0
109	KC_AB2	CG42855	-0.0887202949307342	0.96200897691965	1	pseudobulk	DESeq2	LRT	0
109	KC_AB2	CG4287	-0.545599946473549	0.70247000596938	1	pseudobulk	DESeq2	LRT	0
109	KC_AB2	CG4291	-1.54119476971891	0.435208284687902	1	pseudobulk	DESeq2	LRT	0
109	KC_AB2	CG4293	-0.856512708793407	0.701532976720232	1	pseudobulk	DESeq2	LRT	0
109	KC_AB2	CG4294	-0.0588313340146728	0.803579959775804	1	pseudobulk	DESeq2	LRT	0
109	KC_AB2	CG4297	-0.318323821446687	0.544143368799997	1	pseudobulk	DESeq2	LRT	0
109	KC_AB2	CG43051	1.5243995994652	0.741477491078038	1	pseudobulk	DESeq2	LRT	0
109	KC_AB2	CG43056	1.94691684546882	0.398076844044942	1	pseudobulk	DESeq2	LRT	0
109	KC_AB2	CG43066	-0.80975874360133	0.284497256914294	1	pseudobulk	DESeq2	LRT	0
109	KC_AB2	CG43078	0.538471319565493	0.526785353183381	1	pseudobulk	DESeq2	LRT	0
109	KC_AB2	CG43102	-0.16411581895756	0.731845387398181	1	pseudobulk	DESeq2	LRT	0
109	KC_AB2	CG43111	1.5243995994652	0.741477491078038	1	pseudobulk	DESeq2	LRT	0
109	KC_AB2	CG43117	2.1817927492653	0.458599861292202	1	pseudobulk	DESeq2	LRT	0
109	KC_AB2	CG4313	2.72066226053615	0.290765320578625	1	pseudobulk	DESeq2	LRT	0
109	KC_AB2	CG43175	-0.600547221779133	0.841940696994101	1	pseudobulk	DESeq2	LRT	0
109	KC_AB2	CG43219	1.53811683574911	0.54844951579854	1	pseudobulk	DESeq2	LRT	0
109	KC_AB2	CG43222	-0.0692126845315412	0.780227678416275	1	pseudobulk	DESeq2	LRT	0
109	KC_AB2	CG4329	-0.427431139129811	1	1	pseudobulk	DESeq2	LRT	0
109	KC_AB2	CG4332	0.864341462967788	0.925947094335722	1	pseudobulk	DESeq2	LRT	0
109	KC_AB2	CG43322	-0.427431139129811	1	1	pseudobulk	DESeq2	LRT	0
109	KC_AB2	CG43324	0.229007506996085	0.388774248038354	1	pseudobulk	DESeq2	LRT	0
109	KC_AB2	CG43347	0.399504727911412	0.424943158925544	1	pseudobulk	DESeq2	LRT	0
109	KC_AB2	CG43366	-1.40673172715063	0.587364787535786	1	pseudobulk	DESeq2	LRT	0
109	KC_AB2	CG43367	1.1532246091464	0.453190838347567	1	pseudobulk	DESeq2	LRT	0
109	KC_AB2	CG43373	0.669045118277475	0.232219479376289	1	pseudobulk	DESeq2	LRT	0
109	KC_AB2	CG4338	1.0547002356457	0.651955074600822	1	pseudobulk	DESeq2	LRT	0
109	KC_AB2	CG43394	-0.592481036256484	0.706847545932384	1	pseudobulk	DESeq2	LRT	0

109	KC_AB2	CG4341	-0.573633309023843	0.495995627993216	1	pseudobulk	DESeq2	LRT		0
109	KC_AB2	CG43428	-0.427431139129811	1	1	pseudobulk	DESeq2	LRT		0
109	KC_AB2	CG4360	-0.0172512409931348	0.952233790495482	1	pseudobulk	DESeq2	LRT		0
109	KC_AB2	CG4364	0.791468033138705	0.699865413482108	1	pseudobulk	DESeq2	LRT		0
109	KC_AB2	CG43658	0.111037674321444	0.761126656238745	1	pseudobulk	DESeq2	LRT		0
109	KC_AB2	CG43680	-1.12475160732157	0.574429473125171	1	pseudobulk	DESeq2	LRT		0
109	KC_AB2	CG43693	-0.841882716738579	1	1	pseudobulk	DESeq2	LRT		0
109	KC_AB2	CG43707	-0.0820536141287358	0.900245238001964	1	pseudobulk	DESeq2	LRT		0
109	KC_AB2	CG43736	0.758659449628763	0.0319691418919381	1	pseudobulk	DESeq2	LRT		0
109	KC_AB2	CG43737	0.0748671854354359	0.74496582004172	1	pseudobulk	DESeq2	LRT		0
109	KC_AB2	CG43740	-3.50276997234884	0.0652912702263281	1	pseudobulk	DESeq2	LRT		0
109	KC_AB2	CG43759	0.104187425236162	0.645646465446963	1	pseudobulk	DESeq2	LRT		0
109	KC_AB2	CG43778	0.408304428732225	0.412705532138989	1	pseudobulk	DESeq2	LRT		0
109	KC_AB2	CG43783	-1.54132252871259	0.432696240183291	1	pseudobulk	DESeq2	LRT		0
109	KC_AB2	CG43788	-0.427431139129811	1	1	pseudobulk	DESeq2	LRT		0
109	KC_AB2	CG43789	0.674459942220112	0.74558757773504	1	pseudobulk	DESeq2	LRT		0
109	KC_AB2	CG43795	0.0556895672724125	0.891353011627802	1	pseudobulk	DESeq2	LRT		0
109	KC_AB2	CG43800	2.3820677681374	0.290097649087942	1	pseudobulk	DESeq2	LRT		0
109	KC_AB2	CG43845	-0.600547221779134	1	1	pseudobulk	DESeq2	LRT		0
109	KC_AB2	CG43850	1.70225486324286	0.321432429322056	1	pseudobulk	DESeq2	LRT		0
109	KC_AB2	CG43861	-0.221186036809648	0.76296877035746	1	pseudobulk	DESeq2	LRT		0
109	KC_AB2	CG43867	0.258659121662507	0.552572856960895	1	pseudobulk	DESeq2	LRT		0
109	KC_AB2	CG43897	-0.427431139129811	1	1	pseudobulk	DESeq2	LRT		0
109	KC_AB2	CG4390	-0.979798426066822	0.464361094835081	1	pseudobulk	DESeq2	LRT		0
109	KC_AB2	CG43901	0.360131507997527	0.626924292511555	1	pseudobulk	DESeq2	LRT		0
109	KC_AB2	CG43902	-2.63419818779401	0.222714263443553	1	pseudobulk	DESeq2	LRT		0
109	KC_AB2	CG43925	2.1817927492653	0.458599861292202	1	pseudobulk	DESeq2	LRT		0
109	KC_AB2	CG4393	1.09901490132735	0.502734648474513	1	pseudobulk	DESeq2	LRT		0
109	KC_AB2	CG44002	1.5243995994652	0.741477491078038	1	pseudobulk	DESeq2	LRT		0
109	KC_AB2	CG44004	2.12684015898615	0.445939223035924	1	pseudobulk	DESeq2	LRT		0
110	KC_AB2	CG44008	-1.63925793886161	0.546290859679341	1	pseudobulk	DESeq2	LRT		0
110	KC_AB2	CG4404	-0.63443752429291	0.49554172800905	1	pseudobulk	DESeq2	LRT		0
110	KC_AB2	CG4407	-0.94428595126125	0.54691726661449	1	pseudobulk	DESeq2	LRT		0
110	KC_AB2	CG4409	1.53811683574911	0.54844951579854	1	pseudobulk	DESeq2	LRT		0
110	KC_AB2	CG44098	-1.25295720858312	0.571615770830719	1	pseudobulk	DESeq2	LRT		0
110	KC_AB2	CG44153	0.605309502476111	0.222953732532843	1	pseudobulk	DESeq2	LRT		0
110	KC_AB2	CG44158	-0.427431139129811	1	1	pseudobulk	DESeq2	LRT		0
110	KC_AB2	CG44195	-0.427431139129811	1	1	pseudobulk	DESeq2	LRT		0
110	KC_AB2	CG4424	-0.427431139129811	1	1	pseudobulk	DESeq2	LRT		0
110	KC_AB2	CG44242	0.754432053004484	0.164300975669945	1	pseudobulk	DESeq2	LRT		0
110	KC_AB2	CG44243	-0.894819169487389	0.638536505602624	1	pseudobulk	DESeq2	LRT		0
110	KC_AB2	CG44245	-1.07776915880798	0.775491075088671	1	pseudobulk	DESeq2	LRT		0
110	KC_AB2	CG44247	-0.188299889049511	0.599270760010711	1	pseudobulk	DESeq2	LRT		0
110	KC_AB2	CG44249	-1.12785316439435	0.661319045116135	1	pseudobulk	DESeq2	LRT		0
110	KC_AB2	CG44325	1.5243995994652	0.741477491078038	1	pseudobulk	DESeq2	LRT		0
110	KC_AB2	CG4438	-0.600547221779134	1	1	pseudobulk	DESeq2	LRT		0
110	KC_AB2	CG44405	-0.600547221779133	0.841940696994101	1	pseudobulk	DESeq2	LRT		0
110	KC_AB2	CG44422	0.670724460234433	0.235107024317072	1	pseudobulk	DESeq2	LRT		0
110	KC_AB2	CG4452	1.06667224609914	0.300993785108145	1	pseudobulk	DESeq2	LRT		0
110	KC_AB2	CG44521	-0.427431139129811	1	1	pseudobulk	DESeq2	LRT		0
110	KC_AB2	CG4467	0.173564158176379	0.472456511404001	1	pseudobulk	DESeq2	LRT		0
110	KC_AB2	CG4476	0.443892390635762	1	1	pseudobulk	DESeq2	LRT		0
110	KC_AB2	CG44774	-0.128790443190431	0.751909342804284	1	pseudobulk	DESeq2	LRT		0

110	KC_AB2	CG44815	-0.427431139129811	1	1	pseudobulk	DESeq2	LRT	0
110	KC_AB2	CG44837	0.0651705653607153	0.822821083547426	1	pseudobulk	DESeq2	LRT	0
110	KC_AB2	CG44838	0.604126014006175	0.314472256470564	1	pseudobulk	DESeq2	LRT	0
110	KC_AB2	CG4497	1.81903112764766	0.101580434953634	1	pseudobulk	DESeq2	LRT	0
110	KC_AB2	CG45002	0.943586647867311	0.690632779168748	1	pseudobulk	DESeq2	LRT	0
110	KC_AB2	CG4502	-0.0745779422989174	0.76909730932997	1	pseudobulk	DESeq2	LRT	0
110	KC_AB2	CG45049	-0.241626647429234	0.610972960602092	1	pseudobulk	DESeq2	LRT	0
110	KC_AB2	CG45050	-0.0094248640999674	0.842287526011519	1	pseudobulk	DESeq2	LRT	0
110	KC_AB2	CG45071	0.733956534065321	0.611475844941125	1	pseudobulk	DESeq2	LRT	0
110	KC_AB2	CG45081	1.5243995994652	0.741477491078038	1	pseudobulk	DESeq2	LRT	0
110	KC_AB2	CG4511	-0.0505407145740365	0.820501965154551	1	pseudobulk	DESeq2	LRT	0
110	KC_AB2	CG45218	0.222396124134733	1	1	pseudobulk	DESeq2	LRT	0
110	KC_AB2	CG45263	0.137568840867594	0.520352774148041	1	pseudobulk	DESeq2	LRT	0
110	KC_AB2	CG4537	-0.841882716738579	1	1	pseudobulk	DESeq2	LRT	0
110	KC_AB2	CG4538	0.241915439487958	0.848227801989072	1	pseudobulk	DESeq2	LRT	0
110	KC_AB2	CG4552	0.506662102707318	0.782529221356027	1	pseudobulk	DESeq2	LRT	0
110	KC_AB2	CG4554	-0.427431139129811	1	1	pseudobulk	DESeq2	LRT	0
110	KC_AB2	CG4557	2.28495489614998	0.177173746971051	1	pseudobulk	DESeq2	LRT	0
110	KC_AB2	CG4558	-0.841882716738579	1	1	pseudobulk	DESeq2	LRT	0
110	KC_AB2	CG4562	-1.24192072029347	0.370878220384156	1	pseudobulk	DESeq2	LRT	0
110	KC_AB2	CG4565	0.864341462967788	0.925947094335722	1	pseudobulk	DESeq2	LRT	0
110	KC_AB2	CG4577	-0.215324932096612	0.403247589438581	1	pseudobulk	DESeq2	LRT	0
110	KC_AB2	CG4587	0.231708522394449	0.617133916721978	1	pseudobulk	DESeq2	LRT	0
110	KC_AB2	CG4593	0.228215696441444	0.864464959897697	1	pseudobulk	DESeq2	LRT	0
110	KC_AB2	CG4596	-0.948535895116573	0.262306059551517	1	pseudobulk	DESeq2	LRT	0
110	KC_AB2	CG4598	-1.6150095599849	0.519549602003096	1	pseudobulk	DESeq2	LRT	0
110	KC_AB2	CG4603	0.175817624971804	0.825303574639292	1	pseudobulk	DESeq2	LRT	0
110	KC_AB2	CG4607	1.41524016620164	0.505408490038387	1	pseudobulk	DESeq2	LRT	0
110	KC_AB2	CG4610	-1.29244479833796	0.693295472370086	1	pseudobulk	DESeq2	LRT	0
110	KC_AB2	CG4612	-0.362676066687106	0.366838364504575	1	pseudobulk	DESeq2	LRT	0
110	KC_AB2	CG4615	-0.615476001753369	0.648444773010085	1	pseudobulk	DESeq2	LRT	0
110	KC_AB2	CG4617	-0.748617696849439	0.641417342339418	1	pseudobulk	DESeq2	LRT	0
110	KC_AB2	CG4619	0.108468324554008	1	1	pseudobulk	DESeq2	LRT	0
110	KC_AB2	CG4622	0.222396124134733	1	1	pseudobulk	DESeq2	LRT	0
110	KC_AB2	CG4627	-2.36690004770124	0.282527465979357	1	pseudobulk	DESeq2	LRT	0
110	KC_AB2	CG46280	-0.309281921245926	0.716656566862482	1	pseudobulk	DESeq2	LRT	0
110	KC_AB2	CG46281	-0.592481036256484	0.706847545932384	1	pseudobulk	DESeq2	LRT	0
110	KC_AB2	CG4629	-0.0764721310898858	0.855011796632645	1	pseudobulk	DESeq2	LRT	0
110	KC_AB2	CG46301	0.246903948353797	0.515093637125026	1	pseudobulk	DESeq2	LRT	0
110	KC_AB2	CG4631	1.23085716405798	0.614623732585597	1	pseudobulk	DESeq2	LRT	0
110	KC_AB2	CG46338	0.896799289469375	0.344319298317265	1	pseudobulk	DESeq2	LRT	0
110	KC_AB2	CG46339	-0.43733367647324	0.108706031028084	1	pseudobulk	DESeq2	LRT	0
110	KC_AB2	CG46385	-0.122626912069178	0.65426255455341	1	pseudobulk	DESeq2	LRT	0
110	KC_AB2	CG46394	-0.600547221779134	1	1	pseudobulk	DESeq2	LRT	0
110	KC_AB2	CG46397	-0.427431139129811	1	1	pseudobulk	DESeq2	LRT	0
110	KC_AB2	CG46428	-0.600547221779134	1	1	pseudobulk	DESeq2	LRT	0
110	KC_AB2	CG46448	-0.427431139129811	1	1	pseudobulk	DESeq2	LRT	0
110	KC_AB2	CG4645	-0.911833912158456	0.581367326605306	1	pseudobulk	DESeq2	LRT	0
110	KC_AB2	CG4646	1.10561057146114	0.409820577604984	1	pseudobulk	DESeq2	LRT	0
110	KC_AB2	CG4658	0.0076705880477111	0.895370317399527	1	pseudobulk	DESeq2	LRT	0
110	KC_AB2	CG4660	0.27411322529285	0.546196989795417	1	pseudobulk	DESeq2	LRT	0
110	KC_AB2	CG4676	0.457755086869544	0.804615396056288	1	pseudobulk	DESeq2	LRT	0
110	KC_AB2	CG4678	1.99541469152279	0.0860575829512071	1	pseudobulk	DESeq2	LRT	0

110	KC_AB2	CG4679	-0.638893724814917	0.818738110341823	1	pseudobulk	DESeq2	LRT	0
110	KC_AB2	CG4686	0.330878883704482	0.681594599234241	1	pseudobulk	DESeq2	LRT	0
110	KC_AB2	CG4707	-1.3929484167291	0.512855059074155	1	pseudobulk	DESeq2	LRT	0
110	KC_AB2	CG4709	0.183555857030446	1	1	pseudobulk	DESeq2	LRT	0
110	KC_AB2	CG4730	2.12684015898615	0.445939223035924	1	pseudobulk	DESeq2	LRT	0
110	KC_AB2	CG4743	-0.600547221779134	1	1	pseudobulk	DESeq2	LRT	0
110	KC_AB2	CG4751	-0.828178378785339	0.673584255563891	1	pseudobulk	DESeq2	LRT	0
110	KC_AB2	CG4763	-0.600547221779134	1	1	pseudobulk	DESeq2	LRT	0
110	KC_AB2	CG4768	0.234151575562176	0.733506030854043	1	pseudobulk	DESeq2	LRT	0
110	KC_AB2	CG4783	2.12684015898615	0.445939223035924	1	pseudobulk	DESeq2	LRT	0
110	KC_AB2	CG4788	0.751456027923628	0.684049070099348	1	pseudobulk	DESeq2	LRT	0
110	KC_AB2	CG4789	0.288635524703451	0.754647900367946	1	pseudobulk	DESeq2	LRT	0
110	KC_AB2	CG4806	0.714342384588752	0.585496703894613	1	pseudobulk	DESeq2	LRT	0
110	KC_AB2	CG4813	-1.12785316439435	0.661319045116135	1	pseudobulk	DESeq2	LRT	0
110	KC_AB2	CG4820	-2.63419818779401	0.222714263443553	1	pseudobulk	DESeq2	LRT	0
110	KC_AB2	CG4825	0.203196037706383	0.886378224616078	1	pseudobulk	DESeq2	LRT	0
110	KC_AB2	CG4829	0.202945968446639	0.785941931962241	1	pseudobulk	DESeq2	LRT	0
110	KC_AB2	CG4839	-0.427431139129811	1	1	pseudobulk	DESeq2	LRT	0
110	KC_AB2	CG4849	-2.3627362995981	0.281646618575938	1	pseudobulk	DESeq2	LRT	0
110	KC_AB2	CG4853	0.302877789019481	0.873574745505245	1	pseudobulk	DESeq2	LRT	0
110	KC_AB2	CG4854	-1.9379626606486	0.399953775626921	1	pseudobulk	DESeq2	LRT	0
110	KC_AB2	CG4858	1.17471908769491	0.383011750371927	1	pseudobulk	DESeq2	LRT	0
110	KC_AB2	CG4866	-2.25026703842057	0.308244700817443	1	pseudobulk	DESeq2	LRT	0
110	KC_AB2	CG4882	-2.12967468770992	0.340688355153583	1	pseudobulk	DESeq2	LRT	0
111	KC_AB2	CG4887	0.692771440623899	0.500598352083586	1	pseudobulk	DESeq2	LRT	0
111	KC_AB2	CG4896	0.267993215325217	0.744265958416334	1	pseudobulk	DESeq2	LRT	0
111	KC_AB2	CG4901	-1.6150095599849	0.519549602003096	1	pseudobulk	DESeq2	LRT	0
111	KC_AB2	CG4908	-4.135098989595	0.018702226781719	1	pseudobulk	DESeq2	LRT	0
111	KC_AB2	CG4911	0.578660811882861	0.6200690630829	1	pseudobulk	DESeq2	LRT	0
111	KC_AB2	CG4935	0.864341462967788	0.925947094335722	1	pseudobulk	DESeq2	LRT	0
111	KC_AB2	CG4936	-0.0671971907115817	0.936397765751979	1	pseudobulk	DESeq2	LRT	0
111	KC_AB2	CG4942	1.94391099175295	0.438511443346836	1	pseudobulk	DESeq2	LRT	0
111	KC_AB2	CG4951	-0.841882716738579	1	1	pseudobulk	DESeq2	LRT	0
111	KC_AB2	CG4953	1.39827770914961	0.587246212652858	1	pseudobulk	DESeq2	LRT	0
111	KC_AB2	CG4957	0.0456926662205872	0.981608052892825	1	pseudobulk	DESeq2	LRT	0
111	KC_AB2	CG4968	0.317693052235986	0.752955339439491	1	pseudobulk	DESeq2	LRT	0
111	KC_AB2	CG4972	1.14814388261213	0.568837005180859	1	pseudobulk	DESeq2	LRT	0
111	KC_AB2	CG4984	-0.427431139129811	1	1	pseudobulk	DESeq2	LRT	0
111	KC_AB2	CG5003	-1.16860914851274	0.560500482574758	1	pseudobulk	DESeq2	LRT	0
111	KC_AB2	CG5004	-2.06287163173521	0.257004929899456	1	pseudobulk	DESeq2	LRT	0
111	KC_AB2	CG5009	-0.70727250916738	0.705390475165233	1	pseudobulk	DESeq2	LRT	0
111	KC_AB2	CG5013	1.5243995994652	0.741477491078038	1	pseudobulk	DESeq2	LRT	0
111	KC_AB2	CG5021	-0.137982625176737	0.788773756229197	1	pseudobulk	DESeq2	LRT	0
111	KC_AB2	CG5022	-0.0983769855968567	0.8859970026996	1	pseudobulk	DESeq2	LRT	0
111	KC_AB2	CG5026	0.943586647867311	0.690632779168748	1	pseudobulk	DESeq2	LRT	0
111	KC_AB2	CG5027	0.393974072510627	0.676012038362289	1	pseudobulk	DESeq2	LRT	0
111	KC_AB2	CG5028	-0.199306067769025	0.726696142662628	1	pseudobulk	DESeq2	LRT	0
111	KC_AB2	CG5037	-0.213679798382135	0.749897926301937	1	pseudobulk	DESeq2	LRT	0
111	KC_AB2	CG5038	-0.600547221779133	0.841940696994101	1	pseudobulk	DESeq2	LRT	0
111	KC_AB2	CG5044	0.0563146874970796	0.877574419167179	1	pseudobulk	DESeq2	LRT	0
111	KC_AB2	CG5056	0.045795175916095	0.835391711294413	1	pseudobulk	DESeq2	LRT	0
111	KC_AB2	CG5059	1.00163658160155	0.398423969853148	1	pseudobulk	DESeq2	LRT	0
111	KC_AB2	CG5065	0.52324866491773	0.259649324603585	1	pseudobulk	DESeq2	LRT	0

111	KC_AB2	CG5068	0.481659461415551	0.537224502051717	1	pseudobulk	DESeq2	LRT		0
111	KC_AB2	CG5071	-1.29244479833796	0.693295472370086	1	pseudobulk	DESeq2	LRT		0
111	KC_AB2	CG5087	-1.18294349965014	0.473475374861373	1	pseudobulk	DESeq2	LRT		0
111	KC_AB2	CG5098	-2.15505281559356	0.143387224730511	1	pseudobulk	DESeq2	LRT		0
111	KC_AB2	CG5110	0.628957457777389	0.524764449944787	1	pseudobulk	DESeq2	LRT		0
111	KC_AB2	CG5114	-0.427431139129811	1	1	pseudobulk	DESeq2	LRT		0
111	KC_AB2	CG5116	-0.427431139129811	1	1	pseudobulk	DESeq2	LRT		0
111	KC_AB2	CG5126	-0.600547221779134	1	1	pseudobulk	DESeq2	LRT		0
111	KC_AB2	CG5131	-0.592481036256484	0.706847545932384	1	pseudobulk	DESeq2	LRT		0
111	KC_AB2	CG5149	0.43953467560905	0.71165712055907	1	pseudobulk	DESeq2	LRT		0
111	KC_AB2	CG5151	-0.0036183033497339	0.801477631040693	1	pseudobulk	DESeq2	LRT		0
111	KC_AB2	CG5160	-0.600547221779134	1	1	pseudobulk	DESeq2	LRT		0
111	KC_AB2	CG5174	-0.456338493784963	0.405899311042038	1	pseudobulk	DESeq2	LRT		0
111	KC_AB2	CG5177	0.222396124134733	1	1	pseudobulk	DESeq2	LRT		0
111	KC_AB2	CG5180	0.220861813392571	0.886247699003982	1	pseudobulk	DESeq2	LRT		0
111	KC_AB2	CG5181	-0.0739116589443354	0.853028101269376	1	pseudobulk	DESeq2	LRT		0
111	KC_AB2	CG5188	-0.39917336535616	0.810147819664692	1	pseudobulk	DESeq2	LRT		0
111	KC_AB2	CG5191	0.703141171373176	0.67065453296144	1	pseudobulk	DESeq2	LRT		0
111	KC_AB2	CG5196	-0.43568096683045	0.532091859491258	1	pseudobulk	DESeq2	LRT		0
111	KC_AB2	CG5199	-0.600547221779133	0.841940696994101	1	pseudobulk	DESeq2	LRT		0
111	KC_AB2	CG5214	0.220966100756256	0.692808887205635	1	pseudobulk	DESeq2	LRT		0
111	KC_AB2	CG5220	-0.39917336535616	0.810147819664692	1	pseudobulk	DESeq2	LRT		0
111	KC_AB2	CG5262	-0.841882716738579	1	1	pseudobulk	DESeq2	LRT		0
111	KC_AB2	CG5267	-0.600547221779133	0.841940696994101	1	pseudobulk	DESeq2	LRT		0
111	KC_AB2	CG5270	-0.382929749762112	0.851961810578875	1	pseudobulk	DESeq2	LRT		0
111	KC_AB2	CG5273	0.0028106827283675	0.848279568284041	1	pseudobulk	DESeq2	LRT		0
111	KC_AB2	CG5274	-1.02423531380596	0.548801874948105	1	pseudobulk	DESeq2	LRT		0
111	KC_AB2	CG5276	-2.24425718503641	0.335238867220625	1	pseudobulk	DESeq2	LRT		0
111	KC_AB2	CG5281	-0.55226039283892	0.543736550127745	1	pseudobulk	DESeq2	LRT		0
111	KC_AB2	CG5282	0.14113812901108	0.818074653142095	1	pseudobulk	DESeq2	LRT		0
111	KC_AB2	CG5285	-2.31658951096177	0.321432784490917	1	pseudobulk	DESeq2	LRT		0
111	KC_AB2	CG5287	-0.556718312352294	0.752982669933181	1	pseudobulk	DESeq2	LRT		0
111	KC_AB2	CG5290	-0.600547221779134	1	1	pseudobulk	DESeq2	LRT		0
111	KC_AB2	CG5292	0.55657384749337	0.716688097080595	1	pseudobulk	DESeq2	LRT		0
111	KC_AB2	CG5316	-0.670833611710101	0.682559952282501	1	pseudobulk	DESeq2	LRT		0
111	KC_AB2	CG5323	-1.64305208505983	0.406280385408283	1	pseudobulk	DESeq2	LRT		0
111	KC_AB2	CG5335	-0.600547221779133	0.841940696994101	1	pseudobulk	DESeq2	LRT		0
111	KC_AB2	CG5339	-0.0858057061782988	0.88890881703361	1	pseudobulk	DESeq2	LRT		0
111	KC_AB2	CG5348	0.222396124134733	1	1	pseudobulk	DESeq2	LRT		0
111	KC_AB2	CG5355	0.870430983267102	0.550064673604609	1	pseudobulk	DESeq2	LRT		0
111	KC_AB2	CG5359	-2.26446750979222	0.186619909465498	1	pseudobulk	DESeq2	LRT		0
111	KC_AB2	CG5380	2.03955752684245	0.330463272096619	1	pseudobulk	DESeq2	LRT		0
111	KC_AB2	CG5382	-1.21325885396279	0.540389556718898	1	pseudobulk	DESeq2	LRT		0
111	KC_AB2	CG5412	0.220215791696238	0.843636612230988	1	pseudobulk	DESeq2	LRT		0
111	KC_AB2	CG5435	1.5243995994652	0.741477491078038	1	pseudobulk	DESeq2	LRT		0
111	KC_AB2	CG5439	0.546336468444212	0.778548216578298	1	pseudobulk	DESeq2	LRT		0
111	KC_AB2	CG5445	0.714747346035283	0.589790492455837	1	pseudobulk	DESeq2	LRT		0
111	KC_AB2	CG5446	-0.363965885382413	0.641704648813311	1	pseudobulk	DESeq2	LRT		0
111	KC_AB2	CG5447	0.434972159824309	0.635249796575949	1	pseudobulk	DESeq2	LRT		0
111	KC_AB2	CG5455	-1.17131621050916	0.252676126947167	1	pseudobulk	DESeq2	LRT		0
111	KC_AB2	CG5466	0.395222676535319	0.653461462197177	1	pseudobulk	DESeq2	LRT		0
111	KC_AB2	CG5498	1.5243995994652	0.741477491078038	1	pseudobulk	DESeq2	LRT		0
111	KC_AB2	CG5500	-1.00234626541131	0.465097829109232	1	pseudobulk	DESeq2	LRT		0

111	KC_AB2	CG5504	-0.365567848351684	0.732417329047909	1	pseudobulk	DESeq2	LRT	0
111	KC_AB2	CG5510	1.03472291668693	0.388553708318068	1	pseudobulk	DESeq2	LRT	0
111	KC_AB2	CG5515	-0.0573910477234192	0.894539634018585	1	pseudobulk	DESeq2	LRT	0
111	KC_AB2	CG5516	0.28651779060963	0.766863151084453	1	pseudobulk	DESeq2	LRT	0
111	KC_AB2	CG5521	-2.00381106404089	0.0930869602044428	1	pseudobulk	DESeq2	LRT	0
111	KC_AB2	CG5522	-0.339891687896997	0.659132103481316	1	pseudobulk	DESeq2	LRT	0
111	KC_AB2	CG5532	-0.597799077865952	0.421261253036538	1	pseudobulk	DESeq2	LRT	0
111	KC_AB2	CG5535	-1.05822928322422	0.511814990727336	1	pseudobulk	DESeq2	LRT	0
111	KC_AB2	CG5541	-0.427431139129811	1	1	pseudobulk	DESeq2	LRT	0
111	KC_AB2	CG5554	0.169958040896234	0.875453473313622	1	pseudobulk	DESeq2	LRT	0
111	KC_AB2	CG5555	0.504028533995044	0.539937078196185	1	pseudobulk	DESeq2	LRT	0
111	KC_AB2	CG5567	2.1542884907318	0.280930633341219	1	pseudobulk	DESeq2	LRT	0
111	KC_AB2	CG5569	-1.58452664731304	0.27658197292134	1	pseudobulk	DESeq2	LRT	0
111	KC_AB2	CG5590	-0.573109354659945	0.739419341582026	1	pseudobulk	DESeq2	LRT	0
111	KC_AB2	CG5608	-1.16116875819275	0.736255625566845	1	pseudobulk	DESeq2	LRT	0
111	KC_AB2	CG5611	-0.427431139129811	1	1	pseudobulk	DESeq2	LRT	0
111	KC_AB2	CG5613	2.12684015898615	0.445939223035924	1	pseudobulk	DESeq2	LRT	0
111	KC_AB2	CG5618	-0.696227463937078	0.72129298259466	1	pseudobulk	DESeq2	LRT	0
112	KC_AB2	CG5641	-0.402739887666213	0.637479129114146	1	pseudobulk	DESeq2	LRT	0
112	KC_AB2	CG5644	0.28745556717078	0.641847569483363	1	pseudobulk	DESeq2	LRT	0
112	KC_AB2	CG5645	-0.600547221779134	1	1	pseudobulk	DESeq2	LRT	0
112	KC_AB2	CG5646	0.191630512209535	0.793910079638453	1	pseudobulk	DESeq2	LRT	0
112	KC_AB2	CG5664	-0.841882716738579	1	1	pseudobulk	DESeq2	LRT	0
112	KC_AB2	CG5674	-0.675494073661762	0.652148331789657	1	pseudobulk	DESeq2	LRT	0
112	KC_AB2	CG5676	-0.150832540953811	0.809717235395548	1	pseudobulk	DESeq2	LRT	0
112	KC_AB2	CG5708	-0.247073042912107	0.666344194349969	1	pseudobulk	DESeq2	LRT	0
112	KC_AB2	CG5721	-1.14995976321342	0.586703842885709	1	pseudobulk	DESeq2	LRT	0
112	KC_AB2	CG5726	0.638905045933291	0.644840892047998	1	pseudobulk	DESeq2	LRT	0
112	KC_AB2	CG5727	-1.39989265143196	0.376230101124022	1	pseudobulk	DESeq2	LRT	0
112	KC_AB2	CG5728	-1.07776915880798	0.775491075088671	1	pseudobulk	DESeq2	LRT	0
112	KC_AB2	CG5742	0.384015257232666	0.705670276252077	1	pseudobulk	DESeq2	LRT	0
112	KC_AB2	CG5745	-1.09966906943744	0.642779674562321	1	pseudobulk	DESeq2	LRT	0
112	KC_AB2	CG5746	0.314966479125942	0.538397592692918	1	pseudobulk	DESeq2	LRT	0
112	KC_AB2	CG5758	-0.0594325935190651	0.936050883379269	1	pseudobulk	DESeq2	LRT	0
112	KC_AB2	CG5776	-0.600547221779134	1	1	pseudobulk	DESeq2	LRT	0
112	KC_AB2	CG5787	1.12195683768343	0.443528813831251	1	pseudobulk	DESeq2	LRT	0
112	KC_AB2	CG5789	0.183555857030446	1	1	pseudobulk	DESeq2	LRT	0
112	KC_AB2	CG5793	-1.12785316439435	0.661319045116135	1	pseudobulk	DESeq2	LRT	0
112	KC_AB2	CG5800	-0.427431139129811	1	1	pseudobulk	DESeq2	LRT	0
112	KC_AB2	CG5805	0.14563521552446	0.784923408576626	1	pseudobulk	DESeq2	LRT	0
112	KC_AB2	CG5815	1.39827770914961	0.587246212652858	1	pseudobulk	DESeq2	LRT	0
112	KC_AB2	CG5823	0.352740382489702	0.681134904235245	1	pseudobulk	DESeq2	LRT	0
112	KC_AB2	CG5828	-0.57063622889741	0.720682356245298	1	pseudobulk	DESeq2	LRT	0
112	KC_AB2	CG5830	-0.243753017811245	0.257192680809864	1	pseudobulk	DESeq2	LRT	0
112	KC_AB2	CG5835	-0.427431139129811	1	1	pseudobulk	DESeq2	LRT	0
112	KC_AB2	CG5846	-1.16414686721441	0.585027317672762	1	pseudobulk	DESeq2	LRT	0
112	KC_AB2	CG5850	1.54127986095618	0.432045425768347	1	pseudobulk	DESeq2	LRT	0
112	KC_AB2	CG5854	-1.16116875819275	0.736255625566845	1	pseudobulk	DESeq2	LRT	0
112	KC_AB2	CG5861	0.658784917195005	0.579448724656056	1	pseudobulk	DESeq2	LRT	0
112	KC_AB2	CG5862	0.81524216254894	0.642302155115874	1	pseudobulk	DESeq2	LRT	0
112	KC_AB2	CG5867	-2.57659675686206	0.241930649376094	1	pseudobulk	DESeq2	LRT	0
112	KC_AB2	CG5877	-1.3723417613553	0.619532036522	1	pseudobulk	DESeq2	LRT	0
112	KC_AB2	CG5880	0.943586647867311	0.690632779168748	1	pseudobulk	DESeq2	LRT	0

112	KC_AB2	CG5885	0.692998516844685	0.490685510922664	1	pseudobulk	DESeq2	LRT		0
112	KC_AB2	CG5886	0.864341462967788	0.925947094335722	1	pseudobulk	DESeq2	LRT		0
112	KC_AB2	CG5888	-2.63419818779401	0.222714263443553	1	pseudobulk	DESeq2	LRT		0
112	KC_AB2	CG5890	-0.358479787400826	0.290507146927929	1	pseudobulk	DESeq2	LRT		0
112	KC_AB2	CG5895	-0.600547221779134	1	1	pseudobulk	DESeq2	LRT		0
112	KC_AB2	CG5902	-0.464475921457532	0.642362015828144	1	pseudobulk	DESeq2	LRT		0
112	KC_AB2	CG5903	-0.10860877359423	0.787315902210494	1	pseudobulk	DESeq2	LRT		0
112	KC_AB2	CG5913	2.1817927492653	0.458599861292202	1	pseudobulk	DESeq2	LRT		0
112	KC_AB2	CG5916	0.516933238294497	0.459618349294032	1	pseudobulk	DESeq2	LRT		0
112	KC_AB2	CG5934	-0.405620740476281	0.47424114011669	1	pseudobulk	DESeq2	LRT		0
112	KC_AB2	CG5937	0.220072983780277	0.705661579775397	1	pseudobulk	DESeq2	LRT		0
112	KC_AB2	CG5938	0.379107749040932	0.55813999766743	1	pseudobulk	DESeq2	LRT		0
112	KC_AB2	CG5946	-0.889311537508886	0.149862572585204	1	pseudobulk	DESeq2	LRT		0
112	KC_AB2	CG5953	-2.40840791510765	0.270970617005561	1	pseudobulk	DESeq2	LRT		0
112	KC_AB2	CG5961	-0.660579026126204	0.690917737214444	1	pseudobulk	DESeq2	LRT		0
112	KC_AB2	CG5968	1.46667553000072	0.486649107648129	1	pseudobulk	DESeq2	LRT		0
112	KC_AB2	CG5969	-0.0060326929249164	0.938022512061947	1	pseudobulk	DESeq2	LRT		0
112	KC_AB2	CG5986	0.822896064850214	0.405006025418883	1	pseudobulk	DESeq2	LRT		0
112	KC_AB2	CG5989	-0.988209655533652	0.688142208083301	1	pseudobulk	DESeq2	LRT		0
112	KC_AB2	CG6005	0.768297749882306	0.595383822997973	1	pseudobulk	DESeq2	LRT		0
112	KC_AB2	CG6013	-0.843391163509558	0.401340755230735	1	pseudobulk	DESeq2	LRT		0
112	KC_AB2	CG6024	0.231608522451114	0.617003821563921	1	pseudobulk	DESeq2	LRT		0
112	KC_AB2	CG6028	0.573760393110584	0.792134620722927	1	pseudobulk	DESeq2	LRT		0
112	KC_AB2	CG6040	-0.841882716738579	1	1	pseudobulk	DESeq2	LRT		0
112	KC_AB2	CG6041	-0.985215116318253	0.577381924121708	1	pseudobulk	DESeq2	LRT		0
112	KC_AB2	CG6044	0.109308655825009	0.676645528797986	1	pseudobulk	DESeq2	LRT		0
112	KC_AB2	CG6051	-1.33722881775036	0.233030602069457	1	pseudobulk	DESeq2	LRT		0
112	KC_AB2	CG6052	-0.600547221779134	1	1	pseudobulk	DESeq2	LRT		0
112	KC_AB2	CG6066	-1.54470851095603	0.55301747381519	1	pseudobulk	DESeq2	LRT		0
112	KC_AB2	CG6071	1.5243995994652	0.741477491078038	1	pseudobulk	DESeq2	LRT		0
112	KC_AB2	CG6083	-1.51812168653838	0.552401469179882	1	pseudobulk	DESeq2	LRT		0
112	KC_AB2	CG6084	0.183555857030446	1	1	pseudobulk	DESeq2	LRT		0
112	KC_AB2	CG6094	2.18200495165647	0.262291744542634	1	pseudobulk	DESeq2	LRT		0
112	KC_AB2	CG6115	-0.0397882088128988	0.906931878843786	1	pseudobulk	DESeq2	LRT		0
112	KC_AB2	CG6123	2.12684015898615	0.445939223035924	1	pseudobulk	DESeq2	LRT		0
112	KC_AB2	CG6126	-0.427431139129811	1	1	pseudobulk	DESeq2	LRT		0
112	KC_AB2	CG6136	-1.43450874381432	0.6168250034656	1	pseudobulk	DESeq2	LRT		0
112	KC_AB2	CG6144	0.142116309242583	0.819992377230745	1	pseudobulk	DESeq2	LRT		0
112	KC_AB2	CG6145	-0.454932278062884	0.762190045481453	1	pseudobulk	DESeq2	LRT		0
112	KC_AB2	CG6153	1.10494336405649	0.567012804673765	1	pseudobulk	DESeq2	LRT		0
112	KC_AB2	CG6154	-2.42531221611559	0.155290777600024	1	pseudobulk	DESeq2	LRT		0
112	KC_AB2	CG6171	0.997108555720206	0.400971963213609	1	pseudobulk	DESeq2	LRT		0
112	KC_AB2	CG6178	-0.255481912470171	0.858763198530661	1	pseudobulk	DESeq2	LRT		0
112	KC_AB2	CG6179	-0.93212148301064	0.654346170772296	1	pseudobulk	DESeq2	LRT		0
112	KC_AB2	CG6180	-2.14686882065105	0.13561734294211	1	pseudobulk	DESeq2	LRT		0
112	KC_AB2	CG6184	0.0541266142353729	0.841388216173578	1	pseudobulk	DESeq2	LRT		0
112	KC_AB2	CG6191	0.263560706315105	0.723120147749975	1	pseudobulk	DESeq2	LRT		0
112	KC_AB2	CG6195	-0.289469814412896	0.828762874114481	1	pseudobulk	DESeq2	LRT		0
112	KC_AB2	CG6201	-1.12181309299261	0.580924140803933	1	pseudobulk	DESeq2	LRT		0
112	KC_AB2	CG6204	0.16620837009559	0.871696729574946	1	pseudobulk	DESeq2	LRT		0
112	KC_AB2	CG6218	0.575355945103283	0.695304589641762	1	pseudobulk	DESeq2	LRT		0
112	KC_AB2	CG6227	0.669718686959781	0.744188515542868	1	pseudobulk	DESeq2	LRT		0
112	KC_AB2	CG6230	-0.621805355124546	0.74877258313458	1	pseudobulk	DESeq2	LRT		0

112	KC_AB2	CG6231	-0.621805355124546	0.74877258313458	1	pseudobulk	DESeq2	LRT		0
112	KC_AB2	CG6236	2.00575794562272	0.172970788994107	1	pseudobulk	DESeq2	LRT		0
112	KC_AB2	CG6254	0.0163320842711025	1	1	pseudobulk	DESeq2	LRT		0
112	KC_AB2	CG6276	-0.375787414320961	0.763535714211207	1	pseudobulk	DESeq2	LRT		0
112	KC_AB2	CG6282	-0.142695388119805	0.71385230927838	1	pseudobulk	DESeq2	LRT		0
112	KC_AB2	CG6293	2.1817927492653	0.458599861292202	1	pseudobulk	DESeq2	LRT		0
112	KC_AB2	CG6325	-0.214559818468328	0.83527205011595	1	pseudobulk	DESeq2	LRT		0
112	KC_AB2	CG6329	-0.0205233139298836	0.899941978410914	1	pseudobulk	DESeq2	LRT		0
112	KC_AB2	CG6330	-0.578562211855164	0.515220187564607	1	pseudobulk	DESeq2	LRT		0
112	KC_AB2	CG6340	0.316861911762254	0.589965588636859	1	pseudobulk	DESeq2	LRT		0
112	KC_AB2	CG6345	-0.427431139129811	1	1	pseudobulk	DESeq2	LRT		0
112	KC_AB2	CG6356	3.17100843528264	0.0615344274129858	1	pseudobulk	DESeq2	LRT		0
113	KC_AB2	CG6379	1.06949565642146	0.453616777336345	1	pseudobulk	DESeq2	LRT		0
113	KC_AB2	CG6388	-1.7824418856176	0.467224294431682	1	pseudobulk	DESeq2	LRT		0
113	KC_AB2	CG6418	-3.27939037134403	0.021846875950203	1	pseudobulk	DESeq2	LRT		0
113	KC_AB2	CG6420	1.97932614544327	0.238813401159128	1	pseudobulk	DESeq2	LRT		0
113	KC_AB2	CG6425	-1.07776915880798	0.775491075088671	1	pseudobulk	DESeq2	LRT		0
113	KC_AB2	CG6428	0.312420435075908	0.852318211916589	1	pseudobulk	DESeq2	LRT		0
113	KC_AB2	CG6435	-0.600547221779133	0.841940696994101	1	pseudobulk	DESeq2	LRT		0
113	KC_AB2	CG6443	1.76864279996931	0.0900068292703597	1	pseudobulk	DESeq2	LRT		0
113	KC_AB2	CG6454	0.147426379928402	0.859798164888867	1	pseudobulk	DESeq2	LRT		0
113	KC_AB2	CG6472	-0.584088793534536	0.669515008258558	1	pseudobulk	DESeq2	LRT		0
113	KC_AB2	CG6481	-2.56828513802066	0.219713238754051	1	pseudobulk	DESeq2	LRT		0
113	KC_AB2	CG6495	-0.0291547266356148	0.916629422948101	1	pseudobulk	DESeq2	LRT		0
113	KC_AB2	CG6511	0.779110570428844	0.722427818657622	1	pseudobulk	DESeq2	LRT		0
113	KC_AB2	CG6512	0.477598032164933	0.631925621551469	1	pseudobulk	DESeq2	LRT		0
113	KC_AB2	CG6523	-0.329046417218003	0.691919455149487	1	pseudobulk	DESeq2	LRT		0
113	KC_AB2	CG6550	2.76205395729416	0.251829971474322	1	pseudobulk	DESeq2	LRT		0
113	KC_AB2	CG6565	-2.17956330567484	0.32574827264182	1	pseudobulk	DESeq2	LRT		0
113	KC_AB2	CG6567	0.674459942220112	0.74558757773504	1	pseudobulk	DESeq2	LRT		0
113	KC_AB2	CG6574	1.3067515803171	0.550895625207199	1	pseudobulk	DESeq2	LRT		0
113	KC_AB2	CG6583	0.0952695066566691	0.802682717050958	1	pseudobulk	DESeq2	LRT		0
113	KC_AB2	CG6607	0.429401558487958	0.672103405576168	1	pseudobulk	DESeq2	LRT		0
113	KC_AB2	CG6610	-2.68462049743772	0.211340580653156	1	pseudobulk	DESeq2	LRT		0
113	KC_AB2	CG6617	-0.172580322546172	0.82571512233296	1	pseudobulk	DESeq2	LRT		0
113	KC_AB2	CG6621	0.600647614362868	0.606069368041406	1	pseudobulk	DESeq2	LRT		0
113	KC_AB2	CG6628	1.23085716405798	0.614623732585597	1	pseudobulk	DESeq2	LRT		0
113	KC_AB2	CG6650	0.920212920242661	0.67286879185691	1	pseudobulk	DESeq2	LRT		0
113	KC_AB2	CG6659	0.0456926662205872	0.981608052892825	1	pseudobulk	DESeq2	LRT		0
113	KC_AB2	CG6664	0.699069409719388	0.519300318109204	1	pseudobulk	DESeq2	LRT		0
113	KC_AB2	CG6665	-2.48435623267434	0.256496613429614	1	pseudobulk	DESeq2	LRT		0
113	KC_AB2	CG6674	0.179313863751659	0.903654772145523	1	pseudobulk	DESeq2	LRT		0
113	KC_AB2	CG6683	-0.600547221779133	0.841940696994101	1	pseudobulk	DESeq2	LRT		0
113	KC_AB2	CG6685	0.450446071537416	0.755989050577903	1	pseudobulk	DESeq2	LRT		0
113	KC_AB2	CG6686	0.816098965297459	0.338182522975364	1	pseudobulk	DESeq2	LRT		0
113	KC_AB2	CG6689	0.790118374229576	0.657873015575783	1	pseudobulk	DESeq2	LRT		0
113	KC_AB2	CG6693	-0.515146352484918	0.685008096372994	1	pseudobulk	DESeq2	LRT		0
113	KC_AB2	CG6695	-0.841882716738579	1	1	pseudobulk	DESeq2	LRT		0
113	KC_AB2	CG6700	0.177097207611263	0.617481146937382	1	pseudobulk	DESeq2	LRT		0
113	KC_AB2	CG6707	-0.430000125487946	0.449031627250677	1	pseudobulk	DESeq2	LRT		0
113	KC_AB2	CG6712	-0.477830850170109	0.75583601646087	1	pseudobulk	DESeq2	LRT		0
113	KC_AB2	CG6723	-0.631578663457669	0.750664130745045	1	pseudobulk	DESeq2	LRT		0
113	KC_AB2	CG6724	-0.525980220958878	0.813340603474339	1	pseudobulk	DESeq2	LRT		0

113	KC_AB2	CG6729	0.522867664761254	0.789441383158121	1	pseudobulk	DESeq2	LRT		0
113	KC_AB2	CG6752	0.670379002386476	0.715721827084457	1	pseudobulk	DESeq2	LRT		0
113	KC_AB2	CG6758	-1.24845401291395	0.444685055945064	1	pseudobulk	DESeq2	LRT		0
113	KC_AB2	CG6762	-0.527534172041629	0.747454425625748	1	pseudobulk	DESeq2	LRT		0
113	KC_AB2	CG6765	0.697485200267237	0.373178909628211	1	pseudobulk	DESeq2	LRT		0
113	KC_AB2	CG6766	-0.981872358369453	0.459122070301772	1	pseudobulk	DESeq2	LRT		0
113	KC_AB2	CG6767	-0.0112596802657905	0.864295306790447	1	pseudobulk	DESeq2	LRT		0
113	KC_AB2	CG6769	-0.293694200456602	0.813028787207985	1	pseudobulk	DESeq2	LRT		0
113	KC_AB2	CG6770	-0.0113919084486102	0.793603177698708	1	pseudobulk	DESeq2	LRT		0
113	KC_AB2	CG6791	0.368595265966847	0.787870002362015	1	pseudobulk	DESeq2	LRT		0
113	KC_AB2	CG6805	-2.43175598261552	0.265266490111144	1	pseudobulk	DESeq2	LRT		0
113	KC_AB2	CG6808	-0.841882716738579	1	1	pseudobulk	DESeq2	LRT		0
113	KC_AB2	CG6813	-1.62117873066028	0.447780352838782	1	pseudobulk	DESeq2	LRT		0
113	KC_AB2	CG6833	1.65689834889508	0.294272870748911	1	pseudobulk	DESeq2	LRT		0
113	KC_AB2	CG6841	0.791468033138705	0.699865413482108	1	pseudobulk	DESeq2	LRT		0
113	KC_AB2	CG6843	1.61902509906088	0.197141226640221	1	pseudobulk	DESeq2	LRT		0
113	KC_AB2	CG6847	-0.600547221779134	1	1	pseudobulk	DESeq2	LRT		0
113	KC_AB2	CG6867	-0.242871905137179	0.778102010603814	1	pseudobulk	DESeq2	LRT		0
113	KC_AB2	CG6878	-0.928951618758869	0.380298029035388	1	pseudobulk	DESeq2	LRT		0
113	KC_AB2	CG6903	0.0268385055383861	0.849611283632849	1	pseudobulk	DESeq2	LRT		0
113	KC_AB2	CG6907	0.222396124134733	1	1	pseudobulk	DESeq2	LRT		0
113	KC_AB2	CG6908	-0.600547221779134	1	1	pseudobulk	DESeq2	LRT		0
113	KC_AB2	CG6912	-0.592481036256484	0.706847545932384	1	pseudobulk	DESeq2	LRT		0
113	KC_AB2	CG6923	2.1737802398166	0.175385277600906	1	pseudobulk	DESeq2	LRT		0
113	KC_AB2	CG6937	-0.744585108800119	0.775294067925548	1	pseudobulk	DESeq2	LRT		0
113	KC_AB2	CG6951	-0.600547221779134	1	1	pseudobulk	DESeq2	LRT		0
113	KC_AB2	CG6961	-1.52885944524348	0.436949674193118	1	pseudobulk	DESeq2	LRT		0
113	KC_AB2	CG6962	-0.600547221779133	0.841940696994101	1	pseudobulk	DESeq2	LRT		0
113	KC_AB2	CG6966	0.417065627274344	0.525403529001949	1	pseudobulk	DESeq2	LRT		0
113	KC_AB2	CG6983	0.247748260068024	0.748472037590895	1	pseudobulk	DESeq2	LRT		0
113	KC_AB2	CG6984	-0.600547221779133	0.841940696994101	1	pseudobulk	DESeq2	LRT		0
113	KC_AB2	CG7006	0.713982351041985	0.921569637923553	1	pseudobulk	DESeq2	LRT		0
113	KC_AB2	CG7009	0.729466899214462	0.64577050287978	1	pseudobulk	DESeq2	LRT		0
113	KC_AB2	CG7011	0.401733800039527	0.836927022098515	1	pseudobulk	DESeq2	LRT		0
113	KC_AB2	CG7028	-1.7837640861897	0.137718316964217	1	pseudobulk	DESeq2	LRT		0
113	KC_AB2	CG7029	-0.333933009738481	0.551940293266496	1	pseudobulk	DESeq2	LRT		0
113	KC_AB2	CG7044	-0.427431139129811	1	1	pseudobulk	DESeq2	LRT		0
113	KC_AB2	CG7049	1.50414049992764	0.421618308250985	1	pseudobulk	DESeq2	LRT		0
113	KC_AB2	CG7058	0.426684700398808	0.751903960076585	1	pseudobulk	DESeq2	LRT		0
113	KC_AB2	CG7059	-1.90464030247338	0.293003351996295	1	pseudobulk	DESeq2	LRT		0
113	KC_AB2	CG7065	-0.298719875642213	0.790780866031668	1	pseudobulk	DESeq2	LRT		0
113	KC_AB2	CG7069	-0.525980220958878	0.813340603474339	1	pseudobulk	DESeq2	LRT		0
113	KC_AB2	CG7079	-0.600547221779133	0.841940696994101	1	pseudobulk	DESeq2	LRT		0
113	KC_AB2	CG7083	0.322206144036317	0.814254108427995	1	pseudobulk	DESeq2	LRT		0
113	KC_AB2	CG7084	1.23085716405798	0.614623732585597	1	pseudobulk	DESeq2	LRT		0
113	KC_AB2	CG7101	-1.7824418856176	0.467224294431682	1	pseudobulk	DESeq2	LRT		0
113	KC_AB2	CG7115	0.0385387705313123	0.942018666160123	1	pseudobulk	DESeq2	LRT		0
113	KC_AB2	CG7120	-0.471774414144539	0.575792540081284	1	pseudobulk	DESeq2	LRT		0
113	KC_AB2	CG7133	0.694688426821718	0.631344205713829	1	pseudobulk	DESeq2	LRT		0
113	KC_AB2	CG7137	-1.07776915880798	0.775491075088671	1	pseudobulk	DESeq2	LRT		0
113	KC_AB2	CG7139	0.751456027923628	0.684049070099348	1	pseudobulk	DESeq2	LRT		0
113	KC_AB2	CG7148	0.189981992999488	0.844679022078182	1	pseudobulk	DESeq2	LRT		0
113	KC_AB2	CG7149	-0.600547221779134	1	1	pseudobulk	DESeq2	LRT		0

113	KC_AB2	CG7154	0.173614304529185	0.853610008588845	1	pseudobulk	DESeq2	LRT	0
113	KC_AB2	CG7156	0.443892390635762	1	1	pseudobulk	DESeq2	LRT	0
113	KC_AB2	CG7166	0.281136170283501	0.533495760464049	1	pseudobulk	DESeq2	LRT	0
113	KC_AB2	CG7168	1.35968315642719	0.485168225381963	1	pseudobulk	DESeq2	LRT	0
113	KC_AB2	CG7182	0.0160181127822205	0.986307233441684	1	pseudobulk	DESeq2	LRT	0
113	KC_AB2	CG7183	-0.214559818468328	0.83527205011595	1	pseudobulk	DESeq2	LRT	0
114	KC_AB2	CG7203	-0.727239665042655	0.76302943878648	1	pseudobulk	DESeq2	LRT	0
114	KC_AB2	CG7206	0.351750450636666	0.791092172206587	1	pseudobulk	DESeq2	LRT	0
114	KC_AB2	CG7218	-0.610130018927353	0.706945694269588	1	pseudobulk	DESeq2	LRT	0
114	KC_AB2	CG7220	0.10810520255519	0.860561504607232	1	pseudobulk	DESeq2	LRT	0
114	KC_AB2	CG7222	0.500388447832125	0.607334288974238	1	pseudobulk	DESeq2	LRT	0
114	KC_AB2	CG7231	0.10119185636653	0.87599843293986	1	pseudobulk	DESeq2	LRT	0
114	KC_AB2	CG7239	1.89152654309809	0.198731168661213	1	pseudobulk	DESeq2	LRT	0
114	KC_AB2	CG7246	-0.841882716738579	1	1	pseudobulk	DESeq2	LRT	0
114	KC_AB2	CG7265	-1.1695175843879	0.576121114907499	1	pseudobulk	DESeq2	LRT	0
114	KC_AB2	CG7272	0.864341462967788	0.925947094335722	1	pseudobulk	DESeq2	LRT	0
114	KC_AB2	CG7275	-1.85733156562156	0.448702818121367	1	pseudobulk	DESeq2	LRT	0
114	KC_AB2	CG7289	1.08786685986457	0.434775801983734	1	pseudobulk	DESeq2	LRT	0
114	KC_AB2	CG7324	0.445925202145231	0.621393239812431	1	pseudobulk	DESeq2	LRT	0
114	KC_AB2	CG7326	0.878020331102202	0.638254468045017	1	pseudobulk	DESeq2	LRT	0
114	KC_AB2	CG7332	-0.0418977545722236	0.928382556228727	1	pseudobulk	DESeq2	LRT	0
114	KC_AB2	CG7339	-0.747852219079764	0.708491663089672	1	pseudobulk	DESeq2	LRT	0
114	KC_AB2	CG7365	1.5243995994652	0.741477491078038	1	pseudobulk	DESeq2	LRT	0
114	KC_AB2	CG7368	0.760842608930612	0.345355435320048	1	pseudobulk	DESeq2	LRT	0
114	KC_AB2	CG7369	2.28513114355826	0.109078094725394	1	pseudobulk	DESeq2	LRT	0
114	KC_AB2	CG7376	-1.12785316439435	0.661319045116135	1	pseudobulk	DESeq2	LRT	0
114	KC_AB2	CG7378	-0.509917358831742	0.0415718734985892	1	pseudobulk	DESeq2	LRT	0
114	KC_AB2	CG7379	-0.487302621246147	0.656283661666888	1	pseudobulk	DESeq2	LRT	0
114	KC_AB2	CG7381	1.56413494198648	0.24936052742936	1	pseudobulk	DESeq2	LRT	0
114	KC_AB2	CG7382	0.271946276389839	0.78772498636642	1	pseudobulk	DESeq2	LRT	0
114	KC_AB2	CG7394	-0.945088217876165	0.375244537295726	1	pseudobulk	DESeq2	LRT	0
114	KC_AB2	CG7407	-0.496555530213266	0.365679393031719	1	pseudobulk	DESeq2	LRT	0
114	KC_AB2	CG7414	0.177244406786774	0.8416546449296	1	pseudobulk	DESeq2	LRT	0
114	KC_AB2	CG7420	-1.07776915880798	0.775491075088671	1	pseudobulk	DESeq2	LRT	0
114	KC_AB2	CG7430	-0.0539375505623002	0.849946269126923	1	pseudobulk	DESeq2	LRT	0
114	KC_AB2	CG7453	1.35862143018359	0.42143515793386	1	pseudobulk	DESeq2	LRT	0
114	KC_AB2	CG7461	2.37663483354862	0.318758708170588	1	pseudobulk	DESeq2	LRT	0
114	KC_AB2	CG7483	0.272041250411024	0.812459143169798	1	pseudobulk	DESeq2	LRT	0
114	KC_AB2	CG7484	-0.526634841516336	0.737873236698189	1	pseudobulk	DESeq2	LRT	0
114	KC_AB2	CG7488	-1.13662642782107	0.49188190542785	1	pseudobulk	DESeq2	LRT	0
114	KC_AB2	CG7492	-1.13963237909365	0.204585799268004	1	pseudobulk	DESeq2	LRT	0
114	KC_AB2	CG7504	0.613659617664411	0.76686169970757	1	pseudobulk	DESeq2	LRT	0
114	KC_AB2	CG7506	0.395894754863932	0.667798836489968	1	pseudobulk	DESeq2	LRT	0
114	KC_AB2	CG7518	0.718872192452052	0.425242856589595	1	pseudobulk	DESeq2	LRT	0
114	KC_AB2	CG7519	0.499895348356637	0.706655715482722	1	pseudobulk	DESeq2	LRT	0
114	KC_AB2	CG7523	-1.07776915880798	0.775491075088671	1	pseudobulk	DESeq2	LRT	0
114	KC_AB2	CG7530	-0.427431139129811	1	1	pseudobulk	DESeq2	LRT	0
114	KC_AB2	CG7536	-0.841882716738579	1	1	pseudobulk	DESeq2	LRT	0
114	KC_AB2	CG7546	0.245168531270132	0.77943040189425	1	pseudobulk	DESeq2	LRT	0
114	KC_AB2	CG7550	0.781403300329945	0.721602784448507	1	pseudobulk	DESeq2	LRT	0
114	KC_AB2	CG7556	-0.8626106243943	0.558154777687958	1	pseudobulk	DESeq2	LRT	0
114	KC_AB2	CG7560	1.5243995994652	0.741477491078038	1	pseudobulk	DESeq2	LRT	0
114	KC_AB2	CG7564	-0.638964113774801	0.488406419425863	1	pseudobulk	DESeq2	LRT	0

114	KC_AB2	CG7565	0.679435809601316	0.578456341981934	1	pseudobulk	DESeq2	LRT	0
114	KC_AB2	CG7568	-1.40673172715063	0.587364787535786	1	pseudobulk	DESeq2	LRT	0
114	KC_AB2	CG7582	-0.567957150623305	0.469485834356396	1	pseudobulk	DESeq2	LRT	0
114	KC_AB2	CG7600	0.554639993769006	0.74261267437262	1	pseudobulk	DESeq2	LRT	0
114	KC_AB2	CG7601	-1.29244479833796	0.693295472370086	1	pseudobulk	DESeq2	LRT	0
114	KC_AB2	CG7607	-0.382929749762112	0.851961810578875	1	pseudobulk	DESeq2	LRT	0
114	KC_AB2	CG7611	-0.0592202969631771	0.853625506650754	1	pseudobulk	DESeq2	LRT	0
114	KC_AB2	CG7627	-0.427431139129811	1	1	pseudobulk	DESeq2	LRT	0
114	KC_AB2	CG7630	-0.391139673235612	0.206518756230204	1	pseudobulk	DESeq2	LRT	0
114	KC_AB2	CG7632	-1.90937835128834	0.377732797313411	1	pseudobulk	DESeq2	LRT	0
114	KC_AB2	CG7637	-2.16391070247733	0.211761232029068	1	pseudobulk	DESeq2	LRT	0
114	KC_AB2	CG7638	-0.657909890970536	0.593041011890107	1	pseudobulk	DESeq2	LRT	0
114	KC_AB2	CG7639	-1.0272091282053	0.212469788636765	1	pseudobulk	DESeq2	LRT	0
114	KC_AB2	CG7646	0.0512900719931879	0.782872548070885	1	pseudobulk	DESeq2	LRT	0
114	KC_AB2	CG7650	-1.6150095599849	0.519549602003096	1	pseudobulk	DESeq2	LRT	0
114	KC_AB2	CG7655	-2.3627362995981	0.281646618575938	1	pseudobulk	DESeq2	LRT	0
114	KC_AB2	CG7656	-0.286379382745027	0.696717290121104	1	pseudobulk	DESeq2	LRT	0
114	KC_AB2	CG7668	-0.600547221779133	0.841940696994101	1	pseudobulk	DESeq2	LRT	0
114	KC_AB2	CG7675	1.5243995994652	0.741477491078038	1	pseudobulk	DESeq2	LRT	0
114	KC_AB2	CG7685	-0.573109354659945	0.739419341582026	1	pseudobulk	DESeq2	LRT	0
114	KC_AB2	CG7692	2.1817927492653	0.458599861292202	1	pseudobulk	DESeq2	LRT	0
114	KC_AB2	CG7694	-1.69144362911439	0.144093369050208	1	pseudobulk	DESeq2	LRT	0
114	KC_AB2	CG7706	0.941219035699073	0.538581349183844	1	pseudobulk	DESeq2	LRT	0
114	KC_AB2	CG7728	1.23085716405798	0.614623732585597	1	pseudobulk	DESeq2	LRT	0
114	KC_AB2	CG7737	0.791286025443143	0.690540380845434	1	pseudobulk	DESeq2	LRT	0
114	KC_AB2	CG7739	-0.446472139125649	0.481546831995592	1	pseudobulk	DESeq2	LRT	0
114	KC_AB2	CG7741	-2.35482794471587	0.312286863604367	1	pseudobulk	DESeq2	LRT	0
114	KC_AB2	CG7744	1.94691684546882	0.398076844044942	1	pseudobulk	DESeq2	LRT	0
114	KC_AB2	CG7745	0.344848465703798	0.771318976737493	1	pseudobulk	DESeq2	LRT	0
114	KC_AB2	CG7747	-0.109785922225664	0.939774912519229	1	pseudobulk	DESeq2	LRT	0
114	KC_AB2	CG7766	-0.886360890895607	0.280798985825642	1	pseudobulk	DESeq2	LRT	0
114	KC_AB2	CG7772	0.791286025443143	0.690540380845434	1	pseudobulk	DESeq2	LRT	0
114	KC_AB2	CG7781	0.0713068968423581	0.740944529547661	1	pseudobulk	DESeq2	LRT	0
114	KC_AB2	CG7785	-2.85012482474053	0.0687493578711481	1	pseudobulk	DESeq2	LRT	0
114	KC_AB2	CG7789	0.751192268922472	0.593871128406335	1	pseudobulk	DESeq2	LRT	0
114	KC_AB2	CG7791	-0.965895092355276	0.70584537186792	1	pseudobulk	DESeq2	LRT	0
114	KC_AB2	CG7810	0.0471936707755822	0.953916869426286	1	pseudobulk	DESeq2	LRT	0
114	KC_AB2	CG7834	1.36027594143576	0.252111765977782	1	pseudobulk	DESeq2	LRT	0
114	KC_AB2	CG7839	1.53811683574911	0.54844951579854	1	pseudobulk	DESeq2	LRT	0
114	KC_AB2	CG7840	1.5243995994652	0.741477491078038	1	pseudobulk	DESeq2	LRT	0
114	KC_AB2	CG7841	-1.13203571182402	0.515128055582874	1	pseudobulk	DESeq2	LRT	0
114	KC_AB2	CG7849	-1.04445428854771	0.603034233836386	1	pseudobulk	DESeq2	LRT	0
114	KC_AB2	CG7857	-1.26643386773811	0.407195538709078	1	pseudobulk	DESeq2	LRT	0
114	KC_AB2	CG7872	-0.120341634347934	0.796922980054235	1	pseudobulk	DESeq2	LRT	0
114	KC_AB2	CG7878	-0.733799092210619	0.595581324799525	1	pseudobulk	DESeq2	LRT	0
114	KC_AB2	CG7879	-0.245194272465198	0.681848305246622	1	pseudobulk	DESeq2	LRT	0
114	KC_AB2	CG7888	-0.211959943697087	0.913646816097592	1	pseudobulk	DESeq2	LRT	0
114	KC_AB2	CG7889	-0.70727250916738	0.705390475165233	1	pseudobulk	DESeq2	LRT	0
114	KC_AB2	CG7903	-0.990173036589667	0.255758956713904	1	pseudobulk	DESeq2	LRT	0
114	KC_AB2	CG7920	0.0735617882363174	0.752628965524182	1	pseudobulk	DESeq2	LRT	0
114	KC_AB2	CG7943	-1.59444973324989	0.132661627577084	1	pseudobulk	DESeq2	LRT	0
114	KC_AB2	CG7945	-0.282138620778927	0.693747837167961	1	pseudobulk	DESeq2	LRT	0
114	KC_AB2	CG7946	-0.140714024380289	0.871607324488109	1	pseudobulk	DESeq2	LRT	0

115	KC_AB2	CG7956	-0.0316021269790819	0.88501217935157	1	pseudobulk	DESeq2	LRT		0
115	KC_AB2	CG7970	-0.0852743682753834	0.917718671153184	1	pseudobulk	DESeq2	LRT		0
115	KC_AB2	CG7971	-0.0365248090813123	0.815920902260267	1	pseudobulk	DESeq2	LRT		0
115	KC_AB2	CG7974	0.943586647867311	0.690632779168748	1	pseudobulk	DESeq2	LRT		0
115	KC_AB2	CG7985	0.280362366614727	0.731018725580071	1	pseudobulk	DESeq2	LRT		0
115	KC_AB2	CG7987	-0.572699501043507	0.646702903541501	1	pseudobulk	DESeq2	LRT		0
115	KC_AB2	CG7988	0.70378358684898	0.932301451973468	1	pseudobulk	DESeq2	LRT		0
115	KC_AB2	CG7990	-0.23600120215621	0.638589157132045	1	pseudobulk	DESeq2	LRT		0
115	KC_AB2	CG7991	-1.70614408517477	0.52816093424714	1	pseudobulk	DESeq2	LRT		0
115	KC_AB2	CG8001	1.91940128222308	0.325447237254087	1	pseudobulk	DESeq2	LRT		0
115	KC_AB2	CG8003	0.160783517120652	0.891450558630448	1	pseudobulk	DESeq2	LRT		0
115	KC_AB2	CG8004	0.435764134493322	0.599361059572416	1	pseudobulk	DESeq2	LRT		0
115	KC_AB2	CG8005	1.5243995994652	0.741477491078038	1	pseudobulk	DESeq2	LRT		0
115	KC_AB2	CG8009	-0.881712936649673	0.373286288711223	1	pseudobulk	DESeq2	LRT		0
115	KC_AB2	CG8010	1.23571851286199	0.575848719421412	1	pseudobulk	DESeq2	LRT		0
115	KC_AB2	CG8012	-0.427431139129811	1	1	pseudobulk	DESeq2	LRT		0
115	KC_AB2	CG8031	1.41392113638259	0.617674323943697	1	pseudobulk	DESeq2	LRT		0
115	KC_AB2	CG8032	0.10269109923854	0.920310179743496	1	pseudobulk	DESeq2	LRT		0
115	KC_AB2	CG8034	-1.16116875819275	0.736255625566845	1	pseudobulk	DESeq2	LRT		0
115	KC_AB2	CG8036	-0.209808076863057	0.877444409505505	1	pseudobulk	DESeq2	LRT		0
115	KC_AB2	CG8042	-0.189058878747218	0.810544405147214	1	pseudobulk	DESeq2	LRT		0
115	KC_AB2	CG8043	-0.187579055727378	0.895795928577846	1	pseudobulk	DESeq2	LRT		0
115	KC_AB2	CG8060	1.94691684546882	0.398076844044942	1	pseudobulk	DESeq2	LRT		0
115	KC_AB2	CG8064	2.12684015898615	0.445939223035924	1	pseudobulk	DESeq2	LRT		0
115	KC_AB2	CG8066	-0.600547221779134	1	1	pseudobulk	DESeq2	LRT		0
115	KC_AB2	CG8067	-0.975846932608671	0.648794276036568	1	pseudobulk	DESeq2	LRT		0
115	KC_AB2	CG8078	-1.99338034826303	0.401583286802675	1	pseudobulk	DESeq2	LRT		0
115	KC_AB2	CG8079	0.626381759533374	0.656814767552552	1	pseudobulk	DESeq2	LRT		0
115	KC_AB2	CG8080	-0.70727250916738	0.705390475165233	1	pseudobulk	DESeq2	LRT		0
115	KC_AB2	CG8089	1.5243995994652	0.741477491078038	1	pseudobulk	DESeq2	LRT		0
115	KC_AB2	CG8097	-0.841882716738579	1	1	pseudobulk	DESeq2	LRT		0
115	KC_AB2	CG8108	0.0927364393328547	0.738736675738308	1	pseudobulk	DESeq2	LRT		0
115	KC_AB2	CG8111	0.905299431108869	0.481220780960401	1	pseudobulk	DESeq2	LRT		0
115	KC_AB2	CG8132	0.124469533230075	0.879106681361008	1	pseudobulk	DESeq2	LRT		0
115	KC_AB2	CG8134	-0.90780790323272	0.42129539222171	1	pseudobulk	DESeq2	LRT		0
115	KC_AB2	CG8149	-0.0729924660251299	0.883103133394531	1	pseudobulk	DESeq2	LRT		0
115	KC_AB2	CG8152	0.522867664761254	0.789441383158121	1	pseudobulk	DESeq2	LRT		0
115	KC_AB2	CG8155	-0.500443775160262	0.779866416934443	1	pseudobulk	DESeq2	LRT		0
115	KC_AB2	CG8159	1.41524016620164	0.505408490038387	1	pseudobulk	DESeq2	LRT		0
115	KC_AB2	CG8173	0.915907466393497	0.431216006125332	1	pseudobulk	DESeq2	LRT		0
115	KC_AB2	CG8176	0.581500382565273	0.535094871493449	1	pseudobulk	DESeq2	LRT		0
115	KC_AB2	CG8177	0.0922900981527821	0.836373534787894	1	pseudobulk	DESeq2	LRT		0
115	KC_AB2	CG8187	0.139725873750893	0.895323740949644	1	pseudobulk	DESeq2	LRT		0
115	KC_AB2	CG8188	-0.349177216494308	0.559928133790087	1	pseudobulk	DESeq2	LRT		0
115	KC_AB2	CG8195	0.172065817198206	0.847936229673091	1	pseudobulk	DESeq2	LRT		0
115	KC_AB2	CG8206	-0.421745386842927	0.705422721674368	1	pseudobulk	DESeq2	LRT		0
115	KC_AB2	CG8207	-0.167620063280405	0.874493033115399	1	pseudobulk	DESeq2	LRT		0
115	KC_AB2	CG8209	-1.07842346057085	0.406311492678767	1	pseudobulk	DESeq2	LRT		0
115	KC_AB2	CG8219	1.5243995994652	0.741477491078038	1	pseudobulk	DESeq2	LRT		0
115	KC_AB2	CG8223	-0.195680433958221	0.836610256099757	1	pseudobulk	DESeq2	LRT		0
115	KC_AB2	CG8229	-0.0513036781903953	0.953539809697205	1	pseudobulk	DESeq2	LRT		0
115	KC_AB2	CG8230	1.46667553000072	0.486649107648129	1	pseudobulk	DESeq2	LRT		0
115	KC_AB2	CG8237	-0.129169751264268	0.819008942738722	1	pseudobulk	DESeq2	LRT		0

115	KC_AB2	CG8243	0.878400519296987	0.266972796164283	1	pseudobulk	DESeq2	LRT		0
115	KC_AB2	CG8245	-0.0132064388658926	0.944993928510436	1	pseudobulk	DESeq2	LRT		0
115	KC_AB2	CG8248	0.0996159567950134	0.851927989736722	1	pseudobulk	DESeq2	LRT		0
115	KC_AB2	CG8270	0.0471936707755822	0.953916869426286	1	pseudobulk	DESeq2	LRT		0
115	KC_AB2	CG8272	-0.266975112354047	0.883405790241326	1	pseudobulk	DESeq2	LRT		0
115	KC_AB2	CG8281	-1.16664752369898	0.477498709706391	1	pseudobulk	DESeq2	LRT		0
115	KC_AB2	CG8289	0.560366119054685	0.502751957293823	1	pseudobulk	DESeq2	LRT		0
115	KC_AB2	CG8298	-0.231439034919415	0.80021088014829	1	pseudobulk	DESeq2	LRT		0
115	KC_AB2	CG8300	0.119184245502257	0.867041662569587	1	pseudobulk	DESeq2	LRT		0
115	KC_AB2	CG8301	-0.0714250666109256	0.858174176030784	1	pseudobulk	DESeq2	LRT		0
115	KC_AB2	CG8311	-1.00040120329973	0.385904636843468	1	pseudobulk	DESeq2	LRT		0
115	KC_AB2	CG8312	1.5243995994652	0.741477491078038	1	pseudobulk	DESeq2	LRT		0
115	KC_AB2	CG8314	0.291588086455099	0.73713937053781	1	pseudobulk	DESeq2	LRT		0
115	KC_AB2	CG8319	0.506662102707318	0.782529221356027	1	pseudobulk	DESeq2	LRT		0
115	KC_AB2	CG8320	-1.12569474978191	0.563105123273263	1	pseudobulk	DESeq2	LRT		0
115	KC_AB2	CG8321	-0.206737450297679	0.839710750947916	1	pseudobulk	DESeq2	LRT		0
115	KC_AB2	CG8323	-1.99338034826303	0.401583286802675	1	pseudobulk	DESeq2	LRT		0
115	KC_AB2	CG8326	-1.07776915880798	0.775491075088671	1	pseudobulk	DESeq2	LRT		0
115	KC_AB2	CG8336	-1.34407461458031	0.584057515591355	1	pseudobulk	DESeq2	LRT		0
115	KC_AB2	CG8353	-0.0852743682753834	0.917718671153184	1	pseudobulk	DESeq2	LRT		0
115	KC_AB2	CG8360	-1.17605181232094	0.305064272881729	1	pseudobulk	DESeq2	LRT		0
115	KC_AB2	CG8366	-0.710748485197251	0.690315254689061	1	pseudobulk	DESeq2	LRT		0
115	KC_AB2	CG8369	0.423094842727727	0.491552091975305	1	pseudobulk	DESeq2	LRT		0
115	KC_AB2	CG8370	-0.938817101015206	0.629705053774855	1	pseudobulk	DESeq2	LRT		0
115	KC_AB2	CG8372	-0.264028253434503	0.735432777295175	1	pseudobulk	DESeq2	LRT		0
115	KC_AB2	CG8379	0.674297994991685	0.677663040711554	1	pseudobulk	DESeq2	LRT		0
115	KC_AB2	CG8388	-1.6150095599849	0.519549602003096	1	pseudobulk	DESeq2	LRT		0
115	KC_AB2	CG8389	2.56928843919784	0.100720093526046	1	pseudobulk	DESeq2	LRT		0
115	KC_AB2	CG8397	0.502689766770221	0.531540208857615	1	pseudobulk	DESeq2	LRT		0
115	KC_AB2	CG8399	1.5243995994652	0.741477491078038	1	pseudobulk	DESeq2	LRT		0
115	KC_AB2	CG8405	-0.205129176494172	0.829702602891631	1	pseudobulk	DESeq2	LRT		0
115	KC_AB2	CG8412	-0.666462228453646	0.57622260148614	1	pseudobulk	DESeq2	LRT		0
115	KC_AB2	CG8414	-0.600547221779134	1	1	pseudobulk	DESeq2	LRT		0
115	KC_AB2	CG8435	0.0586988282336896	0.836674359608016	1	pseudobulk	DESeq2	LRT		0
115	KC_AB2	CG8441	0.107479031250535	0.88712690359169	1	pseudobulk	DESeq2	LRT		0
115	KC_AB2	CG8460	2.1817927492653	0.458599861292202	1	pseudobulk	DESeq2	LRT		0
115	KC_AB2	CG8461	-1.54470851095603	0.55301747381519	1	pseudobulk	DESeq2	LRT		0
115	KC_AB2	CG8475	0.701184840342706	0.511078110420118	1	pseudobulk	DESeq2	LRT		0
115	KC_AB2	CG8485	-0.173446535018959	0.777082273297872	1	pseudobulk	DESeq2	LRT		0
115	KC_AB2	CG8490	-1.52885944524348	0.436949674193118	1	pseudobulk	DESeq2	LRT		0
115	KC_AB2	CG8500	-0.0775425601282223	0.832751896539071	1	pseudobulk	DESeq2	LRT		0
115	KC_AB2	CG8507	-0.797464827647013	0.680572496132846	1	pseudobulk	DESeq2	LRT		0
115	KC_AB2	CG8516	-0.211959943697087	0.913646816097592	1	pseudobulk	DESeq2	LRT		0
115	KC_AB2	CG8519	1.64759826860276	0.469402419161652	1	pseudobulk	DESeq2	LRT		0
115	KC_AB2	CG8520	-1.24008539618013	0.475175960720631	1	pseudobulk	DESeq2	LRT		0
115	KC_AB2	CG8531	-1.3723417613553	0.619532036522	1	pseudobulk	DESeq2	LRT		0
115	KC_AB2	CG8547	-0.427431139129811	1	1	pseudobulk	DESeq2	LRT		0
116	KC_AB2	CG8549	-0.204032266913788	0.8225074162257	1	pseudobulk	DESeq2	LRT		0
116	KC_AB2	CG8569	0.674459942220112	0.74558757773504	1	pseudobulk	DESeq2	LRT		0
116	KC_AB2	CG8578	-0.0346299205288152	0.960816705563255	1	pseudobulk	DESeq2	LRT		0
116	KC_AB2	CG8611	1.41392113638259	0.617674323943697	1	pseudobulk	DESeq2	LRT		0
116	KC_AB2	CG8613	-0.256842571784142	0.695576586955885	1	pseudobulk	DESeq2	LRT		0
116	KC_AB2	CG8617	0.386277826735825	0.636750798782711	1	pseudobulk	DESeq2	LRT		0

116	KC_AB2	CG8635	-0.264893241223518	0.794736650361695	1	pseudobulk	DESeq2	LRT		0
116	KC_AB2	CG8641	-3.35516597267843	0.35700766446396	1	pseudobulk	DESeq2	LRT		0
116	KC_AB2	CG8668	-0.421805629491798	0.556935873618308	1	pseudobulk	DESeq2	LRT		0
116	KC_AB2	CG8671	0.153194126246611	0.750510155274806	1	pseudobulk	DESeq2	LRT		0
116	KC_AB2	CG8673	1.5243995994652	0.741477491078038	1	pseudobulk	DESeq2	LRT		0
116	KC_AB2	CG8675	2.07908843388644	0.372593257917908	1	pseudobulk	DESeq2	LRT		0
116	KC_AB2	CG8677	-0.217198129923736	0.709794660316719	1	pseudobulk	DESeq2	LRT		0
116	KC_AB2	CG8712	-1.51928970377954	0.486908946651791	1	pseudobulk	DESeq2	LRT		0
116	KC_AB2	CG8726	0.526581258016316	0.201741099788804	1	pseudobulk	DESeq2	LRT		0
116	KC_AB2	CG8728	-1.49135010060466	0.247553067119926	1	pseudobulk	DESeq2	LRT		0
116	KC_AB2	CG8745	-1.31605706048848	0.32617678882416	1	pseudobulk	DESeq2	LRT		0
116	KC_AB2	CG8757	3.20650492957429	0.177944556633249	1	pseudobulk	DESeq2	LRT		0
116	KC_AB2	CG8765	1.03433320541275	0.508458926979953	1	pseudobulk	DESeq2	LRT		0
116	KC_AB2	CG8768	-0.74166767034759	0.710107880048253	1	pseudobulk	DESeq2	LRT		0
116	KC_AB2	CG8777	-1.93220872672389	0.398686710033904	1	pseudobulk	DESeq2	LRT		0
116	KC_AB2	CG8778	-0.132376408518421	0.803853205135446	1	pseudobulk	DESeq2	LRT		0
116	KC_AB2	CG8785	-0.427431139129811	1	1	pseudobulk	DESeq2	LRT		0
116	KC_AB2	CG8814	-0.97823833072745	0.49768557370507	1	pseudobulk	DESeq2	LRT		0
116	KC_AB2	CG8818	-0.448505397966453	0.534244911948061	1	pseudobulk	DESeq2	LRT		0
116	KC_AB2	CG8833	-0.607142276902872	0.748306560670997	1	pseudobulk	DESeq2	LRT		0
116	KC_AB2	CG8839	1.40241945196173	0.57265124481703	1	pseudobulk	DESeq2	LRT		0
116	KC_AB2	CG8841	-0.427431139129811	1	1	pseudobulk	DESeq2	LRT		0
116	KC_AB2	CG8850	-0.600547221779133	0.841940696994101	1	pseudobulk	DESeq2	LRT		0
116	KC_AB2	CG8858	-0.841882716738579	1	1	pseudobulk	DESeq2	LRT		0
116	KC_AB2	CG8860	-0.854780544062925	0.322035644668713	1	pseudobulk	DESeq2	LRT		0
116	KC_AB2	CG8861	1.5243995994652	0.741477491078038	1	pseudobulk	DESeq2	LRT		0
116	KC_AB2	CG8870	-0.600547221779134	1	1	pseudobulk	DESeq2	LRT		0
116	KC_AB2	CG8878	0.30070001772876	0.711703067475664	1	pseudobulk	DESeq2	LRT		0
116	KC_AB2	CG8891	1.5243995994652	0.741477491078038	1	pseudobulk	DESeq2	LRT		0
116	KC_AB2	CG8892	-1.26581990428552	0.438039698892386	1	pseudobulk	DESeq2	LRT		0
116	KC_AB2	CG8910	-0.923187930790853	0.011289792899475	1	pseudobulk	DESeq2	LRT		0
116	KC_AB2	CG8915	-1.54470851095603	0.55301747381519	1	pseudobulk	DESeq2	LRT		0
116	KC_AB2	CG8924	0.378473117141395	0.684005709625445	1	pseudobulk	DESeq2	LRT		0
116	KC_AB2	CG8929	-0.578883641400771	0.357407869344698	1	pseudobulk	DESeq2	LRT		0
116	KC_AB2	CG8939	-0.600547221779134	1	1	pseudobulk	DESeq2	LRT		0
116	KC_AB2	CG8944	-0.338771435603146	0.816656863441963	1	pseudobulk	DESeq2	LRT		0
116	KC_AB2	CG8950	0.0163320842711025	1	1	pseudobulk	DESeq2	LRT		0
116	KC_AB2	CG8963	-0.0370627279326144	0.948916702147431	1	pseudobulk	DESeq2	LRT		0
116	KC_AB2	CG8974	-0.66662670075323	0.291269167354018	1	pseudobulk	DESeq2	LRT		0
116	KC_AB2	CG8993	-1.0311426436512	0.117034576921561	1	pseudobulk	DESeq2	LRT		0
116	KC_AB2	CG9003	0.945277482873228	0.198658611167567	1	pseudobulk	DESeq2	LRT		0
116	KC_AB2	CG9004	0.70378358684898	0.932301451973468	1	pseudobulk	DESeq2	LRT		0
116	KC_AB2	CG9005	-0.0542497144667972	0.857492073852923	1	pseudobulk	DESeq2	LRT		0
116	KC_AB2	CG9008	-0.114665067074464	0.796190723332388	1	pseudobulk	DESeq2	LRT		0
116	KC_AB2	CG9018	-0.590530304575484	0.665583425160295	1	pseudobulk	DESeq2	LRT		0
116	KC_AB2	CG9034	-1.24082342278331	0.0194585695669682	1	pseudobulk	DESeq2	LRT		0
116	KC_AB2	CG9044	-0.661981047763185	0.673259890353592	1	pseudobulk	DESeq2	LRT		0
116	KC_AB2	CG9062	-0.796646902391464	0.865131871797536	1	pseudobulk	DESeq2	LRT		0
116	KC_AB2	CG9065	-1.04855790509279	0.0899636932025314	1	pseudobulk	DESeq2	LRT		0
116	KC_AB2	CG9067	0.233567218377804	0.870856688286003	1	pseudobulk	DESeq2	LRT		0
116	KC_AB2	CG9107	-2.06407076736304	0.362444518317458	1	pseudobulk	DESeq2	LRT		0
116	KC_AB2	CG9114	-0.427431139129811	1	1	pseudobulk	DESeq2	LRT		0
116	KC_AB2	CG9121	1.67771847552954	0.204889995302086	1	pseudobulk	DESeq2	LRT		0

116	KC_AB2	CG9123	-0.388706041365385	0.719248289460076	1	pseudobulk	DESeq2	LRT		0
116	KC_AB2	CG9125	0.183555857030446	1	1	pseudobulk	DESeq2	LRT		0
116	KC_AB2	CG9132	0.0657449816609969	0.889463457135824	1	pseudobulk	DESeq2	LRT		0
116	KC_AB2	CG9135	-1.10708192968218	0.475910309996732	1	pseudobulk	DESeq2	LRT		0
116	KC_AB2	CG9143	-1.41185216024919	0.479262923708383	1	pseudobulk	DESeq2	LRT		0
116	KC_AB2	CG9147	-1.7824418856176	0.467224294431682	1	pseudobulk	DESeq2	LRT		0
116	KC_AB2	CG9149	-0.954402825835707	0.71434914773897	1	pseudobulk	DESeq2	LRT		0
116	KC_AB2	CG9154	-0.338771435603146	0.816656863441963	1	pseudobulk	DESeq2	LRT		0
116	KC_AB2	CG9164	-1.18234336368098	0.539222050855113	1	pseudobulk	DESeq2	LRT		0
116	KC_AB2	CG9170	-0.0117286812516789	0.926295867741721	1	pseudobulk	DESeq2	LRT		0
116	KC_AB2	CG9171	-0.304126844841612	0.415405553179891	1	pseudobulk	DESeq2	LRT		0
116	KC_AB2	CG9175	-1.03879166146466	0.452281684916054	1	pseudobulk	DESeq2	LRT		0
116	KC_AB2	CG9205	-1.67208166987408	0.128109985527051	1	pseudobulk	DESeq2	LRT		0
116	KC_AB2	CG9213	-0.167620063280405	0.874493033115399	1	pseudobulk	DESeq2	LRT		0
116	KC_AB2	CG9215	0.562241701254933	0.70370164834238	1	pseudobulk	DESeq2	LRT		0
116	KC_AB2	CG9231	-0.736732886329631	0.410336883872476	1	pseudobulk	DESeq2	LRT		0
116	KC_AB2	CG9240	1.5563381047723	0.327237453727378	1	pseudobulk	DESeq2	LRT		0
116	KC_AB2	CG9246	-1.70380348762697	0.473446675363872	1	pseudobulk	DESeq2	LRT		0
116	KC_AB2	CG9248	-0.427431139129811	1	1	pseudobulk	DESeq2	LRT		0
116	KC_AB2	CG9253	0.757219824578072	0.665134175478325	1	pseudobulk	DESeq2	LRT		0
116	KC_AB2	CG9254	-0.427431139129811	1	1	pseudobulk	DESeq2	LRT		0
116	KC_AB2	CG9257	-0.901837023554483	0.505426351837533	1	pseudobulk	DESeq2	LRT		0
116	KC_AB2	CG9265	-0.942546179946792	0.331592775089447	1	pseudobulk	DESeq2	LRT		0
116	KC_AB2	CG9272	-1.60499793386288	0.434358912762731	1	pseudobulk	DESeq2	LRT		0
116	KC_AB2	CG9281	0.188570594075831	0.78615416297804	1	pseudobulk	DESeq2	LRT		0
116	KC_AB2	CG9286	0.222396124134733	1	1	pseudobulk	DESeq2	LRT		0
116	KC_AB2	CG9300	0.49522746739306	0.807638127459475	1	pseudobulk	DESeq2	LRT		0
116	KC_AB2	CG9302	-1.16116875819275	0.736255625566845	1	pseudobulk	DESeq2	LRT		0
116	KC_AB2	CG9304	-1.78094334699383	0.470930349923561	1	pseudobulk	DESeq2	LRT		0
116	KC_AB2	CG9313	1.5243995994652	0.741477491078038	1	pseudobulk	DESeq2	LRT		0
116	KC_AB2	CG9328	-0.402771929862888	0.225793454449009	1	pseudobulk	DESeq2	LRT		0
116	KC_AB2	CG9330	-0.512482960385716	0.624092930078225	1	pseudobulk	DESeq2	LRT		0
116	KC_AB2	CG9336	-0.600547221779133	0.841940696994101	1	pseudobulk	DESeq2	LRT		0
116	KC_AB2	CG9344	0.296024567156581	0.809213472782919	1	pseudobulk	DESeq2	LRT		0
116	KC_AB2	CG9346	0.67198465404655	0.644009148297803	1	pseudobulk	DESeq2	LRT		0
116	KC_AB2	CG9356	0.183555857030446	1	1	pseudobulk	DESeq2	LRT		0
116	KC_AB2	CG9368	-0.327526338404882	0.439022568122681	1	pseudobulk	DESeq2	LRT		0
116	KC_AB2	CG9380	1.40241945196173	0.57265124481703	1	pseudobulk	DESeq2	LRT		0
116	KC_AB2	CG9384	-2.6623779188682	0.0969884494388077	1	pseudobulk	DESeq2	LRT		0
116	KC_AB2	CG9386	0.49522746739306	0.807638127459475	1	pseudobulk	DESeq2	LRT		0
116	KC_AB2	CG9391	-1.03273984421759	0.159452768063864	1	pseudobulk	DESeq2	LRT		0
117	KC_AB2	CG9393	-1.04077127086163	0.605194231525991	1	pseudobulk	DESeq2	LRT		0
117	KC_AB2	CG9394	-1.07776915880798	0.775491075088671	1	pseudobulk	DESeq2	LRT		0
117	KC_AB2	CG9395	-0.420330008058464	0.760607148862182	1	pseudobulk	DESeq2	LRT		0
117	KC_AB2	CG9399	-0.0822393786369241	0.945088287701258	1	pseudobulk	DESeq2	LRT		0
117	KC_AB2	CG9410	0.0615981262762758	0.891056019088381	1	pseudobulk	DESeq2	LRT		0
117	KC_AB2	CG9422	-0.265566251458151	0.79369727795903	1	pseudobulk	DESeq2	LRT		0
117	KC_AB2	CG9425	-0.0469752732238029	0.866278039964915	1	pseudobulk	DESeq2	LRT		0
117	KC_AB2	CG9426	-0.0412605369877587	0.935472558345137	1	pseudobulk	DESeq2	LRT		0
117	KC_AB2	CG9467	-0.131394265114284	0.912761082955056	1	pseudobulk	DESeq2	LRT		0
117	KC_AB2	CG9471	-1.29244479833796	0.693295472370086	1	pseudobulk	DESeq2	LRT		0
117	KC_AB2	CG9485	1.21092382796727	0.637125262974216	1	pseudobulk	DESeq2	LRT		0
117	KC_AB2	CG9515	-0.841882716738579	1	1	pseudobulk	DESeq2	LRT		0

117	KC_AB2	CG9518	-0.427431139129811	1	1	pseudobulk	DESeq2	LRT	0
117	KC_AB2	CG9521	-0.427431139129811	1	1	pseudobulk	DESeq2	LRT	0
117	KC_AB2	CG9527	1.6586891832962	0.455669746689673	1	pseudobulk	DESeq2	LRT	0
117	KC_AB2	CG9536	0.920707161609556	0.66023874320038	1	pseudobulk	DESeq2	LRT	0
117	KC_AB2	CG9570	-0.600547221779134	1	1	pseudobulk	DESeq2	LRT	0
117	KC_AB2	CG9577	1.39827770914961	0.587246212652858	1	pseudobulk	DESeq2	LRT	0
117	KC_AB2	CG9578	-0.246880080867625	0.883966189422952	1	pseudobulk	DESeq2	LRT	0
117	KC_AB2	CG9581	-1.16116875819275	0.736255625566845	1	pseudobulk	DESeq2	LRT	0
117	KC_AB2	CG9586	-1.81908534540922	0.201688772560066	1	pseudobulk	DESeq2	LRT	0
117	KC_AB2	CG9588	1.68085412209716	0.397337885826871	1	pseudobulk	DESeq2	LRT	0
117	KC_AB2	CG9590	1.62063118311225	0.212389735341338	1	pseudobulk	DESeq2	LRT	0
117	KC_AB2	CG9596	0.183555857030446	1	1	pseudobulk	DESeq2	LRT	0
117	KC_AB2	CG9601	2.08525124271099	0.198874418179649	1	pseudobulk	DESeq2	LRT	0
117	KC_AB2	CG9609	0.136957844448697	0.85600562066025	1	pseudobulk	DESeq2	LRT	0
117	KC_AB2	CG9628	-0.427431139129811	1	1	pseudobulk	DESeq2	LRT	0
117	KC_AB2	CG9636	0.318992570162381	0.718855596926143	1	pseudobulk	DESeq2	LRT	0
117	KC_AB2	CG9641	-0.0043534154198402	0.970644561615216	1	pseudobulk	DESeq2	LRT	0
117	KC_AB2	CG9643	0.222396124134733	1	1	pseudobulk	DESeq2	LRT	0
117	KC_AB2	CG9646	-0.111904246716299	0.78404063631332	1	pseudobulk	DESeq2	LRT	0
117	KC_AB2	CG9657	-0.841882716738579	1	1	pseudobulk	DESeq2	LRT	0
117	KC_AB2	CG9662	1.18344865139507	0.266315848497431	1	pseudobulk	DESeq2	LRT	0
117	KC_AB2	CG9667	-2.07054128397783	0.262558868429793	1	pseudobulk	DESeq2	LRT	0
117	KC_AB2	CG9674	0.023081105835085	0.867796675570713	1	pseudobulk	DESeq2	LRT	0
117	KC_AB2	CG9686	-1.48127553173043	0.150608502257625	1	pseudobulk	DESeq2	LRT	0
117	KC_AB2	CG9689	-0.427431139129811	1	1	pseudobulk	DESeq2	LRT	0
117	KC_AB2	CG9691	2.1817927492653	0.458599861292202	1	pseudobulk	DESeq2	LRT	0
117	KC_AB2	CG9705	0.0867297377308415	0.871318349919412	1	pseudobulk	DESeq2	LRT	0
117	KC_AB2	CG9723	3.10268509626818	0.129683532612523	1	pseudobulk	DESeq2	LRT	0
117	KC_AB2	CG9727	-2.03791470499407	0.269025214028471	1	pseudobulk	DESeq2	LRT	0
117	KC_AB2	CG9760	-0.182203849035823	0.920257233805031	1	pseudobulk	DESeq2	LRT	0
117	KC_AB2	CG9766	-0.828178378785339	0.673584255563891	1	pseudobulk	DESeq2	LRT	0
117	KC_AB2	CG9773	0.989503050128857	0.435504869099892	1	pseudobulk	DESeq2	LRT	0
117	KC_AB2	CG9775	0.227845188584872	0.640882027103813	1	pseudobulk	DESeq2	LRT	0
117	KC_AB2	CG9776	-0.201540191167662	0.829657879956758	1	pseudobulk	DESeq2	LRT	0
117	KC_AB2	CG9782	-0.427431139129811	1	1	pseudobulk	DESeq2	LRT	0
117	KC_AB2	CG9784	-0.583690020715589	0.597341688165409	1	pseudobulk	DESeq2	LRT	0
117	KC_AB2	CG9795	-0.581353014112081	0.55803336900707	1	pseudobulk	DESeq2	LRT	0
117	KC_AB2	CG9799	0.329740041077442	0.855811003303737	1	pseudobulk	DESeq2	LRT	0
117	KC_AB2	CG9801	1.85888970213998	0.270281685476271	1	pseudobulk	DESeq2	LRT	0
117	KC_AB2	CG9804	0.190393219485123	0.893175556614257	1	pseudobulk	DESeq2	LRT	0
117	KC_AB2	CG9813	-0.0534338981983907	0.77612639162835	1	pseudobulk	DESeq2	LRT	0
117	KC_AB2	CG9837	0.250462962869936	0.777863086403558	1	pseudobulk	DESeq2	LRT	0
117	KC_AB2	CG9839	1.26307719581879	0.569064211306759	1	pseudobulk	DESeq2	LRT	0
117	KC_AB2	CG9849	-0.843996296624293	0.67626831722335	1	pseudobulk	DESeq2	LRT	0
117	KC_AB2	CG9853	1.03314595604148	0.324382799413015	1	pseudobulk	DESeq2	LRT	0
117	KC_AB2	CG9855	-0.528672640674623	0.70665773831746	1	pseudobulk	DESeq2	LRT	0
117	KC_AB2	CG9886	0.0163320842711025	1	1	pseudobulk	DESeq2	LRT	0
117	KC_AB2	CG9890	0.725498497157359	0.93879047033739	1	pseudobulk	DESeq2	LRT	0
117	KC_AB2	CG9902	-0.427431139129811	1	1	pseudobulk	DESeq2	LRT	0
117	KC_AB2	CG9911	-0.412679127629717	0.658684360487684	1	pseudobulk	DESeq2	LRT	0
117	KC_AB2	CG9915	-0.502815823857295	0.699532572169496	1	pseudobulk	DESeq2	LRT	0
117	KC_AB2	CG9917	0.0731252952909873	0.947700872494963	1	pseudobulk	DESeq2	LRT	0
117	KC_AB2	CG9919	-0.320391866268249	0.630109414402502	1	pseudobulk	DESeq2	LRT	0

117	KC_AB2	CG9921	-0.165985692405058	0.794289609038726	1	pseudobulk	DESeq2	LRT		0
117	KC_AB2	CG9922	0.535194229047867	0.548033525923725	1	pseudobulk	DESeq2	LRT		0
117	KC_AB2	CG9932	0.129467370471053	0.756490047498992	1	pseudobulk	DESeq2	LRT		0
117	KC_AB2	CG9934	1.27496040324747	0.501655537161972	1	pseudobulk	DESeq2	LRT		0
117	KC_AB2	CG9941	-0.224292596433285	0.814963136352509	1	pseudobulk	DESeq2	LRT		0
117	KC_AB2	CG9945	-2.30493359034205	0.318713340463956	1	pseudobulk	DESeq2	LRT		0
117	KC_AB2	CG9948	-1.12475160732157	0.574429473125171	1	pseudobulk	DESeq2	LRT		0
117	KC_AB2	CG9951	-0.798697133454274	0.699707530640512	1	pseudobulk	DESeq2	LRT		0
117	KC_AB2	CG9953	1.02427748641037	0.399772769019921	1	pseudobulk	DESeq2	LRT		0
117	KC_AB2	CG9967	-0.529556196993598	0.843734517510881	1	pseudobulk	DESeq2	LRT		0
117	KC_AB2	CG9986	-2.57029135982524	0.22992528693203	1	pseudobulk	DESeq2	LRT		0
117	KC_AB2	CG9992	-1.37002769466724	0.479308071448389	1	pseudobulk	DESeq2	LRT		0
117	KC_AB2	Charon	-1.7824418856176	0.467224294431682	1	pseudobulk	DESeq2	LRT		0
117	KC_AB2	chas	2.12684015898615	0.445939223035924	1	pseudobulk	DESeq2	LRT		0
117	KC_AB2	ChAT	0.0583814011176656	0.915595560669605	1	pseudobulk	DESeq2	LRT		0
117	KC_AB2	chb	-0.683098974020512	0.526856165288386	1	pseudobulk	DESeq2	LRT		0
117	KC_AB2	Chc	0.394964531066221	0.502767823796903	1	pseudobulk	DESeq2	LRT		0
117	KC_AB2	Chchd2	-0.304150259546675	0.340132433810038	1	pseudobulk	DESeq2	LRT		0
117	KC_AB2	Chchd3	0.169664330035774	0.782696621666989	1	pseudobulk	DESeq2	LRT		0
117	KC_AB2	Chd1	0.548108210678744	0.52846523237125	1	pseudobulk	DESeq2	LRT		0
117	KC_AB2	Chd64	-0.167679005593927	0.896089390589033	1	pseudobulk	DESeq2	LRT		0
117	KC_AB2	CheB42c	-0.621805355124546	0.74877258313458	1	pseudobulk	DESeq2	LRT		0
117	KC_AB2	CheB53a	-0.427431139129811	1	1	pseudobulk	DESeq2	LRT		0
117	KC_AB2	CHES-1-like	0.613811270709083	0.34984762601942	1	pseudobulk	DESeq2	LRT		0
117	KC_AB2	Chi	-0.669080214490743	0.486079389964222	1	pseudobulk	DESeq2	LRT		0
117	KC_AB2	chic	-0.266973317063429	0.591981909264811	1	pseudobulk	DESeq2	LRT		0
117	KC_AB2	chico	0.0951652990582188	0.829122231837765	1	pseudobulk	DESeq2	LRT		0
117	KC_AB2	chif	-0.475720843230228	0.601915854455432	1	pseudobulk	DESeq2	LRT		0
117	KC_AB2	chinmo	-0.307089419979504	0.259240911522031	1	pseudobulk	DESeq2	LRT		0
117	KC_AB2	CHKov1	-2.18471391069127	0.356826310757868	1	pseudobulk	DESeq2	LRT		0
117	KC_AB2	chm	0.864341462967788	0.925947094335722	1	pseudobulk	DESeq2	LRT		0
117	KC_AB2	Chmp1	0.585637392583363	0.391006283642667	1	pseudobulk	DESeq2	LRT		0
117	KC_AB2	CHMP2B	-1.24424178848872	0.585354168105637	1	pseudobulk	DESeq2	LRT		0
117	KC_AB2	chn	-0.417909920074897	0.177094489453521	1	pseudobulk	DESeq2	LRT		0
117	KC_AB2	CHOp24	0.519130536701763	0.478071597710617	1	pseudobulk	DESeq2	LRT		0
118	KC_AB2	CHORD	2.04515758669385	0.346545205091149	1	pseudobulk	DESeq2	LRT		0
118	KC_AB2	Chrac-14	-0.600547221779134	1	1	pseudobulk	DESeq2	LRT		0
118	KC_AB2	Chrac-16	-0.334845229701366	0.850730256510717	1	pseudobulk	DESeq2	LRT		0
118	KC_AB2	chrb	-0.241955329109855	0.346148973159949	1	pseudobulk	DESeq2	LRT		0
118	KC_AB2	Chro	0.532385279135309	0.495130617492723	1	pseudobulk	DESeq2	LRT		0
118	KC_AB2	ChT	-0.199236179913843	0.584663916668884	1	pseudobulk	DESeq2	LRT		0
118	KC_AB2	Cht11	0.864341462967788	0.925947094335722	1	pseudobulk	DESeq2	LRT		0
118	KC_AB2	Cht6	-0.427431139129811	1	1	pseudobulk	DESeq2	LRT		0
118	KC_AB2	CIA30	-0.598123821701242	0.71217014349458	1	pseudobulk	DESeq2	LRT		0
118	KC_AB2	Ciao1	-1.13898311207644	0.50341302473168	1	pseudobulk	DESeq2	LRT		0
118	KC_AB2	CIAPIN1	-0.167679005593927	0.896089390589033	1	pseudobulk	DESeq2	LRT		0
118	KC_AB2	cib	2.17724274818058	0.171016838883652	1	pseudobulk	DESeq2	LRT		0
118	KC_AB2	cic	0.0532371323540448	0.883518818225023	1	pseudobulk	DESeq2	LRT		0
118	KC_AB2	cin	-2.09422833385209	0.238356525220853	1	pseudobulk	DESeq2	LRT		0
118	KC_AB2	cindr	-0.520942599875343	0.546728131740213	1	pseudobulk	DESeq2	LRT		0
118	KC_AB2	Cip4	-0.169949746977439	0.616326483749852	1	pseudobulk	DESeq2	LRT		0
118	KC_AB2	Cirl	0.128698250680627	0.687515389274841	1	pseudobulk	DESeq2	LRT		0
118	KC_AB2	Cisd2	-0.0243805637060782	0.957154020973192	1	pseudobulk	DESeq2	LRT		0

118	KC_AB2	Cka	0.134238102280977	0.795714960657275	1	pseudobulk	DESeq2	LRT	0
118	KC_AB2	ckd	-0.427431139129811	1	1	pseudobulk	DESeq2	LRT	0
118	KC_AB2	CkIalpha	-0.295784115021746	0.428892808215396	1	pseudobulk	DESeq2	LRT	0
118	KC_AB2	CkIIalpha	0.0854818887791494	0.805067343309464	1	pseudobulk	DESeq2	LRT	0
118	KC_AB2	CkIIalpha-i1	-0.211959943697087	0.913646816097592	1	pseudobulk	DESeq2	LRT	0
118	KC_AB2	CkIIalpha-i3	2.42552382861566	0.181202941708966	1	pseudobulk	DESeq2	LRT	0
118	KC_AB2	CkIIbeta	0.287120385341896	0.51479602575996	1	pseudobulk	DESeq2	LRT	0
118	KC_AB2	ckn	0.0667921956098862	0.87643428909792	1	pseudobulk	DESeq2	LRT	0
118	KC_AB2	Cks85A	0.184099623703983	0.800846503475085	1	pseudobulk	DESeq2	LRT	0
118	KC_AB2	cl	-0.0262152719221834	0.976635973692288	1	pseudobulk	DESeq2	LRT	0
118	KC_AB2	Clamp	-0.607579818276817	0.450531309521651	1	pseudobulk	DESeq2	LRT	0
118	KC_AB2	Clbn	-0.684909070287135	0.624083564370043	1	pseudobulk	DESeq2	LRT	0
118	KC_AB2	Clc	-0.553477203338454	0.371567517793319	1	pseudobulk	DESeq2	LRT	0
118	KC_AB2	ClC-b	-0.259134671513485	1	1	pseudobulk	DESeq2	LRT	0
118	KC_AB2	ClC-c	0.608584640956804	0.497955586825163	1	pseudobulk	DESeq2	LRT	0
118	KC_AB2	Clic	0.40720097936403	0.687964667399847	1	pseudobulk	DESeq2	LRT	0
118	KC_AB2	CLIP-190	-0.439906133544971	0.0335705856311687	1	pseudobulk	DESeq2	LRT	0
118	KC_AB2	Clp	-1.38368758155045	0.462536107716416	1	pseudobulk	DESeq2	LRT	0
118	KC_AB2	ClpP	-0.64734115546371	0.528510250465673	1	pseudobulk	DESeq2	LRT	0
118	KC_AB2	CLS	6.03027979003313E-0	0.942364063697085	1	pseudobulk	DESeq2	LRT	0
118	KC_AB2	clt	-0.427431139129811	1	1	pseudobulk	DESeq2	LRT	0
118	KC_AB2	clu	0.11633794598805	0.883891211060209	1	pseudobulk	DESeq2	LRT	0
118	KC_AB2	cm	0.498593948417392	0.805619674491474	1	pseudobulk	DESeq2	LRT	0
118	KC_AB2	cmb	-1.51812168653838	0.552401469179882	1	pseudobulk	DESeq2	LRT	0
118	KC_AB2	cmpy	0.209748528404733	0.668634614498059	1	pseudobulk	DESeq2	LRT	0
118	KC_AB2	cN-IIIB	-0.482173055655404	0.804949101714764	1	pseudobulk	DESeq2	LRT	0
118	KC_AB2	CNBP	-0.700076567279655	0.253852605834855	1	pseudobulk	DESeq2	LRT	0
118	KC_AB2	cnc	1.26936311270713	0.252768314482512	1	pseudobulk	DESeq2	LRT	0
118	KC_AB2	Cndp2	1.08480356307325	0.564615346523986	1	pseudobulk	DESeq2	LRT	0
118	KC_AB2	CngA	-0.542731118099782	0.588424217960642	1	pseudobulk	DESeq2	LRT	0
118	KC_AB2	CngB	-0.232060037414333	0.770252413941975	1	pseudobulk	DESeq2	LRT	0
118	KC_AB2	CngI	0.305983167222807	0.127341830090812	1	pseudobulk	DESeq2	LRT	0
118	KC_AB2	cni	0.177840025492907	0.789027121114321	1	pseudobulk	DESeq2	LRT	0
118	KC_AB2	cnir	0.676125958829793	0.700351457292265	1	pseudobulk	DESeq2	LRT	0
118	KC_AB2	cnk	-0.073870656886408	0.963551931599223	1	pseudobulk	DESeq2	LRT	0
118	KC_AB2	CNMaR	1.8462245261508	0.111196904615791	1	pseudobulk	DESeq2	LRT	0
118	KC_AB2	cnn	1.19363808316145	0.496725974479902	1	pseudobulk	DESeq2	LRT	0
118	KC_AB2	cno	-0.0393423812329166	0.76617253522968	1	pseudobulk	DESeq2	LRT	0
118	KC_AB2	Cnot4	-0.954378434587	0.195750854812441	1	pseudobulk	DESeq2	LRT	0
118	KC_AB2	CNPYb	0.861406466225554	0.587973208811527	1	pseudobulk	DESeq2	LRT	0
118	KC_AB2	Cnx14D	-0.600547221779134	1	1	pseudobulk	DESeq2	LRT	0
118	KC_AB2	Cnx99A	0.0567164161300313	0.91242517709211	1	pseudobulk	DESeq2	LRT	0
118	KC_AB2	Coa7	-0.721456637161026	0.499289611851749	1	pseudobulk	DESeq2	LRT	0
118	KC_AB2	Cog1	-1.63925793886161	0.546290859679341	1	pseudobulk	DESeq2	LRT	0
118	KC_AB2	Cog2	-2.47242271668572	0.279151826981215	1	pseudobulk	DESeq2	LRT	0
118	KC_AB2	Cog3	1.3067515803171	0.550895625207199	1	pseudobulk	DESeq2	LRT	0
118	KC_AB2	Cog4	0.19513139207807	0.91132214607951	1	pseudobulk	DESeq2	LRT	0
118	KC_AB2	Cog6	-0.93212148301064	0.654346170772296	1	pseudobulk	DESeq2	LRT	0
118	KC_AB2	Cog7	-1.02449375894128	0.52208429729139	1	pseudobulk	DESeq2	LRT	0
118	KC_AB2	Cog8	-1.99338034826303	0.401583286802675	1	pseudobulk	DESeq2	LRT	0
118	KC_AB2	coil	1.94691684546882	0.398076844044942	1	pseudobulk	DESeq2	LRT	0
118	KC_AB2	Col4a1	3.45541309326679	0.127050452367876	1	pseudobulk	DESeq2	LRT	0
118	KC_AB2	cold	0.0471936707755822	0.953916869426286	1	pseudobulk	DESeq2	LRT	0

118	KC_AB2	colt	0.70378358684898	0.932301451973468	1	pseudobulk	DESeq2	LRT		0
118	KC_AB2	comm	-0.600514111649307	0.277867233351968	1	pseudobulk	DESeq2	LRT		0
118	KC_AB2	comt	-0.0661125549807229	0.89513008990748	1	pseudobulk	DESeq2	LRT		0
118	KC_AB2	Con	-0.0042455613138227	0.921980285969667	1	pseudobulk	DESeq2	LRT		0
118	KC_AB2	Cont	-0.621805355124546	0.74877258313458	1	pseudobulk	DESeq2	LRT		0
118	KC_AB2	conu	0.43910054708556	0.495500404467868	1	pseudobulk	DESeq2	LRT		0
118	KC_AB2	Coprox	-1.54681640618506	0.429504009228708	1	pseudobulk	DESeq2	LRT		0
118	KC_AB2	Coq2	-1.44810186689952	0.454949999368433	1	pseudobulk	DESeq2	LRT		0
118	KC_AB2	Coq3	-1.16116875819275	0.736255625566845	1	pseudobulk	DESeq2	LRT		0
118	KC_AB2	Coq5	-0.427431139129811	1	1	pseudobulk	DESeq2	LRT		0
118	KC_AB2	Coq6	-2.8438335749426	0.201235480146964	1	pseudobulk	DESeq2	LRT		0
118	KC_AB2	Coq7	-1.03822088107615	0.434677969445257	1	pseudobulk	DESeq2	LRT		0
118	KC_AB2	Coq8	1.39827770914961	0.587246212652858	1	pseudobulk	DESeq2	LRT		0
118	KC_AB2	Coq9	0.216039610998433	0.882275917368053	1	pseudobulk	DESeq2	LRT		0
118	KC_AB2	cora	-0.466241113710562	0.230898733244461	1	pseudobulk	DESeq2	LRT		0
118	KC_AB2	CoRest	0.338550026104615	0.632265959013446	1	pseudobulk	DESeq2	LRT		0
118	KC_AB2	Corin	-1.54340222994928	0.220194974374229	1	pseudobulk	DESeq2	LRT		0
118	KC_AB2	corn	0.486536244990472	0.275070833962462	1	pseudobulk	DESeq2	LRT		0
118	KC_AB2	coro	-1.5847653577014	0.0721719962857278	1	pseudobulk	DESeq2	LRT		0
118	KC_AB2	Cortactin	-3.18391272912889	0.117745575300102	1	pseudobulk	DESeq2	LRT		0
118	KC_AB2	corto	-0.264423151307326	0.553501639520662	1	pseudobulk	DESeq2	LRT		0
118	KC_AB2	cos	1.03525233079433	0.233042673972029	1	pseudobulk	DESeq2	LRT		0
118	KC_AB2	Cow	0.36690381794648	0.448315294301948	1	pseudobulk	DESeq2	LRT		0
118	KC_AB2	COX4	-0.0558839138131536	0.77380961319108	1	pseudobulk	DESeq2	LRT		0
118	KC_AB2	COX5A	-0.333072923244414	0.297056632666904	1	pseudobulk	DESeq2	LRT		0
118	KC_AB2	COX5B	-0.274142201652341	0.433687219118861	1	pseudobulk	DESeq2	LRT		0
118	KC_AB2	COX6B	-0.104275135372817	0.702731467965308	1	pseudobulk	DESeq2	LRT		0
118	KC_AB2	COX7A	-0.102711201496674	0.648687951731485	1	pseudobulk	DESeq2	LRT		0
118	KC_AB2	COX7C	-0.195664694896755	0.551864341508415	1	pseudobulk	DESeq2	LRT		0
119	KC_AB2	COX8	-0.164778638951718	0.549632443517494	1	pseudobulk	DESeq2	LRT		0
119	KC_AB2	Cp1	-1.29837604172451	0.0926629949756199	1	pseudobulk	DESeq2	LRT		0
119	KC_AB2	Cp110	-0.0540516421973474	0.818528979448858	1	pseudobulk	DESeq2	LRT		0
119	KC_AB2	Cp18	-0.600547221779134	1	1	pseudobulk	DESeq2	LRT		0
119	KC_AB2	Cp190	0.863795093071214	0.27629699199102	1	pseudobulk	DESeq2	LRT		0
119	KC_AB2	Cp7Fb	-0.600547221779134	1	1	pseudobulk	DESeq2	LRT		0
119	KC_AB2	Cp7Fc	-0.841882716738579	1	1	pseudobulk	DESeq2	LRT		0
119	KC_AB2	cpa	0.454876971406626	0.733352390230567	1	pseudobulk	DESeq2	LRT		0
119	KC_AB2	cpb	0.595958215258478	0.574470825867666	1	pseudobulk	DESeq2	LRT		0
119	KC_AB2	Cpes	-2.17489218640147	0.354960305801642	1	pseudobulk	DESeq2	LRT		0
119	KC_AB2	cpo	0.0624970506364289	0.756500403043925	1	pseudobulk	DESeq2	LRT		0
119	KC_AB2	Cpr11A	-0.592481036256484	0.706847545932384	1	pseudobulk	DESeq2	LRT		0
119	KC_AB2	Cpsf100	1.40241945196173	0.57265124481703	1	pseudobulk	DESeq2	LRT		0
119	KC_AB2	Cpsf160	0.573760393110584	0.792134620722927	1	pseudobulk	DESeq2	LRT		0
119	KC_AB2	Cpsf5	-0.129057720235316	0.804544507263128	1	pseudobulk	DESeq2	LRT		0
119	KC_AB2	Cpsf6	-0.0957102964841632	0.846886791591176	1	pseudobulk	DESeq2	LRT		0
119	KC_AB2	Cpsf73	-1.78094334699383	0.470930349923561	1	pseudobulk	DESeq2	LRT		0
119	KC_AB2	cpx	0.0093470286078874	0.934994160325386	1	pseudobulk	DESeq2	LRT		0
119	KC_AB2	CR12842	0.613437827066099	0.732234776459011	1	pseudobulk	DESeq2	LRT		0
119	KC_AB2	CR15345	0.772278413675747	0.668572171405162	1	pseudobulk	DESeq2	LRT		0
119	KC_AB2	CR18166	-1.21728107964896	0.459229713945245	1	pseudobulk	DESeq2	LRT		0
119	KC_AB2	CR18217	-0.427431139129811	1	1	pseudobulk	DESeq2	LRT		0
119	KC_AB2	CR18228	-0.427431139129811	1	1	pseudobulk	DESeq2	LRT		0
119	KC_AB2	CR18275	1.5243995994652	0.741477491078038	1	pseudobulk	DESeq2	LRT		0

119	KC_AB2	CR31032	-0.211959943697087	0.913646816097592	1	pseudobulk	DESeq2	LRT	0
119	KC_AB2	CR32010	-1.40673172715063	0.587364787535786	1	pseudobulk	DESeq2	LRT	0
119	KC_AB2	CR32745	-0.825312539513664	0.666826652877566	1	pseudobulk	DESeq2	LRT	0
119	KC_AB2	CR41320	0.222396124134733	1	1	pseudobulk	DESeq2	LRT	0
119	KC_AB2	CR41597	0.101800854924797	0.864586819803219	1	pseudobulk	DESeq2	LRT	0
119	KC_AB2	CR43281	-0.0092498888606584	0.971103347668572	1	pseudobulk	DESeq2	LRT	0
119	KC_AB2	CR43361	-0.427431139129811	1	1	pseudobulk	DESeq2	LRT	0
119	KC_AB2	CR43670	-1.77537130143107	0.329901177614655	1	pseudobulk	DESeq2	LRT	0
119	KC_AB2	CR43972	1.5243995994652	0.741477491078038	1	pseudobulk	DESeq2	LRT	0
119	KC_AB2	CR45391	2.12684015898615	0.445939223035924	1	pseudobulk	DESeq2	LRT	0
119	KC_AB2	CR46405	-0.427431139129811	1	1	pseudobulk	DESeq2	LRT	0
119	KC_AB2	CR7448	-0.600547221779133	0.841940696994101	1	pseudobulk	DESeq2	LRT	0
119	KC_AB2	Crag	-0.711243604785863	0.57198010007847	1	pseudobulk	DESeq2	LRT	0
119	KC_AB2	CRAT	-0.338771435603146	0.816656863441963	1	pseudobulk	DESeq2	LRT	0
119	KC_AB2	crb	-0.175711721458146	0.54844223792385	1	pseudobulk	DESeq2	LRT	0
119	KC_AB2	crc	-0.209468778801633	0.787066275879536	1	pseudobulk	DESeq2	LRT	0
119	KC_AB2	CrebA	-2.19077380021023	0.026299260935213	1	pseudobulk	DESeq2	LRT	0
119	KC_AB2	CrebB	0.35570043631343	0.691962348038048	1	pseudobulk	DESeq2	LRT	0
119	KC_AB2	CREG	2.12684015898615	0.445939223035924	1	pseudobulk	DESeq2	LRT	0
119	KC_AB2	Crg-1	0.725498497157359	0.93879047033739	1	pseudobulk	DESeq2	LRT	0
119	KC_AB2	CRIF	-0.507887091766677	0.763532594123782	1	pseudobulk	DESeq2	LRT	0
119	KC_AB2	crim	1.82245639658508	0.285345440158223	1	pseudobulk	DESeq2	LRT	0
119	KC_AB2	Crk	-0.453908672647451	0.387672836799415	1	pseudobulk	DESeq2	LRT	0
119	KC_AB2	crm	2.12684015898615	0.445939223035924	1	pseudobulk	DESeq2	LRT	0
119	KC_AB2	CRMP	0.005903797160809	0.94159990358605	1	pseudobulk	DESeq2	LRT	0
119	KC_AB2	crn	-0.828178378785339	0.673584255563891	1	pseudobulk	DESeq2	LRT	0
119	KC_AB2	crol	0.120712033357461	0.748645432485431	1	pseudobulk	DESeq2	LRT	0
119	KC_AB2	CROT	-0.841882716738579	1	1	pseudobulk	DESeq2	LRT	0
119	KC_AB2	crp	-0.0865596290750653	0.835903746912431	1	pseudobulk	DESeq2	LRT	0
119	KC_AB2	crq	-1.01749654653381	0.377875770084181	1	pseudobulk	DESeq2	LRT	0
119	KC_AB2	Crtc	-0.234570111187162	0.654124385490527	1	pseudobulk	DESeq2	LRT	0
119	KC_AB2	cry	-0.592481036256484	0.706847545932384	1	pseudobulk	DESeq2	LRT	0
119	KC_AB2	Cse1	0.49522746739306	0.807638127459475	1	pseudobulk	DESeq2	LRT	0
119	KC_AB2	Csk	-0.584419080604014	0.281460870488278	1	pseudobulk	DESeq2	LRT	0
119	KC_AB2	Csl4	0.70378358684898	0.932301451973468	1	pseudobulk	DESeq2	LRT	0
119	KC_AB2	CSN1b	-0.427431139129811	1	1	pseudobulk	DESeq2	LRT	0
119	KC_AB2	CSN3	1.15905227737812	0.43324191879043	1	pseudobulk	DESeq2	LRT	0
119	KC_AB2	CSN4	0.282778851189353	0.767755954672442	1	pseudobulk	DESeq2	LRT	0
119	KC_AB2	CSN5	-0.71627738352641	0.624159406619319	1	pseudobulk	DESeq2	LRT	0
119	KC_AB2	CSN6	-2.33635626336857	0.190922449030435	1	pseudobulk	DESeq2	LRT	0
119	KC_AB2	CSN7	-0.714456462293353	0.426738816735731	1	pseudobulk	DESeq2	LRT	0
119	KC_AB2	CSN8	0.135144032684185	0.883874997571851	1	pseudobulk	DESeq2	LRT	0
119	KC_AB2	Csp	-0.0994822542406888	0.790647383058906	1	pseudobulk	DESeq2	LRT	0
119	KC_AB2	CstF50	-0.427431139129811	1	1	pseudobulk	DESeq2	LRT	0
119	KC_AB2	CstF64	-1.65391840723379	0.392847699484482	1	pseudobulk	DESeq2	LRT	0
119	KC_AB2	csul	-0.600547221779134	1	1	pseudobulk	DESeq2	LRT	0
119	KC_AB2	csw	-0.223616310734466	0.572773579725621	1	pseudobulk	DESeq2	LRT	0
119	KC_AB2	ct	1.5243995994652	0.741477491078038	1	pseudobulk	DESeq2	LRT	0
119	KC_AB2	cta	-0.507917670681657	0.569552240315008	1	pseudobulk	DESeq2	LRT	0
119	KC_AB2	CtBP	-0.139246634996063	0.634399291212725	1	pseudobulk	DESeq2	LRT	0
119	KC_AB2	CTCF	1.50255200571414	0.44025635980316	1	pseudobulk	DESeq2	LRT	0
119	KC_AB2	CtIP	1.19722905929633	0.568136033732323	1	pseudobulk	DESeq2	LRT	0
119	KC_AB2	Ctl1	0.441525463992166	0.6777452085166	1	pseudobulk	DESeq2	LRT	0

119	KC_AB2	Ctl2	0.519029044305671	0.724845399681633	1	pseudobulk	DESeq2	LRT	0
119	KC_AB2	ctp	-0.205000767301525	0.208382061856181	1	pseudobulk	DESeq2	LRT	0
119	KC_AB2	CTPsyn	-0.319733965796993	0.717703651564624	1	pseudobulk	DESeq2	LRT	0
119	KC_AB2	Ctr1A	-1.05021027530155	0.420691609259227	1	pseudobulk	DESeq2	LRT	0
119	KC_AB2	Ctr9	0.591339587784036	0.380510672044476	1	pseudobulk	DESeq2	LRT	0
119	KC_AB2	ctrip	0.0982359730714619	0.856950934109628	1	pseudobulk	DESeq2	LRT	0
119	KC_AB2	CtsB1	0.399632969839057	0.608126348368276	1	pseudobulk	DESeq2	LRT	0
119	KC_AB2	cu	-1.40673172715063	0.587364787535786	1	pseudobulk	DESeq2	LRT	0
119	KC_AB2	cue	-0.464788641252857	0.630007003844332	1	pseudobulk	DESeq2	LRT	0
119	KC_AB2	Cul1	-0.197159207196904	0.752000737010642	1	pseudobulk	DESeq2	LRT	0
119	KC_AB2	Cul2	-0.711008860042205	0.434680961073363	1	pseudobulk	DESeq2	LRT	0
119	KC_AB2	Cul3	-0.460347560028975	0.613652501109707	1	pseudobulk	DESeq2	LRT	0
119	KC_AB2	Cul4	-1.59936180846964	0.410112889830765	1	pseudobulk	DESeq2	LRT	0
119	KC_AB2	Cul5	-0.354041112671957	0.834274804934616	1	pseudobulk	DESeq2	LRT	0
119	KC_AB2	cv-c	0.383229411456099	0.414402096046463	1	pseudobulk	DESeq2	LRT	0
119	KC_AB2	Cwc25	-0.0621821072351001	0.95465565417662	1	pseudobulk	DESeq2	LRT	0
119	KC_AB2	cwo	-0.210094072682091	0.779635081136821	1	pseudobulk	DESeq2	LRT	0
119	KC_AB2	cyc	-0.214235388246561	0.818458328720721	1	pseudobulk	DESeq2	LRT	0
119	KC_AB2	CycC	0.0952695066566691	0.802682717050958	1	pseudobulk	DESeq2	LRT	0
119	KC_AB2	CycG	-0.0426612377143517	0.875870514382886	1	pseudobulk	DESeq2	LRT	0
119	KC_AB2	CycH	-0.841882716738579	1	1	pseudobulk	DESeq2	LRT	0
119	KC_AB2	CycJ	2.12684015898615	0.445939223035924	1	pseudobulk	DESeq2	LRT	0
119	KC_AB2	CycK	-0.0844444028837079	0.928913877037491	1	pseudobulk	DESeq2	LRT	0
120	KC_AB2	CycT	-0.192671271658806	0.687759543164065	1	pseudobulk	DESeq2	LRT	0
120	KC_AB2	CycY	-0.0438957303713566	0.937830898191074	1	pseudobulk	DESeq2	LRT	0
120	KC_AB2	CYLD	1.81089901712153	0.32637037899926	1	pseudobulk	DESeq2	LRT	0
120	KC_AB2	Cyp1	-0.29761349749453	0.409042065786872	1	pseudobulk	DESeq2	LRT	0
120	KC_AB2	Cyp12b2	-0.481993539656925	0.883279710820779	1	pseudobulk	DESeq2	LRT	0
120	KC_AB2	Cyp28a5	-0.600547221779133	0.841940696994101	1	pseudobulk	DESeq2	LRT	0
120	KC_AB2	Cyp311a1	-0.427431139129811	1	1	pseudobulk	DESeq2	LRT	0
120	KC_AB2	cyp33	-2.22988039125053	0.338836482281886	1	pseudobulk	DESeq2	LRT	0
120	KC_AB2	Cyp49a1	1.14619702729628	0.262441433819793	1	pseudobulk	DESeq2	LRT	0
120	KC_AB2	Cyp4ae1	-0.427431139129811	1	1	pseudobulk	DESeq2	LRT	0
120	KC_AB2	Cyp4d2	-0.427431139129811	1	1	pseudobulk	DESeq2	LRT	0
120	KC_AB2	Cyp4g1	-0.427431139129811	1	1	pseudobulk	DESeq2	LRT	0
120	KC_AB2	Cyp4p2	-0.427431139129811	1	1	pseudobulk	DESeq2	LRT	0
120	KC_AB2	Cyp6a14	-0.427431139129811	1	1	pseudobulk	DESeq2	LRT	0
120	KC_AB2	Cyp6d5	-0.592481036256484	0.706847545932384	1	pseudobulk	DESeq2	LRT	0
120	KC_AB2	Cyp6g2	0.864341462967788	0.925947094335722	1	pseudobulk	DESeq2	LRT	0
120	KC_AB2	Cyp6u1	0.0163284183731416	0.882597754518706	1	pseudobulk	DESeq2	LRT	0
120	KC_AB2	Cyp9f3	0.183555857030446	1	1	pseudobulk	DESeq2	LRT	0
120	KC_AB2	cype	-0.248388806370101	0.29313343613304	1	pseudobulk	DESeq2	LRT	0
120	KC_AB2	Cypl	-3.28978076107524	0.0902833784282089	1	pseudobulk	DESeq2	LRT	0
120	KC_AB2	Cys	-1.61525285302842	0.415003765220057	1	pseudobulk	DESeq2	LRT	0
120	KC_AB2	CysRS	0.158885872213844	0.913023095756665	1	pseudobulk	DESeq2	LRT	0
120	KC_AB2	CysRS-m	0.674459942220112	0.74558757773504	1	pseudobulk	DESeq2	LRT	0
120	KC_AB2	Cyt-b5	-0.320198365950336	0.619387016101827	1	pseudobulk	DESeq2	LRT	0
120	KC_AB2	Cyt-c-d	-0.600547221779134	1	1	pseudobulk	DESeq2	LRT	0
120	KC_AB2	Cyt-c-p	-0.164208433782719	0.522226422279671	1	pseudobulk	DESeq2	LRT	0
120	KC_AB2	Cyt-c1	-0.160930089543392	0.682506999125723	1	pseudobulk	DESeq2	LRT	0
120	KC_AB2	D1	-0.875747364248121	0.119808932556177	1	pseudobulk	DESeq2	LRT	0
120	KC_AB2	D12	-0.504597849832364	0.809397978921941	1	pseudobulk	DESeq2	LRT	0
120	KC_AB2	D19A	-0.805878497061905	0.632661607079542	1	pseudobulk	DESeq2	LRT	0

120	KC_AB2	D19B	-1.91448785306366	0.338979826000835	1	pseudobulk	DESeq2	LRT		0
120	KC_AB2	D2hgdh	-0.382929749762112	0.851961810578875	1	pseudobulk	DESeq2	LRT		0
120	KC_AB2	d4	0.237386596250663	0.737680763523135	1	pseudobulk	DESeq2	LRT		0
120	KC_AB2	da	0.166190673672238	0.742750893278559	1	pseudobulk	DESeq2	LRT		0
120	KC_AB2	DAAM	1.20329062951446	0.172492324962515	1	pseudobulk	DESeq2	LRT		0
120	KC_AB2	Dab	-0.0253517819643971	0.857102319424834	1	pseudobulk	DESeq2	LRT		0
120	KC_AB2	dac	-0.458858362560531	0.130468199750548	1	pseudobulk	DESeq2	LRT		0
120	KC_AB2	Dad	-1.91688326235661	0.322858002756985	1	pseudobulk	DESeq2	LRT		0
120	KC_AB2	Dad1	-0.596598117972573	0.412116242190854	1	pseudobulk	DESeq2	LRT		0
120	KC_AB2	daed	-0.600547221779134	1	1	pseudobulk	DESeq2	LRT		0
120	KC_AB2	Dak1	0.651578093451697	0.208131183126983	1	pseudobulk	DESeq2	LRT		0
120	KC_AB2	dally	1.41524016620164	0.505408490038387	1	pseudobulk	DESeq2	LRT		0
120	KC_AB2	dao	0.237681289134807	0.742383533208499	1	pseudobulk	DESeq2	LRT		0
120	KC_AB2	Dap160	0.43302514741587	0.380367083782061	1	pseudobulk	DESeq2	LRT		0
120	KC_AB2	dare	-1.01337037550694	0.608094630643134	1	pseudobulk	DESeq2	LRT		0
120	KC_AB2	Dark	-0.153242631672537	0.884856181571465	1	pseudobulk	DESeq2	LRT		0
120	KC_AB2	dati	0.469530501224965	0.100217760721431	1	pseudobulk	DESeq2	LRT		0
120	KC_AB2	Daxx	1.12907856497414	0.234240613778822	1	pseudobulk	DESeq2	LRT		0
120	KC_AB2	dbe	2.1817927492653	0.458599861292202	1	pseudobulk	DESeq2	LRT		0
120	KC_AB2	dbo	-0.501649970053799	0.519086528387965	1	pseudobulk	DESeq2	LRT		0
120	KC_AB2	Dbp45A	-1.78094334699383	0.470930349923561	1	pseudobulk	DESeq2	LRT		0
120	KC_AB2	Dbp73D	-0.427431139129811	1	1	pseudobulk	DESeq2	LRT		0
120	KC_AB2	Dbp80	-0.151529067182957	0.67189039758251	1	pseudobulk	DESeq2	LRT		0
120	KC_AB2	dbr	-0.427431139129811	1	1	pseudobulk	DESeq2	LRT		0
120	KC_AB2	DCAF12	-2.53693580180331	0.244367126599884	1	pseudobulk	DESeq2	LRT		0
120	KC_AB2	Dci	-1.51928970377954	0.486908946651791	1	pseudobulk	DESeq2	LRT		0
120	KC_AB2	dco	-0.23861039720679	0.698738232225233	1	pseudobulk	DESeq2	LRT		0
120	KC_AB2	Dcp-1	-1.46820840365121	0.491097147297708	1	pseudobulk	DESeq2	LRT		0
120	KC_AB2	DCP1	0.122161731853065	0.900663996560905	1	pseudobulk	DESeq2	LRT		0
120	KC_AB2	DCP2	0.0516279911633075	0.924138734254097	1	pseudobulk	DESeq2	LRT		0
120	KC_AB2	Dcr-1	-0.841882716738579	1	1	pseudobulk	DESeq2	LRT		0
120	KC_AB2	Dcr-2	-0.614452410037855	0.666477852326312	1	pseudobulk	DESeq2	LRT		0
120	KC_AB2	DCTN1-p150	-0.30809985320849	0.725833313481273	1	pseudobulk	DESeq2	LRT		0
120	KC_AB2	DCTN2-p50	-0.338296831740091	0.745475271625083	1	pseudobulk	DESeq2	LRT		0
120	KC_AB2	DCTN3-p24	0.0686595713970781	0.907631373090399	1	pseudobulk	DESeq2	LRT		0
120	KC_AB2	DCTN4-p62	1.22108044457171	0.37539025889362	1	pseudobulk	DESeq2	LRT		0
120	KC_AB2	DCTN5-p25	-0.380396221655848	0.800275859940725	1	pseudobulk	DESeq2	LRT		0
120	KC_AB2	DCTN6-p27	-2.67485351907516	0.0955610496616036	1	pseudobulk	DESeq2	LRT		0
120	KC_AB2	Dd	0.420856780863074	0.451957278212315	1	pseudobulk	DESeq2	LRT		0
120	KC_AB2	Ddc	-0.152265998015665	0.731635048845386	1	pseudobulk	DESeq2	LRT		0
120	KC_AB2	Ddx1	-0.493357239699781	0.783807521297462	1	pseudobulk	DESeq2	LRT		0
120	KC_AB2	Deaf1	-0.236797287709064	0.648196181826079	1	pseudobulk	DESeq2	LRT		0
120	KC_AB2	DEF8	0.108468324554008	1	1	pseudobulk	DESeq2	LRT		0
120	KC_AB2	defl	-0.600547221779134	1	1	pseudobulk	DESeq2	LRT		0
120	KC_AB2	Dek	-0.123341812930291	0.787191702529023	1	pseudobulk	DESeq2	LRT		0
120	KC_AB2	deltaCOP	-1.03651423229083	0.349432750981383	1	pseudobulk	DESeq2	LRT		0
120	KC_AB2	Den1	-0.28196348260935	0.715260907857326	1	pseudobulk	DESeq2	LRT		0
120	KC_AB2	DENR	-1.08691172363926	0.369448108763893	1	pseudobulk	DESeq2	LRT		0
120	KC_AB2	Der-1	-4.13940488083952	0.0181647370950291	1	pseudobulk	DESeq2	LRT		0
120	KC_AB2	Der-2	0.665082985379829	0.458785176302491	1	pseudobulk	DESeq2	LRT		0
120	KC_AB2	Desat1	-0.313571490880682	0.431336892692083	1	pseudobulk	DESeq2	LRT		0
120	KC_AB2	Df31	-0.345938976438451	0.249565818927898	1	pseudobulk	DESeq2	LRT		0
120	KC_AB2	Dg	0.117926997406792	0.768616595203507	1	pseudobulk	DESeq2	LRT		0

120	KC_AB2	Dgk	0.016299533416427	0.923011251435594	1	pseudobulk	DESeq2	LRT		0
120	KC_AB2	Dgkepsilon	-0.465913631705064	0.638418876952189	1	pseudobulk	DESeq2	LRT		0
120	KC_AB2	Dgp-1	0.361198079552773	0.752193761529442	1	pseudobulk	DESeq2	LRT		0
120	KC_AB2	dgrn	-1.54470851095603	0.55301747381519	1	pseudobulk	DESeq2	LRT		0
120	KC_AB2	dgt1	0.195251340687946	0.79013788052411	1	pseudobulk	DESeq2	LRT		0
120	KC_AB2	Dh31-R	-1.85733156562156	0.448702818121367	1	pseudobulk	DESeq2	LRT		0
120	KC_AB2	Dh44-R1	-0.876580544286109	0.44848698529904	1	pseudobulk	DESeq2	LRT		0
120	KC_AB2	Dhap-at	1.35740993864998	0.237362180612232	1	pseudobulk	DESeq2	LRT		0
120	KC_AB2	Dhc64C	-0.320251663995399	0.616194089624009	1	pseudobulk	DESeq2	LRT		0
120	KC_AB2	Dhit	-0.0060434453652607	0.921188279060026	1	pseudobulk	DESeq2	LRT		0
120	KC_AB2	Dhod	-0.406746729298491	0.800090602086552	1	pseudobulk	DESeq2	LRT		0
120	KC_AB2	DhpD	-0.427431139129811	1	1	pseudobulk	DESeq2	LRT		0
120	KC_AB2	Dhpr	-0.300722122403753	0.620112529849684	1	pseudobulk	DESeq2	LRT		0
120	KC_AB2	Dhx15	-0.493357239699781	0.783807521297462	1	pseudobulk	DESeq2	LRT		0
120	KC_AB2	dia	-0.427431139129811	1	1	pseudobulk	DESeq2	LRT		0
120	KC_AB2	Diap1	-0.0533399030364768	0.908459917236253	1	pseudobulk	DESeq2	LRT		0
120	KC_AB2	Diap2	-0.0936538773992154	0.887312165088227	1	pseudobulk	DESeq2	LRT		0
121	KC_AB2	Dic1	-0.682270643975866	0.259857435980421	1	pseudobulk	DESeq2	LRT		0
121	KC_AB2	didum	-1.18724001155315	0.398623411458916	1	pseudobulk	DESeq2	LRT		0
121	KC_AB2	Dif	0.0323272163245606	0.944644194836955	1	pseudobulk	DESeq2	LRT		0
121	KC_AB2	dikar	-0.158608205789592	0.701524398268023	1	pseudobulk	DESeq2	LRT		0
121	KC_AB2	Dim1	1.67948111813869	0.235108536711592	1	pseudobulk	DESeq2	LRT		0
121	KC_AB2	dind	-0.721011057183819	0.707719845314038	1	pseudobulk	DESeq2	LRT		0
121	KC_AB2	DIP-alpha	-1.16414686721441	0.585027317672762	1	pseudobulk	DESeq2	LRT		0
121	KC_AB2	Dip-B	-0.427431139129811	1	1	pseudobulk	DESeq2	LRT		0
121	KC_AB2	Dip-C	-0.181031246196817	0.840970667470491	1	pseudobulk	DESeq2	LRT		0
121	KC_AB2	DIP-gamma	3.13444977473788	0.190512924459537	1	pseudobulk	DESeq2	LRT		0
121	KC_AB2	DIP-kappa	-0.175500075243962	0.375299814643536	1	pseudobulk	DESeq2	LRT		0
121	KC_AB2	DIP-lambda	-3.04547355004204	0.142660039361559	1	pseudobulk	DESeq2	LRT		0
121	KC_AB2	DIP1	-0.596287698028033	0.276943386668887	1	pseudobulk	DESeq2	LRT		0
121	KC_AB2	DIP2	0.174275941098506	0.791279195021647	1	pseudobulk	DESeq2	LRT		0
121	KC_AB2	Dis3	-0.600547221779134	1	1	pseudobulk	DESeq2	LRT		0
121	KC_AB2	Dis3l2	-1.50331603016663	0.431246352444201	1	pseudobulk	DESeq2	LRT		0
121	KC_AB2	disp	-1.49699969891508	0.440827628718793	1	pseudobulk	DESeq2	LRT		0
121	KC_AB2	DJ-1alpha	-0.365479990214618	0.841166477011201	1	pseudobulk	DESeq2	LRT		0
121	KC_AB2	dj-1beta	-1.19904527440385	0.229461214427496	1	pseudobulk	DESeq2	LRT		0
121	KC_AB2	dl	-0.702294577929092	0.559979538605807	1	pseudobulk	DESeq2	LRT		0
121	KC_AB2	Dlc90F	-0.0643828975900397	0.900470092348802	1	pseudobulk	DESeq2	LRT		0
121	KC_AB2	dlg1	-0.123496870522055	0.574469591627343	1	pseudobulk	DESeq2	LRT		0
121	KC_AB2	Dlg5	-0.0265825936398205	0.900663231042675	1	pseudobulk	DESeq2	LRT		0
121	KC_AB2	Dlic	1.03737886323914	0.0541593437486653	1	pseudobulk	DESeq2	LRT		0
121	KC_AB2	Dlip1	-1.3929484167291	0.512855059074155	1	pseudobulk	DESeq2	LRT		0
121	KC_AB2	Dlip2	1.19851019041684	0.408492760201926	1	pseudobulk	DESeq2	LRT		0
121	KC_AB2	Dlip3	0.0711560446452259	0.946468370626269	1	pseudobulk	DESeq2	LRT		0
121	KC_AB2	dlp	-1.29244479833796	0.693295472370086	1	pseudobulk	DESeq2	LRT		0
121	KC_AB2	dlt	-2.12967468770992	0.340688355153583	1	pseudobulk	DESeq2	LRT		0
121	KC_AB2	DMAP1	-0.732030197893322	0.623528320369286	1	pseudobulk	DESeq2	LRT		0
121	KC_AB2	dmpd	0.144475057009906	0.822168358729528	1	pseudobulk	DESeq2	LRT		0
121	KC_AB2	dmrt11E	-0.600547221779134	1	1	pseudobulk	DESeq2	LRT		0
121	KC_AB2	dmt	-0.109785922225664	0.939774912519229	1	pseudobulk	DESeq2	LRT		0
121	KC_AB2	Dmtn	1.24170260050305	0.173666793630341	1	pseudobulk	DESeq2	LRT		0
121	KC_AB2	DnaJ-1	0.195648585454767	0.746983088554578	1	pseudobulk	DESeq2	LRT		0
121	KC_AB2	DnaJ-H	-0.268555798249347	0.7568566117393	1	pseudobulk	DESeq2	LRT		0

121	KC_AB2	DNAlig3	-1.3723417613553	0.619532036522	1	pseudobulk	DESeq2	LRT		0
121	KC_AB2	DNAlig4	-1.40673172715063	0.587364787535786	1	pseudobulk	DESeq2	LRT		0
121	KC_AB2	DNApol-eta	-0.427431139129811	1	1	pseudobulk	DESeq2	LRT		0
121	KC_AB2	DNApol-iota	1.62571710983431	0.470438722277963	1	pseudobulk	DESeq2	LRT		0
121	KC_AB2	DNApol-theta	-0.592481036256484	0.706847545932384	1	pseudobulk	DESeq2	LRT		0
121	KC_AB2	DNApol-zeta	-1.3723417613553	0.619532036522	1	pseudobulk	DESeq2	LRT		0
121	KC_AB2	dnc	0.210470513811503	0.27343378727475	1	pseudobulk	DESeq2	LRT		0
121	KC_AB2	dnk	1.58773285193391	0.368307560267092	1	pseudobulk	DESeq2	LRT		0
121	KC_AB2	dnr1	-0.427431139129811	1	1	pseudobulk	DESeq2	LRT		0
121	KC_AB2	dnt	-0.338771435603146	0.816656863441963	1	pseudobulk	DESeq2	LRT		0
121	KC_AB2	Dnz1	-0.199186737838788	0.82333340650874	1	pseudobulk	DESeq2	LRT		0
121	KC_AB2	Doa	-0.308836701126105	0.656745675490445	1	pseudobulk	DESeq2	LRT		0
121	KC_AB2	Doc3	1.5243995994652	0.741477491078038	1	pseudobulk	DESeq2	LRT		0
121	KC_AB2	dock	-0.292676365190634	0.583830825763543	1	pseudobulk	DESeq2	LRT		0
121	KC_AB2	dod	0.29081666754322	0.678152817430464	1	pseudobulk	DESeq2	LRT		0
121	KC_AB2	dom	-1.24917511784899	0.204992555649378	1	pseudobulk	DESeq2	LRT		0
121	KC_AB2	dome	1.40976310667017	0.385924033809312	1	pseudobulk	DESeq2	LRT		0
121	KC_AB2	dop	0.654928762448402	0.501464281816408	1	pseudobulk	DESeq2	LRT		0
121	KC_AB2	Dop1R1	-0.0872130092392679	0.723065745507083	1	pseudobulk	DESeq2	LRT		0
121	KC_AB2	Dop1R2	0.0933511809352207	0.713023167947396	1	pseudobulk	DESeq2	LRT		0
121	KC_AB2	Dop2R	-0.03929356787539	0.846887328021077	1	pseudobulk	DESeq2	LRT		0
121	KC_AB2	DopEcR	-0.0304667468480023	0.929365987153194	1	pseudobulk	DESeq2	LRT		0
121	KC_AB2	dor	0.864341462967788	0.925947094335722	1	pseudobulk	DESeq2	LRT		0
121	KC_AB2	DOR	-0.21305576025907	0.476539439560882	1	pseudobulk	DESeq2	LRT		0
121	KC_AB2	dos	0.324325556848176	0.757697093383537	1	pseudobulk	DESeq2	LRT		0
121	KC_AB2	Dp	0.506477883881803	0.463295919175569	1	pseudobulk	DESeq2	LRT		0
121	KC_AB2	Dp1	0.754262023369963	0.545686594045592	1	pseudobulk	DESeq2	LRT		0
121	KC_AB2	Dpck	-4.11631816360254	0.0222164849145218	1	pseudobulk	DESeq2	LRT		0
121	KC_AB2	DPCoAC	0.651869231857958	0.64198904947059	1	pseudobulk	DESeq2	LRT		0
121	KC_AB2	Dph1	-0.427431139129811	1	1	pseudobulk	DESeq2	LRT		0
121	KC_AB2	Dph5	-1.13000773893157	0.409333070135471	1	pseudobulk	DESeq2	LRT		0
121	KC_AB2	Dpit47	0.458061275320087	0.802259721126042	1	pseudobulk	DESeq2	LRT		0
121	KC_AB2	DppIII	-0.41404693822881	0.672715903825973	1	pseudobulk	DESeq2	LRT		0
121	KC_AB2	dpr1	0.920212920242661	0.67286879185691	1	pseudobulk	DESeq2	LRT		0
121	KC_AB2	dpr10	-0.10986650703912	0.664287798866885	1	pseudobulk	DESeq2	LRT		0
121	KC_AB2	dpr12	0.229273347125603	0.449547091906266	1	pseudobulk	DESeq2	LRT		0
121	KC_AB2	dpr14	0.0654865496343039	0.784571952835369	1	pseudobulk	DESeq2	LRT		0
121	KC_AB2	dpr16	-0.478339813047823	0.649560839608111	1	pseudobulk	DESeq2	LRT		0
121	KC_AB2	dpr17	0.209952830249259	0.625510657408062	1	pseudobulk	DESeq2	LRT		0
121	KC_AB2	dpr19	0.0646691241544207	0.889144694375738	1	pseudobulk	DESeq2	LRT		0
121	KC_AB2	dpr2	-1.78766527924797	0.333235446164714	1	pseudobulk	DESeq2	LRT		0
121	KC_AB2	dpr20	2.01066821707654	0.222449972214956	1	pseudobulk	DESeq2	LRT		0
121	KC_AB2	dpr21	-0.195249879459363	0.517209616763573	1	pseudobulk	DESeq2	LRT		0
121	KC_AB2	dpr3	-0.480290087023551	0.7714263901498	1	pseudobulk	DESeq2	LRT		0
121	KC_AB2	dpr4	-0.0114319618324095	0.830130999980748	1	pseudobulk	DESeq2	LRT		0
121	KC_AB2	dpr5	0.251734229871675	0.697798752738186	1	pseudobulk	DESeq2	LRT		0
121	KC_AB2	dpr6	0.0237614114784502	0.780712038665947	1	pseudobulk	DESeq2	LRT		0
121	KC_AB2	dpr7	0.0769953972318993	0.830185057462144	1	pseudobulk	DESeq2	LRT		0
121	KC_AB2	dpr8	-0.0039648998756262	0.929190031517746	1	pseudobulk	DESeq2	LRT		0
121	KC_AB2	dpr9	0.327821973017797	0.282313443352145	1	pseudobulk	DESeq2	LRT		0
121	KC_AB2	dpy	-0.841882716738579	1	1	pseudobulk	DESeq2	LRT		0
121	KC_AB2	Dpy-30L1	-0.987177826797544	0.309852413866131	1	pseudobulk	DESeq2	LRT		0
121	KC_AB2	Drak	-0.480626224772412	0.475592497715518	1	pseudobulk	DESeq2	LRT		0

121	KC_AB2	Drat	0.296717398933936	0.41371082275446	1	pseudobulk	DESeq2	LRT	0
121	KC_AB2	dre4	0.938309195058695	0.521142847958191	1	pseudobulk	DESeq2	LRT	0
121	KC_AB2	Dref	0.119304913847595	0.875786357402216	1	pseudobulk	DESeq2	LRT	0
121	KC_AB2	Drep1	-0.869983840300699	0.681381135624691	1	pseudobulk	DESeq2	LRT	0
121	KC_AB2	Drep2	0.133479974514697	0.692619705753813	1	pseudobulk	DESeq2	LRT	0
121	KC_AB2	Drep4	-0.55127884429609	0.742675701031897	1	pseudobulk	DESeq2	LRT	0
121	KC_AB2	drk	0.0267617779862794	0.975500551600342	1	pseudobulk	DESeq2	LRT	0
121	KC_AB2	drl	1.56883432319406	0.279533125818941	1	pseudobulk	DESeq2	LRT	0
121	KC_AB2	Drl-2	-0.519594806869021	0.313343826093645	1	pseudobulk	DESeq2	LRT	0
121	KC_AB2	Droj2	-0.258210891673728	0.568455208349621	1	pseudobulk	DESeq2	LRT	0
121	KC_AB2	Dronc	-1.12785316439435	0.661319045116135	1	pseudobulk	DESeq2	LRT	0
122	KC_AB2	drongo	1.83202863219247	0.154245757274926	1	pseudobulk	DESeq2	LRT	0
122	KC_AB2	drosha	-2.43175598261552	0.265266490111144	1	pseudobulk	DESeq2	LRT	0
122	KC_AB2	Drp1	0.163675511293735	0.801125987641584	1	pseudobulk	DESeq2	LRT	0
122	KC_AB2	drpr	-0.492147284953816	0.671269925425552	1	pseudobulk	DESeq2	LRT	0
122	KC_AB2	Dscam1	-0.25492796776137	0.578606011234402	1	pseudobulk	DESeq2	LRT	0
122	KC_AB2	Dscam2	0.208229764319171	0.638678428393481	1	pseudobulk	DESeq2	LRT	0
122	KC_AB2	Dscam3	-0.877256597174531	0.0466820300055213	1	pseudobulk	DESeq2	LRT	0
122	KC_AB2	Dscam4	-0.681379873551133	0.290485960601046	1	pseudobulk	DESeq2	LRT	0
122	KC_AB2	dsd	0.683793084656692	0.243104286370579	1	pseudobulk	DESeq2	LRT	0
122	KC_AB2	dsh	-0.178195278469443	0.763850720302089	1	pseudobulk	DESeq2	LRT	0
122	KC_AB2	Dsor1	-0.848743926576292	0.392475684567127	1	pseudobulk	DESeq2	LRT	0
122	KC_AB2	Dsp1	0.220930564528768	0.501929193023027	1	pseudobulk	DESeq2	LRT	0
122	KC_AB2	dsx-c73A	-0.600547221779134	1	1	pseudobulk	DESeq2	LRT	0
122	KC_AB2	Dtd	-0.600547221779134	1	1	pseudobulk	DESeq2	LRT	0
122	KC_AB2	dtn	-0.292475852858162	0.457926862274608	1	pseudobulk	DESeq2	LRT	0
122	KC_AB2	Duba	-1.35300192000393	0.223572371475824	1	pseudobulk	DESeq2	LRT	0
122	KC_AB2	DUBAI	-0.285094147796605	0.833800287812328	1	pseudobulk	DESeq2	LRT	0
122	KC_AB2	dunk	0.402801018475984	0.810935419177084	1	pseudobulk	DESeq2	LRT	0
122	KC_AB2	dup	1.67530465152129	0.416998667908363	1	pseudobulk	DESeq2	LRT	0
122	KC_AB2	dwg	0.906356398385783	0.754027117686013	1	pseudobulk	DESeq2	LRT	0
122	KC_AB2	Dyb	-0.141443230048353	0.658395395557497	1	pseudobulk	DESeq2	LRT	0
122	KC_AB2	Dyrk2	0.392859523919872	0.445037333794519	1	pseudobulk	DESeq2	LRT	0
122	KC_AB2	Dyrk3	0.0316769401710274	0.861594147714225	1	pseudobulk	DESeq2	LRT	0
122	KC_AB2	Dys	0.0852184594700659	0.839218954497592	1	pseudobulk	DESeq2	LRT	0
122	KC_AB2	Dysb	-0.600547221779134	1	1	pseudobulk	DESeq2	LRT	0
122	KC_AB2	dysc	0.0439842312509106	0.795008380164503	1	pseudobulk	DESeq2	LRT	0
122	KC_AB2	E(bx)	0.177785143789161	0.723487034119403	1	pseudobulk	DESeq2	LRT	0
122	KC_AB2	E(Pc)	-0.228380182516489	0.631564814906732	1	pseudobulk	DESeq2	LRT	0
122	KC_AB2	e(r)	-0.278885508413632	0.593931515612941	1	pseudobulk	DESeq2	LRT	0
122	KC_AB2	E(var)3-9	-0.659832019250431	0.687961807714689	1	pseudobulk	DESeq2	LRT	0
122	KC_AB2	e(y)1	-2.43175598261552	0.265266490111144	1	pseudobulk	DESeq2	LRT	0
122	KC_AB2	e(y)2	-0.293694200456602	0.813028787207985	1	pseudobulk	DESeq2	LRT	0
122	KC_AB2	e(y)3	-0.587725466860654	0.46376355376167	1	pseudobulk	DESeq2	LRT	0
122	KC_AB2	E(z)	-0.670833611710101	0.682559952282501	1	pseudobulk	DESeq2	LRT	0
122	KC_AB2	E2f1	0.375568163168146	0.754255770281326	1	pseudobulk	DESeq2	LRT	0
122	KC_AB2	E2f2	-1.05540235670195	0.599449538772552	1	pseudobulk	DESeq2	LRT	0
122	KC_AB2	Eaat2	0.685286179123795	0.348605997579528	1	pseudobulk	DESeq2	LRT	0
122	KC_AB2	Eaf	-1.24522584101331	0.423417190931214	1	pseudobulk	DESeq2	LRT	0
122	KC_AB2	Eaf6	-1.29244479833796	0.693295472370086	1	pseudobulk	DESeq2	LRT	0
122	KC_AB2	eag	0.236064565623629	0.692819299509479	1	pseudobulk	DESeq2	LRT	0
122	KC_AB2	ear	0.16229071933367	0.85275486439557	1	pseudobulk	DESeq2	LRT	0
122	KC_AB2	eas	0.51414687023645	0.488891278722226	1	pseudobulk	DESeq2	LRT	0

122	KC_AB2	east	-0.0682821727239142	0.897583679907326	1	pseudobulk	DESeq2	LRT		0
122	KC_AB2	Eato	0.195179088529276	0.926055845321122	1	pseudobulk	DESeq2	LRT		0
122	KC_AB2	Eb1	0.147190963120321	0.719372319889174	1	pseudobulk	DESeq2	LRT		0
122	KC_AB2	ebd1	-0.825312539513664	0.666826652877566	1	pseudobulk	DESeq2	LRT		0
122	KC_AB2	ebd2	-0.919072033633204	0.474824459847445	1	pseudobulk	DESeq2	LRT		0
122	KC_AB2	ebi	0.240831253235064	0.850280351115472	1	pseudobulk	DESeq2	LRT		0
122	KC_AB2	ebo	-0.357354465800576	0.852988665057457	1	pseudobulk	DESeq2	LRT		0
122	KC_AB2	EbpIII	-2.05746818172871	0.352594693121816	1	pseudobulk	DESeq2	LRT		0
122	KC_AB2	eca	-0.0301584626951744	0.959208517610375	1	pseudobulk	DESeq2	LRT		0
122	KC_AB2	ecd	0.914818958172866	0.636986897982382	1	pseudobulk	DESeq2	LRT		0
122	KC_AB2	Echs1	-3.14999549719461	0.130265118305109	1	pseudobulk	DESeq2	LRT		0
122	KC_AB2	EcR	-0.188430293371932	0.663540802994145	1	pseudobulk	DESeq2	LRT		0
122	KC_AB2	ECSIT	0.222396124134733	1	1	pseudobulk	DESeq2	LRT		0
122	KC_AB2	Ect3	-0.592481036256484	0.706847545932384	1	pseudobulk	DESeq2	LRT		0
122	KC_AB2	Ect4	-0.592481036256484	0.706847545932384	1	pseudobulk	DESeq2	LRT		0
122	KC_AB2	ed	0.834949068306991	0.407900687052682	1	pseudobulk	DESeq2	LRT		0
122	KC_AB2	Edc3	0.208682867171892	0.77742768168426	1	pseudobulk	DESeq2	LRT		0
122	KC_AB2	Edem1	1.61170053689188	0.426743794296421	1	pseudobulk	DESeq2	LRT		0
122	KC_AB2	Edem2	-1.47385083621876	0.345544290516083	1	pseudobulk	DESeq2	LRT		0
122	KC_AB2	EDTP	0.0994650467072686	0.935070622509849	1	pseudobulk	DESeq2	LRT		0
122	KC_AB2	eEF1alpha1	-0.266718490789834	0.817742134122333	1	pseudobulk	DESeq2	LRT		0
122	KC_AB2	eEF1alpha2	-0.115668509689407	0.678341658398037	1	pseudobulk	DESeq2	LRT		0
122	KC_AB2	eEF1beta	-0.77068366514966	0.366276832526859	1	pseudobulk	DESeq2	LRT		0
122	KC_AB2	eEF1delta	-0.128925572758013	0.757750799005408	1	pseudobulk	DESeq2	LRT		0
122	KC_AB2	eEF1gamma	-0.128523056430433	0.881902950163164	1	pseudobulk	DESeq2	LRT		0
122	KC_AB2	eEF2	-0.133826726187696	0.736981065694071	1	pseudobulk	DESeq2	LRT		0
122	KC_AB2	eEF5	-0.199973901869861	0.594208741964097	1	pseudobulk	DESeq2	LRT		0
122	KC_AB2	eEFSec	-0.592481036256484	0.706847545932384	1	pseudobulk	DESeq2	LRT		0
122	KC_AB2	Efa6	0.800964845515188	0.341581515959305	1	pseudobulk	DESeq2	LRT		0
122	KC_AB2	eff	-0.14674985540912	0.564527722747738	1	pseudobulk	DESeq2	LRT		0
122	KC_AB2	Efr	0.0987291371049698	0.889956536318748	1	pseudobulk	DESeq2	LRT		0
122	KC_AB2	egg	0.791468033138705	0.699865413482108	1	pseudobulk	DESeq2	LRT		0
122	KC_AB2	egh	0.359045663297766	0.337937385362636	1	pseudobulk	DESeq2	LRT		0
122	KC_AB2	egl	-0.252863568825607	0.767769675474654	1	pseudobulk	DESeq2	LRT		0
122	KC_AB2	Egm	0.329740041077442	0.855811003303737	1	pseudobulk	DESeq2	LRT		0
122	KC_AB2	Ehbp1	0.196169996999767	0.710802516005037	1	pseudobulk	DESeq2	LRT		0
122	KC_AB2	eIF1	-0.037601033379281	0.930638011141085	1	pseudobulk	DESeq2	LRT		0
122	KC_AB2	eIF1A	-0.56445796099863	0.111293632108942	1	pseudobulk	DESeq2	LRT		0
122	KC_AB2	eIF2alpha	-0.3323151401716	0.694620046186085	1	pseudobulk	DESeq2	LRT		0
122	KC_AB2	eIF2Balpha	1.5243995994652	0.741477491078038	1	pseudobulk	DESeq2	LRT		0
122	KC_AB2	eIF2Bbeta	-2.12967468770992	0.340688355153583	1	pseudobulk	DESeq2	LRT		0
122	KC_AB2	eIF2Bdelta	-0.617874106857047	0.716176596915688	1	pseudobulk	DESeq2	LRT		0
122	KC_AB2	eIF2Bepsilon	2.12684015898615	0.445939223035924	1	pseudobulk	DESeq2	LRT		0
122	KC_AB2	eIF2beta	-0.83241515200314	0.23012899248702	1	pseudobulk	DESeq2	LRT		0
122	KC_AB2	eIF2Bgamma	-3.09367200686729	0.12439071639243	1	pseudobulk	DESeq2	LRT		0
122	KC_AB2	eIF2D	-0.289750769262794	0.829335057497959	1	pseudobulk	DESeq2	LRT		0
122	KC_AB2	eIF2gamma	0.128772301688567	0.909079681526169	1	pseudobulk	DESeq2	LRT		0
122	KC_AB2	eIF3a	-0.519567061871295	0.464651216298289	1	pseudobulk	DESeq2	LRT		0
122	KC_AB2	eIF3b	-0.348672976821052	0.582283266112843	1	pseudobulk	DESeq2	LRT		0
122	KC_AB2	eIF3c	-0.508980186237928	0.45694254781602	1	pseudobulk	DESeq2	LRT		0
122	KC_AB2	eIF3d1	0.283979982850431	0.692806993670359	1	pseudobulk	DESeq2	LRT		0
122	KC_AB2	eIF3e	0.333283799929909	0.660499078663551	1	pseudobulk	DESeq2	LRT		0
122	KC_AB2	eIF3f1	-0.531267736055382	0.412160851141103	1	pseudobulk	DESeq2	LRT		0

122	KC_AB2	eIF3g1	-0.180803933559429	0.79000579808155	1	pseudobulk	DESeq2	LRT		0
122	KC_AB2	eIF3h	-0.34421320520951	0.598913801025882	1	pseudobulk	DESeq2	LRT		0
122	KC_AB2	eIF3i	-0.386616839938841	0.57738282680047	1	pseudobulk	DESeq2	LRT		0
122	KC_AB2	eIF3j	0.531374426021462	0.459907851424614	1	pseudobulk	DESeq2	LRT		0
122	KC_AB2	eIF3k	0.426668242679434	0.63542838958605	1	pseudobulk	DESeq2	LRT		0
123	KC_AB2	eIF3l	-0.627673059029692	0.269056270450673	1	pseudobulk	DESeq2	LRT		0
123	KC_AB2	eIF3m	-0.160670560325844	0.788823112496021	1	pseudobulk	DESeq2	LRT		0
123	KC_AB2	eIF4A	0.0942783214244157	0.706822469771623	1	pseudobulk	DESeq2	LRT		0
123	KC_AB2	eIF4B	0.439054004263391	0.431977397566974	1	pseudobulk	DESeq2	LRT		0
123	KC_AB2	eIF4E1	0.136170119195975	0.759703336900456	1	pseudobulk	DESeq2	LRT		0
123	KC_AB2	eIF4EHP	0.105508484712962	0.698605916114562	1	pseudobulk	DESeq2	LRT		0
123	KC_AB2	eIF4G1	-0.248810894228378	0.658901234273617	1	pseudobulk	DESeq2	LRT		0
123	KC_AB2	eIF4G2	0.224430192564035	0.664745244265777	1	pseudobulk	DESeq2	LRT		0
123	KC_AB2	eIF4H1	-0.0540265252319272	0.881866483418954	1	pseudobulk	DESeq2	LRT		0
123	KC_AB2	eIF5	0.525156308232917	0.362472767064956	1	pseudobulk	DESeq2	LRT		0
123	KC_AB2	eIF5B	0.0865104497721783	0.865017669304243	1	pseudobulk	DESeq2	LRT		0
123	KC_AB2	eIF6	-1.85733156562156	0.448702818121367	1	pseudobulk	DESeq2	LRT		0
123	KC_AB2	Eip55E	-1.78094334699383	0.470930349923561	1	pseudobulk	DESeq2	LRT		0
123	KC_AB2	Eip63E	-0.282220557982862	0.602517704592107	1	pseudobulk	DESeq2	LRT		0
123	KC_AB2	Eip63F-1	-1.40673172715063	0.587364787535786	1	pseudobulk	DESeq2	LRT		0
123	KC_AB2	Eip71CD	1.74949131031298	0.0658061966663312	1	pseudobulk	DESeq2	LRT		0
123	KC_AB2	Eip74EF	0.965689973620295	0.613275257848467	1	pseudobulk	DESeq2	LRT		0
123	KC_AB2	Eip75B	-0.258408681682747	0.378361582852821	1	pseudobulk	DESeq2	LRT		0
123	KC_AB2	Eip78C	0.120468002953641	0.877209770312458	1	pseudobulk	DESeq2	LRT		0
123	KC_AB2	Eip93F	0.0936561185674126	0.62275647396783	1	pseudobulk	DESeq2	LRT		0
123	KC_AB2	elav	0.0987818760937718	0.766415038082294	1	pseudobulk	DESeq2	LRT		0
123	KC_AB2	elB	0.179303920438641	0.813747538409089	1	pseudobulk	DESeq2	LRT		0
123	KC_AB2	elgi	-0.305652093479492	0.846052208680978	1	pseudobulk	DESeq2	LRT		0
123	KC_AB2	Elk	-0.601224400772064	0.074813927116288	1	pseudobulk	DESeq2	LRT		0
123	KC_AB2	elm	0.577002077814595	0.33931290618102	1	pseudobulk	DESeq2	LRT		0
123	KC_AB2	EloA	0.343376874783972	0.702237810623515	1	pseudobulk	DESeq2	LRT		0
123	KC_AB2	EloB	1.04898121615571	0.292722283942885	1	pseudobulk	DESeq2	LRT		0
123	KC_AB2	EloC	-0.541481859657059	0.31765330681763	1	pseudobulk	DESeq2	LRT		0
123	KC_AB2	Elp1	-1.7824418856176	0.467224294431682	1	pseudobulk	DESeq2	LRT		0
123	KC_AB2	Elp2	1.21092382796727	0.637125262974216	1	pseudobulk	DESeq2	LRT		0
123	KC_AB2	Elp3	0.506662102707318	0.782529221356027	1	pseudobulk	DESeq2	LRT		0
123	KC_AB2	Elys	-0.2488904916109	0.790972801778357	1	pseudobulk	DESeq2	LRT		0
123	KC_AB2	ema	0.0399248009710874	0.953621992354699	1	pseudobulk	DESeq2	LRT		0
123	KC_AB2	emb	0.422857589316459	0.598482663891757	1	pseudobulk	DESeq2	LRT		0
123	KC_AB2	emc	0.0491063675128921	0.873030558752776	1	pseudobulk	DESeq2	LRT		0
123	KC_AB2	EMC1	-0.676788157317206	0.608016060975217	1	pseudobulk	DESeq2	LRT		0
123	KC_AB2	EMC3	-0.284164906401899	0.823749586966724	1	pseudobulk	DESeq2	LRT		0
123	KC_AB2	EMC8-9	-1.26206596011159	0.113898907864921	1	pseudobulk	DESeq2	LRT		0
123	KC_AB2	EMRE	-0.472997365871795	0.477498676439511	1	pseudobulk	DESeq2	LRT		0
123	KC_AB2	ena	-1.15343358598114	0.334691668348747	1	pseudobulk	DESeq2	LRT		0
123	KC_AB2	enc	0.166886203542644	0.562016168429744	1	pseudobulk	DESeq2	LRT		0
123	KC_AB2	EndoA	0.140643348256542	0.641570748836874	1	pseudobulk	DESeq2	LRT		0
123	KC_AB2	EndoB	-0.0830780138128019	0.897999674841018	1	pseudobulk	DESeq2	LRT		0
123	KC_AB2	EndoG	0.029740537311628	0.95761367379353	1	pseudobulk	DESeq2	LRT		0
123	KC_AB2	EndoGI	1.5243995994652	0.741477491078038	1	pseudobulk	DESeq2	LRT		0
123	KC_AB2	endos	-0.437089834171455	0.399642300669076	1	pseudobulk	DESeq2	LRT		0
123	KC_AB2	Eno	0.214154860203105	0.444095657201113	1	pseudobulk	DESeq2	LRT		0
123	KC_AB2	enok	0.29783557724074	0.770590927812463	1	pseudobulk	DESeq2	LRT		0

123	KC_AB2	ens	0.656939359814901	0.163899311854226	1	pseudobulk	DESeq2	LRT		0
123	KC_AB2	Ent1	2.37962608852272	0.268085683686126	1	pseudobulk	DESeq2	LRT		0
123	KC_AB2	Ent3	0.872796431322145	0.186962351440378	1	pseudobulk	DESeq2	LRT		0
123	KC_AB2	Eogt	-1.3929484167291	0.512855059074155	1	pseudobulk	DESeq2	LRT		0
123	KC_AB2	Epac	0.725498497157359	0.93879047033739	1	pseudobulk	DESeq2	LRT		0
123	KC_AB2	Epg5	2.48629812612306	0.259054692310381	1	pseudobulk	DESeq2	LRT		0
123	KC_AB2	Eph	-0.303120330366847	0.501230308762854	1	pseudobulk	DESeq2	LRT		0
123	KC_AB2	Ephrin	0.171514532691996	0.760458761695376	1	pseudobulk	DESeq2	LRT		0
123	KC_AB2	Eps-15	-0.594162220505661	0.338650123887761	1	pseudobulk	DESeq2	LRT		0
123	KC_AB2	epsilonCOP	0.24325151163626	0.828530887290091	1	pseudobulk	DESeq2	LRT		0
123	KC_AB2	eRF1	-0.522593135055599	0.449868619741679	1	pseudobulk	DESeq2	LRT		0
123	KC_AB2	eRF3	-3.01459641366802	0.149509045856783	1	pseudobulk	DESeq2	LRT		0
123	KC_AB2	ergic53	-1.00711362121993	0.567250614423781	1	pseudobulk	DESeq2	LRT		0
123	KC_AB2	Ero1L	0.683176619872214	0.597039999452256	1	pseudobulk	DESeq2	LRT		0
123	KC_AB2	ERp60	0.203756491678228	0.847837582644861	1	pseudobulk	DESeq2	LRT		0
123	KC_AB2	ERR	0.191636765943703	0.862405194752902	1	pseudobulk	DESeq2	LRT		0
123	KC_AB2	Es2	1.48010047180759	0.482153739285344	1	pseudobulk	DESeq2	LRT		0
123	KC_AB2	esc	-0.600547221779133	0.841940696994101	1	pseudobulk	DESeq2	LRT		0
123	KC_AB2	Est-6	2.12684015898615	0.445939223035924	1	pseudobulk	DESeq2	LRT		0
123	KC_AB2	Est-Q	-0.600547221779133	0.841940696994101	1	pseudobulk	DESeq2	LRT		0
123	KC_AB2	Esyt2	0.629301467947601	0.281876320153485	1	pseudobulk	DESeq2	LRT		0
123	KC_AB2	Etf-QO	0.183555857030446	1	1	pseudobulk	DESeq2	LRT		0
123	KC_AB2	Etl1	-0.255481912470171	0.858763198530661	1	pseudobulk	DESeq2	LRT		0
123	KC_AB2	Ets97D	0.864341462967788	0.925947094335722	1	pseudobulk	DESeq2	LRT		0
123	KC_AB2	Evi5	0.0904588328466478	0.855701572324927	1	pseudobulk	DESeq2	LRT		0
123	KC_AB2	ewg	0.711923955616448	0.310387383059789	1	pseudobulk	DESeq2	LRT		0
123	KC_AB2	exd	-0.914701940598279	0.286102767516317	1	pseudobulk	DESeq2	LRT		0
123	KC_AB2	Exn	0.401642815078518	0.681728825078417	1	pseudobulk	DESeq2	LRT		0
123	KC_AB2	Exo70	-1.86119559582179	0.450127557232397	1	pseudobulk	DESeq2	LRT		0
123	KC_AB2	Exo84	1.26334827431191	0.462883082783822	1	pseudobulk	DESeq2	LRT		0
123	KC_AB2	ey	-0.0146957850694834	0.848991670579447	1	pseudobulk	DESeq2	LRT		0
123	KC_AB2	eys	-0.134466422115222	0.74506743014215	1	pseudobulk	DESeq2	LRT		0
123	KC_AB2	f-cup	-1.29035868264066	0.50372094761382	1	pseudobulk	DESeq2	LRT		0
123	KC_AB2	fabp	-0.36432503074311	0.686680955964425	1	pseudobulk	DESeq2	LRT		0
123	KC_AB2	Fadd	-0.427431139129811	1	1	pseudobulk	DESeq2	LRT		0
123	KC_AB2	faf	0.400064904240639	0.478024145100071	1	pseudobulk	DESeq2	LRT		0
123	KC_AB2	Faf2	0.163035815070398	0.853057628677246	1	pseudobulk	DESeq2	LRT		0
123	KC_AB2	Fak	-0.515429497223693	0.447173688029354	1	pseudobulk	DESeq2	LRT		0
123	KC_AB2	fal	0.150716830215252	0.873698872950822	1	pseudobulk	DESeq2	LRT		0
123	KC_AB2	FAM21	-0.70727250916738	0.705390475165233	1	pseudobulk	DESeq2	LRT		0
123	KC_AB2	Fancd2	-0.427431139129811	1	1	pseudobulk	DESeq2	LRT		0
123	KC_AB2	fand	-0.290063816371847	0.881698537689548	1	pseudobulk	DESeq2	LRT		0
123	KC_AB2	Fas1	-0.071951938524009	0.74981690974178	1	pseudobulk	DESeq2	LRT		0
123	KC_AB2	Fas2	0.155445597119989	0.50103285138473	1	pseudobulk	DESeq2	LRT		0
123	KC_AB2	Fas3	0.0069501800993374	0.913052845775069	1	pseudobulk	DESeq2	LRT		0
123	KC_AB2	FASN1	0.279016383659005	0.621098018995535	1	pseudobulk	DESeq2	LRT		0
123	KC_AB2	Fatp1	1.04808974416922	0.492159069841738	1	pseudobulk	DESeq2	LRT		0
123	KC_AB2	fax	0.0522694046331931	0.810279793345579	1	pseudobulk	DESeq2	LRT		0
123	KC_AB2	fbl	0.120099014797999	0.922615969474866	1	pseudobulk	DESeq2	LRT		0
123	KC_AB2	Fbl6	0.210612382898966	0.813152052213742	1	pseudobulk	DESeq2	LRT		0
123	KC_AB2	Fbw5	1.8688760377645	0.356303822006979	1	pseudobulk	DESeq2	LRT		0
123	KC_AB2	Fbxl4	-0.828178378785339	0.673584255563891	1	pseudobulk	DESeq2	LRT		0
124	KC_AB2	FBXO11	-0.882523945751818	0.286223513141228	1	pseudobulk	DESeq2	LRT		0

124	KC_AB2	Fcp1	-0.47841585496464	0.688406081840727	1	pseudobulk	DESeq2	LRT	0
124	KC_AB2	fdl	-0.598229230258589	0.549996341521615	1	pseudobulk	DESeq2	LRT	0
124	KC_AB2	Fdx1	-0.509007437760716	0.731624785814057	1	pseudobulk	DESeq2	LRT	0
124	KC_AB2	Fdx2	-0.425347426251263	0.74574460353644	1	pseudobulk	DESeq2	LRT	0
124	KC_AB2	FeCH	-2.16906696862276	0.324569721711647	1	pseudobulk	DESeq2	LRT	0
124	KC_AB2	Fem-1	-0.963734693398155	0.466920588071799	1	pseudobulk	DESeq2	LRT	0
124	KC_AB2	fend	-0.12588378621344	0.64766547921515	1	pseudobulk	DESeq2	LRT	0
124	KC_AB2	FER	-1.70614408517477	0.52816093424714	1	pseudobulk	DESeq2	LRT	0
124	KC_AB2	Fer1HCH	0.0675627417116836	0.792420566184173	1	pseudobulk	DESeq2	LRT	0
124	KC_AB2	Fer2LCH	-0.0974051402134653	0.744611999035017	1	pseudobulk	DESeq2	LRT	0
124	KC_AB2	fest	-1.28379628855895	0.344847161445576	1	pseudobulk	DESeq2	LRT	0
124	KC_AB2	Fgop2	-1.72543571481447	0.362940138597624	1	pseudobulk	DESeq2	LRT	0
124	KC_AB2	fh	-0.493357239699781	0.783807521297462	1	pseudobulk	DESeq2	LRT	0
124	KC_AB2	Fibp	-0.953311341354687	0.40018366925231	1	pseudobulk	DESeq2	LRT	0
124	KC_AB2	Fic	0.458061275320087	0.802259721126042	1	pseudobulk	DESeq2	LRT	0
124	KC_AB2	fid	-0.23582094641692	0.827863393159815	1	pseudobulk	DESeq2	LRT	0
124	KC_AB2	fidipidine	-2.72031933562543	0.207919053002488	1	pseudobulk	DESeq2	LRT	0
124	KC_AB2	Fie	1.26601742838262	0.230633419561289	1	pseudobulk	DESeq2	LRT	0
124	KC_AB2	Fife	-0.267784025300524	0.525789914748047	1	pseudobulk	DESeq2	LRT	0
124	KC_AB2	fig	1.5243995994652	0.741477491078038	1	pseudobulk	DESeq2	LRT	0
124	KC_AB2	Fim	0.521726433814198	0.460366367801633	1	pseudobulk	DESeq2	LRT	0
124	KC_AB2	Fip1	-1.47177386068916	0.26129967789484	1	pseudobulk	DESeq2	LRT	0
124	KC_AB2	fipi	0.808179083777674	0.197621155247888	1	pseudobulk	DESeq2	LRT	0
124	KC_AB2	Fis1	-0.130537181714795	0.761499989685802	1	pseudobulk	DESeq2	LRT	0
124	KC_AB2	Fit1	0.960213147590235	0.56217518180866	1	pseudobulk	DESeq2	LRT	0
124	KC_AB2	Fitm	1.5243995994652	0.741477491078038	1	pseudobulk	DESeq2	LRT	0
124	KC_AB2	Fkbp12	-0.303547927970318	0.164618928418265	1	pseudobulk	DESeq2	LRT	0
124	KC_AB2	Fkbp14	-0.841882716738579	1	1	pseudobulk	DESeq2	LRT	0
124	KC_AB2	Fkbp39	0.0866726802508225	0.821590878977733	1	pseudobulk	DESeq2	LRT	0
124	KC_AB2	Fkbp59	-0.0124015906134846	0.907093915979133	1	pseudobulk	DESeq2	LRT	0
124	KC_AB2	fl(2)d	0.193199252467521	0.724188231579178	1	pseudobulk	DESeq2	LRT	0
124	KC_AB2	Flacc	0.0289683936047307	0.878693302983797	1	pseudobulk	DESeq2	LRT	0
124	KC_AB2	FLASH	-0.600547221779134	1	1	pseudobulk	DESeq2	LRT	0
124	KC_AB2	flfl	-0.488712560272099	0.491467946269834	1	pseudobulk	DESeq2	LRT	0
124	KC_AB2	fliI	0.657254067967356	0.701168720187778	1	pseudobulk	DESeq2	LRT	0
124	KC_AB2	Flo1	-0.508199779263114	0.358009408818774	1	pseudobulk	DESeq2	LRT	0
124	KC_AB2	Flo2	0.129504747039755	0.822496266382615	1	pseudobulk	DESeq2	LRT	0
124	KC_AB2	flr	0.70378358684898	0.932301451973468	1	pseudobulk	DESeq2	LRT	0
124	KC_AB2	flw	-0.680301507220621	0.176092413020392	1	pseudobulk	DESeq2	LRT	0
124	KC_AB2	Fmr1	0.132157076739892	0.831519424104122	1	pseudobulk	DESeq2	LRT	0
124	KC_AB2	fmt	0.145400742129114	0.82667730171835	1	pseudobulk	DESeq2	LRT	0
124	KC_AB2	fne	0.126376460276366	0.585724967364231	1	pseudobulk	DESeq2	LRT	0
124	KC_AB2	fng	-0.600547221779134	1	1	pseudobulk	DESeq2	LRT	0
124	KC_AB2	Fnta	1.40919686118418	0.389843538833747	1	pseudobulk	DESeq2	LRT	0
124	KC_AB2	fog	1.5243995994652	0.741477491078038	1	pseudobulk	DESeq2	LRT	0
124	KC_AB2	foi	0.146498060900338	0.869074505610287	1	pseudobulk	DESeq2	LRT	0
124	KC_AB2	fok	-0.038132967848035	0.867813253960719	1	pseudobulk	DESeq2	LRT	0
124	KC_AB2	form3	0.228215696441444	0.864464959897697	1	pseudobulk	DESeq2	LRT	0
124	KC_AB2	FoxK	-0.210391003928696	0.672995578219105	1	pseudobulk	DESeq2	LRT	0
124	KC_AB2	FoxP	-0.0008463401703238	0.848919206720493	1	pseudobulk	DESeq2	LRT	0
124	KC_AB2	Fpps	1.23085716405798	0.614623732585597	1	pseudobulk	DESeq2	LRT	0
124	KC_AB2	fra	-1.92942763539083	0.427765257981976	1	pseudobulk	DESeq2	LRT	0
124	KC_AB2	fray	-3.06333495211286	0.123567477020264	1	pseudobulk	DESeq2	LRT	0

124	KC_AB2	FRG1	6.03027979003313E-0	0.942364063697085	1	pseudobulk	DESeq2	LRT		0
124	KC_AB2	frj	0.176151359390506	0.804757728006748	1	pseudobulk	DESeq2	LRT		0
124	KC_AB2	Frl	0.708402871905741	0.142135854771583	1	pseudobulk	DESeq2	LRT		0
124	KC_AB2	Frq1	0.100101303196065	0.773382477806774	1	pseudobulk	DESeq2	LRT		0
124	KC_AB2	Frq2	-0.701368061918147	0.351433040422279	1	pseudobulk	DESeq2	LRT		0
124	KC_AB2	fru	-1.91869873223882	0.201109817308461	1	pseudobulk	DESeq2	LRT		0
124	KC_AB2	fry	1.07282639016441	0.323766760947449	1	pseudobulk	DESeq2	LRT		0
124	KC_AB2	fs(1)h	0.278038647910374	0.271600604958161	1	pseudobulk	DESeq2	LRT		0
124	KC_AB2	fs(1)K10	1.39827770914961	0.587246212652858	1	pseudobulk	DESeq2	LRT		0
124	KC_AB2	Fs(2)Ket	-0.267440353630711	0.335346896843422	1	pseudobulk	DESeq2	LRT		0
124	KC_AB2	fs(2)ltoPP43	0.318764816980533	0.842241734994733	1	pseudobulk	DESeq2	LRT		0
124	KC_AB2	fsd	0.70378358684898	0.932301451973468	1	pseudobulk	DESeq2	LRT		0
124	KC_AB2	Fsn	0.658076648527647	0.568923566282754	1	pseudobulk	DESeq2	LRT		0
124	KC_AB2	ftz-f1	-0.67276770217407	0.283145379940176	1	pseudobulk	DESeq2	LRT		0
124	KC_AB2	fu	0.506662102707318	0.782529221356027	1	pseudobulk	DESeq2	LRT		0
124	KC_AB2	FucT6	-0.234534897929976	0.839176928637056	1	pseudobulk	DESeq2	LRT		0
124	KC_AB2	FucTA	0.088470719838257	0.922736771837542	1	pseudobulk	DESeq2	LRT		0
124	KC_AB2	FucTB	1.5243995994652	0.741477491078038	1	pseudobulk	DESeq2	LRT		0
124	KC_AB2	Fum1	-0.426405869482572	0.53936737179064	1	pseudobulk	DESeq2	LRT		0
124	KC_AB2	Fur1	0.321696180882861	0.658582011297312	1	pseudobulk	DESeq2	LRT		0
124	KC_AB2	Fur2	-0.189226069253547	0.776632885558913	1	pseudobulk	DESeq2	LRT		0
124	KC_AB2	fuss	-0.427431139129811	1	1	pseudobulk	DESeq2	LRT		0
124	KC_AB2	futsch	-0.0082298730376397	0.953915024090844	1	pseudobulk	DESeq2	LRT		0
124	KC_AB2	fwd	0.455042240767849	0.544067047851605	1	pseudobulk	DESeq2	LRT		0
124	KC_AB2	fwe	-0.166291446711726	0.572918741406483	1	pseudobulk	DESeq2	LRT		0
124	KC_AB2	fws	-0.825973974745624	0.678387419789199	1	pseudobulk	DESeq2	LRT		0
124	KC_AB2	fy	1.41524016620164	0.505408490038387	1	pseudobulk	DESeq2	LRT		0
124	KC_AB2	fz	0.0469939316551205	0.825437002505073	1	pseudobulk	DESeq2	LRT		0
124	KC_AB2	fz2	0.327719644455895	0.758977391192572	1	pseudobulk	DESeq2	LRT		0
124	KC_AB2	fz3	1.50386349922033	0.317448777274538	1	pseudobulk	DESeq2	LRT		0
124	KC_AB2	fz4	-0.382929749762112	0.851961810578875	1	pseudobulk	DESeq2	LRT		0
124	KC_AB2	fzr	-0.038893292174732	0.801833047034809	1	pseudobulk	DESeq2	LRT		0
124	KC_AB2	g	0.219820046666602	0.560359419309027	1	pseudobulk	DESeq2	LRT		0
124	KC_AB2	G6P	2.47819260666218	0.0850297977385261	1	pseudobulk	DESeq2	LRT		0
124	KC_AB2	G9a	0.639244761700723	0.393044104645134	1	pseudobulk	DESeq2	LRT		0
124	KC_AB2	GAA1	-0.98094877798171	0.534102984607842	1	pseudobulk	DESeq2	LRT		0
124	KC_AB2	GABA-B-R1	0.462546792259296	0.256205448513901	1	pseudobulk	DESeq2	LRT		0
124	KC_AB2	GABA-B-R2	0.450214260786584	0.385832392439719	1	pseudobulk	DESeq2	LRT		0
124	KC_AB2	Gabat	-0.448541444934387	0.547580673828284	1	pseudobulk	DESeq2	LRT		0
124	KC_AB2	GABPI	0.297785129602179	0.72593635049192	1	pseudobulk	DESeq2	LRT		0
124	KC_AB2	Gale	0.892659559600706	0.712043317464255	1	pseudobulk	DESeq2	LRT		0
124	KC_AB2	galectin	1.13735825725892	0.206153758013327	1	pseudobulk	DESeq2	LRT		0
124	KC_AB2	Galk	-2.41610217722532	0.272481539699592	1	pseudobulk	DESeq2	LRT		0
124	KC_AB2	galla-1	0.0984370962831134	0.855954115769961	1	pseudobulk	DESeq2	LRT		0
124	KC_AB2	galla-2	-0.0430600133583834	0.943191527102359	1	pseudobulk	DESeq2	LRT		0
124	KC_AB2	Galphai	-0.841882716738579	1	1	pseudobulk	DESeq2	LRT		0
125	KC_AB2	Galphao	0.0611241009119206	0.786611055539885	1	pseudobulk	DESeq2	LRT		0
125	KC_AB2	Galphaq	0.183557114487127	0.697213276546694	1	pseudobulk	DESeq2	LRT		0
125	KC_AB2	Galphas	-0.234494888363473	0.534246597629265	1	pseudobulk	DESeq2	LRT		0
125	KC_AB2	Galt	-0.0715309630154252	1	1	pseudobulk	DESeq2	LRT		0
125	KC_AB2	GalT1	-1.41551919808929	0.477652937997659	1	pseudobulk	DESeq2	LRT		0
125	KC_AB2	gammaCOP	-0.591007481057462	0.631408170104267	1	pseudobulk	DESeq2	LRT		0
125	KC_AB2	gammaSnap1	-0.128462322350279	0.718765050492753	1	pseudobulk	DESeq2	LRT		0

125	KC_AB2	gammaSnap2	0.803175418184768	0.374757372010469	1	pseudobulk	DESeq2	LRT	0
125	KC_AB2	gammaTub23C	-2.63181173635671	0.221727059081464	1	pseudobulk	DESeq2	LRT	0
125	KC_AB2	GAPcenA	0.62857471563027	0.428171640721153	1	pseudobulk	DESeq2	LRT	0
125	KC_AB2	Gapdh1	0.499667619785103	0.18125370198039	1	pseudobulk	DESeq2	LRT	0
125	KC_AB2	Gapdh2	0.149104261217978	0.59270101242347	1	pseudobulk	DESeq2	LRT	0
125	KC_AB2	GAPsec	-0.635253761627116	0.725756335940405	1	pseudobulk	DESeq2	LRT	0
125	KC_AB2	Gapvd1	-0.119059215914671	0.784626822235289	1	pseudobulk	DESeq2	LRT	0
125	KC_AB2	garz	-0.496445403952564	0.691667578770936	1	pseudobulk	DESeq2	LRT	0
125	KC_AB2	Gas41	0.0806715714550146	0.88136911310611	1	pseudobulk	DESeq2	LRT	0
125	KC_AB2	Gasz	0.225824877348164	0.890981852394266	1	pseudobulk	DESeq2	LRT	0
125	KC_AB2	Gat	-0.592481036256484	0.706847545932384	1	pseudobulk	DESeq2	LRT	0
125	KC_AB2	GatA	0.697139614205724	0.732716879498292	1	pseudobulk	DESeq2	LRT	0
125	KC_AB2	GATAd	-0.589376301107307	0.587656463464292	1	pseudobulk	DESeq2	LRT	0
125	KC_AB2	GatB	0.443892390635762	1	1	pseudobulk	DESeq2	LRT	0
125	KC_AB2	gb	-2.84060426811147	0.179931719409684	1	pseudobulk	DESeq2	LRT	0
125	KC_AB2	Gba1a	-0.427431139129811	1	1	pseudobulk	DESeq2	LRT	0
125	KC_AB2	Gbeta13F	-0.0470473755403767	0.807218922406366	1	pseudobulk	DESeq2	LRT	0
125	KC_AB2	Gbeta5	0.326167069449252	0.479093909993078	1	pseudobulk	DESeq2	LRT	0
125	KC_AB2	Gbs-70E	1.5243995994652	0.741477491078038	1	pseudobulk	DESeq2	LRT	0
125	KC_AB2	Gbs-76A	0.435232983853471	0.537291769338211	1	pseudobulk	DESeq2	LRT	0
125	KC_AB2	GC	-0.412042094725368	0.474376528675464	1	pseudobulk	DESeq2	LRT	0
125	KC_AB2	GC1	-0.929271649313813	0.409982684611538	1	pseudobulk	DESeq2	LRT	0
125	KC_AB2	GCC185	1.35370955176771	0.394160645568769	1	pseudobulk	DESeq2	LRT	0
125	KC_AB2	GCC88	-0.61332455345565	0.669078188419777	1	pseudobulk	DESeq2	LRT	0
125	KC_AB2	gce	0.494547001361974	0.312900214081384	1	pseudobulk	DESeq2	LRT	0
125	KC_AB2	GckIII	0.563607323742741	0.573407646495623	1	pseudobulk	DESeq2	LRT	0
125	KC_AB2	gcl	-0.270270374853165	0.815512565658368	1	pseudobulk	DESeq2	LRT	0
125	KC_AB2	Gclc	-0.0956413060175604	0.843415947104099	1	pseudobulk	DESeq2	LRT	0
125	KC_AB2	Gclm	-0.0563853817037642	0.825692678661322	1	pseudobulk	DESeq2	LRT	0
125	KC_AB2	Gcn2	0.92191528491681	0.613371057887787	1	pseudobulk	DESeq2	LRT	0
125	KC_AB2	Gcn5	-0.183413879984093	0.819306809345333	1	pseudobulk	DESeq2	LRT	0
125	KC_AB2	GCS1	0.0863993518661371	0.888400731936336	1	pseudobulk	DESeq2	LRT	0
125	KC_AB2	GCS2alpha	-0.0981078313107315	0.866913277270783	1	pseudobulk	DESeq2	LRT	0
125	KC_AB2	GCS2beta	0.754666397831388	0.665136388583116	1	pseudobulk	DESeq2	LRT	0
125	KC_AB2	Gdap2	-0.023092871403788	0.864436570615413	1	pseudobulk	DESeq2	LRT	0
125	KC_AB2	Gdh	-0.414525272836544	0.32510803956126	1	pseudobulk	DESeq2	LRT	0
125	KC_AB2	Gdi	-0.0894001378607229	0.771147502939397	1	pseudobulk	DESeq2	LRT	0
125	KC_AB2	gdl	0.713982351041985	0.921569637923553	1	pseudobulk	DESeq2	LRT	0
125	KC_AB2	Gdn1	0.864341462967788	0.925947094335722	1	pseudobulk	DESeq2	LRT	0
125	KC_AB2	Ge-1	-0.572189861627776	0.621377122432926	1	pseudobulk	DESeq2	LRT	0
125	KC_AB2	gek	0.715887109657438	0.574939498070327	1	pseudobulk	DESeq2	LRT	0
125	KC_AB2	Gem3	1.23085716405798	0.614623732585597	1	pseudobulk	DESeq2	LRT	0
125	KC_AB2	geminin	-1.29571315406631	0.418207159025623	1	pseudobulk	DESeq2	LRT	0
125	KC_AB2	Gfat1	-0.600547221779133	0.841940696994101	1	pseudobulk	DESeq2	LRT	0
125	KC_AB2	Gfat2	-0.454310905096637	0.707820794204216	1	pseudobulk	DESeq2	LRT	0
125	KC_AB2	Gfrl	0.255222559680181	0.233299880943535	1	pseudobulk	DESeq2	LRT	0
125	KC_AB2	gfzf	1.5243995994652	0.741477491078038	1	pseudobulk	DESeq2	LRT	0
125	KC_AB2	Gga	-0.277092198266556	0.807866766712997	1	pseudobulk	DESeq2	LRT	0
125	KC_AB2	Ggamma1	-0.0659858705910878	0.766244773172213	1	pseudobulk	DESeq2	LRT	0
125	KC_AB2	Ggamma30A	-0.180668104847062	0.306414539547291	1	pseudobulk	DESeq2	LRT	0
125	KC_AB2	ghi	-0.411597189621852	0.472574872138712	1	pseudobulk	DESeq2	LRT	0
125	KC_AB2	gig	0.920212920242661	0.67286879185691	1	pseudobulk	DESeq2	LRT	0
125	KC_AB2	GIIIspla2	-0.260537006881542	0.858023771874749	1	pseudobulk	DESeq2	LRT	0

125	KC_AB2	GILT1	-2.09813727732686	0.112613155933715	1	pseudobulk	DESeq2	LRT		0
125	KC_AB2	GILT3	-0.600547221779133	0.841940696994101	1	pseudobulk	DESeq2	LRT		0
125	KC_AB2	Gip	0.713982351041985	0.921569637923553	1	pseudobulk	DESeq2	LRT		0
125	KC_AB2	Girdin	0.597815607188837	0.693139884449045	1	pseudobulk	DESeq2	LRT		0
125	KC_AB2	gish	0.191991298807777	0.4950588702385	1	pseudobulk	DESeq2	LRT		0
125	KC_AB2	Git	-1.01440980512594	0.597957154844041	1	pseudobulk	DESeq2	LRT		0
125	KC_AB2	Gk1	1.82579090108959	0.320739368286303	1	pseudobulk	DESeq2	LRT		0
125	KC_AB2	Gk2	-0.427431139129811	1	1	pseudobulk	DESeq2	LRT		0
125	KC_AB2	gkt	-1.07776915880798	0.775491075088671	1	pseudobulk	DESeq2	LRT		0
125	KC_AB2	GLaz	-1.07776915880798	0.775491075088671	1	pseudobulk	DESeq2	LRT		0
125	KC_AB2	GlcAT-I	-0.302853552919641	0.84048250228301	1	pseudobulk	DESeq2	LRT		0
125	KC_AB2	GlcAT-P	0.167067317392046	0.55555335886342	1	pseudobulk	DESeq2	LRT		0
125	KC_AB2	GlcAT-S	-0.415869829212112	0.583761055640191	1	pseudobulk	DESeq2	LRT		0
125	KC_AB2	GlcT	-2.02285942584217	0.357240028002837	1	pseudobulk	DESeq2	LRT		0
125	KC_AB2	Gld2	1.5243995994652	0.741477491078038	1	pseudobulk	DESeq2	LRT		0
125	KC_AB2	Gle1	0.836620822732844	0.683104133922741	1	pseudobulk	DESeq2	LRT		0
125	KC_AB2	glec	0.0635304636240189	0.930645916521742	1	pseudobulk	DESeq2	LRT		0
125	KC_AB2	Glg1	-0.122984295346838	0.825208228914231	1	pseudobulk	DESeq2	LRT		0
125	KC_AB2	GlnRS	0.892659559600706	0.712043317464255	1	pseudobulk	DESeq2	LRT		0
125	KC_AB2	glo	-0.306007092398457	0.489818264194457	1	pseudobulk	DESeq2	LRT		0
125	KC_AB2	Glo1	0.123586158277173	0.894010826088748	1	pseudobulk	DESeq2	LRT		0
125	KC_AB2	Glos	1.5243995994652	0.741477491078038	1	pseudobulk	DESeq2	LRT		0
125	KC_AB2	GLS	0.20470245838232	0.868205445584788	1	pseudobulk	DESeq2	LRT		0
125	KC_AB2	glu	0.864341462967788	0.925947094335722	1	pseudobulk	DESeq2	LRT		0
125	KC_AB2	GluClalpha	0.192524602502883	0.621613916471545	1	pseudobulk	DESeq2	LRT		0
125	KC_AB2	GluProRS	0.505050627557051	0.709478192103638	1	pseudobulk	DESeq2	LRT		0
125	KC_AB2	GluRIA	-0.107042506569826	0.770028297242806	1	pseudobulk	DESeq2	LRT		0
125	KC_AB2	GluRIB	-0.0694408353306945	0.799565081188784	1	pseudobulk	DESeq2	LRT		0
125	KC_AB2	GluRS-m	1.5243995994652	0.741477491078038	1	pseudobulk	DESeq2	LRT		0
125	KC_AB2	Glut1	0.644217325640914	0.189011569050759	1	pseudobulk	DESeq2	LRT		0
125	KC_AB2	Glut4EF	-0.573939717119207	0.0514316199165381	1	pseudobulk	DESeq2	LRT		0
125	KC_AB2	GlyP	0.467754932124887	0.325855784449964	1	pseudobulk	DESeq2	LRT		0
125	KC_AB2	GlyRS	-0.455085352080452	0.720086903532819	1	pseudobulk	DESeq2	LRT		0
125	KC_AB2	GlyS	0.20702534630372	0.822965380923059	1	pseudobulk	DESeq2	LRT		0
125	KC_AB2	GlyT	0.164492398323918	0.635591612250784	1	pseudobulk	DESeq2	LRT		0
125	KC_AB2	GM130	0.870051735857077	0.502701162282739	1	pseudobulk	DESeq2	LRT		0
125	KC_AB2	Gmap	0.401994487214901	0.759902208140886	1	pseudobulk	DESeq2	LRT		0
125	KC_AB2	Gmd	-0.600547221779134	1	1	pseudobulk	DESeq2	LRT		0
125	KC_AB2	Gmer	-0.427431139129811	1	1	pseudobulk	DESeq2	LRT		0
125	KC_AB2	GMF	2.11445558630655	0.294437352818674	1	pseudobulk	DESeq2	LRT		0
126	KC_AB2	Gnf1	-0.419209304436447	0.791208571196609	1	pseudobulk	DESeq2	LRT		0
126	KC_AB2	Gnpnat	0.107479031250535	0.88712690359169	1	pseudobulk	DESeq2	LRT		0
126	KC_AB2	gny	0.183555857030446	1	1	pseudobulk	DESeq2	LRT		0
126	KC_AB2	gogo	-1.49872568482588	0.456371830192787	1	pseudobulk	DESeq2	LRT		0
126	KC_AB2	gol	0.633879447274141	0.314016544685704	1	pseudobulk	DESeq2	LRT		0
126	KC_AB2	Golgin104	-0.592481036256484	0.706847545932384	1	pseudobulk	DESeq2	LRT		0
126	KC_AB2	Golgin245	-1.06173487873955	0.338934051354025	1	pseudobulk	DESeq2	LRT		0
126	KC_AB2	Golgin84	-1.61461009685556	0.408795423318369	1	pseudobulk	DESeq2	LRT		0
126	KC_AB2	gom	1.35886015794921	0.316481930815885	1	pseudobulk	DESeq2	LRT		0
126	KC_AB2	Gorab	1.17792980580552	0.54998789412272	1	pseudobulk	DESeq2	LRT		0
126	KC_AB2	Gos28	0.278943025585246	0.759767524445483	1	pseudobulk	DESeq2	LRT		0
126	KC_AB2	Got1	-0.243589332410506	0.785859078659454	1	pseudobulk	DESeq2	LRT		0
126	KC_AB2	Got2	-0.0260564731435125	0.958647397491783	1	pseudobulk	DESeq2	LRT		0

126	KC_AB2	Gp210	-0.727239665042655	0.76302943878648	1	pseudobulk	DESeq2	LRT		0
126	KC_AB2	Gp93	0.906410378740576	0.546266905361032	1	pseudobulk	DESeq2	LRT		0
126	KC_AB2	Gpat4	0.692434534409052	0.518304106300836	1	pseudobulk	DESeq2	LRT		0
126	KC_AB2	Gpb5	-0.537638909370627	0.73575551347254	1	pseudobulk	DESeq2	LRT		0
126	KC_AB2	Gpdh1	-0.392550113673122	0.25548391231879	1	pseudobulk	DESeq2	LRT		0
126	KC_AB2	Gpdh3	1.5243995994652	0.741477491078038	1	pseudobulk	DESeq2	LRT		0
126	KC_AB2	GPHR	-1.77538897748067	0.365159075741735	1	pseudobulk	DESeq2	LRT		0
126	KC_AB2	Gpo1	-0.24251545383888	0.796823630871796	1	pseudobulk	DESeq2	LRT		0
126	KC_AB2	gpp	0.160723022134364	0.702951680630132	1	pseudobulk	DESeq2	LRT		0
126	KC_AB2	Gprk1	0.0555229829341158	0.829790727768158	1	pseudobulk	DESeq2	LRT		0
126	KC_AB2	Gprk2	0.133148552402058	0.663555242949364	1	pseudobulk	DESeq2	LRT		0
126	KC_AB2	gprs	0.0496682414798919	0.862983807203094	1	pseudobulk	DESeq2	LRT		0
126	KC_AB2	Gr2a	-0.600547221779133	0.841940696994101	1	pseudobulk	DESeq2	LRT		0
126	KC_AB2	Gr47b	-0.600547221779134	1	1	pseudobulk	DESeq2	LRT		0
126	KC_AB2	Graf	-0.763256586262921	0.210702067446288	1	pseudobulk	DESeq2	LRT		0
126	KC_AB2	GramD1B	0.677398811411627	0.27511559741124	1	pseudobulk	DESeq2	LRT		0
126	KC_AB2	Grasp65	-0.426681981647068	0.769139358708283	1	pseudobulk	DESeq2	LRT		0
126	KC_AB2	grau	-0.328934802630227	0.853923270108708	1	pseudobulk	DESeq2	LRT		0
126	KC_AB2	Grik	-2.41610217722532	0.272481539699592	1	pseudobulk	DESeq2	LRT		0
126	KC_AB2	grim	-0.592481036256484	0.706847545932384	1	pseudobulk	DESeq2	LRT		0
126	KC_AB2	Grip128	1.5243995994652	0.741477491078038	1	pseudobulk	DESeq2	LRT		0
126	KC_AB2	Grip163	2.05658823775573	0.17614017235914	1	pseudobulk	DESeq2	LRT		0
126	KC_AB2	Grip75	0.186298416894564	0.947947908360033	1	pseudobulk	DESeq2	LRT		0
126	KC_AB2	Grip84	-0.209808076863057	0.877444409505505	1	pseudobulk	DESeq2	LRT		0
126	KC_AB2	Grip91	-1.6150095599849	0.519549602003096	1	pseudobulk	DESeq2	LRT		0
126	KC_AB2	grk	0.864341462967788	0.925947094335722	1	pseudobulk	DESeq2	LRT		0
126	KC_AB2	gro	0.113730773819351	0.767681816893505	1	pseudobulk	DESeq2	LRT		0
126	KC_AB2	grp	-0.446525858485042	0.295471156960257	1	pseudobulk	DESeq2	LRT		0
126	KC_AB2	grsm	-0.525980220958878	0.813340603474339	1	pseudobulk	DESeq2	LRT		0
126	KC_AB2	Grx1	0.566035946798111	0.336789334312592	1	pseudobulk	DESeq2	LRT		0
126	KC_AB2	gry	0.538439070450221	0.740015012569577	1	pseudobulk	DESeq2	LRT		0
126	KC_AB2	Gs1	-0.37230611861157	0.472582999542096	1	pseudobulk	DESeq2	LRT		0
126	KC_AB2	Gs1l	0.166952224751067	0.732905608302048	1	pseudobulk	DESeq2	LRT		0
126	KC_AB2	Gs2	1.15572398393975	0.592367151364093	1	pseudobulk	DESeq2	LRT		0
126	KC_AB2	GstD1	-0.3973606860017	0.4032841235342	1	pseudobulk	DESeq2	LRT		0
126	KC_AB2	GstD11	0.493460161786657	0.617037816302091	1	pseudobulk	DESeq2	LRT		0
126	KC_AB2	GstD9	1.62571710983431	0.470438722277963	1	pseudobulk	DESeq2	LRT		0
126	KC_AB2	GstE1	-0.427431139129811	1	1	pseudobulk	DESeq2	LRT		0
126	KC_AB2	GstE12	0.49522746739306	0.807638127459475	1	pseudobulk	DESeq2	LRT		0
126	KC_AB2	GstE2	-0.600547221779133	0.841940696994101	1	pseudobulk	DESeq2	LRT		0
126	KC_AB2	GstE8	1.5243995994652	0.741477491078038	1	pseudobulk	DESeq2	LRT		0
126	KC_AB2	GstO2	0.197374195493975	0.910762076758849	1	pseudobulk	DESeq2	LRT		0
126	KC_AB2	GstS1	-0.592481036256484	0.706847545932384	1	pseudobulk	DESeq2	LRT		0
126	KC_AB2	GstT1	0.713982351041985	0.921569637923553	1	pseudobulk	DESeq2	LRT		0
126	KC_AB2	GstZ2	-0.39917336535616	0.810147819664692	1	pseudobulk	DESeq2	LRT		0
126	KC_AB2	gt	0.70378358684898	0.932301451973468	1	pseudobulk	DESeq2	LRT		0
126	KC_AB2	Gtp-bp	1.5243995994652	0.741477491078038	1	pseudobulk	DESeq2	LRT		0
126	KC_AB2	Gug	-0.190325645628325	0.568715199997586	1	pseudobulk	DESeq2	LRT		0
126	KC_AB2	gukh	-2.68070056372077	0.245377659489442	1	pseudobulk	DESeq2	LRT		0
126	KC_AB2	gus	-0.362195726278604	0.420402797780685	1	pseudobulk	DESeq2	LRT		0
126	KC_AB2	gw	-0.0827662627852443	0.753820517647738	1	pseudobulk	DESeq2	LRT		0
126	KC_AB2	GXIVsPLA2	-0.304040176581622	0.842181568337431	1	pseudobulk	DESeq2	LRT		0
126	KC_AB2	Gyc88E	-0.0684933057704741	0.778831821354924	1	pseudobulk	DESeq2	LRT		0

126	KC_AB2	Gyc89Db	-0.933620200863253	0.585674237831869	1	pseudobulk	DESeq2	LRT	0
126	KC_AB2	Gycalpha99B	-0.131396272206664	0.737962737949266	1	pseudobulk	DESeq2	LRT	0
126	KC_AB2	Gycbeta100B	0.697801770160348	0.255112799978951	1	pseudobulk	DESeq2	LRT	0
126	KC_AB2	Gyf	-0.184752034784379	0.747047090909988	1	pseudobulk	DESeq2	LRT	0
126	KC_AB2	Gyg	-0.592481036256484	0.706847545932384	1	pseudobulk	DESeq2	LRT	0
126	KC_AB2	gzl	0.139928766745509	0.784842497341605	1	pseudobulk	DESeq2	LRT	0
126	KC_AB2	h	-0.592481036256484	0.706847545932384	1	pseudobulk	DESeq2	LRT	0
126	KC_AB2	H	-0.0736599680354628	0.888735130997722	1	pseudobulk	DESeq2	LRT	0
126	KC_AB2	h-cup	2.12684015898615	0.445939223035924	1	pseudobulk	DESeq2	LRT	0
126	KC_AB2	Hacd1	0.0340158931069051	0.898596776407196	1	pseudobulk	DESeq2	LRT	0
126	KC_AB2	Hacd2	-0.833787682492007	0.444386632344323	1	pseudobulk	DESeq2	LRT	0
126	KC_AB2	Hacl	1.10817761709104	0.660411427470312	1	pseudobulk	DESeq2	LRT	0
126	KC_AB2	haf	-0.427431139129811	1	1	pseudobulk	DESeq2	LRT	0
126	KC_AB2	Hakai	-0.828923104362394	0.596880306558244	1	pseudobulk	DESeq2	LRT	0
126	KC_AB2	hang	-0.297164704268427	0.580567482206556	1	pseudobulk	DESeq2	LRT	0
126	KC_AB2	Hasp	0.229026936125336	0.521998462722575	1	pseudobulk	DESeq2	LRT	0
126	KC_AB2	Haspin	-0.931176001634616	0.255045169979352	1	pseudobulk	DESeq2	LRT	0
126	KC_AB2	Hat1	-0.337040242503989	0.807622671934007	1	pseudobulk	DESeq2	LRT	0
126	KC_AB2	hay	0.329740041077442	0.855811003303737	1	pseudobulk	DESeq2	LRT	0
126	KC_AB2	HBS1	0.0340158931069051	0.898596776407196	1	pseudobulk	DESeq2	LRT	0
126	KC_AB2	Hcf	0.441781874757909	0.566050811109473	1	pseudobulk	DESeq2	LRT	0
126	KC_AB2	Hcs	-0.116411347810869	0.916848826069363	1	pseudobulk	DESeq2	LRT	0
126	KC_AB2	HDAC1	1.27496040324747	0.501655537161972	1	pseudobulk	DESeq2	LRT	0
126	KC_AB2	HDAC11	-2.42100284528813	0.295221564390726	1	pseudobulk	DESeq2	LRT	0
126	KC_AB2	HDAC4	-0.353894178214124	0.266276111848203	1	pseudobulk	DESeq2	LRT	0
126	KC_AB2	HDAC6	0.373000211715533	0.688678984271617	1	pseudobulk	DESeq2	LRT	0
126	KC_AB2	hdc	-0.182328666103502	0.886848451451546	1	pseudobulk	DESeq2	LRT	0
126	KC_AB2	hdly	-1.29244479833796	0.693295472370086	1	pseudobulk	DESeq2	LRT	0
126	KC_AB2	hdm	2.07946313289983	0.357635956314506	1	pseudobulk	DESeq2	LRT	0
126	KC_AB2	hec	-0.592481036256484	0.706847545932384	1	pseudobulk	DESeq2	LRT	0
126	KC_AB2	heix	0.522867664761254	0.789441383158121	1	pseudobulk	DESeq2	LRT	0
126	KC_AB2	Hel25E	-0.18458695974727	0.815338991311041	1	pseudobulk	DESeq2	LRT	0
126	KC_AB2	Hel89B	0.976771638689801	0.254281953123987	1	pseudobulk	DESeq2	LRT	0
126	KC_AB2	Hem	0.747275992236611	0.683032388442688	1	pseudobulk	DESeq2	LRT	0
127	KC_AB2	HemK1	0.0041040930466438	0.898171824806643	1	pseudobulk	DESeq2	LRT	0
127	KC_AB2	Hen1	1.5243995994652	0.741477491078038	1	pseudobulk	DESeq2	LRT	0
127	KC_AB2	hep	0.91374837495475	0.265374639692498	1	pseudobulk	DESeq2	LRT	0
127	KC_AB2	heph	0.24902804188566	0.754049630183503	1	pseudobulk	DESeq2	LRT	0
127	KC_AB2	her	-1.12785316439435	0.661319045116135	1	pseudobulk	DESeq2	LRT	0
127	KC_AB2	HERC2	0.324974988488897	0.627756712897923	1	pseudobulk	DESeq2	LRT	0
127	KC_AB2	Herc4	-0.431959627502394	0.59123907403764	1	pseudobulk	DESeq2	LRT	0
127	KC_AB2	Herp	1.40017261593459	0.357373907126391	1	pseudobulk	DESeq2	LRT	0
127	KC_AB2	Hers	-0.131361830825011	0.780863035809059	1	pseudobulk	DESeq2	LRT	0
127	KC_AB2	Hesr	2.12684015898615	0.445939223035924	1	pseudobulk	DESeq2	LRT	0
127	KC_AB2	Hex-A	-0.25570710947021	0.550463604606737	1	pseudobulk	DESeq2	LRT	0
127	KC_AB2	Hexim	0.793537507497247	0.615018765811704	1	pseudobulk	DESeq2	LRT	0
127	KC_AB2	Hexo1	1.82013411219004	0.375922530320745	1	pseudobulk	DESeq2	LRT	0
127	KC_AB2	Hey	-0.763778185829747	0.620882400998576	1	pseudobulk	DESeq2	LRT	0
127	KC_AB2	hfp	-0.462012936098155	0.268260434200176	1	pseudobulk	DESeq2	LRT	0
127	KC_AB2	hfw	0.0163320842711025	1	1	pseudobulk	DESeq2	LRT	0
127	KC_AB2	HHEX	-0.427431139129811	1	1	pseudobulk	DESeq2	LRT	0
127	KC_AB2	hig	-0.427431139129811	1	1	pseudobulk	DESeq2	LRT	0
127	KC_AB2	HINT1	-0.618044064798282	0.417320666367856	1	pseudobulk	DESeq2	LRT	0

127	KC_AB2	HIP	-0.308736534669881	0.801370761474978	1	pseudobulk	DESeq2	LRT	0
127	KC_AB2	HIP-R	-0.333259514131327	0.685632529111592	1	pseudobulk	DESeq2	LRT	0
127	KC_AB2	Hip1	-2.66938810389172	0.212278219588769	1	pseudobulk	DESeq2	LRT	0
127	KC_AB2	Hip14	0.186418534070639	0.857206049080101	1	pseudobulk	DESeq2	LRT	0
127	KC_AB2	Hipk	0.132368412170588	0.739126194191743	1	pseudobulk	DESeq2	LRT	0
127	KC_AB2	HIPP1	0.0165559748477986	0.986060856888164	1	pseudobulk	DESeq2	LRT	0
127	KC_AB2	His2Av	0.0619961124661184	0.913345366293851	1	pseudobulk	DESeq2	LRT	0
127	KC_AB2	His3.3A	-0.097799514148536	0.803857703460506	1	pseudobulk	DESeq2	LRT	0
127	KC_AB2	His3.3B	-0.208953142337048	0.365516479630476	1	pseudobulk	DESeq2	LRT	0
127	KC_AB2	His4r	-0.345890423007503	0.255406156090357	1	pseudobulk	DESeq2	LRT	0
127	KC_AB2	HisCl1	-0.600547221779134	1	1	pseudobulk	DESeq2	LRT	0
127	KC_AB2	HisRS	-0.570600887023361	0.689915798370851	1	pseudobulk	DESeq2	LRT	0
127	KC_AB2	hiw	0.132697429481832	0.788783456996094	1	pseudobulk	DESeq2	LRT	0
127	KC_AB2	Hk	0.501997514445302	0.299567690797064	1	pseudobulk	DESeq2	LRT	0
127	KC_AB2	Hlc	-1.16892643066281	0.421273499023634	1	pseudobulk	DESeq2	LRT	0
127	KC_AB2	Hmg-2	-2.53564472550049	0.241603852619635	1	pseudobulk	DESeq2	LRT	0
127	KC_AB2	Hmgcr	0.488042427773022	0.636105181039713	1	pseudobulk	DESeq2	LRT	0
127	KC_AB2	HmgD	-0.0028870217308099	0.890856336301563	1	pseudobulk	DESeq2	LRT	0
127	KC_AB2	Hmgs	1.00336427934559	0.366112025866822	1	pseudobulk	DESeq2	LRT	0
127	KC_AB2	HmgZ	-0.0309845785725577	0.835687383148	1	pseudobulk	DESeq2	LRT	0
127	KC_AB2	Hmr	1.5243995994652	0.741477491078038	1	pseudobulk	DESeq2	LRT	0
127	KC_AB2	Hmt-1	-3.17008147600632	0.106860491666981	1	pseudobulk	DESeq2	LRT	0
127	KC_AB2	Hmt4-20	0.0647099371689184	0.926629434186446	1	pseudobulk	DESeq2	LRT	0
127	KC_AB2	Hmu	1.82022151615841	0.392550578096768	1	pseudobulk	DESeq2	LRT	0
127	KC_AB2	Hn	-0.600547221779134	1	1	pseudobulk	DESeq2	LRT	0
127	KC_AB2	hng1	0.588786731209445	0.724276480055672	1	pseudobulk	DESeq2	LRT	0
127	KC_AB2	hng2	0.864341462967788	0.925947094335722	1	pseudobulk	DESeq2	LRT	0
127	KC_AB2	hng3	-0.600547221779133	0.841940696994101	1	pseudobulk	DESeq2	LRT	0
127	KC_AB2	HnRNP-K	-0.562345723374669	0.238074579749289	1	pseudobulk	DESeq2	LRT	0
127	KC_AB2	Ho	-0.906162109315661	0.57104918975774	1	pseudobulk	DESeq2	LRT	0
127	KC_AB2	hob	-0.0852743682753834	0.917718671153184	1	pseudobulk	DESeq2	LRT	0
127	KC_AB2	hoe1	-0.0439281121698518	0.813523401003685	1	pseudobulk	DESeq2	LRT	0
127	KC_AB2	hoe2	0.0686101167758565	0.918935863850441	1	pseudobulk	DESeq2	LRT	0
127	KC_AB2	hoip	-0.711801656212134	0.14600417146425	1	pseudobulk	DESeq2	LRT	0
127	KC_AB2	holn1	0.190393219485123	0.893175556614257	1	pseudobulk	DESeq2	LRT	0
127	KC_AB2	homer	0.461458211312255	0.667002377706727	1	pseudobulk	DESeq2	LRT	0
127	KC_AB2	hook	0.0973439731124593	0.829124919426765	1	pseudobulk	DESeq2	LRT	0
127	KC_AB2	hop	0.0863993518661371	0.888400731936336	1	pseudobulk	DESeq2	LRT	0
127	KC_AB2	HP1b	-0.763865388434088	0.483932172997358	1	pseudobulk	DESeq2	LRT	0
127	KC_AB2	HP1c	-0.177384942049574	0.831499107555631	1	pseudobulk	DESeq2	LRT	0
127	KC_AB2	HP4	-0.40013000197214	0.833508988348	1	pseudobulk	DESeq2	LRT	0
127	KC_AB2	HP5	-0.0859337711465027	0.88743864336249	1	pseudobulk	DESeq2	LRT	0
127	KC_AB2	HP6	-0.427431139129811	1	1	pseudobulk	DESeq2	LRT	0
127	KC_AB2	Hpd	-0.600547221779133	0.841940696994101	1	pseudobulk	DESeq2	LRT	0
127	KC_AB2	Hph	-0.154787576538485	0.846203132822955	1	pseudobulk	DESeq2	LRT	0
127	KC_AB2	hpo	-0.878330035737122	0.609773395849331	1	pseudobulk	DESeq2	LRT	0
127	KC_AB2	hppy	-0.235615162263972	0.627398684956699	1	pseudobulk	DESeq2	LRT	0
127	KC_AB2	Hpr1	-1.3723417613553	0.619532036522	1	pseudobulk	DESeq2	LRT	0
127	KC_AB2	hpRNA:CR18854	-0.14927504145562	0.790910637011448	1	pseudobulk	DESeq2	LRT	0
127	KC_AB2	hpRNA:CR32207	1.5243995994652	0.741477491078038	1	pseudobulk	DESeq2	LRT	0
127	KC_AB2	hpRNA:CR46342	0.127482371476527	0.801370755757578	1	pseudobulk	DESeq2	LRT	0
127	KC_AB2	HPS1	-0.732681375701997	0.585016317854019	1	pseudobulk	DESeq2	LRT	0
127	KC_AB2	HPS4	-0.821704131336734	0.392980616796182	1	pseudobulk	DESeq2	LRT	0

127	KC_AB2	Hr3	-0.666463106437031	0.300424883104591	1	pseudobulk	DESeq2	LRT		0
127	KC_AB2	Hr38	-0.688664682115293	0.175648595830718	1	pseudobulk	DESeq2	LRT		0
127	KC_AB2	Hr39	-0.879625336549345	0.218936713372063	1	pseudobulk	DESeq2	LRT		0
127	KC_AB2	Hr4	0.613659617664411	0.76686169970757	1	pseudobulk	DESeq2	LRT		0
127	KC_AB2	Hr51	0.221310759318131	0.673434662768025	1	pseudobulk	DESeq2	LRT		0
127	KC_AB2	Hr78	-0.0405360282642227	0.898948866316063	1	pseudobulk	DESeq2	LRT		0
127	KC_AB2	Hr96	0.791468033138705	0.699865413482108	1	pseudobulk	DESeq2	LRT		0
127	KC_AB2	Hrb27C	-0.118441948899757	0.653279584493627	1	pseudobulk	DESeq2	LRT		0
127	KC_AB2	Hrb87F	-0.240834793923204	0.75851568131018	1	pseudobulk	DESeq2	LRT		0
127	KC_AB2	Hrb98DE	-0.0944544825989614	0.778380914447665	1	pseudobulk	DESeq2	LRT		0
127	KC_AB2	Hrd3	-2.45405661295427	0.15243390837396	1	pseudobulk	DESeq2	LRT		0
127	KC_AB2	hrg	0.61150655417181	0.284776595952147	1	pseudobulk	DESeq2	LRT		0
127	KC_AB2	hrm	-0.0250406053975498	0.90729834402634	1	pseudobulk	DESeq2	LRT		0
127	KC_AB2	Hrs	-1.35157211129192	0.396167000887802	1	pseudobulk	DESeq2	LRT		0
127	KC_AB2	Hs2st	0.835812636789871	0.532102017814907	1	pseudobulk	DESeq2	LRT		0
127	KC_AB2	Hs3st-A	0.495895304283973	0.0439980204410641	1	pseudobulk	DESeq2	LRT		0
127	KC_AB2	Hs3st-B	-0.573109354659945	0.739419341582026	1	pseudobulk	DESeq2	LRT		0
127	KC_AB2	Hs6st	0.108681086194343	0.820580729088429	1	pseudobulk	DESeq2	LRT		0
127	KC_AB2	Hsc20	0.920212920242661	0.67286879185691	1	pseudobulk	DESeq2	LRT		0
127	KC_AB2	Hsc70-2	1.5243995994652	0.741477491078038	1	pseudobulk	DESeq2	LRT		0
127	KC_AB2	Hsc70-3	-0.584293399521548	0.394310414000321	1	pseudobulk	DESeq2	LRT		0
127	KC_AB2	Hsc70-4	0.207170016072283	0.609624574450845	1	pseudobulk	DESeq2	LRT		0
127	KC_AB2	Hsc70-5	0.180181821712607	0.759040499124491	1	pseudobulk	DESeq2	LRT		0
127	KC_AB2	Hsc70Cb	0.803716364826546	0.093096488618506	1	pseudobulk	DESeq2	LRT		0
127	KC_AB2	Hsepi	-2.08021320860378	0.237423930346101	1	pseudobulk	DESeq2	LRT		0
127	KC_AB2	Hsf	0.559294458405355	0.488226283472159	1	pseudobulk	DESeq2	LRT		0
127	KC_AB2	Hsl	1.39827770914961	0.587246212652858	1	pseudobulk	DESeq2	LRT		0
127	KC_AB2	Hsp23	0.774857026805695	0.07338513658934	1	pseudobulk	DESeq2	LRT		0
128	KC_AB2	Hsp26	-0.565436216698217	0.43304961112515	1	pseudobulk	DESeq2	LRT		0
128	KC_AB2	Hsp27	0.252783123410522	0.716102237320835	1	pseudobulk	DESeq2	LRT		0
128	KC_AB2	Hsp60A	0.709047800055144	0.289702282381873	1	pseudobulk	DESeq2	LRT		0
128	KC_AB2	Hsp67Bc	-2.16667937655366	0.239573963782881	1	pseudobulk	DESeq2	LRT		0
128	KC_AB2	Hsp68	0.440731473579088	0.617661210007752	1	pseudobulk	DESeq2	LRT		0
128	KC_AB2	Hsp70Aa	0.929733572386919	0.571040056243757	1	pseudobulk	DESeq2	LRT		0
128	KC_AB2	Hsp70Ab	0.490734853312675	0.40174823637547	1	pseudobulk	DESeq2	LRT		0
128	KC_AB2	Hsp70Ba	-0.501977021014657	0.755002804868743	1	pseudobulk	DESeq2	LRT		0
128	KC_AB2	Hsp70Bb	0.0856212817256728	0.84627912862552	1	pseudobulk	DESeq2	LRT		0
128	KC_AB2	Hsp70Bbb	-0.427431139129811	1	1	pseudobulk	DESeq2	LRT		0
128	KC_AB2	Hsp70Bc	-0.130547543654306	0.897644805188164	1	pseudobulk	DESeq2	LRT		0
128	KC_AB2	Hsp83	0.23906934205266	0.385300057991857	1	pseudobulk	DESeq2	LRT		0
128	KC_AB2	HSPBAP1	-1.07776915880798	0.775491075088671	1	pseudobulk	DESeq2	LRT		0
128	KC_AB2	HSPC300	-0.760016385057274	0.482874769040579	1	pseudobulk	DESeq2	LRT		0
128	KC_AB2	hth	-0.697599397124872	0.378935082613636	1	pseudobulk	DESeq2	LRT		0
128	KC_AB2	htk	-0.733833395582818	0.356801470266436	1	pseudobulk	DESeq2	LRT		0
128	KC_AB2	HtrA2	-0.600547221779134	1	1	pseudobulk	DESeq2	LRT		0
128	KC_AB2	hts	0.188271282083628	0.707360154670392	1	pseudobulk	DESeq2	LRT		0
128	KC_AB2	htt	-0.497171711025193	0.67984019751363	1	pseudobulk	DESeq2	LRT		0
128	KC_AB2	Hug	-2.12214992510756	0.367174323489033	1	pseudobulk	DESeq2	LRT		0
128	KC_AB2	HUWE1	0.245967714933849	0.684159627163672	1	pseudobulk	DESeq2	LRT		0
128	KC_AB2	hwt	-0.549232497077579	0.126358593543774	1	pseudobulk	DESeq2	LRT		0
128	KC_AB2	Hyccin	-0.234829744605693	0.782025091106523	1	pseudobulk	DESeq2	LRT		0
128	KC_AB2	hyd	-0.16591980908048	0.818241509413464	1	pseudobulk	DESeq2	LRT		0
128	KC_AB2	Hydr1	-1.51812168653838	0.552401469179882	1	pseudobulk	DESeq2	LRT		0

128	KC_AB2	Hydr2	0.0813323910969541	0.920494654345338	1	pseudobulk	DESeq2	LRT	0
128	KC_AB2	hyx	-0.119831229562752	1	1	pseudobulk	DESeq2	LRT	0
128	KC_AB2	I-2	0.183606084085621	0.724605917247734	1	pseudobulk	DESeq2	LRT	0
128	KC_AB2	I-3	-0.600547221779133	0.841940696994101	1	pseudobulk	DESeq2	LRT	0
128	KC_AB2	IA-2	0.118481910125155	0.60524471972045	1	pseudobulk	DESeq2	LRT	0
128	KC_AB2	Ibf1	-1.04864124217227	0.536021979369573	1	pseudobulk	DESeq2	LRT	0
128	KC_AB2	Ibf2	-0.841882716738579	1	1	pseudobulk	DESeq2	LRT	0
128	KC_AB2	ICA69	-0.721316873467424	0.354761086245677	1	pseudobulk	DESeq2	LRT	0
128	KC_AB2	Ice1	0.70378358684898	0.932301451973468	1	pseudobulk	DESeq2	LRT	0
128	KC_AB2	Ice2	1.94691684546882	0.398076844044942	1	pseudobulk	DESeq2	LRT	0
128	KC_AB2	ics	-0.0702115793368272	0.896012713963835	1	pseudobulk	DESeq2	LRT	0
128	KC_AB2	ida	0.458061275320087	0.802259721126042	1	pseudobulk	DESeq2	LRT	0
128	KC_AB2	Ide	-0.826817549904682	0.427270818728062	1	pseudobulk	DESeq2	LRT	0
128	KC_AB2	Idgf2	1.5243995994652	0.741477491078038	1	pseudobulk	DESeq2	LRT	0
128	KC_AB2	Idgf3	0.864341462967788	0.925947094335722	1	pseudobulk	DESeq2	LRT	0
128	KC_AB2	Idgf4	-0.427431139129811	1	1	pseudobulk	DESeq2	LRT	0
128	KC_AB2	Idgf6	0.443892390635762	1	1	pseudobulk	DESeq2	LRT	0
128	KC_AB2	Idh	-2.49273365458565	0.14945798738764	1	pseudobulk	DESeq2	LRT	0
128	KC_AB2	Idh3a	-0.580776191675759	0.261278151947563	1	pseudobulk	DESeq2	LRT	0
128	KC_AB2	Idh3b	-0.473620548682135	0.41317832635205	1	pseudobulk	DESeq2	LRT	0
128	KC_AB2	Idi	1.02248357785599	0.556541519223439	1	pseudobulk	DESeq2	LRT	0
128	KC_AB2	ifc	0.311074945364061	0.80926918288763	1	pseudobulk	DESeq2	LRT	0
128	KC_AB2	IFT43	-1.90937835128834	0.377732797313411	1	pseudobulk	DESeq2	LRT	0
128	KC_AB2	igl	0.139612617144394	0.56737465182096	1	pseudobulk	DESeq2	LRT	0
128	KC_AB2	Ih	0.258044749091606	0.35972003257801	1	pseudobulk	DESeq2	LRT	0
128	KC_AB2	IKKepsilon	2.49750470281195	0.235076905519563	1	pseudobulk	DESeq2	LRT	0
128	KC_AB2	IleRS	-3.04139207120202	0.129920864932141	1	pseudobulk	DESeq2	LRT	0
128	KC_AB2	Ilk	0.883706851581311	0.592957842201542	1	pseudobulk	DESeq2	LRT	0
128	KC_AB2	IM14	-0.427431139129811	1	1	pseudobulk	DESeq2	LRT	0
128	KC_AB2	IM33	1.23085716405798	0.614623732585597	1	pseudobulk	DESeq2	LRT	0
128	KC_AB2	IM4	-0.600547221779133	0.841940696994101	1	pseudobulk	DESeq2	LRT	0
128	KC_AB2	imd	-1.29244479833796	0.693295472370086	1	pseudobulk	DESeq2	LRT	0
128	KC_AB2	Iml1	1.3020478033011	0.329906495561127	1	pseudobulk	DESeq2	LRT	0
128	KC_AB2	Imp	0.600912622655445	0.657060913731537	1	pseudobulk	DESeq2	LRT	0
128	KC_AB2	Impbeta11	-0.382929749762112	0.851961810578875	1	pseudobulk	DESeq2	LRT	0
128	KC_AB2	ImpL2	0.443892390635762	1	1	pseudobulk	DESeq2	LRT	0
128	KC_AB2	inaE	0.616995147895006	0.314016485488488	1	pseudobulk	DESeq2	LRT	0
128	KC_AB2	inc	-0.419304456378038	0.687677176696803	1	pseudobulk	DESeq2	LRT	0
128	KC_AB2	ind	-0.600547221779134	1	1	pseudobulk	DESeq2	LRT	0
128	KC_AB2	Ing3	-0.498192533448519	0.74054356744874	1	pseudobulk	DESeq2	LRT	0
128	KC_AB2	Ing5	0.609599016649936	0.749620012955861	1	pseudobulk	DESeq2	LRT	0
128	KC_AB2	Ino80	-1.78094334699383	0.470930349923561	1	pseudobulk	DESeq2	LRT	0
128	KC_AB2	Inos	0.168428188685829	0.753895994918898	1	pseudobulk	DESeq2	LRT	0
128	KC_AB2	INPP5E	-0.30109498463501	0.866895752769536	1	pseudobulk	DESeq2	LRT	0
128	KC_AB2	InR	0.914818958172866	0.636986897982382	1	pseudobulk	DESeq2	LRT	0
128	KC_AB2	IntS1	1.29699351328743	0.46055405427585	1	pseudobulk	DESeq2	LRT	0
128	KC_AB2	IntS11	-0.539665365830432	0.805145917072468	1	pseudobulk	DESeq2	LRT	0
128	KC_AB2	IntS14	-1.07776915880798	0.775491075088671	1	pseudobulk	DESeq2	LRT	0
128	KC_AB2	IntS2	-1.6150095599849	0.519549602003096	1	pseudobulk	DESeq2	LRT	0
128	KC_AB2	IntS3	0.203469047808105	0.856389733705977	1	pseudobulk	DESeq2	LRT	0
128	KC_AB2	IntS4	-1.54470851095603	0.55301747381519	1	pseudobulk	DESeq2	LRT	0
128	KC_AB2	IntS6	0.658018617002382	0.465813084037764	1	pseudobulk	DESeq2	LRT	0
128	KC_AB2	IntS8	0.70378358684898	0.932301451973468	1	pseudobulk	DESeq2	LRT	0

128	KC_AB2	IntS9	-0.427431139129811	1	1	pseudobulk	DESeq2	LRT	0
128	KC_AB2	Invadolysin	-1.98396100792701	0.295323175485782	1	pseudobulk	DESeq2	LRT	0
128	KC_AB2	Inx2	2.76205395729416	0.251829971474322	1	pseudobulk	DESeq2	LRT	0
128	KC_AB2	Inx3	-0.427431139129811	1	1	pseudobulk	DESeq2	LRT	0
128	KC_AB2	Ip259	-0.514850023206522	0.280545417607376	1	pseudobulk	DESeq2	LRT	0
128	KC_AB2	IP3K1	-0.600547221779134	1	1	pseudobulk	DESeq2	LRT	0
128	KC_AB2	IPIP	-0.244467432701586	0.781069281087492	1	pseudobulk	DESeq2	LRT	0
128	KC_AB2	Ipk2	-1.07776915880798	0.775491075088671	1	pseudobulk	DESeq2	LRT	0
128	KC_AB2	iPLA2-VIA	0.971240930922287	0.381736486152222	1	pseudobulk	DESeq2	LRT	0
128	KC_AB2	Ipod	-0.427431139129811	1	1	pseudobulk	DESeq2	LRT	0
128	KC_AB2	Ipp	-0.335518339275227	0.54220765876366	1	pseudobulk	DESeq2	LRT	0
128	KC_AB2	Ir41a	-0.600547221779133	0.841940696994101	1	pseudobulk	DESeq2	LRT	0
128	KC_AB2	Ir56a	0.941771475342785	0.508586688904413	1	pseudobulk	DESeq2	LRT	0
128	KC_AB2	Ir68a	1.5243995994652	0.741477491078038	1	pseudobulk	DESeq2	LRT	0
128	KC_AB2	Ir93a	1.5243995994652	0.741477491078038	1	pseudobulk	DESeq2	LRT	0
128	KC_AB2	Irbp18	-0.546131899217828	0.672465332021014	1	pseudobulk	DESeq2	LRT	0
128	KC_AB2	Irc	-0.600547221779133	0.841940696994101	1	pseudobulk	DESeq2	LRT	0
128	KC_AB2	Ire1	-0.481993539656925	0.883279710820779	1	pseudobulk	DESeq2	LRT	0
128	KC_AB2	Irk2	-1.3723417613553	0.619532036522	1	pseudobulk	DESeq2	LRT	0
128	KC_AB2	Irk3	-0.600547221779133	0.841940696994101	1	pseudobulk	DESeq2	LRT	0
128	KC_AB2	Irp-1A	-0.219379579169058	0.766088261357595	1	pseudobulk	DESeq2	LRT	0
128	KC_AB2	Irp-1B	-2.6192699331157	0.290273891074663	1	pseudobulk	DESeq2	LRT	0
129	KC_AB2	IRSp53	1.63976177124413	0.341478127257815	1	pseudobulk	DESeq2	LRT	0
129	KC_AB2	Iru	-1.81191247704444	0.12954513608111	1	pseudobulk	DESeq2	LRT	0
129	KC_AB2	IscU	-0.0904526703342653	0.777277701769331	1	pseudobulk	DESeq2	LRT	0
129	KC_AB2	isopeptidase-T-3	1.36390873566401	0.513938015567603	1	pseudobulk	DESeq2	LRT	0
129	KC_AB2	isoQC	-2.47736320023278	0.259633277663633	1	pseudobulk	DESeq2	LRT	0
129	KC_AB2	Iswi	0.0893155117674409	0.899489680639082	1	pseudobulk	DESeq2	LRT	0
129	KC_AB2	Itpr	2.02152236800105	0.112310939828992	1	pseudobulk	DESeq2	LRT	0
129	KC_AB2	ix	-1.01440980512594	0.597957154844041	1	pseudobulk	DESeq2	LRT	0
129	KC_AB2	Jafrac1	-0.186888390358502	0.745482598099033	1	pseudobulk	DESeq2	LRT	0
129	KC_AB2	Jafrac2	-0.841882716738579	1	1	pseudobulk	DESeq2	LRT	0
129	KC_AB2	jagn	-0.585539635335735	0.509975935232589	1	pseudobulk	DESeq2	LRT	0
129	KC_AB2	janA	-1.02423531380596	0.548801874948105	1	pseudobulk	DESeq2	LRT	0
129	KC_AB2	jar	1.13905409870282	0.123278993788626	1	pseudobulk	DESeq2	LRT	0
129	KC_AB2	Jarid2	0.0854552315919127	0.862690360022682	1	pseudobulk	DESeq2	LRT	0
129	KC_AB2	jdp	0.187449698671968	0.362972161079708	1	pseudobulk	DESeq2	LRT	0
129	KC_AB2	jeb	-1.7808893131519	0.0023653608430607	1	pseudobulk	DESeq2	LRT	0
129	KC_AB2	jef	0.254736286664599	0.518680452459167	1	pseudobulk	DESeq2	LRT	0
129	KC_AB2	jet	-0.533946064220369	0.73906067333976	1	pseudobulk	DESeq2	LRT	0
129	KC_AB2	JHDM2	-1.12475160732157	0.574429473125171	1	pseudobulk	DESeq2	LRT	0
129	KC_AB2	Jheh2	2.12684015898615	0.445939223035924	1	pseudobulk	DESeq2	LRT	0
129	KC_AB2	JhI-21	-0.600547221779133	0.841940696994101	1	pseudobulk	DESeq2	LRT	0
129	KC_AB2	jigr1	1.05913718107842	0.210371089984874	1	pseudobulk	DESeq2	LRT	0
129	KC_AB2	JIL-1	-0.0521586313276094	0.918912483220091	1	pseudobulk	DESeq2	LRT	0
129	KC_AB2	jim	-0.0557087643109814	0.827144656697125	1	pseudobulk	DESeq2	LRT	0
129	KC_AB2	jing	-0.965983307824617	0.446011010651107	1	pseudobulk	DESeq2	LRT	0
129	KC_AB2	JMJD4	-0.055716669860236	0.908649369064948	1	pseudobulk	DESeq2	LRT	0
129	KC_AB2	JMJD5	0.791286025443143	0.690540380845434	1	pseudobulk	DESeq2	LRT	0
129	KC_AB2	JMJD7	1.13203714719002	0.570133461330773	1	pseudobulk	DESeq2	LRT	0
129	KC_AB2	jnj	-1.7824418856176	0.467224294431682	1	pseudobulk	DESeq2	LRT	0
129	KC_AB2	jp	0.211556021990153	0.811664623395253	1	pseudobulk	DESeq2	LRT	0
129	KC_AB2	Jra	-0.282242283816103	0.828339932930307	1	pseudobulk	DESeq2	LRT	0

129	KC_AB2	JTBR	0.555357365394912	0.764907956103394	1	pseudobulk	DESeq2	LRT	0
129	KC_AB2	jub	1.400777848138	0.492920641857113	1	pseudobulk	DESeq2	LRT	0
129	KC_AB2	jumu	0.70378358684898	0.932301451973468	1	pseudobulk	DESeq2	LRT	0
129	KC_AB2	Jupiter	-0.0912239626898237	0.744420035318862	1	pseudobulk	DESeq2	LRT	0
129	KC_AB2	jus	0.125841503475851	0.566328774064052	1	pseudobulk	DESeq2	LRT	0
129	KC_AB2	jv	2.70714859398546	0.251391037333528	1	pseudobulk	DESeq2	LRT	0
129	KC_AB2	jvl	0.141558605057191	0.683271470127439	1	pseudobulk	DESeq2	LRT	0
129	KC_AB2	Jwa	-0.430904989684069	0.634928453227025	1	pseudobulk	DESeq2	LRT	0
129	KC_AB2	KaiR1D	-0.80369631077251	0.53994768074258	1	pseudobulk	DESeq2	LRT	0
129	KC_AB2	Kank	1.23803699132853	0.0948414947251383	1	pseudobulk	DESeq2	LRT	0
129	KC_AB2	Kap-alpha1	0.145093299634932	0.79702653066578	1	pseudobulk	DESeq2	LRT	0
129	KC_AB2	Kap-alpha3	0.269530645371225	0.621532469642519	1	pseudobulk	DESeq2	LRT	0
129	KC_AB2	Kap3	0.45162989537545	0.529311037287488	1	pseudobulk	DESeq2	LRT	0
129	KC_AB2	kappaB-Ras	-1.12785316439435	0.661319045116135	1	pseudobulk	DESeq2	LRT	0
129	KC_AB2	Karybeta3	-0.69292784128696	0.443482397279015	1	pseudobulk	DESeq2	LRT	0
129	KC_AB2	kat-60L1	-0.0018266188148332	0.981994009853847	1	pseudobulk	DESeq2	LRT	0
129	KC_AB2	Kat60	0.281691990941474	0.839420921486687	1	pseudobulk	DESeq2	LRT	0
129	KC_AB2	kat80	0.107479031250535	0.88712690359169	1	pseudobulk	DESeq2	LRT	0
129	KC_AB2	kay	1.03480469826232	0.413579575241544	1	pseudobulk	DESeq2	LRT	0
129	KC_AB2	Kaz1-ORFB	-0.427431139129811	1	1	pseudobulk	DESeq2	LRT	0
129	KC_AB2	kcc	0.455584405413938	0.342574762597531	1	pseudobulk	DESeq2	LRT	0
129	KC_AB2	Kcmf1	0.889987226830469	0.199575173942618	1	pseudobulk	DESeq2	LRT	0
129	KC_AB2	KCNQ	0.93185634766669	0.0250617892111058	1	pseudobulk	DESeq2	LRT	0
129	KC_AB2	KdelR	-0.267057931729429	0.731277526598001	1	pseudobulk	DESeq2	LRT	0
129	KC_AB2	Kdm2	0.457195565707855	0.714913793677025	1	pseudobulk	DESeq2	LRT	0
129	KC_AB2	Kdm4A	-0.360333436166438	0.773817512094502	1	pseudobulk	DESeq2	LRT	0
129	KC_AB2	Kdm4B	0.363188562560769	0.449579006717189	1	pseudobulk	DESeq2	LRT	0
129	KC_AB2	kdn	0.443144627616241	0.548769716083623	1	pseudobulk	DESeq2	LRT	0
129	KC_AB2	Keap1	-0.0202042749192286	0.956236388784323	1	pseudobulk	DESeq2	LRT	0
129	KC_AB2	kek1	-0.0853886001146628	0.71933866971253	1	pseudobulk	DESeq2	LRT	0
129	KC_AB2	kek2	-0.462420751547057	0.499002873842924	1	pseudobulk	DESeq2	LRT	0
129	KC_AB2	kek3	-0.338468037999923	0.685008238257065	1	pseudobulk	DESeq2	LRT	0
129	KC_AB2	kek4	0.15122146245013	0.894393376257336	1	pseudobulk	DESeq2	LRT	0
129	KC_AB2	kek5	1.39571658190498	0.531907471385237	1	pseudobulk	DESeq2	LRT	0
129	KC_AB2	kek6	0.142049503829583	0.872341848136429	1	pseudobulk	DESeq2	LRT	0
129	KC_AB2	kel	0.984515255073584	0.531461569855196	1	pseudobulk	DESeq2	LRT	0
129	KC_AB2	key	-1.57995942323514	0.26455084889652	1	pseudobulk	DESeq2	LRT	0
129	KC_AB2	Khc	0.333613645286228	0.692845121734527	1	pseudobulk	DESeq2	LRT	0
129	KC_AB2	Khc-73	0.477899803432384	0.332844055714605	1	pseudobulk	DESeq2	LRT	0
129	KC_AB2	Kif3C	1.84998908200093	0.351217765903382	1	pseudobulk	DESeq2	LRT	0
129	KC_AB2	kin17	0.857840141808107	0.598254214434548	1	pseudobulk	DESeq2	LRT	0
129	KC_AB2	kirre	-0.544889027249409	0.443414511141829	1	pseudobulk	DESeq2	LRT	0
129	KC_AB2	kis	0.0562089760586487	0.801156356360388	1	pseudobulk	DESeq2	LRT	0
129	KC_AB2	klar	-0.223195281446454	0.74875518472102	1	pseudobulk	DESeq2	LRT	0
129	KC_AB2	Klc	-0.493690927809784	0.560897762208194	1	pseudobulk	DESeq2	LRT	0
129	KC_AB2	klg	2.08039342425865	0.172607459160389	1	pseudobulk	DESeq2	LRT	0
129	KC_AB2	KLHL18	-0.381520986379041	0.718150260974494	1	pseudobulk	DESeq2	LRT	0
129	KC_AB2	Klp10A	0.864684364700415	0.417904498213134	1	pseudobulk	DESeq2	LRT	0
129	KC_AB2	Klp31E	0.428118970844364	0.8279364275092	1	pseudobulk	DESeq2	LRT	0
129	KC_AB2	Klp54D	-0.211959943697087	0.913646816097592	1	pseudobulk	DESeq2	LRT	0
129	KC_AB2	Klp64D	-1.0102493692505	0.407494453713185	1	pseudobulk	DESeq2	LRT	0
129	KC_AB2	Klp68D	-0.0892071691295749	0.843001872504777	1	pseudobulk	DESeq2	LRT	0
129	KC_AB2	Klp98A	1.18508343102242	0.0922341571790139	1	pseudobulk	DESeq2	LRT	0

129	KC_AB2	Kmn1	-2.17734931898309	0.348712604149285	1	pseudobulk	DESeq2	LRT		0
129	KC_AB2	kmr	-0.665025327898426	0.280480158879399	1	pseudobulk	DESeq2	LRT		0
129	KC_AB2	ko	-0.841882716738579	1	1	pseudobulk	DESeq2	LRT		0
129	KC_AB2	koi	1.01513505174573	0.370928256975693	1	pseudobulk	DESeq2	LRT		0
129	KC_AB2	Kr-h1	-0.473797945270466	0.553026040832311	1	pseudobulk	DESeq2	LRT		0
129	KC_AB2	Kr-h2	-0.869247041302839	0.381052355360376	1	pseudobulk	DESeq2	LRT		0
129	KC_AB2	kra	-0.189081896840082	0.573092825499587	1	pseudobulk	DESeq2	LRT		0
129	KC_AB2	kraken	0.274199773741423	0.757197004379149	1	pseudobulk	DESeq2	LRT		0
129	KC_AB2	kri	0.04114377027796	0.961695537918712	1	pseudobulk	DESeq2	LRT		0
129	KC_AB2	Krn	-1.45972214055569	0.452659000139653	1	pseudobulk	DESeq2	LRT		0
129	KC_AB2	KrT95D	0.64581421859438	0.283685053230559	1	pseudobulk	DESeq2	LRT		0
129	KC_AB2	krz	0.189562049781634	0.734185853329152	1	pseudobulk	DESeq2	LRT		0
129	KC_AB2	ksh	0.225254912387131	0.802279976223733	1	pseudobulk	DESeq2	LRT		0
129	KC_AB2	ksr	-0.0285198573065885	0.948439834289392	1	pseudobulk	DESeq2	LRT		0
129	KC_AB2	Ktl	0.291314978639595	0.452958904672601	1	pseudobulk	DESeq2	LRT		0
129	KC_AB2	kto	-1.93220872672389	0.398686710033904	1	pseudobulk	DESeq2	LRT		0
130	KC_AB2	ktub	0.473308979923015	0.756796954469045	1	pseudobulk	DESeq2	LRT		0
130	KC_AB2	Kua	-1.78094334699383	0.470930349923561	1	pseudobulk	DESeq2	LRT		0
130	KC_AB2	kud	-0.0474041421947227	0.952466776134269	1	pseudobulk	DESeq2	LRT		0
130	KC_AB2	kug	-1.62647981469485	0.676101982919167	1	pseudobulk	DESeq2	LRT		0
130	KC_AB2	kuk	-0.457867327977596	0.656578504458111	1	pseudobulk	DESeq2	LRT		0
130	KC_AB2	Kul	-2.62007326210819	0.112813611116877	1	pseudobulk	DESeq2	LRT		0
130	KC_AB2	kune	-0.362179350257902	0.730583715996031	1	pseudobulk	DESeq2	LRT		0
130	KC_AB2	kuz	-0.0379915183232196	0.894720396394923	1	pseudobulk	DESeq2	LRT		0
130	KC_AB2	Kyat	0.222396124134733	1	1	pseudobulk	DESeq2	LRT		0
130	KC_AB2	kz	-0.600547221779133	0.841940696994101	1	pseudobulk	DESeq2	LRT		0
130	KC_AB2	l(1)10Bb	0.717701898513957	0.495378299386351	1	pseudobulk	DESeq2	LRT		0
130	KC_AB2	l(1)G0004	0.260291521188346	0.813396556127474	1	pseudobulk	DESeq2	LRT		0
130	KC_AB2	l(1)G0007	0.498653282344605	0.663082117676583	1	pseudobulk	DESeq2	LRT		0
130	KC_AB2	l(1)G0193	-1.55303551654732	0.094164956270554	1	pseudobulk	DESeq2	LRT		0
130	KC_AB2	l(1)G0196	0.161748964225241	0.777923858736897	1	pseudobulk	DESeq2	LRT		0
130	KC_AB2	l(1)G0289	-0.26796215041398	0.517976317228178	1	pseudobulk	DESeq2	LRT		0
130	KC_AB2	l(1)G0320	-0.825960637729846	0.303443009279522	1	pseudobulk	DESeq2	LRT		0
130	KC_AB2	l(1)G0469	-0.932096530697492	0.219192004543843	1	pseudobulk	DESeq2	LRT		0
130	KC_AB2	l(2)05287	-2.41610217722532	0.272481539699592	1	pseudobulk	DESeq2	LRT		0
130	KC_AB2	l(2)05714	0.536038856924057	0.762696974399166	1	pseudobulk	DESeq2	LRT		0
130	KC_AB2	l(2)09851	0.573760393110584	0.792134620722927	1	pseudobulk	DESeq2	LRT		0
130	KC_AB2	l(2)10685	0.498593948417392	0.805619674491474	1	pseudobulk	DESeq2	LRT		0
130	KC_AB2	l(2)34Fd	-0.841882716738579	1	1	pseudobulk	DESeq2	LRT		0
130	KC_AB2	l(2)35Be	0.272860557109916	0.841150084609554	1	pseudobulk	DESeq2	LRT		0
130	KC_AB2	l(2)37Bb	-1.12785316439435	0.661319045116135	1	pseudobulk	DESeq2	LRT		0
130	KC_AB2	l(2)37Cb	-0.209808076863057	0.877444409505505	1	pseudobulk	DESeq2	LRT		0
130	KC_AB2	l(2)37Cc	-0.643202663464401	0.504461456538739	1	pseudobulk	DESeq2	LRT		0
130	KC_AB2	l(2)37Cg	-2.48435623267434	0.256496613429614	1	pseudobulk	DESeq2	LRT		0
130	KC_AB2	l(2)41Ab	-1.07776915880798	0.775491075088671	1	pseudobulk	DESeq2	LRT		0
130	KC_AB2	l(2)efl	0.458718245521617	0.813205231836982	1	pseudobulk	DESeq2	LRT		0
130	KC_AB2	l(2)gd1	2.49750470281195	0.235076905519563	1	pseudobulk	DESeq2	LRT		0
130	KC_AB2	l(2)k01209	0.219984901460059	0.796146570319975	1	pseudobulk	DESeq2	LRT		0
130	KC_AB2	l(2)k05819	0.410913051743615	0.655204625966063	1	pseudobulk	DESeq2	LRT		0
130	KC_AB2	l(2)k09022	-0.427431139129811	1	1	pseudobulk	DESeq2	LRT		0
130	KC_AB2	l(2)k09848	0.189813678325555	1	1	pseudobulk	DESeq2	LRT		0
130	KC_AB2	l(2)k09913	0.0050659713026831	0.89533420743982	1	pseudobulk	DESeq2	LRT		0
130	KC_AB2	l(2)k10201	1.5243995994652	0.741477491078038	1	pseudobulk	DESeq2	LRT		0

130	KC_AB2	l(2)k14505	-0.148041889400913	0.852937619861225	1	pseudobulk	DESeq2	LRT	0
130	KC_AB2	l(2)SH0834	-0.674404379499631	0.426620071510247	1	pseudobulk	DESeq2	LRT	0
130	KC_AB2	l(3)02640	0.763889976210619	0.380516330767794	1	pseudobulk	DESeq2	LRT	0
130	KC_AB2	l(3)04053	1.70053690678249	0.319223901856326	1	pseudobulk	DESeq2	LRT	0
130	KC_AB2	l(3)05822	0.739782685177043	0.39761071899441	1	pseudobulk	DESeq2	LRT	0
130	KC_AB2	l(3)07882	0.873856633876596	0.589642654681684	1	pseudobulk	DESeq2	LRT	0
130	KC_AB2	l(3)72Ab	1.55951432076123	0.324397497404608	1	pseudobulk	DESeq2	LRT	0
130	KC_AB2	l(3)72Dn	0.70378358684898	0.932301451973468	1	pseudobulk	DESeq2	LRT	0
130	KC_AB2	l(3)72Dp	-0.592481036256484	0.706847545932384	1	pseudobulk	DESeq2	LRT	0
130	KC_AB2	l(3)72Dr	-2.13580979111316	0.396045572646122	1	pseudobulk	DESeq2	LRT	0
130	KC_AB2	l(3)73Ah	-1.08957696563443	0.256835332133831	1	pseudobulk	DESeq2	LRT	0
130	KC_AB2	l(3)76BDm	-0.844342502731934	0.648507321983482	1	pseudobulk	DESeq2	LRT	0
130	KC_AB2	l(3)80Fg	0.0669086029258848	0.783344555380663	1	pseudobulk	DESeq2	LRT	0
130	KC_AB2	l(3)80Fj	-0.126964277864619	0.668430717755426	1	pseudobulk	DESeq2	LRT	0
130	KC_AB2	l(3)87Df	0.619344519642537	0.606853470016108	1	pseudobulk	DESeq2	LRT	0
130	KC_AB2	l(3)L1231	-0.598496602658783	0.241913057462633	1	pseudobulk	DESeq2	LRT	0
130	KC_AB2	l(3)mbt	1.67530465152129	0.416998667908363	1	pseudobulk	DESeq2	LRT	0
130	KC_AB2	l(3)neo38	-0.0127477663874723	0.893134202759116	1	pseudobulk	DESeq2	LRT	0
130	KC_AB2	l(3)neo43	-0.42561018199242	0.636030697910665	1	pseudobulk	DESeq2	LRT	0
130	KC_AB2	l(3)psg2	-0.651939325008421	0.599560511761349	1	pseudobulk	DESeq2	LRT	0
130	KC_AB2	L2HGDH	0.467089618589023	0.708071239338366	1	pseudobulk	DESeq2	LRT	0
130	KC_AB2	La	0.531918207622378	0.635168323621341	1	pseudobulk	DESeq2	LRT	0
130	KC_AB2	Lac	-0.592481036256484	0.706847545932384	1	pseudobulk	DESeq2	LRT	0
130	KC_AB2	lace	0.875055538995535	0.483448142512462	1	pseudobulk	DESeq2	LRT	0
130	KC_AB2	Lam	1.30649777487861	0.35925821775819	1	pseudobulk	DESeq2	LRT	0
130	KC_AB2	LamC	2.1817927492653	0.458599861292202	1	pseudobulk	DESeq2	LRT	0
130	KC_AB2	Lamp1	0.173420323341887	0.690934143660282	1	pseudobulk	DESeq2	LRT	0
130	KC_AB2	lap	0.0141642029724931	0.880238413237686	1	pseudobulk	DESeq2	LRT	0
130	KC_AB2	Lap1	-0.406746729298491	0.800090602086552	1	pseudobulk	DESeq2	LRT	0
130	KC_AB2	Lar	0.200375350637351	0.443111230634569	1	pseudobulk	DESeq2	LRT	0
130	KC_AB2	lark	0.391141242849863	0.543468402468036	1	pseudobulk	DESeq2	LRT	0
130	KC_AB2	larp	0.215756264670742	0.67342535588493	1	pseudobulk	DESeq2	LRT	0
130	KC_AB2	Larp4B	0.0131296471904398	0.834542998463449	1	pseudobulk	DESeq2	LRT	0
130	KC_AB2	Larp7	-2.63419818779401	0.222714263443553	1	pseudobulk	DESeq2	LRT	0
130	KC_AB2	Las	0.976287672858323	0.478455557399567	1	pseudobulk	DESeq2	LRT	0
130	KC_AB2	lawc	0.276820153807548	0.565871158680728	1	pseudobulk	DESeq2	LRT	0
130	KC_AB2	lbk	0.343233922747934	0.396330995204625	1	pseudobulk	DESeq2	LRT	0
130	KC_AB2	lbm	-0.799634786835583	0.271150956164558	1	pseudobulk	DESeq2	LRT	0
130	KC_AB2	Lcch3	0.641460474743327	0.36820084417817	1	pseudobulk	DESeq2	LRT	0
130	KC_AB2	Ldh	1.46148027171274	0.158588261416808	1	pseudobulk	DESeq2	LRT	0
130	KC_AB2	Leash	0.522867664761254	0.789441383158121	1	pseudobulk	DESeq2	LRT	0
130	KC_AB2	Lerp	-1.0327096002559	0.284347149737064	1	pseudobulk	DESeq2	LRT	0
130	KC_AB2	Letm1	-1.2609532936122	0.446703074884471	1	pseudobulk	DESeq2	LRT	0
130	KC_AB2	LeuRS	0.38377126462555	0.763541203839965	1	pseudobulk	DESeq2	LRT	0
130	KC_AB2	LeuRS-m	1.53811683574911	0.54844951579854	1	pseudobulk	DESeq2	LRT	0
130	KC_AB2	levy	-0.322941585332346	0.318334910999461	1	pseudobulk	DESeq2	LRT	0
130	KC_AB2	Lgr1	0.192821180391777	0.741906244755465	1	pseudobulk	DESeq2	LRT	0
130	KC_AB2	Lgr3	-0.841882716738579	1	1	pseudobulk	DESeq2	LRT	0
130	KC_AB2	lgs	0.0964569110297667	0.82324900018823	1	pseudobulk	DESeq2	LRT	0
130	KC_AB2	Lhr	0.920212920242661	0.67286879185691	1	pseudobulk	DESeq2	LRT	0
130	KC_AB2	lic	-1.59387851123624	0.452768286821797	1	pseudobulk	DESeq2	LRT	0
130	KC_AB2	lid	-0.676262778502868	0.258860888825547	1	pseudobulk	DESeq2	LRT	0
130	KC_AB2	lig	0.779019535100111	0.225301216629653	1	pseudobulk	DESeq2	LRT	0

130	KC_AB2	lili	0.346589616049749	0.84654468695694	1	pseudobulk	DESeq2	LRT	0
130	KC_AB2	lilli	0.123258219891743	0.706156548909557	1	pseudobulk	DESeq2	LRT	0
130	KC_AB2	LIMK1	0.490490085969749	0.591432835421219	1	pseudobulk	DESeq2	LRT	0
130	KC_AB2	lin	0.292346552234682	0.842418166025389	1	pseudobulk	DESeq2	LRT	0
130	KC_AB2	lin-52	2.12740730124952	0.150083246817168	1	pseudobulk	DESeq2	LRT	0
130	KC_AB2	link	3.20650492957429	0.177944556633249	1	pseudobulk	DESeq2	LRT	0
130	KC_AB2	Lint-1	-0.93816188680777	0.429731233283866	1	pseudobulk	DESeq2	LRT	0
130	KC_AB2	Liprin-alpha	-0.940586329507494	0.298857994777992	1	pseudobulk	DESeq2	LRT	0
130	KC_AB2	Liprin-beta	0.0586988282336896	0.836674359608016	1	pseudobulk	DESeq2	LRT	0
130	KC_AB2	Liprin-gamma	-0.248522571718141	0.485780718997438	1	pseudobulk	DESeq2	LRT	0
131	KC_AB2	Lis-1	-0.0866426663634679	0.705847929869295	1	pseudobulk	DESeq2	LRT	0
131	KC_AB2	List	-1.51812168653838	0.552401469179882	1	pseudobulk	DESeq2	LRT	0
131	KC_AB2	Lk6	0.36599884421087	0.249999501181623	1	pseudobulk	DESeq2	LRT	0
131	KC_AB2	Lkb1	0.786388361225653	0.533472287638011	1	pseudobulk	DESeq2	LRT	0
131	KC_AB2	LKRSDH	-1.85733156562156	0.448702818121367	1	pseudobulk	DESeq2	LRT	0
131	KC_AB2	Lmpt	0.114014192724552	0.796643370917252	1	pseudobulk	DESeq2	LRT	0
131	KC_AB2	lms	0.725498497157359	0.93879047033739	1	pseudobulk	DESeq2	LRT	0
131	KC_AB2	lncRNA:alphagamr	-0.59613285535622	0.43227351613767	1	pseudobulk	DESeq2	LRT	0
131	KC_AB2	lncRNA:cherub	2.9858520074866	0.150088943043272	1	pseudobulk	DESeq2	LRT	0
131	KC_AB2	lncRNA:CR30055	-0.191746134789014	0.763462450460114	1	pseudobulk	DESeq2	LRT	0
131	KC_AB2	lncRNA:CR31044	-0.600547221779134	1	1	pseudobulk	DESeq2	LRT	0
131	KC_AB2	lncRNA:CR31451	-0.370471341091139	0.179620093101046	1	pseudobulk	DESeq2	LRT	0
131	KC_AB2	lncRNA:CR32027	-0.427431139129811	1	1	pseudobulk	DESeq2	LRT	0
131	KC_AB2	lncRNA:CR32218	0.0951652990582188	0.829122231837765	1	pseudobulk	DESeq2	LRT	0
131	KC_AB2	lncRNA:CR32582	-0.427431139129811	1	1	pseudobulk	DESeq2	LRT	0
131	KC_AB2	lncRNA:CR32730	2.12684015898615	0.445939223035924	1	pseudobulk	DESeq2	LRT	0
131	KC_AB2	lncRNA:CR32773	-1.51928970377954	0.486908946651791	1	pseudobulk	DESeq2	LRT	0
131	KC_AB2	lncRNA:CR34335	-0.358951098584123	0.117586788665763	1	pseudobulk	DESeq2	LRT	0
131	KC_AB2	lncRNA:CR40469	-0.600547221779134	1	1	pseudobulk	DESeq2	LRT	0
131	KC_AB2	lncRNA:CR42491	1.5243995994652	0.741477491078038	1	pseudobulk	DESeq2	LRT	0
131	KC_AB2	lncRNA:CR42549	-0.0852743682753834	0.917718671153184	1	pseudobulk	DESeq2	LRT	0
131	KC_AB2	lncRNA:CR42715	-1.59432579116312	0.459120231439715	1	pseudobulk	DESeq2	LRT	0
131	KC_AB2	lncRNA:CR42861	0.486115329164496	0.40510037281041	1	pseudobulk	DESeq2	LRT	0
131	KC_AB2	lncRNA:CR42862	0.10114389210432	0.656690356927183	1	pseudobulk	DESeq2	LRT	0
131	KC_AB2	lncRNA:CR43130	-0.592481036256484	0.706847545932384	1	pseudobulk	DESeq2	LRT	0
131	KC_AB2	lncRNA:CR43146	-0.600547221779133	0.841940696994101	1	pseudobulk	DESeq2	LRT	0
131	KC_AB2	lncRNA:CR43148	-1.29244479833796	0.693295472370086	1	pseudobulk	DESeq2	LRT	0
131	KC_AB2	lncRNA:CR43256	0.713982351041985	0.921569637923553	1	pseudobulk	DESeq2	LRT	0
131	KC_AB2	lncRNA:CR43264	-3.01459641366802	0.149509045856783	1	pseudobulk	DESeq2	LRT	0
131	KC_AB2	lncRNA:CR43287	-0.592481036256484	0.706847545932384	1	pseudobulk	DESeq2	LRT	0
131	KC_AB2	lncRNA:CR43334	-0.347045898668302	0.755825692028621	1	pseudobulk	DESeq2	LRT	0
131	KC_AB2	lncRNA:CR43408	-1.70380348762697	0.473446675363872	1	pseudobulk	DESeq2	LRT	0
131	KC_AB2	lncRNA:CR43459	0.851114050509941	0.266833919660622	1	pseudobulk	DESeq2	LRT	0
131	KC_AB2	lncRNA:CR43492	-0.621805355124546	0.74877258313458	1	pseudobulk	DESeq2	LRT	0
131	KC_AB2	lncRNA:CR43496	2.97439820060169	0.149184448246935	1	pseudobulk	DESeq2	LRT	0
131	KC_AB2	lncRNA:CR43651	0.439455996901645	0.701246557346907	1	pseudobulk	DESeq2	LRT	0
131	KC_AB2	lncRNA:CR43685	0.964480011048761	0.36005757583551	1	pseudobulk	DESeq2	LRT	0
131	KC_AB2	lncRNA:CR43700	1.5243995994652	0.741477491078038	1	pseudobulk	DESeq2	LRT	0
131	KC_AB2	lncRNA:CR43801	-0.600547221779134	1	1	pseudobulk	DESeq2	LRT	0
131	KC_AB2	lncRNA:CR43836	-0.340392739454829	0.813455907448286	1	pseudobulk	DESeq2	LRT	0
131	KC_AB2	lncRNA:CR43856	-1.90937835128834	0.377732797313411	1	pseudobulk	DESeq2	LRT	0
131	KC_AB2	lncRNA:CR43870	-0.600547221779133	0.841940696994101	1	pseudobulk	DESeq2	LRT	0
131	KC_AB2	lncRNA:CR43878	-0.174678693229703	0.860716007308159	1	pseudobulk	DESeq2	LRT	0

131	KC_AB2	lncRNA:CR43890	-0.427431139129811	1	1	pseudobulk	DESeq2	LRT	0
131	KC_AB2	lncRNA:CR43900	2.12684015898615	0.445939223035924	1	pseudobulk	DESeq2	LRT	0
131	KC_AB2	lncRNA:CR43910	0.864341462967788	0.925947094335722	1	pseudobulk	DESeq2	LRT	0
131	KC_AB2	lncRNA:CR43943	0.864341462967788	0.925947094335722	1	pseudobulk	DESeq2	LRT	0
131	KC_AB2	lncRNA:CR43951	-0.592481036256484	0.706847545932384	1	pseudobulk	DESeq2	LRT	0
131	KC_AB2	lncRNA:CR43958	-1.70614408517477	0.52816093424714	1	pseudobulk	DESeq2	LRT	0
131	KC_AB2	lncRNA:CR43963	1.40241945196173	0.57265124481703	1	pseudobulk	DESeq2	LRT	0
131	KC_AB2	lncRNA:CR43995	-0.405479754122697	0.85323682425868	1	pseudobulk	DESeq2	LRT	0
131	KC_AB2	lncRNA:CR44022	-0.841882716738579	1	1	pseudobulk	DESeq2	LRT	0
131	KC_AB2	lncRNA:CR44023	-0.214559818468328	0.83527205011595	1	pseudobulk	DESeq2	LRT	0
131	KC_AB2	lncRNA:CR44024	0.0700919807887279	0.810744872853448	1	pseudobulk	DESeq2	LRT	0
131	KC_AB2	lncRNA:CR44043	-2.2477182984077	0.33075850732842	1	pseudobulk	DESeq2	LRT	0
131	KC_AB2	lncRNA:CR44050	-1.99153573919778	0.406760593108112	1	pseudobulk	DESeq2	LRT	0
131	KC_AB2	lncRNA:CR44092	-1.10405600102669	0.617983244849634	1	pseudobulk	DESeq2	LRT	0
131	KC_AB2	lncRNA:CR44126	-0.600547221779133	0.841940696994101	1	pseudobulk	DESeq2	LRT	0
131	KC_AB2	lncRNA:CR44127	1.40241945196173	0.57265124481703	1	pseudobulk	DESeq2	LRT	0
131	KC_AB2	lncRNA:CR44129	-1.16116875819275	0.736255625566845	1	pseudobulk	DESeq2	LRT	0
131	KC_AB2	lncRNA:CR44156	-0.427431139129811	1	1	pseudobulk	DESeq2	LRT	0
131	KC_AB2	lncRNA:CR44178	1.8688760377645	0.356303822006979	1	pseudobulk	DESeq2	LRT	0
131	KC_AB2	lncRNA:CR44187	0.0180880210312664	0.990469689325653	1	pseudobulk	DESeq2	LRT	0
131	KC_AB2	lncRNA:CR44188	2.12684015898615	0.445939223035924	1	pseudobulk	DESeq2	LRT	0
131	KC_AB2	lncRNA:CR44206	1.79673692932728	0.295178962685896	1	pseudobulk	DESeq2	LRT	0
131	KC_AB2	lncRNA:CR44272	-2.47950523487676	0.289933331352224	1	pseudobulk	DESeq2	LRT	0
131	KC_AB2	lncRNA:CR44347	-0.716210759982969	0.496154550066123	1	pseudobulk	DESeq2	LRT	0
131	KC_AB2	lncRNA:CR44348	-1.70380348762697	0.473446675363872	1	pseudobulk	DESeq2	LRT	0
131	KC_AB2	lncRNA:CR44357	-0.631578663457669	0.750664130745045	1	pseudobulk	DESeq2	LRT	0
131	KC_AB2	lncRNA:CR44384	-0.573109354659945	0.739419341582026	1	pseudobulk	DESeq2	LRT	0
131	KC_AB2	lncRNA:CR44409	-0.427431139129811	1	1	pseudobulk	DESeq2	LRT	0
131	KC_AB2	lncRNA:CR44417	-0.436440550484611	0.49262926886291	1	pseudobulk	DESeq2	LRT	0
131	KC_AB2	lncRNA:CR44444	-2.3076113135058	0.325130952902757	1	pseudobulk	DESeq2	LRT	0
131	KC_AB2	lncRNA:CR44445	-0.600547221779134	1	1	pseudobulk	DESeq2	LRT	0
131	KC_AB2	lncRNA:CR44464	1.5243995994652	0.741477491078038	1	pseudobulk	DESeq2	LRT	0
131	KC_AB2	lncRNA:CR44472	-0.600547221779134	1	1	pseudobulk	DESeq2	LRT	0
131	KC_AB2	lncRNA:CR44506	-2.93014069247264	0.149341146677298	1	pseudobulk	DESeq2	LRT	0
131	KC_AB2	lncRNA:CR44564	-0.600547221779133	0.841940696994101	1	pseudobulk	DESeq2	LRT	0
131	KC_AB2	lncRNA:CR44589	1.5243995994652	0.741477491078038	1	pseudobulk	DESeq2	LRT	0
131	KC_AB2	lncRNA:CR44595	1.53811683574911	0.54844951579854	1	pseudobulk	DESeq2	LRT	0
131	KC_AB2	lncRNA:CR44596	-0.600547221779134	1	1	pseudobulk	DESeq2	LRT	0
131	KC_AB2	lncRNA:CR44597	1.54053875912195	0.39238818226048	1	pseudobulk	DESeq2	LRT	0
131	KC_AB2	lncRNA:CR44598	-1.65391840723379	0.392847699484482	1	pseudobulk	DESeq2	LRT	0
131	KC_AB2	lncRNA:CR44603	-0.427431139129811	1	1	pseudobulk	DESeq2	LRT	0
131	KC_AB2	lncRNA:CR44642	1.5243995994652	0.741477491078038	1	pseudobulk	DESeq2	LRT	0
131	KC_AB2	lncRNA:CR44662	-0.214559818468328	0.83527205011595	1	pseudobulk	DESeq2	LRT	0
131	KC_AB2	lncRNA:CR44663	-0.427431139129811	1	1	pseudobulk	DESeq2	LRT	0
131	KC_AB2	lncRNA:CR44708	0.195251340687946	0.79013788052411	1	pseudobulk	DESeq2	LRT	0
131	KC_AB2	lncRNA:CR44716	2.76205395729416	0.251829971474322	1	pseudobulk	DESeq2	LRT	0
131	KC_AB2	lncRNA:CR44718	1.5243995994652	0.741477491078038	1	pseudobulk	DESeq2	LRT	0
131	KC_AB2	lncRNA:CR44779	-0.427431139129811	1	1	pseudobulk	DESeq2	LRT	0
131	KC_AB2	lncRNA:CR44805	1.21092382796727	0.637125262974216	1	pseudobulk	DESeq2	LRT	0
131	KC_AB2	lncRNA:CR44809	-0.427431139129811	1	1	pseudobulk	DESeq2	LRT	0
131	KC_AB2	lncRNA:CR44811	-0.600547221779134	1	1	pseudobulk	DESeq2	LRT	0
131	KC_AB2	lncRNA:CR44814	2.1817927492653	0.458599861292202	1	pseudobulk	DESeq2	LRT	0
131	KC_AB2	lncRNA:CR44816	1.48010047180759	0.482153739285344	1	pseudobulk	DESeq2	LRT	0

131	KC_AB2	lncRNA:CR44833	2.12684015898615	0.445939223035924	1	pseudobulk	DESeq2	LRT	0
131	KC_AB2	lncRNA:CR44848	-0.427431139129811	1	1	pseudobulk	DESeq2	LRT	0
131	KC_AB2	lncRNA:CR44864	1.5243995994652	0.741477491078038	1	pseudobulk	DESeq2	LRT	0
131	KC_AB2	lncRNA:CR44886	-0.5010478185981	0.457358697977617	1	pseudobulk	DESeq2	LRT	0
132	KC_AB2	lncRNA:CR44922	-0.165714785611847	0.847930715540501	1	pseudobulk	DESeq2	LRT	0
132	KC_AB2	lncRNA:CR44951	-0.600547221779134	1	1	pseudobulk	DESeq2	LRT	0
132	KC_AB2	lncRNA:CR44978	-1.07776915880798	0.775491075088671	1	pseudobulk	DESeq2	LRT	0
132	KC_AB2	lncRNA:CR44986	0.183555857030446	1	1	pseudobulk	DESeq2	LRT	0
132	KC_AB2	lncRNA:CR44997	1.13398697935673	0.313414065783907	1	pseudobulk	DESeq2	LRT	0
132	KC_AB2	lncRNA:CR44999	-0.167620063280405	0.874493033115399	1	pseudobulk	DESeq2	LRT	0
132	KC_AB2	lncRNA:CR45025	0.725498497157359	0.93879047033739	1	pseudobulk	DESeq2	LRT	0
132	KC_AB2	lncRNA:CR45030	0.864341462967788	0.925947094335722	1	pseudobulk	DESeq2	LRT	0
132	KC_AB2	lncRNA:CR45035	-0.828178378785339	0.673584255563891	1	pseudobulk	DESeq2	LRT	0
132	KC_AB2	lncRNA:CR45054	0.38899558150697	0.323375749523746	1	pseudobulk	DESeq2	LRT	0
132	KC_AB2	lncRNA:CR45102	0.586933541760409	0.472181789707187	1	pseudobulk	DESeq2	LRT	0
132	KC_AB2	lncRNA:CR45138	-0.427431139129811	1	1	pseudobulk	DESeq2	LRT	0
132	KC_AB2	lncRNA:CR45156	1.40241945196173	0.57265124481703	1	pseudobulk	DESeq2	LRT	0
132	KC_AB2	lncRNA:CR45159	-0.427431139129811	1	1	pseudobulk	DESeq2	LRT	0
132	KC_AB2	lncRNA:CR45170	-1.07776915880798	0.775491075088671	1	pseudobulk	DESeq2	LRT	0
132	KC_AB2	lncRNA:CR45178	-0.600547221779133	0.841940696994101	1	pseudobulk	DESeq2	LRT	0
132	KC_AB2	lncRNA:CR45179	-0.427431139129811	1	1	pseudobulk	DESeq2	LRT	0
132	KC_AB2	lncRNA:CR45187	-0.427431139129811	1	1	pseudobulk	DESeq2	LRT	0
132	KC_AB2	lncRNA:CR45196	1.40241945196173	0.57265124481703	1	pseudobulk	DESeq2	LRT	0
132	KC_AB2	lncRNA:CR45217	-0.600547221779134	1	1	pseudobulk	DESeq2	LRT	0
132	KC_AB2	lncRNA:CR45224	0.70378358684898	0.932301451973468	1	pseudobulk	DESeq2	LRT	0
132	KC_AB2	lncRNA:CR45251	2.12684015898615	0.445939223035924	1	pseudobulk	DESeq2	LRT	0
132	KC_AB2	lncRNA:CR45262	-0.600547221779134	1	1	pseudobulk	DESeq2	LRT	0
132	KC_AB2	lncRNA:CR45312	-0.427431139129811	1	1	pseudobulk	DESeq2	LRT	0
132	KC_AB2	lncRNA:CR45318	-0.600547221779134	1	1	pseudobulk	DESeq2	LRT	0
132	KC_AB2	lncRNA:CR45388	2.48629812612306	0.259054692310381	1	pseudobulk	DESeq2	LRT	0
132	KC_AB2	lncRNA:CR45409	-1.45100191296786	0.365490864520672	1	pseudobulk	DESeq2	LRT	0
132	KC_AB2	lncRNA:CR45418	-0.427431139129811	1	1	pseudobulk	DESeq2	LRT	0
132	KC_AB2	lncRNA:CR45473	1.11471276021981	0.644913768495318	1	pseudobulk	DESeq2	LRT	0
132	KC_AB2	lncRNA:CR45474	1.5243995994652	0.741477491078038	1	pseudobulk	DESeq2	LRT	0
132	KC_AB2	lncRNA:CR45504	-0.427431139129811	1	1	pseudobulk	DESeq2	LRT	0
132	KC_AB2	lncRNA:CR45522	1.36390873566401	0.513938015567603	1	pseudobulk	DESeq2	LRT	0
132	KC_AB2	lncRNA:CR45527	-0.427431139129811	1	1	pseudobulk	DESeq2	LRT	0
132	KC_AB2	lncRNA:CR45530	2.72066226053615	0.290765320578625	1	pseudobulk	DESeq2	LRT	0
132	KC_AB2	lncRNA:CR45531	-1.54470851095603	0.55301747381519	1	pseudobulk	DESeq2	LRT	0
132	KC_AB2	lncRNA:CR45539	1.5243995994652	0.741477491078038	1	pseudobulk	DESeq2	LRT	0
132	KC_AB2	lncRNA:CR45634	-0.427431139129811	1	1	pseudobulk	DESeq2	LRT	0
132	KC_AB2	lncRNA:CR45643	-1.40673172715063	0.587364787535786	1	pseudobulk	DESeq2	LRT	0
132	KC_AB2	lncRNA:CR45660	-2.09895100891983	0.23147183767828	1	pseudobulk	DESeq2	LRT	0
132	KC_AB2	lncRNA:CR45668	-0.250980589646313	0.863293969735939	1	pseudobulk	DESeq2	LRT	0
132	KC_AB2	lncRNA:CR45669	-0.427431139129811	1	1	pseudobulk	DESeq2	LRT	0
132	KC_AB2	lncRNA:CR45684	-0.531428829108344	0.702546908292458	1	pseudobulk	DESeq2	LRT	0
132	KC_AB2	lncRNA:CR45736	-0.600547221779134	1	1	pseudobulk	DESeq2	LRT	0
132	KC_AB2	lncRNA:CR45740	1.5243995994652	0.741477491078038	1	pseudobulk	DESeq2	LRT	0
132	KC_AB2	lncRNA:CR45758	0.258520538952062	0.74301437419219	1	pseudobulk	DESeq2	LRT	0
132	KC_AB2	lncRNA:CR45825	0.345399645638984	0.819123986838626	1	pseudobulk	DESeq2	LRT	0
132	KC_AB2	lncRNA:CR45910	2.07946313289983	0.357635956314506	1	pseudobulk	DESeq2	LRT	0
132	KC_AB2	lncRNA:CR45911	0.108468324554008	1	1	pseudobulk	DESeq2	LRT	0
132	KC_AB2	lncRNA:CR45918	0.506662102707318	0.782529221356027	1	pseudobulk	DESeq2	LRT	0

132	KC_AB2	lncRNA:CR45919	2.49750470281195	0.235076905519563	1	pseudobulk	DESeq2	LRT		0
132	KC_AB2	lncRNA:CR45922	1.5243995994652	0.741477491078038	1	pseudobulk	DESeq2	LRT		0
132	KC_AB2	lncRNA:CR45938	1.5243995994652	0.741477491078038	1	pseudobulk	DESeq2	LRT		0
132	KC_AB2	lncRNA:CR45939	1.5243995994652	0.741477491078038	1	pseudobulk	DESeq2	LRT		0
132	KC_AB2	lncRNA:CR45959	1.5243995994652	0.741477491078038	1	pseudobulk	DESeq2	LRT		0
132	KC_AB2	lncRNA:CR45961	-0.427431139129811	1	1	pseudobulk	DESeq2	LRT		0
132	KC_AB2	lncRNA:CR45962	0.222396124134733	1	1	pseudobulk	DESeq2	LRT		0
132	KC_AB2	lncRNA:CR45971	-0.427431139129811	1	1	pseudobulk	DESeq2	LRT		0
132	KC_AB2	lncRNA:CR45972	0.864341462967788	0.925947094335722	1	pseudobulk	DESeq2	LRT		0
132	KC_AB2	lncRNA:CR45973	-0.592481036256484	0.706847545932384	1	pseudobulk	DESeq2	LRT		0
132	KC_AB2	lncRNA:CR45979	-0.697991246043347	0.723097050254034	1	pseudobulk	DESeq2	LRT		0
132	KC_AB2	lncRNA:CR45982	-0.427431139129811	1	1	pseudobulk	DESeq2	LRT		0
132	KC_AB2	lncRNA:CR45993	-1.66828165461268	0.531269883399465	1	pseudobulk	DESeq2	LRT		0
132	KC_AB2	lncRNA:CR45994	0.221584967840537	0.835428947919425	1	pseudobulk	DESeq2	LRT		0
132	KC_AB2	lncRNA:CR45996	-0.427431139129811	1	1	pseudobulk	DESeq2	LRT		0
132	KC_AB2	lncRNA:CR46003	1.5243995994652	0.741477491078038	1	pseudobulk	DESeq2	LRT		0
132	KC_AB2	lncRNA:CR46006	-0.0009002208706172	0.91562704263891	1	pseudobulk	DESeq2	LRT		0
132	KC_AB2	lncRNA:CR46016	-0.600547221779134	1	1	pseudobulk	DESeq2	LRT		0
132	KC_AB2	lncRNA:CR46064	-0.424943148415755	0.678570548493631	1	pseudobulk	DESeq2	LRT		0
132	KC_AB2	lncRNA:CR46079	-0.427431139129811	1	1	pseudobulk	DESeq2	LRT		0
132	KC_AB2	lncRNA:CR46085	2.12684015898615	0.445939223035924	1	pseudobulk	DESeq2	LRT		0
132	KC_AB2	lncRNA:CR46095	1.5243995994652	0.741477491078038	1	pseudobulk	DESeq2	LRT		0
132	KC_AB2	lncRNA:CR46208	-1.28432163939267	0.047286775863051	1	pseudobulk	DESeq2	LRT		0
132	KC_AB2	lncRNA:CR46228	-0.600547221779134	1	1	pseudobulk	DESeq2	LRT		0
132	KC_AB2	lncRNA:CR46231	-0.600547221779133	0.841940696994101	1	pseudobulk	DESeq2	LRT		0
132	KC_AB2	lncRNA:CR46268	-0.799431675787062	0.653930468662593	1	pseudobulk	DESeq2	LRT		0
132	KC_AB2	lncRNA:CR46271	2.12684015898615	0.445939223035924	1	pseudobulk	DESeq2	LRT		0
132	KC_AB2	lncRNA:CR46348	-1.48432288931349	0.443053405298839	1	pseudobulk	DESeq2	LRT		0
132	KC_AB2	lncRNA:CR9162	2.12684015898615	0.445939223035924	1	pseudobulk	DESeq2	LRT		0
132	KC_AB2	lncRNA:flam	0.222396124134733	1	1	pseudobulk	DESeq2	LRT		0
132	KC_AB2	lncRNA:Hsromega	0.0978190879680267	0.634432492704589	1	pseudobulk	DESeq2	LRT		0
132	KC_AB2	lncRNA:let7C	0.531836773232437	0.595415521367879	1	pseudobulk	DESeq2	LRT		0
132	KC_AB2	lncRNA:noe	-0.132004118392533	0.429040212548885	1	pseudobulk	DESeq2	LRT		0
132	KC_AB2	lncRNA:roX1	6.45904498607093	0.111483264368721	1	pseudobulk	DESeq2	LRT		0
132	KC_AB2	lncRNA:roX2	4.65143943647405	0.188196754228112	1	pseudobulk	DESeq2	LRT		0
132	KC_AB2	Lnk	0.931434229517312	0.242390866327095	1	pseudobulk	DESeq2	LRT		0
132	KC_AB2	Lnpk	-0.609256093348873	0.729816784107073	1	pseudobulk	DESeq2	LRT		0
132	KC_AB2	loj	0.054943684426131	0.948321331521485	1	pseudobulk	DESeq2	LRT		0
132	KC_AB2	lola	-0.0025984164788367	0.920714800381258	1	pseudobulk	DESeq2	LRT		0
132	KC_AB2	lolal	0.142829063203462	0.660656742440626	1	pseudobulk	DESeq2	LRT		0
132	KC_AB2	Lon	1.04775789966643	0.544260005408745	1	pseudobulk	DESeq2	LRT		0
132	KC_AB2	loqs	-0.0356984537085592	0.873182574243248	1	pseudobulk	DESeq2	LRT		0
132	KC_AB2	lost	-0.141153006793013	0.838680458548909	1	pseudobulk	DESeq2	LRT		0
132	KC_AB2	lovit	-1.05055587798362	0.124667808048896	1	pseudobulk	DESeq2	LRT		0
132	KC_AB2	LPCAT	0.159348325227285	0.881553691053942	1	pseudobulk	DESeq2	LRT		0
132	KC_AB2	Lpin	-0.311590475313994	0.653830541049291	1	pseudobulk	DESeq2	LRT		0
132	KC_AB2	LpR1	1.5243995994652	0.741477491078038	1	pseudobulk	DESeq2	LRT		0
132	KC_AB2	LpR2	0.892659559600706	0.712043317464255	1	pseudobulk	DESeq2	LRT		0
132	KC_AB2	Lpt	1.82579090108959	0.320739368286303	1	pseudobulk	DESeq2	LRT		0
132	KC_AB2	lqf	0.217724509397688	0.65781731430632	1	pseudobulk	DESeq2	LRT		0
132	KC_AB2	lqfR	0.723565243103268	0.427968938299247	1	pseudobulk	DESeq2	LRT		0
133	KC_AB2	Lrch	1.16575479028852	0.116115510533305	1	pseudobulk	DESeq2	LRT		0
133	KC_AB2	LRP1	1.59269308985321	0.168718156232942	1	pseudobulk	DESeq2	LRT		0

133	KC_AB2	Lrp4	0.21394024370622	0.624710313823942	1	pseudobulk	DESeq2	LRT		0
133	KC_AB2	Lrpprc2	-0.841882716738579	1	1	pseudobulk	DESeq2	LRT		0
133	KC_AB2	LRR	0.0182905127220195	0.848641515352229	1	pseudobulk	DESeq2	LRT		0
133	KC_AB2	Lrr47	1.5243995994652	0.741477491078038	1	pseudobulk	DESeq2	LRT		0
133	KC_AB2	Lrrk	-0.0852743682753834	0.917718671153184	1	pseudobulk	DESeq2	LRT		0
133	KC_AB2	Lsd-2	-1.12475160732157	0.574429473125171	1	pseudobulk	DESeq2	LRT		0
133	KC_AB2	LSm1	0.6546639689275	0.714019207783944	1	pseudobulk	DESeq2	LRT		0
133	KC_AB2	Lsm11	-0.427431139129811	1	1	pseudobulk	DESeq2	LRT		0
133	KC_AB2	Lsm12a	0.184065985572935	0.821273152656249	1	pseudobulk	DESeq2	LRT		0
133	KC_AB2	LSm3	0.258542061416931	0.861296075417193	1	pseudobulk	DESeq2	LRT		0
133	KC_AB2	LSm7	-1.18294349965014	0.473475374861373	1	pseudobulk	DESeq2	LRT		0
133	KC_AB2	lsn	2.44897796029457	0.170391733097837	1	pseudobulk	DESeq2	LRT		0
133	KC_AB2	Lst8	-0.455360268031223	0.811447118210737	1	pseudobulk	DESeq2	LRT		0
133	KC_AB2	lt	0.726108765548345	0.492564983938801	1	pseudobulk	DESeq2	LRT		0
133	KC_AB2	Ltn1	0.188639526570825	0.851433643422401	1	pseudobulk	DESeq2	LRT		0
133	KC_AB2	LTV1	0.378315364652663	0.772440255886467	1	pseudobulk	DESeq2	LRT		0
133	KC_AB2	luna	0.295050977423338	0.871457484961373	1	pseudobulk	DESeq2	LRT		0
133	KC_AB2	lute	-0.14655948955193	0.771136768138731	1	pseudobulk	DESeq2	LRT		0
133	KC_AB2	lva	-1.02044505369008	0.413667471920581	1	pseudobulk	DESeq2	LRT		0
133	KC_AB2	lwr	-0.548040660031616	0.31405520064462	1	pseudobulk	DESeq2	LRT		0
133	KC_AB2	LysRS	-0.137953738718812	0.84000137196207	1	pseudobulk	DESeq2	LRT		0
133	KC_AB2	Lztr1	-0.43918808875889	0.689313289081828	1	pseudobulk	DESeq2	LRT		0
133	KC_AB2	m-cup	0.368753935668516	0.755896670960234	1	pseudobulk	DESeq2	LRT		0
133	KC_AB2	M1BP	-0.971629442026314	0.560257700325674	1	pseudobulk	DESeq2	LRT		0
133	KC_AB2	M6	1.08573957026188	0.300994950097891	1	pseudobulk	DESeq2	LRT		0
133	KC_AB2	mAChR-A	-0.198551094259947	0.624092282923625	1	pseudobulk	DESeq2	LRT		0
133	KC_AB2	mAChR-B	0.368366207030571	0.331457794763945	1	pseudobulk	DESeq2	LRT		0
133	KC_AB2	mAcon1	-0.289733689507666	0.63638751760268	1	pseudobulk	DESeq2	LRT		0
133	KC_AB2	Mad	-0.533505793334084	0.524956304237616	1	pseudobulk	DESeq2	LRT		0
133	KC_AB2	Mad1	0.171764425832421	0.839867099702031	1	pseudobulk	DESeq2	LRT		0
133	KC_AB2	Madm	0.315461791510543	0.78738660309588	1	pseudobulk	DESeq2	LRT		0
133	KC_AB2	mael	-1.63925793886161	0.546290859679341	1	pseudobulk	DESeq2	LRT		0
133	KC_AB2	maf-S	0.0853279736507493	0.888067274687404	1	pseudobulk	DESeq2	LRT		0
133	KC_AB2	Maf1	-0.339958749940647	0.471686550913787	1	pseudobulk	DESeq2	LRT		0
133	KC_AB2	Magi	-0.671361547854083	0.739100711960565	1	pseudobulk	DESeq2	LRT		0
133	KC_AB2	mago	-0.0045410720579754	0.86174880889105	1	pseudobulk	DESeq2	LRT		0
133	KC_AB2	MagR	0.0510680081036701	0.892558419606	1	pseudobulk	DESeq2	LRT		0
133	KC_AB2	mahe	0.347231528678291	0.419481187570954	1	pseudobulk	DESeq2	LRT		0
133	KC_AB2	mahj	0.0471936707755822	0.953916869426286	1	pseudobulk	DESeq2	LRT		0
133	KC_AB2	Mal-A5	-1.12785316439435	0.661319045116135	1	pseudobulk	DESeq2	LRT		0
133	KC_AB2	Mal-B2	0.864341462967788	0.925947094335722	1	pseudobulk	DESeq2	LRT		0
133	KC_AB2	mam	-0.398898123015467	0.477509841685975	1	pseudobulk	DESeq2	LRT		0
133	KC_AB2	mamo	-1.23686842869218	0.129837647255407	1	pseudobulk	DESeq2	LRT		0
133	KC_AB2	MAN1	0.443892390635762	1	1	pseudobulk	DESeq2	LRT		0
133	KC_AB2	Manf	-0.574970558483856	0.557210202774007	1	pseudobulk	DESeq2	LRT		0
133	KC_AB2	Map205	-0.352151802735936	0.729941717699033	1	pseudobulk	DESeq2	LRT		0
133	KC_AB2	Map60	-1.04119268392061	0.402938079033242	1	pseudobulk	DESeq2	LRT		0
133	KC_AB2	MAPk-Ak2	0.373698706956753	0.739852079955752	1	pseudobulk	DESeq2	LRT		0
133	KC_AB2	Mapmodulin	-0.554622298029305	0.152524518362896	1	pseudobulk	DESeq2	LRT		0
133	KC_AB2	Marcal1	1.5243995994652	0.741477491078038	1	pseudobulk	DESeq2	LRT		0
133	KC_AB2	Marf	-0.585069418237187	0.350299946494343	1	pseudobulk	DESeq2	LRT		0
133	KC_AB2	Marf1	-0.600547221779134	1	1	pseudobulk	DESeq2	LRT		0
133	KC_AB2	mask	-0.310995536580544	0.508913550290986	1	pseudobulk	DESeq2	LRT		0

133	KC_AB2	Mat1	-0.164156875967399	0.913361297797966	1	pseudobulk	DESeq2	LRT		0
133	KC_AB2	Mat89Ba	-1.45185768489783	0.445407259051521	1	pseudobulk	DESeq2	LRT		0
133	KC_AB2	mats	-0.63948267899906	0.580039254948089	1	pseudobulk	DESeq2	LRT		0
133	KC_AB2	Max	0.480382453293244	0.608102604195663	1	pseudobulk	DESeq2	LRT		0
133	KC_AB2	mbc	-1.42206995787549	0.146965117339167	1	pseudobulk	DESeq2	LRT		0
133	KC_AB2	MBD-like	-0.413378179052979	0.641399835788817	1	pseudobulk	DESeq2	LRT		0
133	KC_AB2	MBD-R2	0.678558942019409	0.609181116814196	1	pseudobulk	DESeq2	LRT		0
133	KC_AB2	mbf1	-0.463190455371089	0.417496482183785	1	pseudobulk	DESeq2	LRT		0
133	KC_AB2	mbl	-0.493357239699781	0.783807521297462	1	pseudobulk	DESeq2	LRT		0
133	KC_AB2	mbm	-0.427431139129811	1	1	pseudobulk	DESeq2	LRT		0
133	KC_AB2	mbo	-0.427431139129811	1	1	pseudobulk	DESeq2	LRT		0
133	KC_AB2	Mbs	-0.0208029147953396	0.972198654519593	1	pseudobulk	DESeq2	LRT		0
133	KC_AB2	mbt	0.283662283632936	0.734611935973805	1	pseudobulk	DESeq2	LRT		0
133	KC_AB2	Mccc1	-0.427431139129811	1	1	pseudobulk	DESeq2	LRT		0
133	KC_AB2	Mcr	-0.381022595176465	0.906491838131301	1	pseudobulk	DESeq2	LRT		0
133	KC_AB2	Mct1	0.713982351041985	0.921569637923553	1	pseudobulk	DESeq2	LRT		0
133	KC_AB2	Mctp	0.702648799769207	0.551967693452151	1	pseudobulk	DESeq2	LRT		0
133	KC_AB2	MCTS1	-0.600395529648141	0.474693132854307	1	pseudobulk	DESeq2	LRT		0
133	KC_AB2	MCU	0.224200267093338	0.775767128449566	1	pseudobulk	DESeq2	LRT		0
133	KC_AB2	Mdh1	0.191064673265439	0.810851953948891	1	pseudobulk	DESeq2	LRT		0
133	KC_AB2	Mdh2	-0.262660644981274	0.551718060802411	1	pseudobulk	DESeq2	LRT		0
133	KC_AB2	mdlc	2.1817927492653	0.458599861292202	1	pseudobulk	DESeq2	LRT		0
133	KC_AB2	me31B	-0.137686982380084	0.757072690763811	1	pseudobulk	DESeq2	LRT		0
133	KC_AB2	Med	-0.89791013962584	0.36113713978763	1	pseudobulk	DESeq2	LRT		0
133	KC_AB2	MED1	-0.129169751264268	0.819008942738722	1	pseudobulk	DESeq2	LRT		0
133	KC_AB2	MED10	0.767876690138535	0.477295248351988	1	pseudobulk	DESeq2	LRT		0
133	KC_AB2	MED11	0.0686595713970781	0.907631373090399	1	pseudobulk	DESeq2	LRT		0
133	KC_AB2	MED14	-0.518850182615239	0.662815356196604	1	pseudobulk	DESeq2	LRT		0
133	KC_AB2	MED15	-0.621805355124546	0.74877258313458	1	pseudobulk	DESeq2	LRT		0
133	KC_AB2	MED16	-1.12785316439435	0.661319045116135	1	pseudobulk	DESeq2	LRT		0
133	KC_AB2	MED17	-1.04445428854771	0.603034233836386	1	pseudobulk	DESeq2	LRT		0
133	KC_AB2	MED18	-1.36771957505847	0.50359885895216	1	pseudobulk	DESeq2	LRT		0
133	KC_AB2	MED19	0.132078006807538	0.85302397074508	1	pseudobulk	DESeq2	LRT		0
133	KC_AB2	MED20	0.365414834050204	0.83648747380843	1	pseudobulk	DESeq2	LRT		0
133	KC_AB2	MED21	-0.558174616540899	0.343440243983401	1	pseudobulk	DESeq2	LRT		0
133	KC_AB2	MED22	0.385232186715864	0.721851991576141	1	pseudobulk	DESeq2	LRT		0
133	KC_AB2	MED24	0.970722231051727	0.452699010682964	1	pseudobulk	DESeq2	LRT		0
133	KC_AB2	MED25	0.16620837009559	0.871696729574946	1	pseudobulk	DESeq2	LRT		0
133	KC_AB2	MED26	-0.540077085409921	0.269755097241767	1	pseudobulk	DESeq2	LRT		0
133	KC_AB2	MED27	1.08480356307325	0.564615346523986	1	pseudobulk	DESeq2	LRT		0
133	KC_AB2	MED28	-0.285256418039142	0.845250603657592	1	pseudobulk	DESeq2	LRT		0
133	KC_AB2	MED30	-0.455360268031223	0.811447118210737	1	pseudobulk	DESeq2	LRT		0
133	KC_AB2	MED31	-0.664796076574542	0.548460674423375	1	pseudobulk	DESeq2	LRT		0
133	KC_AB2	MED4	-0.930993964298746	0.559205071649537	1	pseudobulk	DESeq2	LRT		0
133	KC_AB2	MED6	-0.526634841516336	0.737873236698189	1	pseudobulk	DESeq2	LRT		0
134	KC_AB2	MED7	-0.244896684027766	0.834318868085478	1	pseudobulk	DESeq2	LRT		0
134	KC_AB2	MED8	-0.286308310121072	0.841262199863501	1	pseudobulk	DESeq2	LRT		0
134	KC_AB2	Mef2	0.182025195301277	0.449854059689629	1	pseudobulk	DESeq2	LRT		0
134	KC_AB2	mEFG1	-0.0852743682753834	0.917718671153184	1	pseudobulk	DESeq2	LRT		0
134	KC_AB2	mEFTs	-1.20939994045982	0.128981500480434	1	pseudobulk	DESeq2	LRT		0
134	KC_AB2	mEFTu1	-0.211777242518098	0.781546412488112	1	pseudobulk	DESeq2	LRT		0
134	KC_AB2	mEFTu2	1.94559924579109	0.42325307792885	1	pseudobulk	DESeq2	LRT		0
134	KC_AB2	Megf8	0.787572202125122	0.489326229691872	1	pseudobulk	DESeq2	LRT		0

134	KC_AB2	mei-9	-0.600547221779133	0.841940696994101	1	pseudobulk	DESeq2	LRT		0
134	KC_AB2	mei-P26	-0.0413794816520677	0.817731040633882	1	pseudobulk	DESeq2	LRT		0
134	KC_AB2	mei-S332	1.5243995994652	0.741477491078038	1	pseudobulk	DESeq2	LRT		0
134	KC_AB2	mei-W68	2.12684015898615	0.445939223035924	1	pseudobulk	DESeq2	LRT		0
134	KC_AB2	Meics	3.13444977473788	0.190512924459537	1	pseudobulk	DESeq2	LRT		0
134	KC_AB2	meigo	0.864341462967788	0.925947094335722	1	pseudobulk	DESeq2	LRT		0
134	KC_AB2	Mekk1	0.72945670119806	0.577941880761715	1	pseudobulk	DESeq2	LRT		0
134	KC_AB2	Membrin	-0.843996296624293	0.67626831722335	1	pseudobulk	DESeq2	LRT		0
134	KC_AB2	Men	-0.234515718328375	0.789550334968822	1	pseudobulk	DESeq2	LRT		0
134	KC_AB2	Men-b	0.156716407449142	0.688960847313694	1	pseudobulk	DESeq2	LRT		0
134	KC_AB2	meng	-0.6342012729314	0.545556343477092	1	pseudobulk	DESeq2	LRT		0
134	KC_AB2	MEP-1	1.05589189169086	0.480639433559118	1	pseudobulk	DESeq2	LRT		0
134	KC_AB2	Mer	1.2224385214167	0.489015734827038	1	pseudobulk	DESeq2	LRT		0
134	KC_AB2	Mes4	-0.434057374494302	0.709511973043299	1	pseudobulk	DESeq2	LRT		0
134	KC_AB2	MESK2	-0.309331662158679	0.345621098563364	1	pseudobulk	DESeq2	LRT		0
134	KC_AB2	MESR3	2.70206512243748	0.189689513649223	1	pseudobulk	DESeq2	LRT		0
134	KC_AB2	MESR4	-2.89930553040925	0.0713499585686287	1	pseudobulk	DESeq2	LRT		0
134	KC_AB2	MESR6	-0.841882716738579	1	1	pseudobulk	DESeq2	LRT		0
134	KC_AB2	Met	-0.419977648489968	0.589609947011513	1	pseudobulk	DESeq2	LRT		0
134	KC_AB2	metl	-0.820663486926854	0.550461150069302	1	pseudobulk	DESeq2	LRT		0
134	KC_AB2	metro	0.335069082276988	0.416869539076726	1	pseudobulk	DESeq2	LRT		0
134	KC_AB2	MetRS	0.522146876767621	0.704558696720362	1	pseudobulk	DESeq2	LRT		0
134	KC_AB2	MetRS-m	0.0163320842711025	1	1	pseudobulk	DESeq2	LRT		0
134	KC_AB2	Mettl14	-0.600547221779133	0.841940696994101	1	pseudobulk	DESeq2	LRT		0
134	KC_AB2	Mettl3	-0.209808076863057	0.877444409505505	1	pseudobulk	DESeq2	LRT		0
134	KC_AB2	mew	-1.72451398333654	0.381278133120475	1	pseudobulk	DESeq2	LRT		0
134	KC_AB2	mex1	-0.592481036256484	0.706847545932384	1	pseudobulk	DESeq2	LRT		0
134	KC_AB2	Mf	0.498593948417392	0.805619674491474	1	pseudobulk	DESeq2	LRT		0
134	KC_AB2	Mfap1	-0.919308131532992	0.540096183948873	1	pseudobulk	DESeq2	LRT		0
134	KC_AB2	Mfe2	0.669718686959781	0.744188515542868	1	pseudobulk	DESeq2	LRT		0
134	KC_AB2	mfrn	0.309463605212933	0.765214482773298	1	pseudobulk	DESeq2	LRT		0
134	KC_AB2	MFS10	1.62589665779324	0.261782883425198	1	pseudobulk	DESeq2	LRT		0
134	KC_AB2	MFS15	-1.33513207944511	0.415043438941844	1	pseudobulk	DESeq2	LRT		0
134	KC_AB2	MFS16	0.864341462967788	0.925947094335722	1	pseudobulk	DESeq2	LRT		0
134	KC_AB2	MFS17	-0.0008274970777940	0.988360690711738	1	pseudobulk	DESeq2	LRT		0
134	KC_AB2	MFS18	-2.22993811639915	0.206376315490151	1	pseudobulk	DESeq2	LRT		0
134	KC_AB2	MFS9	-1.07776915880798	0.775491075088671	1	pseudobulk	DESeq2	LRT		0
134	KC_AB2	Mgat1	-0.90686020782017	0.584252301074776	1	pseudobulk	DESeq2	LRT		0
134	KC_AB2	Mgat2	0.27407999832563	0.733817579112529	1	pseudobulk	DESeq2	LRT		0
134	KC_AB2	mge	-0.618551796144127	0.244533465803221	1	pseudobulk	DESeq2	LRT		0
134	KC_AB2	mgl	-1.33596340965229	0.0490426139854694	1	pseudobulk	DESeq2	LRT		0
134	KC_AB2	mGluR	-2.76454913147266	0.219782699481157	1	pseudobulk	DESeq2	LRT		0
134	KC_AB2	mgr	-0.883767462251061	0.43391564657364	1	pseudobulk	DESeq2	LRT		0
134	KC_AB2	MgstI	-0.382929749762112	0.851961810578875	1	pseudobulk	DESeq2	LRT		0
134	KC_AB2	MhcI	-0.0365499604749836	0.94901219410694	1	pseudobulk	DESeq2	LRT		0
134	KC_AB2	mi	0.205070454022938	0.807210387142747	1	pseudobulk	DESeq2	LRT		0
134	KC_AB2	Mi-2	0.171270009110499	0.73551440102364	1	pseudobulk	DESeq2	LRT		0
134	KC_AB2	mib1	-0.329892833217003	0.817835057965816	1	pseudobulk	DESeq2	LRT		0
134	KC_AB2	mib2	1.49348098191083	0.123503521094681	1	pseudobulk	DESeq2	LRT		0
134	KC_AB2	Mical	-0.154171177797893	0.86132040797713	1	pseudobulk	DESeq2	LRT		0
134	KC_AB2	MICAL-like	0.522867664761254	0.789441383158121	1	pseudobulk	DESeq2	LRT		0
134	KC_AB2	MICU1	0.215434014383363	0.863331331771034	1	pseudobulk	DESeq2	LRT		0
134	KC_AB2	MICU3	-0.283114926521931	0.722414891486832	1	pseudobulk	DESeq2	LRT		0

134	KC_AB2	Mid1	0.179892430220306	0.597221138381754	1	pseudobulk	DESeq2	LRT		0
134	KC_AB2	mIF3	0.356878814236573	0.847328447129494	1	pseudobulk	DESeq2	LRT		0
134	KC_AB2	Miga	-0.224138313958674	0.823500308383405	1	pseudobulk	DESeq2	LRT		0
134	KC_AB2	milt	0.396747651925421	0.480015237004628	1	pseudobulk	DESeq2	LRT		0
134	KC_AB2	mim	-0.768605432721702	0.153746769860779	1	pseudobulk	DESeq2	LRT		0
134	KC_AB2	mio	1.39827770914961	0.587246212652858	1	pseudobulk	DESeq2	LRT		0
134	KC_AB2	mip120	0.554639993769006	0.74261267437262	1	pseudobulk	DESeq2	LRT		0
134	KC_AB2	mip130	-1.43450874381432	0.6168250034656	1	pseudobulk	DESeq2	LRT		0
134	KC_AB2	mip40	0.0134578055717764	0.876588281214639	1	pseudobulk	DESeq2	LRT		0
134	KC_AB2	miple1	-0.138691555454314	0.562535017236617	1	pseudobulk	DESeq2	LRT		0
134	KC_AB2	miple2	0.652091318848626	0.725625206502254	1	pseudobulk	DESeq2	LRT		0
134	KC_AB2	Mipp2	1.5243995994652	0.741477491078038	1	pseudobulk	DESeq2	LRT		0
134	KC_AB2	Miro	0.786721545474008	0.53333493756481	1	pseudobulk	DESeq2	LRT		0
134	KC_AB2	Mis12	-1.94046878246228	0.29906775385933	1	pseudobulk	DESeq2	LRT		0
134	KC_AB2	Mitf	0.0411987503127017	0.971889220468077	1	pseudobulk	DESeq2	LRT		0
134	KC_AB2	Mitofilin	0.121784444298643	0.820906207755469	1	pseudobulk	DESeq2	LRT		0
134	KC_AB2	Mkk4	-0.809624884086304	0.323111653680469	1	pseudobulk	DESeq2	LRT		0
134	KC_AB2	MKP-4	0.328107180976296	0.745075903140054	1	pseudobulk	DESeq2	LRT		0
134	KC_AB2	Mkp3	-0.361798167207617	0.171904642464295	1	pseudobulk	DESeq2	LRT		0
134	KC_AB2	Mkrn1	0.512296370759952	0.574559913488771	1	pseudobulk	DESeq2	LRT		0
134	KC_AB2	Mlc-c	0.483311233125768	0.654420273164299	1	pseudobulk	DESeq2	LRT		0
134	KC_AB2	mld	1.0480288610975	0.145437269209303	1	pseudobulk	DESeq2	LRT		0
134	KC_AB2	mldr	-0.427431139129811	1	1	pseudobulk	DESeq2	LRT		0
134	KC_AB2	mle	-0.265131337090123	0.693787077094237	1	pseudobulk	DESeq2	LRT		0
134	KC_AB2	Mlf	0.130682308570667	0.72252867356888	1	pseudobulk	DESeq2	LRT		0
134	KC_AB2	Mlp84B	-2.02285942584217	0.357240028002837	1	pseudobulk	DESeq2	LRT		0
134	KC_AB2	mlt	0.15497228959516	0.824123674930366	1	pseudobulk	DESeq2	LRT		0
134	KC_AB2	mmd	0.119374855270968	0.61265895164472	1	pseudobulk	DESeq2	LRT		0
134	KC_AB2	MME1	1.5243995994652	0.741477491078038	1	pseudobulk	DESeq2	LRT		0
134	KC_AB2	Mmp1	-0.592481036256484	0.706847545932384	1	pseudobulk	DESeq2	LRT		0
134	KC_AB2	Mmp2	0.657450125667262	0.110520893730966	1	pseudobulk	DESeq2	LRT		0
134	KC_AB2	Mms19	0.183555857030446	1	1	pseudobulk	DESeq2	LRT		0
134	KC_AB2	mmy	0.0210680144448556	0.977070292138014	1	pseudobulk	DESeq2	LRT		0
134	KC_AB2	mnb	0.692002142250277	0.258346485442666	1	pseudobulk	DESeq2	LRT		0
134	KC_AB2	mnd	1.40241945196173	0.57265124481703	1	pseudobulk	DESeq2	LRT		0
134	KC_AB2	Mnn1	-0.370296746720964	0.288059301470213	1	pseudobulk	DESeq2	LRT		0
134	KC_AB2	Mnt	0.545098747949602	0.213459368079939	1	pseudobulk	DESeq2	LRT		0
134	KC_AB2	Mo25	-0.376700703915586	0.66180721937486	1	pseudobulk	DESeq2	LRT		0
134	KC_AB2	Mob2	0.301885599820182	0.354324390391346	1	pseudobulk	DESeq2	LRT		0
135	KC_AB2	Mob3	0.47044400423197	0.690501138933685	1	pseudobulk	DESeq2	LRT		0
135	KC_AB2	Mob4	0.103961484003466	0.848424513071568	1	pseudobulk	DESeq2	LRT		0
135	KC_AB2	Moca-cyp	0.558835348694096	0.512309147894169	1	pseudobulk	DESeq2	LRT		0
135	KC_AB2	mod	-0.111419678928907	0.869601204143719	1	pseudobulk	DESeq2	LRT		0
135	KC_AB2	Moe	-0.086201983814234	0.823656104556311	1	pseudobulk	DESeq2	LRT		0
135	KC_AB2	mof	0.346589616049749	0.84654468695694	1	pseudobulk	DESeq2	LRT		0
135	KC_AB2	Mon1	-1.60499793386288	0.434358912762731	1	pseudobulk	DESeq2	LRT		0
135	KC_AB2	mon2	-0.873181008678992	0.49642904553292	1	pseudobulk	DESeq2	LRT		0
135	KC_AB2	Mondo	0.827780853931199	0.521162245217912	1	pseudobulk	DESeq2	LRT		0
135	KC_AB2	moody	-0.600547221779134	1	1	pseudobulk	DESeq2	LRT		0
135	KC_AB2	mop	-1.5962074891574	0.297694319415404	1	pseudobulk	DESeq2	LRT		0
135	KC_AB2	mor	-0.147993678385652	0.883412024001724	1	pseudobulk	DESeq2	LRT		0
135	KC_AB2	morgue	0.944456141247415	0.433113269654549	1	pseudobulk	DESeq2	LRT		0
135	KC_AB2	Mp	0.14777998531672	0.681134390386957	1	pseudobulk	DESeq2	LRT		0

135	KC_AB2	MP1	-0.600547221779134	1	1	pseudobulk	DESeq2	LRT		0
135	KC_AB2	Mpc1	0.873593731713	0.400793281044873	1	pseudobulk	DESeq2	LRT		0
135	KC_AB2	Mpcp2	-0.214102391774068	0.477836662455624	1	pseudobulk	DESeq2	LRT		0
135	KC_AB2	Mpi	0.522867664761254	0.789441383158121	1	pseudobulk	DESeq2	LRT		0
135	KC_AB2	Mpp6	-2.11277905327326	0.262770194613923	1	pseudobulk	DESeq2	LRT		0
135	KC_AB2	Mppe	0.222396124134733	1	1	pseudobulk	DESeq2	LRT		0
135	KC_AB2	mr	1.22036108458351	0.522938943418067	1	pseudobulk	DESeq2	LRT		0
135	KC_AB2	mre11	1.25230092358671	0.476991461296781	1	pseudobulk	DESeq2	LRT		0
135	KC_AB2	mRF1	-1.07776915880798	0.775491075088671	1	pseudobulk	DESeq2	LRT		0
135	KC_AB2	MRG15	-0.840907844409189	0.510346442813579	1	pseudobulk	DESeq2	LRT		0
135	KC_AB2	MrgBP	-0.56746246236304	0.50676974769889	1	pseudobulk	DESeq2	LRT		0
135	KC_AB2	mri	1.13640606304527	0.325477501449365	1	pseudobulk	DESeq2	LRT		0
135	KC_AB2	mrj	0.114174385360425	0.797592172710422	1	pseudobulk	DESeq2	LRT		0
135	KC_AB2	mrn	-1.3723417613553	0.619532036522	1	pseudobulk	DESeq2	LRT		0
135	KC_AB2	mRNA-cap	-0.500194289414117	0.663731419879204	1	pseudobulk	DESeq2	LRT		0
135	KC_AB2	MRP	0.015571344034631	0.887476513364654	1	pseudobulk	DESeq2	LRT		0
135	KC_AB2	Mrp4	1.23085716405798	0.614623732585597	1	pseudobulk	DESeq2	LRT		0
135	KC_AB2	mRpL1	0.278315791291818	0.795036033031111	1	pseudobulk	DESeq2	LRT		0
135	KC_AB2	mRpL10	0.702881756797625	0.522943984566964	1	pseudobulk	DESeq2	LRT		0
135	KC_AB2	mRpL11	-0.678490890316833	0.616400682332678	1	pseudobulk	DESeq2	LRT		0
135	KC_AB2	mRpL12	-0.0667597502839618	0.911936880827519	1	pseudobulk	DESeq2	LRT		0
135	KC_AB2	mRpL13	-2.66874423905794	0.10991722734842	1	pseudobulk	DESeq2	LRT		0
135	KC_AB2	mRpL14	0.220861813392571	0.886247699003982	1	pseudobulk	DESeq2	LRT		0
135	KC_AB2	mRpL15	0.285087501243503	0.810731832073941	1	pseudobulk	DESeq2	LRT		0
135	KC_AB2	mRpL16	-0.187862592583862	0.793851680103338	1	pseudobulk	DESeq2	LRT		0
135	KC_AB2	mRpL17	-0.0352321977114399	0.959555459810122	1	pseudobulk	DESeq2	LRT		0
135	KC_AB2	mRpL18	-1.13852612675049	0.521328413681372	1	pseudobulk	DESeq2	LRT		0
135	KC_AB2	mRpL19	-0.494818389228842	0.729404113179771	1	pseudobulk	DESeq2	LRT		0
135	KC_AB2	mRpL2	0.388044739521178	0.724978328344578	1	pseudobulk	DESeq2	LRT		0
135	KC_AB2	mRpL20	1.5312126991267	0.288078541523957	1	pseudobulk	DESeq2	LRT		0
135	KC_AB2	mRpL21	-0.398178175112781	0.703390654914779	1	pseudobulk	DESeq2	LRT		0
135	KC_AB2	mRpL22	-0.362740182550431	0.712888023420834	1	pseudobulk	DESeq2	LRT		0
135	KC_AB2	mRpL23	-0.416793263886129	0.690263525636451	1	pseudobulk	DESeq2	LRT		0
135	KC_AB2	mRpL24	-0.666929281218115	0.42189295081689	1	pseudobulk	DESeq2	LRT		0
135	KC_AB2	mRpL27	-0.894614181606271	0.442586075483094	1	pseudobulk	DESeq2	LRT		0
135	KC_AB2	mRpL28	0.232632893942468	0.807259725135417	1	pseudobulk	DESeq2	LRT		0
135	KC_AB2	mRpL3	0.427408177268532	0.783010727256973	1	pseudobulk	DESeq2	LRT		0
135	KC_AB2	mRpL30	1.8937289938237	0.0984465827287656	1	pseudobulk	DESeq2	LRT		0
135	KC_AB2	mRpL32	-2.81801801860298	0.185041356891099	1	pseudobulk	DESeq2	LRT		0
135	KC_AB2	mRpL33	-0.691955717655139	0.366131194100861	1	pseudobulk	DESeq2	LRT		0
135	KC_AB2	mRpL34	-1.04892759016556	0.275790801074885	1	pseudobulk	DESeq2	LRT		0
135	KC_AB2	mRpL35	0.990611593100534	0.282119743528764	1	pseudobulk	DESeq2	LRT		0
135	KC_AB2	mRpL36	0.397238939072719	0.673217278075783	1	pseudobulk	DESeq2	LRT		0
135	KC_AB2	mRpL37	-0.831304672821689	0.614141931635772	1	pseudobulk	DESeq2	LRT		0
135	KC_AB2	mRpL38	-1.66844842576351	0.379982267319401	1	pseudobulk	DESeq2	LRT		0
135	KC_AB2	mRpL39	1.22608028368917	0.398389874370337	1	pseudobulk	DESeq2	LRT		0
135	KC_AB2	mRpL4	0.0186816764350967	0.955880557580993	1	pseudobulk	DESeq2	LRT		0
135	KC_AB2	mRpL40	1.86965641993578	0.214152286337574	1	pseudobulk	DESeq2	LRT		0
135	KC_AB2	mRpL41	-1.4578055514909	0.26091616130325	1	pseudobulk	DESeq2	LRT		0
135	KC_AB2	mRpL42	-0.198631420284981	0.829646901428885	1	pseudobulk	DESeq2	LRT		0
135	KC_AB2	mRpL43	1.20017382039488	0.348668961813694	1	pseudobulk	DESeq2	LRT		0
135	KC_AB2	mRpL44	-0.0927107588322969	0.92460955304492	1	pseudobulk	DESeq2	LRT		0
135	KC_AB2	mRpL45	-0.187715450427201	0.902906610282683	1	pseudobulk	DESeq2	LRT		0

135	KC_AB2	mRpL46	-1.58845913170859	0.408579551508681	1	pseudobulk	DESeq2	LRT	0
135	KC_AB2	mRpL47	-0.494747959441996	0.617644613487054	1	pseudobulk	DESeq2	LRT	0
135	KC_AB2	mRpL48	-1.18497645265394	0.163875621239897	1	pseudobulk	DESeq2	LRT	0
135	KC_AB2	mRpL49	0.447982566048601	0.641342486557856	1	pseudobulk	DESeq2	LRT	0
135	KC_AB2	mRpL50	1.14759657771276	0.335310606805059	1	pseudobulk	DESeq2	LRT	0
135	KC_AB2	mRpL51	-1.07308208295601	0.580312703940698	1	pseudobulk	DESeq2	LRT	0
135	KC_AB2	mRpL52	-1.39821263454541	0.35038876373669	1	pseudobulk	DESeq2	LRT	0
135	KC_AB2	mRpL54	-2.75272692798437	0.09552204451794	1	pseudobulk	DESeq2	LRT	0
135	KC_AB2	mRpL55	-1.14610452695596	0.134498265707443	1	pseudobulk	DESeq2	LRT	0
135	KC_AB2	mRpL9	-1.58613947610827	0.31636484042437	1	pseudobulk	DESeq2	LRT	0
135	KC_AB2	mRpS10	0.495185670953266	0.610619411947877	1	pseudobulk	DESeq2	LRT	0
135	KC_AB2	mRpS11	0.433783814367627	0.630510015092203	1	pseudobulk	DESeq2	LRT	0
135	KC_AB2	mRpS14	-0.923872600884323	0.461542171839644	1	pseudobulk	DESeq2	LRT	0
135	KC_AB2	mRpS16	-1.06207491674101	0.285074262746268	1	pseudobulk	DESeq2	LRT	0
135	KC_AB2	mRpS17	-0.165976136383899	0.918001317430575	1	pseudobulk	DESeq2	LRT	0
135	KC_AB2	mRpS18A	-0.816709010482543	0.501135512491443	1	pseudobulk	DESeq2	LRT	0
135	KC_AB2	mRpS18B	-0.493357239699781	0.783807521297462	1	pseudobulk	DESeq2	LRT	0
135	KC_AB2	mRpS18C	1.52087502197186	0.161216747640101	1	pseudobulk	DESeq2	LRT	0
135	KC_AB2	mRpS2	0.178361519141227	0.895433560431735	1	pseudobulk	DESeq2	LRT	0
135	KC_AB2	mRpS21	0.734069704290704	0.607245892089807	1	pseudobulk	DESeq2	LRT	0
135	KC_AB2	mRpS22	2.70206512243748	0.189689513649223	1	pseudobulk	DESeq2	LRT	0
135	KC_AB2	mRpS23	-1.40939030311964	0.303692686841006	1	pseudobulk	DESeq2	LRT	0
135	KC_AB2	mRpS24	0.0146196520177219	0.831533919136137	1	pseudobulk	DESeq2	LRT	0
135	KC_AB2	mRpS25	-0.413836594337482	0.719590964767333	1	pseudobulk	DESeq2	LRT	0
135	KC_AB2	mRpS26	-1.47347959343745	0.347055402000811	1	pseudobulk	DESeq2	LRT	0
135	KC_AB2	mRpS28	0.100360153279958	0.938844530780903	1	pseudobulk	DESeq2	LRT	0
135	KC_AB2	mRpS29	-0.172929640286733	0.847999519004836	1	pseudobulk	DESeq2	LRT	0
135	KC_AB2	mRpS30	-2.17489218640147	0.354960305801642	1	pseudobulk	DESeq2	LRT	0
135	KC_AB2	mRpS31	-0.720805124487304	0.630409341769778	1	pseudobulk	DESeq2	LRT	0
135	KC_AB2	mRpS33	-1.3247619863844	0.253979427791386	1	pseudobulk	DESeq2	LRT	0
135	KC_AB2	mRpS34	-2.40086906442185	0.153166831774015	1	pseudobulk	DESeq2	LRT	0
135	KC_AB2	mRpS35	-0.163479557912485	0.8110095872151	1	pseudobulk	DESeq2	LRT	0
135	KC_AB2	mRpS5	0.0366452328433107	0.872993639005023	1	pseudobulk	DESeq2	LRT	0
136	KC_AB2	mRpS6	-1.13926358083461	0.342310935830609	1	pseudobulk	DESeq2	LRT	0
136	KC_AB2	mRpS7	-0.988717747414914	0.407354664731213	1	pseudobulk	DESeq2	LRT	0
136	KC_AB2	mRpS9	0.0181111506852413	0.890719139095754	1	pseudobulk	DESeq2	LRT	0
136	KC_AB2	mRRF1	-1.99748597705924	0.407706590597114	1	pseudobulk	DESeq2	LRT	0
136	KC_AB2	mRRF2	-0.600547221779134	1	1	pseudobulk	DESeq2	LRT	0
136	KC_AB2	mrt	0.190621536209891	0.801748783248777	1	pseudobulk	DESeq2	LRT	0
136	KC_AB2	Mrtf	0.922261158662684	0.28388454926008	1	pseudobulk	DESeq2	LRT	0
136	KC_AB2	mrva	-0.0166377257629695	0.878260285357242	1	pseudobulk	DESeq2	LRT	0
136	KC_AB2	MSBP	-0.0615260645013771	0.755994633669946	1	pseudobulk	DESeq2	LRT	0
136	KC_AB2	Msh6	1.5243995994652	0.741477491078038	1	pseudobulk	DESeq2	LRT	0
136	KC_AB2	msi	-0.665528308711542	0.890090109320244	1	pseudobulk	DESeq2	LRT	0
136	KC_AB2	msk	1.10266287134534	0.395557708772626	1	pseudobulk	DESeq2	LRT	0
136	KC_AB2	msl-1	-0.284629840655205	0.81493874701301	1	pseudobulk	DESeq2	LRT	0
136	KC_AB2	msl-2	0.701502479899559	0.628925317970038	1	pseudobulk	DESeq2	LRT	0
136	KC_AB2	msl-3	-1.40673172715063	0.587364787535786	1	pseudobulk	DESeq2	LRT	0
136	KC_AB2	msn	0.0350964133794572	0.884393849248045	1	pseudobulk	DESeq2	LRT	0
136	KC_AB2	Msp300	0.379477187371622	0.133187497524548	1	pseudobulk	DESeq2	LRT	0
136	KC_AB2	mspo	-0.0931646002557316	0.766602070383828	1	pseudobulk	DESeq2	LRT	0
136	KC_AB2	msps	-0.064392725441588	0.92766387425073	1	pseudobulk	DESeq2	LRT	0
136	KC_AB2	Msr-110	-0.841882716738579	1	1	pseudobulk	DESeq2	LRT	0

136	KC_AB2	Mst36Fb	-0.427431139129811	1	1	pseudobulk	DESeq2	LRT	0
136	KC_AB2	Mst77F	0.0917494523913061	0.948623987030242	1	pseudobulk	DESeq2	LRT	0
136	KC_AB2	Mst85C	-1.80106903453409	0.373638451173457	1	pseudobulk	DESeq2	LRT	0
136	KC_AB2	mt:ATPase6	0.0562445670488656	0.863570628077936	1	pseudobulk	DESeq2	LRT	0
136	KC_AB2	mt:ATPase8	-1.67369493986616	0.40165972659436	1	pseudobulk	DESeq2	LRT	0
136	KC_AB2	mt:CoI	0.359913623872222	0.282820900190723	1	pseudobulk	DESeq2	LRT	0
136	KC_AB2	mt:CoII	-0.174111819505498	0.593963716583949	1	pseudobulk	DESeq2	LRT	0
136	KC_AB2	mt:CoIII	0.265407792986356	0.379984557198956	1	pseudobulk	DESeq2	LRT	0
136	KC_AB2	mt:Cyt-b	0.525196100140983	0.193928719889304	1	pseudobulk	DESeq2	LRT	0
136	KC_AB2	mt:lrRNA	0.208025491116418	0.257628186906285	1	pseudobulk	DESeq2	LRT	0
136	KC_AB2	mt:ND1	0.051238067889074	0.786986582187324	1	pseudobulk	DESeq2	LRT	0
136	KC_AB2	mt:ND2	-0.0970721526331735	0.799907016746944	1	pseudobulk	DESeq2	LRT	0
136	KC_AB2	mt:ND3	-0.147097869205307	0.792235680753718	1	pseudobulk	DESeq2	LRT	0
136	KC_AB2	mt:ND4	-0.140880434120005	0.70621637034253	1	pseudobulk	DESeq2	LRT	0
136	KC_AB2	mt:ND4L	-0.427431139129811	1	1	pseudobulk	DESeq2	LRT	0
136	KC_AB2	mt:ND5	-0.0420376389213594	0.773825084055618	1	pseudobulk	DESeq2	LRT	0
136	KC_AB2	mt:ND6	0.68118912326298	0.224487769677221	1	pseudobulk	DESeq2	LRT	0
136	KC_AB2	mt:srRNA	0.234640354893732	0.655266488774922	1	pseudobulk	DESeq2	LRT	0
136	KC_AB2	mt:tRNA:Ala-TGC	1.40241945196173	0.57265124481703	1	pseudobulk	DESeq2	LRT	0
136	KC_AB2	MTA1-like	0.413437168155649	0.665894873232463	1	pseudobulk	DESeq2	LRT	0
136	KC_AB2	Mtap	0.293291082179477	0.808821358193447	1	pseudobulk	DESeq2	LRT	0
136	KC_AB2	Mtch	-0.772516061261533	0.290554050791161	1	pseudobulk	DESeq2	LRT	0
136	KC_AB2	mtd	-0.0019019783705710	0.992346209586096	1	pseudobulk	DESeq2	LRT	0
136	KC_AB2	mTerf3	-2.51763403918309	0.148414412721342	1	pseudobulk	DESeq2	LRT	0
136	KC_AB2	mTerf5	-0.539665365830432	0.805145917072468	1	pseudobulk	DESeq2	LRT	0
136	KC_AB2	MTF-1	-0.66365663963907	0.71350233507668	1	pseudobulk	DESeq2	LRT	0
136	KC_AB2	mtg	-0.427431139129811	1	1	pseudobulk	DESeq2	LRT	0
136	KC_AB2	mtgo	-0.503085148519203	0.126103494597734	1	pseudobulk	DESeq2	LRT	0
136	KC_AB2	mthl11	-0.427431139129811	1	1	pseudobulk	DESeq2	LRT	0
136	KC_AB2	mthl13	-0.427431139129811	1	1	pseudobulk	DESeq2	LRT	0
136	KC_AB2	mthl14	-0.427431139129811	1	1	pseudobulk	DESeq2	LRT	0
136	KC_AB2	mthl8	3.17195708651769	0.15530090920866	1	pseudobulk	DESeq2	LRT	0
136	KC_AB2	Mtl	-1.02733576719822	0.542948041972163	1	pseudobulk	DESeq2	LRT	0
136	KC_AB2	mtm	-1.73514905896867	0.352556377341915	1	pseudobulk	DESeq2	LRT	0
136	KC_AB2	MtnA	0.509624438896496	0.676940047412069	1	pseudobulk	DESeq2	LRT	0
136	KC_AB2	MtnB	-0.600547221779133	0.841940696994101	1	pseudobulk	DESeq2	LRT	0
136	KC_AB2	MtnE	-0.427431139129811	1	1	pseudobulk	DESeq2	LRT	0
136	KC_AB2	Mtor	1.47129758695777	0.269798101882687	1	pseudobulk	DESeq2	LRT	0
136	KC_AB2	Mtp	-0.600547221779134	1	1	pseudobulk	DESeq2	LRT	0
136	KC_AB2	Mtpalpha	-0.0745492687879989	0.920659452974254	1	pseudobulk	DESeq2	LRT	0
136	KC_AB2	MTPAP	0.613659617664411	0.76686169970757	1	pseudobulk	DESeq2	LRT	0
136	KC_AB2	Mtpbeta	-1.94180428862563	0.287963983973738	1	pseudobulk	DESeq2	LRT	0
136	KC_AB2	Mtr3	0.70378358684898	0.932301451973468	1	pseudobulk	DESeq2	LRT	0
136	KC_AB2	Mtr4	-0.0852743682753834	0.917718671153184	1	pseudobulk	DESeq2	LRT	0
136	KC_AB2	mtRNApol	-0.825973974745624	0.678387419789199	1	pseudobulk	DESeq2	LRT	0
136	KC_AB2	mts	-0.183741823832172	0.515778209059599	1	pseudobulk	DESeq2	LRT	0
136	KC_AB2	mtSSB	-2.25375902476868	0.307909557410438	1	pseudobulk	DESeq2	LRT	0
136	KC_AB2	mtt	0.683096823716164	0.441995550302961	1	pseudobulk	DESeq2	LRT	0
136	KC_AB2	mTTF	-0.938817101015206	0.629705053774855	1	pseudobulk	DESeq2	LRT	0
136	KC_AB2	mub	0.11558400089974	0.528832829116477	1	pseudobulk	DESeq2	LRT	0
136	KC_AB2	muc	-0.114734722271535	0.797176093675704	1	pseudobulk	DESeq2	LRT	0
136	KC_AB2	Mul1	-0.427431139129811	1	1	pseudobulk	DESeq2	LRT	0
136	KC_AB2	Mulk	-1.44810186689952	0.454949999368433	1	pseudobulk	DESeq2	LRT	0

136	KC_AB2	Mur2B	-0.128546610744038	0.86236999898321	1	pseudobulk	DESeq2	LRT		0
136	KC_AB2	mura	-0.0430884568035195	0.88786171981659	1	pseudobulk	DESeq2	LRT		0
136	KC_AB2	mus201	2.1817927492653	0.458599861292202	1	pseudobulk	DESeq2	LRT		0
136	KC_AB2	mus304	1.5243995994652	0.741477491078038	1	pseudobulk	DESeq2	LRT		0
136	KC_AB2	muskelin	-0.600547221779134	1	1	pseudobulk	DESeq2	LRT		0
136	KC_AB2	mute	-1.85733156562156	0.448702818121367	1	pseudobulk	DESeq2	LRT		0
136	KC_AB2	mv	0.920212920242661	0.67286879185691	1	pseudobulk	DESeq2	LRT		0
136	KC_AB2	Mvb12	-0.921299111484392	0.592147118101527	1	pseudobulk	DESeq2	LRT		0
136	KC_AB2	Mvd	-0.747852219079764	0.708491663089672	1	pseudobulk	DESeq2	LRT		0
136	KC_AB2	Mvl	-0.753328766976502	0.600098875330801	1	pseudobulk	DESeq2	LRT		0
136	KC_AB2	mxc	0.573760393110584	0.792134620722927	1	pseudobulk	DESeq2	LRT		0
136	KC_AB2	mxt	0.229851567609132	0.8230718080813	1	pseudobulk	DESeq2	LRT		0
136	KC_AB2	Myb	-1.51928970377954	0.486908946651791	1	pseudobulk	DESeq2	LRT		0
136	KC_AB2	Mybbp1A	1.21092382796727	0.637125262974216	1	pseudobulk	DESeq2	LRT		0
136	KC_AB2	Myc	1.10972745887804	0.348526004534859	1	pseudobulk	DESeq2	LRT		0
136	KC_AB2	myo	-2.06407076736304	0.362444518317458	1	pseudobulk	DESeq2	LRT		0
136	KC_AB2	Myo28B1	-1.50114091429564	0.333402527131703	1	pseudobulk	DESeq2	LRT		0
136	KC_AB2	Myo31DF	-1.51928970377954	0.486908946651791	1	pseudobulk	DESeq2	LRT		0
136	KC_AB2	Myo81F	0.242823216367157	0.728447310341214	1	pseudobulk	DESeq2	LRT		0
136	KC_AB2	Myo95E	-0.312496502419637	0.790851861748556	1	pseudobulk	DESeq2	LRT		0
136	KC_AB2	MYPT-75D	-0.929356063124713	0.0445769475409934	1	pseudobulk	DESeq2	LRT		0
136	KC_AB2	mys	0.225910848061325	0.717864331824292	1	pseudobulk	DESeq2	LRT		0
136	KC_AB2	Mys45A	-0.802510175100082	0.722476174078508	1	pseudobulk	DESeq2	LRT		0
136	KC_AB2	Myt1	0.222396124134733	1	1	pseudobulk	DESeq2	LRT		0
136	KC_AB2	N	-0.600547221779133	0.841940696994101	1	pseudobulk	DESeq2	LRT		0
136	KC_AB2	na	0.581414406708992	0.40512451009484	1	pseudobulk	DESeq2	LRT		0
136	KC_AB2	Naa15-16	0.25896007168824	0.8362656789564	1	pseudobulk	DESeq2	LRT		0
137	KC_AB2	Naa20A	-2.36253749349733	0.180207496996792	1	pseudobulk	DESeq2	LRT		0
137	KC_AB2	Naa30A	0.591423740465911	0.488212322070693	1	pseudobulk	DESeq2	LRT		0
137	KC_AB2	Naa35	0.84640264542371	0.530759930553588	1	pseudobulk	DESeq2	LRT		0
137	KC_AB2	Naa60	-1.79169692014535	0.135557414827749	1	pseudobulk	DESeq2	LRT		0
137	KC_AB2	Naa80	-1.23328997901577	0.274138862996637	1	pseudobulk	DESeq2	LRT		0
137	KC_AB2	Nab2	-0.902515971313935	0.6111166515708	1	pseudobulk	DESeq2	LRT		0
137	KC_AB2	Nacalpha	-0.2180127413136	0.509339328869546	1	pseudobulk	DESeq2	LRT		0
137	KC_AB2	nAChRalpha1	0.366101287566174	0.283921339565668	1	pseudobulk	DESeq2	LRT		0
137	KC_AB2	nAChRalpha2	-0.956495114100261	0.179173461707418	1	pseudobulk	DESeq2	LRT		0
137	KC_AB2	nAChRalpha3	0.521875580198549	0.642830423379037	1	pseudobulk	DESeq2	LRT		0
137	KC_AB2	nAChRalpha4	-0.231280669639946	0.603612220035769	1	pseudobulk	DESeq2	LRT		0
137	KC_AB2	nAChRalpha5	-0.0851192475958414	0.723342535183449	1	pseudobulk	DESeq2	LRT		0
137	KC_AB2	nAChRalpha6	0.451290067443304	0.0783715425885085	1	pseudobulk	DESeq2	LRT		0
137	KC_AB2	nAChRalpha7	1.20182681204903	0.291621791596076	1	pseudobulk	DESeq2	LRT		0
137	KC_AB2	nAChRbeta1	0.28699360958961	0.248293353521316	1	pseudobulk	DESeq2	LRT		0
137	KC_AB2	nAChRbeta2	0.564431866527572	0.399499470706232	1	pseudobulk	DESeq2	LRT		0
137	KC_AB2	NaCP60E	-0.601298682025629	0.175596514258721	1	pseudobulk	DESeq2	LRT		0
137	KC_AB2	Nadsyn	-0.395589752174231	0.588590245250983	1	pseudobulk	DESeq2	LRT		0
137	KC_AB2	Nak	0.461670283744434	0.578890693381665	1	pseudobulk	DESeq2	LRT		0
137	KC_AB2	Nap1	0.100798276689856	0.836820837734346	1	pseudobulk	DESeq2	LRT		0
137	KC_AB2	NaPi-III	-0.369517003560227	0.307791511344715	1	pseudobulk	DESeq2	LRT		0
137	KC_AB2	NaPi-T	-0.600547221779134	1	1	pseudobulk	DESeq2	LRT		0
137	KC_AB2	Naprt	1.39125031187613	0.225412147840916	1	pseudobulk	DESeq2	LRT		0
137	KC_AB2	NAT1	0.0127385550445615	0.897222340662054	1	pseudobulk	DESeq2	LRT		0
137	KC_AB2	Naus	0.652368767905626	0.474970301523794	1	pseudobulk	DESeq2	LRT		0
137	KC_AB2	Naxd	-2.52691149003434	0.278972704083546	1	pseudobulk	DESeq2	LRT		0

137	KC_AB2	Naxe	2.70206512243748	0.189689513649223	1	pseudobulk	DESeq2	LRT	0
137	KC_AB2	Nbr	1.28350017047366	0.376443193878839	1	pseudobulk	DESeq2	LRT	0
137	KC_AB2	nbs	-1.51928970377954	0.486908946651791	1	pseudobulk	DESeq2	LRT	0
137	KC_AB2	NC2alpha	0.659500443735188	0.719753687728381	1	pseudobulk	DESeq2	LRT	0
137	KC_AB2	NC2beta	-0.649743242931105	0.563026653387119	1	pseudobulk	DESeq2	LRT	0
137	KC_AB2	Nc73EF	-0.111855237054049	0.81269720836262	1	pseudobulk	DESeq2	LRT	0
137	KC_AB2	Nca	-0.948307543459416	0.0455455563690812	1	pseudobulk	DESeq2	LRT	0
137	KC_AB2	Nckx30C	0.145472100516342	0.586698406733149	1	pseudobulk	DESeq2	LRT	0
137	KC_AB2	nclb	-0.539665365830432	0.805145917072468	1	pseudobulk	DESeq2	LRT	0
137	KC_AB2	ncm	-0.834292552741443	0.593644801390953	1	pseudobulk	DESeq2	LRT	0
137	KC_AB2	Ncoa6	0.111192029687774	0.768924941377052	1	pseudobulk	DESeq2	LRT	0
137	KC_AB2	Nct	0.0018259729971447	0.847163615510628	1	pseudobulk	DESeq2	LRT	0
137	KC_AB2	ND-13A	-0.116813858399597	0.763321357855343	1	pseudobulk	DESeq2	LRT	0
137	KC_AB2	ND-13B	-0.104831803781134	0.833114995787573	1	pseudobulk	DESeq2	LRT	0
137	KC_AB2	ND-15	-0.34641672552803	0.446919964793672	1	pseudobulk	DESeq2	LRT	0
137	KC_AB2	ND-18	-0.304485974036046	0.561887687977845	1	pseudobulk	DESeq2	LRT	0
137	KC_AB2	ND-19	0.349349185522808	0.536070298069591	1	pseudobulk	DESeq2	LRT	0
137	KC_AB2	ND-20	-1.04716166771804	0.0887294962314196	1	pseudobulk	DESeq2	LRT	0
137	KC_AB2	ND-23	-0.362279695664296	0.54818489678914	1	pseudobulk	DESeq2	LRT	0
137	KC_AB2	ND-24	0.371605144973046	0.625345015202246	1	pseudobulk	DESeq2	LRT	0
137	KC_AB2	ND-30	-0.163876600944922	0.758341080679322	1	pseudobulk	DESeq2	LRT	0
137	KC_AB2	ND-39	-0.0075398288478652	0.889237303410348	1	pseudobulk	DESeq2	LRT	0
137	KC_AB2	ND-42	-0.513265904062111	0.715664821674673	1	pseudobulk	DESeq2	LRT	0
137	KC_AB2	ND-49	0.105243049386775	0.777874116754278	1	pseudobulk	DESeq2	LRT	0
137	KC_AB2	ND-51	-0.0504269032009459	0.875740755701884	1	pseudobulk	DESeq2	LRT	0
137	KC_AB2	ND-75	-0.870495515524991	0.206771647480713	1	pseudobulk	DESeq2	LRT	0
137	KC_AB2	ND-ACP	-0.116710647514144	0.772770349864164	1	pseudobulk	DESeq2	LRT	0
137	KC_AB2	ND-AGGG	-0.196324645520888	0.691227510828706	1	pseudobulk	DESeq2	LRT	0
137	KC_AB2	ND-ASHI	-0.901424454393627	0.203914977633438	1	pseudobulk	DESeq2	LRT	0
137	KC_AB2	ND-B12	-0.403274766045063	0.336855052070041	1	pseudobulk	DESeq2	LRT	0
137	KC_AB2	ND-B14	-0.0863314633427491	0.836169889717704	1	pseudobulk	DESeq2	LRT	0
137	KC_AB2	ND-B14.5A	0.637674275685648	0.290923467018997	1	pseudobulk	DESeq2	LRT	0
137	KC_AB2	ND-B14.5B	0.0470001428328547	0.812362463331001	1	pseudobulk	DESeq2	LRT	0
137	KC_AB2	ND-B14.7	-0.80815359222405	0.162565552537952	1	pseudobulk	DESeq2	LRT	0
137	KC_AB2	ND-B15	-0.419235159466462	0.36878733237966	1	pseudobulk	DESeq2	LRT	0
137	KC_AB2	ND-B16.6	-0.15297789502916	0.678966378232508	1	pseudobulk	DESeq2	LRT	0
137	KC_AB2	ND-B17	-0.328548973276171	0.607641969349121	1	pseudobulk	DESeq2	LRT	0
137	KC_AB2	ND-B17.2	-0.648607181913504	0.244592728781307	1	pseudobulk	DESeq2	LRT	0
137	KC_AB2	ND-B18	0.0776688473992343	0.858020256044024	1	pseudobulk	DESeq2	LRT	0
137	KC_AB2	ND-B22	-0.756473445729472	0.110365747628172	1	pseudobulk	DESeq2	LRT	0
137	KC_AB2	ND-MLRQ	-0.134843104249246	0.658666826609659	1	pseudobulk	DESeq2	LRT	0
137	KC_AB2	ND-MNLL	-0.493357239699781	0.783807521297462	1	pseudobulk	DESeq2	LRT	0
137	KC_AB2	ND-MWFE	-0.525970713962231	0.252411003440371	1	pseudobulk	DESeq2	LRT	0
137	KC_AB2	ND-PDSW	-0.252113509327483	0.613686568750515	1	pseudobulk	DESeq2	LRT	0
137	KC_AB2	ND-SGDH	-0.158878059149048	0.722130392435697	1	pseudobulk	DESeq2	LRT	0
137	KC_AB2	Ndae1	0.204257718976819	0.600278733215708	1	pseudobulk	DESeq2	LRT	0
137	KC_AB2	Ndf	0.529236797396531	0.40581889959161	1	pseudobulk	DESeq2	LRT	0
137	KC_AB2	Ndfip	-0.899432624776812	0.215550280132114	1	pseudobulk	DESeq2	LRT	0
137	KC_AB2	Neb-cGP	-0.344747024652812	0.283645119524089	1	pseudobulk	DESeq2	LRT	0
137	KC_AB2	Nedd4	-0.06620111698583	0.863087108958921	1	pseudobulk	DESeq2	LRT	0
137	KC_AB2	Nedd8	0.0237085907554979	0.936700897337025	1	pseudobulk	DESeq2	LRT	0
137	KC_AB2	nej	0.282211736364564	0.500023056418877	1	pseudobulk	DESeq2	LRT	0
137	KC_AB2	Nelf-A	1.34354710194488	0.249725489060861	1	pseudobulk	DESeq2	LRT	0

137	KC_AB2	NELF-B	1.14814388261213	0.568837005180859	1	pseudobulk	DESeq2	LRT	0
137	KC_AB2	Nelf-E	-0.866156958692976	0.512982362366018	1	pseudobulk	DESeq2	LRT	0
137	KC_AB2	nemy	-3.18817015137688	0.117532970725704	1	pseudobulk	DESeq2	LRT	0
137	KC_AB2	Neos	1.58071543686757	0.287243678453588	1	pseudobulk	DESeq2	LRT	0
137	KC_AB2	Nep1	-0.113557199603088	0.69714463533337	1	pseudobulk	DESeq2	LRT	0
137	KC_AB2	Nep3	-0.0320481032925977	0.967812498775885	1	pseudobulk	DESeq2	LRT	0
137	KC_AB2	Nep4	1.5243995994652	0.741477491078038	1	pseudobulk	DESeq2	LRT	0
137	KC_AB2	Nepl16	0.450208219041879	0.637908231788607	1	pseudobulk	DESeq2	LRT	0
137	KC_AB2	Nepl5	1.50255200571414	0.44025635980316	1	pseudobulk	DESeq2	LRT	0
137	KC_AB2	nero	1.57169768352932	0.343670787809291	1	pseudobulk	DESeq2	LRT	0
137	KC_AB2	nes	0.509304900271282	0.681061033012896	1	pseudobulk	DESeq2	LRT	0
137	KC_AB2	net	2.12684015898615	0.445939223035924	1	pseudobulk	DESeq2	LRT	0
137	KC_AB2	NetB	0.286490697235159	0.446895044517001	1	pseudobulk	DESeq2	LRT	0
137	KC_AB2	Neto	-0.427431139129811	1	1	pseudobulk	DESeq2	LRT	0
137	KC_AB2	neur	-0.997739058959085	0.155118673560174	1	pseudobulk	DESeq2	LRT	0
137	KC_AB2	Neurl4	-0.780898413398748	0.547910706100862	1	pseudobulk	DESeq2	LRT	0
137	KC_AB2	Nf-YA	-1.72864481639911	0.372132044717094	1	pseudobulk	DESeq2	LRT	0
137	KC_AB2	Nf-YB	-1.50331603016663	0.431246352444201	1	pseudobulk	DESeq2	LRT	0
137	KC_AB2	Nf-YC	0.421827822972071	0.723597535201216	1	pseudobulk	DESeq2	LRT	0
137	KC_AB2	Nf1	-0.356700742159706	0.661625040961186	1	pseudobulk	DESeq2	LRT	0
137	KC_AB2	NFAT	0.337312268006882	0.404027490214881	1	pseudobulk	DESeq2	LRT	0
138	KC_AB2	NfI	0.32993857876777	0.534971777080502	1	pseudobulk	DESeq2	LRT	0
138	KC_AB2	Nfs1	0.633467873549439	0.537605351485196	1	pseudobulk	DESeq2	LRT	0
138	KC_AB2	Nhe1	1.86597025831111	0.254048387265776	1	pseudobulk	DESeq2	LRT	0
138	KC_AB2	Nhe2	0.603304359620783	0.595075184532444	1	pseudobulk	DESeq2	LRT	0
138	KC_AB2	Nhe3	0.18369545361529	0.699885371330431	1	pseudobulk	DESeq2	LRT	0
138	KC_AB2	NHP2	-0.290063816371847	0.881698537689548	1	pseudobulk	DESeq2	LRT	0
138	KC_AB2	NijA	1.5243995994652	0.741477491078038	1	pseudobulk	DESeq2	LRT	0
138	KC_AB2	NimC2	1.5243995994652	0.741477491078038	1	pseudobulk	DESeq2	LRT	0
138	KC_AB2	NimC4	-0.427431139129811	1	1	pseudobulk	DESeq2	LRT	0
138	KC_AB2	NiPp1	-0.728389096369757	0.607923560097685	1	pseudobulk	DESeq2	LRT	0
138	KC_AB2	Nipped-A	0.0072367037943029	0.953483249866502	1	pseudobulk	DESeq2	LRT	0
138	KC_AB2	Nipped-B	0.156927541768972	0.659819585035806	1	pseudobulk	DESeq2	LRT	0
138	KC_AB2	Nipsnap	-0.16243038993537	0.830445962353558	1	pseudobulk	DESeq2	LRT	0
138	KC_AB2	NitFhit	-0.600547221779134	1	1	pseudobulk	DESeq2	LRT	0
138	KC_AB2	nito	0.292036936660594	0.80614001308019	1	pseudobulk	DESeq2	LRT	0
138	KC_AB2	NK7.1	0.609347991096522	0.577343538664901	1	pseudobulk	DESeq2	LRT	0
138	KC_AB2	NKAIN	-0.850279313199458	0.391337237596017	1	pseudobulk	DESeq2	LRT	0
138	KC_AB2	nkd	0.0960107080639295	0.848693253842948	1	pseudobulk	DESeq2	LRT	0
138	KC_AB2	Nle	-1.51812168653838	0.552401469179882	1	pseudobulk	DESeq2	LRT	0
138	KC_AB2	Nlg2	-0.0104417523224997	0.911797422040752	1	pseudobulk	DESeq2	LRT	0
138	KC_AB2	Nlg3	-0.146561995671984	0.571358080025414	1	pseudobulk	DESeq2	LRT	0
138	KC_AB2	Nlp	-0.261773998647603	0.701952686222127	1	pseudobulk	DESeq2	LRT	0
138	KC_AB2	nmd	-3.85831027536646	0.036227360996646	1	pseudobulk	DESeq2	LRT	0
138	KC_AB2	Nmd3	-1.12785316439435	0.661319045116135	1	pseudobulk	DESeq2	LRT	0
138	KC_AB2	Nmda1	-0.546598503763039	0.383417596116904	1	pseudobulk	DESeq2	LRT	0
138	KC_AB2	Nmdar1	0.748086487412103	0.429458888512478	1	pseudobulk	DESeq2	LRT	0
138	KC_AB2	Nmdar2	0.495007378129391	0.587944360210347	1	pseudobulk	DESeq2	LRT	0
138	KC_AB2	nmdyn-D6	-0.841882716738579	1	1	pseudobulk	DESeq2	LRT	0
138	KC_AB2	Nmnat	-0.611320558426458	0.633497294774128	1	pseudobulk	DESeq2	LRT	0
138	KC_AB2	nmo	-0.041415278721415	0.838136862511538	1	pseudobulk	DESeq2	LRT	0
138	KC_AB2	Nmt	-0.248014251067743	0.888879863269151	1	pseudobulk	DESeq2	LRT	0
138	KC_AB2	Nna1	-0.351962840701728	0.942720338310845	1	pseudobulk	DESeq2	LRT	0

138	KC_AB2	Nnp-1	-0.884246631417692	0.652530686825729	1	pseudobulk	DESeq2	LRT	0
138	KC_AB2	NO66	-1.3723417613553	0.619532036522	1	pseudobulk	DESeq2	LRT	0
138	KC_AB2	Noa36	0.0980722876466859	0.906038791847231	1	pseudobulk	DESeq2	LRT	0
138	KC_AB2	noc	-0.600547221779134	1	1	pseudobulk	DESeq2	LRT	0
138	KC_AB2	nocte	0.202931839487322	0.653232347357409	1	pseudobulk	DESeq2	LRT	0
138	KC_AB2	noi	0.222396124134733	1	1	pseudobulk	DESeq2	LRT	0
138	KC_AB2	nolo	-0.427431139129811	1	1	pseudobulk	DESeq2	LRT	0
138	KC_AB2	nom	-1.29244479833796	0.693295472370086	1	pseudobulk	DESeq2	LRT	0
138	KC_AB2	Non1	-0.0445989742648973	0.87025568912662	1	pseudobulk	DESeq2	LRT	0
138	KC_AB2	Non2	0.0911013198698453	0.903398749104694	1	pseudobulk	DESeq2	LRT	0
138	KC_AB2	Non3	-0.675494073661762	0.652148331789657	1	pseudobulk	DESeq2	LRT	0
138	KC_AB2	nonA	0.0855754075467004	0.843642178146906	1	pseudobulk	DESeq2	LRT	0
138	KC_AB2	nonA-l	0.075332578277245	0.926270909114066	1	pseudobulk	DESeq2	LRT	0
138	KC_AB2	nonC	0.779110570428844	0.722427818657622	1	pseudobulk	DESeq2	LRT	0
138	KC_AB2	Nop17l	0.559636363658312	0.627283621480446	1	pseudobulk	DESeq2	LRT	0
138	KC_AB2	Nop56	-1.42074207964214	0.374467479351221	1	pseudobulk	DESeq2	LRT	0
138	KC_AB2	Nop60B	0.864341462967788	0.925947094335722	1	pseudobulk	DESeq2	LRT	0
138	KC_AB2	Nopp140	-0.167620063280405	0.874493033115399	1	pseudobulk	DESeq2	LRT	0
138	KC_AB2	nord	-1.51928970377954	0.486908946651791	1	pseudobulk	DESeq2	LRT	0
138	KC_AB2	norpA	0.741446016531967	0.500617553502749	1	pseudobulk	DESeq2	LRT	0
138	KC_AB2	Nos	-2.3716367212675	0.286085607033758	1	pseudobulk	DESeq2	LRT	0
138	KC_AB2	Nost	-0.504597849832364	0.809397978921941	1	pseudobulk	DESeq2	LRT	0
138	KC_AB2	not	0.101932216715318	0.899420449412345	1	pseudobulk	DESeq2	LRT	0
138	KC_AB2	Not1	0.0423427895162124	0.874957032885267	1	pseudobulk	DESeq2	LRT	0
138	KC_AB2	Not10	-1.29035868264066	0.50372094761382	1	pseudobulk	DESeq2	LRT	0
138	KC_AB2	Not11	-0.299517699571872	0.819591966188451	1	pseudobulk	DESeq2	LRT	0
138	KC_AB2	Not3	0.513223663034042	0.386989455025977	1	pseudobulk	DESeq2	LRT	0
138	KC_AB2	NP15.6	-0.075795270982864	0.841239414606718	1	pseudobulk	DESeq2	LRT	0
138	KC_AB2	Npc1a	0.61859935867775	0.610056249598164	1	pseudobulk	DESeq2	LRT	0
138	KC_AB2	Npc2a	1.23571851286199	0.575848719421412	1	pseudobulk	DESeq2	LRT	0
138	KC_AB2	Npc2b	-0.600547221779133	0.841940696994101	1	pseudobulk	DESeq2	LRT	0
138	KC_AB2	Nph	-1.04343645360733	0.275352207086509	1	pseudobulk	DESeq2	LRT	0
138	KC_AB2	Npl4	0.888527035742451	0.580703735096284	1	pseudobulk	DESeq2	LRT	0
138	KC_AB2	Nplp1	0.212754629509105	0.843360808043461	1	pseudobulk	DESeq2	LRT	0
138	KC_AB2	Nplp2	0.864341462967788	0.925947094335722	1	pseudobulk	DESeq2	LRT	0
138	KC_AB2	Nplp3	-1.72976330140494	0.41918502381035	1	pseudobulk	DESeq2	LRT	0
138	KC_AB2	Nprl2	0.183555857030446	1	1	pseudobulk	DESeq2	LRT	0
138	KC_AB2	Nprl3	-0.354834447225471	0.927434619009882	1	pseudobulk	DESeq2	LRT	0
138	KC_AB2	Nrd1	0.899269135956093	0.642144625480232	1	pseudobulk	DESeq2	LRT	0
138	KC_AB2	Nrg	0.522028912426819	0.117385783536197	1	pseudobulk	DESeq2	LRT	0
138	KC_AB2	Nrk	1.8688760377645	0.356303822006979	1	pseudobulk	DESeq2	LRT	0
138	KC_AB2	nrm	-0.689833854726931	0.178224937095636	1	pseudobulk	DESeq2	LRT	0
138	KC_AB2	Nrt	0.135203166095218	0.82077028899126	1	pseudobulk	DESeq2	LRT	0
138	KC_AB2	nrv1	1.5243995994652	0.741477491078038	1	pseudobulk	DESeq2	LRT	0
138	KC_AB2	nrv2	0.250985186978641	0.70694963693742	1	pseudobulk	DESeq2	LRT	0
138	KC_AB2	nrv3	0.0914340611951555	0.583617475257151	1	pseudobulk	DESeq2	LRT	0
138	KC_AB2	Nrx-1	-0.118117773482571	0.673810299675324	1	pseudobulk	DESeq2	LRT	0
138	KC_AB2	Nrx-IV	0.111295718192756	0.898346666030213	1	pseudobulk	DESeq2	LRT	0
138	KC_AB2	Ns1	-0.0701070191740634	0.887142187656793	1	pseudobulk	DESeq2	LRT	0
138	KC_AB2	Ns2	0.376434368108162	0.795640043961019	1	pseudobulk	DESeq2	LRT	0
138	KC_AB2	Ns3	-2.57058714643175	0.234063990386298	1	pseudobulk	DESeq2	LRT	0
138	KC_AB2	Ns4	-1.33633290695479	0.429904578428862	1	pseudobulk	DESeq2	LRT	0
138	KC_AB2	NSD	1.94559924579109	0.42325307792885	1	pseudobulk	DESeq2	LRT	0

138	KC_AB2	Nsf2	-0.809780800925021	0.580818005169973	1	pseudobulk	DESeq2	LRT			0
138	KC_AB2	nsl1	0.300984235283639	0.563475375941917	1	pseudobulk	DESeq2	LRT			0
138	KC_AB2	nSMase	-1.16228494870803	0.329555089346871	1	pseudobulk	DESeq2	LRT			0
138	KC_AB2	nst	-0.322708466881273	0.786632685202524	1	pseudobulk	DESeq2	LRT			0
138	KC_AB2	Nsun2	-0.0852743682753834	0.917718671153184	1	pseudobulk	DESeq2	LRT			0
138	KC_AB2	Nsun5	-0.0715309630154252	1	1	pseudobulk	DESeq2	LRT			0
138	KC_AB2	nSyb	-0.182366269890474	0.433565613918747	1	pseudobulk	DESeq2	LRT			0
138	KC_AB2	NT5E-2	-0.427431139129811	1	1	pseudobulk	DESeq2	LRT			0
138	KC_AB2	Ntan1	0.806220781436531	0.621489750049961	1	pseudobulk	DESeq2	LRT			0
138	KC_AB2	ntc	-1.78094334699383	0.470930349923561	1	pseudobulk	DESeq2	LRT			0
138	KC_AB2	Ntf-2	-0.213644153284096	0.670463560709742	1	pseudobulk	DESeq2	LRT			0
138	KC_AB2	Ntmt	-0.600547221779133	0.841940696994101	1	pseudobulk	DESeq2	LRT			0
138	KC_AB2	NTPase	-1.3723417613553	0.619532036522	1	pseudobulk	DESeq2	LRT			0
138	KC_AB2	Nuak1	-0.538037652793241	0.363186907330264	1	pseudobulk	DESeq2	LRT			0
138	KC_AB2	NUCB1	0.494811846403784	0.510060071542497	1	pseudobulk	DESeq2	LRT			0
139	KC_AB2	nudC	1.47589109688499	0.418170998376279	1	pseudobulk	DESeq2	LRT			0
139	KC_AB2	nudE	-1.59695243204079	0.0788669478192014	1	pseudobulk	DESeq2	LRT			0
139	KC_AB2	nuf	-1.07776915880798	0.775491075088671	1	pseudobulk	DESeq2	LRT			0
139	KC_AB2	Nufip	1.53811683574911	0.54844951579854	1	pseudobulk	DESeq2	LRT			0
139	KC_AB2	Nulp1	0.879468944562913	0.428070611252716	1	pseudobulk	DESeq2	LRT			0
139	KC_AB2	numb	0.266108184074921	0.656432050177745	1	pseudobulk	DESeq2	LRT			0
139	KC_AB2	Nup107	0.609599016649936	0.749620012955861	1	pseudobulk	DESeq2	LRT			0
139	KC_AB2	Nup133	-1.16116875819275	0.736255625566845	1	pseudobulk	DESeq2	LRT			0
139	KC_AB2	Nup153	-0.231062952139379	0.756667125333908	1	pseudobulk	DESeq2	LRT			0
139	KC_AB2	Nup154	0.443892390635762	1	1	pseudobulk	DESeq2	LRT			0
139	KC_AB2	Nup160	-0.427431139129811	1	1	pseudobulk	DESeq2	LRT			0
139	KC_AB2	Nup188	2.49750470281195	0.235076905519563	1	pseudobulk	DESeq2	LRT			0
139	KC_AB2	Nup205	-0.427431139129811	1	1	pseudobulk	DESeq2	LRT			0
139	KC_AB2	Nup214	2.03955752684245	0.330463272096619	1	pseudobulk	DESeq2	LRT			0
139	KC_AB2	Nup35	-0.382929749762112	0.851961810578875	1	pseudobulk	DESeq2	LRT			0
139	KC_AB2	Nup358	0.677169616806547	0.464521444304516	1	pseudobulk	DESeq2	LRT			0
139	KC_AB2	Nup37	-1.70380348762697	0.473446675363872	1	pseudobulk	DESeq2	LRT			0
139	KC_AB2	Nup43	-0.843927729434532	0.756465370408908	1	pseudobulk	DESeq2	LRT			0
139	KC_AB2	Nup44A	-1.12352540566122	0.562479211609495	1	pseudobulk	DESeq2	LRT			0
139	KC_AB2	Nup50	-1.01440980512594	0.597957154844041	1	pseudobulk	DESeq2	LRT			0
139	KC_AB2	Nup54	0.189813678325555	1	1	pseudobulk	DESeq2	LRT			0
139	KC_AB2	Nup58	-1.04445428854771	0.603034233836386	1	pseudobulk	DESeq2	LRT			0
139	KC_AB2	Nup62	0.34005440178435	0.820055977427063	1	pseudobulk	DESeq2	LRT			0
139	KC_AB2	Nup93-1	1.21092382796727	0.637125262974216	1	pseudobulk	DESeq2	LRT			0
139	KC_AB2	Nup93-2	-0.0715309630154252	1	1	pseudobulk	DESeq2	LRT			0
139	KC_AB2	Nup98-96	-1.22969237909868	0.454617699680033	1	pseudobulk	DESeq2	LRT			0
139	KC_AB2	Nurf-38	-0.144447067668686	0.85030742595973	1	pseudobulk	DESeq2	LRT			0
139	KC_AB2	nvy	1.41524016620164	0.505408490038387	1	pseudobulk	DESeq2	LRT			0
139	KC_AB2	nwk	0.766398252566265	0.158973264544251	1	pseudobulk	DESeq2	LRT			0
139	KC_AB2	Nxf3	0.190393219485123	0.893175556614257	1	pseudobulk	DESeq2	LRT			0
139	KC_AB2	Nxt1	-0.568652618648196	0.644483735441664	1	pseudobulk	DESeq2	LRT			0
139	KC_AB2	O-fut1	-1.7824418856176	0.467224294431682	1	pseudobulk	DESeq2	LRT			0
139	KC_AB2	oaf	1.64759826860276	0.469402419161652	1	pseudobulk	DESeq2	LRT			0
139	KC_AB2	Oamb	-0.085265859653884	0.780502909900335	1	pseudobulk	DESeq2	LRT			0
139	KC_AB2	Oatp30B	0.281189802484751	0.785311600438972	1	pseudobulk	DESeq2	LRT			0
139	KC_AB2	obe	1.53811683574911	0.54844951579854	1	pseudobulk	DESeq2	LRT			0
139	KC_AB2	Obp18a	0.864341462967788	0.925947094335722	1	pseudobulk	DESeq2	LRT			0
139	KC_AB2	Obp44a	0.312094423945427	0.42877766645752	1	pseudobulk	DESeq2	LRT			0

139	KC_AB2	Obp83b	-0.427431139129811	1	1	pseudobulk	DESeq2	LRT			0
139	KC_AB2	Obp85a	0.108468324554008	1	1	pseudobulk	DESeq2	LRT			0
139	KC_AB2	Obp99c	-0.427431139129811	1	1	pseudobulk	DESeq2	LRT			0
139	KC_AB2	obst-E	1.40241945196173	0.57265124481703	1	pseudobulk	DESeq2	LRT			0
139	KC_AB2	ocm	-2.88282416205009	0.20359945545086	1	pseudobulk	DESeq2	LRT			0
139	KC_AB2	Ocrl	-0.3761765591	0.728318857184168	1	pseudobulk	DESeq2	LRT			0
139	KC_AB2	Octalpha2R	0.503294517473805	0.303142208918103	1	pseudobulk	DESeq2	LRT			0
139	KC_AB2	Octbeta1R	-0.283561269135707	0.328307298595399	1	pseudobulk	DESeq2	LRT			0
139	KC_AB2	Octbeta2R	0.369669687008046	0.737316542523516	1	pseudobulk	DESeq2	LRT			0
139	KC_AB2	Octbeta3R	-0.22396660257469	0.467114206817787	1	pseudobulk	DESeq2	LRT			0
139	KC_AB2	Oda	0.0596375927461806	0.808129653769793	1	pseudobulk	DESeq2	LRT			0
139	KC_AB2	Odj	-0.255966909976249	0.882151381391145	1	pseudobulk	DESeq2	LRT			0
139	KC_AB2	Oga	-0.493357239699781	0.783807521297462	1	pseudobulk	DESeq2	LRT			0
139	KC_AB2	Ogg1	1.5243995994652	0.741477491078038	1	pseudobulk	DESeq2	LRT			0
139	KC_AB2	ogre	-1.12785316439435	0.661319045116135	1	pseudobulk	DESeq2	LRT			0
139	KC_AB2	ohgt	-0.0387554433263296	0.971589779765693	1	pseudobulk	DESeq2	LRT			0
139	KC_AB2	olf186-F	-0.302999951891662	0.749438459620695	1	pseudobulk	DESeq2	LRT			0
139	KC_AB2	olf413	-0.169261348089845	0.559299635753693	1	pseudobulk	DESeq2	LRT			0
139	KC_AB2	Oli	-0.426573599359599	0.605273276374913	1	pseudobulk	DESeq2	LRT			0
139	KC_AB2	omd	-0.382929749762112	0.851961810578875	1	pseudobulk	DESeq2	LRT			0
139	KC_AB2	ome	-0.0886920777559678	0.735413871187214	1	pseudobulk	DESeq2	LRT			0
139	KC_AB2	onecut	-0.353489433672746	0.474623476069352	1	pseudobulk	DESeq2	LRT			0
139	KC_AB2	Opa1	-0.175861086577701	0.762821751593687	1	pseudobulk	DESeq2	LRT			0
139	KC_AB2	Opbp	-2.30837701085768	0.307948306969936	1	pseudobulk	DESeq2	LRT			0
139	KC_AB2	opm	1.26334827431191	0.462883082783822	1	pseudobulk	DESeq2	LRT			0
139	KC_AB2	or	-0.871972041609003	0.458125022229204	1	pseudobulk	DESeq2	LRT			0
139	KC_AB2	Or43b	-0.237187092233211	0.858867931105627	1	pseudobulk	DESeq2	LRT			0
139	KC_AB2	Or45a	-0.669774949668414	0.69553201717938	1	pseudobulk	DESeq2	LRT			0
139	KC_AB2	orb	0.738048013655795	0.524158913560311	1	pseudobulk	DESeq2	LRT			0
139	KC_AB2	orb2	0.0367839023069273	0.821517688536909	1	pseudobulk	DESeq2	LRT			0
139	KC_AB2	Orc4	-0.600547221779134	1	1	pseudobulk	DESeq2	LRT			0
139	KC_AB2	Orct	-0.600547221779134	1	1	pseudobulk	DESeq2	LRT			0
139	KC_AB2	Orct2	-0.427431139129811	1	1	pseudobulk	DESeq2	LRT			0
139	KC_AB2	Ork1	-0.600547221779134	1	1	pseudobulk	DESeq2	LRT			0
139	KC_AB2	ORMDL	0.843452047529779	0.597826993365527	1	pseudobulk	DESeq2	LRT			0
139	KC_AB2	ORY	1.5243995994652	0.741477491078038	1	pseudobulk	DESeq2	LRT			0
139	KC_AB2	osa	-0.0694586682983078	0.835153548220066	1	pseudobulk	DESeq2	LRT			0
139	KC_AB2	Osbp	-0.431114476012985	0.759673330165406	1	pseudobulk	DESeq2	LRT			0
139	KC_AB2	Oscillin	-0.592481036256484	0.706847545932384	1	pseudobulk	DESeq2	LRT			0
139	KC_AB2	Oseg1	-0.841882716738579	1	1	pseudobulk	DESeq2	LRT			0
139	KC_AB2	Osi2	-0.427431139129811	1	1	pseudobulk	DESeq2	LRT			0
139	KC_AB2	osp	-0.94307066344797	0.629943854378022	1	pseudobulk	DESeq2	LRT			0
139	KC_AB2	Ost48	1.42752897061888	0.172916602965108	1	pseudobulk	DESeq2	LRT			0
139	KC_AB2	OstDelta	-1.1310773948707	0.606203352870057	1	pseudobulk	DESeq2	LRT			0
139	KC_AB2	Ostgamma	-0.45904558516651	0.699509839684356	1	pseudobulk	DESeq2	LRT			0
139	KC_AB2	otk	-0.366451721714537	0.743377535895637	1	pseudobulk	DESeq2	LRT			0
139	KC_AB2	otk2	0.443892390635762	1	1	pseudobulk	DESeq2	LRT			0
139	KC_AB2	OtopLa	-0.135738547963862	0.636614711267702	1	pseudobulk	DESeq2	LRT			0
139	KC_AB2	OtopLc	-0.219378255322083	0.696517573890349	1	pseudobulk	DESeq2	LRT			0
139	KC_AB2	out	-0.592481036256484	0.706847545932384	1	pseudobulk	DESeq2	LRT			0
139	KC_AB2	ova	-0.304288815303329	0.702975690671266	1	pseudobulk	DESeq2	LRT			0
139	KC_AB2	ox	-0.0531524585303097	0.840700414122578	1	pseudobulk	DESeq2	LRT			0
139	KC_AB2	OXA1L	-0.686610535511796	0.699722356464793	1	pseudobulk	DESeq2	LRT			0

139	KC_AB2	oxt	0.171334172539735	0.840983434469093	1	pseudobulk	DESeq2	LRT		0
139	KC_AB2	oys	1.44418837185514	0.424073447958998	1	pseudobulk	DESeq2	LRT		0
139	KC_AB2	p	-0.340171672362751	0.676798621123319	1	pseudobulk	DESeq2	LRT		0
139	KC_AB2	p115	-1.92942763539083	0.427765257981976	1	pseudobulk	DESeq2	LRT		0
139	KC_AB2	p120ctn	-0.0920817422114577	0.728674406846811	1	pseudobulk	DESeq2	LRT		0
139	KC_AB2	p130CAS	0.544420528331827	0.471728630367609	1	pseudobulk	DESeq2	LRT		0
139	KC_AB2	p23	-0.302549576850105	0.516461903128337	1	pseudobulk	DESeq2	LRT		0
139	KC_AB2	p24-1	-0.804487418562396	0.324762984263225	1	pseudobulk	DESeq2	LRT		0
139	KC_AB2	P32	0.852046578724701	0.338276790673415	1	pseudobulk	DESeq2	LRT		0
140	KC_AB2	p38b	-0.286072753987597	0.69909791744679	1	pseudobulk	DESeq2	LRT		0
140	KC_AB2	p47	-0.729678337606263	0.554054868957622	1	pseudobulk	DESeq2	LRT		0
140	KC_AB2	P58IPK	-1.11954039051625	0.638853401075788	1	pseudobulk	DESeq2	LRT		0
140	KC_AB2	P5CDh1	-0.956446436913917	0.0934178475066077	1	pseudobulk	DESeq2	LRT		0
140	KC_AB2	P5cr	-0.508031291213997	0.627384081862923	1	pseudobulk	DESeq2	LRT		0
140	KC_AB2	Pa1	0.167051830934172	0.764754805986588	1	pseudobulk	DESeq2	LRT		0
140	KC_AB2	pAbp	-0.449621661858999	0.0885159401658376	1	pseudobulk	DESeq2	LRT		0
140	KC_AB2	Pabp2	0.269334516010148	0.720802767797076	1	pseudobulk	DESeq2	LRT		0
140	KC_AB2	pad	-1.45439488874766	0.456946233886225	1	pseudobulk	DESeq2	LRT		0
140	KC_AB2	Paf-AHalpha	0.234997810618992	0.770038472787887	1	pseudobulk	DESeq2	LRT		0
140	KC_AB2	Paics	-0.70727250916738	0.705390475165233	1	pseudobulk	DESeq2	LRT		0
140	KC_AB2	Paip2	1.0321932169016	0.0735408160791439	1	pseudobulk	DESeq2	LRT		0
140	KC_AB2	Pak	0.545340372025022	0.460468192374943	1	pseudobulk	DESeq2	LRT		0
140	KC_AB2	Pak3	-0.519667034314251	0.780427814827476	1	pseudobulk	DESeq2	LRT		0
140	KC_AB2	Pal1	0.0955798640580949	0.913506477312478	1	pseudobulk	DESeq2	LRT		0
140	KC_AB2	Pal2	-1.0194904584144	0.0500989485983597	1	pseudobulk	DESeq2	LRT		0
140	KC_AB2	pall	0.452476254173715	0.685832629653713	1	pseudobulk	DESeq2	LRT		0
140	KC_AB2	pan	0.176947923326098	0.390637645354116	1	pseudobulk	DESeq2	LRT		0
140	KC_AB2	PAN2	0.65360470678452	0.777547207962487	1	pseudobulk	DESeq2	LRT		0
140	KC_AB2	Panx	-0.681816697621939	0.692429626715667	1	pseudobulk	DESeq2	LRT		0
140	KC_AB2	papi	0.976909465230314	0.598676537442	1	pseudobulk	DESeq2	LRT		0
140	KC_AB2	PAPLA1	0.584922016033877	0.522435455749144	1	pseudobulk	DESeq2	LRT		0
140	KC_AB2	Papss	-1.13924737342506	0.632441448931001	1	pseudobulk	DESeq2	LRT		0
140	KC_AB2	Papst2	-0.15878027965947	0.851063766983005	1	pseudobulk	DESeq2	LRT		0
140	KC_AB2	par-1	-0.274210140914486	0.439118333620715	1	pseudobulk	DESeq2	LRT		0
140	KC_AB2	par-6	0.321926475910918	0.793287515975983	1	pseudobulk	DESeq2	LRT		0
140	KC_AB2	para	0.0983764939943631	0.581192784692819	1	pseudobulk	DESeq2	LRT		0
140	KC_AB2	Parg	-1.12785316439435	0.661319045116135	1	pseudobulk	DESeq2	LRT		0
140	KC_AB2	Parp	0.29119147577517	0.539965999690259	1	pseudobulk	DESeq2	LRT		0
140	KC_AB2	parvin	-1.65984791301014	0.268650606271326	1	pseudobulk	DESeq2	LRT		0
140	KC_AB2	pasha	-1.84905028965745	0.445198155344163	1	pseudobulk	DESeq2	LRT		0
140	KC_AB2	pasi1	-0.381022595176465	0.906491838131301	1	pseudobulk	DESeq2	LRT		0
140	KC_AB2	pasi2	-0.976083383606719	0.568582589468907	1	pseudobulk	DESeq2	LRT		0
140	KC_AB2	Pask	-0.799431675787062	0.653930468662593	1	pseudobulk	DESeq2	LRT		0
140	KC_AB2	Past1	-0.400833346343329	0.469418952910047	1	pseudobulk	DESeq2	LRT		0
140	KC_AB2	Pat1	-0.737933746874084	0.339101343096483	1	pseudobulk	DESeq2	LRT		0
140	KC_AB2	path	0.71233793349382	0.676758006984577	1	pseudobulk	DESeq2	LRT		0
140	KC_AB2	Patj	1.35968315642719	0.485168225381963	1	pseudobulk	DESeq2	LRT		0
140	KC_AB2	Patr-1	0.631329516938683	0.600275494861476	1	pseudobulk	DESeq2	LRT		0
140	KC_AB2	Patronin	-0.0136208729554742	0.984855136007003	1	pseudobulk	DESeq2	LRT		0
140	KC_AB2	Patsas	2.41137886961621	0.0681396431483553	1	pseudobulk	DESeq2	LRT		0
140	KC_AB2	Pax	0.818049755224763	0.631984103515073	1	pseudobulk	DESeq2	LRT		0
140	KC_AB2	pb	-0.600547221779134	1	1	pseudobulk	DESeq2	LRT		0
140	KC_AB2	Pbgs	2.07946313289983	0.357635956314506	1	pseudobulk	DESeq2	LRT		0

140	KC_AB2	Pbp95	-0.600547221779134	1	1	pseudobulk	DESeq2	LRT	0
140	KC_AB2	Pc	0.426836539188505	0.627053929973327	1	pseudobulk	DESeq2	LRT	0
140	KC_AB2	PCB	0.632897161759667	0.525341652279041	1	pseudobulk	DESeq2	LRT	0
140	KC_AB2	Pcd	-0.25799485577727	0.489348407942611	1	pseudobulk	DESeq2	LRT	0
140	KC_AB2	Pcf11	0.361270165062673	0.513527991105294	1	pseudobulk	DESeq2	LRT	0
140	KC_AB2	PCID2	2.03955752684245	0.330463272096619	1	pseudobulk	DESeq2	LRT	0
140	KC_AB2	pck	0.298742243500728	0.836058134674997	1	pseudobulk	DESeq2	LRT	0
140	KC_AB2	Pcl	-0.108284296267357	0.792685304101195	1	pseudobulk	DESeq2	LRT	0
140	KC_AB2	pcm	0.337910611974924	0.840375118616518	1	pseudobulk	DESeq2	LRT	0
140	KC_AB2	Pcmt	-0.999923188550977	0.26731690841254	1	pseudobulk	DESeq2	LRT	0
140	KC_AB2	PCNA2	-0.427431139129811	1	1	pseudobulk	DESeq2	LRT	0
140	KC_AB2	pcs	-0.445146932868256	0.311599883591772	1	pseudobulk	DESeq2	LRT	0
140	KC_AB2	pcx	0.0130748218608949	0.914094617532487	1	pseudobulk	DESeq2	LRT	0
140	KC_AB2	Pcyt1	-0.291418150617629	0.5806979700747	1	pseudobulk	DESeq2	LRT	0
140	KC_AB2	Pcyt2	-1.94611228835265	0.327954192366983	1	pseudobulk	DESeq2	LRT	0
140	KC_AB2	PDCD-5	-0.40518048428456	0.572704950367314	1	pseudobulk	DESeq2	LRT	0
140	KC_AB2	Pdcd4	0.429587857727989	0.290438678527512	1	pseudobulk	DESeq2	LRT	0
140	KC_AB2	Pde11	1.5243995994652	0.741477491078038	1	pseudobulk	DESeq2	LRT	0
140	KC_AB2	Pde1c	-0.0653215737470552	0.741924158586636	1	pseudobulk	DESeq2	LRT	0
140	KC_AB2	Pde6	0.284880902614507	0.678610618732557	1	pseudobulk	DESeq2	LRT	0
140	KC_AB2	Pde8	-0.0706015715197795	0.82999432817578	1	pseudobulk	DESeq2	LRT	0
140	KC_AB2	Pde9	-0.0535461769103883	0.798405557839002	1	pseudobulk	DESeq2	LRT	0
140	KC_AB2	Pdfr	-0.600547221779133	0.841940696994101	1	pseudobulk	DESeq2	LRT	0
140	KC_AB2	pdgy	-0.427431139129811	1	1	pseudobulk	DESeq2	LRT	0
140	KC_AB2	Pdha	-0.673009417891745	0.276274590632628	1	pseudobulk	DESeq2	LRT	0
140	KC_AB2	Pdhb	-0.189841537662668	0.768850921055588	1	pseudobulk	DESeq2	LRT	0
140	KC_AB2	Pdi	-0.360958607797462	0.669037672257093	1	pseudobulk	DESeq2	LRT	0
140	KC_AB2	Pdk	0.216273639674597	0.721105073172592	1	pseudobulk	DESeq2	LRT	0
140	KC_AB2	Pdk1	-0.963671611224479	0.283066984991551	1	pseudobulk	DESeq2	LRT	0
140	KC_AB2	pdm3	0.0471936707755822	0.953916869426286	1	pseudobulk	DESeq2	LRT	0
140	KC_AB2	Pdp	-0.38286866403537	0.788072627095774	1	pseudobulk	DESeq2	LRT	0
140	KC_AB2	Pdp1	0.13072590456438	0.537864839747102	1	pseudobulk	DESeq2	LRT	0
140	KC_AB2	pds5	1.73005688870496	0.145435662937423	1	pseudobulk	DESeq2	LRT	0
140	KC_AB2	Pdss2	0.494823716700669	0.563192752449203	1	pseudobulk	DESeq2	LRT	0
140	KC_AB2	Pdxk	0.428118970844364	0.8279364275092	1	pseudobulk	DESeq2	LRT	0
140	KC_AB2	PDZ-GEF	0.513364006853237	0.329847775184968	1	pseudobulk	DESeq2	LRT	0
140	KC_AB2	pea	0.662917340385659	0.664426007192919	1	pseudobulk	DESeq2	LRT	0
140	KC_AB2	Pect	-0.583275968168436	0.181326027498432	1	pseudobulk	DESeq2	LRT	0
140	KC_AB2	PEK	-0.338771435603146	0.816656863441963	1	pseudobulk	DESeq2	LRT	0
140	KC_AB2	pelo	-1.13272462164137	0.548712555145712	1	pseudobulk	DESeq2	LRT	0
140	KC_AB2	pen-2	-1.00387043975721	0.411048772902851	1	pseudobulk	DESeq2	LRT	0
140	KC_AB2	peng	1.25670364800945	0.496186024109763	1	pseudobulk	DESeq2	LRT	0
140	KC_AB2	Pep	-0.281561879207472	0.406150911887078	1	pseudobulk	DESeq2	LRT	0
140	KC_AB2	per	0.27536364069594	0.846147898112772	1	pseudobulk	DESeq2	LRT	0
140	KC_AB2	pes	1.08606695966652	0.544050026752335	1	pseudobulk	DESeq2	LRT	0
140	KC_AB2	Pex1	1.5243995994652	0.741477491078038	1	pseudobulk	DESeq2	LRT	0
140	KC_AB2	Pex10	-0.0852743682753834	0.917718671153184	1	pseudobulk	DESeq2	LRT	0
140	KC_AB2	Pex11	0.662917340385659	0.664426007192919	1	pseudobulk	DESeq2	LRT	0
140	KC_AB2	Pex12	-0.600547221779133	0.841940696994101	1	pseudobulk	DESeq2	LRT	0
140	KC_AB2	Pex13	0.0596210017019056	0.909440864461356	1	pseudobulk	DESeq2	LRT	0
140	KC_AB2	Pex14	-2.32734388697589	0.172110711741461	1	pseudobulk	DESeq2	LRT	0
140	KC_AB2	Pex16	0.892659559600706	0.712043317464255	1	pseudobulk	DESeq2	LRT	0
140	KC_AB2	Pex19	-1.1662311993738	0.147497519597047	1	pseudobulk	DESeq2	LRT	0

140	KC_AB2	Pex2	-0.0130439207929849	0.914160278921935	1	pseudobulk	DESeq2	LRT	0
140	KC_AB2	Pex23	-0.180304058419429	0.781180237618353	1	pseudobulk	DESeq2	LRT	0
140	KC_AB2	Pex3	-0.654881413933017	0.62628495298881	1	pseudobulk	DESeq2	LRT	0
141	KC_AB2	Pex5	-3.08716144587932	0.137723309452189	1	pseudobulk	DESeq2	LRT	0
141	KC_AB2	Pex6	2.12684015898615	0.445939223035924	1	pseudobulk	DESeq2	LRT	0
141	KC_AB2	Pfdn1	-1.40201901932004	0.248357336742015	1	pseudobulk	DESeq2	LRT	0
141	KC_AB2	Pfdn2	-0.0184220849426998	0.90178543102987	1	pseudobulk	DESeq2	LRT	0
141	KC_AB2	Pfdn4	-0.271789600028874	0.754858785393651	1	pseudobulk	DESeq2	LRT	0
141	KC_AB2	Pfdn5	-0.876830476764253	0.494186550200166	1	pseudobulk	DESeq2	LRT	0
141	KC_AB2	Pfdn6	-0.765612655230923	0.240831707065541	1	pseudobulk	DESeq2	LRT	0
141	KC_AB2	Pfk	0.382274249645426	0.533072765530416	1	pseudobulk	DESeq2	LRT	0
141	KC_AB2	Pfrx	-0.245500767454167	0.704945969509183	1	pseudobulk	DESeq2	LRT	0
141	KC_AB2	Pgam5	-0.971629442026314	0.560257700325674	1	pseudobulk	DESeq2	LRT	0
141	KC_AB2	Pgant1	0.0688585299112319	0.935178439469155	1	pseudobulk	DESeq2	LRT	0
141	KC_AB2	Pgant2	-0.0520114340239729	0.842861873389795	1	pseudobulk	DESeq2	LRT	0
141	KC_AB2	Pgant3	-0.854434281129776	0.735028022799924	1	pseudobulk	DESeq2	LRT	0
141	KC_AB2	Pgant35A	-1.22611802355401	0.528829772451552	1	pseudobulk	DESeq2	LRT	0
141	KC_AB2	Pgant5	0.611585578227072	0.362125321898511	1	pseudobulk	DESeq2	LRT	0
141	KC_AB2	Pgant6	-0.0852743682753834	0.917718671153184	1	pseudobulk	DESeq2	LRT	0
141	KC_AB2	Pgant7	-0.935388289097994	0.665012597320941	1	pseudobulk	DESeq2	LRT	0
141	KC_AB2	PGAP1	-0.427431139129811	1	1	pseudobulk	DESeq2	LRT	0
141	KC_AB2	PGAP2	1.75425971630948	0.141413306915365	1	pseudobulk	DESeq2	LRT	0
141	KC_AB2	PGAP3	-0.167679005593927	0.896089390589033	1	pseudobulk	DESeq2	LRT	0
141	KC_AB2	PGAP5	1.30608177625448	0.421375825593331	1	pseudobulk	DESeq2	LRT	0
141	KC_AB2	Pgd	-0.600547221779133	0.841940696994101	1	pseudobulk	DESeq2	LRT	0
141	KC_AB2	Pgi	0.48070504154081	0.342945902651442	1	pseudobulk	DESeq2	LRT	0
141	KC_AB2	Pgk	0.0756830909332762	0.755642665129559	1	pseudobulk	DESeq2	LRT	0
141	KC_AB2	Pglym78	0.309274494063618	0.423210424928229	1	pseudobulk	DESeq2	LRT	0
141	KC_AB2	Pgm1	-0.229300373679092	0.785044957767548	1	pseudobulk	DESeq2	LRT	0
141	KC_AB2	Pgm2a	-2.43705776276907	0.162689925814735	1	pseudobulk	DESeq2	LRT	0
141	KC_AB2	PGRP-LE	-2.36899097638582	0.27901845799825	1	pseudobulk	DESeq2	LRT	0
141	KC_AB2	ph-d	0.0840286645147261	0.947402339222577	1	pseudobulk	DESeq2	LRT	0
141	KC_AB2	ph-p	-0.0842035680160362	0.95034678905991	1	pseudobulk	DESeq2	LRT	0
141	KC_AB2	PH4alphaEFB	-0.182511858789562	0.899384007464213	1	pseudobulk	DESeq2	LRT	0
141	KC_AB2	PH4alphaNE1	1.94691684546882	0.398076844044942	1	pseudobulk	DESeq2	LRT	0
141	KC_AB2	Phax	-1.10584623890457	0.503118158149943	1	pseudobulk	DESeq2	LRT	0
141	KC_AB2	Phb2	-0.437615219693744	0.5269394583855	1	pseudobulk	DESeq2	LRT	0
141	KC_AB2	pHCl-1	0.158166548449949	0.55137468803755	1	pseudobulk	DESeq2	LRT	0
141	KC_AB2	PheRS-m	1.14814388261213	0.568837005180859	1	pseudobulk	DESeq2	LRT	0
141	KC_AB2	Phf5a	-0.15827417670658	0.893906289952732	1	pseudobulk	DESeq2	LRT	0
141	KC_AB2	PHGPx	-0.11367015835869	0.819466794982183	1	pseudobulk	DESeq2	LRT	0
141	KC_AB2	PhKgamma	-0.0854979597366741	0.843037023812483	1	pseudobulk	DESeq2	LRT	0
141	KC_AB2	Phm	-0.461532435771562	0.332269449636795	1	pseudobulk	DESeq2	LRT	0
141	KC_AB2	pho	0.346764798589106	0.625556514611557	1	pseudobulk	DESeq2	LRT	0
141	KC_AB2	phol	1.02724590699198	0.450340186740685	1	pseudobulk	DESeq2	LRT	0
141	KC_AB2	phtf	0.0535158461294116	0.92257176130014	1	pseudobulk	DESeq2	LRT	0
141	KC_AB2	phyl	-0.187103137636239	0.622632196139942	1	pseudobulk	DESeq2	LRT	0
141	KC_AB2	PI31	0.249082995865248	0.726392379616484	1	pseudobulk	DESeq2	LRT	0
141	KC_AB2	Pi3K21B	0.0620357936080025	0.893426410743259	1	pseudobulk	DESeq2	LRT	0
141	KC_AB2	Pi3K59F	0.601351318444478	0.763393428383397	1	pseudobulk	DESeq2	LRT	0
141	KC_AB2	Pi3K68D	1.06828113921183	0.270993427384108	1	pseudobulk	DESeq2	LRT	0
141	KC_AB2	Pi3K92E	0.573760393110584	0.792134620722927	1	pseudobulk	DESeq2	LRT	0
141	KC_AB2	Pi4KIIalpha	-0.256783256320307	0.767044740116145	1	pseudobulk	DESeq2	LRT	0

141	KC_AB2	PI4KIIIalpha	-0.565111192842338	0.407503360125973	1	pseudobulk	DESeq2	LRT	0
141	KC_AB2	pic	0.341286121848233	0.813452069665491	1	pseudobulk	DESeq2	LRT	0
141	KC_AB2	PICK1	0.280040063401579	0.673834416270937	1	pseudobulk	DESeq2	LRT	0
141	KC_AB2	pico	0.164573591545853	0.83516785936626	1	pseudobulk	DESeq2	LRT	0
141	KC_AB2	Picot	-0.191498323782897	0.873431633130813	1	pseudobulk	DESeq2	LRT	0
141	KC_AB2	Piezo	1.61825668365391	0.444984663888389	1	pseudobulk	DESeq2	LRT	0
141	KC_AB2	Pif1A	1.5243995994652	0.741477491078038	1	pseudobulk	DESeq2	LRT	0
141	KC_AB2	PIG-A	1.39827770914961	0.587246212652858	1	pseudobulk	DESeq2	LRT	0
141	KC_AB2	PIG-B	-0.256313347368378	0.817550786634054	1	pseudobulk	DESeq2	LRT	0
141	KC_AB2	PIG-C	-0.600547221779134	1	1	pseudobulk	DESeq2	LRT	0
141	KC_AB2	PIG-F	0.487419270992043	0.575135821305232	1	pseudobulk	DESeq2	LRT	0
141	KC_AB2	PIG-G	-0.427431139129811	1	1	pseudobulk	DESeq2	LRT	0
141	KC_AB2	PIG-H	0.158885872213844	0.913023095756665	1	pseudobulk	DESeq2	LRT	0
141	KC_AB2	PIG-K	-0.086811296787004	0.939306315852829	1	pseudobulk	DESeq2	LRT	0
141	KC_AB2	PIG-L	1.24347540705841	0.417858737985157	1	pseudobulk	DESeq2	LRT	0
141	KC_AB2	PIG-N	-1.07776915880798	0.775491075088671	1	pseudobulk	DESeq2	LRT	0
141	KC_AB2	PIG-O	2.48629812612306	0.259054692310381	1	pseudobulk	DESeq2	LRT	0
141	KC_AB2	PIG-Q	-0.427431139129811	1	1	pseudobulk	DESeq2	LRT	0
141	KC_AB2	PIG-S	-2.71632541924797	0.187022462241557	1	pseudobulk	DESeq2	LRT	0
141	KC_AB2	PIG-T	0.124939560397729	0.781337868463629	1	pseudobulk	DESeq2	LRT	0
141	KC_AB2	PIG-U	0.791286025443143	0.690540380845434	1	pseudobulk	DESeq2	LRT	0
141	KC_AB2	PIG-V	1.41524016620164	0.505408490038387	1	pseudobulk	DESeq2	LRT	0
141	KC_AB2	PIG-X	-1.54470851095603	0.55301747381519	1	pseudobulk	DESeq2	LRT	0
141	KC_AB2	pigeon	0.948979797867376	0.532135925174789	1	pseudobulk	DESeq2	LRT	0
141	KC_AB2	pigs	0.153694306840029	0.767456185465405	1	pseudobulk	DESeq2	LRT	0
141	KC_AB2	Pih1D1	0.392112224961027	0.72884431536444	1	pseudobulk	DESeq2	LRT	0
141	KC_AB2	Pink1	0.471774042477505	0.634746292878303	1	pseudobulk	DESeq2	LRT	0
141	KC_AB2	Pino	0.320325255278458	0.675443853164203	1	pseudobulk	DESeq2	LRT	0
141	KC_AB2	pins	-0.901045179657486	0.530350745152383	1	pseudobulk	DESeq2	LRT	0
141	KC_AB2	pinta	3.20650492957429	0.177944556633249	1	pseudobulk	DESeq2	LRT	0
141	KC_AB2	PIP4K	0.043545266762603	0.833962375454194	1	pseudobulk	DESeq2	LRT	0
141	KC_AB2	PIP5K59B	-0.415973185972788	0.414277741106415	1	pseudobulk	DESeq2	LRT	0
141	KC_AB2	Pis	0.0031419626335560	0.886573773476636	1	pseudobulk	DESeq2	LRT	0
141	KC_AB2	Pisd	-0.573109354659945	0.739419341582026	1	pseudobulk	DESeq2	LRT	0
141	KC_AB2	pit	-0.841882716738579	1	1	pseudobulk	DESeq2	LRT	0
141	KC_AB2	pita	1.5243995994652	0.741477491078038	1	pseudobulk	DESeq2	LRT	0
141	KC_AB2	Pits	0.266332553372529	0.40544604202701	1	pseudobulk	DESeq2	LRT	0
141	KC_AB2	Pitslre	-0.145210868714325	0.6147668967535	1	pseudobulk	DESeq2	LRT	0
141	KC_AB2	pix	-1.85070952880773	0.136396352683598	1	pseudobulk	DESeq2	LRT	0
141	KC_AB2	Pka-C1	-0.11762559923949	0.471422568524014	1	pseudobulk	DESeq2	LRT	0
141	KC_AB2	Pka-C3	-2.53282044847072	0.244380613967081	1	pseudobulk	DESeq2	LRT	0
141	KC_AB2	Pka-R1	-0.135128644624641	0.560482200863871	1	pseudobulk	DESeq2	LRT	0
141	KC_AB2	Pka-R2	-0.0891382330214736	0.583531822495725	1	pseudobulk	DESeq2	LRT	0
141	KC_AB2	pkaap	0.443892390635762	1	1	pseudobulk	DESeq2	LRT	0
141	KC_AB2	Pkc53E	-0.0791097598428381	0.683328750935144	1	pseudobulk	DESeq2	LRT	0
141	KC_AB2	Pkc98E	-0.136969026526682	0.630991155408095	1	pseudobulk	DESeq2	LRT	0
141	KC_AB2	PKD	2.12684015898615	0.445939223035924	1	pseudobulk	DESeq2	LRT	0
141	KC_AB2	Pkn	-0.670146173175947	0.495628743576936	1	pseudobulk	DESeq2	LRT	0
141	KC_AB2	Plap	1.64759826860276	0.469402419161652	1	pseudobulk	DESeq2	LRT	0
141	KC_AB2	Plc21C	0.177399855298476	0.521986609181603	1	pseudobulk	DESeq2	LRT	0
142	KC_AB2	PLCXD	1.5243995994652	0.741477491078038	1	pseudobulk	DESeq2	LRT	0
142	KC_AB2	Pldn	0.200916651495238	0.852819417240572	1	pseudobulk	DESeq2	LRT	0
142	KC_AB2	Plekhm1	-1.98716608866395	0.290381450420067	1	pseudobulk	DESeq2	LRT	0

142	KC_AB2	PlexA	0.369791219937964	0.322443922241969	1	pseudobulk	DESeq2	LRT	0
142	KC_AB2	PlexB	-0.405087083967503	0.507485248615681	1	pseudobulk	DESeq2	LRT	0
142	KC_AB2	Pli	-0.0597727485014661	0.842752652318289	1	pseudobulk	DESeq2	LRT	0
142	KC_AB2	pll	1.5243995994652	0.741477491078038	1	pseudobulk	DESeq2	LRT	0
142	KC_AB2	Plod	-1.07776915880798	0.775491075088671	1	pseudobulk	DESeq2	LRT	0
142	KC_AB2	Plp	-0.88481980846979	0.579476261608411	1	pseudobulk	DESeq2	LRT	0
142	KC_AB2	plum	0.609599016649936	0.749620012955861	1	pseudobulk	DESeq2	LRT	0
142	KC_AB2	plx	-0.0045372710416868	0.956505177692886	1	pseudobulk	DESeq2	LRT	0
142	KC_AB2	Plzf	-1.29244479833796	0.693295472370086	1	pseudobulk	DESeq2	LRT	0
142	KC_AB2	PMCA	0.0229587840694848	0.813281948080066	1	pseudobulk	DESeq2	LRT	0
142	KC_AB2	Pmi	-0.427431139129811	1	1	pseudobulk	DESeq2	LRT	0
142	KC_AB2	Pmm2	-0.211959943697087	0.913646816097592	1	pseudobulk	DESeq2	LRT	0
142	KC_AB2	PMP34	0.288600477117746	0.704718891061802	1	pseudobulk	DESeq2	LRT	0
142	KC_AB2	Pmp70	-0.522607582689706	0.720316240022395	1	pseudobulk	DESeq2	LRT	0
142	KC_AB2	pn	-0.631578663457669	0.750664130745045	1	pseudobulk	DESeq2	LRT	0
142	KC_AB2	Pngl	0.215836260682646	0.876157071353986	1	pseudobulk	DESeq2	LRT	0
142	KC_AB2	Pnn	-0.253422194790992	0.745787383675581	1	pseudobulk	DESeq2	LRT	0
142	KC_AB2	PNPase	2.12684015898615	0.445939223035924	1	pseudobulk	DESeq2	LRT	0
142	KC_AB2	pns	-0.104679192158986	0.837972136004495	1	pseudobulk	DESeq2	LRT	0
142	KC_AB2	pnut	-0.0570785015510141	0.938444679394851	1	pseudobulk	DESeq2	LRT	0
142	KC_AB2	PNUTS	0.179181173051903	0.634809959956365	1	pseudobulk	DESeq2	LRT	0
142	KC_AB2	pod1	-0.227646907295823	0.785322758953991	1	pseudobulk	DESeq2	LRT	0
142	KC_AB2	poe	0.818430670968312	0.210190893853285	1	pseudobulk	DESeq2	LRT	0
142	KC_AB2	Pof	-0.246880080867625	0.883966189422952	1	pseudobulk	DESeq2	LRT	0
142	KC_AB2	Pol32	0.609599016649936	0.749620012955861	1	pseudobulk	DESeq2	LRT	0
142	KC_AB2	POLDIP2	-1.60615111953864	0.203181527303407	1	pseudobulk	DESeq2	LRT	0
142	KC_AB2	poly	-0.664271364064587	0.493754569986603	1	pseudobulk	DESeq2	LRT	0
142	KC_AB2	polybromo	0.339931626074048	0.902536732977733	1	pseudobulk	DESeq2	LRT	0
142	KC_AB2	Pomp	-0.175852213971612	0.784269540175922	1	pseudobulk	DESeq2	LRT	0
142	KC_AB2	pont	2.03955752684245	0.330463272096619	1	pseudobulk	DESeq2	LRT	0
142	KC_AB2	Pop2	0.0645745602772531	0.891841278248771	1	pseudobulk	DESeq2	LRT	0
142	KC_AB2	por	0.906356398385783	0.754027117686013	1	pseudobulk	DESeq2	LRT	0
142	KC_AB2	porin	-0.315679975378795	0.231506298710736	1	pseudobulk	DESeq2	LRT	0
142	KC_AB2	POSH	1.18889395892318	0.651704638100559	1	pseudobulk	DESeq2	LRT	0
142	KC_AB2	Poxn	-0.592481036256484	0.706847545932384	1	pseudobulk	DESeq2	LRT	0
142	KC_AB2	Pp1-87B	-0.184230674441423	0.629617609624807	1	pseudobulk	DESeq2	LRT	0
142	KC_AB2	Pp1alpha-96A	0.236250831344659	0.625443567600794	1	pseudobulk	DESeq2	LRT	0
142	KC_AB2	Pp2A-29B	-0.467682695143844	0.361092972798696	1	pseudobulk	DESeq2	LRT	0
142	KC_AB2	Pp2B-14D	0.0797421814641493	0.814138157560125	1	pseudobulk	DESeq2	LRT	0
142	KC_AB2	Pp2C1	-0.0237627911598527	0.915227555385339	1	pseudobulk	DESeq2	LRT	0
142	KC_AB2	Pp4-19C	0.428707835275847	0.696448943286529	1	pseudobulk	DESeq2	LRT	0
142	KC_AB2	Ppa	2.34817442587308	0.244503819929596	1	pseudobulk	DESeq2	LRT	0
142	KC_AB2	ppan	-1.12785316439435	0.661319045116135	1	pseudobulk	DESeq2	LRT	0
142	KC_AB2	Ppat-Dpck	0.189813678325555	1	1	pseudobulk	DESeq2	LRT	0
142	KC_AB2	Ppcs	0.191169358194762	0.486610670115197	1	pseudobulk	DESeq2	LRT	0
142	KC_AB2	PpD3	-0.206071851008905	0.969636392453816	1	pseudobulk	DESeq2	LRT	0
142	KC_AB2	ppk28	-0.427431139129811	1	1	pseudobulk	DESeq2	LRT	0
142	KC_AB2	ppk8	1.5243995994652	0.741477491078038	1	pseudobulk	DESeq2	LRT	0
142	KC_AB2	ppl	-2.11639354481639	0.24187951884996	1	pseudobulk	DESeq2	LRT	0
142	KC_AB2	PPO2	-0.600547221779134	1	1	pseudobulk	DESeq2	LRT	0
142	KC_AB2	Ppox	-0.427431139129811	1	1	pseudobulk	DESeq2	LRT	0
142	KC_AB2	PPP1R15	0.115058829077874	0.790396875344879	1	pseudobulk	DESeq2	LRT	0
142	KC_AB2	PPP4R2r	0.38580367708627	0.66592510178242	1	pseudobulk	DESeq2	LRT	0

142	KC_AB2	pps	0.369286138696588	0.790889369923269	1	pseudobulk	DESeq2	LRT	0
142	KC_AB2	Ppt2	2.06147774369801	0.246400384346089	1	pseudobulk	DESeq2	LRT	0
142	KC_AB2	PpV	0.135106408533742	0.899177244737705	1	pseudobulk	DESeq2	LRT	0
142	KC_AB2	PQBP1	-1.77049677143504	0.34188273241158	1	pseudobulk	DESeq2	LRT	0
142	KC_AB2	pr	-0.378695292153294	0.771589887213337	1	pseudobulk	DESeq2	LRT	0
142	KC_AB2	PR-Set7	-0.200563908122188	0.767274134494794	1	pseudobulk	DESeq2	LRT	0
142	KC_AB2	prage	0.537633387514518	0.454586687026595	1	pseudobulk	DESeq2	LRT	0
142	KC_AB2	PRAS40	-1.04347484124768	0.468861227305465	1	pseudobulk	DESeq2	LRT	0
142	KC_AB2	PrBP	0.383019339193549	0.556766515505326	1	pseudobulk	DESeq2	LRT	0
142	KC_AB2	prd1	0.232790825004077	0.751903433656833	1	pseudobulk	DESeq2	LRT	0
142	KC_AB2	pre-mod(mdg4)-AE	2.12684015898615	0.445939223035924	1	pseudobulk	DESeq2	LRT	0
142	KC_AB2	pre-rRNA:CR4584	-0.152295027939677	0.864594408913739	1	pseudobulk	DESeq2	LRT	0
142	KC_AB2	prel	0.380219996430611	0.509776627033313	1	pseudobulk	DESeq2	LRT	0
142	KC_AB2	Prestin	-0.670649182913001	0.657570248402529	1	pseudobulk	DESeq2	LRT	0
142	KC_AB2	prg	-0.855909128841314	0.703076219237071	1	pseudobulk	DESeq2	LRT	0
142	KC_AB2	prim	-0.600547221779134	1	1	pseudobulk	DESeq2	LRT	0
142	KC_AB2	PRL-1	0.234461439569085	0.772236512922343	1	pseudobulk	DESeq2	LRT	0
142	KC_AB2	prod	-0.198651485007679	0.817568345449257	1	pseudobulk	DESeq2	LRT	0
142	KC_AB2	prom	3.98183091440589	0.0383060496509873	1	pseudobulk	DESeq2	LRT	0
142	KC_AB2	prominin-like	0.260821099540257	0.531268149435178	1	pseudobulk	DESeq2	LRT	0
142	KC_AB2	ProRS-m	0.339931626074048	0.902536732977733	1	pseudobulk	DESeq2	LRT	0
142	KC_AB2	pros	-0.0451873807130264	0.75026904762287	1	pseudobulk	DESeq2	LRT	0
142	KC_AB2	Prosalpha1	-0.600547221779133	0.841940696994101	1	pseudobulk	DESeq2	LRT	0
142	KC_AB2	Prosalpha2	0.684952212061518	0.438586729072153	1	pseudobulk	DESeq2	LRT	0
142	KC_AB2	Prosalpha3	-0.832117226600428	0.460059605685217	1	pseudobulk	DESeq2	LRT	0
142	KC_AB2	Prosalpha4	-0.44634541168156	0.699241121424482	1	pseudobulk	DESeq2	LRT	0
142	KC_AB2	Prosalpha5	0.528620241172598	0.644659226708899	1	pseudobulk	DESeq2	LRT	0
142	KC_AB2	Prosalpha6	0.510940814948466	0.708845308829595	1	pseudobulk	DESeq2	LRT	0
142	KC_AB2	Prosalpha7	0.190970966396427	0.871545862598261	1	pseudobulk	DESeq2	LRT	0
142	KC_AB2	Prosbeta1	-0.711322042331042	0.574209162191682	1	pseudobulk	DESeq2	LRT	0
142	KC_AB2	Prosbeta2	-0.0696928506773508	0.847734026888657	1	pseudobulk	DESeq2	LRT	0
142	KC_AB2	Prosbeta3	-0.876923970182888	0.514741558304968	1	pseudobulk	DESeq2	LRT	0
142	KC_AB2	Prosbeta4	-0.999882855325452	0.33990651705814	1	pseudobulk	DESeq2	LRT	0
142	KC_AB2	Prosbeta5	-0.658123510469839	0.396838732532308	1	pseudobulk	DESeq2	LRT	0
142	KC_AB2	Prosbeta6	-0.929222392145845	0.516812384715486	1	pseudobulk	DESeq2	LRT	0
142	KC_AB2	Prosbeta7	-1.32204163094206	0.425769636141347	1	pseudobulk	DESeq2	LRT	0
142	KC_AB2	Prp18	0.507665260063317	0.752319609055736	1	pseudobulk	DESeq2	LRT	0
142	KC_AB2	Prp19	-0.26487544492482	0.844340958692425	1	pseudobulk	DESeq2	LRT	0
142	KC_AB2	Prp3	1.00909773181456	0.372954761117169	1	pseudobulk	DESeq2	LRT	0
142	KC_AB2	Prp31	-0.126703431426612	0.90541463252898	1	pseudobulk	DESeq2	LRT	0
142	KC_AB2	Prp38	1.08480356307325	0.564615346523986	1	pseudobulk	DESeq2	LRT	0
142	KC_AB2	Prp8	0.484927681099921	0.737950345939502	1	pseudobulk	DESeq2	LRT	0
142	KC_AB2	Prpk	0.304567174146663	0.872999095445002	1	pseudobulk	DESeq2	LRT	0
142	KC_AB2	prt	-0.494616113541613	0.0350187904142958	1	pseudobulk	DESeq2	LRT	0
143	KC_AB2	prtp	0.461762134775536	0.556529194043142	1	pseudobulk	DESeq2	LRT	0
143	KC_AB2	Prx2540-1	-0.600547221779134	1	1	pseudobulk	DESeq2	LRT	0
143	KC_AB2	Prx3	-0.472211767148692	0.531785110680159	1	pseudobulk	DESeq2	LRT	0
143	KC_AB2	Prx6005	1.5243995994652	0.741477491078038	1	pseudobulk	DESeq2	LRT	0
143	KC_AB2	ps	-0.230786496393762	0.265722267701663	1	pseudobulk	DESeq2	LRT	0
143	KC_AB2	Psa	0.426780340420859	0.672099091061944	1	pseudobulk	DESeq2	LRT	0
143	KC_AB2	Psc	2.23928592769258	0.0262632924866637	1	pseudobulk	DESeq2	LRT	0
143	KC_AB2	Psf3	-0.282245504108145	0.845967581318217	1	pseudobulk	DESeq2	LRT	0
143	KC_AB2	PsGEF	0.304314732664507	0.677005927457614	1	pseudobulk	DESeq2	LRT	0

143	KC_AB2	Psi	-0.555072791330363	0.28814021881018	1	pseudobulk	DESeq2	LRT	0
143	KC_AB2	psidin	2.08701327791959	0.31723996803431	1	pseudobulk	DESeq2	LRT	0
143	KC_AB2	PSMG1	-1.09470786763053	0.519949606495745	1	pseudobulk	DESeq2	LRT	0
143	KC_AB2	Psn	0.233138099216394	0.818108880875556	1	pseudobulk	DESeq2	LRT	0
143	KC_AB2	psq	0.382590732710139	0.20645465633853	1	pseudobulk	DESeq2	LRT	0
143	KC_AB2	PSR	-0.509007437760716	0.731624785814057	1	pseudobulk	DESeq2	LRT	0
143	KC_AB2	Pten	-0.0075120922740926	0.954035102182813	1	pseudobulk	DESeq2	LRT	0
143	KC_AB2	Ptip	0.8712537960504	0.586368680797307	1	pseudobulk	DESeq2	LRT	0
143	KC_AB2	PTP-ER	0.479169607207848	0.59979597742678	1	pseudobulk	DESeq2	LRT	0
143	KC_AB2	Ptp10D	-0.841882716738579	1	1	pseudobulk	DESeq2	LRT	0
143	KC_AB2	Ptp36E	0.617824905942191	0.247491904199914	1	pseudobulk	DESeq2	LRT	0
143	KC_AB2	Ptp4E	0.0397505248861973	0.98242400247297	1	pseudobulk	DESeq2	LRT	0
143	KC_AB2	Ptp61F	0.650271336012993	0.581894957229923	1	pseudobulk	DESeq2	LRT	0
143	KC_AB2	Ptp69D	-0.843996296624293	0.67626831722335	1	pseudobulk	DESeq2	LRT	0
143	KC_AB2	Ptp99A	0.241416216545917	0.375948171479242	1	pseudobulk	DESeq2	LRT	0
143	KC_AB2	Ptpa	0.0699321013427868	0.914382103562725	1	pseudobulk	DESeq2	LRT	0
143	KC_AB2	Ptpmeg	1.81479439944007	0.0422288601345751	1	pseudobulk	DESeq2	LRT	0
143	KC_AB2	Ptpmeg2	0.0440560687464759	0.898101375319403	1	pseudobulk	DESeq2	LRT	0
143	KC_AB2	PTPMT1	0.63066469473651	0.648267601377665	1	pseudobulk	DESeq2	LRT	0
143	KC_AB2	Pu	1.50255200571414	0.44025635980316	1	pseudobulk	DESeq2	LRT	0
143	KC_AB2	puc	0.680920029341156	0.386846191923298	1	pseudobulk	DESeq2	LRT	0
143	KC_AB2	puf	0.368439486395861	0.749666704434883	1	pseudobulk	DESeq2	LRT	0
143	KC_AB2	pum	0.236234682654704	0.175249082941478	1	pseudobulk	DESeq2	LRT	0
143	KC_AB2	Pur-alpha	0.217152780416916	0.442852155495263	1	pseudobulk	DESeq2	LRT	0
143	KC_AB2	Pus1	1.23085716405798	0.614623732585597	1	pseudobulk	DESeq2	LRT	0
143	KC_AB2	Pus7	-0.94307066344797	0.629943854378022	1	pseudobulk	DESeq2	LRT	0
143	KC_AB2	put	-0.104933267079698	0.898423946239756	1	pseudobulk	DESeq2	LRT	0
143	KC_AB2	Pvf1	-1.12785316439435	0.661319045116135	1	pseudobulk	DESeq2	LRT	0
143	KC_AB2	Pvf3	0.9274392344303	0.19156231889622	1	pseudobulk	DESeq2	LRT	0
143	KC_AB2	Pvr	-0.427431139129811	1	1	pseudobulk	DESeq2	LRT	0
143	KC_AB2	PVRAP	1.10260939016296	0.0128961358304558	1	pseudobulk	DESeq2	LRT	0
143	KC_AB2	px	0.245967423467996	0.612239364387397	1	pseudobulk	DESeq2	LRT	0
143	KC_AB2	pxb	-0.640988538397006	0.33872641116196	1	pseudobulk	DESeq2	LRT	0
143	KC_AB2	Pxn	0.0257659937429937	0.886603349616938	1	pseudobulk	DESeq2	LRT	0
143	KC_AB2	pyd	0.21483265196118	0.650810435546484	1	pseudobulk	DESeq2	LRT	0
143	KC_AB2	pygo	-0.53104092268931	0.727059907025215	1	pseudobulk	DESeq2	LRT	0
143	KC_AB2	PyK	-0.0113218036074332	0.822073651150566	1	pseudobulk	DESeq2	LRT	0
143	KC_AB2	Pym	-1.40774689980751	0.332848109619081	1	pseudobulk	DESeq2	LRT	0
143	KC_AB2	Pyroxd1	0.186298416894564	0.947947908360033	1	pseudobulk	DESeq2	LRT	0
143	KC_AB2	pzg	-0.617084615209845	0.65164762963765	1	pseudobulk	DESeq2	LRT	0
143	KC_AB2	Pzl	0.906356398385783	0.754027117686013	1	pseudobulk	DESeq2	LRT	0
143	KC_AB2	QIL1	-0.368834478350219	0.597282268830822	1	pseudobulk	DESeq2	LRT	0
143	KC_AB2	qin	-0.841882716738579	1	1	pseudobulk	DESeq2	LRT	0
143	KC_AB2	qkr54B	0.412220630071304	0.573146995337937	1	pseudobulk	DESeq2	LRT	0
143	KC_AB2	qkr58E-1	0.257953394521918	0.585164889710706	1	pseudobulk	DESeq2	LRT	0
143	KC_AB2	qkr58E-2	0.741891766573645	0.486601153540052	1	pseudobulk	DESeq2	LRT	0
143	KC_AB2	qkr58E-3	-0.377033138837431	0.293994504535811	1	pseudobulk	DESeq2	LRT	0
143	KC_AB2	qless	-0.625417484011685	0.660237408124357	1	pseudobulk	DESeq2	LRT	0
143	KC_AB2	qm	-0.0641515081365676	0.803709812403159	1	pseudobulk	DESeq2	LRT	0
143	KC_AB2	Qsox1	-1.13898311207644	0.50341302473168	1	pseudobulk	DESeq2	LRT	0
143	KC_AB2	Qsox2	-0.427431139129811	1	1	pseudobulk	DESeq2	LRT	0
143	KC_AB2	qtc	-0.196073366291236	0.696497742763948	1	pseudobulk	DESeq2	LRT	0
143	KC_AB2	Qtzl	-0.529889471220762	0.616484504617999	1	pseudobulk	DESeq2	LRT	0

143	KC_AB2	qvr	0.4002768315679	0.261570246898739	1	pseudobulk	DESeq2	LRT		0
143	KC_AB2	r-l	3.20650492957429	0.177944556633249	1	pseudobulk	DESeq2	LRT		0
143	KC_AB2	r2d2	-0.608977756068493	0.62439858396654	1	pseudobulk	DESeq2	LRT		0
143	KC_AB2	Rab1	-0.383414554633958	0.416584621485868	1	pseudobulk	DESeq2	LRT		0
143	KC_AB2	Rab10	-0.200464146750203	0.822509015932511	1	pseudobulk	DESeq2	LRT		0
143	KC_AB2	Rab11	-0.0915924219999116	0.816669648706086	1	pseudobulk	DESeq2	LRT		0
143	KC_AB2	Rab14	0.281751296671491	0.570089512422689	1	pseudobulk	DESeq2	LRT		0
143	KC_AB2	Rab18	-0.708774023689912	0.50524236029526	1	pseudobulk	DESeq2	LRT		0
143	KC_AB2	Rab19	-0.017668334989552	0.940176589391492	1	pseudobulk	DESeq2	LRT		0
143	KC_AB2	Rab2	0.442041836094002	0.199761852657013	1	pseudobulk	DESeq2	LRT		0
143	KC_AB2	Rab21	0.323095707938641	0.661919509823983	1	pseudobulk	DESeq2	LRT		0
143	KC_AB2	Rab23	-2.32643960722241	0.0729156141315484	1	pseudobulk	DESeq2	LRT		0
143	KC_AB2	Rab26	-0.100745241011393	0.644700877011758	1	pseudobulk	DESeq2	LRT		0
143	KC_AB2	Rab27	-0.0057231715112681	0.981786369279254	1	pseudobulk	DESeq2	LRT		0
143	KC_AB2	Rab3	-0.0041114105970816	0.95432331357812	1	pseudobulk	DESeq2	LRT		0
143	KC_AB2	Rab3-GAP	-1.90937835128834	0.377732797313411	1	pseudobulk	DESeq2	LRT		0
143	KC_AB2	Rab3-GEF	0.430486838094487	0.290901369257488	1	pseudobulk	DESeq2	LRT		0
143	KC_AB2	Rab30	0.767766296326207	0.555614093577066	1	pseudobulk	DESeq2	LRT		0
143	KC_AB2	Rab35	0.417551634989872	0.464494111057714	1	pseudobulk	DESeq2	LRT		0
143	KC_AB2	Rab39	-0.565703269739289	0.677726954256297	1	pseudobulk	DESeq2	LRT		0
143	KC_AB2	Rab4	0.0104731047025651	0.966422569916301	1	pseudobulk	DESeq2	LRT		0
143	KC_AB2	Rab40	0.454323854478325	0.700614112029585	1	pseudobulk	DESeq2	LRT		0
143	KC_AB2	Rab5	0.191228169312989	0.65788600198027	1	pseudobulk	DESeq2	LRT		0
143	KC_AB2	Rab6	-0.544036168300049	0.616841550508456	1	pseudobulk	DESeq2	LRT		0
143	KC_AB2	Rab7	-0.711974633588198	0.427890101130703	1	pseudobulk	DESeq2	LRT		0
143	KC_AB2	Rab8	0.205064951219699	0.81169872745653	1	pseudobulk	DESeq2	LRT		0
143	KC_AB2	Rab9	1.5243995994652	0.741477491078038	1	pseudobulk	DESeq2	LRT		0
143	KC_AB2	Rabex-5	-0.258646132445231	0.867415245385041	1	pseudobulk	DESeq2	LRT		0
143	KC_AB2	RabX1	0.843452047529779	0.597826993365527	1	pseudobulk	DESeq2	LRT		0
143	KC_AB2	RabX4	0.281547584973309	0.643151652198032	1	pseudobulk	DESeq2	LRT		0
143	KC_AB2	RabX6	0.892659559600706	0.712043317464255	1	pseudobulk	DESeq2	LRT		0
143	KC_AB2	Rac1	0.484076517795609	0.327165337769571	1	pseudobulk	DESeq2	LRT		0
143	KC_AB2	Rack1	-0.0140689199112666	0.850867796376192	1	pseudobulk	DESeq2	LRT		0
143	KC_AB2	rad	0.232717609268629	0.240258440597652	1	pseudobulk	DESeq2	LRT		0
143	KC_AB2	Rad17	0.713982351041985	0.921569637923553	1	pseudobulk	DESeq2	LRT		0
143	KC_AB2	Rad23	-0.739532029124786	0.302665617416758	1	pseudobulk	DESeq2	LRT		0
143	KC_AB2	Rad51D	-0.600547221779134	1	1	pseudobulk	DESeq2	LRT		0
143	KC_AB2	Rad60	-0.979560107551823	0.588555853813166	1	pseudobulk	DESeq2	LRT		0
144	KC_AB2	Rad9	-0.427431139129811	1	1	pseudobulk	DESeq2	LRT		0
144	KC_AB2	Rae1	-1.72976330140494	0.41918502381035	1	pseudobulk	DESeq2	LRT		0
144	KC_AB2	Raf	-1.18294349965014	0.473475374861373	1	pseudobulk	DESeq2	LRT		0
144	KC_AB2	RAF2	-1.63993462454723	0.377201316569869	1	pseudobulk	DESeq2	LRT		0
144	KC_AB2	RagA-B	-1.54470851095603	0.55301747381519	1	pseudobulk	DESeq2	LRT		0
144	KC_AB2	RagC-D	-1.33852991308412	0.329156225373887	1	pseudobulk	DESeq2	LRT		0
144	KC_AB2	Rala	-0.108351621379948	0.895875130673234	1	pseudobulk	DESeq2	LRT		0
144	KC_AB2	Ran	-0.103871633042182	0.762381504485724	1	pseudobulk	DESeq2	LRT		0
144	KC_AB2	Ranbp16	0.852066995623382	0.534189055536773	1	pseudobulk	DESeq2	LRT		0
144	KC_AB2	Ranbp21	0.864341462967788	0.925947094335722	1	pseudobulk	DESeq2	LRT		0
144	KC_AB2	RanBP3	-2.60774669105106	0.109011996814322	1	pseudobulk	DESeq2	LRT		0
144	KC_AB2	RanBPM	0.146292388798731	0.851343093020859	1	pseudobulk	DESeq2	LRT		0
144	KC_AB2	ranshi	-0.600547221779133	0.841940696994101	1	pseudobulk	DESeq2	LRT		0
144	KC_AB2	Rap1	-0.301977443991561	0.322738862133261	1	pseudobulk	DESeq2	LRT		0
144	KC_AB2	Rap2l	1.0769749161296	0.093839841568813	1	pseudobulk	DESeq2	LRT		0

144	KC_AB2	RapGAP1	-1.7824418856176	0.467224294431682	1	pseudobulk	DESeq2	LRT	0
144	KC_AB2	raptor	-0.150021717358947	0.811821229496557	1	pseudobulk	DESeq2	LRT	0
144	KC_AB2	ras	-0.111236925708119	0.876706260332183	1	pseudobulk	DESeq2	LRT	0
144	KC_AB2	Ras64B	-0.737265930544148	0.681650346788831	1	pseudobulk	DESeq2	LRT	0
144	KC_AB2	Ras85D	-0.807622174500492	0.387862050685561	1	pseudobulk	DESeq2	LRT	0
144	KC_AB2	RasGAP1	-0.0285764127091088	0.897963240630961	1	pseudobulk	DESeq2	LRT	0
144	KC_AB2	raskol	0.533064518986454	0.247170127425301	1	pseudobulk	DESeq2	LRT	0
144	KC_AB2	Rassf	-0.841882716738579	1	1	pseudobulk	DESeq2	LRT	0
144	KC_AB2	RASSF8	-0.402776020007627	0.606801583049308	1	pseudobulk	DESeq2	LRT	0
144	KC_AB2	Rat1	-1.15065264789287	0.569010514080496	1	pseudobulk	DESeq2	LRT	0
144	KC_AB2	Ravus	1.99856294838389	0.254604573755456	1	pseudobulk	DESeq2	LRT	0
144	KC_AB2	raw	-0.367742280668506	0.613318545963972	1	pseudobulk	DESeq2	LRT	0
144	KC_AB2	rb	-1.93743737711859	0.0952763731019832	1	pseudobulk	DESeq2	LRT	0
144	KC_AB2	Rb97D	0.802081467473345	0.401233262710229	1	pseudobulk	DESeq2	LRT	0
144	KC_AB2	Rbbp5	0.674829464434859	0.816518190918181	1	pseudobulk	DESeq2	LRT	0
144	KC_AB2	Rbcn-3A	0.284244672891867	0.609965409911741	1	pseudobulk	DESeq2	LRT	0
144	KC_AB2	Rbcn-3B	-0.062022962662061	0.934536553754523	1	pseudobulk	DESeq2	LRT	0
144	KC_AB2	Rbf	-0.90686020782017	0.584252301074776	1	pseudobulk	DESeq2	LRT	0
144	KC_AB2	Rbfox1	0.145448384284336	0.47650789521755	1	pseudobulk	DESeq2	LRT	0
144	KC_AB2	Rbp	-0.110346381789248	0.594881264165342	1	pseudobulk	DESeq2	LRT	0
144	KC_AB2	Rbp1	0.452370673591278	0.487572104759166	1	pseudobulk	DESeq2	LRT	0
144	KC_AB2	Rbp1-like	0.0556371635262924	0.806875333496955	1	pseudobulk	DESeq2	LRT	0
144	KC_AB2	Rbp6	-2.45250621188369	0.141963478816846	1	pseudobulk	DESeq2	LRT	0
144	KC_AB2	Rbp9	-0.849606914821755	0.260275292006809	1	pseudobulk	DESeq2	LRT	0
144	KC_AB2	Rbpn-5	0.527318205539083	0.72729972774699	1	pseudobulk	DESeq2	LRT	0
144	KC_AB2	Rbsn-5	0.447818675064749	0.754791473935567	1	pseudobulk	DESeq2	LRT	0
144	KC_AB2	Rca1	0.12520760649288	0.827086928021371	1	pseudobulk	DESeq2	LRT	0
144	KC_AB2	Rcc1	-0.0966886633262495	0.876098901042912	1	pseudobulk	DESeq2	LRT	0
144	KC_AB2	Rcd-1	0.161539543263495	0.818391185739612	1	pseudobulk	DESeq2	LRT	0
144	KC_AB2	Rcd1	0.476303409939298	0.676364037298462	1	pseudobulk	DESeq2	LRT	0
144	KC_AB2	Rcd4	-3.40300861945852	0.0791728917564322	1	pseudobulk	DESeq2	LRT	0
144	KC_AB2	Rcd5	0.618187857193966	0.635065139509991	1	pseudobulk	DESeq2	LRT	0
144	KC_AB2	Rcp	-1.6150095599849	0.519549602003096	1	pseudobulk	DESeq2	LRT	0
144	KC_AB2	rdgA	-0.539038654168299	0.261699301054318	1	pseudobulk	DESeq2	LRT	0
144	KC_AB2	rdgB	0.281189911106867	0.36546070190363	1	pseudobulk	DESeq2	LRT	0
144	KC_AB2	rdgBbeta	-0.150418203005639	0.801362752321997	1	pseudobulk	DESeq2	LRT	0
144	KC_AB2	rdgC	0.990004945110479	0.406269281969864	1	pseudobulk	DESeq2	LRT	0
144	KC_AB2	Rdh	-0.255191073019821	0.402942827316327	1	pseudobulk	DESeq2	LRT	0
144	KC_AB2	Rdl	-0.0751099854601523	0.671841472681597	1	pseudobulk	DESeq2	LRT	0
144	KC_AB2	rdo	3.20650492957429	0.177944556633249	1	pseudobulk	DESeq2	LRT	0
144	KC_AB2	rdog	2.07908843388644	0.372593257917908	1	pseudobulk	DESeq2	LRT	0
144	KC_AB2	rdx	0.0926026714864936	0.806463810276871	1	pseudobulk	DESeq2	LRT	0
144	KC_AB2	rec	-0.621805355124546	0.74877258313458	1	pseudobulk	DESeq2	LRT	0
144	KC_AB2	Reck	-0.427431139129811	1	1	pseudobulk	DESeq2	LRT	0
144	KC_AB2	RecQ5	-0.757562933662594	0.73082653074729	1	pseudobulk	DESeq2	LRT	0
144	KC_AB2	red	-0.670983469109568	0.514311035113253	1	pseudobulk	DESeq2	LRT	0
144	KC_AB2	ReepA	0.0851757542840683	0.867108230084608	1	pseudobulk	DESeq2	LRT	0
144	KC_AB2	ReepB	-1.04734531418653	0.0294492411032714	1	pseudobulk	DESeq2	LRT	0
144	KC_AB2	ref(2)P	-0.710102926764796	0.31507074814824	1	pseudobulk	DESeq2	LRT	0
144	KC_AB2	Ref1	-0.155294951253254	0.808731128220211	1	pseudobulk	DESeq2	LRT	0
144	KC_AB2	REG	-0.348235344508345	0.710422922988067	1	pseudobulk	DESeq2	LRT	0
144	KC_AB2	regucalcin	-1.99748597705924	0.407706590597114	1	pseudobulk	DESeq2	LRT	0
144	KC_AB2	Rel	-0.600547221779134	1	1	pseudobulk	DESeq2	LRT	0

144	KC_AB2	Rep	-3.81070780733795	0.0362787911052072	1	pseudobulk	DESeq2	LRT		0
144	KC_AB2	Reph	-0.145991546893279	0.689614880077487	1	pseudobulk	DESeq2	LRT		0
144	KC_AB2	Reps	-0.792406643518554	0.545046405465154	1	pseudobulk	DESeq2	LRT		0
144	KC_AB2	rept	-0.841882716738579	1	1	pseudobulk	DESeq2	LRT		0
144	KC_AB2	REPTOR	2.1248984859322	0.0146978891239963	1	pseudobulk	DESeq2	LRT		0
144	KC_AB2	REPTOR-BP	-0.156064429896365	0.792150184605616	1	pseudobulk	DESeq2	LRT		0
144	KC_AB2	retm	0.882536735142234	0.321668816611366	1	pseudobulk	DESeq2	LRT		0
144	KC_AB2	retn	-0.600547221779133	0.841940696994101	1	pseudobulk	DESeq2	LRT		0
144	KC_AB2	Rexo5	-0.339200823106451	0.847510851734862	1	pseudobulk	DESeq2	LRT		0
144	KC_AB2	RfC3	-1.84905028965745	0.445198155344163	1	pseudobulk	DESeq2	LRT		0
144	KC_AB2	RfC38	1.41392113638259	0.617674323943697	1	pseudobulk	DESeq2	LRT		0
144	KC_AB2	RfC4	-0.463052496301942	0.794041648114126	1	pseudobulk	DESeq2	LRT		0
144	KC_AB2	RFeSP	-0.108789462863555	0.753798937704728	1	pseudobulk	DESeq2	LRT		0
144	KC_AB2	Rfx	-1.70380348762697	0.473446675363872	1	pseudobulk	DESeq2	LRT		0
144	KC_AB2	rg	-0.522672834547271	0.823382745807782	1	pseudobulk	DESeq2	LRT		0
144	KC_AB2	Rga	-0.0367029303495185	0.9437937594373	1	pseudobulk	DESeq2	LRT		0
144	KC_AB2	Rgk1	-0.231176581429679	0.225707223409444	1	pseudobulk	DESeq2	LRT		0
144	KC_AB2	Rgk2	0.804698059333956	0.35600750905435	1	pseudobulk	DESeq2	LRT		0
144	KC_AB2	Rgk3	-0.345927674022578	0.27939461134015	1	pseudobulk	DESeq2	LRT		0
144	KC_AB2	Rgl	-0.323536217808295	0.521375827355451	1	pseudobulk	DESeq2	LRT		0
144	KC_AB2	rgn	-1.31366174362008	0.00726750614041334	1	pseudobulk	DESeq2	LRT		0
144	KC_AB2	Rh50	1.5243995994652	0.741477491078038	1	pseudobulk	DESeq2	LRT		0
144	KC_AB2	Rh7	-2.18348597357589	0.114169445524889	1	pseudobulk	DESeq2	LRT		0
144	KC_AB2	Rhau	0.443892390635762	1	1	pseudobulk	DESeq2	LRT		0
144	KC_AB2	rhea	-0.3452561223723	0.664638081856502	1	pseudobulk	DESeq2	LRT		0
144	KC_AB2	Rheb	1.19187081095067	0.238576819270661	1	pseudobulk	DESeq2	LRT		0
144	KC_AB2	rho	-0.402780480652741	0.701734776607761	1	pseudobulk	DESeq2	LRT		0
144	KC_AB2	rho-4	-1.54470851095603	0.55301747381519	1	pseudobulk	DESeq2	LRT		0
144	KC_AB2	rho-5	0.383266201686245	0.545575128723184	1	pseudobulk	DESeq2	LRT		0
144	KC_AB2	rho-7	-1.1439706327711	0.497490875751088	1	pseudobulk	DESeq2	LRT		0
144	KC_AB2	Rho1	-0.181590238992386	0.711293328331503	1	pseudobulk	DESeq2	LRT		0
144	KC_AB2	RhoBTB	-0.833301860416351	0.403347075913666	1	pseudobulk	DESeq2	LRT		0
145	KC_AB2	RhoGAP100F	0.0228274606667306	0.954447842284158	1	pseudobulk	DESeq2	LRT		0
145	KC_AB2	RhoGAP102A	0.818097236913285	0.642607168556225	1	pseudobulk	DESeq2	LRT		0
145	KC_AB2	RhoGAP16F	-1.3929484167291	0.512855059074155	1	pseudobulk	DESeq2	LRT		0
145	KC_AB2	RhoGAP19D	0.30880348046746	0.446172152204115	1	pseudobulk	DESeq2	LRT		0
145	KC_AB2	RhoGAP1A	-1.55150556642147	0.187896183407092	1	pseudobulk	DESeq2	LRT		0
145	KC_AB2	RhoGAP5A	0.70378358684898	0.932301451973468	1	pseudobulk	DESeq2	LRT		0
145	KC_AB2	RhoGAP68F	0.217832622598037	0.776958697778748	1	pseudobulk	DESeq2	LRT		0
145	KC_AB2	RhoGAP71E	-0.0605864297562415	0.860351047615426	1	pseudobulk	DESeq2	LRT		0
145	KC_AB2	RhoGAP92B	0.911289895041351	0.499420535715043	1	pseudobulk	DESeq2	LRT		0
145	KC_AB2	RhoGAP93B	0.462042942368677	0.268016074958103	1	pseudobulk	DESeq2	LRT		0
145	KC_AB2	RhoGAPp190	0.0688509288806758	0.848790714900359	1	pseudobulk	DESeq2	LRT		0
145	KC_AB2	RhoGDI	-0.0383332041373627	0.797210483528744	1	pseudobulk	DESeq2	LRT		0
145	KC_AB2	RhoGEF2	-0.600547221779134	1	1	pseudobulk	DESeq2	LRT		0
145	KC_AB2	RhoGEF3	1.02538636693325	0.222567984930313	1	pseudobulk	DESeq2	LRT		0
145	KC_AB2	RhoGEF64C	1.5243995994652	0.741477491078038	1	pseudobulk	DESeq2	LRT		0
145	KC_AB2	RhoU	-0.621033250316403	0.810980097606566	1	pseudobulk	DESeq2	LRT		0
145	KC_AB2	Rhp	-0.427431139129811	1	1	pseudobulk	DESeq2	LRT		0
145	KC_AB2	Ric	0.0605236468702611	0.779070424440259	1	pseudobulk	DESeq2	LRT		0
145	KC_AB2	RIC-3	0.743769442871024	0.3396561628279	1	pseudobulk	DESeq2	LRT		0
145	KC_AB2	ric8a	1.54053875912195	0.39238818226048	1	pseudobulk	DESeq2	LRT		0
145	KC_AB2	Rich	0.108468324554008	1	1	pseudobulk	DESeq2	LRT		0

145	KC_AB2	rictor	2.38997178505017	0.0772009528586821	1	pseudobulk	DESeq2	LRT		0
145	KC_AB2	Rift	-2.77125686148606	0.191776729603111	1	pseudobulk	DESeq2	LRT		0
145	KC_AB2	rig	-1.44722825767989	0.460274013973674	1	pseudobulk	DESeq2	LRT		0
145	KC_AB2	Rilpl	0.485922779332728	0.618770223542529	1	pseudobulk	DESeq2	LRT		0
145	KC_AB2	Rim	0.0233165289837758	0.873790396000863	1	pseudobulk	DESeq2	LRT		0
145	KC_AB2	Rim2	-0.483749671172354	0.565980206818257	1	pseudobulk	DESeq2	LRT		0
145	KC_AB2	rin	-0.0372827121735307	0.924139482056003	1	pseudobulk	DESeq2	LRT		0
145	KC_AB2	ringer	-0.697306223889345	0.164151245977421	1	pseudobulk	DESeq2	LRT		0
145	KC_AB2	Rint1	0.892659559600706	0.712043317464255	1	pseudobulk	DESeq2	LRT		0
145	KC_AB2	RIOK1	-0.427431139129811	1	1	pseudobulk	DESeq2	LRT		0
145	KC_AB2	RIOK2	-2.62177086959622	0.246089746835538	1	pseudobulk	DESeq2	LRT		0
145	KC_AB2	Rip11	-0.652236316281899	0.618325600943115	1	pseudobulk	DESeq2	LRT		0
145	KC_AB2	Ripalpha	-2.55708532772848	0.266007159966735	1	pseudobulk	DESeq2	LRT		0
145	KC_AB2	rk	-0.507984909432264	0.526605112218172	1	pseudobulk	DESeq2	LRT		0
145	KC_AB2	rl	-0.115085256716463	0.758379454088839	1	pseudobulk	DESeq2	LRT		0
145	KC_AB2	Rlb1	-1.49049408144637	0.451343952443291	1	pseudobulk	DESeq2	LRT		0
145	KC_AB2	Rlip	0.423917622572907	0.577955001536752	1	pseudobulk	DESeq2	LRT		0
145	KC_AB2	RluA-2	-1.93220872672389	0.398686710033904	1	pseudobulk	DESeq2	LRT		0
145	KC_AB2	Rm62	0.258749570771749	0.457629604735174	1	pseudobulk	DESeq2	LRT		0
145	KC_AB2	Rme-8	-0.322490802744433	0.820336220082175	1	pseudobulk	DESeq2	LRT		0
145	KC_AB2	rn	-0.235388946260941	0.254662921906964	1	pseudobulk	DESeq2	LRT		0
145	KC_AB2	RN-tre	-0.438833519384627	0.68761542334612	1	pseudobulk	DESeq2	LRT		0
145	KC_AB2	RNASEK	-0.0152479120177638	0.809120608851409	1	pseudobulk	DESeq2	LRT		0
145	KC_AB2	RNaseMRP:RNA	-1.85733156562156	0.448702818121367	1	pseudobulk	DESeq2	LRT		0
145	KC_AB2	RNaseP:RNA	-0.600547221779133	0.841940696994101	1	pseudobulk	DESeq2	LRT		0
145	KC_AB2	RNaseX25	-0.539576916214097	0.747924312632912	1	pseudobulk	DESeq2	LRT		0
145	KC_AB2	RNaseZ	-0.600547221779133	0.841940696994101	1	pseudobulk	DESeq2	LRT		0
145	KC_AB2	Rnb	-1.12785316439435	0.661319045116135	1	pseudobulk	DESeq2	LRT		0
145	KC_AB2	Rnf146	-0.894819169487389	0.638536505602624	1	pseudobulk	DESeq2	LRT		0
145	KC_AB2	rngo	1.11471276021981	0.644913768495318	1	pseudobulk	DESeq2	LRT		0
145	KC_AB2	rnh1	-0.600547221779133	0.841940696994101	1	pseudobulk	DESeq2	LRT		0
145	KC_AB2	Rnmt	-0.664801898013526	0.760993114160489	1	pseudobulk	DESeq2	LRT		0
145	KC_AB2	rno	-0.441234963400661	0.547077395983677	1	pseudobulk	DESeq2	LRT		0
145	KC_AB2	Rnp4F	-1.58051390927725	0.410037109502309	1	pseudobulk	DESeq2	LRT		0
145	KC_AB2	RnpS1	-2.45666582461286	0.157256469796051	1	pseudobulk	DESeq2	LRT		0
145	KC_AB2	RnrL	-0.600547221779134	1	1	pseudobulk	DESeq2	LRT		0
145	KC_AB2	robl	-0.081424674128515	0.816941968448621	1	pseudobulk	DESeq2	LRT		0
145	KC_AB2	robo1	-0.333474462771222	0.666190326541326	1	pseudobulk	DESeq2	LRT		0
145	KC_AB2	robo2	-0.427431139129811	1	1	pseudobulk	DESeq2	LRT		0
145	KC_AB2	Roc1a	-0.187399057056046	0.8028367944344731	1	pseudobulk	DESeq2	LRT		0
145	KC_AB2	Roc2	-0.647209785783459	0.432468329115857	1	pseudobulk	DESeq2	LRT		0
145	KC_AB2	Roe1	-0.428558046168737	0.605946407520881	1	pseudobulk	DESeq2	LRT		0
145	KC_AB2	rogdi	0.0680554358757914	0.905446250808809	1	pseudobulk	DESeq2	LRT		0
145	KC_AB2	roh	0.0353276582118417	0.879530434995575	1	pseudobulk	DESeq2	LRT		0
145	KC_AB2	Rok	0.653496285092216	0.582943933810734	1	pseudobulk	DESeq2	LRT		0
145	KC_AB2	rols	-0.600547221779134	1	1	pseudobulk	DESeq2	LRT		0
145	KC_AB2	Rop	-0.0153997394446281	0.964350729003637	1	pseudobulk	DESeq2	LRT		0
145	KC_AB2	roq	-0.600596594310743	0.529512987472861	1	pseudobulk	DESeq2	LRT		0
145	KC_AB2	Ror	-1.69408872708848	0.422509811434123	1	pseudobulk	DESeq2	LRT		0
145	KC_AB2	row	0.935167643273841	0.336077304044018	1	pseudobulk	DESeq2	LRT		0
145	KC_AB2	Rox8	0.461676765614646	0.487083066340798	1	pseudobulk	DESeq2	LRT		0
145	KC_AB2	RpA-70	-1.84905028965745	0.445198155344163	1	pseudobulk	DESeq2	LRT		0
145	KC_AB2	RPA3	-0.600547221779134	1	1	pseudobulk	DESeq2	LRT		0

145	KC_AB2	Rpb10	-0.471535874851684	0.552471810946789	1	pseudobulk	DESeq2	LRT		0
145	KC_AB2	Rpb11	-0.233291439837028	0.811545588167701	1	pseudobulk	DESeq2	LRT		0
145	KC_AB2	Rpb12	0.124746571798526	0.876065575179117	1	pseudobulk	DESeq2	LRT		0
145	KC_AB2	Rpb4	-0.600547221779134	1	1	pseudobulk	DESeq2	LRT		0
145	KC_AB2	Rpb5	-0.747852219079764	0.708491663089672	1	pseudobulk	DESeq2	LRT		0
145	KC_AB2	Rpb7	0.886490225959685	0.344874476476079	1	pseudobulk	DESeq2	LRT		0
145	KC_AB2	Rpb8	-0.686610535511796	0.699722356464793	1	pseudobulk	DESeq2	LRT		0
145	KC_AB2	Rph	0.06187200490928	0.825236688120372	1	pseudobulk	DESeq2	LRT		0
145	KC_AB2	Rpi	-0.592481036256484	0.706847545932384	1	pseudobulk	DESeq2	LRT		0
145	KC_AB2	RpI1	-0.427431139129811	1	1	pseudobulk	DESeq2	LRT		0
145	KC_AB2	RpI135	-0.600547221779134	1	1	pseudobulk	DESeq2	LRT		0
145	KC_AB2	RpII140	-0.737265930544148	0.681650346788831	1	pseudobulk	DESeq2	LRT		0
145	KC_AB2	RpII15	0.46153293020083	0.749397723842613	1	pseudobulk	DESeq2	LRT		0
145	KC_AB2	RpII18	-0.406893991304522	0.638311635506645	1	pseudobulk	DESeq2	LRT		0
145	KC_AB2	RpII215	-0.291636354710978	0.667096100164633	1	pseudobulk	DESeq2	LRT		0
145	KC_AB2	RpII33	-1.50991954569714	0.23937039231387	1	pseudobulk	DESeq2	LRT		0
145	KC_AB2	RpIII128	0.864341462967788	0.925947094335722	1	pseudobulk	DESeq2	LRT		0
145	KC_AB2	RpIIIC160	-0.600547221779133	0.841940696994101	1	pseudobulk	DESeq2	LRT		0
145	KC_AB2	RpIIIC53	0.222396124134733	1	1	pseudobulk	DESeq2	LRT		0
145	KC_AB2	RpL10	-0.431671424265906	0.156993033141159	1	pseudobulk	DESeq2	LRT		0
145	KC_AB2	RpL10Ab	-0.703293655412627	0.0917054824025312	1	pseudobulk	DESeq2	LRT		0
145	KC_AB2	RpL11	-0.629429043132488	0.316569807233658	1	pseudobulk	DESeq2	LRT		0
145	KC_AB2	RpL12	0.212600034560886	0.593886572472804	1	pseudobulk	DESeq2	LRT		0
145	KC_AB2	RpL13	-0.39290543677037	0.248162561524482	1	pseudobulk	DESeq2	LRT		0
145	KC_AB2	RpL13A	-0.525836699998224	0.231936399794891	1	pseudobulk	DESeq2	LRT		0
145	KC_AB2	RpL14	-0.0443743120857706	0.837536296738473	1	pseudobulk	DESeq2	LRT		0
146	KC_AB2	RpL15	-0.291295045520609	0.255868241962955	1	pseudobulk	DESeq2	LRT		0
146	KC_AB2	RpL17	-0.438426701858639	0.220215893962366	1	pseudobulk	DESeq2	LRT		0
146	KC_AB2	RpL18	-0.393298462394258	0.394653149284162	1	pseudobulk	DESeq2	LRT		0
146	KC_AB2	RpL18A	0.0126106160469026	0.843415370211858	1	pseudobulk	DESeq2	LRT		0
146	KC_AB2	RpL19	-0.625527295685314	0.0936211438300264	1	pseudobulk	DESeq2	LRT		0
146	KC_AB2	RpL21	-0.341007314383804	0.242491270621992	1	pseudobulk	DESeq2	LRT		0
146	KC_AB2	RpL22	-0.469991011898808	0.504433269164299	1	pseudobulk	DESeq2	LRT		0
146	KC_AB2	RpL23	-0.319111296580072	0.382379792193792	1	pseudobulk	DESeq2	LRT		0
146	KC_AB2	RpL23A	-0.158339863415276	0.730993930230004	1	pseudobulk	DESeq2	LRT		0
146	KC_AB2	RpL24	-0.123849756194302	0.728435711856969	1	pseudobulk	DESeq2	LRT		0
146	KC_AB2	RpL24-like	0.204926689490537	0.660042921208678	1	pseudobulk	DESeq2	LRT		0
146	KC_AB2	RpL26	-0.12565678598549	0.729562755275356	1	pseudobulk	DESeq2	LRT		0
146	KC_AB2	RpL27	-0.496266323353882	0.272140905820336	1	pseudobulk	DESeq2	LRT		0
146	KC_AB2	RpL27A	-0.472126528652958	0.147384227727511	1	pseudobulk	DESeq2	LRT		0
146	KC_AB2	RpL28	-0.102353953293332	0.732648857699826	1	pseudobulk	DESeq2	LRT		0
146	KC_AB2	RpL29	-0.121811399099994	0.688082666519947	1	pseudobulk	DESeq2	LRT		0
146	KC_AB2	RpL3	0.164004771273299	0.722988181039722	1	pseudobulk	DESeq2	LRT		0
146	KC_AB2	RpL30	0.306497790460555	0.653820151105548	1	pseudobulk	DESeq2	LRT		0
146	KC_AB2	RpL31	-0.571230781615134	0.25188551253268	1	pseudobulk	DESeq2	LRT		0
146	KC_AB2	RpL32	-0.507368041658898	0.223781409618111	1	pseudobulk	DESeq2	LRT		0
146	KC_AB2	RpL34a	0.0108603502265348	0.925799477066812	1	pseudobulk	DESeq2	LRT		0
146	KC_AB2	RpL34b	0.0177300271901299	0.870261134055235	1	pseudobulk	DESeq2	LRT		0
146	KC_AB2	RpL35	-0.0936551207547132	0.794332366309874	1	pseudobulk	DESeq2	LRT		0
146	KC_AB2	RpL35A	-0.427503362277117	0.337501444906353	1	pseudobulk	DESeq2	LRT		0
146	KC_AB2	RpL36	-0.0578998195493611	0.776359520392294	1	pseudobulk	DESeq2	LRT		0
146	KC_AB2	RpL36A	-0.571359718376907	0.124329352348207	1	pseudobulk	DESeq2	LRT		0
146	KC_AB2	RpL37a	-0.152089065224008	0.635143374145494	1	pseudobulk	DESeq2	LRT		0

146	KC_AB2	RpL37A	-0.0001093344568630	0.829007852477132	1	pseudobulk	DESeq2	LRT	0
146	KC_AB2	RpL38	-0.272937769964565	0.387184349217801	1	pseudobulk	DESeq2	LRT	0
146	KC_AB2	RpL39	-0.687823766995457	0.0702278360019594	1	pseudobulk	DESeq2	LRT	0
146	KC_AB2	RpL4	-0.232735821920765	0.635794896389905	1	pseudobulk	DESeq2	LRT	0
146	KC_AB2	RpL40	0.0370811317377151	0.87225245612249	1	pseudobulk	DESeq2	LRT	0
146	KC_AB2	RpL41	0.306730619684528	0.220161328874858	1	pseudobulk	DESeq2	LRT	0
146	KC_AB2	RpL5	-0.878470634205275	0.0849694300127347	1	pseudobulk	DESeq2	LRT	0
146	KC_AB2	RpL6	-0.637229112908092	0.0543114010953496	1	pseudobulk	DESeq2	LRT	0
146	KC_AB2	RpL7	-0.678439026477984	0.0517385298302141	1	pseudobulk	DESeq2	LRT	0
146	KC_AB2	RpL7A	-0.144733685285531	0.710047981078571	1	pseudobulk	DESeq2	LRT	0
146	KC_AB2	RpL8	0.0685521160538304	0.754070597545999	1	pseudobulk	DESeq2	LRT	0
146	KC_AB2	RpL9	-0.352170814143427	0.336998158346715	1	pseudobulk	DESeq2	LRT	0
146	KC_AB2	RpLP0	-0.606905955696539	0.166279631346636	1	pseudobulk	DESeq2	LRT	0
146	KC_AB2	RpLP0-like	-2.68080992657177	0.213054001326578	1	pseudobulk	DESeq2	LRT	0
146	KC_AB2	RpLP1	-0.106320248342443	0.711706390617348	1	pseudobulk	DESeq2	LRT	0
146	KC_AB2	RpLP2	0.18230893433545	0.649349844310905	1	pseudobulk	DESeq2	LRT	0
146	KC_AB2	Rpn1	-1.26241275038835	0.353001769994644	1	pseudobulk	DESeq2	LRT	0
146	KC_AB2	Rpn10	-0.0186746041439121	0.899903351409633	1	pseudobulk	DESeq2	LRT	0
146	KC_AB2	Rpn11	0.511246039934043	0.652068836324889	1	pseudobulk	DESeq2	LRT	0
146	KC_AB2	Rpn12	0.0890976047304909	0.925368015008618	1	pseudobulk	DESeq2	LRT	0
146	KC_AB2	Rpn13	-0.577199877003177	0.57166383081447	1	pseudobulk	DESeq2	LRT	0
146	KC_AB2	Rpn13R	-0.600547221779134	1	1	pseudobulk	DESeq2	LRT	0
146	KC_AB2	Rpn2	-0.0087944767283343	0.948498578569082	1	pseudobulk	DESeq2	LRT	0
146	KC_AB2	Rpn3	0.0920390296490068	0.921929622402554	1	pseudobulk	DESeq2	LRT	0
146	KC_AB2	Rpn5	-0.109531422305612	0.89259530520336	1	pseudobulk	DESeq2	LRT	0
146	KC_AB2	Rpn6	-0.0071171347245176	0.935071936909975	1	pseudobulk	DESeq2	LRT	0
146	KC_AB2	Rpn7	0.599425508393726	0.602725562161516	1	pseudobulk	DESeq2	LRT	0
146	KC_AB2	Rpn8	-0.730560719529987	0.41043708961647	1	pseudobulk	DESeq2	LRT	0
146	KC_AB2	Rpn9	-0.73568661792243	0.432037917102874	1	pseudobulk	DESeq2	LRT	0
146	KC_AB2	Rpp30	0.222396124134733	1	1	pseudobulk	DESeq2	LRT	0
146	KC_AB2	rpr	-2.05961209064392	0.38565757104123	1	pseudobulk	DESeq2	LRT	0
146	KC_AB2	RpS10b	-0.182698817091172	0.570898849284874	1	pseudobulk	DESeq2	LRT	0
146	KC_AB2	RpS11	-0.355211196785246	0.306301239269922	1	pseudobulk	DESeq2	LRT	0
146	KC_AB2	RpS12	-0.554224060539912	0.2292752950625	1	pseudobulk	DESeq2	LRT	0
146	KC_AB2	RpS13	-0.574738073629435	0.123022583756947	1	pseudobulk	DESeq2	LRT	0
146	KC_AB2	RpS14a	-0.316680735408082	0.40630791858347	1	pseudobulk	DESeq2	LRT	0
146	KC_AB2	RpS14b	-0.209792508804025	0.737260614243899	1	pseudobulk	DESeq2	LRT	0
146	KC_AB2	RpS15	0.0982179830830188	0.741485096522526	1	pseudobulk	DESeq2	LRT	0
146	KC_AB2	RpS15Aa	-0.248508189024865	0.521041554421212	1	pseudobulk	DESeq2	LRT	0
146	KC_AB2	RpS15Ab	0.108468324554008	1	1	pseudobulk	DESeq2	LRT	0
146	KC_AB2	RpS16	-0.597661793650447	0.243382851399921	1	pseudobulk	DESeq2	LRT	0
146	KC_AB2	RpS17	0.121265628903185	0.732885721114284	1	pseudobulk	DESeq2	LRT	0
146	KC_AB2	RpS18	-0.15957497391322	0.598478268366652	1	pseudobulk	DESeq2	LRT	0
146	KC_AB2	RpS19a	-0.408070537460254	0.468226348701804	1	pseudobulk	DESeq2	LRT	0
146	KC_AB2	RpS2	-0.404492618759207	0.257466758006932	1	pseudobulk	DESeq2	LRT	0
146	KC_AB2	RpS20	-0.595334211119563	0.0829542744974926	1	pseudobulk	DESeq2	LRT	0
146	KC_AB2	RpS21	-0.209949586623567	0.62537118812014	1	pseudobulk	DESeq2	LRT	0
146	KC_AB2	RpS23	-0.422795638148079	0.279642721304701	1	pseudobulk	DESeq2	LRT	0
146	KC_AB2	RpS24	-0.542888668638371	0.210771709244584	1	pseudobulk	DESeq2	LRT	0
146	KC_AB2	RpS25	-0.355431411558603	0.251776250504946	1	pseudobulk	DESeq2	LRT	0
146	KC_AB2	RpS26	-0.191583361494424	0.456013491404386	1	pseudobulk	DESeq2	LRT	0
146	KC_AB2	RpS27	-0.266541148031695	0.447834976332534	1	pseudobulk	DESeq2	LRT	0
146	KC_AB2	RpS27A	-0.159938383279531	0.575034841907689	1	pseudobulk	DESeq2	LRT	0

146	KC_AB2	RpS28b	-0.35325446189692	0.308179760813445	1	pseudobulk	DESeq2	LRT	0
146	KC_AB2	RpS29	-0.450733085452111	0.170726855881825	1	pseudobulk	DESeq2	LRT	0
146	KC_AB2	RpS3	-0.213434745924101	0.556288599440336	1	pseudobulk	DESeq2	LRT	0
146	KC_AB2	RpS30	-0.376023397190379	0.190819261453089	1	pseudobulk	DESeq2	LRT	0
146	KC_AB2	RpS3A	-0.477186353360501	0.136099219924177	1	pseudobulk	DESeq2	LRT	0
146	KC_AB2	RpS4	-0.426087579576453	0.244588551297282	1	pseudobulk	DESeq2	LRT	0
146	KC_AB2	RpS5a	-0.575788109889263	0.322130353848212	1	pseudobulk	DESeq2	LRT	0
146	KC_AB2	RpS6	-0.635558003975641	0.0880996220959544	1	pseudobulk	DESeq2	LRT	0
146	KC_AB2	RpS7	-0.311012339750503	0.505443592817673	1	pseudobulk	DESeq2	LRT	0
146	KC_AB2	RpS8	0.266336571172977	0.400951631111085	1	pseudobulk	DESeq2	LRT	0
146	KC_AB2	RpS9	-0.305045505592766	0.45698442781846	1	pseudobulk	DESeq2	LRT	0
146	KC_AB2	Rpt1	-0.0589664954125515	0.870075034424347	1	pseudobulk	DESeq2	LRT	0
146	KC_AB2	Rpt2	-0.170843256156947	0.888057795191991	1	pseudobulk	DESeq2	LRT	0
146	KC_AB2	Rpt3	0.858882957183383	0.387019311430509	1	pseudobulk	DESeq2	LRT	0
146	KC_AB2	Rpt4	-0.75708990899747	0.419538076334217	1	pseudobulk	DESeq2	LRT	0
146	KC_AB2	Rpt5	-0.510892886397289	0.645141523852915	1	pseudobulk	DESeq2	LRT	0
146	KC_AB2	Rpt6	-0.211061380758303	0.778269302583372	1	pseudobulk	DESeq2	LRT	0
146	KC_AB2	Rrp1	0.864341462967788	0.925947094335722	1	pseudobulk	DESeq2	LRT	0
146	KC_AB2	Rrp4	-0.600547221779133	0.841940696994101	1	pseudobulk	DESeq2	LRT	0
146	KC_AB2	Rrp40	1.5243995994652	0.741477491078038	1	pseudobulk	DESeq2	LRT	0
147	KC_AB2	Rrp42	-1.07776915880798	0.775491075088671	1	pseudobulk	DESeq2	LRT	0
147	KC_AB2	Rrp46	1.5243995994652	0.741477491078038	1	pseudobulk	DESeq2	LRT	0
147	KC_AB2	Rrp47	0.836755521754435	0.395665973565795	1	pseudobulk	DESeq2	LRT	0
147	KC_AB2	Rrp6	1.54127986095618	0.432045425768347	1	pseudobulk	DESeq2	LRT	0
147	KC_AB2	Rs1	-0.119831229562752	1	1	pseudobulk	DESeq2	LRT	0
147	KC_AB2	Rsf1	0.0663643293919829	0.80687951119257	1	pseudobulk	DESeq2	LRT	0
147	KC_AB2	RSG7	-0.226016594427512	0.512866573280356	1	pseudobulk	DESeq2	LRT	0
147	KC_AB2	rswl	0.831378082847564	0.569973648010357	1	pseudobulk	DESeq2	LRT	0
147	KC_AB2	rt	-0.600547221779133	0.841940696994101	1	pseudobulk	DESeq2	LRT	0
147	KC_AB2	Rtc1	0.920212920242661	0.67286879185691	1	pseudobulk	DESeq2	LRT	0
147	KC_AB2	Rtca	0.555133184826415	0.603168731601564	1	pseudobulk	DESeq2	LRT	0
147	KC_AB2	RtcB	-0.0852743682753834	0.917718671153184	1	pseudobulk	DESeq2	LRT	0
147	KC_AB2	rtet	-0.600547221779133	0.841940696994101	1	pseudobulk	DESeq2	LRT	0
147	KC_AB2	Rtf1	-0.117742688079838	0.930942295908294	1	pseudobulk	DESeq2	LRT	0
147	KC_AB2	RtGEF	0.492301634826656	0.721389745561349	1	pseudobulk	DESeq2	LRT	0
147	KC_AB2	Rtnl1	-0.460329584205102	0.0710530023817435	1	pseudobulk	DESeq2	LRT	0
147	KC_AB2	Rubicon	0.837740692476529	0.563206978109164	1	pseudobulk	DESeq2	LRT	0
147	KC_AB2	rudhira	0.340202179347574	0.388794768295701	1	pseudobulk	DESeq2	LRT	0
147	KC_AB2	rumi	0.537141908207746	0.858524852458587	1	pseudobulk	DESeq2	LRT	0
147	KC_AB2	rump	-0.0741341341616969	0.903634085434184	1	pseudobulk	DESeq2	LRT	0
147	KC_AB2	rush	-0.305778643438596	0.791123876248923	1	pseudobulk	DESeq2	LRT	0
147	KC_AB2	rut	0.123312402697438	0.546886027158415	1	pseudobulk	DESeq2	LRT	0
147	KC_AB2	rux	-1.5063609558842	0.342778152834779	1	pseudobulk	DESeq2	LRT	0
147	KC_AB2	RYBP	1.07540368647167	0.383524009134959	1	pseudobulk	DESeq2	LRT	0
147	KC_AB2	RyR	-0.910803431462542	0.477275214481585	1	pseudobulk	DESeq2	LRT	0
147	KC_AB2	S	0.12219127889181	0.811112214683751	1	pseudobulk	DESeq2	LRT	0
147	KC_AB2	s-cup	-1.3723417613553	0.619532036522	1	pseudobulk	DESeq2	LRT	0
147	KC_AB2	S-Lap1	-1.3723417613553	0.619532036522	1	pseudobulk	DESeq2	LRT	0
147	KC_AB2	S1P	-0.427431139129811	1	1	pseudobulk	DESeq2	LRT	0
147	KC_AB2	S2P	1.95121062543026	0.411198785415779	1	pseudobulk	DESeq2	LRT	0
147	KC_AB2	S6k	1.16000307696513	0.194945794474876	1	pseudobulk	DESeq2	LRT	0
147	KC_AB2	S6kII	-0.967294788998528	0.40687943354659	1	pseudobulk	DESeq2	LRT	0
147	KC_AB2	S6KL	-0.780831702126752	0.568378425506171	1	pseudobulk	DESeq2	LRT	0

147	KC_AB2	SA	0.292346552234682	0.842418166025389	1	pseudobulk	DESeq2	LRT	0
147	KC_AB2	Sac1	1.24310458700474	0.400115812040422	1	pseudobulk	DESeq2	LRT	0
147	KC_AB2	Saf-B	-0.0752919168376064	0.774243470406697	1	pseudobulk	DESeq2	LRT	0
147	KC_AB2	Saf6	-0.427431139129811	1	1	pseudobulk	DESeq2	LRT	0
147	KC_AB2	sage	-0.623496651300123	0.726179083076297	1	pseudobulk	DESeq2	LRT	0
147	KC_AB2	SAK	-0.39917336535616	0.810147819664692	1	pseudobulk	DESeq2	LRT	0
147	KC_AB2	sals	0.432167782334268	0.448183342358026	1	pseudobulk	DESeq2	LRT	0
147	KC_AB2	Sam-S	0.0656726148229677	0.805588615871272	1	pseudobulk	DESeq2	LRT	0
147	KC_AB2	SamDC	0.00676388720338980	0.935777843883858	1	pseudobulk	DESeq2	LRT	0
147	KC_AB2	Samtor	-1.12785316439435	0.661319045116135	1	pseudobulk	DESeq2	LRT	0
147	KC_AB2	Samuel	0.864341462967788	0.925947094335722	1	pseudobulk	DESeq2	LRT	0
147	KC_AB2	san	0.471486335346162	0.50273304341032	1	pseudobulk	DESeq2	LRT	0
147	KC_AB2	sano	2.72066226053615	0.290765320578625	1	pseudobulk	DESeq2	LRT	0
147	KC_AB2	santa-maria	1.40241945196173	0.57265124481703	1	pseudobulk	DESeq2	LRT	0
147	KC_AB2	Sap-r	-0.451106140808832	0.609134856698594	1	pseudobulk	DESeq2	LRT	0
147	KC_AB2	Sap130	0.596788699944113	0.546156995752397	1	pseudobulk	DESeq2	LRT	0
147	KC_AB2	Sap30	0.997249947847885	0.601888101051434	1	pseudobulk	DESeq2	LRT	0
147	KC_AB2	Sap47	-0.0002492232287859	0.898570885838415	1	pseudobulk	DESeq2	LRT	0
147	KC_AB2	Sar1	-0.0067761743729918	0.868961651995424	1	pseudobulk	DESeq2	LRT	0
147	KC_AB2	Sara	0.444243853278005	0.750341133262179	1	pseudobulk	DESeq2	LRT	0
147	KC_AB2	Sas10	-1.24330161354718	0.542016636259656	1	pseudobulk	DESeq2	LRT	0
147	KC_AB2	sau	-0.639082990507881	0.44054498528664	1	pseudobulk	DESeq2	LRT	0
147	KC_AB2	sax	0.34386446062318	0.784606392346515	1	pseudobulk	DESeq2	LRT	0
147	KC_AB2	sba	0.655425205123234	0.193888904574722	1	pseudobulk	DESeq2	LRT	0
147	KC_AB2	Sbat	0.0298804629141662	0.942015162648043	1	pseudobulk	DESeq2	LRT	0
147	KC_AB2	sbb	0.31202549049355	0.261793670761103	1	pseudobulk	DESeq2	LRT	0
147	KC_AB2	Sbf	0.368887526275833	0.700543803945114	1	pseudobulk	DESeq2	LRT	0
147	KC_AB2	sbm	0.472739025269434	0.394114606188865	1	pseudobulk	DESeq2	LRT	0
147	KC_AB2	Sbp2	-0.894819169487389	0.638536505602624	1	pseudobulk	DESeq2	LRT	0
147	KC_AB2	sbr	0.142261533529056	0.779663615807366	1	pseudobulk	DESeq2	LRT	0
147	KC_AB2	Sc2	0.302229930937595	0.776415738704687	1	pseudobulk	DESeq2	LRT	0
147	KC_AB2	SC35	-0.52604116933924	0.393052387869211	1	pseudobulk	DESeq2	LRT	0
147	KC_AB2	sca	-1.1104292459921	0.135972122721141	1	pseudobulk	DESeq2	LRT	0
147	KC_AB2	scaf6	-0.0143978767906023	0.872831872248864	1	pseudobulk	DESeq2	LRT	0
147	KC_AB2	SCaMC	0.0704222248439911	0.91893865828682	1	pseudobulk	DESeq2	LRT	0
147	KC_AB2	Scamp	-0.346748581713499	0.558239267156281	1	pseudobulk	DESeq2	LRT	0
147	KC_AB2	SCAP	-0.632403201200654	0.256306765724097	1	pseudobulk	DESeq2	LRT	0
147	KC_AB2	SCAR	0.352091737256133	0.631214309733837	1	pseudobulk	DESeq2	LRT	0
147	KC_AB2	scat	-0.382929749762112	0.851961810578875	1	pseudobulk	DESeq2	LRT	0
147	KC_AB2	scb	-1.63925793886161	0.546290859679341	1	pseudobulk	DESeq2	LRT	0
147	KC_AB2	Sccpdh1	-0.482173055655404	0.804949101714764	1	pseudobulk	DESeq2	LRT	0
147	KC_AB2	SCCRO	-0.153658068866786	0.855625557662143	1	pseudobulk	DESeq2	LRT	0
147	KC_AB2	SCCRO3	-0.181015942704333	0.795349041024612	1	pseudobulk	DESeq2	LRT	0
147	KC_AB2	SCCRO4	-0.273967271050399	0.71928421222874	1	pseudobulk	DESeq2	LRT	0
147	KC_AB2	Sce	0.485366027558762	0.700218056002204	1	pseudobulk	DESeq2	LRT	0
147	KC_AB2	sced	0.0927096185524118	0.862287637997264	1	pseudobulk	DESeq2	LRT	0
147	KC_AB2	scf	0.00557252249446438	0.979040317850745	1	pseudobulk	DESeq2	LRT	0
147	KC_AB2	Scgalpha	-0.101074278150044	0.89332214686374	1	pseudobulk	DESeq2	LRT	0
147	KC_AB2	Scgbeta	-0.540437122883399	0.284041163103701	1	pseudobulk	DESeq2	LRT	0
147	KC_AB2	Scgdelta	-0.427431139129811	1	1	pseudobulk	DESeq2	LRT	0
147	KC_AB2	Schip1	-0.483364814732502	0.39775202133368	1	pseudobulk	DESeq2	LRT	0
147	KC_AB2	schlank	-0.187277739947587	0.793790178546506	1	pseudobulk	DESeq2	LRT	0
147	KC_AB2	Scm	-0.0030604664205696	0.831977160071028	1	pseudobulk	DESeq2	LRT	0

147	KC_AB2	scny	0.29335586098125	0.775738332610004	1	pseudobulk	DESeq2	LRT	0
147	KC_AB2	SCOT	-1.3723417613553	0.619532036522	1	pseudobulk	DESeq2	LRT	0
147	KC_AB2	Scox	-1.40607187404674	0.374996996131114	1	pseudobulk	DESeq2	LRT	0
147	KC_AB2	ScpX	-1.69577322030563	0.369299286678392	1	pseudobulk	DESeq2	LRT	0
147	KC_AB2	scramb1	0.314887250043695	0.743531284607975	1	pseudobulk	DESeq2	LRT	0
147	KC_AB2	scramb2	-0.153523371929089	0.845725782539323	1	pseudobulk	DESeq2	LRT	0
147	KC_AB2	scrib	0.135028813707469	0.590919564926823	1	pseudobulk	DESeq2	LRT	0
147	KC_AB2	scro	-1.12475160732157	0.574429473125171	1	pseudobulk	DESeq2	LRT	0
147	KC_AB2	scrt	0.464037595269305	0.186231461338878	1	pseudobulk	DESeq2	LRT	0
147	KC_AB2	Scsalpha1	0.0979690043782391	0.795511343367405	1	pseudobulk	DESeq2	LRT	0
147	KC_AB2	ScsbetaA	-0.222457654050536	0.61503564162945	1	pseudobulk	DESeq2	LRT	0
147	KC_AB2	ScsbetaG	-0.427431139129811	1	1	pseudobulk	DESeq2	LRT	0
147	KC_AB2	scu	-1.04997576528788	0.542822999158155	1	pseudobulk	DESeq2	LRT	0
147	KC_AB2	scyl	-0.646386878648621	0.0421593507893674	1	pseudobulk	DESeq2	LRT	0
148	KC_AB2	sd	-0.0375758733994619	0.938618226889982	1	pseudobulk	DESeq2	LRT	0
148	KC_AB2	Sdc	-0.0094128760874497	0.93173735716452	1	pseudobulk	DESeq2	LRT	0
148	KC_AB2	SdhA	-0.149055614555643	0.776557477361266	1	pseudobulk	DESeq2	LRT	0
148	KC_AB2	Sdhaf3	-1.14733297160428	0.508767252516577	1	pseudobulk	DESeq2	LRT	0
148	KC_AB2	SdhB	0.111122815519708	0.830377840098649	1	pseudobulk	DESeq2	LRT	0
148	KC_AB2	SdhC	0.0783016339250287	0.783423940504126	1	pseudobulk	DESeq2	LRT	0
148	KC_AB2	SdhD	-0.376324716537051	0.429753438155598	1	pseudobulk	DESeq2	LRT	0
148	KC_AB2	sds22	-0.792485885433839	0.683191783669831	1	pseudobulk	DESeq2	LRT	0
148	KC_AB2	sdt	-0.142493030477835	0.484890711577318	1	pseudobulk	DESeq2	LRT	0
148	KC_AB2	Sec10	-1.39789307557073	0.488037514210405	1	pseudobulk	DESeq2	LRT	0
148	KC_AB2	Sec13	-0.248684006771773	0.764072064082403	1	pseudobulk	DESeq2	LRT	0
148	KC_AB2	Sec15	-0.841882716738579	1	1	pseudobulk	DESeq2	LRT	0
148	KC_AB2	Sec16	0.101982798826678	0.888393239741434	1	pseudobulk	DESeq2	LRT	0
148	KC_AB2	Sec22	-0.262670794631894	0.780424537401321	1	pseudobulk	DESeq2	LRT	0
148	KC_AB2	Sec23	-0.906080737140215	0.394185138547892	1	pseudobulk	DESeq2	LRT	0
148	KC_AB2	Sec24AB	-0.607783240243772	0.573964742343888	1	pseudobulk	DESeq2	LRT	0
148	KC_AB2	Sec24CD	0.468723696978071	0.634682120331201	1	pseudobulk	DESeq2	LRT	0
148	KC_AB2	Sec3	-0.427431139129811	1	1	pseudobulk	DESeq2	LRT	0
148	KC_AB2	Sec31	-0.134369194205879	0.832208605970988	1	pseudobulk	DESeq2	LRT	0
148	KC_AB2	Sec5	0.0111265215899964	0.948689921659074	1	pseudobulk	DESeq2	LRT	0
148	KC_AB2	Sec6	-0.251731352200388	0.812439362653395	1	pseudobulk	DESeq2	LRT	0
148	KC_AB2	Sec61alpha	-0.854295117945873	0.558217297616779	1	pseudobulk	DESeq2	LRT	0
148	KC_AB2	Sec61beta	-0.206284337734469	0.805330374299526	1	pseudobulk	DESeq2	LRT	0
148	KC_AB2	Sec61gamma	-1.92630590995644	0.0414583459906354	1	pseudobulk	DESeq2	LRT	0
148	KC_AB2	Sec63	-0.84234303800674	0.497544594610959	1	pseudobulk	DESeq2	LRT	0
148	KC_AB2	Sec71	-2.25026703842057	0.308244700817443	1	pseudobulk	DESeq2	LRT	0
148	KC_AB2	Sec8	0.914462297515877	0.47279058445707	1	pseudobulk	DESeq2	LRT	0
148	KC_AB2	Secp43	1.03797333083192	0.383614717313534	1	pseudobulk	DESeq2	LRT	0
148	KC_AB2	SecS	-0.567201655705209	0.577199917475237	1	pseudobulk	DESeq2	LRT	0
148	KC_AB2	sei	1.11864239324499	0.258516548333116	1	pseudobulk	DESeq2	LRT	0
148	KC_AB2	SelG	0.151516921185839	0.878709683775074	1	pseudobulk	DESeq2	LRT	0
148	KC_AB2	SelR	-0.475119047766557	0.297020660175348	1	pseudobulk	DESeq2	LRT	0
148	KC_AB2	SelT	-0.0327556975929517	0.946093480563323	1	pseudobulk	DESeq2	LRT	0
148	KC_AB2	Sem1	-0.241840631018037	0.826922114844876	1	pseudobulk	DESeq2	LRT	0
148	KC_AB2	Sema1a	0.538104322939992	0.174629322963327	1	pseudobulk	DESeq2	LRT	0
148	KC_AB2	Sema1b	0.701824352663177	0.533168389782615	1	pseudobulk	DESeq2	LRT	0
148	KC_AB2	Sema2a	0.157912715188726	0.792417533298631	1	pseudobulk	DESeq2	LRT	0
148	KC_AB2	Sema2b	2.27464007273167	0.156658886551626	1	pseudobulk	DESeq2	LRT	0
148	KC_AB2	senju	-2.46542249222033	0.239120144068241	1	pseudobulk	DESeq2	LRT	0

148	KC_AB2	Sep1	-0.932853220605373	0.426221301108562	1	pseudobulk	DESeq2	LRT		0
148	KC_AB2	Sep2	0.764600416013492	0.425108317293397	1	pseudobulk	DESeq2	LRT		0
148	KC_AB2	Sep4	-0.657354883501796	0.296209495785531	1	pseudobulk	DESeq2	LRT		0
148	KC_AB2	SERCA	0.212127378052893	0.689629282244656	1	pseudobulk	DESeq2	LRT		0
148	KC_AB2	SerRS	1.10817761709104	0.660411427470312	1	pseudobulk	DESeq2	LRT		0
148	KC_AB2	SerRS-m	0.773551146205319	0.702323084538382	1	pseudobulk	DESeq2	LRT		0
148	KC_AB2	sesB	-0.274995252924703	0.291688940204227	1	pseudobulk	DESeq2	LRT		0
148	KC_AB2	Sesn	1.23085716405798	0.614623732585597	1	pseudobulk	DESeq2	LRT		0
148	KC_AB2	Set	0.514466217683027	0.563451969179513	1	pseudobulk	DESeq2	LRT		0
148	KC_AB2	Set1	-0.272887115286231	0.685819857839243	1	pseudobulk	DESeq2	LRT		0
148	KC_AB2	Set2	0.583265811995711	0.712413120111036	1	pseudobulk	DESeq2	LRT		0
148	KC_AB2	Setd3	0.703141171373176	0.67065453296144	1	pseudobulk	DESeq2	LRT		0
148	KC_AB2	SF1	0.781516587862844	0.537748587349733	1	pseudobulk	DESeq2	LRT		0
148	KC_AB2	SF2	-0.401047512198975	0.473320808096409	1	pseudobulk	DESeq2	LRT		0
148	KC_AB2	Sf3a1	1.56063818203459	0.222219747064702	1	pseudobulk	DESeq2	LRT		0
148	KC_AB2	Sf3a2	0.855762156883605	0.640245416406156	1	pseudobulk	DESeq2	LRT		0
148	KC_AB2	Sf3b1	-0.657707174049842	0.638137169872918	1	pseudobulk	DESeq2	LRT		0
148	KC_AB2	Sf3b2	-1.32094497785632	0.227500268841186	1	pseudobulk	DESeq2	LRT		0
148	KC_AB2	Sf3b3	-2.16906696862276	0.324569721711647	1	pseudobulk	DESeq2	LRT		0
148	KC_AB2	Sf3b5	1.74768074483375	0.0303433608446354	1	pseudobulk	DESeq2	LRT		0
148	KC_AB2	Sf3b6	-0.0935663029022267	0.898912310472949	1	pseudobulk	DESeq2	LRT		0
148	KC_AB2	sff	0.368145171269802	0.495386960968192	1	pseudobulk	DESeq2	LRT		0
148	KC_AB2	sfl	-0.127496091024648	0.824747837211569	1	pseudobulk	DESeq2	LRT		0
148	KC_AB2	Sfmbt	-0.678521272049388	0.5688666287053	1	pseudobulk	DESeq2	LRT		0
148	KC_AB2	Sfxn1-3	-0.140489984033679	0.851859385926505	1	pseudobulk	DESeq2	LRT		0
148	KC_AB2	Sfxn2	-0.620344921270263	0.638440342884503	1	pseudobulk	DESeq2	LRT		0
148	KC_AB2	Sgf11	-1.37002769466724	0.479308071448389	1	pseudobulk	DESeq2	LRT		0
148	KC_AB2	Sgf29	0.134982074471121	0.968421605092439	1	pseudobulk	DESeq2	LRT		0
148	KC_AB2	sgg	0.0355671634163114	0.812518574997771	1	pseudobulk	DESeq2	LRT		0
148	KC_AB2	sgl	0.346589616049749	0.84654468695694	1	pseudobulk	DESeq2	LRT		0
148	KC_AB2	sgll	2.07946313289983	0.357635956314506	1	pseudobulk	DESeq2	LRT		0
148	KC_AB2	Sgt	-0.0853077603626937	0.846523120205619	1	pseudobulk	DESeq2	LRT		0
148	KC_AB2	Sgt1	0.388044739521178	0.724978328344578	1	pseudobulk	DESeq2	LRT		0
148	KC_AB2	Sh	0.0130598262549837	0.911302906753494	1	pseudobulk	DESeq2	LRT		0
148	KC_AB2	Sh3beta	-0.473452093178147	0.482504996512904	1	pseudobulk	DESeq2	LRT		0
148	KC_AB2	SH3PX1	-0.868279001492672	0.554397223183727	1	pseudobulk	DESeq2	LRT		0
148	KC_AB2	Shab	-0.114710867860568	0.745438951714778	1	pseudobulk	DESeq2	LRT		0
148	KC_AB2	shakB	-0.0416402312198016	0.875461738369318	1	pseudobulk	DESeq2	LRT		0
148	KC_AB2	Shal	-0.168859305354081	0.664313345280835	1	pseudobulk	DESeq2	LRT		0
148	KC_AB2	shams	1.37302911771381	0.54957623980966	1	pseudobulk	DESeq2	LRT		0
148	KC_AB2	Shaw	0.274821401160593	0.668639667635597	1	pseudobulk	DESeq2	LRT		0
148	KC_AB2	Shawl	0.36188826123571	0.344823540888847	1	pseudobulk	DESeq2	LRT		0
148	KC_AB2	Shc	-1.18349517086566	0.609141822494309	1	pseudobulk	DESeq2	LRT		0
148	KC_AB2	shep	-0.429690214713783	0.088256313079118	1	pseudobulk	DESeq2	LRT		0
148	KC_AB2	shg	-1.07776915880798	0.775491075088671	1	pseudobulk	DESeq2	LRT		0
148	KC_AB2	shi	0.0422629155358708	0.828655497799457	1	pseudobulk	DESeq2	LRT		0
148	KC_AB2	shn	2.04515758669385	0.346545205091149	1	pseudobulk	DESeq2	LRT		0
148	KC_AB2	shop	1.37785179899409	0.533959925164643	1	pseudobulk	DESeq2	LRT		0
148	KC_AB2	shot	-0.211960119238534	0.507045465933707	1	pseudobulk	DESeq2	LRT		0
148	KC_AB2	shrb	-0.814126304385231	0.416250675245337	1	pseudobulk	DESeq2	LRT		0
148	KC_AB2	shv	-2.06407076736304	0.362444518317458	1	pseudobulk	DESeq2	LRT		0
148	KC_AB2	sicily	-0.465605661365784	0.791706020110304	1	pseudobulk	DESeq2	LRT		0
148	KC_AB2	sick	-0.0321090655462827	0.938278809623878	1	pseudobulk	DESeq2	LRT		0

148	KC_AB2	side	-0.427431139129811	1	1	pseudobulk	DESeq2	LRT		0
148	KC_AB2	side-II	-0.135372794959915	0.601114955454053	1	pseudobulk	DESeq2	LRT		0
148	KC_AB2	side-III	-0.13145080507252	0.719288979179322	1	pseudobulk	DESeq2	LRT		0
148	KC_AB2	side-IV	-1.12245224691824	0.563423324403402	1	pseudobulk	DESeq2	LRT		0
148	KC_AB2	side-V	0.244458660221086	0.795894662646242	1	pseudobulk	DESeq2	LRT		0
148	KC_AB2	side-VI	-0.0608172766795996	0.844169516330044	1	pseudobulk	DESeq2	LRT		0
148	KC_AB2	side-VII	0.113715634899483	0.8236940236634	1	pseudobulk	DESeq2	LRT		0
148	KC_AB2	side-VIII	-0.600547221779134	1	1	pseudobulk	DESeq2	LRT		0
149	KC_AB2	SIDL	-0.209984574829262	0.825826756920249	1	pseudobulk	DESeq2	LRT		0
149	KC_AB2	Sidpn	-0.756402310184519	0.558387592289531	1	pseudobulk	DESeq2	LRT		0
149	KC_AB2	sif	-0.244261278388677	0.515113316889736	1	pseudobulk	DESeq2	LRT		0
149	KC_AB2	SIFa	-0.600547221779133	0.841940696994101	1	pseudobulk	DESeq2	LRT		0
149	KC_AB2	SIFaR	0.0859966171561038	0.888750583775208	1	pseudobulk	DESeq2	LRT		0
149	KC_AB2	sigmar	0.360739644253486	0.65012919496741	1	pseudobulk	DESeq2	LRT		0
149	KC_AB2	Sik2	0.629312849717786	0.606510833949087	1	pseudobulk	DESeq2	LRT		0
149	KC_AB2	Sik3	-0.434705273607504	0.60599466592259	1	pseudobulk	DESeq2	LRT		0
149	KC_AB2	Sil1	-2.48435623267434	0.256496613429614	1	pseudobulk	DESeq2	LRT		0
149	KC_AB2	sima	0.744098347273929	0.155903660557879	1	pseudobulk	DESeq2	LRT		0
149	KC_AB2	simj	0.0984953874726454	0.756925117838912	1	pseudobulk	DESeq2	LRT		0
149	KC_AB2	Sin	-0.427431139129811	1	1	pseudobulk	DESeq2	LRT		0
149	KC_AB2	Sin1	-1.88866155730992	0.344193058535892	1	pseudobulk	DESeq2	LRT		0
149	KC_AB2	Sin3A	0.383070672071391	0.410274314345683	1	pseudobulk	DESeq2	LRT		0
149	KC_AB2	sina	-0.0388431936999803	0.930994171914437	1	pseudobulk	DESeq2	LRT		0
149	KC_AB2	sinu	-2.71632541924797	0.187022462241557	1	pseudobulk	DESeq2	LRT		0
149	KC_AB2	sip1	2.12684015898615	0.445939223035924	1	pseudobulk	DESeq2	LRT		0
149	KC_AB2	sip3	-0.286290266094793	0.957909532056083	1	pseudobulk	DESeq2	LRT		0
149	KC_AB2	Sirt1	0.0428569747896205	0.871588520995679	1	pseudobulk	DESeq2	LRT		0
149	KC_AB2	Sirt2	0.15062799533246	0.865283858944065	1	pseudobulk	DESeq2	LRT		0
149	KC_AB2	Sirt4	0.421346619905412	0.670414220891648	1	pseudobulk	DESeq2	LRT		0
149	KC_AB2	Sirt6	1.23085716405798	0.614623732585597	1	pseudobulk	DESeq2	LRT		0
149	KC_AB2	Sirt7	-2.25375902476868	0.307909557410438	1	pseudobulk	DESeq2	LRT		0
149	KC_AB2	Sirup	0.72715759396364	0.376403920743398	1	pseudobulk	DESeq2	LRT		0
149	KC_AB2	sisRNA:1	1.5243995994652	0.741477491078038	1	pseudobulk	DESeq2	LRT		0
149	KC_AB2	siz	-0.0076286783309176	0.970059541913016	1	pseudobulk	DESeq2	LRT		0
149	KC_AB2	SK	-0.742646209075999	0.013794650020308	1	pseudobulk	DESeq2	LRT		0
149	KC_AB2	Sk2	2.12684015898615	0.445939223035924	1	pseudobulk	DESeq2	LRT		0
149	KC_AB2	skd	-0.0734649039841316	0.860885666088572	1	pseudobulk	DESeq2	LRT		0
149	KC_AB2	Ski6	-0.600547221779133	0.841940696994101	1	pseudobulk	DESeq2	LRT		0
149	KC_AB2	SKIP	-2.67303973337015	0.241708341257578	1	pseudobulk	DESeq2	LRT		0
149	KC_AB2	skl	0.692528074755167	0.613663031098661	1	pseudobulk	DESeq2	LRT		0
149	KC_AB2	SkpA	-0.2126693209516	0.59832738578539	1	pseudobulk	DESeq2	LRT		0
149	KC_AB2	sky	-0.21778476708315	0.513333259155127	1	pseudobulk	DESeq2	LRT		0
149	KC_AB2	sl	-0.482173055655404	0.804949101714764	1	pseudobulk	DESeq2	LRT		0
149	KC_AB2	Slbp	-0.789760659376432	0.557392363010031	1	pseudobulk	DESeq2	LRT		0
149	KC_AB2	SLC22A	-0.284260476544379	0.906742499605733	1	pseudobulk	DESeq2	LRT		0
149	KC_AB2	Slc25A46a	2.11979463626023	0.282224548963575	1	pseudobulk	DESeq2	LRT		0
149	KC_AB2	SLC5A11	1.5243995994652	0.741477491078038	1	pseudobulk	DESeq2	LRT		0
149	KC_AB2	sle	-0.427431139129811	1	1	pseudobulk	DESeq2	LRT		0
149	KC_AB2	slgA	0.149996006977979	0.719256845433151	1	pseudobulk	DESeq2	LRT		0
149	KC_AB2	Slh	0.674568040846456	0.734987411917881	1	pseudobulk	DESeq2	LRT		0
149	KC_AB2	slif	-0.427431139129811	1	1	pseudobulk	DESeq2	LRT		0
149	KC_AB2	Slik	-0.0365097624987977	0.938149445961221	1	pseudobulk	DESeq2	LRT		0
149	KC_AB2	slim	0.25932083257705	0.811791993044166	1	pseudobulk	DESeq2	LRT		0

149	KC_AB2	Slip1	-0.0342312074903758	0.862420476272669	1	pseudobulk	DESeq2	LRT		0
149	KC_AB2	SLIRP2	-0.482173055655404	0.804949101714764	1	pseudobulk	DESeq2	LRT		0
149	KC_AB2	sll	-0.812631785046283	0.64219411005506	1	pseudobulk	DESeq2	LRT		0
149	KC_AB2	Slmap	-1.16299439413838	0.287759174699778	1	pseudobulk	DESeq2	LRT		0
149	KC_AB2	slmb	-0.0247984049370691	0.959540607765827	1	pseudobulk	DESeq2	LRT		0
149	KC_AB2	slmo	1.06949565642146	0.453616777336345	1	pseudobulk	DESeq2	LRT		0
149	KC_AB2	Sln	1.54932789866524	0.104488955811136	1	pseudobulk	DESeq2	LRT		0
149	KC_AB2	slo	-0.238597674213242	0.194748127914398	1	pseudobulk	DESeq2	LRT		0
149	KC_AB2	SLO2	0.133375867651723	0.736832131398223	1	pseudobulk	DESeq2	LRT		0
149	KC_AB2	Slob	0.323850076693862	0.306464438562827	1	pseudobulk	DESeq2	LRT		0
149	KC_AB2	slpr	0.775255968508184	0.281020665480519	1	pseudobulk	DESeq2	LRT		0
149	KC_AB2	Slu7	1.24836961907137	0.503455340579622	1	pseudobulk	DESeq2	LRT		0
149	KC_AB2	slv	0.126804421584346	0.915782912410038	1	pseudobulk	DESeq2	LRT		0
149	KC_AB2	slx1	-0.427431139129811	1	1	pseudobulk	DESeq2	LRT		0
149	KC_AB2	sm	0.184871195441438	0.648467967731206	1	pseudobulk	DESeq2	LRT		0
149	KC_AB2	smal	0.864341462967788	0.925947094335722	1	pseudobulk	DESeq2	LRT		0
149	KC_AB2	smash	-0.382551643265762	0.583408084345976	1	pseudobulk	DESeq2	LRT		0
149	KC_AB2	SmB	-0.862236176772297	0.511983604062217	1	pseudobulk	DESeq2	LRT		0
149	KC_AB2	SMC1	1.32711916219809	0.2797339135593	1	pseudobulk	DESeq2	LRT		0
149	KC_AB2	SMC2	-0.600547221779133	0.841940696994101	1	pseudobulk	DESeq2	LRT		0
149	KC_AB2	SMC3	0.650234962624991	0.65385776971345	1	pseudobulk	DESeq2	LRT		0
149	KC_AB2	SMC5	0.864341462967788	0.925947094335722	1	pseudobulk	DESeq2	LRT		0
149	KC_AB2	SmD1	0.709047187369859	0.629907003768152	1	pseudobulk	DESeq2	LRT		0
149	KC_AB2	SmD2	-0.20859155704074	0.831428236835744	1	pseudobulk	DESeq2	LRT		0
149	KC_AB2	SmD3	-1.36590951444543	0.239860239838382	1	pseudobulk	DESeq2	LRT		0
149	KC_AB2	SmE	-0.484919589746807	0.538394962046603	1	pseudobulk	DESeq2	LRT		0
149	KC_AB2	SmF	-0.257437497957249	0.815297834418979	1	pseudobulk	DESeq2	LRT		0
149	KC_AB2	smg	0.649934471595783	0.45657758884474	1	pseudobulk	DESeq2	LRT		0
149	KC_AB2	Smg5	-0.576203044970329	0.397890683559266	1	pseudobulk	DESeq2	LRT		0
149	KC_AB2	Smg6	0.49522746739306	0.807638127459475	1	pseudobulk	DESeq2	LRT		0
149	KC_AB2	smid	0.462270394540643	0.776433198419781	1	pseudobulk	DESeq2	LRT		0
149	KC_AB2	Smn	1.02248357785599	0.556541519223439	1	pseudobulk	DESeq2	LRT		0
149	KC_AB2	smo	0.0806715714550146	0.88136911310611	1	pseudobulk	DESeq2	LRT		0
149	KC_AB2	smog	0.0016556538234533	0.894365917683186	1	pseudobulk	DESeq2	LRT		0
149	KC_AB2	Smox	-0.133040860723527	0.721194187470386	1	pseudobulk	DESeq2	LRT		0
149	KC_AB2	smp-30	-0.246880080867625	0.883966189422952	1	pseudobulk	DESeq2	LRT		0
149	KC_AB2	Smr	-0.0582248589810104	0.806960926195767	1	pseudobulk	DESeq2	LRT		0
149	KC_AB2	Sms	-0.238943413428053	0.694385453546917	1	pseudobulk	DESeq2	LRT		0
149	KC_AB2	SMSr	0.245633957507417	0.863027200132824	1	pseudobulk	DESeq2	LRT		0
149	KC_AB2	smt3	-0.550926257056684	0.120935757530968	1	pseudobulk	DESeq2	LRT		0
149	KC_AB2	Smu1	-1.22611802355401	0.528829772451552	1	pseudobulk	DESeq2	LRT		0
149	KC_AB2	Smurf	-0.39517987970357	0.683830678221099	1	pseudobulk	DESeq2	LRT		0
149	KC_AB2	Smyd3	0.640757114944055	0.724386129653129	1	pseudobulk	DESeq2	LRT		0
149	KC_AB2	Smyd4-4	-0.76382388514627	0.651762467548631	1	pseudobulk	DESeq2	LRT		0
149	KC_AB2	SmydA-1	1.5243995994652	0.741477491078038	1	pseudobulk	DESeq2	LRT		0
149	KC_AB2	SmydA-3	-0.671196855135827	0.716569806744831	1	pseudobulk	DESeq2	LRT		0
149	KC_AB2	SmydA-7	-0.841882716738579	1	1	pseudobulk	DESeq2	LRT		0
149	KC_AB2	sn	1.14186374083846	0.300158783969307	1	pseudobulk	DESeq2	LRT		0
149	KC_AB2	snama	-2.01192601719761	0.25652455100293	1	pseudobulk	DESeq2	LRT		0
149	KC_AB2	Snap24	-0.0549980703544417	0.908101282166394	1	pseudobulk	DESeq2	LRT		0
149	KC_AB2	Snap25	0.0508935770452194	0.811719952327772	1	pseudobulk	DESeq2	LRT		0
149	KC_AB2	Snap29	0.0166662253408525	0.96766857979316	1	pseudobulk	DESeq2	LRT		0
149	KC_AB2	snf	-0.225039748954947	0.749775303383409	1	pseudobulk	DESeq2	LRT		0

149	KC_AB2	SNF4Agamma	-0.469630848325639	0.540319259288127	1	pseudobulk	DESeq2	LRT		0
149	KC_AB2	sni	0.0254364124610951	0.9853505695896	1	pseudobulk	DESeq2	LRT		0
150	KC_AB2	Snm1	-0.427431139129811	1	1	pseudobulk	DESeq2	LRT		0
150	KC_AB2	sno	0.665413478880216	0.218422577508945	1	pseudobulk	DESeq2	LRT		0
150	KC_AB2	Snoo	0.16670331147182	0.675954266632523	1	pseudobulk	DESeq2	LRT		0
150	KC_AB2	snoRNA:lola-c	1.5243995994652	0.741477491078038	1	pseudobulk	DESeq2	LRT		0
150	KC_AB2	snoRNA:Me28S-C	-0.427431139129811	1	1	pseudobulk	DESeq2	LRT		0
150	KC_AB2	snoRNA:Psi28S-1	1.5243995994652	0.741477491078038	1	pseudobulk	DESeq2	LRT		0
150	KC_AB2	snoRNA:Psi28S-3	-0.427431139129811	1	1	pseudobulk	DESeq2	LRT		0
150	KC_AB2	Snp	-2.12929393505405	0.232428770089952	1	pseudobulk	DESeq2	LRT		0
150	KC_AB2	sNPF	0.046801505789197	0.803336122670704	1	pseudobulk	DESeq2	LRT		0
150	KC_AB2	Snr1	-0.123944117872924	0.890874111284181	1	pseudobulk	DESeq2	LRT		0
150	KC_AB2	snRNA:7SK	-0.942785371791517	0.301740384417529	1	pseudobulk	DESeq2	LRT		0
150	KC_AB2	snRNA:U12	-1.51928970377954	0.486908946651791	1	pseudobulk	DESeq2	LRT		0
150	KC_AB2	snRNA:U6:96Ab	-0.592481036256484	0.706847545932384	1	pseudobulk	DESeq2	LRT		0
150	KC_AB2	snRNP-U1-70K	0.396750716786301	0.568012198508839	1	pseudobulk	DESeq2	LRT		0
150	KC_AB2	snRNP-U1-C	-0.711243604785863	0.57198010007847	1	pseudobulk	DESeq2	LRT		0
150	KC_AB2	SNRPG	-0.442574377008828	0.521463299737893	1	pseudobulk	DESeq2	LRT		0
150	KC_AB2	snu	-0.427431139129811	1	1	pseudobulk	DESeq2	LRT		0
150	KC_AB2	Snx1	-0.768778191978599	0.643910962984695	1	pseudobulk	DESeq2	LRT		0
150	KC_AB2	Snx16	-0.856078077825008	0.623769054102708	1	pseudobulk	DESeq2	LRT		0
150	KC_AB2	Snx17	0.500691504109503	0.447265524352311	1	pseudobulk	DESeq2	LRT		0
150	KC_AB2	Snx21	0.791468033138705	0.699865413482108	1	pseudobulk	DESeq2	LRT		0
150	KC_AB2	Snx27	0.0413214594878537	0.864176557866663	1	pseudobulk	DESeq2	LRT		0
150	KC_AB2	Snx3	-0.0800683275229915	0.914085052917092	1	pseudobulk	DESeq2	LRT		0
150	KC_AB2	Snx6	0.478841732173452	0.612815798259679	1	pseudobulk	DESeq2	LRT		0
150	KC_AB2	snz	-0.475738693876102	0.699442630538439	1	pseudobulk	DESeq2	LRT		0
150	KC_AB2	Socs16D	0.444945713422909	0.463285012616213	1	pseudobulk	DESeq2	LRT		0
150	KC_AB2	Socs36E	0.381558382284351	0.487209588959741	1	pseudobulk	DESeq2	LRT		0
150	KC_AB2	Socs44A	-0.696583899755645	0.498822459087656	1	pseudobulk	DESeq2	LRT		0
150	KC_AB2	Sod1	-0.299255740432345	0.342992255460715	1	pseudobulk	DESeq2	LRT		0
150	KC_AB2	Sod2	-0.300908721696564	0.681221122154521	1	pseudobulk	DESeq2	LRT		0
150	KC_AB2	Sod3	1.23687360539747	0.522258627543994	1	pseudobulk	DESeq2	LRT		0
150	KC_AB2	sol	-0.090322596421031	0.886078272476845	1	pseudobulk	DESeq2	LRT		0
150	KC_AB2	Son	0.688004250342319	0.495712757581536	1	pseudobulk	DESeq2	LRT		0
150	KC_AB2	Sos	1.41071272988911	0.330607993616981	1	pseudobulk	DESeq2	LRT		0
150	KC_AB2	sotv	-0.408208341425708	0.706213826421829	1	pseudobulk	DESeq2	LRT		0
150	KC_AB2	sov	-0.373292324527099	0.540971019228042	1	pseudobulk	DESeq2	LRT		0
150	KC_AB2	sowah	1.2626208478484	0.258205507563006	1	pseudobulk	DESeq2	LRT		0
150	KC_AB2	Sox14	-0.600547221779134	1	1	pseudobulk	DESeq2	LRT		0
150	KC_AB2	SP1173	0.48537355354838	0.164367264619242	1	pseudobulk	DESeq2	LRT		0
150	KC_AB2	SP2353	0.669718686959781	0.744188515542868	1	pseudobulk	DESeq2	LRT		0
150	KC_AB2	spab	-0.0049552196745380	0.984369533866969	1	pseudobulk	DESeq2	LRT		0
150	KC_AB2	spag	-0.772768427838894	0.67287259907463	1	pseudobulk	DESeq2	LRT		0
150	KC_AB2	SPARC	0.725498497157359	0.93879047033739	1	pseudobulk	DESeq2	LRT		0
150	KC_AB2	spartin	-1.18529493787286	0.474759895137805	1	pseudobulk	DESeq2	LRT		0
150	KC_AB2	spas	1.06178370466418	0.318236659512875	1	pseudobulk	DESeq2	LRT		0
150	KC_AB2	Spase12	0.502259906340665	0.601283337675103	1	pseudobulk	DESeq2	LRT		0
150	KC_AB2	Spase22-23	-1.10646770029314	0.337722749531352	1	pseudobulk	DESeq2	LRT		0
150	KC_AB2	Spase25	0.384756694967145	0.657856722207619	1	pseudobulk	DESeq2	LRT		0
150	KC_AB2	spdo	0.70378358684898	0.932301451973468	1	pseudobulk	DESeq2	LRT		0
150	KC_AB2	SpdS	-0.144283299427928	0.851536972013326	1	pseudobulk	DESeq2	LRT		0
150	KC_AB2	Spec2	0.290677432798505	0.842113648321692	1	pseudobulk	DESeq2	LRT		0

150	KC_AB2	spel1	-1.6150095599849	0.519549602003096	1	pseudobulk	DESeq2	LRT		0
150	KC_AB2	spen	-0.112207035987921	0.601768067970126	1	pseudobulk	DESeq2	LRT		0
150	KC_AB2	Spf45	0.0713417329599653	0.849101539243196	1	pseudobulk	DESeq2	LRT		0
150	KC_AB2	spg	2.60449690258161	0.170888565624672	1	pseudobulk	DESeq2	LRT		0
150	KC_AB2	Spg7	-0.400704878443422	0.808750298973408	1	pseudobulk	DESeq2	LRT		0
150	KC_AB2	spi	0.0578520043204766	0.912466337090332	1	pseudobulk	DESeq2	LRT		0
150	KC_AB2	spict	1.50255200571414	0.44025635980316	1	pseudobulk	DESeq2	LRT		0
150	KC_AB2	spidey	-0.560244940354852	0.386210387865086	1	pseudobulk	DESeq2	LRT		0
150	KC_AB2	spin	-1.16159699984361	0.496963930542756	1	pseudobulk	DESeq2	LRT		0
150	KC_AB2	spir	0.205060749391038	0.548183647654637	1	pseudobulk	DESeq2	LRT		0
150	KC_AB2	sPLA2	-0.119831229562752	1	1	pseudobulk	DESeq2	LRT		0
150	KC_AB2	Sply	-1.06582837126129	0.419472684586104	1	pseudobulk	DESeq2	LRT		0
150	KC_AB2	Spn	-0.231858399682667	0.486551136762025	1	pseudobulk	DESeq2	LRT		0
150	KC_AB2	spn-F	0.997950432548483	0.520665954048796	1	pseudobulk	DESeq2	LRT		0
150	KC_AB2	Spn42Da	-1.22254618054459	0.544834385284281	1	pseudobulk	DESeq2	LRT		0
150	KC_AB2	Spn43Ab	1.82022151615841	0.392550578096768	1	pseudobulk	DESeq2	LRT		0
150	KC_AB2	SPoCk	-0.0918957600937776	0.824470644772677	1	pseudobulk	DESeq2	LRT		0
150	KC_AB2	spoon	-1.88775788947444	0.190493294567213	1	pseudobulk	DESeq2	LRT		0
150	KC_AB2	Spp	-3.58211612529554	0.0574393812091751	1	pseudobulk	DESeq2	LRT		0
150	KC_AB2	SppL	0.308811238907445	0.757307451616507	1	pseudobulk	DESeq2	LRT		0
150	KC_AB2	Spps	2.44288728698702	0.174517898007887	1	pseudobulk	DESeq2	LRT		0
150	KC_AB2	SPR	-2.04938525072738	0.386701250685179	1	pseudobulk	DESeq2	LRT		0
150	KC_AB2	Spred	-0.199498192175956	0.506154222829786	1	pseudobulk	DESeq2	LRT		0
150	KC_AB2	spri	-1.0959887080913	0.548011973135596	1	pseudobulk	DESeq2	LRT		0
150	KC_AB2	Sps1	-0.055716669860236	0.908649369064948	1	pseudobulk	DESeq2	LRT		0
150	KC_AB2	Sps2	-0.427431139129811	1	1	pseudobulk	DESeq2	LRT		0
150	KC_AB2	Spt	1.8688760377645	0.356303822006979	1	pseudobulk	DESeq2	LRT		0
150	KC_AB2	Spt-I	1.29277529338799	0.188457826635709	1	pseudobulk	DESeq2	LRT		0
150	KC_AB2	Spt20	0.0826619549893501	0.875929861588074	1	pseudobulk	DESeq2	LRT		0
150	KC_AB2	Spt3	2.07946313289983	0.357635956314506	1	pseudobulk	DESeq2	LRT		0
150	KC_AB2	spt4	0.674325408286308	0.528525708812258	1	pseudobulk	DESeq2	LRT		0
150	KC_AB2	Spt5	0.0382050993768415	0.949871042757746	1	pseudobulk	DESeq2	LRT		0
150	KC_AB2	Spt6	0.72911530581051	0.455853687265109	1	pseudobulk	DESeq2	LRT		0
150	KC_AB2	Spt7	-1.12475160732157	0.574429473125171	1	pseudobulk	DESeq2	LRT		0
150	KC_AB2	Sptr	-0.756316049197303	0.451411656385784	1	pseudobulk	DESeq2	LRT		0
150	KC_AB2	Spx	-2.57659675686206	0.241930649376094	1	pseudobulk	DESeq2	LRT		0
150	KC_AB2	spz	1.85537185660574	0.410490945917095	1	pseudobulk	DESeq2	LRT		0
150	KC_AB2	sqd	-0.030672912681357	0.795453907148653	1	pseudobulk	DESeq2	LRT		0
150	KC_AB2	sqh	-0.682990204197339	0.260996352911528	1	pseudobulk	DESeq2	LRT		0
150	KC_AB2	sqz	1.43191469273003	0.651609831456347	1	pseudobulk	DESeq2	LRT		0
150	KC_AB2	sra	-0.541076959761417	0.141311106394838	1	pseudobulk	DESeq2	LRT		0
150	KC_AB2	Sra-1	-2.36690004770124	0.282527465979357	1	pseudobulk	DESeq2	LRT		0
150	KC_AB2	Sras	0.555357365394912	0.764907956103394	1	pseudobulk	DESeq2	LRT		0
150	KC_AB2	Src42A	-0.190865788144741	0.749788486349735	1	pseudobulk	DESeq2	LRT		0
150	KC_AB2	Src64B	-0.307686135300344	0.625123692964751	1	pseudobulk	DESeq2	LRT		0
150	KC_AB2	SREBP	0.090922731717536	0.819517313553611	1	pseudobulk	DESeq2	LRT		0
150	KC_AB2	Srlp	-0.052527168753783	0.971722578397277	1	pseudobulk	DESeq2	LRT		0
150	KC_AB2	SRm160	-0.396038435332246	0.58994111227296	1	pseudobulk	DESeq2	LRT		0
150	KC_AB2	srp	-0.427431139129811	1	1	pseudobulk	DESeq2	LRT		0
151	KC_AB2	Srp14	-1.29340579945306	0.300058502341897	1	pseudobulk	DESeq2	LRT		0
151	KC_AB2	Srp19	-0.155441980429581	0.859165964626813	1	pseudobulk	DESeq2	LRT		0
151	KC_AB2	Srp54	0.761887215797067	0.353236299989179	1	pseudobulk	DESeq2	LRT		0
151	KC_AB2	Srp54k	0.312836475827802	0.823736929110263	1	pseudobulk	DESeq2	LRT		0

151	KC_AB2	Srp68	-1.54470851095603	0.55301747381519	1	pseudobulk	DESeq2	LRT	0
151	KC_AB2	Srp72	-0.525980220958878	0.813340603474339	1	pseudobulk	DESeq2	LRT	0
151	KC_AB2	Srp9	1.05321418427322	0.435014491640701	1	pseudobulk	DESeq2	LRT	0
151	KC_AB2	SRPK	-0.0287354624282611	0.86088294040186	1	pseudobulk	DESeq2	LRT	0
151	KC_AB2	Srpk79D	-0.43383149620722	0.723000568634778	1	pseudobulk	DESeq2	LRT	0
151	KC_AB2	SrpRbeta	1.36311188082674	0.403360363733115	1	pseudobulk	DESeq2	LRT	0
151	KC_AB2	Sry-delta	-0.0852743682753834	0.917718671153184	1	pseudobulk	DESeq2	LRT	0
151	KC_AB2	Ssadh	-0.710086369187933	0.474324652706969	1	pseudobulk	DESeq2	LRT	0
151	KC_AB2	Ssb-c31a	-0.757597497975363	0.525092881902072	1	pseudobulk	DESeq2	LRT	0
151	KC_AB2	Ssdp	-0.10989736227281	0.740188397943603	1	pseudobulk	DESeq2	LRT	0
151	KC_AB2	ssh	0.84192686591552	0.355578955877213	1	pseudobulk	DESeq2	LRT	0
151	KC_AB2	Ssk	-0.427431139129811	1	1	pseudobulk	DESeq2	LRT	0
151	KC_AB2	Ssl1	0.864341462967788	0.925947094335722	1	pseudobulk	DESeq2	LRT	0
151	KC_AB2	ssp	-0.427431139129811	1	1	pseudobulk	DESeq2	LRT	0
151	KC_AB2	ssp3	0.390058007138359	0.688489199640648	1	pseudobulk	DESeq2	LRT	0
151	KC_AB2	ssp6	-0.841882716738579	1	1	pseudobulk	DESeq2	LRT	0
151	KC_AB2	SsRbeta	-0.453966811185393	0.670965558149812	1	pseudobulk	DESeq2	LRT	0
151	KC_AB2	Ssrp	-0.397720808073528	0.939555995513386	1	pseudobulk	DESeq2	LRT	0
151	KC_AB2	sstn	-2.12214992510756	0.367174323489033	1	pseudobulk	DESeq2	LRT	0
151	KC_AB2	Ssu72	0.892659559600706	0.712043317464255	1	pseudobulk	DESeq2	LRT	0
151	KC_AB2	ssx	0.0677610757598425	0.846792922136995	1	pseudobulk	DESeq2	LRT	0
151	KC_AB2	St1	-0.427431139129811	1	1	pseudobulk	DESeq2	LRT	0
151	KC_AB2	St3	-1.05525863138807	0.126643434831785	1	pseudobulk	DESeq2	LRT	0
151	KC_AB2	sta	-0.797320151695735	0.0745494211784662	1	pseudobulk	DESeq2	LRT	0
151	KC_AB2	stac	-0.600547221779133	0.841940696994101	1	pseudobulk	DESeq2	LRT	0
151	KC_AB2	Stacl	-0.514207156755239	0.00839411479228829	1	pseudobulk	DESeq2	LRT	0
151	KC_AB2	stai	-0.145092110703501	0.464244944260213	1	pseudobulk	DESeq2	LRT	0
151	KC_AB2	Stam	-0.685271178276758	0.592584814531523	1	pseudobulk	DESeq2	LRT	0
151	KC_AB2	stan	2.71015416549738	0.182821022008861	1	pseudobulk	DESeq2	LRT	0
151	KC_AB2	Start1	1.9758607418779	0.322720287118553	1	pseudobulk	DESeq2	LRT	0
151	KC_AB2	stas	0.946967744696415	0.0995015931947653	1	pseudobulk	DESeq2	LRT	0
151	KC_AB2	Stat92E	-0.0773249282415757	0.848461281722304	1	pseudobulk	DESeq2	LRT	0
151	KC_AB2	stau	0.205015517692971	0.732870342191858	1	pseudobulk	DESeq2	LRT	0
151	KC_AB2	stc	-1.3723417613553	0.619532036522	1	pseudobulk	DESeq2	LRT	0
151	KC_AB2	stck	-0.787881808385349	0.361904980738928	1	pseudobulk	DESeq2	LRT	0
151	KC_AB2	ste14	1.14814388261213	0.568837005180859	1	pseudobulk	DESeq2	LRT	0
151	KC_AB2	ste24a	0.602594940100271	0.693985196153841	1	pseudobulk	DESeq2	LRT	0
151	KC_AB2	step	0.0465906369421993	0.912818624993874	1	pseudobulk	DESeq2	LRT	0
151	KC_AB2	stet	-1.12475160732157	0.574429473125171	1	pseudobulk	DESeq2	LRT	0
151	KC_AB2	Stim	-1.36193163158604	0.299792882604197	1	pseudobulk	DESeq2	LRT	0
151	KC_AB2	Stip1	-0.840584875608836	0.599252166171352	1	pseudobulk	DESeq2	LRT	0
151	KC_AB2	stj	0.600902004192456	0.191271411381872	1	pseudobulk	DESeq2	LRT	0
151	KC_AB2	Stlk	-0.745702126435306	0.245433064452746	1	pseudobulk	DESeq2	LRT	0
151	KC_AB2	stmA	0.0219089764279216	0.830012772378758	1	pseudobulk	DESeq2	LRT	0
151	KC_AB2	Stoml2	0.196423475656606	0.747218017079152	1	pseudobulk	DESeq2	LRT	0
151	KC_AB2	stops	-0.600547221779134	1	1	pseudobulk	DESeq2	LRT	0
151	KC_AB2	strat	-1.16116875819275	0.736255625566845	1	pseudobulk	DESeq2	LRT	0
151	KC_AB2	Strip	-0.335096608340763	0.798925799621256	1	pseudobulk	DESeq2	LRT	0
151	KC_AB2	Strump	-1.25727291634246	0.518975364824881	1	pseudobulk	DESeq2	LRT	0
151	KC_AB2	Stt3A	0.589670356630379	0.560817520568532	1	pseudobulk	DESeq2	LRT	0
151	KC_AB2	Stt3B	1.71900246419973	0.263350655668293	1	pseudobulk	DESeq2	LRT	0
151	KC_AB2	STUB1	-0.538142503208313	0.452640982106824	1	pseudobulk	DESeq2	LRT	0
151	KC_AB2	sturkopf	1.3231412480248	0.245414852140701	1	pseudobulk	DESeq2	LRT	0

151	KC_AB2	stv	-0.096611867582802	0.713752397250702	1	pseudobulk	DESeq2	LRT	0
151	KC_AB2	stw	-0.427431139129811	1	1	pseudobulk	DESeq2	LRT	0
151	KC_AB2	stwl	1.22036108458351	0.522938943418067	1	pseudobulk	DESeq2	LRT	0
151	KC_AB2	stx	0.403684101150355	0.507675301049159	1	pseudobulk	DESeq2	LRT	0
151	KC_AB2	sty	-0.764474036508456	0.0118126803478561	1	pseudobulk	DESeq2	LRT	0
151	KC_AB2	Su(dx)	-0.743320870403689	0.499940583233123	1	pseudobulk	DESeq2	LRT	0
151	KC_AB2	su(f)	0.671599090397225	0.592529805208347	1	pseudobulk	DESeq2	LRT	0
151	KC_AB2	Su(fu)	-0.352770762553185	0.871009546934938	1	pseudobulk	DESeq2	LRT	0
151	KC_AB2	Su(H)	-0.587750204858197	0.663823170187332	1	pseudobulk	DESeq2	LRT	0
151	KC_AB2	su(Hw)	0.93182432838831	0.502006927804655	1	pseudobulk	DESeq2	LRT	0
151	KC_AB2	Su(P)	-0.967680389784645	0.331644246406534	1	pseudobulk	DESeq2	LRT	0
151	KC_AB2	su(sable)	-0.868205810020992	0.471224502081362	1	pseudobulk	DESeq2	LRT	0
151	KC_AB2	Su(Tpl)	-0.0351376634626466	0.858713610902551	1	pseudobulk	DESeq2	LRT	0
151	KC_AB2	Su(var)2-10	0.241838544510199	0.737760165247827	1	pseudobulk	DESeq2	LRT	0
151	KC_AB2	Su(var)2-HP2	0.29435936216808	0.752872077465477	1	pseudobulk	DESeq2	LRT	0
151	KC_AB2	Su(var)205	-0.260925383107486	0.724050038262146	1	pseudobulk	DESeq2	LRT	0
151	KC_AB2	Su(var)3-3	0.997950432548483	0.520665954048796	1	pseudobulk	DESeq2	LRT	0
151	KC_AB2	Su(var)3-7	0.751456027923628	0.684049070099348	1	pseudobulk	DESeq2	LRT	0
151	KC_AB2	su(w[a])	1.0062360180426	0.291860893515915	1	pseudobulk	DESeq2	LRT	0
151	KC_AB2	Su(z)12	-0.273404349803985	0.772303394568527	1	pseudobulk	DESeq2	LRT	0
151	KC_AB2	Su(z)2	0.145457002369979	0.831305931236695	1	pseudobulk	DESeq2	LRT	0
151	KC_AB2	subdued	-1.02236307652192	0.679402188422793	1	pseudobulk	DESeq2	LRT	0
151	KC_AB2	sud1	2.1817927492653	0.458599861292202	1	pseudobulk	DESeq2	LRT	0
151	KC_AB2	Sugb	-1.07776915880798	0.775491075088671	1	pseudobulk	DESeq2	LRT	0
151	KC_AB2	Sulf1	-0.600547221779134	1	1	pseudobulk	DESeq2	LRT	0
151	KC_AB2	sun	0.112071777308059	0.692917670814776	1	pseudobulk	DESeq2	LRT	0
151	KC_AB2	Sur-8	-0.161902303459113	0.786264445248996	1	pseudobulk	DESeq2	LRT	0
151	KC_AB2	Surf1	-2.08017178210869	0.263646787767044	1	pseudobulk	DESeq2	LRT	0
151	KC_AB2	Surf4	-0.639319674106996	0.438276486672959	1	pseudobulk	DESeq2	LRT	0
151	KC_AB2	Surf6	0.725498497157359	0.93879047033739	1	pseudobulk	DESeq2	LRT	0
151	KC_AB2	Suv3	-1.1937695127578	0.437627710103042	1	pseudobulk	DESeq2	LRT	0
151	KC_AB2	sv	-0.600547221779133	0.841940696994101	1	pseudobulk	DESeq2	LRT	0
151	KC_AB2	Svil	0.36013817800528	0.472403598690751	1	pseudobulk	DESeq2	LRT	0
151	KC_AB2	svr	-0.295852289319919	0.422802479419164	1	pseudobulk	DESeq2	LRT	0
151	KC_AB2	sw	-0.587771582832088	0.50844950516619	1	pseudobulk	DESeq2	LRT	0
151	KC_AB2	Swip-1	0.0475131735964229	0.897875824841034	1	pseudobulk	DESeq2	LRT	0
151	KC_AB2	swm	0.421949989992875	0.401297349942326	1	pseudobulk	DESeq2	LRT	0
151	KC_AB2	sws	-0.166524302311379	0.858565957196421	1	pseudobulk	DESeq2	LRT	0
151	KC_AB2	sxc	0.395030130286887	0.445320759971767	1	pseudobulk	DESeq2	LRT	0
151	KC_AB2	sxe2	-0.427431139129811	1	1	pseudobulk	DESeq2	LRT	0
151	KC_AB2	Sxl	-0.405573272780074	0.222150165836753	1	pseudobulk	DESeq2	LRT	0
151	KC_AB2	Syb	-0.592481036256484	0.706847545932384	1	pseudobulk	DESeq2	LRT	0
151	KC_AB2	syd	0.215393943853972	0.717450481566875	1	pseudobulk	DESeq2	LRT	0
152	KC_AB2	Sym	1.5243995994652	0.741477491078038	1	pseudobulk	DESeq2	LRT	0
152	KC_AB2	Syn	-0.239390406601736	0.482825564351639	1	pseudobulk	DESeq2	LRT	0
152	KC_AB2	Syn1	-0.0808812337334512	0.804860467832097	1	pseudobulk	DESeq2	LRT	0
152	KC_AB2	Syn2	0.0855296194818611	0.910813950083558	1	pseudobulk	DESeq2	LRT	0
152	KC_AB2	Synd	0.564655444561182	0.565891299347443	1	pseudobulk	DESeq2	LRT	0
152	KC_AB2	Syngr	0.165360774729687	0.704321120702042	1	pseudobulk	DESeq2	LRT	0
152	KC_AB2	Synj	0.0788359772346402	0.853790799449602	1	pseudobulk	DESeq2	LRT	0
152	KC_AB2	Syp	0.161272886956249	0.69222787381386	1	pseudobulk	DESeq2	LRT	0
152	KC_AB2	Syt1	0.0957143845146452	0.682727804146357	1	pseudobulk	DESeq2	LRT	0
152	KC_AB2	Syt12	-0.0479091659340418	0.935595607439232	1	pseudobulk	DESeq2	LRT	0

152	KC_AB2	Syt14	0.0529326125895884	0.925972187775333	1	pseudobulk	DESeq2	LRT		0
152	KC_AB2	Syt4	0.323468428023559	0.326475419691953	1	pseudobulk	DESeq2	LRT		0
152	KC_AB2	Syt7	0.0059868320270298	0.86481328483386	1	pseudobulk	DESeq2	LRT		0
152	KC_AB2	Sytalpha	0.294860419949644	0.389249926749981	1	pseudobulk	DESeq2	LRT		0
152	KC_AB2	Sytbeta	-0.373048008540101	0.382697368555759	1	pseudobulk	DESeq2	LRT		0
152	KC_AB2	Syx13	0.144620766540175	0.887322179538637	1	pseudobulk	DESeq2	LRT		0
152	KC_AB2	Syx16	0.180496912761357	0.831033343194765	1	pseudobulk	DESeq2	LRT		0
152	KC_AB2	Syx17	-1.26740980607835	0.411245004785498	1	pseudobulk	DESeq2	LRT		0
152	KC_AB2	Syx18	-2.04523516886996	0.249152927586553	1	pseudobulk	DESeq2	LRT		0
152	KC_AB2	Syx1A	-0.251241639108078	0.280450526638772	1	pseudobulk	DESeq2	LRT		0
152	KC_AB2	Syx5	-0.104043763877702	0.943468686780237	1	pseudobulk	DESeq2	LRT		0
152	KC_AB2	Syx6	0.0059596278344329	0.836709924827228	1	pseudobulk	DESeq2	LRT		0
152	KC_AB2	Syx7	-0.387522694501145	0.578441862645262	1	pseudobulk	DESeq2	LRT		0
152	KC_AB2	Syx8	0.790118374229576	0.657873015575783	1	pseudobulk	DESeq2	LRT		0
152	KC_AB2	tacc	-0.585840077978844	0.239405403326828	1	pseudobulk	DESeq2	LRT		0
152	KC_AB2	Tace	0.592628385408796	0.43981921591076	1	pseudobulk	DESeq2	LRT		0
152	KC_AB2	tadr	1.18330892943219	0.39051962766292	1	pseudobulk	DESeq2	LRT		0
152	KC_AB2	Taf1	1.53811683574911	0.54844951579854	1	pseudobulk	DESeq2	LRT		0
152	KC_AB2	Taf10	1.00421226643646	0.353776021491994	1	pseudobulk	DESeq2	LRT		0
152	KC_AB2	Taf10b	-0.83816800839172	0.303643089200705	1	pseudobulk	DESeq2	LRT		0
152	KC_AB2	Taf11	-0.759250734475864	0.739101190086705	1	pseudobulk	DESeq2	LRT		0
152	KC_AB2	Taf12	0.123168180477062	0.853848023480161	1	pseudobulk	DESeq2	LRT		0
152	KC_AB2	Taf13	-0.284251481678241	0.845436017905768	1	pseudobulk	DESeq2	LRT		0
152	KC_AB2	TAF1C-like	1.5243995994652	0.741477491078038	1	pseudobulk	DESeq2	LRT		0
152	KC_AB2	Taf2	-0.493357239699781	0.783807521297462	1	pseudobulk	DESeq2	LRT		0
152	KC_AB2	Taf4	-0.207387698433629	0.778776351174453	1	pseudobulk	DESeq2	LRT		0
152	KC_AB2	Taf5	1.64759826860276	0.469402419161652	1	pseudobulk	DESeq2	LRT		0
152	KC_AB2	Taf6	-3.2163692725146	0.11487627323699	1	pseudobulk	DESeq2	LRT		0
152	KC_AB2	Taf7	1.97353325796147	0.191659774940606	1	pseudobulk	DESeq2	LRT		0
152	KC_AB2	Taf8	-0.525980220958878	0.813340603474339	1	pseudobulk	DESeq2	LRT		0
152	KC_AB2	tai	0.0412099363925817	0.888415731601542	1	pseudobulk	DESeq2	LRT		0
152	KC_AB2	Tailor	-0.0390284161562194	0.950457610740483	1	pseudobulk	DESeq2	LRT		0
152	KC_AB2	Tak1	0.0884365751276113	0.916523050296656	1	pseudobulk	DESeq2	LRT		0
152	KC_AB2	tamo	-0.424137573795616	0.710315232552586	1	pseudobulk	DESeq2	LRT		0
152	KC_AB2	Tango1	-0.784657099438954	0.473914199263812	1	pseudobulk	DESeq2	LRT		0
152	KC_AB2	Tango10	1.44980254757427	0.49719293000444	1	pseudobulk	DESeq2	LRT		0
152	KC_AB2	Tango11	-0.210214265520897	0.751721980907191	1	pseudobulk	DESeq2	LRT		0
152	KC_AB2	Tango14	1.29908447958025	0.158197186448919	1	pseudobulk	DESeq2	LRT		0
152	KC_AB2	Tango2	0.920707161609556	0.66023874320038	1	pseudobulk	DESeq2	LRT		0
152	KC_AB2	Tango4	-1.26727443882866	0.537816608126716	1	pseudobulk	DESeq2	LRT		0
152	KC_AB2	Tango5	-0.0450375894193117	0.901270000876503	1	pseudobulk	DESeq2	LRT		0
152	KC_AB2	Tango6	2.76205395729416	0.251829971474322	1	pseudobulk	DESeq2	LRT		0
152	KC_AB2	Tango9	-1.35893442953486	0.278695252819191	1	pseudobulk	DESeq2	LRT		0
152	KC_AB2	tank	0.674459942220112	0.74558757773504	1	pseudobulk	DESeq2	LRT		0
152	KC_AB2	tant	0.880731037017389	0.517426418884505	1	pseudobulk	DESeq2	LRT		0
152	KC_AB2	Tao	0.026738691724264	0.768246062726305	1	pseudobulk	DESeq2	LRT		0
152	KC_AB2	Tap42	1.9522356932086	0.265846497135762	1	pseudobulk	DESeq2	LRT		0
152	KC_AB2	tapas	0.73928193184843	0.383916961898669	1	pseudobulk	DESeq2	LRT		0
152	KC_AB2	Tapdelta	0.266842321812367	0.776128566947663	1	pseudobulk	DESeq2	LRT		0
152	KC_AB2	tara	0.0874370058212098	0.850250637891091	1	pseudobulk	DESeq2	LRT		0
152	KC_AB2	Task6	-0.219091518423832	0.52027450268627	1	pseudobulk	DESeq2	LRT		0
152	KC_AB2	Task7	-0.240229410557227	0.736932087021365	1	pseudobulk	DESeq2	LRT		0
152	KC_AB2	Tasp1	1.5243995994652	0.741477491078038	1	pseudobulk	DESeq2	LRT		0

152	KC_AB2	Tat	-0.26466215320498	0.596220615278591	1	pseudobulk	DESeq2	LRT		0
152	KC_AB2	tau	-0.0230702221613317	0.868215122872331	1	pseudobulk	DESeq2	LRT		0
152	KC_AB2	tay	0.0352592441685616	0.89467462692611	1	pseudobulk	DESeq2	LRT		0
152	KC_AB2	Taz	0.864341462967788	0.925947094335722	1	pseudobulk	DESeq2	LRT		0
152	KC_AB2	Tbc1d15-17	-1.12475160732157	0.574429473125171	1	pseudobulk	DESeq2	LRT		0
152	KC_AB2	TBC1D16	-0.0369412729519829	0.919236515448467	1	pseudobulk	DESeq2	LRT		0
152	KC_AB2	TBC1D5	-1.36192722140614	0.504761005035368	1	pseudobulk	DESeq2	LRT		0
152	KC_AB2	TBC1d7	-0.983082814601118	0.644813365356324	1	pseudobulk	DESeq2	LRT		0
152	KC_AB2	TBCB	-0.413914861616268	0.694921935040489	1	pseudobulk	DESeq2	LRT		0
152	KC_AB2	TBCC	0.516211129282802	0.603463742359345	1	pseudobulk	DESeq2	LRT		0
152	KC_AB2	TBCD	-0.0522601304697812	0.97733020544061	1	pseudobulk	DESeq2	LRT		0
152	KC_AB2	Tbce	-2.69654863153377	0.0900388607903439	1	pseudobulk	DESeq2	LRT		0
152	KC_AB2	Tbh	-0.592481036256484	0.706847545932384	1	pseudobulk	DESeq2	LRT		0
152	KC_AB2	Tbp	-0.250351096847887	0.877669731143621	1	pseudobulk	DESeq2	LRT		0
152	KC_AB2	TBPH	-0.663835341901522	0.1338782275627	1	pseudobulk	DESeq2	LRT		0
152	KC_AB2	Tcs3	1.80916697429563	0.29381379198604	1	pseudobulk	DESeq2	LRT		0
152	KC_AB2	Tctp	-0.249984523487025	0.546455208966092	1	pseudobulk	DESeq2	LRT		0
152	KC_AB2	Tdrd3	-0.317944362370968	0.851140486993993	1	pseudobulk	DESeq2	LRT		0
152	KC_AB2	tea	0.713982351041985	0.921569637923553	1	pseudobulk	DESeq2	LRT		0
152	KC_AB2	tef	1.5243995994652	0.741477491078038	1	pseudobulk	DESeq2	LRT		0
152	KC_AB2	Teh1	-0.245867490430957	0.843546090929354	1	pseudobulk	DESeq2	LRT		0
152	KC_AB2	Teh2	-0.0606307852335845	0.79226688703919	1	pseudobulk	DESeq2	LRT		0
152	KC_AB2	Teh3	0.864341462967788	0.925947094335722	1	pseudobulk	DESeq2	LRT		0
152	KC_AB2	Teh4	-0.138217473594577	0.924397299256972	1	pseudobulk	DESeq2	LRT		0
152	KC_AB2	Tektin-C	-0.600547221779134	1	1	pseudobulk	DESeq2	LRT		0
152	KC_AB2	temp	-0.533946064220369	0.73906067333976	1	pseudobulk	DESeq2	LRT		0
152	KC_AB2	Ten-a	-0.249937790903047	0.131674749969977	1	pseudobulk	DESeq2	LRT		0
152	KC_AB2	Ten-m	-1.25046665961962	0.11283744382775	1	pseudobulk	DESeq2	LRT		0
152	KC_AB2	teq	1.5243995994652	0.741477491078038	1	pseudobulk	DESeq2	LRT		0
152	KC_AB2	TER94	0.464968636918403	0.609758075505138	1	pseudobulk	DESeq2	LRT		0
152	KC_AB2	Tet	0.144540623228219	0.825712795900683	1	pseudobulk	DESeq2	LRT		0
152	KC_AB2	TFAM	0.426106283215778	0.74196633237327	1	pseudobulk	DESeq2	LRT		0
152	KC_AB2	Tfb1	1.16332191685055	0.554022108806959	1	pseudobulk	DESeq2	LRT		0
152	KC_AB2	Tfb4	-1.12475160732157	0.574429473125171	1	pseudobulk	DESeq2	LRT		0
152	KC_AB2	TfIIA-L	-0.683135170053594	0.558118171492291	1	pseudobulk	DESeq2	LRT		0
152	KC_AB2	TfIIA-S	-0.504413242017186	0.374817132541106	1	pseudobulk	DESeq2	LRT		0
152	KC_AB2	TfIIB	0.467818615554021	0.735554993715942	1	pseudobulk	DESeq2	LRT		0
153	KC_AB2	TfIIEalpha	1.33934673797804	0.451769783817336	1	pseudobulk	DESeq2	LRT		0
153	KC_AB2	TfIIEbeta	2.03955752684245	0.330463272096619	1	pseudobulk	DESeq2	LRT		0
153	KC_AB2	TfIIFalpha	-0.208129026640825	0.78424641520296	1	pseudobulk	DESeq2	LRT		0
153	KC_AB2	TfIIFbeta	-3.31189987428711	0.10274916671603	1	pseudobulk	DESeq2	LRT		0
153	KC_AB2	TfIIS	0.0944152646999865	0.821812400886879	1	pseudobulk	DESeq2	LRT		0
153	KC_AB2	Tgi	0.649675493186657	0.493844017111006	1	pseudobulk	DESeq2	LRT		0
153	KC_AB2	Tgt	0.920212920242661	0.67286879185691	1	pseudobulk	DESeq2	LRT		0
153	KC_AB2	TH1	-0.357354465800576	0.852988665057457	1	pseudobulk	DESeq2	LRT		0
153	KC_AB2	Thd1	-0.0980907189669415	0.80995966651311	1	pseudobulk	DESeq2	LRT		0
153	KC_AB2	Theg	-1.63925793886161	0.546290859679341	1	pseudobulk	DESeq2	LRT		0
153	KC_AB2	THG	-1.70380348762697	0.473446675363872	1	pseudobulk	DESeq2	LRT		0
153	KC_AB2	tho2	-0.856525941633148	0.465253260224506	1	pseudobulk	DESeq2	LRT		0
153	KC_AB2	thoc5	-1.43450874381432	0.6168250034656	1	pseudobulk	DESeq2	LRT		0
153	KC_AB2	thoc6	0.642870763476604	0.702407913000129	1	pseudobulk	DESeq2	LRT		0
153	KC_AB2	thoc7	-1.22101204004314	0.318929645704868	1	pseudobulk	DESeq2	LRT		0
153	KC_AB2	ThrRS	-0.0126961264816158	0.944070883210312	1	pseudobulk	DESeq2	LRT		0

153	KC_AB2	ths	-0.219388914041305	0.726995995903411	1	pseudobulk	DESeq2	LRT	0
153	KC_AB2	Tie	-1.70614408517477	0.52816093424714	1	pseudobulk	DESeq2	LRT	0
153	KC_AB2	Tif-IA	-0.236709784797297	0.824263927821858	1	pseudobulk	DESeq2	LRT	0
153	KC_AB2	Tim17b	-0.0469110465976503	0.908097162216097	1	pseudobulk	DESeq2	LRT	0
153	KC_AB2	Tim17b1	0.864341462967788	0.925947094335722	1	pseudobulk	DESeq2	LRT	0
153	KC_AB2	Tim23	0.232445642327317	0.722478582463933	1	pseudobulk	DESeq2	LRT	0
153	KC_AB2	Tim8	-0.343150157441329	0.49646100678396	1	pseudobulk	DESeq2	LRT	0
153	KC_AB2	Tim9a	-0.449374240308054	0.711715615522065	1	pseudobulk	DESeq2	LRT	0
153	KC_AB2	timeout	-1.12569474978191	0.563105123273263	1	pseudobulk	DESeq2	LRT	0
153	KC_AB2	Timp	-0.573109354659945	0.739419341582026	1	pseudobulk	DESeq2	LRT	0
153	KC_AB2	Tina-1	-1.57986273853982	0.0399798327717135	1	pseudobulk	DESeq2	LRT	0
153	KC_AB2	tinc	0.374564527310641	0.423657494609602	1	pseudobulk	DESeq2	LRT	0
153	KC_AB2	Tip60	-1.93220872672389	0.398686710033904	1	pseudobulk	DESeq2	LRT	0
153	KC_AB2	tipE	-0.356292855760168	0.565687194882242	1	pseudobulk	DESeq2	LRT	0
153	KC_AB2	tko	-0.581659748182966	0.5597860223508	1	pseudobulk	DESeq2	LRT	0
153	KC_AB2	TkR86C	-0.338771435603146	0.816656863441963	1	pseudobulk	DESeq2	LRT	0
153	KC_AB2	tkv	0.53306576513325	0.577588280825965	1	pseudobulk	DESeq2	LRT	0
153	KC_AB2	Tl	-0.0869817254159862	0.813433130718282	1	pseudobulk	DESeq2	LRT	0
153	KC_AB2	tld	2.12684015898615	0.445939223035924	1	pseudobulk	DESeq2	LRT	0
153	KC_AB2	Tlk	0.165757555084356	0.589747612708835	1	pseudobulk	DESeq2	LRT	0
153	KC_AB2	Tm1	-0.104907606856824	0.733617566158061	1	pseudobulk	DESeq2	LRT	0
153	KC_AB2	TM4SF	0.208774235257162	0.422045172281955	1	pseudobulk	DESeq2	LRT	0
153	KC_AB2	TM9SF2	-1.03322392399594	0.314180860651956	1	pseudobulk	DESeq2	LRT	0
153	KC_AB2	TM9SF3	1.70520855891462	0.276021814891883	1	pseudobulk	DESeq2	LRT	0
153	KC_AB2	TM9SF4	-1.15743744844182	0.245636792547946	1	pseudobulk	DESeq2	LRT	0
153	KC_AB2	Tmc	1.23085716405798	0.614623732585597	1	pseudobulk	DESeq2	LRT	0
153	KC_AB2	Tmem18	0.645593088882016	0.712081648215049	1	pseudobulk	DESeq2	LRT	0
153	KC_AB2	TMEM216	1.20140613998183	0.1953800467893	1	pseudobulk	DESeq2	LRT	0
153	KC_AB2	Tmem63	0.671599090397225	0.592529805208347	1	pseudobulk	DESeq2	LRT	0
153	KC_AB2	tmod	-0.497464808836101	0.520796617126868	1	pseudobulk	DESeq2	LRT	0
153	KC_AB2	TMS1	0.170578599205053	0.64841437884956	1	pseudobulk	DESeq2	LRT	0
153	KC_AB2	Tmtc3	0.0373453070339051	0.964087792763343	1	pseudobulk	DESeq2	LRT	0
153	KC_AB2	tna	-0.426448895381245	0.137311314928427	1	pseudobulk	DESeq2	LRT	0
153	KC_AB2	tnc	-2.92995403667278	0.159449090754912	1	pseudobulk	DESeq2	LRT	0
153	KC_AB2	Tnks	0.0418650542414348	0.972476924292918	1	pseudobulk	DESeq2	LRT	0
153	KC_AB2	Tnpo	-0.302740347972979	0.59804072441698	1	pseudobulk	DESeq2	LRT	0
153	KC_AB2	Tnpo-SR	0.178361519141227	0.895433560431735	1	pseudobulk	DESeq2	LRT	0
153	KC_AB2	Tob	-0.101460290856298	0.623862565059781	1	pseudobulk	DESeq2	LRT	0
153	KC_AB2	toc	0.339930628019869	0.341436995116215	1	pseudobulk	DESeq2	LRT	0
153	KC_AB2	tok	1.69515175799364	0.368369504470473	1	pseudobulk	DESeq2	LRT	0
153	KC_AB2	Toll-6	-0.51835155902916	0.604141735581813	1	pseudobulk	DESeq2	LRT	0
153	KC_AB2	Toll-7	0.0163320842711025	1	1	pseudobulk	DESeq2	LRT	0
153	KC_AB2	Tom20	-0.371763402489128	0.340601258015513	1	pseudobulk	DESeq2	LRT	0
153	KC_AB2	Tom40	0.605456587169172	0.49027917762623	1	pseudobulk	DESeq2	LRT	0
153	KC_AB2	Tom7	-0.401566861942346	0.340735543231932	1	pseudobulk	DESeq2	LRT	0
153	KC_AB2	Tom70	-0.717515093472411	0.584077843609493	1	pseudobulk	DESeq2	LRT	0
153	KC_AB2	Tomosyn	0.233713562360715	0.33971165416643	1	pseudobulk	DESeq2	LRT	0
153	KC_AB2	Top1	-0.0354946309126528	0.896962162704371	1	pseudobulk	DESeq2	LRT	0
153	KC_AB2	Top2	-0.174687281224032	0.871690317704922	1	pseudobulk	DESeq2	LRT	0
153	KC_AB2	Top3alpha	1.5243995994652	0.741477491078038	1	pseudobulk	DESeq2	LRT	0
153	KC_AB2	Top3beta	-1.85734671293876	0.323831233144879	1	pseudobulk	DESeq2	LRT	0
153	KC_AB2	Topors	-1.0615858373902	0.551870476649274	1	pseudobulk	DESeq2	LRT	0
153	KC_AB2	Tor	0.980806052910289	0.518144919068054	1	pseudobulk	DESeq2	LRT	0

153	KC_AB2	TORIP	0.676903219759297	0.708836762102949	1	pseudobulk	DESeq2	LRT	0
153	KC_AB2	Torsin	0.754546145017482	0.479445224111638	1	pseudobulk	DESeq2	LRT	0
153	KC_AB2	TotA	0.920707161609556	0.66023874320038	1	pseudobulk	DESeq2	LRT	0
153	KC_AB2	tou	0.0034062997846064	0.964430664273578	1	pseudobulk	DESeq2	LRT	0
153	KC_AB2	tow	-0.100164140799184	0.73334430951821	1	pseudobulk	DESeq2	LRT	0
153	KC_AB2	toy	0.175644186353538	0.492399260934579	1	pseudobulk	DESeq2	LRT	0
153	KC_AB2	Tpc1	-1.45185768489783	0.445407259051521	1	pseudobulk	DESeq2	LRT	0
153	KC_AB2	Tpi	0.202628476197608	0.521552324792404	1	pseudobulk	DESeq2	LRT	0
153	KC_AB2	TppII	-0.421438723273008	0.779589632058169	1	pseudobulk	DESeq2	LRT	0
153	KC_AB2	Tpr2	-0.400615497946105	0.521479080203072	1	pseudobulk	DESeq2	LRT	0
153	KC_AB2	Tpst	0.379378645244074	0.822219735623116	1	pseudobulk	DESeq2	LRT	0
153	KC_AB2	tra	-1.56605639796142	0.275919236362101	1	pseudobulk	DESeq2	LRT	0
153	KC_AB2	tra2	-0.155097891520823	0.85349537922521	1	pseudobulk	DESeq2	LRT	0
153	KC_AB2	Traf6	-0.843441052194794	0.620862592897087	1	pseudobulk	DESeq2	LRT	0
153	KC_AB2	tral	0.215981133925366	0.602439278262659	1	pseudobulk	DESeq2	LRT	0
153	KC_AB2	TRAM	0.19117119482715	0.864279669657469	1	pseudobulk	DESeq2	LRT	0
153	KC_AB2	Trap1	-0.0314136140345526	0.915028691227536	1	pseudobulk	DESeq2	LRT	0
153	KC_AB2	Trax	0.864341462967788	0.925947094335722	1	pseudobulk	DESeq2	LRT	0
153	KC_AB2	trbd	0.0609887132040365	0.933560495389128	1	pseudobulk	DESeq2	LRT	0
153	KC_AB2	trc	0.57320000846352	0.497344772610926	1	pseudobulk	DESeq2	LRT	0
153	KC_AB2	Trc8	-1.10405600102669	0.617983244849634	1	pseudobulk	DESeq2	LRT	0
153	KC_AB2	Treh	0.613659617664411	0.76686169970757	1	pseudobulk	DESeq2	LRT	0
153	KC_AB2	trem	-1.70380348762697	0.473446675363872	1	pseudobulk	DESeq2	LRT	0
153	KC_AB2	Trf	0.864341462967788	0.925947094335722	1	pseudobulk	DESeq2	LRT	0
153	KC_AB2	Trf2	-0.0170916349383187	0.867314323109199	1	pseudobulk	DESeq2	LRT	0
153	KC_AB2	Trf4-1	0.0122000185342777	0.851954635680048	1	pseudobulk	DESeq2	LRT	0
153	KC_AB2	trio	-0.0878761533205498	0.857529057868759	1	pseudobulk	DESeq2	LRT	0
153	KC_AB2	Trissin	2.72066226053615	0.290765320578625	1	pseudobulk	DESeq2	LRT	0
153	KC_AB2	Trl	-0.338771435603146	0.816656863441963	1	pseudobulk	DESeq2	LRT	0
153	KC_AB2	trn	0.331589896610526	0.533282436096468	1	pseudobulk	DESeq2	LRT	0
153	KC_AB2	tRNA:Arg-TCG-2-4	-0.600547221779134	1	1	pseudobulk	DESeq2	LRT	0
154	KC_AB2	tRNA:Cys-GCA-4-	1.23085716405798	0.614623732585597	1	pseudobulk	DESeq2	LRT	0
154	KC_AB2	tRNA:Leu-AAG-1-	-0.427431139129811	1	1	pseudobulk	DESeq2	LRT	0
154	KC_AB2	trol	-0.600547221779133	0.841940696994101	1	pseudobulk	DESeq2	LRT	0
154	KC_AB2	Trp1	0.0443190496481355	0.878922360647589	1	pseudobulk	DESeq2	LRT	0
154	KC_AB2	Trpm	0.165802563644429	0.731026792243318	1	pseudobulk	DESeq2	LRT	0
154	KC_AB2	TrpRS	0.197666323965435	0.81675096270993	1	pseudobulk	DESeq2	LRT	0
154	KC_AB2	trr	0.814241159408071	0.416625741165309	1	pseudobulk	DESeq2	LRT	0
154	KC_AB2	Trs20	0.37195685527863	0.690480093508339	1	pseudobulk	DESeq2	LRT	0
154	KC_AB2	Trs23	1.00094217107513	0.50766339531821	1	pseudobulk	DESeq2	LRT	0
154	KC_AB2	Trs31	-1.42671777446436	0.273017699862554	1	pseudobulk	DESeq2	LRT	0
154	KC_AB2	Trs33	0.183555857030446	1	1	pseudobulk	DESeq2	LRT	0
154	KC_AB2	trsn	-0.520592429962075	0.695121715665943	1	pseudobulk	DESeq2	LRT	0
154	KC_AB2	trus	-0.719634635163849	0.67079137559935	1	pseudobulk	DESeq2	LRT	0
154	KC_AB2	trv	0.096645961711726	0.673816523184798	1	pseudobulk	DESeq2	LRT	0
154	KC_AB2	trx	0.319744999266355	0.554211184051965	1	pseudobulk	DESeq2	LRT	0
154	KC_AB2	Trx-2	0.992464721094184	0.0502861229883489	1	pseudobulk	DESeq2	LRT	0
154	KC_AB2	Trxr-1	0.744594681291422	0.326415487341341	1	pseudobulk	DESeq2	LRT	0
154	KC_AB2	Try29F	-0.841882716738579	1	1	pseudobulk	DESeq2	LRT	0
154	KC_AB2	Tsc1	0.212754629509105	0.843360808043461	1	pseudobulk	DESeq2	LRT	0
154	KC_AB2	Tsen15	-0.600547221779134	1	1	pseudobulk	DESeq2	LRT	0
154	KC_AB2	Tsen2	3.20650492957429	0.177944556633249	1	pseudobulk	DESeq2	LRT	0
154	KC_AB2	Tsen34	0.689969214341557	0.693684221059153	1	pseudobulk	DESeq2	LRT	0

154	KC_AB2	Tsf1	0.222396124134733	1	1	pseudobulk	DESeq2	LRT	0
154	KC_AB2	Tsf2	1.39701197906864	0.475961471361343	1	pseudobulk	DESeq2	LRT	0
154	KC_AB2	TSG101	0.60252149952049	0.625235872790051	1	pseudobulk	DESeq2	LRT	0
154	KC_AB2	tsl	1.5243995994652	0.741477491078038	1	pseudobulk	DESeq2	LRT	0
154	KC_AB2	Tsp26A	0.92971423364828	0.601507154303804	1	pseudobulk	DESeq2	LRT	0
154	KC_AB2	Tsp39D	-0.0137872345846508	0.872811756628317	1	pseudobulk	DESeq2	LRT	0
154	KC_AB2	Tsp3A	-0.750151825203354	0.775928864461506	1	pseudobulk	DESeq2	LRT	0
154	KC_AB2	Tsp42Ea	-1.59432579116312	0.459120231439715	1	pseudobulk	DESeq2	LRT	0
154	KC_AB2	Tsp42Ee	-1.50800995683703	0.0907736902357217	1	pseudobulk	DESeq2	LRT	0
154	KC_AB2	Tsp42Ej	-0.206862144548391	0.523957509638362	1	pseudobulk	DESeq2	LRT	0
154	KC_AB2	Tsp42Ek	0.10071598675693	0.924250722482424	1	pseudobulk	DESeq2	LRT	0
154	KC_AB2	Tsp42El	-0.600547221779134	1	1	pseudobulk	DESeq2	LRT	0
154	KC_AB2	Tsp42En	-0.241732835866678	0.662296036713067	1	pseudobulk	DESeq2	LRT	0
154	KC_AB2	Tsp42Eo	0.70378358684898	0.932301451973468	1	pseudobulk	DESeq2	LRT	0
154	KC_AB2	Tsp42Ep	1.5243995994652	0.741477491078038	1	pseudobulk	DESeq2	LRT	0
154	KC_AB2	Tsp5D	-0.427431139129811	1	1	pseudobulk	DESeq2	LRT	0
154	KC_AB2	Tsp66E	-0.312307183738104	0.457000824668406	1	pseudobulk	DESeq2	LRT	0
154	KC_AB2	Tsp86D	0.0649494718547162	0.874015228642256	1	pseudobulk	DESeq2	LRT	0
154	KC_AB2	Tsp96F	-0.0107889455447512	0.974171992617657	1	pseudobulk	DESeq2	LRT	0
154	KC_AB2	Tsp97E	0.235355228768033	0.62284032755261	1	pseudobulk	DESeq2	LRT	0
154	KC_AB2	Tspo	0.703141171373176	0.67065453296144	1	pseudobulk	DESeq2	LRT	0
154	KC_AB2	tsr	0.258305330136432	0.542462720957912	1	pseudobulk	DESeq2	LRT	0
154	KC_AB2	Tsr1	2.81818065048002	0.157341165725105	1	pseudobulk	DESeq2	LRT	0
154	KC_AB2	tst	1.82821192274456	0.389528582233255	1	pseudobulk	DESeq2	LRT	0
154	KC_AB2	tsu	0.592311236951025	0.588344737105246	1	pseudobulk	DESeq2	LRT	0
154	KC_AB2	Ttc19	-1.03516737144052	0.506656490110997	1	pseudobulk	DESeq2	LRT	0
154	KC_AB2	Ttc7	0.333404261163793	0.718304148890576	1	pseudobulk	DESeq2	LRT	0
154	KC_AB2	Ttd14	0.203656147886541	0.661885226925767	1	pseudobulk	DESeq2	LRT	0
154	KC_AB2	tth	-0.107956053860867	0.808567676918379	1	pseudobulk	DESeq2	LRT	0
154	KC_AB2	TTLL12	-1.07776915880798	0.775491075088671	1	pseudobulk	DESeq2	LRT	0
154	KC_AB2	TTLL4A	0.277690278357275	0.777169137958755	1	pseudobulk	DESeq2	LRT	0
154	KC_AB2	TTLL5	1.07173252967008	0.608626529368362	1	pseudobulk	DESeq2	LRT	0
154	KC_AB2	ttm50	-0.497363880403185	0.619658101448079	1	pseudobulk	DESeq2	LRT	0
154	KC_AB2	ttv	0.383591055696452	0.589497471808218	1	pseudobulk	DESeq2	LRT	0
154	KC_AB2	tty	0.459347808208211	0.372996272641809	1	pseudobulk	DESeq2	LRT	0
154	KC_AB2	tub	0.101412919525821	0.890432657988466	1	pseudobulk	DESeq2	LRT	0
154	KC_AB2	tud	0.0641849185582838	0.931208111185842	1	pseudobulk	DESeq2	LRT	0
154	KC_AB2	Tudor-SN	-0.833034136094896	0.556026089962546	1	pseudobulk	DESeq2	LRT	0
154	KC_AB2	tum	1.5243995994652	0.741477491078038	1	pseudobulk	DESeq2	LRT	0
154	KC_AB2	tun	0.208998467918682	0.680655226495114	1	pseudobulk	DESeq2	LRT	0
154	KC_AB2	Tusp	0.10326398713109	0.911674850577506	1	pseudobulk	DESeq2	LRT	0
154	KC_AB2	tut	1.8449248580274	0.384050474907707	1	pseudobulk	DESeq2	LRT	0
154	KC_AB2	tutl	0.0853445119447619	0.744624255922709	1	pseudobulk	DESeq2	LRT	0
154	KC_AB2	tw	1.21092382796727	0.637125262974216	1	pseudobulk	DESeq2	LRT	0
154	KC_AB2	tweek	0.0926368308587724	0.839143529195378	1	pseudobulk	DESeq2	LRT	0
154	KC_AB2	twf	0.485566018034066	0.705538960248742	1	pseudobulk	DESeq2	LRT	0
154	KC_AB2	twin	-0.38288550103371	0.570989385674351	1	pseudobulk	DESeq2	LRT	0
154	KC_AB2	twit	0.725498497157359	0.93879047033739	1	pseudobulk	DESeq2	LRT	0
154	KC_AB2	twr	0.296059779513583	0.698465580715689	1	pseudobulk	DESeq2	LRT	0
154	KC_AB2	tws	1.24132926355141	0.106072085246427	1	pseudobulk	DESeq2	LRT	0
154	KC_AB2	twz	0.443892390635762	1	1	pseudobulk	DESeq2	LRT	0
154	KC_AB2	Txl	0.880224011845218	0.459020848819171	1	pseudobulk	DESeq2	LRT	0
154	KC_AB2	tyf	-0.112291343196545	0.735374693640992	1	pseudobulk	DESeq2	LRT	0

154	KC_AB2	tyn	0.580213801297725	0.640951929528777	1	pseudobulk	DESeq2	LRT		0
154	KC_AB2	TyrR	0.827759717644438	0.418198407495005	1	pseudobulk	DESeq2	LRT		0
154	KC_AB2	TyrRII	-0.427431139129811	1	1	pseudobulk	DESeq2	LRT		0
154	KC_AB2	TyrRS	-2.30899283344184	0.188917792400299	1	pseudobulk	DESeq2	LRT		0
154	KC_AB2	TyrRS-m	1.40241945196173	0.57265124481703	1	pseudobulk	DESeq2	LRT		0
154	KC_AB2	tzn	0.661528971237473	0.454354016406713	1	pseudobulk	DESeq2	LRT		0
154	KC_AB2	U2A	-2.93014069247264	0.149341146677298	1	pseudobulk	DESeq2	LRT		0
154	KC_AB2	U2af38	-0.205897169561044	0.844427006052769	1	pseudobulk	DESeq2	LRT		0
154	KC_AB2	U2af50	0.466312798346005	0.556434035757448	1	pseudobulk	DESeq2	LRT		0
154	KC_AB2	U4-U6-60K	-1.78094334699383	0.470930349923561	1	pseudobulk	DESeq2	LRT		0
154	KC_AB2	Uba1	-0.0316494388230611	0.897944638895402	1	pseudobulk	DESeq2	LRT		0
154	KC_AB2	Uba2	0.407492304335524	0.69276156913511	1	pseudobulk	DESeq2	LRT		0
154	KC_AB2	Uba3	-0.841882716738579	1	1	pseudobulk	DESeq2	LRT		0
154	KC_AB2	Uba5	-1.70380348762697	0.473446675363872	1	pseudobulk	DESeq2	LRT		0
154	KC_AB2	Ubc10	-0.0474138080746483	0.854891707799981	1	pseudobulk	DESeq2	LRT		0
154	KC_AB2	Ubc2	0.401359745895974	0.685056579055932	1	pseudobulk	DESeq2	LRT		0
154	KC_AB2	Ubc4	0.49283345638395	0.575685602141032	1	pseudobulk	DESeq2	LRT		0
154	KC_AB2	Ubc6	0.499180072383992	0.355441401472097	1	pseudobulk	DESeq2	LRT		0
154	KC_AB2	Ubc7	0.538484268013297	0.672027325915104	1	pseudobulk	DESeq2	LRT		0
154	KC_AB2	UbcE2H	0.27675269131357	0.577779838885266	1	pseudobulk	DESeq2	LRT		0
154	KC_AB2	UbcE2M	-0.808386815777465	0.383290969669127	1	pseudobulk	DESeq2	LRT		0
154	KC_AB2	Ube3a	-1.59873081836234	0.427035120320898	1	pseudobulk	DESeq2	LRT		0
154	KC_AB2	Ubi-p5E	0.511787510426142	0.232033329568972	1	pseudobulk	DESeq2	LRT		0
154	KC_AB2	Ubi-p63E	0.149826108238598	0.7301746166674	1	pseudobulk	DESeq2	LRT		0
154	KC_AB2	ubl	-1.61663140868654	0.288524125234832	1	pseudobulk	DESeq2	LRT		0
155	KC_AB2	UBL3	-0.177151973932777	0.609539117638686	1	pseudobulk	DESeq2	LRT		0
155	KC_AB2	Ublcp1	0.269847418522668	0.867699263195371	1	pseudobulk	DESeq2	LRT		0
155	KC_AB2	Ubqn	0.670489988417314	0.345604729249669	1	pseudobulk	DESeq2	LRT		0
155	KC_AB2	Ubr1	0.270914357111368	0.625849102745654	1	pseudobulk	DESeq2	LRT		0
155	KC_AB2	Ubr3	-0.0538789317416932	0.893584498077416	1	pseudobulk	DESeq2	LRT		0
155	KC_AB2	Uch	0.401733800039527	0.836927022098515	1	pseudobulk	DESeq2	LRT		0
155	KC_AB2	Uch-L5	0.739990113073177	0.553420264191784	1	pseudobulk	DESeq2	LRT		0
155	KC_AB2	Uck	0.136999057689807	0.758561920891292	1	pseudobulk	DESeq2	LRT		0
155	KC_AB2	Ucp4A	0.0932402964374178	0.87440732791416	1	pseudobulk	DESeq2	LRT		0
155	KC_AB2	udd	-0.314805936676809	0.847697048432822	1	pseudobulk	DESeq2	LRT		0
155	KC_AB2	udt	0.107479031250535	0.88712690359169	1	pseudobulk	DESeq2	LRT		0
155	KC_AB2	Uev1A	-0.734496798852986	0.268903031042296	1	pseudobulk	DESeq2	LRT		0
155	KC_AB2	uex	-0.298455388031891	0.450440146925663	1	pseudobulk	DESeq2	LRT		0
155	KC_AB2	Ufc1	-1.22781971198069	0.427358624895997	1	pseudobulk	DESeq2	LRT		0
155	KC_AB2	Ufd1-like	-0.454152727407029	0.653043368424647	1	pseudobulk	DESeq2	LRT		0
155	KC_AB2	Ufd4	0.155572811242138	0.817481168346406	1	pseudobulk	DESeq2	LRT		0
155	KC_AB2	Ufl1	-0.825312539513664	0.666826652877566	1	pseudobulk	DESeq2	LRT		0
155	KC_AB2	Ufm1	-0.157228498215796	0.820510010636793	1	pseudobulk	DESeq2	LRT		0
155	KC_AB2	Ugalt	-3.03739831206252	0.144197076342415	1	pseudobulk	DESeq2	LRT		0
155	KC_AB2	Uggt	-0.828178378785339	0.673584255563891	1	pseudobulk	DESeq2	LRT		0
155	KC_AB2	UGP	-0.427431139129811	1	1	pseudobulk	DESeq2	LRT		0
155	KC_AB2	Ugt316A1	-0.599429608920071	0.56533435877518	1	pseudobulk	DESeq2	LRT		0
155	KC_AB2	Ugt317A1	1.5243995994652	0.741477491078038	1	pseudobulk	DESeq2	LRT		0
155	KC_AB2	Ugt35A1	-0.600547221779133	0.841940696994101	1	pseudobulk	DESeq2	LRT		0
155	KC_AB2	Ugt35B1	-1.59432579116312	0.459120231439715	1	pseudobulk	DESeq2	LRT		0
155	KC_AB2	Ugt35C1	2.1817927492653	0.458599861292202	1	pseudobulk	DESeq2	LRT		0
155	KC_AB2	Uhg1	-0.48458787722798	0.721242982419358	1	pseudobulk	DESeq2	LRT		0
155	KC_AB2	Uhg2	-1.12785316439435	0.661319045116135	1	pseudobulk	DESeq2	LRT		0

155	KC_AB2	Uhg3	-0.199031949864857	0.760460833624344	1	pseudobulk	DESeq2	LRT		0
155	KC_AB2	Uhg4	1.43191469273003	0.651609831456347	1	pseudobulk	DESeq2	LRT		0
155	KC_AB2	Uhg5	-1.61357645155839	0.216389710845685	1	pseudobulk	DESeq2	LRT		0
155	KC_AB2	Ulp1	0.700793584501144	0.527127444654113	1	pseudobulk	DESeq2	LRT		0
155	KC_AB2	unc-104	0.283503145612709	0.331814593157882	1	pseudobulk	DESeq2	LRT		0
155	KC_AB2	Unc-115a	-0.126863985931054	0.897464632831304	1	pseudobulk	DESeq2	LRT		0
155	KC_AB2	unc-119	0.217550884498573	0.666581763458371	1	pseudobulk	DESeq2	LRT		0
155	KC_AB2	unc-13	0.0830504270502324	0.685705866223974	1	pseudobulk	DESeq2	LRT		0
155	KC_AB2	unc-13-4A	0.107170001988896	0.795881128298372	1	pseudobulk	DESeq2	LRT		0
155	KC_AB2	unc-45	1.41524016620164	0.505408490038387	1	pseudobulk	DESeq2	LRT		0
155	KC_AB2	unc-5	0.386562633577314	0.688455097085084	1	pseudobulk	DESeq2	LRT		0
155	KC_AB2	Unc-76	-0.546625559976719	0.13497409788228	1	pseudobulk	DESeq2	LRT		0
155	KC_AB2	unc79	-0.217189741540875	0.599760861283391	1	pseudobulk	DESeq2	LRT		0
155	KC_AB2	unc80	-0.47583607067264	0.326146314573338	1	pseudobulk	DESeq2	LRT		0
155	KC_AB2	und	-0.542696573385532	0.63159847645232	1	pseudobulk	DESeq2	LRT		0
155	KC_AB2	unk	-0.78800562760776	0.335192094487275	1	pseudobulk	DESeq2	LRT		0
155	KC_AB2	Unr	0.13261734801786	0.57363826605944	1	pseudobulk	DESeq2	LRT		0
155	KC_AB2	Updo	-0.524840464341894	0.476179499328416	1	pseudobulk	DESeq2	LRT		0
155	KC_AB2	Upf1	-0.319604581482846	0.837100547724082	1	pseudobulk	DESeq2	LRT		0
155	KC_AB2	Upf2	-1.18234336368098	0.539222050855113	1	pseudobulk	DESeq2	LRT		0
155	KC_AB2	Upf3	0.864341462967788	0.925947094335722	1	pseudobulk	DESeq2	LRT		0
155	KC_AB2	upSET	-0.0848880610567328	0.853554148841588	1	pseudobulk	DESeq2	LRT		0
155	KC_AB2	UQCR-11	-0.329501338318838	0.264212683903029	1	pseudobulk	DESeq2	LRT		0
155	KC_AB2	UQCR-14	-0.322109194946375	0.38505320206606	1	pseudobulk	DESeq2	LRT		0
155	KC_AB2	UQCR-6.4	-0.258515618813998	0.429490717852774	1	pseudobulk	DESeq2	LRT		0
155	KC_AB2	UQCR-C1	-0.333043983486189	0.435439242271455	1	pseudobulk	DESeq2	LRT		0
155	KC_AB2	UQCR-C2	-0.27974872686596	0.43131260279031	1	pseudobulk	DESeq2	LRT		0
155	KC_AB2	UQCR-Q	0.147053178083831	0.621109265389994	1	pseudobulk	DESeq2	LRT		0
155	KC_AB2	uri	1.5243995994652	0.741477491078038	1	pseudobulk	DESeq2	LRT		0
155	KC_AB2	Urm1	0.222396124134733	1	1	pseudobulk	DESeq2	LRT		0
155	KC_AB2	Uros1	1.45570207067196	0.55359324528723	1	pseudobulk	DESeq2	LRT		0
155	KC_AB2	Use1	-0.938817101015206	0.629705053774855	1	pseudobulk	DESeq2	LRT		0
155	KC_AB2	Usf	-0.118559348300938	0.874596221870495	1	pseudobulk	DESeq2	LRT		0
155	KC_AB2	ush	2.12684015898615	0.445939223035924	1	pseudobulk	DESeq2	LRT		0
155	KC_AB2	usp	-0.661067661642958	0.44659255967884	1	pseudobulk	DESeq2	LRT		0
155	KC_AB2	Usp1	0.641356531556062	0.406626472316589	1	pseudobulk	DESeq2	LRT		0
155	KC_AB2	Usp10	-2.70032715096736	0.112886382909509	1	pseudobulk	DESeq2	LRT		0
155	KC_AB2	Usp12-46	1.98712623954142	0.308140074318694	1	pseudobulk	DESeq2	LRT		0
155	KC_AB2	Usp14	0.139572136578075	0.924591848952663	1	pseudobulk	DESeq2	LRT		0
155	KC_AB2	Usp15-31	-1.2260614605833	0.46015475607216	1	pseudobulk	DESeq2	LRT		0
155	KC_AB2	Usp16-45	0.305646097332501	0.865220320325434	1	pseudobulk	DESeq2	LRT		0
155	KC_AB2	Usp2	-0.392471581975463	0.428865587568824	1	pseudobulk	DESeq2	LRT		0
155	KC_AB2	Usp20-33	0.379378645244074	0.822219735623116	1	pseudobulk	DESeq2	LRT		0
155	KC_AB2	Usp30	0.117047283227852	0.90330921080474	1	pseudobulk	DESeq2	LRT		0
155	KC_AB2	Usp32	-0.540617023457509	0.718487718532927	1	pseudobulk	DESeq2	LRT		0
155	KC_AB2	Usp39	-1.39722000842079	0.483349391143715	1	pseudobulk	DESeq2	LRT		0
155	KC_AB2	Usp47	-0.0476558998067625	0.889298329798761	1	pseudobulk	DESeq2	LRT		0
155	KC_AB2	Usp5	-0.637598692711417	0.709430193736439	1	pseudobulk	DESeq2	LRT		0
155	KC_AB2	Usp7	0.103600782497946	0.820109747644254	1	pseudobulk	DESeq2	LRT		0
155	KC_AB2	Usp8	-1.56756252506593	0.405780967349043	1	pseudobulk	DESeq2	LRT		0
155	KC_AB2	Utx	-0.0295880465691438	0.920395220967788	1	pseudobulk	DESeq2	LRT		0
155	KC_AB2	Uvrag	2.16652488181969	0.18211645475461	1	pseudobulk	DESeq2	LRT		0
155	KC_AB2	Uxs	1.5243995994652	0.741477491078038	1	pseudobulk	DESeq2	LRT		0

155	KC_AB2	Uxt	1.44252766211864	0.444521560312303	1	pseudobulk	DESeq2	LRT	0
155	KC_AB2	uzip	-0.947321105171273	0.203398651907543	1	pseudobulk	DESeq2	LRT	0
155	KC_AB2	VAChT	0.0196872274640679	0.862950683363852	1	pseudobulk	DESeq2	LRT	0
155	KC_AB2	ValRS	1.3067515803171	0.550895625207199	1	pseudobulk	DESeq2	LRT	0
155	KC_AB2	Vamp7	-0.210810463534994	0.761020460657186	1	pseudobulk	DESeq2	LRT	0
155	KC_AB2	Vang	-0.586387722784735	0.624669083112908	1	pseudobulk	DESeq2	LRT	0
155	KC_AB2	vap	-0.0359987322740939	0.905305026644105	1	pseudobulk	DESeq2	LRT	0
155	KC_AB2	Vap33	-0.0441584506362803	0.783683044844521	1	pseudobulk	DESeq2	LRT	0
155	KC_AB2	vari	-0.600547221779133	0.841940696994101	1	pseudobulk	DESeq2	LRT	0
155	KC_AB2	Vav	-0.298260255434763	0.807794722632849	1	pseudobulk	DESeq2	LRT	0
155	KC_AB2	veli	-0.0518785787422064	0.869459979729129	1	pseudobulk	DESeq2	LRT	0
155	KC_AB2	velo	0.815087488476331	0.499379911855448	1	pseudobulk	DESeq2	LRT	0
155	KC_AB2	VepD	0.32900059684234	0.442098044994009	1	pseudobulk	DESeq2	LRT	0
155	KC_AB2	vers	0.50416862877443	0.67029623210264	1	pseudobulk	DESeq2	LRT	0
155	KC_AB2	VGAT	-0.456546978094992	0.605381382067614	1	pseudobulk	DESeq2	LRT	0
155	KC_AB2	Vha100-1	-0.340496395602242	0.409148454502903	1	pseudobulk	DESeq2	LRT	0
155	KC_AB2	Vha13	0.136062482298761	0.591226443880706	1	pseudobulk	DESeq2	LRT	0
155	KC_AB2	Vha14-1	-0.0875821980052605	0.728213747545446	1	pseudobulk	DESeq2	LRT	0
155	KC_AB2	Vha16-1	-0.201917854527414	0.251569635826955	1	pseudobulk	DESeq2	LRT	0
156	KC_AB2	Vha26	-0.150246350993212	0.666370021393938	1	pseudobulk	DESeq2	LRT	0
156	KC_AB2	Vha36-1	-0.325265645013808	0.302710285756153	1	pseudobulk	DESeq2	LRT	0
156	KC_AB2	Vha44	-0.0676110322230568	0.810309719434452	1	pseudobulk	DESeq2	LRT	0
156	KC_AB2	Vha55	-0.302779980092676	0.396709021583521	1	pseudobulk	DESeq2	LRT	0
156	KC_AB2	Vha68-1	-0.44522216475833	0.42347916167764	1	pseudobulk	DESeq2	LRT	0
156	KC_AB2	VhaAC39-1	-0.121187925792228	0.759279527536874	1	pseudobulk	DESeq2	LRT	0
156	KC_AB2	VhaAC45	0.0907405137552186	0.919411645163095	1	pseudobulk	DESeq2	LRT	0
156	KC_AB2	VhaM9.7-a	0.139937279903248	0.627310938223315	1	pseudobulk	DESeq2	LRT	0
156	KC_AB2	VhaM9.7-b	0.624919048339518	0.265331874677843	1	pseudobulk	DESeq2	LRT	0
156	KC_AB2	VhaM9.7-c	0.169958040896234	0.875453473313622	1	pseudobulk	DESeq2	LRT	0
156	KC_AB2	VhaPPA1-1	0.0329003662573126	0.863624184320151	1	pseudobulk	DESeq2	LRT	0
156	KC_AB2	VhaSFD	-0.278601742829411	0.603075231546777	1	pseudobulk	DESeq2	LRT	0
156	KC_AB2	Vhl	-0.253840868072933	0.732207567064366	1	pseudobulk	DESeq2	LRT	0
156	KC_AB2	viaf	-1.12238728857605	0.424738315523382	1	pseudobulk	DESeq2	LRT	0
156	KC_AB2	vib	0.552380938383226	0.285599039160299	1	pseudobulk	DESeq2	LRT	0
156	KC_AB2	vig	-0.284614186495953	0.324808656095811	1	pseudobulk	DESeq2	LRT	0
156	KC_AB2	vig2	-0.0890476647093872	0.755081347396305	1	pseudobulk	DESeq2	LRT	0
156	KC_AB2	vih	0.207703581742199	0.742446648318617	1	pseudobulk	DESeq2	LRT	0
156	KC_AB2	vimar	0.2565836676915	0.769192808960835	1	pseudobulk	DESeq2	LRT	0
156	KC_AB2	vir	0.70378358684898	0.932301451973468	1	pseudobulk	DESeq2	LRT	0
156	KC_AB2	vir-1	0.189813678325555	1	1	pseudobulk	DESeq2	LRT	0
156	KC_AB2	vito	2.08523640814881	0.23466677372855	1	pseudobulk	DESeq2	LRT	0
156	KC_AB2	vlc	0.444020982686296	0.673115750844229	1	pseudobulk	DESeq2	LRT	0
156	KC_AB2	Vlet	0.266691076630452	0.822872232270994	1	pseudobulk	DESeq2	LRT	0
156	KC_AB2	vls	-2.12214992510756	0.367174323489033	1	pseudobulk	DESeq2	LRT	0
156	KC_AB2	vnd	2.12684015898615	0.445939223035924	1	pseudobulk	DESeq2	LRT	0
156	KC_AB2	Vps11	0.3912540463768	0.617009535409255	1	pseudobulk	DESeq2	LRT	0
156	KC_AB2	Vps13	0.0685005292199844	0.82499705387034	1	pseudobulk	DESeq2	LRT	0
156	KC_AB2	Vps13B	-0.600547221779134	1	1	pseudobulk	DESeq2	LRT	0
156	KC_AB2	Vps13D	0.70378358684898	0.932301451973468	1	pseudobulk	DESeq2	LRT	0
156	KC_AB2	Vps15	-0.427431139129811	1	1	pseudobulk	DESeq2	LRT	0
156	KC_AB2	Vps16A	0.351295118344814	0.781390979432627	1	pseudobulk	DESeq2	LRT	0
156	KC_AB2	Vps16B	0.864341462967788	0.925947094335722	1	pseudobulk	DESeq2	LRT	0
156	KC_AB2	Vps2	-0.550628223667391	0.538639407266552	1	pseudobulk	DESeq2	LRT	0

156	KC_AB2	Vps20	1.72742152556296	0.272412152579715	1	pseudobulk	DESeq2	LRT	0
156	KC_AB2	Vps24	0.304755353991572	0.598776367998021	1	pseudobulk	DESeq2	LRT	0
156	KC_AB2	Vps25	-1.03087415997487	0.46657664367539	1	pseudobulk	DESeq2	LRT	0
156	KC_AB2	Vps26	-0.950413557436179	0.498840713450416	1	pseudobulk	DESeq2	LRT	0
156	KC_AB2	Vps28	0.263947196125433	0.796440575028523	1	pseudobulk	DESeq2	LRT	0
156	KC_AB2	Vps29	0.711297827488353	0.522726138498134	1	pseudobulk	DESeq2	LRT	0
156	KC_AB2	Vps33B	-1.6150095599849	0.519549602003096	1	pseudobulk	DESeq2	LRT	0
156	KC_AB2	Vps35	-0.181160014612177	0.90177485351735	1	pseudobulk	DESeq2	LRT	0
156	KC_AB2	Vps36	0.0163320842711025	1	1	pseudobulk	DESeq2	LRT	0
156	KC_AB2	Vps37B	-0.970444244373282	0.422985283984942	1	pseudobulk	DESeq2	LRT	0
156	KC_AB2	Vps39	1.23085716405798	0.614623732585597	1	pseudobulk	DESeq2	LRT	0
156	KC_AB2	Vps4	0.246498142994955	0.780680914805761	1	pseudobulk	DESeq2	LRT	0
156	KC_AB2	Vps45	-1.07816229823851	0.519548981943782	1	pseudobulk	DESeq2	LRT	0
156	KC_AB2	Vps50	1.20687884901259	0.374048324615753	1	pseudobulk	DESeq2	LRT	0
156	KC_AB2	Vps51	-3.19605601759	0.106710547003866	1	pseudobulk	DESeq2	LRT	0
156	KC_AB2	Vps52	-0.556718312352294	0.752982669933181	1	pseudobulk	DESeq2	LRT	0
156	KC_AB2	Vps53	1.26307719581879	0.569064211306759	1	pseudobulk	DESeq2	LRT	0
156	KC_AB2	Vps60	-0.591218612219388	0.550631994772333	1	pseudobulk	DESeq2	LRT	0
156	KC_AB2	Vps8	1.5243995994652	0.741477491078038	1	pseudobulk	DESeq2	LRT	0
156	KC_AB2	Vrp1	0.876752804701651	0.141403528512209	1	pseudobulk	DESeq2	LRT	0
156	KC_AB2	vsg	0.233739000108935	0.662078153219029	1	pseudobulk	DESeq2	LRT	0
156	KC_AB2	Vsp37A	-1.09833875266235	0.431070928040004	1	pseudobulk	DESeq2	LRT	0
156	KC_AB2	Vsx1	0.248834901942451	0.762801894759051	1	pseudobulk	DESeq2	LRT	0
156	KC_AB2	Vta1	0.857846916658437	0.25724774078885	1	pseudobulk	DESeq2	LRT	0
156	KC_AB2	vtd	-0.397385786595157	0.596496128511646	1	pseudobulk	DESeq2	LRT	0
156	KC_AB2	Vti1a	-0.0859968649942006	0.921378717454024	1	pseudobulk	DESeq2	LRT	0
156	KC_AB2	Vti1b	-0.589512561427412	0.518748177164843	1	pseudobulk	DESeq2	LRT	0
156	KC_AB2	w	-1.35840781602219	0.290418551764789	1	pseudobulk	DESeq2	LRT	0
156	KC_AB2	wac	-1.54470851095603	0.55301747381519	1	pseudobulk	DESeq2	LRT	0
156	KC_AB2	wake	0.669702595993445	0.193571248156698	1	pseudobulk	DESeq2	LRT	0
156	KC_AB2	wal	-2.52691149003434	0.278972704083546	1	pseudobulk	DESeq2	LRT	0
156	KC_AB2	wap	-3.42607408998142	0.0934853466536962	1	pseudobulk	DESeq2	LRT	0
156	KC_AB2	wapl	0.978547451180442	0.32854481005592	1	pseudobulk	DESeq2	LRT	0
156	KC_AB2	wash	0.522867664761254	0.789441383158121	1	pseudobulk	DESeq2	LRT	0
156	KC_AB2	WASp	-0.0626881815629982	0.784708348266931	1	pseudobulk	DESeq2	LRT	0
156	KC_AB2	Wbp2	-0.341936253946889	0.763887791197764	1	pseudobulk	DESeq2	LRT	0
156	KC_AB2	wcy	0.00492159649217425	0.944323147352474	1	pseudobulk	DESeq2	LRT	0
156	KC_AB2	wda	-0.0715309630154252	1	1	pseudobulk	DESeq2	LRT	0
156	KC_AB2	wdb	0.0245535704770078	0.913958524513045	1	pseudobulk	DESeq2	LRT	0
156	KC_AB2	wde	-1.46249657635653	0.155178265908306	1	pseudobulk	DESeq2	LRT	0
156	KC_AB2	Wdfy2	-0.89950704653138	0.584324115830211	1	pseudobulk	DESeq2	LRT	0
156	KC_AB2	wdn	-0.211959943697087	0.913646816097592	1	pseudobulk	DESeq2	LRT	0
156	KC_AB2	Wdr24	0.725498497157359	0.93879047033739	1	pseudobulk	DESeq2	LRT	0
156	KC_AB2	Wdr33	-1.69826355674084	0.218759624837469	1	pseudobulk	DESeq2	LRT	0
156	KC_AB2	Wdr59	1.5243995994652	0.741477491078038	1	pseudobulk	DESeq2	LRT	0
156	KC_AB2	Wdr62	-0.112892013780052	0.737129884812904	1	pseudobulk	DESeq2	LRT	0
156	KC_AB2	Wdr81	0.508489170660969	0.863841523886068	1	pseudobulk	DESeq2	LRT	0
156	KC_AB2	Wdr82	-0.727230994904562	0.663287456147374	1	pseudobulk	DESeq2	LRT	0
156	KC_AB2	Wdr92	-1.7824418856176	0.467224294431682	1	pseudobulk	DESeq2	LRT	0
156	KC_AB2	wds	-0.739560676340517	0.4577449065806	1	pseudobulk	DESeq2	LRT	0
156	KC_AB2	WDY	-1.72976330140494	0.41918502381035	1	pseudobulk	DESeq2	LRT	0
156	KC_AB2	wech	1.35650669499504	0.094810234590879	1	pseudobulk	DESeq2	LRT	0
156	KC_AB2	Wee1	0.92191528491681	0.613371057887787	1	pseudobulk	DESeq2	LRT	0

156	KC_AB2	wek	-0.556718312352294	0.752982669933181	1	pseudobulk	DESeq2	LRT	0
156	KC_AB2	wfs1	-1.07776915880798	0.775491075088671	1	pseudobulk	DESeq2	LRT	0
156	KC_AB2	wge	-0.296038257706837	0.627738101556629	1	pseudobulk	DESeq2	LRT	0
156	KC_AB2	whd	-0.600547221779133	0.841940696994101	1	pseudobulk	DESeq2	LRT	0
156	KC_AB2	wit	1.00360044973034	0.380036614146875	1	pseudobulk	DESeq2	LRT	0
156	KC_AB2	wkd	0.877883818172883	0.42914122008957	1	pseudobulk	DESeq2	LRT	0
156	KC_AB2	wls	-0.482173055655404	0.804949101714764	1	pseudobulk	DESeq2	LRT	0
156	KC_AB2	wmd	0.114737113112732	0.787948201682373	1	pseudobulk	DESeq2	LRT	0
156	KC_AB2	wnd	0.180142048024484	0.764719401202665	1	pseudobulk	DESeq2	LRT	0
156	KC_AB2	Wnk	-0.897344679515137	0.346310669067056	1	pseudobulk	DESeq2	LRT	0
156	KC_AB2	Wnt5	0.318963395825347	0.806796907207111	1	pseudobulk	DESeq2	LRT	0
156	KC_AB2	woc	0.227905882639659	0.803289879696577	1	pseudobulk	DESeq2	LRT	0
156	KC_AB2	wol	-0.116411347810869	0.916848826069363	1	pseudobulk	DESeq2	LRT	0
157	KC_AB2	wrd	0.172266549638538	0.653800372281082	1	pseudobulk	DESeq2	LRT	0
157	KC_AB2	wry	-0.684026408158203	0.562989995832285	1	pseudobulk	DESeq2	LRT	0
157	KC_AB2	wtrw	1.23085716405798	0.614623732585597	1	pseudobulk	DESeq2	LRT	0
157	KC_AB2	wts	-0.186864774934177	0.747166482602722	1	pseudobulk	DESeq2	LRT	0
157	KC_AB2	wuho	1.28776801764004	0.416248506335861	1	pseudobulk	DESeq2	LRT	0
157	KC_AB2	wun	-0.293602242152435	0.810926304210499	1	pseudobulk	DESeq2	LRT	0
157	KC_AB2	wun2	1.5243995994652	0.741477491078038	1	pseudobulk	DESeq2	LRT	0
157	KC_AB2	Wwox	-1.3753987059131	0.483595520004958	1	pseudobulk	DESeq2	LRT	0
157	KC_AB2	X11L	0.487948414248501	0.345057422631203	1	pseudobulk	DESeq2	LRT	0
157	KC_AB2	X11Lbeta	0.0342075517578269	0.921479874352763	1	pseudobulk	DESeq2	LRT	0
157	KC_AB2	x16	-0.256915189068941	0.732128125252048	1	pseudobulk	DESeq2	LRT	0
157	KC_AB2	Xbp1	-0.44039857340956	0.536686357107995	1	pseudobulk	DESeq2	LRT	0
157	KC_AB2	Xe7	0.342244124105219	0.367171475430411	1	pseudobulk	DESeq2	LRT	0
157	KC_AB2	xit	-0.600547221779133	0.841940696994101	1	pseudobulk	DESeq2	LRT	0
157	KC_AB2	xmas	-0.427431139129811	1	1	pseudobulk	DESeq2	LRT	0
157	KC_AB2	XNP	-0.255825824165206	0.665330605817022	1	pseudobulk	DESeq2	LRT	0
157	KC_AB2	Xpac	0.19117119482715	0.864279669657469	1	pseudobulk	DESeq2	LRT	0
157	KC_AB2	Xpc	-0.1401263291353	0.79195328281074	1	pseudobulk	DESeq2	LRT	0
157	KC_AB2	XRCC1	-0.427431139129811	1	1	pseudobulk	DESeq2	LRT	0
157	KC_AB2	Xrp1	0.0172534455025519	0.79963145742486	1	pseudobulk	DESeq2	LRT	0
157	KC_AB2	Xxylt	-0.465598229168291	0.53915692742173	1	pseudobulk	DESeq2	LRT	0
157	KC_AB2	yata	0.522331483801781	0.690678989627914	1	pseudobulk	DESeq2	LRT	0
157	KC_AB2	yem	0.892659559600706	0.712043317464255	1	pseudobulk	DESeq2	LRT	0
157	KC_AB2	Yeti	-0.114740965689562	0.793750032567118	1	pseudobulk	DESeq2	LRT	0
157	KC_AB2	Yif1	-0.338771435603146	0.816656863441963	1	pseudobulk	DESeq2	LRT	0
157	KC_AB2	Yip1d1	0.0936344474399038	0.93776006455204	1	pseudobulk	DESeq2	LRT	0
157	KC_AB2	Yippee	-0.149883624602596	0.798992450738469	1	pseudobulk	DESeq2	LRT	0
157	KC_AB2	yki	-0.666504981783592	0.587684468074128	1	pseudobulk	DESeq2	LRT	0
157	KC_AB2	Ykt6	-0.371539460633835	0.588174154872352	1	pseudobulk	DESeq2	LRT	0
157	KC_AB2	YL-1	0.756080480905839	0.527350547464786	1	pseudobulk	DESeq2	LRT	0
157	KC_AB2	YME1L	-0.774486632812475	0.44279975851212	1	pseudobulk	DESeq2	LRT	0
157	KC_AB2	yps	-0.312190880289449	0.629784154292326	1	pseudobulk	DESeq2	LRT	0
157	KC_AB2	yrt	0.0384181819372737	0.79984777507014	1	pseudobulk	DESeq2	LRT	0
157	KC_AB2	Ythdc1	-0.0320323762836028	0.854041924694869	1	pseudobulk	DESeq2	LRT	0
157	KC_AB2	Ythdf	-0.464073360399862	0.570038639975635	1	pseudobulk	DESeq2	LRT	0
157	KC_AB2	ytr	1.18134661602927	0.493683637475394	1	pseudobulk	DESeq2	LRT	0
157	KC_AB2	z	0.562835146681691	0.623828919851401	1	pseudobulk	DESeq2	LRT	0
157	KC_AB2	ZAP3	-0.88041718667096	0.304859740104201	1	pseudobulk	DESeq2	LRT	0
157	KC_AB2	Zasp52	0.343239532370367	0.84050451468094	1	pseudobulk	DESeq2	LRT	0
157	KC_AB2	ZC3H3	0.0159250018946664	0.954942357683787	1	pseudobulk	DESeq2	LRT	0

157	KC_AB2	Zcchc7	0.691409358157237	0.491220786540543	1	pseudobulk	DESeq2	LRT	0
157	KC_AB2	zda	0.0829868190028312	0.898594563580016	1	pseudobulk	DESeq2	LRT	0
157	KC_AB2	Zdhhc8	-0.104009337328751	0.756858731764373	1	pseudobulk	DESeq2	LRT	0
157	KC_AB2	zetaCOP	0.364361076034095	0.637482109549288	1	pseudobulk	DESeq2	LRT	0
157	KC_AB2	zf30C	-0.443340786010224	0.751660177548101	1	pseudobulk	DESeq2	LRT	0
157	KC_AB2	zfh2	0.550072370777543	0.749664101164057	1	pseudobulk	DESeq2	LRT	0
157	KC_AB2	Zfrp8	-1.69146138677642	0.37467917521115	1	pseudobulk	DESeq2	LRT	0
157	KC_AB2	Zif	0.111196987693089	0.785136067767327	1	pseudobulk	DESeq2	LRT	0
157	KC_AB2	zip	0.110458010431383	0.845706917956732	1	pseudobulk	DESeq2	LRT	0
157	KC_AB2	Zip102B	-0.604824710046892	0.447385371367111	1	pseudobulk	DESeq2	LRT	0
157	KC_AB2	Zip48C	-2.71632541924797	0.187022462241557	1	pseudobulk	DESeq2	LRT	0
157	KC_AB2	Zip71B	-0.600547221779134	1	1	pseudobulk	DESeq2	LRT	0
157	KC_AB2	Zip88E	1.82245639658508	0.285345440158223	1	pseudobulk	DESeq2	LRT	0
157	KC_AB2	Zip99C	2.08523640814881	0.23466677372855	1	pseudobulk	DESeq2	LRT	0
157	KC_AB2	ZIPIC	-1.16116875819275	0.736255625566845	1	pseudobulk	DESeq2	LRT	0
157	KC_AB2	Zir	0.481272463019833	0.708030183232448	1	pseudobulk	DESeq2	LRT	0
157	KC_AB2	Ziz	0.573887066626135	0.403693654237589	1	pseudobulk	DESeq2	LRT	0
157	KC_AB2	Zn72D	-0.0550778639102491	0.777473542126272	1	pseudobulk	DESeq2	LRT	0
157	KC_AB2	ZnT33D	-0.841882716738579	1	1	pseudobulk	DESeq2	LRT	0
157	KC_AB2	ZnT49B	-0.262775192911593	0.779051254108786	1	pseudobulk	DESeq2	LRT	0
157	KC_AB2	ZnT63C	0.292272102673992	0.605094643452976	1	pseudobulk	DESeq2	LRT	0
157	KC_AB2	Zpr1	0.892659559600706	0.712043317464255	1	pseudobulk	DESeq2	LRT	0
157	KC_AB2	Zw10	-1.84423614433138	0.419369668145802	1	pseudobulk	DESeq2	LRT	0
157	KC_AB2	zyd	1.5243995994652	0.741477491078038	1	pseudobulk	DESeq2	LRT	0
157	KC_AB2	Zyx	-0.51253072733829	0.395534143219877	1	pseudobulk	DESeq2	LRT	0
157	KC_AB3	128up	2.1034514528998	0.227376198540873	1	pseudobulk	DESeq2	LRT	0
157	KC_AB3	14-3-3epsilon	-0.0825969274244987	0.59803103747755	1	pseudobulk	DESeq2	LRT	0
157	KC_AB3	14-3-3zeta	-0.0069215406044722	0.920137413615311	1	pseudobulk	DESeq2	LRT	0
157	KC_AB3	140up	1.92721325997777	0.226827643907813	1	pseudobulk	DESeq2	LRT	0
157	KC_AB3	18SrRNA-Psi:CR4	1.31969784008702	0.482872192463621	1	pseudobulk	DESeq2	LRT	0
157	KC_AB3	18w	-1.04617605080021	0.562887713614191	1	pseudobulk	DESeq2	LRT	0
157	KC_AB3	26-29-p	0.378289046911646	0.714323504655029	1	pseudobulk	DESeq2	LRT	0
157	KC_AB3	28SrRNA-Psi:CR4	-0.530867992313282	0.584692936028896	1	pseudobulk	DESeq2	LRT	0
157	KC_AB3	28SrRNA-Psi:CR4	1.20132755712293	1	1	pseudobulk	DESeq2	LRT	0
157	KC_AB3	28SrRNA-Psi:CR4	0.632774104902944	1	1	pseudobulk	DESeq2	LRT	0
157	KC_AB3	28SrRNA-Psi:CR4	1.59359598433783	0.86377329553208	1	pseudobulk	DESeq2	LRT	0
157	KC_AB3	28SrRNA-Psi:CR4	-0.509737198953624	0.601660606853334	1	pseudobulk	DESeq2	LRT	0
157	KC_AB3	28SrRNA-Psi:CR4	2.4096641859508	0.428875287944006	1	pseudobulk	DESeq2	LRT	0
157	KC_AB3	2mit	-0.340583797188414	0.406698617283029	1	pseudobulk	DESeq2	LRT	0
157	KC_AB3	312	0.453755346622915	0.777427083113816	1	pseudobulk	DESeq2	LRT	0
157	KC_AB3	4E-T	0.0418559024359814	0.66350315279894	1	pseudobulk	DESeq2	LRT	0
157	KC_AB3	5-HT1A	0.142535510580861	0.596931581371877	1	pseudobulk	DESeq2	LRT	0
157	KC_AB3	5-HT1B	0.681493368394947	0.248173279995031	1	pseudobulk	DESeq2	LRT	0
157	KC_AB3	5-HT2A	-0.668697628914053	0.766626548836875	1	pseudobulk	DESeq2	LRT	0
157	KC_AB3	5-HT2B	-0.589020618761755	0.625450060902649	1	pseudobulk	DESeq2	LRT	0
157	KC_AB3	5.8SrRNA-Psi:CR4	-0.0327300486906476	1	1	pseudobulk	DESeq2	LRT	0
157	KC_AB3	5PtaseI	-0.548626815401189	0.321042200432158	1	pseudobulk	DESeq2	LRT	0
157	KC_AB3	7B2	-0.14098340020423	0.513150591257573	1	pseudobulk	DESeq2	LRT	0
157	KC_AB3	a	0.26261345049196	0.35783233651643	1	pseudobulk	DESeq2	LRT	0
157	KC_AB3	A16	0.131035839239545	0.914185392090952	1	pseudobulk	DESeq2	LRT	0
157	KC_AB3	a6	0.34581048266371	0.59318300474629	1	pseudobulk	DESeq2	LRT	0
157	KC_AB3	AANAT1	-0.268458218065157	0.4973460328515	1	pseudobulk	DESeq2	LRT	0
157	KC_AB3	AANATL7	0.173042533477813	1	1	pseudobulk	DESeq2	LRT	0

157	KC_AB3	Aasdh	0.664198150190449	0.806077681778353	1	pseudobulk	DESeq2	LRT	0
157	KC_AB3	Aatf	-0.822539571735454	0.515085703764047	1	pseudobulk	DESeq2	LRT	0
157	KC_AB3	aay	-1.22367607012006	0.454447138642056	1	pseudobulk	DESeq2	LRT	0
157	KC_AB3	ab	0.73570233426401	0.405212723699558	1	pseudobulk	DESeq2	LRT	0
157	KC_AB3	ABCA	0.340121983939124	0.550329000089865	1	pseudobulk	DESeq2	LRT	0
157	KC_AB3	ABCB7	-0.958141494675581	0.360842860008912	1	pseudobulk	DESeq2	LRT	0
157	KC_AB3	ABCD	0.0421332882594753	0.632066222392142	1	pseudobulk	DESeq2	LRT	0
158	KC_AB3	Abi	-0.197287863695633	0.6591668050122	1	pseudobulk	DESeq2	LRT	0
158	KC_AB3	Abl	0.573443707036118	0.097480401924191	1	pseudobulk	DESeq2	LRT	0
158	KC_AB3	abo	1.23133864869565	1	1	pseudobulk	DESeq2	LRT	0
158	KC_AB3	Abp1	0.179953442668497	0.572718675908122	1	pseudobulk	DESeq2	LRT	0
158	KC_AB3	abs	0.732079652272449	0.753859428914587	1	pseudobulk	DESeq2	LRT	0
158	KC_AB3	Ac3	-0.26484272529135	0.499775159449819	1	pseudobulk	DESeq2	LRT	0
158	KC_AB3	Ac76E	2.62817135292692	0.54954870385793	1	pseudobulk	DESeq2	LRT	0
158	KC_AB3	Ac78C	-1.04220261748592	0.411126246800301	1	pseudobulk	DESeq2	LRT	0
158	KC_AB3	Acbp1	-0.279449608401311	0.46186034988127	1	pseudobulk	DESeq2	LRT	0
158	KC_AB3	Acbp2	-0.185594513059781	1	1	pseudobulk	DESeq2	LRT	0
158	KC_AB3	ACC	-1.1489133165393	0.188818751442373	1	pseudobulk	DESeq2	LRT	0
158	KC_AB3	AcCoAS	-0.0247701624073405	0.823724131038178	1	pseudobulk	DESeq2	LRT	0
158	KC_AB3	Ace	0.215758451781604	0.64225499349473	1	pseudobulk	DESeq2	LRT	0
158	KC_AB3	Acer	1.16455392970854	0.70736672153235	1	pseudobulk	DESeq2	LRT	0
158	KC_AB3	Acf	-0.126299942566893	0.810155311309263	1	pseudobulk	DESeq2	LRT	0
158	KC_AB3	achi	0.64039360257429	1	1	pseudobulk	DESeq2	LRT	0
158	KC_AB3	Ack	-2.91272750140726	0.0252150633619007	1	pseudobulk	DESeq2	LRT	0
158	KC_AB3	Ack-like	0.116512609411292	0.799491149301925	1	pseudobulk	DESeq2	LRT	0
158	KC_AB3	Acn	0.409683604573023	0.484050354014942	1	pseudobulk	DESeq2	LRT	0
158	KC_AB3	Acph-1	0.623455309939184	1	1	pseudobulk	DESeq2	LRT	0
158	KC_AB3	Acsl	0.0255245850421933	0.924074900566825	1	pseudobulk	DESeq2	LRT	0
158	KC_AB3	Act42A	-0.293259724966844	0.697994185731577	1	pseudobulk	DESeq2	LRT	0
158	KC_AB3	Act5C	0.698820083775527	0.0261348068891063	1	pseudobulk	DESeq2	LRT	0
158	KC_AB3	Actbeta	-0.120906908702915	0.597833475543989	1	pseudobulk	DESeq2	LRT	0
158	KC_AB3	Actn	-0.269891394701783	0.750155631517377	1	pseudobulk	DESeq2	LRT	0
158	KC_AB3	ACXC	3.2740078253729	0.271992502367972	1	pseudobulk	DESeq2	LRT	0
158	KC_AB3	ACXD	1.95267092137636	1	1	pseudobulk	DESeq2	LRT	0
158	KC_AB3	Acyp2	-0.370784069073909	0.573561066179728	1	pseudobulk	DESeq2	LRT	0
158	KC_AB3	Ada1-1	1.93443687997187	0.65822970246154	1	pseudobulk	DESeq2	LRT	0
158	KC_AB3	Ada1-2	0.263237126093928	1	1	pseudobulk	DESeq2	LRT	0
158	KC_AB3	Ada2a	0.632774104902943	1	1	pseudobulk	DESeq2	LRT	0
158	KC_AB3	Ada2b	-0.361464312526204	0.647627964778316	1	pseudobulk	DESeq2	LRT	0
158	KC_AB3	Ada3	-1.29036345861895	0.310112703855883	1	pseudobulk	DESeq2	LRT	0
158	KC_AB3	Adar	-0.523992142203262	0.376796694508666	1	pseudobulk	DESeq2	LRT	0
158	KC_AB3	Adat1	1.59359598433783	0.86377329553208	1	pseudobulk	DESeq2	LRT	0
158	KC_AB3	Adck5	0.272714256234976	1	1	pseudobulk	DESeq2	LRT	0
158	KC_AB3	ADD1	1.15441309488324	0.125585921462864	1	pseudobulk	DESeq2	LRT	0
158	KC_AB3	AdenoK	-0.172022708331666	0.766042402892218	1	pseudobulk	DESeq2	LRT	0
158	KC_AB3	Adf1	-0.508246671045843	0.464455705985634	1	pseudobulk	DESeq2	LRT	0
158	KC_AB3	Adgf-D	1.23133864869565	1	1	pseudobulk	DESeq2	LRT	0
158	KC_AB3	Adi1	0.211120208429822	1	1	pseudobulk	DESeq2	LRT	0
158	KC_AB3	Adk1	-0.872947321987862	0.151517542221906	1	pseudobulk	DESeq2	LRT	0
158	KC_AB3	Adk2	-0.225241564615897	0.438804328027654	1	pseudobulk	DESeq2	LRT	0
158	KC_AB3	Adk3	1.23133864869565	1	1	pseudobulk	DESeq2	LRT	0
158	KC_AB3	adp	0.645298529048894	0.739440416747824	1	pseudobulk	DESeq2	LRT	0
158	KC_AB3	ADPS	0.36005837911044	0.652015408605569	1	pseudobulk	DESeq2	LRT	0

158	KC_AB3	AdSL	0.588111979197183	0.771359550789339	1	pseudobulk	DESeq2	LRT	0
158	KC_AB3	AdSS	-1.27939397934915	0.11688266851362	1	pseudobulk	DESeq2	LRT	0
158	KC_AB3	Aduk	-0.645207318417909	0.501730593650749	1	pseudobulk	DESeq2	LRT	0
158	KC_AB3	Ady43A	-0.621109663664844	0.58084698479483	1	pseudobulk	DESeq2	LRT	0
158	KC_AB3	Aef1	-1.01121089986192	0.140728020635054	1	pseudobulk	DESeq2	LRT	0
158	KC_AB3	aft	1.95267092137636	1	1	pseudobulk	DESeq2	LRT	0
158	KC_AB3	Afti	-0.920680324446148	0.39680805197194	1	pseudobulk	DESeq2	LRT	0
158	KC_AB3	Ag5r	1.12059961403812	1	1	pseudobulk	DESeq2	LRT	0
158	KC_AB3	AGBE	0.189700419481903	1	1	pseudobulk	DESeq2	LRT	0
158	KC_AB3	ago	0.324182559248046	0.549328304914236	1	pseudobulk	DESeq2	LRT	0
158	KC_AB3	AGO1	-0.13545277413223	0.563466785083415	1	pseudobulk	DESeq2	LRT	0
158	KC_AB3	AGO2	0.521144641166448	0.489634319463858	1	pseudobulk	DESeq2	LRT	0
158	KC_AB3	AGO3	-0.680094168819758	0.737706015643587	1	pseudobulk	DESeq2	LRT	0
158	KC_AB3	Agpat1	-0.664171197338023	0.647602868014152	1	pseudobulk	DESeq2	LRT	0
158	KC_AB3	Agpat2	0.632774104902944	1	1	pseudobulk	DESeq2	LRT	0
158	KC_AB3	Agpat3	-0.315313359624693	0.622185408760881	1	pseudobulk	DESeq2	LRT	0
158	KC_AB3	agt	0.632774104902943	1	1	pseudobulk	DESeq2	LRT	0
158	KC_AB3	Ahcy	0.165250055969276	1	1	pseudobulk	DESeq2	LRT	0
158	KC_AB3	AhcyL1	-0.501412534833069	0.618393572966464	1	pseudobulk	DESeq2	LRT	0
158	KC_AB3	AIF	0.512323415655317	0.735588920816918	1	pseudobulk	DESeq2	LRT	0
158	KC_AB3	AIMP1	0.907335125402267	0.313717874363617	1	pseudobulk	DESeq2	LRT	0
158	KC_AB3	AIMP2	0.271753469891261	0.706420686209992	1	pseudobulk	DESeq2	LRT	0
158	KC_AB3	AIMP3	0.436885843420482	0.426752367789451	1	pseudobulk	DESeq2	LRT	0
158	KC_AB3	Ak6	0.335884300763519	1	1	pseudobulk	DESeq2	LRT	0
158	KC_AB3	Akap200	-0.693529684616306	0.504194367911557	1	pseudobulk	DESeq2	LRT	0
158	KC_AB3	Akh	0.632774104902944	1	1	pseudobulk	DESeq2	LRT	0
158	KC_AB3	akirin	-0.010266625434571	0.799919904301042	1	pseudobulk	DESeq2	LRT	0
158	KC_AB3	Akt1	0.0939286451796076	0.877589954283217	1	pseudobulk	DESeq2	LRT	0
158	KC_AB3	Aladin	-0.184744780581018	1	1	pseudobulk	DESeq2	LRT	0
158	KC_AB3	AlaRS	-0.0217122226621366	1	1	pseudobulk	DESeq2	LRT	0
158	KC_AB3	AlaRS-m	0.178895417472062	0.85211653540939	1	pseudobulk	DESeq2	LRT	0
158	KC_AB3	Alas	-0.609495787648466	0.198920228740618	1	pseudobulk	DESeq2	LRT	0
158	KC_AB3	alc	0.0100980430075596	0.950927243566797	1	pseudobulk	DESeq2	LRT	0
158	KC_AB3	Ald1	-0.388201974249828	0.197400798806048	1	pseudobulk	DESeq2	LRT	0
158	KC_AB3	Aldh	0.632774104902944	1	1	pseudobulk	DESeq2	LRT	0
158	KC_AB3	Aldh-III	-0.688892104319043	0.438252544266604	1	pseudobulk	DESeq2	LRT	0
158	KC_AB3	Alg-2	-0.188467787304251	0.672427868981574	1	pseudobulk	DESeq2	LRT	0
158	KC_AB3	Alg1	0.593667095203339	1	1	pseudobulk	DESeq2	LRT	0
158	KC_AB3	Alg10	1.53928661184321	0.387229872623441	1	pseudobulk	DESeq2	LRT	0
158	KC_AB3	Alg11	-0.397311304424017	0.577545079101778	1	pseudobulk	DESeq2	LRT	0
158	KC_AB3	Alg14	0.263352619573195	1	1	pseudobulk	DESeq2	LRT	0
158	KC_AB3	Alg2	0.0542092317277189	1	1	pseudobulk	DESeq2	LRT	0
158	KC_AB3	Alg3	0.613794752365723	0.787707956402627	1	pseudobulk	DESeq2	LRT	0
158	KC_AB3	Alg9	0.632774104902944	1	1	pseudobulk	DESeq2	LRT	0
158	KC_AB3	Alh	0.187688347135432	0.666852985286788	1	pseudobulk	DESeq2	LRT	0
158	KC_AB3	alien	-0.454838436879585	0.641121438523392	1	pseudobulk	DESeq2	LRT	0
158	KC_AB3	ALiX	-0.11525905931824	0.512456357180229	1	pseudobulk	DESeq2	LRT	0
158	KC_AB3	Alk	-0.0685976537409065	0.632526389151518	1	pseudobulk	DESeq2	LRT	0
158	KC_AB3	Alp12	1.95267092137636	1	1	pseudobulk	DESeq2	LRT	0
158	KC_AB3	Alp5	1.95267092137636	1	1	pseudobulk	DESeq2	LRT	0
158	KC_AB3	Alp6	-0.290449198923282	0.852821004587482	1	pseudobulk	DESeq2	LRT	0
158	KC_AB3	alph	-0.0355189957943396	0.927461272076136	1	pseudobulk	DESeq2	LRT	0
158	KC_AB3	alpha-Cat	0.172187288258497	0.628459137516601	1	pseudobulk	DESeq2	LRT	0

158	KC_AB3	alpha-Catr	-0.0248756097262117	0.697518359419973	1	pseudobulk	DESeq2	LRT	0
159	KC_AB3	alpha-Est8	0.0992662063214884	0.680649295342411	1	pseudobulk	DESeq2	LRT	0
159	KC_AB3	alpha-Est9	0.139441553163555	0.784212936154368	1	pseudobulk	DESeq2	LRT	0
159	KC_AB3	alpha-Man-Ia	-0.0594229906419988	0.860484838861797	1	pseudobulk	DESeq2	LRT	0
159	KC_AB3	alpha-Man-Ib	0.328440454284809	0.410411631211332	1	pseudobulk	DESeq2	LRT	0
159	KC_AB3	alpha-Man-IIa	-0.185700748152894	0.673777701593843	1	pseudobulk	DESeq2	LRT	0
159	KC_AB3	alpha-Man-IIb	0.356766182173	0.592244235402523	1	pseudobulk	DESeq2	LRT	0
159	KC_AB3	alpha-PheRS	-0.283160869221142	0.682547681579316	1	pseudobulk	DESeq2	LRT	0
159	KC_AB3	alpha-Spec	-0.210906928918682	0.59199973383675	1	pseudobulk	DESeq2	LRT	0
159	KC_AB3	alpha4GT1	0.362571124435348	0.721659602517408	1	pseudobulk	DESeq2	LRT	0
159	KC_AB3	alphaCOP	-0.0130773269229125	0.742080582661966	1	pseudobulk	DESeq2	LRT	0
159	KC_AB3	alphaSnap	-0.385766315720643	0.272330510170392	1	pseudobulk	DESeq2	LRT	0
159	KC_AB3	alphaTub67C	-0.0141636105926055	0.883300074160999	1	pseudobulk	DESeq2	LRT	0
159	KC_AB3	alphaTub84B	0.0807176672101168	0.579216883345827	1	pseudobulk	DESeq2	LRT	0
159	KC_AB3	alphaTub84D	-0.121067215259842	0.675893963198814	1	pseudobulk	DESeq2	LRT	0
159	KC_AB3	Alr	0.811988120878673	0.419506282791516	1	pseudobulk	DESeq2	LRT	0
159	KC_AB3	alrm	-0.242705193805337	0.807323308907096	1	pseudobulk	DESeq2	LRT	0
159	KC_AB3	Als2	0.315482641211247	0.907421536285001	1	pseudobulk	DESeq2	LRT	0
159	KC_AB3	alt	-0.426651945809671	0.452850754892207	1	pseudobulk	DESeq2	LRT	0
159	KC_AB3	Amacr	0.432109301296454	0.757208074723755	1	pseudobulk	DESeq2	LRT	0
159	KC_AB3	amn	1.59575615951652	0.886898266337347	1	pseudobulk	DESeq2	LRT	0
159	KC_AB3	Amnionless	0.711634880763962	0.304223008814798	1	pseudobulk	DESeq2	LRT	0
159	KC_AB3	amon	-0.155056075484873	0.441812776542579	1	pseudobulk	DESeq2	LRT	0
159	KC_AB3	AMPdeam	0.580166878578615	0.36067896218531	1	pseudobulk	DESeq2	LRT	0
159	KC_AB3	Amph	-0.612053827386078	0.404862454737045	1	pseudobulk	DESeq2	LRT	0
159	KC_AB3	AMPKalpha	-0.580165335704182	0.35721996593209	1	pseudobulk	DESeq2	LRT	0
159	KC_AB3	Amun	-0.437183385374386	0.487852853007422	1	pseudobulk	DESeq2	LRT	0
159	KC_AB3	amx	-0.0450246493562332	0.724920469236234	1	pseudobulk	DESeq2	LRT	0
159	KC_AB3	Amyrel	-0.0327300486906476	1	1	pseudobulk	DESeq2	LRT	0
159	KC_AB3	ana	0.632774104902944	1	1	pseudobulk	DESeq2	LRT	0
159	KC_AB3	ana2	1.95267092137636	1	1	pseudobulk	DESeq2	LRT	0
159	KC_AB3	anchor	0.571657766026868	0.421316432625177	1	pseudobulk	DESeq2	LRT	0
159	KC_AB3	angel	-0.657534285283636	0.564603183830293	1	pseudobulk	DESeq2	LRT	0
159	KC_AB3	Ank	0.415888756713648	0.363140314526821	1	pseudobulk	DESeq2	LRT	0
159	KC_AB3	Ank2	-0.210622764001239	0.395003027469715	1	pseudobulk	DESeq2	LRT	0
159	KC_AB3	Ankle2	0.257885424941626	0.931562283065932	1	pseudobulk	DESeq2	LRT	0
159	KC_AB3	anne	-0.14098516902634	0.428286906586419	1	pseudobulk	DESeq2	LRT	0
159	KC_AB3	Ant2	0.161515868851812	1	1	pseudobulk	DESeq2	LRT	0
159	KC_AB3	AnxB10	-0.361056006271045	0.702335745239009	1	pseudobulk	DESeq2	LRT	0
159	KC_AB3	AnxB11	0.101117385634039	0.648063726064471	1	pseudobulk	DESeq2	LRT	0
159	KC_AB3	AnxB9	0.632774104902944	1	1	pseudobulk	DESeq2	LRT	0
159	KC_AB3	aop	-0.287771893734738	0.65528845616138	1	pseudobulk	DESeq2	LRT	0
159	KC_AB3	Aos1	-1.16243044537979	0.252219747748475	1	pseudobulk	DESeq2	LRT	0
159	KC_AB3	AOX3	0.632774104902943	1	1	pseudobulk	DESeq2	LRT	0
159	KC_AB3	AP-1-2beta	0.40491405309149	0.506189571882414	1	pseudobulk	DESeq2	LRT	0
159	KC_AB3	AP-1gamma	-0.12375358761626	0.733152078942389	1	pseudobulk	DESeq2	LRT	0
159	KC_AB3	AP-1mu	0.0897034271071676	0.618564665568903	1	pseudobulk	DESeq2	LRT	0
159	KC_AB3	AP-1sigma	-0.358019735570494	0.378552102681388	1	pseudobulk	DESeq2	LRT	0
159	KC_AB3	AP-2alpha	0.110645251475469	0.736167755922328	1	pseudobulk	DESeq2	LRT	0
159	KC_AB3	AP-2mu	0.0704423926291706	0.516005221006846	1	pseudobulk	DESeq2	LRT	0
159	KC_AB3	AP-2sigma	-0.10854923970183	0.451570065544386	1	pseudobulk	DESeq2	LRT	0
159	KC_AB3	Apc	0.153840596834489	0.812935436979561	1	pseudobulk	DESeq2	LRT	0
159	KC_AB3	APC10	0.30717719652288	0.721862753979333	1	pseudobulk	DESeq2	LRT	0

159	KC_AB3	APC4	0.111792608872726	1	1	pseudobulk	DESeq2	LRT	0
159	KC_AB3	ApepP	0.541152198903338	1	1	pseudobulk	DESeq2	LRT	0
159	KC_AB3	Apf	-1.31033812278169	0.305294878085323	1	pseudobulk	DESeq2	LRT	0
159	KC_AB3	aph-1	0.273587266434216	0.691886371969922	1	pseudobulk	DESeq2	LRT	0
159	KC_AB3	aPKC	0.451825077292916	0.346778004295808	1	pseudobulk	DESeq2	LRT	0
159	KC_AB3	Apl	0.238162730231771	0.329288039165875	1	pseudobulk	DESeq2	LRT	0
159	KC_AB3	Aplip1	0.0515698338645859	0.624839632950388	1	pseudobulk	DESeq2	LRT	0
159	KC_AB3	apolpp	1.95267092137636	1	1	pseudobulk	DESeq2	LRT	0
159	KC_AB3	Apoltp	1.45422967012522	0.753390297571937	1	pseudobulk	DESeq2	LRT	0
159	KC_AB3	app	0.632774104902944	1	1	pseudobulk	DESeq2	LRT	0
159	KC_AB3	APP-BP1	0.431760236074716	0.945155314406571	1	pseudobulk	DESeq2	LRT	0
159	KC_AB3	Appl	-0.256613932121761	0.244103411806982	1	pseudobulk	DESeq2	LRT	0
159	KC_AB3	Aprt	1.37794690317769	0.597397478581202	1	pseudobulk	DESeq2	LRT	0
159	KC_AB3	Aps	-0.350599886580618	0.418721834446446	1	pseudobulk	DESeq2	LRT	0
159	KC_AB3	aqz	-0.11563358179596	0.536765570363717	1	pseudobulk	DESeq2	LRT	0
159	KC_AB3	aralar1	0.452468522172246	0.554428922136253	1	pseudobulk	DESeq2	LRT	0
159	KC_AB3	Arc1	2.36668504463107	0.433543977456183	1	pseudobulk	DESeq2	LRT	0
159	KC_AB3	Arc42	1.23133864869565	1	1	pseudobulk	DESeq2	LRT	0
159	KC_AB3	Archease	1.30732999830804	0.760961512337506	1	pseudobulk	DESeq2	LRT	0
159	KC_AB3	Arf102F	-0.128535549302338	0.807189231646074	1	pseudobulk	DESeq2	LRT	0
159	KC_AB3	Arf51F	0.957164338620938	0.225509384614056	1	pseudobulk	DESeq2	LRT	0
159	KC_AB3	Arf79F	-0.159503587462452	0.523631877155219	1	pseudobulk	DESeq2	LRT	0
159	KC_AB3	ArfGAP1	-0.589852403250211	0.526847023012716	1	pseudobulk	DESeq2	LRT	0
159	KC_AB3	ArfGAP3	1.35106924840207	0.236583674840585	1	pseudobulk	DESeq2	LRT	0
159	KC_AB3	Arfip	0.291172144026235	0.541299083408356	1	pseudobulk	DESeq2	LRT	0
159	KC_AB3	Arfrp1	-1.05396012854827	0.460675870249541	1	pseudobulk	DESeq2	LRT	0
159	KC_AB3	arg	-0.853854037925779	0.611355273229667	1	pseudobulk	DESeq2	LRT	0
159	KC_AB3	Argk	-0.244632962264756	0.30000721788312	1	pseudobulk	DESeq2	LRT	0
159	KC_AB3	Argl	0.161515868851812	1	1	pseudobulk	DESeq2	LRT	0
159	KC_AB3	ArgRS	-0.987189378304843	0.481223954187462	1	pseudobulk	DESeq2	LRT	0
159	KC_AB3	ArgRS-m	0.327507071673806	1	1	pseudobulk	DESeq2	LRT	0
159	KC_AB3	ari-1	-0.214562373365004	0.567527453075748	1	pseudobulk	DESeq2	LRT	0
159	KC_AB3	ari-2	0.235579569755507	0.44832794692756	1	pseudobulk	DESeq2	LRT	0
159	KC_AB3	Arl1	0.443596085371972	0.484782164037806	1	pseudobulk	DESeq2	LRT	0
159	KC_AB3	Arl2	1.2544410254207	0.220523565289475	1	pseudobulk	DESeq2	LRT	0
159	KC_AB3	Arl4	0.06198569076777	0.803413604734001	1	pseudobulk	DESeq2	LRT	0
159	KC_AB3	Arl5	0.258348313617643	0.667643151220165	1	pseudobulk	DESeq2	LRT	0
159	KC_AB3	Arl6IP1	-0.105075368959956	0.607246194268743	1	pseudobulk	DESeq2	LRT	0
159	KC_AB3	Arl8	0.271925650217855	0.473562276411234	1	pseudobulk	DESeq2	LRT	0
159	KC_AB3	arm	0.0297392890195248	0.751308524278692	1	pseudobulk	DESeq2	LRT	0
159	KC_AB3	armi	0.632774104902944	1	1	pseudobulk	DESeq2	LRT	0
159	KC_AB3	Arp1	-0.124409514484486	0.55982961305223	1	pseudobulk	DESeq2	LRT	0
159	KC_AB3	Arp10	0.713281307285132	0.84315173270398	1	pseudobulk	DESeq2	LRT	0
159	KC_AB3	Arp3	-0.452944890850635	0.584259925405398	1	pseudobulk	DESeq2	LRT	0
159	KC_AB3	Arp5	-0.529754410077637	0.652685447816764	1	pseudobulk	DESeq2	LRT	0
159	KC_AB3	Arp6	1.55564881408874	0.310957424463763	1	pseudobulk	DESeq2	LRT	0
159	KC_AB3	Arp8	-0.714227006634363	0.576843796398186	1	pseudobulk	DESeq2	LRT	0
159	KC_AB3	Arpc1	-0.0511116479399921	0.892006190967947	1	pseudobulk	DESeq2	LRT	0
160	KC_AB3	Arpc2	0.539722422807458	0.691377817850711	1	pseudobulk	DESeq2	LRT	0
160	KC_AB3	Arpc3A	0.980389758009687	0.312613909560452	1	pseudobulk	DESeq2	LRT	0
160	KC_AB3	Arpc4	-0.124306969338955	0.739014703454478	1	pseudobulk	DESeq2	LRT	0
160	KC_AB3	Arpc5	-0.494335028783138	0.529721174873983	1	pseudobulk	DESeq2	LRT	0
160	KC_AB3	arr	-0.333931685992957	0.525842675682136	1	pseudobulk	DESeq2	LRT	0

160	KC_AB3	Ars2	0.372428686657825	0.591693341971843	1	pseudobulk	DESeq2	LRT	0
160	KC_AB3	Art1	1.28191915596376	0.363841059134373	1	pseudobulk	DESeq2	LRT	0
160	KC_AB3	Art3	1.84671504053046	1	1	pseudobulk	DESeq2	LRT	0
160	KC_AB3	Art4	1.86689087684802	0.535325076131883	1	pseudobulk	DESeq2	LRT	0
160	KC_AB3	Art7	0.632774104902943	1	1	pseudobulk	DESeq2	LRT	0
160	KC_AB3	Arts	-0.560477492866218	0.784070523163114	1	pseudobulk	DESeq2	LRT	0
160	KC_AB3	Arv1	1.14894055867507	0.234431502274382	1	pseudobulk	DESeq2	LRT	0
160	KC_AB3	arx	2.62817135292692	0.54954870385793	1	pseudobulk	DESeq2	LRT	0
160	KC_AB3	Asap	-0.194812397447452	0.702886172613099	1	pseudobulk	DESeq2	LRT	0
160	KC_AB3	Asator	-0.294759856644254	0.336773701839126	1	pseudobulk	DESeq2	LRT	0
160	KC_AB3	Asciz	0.649715915883756	0.780910159678579	1	pseudobulk	DESeq2	LRT	0
160	KC_AB3	asf1	-0.117316627149919	0.770360069477503	1	pseudobulk	DESeq2	LRT	0
160	KC_AB3	ash1	-0.194013586176682	0.635796924185803	1	pseudobulk	DESeq2	LRT	0
160	KC_AB3	ash2	0.4135959724801	1	1	pseudobulk	DESeq2	LRT	0
160	KC_AB3	Ask1	-1.53642641152681	0.378146177966233	1	pseudobulk	DESeq2	LRT	0
160	KC_AB3	asl	1.23133864869565	1	1	pseudobulk	DESeq2	LRT	0
160	KC_AB3	AsnRS	0.687958262823875	0.396178035085642	1	pseudobulk	DESeq2	LRT	0
160	KC_AB3	AsnRS-m	-0.232714443854297	0.839472778407846	1	pseudobulk	DESeq2	LRT	0
160	KC_AB3	AspRS	-0.909965179097225	0.531095342261223	1	pseudobulk	DESeq2	LRT	0
160	KC_AB3	AspRS-m	0.0897954941213223	0.862944518564239	1	pseudobulk	DESeq2	LRT	0
160	KC_AB3	asrij	-0.102903607211275	0.870011405128367	1	pseudobulk	DESeq2	LRT	0
160	KC_AB3	asRNA:CR30267	-0.0327300486906476	1	1	pseudobulk	DESeq2	LRT	0
160	KC_AB3	asRNA:CR31144	3.74944488710582	0.210867092758549	1	pseudobulk	DESeq2	LRT	0
160	KC_AB3	asRNA:CR31514	1.95267092137636	1	1	pseudobulk	DESeq2	LRT	0
160	KC_AB3	asRNA:CR31912	1.95267092137636	1	1	pseudobulk	DESeq2	LRT	0
160	KC_AB3	asRNA:CR42254	-0.36569053469663	0.713295621838618	1	pseudobulk	DESeq2	LRT	0
160	KC_AB3	asRNA:CR42547	0.414593131727451	1	1	pseudobulk	DESeq2	LRT	0
160	KC_AB3	asRNA:CR43259	-0.185594513059781	1	1	pseudobulk	DESeq2	LRT	0
160	KC_AB3	asRNA:CR43425	0.0657424083752195	0.80914544131957	1	pseudobulk	DESeq2	LRT	0
160	KC_AB3	asRNA:CR43426	2.62817135292692	0.54954870385793	1	pseudobulk	DESeq2	LRT	0
160	KC_AB3	asRNA:CR43460	-0.185594513059781	1	1	pseudobulk	DESeq2	LRT	0
160	KC_AB3	asRNA:CR43629	0.845629988421954	0.723616189098383	1	pseudobulk	DESeq2	LRT	0
160	KC_AB3	asRNA:CR43906	1.4425790546433	0.553702792681862	1	pseudobulk	DESeq2	LRT	0
160	KC_AB3	asRNA:CR43933	0.0020939056309239	1	1	pseudobulk	DESeq2	LRT	0
160	KC_AB3	asRNA:CR43944	1.23133864869565	1	1	pseudobulk	DESeq2	LRT	0
160	KC_AB3	asRNA:CR43957	1.87518227264439	0.766977424371083	1	pseudobulk	DESeq2	LRT	0
160	KC_AB3	asRNA:CR43959	-0.185594513059781	1	1	pseudobulk	DESeq2	LRT	0
160	KC_AB3	asRNA:CR44018	-0.803374324112755	0.466454060568476	1	pseudobulk	DESeq2	LRT	0
160	KC_AB3	asRNA:CR44027	-0.606508186280786	0.58544958215239	1	pseudobulk	DESeq2	LRT	0
160	KC_AB3	asRNA:CR44029	-0.87743297920362	0.458906734783461	1	pseudobulk	DESeq2	LRT	0
160	KC_AB3	asRNA:CR44030	1.12549028440491	0.261885257545601	1	pseudobulk	DESeq2	LRT	0
160	KC_AB3	asRNA:CR44031	-0.607183838294338	0.644544647637467	1	pseudobulk	DESeq2	LRT	0
160	KC_AB3	asRNA:CR44046	1.86689087684802	0.535325076131883	1	pseudobulk	DESeq2	LRT	0
160	KC_AB3	asRNA:CR44049	2.62817135292692	0.54954870385793	1	pseudobulk	DESeq2	LRT	0
160	KC_AB3	asRNA:CR44093	0.0025701398975595	1	1	pseudobulk	DESeq2	LRT	0
160	KC_AB3	asRNA:CR44107	-0.778630514777685	0.6329320942177	1	pseudobulk	DESeq2	LRT	0
160	KC_AB3	asRNA:CR44114	0.632774104902944	1	1	pseudobulk	DESeq2	LRT	0
160	KC_AB3	asRNA:CR44179	0.4135959724801	1	1	pseudobulk	DESeq2	LRT	0
160	KC_AB3	asRNA:CR44228	0.632774104902943	1	1	pseudobulk	DESeq2	LRT	0
160	KC_AB3	asRNA:CR44340	1.23133864869565	1	1	pseudobulk	DESeq2	LRT	0
160	KC_AB3	asRNA:CR44512	1.59576167909336	0.887029991980463	1	pseudobulk	DESeq2	LRT	0
160	KC_AB3	asRNA:CR44690	0.632774104902944	1	1	pseudobulk	DESeq2	LRT	0
160	KC_AB3	asRNA:CR44840	1.31841240790001	0.766867661691019	1	pseudobulk	DESeq2	LRT	0

160	KC_AB3	asRNA:CR44843	0.161515868851812	1	1	pseudobulk	DESeq2	LRT		0
160	KC_AB3	asRNA:CR44892	-0.52878559648638	0.686420589858048	1	pseudobulk	DESeq2	LRT		0
160	KC_AB3	asRNA:CR44894	-0.269607325030506	0.685661544360775	1	pseudobulk	DESeq2	LRT		0
160	KC_AB3	asRNA:CR44907	0.632774104902944	1	1	pseudobulk	DESeq2	LRT		0
160	KC_AB3	asRNA:CR44989	1.12059961403812	1	1	pseudobulk	DESeq2	LRT		0
160	KC_AB3	asRNA:CR45000	-0.186961738271884	1	1	pseudobulk	DESeq2	LRT		0
160	KC_AB3	asRNA:CR45009	-0.782073562885836	0.539450763524473	1	pseudobulk	DESeq2	LRT		0
160	KC_AB3	asRNA:CR45026	1.95267092137636	1	1	pseudobulk	DESeq2	LRT		0
160	KC_AB3	asRNA:CR45056	0.632774104902943	1	1	pseudobulk	DESeq2	LRT		0
160	KC_AB3	asRNA:CR45124	-1.2395315917889	0.5025052194406	1	pseudobulk	DESeq2	LRT		0
160	KC_AB3	asRNA:CR45126	-0.725379765710518	0.577904944341689	1	pseudobulk	DESeq2	LRT		0
160	KC_AB3	asRNA:CR45129	-0.120528428548273	0.914416877354382	1	pseudobulk	DESeq2	LRT		0
160	KC_AB3	asRNA:CR45131	0.632774104902943	1	1	pseudobulk	DESeq2	LRT		0
160	KC_AB3	asRNA:CR45134	0.411145774616137	1	1	pseudobulk	DESeq2	LRT		0
160	KC_AB3	asRNA:CR45137	0.632774104902943	1	1	pseudobulk	DESeq2	LRT		0
160	KC_AB3	asRNA:CR45145	-0.866520209357606	0.555976048648481	1	pseudobulk	DESeq2	LRT		0
160	KC_AB3	asRNA:CR45151	2.62817135292692	0.54954870385793	1	pseudobulk	DESeq2	LRT		0
160	KC_AB3	asRNA:CR45162	0.632774104902944	1	1	pseudobulk	DESeq2	LRT		0
160	KC_AB3	asRNA:CR45164	-0.562114017828961	0.802765549578463	1	pseudobulk	DESeq2	LRT		0
160	KC_AB3	asRNA:CR45182	1.8751573179585	0.767073395253422	1	pseudobulk	DESeq2	LRT		0
160	KC_AB3	asRNA:CR45198	0.594117478581602	1	1	pseudobulk	DESeq2	LRT		0
160	KC_AB3	asRNA:CR45208	0.632774104902944	1	1	pseudobulk	DESeq2	LRT		0
160	KC_AB3	asRNA:CR45281	1.23133864869565	1	1	pseudobulk	DESeq2	LRT		0
160	KC_AB3	asRNA:CR45350	0.632774104902944	1	1	pseudobulk	DESeq2	LRT		0
160	KC_AB3	asRNA:CR45384	1.95267092137636	1	1	pseudobulk	DESeq2	LRT		0
160	KC_AB3	asRNA:CR45469	-0.186961738271884	1	1	pseudobulk	DESeq2	LRT		0
160	KC_AB3	asRNA:CR45600	1.31558647470709	0.624964258876066	1	pseudobulk	DESeq2	LRT		0
160	KC_AB3	asRNA:CR45620	-0.0327300486906476	1	1	pseudobulk	DESeq2	LRT		0
160	KC_AB3	asRNA:CR45683	0.632774104902944	1	1	pseudobulk	DESeq2	LRT		0
160	KC_AB3	asRNA:CR45789	-0.13718526997977	1	1	pseudobulk	DESeq2	LRT		0
160	KC_AB3	asRNA:CR45804	0.632774104902944	1	1	pseudobulk	DESeq2	LRT		0
160	KC_AB3	asRNA:CR45813	0.632774104902943	1	1	pseudobulk	DESeq2	LRT		0
160	KC_AB3	asRNA:CR45815	0.632774104902944	1	1	pseudobulk	DESeq2	LRT		0
160	KC_AB3	asRNA:CR45822	0.0844828759738395	1	1	pseudobulk	DESeq2	LRT		0
160	KC_AB3	asRNA:CR45832	1.95267092137636	1	1	pseudobulk	DESeq2	LRT		0
160	KC_AB3	asRNA:CR45835	-0.111243483731708	0.918746075657284	1	pseudobulk	DESeq2	LRT		0
160	KC_AB3	asRNA:CR45865	2.3149273646917	0.611236276445972	1	pseudobulk	DESeq2	LRT		0
160	KC_AB3	asRNA:CR45873	1.95267092137636	1	1	pseudobulk	DESeq2	LRT		0
160	KC_AB3	asRNA:CR45874	0.0033107670165597	0.730543241492708	1	pseudobulk	DESeq2	LRT		0
160	KC_AB3	asRNA:CR45889	2.3149273646917	0.611236276445972	1	pseudobulk	DESeq2	LRT		0
160	KC_AB3	asRNA:CR45912	3.07272693214569	0.238940395988569	1	pseudobulk	DESeq2	LRT		0
160	KC_AB3	asRNA:CR45925	2.3149273646917	0.611236276445972	1	pseudobulk	DESeq2	LRT		0
161	KC_AB3	asRNA:CR45943	0.632774104902944	1	1	pseudobulk	DESeq2	LRT		0
161	KC_AB3	asRNA:CR45951	0.161515868851812	1	1	pseudobulk	DESeq2	LRT		0
161	KC_AB3	asRNA:CR45960	0.632774104902944	1	1	pseudobulk	DESeq2	LRT		0
161	KC_AB3	asRNA:CR46066	1.95267092137636	1	1	pseudobulk	DESeq2	LRT		0
161	KC_AB3	asRNA:CR46134	2.62817135292692	0.54954870385793	1	pseudobulk	DESeq2	LRT		0
161	KC_AB3	asRNA:CR46135	2.3149273646917	0.611236276445972	1	pseudobulk	DESeq2	LRT		0
161	KC_AB3	asRNA:CR46137	1.95267092137636	1	1	pseudobulk	DESeq2	LRT		0
161	KC_AB3	asRNA:CR46262	1.95267092137636	1	1	pseudobulk	DESeq2	LRT		0
161	KC_AB3	asRNA:CR46354	1.23133864869565	1	1	pseudobulk	DESeq2	LRT		0
161	KC_AB3	ast	1.20132755712293	1	1	pseudobulk	DESeq2	LRT		0
161	KC_AB3	AstA-R1	0.632774104902944	1	1	pseudobulk	DESeq2	LRT		0

161	KC_AB3	AstA-R2	0.632774104902944	1	1	pseudobulk	DESeq2	LRT		0
161	KC_AB3	AstC-R1	1.95267092137636	1	1	pseudobulk	DESeq2	LRT		0
161	KC_AB3	asun	1.95267092137636	1	1	pseudobulk	DESeq2	LRT		0
161	KC_AB3	Asx	0.0969040527365026	0.715282025341484	1	pseudobulk	DESeq2	LRT		0
161	KC_AB3	Atac1	0.053197396997799	1	1	pseudobulk	DESeq2	LRT		0
161	KC_AB3	Atac2	-1.6467745047992	0.310718317725107	1	pseudobulk	DESeq2	LRT		0
161	KC_AB3	Atac3	-1.32559719764957	0.276270639615157	1	pseudobulk	DESeq2	LRT		0
161	KC_AB3	ATbp	1.11479939981603	0.68366681357092	1	pseudobulk	DESeq2	LRT		0
161	KC_AB3	Ate1	0.254152020748798	0.670067684414011	1	pseudobulk	DESeq2	LRT		0
161	KC_AB3	Atet	-0.21506369185466	0.542497139743653	1	pseudobulk	DESeq2	LRT		0
161	KC_AB3	Atf-2	1.82121009152559	0.16101498303951	1	pseudobulk	DESeq2	LRT		0
161	KC_AB3	Atf3	-1.77145074240478	0.289779081311803	1	pseudobulk	DESeq2	LRT		0
161	KC_AB3	Atf6	-0.261968347706893	0.518876780401787	1	pseudobulk	DESeq2	LRT		0
161	KC_AB3	Atg1	-0.32578171759695	0.340177777651017	1	pseudobulk	DESeq2	LRT		0
161	KC_AB3	Atg10	1.95267092137636	1	1	pseudobulk	DESeq2	LRT		0
161	KC_AB3	Atg101	-0.322659253654475	0.624550614293918	1	pseudobulk	DESeq2	LRT		0
161	KC_AB3	Atg12	0.12429285117339	0.656400299632917	1	pseudobulk	DESeq2	LRT		0
161	KC_AB3	Atg13	0.440829071215768	0.509527703600758	1	pseudobulk	DESeq2	LRT		0
161	KC_AB3	Atg14	-0.553562788404803	0.483983218143555	1	pseudobulk	DESeq2	LRT		0
161	KC_AB3	Atg16	1.70764620409393	0.406718663428213	1	pseudobulk	DESeq2	LRT		0
161	KC_AB3	Atg17	-0.418595046190017	0.447056252784917	1	pseudobulk	DESeq2	LRT		0
161	KC_AB3	Atg18a	-0.965744305877935	0.319214705837611	1	pseudobulk	DESeq2	LRT		0
161	KC_AB3	Atg18b	0.644227363972446	0.338625492878516	1	pseudobulk	DESeq2	LRT		0
161	KC_AB3	Atg2	0.00375717646746098	0.837291772644316	1	pseudobulk	DESeq2	LRT		0
161	KC_AB3	Atg3	0.77309510565042	0.448449379375469	1	pseudobulk	DESeq2	LRT		0
161	KC_AB3	Atg4a	1.15399224695635	0.274394084173382	1	pseudobulk	DESeq2	LRT		0
161	KC_AB3	Atg4b	0.0542213283300749	0.937446228327618	1	pseudobulk	DESeq2	LRT		0
161	KC_AB3	Atg5	-0.77488500894959	0.471304097901887	1	pseudobulk	DESeq2	LRT		0
161	KC_AB3	Atg6	-0.343246998242847	0.33700584897033	1	pseudobulk	DESeq2	LRT		0
161	KC_AB3	Atg7	2.19539902116932	0.526642538463226	1	pseudobulk	DESeq2	LRT		0
161	KC_AB3	Atg8a	-0.0148892076037111	0.604727916575263	1	pseudobulk	DESeq2	LRT		0
161	KC_AB3	Atg9	-0.152692314373808	0.850051925812696	1	pseudobulk	DESeq2	LRT		0
161	KC_AB3	atl	-0.763966404596712	0.417419614200662	1	pseudobulk	DESeq2	LRT		0
161	KC_AB3	atms	1.29692595799497	0.512134704843688	1	pseudobulk	DESeq2	LRT		0
161	KC_AB3	Atox1	-0.071360440000151	0.621877218748451	1	pseudobulk	DESeq2	LRT		0
161	KC_AB3	ATP6AP2	-0.0450939815196353	0.459515004396105	1	pseudobulk	DESeq2	LRT		0
161	KC_AB3	ATP7	-1.11571707559185	0.514634393193615	1	pseudobulk	DESeq2	LRT		0
161	KC_AB3	ATP8A	-0.398915128510825	0.453899209606393	1	pseudobulk	DESeq2	LRT		0
161	KC_AB3	ATP8B	1.95267092137636	1	1	pseudobulk	DESeq2	LRT		0
161	KC_AB3	Atpalpha	-0.200139917542795	0.374819261213052	1	pseudobulk	DESeq2	LRT		0
161	KC_AB3	ATPCL	0.171013268806675	0.648556571608197	1	pseudobulk	DESeq2	LRT		0
161	KC_AB3	ATPsynB	-0.178990047232778	0.390552721568414	1	pseudobulk	DESeq2	LRT		0
161	KC_AB3	ATPsynbeta	-0.126931543392508	0.52771571564521	1	pseudobulk	DESeq2	LRT		0
161	KC_AB3	ATPsynC	-0.151933931539204	0.455659110394552	1	pseudobulk	DESeq2	LRT		0
161	KC_AB3	ATPsynCF6	-0.0052216389398337	0.610643731636707	1	pseudobulk	DESeq2	LRT		0
161	KC_AB3	ATPsynD	0.0485601808004583	0.592978030311549	1	pseudobulk	DESeq2	LRT		0
161	KC_AB3	ATPsyndelta	0.226862246755975	0.500320810530025	1	pseudobulk	DESeq2	LRT		0
161	KC_AB3	ATPsynE	-0.0027903167005029	0.845375370169348	1	pseudobulk	DESeq2	LRT		0
161	KC_AB3	ATPsynF	-0.156594194153679	0.427327077208073	1	pseudobulk	DESeq2	LRT		0
161	KC_AB3	ATPsynG	-0.152218265305566	0.463606850359872	1	pseudobulk	DESeq2	LRT		0
161	KC_AB3	ATPsyngamma	-0.208434366413242	0.355730553777605	1	pseudobulk	DESeq2	LRT		0
161	KC_AB3	ATPsynO	0.0617775789515917	0.71506883153162	1	pseudobulk	DESeq2	LRT		0
161	KC_AB3	Atu	-0.0786163856735961	0.830514617041283	1	pseudobulk	DESeq2	LRT		0

161	KC_AB3	Atx2	-0.23406061099751	0.571879408186009	1	pseudobulk	DESeq2	LRT		0
161	KC_AB3	Atxn7	-0.217963601131697	0.567362112536363	1	pseudobulk	DESeq2	LRT		0
161	KC_AB3	aub	0.632774104902943	1	1	pseudobulk	DESeq2	LRT		0
161	KC_AB3	aux	0.15343856744651	0.651832823763353	1	pseudobulk	DESeq2	LRT		0
161	KC_AB3	ave	-1.40301114808949	0.221852218859231	1	pseudobulk	DESeq2	LRT		0
161	KC_AB3	Aven	-2.1268894925632	0.198969884341887	1	pseudobulk	DESeq2	LRT		0
161	KC_AB3	awd	0.195293073447063	0.543810277729169	1	pseudobulk	DESeq2	LRT		0
161	KC_AB3	axed	0.0089308468791886	0.669618046545462	1	pseudobulk	DESeq2	LRT		0
161	KC_AB3	Axn	0.259371265214543	0.787828602415775	1	pseudobulk	DESeq2	LRT		0
161	KC_AB3	axo	-1.9500697671193	0.150422093646677	1	pseudobulk	DESeq2	LRT		0
161	KC_AB3	Axud1	-0.336938560182753	0.601276328079841	1	pseudobulk	DESeq2	LRT		0
161	KC_AB3	az2	0.644528576631096	0.678650531910201	1	pseudobulk	DESeq2	LRT		0
161	KC_AB3	B4	-0.0095960082669617	0.486069494337694	1	pseudobulk	DESeq2	LRT		0
161	KC_AB3	B52	-0.29627248894001	0.381940943284792	1	pseudobulk	DESeq2	LRT		0
161	KC_AB3	b6	0.632774104902944	1	1	pseudobulk	DESeq2	LRT		0
161	KC_AB3	bab1	1.95267092137636	1	1	pseudobulk	DESeq2	LRT		0
161	KC_AB3	babo	-0.255072551110692	0.580389546949976	1	pseudobulk	DESeq2	LRT		0
161	KC_AB3	Bacc	-0.436532616614367	0.0801892586815047	1	pseudobulk	DESeq2	LRT		0
161	KC_AB3	baf	0.696780851856741	0.65603821659246	1	pseudobulk	DESeq2	LRT		0
161	KC_AB3	bai	-0.394929036548837	0.587695015601737	1	pseudobulk	DESeq2	LRT		0
161	KC_AB3	Baldspot	-0.533372321465188	0.280831488029764	1	pseudobulk	DESeq2	LRT		0
161	KC_AB3	Bap111	-0.713337916521258	0.521031798733589	1	pseudobulk	DESeq2	LRT		0
161	KC_AB3	Bap170	1.78485899047987	0.50666947357673	1	pseudobulk	DESeq2	LRT		0
161	KC_AB3	Bap55	0.206034846674913	0.791880839872442	1	pseudobulk	DESeq2	LRT		0
161	KC_AB3	Bap60	-0.240735995890137	0.735730954479599	1	pseudobulk	DESeq2	LRT		0
161	KC_AB3	barc	-0.542930834967733	0.427322378230893	1	pseudobulk	DESeq2	LRT		0
161	KC_AB3	bbc	0.0259951870308109	0.709422758703836	1	pseudobulk	DESeq2	LRT		0
161	KC_AB3	bbx	1.93443687997187	0.65822970246154	1	pseudobulk	DESeq2	LRT		0
161	KC_AB3	bc10	-0.193139491987073	0.634596857014243	1	pseudobulk	DESeq2	LRT		0
161	KC_AB3	BCAS2	-1.15388808035748	0.391684672906999	1	pseudobulk	DESeq2	LRT		0
161	KC_AB3	bchs	0.489881011342083	0.45467739790501	1	pseudobulk	DESeq2	LRT		0
161	KC_AB3	BCL7-like	1.59575615951652	0.886898266337347	1	pseudobulk	DESeq2	LRT		0
161	KC_AB3	bcn92	0.0360202817986889	0.882766866859659	1	pseudobulk	DESeq2	LRT		0
161	KC_AB3	Bdbt	0.223947528638243	0.658346308320237	1	pseudobulk	DESeq2	LRT		0
161	KC_AB3	bdg	3.11651768969717	0.314680495027058	1	pseudobulk	DESeq2	LRT		0
161	KC_AB3	Bdp1	-0.962336549607539	0.567620274858258	1	pseudobulk	DESeq2	LRT		0
162	KC_AB3	be	1.23133864869565	1	1	pseudobulk	DESeq2	LRT		0
162	KC_AB3	BEAF-32	-0.425804543304021	0.485319958491379	1	pseudobulk	DESeq2	LRT		0
162	KC_AB3	beag	-0.997052251591004	0.300564590061834	1	pseudobulk	DESeq2	LRT		0
162	KC_AB3	beat-Ia	0.632774104902944	1	1	pseudobulk	DESeq2	LRT		0
162	KC_AB3	beat-Ic	0.632774104902944	1	1	pseudobulk	DESeq2	LRT		0
162	KC_AB3	beat-IIa	0.632774104902943	1	1	pseudobulk	DESeq2	LRT		0
162	KC_AB3	beat-IIIb	0.123195005433465	0.534653146411771	1	pseudobulk	DESeq2	LRT		0
162	KC_AB3	beat-IV	-0.551103400984568	0.367366106832092	1	pseudobulk	DESeq2	LRT		0
162	KC_AB3	beat-Vb	0.632774104902944	1	1	pseudobulk	DESeq2	LRT		0
162	KC_AB3	beat-Vc	0.632774104902944	1	1	pseudobulk	DESeq2	LRT		0
162	KC_AB3	beat-VI	2.1954058311005	0.52671549486106	1	pseudobulk	DESeq2	LRT		0
162	KC_AB3	beat-VII	0.0453407421063638	0.855673108671476	1	pseudobulk	DESeq2	LRT		0
162	KC_AB3	beg	0.414412066932177	1	1	pseudobulk	DESeq2	LRT		0
162	KC_AB3	bel	-0.188276377343361	0.420575578751407	1	pseudobulk	DESeq2	LRT		0
162	KC_AB3	Bem46	0.584818197894999	0.642759073408518	1	pseudobulk	DESeq2	LRT		0
162	KC_AB3	ben	-0.113212301773832	0.432282570563348	1	pseudobulk	DESeq2	LRT		0
162	KC_AB3	Best1	-2.34213842332754	0.139198297581164	1	pseudobulk	DESeq2	LRT		0

162	KC_AB3	Bet1	-0.0465124021153616	0.707955281874445	1	pseudobulk	DESeq2	LRT		0
162	KC_AB3	Bet3	0.297395080524754	0.67973923165494	1	pseudobulk	DESeq2	LRT		0
162	KC_AB3	Bet5	0.313285445378211	0.674712727140925	1	pseudobulk	DESeq2	LRT		0
162	KC_AB3	beta-PheRS	1.23175250554525	0.336994560974075	1	pseudobulk	DESeq2	LRT		0
162	KC_AB3	beta-Spec	-0.0552619217212668	0.493054892199097	1	pseudobulk	DESeq2	LRT		0
162	KC_AB3	beta'COP	-0.0475767367362991	0.718259739325281	1	pseudobulk	DESeq2	LRT		0
162	KC_AB3	beta3GalTII	1.95267092137636	1	1	pseudobulk	DESeq2	LRT		0
162	KC_AB3	beta4GalNAcTA	1.05144796740111	0.487966928542661	1	pseudobulk	DESeq2	LRT		0
162	KC_AB3	beta4GalNAcTB	-0.559055794815147	0.672304419242972	1	pseudobulk	DESeq2	LRT		0
162	KC_AB3	beta4GalT7	0.659552729514226	0.726736773849902	1	pseudobulk	DESeq2	LRT		0
162	KC_AB3	betaCOP	-0.292618296270932	0.695492058716959	1	pseudobulk	DESeq2	LRT		0
162	KC_AB3	betaggt-I	-0.277279196760575	0.822479731035358	1	pseudobulk	DESeq2	LRT		0
162	KC_AB3	betaggt-II	-0.0703071441296001	0.866299575128811	1	pseudobulk	DESeq2	LRT		0
162	KC_AB3	betaNACtes6	1.95267092137636	1	1	pseudobulk	DESeq2	LRT		0
162	KC_AB3	betaTub56D	-0.670500928376138	0.0370399036095641	1	pseudobulk	DESeq2	LRT		0
162	KC_AB3	betaTub85D	1.60186822711471	1	1	pseudobulk	DESeq2	LRT		0
162	KC_AB3	Bgb	-0.330388678531992	0.434420669202357	1	pseudobulk	DESeq2	LRT		0
162	KC_AB3	BHD	-0.399671897798429	0.965137949353374	1	pseudobulk	DESeq2	LRT		0
162	KC_AB3	BI-1	0.0550065470409043	0.591575960357808	1	pseudobulk	DESeq2	LRT		0
162	KC_AB3	bic	0.185387956925123	0.553813951077335	1	pseudobulk	DESeq2	LRT		0
162	KC_AB3	BicD	-0.381199808273598	0.448591481330448	1	pseudobulk	DESeq2	LRT		0
162	KC_AB3	bif	0.0898616596203199	0.796680298982811	1	pseudobulk	DESeq2	LRT		0
162	KC_AB3	bigmax	0.732571253900474	0.326797780355901	1	pseudobulk	DESeq2	LRT		0
162	KC_AB3	Bili	-0.106758870119052	0.711363906503228	1	pseudobulk	DESeq2	LRT		0
162	KC_AB3	Bin1	-1.48972938277733	0.243706897139525	1	pseudobulk	DESeq2	LRT		0
162	KC_AB3	bin3	-1.0401573513108	0.105312900225154	1	pseudobulk	DESeq2	LRT		0
162	KC_AB3	bip2	-0.0412766208693862	0.786913267321906	1	pseudobulk	DESeq2	LRT		0
162	KC_AB3	Bka	-0.537409388618661	0.623442091605529	1	pseudobulk	DESeq2	LRT		0
162	KC_AB3	Blimp-1	-0.645118383190787	0.198455378110077	1	pseudobulk	DESeq2	LRT		0
162	KC_AB3	Blm	0.726041592174944	1	1	pseudobulk	DESeq2	LRT		0
162	KC_AB3	Blos1	0.228630623567217	0.47942510054899	1	pseudobulk	DESeq2	LRT		0
162	KC_AB3	Blos2	-0.333759913749796	0.728415011089192	1	pseudobulk	DESeq2	LRT		0
162	KC_AB3	Blos3	-0.301414492939807	0.608839121916363	1	pseudobulk	DESeq2	LRT		0
162	KC_AB3	Blos4	-0.650216022954674	0.551814643817503	1	pseudobulk	DESeq2	LRT		0
162	KC_AB3	blp	-0.0733412520064038	0.809434682353699	1	pseudobulk	DESeq2	LRT		0
162	KC_AB3	blw	-0.240063503546636	0.313362289185332	1	pseudobulk	DESeq2	LRT		0
162	KC_AB3	bma	-0.213049675208233	0.474415528735214	1	pseudobulk	DESeq2	LRT		0
162	KC_AB3	Bmcp	-0.0967154255057026	0.669718115178546	1	pseudobulk	DESeq2	LRT		0
162	KC_AB3	bmm	0.0689155172258351	0.806807210557681	1	pseudobulk	DESeq2	LRT		0
162	KC_AB3	bnb	0.506462310588313	1	1	pseudobulk	DESeq2	LRT		0
162	KC_AB3	boca	0.937644155186641	0.351847461924212	1	pseudobulk	DESeq2	LRT		0
162	KC_AB3	bocks	0.132748853053384	0.912856434500841	1	pseudobulk	DESeq2	LRT		0
162	KC_AB3	BOD1	-0.543108151775076	0.590416837626442	1	pseudobulk	DESeq2	LRT		0
162	KC_AB3	boi	-0.184744780581018	1	1	pseudobulk	DESeq2	LRT		0
162	KC_AB3	bol	-0.322005922382447	0.202778788385596	1	pseudobulk	DESeq2	LRT		0
162	KC_AB3	Bom065	0.632774104902944	1	1	pseudobulk	DESeq2	LRT		0
162	KC_AB3	Bom067	1.95267092137636	1	1	pseudobulk	DESeq2	LRT		0
162	KC_AB3	Bom2	1.44166967198646	0.650262880734675	1	pseudobulk	DESeq2	LRT		0
162	KC_AB3	Bom3	1.95267092137636	1	1	pseudobulk	DESeq2	LRT		0
162	KC_AB3	Bom836	0.632774104902944	1	1	pseudobulk	DESeq2	LRT		0
162	KC_AB3	bon	-0.0613972062533948	0.903873224513327	1	pseudobulk	DESeq2	LRT		0
162	KC_AB3	bonsai	-0.126491475400312	0.729195604649892	1	pseudobulk	DESeq2	LRT		0
162	KC_AB3	bor	0.467526691652125	0.786085165185825	1	pseudobulk	DESeq2	LRT		0

162	KC_AB3	BORCS5	0.276928663562055	0.564650349725485	1	pseudobulk	DESeq2	LRT	0
162	KC_AB3	BORCS6	-0.200090049738972	0.753880289740332	1	pseudobulk	DESeq2	LRT	0
162	KC_AB3	botv	-0.925248859526233	0.477526763039245	1	pseudobulk	DESeq2	LRT	0
162	KC_AB3	bou	1.23133864869565	1	1	pseudobulk	DESeq2	LRT	0
162	KC_AB3	br	-3.5832038273314	0.0144215250989558	1	pseudobulk	DESeq2	LRT	0
162	KC_AB3	Br140	-1.24487909952566	0.439599138109549	1	pseudobulk	DESeq2	LRT	0
162	KC_AB3	brat	-0.816460811574095	0.0336146588173669	1	pseudobulk	DESeq2	LRT	0
162	KC_AB3	Brca2	0.969051891352446	1	1	pseudobulk	DESeq2	LRT	0
162	KC_AB3	Brd8	1.90181948988857	0.350418999829868	1	pseudobulk	DESeq2	LRT	0
162	KC_AB3	Bre1	-0.395585635918148	0.626597615551456	1	pseudobulk	DESeq2	LRT	0
162	KC_AB3	Brf	0.613615002834075	0.67866519946023	1	pseudobulk	DESeq2	LRT	0
162	KC_AB3	brk	1.23133864869565	1	1	pseudobulk	DESeq2	LRT	0
162	KC_AB3	brm	-0.67962969595796	0.389860786232439	1	pseudobulk	DESeq2	LRT	0
162	KC_AB3	Brms1	-0.402784076728567	0.636547937235672	1	pseudobulk	DESeq2	LRT	0
162	KC_AB3	brn	0.0025701398975595	1	1	pseudobulk	DESeq2	LRT	0
162	KC_AB3	brp	-0.250354609748664	0.25996876771006	1	pseudobulk	DESeq2	LRT	0
162	KC_AB3	bru1	-0.423627550077266	0.343096470627521	1	pseudobulk	DESeq2	LRT	0
162	KC_AB3	bru2	-0.0644388768301007	0.696695902061361	1	pseudobulk	DESeq2	LRT	0
162	KC_AB3	bru3	-0.328097290357094	0.148872491640798	1	pseudobulk	DESeq2	LRT	0
162	KC_AB3	Bruce	0.117848654387001	0.797863655929879	1	pseudobulk	DESeq2	LRT	0
162	KC_AB3	brun	-0.0232436313515165	0.826692965106567	1	pseudobulk	DESeq2	LRT	0
162	KC_AB3	BRWD3	-0.671520500560721	0.427583084210116	1	pseudobulk	DESeq2	LRT	0
162	KC_AB3	brwl	0.20064383659574	0.760906262461486	1	pseudobulk	DESeq2	LRT	0
162	KC_AB3	bs	-0.337723533647137	0.730822861362682	1	pseudobulk	DESeq2	LRT	0
162	KC_AB3	bsf	0.120050935741622	0.475044958809032	1	pseudobulk	DESeq2	LRT	0
162	KC_AB3	Bsg	-0.295771959314505	0.239324896309089	1	pseudobulk	DESeq2	LRT	0
162	KC_AB3	Bsg25D	-0.997662163623566	0.295667995360751	1	pseudobulk	DESeq2	LRT	0
162	KC_AB3	bsk	-0.210383516638781	0.508571072502833	1	pseudobulk	DESeq2	LRT	0
162	KC_AB3	bt	-1.54139333493767	0.210901668031761	1	pseudobulk	DESeq2	LRT	0
162	KC_AB3	BTBD9	0.206058518631036	0.950381353756269	1	pseudobulk	DESeq2	LRT	0
163	KC_AB3	BtbVII	-0.0099527181844691	0.655944601742793	1	pseudobulk	DESeq2	LRT	0
163	KC_AB3	Btk29A	0.632774104902944	1	1	pseudobulk	DESeq2	LRT	0
163	KC_AB3	btsz	0.359363799040603	0.353424763798947	1	pseudobulk	DESeq2	LRT	0
163	KC_AB3	btz	0.191819868789025	0.780965422854986	1	pseudobulk	DESeq2	LRT	0
163	KC_AB3	Bub1	0.182487529225684	0.798786850842039	1	pseudobulk	DESeq2	LRT	0
163	KC_AB3	Bub3	0.0936535759542421	0.832100967556664	1	pseudobulk	DESeq2	LRT	0
163	KC_AB3	bug	1.04627162934463	0.710972742800829	1	pseudobulk	DESeq2	LRT	0
163	KC_AB3	Bug22	0.461024846984779	0.490537243509975	1	pseudobulk	DESeq2	LRT	0
163	KC_AB3	BuGZ	0.144813493878513	0.468939321801741	1	pseudobulk	DESeq2	LRT	0
163	KC_AB3	bun	-0.0991356745824812	0.624839690221307	1	pseudobulk	DESeq2	LRT	0
163	KC_AB3	bur	-1.23265105741995	0.276826505039786	1	pseudobulk	DESeq2	LRT	0
163	KC_AB3	bves	0.137773735679169	0.655026826957531	1	pseudobulk	DESeq2	LRT	0
163	KC_AB3	bw	0.632774104902944	1	1	pseudobulk	DESeq2	LRT	0
163	KC_AB3	bwa	-1.87169847415074	0.107593052450966	1	pseudobulk	DESeq2	LRT	0
163	KC_AB3	Bx	0.0029262859571499	0.927004625198395	1	pseudobulk	DESeq2	LRT	0
163	KC_AB3	Bx42	-0.603253155103673	0.454541015619562	1	pseudobulk	DESeq2	LRT	0
163	KC_AB3	bys	-1.51592992184354	0.363842024924771	1	pseudobulk	DESeq2	LRT	0
163	KC_AB3	c(3)G	0.632774104902944	1	1	pseudobulk	DESeq2	LRT	0
163	KC_AB3	c11.1	-0.392921637502232	0.580778927561424	1	pseudobulk	DESeq2	LRT	0
163	KC_AB3	c12.1	0.425032010418372	0.542063304476545	1	pseudobulk	DESeq2	LRT	0
163	KC_AB3	c12.2	0.103184801705115	0.767365932456956	1	pseudobulk	DESeq2	LRT	0
163	KC_AB3	C1GalTA	0.659489974814733	0.192197607100019	1	pseudobulk	DESeq2	LRT	0
163	KC_AB3	C3G	0.492355504612696	0.441007727459003	1	pseudobulk	DESeq2	LRT	0

163	KC_AB3	C901	-0.399671897798429	0.965137949353374	1	pseudobulk	DESeq2	LRT	0
163	KC_AB3	ca	-0.041545016882418	0.765028396576869	1	pseudobulk	DESeq2	LRT	0
163	KC_AB3	Ca-alpha1D	0.00061490524255252	0.843257195474673	1	pseudobulk	DESeq2	LRT	0
163	KC_AB3	Ca-alpha1T	-0.548767284186772	0.0618797655685048	1	pseudobulk	DESeq2	LRT	0
163	KC_AB3	Ca-beta	0.0157168574773066	0.584426933240741	1	pseudobulk	DESeq2	LRT	0
163	KC_AB3	Ca-Ma2d	-0.672791042608242	0.364448459549658	1	pseudobulk	DESeq2	LRT	0
163	KC_AB3	CaBP1	-0.54722748243532	0.593480446656405	1	pseudobulk	DESeq2	LRT	0
163	KC_AB3	cac	-0.131889790678656	0.598666883124277	1	pseudobulk	DESeq2	LRT	0
163	KC_AB3	cact	-0.501896187020204	0.238653595746032	1	pseudobulk	DESeq2	LRT	0
163	KC_AB3	cactin	0.0124993148778237	0.806948074952024	1	pseudobulk	DESeq2	LRT	0
163	KC_AB3	Cad86C	0.632774104902944	1	1	pseudobulk	DESeq2	LRT	0
163	KC_AB3	Cad87A	-2.32994845330428	0.17346026912558	1	pseudobulk	DESeq2	LRT	0
163	KC_AB3	Cad96Ca	1.84671504053046	1	1	pseudobulk	DESeq2	LRT	0
163	KC_AB3	CadN	0.467025881090376	0.434753318173603	1	pseudobulk	DESeq2	LRT	0
163	KC_AB3	CadN2	-0.680094168819758	0.737706015643587	1	pseudobulk	DESeq2	LRT	0
163	KC_AB3	Cadps	-0.291381660050456	0.340635329329589	1	pseudobulk	DESeq2	LRT	0
163	KC_AB3	Caf1-105	-0.399671897798429	0.965137949353374	1	pseudobulk	DESeq2	LRT	0
163	KC_AB3	Caf1-55	-0.0226924515209506	0.706439118313955	1	pseudobulk	DESeq2	LRT	0
163	KC_AB3	cag	1.18975651112157	0.578844528374702	1	pseudobulk	DESeq2	LRT	0
163	KC_AB3	CAH1	-0.0591601901315235	1	1	pseudobulk	DESeq2	LRT	0
163	KC_AB3	CAH2	-1.5690554751454	0.391197597288674	1	pseudobulk	DESeq2	LRT	0
163	KC_AB3	CAH3	-0.649651857322063	0.35256604917346	1	pseudobulk	DESeq2	LRT	0
163	KC_AB3	CAH7	1.23133864869565	1	1	pseudobulk	DESeq2	LRT	0
163	KC_AB3	CAH8	-0.314262020399948	0.650078803123863	1	pseudobulk	DESeq2	LRT	0
163	KC_AB3	CAHbeta	0.191805819003978	0.759766065418019	1	pseudobulk	DESeq2	LRT	0
163	KC_AB3	cal1	0.174367281879518	0.745882498720536	1	pseudobulk	DESeq2	LRT	0
163	KC_AB3	CalpA	0.883736438436388	0.555754465269734	1	pseudobulk	DESeq2	LRT	0
163	KC_AB3	CalpB	-1.04010885937676	0.478281079323182	1	pseudobulk	DESeq2	LRT	0
163	KC_AB3	Calr	-0.543790380665069	0.388714428435519	1	pseudobulk	DESeq2	LRT	0
163	KC_AB3	Cals	-0.0897276491848957	0.545259579387166	1	pseudobulk	DESeq2	LRT	0
163	KC_AB3	Calx	-0.255472207752789	0.569648168240629	1	pseudobulk	DESeq2	LRT	0
163	KC_AB3	calypso	-0.045903884431227	0.778998179661108	1	pseudobulk	DESeq2	LRT	0
163	KC_AB3	Cam	0.285550635703753	0.147687932649411	1	pseudobulk	DESeq2	LRT	0
163	KC_AB3	CaMKI	-0.294846122372823	0.291843169926845	1	pseudobulk	DESeq2	LRT	0
163	KC_AB3	CaMKII	-0.126252475254136	0.531359650701338	1	pseudobulk	DESeq2	LRT	0
163	KC_AB3	Camp	0.0750787629484365	0.833382064350496	1	pseudobulk	DESeq2	LRT	0
163	KC_AB3	Camta	0.263373825822323	0.564749196522484	1	pseudobulk	DESeq2	LRT	0
163	KC_AB3	CanA-14F	0.323966568516867	0.246013834993296	1	pseudobulk	DESeq2	LRT	0
163	KC_AB3	CanB	-0.322998753563607	0.204334629620733	1	pseudobulk	DESeq2	LRT	0
163	KC_AB3	CanB2	0.0074671361327671	0.580352945620078	1	pseudobulk	DESeq2	LRT	0
163	KC_AB3	Cand1	0.232527667053657	0.722543971566477	1	pseudobulk	DESeq2	LRT	0
163	KC_AB3	CAP	0.605105923301435	0.252792082065005	1	pseudobulk	DESeq2	LRT	0
163	KC_AB3	Cap-D3	1.93210469959844	0.634076921097483	1	pseudobulk	DESeq2	LRT	0
163	KC_AB3	Cap-G	0.449389554816353	1	1	pseudobulk	DESeq2	LRT	0
163	KC_AB3	Cap-H2	-0.197633536150048	0.823421426195676	1	pseudobulk	DESeq2	LRT	0
163	KC_AB3	Caper	-0.281714500317799	0.369187042498613	1	pseudobulk	DESeq2	LRT	0
163	KC_AB3	Capr	0.566785539611343	0.423353682187887	1	pseudobulk	DESeq2	LRT	0
163	KC_AB3	caps	-0.337603852883488	0.383481409711057	1	pseudobulk	DESeq2	LRT	0
163	KC_AB3	capt	-0.761118015219875	0.301644401150779	1	pseudobulk	DESeq2	LRT	0
163	KC_AB3	capu	-0.334231746700007	0.412335409711768	1	pseudobulk	DESeq2	LRT	0
163	KC_AB3	car	0.17742914264977	0.68498288854684	1	pseudobulk	DESeq2	LRT	0
163	KC_AB3	CARPB	2.64926057711297	0.482244576496623	1	pseudobulk	DESeq2	LRT	0
163	KC_AB3	CASK	-0.0483938175601974	0.569236827162879	1	pseudobulk	DESeq2	LRT	0

163	KC_AB3	casp	0.766488401379538	0.343366060131193	1	pseudobulk	DESeq2	LRT	0
163	KC_AB3	cass	0.27425472371939	0.372271041371021	1	pseudobulk	DESeq2	LRT	0
163	KC_AB3	Cat	0.403854031563585	0.787205780198323	1	pseudobulk	DESeq2	LRT	0
163	KC_AB3	cathD	-0.665906816383382	0.500793086231775	1	pseudobulk	DESeq2	LRT	0
163	KC_AB3	Catsup	-0.439885213879082	0.748575087539768	1	pseudobulk	DESeq2	LRT	0
163	KC_AB3	cav	1.16463472133474	0.690308255793063	1	pseudobulk	DESeq2	LRT	0
163	KC_AB3	caz	-0.785308833018459	0.218211817607322	1	pseudobulk	DESeq2	LRT	0
163	KC_AB3	cbc	1.40851779650944	0.659837892379742	1	pseudobulk	DESeq2	LRT	0
163	KC_AB3	Cbl	0.505612179175236	0.402571142844352	1	pseudobulk	DESeq2	LRT	0
163	KC_AB3	Cbp20	-0.0989407100719638	0.760642090309447	1	pseudobulk	DESeq2	LRT	0
163	KC_AB3	Cbp80	0.62824034328027	0.632689745364045	1	pseudobulk	DESeq2	LRT	0
163	KC_AB3	cbs	0.585259553436878	0.499758996254626	1	pseudobulk	DESeq2	LRT	0
163	KC_AB3	Cbs	0.907455377505268	0.475496759794951	1	pseudobulk	DESeq2	LRT	0
163	KC_AB3	CCAP-R	0.81849124607723	0.540224370792947	1	pseudobulk	DESeq2	LRT	0
163	KC_AB3	CCDC53	-0.699244079479669	0.607361376705186	1	pseudobulk	DESeq2	LRT	0
163	KC_AB3	Ccdc58	0.773972736865883	0.616158151994724	1	pseudobulk	DESeq2	LRT	0
163	KC_AB3	Cchl	-0.142925750837248	0.704257142488507	1	pseudobulk	DESeq2	LRT	0
163	KC_AB3	CCKLR-17D1	0.20523102870836	0.844456356449324	1	pseudobulk	DESeq2	LRT	0
163	KC_AB3	CCKLR-17D3	0.892479428176078	0.740134815117468	1	pseudobulk	DESeq2	LRT	0
163	KC_AB3	Ccm3	-0.0675859536768368	0.644064075637331	1	pseudobulk	DESeq2	LRT	0
163	KC_AB3	Ccn	-0.0954847706600117	0.692374335010274	1	pseudobulk	DESeq2	LRT	0
163	KC_AB3	Ccs	-1.51588276053546	0.296466430058775	1	pseudobulk	DESeq2	LRT	0
163	KC_AB3	CCT1	-1.12719799670921	0.31520561557378	1	pseudobulk	DESeq2	LRT	0
163	KC_AB3	CCT2	-0.529189725414391	0.604396652467442	1	pseudobulk	DESeq2	LRT	0
164	KC_AB3	CCT3	0.412736648754519	0.701242730732049	1	pseudobulk	DESeq2	LRT	0
164	KC_AB3	CCT4	0.241650978897518	0.66666557782387	1	pseudobulk	DESeq2	LRT	0
164	KC_AB3	CCT5	0.912744972921561	0.504876870781839	1	pseudobulk	DESeq2	LRT	0
164	KC_AB3	CCT6	-0.0928682474990108	0.699725425477551	1	pseudobulk	DESeq2	LRT	0
164	KC_AB3	CCT7	0.8259375621382	0.738437659341897	1	pseudobulk	DESeq2	LRT	0
164	KC_AB3	CCT8	0.0404472444159782	0.871398329480487	1	pseudobulk	DESeq2	LRT	0
164	KC_AB3	Ccz1	1.22271830810254	0.392903848747017	1	pseudobulk	DESeq2	LRT	0
164	KC_AB3	CD98hc	0.422346297826886	0.586543046156854	1	pseudobulk	DESeq2	LRT	0
164	KC_AB3	Cda5	1.23957054230214	0.311222954948824	1	pseudobulk	DESeq2	LRT	0
164	KC_AB3	cdc14	-0.344811271787114	0.516682329729708	1	pseudobulk	DESeq2	LRT	0
164	KC_AB3	Cdc16	-0.441995100341736	0.70575945438678	1	pseudobulk	DESeq2	LRT	0
164	KC_AB3	Cdc27	-0.636314472467052	0.396418816695005	1	pseudobulk	DESeq2	LRT	0
164	KC_AB3	Cdc2rk	0.894215782633011	0.846184688964882	1	pseudobulk	DESeq2	LRT	0
164	KC_AB3	Cdc37	-0.0158671798599925	0.732556471585701	1	pseudobulk	DESeq2	LRT	0
164	KC_AB3	Cdc42	-0.730004318899559	0.219401343145138	1	pseudobulk	DESeq2	LRT	0
164	KC_AB3	CDC45L	-1.13441954721443	0.538223795140586	1	pseudobulk	DESeq2	LRT	0
164	KC_AB3	Cdc5	-0.4154421904041	0.605637796274667	1	pseudobulk	DESeq2	LRT	0
164	KC_AB3	CDC50	-0.0375230188786346	0.739200395045713	1	pseudobulk	DESeq2	LRT	0
164	KC_AB3	Cdc7	0.142023179407426	0.814769287922582	1	pseudobulk	DESeq2	LRT	0
164	KC_AB3	Cdep	-0.614422947380587	0.154432766969797	1	pseudobulk	DESeq2	LRT	0
164	KC_AB3	CdGAPr	-0.484722778155604	0.485757633337676	1	pseudobulk	DESeq2	LRT	0
164	KC_AB3	cdi	-0.921528292713723	0.270744696439929	1	pseudobulk	DESeq2	LRT	0
164	KC_AB3	Cdk12	0.793813479517356	0.329894523269429	1	pseudobulk	DESeq2	LRT	0
164	KC_AB3	Cdk2	-1.61325709380652	0.221672883478116	1	pseudobulk	DESeq2	LRT	0
164	KC_AB3	CDK2AP1	0.609543746498012	0.54896648773894	1	pseudobulk	DESeq2	LRT	0
164	KC_AB3	Cdk5	-0.181702536044748	0.719298954806377	1	pseudobulk	DESeq2	LRT	0
164	KC_AB3	Cdk5alpha	-0.0913959580878942	0.424799189963896	1	pseudobulk	DESeq2	LRT	0
164	KC_AB3	Cdk7	-0.154365425666664	0.864050468757873	1	pseudobulk	DESeq2	LRT	0
164	KC_AB3	Cdk8	-0.71442583789326	0.699995732773256	1	pseudobulk	DESeq2	LRT	0

164	KC_AB3	Cdk9	0.728669180149423	0.746980596566024	1	pseudobulk	DESeq2	LRT		0
164	KC_AB3	cdm	-0.695868808899487	0.332984883502771	1	pseudobulk	DESeq2	LRT		0
164	KC_AB3	Cds	-0.324524838998247	0.333310912662865	1	pseudobulk	DESeq2	LRT		0
164	KC_AB3	Ced-12	-0.467629153535307	0.648712924779321	1	pseudobulk	DESeq2	LRT		0
164	KC_AB3	ced-6	0.632774104902943	1	1	pseudobulk	DESeq2	LRT		0
164	KC_AB3	Cen	0.356418373744227	0.627946775031282	1	pseudobulk	DESeq2	LRT		0
164	KC_AB3	CenB1A	0.0092247012210650	0.766939423345843	1	pseudobulk	DESeq2	LRT		0
164	KC_AB3	CenG1A	-0.579558159845711	0.492501985062664	1	pseudobulk	DESeq2	LRT		0
164	KC_AB3	Cep97	0.0458270506077295	0.716940926469669	1	pseudobulk	DESeq2	LRT		0
164	KC_AB3	cer	-0.855389100564504	0.336019282935844	1	pseudobulk	DESeq2	LRT		0
164	KC_AB3	Cerk	0.59942375923279	0.398494726972054	1	pseudobulk	DESeq2	LRT		0
164	KC_AB3	cert	0.0056899630322873	0.958954086682887	1	pseudobulk	DESeq2	LRT		0
164	KC_AB3	cerv	0.414593131727451	1	1	pseudobulk	DESeq2	LRT		0
164	KC_AB3	Cf2	-0.444706031712651	0.509648947940231	1	pseudobulk	DESeq2	LRT		0
164	KC_AB3	Cfp1	0.885396802369744	0.878317732038515	1	pseudobulk	DESeq2	LRT		0
164	KC_AB3	cg	-0.766550820385798	0.0603889558588202	1	pseudobulk	DESeq2	LRT		0
164	KC_AB3	CG10011	0.476633565945587	0.215040495308747	1	pseudobulk	DESeq2	LRT		0
164	KC_AB3	CG10038	-1.33633141142357	0.361107414491829	1	pseudobulk	DESeq2	LRT		0
164	KC_AB3	CG10050	0.632774104902944	1	1	pseudobulk	DESeq2	LRT		0
164	KC_AB3	CG10051	1.95267092137636	1	1	pseudobulk	DESeq2	LRT		0
164	KC_AB3	CG10053	-0.160804957331434	0.818391168986531	1	pseudobulk	DESeq2	LRT		0
164	KC_AB3	CG10055	1.68573136831825	0.324050937667757	1	pseudobulk	DESeq2	LRT		0
164	KC_AB3	CG10068	0.0671033170132627	0.847548627409205	1	pseudobulk	DESeq2	LRT		0
164	KC_AB3	CG10075	0.115422257585446	0.653081401635755	1	pseudobulk	DESeq2	LRT		0
164	KC_AB3	CG10077	-0.399809452846424	0.0974000365527542	1	pseudobulk	DESeq2	LRT		0
164	KC_AB3	CG10082	0.0766130912865479	0.637215735800201	1	pseudobulk	DESeq2	LRT		0
164	KC_AB3	CG10089	0.227179259352197	0.580093718072296	1	pseudobulk	DESeq2	LRT		0
164	KC_AB3	CG10098	-0.0219709282255211	0.972832138910771	1	pseudobulk	DESeq2	LRT		0
164	KC_AB3	CG10103	0.10595678740467	0.724742531947088	1	pseudobulk	DESeq2	LRT		0
164	KC_AB3	CG10126	0.632774104902943	1	1	pseudobulk	DESeq2	LRT		0
164	KC_AB3	CG10132	-0.155353009152432	0.707312103669317	1	pseudobulk	DESeq2	LRT		0
164	KC_AB3	CG10137	-0.158819997464634	0.72108890206451	1	pseudobulk	DESeq2	LRT		0
164	KC_AB3	CG10147	0.678817485266327	0.763903043601644	1	pseudobulk	DESeq2	LRT		0
164	KC_AB3	CG10151	0.97111516858185	0.420154117322009	1	pseudobulk	DESeq2	LRT		0
164	KC_AB3	CG10165	0.893903990774454	0.846314690533615	1	pseudobulk	DESeq2	LRT		0
164	KC_AB3	CG10166	0.857037594830445	0.786425553887833	1	pseudobulk	DESeq2	LRT		0
164	KC_AB3	CG10188	-0.235547477588225	0.434739115329598	1	pseudobulk	DESeq2	LRT		0
164	KC_AB3	CG10189	-0.566641765370198	0.674958268335822	1	pseudobulk	DESeq2	LRT		0
164	KC_AB3	CG10194	-0.278184143944042	0.792470253096558	1	pseudobulk	DESeq2	LRT		0
164	KC_AB3	CG10208	-0.302794075441951	0.851617925917851	1	pseudobulk	DESeq2	LRT		0
164	KC_AB3	CG10209	0.58428516015886	0.584986914408029	1	pseudobulk	DESeq2	LRT		0
164	KC_AB3	CG10214	-0.767158408362354	0.465469855052962	1	pseudobulk	DESeq2	LRT		0
164	KC_AB3	CG10222	-0.185594513059781	1	1	pseudobulk	DESeq2	LRT		0
164	KC_AB3	CG1024	0.64988012405529	0.791050199243947	1	pseudobulk	DESeq2	LRT		0
164	KC_AB3	CG10252	0.497004037970397	0.227139112850458	1	pseudobulk	DESeq2	LRT		0
164	KC_AB3	CG10254	0.65400233854438	0.422869152717941	1	pseudobulk	DESeq2	LRT		0
164	KC_AB3	CG10257	-1.64465438768723	0.0978136823169896	1	pseudobulk	DESeq2	LRT		0
164	KC_AB3	CG10265	-0.197184577653855	0.777647786933874	1	pseudobulk	DESeq2	LRT		0
164	KC_AB3	CG10268	0.414593131727451	1	1	pseudobulk	DESeq2	LRT		0
164	KC_AB3	CG10274	-0.399671897798429	0.965137949353374	1	pseudobulk	DESeq2	LRT		0
164	KC_AB3	CG10283	-0.110369332690745	0.919442321556733	1	pseudobulk	DESeq2	LRT		0
164	KC_AB3	CG10286	-0.471298305621752	0.700561369951974	1	pseudobulk	DESeq2	LRT		0
164	KC_AB3	CG10321	0.178665958871273	0.803240879575047	1	pseudobulk	DESeq2	LRT		0

164	KC_AB3	CG10324	-0.345816202246257	0.682665746974378	1	pseudobulk	DESeq2	LRT	0
164	KC_AB3	CG10333	0.0691759094063989	0.688200445635051	1	pseudobulk	DESeq2	LRT	0
164	KC_AB3	CG10338	0.781683616896023	0.872248030431456	1	pseudobulk	DESeq2	LRT	0
164	KC_AB3	CG10340	-0.225646530996484	0.572002516301001	1	pseudobulk	DESeq2	LRT	0
164	KC_AB3	CG10341	0.0478638348083297	1	1	pseudobulk	DESeq2	LRT	0
164	KC_AB3	CG10343	-1.09919459729583	0.35878996191524	1	pseudobulk	DESeq2	LRT	0
164	KC_AB3	CG10347	-0.460918900452187	0.690281391365899	1	pseudobulk	DESeq2	LRT	0
164	KC_AB3	CG10352	2.3149273646917	0.611236276445972	1	pseudobulk	DESeq2	LRT	0
164	KC_AB3	CG10353	-0.480985321099136	0.527690277814733	1	pseudobulk	DESeq2	LRT	0
164	KC_AB3	CG10362	-0.812561353856297	0.465183143159444	1	pseudobulk	DESeq2	LRT	0
164	KC_AB3	CG10365	-1.24487909952566	0.439599138109549	1	pseudobulk	DESeq2	LRT	0
164	KC_AB3	CG10366	-0.197184577653855	0.777647786933874	1	pseudobulk	DESeq2	LRT	0
164	KC_AB3	CG10375	-0.991665393805924	0.210635485588851	1	pseudobulk	DESeq2	LRT	0
164	KC_AB3	CG10376	0.0019407554618122	0.836136568669204	1	pseudobulk	DESeq2	LRT	0
164	KC_AB3	CG10383	0.137889284842245	1	1	pseudobulk	DESeq2	LRT	0
164	KC_AB3	CG10384	1.95267092137636	1	1	pseudobulk	DESeq2	LRT	0
164	KC_AB3	CG10395	-1.13558493864421	0.199872772499063	1	pseudobulk	DESeq2	LRT	0
164	KC_AB3	CG10413	-0.71303359574103	0.497751251225614	1	pseudobulk	DESeq2	LRT	0
165	KC_AB3	CG10417	-0.203529205182254	0.709257891624057	1	pseudobulk	DESeq2	LRT	0
165	KC_AB3	CG10418	0.255820646246031	0.665995396565919	1	pseudobulk	DESeq2	LRT	0
165	KC_AB3	CG10425	1.93210469959844	0.634076921097483	1	pseudobulk	DESeq2	LRT	0
165	KC_AB3	CG10428	0.781683616896023	0.872248030431456	1	pseudobulk	DESeq2	LRT	0
165	KC_AB3	CG10431	0.632774104902943	1	1	pseudobulk	DESeq2	LRT	0
165	KC_AB3	CG10435	0.0149899547680803	0.700071520989696	1	pseudobulk	DESeq2	LRT	0
165	KC_AB3	CG10444	-0.352544196385097	0.799575396616172	1	pseudobulk	DESeq2	LRT	0
165	KC_AB3	CG10463	-0.565055821286187	0.639171913464476	1	pseudobulk	DESeq2	LRT	0
165	KC_AB3	CG10465	-0.118429978554478	0.59761280436752	1	pseudobulk	DESeq2	LRT	0
165	KC_AB3	CG10466	-0.0011133105143748	0.408820866372532	1	pseudobulk	DESeq2	LRT	0
165	KC_AB3	CG10470	-0.578155976131731	0.399937379551261	1	pseudobulk	DESeq2	LRT	0
165	KC_AB3	CG10483	-0.321351553965092	0.563108633369863	1	pseudobulk	DESeq2	LRT	0
165	KC_AB3	CG10492	-0.16434299992274	0.783455827472688	1	pseudobulk	DESeq2	LRT	0
165	KC_AB3	CG10494	-0.438647368034814	0.661916125305762	1	pseudobulk	DESeq2	LRT	0
165	KC_AB3	CG10495	-0.782209672252057	0.539409308844827	1	pseudobulk	DESeq2	LRT	0
165	KC_AB3	CG10508	0.0654090815746387	0.555496627119491	1	pseudobulk	DESeq2	LRT	0
165	KC_AB3	CG10512	0.872272709668745	1	1	pseudobulk	DESeq2	LRT	0
165	KC_AB3	CG10513	1.23133864869565	1	1	pseudobulk	DESeq2	LRT	0
165	KC_AB3	CG10516	-0.785834882197716	0.579556193254081	1	pseudobulk	DESeq2	LRT	0
165	KC_AB3	CG10543	-0.107940245347137	0.640047188224496	1	pseudobulk	DESeq2	LRT	0
165	KC_AB3	CG10555	0.286043825923718	0.658267626086068	1	pseudobulk	DESeq2	LRT	0
165	KC_AB3	CG10561	0.547763272546784	0.599774184214803	1	pseudobulk	DESeq2	LRT	0
165	KC_AB3	CG10562	2.62817135292692	0.54954870385793	1	pseudobulk	DESeq2	LRT	0
165	KC_AB3	CG10565	-0.637785555002194	0.393802047781957	1	pseudobulk	DESeq2	LRT	0
165	KC_AB3	CG10576	0.170968110622797	0.665794170561524	1	pseudobulk	DESeq2	LRT	0
165	KC_AB3	CG10581	4.1197552329049	0.183263749029345	1	pseudobulk	DESeq2	LRT	0
165	KC_AB3	CG10600	0.630066268388112	0.457049460032906	1	pseudobulk	DESeq2	LRT	0
165	KC_AB3	CG10602	1.3137820482232	0.51943250695904	1	pseudobulk	DESeq2	LRT	0
165	KC_AB3	CG10616	1.46833011305735	0.320502023472851	1	pseudobulk	DESeq2	LRT	0
165	KC_AB3	CG10628	1.91601206439215	0.439574641803634	1	pseudobulk	DESeq2	LRT	0
165	KC_AB3	CG10631	0.523889204759387	0.513450810952715	1	pseudobulk	DESeq2	LRT	0
165	KC_AB3	CG10638	0.680080667470291	0.567763523120082	1	pseudobulk	DESeq2	LRT	0
165	KC_AB3	CG10646	2.21525873316417	0.0631532407492379	1	pseudobulk	DESeq2	LRT	0
165	KC_AB3	CG10654	1.49048409407206	0.503211709004343	1	pseudobulk	DESeq2	LRT	0
165	KC_AB3	CG10669	0.969051891352446	1	1	pseudobulk	DESeq2	LRT	0

165	KC_AB3	CG10672	-1.71224324652168	0.2270593323089	1	pseudobulk	DESeq2	LRT	0
165	KC_AB3	CG10674	0.47444370577921	0.430310406415839	1	pseudobulk	DESeq2	LRT	0
165	KC_AB3	CG10681	-0.0890487677932199	0.74219778773039	1	pseudobulk	DESeq2	LRT	0
165	KC_AB3	CG10694	0.632774104902944	1	1	pseudobulk	DESeq2	LRT	0
165	KC_AB3	CG10713	-0.515642828760177	0.591640683132663	1	pseudobulk	DESeq2	LRT	0
165	KC_AB3	CG10731	2.12834645750088	0.356132304492531	1	pseudobulk	DESeq2	LRT	0
165	KC_AB3	CG10737	0.0959437178933617	0.784229864668062	1	pseudobulk	DESeq2	LRT	0
165	KC_AB3	CG10738	1.35432420877493	0.234418330342169	1	pseudobulk	DESeq2	LRT	0
165	KC_AB3	CG1074	0.465023431113697	0.687111447298668	1	pseudobulk	DESeq2	LRT	0
165	KC_AB3	CG10747	0.147913866045494	1	1	pseudobulk	DESeq2	LRT	0
165	KC_AB3	CG10778	1.49336508771291	0.543534243454597	1	pseudobulk	DESeq2	LRT	0
165	KC_AB3	CG10795	1.77809910944096	0.602511884574285	1	pseudobulk	DESeq2	LRT	0
165	KC_AB3	CG10802	0.226773299331124	0.800966540906719	1	pseudobulk	DESeq2	LRT	0
165	KC_AB3	CG10803	0.146865992332821	0.667005727909821	1	pseudobulk	DESeq2	LRT	0
165	KC_AB3	CG10804	0.0734197709264998	0.86578993623821	1	pseudobulk	DESeq2	LRT	0
165	KC_AB3	CG10809	-1.16258756595303	0.133705900989617	1	pseudobulk	DESeq2	LRT	0
165	KC_AB3	CG10841	3.07272693214569	0.238940395988569	1	pseudobulk	DESeq2	LRT	0
165	KC_AB3	CG10863	1.23133864869565	1	1	pseudobulk	DESeq2	LRT	0
165	KC_AB3	CG10866	0.193766454092491	1	1	pseudobulk	DESeq2	LRT	0
165	KC_AB3	CG10869	1.22707713470494	1	1	pseudobulk	DESeq2	LRT	0
165	KC_AB3	CG10874	-0.184890010561551	0.838346086191325	1	pseudobulk	DESeq2	LRT	0
165	KC_AB3	CG10889	-0.382003564959115	0.734082631073315	1	pseudobulk	DESeq2	LRT	0
165	KC_AB3	CG10898	-1.440143368582	0.347598984508527	1	pseudobulk	DESeq2	LRT	0
165	KC_AB3	CG1090	0.0838182775848913	0.655316027283794	1	pseudobulk	DESeq2	LRT	0
165	KC_AB3	CG10903	0.247739906366514	1	1	pseudobulk	DESeq2	LRT	0
165	KC_AB3	CG10904	-0.225834624576238	0.741086043001516	1	pseudobulk	DESeq2	LRT	0
165	KC_AB3	CG10907	-1.05016648806908	0.492025098252198	1	pseudobulk	DESeq2	LRT	0
165	KC_AB3	CG10914	0.272383645871586	1	1	pseudobulk	DESeq2	LRT	0
165	KC_AB3	CG10916	0.249985534354146	1	1	pseudobulk	DESeq2	LRT	0
165	KC_AB3	CG10927	0.632774104902944	1	1	pseudobulk	DESeq2	LRT	0
165	KC_AB3	CG10932	-0.699337449500035	0.608828847364277	1	pseudobulk	DESeq2	LRT	0
165	KC_AB3	CG10939	1.95267092137636	1	1	pseudobulk	DESeq2	LRT	0
165	KC_AB3	CG10947	0.863955114233012	0.0121528933569629	1	pseudobulk	DESeq2	LRT	0
165	KC_AB3	CG10948	-0.810589969397839	0.240682874882658	1	pseudobulk	DESeq2	LRT	0
165	KC_AB3	CG10949	-0.373834823632096	0.740680855322966	1	pseudobulk	DESeq2	LRT	0
165	KC_AB3	CG10958	0.593667095203339	1	1	pseudobulk	DESeq2	LRT	0
165	KC_AB3	CG10959	1.39709787522791	0.224538106650414	1	pseudobulk	DESeq2	LRT	0
165	KC_AB3	CG10960	-1.02822388270595	0.276703386007925	1	pseudobulk	DESeq2	LRT	0
165	KC_AB3	CG10973	-0.2720488678935	0.7741310084659	1	pseudobulk	DESeq2	LRT	0
165	KC_AB3	CG10979	-0.0203007981365936	0.740767623746588	1	pseudobulk	DESeq2	LRT	0
165	KC_AB3	CG10984	0.245095482064958	0.672316528027001	1	pseudobulk	DESeq2	LRT	0
165	KC_AB3	CG11000	-0.387575394852838	0.356347059557243	1	pseudobulk	DESeq2	LRT	0
165	KC_AB3	CG11007	-0.0115873920192364	0.837900795998945	1	pseudobulk	DESeq2	LRT	0
165	KC_AB3	CG1103	0.7100906250926	0.636025035487895	1	pseudobulk	DESeq2	LRT	0
165	KC_AB3	CG11030	0.257194689090207	0.743868763626194	1	pseudobulk	DESeq2	LRT	0
165	KC_AB3	CG11035	0.802713901182994	0.599167944696354	1	pseudobulk	DESeq2	LRT	0
165	KC_AB3	CG11041	0.632774104902944	1	1	pseudobulk	DESeq2	LRT	0
165	KC_AB3	CG1105	0.511577859797879	0.618148142269363	1	pseudobulk	DESeq2	LRT	0
165	KC_AB3	CG11050	0.484219930044499	0.271067916510084	1	pseudobulk	DESeq2	LRT	0
165	KC_AB3	CG11070	0.7970846057117	0.472075953389538	1	pseudobulk	DESeq2	LRT	0
165	KC_AB3	CG11076	0.199613651378105	0.576088464982432	1	pseudobulk	DESeq2	LRT	0
165	KC_AB3	CG11077	0.109982637792967	0.73297949194779	1	pseudobulk	DESeq2	LRT	0
165	KC_AB3	CG11089	-0.186961738271884	1	1	pseudobulk	DESeq2	LRT	0

165	KC_AB3	CG11095	0.0482468039015262	0.798735740711775	1	pseudobulk	DESeq2	LRT	0
165	KC_AB3	CG11099	2.07930641071614	0.045434807139275	1	pseudobulk	DESeq2	LRT	0
165	KC_AB3	CG11103	0.927913762594861	0.586994361579733	1	pseudobulk	DESeq2	LRT	0
165	KC_AB3	CG11109	0.629169735864046	0.722585832160676	1	pseudobulk	DESeq2	LRT	0
165	KC_AB3	CG11110	0.38127044160078	0.695589427555323	1	pseudobulk	DESeq2	LRT	0
165	KC_AB3	CG11122	-0.360059996767399	0.467846337729958	1	pseudobulk	DESeq2	LRT	0
165	KC_AB3	CG11123	0.593667095203339	1	1	pseudobulk	DESeq2	LRT	0
165	KC_AB3	CG11127	0.977358746496936	0.721706460915285	1	pseudobulk	DESeq2	LRT	0
165	KC_AB3	CG1113	1.23133864869565	1	1	pseudobulk	DESeq2	LRT	0
165	KC_AB3	CG11134	1.23133864869565	1	1	pseudobulk	DESeq2	LRT	0
165	KC_AB3	CG11137	-0.115520875119493	0.720109617824991	1	pseudobulk	DESeq2	LRT	0
165	KC_AB3	CG11141	0.0932431688908785	0.798195846991818	1	pseudobulk	DESeq2	LRT	0
166	KC_AB3	CG11151	-0.170967035278569	0.638529200343948	1	pseudobulk	DESeq2	LRT	0
166	KC_AB3	CG11155	-0.179763332643068	0.419374687107109	1	pseudobulk	DESeq2	LRT	0
166	KC_AB3	CG11159	2.62817135292692	0.54954870385793	1	pseudobulk	DESeq2	LRT	0
166	KC_AB3	CG1116	-0.20671116229885	0.813258502655378	1	pseudobulk	DESeq2	LRT	0
166	KC_AB3	CG11170	0.161515868851812	1	1	pseudobulk	DESeq2	LRT	0
166	KC_AB3	CG11178	-0.528420466475746	0.617666467890503	1	pseudobulk	DESeq2	LRT	0
166	KC_AB3	CG11180	0.632774104902944	1	1	pseudobulk	DESeq2	LRT	0
166	KC_AB3	CG11191	-0.0595829524034119	0.649333033545287	1	pseudobulk	DESeq2	LRT	0
166	KC_AB3	CG11200	0.0025701398975595	1	1	pseudobulk	DESeq2	LRT	0
166	KC_AB3	CG11241	0.632774104902944	1	1	pseudobulk	DESeq2	LRT	0
166	KC_AB3	CG11247	-0.0177869189272764	0.764246082019904	1	pseudobulk	DESeq2	LRT	0
166	KC_AB3	CG11248	1.22881132671401	0.529705441539229	1	pseudobulk	DESeq2	LRT	0
166	KC_AB3	CG11257	-0.0781543137615753	0.830613034175565	1	pseudobulk	DESeq2	LRT	0
166	KC_AB3	CG11267	0.0486480463480114	0.467221748450376	1	pseudobulk	DESeq2	LRT	0
166	KC_AB3	CG11269	0.632774104902944	1	1	pseudobulk	DESeq2	LRT	0
166	KC_AB3	CG11279	-0.0235498729353205	0.743021295505515	1	pseudobulk	DESeq2	LRT	0
166	KC_AB3	CG1129	-0.570346016758453	0.483257635672925	1	pseudobulk	DESeq2	LRT	0
166	KC_AB3	CG11307	1.59576167909336	0.887029991980463	1	pseudobulk	DESeq2	LRT	0
166	KC_AB3	CG11309	0.440578571731502	1	1	pseudobulk	DESeq2	LRT	0
166	KC_AB3	CG11317	0.187737212201082	0.596545347042973	1	pseudobulk	DESeq2	LRT	0
166	KC_AB3	CG11319	-0.132802475651209	0.618905970517118	1	pseudobulk	DESeq2	LRT	0
166	KC_AB3	CG11334	0.245792428733689	0.551864406473025	1	pseudobulk	DESeq2	LRT	0
166	KC_AB3	CG11342	0.632774104902944	1	1	pseudobulk	DESeq2	LRT	0
166	KC_AB3	CG11357	-0.31890318356989	0.622329568899268	1	pseudobulk	DESeq2	LRT	0
166	KC_AB3	CG11360	0.488317672682195	0.452938943692558	1	pseudobulk	DESeq2	LRT	0
166	KC_AB3	CG11367	1.22043625251371	0.41950916817146	1	pseudobulk	DESeq2	LRT	0
166	KC_AB3	CG11374	-0.560477492866218	0.784070523163114	1	pseudobulk	DESeq2	LRT	0
166	KC_AB3	CG11377	-1.68393536535912	0.256024850209544	1	pseudobulk	DESeq2	LRT	0
166	KC_AB3	CG11396	-0.354819360064499	0.67993835569756	1	pseudobulk	DESeq2	LRT	0
166	KC_AB3	CG11398	-1.30218587404643	0.440980805989893	1	pseudobulk	DESeq2	LRT	0
166	KC_AB3	CG11399	-0.687549062856548	0.501897470695214	1	pseudobulk	DESeq2	LRT	0
166	KC_AB3	CG11403	-0.302218176137793	0.851957073307525	1	pseudobulk	DESeq2	LRT	0
166	KC_AB3	CG11406	-1.19034330638712	0.135081648436826	1	pseudobulk	DESeq2	LRT	0
166	KC_AB3	CG11407	1.23133864869565	1	1	pseudobulk	DESeq2	LRT	0
166	KC_AB3	CG11413	1.59575615951652	0.886898266337347	1	pseudobulk	DESeq2	LRT	0
166	KC_AB3	CG11414	-0.306047630985897	0.513849095382522	1	pseudobulk	DESeq2	LRT	0
166	KC_AB3	CG11417	-0.155684407748557	0.884080788772732	1	pseudobulk	DESeq2	LRT	0
166	KC_AB3	CG1142	0.916928608220278	0.699839404516802	1	pseudobulk	DESeq2	LRT	0
166	KC_AB3	CG11436	1.51046140671095	0.502297228740925	1	pseudobulk	DESeq2	LRT	0
166	KC_AB3	CG11444	0.0822921954528574	0.649910359248638	1	pseudobulk	DESeq2	LRT	0
166	KC_AB3	CG11447	0.0097177289825372	0.768563692611494	1	pseudobulk	DESeq2	LRT	0

166	KC_AB3	CG11454	0.201614715421947	0.733165484390725	1	pseudobulk	DESeq2	LRT	0
166	KC_AB3	CG11456	-1.11132293463423	0.310763750265423	1	pseudobulk	DESeq2	LRT	0
166	KC_AB3	CG1146	-0.302794075441951	0.851617925917851	1	pseudobulk	DESeq2	LRT	0
166	KC_AB3	CG11474	0.541882151162954	0.493356014370486	1	pseudobulk	DESeq2	LRT	0
166	KC_AB3	CG11486	0.078896771855832	0.670774474750098	1	pseudobulk	DESeq2	LRT	0
166	KC_AB3	CG11504	-0.596031722806557	0.294195744042751	1	pseudobulk	DESeq2	LRT	0
166	KC_AB3	CG11523	-1.04714795039191	0.222320976679148	1	pseudobulk	DESeq2	LRT	0
166	KC_AB3	CG11537	-0.0478743855843229	0.884302076870346	1	pseudobulk	DESeq2	LRT	0
166	KC_AB3	CG11539	1.24448570466626	0.941156201050192	1	pseudobulk	DESeq2	LRT	0
166	KC_AB3	CG11555	0.49023817916048	0.669421735168341	1	pseudobulk	DESeq2	LRT	0
166	KC_AB3	CG11560	0.946628173973207	0.505565659704944	1	pseudobulk	DESeq2	LRT	0
166	KC_AB3	CG11562	-0.760524262661345	0.582329072507387	1	pseudobulk	DESeq2	LRT	0
166	KC_AB3	CG11563	-1.460635393447	0.180883325593548	1	pseudobulk	DESeq2	LRT	0
166	KC_AB3	CG11583	0.447170752511285	0.680420877009769	1	pseudobulk	DESeq2	LRT	0
166	KC_AB3	CG11590	2.04059835111115	0.12925558384197	1	pseudobulk	DESeq2	LRT	0
166	KC_AB3	CG11593	0.822933985690462	0.36434860352539	1	pseudobulk	DESeq2	LRT	0
166	KC_AB3	CG11596	-0.372198058015524	0.692132235527108	1	pseudobulk	DESeq2	LRT	0
166	KC_AB3	CG11597	-0.197933275668192	0.778355419202257	1	pseudobulk	DESeq2	LRT	0
166	KC_AB3	CG11601	0.632774104902943	1	1	pseudobulk	DESeq2	LRT	0
166	KC_AB3	CG1161	0.0206836492420759	0.612720878369071	1	pseudobulk	DESeq2	LRT	0
166	KC_AB3	CG11626	0.632774104902943	1	1	pseudobulk	DESeq2	LRT	0
166	KC_AB3	CG11638	-0.835071691045028	0.645830720505476	1	pseudobulk	DESeq2	LRT	0
166	KC_AB3	CG11658	0.22384954696757	0.618391941895903	1	pseudobulk	DESeq2	LRT	0
166	KC_AB3	CG11695	1.16463472133474	0.690308255793063	1	pseudobulk	DESeq2	LRT	0
166	KC_AB3	CG11696	0.979246191006338	0.680452881045326	1	pseudobulk	DESeq2	LRT	0
166	KC_AB3	CG11699	-0.327970668651142	0.490345477991379	1	pseudobulk	DESeq2	LRT	0
166	KC_AB3	CG11710	0.792874578103278	0.586945408675634	1	pseudobulk	DESeq2	LRT	0
166	KC_AB3	CG1172	-0.161667427544923	0.74878124538277	1	pseudobulk	DESeq2	LRT	0
166	KC_AB3	CG11722	0.389555171649491	0.590163916820661	1	pseudobulk	DESeq2	LRT	0
166	KC_AB3	CG11723	0.528944375578886	0.79163134880768	1	pseudobulk	DESeq2	LRT	0
166	KC_AB3	CG11737	-1.21952962325897	0.4791438739141	1	pseudobulk	DESeq2	LRT	0
166	KC_AB3	CG11752	0.207011068324316	0.459918704527053	1	pseudobulk	DESeq2	LRT	0
166	KC_AB3	CG11753	-0.448684818612628	0.517338542648129	1	pseudobulk	DESeq2	LRT	0
166	KC_AB3	CG11755	2.18824795234538	0.327873701643271	1	pseudobulk	DESeq2	LRT	0
166	KC_AB3	CG11760	-0.73329451625302	0.55526094352985	1	pseudobulk	DESeq2	LRT	0
166	KC_AB3	CG11768	0.123708985517264	0.496206955792235	1	pseudobulk	DESeq2	LRT	0
166	KC_AB3	CG11771	-0.58029643579009	0.577739214692297	1	pseudobulk	DESeq2	LRT	0
166	KC_AB3	CG11777	1.11916865944137	0.504910440053014	1	pseudobulk	DESeq2	LRT	0
166	KC_AB3	CG11779	0.633591092559079	0.411531170347182	1	pseudobulk	DESeq2	LRT	0
166	KC_AB3	CG11781	0.019295759374219	0.657022948409598	1	pseudobulk	DESeq2	LRT	0
166	KC_AB3	CG11788	1.93210469959844	0.634076921097483	1	pseudobulk	DESeq2	LRT	0
166	KC_AB3	CG11790	-0.472442822428732	0.731566611637119	1	pseudobulk	DESeq2	LRT	0
166	KC_AB3	CG11791	0.4135959724801	1	1	pseudobulk	DESeq2	LRT	0
166	KC_AB3	CG11807	0.865494092374218	0.334546041824826	1	pseudobulk	DESeq2	LRT	0
166	KC_AB3	CG11808	0.649715915883756	0.780910159678579	1	pseudobulk	DESeq2	LRT	0
166	KC_AB3	CG11811	0.929788187689533	0.391142822067732	1	pseudobulk	DESeq2	LRT	0
166	KC_AB3	CG11835	-1.93493071358919	0.174475460249944	1	pseudobulk	DESeq2	LRT	0
166	KC_AB3	CG11837	-0.184744780581018	1	1	pseudobulk	DESeq2	LRT	0
166	KC_AB3	CG11839	2.03113667946177	0.349867032982222	1	pseudobulk	DESeq2	LRT	0
166	KC_AB3	CG11857	-0.0785881951438953	0.633680395548195	1	pseudobulk	DESeq2	LRT	0
166	KC_AB3	CG11858	0.31623622981122	0.692388675063227	1	pseudobulk	DESeq2	LRT	0
166	KC_AB3	CG11873	0.209027313331282	0.498130771241282	1	pseudobulk	DESeq2	LRT	0
166	KC_AB3	CG11882	0.928776612232151	0.96148233279097	1	pseudobulk	DESeq2	LRT	0

166	KC_AB3	CG11883	-0.639867683215499	0.211747743527473	1	pseudobulk	DESeq2	LRT	0
166	KC_AB3	CG11885	2.01986679378286	0.271017251249341	1	pseudobulk	DESeq2	LRT	0
166	KC_AB3	CG11898	0.0020939056309239	1	1	pseudobulk	DESeq2	LRT	0
166	KC_AB3	CG11902	0.916597216976181	0.710796605042257	1	pseudobulk	DESeq2	LRT	0
166	KC_AB3	CG11906	1.13720220944247	0.539202440585012	1	pseudobulk	DESeq2	LRT	0
166	KC_AB3	CG11911	-0.032730048690647	1	1	pseudobulk	DESeq2	LRT	0
167	KC_AB3	CG11920	2.62817135292692	0.54954870385793	1	pseudobulk	DESeq2	LRT	0
167	KC_AB3	CG11927	1.20182930024029	0.398145184044889	1	pseudobulk	DESeq2	LRT	0
167	KC_AB3	CG11961	0.632774104902944	1	1	pseudobulk	DESeq2	LRT	0
167	KC_AB3	CG11964	0.724048602294202	0.702548510373228	1	pseudobulk	DESeq2	LRT	0
167	KC_AB3	CG11970	0.313553183536344	1	1	pseudobulk	DESeq2	LRT	0
167	KC_AB3	CG11975	0.263617723803305	0.293725129311351	1	pseudobulk	DESeq2	LRT	0
167	KC_AB3	CG11980	0.356752343902978	0.659099271295773	1	pseudobulk	DESeq2	LRT	0
167	KC_AB3	CG11999	-0.562988699215838	0.601547410214347	1	pseudobulk	DESeq2	LRT	0
167	KC_AB3	CG12004	0.0418974922344863	0.600461299014262	1	pseudobulk	DESeq2	LRT	0
167	KC_AB3	CG12007	-0.369844920127649	0.682370839755267	1	pseudobulk	DESeq2	LRT	0
167	KC_AB3	CG12012	-0.453679024737525	0.594169915560329	1	pseudobulk	DESeq2	LRT	0
167	KC_AB3	CG12024	-0.468742913271385	0.441663079099493	1	pseudobulk	DESeq2	LRT	0
167	KC_AB3	CG12025	0.0904099918457391	0.821402558623854	1	pseudobulk	DESeq2	LRT	0
167	KC_AB3	CG12038	-0.864693823158537	0.160537618888942	1	pseudobulk	DESeq2	LRT	0
167	KC_AB3	CG12054	-0.458034101285346	0.225457291460029	1	pseudobulk	DESeq2	LRT	0
167	KC_AB3	CG12056	1.76606778079447	0.509746345076376	1	pseudobulk	DESeq2	LRT	0
167	KC_AB3	CG12065	0.632774104902944	1	1	pseudobulk	DESeq2	LRT	0
167	KC_AB3	CG12071	-0.279932483903995	0.431971267813581	1	pseudobulk	DESeq2	LRT	0
167	KC_AB3	CG12081	0.165081983888354	0.726038938554368	1	pseudobulk	DESeq2	LRT	0
167	KC_AB3	CG12084	0.0215491113722007	0.734675042796983	1	pseudobulk	DESeq2	LRT	0
167	KC_AB3	CG12091	0.0068750921979310	0.755053175055845	1	pseudobulk	DESeq2	LRT	0
167	KC_AB3	CG12093	-1.09474263775169	0.306475898296982	1	pseudobulk	DESeq2	LRT	0
167	KC_AB3	CG12096	-0.447987724091539	0.661257937690023	1	pseudobulk	DESeq2	LRT	0
167	KC_AB3	CG12099	0.0914111098410633	0.868017401999791	1	pseudobulk	DESeq2	LRT	0
167	KC_AB3	CG12104	0.973297877578351	0.664324161260865	1	pseudobulk	DESeq2	LRT	0
167	KC_AB3	CG12106	0.156113083159112	0.812909354323055	1	pseudobulk	DESeq2	LRT	0
167	KC_AB3	CG12107	1.07666942661763	0.17954669399681	1	pseudobulk	DESeq2	LRT	0
167	KC_AB3	CG12112	0.632774104902943	1	1	pseudobulk	DESeq2	LRT	0
167	KC_AB3	CG12118	0.87750923882559	0.280348856784298	1	pseudobulk	DESeq2	LRT	0
167	KC_AB3	CG12121	-0.149628175672368	0.692924898510009	1	pseudobulk	DESeq2	LRT	0
167	KC_AB3	CG12123	-1.42593334856544	0.177502113276523	1	pseudobulk	DESeq2	LRT	0
167	KC_AB3	CG12128	-0.400046519007236	0.964129537865033	1	pseudobulk	DESeq2	LRT	0
167	KC_AB3	CG12129	0.952256340296599	0.493489136227584	1	pseudobulk	DESeq2	LRT	0
167	KC_AB3	CG12134	1.22707713470494	1	1	pseudobulk	DESeq2	LRT	0
167	KC_AB3	CG12155	0.628911710196484	0.53286026140891	1	pseudobulk	DESeq2	LRT	0
167	KC_AB3	CG12158	-0.560477492866218	0.784070523163114	1	pseudobulk	DESeq2	LRT	0
167	KC_AB3	CG12159	0.0253973618051395	0.685746793769862	1	pseudobulk	DESeq2	LRT	0
167	KC_AB3	CG12163	-0.11055066055452	0.77673336048608	1	pseudobulk	DESeq2	LRT	0
167	KC_AB3	CG12170	0.259241847397765	0.78787667173433	1	pseudobulk	DESeq2	LRT	0
167	KC_AB3	CG12171	1.93443109363625	0.658150581914386	1	pseudobulk	DESeq2	LRT	0
167	KC_AB3	CG12173	1.07058553669184	0.370271559544941	1	pseudobulk	DESeq2	LRT	0
167	KC_AB3	CG12177	-0.184744780581018	1	1	pseudobulk	DESeq2	LRT	0
167	KC_AB3	CG12179	1.84671504053046	1	1	pseudobulk	DESeq2	LRT	0
167	KC_AB3	CG1218	-0.378276245906696	0.706290686047516	1	pseudobulk	DESeq2	LRT	0
167	KC_AB3	CG12182	0.328024391226458	1	1	pseudobulk	DESeq2	LRT	0
167	KC_AB3	CG12184	0.632774104902944	1	1	pseudobulk	DESeq2	LRT	0
167	KC_AB3	CG12194	-0.253786315811563	0.439852449231296	1	pseudobulk	DESeq2	LRT	0

167	KC_AB3	CG12204	-0.288175978692328	0.690529039158236	1	pseudobulk	DESeq2	LRT	0
167	KC_AB3	CG12213	0.0380389989946216	0.63480048519496	1	pseudobulk	DESeq2	LRT	0
167	KC_AB3	CG12219	-0.231710483441688	0.742064284812227	1	pseudobulk	DESeq2	LRT	0
167	KC_AB3	CG12236	-0.97468518284459	0.270109489658043	1	pseudobulk	DESeq2	LRT	0
167	KC_AB3	CG12237	-0.0157807354849895	0.72657180700687	1	pseudobulk	DESeq2	LRT	0
167	KC_AB3	CG12241	-0.988277721451467	0.361486037866545	1	pseudobulk	DESeq2	LRT	0
167	KC_AB3	CG12253	0.545378960070763	0.591710375106382	1	pseudobulk	DESeq2	LRT	0
167	KC_AB3	CG12259	-0.880952807626271	0.518778193463119	1	pseudobulk	DESeq2	LRT	0
167	KC_AB3	CG12267	1.88513752152589	0.739152646872234	1	pseudobulk	DESeq2	LRT	0
167	KC_AB3	CG1227	2.00590356873193	0.478745330999366	1	pseudobulk	DESeq2	LRT	0
167	KC_AB3	CG12279	0.0786116186241126	0.689309551899095	1	pseudobulk	DESeq2	LRT	0
167	KC_AB3	CG12288	0.632774104902944	1	1	pseudobulk	DESeq2	LRT	0
167	KC_AB3	CG12299	-0.378669409605776	0.491919353211517	1	pseudobulk	DESeq2	LRT	0
167	KC_AB3	CG12301	-0.388525681808363	0.682330209171299	1	pseudobulk	DESeq2	LRT	0
167	KC_AB3	CG1231	0.294333387895249	0.895696506246484	1	pseudobulk	DESeq2	LRT	0
167	KC_AB3	CG12316	-0.472022559665467	0.62703757644888	1	pseudobulk	DESeq2	LRT	0
167	KC_AB3	CG12320	-1.04776935228448	0.559994920957393	1	pseudobulk	DESeq2	LRT	0
167	KC_AB3	CG12321	0.729708735861348	0.478005536782924	1	pseudobulk	DESeq2	LRT	0
167	KC_AB3	CG12325	1.23133864869565	1	1	pseudobulk	DESeq2	LRT	0
167	KC_AB3	CG1233	-0.242705193805337	0.807323308907096	1	pseudobulk	DESeq2	LRT	0
167	KC_AB3	CG12333	0.494922349121128	0.427452391033103	1	pseudobulk	DESeq2	LRT	0
167	KC_AB3	CG12338	0.632774104902943	1	1	pseudobulk	DESeq2	LRT	0
167	KC_AB3	CG1234	0.318500409296569	0.732993974121825	1	pseudobulk	DESeq2	LRT	0
167	KC_AB3	CG12341	0.304219873897165	0.667377263299376	1	pseudobulk	DESeq2	LRT	0
167	KC_AB3	CG12355	0.137244394432208	0.75296439218821	1	pseudobulk	DESeq2	LRT	0
167	KC_AB3	CG1236	-0.779009454654304	0.632803062798981	1	pseudobulk	DESeq2	LRT	0
167	KC_AB3	CG12375	0.391324421376436	0.351854474170148	1	pseudobulk	DESeq2	LRT	0
167	KC_AB3	CG12379	0.0538103424110901	1	1	pseudobulk	DESeq2	LRT	0
167	KC_AB3	CG12384	0.144450621419211	0.662183166042394	1	pseudobulk	DESeq2	LRT	0
167	KC_AB3	CG1239	-2.23800037066536	0.105378242398029	1	pseudobulk	DESeq2	LRT	0
167	KC_AB3	CG12391	0.65205135781531	0.287366019613057	1	pseudobulk	DESeq2	LRT	0
167	KC_AB3	CG12402	0.0784418212390482	0.811037749850146	1	pseudobulk	DESeq2	LRT	0
167	KC_AB3	CG12499	0.311862295904005	0.779571720159938	1	pseudobulk	DESeq2	LRT	0
167	KC_AB3	CG12502	-0.685892053704454	0.545231978444544	1	pseudobulk	DESeq2	LRT	0
167	KC_AB3	CG12531	-0.0945889242813537	0.721437486665988	1	pseudobulk	DESeq2	LRT	0
167	KC_AB3	CG12535	-0.0355517206969949	0.906574025627152	1	pseudobulk	DESeq2	LRT	0
167	KC_AB3	CG12540	0.632774104902944	1	1	pseudobulk	DESeq2	LRT	0
167	KC_AB3	CG12541	0.208588313721779	0.443895053392364	1	pseudobulk	DESeq2	LRT	0
167	KC_AB3	CG12547	-0.697712469510089	0.374700410721976	1	pseudobulk	DESeq2	LRT	0
167	KC_AB3	CG12560	0.692653999526471	0.683194363527121	1	pseudobulk	DESeq2	LRT	0
167	KC_AB3	CG12567	-0.479307712382129	0.514144533253295	1	pseudobulk	DESeq2	LRT	0
167	KC_AB3	CG12576	-0.270382350330085	0.678668665582	1	pseudobulk	DESeq2	LRT	0
167	KC_AB3	CG12594	1.95267092137636	1	1	pseudobulk	DESeq2	LRT	0
167	KC_AB3	CG12605	-0.384838814837188	0.297590023846403	1	pseudobulk	DESeq2	LRT	0
167	KC_AB3	CG12608	1.23133864869565	1	1	pseudobulk	DESeq2	LRT	0
167	KC_AB3	CG1265	-0.0492775568650978	0.892605006991737	1	pseudobulk	DESeq2	LRT	0
167	KC_AB3	CG12659	-0.584602167601671	0.636282344286611	1	pseudobulk	DESeq2	LRT	0
167	KC_AB3	CG12672	1.09402331202049	0.173387445410074	1	pseudobulk	DESeq2	LRT	0
167	KC_AB3	CG12698	1.95267092137636	1	1	pseudobulk	DESeq2	LRT	0
167	KC_AB3	CG1271	-0.972472888132715	0.580553414221232	1	pseudobulk	DESeq2	LRT	0
167	KC_AB3	CG12713	0.77554105530017	1	1	pseudobulk	DESeq2	LRT	0
167	KC_AB3	CG12715	2.1445051383883	0.530068988727821	1	pseudobulk	DESeq2	LRT	0
167	KC_AB3	CG12717	0.122016677210207	0.982950981684759	1	pseudobulk	DESeq2	LRT	0

168	KC_AB3	CG12728	-0.251148348845382	0.793024296437907	1	pseudobulk	DESeq2	LRT	0
168	KC_AB3	CG12730	-0.0539381888640068	0.660689780871928	1	pseudobulk	DESeq2	LRT	0
168	KC_AB3	CG12746	-1.87768827829215	0.179514913713745	1	pseudobulk	DESeq2	LRT	0
168	KC_AB3	CG1275	0.252417586026418	0.64435785538929	1	pseudobulk	DESeq2	LRT	0
168	KC_AB3	CG12768	-0.471154001643531	0.766828223954262	1	pseudobulk	DESeq2	LRT	0
168	KC_AB3	CG12769	-1.42817185934463	0.106986844890164	1	pseudobulk	DESeq2	LRT	0
168	KC_AB3	CG12773	0.0153055030197099	0.958026781585604	1	pseudobulk	DESeq2	LRT	0
168	KC_AB3	CG12795	-0.166341146343636	0.676462885779691	1	pseudobulk	DESeq2	LRT	0
168	KC_AB3	CG12814	-0.547361577986966	0.814803379895176	1	pseudobulk	DESeq2	LRT	0
168	KC_AB3	CG12817	-1.12728210694406	0.283212937478189	1	pseudobulk	DESeq2	LRT	0
168	KC_AB3	CG12818	0.261291470593178	0.821346588549822	1	pseudobulk	DESeq2	LRT	0
168	KC_AB3	CG12822	-0.36101342556198	0.47717574887463	1	pseudobulk	DESeq2	LRT	0
168	KC_AB3	CG12825	1.95267092137636	1	1	pseudobulk	DESeq2	LRT	0
168	KC_AB3	CG12836	0.161515868851812	1	1	pseudobulk	DESeq2	LRT	0
168	KC_AB3	CG12848	-0.0258075529254765	0.7477608192291	1	pseudobulk	DESeq2	LRT	0
168	KC_AB3	CG12851	-0.887387801030106	0.444172125243372	1	pseudobulk	DESeq2	LRT	0
168	KC_AB3	CG12863	1.95267092137636	1	1	pseudobulk	DESeq2	LRT	0
168	KC_AB3	CG12868	-0.0590343839267669	0.784344880056355	1	pseudobulk	DESeq2	LRT	0
168	KC_AB3	CG12877	-0.430129648694603	0.696443201764628	1	pseudobulk	DESeq2	LRT	0
168	KC_AB3	CG12883	0.857037594830445	0.786425553887833	1	pseudobulk	DESeq2	LRT	0
168	KC_AB3	CG12895	0.440957827750075	0.52892144162201	1	pseudobulk	DESeq2	LRT	0
168	KC_AB3	CG12896	1.95267092137636	1	1	pseudobulk	DESeq2	LRT	0
168	KC_AB3	CG12909	-0.120255328429205	0.914604900667141	1	pseudobulk	DESeq2	LRT	0
168	KC_AB3	CG12910	-0.370378916918409	0.501412388815267	1	pseudobulk	DESeq2	LRT	0
168	KC_AB3	CG12913	-0.991363335647687	0.387838527526473	1	pseudobulk	DESeq2	LRT	0
168	KC_AB3	CG12929	-0.754818909547871	0.547648728588812	1	pseudobulk	DESeq2	LRT	0
168	KC_AB3	CG12935	-0.60634467351113	0.617175283349625	1	pseudobulk	DESeq2	LRT	0
168	KC_AB3	CG12942	1.47800220437468	0.708296824700333	1	pseudobulk	DESeq2	LRT	0
168	KC_AB3	CG12947	-3.40741491832534	0.321881169879726	1	pseudobulk	DESeq2	LRT	0
168	KC_AB3	CG12948	0.988618733083305	0.582225246346962	1	pseudobulk	DESeq2	LRT	0
168	KC_AB3	CG12963	0.632774104902944	1	1	pseudobulk	DESeq2	LRT	0
168	KC_AB3	CG12975	-0.726333667440566	0.37902286666301	1	pseudobulk	DESeq2	LRT	0
168	KC_AB3	CG12990	1.95267092137636	1	1	pseudobulk	DESeq2	LRT	0
168	KC_AB3	CG12991	-0.215474489990063	0.522198146121027	1	pseudobulk	DESeq2	LRT	0
168	KC_AB3	CG12992	0.632774104902944	1	1	pseudobulk	DESeq2	LRT	0
168	KC_AB3	CG12994	1.23133864869565	1	1	pseudobulk	DESeq2	LRT	0
168	KC_AB3	CG13001	0.140111042078886	0.736841819708221	1	pseudobulk	DESeq2	LRT	0
168	KC_AB3	CG13005	2.36933983601176	0.406024215854308	1	pseudobulk	DESeq2	LRT	0
168	KC_AB3	CG13014	0.263237126093928	1	1	pseudobulk	DESeq2	LRT	0
168	KC_AB3	CG13016	1.17410956900638	0.63430400331941	1	pseudobulk	DESeq2	LRT	0
168	KC_AB3	CG13018	1.01298092357986	0.424146075432454	1	pseudobulk	DESeq2	LRT	0
168	KC_AB3	CG13025	-0.53615682788415	0.646459679759324	1	pseudobulk	DESeq2	LRT	0
168	KC_AB3	CG13054	0.327369208630244	0.777002822515658	1	pseudobulk	DESeq2	LRT	0
168	KC_AB3	CG13055	-0.600395973949122	0.18462989995946	1	pseudobulk	DESeq2	LRT	0
168	KC_AB3	CG1307	0.396919793942589	0.57264983468754	1	pseudobulk	DESeq2	LRT	0
168	KC_AB3	CG13085	-0.583057444465563	0.678110339883318	1	pseudobulk	DESeq2	LRT	0
168	KC_AB3	CG1309	0.241738398927704	0.86896180745417	1	pseudobulk	DESeq2	LRT	0
168	KC_AB3	CG13096	-0.229039602237312	1	1	pseudobulk	DESeq2	LRT	0
168	KC_AB3	CG13097	1.59575615951652	0.886898266337347	1	pseudobulk	DESeq2	LRT	0
168	KC_AB3	CG13108	-0.253363846894497	0.601019742694745	1	pseudobulk	DESeq2	LRT	0
168	KC_AB3	CG13123	1.339264475551	0.544484663073387	1	pseudobulk	DESeq2	LRT	0
168	KC_AB3	CG13124	1.23133864869565	1	1	pseudobulk	DESeq2	LRT	0
168	KC_AB3	CG13126	1.95267092137636	1	1	pseudobulk	DESeq2	LRT	0

168	KC_AB3	CG13148	0.154100118446557	0.839985685034188	1	pseudobulk	DESeq2	LRT	0
168	KC_AB3	CG13151	0.64039360257429	1	1	pseudobulk	DESeq2	LRT	0
168	KC_AB3	CG1316	-0.300603568370109	0.434280462360724	1	pseudobulk	DESeq2	LRT	0
168	KC_AB3	CG13168	-0.0327300486906476	1	1	pseudobulk	DESeq2	LRT	0
168	KC_AB3	CG1317	-0.810191968370852	0.340319587954585	1	pseudobulk	DESeq2	LRT	0
168	KC_AB3	CG13185	0.632774104902943	1	1	pseudobulk	DESeq2	LRT	0
168	KC_AB3	CG13192	-0.445967656110026	0.62927467044644	1	pseudobulk	DESeq2	LRT	0
168	KC_AB3	CG13197	-0.0989496420564344	0.748780952898415	1	pseudobulk	DESeq2	LRT	0
168	KC_AB3	CG13204	0.0231221554928008	0.737751796576303	1	pseudobulk	DESeq2	LRT	0
168	KC_AB3	CG13220	-1.84374901858591	0.105561574434916	1	pseudobulk	DESeq2	LRT	0
168	KC_AB3	CG13239	2.62817135292692	0.54954870385793	1	pseudobulk	DESeq2	LRT	0
168	KC_AB3	CG13248	0.307245020229349	0.553808810881021	1	pseudobulk	DESeq2	LRT	0
168	KC_AB3	CG13255	1.95267092137636	1	1	pseudobulk	DESeq2	LRT	0
168	KC_AB3	CG13284	1.8751573179585	0.767073395253422	1	pseudobulk	DESeq2	LRT	0
168	KC_AB3	CG13293	-0.615067937003899	0.238536600197157	1	pseudobulk	DESeq2	LRT	0
168	KC_AB3	CG13295	-0.714227006634363	0.576843796398186	1	pseudobulk	DESeq2	LRT	0
168	KC_AB3	CG13300	1.95267092137636	1	1	pseudobulk	DESeq2	LRT	0
168	KC_AB3	CG13315	0.632774104902944	1	1	pseudobulk	DESeq2	LRT	0
168	KC_AB3	CG13319	0.632774104902944	1	1	pseudobulk	DESeq2	LRT	0
168	KC_AB3	CG13325	0.632774104902944	1	1	pseudobulk	DESeq2	LRT	0
168	KC_AB3	CG13339	-0.190065418326364	0.726778968878481	1	pseudobulk	DESeq2	LRT	0
168	KC_AB3	CG13344	0.637535876772942	0.297461221106308	1	pseudobulk	DESeq2	LRT	0
168	KC_AB3	CG13364	0.0030402525562138	0.971828813615163	1	pseudobulk	DESeq2	LRT	0
168	KC_AB3	CG13365	0.473676590382024	0.353156218866401	1	pseudobulk	DESeq2	LRT	0
168	KC_AB3	CG13366	1.00038445566365	0.303671286297671	1	pseudobulk	DESeq2	LRT	0
168	KC_AB3	CG13367	1.59576167909336	0.887029991980463	1	pseudobulk	DESeq2	LRT	0
168	KC_AB3	CG13369	1.05451978822819	0.444295253880801	1	pseudobulk	DESeq2	LRT	0
168	KC_AB3	CG13375	-1.04901195946427	0.228582131337305	1	pseudobulk	DESeq2	LRT	0
168	KC_AB3	CG13377	2.64926057711297	0.482244576496623	1	pseudobulk	DESeq2	LRT	0
168	KC_AB3	CG13384	0.0902555591204802	0.613522251372767	1	pseudobulk	DESeq2	LRT	0
168	KC_AB3	CG13390	0.453013475402082	0.709857402749621	1	pseudobulk	DESeq2	LRT	0
168	KC_AB3	CG13392	0.0226638642085793	0.888631589930686	1	pseudobulk	DESeq2	LRT	0
168	KC_AB3	CG13398	-0.47178560816757	0.700413808129884	1	pseudobulk	DESeq2	LRT	0
168	KC_AB3	CG13404	0.120545968634218	0.70814193173516	1	pseudobulk	DESeq2	LRT	0
168	KC_AB3	CG13409	1.03493850294572	0.237914396513699	1	pseudobulk	DESeq2	LRT	0
168	KC_AB3	CG1344	0.311208023881363	0.745982035500929	1	pseudobulk	DESeq2	LRT	0
168	KC_AB3	CG13482	1.59575615951652	0.886898266337347	1	pseudobulk	DESeq2	LRT	0
168	KC_AB3	CG13484	-0.529441097642397	0.593306711545385	1	pseudobulk	DESeq2	LRT	0
168	KC_AB3	CG13506	-1.07735855611271	0.189662495779306	1	pseudobulk	DESeq2	LRT	0
168	KC_AB3	CG13526	2.3149273646917	0.611236276445972	1	pseudobulk	DESeq2	LRT	0
168	KC_AB3	CG13531	0.438392768241677	1	1	pseudobulk	DESeq2	LRT	0
168	KC_AB3	CG13532	-0.517404121894957	0.575260492667725	1	pseudobulk	DESeq2	LRT	0
168	KC_AB3	CG1354	-0.952166424129325	0.441827234478913	1	pseudobulk	DESeq2	LRT	0
168	KC_AB3	CG13551	0.458957767754155	0.231187782442566	1	pseudobulk	DESeq2	LRT	0
168	KC_AB3	CG13578	1.27366152380294	0.222235175982589	1	pseudobulk	DESeq2	LRT	0
168	KC_AB3	CG1358	-0.508775120760443	0.379000607459617	1	pseudobulk	DESeq2	LRT	0
168	KC_AB3	CG13585	-0.22052580393254	0.468283307496054	1	pseudobulk	DESeq2	LRT	0
169	KC_AB3	CG1360	0.540278194156591	1	1	pseudobulk	DESeq2	LRT	0
169	KC_AB3	CG13601	-0.347892647000127	0.656682554038221	1	pseudobulk	DESeq2	LRT	0
169	KC_AB3	CG13603	-0.242650257806263	0.727337744936791	1	pseudobulk	DESeq2	LRT	0
169	KC_AB3	CG13604	1.20175497323432	1	1	pseudobulk	DESeq2	LRT	0
169	KC_AB3	CG13605	0.440647598459493	0.815020090178143	1	pseudobulk	DESeq2	LRT	0
169	KC_AB3	CG13609	1.23133864869565	1	1	pseudobulk	DESeq2	LRT	0

169	KC_AB3	CG13623	-0.256018021349231	0.570323892628107	1	pseudobulk	DESeq2	LRT	0
169	KC_AB3	CG13625	1.27778455479166	0.373852818752167	1	pseudobulk	DESeq2	LRT	0
169	KC_AB3	CG13630	-0.308355688787654	0.653641030460637	1	pseudobulk	DESeq2	LRT	0
169	KC_AB3	CG13631	-0.229039602237312	1	1	pseudobulk	DESeq2	LRT	0
169	KC_AB3	CG13654	0.658530332580915	0.564629257609608	1	pseudobulk	DESeq2	LRT	0
169	KC_AB3	CG13663	-0.39963712745553	0.672708156928523	1	pseudobulk	DESeq2	LRT	0
169	KC_AB3	CG13671	1.05674580342163	0.593440799020684	1	pseudobulk	DESeq2	LRT	0
169	KC_AB3	CG13674	-1.77406713908084	0.326549391762558	1	pseudobulk	DESeq2	LRT	0
169	KC_AB3	CG13689	-0.509943271867356	0.601616566974048	1	pseudobulk	DESeq2	LRT	0
169	KC_AB3	CG1371	-0.660047485563789	0.49347836527466	1	pseudobulk	DESeq2	LRT	0
169	KC_AB3	CG13737	-0.246432117623312	0.692906540971832	1	pseudobulk	DESeq2	LRT	0
169	KC_AB3	CG13739	-0.126609603173014	0.524149465979868	1	pseudobulk	DESeq2	LRT	0
169	KC_AB3	CG13743	2.16885543174516	0.112939220317245	1	pseudobulk	DESeq2	LRT	0
169	KC_AB3	CG13751	-0.400541149137932	0.413054786390577	1	pseudobulk	DESeq2	LRT	0
169	KC_AB3	CG13760	0.577397005249272	0.662529173681119	1	pseudobulk	DESeq2	LRT	0
169	KC_AB3	CG13766	0.818124421089535	0.44827160715981	1	pseudobulk	DESeq2	LRT	0
169	KC_AB3	CG13773	2.1445051383883	0.530068988727821	1	pseudobulk	DESeq2	LRT	0
169	KC_AB3	CG13775	0.632774104902944	1	1	pseudobulk	DESeq2	LRT	0
169	KC_AB3	CG13784	-0.219664578842435	0.460907963901763	1	pseudobulk	DESeq2	LRT	0
169	KC_AB3	CG13807	0.728850425851573	0.74689437451984	1	pseudobulk	DESeq2	LRT	0
169	KC_AB3	CG13827	0.632774104902943	1	1	pseudobulk	DESeq2	LRT	0
169	KC_AB3	CG13850	0.893903990774454	0.846314690533615	1	pseudobulk	DESeq2	LRT	0
169	KC_AB3	CG13868	0.211015894737439	0.507946687783299	1	pseudobulk	DESeq2	LRT	0
169	KC_AB3	CG13870	0.632774104902943	1	1	pseudobulk	DESeq2	LRT	0
169	KC_AB3	CG13876	1.12059961403812	1	1	pseudobulk	DESeq2	LRT	0
169	KC_AB3	CG13887	0.27412394815695	0.547733226845131	1	pseudobulk	DESeq2	LRT	0
169	KC_AB3	CG13893	-0.792919478540393	0.58155750177774	1	pseudobulk	DESeq2	LRT	0
169	KC_AB3	CG13895	-0.400046519007236	0.964129537865033	1	pseudobulk	DESeq2	LRT	0
169	KC_AB3	CG13896	0.632774104902944	1	1	pseudobulk	DESeq2	LRT	0
169	KC_AB3	CG13898	0.96002182142999	1	1	pseudobulk	DESeq2	LRT	0
169	KC_AB3	CG13901	-0.502309390449855	0.612783922765368	1	pseudobulk	DESeq2	LRT	0
169	KC_AB3	CG13907	-0.279002443147119	0.630625505981265	1	pseudobulk	DESeq2	LRT	0
169	KC_AB3	CG13917	0.194184088496575	0.538456564371334	1	pseudobulk	DESeq2	LRT	0
169	KC_AB3	CG13919	-0.424270869144227	0.694523460002138	1	pseudobulk	DESeq2	LRT	0
169	KC_AB3	CG13920	-0.135977671272923	0.431535042811814	1	pseudobulk	DESeq2	LRT	0
169	KC_AB3	CG13921	-0.175681150326113	0.486297557037349	1	pseudobulk	DESeq2	LRT	0
169	KC_AB3	CG13926	0.0226924283321225	0.696733192875928	1	pseudobulk	DESeq2	LRT	0
169	KC_AB3	CG13928	-0.114997870646976	0.541750211764172	1	pseudobulk	DESeq2	LRT	0
169	KC_AB3	CG13933	-1.79346920085183	0.125095591768776	1	pseudobulk	DESeq2	LRT	0
169	KC_AB3	CG13937	3.17937028801111	0.0261430943267005	1	pseudobulk	DESeq2	LRT	0
169	KC_AB3	CG13954	0.114584109231903	0.710509657930262	1	pseudobulk	DESeq2	LRT	0
169	KC_AB3	CG13982	1.59359598433783	0.86377329553208	1	pseudobulk	DESeq2	LRT	0
169	KC_AB3	CG13994	-0.466812947692787	0.594705637235522	1	pseudobulk	DESeq2	LRT	0
169	KC_AB3	CG13999	-0.672656262745224	0.290663924639542	1	pseudobulk	DESeq2	LRT	0
169	KC_AB3	CG14005	-0.118059581015957	0.842472551369135	1	pseudobulk	DESeq2	LRT	0
169	KC_AB3	CG14015	0.021820171128364	0.792504127149845	1	pseudobulk	DESeq2	LRT	0
169	KC_AB3	CG14024	0.320364589912232	0.558010050279344	1	pseudobulk	DESeq2	LRT	0
169	KC_AB3	CG14043	-0.447371970469025	0.59599027563963	1	pseudobulk	DESeq2	LRT	0
169	KC_AB3	CG14053	1.95267092137636	1	1	pseudobulk	DESeq2	LRT	0
169	KC_AB3	CG14057	-0.962336549607539	0.567620274858258	1	pseudobulk	DESeq2	LRT	0
169	KC_AB3	CG1407	0.254025051602519	0.722590296475989	1	pseudobulk	DESeq2	LRT	0
169	KC_AB3	CG14073	-0.113421425034908	0.62651968067425	1	pseudobulk	DESeq2	LRT	0
169	KC_AB3	CG14074	0.135576260427557	0.844845202746717	1	pseudobulk	DESeq2	LRT	0

169	KC_AB3	CG14082	0.227788323531277	0.869818105991501	1	pseudobulk	DESeq2	LRT		0
169	KC_AB3	CG14100	0.597949772665551	0.768871729683162	1	pseudobulk	DESeq2	LRT		0
169	KC_AB3	CG14102	1.02673249970624	1	1	pseudobulk	DESeq2	LRT		0
169	KC_AB3	CG14104	0.632774104902944	1	1	pseudobulk	DESeq2	LRT		0
169	KC_AB3	CG14130	1.92580984707296	0.339458269934877	1	pseudobulk	DESeq2	LRT		0
169	KC_AB3	CG14135	0.560984869422946	0.644576738596891	1	pseudobulk	DESeq2	LRT		0
169	KC_AB3	CG14142	0.632774104902944	1	1	pseudobulk	DESeq2	LRT		0
169	KC_AB3	CG14153	-0.838505508417818	0.664237228246735	1	pseudobulk	DESeq2	LRT		0
169	KC_AB3	CG14154	0.632774104902944	1	1	pseudobulk	DESeq2	LRT		0
169	KC_AB3	CG1416	-0.0861396885831601	0.535662896023074	1	pseudobulk	DESeq2	LRT		0
169	KC_AB3	CG14174	1.7068524709271	0.575952835840995	1	pseudobulk	DESeq2	LRT		0
169	KC_AB3	CG1418	-0.306225666541489	0.672277511666004	1	pseudobulk	DESeq2	LRT		0
169	KC_AB3	CG14182	0.713281307285132	0.84315173270398	1	pseudobulk	DESeq2	LRT		0
169	KC_AB3	CG14183	0.0020939056309239	1	1	pseudobulk	DESeq2	LRT		0
169	KC_AB3	CG14184	0.611648330765222	0.401985507430522	1	pseudobulk	DESeq2	LRT		0
169	KC_AB3	CG14186	-0.307675410224058	0.532169887925828	1	pseudobulk	DESeq2	LRT		0
169	KC_AB3	CG14194	0.270405163782269	0.701798093708255	1	pseudobulk	DESeq2	LRT		0
169	KC_AB3	CG14207	0.194325104363031	0.562592217638431	1	pseudobulk	DESeq2	LRT		0
169	KC_AB3	CG14210	-0.391685377871105	0.739139478641909	1	pseudobulk	DESeq2	LRT		0
169	KC_AB3	CG14220	-0.752582225082204	0.606307740823456	1	pseudobulk	DESeq2	LRT		0
169	KC_AB3	CG14229	-0.756911065026489	0.408211435955467	1	pseudobulk	DESeq2	LRT		0
169	KC_AB3	CG14230	1.59359598433783	0.86377329553208	1	pseudobulk	DESeq2	LRT		0
169	KC_AB3	CG14231	0.633245595655224	0.764780248577245	1	pseudobulk	DESeq2	LRT		0
169	KC_AB3	CG14232	-0.297839981263271	0.697670299900335	1	pseudobulk	DESeq2	LRT		0
169	KC_AB3	CG14234	-0.99487043278089	0.215183393486631	1	pseudobulk	DESeq2	LRT		0
169	KC_AB3	CG14252	-1.33713774668078	0.356601341569333	1	pseudobulk	DESeq2	LRT		0
169	KC_AB3	CG14270	-0.500365506369729	0.645996109474125	1	pseudobulk	DESeq2	LRT		0
169	KC_AB3	CG14286	-0.962586053545908	0.567525062839105	1	pseudobulk	DESeq2	LRT		0
169	KC_AB3	CG14304	1.95267092137636	1	1	pseudobulk	DESeq2	LRT		0
169	KC_AB3	CG14312	0.325450455351058	0.530106087582812	1	pseudobulk	DESeq2	LRT		0
169	KC_AB3	CG14314	-0.107023776504053	0.735944302585278	1	pseudobulk	DESeq2	LRT		0
169	KC_AB3	CG14321	0.319086227871377	0.509017357854246	1	pseudobulk	DESeq2	LRT		0
169	KC_AB3	CG14322	-0.972472888132715	0.580553414221232	1	pseudobulk	DESeq2	LRT		0
169	KC_AB3	CG1434	-0.560477492866218	0.784070523163114	1	pseudobulk	DESeq2	LRT		0
169	KC_AB3	CG14340	-0.357607384325327	0.790157014682946	1	pseudobulk	DESeq2	LRT		0
169	KC_AB3	CG14341	-0.376500590350636	0.607222892446624	1	pseudobulk	DESeq2	LRT		0
169	KC_AB3	CG14352	0.4135959724801	1	1	pseudobulk	DESeq2	LRT		0
169	KC_AB3	CG14367	0.632774104902944	1	1	pseudobulk	DESeq2	LRT		0
169	KC_AB3	CG1440	-0.62083977388827	0.486681684300721	1	pseudobulk	DESeq2	LRT		0
169	KC_AB3	CG14407	-0.152126844184306	0.590219006132328	1	pseudobulk	DESeq2	LRT		0
169	KC_AB3	CG14408	0.431448549004531	0.331361880888957	1	pseudobulk	DESeq2	LRT		0
170	KC_AB3	CG14410	0.0025701398975595	1	1	pseudobulk	DESeq2	LRT		0
170	KC_AB3	CG14411	0.718355958628294	0.455456278459856	1	pseudobulk	DESeq2	LRT		0
170	KC_AB3	CG14414	0.113343065797539	0.78772549484892	1	pseudobulk	DESeq2	LRT		0
170	KC_AB3	CG14431	-0.0932330577257072	0.649031322946095	1	pseudobulk	DESeq2	LRT		0
170	KC_AB3	CG14434	-1.60329424728781	0.177632541896628	1	pseudobulk	DESeq2	LRT		0
170	KC_AB3	CG14435	-1.42892340034595	0.154021746950298	1	pseudobulk	DESeq2	LRT		0
170	KC_AB3	CG14440	-0.0220873485683429	0.755703748034669	1	pseudobulk	DESeq2	LRT		0
170	KC_AB3	CG14441	1.95267092137636	1	1	pseudobulk	DESeq2	LRT		0
170	KC_AB3	CG14442	-0.325292372373408	0.40450892463123	1	pseudobulk	DESeq2	LRT		0
170	KC_AB3	CG14450	0.269354051384433	0.738650050168586	1	pseudobulk	DESeq2	LRT		0
170	KC_AB3	CG14459	2.62817135292692	0.54954870385793	1	pseudobulk	DESeq2	LRT		0
170	KC_AB3	CG14464	0.0676274146190372	0.628400004847922	1	pseudobulk	DESeq2	LRT		0

170	KC_AB3	CG14478	0.0594708833953574	0.75524923368413	1	pseudobulk	DESeq2	LRT		0
170	KC_AB3	CG14483	-0.446195868439551	0.547236502596298	1	pseudobulk	DESeq2	LRT		0
170	KC_AB3	CG14502	-0.61551279743889	0.673825799139955	1	pseudobulk	DESeq2	LRT		0
170	KC_AB3	CG14512	0.440578571731502	1	1	pseudobulk	DESeq2	LRT		0
170	KC_AB3	CG14535	-0.446954179850258	0.574605036961353	1	pseudobulk	DESeq2	LRT		0
170	KC_AB3	CG14543	-0.238060661747802	0.716853221665401	1	pseudobulk	DESeq2	LRT		0
170	KC_AB3	CG14544	0.632774104902944	1	1	pseudobulk	DESeq2	LRT		0
170	KC_AB3	CG14562	0.18330128845266	0.72060690164694	1	pseudobulk	DESeq2	LRT		0
170	KC_AB3	CG14563	-1.57123716123006	0.309494897701156	1	pseudobulk	DESeq2	LRT		0
170	KC_AB3	CG14589	0.632774104902944	1	1	pseudobulk	DESeq2	LRT		0
170	KC_AB3	CG14591	-0.178965713855249	0.614786472793853	1	pseudobulk	DESeq2	LRT		0
170	KC_AB3	CG14609	0.632774104902944	1	1	pseudobulk	DESeq2	LRT		0
170	KC_AB3	CG1461	0.632774104902944	1	1	pseudobulk	DESeq2	LRT		0
170	KC_AB3	CG14613	0.353103013381196	0.676268419183526	1	pseudobulk	DESeq2	LRT		0
170	KC_AB3	CG14615	1.95267092137636	1	1	pseudobulk	DESeq2	LRT		0
170	KC_AB3	CG14618	0.263495039620072	1	1	pseudobulk	DESeq2	LRT		0
170	KC_AB3	CG14621	0.317800582647856	0.691614583809659	1	pseudobulk	DESeq2	LRT		0
170	KC_AB3	CG1463	-1.40625109827745	0.234107655337364	1	pseudobulk	DESeq2	LRT		0
170	KC_AB3	CG14641	1.11020374211973	0.178525159486859	1	pseudobulk	DESeq2	LRT		0
170	KC_AB3	CG14646	-0.671322241244501	0.564467518419154	1	pseudobulk	DESeq2	LRT		0
170	KC_AB3	CG14647	0.449963293671406	0.762921929444907	1	pseudobulk	DESeq2	LRT		0
170	KC_AB3	CG14650	-1.47061801868006	0.235552441050247	1	pseudobulk	DESeq2	LRT		0
170	KC_AB3	CG14657	-1.18177296471571	0.223526106233395	1	pseudobulk	DESeq2	LRT		0
170	KC_AB3	CG14667	-0.21784418791863	0.743818979259508	1	pseudobulk	DESeq2	LRT		0
170	KC_AB3	CG14671	0.985125689142457	0.40275118100292	1	pseudobulk	DESeq2	LRT		0
170	KC_AB3	CG14683	2.56062641309819	0.317483369693272	1	pseudobulk	DESeq2	LRT		0
170	KC_AB3	CG14687	0.632774104902943	1	1	pseudobulk	DESeq2	LRT		0
170	KC_AB3	CG14692	0.632774104902944	1	1	pseudobulk	DESeq2	LRT		0
170	KC_AB3	CG14694	0.632774104902944	1	1	pseudobulk	DESeq2	LRT		0
170	KC_AB3	CG14701	2.19539902116932	0.526642538463226	1	pseudobulk	DESeq2	LRT		0
170	KC_AB3	CG14710	-0.120293723421006	0.768115705246508	1	pseudobulk	DESeq2	LRT		0
170	KC_AB3	CG14711	0.961446261994835	0.777066013571642	1	pseudobulk	DESeq2	LRT		0
170	KC_AB3	CG14712	0.272714256234976	1	1	pseudobulk	DESeq2	LRT		0
170	KC_AB3	CG14715	-0.354784143187371	0.559982965493059	1	pseudobulk	DESeq2	LRT		0
170	KC_AB3	CG14721	0.130020638491525	0.76823500365879	1	pseudobulk	DESeq2	LRT		0
170	KC_AB3	CG14722	-1.32058613967016	0.304766566001177	1	pseudobulk	DESeq2	LRT		0
170	KC_AB3	CG14757	-0.850062101308309	0.453477624495929	1	pseudobulk	DESeq2	LRT		0
170	KC_AB3	CG14762	-0.162472250471566	0.472823081765163	1	pseudobulk	DESeq2	LRT		0
170	KC_AB3	CG14764	-0.0879061017960969	0.494122206744635	1	pseudobulk	DESeq2	LRT		0
170	KC_AB3	CG14767	-0.470969511338412	0.25057793846117	1	pseudobulk	DESeq2	LRT		0
170	KC_AB3	CG14777	0.00633078653300548	0.82075260966876	1	pseudobulk	DESeq2	LRT		0
170	KC_AB3	CG14795	2.3149273646917	0.611236276445972	1	pseudobulk	DESeq2	LRT		0
170	KC_AB3	CG14797	0.632774104902944	1	1	pseudobulk	DESeq2	LRT		0
170	KC_AB3	CG14803	-0.302794075441951	0.851617925917851	1	pseudobulk	DESeq2	LRT		0
170	KC_AB3	CG14805	-0.0861210374158044	0.795337134956882	1	pseudobulk	DESeq2	LRT		0
170	KC_AB3	CG14806	-0.551549715924555	0.342934572880967	1	pseudobulk	DESeq2	LRT		0
170	KC_AB3	CG14810	1.95267092137636	1	1	pseudobulk	DESeq2	LRT		0
170	KC_AB3	CG14812	-0.514940711154507	0.469938633232268	1	pseudobulk	DESeq2	LRT		0
170	KC_AB3	CG14817	-0.40299543699518	0.408246355124909	1	pseudobulk	DESeq2	LRT		0
170	KC_AB3	CG14818	-0.0290002681166724	0.816371710622125	1	pseudobulk	DESeq2	LRT		0
170	KC_AB3	CG14820	1.95267092137636	1	1	pseudobulk	DESeq2	LRT		0
170	KC_AB3	CG14834	1.95267092137636	1	1	pseudobulk	DESeq2	LRT		0
170	KC_AB3	CG14853	-0.309550910600025	0.615559907580828	1	pseudobulk	DESeq2	LRT		0

170	KC_AB3	CG1486	-0.379244922806191	0.505259811321846	1	pseudobulk	DESeq2	LRT	0
170	KC_AB3	CG14868	0.299758434733622	0.561103867909214	1	pseudobulk	DESeq2	LRT	0
170	KC_AB3	CG14881	0.40850766128287	0.846518495087318	1	pseudobulk	DESeq2	LRT	0
170	KC_AB3	CG14882	-0.800304379643462	0.546567485783789	1	pseudobulk	DESeq2	LRT	0
170	KC_AB3	CG14883	-0.227214078846891	0.70165027689638	1	pseudobulk	DESeq2	LRT	0
170	KC_AB3	CG14894	-0.691854621698618	0.394526570973032	1	pseudobulk	DESeq2	LRT	0
170	KC_AB3	CG14903	-0.231661348032844	0.668274984453097	1	pseudobulk	DESeq2	LRT	0
170	KC_AB3	CG14906	-0.302218176137793	0.851957073307525	1	pseudobulk	DESeq2	LRT	0
170	KC_AB3	CG1492	-0.463955421386414	0.662248360131555	1	pseudobulk	DESeq2	LRT	0
170	KC_AB3	CG14937	1.95267092137636	1	1	pseudobulk	DESeq2	LRT	0
170	KC_AB3	CG14965	0.158199335652497	0.922848984931983	1	pseudobulk	DESeq2	LRT	0
170	KC_AB3	CG14966	-0.37164714611046	0.641552753756197	1	pseudobulk	DESeq2	LRT	0
170	KC_AB3	CG14969	-0.208348942260732	0.672193708306151	1	pseudobulk	DESeq2	LRT	0
170	KC_AB3	CG14971	0.40850766128287	0.846518495087318	1	pseudobulk	DESeq2	LRT	0
170	KC_AB3	CG14977	-0.254696261032022	0.606656683373289	1	pseudobulk	DESeq2	LRT	0
170	KC_AB3	CG14982	0.336928135774495	0.608050249702933	1	pseudobulk	DESeq2	LRT	0
170	KC_AB3	CG14984	2.64926057711297	0.482244576496623	1	pseudobulk	DESeq2	LRT	0
170	KC_AB3	CG14989	-1.18273365443304	0.505456699009851	1	pseudobulk	DESeq2	LRT	0
170	KC_AB3	CG14995	-0.721535836884894	0.488385123784693	1	pseudobulk	DESeq2	LRT	0
170	KC_AB3	CG14997	1.59359598433783	0.86377329553208	1	pseudobulk	DESeq2	LRT	0
170	KC_AB3	CG15011	0.759758178017103	1	1	pseudobulk	DESeq2	LRT	0
170	KC_AB3	CG15012	-0.301071331939631	0.546491444662786	1	pseudobulk	DESeq2	LRT	0
170	KC_AB3	CG15014	0.398057110078313	0.899247232904305	1	pseudobulk	DESeq2	LRT	0
170	KC_AB3	CG15019	-1.05793885599925	0.282119658112533	1	pseudobulk	DESeq2	LRT	0
170	KC_AB3	CG15027	-1.41621789235643	0.354222802318319	1	pseudobulk	DESeq2	LRT	0
170	KC_AB3	CG1504	0.161515868851812	1	1	pseudobulk	DESeq2	LRT	0
170	KC_AB3	CG15073	0.309898311549018	0.883425311467092	1	pseudobulk	DESeq2	LRT	0
170	KC_AB3	CG15084	0.0020939056309239	1	1	pseudobulk	DESeq2	LRT	0
170	KC_AB3	CG15097	-0.409384557539862	0.33262721926173	1	pseudobulk	DESeq2	LRT	0
170	KC_AB3	CG15098	-1.07854013385922	0.429115506043122	1	pseudobulk	DESeq2	LRT	0
170	KC_AB3	CG15099	0.670548920892758	0.446619768228803	1	pseudobulk	DESeq2	LRT	0
170	KC_AB3	CG15107	-0.277673757398893	0.822254681221855	1	pseudobulk	DESeq2	LRT	0
170	KC_AB3	CG15109	0.632774104902944	1	1	pseudobulk	DESeq2	LRT	0
170	KC_AB3	CG15111	-0.0015190806058047	0.692426893001474	1	pseudobulk	DESeq2	LRT	0
170	KC_AB3	CG15118	-0.347058079715588	0.628693730224105	1	pseudobulk	DESeq2	LRT	0
171	KC_AB3	CG1513	-0.215219896178915	0.491952992776848	1	pseudobulk	DESeq2	LRT	0
171	KC_AB3	CG15141	0.973826137036694	0.42504968205626	1	pseudobulk	DESeq2	LRT	0
171	KC_AB3	CG15160	-0.412055202951741	0.639197061809653	1	pseudobulk	DESeq2	LRT	0
171	KC_AB3	CG15168	0.400878275935616	0.525581470994295	1	pseudobulk	DESeq2	LRT	0
171	KC_AB3	CG15201	-0.292959248872863	0.698040547927549	1	pseudobulk	DESeq2	LRT	0
171	KC_AB3	CG15202	-1.31222595516382	0.477398539794385	1	pseudobulk	DESeq2	LRT	0
171	KC_AB3	CG15209	-0.295010914744155	0.765520559953197	1	pseudobulk	DESeq2	LRT	0
171	KC_AB3	CG1523	-0.584383604364157	0.636344708343529	1	pseudobulk	DESeq2	LRT	0
171	KC_AB3	CG15237	0.55864358073981	0.415435018581144	1	pseudobulk	DESeq2	LRT	0
171	KC_AB3	CG15270	0.552415437285346	0.699215006594431	1	pseudobulk	DESeq2	LRT	0
171	KC_AB3	CG1529	1.95267092137636	1	1	pseudobulk	DESeq2	LRT	0
171	KC_AB3	CG15309	-0.639162977313316	0.254135073262679	1	pseudobulk	DESeq2	LRT	0
171	KC_AB3	CG15312	0.694626215104327	0.14735972567108	1	pseudobulk	DESeq2	LRT	0
171	KC_AB3	CG15317	-1.21905386764267	0.479301953770297	1	pseudobulk	DESeq2	LRT	0
171	KC_AB3	CG1532	0.160522996318193	0.898559221213049	1	pseudobulk	DESeq2	LRT	0
171	KC_AB3	CG15332	1.22707713470494	1	1	pseudobulk	DESeq2	LRT	0
171	KC_AB3	CG15356	0.258301758416754	0.777032992501228	1	pseudobulk	DESeq2	LRT	0
171	KC_AB3	CG15385	0.454277236228061	0.57669587340752	1	pseudobulk	DESeq2	LRT	0

171	KC_AB3	CG15387	-0.692704223109104	0.504706155948768	1	pseudobulk	DESeq2	LRT	0
171	KC_AB3	CG15390	1.30772724211669	0.760828999410225	1	pseudobulk	DESeq2	LRT	0
171	KC_AB3	CG1542	2.83964872000554	0.240684919262511	1	pseudobulk	DESeq2	LRT	0
171	KC_AB3	CG15431	-0.336622690616646	0.278600424536762	1	pseudobulk	DESeq2	LRT	0
171	KC_AB3	CG15432	-0.338065273089582	0.581260025925106	1	pseudobulk	DESeq2	LRT	0
171	KC_AB3	CG15439	-0.357607384325327	0.790157014682946	1	pseudobulk	DESeq2	LRT	0
171	KC_AB3	CG15443	0.225119131900571	0.695530405325209	1	pseudobulk	DESeq2	LRT	0
171	KC_AB3	CG15446	1.95267092137636	1	1	pseudobulk	DESeq2	LRT	0
171	KC_AB3	CG15449	0.12205886208975	0.952632742998906	1	pseudobulk	DESeq2	LRT	0
171	KC_AB3	CG1545	-0.6797894546527	0.737860377211966	1	pseudobulk	DESeq2	LRT	0
171	KC_AB3	CG15456	0.227359795751061	0.571582503926894	1	pseudobulk	DESeq2	LRT	0
171	KC_AB3	CG15473	0.161515868851812	1	1	pseudobulk	DESeq2	LRT	0
171	KC_AB3	CG15514	-0.134656696070653	0.640306112104452	1	pseudobulk	DESeq2	LRT	0
171	KC_AB3	CG1552	-1.19352368870283	0.315711333431014	1	pseudobulk	DESeq2	LRT	0
171	KC_AB3	CG15522	2.88619786524384	0.39214769278579	1	pseudobulk	DESeq2	LRT	0
171	KC_AB3	CG15525	-1.12781261279613	0.378869712099569	1	pseudobulk	DESeq2	LRT	0
171	KC_AB3	CG15535	0.621136537657909	0.276905921883901	1	pseudobulk	DESeq2	LRT	0
171	KC_AB3	CG15536	3.28481501080097	0.219670351486969	1	pseudobulk	DESeq2	LRT	0
171	KC_AB3	CG15537	0.632774104902943	1	1	pseudobulk	DESeq2	LRT	0
171	KC_AB3	CG15539	2.3149273646917	0.611236276445972	1	pseudobulk	DESeq2	LRT	0
171	KC_AB3	CG15547	-0.560477492866218	0.784070523163114	1	pseudobulk	DESeq2	LRT	0
171	KC_AB3	CG15561	2.58766452360674	0.057129981722305	1	pseudobulk	DESeq2	LRT	0
171	KC_AB3	CG15579	0.632774104902943	1	1	pseudobulk	DESeq2	LRT	0
171	KC_AB3	CG15601	0.28941340686042	0.909432039790922	1	pseudobulk	DESeq2	LRT	0
171	KC_AB3	CG15602	0.733248440000269	0.728526548402541	1	pseudobulk	DESeq2	LRT	0
171	KC_AB3	CG15614	0.632774104902944	1	1	pseudobulk	DESeq2	LRT	0
171	KC_AB3	CG15628	0.0549045754109176	0.700823770929089	1	pseudobulk	DESeq2	LRT	0
171	KC_AB3	CG15642	-0.814879100666134	0.432124659079958	1	pseudobulk	DESeq2	LRT	0
171	KC_AB3	CG15643	1.03912517029793	0.277750016109625	1	pseudobulk	DESeq2	LRT	0
171	KC_AB3	CG15651	-0.155325307394222	0.627490218245006	1	pseudobulk	DESeq2	LRT	0
171	KC_AB3	CG15653	-0.54519205879466	0.637823061221008	1	pseudobulk	DESeq2	LRT	0
171	KC_AB3	CG15706	1.23133864869565	1	1	pseudobulk	DESeq2	LRT	0
171	KC_AB3	CG15715	0.835365916415185	0.259876347660944	1	pseudobulk	DESeq2	LRT	0
171	KC_AB3	CG15719	1.95267092137636	1	1	pseudobulk	DESeq2	LRT	0
171	KC_AB3	CG1572	1.95267092137636	1	1	pseudobulk	DESeq2	LRT	0
171	KC_AB3	CG15725	0.632774104902944	1	1	pseudobulk	DESeq2	LRT	0
171	KC_AB3	CG15743	-0.352320779585994	0.250758409735465	1	pseudobulk	DESeq2	LRT	0
171	KC_AB3	CG15744	2.3149273646917	0.611236276445972	1	pseudobulk	DESeq2	LRT	0
171	KC_AB3	CG15747	-0.152459412153519	0.717411430476239	1	pseudobulk	DESeq2	LRT	0
171	KC_AB3	CG1575	0.632774104902944	1	1	pseudobulk	DESeq2	LRT	0
171	KC_AB3	CG15760	0.0480576933399466	0.450677300477663	1	pseudobulk	DESeq2	LRT	0
171	KC_AB3	CG15765	-0.499375858986611	0.168215077672814	1	pseudobulk	DESeq2	LRT	0
171	KC_AB3	CG15771	0.675516197976904	0.40480038602444	1	pseudobulk	DESeq2	LRT	0
171	KC_AB3	CG15772	-0.109759573779601	0.674207903614131	1	pseudobulk	DESeq2	LRT	0
171	KC_AB3	CG1578	0.361139927182469	0.496604191159154	1	pseudobulk	DESeq2	LRT	0
171	KC_AB3	CG15812	1.70400726959938	0.298823946899066	1	pseudobulk	DESeq2	LRT	0
171	KC_AB3	CG15814	-0.323951008010639	0.655168681619063	1	pseudobulk	DESeq2	LRT	0
171	KC_AB3	CG1582	0.190037944770009	1	1	pseudobulk	DESeq2	LRT	0
171	KC_AB3	CG15877	0.971815092004695	0.431099926099092	1	pseudobulk	DESeq2	LRT	0
171	KC_AB3	CG15890	1.95267092137636	1	1	pseudobulk	DESeq2	LRT	0
171	KC_AB3	CG15891	0.11649965671703	0.647756066580148	1	pseudobulk	DESeq2	LRT	0
171	KC_AB3	CG15894	-0.903137655480761	0.0324937212058572	1	pseudobulk	DESeq2	LRT	0
171	KC_AB3	CG15908	-0.910045228482974	0.433396985114417	1	pseudobulk	DESeq2	LRT	0

171	KC_AB3	CG15912	0.178895417472062	0.85211653540939	1	pseudobulk	DESeq2	LRT	0
171	KC_AB3	CG15916	0.459147960726468	0.274108115798136	1	pseudobulk	DESeq2	LRT	0
171	KC_AB3	CG15922	-0.073005151732119	0.642581366901993	1	pseudobulk	DESeq2	LRT	0
171	KC_AB3	CG15923	1.95267092137636	1	1	pseudobulk	DESeq2	LRT	0
171	KC_AB3	CG1598	-0.112478921209355	0.775076711711196	1	pseudobulk	DESeq2	LRT	0
171	KC_AB3	CG1602	-0.185594513059781	1	1	pseudobulk	DESeq2	LRT	0
171	KC_AB3	CG1603	0.790010899828778	1	1	pseudobulk	DESeq2	LRT	0
171	KC_AB3	CG1607	-0.556263725866787	0.543236337849645	1	pseudobulk	DESeq2	LRT	0
171	KC_AB3	CG1620	-0.271442528140787	0.675966923603257	1	pseudobulk	DESeq2	LRT	0
171	KC_AB3	CG1622	0.172274803242538	0.643288245523298	1	pseudobulk	DESeq2	LRT	0
171	KC_AB3	CG1635	-0.263698647348123	0.702632944676525	1	pseudobulk	DESeq2	LRT	0
171	KC_AB3	CG1636	-0.292309537611238	0.483591469390403	1	pseudobulk	DESeq2	LRT	0
171	KC_AB3	CG1637	0.358823137024954	0.525155703737891	1	pseudobulk	DESeq2	LRT	0
171	KC_AB3	CG1640	0.215191965879687	0.686933738835525	1	pseudobulk	DESeq2	LRT	0
171	KC_AB3	CG1646	-0.162538556378477	0.625812587478447	1	pseudobulk	DESeq2	LRT	0
171	KC_AB3	CG1647	0.415245410763795	0.791987892114859	1	pseudobulk	DESeq2	LRT	0
171	KC_AB3	CG1648	-1.14096654355616	0.0671297513928088	1	pseudobulk	DESeq2	LRT	0
171	KC_AB3	CG1662	0.730229754789315	1	1	pseudobulk	DESeq2	LRT	0
171	KC_AB3	CG1663	0.567057586408852	0.841964833167075	1	pseudobulk	DESeq2	LRT	0
171	KC_AB3	CG16711	0.060285799359057	0.788774329570324	1	pseudobulk	DESeq2	LRT	0
171	KC_AB3	CG16717	-0.255079748909389	0.651548517549133	1	pseudobulk	DESeq2	LRT	0
171	KC_AB3	CG16721	-0.498152370024956	0.561271504911816	1	pseudobulk	DESeq2	LRT	0
171	KC_AB3	CG1674	0.632774104902944	1	1	pseudobulk	DESeq2	LRT	0
171	KC_AB3	CG16753	-0.176487229948032	0.83049434310875	1	pseudobulk	DESeq2	LRT	0
171	KC_AB3	CG1677	-0.354265597588684	0.388767262110406	1	pseudobulk	DESeq2	LRT	0
171	KC_AB3	CG16779	-0.0373326116300345	0.885657603176628	1	pseudobulk	DESeq2	LRT	0
171	KC_AB3	CG16787	0.0497129240136997	0.52175525268568	1	pseudobulk	DESeq2	LRT	0
171	KC_AB3	CG16789	0.632774104902943	1	1	pseudobulk	DESeq2	LRT	0
171	KC_AB3	CG16790	1.95267092137636	1	1	pseudobulk	DESeq2	LRT	0
172	KC_AB3	CG16791	-0.0029256203483938	0.728857964557993	1	pseudobulk	DESeq2	LRT	0
172	KC_AB3	CG16798	0.593667095203339	1	1	pseudobulk	DESeq2	LRT	0
172	KC_AB3	CG16812	0.0491369726087927	0.613354554444424	1	pseudobulk	DESeq2	LRT	0
172	KC_AB3	CG16854	-0.423379057312453	0.770809804625243	1	pseudobulk	DESeq2	LRT	0
172	KC_AB3	CG16863	0.161515868851812	1	1	pseudobulk	DESeq2	LRT	0
172	KC_AB3	CG16865	0.263495039620072	1	1	pseudobulk	DESeq2	LRT	0
172	KC_AB3	CG16868	-0.838167990006469	0.55551573898003	1	pseudobulk	DESeq2	LRT	0
172	KC_AB3	CG1688	-1.32323489164364	0.171916019107388	1	pseudobulk	DESeq2	LRT	0
172	KC_AB3	CG16890	-0.184744780581018	1	1	pseudobulk	DESeq2	LRT	0
172	KC_AB3	CG16896	1.4936284640642	0.543447776174216	1	pseudobulk	DESeq2	LRT	0
172	KC_AB3	CG16903	0.626700690459298	0.430091317873522	1	pseudobulk	DESeq2	LRT	0
172	KC_AB3	CG16935	0.602184372028392	0.474538911188358	1	pseudobulk	DESeq2	LRT	0
172	KC_AB3	CG1695	-0.586119157670662	0.299214289198302	1	pseudobulk	DESeq2	LRT	0
172	KC_AB3	CG16952	0.0230497640954334	0.835807023588562	1	pseudobulk	DESeq2	LRT	0
172	KC_AB3	CG16953	0.327638228987846	0.579227837627878	1	pseudobulk	DESeq2	LRT	0
172	KC_AB3	CG16972	-0.262933615016695	0.779276997073526	1	pseudobulk	DESeq2	LRT	0
172	KC_AB3	CG16974	0.046906392887572	0.855977881782865	1	pseudobulk	DESeq2	LRT	0
172	KC_AB3	CG16979	0.161515868851812	1	1	pseudobulk	DESeq2	LRT	0
172	KC_AB3	CG16984	1.95267092137636	1	1	pseudobulk	DESeq2	LRT	0
172	KC_AB3	CG16985	1.30732999830804	0.760961512337506	1	pseudobulk	DESeq2	LRT	0
172	KC_AB3	CG16986	0.418930758491869	0.778743772945424	1	pseudobulk	DESeq2	LRT	0
172	KC_AB3	CG16989	1.00428465509825	0.543457431242283	1	pseudobulk	DESeq2	LRT	0
172	KC_AB3	CG17002	-1.14790722909473	0.369877980673102	1	pseudobulk	DESeq2	LRT	0
172	KC_AB3	CG17019	0.652534459439716	0.396706938657009	1	pseudobulk	DESeq2	LRT	0

172	KC_AB3	CG17026	1.9787018141276	0.200917775347308	1	pseudobulk	DESeq2	LRT	0
172	KC_AB3	CG17029	1.06775633057668	0.643950806051851	1	pseudobulk	DESeq2	LRT	0
172	KC_AB3	CG1703	-0.266950836216236	0.663083629984173	1	pseudobulk	DESeq2	LRT	0
172	KC_AB3	CG17030	0.632774104902944	1	1	pseudobulk	DESeq2	LRT	0
172	KC_AB3	CG17059	0.0208099653460467	0.698688358958746	1	pseudobulk	DESeq2	LRT	0
172	KC_AB3	CG17065	-0.0443337079499378	0.601187011548113	1	pseudobulk	DESeq2	LRT	0
172	KC_AB3	CG17068	0.905162277928588	0.863434859051401	1	pseudobulk	DESeq2	LRT	0
172	KC_AB3	CG17075	-0.580409176271756	0.501553192184206	1	pseudobulk	DESeq2	LRT	0
172	KC_AB3	CG17078	0.132142501558205	0.73395339726029	1	pseudobulk	DESeq2	LRT	0
172	KC_AB3	CG17111	0.0478638348083297	1	1	pseudobulk	DESeq2	LRT	0
172	KC_AB3	CG17124	-0.446660548666667	0.198201772446283	1	pseudobulk	DESeq2	LRT	0
172	KC_AB3	CG17141	-0.97272493938239	0.58046351237677	1	pseudobulk	DESeq2	LRT	0
172	KC_AB3	CG17162	0.438392768241677	1	1	pseudobulk	DESeq2	LRT	0
172	KC_AB3	CG17163	-0.534812572367812	0.504002441501655	1	pseudobulk	DESeq2	LRT	0
172	KC_AB3	CG17167	0.632774104902943	1	1	pseudobulk	DESeq2	LRT	0
172	KC_AB3	CG17168	0.155168424613209	0.845562655683241	1	pseudobulk	DESeq2	LRT	0
172	KC_AB3	CG17169	2.62817135292692	0.54954870385793	1	pseudobulk	DESeq2	LRT	0
172	KC_AB3	CG17186	2.3149273646917	0.611236276445972	1	pseudobulk	DESeq2	LRT	0
172	KC_AB3	CG17187	-0.120528428548273	0.914416877354382	1	pseudobulk	DESeq2	LRT	0
172	KC_AB3	CG17189	-1.29893225424569	0.497838359546512	1	pseudobulk	DESeq2	LRT	0
172	KC_AB3	CG17202	0.320387148227339	0.458604507246557	1	pseudobulk	DESeq2	LRT	0
172	KC_AB3	CG17221	1.93443687997187	0.65822970246154	1	pseudobulk	DESeq2	LRT	0
172	KC_AB3	CG17230	0.661329417287926	0.561190991919634	1	pseudobulk	DESeq2	LRT	0
172	KC_AB3	CG17233	-1.013357059685	0.260769541541651	1	pseudobulk	DESeq2	LRT	0
172	KC_AB3	CG17249	-0.908452148604833	0.450711808564713	1	pseudobulk	DESeq2	LRT	0
172	KC_AB3	CG17260	-1.18273365443304	0.505456699009851	1	pseudobulk	DESeq2	LRT	0
172	KC_AB3	CG17266	1.48461250509522	0.557313588000571	1	pseudobulk	DESeq2	LRT	0
172	KC_AB3	CG17270	-0.341217889954974	0.605551495237616	1	pseudobulk	DESeq2	LRT	0
172	KC_AB3	CG17271	-0.3595110618865	0.667495360352661	1	pseudobulk	DESeq2	LRT	0
172	KC_AB3	CG17272	-0.312077396904282	0.394618940890397	1	pseudobulk	DESeq2	LRT	0
172	KC_AB3	CG17279	0.632774104902944	1	1	pseudobulk	DESeq2	LRT	0
172	KC_AB3	CG17294	0.562067571493137	0.47158685307637	1	pseudobulk	DESeq2	LRT	0
172	KC_AB3	CG17321	-0.0678246832738073	0.666441215754308	1	pseudobulk	DESeq2	LRT	0
172	KC_AB3	CG17328	-1.21594498872333	0.358790227836571	1	pseudobulk	DESeq2	LRT	0
172	KC_AB3	CG17343	-0.658089723696325	0.521470354219685	1	pseudobulk	DESeq2	LRT	0
172	KC_AB3	CG17359	1.24080369477374	0.455733013068078	1	pseudobulk	DESeq2	LRT	0
172	KC_AB3	CG17361	-0.851238408205157	0.504383074933176	1	pseudobulk	DESeq2	LRT	0
172	KC_AB3	CG17364	-0.526226324772707	0.470019202999314	1	pseudobulk	DESeq2	LRT	0
172	KC_AB3	CG1737	0.00325612148592230	0.818913051468189	1	pseudobulk	DESeq2	LRT	0
172	KC_AB3	CG17378	-0.274242359635588	0.620315489090416	1	pseudobulk	DESeq2	LRT	0
172	KC_AB3	CG1738	-0.90313917091705	0.461855814846249	1	pseudobulk	DESeq2	LRT	0
172	KC_AB3	CG17385	-0.0619843743896591	0.892366414096876	1	pseudobulk	DESeq2	LRT	0
172	KC_AB3	CG17454	-0.0726490401178968	0.524328979974245	1	pseudobulk	DESeq2	LRT	0
172	KC_AB3	CG17486	-0.322195278040081	0.539693563114059	1	pseudobulk	DESeq2	LRT	0
172	KC_AB3	CG17493	0.114172799649364	0.756583453201071	1	pseudobulk	DESeq2	LRT	0
172	KC_AB3	CG1750	-0.991086464329007	0.44907438838533	1	pseudobulk	DESeq2	LRT	0
172	KC_AB3	CG17508	-0.327100375437592	0.533212778917636	1	pseudobulk	DESeq2	LRT	0
172	KC_AB3	CG17528	0.507195387334292	0.512412996647531	1	pseudobulk	DESeq2	LRT	0
172	KC_AB3	CG17544	-0.0327300486906476	1	1	pseudobulk	DESeq2	LRT	0
172	KC_AB3	CG17549	1.95267092137636	1	1	pseudobulk	DESeq2	LRT	0
172	KC_AB3	CG17556	-1.34643677058769	0.246475020525689	1	pseudobulk	DESeq2	LRT	0
172	KC_AB3	CG17565	-1.67960979830629	0.120387075080264	1	pseudobulk	DESeq2	LRT	0
172	KC_AB3	CG17568	-0.044519754582785	0.917596762614691	1	pseudobulk	DESeq2	LRT	0

172	KC_AB3	CG17574	0.0581715578916655	0.799594457130519	1	pseudobulk	DESeq2	LRT	0
172	KC_AB3	CG17593	-0.165990699993745	0.601895853790255	1	pseudobulk	DESeq2	LRT	0
172	KC_AB3	CG17598	0.52454872872563	0.669451438054626	1	pseudobulk	DESeq2	LRT	0
172	KC_AB3	CG17600	1.00870819599921	0.468219703145863	1	pseudobulk	DESeq2	LRT	0
172	KC_AB3	CG17612	0.669936905937546	0.294299517562296	1	pseudobulk	DESeq2	LRT	0
172	KC_AB3	CG17637	0.632774104902944	1	1	pseudobulk	DESeq2	LRT	0
172	KC_AB3	CG1764	-0.368617351648105	0.573715765865139	1	pseudobulk	DESeq2	LRT	0
172	KC_AB3	CG17646	0.632774104902944	1	1	pseudobulk	DESeq2	LRT	0
172	KC_AB3	CG17652	-0.535377951314043	0.579928890476657	1	pseudobulk	DESeq2	LRT	0
172	KC_AB3	CG17660	-0.243870325364918	0.744013434604075	1	pseudobulk	DESeq2	LRT	0
172	KC_AB3	CG17669	-0.583057444465563	0.678110339883318	1	pseudobulk	DESeq2	LRT	0
172	KC_AB3	CG17683	0.401696364439027	0.576726803319783	1	pseudobulk	DESeq2	LRT	0
172	KC_AB3	CG17684	-0.536005838474323	0.123258041891968	1	pseudobulk	DESeq2	LRT	0
172	KC_AB3	CG17691	-0.653376239202093	0.553486564290712	1	pseudobulk	DESeq2	LRT	0
172	KC_AB3	CG17698	-0.2075418682615	0.518825186123678	1	pseudobulk	DESeq2	LRT	0
172	KC_AB3	CG17712	2.33823942106448	0.0953828089854053	1	pseudobulk	DESeq2	LRT	0
172	KC_AB3	CG17715	-0.109979700786429	0.757717223494868	1	pseudobulk	DESeq2	LRT	0
172	KC_AB3	CG17716	0.632774104902944	1	1	pseudobulk	DESeq2	LRT	0
172	KC_AB3	CG17721	1.9156821579595	0.439653312517014	1	pseudobulk	DESeq2	LRT	0
172	KC_AB3	CG17724	0.124910067239709	0.600291647693791	1	pseudobulk	DESeq2	LRT	0
172	KC_AB3	CG17726	-0.931079887531931	0.582278950522693	1	pseudobulk	DESeq2	LRT	0
172	KC_AB3	CG17734	-0.411133756826876	0.367391170832167	1	pseudobulk	DESeq2	LRT	0
172	KC_AB3	CG17739	1.95267092137636	1	1	pseudobulk	DESeq2	LRT	0
173	KC_AB3	CG17746	0.922171622420025	0.450544667305007	1	pseudobulk	DESeq2	LRT	0
173	KC_AB3	CG17754	0.935500440483277	0.431445839926249	1	pseudobulk	DESeq2	LRT	0
173	KC_AB3	CG17760	0.632774104902944	1	1	pseudobulk	DESeq2	LRT	0
173	KC_AB3	CG17765	0.0161779044145034	0.822557885349564	1	pseudobulk	DESeq2	LRT	0
173	KC_AB3	CG17776	-0.445549330231167	0.449075794573634	1	pseudobulk	DESeq2	LRT	0
173	KC_AB3	CG17778	-0.468331557135828	0.480283756914072	1	pseudobulk	DESeq2	LRT	0
173	KC_AB3	CG17782	0.632774104902944	1	1	pseudobulk	DESeq2	LRT	0
173	KC_AB3	CG17786	3.59085358380424	0.129916963966056	1	pseudobulk	DESeq2	LRT	0
173	KC_AB3	CG17802	0.518828014541643	0.842410901216243	1	pseudobulk	DESeq2	LRT	0
173	KC_AB3	CG17803	-0.931079887531931	0.582278950522693	1	pseudobulk	DESeq2	LRT	0
173	KC_AB3	CG17806	0.414412066932177	1	1	pseudobulk	DESeq2	LRT	0
173	KC_AB3	CG17807	0.6229941287632	1	1	pseudobulk	DESeq2	LRT	0
173	KC_AB3	CG17816	-0.117919520643292	0.568828242287962	1	pseudobulk	DESeq2	LRT	0
173	KC_AB3	CG17829	-0.40463871694335	0.635343377210531	1	pseudobulk	DESeq2	LRT	0
173	KC_AB3	CG17839	0.920642459949693	0.463172053506259	1	pseudobulk	DESeq2	LRT	0
173	KC_AB3	CG17841	-0.164232363091661	0.548886762693823	1	pseudobulk	DESeq2	LRT	0
173	KC_AB3	CG1785	2.12302691045992	0.658751388073158	1	pseudobulk	DESeq2	LRT	0
173	KC_AB3	CG17883	-0.457543382008328	0.364151505803043	1	pseudobulk	DESeq2	LRT	0
173	KC_AB3	CG1789	-0.573150413219913	0.567844859145405	1	pseudobulk	DESeq2	LRT	0
173	KC_AB3	CG17896	-0.399671897798429	0.965137949353374	1	pseudobulk	DESeq2	LRT	0
173	KC_AB3	CG17904	-0.756334224305331	0.433768621191181	1	pseudobulk	DESeq2	LRT	0
173	KC_AB3	CG1792	0.0457105858314303	0.806386167399349	1	pseudobulk	DESeq2	LRT	0
173	KC_AB3	CG17931	-0.520369424381307	0.392432086949296	1	pseudobulk	DESeq2	LRT	0
173	KC_AB3	CG17977	0.206412229540432	0.767006271849778	1	pseudobulk	DESeq2	LRT	0
173	KC_AB3	CG17982	0.053197396997799	1	1	pseudobulk	DESeq2	LRT	0
173	KC_AB3	CG17985	1.25271944562072	0.436140925059487	1	pseudobulk	DESeq2	LRT	0
173	KC_AB3	CG17996	-0.437776622458925	0.491986550334127	1	pseudobulk	DESeq2	LRT	0
173	KC_AB3	CG18004	-0.605115802663449	0.505190439929926	1	pseudobulk	DESeq2	LRT	0
173	KC_AB3	CG18048	0.281180211939512	1	1	pseudobulk	DESeq2	LRT	0
173	KC_AB3	CG18067	3.2740078253729	0.271992502367972	1	pseudobulk	DESeq2	LRT	0

173	KC_AB3	CG18081	0.624362593130619	0.534806993462221	1	pseudobulk	DESeq2	LRT	0
173	KC_AB3	CG1812	2.84131774307863	0.219167761053558	1	pseudobulk	DESeq2	LRT	0
173	KC_AB3	CG18135	-0.0081517506682789	0.702899189933449	1	pseudobulk	DESeq2	LRT	0
173	KC_AB3	CG1814	-0.280344767381296	0.489628261229864	1	pseudobulk	DESeq2	LRT	0
173	KC_AB3	CG1815	-0.186177642634338	0.805829253893955	1	pseudobulk	DESeq2	LRT	0
173	KC_AB3	CG18178	-0.817737327496598	0.493125500729708	1	pseudobulk	DESeq2	LRT	0
173	KC_AB3	CG1824	0.121951644622358	0.952673032511186	1	pseudobulk	DESeq2	LRT	0
173	KC_AB3	CG18259	0.619427358442613	0.87913278397031	1	pseudobulk	DESeq2	LRT	0
173	KC_AB3	CG18262	-0.836692461963401	0.536881793733203	1	pseudobulk	DESeq2	LRT	0
173	KC_AB3	CG18265	-0.939428634062326	0.213590730693682	1	pseudobulk	DESeq2	LRT	0
173	KC_AB3	CG1827	1.59359598433783	0.86377329553208	1	pseudobulk	DESeq2	LRT	0
173	KC_AB3	CG18273	-0.667766866554716	0.767102143892226	1	pseudobulk	DESeq2	LRT	0
173	KC_AB3	CG18281	0.632774104902944	1	1	pseudobulk	DESeq2	LRT	0
173	KC_AB3	CG18324	1.74936205277208	0.121629999394243	1	pseudobulk	DESeq2	LRT	0
173	KC_AB3	CG18327	1.95267092137636	1	1	pseudobulk	DESeq2	LRT	0
173	KC_AB3	CG18335	2.62817135292692	0.54954870385793	1	pseudobulk	DESeq2	LRT	0
173	KC_AB3	CG1840	0.401707351387074	0.603361983828192	1	pseudobulk	DESeq2	LRT	0
173	KC_AB3	CG18428	0.167787607173801	0.816041603803586	1	pseudobulk	DESeq2	LRT	0
173	KC_AB3	CG18467	0.220236734116472	0.560079512086936	1	pseudobulk	DESeq2	LRT	0
173	KC_AB3	CG1847	0.556859692590382	0.475098417477768	1	pseudobulk	DESeq2	LRT	0
173	KC_AB3	CG18476	0.0542213283300749	0.937446228327618	1	pseudobulk	DESeq2	LRT	0
173	KC_AB3	CG18508	0.190912139291121	0.616630797751482	1	pseudobulk	DESeq2	LRT	0
173	KC_AB3	CG18528	1.29264301881215	0.740627414289582	1	pseudobulk	DESeq2	LRT	0
173	KC_AB3	CG18549	0.192606993365598	0.826820815081025	1	pseudobulk	DESeq2	LRT	0
173	KC_AB3	CG18596	0.263237126093928	1	1	pseudobulk	DESeq2	LRT	0
173	KC_AB3	CG18598	0.594117478581602	1	1	pseudobulk	DESeq2	LRT	0
173	KC_AB3	CG18600	1.59575615951652	0.886898266337347	1	pseudobulk	DESeq2	LRT	0
173	KC_AB3	CG18628	-0.6797894546527	0.737860377211966	1	pseudobulk	DESeq2	LRT	0
173	KC_AB3	CG18636	1.95267092137636	1	1	pseudobulk	DESeq2	LRT	0
173	KC_AB3	CG18659	-0.219734640091928	0.633841377706045	1	pseudobulk	DESeq2	LRT	0
173	KC_AB3	CG18661	0.295747261864208	0.800393531838992	1	pseudobulk	DESeq2	LRT	0
173	KC_AB3	CG18675	2.19269259199673	0.500800656633403	1	pseudobulk	DESeq2	LRT	0
173	KC_AB3	CG18731	0.216685152057129	0.726945642348747	1	pseudobulk	DESeq2	LRT	0
173	KC_AB3	CG18764	-0.400046519007236	0.964129537865033	1	pseudobulk	DESeq2	LRT	0
173	KC_AB3	CG18766	-0.0199632993815967	0.631701050876708	1	pseudobulk	DESeq2	LRT	0
173	KC_AB3	CG18787	2.3149273646917	0.611236276445972	1	pseudobulk	DESeq2	LRT	0
173	KC_AB3	CG18789	1.95267092137636	1	1	pseudobulk	DESeq2	LRT	0
173	KC_AB3	CG18809	-0.404223691134559	0.695596630421348	1	pseudobulk	DESeq2	LRT	0
173	KC_AB3	CG18815	-0.0241092582356948	0.783586409509897	1	pseudobulk	DESeq2	LRT	0
173	KC_AB3	CG1882	-0.604707194004345	0.444695409108475	1	pseudobulk	DESeq2	LRT	0
173	KC_AB3	CG18858	3.07272693214569	0.238940395988569	1	pseudobulk	DESeq2	LRT	0
173	KC_AB3	CG18870	-1.84747171445036	0.202439450877248	1	pseudobulk	DESeq2	LRT	0
173	KC_AB3	CG1888	0.906903073936767	0.4377217435346	1	pseudobulk	DESeq2	LRT	0
173	KC_AB3	CG1890	0.427124437953525	0.866081845538235	1	pseudobulk	DESeq2	LRT	0
173	KC_AB3	CG1896	0.708220363202946	0.460788822683184	1	pseudobulk	DESeq2	LRT	0
173	KC_AB3	CG1902	0.758379315862171	0.479850565797189	1	pseudobulk	DESeq2	LRT	0
173	KC_AB3	CG1907	-0.386536885158196	0.552902010786584	1	pseudobulk	DESeq2	LRT	0
173	KC_AB3	CG1909	-0.312725128216625	0.318296164134534	1	pseudobulk	DESeq2	LRT	0
173	KC_AB3	CG1910	-0.0536245402987231	0.587789322255417	1	pseudobulk	DESeq2	LRT	0
173	KC_AB3	CG1927	-0.111283422557462	0.725776663038531	1	pseudobulk	DESeq2	LRT	0
173	KC_AB3	CG1941	1.95267092137636	1	1	pseudobulk	DESeq2	LRT	0
173	KC_AB3	CG1943	0.153464971645155	0.648084570316528	1	pseudobulk	DESeq2	LRT	0
173	KC_AB3	CG1951	-0.77991682801454	0.444908601443947	1	pseudobulk	DESeq2	LRT	0

173	KC_AB3	CG1965	-0.134282605588538	0.835720789165189	1	pseudobulk	DESeq2	LRT		0
173	KC_AB3	CG1983	0.941308804896449	0.416471446873102	1	pseudobulk	DESeq2	LRT		0
173	KC_AB3	CG1998	-0.148411839540142	0.580297033793837	1	pseudobulk	DESeq2	LRT		0
173	KC_AB3	CG2003	0.0025701398975595	1	1	pseudobulk	DESeq2	LRT		0
173	KC_AB3	CG2006	1.93443109363625	0.658150581914386	1	pseudobulk	DESeq2	LRT		0
173	KC_AB3	CG2017	-0.350378393289556	0.478985931295515	1	pseudobulk	DESeq2	LRT		0
173	KC_AB3	CG2021	0.312516267329773	0.633677195235915	1	pseudobulk	DESeq2	LRT		0
173	KC_AB3	CG2023	-0.562094181704424	0.653961950305913	1	pseudobulk	DESeq2	LRT		0
173	KC_AB3	CG2034	-0.606729092054646	0.619603098811507	1	pseudobulk	DESeq2	LRT		0
173	KC_AB3	CG2059	0.152349458991939	0.713588467813157	1	pseudobulk	DESeq2	LRT		0
173	KC_AB3	CG2061	-0.799642152025136	0.323813947628976	1	pseudobulk	DESeq2	LRT		0
173	KC_AB3	CG2063	-1.33099102455312	0.17450419051733	1	pseudobulk	DESeq2	LRT		0
173	KC_AB3	CG2064	0.632774104902943	1	1	pseudobulk	DESeq2	LRT		0
173	KC_AB3	CG2076	0.160954166740859	0.871247277518719	1	pseudobulk	DESeq2	LRT		0
173	KC_AB3	CG2082	1.23133864869565	1	1	pseudobulk	DESeq2	LRT		0
173	KC_AB3	CG2091	0.241601448826196	0.82083386868245	1	pseudobulk	DESeq2	LRT		0
173	KC_AB3	CG2100	0.632774104902944	1	1	pseudobulk	DESeq2	LRT		0
174	KC_AB3	CG2111	2.3149273646917	0.611236276445972	1	pseudobulk	DESeq2	LRT		0
174	KC_AB3	CG2116	0.60429312577504	0.672678763810949	1	pseudobulk	DESeq2	LRT		0
174	KC_AB3	CG2121	0.632774104902944	1	1	pseudobulk	DESeq2	LRT		0
174	KC_AB3	CG2124	0.632774104902944	1	1	pseudobulk	DESeq2	LRT		0
174	KC_AB3	CG2126	-0.709168666620783	0.52893559585128	1	pseudobulk	DESeq2	LRT		0
174	KC_AB3	CG2129	0.632774104902943	1	1	pseudobulk	DESeq2	LRT		0
174	KC_AB3	CG2147	1.73724899717076	0.229237235804085	1	pseudobulk	DESeq2	LRT		0
174	KC_AB3	CG2162	-0.379178832055594	0.598921553580981	1	pseudobulk	DESeq2	LRT		0
174	KC_AB3	CG2182	-0.460557625439154	0.470252853644323	1	pseudobulk	DESeq2	LRT		0
174	KC_AB3	CG2186	-0.559452045984581	0.40378365616181	1	pseudobulk	DESeq2	LRT		0
174	KC_AB3	CG2199	-1.06224622075458	0.392717158012781	1	pseudobulk	DESeq2	LRT		0
174	KC_AB3	CG2200	0.643786716084918	0.654439471174531	1	pseudobulk	DESeq2	LRT		0
174	KC_AB3	CG2201	-1.05766720415196	0.422777534727199	1	pseudobulk	DESeq2	LRT		0
174	KC_AB3	CG2202	0.825455030122932	0.700102658338621	1	pseudobulk	DESeq2	LRT		0
174	KC_AB3	CG2209	1.23133864869565	1	1	pseudobulk	DESeq2	LRT		0
174	KC_AB3	CG2211	0.161515868851812	1	1	pseudobulk	DESeq2	LRT		0
174	KC_AB3	CG2218	0.834796128686501	0.673902056670653	1	pseudobulk	DESeq2	LRT		0
174	KC_AB3	CG2224	-1.6499100163518E-0	0.731960929149871	1	pseudobulk	DESeq2	LRT		0
174	KC_AB3	CG2225	-0.823978994395673	0.192172920285499	1	pseudobulk	DESeq2	LRT		0
174	KC_AB3	CG2246	-0.111860781286156	0.598977107460426	1	pseudobulk	DESeq2	LRT		0
174	KC_AB3	CG2247	0.587801072610362	0.844815809239213	1	pseudobulk	DESeq2	LRT		0
174	KC_AB3	CG2256	-0.229039602237312	1	1	pseudobulk	DESeq2	LRT		0
174	KC_AB3	CG2258	0.093493392428045	0.605200929347887	1	pseudobulk	DESeq2	LRT		0
174	KC_AB3	CG2260	0.857037594830445	0.786425553887833	1	pseudobulk	DESeq2	LRT		0
174	KC_AB3	CG2269	0.500586064331697	0.0594319360441238	1	pseudobulk	DESeq2	LRT		0
174	KC_AB3	CG2277	-0.743090580390045	0.452450664948349	1	pseudobulk	DESeq2	LRT		0
174	KC_AB3	CG2278	-0.359797574434788	0.607164020244882	1	pseudobulk	DESeq2	LRT		0
174	KC_AB3	CG2321	-1.04776935228448	0.559994920957393	1	pseudobulk	DESeq2	LRT		0
174	KC_AB3	CG2519	-0.696898817106633	0.363880668727018	1	pseudobulk	DESeq2	LRT		0
174	KC_AB3	CG2540	1.24749025498703	0.537738777056439	1	pseudobulk	DESeq2	LRT		0
174	KC_AB3	CG2608	-0.177146327547878	0.744439433789593	1	pseudobulk	DESeq2	LRT		0
174	KC_AB3	CG2611	0.138370705973553	0.862212208257137	1	pseudobulk	DESeq2	LRT		0
174	KC_AB3	CG2614	0.730229754789315	1	1	pseudobulk	DESeq2	LRT		0
174	KC_AB3	CG2616	0.632774104902943	1	1	pseudobulk	DESeq2	LRT		0
174	KC_AB3	CG2617	-0.987189378304843	0.481223954187462	1	pseudobulk	DESeq2	LRT		0
174	KC_AB3	CG2656	-0.987472621393295	0.475551251793775	1	pseudobulk	DESeq2	LRT		0

174	KC_AB3	CG2662	1.47837773392725	0.70817076420814	1	pseudobulk	DESeq2	LRT		0
174	KC_AB3	CG2680	0.0690460583567777	0.806788977627479	1	pseudobulk	DESeq2	LRT		0
174	KC_AB3	CG2685	1.2051623378688	0.672483779381494	1	pseudobulk	DESeq2	LRT		0
174	KC_AB3	CG2691	0.593667095203339	1	1	pseudobulk	DESeq2	LRT		0
174	KC_AB3	CG2698	0.175522871329191	0.402296721624952	1	pseudobulk	DESeq2	LRT		0
174	KC_AB3	CG2701	-0.935360889494124	0.36863022484023	1	pseudobulk	DESeq2	LRT		0
174	KC_AB3	CG2712	0.968634025215337	1	1	pseudobulk	DESeq2	LRT		0
174	KC_AB3	CG2747	-0.30191923336519	0.533418282999532	1	pseudobulk	DESeq2	LRT		0
174	KC_AB3	CG2765	0.114144477435883	0.717248278072924	1	pseudobulk	DESeq2	LRT		0
174	KC_AB3	CG2767	1.23133864869565	1	1	pseudobulk	DESeq2	LRT		0
174	KC_AB3	CG2772	-1.27647028137981	0.135335354163886	1	pseudobulk	DESeq2	LRT		0
174	KC_AB3	CG2790	-0.186501019639372	0.556522628238914	1	pseudobulk	DESeq2	LRT		0
174	KC_AB3	CG2794	0.593667095203339	1	1	pseudobulk	DESeq2	LRT		0
174	KC_AB3	CG2811	-0.277856021576961	0.630364349083681	1	pseudobulk	DESeq2	LRT		0
174	KC_AB3	CG2812	1.95267092137636	1	1	pseudobulk	DESeq2	LRT		0
174	KC_AB3	CG2818	1.11624962829583	0.142979222760117	1	pseudobulk	DESeq2	LRT		0
174	KC_AB3	CG2841	2.3149273646917	0.611236276445972	1	pseudobulk	DESeq2	LRT		0
174	KC_AB3	CG2846	-0.108831053939639	0.782638561788784	1	pseudobulk	DESeq2	LRT		0
174	KC_AB3	CG2852	0.342253944579196	0.427757236916368	1	pseudobulk	DESeq2	LRT		0
174	KC_AB3	CG2865	-0.388520036342306	0.279698335546945	1	pseudobulk	DESeq2	LRT		0
174	KC_AB3	CG2889	-1.15316212513758	0.34521957676733	1	pseudobulk	DESeq2	LRT		0
174	KC_AB3	CG2906	2.36669165384951	0.433474334598526	1	pseudobulk	DESeq2	LRT		0
174	KC_AB3	CG2909	-0.0875233604786949	0.752794019802608	1	pseudobulk	DESeq2	LRT		0
174	KC_AB3	CG2911	1.95267092137636	1	1	pseudobulk	DESeq2	LRT		0
174	KC_AB3	CG2915	2.40965645281937	0.428807735193828	1	pseudobulk	DESeq2	LRT		0
174	KC_AB3	CG2918	-0.179664689044544	0.71645188194776	1	pseudobulk	DESeq2	LRT		0
174	KC_AB3	CG2921	-1.04293917322264	0.251906362361745	1	pseudobulk	DESeq2	LRT		0
174	KC_AB3	CG2924	0.123864865805856	0.802206537612707	1	pseudobulk	DESeq2	LRT		0
174	KC_AB3	CG2926	0.138474246571173	0.541926313949181	1	pseudobulk	DESeq2	LRT		0
174	KC_AB3	CG2931	-0.386993229478757	0.513831411597969	1	pseudobulk	DESeq2	LRT		0
174	KC_AB3	CG2938	0.467229084903379	0.490324167411377	1	pseudobulk	DESeq2	LRT		0
174	KC_AB3	CG2972	0.319549864447076	1	1	pseudobulk	DESeq2	LRT		0
174	KC_AB3	CG2991	0.576597360307705	0.393418002004032	1	pseudobulk	DESeq2	LRT		0
174	KC_AB3	CG2993	-0.273599232763731	0.438420865953015	1	pseudobulk	DESeq2	LRT		0
174	KC_AB3	CG30010	-0.399671897798429	0.965137949353374	1	pseudobulk	DESeq2	LRT		0
174	KC_AB3	CG30015	0.593667095203339	1	1	pseudobulk	DESeq2	LRT		0
174	KC_AB3	CG30020	0.0617010032752113	0.823091830198943	1	pseudobulk	DESeq2	LRT		0
174	KC_AB3	CG30054	1.22707713470494	1	1	pseudobulk	DESeq2	LRT		0
174	KC_AB3	CG30060	1.23133864869565	1	1	pseudobulk	DESeq2	LRT		0
174	KC_AB3	CG3008	0.588753687422647	0.610798817591351	1	pseudobulk	DESeq2	LRT		0
174	KC_AB3	CG30089	0.186241848521592	0.741663955041616	1	pseudobulk	DESeq2	LRT		0
174	KC_AB3	CG3009	-0.0514272703028709	0.69331461666687	1	pseudobulk	DESeq2	LRT		0
174	KC_AB3	CG30094	-0.667639835463386	0.440498709735264	1	pseudobulk	DESeq2	LRT		0
174	KC_AB3	CG30096	-0.486249626956945	0.670575327420336	1	pseudobulk	DESeq2	LRT		0
174	KC_AB3	CG30109	-0.286442588166848	0.685530966747091	1	pseudobulk	DESeq2	LRT		0
174	KC_AB3	CG30110	0.632774104902944	1	1	pseudobulk	DESeq2	LRT		0
174	KC_AB3	CG30116	-0.326321182799817	0.252095522500866	1	pseudobulk	DESeq2	LRT		0
174	KC_AB3	CG30122	-0.443954592491569	0.452588488777935	1	pseudobulk	DESeq2	LRT		0
174	KC_AB3	CG30156	0.398203167227038	0.899159112157427	1	pseudobulk	DESeq2	LRT		0
174	KC_AB3	CG30158	-0.328564049764258	0.297013165024164	1	pseudobulk	DESeq2	LRT		0
174	KC_AB3	CG30172	0.117930328053122	0.680331738085291	1	pseudobulk	DESeq2	LRT		0
174	KC_AB3	CG30178	0.632774104902944	1	1	pseudobulk	DESeq2	LRT		0
174	KC_AB3	CG30183	-0.142978647936007	0.8821059731563	1	pseudobulk	DESeq2	LRT		0

174	KC_AB3	CG30197	1.59359598433783	0.86377329553208	1	pseudobulk	DESeq2	LRT	0
174	KC_AB3	CG3021	0.580659916124113	0.820602155286262	1	pseudobulk	DESeq2	LRT	0
174	KC_AB3	CG30291	-0.179661781216491	0.752912749995518	1	pseudobulk	DESeq2	LRT	0
174	KC_AB3	CG3032	2.62817135292692	0.54954870385793	1	pseudobulk	DESeq2	LRT	0
174	KC_AB3	CG30334	2.62817135292692	0.54954870385793	1	pseudobulk	DESeq2	LRT	0
174	KC_AB3	CG30344	1.22881132671401	0.529705441539229	1	pseudobulk	DESeq2	LRT	0
174	KC_AB3	CG30345	0.632774104902943	1	1	pseudobulk	DESeq2	LRT	0
174	KC_AB3	CG30349	0.263237126093928	1	1	pseudobulk	DESeq2	LRT	0
174	KC_AB3	CG30357	0.632774104902944	1	1	pseudobulk	DESeq2	LRT	0
174	KC_AB3	CG3036	2.62817135292692	0.54954870385793	1	pseudobulk	DESeq2	LRT	0
174	KC_AB3	CG30373	-1.10730914162238	0.259640984777324	1	pseudobulk	DESeq2	LRT	0
175	KC_AB3	CG30389	-0.146828285417757	0.620268093394584	1	pseudobulk	DESeq2	LRT	0
175	KC_AB3	CG30392	-0.315116661553355	0.565932107098084	1	pseudobulk	DESeq2	LRT	0
175	KC_AB3	CG30394	1.12025613006486	0.757144729274267	1	pseudobulk	DESeq2	LRT	0
175	KC_AB3	CG3040	1.80360358894928	0.0637389940925394	1	pseudobulk	DESeq2	LRT	0
175	KC_AB3	CG30419	-0.349296896062859	0.389097301339074	1	pseudobulk	DESeq2	LRT	0
175	KC_AB3	CG30423	-0.369829366523528	0.342732156282376	1	pseudobulk	DESeq2	LRT	0
175	KC_AB3	CG30424	1.95267092137636	1	1	pseudobulk	DESeq2	LRT	0
175	KC_AB3	CG30428	-0.517911218271462	0.327008798583743	1	pseudobulk	DESeq2	LRT	0
175	KC_AB3	CG3045	-1.11546323953652	0.514722260743204	1	pseudobulk	DESeq2	LRT	0
175	KC_AB3	CG30460	0.929039064969518	1	1	pseudobulk	DESeq2	LRT	0
175	KC_AB3	CG30467	-0.0716602429827649	0.796505468216085	1	pseudobulk	DESeq2	LRT	0
175	KC_AB3	CG30491	0.0966130851822739	0.884394408158738	1	pseudobulk	DESeq2	LRT	0
175	KC_AB3	CG30495	0.161515868851812	1	1	pseudobulk	DESeq2	LRT	0
175	KC_AB3	CG30496	0.632774104902944	1	1	pseudobulk	DESeq2	LRT	0
175	KC_AB3	CG3061	-0.0440880714901925	0.582623163796726	1	pseudobulk	DESeq2	LRT	0
175	KC_AB3	CG3065	-0.0670881187967513	0.625826879996404	1	pseudobulk	DESeq2	LRT	0
175	KC_AB3	CG3071	1.796136236207	0.45032995637859	1	pseudobulk	DESeq2	LRT	0
175	KC_AB3	CG3078	0.580506127047156	0.450162745575419	1	pseudobulk	DESeq2	LRT	0
175	KC_AB3	CG31030	0.619783921462036	0.0774789430215389	1	pseudobulk	DESeq2	LRT	0
175	KC_AB3	CG31038	-0.185594513059781	1	1	pseudobulk	DESeq2	LRT	0
175	KC_AB3	CG3104	0.0409357904441806	0.547666955509259	1	pseudobulk	DESeq2	LRT	0
175	KC_AB3	CG31041	0.632774104902944	1	1	pseudobulk	DESeq2	LRT	0
175	KC_AB3	CG31051	-0.254487665770912	0.741338667556786	1	pseudobulk	DESeq2	LRT	0
175	KC_AB3	CG31064	0.408180095555835	0.292172175975768	1	pseudobulk	DESeq2	LRT	0
175	KC_AB3	CG3107	0.377223097448226	0.482697187389221	1	pseudobulk	DESeq2	LRT	0
175	KC_AB3	CG31098	0.77838161290934	0.933412204585332	1	pseudobulk	DESeq2	LRT	0
175	KC_AB3	CG31100	0.632774104902943	1	1	pseudobulk	DESeq2	LRT	0
175	KC_AB3	CG31111	0.904857485982405	0.863591387425197	1	pseudobulk	DESeq2	LRT	0
175	KC_AB3	CG31122	-0.26141650090311	0.712978812792549	1	pseudobulk	DESeq2	LRT	0
175	KC_AB3	CG31125	-1.4710085459164	0.312242447284166	1	pseudobulk	DESeq2	LRT	0
175	KC_AB3	CG31126	0.727672353128284	0.428800175423015	1	pseudobulk	DESeq2	LRT	0
175	KC_AB3	CG31140	-0.375286506071444	0.222097147890876	1	pseudobulk	DESeq2	LRT	0
175	KC_AB3	CG31142	-0.959898552097931	0.520483131482047	1	pseudobulk	DESeq2	LRT	0
175	KC_AB3	CG31156	-0.269891394701783	0.750155631517377	1	pseudobulk	DESeq2	LRT	0
175	KC_AB3	CG31174	0.759758178017103	1	1	pseudobulk	DESeq2	LRT	0
175	KC_AB3	CG31191	-0.380198530974799	0.36722612477704	1	pseudobulk	DESeq2	LRT	0
175	KC_AB3	CG31195	0.612233504036476	1	1	pseudobulk	DESeq2	LRT	0
175	KC_AB3	CG31211	0.213609877738797	0.623311614312194	1	pseudobulk	DESeq2	LRT	0
175	KC_AB3	CG31221	-0.0038008202211403	0.773800134311512	1	pseudobulk	DESeq2	LRT	0
175	KC_AB3	CG31223	0.759758178017103	1	1	pseudobulk	DESeq2	LRT	0
175	KC_AB3	CG31224	-0.145018164848092	0.832588177259364	1	pseudobulk	DESeq2	LRT	0
175	KC_AB3	CG31229	1.34167906582601	0.0719522396294142	1	pseudobulk	DESeq2	LRT	0

175	KC_AB3	CG31248	0.632774104902944	1	1	pseudobulk	DESeq2	LRT	0
175	KC_AB3	CG31249	0.852673720941954	0.292743824631158	1	pseudobulk	DESeq2	LRT	0
175	KC_AB3	CG31251	-0.186961738271884	1	1	pseudobulk	DESeq2	LRT	0
175	KC_AB3	CG31278	0.494472967217822	1	1	pseudobulk	DESeq2	LRT	0
175	KC_AB3	CG31301	-0.605492699024018	0.505694042111395	1	pseudobulk	DESeq2	LRT	0
175	KC_AB3	CG31324	0.263439232431046	0.687326633966649	1	pseudobulk	DESeq2	LRT	0
175	KC_AB3	CG31342	-0.071880381325418	0.627399292851498	1	pseudobulk	DESeq2	LRT	0
175	KC_AB3	CG31344	-0.61367743215715	0.374952552363339	1	pseudobulk	DESeq2	LRT	0
175	KC_AB3	CG31345	0.790010899828778	1	1	pseudobulk	DESeq2	LRT	0
175	KC_AB3	CG31357	-0.422961438985012	0.524132755151518	1	pseudobulk	DESeq2	LRT	0
175	KC_AB3	CG31360	0.456596241701492	0.688835468423211	1	pseudobulk	DESeq2	LRT	0
175	KC_AB3	CG31365	1.37794690317769	0.597397478581202	1	pseudobulk	DESeq2	LRT	0
175	KC_AB3	CG31368	1.36937874215905	0.203960784618716	1	pseudobulk	DESeq2	LRT	0
175	KC_AB3	CG31371	1.22707713470494	1	1	pseudobulk	DESeq2	LRT	0
175	KC_AB3	CG31381	2.62817135292692	0.54954870385793	1	pseudobulk	DESeq2	LRT	0
175	KC_AB3	CG31388	-0.0371482244791053	0.723324627893922	1	pseudobulk	DESeq2	LRT	0
175	KC_AB3	CG31415	0.632774104902944	1	1	pseudobulk	DESeq2	LRT	0
175	KC_AB3	CG31441	2.3149273646917	0.611236276445972	1	pseudobulk	DESeq2	LRT	0
175	KC_AB3	CG31457	0.696940492117491	0.693678485472956	1	pseudobulk	DESeq2	LRT	0
175	KC_AB3	CG31460	-0.454383677326658	0.663417339588169	1	pseudobulk	DESeq2	LRT	0
175	KC_AB3	CG31469	0.632774104902944	1	1	pseudobulk	DESeq2	LRT	0
175	KC_AB3	CG31475	-0.739645556791374	0.210559261499792	1	pseudobulk	DESeq2	LRT	0
175	KC_AB3	CG3149	1.3987583341885	0.187434541974073	1	pseudobulk	DESeq2	LRT	0
175	KC_AB3	CG31510	-0.0047416069882952	0.74054901994678	1	pseudobulk	DESeq2	LRT	0
175	KC_AB3	CG31522	0.243956460483156	0.61656241449329	1	pseudobulk	DESeq2	LRT	0
175	KC_AB3	CG31523	-0.893928238384527	0.170473408427137	1	pseudobulk	DESeq2	LRT	0
175	KC_AB3	CG31538	-0.229039602237312	1	1	pseudobulk	DESeq2	LRT	0
175	KC_AB3	CG31550	0.342243544065018	0.623195522447152	1	pseudobulk	DESeq2	LRT	0
175	KC_AB3	CG3156	-1.33713774668078	0.356601341569333	1	pseudobulk	DESeq2	LRT	0
175	KC_AB3	CG31612	1.04436316651002	1	1	pseudobulk	DESeq2	LRT	0
175	KC_AB3	CG31626	1.95267092137636	1	1	pseudobulk	DESeq2	LRT	0
175	KC_AB3	CG3163	-0.853784057254757	0.427271371792688	1	pseudobulk	DESeq2	LRT	0
175	KC_AB3	CG31633	-1.27979282340932	0.373300441956348	1	pseudobulk	DESeq2	LRT	0
175	KC_AB3	CG31635	0.29737259646697	0.398029821837948	1	pseudobulk	DESeq2	LRT	0
175	KC_AB3	CG31637	-0.2615228063758	0.500969390105862	1	pseudobulk	DESeq2	LRT	0
175	KC_AB3	CG31638	0.0944623826337334	0.735359862793406	1	pseudobulk	DESeq2	LRT	0
175	KC_AB3	CG3164	0.0721354725116212	0.785668746374725	1	pseudobulk	DESeq2	LRT	0
175	KC_AB3	CG31642	0.632774104902944	1	1	pseudobulk	DESeq2	LRT	0
175	KC_AB3	CG31648	0.517512004815322	0.725186735222178	1	pseudobulk	DESeq2	LRT	0
175	KC_AB3	CG3165	0.259621378147966	0.511834104357676	1	pseudobulk	DESeq2	LRT	0
175	KC_AB3	CG31650	-0.281142106515068	0.605880439173947	1	pseudobulk	DESeq2	LRT	0
175	KC_AB3	CG31664	1.95267092137636	1	1	pseudobulk	DESeq2	LRT	0
175	KC_AB3	CG31673	0.632774104902944	1	1	pseudobulk	DESeq2	LRT	0
175	KC_AB3	CG31676	0.790010899828778	1	1	pseudobulk	DESeq2	LRT	0
175	KC_AB3	CG31678	-0.105581846590776	0.797297744598995	1	pseudobulk	DESeq2	LRT	0
175	KC_AB3	CG3168	2.3149273646917	0.611236276445972	1	pseudobulk	DESeq2	LRT	0
175	KC_AB3	CG31687	0.682734067881938	0.485372459881193	1	pseudobulk	DESeq2	LRT	0
175	KC_AB3	CG31688	0.12085905718906	0.489796649674568	1	pseudobulk	DESeq2	LRT	0
175	KC_AB3	CG31690	1.69067249796896	0.0708427352891645	1	pseudobulk	DESeq2	LRT	0
175	KC_AB3	CG31694	-0.842142642559827	0.215190403707386	1	pseudobulk	DESeq2	LRT	0
175	KC_AB3	CG31705	-0.0327300486906476	1	1	pseudobulk	DESeq2	LRT	0
175	KC_AB3	CG31706	2.17856268118853	0.399053277596756	1	pseudobulk	DESeq2	LRT	0
175	KC_AB3	CG31712	0.0738250635987759	0.626142420509164	1	pseudobulk	DESeq2	LRT	0

175	KC_AB3	CG31715	-0.224049095890743	0.471156091601389	1	pseudobulk	DESeq2	LRT		0
175	KC_AB3	CG31717	-0.337033087782754	0.591248695427109	1	pseudobulk	DESeq2	LRT		0
175	KC_AB3	CG31729	0.523639690540389	0.508300094310106	1	pseudobulk	DESeq2	LRT		0
175	KC_AB3	CG31751	-0.185594513059781	1	1	pseudobulk	DESeq2	LRT		0
175	KC_AB3	CG3176	0.319019293298944	1	1	pseudobulk	DESeq2	LRT		0
176	KC_AB3	CG31760	-0.201602190570274	0.523256229177891	1	pseudobulk	DESeq2	LRT		0
176	KC_AB3	CG31788	0.632774104902943	1	1	pseudobulk	DESeq2	LRT		0
176	KC_AB3	CG31793	0.570858663621144	1	1	pseudobulk	DESeq2	LRT		0
176	KC_AB3	CG31800	-0.429697416871068	0.612622122643281	1	pseudobulk	DESeq2	LRT		0
176	KC_AB3	CG31808	-0.0375723326432688	0.633787291516065	1	pseudobulk	DESeq2	LRT		0
176	KC_AB3	CG31815	-0.186961738271884	1	1	pseudobulk	DESeq2	LRT		0
176	KC_AB3	CG31817	0.632774104902944	1	1	pseudobulk	DESeq2	LRT		0
176	KC_AB3	CG3184	-1.4184720985282	0.364283321778696	1	pseudobulk	DESeq2	LRT		0
176	KC_AB3	CG31849	1.23133864869565	1	1	pseudobulk	DESeq2	LRT		0
176	KC_AB3	CG31855	-0.541010882127808	0.445378844430052	1	pseudobulk	DESeq2	LRT		0
176	KC_AB3	CG31886	-0.0327300486906476	1	1	pseudobulk	DESeq2	LRT		0
176	KC_AB3	CG31897	0.632774104902944	1	1	pseudobulk	DESeq2	LRT		0
176	KC_AB3	CG31908	0.0008966518262808	0.771478529381627	1	pseudobulk	DESeq2	LRT		0
176	KC_AB3	CG31915	1.59359598433783	0.86377329553208	1	pseudobulk	DESeq2	LRT		0
176	KC_AB3	CG31921	1.93443109363625	0.658150581914386	1	pseudobulk	DESeq2	LRT		0
176	KC_AB3	CG31922	0.596460669567726	0.473000840871023	1	pseudobulk	DESeq2	LRT		0
176	KC_AB3	CG31935	1.10118349162554	0.289993016206351	1	pseudobulk	DESeq2	LRT		0
176	KC_AB3	CG31937	0.594117478581602	1	1	pseudobulk	DESeq2	LRT		0
176	KC_AB3	CG31957	-0.803176581656176	0.532801752108079	1	pseudobulk	DESeq2	LRT		0
176	KC_AB3	CG3198	0.295569185623157	0.338096090950505	1	pseudobulk	DESeq2	LRT		0
176	KC_AB3	CG31997	-0.0555186001540784	0.517434333637075	1	pseudobulk	DESeq2	LRT		0
176	KC_AB3	CG31998	0.117302348705751	0.756778612413897	1	pseudobulk	DESeq2	LRT		0
176	KC_AB3	CG32017	0.492196798986393	0.231369745018232	1	pseudobulk	DESeq2	LRT		0
176	KC_AB3	CG32022	0.237943505867968	0.773768492796373	1	pseudobulk	DESeq2	LRT		0
176	KC_AB3	CG32036	0.632774104902944	1	1	pseudobulk	DESeq2	LRT		0
176	KC_AB3	CG32039	0.054553748843664	0.61284513760043	1	pseudobulk	DESeq2	LRT		0
176	KC_AB3	CG32052	-0.352544196385097	0.799575396616172	1	pseudobulk	DESeq2	LRT		0
176	KC_AB3	CG32066	-0.150707018885777	0.482475991176134	1	pseudobulk	DESeq2	LRT		0
176	KC_AB3	CG32069	-0.0564327608092152	0.690677886613852	1	pseudobulk	DESeq2	LRT		0
176	KC_AB3	CG32075	0.612233504036476	1	1	pseudobulk	DESeq2	LRT		0
176	KC_AB3	CG32085	-0.0786262398495979	0.84319872400176	1	pseudobulk	DESeq2	LRT		0
176	KC_AB3	CG32095	0.678325774722761	0.764141355095609	1	pseudobulk	DESeq2	LRT		0
176	KC_AB3	CG32099	0.222895129765467	0.910723611350132	1	pseudobulk	DESeq2	LRT		0
176	KC_AB3	CG32100	0.870167573090808	0.278138929337111	1	pseudobulk	DESeq2	LRT		0
176	KC_AB3	CG32104	1.59575615951652	0.886898266337347	1	pseudobulk	DESeq2	LRT		0
176	KC_AB3	CG32109	-0.573681430857463	0.494943844045993	1	pseudobulk	DESeq2	LRT		0
176	KC_AB3	CG32112	0.659167386665375	0.722968174827344	1	pseudobulk	DESeq2	LRT		0
176	KC_AB3	CG32115	0.161515868851812	1	1	pseudobulk	DESeq2	LRT		0
176	KC_AB3	CG32137	0.632774104902944	1	1	pseudobulk	DESeq2	LRT		0
176	KC_AB3	CG32147	1.71724348734451	0.31526579154323	1	pseudobulk	DESeq2	LRT		0
176	KC_AB3	CG32163	0.757229211699233	0.770485041699765	1	pseudobulk	DESeq2	LRT		0
176	KC_AB3	CG32176	-0.529372639386131	0.65282455572652	1	pseudobulk	DESeq2	LRT		0
176	KC_AB3	CG32187	0.568710977583255	1	1	pseudobulk	DESeq2	LRT		0
176	KC_AB3	CG32195	0.632774104902944	1	1	pseudobulk	DESeq2	LRT		0
176	KC_AB3	CG32196	0.632774104902944	1	1	pseudobulk	DESeq2	LRT		0
176	KC_AB3	CG32202	0.817612402088146	0.440867053773351	1	pseudobulk	DESeq2	LRT		0
176	KC_AB3	CG32204	-0.101857644368807	0.668213860411388	1	pseudobulk	DESeq2	LRT		0
176	KC_AB3	CG32206	0.187325800667422	0.518775225159632	1	pseudobulk	DESeq2	LRT		0

176	KC_AB3	CG3222	1.23133864869565	1	1	pseudobulk	DESeq2	LRT	0
176	KC_AB3	CG32221	-1.13259113815761	0.486173583538128	1	pseudobulk	DESeq2	LRT	0
176	KC_AB3	CG32225	0.674072247112546	0.195680824440837	1	pseudobulk	DESeq2	LRT	0
176	KC_AB3	CG3223	0.0254496459441501	0.822753554192357	1	pseudobulk	DESeq2	LRT	0
176	KC_AB3	CG32232	0.632774104902944	1	1	pseudobulk	DESeq2	LRT	0
176	KC_AB3	CG3224	-1.220473180656	0.186543935860507	1	pseudobulk	DESeq2	LRT	0
176	KC_AB3	CG32243	0.188745609088196	0.760360688729613	1	pseudobulk	DESeq2	LRT	0
176	KC_AB3	CG3226	-0.43105940936121	0.610096660311935	1	pseudobulk	DESeq2	LRT	0
176	KC_AB3	CG32262	0.326455573676792	0.521065585745401	1	pseudobulk	DESeq2	LRT	0
176	KC_AB3	CG32264	-0.227200321379302	0.413070344928123	1	pseudobulk	DESeq2	LRT	0
176	KC_AB3	CG32267	0.118670277757234	0.630962306861421	1	pseudobulk	DESeq2	LRT	0
176	KC_AB3	CG32276	0.0275415286713699	0.742830232646862	1	pseudobulk	DESeq2	LRT	0
176	KC_AB3	CG32278	0.0247978361406234	0.930198880991201	1	pseudobulk	DESeq2	LRT	0
176	KC_AB3	CG32280	-0.512334399653183	0.527306290464096	1	pseudobulk	DESeq2	LRT	0
176	KC_AB3	CG32281	1.19736109821733	0.191649992032982	1	pseudobulk	DESeq2	LRT	0
176	KC_AB3	CG32333	1.23133864869565	1	1	pseudobulk	DESeq2	LRT	0
176	KC_AB3	CG32344	1.60186822711471	1	1	pseudobulk	DESeq2	LRT	0
176	KC_AB3	CG32365	1.59576167909336	0.887029991980463	1	pseudobulk	DESeq2	LRT	0
176	KC_AB3	CG32368	-0.962586053545908	0.567525062839105	1	pseudobulk	DESeq2	LRT	0
176	KC_AB3	CG32369	0.177085872442241	0.495941551033723	1	pseudobulk	DESeq2	LRT	0
176	KC_AB3	CG32373	-1.10622412649225	0.37470585674303	1	pseudobulk	DESeq2	LRT	0
176	KC_AB3	CG32392	-1.54234221000846	0.40242154757046	1	pseudobulk	DESeq2	LRT	0
176	KC_AB3	CG32396	1.95267092137636	1	1	pseudobulk	DESeq2	LRT	0
176	KC_AB3	CG32409	0.4135959724801	1	1	pseudobulk	DESeq2	LRT	0
176	KC_AB3	CG32425	0.596332555899076	0.299722993264659	1	pseudobulk	DESeq2	LRT	0
176	KC_AB3	CG32428	-0.998550059179721	0.130624093591208	1	pseudobulk	DESeq2	LRT	0
176	KC_AB3	CG32432	0.264009873413346	0.433138707339083	1	pseudobulk	DESeq2	LRT	0
176	KC_AB3	CG32441	-0.526021049587405	0.344751692673029	1	pseudobulk	DESeq2	LRT	0
176	KC_AB3	CG32448	-0.512551963678559	0.484889267062569	1	pseudobulk	DESeq2	LRT	0
176	KC_AB3	CG32452	1.4351580737666	0.515946410254263	1	pseudobulk	DESeq2	LRT	0
176	KC_AB3	CG32457	0.632774104902944	1	1	pseudobulk	DESeq2	LRT	0
176	KC_AB3	CG32459	0.632774104902944	1	1	pseudobulk	DESeq2	LRT	0
176	KC_AB3	CG32473	-0.269742198537673	0.750195831134786	1	pseudobulk	DESeq2	LRT	0
176	KC_AB3	CG32485	-0.336197674363645	0.489406511046681	1	pseudobulk	DESeq2	LRT	0
176	KC_AB3	CG32486	-0.473157581228378	0.141029417166274	1	pseudobulk	DESeq2	LRT	0
176	KC_AB3	CG32506	0.980425227371519	0.400440305450434	1	pseudobulk	DESeq2	LRT	0
176	KC_AB3	CG32512	-0.599327084947638	0.714415237876582	1	pseudobulk	DESeq2	LRT	0
176	KC_AB3	CG32521	-0.471154001643531	0.766828223954262	1	pseudobulk	DESeq2	LRT	0
176	KC_AB3	CG3253	0.191355244004072	0.756938232175614	1	pseudobulk	DESeq2	LRT	0
176	KC_AB3	CG32533	0.0538103424110901	1	1	pseudobulk	DESeq2	LRT	0
176	KC_AB3	CG32537	0.687916138902321	0.738972459068669	1	pseudobulk	DESeq2	LRT	0
176	KC_AB3	CG32544	-0.0476200800454616	0.732054869768393	1	pseudobulk	DESeq2	LRT	0
176	KC_AB3	CG32547	-0.434913279458111	0.419231974634561	1	pseudobulk	DESeq2	LRT	0
176	KC_AB3	CG32549	-0.648584151756449	0.430664702901297	1	pseudobulk	DESeq2	LRT	0
176	KC_AB3	CG32554	1.22707713470494	1	1	pseudobulk	DESeq2	LRT	0
176	KC_AB3	CG3257	-0.0094354097396922	0.646366426775128	1	pseudobulk	DESeq2	LRT	0
176	KC_AB3	CG32576	-1.57999084568954	0.233719920207458	1	pseudobulk	DESeq2	LRT	0
176	KC_AB3	CG32581	4.78108412938824	0.166989680375175	1	pseudobulk	DESeq2	LRT	0
176	KC_AB3	CG32590	0.0308961192537421	0.782981733878744	1	pseudobulk	DESeq2	LRT	0
176	KC_AB3	CG32591	0.632774104902943	1	1	pseudobulk	DESeq2	LRT	0
176	KC_AB3	CG32638	2.62817135292692	0.54954870385793	1	pseudobulk	DESeq2	LRT	0
176	KC_AB3	CG32643	1.95267092137636	1	1	pseudobulk	DESeq2	LRT	0
177	KC_AB3	CG32647	-0.0194436340766834	0.779239929481112	1	pseudobulk	DESeq2	LRT	0

177	KC_AB3	CG32655	1.23133864869565	1	1	pseudobulk	DESeq2	LRT	0
177	KC_AB3	CG32683	-0.403886986427487	0.268320377193368	1	pseudobulk	DESeq2	LRT	0
177	KC_AB3	CG32686	0.632774104902944	1	1	pseudobulk	DESeq2	LRT	0
177	KC_AB3	CG3270	0.632774104902943	1	1	pseudobulk	DESeq2	LRT	0
177	KC_AB3	CG32700	-0.155485571820322	0.440964163779395	1	pseudobulk	DESeq2	LRT	0
177	KC_AB3	CG32708	-0.356231852576693	0.685536536187171	1	pseudobulk	DESeq2	LRT	0
177	KC_AB3	CG32756	0.68610001984073	0.616136432518564	1	pseudobulk	DESeq2	LRT	0
177	KC_AB3	CG32762	1.95267092137636	1	1	pseudobulk	DESeq2	LRT	0
177	KC_AB3	CG32767	-0.280106781924119	0.287960234691005	1	pseudobulk	DESeq2	LRT	0
177	KC_AB3	CG32772	-0.138434338276455	0.800015831222569	1	pseudobulk	DESeq2	LRT	0
177	KC_AB3	CG32795	-0.5678942341508	0.360588457664479	1	pseudobulk	DESeq2	LRT	0
177	KC_AB3	CG32803	-1.08110023483159	0.435202436595456	1	pseudobulk	DESeq2	LRT	0
177	KC_AB3	CG32808	-0.633495181115116	0.625593166611771	1	pseudobulk	DESeq2	LRT	0
177	KC_AB3	CG32809	-0.468476734166483	0.301446578351468	1	pseudobulk	DESeq2	LRT	0
177	KC_AB3	CG3281	-1.59030081952146	0.352608792034627	1	pseudobulk	DESeq2	LRT	0
177	KC_AB3	CG32813	-0.221855867082626	0.726563408161688	1	pseudobulk	DESeq2	LRT	0
177	KC_AB3	CG32815	-0.3130265578028	0.436952073264597	1	pseudobulk	DESeq2	LRT	0
177	KC_AB3	CG32816	-0.184744780581018	1	1	pseudobulk	DESeq2	LRT	0
177	KC_AB3	CG32850	0.0426059387284843	0.561237674653677	1	pseudobulk	DESeq2	LRT	0
177	KC_AB3	CG32856	-0.468825204411133	0.630393017022748	1	pseudobulk	DESeq2	LRT	0
177	KC_AB3	CG3288	0.632774104902943	1	1	pseudobulk	DESeq2	LRT	0
177	KC_AB3	CG3294	-1.45112911319816	0.403437655324967	1	pseudobulk	DESeq2	LRT	0
177	KC_AB3	CG32944	0.632774104902944	1	1	pseudobulk	DESeq2	LRT	0
177	KC_AB3	CG3295	-1.06305793782191	0.29664506063596	1	pseudobulk	DESeq2	LRT	0
177	KC_AB3	CG32971	-0.229039602237312	1	1	pseudobulk	DESeq2	LRT	0
177	KC_AB3	CG32982	-0.228403721872776	0.682551333388669	1	pseudobulk	DESeq2	LRT	0
177	KC_AB3	CG33054	-0.94445855398478	0.602978626154504	1	pseudobulk	DESeq2	LRT	0
177	KC_AB3	CG33056	-0.962336549607539	0.567620274858258	1	pseudobulk	DESeq2	LRT	0
177	KC_AB3	CG3308	1.65426315697425	0.358292769251671	1	pseudobulk	DESeq2	LRT	0
177	KC_AB3	CG33082	0.632774104902943	1	1	pseudobulk	DESeq2	LRT	0
177	KC_AB3	CG3309	-0.871797925428329	0.501199079669871	1	pseudobulk	DESeq2	LRT	0
177	KC_AB3	CG33090	-0.282374404897597	0.395346920474211	1	pseudobulk	DESeq2	LRT	0
177	KC_AB3	CG33096	1.0752842321091	1	1	pseudobulk	DESeq2	LRT	0
177	KC_AB3	CG33098	0.730229754789315	1	1	pseudobulk	DESeq2	LRT	0
177	KC_AB3	CG33107	1.09999655962092	0.394708831034653	1	pseudobulk	DESeq2	LRT	0
177	KC_AB3	CG33108	0.594117478581602	1	1	pseudobulk	DESeq2	LRT	0
177	KC_AB3	CG33111	0.969051891352446	1	1	pseudobulk	DESeq2	LRT	0
177	KC_AB3	CG33116	-0.0487954923896253	0.704525983275249	1	pseudobulk	DESeq2	LRT	0
177	KC_AB3	CG33127	0.0025701398975595	1	1	pseudobulk	DESeq2	LRT	0
177	KC_AB3	CG33129	0.0610140304135931	0.529427175514887	1	pseudobulk	DESeq2	LRT	0
177	KC_AB3	CG33144	-1.02568709193529	0.17193893613398	1	pseudobulk	DESeq2	LRT	0
177	KC_AB3	CG33155	-1.95849092986586	0.24842432905633	1	pseudobulk	DESeq2	LRT	0
177	KC_AB3	CG33156	0.669445033150068	0.592854415811761	1	pseudobulk	DESeq2	LRT	0
177	KC_AB3	CG33158	-0.94445855398478	0.602978626154504	1	pseudobulk	DESeq2	LRT	0
177	KC_AB3	CG33169	0.0827000031677724	0.685493539266383	1	pseudobulk	DESeq2	LRT	0
177	KC_AB3	CG33170	0.093908475737199	0.594937948199399	1	pseudobulk	DESeq2	LRT	0
177	KC_AB3	CG33181	-0.318012347406355	0.275627113032216	1	pseudobulk	DESeq2	LRT	0
177	KC_AB3	CG33189	1.95267092137636	1	1	pseudobulk	DESeq2	LRT	0
177	KC_AB3	CG33199	0.314050116421218	0.508188737695478	1	pseudobulk	DESeq2	LRT	0
177	KC_AB3	CG33203	0.142628566475099	0.69858608494924	1	pseudobulk	DESeq2	LRT	0
177	KC_AB3	CG33213	0.593667095203339	1	1	pseudobulk	DESeq2	LRT	0
177	KC_AB3	CG33217	-0.636311655702943	0.531797552684523	1	pseudobulk	DESeq2	LRT	0
177	KC_AB3	CG33228	0.914096488179428	0.634485616740349	1	pseudobulk	DESeq2	LRT	0

177	KC_AB3	CG33229	0.342003710897831	0.326543697697279	1	pseudobulk	DESeq2	LRT	0
177	KC_AB3	CG33230	1.19794739722554	0.641287708553736	1	pseudobulk	DESeq2	LRT	0
177	KC_AB3	CG33253	0.632774104902943	1	1	pseudobulk	DESeq2	LRT	0
177	KC_AB3	CG33267	-1.11571707559185	0.514634393193615	1	pseudobulk	DESeq2	LRT	0
177	KC_AB3	CG33298	0.610583717214136	0.41474974899434	1	pseudobulk	DESeq2	LRT	0
177	KC_AB3	CG33303	0.724917236032741	0.218430651554117	1	pseudobulk	DESeq2	LRT	0
177	KC_AB3	CG33310	-0.191505755986233	0.759393716956969	1	pseudobulk	DESeq2	LRT	0
177	KC_AB3	CG33331	-0.116334061785151	0.825820151751704	1	pseudobulk	DESeq2	LRT	0
177	KC_AB3	CG33332	1.88513752152589	0.739152646872234	1	pseudobulk	DESeq2	LRT	0
177	KC_AB3	CG3335	0.0772739847616278	1	1	pseudobulk	DESeq2	LRT	0
177	KC_AB3	CG3337	-0.0897588181973639	0.644411439306937	1	pseudobulk	DESeq2	LRT	0
177	KC_AB3	CG3342	-0.343859627219784	0.761749936319153	1	pseudobulk	DESeq2	LRT	0
177	KC_AB3	CG33475	2.62817135292692	0.54954870385793	1	pseudobulk	DESeq2	LRT	0
177	KC_AB3	CG33493	-0.779009454654304	0.632803062798981	1	pseudobulk	DESeq2	LRT	0
177	KC_AB3	CG33494	0.632774104902944	1	1	pseudobulk	DESeq2	LRT	0
177	KC_AB3	CG33506	-0.314710380933266	0.758977579311252	1	pseudobulk	DESeq2	LRT	0
177	KC_AB3	CG33521	-0.120070896861481	0.763645855418528	1	pseudobulk	DESeq2	LRT	0
177	KC_AB3	CG33523	-0.0409897930852614	0.818969228836876	1	pseudobulk	DESeq2	LRT	0
177	KC_AB3	CG33543	1.04961872769432	0.859434436492976	1	pseudobulk	DESeq2	LRT	0
177	KC_AB3	CG3356	0.0332750178608141	0.824679112563425	1	pseudobulk	DESeq2	LRT	0
177	KC_AB3	CG3358	0.0600259639897171	0.869760739365768	1	pseudobulk	DESeq2	LRT	0
177	KC_AB3	CG33635	0.614211094879669	0.50377222317596	1	pseudobulk	DESeq2	LRT	0
177	KC_AB3	CG33642	0.632774104902944	1	1	pseudobulk	DESeq2	LRT	0
177	KC_AB3	CG33679	0.632774104902944	1	1	pseudobulk	DESeq2	LRT	0
177	KC_AB3	CG3368	-0.264376335180536	0.753755184517366	1	pseudobulk	DESeq2	LRT	0
177	KC_AB3	CG33695	0.0272846188272494	0.893125592597165	1	pseudobulk	DESeq2	LRT	0
177	KC_AB3	CG33700	0.632774104902944	1	1	pseudobulk	DESeq2	LRT	0
177	KC_AB3	CG33774	-0.531398806710843	0.453075505970047	1	pseudobulk	DESeq2	LRT	0
177	KC_AB3	CG33777	1.95267092137636	1	1	pseudobulk	DESeq2	LRT	0
177	KC_AB3	CG3386	-0.518449774887281	0.38425011956268	1	pseudobulk	DESeq2	LRT	0
177	KC_AB3	CG33927	0.632774104902944	1	1	pseudobulk	DESeq2	LRT	0
177	KC_AB3	CG33941	1.79428039147285	0.127333215710617	1	pseudobulk	DESeq2	LRT	0
177	KC_AB3	CG33947	-0.610068482242663	0.590431891627951	1	pseudobulk	DESeq2	LRT	0
177	KC_AB3	CG33969	-0.570684232199612	0.649182633395601	1	pseudobulk	DESeq2	LRT	0
177	KC_AB3	CG33970	-2.32240378246485	0.0515205004610801	1	pseudobulk	DESeq2	LRT	0
177	KC_AB3	CG33977	0.297192726498804	0.682284669337937	1	pseudobulk	DESeq2	LRT	0
177	KC_AB3	CG33978	-0.977406950181465	0.459265811735426	1	pseudobulk	DESeq2	LRT	0
177	KC_AB3	CG33993	-1.92515400829908	0.130600499012497	1	pseudobulk	DESeq2	LRT	0
177	KC_AB3	CG33998	0.170564247746436	0.556408483567885	1	pseudobulk	DESeq2	LRT	0
177	KC_AB3	CG34001	2.13877048085486	0.558952959988922	1	pseudobulk	DESeq2	LRT	0
177	KC_AB3	CG34008	1.23133864869565	1	1	pseudobulk	DESeq2	LRT	0
177	KC_AB3	CG34015	0.166068412595862	0.600803869643277	1	pseudobulk	DESeq2	LRT	0
177	KC_AB3	CG3402	0.775560846894614	0.399800825922565	1	pseudobulk	DESeq2	LRT	0
177	KC_AB3	CG34028	-1.36278797023253	0.317338139343912	1	pseudobulk	DESeq2	LRT	0
177	KC_AB3	CG34039	-0.267312703694252	0.736869926653066	1	pseudobulk	DESeq2	LRT	0
177	KC_AB3	CG34045	1.23133864869565	1	1	pseudobulk	DESeq2	LRT	0
178	KC_AB3	CG3407	1.88513752152589	0.739152646872234	1	pseudobulk	DESeq2	LRT	0
178	KC_AB3	CG3408	0.632774104902944	1	1	pseudobulk	DESeq2	LRT	0
178	KC_AB3	CG34116	-0.853854037925779	0.611355273229667	1	pseudobulk	DESeq2	LRT	0
178	KC_AB3	CG34117	-0.526600291269793	0.538473587087948	1	pseudobulk	DESeq2	LRT	0
178	KC_AB3	CG34126	0.157823089756238	0.591666753833048	1	pseudobulk	DESeq2	LRT	0
178	KC_AB3	CG34132	-0.144971397424108	0.715395853006999	1	pseudobulk	DESeq2	LRT	0
178	KC_AB3	CG34133	0.551830233839872	0.671984357608583	1	pseudobulk	DESeq2	LRT	0

178	KC_AB3	CG34134	0.599313146512024	0.751341704472417	1	pseudobulk	DESeq2	LRT	0
178	KC_AB3	CG34136	0.0020939056309239	1	1	pseudobulk	DESeq2	LRT	0
178	KC_AB3	CG34148	0.601885489520844	0.645591820815563	1	pseudobulk	DESeq2	LRT	0
178	KC_AB3	CG34149	0.0025701398975595	1	1	pseudobulk	DESeq2	LRT	0
178	KC_AB3	CG34150	0.799261058968887	0.492114129555808	1	pseudobulk	DESeq2	LRT	0
178	KC_AB3	CG34159	-0.402419406901664	0.342932671045535	1	pseudobulk	DESeq2	LRT	0
178	KC_AB3	CG34163	-0.343630862229391	0.761839396452642	1	pseudobulk	DESeq2	LRT	0
178	KC_AB3	CG34174	-1.15124768052744	0.372656915295434	1	pseudobulk	DESeq2	LRT	0
178	KC_AB3	CG34183	-0.513689897270525	0.575140543961917	1	pseudobulk	DESeq2	LRT	0
178	KC_AB3	CG34188	0.632774104902944	1	1	pseudobulk	DESeq2	LRT	0
178	KC_AB3	CG34195	0.756181798238401	0.810198154984395	1	pseudobulk	DESeq2	LRT	0
178	KC_AB3	CG34198	-0.184744780581018	1	1	pseudobulk	DESeq2	LRT	0
178	KC_AB3	CG3420	-0.300038825835773	0.632444787242924	1	pseudobulk	DESeq2	LRT	0
178	KC_AB3	CG34200	-0.186384992893844	0.517143532441446	1	pseudobulk	DESeq2	LRT	0
178	KC_AB3	CG34213	0.593667095203339	1	1	pseudobulk	DESeq2	LRT	0
178	KC_AB3	CG34219	-0.268747256859248	0.429405398464728	1	pseudobulk	DESeq2	LRT	0
178	KC_AB3	CG34222	0.632774104902944	1	1	pseudobulk	DESeq2	LRT	0
178	KC_AB3	CG34228	0.481407607299904	0.763581664559315	1	pseudobulk	DESeq2	LRT	0
178	KC_AB3	CG34229	0.485969923650358	0.69147449433061	1	pseudobulk	DESeq2	LRT	0
178	KC_AB3	CG34231	0.632774104902943	1	1	pseudobulk	DESeq2	LRT	0
178	KC_AB3	CG34232	1.23133864869565	1	1	pseudobulk	DESeq2	LRT	0
178	KC_AB3	CG34234	0.632774104902944	1	1	pseudobulk	DESeq2	LRT	0
178	KC_AB3	CG34242	-0.176842476196496	0.69684229563195	1	pseudobulk	DESeq2	LRT	0
178	KC_AB3	CG34250	-0.160788902751239	0.525889125263399	1	pseudobulk	DESeq2	LRT	0
178	KC_AB3	CG34256	1.95267092137636	1	1	pseudobulk	DESeq2	LRT	0
178	KC_AB3	CG34261	0.632774104902944	1	1	pseudobulk	DESeq2	LRT	0
178	KC_AB3	CG34286	1.23133864869565	1	1	pseudobulk	DESeq2	LRT	0
178	KC_AB3	CG34293	0.225040993434723	0.695572762491664	1	pseudobulk	DESeq2	LRT	0
178	KC_AB3	CG34309	0.632774104902944	1	1	pseudobulk	DESeq2	LRT	0
178	KC_AB3	CG34310	0.632774104902944	1	1	pseudobulk	DESeq2	LRT	0
178	KC_AB3	CG34325	0.632774104902944	1	1	pseudobulk	DESeq2	LRT	0
178	KC_AB3	CG34330	0.699757255263309	0.93897754694551	1	pseudobulk	DESeq2	LRT	0
178	KC_AB3	CG3434	1.71622639861318	0.549687911731452	1	pseudobulk	DESeq2	LRT	0
178	KC_AB3	CG34347	0.735715274553857	0.294096133057056	1	pseudobulk	DESeq2	LRT	0
178	KC_AB3	CG34348	-0.343859627219784	0.761749936319153	1	pseudobulk	DESeq2	LRT	0
178	KC_AB3	CG34351	0.429656984638018	0.693484292559537	1	pseudobulk	DESeq2	LRT	0
178	KC_AB3	CG34353	0.0388008779506432	0.732158546395441	1	pseudobulk	DESeq2	LRT	0
178	KC_AB3	CG34354	0.0978846209329206	0.642591325753334	1	pseudobulk	DESeq2	LRT	0
178	KC_AB3	CG34355	0.242817198850452	0.566334343800081	1	pseudobulk	DESeq2	LRT	0
178	KC_AB3	CG34357	0.0711925182565191	0.865530936244224	1	pseudobulk	DESeq2	LRT	0
178	KC_AB3	CG3436	-0.442627108409067	0.525037487893853	1	pseudobulk	DESeq2	LRT	0
178	KC_AB3	CG3437	-1.34314763376743	0.385543298192428	1	pseudobulk	DESeq2	LRT	0
178	KC_AB3	CG34370	-0.375059674301881	0.667733491890829	1	pseudobulk	DESeq2	LRT	0
178	KC_AB3	CG34376	-0.498687107133019	0.633853642756842	1	pseudobulk	DESeq2	LRT	0
178	KC_AB3	CG34377	-0.562114017828961	0.802765549578463	1	pseudobulk	DESeq2	LRT	0
178	KC_AB3	CG34384	-0.178176645679152	0.606283307135744	1	pseudobulk	DESeq2	LRT	0
178	KC_AB3	CG34401	-0.706980037376191	0.137787082822802	1	pseudobulk	DESeq2	LRT	0
178	KC_AB3	CG34402	-0.145272363997709	0.603852048001954	1	pseudobulk	DESeq2	LRT	0
178	KC_AB3	CG34404	0.551964922674103	0.515623817954298	1	pseudobulk	DESeq2	LRT	0
178	KC_AB3	CG34408	-0.346079684333652	0.693503217072141	1	pseudobulk	DESeq2	LRT	0
178	KC_AB3	CG34425	0.632774104902944	1	1	pseudobulk	DESeq2	LRT	0
178	KC_AB3	CG34456	0.632774104902944	1	1	pseudobulk	DESeq2	LRT	0
178	KC_AB3	CG3448	0.632774104902944	1	1	pseudobulk	DESeq2	LRT	0

178	KC_AB3	CG3500	-0.811498976005764	0.526338635124398	1	pseudobulk	DESeq2	LRT	0
178	KC_AB3	CG3511	1.32032676431938	0.427750132545264	1	pseudobulk	DESeq2	LRT	0
178	KC_AB3	CG3517	0.632774104902944	1	1	pseudobulk	DESeq2	LRT	0
178	KC_AB3	CG3527	0.692653999526471	0.683194363527121	1	pseudobulk	DESeq2	LRT	0
178	KC_AB3	CG3529	-0.283544091756824	0.622458947405618	1	pseudobulk	DESeq2	LRT	0
178	KC_AB3	CG3530	0.0419615854455778	0.513055951337887	1	pseudobulk	DESeq2	LRT	0
178	KC_AB3	CG3534	0.632774104902943	1	1	pseudobulk	DESeq2	LRT	0
178	KC_AB3	CG3542	0.0237052808445643	0.87404921247068	1	pseudobulk	DESeq2	LRT	0
178	KC_AB3	CG3548	0.181332464159774	0.636995681763363	1	pseudobulk	DESeq2	LRT	0
178	KC_AB3	CG3552	0.494110497880344	0.897268430762443	1	pseudobulk	DESeq2	LRT	0
178	KC_AB3	CG3556	-0.576975371615495	0.385075174349297	1	pseudobulk	DESeq2	LRT	0
178	KC_AB3	CG3558	-0.0699404782486616	0.659596910802238	1	pseudobulk	DESeq2	LRT	0
178	KC_AB3	CG3566	-0.0393018067525385	0.8998991403285	1	pseudobulk	DESeq2	LRT	0
178	KC_AB3	CG3568	0.457862016213519	0.425316339199986	1	pseudobulk	DESeq2	LRT	0
178	KC_AB3	CG3587	-0.52878559648638	0.686420589858048	1	pseudobulk	DESeq2	LRT	0
178	KC_AB3	CG3589	-0.792800206967055	0.5642994928178	1	pseudobulk	DESeq2	LRT	0
178	KC_AB3	CG3594	0.488984669381636	0.907827351240307	1	pseudobulk	DESeq2	LRT	0
178	KC_AB3	CG3609	1.23133864869565	1	1	pseudobulk	DESeq2	LRT	0
178	KC_AB3	CG3618	-0.208524840818113	0.749338456231829	1	pseudobulk	DESeq2	LRT	0
178	KC_AB3	CG3625	0.0114112788581564	0.639243613278861	1	pseudobulk	DESeq2	LRT	0
178	KC_AB3	CG3626	0.0025701398975595	1	1	pseudobulk	DESeq2	LRT	0
178	KC_AB3	CG3631	2.64926057711297	0.482244576496623	1	pseudobulk	DESeq2	LRT	0
178	KC_AB3	CG3632	0.34265342201782	0.630137698404256	1	pseudobulk	DESeq2	LRT	0
178	KC_AB3	CG3634	-0.304395941915412	0.627287837164189	1	pseudobulk	DESeq2	LRT	0
178	KC_AB3	CG3638	-0.48839433213506	0.392898713418103	1	pseudobulk	DESeq2	LRT	0
178	KC_AB3	CG3645	-0.244723912741144	0.770578184926794	1	pseudobulk	DESeq2	LRT	0
178	KC_AB3	CG3651	-0.104994555301603	0.679633049988773	1	pseudobulk	DESeq2	LRT	0
178	KC_AB3	CG3652	-0.21219254102173	0.753842547588505	1	pseudobulk	DESeq2	LRT	0
178	KC_AB3	CG3655	2.64926057711297	0.482244576496623	1	pseudobulk	DESeq2	LRT	0
178	KC_AB3	CG3662	0.142397174617903	0.556938325610369	1	pseudobulk	DESeq2	LRT	0
178	KC_AB3	CG3678	0.393349617217635	0.586901323238538	1	pseudobulk	DESeq2	LRT	0
178	KC_AB3	CG3700	-0.184744780581018	1	1	pseudobulk	DESeq2	LRT	0
178	KC_AB3	CG3702	-0.625217556102004	0.505860602601014	1	pseudobulk	DESeq2	LRT	0
178	KC_AB3	CG3703	0.103889763964527	0.575006671034016	1	pseudobulk	DESeq2	LRT	0
178	KC_AB3	CG3704	0.318750620495719	1	1	pseudobulk	DESeq2	LRT	0
178	KC_AB3	CG3719	0.170447932669831	0.608061273056703	1	pseudobulk	DESeq2	LRT	0
178	KC_AB3	CG3726	1.12059961403812	1	1	pseudobulk	DESeq2	LRT	0
178	KC_AB3	CG3732	0.341832016876565	0.577980564175023	1	pseudobulk	DESeq2	LRT	0
178	KC_AB3	CG3735	-0.184744780581018	1	1	pseudobulk	DESeq2	LRT	0
178	KC_AB3	CG3740	0.996493299826418	0.727730352663394	1	pseudobulk	DESeq2	LRT	0
179	KC_AB3	CG3744	-0.129306752015469	0.668818950531644	1	pseudobulk	DESeq2	LRT	0
179	KC_AB3	CG3756	0.517512004815322	0.725186735222178	1	pseudobulk	DESeq2	LRT	0
179	KC_AB3	CG3760	0.258051131782241	0.567630394563783	1	pseudobulk	DESeq2	LRT	0
179	KC_AB3	CG3764	0.0483818348975595	0.444215340896714	1	pseudobulk	DESeq2	LRT	0
179	KC_AB3	CG3776	-0.154661116978062	0.739916598803265	1	pseudobulk	DESeq2	LRT	0
179	KC_AB3	CG3777	0.632774104902944	1	1	pseudobulk	DESeq2	LRT	0
179	KC_AB3	CG3781	0.0911271160570854	0.734546292070014	1	pseudobulk	DESeq2	LRT	0
179	KC_AB3	CG3792	-0.842756156788523	0.386225977846051	1	pseudobulk	DESeq2	LRT	0
179	KC_AB3	CG3793	0.176027774583888	0.515891764437746	1	pseudobulk	DESeq2	LRT	0
179	KC_AB3	CG3803	0.148696213889022	0.812878060756928	1	pseudobulk	DESeq2	LRT	0
179	KC_AB3	CG3808	-0.933347948759194	0.604424649436326	1	pseudobulk	DESeq2	LRT	0
179	KC_AB3	CG3815	0.185409568099504	0.933599900628393	1	pseudobulk	DESeq2	LRT	0
179	KC_AB3	CG3817	-1.75169288298133	0.25300301904527	1	pseudobulk	DESeq2	LRT	0

179	KC_AB3	CG3819	0.305859900679973	1	1	pseudobulk	DESeq2	LRT	0
179	KC_AB3	CG3823	-0.383909528978698	0.756397128007823	1	pseudobulk	DESeq2	LRT	0
179	KC_AB3	CG3842	0.632774104902943	1	1	pseudobulk	DESeq2	LRT	0
179	KC_AB3	CG3847	-0.0842565066527362	0.8976062623206	1	pseudobulk	DESeq2	LRT	0
179	KC_AB3	CG3857	-2.27297904757785	0.154174513070766	1	pseudobulk	DESeq2	LRT	0
179	KC_AB3	CG3860	-0.365520883600099	0.55453863444955	1	pseudobulk	DESeq2	LRT	0
179	KC_AB3	CG3862	-1.89239420540325	0.18474025002941	1	pseudobulk	DESeq2	LRT	0
179	KC_AB3	CG3894	0.125546648879455	0.844846888277443	1	pseudobulk	DESeq2	LRT	0
179	KC_AB3	CG3902	1.95267092137636	1	1	pseudobulk	DESeq2	LRT	0
179	KC_AB3	CG3907	0.0744558301303237	0.752873659911079	1	pseudobulk	DESeq2	LRT	0
179	KC_AB3	CG3909	-0.0593883633694943	1	1	pseudobulk	DESeq2	LRT	0
179	KC_AB3	CG3918	-1.02496839252085	0.408971384717142	1	pseudobulk	DESeq2	LRT	0
179	KC_AB3	CG3919	0.632774104902944	1	1	pseudobulk	DESeq2	LRT	0
179	KC_AB3	CG3939	0.632774104902944	1	1	pseudobulk	DESeq2	LRT	0
179	KC_AB3	CG3955	-0.140462757295905	0.558239296359747	1	pseudobulk	DESeq2	LRT	0
179	KC_AB3	CG3982	0.632774104902944	1	1	pseudobulk	DESeq2	LRT	0
179	KC_AB3	CG3995	-0.378258474914136	0.706010934244435	1	pseudobulk	DESeq2	LRT	0
179	KC_AB3	CG3999	2.62817135292692	0.54954870385793	1	pseudobulk	DESeq2	LRT	0
179	KC_AB3	CG4000	-1.36652911181544	0.338707422956066	1	pseudobulk	DESeq2	LRT	0
179	KC_AB3	CG4004	0.115878321264628	0.865742593175529	1	pseudobulk	DESeq2	LRT	0
179	KC_AB3	CG40045	-0.0827479126999049	0.624904511030127	1	pseudobulk	DESeq2	LRT	0
179	KC_AB3	CG40191	-0.196010832592413	0.594287667360851	1	pseudobulk	DESeq2	LRT	0
179	KC_AB3	CG4022	-0.227145562289931	0.712659328415209	1	pseudobulk	DESeq2	LRT	0
179	KC_AB3	CG40228	0.0345910070252137	0.772206140928439	1	pseudobulk	DESeq2	LRT	0
179	KC_AB3	CG4025	0.20310445231258	0.802269556951236	1	pseudobulk	DESeq2	LRT	0
179	KC_AB3	CG4036	-0.205384879325428	0.770248427511297	1	pseudobulk	DESeq2	LRT	0
179	KC_AB3	CG4038	2.4096641859508	0.428875287944006	1	pseudobulk	DESeq2	LRT	0
179	KC_AB3	CG4041	-0.297871320590166	0.700398233200931	1	pseudobulk	DESeq2	LRT	0
179	KC_AB3	CG4042	-0.1720841719711	0.823357092762867	1	pseudobulk	DESeq2	LRT	0
179	KC_AB3	CG40439	1.37411035363009	0.556967526275709	1	pseudobulk	DESeq2	LRT	0
179	KC_AB3	CG4045	-0.263762666188106	0.779041324104227	1	pseudobulk	DESeq2	LRT	0
179	KC_AB3	CG40470	0.639786027981832	1	1	pseudobulk	DESeq2	LRT	0
179	KC_AB3	CG40472	-0.545136593466226	0.449501875044685	1	pseudobulk	DESeq2	LRT	0
179	KC_AB3	CG4049	-0.258665707410761	0.623221953359058	1	pseudobulk	DESeq2	LRT	0
179	KC_AB3	CG40498	-0.0601163610797672	0.819420203813018	1	pseudobulk	DESeq2	LRT	0
179	KC_AB3	CG4069	0.632774104902944	1	1	pseudobulk	DESeq2	LRT	0
179	KC_AB3	CG4074	1.21262516072997	0.469560971513786	1	pseudobulk	DESeq2	LRT	0
179	KC_AB3	CG4078	1.95267092137636	1	1	pseudobulk	DESeq2	LRT	0
179	KC_AB3	CG4080	0.218781390075057	0.581924846225218	1	pseudobulk	DESeq2	LRT	0
179	KC_AB3	CG4101	0.354956862410301	0.493741139466525	1	pseudobulk	DESeq2	LRT	0
179	KC_AB3	CG41099	-0.0800989763315056	0.613422897522826	1	pseudobulk	DESeq2	LRT	0
179	KC_AB3	CG41128	0.0848498328012019	0.57642409409713	1	pseudobulk	DESeq2	LRT	0
179	KC_AB3	CG4115	0.632774104902944	1	1	pseudobulk	DESeq2	LRT	0
179	KC_AB3	CG4119	0.335493346178734	0.492573836251112	1	pseudobulk	DESeq2	LRT	0
179	KC_AB3	CG4133	-0.0615107795974223	0.907452958466066	1	pseudobulk	DESeq2	LRT	0
179	KC_AB3	CG4186	0.77554105530017	1	1	pseudobulk	DESeq2	LRT	0
179	KC_AB3	CG4199	0.130020638491525	0.76823500365879	1	pseudobulk	DESeq2	LRT	0
179	KC_AB3	CG42231	1.47800220437468	0.708296824700333	1	pseudobulk	DESeq2	LRT	0
179	KC_AB3	CG42232	-0.763295935445257	0.413527165243838	1	pseudobulk	DESeq2	LRT	0
179	KC_AB3	CG42233	-0.931079887531931	0.582278950522693	1	pseudobulk	DESeq2	LRT	0
179	KC_AB3	CG42235	0.632774104902944	1	1	pseudobulk	DESeq2	LRT	0
179	KC_AB3	CG42238	-0.465436501905226	0.1342520400669	1	pseudobulk	DESeq2	LRT	0
179	KC_AB3	CG42239	-1.13259113815761	0.486173583538128	1	pseudobulk	DESeq2	LRT	0

179	KC_AB3	CG42240	0.959823741252395	0.269068833738717	1	pseudobulk	DESeq2	LRT	0
179	KC_AB3	CG42258	0.0494815095267884	0.510798646323268	1	pseudobulk	DESeq2	LRT	0
179	KC_AB3	CG42260	-0.216763150410124	0.356159536113706	1	pseudobulk	DESeq2	LRT	0
179	KC_AB3	CG42268	0.587795338344227	0.10552523494569	1	pseudobulk	DESeq2	LRT	0
179	KC_AB3	CG42271	1.46301486741067	0.28533031843481	1	pseudobulk	DESeq2	LRT	0
179	KC_AB3	CG4230	0.26286299695464	0.469491088338489	1	pseudobulk	DESeq2	LRT	0
179	KC_AB3	CG42307	1.14188260147974	0.82064435497816	1	pseudobulk	DESeq2	LRT	0
179	KC_AB3	CG42322	-0.188956460703771	0.633619357365005	1	pseudobulk	DESeq2	LRT	0
179	KC_AB3	CG42323	0.632774104902944	1	1	pseudobulk	DESeq2	LRT	0
179	KC_AB3	CG42324	0.134095593508438	0.711451932888478	1	pseudobulk	DESeq2	LRT	0
179	KC_AB3	CG42327	0.632774104902944	1	1	pseudobulk	DESeq2	LRT	0
179	KC_AB3	CG42336	0.306220691727479	0.676187501613157	1	pseudobulk	DESeq2	LRT	0
179	KC_AB3	CG42337	-0.615723014675993	0.103365812865233	1	pseudobulk	DESeq2	LRT	0
179	KC_AB3	CG42339	-0.203066591326934	0.478313910367669	1	pseudobulk	DESeq2	LRT	0
179	KC_AB3	CG42340	0.184726517648061	0.616501656166398	1	pseudobulk	DESeq2	LRT	0
179	KC_AB3	CG42342	0.329751249467339	0.575615140584971	1	pseudobulk	DESeq2	LRT	0
179	KC_AB3	CG42346	-0.66047346058956	0.352812636780514	1	pseudobulk	DESeq2	LRT	0
179	KC_AB3	CG42354	0.632774104902944	1	1	pseudobulk	DESeq2	LRT	0
179	KC_AB3	CG42359	0.164412683240909	0.795629240603799	1	pseudobulk	DESeq2	LRT	0
179	KC_AB3	CG42360	-0.380004576913793	0.618984784768229	1	pseudobulk	DESeq2	LRT	0
179	KC_AB3	CG42361	-0.278607317335475	0.354835948165855	1	pseudobulk	DESeq2	LRT	0
179	KC_AB3	CG42365	2.1445051383883	0.530068988727821	1	pseudobulk	DESeq2	LRT	0
179	KC_AB3	CG42376	-1.02093470650792	0.211021607278251	1	pseudobulk	DESeq2	LRT	0
179	KC_AB3	CG4238	0.178693191689334	0.728293315399408	1	pseudobulk	DESeq2	LRT	0
179	KC_AB3	CG42382	-0.254519419750211	0.734388775587942	1	pseudobulk	DESeq2	LRT	0
179	KC_AB3	CG4239	-1.36133428118551	0.344716768535403	1	pseudobulk	DESeq2	LRT	0
179	KC_AB3	CG42391	1.95267092137636	1	1	pseudobulk	DESeq2	LRT	0
179	KC_AB3	CG42392	-1.30693688696187	0.00188059255949192	1	pseudobulk	DESeq2	LRT	0
179	KC_AB3	CG42394	0.34057029593479	0.893503742282232	1	pseudobulk	DESeq2	LRT	0
179	KC_AB3	CG42399	-1.90757337143006	0.187915945935126	1	pseudobulk	DESeq2	LRT	0
179	KC_AB3	CG42402	0.00248559249843968	0.697465089428622	1	pseudobulk	DESeq2	LRT	0
179	KC_AB3	CG42404	-0.64971597424336	0.301288722137609	1	pseudobulk	DESeq2	LRT	0
179	KC_AB3	CG42450	-0.170770190263691	0.474708675831219	1	pseudobulk	DESeq2	LRT	0
179	KC_AB3	CG42495	-0.191268081609981	0.75401497613057	1	pseudobulk	DESeq2	LRT	0
180	KC_AB3	CG4250	0.632774104902944	1	1	pseudobulk	DESeq2	LRT	0
180	KC_AB3	CG42514	-0.120255328429205	0.914604900667141	1	pseudobulk	DESeq2	LRT	0
180	KC_AB3	CG42518	1.23133864869565	1	1	pseudobulk	DESeq2	LRT	0
180	KC_AB3	CG42526	2.19539902116932	0.526642538463226	1	pseudobulk	DESeq2	LRT	0
180	KC_AB3	CG42540	-0.742498390188323	0.0234906270213543	1	pseudobulk	DESeq2	LRT	0
180	KC_AB3	CG42541	0.247606881481669	0.315227792814473	1	pseudobulk	DESeq2	LRT	0
180	KC_AB3	CG42542	0.0213352143625378	0.745486899816175	1	pseudobulk	DESeq2	LRT	0
180	KC_AB3	CG42553	1.0989137265887	1	1	pseudobulk	DESeq2	LRT	0
180	KC_AB3	CG42554	-0.426680724027462	0.640143244624417	1	pseudobulk	DESeq2	LRT	0
180	KC_AB3	CG42588	1.01851300484389	0.279600875207868	1	pseudobulk	DESeq2	LRT	0
180	KC_AB3	CG42594	-2.40861242475001	0.0951595587900898	1	pseudobulk	DESeq2	LRT	0
180	KC_AB3	CG42613	-0.507283121477269	0.115512932686407	1	pseudobulk	DESeq2	LRT	0
180	KC_AB3	CG4266	0.229622091431227	0.599444248979402	1	pseudobulk	DESeq2	LRT	0
180	KC_AB3	CG42668	-0.406217953312432	0.279549908611038	1	pseudobulk	DESeq2	LRT	0
180	KC_AB3	CG42671	-0.268966582727044	0.671885435214852	1	pseudobulk	DESeq2	LRT	0
180	KC_AB3	CG42672	-0.143794705879956	0.715710214966878	1	pseudobulk	DESeq2	LRT	0
180	KC_AB3	CG42673	-0.0812219478469341	0.537306941523986	1	pseudobulk	DESeq2	LRT	0
180	KC_AB3	CG42674	-2.01852465620326	0.0405009190115801	1	pseudobulk	DESeq2	LRT	0
180	KC_AB3	CG42676	0.141662274479138	0.732010145113433	1	pseudobulk	DESeq2	LRT	0

180	KC_AB3	CG42692	1.95267092137636	1	1	pseudobulk	DESeq2	LRT	0
180	KC_AB3	CG42699	-0.292900171275135	0.714815654735109	1	pseudobulk	DESeq2	LRT	0
180	KC_AB3	CG42709	-0.843964045852311	0.366010733384235	1	pseudobulk	DESeq2	LRT	0
180	KC_AB3	CG42724	-0.0438515059251254	0.569577686121359	1	pseudobulk	DESeq2	LRT	0
180	KC_AB3	CG42748	-0.177617075092515	0.623517508702781	1	pseudobulk	DESeq2	LRT	0
180	KC_AB3	CG42750	0.638153664234818	0.0882087914359355	1	pseudobulk	DESeq2	LRT	0
180	KC_AB3	CG42751	1.95267092137636	1	1	pseudobulk	DESeq2	LRT	0
180	KC_AB3	CG4278	-1.05565013484538	0.47760875712905	1	pseudobulk	DESeq2	LRT	0
180	KC_AB3	CG42784	0.162291051050309	0.724057307882583	1	pseudobulk	DESeq2	LRT	0
180	KC_AB3	CG42788	0.140827226891782	0.693164439625607	1	pseudobulk	DESeq2	LRT	0
180	KC_AB3	CG42795	-0.227309305641556	0.574305414895526	1	pseudobulk	DESeq2	LRT	0
180	KC_AB3	CG42797	-1.13170491319012	0.336596199893676	1	pseudobulk	DESeq2	LRT	0
180	KC_AB3	CG42806	-3.15288704353942	0.0375158852012663	1	pseudobulk	DESeq2	LRT	0
180	KC_AB3	CG42809	0.594117478581602	1	1	pseudobulk	DESeq2	LRT	0
180	KC_AB3	CG4281	0.673455070620377	0.527560519668146	1	pseudobulk	DESeq2	LRT	0
180	KC_AB3	CG42813	0.140216758442117	0.540285593791095	1	pseudobulk	DESeq2	LRT	0
180	KC_AB3	CG4282	0.798131357004223	1	1	pseudobulk	DESeq2	LRT	0
180	KC_AB3	CG42855	1.4045634242129	0.452522445804646	1	pseudobulk	DESeq2	LRT	0
180	KC_AB3	CG4287	0.473657950296036	0.560217794911391	1	pseudobulk	DESeq2	LRT	0
180	KC_AB3	CG4291	-0.82460050656748	0.555962461799476	1	pseudobulk	DESeq2	LRT	0
180	KC_AB3	CG4293	1.22707713470494	1	1	pseudobulk	DESeq2	LRT	0
180	KC_AB3	CG4297	0.190678625198724	0.499378026383598	1	pseudobulk	DESeq2	LRT	0
180	KC_AB3	CG43049	0.632774104902943	1	1	pseudobulk	DESeq2	LRT	0
180	KC_AB3	CG43051	2.3149273646917	0.611236276445972	1	pseudobulk	DESeq2	LRT	0
180	KC_AB3	CG43056	0.632774104902943	1	1	pseudobulk	DESeq2	LRT	0
180	KC_AB3	CG43066	-0.217611787833054	0.559211621190781	1	pseudobulk	DESeq2	LRT	0
180	KC_AB3	CG43078	-0.054149994828589	0.58743779346569	1	pseudobulk	DESeq2	LRT	0
180	KC_AB3	CG43092	1.95267092137636	1	1	pseudobulk	DESeq2	LRT	0
180	KC_AB3	CG43093	1.95267092137636	1	1	pseudobulk	DESeq2	LRT	0
180	KC_AB3	CG43095	0.632774104902943	1	1	pseudobulk	DESeq2	LRT	0
180	KC_AB3	CG43102	-0.039483688566679	0.802754278681206	1	pseudobulk	DESeq2	LRT	0
180	KC_AB3	CG43117	0.568710977583255	1	1	pseudobulk	DESeq2	LRT	0
180	KC_AB3	CG43124	0.632774104902943	1	1	pseudobulk	DESeq2	LRT	0
180	KC_AB3	CG4313	0.759758178017103	1	1	pseudobulk	DESeq2	LRT	0
180	KC_AB3	CG43163	2.62817135292692	0.54954870385793	1	pseudobulk	DESeq2	LRT	0
180	KC_AB3	CG43191	0.632774104902944	1	1	pseudobulk	DESeq2	LRT	0
180	KC_AB3	CG43219	-0.547361577986966	0.814803379895176	1	pseudobulk	DESeq2	LRT	0
180	KC_AB3	CG43222	0.925564937598536	0.0894475887853055	1	pseudobulk	DESeq2	LRT	0
180	KC_AB3	CG43245	0.632774104902944	1	1	pseudobulk	DESeq2	LRT	0
180	KC_AB3	CG4329	0.0020939056309239	1	1	pseudobulk	DESeq2	LRT	0
180	KC_AB3	CG43313	0.632774104902944	1	1	pseudobulk	DESeq2	LRT	0
180	KC_AB3	CG4332	3.07272693214569	0.238940395988569	1	pseudobulk	DESeq2	LRT	0
180	KC_AB3	CG43324	0.0525051002344222	0.58085167245776	1	pseudobulk	DESeq2	LRT	0
180	KC_AB3	CG43342	1.23133864869565	1	1	pseudobulk	DESeq2	LRT	0
180	KC_AB3	CG43347	-0.0601489946313261	0.767108189958413	1	pseudobulk	DESeq2	LRT	0
180	KC_AB3	CG4335	-0.0327300486906476	1	1	pseudobulk	DESeq2	LRT	0
180	KC_AB3	CG43367	0.451062704628657	0.226578203419968	1	pseudobulk	DESeq2	LRT	0
180	KC_AB3	CG43373	0.598314265251263	0.216932014556292	1	pseudobulk	DESeq2	LRT	0
180	KC_AB3	CG43376	0.632774104902944	1	1	pseudobulk	DESeq2	LRT	0
180	KC_AB3	CG4338	0.655693739600423	0.657269919913425	1	pseudobulk	DESeq2	LRT	0
180	KC_AB3	CG43394	1.06106285917103	0.754249488000365	1	pseudobulk	DESeq2	LRT	0
180	KC_AB3	CG4341	-0.0710571709348161	0.707986398964215	1	pseudobulk	DESeq2	LRT	0
180	KC_AB3	CG43445	0.632774104902944	1	1	pseudobulk	DESeq2	LRT	0

180	KC_AB3	CG4360	0.321021416682947	0.618934287856948	1	pseudobulk	DESeq2	LRT		0
180	KC_AB3	CG4364	-0.265571325951155	0.732874865298341	1	pseudobulk	DESeq2	LRT		0
180	KC_AB3	CG43658	1.59359598433783	0.86377329553208	1	pseudobulk	DESeq2	LRT		0
180	KC_AB3	CG43693	1.95267092137636	1	1	pseudobulk	DESeq2	LRT		0
180	KC_AB3	CG43707	-0.15797149498393	0.57694308759313	1	pseudobulk	DESeq2	LRT		0
180	KC_AB3	CG43736	0.427647743407606	0.250384184140391	1	pseudobulk	DESeq2	LRT		0
180	KC_AB3	CG43737	-0.750490732242671	0.0339547812215677	1	pseudobulk	DESeq2	LRT		0
180	KC_AB3	CG43740	-0.739570937575533	0.503604988696696	1	pseudobulk	DESeq2	LRT		0
180	KC_AB3	CG43759	-0.344293176856248	0.203786078353188	1	pseudobulk	DESeq2	LRT		0
180	KC_AB3	CG43778	-0.256824289125403	0.575759279989534	1	pseudobulk	DESeq2	LRT		0
180	KC_AB3	CG43783	-0.838840274407195	0.485417284465545	1	pseudobulk	DESeq2	LRT		0
180	KC_AB3	CG43788	0.632774104902944	1	1	pseudobulk	DESeq2	LRT		0
180	KC_AB3	CG43789	0.632774104902943	1	1	pseudobulk	DESeq2	LRT		0
180	KC_AB3	CG43800	-0.560477492866218	0.784070523163114	1	pseudobulk	DESeq2	LRT		0
180	KC_AB3	CG43843	0.632774104902943	1	1	pseudobulk	DESeq2	LRT		0
180	KC_AB3	CG43850	-0.185513719742512	0.607075965433349	1	pseudobulk	DESeq2	LRT		0
180	KC_AB3	CG43861	-0.974835309593201	0.162262028045667	1	pseudobulk	DESeq2	LRT		0
180	KC_AB3	CG43867	0.0446153264042911	0.797316776319406	1	pseudobulk	DESeq2	LRT		0
180	KC_AB3	CG4390	-0.156904206644792	0.7089281239119	1	pseudobulk	DESeq2	LRT		0
180	KC_AB3	CG43901	0.0126999214590861	0.818963366102096	1	pseudobulk	DESeq2	LRT		0
180	KC_AB3	CG43902	2.19539902116932	0.526642538463226	1	pseudobulk	DESeq2	LRT		0
180	KC_AB3	CG4393	-1.0897553668535	0.356242285496338	1	pseudobulk	DESeq2	LRT		0
180	KC_AB3	CG44002	-0.185594513059781	1	1	pseudobulk	DESeq2	LRT		0
180	KC_AB3	CG44008	0.632774104902944	1	1	pseudobulk	DESeq2	LRT		0
180	KC_AB3	CG4404	-0.57840135831899	0.318268123882172	1	pseudobulk	DESeq2	LRT		0
180	KC_AB3	CG4407	0.756906830234527	0.603723706113333	1	pseudobulk	DESeq2	LRT		0
180	KC_AB3	CG4409	1.8751573179585	0.767073395253422	1	pseudobulk	DESeq2	LRT		0
180	KC_AB3	CG44098	1.95267092137636	1	1	pseudobulk	DESeq2	LRT		0
181	KC_AB3	CG44153	2.90025147168724	0.00538638874050917	1	pseudobulk	DESeq2	LRT		0
181	KC_AB3	CG44158	-1.05656802886995	0.454869360732975	1	pseudobulk	DESeq2	LRT		0
181	KC_AB3	CG4424	-1.06012604562781	0.449194148504691	1	pseudobulk	DESeq2	LRT		0
181	KC_AB3	CG44242	0.270803121222033	0.436501271711189	1	pseudobulk	DESeq2	LRT		0
181	KC_AB3	CG44243	-0.26477300364164	0.753660588932368	1	pseudobulk	DESeq2	LRT		0
181	KC_AB3	CG44245	0.632774104902944	1	1	pseudobulk	DESeq2	LRT		0
181	KC_AB3	CG44247	-0.30499506913705	0.443794037475764	1	pseudobulk	DESeq2	LRT		0
181	KC_AB3	CG4438	1.95267092137636	1	1	pseudobulk	DESeq2	LRT		0
181	KC_AB3	CG44422	2.242750022971	0.00423939393568874	1	pseudobulk	DESeq2	LRT		0
181	KC_AB3	CG4452	0.419385628014616	0.478029279380379	1	pseudobulk	DESeq2	LRT		0
181	KC_AB3	CG4467	0.0668216331852149	0.617104973396338	1	pseudobulk	DESeq2	LRT		0
181	KC_AB3	CG4476	0.632774104902943	1	1	pseudobulk	DESeq2	LRT		0
181	KC_AB3	CG44774	0.0963671417841098	0.570599395477911	1	pseudobulk	DESeq2	LRT		0
181	KC_AB3	CG44837	2.3149273646917	0.611236276445972	1	pseudobulk	DESeq2	LRT		0
181	KC_AB3	CG44838	-0.331649382027607	0.337467171052994	1	pseudobulk	DESeq2	LRT		0
181	KC_AB3	CG4497	0.316380108658924	0.525316581485782	1	pseudobulk	DESeq2	LRT		0
181	KC_AB3	CG45002	4.66747138185434	0.106528671559646	1	pseudobulk	DESeq2	LRT		0
181	KC_AB3	CG4502	0.119208990183563	0.538355134899542	1	pseudobulk	DESeq2	LRT		0
181	KC_AB3	CG45049	0.0765488575467977	0.593863193364781	1	pseudobulk	DESeq2	LRT		0
181	KC_AB3	CG45050	-0.0420994210361924	0.606978936894711	1	pseudobulk	DESeq2	LRT		0
181	KC_AB3	CG45071	-0.0923530970613437	0.632410074552859	1	pseudobulk	DESeq2	LRT		0
181	KC_AB3	CG45081	1.59576167909336	0.887029991980463	1	pseudobulk	DESeq2	LRT		0
181	KC_AB3	CG4511	-1.98431294122278	0.132235518767603	1	pseudobulk	DESeq2	LRT		0
181	KC_AB3	CG4520	0.632774104902944	1	1	pseudobulk	DESeq2	LRT		0
181	KC_AB3	CG45263	-0.242240624412691	0.296062294971969	1	pseudobulk	DESeq2	LRT		0

181	KC_AB3	CG4537	0.149133779918015	1	1	pseudobulk	DESeq2	LRT		0
181	KC_AB3	CG4538	-0.248640101878239	0.675867172779057	1	pseudobulk	DESeq2	LRT		0
181	KC_AB3	CG45428	2.62817135292692	0.54954870385793	1	pseudobulk	DESeq2	LRT		0
181	KC_AB3	CG4552	-0.3709882719148	0.63544504983749	1	pseudobulk	DESeq2	LRT		0
181	KC_AB3	CG4553	1.06081529894343	0.754356232755953	1	pseudobulk	DESeq2	LRT		0
181	KC_AB3	CG4554	0.0020939056309239	1	1	pseudobulk	DESeq2	LRT		0
181	KC_AB3	CG4557	0.850850710489179	0.632720440542834	1	pseudobulk	DESeq2	LRT		0
181	KC_AB3	CG4558	0.381267561741905	0.690575971932215	1	pseudobulk	DESeq2	LRT		0
181	KC_AB3	CG45603	0.0020939056309239	1	1	pseudobulk	DESeq2	LRT		0
181	KC_AB3	CG4562	0.780995816113069	0.496070926842921	1	pseudobulk	DESeq2	LRT		0
181	KC_AB3	CG4565	0.790010899828778	1	1	pseudobulk	DESeq2	LRT		0
181	KC_AB3	CG4572	1.95267092137636	1	1	pseudobulk	DESeq2	LRT		0
181	KC_AB3	CG4577	-0.292061666097565	0.321859071728883	1	pseudobulk	DESeq2	LRT		0
181	KC_AB3	CG4587	-0.212919943765941	0.593922088000376	1	pseudobulk	DESeq2	LRT		0
181	KC_AB3	CG4593	-1.88696057335282	0.241297186219987	1	pseudobulk	DESeq2	LRT		0
181	KC_AB3	CG4596	0.0394798626901713	0.952537527870738	1	pseudobulk	DESeq2	LRT		0
181	KC_AB3	CG4598	-0.853854037925779	0.611355273229667	1	pseudobulk	DESeq2	LRT		0
181	KC_AB3	CG4603	-0.0387478417558035	0.786997082497757	1	pseudobulk	DESeq2	LRT		0
181	KC_AB3	CG4610	1.32934523627547	0.192776272019874	1	pseudobulk	DESeq2	LRT		0
181	KC_AB3	CG4611	-0.400046519007236	0.964129537865033	1	pseudobulk	DESeq2	LRT		0
181	KC_AB3	CG4612	-0.246238053807994	0.370583127005166	1	pseudobulk	DESeq2	LRT		0
181	KC_AB3	CG4615	0.37072141629083	0.649823934198553	1	pseudobulk	DESeq2	LRT		0
181	KC_AB3	CG4617	0.115216433565035	0.615750046027938	1	pseudobulk	DESeq2	LRT		0
181	KC_AB3	CG4619	1.84671504053046	1	1	pseudobulk	DESeq2	LRT		0
181	KC_AB3	CG4622	0.0787646459241481	0.830631241595568	1	pseudobulk	DESeq2	LRT		0
181	KC_AB3	CG4627	-0.294622816774992	0.772314250939593	1	pseudobulk	DESeq2	LRT		0
181	KC_AB3	CG46280	0.475542826683752	0.517347349407635	1	pseudobulk	DESeq2	LRT		0
181	KC_AB3	CG46281	-0.0711933168887147	1	1	pseudobulk	DESeq2	LRT		0
181	KC_AB3	CG4629	-0.991288476054954	0.318286532803131	1	pseudobulk	DESeq2	LRT		0
181	KC_AB3	CG46301	-0.0065512130899609	0.562202315102067	1	pseudobulk	DESeq2	LRT		0
181	KC_AB3	CG4631	0.632774104902943	1	1	pseudobulk	DESeq2	LRT		0
181	KC_AB3	CG46338	-0.0659625599385247	0.863851856862139	1	pseudobulk	DESeq2	LRT		0
181	KC_AB3	CG46339	-0.933053153416052	0.0103587058157215	1	pseudobulk	DESeq2	LRT		0
181	KC_AB3	CG46385	-0.104547101357469	0.549604248026351	1	pseudobulk	DESeq2	LRT		0
181	KC_AB3	CG46387	0.632774104902943	1	1	pseudobulk	DESeq2	LRT		0
181	KC_AB3	CG4645	-0.416776998096369	0.616701081269048	1	pseudobulk	DESeq2	LRT		0
181	KC_AB3	CG4646	0.480378821404291	0.534998228432889	1	pseudobulk	DESeq2	LRT		0
181	KC_AB3	CG4658	0.148647346056614	0.537862181038975	1	pseudobulk	DESeq2	LRT		0
181	KC_AB3	CG4660	-0.772482229006561	0.123424166741411	1	pseudobulk	DESeq2	LRT		0
181	KC_AB3	CG4661	1.95267092137636	1	1	pseudobulk	DESeq2	LRT		0
181	KC_AB3	CG4676	0.191108455713932	0.808368180717691	1	pseudobulk	DESeq2	LRT		0
181	KC_AB3	CG4678	0.388398606658397	0.407282930338494	1	pseudobulk	DESeq2	LRT		0
181	KC_AB3	CG4679	-0.50333114794564	0.616130100237568	1	pseudobulk	DESeq2	LRT		0
181	KC_AB3	CG4686	1.20789915773961	0.124369420186113	1	pseudobulk	DESeq2	LRT		0
181	KC_AB3	CG4707	0.621208378560518	1	1	pseudobulk	DESeq2	LRT		0
181	KC_AB3	CG4709	-0.13718526997977	1	1	pseudobulk	DESeq2	LRT		0
181	KC_AB3	CG4730	0.632774104902944	1	1	pseudobulk	DESeq2	LRT		0
181	KC_AB3	CG4743	0.4135959724801	1	1	pseudobulk	DESeq2	LRT		0
181	KC_AB3	CG4751	-1.21885025268643	0.392245376508061	1	pseudobulk	DESeq2	LRT		0
181	KC_AB3	CG4763	0.632774104902943	1	1	pseudobulk	DESeq2	LRT		0
181	KC_AB3	CG4768	0.639915054695206	0.345544683972065	1	pseudobulk	DESeq2	LRT		0
181	KC_AB3	CG4788	-0.189034062112624	0.792414468453207	1	pseudobulk	DESeq2	LRT		0
181	KC_AB3	CG4789	-0.79666018628099	0.296403184417948	1	pseudobulk	DESeq2	LRT		0

181	KC_AB3	CG4806	0.574922256784116	0.54759881296145	1	pseudobulk	DESeq2	LRT		0
181	KC_AB3	CG4813	-0.279787604946107	0.85069427849287	1	pseudobulk	DESeq2	LRT		0
181	KC_AB3	CG4820	-0.174000362326889	0.802917580604449	1	pseudobulk	DESeq2	LRT		0
181	KC_AB3	CG4825	0.620954807881443	0.796066846017661	1	pseudobulk	DESeq2	LRT		0
181	KC_AB3	CG4829	-1.18348062730302	0.505231178661888	1	pseudobulk	DESeq2	LRT		0
181	KC_AB3	CG4849	0.875145527921513	0.741029504952822	1	pseudobulk	DESeq2	LRT		0
181	KC_AB3	CG4853	0.440015442969718	0.777701196310368	1	pseudobulk	DESeq2	LRT		0
181	KC_AB3	CG4854	-0.186961738271884	1	1	pseudobulk	DESeq2	LRT		0
181	KC_AB3	CG4858	0.646575238773626	0.651166205789619	1	pseudobulk	DESeq2	LRT		0
181	KC_AB3	CG4866	-1.6110818117999	0.28467798399635	1	pseudobulk	DESeq2	LRT		0
181	KC_AB3	CG4880	0.0020939056309239	1	1	pseudobulk	DESeq2	LRT		0
181	KC_AB3	CG4882	-0.606508186280786	0.58544958215239	1	pseudobulk	DESeq2	LRT		0
181	KC_AB3	CG4887	-1.46049442939739	0.126723491434661	1	pseudobulk	DESeq2	LRT		0
181	KC_AB3	CG4896	-0.0913305610489847	0.700806932166687	1	pseudobulk	DESeq2	LRT		0
181	KC_AB3	CG4901	0.467708815235283	1	1	pseudobulk	DESeq2	LRT		0
181	KC_AB3	CG4908	0.65762966094718	0.621234112679855	1	pseudobulk	DESeq2	LRT		0
181	KC_AB3	CG4911	0.549063178847013	0.501302347214806	1	pseudobulk	DESeq2	LRT		0
181	KC_AB3	CG4935	-0.229039602237312	1	1	pseudobulk	DESeq2	LRT		0
181	KC_AB3	CG4936	-0.0762491920305812	0.703350345193443	1	pseudobulk	DESeq2	LRT		0
181	KC_AB3	CG4942	-0.291105566108764	0.852442434704911	1	pseudobulk	DESeq2	LRT		0
181	KC_AB3	CG4949	1.23133864869565	1	1	pseudobulk	DESeq2	LRT		0
181	KC_AB3	CG4951	1.16099758470784	0.570843610789316	1	pseudobulk	DESeq2	LRT		0
182	KC_AB3	CG4953	0.49408580417248	0.88350040777222	1	pseudobulk	DESeq2	LRT		0
182	KC_AB3	CG4957	-0.616071951939002	0.55438820102709	1	pseudobulk	DESeq2	LRT		0
182	KC_AB3	CG4968	0.174108696331848	0.631603905670767	1	pseudobulk	DESeq2	LRT		0
182	KC_AB3	CG4972	1.91601206439215	0.439574641803634	1	pseudobulk	DESeq2	LRT		0
182	KC_AB3	CG4984	0.632774104902943	1	1	pseudobulk	DESeq2	LRT		0
182	KC_AB3	CG5003	0.512901577540203	0.74287803907252	1	pseudobulk	DESeq2	LRT		0
182	KC_AB3	CG5004	-1.23901582083763	0.338102353370084	1	pseudobulk	DESeq2	LRT		0
182	KC_AB3	CG5009	0.740076558422264	0.771979400218668	1	pseudobulk	DESeq2	LRT		0
182	KC_AB3	CG5013	-0.606050694161965	0.606945043299773	1	pseudobulk	DESeq2	LRT		0
182	KC_AB3	CG5021	0.0325431033711839	0.872935604460552	1	pseudobulk	DESeq2	LRT		0
182	KC_AB3	CG5022	0.421517269754874	0.505660566411619	1	pseudobulk	DESeq2	LRT		0
182	KC_AB3	CG5027	-0.275406750233187	0.707475074849765	1	pseudobulk	DESeq2	LRT		0
182	KC_AB3	CG5028	0.0531187313643124	0.680996690096029	1	pseudobulk	DESeq2	LRT		0
182	KC_AB3	CG5033	2.72820644206127	0.273632857301596	1	pseudobulk	DESeq2	LRT		0
182	KC_AB3	CG5037	-0.455496496939574	0.586155818740325	1	pseudobulk	DESeq2	LRT		0
182	KC_AB3	CG5038	-0.184744780581018	1	1	pseudobulk	DESeq2	LRT		0
182	KC_AB3	CG5039	0.632774104902944	1	1	pseudobulk	DESeq2	LRT		0
182	KC_AB3	CG5044	-0.287665305735437	0.655295377236347	1	pseudobulk	DESeq2	LRT		0
182	KC_AB3	CG5050	1.95267092137636	1	1	pseudobulk	DESeq2	LRT		0
182	KC_AB3	CG5056	0.172255584489795	0.609631830520667	1	pseudobulk	DESeq2	LRT		0
182	KC_AB3	CG5059	0.429364427675496	0.608988518291925	1	pseudobulk	DESeq2	LRT		0
182	KC_AB3	CG5065	-0.382036360074466	0.33199839446605	1	pseudobulk	DESeq2	LRT		0
182	KC_AB3	CG5068	0.684010846060335	0.285455369122006	1	pseudobulk	DESeq2	LRT		0
182	KC_AB3	CG5071	-0.185594513059781	1	1	pseudobulk	DESeq2	LRT		0
182	KC_AB3	CG5079	1.95267092137636	1	1	pseudobulk	DESeq2	LRT		0
182	KC_AB3	CG5087	-0.344761467885438	0.701509238363084	1	pseudobulk	DESeq2	LRT		0
182	KC_AB3	CG5098	-0.603880717310531	0.542830803747347	1	pseudobulk	DESeq2	LRT		0
182	KC_AB3	CG5104	0.414593131727451	1	1	pseudobulk	DESeq2	LRT		0
182	KC_AB3	CG5110	0.037139520669311	0.864250762800856	1	pseudobulk	DESeq2	LRT		0
182	KC_AB3	CG5114	0.438973952551762	1	1	pseudobulk	DESeq2	LRT		0
182	KC_AB3	CG5116	0.632774104902944	1	1	pseudobulk	DESeq2	LRT		0

182	KC_AB3	CG5126	-0.186961738271884	1	1	pseudobulk	DESeq2	LRT	0
182	KC_AB3	CG5131	0.305680611015153	1	1	pseudobulk	DESeq2	LRT	0
182	KC_AB3	CG5149	0.35122455329731	0.658405200994704	1	pseudobulk	DESeq2	LRT	0
182	KC_AB3	CG5151	-0.0059764632877730	0.690625041630114	1	pseudobulk	DESeq2	LRT	0
182	KC_AB3	CG5157	0.632774104902944	1	1	pseudobulk	DESeq2	LRT	0
182	KC_AB3	CG5174	-0.198910379246797	0.61446643412451	1	pseudobulk	DESeq2	LRT	0
182	KC_AB3	CG5177	2.62817135292692	0.54954870385793	1	pseudobulk	DESeq2	LRT	0
182	KC_AB3	CG5180	-1.32193495440625	0.330348554290152	1	pseudobulk	DESeq2	LRT	0
182	KC_AB3	CG5181	-0.34383042184164	0.660996340579644	1	pseudobulk	DESeq2	LRT	0
182	KC_AB3	CG5188	-1.58973829274406	0.352754719714348	1	pseudobulk	DESeq2	LRT	0
182	KC_AB3	CG5191	0.528914650833881	1	1	pseudobulk	DESeq2	LRT	0
182	KC_AB3	CG5196	-0.152598669628575	0.738626033725355	1	pseudobulk	DESeq2	LRT	0
182	KC_AB3	CG5199	0.0576455966116859	0.91661042110746	1	pseudobulk	DESeq2	LRT	0
182	KC_AB3	CG5214	-0.1381724880532	0.659302277758156	1	pseudobulk	DESeq2	LRT	0
182	KC_AB3	CG5220	-0.848976662347701	0.466151105807721	1	pseudobulk	DESeq2	LRT	0
182	KC_AB3	CG5262	-0.286250330328022	0.782280967605218	1	pseudobulk	DESeq2	LRT	0
182	KC_AB3	CG5270	1.59359598433783	0.86377329553208	1	pseudobulk	DESeq2	LRT	0
182	KC_AB3	CG5273	0.69792702464193	0.502171269222088	1	pseudobulk	DESeq2	LRT	0
182	KC_AB3	CG5274	0.211402989800783	0.841247100694194	1	pseudobulk	DESeq2	LRT	0
182	KC_AB3	CG5276	1.12059961403812	1	1	pseudobulk	DESeq2	LRT	0
182	KC_AB3	CG5281	-0.087031105113558	0.634565320282223	1	pseudobulk	DESeq2	LRT	0
182	KC_AB3	CG5282	0.0278670032891879	0.817134797173191	1	pseudobulk	DESeq2	LRT	0
182	KC_AB3	CG5285	0.810807113092937	0.644088181038271	1	pseudobulk	DESeq2	LRT	0
182	KC_AB3	CG5287	0.790402236745665	0.286225950082386	1	pseudobulk	DESeq2	LRT	0
182	KC_AB3	CG5290	2.3149273646917	0.611236276445972	1	pseudobulk	DESeq2	LRT	0
182	KC_AB3	CG5292	-0.633342702137624	0.478107885321154	1	pseudobulk	DESeq2	LRT	0
182	KC_AB3	CG5316	-0.554299888438616	0.485417109408712	1	pseudobulk	DESeq2	LRT	0
182	KC_AB3	CG5321	0.632774104902943	1	1	pseudobulk	DESeq2	LRT	0
182	KC_AB3	CG5323	-0.561340870932967	0.534627100011094	1	pseudobulk	DESeq2	LRT	0
182	KC_AB3	CG5339	0.1721942287818	0.798879841216573	1	pseudobulk	DESeq2	LRT	0
182	KC_AB3	CG5348	-0.560477492866218	0.784070523163114	1	pseudobulk	DESeq2	LRT	0
182	KC_AB3	CG5355	0.29806380777653	0.707406520397871	1	pseudobulk	DESeq2	LRT	0
182	KC_AB3	CG5359	0.575256600809562	0.332270002787766	1	pseudobulk	DESeq2	LRT	0
182	KC_AB3	CG5380	1.86689087684802	0.535325076131883	1	pseudobulk	DESeq2	LRT	0
182	KC_AB3	CG5382	-0.783554431668194	0.495521370490855	1	pseudobulk	DESeq2	LRT	0
182	KC_AB3	CG5412	0.951144707177691	0.489490586530597	1	pseudobulk	DESeq2	LRT	0
182	KC_AB3	CG5439	-0.483201243060734	0.645843804054382	1	pseudobulk	DESeq2	LRT	0
182	KC_AB3	CG5445	-0.576147694538189	0.490172491172336	1	pseudobulk	DESeq2	LRT	0
182	KC_AB3	CG5446	-0.607666744569285	0.41403052111802	1	pseudobulk	DESeq2	LRT	0
182	KC_AB3	CG5447	0.900229985692037	0.146113288697288	1	pseudobulk	DESeq2	LRT	0
182	KC_AB3	CG5455	0.230046633916169	0.66875299754153	1	pseudobulk	DESeq2	LRT	0
182	KC_AB3	CG5458	0.161515868851812	1	1	pseudobulk	DESeq2	LRT	0
182	KC_AB3	CG5466	0.615361688505515	0.335225019091054	1	pseudobulk	DESeq2	LRT	0
182	KC_AB3	CG5498	0.632774104902943	1	1	pseudobulk	DESeq2	LRT	0
182	KC_AB3	CG5500	-0.289425880855084	0.651754351617389	1	pseudobulk	DESeq2	LRT	0
182	KC_AB3	CG5504	-0.880850376253346	0.307266265637899	1	pseudobulk	DESeq2	LRT	0
182	KC_AB3	CG5509	0.632774104902944	1	1	pseudobulk	DESeq2	LRT	0
182	KC_AB3	CG5510	-0.884270048467637	0.440892102409407	1	pseudobulk	DESeq2	LRT	0
182	KC_AB3	CG5515	-0.186822819050973	0.685925381872981	1	pseudobulk	DESeq2	LRT	0
182	KC_AB3	CG5516	0.0465749275831391	0.736645601248423	1	pseudobulk	DESeq2	LRT	0
182	KC_AB3	CG5521	0.632537776013729	0.447362990726305	1	pseudobulk	DESeq2	LRT	0
182	KC_AB3	CG5522	-0.032092985386282	0.905422117157762	1	pseudobulk	DESeq2	LRT	0
182	KC_AB3	CG5532	-0.782129215199889	0.279719963930886	1	pseudobulk	DESeq2	LRT	0

182	KC_AB3	CG5535	-0.754569156587427	0.499437304706856	1	pseudobulk	DESeq2	LRT	0
182	KC_AB3	CG5543	1.93210469959844	0.634076921097483	1	pseudobulk	DESeq2	LRT	0
182	KC_AB3	CG5554	-0.369566415768981	0.60458613417599	1	pseudobulk	DESeq2	LRT	0
182	KC_AB3	CG5555	-0.362978680040443	0.586817290500179	1	pseudobulk	DESeq2	LRT	0
182	KC_AB3	CG5567	-1.01109393112947	0.426889153349125	1	pseudobulk	DESeq2	LRT	0
182	KC_AB3	CG5569	-0.166389448180126	0.747692675017338	1	pseudobulk	DESeq2	LRT	0
182	KC_AB3	CG5589	1.23133864869565	1	1	pseudobulk	DESeq2	LRT	0
182	KC_AB3	CG5590	0.196331120591073	1	1	pseudobulk	DESeq2	LRT	0
182	KC_AB3	CG5599	1.59359598433783	0.86377329553208	1	pseudobulk	DESeq2	LRT	0
182	KC_AB3	CG5608	-0.749757324245205	0.523013300962106	1	pseudobulk	DESeq2	LRT	0
182	KC_AB3	CG5611	-0.751467251652363	0.610657777502579	1	pseudobulk	DESeq2	LRT	0
182	KC_AB3	CG5612	1.95267092137636	1	1	pseudobulk	DESeq2	LRT	0
182	KC_AB3	CG5618	0.904857485982405	0.863591387425197	1	pseudobulk	DESeq2	LRT	0
182	KC_AB3	CG5641	0.00416632571704587	0.614146833708307	1	pseudobulk	DESeq2	LRT	0
182	KC_AB3	CG5644	0.407838159947076	0.442756876192909	1	pseudobulk	DESeq2	LRT	0
182	KC_AB3	CG5646	0.278551142201039	0.819496371689126	1	pseudobulk	DESeq2	LRT	0
183	KC_AB3	CG5664	1.44807872909241	0.846133773817643	1	pseudobulk	DESeq2	LRT	0
183	KC_AB3	CG5674	0.236249650473432	0.766254919380136	1	pseudobulk	DESeq2	LRT	0
183	KC_AB3	CG5676	-0.192690393484668	0.613651082335095	1	pseudobulk	DESeq2	LRT	0
183	KC_AB3	CG5708	0.3406211501163	0.424116539778033	1	pseudobulk	DESeq2	LRT	0
183	KC_AB3	CG5721	-0.0251883360732537	0.895916609941625	1	pseudobulk	DESeq2	LRT	0
183	KC_AB3	CG5726	-0.966686778168707	0.414764470707044	1	pseudobulk	DESeq2	LRT	0
183	KC_AB3	CG5727	0.0637416529919669	0.630317475999822	1	pseudobulk	DESeq2	LRT	0
183	KC_AB3	CG5728	1.12059961403812	1	1	pseudobulk	DESeq2	LRT	0
183	KC_AB3	CG5742	-1.018256842474	0.360153825943409	1	pseudobulk	DESeq2	LRT	0
183	KC_AB3	CG5745	-0.13718526997977	1	1	pseudobulk	DESeq2	LRT	0
183	KC_AB3	CG5746	-0.333819598240423	0.405868701947521	1	pseudobulk	DESeq2	LRT	0
183	KC_AB3	CG5758	0.0573734686146666	0.860561659815083	1	pseudobulk	DESeq2	LRT	0
183	KC_AB3	CG5787	-0.00543091883071163	0.682107385300686	1	pseudobulk	DESeq2	LRT	0
183	KC_AB3	CG5789	1.95267092137636	1	1	pseudobulk	DESeq2	LRT	0
183	KC_AB3	CG5793	1.23133864869565	1	1	pseudobulk	DESeq2	LRT	0
183	KC_AB3	CG5805	-0.157439079755098	0.748808608274045	1	pseudobulk	DESeq2	LRT	0
183	KC_AB3	CG5808	-0.835071691045028	0.645830720505476	1	pseudobulk	DESeq2	LRT	0
183	KC_AB3	CG5815	0.435279348735246	1	1	pseudobulk	DESeq2	LRT	0
183	KC_AB3	CG5823	-0.0146289951347212	0.541373722523632	1	pseudobulk	DESeq2	LRT	0
183	KC_AB3	CG5828	0.293640150203267	0.704824080735809	1	pseudobulk	DESeq2	LRT	0
183	KC_AB3	CG5830	-0.134635593551583	0.539389792018937	1	pseudobulk	DESeq2	LRT	0
183	KC_AB3	CG5846	0.179292784526877	0.830944558671915	1	pseudobulk	DESeq2	LRT	0
183	KC_AB3	CG5850	1.3297418896474	0.192708768022253	1	pseudobulk	DESeq2	LRT	0
183	KC_AB3	CG5854	0.632774104902944	1	1	pseudobulk	DESeq2	LRT	0
183	KC_AB3	CG5861	0.330874242415946	0.566578345425432	1	pseudobulk	DESeq2	LRT	0
183	KC_AB3	CG5862	-0.573246682136261	0.596219757359087	1	pseudobulk	DESeq2	LRT	0
183	KC_AB3	CG5867	0.00209390563092397	1	1	pseudobulk	DESeq2	LRT	0
183	KC_AB3	CG5877	-1.33451458674231	0.422357959515779	1	pseudobulk	DESeq2	LRT	0
183	KC_AB3	CG5880	0.998556473833416	0.215198117736233	1	pseudobulk	DESeq2	LRT	0
183	KC_AB3	CG5885	-0.0385237941261412	0.63950549508353	1	pseudobulk	DESeq2	LRT	0
183	KC_AB3	CG5886	0.0561716111586602	0.935900289616787	1	pseudobulk	DESeq2	LRT	0
183	KC_AB3	CG5888	-0.107023776504053	0.735944302585278	1	pseudobulk	DESeq2	LRT	0
183	KC_AB3	CG5890	-0.571051576504697	0.162680086971442	1	pseudobulk	DESeq2	LRT	0
183	KC_AB3	CG5902	0.517925341452983	0.53007837081017	1	pseudobulk	DESeq2	LRT	0
183	KC_AB3	CG5903	-0.289437963224524	0.34182554096207	1	pseudobulk	DESeq2	LRT	0
183	KC_AB3	CG5913	-0.987189378304843	0.481223954187462	1	pseudobulk	DESeq2	LRT	0
183	KC_AB3	CG5916	0.941658869927879	0.170065209679306	1	pseudobulk	DESeq2	LRT	0

183	KC_AB3	CG5934	0.432159045155965	0.333671311215585	1	pseudobulk	DESeq2	LRT	0
183	KC_AB3	CG5937	0.617805002527226	0.361633737701377	1	pseudobulk	DESeq2	LRT	0
183	KC_AB3	CG5938	-0.108981178476715	0.527853416722406	1	pseudobulk	DESeq2	LRT	0
183	KC_AB3	CG5946	0.288971180304047	0.447303339780727	1	pseudobulk	DESeq2	LRT	0
183	KC_AB3	CG5953	-0.185594513059781	1	1	pseudobulk	DESeq2	LRT	0
183	KC_AB3	CG5955	2.3149273646917	0.611236276445972	1	pseudobulk	DESeq2	LRT	0
183	KC_AB3	CG5961	0.195664704886625	0.798926539671751	1	pseudobulk	DESeq2	LRT	0
183	KC_AB3	CG5968	1.04436316651002	1	1	pseudobulk	DESeq2	LRT	0
183	KC_AB3	CG5969	-0.300943866051969	0.664892458181483	1	pseudobulk	DESeq2	LRT	0
183	KC_AB3	CG5986	-0.136169268687392	0.669621802576695	1	pseudobulk	DESeq2	LRT	0
183	KC_AB3	CG5989	-0.443880810522208	0.646451158566482	1	pseudobulk	DESeq2	LRT	0
183	KC_AB3	CG6000	1.95267092137636	1	1	pseudobulk	DESeq2	LRT	0
183	KC_AB3	CG6005	0.608236637143085	0.579746287233232	1	pseudobulk	DESeq2	LRT	0
183	KC_AB3	CG6006	-1.11937801958418	0.295968198931491	1	pseudobulk	DESeq2	LRT	0
183	KC_AB3	CG6013	-0.405528366579653	0.539693454634178	1	pseudobulk	DESeq2	LRT	0
183	KC_AB3	CG6023	0.632774104902943	1	1	pseudobulk	DESeq2	LRT	0
183	KC_AB3	CG6024	-0.328926297661309	0.397782427696432	1	pseudobulk	DESeq2	LRT	0
183	KC_AB3	CG6028	1.05852712208531	0.521487993805385	1	pseudobulk	DESeq2	LRT	0
183	KC_AB3	CG6036	0.632774104902944	1	1	pseudobulk	DESeq2	LRT	0
183	KC_AB3	CG6040	-0.186961738271884	1	1	pseudobulk	DESeq2	LRT	0
183	KC_AB3	CG6041	-2.1266272220067	0.199019891939749	1	pseudobulk	DESeq2	LRT	0
183	KC_AB3	CG6044	-0.247712926595739	0.310955946271604	1	pseudobulk	DESeq2	LRT	0
183	KC_AB3	CG6051	0.555679313765181	0.41849333369307	1	pseudobulk	DESeq2	LRT	0
183	KC_AB3	CG6066	0.785641151849316	0.773726274845071	1	pseudobulk	DESeq2	LRT	0
183	KC_AB3	CG6073	0.632774104902944	1	1	pseudobulk	DESeq2	LRT	0
183	KC_AB3	CG6083	1.59576167909336	0.887029991980463	1	pseudobulk	DESeq2	LRT	0
183	KC_AB3	CG6084	-1.00730731534605	0.546390305747609	1	pseudobulk	DESeq2	LRT	0
183	KC_AB3	CG6094	-0.725660095085314	0.533400183317757	1	pseudobulk	DESeq2	LRT	0
183	KC_AB3	CG6115	0.206799734715613	0.502508297452192	1	pseudobulk	DESeq2	LRT	0
183	KC_AB3	CG6123	-0.185594513059781	1	1	pseudobulk	DESeq2	LRT	0
183	KC_AB3	CG6136	1.63890065715102	0.438548372038653	1	pseudobulk	DESeq2	LRT	0
183	KC_AB3	CG6144	-0.647943047915683	0.366510657285829	1	pseudobulk	DESeq2	LRT	0
183	KC_AB3	CG6145	-0.0916327195660743	0.705902053506193	1	pseudobulk	DESeq2	LRT	0
183	KC_AB3	CG6153	2.1445051383883	0.530068988727821	1	pseudobulk	DESeq2	LRT	0
183	KC_AB3	CG6154	-0.618426225750661	0.121577801403571	1	pseudobulk	DESeq2	LRT	0
183	KC_AB3	CG6163	0.594117478581602	1	1	pseudobulk	DESeq2	LRT	0
183	KC_AB3	CG6171	0.374212320008472	0.757120746656975	1	pseudobulk	DESeq2	LRT	0
183	KC_AB3	CG6178	2.69211178539882	0.142008410636692	1	pseudobulk	DESeq2	LRT	0
183	KC_AB3	CG6179	0.789779005847331	0.583006212839495	1	pseudobulk	DESeq2	LRT	0
183	KC_AB3	CG6180	0.0604004394389187	0.661243601856734	1	pseudobulk	DESeq2	LRT	0
183	KC_AB3	CG6184	0.332652779265186	0.483748337635045	1	pseudobulk	DESeq2	LRT	0
183	KC_AB3	CG6191	-0.202724715612693	0.730544388649832	1	pseudobulk	DESeq2	LRT	0
183	KC_AB3	CG6195	0.42139485829852	0.680641833060975	1	pseudobulk	DESeq2	LRT	0
183	KC_AB3	CG6201	1.4630841789279	0.172922048074693	1	pseudobulk	DESeq2	LRT	0
183	KC_AB3	CG6204	-0.0672673788660764	1	1	pseudobulk	DESeq2	LRT	0
183	KC_AB3	CG6218	-0.0111749369273221	0.409516219957737	1	pseudobulk	DESeq2	LRT	0
183	KC_AB3	CG6227	-1.61894743278388	0.259373573668413	1	pseudobulk	DESeq2	LRT	0
183	KC_AB3	CG6230	0.639786027981832	1	1	pseudobulk	DESeq2	LRT	0
183	KC_AB3	CG6231	1.23133864869565	1	1	pseudobulk	DESeq2	LRT	0
183	KC_AB3	CG6236	0.568268985276844	0.912357815758873	1	pseudobulk	DESeq2	LRT	0
183	KC_AB3	CG6254	-0.435984073754947	0.706830553871167	1	pseudobulk	DESeq2	LRT	0
183	KC_AB3	CG6276	0.366692072894908	0.596627090045627	1	pseudobulk	DESeq2	LRT	0
183	KC_AB3	CG6282	-0.189595343626083	0.436160322343963	1	pseudobulk	DESeq2	LRT	0

183	KC_AB3	CG6287	1.95267092137636	1	1	pseudobulk	DESeq2	LRT	0
183	KC_AB3	CG6293	-0.186961738271884	1	1	pseudobulk	DESeq2	LRT	0
183	KC_AB3	CG6325	0.527439668447581	1	1	pseudobulk	DESeq2	LRT	0
183	KC_AB3	CG6329	-0.146845975855187	0.485405715542807	1	pseudobulk	DESeq2	LRT	0
183	KC_AB3	CG6330	-0.0881048000636072	0.40884797304645	1	pseudobulk	DESeq2	LRT	0
183	KC_AB3	CG6340	-0.0779400799444708	0.695865035163101	1	pseudobulk	DESeq2	LRT	0
183	KC_AB3	CG6345	0.632774104902944	1	1	pseudobulk	DESeq2	LRT	0
183	KC_AB3	CG6356	1.14188260147974	0.82064435497816	1	pseudobulk	DESeq2	LRT	0
183	KC_AB3	CG6357	2.62817135292692	0.54954870385793	1	pseudobulk	DESeq2	LRT	0
183	KC_AB3	CG6379	-0.232778749665896	0.674497845629219	1	pseudobulk	DESeq2	LRT	0
184	KC_AB3	CG6418	0.463945077915346	0.532759900745365	1	pseudobulk	DESeq2	LRT	0
184	KC_AB3	CG6420	-1.03990474527781	0.381227846075073	1	pseudobulk	DESeq2	LRT	0
184	KC_AB3	CG6424	0.632774104902944	1	1	pseudobulk	DESeq2	LRT	0
184	KC_AB3	CG6425	0.249787394738164	1	1	pseudobulk	DESeq2	LRT	0
184	KC_AB3	CG6428	0.34469165666001	0.809953566646887	1	pseudobulk	DESeq2	LRT	0
184	KC_AB3	CG6443	-0.0241516252653703	0.609478097532629	1	pseudobulk	DESeq2	LRT	0
184	KC_AB3	CG6454	-0.0500599555697147	0.618079337882718	1	pseudobulk	DESeq2	LRT	0
184	KC_AB3	CG6470	1.95267092137636	1	1	pseudobulk	DESeq2	LRT	0
184	KC_AB3	CG6472	0.632774104902944	1	1	pseudobulk	DESeq2	LRT	0
184	KC_AB3	CG6479	0.632774104902944	1	1	pseudobulk	DESeq2	LRT	0
184	KC_AB3	CG6481	0.632774104902944	1	1	pseudobulk	DESeq2	LRT	0
184	KC_AB3	CG6495	-0.604095312187447	0.459036814332985	1	pseudobulk	DESeq2	LRT	0
184	KC_AB3	CG6511	1.12353287855391	0.562368820459146	1	pseudobulk	DESeq2	LRT	0
184	KC_AB3	CG6512	0.32272741551768	0.667093217965998	1	pseudobulk	DESeq2	LRT	0
184	KC_AB3	CG6523	-0.341185086693287	0.480153192175411	1	pseudobulk	DESeq2	LRT	0
184	KC_AB3	CG6527	0.632774104902944	1	1	pseudobulk	DESeq2	LRT	0
184	KC_AB3	CG6550	0.968634025215337	1	1	pseudobulk	DESeq2	LRT	0
184	KC_AB3	CG6565	0.760168845856676	0.331184981956681	1	pseudobulk	DESeq2	LRT	0
184	KC_AB3	CG6567	1.66915607199259	0.414453879754371	1	pseudobulk	DESeq2	LRT	0
184	KC_AB3	CG6574	2.83964872000554	0.240684919262511	1	pseudobulk	DESeq2	LRT	0
184	KC_AB3	CG6583	-0.380538684826987	0.692347997580521	1	pseudobulk	DESeq2	LRT	0
184	KC_AB3	CG6607	-0.693623256459429	0.428553317687445	1	pseudobulk	DESeq2	LRT	0
184	KC_AB3	CG6610	-0.0711933168887147	1	1	pseudobulk	DESeq2	LRT	0
184	KC_AB3	CG6617	-0.586637624673893	0.387279818220625	1	pseudobulk	DESeq2	LRT	0
184	KC_AB3	CG6621	1.14400346292199	0.259371368514991	1	pseudobulk	DESeq2	LRT	0
184	KC_AB3	CG6628	0.632774104902944	1	1	pseudobulk	DESeq2	LRT	0
184	KC_AB3	CG6638	0.632774104902944	1	1	pseudobulk	DESeq2	LRT	0
184	KC_AB3	CG6650	0.0578630818178232	0.916666767587641	1	pseudobulk	DESeq2	LRT	0
184	KC_AB3	CG6654	-0.120528428548273	0.914416877354382	1	pseudobulk	DESeq2	LRT	0
184	KC_AB3	CG6659	-1.28074813721928	0.341123972061916	1	pseudobulk	DESeq2	LRT	0
184	KC_AB3	CG6664	0.247186730884088	0.695232845474547	1	pseudobulk	DESeq2	LRT	0
184	KC_AB3	CG6665	-0.0626788152302253	0.82829540026287	1	pseudobulk	DESeq2	LRT	0
184	KC_AB3	CG6674	0.544029429858645	0.503329103825136	1	pseudobulk	DESeq2	LRT	0
184	KC_AB3	CG6685	1.71062829653273	0.614680951739819	1	pseudobulk	DESeq2	LRT	0
184	KC_AB3	CG6686	0.209638942996982	0.574925795188171	1	pseudobulk	DESeq2	LRT	0
184	KC_AB3	CG6689	0.336728598231627	0.734063263004444	1	pseudobulk	DESeq2	LRT	0
184	KC_AB3	CG6693	0.934982385769195	0.351095649259476	1	pseudobulk	DESeq2	LRT	0
184	KC_AB3	CG6695	0.438392768241677	1	1	pseudobulk	DESeq2	LRT	0
184	KC_AB3	CG6700	-0.0347019633805591	0.672327356456611	1	pseudobulk	DESeq2	LRT	0
184	KC_AB3	CG6707	0.0526169735753306	0.880922451974468	1	pseudobulk	DESeq2	LRT	0
184	KC_AB3	CG6712	-0.105581846590776	0.797297744598995	1	pseudobulk	DESeq2	LRT	0
184	KC_AB3	CG6723	2.64926057711297	0.482244576496623	1	pseudobulk	DESeq2	LRT	0
184	KC_AB3	CG6724	0.530929063645009	0.766900988264411	1	pseudobulk	DESeq2	LRT	0

184	KC_AB3	CG6729	-0.963850792303616	0.481278563360969	1	pseudobulk	DESeq2	LRT		0
184	KC_AB3	CG6752	2.36698786238084	0.294790555962472	1	pseudobulk	DESeq2	LRT		0
184	KC_AB3	CG6758	-0.221703057450068	0.565484294108746	1	pseudobulk	DESeq2	LRT		0
184	KC_AB3	CG6762	0.2160908195545	0.783101517602521	1	pseudobulk	DESeq2	LRT		0
184	KC_AB3	CG6763	1.95267092137636	1	1	pseudobulk	DESeq2	LRT		0
184	KC_AB3	CG6765	-0.444515809318334	0.47631907891685	1	pseudobulk	DESeq2	LRT		0
184	KC_AB3	CG6766	-1.13832017321223	0.284157989991642	1	pseudobulk	DESeq2	LRT		0
184	KC_AB3	CG6767	0.122417781845789	0.574547765882097	1	pseudobulk	DESeq2	LRT		0
184	KC_AB3	CG6769	0.0801735577577996	0.900156332381356	1	pseudobulk	DESeq2	LRT		0
184	KC_AB3	CG6770	-0.442166935168332	0.171446475211112	1	pseudobulk	DESeq2	LRT		0
184	KC_AB3	CG6791	-0.398037776036676	0.610043183880959	1	pseudobulk	DESeq2	LRT		0
184	KC_AB3	CG6805	-0.0427682598512494	0.353578325480778	1	pseudobulk	DESeq2	LRT		0
184	KC_AB3	CG6808	-0.184744780581018	1	1	pseudobulk	DESeq2	LRT		0
184	KC_AB3	CG6813	0.209554561423276	0.715275355595908	1	pseudobulk	DESeq2	LRT		0
184	KC_AB3	CG6833	-1.68849875795641	0.246642804813056	1	pseudobulk	DESeq2	LRT		0
184	KC_AB3	CG6836	1.95267092137636	1	1	pseudobulk	DESeq2	LRT		0
184	KC_AB3	CG6841	-0.331488439761303	0.63690233252083	1	pseudobulk	DESeq2	LRT		0
184	KC_AB3	CG6843	0.521318909998902	0.529035372579114	1	pseudobulk	DESeq2	LRT		0
184	KC_AB3	CG6867	0.0409576754284232	0.716608698443314	1	pseudobulk	DESeq2	LRT		0
184	KC_AB3	CG6878	0.512418712050764	0.221281357975433	1	pseudobulk	DESeq2	LRT		0
184	KC_AB3	CG6903	0.757229211699233	0.770485041699765	1	pseudobulk	DESeq2	LRT		0
184	KC_AB3	CG6907	1.13212249605286	0.867286905232711	1	pseudobulk	DESeq2	LRT		0
184	KC_AB3	CG6912	1.97890667062975	0.390732853722831	1	pseudobulk	DESeq2	LRT		0
184	KC_AB3	CG6923	-0.405913659466425	0.721926065672244	1	pseudobulk	DESeq2	LRT		0
184	KC_AB3	CG6928	2.62817135292692	0.54954870385793	1	pseudobulk	DESeq2	LRT		0
184	KC_AB3	CG6937	-0.0898663316387701	0.855031762958412	1	pseudobulk	DESeq2	LRT		0
184	KC_AB3	CG6938	0.632774104902944	1	1	pseudobulk	DESeq2	LRT		0
184	KC_AB3	CG6961	0.146246774579159	0.8097538400014	1	pseudobulk	DESeq2	LRT		0
184	KC_AB3	CG6962	0.759758178017103	1	1	pseudobulk	DESeq2	LRT		0
184	KC_AB3	CG6966	0.463867190334246	0.237944624568662	1	pseudobulk	DESeq2	LRT		0
184	KC_AB3	CG6983	-0.550105901315351	0.436725717875306	1	pseudobulk	DESeq2	LRT		0
184	KC_AB3	CG6984	2.08873676009282	0.356171360275953	1	pseudobulk	DESeq2	LRT		0
184	KC_AB3	CG7006	0.414593131727451	1	1	pseudobulk	DESeq2	LRT		0
184	KC_AB3	CG7009	-0.79318133048928	0.522367741514103	1	pseudobulk	DESeq2	LRT		0
184	KC_AB3	CG7011	-0.0116155408580918	0.751876471414558	1	pseudobulk	DESeq2	LRT		0
184	KC_AB3	CG7028	-0.0491022866338932	0.7120845115514	1	pseudobulk	DESeq2	LRT		0
184	KC_AB3	CG7029	0.139003113817122	0.51698348101045	1	pseudobulk	DESeq2	LRT		0
184	KC_AB3	CG7044	-1.98044701123315	0.489192348139976	1	pseudobulk	DESeq2	LRT		0
184	KC_AB3	CG7049	0.482432338015589	1	1	pseudobulk	DESeq2	LRT		0
184	KC_AB3	CG7058	-0.331884564221727	0.593033295817603	1	pseudobulk	DESeq2	LRT		0
184	KC_AB3	CG7059	0.366134831381199	0.958550265698161	1	pseudobulk	DESeq2	LRT		0
184	KC_AB3	CG7065	0.141459867786903	0.646308297822707	1	pseudobulk	DESeq2	LRT		0
184	KC_AB3	CG7069	0.438973952551762	1	1	pseudobulk	DESeq2	LRT		0
184	KC_AB3	CG7083	-0.306378104066181	0.656770989566556	1	pseudobulk	DESeq2	LRT		0
184	KC_AB3	CG7084	1.95267092137636	1	1	pseudobulk	DESeq2	LRT		0
184	KC_AB3	CG7099	-0.186961738271884	1	1	pseudobulk	DESeq2	LRT		0
184	KC_AB3	CG7101	1.50187980871111	0.691691001584769	1	pseudobulk	DESeq2	LRT		0
184	KC_AB3	CG7102	0.632774104902944	1	1	pseudobulk	DESeq2	LRT		0
184	KC_AB3	CG7115	0.0088882523557927	0.752146660948418	1	pseudobulk	DESeq2	LRT		0
184	KC_AB3	CG7120	-0.786590095973747	0.325771932445572	1	pseudobulk	DESeq2	LRT		0
184	KC_AB3	CG7133	-0.518840907275457	0.603006660217849	1	pseudobulk	DESeq2	LRT		0
184	KC_AB3	CG7137	0.632774104902944	1	1	pseudobulk	DESeq2	LRT		0
184	KC_AB3	CG7139	0.315436404321479	0.693506076908165	1	pseudobulk	DESeq2	LRT		0

184	KC_AB3	CG7148	-0.613355387553881	0.516012907504236	1	pseudobulk	DESeq2	LRT		0
184	KC_AB3	CG7149	2.13877048085486	0.558952959988922	1	pseudobulk	DESeq2	LRT		0
184	KC_AB3	CG7154	1.46806994051593	0.0582608738928631	1	pseudobulk	DESeq2	LRT		0
184	KC_AB3	CG7156	-0.0591601901315235	1	1	pseudobulk	DESeq2	LRT		0
185	KC_AB3	CG7166	-0.217162823938749	0.458665878314298	1	pseudobulk	DESeq2	LRT		0
185	KC_AB3	CG7168	1.37201545480514	0.369334702113428	1	pseudobulk	DESeq2	LRT		0
185	KC_AB3	CG7182	0.964632078822801	0.613356434008034	1	pseudobulk	DESeq2	LRT		0
185	KC_AB3	CG7183	-0.908116463842773	0.45392176452227	1	pseudobulk	DESeq2	LRT		0
185	KC_AB3	CG7203	0.340432129965576	1	1	pseudobulk	DESeq2	LRT		0
185	KC_AB3	CG7206	0.154397908580869	0.769008121183113	1	pseudobulk	DESeq2	LRT		0
185	KC_AB3	CG7218	0.146576789517357	0.809706489058964	1	pseudobulk	DESeq2	LRT		0
185	KC_AB3	CG7220	0.233010546514578	0.726897465166909	1	pseudobulk	DESeq2	LRT		0
185	KC_AB3	CG7222	0.126501230080225	0.670038276513845	1	pseudobulk	DESeq2	LRT		0
185	KC_AB3	CG7231	-0.475374529079869	0.469356308409863	1	pseudobulk	DESeq2	LRT		0
185	KC_AB3	CG7239	-0.188621119954309	0.7386806244516	1	pseudobulk	DESeq2	LRT		0
185	KC_AB3	CG7265	0.690479044565549	0.767775667127486	1	pseudobulk	DESeq2	LRT		0
185	KC_AB3	CG7272	0.75572823498085	0.810389204999521	1	pseudobulk	DESeq2	LRT		0
185	KC_AB3	CG7275	1.95267092137636	1	1	pseudobulk	DESeq2	LRT		0
185	KC_AB3	CG7289	-0.871427859970374	0.535486977559646	1	pseudobulk	DESeq2	LRT		0
185	KC_AB3	CG7324	-0.773685135988182	0.45957490542214	1	pseudobulk	DESeq2	LRT		0
185	KC_AB3	CG7326	0.130807371119026	0.835818474282739	1	pseudobulk	DESeq2	LRT		0
185	KC_AB3	CG7332	-0.0488193697080191	0.584393483245311	1	pseudobulk	DESeq2	LRT		0
185	KC_AB3	CG7339	0.10279142284778	0.849937297831246	1	pseudobulk	DESeq2	LRT		0
185	KC_AB3	CG7352	0.632774104902943	1	1	pseudobulk	DESeq2	LRT		0
185	KC_AB3	CG7368	-3.15860973476799	0.0124240091768369	1	pseudobulk	DESeq2	LRT		0
185	KC_AB3	CG7369	0.460746461200331	0.742950108219176	1	pseudobulk	DESeq2	LRT		0
185	KC_AB3	CG7376	1.95267092137636	1	1	pseudobulk	DESeq2	LRT		0
185	KC_AB3	CG7378	-0.22498594538551	0.322305801033053	1	pseudobulk	DESeq2	LRT		0
185	KC_AB3	CG7379	-0.0869585200485398	0.700959820168875	1	pseudobulk	DESeq2	LRT		0
185	KC_AB3	CG7381	1.95267092137636	1	1	pseudobulk	DESeq2	LRT		0
185	KC_AB3	CG7382	-0.102858096952832	0.660542186104723	1	pseudobulk	DESeq2	LRT		0
185	KC_AB3	CG7394	-0.182232093433964	0.609382003110453	1	pseudobulk	DESeq2	LRT		0
185	KC_AB3	CG7407	-0.305679305518367	0.424872014477616	1	pseudobulk	DESeq2	LRT		0
185	KC_AB3	CG7414	-0.52780460160628	0.428381921803712	1	pseudobulk	DESeq2	LRT		0
185	KC_AB3	CG7420	0.077761817165399	1	1	pseudobulk	DESeq2	LRT		0
185	KC_AB3	CG7430	-0.0329100328621097	0.792369701153776	1	pseudobulk	DESeq2	LRT		0
185	KC_AB3	CG7453	0.11946792512888	0.765076885369135	1	pseudobulk	DESeq2	LRT		0
185	KC_AB3	CG7461	0.343530455593162	1	1	pseudobulk	DESeq2	LRT		0
185	KC_AB3	CG7483	-0.358914585075137	0.616319148909737	1	pseudobulk	DESeq2	LRT		0
185	KC_AB3	CG7484	0.514278715180982	0.592850655731506	1	pseudobulk	DESeq2	LRT		0
185	KC_AB3	CG7488	0.650907446221134	0.785362401778475	1	pseudobulk	DESeq2	LRT		0
185	KC_AB3	CG7492	-0.852185728862826	0.29556763078639	1	pseudobulk	DESeq2	LRT		0
185	KC_AB3	CG7504	0.594117478581602	1	1	pseudobulk	DESeq2	LRT		0
185	KC_AB3	CG7506	1.13411758494726	0.195433259238941	1	pseudobulk	DESeq2	LRT		0
185	KC_AB3	CG7518	-0.223081095985168	0.764610030952552	1	pseudobulk	DESeq2	LRT		0
185	KC_AB3	CG7519	0.399956047155185	0.579915285630964	1	pseudobulk	DESeq2	LRT		0
185	KC_AB3	CG7523	-0.379402940001048	0.708351494558668	1	pseudobulk	DESeq2	LRT		0
185	KC_AB3	CG7530	1.23133864869565	1	1	pseudobulk	DESeq2	LRT		0
185	KC_AB3	CG7536	2.12302691045992	0.658751388073158	1	pseudobulk	DESeq2	LRT		0
185	KC_AB3	CG7546	0.0803521383996509	0.715565285976633	1	pseudobulk	DESeq2	LRT		0
185	KC_AB3	CG7550	-0.787941446947624	0.558718244729915	1	pseudobulk	DESeq2	LRT		0
185	KC_AB3	CG7556	-0.54512114909935	0.635517535082833	1	pseudobulk	DESeq2	LRT		0
185	KC_AB3	CG7564	-0.129726711405961	0.676847265068312	1	pseudobulk	DESeq2	LRT		0

185	KC_AB3	CG7565	-0.0636807735290782	0.613454693082198	1	pseudobulk	DESeq2	LRT	0
185	KC_AB3	CG7582	0.295424368377986	0.492888660972905	1	pseudobulk	DESeq2	LRT	0
185	KC_AB3	CG7600	0.932377568357744	0.576702519133407	1	pseudobulk	DESeq2	LRT	0
185	KC_AB3	CG7601	1.04515988920267	1	1	pseudobulk	DESeq2	LRT	0
185	KC_AB3	CG7607	-0.373868179296869	0.745931138008953	1	pseudobulk	DESeq2	LRT	0
185	KC_AB3	CG7611	-0.46807617595448	0.509999734455736	1	pseudobulk	DESeq2	LRT	0
185	KC_AB3	CG7630	-0.15431178823534	0.431583500019194	1	pseudobulk	DESeq2	LRT	0
185	KC_AB3	CG7632	0.414593131727451	1	1	pseudobulk	DESeq2	LRT	0
185	KC_AB3	CG7637	-0.479601184617365	0.439565095653239	1	pseudobulk	DESeq2	LRT	0
185	KC_AB3	CG7638	0.612850078279868	0.562759824328918	1	pseudobulk	DESeq2	LRT	0
185	KC_AB3	CG7639	-0.813755364681178	0.363489829637959	1	pseudobulk	DESeq2	LRT	0
185	KC_AB3	CG7646	0.152941826371908	0.497930587169076	1	pseudobulk	DESeq2	LRT	0
185	KC_AB3	CG7650	-0.911872778350846	0.564492477106869	1	pseudobulk	DESeq2	LRT	0
185	KC_AB3	CG7655	-0.386557073875609	0.717828815305475	1	pseudobulk	DESeq2	LRT	0
185	KC_AB3	CG7656	0.376733293999498	0.44313666410582	1	pseudobulk	DESeq2	LRT	0
185	KC_AB3	CG7668	-1.95673356104399	0.202341422403298	1	pseudobulk	DESeq2	LRT	0
185	KC_AB3	CG7685	-1.7560203900089	0.190362669290659	1	pseudobulk	DESeq2	LRT	0
185	KC_AB3	CG7692	0.798131357004223	1	1	pseudobulk	DESeq2	LRT	0
185	KC_AB3	CG7694	0.198561441563418	0.774832459766625	1	pseudobulk	DESeq2	LRT	0
185	KC_AB3	CG7706	0.0981895386498904	0.821931433018798	1	pseudobulk	DESeq2	LRT	0
185	KC_AB3	CG7718	0.161515868851812	1	1	pseudobulk	DESeq2	LRT	0
185	KC_AB3	CG7728	1.23133864869565	1	1	pseudobulk	DESeq2	LRT	0
185	KC_AB3	CG7737	1.02009234801989	0.246359212513055	1	pseudobulk	DESeq2	LRT	0
185	KC_AB3	CG7739	-0.063800834998337	0.636005380147606	1	pseudobulk	DESeq2	LRT	0
185	KC_AB3	CG7741	-0.160804957331434	0.818391168986531	1	pseudobulk	DESeq2	LRT	0
185	KC_AB3	CG7744	1.95267092137636	1	1	pseudobulk	DESeq2	LRT	0
185	KC_AB3	CG7745	-0.0210094075817855	0.816235300954536	1	pseudobulk	DESeq2	LRT	0
185	KC_AB3	CG7747	-2.42385808264761	0.0803817520246616	1	pseudobulk	DESeq2	LRT	0
185	KC_AB3	CG7766	0.174644851103139	0.766113782982718	1	pseudobulk	DESeq2	LRT	0
185	KC_AB3	CG7772	-0.144374127620475	0.812302221232896	1	pseudobulk	DESeq2	LRT	0
185	KC_AB3	CG7781	-0.165608975869349	0.397532104872141	1	pseudobulk	DESeq2	LRT	0
185	KC_AB3	CG7785	0.208165675947382	0.657643343021798	1	pseudobulk	DESeq2	LRT	0
185	KC_AB3	CG7791	0.408201027199079	0.824090789015403	1	pseudobulk	DESeq2	LRT	0
185	KC_AB3	CG7806	-0.232714443854297	0.839472778407846	1	pseudobulk	DESeq2	LRT	0
185	KC_AB3	CG7810	0.601923922666198	0.674894710795899	1	pseudobulk	DESeq2	LRT	0
185	KC_AB3	CG7824	0.632774104902944	1	1	pseudobulk	DESeq2	LRT	0
185	KC_AB3	CG7834	-0.558370635945677	0.46183333337106	1	pseudobulk	DESeq2	LRT	0
185	KC_AB3	CG7839	-0.227302102825738	0.712641564533483	1	pseudobulk	DESeq2	LRT	0
185	KC_AB3	CG7840	-1.48995456299667	0.407892516841853	1	pseudobulk	DESeq2	LRT	0
185	KC_AB3	CG7841	0.757168229264176	0.603596969657716	1	pseudobulk	DESeq2	LRT	0
185	KC_AB3	CG7849	0.878533177611258	0.776982044426051	1	pseudobulk	DESeq2	LRT	0
185	KC_AB3	CG7857	-0.7189773223923	0.560289649639123	1	pseudobulk	DESeq2	LRT	0
185	KC_AB3	CG7872	-0.0619589632727531	0.925641565560052	1	pseudobulk	DESeq2	LRT	0
185	KC_AB3	CG7878	-0.808207985799935	0.350589906896549	1	pseudobulk	DESeq2	LRT	0
185	KC_AB3	CG7879	-0.15252799505071	0.563140166834699	1	pseudobulk	DESeq2	LRT	0
185	KC_AB3	CG7888	0.593667095203339	1	1	pseudobulk	DESeq2	LRT	0
185	KC_AB3	CG7889	-0.546690377232218	0.815223005364861	1	pseudobulk	DESeq2	LRT	0
185	KC_AB3	CG7914	1.59576167909336	0.887029991980463	1	pseudobulk	DESeq2	LRT	0
185	KC_AB3	CG7920	0.0066038027525489	0.498510253747948	1	pseudobulk	DESeq2	LRT	0
185	KC_AB3	CG7943	-0.179328274333204	0.631512953888806	1	pseudobulk	DESeq2	LRT	0
185	KC_AB3	CG7945	0.174214580765533	0.586247266325171	1	pseudobulk	DESeq2	LRT	0
186	KC_AB3	CG7946	-0.414802063063469	0.634077447598136	1	pseudobulk	DESeq2	LRT	0
186	KC_AB3	CG7956	0.336255160872334	0.606043550233924	1	pseudobulk	DESeq2	LRT	0

186	KC_AB3	CG7970	0.428997237408529	0.700820042179921	1	pseudobulk	DESeq2	LRT	0
186	KC_AB3	CG7971	-0.0185166080364751	0.590573138382095	1	pseudobulk	DESeq2	LRT	0
186	KC_AB3	CG7974	0.516108242369322	0.801500446539243	1	pseudobulk	DESeq2	LRT	0
186	KC_AB3	CG7985	-0.291418598864274	0.342679966896877	1	pseudobulk	DESeq2	LRT	0
186	KC_AB3	CG7987	-0.403972587711391	0.626083643351971	1	pseudobulk	DESeq2	LRT	0
186	KC_AB3	CG7988	-0.0781543137615753	0.830613034175565	1	pseudobulk	DESeq2	LRT	0
186	KC_AB3	CG7990	0.223259226637434	0.479913498035524	1	pseudobulk	DESeq2	LRT	0
186	KC_AB3	CG7997	0.632774104902944	1	1	pseudobulk	DESeq2	LRT	0
186	KC_AB3	CG8001	0.899002080771093	0.667309241747911	1	pseudobulk	DESeq2	LRT	0
186	KC_AB3	CG8003	1.49830550591567	0.455083957815813	1	pseudobulk	DESeq2	LRT	0
186	KC_AB3	CG8004	-0.183613478865637	0.596924815091275	1	pseudobulk	DESeq2	LRT	0
186	KC_AB3	CG8005	1.59359598433783	0.86377329553208	1	pseudobulk	DESeq2	LRT	0
186	KC_AB3	CG8009	-0.218598703569895	0.613321901346734	1	pseudobulk	DESeq2	LRT	0
186	KC_AB3	CG8010	2.38376667944252	0.145368995206195	1	pseudobulk	DESeq2	LRT	0
186	KC_AB3	CG8012	-0.399671897798429	0.965137949353374	1	pseudobulk	DESeq2	LRT	0
186	KC_AB3	CG8027	2.36933983601176	0.406024215854308	1	pseudobulk	DESeq2	LRT	0
186	KC_AB3	CG8031	-0.139874607127884	0.700241231453086	1	pseudobulk	DESeq2	LRT	0
186	KC_AB3	CG8032	0.1788598213959	0.685759312569942	1	pseudobulk	DESeq2	LRT	0
186	KC_AB3	CG8036	2.1445051383883	0.530068988727821	1	pseudobulk	DESeq2	LRT	0
186	KC_AB3	CG8038	2.88619786524384	0.39214769278579	1	pseudobulk	DESeq2	LRT	0
186	KC_AB3	CG8042	0.930087007104492	0.460767626580454	1	pseudobulk	DESeq2	LRT	0
186	KC_AB3	CG8043	1.56560767662223	0.374584732577098	1	pseudobulk	DESeq2	LRT	0
186	KC_AB3	CG8060	1.60186822711471	1	1	pseudobulk	DESeq2	LRT	0
186	KC_AB3	CG8064	-0.782080318363963	0.603186949050577	1	pseudobulk	DESeq2	LRT	0
186	KC_AB3	CG8066	0.594117478581602	1	1	pseudobulk	DESeq2	LRT	0
186	KC_AB3	CG8067	2.13877048085486	0.558952959988922	1	pseudobulk	DESeq2	LRT	0
186	KC_AB3	CG8078	-0.0647180247789504	0.956798342967774	1	pseudobulk	DESeq2	LRT	0
186	KC_AB3	CG8079	0.239044860450072	0.537930390209698	1	pseudobulk	DESeq2	LRT	0
186	KC_AB3	CG8080	0.869643386263749	0.603447538137028	1	pseudobulk	DESeq2	LRT	0
186	KC_AB3	CG8097	0.632774104902943	1	1	pseudobulk	DESeq2	LRT	0
186	KC_AB3	CG8108	0.141144994837707	0.574979541277858	1	pseudobulk	DESeq2	LRT	0
186	KC_AB3	CG8111	0.0995077948314781	0.775495239352071	1	pseudobulk	DESeq2	LRT	0
186	KC_AB3	CG8132	0.529276856809442	0.615433272967524	1	pseudobulk	DESeq2	LRT	0
186	KC_AB3	CG8134	-0.100992002061178	0.69445911105956	1	pseudobulk	DESeq2	LRT	0
186	KC_AB3	CG8149	-0.388665012489528	0.615881089685835	1	pseudobulk	DESeq2	LRT	0
186	KC_AB3	CG8152	0.810682882162716	0.632551983829153	1	pseudobulk	DESeq2	LRT	0
186	KC_AB3	CG8155	0.552253797824836	0.611425670877856	1	pseudobulk	DESeq2	LRT	0
186	KC_AB3	CG8159	2.22120548490942	0.356587304491996	1	pseudobulk	DESeq2	LRT	0
186	KC_AB3	CG8173	0.318750620495719	1	1	pseudobulk	DESeq2	LRT	0
186	KC_AB3	CG8176	0.633472183666253	0.397615808366429	1	pseudobulk	DESeq2	LRT	0
186	KC_AB3	CG8177	0.174667009778706	0.537365536362438	1	pseudobulk	DESeq2	LRT	0
186	KC_AB3	CG8187	0.214287277258648	0.730386998480424	1	pseudobulk	DESeq2	LRT	0
186	KC_AB3	CG8188	-0.421700005177365	0.434596181020267	1	pseudobulk	DESeq2	LRT	0
186	KC_AB3	CG8195	0.575506394978992	0.433016602447368	1	pseudobulk	DESeq2	LRT	0
186	KC_AB3	CG8199	1.95267092137636	1	1	pseudobulk	DESeq2	LRT	0
186	KC_AB3	CG8202	-0.185594513059781	1	1	pseudobulk	DESeq2	LRT	0
186	KC_AB3	CG8204	1.74823883803592	0.505032606439544	1	pseudobulk	DESeq2	LRT	0
186	KC_AB3	CG8206	-0.115393607242874	0.608895109434499	1	pseudobulk	DESeq2	LRT	0
186	KC_AB3	CG8207	1.59359598433783	0.86377329553208	1	pseudobulk	DESeq2	LRT	0
186	KC_AB3	CG8209	0.604335867958414	0.441683053673863	1	pseudobulk	DESeq2	LRT	0
186	KC_AB3	CG8223	-0.586025066206254	0.44342480414383	1	pseudobulk	DESeq2	LRT	0
186	KC_AB3	CG8229	0.0460327519186914	0.704075325501677	1	pseudobulk	DESeq2	LRT	0
186	KC_AB3	CG8230	0.982185367396689	0.665800941285838	1	pseudobulk	DESeq2	LRT	0

186	KC_AB3	CG8237	-0.628129780388926	0.614965565353609	1	pseudobulk	DESeq2	LRT		0
186	KC_AB3	CG8243	0.0382294334409521	0.589532515457811	1	pseudobulk	DESeq2	LRT		0
186	KC_AB3	CG8245	-0.0512507445488176	0.79815403821392	1	pseudobulk	DESeq2	LRT		0
186	KC_AB3	CG8248	-0.0371747350718286	0.751106682611074	1	pseudobulk	DESeq2	LRT		0
186	KC_AB3	CG8270	-0.231710483441688	0.742064284812227	1	pseudobulk	DESeq2	LRT		0
186	KC_AB3	CG8272	0.221589904233725	0.325228708922885	1	pseudobulk	DESeq2	LRT		0
186	KC_AB3	CG8281	-0.368068338089008	0.678734984782294	1	pseudobulk	DESeq2	LRT		0
186	KC_AB3	CG8289	0.294184731035589	0.681529976248757	1	pseudobulk	DESeq2	LRT		0
186	KC_AB3	CG8298	-0.0355517206969949	0.906574025627152	1	pseudobulk	DESeq2	LRT		0
186	KC_AB3	CG8300	0.483863103974227	0.588077539505303	1	pseudobulk	DESeq2	LRT		0
186	KC_AB3	CG8301	-0.742461273923017	0.2847024284627	1	pseudobulk	DESeq2	LRT		0
186	KC_AB3	CG8311	-0.905393277557758	0.475166983847038	1	pseudobulk	DESeq2	LRT		0
186	KC_AB3	CG8314	0.561592877946436	0.522900352463777	1	pseudobulk	DESeq2	LRT		0
186	KC_AB3	CG8319	-0.584602167601671	0.636282344286611	1	pseudobulk	DESeq2	LRT		0
186	KC_AB3	CG8320	-0.862244268108696	0.529219098969625	1	pseudobulk	DESeq2	LRT		0
186	KC_AB3	CG8321	0.516222197093013	0.397705102408588	1	pseudobulk	DESeq2	LRT		0
186	KC_AB3	CG8323	-1.13441954721443	0.538223795140586	1	pseudobulk	DESeq2	LRT		0
186	KC_AB3	CG8326	-0.436464933976793	0.70311968439253	1	pseudobulk	DESeq2	LRT		0
186	KC_AB3	CG8336	1.58688092033982	0.360997331092037	1	pseudobulk	DESeq2	LRT		0
186	KC_AB3	CG8353	0.131572019914183	0.89473684634783	1	pseudobulk	DESeq2	LRT		0
186	KC_AB3	CG8360	0.332825841511404	0.590059084252766	1	pseudobulk	DESeq2	LRT		0
186	KC_AB3	CG8366	1.7218506292881	0.225605050796102	1	pseudobulk	DESeq2	LRT		0
186	KC_AB3	CG8369	-0.106822200823472	0.737596937841491	1	pseudobulk	DESeq2	LRT		0
186	KC_AB3	CG8370	0.809245658539122	0.767096289129898	1	pseudobulk	DESeq2	LRT		0
186	KC_AB3	CG8372	0.363184842091323	0.448912661369323	1	pseudobulk	DESeq2	LRT		0
186	KC_AB3	CG8379	-0.356771760578194	0.70011815192909	1	pseudobulk	DESeq2	LRT		0
186	KC_AB3	CG8388	-0.793303759873938	0.58142077205866	1	pseudobulk	DESeq2	LRT		0
186	KC_AB3	CG8389	0.300625736059036	0.926672527069091	1	pseudobulk	DESeq2	LRT		0
186	KC_AB3	CG8397	0.269770652227487	0.513108456791714	1	pseudobulk	DESeq2	LRT		0
186	KC_AB3	CG8401	-0.932643767989955	0.60464164473687	1	pseudobulk	DESeq2	LRT		0
186	KC_AB3	CG8405	0.011010357941889	0.729689926731938	1	pseudobulk	DESeq2	LRT		0
186	KC_AB3	CG8412	-0.515448240441259	0.584806760121858	1	pseudobulk	DESeq2	LRT		0
186	KC_AB3	CG8414	1.23133864869565	1	1	pseudobulk	DESeq2	LRT		0
186	KC_AB3	CG8435	0.139350953149196	1	1	pseudobulk	DESeq2	LRT		0
186	KC_AB3	CG8441	1.2163430419165	0.249162148487358	1	pseudobulk	DESeq2	LRT		0
186	KC_AB3	CG8460	-0.560477492866218	0.784070523163114	1	pseudobulk	DESeq2	LRT		0
186	KC_AB3	CG8461	-1.4285828663036	0.391325346644975	1	pseudobulk	DESeq2	LRT		0
186	KC_AB3	CG8475	-0.0441735005079017	0.73397778211895	1	pseudobulk	DESeq2	LRT		0
186	KC_AB3	CG8485	-0.352014906234914	0.406282530097546	1	pseudobulk	DESeq2	LRT		0
186	KC_AB3	CG8490	-0.808570040988548	0.449061683458	1	pseudobulk	DESeq2	LRT		0
186	KC_AB3	CG8500	-0.574580948975329	0.10195965890767	1	pseudobulk	DESeq2	LRT		0
186	KC_AB3	CG8507	0.518392870252062	0.842558225078646	1	pseudobulk	DESeq2	LRT		0
186	KC_AB3	CG8516	2.4096641859508	0.428875287944006	1	pseudobulk	DESeq2	LRT		0
186	KC_AB3	CG8519	0.219983815863151	0.904797812719685	1	pseudobulk	DESeq2	LRT		0
186	KC_AB3	CG8520	-0.460504470666176	0.648843675159369	1	pseudobulk	DESeq2	LRT		0
187	KC_AB3	CG8531	1.35664030210559	0.33251727790701	1	pseudobulk	DESeq2	LRT		0
187	KC_AB3	CG8547	1.95267092137636	1	1	pseudobulk	DESeq2	LRT		0
187	KC_AB3	CG8549	-0.292279340932043	0.727642614773491	1	pseudobulk	DESeq2	LRT		0
187	KC_AB3	CG8569	-0.155252150225445	0.884340610954591	1	pseudobulk	DESeq2	LRT		0
187	KC_AB3	CG8578	-0.756881292104724	0.360150213098745	1	pseudobulk	DESeq2	LRT		0
187	KC_AB3	CG8611	0.154493176751981	0.95879150424917	1	pseudobulk	DESeq2	LRT		0
187	KC_AB3	CG8613	0.325913192516905	0.438641787032361	1	pseudobulk	DESeq2	LRT		0
187	KC_AB3	CG8617	0.194450070346141	0.771171230158164	1	pseudobulk	DESeq2	LRT		0

187	KC_AB3	CG8635	-0.0103515549276991	0.851386925356876	1	pseudobulk	DESeq2	LRT	0
187	KC_AB3	CG8641	-0.668697628914053	0.766626548836875	1	pseudobulk	DESeq2	LRT	0
187	KC_AB3	CG8654	1.95267092137636	1	1	pseudobulk	DESeq2	LRT	0
187	KC_AB3	CG8668	-0.160699017214649	0.685504924920914	1	pseudobulk	DESeq2	LRT	0
187	KC_AB3	CG8671	0.167325414751816	0.678950728707358	1	pseudobulk	DESeq2	LRT	0
187	KC_AB3	CG8675	-1.20671536172949	0.466441833188037	1	pseudobulk	DESeq2	LRT	0
187	KC_AB3	CG8677	-0.510477673857244	0.296092088933205	1	pseudobulk	DESeq2	LRT	0
187	KC_AB3	CG8679	1.23133864869565	1	1	pseudobulk	DESeq2	LRT	0
187	KC_AB3	CG8712	0.0478638348083297	1	1	pseudobulk	DESeq2	LRT	0
187	KC_AB3	CG8726	-0.0489115783158671	0.897364087330806	1	pseudobulk	DESeq2	LRT	0
187	KC_AB3	CG8728	-1.26784661947391	0.243135747570527	1	pseudobulk	DESeq2	LRT	0
187	KC_AB3	CG8745	1.23133864869565	1	1	pseudobulk	DESeq2	LRT	0
187	KC_AB3	CG8757	1.95267092137636	1	1	pseudobulk	DESeq2	LRT	0
187	KC_AB3	CG8765	-1.13856009304031	0.37253964191772	1	pseudobulk	DESeq2	LRT	0
187	KC_AB3	CG8768	0.645298529048894	0.739440416747824	1	pseudobulk	DESeq2	LRT	0
187	KC_AB3	CG8777	1.23133864869565	1	1	pseudobulk	DESeq2	LRT	0
187	KC_AB3	CG8778	1.51619375775369	0.372030413732992	1	pseudobulk	DESeq2	LRT	0
187	KC_AB3	CG8814	-0.231060126869779	0.704664194571032	1	pseudobulk	DESeq2	LRT	0
187	KC_AB3	CG8818	-0.59820885078278	0.32728214844284	1	pseudobulk	DESeq2	LRT	0
187	KC_AB3	CG8833	-0.865892235443794	0.478713487488244	1	pseudobulk	DESeq2	LRT	0
187	KC_AB3	CG8839	1.23133864869565	1	1	pseudobulk	DESeq2	LRT	0
187	KC_AB3	CG8841	-0.0067121834966269	0.863212699206267	1	pseudobulk	DESeq2	LRT	0
187	KC_AB3	CG8858	-0.0181238182178296	0.833004489073384	1	pseudobulk	DESeq2	LRT	0
187	KC_AB3	CG8860	-0.0075521692858477	0.602163400665287	1	pseudobulk	DESeq2	LRT	0
187	KC_AB3	CG8861	0.632774104902944	1	1	pseudobulk	DESeq2	LRT	0
187	KC_AB3	CG8878	-0.903815086723899	0.26986324821051	1	pseudobulk	DESeq2	LRT	0
187	KC_AB3	CG8891	-1.27687747850153	0.398995671513468	1	pseudobulk	DESeq2	LRT	0
187	KC_AB3	CG8892	-0.323806791730673	0.692006808549666	1	pseudobulk	DESeq2	LRT	0
187	KC_AB3	CG8910	-0.130800691438644	0.653807711853249	1	pseudobulk	DESeq2	LRT	0
187	KC_AB3	CG8915	2.19269259199673	0.500800656633403	1	pseudobulk	DESeq2	LRT	0
187	KC_AB3	CG8924	-0.164811248155521	0.665306228824618	1	pseudobulk	DESeq2	LRT	0
187	KC_AB3	CG8929	0.0605058465124698	0.746306620509048	1	pseudobulk	DESeq2	LRT	0
187	KC_AB3	CG8939	1.23133864869565	1	1	pseudobulk	DESeq2	LRT	0
187	KC_AB3	CG8944	-1.45591444269215	0.256428943034281	1	pseudobulk	DESeq2	LRT	0
187	KC_AB3	CG8950	0.632774104902943	1	1	pseudobulk	DESeq2	LRT	0
187	KC_AB3	CG8963	0.350570361772232	0.570949225244195	1	pseudobulk	DESeq2	LRT	0
187	KC_AB3	CG8974	-0.147105734889826	0.650930463341488	1	pseudobulk	DESeq2	LRT	0
187	KC_AB3	CG8993	0.118973064137583	0.643408590396441	1	pseudobulk	DESeq2	LRT	0
187	KC_AB3	CG9003	-0.304816162030613	0.481441420070855	1	pseudobulk	DESeq2	LRT	0
187	KC_AB3	CG9004	0.258605747001153	0.900905873167643	1	pseudobulk	DESeq2	LRT	0
187	KC_AB3	CG9005	0.36110131955788	0.187475571670597	1	pseudobulk	DESeq2	LRT	0
187	KC_AB3	CG9018	-0.991243080111066	0.324622182256026	1	pseudobulk	DESeq2	LRT	0
187	KC_AB3	CG9034	0.114581155192159	0.608628981345567	1	pseudobulk	DESeq2	LRT	0
187	KC_AB3	CG9044	-1.70940920267674	0.15659315203368	1	pseudobulk	DESeq2	LRT	0
187	KC_AB3	CG9062	-0.621180125864591	0.580211361935285	1	pseudobulk	DESeq2	LRT	0
187	KC_AB3	CG9065	0.314000545265312	0.409599497432676	1	pseudobulk	DESeq2	LRT	0
187	KC_AB3	CG9067	-0.307498844133879	0.703344016120614	1	pseudobulk	DESeq2	LRT	0
187	KC_AB3	CG9098	0.494938702510773	1	1	pseudobulk	DESeq2	LRT	0
187	KC_AB3	CG9107	0.438973952551762	1	1	pseudobulk	DESeq2	LRT	0
187	KC_AB3	CG9117	0.632774104902944	1	1	pseudobulk	DESeq2	LRT	0
187	KC_AB3	CG9121	-0.147790268845311	0.799475349492863	1	pseudobulk	DESeq2	LRT	0
187	KC_AB3	CG9123	0.0080182395117345	0.633425348052334	1	pseudobulk	DESeq2	LRT	0
187	KC_AB3	CG9125	0.979052132485495	0.680540652806045	1	pseudobulk	DESeq2	LRT	0

187	KC_AB3	CG9132	0.0389439552637273	0.530051939272162	1	pseudobulk	DESeq2	LRT	0
187	KC_AB3	CG9135	0.73608811497376	0.547322692433948	1	pseudobulk	DESeq2	LRT	0
187	KC_AB3	CG9143	0.111944443974979	0.928128343525443	1	pseudobulk	DESeq2	LRT	0
187	KC_AB3	CG9147	-1.15956503034481	0.361829520668106	1	pseudobulk	DESeq2	LRT	0
187	KC_AB3	CG9149	-0.683994382128744	0.471742106735048	1	pseudobulk	DESeq2	LRT	0
187	KC_AB3	CG9154	-1.26223204026894	0.352084354733136	1	pseudobulk	DESeq2	LRT	0
187	KC_AB3	CG9164	0.757229211699233	0.770485041699765	1	pseudobulk	DESeq2	LRT	0
187	KC_AB3	CG9170	-0.590284364773335	0.213401123277854	1	pseudobulk	DESeq2	LRT	0
187	KC_AB3	CG9171	-0.172995725678702	0.549264093350095	1	pseudobulk	DESeq2	LRT	0
187	KC_AB3	CG9175	-0.0440316638613436	0.781708219068145	1	pseudobulk	DESeq2	LRT	0
187	KC_AB3	CG9205	-0.494386851785604	0.422930788344391	1	pseudobulk	DESeq2	LRT	0
187	KC_AB3	CG9213	0.0409483039249518	0.839610338517549	1	pseudobulk	DESeq2	LRT	0
187	KC_AB3	CG9215	0.442896448351053	0.821592201731171	1	pseudobulk	DESeq2	LRT	0
187	KC_AB3	CG9231	0.292183358794297	0.26072444704228	1	pseudobulk	DESeq2	LRT	0
187	KC_AB3	CG9240	0.545821433798159	0.509444009735531	1	pseudobulk	DESeq2	LRT	0
187	KC_AB3	CG9246	0.0578630818178232	0.916666767587641	1	pseudobulk	DESeq2	LRT	0
187	KC_AB3	CG9253	0.7100906250926	0.636025035487895	1	pseudobulk	DESeq2	LRT	0
187	KC_AB3	CG9257	-0.245463330923494	0.71276096103457	1	pseudobulk	DESeq2	LRT	0
187	KC_AB3	CG9263	0.632774104902944	1	1	pseudobulk	DESeq2	LRT	0
187	KC_AB3	CG9265	-0.361875638855628	0.648103767471187	1	pseudobulk	DESeq2	LRT	0
187	KC_AB3	CG9272	-0.0327300486906476	1	1	pseudobulk	DESeq2	LRT	0
187	KC_AB3	CG9281	-0.137883977604759	0.736322427895569	1	pseudobulk	DESeq2	LRT	0
187	KC_AB3	CG9286	0.118531522098994	0.28394856592771	1	pseudobulk	DESeq2	LRT	0
187	KC_AB3	CG9300	-0.779009454654304	0.632803062798981	1	pseudobulk	DESeq2	LRT	0
187	KC_AB3	CG9302	1.23133864869565	1	1	pseudobulk	DESeq2	LRT	0
187	KC_AB3	CG9304	0.154493176751981	0.95879150424917	1	pseudobulk	DESeq2	LRT	0
187	KC_AB3	CG9313	0.632774104902943	1	1	pseudobulk	DESeq2	LRT	0
187	KC_AB3	CG9328	-0.295097553308893	0.337495140680994	1	pseudobulk	DESeq2	LRT	0
187	KC_AB3	CG9330	0.194676320595781	0.645113858053771	1	pseudobulk	DESeq2	LRT	0
187	KC_AB3	CG9331	-0.400046519007236	0.964129537865033	1	pseudobulk	DESeq2	LRT	0
187	KC_AB3	CG9336	0.632774104902944	1	1	pseudobulk	DESeq2	LRT	0
187	KC_AB3	CG9344	0.375571402851085	0.604436022958612	1	pseudobulk	DESeq2	LRT	0
187	KC_AB3	CG9346	-0.171866260925912	0.704571955340064	1	pseudobulk	DESeq2	LRT	0
187	KC_AB3	CG9356	1.12059961403812	1	1	pseudobulk	DESeq2	LRT	0
187	KC_AB3	CG9368	-0.638367737521228	0.16883876672363	1	pseudobulk	DESeq2	LRT	0
187	KC_AB3	CG9372	1.95267092137636	1	1	pseudobulk	DESeq2	LRT	0
187	KC_AB3	CG9380	0.104148799864746	1	1	pseudobulk	DESeq2	LRT	0
187	KC_AB3	CG9384	0.771699635722567	0.478180651720687	1	pseudobulk	DESeq2	LRT	0
187	KC_AB3	CG9386	-0.343859627219784	0.761749936319153	1	pseudobulk	DESeq2	LRT	0
188	KC_AB3	CG9391	0.0609924729923474	0.573583815019395	1	pseudobulk	DESeq2	LRT	0
188	KC_AB3	CG9393	0.165594017070599	0.89667352792462	1	pseudobulk	DESeq2	LRT	0
188	KC_AB3	CG9394	1.56961836772998	0.613510712230331	1	pseudobulk	DESeq2	LRT	0
188	KC_AB3	CG9395	-0.359238669406361	0.682074794699393	1	pseudobulk	DESeq2	LRT	0
188	KC_AB3	CG9399	0.670929846468992	0.42991692270441	1	pseudobulk	DESeq2	LRT	0
188	KC_AB3	CG9410	0.199104087025059	0.569172602837681	1	pseudobulk	DESeq2	LRT	0
188	KC_AB3	CG9422	-0.447321976558867	0.541640491889574	1	pseudobulk	DESeq2	LRT	0
188	KC_AB3	CG9425	-0.152324919666023	0.611316675795152	1	pseudobulk	DESeq2	LRT	0
188	KC_AB3	CG9426	-0.251060215337139	0.657282732373552	1	pseudobulk	DESeq2	LRT	0
188	KC_AB3	CG9467	0.932604532528152	0.441628004885274	1	pseudobulk	DESeq2	LRT	0
188	KC_AB3	CG9471	0.243625595260693	1	1	pseudobulk	DESeq2	LRT	0
188	KC_AB3	CG9485	1.95267092137636	1	1	pseudobulk	DESeq2	LRT	0
188	KC_AB3	CG9497	1.95267092137636	1	1	pseudobulk	DESeq2	LRT	0
188	KC_AB3	CG9503	0.632774104902943	1	1	pseudobulk	DESeq2	LRT	0

188	KC_AB3	CG9514	0.632774104902944	1	1	pseudobulk	DESeq2	LRT	0
188	KC_AB3	CG9515	0.440578571731502	1	1	pseudobulk	DESeq2	LRT	0
188	KC_AB3	CG9518	-0.186961738271884	1	1	pseudobulk	DESeq2	LRT	0
188	KC_AB3	CG9527	0.494110497880344	0.897268430762443	1	pseudobulk	DESeq2	LRT	0
188	KC_AB3	CG9536	2.21793839360199	0.0323239928719862	1	pseudobulk	DESeq2	LRT	0
188	KC_AB3	CG9541	-0.120255328429205	0.914604900667141	1	pseudobulk	DESeq2	LRT	0
188	KC_AB3	CG9570	1.22707713470494	1	1	pseudobulk	DESeq2	LRT	0
188	KC_AB3	CG9577	0.215823428609594	1	1	pseudobulk	DESeq2	LRT	0
188	KC_AB3	CG9578	-1.37946637138667	0.27578230977953	1	pseudobulk	DESeq2	LRT	0
188	KC_AB3	CG9581	1.17433176602323	0.634218418131115	1	pseudobulk	DESeq2	LRT	0
188	KC_AB3	CG9586	0.0140241300483856	0.707138336294272	1	pseudobulk	DESeq2	LRT	0
188	KC_AB3	CG9588	0.743407850345272	0.664152772270378	1	pseudobulk	DESeq2	LRT	0
188	KC_AB3	CG9590	-0.456860757659888	0.606223640133622	1	pseudobulk	DESeq2	LRT	0
188	KC_AB3	CG9596	0.00209390563092391	1	1	pseudobulk	DESeq2	LRT	0
188	KC_AB3	CG9601	-0.310690119234561	0.663761358881422	1	pseudobulk	DESeq2	LRT	0
188	KC_AB3	CG9609	-0.424498635897231	0.481024954734412	1	pseudobulk	DESeq2	LRT	0
188	KC_AB3	CG9628	0.632774104902944	1	1	pseudobulk	DESeq2	LRT	0
188	KC_AB3	CG9630	-0.0327300486906476	1	1	pseudobulk	DESeq2	LRT	0
188	KC_AB3	CG9641	0.64978338680298	0.378474939587198	1	pseudobulk	DESeq2	LRT	0
188	KC_AB3	CG9643	0.837103878277954	0.560333990980021	1	pseudobulk	DESeq2	LRT	0
188	KC_AB3	CG9646	0.212941164195306	0.620654979413717	1	pseudobulk	DESeq2	LRT	0
188	KC_AB3	CG9650	0.328024391226458	1	1	pseudobulk	DESeq2	LRT	0
188	KC_AB3	CG9657	1.95267092137636	1	1	pseudobulk	DESeq2	LRT	0
188	KC_AB3	CG9662	0.647302913507952	0.46254487792741	1	pseudobulk	DESeq2	LRT	0
188	KC_AB3	CG9663	-0.668697628914053	0.766626548836875	1	pseudobulk	DESeq2	LRT	0
188	KC_AB3	CG9667	-0.365239890196305	0.736232276309201	1	pseudobulk	DESeq2	LRT	0
188	KC_AB3	CG9674	-0.170439683534254	0.687155940280044	1	pseudobulk	DESeq2	LRT	0
188	KC_AB3	CG9686	0.0347424623850317	0.848034263421424	1	pseudobulk	DESeq2	LRT	0
188	KC_AB3	CG9691	-0.184744780581018	1	1	pseudobulk	DESeq2	LRT	0
188	KC_AB3	CG9705	0.553357045481942	0.486978909575395	1	pseudobulk	DESeq2	LRT	0
188	KC_AB3	CG9706	0.632774104902943	1	1	pseudobulk	DESeq2	LRT	0
188	KC_AB3	CG9723	0.13333664881385	1	1	pseudobulk	DESeq2	LRT	0
188	KC_AB3	CG9727	-0.5713422305168	0.667123283894121	1	pseudobulk	DESeq2	LRT	0
188	KC_AB3	CG9752	0.759758178017103	1	1	pseudobulk	DESeq2	LRT	0
188	KC_AB3	CG9760	0.594117478581602	1	1	pseudobulk	DESeq2	LRT	0
188	KC_AB3	CG9766	1.23133864869565	1	1	pseudobulk	DESeq2	LRT	0
188	KC_AB3	CG9773	0.519451322955217	0.656814323080258	1	pseudobulk	DESeq2	LRT	0
188	KC_AB3	CG9775	0.179376126170198	0.570199355028194	1	pseudobulk	DESeq2	LRT	0
188	KC_AB3	CG9776	-0.0800943156284745	0.783065960637036	1	pseudobulk	DESeq2	LRT	0
188	KC_AB3	CG9782	0.632774104902944	1	1	pseudobulk	DESeq2	LRT	0
188	KC_AB3	CG9784	-0.10042310906194	0.683388990683901	1	pseudobulk	DESeq2	LRT	0
188	KC_AB3	CG9795	0.756704312750417	0.412255751602657	1	pseudobulk	DESeq2	LRT	0
188	KC_AB3	CG9799	2.36933983601176	0.406024215854308	1	pseudobulk	DESeq2	LRT	0
188	KC_AB3	CG9801	-1.26246196555178	0.416659513356951	1	pseudobulk	DESeq2	LRT	0
188	KC_AB3	CG9804	0.395366799811593	0.800062570481079	1	pseudobulk	DESeq2	LRT	0
188	KC_AB3	CG9806	1.95267092137636	1	1	pseudobulk	DESeq2	LRT	0
188	KC_AB3	CG9813	-0.0299222790321358	0.489181850297649	1	pseudobulk	DESeq2	LRT	0
188	KC_AB3	CG9837	0.123611894036401	0.760579339841659	1	pseudobulk	DESeq2	LRT	0
188	KC_AB3	CG9839	0.8259375621382	0.738437659341897	1	pseudobulk	DESeq2	LRT	0
188	KC_AB3	CG9849	-3.09916003406584	0.0378658665390271	1	pseudobulk	DESeq2	LRT	0
188	KC_AB3	CG9853	-0.103178263317996	0.703955790272384	1	pseudobulk	DESeq2	LRT	0
188	KC_AB3	CG9855	0.160115363786658	0.687603166714145	1	pseudobulk	DESeq2	LRT	0
188	KC_AB3	CG9886	0.422484797186058	1	1	pseudobulk	DESeq2	LRT	0

188	KC_AB3	CG9890	1.56961836772998	0.613510712230331	1	pseudobulk	DESeq2	LRT	0
188	KC_AB3	CG9902	0.288995321767672	1	1	pseudobulk	DESeq2	LRT	0
188	KC_AB3	CG9911	-0.629723145962398	0.515048669087113	1	pseudobulk	DESeq2	LRT	0
188	KC_AB3	CG9915	0.753738073806696	0.463122912406461	1	pseudobulk	DESeq2	LRT	0
188	KC_AB3	CG9917	0.395503896531145	0.60839030046356	1	pseudobulk	DESeq2	LRT	0
188	KC_AB3	CG9919	-0.0747394288485938	0.862384869572553	1	pseudobulk	DESeq2	LRT	0
188	KC_AB3	CG9921	-0.598633218336334	0.484409038715871	1	pseudobulk	DESeq2	LRT	0
188	KC_AB3	CG9922	0.297068978867681	0.632464070586927	1	pseudobulk	DESeq2	LRT	0
188	KC_AB3	CG9932	0.555116039874619	0.503526803430332	1	pseudobulk	DESeq2	LRT	0
188	KC_AB3	CG9934	-0.961271553848476	0.416426317097351	1	pseudobulk	DESeq2	LRT	0
188	KC_AB3	CG9941	-0.344677282933271	0.588891870467728	1	pseudobulk	DESeq2	LRT	0
188	KC_AB3	CG9945	0.166979068201353	0.849183831668771	1	pseudobulk	DESeq2	LRT	0
188	KC_AB3	CG9948	0.558549567513924	0.748150623531404	1	pseudobulk	DESeq2	LRT	0
188	KC_AB3	CG9951	-0.40555469570435	0.711937694371915	1	pseudobulk	DESeq2	LRT	0
188	KC_AB3	CG9953	0.484822397679239	0.689139538766372	1	pseudobulk	DESeq2	LRT	0
188	KC_AB3	CG9967	1.23133864869565	1	1	pseudobulk	DESeq2	LRT	0
188	KC_AB3	CG9986	1.13162262164917	0.518376245457657	1	pseudobulk	DESeq2	LRT	0
188	KC_AB3	CG9992	-0.0023101677929702	0.876884830246096	1	pseudobulk	DESeq2	LRT	0
188	KC_AB3	Charon	0.053197396997799	1	1	pseudobulk	DESeq2	LRT	0
188	KC_AB3	chas	0.79078709321265	1	1	pseudobulk	DESeq2	LRT	0
188	KC_AB3	ChAT	0.26466529831404	0.53933488624675	1	pseudobulk	DESeq2	LRT	0
188	KC_AB3	chb	-0.240255799834831	0.661948528478642	1	pseudobulk	DESeq2	LRT	0
188	KC_AB3	Chc	-0.0805538649448459	0.486997167825797	1	pseudobulk	DESeq2	LRT	0
188	KC_AB3	Chchd2	0.086134405067924	0.695218147532642	1	pseudobulk	DESeq2	LRT	0
188	KC_AB3	Chchd3	0.224738015832087	0.7059209233539	1	pseudobulk	DESeq2	LRT	0
188	KC_AB3	Chd1	-1.30968932362448	0.102986773594864	1	pseudobulk	DESeq2	LRT	0
188	KC_AB3	Chd64	1.59576167909336	0.887029991980463	1	pseudobulk	DESeq2	LRT	0
188	KC_AB3	CheB42c	-0.835071691045028	0.645830720505476	1	pseudobulk	DESeq2	LRT	0
188	KC_AB3	CheB53a	1.87518227264439	0.766977424371083	1	pseudobulk	DESeq2	LRT	0
188	KC_AB3	cher	0.632774104902944	1	1	pseudobulk	DESeq2	LRT	0
188	KC_AB3	CHES-1-like	-0.055637098429167	0.610440436656008	1	pseudobulk	DESeq2	LRT	0
188	KC_AB3	Chi	0.168921571357883	0.806441525847782	1	pseudobulk	DESeq2	LRT	0
188	KC_AB3	chic	-0.336252700647693	0.424246778452771	1	pseudobulk	DESeq2	LRT	0
189	KC_AB3	chico	-0.571944505800108	0.50718790525503	1	pseudobulk	DESeq2	LRT	0
189	KC_AB3	chif	-0.189805250382421	0.711371763161794	1	pseudobulk	DESeq2	LRT	0
189	KC_AB3	chinmo	-0.410224580240742	0.186931712311938	1	pseudobulk	DESeq2	LRT	0
189	KC_AB3	CHKov1	0.632774104902944	1	1	pseudobulk	DESeq2	LRT	0
189	KC_AB3	chm	-0.096614988217072	0.803842219719722	1	pseudobulk	DESeq2	LRT	0
189	KC_AB3	Chmp1	0.476761229745507	0.360698220185482	1	pseudobulk	DESeq2	LRT	0
189	KC_AB3	CHMP2B	-2.35589671399064	0.0966652016417432	1	pseudobulk	DESeq2	LRT	0
189	KC_AB3	chn	-0.17762262455285	0.513193302595845	1	pseudobulk	DESeq2	LRT	0
189	KC_AB3	CHOp24	-0.0441484902680621	0.61816870349277	1	pseudobulk	DESeq2	LRT	0
189	KC_AB3	CHORD	0.88302330485018	0.47398462356264	1	pseudobulk	DESeq2	LRT	0
189	KC_AB3	Chrac-14	-0.933347948759194	0.604424649436326	1	pseudobulk	DESeq2	LRT	0
189	KC_AB3	Chrac-16	-1.65017442983054	0.363940058183393	1	pseudobulk	DESeq2	LRT	0
189	KC_AB3	chrb	-0.0845131875497485	0.680349616564839	1	pseudobulk	DESeq2	LRT	0
189	KC_AB3	ChT	0.261504463934083	0.419228064168218	1	pseudobulk	DESeq2	LRT	0
189	KC_AB3	Cht11	0.0478638348083297	1	1	pseudobulk	DESeq2	LRT	0
189	KC_AB3	Cht2	2.62817135292692	0.54954870385793	1	pseudobulk	DESeq2	LRT	0
189	KC_AB3	CIA30	0.20944177103935	0.842322867081808	1	pseudobulk	DESeq2	LRT	0
189	KC_AB3	Ciao1	-0.119956464134588	0.768151687173359	1	pseudobulk	DESeq2	LRT	0
189	KC_AB3	CIAPIN1	1.65720336841038	0.200111493997414	1	pseudobulk	DESeq2	LRT	0
189	KC_AB3	cib	-0.0344801279775842	0.885771527404421	1	pseudobulk	DESeq2	LRT	0

189	KC_AB3	cic	0.0799024308879618	0.586915710245263	1	pseudobulk	DESeq2	LRT	0
189	KC_AB3	cin	0.755590266069045	0.452007553075763	1	pseudobulk	DESeq2	LRT	0
189	KC_AB3	cindr	0.182716270978957	0.81031314628568	1	pseudobulk	DESeq2	LRT	0
189	KC_AB3	Cip4	-0.199062348678402	0.485482071793273	1	pseudobulk	DESeq2	LRT	0
189	KC_AB3	Cirl	0.0582962870844569	0.822289245675264	1	pseudobulk	DESeq2	LRT	0
189	KC_AB3	Cisd2	0.0883346589508785	0.649249356999643	1	pseudobulk	DESeq2	LRT	0
189	KC_AB3	Cka	0.386292711203953	0.323971930104042	1	pseudobulk	DESeq2	LRT	0
189	KC_AB3	CkIalpha	-0.186145284012549	0.398649737082112	1	pseudobulk	DESeq2	LRT	0
189	KC_AB3	CkIIalpha	-0.152332332904646	0.578090123246453	1	pseudobulk	DESeq2	LRT	0
189	KC_AB3	CkIIalpha-i1	-0.782080318363963	0.603186949050577	1	pseudobulk	DESeq2	LRT	0
189	KC_AB3	CkIIalpha-i3	0.542438032328221	0.362587115796233	1	pseudobulk	DESeq2	LRT	0
189	KC_AB3	CkIIbeta	-0.0899953069761583	0.726751669179914	1	pseudobulk	DESeq2	LRT	0
189	KC_AB3	ckn	0.269682468187268	0.531603831460282	1	pseudobulk	DESeq2	LRT	0
189	KC_AB3	Cks85A	-0.0935302492521268	0.485641219168413	1	pseudobulk	DESeq2	LRT	0
189	KC_AB3	cl	-0.274485254653012	0.672443462758148	1	pseudobulk	DESeq2	LRT	0
189	KC_AB3	Clamp	0.0834744844753686	0.593907892107935	1	pseudobulk	DESeq2	LRT	0
189	KC_AB3	Clbn	-0.0094219554617447	0.67838471695385	1	pseudobulk	DESeq2	LRT	0
189	KC_AB3	Clc	0.0990070826907721	0.720541330599103	1	pseudobulk	DESeq2	LRT	0
189	KC_AB3	ClC-b	1.23133864869565	1	1	pseudobulk	DESeq2	LRT	0
189	KC_AB3	ClC-c	-0.193010947317402	0.65342365341653	1	pseudobulk	DESeq2	LRT	0
189	KC_AB3	Clic	0.656803352224507	0.425984284683716	1	pseudobulk	DESeq2	LRT	0
189	KC_AB3	CLIP-190	-0.413493103310933	0.089100097619077	1	pseudobulk	DESeq2	LRT	0
189	KC_AB3	clos	0.632774104902943	1	1	pseudobulk	DESeq2	LRT	0
189	KC_AB3	Clp	-1.3042068017593	0.437143742310806	1	pseudobulk	DESeq2	LRT	0
189	KC_AB3	ClpP	0.40199021488198	0.520930325411608	1	pseudobulk	DESeq2	LRT	0
189	KC_AB3	CLS	0.811063320360319	0.643966296350457	1	pseudobulk	DESeq2	LRT	0
189	KC_AB3	clt	0.161515868851812	1	1	pseudobulk	DESeq2	LRT	0
189	KC_AB3	clu	0.183124536045944	0.758282778387474	1	pseudobulk	DESeq2	LRT	0
189	KC_AB3	cm	-0.173379687976341	0.652944595538333	1	pseudobulk	DESeq2	LRT	0
189	KC_AB3	cmb	-0.871427859970374	0.535486977559646	1	pseudobulk	DESeq2	LRT	0
189	KC_AB3	cmpy	0.570216234470827	0.641296494771115	1	pseudobulk	DESeq2	LRT	0
189	KC_AB3	cN-IIIB	0.759758178017103	1	1	pseudobulk	DESeq2	LRT	0
189	KC_AB3	CNBP	-0.0558486580199474	0.821041307496625	1	pseudobulk	DESeq2	LRT	0
189	KC_AB3	cnc	0.788024079550899	0.307915872585747	1	pseudobulk	DESeq2	LRT	0
189	KC_AB3	Cndp2	1.00211067683036	0.63428698707805	1	pseudobulk	DESeq2	LRT	0
189	KC_AB3	CngA	-2.09979078353538	0.472208093691409	1	pseudobulk	DESeq2	LRT	0
189	KC_AB3	CngB	1.57912044421911	0.0204825729902337	1	pseudobulk	DESeq2	LRT	0
189	KC_AB3	CngI	0.500305893815234	0.12955331044548	1	pseudobulk	DESeq2	LRT	0
189	KC_AB3	cni	-0.141879718164885	0.564603490013176	1	pseudobulk	DESeq2	LRT	0
189	KC_AB3	cnir	-0.820190479038809	0.484659526860041	1	pseudobulk	DESeq2	LRT	0
189	KC_AB3	cnk	-0.120528428548273	0.914416877354382	1	pseudobulk	DESeq2	LRT	0
189	KC_AB3	cnn	0.17559177476494	0.800627054211262	1	pseudobulk	DESeq2	LRT	0
189	KC_AB3	cno	0.660648519933382	1	1	pseudobulk	DESeq2	LRT	0
189	KC_AB3	Cnot4	0.283909768710082	0.654119817340155	1	pseudobulk	DESeq2	LRT	0
189	KC_AB3	CNPYb	-0.72021366407741	0.507549776248019	1	pseudobulk	DESeq2	LRT	0
189	KC_AB3	Cnx14D	0.632774104902944	1	1	pseudobulk	DESeq2	LRT	0
189	KC_AB3	Cnx99A	0.402984365146428	0.506066087674018	1	pseudobulk	DESeq2	LRT	0
189	KC_AB3	Coa7	0.15091944333398	0.750898475557707	1	pseudobulk	DESeq2	LRT	0
189	KC_AB3	Cog1	0.426069958552585	0.657368451489234	1	pseudobulk	DESeq2	LRT	0
189	KC_AB3	Cog2	0.972582044658406	0.764620665402759	1	pseudobulk	DESeq2	LRT	0
189	KC_AB3	Cog3	2.09688062235324	0.0937870004872279	1	pseudobulk	DESeq2	LRT	0
189	KC_AB3	Cog4	-0.293214933285008	0.693356788021767	1	pseudobulk	DESeq2	LRT	0
189	KC_AB3	Cog6	-0.53615682788415	0.646459679759324	1	pseudobulk	DESeq2	LRT	0

189	KC_AB3	Cog7	0.254937961023794	0.7736910169797		1	pseudobulk	DESeq2	LRT		0
189	KC_AB3	Cog8	-1.35977450104929	0.371128411661412		1	pseudobulk	DESeq2	LRT		0
189	KC_AB3	coil	-1.22367607012006	0.454447138642056		1	pseudobulk	DESeq2	LRT		0
189	KC_AB3	Col4a1	1.95267092137636	1		1	pseudobulk	DESeq2	LRT		0
189	KC_AB3	cold	-1.06857621727234	0.441294925941392		1	pseudobulk	DESeq2	LRT		0
189	KC_AB3	colt	1.79748203149538	0.291923601627993		1	pseudobulk	DESeq2	LRT		0
189	KC_AB3	comm	-0.473057609498081	0.278472102913897		1	pseudobulk	DESeq2	LRT		0
189	KC_AB3	comt	-0.264296230013906	0.531499122186848		1	pseudobulk	DESeq2	LRT		0
189	KC_AB3	Con	0.0194311873990592	0.686023833259376		1	pseudobulk	DESeq2	LRT		0
189	KC_AB3	Cont	1.06705346603883	0.678124001495716		1	pseudobulk	DESeq2	LRT		0
189	KC_AB3	conu	-0.0144633789646438	0.629228260311528		1	pseudobulk	DESeq2	LRT		0
189	KC_AB3	Coprox	2.13877048085486	0.558952959988922		1	pseudobulk	DESeq2	LRT		0
189	KC_AB3	Coq2	0.488826768100758	0.694136905097828		1	pseudobulk	DESeq2	LRT		0
189	KC_AB3	Coq3	1.23133864869565	1		1	pseudobulk	DESeq2	LRT		0
189	KC_AB3	Coq5	1.8751573179585	0.767073395253422		1	pseudobulk	DESeq2	LRT		0
189	KC_AB3	Coq6	1.10874899312791	0.453968207176723		1	pseudobulk	DESeq2	LRT		0
189	KC_AB3	Coq7	-0.662515187288742	0.418887769687511		1	pseudobulk	DESeq2	LRT		0
189	KC_AB3	Coq8	1.56961836772998	0.613510712230331		1	pseudobulk	DESeq2	LRT		0
189	KC_AB3	Coq9	0.0774553719004063	0.943232852563786		1	pseudobulk	DESeq2	LRT		0
189	KC_AB3	cora	-0.071876730186554	0.525296041946283		1	pseudobulk	DESeq2	LRT		0
189	KC_AB3	CoRest	0.295807824965799	0.6294147030889		1	pseudobulk	DESeq2	LRT		0
189	KC_AB3	Corin	-0.792110573353516	0.29425205981281		1	pseudobulk	DESeq2	LRT		0
189	KC_AB3	corn	-0.0834429454811653	0.652886896569913		1	pseudobulk	DESeq2	LRT		0
189	KC_AB3	coro	0.558465278193285	0.367522187528963		1	pseudobulk	DESeq2	LRT		0
189	KC_AB3	Cortactin	-0.279016971343098	0.670427421487783		1	pseudobulk	DESeq2	LRT		0
189	KC_AB3	corto	-0.207701082662435	0.469687224645721		1	pseudobulk	DESeq2	LRT		0
189	KC_AB3	cos	-1.35398243001257	0.202287565192484		1	pseudobulk	DESeq2	LRT		0
190	KC_AB3	Cow	-0.0631552951003025	0.820501362916984		1	pseudobulk	DESeq2	LRT		0
190	KC_AB3	COX4	-0.215739123661605	0.331332891250756		1	pseudobulk	DESeq2	LRT		0
190	KC_AB3	COX5A	0.0761255988493025	0.645010352116315		1	pseudobulk	DESeq2	LRT		0
190	KC_AB3	COX5B	0.0675597459054557	0.493921924545253		1	pseudobulk	DESeq2	LRT		0
190	KC_AB3	COX6B	0.0658251491501091	0.714355105760169		1	pseudobulk	DESeq2	LRT		0
190	KC_AB3	COX7A	-0.20825079724379	0.332903305932875		1	pseudobulk	DESeq2	LRT		0
190	KC_AB3	COX7C	-0.0096907519751081	0.643121470510502		1	pseudobulk	DESeq2	LRT		0
190	KC_AB3	COX8	-0.0362327676605908	0.536820760004362		1	pseudobulk	DESeq2	LRT		0
190	KC_AB3	Cp1	-1.03743119331131	0.127119111113086		1	pseudobulk	DESeq2	LRT		0
190	KC_AB3	Cp110	-0.169142697333507	0.500711657011743		1	pseudobulk	DESeq2	LRT		0
190	KC_AB3	Cp190	-0.218390086052462	0.549468183774799		1	pseudobulk	DESeq2	LRT		0
190	KC_AB3	Cp38	0.632774104902944	1		1	pseudobulk	DESeq2	LRT		0
190	KC_AB3	cpa	-0.610578460315976	0.528215359101114		1	pseudobulk	DESeq2	LRT		0
190	KC_AB3	cpb	0.224751463586629	0.684487497456755		1	pseudobulk	DESeq2	LRT		0
190	KC_AB3	Cpes	-0.453244014930205	0.610779489252405		1	pseudobulk	DESeq2	LRT		0
190	KC_AB3	cpo	-0.16221777445678	0.457997488729594		1	pseudobulk	DESeq2	LRT		0
190	KC_AB3	Cpr	0.0206177966980715	1		1	pseudobulk	DESeq2	LRT		0
190	KC_AB3	Cpr64Aa	1.95267092137636	1		1	pseudobulk	DESeq2	LRT		0
190	KC_AB3	Cpr64Ad	1.95267092137636	1		1	pseudobulk	DESeq2	LRT		0
190	KC_AB3	Cpr67B	1.95267092137636	1		1	pseudobulk	DESeq2	LRT		0
190	KC_AB3	Cpsf100	0.759758178017103	1		1	pseudobulk	DESeq2	LRT		0
190	KC_AB3	Cpsf160	-0.570658062786698	0.667334754531759		1	pseudobulk	DESeq2	LRT		0
190	KC_AB3	Cpsf5	0.44008405676691	0.409557210388574		1	pseudobulk	DESeq2	LRT		0
190	KC_AB3	Cpsf6	0.252553021254203	0.655526822656017		1	pseudobulk	DESeq2	LRT		0
190	KC_AB3	Cpsf73	0.621446144393834	0.698506831550887		1	pseudobulk	DESeq2	LRT		0
190	KC_AB3	cpx	-0.328988989695991	0.190838734805097		1	pseudobulk	DESeq2	LRT		0

190	KC_AB3	CR12842	-0.46164190455924	0.690085796704699	1	pseudobulk	DESeq2	LRT	0
190	KC_AB3	CR14798	1.23133864869565	1	1	pseudobulk	DESeq2	LRT	0
190	KC_AB3	CR15345	-1.80445910016406	0.324181016964984	1	pseudobulk	DESeq2	LRT	0
190	KC_AB3	CR18166	-0.251480667064896	0.844013238218174	1	pseudobulk	DESeq2	LRT	0
190	KC_AB3	CR18228	-0.186961738271884	1	1	pseudobulk	DESeq2	LRT	0
190	KC_AB3	CR18275	2.3149273646917	0.611236276445972	1	pseudobulk	DESeq2	LRT	0
190	KC_AB3	CR31032	2.4630830249376	0.263991262143279	1	pseudobulk	DESeq2	LRT	0
190	KC_AB3	CR32745	-1.09727042159967	0.372163664913021	1	pseudobulk	DESeq2	LRT	0
190	KC_AB3	CR40190	0.632774104902944	1	1	pseudobulk	DESeq2	LRT	0
190	KC_AB3	CR41320	0.632774104902944	1	1	pseudobulk	DESeq2	LRT	0
190	KC_AB3	CR41597	-0.265176665027752	0.723329782365753	1	pseudobulk	DESeq2	LRT	0
190	KC_AB3	CR43170	1.95267092137636	1	1	pseudobulk	DESeq2	LRT	0
190	KC_AB3	CR43212	2.62817135292692	0.54954870385793	1	pseudobulk	DESeq2	LRT	0
190	KC_AB3	CR43281	-0.667633140246005	0.365089405382273	1	pseudobulk	DESeq2	LRT	0
190	KC_AB3	CR43361	-0.560477492866218	0.784070523163114	1	pseudobulk	DESeq2	LRT	0
190	KC_AB3	CR43670	-0.0849465925734159	0.799433149880382	1	pseudobulk	DESeq2	LRT	0
190	KC_AB3	CR43972	0.00257013989755951	1	1	pseudobulk	DESeq2	LRT	0
190	KC_AB3	CR4575	0.632774104902944	1	1	pseudobulk	DESeq2	LRT	0
190	KC_AB3	CR46405	1.95267092137636	1	1	pseudobulk	DESeq2	LRT	0
190	KC_AB3	CR6900	1.22707713470494	1	1	pseudobulk	DESeq2	LRT	0
190	KC_AB3	CR7448	1.59575615951652	0.886898266337347	1	pseudobulk	DESeq2	LRT	0
190	KC_AB3	Crag	0.444798487865606	0.663560973702201	1	pseudobulk	DESeq2	LRT	0
190	KC_AB3	CRAT	0.333564712483826	1	1	pseudobulk	DESeq2	LRT	0
190	KC_AB3	crb	-0.307315680218782	0.300316528225384	1	pseudobulk	DESeq2	LRT	0
190	KC_AB3	crc	-0.414937282955942	0.4289939337549	1	pseudobulk	DESeq2	LRT	0
190	KC_AB3	CrebA	-1.19440690536666	0.331793839642941	1	pseudobulk	DESeq2	LRT	0
190	KC_AB3	CrebB	0.504426006394316	0.499719623236283	1	pseudobulk	DESeq2	LRT	0
190	KC_AB3	CREG	0.161515868851812	1	1	pseudobulk	DESeq2	LRT	0
190	KC_AB3	Crg-1	0.0576455966116859	0.91661042110746	1	pseudobulk	DESeq2	LRT	0
190	KC_AB3	CRIF	0.706484286529483	0.394780000039313	1	pseudobulk	DESeq2	LRT	0
190	KC_AB3	crim	-0.800304379643462	0.546567485783789	1	pseudobulk	DESeq2	LRT	0
190	KC_AB3	Crk	-0.337281506761522	0.33669406290845	1	pseudobulk	DESeq2	LRT	0
190	KC_AB3	crm	0.514394343564188	1	1	pseudobulk	DESeq2	LRT	0
190	KC_AB3	CRMP	0.422666423252952	0.393506511817289	1	pseudobulk	DESeq2	LRT	0
190	KC_AB3	crn	-0.810588416937337	0.439631042317429	1	pseudobulk	DESeq2	LRT	0
190	KC_AB3	crol	0.254014060103962	0.478663667197907	1	pseudobulk	DESeq2	LRT	0
190	KC_AB3	CROT	2.62817135292692	0.54954870385793	1	pseudobulk	DESeq2	LRT	0
190	KC_AB3	crp	0.326166438055957	0.439813865695246	1	pseudobulk	DESeq2	LRT	0
190	KC_AB3	crq	-0.0844157292356786	0.691003113928477	1	pseudobulk	DESeq2	LRT	0
190	KC_AB3	Crtc	-0.0128822640086336	0.95068152052978	1	pseudobulk	DESeq2	LRT	0
190	KC_AB3	Csas	0.632774104902944	1	1	pseudobulk	DESeq2	LRT	0
190	KC_AB3	Cse1	1.23133864869565	1	1	pseudobulk	DESeq2	LRT	0
190	KC_AB3	Csk	-0.520352052584935	0.322671483038322	1	pseudobulk	DESeq2	LRT	0
190	KC_AB3	Csl4	-0.0166879496878919	0.886676062724866	1	pseudobulk	DESeq2	LRT	0
190	KC_AB3	CSN1b	-0.330482561265342	0.742573954394208	1	pseudobulk	DESeq2	LRT	0
190	KC_AB3	CSN3	0.177030825780547	0.830340409244393	1	pseudobulk	DESeq2	LRT	0
190	KC_AB3	CSN4	0.467149215616015	0.570545431218723	1	pseudobulk	DESeq2	LRT	0
190	KC_AB3	CSN5	-0.115011513103154	0.659022529179736	1	pseudobulk	DESeq2	LRT	0
190	KC_AB3	CSN6	-0.209607625678395	0.723416567394953	1	pseudobulk	DESeq2	LRT	0
190	KC_AB3	CSN7	0.232615714490651	0.585458162876511	1	pseudobulk	DESeq2	LRT	0
190	KC_AB3	CSN8	-0.357341761176704	0.559244209135146	1	pseudobulk	DESeq2	LRT	0
190	KC_AB3	Csp	0.0817165343363959	0.718125050533056	1	pseudobulk	DESeq2	LRT	0
190	KC_AB3	CstF50	0.759758178017103	1	1	pseudobulk	DESeq2	LRT	0

190	KC_AB3	CstF64	-0.548265633952441	0.608076033575423	1	pseudobulk	DESeq2	LRT	0
190	KC_AB3	csul	-0.792919478540393	0.58155750177774	1	pseudobulk	DESeq2	LRT	0
190	KC_AB3	csw	0.0438828687094785	0.866296456958677	1	pseudobulk	DESeq2	LRT	0
190	KC_AB3	cta	-0.395759294724055	0.497941916287963	1	pseudobulk	DESeq2	LRT	0
190	KC_AB3	CtBP	0.087218288320254	0.66421834854077	1	pseudobulk	DESeq2	LRT	0
190	KC_AB3	CTCF	-0.450861581012841	0.675772710473606	1	pseudobulk	DESeq2	LRT	0
190	KC_AB3	CtIP	-0.269742198537673	0.750195831134786	1	pseudobulk	DESeq2	LRT	0
190	KC_AB3	Ctl1	-1.52427471832351	0.111899809233445	1	pseudobulk	DESeq2	LRT	0
190	KC_AB3	Ctl2	-1.3609185784394	0.287332342560009	1	pseudobulk	DESeq2	LRT	0
190	KC_AB3	ctp	0.137366549786732	0.500261456956303	1	pseudobulk	DESeq2	LRT	0
190	KC_AB3	CTPsyn	0.0405582049909724	0.741868213645275	1	pseudobulk	DESeq2	LRT	0
190	KC_AB3	Ctr1A	-0.567542028396408	0.545545108627002	1	pseudobulk	DESeq2	LRT	0
190	KC_AB3	Ctr9	0.140980740873142	0.552277213402373	1	pseudobulk	DESeq2	LRT	0
190	KC_AB3	ctrip	-0.098982851599188	0.576326448303868	1	pseudobulk	DESeq2	LRT	0
190	KC_AB3	CtsB1	0.158537264273913	0.641453058654887	1	pseudobulk	DESeq2	LRT	0
190	KC_AB3	Cubn	0.632774104902944	1	1	pseudobulk	DESeq2	LRT	0
190	KC_AB3	Cul1	0.460916913559792	0.412668472389678	1	pseudobulk	DESeq2	LRT	0
190	KC_AB3	Cul2	-0.333845833060708	0.479978954562992	1	pseudobulk	DESeq2	LRT	0
190	KC_AB3	Cul3	0.145037508236171	0.268169589665282	1	pseudobulk	DESeq2	LRT	0
190	KC_AB3	Cul4	-0.642571256433004	0.555745222187765	1	pseudobulk	DESeq2	LRT	0
190	KC_AB3	Cul5	0.132878861451819	0.775392501529689	1	pseudobulk	DESeq2	LRT	0
191	KC_AB3	cv	2.62817135292692	0.54954870385793	1	pseudobulk	DESeq2	LRT	0
191	KC_AB3	cv-c	-0.034673239283601	0.886418964802422	1	pseudobulk	DESeq2	LRT	0
191	KC_AB3	Cwc25	0.0289787276062662	0.739048719911041	1	pseudobulk	DESeq2	LRT	0
191	KC_AB3	cwo	-1.21160256659715	0.0899082691244359	1	pseudobulk	DESeq2	LRT	0
191	KC_AB3	cyc	0.532321640605154	0.328523742857463	1	pseudobulk	DESeq2	LRT	0
191	KC_AB3	CycC	-0.918300592410324	0.382412243496395	1	pseudobulk	DESeq2	LRT	0
191	KC_AB3	CycD	0.632774104902943	1	1	pseudobulk	DESeq2	LRT	0
191	KC_AB3	CycG	-0.050733141219374	0.796034595392749	1	pseudobulk	DESeq2	LRT	0
191	KC_AB3	CycH	0.969051891352446	1	1	pseudobulk	DESeq2	LRT	0
191	KC_AB3	CycJ	1.95267092137636	1	1	pseudobulk	DESeq2	LRT	0
191	KC_AB3	CycK	0.249810122877118	0.589426979138351	1	pseudobulk	DESeq2	LRT	0
191	KC_AB3	CycT	-0.177295060801288	0.615377892066351	1	pseudobulk	DESeq2	LRT	0
191	KC_AB3	CycY	0.0071907971694662	0.821008407559074	1	pseudobulk	DESeq2	LRT	0
191	KC_AB3	CYLD	-0.247761122023114	0.629994081661077	1	pseudobulk	DESeq2	LRT	0
191	KC_AB3	Cyp1	0.189028329483408	0.513247467344863	1	pseudobulk	DESeq2	LRT	0
191	KC_AB3	Cyp12b2	0.51266881388979	0.663311849411687	1	pseudobulk	DESeq2	LRT	0
191	KC_AB3	Cyp28a5	0.632774104902944	1	1	pseudobulk	DESeq2	LRT	0
191	KC_AB3	Cyp28d1	-0.186961738271884	1	1	pseudobulk	DESeq2	LRT	0
191	KC_AB3	Cyp310a1	0.632774104902943	1	1	pseudobulk	DESeq2	LRT	0
191	KC_AB3	cyp33	-0.325726381157459	0.640950670863524	1	pseudobulk	DESeq2	LRT	0
191	KC_AB3	Cyp49a1	-0.44306953104342	0.554896454245953	1	pseudobulk	DESeq2	LRT	0
191	KC_AB3	Cyp4ae1	0.272383645871586	1	1	pseudobulk	DESeq2	LRT	0
191	KC_AB3	Cyp4p2	1.59576167909336	0.887029991980463	1	pseudobulk	DESeq2	LRT	0
191	KC_AB3	Cyp4s3	1.95267092137636	1	1	pseudobulk	DESeq2	LRT	0
191	KC_AB3	Cyp6d5	-1.22959954014606	0.473415960731832	1	pseudobulk	DESeq2	LRT	0
191	KC_AB3	Cyp6g1	1.95267092137636	1	1	pseudobulk	DESeq2	LRT	0
191	KC_AB3	Cyp6g2	0.632774104902944	1	1	pseudobulk	DESeq2	LRT	0
191	KC_AB3	Cyp6u1	-1.29226487611801	0.307051117337482	1	pseudobulk	DESeq2	LRT	0
191	KC_AB3	Cyp9c1	1.95267092137636	1	1	pseudobulk	DESeq2	LRT	0
191	KC_AB3	Cyp9f2	0.632774104902944	1	1	pseudobulk	DESeq2	LRT	0
191	KC_AB3	Cyp9f3	1.8751573179585	0.767073395253422	1	pseudobulk	DESeq2	LRT	0
191	KC_AB3	cype	-0.009138918950983	0.636406326290324	1	pseudobulk	DESeq2	LRT	0

191	KC_AB3	Cypl	-0.0733944103731382	0.876697692971536	1	pseudobulk	DESeq2	LRT		0
191	KC_AB3	Cys	0.45117709139799	0.757522924414908	1	pseudobulk	DESeq2	LRT		0
191	KC_AB3	CysRS	-0.136393889501474	0.831064221966965	1	pseudobulk	DESeq2	LRT		0
191	KC_AB3	CysRS-m	1.11479939981603	0.68366681357092	1	pseudobulk	DESeq2	LRT		0
191	KC_AB3	Cyt-b5	-0.857812466557965	0.115363190559456	1	pseudobulk	DESeq2	LRT		0
191	KC_AB3	Cyt-c-d	0.632774104902944	1	1	pseudobulk	DESeq2	LRT		0
191	KC_AB3	Cyt-c-p	-0.0050462691999817	0.745576047895331	1	pseudobulk	DESeq2	LRT		0
191	KC_AB3	Cyt-c1	-0.119188308699877	0.588086238446963	1	pseudobulk	DESeq2	LRT		0
191	KC_AB3	D1	0.0771796236666864	0.761213542852852	1	pseudobulk	DESeq2	LRT		0
191	KC_AB3	D12	-0.120528428548273	0.914416877354382	1	pseudobulk	DESeq2	LRT		0
191	KC_AB3	D19A	-0.47097215059727	0.58524962061999	1	pseudobulk	DESeq2	LRT		0
191	KC_AB3	D19B	-0.441145035036254	0.570852377776819	1	pseudobulk	DESeq2	LRT		0
191	KC_AB3	D2hgdh	1.95267092137636	1	1	pseudobulk	DESeq2	LRT		0
191	KC_AB3	d4	-0.148604884370088	0.553490745379272	1	pseudobulk	DESeq2	LRT		0
191	KC_AB3	da	0.453739384260403	0.351424423618402	1	pseudobulk	DESeq2	LRT		0
191	KC_AB3	DAAM	-0.083785983471189	0.820800246176097	1	pseudobulk	DESeq2	LRT		0
191	KC_AB3	Dab	0.0794032934557446	0.798683001764314	1	pseudobulk	DESeq2	LRT		0
191	KC_AB3	dac	-0.276266151937272	0.2622378052133	1	pseudobulk	DESeq2	LRT		0
191	KC_AB3	Dad	-1.21837604790465	0.323073810627626	1	pseudobulk	DESeq2	LRT		0
191	KC_AB3	Dad1	-0.507133332512631	0.422117125511465	1	pseudobulk	DESeq2	LRT		0
191	KC_AB3	daed	-1.68896856489137	0.273317896312829	1	pseudobulk	DESeq2	LRT		0
191	KC_AB3	Dak1	0.687015775626698	0.128932593120605	1	pseudobulk	DESeq2	LRT		0
191	KC_AB3	dally	0.632774104902944	1	1	pseudobulk	DESeq2	LRT		0
191	KC_AB3	dao	-0.739309412397808	0.357546136219744	1	pseudobulk	DESeq2	LRT		0
191	KC_AB3	Dap160	0.14249125704556	0.596411841439252	1	pseudobulk	DESeq2	LRT		0
191	KC_AB3	dare	0.410241721489702	1	1	pseudobulk	DESeq2	LRT		0
191	KC_AB3	Dark	-0.827050722750757	0.355205478692984	1	pseudobulk	DESeq2	LRT		0
191	KC_AB3	dati	-0.36885404842535	0.165815729542183	1	pseudobulk	DESeq2	LRT		0
191	KC_AB3	Daxx	-0.0337213332470579	0.788810042292737	1	pseudobulk	DESeq2	LRT		0
191	KC_AB3	dbe	-0.908452148604833	0.450711808564713	1	pseudobulk	DESeq2	LRT		0
191	KC_AB3	dbo	-0.135309259581979	0.599819134248274	1	pseudobulk	DESeq2	LRT		0
191	KC_AB3	Dbp21E2	-0.0327300486906476	1	1	pseudobulk	DESeq2	LRT		0
191	KC_AB3	Dbp45A	0.627858524366338	0.743106941822197	1	pseudobulk	DESeq2	LRT		0
191	KC_AB3	Dbp73D	0.593667095203339	1	1	pseudobulk	DESeq2	LRT		0
191	KC_AB3	Dbp80	-0.123436392513353	0.522395616122025	1	pseudobulk	DESeq2	LRT		0
191	KC_AB3	dbr	-0.155252150225445	0.884340610954591	1	pseudobulk	DESeq2	LRT		0
191	KC_AB3	DCAF12	1.01965553741436	0.79973234753861	1	pseudobulk	DESeq2	LRT		0
191	KC_AB3	Dci	1.59359598433783	0.86377329553208	1	pseudobulk	DESeq2	LRT		0
191	KC_AB3	dco	0.733202150631919	0.204454788283082	1	pseudobulk	DESeq2	LRT		0
191	KC_AB3	Dcp-1	-0.297671726688543	0.696585662248982	1	pseudobulk	DESeq2	LRT		0
191	KC_AB3	DCP1	0.233793645933506	0.783068072756088	1	pseudobulk	DESeq2	LRT		0
191	KC_AB3	DCP2	-0.0501613016486688	0.55999669170872	1	pseudobulk	DESeq2	LRT		0
191	KC_AB3	Dcr-1	1.95267092137636	1	1	pseudobulk	DESeq2	LRT		0
191	KC_AB3	Dcr-2	-0.201388973929632	0.858218381574578	1	pseudobulk	DESeq2	LRT		0
191	KC_AB3	DCTN1-p150	-0.673961573867215	0.456582211385261	1	pseudobulk	DESeq2	LRT		0
191	KC_AB3	DCTN2-p50	0.524136809926478	0.486818742019194	1	pseudobulk	DESeq2	LRT		0
191	KC_AB3	DCTN3-p24	-0.420060397006392	0.641415338791624	1	pseudobulk	DESeq2	LRT		0
191	KC_AB3	DCTN4-p62	0.619787373658983	0.598040396664112	1	pseudobulk	DESeq2	LRT		0
191	KC_AB3	DCTN5-p25	-1.91335485720236	0.180219017995142	1	pseudobulk	DESeq2	LRT		0
191	KC_AB3	DCTN6-p27	0.518501132431854	0.537573011737296	1	pseudobulk	DESeq2	LRT		0
191	KC_AB3	Dd	-0.354688389603966	0.392706771951579	1	pseudobulk	DESeq2	LRT		0
191	KC_AB3	Ddc	0.222169167848533	0.617497860880192	1	pseudobulk	DESeq2	LRT		0
191	KC_AB3	Ddx1	-0.440883704044826	0.657765275716919	1	pseudobulk	DESeq2	LRT		0

191	KC_AB3	Deaf1	0.729373364508502	0.163487347736569	1	pseudobulk	DESeq2	LRT	0
191	KC_AB3	Debcl	1.95267092137636	1	1	pseudobulk	DESeq2	LRT	0
191	KC_AB3	DEF8	1.23360551849754	0.540823118759473	1	pseudobulk	DESeq2	LRT	0
191	KC_AB3	defl	0.632774104902944	1	1	pseudobulk	DESeq2	LRT	0
191	KC_AB3	Dek	-0.184863561613326	0.472125162113033	1	pseudobulk	DESeq2	LRT	0
191	KC_AB3	del	2.3149273646917	0.611236276445972	1	pseudobulk	DESeq2	LRT	0
191	KC_AB3	deltaCOP	0.203720458276757	0.719113487536973	1	pseudobulk	DESeq2	LRT	0
191	KC_AB3	Den1	0.0034659987013165	0.962291363994379	1	pseudobulk	DESeq2	LRT	0
191	KC_AB3	DENR	-0.50765669579068	0.515968702096091	1	pseudobulk	DESeq2	LRT	0
191	KC_AB3	Der-1	0.506179909988339	0.519771353860199	1	pseudobulk	DESeq2	LRT	0
191	KC_AB3	Der-2	0.7308894650361	0.423230803603242	1	pseudobulk	DESeq2	LRT	0
191	KC_AB3	Desat1	-0.0067839703602448	0.868335061767806	1	pseudobulk	DESeq2	LRT	0
191	KC_AB3	Det	1.95267092137636	1	1	pseudobulk	DESeq2	LRT	0
191	KC_AB3	Df31	-0.162335516021112	0.415294637327626	1	pseudobulk	DESeq2	LRT	0
191	KC_AB3	Dg	0.290522526751887	0.502824125995993	1	pseudobulk	DESeq2	LRT	0
192	KC_AB3	Dgk	-1.21946350542898	0.199711276989432	1	pseudobulk	DESeq2	LRT	0
192	KC_AB3	Dgkepsilon	0.700431739644163	0.359198657621963	1	pseudobulk	DESeq2	LRT	0
192	KC_AB3	Dgp-1	-1.29058801484972	0.149247287330741	1	pseudobulk	DESeq2	LRT	0
192	KC_AB3	dgrn	-0.782209672252057	0.539409308844827	1	pseudobulk	DESeq2	LRT	0
192	KC_AB3	dgt1	2.15298148099863	0.159817885685376	1	pseudobulk	DESeq2	LRT	0
192	KC_AB3	Dh44-R1	-0.961420841970914	0.212169292698359	1	pseudobulk	DESeq2	LRT	0
192	KC_AB3	Dh44-R2	-0.762621658570846	0.243255797021264	1	pseudobulk	DESeq2	LRT	0
192	KC_AB3	Dhap-at	0.293707903496883	0.651606388668597	1	pseudobulk	DESeq2	LRT	0
192	KC_AB3	Dhc64C	0.186673882293889	0.728484989532454	1	pseudobulk	DESeq2	LRT	0
192	KC_AB3	Dhit	-0.227835014536364	0.345465695842192	1	pseudobulk	DESeq2	LRT	0
192	KC_AB3	Dhod	1.39179995562774	0.484003730663405	1	pseudobulk	DESeq2	LRT	0
192	KC_AB3	DhpD	0.190037944770009	1	1	pseudobulk	DESeq2	LRT	0
192	KC_AB3	Dhpr	-0.152942310098637	0.63650336897632	1	pseudobulk	DESeq2	LRT	0
192	KC_AB3	Dhx15	-0.716786462302426	0.538828825560974	1	pseudobulk	DESeq2	LRT	0
192	KC_AB3	dia	-0.184744780581018	1	1	pseudobulk	DESeq2	LRT	0
192	KC_AB3	Diap1	0.0177673179088491	0.469098912781503	1	pseudobulk	DESeq2	LRT	0
192	KC_AB3	Diap2	-0.0824486813151131	0.657729979405676	1	pseudobulk	DESeq2	LRT	0
192	KC_AB3	Dic1	-0.304139222509758	0.50816208129364	1	pseudobulk	DESeq2	LRT	0
192	KC_AB3	didum	0.541312110263125	0.509495821125966	1	pseudobulk	DESeq2	LRT	0
192	KC_AB3	Dif	-0.413308068501668	0.334909270818748	1	pseudobulk	DESeq2	LRT	0
192	KC_AB3	dikar	-0.120450299255542	0.615080761167964	1	pseudobulk	DESeq2	LRT	0
192	KC_AB3	Dim1	-0.934865717527165	0.433191839964508	1	pseudobulk	DESeq2	LRT	0
192	KC_AB3	dind	0.545373413548383	0.712313707369304	1	pseudobulk	DESeq2	LRT	0
192	KC_AB3	DIP-alpha	1.22707713470494	1	1	pseudobulk	DESeq2	LRT	0
192	KC_AB3	Dip-C	-0.144364521447579	0.726878247642944	1	pseudobulk	DESeq2	LRT	0
192	KC_AB3	DIP-gamma	1.23133864869565	1	1	pseudobulk	DESeq2	LRT	0
192	KC_AB3	DIP-kappa	-0.173505850170689	0.455116260841875	1	pseudobulk	DESeq2	LRT	0
192	KC_AB3	DIP-lambda	0.621922437238901	1	1	pseudobulk	DESeq2	LRT	0
192	KC_AB3	DIP1	-0.298433493438745	0.536427858683652	1	pseudobulk	DESeq2	LRT	0
192	KC_AB3	DIP2	-0.562584193984567	0.463048547287781	1	pseudobulk	DESeq2	LRT	0
192	KC_AB3	Dis3	0.0020939056309239	1	1	pseudobulk	DESeq2	LRT	0
192	KC_AB3	Dis3l2	0.940942981041711	0.494978527689483	1	pseudobulk	DESeq2	LRT	0
192	KC_AB3	disp	0.494110497880344	0.897268430762443	1	pseudobulk	DESeq2	LRT	0
192	KC_AB3	DJ-1alpha	0.367934540426187	1	1	pseudobulk	DESeq2	LRT	0
192	KC_AB3	dj-1beta	0.167563659819235	0.689032375247709	1	pseudobulk	DESeq2	LRT	0
192	KC_AB3	dl	-0.400046519007236	0.964129537865033	1	pseudobulk	DESeq2	LRT	0
192	KC_AB3	Dlc90F	-0.15893883673523	0.643506417972202	1	pseudobulk	DESeq2	LRT	0
192	KC_AB3	dlg1	-0.307071193628079	0.197648866986779	1	pseudobulk	DESeq2	LRT	0

192	KC_AB3	Dlg5	-0.843639498490756	0.38810872687464	1	pseudobulk	DESeq2	LRT		0
192	KC_AB3	Dlic	0.0493364110459447	0.612615154263379	1	pseudobulk	DESeq2	LRT		0
192	KC_AB3	Dlip1	-0.155252150225445	0.884340610954591	1	pseudobulk	DESeq2	LRT		0
192	KC_AB3	Dlip2	0.0599167040207206	0.759091061745381	1	pseudobulk	DESeq2	LRT		0
192	KC_AB3	Dlip3	0.1175664906256	0.725591746891884	1	pseudobulk	DESeq2	LRT		0
192	KC_AB3	Dll	0.161515868851812	1	1	pseudobulk	DESeq2	LRT		0
192	KC_AB3	dlp	1.95267092137636	1	1	pseudobulk	DESeq2	LRT		0
192	KC_AB3	DMAP1	-0.74147812548102	0.447688438094979	1	pseudobulk	DESeq2	LRT		0
192	KC_AB3	dmrt11E	-0.302794075441951	0.851617925917851	1	pseudobulk	DESeq2	LRT		0
192	KC_AB3	dmt	1.23133864869565	1	1	pseudobulk	DESeq2	LRT		0
192	KC_AB3	Dmtn	-0.495563134254374	0.532411085142365	1	pseudobulk	DESeq2	LRT		0
192	KC_AB3	DnaJ-H	-0.666576503814084	0.423181370799434	1	pseudobulk	DESeq2	LRT		0
192	KC_AB3	DNAlig1	2.62817135292692	0.54954870385793	1	pseudobulk	DESeq2	LRT		0
192	KC_AB3	DNAlig3	0.0222020225849519	0.891684819584291	1	pseudobulk	DESeq2	LRT		0
192	KC_AB3	DNAlig4	1.47837773392725	0.70817076420814	1	pseudobulk	DESeq2	LRT		0
192	KC_AB3	DNApol-delta	1.23133864869565	1	1	pseudobulk	DESeq2	LRT		0
192	KC_AB3	DNApol-eta	1.45422967012522	0.753390297571937	1	pseudobulk	DESeq2	LRT		0
192	KC_AB3	DNApol-iota	-1.5552356773072	0.36454521810638	1	pseudobulk	DESeq2	LRT		0
192	KC_AB3	DNApol-zeta	2.88619786524384	0.39214769278579	1	pseudobulk	DESeq2	LRT		0
192	KC_AB3	DNaseII	1.95267092137636	1	1	pseudobulk	DESeq2	LRT		0
192	KC_AB3	dnc	-0.115119110103772	0.554537662152881	1	pseudobulk	DESeq2	LRT		0
192	KC_AB3	dnk	0.730084804452018	0.482854176111558	1	pseudobulk	DESeq2	LRT		0
192	KC_AB3	dnt	-0.32882510184214	0.693608329500188	1	pseudobulk	DESeq2	LRT		0
192	KC_AB3	Dnz1	-0.670320484090285	0.436399602198538	1	pseudobulk	DESeq2	LRT		0
192	KC_AB3	Doa	0.105816159749768	0.736052098798702	1	pseudobulk	DESeq2	LRT		0
192	KC_AB3	dock	0.0214017323661617	0.642981256277915	1	pseudobulk	DESeq2	LRT		0
192	KC_AB3	dod	-0.0270229904992175	0.919091758041502	1	pseudobulk	DESeq2	LRT		0
192	KC_AB3	dom	0.135033941941662	0.819823733109993	1	pseudobulk	DESeq2	LRT		0
192	KC_AB3	dome	0.415662560627221	0.687242545067049	1	pseudobulk	DESeq2	LRT		0
192	KC_AB3	dop	-0.0248462012250037	0.683025522804697	1	pseudobulk	DESeq2	LRT		0
192	KC_AB3	Dop1R1	-0.549459068546068	0.098413702239373	1	pseudobulk	DESeq2	LRT		0
192	KC_AB3	Dop1R2	-0.0409259807599078	0.665206311004048	1	pseudobulk	DESeq2	LRT		0
192	KC_AB3	Dop2R	-0.228365599592812	0.3951983608494	1	pseudobulk	DESeq2	LRT		0
192	KC_AB3	DopEcR	-0.272986781010615	0.394799886168623	1	pseudobulk	DESeq2	LRT		0
192	KC_AB3	dor	1.10376293347703	0.538862154714827	1	pseudobulk	DESeq2	LRT		0
192	KC_AB3	DOR	-0.155165691329484	0.55289796736944	1	pseudobulk	DESeq2	LRT		0
192	KC_AB3	dos	0.112681898577806	0.864603433499802	1	pseudobulk	DESeq2	LRT		0
192	KC_AB3	Dp	-0.450583614578971	0.394259007028718	1	pseudobulk	DESeq2	LRT		0
192	KC_AB3	Dp1	-0.535522774328875	0.57946498025626	1	pseudobulk	DESeq2	LRT		0
192	KC_AB3	Dpck	-0.958432651310066	0.425204235713926	1	pseudobulk	DESeq2	LRT		0
192	KC_AB3	DPCoAC	-0.546690377232218	0.815223005364861	1	pseudobulk	DESeq2	LRT		0
192	KC_AB3	Dph5	1.17664402231334	0.186846788810512	1	pseudobulk	DESeq2	LRT		0
192	KC_AB3	Dpit47	0.440578571731502	1	1	pseudobulk	DESeq2	LRT		0
192	KC_AB3	DppIII	-0.491189162264626	0.489782973204773	1	pseudobulk	DESeq2	LRT		0
192	KC_AB3	dpr1	0.143168755314923	0.725402802740768	1	pseudobulk	DESeq2	LRT		0
192	KC_AB3	dpr10	0.168883666220578	0.615766667335092	1	pseudobulk	DESeq2	LRT		0
192	KC_AB3	dpr11	1.95267092137636	1	1	pseudobulk	DESeq2	LRT		0
192	KC_AB3	dpr12	-0.42408981203207	0.193725222749549	1	pseudobulk	DESeq2	LRT		0
192	KC_AB3	dpr13	0.632774104902943	1	1	pseudobulk	DESeq2	LRT		0
192	KC_AB3	dpr14	-0.0252047823775787	0.711963084179479	1	pseudobulk	DESeq2	LRT		0
192	KC_AB3	dpr19	-0.0174193433569486	0.827722192556995	1	pseudobulk	DESeq2	LRT		0
192	KC_AB3	dpr2	-1.18648839128819	0.394644300930517	1	pseudobulk	DESeq2	LRT		0
192	KC_AB3	dpr20	-0.813026363773094	0.443192946962325	1	pseudobulk	DESeq2	LRT		0

192	KC_AB3	dpr21	-0.519102160319712	0.12484594942924	1	pseudobulk	DESeq2	LRT		0
192	KC_AB3	dpr3	0.247739906366514	1	1	pseudobulk	DESeq2	LRT		0
192	KC_AB3	dpr4	-0.572802673316773	0.268948165663312	1	pseudobulk	DESeq2	LRT		0
192	KC_AB3	dpr5	-1.30639013240762	0.0868033450649714	1	pseudobulk	DESeq2	LRT		0
192	KC_AB3	dpr7	0.245839232660933	0.459938078272417	1	pseudobulk	DESeq2	LRT		0
192	KC_AB3	dpr8	-0.114336077561529	0.69758342479687	1	pseudobulk	DESeq2	LRT		0
192	KC_AB3	dpr9	-0.16099117807226	0.447177043291017	1	pseudobulk	DESeq2	LRT		0
192	KC_AB3	dpy	3.11651768969717	0.314680495027058	1	pseudobulk	DESeq2	LRT		0
192	KC_AB3	Dpy-30L1	-0.614272451670413	0.354649083861219	1	pseudobulk	DESeq2	LRT		0
193	KC_AB3	Drak	0.211239302704068	0.384572617040992	1	pseudobulk	DESeq2	LRT		0
193	KC_AB3	Drat	-0.250287049182525	0.321842537860097	1	pseudobulk	DESeq2	LRT		0
193	KC_AB3	dre4	-1.30530486032859	0.267489318650405	1	pseudobulk	DESeq2	LRT		0
193	KC_AB3	Dredd	0.632774104902944	1	1	pseudobulk	DESeq2	LRT		0
193	KC_AB3	Dref	-0.242258415151571	0.701679299444786	1	pseudobulk	DESeq2	LRT		0
193	KC_AB3	Drep1	2.12493143161906	0.332053268466731	1	pseudobulk	DESeq2	LRT		0
193	KC_AB3	Drep2	-0.332929282920979	0.313739743829708	1	pseudobulk	DESeq2	LRT		0
193	KC_AB3	Drep4	0.334259228010399	0.651362406164982	1	pseudobulk	DESeq2	LRT		0
193	KC_AB3	Drice	0.319019293298944	1	1	pseudobulk	DESeq2	LRT		0
193	KC_AB3	drk	-0.429435824283464	0.545234944937719	1	pseudobulk	DESeq2	LRT		0
193	KC_AB3	drl	0.125481105743871	0.670920765901682	1	pseudobulk	DESeq2	LRT		0
193	KC_AB3	Drl-2	-0.680094168819758	0.737706015643587	1	pseudobulk	DESeq2	LRT		0
193	KC_AB3	Droj2	-0.215746927668408	0.397100615334697	1	pseudobulk	DESeq2	LRT		0
193	KC_AB3	Dronc	0.632774104902944	1	1	pseudobulk	DESeq2	LRT		0
193	KC_AB3	drongo	0.536126703182003	0.494349858591229	1	pseudobulk	DESeq2	LRT		0
193	KC_AB3	drosha	1.37780100060845	0.581529761941749	1	pseudobulk	DESeq2	LRT		0
193	KC_AB3	Drp1	-0.222307897067507	0.648310498088089	1	pseudobulk	DESeq2	LRT		0
193	KC_AB3	drpr	-0.764573875616638	0.42354299506577	1	pseudobulk	DESeq2	LRT		0
193	KC_AB3	Drsl2	0.632774104902944	1	1	pseudobulk	DESeq2	LRT		0
193	KC_AB3	Dscam1	-0.572079318294063	0.258605917501572	1	pseudobulk	DESeq2	LRT		0
193	KC_AB3	Dscam2	0.0555067898314693	0.718730665006114	1	pseudobulk	DESeq2	LRT		0
193	KC_AB3	Dscam3	1.59576167909336	0.887029991980463	1	pseudobulk	DESeq2	LRT		0
193	KC_AB3	Dscam4	-0.497683215675358	0.287459333627282	1	pseudobulk	DESeq2	LRT		0
193	KC_AB3	dsd	-0.110787807575309	0.700955978382611	1	pseudobulk	DESeq2	LRT		0
193	KC_AB3	dsh	-0.843631967701306	0.217458715733568	1	pseudobulk	DESeq2	LRT		0
193	KC_AB3	Dsor1	0.670728192152295	0.212280623733872	1	pseudobulk	DESeq2	LRT		0
193	KC_AB3	Dsp1	-0.0459098553750395	0.824060756212825	1	pseudobulk	DESeq2	LRT		0
193	KC_AB3	Dtd	1.32659218078634	0.784398264662993	1	pseudobulk	DESeq2	LRT		0
193	KC_AB3	dtn	-0.0213560342734124	0.838578982402009	1	pseudobulk	DESeq2	LRT		0
193	KC_AB3	Duba	-0.148957193772445	0.630072319275254	1	pseudobulk	DESeq2	LRT		0
193	KC_AB3	DUBAI	-0.839248560128019	0.407541083654309	1	pseudobulk	DESeq2	LRT		0
193	KC_AB3	dunk	-0.205004760353214	0.742145652423133	1	pseudobulk	DESeq2	LRT		0
193	KC_AB3	dup	0.829888880200502	0.283065367792505	1	pseudobulk	DESeq2	LRT		0
193	KC_AB3	dUTPase	0.632774104902944	1	1	pseudobulk	DESeq2	LRT		0
193	KC_AB3	dve	1.22707713470494	1	1	pseudobulk	DESeq2	LRT		0
193	KC_AB3	dwg	-0.232209729512227	0.839714225908955	1	pseudobulk	DESeq2	LRT		0
193	KC_AB3	Dyb	0.0004821616099894	0.582740536558723	1	pseudobulk	DESeq2	LRT		0
193	KC_AB3	Dyrk2	0.201500559409647	0.467649206263491	1	pseudobulk	DESeq2	LRT		0
193	KC_AB3	Dyrk3	0.399180068818364	0.278497699609777	1	pseudobulk	DESeq2	LRT		0
193	KC_AB3	Dys	-0.66271693525548	0.170875775794421	1	pseudobulk	DESeq2	LRT		0
193	KC_AB3	dysc	-0.263425241756309	0.191475551552308	1	pseudobulk	DESeq2	LRT		0
193	KC_AB3	E(bx)	-0.327781080675535	0.502025955091802	1	pseudobulk	DESeq2	LRT		0
193	KC_AB3	E(Pc)	-0.0594491790372997	0.492658402944704	1	pseudobulk	DESeq2	LRT		0
193	KC_AB3	e(r)	-0.0423265861355487	0.600880593019021	1	pseudobulk	DESeq2	LRT		0

193	KC_AB3	E(var)3-9	-0.531980511788713	0.529349271462236	1	pseudobulk	DESeq2	LRT	0
193	KC_AB3	e(y)1	0.452860999606561	0.800970496636225	1	pseudobulk	DESeq2	LRT	0
193	KC_AB3	e(y)2	-0.555518319575159	0.586314296808802	1	pseudobulk	DESeq2	LRT	0
193	KC_AB3	e(y)2b	1.95267092137636	1	1	pseudobulk	DESeq2	LRT	0
193	KC_AB3	e(y)3	-0.219539223273481	0.658721550007622	1	pseudobulk	DESeq2	LRT	0
193	KC_AB3	E(z)	2.38879264335236	0.160202081103864	1	pseudobulk	DESeq2	LRT	0
193	KC_AB3	E2f1	-0.0644986837341558	0.814813418239616	1	pseudobulk	DESeq2	LRT	0
193	KC_AB3	E2f2	-0.514182193229933	0.608805512776193	1	pseudobulk	DESeq2	LRT	0
193	KC_AB3	Eaat1	1.23133864869565	1	1	pseudobulk	DESeq2	LRT	0
193	KC_AB3	Eaat2	0.152358308910688	0.653998209876859	1	pseudobulk	DESeq2	LRT	0
193	KC_AB3	Eaf	-0.26757850652982	0.597105141836608	1	pseudobulk	DESeq2	LRT	0
193	KC_AB3	Eaf6	0.243753880193116	0.933376358709583	1	pseudobulk	DESeq2	LRT	0
193	KC_AB3	eag	-0.673483909228962	0.182496294930056	1	pseudobulk	DESeq2	LRT	0
193	KC_AB3	ear	-0.808721459642031	0.345872365511705	1	pseudobulk	DESeq2	LRT	0
193	KC_AB3	eas	-0.691162344520948	0.276446370254804	1	pseudobulk	DESeq2	LRT	0
193	KC_AB3	east	-0.12936442420912	0.472679417406291	1	pseudobulk	DESeq2	LRT	0
193	KC_AB3	eater	0.632774104902944	1	1	pseudobulk	DESeq2	LRT	0
193	KC_AB3	Eato	0.741719359423478	0.284646990190188	1	pseudobulk	DESeq2	LRT	0
193	KC_AB3	Eb1	-0.0003403050606214	0.70381212400672	1	pseudobulk	DESeq2	LRT	0
193	KC_AB3	ebd1	-0.0908446882493655	0.805552398592029	1	pseudobulk	DESeq2	LRT	0
193	KC_AB3	ebd2	-0.411633181907479	0.626962855462103	1	pseudobulk	DESeq2	LRT	0
193	KC_AB3	ebi	-1.41068138223411	0.333907993792559	1	pseudobulk	DESeq2	LRT	0
193	KC_AB3	ebo	0.077761817165399	1	1	pseudobulk	DESeq2	LRT	0
193	KC_AB3	EbpIII	0.632774104902943	1	1	pseudobulk	DESeq2	LRT	0
193	KC_AB3	eca	-0.326208254272563	0.577057015919849	1	pseudobulk	DESeq2	LRT	0
193	KC_AB3	ecd	0.38792488359198	0.438241344764949	1	pseudobulk	DESeq2	LRT	0
193	KC_AB3	Echs1	-0.163650770166386	0.720799371478901	1	pseudobulk	DESeq2	LRT	0
193	KC_AB3	EcR	-0.37743907103126	0.306765111581052	1	pseudobulk	DESeq2	LRT	0
193	KC_AB3	ECSIT	0.0025701398975595	1	1	pseudobulk	DESeq2	LRT	0
193	KC_AB3	Ect4	-0.673478386686469	0.610865403135094	1	pseudobulk	DESeq2	LRT	0
193	KC_AB3	ed	-2.46028502627838	0.130338641704723	1	pseudobulk	DESeq2	LRT	0
193	KC_AB3	Edc3	-0.589818529136297	0.376732854740041	1	pseudobulk	DESeq2	LRT	0
193	KC_AB3	Edem1	-0.566641765370198	0.674958268335822	1	pseudobulk	DESeq2	LRT	0
193	KC_AB3	Edem2	0.578414581127016	0.676060816260264	1	pseudobulk	DESeq2	LRT	0
193	KC_AB3	Edg78E	1.95267092137636	1	1	pseudobulk	DESeq2	LRT	0
193	KC_AB3	EDTP	0.972582044658406	0.764620665402759	1	pseudobulk	DESeq2	LRT	0
193	KC_AB3	eEF1alpha1	0.13041059735751	0.802826601182421	1	pseudobulk	DESeq2	LRT	0
193	KC_AB3	eEF1alpha2	-0.0137507118696095	0.808501763642247	1	pseudobulk	DESeq2	LRT	0
193	KC_AB3	eEF1beta	-0.431912616836832	0.564378075083131	1	pseudobulk	DESeq2	LRT	0
193	KC_AB3	eEF1delta	0.183393637775778	0.645682767093696	1	pseudobulk	DESeq2	LRT	0
193	KC_AB3	eEF1gamma	0.13732610939184	0.686142169704968	1	pseudobulk	DESeq2	LRT	0
193	KC_AB3	eEF2	0.147332149148769	0.58127489832943	1	pseudobulk	DESeq2	LRT	0
193	KC_AB3	eEF5	0.1192379189071	0.454106513740888	1	pseudobulk	DESeq2	LRT	0
193	KC_AB3	eEFSec	-0.186961738271884	1	1	pseudobulk	DESeq2	LRT	0
193	KC_AB3	Efa6	-0.523543891170804	0.558349314144585	1	pseudobulk	DESeq2	LRT	0
193	KC_AB3	eff	0.0038359423988420	0.755937425415315	1	pseudobulk	DESeq2	LRT	0
193	KC_AB3	Efr	-0.395859129495344	0.702258923937399	1	pseudobulk	DESeq2	LRT	0
193	KC_AB3	egg	0.396473548104208	0.42663346003763	1	pseudobulk	DESeq2	LRT	0
193	KC_AB3	egh	0.150160398452491	0.429735096934973	1	pseudobulk	DESeq2	LRT	0
193	KC_AB3	egl	-0.389209656049491	0.608923824326108	1	pseudobulk	DESeq2	LRT	0
193	KC_AB3	Eglp4	1.95267092137636	1	1	pseudobulk	DESeq2	LRT	0
193	KC_AB3	Egm	0.832170875378037	0.244862329317112	1	pseudobulk	DESeq2	LRT	0
193	KC_AB3	Ehbp1	-0.21891079906195	0.534542742882653	1	pseudobulk	DESeq2	LRT	0

193	KC_AB3	eIF1	0.171684191179045	0.629423129544295	1	pseudobulk	DESeq2	LRT	0
193	KC_AB3	eIF1A	0.0622733366334913	0.642303062497397	1	pseudobulk	DESeq2	LRT	0
193	KC_AB3	eIF2alpha	-1.52747241598951	0.0628825344942757	1	pseudobulk	DESeq2	LRT	0
194	KC_AB3	eIF2Balpha	-0.626986988173465	0.591424118579615	1	pseudobulk	DESeq2	LRT	0
194	KC_AB3	eIF2Bbeta	-0.809408161560303	0.387109279516248	1	pseudobulk	DESeq2	LRT	0
194	KC_AB3	eIF2Bdelta	0.124710800631261	0.746981448163527	1	pseudobulk	DESeq2	LRT	0
194	KC_AB3	eIF2Bepsilon	0.191517849086895	0.909082721946966	1	pseudobulk	DESeq2	LRT	0
194	KC_AB3	eIF2beta	-0.218299772610402	0.446717185135358	1	pseudobulk	DESeq2	LRT	0
194	KC_AB3	eIF2Bgamma	0.601654341559522	1	1	pseudobulk	DESeq2	LRT	0
194	KC_AB3	eIF2D	-0.0191307814769492	0.818839377728779	1	pseudobulk	DESeq2	LRT	0
194	KC_AB3	eIF2gamma	0.125998768512386	0.757127566067373	1	pseudobulk	DESeq2	LRT	0
194	KC_AB3	eIF3a	-0.152513911730373	0.573821029490401	1	pseudobulk	DESeq2	LRT	0
194	KC_AB3	eIF3b	-0.344894326300549	0.422920358164151	1	pseudobulk	DESeq2	LRT	0
194	KC_AB3	eIF3c	0.503250929308526	0.369738219551175	1	pseudobulk	DESeq2	LRT	0
194	KC_AB3	eIF3d1	-0.519402655020032	0.388453064516899	1	pseudobulk	DESeq2	LRT	0
194	KC_AB3	eIF3e	-0.379783854661097	0.399750461894792	1	pseudobulk	DESeq2	LRT	0
194	KC_AB3	eIF3f1	-0.0449406319281456	0.547435065659822	1	pseudobulk	DESeq2	LRT	0
194	KC_AB3	eIF3g1	0.478171578248502	0.240095813971036	1	pseudobulk	DESeq2	LRT	0
194	KC_AB3	eIF3h	0.0120232775137737	0.599406702848764	1	pseudobulk	DESeq2	LRT	0
194	KC_AB3	eIF3i	0.3317478022484	0.536747340674492	1	pseudobulk	DESeq2	LRT	0
194	KC_AB3	eIF3j	-0.0566598752127753	0.906917962034617	1	pseudobulk	DESeq2	LRT	0
194	KC_AB3	eIF3k	-0.0899347480755525	0.432404518315001	1	pseudobulk	DESeq2	LRT	0
194	KC_AB3	eIF3l	0.102674972024668	0.770576557285545	1	pseudobulk	DESeq2	LRT	0
194	KC_AB3	eIF3m	-0.382192040618514	0.337744020869372	1	pseudobulk	DESeq2	LRT	0
194	KC_AB3	eIF4A	-0.0964157085049879	0.612863657687086	1	pseudobulk	DESeq2	LRT	0
194	KC_AB3	eIF4B	0.160950686873567	0.705216069692338	1	pseudobulk	DESeq2	LRT	0
194	KC_AB3	eIF4E1	-0.106529024881446	0.579606990671989	1	pseudobulk	DESeq2	LRT	0
194	KC_AB3	eIF4EHP	-0.166590388004442	0.503223238097551	1	pseudobulk	DESeq2	LRT	0
194	KC_AB3	eIF4G1	0.151757491553193	0.507874518964201	1	pseudobulk	DESeq2	LRT	0
194	KC_AB3	eIF4G2	-0.36998503794415	0.342896536388983	1	pseudobulk	DESeq2	LRT	0
194	KC_AB3	eIF4H1	-0.605854870556787	0.234788646156958	1	pseudobulk	DESeq2	LRT	0
194	KC_AB3	eIF5	-0.545750574844096	0.296110879292908	1	pseudobulk	DESeq2	LRT	0
194	KC_AB3	eIF5B	0.209887829868708	0.485723705787336	1	pseudobulk	DESeq2	LRT	0
194	KC_AB3	eIF6	1.02636432098494	0.303340631462557	1	pseudobulk	DESeq2	LRT	0
194	KC_AB3	Eip55E	0.156657196974426	1	1	pseudobulk	DESeq2	LRT	0
194	KC_AB3	Eip63E	-0.372166598828142	0.427156532827249	1	pseudobulk	DESeq2	LRT	0
194	KC_AB3	Eip63F-1	3.01949397490798	0.184485083798484	1	pseudobulk	DESeq2	LRT	0
194	KC_AB3	Eip71CD	0.568048305633189	0.467198538261574	1	pseudobulk	DESeq2	LRT	0
194	KC_AB3	Eip74EF	-0.213723255168016	0.66431725317016	1	pseudobulk	DESeq2	LRT	0
194	KC_AB3	Eip75B	-0.105087635675139	0.666955854081308	1	pseudobulk	DESeq2	LRT	0
194	KC_AB3	Eip78C	-0.654561083865305	0.327796122084568	1	pseudobulk	DESeq2	LRT	0
194	KC_AB3	Eip93F	-0.225091792418975	0.26849603614947	1	pseudobulk	DESeq2	LRT	0
194	KC_AB3	elav	-0.407468864703836	0.179200407552728	1	pseudobulk	DESeq2	LRT	0
194	KC_AB3	elB	-0.33535178735708	0.533117259511702	1	pseudobulk	DESeq2	LRT	0
194	KC_AB3	elgi	-0.1421222727858	0.744199793050744	1	pseudobulk	DESeq2	LRT	0
194	KC_AB3	Elk	-0.127728988654287	0.645111633063002	1	pseudobulk	DESeq2	LRT	0
194	KC_AB3	elm	-0.38425865722593	0.372062787619781	1	pseudobulk	DESeq2	LRT	0
194	KC_AB3	EloA	1.08741152645441	0.190168409889097	1	pseudobulk	DESeq2	LRT	0
194	KC_AB3	EloB	-0.136528940495737	0.426404683232254	1	pseudobulk	DESeq2	LRT	0
194	KC_AB3	EloC	-0.571499935852794	0.19335853634283	1	pseudobulk	DESeq2	LRT	0
194	KC_AB3	ELOVL	0.632774104902944	1	1	pseudobulk	DESeq2	LRT	0
194	KC_AB3	Elp1	-0.0188092059439452	0.900072384019603	1	pseudobulk	DESeq2	LRT	0
194	KC_AB3	Elp3	0.506673703470505	1	1	pseudobulk	DESeq2	LRT	0

194	KC_AB3	Elys	0.71219336951787	0.663979703322961	1	pseudobulk	DESeq2	LRT		0
194	KC_AB3	ema	-0.787992255552014	0.467041403080077	1	pseudobulk	DESeq2	LRT		0
194	KC_AB3	emb	-0.488521052910232	0.373556541143096	1	pseudobulk	DESeq2	LRT		0
194	KC_AB3	emc	0.0594951115870713	0.645533805037512	1	pseudobulk	DESeq2	LRT		0
194	KC_AB3	EMC1	-1.0230966244732	0.41062199456604	1	pseudobulk	DESeq2	LRT		0
194	KC_AB3	EMC3	-0.362590742983778	0.637555946218391	1	pseudobulk	DESeq2	LRT		0
194	KC_AB3	EMC8-9	0.0023857313267285	0.960238068836215	1	pseudobulk	DESeq2	LRT		0
194	KC_AB3	EMRE	-0.444377126809552	0.30622675451403	1	pseudobulk	DESeq2	LRT		0
194	KC_AB3	ena	0.298702707721466	0.607101216022488	1	pseudobulk	DESeq2	LRT		0
194	KC_AB3	enc	0.0342225879897054	0.869011114580677	1	pseudobulk	DESeq2	LRT		0
194	KC_AB3	EndoA	-0.0758684755419262	0.460198224555669	1	pseudobulk	DESeq2	LRT		0
194	KC_AB3	EndoB	-0.0502529947708308	0.720592271759228	1	pseudobulk	DESeq2	LRT		0
194	KC_AB3	EndoG	-0.776076449332054	0.499450566837674	1	pseudobulk	DESeq2	LRT		0
194	KC_AB3	EndoGI	0.928226709021685	0.962506701600556	1	pseudobulk	DESeq2	LRT		0
194	KC_AB3	endos	-0.0278310791289679	0.620616456061715	1	pseudobulk	DESeq2	LRT		0
194	KC_AB3	Eno	-0.0233510181754932	0.713008564458994	1	pseudobulk	DESeq2	LRT		0
194	KC_AB3	ens	-0.0979840681566663	0.772311556691433	1	pseudobulk	DESeq2	LRT		0
194	KC_AB3	Ent1	0.161515868851812	1	1	pseudobulk	DESeq2	LRT		0
194	KC_AB3	Ent2	-0.977688999858576	0.518234836155577	1	pseudobulk	DESeq2	LRT		0
194	KC_AB3	Ent3	0.544907688776289	0.411182431271429	1	pseudobulk	DESeq2	LRT		0
194	KC_AB3	Eogt	-0.673478386686469	0.610865403135094	1	pseudobulk	DESeq2	LRT		0
194	KC_AB3	Epac	0.632774104902944	1	1	pseudobulk	DESeq2	LRT		0
194	KC_AB3	Epg5	-1.27972904618749	0.37417080019014	1	pseudobulk	DESeq2	LRT		0
194	KC_AB3	Eph	-0.224515732946812	0.504987375019664	1	pseudobulk	DESeq2	LRT		0
194	KC_AB3	Ephrin	0.0182456927210819	0.710329052369808	1	pseudobulk	DESeq2	LRT		0
194	KC_AB3	Eps-15	0.329199267520726	0.460127514387662	1	pseudobulk	DESeq2	LRT		0
194	KC_AB3	epsilonCOP	0.171441393265944	0.721625501534262	1	pseudobulk	DESeq2	LRT		0
194	KC_AB3	Ercc1	1.95267092137636	1	1	pseudobulk	DESeq2	LRT		0
194	KC_AB3	eRF1	0.531871676566788	0.267355261137204	1	pseudobulk	DESeq2	LRT		0
194	KC_AB3	eRF3	0.421833669242858	0.652783445249822	1	pseudobulk	DESeq2	LRT		0
194	KC_AB3	ergic53	-0.93184075257869	0.469062512561015	1	pseudobulk	DESeq2	LRT		0
194	KC_AB3	Ero1L	-0.510962930340367	0.658059782473397	1	pseudobulk	DESeq2	LRT		0
194	KC_AB3	ERp60	0.357074008899482	0.675139689387204	1	pseudobulk	DESeq2	LRT		0
194	KC_AB3	ERR	-0.236138073477934	0.694247983143448	1	pseudobulk	DESeq2	LRT		0
194	KC_AB3	Es2	0.760007492578728	0.910699105205813	1	pseudobulk	DESeq2	LRT		0
194	KC_AB3	escl	1.20132755712293	1	1	pseudobulk	DESeq2	LRT		0
194	KC_AB3	esn	0.857589868272425	0.779075846623175	1	pseudobulk	DESeq2	LRT		0
194	KC_AB3	Est-Q	0.632774104902944	1	1	pseudobulk	DESeq2	LRT		0
194	KC_AB3	Esyt2	-0.232179101871661	0.710132803586979	1	pseudobulk	DESeq2	LRT		0
194	KC_AB3	Etf-QO	-0.583057444465563	0.678110339883318	1	pseudobulk	DESeq2	LRT		0
194	KC_AB3	Etl1	-0.774396775788032	0.517294051928045	1	pseudobulk	DESeq2	LRT		0
194	KC_AB3	Ets65A	1.95267092137636	1	1	pseudobulk	DESeq2	LRT		0
194	KC_AB3	Ets97D	0.0578630818178232	0.916666767587641	1	pseudobulk	DESeq2	LRT		0
194	KC_AB3	euc	1.95267092137636	1	1	pseudobulk	DESeq2	LRT		0
194	KC_AB3	Evi5	0.0711573770196076	0.57287738034674	1	pseudobulk	DESeq2	LRT		0
194	KC_AB3	ewg	-0.234223696117723	0.532240776930056	1	pseudobulk	DESeq2	LRT		0
194	KC_AB3	Exd2	-0.207400534640008	0.852727734577056	1	pseudobulk	DESeq2	LRT		0
194	KC_AB3	Exn	-0.089278810270091	0.671045972149616	1	pseudobulk	DESeq2	LRT		0
194	KC_AB3	Exo70	-1.47247754063827	0.310298146990612	1	pseudobulk	DESeq2	LRT		0
194	KC_AB3	Exo84	-0.596382824510968	0.562856757238182	1	pseudobulk	DESeq2	LRT		0
195	KC_AB3	ey	-0.149656039815493	0.504754581629093	1	pseudobulk	DESeq2	LRT		0
195	KC_AB3	eys	0.352779903168263	0.386649289758271	1	pseudobulk	DESeq2	LRT		0
195	KC_AB3	f-cup	-0.160804957331434	0.818391168986531	1	pseudobulk	DESeq2	LRT		0

195	KC_AB3	fabp	0.556636655385653	0.5170498958734	1	pseudobulk	DESeq2	LRT	0
195	KC_AB3	Fadd	0.632774104902943	1	1	pseudobulk	DESeq2	LRT	0
195	KC_AB3	faf	0.139494232060395	0.473632282064104	1	pseudobulk	DESeq2	LRT	0
195	KC_AB3	Faf2	-1.33170218879083	0.203849173125787	1	pseudobulk	DESeq2	LRT	0
195	KC_AB3	Fak	0.0417607589585211	0.812036620530399	1	pseudobulk	DESeq2	LRT	0
195	KC_AB3	fal	0.0037571764674609	0.837291772644316	1	pseudobulk	DESeq2	LRT	0
195	KC_AB3	FAM21	0.256578072384352	0.76493949824124	1	pseudobulk	DESeq2	LRT	0
195	KC_AB3	fand	0.189700419481903	1	1	pseudobulk	DESeq2	LRT	0
195	KC_AB3	Fas1	-0.0729520934070142	0.775316990739022	1	pseudobulk	DESeq2	LRT	0
195	KC_AB3	Fas2	0.0316558449142003	0.807373192370764	1	pseudobulk	DESeq2	LRT	0
195	KC_AB3	Fas3	2.64926057711297	0.482244576496623	1	pseudobulk	DESeq2	LRT	0
195	KC_AB3	FASN1	-0.0605365043843152	0.816439973872874	1	pseudobulk	DESeq2	LRT	0
195	KC_AB3	Fatp1	0.121325815299645	0.761606741894337	1	pseudobulk	DESeq2	LRT	0
195	KC_AB3	fax	-0.157065307248799	0.475745721161042	1	pseudobulk	DESeq2	LRT	0
195	KC_AB3	fbl	-0.786797700653312	0.329562054015482	1	pseudobulk	DESeq2	LRT	0
195	KC_AB3	Fbl6	0.0193867445251214	0.772092153727894	1	pseudobulk	DESeq2	LRT	0
195	KC_AB3	fbp	-0.547361577986966	0.814803379895176	1	pseudobulk	DESeq2	LRT	0
195	KC_AB3	Fbw5	0.465192970281754	1	1	pseudobulk	DESeq2	LRT	0
195	KC_AB3	Fbxl4	-0.437417274422759	0.632342503955613	1	pseudobulk	DESeq2	LRT	0
195	KC_AB3	FBXO11	-0.188552282520594	0.798111113113511	1	pseudobulk	DESeq2	LRT	0
195	KC_AB3	Fcp1	0.0676363105142286	0.743708601708961	1	pseudobulk	DESeq2	LRT	0
195	KC_AB3	Fdh	2.62817135292692	0.54954870385793	1	pseudobulk	DESeq2	LRT	0
195	KC_AB3	fdl	-0.998882578168065	0.19391840482742	1	pseudobulk	DESeq2	LRT	0
195	KC_AB3	Fdx1	0.980022187343114	0.397458166060575	1	pseudobulk	DESeq2	LRT	0
195	KC_AB3	Fdx2	-0.365807654019869	0.646406525349412	1	pseudobulk	DESeq2	LRT	0
195	KC_AB3	FeCH	1.07123590504056	0.536749416768919	1	pseudobulk	DESeq2	LRT	0
195	KC_AB3	Fem-1	0.66653510725646	0.447265865459457	1	pseudobulk	DESeq2	LRT	0
195	KC_AB3	Fen1	1.95267092137636	1	1	pseudobulk	DESeq2	LRT	0
195	KC_AB3	fend	-0.465432174447855	0.127435150134487	1	pseudobulk	DESeq2	LRT	0
195	KC_AB3	Fer1HCH	-0.38462828620614	0.282837475461992	1	pseudobulk	DESeq2	LRT	0
195	KC_AB3	Fer2LCH	-0.104719117620122	0.52281137426446	1	pseudobulk	DESeq2	LRT	0
195	KC_AB3	fest	0.820715238358767	0.73243517086529	1	pseudobulk	DESeq2	LRT	0
195	KC_AB3	Fgop2	0.959315139564569	0.476016924844424	1	pseudobulk	DESeq2	LRT	0
195	KC_AB3	fh	-0.441822610478521	0.705822253611359	1	pseudobulk	DESeq2	LRT	0
195	KC_AB3	Fhos	-1.23593561070319	0.667875401791453	1	pseudobulk	DESeq2	LRT	0
195	KC_AB3	Fib	-0.0620594830854087	0.926875593468138	1	pseudobulk	DESeq2	LRT	0
195	KC_AB3	Fibp	-0.969109958741019	0.193486719253823	1	pseudobulk	DESeq2	LRT	0
195	KC_AB3	Fic	1.13737085234098	0.539134505588107	1	pseudobulk	DESeq2	LRT	0
195	KC_AB3	fid	0.207645712646542	0.795929395616769	1	pseudobulk	DESeq2	LRT	0
195	KC_AB3	fidipidine	0.0772739847616278	1	1	pseudobulk	DESeq2	LRT	0
195	KC_AB3	Fie	-0.458325069155451	0.569605771985534	1	pseudobulk	DESeq2	LRT	0
195	KC_AB3	Fife	-0.154068864602986	0.615080597186066	1	pseudobulk	DESeq2	LRT	0
195	KC_AB3	FIG4	1.08720378351341	0.691002137169636	1	pseudobulk	DESeq2	LRT	0
195	KC_AB3	Fim	0.250752684950436	0.474184529018338	1	pseudobulk	DESeq2	LRT	0
195	KC_AB3	Fip1	-0.658088388868546	0.463696655255562	1	pseudobulk	DESeq2	LRT	0
195	KC_AB3	fipi	-0.554138034299476	0.494219593262253	1	pseudobulk	DESeq2	LRT	0
195	KC_AB3	Fis1	0.0344507303071478	0.511290436539883	1	pseudobulk	DESeq2	LRT	0
195	KC_AB3	Fit1	0.29976925407201	0.324954744154514	1	pseudobulk	DESeq2	LRT	0
195	KC_AB3	Fitm	-0.191106115929269	0.754041042749638	1	pseudobulk	DESeq2	LRT	0
195	KC_AB3	fiz	0.632774104902944	1	1	pseudobulk	DESeq2	LRT	0
195	KC_AB3	Fkbp12	-0.0344752337778454	0.674601804554209	1	pseudobulk	DESeq2	LRT	0
195	KC_AB3	Fkbp14	1.95267092137636	1	1	pseudobulk	DESeq2	LRT	0
195	KC_AB3	Fkbp39	-0.0083443510753618	0.716387343112939	1	pseudobulk	DESeq2	LRT	0

195	KC_AB3	Fkbp59	-0.524056981233012	0.464758023104062	1	pseudobulk	DESeq2	LRT		0
195	KC_AB3	fl(2)d	-6.09958355632806E-	0.930840580116105	1	pseudobulk	DESeq2	LRT		0
195	KC_AB3	Flacc	-0.324351060026784	0.559664646294375	1	pseudobulk	DESeq2	LRT		0
195	KC_AB3	FLASH	1.59576167909336	0.887029991980463	1	pseudobulk	DESeq2	LRT		0
195	KC_AB3	flfl	0.321728986277246	0.456738123719092	1	pseudobulk	DESeq2	LRT		0
195	KC_AB3	fliI	-1.57712561876955	0.240056568317235	1	pseudobulk	DESeq2	LRT		0
195	KC_AB3	Flo1	-0.358575542834816	0.343346723186099	1	pseudobulk	DESeq2	LRT		0
195	KC_AB3	Flo2	-1.01211061906374	0.0886792125512357	1	pseudobulk	DESeq2	LRT		0
195	KC_AB3	flr	-0.4954122127714	0.725533633966505	1	pseudobulk	DESeq2	LRT		0
195	KC_AB3	flw	-0.307578778704045	0.348595766368224	1	pseudobulk	DESeq2	LRT		0
195	KC_AB3	Fmr1	-0.284443599388423	0.527869479264792	1	pseudobulk	DESeq2	LRT		0
195	KC_AB3	FMRFaR	-0.184744780581018	1	1	pseudobulk	DESeq2	LRT		0
195	KC_AB3	fne	-0.0725241907174682	0.611555520087165	1	pseudobulk	DESeq2	LRT		0
195	KC_AB3	fng	1.95267092137636	1	1	pseudobulk	DESeq2	LRT		0
195	KC_AB3	Fnta	0.102422156719503	0.738477311527226	1	pseudobulk	DESeq2	LRT		0
195	KC_AB3	fog	-1.33700612181291	0.356638175499019	1	pseudobulk	DESeq2	LRT		0
195	KC_AB3	foi	1.06684506113302	0.258955822424966	1	pseudobulk	DESeq2	LRT		0
195	KC_AB3	fok	-0.0292238255355704	0.554566899838463	1	pseudobulk	DESeq2	LRT		0
195	KC_AB3	FoxK	0.208872210346684	0.649332932996479	1	pseudobulk	DESeq2	LRT		0
195	KC_AB3	FoxP	0.129337094591281	0.690455055823937	1	pseudobulk	DESeq2	LRT		0
195	KC_AB3	fra	-0.838505508417818	0.664237228246735	1	pseudobulk	DESeq2	LRT		0
195	KC_AB3	fray	1.3091801516239	0.198608309414754	1	pseudobulk	DESeq2	LRT		0
195	KC_AB3	FRG1	0.517865899055647	0.561139145554515	1	pseudobulk	DESeq2	LRT		0
195	KC_AB3	frj	-0.157170511894885	0.668945719785562	1	pseudobulk	DESeq2	LRT		0
195	KC_AB3	Frl	0.235494344552654	0.57202856791267	1	pseudobulk	DESeq2	LRT		0
195	KC_AB3	Frq1	-0.544641519774349	0.393090880357819	1	pseudobulk	DESeq2	LRT		0
195	KC_AB3	Frq2	0.685098036874194	0.603061705223628	1	pseudobulk	DESeq2	LRT		0
195	KC_AB3	frtz	0.632774104902944	1	1	pseudobulk	DESeq2	LRT		0
195	KC_AB3	fru	-0.123376559345259	0.592458122419677	1	pseudobulk	DESeq2	LRT		0
195	KC_AB3	fry	-0.168796674236737	0.685903587118889	1	pseudobulk	DESeq2	LRT		0
195	KC_AB3	fs(1)h	0.0198138788783094	0.820150457142529	1	pseudobulk	DESeq2	LRT		0
195	KC_AB3	fs(1)K10	0.368867458998006	1	1	pseudobulk	DESeq2	LRT		0
195	KC_AB3	Fs(2)Ket	-0.248650682963611	0.419782828967013	1	pseudobulk	DESeq2	LRT		0
195	KC_AB3	fs(2)ltoPP43	1.30939190163493	0.198508792375476	1	pseudobulk	DESeq2	LRT		0
195	KC_AB3	fsd	-0.103929897503816	0.881449403958175	1	pseudobulk	DESeq2	LRT		0
195	KC_AB3	Fsn	-0.935864561541796	0.379976158691727	1	pseudobulk	DESeq2	LRT		0
195	KC_AB3	ftz-f1	-1.30541470757479	0.0746033653044787	1	pseudobulk	DESeq2	LRT		0
195	KC_AB3	fu	-2.40509757930165	0.160737983205612	1	pseudobulk	DESeq2	LRT		0
195	KC_AB3	FucT6	0.384545991542954	0.684812640130254	1	pseudobulk	DESeq2	LRT		0
195	KC_AB3	FucTA	-0.407219234935362	0.550524674731991	1	pseudobulk	DESeq2	LRT		0
195	KC_AB3	FucTB	0.632774104902944	1	1	pseudobulk	DESeq2	LRT		0
195	KC_AB3	Fum1	0.0838391938609013	0.636934190575909	1	pseudobulk	DESeq2	LRT		0
195	KC_AB3	Fur1	0.416711605191293	0.172729882186542	1	pseudobulk	DESeq2	LRT		0
195	KC_AB3	Fur2	0.189602655849656	0.591395774619032	1	pseudobulk	DESeq2	LRT		0
196	KC_AB3	fuss	2.88619786524384	0.39214769278579	1	pseudobulk	DESeq2	LRT		0
196	KC_AB3	futsch	-0.386452308381159	0.0806238112190234	1	pseudobulk	DESeq2	LRT		0
196	KC_AB3	fwd	0.297413712064788	0.533069528235319	1	pseudobulk	DESeq2	LRT		0
196	KC_AB3	fwe	0.158878678825039	0.429468707423922	1	pseudobulk	DESeq2	LRT		0
196	KC_AB3	fws	-0.452217308885951	0.708614273813318	1	pseudobulk	DESeq2	LRT		0
196	KC_AB3	fy	-0.186961738271884	1	1	pseudobulk	DESeq2	LRT		0
196	KC_AB3	fz	-0.434836550242003	0.170310076062729	1	pseudobulk	DESeq2	LRT		0
196	KC_AB3	fz2	-0.346475107401357	0.661156438665172	1	pseudobulk	DESeq2	LRT		0
196	KC_AB3	fz3	-1.29849500942567	0.371866854679707	1	pseudobulk	DESeq2	LRT		0

196	KC_AB3	fz4	0.759758178017103	1	1	pseudobulk	DESeq2	LRT			0
196	KC_AB3	fzr	0.0718037594900806	0.768700873186415	1	pseudobulk	DESeq2	LRT			0
196	KC_AB3	fzy	1.95267092137636	1	1	pseudobulk	DESeq2	LRT			0
196	KC_AB3	g	-0.244275668956803	0.510988460758594	1	pseudobulk	DESeq2	LRT			0
196	KC_AB3	G6P	1.37446071174073	0.594555113706268	1	pseudobulk	DESeq2	LRT			0
196	KC_AB3	G9a	0.0039293620100398	0.96354946370737	1	pseudobulk	DESeq2	LRT			0
196	KC_AB3	GAA1	1.58405176669631	0.255779894640102	1	pseudobulk	DESeq2	LRT			0
196	KC_AB3	GABA-B-R1	-0.0842961312207737	0.575464753839881	1	pseudobulk	DESeq2	LRT			0
[truncated: 588,097 more chars]
