# Supplementary material for: Transcriptional changes in specific subsets of Drosophila neurons following inhibition of the serotonin transporter
Source: Transl Psychiatry. 2023 Jun 24;13:226. doi: 10.1038/s41398-023-02521-3 (PMC10290657; doi:10.1038/s41398-023-02521-3)
Supplement: Supplementary file 4 — Supplemental Table 3 [file 41398_2023_2521_MOESM4_ESM.pdf]

scRNA-seq_DEGs_dSERT-TMKO_v_WT_Day0

	cell_type	gene	avg_logFC	p_val	p_val_adj	de_family	de_method	de_type	minuslogP
1	KC_G1	Hsp23	-3.24187443131255	3.93871489315845E-11	3.63779707532115E-07	pseudobulk	DESeq2	LRT	10.404645454786
2	KC_G1	CG30419	1.31536181318902	9.01300342634707E-09	3.640122437842E-05	pseudobulk	DESeq2	LRT	8.04513046383385
3	KC_G1	CG42392	-0.752243373111456	1.18236978275509E-08	3.640122437842E-05	pseudobulk	DESeq2	LRT	7.9272466778586
4	KC_G2	LysRS	-1.91095535345546	4.98342837633404E-09	4.08740795426918E-05	pseudobulk	DESeq2	LRT	8.30247177919825
5	KC_G2	CG42392	-0.987712641972316	6.53066770339196E-08	0.000267822682516104	pseudobulk	DESeq2	LRT	7.18504241365996
6	KC_ABp1	dpr1	6.30116010428411	8.77049662101269E-08	0.000652086423772294	pseudobulk	DESeq2	LRT	7.05697581442655
7	KC_G4	DIP-kappa	-3.77134090827105	5.93364390382408E-07	0.00233844906249707	pseudobulk	DESeq2	LRT	6.22667852058147
8	KC_G4	Scp2	6.48054762497124	5.74463608604085E-07	0.00233844906249707	pseudobulk	DESeq2	LRT	6.24073747799923
9	KC_G2	mamo	-0.716736800326647	1.42879846208731E-06	0.00390633499534671	pseudobulk	DESeq2	LRT	5.84502902591588
10	KC_G1	LysRS	-0.879101333795579	8.28813189591225E-06	0.0191372965476614	pseudobulk	DESeq2	LRT	5.08154334625581
11	KC_G1	CG9801	-1.03090317688931	1.38458388504591E-05	0.0213133612704734	pseudobulk	DESeq2	LRT	4.85868072738034
12	KC_G1	Rab27	0.64406074222323	1.20775680960027E-05	0.0213133612704734	pseudobulk	DESeq2	LRT	4.9180205051861
13	KC_G1	CG31221	-0.451369168978424	3.71977404542638E-05	0.0429447913544476	pseudobulk	DESeq2	LRT	4.42948344017317
14	KC_G1	nAChRalpha1	-0.523820115333995	3.62574065517932E-05	0.0429447913544476	pseudobulk	DESeq2	LRT	4.44060326368581
15	KC_G1	CG5131	-4.22210819378357	4.73832141149782E-05	0.0486257072851042	pseudobulk	DESeq2	LRT	4.3243754833968
16	KC_G1	Ddc	0.855749764822871	6.42180108721991E-05	0.0593117548415631	pseudobulk	DESeq2	LRT	4.19234315066311
17	KC_G2	CG10384	-1.3300343816847	3.14051227433546E-05	0.0643962041852486	pseudobulk	DESeq2	LRT	4.50299950486595
18	KC_G2	Rh7	1.78716199408394	0.000153760349537097	0.252228477380655	pseudobulk	DESeq2	LRT	3.81315564241057
19	KC_ABp1	CG34353	1.69992366862786	7.82440183961733E-05	0.262766109682428	pseudobulk	DESeq2	LRT	4.10654885348541
20	KC_ABp1	CG42392	-0.864418571861911	0.000141367106755846	0.262766109682428	pseudobulk	DESeq2	LRT	3.84965163025803
21	KC_ABp1	Imp	1.67070432244435	0.000127553440295314	0.262766109682428	pseudobulk	DESeq2	LRT	3.89430782336449
22	KC_G1	Hsp27	-1.35393842867096	0.000342563123156482	0.273381979046123	pseudobulk	DESeq2	LRT	3.46525939055803
23	KC_G1	mthl8	4.37208393146944	0.000355195295425886	0.273381979046123	pseudobulk	DESeq2	LRT	3.44953279510095
24	KC_G1	Octbeta3R	2.49487146398693	0.000520265476693276	0.349908370789327	pseudobulk	DESeq2	LRT	3.28377499166893
25	KC_G1	Pgant9	0.429655532680339	0.000530393805873817	0.349908370789327	pseudobulk	DESeq2	LRT	3.27540155641801
26	KC_G1	Octbeta2R	1.71887403319085	0.000569206266899203	0.350479272072069	pseudobulk	DESeq2	LRT	3.24473032702633
27	KC_G1	Sema2b	-0.476565837727733	0.000680993081118532	0.393103256075672	pseudobulk	DESeq2	LRT	3.16685730049148
28	KC_AB1	CG42392	-0.887508076027822	0.00022156453718505	0.396999337728173	pseudobulk	DESeq2	LRT	3.65449974997844
29	KC_AB1	CG7272	3.53311073598857	0.000215227820717946	0.396999337728173	pseudobulk	DESeq2	LRT	3.6671015917261
30	KC_AB1	drd	6.72197266369935	0.000170751973329347	0.396999337728173	pseudobulk	DESeq2	LRT	3.76763426860635
31	KC_AB1	lbm	-2.94180302561107	0.000114233005437734	0.396999337728173	pseudobulk	DESeq2	LRT	3.94220839689138
32	KC_AB1	Pgant9	0.688727653013633	0.000217312779815088	0.396999337728173	pseudobulk	DESeq2	LRT	3.66291473276843
33	KC_G1	gammaSnap1	0.545834076025494	0.000751350257953224	0.40177255880841	pseudobulk	DESeq2	LRT	3.124157560153
34	KC_G1	Hsp68	-1.39210929768907	0.000806340204020574	0.40177255880841	pseudobulk	DESeq2	LRT	3.09348168579287
35	KC_G1	mt:ND1	-0.430135987605034	0.000826513492568189	0.40177255880841	pseudobulk	DESeq2	LRT	3.08275005231627
36	KC_G1	CG17691	1.17154129731987	0.00091572070958317	0.402742689224293	pseudobulk	DESeq2	LRT	3.03823696386718
37	KC_G1	fz	-0.407987651424798	0.00088855181733619	0.402742689224293	pseudobulk	DESeq2	LRT	3.05131724053512
38	KC_ABp1	CG34370	-1.01946046572743	0.000287065111168518	0.426865820307586	pseudobulk	DESeq2	LRT	3.54201958684072
39	KC_G1	Idh3a	-0.511546118459666	0.00103912439862111	0.436243315712025	pseudobulk	DESeq2	LRT	2.98333245783191
40	KC_AB1	LysRS	-0.981039577484016	0.000293614177683141	0.43841490297721	pseudobulk	DESeq2	LRT	3.53222297756764
41	KC_ABp1	Cbp53E	-2.28579557069591	0.000365930392859983	0.453448745152329	pseudobulk	DESeq2	LRT	3.43660151808045
42	KC_G2	sty	1.04967697855718	0.000339617921932458	0.464257699281671	pseudobulk	DESeq2	LRT	3.46900939972667
43	KC_G1	CG10384	-0.935075538319747	0.00123809688095104	0.497176643150601	pseudobulk	DESeq2	LRT	2.90724537048858
44	KC_AB1	CG8012	5.64970057700276	0.000398137440575902	0.509559047159929	pseudobulk	DESeq2	LRT	3.39996697973582
45	KC_G1	CG8012	1.04369141856912	0.00140593789693087	0.541051767335562	pseudobulk	DESeq2	LRT	2.8520338625423
46	KC_ABp1	klg	1.43751719603912	0.000715323722171566	0.75977598204937	pseudobulk	DESeq2	LRT	3.14549737227504
47	KC_G1	CG42541	0.549865926656045	0.0024348158897319	0.899518382302551	pseudobulk	DESeq2	LRT	2.61353387268198
48	KC_AB1	CG4297	1.16166578559822	0.000811771532167226	0.909082644585773	pseudobulk	DESeq2	LRT	3.09056618292692
49	KC_G1	CG14762	-0.329329579165035	0.00269173470271704	0.934256465193803	pseudobulk	DESeq2	LRT	2.56996774638854
50	KC_G1	eEF1beta	-0.54565683118303	0.00273115250760423	0.934256465193803	pseudobulk	DESeq2	LRT	2.56365404817162
51	KC_AB1	fz	-0.599075045265445	0.000949381637504819	0.945056676711741	pseudobulk	DESeq2	LRT	3.02255917245698
52	KC_G1	Hsp70Ab	-1.05530583001863	0.00291301086039655	0.960877439522235	pseudobulk	DESeq2	LRT	2.53565789621631
53	KC_G1	CG30356	4.72485092619246	0.00321095569285448	0.988546225973465	pseudobulk	DESeq2	LRT	2.49336568710917
54	KC_G1	dpr1	3.29043781221565	0.00320590090793371	0.988546225973465	pseudobulk	DESeq2	LRT	2.49404990550111

55	KC_G1	128up	0.0442714725283474	0.909776945759417	0.998043617620652	pseudobulk	DESeq2	LRT	0.0410650726240319
56	KC_G1	14-3-3epsilon	-0.0622451673872919	0.503483285495167	0.998043617620652	pseudobulk	DESeq2	LRT	0.298014942464299
57	KC_G1	14-3-3zeta	-0.0726876035503139	0.571073558599065	0.998043617620652	pseudobulk	DESeq2	LRT	0.243307947732904
58	KC_G1	140up	0.163779390335249	0.733561489026392	0.998043617620652	pseudobulk	DESeq2	LRT	0.134563476605137
59	KC_G1	18SrRNA-Psi:CR41602	-0.344493671980441	0.61545554781105	0.998043617620652	pseudobulk	DESeq2	LRT	0.210803309162447
60	KC_G1	18w	-0.358686124263966	0.0934350882896702	0.998043617620652	pseudobulk	DESeq2	LRT	1.02948999969186
61	KC_G1	26-29-p	-0.0159509752798576	0.956939161986823	0.998043617620652	pseudobulk	DESeq2	LRT	0.0191156718922058
62	KC_G1	28SrRNA-Psi:CR40596	-0.0523405849450794	0.91674573698819	0.998043617620652	pseudobulk	DESeq2	LRT	0.0377511009002858
63	KC_G1	28SrRNA-Psi:CR40741	0.178740243421447	0.81100891359383	0.998043617620652	pseudobulk	DESeq2	LRT	0.0909743725418143
64	KC_G1	28SrRNA-Psi:CR41609	-0.563823563234898	0.761788571487294	0.998043617620652	pseudobulk	DESeq2	LRT	0.118165547003551
65	KC_G1	28SrRNA-Psi:CR45851	0.173116475207952	0.97305562682953	0.998043617620652	pseudobulk	DESeq2	LRT	0.011862331638572
66	KC_G1	28SrRNA-Psi:CR45853	-0.794610277746993	0.821479263487599	0.998043617620652	pseudobulk	DESeq2	LRT	0.085403394939296
67	KC_G1	28SrRNA-Psi:CR45855	-1.39542401216867	0.462167035373762	0.998043617620652	pseudobulk	DESeq2	LRT	0.335201034334682
68	KC_G1	28SrRNA-Psi:CR45859	-0.0747417676625034	0.876291687468726	0.998043617620652	pseudobulk	DESeq2	LRT	0.0573513080187422
69	KC_G1	28SrRNA-Psi:CR45860	-0.0162909046050816	0.973166358213313	0.998043617620652	pseudobulk	DESeq2	LRT	0.0118129127856639
70	KC_G1	2mit	0.0209719994191228	0.88277663861153	0.998043617620652	pseudobulk	DESeq2	LRT	0.0541491683245497
71	KC_G1	312	-0.183998343048626	0.634126254218427	0.998043617620652	pseudobulk	DESeq2	LRT	0.197824265696257
72	KC_G1	4E-T	-0.0504452004517944	0.787643082889029	0.998043617620652	pseudobulk	DESeq2	LRT	0.103670536630875
73	KC_G1	5-HT1A	-0.0250144992198458	0.858012130237436	0.998043617620652	pseudobulk	DESeq2	LRT	0.0665065722236215
74	KC_G1	5-HT1B	-0.0315123603583889	0.859632564969552	0.998043617620652	pseudobulk	DESeq2	LRT	0.0656871407391158
75	KC_G1	5-HT2A	0.38160494332686	0.48031430313018	0.998043617620652	pseudobulk	DESeq2	LRT	0.318474480448231
76	KC_G1	5-HT2B	3.13633979828739	0.129423850759527	0.998043617620652	pseudobulk	DESeq2	LRT	0.887985682718614
77	KC_G1	5-HT7	1.2696631304081	0.378697559696997	0.998043617620652	pseudobulk	DESeq2	LRT	0.421707493436192
78	KC_G1	5.8SrRNA-Psi:CR45849	1.12508065046033	0.900770125530981	0.998043617620652	pseudobulk	DESeq2	LRT	0.0453860258366365
79	KC_G1	5.8SrRNA-Psi:CR45854	2.04147257822512	0.408147672536479	0.998043617620652	pseudobulk	DESeq2	LRT	0.389182675723292
80	KC_G1	5.8SrRNA-Psi:CR45863	-0.794610277746993	0.821479263487599	0.998043617620652	pseudobulk	DESeq2	LRT	0.085403394939296
81	KC_G1	5PtaseI	0.0740810986392507	0.680399617154483	0.998043617620652	pseudobulk	DESeq2	LRT	0.167235939427109
82	KC_G1	7B2	-0.0037622641497086	0.967990217077581	0.998043617620652	pseudobulk	DESeq2	LRT	0.0141290318348839
83	KC_G1	a	-0.156224253656615	0.288282342478592	0.998043617620652	pseudobulk	DESeq2	LRT	0.540181957677556
84	KC_G1	A16	0.222138188478818	0.667340246665273	0.998043617620652	pseudobulk	DESeq2	LRT	0.175652682465931
85	KC_G1	a6	0.284466546727604	0.506216991257572	0.998043617620652	pseudobulk	DESeq2	LRT	0.295663281766542
86	KC_G1	AANAT1	-0.0097706061111957	0.950679446202888	0.998043617620652	pseudobulk	DESeq2	LRT	0.021965895484338
87	KC_G1	AANATL2	-0.117540371687341	0.849687351385237	0.998043617620652	pseudobulk	DESeq2	LRT	0.0707408466927762
88	KC_G1	Aasdh	0.354791155372515	0.629935938325628	0.998043617620652	pseudobulk	DESeq2	LRT	0.200703614111976
89	KC_G1	Aatf	0.569077307416035	0.092661350192269	0.998043617620652	pseudobulk	DESeq2	LRT	1.03310137587062
90	KC_G1	aay	0.140220863955476	0.682498860104318	0.998043617620652	pseudobulk	DESeq2	LRT	0.16589806963639
91	KC_G1	ab	-0.220648239760165	0.0496629983103018	0.998043617620652	pseudobulk	DESeq2	LRT	1.3039670642758
92	KC_G1	ABCA	0.222690932034628	0.483013099170778	0.998043617620652	pseudobulk	DESeq2	LRT	0.316041091152328
93	KC_G1	ABCB7	-0.0429714630040045	0.930306418946329	0.998043617620652	pseudobulk	DESeq2	LRT	0.0313739824791575
94	KC_G1	ABCD	0.0820084697802875	0.578189076892818	0.998043617620652	pseudobulk	DESeq2	LRT	0.237930117248523
95	KC_G1	Abd-B	-1.90559848246913	0.302272868332606	0.998043617620652	pseudobulk	DESeq2	LRT	0.519600832849436
96	KC_G1	Abi	0.096624139778761	0.726078940651678	0.998043617620652	pseudobulk	DESeq2	LRT	0.139016159429835
97	KC_G1	Abl	-0.0965268280984021	0.428897473154192	0.998043617620652	pseudobulk	DESeq2	LRT	0.367646512395629
98	KC_G1	abo	-0.22908868502699	0.863525920438116	0.998043617620652	pseudobulk	DESeq2	LRT	0.0637246216931771
99	KC_G1	Abp1	-0.15953479904078	0.606151224441078	0.998043617620652	pseudobulk	DESeq2	LRT	0.217419013214751
100	KC_G1	abs	-0.214612349509717	0.714815965558339	0.998043617620652	pseudobulk	DESeq2	LRT	0.145805756003477
101	KC_G1	Ac13E	1.13582588427274	0.633424328955532	0.998043617620652	pseudobulk	DESeq2	LRT	0.198305259991224
102	KC_G1	Ac3	0.0987002536358512	0.656517686279144	0.998043617620652	pseudobulk	DESeq2	LRT	0.182753569726863
103	KC_G1	Ac76E	0.618610524744411	0.173277717461146	0.998043617620652	pseudobulk	DESeq2	LRT	0.761257281520682
104	KC_G1	Ac78C	0.0325391209564611	0.940168385581369	0.998043617620652	pseudobulk	DESeq2	LRT	0.0267943566346856
105	KC_G1	Acbp1	-0.136798244656586	0.431348848746504	0.998043617620652	pseudobulk	DESeq2	LRT	0.365171356739832
106	KC_G1	Acbp2	-0.0133331902194689	0.943951730298195	0.998043617620652	pseudobulk	DESeq2	LRT	0.0250502131183656
107	KC_G1	ACC	-0.447974118038474	0.112616322022216	0.998043617620652	pseudobulk	DESeq2	LRT	0.94839866054624
108	KC_G1	AcCoAS	-0.102767521216008	0.531519665311107	0.998043617620652	pseudobulk	DESeq2	LRT	0.274480662696462
109	KC_G1	Ace	-0.0734529485408571	0.674182253843792	0.998043617620652	pseudobulk	DESeq2	LRT	0.17122268337712
110	KC_G1	Acer	0.117392999797369	0.81819193459079	0.998043617620652	pseudobulk	DESeq2	LRT	0.0871448059180391

111	KC_G1	Acf	-0.130930478939202	0.643902869088639	0.998043617620652	pseudobulk	DESeq2	LRT	0.191179639783496
112	KC_G1	achi	0.222057805416336	0.718272838888545	0.998043617620652	pseudobulk	DESeq2	LRT	0.143710555874724
113	KC_G1	Ack	0.19766692945479	0.6458715686944	0.998043617620652	pseudobulk	DESeq2	LRT	0.189853832705498
114	KC_G1	Ack-like	-0.091753190366135	0.621167474472888	0.998043617620652	pseudobulk	DESeq2	LRT	0.2067912928449
115	KC_G1	Acn	-0.0717048837828648	0.652413329233762	0.998043617620652	pseudobulk	DESeq2	LRT	0.185477174618985
116	KC_G1	Acox57D-d	1.13582588427274	0.704684208645453	0.998043617620652	pseudobulk	DESeq2	LRT	0.152005460551806
117	KC_G1	Acox57D-p	1.13582588427274	0.633424328955532	0.998043617620652	pseudobulk	DESeq2	LRT	0.198305259991224
118	KC_G1	Acp1	-0.404642012047016	0.766590301789625	0.998043617620652	pseudobulk	DESeq2	LRT	0.115436679349724
119	KC_G1	Acp53C14a	-2.27131838799805	0.327405917019026	0.998043617620652	pseudobulk	DESeq2	LRT	0.484913476098507
120	KC_G1	Acp53C14c	-0.794610277746993	0.815039924133852	0.998043617620652	pseudobulk	DESeq2	LRT	0.0888211171417967
121	KC_G1	Acp53Ea	1.12508065046033	0.900770125530981	0.998043617620652	pseudobulk	DESeq2	LRT	0.0453860258366365
122	KC_G1	Acp95EF	1.90250740646012	0.460774913678782	0.998043617620652	pseudobulk	DESeq2	LRT	0.336511173568024
123	KC_G1	Acph-1	-0.154522950866164	0.819455216924188	0.998043617620652	pseudobulk	DESeq2	LRT	0.086474775557962
124	KC_G1	Acsl	-0.111499352112635	0.602099331954977	0.998043617620652	pseudobulk	DESeq2	LRT	0.220331854653719
125	KC_G1	Act42A	0.402626998487333	0.310241684147394	0.998043617620652	pseudobulk	DESeq2	LRT	0.5082998506908
126	KC_G1	Act5C	-0.101261994661453	0.502974254201712	0.998043617620652	pseudobulk	DESeq2	LRT	0.298454244654395
127	KC_G1	Act79B	-0.552868473583876	0.340085360767493	0.998043617620652	pseudobulk	DESeq2	LRT	0.468412062200568
128	KC_G1	Actbeta	-0.0605687085352316	0.727527275150996	0.998043617620652	pseudobulk	DESeq2	LRT	0.138150720245115
129	KC_G1	Actn	0.384325723369749	0.536211041398121	0.998043617620652	pseudobulk	DESeq2	LRT	0.270664247479388
130	KC_G1	Acyp2	0.03952754173232	0.892275758903905	0.998043617620652	pseudobulk	DESeq2	LRT	0.0495009056436975
131	KC_G1	Ada	1.13582588427274	0.633424328955532	0.998043617620652	pseudobulk	DESeq2	LRT	0.198305259991224
132	KC_G1	Ada1-2	-0.683248847121236	0.403849544879647	0.998043617620652	pseudobulk	DESeq2	LRT	0.393780402212811
133	KC_G1	Ada2a	1.93605158052896	0.300483392561826	0.998043617620652	pseudobulk	DESeq2	LRT	0.52217952605082
134	KC_G1	Ada2b	-0.0278362414836836	0.922940674825812	0.998043617620652	pseudobulk	DESeq2	LRT	0.0348262138428028
135	KC_G1	Ada3	0.221785324338922	0.629507461989501	0.998043617620652	pseudobulk	DESeq2	LRT	0.200999117531246
136	KC_G1	AdamTS-A	-0.758144072848717	0.770267554036614	0.998043617620652	pseudobulk	DESeq2	LRT	0.113358395530507
137	KC_G1	AdamTS-B	2.97392976749169	0.209628783309326	0.998043617620652	pseudobulk	DESeq2	LRT	0.678549086315318
138	KC_G1	Adar	-0.0168440716000954	0.922794063915767	0.998043617620652	pseudobulk	DESeq2	LRT	0.0348952078407123
139	KC_G1	Adat1	0.554543440626045	0.504193585642591	0.998043617620652	pseudobulk	DESeq2	LRT	0.297402683724523
140	KC_G1	Adck1	0.0706046966190722	0.953350214593764	0.998043617620652	pseudobulk	DESeq2	LRT	0.0207475313388619
141	KC_G1	Adck5	0.192632781595559	0.795407114027854	0.998043617620652	pseudobulk	DESeq2	LRT	0.0994105290538044
142	KC_G1	ADD1	-0.113618495639417	0.686881752218784	0.998043617620652	pseudobulk	DESeq2	LRT	0.163118020988474
143	KC_G1	AdenoK	-0.270833416097464	0.551506033354394	0.998043617620652	pseudobulk	DESeq2	LRT	0.258449732111936
144	KC_G1	Adf1	0.0457151349223558	0.835766652676282	0.998043617620652	pseudobulk	DESeq2	LRT	0.0779149613182631
145	KC_G1	Adgf-C	-2.02477450664369	0.298464370325329	0.998043617620652	pseudobulk	DESeq2	LRT	0.52510750605779
146	KC_G1	Adgf-D	0.0320799782026312	0.912418115103082	0.998043617620652	pseudobulk	DESeq2	LRT	0.0398061008491279
147	KC_G1	Adh	2.16335699899024	0.30673159768659	0.998043617620652	pseudobulk	DESeq2	LRT	0.513241483247751
148	KC_G1	Adi1	-0.0448139063735047	0.916937636232297	0.998043617620652	pseudobulk	DESeq2	LRT	0.0376602010379447
149	KC_G1	AdipoR	-0.136826261695906	0.459383433612248	0.998043617620652	pseudobulk	DESeq2	LRT	0.337824670482988
150	KC_G1	Adk1	-0.00426153237381703	0.970944990060442	0.998043617620652	pseudobulk	DESeq2	LRT	0.0128053748190378
151	KC_G1	Adk2	0.153214620682008	0.34556942926194	0.998043617620652	pseudobulk	DESeq2	LRT	0.461464684277897
152	KC_G1	Adk3	-0.354079813708319	0.712258820803115	0.998043617620652	pseudobulk	DESeq2	LRT	0.14736216363654
153	KC_G1	adp	0.16983762362791	0.76948171686781	0.998043617620652	pseudobulk	DESeq2	LRT	0.113801694685982
154	KC_G1	ADPS	0.0169484260593226	0.930371849565634	0.998043617620652	pseudobulk	DESeq2	LRT	0.0313434386098554
155	KC_G1	AdSL	0.0474596835875231	0.921329662632722	0.998043617620652	pseudobulk	DESeq2	LRT	0.035584946301361
156	KC_G1	AdSS	0.054073439203608	0.824546275557104	0.998043617620652	pseudobulk	DESeq2	LRT	0.0837849656651458
157	KC_G1	Aduk	0.0162835005574932	0.958468628471085	0.998043617620652	pseudobulk	DESeq2	LRT	0.0184220973971496
158	KC_G1	Ady43A	-0.384142376436038	0.307723107768526	0.998043617620652	pseudobulk	DESeq2	LRT	0.511839890169341
159	KC_G1	Aef1	-0.0435547782135238	0.795640524489301	0.998043617620652	pseudobulk	DESeq2	LRT	0.0992831049922011
160	KC_G1	aft	-0.171552556299732	0.855776136285363	0.998043617620652	pseudobulk	DESeq2	LRT	0.0676398281863745
161	KC_G1	Afti	-0.113207035735908	0.616574623846363	0.998043617620652	pseudobulk	DESeq2	LRT	0.210014353353074
162	KC_G1	Ag5r	-0.0512899867636406	0.952039363798706	0.998043617620652	pseudobulk	DESeq2	LRT	0.0213450945492041
163	KC_G1	AGBE	-0.604508069264128	0.440726288134457	0.998043617620652	pseudobulk	DESeq2	LRT	0.355831044222922
164	KC_G1	AgmNAT	-0.794610277746993	0.821479263487599	0.998043617620652	pseudobulk	DESeq2	LRT	0.085403394939296
165	KC_G1	ago	0.067545479577526	0.806901328088215	0.998043617620652	pseudobulk	DESeq2	LRT	0.093179569722563
166	KC_G1	AGO1	-0.0752433990797048	0.445849023197719	0.998043617620652	pseudobulk	DESeq2	LRT	0.350812180508728

167	KC_G1	AGO2	-0.296455584978296	0.250113748299122	0.998043617620652	pseudobulk	DESeq2	LRT	0.601862435233356
168	KC_G1	AGO3	-0.423064214241167	0.698422214086836	0.998043617620652	pseudobulk	DESeq2	LRT	0.155881955869056
169	KC_G1	Agpat1	0.525511012711615	0.354733880165033	0.998043617620652	pseudobulk	DESeq2	LRT	0.450097330653962
170	KC_G1	Agpat2	0.562077451288907	0.794187709521711	0.998043617620652	pseudobulk	DESeq2	LRT	0.100076838158817
171	KC_G1	Agpat3	-0.228033393827545	0.319376338721657	0.998043617620652	pseudobulk	DESeq2	LRT	0.495697262096289
172	KC_G1	agt	-0.355183270625803	0.815163518525073	0.998043617620652	pseudobulk	DESeq2	LRT	0.0887552647938018
173	KC_G1	Ahcy	0.124134862578147	0.809494029091437	0.998043617620652	pseudobulk	DESeq2	LRT	0.0917863502981362
174	KC_G1	AhcyL1	-0.114000778748181	0.704377117665125	0.998043617620652	pseudobulk	DESeq2	LRT	0.152194760929044
175	KC_G1	AIF	0.163481322269861	0.761765452517093	0.998043617620652	pseudobulk	DESeq2	LRT	0.11817872729257
176	KC_G1	AIMP1	0.358108880450945	0.400478690841738	0.998043617620652	pseudobulk	DESeq2	LRT	0.397420587435059
177	KC_G1	AIMP2	0.12480615137942	0.739348812686722	0.998043617620652	pseudobulk	DESeq2	LRT	0.131150620218443
178	KC_G1	AIMP3	0.0883513030100808	0.628686380954391	0.998043617620652	pseudobulk	DESeq2	LRT	0.201565947539777
179	KC_G1	Ak6	-0.6845303099488	0.133527907085191	0.998043617620652	pseudobulk	DESeq2	LRT	0.874427958070953
180	KC_G1	Akap200	-0.0215929804262652	0.92951682331324	0.998043617620652	pseudobulk	DESeq2	LRT	0.0317427455306098
181	KC_G1	Akh	2.2628183737297	0.276020242822815	0.998043617620652	pseudobulk	DESeq2	LRT	0.559059066399029
182	KC_G1	akirin	-0.162712715515595	0.296778455885783	0.998043617620652	pseudobulk	DESeq2	LRT	0.52756762909895
183	KC_G1	Akt1	-0.0500089304125038	0.857057544848191	0.998043617620652	pseudobulk	DESeq2	LRT	0.0669900175505224
184	KC_G1	Aladin	0.549001127454919	0.570773272696626	0.998043617620652	pseudobulk	DESeq2	LRT	0.243536371563237
185	KC_G1	AlaRS	-0.310817938320837	0.653705932113081	0.998043617620652	pseudobulk	DESeq2	LRT	0.184617573971993
186	KC_G1	AlaRS-m	0.218645378030897	0.738996825362969	0.998043617620652	pseudobulk	DESeq2	LRT	0.131357427275413
187	KC_G1	Alas	-0.240482430370542	0.257007010628878	0.998043617620652	pseudobulk	DESeq2	LRT	0.590055029836372
188	KC_G1	alc	0.232188232526593	0.256287518878777	0.998043617620652	pseudobulk	DESeq2	LRT	0.591272543304874
189	KC_G1	Ald1	-0.0960380505476322	0.792319948181426	0.998043617620652	pseudobulk	DESeq2	LRT	0.101099409735337
190	KC_G1	Aldh	-0.340484337882043	0.544942578431114	0.998043617620652	pseudobulk	DESeq2	LRT	0.263649257694619
191	KC_G1	Aldh-III	-0.0425887435488357	0.89441167519006	0.998043617620652	pseudobulk	DESeq2	LRT	0.0484625403638188
192	KC_G1	Aldh7A1	-0.711364822669339	0.836431126230485	0.998043617620652	pseudobulk	DESeq2	LRT	0.0775698140913142
193	KC_G1	Alg-2	-0.162241674566228	0.401121358866465	0.998043617620652	pseudobulk	DESeq2	LRT	0.396724212137335
194	KC_G1	Alg1	-0.0493458714386826	0.919428872975326	0.998043617620652	pseudobulk	DESeq2	LRT	0.036481862150893
195	KC_G1	Alg10	-0.0525748259844291	0.932485985569024	0.998043617620652	pseudobulk	DESeq2	LRT	0.0303576865281503
196	KC_G1	Alg11	0.0123630930102996	0.947564214249561	0.998043617620652	pseudobulk	DESeq2	LRT	0.0233913492234537
197	KC_G1	Alg14	0.878462841443372	0.150251765058391	0.998043617620652	pseudobulk	DESeq2	LRT	0.823180417490834
198	KC_G1	Alg2	-0.020156341022049	0.957817538401724	0.998043617620652	pseudobulk	DESeq2	LRT	0.0187172149428635
199	KC_G1	Alg3	-0.131081622636029	0.800324426879632	0.998043617620652	pseudobulk	DESeq2	LRT	0.0967339277053902
200	KC_G1	Alg9	0.0805875210833093	0.894946652952291	0.998043617620652	pseudobulk	DESeq2	LRT	0.0482028518561046
201	KC_G1	Alh	-0.0330288139574484	0.867565370172272	0.998043617620652	pseudobulk	DESeq2	LRT	0.0616977916546717
202	KC_G1	alien	-0.00155848314394179	0.955943223426019	0.998043617620652	pseudobulk	DESeq2	LRT	0.0195679011182713
203	KC_G1	ALiX	-0.0319796414647243	0.910351110303877	0.998043617620652	pseudobulk	DESeq2	LRT	0.0407910737690566
204	KC_G1	Alk	-0.307169703111629	0.0053501972042695	0.998043617620652	pseudobulk	DESeq2	LRT	2.27163020991379
205	KC_G1	AlkB	-1.76538389030833	0.441822179843785	0.998043617620652	pseudobulk	DESeq2	LRT	0.354752485970289
206	KC_G1	Alp12	1.4002337853638	0.479004357255816	0.998043617620652	pseudobulk	DESeq2	LRT	0.319660536014351
207	KC_G1	Alp4	0.6030885330520 36	0.791040718043767	0.998043617620652	pseudobulk	DESeq2	LRT	0.101801161045192
208	KC_G1	Alp6	2.63732022130093	0.22943645835378	0.998043617620652	pseudobulk	DESeq2	LRT	0.639337569853304
209	KC_G1	Alp8	1.13582588427274	0.633424328955532	0.998043617620652	pseudobulk	DESeq2	LRT	0.198305259991224
210	KC_G1	alph	0.0489462221041925	0.771047894955404	0.998043617620652	pseudobulk	DESeq2	LRT	0.112918644168498
211	KC_G1	alpha-Cat	-0.0836694829832159	0.533732312116892	0.998043617620652	pseudobulk	DESeq2	LRT	0.272676504242921
212	KC_G1	alpha-Catr	0.304441534876011	0.252137284605889	0.998043617620652	pseudobulk	DESeq2	LRT	0.598362928614265
213	KC_G1	alpha-Est10	-2.27643791964046	0.298906309922724	0.998043617620652	pseudobulk	DESeq2	LRT	0.52446491689215
214	KC_G1	alpha-Est2	2.74764263117927	0.228409304049609	0.998043617620652	pseudobulk	DESeq2	LRT	0.64128620946688
215	KC_G1	alpha-Est3	-1.7392816361115	0.38609804243622	0.998043617620652	pseudobulk	DESeq2	LRT	0.413302400295928
216	KC_G1	alpha-Est5	-1.73907471612892	0.507597351592657	0.998043617620652	pseudobulk	DESeq2	LRT	0.294480652518419
217	KC_G1	alpha-Est8	-0.470499821659928	0.328761874968778	0.998043617620652	pseudobulk	DESeq2	LRT	0.483118551383489
218	KC_G1	alpha-Est9	0.0129978848835509	0.935787838387242	0.998043617620652	pseudobulk	DESeq2	LRT	0.0288226032511615
219	KC_G1	alpha-Man-Ia	0.0624032449384473	0.700344808786352	0.998043617620652	pseudobulk	DESeq2	LRT	0.154688086152226
220	KC_G1	alpha-Man-Ib	0.0360674752074624	0.888976435508176	0.998043617620652	pseudobulk	DESeq2	LRT	0.0511097509132561
221	KC_G1	alpha-Man-IIa	0.0920586421482884	0.888115741051654	0.998043617620652	pseudobulk	DESeq2	LRT	0.0515304323919435
222	KC_G1	alpha-Man-IIb	0.0304256416881722	0.896940528421726	0.998043617620652	pseudobulk	DESeq2	LRT	0.0472363518661902

223	KC_G1	alpha-PheRS	0.456602696462514	0.329741181228968	0.998043617620652	pseudobulk	DESeq2	LRT	0.481826810625603
224	KC_G1	alpha-Spec	-0.168356286800399	0.226083678099307	0.998043617620652	pseudobulk	DESeq2	LRT	0.645730790008789
225	KC_G1	alpha4GT1	-0.282116759547492	0.343930195334498	0.998043617620652	pseudobulk	DESeq2	LRT	0.463529693641558
226	KC_G1	alphaCOP	-0.195476247290613	0.614538035759879	0.998043617620652	pseudobulk	DESeq2	LRT	0.211451232046166
227	KC_G1	alphaKap4	1.12508065046033	0.900770125530981	0.998043617620652	pseudobulk	DESeq2	LRT	0.0453860258366365
228	KC_G1	alphaSnap	-0.0181511104574583	0.885379120882843	0.998043617620652	pseudobulk	DESeq2	LRT	0.0528707238212381
229	KC_G1	alphaTub67C	0.857717870277898	0.254915994458312	0.998043617620652	pseudobulk	DESeq2	LRT	0.593602914287257
230	KC_G1	alphaTub84B	-0.13697019526467	0.262735740467988	0.998043617620652	pseudobulk	DESeq2	LRT	0.580480845248786
231	KC_G1	alphaTub84D	0.178611640560731	0.342914814390655	0.998043617620652	pseudobulk	DESeq2	LRT	0.464813752362054
232	KC_G1	Alr	0.203947542434355	0.626612648088626	0.998043617620652	pseudobulk	DESeq2	LRT	0.203000843192489
233	KC_G1	alrm	0.126310890109921	0.556022474166512	0.998043617620652	pseudobulk	DESeq2	LRT	0.254907654084771
234	KC_G1	Als2	-0.185147469666837	0.659588527976329	0.998043617620652	pseudobulk	DESeq2	LRT	0.180726906514334
235	KC_G1	alt	-0.0864982756289123	0.605834085051227	0.998043617620652	pseudobulk	DESeq2	LRT	0.217646296316405
236	KC_G1	Ama	1.13582588427274	0.633424328955532	0.998043617620652	pseudobulk	DESeq2	LRT	0.198305259991224
237	KC_G1	Amacr	-0.312113786930802	0.431478963241042	0.998043617620652	pseudobulk	DESeq2	LRT	0.365040373462203
238	KC_G1	amn	0.705912553639422	0.427393929450614	0.998043617620652	pseudobulk	DESeq2	LRT	0.369171650690489
239	KC_G1	Amnionless	-0.859989255792865	0.564247820111202	0.998043617620652	pseudobulk	DESeq2	LRT	0.248530110080071
240	KC_G1	amon	-0.0204365789726238	0.870540886458876	0.998043617620652	pseudobulk	DESeq2	LRT	0.0602108266759096
241	KC_G1	AMPdeam	0.184916274804752	0.628016385135779	0.998043617620652	pseudobulk	DESeq2	LRT	0.202029025241746
242	KC_G1	Amph	-0.049063055309061	0.814496146152798	0.998043617620652	pseudobulk	DESeq2	LRT	0.0891109662462433
243	KC_G1	AMPKalpha	-0.0847250382795202	0.736129887991011	0.998043617620652	pseudobulk	DESeq2	LRT	0.133045548895096
244	KC_G1	Amun	-0.0811233672224498	0.616589028461999	0.998043617620652	pseudobulk	DESeq2	LRT	0.210004207343543
245	KC_G1	amx	-0.635699913421015	0.298016253137753	0.998043617620652	pseudobulk	DESeq2	LRT	0.525760049831279
246	KC_G1	Amyrel	-0.794610277746993	0.879485534599449	0.998043617620652	pseudobulk	DESeq2	LRT	0.0557712992369932
247	KC_G1	ana	-0.345004413671218	0.716805526441974	0.998043617620652	pseudobulk	DESeq2	LRT	0.144598655000505
248	KC_G1	ana1	0.949247102466247	0.731817859237848	0.998043617620652	pseudobulk	DESeq2	LRT	0.135596996224368
249	KC_G1	ana2	1.75162781140343	0.300412726727111	0.998043617620652	pseudobulk	DESeq2	LRT	0.522281672765476
250	KC_G1	ana3	-0.794610277746993	0.879485534599449	0.998043617620652	pseudobulk	DESeq2	LRT	0.0557712992369932
251	KC_G1	Ance-3	1.12508065046033	0.900770125530981	0.998043617620652	pseudobulk	DESeq2	LRT	0.0453860258366365
252	KC_G1	Ance-4	1.12508065046033	0.900770125530981	0.998043617620652	pseudobulk	DESeq2	LRT	0.0453860258366365
253	KC_G1	angel	0.161697602818709	0.77565275722218	0.998043617620652	pseudobulk	DESeq2	LRT	0.11033265937769
254	KC_G1	Ank	0.0466731588017967	0.750450343822037	0.998043617620652	pseudobulk	DESeq2	LRT	0.124678039086988
255	KC_G1	Ank2	-0.139924635969454	0.142863924622556	0.998043617620652	pseudobulk	DESeq2	LRT	0.845077423520536
256	KC_G1	Ankle2	-0.378480324300878	0.374321033252946	0.998043617620652	pseudobulk	DESeq2	LRT	0.426755769014226
257	KC_G1	anne	0.0280277035778666	0.834208736708285	0.998043617620652	pseudobulk	DESeq2	LRT	0.0787252660793396
258	KC_G1	Ant2	0.00714171957018244	0.970116012434543	0.998043617620652	pseudobulk	DESeq2	LRT	0.0131763270250475
259	KC_G1	AnxB10	0.104550321090633	0.825891556454698	0.998043617620652	pseudobulk	DESeq2	LRT	0.0830769738965449
260	KC_G1	AnxB11	-0.0387640936309433	0.917206700762291	0.998043617620652	pseudobulk	DESeq2	LRT	0.0375327811406841
261	KC_G1	AnxB9	0.794104264087035	0.169981923136716	0.998043617620652	pseudobulk	DESeq2	LRT	0.769597261559391
262	KC_G1	aop	-0.145735881198615	0.59919088576929	0.998043617620652	pseudobulk	DESeq2	LRT	0.222434801266882
263	KC_G1	aos	2.63732022130093	0.22943645835378	0.998043617620652	pseudobulk	DESeq2	LRT	0.639337569853304
264	KC_G1	Aos1	-0.265845402254652	0.384699817751206	0.998043617620652	pseudobulk	DESeq2	LRT	0.414878019437048
265	KC_G1	ap	-1.08158947309595	0.499279094021967	0.998043617620652	pseudobulk	DESeq2	LRT	0.301656618485212
266	KC_G1	AP-1-2beta	-0.231475153692945	0.273355380177623	0.998043617620652	pseudobulk	DESeq2	LRT	0.56327237390903
267	KC_G1	AP-1gamma	0.0444688403563211	0.780590717720087	0.998043617620652	pseudobulk	DESeq2	LRT	0.107576617373133
268	KC_G1	AP-1mu	-0.0682804626788829	0.786806406886652	0.998043617620652	pseudobulk	DESeq2	LRT	0.104132112321346
269	KC_G1	AP-1sigma	-0.188382048104489	0.203532720425901	0.998043617620652	pseudobulk	DESeq2	LRT	0.691365762565977
270	KC_G1	AP-2alpha	-0.0737627438100813	0.605335036881466	0.998043617620652	pseudobulk	DESeq2	LRT	0.218004188331106
271	KC_G1	AP-2mu	0.0203073669894765	0.910566565058809	0.998043617620652	pseudobulk	DESeq2	LRT	0.0406883005214032
272	KC_G1	AP-2sigma	-0.022771859202226	0.870703113408749	0.998043617620652	pseudobulk	DESeq2	LRT	0.0601299026131648
273	KC_G1	Apc	-0.05540509940523	0.757993292176989	0.998043617620652	pseudobulk	DESeq2	LRT	0.120334637617826
274	KC_G1	APC10	-0.0646547052496088	0.853615169201997	0.998043617620652	pseudobulk	DESeq2	LRT	0.0687378758678105
275	KC_G1	APC4	0.255116739709697	0.797200887286264	0.998043617620652	pseudobulk	DESeq2	LRT	0.0984322266006093
276	KC_G1	ApepP	0.999626947038581	0.159005729644052	0.998043617620652	pseudobulk	DESeq2	LRT	0.798587225943942
277	KC_G1	Apf	0.570019374641647	0.138292416663722	0.998043617620652	pseudobulk	DESeq2	LRT	0.859201634000566
278	KC_G1	aph-1	0.0876116663304104	0.740822384205511	0.998043617620652	pseudobulk	DESeq2	LRT	0.130285903769336

279	KC_G1	aPKC	0.111643383558015	0.462405580950036	0.998043617620652	pseudobulk	DESeq2	LRT	0.334976932889479
280	KC_G1	Apl	-0.325474091048357	0.41807577797467	0.998043617620652	pseudobulk	DESeq2	LRT	0.378744993407894
281	KC_G1	Aplip1	-0.100319039209601	0.481014665688543	0.998043617620652	pseudobulk	DESeq2	LRT	0.317841682190595
282	KC_G1	apolpp	-0.130916214233939	0.82200917030645	0.998043617620652	pseudobulk	DESeq2	LRT	0.0851233374584002
283	KC_G1	Apoltp	-1.42071082011206	0.11761934971901	0.998043617620652	pseudobulk	DESeq2	LRT	0.929521226007152
284	KC_G1	app	-0.582258242448631	0.619998724477337	0.998043617620652	pseudobulk	DESeq2	LRT	0.207609203974365
285	KC_G1	APP-BP1	-0.628771153138979	0.332346504539454	0.998043617620652	pseudobulk	DESeq2	LRT	0.478408884566115
286	KC_G1	Appl	-0.0890077110848131	0.530766761400942	0.998043617620652	pseudobulk	DESeq2	LRT	0.275096282100265
287	KC_G1	Aprt	-0.107411173880891	0.876236718773325	0.998043617620652	pseudobulk	DESeq2	LRT	0.057378551629759
288	KC_G1	Aps	0.0397246074780902	0.828436517677383	0.998043617620652	pseudobulk	DESeq2	LRT	0.0817407655532945
289	KC_G1	apt	1.13582588427274	0.633424328955532	0.998043617620652	pseudobulk	DESeq2	LRT	0.198305259991224
290	KC_G1	aqz	-0.0645082293860361	0.493461911385489	0.998043617620652	pseudobulk	DESeq2	LRT	0.306746363386414
291	KC_G1	aralar1	-0.340732010573616	0.28689287624547	0.998043617620652	pseudobulk	DESeq2	LRT	0.542280235461541
292	KC_G1	Arc1	-1.4587746945059	0.405547325367764	0.998043617620652	pseudobulk	DESeq2	LRT	0.391958458476742
293	KC_G1	Arc2	0.1729159612032	0.899814506689544	0.998043617620652	pseudobulk	DESeq2	LRT	0.0458470094762598
294	KC_G1	Arc42	1.11979036081061	0.207827230208715	0.998043617620652	pseudobulk	DESeq2	LRT	0.682297550356242
295	KC_G1	Archease	-0.116790256023461	0.771289439967023	0.998043617620652	pseudobulk	DESeq2	LRT	0.112782614696657
296	KC_G1	Arf102F	0.0690519529656847	0.696220745607385	0.998043617620652	pseudobulk	DESeq2	LRT	0.157253039987989
297	KC_G1	Arf51F	0.0941270298321533	0.662867096343813	0.998043617620652	pseudobulk	DESeq2	LRT	0.178573538126796
298	KC_G1	Arf79F	-0.0663509125771939	0.583990396978534	0.998043617620652	pseudobulk	DESeq2	LRT	0.233594294280617
299	KC_G1	ArfGAP1	0.146851422553806	0.715167366367009	0.998043617620652	pseudobulk	DESeq2	LRT	0.145592310948829
300	KC_G1	ArfGAP3	0.238113914868796	0.55448934272273	0.998043617620652	pseudobulk	DESeq2	LRT	0.256106796568363
301	KC_G1	Arfip	-0.061113007868831	0.83068287418601	0.998043617620652	pseudobulk	DESeq2	LRT	0.0805647430958356
302	KC_G1	Arfrp1	0.27179174126563	0.6374368289058	0.998043617620652	pseudobulk	DESeq2	LRT	0.195562848097149
303	KC_G1	arg	-0.190987672464664	0.780541549263349	0.998043617620652	pseudobulk	DESeq2	LRT	0.107603973914156
304	KC_G1	Argk	0.0607321394611515	0.702194281834151	0.998043617620652	pseudobulk	DESeq2	LRT	0.153542711438577
305	KC_G1	Argl	1.13582588427274	0.633424328955532	0.998043617620652	pseudobulk	DESeq2	LRT	0.198305259991224
306	KC_G1	ArgRS	0.389337008070627	0.528499407048936	0.998043617620652	pseudobulk	DESeq2	LRT	0.276955495613924
307	KC_G1	ArgRS-m	-0.0456494002123528	0.953056715515791	0.998043617620652	pseudobulk	DESeq2	LRT	0.0208812541332993
308	KC_G1	ari-1	0.0750890421554346	0.632799154779269	0.998043617620652	pseudobulk	DESeq2	LRT	0.198734109590887
309	KC_G1	ari-2	-0.00589963206069752	0.959375568322481	0.998043617620652	pseudobulk	DESeq2	LRT	0.0180113455718457
310	KC_G1	Arl1	0.105487484214111	0.685303237853746	0.998043617620652	pseudobulk	DESeq2	LRT	0.164117216265645
311	KC_G1	Arl2	-0.432165050928414	0.264368205965086	0.998043617620652	pseudobulk	DESeq2	LRT	0.577790776131139
312	KC_G1	Arl4	0.0377345447726348	0.793956843279858	0.998043617620652	pseudobulk	DESeq2	LRT	0.100203103662073
313	KC_G1	Arl5	0.126898509782542	0.698215298436795	0.998043617620652	pseudobulk	DESeq2	LRT	0.156010639689853
314	KC_G1	Arl6IP1	-0.148601023188905	0.278229211096077	0.998043617620652	pseudobulk	DESeq2	LRT	0.555597275667284
315	KC_G1	Arl8	-0.145759790589713	0.400323911250459	0.998043617620652	pseudobulk	DESeq2	LRT	0.397588468815727
316	KC_G1	arm	-0.120341146561482	0.372895883977087	0.998043617620652	pseudobulk	DESeq2	LRT	0.428412410352568
317	KC_G1	armi	1.06832895747179	0.310031259345974	0.998043617620652	pseudobulk	DESeq2	LRT	0.508594515594619
318	KC_G1	Arp1	-0.0221129026464935	0.905435455029667	0.998043617620652	pseudobulk	DESeq2	LRT	0.0431425034101717
319	KC_G1	Arp10	0.0722762013604519	0.866248434416184	0.998043617620652	pseudobulk	DESeq2	LRT	0.0623575372863726
320	KC_G1	Arp2	-0.122951685505623	0.615955748955662	0.998043617620652	pseudobulk	DESeq2	LRT	0.210450486982783
321	KC_G1	Arp3	0.115776334916933	0.691188107154046	0.998043617620652	pseudobulk	DESeq2	LRT	0.160403743101222
322	KC_G1	Arp5	0.151080122666865	0.747393078035097	0.998043617620652	pseudobulk	DESeq2	LRT	0.126450928676751
323	KC_G1	Arp6	-0.0842961852564765	0.825261152755738	0.998043617620652	pseudobulk	DESeq2	LRT	0.0834085978094699
324	KC_G1	Arp8	0.0452584054500838	0.944473796850319	0.998043617620652	pseudobulk	DESeq2	LRT	0.0248100864887576
325	KC_G1	Arpc1	-0.0783974212883261	0.67495721025726	0.998043617620652	pseudobulk	DESeq2	LRT	0.17072375892927
326	KC_G1	Arpc2	-0.091690569507205	0.764310844661744	0.998043617620652	pseudobulk	DESeq2	LRT	0.116729978249201
327	KC_G1	Arpc3A	0.0730101657324401	0.838417997960006	0.998043617620652	pseudobulk	DESeq2	LRT	0.0765394074466798
328	KC_G1	Arpc5	-0.259374298863598	0.294157168649725	0.998043617620652	pseudobulk	DESeq2	LRT	0.531420563332689
329	KC_G1	arr	-0.0862722066570969	0.81348236604135	0.998043617620652	pseudobulk	DESeq2	LRT	0.0896518568807062
330	KC_G1	Arr1	1.69981218657454	0.566400560818652	0.998043617620652	pseudobulk	DESeq2	LRT	0.246876325303589
331	KC_G1	Ars2	-0.0878361574601772	0.642787969034721	0.998043617620652	pseudobulk	DESeq2	LRT	0.191932260456811
332	KC_G1	Art1	0.075735133822963	0.867031374176907	0.998043617620652	pseudobulk	DESeq2	LRT	0.0619651869697239
333	KC_G1	Art2	-0.65817001371651	0.770857516266391	0.998043617620652	pseudobulk	DESeq2	LRT	0.113025888638787
334	KC_G1	Art3	0.0481142042679847	0.940153117789325	0.998043617620652	pseudobulk	DESeq2	LRT	0.0268014093837221

335	KC_G1	Art4	0.353763282037009	0.561613661238475	0.998043617620652	pseudobulk	DESeq2	LRT	0.250562336547188
336	KC_G1	Art7	1.12870130307672	0.256198910637517	0.998043617620652	pseudobulk	DESeq2	LRT	0.591422721215536
337	KC_G1	Art8	-0.466304985047353	0.73701340603124	0.998043617620652	pseudobulk	DESeq2	LRT	0.13252461239544
338	KC_G1	Arts	-0.274831407758838	0.700569007176489	0.998043617620652	pseudobulk	DESeq2	LRT	0.154549079564654
339	KC_G1	aru	0.737875864562409	0.679733946854191	0.998043617620652	pseudobulk	DESeq2	LRT	0.167661040268549
340	KC_G1	Arv1	0.0639372835245807	0.880695485952369	0.998043617620652	pseudobulk	DESeq2	LRT	0.0551742296486099
341	KC_G1	arx	-0.40820863760091	0.798051598365383	0.998043617620652	pseudobulk	DESeq2	LRT	0.0979690282471183
342	KC_G1	Asap	0.054201889630427	0.83838660591005	0.998043617620652	pseudobulk	DESeq2	LRT	0.0765556686067956
343	KC_G1	Asator	-0.108679152025371	0.377554572125917	0.998043617620652	pseudobulk	DESeq2	LRT	0.423020266126897
344	KC_G1	Asciz	0.204963257274342	0.745801967011413	0.998043617620652	pseudobulk	DESeq2	LRT	0.12737647554554
345	KC_G1	ase	1.12508065046033	0.900770125530981	0.998043617620652	pseudobulk	DESeq2	LRT	0.0453860258366365
346	KC_G1	asf1	-0.16604494409291	0.594226582482208	0.998043617620652	pseudobulk	DESeq2	LRT	0.226047924112442
347	KC_G1	ash1	-0.0709448123299459	0.800832239556171	0.998043617620652	pseudobulk	DESeq2	LRT	0.0964584515383431
348	KC_G1	ash2	-0.544688302596257	0.370328099550544	0.998043617620652	pseudobulk	DESeq2	LRT	0.431413333543998
349	KC_G1	Ask1	-0.110229460450812	0.84274463623033	0.998043617620652	pseudobulk	DESeq2	LRT	0.0743040029280026
350	KC_G1	asl	0.849829402468815	0.441024733486524	0.998043617620652	pseudobulk	DESeq2	LRT	0.35553705380766
351	KC_G1	AsnRS	-0.31112213690488	0.389719892612515	0.998043617620652	pseudobulk	DESeq2	LRT	0.409247425792237
352	KC_G1	AsnRS-m	-0.431508043548676	0.521849681474471	0.998043617620652	pseudobulk	DESeq2	LRT	0.282454577280416
353	KC_G1	AsnS	1.30952621665888	0.518969544296517	0.998043617620652	pseudobulk	DESeq2	LRT	0.284858127955132
354	KC_G1	asp	1.12508065046033	0.900770125530981	0.998043617620652	pseudobulk	DESeq2	LRT	0.0453860258366365
355	KC_G1	Asph	-0.112994600819857	0.878360781001505	0.998043617620652	pseudobulk	DESeq2	LRT	0.0563270638034563
356	KC_G1	ASPP	0.1729159612032	0.899814506689544	0.998043617620652	pseudobulk	DESeq2	LRT	0.0458470094762598
357	KC_G1	AspRS	-0.0161078987171409	0.960514909972719	0.998043617620652	pseudobulk	DESeq2	LRT	0.0174958892420384
358	KC_G1	AspRS-m	-0.0112309503139273	0.937695326398924	0.998043617620652	pseudobulk	DESeq2	LRT	0.027938248569365
359	KC_G1	asrij	-0.326601880749456	0.304802110585792	0.998043617620652	pseudobulk	DESeq2	LRT	0.515982030073286
360	KC_G1	asRNA:CR17025	-2.27643791964046	0.298906309922724	0.998043617620652	pseudobulk	DESeq2	LRT	0.52446491689215
361	KC_G1	asRNA:CR30267	-0.794610277746993	0.815039924133852	0.998043617620652	pseudobulk	DESeq2	LRT	0.0888211171417967
362	KC_G1	asRNA:CR31144	0.660223260418914	0.760341410401239	0.998043617620652	pseudobulk	DESeq2	LRT	0.118991355930445
363	KC_G1	asRNA:CR31845	1.13582588427274	0.704684208645453	0.998043617620652	pseudobulk	DESeq2	LRT	0.152005460551806
364	KC_G1	asRNA:CR31912	0.948682853426738	0.716182290134517	0.998043617620652	pseudobulk	DESeq2	LRT	0.144976422491876
365	KC_G1	asRNA:CR42254	0.817981482362889	0.127492284188479	0.998043617620652	pseudobulk	DESeq2	LRT	0.894516097863469
366	KC_G1	asRNA:CR42547	-2.37182653168279	0.0283358628461466	0.998043617620652	pseudobulk	DESeq2	LRT	1.54766355826107
367	KC_G1	asRNA:CR42860	-0.794610277746993	0.821479263487599	0.998043617620652	pseudobulk	DESeq2	LRT	0.085403394939296
368	KC_G1	asRNA:CR43152	-0.472759662961788	0.788665429949699	0.998043617620652	pseudobulk	DESeq2	LRT	0.10310719545029
369	KC_G1	asRNA:CR43159	-0.65817001371651	0.770857516266391	0.998043617620652	pseudobulk	DESeq2	LRT	0.113025888638787
370	KC_G1	asRNA:CR43259	0.665188601046461	0.522590844870456	0.998043617620652	pseudobulk	DESeq2	LRT	0.281838202831175
371	KC_G1	asRNA:CR43425	-0.404581148302586	0.686795184237567	0.998043617620652	pseudobulk	DESeq2	LRT	0.163172758744644
372	KC_G1	asRNA:CR43426	-0.65817001371651	0.770857516266391	0.998043617620652	pseudobulk	DESeq2	LRT	0.113025888638787
373	KC_G1	asRNA:CR43460	0.949247102466247	0.731817859237848	0.998043617620652	pseudobulk	DESeq2	LRT	0.135596996224368
374	KC_G1	asRNA:CR43464	-0.794610277746993	0.821479263487599	0.998043617620652	pseudobulk	DESeq2	LRT	0.085403394939296
375	KC_G1	asRNA:CR43466	1.12508065046033	0.900770125530981	0.998043617620652	pseudobulk	DESeq2	LRT	0.0453860258366365
376	KC_G1	asRNA:CR43480	-1.73907471612892	0.507597351592657	0.998043617620652	pseudobulk	DESeq2	LRT	0.294480652518419
377	KC_G1	asRNA:CR43481	1.13582588427274	0.704684208645453	0.998043617620652	pseudobulk	DESeq2	LRT	0.152005460551806
378	KC_G1	asRNA:CR43629	0.204916177109238	0.715108192605771	0.998043617620652	pseudobulk	DESeq2	LRT	0.145628246454869
379	KC_G1	asRNA:CR43683	-0.794610277746993	0.815039924133852	0.998043617620652	pseudobulk	DESeq2	LRT	0.0888211171417967
380	KC_G1	asRNA:CR43730	-1.07047731052272	0.422150512834144	0.998043617620652	pseudobulk	DESeq2	LRT	0.374532678803639
381	KC_G1	asRNA:CR43767	-0.263674512857996	0.640318115235732	0.998043617620652	pseudobulk	DESeq2	LRT	0.193604211379599
382	KC_G1	asRNA:CR43786	0.173116475207952	0.97305562682953	0.998043617620652	pseudobulk	DESeq2	LRT	0.011862331638572
383	KC_G1	asRNA:CR43787	1.13582588427274	0.704684208645453	0.998043617620652	pseudobulk	DESeq2	LRT	0.152005460551806
384	KC_G1	asRNA:CR43806	1.12508065046033	0.900770125530981	0.998043617620652	pseudobulk	DESeq2	LRT	0.0453860258366365
385	KC_G1	asRNA:CR43809	2.04147257822512	0.408147672536479	0.998043617620652	pseudobulk	DESeq2	LRT	0.389182675723292
386	KC_G1	asRNA:CR43820	0.172834960772825	0.908610676445368	0.998043617620652	pseudobulk	DESeq2	LRT	0.0416221644022337
387	KC_G1	asRNA:CR43833	-0.794610277746993	0.815039924133852	0.998043617620652	pseudobulk	DESeq2	LRT	0.0888211171417967
388	KC_G1	asRNA:CR43865	0.228570564635066	0.925908451678216	0.998043617620652	pseudobulk	DESeq2	LRT	0.0334319516511941
389	KC_G1	asRNA:CR43868	-0.794610277746993	0.815039924133852	0.998043617620652	pseudobulk	DESeq2	LRT	0.0888211171417967
390	KC_G1	asRNA:CR43872	-0.794610277746993	0.821479263487599	0.998043617620652	pseudobulk	DESeq2	LRT	0.085403394939296

391	KC_G1	asRNA:CR43885	1.12508065046033	0.900770125530981	0.998043617620652	pseudobulk	DESeq2	LRT	0.0453860258366365
392	KC_G1	asRNA:CR43889	-0.323009177088943	0.862831841642416	0.998043617620652	pseudobulk	DESeq2	LRT	0.0640738362231444
393	KC_G1	asRNA:CR43906	-0.214727010679646	0.668145450746112	0.998043617620652	pseudobulk	DESeq2	LRT	0.175128984260602
394	KC_G1	asRNA:CR43916	1.13582588427274	0.704684208645453	0.998043617620652	pseudobulk	DESeq2	LRT	0.152005460551806
395	KC_G1	asRNA:CR43933	0.307661268876215	0.720534972615921	0.998043617620652	pseudobulk	DESeq2	LRT	0.142344934981142
396	KC_G1	asRNA:CR43936	-0.410204446226527	0.854948780430877	0.998043617620652	pseudobulk	DESeq2	LRT	0.0680599028654498
397	KC_G1	asRNA:CR43944	-0.855160783576656	0.446970372426559	0.998043617620652	pseudobulk	DESeq2	LRT	0.349721263262019
398	KC_G1	asRNA:CR43957	-2.73425569144472	0.216926985303784	0.998043617620652	pseudobulk	DESeq2	LRT	0.663686419218864
399	KC_G1	asRNA:CR43959	0.728069516433298	0.488954698938771	0.998043617620652	pseudobulk	DESeq2	LRT	0.310731375870805
400	KC_G1	asRNA:CR44018	0.0330351215975759	0.930744500016856	0.998043617620652	pseudobulk	DESeq2	LRT	0.0311695214478858
401	KC_G1	asRNA:CR44029	0.283910397840822	0.603312399402884	0.998043617620652	pseudobulk	DESeq2	LRT	0.219457748880655
402	KC_G1	asRNA:CR44030	-0.420990414439415	0.181633680046375	0.998043617620652	pseudobulk	DESeq2	LRT	0.740803617807935
403	KC_G1	asRNA:CR44031	0.228633990858825	0.83148684961927	0.998043617620652	pseudobulk	DESeq2	LRT	0.0801446149747661
404	KC_G1	asRNA:CR44046	-0.868711691328719	0.389444753556272	0.998043617620652	pseudobulk	DESeq2	LRT	0.409554142426356
405	KC_G1	asRNA:CR44047	2.38466886841527	0.34619303318143	0.998043617620652	pseudobulk	DESeq2	LRT	0.460681676152437
406	KC_G1	asRNA:CR44054	1.13582588427274	0.633424328955532	0.998043617620652	pseudobulk	DESeq2	LRT	0.198305259991224
407	KC_G1	asRNA:CR44067	0.173195945230598	0.976215465741119	0.998043617620652	pseudobulk	DESeq2	LRT	0.0104543162934892
408	KC_G1	asRNA:CR44069	0.173116475207952	0.97305562682953	0.998043617620652	pseudobulk	DESeq2	LRT	0.011862331638572
409	KC_G1	asRNA:CR44093	-1.07948179811012	0.418276943510993	0.998043617620652	pseudobulk	DESeq2	LRT	0.378536074176057
410	KC_G1	asRNA:CR44106	-0.587599495628208	0.63952875930746	0.998043617620652	pseudobulk	DESeq2	LRT	0.194139920727635
411	KC_G1	asRNA:CR44114	1.69981218657454	0.566400560818652	0.998043617620652	pseudobulk	DESeq2	LRT	0.246876325303589
412	KC_G1	asRNA:CR44116	1.11121460044925	0.691240116375203	0.998043617620652	pseudobulk	DESeq2	LRT	0.16037106535726
413	KC_G1	asRNA:CR44119	1.01144400189899	0.358416305713217	0.998043617620652	pseudobulk	DESeq2	LRT	0.445612240862062
414	KC_G1	asRNA:CR44137	-1.1945855544474	0.480023068708373	0.998043617620652	pseudobulk	DESeq2	LRT	0.318737891016054
415	KC_G1	asRNA:CR44169	-1.72343437992478	0.512120560562093	0.998043617620652	pseudobulk	DESeq2	LRT	0.290627787806665
416	KC_G1	asRNA:CR44179	-0.432935376641644	0.80600096134232	0.998043617620652	pseudobulk	DESeq2	LRT	0.0936644401981157
417	KC_G1	asRNA:CR44228	0.910672333913252	0.602027119915213	0.998043617620652	pseudobulk	DESeq2	LRT	0.220383944349756
418	KC_G1	asRNA:CR44291	1.13582588427274	0.633424328955532	0.998043617620652	pseudobulk	DESeq2	LRT	0.198305259991224
419	KC_G1	asRNA:CR44340	0.707339928202551	0.642654965576214	0.998043617620652	pseudobulk	DESeq2	LRT	0.192022132463968
420	KC_G1	asRNA:CR44351	1.12508065046033	0.900770125530981	0.998043617620652	pseudobulk	DESeq2	LRT	0.0453860258366365
421	KC_G1	asRNA:CR44368	-0.71088171110674	0.784645801042283	0.998043617620652	pseudobulk	DESeq2	LRT	0.105326344996467
422	KC_G1	asRNA:CR44429	-0.139046923079547	0.930850499186999	0.998043617620652	pseudobulk	DESeq2	LRT	0.0311200640151481
423	KC_G1	asRNA:CR44431	-0.718676691072112	0.739205546127982	0.998043617620652	pseudobulk	DESeq2	LRT	0.131234783341328
424	KC_G1	asRNA:CR44690	-0.518923163042321	0.767545638476955	0.998043617620652	pseudobulk	DESeq2	LRT	0.114895791812437
425	KC_G1	asRNA:CR44806	2.18876425250967	0.402105238436886	0.998043617620652	pseudobulk	DESeq2	LRT	0.395660269077107
426	KC_G1	asRNA:CR44840	-0.764352369264947	0.597803010079667	0.998043617620652	pseudobulk	DESeq2	LRT	0.22344190251571
427	KC_G1	asRNA:CR44841	-2.31763425292248	0.315578515403755	0.998043617620652	pseudobulk	DESeq2	LRT	0.50089257123956
428	KC_G1	asRNA:CR44843	-0.265978753400783	0.894647009539557	0.998043617620652	pseudobulk	DESeq2	LRT	0.0483482854085724
429	KC_G1	asRNA:CR44892	-0.157040946914118	0.84830473727543	0.998043617620652	pseudobulk	DESeq2	LRT	0.0714481077162027
430	KC_G1	asRNA:CR44894	-0.817156062867586	0.0245415236590703	0.998043617620652	pseudobulk	DESeq2	LRT	1.6100984776243
431	KC_G1	asRNA:CR44897	-0.794610277746993	0.821479263487599	0.998043617620652	pseudobulk	DESeq2	LRT	0.085403394939296
432	KC_G1	asRNA:CR44911	1.69981218657454	0.566400560818652	0.998043617620652	pseudobulk	DESeq2	LRT	0.246876325303589
433	KC_G1	asRNA:CR44960	-0.685508625450168	0.487915806769467	0.998043617620652	pseudobulk	DESeq2	LRT	0.311655112034162
434	KC_G1	asRNA:CR44976	1.13582588427274	0.704684208645453	0.998043617620652	pseudobulk	DESeq2	LRT	0.152005460551806
435	KC_G1	asRNA:CR44981	-1.76164463706579	0.438917041178301	0.998043617620652	pseudobulk	DESeq2	LRT	0.35761755712261
436	KC_G1	asRNA:CR44984	-1.73907471612892	0.507597351592657	0.998043617620652	pseudobulk	DESeq2	LRT	0.294480652518419
437	KC_G1	asRNA:CR44987	-0.698828349056341	0.53190580572866	0.998043617620652	pseudobulk	DESeq2	LRT	0.27416526934823
438	KC_G1	asRNA:CR44989	0.207131536130541	0.85447114735865	0.998043617620652	pseudobulk	DESeq2	LRT	0.0683025973721285
439	KC_G1	asRNA:CR44990	-0.794610277746993	0.821479263487599	0.998043617620652	pseudobulk	DESeq2	LRT	0.085403394939296
440	KC_G1	asRNA:CR44992	1.69981218657454	0.566400560818652	0.998043617620652	pseudobulk	DESeq2	LRT	0.246876325303589
441	KC_G1	asRNA:CR44995	1.12508065046033	0.900770125530981	0.998043617620652	pseudobulk	DESeq2	LRT	0.0453860258366365
442	KC_G1	asRNA:CR44998	-1.41151038767523	0.280086422846881	0.998043617620652	pseudobulk	DESeq2	LRT	0.552707943035028
443	KC_G1	asRNA:CR45000	1.85122539377412	0.196138286139441	0.998043617620652	pseudobulk	DESeq2	LRT	0.707437623893922
444	KC_G1	asRNA:CR45007	-0.6685935744981 23	0.744279897808527	0.998043617620652	pseudobulk	DESeq2	LRT	0.128263710671876
445	KC_G1	asRNA:CR45009	-0.692279278230738	0.270572045201859	0.998043617620652	pseudobulk	DESeq2	LRT	0.567717075593984
446	KC_G1	asRNA:CR45026	1.13582588427274	0.633424328955532	0.998043617620652	pseudobulk	DESeq2	LRT	0.198305259991224

447	KC_G1	asRNA:CR45041	-0.794610277746993	0.879485534599449	0.998043617620652	pseudobulk	DESeq2	LRT	0.0557712992369932
448	KC_G1	asRNA:CR45104	-1.73907471612892	0.507597351592657	0.998043617620652	pseudobulk	DESeq2	LRT	0.294480652518419
449	KC_G1	asRNA:CR45108	-0.794610277746993	0.879485534599449	0.998043617620652	pseudobulk	DESeq2	LRT	0.0557712992369932
450	KC_G1	asRNA:CR45121	1.13582588427274	0.633424328955532	0.998043617620652	pseudobulk	DESeq2	LRT	0.198305259991224
451	KC_G1	asRNA:CR45124	1.13582588427274	0.704684208645453	0.998043617620652	pseudobulk	DESeq2	LRT	0.152005460551806
452	KC_G1	asRNA:CR45126	0.0913439104287783	0.920782600105165	0.998043617620652	pseudobulk	DESeq2	LRT	0.035842896100366
453	KC_G1	asRNA:CR45129	0.0326264548033904	0.943493085087472	0.998043617620652	pseudobulk	DESeq2	LRT	0.025261278455445
454	KC_G1	asRNA:CR45131	0.136467190582412	0.873925659865826	0.998043617620652	pseudobulk	DESeq2	LRT	0.0585255088795484
455	KC_G1	asRNA:CR45133	-0.794610277746993	0.879485534599449	0.998043617620652	pseudobulk	DESeq2	LRT	0.0557712992369932
456	KC_G1	asRNA:CR45134	-0.525461591595144	0.167930306623895	0.998043617620652	pseudobulk	DESeq2	LRT	0.774870919039027
457	KC_G1	asRNA:CR45137	1.06119553529735	0.439679793381025	0.998043617620652	pseudobulk	DESeq2	LRT	0.356863493044767
458	KC_G1	asRNA:CR45139	1.13582588427274	0.633424328955532	0.998043617620652	pseudobulk	DESeq2	LRT	0.198305259991224
459	KC_G1	asRNA:CR45140	-0.794610277746993	0.815039924133852	0.998043617620652	pseudobulk	DESeq2	LRT	0.0888211171417967
460	KC_G1	asRNA:CR45141	1.12508065046033	0.900770125530981	0.998043617620652	pseudobulk	DESeq2	LRT	0.0453860258366365
461	KC_G1	asRNA:CR45145	-0.344484866141779	0.752192273455876	0.998043617620652	pseudobulk	DESeq2	LRT	0.123671131978113
462	KC_G1	asRNA:CR45146	1.12508065046033	0.900770125530981	0.998043617620652	pseudobulk	DESeq2	LRT	0.0453860258366365
463	KC_G1	asRNA:CR45151	3.05843862762105	0.143087677362356	0.998043617620652	pseudobulk	DESeq2	LRT	0.844397765848649
464	KC_G1	asRNA:CR45161	-0.72844370850556	0.2973172449646	0.998043617620652	pseudobulk	DESeq2	LRT	0.526779900160138
465	KC_G1	asRNA:CR45162	-2.27643791964046	0.298906309922724	0.998043617620652	pseudobulk	DESeq2	LRT	0.52446491689215
466	KC_G1	asRNA:CR45164	-0.349876657302537	0.807067817500839	0.998043617620652	pseudobulk	DESeq2	LRT	0.0930899701987404
467	KC_G1	asRNA:CR45175	1.13582588427274	0.704684208645453	0.998043617620652	pseudobulk	DESeq2	LRT	0.152005460551806
468	KC_G1	asRNA:CR45182	0.067976935513013	0.942577856778656	0.998043617620652	pseudobulk	DESeq2	LRT	0.0256827669865239
469	KC_G1	asRNA:CR45198	-0.794610277746993	0.821479263487599	0.998043617620652	pseudobulk	DESeq2	LRT	0.085403394939296
470	KC_G1	asRNA:CR45202	-0.794610277746993	0.815039924133852	0.998043617620652	pseudobulk	DESeq2	LRT	0.0888211171417967
471	KC_G1	asRNA:CR45208	0.487489955399504	0.783388322009348	0.998043617620652	pseudobulk	DESeq2	LRT	0.106022906779347
472	KC_G1	asRNA:CR45214	-0.794610277746993	0.879485534599449	0.998043617620652	pseudobulk	DESeq2	LRT	0.0557712992369932
473	KC_G1	asRNA:CR45275	1.13582588427274	0.633424328955532	0.998043617620652	pseudobulk	DESeq2	LRT	0.198305259991224
474	KC_G1	asRNA:CR45281	-0.307135509172264	0.779667895868597	0.998043617620652	pseudobulk	DESeq2	LRT	0.108090348213808
475	KC_G1	asRNA:CR45350	1.53295723161659	0.396145056946214	0.998043617620652	pseudobulk	DESeq2	LRT	0.402145758781636
476	KC_G1	asRNA:CR45384	-0.704123705228411	0.78667415397905	0.998043617620652	pseudobulk	DESeq2	LRT	0.104205118254245
477	KC_G1	asRNA:CR45396	0.173195945230598	0.976215465741119	0.998043617620652	pseudobulk	DESeq2	LRT	0.0104543162934892
478	KC_G1	asRNA:CR45445	1.12508065046033	0.900770125530981	0.998043617620652	pseudobulk	DESeq2	LRT	0.0453860258366365
479	KC_G1	asRNA:CR45467	0.172845155749872	0.90730697492154	0.998043617620652	pseudobulk	DESeq2	LRT	0.0422457504714555
480	KC_G1	asRNA:CR45468	-0.794610277746993	0.815039924133852	0.998043617620652	pseudobulk	DESeq2	LRT	0.0888211171417967
481	KC_G1	asRNA:CR45469	0.445697832867451	0.576926774240005	0.998043617620652	pseudobulk	DESeq2	LRT	0.238879305668533
482	KC_G1	asRNA:CR45479	-1.73634999673762	0.424993395565854	0.998043617620652	pseudobulk	DESeq2	LRT	0.371617818871082
483	KC_G1	asRNA:CR45485	1.1112567833086	0.689254921431598	0.998043617620652	pseudobulk	DESeq2	LRT	0.161620124253084
484	KC_G1	asRNA:CR45501	-0.794610277746993	0.821479263487599	0.998043617620652	pseudobulk	DESeq2	LRT	0.085403394939296
485	KC_G1	asRNA:CR45520	-0.0233406188878374	0.967822561333557	0.998043617620652	pseudobulk	DESeq2	LRT	0.0142042580814335
486	KC_G1	asRNA:CR45528	-0.302208931826685	0.90049343027991	0.998043617620652	pseudobulk	DESeq2	LRT	0.0455194513104007
487	KC_G1	asRNA:CR45535	-1.60891734856068	0.545249891924755	0.998043617620652	pseudobulk	DESeq2	LRT	0.263404411831769
488	KC_G1	asRNA:CR45549	1.13582588427274	0.704684208645453	0.998043617620652	pseudobulk	DESeq2	LRT	0.152005460551806
489	KC_G1	asRNA:CR45604	2.49458669251252	0.224065452590139	0.998043617620652	pseudobulk	DESeq2	LRT	0.649625099761591
490	KC_G1	asRNA:CR45620	-0.0506284562553183	0.916951443327909	0.998043617620652	pseudobulk	DESeq2	LRT	0.0376536615525396
491	KC_G1	asRNA:CR45623	2.07738706174348	0.431998797123676	0.998043617620652	pseudobulk	DESeq2	LRT	0.364517462451933
492	KC_G1	asRNA:CR45683	0.369226006300281	0.835642362134648	0.998043617620652	pseudobulk	DESeq2	LRT	0.0779795519672372
493	KC_G1	asRNA:CR45713	-2.31763425292248	0.315578515403755	0.998043617620652	pseudobulk	DESeq2	LRT	0.50089257123956
494	KC_G1	asRNA:CR45789	-0.794610277746993	0.815039924133852	0.998043617620652	pseudobulk	DESeq2	LRT	0.0888211171417967
495	KC_G1	asRNA:CR45798	-0.794610277746993	0.815039924133852	0.998043617620652	pseudobulk	DESeq2	LRT	0.0888211171417967
496	KC_G1	asRNA:CR45804	1.06585281705208	0.683859663547483	0.998043617620652	pseudobulk	DESeq2	LRT	0.1650330117303
497	KC_G1	asRNA:CR45812	-0.794610277746993	0.879485534599449	0.998043617620652	pseudobulk	DESeq2	LRT	0.0557712992369932
498	KC_G1	asRNA:CR45815	0.416431064791215	0.848864097724124	0.998043617620652	pseudobulk	DESeq2	LRT	0.0711618342941965
499	KC_G1	asRNA:CR45818	-0.794610277746993	0.815039924133852	0.998043617620652	pseudobulk	DESeq2	LRT	0.0888211171417967
500	KC_G1	asRNA:CR45822	0.546893190128194	0.35976973994644	0.998043617620652	pseudobulk	DESeq2	LRT	0.443975367746837
501	KC_G1	asRNA:CR45828	2.07738706174348	0.431998797123676	0.998043617620652	pseudobulk	DESeq2	LRT	0.364517462451933
502	KC_G1	asRNA:CR45830	-0.794610277746993	0.821479263487599	0.998043617620652	pseudobulk	DESeq2	LRT	0.085403394939296

503	KC_G1	asRNA:CR45835	-0.376751230461358	0.629858675434629	0.998043617620652	pseudobulk	DESeq2	LRT	0.200756884457621
504	KC_G1	asRNA:CR45865	-0.794610277746993	0.879485534599449	0.998043617620652	pseudobulk	DESeq2	LRT	0.0557712992369932
505	KC_G1	asRNA:CR45868	1.12508065046033	0.900770125530981	0.998043617620652	pseudobulk	DESeq2	LRT	0.0453860258366365
506	KC_G1	asRNA:CR45870	1.12508065046033	0.900770125530981	0.998043617620652	pseudobulk	DESeq2	LRT	0.0453860258366365
507	KC_G1	asRNA:CR45873	0.842924259868325	0.698561538795125	0.998043617620652	pseudobulk	DESeq2	LRT	0.155795329303674
508	KC_G1	asRNA:CR45874	0.598390935835252	0.200273899641857	0.998043617620652	pseudobulk	DESeq2	LRT	0.698375645714453
509	KC_G1	asRNA:CR45875	1.66849878552847	0.468694210005612	0.998043617620652	pseudobulk	DESeq2	LRT	0.329110411485433
510	KC_G1	asRNA:CR45882	-0.794610277746993	0.815039924133852	0.998043617620652	pseudobulk	DESeq2	LRT	0.0888211171417967
511	KC_G1	asRNA:CR45889	-1.12994895298108	0.493263939505566	0.998043617620652	pseudobulk	DESeq2	LRT	0.306920632857714
512	KC_G1	asRNA:CR45891	2.18724823153059	0.328512928754358	0.998043617620652	pseudobulk	DESeq2	LRT	0.48344753393925
513	KC_G1	asRNA:CR45896	-1.73620836937294	0.411085278424018	0.998043617620652	pseudobulk	DESeq2	LRT	0.386068075673294
514	KC_G1	asRNA:CR45907	-0.832116511972304	0.497700324246774	0.998043617620652	pseudobulk	DESeq2	LRT	0.303032076317382
515	KC_G1	asRNA:CR45925	-2.75734044624503	0.184608089169037	0.998043617620652	pseudobulk	DESeq2	LRT	0.733749272949832
516	KC_G1	asRNA:CR45926	1.13582588427274	0.704684208645453	0.998043617620652	pseudobulk	DESeq2	LRT	0.152005460551806
517	KC_G1	asRNA:CR45928	2.06298032700733	0.206017225689272	0.998043617620652	pseudobulk	DESeq2	LRT	0.68609646550932
518	KC_G1	asRNA:CR45943	-0.766436826297259	0.781543751736021	0.998043617620652	pseudobulk	DESeq2	LRT	0.107046704649702
519	KC_G1	asRNA:CR45944	1.12508065046033	0.900770125530981	0.998043617620652	pseudobulk	DESeq2	LRT	0.0453860258366365
520	KC_G1	asRNA:CR45951	-0.537865779720713	0.594646067298683	0.998043617620652	pseudobulk	DESeq2	LRT	0.225741448984713
521	KC_G1	asRNA:CR45952	1.13582588427274	0.704684208645453	0.998043617620652	pseudobulk	DESeq2	LRT	0.152005460551806
522	KC_G1	asRNA:CR45960	-1.33907347264396	0.653093062441393	0.998043617620652	pseudobulk	DESeq2	LRT	0.185024929559445
523	KC_G1	asRNA:CR45970	-0.794610277746993	0.815039924133852	0.998043617620652	pseudobulk	DESeq2	LRT	0.0888211171417967
524	KC_G1	asRNA:CR45999	3.05843862762105	0.143087677362356	0.998043617620652	pseudobulk	DESeq2	LRT	0.844397765848649
525	KC_G1	asRNA:CR46020	-0.794610277746993	0.821479263487599	0.998043617620652	pseudobulk	DESeq2	LRT	0.085403394939296
526	KC_G1	asRNA:CR46029	-0.794610277746993	0.815039924133852	0.998043617620652	pseudobulk	DESeq2	LRT	0.0888211171417967
527	KC_G1	asRNA:CR46033	-3.03164023420374	0.134548679417994	0.998043617620652	pseudobulk	DESeq2	LRT	0.871120560436775
528	KC_G1	asRNA:CR46050	1.06585281705208	0.683859663547483	0.998043617620652	pseudobulk	DESeq2	LRT	0.1650330117303
529	KC_G1	asRNA:CR46058	1.13582588427274	0.704684208645453	0.998043617620652	pseudobulk	DESeq2	LRT	0.152005460551806
530	KC_G1	asRNA:CR46066	-0.794610277746993	0.821479263487599	0.998043617620652	pseudobulk	DESeq2	LRT	0.085403394939296
531	KC_G1	asRNA:CR46067	-1.67593316621313	0.495877122847873	0.998043617620652	pseudobulk	DESeq2	LRT	0.304625927300966
532	KC_G1	asRNA:CR46082	-0.794610277746993	0.821479263487599	0.998043617620652	pseudobulk	DESeq2	LRT	0.085403394939296
533	KC_G1	asRNA:CR46101	1.12508065046033	0.900770125530981	0.998043617620652	pseudobulk	DESeq2	LRT	0.0453860258366365
534	KC_G1	asRNA:CR46104	2.04147257822512	0.408147672536479	0.998043617620652	pseudobulk	DESeq2	LRT	0.389182675723292
535	KC_G1	asRNA:CR46109	1.12508065046033	0.900770125530981	0.998043617620652	pseudobulk	DESeq2	LRT	0.0453860258366365
536	KC_G1	asRNA:CR46128	1.13582588427274	0.633424328955532	0.998043617620652	pseudobulk	DESeq2	LRT	0.198305259991224
537	KC_G1	asRNA:CR46133	1.13582588427274	0.633424328955532	0.998043617620652	pseudobulk	DESeq2	LRT	0.198305259991224
538	KC_G1	asRNA:CR46134	1.12508065046033	0.900770125530981	0.998043617620652	pseudobulk	DESeq2	LRT	0.0453860258366365
539	KC_G1	asRNA:CR46135	-0.0865494580772328	0.941792034383193	0.998043617620652	pseudobulk	DESeq2	LRT	0.0260449871127209
540	KC_G1	asRNA:CR46137	-0.794610277746993	0.879485534599449	0.998043617620652	pseudobulk	DESeq2	LRT	0.0557712992369932
541	KC_G1	asRNA:CR46139	-0.794610277746993	0.879485534599449	0.998043617620652	pseudobulk	DESeq2	LRT	0.0557712992369932
542	KC_G1	asRNA:CR46251	-0.794610277746993	0.879485534599449	0.998043617620652	pseudobulk	DESeq2	LRT	0.0557712992369932
543	KC_G1	asRNA:CR46262	1.69981218657454	0.566400560818652	0.998043617620652	pseudobulk	DESeq2	LRT	0.246876325303589
544	KC_G1	asRNA:CR46263	1.56852847737218	0.523617788648374	0.998043617620652	pseudobulk	DESeq2	LRT	0.280985607765667
545	KC_G1	asRNA:CR46354	0.136596810046371	0.860292318204677	0.998043617620652	pseudobulk	DESeq2	LRT	0.065353955021194
546	KC_G1	ast	-0.172947571338098	0.823570317828666	0.998043617620652	pseudobulk	DESeq2	LRT	0.084299314112671
547	KC_G1	AstA	-0.794610277746993	0.821479263487599	0.998043617620652	pseudobulk	DESeq2	LRT	0.085403394939296
548	KC_G1	AstA-R1	1.30097290456257	0.185286426420925	0.998043617620652	pseudobulk	DESeq2	LRT	0.732156394746995
549	KC_G1	asun	1.01810567267524	0.278604801743867	0.998043617620652	pseudobulk	DESeq2	LRT	0.555011402797575
550	KC_G1	Asx	0.0335398624430162	0.877554956412203	0.998043617620652	pseudobulk	DESeq2	LRT	0.0567256765496462
551	KC_G1	Atac2	0.262540226784751	0.778860192224457	0.998043617620652	pseudobulk	DESeq2	LRT	0.108540492513096
552	KC_G1	Atac3	-0.24852961368905	0.528307042132089	0.998043617620652	pseudobulk	DESeq2	LRT	0.277113600294087
553	KC_G1	ATbp	-0.241631539965661	0.725641144975686	0.998043617620652	pseudobulk	DESeq2	LRT	0.139278100080696
554	KC_G1	Ate1	-0.0204261454732534	0.944783677456037	0.998043617620652	pseudobulk	DESeq2	LRT	0.0246676184131784
555	KC_G1	Atet	0.0947717447270565	0.556889247043437	0.998043617620652	pseudobulk	DESeq2	LRT	0.254231167807467
556	KC_G1	Atf-2	0.131752657722897	0.813220337355584	0.998043617620652	pseudobulk	DESeq2	LRT	0.0897917688794694
557	KC_G1	Atf3	-0.442209196756417	0.432046179195736	0.998043617620652	pseudobulk	DESeq2	LRT	0.364469831198882
558	KC_G1	Atf6	0.0597256084507872	0.716463492511123	0.998043617620652	pseudobulk	DESeq2	LRT	0.144805934233709

559	KC_G1	Atg1	-0.106435741435197	0.451598240985007	0.998043617620652	pseudobulk	DESeq2	LRT	0.3452477583591
560	KC_G1	Atg10	-0.755137904119269	0.584110875225483	0.998043617620652	pseudobulk	DESeq2	LRT	0.233504707805078
561	KC_G1	Atg101	0.0864500286922066	0.756833177167107	0.998043617620652	pseudobulk	DESeq2	LRT	0.120999838095719
562	KC_G1	Atg12	0.299300262706079	0.207995145431459	0.998043617620652	pseudobulk	DESeq2	LRT	0.681946801272494
563	KC_G1	Atg13	0.115575691538553	0.716729044191177	0.998043617620652	pseudobulk	DESeq2	LRT	0.144644996149434
564	KC_G1	Atg14	0.0831537169929172	0.793719617584414	0.998043617620652	pseudobulk	DESeq2	LRT	0.100332885533635
565	KC_G1	Atg16	0.498476204851814	0.317915750679371	0.998043617620652	pseudobulk	DESeq2	LRT	0.497687955055707
566	KC_G1	Atg17	0.0417952658327196	0.825201144384338	0.998043617620652	pseudobulk	DESeq2	LRT	0.0834401784213067
567	KC_G1	Atg18b	0.17884280298228	0.457802308031241	0.998043617620652	pseudobulk	DESeq2	LRT	0.339322022140494
568	KC_G1	Atg2	0.0303061530673016	0.949440654491344	0.998043617620652	pseudobulk	DESeq2	LRT	0.0225321759847961
569	KC_G1	Atg3	0.193554691059479	0.500396032101143	0.998043617620652	pseudobulk	DESeq2	LRT	0.300686142710453
570	KC_G1	Atg4a	-0.276043192552969	0.473004553233571	0.998043617620652	pseudobulk	DESeq2	LRT	0.32513467863915
571	KC_G1	Atg4b	0.164114381269954	0.748248102480616	0.998043617620652	pseudobulk	DESeq2	LRT	0.125954375835238
572	KC_G1	Atg5	0.147241035211673	0.679442057633794	0.998043617620652	pseudobulk	DESeq2	LRT	0.167847573696561
573	KC_G1	Atg6	0.127433372711819	0.756118237913237	0.998043617620652	pseudobulk	DESeq2	LRT	0.121410286432484
574	KC_G1	Atg7	-0.155408017263686	0.829197321559421	0.998043617620652	pseudobulk	DESeq2	LRT	0.0813421094168672
575	KC_G1	Atg8a	0.00857476662775531	0.934871109828892	0.998043617620652	pseudobulk	DESeq2	LRT	0.0292482609441848
576	KC_G1	Atg9	-0.881366382701399	0.160032658491111	0.998043617620652	pseudobulk	DESeq2	LRT	0.795791380124405
577	KC_G1	atilla	1.13582588427274	0.704684208645453	0.998043617620652	pseudobulk	DESeq2	LRT	0.152005460551806
578	KC_G1	atl	0.042756416433104	0.880587596091991	0.998043617620652	pseudobulk	DESeq2	LRT	0.0552274362813786
579	KC_G1	atms	0.314127423943092	0.248230158130898	0.998043617620652	pseudobulk	DESeq2	LRT	0.605145456059788
580	KC_G1	Atox1	0.119300480464056	0.382507964976261	0.998043617620652	pseudobulk	DESeq2	LRT	0.417359517087557
581	KC_G1	ATP6AP2	-0.0651972365951239	0.649318090222216	0.998043617620652	pseudobulk	DESeq2	LRT	0.187542497300246
582	KC_G1	ATP7	0.0118817857116433	0.944779444623738	0.998043617620652	pseudobulk	DESeq2	LRT	0.0246695641494059
583	KC_G1	ATP8A	0.119244252145491	0.523777039455434	0.998043617620652	pseudobulk	DESeq2	LRT	0.280853543437255
584	KC_G1	ATP8B	0.0196949175123153	0.927963429359678	0.998043617620652	pseudobulk	DESeq2	LRT	0.032469138802956
585	KC_G1	Atpalpha	-0.0971353560444387	0.405315862516824	0.998043617620652	pseudobulk	DESeq2	LRT	0.392206399296256
586	KC_G1	ATPCL	-0.123680305824625	0.465734825306689	0.998043617620652	pseudobulk	DESeq2	LRT	0.331861286497058
587	KC_G1	ATPsynB	-0.0469073998413463	0.670385987184913	0.998043617620652	pseudobulk	DESeq2	LRT	0.173675072169951
588	KC_G1	ATPsynbeta	-0.0566335563593963	0.595189099225806	0.998043617620652	pseudobulk	DESeq2	LRT	0.225345031409478
589	KC_G1	ATPsynC	-0.0569246420674424	0.606187868595726	0.998043617620652	pseudobulk	DESeq2	LRT	0.217392759249579
590	KC_G1	ATPsynCF6	-0.0715292498561399	0.565557984791527	0.998043617620652	pseudobulk	DESeq2	LRT	0.247522861677496
591	KC_G1	ATPsynCF6L	0.123111392995658	0.947458394331763	0.998043617620652	pseudobulk	DESeq2	LRT	0.0234398520816413
592	KC_G1	ATPsynD	-0.0611026818164002	0.617410913236443	0.998043617620652	pseudobulk	DESeq2	LRT	0.209425698280424
593	KC_G1	ATPsyndelta	-0.14462430307668	0.272103022293035	0.998043617620652	pseudobulk	DESeq2	LRT	0.565266634413193
594	KC_G1	ATPsynE	-0.0943859891925087	0.456262761816613	0.998043617620652	pseudobulk	DESeq2	LRT	0.340784975008845
595	KC_G1	ATPsynF	-0.096319919181623	0.420907506066159	0.998043617620652	pseudobulk	DESeq2	LRT	0.375813329386102
596	KC_G1	ATPsynG	-0.00589091415358877	0.957460143955	0.998043617620652	pseudobulk	DESeq2	LRT	0.0188792952921583
597	KC_G1	ATPsyngamma	-0.0204692535577633	0.875813298230486	0.998043617620652	pseudobulk	DESeq2	LRT	0.0575884648252443
598	KC_G1	AttA	2.18876425250967	0.402105238436886	0.998043617620652	pseudobulk	DESeq2	LRT	0.395660269077107
599	KC_G1	Atu	0.400868008621807	0.526860628036394	0.998043617620652	pseudobulk	DESeq2	LRT	0.2783042547683
600	KC_G1	Atx2	-0.0570736674580347	0.686798442264476	0.998043617620652	pseudobulk	DESeq2	LRT	0.163170698538362
601	KC_G1	Atxn7	-0.0622293648224713	0.780755019414649	0.998043617620652	pseudobulk	DESeq2	LRT	0.10748521504259
602	KC_G1	aub	1.69981218657454	0.566400560818652	0.998043617620652	pseudobulk	DESeq2	LRT	0.246876325303589
603	KC_G1	aurA	1.13582588427274	0.633424328955532	0.998043617620652	pseudobulk	DESeq2	LRT	0.198305259991224
604	KC_G1	aux	-0.0684144655727019	0.835962886471474	0.998043617620652	pseudobulk	DESeq2	LRT	0.0778130031332691
605	KC_G1	ave	0.0364997018985058	0.898759318891527	0.998043617620652	pseudobulk	DESeq2	LRT	0.0463565935523867
606	KC_G1	Aven	0.0930263617700074	0.913413878597827	0.998043617620652	pseudobulk	DESeq2	LRT	0.0393323939194475
607	KC_G1	awd	-0.0329660700140809	0.800968955521299	0.998043617620652	pseudobulk	DESeq2	LRT	0.0963843162591715
608	KC_G1	axed	-0.109901357029411	0.604949913794679	0.998043617620652	pseudobulk	DESeq2	LRT	0.21828058082363
609	KC_G1	Axn	-0.0480131540425767	0.888132826096999	0.998043617620652	pseudobulk	DESeq2	LRT	0.0515220777719268
610	KC_G1	axo	-0.513544332632883	0.0844760332456324	0.998043617620652	pseudobulk	DESeq2	LRT	1.07326648756571
611	KC_G1	Axud1	-0.165018076248272	0.503251201680239	0.998043617620652	pseudobulk	DESeq2	LRT	0.298215179415018
612	KC_G1	az2	0.581265909023747	0.288663325719443	0.998043617620652	pseudobulk	DESeq2	LRT	0.539608389134209
613	KC_G1	azot	0.763164787219597	0.794486984020146	0.998043617620652	pseudobulk	DESeq2	LRT	0.0999132133899323
614	KC_G1	b	1.12508065046033	0.900770125530981	0.998043617620652	pseudobulk	DESeq2	LRT	0.0453860258366365

615	KC_G1	B-H1	1.13582588427274	0.704684208645453	0.998043617620652	pseudobulk	DESeq2	LRT	0.152005460551806
616	KC_G1	B4	-0.214622077929902	0.227625031830146	0.998043617620652	pseudobulk	DESeq2	LRT	0.642779980467069
617	KC_G1	B52	-0.109477834454903	0.360088332211737	0.998043617620652	pseudobulk	DESeq2	LRT	0.443590950659133
618	KC_G1	bab1	2.04147257822512	0.408147672536479	0.998043617620652	pseudobulk	DESeq2	LRT	0.389182675723292
619	KC_G1	bab2	0.172845155749872	0.90730697492154	0.998043617620652	pseudobulk	DESeq2	LRT	0.0422457504714555
620	KC_G1	babo	-0.0769307210896123	0.699093945705404	0.998043617620652	pseudobulk	DESeq2	LRT	0.155464458932529
621	KC_G1	babos	-0.707615150921207	0.723087320054014	0.998043617620652	pseudobulk	DESeq2	LRT	0.14080925411936
622	KC_G1	Bacc	-0.0405811489819743	0.639535034539177	0.998043617620652	pseudobulk	DESeq2	LRT	0.194135659331886
623	KC_G1	baf	0.684556653614966	0.163028093382886	0.998043617620652	pseudobulk	DESeq2	LRT	0.787737550504816
624	KC_G1	bai	-0.183556338742341	0.493436016918336	0.998043617620652	pseudobulk	DESeq2	LRT	0.306769153634274
625	KC_G1	Balat	-0.298245620303751	0.888274138240211	0.998043617620652	pseudobulk	DESeq2	LRT	0.051452982023729
626	KC_G1	Baldspot	0.0108279228967956	0.934275929446139	0.998043617620652	pseudobulk	DESeq2	LRT	0.029524840110266
627	KC_G1	ball	-1.11843491036606	0.229893705444349	0.998043617620652	pseudobulk	DESeq2	LRT	0.638472919675521
628	KC_G1	bam	1.13582588427274	0.704684208645453	0.998043617620652	pseudobulk	DESeq2	LRT	0.152005460551806
629	KC_G1	Bap111	-0.300211226008438	0.378047222731978	0.998043617620652	pseudobulk	DESeq2	LRT	0.422453948070237
630	KC_G1	Bap170	-0.396166228963469	0.513577916677814	0.998043617620652	pseudobulk	DESeq2	LRT	0.289393658753765
631	KC_G1	Bap55	0.0446910362337628	0.875130807241418	0.998043617620652	pseudobulk	DESeq2	LRT	0.05792702741509
632	KC_G1	Bap60	0.159293052738587	0.484298331093416	0.998043617620652	pseudobulk	DESeq2	LRT	0.314887027542701
633	KC_G1	barc	-0.124236314019794	0.54989969303718	0.998043617620652	pseudobulk	DESeq2	LRT	0.259716522748192
634	KC_G1	baz	-1.68732172404213	0.425379511022056	0.998043617620652	pseudobulk	DESeq2	LRT	0.371223432309067
635	KC_G1	bbc	-0.165509205079434	0.336763743145996	0.998043617620652	pseudobulk	DESeq2	LRT	0.472674671920697
636	KC_G1	bbg	-0.794610277746993	0.815039924133852	0.998043617620652	pseudobulk	DESeq2	LRT	0.0888211171417967
637	KC_G1	BBS1	1.12508065046033	0.900770125530981	0.998043617620652	pseudobulk	DESeq2	LRT	0.0453860258366365
638	KC_G1	BBS5	0.172834960772825	0.908610676445368	0.998043617620652	pseudobulk	DESeq2	LRT	0.0416221644022337
639	KC_G1	bbx	-1.70267869630403	0.271986817687931	0.998043617620652	pseudobulk	DESeq2	LRT	0.565452144289787
640	KC_G1	bc10	0.217133400943658	0.473141316695977	0.998043617620652	pseudobulk	DESeq2	LRT	0.325009125869011
641	KC_G1	BCAS2	-0.0678237601345346	0.854060057757466	0.998043617620652	pseudobulk	DESeq2	LRT	0.0685115885199319
642	KC_G1	bchs	-0.0594667747909643	0.733467994303775	0.998043617620652	pseudobulk	DESeq2	LRT	0.134618832332661
643	KC_G1	BCL7-like	0.118288293142384	0.807849489626972	0.998043617620652	pseudobulk	DESeq2	LRT	0.0926695450586412
644	KC_G1	bcn92	0.238496376525154	0.265056802752215	0.998043617620652	pseudobulk	DESeq2	LRT	0.576661045012972
645	KC_G1	Bdbt	-0.0393169590705839	0.895638208904867	0.998043617620652	pseudobulk	DESeq2	LRT	0.0478673872205409
646	KC_G1	bdg	0.208907451323622	0.658240951206472	0.998043617620652	pseudobulk	DESeq2	LRT	0.181615102397525
647	KC_G1	bdl	-0.52059739284683	0.165645284305617	0.998043617620652	pseudobulk	DESeq2	LRT	0.780820923375143
648	KC_G1	Bdp1	0.229107449425923	0.774728551990518	0.998043617620652	pseudobulk	DESeq2	LRT	0.110850438168514
649	KC_G1	be	0.425465596079142	0.652340831992687	0.998043617620652	pseudobulk	DESeq2	LRT	0.185525436816907
650	KC_G1	BEAF-32	-0.186321578942984	0.473595364617648	0.998043617620652	pseudobulk	DESeq2	LRT	0.324592556983677
651	KC_G1	beag	-0.101113223383037	0.719673345715954	0.998043617620652	pseudobulk	DESeq2	LRT	0.14286458182389
652	KC_G1	beat-Ia	-1.18122608037593	0.461504055220097	0.998043617620652	pseudobulk	DESeq2	LRT	0.335824478490781
653	KC_G1	beat-Ib	-0.661372730573582	0.56437425993483	0.998043617620652	pseudobulk	DESeq2	LRT	0.248432801833325
654	KC_G1	beat-Ic	-0.447660703894821	0.78115731765655	0.998043617620652	pseudobulk	DESeq2	LRT	0.107261494535037
655	KC_G1	beat-IIa	0.12721499062249	0.945621294416435	0.998043617620652	pseudobulk	DESeq2	LRT	0.0242827564888097
656	KC_G1	beat-IIb	0.0984654298566411	0.959322147339849	0.998043617620652	pseudobulk	DESeq2	LRT	0.0180355290977775
657	KC_G1	beat-IIIb	0.139843650801819	0.556847235699144	0.998043617620652	pseudobulk	DESeq2	LRT	0.25426393192231
658	KC_G1	beat-IIIc	-0.272843129514691	0.841327858594814	0.998043617620652	pseudobulk	DESeq2	LRT	0.0750347302051704
659	KC_G1	beat-IV	-0.151190962425112	0.447206639717243	0.998043617620652	pseudobulk	DESeq2	LRT	0.349491757064492
660	KC_G1	beat-Va	-1.30542545798382	0.592980516839761	0.998043617620652	pseudobulk	DESeq2	LRT	0.226959575721717
661	KC_G1	beat-Vb	-0.550077512215963	0.576439405913131	0.998043617620652	pseudobulk	DESeq2	LRT	0.23924633808418
662	KC_G1	beat-Vc	-1.71430442789954	0.393403271836839	0.998043617620652	pseudobulk	DESeq2	LRT	0.405162032482834
663	KC_G1	beat-VI	-0.463458673305872	0.37535791770476	0.998043617620652	pseudobulk	DESeq2	LRT	0.425554418803271
664	KC_G1	beat-VII	0.631015747624789	0.422144157214347	0.998043617620652	pseudobulk	DESeq2	LRT	0.374539217304247
665	KC_G1	beg	0.111509325034037	0.824793415777319	0.998043617620652	pseudobulk	DESeq2	LRT	0.0836548146292335
666	KC_G1	bel	-0.0987827713177155	0.423264265878229	0.998043617620652	pseudobulk	DESeq2	LRT	0.37338839532704
667	KC_G1	Bem46	-0.213822403055003	0.632569411365742	0.998043617620652	pseudobulk	DESeq2	LRT	0.198891812730532
668	KC_G1	ben	-0.105316953686136	0.428294633178806	0.998043617620652	pseudobulk	DESeq2	LRT	0.368257367572688
669	KC_G1	Best1	-0.30900521978866	0.388488896325697	0.998043617620652	pseudobulk	DESeq2	LRT	0.410621389561631
670	KC_G1	Best2	-0.794610277746993	0.879485534599449	0.998043617620652	pseudobulk	DESeq2	LRT	0.0557712992369932

671	KC_G1	Bet1	0.127860846684691	0.627469858943275	0.998043617620652	pseudobulk	DESeq2	LRT	0.202407131056496
672	KC_G1	Bet3	0.0918050260917421	0.729192765547356	0.998043617620652	pseudobulk	DESeq2	LRT	0.137157648709393
673	KC_G1	Bet5	-0.164125256508824	0.587415635306257	0.998043617620652	pseudobulk	DESeq2	LRT	0.231054497995615
674	KC_G1	beta-Man	-0.425614289477036	0.845154747277466	0.998043617620652	pseudobulk	DESeq2	LRT	0.0730637647359404
675	KC_G1	beta-PheRS	0.657415213180209	0.175584958101312	0.998043617620652	pseudobulk	DESeq2	LRT	0.755512691695322
676	KC_G1	beta-Spec	-0.0330632633132701	0.813805827301528	0.998043617620652	pseudobulk	DESeq2	LRT	0.0894792046819998
677	KC_G1	beta'COP	0.250105514043328	0.459660909945068	0.998043617620652	pseudobulk	DESeq2	LRT	0.337562427546161
678	KC_G1	beta3GalTII	0.0673570690605041	0.939969486841423	0.998043617620652	pseudobulk	DESeq2	LRT	0.0268862441784722
679	KC_G1	beta4GalNAcTA	0.0703271095533354	0.835614653087171	0.998043617620652	pseudobulk	DESeq2	LRT	0.0779939529675732
680	KC_G1	beta4GalNAcTB	-0.435492300750321	0.417977192631782	0.998043617620652	pseudobulk	DESeq2	LRT	0.378847415316832
681	KC_G1	beta4GalT7	-0.374106694029133	0.43653837344364	0.998043617620652	pseudobulk	DESeq2	LRT	0.359977574083665
682	KC_G1	betaCOP	-0.28780183090084	0.43220973461682	0.998043617620652	pseudobulk	DESeq2	LRT	0.364305455771245
683	KC_G1	betaggt-I	-0.3527399364518	0.506533338089989	0.998043617620652	pseudobulk	DESeq2	LRT	0.295391965758691
684	KC_G1	betaggt-II	0.417738242163448	0.282554564771288	0.998043617620652	pseudobulk	DESeq2	LRT	0.548897672124823
685	KC_G1	betaGlu	-0.730022880232445	0.503528807918411	0.998043617620652	pseudobulk	DESeq2	LRT	0.297975677519299
686	KC_G1	betaNACtes2	-0.794610277746993	0.821479263487599	0.998043617620652	pseudobulk	DESeq2	LRT	0.085403394939296
687	KC_G1	betaNACtes3	-1.66808346769027	0.498191247612582	0.998043617620652	pseudobulk	DESeq2	LRT	0.302603906560549
688	KC_G1	betaNACtes4	1.13582588427274	0.633424328955532	0.998043617620652	pseudobulk	DESeq2	LRT	0.198305259991224
689	KC_G1	betaNACtes6	-0.794610277746993	0.879485534599449	0.998043617620652	pseudobulk	DESeq2	LRT	0.0557712992369932
690	KC_G1	betaTub56D	-0.240053351962385	0.129804205062663	0.998043617620652	pseudobulk	DESeq2	LRT	0.886711238152526
691	KC_G1	betaTub85D	-0.386614198206602	0.782434201673726	0.998043617620652	pseudobulk	DESeq2	LRT	0.10655217397515
692	KC_G1	betaTub97EF	0.688312126427135	0.483321050510327	0.998043617620652	pseudobulk	DESeq2	LRT	0.315764289236485
693	KC_G1	Bgb	-0.0201804787853941	0.918714221740664	0.998043617620652	pseudobulk	DESeq2	LRT	0.036819560673091
694	KC_G1	BHD	0.295820256417478	0.631345437543517	0.998043617620652	pseudobulk	DESeq2	LRT	0.199732953640636
695	KC_G1	bi	-2.72388227859124	0.250667797322887	0.998043617620652	pseudobulk	DESeq2	LRT	0.600901455205215
696	KC_G1	bic	-0.190343424352761	0.0736852439131	0.998043617620652	pseudobulk	DESeq2	LRT	1.13261947453917
697	KC_G1	BicD	0.22479501616922	0.115278002075109	0.998043617620652	pseudobulk	DESeq2	LRT	0.938253559044123
698	KC_G1	bif	-0.0835700073776621	0.669768079385898	0.998043617620652	pseudobulk	DESeq2	LRT	0.174075554432621
699	KC_G1	bigmax	-0.371721127377095	0.422094320915947	0.998043617620652	pseudobulk	DESeq2	LRT	0.374590491037865
700	KC_G1	Bili	-0.141623929351949	0.439754840138393	0.998043617620652	pseudobulk	DESeq2	LRT	0.356789371804946
701	KC_G1	Bin1	-0.0780900034411897	0.793614759035673	0.998043617620652	pseudobulk	DESeq2	LRT	0.100390264104698
702	KC_G1	bin3	-0.137862447012463	0.407965194788235	0.998043617620652	pseudobulk	DESeq2	LRT	0.38937688680273
703	KC_G1	bip1	0.332704861715976	0.83017748257433	0.998043617620652	pseudobulk	DESeq2	LRT	0.0808290504399582
704	KC_G1	bip2	-0.0739400036546751	0.557497004568185	0.998043617620652	pseudobulk	DESeq2	LRT	0.253757461738103
705	KC_G1	Bka	0.279266000470052	0.513022919569335	0.998043617620652	pseudobulk	DESeq2	LRT	0.28986323211988
706	KC_G1	Blimp-1	-0.276960049943006	0.057823776442198	0.998043617620652	pseudobulk	DESeq2	LRT	1.23789354818162
707	KC_G1	Blm	0.156559326498369	0.814999367160298	0.998043617620652	pseudobulk	DESeq2	LRT	0.0888427284856644
708	KC_G1	Blos1	0.243555499571434	0.389478092403867	0.998043617620652	pseudobulk	DESeq2	LRT	0.40951696575956
709	KC_G1	Blos2	0.0596098991531972	0.88869922536323	0.998043617620652	pseudobulk	DESeq2	LRT	0.0512451983853717
710	KC_G1	Blos3	-0.0523594787220867	0.852329939835349	0.998043617620652	pseudobulk	DESeq2	LRT	0.0693922558060168
711	KC_G1	Blos4	-0.241389935366892	0.615391485594402	0.998043617620652	pseudobulk	DESeq2	LRT	0.210848516838883
712	KC_G1	blot	0.184618432620888	0.899541489918007	0.998043617620652	pseudobulk	DESeq2	LRT	0.0459788007154356
713	KC_G1	blow	-1.06136067336851	0.608964828463263	0.998043617620652	pseudobulk	DESeq2	LRT	0.215407789871927
714	KC_G1	blp	0.161398259000867	0.667025880766018	0.998043617620652	pseudobulk	DESeq2	LRT	0.175857315025263
715	KC_G1	blw	0.0141087288376081	0.904676339647959	0.998043617620652	pseudobulk	DESeq2	LRT	0.0435067678072115
716	KC_G1	bma	-0.0896097207318726	0.504496045279746	0.998043617620652	pseudobulk	DESeq2	LRT	0.2971422338273
717	KC_G1	Bmcp	0.296203368132054	0.366983104570975	0.998043617620652	pseudobulk	DESeq2	LRT	0.435353929645728
718	KC_G1	bmm	-0.245184352341483	0.627346119943815	0.998043617620652	pseudobulk	DESeq2	LRT	0.202492783719102
719	KC_G1	bnb	0.10987551767431	0.664542104232254	0.998043617620652	pseudobulk	DESeq2	LRT	0.177477497706279
720	KC_G1	boca	0.200928751205474	0.496795456794369	0.998043617620652	pseudobulk	DESeq2	LRT	0.303822384446126
721	KC_G1	bocks	0.380706693642824	0.419248576410673	0.998043617620652	pseudobulk	DESeq2	LRT	0.377528403398177
722	KC_G1	BOD1	0.128428797661024	0.702659689156747	0.998043617620652	pseudobulk	DESeq2	LRT	0.153254960763263
723	KC_G1	bol	0.1096839832605	0.384745895623436	0.998043617620652	pseudobulk	DESeq2	LRT	0.414826004420723
724	KC_G1	Bom068	-0.794610277746993	0.879485534599449	0.998043617620652	pseudobulk	DESeq2	LRT	0.0557712992369932
725	KC_G1	Bom107	-0.794610277746993	0.821479263487599	0.998043617620652	pseudobulk	DESeq2	LRT	0.085403394939296
726	KC_G1	Bom3	0.541402693285434	0.822604845289713	0.998043617620652	pseudobulk	DESeq2	LRT	0.0848087367471106

727	KC_G1	Bom836	-0.794610277746993	0.821479263487599	0.998043617620652	pseudobulk	DESeq2	LRT	0.085403394939296
728	KC_G1	bon	-0.135617570742297	0.510252451666196	0.998043617620652	pseudobulk	DESeq2	LRT	0.292214899904313
729	KC_G1	bonsai	-0.396781421803602	0.113976530699063	0.998043617620652	pseudobulk	DESeq2	LRT	0.943184566533832
730	KC_G1	bor	-0.0362646665007442	0.920820471283408	0.998043617620652	pseudobulk	DESeq2	LRT	0.0358250342234042
731	KC_G1	BORCS6	-0.286822075506953	0.430776605654004	0.998043617620652	pseudobulk	DESeq2	LRT	0.365747890126265
732	KC_G1	botv	0.589996791998383	0.231242832503463	0.998043617620652	pseudobulk	DESeq2	LRT	0.635931719572438
733	KC_G1	bou	-0.696476476856433	0.490553536459344	0.998043617620652	pseudobulk	DESeq2	LRT	0.309313589058541
734	KC_G1	br	0.569647355621788	0.617659087530122	0.998043617620652	pseudobulk	DESeq2	LRT	0.209251164484479
735	KC_G1	Br140	-0.0249474251116909	0.937813512858715	0.998043617620652	pseudobulk	DESeq2	LRT	0.0278835138474416
736	KC_G1	brat	-0.214597366017086	0.069524680092839	0.998043617620652	pseudobulk	DESeq2	LRT	1.15786100079871
737	KC_G1	Brca2	-0.333208873692188	0.611932406095085	0.998043617620652	pseudobulk	DESeq2	LRT	0.213296547268107
738	KC_G1	Brd8	-0.418316755282282	0.52708801840212	0.998043617620652	pseudobulk	DESeq2	LRT	0.278116855917439
739	KC_G1	Bre1	-0.0737905772153928	0.793670191278666	0.998043617620652	pseudobulk	DESeq2	LRT	0.100359930651015
740	KC_G1	Brf	0.210186731623035	0.609317613677741	0.998043617620652	pseudobulk	DESeq2	LRT	0.215156267448344
741	KC_G1	brk	-0.557341824171769	0.683495504577138	0.998043617620652	pseudobulk	DESeq2	LRT	0.165264337487912
742	KC_G1	brm	-0.147595487293453	0.581427703256578	0.998043617620652	pseudobulk	DESeq2	LRT	0.235504279265231
743	KC_G1	Brms1	0.0439578803391243	0.869878309780701	0.998043617620652	pseudobulk	DESeq2	LRT	0.0605414980563364
744	KC_G1	brn	-0.550648786386358	0.693357853739098	0.998043617620652	pseudobulk	DESeq2	LRT	0.159042560782937
745	KC_G1	brp	-0.17652815383439	0.0732432006317871	0.998043617620652	pseudobulk	DESeq2	LRT	1.13523268585652
746	KC_G1	bru1	-0.214509228856757	0.145526499847742	0.998043617620652	pseudobulk	DESeq2	LRT	0.837057916026992
747	KC_G1	bru2	0.111901476437524	0.398275133050388	0.998043617620652	pseudobulk	DESeq2	LRT	0.399816808619886
748	KC_G1	bru3	-0.157108058354254	0.114017196279905	0.998043617620652	pseudobulk	DESeq2	LRT	0.943029642643734
749	KC_G1	Bruce	-0.0321172137144095	0.875603028827528	0.998043617620652	pseudobulk	DESeq2	LRT	0.0576927448190364
750	KC_G1	brun	-0.609248829280893	0.234958034070345	0.998043617620652	pseudobulk	DESeq2	LRT	0.629009700278127
751	KC_G1	BRWD3	-0.0788862194458023	0.789670597659302	0.998043617620652	pseudobulk	DESeq2	LRT	0.102554032066766
752	KC_G1	brwl	0.0711950523811602	0.838191383453959	0.998043617620652	pseudobulk	DESeq2	LRT	0.0766568079946978
753	KC_G1	bs	0.071785269662891	0.875594555825305	0.998043617620652	pseudobulk	DESeq2	LRT	0.0576969474037622
754	KC_G1	bsf	-0.258932778379017	0.47414047686691	0.998043617620652	pseudobulk	DESeq2	LRT	0.324092967841606
755	KC_G1	Bsg	-0.0416359005405368	0.730661016469193	0.998043617620652	pseudobulk	DESeq2	LRT	0.136284063291098
756	KC_G1	Bsg25D	0.271015678678834	0.361853215178744	0.998043617620652	pseudobulk	DESeq2	LRT	0.441467564178778
757	KC_G1	bt	0.242907451224626	0.764786657571825	0.998043617620652	pseudobulk	DESeq2	LRT	0.116459697344766
758	KC_G1	BTBD9	-0.057606192004319	0.911125907418107	0.998043617620652	pseudobulk	DESeq2	LRT	0.040421604236007
759	KC_G1	BtbVII	-0.0351163895161682	0.80582549438858	0.998043617620652	pseudobulk	DESeq2	LRT	0.0937589966930731
760	KC_G1	btd	1.12508065046033	0.900770125530981	0.998043617620652	pseudobulk	DESeq2	LRT	0.0453860258366365
761	KC_G1	Btk29A	0.271795986553001	0.813947744062564	0.998043617620652	pseudobulk	DESeq2	LRT	0.0894034761842125
762	KC_G1	btsz	-0.118395711606265	0.325686943727955	0.998043617620652	pseudobulk	DESeq2	LRT	0.487199651261832
763	KC_G1	btv	1.13582588427274	0.704684208645453	0.998043617620652	pseudobulk	DESeq2	LRT	0.152005460551806
764	KC_G1	btz	0.300992333656256	0.449509911781016	0.998043617620652	pseudobulk	DESeq2	LRT	0.347260727547344
765	KC_G1	Bub1	0.133279122858575	0.750409984748078	0.998043617620652	pseudobulk	DESeq2	LRT	0.124701395988074
766	KC_G1	Bub3	-0.288159125029788	0.248592541856103	0.998043617620652	pseudobulk	DESeq2	LRT	0.604511904975658
767	KC_G1	BubR1	1.26590296883009	0.529670137574823	0.998043617620652	pseudobulk	DESeq2	LRT	0.275994511587826
768	KC_G1	Buffy	1.12508065046033	0.900770125530981	0.998043617620652	pseudobulk	DESeq2	LRT	0.0453860258366365
769	KC_G1	bug	-0.0333659853002712	0.949917471301928	0.998043617620652	pseudobulk	DESeq2	LRT	0.0223141245165097
770	KC_G1	Bug22	0.325548362101126	0.304884293351438	0.998043617620652	pseudobulk	DESeq2	LRT	0.515864948499502
771	KC_G1	BuGZ	-0.00283524928539782	0.945662366888222	0.998043617620652	pseudobulk	DESeq2	LRT	0.0242638935882061
772	KC_G1	bun	-0.0360726914364008	0.727096264100036	0.998043617620652	pseudobulk	DESeq2	LRT	0.138408086802851
773	KC_G1	bur	-0.0351793656120706	0.900453753126815	0.998043617620652	pseudobulk	DESeq2	LRT	0.0455385874281154
774	KC_G1	bw	-1.67593316621313	0.495877122847873	0.998043617620652	pseudobulk	DESeq2	LRT	0.304625927300966
775	KC_G1	bwa	-0.704743723785135	0.292090374664307	0.998043617620652	pseudobulk	DESeq2	LRT	0.534482754218005
776	KC_G1	Bx	0.0355965423999218	0.731623553137461	0.998043617620652	pseudobulk	DESeq2	LRT	0.135712321739937
777	KC_G1	Bx42	0.031009770274344	0.899228507015306	0.998043617620652	pseudobulk	DESeq2	LRT	0.0461299337109561
778	KC_G1	by	-0.718676691072112	0.739205546127982	0.998043617620652	pseudobulk	DESeq2	LRT	0.131234783341328
779	KC_G1	bys	0.42962044299195	0.458974335397815	0.998043617620652	pseudobulk	DESeq2	LRT	0.338211598355398
780	KC_G1	c(3)G	-0.162116592667723	0.877879739758788	0.998043617620652	pseudobulk	DESeq2	LRT	0.056564973770838
781	KC_G1	c11.1	0.17221948003018	0.607096332019266	0.998043617620652	pseudobulk	DESeq2	LRT	0.216742391058207
782	KC_G1	c12.1	0.0737483418491774	0.760966710217229	0.998043617620652	pseudobulk	DESeq2	LRT	0.118634341764502

783	KC_G1	c12.2	-0.0953176598543454	0.827086176688381	0.998043617620652	pseudobulk	DESeq2	LRT	0.0824492375932864
784	KC_G1	C1GalTA	-0.109477352287471	0.623957234871392	0.998043617620652	pseudobulk	DESeq2	LRT	0.204845175214739
785	KC_G1	C3G	-0.0709411975941592	0.649305837734901	0.998043617620652	pseudobulk	DESeq2	LRT	0.18755069248431
786	KC_G1	C901	1.59649238946975	0.275318044813397	0.998043617620652	pseudobulk	DESeq2	LRT	0.560165323274079
787	KC_G1	ca	-0.0488315087039672	0.879450782964804	0.998043617620652	pseudobulk	DESeq2	LRT	0.0557884601120228
788	KC_G1	Ca-alpha1D	-0.128971065929225	0.292680565557834	0.998043617620652	pseudobulk	DESeq2	LRT	0.533606114434021
789	KC_G1	Ca-alpha1T	0.503345148516997	0.128756872397272	0.998043617620652	pseudobulk	DESeq2	LRT	0.890229581202822
790	KC_G1	Ca-beta	-0.0703767624562363	0.544998670875105	0.998043617620652	pseudobulk	DESeq2	LRT	0.263604556865214
791	KC_G1	Ca-Ma2d	-0.341681237513521	0.0350053246750062	0.998043617620652	pseudobulk	DESeq2	LRT	1.45586588990435
792	KC_G1	CaBP1	0.223963739755235	0.68332595605067	0.998043617620652	pseudobulk	DESeq2	LRT	0.165372082342611
793	KC_G1	cac	-0.116876294231464	0.259407099997441	0.998043617620652	pseudobulk	DESeq2	LRT	0.586018141407251
794	KC_G1	cact	-0.054342257868894	0.718921799112489	0.998043617620652	pseudobulk	DESeq2	LRT	0.143318347532253
795	KC_G1	cactin	-0.267564471088059	0.546979583337989	0.998043617620652	pseudobulk	DESeq2	LRT	0.262028883920922
796	KC_G1	Cad86C	1.13582588427274	0.633424328955532	0.998043617620652	pseudobulk	DESeq2	LRT	0.198305259991224
797	KC_G1	Cad87A	0.860878589929652	0.14499280236392	0.998043617620652	pseudobulk	DESeq2	LRT	0.838653556187218
798	KC_G1	Cad96Ca	-1.15339046372897	0.369189822086965	0.998043617620652	pseudobulk	DESeq2	LRT	0.432750280214913
799	KC_G1	Cad96Cb	-0.239514771750807	0.90566796171396	0.998043617620652	pseudobulk	DESeq2	LRT	0.0430309952879792
800	KC_G1	CadN	-0.179265025622772	0.227496274840384	0.998043617620652	pseudobulk	DESeq2	LRT	0.643025710345379
801	KC_G1	CadN2	-0.784444463566244	0.543593603474333	0.998043617620652	pseudobulk	DESeq2	LRT	0.264725662300369
802	KC_G1	Cadps	-0.0640521474380554	0.558010662837571	0.998043617620652	pseudobulk	DESeq2	LRT	0.253357502196339
803	KC_G1	Caf1-105	-0.885197819185439	0.234200589206437	0.998043617620652	pseudobulk	DESeq2	LRT	0.630412016655703
804	KC_G1	Caf1-180	1.13582588427274	0.704684208645453	0.998043617620652	pseudobulk	DESeq2	LRT	0.152005460551806
805	KC_G1	Caf1-55	0.0337562767746913	0.910280939547092	0.998043617620652	pseudobulk	DESeq2	LRT	0.0408245509040771
806	KC_G1	cag	0.21766908601816	0.587289521821202	0.998043617620652	pseudobulk	DESeq2	LRT	0.231147747591961
807	KC_G1	CAH1	-0.327297117894943	0.0977828116871019	0.998043617620652	pseudobulk	DESeq2	LRT	1.00973747901085
808	KC_G1	CAH2	-0.046351133097069	0.917379939581994	0.998043617620652	pseudobulk	DESeq2	LRT	0.0374507608511893
809	KC_G1	CAH3	0.0869007499887413	0.693214541375948	0.998043617620652	pseudobulk	DESeq2	LRT	0.159132335783429
810	KC_G1	CAH7	-0.212033748835506	0.383544599597139	0.998043617620652	pseudobulk	DESeq2	LRT	0.416184127848694
811	KC_G1	CAH8	-0.343566162881417	0.350595999770043	0.998043617620652	pseudobulk	DESeq2	LRT	0.455193043426786
812	KC_G1	CAHbeta	0.673966965875621	0.313218771506587	0.998043617620652	pseudobulk	DESeq2	LRT	0.504152218142085
813	KC_G1	cal1	-0.153611898294448	0.689148701064463	0.998043617620652	pseudobulk	DESeq2	LRT	0.16168705808676
814	KC_G1	CalpA	-0.0855379978320914	0.85175547610297	0.998043617620652	pseudobulk	DESeq2	LRT	0.0696850655867277
815	KC_G1	CalpB	0.110214163426226	0.789980269263546	0.998043617620652	pseudobulk	DESeq2	LRT	0.102383755617122
816	KC_G1	Calr	-0.226903618501145	0.248846237364419	0.998043617620652	pseudobulk	DESeq2	LRT	0.604068921543321
817	KC_G1	Cals	0.0168930704291592	0.868982406976526	0.998043617620652	pseudobulk	DESeq2	LRT	0.0609890159913309
818	KC_G1	Calx	0.0778695228241535	0.593814765835097	0.998043617620652	pseudobulk	DESeq2	LRT	0.226349007411963
819	KC_G1	calypso	0.421338077926431	0.405135442522287	0.998043617620652	pseudobulk	DESeq2	LRT	0.392399761707692
820	KC_G1	Cam	0.0468760749163488	0.691468657998751	0.998043617620652	pseudobulk	DESeq2	LRT	0.160227500249791
821	KC_G1	CaMKI	-0.0953915767398171	0.439796691596429	0.998043617620652	pseudobulk	DESeq2	LRT	0.356748041975727
822	KC_G1	CaMKII	-0.0365400924277421	0.702095123160162	0.998043617620652	pseudobulk	DESeq2	LRT	0.153604043618205
823	KC_G1	Camp	0.185632591936927	0.661313927576485	0.998043617620652	pseudobulk	DESeq2	LRT	0.179592330757504
824	KC_G1	Camta	-0.0765862157468005	0.761135496342207	0.998043617620652	pseudobulk	DESeq2	LRT	0.118538023813336
825	KC_G1	can	1.13582588427274	0.633424328955532	0.998043617620652	pseudobulk	DESeq2	LRT	0.198305259991224
826	KC_G1	cana	-0.794610277746993	0.815039924133852	0.998043617620652	pseudobulk	DESeq2	LRT	0.0888211171417967
827	KC_G1	CanA-14F	-0.0909983090892024	0.426834472073698	0.998043617620652	pseudobulk	DESeq2	LRT	0.369740513270941
828	KC_G1	CanB	-0.0174659353071099	0.882024543851554	0.998043617620652	pseudobulk	DESeq2	LRT	0.0545193297083308
829	KC_G1	CanB2	0.14692109238978	0.478390008231186	0.998043617620652	pseudobulk	DESeq2	LRT	0.320217899703961
830	KC_G1	Cand1	-0.230844284505381	0.508585825361287	0.998043617620652	pseudobulk	DESeq2	LRT	0.293635748079226
831	KC_G1	CAP	-0.901442001026738	0.442174283224981	0.998043617620652	pseudobulk	DESeq2	LRT	0.354406519510042
832	KC_G1	Cap-D2	1.90250740646012	0.460774913678782	0.998043617620652	pseudobulk	DESeq2	LRT	0.336511173568024
833	KC_G1	Cap-D3	0.0298383293599308	0.967808846849632	0.998043617620652	pseudobulk	DESeq2	LRT	0.0142104122744947
834	KC_G1	Cap-G	-0.527804440361887	0.458401668882969	0.998043617620652	pseudobulk	DESeq2	LRT	0.338753809920804
835	KC_G1	Cap-H2	-0.222726145413397	0.603209819763183	0.998043617620652	pseudobulk	DESeq2	LRT	0.219531597121287
836	KC_G1	Capa	0.437611812806754	0.773900533030561	0.998043617620652	pseudobulk	DESeq2	LRT	0.111314854216187
837	KC_G1	Caper	-0.0571784345489422	0.617057433551773	0.998043617620652	pseudobulk	DESeq2	LRT	0.209674411471186
838	KC_G1	Capr	0.0857095562760101	0.698993624567744	0.998043617620652	pseudobulk	DESeq2	LRT	0.155526785381179

839	KC_G1	caps	0.0227376400252796	0.885166340606361	0.998043617620652	pseudobulk	DESeq2	LRT	0.0529751089414045
840	KC_G1	capt	0.0100970615345227	0.960054053820439	0.998043617620652	pseudobulk	DESeq2	LRT	0.0177043142364036
841	KC_G1	capu	-0.199635885620072	0.413566432309089	0.998043617620652	pseudobulk	DESeq2	LRT	0.383454718595326
842	KC_G1	car	-0.334527615539197	0.393846871634488	0.998043617620652	pseudobulk	DESeq2	LRT	0.404672599825933
843	KC_G1	CARPB	0.37154358601471	0.417649023280815	0.998043617620652	pseudobulk	DESeq2	LRT	0.379188529903396
844	KC_G1	CarT	0.763164787219597	0.794486984020146	0.998043617620652	pseudobulk	DESeq2	LRT	0.0999132133899323
845	KC_G1	CASK	-0.0653481869491844	0.566204040616955	0.998043617620652	pseudobulk	DESeq2	LRT	0.247027035692594
846	KC_G1	casp	-0.0437957822110938	0.873524385031011	0.998043617620652	pseudobulk	DESeq2	LRT	0.0587249668841526
847	KC_G1	cass	-0.0167639318088614	0.904524400894547	0.998043617620652	pseudobulk	DESeq2	LRT	0.0435797129048677
848	KC_G1	Cat	0.493979580582422	0.22980581061504	0.998043617620652	pseudobulk	DESeq2	LRT	0.63863899441995
849	KC_G1	cathD	-0.180427138799314	0.539650880575846	0.998043617620652	pseudobulk	DESeq2	LRT	0.267887109942338
850	KC_G1	Catsup	-0.122573023477191	0.818824771149273	0.998043617620652	pseudobulk	DESeq2	LRT	0.0868090275010899
851	KC_G1	cav	-0.774875445618726	0.486781073813365	0.998043617620652	pseudobulk	DESeq2	LRT	0.312666315606735
852	KC_G1	caz	-0.146590113982973	0.452555343052526	0.998043617620652	pseudobulk	DESeq2	LRT	0.344328303281693
853	KC_G1	cbc	-0.214251763357858	0.737655637502617	0.998043617620652	pseudobulk	DESeq2	LRT	0.132146334160088
854	KC_G1	Cbl	0.255585472129594	0.187044192831742	0.998043617620652	pseudobulk	DESeq2	LRT	0.728055770813973
855	KC_G1	Cbp20	0.021708871174234	0.931061480837875	0.998043617620652	pseudobulk	DESeq2	LRT	0.0310216402785444
856	KC_G1	Cbp53E	-0.0130171473304896	0.937161154881693	0.998043617620652	pseudobulk	DESeq2	LRT	0.0281857211102415
857	KC_G1	Cbp80	-0.321212331934705	0.427343111489516	0.998043617620652	pseudobulk	DESeq2	LRT	0.369223292210758
858	KC_G1	cbs	0.17790513984015	0.657726659680924	0.998043617620652	pseudobulk	DESeq2	LRT	0.181954554495218
859	KC_G1	Cbs	-0.506575531603688	0.26901349688466	0.998043617620652	pseudobulk	DESeq2	LRT	0.570225930125904
860	KC_G1	cbt	-0.264810709465367	0.490650670959801	0.998043617620652	pseudobulk	DESeq2	LRT	0.309227602925645
861	KC_G1	CCAP	-0.794610277746993	0.879485534599449	0.998043617620652	pseudobulk	DESeq2	LRT	0.0557712992369932
862	KC_G1	CCDC53	-0.312495356169358	0.613213241255841	0.998043617620652	pseudobulk	DESeq2	LRT	0.212388475894868
863	KC_G1	Ccdc58	-0.0413290332336496	0.915034460408208	0.998043617620652	pseudobulk	DESeq2	LRT	0.0385625499955033
864	KC_G1	Cchl	0.182812239132516	0.328309795498096	0.998043617620652	pseudobulk	DESeq2	LRT	0.483716159404968
865	KC_G1	CCKLR-17D1	-0.0667470814465943	0.797281458751657	0.998043617620652	pseudobulk	DESeq2	LRT	0.0983883355623224
866	KC_G1	CCKLR-17D3	0.336367984852865	0.317810353885524	0.998043617620652	pseudobulk	DESeq2	LRT	0.497831958097485
867	KC_G1	Ccm3	0.0526978003302785	0.82196884705012	0.998043617620652	pseudobulk	DESeq2	LRT	0.0851446420835658
868	KC_G1	Ccp84Ag	0.173195945230598	0.976215465741119	0.998043617620652	pseudobulk	DESeq2	LRT	0.0104543162934892
869	KC_G1	Ccs	0.774750097873375	0.104225787884846	0.998043617620652	pseudobulk	DESeq2	LRT	0.982024813181778
870	KC_G1	CCT1	-0.0749310460622413	0.832888393148138	0.998043617620652	pseudobulk	DESeq2	LRT	0.0794131900545108
871	KC_G1	CCT2	-0.0832003561563361	0.770370477623298	0.998043617620652	pseudobulk	DESeq2	LRT	0.113300368732645
872	KC_G1	CCT3	-0.161476282998003	0.579897780737736	0.998043617620652	pseudobulk	DESeq2	LRT	0.236648553288687
873	KC_G1	CCT4	-0.0980955360438024	0.721601228544418	0.998043617620652	pseudobulk	DESeq2	LRT	0.1417027360681
874	KC_G1	CCT5	-0.263111868233174	0.521545675784017	0.998043617620652	pseudobulk	DESeq2	LRT	0.282707651048953
875	KC_G1	CCT6	-0.195691245645724	0.548874601361581	0.998043617620652	pseudobulk	DESeq2	LRT	0.260526865308241
876	KC_G1	CCT7	-0.513097491188573	0.420674796280658	0.998043617620652	pseudobulk	DESeq2	LRT	0.376053506913653
877	KC_G1	CCT8	-0.0346974462945233	0.913134202122977	0.998043617620652	pseudobulk	DESeq2	LRT	0.0394653900907248
878	KC_G1	Ccz1	0.302821704552111	0.516895227121005	0.998043617620652	pseudobulk	DESeq2	LRT	0.286597477978528
879	KC_G1	CD98hc	-0.099059673534602	0.618460521609383	0.998043617620652	pseudobulk	DESeq2	LRT	0.208688017610296
880	KC_G1	Cda5	-0.177303014163013	0.672083774043231	0.998043617620652	pseudobulk	DESeq2	LRT	0.172576589539784
881	KC_G1	CDase	-0.634162396035478	0.39606361022317	0.998043617620652	pseudobulk	DESeq2	LRT	0.402235058139186
882	KC_G1	cdc14	-0.21613444352725	0.255770727489089	0.998043617620652	pseudobulk	DESeq2	LRT	0.592149161254614
883	KC_G1	Cdc16	-0.0839442792667802	0.917130214735324	0.998043617620652	pseudobulk	DESeq2	LRT	0.0375689985434887
884	KC_G1	Cdc23	-0.468508970898516	0.574505969482892	0.998043617620652	pseudobulk	DESeq2	LRT	0.240705454356008
885	KC_G1	Cdc27	-0.0599210644027356	0.806387318446755	0.998043617620652	pseudobulk	DESeq2	LRT	0.0934563107248185
886	KC_G1	Cdc2rk	0.051857442855107	0.911525747267468	0.998043617620652	pseudobulk	DESeq2	LRT	0.040231059605538
887	KC_G1	Cdc37	-0.00996889661025993	0.954988335498113	0.998043617620652	pseudobulk	DESeq2	LRT	0.0200019329814279
888	KC_G1	Cdc42	-0.112859616355767	0.58511957684052	0.998043617620652	pseudobulk	DESeq2	LRT	0.23275537108843
889	KC_G1	CDC45L	0.551647219045219	0.503375109276932	0.998043617620652	pseudobulk	DESeq2	LRT	0.298108263104177
890	KC_G1	Cdc5	-0.411068993341204	0.310635668318391	0.998043617620652	pseudobulk	DESeq2	LRT	0.507748678470689
891	KC_G1	CDC50	-0.23576117491413	0.405327672361156	0.998043617620652	pseudobulk	DESeq2	LRT	0.392193745275085
892	KC_G1	Cdc7	-0.050046605741117	0.91599033315262	0.998043617620652	pseudobulk	DESeq2	LRT	0.038109109607944
893	KC_G1	Cdep	-0.0732608259564336	0.635403663821452	0.998043617620652	pseudobulk	DESeq2	LRT	0.196950285302306
894	KC_G1	CdGAPr	0.016018262648314	0.953384552413497	0.998043617620652	pseudobulk	DESeq2	LRT	0.0207318891783577

895	KC_G1	cdi	0.228326916138098	0.24409848044431	0.998043617620652	pseudobulk	DESeq2	LRT	0.612434924133426
896	KC_G1	Cdk12	-0.14037306760702	0.546807165053288	0.998043617620652	pseudobulk	DESeq2	LRT	0.262165803316508
897	KC_G1	Cdk2	0.396263052220205	0.435037894201049	0.998043617620652	pseudobulk	DESeq2	LRT	0.361472911951954
898	KC_G1	CDK2AP1	-0.321095564261382	0.260779918344781	0.998043617620652	pseudobulk	DESeq2	LRT	0.583725854994717
899	KC_G1	Cdk4	0.0669206423712383	0.97167197713177	0.998043617620652	pseudobulk	DESeq2	LRT	0.0124803220746587
900	KC_G1	Cdk5	-0.089691833364013	0.780292206487018	0.998043617620652	pseudobulk	DESeq2	LRT	0.107742730770721
901	KC_G1	Cdk5alpha	-0.0353311230208036	0.778328774034286	0.998043617620652	pseudobulk	DESeq2	LRT	0.108836913822949
902	KC_G1	Cdk7	0.500171137100044	0.347313307473252	0.998043617620652	pseudobulk	DESeq2	LRT	0.45927857617245
903	KC_G1	Cdk8	0.408007144731585	0.606296114016955	0.998043617620652	pseudobulk	DESeq2	LRT	0.217315215315094
904	KC_G1	Cdk9	0.433354817710034	0.302482044092796	0.998043617620652	pseudobulk	DESeq2	LRT	0.519300400789589
905	KC_G1	cdm	0.011063154333511	0.960557101148841	0.998043617620652	pseudobulk	DESeq2	LRT	0.0174768130232695
906	KC_G1	Cds	0.0567153026712343	0.729910486719359	0.998043617620652	pseudobulk	DESeq2	LRT	0.136730396739115
907	KC_G1	Ced-12	0.219362899225343	0.663772878332224	0.998043617620652	pseudobulk	DESeq2	LRT	0.177980496777207
908	KC_G1	ced-6	-0.413842257547672	0.56372174087469	0.998043617620652	pseudobulk	DESeq2	LRT	0.248935215561513
909	KC_G1	Cen	-0.186720086473752	0.497107665410972	0.998043617620652	pseudobulk	DESeq2	LRT	0.303549539979046
910	KC_G1	CenB1A	-0.354768811213515	0.399626109426506	0.998043617620652	pseudobulk	DESeq2	LRT	0.398346145047085
911	KC_G1	CenG1A	-0.282330008837965	0.287482426838901	0.998043617620652	pseudobulk	DESeq2	LRT	0.541388697618356
912	KC_G1	Cenp-C	-0.794610277746993	0.821479263487599	0.998043617620652	pseudobulk	DESeq2	LRT	0.085403394939296
913	KC_G1	Cep135	1.13582588427274	0.704684208645453	0.998043617620652	pseudobulk	DESeq2	LRT	0.152005460551806
914	KC_G1	cep290	3.84576131503405	0.0523113935289892	0.998043617620652	pseudobulk	DESeq2	LRT	1.2814037105998
915	KC_G1	Cep97	0.00860996107820739	0.964739762098008	0.998043617620652	pseudobulk	DESeq2	LRT	0.0155898215028041
916	KC_G1	cer	0.112710218220809	0.788028754578314	0.998043617620652	pseudobulk	DESeq2	LRT	0.103457935141345
917	KC_G1	Cerk	-0.104255266415219	0.749721883951747	0.998043617620652	pseudobulk	DESeq2	LRT	0.125099812162057
918	KC_G1	cert	-0.331102463645616	0.395475851309099	0.998043617620652	pseudobulk	DESeq2	LRT	0.402880030404983
919	KC_G1	cerv	0.0373940623221593	0.941130776366551	0.998043617620652	pseudobulk	DESeq2	LRT	0.0263500242793096
920	KC_G1	Cf2	-0.188670963666732	0.326259384212801	0.998043617620652	pseudobulk	DESeq2	LRT	0.486436987843015
921	KC_G1	Cfp1	0.0825766464932348	0.818149038021005	0.998043617620652	pseudobulk	DESeq2	LRT	0.087167575919972
922	KC_G1	cg	-0.0126573695718369	0.921898098243434	0.998043617620652	pseudobulk	DESeq2	LRT	0.0353170809163929
923	KC_G1	CG10011	0.136354303391166	0.505329440408793	0.998043617620652	pseudobulk	DESeq2	LRT	0.296425399100723
924	KC_G1	CG10019	-0.0977097309712541	0.688899869391745	0.998043617620652	pseudobulk	DESeq2	LRT	0.161843897585564
925	KC_G1	CG10035	0.0592732950682391	0.928443181076855	0.998043617620652	pseudobulk	DESeq2	LRT	0.0322446690915031
926	KC_G1	CG10038	-0.276493411170116	0.686836840153765	0.998043617620652	pseudobulk	DESeq2	LRT	0.163146418452298
927	KC_G1	CG10041	-1.72343437992478	0.512120560562093	0.998043617620652	pseudobulk	DESeq2	LRT	0.290627787806665
928	KC_G1	CG10050	-0.489721899687746	0.613149638953277	0.998043617620652	pseudobulk	DESeq2	LRT	0.212433523130903
929	KC_G1	CG10051	-0.794610277746993	0.815039924133852	0.998043617620652	pseudobulk	DESeq2	LRT	0.0888211171417967
930	KC_G1	CG10053	-0.14684746547212	0.786949116907134	0.998043617620652	pseudobulk	DESeq2	LRT	0.104053347642029
931	KC_G1	CG10055	-0.212671105148487	0.604529573693858	0.998043617620652	pseudobulk	DESeq2	LRT	0.218582448520515
932	KC_G1	CG10063	1.13582588427274	0.633424328955532	0.998043617620652	pseudobulk	DESeq2	LRT	0.198305259991224
933	KC_G1	CG10068	-0.070937819686456	0.89241614295695	0.998043617620652	pseudobulk	DESeq2	LRT	0.0494325823441443
934	KC_G1	CG10075	0.175425943705161	0.611169964491374	0.998043617620652	pseudobulk	DESeq2	LRT	0.213837996998102
935	KC_G1	CG10077	-0.349418899349522	0.0119576197202361	0.998043617620652	pseudobulk	DESeq2	LRT	1.92235526225799
936	KC_G1	CG10082	-0.14024317184221	0.295268763902753	0.998043617620652	pseudobulk	DESeq2	LRT	0.529782494051488
937	KC_G1	CG10089	0.00599321092915003	0.964538508289907	0.998043617620652	pseudobulk	DESeq2	LRT	0.0156804288765341
938	KC_G1	CG10098	-0.365657356633445	0.174554809675542	0.998043617620652	pseudobulk	DESeq2	LRT	0.758068180153899
939	KC_G1	CG10103	-0.0120103919922435	0.961683283525614	0.998043617620652	pseudobulk	DESeq2	LRT	0.0169679330187621
940	KC_G1	CG10116	1.13582588427274	0.633424328955532	0.998043617620652	pseudobulk	DESeq2	LRT	0.198305259991224
941	KC_G1	CG10126	0.296252136149598	0.873843293967724	0.998043617620652	pseudobulk	DESeq2	LRT	0.0585664422706479
942	KC_G1	CG10132	-0.0081840113329081	0.957857045302182	0.998043617620652	pseudobulk	DESeq2	LRT	0.0186993020582697
943	KC_G1	CG10137	-0.0796233439688025	0.747523002099137	0.998043617620652	pseudobulk	DESeq2	LRT	0.126375439082424
944	KC_G1	CG10139	-0.0295324380477951	0.893148327118198	0.998043617620652	pseudobulk	DESeq2	LRT	0.0490764108766685
945	KC_G1	CG10147	-0.36115980890297	0.543321412495795	0.998043617620652	pseudobulk	DESeq2	LRT	0.264943178924461
946	KC_G1	CG10151	0.00185455751804985	0.956933415336394	0.998043617620652	pseudobulk	DESeq2	LRT	0.0191182799431288
947	KC_G1	CG10165	0.406110867039094	0.824151088752212	0.998043617620652	pseudobulk	DESeq2	LRT	0.0839931633073423
948	KC_G1	CG10166	0.393637096687165	0.397517007280145	0.998043617620652	pseudobulk	DESeq2	LRT	0.400644285850498
949	KC_G1	CG10171	1.11719180756322	0.139438244863927	0.998043617620652	pseudobulk	DESeq2	LRT	0.855618092412039
950	KC_G1	CG10177	0.912534563407916	0.581905844801101	0.998043617620652	pseudobulk	DESeq2	LRT	0.235147280627318

951	KC_G1	CG10184	-0.765625368254185	0.836625948066609	0.998043617620652	pseudobulk	DESeq2	LRT	0.0774686698445758
952	KC_G1	CG10185	-0.794610277746993	0.821479263487599	0.998043617620652	pseudobulk	DESeq2	LRT	0.085403394939296
953	KC_G1	CG10188	-0.143703989297649	0.605335255804588	0.998043617620652	pseudobulk	DESeq2	LRT	0.218004031265876
954	KC_G1	CG10189	-0.34938677535589	0.488877059437396	0.998043617620652	pseudobulk	DESeq2	LRT	0.310800341532448
955	KC_G1	CG10194	0.0295671945359782	0.951667889617776	0.998043617620652	pseudobulk	DESeq2	LRT	0.0215145840426183
956	KC_G1	CG10195	0.949247102466247	0.731817859237848	0.998043617620652	pseudobulk	DESeq2	LRT	0.135596996224368
957	KC_G1	CG10208	0.46096068960305	0.679199811222299	0.998043617620652	pseudobulk	DESeq2	LRT	0.168002443472474
958	KC_G1	CG10209	0.42176358355002	0.270274921384128	0.998043617620652	pseudobulk	DESeq2	LRT	0.568194250304896
959	KC_G1	CG10214	0.110145622076374	0.748325330781105	0.998043617620652	pseudobulk	DESeq2	LRT	0.125909553678125
960	KC_G1	CG10222	0.195446171707795	0.847400562205357	0.998043617620652	pseudobulk	DESeq2	LRT	0.0719112522035642
961	KC_G1	CG10226	-2.58457033458099	0.577134559119084	0.998043617620652	pseudobulk	DESeq2	LRT	0.238722919132092
962	KC_G1	CG10232	0.173116475207952	0.97305562682953	0.998043617620652	pseudobulk	DESeq2	LRT	0.011862331638572
963	KC_G1	CG10237	-0.25143610382357	0.887110069468294	0.998043617620652	pseudobulk	DESeq2	LRT	0.0520224911073972
964	KC_G1	CG1024	-0.356413076049023	0.55091157277775	0.998043617620652	pseudobulk	DESeq2	LRT	0.258918104481413
965	KC_G1	CG10252	-0.971172200646484	0.208574464421488	0.998043617620652	pseudobulk	DESeq2	LRT	0.680738862926165
966	KC_G1	CG10254	-0.0758039986595222	0.802142930272058	0.998043617620652	pseudobulk	DESeq2	LRT	0.0957482398233254
967	KC_G1	CG10257	0.424679416333412	0.144009601510131	0.998043617620652	pseudobulk	DESeq2	LRT	0.841608551350196
968	KC_G1	CG10265	0.0255709395495028	0.929533521701683	0.998043617620652	pseudobulk	DESeq2	LRT	0.0317349436784684
969	KC_G1	CG10268	-0.317579647471976	0.607591623407912	0.998043617620652	pseudobulk	DESeq2	LRT	0.21638822219042
970	KC_G1	CG10274	-0.337198192266385	0.648556824612133	0.998043617620652	pseudobulk	DESeq2	LRT	0.188051966327286
971	KC_G1	CG10283	-0.21222318789464	0.465418895520556	0.998043617620652	pseudobulk	DESeq2	LRT	0.332155988789457
972	KC_G1	CG10286	-0.264715260033403	0.762072579192297	0.998043617620652	pseudobulk	DESeq2	LRT	0.118003664826197
973	KC_G1	CG10311	1.59184682593427	0.0854303405875094	0.998043617620652	pseudobulk	DESeq2	LRT	1.06838786221972
974	KC_G1	CG10321	-0.2656605159762	0.399557019398133	0.998043617620652	pseudobulk	DESeq2	LRT	0.398421235266259
975	KC_G1	CG10324	0.370922991516507	0.503052777287705	0.998043617620652	pseudobulk	DESeq2	LRT	0.298386448975053
976	KC_G1	CG10333	-0.370140989484179	0.173321073076657	0.998043617620652	pseudobulk	DESeq2	LRT	0.761148630797206
977	KC_G1	CG10336	-0.65817001371651	0.770857516266391	0.998043617620652	pseudobulk	DESeq2	LRT	0.113025888638787
978	KC_G1	CG10337	-1.73907471612892	0.507597351592657	0.998043617620652	pseudobulk	DESeq2	LRT	0.294480652518419
979	KC_G1	CG10338	0.286591024494634	0.762710989807572	0.998043617620652	pseudobulk	DESeq2	LRT	0.117639995871152
980	KC_G1	CG10340	0.079457140555495	0.719382531387074	0.998043617620652	pseudobulk	DESeq2	LRT	0.143040112269955
981	KC_G1	CG10341	0.439808112487099	0.463733356765532	0.998043617620652	pseudobulk	DESeq2	LRT	0.333731663780338
982	KC_G1	CG10343	-0.258221400624165	0.536493256658847	0.998043617620652	pseudobulk	DESeq2	LRT	0.270435732438491
983	KC_G1	CG10347	-0.223619445326046	0.701417929807971	0.998043617620652	pseudobulk	DESeq2	LRT	0.154023136776246
984	KC_G1	CG10352	-0.438790272948703	0.702733460533623	0.998043617620652	pseudobulk	DESeq2	LRT	0.153209367112923
985	KC_G1	CG10353	-0.100652772292258	0.705563310784941	0.998043617620652	pseudobulk	DESeq2	LRT	0.151464010554694
986	KC_G1	CG10359	-2.73026776509574	0.214564985109251	0.998043617620652	pseudobulk	DESeq2	LRT	0.668441149166746
987	KC_G1	CG10361	-2.70281465192265	0.197132217389703	0.998043617620652	pseudobulk	DESeq2	LRT	0.705242393015524
988	KC_G1	CG10362	-0.201936475056585	0.496976942886191	0.998043617620652	pseudobulk	DESeq2	LRT	0.303663759776891
989	KC_G1	CG10365	-0.687622984155781	0.297814807330726	0.998043617620652	pseudobulk	DESeq2	LRT	0.526053712956726
990	KC_G1	CG10366	-0.0161670308341067	0.961570215161218	0.998043617620652	pseudobulk	DESeq2	LRT	0.01701899749552
991	KC_G1	CG10375	-0.19369655288984	0.323342629557845	0.998043617620652	pseudobulk	DESeq2	LRT	0.490337034141138
992	KC_G1	CG10376	0.0813753863021138	0.897565083090069	0.998043617620652	pseudobulk	DESeq2	LRT	0.0469340506024662
993	KC_G1	CG10383	0.222838910317956	0.694326160733006	0.998043617620652	pseudobulk	DESeq2	LRT	0.158436471142775
994	KC_G1	CG10395	0.14909742935382	0.517126880450589	0.998043617620652	pseudobulk	DESeq2	LRT	0.286402886849964
995	KC_G1	CG10413	-0.226965680953065	0.609288651533702	0.998043617620652	pseudobulk	DESeq2	LRT	0.215176910866488
996	KC_G1	CG10417	-0.0431405547447168	0.794881954944671	0.998043617620652	pseudobulk	DESeq2	LRT	0.0996973620638377
997	KC_G1	CG10418	-0.217778958725015	0.573884818321593	0.998043617620652	pseudobulk	DESeq2	LRT	0.241175264025246
998	KC_G1	CG10425	0.333615719840422	0.602776566380353	0.998043617620652	pseudobulk	DESeq2	LRT	0.219843639717599
999	KC_G1	CG10428	-0.268017768961988	0.377993133240034	0.998043617620652	pseudobulk	DESeq2	LRT	0.422516089641741
100	KC_G1	CG10431	2.3780703570557	0.117521505788105	0.998043617620652	pseudobulk	DESeq2	LRT	0.929882652622194
100	KC_G1	CG10433	0.904363037177067	0.179768058697249	0.998043617620652	pseudobulk	DESeq2	LRT	0.745287471467325
100	KC_G1	CG10435	-0.0438073971308547	0.911042447552654	0.998043617620652	pseudobulk	DESeq2	LRT	0.0404613877817516
100	KC_G1	CG10444	1.2318790082855	0.171963175605123	0.998043617620652	pseudobulk	DESeq2	LRT	0.764564543462874
100	KC_G1	CG10462	-0.65817001371651	0.770857516266391	0.998043617620652	pseudobulk	DESeq2	LRT	0.113025888638787
100	KC_G1	CG10463	-0.0733775777478372	0.90394021303662	0.998043617620652	pseudobulk	DESeq2	LRT	0.0438602929836404
100	KC_G1	CG10465	0.0222296800547311	0.898101335769744	0.998043617620652	pseudobulk	DESeq2	LRT	0.046674657672726

100	KC_G1	CG10466	0.07652949106193	0.866815629998632	0.998043617620652	pseudobulk	DESeq2	LRT	0.0620732662943534
100	KC_G1	CG10467	3.51433627222488	0.0724261714418414	0.998043617620652	pseudobulk	DESeq2	LRT	1.14010447166285
100	KC_G1	CG10469	2.55520982185551	0.192326789588035	0.998043617620652	pseudobulk	DESeq2	LRT	0.715960217790032
101	KC_G1	CG10470	0.0378964840756575	0.890410199407387	0.998043617620652	pseudobulk	DESeq2	LRT	0.0504098739193306
101	KC_G1	CG10479	-1.55570024235503	0.221498086343823	0.998043617620652	pseudobulk	DESeq2	LRT	0.654630021557879
101	KC_G1	CG10483	-0.23764781603225	0.375149805349571	0.998043617620652	pseudobulk	DESeq2	LRT	0.425795274552026
101	KC_G1	CG10492	-0.0709059741027919	0.849287807475126	0.998043617620652	pseudobulk	DESeq2	LRT	0.0709451106783126
101	KC_G1	CG10494	0.502063725246193	0.391800060717936	0.998043617620652	pseudobulk	DESeq2	LRT	0.406935501037907
101	KC_G1	CG10495	0.310710555986046	0.655430258415682	0.998043617620652	pseudobulk	DESeq2	LRT	0.183473512969475
101	KC_G1	CG10508	-0.0644155928741696	0.707434148865964	0.998043617620652	pseudobulk	DESeq2	LRT	0.150313979994695
101	KC_G1	CG10512	-0.500660832558891	0.4391766646247	0.998043617620652	pseudobulk	DESeq2	LRT	0.357360743908521
101	KC_G1	CG10513	-0.150041486084687	0.651955512380376	0.998043617620652	pseudobulk	DESeq2	LRT	0.185782038297063
101	KC_G1	CG10514	0.448708562484223	0.493208045448363	0.998043617620652	pseudobulk	DESeq2	LRT	0.306969847596724
102	KC_G1	CG10516	-0.103056180253086	0.864716844488791	0.998043617620652	pseudobulk	DESeq2	LRT	0.0631260809835769
102	KC_G1	CG10527	0.504621265382822	0.356897880099456	0.998043617620652	pseudobulk	DESeq2	LRT	0.447456031657236
102	KC_G1	CG10543	-0.0979659099791964	0.477864595187588	0.998043617620652	pseudobulk	DESeq2	LRT	0.320695145005999
102	KC_G1	CG10550	0.475181720440614	0.719033304106904	0.998043617620652	pseudobulk	DESeq2	LRT	0.14325099354635
102	KC_G1	CG10555	-0.107245980857095	0.692538783886062	0.998043617620652	pseudobulk	DESeq2	LRT	0.159555900016507
102	KC_G1	CG10562	0.664759300240333	0.665405290108214	0.998043617620652	pseudobulk	DESeq2	LRT	0.176913750723473
102	KC_G1	CG10565	0.258398019995733	0.403316506185717	0.998043617620652	pseudobulk	DESeq2	LRT	0.394354003634133
102	KC_G1	CG10576	0.054950533007617	0.86828879495989	0.998043617620652	pseudobulk	DESeq2	LRT	0.0613358033980144
102	KC_G1	CG10581	0.723480946103744	0.575395609303193	0.998043617620652	pseudobulk	DESeq2	LRT	0.240033456423222
102	KC_G1	CG10584	2.63732022130093	0.22943645835378	0.998043617620652	pseudobulk	DESeq2	LRT	0.639337569853304
103	KC_G1	CG10600	0.100116949640151	0.668594131881313	0.998043617620652	pseudobulk	DESeq2	LRT	0.174837439443643
103	KC_G1	CG10602	-0.0295636979229679	0.910391868903104	0.998043617620652	pseudobulk	DESeq2	LRT	0.04077162980037
103	KC_G1	CG10616	-0.140486499192645	0.687031749798856	0.998043617620652	pseudobulk	DESeq2	LRT	0.163023192425414
103	KC_G1	CG10621	1.42944759940171	0.552522613276143	0.998043617620652	pseudobulk	DESeq2	LRT	0.25764994276489
103	KC_G1	CG10623	-0.108745743401772	0.92347755490238	0.998043617620652	pseudobulk	DESeq2	LRT	0.0345736556113964
103	KC_G1	CG10628	0.477438921064407	0.282206232577015	0.998043617620652	pseudobulk	DESeq2	LRT	0.54943339900153
103	KC_G1	CG10631	-0.0290246258989002	0.89482746215987	0.998043617620652	pseudobulk	DESeq2	LRT	0.0482606959193098
103	KC_G1	CG10638	0.240404024751497	0.530862560305634	0.998043617620652	pseudobulk	DESeq2	LRT	0.275017902698072
103	KC_G1	CG10646	0.37201297576218	0.438034947501691	0.998043617620652	pseudobulk	DESeq2	LRT	0.358491239035455
103	KC_G1	CG10654	0.147589739767413	0.640206543607459	0.998043617620652	pseudobulk	DESeq2	LRT	0.193679891207057
104	KC_G1	CG10660	-0.0190232237688427	0.958029348895871	0.998043617620652	pseudobulk	DESeq2	LRT	0.0186211862572708
104	KC_G1	CG10669	-0.0578920808385729	0.914013526627296	0.998043617620652	pseudobulk	DESeq2	LRT	0.0390473770275207
104	KC_G1	CG10672	-0.0379655617497213	0.913675571068265	0.998043617620652	pseudobulk	DESeq2	LRT	0.0392079866588564
104	KC_G1	CG10674	0.106978427160075	0.685332503475399	0.998043617620652	pseudobulk	DESeq2	LRT	0.1640986702762
104	KC_G1	CG10681	0.109824834239667	0.81856095944757	0.998043617620652	pseudobulk	DESeq2	LRT	0.0869489724940886
104	KC_G1	CG10702	-0.465932799966057	0.838600399555844	0.998043617620652	pseudobulk	DESeq2	LRT	0.0764449350103697
104	KC_G1	CG10710	-1.66808346769027	0.498191247612582	0.998043617620652	pseudobulk	DESeq2	LRT	0.302603906560549
104	KC_G1	CG10713	0.547763685893279	0.150897846856818	0.998043617620652	pseudobulk	DESeq2	LRT	0.821316957075795
104	KC_G1	CG10731	-0.0461507036698512	0.909465801527229	0.998043617620652	pseudobulk	DESeq2	LRT	0.0412136269878014
104	KC_G1	CG10737	0.105027176460707	0.699128794733355	0.998043617620652	pseudobulk	DESeq2	LRT	0.155442810392415
105	KC_G1	CG10738	-0.704797641189757	0.838804762683723	0.998043617620652	pseudobulk	DESeq2	LRT	0.0763391123013672
105	KC_G1	CG1074	0.0102031022017364	0.964112219787742	0.998043617620652	pseudobulk	DESeq2	LRT	0.0158724125721912
105	KC_G1	CG10747	0.305109686989819	0.567744230735576	0.998043617620652	pseudobulk	DESeq2	LRT	0.245847270269714
105	KC_G1	CG10778	0.272986765557521	0.671123927245084	0.998043617620652	pseudobulk	DESeq2	LRT	0.173197277224143
105	KC_G1	CG10793	-0.794610277746993	0.821479263487599	0.998043617620652	pseudobulk	DESeq2	LRT	0.085403394939296
105	KC_G1	CG10795	-0.505955870814011	0.318851710753831	0.998043617620652	pseudobulk	DESeq2	LRT	0.496411248524756
105	KC_G1	CG10802	0.0859524154455162	0.853137464204846	0.998043617620652	pseudobulk	DESeq2	LRT	0.0689809862583813
105	KC_G1	CG10803	0.022629159479825	0.928312928877101	0.998043617620652	pseudobulk	DESeq2	LRT	0.0323056009623055
105	KC_G1	CG10804	-0.0685382562808775	0.706710891441743	0.998043617620652	pseudobulk	DESeq2	LRT	0.150758215524672
105	KC_G1	CG10809	0.0111202339431688	0.940561937216795	0.998043617620652	pseudobulk	DESeq2	LRT	0.0266126003200964
106	KC_G1	CG10814	2.04147257822512	0.408147672536479	0.998043617620652	pseudobulk	DESeq2	LRT	0.389182675723292
106	KC_G1	CG10863	0.188346708526946	0.761769042901628	0.998043617620652	pseudobulk	DESeq2	LRT	0.118176680362624
106	KC_G1	CG10864	-2.5432294906949	0.155588467237401	0.998043617620652	pseudobulk	DESeq2	LRT	0.808022597582714

106	KC_G1	CG10866	-0.494590253647027	0.484248167556545	0.998043617620652	pseudobulk	DESeq2	LRT	0.314932014019459
106	KC_G1	CG10869	2.23045770445974	0.357070320422792	0.998043617620652	pseudobulk	DESeq2	LRT	0.447246246733247
106	KC_G1	CG10874	-0.0255532872798135	0.942077211266467	0.998043617620652	pseudobulk	DESeq2	LRT	0.025913501609675
106	KC_G1	CG10889	0.683647645357422	0.320203383069603	0.998043617620652	pseudobulk	DESeq2	LRT	0.494574083907353
106	KC_G1	CG10898	0.190139674863275	0.67037359606781	0.998043617620652	pseudobulk	DESeq2	LRT	0.173683099550623
106	KC_G1	CG1090	-0.132945817231206	0.455053970027624	0.998043617620652	pseudobulk	DESeq2	LRT	0.341937092364452
106	KC_G1	CG10903	0.70151948316835	0.218442655023988	0.998043617620652	pseudobulk	DESeq2	LRT	0.660662553546218
107	KC_G1	CG10904	0.0330378512986927	0.94279041573391	0.998043617620652	pseudobulk	DESeq2	LRT	0.0255848410978976
107	KC_G1	CG10907	0.111988113008439	0.850744289299143	0.998043617620652	pseudobulk	DESeq2	LRT	0.0702009574648231
107	KC_G1	CG10914	1.15625789671017	0.082747406942496	0.998043617620652	pseudobulk	DESeq2	LRT	1.08224560683575
107	KC_G1	CG10916	0.240216709234809	0.632297111716492	0.998043617620652	pseudobulk	DESeq2	LRT	0.199078802005309
107	KC_G1	CG10919	-0.794610277746993	0.821479263487599	0.998043617620652	pseudobulk	DESeq2	LRT	0.085403394939296
107	KC_G1	CG10927	1.37180903653937	0.322571597999376	0.998043617620652	pseudobulk	DESeq2	LRT	0.49137387431582
107	KC_G1	CG10932	0.221475018823168	0.742657866291556	0.998043617620652	pseudobulk	DESeq2	LRT	0.129211214506563
107	KC_G1	CG10934	1.12508065046033	0.900770125530981	0.998043617620652	pseudobulk	DESeq2	LRT	0.0453860258366365
107	KC_G1	CG10947	0.0445963815710264	0.823717115957438	0.998043617620652	pseudobulk	DESeq2	LRT	0.0842219097494244
107	KC_G1	CG10948	0.0637576932594892	0.792839549461906	0.998043617620652	pseudobulk	DESeq2	LRT	0.100814693935672
108	KC_G1	CG10949	0.00025218315337179	0.96483504795422	0.998043617620652	pseudobulk	DESeq2	LRT	0.0155469290257935
108	KC_G1	CG10958	0.344469339705085	0.705117635016503	0.998043617620652	pseudobulk	DESeq2	LRT	0.151738423467077
108	KC_G1	CG10959	0.149089725385979	0.81627943579664	0.998043617620652	pseudobulk	DESeq2	LRT	0.0881611443710191
108	KC_G1	CG10960	-0.336206195518662	0.104566266733519	0.998043617620652	pseudobulk	DESeq2	LRT	0.980608397058811
108	KC_G1	CG10973	-0.149260178140963	0.735003331867352	0.998043617620652	pseudobulk	DESeq2	LRT	0.133710692196995
108	KC_G1	CG10979	-0.342010688432133	0.371847315325974	0.998043617620652	pseudobulk	DESeq2	LRT	0.429635349697068
108	KC_G1	CG10984	-0.237037365207361	0.368788988361374	0.998043617620652	pseudobulk	DESeq2	LRT	0.433222054986327
108	KC_G1	CG10996	-0.794610277746993	0.815039924133852	0.998043617620652	pseudobulk	DESeq2	LRT	0.0888211171417967
108	KC_G1	CG11000	0.143332527168508	0.482082696353898	0.998043617620652	pseudobulk	DESeq2	LRT	0.316878456595991
108	KC_G1	CG11007	-0.240553536928729	0.518609560039746	0.998043617620652	pseudobulk	DESeq2	LRT	0.285159481715837
109	KC_G1	CG1103	0.233271129914362	0.503308833799578	0.998043617620652	pseudobulk	DESeq2	LRT	0.298165447038301
109	KC_G1	CG11030	-0.015888200858305	0.952401980219785	0.998043617620652	pseudobulk	DESeq2	LRT	0.0211797102877043
109	KC_G1	CG11035	-0.0638190964188239	0.877855140018244	0.998043617620652	pseudobulk	DESeq2	LRT	0.0565771436396469
109	KC_G1	CG11041	-0.794610277746993	0.815039924133852	0.998043617620652	pseudobulk	DESeq2	LRT	0.0888211171417967
109	KC_G1	CG11044	1.13582588427274	0.633424328955532	0.998043617620652	pseudobulk	DESeq2	LRT	0.198305259991224
109	KC_G1	CG1105	-0.165515146664384	0.662669398704739	0.998043617620652	pseudobulk	DESeq2	LRT	0.17870308415543
109	KC_G1	CG11050	-0.369568225400261	0.0596944101086025	0.998043617620652	pseudobulk	DESeq2	LRT	1.22406633507917
109	KC_G1	CG11076	-0.106041321309143	0.623860550346055	0.998043617620652	pseudobulk	DESeq2	LRT	0.204912475996024
109	KC_G1	CG11077	0.317359481590465	0.20735507065471	0.998043617620652	pseudobulk	DESeq2	LRT	0.683285339945808
109	KC_G1	CG11089	1.85874937412271	0.0576565257359519	0.998043617620652	pseudobulk	DESeq2	LRT	1.23915153085458
110	KC_G1	CG11095	0.761623133860219	0.463295518118585	0.998043617620652	pseudobulk	DESeq2	LRT	0.334141901104382
110	KC_G1	CG11099	0.657399488000757	0.156163298057362	0.998043617620652	pseudobulk	DESeq2	LRT	0.806421027591136
110	KC_G1	CG11103	0.457683696058403	0.381806772382483	0.998043617620652	pseudobulk	DESeq2	LRT	0.418156372479135
110	KC_G1	CG11109	-0.613864592495461	0.330318485393468	0.998043617620652	pseudobulk	DESeq2	LRT	0.48106712149557
110	KC_G1	CG11110	0.682442445030615	0.0630269643644737	0.998043617620652	pseudobulk	DESeq2	LRT	1.20047360976327
110	KC_G1	CG11122	-0.0473612401931916	0.763901761277419	0.998043617620652	pseudobulk	DESeq2	LRT	0.116962488647301
110	KC_G1	CG11123	0.524085468694827	0.59149858320982	0.998043617620652	pseudobulk	DESeq2	LRT	0.228046291281012
110	KC_G1	CG11127	0.357543417006298	0.642210791274358	0.998043617620652	pseudobulk	DESeq2	LRT	0.192322401118424
110	KC_G1	CG1113	-0.0245647992648994	0.943438984215077	0.998043617620652	pseudobulk	DESeq2	LRT	0.0252861820656341
110	KC_G1	CG11134	-0.315675895435408	0.64447227117816	0.998043617620652	pseudobulk	DESeq2	LRT	0.190795763699839
111	KC_G1	CG11137	-0.00061456737446802	0.963246975311404	0.998043617620652	pseudobulk	DESeq2	LRT	0.0162623460394661
111	KC_G1	CG11141	-0.114681318860027	0.802791677427317	0.998043617620652	pseudobulk	DESeq2	LRT	0.095397138509075
111	KC_G1	CG11151	-0.125066392460985	0.510086342648184	0.998043617620652	pseudobulk	DESeq2	LRT	0.292356304369096
111	KC_G1	CG11155	-0.0402325527613955	0.713928281542604	0.998043617620652	pseudobulk	DESeq2	LRT	0.146345413566536
111	KC_G1	CG11159	-0.720862067059996	0.222879022161102	0.998043617620652	pseudobulk	DESeq2	LRT	0.65193080629955
111	KC_G1	CG1116	-0.17267241384756	0.730170485920323	0.998043617620652	pseudobulk	DESeq2	LRT	0.136575725564587
111	KC_G1	CG11168	-1.2611416914115	0.382550382138326	0.998043617620652	pseudobulk	DESeq2	LRT	0.417311359873114
111	KC_G1	CG11170	-0.232981786540842	0.780722780583436	0.998043617620652	pseudobulk	DESeq2	LRT	0.107503148242153
111	KC_G1	CG11178	-0.308558971519073	0.466649655083793	0.998043617620652	pseudobulk	DESeq2	LRT	0.331009050836924

111	KC_G1	CG11180	-0.067539204269033	0.90996588937429	0.998043617620652	pseudobulk	DESeq2	LRT	0.0409748871670093
112	KC_G1	CG11191	0.00953321107455266	0.941371268952017	0.998043617620652	pseudobulk	DESeq2	LRT	0.0262390606778256
112	KC_G1	CG11200	0.555923773859732	0.431814278955298	0.998043617620652	pseudobulk	DESeq2	LRT	0.364703000795298
112	KC_G1	CG1124	-2.92939794663066	0.206617901318947	0.998043617620652	pseudobulk	DESeq2	LRT	0.684832054030145
112	KC_G1	CG11241	2.15958726504788	0.0647010925496348	0.998043617620652	pseudobulk	DESeq2	LRT	1.18908838572587
112	KC_G1	CG11247	-0.0357446395043562	0.854311814540925	0.998043617620652	pseudobulk	DESeq2	LRT	0.0683835876035009
112	KC_G1	CG11248	0.0684724819737687	0.850203162892761	0.998043617620652	pseudobulk	DESeq2	LRT	0.0704772837204391
112	KC_G1	CG11257	0.120076447572526	0.832670217657109	0.998043617620652	pseudobulk	DESeq2	LRT	0.079526968593156
112	KC_G1	CG11267	0.13909276524194	0.335947563591516	0.998043617620652	pseudobulk	DESeq2	LRT	0.473728504217339
112	KC_G1	CG11279	-0.111604204946153	0.777943011720979	0.998043617620652	pseudobulk	DESeq2	LRT	0.109052216122921
112	KC_G1	CG11307	-0.398614732619291	0.460235791415389	0.998043617620652	pseudobulk	DESeq2	LRT	0.33701961033105
113	KC_G1	CG11309	-0.171190167066658	0.774173241603706	0.998043617620652	pseudobulk	DESeq2	LRT	0.111161843640528
113	KC_G1	CG11317	0.47501972031346	0.0952912218639046	0.998043617620652	pseudobulk	DESeq2	LRT	1.02094710431083
113	KC_G1	CG11319	-0.0235317409958672	0.856223087611853	0.998043617620652	pseudobulk	DESeq2	LRT	0.0674130658181742
113	KC_G1	CG11334	0.490672432882794	0.305792694774196	0.998043617620652	pseudobulk	DESeq2	LRT	0.514572893865332
113	KC_G1	CG11342	-0.634175616417158	0.651467665942765	0.998043617620652	pseudobulk	DESeq2	LRT	0.186107134596235
113	KC_G1	CG11357	0.0375042069732088	0.890685290551649	0.998043617620652	pseudobulk	DESeq2	LRT	0.0502757198915462
113	KC_G1	CG11360	0.00925278412203459	0.944006831178229	0.998043617620652	pseudobulk	DESeq2	LRT	0.0250248629770627
113	KC_G1	CG11367	0.431030380071451	0.373898559569352	0.998043617620652	pseudobulk	DESeq2	LRT	0.427246207946308
113	KC_G1	CG11374	0.447302969164969	0.704024304126775	0.998043617620652	pseudobulk	DESeq2	LRT	0.152412348008523
113	KC_G1	CG11377	-0.147897919000623	0.749654737255858	0.998043617620652	pseudobulk	DESeq2	LRT	0.125138710246914
114	KC_G1	CG11378	-0.614175551721446	0.683221246404667	0.998043617620652	pseudobulk	DESeq2	LRT	0.165438636679656
114	KC_G1	CG11391	-0.525809424813515	0.801332005230554	0.998043617620652	pseudobulk	DESeq2	LRT	0.0961875111745507
114	KC_G1	CG11396	0.476126652872973	0.313528878905117	0.998043617620652	pseudobulk	DESeq2	LRT	0.503722450458454
114	KC_G1	CG11398	-1.02086252743698	0.14989678062754	0.998043617620652	pseudobulk	DESeq2	LRT	0.824207694508017
114	KC_G1	CG11399	0.117213872054617	0.71989633114967	0.998043617620652	pseudobulk	DESeq2	LRT	0.142730039751021
114	KC_G1	CG11403	2.01540473478043	0.38050535746174	0.998043617620652	pseudobulk	DESeq2	LRT	0.419639224045338
114	KC_G1	CG11406	1.89247811065421	0.405224567408468	0.998043617620652	pseudobulk	DESeq2	LRT	0.392304232693548
114	KC_G1	CG11407	0.195782197108319	0.748473997784436	0.998043617620652	pseudobulk	DESeq2	LRT	0.12582328258197
114	KC_G1	CG11413	-1.10818068055368	0.249811328892187	0.998043617620652	pseudobulk	DESeq2	LRT	0.602387870350198
114	KC_G1	CG11414	-0.303157847694233	0.402859023345552	0.998043617620652	pseudobulk	DESeq2	LRT	0.394846904465523
115	KC_G1	CG11417	-0.277427000368731	0.695360229949875	0.998043617620652	pseudobulk	DESeq2	LRT	0.157790151741954
115	KC_G1	CG1142	0.740553234841994	0.0934486366571046	0.998043617620652	pseudobulk	DESeq2	LRT	1.02942703025749
115	KC_G1	CG11425	0.777030258285493	0.70316795941598	0.998043617620652	pseudobulk	DESeq2	LRT	0.152940926566052
115	KC_G1	CG11426	-0.0694934868276069	0.940095751540715	0.998043617620652	pseudobulk	DESeq2	LRT	0.0268279099663132
115	KC_G1	CG1143	-1.72984073695683	0.480047293856853	0.998043617620652	pseudobulk	DESeq2	LRT	0.318715974188453
115	KC_G1	CG11436	0.900688551957437	0.132086929626156	0.998043617620652	pseudobulk	DESeq2	LRT	0.879140154921998
115	KC_G1	CG11444	0.0431184994740781	0.844972399073719	0.998043617620652	pseudobulk	DESeq2	LRT	0.0731574769979444
115	KC_G1	CG11447	0.508267358677969	0.246263990688657	0.998043617620652	pseudobulk	DESeq2	LRT	0.608599087099256
115	KC_G1	CG11453	1.13582588427274	0.704684208645453	0.998043617620652	pseudobulk	DESeq2	LRT	0.152005460551806
115	KC_G1	CG11454	0.0912969155721857	0.732064821999657	0.998043617620652	pseudobulk	DESeq2	LRT	0.135450461850976
116	KC_G1	CG11456	0.0173857983188368	0.957795623536756	0.998043617620652	pseudobulk	DESeq2	LRT	0.0187271517141455
116	KC_G1	CG1146	-2.28172252880459	0.324719824317488	0.998043617620652	pseudobulk	DESeq2	LRT	0.488491196656175
116	KC_G1	CG11470	1.12508065046033	0.900770125530981	0.998043617620652	pseudobulk	DESeq2	LRT	0.0453860258366365
116	KC_G1	CG11474	-0.743971612949498	0.0576334508864355	0.998043617620652	pseudobulk	DESeq2	LRT	1.2393253756283
116	KC_G1	CG11475	-0.0942152128166348	0.937123272289403	0.998043617620652	pseudobulk	DESeq2	LRT	0.028203276824364
116	KC_G1	CG11486	0.137807756959398	0.279546314745193	0.998043617620652	pseudobulk	DESeq2	LRT	0.553546228665106
116	KC_G1	CG11504	0.283438490038898	0.32944392946224	0.998043617620652	pseudobulk	DESeq2	LRT	0.482218490621654
116	KC_G1	CG11523	0.183870319586194	0.452946465880819	0.998043617620652	pseudobulk	DESeq2	LRT	0.343953124579757
116	KC_G1	CG11537	-0.0870962392355622	0.521488858456959	0.998043617620652	pseudobulk	DESeq2	LRT	0.282754965784625
116	KC_G1	CG11539	-1.28765143752845	0.291561175672312	0.998043617620652	pseudobulk	DESeq2	LRT	0.53527030721911
117	KC_G1	CG11555	-0.144036754849401	0.701233970533395	0.998043617620652	pseudobulk	DESeq2	LRT	0.154137053134656
117	KC_G1	CG11560	-0.120925988872463	0.825135148681261	0.998043617620652	pseudobulk	DESeq2	LRT	0.0834749126385829
117	KC_G1	CG11562	-0.216494977966835	0.574033955543749	0.998043617620652	pseudobulk	DESeq2	LRT	0.241062417237707
117	KC_G1	CG11563	-0.088338308571395	0.855169429826712	0.998043617620652	pseudobulk	DESeq2	LRT	0.0679478324658677
117	KC_G1	CG11581	1.13582588427274	0.633424328955532	0.998043617620652	pseudobulk	DESeq2	LRT	0.198305259991224

117	KC_G1	CG11583	0.290421504158555	0.488555551680552	0.998043617620652	pseudobulk	DESeq2	LRT	0.311086047264247
117	KC_G1	CG11586	-0.259363346879974	0.296964501907249	0.998043617620652	pseudobulk	DESeq2	LRT	0.52729546161608
117	KC_G1	CG11590	0.126940968389519	0.815035525455191	0.998043617620652	pseudobulk	DESeq2	LRT	0.0888234609865197
117	KC_G1	CG11593	0.334522644673662	0.375772774390462	0.998043617620652	pseudobulk	DESeq2	LRT	0.425074688738724
117	KC_G1	CG11594	-0.242127184317587	0.891594945542019	0.998043617620652	pseudobulk	DESeq2	LRT	0.0498324022032762
118	KC_G1	CG11596	-0.315588036461254	0.509129182505019	0.998043617620652	pseudobulk	DESeq2	LRT	0.293172009154629
118	KC_G1	CG11597	-0.369076813967568	0.564080835535027	0.998043617620652	pseudobulk	DESeq2	LRT	0.248658655039313
118	KC_G1	CG11601	0.750839297108612	0.547525446824315	0.998043617620652	pseudobulk	DESeq2	LRT	0.261595691723342
118	KC_G1	CG1161	-0.326731495269749	0.125605241583469	0.998043617620652	pseudobulk	DESeq2	LRT	0.900992236846604
118	KC_G1	CG11617	1.79196735084865	0.450785457994645	0.998043617620652	pseudobulk	DESeq2	LRT	0.346030102418627
118	KC_G1	CG11619	-0.794610277746993	0.821479263487599	0.998043617620652	pseudobulk	DESeq2	LRT	0.085403394939296
118	KC_G1	CG11626	-0.930842989432155	0.565913018229735	0.998043617620652	pseudobulk	DESeq2	LRT	0.247250315465081
118	KC_G1	CG11638	1.01836555833589	0.308388926942175	0.998043617620652	pseudobulk	DESeq2	LRT	0.510901224182189
118	KC_G1	CG11655	0.390186547525828	0.835556986655899	0.998043617620652	pseudobulk	DESeq2	LRT	0.0780239250091121
118	KC_G1	CG11658	-0.226852019973838	0.433626449193833	0.998043617620652	pseudobulk	DESeq2	LRT	0.362884235721039
119	KC_G1	CG11679	-1.31481767438999	0.589948159507212	0.998043617620652	pseudobulk	DESeq2	LRT	0.229186149424154
119	KC_G1	CG11686	-0.858776732305277	0.658614035084657	0.998043617620652	pseudobulk	DESeq2	LRT	0.181369018697186
119	KC_G1	CG11695	0.358619054238162	0.54513342099818	0.998043617620652	pseudobulk	DESeq2	LRT	0.263497191463128
119	KC_G1	CG11696	0.0288505242384296	0.954987600083853	0.998043617620652	pseudobulk	DESeq2	LRT	0.0200022674216155
119	KC_G1	CG11699	0.131560641265894	0.520740430039039	0.998043617620652	pseudobulk	DESeq2	LRT	0.283378702598512
119	KC_G1	CG11700	1.06667464613488	0.669131470313168	0.998043617620652	pseudobulk	DESeq2	LRT	0.174488544082685
119	KC_G1	CG11710	0.243898397150284	0.559075456420121	0.998043617620652	pseudobulk	DESeq2	LRT	0.252529572980594
119	KC_G1	CG1172	0.135920763251406	0.674236855931415	0.998043617620652	pseudobulk	DESeq2	LRT	0.171187511248223
119	KC_G1	CG11722	-0.0972361873911951	0.771918638418514	0.998043617620652	pseudobulk	DESeq2	LRT	0.112428472653892
119	KC_G1	CG11723	-0.260812849161934	0.633342482493558	0.998043617620652	pseudobulk	DESeq2	LRT	0.198361379975623
120	KC_G1	CG11737	0.0945711896639282	0.89214451131991	0.998043617620652	pseudobulk	DESeq2	LRT	0.049564792052685
120	KC_G1	CG11752	-0.0815852726527426	0.586151934600545	0.998043617620652	pseudobulk	DESeq2	LRT	0.231989797282099
120	KC_G1	CG11753	0.198740896725544	0.462363809839543	0.998043617620652	pseudobulk	DESeq2	LRT	0.335016166374027
120	KC_G1	CG11755	-0.894233077987579	0.211981088648874	0.998043617620652	pseudobulk	DESeq2	LRT	0.673702881815511
120	KC_G1	CG11760	1.11154651156394	0.675883747053588	0.998043617620652	pseudobulk	DESeq2	LRT	0.170127996891038
120	KC_G1	CG11768	-0.388595048880833	0.109958573709371	0.998043617620652	pseudobulk	DESeq2	LRT	0.958770902096732
120	KC_G1	CG11771	0.136671911677574	0.698885885024696	0.998043617620652	pseudobulk	DESeq2	LRT	0.155593730620451
120	KC_G1	CG11777	-0.330818725683493	0.491682989929812	0.998043617620652	pseudobulk	DESeq2	LRT	0.308314816130021
120	KC_G1	CG11779	-0.131209320555458	0.691950635462114	0.998043617620652	pseudobulk	DESeq2	LRT	0.159924887495727
120	KC_G1	CG11781	0.196631420203874	0.448450282595097	0.998043617620652	pseudobulk	DESeq2	LRT	0.348285697974309
121	KC_G1	CG11788	1.94179592464598	0.127020897008269	0.998043617620652	pseudobulk	DESeq2	LRT	0.896124824643978
121	KC_G1	CG11790	0.293599001066269	0.593460447108526	0.998043617620652	pseudobulk	DESeq2	LRT	0.226608220559303
121	KC_G1	CG11791	0.119693522573782	0.877954121909224	0.998043617620652	pseudobulk	DESeq2	LRT	0.0565281778549933
121	KC_G1	CG11807	0.240518703106089	0.496087654257839	0.998043617620652	pseudobulk	DESeq2	LRT	0.304441580773553
121	KC_G1	CG11808	0.200073631069681	0.703161043507577	0.998043617620652	pseudobulk	DESeq2	LRT	0.152945198028634
121	KC_G1	CG11811	0.196392930844501	0.451745038690813	0.998043617620652	pseudobulk	DESeq2	LRT	0.345106608398541
121	KC_G1	CG11835	0.744489654998961	0.62114823709745	0.998043617620652	pseudobulk	DESeq2	LRT	0.20680474302656
121	KC_G1	CG11836	-1.72343437992478	0.512120560562093	0.998043617620652	pseudobulk	DESeq2	LRT	0.290627787806665
121	KC_G1	CG11837	-0.774934993069211	0.336222729842434	0.998043617620652	pseudobulk	DESeq2	LRT	0.473372930065305
121	KC_G1	CG11839	-0.548631631395572	0.315681499129341	0.998043617620652	pseudobulk	DESeq2	LRT	0.500750869680408
122	KC_G1	CG11852	-2.63950342027325	0.237576358078543	0.998043617620652	pseudobulk	DESeq2	LRT	0.624196779460586
122	KC_G1	CG11857	-0.153726016484011	0.563996557251061	0.998043617620652	pseudobulk	DESeq2	LRT	0.248723547029832
122	KC_G1	CG11858	-0.28331784502751	0.408067342161318	0.998043617620652	pseudobulk	DESeq2	LRT	0.389268160646127
122	KC_G1	CG11873	0.0117132873852763	0.929825775624517	0.998043617620652	pseudobulk	DESeq2	LRT	0.0315984189441374
122	KC_G1	CG11878	1.5684833933741	0.521894780878679	0.998043617620652	pseudobulk	DESeq2	LRT	0.282417046212031
122	KC_G1	CG11882	0.213024889557487	0.766335487688989	0.998043617620652	pseudobulk	DESeq2	LRT	0.11558106254113
122	KC_G1	CG11883	0.436586057835532	0.47329447941542	0.998043617620652	pseudobulk	DESeq2	LRT	0.324868561184664
122	KC_G1	CG11885	-0.0844387658708086	0.843996336926027	0.998043617620652	pseudobulk	DESeq2	LRT	0.0736594382751337
122	KC_G1	CG11892	-0.619015293963197	0.785650117817309	0.998043617620652	pseudobulk	DESeq2	LRT	0.104770820031127
122	KC_G1	CG11893	1.13582588427274	0.704684208645453	0.998043617620652	pseudobulk	DESeq2	LRT	0.152005460551806
123	KC_G1	CG11898	1.37557257511177	0.418365201146247	0.998043617620652	pseudobulk	DESeq2	LRT	0.378444446460758

123	KC_G1	CG11899	0.263323413406858	0.834141153574718	0.998043617620652	pseudobulk	DESeq2	LRT	0.0787604517225491
123	KC_G1	CG11902	-0.278302198418813	0.576757896359661	0.998043617620652	pseudobulk	DESeq2	LRT	0.239006450865036
123	KC_G1	CG11906	-0.239753321918316	0.694344448380598	0.998043617620652	pseudobulk	DESeq2	LRT	0.158425032541879
123	KC_G1	CG11920	-0.940754364142899	0.225311534616686	0.998043617620652	pseudobulk	DESeq2	LRT	0.647216574400674
123	KC_G1	CG11927	0.111420329391882	0.790046265660723	0.998043617620652	pseudobulk	DESeq2	LRT	0.102347475376882
123	KC_G1	CG11961	0.818802840604655	0.640550501329631	0.998043617620652	pseudobulk	DESeq2	LRT	0.193446624570064
123	KC_G1	CG11964	0.433640585423541	0.365741030174078	0.998043617620652	pseudobulk	DESeq2	LRT	0.43682631617646
123	KC_G1	CG11970	-0.73510911883494	0.259513364484681	0.998043617620652	pseudobulk	DESeq2	LRT	0.585840271832876
123	KC_G1	CG11975	-0.305788375879968	0.359533966381452	0.998043617620652	pseudobulk	DESeq2	LRT	0.444260074084422
124	KC_G1	CG11980	0.139078087755668	0.719864874535275	0.998043617620652	pseudobulk	DESeq2	LRT	0.142749017111988
124	KC_G1	CG11983	-0.994041909760027	0.560136177617481	0.998043617620652	pseudobulk	DESeq2	LRT	0.251706376568465
124	KC_G1	CG11986	1.08902587940666	0.291955320169285	0.998043617620652	pseudobulk	DESeq2	LRT	0.534683606389363
124	KC_G1	CG11999	0.0400706833998761	0.890582265073847	0.998043617620652	pseudobulk	DESeq2	LRT	0.0503259575960543
124	KC_G1	CG12003	-0.794610277746993	0.815039924133852	0.998043617620652	pseudobulk	DESeq2	LRT	0.0888211171417967
124	KC_G1	CG12004	0.0031130588281449	0.951625184968291	0.998043617620652	pseudobulk	DESeq2	LRT	0.0215340727843931
124	KC_G1	CG12007	0.108915233335403	0.798094650869274	0.998043617620652	pseudobulk	DESeq2	LRT	0.0979455999868503
124	KC_G1	CG12010	0.269816051094929	0.76518404343665	0.998043617620652	pseudobulk	DESeq2	LRT	0.116234094995588
124	KC_G1	CG12011	-0.087837150536357	0.937636323616979	0.998043617620652	pseudobulk	DESeq2	LRT	0.0279655766237108
124	KC_G1	CG12012	0.062267789463266	0.840281082417077	0.998043617620652	pseudobulk	DESeq2	LRT	0.0755754137914792
125	KC_G1	CG12024	0.189776211945202	0.40507460746695	0.998043617620652	pseudobulk	DESeq2	LRT	0.392464980175148
125	KC_G1	CG12025	-0.0773955960663545	0.678351403025385	0.998043617620652	pseudobulk	DESeq2	LRT	0.168545272416861
125	KC_G1	CG12038	0.0534764775146022	0.777697912102769	0.998043617620652	pseudobulk	DESeq2	LRT	0.10918906700021
125	KC_G1	CG12054	-0.0752484415063707	0.572952797362831	0.998043617620652	pseudobulk	DESeq2	LRT	0.241881155849833
125	KC_G1	CG12056	0.422816693802807	0.466651387074277	0.998043617620652	pseudobulk	DESeq2	LRT	0.331007438937063
125	KC_G1	CG12065	0.75798681973923	0.470118617837302	0.998043617620652	pseudobulk	DESeq2	LRT	0.32779254935653
125	KC_G1	CG12071	-0.104814674551042	0.472708908411316	0.998043617620652	pseudobulk	DESeq2	LRT	0.325406213145739
125	KC_G1	CG12075	-0.794610277746993	0.815039924133852	0.998043617620652	pseudobulk	DESeq2	LRT	0.0888211171417967
125	KC_G1	CG1208	-0.794610277746993	0.821479263487599	0.998043617620652	pseudobulk	DESeq2	LRT	0.085403394939296
125	KC_G1	CG12081	-0.104531507426154	0.774439796690355	0.998043617620652	pseudobulk	DESeq2	LRT	0.111012337725835
126	KC_G1	CG12084	0.00092213796039987	0.975341537461328	0.998043617620652	pseudobulk	DESeq2	LRT	0.0108432798279797
126	KC_G1	CG12091	0.0653076736687447	0.716801565738313	0.998043617620652	pseudobulk	DESeq2	LRT	0.144601054698098
126	KC_G1	CG12093	-0.20167517366084	0.657811989054537	0.998043617620652	pseudobulk	DESeq2	LRT	0.181898215479725
126	KC_G1	CG12096	0.126234152747119	0.777850165017706	0.998043617620652	pseudobulk	DESeq2	LRT	0.109104051818751
126	KC_G1	CG12099	0.0428229823157496	0.835916158371296	0.998043617620652	pseudobulk	DESeq2	LRT	0.0778372797173015
126	KC_G1	CG12104	-0.246870103981618	0.638352834105329	0.998043617620652	pseudobulk	DESeq2	LRT	0.194939209133073
126	KC_G1	CG12105	-0.767324608732104	0.724630599467818	0.998043617620652	pseudobulk	DESeq2	LRT	0.139883330666603
126	KC_G1	CG12106	0.0335049202207216	0.932199894288815	0.998043617620652	pseudobulk	DESeq2	LRT	0.0304909506522926
126	KC_G1	CG12107	0.185124587220501	0.511454502208216	0.998043617620652	pseudobulk	DESeq2	LRT	0.291192994051061
126	KC_G1	CG12112	0.332066181466496	0.686634109185038	0.998043617620652	pseudobulk	DESeq2	LRT	0.163274626393953
127	KC_G1	CG12118	-0.232181734796289	0.614733766563081	0.998043617620652	pseudobulk	DESeq2	LRT	0.211312930966898
127	KC_G1	CG12121	-0.0116260491390392	0.964051785356602	0.998043617620652	pseudobulk	DESeq2	LRT	0.0158996367501133
127	KC_G1	CG12123	-0.107519547516533	0.776202767727277	0.998043617620652	pseudobulk	DESeq2	LRT	0.110024813017621
127	KC_G1	CG12128	0.492717488601183	0.3982304302587	0.998043617620652	pseudobulk	DESeq2	LRT	0.399865556994462
127	KC_G1	CG12129	0.204853107062359	0.568553574046331	0.998043617620652	pseudobulk	DESeq2	LRT	0.24522860605724
127	KC_G1	CG1213	-0.303291771980185	0.78946911104989	0.998043617620652	pseudobulk	DESeq2	LRT	0.102664857629111
127	KC_G1	CG12134	1.07914705685927	0.40337803981048	0.998043617620652	pseudobulk	DESeq2	LRT	0.394287748782478
127	KC_G1	CG12147	1.06667464613488	0.669131470313168	0.998043617620652	pseudobulk	DESeq2	LRT	0.174488544082685
127	KC_G1	CG12155	-0.0660886799332305	0.863176106331885	0.998043617620652	pseudobulk	DESeq2	LRT	0.0639005899097555
127	KC_G1	CG12158	1.12508065046033	0.900770125530981	0.998043617620652	pseudobulk	DESeq2	LRT	0.0453860258366365
128	KC_G1	CG12159	0.0898157806575153	0.662543268860775	0.998043617620652	pseudobulk	DESeq2	LRT	0.178785753898548
128	KC_G1	CG12163	-0.121561310997107	0.661467881322185	0.998043617620652	pseudobulk	DESeq2	LRT	0.179491238858481
128	KC_G1	CG12170	-0.0143994054160636	0.953172102861673	0.998043617620652	pseudobulk	DESeq2	LRT	0.020828676932518
128	KC_G1	CG12171	0.0249609511565958	0.922014157396993	0.998043617620652	pseudobulk	DESeq2	LRT	0.0352624103648227
128	KC_G1	CG12173	0.119533119444197	0.716198973397766	0.998043617620652	pseudobulk	DESeq2	LRT	0.144966305842972
128	KC_G1	CG12177	-0.158890058865161	0.645066280324532	0.998043617620652	pseudobulk	DESeq2	LRT	0.190395659472765
128	KC_G1	CG12179	-0.341880050683051	0.726378000637983	0.998043617620652	pseudobulk	DESeq2	LRT	0.138837317496621

128	KC_G1	CG1218	-0.187703132104477	0.713419481737643	0.998043617620652	pseudobulk	DESeq2	LRT	0.146655035317775
128	KC_G1	CG12182	0.0553137960671541	0.924508787430707	0.998043617620652	pseudobulk	DESeq2	LRT	0.0340889565280327
128	KC_G1	CG12194	-0.0745126490759951	0.590546998451291	0.998043617620652	pseudobulk	DESeq2	LRT	0.228745533519011
129	KC_G1	CG12204	-0.307478310909703	0.6075429907566	0.998043617620652	pseudobulk	DESeq2	LRT	0.216422985239501
129	KC_G1	CG12206	0.223908886413978	0.848634630497929	0.998043617620652	pseudobulk	DESeq2	LRT	0.0712792498174768
129	KC_G1	CG12213	0.118197527931886	0.662440598091806	0.998043617620652	pseudobulk	DESeq2	LRT	0.178853059395041
129	KC_G1	CG12219	0.155996080077226	0.774586392240052	0.998043617620652	pseudobulk	DESeq2	LRT	0.110930136870937
129	KC_G1	CG12236	-0.379853716586845	0.238083028493709	0.998043617620652	pseudobulk	DESeq2	LRT	0.623271561731741
129	KC_G1	CG12237	0.0932237419656615	0.736788406946812	0.998043617620652	pseudobulk	DESeq2	LRT	0.132657216196981
129	KC_G1	CG12239	1.12508065046033	0.900770125530981	0.998043617620652	pseudobulk	DESeq2	LRT	0.0453860258366365
129	KC_G1	CG12241	-0.0284966722803478	0.923527672893794	0.998043617620652	pseudobulk	DESeq2	LRT	0.0345500866828491
129	KC_G1	CG12253	-0.411594977673276	0.3226186869332	0.998043617620652	pseudobulk	DESeq2	LRT	0.491310480725952
129	KC_G1	CG12259	0.124001539797486	0.819076444650765	0.998043617620652	pseudobulk	DESeq2	LRT	0.0866755635134634
130	KC_G1	CG12267	0.327849416232603	0.742970067739612	0.998043617620652	pseudobulk	DESeq2	LRT	0.129028682440461
130	KC_G1	CG1227	-0.319538133983604	0.705359919339153	0.998043617620652	pseudobulk	DESeq2	LRT	0.151589221879163
130	KC_G1	CG12279	0.0126672707019889	0.960820266454755	0.998043617620652	pseudobulk	DESeq2	LRT	0.0173578449924928
130	KC_G1	CG12288	-0.545984723714646	0.392316705541679	0.998043617620652	pseudobulk	DESeq2	LRT	0.406363198446813
130	KC_G1	CG12290	-2.98257659561824	0.142657801786736	0.998043617620652	pseudobulk	DESeq2	LRT	0.845704472305002
130	KC_G1	CG12299	0.156835897048375	0.533911846168041	0.998043617620652	pseudobulk	DESeq2	LRT	0.272530443127224
130	KC_G1	CG12301	-0.207471251386683	0.677722473734394	0.998043617620652	pseudobulk	DESeq2	LRT	0.16894811262321
130	KC_G1	CG1231	0.366492653622743	0.57195517395146	0.998043617620652	pseudobulk	DESeq2	LRT	0.242638006990859
130	KC_G1	CG12316	-0.073688256624986	0.834322604412905	0.998043617620652	pseudobulk	DESeq2	LRT	0.0786659898588202
130	KC_G1	CG12320	-0.341044447698048	0.707900990985753	0.998043617620652	pseudobulk	DESeq2	LRT	0.150027479704851
131	KC_G1	CG12321	0.560729691823855	0.181204702370528	0.998043617620652	pseudobulk	DESeq2	LRT	0.741830536311119
131	KC_G1	CG1233	-0.165462352840666	0.793624274847727	0.998043617620652	pseudobulk	DESeq2	LRT	0.100385056742004
131	KC_G1	CG12333	0.0284502275007451	0.913023261973788	0.998043617620652	pseudobulk	DESeq2	LRT	0.0395181573855728
131	KC_G1	CG12338	0.810922193521969	0.28342708282377	0.998043617620652	pseudobulk	DESeq2	LRT	0.547558653174848
131	KC_G1	CG1234	0.0965596965382539	0.828290014383714	0.998043617620652	pseudobulk	DESeq2	LRT	0.0818175743336885
131	KC_G1	CG12341	0.436990122388844	0.233157779396549	0.998043617620652	pseudobulk	DESeq2	LRT	0.632350089569859
131	KC_G1	CG12343	0.0792667090360374	0.776127004013491	0.998043617620652	pseudobulk	DESeq2	LRT	0.110067205766186
131	KC_G1	CG12344	1.03649448874304	0.607834051197003	0.998043617620652	pseudobulk	DESeq2	LRT	0.216214974159449
131	KC_G1	CG12347	-0.794610277746993	0.815039924133852	0.998043617620652	pseudobulk	DESeq2	LRT	0.0888211171417967
131	KC_G1	CG12355	0.480119519263284	0.189897622527934	0.998043617620652	pseudobulk	DESeq2	LRT	0.721480472490274
132	KC_G1	CG1236	-1.13269975252363	0.183359451830926	0.998043617620652	pseudobulk	DESeq2	LRT	0.736696698071996
132	KC_G1	CG12375	-0.0291529397942664	0.911367289252127	0.998043617620652	pseudobulk	DESeq2	LRT	0.040306563149049
132	KC_G1	CG12379	0.824607855069659	0.287781079074782	0.998043617620652	pseudobulk	DESeq2	LRT	0.540937763295665
132	KC_G1	CG12384	-0.0264360499540727	0.874459534600909	0.998043617620652	pseudobulk	DESeq2	LRT	0.0582602825902686
132	KC_G1	CG1239	-0.0269928179811662	0.937021977261143	0.998043617620652	pseudobulk	DESeq2	LRT	0.0282502228888391
132	KC_G1	CG12391	-0.211367237033577	0.354641050783341	0.998043617620652	pseudobulk	DESeq2	LRT	0.450210994914422
132	KC_G1	CG12398	1.13582588427274	0.633424328955532	0.998043617620652	pseudobulk	DESeq2	LRT	0.198305259991224
132	KC_G1	CG12402	-0.169088949675251	0.626263855454335	0.998043617620652	pseudobulk	DESeq2	LRT	0.203242652692286
132	KC_G1	CG12420	0.17172214391994	0.93725366732732	0.998043617620652	pseudobulk	DESeq2	LRT	0.0281428515765614
132	KC_G1	CG12426	2.52420870383484	0.282654181793565	0.998043617620652	pseudobulk	DESeq2	LRT	0.548744584884793
133	KC_G1	CG12446	-0.794610277746993	0.815039924133852	0.998043617620652	pseudobulk	DESeq2	LRT	0.0888211171417967
133	KC_G1	CG12470	1.36169501675078	0.389989232729931	0.998043617620652	pseudobulk	DESeq2	LRT	0.408947383308188
133	KC_G1	CG12499	0.373576178061799	0.662819262407334	0.998043617620652	pseudobulk	DESeq2	LRT	0.178604878895346
133	KC_G1	CG12502	0.540228740443496	0.310200822777467	0.998043617620652	pseudobulk	DESeq2	LRT	0.508357054597076
133	KC_G1	CG12512	-0.0391440311858913	0.937548720852688	0.998043617620652	pseudobulk	DESeq2	LRT	0.0280061543760464
133	KC_G1	CG12523	0.173105107343345	0.973555957903825	0.998043617620652	pseudobulk	DESeq2	LRT	0.0116390811132178
133	KC_G1	CG12531	-0.0825788794259685	0.528111210540519	0.998043617620652	pseudobulk	DESeq2	LRT	0.277274613378366
133	KC_G1	CG12535	-0.268972101525251	0.800777795366305	0.998043617620652	pseudobulk	DESeq2	LRT	0.0964879778409063
133	KC_G1	CG12541	-0.286306701028896	0.336636583270455	0.998043617620652	pseudobulk	DESeq2	LRT	0.472838689758992
133	KC_G1	CG12547	0.0154019627633215	0.939665369541212	0.998043617620652	pseudobulk	DESeq2	LRT	0.0270267783530083
134	KC_G1	CG12560	-0.477373310162741	0.156442222255528	0.998043617620652	pseudobulk	DESeq2	LRT	0.805646023540294
134	KC_G1	CG12567	-0.119882407549322	0.561556741959892	0.998043617620652	pseudobulk	DESeq2	LRT	0.250606354317872
134	KC_G1	CG12576	-0.152572719389658	0.531587249023794	0.998043617620652	pseudobulk	DESeq2	LRT	0.274425444856882

134	KC_G1	CG12581	0.977397752787486	0.520074137772034	0.998043617620652	pseudobulk	DESeq2	LRT	0.283934742268541
134	KC_G1	CG12592	0.172834960772825	0.908610676445368	0.998043617620652	pseudobulk	DESeq2	LRT	0.0416221644022337
134	KC_G1	CG12594	0.759711379153322	0.579871529275129	0.998043617620652	pseudobulk	DESeq2	LRT	0.236668213863002
134	KC_G1	CG12607	-0.794610277746993	0.879485534599449	0.998043617620652	pseudobulk	DESeq2	LRT	0.0557712992369932
134	KC_G1	CG12608	0.23120252517711	0.794817405104129	0.998043617620652	pseudobulk	DESeq2	LRT	0.0997326311722934
134	KC_G1	CG12640	-1.66808346769027	0.498191247612582	0.998043617620652	pseudobulk	DESeq2	LRT	0.302603906560549
134	KC_G1	CG12643	-0.765625368254185	0.836625948066609	0.998043617620652	pseudobulk	DESeq2	LRT	0.0774686698445758
135	KC_G1	CG1265	0.0662801711312953	0.752924926937256	0.998043617620652	pseudobulk	DESeq2	LRT	0.123248324519453
135	KC_G1	CG12655	1.25532895350747	0.542118494533867	0.998043617620652	pseudobulk	DESeq2	LRT	0.265905776381103
135	KC_G1	CG12659	-0.471089690892907	0.368801819322956	0.998043617620652	pseudobulk	DESeq2	LRT	0.433206945210676
135	KC_G1	CG12672	-0.432640699632199	0.431523074999823	0.998043617620652	pseudobulk	DESeq2	LRT	0.364995976131505
135	KC_G1	CG12699	1.12508065046033	0.900770125530981	0.998043617620652	pseudobulk	DESeq2	LRT	0.0453860258366365
135	KC_G1	CG1271	-0.047683549492509	0.939684844809014	0.998043617620652	pseudobulk	DESeq2	LRT	0.0270177773682255
135	KC_G1	CG12713	0.24468236110097	0.791654358574927	0.998043617620652	pseudobulk	DESeq2	LRT	0.101464392815044
135	KC_G1	CG12715	2.23045770445974	0.357070320422792	0.998043617620652	pseudobulk	DESeq2	LRT	0.447246246733247
135	KC_G1	CG12716	0.74965298087183	0.660133504944147	0.998043617620652	pseudobulk	DESeq2	LRT	0.180368224159358
135	KC_G1	CG12717	-0.268910580016355	0.515683123127381	0.998043617620652	pseudobulk	DESeq2	LRT	0.287617081609742
136	KC_G1	CG12728	0.376108616157714	0.390779677779369	0.998043617620652	pseudobulk	DESeq2	LRT	0.408068029543251
136	KC_G1	CG1273	1.4913478704297	0.450294972120288	0.998043617620652	pseudobulk	DESeq2	LRT	0.346502902232282
136	KC_G1	CG12730	0.375485248349901	0.308849917897132	0.998043617620652	pseudobulk	DESeq2	LRT	0.510252509776594
136	KC_G1	CG12746	0.283545500218065	0.571481009995169	0.998043617620652	pseudobulk	DESeq2	LRT	0.242998196399642
136	KC_G1	CG1275	0.40993658208385	0.21457478712897	0.998043617620652	pseudobulk	DESeq2	LRT	0.668421309648939
136	KC_G1	CG12766	-0.704797641189757	0.838804762683723	0.998043617620652	pseudobulk	DESeq2	LRT	0.0763391123013672
136	KC_G1	CG12768	-0.946704773636284	0.166809370690541	0.998043617620652	pseudobulk	DESeq2	LRT	0.777779556067084
136	KC_G1	CG12769	0.0376631272933117	0.881826516177619	0.998043617620652	pseudobulk	DESeq2	LRT	0.0546168462485475
136	KC_G1	CG12773	-0.0744263467082942	0.88925817551434	0.998043617620652	pseudobulk	DESeq2	LRT	0.0509721333792435
136	KC_G1	CG12780	2.09164996791006	0.330172039924601	0.998043617620652	pseudobulk	DESeq2	LRT	0.481259707028997
137	KC_G1	CG12795	0.181798293135126	0.603299246569279	0.998043617620652	pseudobulk	DESeq2	LRT	0.219467217052249
137	KC_G1	CG12811	-0.317796297774579	0.819908356073887	0.998043617620652	pseudobulk	DESeq2	LRT	0.086234687464629
137	KC_G1	CG12814	0.126234972651469	0.933375088027233	0.998043617620652	pseudobulk	DESeq2	LRT	0.029943794705081
137	KC_G1	CG12817	0.115431261217636	0.686634526308764	0.998043617620652	pseudobulk	DESeq2	LRT	0.163274362564243
137	KC_G1	CG12818	-0.308969721491629	0.534100515892194	0.998043617620652	pseudobulk	DESeq2	LRT	0.272377002541956
137	KC_G1	CG12822	0.0560738230222713	0.804710917417212	0.998043617620652	pseudobulk	DESeq2	LRT	0.0943601066115453
137	KC_G1	CG12825	2.76740599570183	0.225374198521499	0.998043617620652	pseudobulk	DESeq2	LRT	0.647095804710182
137	KC_G1	CG12848	0.088390927098351	0.769328097280842	0.998043617620652	pseudobulk	DESeq2	LRT	0.113888406037237
137	KC_G1	CG12851	-0.176121508349322	0.462161039996685	0.998043617620652	pseudobulk	DESeq2	LRT	0.335206668176716
137	KC_G1	CG12857	-1.60891734856068	0.545249891924755	0.998043617620652	pseudobulk	DESeq2	LRT	0.263404411831769
138	KC_G1	CG12863	-1.06069945942148	0.235706821122846	0.998043617620652	pseudobulk	DESeq2	LRT	0.627627849240003
138	KC_G1	CG12869	0.949176147629133	0.729784453976284	0.998043617620652	pseudobulk	DESeq2	LRT	0.136805392306293
138	KC_G1	CG12877	0.132868646525621	0.755254005517256	0.998043617620652	pseudobulk	DESeq2	LRT	0.121906962751897
138	KC_G1	CG12883	0.556879691704966	0.407902752831252	0.998043617620652	pseudobulk	DESeq2	LRT	0.38944336373074
138	KC_G1	CG12885	1.12508065046033	0.900770125530981	0.998043617620652	pseudobulk	DESeq2	LRT	0.0453860258366365
138	KC_G1	CG12895	0.151788086399589	0.468003235019832	0.998043617620652	pseudobulk	DESeq2	LRT	0.329751144903641
138	KC_G1	CG12896	0.780037393225056	0.549542460374809	0.998043617620652	pseudobulk	DESeq2	LRT	0.259998746199424
138	KC_G1	CG12909	0.317102701477563	0.780474725007857	0.998043617620652	pseudobulk	DESeq2	LRT	0.107641156621234
138	KC_G1	CG12910	-0.794610277746993	0.879485534599449	0.998043617620652	pseudobulk	DESeq2	LRT	0.0557712992369932
138	KC_G1	CG12911	-0.794610277746993	0.879485534599449	0.998043617620652	pseudobulk	DESeq2	LRT	0.0557712992369932
139	KC_G1	CG12913	0.0888147176042635	0.820986389531954	0.998043617620652	pseudobulk	DESeq2	LRT	0.0856640426371543
139	KC_G1	CG12923	-0.128283932671325	0.903002705067355	0.998043617620652	pseudobulk	DESeq2	LRT	0.0443109486963882
139	KC_G1	CG12926	-0.721219430810425	0.231955433829733	0.998043617620652	pseudobulk	DESeq2	LRT	0.634595449165368
139	KC_G1	CG12929	0.144666108446849	0.790646957068502	0.998043617620652	pseudobulk	DESeq2	LRT	0.102017396178044
139	KC_G1	CG12935	-0.196696061627264	0.678693127556227	0.998043617620652	pseudobulk	DESeq2	LRT	0.168326548450377
139	KC_G1	CG12942	-0.429443581872051	0.478020051675552	0.998043617620652	pseudobulk	DESeq2	LRT	0.320553885502114
139	KC_G1	CG12945	-0.24593404697965	0.46788817129671	0.998043617620652	pseudobulk	DESeq2	LRT	0.329857934088655
139	KC_G1	CG12947	-6.13959285384539	0.150846108147504	0.998043617620652	pseudobulk	DESeq2	LRT	0.821465890208032
139	KC_G1	CG12970	-0.139046923079547	0.930850499186999	0.998043617620652	pseudobulk	DESeq2	LRT	0.0311200640151481

139	KC_G1	CG12975	-0.159538049870588	0.590126576111659	0.998043617620652	pseudobulk	DESeq2	LRT	0.22905482664426
140	KC_G1	CG1299	-0.794610277746993	0.815039924133852	0.998043617620652	pseudobulk	DESeq2	LRT	0.0888211171417967
140	KC_G1	CG12991	-0.0919134211892751	0.686764153595596	0.998043617620652	pseudobulk	DESeq2	LRT	0.16319238139393
140	KC_G1	CG12992	-1.03741575212789	0.453617295805422	0.998043617620652	pseudobulk	DESeq2	LRT	0.343310394746429
140	KC_G1	CG12994	0.917774228005062	0.0373527219615759	0.998043617620652	pseudobulk	DESeq2	LRT	1.42767774485744
140	KC_G1	CG13001	-0.0931751387575168	0.769242750326766	0.998043617620652	pseudobulk	DESeq2	LRT	0.113936588038473
140	KC_G1	CG13005	-0.0995840840355018	0.902156020589401	0.998043617620652	pseudobulk	DESeq2	LRT	0.0447183482435048
140	KC_G1	CG13008	-1.60891734856068	0.545249891924755	0.998043617620652	pseudobulk	DESeq2	LRT	0.263404411831769
140	KC_G1	CG13012	-1.09518749403862	0.584677327274943	0.998043617620652	pseudobulk	DESeq2	LRT	0.233083746987579
140	KC_G1	CG13014	0.733168551367545	0.334176188544505	0.998043617620652	pseudobulk	DESeq2	LRT	0.476024498644139
140	KC_G1	CG13016	-0.196769603445554	0.711818083268254	0.998043617620652	pseudobulk	DESeq2	LRT	0.147630983227564
141	KC_G1	CG13018	0.0868258730502504	0.819183215930427	0.998043617620652	pseudobulk	DESeq2	LRT	0.0866189544437728
141	KC_G1	CG13021	1.12508065046033	0.900770125530981	0.998043617620652	pseudobulk	DESeq2	LRT	0.0453860258366365
141	KC_G1	CG13025	-0.0828954342138482	0.88528032590921	0.998043617620652	pseudobulk	DESeq2	LRT	0.0529191872476872
141	KC_G1	CG13051	-0.794610277746993	0.879485534599449	0.998043617620652	pseudobulk	DESeq2	LRT	0.0557712992369932
141	KC_G1	CG13054	0.101349108201353	0.832744448158114	0.998043617620652	pseudobulk	DESeq2	LRT	0.0794882540343636
141	KC_G1	CG13055	-0.194808589981496	0.192634085072215	0.998043617620652	pseudobulk	DESeq2	LRT	0.715266865451006
141	KC_G1	CG13067	-0.794610277746993	0.879485534599449	0.998043617620652	pseudobulk	DESeq2	LRT	0.0557712992369932
141	KC_G1	CG13068	-1.66808346769027	0.498191247612582	0.998043617620652	pseudobulk	DESeq2	LRT	0.302603906560549
141	KC_G1	CG13069	-0.794610277746993	0.879485534599449	0.998043617620652	pseudobulk	DESeq2	LRT	0.0557712992369932
141	KC_G1	CG1307	0.241646193915902	0.407103367770613	0.998043617620652	pseudobulk	DESeq2	LRT	0.390295304896101
142	KC_G1	CG13070	1.12508065046033	0.900770125530981	0.998043617620652	pseudobulk	DESeq2	LRT	0.0453860258366365
142	KC_G1	CG13085	0.172530702244785	0.946040718765468	0.998043617620652	pseudobulk	DESeq2	LRT	0.0240901706229663
142	KC_G1	CG13088	-0.0114278553659779	0.942777778064275	0.998043617620652	pseudobulk	DESeq2	LRT	0.0255906626536541
142	KC_G1	CG1309	0.0794466588206886	0.835104096194121	0.998043617620652	pseudobulk	DESeq2	LRT	0.0782593860913075
142	KC_G1	CG13096	0.2260013201079	0.664027619683512	0.998043617620652	pseudobulk	DESeq2	LRT	0.177813856133978
142	KC_G1	CG13097	-0.461988101402731	0.364041237006552	0.998043617620652	pseudobulk	DESeq2	LRT	0.438849418576135
142	KC_G1	CG13108	-0.167973937605876	0.470654281094561	0.998043617620652	pseudobulk	DESeq2	LRT	0.327297986593954
142	KC_G1	CG13123	-0.106468582762456	0.872018871557409	0.998043617620652	pseudobulk	DESeq2	LRT	0.0594741163007318
142	KC_G1	CG13124	0.403114000075856	0.608599524539312	0.998043617620652	pseudobulk	DESeq2	LRT	0.215668391264795
142	KC_G1	CG13126	0.0807794835396991	0.921534949687367	0.998043617620652	pseudobulk	DESeq2	LRT	0.0354881892907689
143	KC_G1	CG1314	-0.752107099832492	0.841251974639758	0.998043617620652	pseudobulk	DESeq2	LRT	0.0750739033633379
143	KC_G1	CG13148	0.140273982529335	0.721858198565214	0.998043617620652	pseudobulk	DESeq2	LRT	0.141548106625624
143	KC_G1	CG13151	0.42893344404834	0.698099544877075	0.998043617620652	pseudobulk	DESeq2	LRT	0.156082645129891
143	KC_G1	CG13154	-0.794610277746993	0.815039924133852	0.998043617620652	pseudobulk	DESeq2	LRT	0.0888211171417967
143	KC_G1	CG1316	-0.00644586202397563	0.964454392045198	0.998043617620652	pseudobulk	DESeq2	LRT	0.0157183048282108
143	KC_G1	CG13168	-0.889114689231764	0.658255756426461	0.998043617620652	pseudobulk	DESeq2	LRT	0.181605334313324
143	KC_G1	CG1317	-0.221670443139378	0.433515809986304	0.998043617620652	pseudobulk	DESeq2	LRT	0.36299505951509
143	KC_G1	CG13185	-0.26384392551259	0.69569272527956	0.998043617620652	pseudobulk	DESeq2	LRT	0.157582537949613
143	KC_G1	CG13192	-0.038998299003506	0.933398209325582	0.998043617620652	pseudobulk	DESeq2	LRT	0.0299330366207414
143	KC_G1	CG13197	-0.27147178178979	0.424017042906061	0.998043617620652	pseudobulk	DESeq2	LRT	0.372616687059849
144	KC_G1	CG13202	-0.794610277746993	0.821479263487599	0.998043617620652	pseudobulk	DESeq2	LRT	0.085403394939296
144	KC_G1	CG13204	-0.148405050735958	0.484282451648117	0.998043617620652	pseudobulk	DESeq2	LRT	0.314901267667206
144	KC_G1	CG13220	0.478072478916167	0.116834614684755	0.998043617620652	pseudobulk	DESeq2	LRT	0.932428469385886
144	KC_G1	CG13229	0.519684134854897	0.562933500883525	0.998043617620652	pseudobulk	DESeq2	LRT	0.249542905157664
144	KC_G1	CG13248	0.0584131960415995	0.786750349588597	0.998043617620652	pseudobulk	DESeq2	LRT	0.10416305543811
144	KC_G1	CG13252	-0.743277948839997	0.3599579252367	0.998043617620652	pseudobulk	DESeq2	LRT	0.443748260081114
144	KC_G1	CG13255	0.209399025233779	0.78295634839419	0.998043617620652	pseudobulk	DESeq2	LRT	0.106262450177032
144	KC_G1	CG13284	-0.0197149174281006	0.950365678984645	0.998043617620652	pseudobulk	DESeq2	LRT	0.0221092559663351
144	KC_G1	CG13288	1.12508065046033	0.900770125530981	0.998043617620652	pseudobulk	DESeq2	LRT	0.0453860258366365
144	KC_G1	CG13293	-0.161854842993441	0.39876137980981	0.998043617620652	pseudobulk	DESeq2	LRT	0.399286909908217
145	KC_G1	CG13295	0.152422935830066	0.817963792024905	0.998043617620652	pseudobulk	DESeq2	LRT	0.0872659203773557
145	KC_G1	CG13300	-0.223229426059071	0.31174378497181	0.998043617620652	pseudobulk	DESeq2	LRT	0.506202196002919
145	KC_G1	CG13315	-0.966461363000173	0.649553099843358	0.998043617620652	pseudobulk	DESeq2	LRT	0.187385340316377
145	KC_G1	CG13319	0.178486044756221	0.77327566291161	0.998043617620652	pseudobulk	DESeq2	LRT	0.111665658035473
145	KC_G1	CG13325	0.420255255059539	0.653834022859783	0.998043617620652	pseudobulk	DESeq2	LRT	0.184532484251496

145	KC_G1	CG13339	0.0560319433821096	0.863302251797999	0.998043617620652	pseudobulk	DESeq2	LRT	0.0638371262935569
145	KC_G1	CG13344	0.00676797117513056	0.965599591090547	0.998043617620652	pseudobulk	DESeq2	LRT	0.0152029268244981
145	KC_G1	CG13364	-0.061137322561833	0.711897345616413	0.998043617620652	pseudobulk	DESeq2	LRT	0.147582626372303
145	KC_G1	CG13365	0.187449711779398	0.495745536125396	0.998043617620652	pseudobulk	DESeq2	LRT	0.304741187651831
145	KC_G1	CG13366	0.0891422312145192	0.713481593346173	0.998043617620652	pseudobulk	DESeq2	LRT	0.14661722648949
146	KC_G1	CG13367	-0.292471238090333	0.560136639173512	0.998043617620652	pseudobulk	DESeq2	LRT	0.251706018706997
146	KC_G1	CG13369	0.30483981356988	0.449504848195869	0.998043617620652	pseudobulk	DESeq2	LRT	0.347265619763103
146	KC_G1	CG13373	1.54235971072306	0.529343359605328	0.998043617620652	pseudobulk	DESeq2	LRT	0.276262530595783
146	KC_G1	CG13375	0.158384188459191	0.392912614396116	0.998043617620652	pseudobulk	DESeq2	LRT	0.405704028010073
146	KC_G1	CG13377	0.740448763324109	0.715148959422093	0.998043617620652	pseudobulk	DESeq2	LRT	0.14560348894416
146	KC_G1	CG13384	-0.265903304947996	0.411272579975974	0.998043617620652	pseudobulk	DESeq2	LRT	0.385870244446798
146	KC_G1	CG13392	-0.457757517028089	0.530836064387669	0.998043617620652	pseudobulk	DESeq2	LRT	0.275039579340953
146	KC_G1	CG13397	-0.258640835184631	0.833732816576773	0.998043617620652	pseudobulk	DESeq2	LRT	0.0789731038913356
146	KC_G1	CG13398	-0.0488544545301973	0.891325109859378	0.998043617620652	pseudobulk	DESeq2	LRT	0.0499638586289654
146	KC_G1	CG13404	0.185364318649252	0.594511473279851	0.998043617620652	pseudobulk	DESeq2	LRT	0.22583975965917
147	KC_G1	CG13408	-0.794610277746993	0.815039924133852	0.998043617620652	pseudobulk	DESeq2	LRT	0.0888211171417967
147	KC_G1	CG13409	0.135415331680334	0.72557850039091	0.998043617620652	pseudobulk	DESeq2	LRT	0.139315594332642
147	KC_G1	CG13436	0.172834960772825	0.908610676445368	0.998043617620652	pseudobulk	DESeq2	LRT	0.0416221644022337
147	KC_G1	CG13437	-2.72612288702591	0.116600254647942	0.998043617620652	pseudobulk	DESeq2	LRT	0.933300501102944
147	KC_G1	CG1344	0.352644057455706	0.378707121339112	0.998043617620652	pseudobulk	DESeq2	LRT	0.421696528179408
147	KC_G1	CG13458	2.24250860368706	0.341325130141026	0.998043617620652	pseudobulk	DESeq2	LRT	0.466831735513337
147	KC_G1	CG13461	-0.794610277746993	0.879485534599449	0.998043617620652	pseudobulk	DESeq2	LRT	0.0557712992369932
147	KC_G1	CG13482	0.133674429160019	0.913455266749963	0.998043617620652	pseudobulk	DESeq2	LRT	0.0393127158312411
147	KC_G1	CG13484	0.0120662204545167	0.949021870231409	0.998043617620652	pseudobulk	DESeq2	LRT	0.022723779130794
147	KC_G1	CG13502	0.173116475207952	0.97305562682953	0.998043617620652	pseudobulk	DESeq2	LRT	0.011862331638572
148	KC_G1	CG13506	-0.150059786908748	0.721180781075073	0.998043617620652	pseudobulk	DESeq2	LRT	0.14195585541644
148	KC_G1	CG13526	-1.12087039990708	0.568235250183439	0.998043617620652	pseudobulk	DESeq2	LRT	0.245471828541097
148	KC_G1	CG13531	-0.746567034073548	0.272387545195538	0.998043617620652	pseudobulk	DESeq2	LRT	0.564812754235436
148	KC_G1	CG13532	0.110797836066591	0.691878099216759	0.998043617620652	pseudobulk	DESeq2	LRT	0.159970416382843
148	KC_G1	CG1354	0.129419006095954	0.707952113620497	0.998043617620652	pseudobulk	DESeq2	LRT	0.149996117301354
148	KC_G1	CG13551	-0.0734850300501835	0.574833877579063	0.998043617620652	pseudobulk	DESeq2	LRT	0.240457644831321
148	KC_G1	CG13563	-0.794610277746993	0.821479263487599	0.998043617620652	pseudobulk	DESeq2	LRT	0.085403394939296
148	KC_G1	CG13578	1.58938098679069	0.316327852176686	0.998043617620652	pseudobulk	DESeq2	LRT	0.499862567445299
148	KC_G1	CG13579	1.21753279351964	0.00536681998670999	0.998043617620652	pseudobulk	DESeq2	LRT	2.27028297152676
148	KC_G1	CG1358	-0.113884086327536	0.619105728976038	0.998043617620652	pseudobulk	DESeq2	LRT	0.208235177167569
149	KC_G1	CG13585	0.0536464161701063	0.732736301208248	0.998043617620652	pseudobulk	DESeq2	LRT	0.135052292118839
149	KC_G1	CG13587	-1.73907471612892	0.507597351592657	0.998043617620652	pseudobulk	DESeq2	LRT	0.294480652518419
149	KC_G1	CG13594	-0.771924361917205	0.668832905440925	0.998043617620652	pseudobulk	DESeq2	LRT	0.174682368493869
149	KC_G1	CG1360	-0.614240326031689	0.41797510875922	0.998043617620652	pseudobulk	DESeq2	LRT	0.378849580546482
149	KC_G1	CG13601	0.126493459120536	0.640383543274868	0.998043617620652	pseudobulk	DESeq2	LRT	0.193559837209871
149	KC_G1	CG13603	0.0665552752752664	0.815175882070984	0.998043617620652	pseudobulk	DESeq2	LRT	0.0887486779202168
149	KC_G1	CG13604	-0.0182245867109544	0.973912700588552	0.998043617620652	pseudobulk	DESeq2	LRT	0.0114799705873632
149	KC_G1	CG13609	-0.496383574798577	0.640809933834094	0.998043617620652	pseudobulk	DESeq2	LRT	0.193270764439593
149	KC_G1	CG13623	0.107095743720032	0.731030973201343	0.998043617620652	pseudobulk	DESeq2	LRT	0.136064221940277
149	KC_G1	CG13625	-0.0216187011156564	0.940746896933551	0.998043617620652	pseudobulk	DESeq2	LRT	0.0265272055319976
150	KC_G1	CG13630	-0.182716788215057	0.701155988249711	0.998043617620652	pseudobulk	DESeq2	LRT	0.154185352504333
150	KC_G1	CG13631	-0.313194606072357	0.420875355861998	0.998043617620652	pseudobulk	DESeq2	LRT	0.375846503395452
150	KC_G1	CG13654	-0.176722527383108	0.627541651434227	0.998043617620652	pseudobulk	DESeq2	LRT	0.202357443731019
150	KC_G1	CG13663	0.0675614987654025	0.901109422108542	0.998043617620652	pseudobulk	DESeq2	LRT	0.0452224692511833
150	KC_G1	CG13667	-0.981473414754324	0.545315187798355	0.998043617620652	pseudobulk	DESeq2	LRT	0.263352406429879
150	KC_G1	CG13671	-0.187573407141863	0.812328688454285	0.998043617620652	pseudobulk	DESeq2	LRT	0.0902682088149221
150	KC_G1	CG1368	0.00122123710914415	0.955537993700262	0.998043617620652	pseudobulk	DESeq2	LRT	0.0197520400309208
150	KC_G1	CG13685	0.198073967660795	0.509094312830182	0.998043617620652	pseudobulk	DESeq2	LRT	0.29320175450512
150	KC_G1	CG13689	-0.274208259380055	0.594896581282371	0.998043617620652	pseudobulk	DESeq2	LRT	0.225558526846539
150	KC_G1	CG13693	1.12508065046033	0.900770125530981	0.998043617620652	pseudobulk	DESeq2	LRT	0.0453860258366365
151	KC_G1	CG13698	-1.18758623933229	0.520801314224088	0.998043617620652	pseudobulk	DESeq2	LRT	0.283327928511391

151	KC_G1	CG13707	0.329331827850317	0.553348795324318	0.998043617620652	pseudobulk	DESeq2	LRT	0.257001031203322
151	KC_G1	CG1371	-0.304928131164912	0.388222899821409	0.998043617620652	pseudobulk	DESeq2	LRT	0.410918850779826
151	KC_G1	CG13716	-0.794610277746993	0.821479263487599	0.998043617620652	pseudobulk	DESeq2	LRT	0.085403394939296
151	KC_G1	CG13737	1.13528146900654	0.22022108417363	0.998043617620652	pseudobulk	DESeq2	LRT	0.657141103611717
151	KC_G1	CG13739	0.0358193729926153	0.798732660409027	0.998043617620652	pseudobulk	DESeq2	LRT	0.0975985567777269
151	KC_G1	CG13741	1.24406349205447	0.626072434068117	0.998043617620652	pseudobulk	DESeq2	LRT	0.203375417754029
151	KC_G1	CG13743	0.215174445684321	0.280049578336264	0.998043617620652	pseudobulk	DESeq2	LRT	0.552765076901228
151	KC_G1	CG13751	-0.100481847476122	0.744908311525563	0.998043617620652	pseudobulk	DESeq2	LRT	0.127897179934996
151	KC_G1	CG13760	-0.196792954647642	0.612053051595378	0.998043617620652	pseudobulk	DESeq2	LRT	0.213210932402531
152	KC_G1	CG13766	0.273377488535584	0.488522191993435	0.998043617620652	pseudobulk	DESeq2	LRT	0.311115702894278
152	KC_G1	CG13773	0.224929378974082	0.750963990857545	0.998043617620652	pseudobulk	DESeq2	LRT	0.124380887159346
152	KC_G1	CG13775	0.591786257214813	0.568189696347563	0.998043617620652	pseudobulk	DESeq2	LRT	0.245506646114614
152	KC_G1	CG13784	-0.0763726628291628	0.642505272793997	0.998043617620652	pseudobulk	DESeq2	LRT	0.192123303894036
152	KC_G1	CG13796	-0.651522973901016	0.8143389309148	0.998043617620652	pseudobulk	DESeq2	LRT	0.0891948024940098
152	KC_G1	CG13807	0.0164878513526583	0.967110723954461	0.998043617620652	pseudobulk	DESeq2	LRT	0.014523800943248
152	KC_G1	CG13827	-0.591865308026349	0.545182542351293	0.998043617620652	pseudobulk	DESeq2	LRT	0.263458059443911
152	KC_G1	CG13847	-0.794610277746993	0.821479263487599	0.998043617620652	pseudobulk	DESeq2	LRT	0.085403394939296
152	KC_G1	CG13850	-0.689451520422483	0.13821223153486	0.998043617620652	pseudobulk	DESeq2	LRT	0.859453520977501
152	KC_G1	CG13868	0.138233737018608	0.294733565758405	0.998043617620652	pseudobulk	DESeq2	LRT	0.530570401667723
153	KC_G1	CG13870	-0.794610277746993	0.821479263487599	0.998043617620652	pseudobulk	DESeq2	LRT	0.085403394939296
153	KC_G1	CG13875	-2.66786719284931	0.228087652304378	0.998043617620652	pseudobulk	DESeq2	LRT	0.64189822494353
153	KC_G1	CG13876	0.194453951434146	0.762030484359982	0.998043617620652	pseudobulk	DESeq2	LRT	0.11802765474372
153	KC_G1	CG13877	2.38466886841527	0.34619303318143	0.998043617620652	pseudobulk	DESeq2	LRT	0.460681676152437
153	KC_G1	CG13887	-0.0400557734632009	0.866695346565614	0.998043617620652	pseudobulk	DESeq2	LRT	0.06213353523065
153	KC_G1	CG13893	-0.0955083647497408	0.78812507033552	0.998043617620652	pseudobulk	DESeq2	LRT	0.103404857324617
153	KC_G1	CG13894	0.428737773138681	0.419529431899438	0.998043617620652	pseudobulk	DESeq2	LRT	0.377237566031596
153	KC_G1	CG13895	-0.643929147853006	0.388999440030424	0.998043617620652	pseudobulk	DESeq2	LRT	0.4100510238462
153	KC_G1	CG13896	0.0635391530204423	0.959977530171732	0.998043617620652	pseudobulk	DESeq2	LRT	0.0177389322069233
153	KC_G1	CG13898	-0.299540469042341	0.870848058508045	0.998043617620652	pseudobulk	DESeq2	LRT	0.0600576120506324
154	KC_G1	CG13901	0.422453494498297	0.421523238536348	0.998043617620652	pseudobulk	DESeq2	LRT	0.375178477765449
154	KC_G1	CG13907	0.3292099563095	0.332071772249075	0.998043617620652	pseudobulk	DESeq2	LRT	0.47876804002189
154	KC_G1	CG13917	-0.0736869938248632	0.528542630969737	0.998043617620652	pseudobulk	DESeq2	LRT	0.276919977801717
154	KC_G1	CG13919	-0.125757903172143	0.747298189098178	0.998043617620652	pseudobulk	DESeq2	LRT	0.126506070153334
154	KC_G1	CG13920	0.0766371992711273	0.642913155798227	0.998043617620652	pseudobulk	DESeq2	LRT	0.191847687269902
154	KC_G1	CG13921	-0.0416677942875149	0.793884125111871	0.998043617620652	pseudobulk	DESeq2	LRT	0.100242882329762
154	KC_G1	CG13924	-1.72984073695683	0.480047293856853	0.998043617620652	pseudobulk	DESeq2	LRT	0.318715974188453
154	KC_G1	CG13928	0.0143754893455154	0.892022757497871	0.998043617620652	pseudobulk	DESeq2	LRT	0.0496240656578771
154	KC_G1	CG13931	3.38422863745497	0.0927384082653015	0.998043617620652	pseudobulk	DESeq2	LRT	1.03274036247869
154	KC_G1	CG13933	-0.180656721708362	0.679112255353874	0.998043617620652	pseudobulk	DESeq2	LRT	0.16805843212413
155	KC_G1	CG13937	0.475908149581365	0.0927363714152713	0.998043617620652	pseudobulk	DESeq2	LRT	1.03274990116347
155	KC_G1	CG13954	0.0156744415498087	0.939452564777175	0.998043617620652	pseudobulk	DESeq2	LRT	0.0271251435843236
155	KC_G1	CG13982	0.0739195145548044	0.887764182659843	0.998043617620652	pseudobulk	DESeq2	LRT	0.0517023808098359
155	KC_G1	CG13994	-0.0861261312992271	0.797658662394163	0.998043617620652	pseudobulk	DESeq2	LRT	0.0981829141030758
155	KC_G1	CG13995	-0.0508699955592098	0.858434085199604	0.998043617620652	pseudobulk	DESeq2	LRT	0.0662930464938616
155	KC_G1	CG13999	-0.0894664707376742	0.768262501175276	0.998043617620652	pseudobulk	DESeq2	LRT	0.114490364169837
155	KC_G1	CG14005	-0.0243062415930269	0.956652065153455	0.998043617620652	pseudobulk	DESeq2	LRT	0.0192459866320215
155	KC_G1	CG14015	0.0266307708580235	0.918467242582219	0.998043617620652	pseudobulk	DESeq2	LRT	0.0369363283287494
155	KC_G1	CG14024	0.220126113091979	0.419329811134316	0.998043617620652	pseudobulk	DESeq2	LRT	0.377444261491227
155	KC_G1	CG14043	0.0275661257905349	0.938198591393879	0.998043617620652	pseudobulk	DESeq2	LRT	0.0277052234356786
156	KC_G1	CG14044	-0.284756322679938	0.660084410361271	0.998043617620652	pseudobulk	DESeq2	LRT	0.180400524139834
156	KC_G1	CG14050	0.11746874488831	0.944061018239047	0.998043617620652	pseudobulk	DESeq2	LRT	0.0249999346975866
156	KC_G1	CG14057	0.606002819973962	0.369415710108611	0.998043617620652	pseudobulk	DESeq2	LRT	0.432484639300755
156	KC_G1	CG1407	-0.0418389410682915	0.909519401057511	0.998043617620652	pseudobulk	DESeq2	LRT	0.041188032518745
156	KC_G1	CG14073	0.0349650944858805	0.833803736267766	0.998043617620652	pseudobulk	DESeq2	LRT	0.0789361631353173
156	KC_G1	CG14074	0.049974902431647	0.899173768022146	0.998043617620652	pseudobulk	DESeq2	LRT	0.0461563714473978
156	KC_G1	CG14086	1.12508065046033	0.900770125530981	0.998043617620652	pseudobulk	DESeq2	LRT	0.0453860258366365

156	KC_G1	CG14100	0.07300295359282	0.882384849765791	0.998043617620652	pseudobulk	DESeq2	LRT	0.0543419571857276
156	KC_G1	CG14102	0.247313107013902	0.695218033593994	0.998043617620652	pseudobulk	DESeq2	LRT	0.157878971040428
156	KC_G1	CG14104	0.0555241302310033	0.938534834834124	0.998043617620652	pseudobulk	DESeq2	LRT	0.0275496033884238
157	KC_G1	CG14117	-0.794610277746993	0.815039924133852	0.998043617620652	pseudobulk	DESeq2	LRT	0.0888211171417967
157	KC_G1	CG14130	-0.240382002389387	0.695882358367661	0.998043617620652	pseudobulk	DESeq2	LRT	0.157464173363719
157	KC_G1	CG14135	0.141354677823271	0.784971831983296	0.998043617620652	pseudobulk	DESeq2	LRT	0.105145927246731
157	KC_G1	CG14137	0.337737908433352	0.875517472491096	0.998043617620652	pseudobulk	DESeq2	LRT	0.0577351823837907
157	KC_G1	CG14141	-0.12235865170517	0.630455487606154	0.998043617620652	pseudobulk	DESeq2	LRT	0.200345570739501
157	KC_G1	CG14153	-0.64277910557112	0.764034577741626	0.998043617620652	pseudobulk	DESeq2	LRT	0.116886986211521
157	KC_G1	CG1416	-0.0656309640514925	0.756332795208416	0.998043617620652	pseudobulk	DESeq2	LRT	0.121287067831772
157	KC_G1	CG14174	0.320376960131881	0.749284248163224	0.998043617620652	pseudobulk	DESeq2	LRT	0.125353397266703
157	KC_G1	CG1418	-0.0614657454861607	0.871124850941168	0.998043617620652	pseudobulk	DESeq2	LRT	0.0599195967844966
157	KC_G1	CG14182	-0.0829621833208937	0.829559268204895	0.998043617620652	pseudobulk	DESeq2	LRT	0.0811525801823341
158	KC_G1	CG14183	0.272216222235083	0.772915459658508	0.998043617620652	pseudobulk	DESeq2	LRT	0.11186800596741
158	KC_G1	CG14184	-0.0904846857988782	0.732819377762149	0.998043617620652	pseudobulk	DESeq2	LRT	0.135003055246448
158	KC_G1	CG14185	1.55905404275586	0.39602511226029	0.998043617620652	pseudobulk	DESeq2	LRT	0.402277274250543
158	KC_G1	CG14186	-0.00703232776822545	0.964053403357441	0.998043617620652	pseudobulk	DESeq2	LRT	0.0158989078595527
158	KC_G1	CG14190	1.12508065046033	0.900770125530981	0.998043617620652	pseudobulk	DESeq2	LRT	0.0453860258366365
158	KC_G1	CG14194	-0.300271709561784	0.404935852557149	0.998043617620652	pseudobulk	DESeq2	LRT	0.392613769592636
158	KC_G1	CG14196	2.04147257822512	0.408147672536479	0.998043617620652	pseudobulk	DESeq2	LRT	0.389182675723292
158	KC_G1	CG14200	0.0273514933459682	0.906670471923501	0.998043617620652	pseudobulk	DESeq2	LRT	0.0425505279664447
158	KC_G1	CG14207	0.0206680743231683	0.924447410251802	0.998043617620652	pseudobulk	DESeq2	LRT	0.0341177898450753
158	KC_G1	CG14210	-0.0779911820592331	0.882669685996774	0.998043617620652	pseudobulk	DESeq2	LRT	0.0542017883672246
159	KC_G1	CG14220	0.334591032056319	0.539938213193617	0.998043617620652	pseudobulk	DESeq2	LRT	0.26765593499988
159	KC_G1	CG14221	1.12508065046033	0.900770125530981	0.998043617620652	pseudobulk	DESeq2	LRT	0.0453860258366365
159	KC_G1	CG14229	-0.0255718555161128	0.918607592151402	0.998043617620652	pseudobulk	DESeq2	LRT	0.0368699695257223
159	KC_G1	CG14230	-0.729676073850113	0.297935504363135	0.998043617620652	pseudobulk	DESeq2	LRT	0.525877739718121
159	KC_G1	CG14231	-0.110875445882783	0.892522590333548	0.998043617620652	pseudobulk	DESeq2	LRT	0.0493807827977933
159	KC_G1	CG14232	0.248246513427864	0.40998895560805	0.998043617620652	pseudobulk	DESeq2	LRT	0.387227842263397
159	KC_G1	CG14234	-0.0683712768688039	0.771030885828351	0.998043617620652	pseudobulk	DESeq2	LRT	0.1129282247037
159	KC_G1	CG14246	1.12508065046033	0.900770125530981	0.998043617620652	pseudobulk	DESeq2	LRT	0.0453860258366365
159	KC_G1	CG14252	0.402331582112521	0.403561507132659	0.998043617620652	pseudobulk	DESeq2	LRT	0.394090264727305
159	KC_G1	CG14270	-0.257193257478229	0.488795050080832	0.998043617620652	pseudobulk	DESeq2	LRT	0.310873200747224
160	KC_G1	CG14274	1.12508065046033	0.900770125530981	0.998043617620652	pseudobulk	DESeq2	LRT	0.0453860258366365
160	KC_G1	CG14275	-0.794610277746993	0.821479263487599	0.998043617620652	pseudobulk	DESeq2	LRT	0.085403394939296
160	KC_G1	CG14282	-0.794610277746993	0.821479263487599	0.998043617620652	pseudobulk	DESeq2	LRT	0.085403394939296
160	KC_G1	CG14285	-1.72984073695683	0.480047293856853	0.998043617620652	pseudobulk	DESeq2	LRT	0.318715974188453
160	KC_G1	CG14286	0.154283330173243	0.839114787557589	0.998043617620652	pseudobulk	DESeq2	LRT	0.0761786253537235
160	KC_G1	CG14298	-0.794610277746993	0.879485534599449	0.998043617620652	pseudobulk	DESeq2	LRT	0.0557712992369932
160	KC_G1	CG14304	-0.579504316232662	0.668509103521631	0.998043617620652	pseudobulk	DESeq2	LRT	0.174892674292362
160	KC_G1	CG14312	-0.186573753424336	0.29423959335589	0.998043617620652	pseudobulk	DESeq2	LRT	0.531298888307926
160	KC_G1	CG14314	-0.287854706506204	0.504711325634662	0.998043617620652	pseudobulk	DESeq2	LRT	0.296956949664074
160	KC_G1	CG14321	-0.158220375281507	0.546667780025017	0.998043617620652	pseudobulk	DESeq2	LRT	0.262276522175204
161	KC_G1	CG14322	-0.166827187583468	0.740632727520445	0.998043617620652	pseudobulk	DESeq2	LRT	0.130397100999112
161	KC_G1	CG1434	-0.453197169785777	0.677988001025432	0.998043617620652	pseudobulk	DESeq2	LRT	0.168777992172104
161	KC_G1	CG14341	0.272392909147832	0.339082127024237	0.998043617620652	pseudobulk	DESeq2	LRT	0.4696951012255
161	KC_G1	CG14352	0.0828745737641046	0.911330382427041	0.998043617620652	pseudobulk	DESeq2	LRT	0.0403241507399376
161	KC_G1	CG14355	-0.794610277746993	0.879485534599449	0.998043617620652	pseudobulk	DESeq2	LRT	0.0557712992369932
161	KC_G1	CG14356	0.337737908433352	0.875517472491096	0.998043617620652	pseudobulk	DESeq2	LRT	0.0577351823837907
161	KC_G1	CG14367	-1.15211873046699	0.450432432854086	0.998043617620652	pseudobulk	DESeq2	LRT	0.346370346170813
161	KC_G1	CG14397	1.13582588427274	0.633424328955532	0.998043617620652	pseudobulk	DESeq2	LRT	0.198305259991224
161	KC_G1	CG1440	-0.146539583765575	0.661041354737435	0.998043617620652	pseudobulk	DESeq2	LRT	0.17977137020806
161	KC_G1	CG14401	0.514906180893407	0.749640754461254	0.998043617620652	pseudobulk	DESeq2	LRT	0.125146810918958
162	KC_G1	CG14407	-0.0246108732296914	0.891011405247869	0.998043617620652	pseudobulk	DESeq2	LRT	0.0501167368110349
162	KC_G1	CG14408	-0.00714245144713716	0.956344568190446	0.998043617620652	pseudobulk	DESeq2	LRT	0.0193856044787646
162	KC_G1	CG14410	-0.794610277746993	0.821479263487599	0.998043617620652	pseudobulk	DESeq2	LRT	0.085403394939296

162	KC_G1	CG14411	0.0346542603194696	0.920409951600162	0.998043617620652	pseudobulk	DESeq2	LRT	0.0360186943257846
162	KC_G1	CG14414	0.168763293106163	0.497396736222292	0.998043617620652	pseudobulk	DESeq2	LRT	0.303297068775169
162	KC_G1	CG14431	0.0183692761540251	0.930040306216481	0.998043617620652	pseudobulk	DESeq2	LRT	0.031498229523391
162	KC_G1	CG14434	-0.015804471185759	0.964698601157068	0.998043617620652	pseudobulk	DESeq2	LRT	0.0156083512157643
162	KC_G1	CG14435	-0.138880641869791	0.527534376857324	0.998043617620652	pseudobulk	DESeq2	LRT	0.277749234242624
162	KC_G1	CG14439	-0.794610277746993	0.815039924133852	0.998043617620652	pseudobulk	DESeq2	LRT	0.0888211171417967
162	KC_G1	CG14440	-0.0783576030413226	0.654878836517365	0.998043617620652	pseudobulk	DESeq2	LRT	0.183839044282206
163	KC_G1	CG14441	0.0643840628390522	0.855723520690237	0.998043617620652	pseudobulk	DESeq2	LRT	0.0676665306896902
163	KC_G1	CG14442	-0.0390740037751327	0.807655408124345	0.998043617620652	pseudobulk	DESeq2	LRT	0.0927738945114388
163	KC_G1	CG14445	-0.794610277746993	0.815039924133852	0.998043617620652	pseudobulk	DESeq2	LRT	0.0888211171417967
163	KC_G1	CG14450	0.467352892242438	0.270901408430979	0.998043617620652	pseudobulk	DESeq2	LRT	0.567188737040615
163	KC_G1	CG14451	-0.794610277746993	0.821479263487599	0.998043617620652	pseudobulk	DESeq2	LRT	0.085403394939296
163	KC_G1	CG14457	1.13582588427274	0.704684208645453	0.998043617620652	pseudobulk	DESeq2	LRT	0.152005460551806
163	KC_G1	CG14459	-0.767324608732104	0.724630599467818	0.998043617620652	pseudobulk	DESeq2	LRT	0.139883330666603
163	KC_G1	CG14464	0.0454742421033873	0.733547131009748	0.998043617620652	pseudobulk	DESeq2	LRT	0.134571977144653
163	KC_G1	CG14478	0.0848308117410141	0.503093060529089	0.998043617620652	pseudobulk	DESeq2	LRT	0.298351673122889
163	KC_G1	CG14483	0.642274361677447	0.0116566112813535	0.998043617620652	pseudobulk	DESeq2	LRT	1.93342768591568
164	KC_G1	CG14490	1.13582588427274	0.704684208645453	0.998043617620652	pseudobulk	DESeq2	LRT	0.152005460551806
164	KC_G1	CG14502	1.4427674025696	0.139512198155921	0.998043617620652	pseudobulk	DESeq2	LRT	0.855387818482114
164	KC_G1	CG14505	0.263463073429412	0.875727114260175	0.998043617620652	pseudobulk	DESeq2	LRT	0.0576312034597602
164	KC_G1	CG14512	-0.695874464249049	0.476799440535222	0.998043617620652	pseudobulk	DESeq2	LRT	0.321664262858022
164	KC_G1	CG14516	1.12508065046033	0.900770125530981	0.998043617620652	pseudobulk	DESeq2	LRT	0.0453860258366365
164	KC_G1	CG14535	-0.00932562655721381	0.947656581969665	0.998043617620652	pseudobulk	DESeq2	LRT	0.0233490166453619
164	KC_G1	CG14543	0.0429092069168226	0.917821108810603	0.998043617620652	pseudobulk	DESeq2	LRT	0.0372419582620358
164	KC_G1	CG14544	0.983615621582388	0.630810344880065	0.998043617620652	pseudobulk	DESeq2	LRT	0.200101193137223
164	KC_G1	CG14561	-0.67266566125028	0.521454072449071	0.998043617620652	pseudobulk	DESeq2	LRT	0.282783936442234
164	KC_G1	CG14562	0.128207460142674	0.668833040539951	0.998043617620652	pseudobulk	DESeq2	LRT	0.174682280769782
165	KC_G1	CG14563	0.833101376971631	0.61780191448524	0.998043617620652	pseudobulk	DESeq2	LRT	0.209150750212154
165	KC_G1	CG14565	1.13582588427274	0.704684208645453	0.998043617620652	pseudobulk	DESeq2	LRT	0.152005460551806
165	KC_G1	CG14566	1.5681698674963	0.509898658126755	0.998043617620652	pseudobulk	DESeq2	LRT	0.292516130940769
165	KC_G1	CG14591	-0.299987120877177	0.417329638213726	0.998043617620652	pseudobulk	DESeq2	LRT	0.379520771157935
165	KC_G1	CG14609	-0.0488165746185401	0.947773425099207	0.998043617620652	pseudobulk	DESeq2	LRT	0.0232954727779719
165	KC_G1	CG1461	0.1729159612032	0.899814506689544	0.998043617620652	pseudobulk	DESeq2	LRT	0.0458470094762598
165	KC_G1	CG14613	-0.255068527318245	0.56798455380529	0.998043617620652	pseudobulk	DESeq2	LRT	0.24566347465578
165	KC_G1	CG14618	0.504033389035506	0.521841197396696	0.998043617620652	pseudobulk	DESeq2	LRT	0.282461637969048
165	KC_G1	CG14621	0.192190013327623	0.667333474971256	0.998043617620652	pseudobulk	DESeq2	LRT	0.175657089399697
165	KC_G1	CG14629	-0.794610277746993	0.815039924133852	0.998043617620652	pseudobulk	DESeq2	LRT	0.0888211171417967
166	KC_G1	CG1463	0.669392006698072	0.235010179889463	0.998043617620652	pseudobulk	DESeq2	LRT	0.628913325072666
166	KC_G1	CG14641	0.0735472474394699	0.758630076489059	0.998043617620652	pseudobulk	DESeq2	LRT	0.119969943343876
166	KC_G1	CG14646	-0.127607900638607	0.822009521873044	0.998043617620652	pseudobulk	DESeq2	LRT	0.0851231517142446
166	KC_G1	CG14647	0.166142524876801	0.620998920041293	0.998043617620652	pseudobulk	DESeq2	LRT	0.206909155089949
166	KC_G1	CG14650	-0.0387938367193354	0.902523176897258	0.998043617620652	pseudobulk	DESeq2	LRT	0.0445416365476864
166	KC_G1	CG14657	0.192053612705332	0.464456755611197	0.998043617620652	pseudobulk	DESeq2	LRT	0.333054715844116
166	KC_G1	CG14662	-0.794610277746993	0.821479263487599	0.998043617620652	pseudobulk	DESeq2	LRT	0.085403394939296
166	KC_G1	CG14667	-0.223034169298053	0.556286418839135	0.998043617620652	pseudobulk	DESeq2	LRT	0.254701542782919
166	KC_G1	CG14669	-0.142064336081891	0.372608795068909	0.998043617620652	pseudobulk	DESeq2	LRT	0.428746898219123
166	KC_G1	CG14671	-0.140516398458027	0.739886238506284	0.998043617620652	pseudobulk	DESeq2	LRT	0.130835050251453
167	KC_G1	CG1468	-0.362803653392214	0.434902513433412	0.998043617620652	pseudobulk	DESeq2	LRT	0.361608082407998
167	KC_G1	CG14683	-0.0634388222471186	0.896350766716417	0.998043617620652	pseudobulk	DESeq2	LRT	0.0475220056963092
167	KC_G1	CG14687	1.0622768627093	0.486919632964593	0.998043617620652	pseudobulk	DESeq2	LRT	0.312542714022012
167	KC_G1	CG14688	0.0404916020358415	0.911235656110995	0.998043617620652	pseudobulk	DESeq2	LRT	0.040369294910867
167	KC_G1	CG14693	1.12508065046033	0.900770125530981	0.998043617620652	pseudobulk	DESeq2	LRT	0.0453860258366365
167	KC_G1	CG14694	-0.794610277746993	0.821479263487599	0.998043617620652	pseudobulk	DESeq2	LRT	0.085403394939296
167	KC_G1	CG14696	1.11121460044925	0.691240116375203	0.998043617620652	pseudobulk	DESeq2	LRT	0.16037106535726
167	KC_G1	CG14701	0.389435671198985	0.555259557550964	0.998043617620652	pseudobulk	DESeq2	LRT	0.255503957307322
167	KC_G1	CG14710	-0.617586561644952	0.285366371094865	0.998043617620652	pseudobulk	DESeq2	LRT	0.54459720749485

167	KC_G1	CG14711	-0.255662671778416	0.648301826692923	0.998043617620652	pseudobulk	DESeq2	LRT	0.188222754701988
168	KC_G1	CG14712	-0.492292584162067	0.548240503189037	0.998043617620652	pseudobulk	DESeq2	LRT	0.26102888258399
168	KC_G1	CG14715	0.652516646533099	0.0725774608593845	0.998043617620652	pseudobulk	DESeq2	LRT	1.13919822977073
168	KC_G1	CG14721	0.339402928557358	0.335612052962446	0.998043617620652	pseudobulk	DESeq2	LRT	0.474162450581463
168	KC_G1	CG14722	0.052931450116127	0.891396130505094	0.998043617620652	pseudobulk	DESeq2	LRT	0.0499292554909607
168	KC_G1	CG14757	0.771612738729661	0.237197591864375	0.998043617620652	pseudobulk	DESeq2	LRT	0.624889724436454
168	KC_G1	CG14760	0.296175279079998	0.885816336538646	0.998043617620652	pseudobulk	DESeq2	LRT	0.0526563145647348
168	KC_G1	CG14763	-0.794610277746993	0.815039924133852	0.998043617620652	pseudobulk	DESeq2	LRT	0.0888211171417967
168	KC_G1	CG14764	0.0220998717393256	0.925026924744336	0.998043617620652	pseudobulk	DESeq2	LRT	0.0338456260742514
168	KC_G1	CG14767	0.168170048766321	0.292723647938531	0.998043617620652	pseudobulk	DESeq2	LRT	0.533542191285515
168	KC_G1	CG14770	-0.794610277746993	0.879485534599449	0.998043617620652	pseudobulk	DESeq2	LRT	0.0557712992369932
169	KC_G1	CG14777	-0.182083942524714	0.497016101369613	0.998043617620652	pseudobulk	DESeq2	LRT	0.303629541603279
169	KC_G1	CG14780	1.12508065046033	0.900770125530981	0.998043617620652	pseudobulk	DESeq2	LRT	0.0453860258366365
169	KC_G1	CG14785	0.948682853426738	0.716182290134517	0.998043617620652	pseudobulk	DESeq2	LRT	0.144976422491876
169	KC_G1	CG14787	1.54219940293975	0.517351981327225	0.998043617620652	pseudobulk	DESeq2	LRT	0.286213883335732
169	KC_G1	CG14795	-0.794610277746993	0.821479263487599	0.998043617620652	pseudobulk	DESeq2	LRT	0.085403394939296
169	KC_G1	CG14797	0.145056840792964	0.936983683603047	0.998043617620652	pseudobulk	DESeq2	LRT	0.0282679717406728
169	KC_G1	CG14803	0.673830154831721	0.455723143151081	0.998043617620652	pseudobulk	DESeq2	LRT	0.341298915925795
169	KC_G1	CG14805	-0.285133492120092	0.432228451768293	0.998043617620652	pseudobulk	DESeq2	LRT	0.364286648745524
169	KC_G1	CG14806	-0.0654781933297844	0.748940086553832	0.998043617620652	pseudobulk	DESeq2	LRT	0.125552923448474
169	KC_G1	CG14810	-0.794610277746993	0.821479263487599	0.998043617620652	pseudobulk	DESeq2	LRT	0.085403394939296
170	KC_G1	CG14811	1.12508065046033	0.900770125530981	0.998043617620652	pseudobulk	DESeq2	LRT	0.0453860258366365
170	KC_G1	CG14812	0.232842178703893	0.35264346495595	0.998043617620652	pseudobulk	DESeq2	LRT	0.452664159912013
170	KC_G1	CG14817	-0.37009360167081	0.0842184171397726	0.998043617620652	pseudobulk	DESeq2	LRT	1.07459292527532
170	KC_G1	CG14818	0.0537143045799659	0.701638567994104	0.998043617620652	pseudobulk	DESeq2	LRT	0.153886546485443
170	KC_G1	CG14830	-0.0656476963659548	0.954054624354956	0.998043617620652	pseudobulk	DESeq2	LRT	0.0204267590728538
170	KC_G1	CG14834	-0.579899724087504	0.720803582145565	0.998043617620652	pseudobulk	DESeq2	LRT	0.142183063729623
170	KC_G1	CG14841	1.12508065046033	0.900770125530981	0.998043617620652	pseudobulk	DESeq2	LRT	0.0453860258366365
170	KC_G1	CG14853	-0.209886979586961	0.357229586997738	0.998043617620652	pseudobulk	DESeq2	LRT	0.447052578513278
170	KC_G1	CG14855	-1.67593316621313	0.495877122847873	0.998043617620652	pseudobulk	DESeq2	LRT	0.304625927300966
170	KC_G1	CG14856	1.13582588427274	0.704684208645453	0.998043617620652	pseudobulk	DESeq2	LRT	0.152005460551806
171	KC_G1	CG14857	1.13582588427274	0.633424328955532	0.998043617620652	pseudobulk	DESeq2	LRT	0.198305259991224
171	KC_G1	CG1486	-0.253508867975036	0.489781186316767	0.998043617620652	pseudobulk	DESeq2	LRT	0.309997901195254
171	KC_G1	CG14861	-0.794610277746993	0.879485534599449	0.998043617620652	pseudobulk	DESeq2	LRT	0.0557712992369932
171	KC_G1	CG14864	-0.794610277746993	0.821479263487599	0.998043617620652	pseudobulk	DESeq2	LRT	0.085403394939296
171	KC_G1	CG14866	1.12508065046033	0.900770125530981	0.998043617620652	pseudobulk	DESeq2	LRT	0.0453860258366365
171	KC_G1	CG14868	0.0550608361683605	0.812676526669937	0.998043617620652	pseudobulk	DESeq2	LRT	0.0900822842178147
171	KC_G1	CG14879	-1.66808346769027	0.498191247612582	0.998043617620652	pseudobulk	DESeq2	LRT	0.302603906560549
171	KC_G1	CG14881	-0.366064752916109	0.460589395926972	0.998043617620652	pseudobulk	DESeq2	LRT	0.336686064946288
171	KC_G1	CG14882	-0.00332176770474426	0.963793344563527	0.998043617620652	pseudobulk	DESeq2	LRT	0.0160160770276663
171	KC_G1	CG14883	0.01685716229783	0.959114444178327	0.998043617620652	pseudobulk	DESeq2	LRT	0.0181295685230827
172	KC_G1	CG14894	0.252113209391485	0.377668016327545	0.998043617620652	pseudobulk	DESeq2	LRT	0.422889792828309
172	KC_G1	CG14903	0.204388318236751	0.480667933960299	0.998043617620652	pseudobulk	DESeq2	LRT	0.318154849305935
172	KC_G1	CG14905	-0.794610277746993	0.815039924133852	0.998043617620652	pseudobulk	DESeq2	LRT	0.0888211171417967
172	KC_G1	CG14906	-0.0134119526422922	0.948206331913866	0.998043617620652	pseudobulk	DESeq2	LRT	0.0230971488841048
172	KC_G1	CG1492	0.243597506708005	0.58656650393726	0.998043617620652	pseudobulk	DESeq2	LRT	0.231682741168909
172	KC_G1	CG14937	-1.67593316621313	0.495877122847873	0.998043617620652	pseudobulk	DESeq2	LRT	0.304625927300966
172	KC_G1	CG1494	-0.794610277746993	0.821479263487599	0.998043617620652	pseudobulk	DESeq2	LRT	0.085403394939296
172	KC_G1	CG14965	0.126019967312852	0.819784501721604	0.998043617620652	pseudobulk	DESeq2	LRT	0.086300296412796
172	KC_G1	CG14966	-0.866461855346612	0.158614798463845	0.998043617620652	pseudobulk	DESeq2	LRT	0.799656296265761
172	KC_G1	CG14968	1.16741254893182	0.478294269015844	0.998043617620652	pseudobulk	DESeq2	LRT	0.320304822869843
173	KC_G1	CG14969	-0.244961330494255	0.642842312159956	0.998043617620652	pseudobulk	DESeq2	LRT	0.191895545516632
173	KC_G1	CG14977	0.0206738508894315	0.933315245995764	0.998043617620652	pseudobulk	DESeq2	LRT	0.0299716397778156
173	KC_G1	CG14982	-0.328541294964847	0.088109904396406	0.998043617620652	pseudobulk	DESeq2	LRT	1.05497526998822
173	KC_G1	CG14984	-0.711364822669339	0.836431126230485	0.998043617620652	pseudobulk	DESeq2	LRT	0.0775698140913142
173	KC_G1	CG14985	0.955548841055612	0.654943884636294	0.998043617620652	pseudobulk	DESeq2	LRT	0.183795908614033

173	KC_G1	CG14989	-0.116053569293545	0.907065493292621	0.998043617620652	pseudobulk	DESeq2	LRT	0.0423613542312974
173	KC_G1	CG14995	-0.112784248502367	0.618551880363712	0.998043617620652	pseudobulk	DESeq2	LRT	0.208623868531616
173	KC_G1	CG14997	0.513476002425719	0.376128069742221	0.998043617620652	pseudobulk	DESeq2	LRT	0.424664254774183
173	KC_G1	CG15011	0.388751368875035	0.612221601645682	0.998043617620652	pseudobulk	DESeq2	LRT	0.21309135080858
173	KC_G1	CG15012	0.127996733518459	0.462705224700945	0.998043617620652	pseudobulk	DESeq2	LRT	0.334695596575358
174	KC_G1	CG15014	0.217599784844547	0.650523346715969	0.998043617620652	pseudobulk	DESeq2	LRT	0.186737112372237
174	KC_G1	CG15019	0.0327604853900475	0.92108098885541	0.998043617620652	pseudobulk	DESeq2	LRT	0.0357021814572142
174	KC_G1	CG15021	-0.794610277746993	0.821479263487599	0.998043617620652	pseudobulk	DESeq2	LRT	0.085403394939296
174	KC_G1	CG15027	-0.0329461013687048	0.953046724577001	0.998043617620652	pseudobulk	DESeq2	LRT	0.0208858068868323
174	KC_G1	CG1504	0.320617551563866	0.182108231206925	0.998043617620652	pseudobulk	DESeq2	LRT	0.739670423853862
174	KC_G1	CG15043	1.13582588427274	0.704684208645453	0.998043617620652	pseudobulk	DESeq2	LRT	0.152005460551806
174	KC_G1	CG15047	0.869192002311134	0.676328661462978	0.998043617620652	pseudobulk	DESeq2	LRT	0.169842207648647
174	KC_G1	CG15073	-0.406567644868135	0.480091743280895	0.998043617620652	pseudobulk	DESeq2	LRT	0.318675763054748
174	KC_G1	CG15083	1.69981218657454	0.566400560818652	0.998043617620652	pseudobulk	DESeq2	LRT	0.246876325303589
174	KC_G1	CG15084	0.0119407751208328	0.975662865059521	0.998043617620652	pseudobulk	DESeq2	LRT	0.0107002244828866
175	KC_G1	CG15097	0.430781678482452	0.0635127417541037	0.998043617620652	pseudobulk	DESeq2	LRT	1.19713913898543
175	KC_G1	CG15098	0.142854817172934	0.659033185851055	0.998043617620652	pseudobulk	DESeq2	LRT	0.181092715801874
175	KC_G1	CG15099	-0.118152540930584	0.676813238764011	0.998043617620652	pseudobulk	DESeq2	LRT	0.169531154895266
175	KC_G1	CG15107	0.261558901844507	0.660379186493112	0.998043617620652	pseudobulk	DESeq2	LRT	0.180206623073744
175	KC_G1	CG15111	0.0930028182187413	0.778902566122809	0.998043617620652	pseudobulk	DESeq2	LRT	0.10851686535917
175	KC_G1	CG15117	0.337737908433352	0.875517472491096	0.998043617620652	pseudobulk	DESeq2	LRT	0.0577351823837907
175	KC_G1	CG15118	-0.349043716131356	0.371183893472755	0.998043617620652	pseudobulk	DESeq2	LRT	0.430410877085899
175	KC_G1	CG1513	-0.0412295547949195	0.844340429453794	0.998043617620652	pseudobulk	DESeq2	LRT	0.0734824149571342
175	KC_G1	CG15141	-0.332823082184252	0.294270398938422	0.998043617620652	pseudobulk	DESeq2	LRT	0.531253421978746
175	KC_G1	CG15143	-0.794610277746993	0.815039924133852	0.998043617620652	pseudobulk	DESeq2	LRT	0.0888211171417967
176	KC_G1	CG15144	-2.27643791964046	0.298906309922724	0.998043617620652	pseudobulk	DESeq2	LRT	0.52446491689215
176	KC_G1	CG15145	1.12508065046033	0.900770125530981	0.998043617620652	pseudobulk	DESeq2	LRT	0.0453860258366365
176	KC_G1	CG15160	-0.299293192815193	0.43037950868244	0.998043617620652	pseudobulk	DESeq2	LRT	0.366148414567496
176	KC_G1	CG15168	0.167279311789948	0.475797071546278	0.998043617620652	pseudobulk	DESeq2	LRT	0.322578235302548
176	KC_G1	CG15172	-1.60891734856068	0.545249891924755	0.998043617620652	pseudobulk	DESeq2	LRT	0.263404411831769
176	KC_G1	CG15196	-0.752107099832492	0.841251974639758	0.998043617620652	pseudobulk	DESeq2	LRT	0.0750739033633379
176	KC_G1	CG15199	-0.794610277746993	0.821479263487599	0.998043617620652	pseudobulk	DESeq2	LRT	0.085403394939296
176	KC_G1	CG15201	-0.109058064842449	0.672535507802734	0.998043617620652	pseudobulk	DESeq2	LRT	0.172284781309647
176	KC_G1	CG15202	-1.76164463706579	0.438917041178301	0.998043617620652	pseudobulk	DESeq2	LRT	0.35761755712261
176	KC_G1	CG15203	0.572142716208998	0.578157654258714	0.998043617620652	pseudobulk	DESeq2	LRT	0.237953720336133
177	KC_G1	CG15209	0.00575354442706918	0.94536418267591	0.998043617620652	pseudobulk	DESeq2	LRT	0.0244008559794545
177	KC_G1	CG15210	-0.148913790667767	0.881553515948577	0.998043617620652	pseudobulk	DESeq2	LRT	0.0547513181057968
177	KC_G1	CG15211	-0.794610277746993	0.815039924133852	0.998043617620652	pseudobulk	DESeq2	LRT	0.0888211171417967
177	KC_G1	CG15221	1.13582588427274	0.704684208645453	0.998043617620652	pseudobulk	DESeq2	LRT	0.152005460551806
177	KC_G1	CG1523	0.637385760558402	0.194600403618285	0.998043617620652	pseudobulk	DESeq2	LRT	0.710856263301932
177	KC_G1	CG15236	0.115121122104599	0.722224347121049	0.998043617620652	pseudobulk	DESeq2	LRT	0.141327875031193
177	KC_G1	CG15237	0.11906358070529	0.606396196631018	0.998043617620652	pseudobulk	DESeq2	LRT	0.217243531299719
177	KC_G1	CG15239	1.91602628087015	0.248916839026812	0.998043617620652	pseudobulk	DESeq2	LRT	0.603945722720226
177	KC_G1	CG15253	0.172834960772825	0.908610676445368	0.998043617620652	pseudobulk	DESeq2	LRT	0.0416221644022337
177	KC_G1	CG15270	0.0763883052825537	0.828759479733065	0.998043617620652	pseudobulk	DESeq2	LRT	0.0815714908982246
178	KC_G1	CG15279	-0.767324608732104	0.724630599467818	0.998043617620652	pseudobulk	DESeq2	LRT	0.139883330666603
178	KC_G1	CG15286	1.13582588427274	0.633424328955532	0.998043617620652	pseudobulk	DESeq2	LRT	0.198305259991224
178	KC_G1	CG15309	0.0265927380284389	0.885697365354695	0.998043617620652	pseudobulk	DESeq2	LRT	0.0527146471971249
178	KC_G1	CG15312	-0.104530727150584	0.555535956126626	0.998043617620652	pseudobulk	DESeq2	LRT	0.255287826836474
178	KC_G1	CG15317	0.865054569917861	0.296006494798782	0.998043617620652	pseudobulk	DESeq2	LRT	0.528698759804821
178	KC_G1	CG1532	0.304513620412608	0.422804756293893	0.998043617620652	pseudobulk	DESeq2	LRT	0.373860135790881
178	KC_G1	CG15322	1.13716456806221	0.573561141908917	0.998043617620652	pseudobulk	DESeq2	LRT	0.241420279282692
178	KC_G1	CG15332	-1.32713783702529	0.499619481043016	0.998043617620652	pseudobulk	DESeq2	LRT	0.30136063606144
178	KC_G1	CG15343	-0.251451490642382	0.881921737886703	0.998043617620652	pseudobulk	DESeq2	LRT	0.0545699526368798
178	KC_G1	CG15356	0.12026580024015	0.736994312068089	0.998043617620652	pseudobulk	DESeq2	LRT	0.132535863900752
179	KC_G1	CG15365	-0.794610277746993	0.879485534599449	0.998043617620652	pseudobulk	DESeq2	LRT	0.0557712992369932

179	KC_G1	CG1537	-0.092003367192106	0.787746096027983	0.998043617620652	pseudobulk	DESeq2	LRT	0.103613740458254
179	KC_G1	CG15373	-3.48571529295288	0.0663180080507057	0.998043617620652	pseudobulk	DESeq2	LRT	1.17836852687242
179	KC_G1	CG15382	-1.23043706161686	0.113398059244988	0.998043617620652	pseudobulk	DESeq2	LRT	0.94539437812746
179	KC_G1	CG15385	-0.24878661860742	0.49671020172778	0.998043617620652	pseudobulk	DESeq2	LRT	0.30389692011635
179	KC_G1	CG15387	0.294127086413549	0.45566370922203	0.998043617620652	pseudobulk	DESeq2	LRT	0.341355558892267
179	KC_G1	CG15390	0.413278941942472	0.525144445558445	0.998043617620652	pseudobulk	DESeq2	LRT	0.279721223678428
179	KC_G1	CG15412	-0.64277910557112	0.764034577741626	0.998043617620652	pseudobulk	DESeq2	LRT	0.116886986211521
179	KC_G1	CG1542	-0.136205023820206	0.770591895555829	0.998043617620652	pseudobulk	DESeq2	LRT	0.113175562846884
179	KC_G1	CG15431	0.540231214186853	0.0176321806225719	0.998043617620652	pseudobulk	DESeq2	LRT	1.75369397392946
180	KC_G1	CG15432	-0.150723011855049	0.653561015865469	0.998043617620652	pseudobulk	DESeq2	LRT	0.184713860850555
180	KC_G1	CG15435	0.616169772574662	0.521878358738154	0.998043617620652	pseudobulk	DESeq2	LRT	0.282430712103097
180	KC_G1	CG15436	0.242079181495221	0.90764721745598	0.998043617620652	pseudobulk	DESeq2	LRT	0.0420829194120625
180	KC_G1	CG15439	-0.262917881886228	0.670323702018924	0.998043617620652	pseudobulk	DESeq2	LRT	0.173715424088238
180	KC_G1	CG1544	-0.794610277746993	0.821479263487599	0.998043617620652	pseudobulk	DESeq2	LRT	0.085403394939296
180	KC_G1	CG15443	-0.232017311076395	0.530144062553556	0.998043617620652	pseudobulk	DESeq2	LRT	0.275606098190848
180	KC_G1	CG15445	0.354068723820256	0.59257672612039	0.998043617620652	pseudobulk	DESeq2	LRT	0.22725540975097
180	KC_G1	CG15446	-1.72984073695683	0.480047293856853	0.998043617620652	pseudobulk	DESeq2	LRT	0.318715974188453
180	KC_G1	CG15449	0.422926021183694	0.432719312782089	0.998043617620652	pseudobulk	DESeq2	LRT	0.363793721284941
180	KC_G1	CG1545	0.0151459723738061	0.953194733078081	0.998043617620652	pseudobulk	DESeq2	LRT	0.0208183660333506
181	KC_G1	CG15450	-0.767324608732104	0.724630599467818	0.998043617620652	pseudobulk	DESeq2	LRT	0.139883330666603
181	KC_G1	CG15456	-0.0602644061991099	0.830413845362676	0.998043617620652	pseudobulk	DESeq2	LRT	0.0807054185099678
181	KC_G1	CG15458	0.896317563024066	0.661500137893293	0.998043617620652	pseudobulk	DESeq2	LRT	0.179470060945429
181	KC_G1	CG15459	0.203378379372544	0.450703568495263	0.998043617620652	pseudobulk	DESeq2	LRT	0.346109003340901
181	KC_G1	CG15473	-0.551480803396935	0.777826693009906	0.998043617620652	pseudobulk	DESeq2	LRT	0.109117157064118
181	KC_G1	CG15497	1.13582588427274	0.704684208645453	0.998043617620652	pseudobulk	DESeq2	LRT	0.152005460551806
181	KC_G1	CG1552	-0.100425689405256	0.450550435110472	0.998043617620652	pseudobulk	DESeq2	LRT	0.346256586562466
181	KC_G1	CG15522	2.09620892556682	0.0622473223170637	0.998043617620652	pseudobulk	DESeq2	LRT	1.20587932580548
181	KC_G1	CG15525	-0.161635217128595	0.691895535328806	0.998043617620652	pseudobulk	DESeq2	LRT	0.15995947180772
181	KC_G1	CG15535	0.410791852597464	0.253525739415949	0.998043617620652	pseudobulk	DESeq2	LRT	0.595977941976238
182	KC_G1	CG15536	0.226840220330038	0.711035537620809	0.998043617620652	pseudobulk	DESeq2	LRT	0.148108692650093
182	KC_G1	CG15537	1.12508065046033	0.900770125530981	0.998043617620652	pseudobulk	DESeq2	LRT	0.0453860258366365
182	KC_G1	CG15539	0.175579629065889	0.920099974205621	0.998043617620652	pseudobulk	DESeq2	LRT	0.0361649814730035
182	KC_G1	CG15546	-0.794610277746993	0.821479263487599	0.998043617620652	pseudobulk	DESeq2	LRT	0.085403394939296
182	KC_G1	CG15547	0.807045152427946	0.467680583670453	0.998043617620652	pseudobulk	DESeq2	LRT	0.330050659982066
182	KC_G1	CG15554	-0.794610277746993	0.879485534599449	0.998043617620652	pseudobulk	DESeq2	LRT	0.0557712992369932
182	KC_G1	CG15561	-0.148422287470225	0.825808249246697	0.998043617620652	pseudobulk	DESeq2	LRT	0.0831207831415948
182	KC_G1	CG15563	-0.794610277746993	0.879485534599449	0.998043617620652	pseudobulk	DESeq2	LRT	0.0557712992369932
182	KC_G1	CG15564	1.13582588427274	0.633424328955532	0.998043617620652	pseudobulk	DESeq2	LRT	0.198305259991224
182	KC_G1	CG15579	-1.56369655074139	0.428693746664107	0.998043617620652	pseudobulk	DESeq2	LRT	0.367852851476722
183	KC_G1	CG15601	-0.467473914908858	0.426229463995041	0.998043617620652	pseudobulk	DESeq2	LRT	0.370356532078001
183	KC_G1	CG15602	0.27487847440822	0.555842337355374	0.998043617620652	pseudobulk	DESeq2	LRT	0.255048376990899
183	KC_G1	CG15611	-0.0190232237688427	0.958029348895871	0.998043617620652	pseudobulk	DESeq2	LRT	0.0186211862572708
183	KC_G1	CG15614	-2.70281465192265	0.197132217389703	0.998043617620652	pseudobulk	DESeq2	LRT	0.705242393015524
183	KC_G1	CG15628	0.129158855040648	0.349892474138548	0.998043617620652	pseudobulk	DESeq2	LRT	0.456065398686714
183	KC_G1	CG15631	-0.436310377435442	0.735173004639891	0.998043617620652	pseudobulk	DESeq2	LRT	0.133610448508751
183	KC_G1	CG15638	-1.73288829970739	0.458469392065673	0.998043617620652	pseudobulk	DESeq2	LRT	0.338689653016193
183	KC_G1	CG15642	0.0268240589078554	0.942299746444148	0.998043617620652	pseudobulk	DESeq2	LRT	0.0258109257426224
183	KC_G1	CG15643	-0.979773751796911	0.0799190746810908	0.998043617620652	pseudobulk	DESeq2	LRT	1.09734955310068
183	KC_G1	CG15651	0.118248803209087	0.649426086588372	0.998043617620652	pseudobulk	DESeq2	LRT	0.187470270323757
184	KC_G1	CG15653	-0.818882110717517	0.132653181014408	0.998043617620652	pseudobulk	DESeq2	LRT	0.877282331204154
184	KC_G1	CG15673	2.38466886841527	0.34619303318143	0.998043617620652	pseudobulk	DESeq2	LRT	0.460681676152437
184	KC_G1	CG15674	-0.794610277746993	0.879485534599449	0.998043617620652	pseudobulk	DESeq2	LRT	0.0557712992369932
184	KC_G1	CG15706	-1.03253164122325	0.266385750645901	0.998043617620652	pseudobulk	DESeq2	LRT	0.574489009912272
184	KC_G1	CG15715	0.0815647835239843	0.708933774889309	0.998043617620652	pseudobulk	DESeq2	LRT	0.149394332579218
184	KC_G1	CG15719	0.173195945230598	0.976215465741119	0.998043617620652	pseudobulk	DESeq2	LRT	0.0104543162934892
184	KC_G1	CG1572	1.13716456806221	0.573561141908917	0.998043617620652	pseudobulk	DESeq2	LRT	0.241420279282692

184	KC_G1	CG15725	1.5681698674963	0.509898658126755	0.998043617620652	pseudobulk	DESeq2	LRT	0.292516130940769
184	KC_G1	CG15743	-0.140786582174024	0.760305434127952	0.998043617620652	pseudobulk	DESeq2	LRT	0.119011905470961
184	KC_G1	CG15744	-0.272401486622492	0.719003503105821	0.998043617620652	pseudobulk	DESeq2	LRT	0.143268993656309
185	KC_G1	CG15747	-0.207259886116527	0.553109017226484	0.998043617620652	pseudobulk	DESeq2	LRT	0.257189261256414
185	KC_G1	CG1575	0.0522003720196657	0.940109454221065	0.998043617620652	pseudobulk	DESeq2	LRT	0.0268215798078334
185	KC_G1	CG15760	0.0206543152642124	0.921098911779376	0.998043617620652	pseudobulk	DESeq2	LRT	0.0356937307874665
185	KC_G1	CG15765	0.112633963534267	0.241354379295738	0.998043617620652	pseudobulk	DESeq2	LRT	0.617344816643429
185	KC_G1	CG15767	1.66893182195367	0.456348242815677	0.998043617620652	pseudobulk	DESeq2	LRT	0.34070361739619
185	KC_G1	CG15771	-0.234105912839474	0.339970892719974	0.998043617620652	pseudobulk	DESeq2	LRT	0.468558264346659
185	KC_G1	CG15772	-0.0871971286233736	0.603372039985856	0.998043617620652	pseudobulk	DESeq2	LRT	0.219414818723842
185	KC_G1	CG1578	0.100863191568983	0.581735985581312	0.998043617620652	pseudobulk	DESeq2	LRT	0.235274070367
185	KC_G1	CG15803	-0.120601752686331	0.759094233986749	0.998043617620652	pseudobulk	DESeq2	LRT	0.119704307424528
185	KC_G1	CG15812	-0.366679863766039	0.443480757670637	0.998043617620652	pseudobulk	DESeq2	LRT	0.353125219166545
186	KC_G1	CG15814	0.156642643559689	0.64666104134844	0.998043617620652	pseudobulk	DESeq2	LRT	0.189323302709989
186	KC_G1	CG1582	0.858781252543905	0.322453317671532	0.998043617620652	pseudobulk	DESeq2	LRT	0.491533150315951
186	KC_G1	CG15861	2.23045770445974	0.357070320422792	0.998043617620652	pseudobulk	DESeq2	LRT	0.447246246733247
186	KC_G1	CG15864	-1.67593316621313	0.495877122847873	0.998043617620652	pseudobulk	DESeq2	LRT	0.304625927300966
186	KC_G1	CG15870	-2.63950342027325	0.237576358078543	0.998043617620652	pseudobulk	DESeq2	LRT	0.624196779460586
186	KC_G1	CG15877	0.312491010681015	0.514642897359284	0.998043617620652	pseudobulk	DESeq2	LRT	0.288494016590575
186	KC_G1	CG15890	0.590441901217191	0.746438914571765	0.998043617620652	pseudobulk	DESeq2	LRT	0.127005727322601
186	KC_G1	CG15891	0.0914318009325434	0.707449242253844	0.998043617620652	pseudobulk	DESeq2	LRT	0.150304714248683
186	KC_G1	CG15892	-3.04799945169531	0.155112902507345	0.998043617620652	pseudobulk	DESeq2	LRT	0.809352075463743
186	KC_G1	CG15894	-0.0907624146437494	0.61835154209065	0.998043617620652	pseudobulk	DESeq2	LRT	0.208764551798695
187	KC_G1	CG15908	-0.170446559967363	0.598996271382003	0.998043617620652	pseudobulk	DESeq2	LRT	0.22257588098842
187	KC_G1	CG15912	0.129083622039656	0.806931020793678	0.998043617620652	pseudobulk	DESeq2	LRT	0.0931635886596655
187	KC_G1	CG15916	-0.0355195459176918	0.849523723296017	0.998043617620652	pseudobulk	DESeq2	LRT	0.0708244887752826
187	KC_G1	CG15922	-0.0283756445297288	0.924170343593944	0.998043617620652	pseudobulk	DESeq2	LRT	0.0342479720001237
187	KC_G1	CG15923	-0.711691946027098	0.741255142397939	0.998043617620652	pseudobulk	DESeq2	LRT	0.130032280734188
187	KC_G1	CG1598	0.0200898502314914	0.924022310457893	0.998043617620652	pseudobulk	DESeq2	LRT	0.0343175426416068
187	KC_G1	CG1602	0.721883423146154	0.368130515982432	0.998043617620652	pseudobulk	DESeq2	LRT	0.433998180452089
187	KC_G1	CG1603	-0.330897118109161	0.639845256692008	0.998043617620652	pseudobulk	DESeq2	LRT	0.19392504522019
187	KC_G1	CG1607	-0.30156073931268	0.190704775594909	0.998043617620652	pseudobulk	DESeq2	LRT	0.71963843129302
187	KC_G1	CG1620	0.327977671480998	0.351586338569589	0.998043617620652	pseudobulk	DESeq2	LRT	0.453968008453529
188	KC_G1	CG1622	-0.161852244180938	0.594666488845915	0.998043617620652	pseudobulk	DESeq2	LRT	0.225726534544888
188	KC_G1	CG1628	-2.3243999702458	0.347033693779396	0.998043617620652	pseudobulk	DESeq2	LRT	0.459628357162795
188	KC_G1	CG1631	0.0781071480641268	0.970967397587672	0.998043617620652	pseudobulk	DESeq2	LRT	0.0127953522603565
188	KC_G1	CG1635	0.0572008211992121	0.863994209516692	0.998043617620652	pseudobulk	DESeq2	LRT	0.0634891681500133
188	KC_G1	CG1636	-0.106989713042055	0.657839896073864	0.998043617620652	pseudobulk	DESeq2	LRT	0.181879791359121
188	KC_G1	CG1637	0.0329300776197605	0.888371252142395	0.998043617620652	pseudobulk	DESeq2	LRT	0.0514055037457286
188	KC_G1	CG1638	1.13582588427274	0.704684208645453	0.998043617620652	pseudobulk	DESeq2	LRT	0.152005460551806
188	KC_G1	CG1640	-0.13121591604315	0.501069295030413	0.998043617620652	pseudobulk	DESeq2	LRT	0.300102209525388
188	KC_G1	CG1646	-0.158928073767501	0.460863549394648	0.998043617620652	pseudobulk	DESeq2	LRT	0.33642763972268
188	KC_G1	CG1647	-0.133626165657075	0.679548871905952	0.998043617620652	pseudobulk	DESeq2	LRT	0.167779304147333
189	KC_G1	CG1648	-0.0105730238298102	0.941795753048552	0.998043617620652	pseudobulk	DESeq2	LRT	0.0260432723045699
189	KC_G1	CG1662	0.0648134604845252	0.906119468653527	0.998043617620652	pseudobulk	DESeq2	LRT	0.0428145383536089
189	KC_G1	CG1663	-0.473493062077566	0.533652624928369	0.998043617620652	pseudobulk	DESeq2	LRT	0.272741350026512
189	KC_G1	CG16700	-1.74720907763611	0.407846032164752	0.998043617620652	pseudobulk	DESeq2	LRT	0.389503758482075
189	KC_G1	CG1671	0.285011887986688	0.824889476960507	0.998043617620652	pseudobulk	DESeq2	LRT	0.0836042366157084
189	KC_G1	CG16711	-0.140404138428496	0.311769798253093	0.998043617620652	pseudobulk	DESeq2	LRT	0.506165958060946
189	KC_G1	CG16717	-0.399180340443711	0.240300532710608	0.998043617620652	pseudobulk	DESeq2	LRT	0.619245266428638
189	KC_G1	CG16721	0.152052170085227	0.711489383265446	0.998043617620652	pseudobulk	DESeq2	LRT	0.147831576006448
189	KC_G1	CG1673	1.13582588427274	0.704684208645453	0.998043617620652	pseudobulk	DESeq2	LRT	0.152005460551806
189	KC_G1	CG16734	-0.384036122302282	0.689207175126989	0.998043617620652	pseudobulk	DESeq2	LRT	0.161650209892266
190	KC_G1	CG16736	-0.794610277746993	0.821479263487599	0.998043617620652	pseudobulk	DESeq2	LRT	0.085403394939296
190	KC_G1	CG16739	1.13582588427274	0.704684208645453	0.998043617620652	pseudobulk	DESeq2	LRT	0.152005460551806
190	KC_G1	CG1674	1.85375174928422	0.190940568703477	0.998043617620652	pseudobulk	DESeq2	LRT	0.719101788252659

190	KC_G1	CG16743	-0.760674823318615	0.27562397291017	0.998043617620652	pseudobulk	DESeq2	LRT	0.559683011547367
190	KC_G1	CG16749	0.869192002311134	0.676328661462978	0.998043617620652	pseudobulk	DESeq2	LRT	0.169842207648647
190	KC_G1	CG16753	0.0503764102718302	0.901053565615598	0.998043617620652	pseudobulk	DESeq2	LRT	0.0452493904196206
190	KC_G1	CG16758	-0.56093400084445	0.489743406599737	0.998043617620652	pseudobulk	DESeq2	LRT	0.310031402186981
190	KC_G1	CG1677	0.00647778316247314	0.962562506949294	0.998043617620652	pseudobulk	DESeq2	LRT	0.0165710586592312
190	KC_G1	CG16779	-0.112868300405484	0.358781429271628	0.998043617620652	pseudobulk	DESeq2	LRT	0.445170044372599
190	KC_G1	CG16787	0.202212512958425	0.580137666359692	0.998043617620652	pseudobulk	DESeq2	LRT	0.236468936357757
191	KC_G1	CG16789	0.343588087965571	0.874039307776167	0.998043617620652	pseudobulk	DESeq2	LRT	0.0584690355962459
191	KC_G1	CG16790	-0.110550642478251	0.873839081830525	0.998043617620652	pseudobulk	DESeq2	LRT	0.0585685356807183
191	KC_G1	CG16791	-0.0499038617577169	0.664365729583553	0.998043617620652	pseudobulk	DESeq2	LRT	0.177592778142107
191	KC_G1	CG16798	0.825496035528663	0.699446040114278	0.998043617620652	pseudobulk	DESeq2	LRT	0.155245784223669
191	KC_G1	CG16799	-1.37661465442354	0.464324000788384	0.998043617620652	pseudobulk	DESeq2	LRT	0.333178867183363
191	KC_G1	CG16812	-0.019816051401646	0.918076951785536	0.998043617620652	pseudobulk	DESeq2	LRT	0.0371209153835105
191	KC_G1	CG16825	-0.794610277746993	0.879485534599449	0.998043617620652	pseudobulk	DESeq2	LRT	0.0557712992369932
191	KC_G1	CG16854	-0.525642863729537	0.24255392434289	0.998043617620652	pseudobulk	DESeq2	LRT	0.615191694417943
191	KC_G1	CG16863	-0.140086119113793	0.876832496364183	0.998043617620652	pseudobulk	DESeq2	LRT	0.0570833631364828
191	KC_G1	CG16865	0.101791730063032	0.869444489771935	0.998043617620652	pseudobulk	DESeq2	LRT	0.060758140579394
192	KC_G1	CG16868	-0.0537135828432103	0.893550434477822	0.998043617620652	pseudobulk	DESeq2	LRT	0.0488809296751146
192	KC_G1	CG1688	-0.504156972434758	0.535907725300756	0.998043617620652	pseudobulk	DESeq2	LRT	0.270909982401607
192	KC_G1	CG16890	0.0377129456523336	0.955107304566019	0.998043617620652	pseudobulk	DESeq2	LRT	0.0199478334811808
192	KC_G1	CG16896	0.00317448488577801	0.953724510426705	0.998043617620652	pseudobulk	DESeq2	LRT	0.0205770559874767
192	KC_G1	CG16903	0.417325191787585	0.251783496037697	0.998043617620652	pseudobulk	DESeq2	LRT	0.598972740529441
192	KC_G1	CG16908	0.175579629065889	0.920099974205621	0.998043617620652	pseudobulk	DESeq2	LRT	0.0361649814730035
192	KC_G1	CG16935	-0.193791583458891	0.519583492728462	0.998043617620652	pseudobulk	DESeq2	LRT	0.284344655002434
192	KC_G1	CG1695	-0.0489700775091382	0.798349629191276	0.998043617620652	pseudobulk	DESeq2	LRT	0.0978068720893907
192	KC_G1	CG16952	-0.121876720733503	0.573675441074066	0.998043617620652	pseudobulk	DESeq2	LRT	0.241333741775764
192	KC_G1	CG16953	-0.0793096838205602	0.783529362786874	0.998043617620652	pseudobulk	DESeq2	LRT	0.105944723691413
193	KC_G1	CG16972	-0.0650901739446371	0.882233071139831	0.998043617620652	pseudobulk	DESeq2	LRT	0.0544166664121356
193	KC_G1	CG16974	0.296641128628302	0.1817921498925	0.998043617620652	pseudobulk	DESeq2	LRT	0.740424874315247
193	KC_G1	CG16979	0.688785577636912	0.312033395126706	0.998043617620652	pseudobulk	DESeq2	LRT	0.505798923471557
193	KC_G1	CG1698	-0.794610277746993	0.821479263487599	0.998043617620652	pseudobulk	DESeq2	LRT	0.085403394939296
193	KC_G1	CG16984	1.12508065046033	0.900770125530981	0.998043617620652	pseudobulk	DESeq2	LRT	0.0453860258366365
193	KC_G1	CG16985	-0.381779387508704	0.492986102781741	0.998043617620652	pseudobulk	DESeq2	LRT	0.30716532325882
193	KC_G1	CG16986	0.741722262149468	0.149090526321139	0.998043617620652	pseudobulk	DESeq2	LRT	0.826549952101465
193	KC_G1	CG16989	0.199169558305021	0.716198548102646	0.998043617620652	pseudobulk	DESeq2	LRT	0.144966563736917
193	KC_G1	CG16995	-1.60891734856068	0.545249891924755	0.998043617620652	pseudobulk	DESeq2	LRT	0.263404411831769
193	KC_G1	CG17002	0.138116593097428	0.704787324249493	0.998043617620652	pseudobulk	DESeq2	LRT	0.151941915404753
194	KC_G1	CG17010	0.251172729898621	0.801147151596158	0.998043617620652	pseudobulk	DESeq2	LRT	0.0962877070658884
194	KC_G1	CG17019	-0.232834966062717	0.475556751928918	0.998043617620652	pseudobulk	DESeq2	LRT	0.322797647856231
194	KC_G1	CG17026	1.17952109454343	0.216258737633469	0.998043617620652	pseudobulk	DESeq2	LRT	0.665026336444238
194	KC_G1	CG17027	2.38466886841527	0.34619303318143	0.998043617620652	pseudobulk	DESeq2	LRT	0.460681676152437
194	KC_G1	CG17029	0.418528852849745	0.551578918852033	0.998043617620652	pseudobulk	DESeq2	LRT	0.258392340757954
194	KC_G1	CG1703	-0.117696639628388	0.703546325948741	0.998043617620652	pseudobulk	DESeq2	LRT	0.152707300586722
194	KC_G1	CG17030	-0.551749390360576	0.792123295354081	0.998043617620652	pseudobulk	DESeq2	LRT	0.101207214465552
194	KC_G1	CG17032	-1.66808346769027	0.498191247612582	0.998043617620652	pseudobulk	DESeq2	LRT	0.302603906560549
194	KC_G1	CG17059	0.00940028189990949	0.940075428501577	0.998043617620652	pseudobulk	DESeq2	LRT	0.0268372986686264
194	KC_G1	CG17065	-0.0451190985674472	0.83091771280774	0.998043617620652	pseudobulk	DESeq2	LRT	0.0804419830065182
195	KC_G1	CG17068	-0.327898131587435	0.629431405828508	0.998043617620652	pseudobulk	DESeq2	LRT	0.201051591518536
195	KC_G1	CG17075	-0.113930007811978	0.696920706723217	0.998043617620652	pseudobulk	DESeq2	LRT	0.156816631645931
195	KC_G1	CG17078	-0.314323907517091	0.272679714698506	0.998043617620652	pseudobulk	DESeq2	LRT	0.564347169070418
195	KC_G1	CG17111	-0.920830913158939	0.522010211820903	0.998043617620652	pseudobulk	DESeq2	LRT	0.28232100103205
195	KC_G1	CG17118	2.95840825636686	0.160769763152811	0.998043617620652	pseudobulk	DESeq2	LRT	0.793795628042056
195	KC_G1	CG17119	1.13582588427274	0.633424328955532	0.998043617620652	pseudobulk	DESeq2	LRT	0.198305259991224
195	KC_G1	CG17121	-0.794610277746993	0.821479263487599	0.998043617620652	pseudobulk	DESeq2	LRT	0.085403394939296
195	KC_G1	CG17124	-0.0286130845240977	0.83775974850164	0.998043617620652	pseudobulk	DESeq2	LRT	0.0768805098420358
195	KC_G1	CG17129	-0.794610277746993	0.815039924133852	0.998043617620652	pseudobulk	DESeq2	LRT	0.0888211171417967

195	KC_G1	CG17141	0.76878934341802	0.194436668883404	0.998043617620652	pseudobulk	DESeq2	LRT	0.711221827928439
196	KC_G1	CG17162	-0.0444111199739965	0.940418120196583	0.998043617620652	pseudobulk	DESeq2	LRT	0.0266790113786904
196	KC_G1	CG17163	0.0809893726385828	0.742953953662803	0.998043617620652	pseudobulk	DESeq2	LRT	0.12903810183814
196	KC_G1	CG17167	-4.0443672005562	0.0243118874665556	0.998043617620652	pseudobulk	DESeq2	LRT	1.61418132316039
196	KC_G1	CG17168	0.22153545757998	0.39558115306091	0.998043617620652	pseudobulk	DESeq2	LRT	0.402764407966236
196	KC_G1	CG17169	-0.711691946027098	0.741255142397939	0.998043617620652	pseudobulk	DESeq2	LRT	0.130032280734188
196	KC_G1	CG17186	0.285695353733717	0.693211393605518	0.998043617620652	pseudobulk	DESeq2	LRT	0.159134307845972
196	KC_G1	CG17187	0.102856002089988	0.87862603857682	0.998043617620652	pseudobulk	DESeq2	LRT	0.0561959303187497
196	KC_G1	CG17193	-1.76124029133935	0.0325769579974183	0.998043617620652	pseudobulk	DESeq2	LRT	1.48708947210289
196	KC_G1	CG17202	-0.114439570664448	0.529861152763167	0.998043617620652	pseudobulk	DESeq2	LRT	0.275837920000748
196	KC_G1	CG17211	2.84706876674335	0.20790991633284	0.998043617620652	pseudobulk	DESeq2	LRT	0.682124796354298
197	KC_G1	CG1722	-0.794610277746993	0.879485534599449	0.998043617620652	pseudobulk	DESeq2	LRT	0.0557712992369932
197	KC_G1	CG17230	-0.145915601460368	0.778097429063637	0.998043617620652	pseudobulk	DESeq2	LRT	0.108966019648473
197	KC_G1	CG17233	0.0702237922898709	0.759812813100372	0.998043617620652	pseudobulk	DESeq2	LRT	0.119293386996266
197	KC_G1	CG17249	0.248095907021707	0.617234311765102	0.998043617620652	pseudobulk	DESeq2	LRT	0.209549939711839
197	KC_G1	CG17258	-0.794610277746993	0.821479263487599	0.998043617620652	pseudobulk	DESeq2	LRT	0.085403394939296
197	KC_G1	CG17260	-0.0608970137250128	0.925378889535425	0.998043617620652	pseudobulk	DESeq2	LRT	0.0336804121873752
197	KC_G1	CG17261	0.173105107343345	0.973555957903825	0.998043617620652	pseudobulk	DESeq2	LRT	0.0116390811132178
197	KC_G1	CG17266	0.448170130013617	0.572286110480164	0.998043617620652	pseudobulk	DESeq2	LRT	0.242386794393937
197	KC_G1	CG17270	-0.0361195108101266	0.888103548483012	0.998043617620652	pseudobulk	DESeq2	LRT	0.0515363946797227
197	KC_G1	CG17271	-0.343154821592659	0.278026924640115	0.998043617620652	pseudobulk	DESeq2	LRT	0.555913144166765
198	KC_G1	CG17272	0.0674176204255251	0.688305428592093	0.998043617620652	pseudobulk	DESeq2	LRT	0.162218805198129
198	KC_G1	CG17279	-0.794610277746993	0.815039924133852	0.998043617620652	pseudobulk	DESeq2	LRT	0.0888211171417967
198	KC_G1	CG17292	1.66893182195367	0.456348242815677	0.998043617620652	pseudobulk	DESeq2	LRT	0.34070361739619
198	KC_G1	CG17294	0.177881177809874	0.555275800903882	0.998043617620652	pseudobulk	DESeq2	LRT	0.255491252806764
198	KC_G1	CG17321	-0.00987534931004158	0.946207163986104	0.998043617620652	pseudobulk	DESeq2	LRT	0.0240137681159728
198	KC_G1	CG17328	0.778855597042053	0.321399170374869	0.998043617620652	pseudobulk	DESeq2	LRT	0.492955248612132
198	KC_G1	CG17329	-0.776296732928036	0.736588270584829	0.998043617620652	pseudobulk	DESeq2	LRT	0.132775201121675
198	KC_G1	CG17333	-1.21194906905591	0.557679189728222	0.998043617620652	pseudobulk	DESeq2	LRT	0.253615561272872
198	KC_G1	CG17343	-0.143065731628115	0.659586010192091	0.998043617620652	pseudobulk	DESeq2	LRT	0.18072856430831
198	KC_G1	CG17359	-0.520820032628421	0.473389863025264	0.998043617620652	pseudobulk	DESeq2	LRT	0.324781046109781
199	KC_G1	CG17361	0.14481414041004	0.780965352179615	0.998043617620652	pseudobulk	DESeq2	LRT	0.107368233332673
199	KC_G1	CG17364	0.0598081243551833	0.789755097196711	0.998043617620652	pseudobulk	DESeq2	LRT	0.10250756241267
199	KC_G1	CG1737	0.00617429376175468	0.97484804631167	0.998043617620652	pseudobulk	DESeq2	LRT	0.0110630743438796
199	KC_G1	CG17378	-0.134292742391194	0.601553012908695	0.998043617620652	pseudobulk	DESeq2	LRT	0.220726093678741
199	KC_G1	CG1738	-0.182439075072676	0.771615640599992	0.998043617620652	pseudobulk	DESeq2	LRT	0.112598977817388
199	KC_G1	CG17385	-0.0416033535827135	0.82738502427096	0.998043617620652	pseudobulk	DESeq2	LRT	0.0822923441310368
199	KC_G1	CG17387	-0.794610277746993	0.879485534599449	0.998043617620652	pseudobulk	DESeq2	LRT	0.0557712992369932
199	KC_G1	CG17440	2.23045770445974	0.357070320422792	0.998043617620652	pseudobulk	DESeq2	LRT	0.447246246733247
199	KC_G1	CG17454	0.0700046704171511	0.668558011991033	0.998043617620652	pseudobulk	DESeq2	LRT	0.174860902247201
199	KC_G1	CG17470	-0.794610277746993	0.815039924133852	0.998043617620652	pseudobulk	DESeq2	LRT	0.0888211171417967
200	KC_G1	CG17486	-0.164605019290469	0.482177095313064	0.998043617620652	pseudobulk	DESeq2	LRT	0.316793423604782
200	KC_G1	CG17490	1.90250740646012	0.460774913678782	0.998043617620652	pseudobulk	DESeq2	LRT	0.336511173568024
200	KC_G1	CG17493	-0.0584671553631118	0.71935542619107	0.998043617620652	pseudobulk	DESeq2	LRT	0.143056476107435
200	KC_G1	CG1750	0.0860971674296491	0.858538496087806	0.998043617620652	pseudobulk	DESeq2	LRT	0.0662402266956341
200	KC_G1	CG17508	0.0276901219408862	0.879162772084234	0.998043617620652	pseudobulk	DESeq2	LRT	0.0559307102805367
200	KC_G1	CG17528	0.00103372289987529	0.971139829057548	0.998043617620652	pseudobulk	DESeq2	LRT	0.0127182339271946
200	KC_G1	CG17544	-0.141605876958388	0.683348863433213	0.998043617620652	pseudobulk	DESeq2	LRT	0.165357523576045
200	KC_G1	CG17549	-0.65817001371651	0.770857516266391	0.998043617620652	pseudobulk	DESeq2	LRT	0.113025888638787
200	KC_G1	CG17556	-0.562661583376517	0.0638471822842815	0.998043617620652	pseudobulk	DESeq2	LRT	1.19485826434247
200	KC_G1	CG17565	-0.0407979431711748	0.893409122367874	0.998043617620652	pseudobulk	DESeq2	LRT	0.0489496173736908
201	KC_G1	CG17568	0.265507238617842	0.429230671380696	0.998043617620652	pseudobulk	DESeq2	LRT	0.367309252373697
201	KC_G1	CG17574	-0.0562659486749294	0.882788668108178	0.998043617620652	pseudobulk	DESeq2	LRT	0.0541432502834615
201	KC_G1	CG17593	0.0350792255886059	0.897136959842	0.998043617620652	pseudobulk	DESeq2	LRT	0.047141251088441
201	KC_G1	CG17597	-0.794610277746993	0.879485534599449	0.998043617620652	pseudobulk	DESeq2	LRT	0.0557712992369932
201	KC_G1	CG17598	0.233019436160004	0.465198088531349	0.998043617620652	pseudobulk	DESeq2	LRT	0.332362078427667

201	KC_G1	CG17600	0.326054647038597	0.371270518289892	0.998043617620652	pseudobulk	DESeq2	LRT	0.430309535698056
201	KC_G1	CG17612	0.00178446417408077	0.958051836719281	0.998043617620652	pseudobulk	DESeq2	LRT	0.018610992182311
201	KC_G1	CG17637	-1.63882223825269	0.36415089042169	0.998043617620652	pseudobulk	DESeq2	LRT	0.438718623771626
201	KC_G1	CG1764	0.0294506976438746	0.912671714650739	0.998043617620652	pseudobulk	DESeq2	LRT	0.0396854088337825
201	KC_G1	CG17646	-0.0687158306847888	0.931156950627796	0.998043617620652	pseudobulk	DESeq2	LRT	0.0309771105904187
202	KC_G1	CG17650	2.86622177009429	0.203230025119761	0.998043617620652	pseudobulk	DESeq2	LRT	0.692012129161051
202	KC_G1	CG17652	0.414149942073611	0.331681099392476	0.998043617620652	pseudobulk	DESeq2	LRT	0.479279275732322
202	KC_G1	CG17660	-0.103559082770964	0.827901263021036	0.998043617620652	pseudobulk	DESeq2	LRT	0.0820214548601079
202	KC_G1	CG17669	-2.07822361679844	0.409775960476986	0.998043617620652	pseudobulk	DESeq2	LRT	0.387453523090571
202	KC_G1	CG17683	0.285300168465401	0.212938284997284	0.998043617620652	pseudobulk	DESeq2	LRT	0.671746248057885
202	KC_G1	CG17684	-0.092675681032614	0.397073022319856	0.998043617620652	pseudobulk	DESeq2	LRT	0.401129618490439
202	KC_G1	CG17698	0.137530420239131	0.418007533805267	0.998043617620652	pseudobulk	DESeq2	LRT	0.378815890807333
202	KC_G1	CG17707	-1.67593316621313	0.495877122847873	0.998043617620652	pseudobulk	DESeq2	LRT	0.304625927300966
202	KC_G1	CG17712	0.149426632883257	0.70998135959628	0.998043617620652	pseudobulk	DESeq2	LRT	0.148753053436906
202	KC_G1	CG17715	0.0635572090200322	0.697530331206767	0.998043617620652	pseudobulk	DESeq2	LRT	0.156436902908378
203	KC_G1	CG17716	0.949176147629133	0.729784453976284	0.998043617620652	pseudobulk	DESeq2	LRT	0.136805392306293
203	KC_G1	CG17721	-0.545294449137642	0.425933413157936	0.998043617620652	pseudobulk	DESeq2	LRT	0.370658289531975
203	KC_G1	CG17724	0.176225850973842	0.458679103309912	0.998043617620652	pseudobulk	DESeq2	LRT	0.338491045182865
203	KC_G1	CG17726	0.090082425058306	0.921052985077794	0.998043617620652	pseudobulk	DESeq2	LRT	0.0357153855848883
203	KC_G1	CG17734	0.140915148375508	0.492327650677398	0.998043617620652	pseudobulk	DESeq2	LRT	0.307745772191108
203	KC_G1	CG17739	0.384126538475826	0.723517625080218	0.998043617620652	pseudobulk	DESeq2	LRT	0.140550884887931
203	KC_G1	CG17746	-0.154959891580469	0.663471475038961	0.998043617620652	pseudobulk	DESeq2	LRT	0.178177744238097
203	KC_G1	CG17754	-0.259658497370513	0.450242894389382	0.998043617620652	pseudobulk	DESeq2	LRT	0.346553132371466
203	KC_G1	CG17760	-0.682453401100201	0.731162551182152	0.998043617620652	pseudobulk	DESeq2	LRT	0.135986060479148
203	KC_G1	CG17765	0.0589163253613398	0.751656008443143	0.998043617620652	pseudobulk	DESeq2	LRT	0.123980866608318
204	KC_G1	CG17776	0.112593066660607	0.674468020700299	0.998043617620652	pseudobulk	DESeq2	LRT	0.171038637189474
204	KC_G1	CG17778	-0.00944690125169356	0.954712545279726	0.998043617620652	pseudobulk	DESeq2	LRT	0.0201273706057661
204	KC_G1	CG17781	1.13582588427274	0.704684208645453	0.998043617620652	pseudobulk	DESeq2	LRT	0.152005460551806
204	KC_G1	CG17782	2.07738706174348	0.431998797123676	0.998043617620652	pseudobulk	DESeq2	LRT	0.364517462451933
204	KC_G1	CG17786	-0.00096999144484016	0.965120431869279	0.998043617620652	pseudobulk	DESeq2	LRT	0.0154184901454845
204	KC_G1	CG17802	0.147107722667862	0.760796096070492	0.998043617620652	pseudobulk	DESeq2	LRT	0.118731724592453
204	KC_G1	CG17803	0.0878684986685263	0.840118626342737	0.998043617620652	pseudobulk	DESeq2	LRT	0.0756593864040537
204	KC_G1	CG17806	-0.279094130635964	0.659320599812426	0.998043617620652	pseudobulk	DESeq2	LRT	0.180903354947784
204	KC_G1	CG17807	0.190263206362351	0.783160996298925	0.998043617620652	pseudobulk	DESeq2	LRT	0.106148949797809
204	KC_G1	CG17816	-0.0163102396855634	0.870355681505529	0.998043617620652	pseudobulk	DESeq2	LRT	0.0603032313495052
205	KC_G1	CG17829	0.321798672255645	0.526065171454064	0.998043617620652	pseudobulk	DESeq2	LRT	0.278960450048518
205	KC_G1	CG17834	-1.60891734856068	0.545249891924755	0.998043617620652	pseudobulk	DESeq2	LRT	0.263404411831769
205	KC_G1	CG17839	-0.223722039911099	0.261683872676812	0.998043617620652	pseudobulk	DESeq2	LRT	0.582223041682966
205	KC_G1	CG17841	0.16545083890527	0.363858247108817	0.998043617620652	pseudobulk	DESeq2	LRT	0.439067777034243
205	KC_G1	CG1785	-0.733425569358845	0.36152208913618	0.998043617620652	pseudobulk	DESeq2	LRT	0.44186516200189
205	KC_G1	CG17883	0.123200021608123	0.554433552369287	0.998043617620652	pseudobulk	DESeq2	LRT	0.256150495625839
205	KC_G1	CG1789	-0.297394021586034	0.376740187589916	0.998043617620652	pseudobulk	DESeq2	LRT	0.423958050310078
205	KC_G1	CG17896	0.594200154488236	0.223087393094271	0.998043617620652	pseudobulk	DESeq2	LRT	0.651524971465563
205	KC_G1	CG17904	-0.0146050020529808	0.956266181608442	0.998043617620652	pseudobulk	DESeq2	LRT	0.0194212027938345
205	KC_G1	CG17917	1.12508065046033	0.900770125530981	0.998043617620652	pseudobulk	DESeq2	LRT	0.0453860258366365
206	KC_G1	CG17919	1.13582588427274	0.704684208645453	0.998043617620652	pseudobulk	DESeq2	LRT	0.152005460551806
206	KC_G1	CG1792	-0.214264469748296	0.677552744027705	0.998043617620652	pseudobulk	DESeq2	LRT	0.169056891525752
206	KC_G1	CG17928	-1.35574846224606	0.532546703611297	0.998043617620652	pseudobulk	DESeq2	LRT	0.273642299192052
206	KC_G1	CG17931	-0.181832171959625	0.436608237976415	0.998043617620652	pseudobulk	DESeq2	LRT	0.359908074229384
206	KC_G1	CG17977	0.180096231429414	0.619183334365693	0.998043617620652	pseudobulk	DESeq2	LRT	0.20818074142468
206	KC_G1	CG17982	-0.0949256903961807	0.913082418372687	0.998043617620652	pseudobulk	DESeq2	LRT	0.0394900195862149
206	KC_G1	CG17985	0.375224613978117	0.383670193756519	0.998043617620652	pseudobulk	DESeq2	LRT	0.416041938591298
206	KC_G1	CG17991	1.13582588427274	0.704684208645453	0.998043617620652	pseudobulk	DESeq2	LRT	0.152005460551806
206	KC_G1	CG17996	0.237092624263902	0.418124985065189	0.998043617620652	pseudobulk	DESeq2	LRT	0.378693880400463
206	KC_G1	CG18004	-0.0629858966604125	0.828199117315404	0.998043617620652	pseudobulk	DESeq2	LRT	0.0818652366991968
207	KC_G1	CG18011	-1.35098152246969	0.367111437904376	0.998043617620652	pseudobulk	DESeq2	LRT	0.435202084192906

207	KC_G1	CG18048	0.340133910302581	0.540575814670092	0.998043617620652	pseudobulk	DESeq2	LRT	0.26714338851203
207	KC_G1	CG18067	-0.794610277746993	0.879485534599449	0.998043617620652	pseudobulk	DESeq2	LRT	0.0557712992369932
207	KC_G1	CG18081	-0.0906726987602602	0.795236023406001	0.998043617620652	pseudobulk	DESeq2	LRT	0.0995039550542867
207	KC_G1	CG18094	1.13582588427274	0.704684208645453	0.998043617620652	pseudobulk	DESeq2	LRT	0.152005460551806
207	KC_G1	CG1812	-0.259101078668678	0.689289837251346	0.998043617620652	pseudobulk	DESeq2	LRT	0.161598124607798
207	KC_G1	CG18135	0.239891191289102	0.254027785367362	0.998043617620652	pseudobulk	DESeq2	LRT	0.595118777979442
207	KC_G1	CG1814	0.343491968186756	0.150283486857157	0.998043617620652	pseudobulk	DESeq2	LRT	0.823088737049864
207	KC_G1	CG1815	-0.250231753593597	0.465633737442807	0.998043617620652	pseudobulk	DESeq2	LRT	0.331955560457764
207	KC_G1	CG18171	-0.35212937651957	0.851498463013365	0.998043617620652	pseudobulk	DESeq2	LRT	0.0698161316184874
208	KC_G1	CG18178	0.233966465640603	0.623722454470118	0.998043617620652	pseudobulk	DESeq2	LRT	0.20500862074456
208	KC_G1	CG18213	-1.67593316621313	0.495877122847873	0.998043617620652	pseudobulk	DESeq2	LRT	0.304625927300966
208	KC_G1	CG1824	-0.599699456889478	0.204091659968156	0.998043617620652	pseudobulk	DESeq2	LRT	0.690174741995411
208	KC_G1	CG18259	0.134086028404047	0.785786170078853	0.998043617620652	pseudobulk	DESeq2	LRT	0.104695619086563
208	KC_G1	CG18262	-0.159282416972728	0.825445090306256	0.998043617620652	pseudobulk	DESeq2	LRT	0.0833118112808297
208	KC_G1	CG18265	-0.348684401098817	0.110407278628328	0.998043617620652	pseudobulk	DESeq2	LRT	0.957002294687263
208	KC_G1	CG18268	0.174619737100021	0.923414878852843	0.998043617620652	pseudobulk	DESeq2	LRT	0.0346031320031716
208	KC_G1	CG1827	0.198425077527751	0.900144795529779	0.998043617620652	pseudobulk	DESeq2	LRT	0.0456876251810912
208	KC_G1	CG18273	-0.792272890808019	0.567705790686858	0.998043617620652	pseudobulk	DESeq2	LRT	0.245876675881117
208	KC_G1	CG18281	-0.0703848829489292	0.92616593652747	0.998043617620652	pseudobulk	DESeq2	LRT	0.0333111959724401
209	KC_G1	CG18324	0.288658871615891	0.44054438412028	0.998043617620652	pseudobulk	DESeq2	LRT	0.356010330594753
209	KC_G1	CG18327	1.24393714063128	0.446418896807342	0.998043617620652	pseudobulk	DESeq2	LRT	0.350257430097681
209	KC_G1	CG18335	0.198599073954778	0.903397137636515	0.998043617620652	pseudobulk	DESeq2	LRT	0.0441212898103076
209	KC_G1	CG18336	-1.06429302687617	0.482802292238221	0.998043617620652	pseudobulk	DESeq2	LRT	0.316230676635899
209	KC_G1	CG18343	2.18876425250967	0.402105238436886	0.998043617620652	pseudobulk	DESeq2	LRT	0.395660269077107
209	KC_G1	CG18367	-0.794610277746993	0.879485534599449	0.998043617620652	pseudobulk	DESeq2	LRT	0.0557712992369932
209	KC_G1	CG18371	1.11154651156394	0.675883747053588	0.998043617620652	pseudobulk	DESeq2	LRT	0.170127996891038
209	KC_G1	CG1840	0.0341619184911333	0.915578658202502	0.998043617620652	pseudobulk	DESeq2	LRT	0.0383043391221239
209	KC_G1	CG18420	1.93548344937275	0.254940397151538	0.998043617620652	pseudobulk	DESeq2	LRT	0.593561341973194
209	KC_G1	CG18428	-0.0856587195649098	0.727814184532804	0.998043617620652	pseudobulk	DESeq2	LRT	0.137979484605382
210	KC_G1	CG18467	0.170615715856648	0.324267211752199	0.998043617620652	pseudobulk	DESeq2	LRT	0.489096962751612
210	KC_G1	CG1847	-0.0925655801400818	0.728719129755197	0.998043617620652	pseudobulk	DESeq2	LRT	0.137439829572127
210	KC_G1	CG18473	0.173105107343345	0.973555957903825	0.998043617620652	pseudobulk	DESeq2	LRT	0.0116390811132178
210	KC_G1	CG18476	0.100591134871221	0.839138613431854	0.998043617620652	pseudobulk	DESeq2	LRT	0.0761662941459165
210	KC_G1	CG18508	-0.0716786679808152	0.729965407426412	0.998043617620652	pseudobulk	DESeq2	LRT	0.136697720317615
210	KC_G1	CG18528	0.751758089926176	0.201168607639902	0.998043617620652	pseudobulk	DESeq2	LRT	0.696439789980251
210	KC_G1	CG18536	1.12508065046033	0.900770125530981	0.998043617620652	pseudobulk	DESeq2	LRT	0.0453860258366365
210	KC_G1	CG18539	-0.794610277746993	0.879485534599449	0.998043617620652	pseudobulk	DESeq2	LRT	0.0557712992369932
210	KC_G1	CG18549	0.146708456525816	0.743289959169049	0.998043617620652	pseudobulk	DESeq2	LRT	0.128841733882008
210	KC_G1	CG18563	-0.711691946027098	0.741255142397939	0.998043617620652	pseudobulk	DESeq2	LRT	0.130032280734188
211	KC_G1	CG18577	1.69981218657454	0.566400560818652	0.998043617620652	pseudobulk	DESeq2	LRT	0.246876325303589
211	KC_G1	CG18596	0.257848044130695	0.79298904421617	0.998043617620652	pseudobulk	DESeq2	LRT	0.100732812769824
211	KC_G1	CG18598	0.284726107083236	0.860469109023263	0.998043617620652	pseudobulk	DESeq2	LRT	0.065264716296966
211	KC_G1	CG18600	-0.479030358961069	0.508076905929938	0.998043617620652	pseudobulk	DESeq2	LRT	0.294070545013207
211	KC_G1	CG18622	-0.864080305918759	0.552954524646391	0.998043617620652	pseudobulk	DESeq2	LRT	0.257310583898244
211	KC_G1	CG18628	0.723271857444286	0.637204399804609	0.998043617620652	pseudobulk	DESeq2	LRT	0.195721234119703
211	KC_G1	CG18659	-0.13108872180069	0.672878095352921	0.998043617620652	pseudobulk	DESeq2	LRT	0.17206360934367
211	KC_G1	CG18661	0.652738640016169	0.170702997710186	0.998043617620652	pseudobulk	DESeq2	LRT	0.767758852186023
211	KC_G1	CG18675	-0.711691946027098	0.741255142397939	0.998043617620652	pseudobulk	DESeq2	LRT	0.130032280734188
211	KC_G1	CG18731	0.174255740590419	0.616044990554948	0.998043617620652	pseudobulk	DESeq2	LRT	0.210387569594909
212	KC_G1	CG18744	1.13582588427274	0.704684208645453	0.998043617620652	pseudobulk	DESeq2	LRT	0.152005460551806
212	KC_G1	CG18764	0.417989945120086	0.706407862952758	0.998043617620652	pseudobulk	DESeq2	LRT	0.150944475316687
212	KC_G1	CG18766	-0.104985061171017	0.600596788288531	0.998043617620652	pseudobulk	DESeq2	LRT	0.221416994535136
212	KC_G1	CG18787	-0.718952744627485	0.301516311394864	0.998043617620652	pseudobulk	DESeq2	LRT	0.52068918847519
212	KC_G1	CG18789	0.175230074097782	0.913962428129121	0.998043617620652	pseudobulk	DESeq2	LRT	0.0390716572110089
212	KC_G1	CG18809	-0.120676847133243	0.717447669987636	0.998043617620652	pseudobulk	DESeq2	LRT	0.144209770502567
212	KC_G1	CG18815	0.0801149869874241	0.549290735636882	0.998043617620652	pseudobulk	DESeq2	LRT	0.260197725747821

212	KC_G1	CG1882	-0.167466062116398	0.568358465331293	0.998043617620652	pseudobulk	DESeq2	LRT	0.245377667086078
212	KC_G1	CG18858	-2.03969107803542	0.322154266402034	0.998043617620652	pseudobulk	DESeq2	LRT	0.491936112765278
212	KC_G1	CG18870	-0.132557205611914	0.736736476201989	0.998043617620652	pseudobulk	DESeq2	LRT	0.132687827469563
213	KC_G1	CG1888	0.426589130331313	0.449449066908323	0.998043617620652	pseudobulk	DESeq2	LRT	0.347319516865054
213	KC_G1	CG1890	1.01072268106893	0.0737847603813882	0.998043617620652	pseudobulk	DESeq2	LRT	1.13203332876934
213	KC_G1	CG1894	-0.794610277746993	0.821479263487599	0.998043617620652	pseudobulk	DESeq2	LRT	0.085403394939296
213	KC_G1	CG1896	-0.100599479383339	0.727905532930165	0.998043617620652	pseudobulk	DESeq2	LRT	0.137924979463381
213	KC_G1	CG1902	0.244513884937098	0.403039990104898	0.998043617620652	pseudobulk	DESeq2	LRT	0.394651860508398
213	KC_G1	CG1907	0.0627238463525275	0.842299017393348	0.998043617620652	pseudobulk	DESeq2	LRT	0.0745337059461157
213	KC_G1	CG1909	-0.0226032694018645	0.871449473803945	0.998043617620652	pseudobulk	DESeq2	LRT	0.0597577879966214
213	KC_G1	CG1910	-0.0324831365958301	0.834775942889916	0.998043617620652	pseudobulk	DESeq2	LRT	0.0784300752039787
213	KC_G1	CG1927	0.385718394105527	0.140027962095385	0.998043617620652	pseudobulk	DESeq2	LRT	0.853785231670653
213	KC_G1	CG1943	0.0879784737574076	0.511946263327388	0.998043617620652	pseudobulk	DESeq2	LRT	0.290775622549885
214	KC_G1	CG1951	-0.0396724112300465	0.911767534975146	0.998043617620652	pseudobulk	DESeq2	LRT	0.0401158756481627
214	KC_G1	CG1965	-0.0756025527453748	0.708223657624312	0.998043617620652	pseudobulk	DESeq2	LRT	0.149829570088373
214	KC_G1	CG1971	-0.794610277746993	0.879485534599449	0.998043617620652	pseudobulk	DESeq2	LRT	0.0557712992369932
214	KC_G1	CG1983	0.103570038518205	0.778648861666478	0.998043617620652	pseudobulk	DESeq2	LRT	0.108658346975029
214	KC_G1	CG1998	0.237750637906939	0.238342872057804	0.998043617620652	pseudobulk	DESeq2	LRT	0.622797831663315
214	KC_G1	CG2003	2.18587959326853	0.0500553206836025	0.998043617620652	pseudobulk	DESeq2	LRT	1.30054975193666
214	KC_G1	CG2004	-0.699883962527198	0.463937325156814	0.998043617620652	pseudobulk	DESeq2	LRT	0.333540685774952
214	KC_G1	CG2006	-0.482751493462052	0.586934670841343	0.998043617620652	pseudobulk	DESeq2	LRT	0.23141023550203
214	KC_G1	CG2016	-0.988565475276454	0.568308669626819	0.998043617620652	pseudobulk	DESeq2	LRT	0.245415718682758
214	KC_G1	CG2017	0.0879303328814696	0.668518722745139	0.998043617620652	pseudobulk	DESeq2	LRT	0.174886425244126
215	KC_G1	CG2021	0.0797267659191513	0.80284390538902	0.998043617620652	pseudobulk	DESeq2	LRT	0.095368885129766
215	KC_G1	CG2023	0.394514678195348	0.436130334485303	0.998043617620652	pseudobulk	DESeq2	LRT	0.360383705481093
215	KC_G1	CG2034	0.143868185234491	0.806503966576585	0.998043617620652	pseudobulk	DESeq2	LRT	0.0933934923071504
215	KC_G1	CG2059	0.067624738427173	0.815121186364541	0.998043617620652	pseudobulk	DESeq2	LRT	0.0887778186749429
215	KC_G1	CG2061	0.116673962297695	0.64875598209395	0.998043617620652	pseudobulk	DESeq2	LRT	0.18791862455248
215	KC_G1	CG2063	0.0952432521532758	0.751878139670222	0.998043617620652	pseudobulk	DESeq2	LRT	0.1238525417984
215	KC_G1	CG2064	1.30652690817936	0.299576491287881	0.998043617620652	pseudobulk	DESeq2	LRT	0.523492270093176
215	KC_G1	CG2076	0.210786060084205	0.512585705063594	0.998043617620652	pseudobulk	DESeq2	LRT	0.290233509541877
215	KC_G1	CG2082	2.51669810348735	0.0462081944473922	0.998043617620652	pseudobulk	DESeq2	LRT	1.33528100090034
215	KC_G1	CG2091	0.156068876451329	0.643125005718032	0.998043617620652	pseudobulk	DESeq2	LRT	0.191704604032174
216	KC_G1	CG2100	1.11750245392061	0.240937445715234	0.998043617620652	pseudobulk	DESeq2	LRT	0.618095698118296
216	KC_G1	CG2104	-2.31763425292248	0.315578515403755	0.998043617620652	pseudobulk	DESeq2	LRT	0.50089257123956
216	KC_G1	CG2111	-2.69906283620862	0.197900615670492	0.998043617620652	pseudobulk	DESeq2	LRT	0.70355285469769
216	KC_G1	CG2116	0.418118119551188	0.408806994197196	0.998043617620652	pseudobulk	DESeq2	LRT	0.38848168255225
216	KC_G1	CG2121	-1.60891734856068	0.545249891924755	0.998043617620652	pseudobulk	DESeq2	LRT	0.263404411831769
216	KC_G1	CG2124	0.360880108250943	0.663132477261577	0.998043617620652	pseudobulk	DESeq2	LRT	0.178399701766633
216	KC_G1	CG2126	-0.0954625389876262	0.855563741137807	0.998043617620652	pseudobulk	DESeq2	LRT	0.0677476291466127
216	KC_G1	CG2129	0.681574073322231	0.621675144086478	0.998043617620652	pseudobulk	DESeq2	LRT	0.206436496315103
216	KC_G1	CG2147	0.0130750594327385	0.962671641450527	0.998043617620652	pseudobulk	DESeq2	LRT	0.0165218215191559
216	KC_G1	CG2162	-0.00621433775205551	0.932286129550828	0.998043617620652	pseudobulk	DESeq2	LRT	0.0304507771160091
217	KC_G1	CG2182	0.0924933171129671	0.660642321499663	0.998043617620652	pseudobulk	DESeq2	LRT	0.180033608354209
217	KC_G1	CG2186	-0.0652953874699101	0.734859348766608	0.998043617620652	pseudobulk	DESeq2	LRT	0.133795776425834
217	KC_G1	CG2199	-0.53212646698927	0.156939345795788	0.998043617620652	pseudobulk	DESeq2	LRT	0.804268162086618
217	KC_G1	CG2200	-0.154771090316007	0.743769866560563	0.998043617620652	pseudobulk	DESeq2	LRT	0.128561420829919
217	KC_G1	CG2201	-0.0219554970962088	0.946242601624625	0.998043617620652	pseudobulk	DESeq2	LRT	0.023997503091513
217	KC_G1	CG2202	-0.601145558717388	0.432319679038595	0.998043617620652	pseudobulk	DESeq2	LRT	0.364194995085476
217	KC_G1	CG2209	-0.129255190630159	0.849119655707351	0.998043617620652	pseudobulk	DESeq2	LRT	0.0710311058080896
217	KC_G1	CG2211	0.435660943807747	0.471421404244264	0.998043617620652	pseudobulk	DESeq2	LRT	0.326590702816914
217	KC_G1	CG2218	0.217884955480778	0.581562767060868	0.998043617620652	pseudobulk	DESeq2	LRT	0.235403405755657
217	KC_G1	CG2224	0.350751838203763	0.51785340721159	0.998043617620652	pseudobulk	DESeq2	LRT	0.28579316197144
218	KC_G1	CG2225	-0.0587156122549238	0.68925054647187	0.998043617620652	pseudobulk	DESeq2	LRT	0.161622880891941
218	KC_G1	CG2246	-0.158329748731989	0.493274676387773	0.998043617620652	pseudobulk	DESeq2	LRT	0.306911179667295
218	KC_G1	CG2247	-0.38010574158865	0.285396494599136	0.998043617620652	pseudobulk	DESeq2	LRT	0.544551365438616

218	KC_G1	CG2256	-1.09825245276387	0.599581104447224	0.998043617620652	pseudobulk	DESeq2	LRT	0.222152062220706
218	KC_G1	CG2258	0.102575581347369	0.658311112796441	0.998043617620652	pseudobulk	DESeq2	LRT	0.181568813622128
218	KC_G1	CG2260	-0.239197704354994	0.723589698233614	0.998043617620652	pseudobulk	DESeq2	LRT	0.140507624830664
218	KC_G1	CG2269	0.00908352318885045	0.932234328323783	0.998043617620652	pseudobulk	DESeq2	LRT	0.0304749087762141
218	KC_G1	CG2277	0.280569763038483	0.399920576317275	0.998043617620652	pseudobulk	DESeq2	LRT	0.398026250402215
218	KC_G1	CG2278	-0.130922391492621	0.710303774879913	0.998043617620652	pseudobulk	DESeq2	LRT	0.148555877281824
218	KC_G1	CG2321	-0.218178536539707	0.856115932549884	0.998043617620652	pseudobulk	DESeq2	LRT	0.0674674205360591
219	KC_G1	CG2444	-2.19332442993981	0.381369935212232	0.998043617620652	pseudobulk	DESeq2	LRT	0.418653546964052
219	KC_G1	CG2493	0.807503097657643	0.361705504642628	0.998043617620652	pseudobulk	DESeq2	LRT	0.44164488184461
219	KC_G1	CG2519	-0.0832839726739839	0.675889255252389	0.998043617620652	pseudobulk	DESeq2	LRT	0.170124457568473
219	KC_G1	CG2528	2.04147257822512	0.408147672536479	0.998043617620652	pseudobulk	DESeq2	LRT	0.389182675723292
219	KC_G1	CG2540	-0.20552538537834	0.75258131428906	0.998043617620652	pseudobulk	DESeq2	LRT	0.123446568904668
219	KC_G1	CG2608	-0.131029509978482	0.72915714226412	0.998043617620652	pseudobulk	DESeq2	LRT	0.137178865833291
219	KC_G1	CG2611	0.339128849401278	0.484831169684422	0.998043617620652	pseudobulk	DESeq2	LRT	0.31440946725184
219	KC_G1	CG2614	-0.719134672100318	0.274731012346433	0.998043617620652	pseudobulk	DESeq2	LRT	0.561092313529132
219	KC_G1	CG2616	1.12508065046033	0.900770125530981	0.998043617620652	pseudobulk	DESeq2	LRT	0.0453860258366365
219	KC_G1	CG2641	0.283855156596841	0.89887010581145	0.998043617620652	pseudobulk	DESeq2	LRT	0.0463030628887369
220	KC_G1	CG2656	0.880996894017322	0.0500815792545901	0.998043617620652	pseudobulk	DESeq2	LRT	1.30032198469499
220	KC_G1	CG2662	-0.336380756159575	0.547251289939097	0.998043617620652	pseudobulk	DESeq2	LRT	0.261813206072919
220	KC_G1	CG2680	-0.273053485242706	0.623942479265717	0.998043617620652	pseudobulk	DESeq2	LRT	0.204855445716526
220	KC_G1	CG2681	1.13582588427274	0.633424328955532	0.998043617620652	pseudobulk	DESeq2	LRT	0.198305259991224
220	KC_G1	CG2685	0.590808391633397	0.240370141184973	0.998043617620652	pseudobulk	DESeq2	LRT	0.6191194814446
220	KC_G1	CG2691	1.11208497179825	0.424807831979712	0.998043617620652	pseudobulk	DESeq2	LRT	0.371807484972218
220	KC_G1	CG2694	2.07738706174348	0.431998797123676	0.998043617620652	pseudobulk	DESeq2	LRT	0.364517462451933
220	KC_G1	CG2698	0.0631629519136863	0.827216286986248	0.998043617620652	pseudobulk	DESeq2	LRT	0.0823809233813941
220	KC_G1	CG2701	0.25588722578344	0.396046832095405	0.998043617620652	pseudobulk	DESeq2	LRT	0.40225345620066
220	KC_G1	CG2712	1.39932215126627	0.203135255114346	0.998043617620652	pseudobulk	DESeq2	LRT	0.692214696128012
221	KC_G1	CG2747	0.0593313241516051	0.784729321470559	0.998043617620652	pseudobulk	DESeq2	LRT	0.105280119641368
221	KC_G1	CG2765	-0.0197583061745721	0.938085068358459	0.998043617620652	pseudobulk	DESeq2	LRT	0.0277577767131701
221	KC_G1	CG2767	0.801966974724336	0.550846697275733	0.998043617620652	pseudobulk	DESeq2	LRT	0.258969250133476
221	KC_G1	CG2772	0.122411619479702	0.720565176153687	0.998043617620652	pseudobulk	DESeq2	LRT	0.142326730514508
221	KC_G1	CG2790	0.193213298701609	0.57549170111595	0.998043617620652	pseudobulk	DESeq2	LRT	0.239960934737118
221	KC_G1	CG2794	-0.159842368815273	0.865003505509559	0.998043617620652	pseudobulk	DESeq2	LRT	0.0629821325116334
221	KC_G1	CG2811	0.402274601332424	0.222519331938746	0.998043617620652	pseudobulk	DESeq2	LRT	0.652632252594915
221	KC_G1	CG2812	-0.711691946027098	0.741255142397939	0.998043617620652	pseudobulk	DESeq2	LRT	0.130032280734188
221	KC_G1	CG2816	-0.794610277746993	0.879485534599449	0.998043617620652	pseudobulk	DESeq2	LRT	0.0557712992369932
221	KC_G1	CG2818	0.0392692623252617	0.904918908467749	0.998043617620652	pseudobulk	DESeq2	LRT	0.0433903370189927
222	KC_G1	CG2841	-2.89721534557419	0.097155027727211	0.998043617620652	pseudobulk	DESeq2	LRT	1.01253471994649
222	KC_G1	CG2846	0.366764347055033	0.269162979304305	0.998043617620652	pseudobulk	DESeq2	LRT	0.569984673235143
222	KC_G1	CG2852	-0.10320855331787	0.446700972460199	0.998043617620652	pseudobulk	DESeq2	LRT	0.349983102075646
222	KC_G1	CG2861	1.12508065046033	0.900770125530981	0.998043617620652	pseudobulk	DESeq2	LRT	0.0453860258366365
222	KC_G1	CG2865	0.400335914303825	0.0216079642601516	0.998043617620652	pseudobulk	DESeq2	LRT	1.66538614714862
222	KC_G1	CG2875	2.61451831136424	0.0276895444059241	0.998043617620652	pseudobulk	DESeq2	LRT	1.5576841899251
222	KC_G1	CG2889	0.191820867814396	0.642192475659138	0.998043617620652	pseudobulk	DESeq2	LRT	0.192334787213708
222	KC_G1	CG2906	0.631499252305635	0.407580309084541	0.998043617620652	pseudobulk	DESeq2	LRT	0.389786805682532
222	KC_G1	CG2909	0.300200243666509	0.464046834991758	0.998043617620652	pseudobulk	DESeq2	LRT	0.333438185065795
222	KC_G1	CG2911	0.256228618776943	0.764540300290222	0.998043617620652	pseudobulk	DESeq2	LRT	0.11659961720736
223	KC_G1	CG2915	0.12628608670823	0.823241134407809	0.998043617620652	pseudobulk	DESeq2	LRT	0.0844729375653401
223	KC_G1	CG2918	-0.0750240633574639	0.770458853205389	0.998043617620652	pseudobulk	DESeq2	LRT	0.113250550070823
223	KC_G1	CG2921	0.139771968638293	0.655376016872581	0.998043617620652	pseudobulk	DESeq2	LRT	0.183509455432242
223	KC_G1	CG2924	-0.144509443170755	0.3589075341312	0.998043617620652	pseudobulk	DESeq2	LRT	0.445017424928775
223	KC_G1	CG2926	-0.117932390514498	0.474555125998457	0.998043617620652	pseudobulk	DESeq2	LRT	0.323713331126352
223	KC_G1	CG2930	-0.718676691072112	0.739205546127982	0.998043617620652	pseudobulk	DESeq2	LRT	0.131234783341328
223	KC_G1	CG2931	-0.088534625909607	0.744960118004164	0.998043617620652	pseudobulk	DESeq2	LRT	0.127866976908545
223	KC_G1	CG2938	-0.191558074976102	0.611206701930194	0.998043617620652	pseudobulk	DESeq2	LRT	0.213811892332655
223	KC_G1	CG2972	-0.124327266201394	0.852241194448318	0.998043617620652	pseudobulk	DESeq2	LRT	0.0694374773061839

223	KC_G1	CG2991	0.0772678835599233	0.759632899951547	0.998043617620652	pseudobulk	DESeq2	LRT	0.119396234090375
224	KC_G1	CG2993	-0.0119108679478075	0.93626222270122	0.998043617620652	pseudobulk	DESeq2	LRT	0.0286024996347527
224	KC_G1	CG30001	-0.35212937651957	0.851498463013365	0.998043617620652	pseudobulk	DESeq2	LRT	0.0698161316184874
224	KC_G1	CG30008	-0.20723929275819	0.847315839460118	0.998043617620652	pseudobulk	DESeq2	LRT	0.0719546749505719
224	KC_G1	CG30010	-0.654891220941253	0.352423775091182	0.998043617620652	pseudobulk	DESeq2	LRT	0.45293480104712
224	KC_G1	CG30015	0.411737569752951	0.280510688364186	0.998043617620652	pseudobulk	DESeq2	LRT	0.552050586068604
224	KC_G1	CG30020	-0.555748533061147	0.263359521924859	0.998043617620652	pseudobulk	DESeq2	LRT	0.579450974839575
224	KC_G1	CG30022	0.0281740060006854	0.953490682514416	0.998043617620652	pseudobulk	DESeq2	LRT	0.0206835465113635
224	KC_G1	CG30054	0.537643718532651	0.690428957513106	0.998043617620652	pseudobulk	DESeq2	LRT	0.160881002019934
224	KC_G1	CG30060	-0.410549528760592	0.569136663180281	0.998043617620652	pseudobulk	DESeq2	LRT	0.244783436700557
224	KC_G1	CG30069	0.0293162571246938	0.929750769866762	0.998043617620652	pseudobulk	DESeq2	LRT	0.0316334533575373
225	KC_G1	CG30076	1.24406349205447	0.626072434068117	0.998043617620652	pseudobulk	DESeq2	LRT	0.203375417754029
225	KC_G1	CG3008	0.0376840950592472	0.91607504305873	0.998043617620652	pseudobulk	DESeq2	LRT	0.03806894832845
225	KC_G1	CG30089	-0.245742084082042	0.261092787959244	0.998043617620652	pseudobulk	DESeq2	LRT	0.583205124319385
225	KC_G1	CG3009	-0.0212330988655314	0.967622115371116	0.998043617620652	pseudobulk	DESeq2	LRT	0.0142942142313216
225	KC_G1	CG30094	0.256874230606254	0.399139917567397	0.998043617620652	pseudobulk	DESeq2	LRT	0.398874836705213
225	KC_G1	CG30096	0.551822002323319	0.411781246480998	0.998043617620652	pseudobulk	DESeq2	LRT	0.385333436094086
225	KC_G1	CG30099	-1.31729413969264	0.525615640677063	0.998043617620652	pseudobulk	DESeq2	LRT	0.279331720010916
225	KC_G1	CG30105	1.69794172976599	0.510063195508441	0.998043617620652	pseudobulk	DESeq2	LRT	0.292376012607326
225	KC_G1	CG30108	-0.794610277746993	0.815039924133852	0.998043617620652	pseudobulk	DESeq2	LRT	0.0888211171417967
225	KC_G1	CG30109	0.273018467358911	0.50082193214661	0.998043617620652	pseudobulk	DESeq2	LRT	0.300316660624934
226	KC_G1	CG30110	0.0721553293344594	0.973275465908855	0.998043617620652	pseudobulk	DESeq2	LRT	0.0117642240762772
226	KC_G1	CG30116	-0.0775459277989001	0.459299791038546	0.998043617620652	pseudobulk	DESeq2	LRT	0.337903752167156
226	KC_G1	CG30122	-0.1106201356754	0.580012299063633	0.998043617620652	pseudobulk	DESeq2	LRT	0.23656279719769
226	KC_G1	CG3014	1.12958862771465	0.28866484649331	0.998043617620652	pseudobulk	DESeq2	LRT	0.539606101133276
226	KC_G1	CG30151	-0.794610277746993	0.815039924133852	0.998043617620652	pseudobulk	DESeq2	LRT	0.0888211171417967
226	KC_G1	CG30156	0.173244859647713	0.774533178898909	0.998043617620652	pseudobulk	DESeq2	LRT	0.110959973509582
226	KC_G1	CG30157	-0.174028959974017	0.902886247296524	0.998043617620652	pseudobulk	DESeq2	LRT	0.0443669620711315
226	KC_G1	CG30158	-0.183214812916229	0.230715515995124	0.998043617620652	pseudobulk	DESeq2	LRT	0.636923197483803
226	KC_G1	CG30159	-1.74278423686085	0.385072726505398	0.998043617620652	pseudobulk	DESeq2	LRT	0.414457240005315
226	KC_G1	CG30172	0.0137805320109307	0.921295541404463	0.998043617620652	pseudobulk	DESeq2	LRT	0.0356010305936176
227	KC_G1	CG30183	-1.54381508316076	0.111351910533643	0.998043617620652	pseudobulk	DESeq2	LRT	0.953302327106815
227	KC_G1	CG30187	2.74581739320548	0.230409412685615	0.998043617620652	pseudobulk	DESeq2	LRT	0.637499783087704
227	KC_G1	CG30197	0.162117192865834	0.535606890956566	0.998043617620652	pseudobulk	DESeq2	LRT	0.271153844122389
227	KC_G1	CG3021	0.757151453350889	0.210590448226506	0.998043617620652	pseudobulk	DESeq2	LRT	0.676561331045102
227	KC_G1	CG30285	-0.266250833227903	0.703438615542226	0.998043617620652	pseudobulk	DESeq2	LRT	0.15277379459658
227	KC_G1	CG30291	-0.547685829637015	0.280930239635246	0.998043617620652	pseudobulk	DESeq2	LRT	0.551401510353863
227	KC_G1	CG3032	1.75080050603218	0.213243316332762	0.998043617620652	pseudobulk	DESeq2	LRT	0.671124572002357
227	KC_G1	CG30324	0.435420593768904	0.849021024519256	0.998043617620652	pseudobulk	DESeq2	LRT	0.0710815550803792
227	KC_G1	CG30334	0.173116475207952	0.97305562682953	0.998043617620652	pseudobulk	DESeq2	LRT	0.011862331638572
227	KC_G1	CG30338	-0.712043909782298	0.577673632950129	0.998043617620652	pseudobulk	DESeq2	LRT	0.238317454728957
228	KC_G1	CG30344	-0.681888687784492	0.324214955247333	0.998043617620652	pseudobulk	DESeq2	LRT	0.489166956077291
228	KC_G1	CG30345	1.13582588427274	0.633424328955532	0.998043617620652	pseudobulk	DESeq2	LRT	0.198305259991224
228	KC_G1	CG30349	-0.25481473775873	0.693332094770895	0.998043617620652	pseudobulk	DESeq2	LRT	0.159058695576102
228	KC_G1	CG30357	1.16252191837046	0.459133477677486	0.998043617620652	pseudobulk	DESeq2	LRT	0.338061039535166
228	KC_G1	CG30373	0.237034511288216	0.483664468751506	0.998043617620652	pseudobulk	DESeq2	LRT	0.315455815840703
228	KC_G1	CG30377	1.89288595231245	0.415717432633965	0.998043617620652	pseudobulk	DESeq2	LRT	0.381201763470702
228	KC_G1	CG30389	0.0717265733272507	0.639968783593017	0.998043617620652	pseudobulk	DESeq2	LRT	0.193841209522264
228	KC_G1	CG30392	-0.0098039610232563	0.965991293664147	0.998043617620652	pseudobulk	DESeq2	LRT	0.0150267877984381
228	KC_G1	CG30393	-0.794610277746993	0.879485534599449	0.998043617620652	pseudobulk	DESeq2	LRT	0.0557712992369932
228	KC_G1	CG30394	0.588531369569705	0.290332701467913	0.998043617620652	pseudobulk	DESeq2	LRT	0.537104044887535
229	KC_G1	CG30395	-1.73907471612892	0.507597351592657	0.998043617620652	pseudobulk	DESeq2	LRT	0.294480652518419
229	KC_G1	CG3040	0.0921208922666317	0.781722091224423	0.998043617620652	pseudobulk	DESeq2	LRT	0.106947614841781
229	KC_G1	CG30403	-0.796124617508433	0.16806235365559	0.998043617620652	pseudobulk	DESeq2	LRT	0.774529558605014
229	KC_G1	CG30414	-0.265978753400783	0.894647009539557	0.998043617620652	pseudobulk	DESeq2	LRT	0.0483482854085724
229	KC_G1	CG30423	0.114379350019485	0.528466663315123	0.998043617620652	pseudobulk	DESeq2	LRT	0.276982403616584

229	KC_G1	CG30424	1.0359015041102	0.528296924249789	0.998043617620652	pseudobulk	DESeq2	LRT	0.27712191777271
229	KC_G1	CG30428	-0.43266726344835	0.0466982099063829	0.998043617620652	pseudobulk	DESeq2	LRT	1.33069976702876
229	KC_G1	CG3045	-0.365037822045014	0.760866788560287	0.998043617620652	pseudobulk	DESeq2	LRT	0.118691372214061
229	KC_G1	CG30460	-0.414547759908913	0.820775937779871	0.998043617620652	pseudobulk	DESeq2	LRT	0.085775384009309
229	KC_G1	CG30466	-1.63492202315836	0.238279253445647	0.998043617620652	pseudobulk	DESeq2	LRT	0.622913769262062
230	KC_G1	CG30467	0.0460511219959633	0.912817575716462	0.998043617620652	pseudobulk	DESeq2	LRT	0.0396160064478721
230	KC_G1	CG30480	0.949176147629133	0.729784453976284	0.998043617620652	pseudobulk	DESeq2	LRT	0.136805392306293
230	KC_G1	CG30484	1.13582588427274	0.633424328955532	0.998043617620652	pseudobulk	DESeq2	LRT	0.198305259991224
230	KC_G1	CG30485	-0.794610277746993	0.815039924133852	0.998043617620652	pseudobulk	DESeq2	LRT	0.0888211171417967
230	KC_G1	CG30491	0.557943190080895	0.336462153812054	0.998043617620652	pseudobulk	DESeq2	LRT	0.47306377932992
230	KC_G1	CG30495	0.302408410779895	0.765090828989244	0.998043617620652	pseudobulk	DESeq2	LRT	0.116287003817292
230	KC_G1	CG30496	2.16392942277661	0.298606777329147	0.998043617620652	pseudobulk	DESeq2	LRT	0.524900339533806
230	KC_G1	CG3061	0.012744998005539	0.928339383568437	0.998043617620652	pseudobulk	DESeq2	LRT	0.0322932247878419
230	KC_G1	CG3065	0.0735607945248659	0.826508052384476	0.998043617620652	pseudobulk	DESeq2	LRT	0.0827529108897407
230	KC_G1	CG3071	-0.592359242155802	0.403638283163421	0.998043617620652	pseudobulk	DESeq2	LRT	0.39400764972417
231	KC_G1	CG3091	0.441477305359681	0.686410468320984	0.998043617620652	pseudobulk	DESeq2	LRT	0.163416101762832
231	KC_G1	CG3097	-2.0655499067062	0.287646871704878	0.998043617620652	pseudobulk	DESeq2	LRT	0.541140344769707
231	KC_G1	CG31030	-0.0404125780016114	0.754471439940334	0.998043617620652	pseudobulk	DESeq2	LRT	0.122357195531965
231	KC_G1	CG31036	-0.794610277746993	0.821479263487599	0.998043617620652	pseudobulk	DESeq2	LRT	0.085403394939296
231	KC_G1	CG31038	0.054803673529863	0.930161948310741	0.998043617620652	pseudobulk	DESeq2	LRT	0.0314414308712542
231	KC_G1	CG3104	-0.117352875006351	0.48784055002995	0.998043617620652	pseudobulk	DESeq2	LRT	0.311722103320533
231	KC_G1	CG31041	1.13582588427274	0.704684208645453	0.998043617620652	pseudobulk	DESeq2	LRT	0.152005460551806
231	KC_G1	CG31051	0.383937154267224	0.12061890047348	0.998043617620652	pseudobulk	DESeq2	LRT	0.918584634748145
231	KC_G1	CG31064	0.0565305865718757	0.764197554436174	0.998043617620652	pseudobulk	DESeq2	LRT	0.116794356462546
231	KC_G1	CG3107	0.0554835087648524	0.761867692571482	0.998043617620652	pseudobulk	DESeq2	LRT	0.118120442538579
232	KC_G1	CG3108	-0.794610277746993	0.879485534599449	0.998043617620652	pseudobulk	DESeq2	LRT	0.0557712992369932
232	KC_G1	CG31098	0.18417300438221	0.741857257891579	0.998043617620652	pseudobulk	DESeq2	LRT	0.129679650076293
232	KC_G1	CG31100	-0.892558278366628	0.657011466924577	0.998043617620652	pseudobulk	DESeq2	LRT	0.182427050564101
232	KC_G1	CG31102	-0.794610277746993	0.815039924133852	0.998043617620652	pseudobulk	DESeq2	LRT	0.0888211171417967
232	KC_G1	CG31109	-0.153278598898756	0.61071529926151	0.998043617620652	pseudobulk	DESeq2	LRT	0.214161200191419
232	KC_G1	CG31111	-0.179937888828948	0.744829228569805	0.998043617620652	pseudobulk	DESeq2	LRT	0.127943289119206
232	KC_G1	CG31115	-0.0594049093653602	0.962257478009936	0.998043617620652	pseudobulk	DESeq2	LRT	0.0167087051792469
232	KC_G1	CG31122	0.0399993346009633	0.858637849022972	0.998043617620652	pseudobulk	DESeq2	LRT	0.0661899715990341
232	KC_G1	CG31125	0.071941513886891	0.878405664432384	0.998043617620652	pseudobulk	DESeq2	LRT	0.0563048723204453
232	KC_G1	CG31126	-0.1061172708523	0.73102040321019	0.998043617620652	pseudobulk	DESeq2	LRT	0.136070501458091
233	KC_G1	CG31140	-0.0399444277544451	0.70299688485778	0.998043617620652	pseudobulk	DESeq2	LRT	0.153046599435473
233	KC_G1	CG31142	-0.33397042267097	0.634664007492399	0.998043617620652	pseudobulk	DESeq2	LRT	0.197456130314794
233	KC_G1	CG31145	-0.467814944552293	0.616427962349299	0.998043617620652	pseudobulk	DESeq2	LRT	0.210117669079896
233	KC_G1	CG31156	-0.0498332030639315	0.924575487659735	0.998043617620652	pseudobulk	DESeq2	LRT	0.0340576247582359
233	KC_G1	CG31157	1.13582588427274	0.633424328955532	0.998043617620652	pseudobulk	DESeq2	LRT	0.198305259991224
233	KC_G1	CG31161	-0.566261236961424	0.701441368135181	0.998043617620652	pseudobulk	DESeq2	LRT	0.154008624791776
233	KC_G1	CG31174	-0.128252646050412	0.885105172997736	0.998043617620652	pseudobulk	DESeq2	LRT	0.0530051210098399
233	KC_G1	CG31183	1.13582588427274	0.704684208645453	0.998043617620652	pseudobulk	DESeq2	LRT	0.152005460551806
233	KC_G1	CG31191	-0.255801169719868	0.0678538058130603	0.998043617620652	pseudobulk	DESeq2	LRT	1.16842578842786
233	KC_G1	CG31195	0.163262815640705	0.811355645843185	0.998043617620652	pseudobulk	DESeq2	LRT	0.0907887374402467
234	KC_G1	CG31199	1.12508065046033	0.900770125530981	0.998043617620652	pseudobulk	DESeq2	LRT	0.0453860258366365
234	KC_G1	CG31200	2.50145379121547	0.286990765027193	0.998043617620652	pseudobulk	DESeq2	LRT	0.542132078047399
234	KC_G1	CG31211	-0.0674485258811495	0.752819779979746	0.998043617620652	pseudobulk	DESeq2	LRT	0.123308978551042
234	KC_G1	CG31213	1.13582588427274	0.633424328955532	0.998043617620652	pseudobulk	DESeq2	LRT	0.198305259991224
234	KC_G1	CG31219	-0.794610277746993	0.879485534599449	0.998043617620652	pseudobulk	DESeq2	LRT	0.0557712992369932
234	KC_G1	CG31220	-1.72343437992478	0.512120560562093	0.998043617620652	pseudobulk	DESeq2	LRT	0.290627787806665
234	KC_G1	CG31223	0.290189590018367	0.623104709056238	0.998043617620652	pseudobulk	DESeq2	LRT	0.205438966592436
234	KC_G1	CG31224	0.108565496538405	0.585792508460602	0.998043617620652	pseudobulk	DESeq2	LRT	0.232256186691678
234	KC_G1	CG31229	-0.127840620554889	0.724011394975127	0.998043617620652	pseudobulk	DESeq2	LRT	0.140254598532307
234	KC_G1	CG31248	0.297994761981535	0.530213985031373	0.998043617620652	pseudobulk	DESeq2	LRT	0.275548821412706
235	KC_G1	CG31249	-0.348108534866223	0.369944834251117	0.998043617620652	pseudobulk	DESeq2	LRT	0.431863032599354

235	KC_G1	CG31251	-0.0794093058147311	0.907032626085078	0.998043617620652	pseudobulk	DESeq2	LRT	0.0423770910282441
235	KC_G1	CG31274	-0.794610277746993	0.879485534599449	0.998043617620652	pseudobulk	DESeq2	LRT	0.0557712992369932
235	KC_G1	CG31278	0.753784765600296	0.323139494274587	0.998043617620652	pseudobulk	DESeq2	LRT	0.490609959044241
235	KC_G1	CG31300	0.499880943206487	0.722731565114273	0.998043617620652	pseudobulk	DESeq2	LRT	0.14102297716855
235	KC_G1	CG31301	0.212079414739367	0.482165540193273	0.998043617620652	pseudobulk	DESeq2	LRT	0.316803831367705
235	KC_G1	CG31323	0.172845155749872	0.90730697492154	0.998043617620652	pseudobulk	DESeq2	LRT	0.0422457504714555
235	KC_G1	CG31324	0.269565013392243	0.378542940397123	0.998043617620652	pseudobulk	DESeq2	LRT	0.4218848487405
235	KC_G1	CG31342	-0.0371163867358381	0.88174208554018	0.998043617620652	pseudobulk	DESeq2	LRT	0.0546584298421036
235	KC_G1	CG31344	-0.285572471957376	0.39463303044335	0.998043617620652	pseudobulk	DESeq2	LRT	0.403806567489972
236	KC_G1	CG31345	0.259395432521758	0.606168547194683	0.998043617620652	pseudobulk	DESeq2	LRT	0.217406602006951
236	KC_G1	CG31357	0.158788112936295	0.695889863111379	0.998043617620652	pseudobulk	DESeq2	LRT	0.157459489739869
236	KC_G1	CG31360	0.232793154707333	0.533437128488039	0.998043617620652	pseudobulk	DESeq2	LRT	0.272916759669192
236	KC_G1	CG31365	0.0996786459557374	0.82378215514017	0.998043617620652	pseudobulk	DESeq2	LRT	0.0841876200126359
236	KC_G1	CG31368	-0.134805586626375	0.781516376974824	0.998043617620652	pseudobulk	DESeq2	LRT	0.107061916742333
236	KC_G1	CG31370	-1.73907471612892	0.507597351592657	0.998043617620652	pseudobulk	DESeq2	LRT	0.294480652518419
236	KC_G1	CG31371	0.0933178325725427	0.851810975216072	0.998043617620652	pseudobulk	DESeq2	LRT	0.0696567685296206
236	KC_G1	CG31373	-0.794610277746993	0.815039924133852	0.998043617620652	pseudobulk	DESeq2	LRT	0.0888211171417967
236	KC_G1	CG31381	-0.0239974597231755	0.944343130551377	0.998043617620652	pseudobulk	DESeq2	LRT	0.0248701745278564
236	KC_G1	CG31388	-0.095867750764451	0.766052270260362	0.998043617620652	pseudobulk	DESeq2	LRT	0.115741596018631
237	KC_G1	CG31391	1.12508065046033	0.900770125530981	0.998043617620652	pseudobulk	DESeq2	LRT	0.0453860258366365
237	KC_G1	CG31415	-0.418397140979882	0.766330674132764	0.998043617620652	pseudobulk	DESeq2	LRT	0.115583790468396
237	KC_G1	CG31431	1.12508065046033	0.900770125530981	0.998043617620652	pseudobulk	DESeq2	LRT	0.0453860258366365
237	KC_G1	CG31441	-0.196341359428331	0.835222216109996	0.998043617620652	pseudobulk	DESeq2	LRT	0.0781979623714332
237	KC_G1	CG31445	-0.794610277746993	0.879485534599449	0.998043617620652	pseudobulk	DESeq2	LRT	0.0557712992369932
237	KC_G1	CG31457	-0.127531344620735	0.86924719912078	0.998043617620652	pseudobulk	DESeq2	LRT	0.0608567000215801
237	KC_G1	CG31460	0.203941872557077	0.626013968606965	0.998043617620652	pseudobulk	DESeq2	LRT	0.203415976020813
237	KC_G1	CG31467	-0.481442092739587	0.741251775960373	0.998043617620652	pseudobulk	DESeq2	LRT	0.130034253103072
237	KC_G1	CG31468	-0.794610277746993	0.821479263487599	0.998043617620652	pseudobulk	DESeq2	LRT	0.085403394939296
237	KC_G1	CG31469	0.603088533052036	0.791040718043767	0.998043617620652	pseudobulk	DESeq2	LRT	0.101801161045192
238	KC_G1	CG31473	0.630147809623973	0.720764674673621	0.998043617620652	pseudobulk	DESeq2	LRT	0.142206506671425
238	KC_G1	CG31475	0.117268170225475	0.905449038192101	0.998043617620652	pseudobulk	DESeq2	LRT	0.0431359882596872
238	KC_G1	CG3149	0.0703715574375232	0.92280656754042	0.998043617620652	pseudobulk	DESeq2	LRT	0.0348893233009069
238	KC_G1	CG31510	0.0133492412809541	0.95736552409747	0.998043617620652	pseudobulk	DESeq2	LRT	0.018922216047528
238	KC_G1	CG31522	-0.105201178215607	0.612956525613528	0.998043617620652	pseudobulk	DESeq2	LRT	0.212570327040633
238	KC_G1	CG31523	-0.213510078145035	0.387855006543178	0.998043617620652	pseudobulk	DESeq2	LRT	0.411330598186427
238	KC_G1	CG31538	2.18876425250967	0.402105238436886	0.998043617620652	pseudobulk	DESeq2	LRT	0.395660269077107
238	KC_G1	CG31548	-0.343597265828537	0.743198798059883	0.998043617620652	pseudobulk	DESeq2	LRT	0.128895001377685
238	KC_G1	CG31549	-1.21642705951363	0.180410950546397	0.998043617620652	pseudobulk	DESeq2	LRT	0.743737105277786
238	KC_G1	CG31550	-0.0657572128911018	0.811902155381123	0.998043617620652	pseudobulk	DESeq2	LRT	0.0904963056584034
239	KC_G1	CG3156	-0.352647071239028	0.565311483537442	0.998043617620652	pseudobulk	DESeq2	LRT	0.247712192345845
239	KC_G1	CG31606	1.12508065046033	0.900770125530981	0.998043617620652	pseudobulk	DESeq2	LRT	0.0453860258366365
239	KC_G1	CG31612	0.999663307776749	0.620079451707173	0.998043617620652	pseudobulk	DESeq2	LRT	0.207552660135146
239	KC_G1	CG31627	0.660333798080835	0.729870210504599	0.998043617620652	pseudobulk	DESeq2	LRT	0.136754361623511
239	KC_G1	CG3163	0.0459483062132372	0.898838536100624	0.998043617620652	pseudobulk	DESeq2	LRT	0.0463183162516997
239	KC_G1	CG31633	-0.11876920395712	0.862435968737958	0.998043617620652	pseudobulk	DESeq2	LRT	0.0642731391022965
239	KC_G1	CG31635	-0.129875366858443	0.400684133926544	0.998043617620652	pseudobulk	DESeq2	LRT	0.397197854184654
239	KC_G1	CG31637	-0.225295694526422	0.58759747591315	0.998043617620652	pseudobulk	DESeq2	LRT	0.230920078435466
239	KC_G1	CG31638	0.162214911965299	0.440484757293811	0.998043617620652	pseudobulk	DESeq2	LRT	0.356069115487401
239	KC_G1	CG3164	-0.174168032622929	0.742823340190991	0.998043617620652	pseudobulk	DESeq2	LRT	0.129114458786722
240	KC_G1	CG31642	0.00611501926575206	0.944846785995025	0.998043617620652	pseudobulk	DESeq2	LRT	0.0246386098945459
240	KC_G1	CG31643	-0.11705807167102	0.903926278003404	0.998043617620652	pseudobulk	DESeq2	LRT	0.0438669880665581
240	KC_G1	CG31648	-0.0939384050397422	0.820797968639039	0.998043617620652	pseudobulk	DESeq2	LRT	0.085763727049447
240	KC_G1	CG3165	0.1970334270105	0.642955434205265	0.998043617620652	pseudobulk	DESeq2	LRT	0.191819128707962
240	KC_G1	CG31650	-0.104317108887657	0.585443694911814	0.998043617620652	pseudobulk	DESeq2	LRT	0.232514866885296
240	KC_G1	CG31663	-0.708922326998699	0.357945729361336	0.998043617620652	pseudobulk	DESeq2	LRT	0.446182814768343
240	KC_G1	CG31664	-0.794610277746993	0.821479263487599	0.998043617620652	pseudobulk	DESeq2	LRT	0.085403394939296

240 KC_G1	CG31673	-0.946288338617494	0.55910046274399	0.998043617620652	pseudobulk	DESeq2	LRT	0.252510148293861
240 KC_G1	CG31675	0.372786625046171	0.349321511142752	0.998043617620652	pseudobulk	DESeq2	LRT	0.456774669779055
240 KC_G1	CG31676	0.0342844745151247	0.915371765555559	0.998043617620652	pseudobulk	DESeq2	LRT	0.0384024874216412
241 KC_G1	CG31678	-0.102957381357708	0.786427527821256	0.998043617620652	pseudobulk	DESeq2	LRT	0.104341293024814
241 KC_G1	CG3168	0.452309505320751	0.423292917627434	0.998043617620652	pseudobulk	DESeq2	LRT	0.373358997911664
241 KC_G1	CG31683	0.534298510021818	0.735530767773287	0.998043617620652	pseudobulk	DESeq2	LRT	0.133399155710512
241 KC_G1	CG31687	-0.0566947356253961	0.859563288187513	0.998043617620652	pseudobulk	DESeq2	LRT	0.0657221414333354
241 KC_G1	CG31688	0.123664823090871	0.392814694711865	0.998043617620652	pseudobulk	DESeq2	LRT	0.405812274161802
241 KC_G1	CG31689	1.13582588427274	0.704684208645453	0.998043617620652	pseudobulk	DESeq2	LRT	0.152005460551806
241 KC_G1	CG31690	-0.636753118508458	0.522409797665496	0.998043617620652	pseudobulk	DESeq2	LRT	0.281988686571882
241 KC_G1	CG31694	0.17582893207108	0.357942462865895	0.998043617620652	pseudobulk	DESeq2	LRT	0.446186778015567
241 KC_G1	CG31697	-0.794610277746993	0.815039924133852	0.998043617620652	pseudobulk	DESeq2	LRT	0.0888211171417967
241 KC_G1	CG31705	-0.0270872744512292	0.903496590101077	0.998043617620652	pseudobulk	DESeq2	LRT	0.0440734821767411
242 KC_G1	CG31706	0.198881862397451	0.366595666433094	0.998043617620652	pseudobulk	DESeq2	LRT	0.435812673185363
242 KC_G1	CG31710	-0.369804922455119	0.712322110106103	0.998043617620652	pseudobulk	DESeq2	LRT	0.147323575172421
242 KC_G1	CG31712	-0.0414409164038533	0.77969136338495	0.998043617620652	pseudobulk	DESeq2	LRT	0.108077276418074
242 KC_G1	CG31715	-0.2694201264527	0.178309294589939	0.998043617620652	pseudobulk	DESeq2	LRT	0.748826018103871
242 KC_G1	CG31717	-0.121632163555713	0.677000972142298	0.998043617620652	pseudobulk	DESeq2	LRT	0.169410707687481
242 KC_G1	CG31729	0.0453542786654305	0.849568228497149	0.998043617620652	pseudobulk	DESeq2	LRT	0.0708017373718032
242 KC_G1	CG31751	-0.184943730361848	0.612085950039551	0.998043617620652	pseudobulk	DESeq2	LRT	0.213187589280954
242 KC_G1	CG31752	-0.794610277746993	0.879485534599449	0.998043617620652	pseudobulk	DESeq2	LRT	0.0557712992369932
242 KC_G1	CG3176	-0.511997862767769	0.500153564222053	0.998043617620652	pseudobulk	DESeq2	LRT	0.300896631954316
242 KC_G1	CG31760	-0.238359662479534	0.0742715722334069	0.998043617620652	pseudobulk	DESeq2	LRT	1.12917738253296
243 KC_G1	CG31788	1.06595574389305	0.681978466270766	0.998043617620652	pseudobulk	DESeq2	LRT	0.166229338140333
243 KC_G1	CG31793	-0.549456732734792	0.598939218169721	0.998043617620652	pseudobulk	DESeq2	LRT	0.222617248650542
243 KC_G1	CG31800	-0.211742248365962	0.537771279289968	0.998043617620652	pseudobulk	DESeq2	LRT	0.269402395825912
243 KC_G1	CG31803	1.12508065046033	0.900770125530981	0.998043617620652	pseudobulk	DESeq2	LRT	0.0453860258366365
243 KC_G1	CG31806	-1.66808346769027	0.498191247612582	0.998043617620652	pseudobulk	DESeq2	LRT	0.302603906560549
243 KC_G1	CG31808	0.160324497297601	0.230519356105483	0.998043617620652	pseudobulk	DESeq2	LRT	0.637292602175126
243 KC_G1	CG31810	1.38896997631979	0.587084398012744	0.998043617620652	pseudobulk	DESeq2	LRT	0.23129946100712
243 KC_G1	CG31815	-0.19849050971464	0.79589660940061	0.998043617620652	pseudobulk	DESeq2	LRT	0.0991433454321232
243 KC_G1	CG31817	-0.758144072848717	0.770267554036614	0.998043617620652	pseudobulk	DESeq2	LRT	0.113358395530507
243 KC_G1	CG3184	0.209445773026905	0.602444058711006	0.998043617620652	pseudobulk	DESeq2	LRT	0.220083274265241
244 KC_G1	CG31849	-0.0719638300404982	0.945573963078013	0.998043617620652	pseudobulk	DESeq2	LRT	0.0243044948460954
244 KC_G1	CG31855	-0.0863899961860319	0.704369752648106	0.998043617620652	pseudobulk	DESeq2	LRT	0.152199301966639
244 KC_G1	CG31861	-1.34713616610433	0.62158483183366	0.998043617620652	pseudobulk	DESeq2	LRT	0.206499591908745
244 KC_G1	CG31869	-0.794610277746993	0.821479263487599	0.998043617620652	pseudobulk	DESeq2	LRT	0.085403394939296
244 KC_G1	CG31882	-0.65817001371651	0.770857516266391	0.998043617620652	pseudobulk	DESeq2	LRT	0.113025888638787
244 KC_G1	CG31883	1.13582588427274	0.704684208645453	0.998043617620652	pseudobulk	DESeq2	LRT	0.152005460551806
244 KC_G1	CG31886	0.560239423694263	0.451511298504634	0.998043617620652	pseudobulk	DESeq2	LRT	0.345331377539132
244 KC_G1	CG31897	0.694607657051468	0.544774430074373	0.998043617620652	pseudobulk	DESeq2	LRT	0.263783284975983
244 KC_G1	CG31898	-0.718676691072112	0.739205546127982	0.998043617620652	pseudobulk	DESeq2	LRT	0.131234783341328
244 KC_G1	CG31904	-0.794610277746993	0.879485534599449	0.998043617620652	pseudobulk	DESeq2	LRT	0.0557712992369932
245 KC_G1	CG31908	0.0779883260522751	0.840223746853781	0.998043617620652	pseudobulk	DESeq2	LRT	0.0756050483612012
245 KC_G1	CG31909	1.13582588427274	0.704684208645453	0.998043617620652	pseudobulk	DESeq2	LRT	0.152005460551806
245 KC_G1	CG3191	-0.0577067187120828	0.933175295470925	0.998043617620652	pseudobulk	DESeq2	LRT	0.0300367670736046
245 KC_G1	CG31910	-0.794610277746993	0.879485534599449	0.998043617620652	pseudobulk	DESeq2	LRT	0.0557712992369932
245 KC_G1	CG31915	-0.0815676406953691	0.85968640797129	0.998043617620652	pseudobulk	DESeq2	LRT	0.0656599395985253
245 KC_G1	CG31921	-2.31679508011858	0.210450581917964	0.998043617620652	pseudobulk	DESeq2	LRT	0.67684986904444
245 KC_G1	CG31922	0.299007195876978	0.564863643146236	0.998043617620652	pseudobulk	DESeq2	LRT	0.248056377271281
245 KC_G1	CG31935	0.114224949585006	0.840236649189619	0.998043617620652	pseudobulk	DESeq2	LRT	0.0755983794587133
245 KC_G1	CG31937	-0.549628740899141	0.479267459251952	0.998043617620652	pseudobulk	DESeq2	LRT	0.319422057232021
245 KC_G1	CG31952	-0.65817001371651	0.770857516266391	0.998043617620652	pseudobulk	DESeq2	LRT	0.113025888638787
246 KC_G1	CG31955	1.13582588427274	0.704684208645453	0.998043617620652	pseudobulk	DESeq2	LRT	0.152005460551806
246 KC_G1	CG31957	0.18705891145904	0.737281718309523	0.998043617620652	pseudobulk	DESeq2	LRT	0.132366534760579
246 KC_G1	CG31974	0.14528603363022	0.773233411086867	0.998043617620652	pseudobulk	DESeq2	LRT	0.11168938855817

246	KC_G1	CG3198	0.0460936202867582	0.775632508842409	0.998043617620652	pseudobulk	DESeq2	LRT	0.110343996763269
246	KC_G1	CG31997	0.0470830395916	0.724625694826452	0.998043617620652	pseudobulk	DESeq2	LRT	0.139886270186259
246	KC_G1	CG31998	-0.180777421503497	0.22304886074533	0.998043617620652	pseudobulk	DESeq2	LRT	0.651599990639269
246	KC_G1	CG31999	0.316834697275294	0.681844330983668	0.998043617620652	pseudobulk	DESeq2	LRT	0.166314765976442
246	KC_G1	CG32017	0.070901023204057	0.609580248016314	0.998043617620652	pseudobulk	DESeq2	LRT	0.214969113709954
246	KC_G1	CG32022	0.700558550324419	0.154003008260042	0.998043617620652	pseudobulk	DESeq2	LRT	0.812470795670185
246	KC_G1	CG32032	-0.175161516709792	0.776164515979763	0.998043617620652	pseudobulk	DESeq2	LRT	0.110046215842901
247	KC_G1	CG32036	1.90250740646012	0.460774913678782	0.998043617620652	pseudobulk	DESeq2	LRT	0.336511173568024
247	KC_G1	CG32039	-0.250717503580475	0.35805529461056	0.998043617620652	pseudobulk	DESeq2	LRT	0.446049899920669
247	KC_G1	CG32052	-0.393046673625117	0.626944640213052	0.998043617620652	pseudobulk	DESeq2	LRT	0.202770806077489
247	KC_G1	CG32055	0.280426721601709	0.834893958279766	0.998043617620652	pseudobulk	DESeq2	LRT	0.0783686817121981
247	KC_G1	CG32066	-0.057873200263501	0.752182166243185	0.998043617620652	pseudobulk	DESeq2	LRT	0.123676967635225
247	KC_G1	CG32069	0.25743320747591	0.52709627747045	0.998043617620652	pseudobulk	DESeq2	LRT	0.278110050906559
247	KC_G1	CG32074	1.13582588427274	0.704684208645453	0.998043617620652	pseudobulk	DESeq2	LRT	0.152005460551806
247	KC_G1	CG32075	0.844089890624759	0.297616694118773	0.998043617620652	pseudobulk	DESeq2	LRT	0.526342711700355
247	KC_G1	CG32085	-0.119111434023229	0.404719772164655	0.998043617620652	pseudobulk	DESeq2	LRT	0.392845578083727
247	KC_G1	CG32091	-0.0475028323496869	0.975925606741748	0.998043617620652	pseudobulk	DESeq2	LRT	0.0105832866498391
248	KC_G1	CG32095	-0.130308940981922	0.828292279671722	0.998043617620652	pseudobulk	DESeq2	LRT	0.0818163865845674
248	KC_G1	CG32099	0.488222488720987	0.374138792349028	0.998043617620652	pseudobulk	DESeq2	LRT	0.426967259912751
248	KC_G1	CG32100	0.112351113115072	0.698503159701436	0.998043617620652	pseudobulk	DESeq2	LRT	0.155831625000344
248	KC_G1	CG32104	-0.461194894785283	0.558817359427403	0.998043617620652	pseudobulk	DESeq2	LRT	0.252730111171976
248	KC_G1	CG32109	0.13503820235999	0.632383642411699	0.998043617620652	pseudobulk	DESeq2	LRT	0.199019372298042
248	KC_G1	CG32110	-0.0190232237688427	0.958029348895871	0.998043617620652	pseudobulk	DESeq2	LRT	0.0186211862572708
248	KC_G1	CG32112	-0.61929972468093	0.122247808932422	0.998043617620652	pseudobulk	DESeq2	LRT	0.912758916065988
248	KC_G1	CG32115	2.04147257822512	0.408147672536479	0.998043617620652	pseudobulk	DESeq2	LRT	0.389182675723292
248	KC_G1	CG32119	1.13582588427274	0.704684208645453	0.998043617620652	pseudobulk	DESeq2	LRT	0.152005460551806
248	KC_G1	CG3213	1.12508065046033	0.900770125530981	0.998043617620652	pseudobulk	DESeq2	LRT	0.0453860258366365
249	KC_G1	CG32137	-0.514779854027406	0.616384538908998	0.998043617620652	pseudobulk	DESeq2	LRT	0.210148263449269
249	KC_G1	CG32147	0.29227867359937	0.52709563566064	0.998043617620652	pseudobulk	DESeq2	LRT	0.278110579718164
249	KC_G1	CG32161	1.13582588427274	0.704684208645453	0.998043617620652	pseudobulk	DESeq2	LRT	0.152005460551806
249	KC_G1	CG32163	-0.513701402518136	0.400438922281515	0.998043617620652	pseudobulk	DESeq2	LRT	0.397463716131418
249	KC_G1	CG32176	0.334395638489511	0.332597453974014	0.998043617620652	pseudobulk	DESeq2	LRT	0.478081079618209
249	KC_G1	CG32187	0.0926942713107266	0.860928513738615	0.998043617620652	pseudobulk	DESeq2	LRT	0.0650329082185341
249	KC_G1	CG32192	2.23045770445974	0.357070320422792	0.998043617620652	pseudobulk	DESeq2	LRT	0.447246246733247
249	KC_G1	CG32195	-0.486172326213909	0.604857485772562	0.998043617620652	pseudobulk	DESeq2	LRT	0.218346940113664
249	KC_G1	CG32196	-1.35021071135406	0.30383382974319	0.998043617620652	pseudobulk	DESeq2	LRT	0.517363872168978
249	KC_G1	CG32202	0.262826820108637	0.419643809742001	0.998043617620652	pseudobulk	DESeq2	LRT	0.377119178876907
250	KC_G1	CG32204	0.0165409219621279	0.886782371820387	0.998043617620652	pseudobulk	DESeq2	LRT	0.0521829487298834
250	KC_G1	CG32206	-0.168869220593967	0.375016930541724	0.998043617620652	pseudobulk	DESeq2	LRT	0.4259491251393
250	KC_G1	CG32221	-0.10065891879874	0.918804173239778	0.998043617620652	pseudobulk	DESeq2	LRT	0.0367770408922365
250	KC_G1	CG32225	0.118668733932687	0.694615171294095	0.998043617620652	pseudobulk	DESeq2	LRT	0.158255735368253
250	KC_G1	CG32228	-2.4391242473011	0.0311912783084702	0.998043617620652	pseudobulk	DESeq2	LRT	1.50596682623873
250	KC_G1	CG3223	0.189048957019185	0.644895444805636	0.998043617620652	pseudobulk	DESeq2	LRT	0.190510690674324
250	KC_G1	CG32232	1.06585281705208	0.683859663547483	0.998043617620652	pseudobulk	DESeq2	LRT	0.1650330117303
250	KC_G1	CG3224	0.090607563448656	0.771441269584459	0.998043617620652	pseudobulk	DESeq2	LRT	0.112697131514341
250	KC_G1	CG32243	0.0729305953387684	0.754914350147991	0.998043617620652	pseudobulk	DESeq2	LRT	0.122102319057112
250	KC_G1	CG3225	0.227951477622838	0.866236052330691	0.998043617620652	pseudobulk	DESeq2	LRT	0.0623637451011555
251	KC_G1	CG3226	-0.0875736628662738	0.740287843329817	0.998043617620652	pseudobulk	DESeq2	LRT	0.130599382345649
251	KC_G1	CG32260	-0.794610277746993	0.821479263487599	0.998043617620652	pseudobulk	DESeq2	LRT	0.085403394939296
251	KC_G1	CG32262	0.167301652103965	0.747068784763797	0.998043617620652	pseudobulk	DESeq2	LRT	0.126639409606188
251	KC_G1	CG32264	-0.008791948931652	0.936625497032041	0.998043617620652	pseudobulk	DESeq2	LRT	0.0284340239299796
251	KC_G1	CG32267	0.02894181842465	0.864439040940534	0.998043617620652	pseudobulk	DESeq2	LRT	0.0632656271634396
251	KC_G1	CG32276	0.0382437485087203	0.815035730389892	0.998043617620652	pseudobulk	DESeq2	LRT	0.0888233517863734
251	KC_G1	CG32278	0.0760620605818049	0.650164414304981	0.998043617620652	pseudobulk	DESeq2	LRT	0.186976804593686
251	KC_G1	CG32280	0.154893220096152	0.5981601185452	0.998043617620652	pseudobulk	DESeq2	LRT	0.223182546291561
251	KC_G1	CG32281	0.41811401573662	0.520085105587008	0.998043617620652	pseudobulk	DESeq2	LRT	0.2839255835526

251	KC_G1	CG32295	-0.794610277746993	0.879485534599449	0.998043617620652	pseudobulk	DESeq2	LRT	0.0557712992369932
252	KC_G1	CG32333	-0.440308182754528	0.0402121379130326	0.998043617620652	pseudobulk	DESeq2	LRT	1.39564283664216
252	KC_G1	CG32335	-0.757449795571951	0.820028655901106	0.998043617620652	pseudobulk	DESeq2	LRT	0.0861709709305825
252	KC_G1	CG32344	-0.356032764734328	0.592899757137077	0.998043617620652	pseudobulk	DESeq2	LRT	0.227018727550211
252	KC_G1	CG32354	-0.667492397753742	0.657623986828699	0.998043617620652	pseudobulk	DESeq2	LRT	0.182022354291982
252	KC_G1	CG32365	-1.26749033679739	0.302646296331368	0.998043617620652	pseudobulk	DESeq2	LRT	0.519064636447255
252	KC_G1	CG32368	-0.864214858694564	0.318843561317958	0.998043617620652	pseudobulk	DESeq2	LRT	0.496422348669874
252	KC_G1	CG32369	0.0977866162689506	0.586367236236124	0.998043617620652	pseudobulk	DESeq2	LRT	0.231830304259463
252	KC_G1	CG32373	-0.188515191937122	0.611963829905108	0.998043617620652	pseudobulk	DESeq2	LRT	0.213274246051884
252	KC_G1	CG32392	0.146752534608298	0.424911392797415	0.998043617620652	pseudobulk	DESeq2	LRT	0.371701624375962
252	KC_G1	CG32396	1.5423829701846	0.531064572395972	0.998043617620652	pseudobulk	DESeq2	LRT	0.274852669633639
253	KC_G1	CG32407	0.242514636009774	0.805145407724434	0.998043617620652	pseudobulk	DESeq2	LRT	0.094125679794069
253	KC_G1	CG32409	0.280990293383558	0.707111887661698	0.998043617620652	pseudobulk	DESeq2	LRT	0.150511861522127
253	KC_G1	CG32425	-0.104417875775087	0.540933158014407	0.998043617620652	pseudobulk	DESeq2	LRT	0.266856396444192
253	KC_G1	CG32428	0.10344856348633	0.592610360583234	0.998043617620652	pseudobulk	DESeq2	LRT	0.227230760036537
253	KC_G1	CG32432	-0.216815799585163	0.111205053152461	0.998043617620652	pseudobulk	DESeq2	LRT	0.953875477984457
253	KC_G1	CG32436	-0.99514509239169	0.516706868782583	0.998043617620652	pseudobulk	DESeq2	LRT	0.286755765172849
253	KC_G1	CG32441	0.267171831981644	0.185686110322793	0.998043617620652	pseudobulk	DESeq2	LRT	0.731220581105514
253	KC_G1	CG32444	1.12508065046033	0.900770125530981	0.998043617620652	pseudobulk	DESeq2	LRT	0.0453860258366365
253	KC_G1	CG32447	1.13582588427274	0.704684208645453	0.998043617620652	pseudobulk	DESeq2	LRT	0.152005460551806
253	KC_G1	CG32448	0.0657788996671069	0.818355713225866	0.998043617620652	pseudobulk	DESeq2	LRT	0.0870578812778681
254	KC_G1	CG32452	0.0192638337370214	0.971494639353914	0.998043617620652	pseudobulk	DESeq2	LRT	0.0125595914671195
254	KC_G1	CG32457	-0.647335375037142	0.762664938290985	0.998043617620652	pseudobulk	DESeq2	LRT	0.117666218809609
254	KC_G1	CG32459	-1.60891734856068	0.545249891924755	0.998043617620652	pseudobulk	DESeq2	LRT	0.263404411831769
254	KC_G1	CG3246	1.90250740646012	0.460774913678782	0.998043617620652	pseudobulk	DESeq2	LRT	0.336511173568024
254	KC_G1	CG32473	0.24248756922379	0.644779447115713	0.998043617620652	pseudobulk	DESeq2	LRT	0.190588814482267
254	KC_G1	CG32485	0.127149097901872	0.511021623234181	0.998043617620652	pseudobulk	DESeq2	LRT	0.291560722854325
254	KC_G1	CG32486	0.0156739067557106	0.906140067662586	0.998043617620652	pseudobulk	DESeq2	LRT	0.0428046655558247
254	KC_G1	CG32506	0.172460700710146	0.264094593361003	0.998043617620652	pseudobulk	DESeq2	LRT	0.578240489734073
254	KC_G1	CG32512	-0.421679554135095	0.780725021978179	0.998043617620652	pseudobulk	DESeq2	LRT	0.107501901418062
254	KC_G1	CG32521	-0.13169894090826	0.764895803538833	0.998043617620652	pseudobulk	DESeq2	LRT	0.116397721748551
255	KC_G1	CG3253	0.252523259200487	0.494586328943242	0.998043617620652	pseudobulk	DESeq2	LRT	0.30575789231473
255	KC_G1	CG32533	0.0992567107087281	0.887045275360455	0.998043617620652	pseudobulk	DESeq2	LRT	0.0520542129333229
255	KC_G1	CG32536	1.38669271937	0.601428581131124	0.998043617620652	pseudobulk	DESeq2	LRT	0.220815937172328
255	KC_G1	CG32537	-0.435727803852733	0.504607506772303	0.998043617620652	pseudobulk	DESeq2	LRT	0.297046293006835
255	KC_G1	CG32544	-0.134755393575574	0.513805222124905	0.998043617620652	pseudobulk	DESeq2	LRT	0.289201486039885
255	KC_G1	CG32547	0.621071281747514	0.566580619583008	0.998043617620652	pseudobulk	DESeq2	LRT	0.246738285008146
255	KC_G1	CG32549	-0.00053399852773782	0.97584165041055	0.998043617620652	pseudobulk	DESeq2	LRT	0.01062064947704
255	KC_G1	CG32554	0.441313879545727	0.590785164105469	0.998043617620652	pseudobulk	DESeq2	LRT	0.228570419297849
255	KC_G1	CG3257	-0.151419073436573	0.518158137209306	0.998043617620652	pseudobulk	DESeq2	LRT	0.285537677250237
255	KC_G1	CG32576	0.0454710114077915	0.927241170133038	0.998043617620652	pseudobulk	DESeq2	LRT	0.0328072936485904
256	KC_G1	CG32579	1.56852847737218	0.523617788648374	0.998043617620652	pseudobulk	DESeq2	LRT	0.280985607765667
256	KC_G1	CG32581	-0.658727103626691	0.676418459647865	0.998043617620652	pseudobulk	DESeq2	LRT	0.169784548895389
256	KC_G1	CG32590	0.0462304415106796	0.907748073083306	0.998043617620652	pseudobulk	DESeq2	LRT	0.0420346643100583
256	KC_G1	CG3262	0.199459690858511	0.313171344627281	0.998043617620652	pseudobulk	DESeq2	LRT	0.504217983011247
256	KC_G1	CG32625	1.2609445879621	0.626159485520488	0.998043617620652	pseudobulk	DESeq2	LRT	0.203315036023015
256	KC_G1	CG32638	0.370103771556828	0.562828378713944	0.998043617620652	pseudobulk	DESeq2	LRT	0.249624012857498
256	KC_G1	CG32641	1.45538390056165	0.354217105149643	0.998043617620652	pseudobulk	DESeq2	LRT	0.450730470552116
256	KC_G1	CG32647	-0.123983965158044	0.373240280007503	0.998043617620652	pseudobulk	DESeq2	LRT	0.428011493433001
256	KC_G1	CG32650	-0.752107099832492	0.841251974639758	0.998043617620652	pseudobulk	DESeq2	LRT	0.0750739033633379
256	KC_G1	CG32655	0.764815221789058	0.808325187140535	0.998043617620652	pseudobulk	DESeq2	LRT	0.0924138885208533
257	KC_G1	CG32683	0.0834861764990808	0.685413603288283	0.998043617620652	pseudobulk	DESeq2	LRT	0.164047280451219
257	KC_G1	CG32687	-0.794610277746993	0.879485534599449	0.998043617620652	pseudobulk	DESeq2	LRT	0.0557712992369932
257	KC_G1	CG3270	-0.515335478270475	0.601676243520469	0.998043617620652	pseudobulk	DESeq2	LRT	0.220637135777167
257	KC_G1	CG32700	-0.109549904693766	0.338406716413401	0.998043617620652	pseudobulk	DESeq2	LRT	0.470561026033925
257	KC_G1	CG32706	0.0781071480641268	0.970967397587672	0.998043617620652	pseudobulk	DESeq2	LRT	0.0127953522603565

257	KC_G1	CG32708	0.155617147109221	0.772774075873963	0.998043617620652	pseudobulk	DESeq2	LRT	0.11194745555443
257	KC_G1	CG32712	-0.794610277746993	0.879485534599449	0.998043617620652	pseudobulk	DESeq2	LRT	0.0557712992369932
257	KC_G1	CG32755	-1.74278423686085	0.385072726505398	0.998043617620652	pseudobulk	DESeq2	LRT	0.414457240005315
257	KC_G1	CG32756	0.269687683521516	0.457179137653042	0.998043617620652	pseudobulk	DESeq2	LRT	0.339913595881493
257	KC_G1	CG32762	-0.794610277746993	0.879485534599449	0.998043617620652	pseudobulk	DESeq2	LRT	0.0557712992369932
258	KC_G1	CG32767	-0.0713430040271436	0.484863395045809	0.998043617620652	pseudobulk	DESeq2	LRT	0.314380601880945
258	KC_G1	CG32772	-0.0199461300574016	0.908566650588816	0.998043617620652	pseudobulk	DESeq2	LRT	0.0416432082335433
258	KC_G1	CG32795	0.285891116778999	0.264240148994816	0.998043617620652	pseudobulk	DESeq2	LRT	0.578001194434686
258	KC_G1	CG32801	-0.794610277746993	0.815039924133852	0.998043617620652	pseudobulk	DESeq2	LRT	0.0888211171417967
258	KC_G1	CG32803	0.292062352272601	0.609760032466139	0.998043617620652	pseudobulk	DESeq2	LRT	0.214841045448278
258	KC_G1	CG32808	-0.116514160312103	0.895001754049659	0.998043617620652	pseudobulk	DESeq2	LRT	0.0481761135406899
258	KC_G1	CG32809	-0.0182804586845457	0.898135026663353	0.998043617620652	pseudobulk	DESeq2	LRT	0.0466583660871725
258	KC_G1	CG3281	-0.297193188665882	0.667584761697095	0.998043617620652	pseudobulk	DESeq2	LRT	0.175493585094042
258	KC_G1	CG32812	1.13582588427274	0.633424328955532	0.998043617620652	pseudobulk	DESeq2	LRT	0.198305259991224
258	KC_G1	CG32813	-0.276494264085372	0.456976142230359	0.998043617620652	pseudobulk	DESeq2	LRT	0.340106472945843
259	KC_G1	CG32814	1.13582588427274	0.633424328955532	0.998043617620652	pseudobulk	DESeq2	LRT	0.198305259991224
259	KC_G1	CG32815	0.0414016639713785	0.844356206348499	0.998043617620652	pseudobulk	DESeq2	LRT	0.0734743000380102
259	KC_G1	CG32816	-0.578563475305425	0.742997347307851	0.998043617620652	pseudobulk	DESeq2	LRT	0.129012736779962
259	KC_G1	CG32817	-1.27180443367556	0.603754725943432	0.998043617620652	pseudobulk	DESeq2	LRT	0.219139456748719
259	KC_G1	CG32829	0.830391780932795	0.516050688603767	0.998043617620652	pseudobulk	DESeq2	LRT	0.287307638101986
259	KC_G1	CG32845	1.69981218657454	0.566400560818652	0.998043617620652	pseudobulk	DESeq2	LRT	0.246876325303589
259	KC_G1	CG32850	0.0149018397257133	0.927120385363084	0.998043617620652	pseudobulk	DESeq2	LRT	0.0328638696264603
259	KC_G1	CG32855	-1.66808346769027	0.498191247612582	0.998043617620652	pseudobulk	DESeq2	LRT	0.302603906560549
259	KC_G1	CG32856	-0.0118696125589163	0.971459676687219	0.998043617620652	pseudobulk	DESeq2	LRT	0.0125752213695776
259	KC_G1	CG3288	0.173195945230598	0.976215465741119	0.998043617620652	pseudobulk	DESeq2	LRT	0.0104543162934892
260	KC_G1	CG3294	0.506674536786517	0.521240970981758	0.998043617620652	pseudobulk	DESeq2	LRT	0.282961454870486
260	KC_G1	CG32944	-0.210104483876649	0.40106444644113	0.998043617620652	pseudobulk	DESeq2	LRT	0.396785835646883
260	KC_G1	CG3295	0.16187585396615	0.67171853285498	0.998043617620652	pseudobulk	DESeq2	LRT	0.17281266926031
260	KC_G1	CG32982	0.369304334254664	0.684146100109578	0.998043617620652	pseudobulk	DESeq2	LRT	0.164851144338428
260	KC_G1	CG3301	-0.794610277746993	0.815039924133852	0.998043617620652	pseudobulk	DESeq2	LRT	0.0888211171417967
260	KC_G1	CG33051	-0.153471471511313	0.883958866108718	0.998043617620652	pseudobulk	DESeq2	LRT	0.0535679438530365
260	KC_G1	CG33054	-0.305340408841692	0.690996289103262	0.998043617620652	pseudobulk	DESeq2	LRT	0.160524284935937
260	KC_G1	CG3308	-0.0463754170796624	0.924826247795243	0.998043617620652	pseudobulk	DESeq2	LRT	0.0339398528953548
260	KC_G1	CG33080	0.156284796289774	0.866424931453122	0.998043617620652	pseudobulk	DESeq2	LRT	0.0622690593412625
260	KC_G1	CG33082	0.899718597334948	0.628936864786202	0.998043617620652	pseudobulk	DESeq2	LRT	0.201392948594713
261	KC_G1	CG3309	0.408295785729509	0.521894820303928	0.998043617620652	pseudobulk	DESeq2	LRT	0.28241701340433
261	KC_G1	CG33090	-0.207819088315143	0.680899467396805	0.998043617620652	pseudobulk	DESeq2	LRT	0.166917005528984
261	KC_G1	CG33096	-0.159241842096286	0.807843450813934	0.998043617620652	pseudobulk	DESeq2	LRT	0.0926727914962707
261	KC_G1	CG33098	0.36054126438253	0.441005812115488	0.998043617620652	pseudobulk	DESeq2	LRT	0.355555686829098
261	KC_G1	CG33107	0.674583549560242	0.103836629035167	0.998043617620652	pseudobulk	DESeq2	LRT	0.983649419303231
261	KC_G1	CG33108	-0.175291221931875	0.798330018767344	0.998043617620652	pseudobulk	DESeq2	LRT	0.0978175401014903
261	KC_G1	CG33111	-0.148936123983571	0.837395903490639	0.998043617620652	pseudobulk	DESeq2	LRT	0.0770691679919129
261	KC_G1	CG33116	0.216731570573109	0.400363284606853	0.998043617620652	pseudobulk	DESeq2	LRT	0.397545756426873
261	KC_G1	CG33127	-1.33937415665543	0.606461532424389	0.998043617620652	pseudobulk	DESeq2	LRT	0.217196741022707
261	KC_G1	CG33129	0.107206370214237	0.554156443459043	0.998043617620652	pseudobulk	DESeq2	LRT	0.256367612636753
262	KC_G1	CG33143	-1.08666507429189	0.491095863778815	0.998043617620652	pseudobulk	DESeq2	LRT	0.308833723668439
262	KC_G1	CG33144	0.0195956831641932	0.937863482047718	0.998043617620652	pseudobulk	DESeq2	LRT	0.0278603741031114
262	KC_G1	CG33155	-0.558066221431484	0.50125940900949	0.998043617620652	pseudobulk	DESeq2	LRT	0.299937462266923
262	KC_G1	CG33156	-0.0122798589116248	0.972500235977348	0.998043617620652	pseudobulk	DESeq2	LRT	0.0121102846206118
262	KC_G1	CG33158	-0.294089968784406	0.66212681663521	0.998043617620652	pseudobulk	DESeq2	LRT	0.179058822508579
262	KC_G1	CG33169	0.802777866050373	0.142258573780483	0.998043617620652	pseudobulk	DESeq2	LRT	0.846921549654554
262	KC_G1	CG33170	0.275495897127514	0.218133042067537	0.998043617620652	pseudobulk	DESeq2	LRT	0.661278543954986
262	KC_G1	CG33172	1.2609445879621	0.626159485520488	0.998043617620652	pseudobulk	DESeq2	LRT	0.203315036023015
262	KC_G1	CG33178	1.69981218657454	0.566400560818652	0.998043617620652	pseudobulk	DESeq2	LRT	0.246876325303589
262	KC_G1	CG33181	0.0528802719058991	0.693187251387356	0.998043617620652	pseudobulk	DESeq2	LRT	0.159149433123493
263	KC_G1	CG33189	1.13582588427274	0.633424328955532	0.998043617620652	pseudobulk	DESeq2	LRT	0.198305259991224

263	KC_G1	CG33199	0.0486738667244977	0.81253467781017	0.998043617620652	pseudobulk	DESeq2	LRT	0.0901580948903569
263	KC_G1	CG33203	-0.341646972617867	0.347561280069327	0.998043617620652	pseudobulk	DESeq2	LRT	0.45896861193821
263	KC_G1	CG33213	0.890320172803047	0.268359880064953	0.998043617620652	pseudobulk	DESeq2	LRT	0.571282410890448
263	KC_G1	CG33217	0.262481186365679	0.470471954657865	0.998043617620652	pseudobulk	DESeq2	LRT	0.327466260228977
263	KC_G1	CG33228	0.234071072780301	0.643804773385197	0.998043617620652	pseudobulk	DESeq2	LRT	0.191245807633062
263	KC_G1	CG33229	-0.177009261329307	0.330301686764837	0.998043617620652	pseudobulk	DESeq2	LRT	0.481089208473919
263	KC_G1	CG33230	0.00586381909954292	0.964731227073539	0.998043617620652	pseudobulk	DESeq2	LRT	0.0155936637103839
263	KC_G1	CG33233	0.649178117974024	0.763052752754713	0.998043617620652	pseudobulk	DESeq2	LRT	0.117445436568943
263	KC_G1	CG33234	1.12508065046033	0.900770125530981	0.998043617620652	pseudobulk	DESeq2	LRT	0.0453860258366365
264	KC_G1	CG33258	1.13582588427274	0.704684208645453	0.998043617620652	pseudobulk	DESeq2	LRT	0.152005460551806
264	KC_G1	CG33267	-0.56082440913009	0.467620546437344	0.998043617620652	pseudobulk	DESeq2	LRT	0.33010641494321
264	KC_G1	CG33269	-0.794610277746993	0.815039924133852	0.998043617620652	pseudobulk	DESeq2	LRT	0.0888211171417967
264	KC_G1	CG33272	-0.794610277746993	0.815039924133852	0.998043617620652	pseudobulk	DESeq2	LRT	0.0888211171417967
264	KC_G1	CG3328	-1.33000077039135	0.413403728004936	0.998043617620652	pseudobulk	DESeq2	LRT	0.383625611296848
264	KC_G1	CG33290	1.12508065046033	0.900770125530981	0.998043617620652	pseudobulk	DESeq2	LRT	0.0453860258366365
264	KC_G1	CG33296	3.55256364824513	0.0279562038308839	0.998043617620652	pseudobulk	DESeq2	LRT	1.5535218017114
264	KC_G1	CG33298	0.0185609561698422	0.926297269365762	0.998043617620652	pseudobulk	DESeq2	LRT	0.0332496162046189
264	KC_G1	CG3330	-0.794610277746993	0.821479263487599	0.998043617620652	pseudobulk	DESeq2	LRT	0.085403394939296
264	KC_G1	CG33303	0.0664992958559351	0.739093345631517	0.998043617620652	pseudobulk	DESeq2	LRT	0.131300707839881
265	KC_G1	CG33307	0.777030258285493	0.70316795941598	0.998043617620652	pseudobulk	DESeq2	LRT	0.152940926566052
265	KC_G1	CG33310	0.0592839927463851	0.893931927830931	0.998043617620652	pseudobulk	DESeq2	LRT	0.0486955511067467
265	KC_G1	CG33331	-0.29235505231747	0.531981182955667	0.998043617620652	pseudobulk	DESeq2	LRT	0.274103729139254
265	KC_G1	CG33332	-0.301579606709756	0.635149125178042	0.998043617620652	pseudobulk	DESeq2	LRT	0.197124295749884
265	KC_G1	CG3335	0.305450534011157	0.599210056600572	0.998043617620652	pseudobulk	DESeq2	LRT	0.222420906440958
265	KC_G1	CG3337	0.0809004701480151	0.826761225508077	0.998043617620652	pseudobulk	DESeq2	LRT	0.0826198996581452
265	KC_G1	CG3339	-0.794610277746993	0.815039924133852	0.998043617620652	pseudobulk	DESeq2	LRT	0.0888211171417967
265	KC_G1	CG3342	-0.152565722590078	0.760959205008464	0.998043617620652	pseudobulk	DESeq2	LRT	0.118638625114591
265	KC_G1	CG3345	0.157190835514101	0.933296657326995	0.998043617620652	pseudobulk	DESeq2	LRT	0.0299802896275867
265	KC_G1	CG33468	-2.23957353771189	0.33560557159327	0.998043617620652	pseudobulk	DESeq2	LRT	0.474170837795283
266	KC_G1	CG33469	-0.794610277746993	0.815039924133852	0.998043617620652	pseudobulk	DESeq2	LRT	0.0888211171417967
266	KC_G1	CG3348	-1.73907471612892	0.507597351592657	0.998043617620652	pseudobulk	DESeq2	LRT	0.294480652518419
266	KC_G1	CG33493	-0.52001232297389	0.361357584174917	0.998043617620652	pseudobulk	DESeq2	LRT	0.442062825875403
266	KC_G1	CG33500	2.04147257822512	0.408147672536479	0.998043617620652	pseudobulk	DESeq2	LRT	0.389182675723292
266	KC_G1	CG33506	0.0813325407432992	0.824058828094058	0.998043617620652	pseudobulk	DESeq2	LRT	0.0840417836855665
266	KC_G1	CG33514	1.11121460044925	0.691240116375203	0.998043617620652	pseudobulk	DESeq2	LRT	0.16037106535726
266	KC_G1	CG33521	-0.350028569708049	0.687749847418329	0.998043617620652	pseudobulk	DESeq2	LRT	0.162569497289257
266	KC_G1	CG33523	0.152347026495676	0.574480192444148	0.998043617620652	pseudobulk	DESeq2	LRT	0.240724940797288
266	KC_G1	CG33543	-0.18212204898337	0.101271617787275	0.998043617620652	pseudobulk	DESeq2	LRT	0.994512252225849
266	KC_G1	CG3356	0.357330844704032	0.393202848329006	0.998043617620652	pseudobulk	DESeq2	LRT	0.405383344835097
267	KC_G1	CG3358	-0.650379404559983	0.25031990924787	0.998043617620652	pseudobulk	DESeq2	LRT	0.601504607312952
267	KC_G1	CG33635	0.351148740107544	0.37742233729933	0.998043617620652	pseudobulk	DESeq2	LRT	0.423172400199801
267	KC_G1	CG33641	1.12508065046033	0.900770125530981	0.998043617620652	pseudobulk	DESeq2	LRT	0.0453860258366365
267	KC_G1	CG33642	-0.794610277746993	0.815039924133852	0.998043617620652	pseudobulk	DESeq2	LRT	0.0888211171417967
267	KC_G1	CG33643	1.12508065046033	0.900770125530981	0.998043617620652	pseudobulk	DESeq2	LRT	0.0453860258366365
267	KC_G1	CG33654	1.12508065046033	0.900770125530981	0.998043617620652	pseudobulk	DESeq2	LRT	0.0453860258366365
267	KC_G1	CG33667	-1.31038467776507	0.572047571456938	0.998043617620652	pseudobulk	DESeq2	LRT	0.242567853790582
267	KC_G1	CG33668	1.13582588427274	0.633424328955532	0.998043617620652	pseudobulk	DESeq2	LRT	0.198305259991224
267	KC_G1	CG33669	-0.794610277746993	0.815039924133852	0.998043617620652	pseudobulk	DESeq2	LRT	0.0888211171417967
267	KC_G1	CG3368	0.0252583135787058	0.954332135309823	0.998043617620652	pseudobulk	DESeq2	LRT	0.0203004518905118
268	KC_G1	CG33695	0.0309066022719239	0.945971603039874	0.998043617620652	pseudobulk	DESeq2	LRT	0.0241219004144752
268	KC_G1	CG3376	-0.0896201333961454	0.921955456513793	0.998043617620652	pseudobulk	DESeq2	LRT	0.0352900610044782
268	KC_G1	CG33774	0.160999362668093	0.60932283272235	0.998043617620652	pseudobulk	DESeq2	LRT	0.215152547561513
268	KC_G1	CG33777	0.173195945230598	0.976215465741119	0.998043617620652	pseudobulk	DESeq2	LRT	0.0104543162934892
268	KC_G1	CG3386	0.456763663275662	0.361219323701522	0.998043617620652	pseudobulk	DESeq2	LRT	0.442229024824579
268	KC_G1	CG33912	-0.794610277746993	0.815039924133852	0.998043617620652	pseudobulk	DESeq2	LRT	0.0888211171417967
268	KC_G1	CG33914	-0.776296732928036	0.736588270584829	0.998043617620652	pseudobulk	DESeq2	LRT	0.132775201121675

268	KC_G1	CG33926	-1.73907471612892	0.507597351592657	0.998043617620652	pseudobulk	DESeq2	LRT	0.294480652518419
268	KC_G1	CG33939	-2.19332442993981	0.381369935212232	0.998043617620652	pseudobulk	DESeq2	LRT	0.418653546964052
268	KC_G1	CG33941	-0.264587433156698	0.482528374768389	0.998043617620652	pseudobulk	DESeq2	LRT	0.316477143163699
269	KC_G1	CG33947	-0.0158846522038783	0.959542678888277	0.998043617620652	pseudobulk	DESeq2	LRT	0.0179357037909811
269	KC_G1	CG33958	-0.974232558287219	0.446249946982362	0.998043617620652	pseudobulk	DESeq2	LRT	0.350421822476992
269	KC_G1	CG33969	-0.378604801284229	0.506344259612191	0.998043617620652	pseudobulk	DESeq2	LRT	0.295554109221424
269	KC_G1	CG3397	-0.794610277746993	0.815039924133852	0.998043617620652	pseudobulk	DESeq2	LRT	0.0888211171417967
269	KC_G1	CG33970	-2.2456240932086	0.037360924922411	0.998043617620652	pseudobulk	DESeq2	LRT	1.42758238073619
269	KC_G1	CG33977	-0.0385638001094654	0.858806691530872	0.998043617620652	pseudobulk	DESeq2	LRT	0.0661045803470935
269	KC_G1	CG33978	-1.37005811993566	0.459991625593743	0.998043617620652	pseudobulk	DESeq2	LRT	0.337250074821758
269	KC_G1	CG33993	0.023619593897041	0.927822726285341	0.998043617620652	pseudobulk	DESeq2	LRT	0.0325349939864347
269	KC_G1	CG33998	0.562077451288907	0.794187709521711	0.998043617620652	pseudobulk	DESeq2	LRT	0.100076838158817
269	KC_G1	CG34001	-0.295631525532069	0.746370835409211	0.998043617620652	pseudobulk	DESeq2	LRT	0.127045339074401
270	KC_G1	CG34008	1.38896997631979	0.587084398012744	0.998043617620652	pseudobulk	DESeq2	LRT	0.23129946100712
270	KC_G1	CG34015	-0.160526113637263	0.635253029637957	0.998043617620652	pseudobulk	DESeq2	LRT	0.197053255042789
270	KC_G1	CG3402	0.181084409980653	0.566348311860543	0.998043617620652	pseudobulk	DESeq2	LRT	0.246916389672233
270	KC_G1	CG34025	-0.794610277746993	0.821479263487599	0.998043617620652	pseudobulk	DESeq2	LRT	0.085403394939296
270	KC_G1	CG34026	-0.156849441454188	0.913846499207056	0.998043617620652	pseudobulk	DESeq2	LRT	0.0391267475330446
270	KC_G1	CG34027	0.611960415939833	0.787106614207464	0.998043617620652	pseudobulk	DESeq2	LRT	0.103966438128821
270	KC_G1	CG34028	-0.0759245831010372	0.921416349089394	0.998043617620652	pseudobulk	DESeq2	LRT	0.0355440861398668
270	KC_G1	CG34031	0.693814139898402	0.747913497313991	0.998043617620652	pseudobulk	DESeq2	LRT	0.126148629156436
270	KC_G1	CG34039	-0.274129819649301	0.525496060560087	0.998043617620652	pseudobulk	DESeq2	LRT	0.279430535360627
270	KC_G1	CG34041	-0.794610277746993	0.879485534599449	0.998043617620652	pseudobulk	DESeq2	LRT	0.0557712992369932
271	KC_G1	CG34045	-1.31275600133313	0.545710131686654	0.998043617620652	pseudobulk	DESeq2	LRT	0.263037983004907
271	KC_G1	CG34056	-0.455449419090036	0.834875862426107	0.998043617620652	pseudobulk	DESeq2	LRT	0.0783780949011207
271	KC_G1	CG34057	0.177622136115678	0.935661853927379	0.998043617620652	pseudobulk	DESeq2	LRT	0.0288810759485907
271	KC_G1	CG3407	-0.359091142662763	0.63656953739622	0.998043617620652	pseudobulk	DESeq2	LRT	0.196154148085847
271	KC_G1	CG3408	-0.123821381663999	0.831955569441561	0.998043617620652	pseudobulk	DESeq2	LRT	0.0798998665717744
271	KC_G1	CG34107	0.172845155749872	0.90730697492154	0.998043617620652	pseudobulk	DESeq2	LRT	0.0422457504714555
271	KC_G1	CG34112	1.89288595231245	0.415717432633965	0.998043617620652	pseudobulk	DESeq2	LRT	0.381201763470702
271	KC_G1	CG34116	-0.347401319342613	0.630435555961577	0.998043617620652	pseudobulk	DESeq2	LRT	0.200359301034914
271	KC_G1	CG34117	-0.266467985792554	0.424449523604211	0.998043617620652	pseudobulk	DESeq2	LRT	0.37217394952742
271	KC_G1	CG34125	0.126892971859058	0.610472394856306	0.998043617620652	pseudobulk	DESeq2	LRT	0.214333969774195
272	KC_G1	CG34126	-0.0362993196768647	0.880681918159452	0.998043617620652	pseudobulk	DESeq2	LRT	0.0551809203414526
272	KC_G1	CG34132	-0.0235790174188849	0.912680811477703	0.998043617620652	pseudobulk	DESeq2	LRT	0.0396810801337681
272	KC_G1	CG34133	-0.0492699809875315	0.892827078828059	0.998043617620652	pseudobulk	DESeq2	LRT	0.0492326463553522
272	KC_G1	CG34134	0.184619331403504	0.713343731021485	0.998043617620652	pseudobulk	DESeq2	LRT	0.1467011510541
272	KC_G1	CG34136	0.794722805723984	0.132022145078	0.998043617620652	pseudobulk	DESeq2	LRT	0.879353215168963
272	KC_G1	CG34148	-0.618555940039177	0.288607103436475	0.998043617620652	pseudobulk	DESeq2	LRT	0.539692983896377
272	KC_G1	CG34149	0.512789722072412	0.597335756862621	0.998043617620652	pseudobulk	DESeq2	LRT	0.223781487356846
272	KC_G1	CG34150	0.408675130825501	0.272780685826853	0.998043617620652	pseudobulk	DESeq2	LRT	0.564186383046973
272	KC_G1	CG34155	-0.765625368254185	0.836625948066609	0.998043617620652	pseudobulk	DESeq2	LRT	0.0774686698445758
272	KC_G1	CG34159	-0.0238157696185706	0.897002828407813	0.998043617620652	pseudobulk	DESeq2	LRT	0.047206187546801
273	KC_G1	CG34163	1.43768900174468	0.0495505032940072	0.998043617620652	pseudobulk	DESeq2	LRT	1.30495192994359
273	KC_G1	CG34171	0.1729159612032	0.899814506689544	0.998043617620652	pseudobulk	DESeq2	LRT	0.0458470094762598
273	KC_G1	CG34174	-0.443633425883022	0.497736052986352	0.998043617620652	pseudobulk	DESeq2	LRT	0.303000900453599
273	KC_G1	CG34183	0.0876967819906437	0.826518444251658	0.998043617620652	pseudobulk	DESeq2	LRT	0.0827474504442089
273	KC_G1	CG34189	0.172845155749872	0.90730697492154	0.998043617620652	pseudobulk	DESeq2	LRT	0.0422457504714555
273	KC_G1	CG34195	-0.249172443092798	0.656863376560644	0.998043617620652	pseudobulk	DESeq2	LRT	0.182524951560487
273	KC_G1	CG3420	0.0319640456310584	0.90070488108335	0.998043617620652	pseudobulk	DESeq2	LRT	0.0454174837291853
273	KC_G1	CG34200	-0.0225848393280046	0.89380202264034	0.998043617620652	pseudobulk	DESeq2	LRT	0.0487586668824794
273	KC_G1	CG34213	0.0442264331856576	0.938779985792534	0.998043617620652	pseudobulk	DESeq2	LRT	0.0274361778639394
273	KC_G1	CG34214	-0.794610277746993	0.879485534599449	0.998043617620652	pseudobulk	DESeq2	LRT	0.0557712992369932
274	KC_G1	CG34220	0.765052904958762	0.810501863366603	0.998043617620652	pseudobulk	DESeq2	LRT	0.0912459823590772
274	KC_G1	CG34221	-0.794610277746993	0.879485534599449	0.998043617620652	pseudobulk	DESeq2	LRT	0.0557712992369932
274	KC_G1	CG34228	0.314454168980401	0.43842882368601	0.998043617620652	pseudobulk	DESeq2	LRT	0.358100901703688

274	KC_G1	CG34229	-0.0182567342897347	0.911527146953031	0.998043617620652	pseudobulk	DESeq2	LRT	0.0402303927288737
274	KC_G1	CG34231	-1.54274276239595	0.201629048573781	0.998043617620652	pseudobulk	DESeq2	LRT	0.695446899178431
274	KC_G1	CG34232	-0.404773775176738	0.674437731323246	0.998043617620652	pseudobulk	DESeq2	LRT	0.171058141160447
274	KC_G1	CG34234	-0.901381917288146	0.577606591214262	0.998043617620652	pseudobulk	DESeq2	LRT	0.238367859566447
274	KC_G1	CG34242	0.452107162737732	0.436927472361245	0.998043617620652	pseudobulk	DESeq2	LRT	0.359590647623244
274	KC_G1	CG34250	0.104431541603219	0.69609810638026	0.998043617620652	pseudobulk	DESeq2	LRT	0.157329547663896
274	KC_G1	CG34251	2.04068961104271	0.280458194940156	0.998043617620652	pseudobulk	DESeq2	LRT	0.552131865459094
275	KC_G1	CG34256	-0.794610277746993	0.821479263487599	0.998043617620652	pseudobulk	DESeq2	LRT	0.085403394939296
275	KC_G1	CG34261	-1.72343437992478	0.512120560562093	0.998043617620652	pseudobulk	DESeq2	LRT	0.290627787806665
275	KC_G1	CG34263	1.13582588427274	0.704684208645453	0.998043617620652	pseudobulk	DESeq2	LRT	0.152005460551806
275	KC_G1	CG34265	1.69981218657454	0.566400560818652	0.998043617620652	pseudobulk	DESeq2	LRT	0.246876325303589
275	KC_G1	CG34279	-0.552721601992173	0.802773118645514	0.998043617620652	pseudobulk	DESeq2	LRT	0.0954071785604611
275	KC_G1	CG34286	-0.794610277746993	0.879485534599449	0.998043617620652	pseudobulk	DESeq2	LRT	0.0557712992369932
275	KC_G1	CG34287	-0.794610277746993	0.815039924133852	0.998043617620652	pseudobulk	DESeq2	LRT	0.0888211171417967
275	KC_G1	CG34290	-0.794610277746993	0.821479263487599	0.998043617620652	pseudobulk	DESeq2	LRT	0.085403394939296
275	KC_G1	CG34293	-0.0881092448426513	0.839525174526603	0.998043617620652	pseudobulk	DESeq2	LRT	0.0759662763048935
275	KC_G1	CG3430	0.299243349670864	0.891861473457015	0.998043617620652	pseudobulk	DESeq2	LRT	0.0497025962799188
276	KC_G1	CG34301	-1.66808346769027	0.498191247612582	0.998043617620652	pseudobulk	DESeq2	LRT	0.302603906560549
276	KC_G1	CG34310	-0.794610277746993	0.815039924133852	0.998043617620652	pseudobulk	DESeq2	LRT	0.0888211171417967
276	KC_G1	CG34328	-0.794610277746993	0.879485534599449	0.998043617620652	pseudobulk	DESeq2	LRT	0.0557712992369932
276	KC_G1	CG34332	1.12508065046033	0.900770125530981	0.998043617620652	pseudobulk	DESeq2	LRT	0.0453860258366365
276	KC_G1	CG34334	-0.65817001371651	0.770857516266391	0.998043617620652	pseudobulk	DESeq2	LRT	0.113025888638787
276	KC_G1	CG3434	0.195927351144896	0.758126116628926	0.998043617620652	pseudobulk	DESeq2	LRT	0.120258542125149
276	KC_G1	CG34347	-1.34713616610433	0.62158483183366	0.998043617620652	pseudobulk	DESeq2	LRT	0.206499591908745
276	KC_G1	CG34348	-0.409466093472898	0.479173329766938	0.998043617620652	pseudobulk	DESeq2	LRT	0.319507362274389
276	KC_G1	CG34351	0.645695198842967	0.472964118716602	0.998043617620652	pseudobulk	DESeq2	LRT	0.325171805635275
276	KC_G1	CG34353	-0.109726991593215	0.439842202941909	0.998043617620652	pseudobulk	DESeq2	LRT	0.356703102338914
277	KC_G1	CG34354	-0.00441048653157058	0.960388876876982	0.998043617620652	pseudobulk	DESeq2	LRT	0.0175528785389197
277	KC_G1	CG34355	-0.0174193860544999	0.903022356733445	0.998043617620652	pseudobulk	DESeq2	LRT	0.0443014974320355
277	KC_G1	CG34357	-0.0873928088094504	0.532273474943039	0.998043617620652	pseudobulk	DESeq2	LRT	0.273865175707574
277	KC_G1	CG3436	-0.208666219938109	0.437347458571206	0.998043617620652	pseudobulk	DESeq2	LRT	0.359173392819203
277	KC_G1	CG34367	-0.472498349438487	0.609400589241268	0.998043617620652	pseudobulk	DESeq2	LRT	0.215097130185371
277	KC_G1	CG3437	-0.25931628721574	0.644828077538087	0.998043617620652	pseudobulk	DESeq2	LRT	0.190556060448662
277	KC_G1	CG34370	0.383649432185371	0.186181429653125	0.998043617620652	pseudobulk	DESeq2	LRT	0.730063639151542
277	KC_G1	CG34376	0.424319374499913	0.385249846931521	0.998043617620652	pseudobulk	DESeq2	LRT	0.414257525164531
277	KC_G1	CG34377	1.12508065046033	0.900770125530981	0.998043617620652	pseudobulk	DESeq2	LRT	0.0453860258366365
277	KC_G1	CG34384	-0.359746722255616	0.154731844819645	0.998043617620652	pseudobulk	DESeq2	LRT	0.810420296473895
278	KC_G1	CG34393	-0.752107099832492	0.841251974639758	0.998043617620652	pseudobulk	DESeq2	LRT	0.0750739033633379
278	KC_G1	CG34401	-0.203768883942824	0.117977705752765	0.998043617620652	pseudobulk	DESeq2	LRT	0.9282000535695
278	KC_G1	CG34402	0.00956730250366632	0.932257426073747	0.998043617620652	pseudobulk	DESeq2	LRT	0.0304641484976228
278	KC_G1	CG34404	-0.0883445747344914	0.707330166229351	0.998043617620652	pseudobulk	DESeq2	LRT	0.150377819723892
278	KC_G1	CG34408	0.11800141136187	0.754500649392909	0.998043617620652	pseudobulk	DESeq2	LRT	0.122340382094308
278	KC_G1	CG34409	0.59893461553148	0.510472673767931	0.998043617620652	pseudobulk	DESeq2	LRT	0.292027501274304
278	KC_G1	CG34417	0.116850092037087	0.911336930935158	0.998043617620652	pseudobulk	DESeq2	LRT	0.0403210300596913
278	KC_G1	CG34425	1.13582588427274	0.704684208645453	0.998043617620652	pseudobulk	DESeq2	LRT	0.152005460551806
278	KC_G1	CG34428	-0.794610277746993	0.821479263487599	0.998043617620652	pseudobulk	DESeq2	LRT	0.085403394939296
278	KC_G1	CG34431	-1.67593316621313	0.495877122847873	0.998043617620652	pseudobulk	DESeq2	LRT	0.304625927300966
279	KC_G1	CG34454	-1.32234376952914	0.542789226792531	0.998043617620652	pseudobulk	DESeq2	LRT	0.265368780745144
279	KC_G1	CG34456	1.13582588427274	0.633424328955532	0.998043617620652	pseudobulk	DESeq2	LRT	0.198305259991224
279	KC_G1	CG34457	-0.794610277746993	0.821479263487599	0.998043617620652	pseudobulk	DESeq2	LRT	0.085403394939296
279	KC_G1	CG3448	-1.3517582607626	0.213943994594297	0.998043617620652	pseudobulk	DESeq2	LRT	0.669699899650545
279	KC_G1	CG3500	0.220419397388562	0.577503975249459	0.998043617620652	pseudobulk	DESeq2	LRT	0.238445021958827
279	KC_G1	CG3511	-0.0940231539707564	0.860851347220384	0.998043617620652	pseudobulk	DESeq2	LRT	0.0650718365320054
279	KC_G1	CG3520	1.24386239920316	0.627869490249816	0.998043617620652	pseudobulk	DESeq2	LRT	0.202130619884913
279	KC_G1	CG3527	0.267245243211937	0.495249363822302	0.998043617620652	pseudobulk	DESeq2	LRT	0.30517607366653
279	KC_G1	CG3529	-0.0429691477976518	0.876898288772816	0.998043617620652	pseudobulk	DESeq2	LRT	0.057050777428031

279	KC_G1	CG3530	-0.0475989060506313	0.793037608943407	0.998043617620652	pseudobulk	DESeq2	LRT	0.100706216252018
280	KC_G1	CG3534	-0.12443173564733	0.88128454023932	0.998043617620652	pseudobulk	DESeq2	LRT	0.054883848336572
280	KC_G1	CG3542	0.142872039408824	0.606328633898034	0.998043617620652	pseudobulk	DESeq2	LRT	0.217291921703558
280	KC_G1	CG3548	-0.160640309266528	0.523028912302285	0.998043617620652	pseudobulk	DESeq2	LRT	0.281474303281323
280	KC_G1	CG3552	0.455744520173259	0.405806981481151	0.998043617620652	pseudobulk	DESeq2	LRT	0.391680485659849
280	KC_G1	CG3556	-0.184469770337837	0.363883236341864	0.998043617620652	pseudobulk	DESeq2	LRT	0.439037951371503
280	KC_G1	CG3558	-0.0954956679417895	0.65101785358713	0.998043617620652	pseudobulk	DESeq2	LRT	0.18640710112758
280	KC_G1	CG3566	0.0294950793464687	0.885500561596196	0.998043617620652	pseudobulk	DESeq2	LRT	0.0528111590385009
280	KC_G1	CG3568	0.493292866698695	0.275461307875722	0.998043617620652	pseudobulk	DESeq2	LRT	0.559939394838207
280	KC_G1	CG3587	0.732537536427264	0.219431302193282	0.998043617620652	pseudobulk	DESeq2	LRT	0.658701419604793
280	KC_G1	CG3589	-0.565068398470157	0.505869529488878	0.998043617620652	pseudobulk	DESeq2	LRT	0.295961479068159
281	KC_G1	CG3594	-0.0180725932280319	0.966615275138823	0.998043617620652	pseudobulk	DESeq2	LRT	0.0147463461140802
281	KC_G1	CG3609	0.69047786584059	0.412037587173289	0.998043617620652	pseudobulk	DESeq2	LRT	0.385063164652855
281	KC_G1	CG3618	0.0160747817919796	0.932824356543592	0.998043617620652	pseudobulk	DESeq2	LRT	0.0302001227754752
281	KC_G1	CG3625	0.162156441318217	0.169109931301621	0.998043617620652	pseudobulk	DESeq2	LRT	0.771830886885748
281	KC_G1	CG3626	0.178590088031276	0.822649082231493	0.998043617620652	pseudobulk	DESeq2	LRT	0.0847853824679997
281	KC_G1	CG3631	1.01333791251557	0.150671541766034	0.998043617620652	pseudobulk	DESeq2	LRT	0.821968767732387
281	KC_G1	CG3632	-0.245614963229664	0.47065286767642	0.998043617620652	pseudobulk	DESeq2	LRT	0.327299290822228
281	KC_G1	CG3634	0.213749470204916	0.614867432659569	0.998043617620652	pseudobulk	DESeq2	LRT	0.211218509379822
281	KC_G1	CG3638	-0.0716411623040632	0.684999145623377	0.998043617620652	pseudobulk	DESeq2	LRT	0.164309970188281
281	KC_G1	CG3645	-0.657124008618499	0.199416947391307	0.998043617620652	pseudobulk	DESeq2	LRT	0.700237936065647
282	KC_G1	CG3651	0.0107412162823013	0.956432582529533	0.998043617620652	pseudobulk	DESeq2	LRT	0.0193456373118558
282	KC_G1	CG3652	-0.277453531510392	0.330519938716617	0.998043617620652	pseudobulk	DESeq2	LRT	0.480802336440335
282	KC_G1	CG3655	-1.06312374245479	0.573752051580979	0.998043617620652	pseudobulk	DESeq2	LRT	0.241275748533192
282	KC_G1	CG3662	0.0784851070669159	0.580761790036793	0.998043617620652	pseudobulk	DESeq2	LRT	0.236001964832167
282	KC_G1	CG3663	0.877284618117695	0.608970597464076	0.998043617620652	pseudobulk	DESeq2	LRT	0.215403675622245
282	KC_G1	CG3678	0.670508112124588	0.0408176093722958	0.998043617620652	pseudobulk	DESeq2	LRT	1.38915243486795
282	KC_G1	CG3700	0.186077333605585	0.796165007243536	0.998043617620652	pseudobulk	DESeq2	LRT	0.098996914286465
282	KC_G1	CG3702	0.14974790127838	0.635697420978931	0.998043617620652	pseudobulk	DESeq2	LRT	0.196749550496381
282	KC_G1	CG3703	-0.109194495834299	0.73905539771054	0.998043617620652	pseudobulk	DESeq2	LRT	0.131323006776653
282	KC_G1	CG3704	0.23735743714669	0.723949507976285	0.998043617620652	pseudobulk	DESeq2	LRT	0.140291722714404
283	KC_G1	CG3709	0.441477305359681	0.686410468320984	0.998043617620652	pseudobulk	DESeq2	LRT	0.163416101762832
283	KC_G1	CG3713	-1.35574846224606	0.532546703611297	0.998043617620652	pseudobulk	DESeq2	LRT	0.273642299192052
283	KC_G1	CG3719	0.375360931696232	0.145510511405189	0.998043617620652	pseudobulk	DESeq2	LRT	0.837105632929747
283	KC_G1	CG3726	0.155448677741218	0.335909147465955	0.998043617620652	pseudobulk	DESeq2	LRT	0.473778169305418
283	KC_G1	CG3732	-0.0182420004892456	0.921334664680979	0.998043617620652	pseudobulk	DESeq2	LRT	0.0355825884525395
283	KC_G1	CG3735	0.032353475832784	0.935259037970539	0.998043617620652	pseudobulk	DESeq2	LRT	0.0290680862607199
283	KC_G1	CG3740	0.875080532224758	0.20513190312909	0.998043617620652	pseudobulk	DESeq2	LRT	0.687966790750908
283	KC_G1	CG3744	-0.1192474436283	0.683023668839794	0.998043617620652	pseudobulk	DESeq2	LRT	0.165564246437926
283	KC_G1	CG3756	0.196939242037806	0.719547595001731	0.998043617620652	pseudobulk	DESeq2	LRT	0.142940474051242
283	KC_G1	CG3760	0.160692259444885	0.610798160405436	0.998043617620652	pseudobulk	DESeq2	LRT	0.214102279616551
284	KC_G1	CG3764	0.418988446735545	0.162020379860257	0.998043617620652	pseudobulk	DESeq2	LRT	0.790430353950133
284	KC_G1	CG3777	0.291808303063657	0.597578514590431	0.998043617620652	pseudobulk	DESeq2	LRT	0.223605025587837
284	KC_G1	CG3781	-0.244271202010805	0.566230156271449	0.998043617620652	pseudobulk	DESeq2	LRT	0.247007004710359
284	KC_G1	CG3788	1.13582588427274	0.704684208645453	0.998043617620652	pseudobulk	DESeq2	LRT	0.152005460551806
284	KC_G1	CG3792	0.0918979068126382	0.743499516015412	0.998043617620652	pseudobulk	DESeq2	LRT	0.128719309848018
284	KC_G1	CG3793	-0.0310572007986885	0.899000105581146	0.998043617620652	pseudobulk	DESeq2	LRT	0.0462402572619811
284	KC_G1	CG3803	0.189941083282045	0.687093404409313	0.998043617620652	pseudobulk	DESeq2	LRT	0.162984220346282
284	KC_G1	CG3808	0.779324925349875	0.588005163230038	0.998043617620652	pseudobulk	DESeq2	LRT	0.230618860399254
284	KC_G1	CG3815	0.0505001252781564	0.912664393300786	0.998043617620652	pseudobulk	DESeq2	LRT	0.0396888927092958
284	KC_G1	CG3817	0.168290886325296	0.739695354580768	0.998043617620652	pseudobulk	DESeq2	LRT	0.130947108736613
285	KC_G1	CG3819	-0.240059903988995	0.673993689640266	0.998043617620652	pseudobulk	DESeq2	LRT	0.171344169588183
285	KC_G1	CG3823	0.92020042215104	0.540436922063598	0.998043617620652	pseudobulk	DESeq2	LRT	0.267254988109074
285	KC_G1	CG3831	-0.71088171110674	0.784645801042283	0.998043617620652	pseudobulk	DESeq2	LRT	0.105326344996467
285	KC_G1	CG3847	-0.880590869005533	0.0618594986033061	0.998043617620652	pseudobulk	DESeq2	LRT	1.20859360445408
285	KC_G1	CG3857	0.696233014393654	0.463031704879176	0.998043617620652	pseudobulk	DESeq2	LRT	0.334389270790561

285	KC_G1	CG3860	0.398010484470288	0.108834258383806	0.998043617620652	pseudobulk	DESeq2	LRT	0.963234377752289
285	KC_G1	CG3862	-0.621456004051334	0.331609502425312	0.998043617620652	pseudobulk	DESeq2	LRT	0.479373033032052
285	KC_G1	CG3868	1.12508065046033	0.900770125530981	0.998043617620652	pseudobulk	DESeq2	LRT	0.0453860258366365
285	KC_G1	CG3894	-0.0466554181288748	0.892623502327111	0.998043617620652	pseudobulk	DESeq2	LRT	0.0493316825900407
285	KC_G1	CG3902	0.0927113553975029	0.940969100369411	0.998043617620652	pseudobulk	DESeq2	LRT	0.0264246377410656
286	KC_G1	CG3907	0.24763312974105	0.325366782880946	0.998043617620652	pseudobulk	DESeq2	LRT	0.487626786825928
286	KC_G1	CG3909	-1.24316859004805	0.0358275015220204	0.998043617620652	pseudobulk	DESeq2	LRT	1.44578347687529
286	KC_G1	CG3918	0.143920486966398	0.630751770375605	0.998043617620652	pseudobulk	DESeq2	LRT	0.200141521843638
286	KC_G1	CG3919	-1.61874678838529	0.368592239892773	0.998043617620652	pseudobulk	DESeq2	LRT	0.433453812381254
286	KC_G1	CG3927	-0.794610277746993	0.815039924133852	0.998043617620652	pseudobulk	DESeq2	LRT	0.0888211171417967
286	KC_G1	CG3939	-0.704123705228411	0.78667415397905	0.998043617620652	pseudobulk	DESeq2	LRT	0.104205118254245
286	KC_G1	CG3955	-0.0134923692305029	0.926846759477895	0.998043617620652	pseudobulk	DESeq2	LRT	0.0329920641451573
286	KC_G1	CG3961	0.266839664138307	0.790749855433362	0.998043617620652	pseudobulk	DESeq2	LRT	0.10196087881091
286	KC_G1	CG3982	-0.794610277746993	0.815039924133852	0.998043617620652	pseudobulk	DESeq2	LRT	0.0888211171417967
286	KC_G1	CG3995	-0.195147454397616	0.570097847495591	0.998043617620652	pseudobulk	DESeq2	LRT	0.244050598747757
287	KC_G1	CG4000	2.07675541120147	0.345218614684515	0.998043617620652	pseudobulk	DESeq2	LRT	0.461905794541979
287	KC_G1	CG40006	-0.794610277746993	0.821479263487599	0.998043617620652	pseudobulk	DESeq2	LRT	0.085403394939296
287	KC_G1	CG4004	0.114301786659326	0.707478557837909	0.998043617620652	pseudobulk	DESeq2	LRT	0.150286718141186
287	KC_G1	CG40045	-0.0923124366631892	0.535937826141225	0.998043617620652	pseudobulk	DESeq2	LRT	0.270885589654159
287	KC_G1	CG40160	-0.794610277746993	0.879485534599449	0.998043617620652	pseudobulk	DESeq2	LRT	0.0557712992369932
287	KC_G1	CG40191	-0.0982873704573318	0.660890311694109	0.998043617620652	pseudobulk	DESeq2	LRT	0.17987061461259
287	KC_G1	CG40198	-0.167308619020355	0.918273107820925	0.998043617620652	pseudobulk	DESeq2	LRT	0.0370281340713204
287	KC_G1	CG4022	-0.297789723648226	0.334430515017768	0.998043617620652	pseudobulk	DESeq2	LRT	0.475694102347844
287	KC_G1	CG40228	0.017529786991189	0.916535094505786	0.998043617620652	pseudobulk	DESeq2	LRT	0.0378509010690255
287	KC_G1	CG4025	-0.365326038049009	0.375042526203692	0.998043617620652	pseudobulk	DESeq2	LRT	0.425919484676381
288	KC_G1	CG4036	0.372474304580808	0.438452804984204	0.998043617620652	pseudobulk	DESeq2	LRT	0.358077147196496
288	KC_G1	CG4038	-0.375767552306878	0.536399233276701	0.998043617620652	pseudobulk	DESeq2	LRT	0.27051185159586
288	KC_G1	CG4041	0.159436796834745	0.788796941198272	0.998043617620652	pseudobulk	DESeq2	LRT	0.103034782173447
288	KC_G1	CG4042	-0.0387773279124211	0.924913613036521	0.998043617620652	pseudobulk	DESeq2	LRT	0.0338988284864326
288	KC_G1	CG40439	-0.37642473000192	0.679700256712671	0.998043617620652	pseudobulk	DESeq2	LRT	0.167682566051145
288	KC_G1	CG4045	0.242813289004197	0.761779863536751	0.998043617620652	pseudobulk	DESeq2	LRT	0.11817051142101
288	KC_G1	CG40470	0.0749497135892303	0.964035483025451	0.998043617620652	pseudobulk	DESeq2	LRT	0.0159069808289603
288	KC_G1	CG40472	-0.66280341405271	0.0905574095771142	0.998043617620652	pseudobulk	DESeq2	LRT	1.04307600910695
288	KC_G1	CG4049	-0.291989329336311	0.31315044868436	0.998043617620652	pseudobulk	DESeq2	LRT	0.504246961701769
288	KC_G1	CG40498	-0.0166420398478858	0.889497752931021	0.998043617620652	pseudobulk	DESeq2	LRT	0.0508551447030361
289	KC_G1	CG4069	2.13352173419733	0.0945344418621308	0.998043617620652	pseudobulk	DESeq2	LRT	1.0244099355593
289	KC_G1	CG4074	0.0608365192908758	0.882912418361414	0.998043617620652	pseudobulk	DESeq2	LRT	0.0540823746883633
289	KC_G1	CG4078	0.816383283827615	0.32144581379115	0.998043617620652	pseudobulk	DESeq2	LRT	0.492892225709744
289	KC_G1	CG4080	-0.115451171228625	0.577496445323564	0.998043617620652	pseudobulk	DESeq2	LRT	0.238450684649865
289	KC_G1	CG4098	1.13582588427274	0.633424328955532	0.998043617620652	pseudobulk	DESeq2	LRT	0.198305259991224
289	KC_G1	CG4101	-0.387800562244528	0.1800593426089	0.998043617620652	pseudobulk	DESeq2	LRT	0.744584339784352
289	KC_G1	CG41099	0.0295140857117122	0.892854408971904	0.998043617620652	pseudobulk	DESeq2	LRT	0.0492193524608435
289	KC_G1	CG41128	-0.020860089204064	0.88516335979977	0.998043617620652	pseudobulk	DESeq2	LRT	0.0529765714349204
289	KC_G1	CG4115	-2.27643791964046	0.298906309922724	0.998043617620652	pseudobulk	DESeq2	LRT	0.52446491689215
289	KC_G1	CG4119	0.0624849812398447	0.808255262948475	0.998043617620652	pseudobulk	DESeq2	LRT	0.0924514588017269
290	KC_G1	CG4133	-0.0329120639805468	0.874840611541973	0.998043617620652	pseudobulk	DESeq2	LRT	0.0580710645013456
290	KC_G1	CG41378	-0.0725334254935761	0.766479734543629	0.998043617620652	pseudobulk	DESeq2	LRT	0.115499323252168
290	KC_G1	CG41520	0.400329425834043	0.865512925764825	0.998043617620652	pseudobulk	DESeq2	LRT	0.0627264418886215
290	KC_G1	CG4186	0.500914308463935	0.379482613957656	0.998043617620652	pseudobulk	DESeq2	LRT	0.420808116567491
290	KC_G1	CG4199	0.833756968347134	0.16234966504606	0.998043617620652	pseudobulk	DESeq2	LRT	0.789548603057678
290	KC_G1	CG4210	-0.711364822669339	0.836431126230485	0.998043617620652	pseudobulk	DESeq2	LRT	0.0775698140913142
290	KC_G1	CG42231	-1.22741217093984	0.297153480839676	0.998043617620652	pseudobulk	DESeq2	LRT	0.527019178074089
290	KC_G1	CG42232	-0.461611163415819	0.0486626725587363	0.998043617620652	pseudobulk	DESeq2	LRT	1.31280404325479
290	KC_G1	CG42233	-0.196262741218607	0.772672110454749	0.998043617620652	pseudobulk	DESeq2	LRT	0.112004763300718
290	KC_G1	CG42235	-0.253133118374914	0.683746578842653	0.998043617620652	pseudobulk	DESeq2	LRT	0.165104833665423
291	KC_G1	CG42237	0.173195945230598	0.976215465741119	0.998043617620652	pseudobulk	DESeq2	LRT	0.0104543162934892

291	KC_G1	CG42238	-0.141942109244247	0.239582664006973	0.998043617620652	pseudobulk	DESeq2	LRT	0.620544610316518
291	KC_G1	CG42239	-0.194068517006497	0.754241264790802	0.998043617620652	pseudobulk	DESeq2	LRT	0.122489710884656
291	KC_G1	CG42240	-0.237731744258426	0.73054278915447	0.998043617620652	pseudobulk	DESeq2	LRT	0.13635434160588
291	KC_G1	CG42246	1.13582588427274	0.704684208645453	0.998043617620652	pseudobulk	DESeq2	LRT	0.152005460551806
291	KC_G1	CG42255	1.90250740646012	0.460774913678782	0.998043617620652	pseudobulk	DESeq2	LRT	0.336511173568024
291	KC_G1	CG42258	-0.0743582223943284	0.600043874690007	0.998043617620652	pseudobulk	DESeq2	LRT	0.221816993217819
291	KC_G1	CG42259	0.772572177872764	0.383245065802266	0.998043617620652	pseudobulk	DESeq2	LRT	0.416523427885571
291	KC_G1	CG42260	-0.27403927918348	0.0147491396485354	0.998043617620652	pseudobulk	DESeq2	LRT	1.83123331234963
291	KC_G1	CG42264	-0.794610277746993	0.821479263487599	0.998043617620652	pseudobulk	DESeq2	LRT	0.085403394939296
292	KC_G1	CG42268	-0.208295381720786	0.144510028903822	0.998043617620652	pseudobulk	DESeq2	LRT	0.840102012101541
292	KC_G1	CG42271	0.0107485643521299	0.967304856229864	0.998043617620652	pseudobulk	DESeq2	LRT	0.014436631901483
292	KC_G1	CG4230	-0.176670648486037	0.218142674353003	0.998043617620652	pseudobulk	DESeq2	LRT	0.661259366869169
292	KC_G1	CG42302	1.38896997631979	0.587084398012744	0.998043617620652	pseudobulk	DESeq2	LRT	0.23129946100712
292	KC_G1	CG42307	-0.962173602710892	0.268446272716822	0.998043617620652	pseudobulk	DESeq2	LRT	0.571142621686791
292	KC_G1	CG42322	-0.0802642910560567	0.626946602635461	0.998043617620652	pseudobulk	DESeq2	LRT	0.202769446678533
292	KC_G1	CG42324	-0.120522061316269	0.399667616234449	0.998043617620652	pseudobulk	DESeq2	LRT	0.398301039782053
292	KC_G1	CG42327	0.215121977666087	0.750185863594419	0.998043617620652	pseudobulk	DESeq2	LRT	0.124831123897349
292	KC_G1	CG42336	-0.228290939765583	0.326101690909006	0.998043617620652	pseudobulk	DESeq2	LRT	0.486646949275776
292	KC_G1	CG42337	0.084027252670548	0.610963353497526	0.998043617620652	pseudobulk	DESeq2	LRT	0.2139848386136
293	KC_G1	CG42339	0.00182826148534157	0.965757861217849	0.998043617620652	pseudobulk	DESeq2	LRT	0.0151317480346691
293	KC_G1	CG42340	-0.0213493319224743	0.885787400130617	0.998043617620652	pseudobulk	DESeq2	LRT	0.0526705016225676
293	KC_G1	CG42342	-0.0257661977097628	0.853957814864687	0.998043617620652	pseudobulk	DESeq2	LRT	0.0685635827348782
293	KC_G1	CG42346	0.0967842669079894	0.577871901807475	0.998043617620652	pseudobulk	DESeq2	LRT	0.238168421966623
293	KC_G1	CG42354	0.918078636166071	0.602374121456212	0.998043617620652	pseudobulk	DESeq2	LRT	0.220133694095172
293	KC_G1	CG42359	0.00268876128157595	0.956616719264765	0.998043617620652	pseudobulk	DESeq2	LRT	0.0192620330177074
293	KC_G1	CG42360	-0.539320065714116	0.212362023742416	0.998043617620652	pseudobulk	DESeq2	LRT	0.672923144623139
293	KC_G1	CG42361	-0.0262556311922226	0.842306959223664	0.998043617620652	pseudobulk	DESeq2	LRT	0.0745296111095422
293	KC_G1	CG42364	2.95840825636686	0.160769763152811	0.998043617620652	pseudobulk	DESeq2	LRT	0.793795628042056
293	KC_G1	CG42365	-0.302361175051433	0.659008231159159	0.998043617620652	pseudobulk	DESeq2	LRT	0.181109160937587
294	KC_G1	CG42376	0.073702201311436	0.776786133705844	0.998043617620652	pseudobulk	DESeq2	LRT	0.109698535557294
294	KC_G1	CG4238	-0.10461336945026	0.683224281196547	0.998043617620652	pseudobulk	DESeq2	LRT	0.165436707596732
294	KC_G1	CG42382	-0.00768257464197359	0.946102998030334	0.998043617620652	pseudobulk	DESeq2	LRT	0.0240615813135624
294	KC_G1	CG4239	-0.525601435068723	0.271588356507809	0.998043617620652	pseudobulk	DESeq2	LRT	0.566088852995372
294	KC_G1	CG42390	1.89288595231245	0.415717432633965	0.998043617620652	pseudobulk	DESeq2	LRT	0.381201763470702
294	KC_G1	CG42391	0.172834960772825	0.908610676445368	0.998043617620652	pseudobulk	DESeq2	LRT	0.0416221644022337
294	KC_G1	CG42393	-0.794610277746993	0.821479263487599	0.998043617620652	pseudobulk	DESeq2	LRT	0.085403394939296
294	KC_G1	CG42394	-0.470173369978413	0.238656136665268	0.998043617620652	pseudobulk	DESeq2	LRT	0.622227393958126
294	KC_G1	CG42399	0.613178434290001	0.301815547694927	0.998043617620652	pseudobulk	DESeq2	LRT	0.52025839178312
294	KC_G1	CG42402	-0.134820946594489	0.225922593947454	0.998043617620652	pseudobulk	DESeq2	LRT	0.646040334214522
295	KC_G1	CG42404	-0.0543435738114376	0.793203589153715	0.998043617620652	pseudobulk	DESeq2	LRT	0.100615329330418
295	KC_G1	CG42445	2.53483764076482	0.205449982952058	0.998043617620652	pseudobulk	DESeq2	LRT	0.687293890439235
295	KC_G1	CG42450	-0.0831622790906071	0.485257676239836	0.998043617620652	pseudobulk	DESeq2	LRT	0.314027585826803
295	KC_G1	CG42458	1.13582588427274	0.633424328955532	0.998043617620652	pseudobulk	DESeq2	LRT	0.198305259991224
295	KC_G1	CG42486	1.69981218657454	0.566400560818652	0.998043617620652	pseudobulk	DESeq2	LRT	0.246876325303589
295	KC_G1	CG42489	-0.668593574498123	0.744279897808527	0.998043617620652	pseudobulk	DESeq2	LRT	0.128263710671876
295	KC_G1	CG42494	1.13582588427274	0.633424328955532	0.998043617620652	pseudobulk	DESeq2	LRT	0.198305259991224
295	KC_G1	CG42495	0.27656786259454	0.603606210133079	0.998043617620652	pseudobulk	DESeq2	LRT	0.219246300684628
295	KC_G1	CG4250	1.13582588427274	0.633424328955532	0.998043617620652	pseudobulk	DESeq2	LRT	0.198305259991224
295	KC_G1	CG42514	1.13582588427274	0.704684208645453	0.998043617620652	pseudobulk	DESeq2	LRT	0.152005460551806
296	KC_G1	CG42518	-1.70666911876302	0.312133549814185	0.998043617620652	pseudobulk	DESeq2	LRT	0.505659548489159
296	KC_G1	CG42521	1.90250740646012	0.460774913678782	0.998043617620652	pseudobulk	DESeq2	LRT	0.336511173568024
296	KC_G1	CG42524	-0.65817001371651	0.770857516266391	0.998043617620652	pseudobulk	DESeq2	LRT	0.113025888638787
296	KC_G1	CG42526	-3.84600490268941	0.0337905079041236	0.998043617620652	pseudobulk	DESeq2	LRT	1.47120528030855
296	KC_G1	CG42534	3.40592015880327	0.0405589903389719	0.998043617620652	pseudobulk	DESeq2	LRT	1.39191286471197
296	KC_G1	CG42540	0.134813074893878	0.39154936497006	0.998043617620652	pseudobulk	DESeq2	LRT	0.407213476051575
296	KC_G1	CG42542	0.0250395031947596	0.921929530941139	0.998043617620652	pseudobulk	DESeq2	LRT	0.0353022736242685

296	KC_G1	CG42553	-0.298507955443341	0.734226331446989	0.998043617620652	pseudobulk	DESeq2	LRT	0.134170044515948
296	KC_G1	CG42554	0.305717419980334	0.463005576180209	0.998043617620652	pseudobulk	DESeq2	LRT	0.334413778550704
296	KC_G1	CG42556	0.166881307022832	0.89502753597113	0.998043617620652	pseudobulk	DESeq2	LRT	0.0481636031909532
297	KC_G1	CG42561	-0.752595479487864	0.785589861204421	0.998043617620652	pseudobulk	DESeq2	LRT	0.104804130174502
297	KC_G1	CG42562	-0.661680365268252	0.695408595117629	0.998043617620652	pseudobulk	DESeq2	LRT	0.157759945822606
297	KC_G1	CG42588	-0.0562936332009761	0.837740006877753	0.998043617620652	pseudobulk	DESeq2	LRT	0.0768907440163957
297	KC_G1	CG42594	-0.0351117930852101	0.967955964874919	0.998043617620652	pseudobulk	DESeq2	LRT	0.0141443995581703
297	KC_G1	CG42613	-0.217334760101556	0.0680283141037146	0.998043617620652	pseudobulk	DESeq2	LRT	1.1673102917107
297	KC_G1	CG4266	-0.117426999890189	0.649415736253716	0.998043617620652	pseudobulk	DESeq2	LRT	0.187477192018374
297	KC_G1	CG42663	1.40702347046371	0.167648897497992	0.998043617620652	pseudobulk	DESeq2	LRT	0.775599298257306
297	KC_G1	CG42668	-0.00611119492580803	0.945991504711442	0.998043617620652	pseudobulk	DESeq2	LRT	0.0241127636759122
297	KC_G1	CG42671	0.068991154820575	0.75241000366242	0.998043617620652	pseudobulk	DESeq2	LRT	0.123545438923751
297	KC_G1	CG42672	-0.137172459642571	0.710709979191709	0.998043617620652	pseudobulk	DESeq2	LRT	0.148307586516956
298	KC_G1	CG42673	1.36628815172265	0.0696463085173097	0.998043617620652	pseudobulk	DESeq2	LRT	1.15710189766139
298	KC_G1	CG42674	0.320135306133013	0.250126912887335	0.998043617620652	pseudobulk	DESeq2	LRT	0.601839577003459
298	KC_G1	CG42676	0.263635072883487	0.58554873918096	0.998043617620652	pseudobulk	DESeq2	LRT	0.232436949820694
298	KC_G1	CG42697	-0.794610277746993	0.815039924133852	0.998043617620652	pseudobulk	DESeq2	LRT	0.0888211171417967
298	KC_G1	CG42699	0.110485338909151	0.666432437904964	0.998043617620652	pseudobulk	DESeq2	LRT	0.176243872255118
298	KC_G1	CG42700	-0.169467037570797	0.585003104720399	0.998043617620652	pseudobulk	DESeq2	LRT	0.232841829030027
298	KC_G1	CG42709	-0.20131138595598	0.47776068974185	0.998043617620652	pseudobulk	DESeq2	LRT	0.320789586964815
298	KC_G1	CG42724	-0.106366859673663	0.564313140229076	0.998043617620652	pseudobulk	DESeq2	LRT	0.24847983691313
298	KC_G1	CG42733	-1.60891734856068	0.545249891924755	0.998043617620652	pseudobulk	DESeq2	LRT	0.263404411831769
298	KC_G1	CG42747	-0.157343186380356	0.561982965346517	0.998043617620652	pseudobulk	DESeq2	LRT	0.250276848431172
299	KC_G1	CG42748	-0.196535573171795	0.157231468668261	0.998043617620652	pseudobulk	DESeq2	LRT	0.803460528899358
299	KC_G1	CG42750	-0.129328268330921	0.254993075705696	0.998043617620652	pseudobulk	DESeq2	LRT	0.593471612599914
299	KC_G1	CG42751	-1.67593316621313	0.495877122847873	0.998043617620652	pseudobulk	DESeq2	LRT	0.304625927300966
299	KC_G1	CG42753	-0.794610277746993	0.815039924133852	0.998043617620652	pseudobulk	DESeq2	LRT	0.0888211171417967
299	KC_G1	CG42762	2.84706876674335	0.20790991633284	0.998043617620652	pseudobulk	DESeq2	LRT	0.682124796354298
299	KC_G1	CG42764	-2.19332442993981	0.381369935212232	0.998043617620652	pseudobulk	DESeq2	LRT	0.418653546964052
299	KC_G1	CG42766	1.12508065046033	0.900770125530981	0.998043617620652	pseudobulk	DESeq2	LRT	0.0453860258366365
299	KC_G1	CG4278	0.95505184424915	0.0849315078579353	0.998043617620652	pseudobulk	DESeq2	LRT	1.07093116546466
299	KC_G1	CG42784	-0.0492762086541727	0.728675581261726	0.998043617620652	pseudobulk	DESeq2	LRT	0.1374657839279
299	KC_G1	CG42788	-0.0542797065699053	0.679131477639614	0.998043617620652	pseudobulk	DESeq2	LRT	0.168046139584251
300	KC_G1	CG42789	2.04147257822512	0.408147672536479	0.998043617620652	pseudobulk	DESeq2	LRT	0.389182675723292
300	KC_G1	CG42795	0.0255335575400797	0.833892931063578	0.998043617620652	pseudobulk	DESeq2	LRT	0.0788897076756026
300	KC_G1	CG42797	-0.0882093122518558	0.800222087747644	0.998043617620652	pseudobulk	DESeq2	LRT	0.0967894653857506
300	KC_G1	CG42806	0.122970050680956	0.774703610735879	0.998043617620652	pseudobulk	DESeq2	LRT	0.110864419870357
300	KC_G1	CG42809	0.572578628085982	0.215846228764614	0.998043617620652	pseudobulk	DESeq2	LRT	0.665855534874408
300	KC_G1	CG4281	0.0303154304320795	0.913670500394245	0.998043617620652	pseudobulk	DESeq2	LRT	0.0392103968927806
300	KC_G1	CG42810	-2.95865450380007	0.171287438686412	0.998043617620652	pseudobulk	DESeq2	LRT	0.766274484723943
300	KC_G1	CG42813	0.111229742614978	0.616941627716658	0.998043617620652	pseudobulk	DESeq2	LRT	0.209755925042613
300	KC_G1	CG42815	-0.794610277746993	0.821479263487599	0.998043617620652	pseudobulk	DESeq2	LRT	0.085403394939296
300	KC_G1	CG4282	-0.116790164281154	0.843810555653964	0.998043617620652	pseudobulk	DESeq2	LRT	0.073755046110341
301	KC_G1	CG42831	-1.12943128263269	0.577145423323916	0.998043617620652	pseudobulk	DESeq2	LRT	0.238714743881233
301	KC_G1	CG42855	-0.263828984088913	0.764315607001181	0.998043617620652	pseudobulk	DESeq2	LRT	0.116727272214988
301	KC_G1	CG42866	-0.794610277746993	0.821479263487599	0.998043617620652	pseudobulk	DESeq2	LRT	0.085403394939296
301	KC_G1	CG4287	0.0298823000801454	0.929915095044997	0.998043617620652	pseudobulk	DESeq2	LRT	0.0315567024533265
301	KC_G1	CG4291	-0.28552482786542	0.542364526516826	0.998043617620652	pseudobulk	DESeq2	LRT	0.265708723347851
301	KC_G1	CG4293	-0.422492263598642	0.442437770411485	0.998043617620652	pseudobulk	DESeq2	LRT	0.354147804892567
301	KC_G1	CG4294	-0.278818544689815	0.24012359760755	0.998043617620652	pseudobulk	DESeq2	LRT	0.619565158530378
301	KC_G1	CG4297	0.455879000863483	0.0693501638417649	0.998043617620652	pseudobulk	DESeq2	LRT	1.15895250855623
301	KC_G1	CG4301	-0.794610277746993	0.815039924133852	0.998043617620652	pseudobulk	DESeq2	LRT	0.0888211171417967
301	KC_G1	CG43049	-1.27659866432867	0.602222436331314	0.998043617620652	pseudobulk	DESeq2	LRT	0.220243068495509
302	KC_G1	CG43051	-1.60891734856068	0.545249891924755	0.998043617620652	pseudobulk	DESeq2	LRT	0.263404411831769
302	KC_G1	CG43055	0.172834960772825	0.908610676445368	0.998043617620652	pseudobulk	DESeq2	LRT	0.0416221644022337
302	KC_G1	CG43060	0.948682853426738	0.716182290134517	0.998043617620652	pseudobulk	DESeq2	LRT	0.144976422491876

302	KC_G1	CG43064	1.12508065046033	0.900770125530981	0.998043617620652	pseudobulk	DESeq2	LRT	0.0453860258366365
302	KC_G1	CG43066	-0.0390445916951264	0.791629978373776	0.998043617620652	pseudobulk	DESeq2	LRT	0.101477767780716
302	KC_G1	CG43068	1.12508065046033	0.900770125530981	0.998043617620652	pseudobulk	DESeq2	LRT	0.0453860258366365
302	KC_G1	CG43078	0.32898396742723	0.608844908114251	0.998043617620652	pseudobulk	DESeq2	LRT	0.215493321699187
302	KC_G1	CG43085	0.173195945230598	0.976215465741119	0.998043617620652	pseudobulk	DESeq2	LRT	0.0104543162934892
302	KC_G1	CG43093	1.12508065046033	0.900770125530981	0.998043617620652	pseudobulk	DESeq2	LRT	0.0453860258366365
302	KC_G1	CG43094	-0.794610277746993	0.821479263487599	0.998043617620652	pseudobulk	DESeq2	LRT	0.085403394939296
303	KC_G1	CG43102	-0.00537529187688929	0.938326596092406	0.998043617620652	pseudobulk	DESeq2	LRT	0.0276459737813867
303	KC_G1	CG43107	0.949176147629133	0.729784453976284	0.998043617620652	pseudobulk	DESeq2	LRT	0.136805392306293
303	KC_G1	CG43111	-0.794610277746993	0.879485534599449	0.998043617620652	pseudobulk	DESeq2	LRT	0.0557712992369932
303	KC_G1	CG43117	0.689772565694032	0.35848326826703	0.998043617620652	pseudobulk	DESeq2	LRT	0.44553110964437
303	KC_G1	CG4313	-0.312186284791358	0.715748320048681	0.998043617620652	pseudobulk	DESeq2	LRT	0.145239662648933
303	KC_G1	CG43137	-0.794610277746993	0.879485534599449	0.998043617620652	pseudobulk	DESeq2	LRT	0.0557712992369932
303	KC_G1	CG43155	-1.72984073695683	0.480047293856853	0.998043617620652	pseudobulk	DESeq2	LRT	0.318715974188453
303	KC_G1	CG43163	0.121483991571076	0.960019907175548	0.998043617620652	pseudobulk	DESeq2	LRT	0.0177197612449608
303	KC_G1	CG43184	1.12508065046033	0.900770125530981	0.998043617620652	pseudobulk	DESeq2	LRT	0.0453860258366365
303	KC_G1	CG43185	-0.794610277746993	0.879485534599449	0.998043617620652	pseudobulk	DESeq2	LRT	0.0557712992369932
304	KC_G1	CG43191	-0.711691946027098	0.741255142397939	0.998043617620652	pseudobulk	DESeq2	LRT	0.130032280734188
304	KC_G1	CG43219	2.01538589160551	0.37910076991012	0.998043617620652	pseudobulk	DESeq2	LRT	0.42124533357046
304	KC_G1	CG43222	0.173664168188545	0.602097674614157	0.998043617620652	pseudobulk	DESeq2	LRT	0.220333050095941
304	KC_G1	CG43229	-0.794610277746993	0.821479263487599	0.998043617620652	pseudobulk	DESeq2	LRT	0.085403394939296
304	KC_G1	CG43232	0.665708931789394	0.758505174577836	0.998043617620652	pseudobulk	DESeq2	LRT	0.120041452078391
304	KC_G1	CG43236	-2.3243999702458	0.347033693779396	0.998043617620652	pseudobulk	DESeq2	LRT	0.459628357162795
304	KC_G1	CG43245	1.38896997631979	0.587084398012744	0.998043617620652	pseudobulk	DESeq2	LRT	0.23129946100712
304	KC_G1	CG43273	0.171798486022727	0.87916192492652	0.998043617620652	pseudobulk	DESeq2	LRT	0.0559311287651556
304	KC_G1	CG43313	0.134151906850277	0.936492948904004	0.998043617620652	pseudobulk	DESeq2	LRT	0.0284954881911579
304	KC_G1	CG43315	-0.794610277746993	0.815039924133852	0.998043617620652	pseudobulk	DESeq2	LRT	0.0888211171417967
305	KC_G1	CG4332	0.792319831603799	0.272232826187019	0.998043617620652	pseudobulk	DESeq2	LRT	0.565059508183631
305	KC_G1	CG43322	0.35836192634256	0.714414901264967	0.998043617620652	pseudobulk	DESeq2	LRT	0.146049495511369
305	KC_G1	CG43324	-0.0642572350553904	0.566310897717405	0.998043617620652	pseudobulk	DESeq2	LRT	0.246945081013421
305	KC_G1	CG43340	-0.794610277746993	0.815039924133852	0.998043617620652	pseudobulk	DESeq2	LRT	0.0888211171417967
305	KC_G1	CG43341	-0.84109762055297	0.460655358682833	0.998043617620652	pseudobulk	DESeq2	LRT	0.336623872438191
305	KC_G1	CG43342	-0.0754356726880258	0.947201198565979	0.998043617620652	pseudobulk	DESeq2	LRT	0.0235577610748489
305	KC_G1	CG43347	-0.0215609054870322	0.883264322319698	0.998043617620652	pseudobulk	DESeq2	LRT	0.0539093116582091
305	KC_G1	CG4335	2.63732022130093	0.22943645835378	0.998043617620652	pseudobulk	DESeq2	LRT	0.639337569853304
305	KC_G1	CG43351	-2.07140175462527	0.153145240118766	0.998043617620652	pseudobulk	DESeq2	LRT	0.814896496880778
305	KC_G1	CG43366	-0.605554807267048	0.698041443522835	0.998043617620652	pseudobulk	DESeq2	LRT	0.156118792048808
306	KC_G1	CG43367	0.112482870259236	0.683677931067334	0.998043617620652	pseudobulk	DESeq2	LRT	0.165148438778283
306	KC_G1	CG43373	1.74317226954561	0.216697429396626	0.998043617620652	pseudobulk	DESeq2	LRT	0.664146240528277
306	KC_G1	CG43376	1.13582588427274	0.704684208645453	0.998043617620652	pseudobulk	DESeq2	LRT	0.152005460551806
306	KC_G1	CG4338	-0.449014017469352	0.273938297155118	0.998043617620652	pseudobulk	DESeq2	LRT	0.562347248211629
306	KC_G1	CG43394	0.397168740705283	0.708187353799837	0.998043617620652	pseudobulk	DESeq2	LRT	0.149851832765686
306	KC_G1	CG43396	-0.794610277746993	0.815039924133852	0.998043617620652	pseudobulk	DESeq2	LRT	0.0888211171417967
306	KC_G1	CG4341	0.747616567591732	0.413681159805743	0.998043617620652	pseudobulk	DESeq2	LRT	0.383334257636566
306	KC_G1	CG43428	0.082790564838356	0.940504976651723	0.998043617620652	pseudobulk	DESeq2	LRT	0.026638902052291
306	KC_G1	CG43445	-0.794610277746993	0.879485534599449	0.998043617620652	pseudobulk	DESeq2	LRT	0.0557712992369932
306	KC_G1	CG43446	-0.794610277746993	0.879485534599449	0.998043617620652	pseudobulk	DESeq2	LRT	0.0557712992369932
307	KC_G1	CG4360	0.393505732375781	0.211008875589408	0.998043617620652	pseudobulk	DESeq2	LRT	0.67569927674764
307	KC_G1	CG4364	0.331413127682818	0.284054807866034	0.998043617620652	pseudobulk	DESeq2	LRT	0.546597855525897
307	KC_G1	CG43658	-0.207439329779438	0.127174083220211	0.998043617620652	pseudobulk	DESeq2	LRT	0.895601384452171
307	KC_G1	CG43675	1.12508065046033	0.900770125530981	0.998043617620652	pseudobulk	DESeq2	LRT	0.0453860258366365
307	KC_G1	CG43693	-0.272664149529452	0.453177607420078	0.998043617620652	pseudobulk	DESeq2	LRT	0.3437315577984
307	KC_G1	CG43707	0.0930860112401646	0.658040765503509	0.998043617620652	pseudobulk	DESeq2	LRT	0.181747201089954
307	KC_G1	CG43736	-0.0601039767984872	0.662498003181377	0.998043617620652	pseudobulk	DESeq2	LRT	0.178815426385292
307	KC_G1	CG43737	-0.194720113905966	0.238259948486932	0.998043617620652	pseudobulk	DESeq2	LRT	0.62294895644924
307	KC_G1	CG43740	-0.0696404166674381	0.791841485743012	0.998043617620652	pseudobulk	DESeq2	LRT	0.101361748659477

307	KC_G1	CG4375	2.38466886841527	0.34619303318143	0.998043617620652	pseudobulk	DESeq2	LRT	0.460681676152437
308	KC_G1	CG43755	0.17172214391994	0.93725366732732	0.998043617620652	pseudobulk	DESeq2	LRT	0.0281428515765614
308	KC_G1	CG43759	1.98795016997694	0.120734980874055	0.998043617620652	pseudobulk	DESeq2	LRT	0.918166882350441
308	KC_G1	CG43778	-0.19289542447742	0.663581535164359	0.998043617620652	pseudobulk	DESeq2	LRT	0.178105707168168
308	KC_G1	CG43783	-0.201640254331645	0.648133323424698	0.998043617620652	pseudobulk	DESeq2	LRT	0.188335648956634
308	KC_G1	CG43788	1.12508065046033	0.900770125530981	0.998043617620652	pseudobulk	DESeq2	LRT	0.0453860258366365
308	KC_G1	CG43789	0.393134124233613	0.712963300720919	0.998043617620652	pseudobulk	DESeq2	LRT	0.14693282457253
308	KC_G1	CG43795	-0.765625368254185	0.836625948066609	0.998043617620652	pseudobulk	DESeq2	LRT	0.0774686698445758
308	KC_G1	CG43800	-0.477705414755638	0.500743980390427	0.998043617620652	pseudobulk	DESeq2	LRT	0.300384262801104
308	KC_G1	CG43813	1.12508065046033	0.900770125530981	0.998043617620652	pseudobulk	DESeq2	LRT	0.0453860258366365
308	KC_G1	CG43845	0.208594480859484	0.854663553836208	0.998043617620652	pseudobulk	DESeq2	LRT	0.0682048156445907
309	KC_G1	CG43850	0.098586083198477	0.657367756550147	0.998043617620652	pseudobulk	DESeq2	LRT	0.18219160158656
309	KC_G1	CG43861	-0.107967040185448	0.534454822754568	0.998043617620652	pseudobulk	DESeq2	LRT	0.272088999636906
309	KC_G1	CG43867	-0.065535294859084	0.645539023944551	0.998043617620652	pseudobulk	DESeq2	LRT	0.190077498755414
309	KC_G1	CG43897	-0.794610277746993	0.821479263487599	0.998043617620652	pseudobulk	DESeq2	LRT	0.085403394939296
309	KC_G1	CG4390	-0.155689916469597	0.683207063353064	0.998043617620652	pseudobulk	DESeq2	LRT	0.165447652331636
309	KC_G1	CG43901	-0.0960186514945898	0.61154171020523	0.998043617620652	pseudobulk	DESeq2	LRT	0.21357391656031
309	KC_G1	CG43902	-0.0245063641291009	0.87999034069773	0.998043617620652	pseudobulk	DESeq2	LRT	0.0555220949006249
309	KC_G1	CG43915	0.173195945230598	0.976215465741119	0.998043617620652	pseudobulk	DESeq2	LRT	0.0104543162934892
309	KC_G1	CG43925	1.9973502670615	0.358574172659641	0.998043617620652	pseudobulk	DESeq2	LRT	0.445420994961641
309	KC_G1	CG4393	0.00119880814808676	0.947557384579548	0.998043617620652	pseudobulk	DESeq2	LRT	0.023394479458476
310	KC_G1	CG43980	-1.52794028948093	0.181089509562519	0.998043617620652	pseudobulk	DESeq2	LRT	0.742106707449995
310	KC_G1	CG44002	1.31785194087057	0.200515403155793	0.998043617620652	pseudobulk	DESeq2	LRT	0.697852260207782
310	KC_G1	CG44006	-1.66808346769027	0.498191247612582	0.998043617620652	pseudobulk	DESeq2	LRT	0.302603906560549
310	KC_G1	CG44008	0.522233684157186	0.699023234321813	0.998043617620652	pseudobulk	DESeq2	LRT	0.1555083888179
310	KC_G1	CG4404	0.404495771015028	0.63939776323565	0.998043617620652	pseudobulk	DESeq2	LRT	0.194228887326317
310	KC_G1	CG4407	-0.351665266783792	0.432075482898267	0.998043617620652	pseudobulk	DESeq2	LRT	0.364440376003227
310	KC_G1	CG44085	0.172834960772825	0.908610676445368	0.998043617620652	pseudobulk	DESeq2	LRT	0.0416221644022337
310	KC_G1	CG4409	0.0675987377756788	0.760851265179966	0.998043617620652	pseudobulk	DESeq2	LRT	0.118700232880987
310	KC_G1	CG44098	-1.15303376885588	0.455269553438317	0.998043617620652	pseudobulk	DESeq2	LRT	0.341731392555558
310	KC_G1	CG4415	-0.794610277746993	0.821479263487599	0.998043617620652	pseudobulk	DESeq2	LRT	0.085403394939296
311	KC_G1	CG44153	-2.330634067508	0.236224937023708	0.998043617620652	pseudobulk	DESeq2	LRT	0.626674258119043
311	KC_G1	CG44158	1.00966396359468	0.539780585959712	0.998043617620652	pseudobulk	DESeq2	LRT	0.267782739568667
311	KC_G1	CG44195	-0.794610277746993	0.815039924133852	0.998043617620652	pseudobulk	DESeq2	LRT	0.0888211171417967
311	KC_G1	CG4424	0.120111951199904	0.862995550981016	0.998043617620652	pseudobulk	DESeq2	LRT	0.0639914432064301
311	KC_G1	CG44242	0.0678333301836899	0.644502448665313	0.998043617620652	pseudobulk	DESeq2	LRT	0.190775428287594
311	KC_G1	CG44243	0.563951173233114	0.390041710359724	0.998043617620652	pseudobulk	DESeq2	LRT	0.408888947818471
311	KC_G1	CG44245	-1.18668897631773	0.278159294865034	0.998043617620652	pseudobulk	DESeq2	LRT	0.555706423261715
311	KC_G1	CG44247	-0.0379449394367361	0.82294480037799	0.998043617620652	pseudobulk	DESeq2	LRT	0.084629294429557
311	KC_G1	CG44249	-0.58692344307014	0.666686540497319	0.998043617620652	pseudobulk	DESeq2	LRT	0.176078312606172
311	KC_G1	CG44251	-0.794610277746993	0.821479263487599	0.998043617620652	pseudobulk	DESeq2	LRT	0.085403394939296
312	KC_G1	CG44257	2.66264011801741	0.281871598697827	0.998043617620652	pseudobulk	DESeq2	LRT	0.549948681312517
312	KC_G1	CG44261	1.12508065046033	0.900770125530981	0.998043617620652	pseudobulk	DESeq2	LRT	0.0453860258366365
312	KC_G1	CG44296	-1.24029575767303	0.590792835712426	0.998043617620652	pseudobulk	DESeq2	LRT	0.22856477982831
312	KC_G1	CG44314	-0.255633568439681	0.65706072356198	0.998043617620652	pseudobulk	DESeq2	LRT	0.182394492404837
312	KC_G1	CG44325	0.852262685763223	0.314659879169999	0.998043617620652	pseudobulk	DESeq2	LRT	0.502158628443229
312	KC_G1	CG4438	-0.375983087817734	0.690853263744361	0.998043617620652	pseudobulk	DESeq2	LRT	0.160614186364406
312	KC_G1	CG44385	0.0781071480641268	0.970967397587672	0.998043617620652	pseudobulk	DESeq2	LRT	0.0127953522603565
312	KC_G1	CG44405	-0.794610277746993	0.879485534599449	0.998043617620652	pseudobulk	DESeq2	LRT	0.0557712992369932
312	KC_G1	CG44422	0.02573136444333	0.959658007841506	0.998043617620652	pseudobulk	DESeq2	LRT	0.0178835083865114
312	KC_G1	CG4452	0.015052051409144	0.961260192513458	0.998043617620652	pseudobulk	DESeq2	LRT	0.0171590422189823
313	KC_G1	CG44521	-0.794610277746993	0.821479263487599	0.998043617620652	pseudobulk	DESeq2	LRT	0.085403394939296
313	KC_G1	CG4455	-0.668593574498123	0.744279897808527	0.998043617620652	pseudobulk	DESeq2	LRT	0.128263710671876
313	KC_G1	CG4459	1.12508065046033	0.900770125530981	0.998043617620652	pseudobulk	DESeq2	LRT	0.0453860258366365
313	KC_G1	CG4462	0.44637100861824	0.791550737207803	0.998043617620652	pseudobulk	DESeq2	LRT	0.101521242288474
313	KC_G1	CG4467	-0.0881561807764028	0.472999329451061	0.998043617620652	pseudobulk	DESeq2	LRT	0.325139474940646

313	KC_G1	CG44774	0.0913518226341063	0.509642339137172	0.998043617620652	pseudobulk	DESeq2	LRT	0.2927344996487
313	KC_G1	CG44815	-1.66808346769027	0.498191247612582	0.998043617620652	pseudobulk	DESeq2	LRT	0.302603906560549
313	KC_G1	CG44836	2.74764263117927	0.228409304049609	0.998043617620652	pseudobulk	DESeq2	LRT	0.64128620946688
313	KC_G1	CG44837	0.360179181001756	0.179674228676826	0.998043617620652	pseudobulk	DESeq2	LRT	0.745514210849214
313	KC_G1	CG44838	-0.2290962301462	0.210921479515891	0.998043617620652	pseudobulk	DESeq2	LRT	0.675879190956476
314	KC_G1	CG4496	1.34336351695162	0.516798666181999	0.998043617620652	pseudobulk	DESeq2	LRT	0.28667861589251
314	KC_G1	CG4497	-0.0393658438885733	0.901622140068345	0.998043617620652	pseudobulk	DESeq2	LRT	0.0449754323751549
314	KC_G1	CG45002	0.81081846470541	0.0306147626814612	0.998043617620652	pseudobulk	DESeq2	LRT	1.51406910276528
314	KC_G1	CG4502	-0.0044743812557943	0.97485610095231	0.998043617620652	pseudobulk	DESeq2	LRT	0.0110594860189653
314	KC_G1	CG45049	0.0389642163064813	0.755980602250876	0.998043617620652	pseudobulk	DESeq2	LRT	0.121489347942557
314	KC_G1	CG45050	-0.0981817091762479	0.374756720577132	0.998043617620652	pseudobulk	DESeq2	LRT	0.426250570131737
314	KC_G1	CG45067	-1.76538389030833	0.441822179843785	0.998043617620652	pseudobulk	DESeq2	LRT	0.354752485970289
314	KC_G1	CG45071	0.239386888733325	0.842256689775341	0.998043617620652	pseudobulk	DESeq2	LRT	0.07455553087106
314	KC_G1	CG45081	1.27876475702852	0.505861275979828	0.998043617620652	pseudobulk	DESeq2	LRT	0.295968564853069
314	KC_G1	CG4511	-0.661410359446452	0.0450330717023615	0.998043617620652	pseudobulk	DESeq2	LRT	1.34646842883347
315	KC_G1	CG45218	0.331218660074237	0.729006869288307	0.998043617620652	pseudobulk	DESeq2	LRT	0.137268379391284
315	KC_G1	CG45263	-0.102014793929598	0.478248898877516	0.998043617620652	pseudobulk	DESeq2	LRT	0.320346021221145
315	KC_G1	CG4537	-0.556010891458667	0.384793848393055	0.998043617620652	pseudobulk	DESeq2	LRT	0.414771879540426
315	KC_G1	CG4538	0.0112716021931116	0.968949248735795	0.998043617620652	pseudobulk	DESeq2	LRT	0.0136989696687509
315	KC_G1	CG4552	0.182760602666535	0.640338562805274	0.998043617620652	pseudobulk	DESeq2	LRT	0.193590343077854
315	KC_G1	CG4553	0.257662269961201	0.729371911561633	0.998043617620652	pseudobulk	DESeq2	LRT	0.137050965574846
315	KC_G1	CG4554	-0.116548425541477	0.90802961112551	0.998043617620652	pseudobulk	DESeq2	LRT	0.0418999887710667
315	KC_G1	CG4557	0.536128414794089	0.276147394578626	0.998043617620652	pseudobulk	DESeq2	LRT	0.558859049943341
315	KC_G1	CG4558	-0.0230534047207256	0.957456558564663	0.998043617620652	pseudobulk	DESeq2	LRT	0.0188809215929126
315	KC_G1	CG45603	1.13582588427274	0.633424328955532	0.998043617620652	pseudobulk	DESeq2	LRT	0.198305259991224
316	KC_G1	CG4562	-0.230820980136182	0.35699556649118	0.998043617620652	pseudobulk	DESeq2	LRT	0.447337177334227
316	KC_G1	CG4565	-0.531923886496257	0.551883175547499	0.998043617620652	pseudobulk	DESeq2	LRT	0.258152845431486
316	KC_G1	CG45676	0.943988612140852	0.569979145552308	0.998043617620652	pseudobulk	DESeq2	LRT	0.244141034041969
316	KC_G1	CG4570	1.12508065046033	0.900770125530981	0.998043617620652	pseudobulk	DESeq2	LRT	0.0453860258366365
316	KC_G1	CG4572	0.740950073180404	0.407467359518893	0.998043617620652	pseudobulk	DESeq2	LRT	0.389907175018701
316	KC_G1	CG4576	0.172834960772825	0.908610676445368	0.998043617620652	pseudobulk	DESeq2	LRT	0.0416221644022337
316	KC_G1	CG4577	0.370805701955401	0.365027921008097	0.998043617620652	pseudobulk	DESeq2	LRT	0.437673915061397
316	KC_G1	CG4582	-0.794610277746993	0.821479263487599	0.998043617620652	pseudobulk	DESeq2	LRT	0.085403394939296
316	KC_G1	CG4587	-0.143230681601049	0.273228378644556	0.998043617620652	pseudobulk	DESeq2	LRT	0.563474195002711
316	KC_G1	CG4592	0.173116475207952	0.97305562682953	0.998043617620652	pseudobulk	DESeq2	LRT	0.011862331638572
317	KC_G1	CG4593	-0.107492012254022	0.793898521401034	0.998043617620652	pseudobulk	DESeq2	LRT	0.100235006908076
317	KC_G1	CG4594	-0.794610277746993	0.821479263487599	0.998043617620652	pseudobulk	DESeq2	LRT	0.085403394939296
317	KC_G1	CG4596	-0.307006969061589	0.181398176250942	0.998043617620652	pseudobulk	DESeq2	LRT	0.741367083583166
317	KC_G1	CG4598	0.627100163349555	0.256139265285325	0.998043617620652	pseudobulk	DESeq2	LRT	0.591523840549064
317	KC_G1	CG4603	0.137508595598915	0.672196259131782	0.998043617620652	pseudobulk	DESeq2	LRT	0.172503908770655
317	KC_G1	CG4607	-0.765625368254185	0.836625948066609	0.998043617620652	pseudobulk	DESeq2	LRT	0.0774686698445758
317	KC_G1	CG4610	0.281940999837055	0.684893147079439	0.998043617620652	pseudobulk	DESeq2	LRT	0.164377179242685
317	KC_G1	CG4611	0.110351806758538	0.915643581496877	0.998043617620652	pseudobulk	DESeq2	LRT	0.0382735445763888
317	KC_G1	CG4612	-0.14513011222345	0.220869416459564	0.998043617620652	pseudobulk	DESeq2	LRT	0.655864416235383
317	KC_G1	CG4615	0.126332239757962	0.671263677956286	0.998043617620652	pseudobulk	DESeq2	LRT	0.173106851831803
318	KC_G1	CG4617	0.127069032338805	0.767361793237191	0.998043617620652	pseudobulk	DESeq2	LRT	0.114999828019294
318	KC_G1	CG4619	0.0829117206148246	0.900939846150167	0.998043617620652	pseudobulk	DESeq2	LRT	0.0453042049778365
318	KC_G1	CG4622	-0.258723072326654	0.501939962146999	0.998043617620652	pseudobulk	DESeq2	LRT	0.299348226415906
318	KC_G1	CG4627	0.162916202721723	0.701710608967581	0.998043617620652	pseudobulk	DESeq2	LRT	0.153841957444037
318	KC_G1	CG46280	0.0168587621520852	0.916800921004988	0.998043617620652	pseudobulk	DESeq2	LRT	0.0377249590904906
318	KC_G1	CG46281	-0.474194224819563	0.602037383392337	0.998043617620652	pseudobulk	DESeq2	LRT	0.220376540474851
318	KC_G1	CG4629	0.221046557636427	0.573414983456518	0.998043617620652	pseudobulk	DESeq2	LRT	0.241530963033296
318	KC_G1	CG46298	-0.794610277746993	0.815039924133852	0.998043617620652	pseudobulk	DESeq2	LRT	0.0888211171417967
318	KC_G1	CG4630	1.23455377930426	0.493278243547076	0.998043617620652	pseudobulk	DESeq2	LRT	0.306908039039824
318	KC_G1	CG46301	0.883746099591406	0.129781652122374	0.998043617620652	pseudobulk	DESeq2	LRT	0.88678670156682
319	KC_G1	CG46304	-0.966816426564127	0.626346344839242	0.998043617620652	pseudobulk	DESeq2	LRT	0.20318545263959

319	KC_G1	CG4631	-0.914089776174506	0.236061784739592	0.998043617620652	pseudobulk	DESeq2	LRT	0.626974313726176
319	KC_G1	CG46338	-0.112069477794465	0.679259510152629	0.998043617620652	pseudobulk	DESeq2	LRT	0.167964272412617
319	KC_G1	CG46339	-0.343352775070228	0.073617925545991	0.998043617620652	pseudobulk	DESeq2	LRT	1.13301642455062
319	KC_G1	CG46385	-0.0908673298144605	0.435463641453066	0.998043617620652	pseudobulk	DESeq2	LRT	0.361048100071891
319	KC_G1	CG46387	3.6320083272158	0.0626231724094982	0.998043617620652	pseudobulk	DESeq2	LRT	1.20326493536105
319	KC_G1	CG46399	-0.794610277746993	0.879485534599449	0.998043617620652	pseudobulk	DESeq2	LRT	0.0557712992369932
319	KC_G1	CG46448	1.01340596649651	0.277040641155813	0.998043617620652	pseudobulk	DESeq2	LRT	0.557456516368682
319	KC_G1	CG4645	0.113579472731992	0.736787721119813	0.998043617620652	pseudobulk	DESeq2	LRT	0.132657620452826
319	KC_G1	CG4646	0.20444970830599	0.493774893552385	0.998043617620652	pseudobulk	DESeq2	LRT	0.306470995953006
320	KC_G1	CG4653	1.12508065046033	0.900770125530981	0.998043617620652	pseudobulk	DESeq2	LRT	0.0453860258366365
320	KC_G1	CG4658	0.0471828219284217	0.833031981883748	0.998043617620652	pseudobulk	DESeq2	LRT	0.0793383247768805
320	KC_G1	CG4660	0.0496432576391387	0.767901671180691	0.998043617620652	pseudobulk	DESeq2	LRT	0.114694387257024
320	KC_G1	CG4661	1.38860756728047	0.313526322602472	0.998043617620652	pseudobulk	DESeq2	LRT	0.503725991416666
320	KC_G1	CG4666	1.12508065046033	0.900770125530981	0.998043617620652	pseudobulk	DESeq2	LRT	0.0453860258366365
320	KC_G1	CG4669	1.13582588427274	0.704684208645453	0.998043617620652	pseudobulk	DESeq2	LRT	0.152005460551806
320	KC_G1	CG4676	0.683323831615833	0.146667805103786	0.998043617620652	pseudobulk	DESeq2	LRT	0.833665207221837
320	KC_G1	CG4678	-0.0308061535561445	0.967159401922863	0.998043617620652	pseudobulk	DESeq2	LRT	0.0145019419766135
320	KC_G1	CG4679	0.200930431247121	0.6454632669247	0.998043617620652	pseudobulk	DESeq2	LRT	0.190128468232319
320	KC_G1	CG4686	0.0121146287226921	0.937854304733203	0.998043617620652	pseudobulk	DESeq2	LRT	0.0278646238437501
321	KC_G1	CG4707	0.30484720358486	0.654332334898255	0.998043617620652	pseudobulk	DESeq2	LRT	0.184201617802044
321	KC_G1	CG4709	0.128312026094832	0.8528910344357	0.998043617620652	pseudobulk	DESeq2	LRT	0.0691064508561258
321	KC_G1	CG4730	1.13975808199278	0.337648815594404	0.998043617620652	pseudobulk	DESeq2	LRT	0.471534769360701
321	KC_G1	CG4743	-0.0387808904162489	0.951793797955809	0.998043617620652	pseudobulk	DESeq2	LRT	0.0214571294619768
321	KC_G1	CG4751	-0.196317547759695	0.654674409365665	0.998043617620652	pseudobulk	DESeq2	LRT	0.183974634927237
321	KC_G1	CG4752	-1.31481767438999	0.589948159507212	0.998043617620652	pseudobulk	DESeq2	LRT	0.229186149424154
321	KC_G1	CG4757	-0.794610277746993	0.879485534599449	0.998043617620652	pseudobulk	DESeq2	LRT	0.0557712992369932
321	KC_G1	CG4763	-0.794610277746993	0.821479263487599	0.998043617620652	pseudobulk	DESeq2	LRT	0.085403394939296
321	KC_G1	CG4768	0.0490753389173889	0.809618196913654	0.998043617620652	pseudobulk	DESeq2	LRT	0.0917197392266387
321	KC_G1	CG4788	-0.027189828052008	0.949794035865759	0.998043617620652	pseudobulk	DESeq2	LRT	0.022370561853098
322	KC_G1	CG4789	0.103798027446643	0.644523079062614	0.998043617620652	pseudobulk	DESeq2	LRT	0.190761526828678
322	KC_G1	CG4797	3.31321079271423	0.0160480592452666	0.998043617620652	pseudobulk	DESeq2	LRT	1.79457748101827
322	KC_G1	CG4806	-0.260994094006632	0.43037864342184	0.998043617620652	pseudobulk	DESeq2	LRT	0.366149287699869
322	KC_G1	CG4813	-0.194851465295695	0.764451680164387	0.998043617620652	pseudobulk	DESeq2	LRT	0.116649960482106
322	KC_G1	CG4820	0.0470749389521011	0.940259619317116	0.998043617620652	pseudobulk	DESeq2	LRT	0.0267522148372584
322	KC_G1	CG4825	0.184873838528927	0.640482068907465	0.998043617620652	pseudobulk	DESeq2	LRT	0.193493024363557
322	KC_G1	CG4829	0.497368886402354	0.262504974511092	0.998043617620652	pseudobulk	DESeq2	LRT	0.580862462230416
322	KC_G1	CG4830	1.90250740646012	0.460774913678782	0.998043617620652	pseudobulk	DESeq2	LRT	0.336511173568024
322	KC_G1	CG4849	-0.187970424256721	0.706541563099859	0.998043617620652	pseudobulk	DESeq2	LRT	0.150862285203979
322	KC_G1	CG4853	0.131697773387761	0.663509465252293	0.998043617620652	pseudobulk	DESeq2	LRT	0.178152877354904
323	KC_G1	CG4854	0.546512047585176	0.554032208022305	0.998043617620652	pseudobulk	DESeq2	LRT	0.256464987329745
323	KC_G1	CG4858	0.17240616017707	0.683091565651375	0.998043617620652	pseudobulk	DESeq2	LRT	0.165521077004062
323	KC_G1	CG4866	0.322844464802347	0.496423139695197	0.998043617620652	pseudobulk	DESeq2	LRT	0.304147982999825
323	KC_G1	CG4880	0.991912089885725	0.425121880874032	0.998043617620652	pseudobulk	DESeq2	LRT	0.371486541473019
323	KC_G1	CG4882	-0.176264345907501	0.661059292559829	0.998043617620652	pseudobulk	DESeq2	LRT	0.179759585481535
323	KC_G1	CG4887	-0.0731228705484543	0.79529672655748	0.998043617620652	pseudobulk	DESeq2	LRT	0.0994708051003048
323	KC_G1	CG4896	0.00978828773116509	0.966827021023734	0.998043617620652	pseudobulk	DESeq2	LRT	0.0146512203687368
323	KC_G1	CG4901	0.253830437337808	0.71710752906121	0.998043617620652	pseudobulk	DESeq2	LRT	0.144415717726183
323	KC_G1	CG4908	0.4642375218505	0.211706194345178	0.998043617620652	pseudobulk	DESeq2	LRT	0.674266434703435
323	KC_G1	CG4911	-0.384681902387523	0.0824401823869306	0.998043617620652	pseudobulk	DESeq2	LRT	1.08386105607829
324	KC_G1	CG4935	0.423384301708948	0.497399503398971	0.998043617620652	pseudobulk	DESeq2	LRT	0.303294652663176
324	KC_G1	CG4936	0.151297098192609	0.723604300452864	0.998043617620652	pseudobulk	DESeq2	LRT	0.140498860748931
324	KC_G1	CG4942	0.178441419194515	0.807858883055982	0.998043617620652	pseudobulk	DESeq2	LRT	0.0926644952433974
324	KC_G1	CG4949	-0.427066833574409	0.608923590606081	0.998043617620652	pseudobulk	DESeq2	LRT	0.21543720040502
324	KC_G1	CG4951	-0.191241147523664	0.724609140175537	0.998043617620652	pseudobulk	DESeq2	LRT	0.139896192102802
324	KC_G1	CG4953	0.641066196527438	0.223616284117579	0.998043617620652	pseudobulk	DESeq2	LRT	0.650496573572288
324	KC_G1	CG4957	0.125442486168445	0.726879603793707	0.998043617620652	pseudobulk	DESeq2	LRT	0.138537517253879

324	KC_G1	CG4968	0.0845123116126314	0.741303935061313	0.998043617620652	pseudobulk	DESeq2	LRT	0.130003694508127
324	KC_G1	CG4972	-0.445861262347555	0.392362790468943	0.998043617620652	pseudobulk	DESeq2	LRT	0.406312185441544
324	KC_G1	CG4975	-0.200549242790737	0.911091335680222	0.998043617620652	pseudobulk	DESeq2	LRT	0.0404380834072498
325	KC_G1	CG4982	1.12508065046033	0.900770125530981	0.998043617620652	pseudobulk	DESeq2	LRT	0.0453860258366365
325	KC_G1	CG4991	-2.73975578120602	0.247094641820741	0.998043617620652	pseudobulk	DESeq2	LRT	0.60713667205205
325	KC_G1	CG5001	1.13582588427274	0.633424328955532	0.998043617620652	pseudobulk	DESeq2	LRT	0.198305259991224
325	KC_G1	CG5003	0.136320730775354	0.739746290630388	0.998043617620652	pseudobulk	DESeq2	LRT	0.13091720387979
325	KC_G1	CG5004	-0.0920147457689124	0.776240597846367	0.998043617620652	pseudobulk	DESeq2	LRT	0.110003647141509
325	KC_G1	CG5009	-0.0795516232213418	0.85999392311215	0.998043617620652	pseudobulk	DESeq2	LRT	0.065504617556647
325	KC_G1	CG5013	-0.185944332225551	0.723439478382604	0.998043617620652	pseudobulk	DESeq2	LRT	0.140597795303595
325	KC_G1	CG5021	0.539390947551569	0.0460898096146289	0.998043617620652	pseudobulk	DESeq2	LRT	1.33639508583267
325	KC_G1	CG5022	0.54360780451459	0.0806835705781939	0.998043617620652	pseudobulk	DESeq2	LRT	1.09321489072583
325	KC_G1	CG5023	1.12508065046033	0.900770125530981	0.998043617620652	pseudobulk	DESeq2	LRT	0.0453860258366365
326	KC_G1	CG5026	-0.794610277746993	0.821479263487599	0.998043617620652	pseudobulk	DESeq2	LRT	0.085403394939296
326	KC_G1	CG5027	0.368872058322033	0.222543751027264	0.998043617620652	pseudobulk	DESeq2	LRT	0.65258459609042
326	KC_G1	CG5028	0.0175718617543971	0.914355981673947	0.998043617620652	pseudobulk	DESeq2	LRT	0.0388846896296787
326	KC_G1	CG5033	-0.676920688445759	0.349000419399389	0.998043617620652	pseudobulk	DESeq2	LRT	0.457174051141878
326	KC_G1	CG5037	-0.110522202459313	0.700812153244652	0.998043617620652	pseudobulk	DESeq2	LRT	0.154398375388315
326	KC_G1	CG5038	0.541549108567689	0.72344854394377	0.998043617620652	pseudobulk	DESeq2	LRT	0.140592353108858
326	KC_G1	CG5039	0.794900551882195	0.586979422589235	0.998043617620652	pseudobulk	DESeq2	LRT	0.231377123305348
326	KC_G1	CG5044	0.116161995049661	0.778957796860748	0.998043617620652	pseudobulk	DESeq2	LRT	0.1084860713223
326	KC_G1	CG5048	-0.807058049232644	0.687348524994713	0.998043617620652	pseudobulk	DESeq2	LRT	0.162822994962566
326	KC_G1	CG5050	-0.158044750338727	0.9319696184822	0.998043617620652	pseudobulk	DESeq2	LRT	0.0305982450930102
327	KC_G1	CG5056	-0.286815118407347	0.394027262032526	0.998043617620652	pseudobulk	DESeq2	LRT	0.404473729086364
327	KC_G1	CG5059	-0.163494555315168	0.543822500607396	0.998043617620652	pseudobulk	DESeq2	LRT	0.264542827481374
327	KC_G1	CG5062	-1.72343437992478	0.512120560562093	0.998043617620652	pseudobulk	DESeq2	LRT	0.290627787806665
327	KC_G1	CG5065	0.135866032537892	0.275021454220736	0.998043617620652	pseudobulk	DESeq2	LRT	0.560633425856116
327	KC_G1	CG5068	0.1563476351191	0.626200698777987	0.998043617620652	pseudobulk	DESeq2	LRT	0.203286452089186
327	KC_G1	CG5071	-0.377805012356367	0.560639998212014	0.998043617620652	pseudobulk	DESeq2	LRT	0.251315921233079
327	KC_G1	CG5078	-0.711364822669339	0.836431126230485	0.998043617620652	pseudobulk	DESeq2	LRT	0.0775698140913142
327	KC_G1	CG5079	0.193710721595243	0.698831577061225	0.998043617620652	pseudobulk	DESeq2	LRT	0.155627479428075
327	KC_G1	CG5080	-0.757449795571951	0.820028655901106	0.998043617620652	pseudobulk	DESeq2	LRT	0.0861709709305825
327	KC_G1	CG5087	0.0760615949506156	0.85704266212645	0.998043617620652	pseudobulk	DESeq2	LRT	0.0669975590978621
328	KC_G1	CG5096	1.13582588427274	0.704684208645453	0.998043617620652	pseudobulk	DESeq2	LRT	0.152005460551806
328	KC_G1	CG5098	-0.129980823452417	0.635790035959577	0.998043617620652	pseudobulk	DESeq2	LRT	0.196686282589352
328	KC_G1	CG5104	3.08479732729302	0.0808662036819806	0.998043617620652	pseudobulk	DESeq2	LRT	1.09223294465576
328	KC_G1	CG5110	0.0567599831617313	0.840104577544353	0.998043617620652	pseudobulk	DESeq2	LRT	0.0756666489101326
328	KC_G1	CG5112	0.463811939104671	0.711496745484859	0.998043617620652	pseudobulk	DESeq2	LRT	0.147827082116871
328	KC_G1	CG5114	0.380373447152195	0.590263158656563	0.998043617620652	pseudobulk	DESeq2	LRT	0.228954322473318
328	KC_G1	CG5116	0.764815221789058	0.808325187140535	0.998043617620652	pseudobulk	DESeq2	LRT	0.0924138885208533
328	KC_G1	CG5126	-0.0395787557864271	0.953002641730215	0.998043617620652	pseudobulk	DESeq2	LRT	0.0209058954925602
328	KC_G1	CG5151	-0.138447848151963	0.48932746526642	0.998043617620652	pseudobulk	DESeq2	LRT	0.310400407213917
328	KC_G1	CG5157	1.1112567833086	0.689254921431598	0.998043617620652	pseudobulk	DESeq2	LRT	0.161620124253084
329	KC_G1	CG5160	0.0120186598493425	0.950969127484125	0.998043617620652	pseudobulk	DESeq2	LRT	0.0218335818858469
329	KC_G1	CG5174	0.0873012463467339	0.618830437189797	0.998043617620652	pseudobulk	DESeq2	LRT	0.208428333662898
329	KC_G1	CG5177	1.90250740646012	0.460774913678782	0.998043617620652	pseudobulk	DESeq2	LRT	0.336511173568024
329	KC_G1	CG5180	-0.467350429006749	0.27938666952018	0.998043617620652	pseudobulk	DESeq2	LRT	0.553794319381248
329	KC_G1	CG5181	0.134354377134936	0.710883894835097	0.998043617620652	pseudobulk	DESeq2	LRT	0.148201324656945
329	KC_G1	CG5188	0.207551726321322	0.696717437221557	0.998043617620652	pseudobulk	DESeq2	LRT	0.156943319945788
329	KC_G1	CG5191	0.0161534038495094	0.970770379212415	0.998043617620652	pseudobulk	DESeq2	LRT	0.012883483618281
329	KC_G1	CG5196	0.388136245180033	0.168495975930521	0.998043617620652	pseudobulk	DESeq2	LRT	0.773410466614827
329	KC_G1	CG5199	0.269981784754178	0.668182615815247	0.998043617620652	pseudobulk	DESeq2	LRT	0.175104827640902
329	KC_G1	CG5214	0.145726357372085	0.420008933723207	0.998043617620652	pseudobulk	DESeq2	LRT	0.376741471922508
330	KC_G1	CG5217	6.16890075404149	0.149281551612153	0.998043617620652	pseudobulk	DESeq2	LRT	0.825993859576313
330	KC_G1	CG5220	0.180577781889687	0.675869467470499	0.998043617620652	pseudobulk	DESeq2	LRT	0.170137172447749
330	KC_G1	CG5245	2.04147257822512	0.408147672536479	0.998043617620652	pseudobulk	DESeq2	LRT	0.389182675723292

330	KC_G1	CG5254	0.475005891246782	0.602328584864405	0.998043617620652	pseudobulk	DESeq2	LRT	0.220166525914096
330	KC_G1	CG5262	0.179115551653596	0.790992163666121	0.998043617620652	pseudobulk	DESeq2	LRT	0.10182781902245
330	KC_G1	CG5267	0.172834960772825	0.908610676445368	0.998043617620652	pseudobulk	DESeq2	LRT	0.0416221644022337
330	KC_G1	CG5270	0.397359301071904	0.632114005234223	0.998043617620652	pseudobulk	DESeq2	LRT	0.199204587257178
330	KC_G1	CG5273	0.0321552900166579	0.906490073217562	0.998043617620652	pseudobulk	DESeq2	LRT	0.0426369474101062
330	KC_G1	CG5274	0.093668567984271	0.843741999646518	0.998043617620652	pseudobulk	DESeq2	LRT	0.0737903321174733
330	KC_G1	CG5276	0.616294513775482	0.338444504194287	0.998043617620652	pseudobulk	DESeq2	LRT	0.470512533767856
331	KC_G1	CG5280	0.173116475207952	0.97305562682953	0.998043617620652	pseudobulk	DESeq2	LRT	0.011862331638572
331	KC_G1	CG5281	0.129220347244691	0.531514181505491	0.998043617620652	pseudobulk	DESeq2	LRT	0.274485143431532
331	KC_G1	CG5282	0.197245480951631	0.336232091203897	0.998043617620652	pseudobulk	DESeq2	LRT	0.473360838286052
331	KC_G1	CG5285	0.422720983747518	0.352601691296337	0.998043617620652	pseudobulk	DESeq2	LRT	0.452715608886088
331	KC_G1	CG5287	-0.304831937852259	0.664017733067527	0.998043617620652	pseudobulk	DESeq2	LRT	0.177820322332979
331	KC_G1	CG5290	0.715551292905367	0.624380244503107	0.998043617620652	pseudobulk	DESeq2	LRT	0.204550846543346
331	KC_G1	CG5292	0.169671016345333	0.627319659188736	0.998043617620652	pseudobulk	DESeq2	LRT	0.202511102159137
331	KC_G1	CG5316	-0.419055063701901	0.165326726785346	0.998043617620652	pseudobulk	DESeq2	LRT	0.781656932531856
331	KC_G1	CG5321	-1.73288829970739	0.458469392065673	0.998043617620652	pseudobulk	DESeq2	LRT	0.338689653016193
331	KC_G1	CG5323	0.182849380785195	0.589580011351754	0.998043617620652	pseudobulk	DESeq2	LRT	0.229457248871509
332	KC_G1	CG5335	-0.88271904440604	0.661655916427707	0.998043617620652	pseudobulk	DESeq2	LRT	0.1793677997526
332	KC_G1	CG5339	-0.190893897357825	0.618979001253155	0.998043617620652	pseudobulk	DESeq2	LRT	0.208324084089044
332	KC_G1	CG5346	1.13582588427274	0.704684208645453	0.998043617620652	pseudobulk	DESeq2	LRT	0.152005460551806
332	KC_G1	CG5348	-0.62780860931913	0.528232514675844	0.998043617620652	pseudobulk	DESeq2	LRT	0.277174869865762
332	KC_G1	CG5355	0.140061050463861	0.679824355951929	0.998043617620652	pseudobulk	DESeq2	LRT	0.167603280079467
332	KC_G1	CG5359	-0.0418268451180233	0.925390243245424	0.998043617620652	pseudobulk	DESeq2	LRT	0.0336750837501173
332	KC_G1	CG5377	-2.73435025898205	0.132292010155107	0.998043617620652	pseudobulk	DESeq2	LRT	0.878466384462013
332	KC_G1	CG5380	0.345758867995752	0.541551061590503	0.998043617620652	pseudobulk	DESeq2	LRT	0.266360588491091
332	KC_G1	CG5381	2.18876425250967	0.402105238436886	0.998043617620652	pseudobulk	DESeq2	LRT	0.395660269077107
332	KC_G1	CG5382	0.443814589680878	0.243676866809464	0.998043617620652	pseudobulk	DESeq2	LRT	0.613185698118787
333	KC_G1	CG5390	-0.172284177355598	0.886511243634999	0.998043617620652	pseudobulk	DESeq2	LRT	0.0523157518649014
333	KC_G1	CG5404	0.43314353030777	0.489646431720087	0.998043617620652	pseudobulk	DESeq2	LRT	0.31011740605103
333	KC_G1	CG5412	0.245693435031723	0.50283923077388	0.998043617620652	pseudobulk	DESeq2	LRT	0.298570846650417
333	KC_G1	CG5435	0.177622136115678	0.935661853927379	0.998043617620652	pseudobulk	DESeq2	LRT	0.0288810759485907
333	KC_G1	CG5439	0.415093539340842	0.331595450477948	0.998043617620652	pseudobulk	DESeq2	LRT	0.479391436643708
333	KC_G1	CG5445	-0.125806531165819	0.660594270665454	0.998043617620652	pseudobulk	DESeq2	LRT	0.180065197264599
333	KC_G1	CG5446	0.0255781520062982	0.918975217769089	0.998043617620652	pseudobulk	DESeq2	LRT	0.0366962001821736
333	KC_G1	CG5447	0.048443780895254	0.833041781373271	0.998043617620652	pseudobulk	DESeq2	LRT	0.0793332159223762
333	KC_G1	CG5455	0.438870785804952	0.258797270985893	0.998043617620652	pseudobulk	DESeq2	LRT	0.587040307609275
333	KC_G1	CG5458	0.173116475207952	0.97305562682953	0.998043617620652	pseudobulk	DESeq2	LRT	0.011862331638572
334	KC_G1	CG5466	-0.159227946683481	0.452121982792176	0.998043617620652	pseudobulk	DESeq2	LRT	0.344744376457763
334	KC_G1	CG5498	0.372295684822618	0.589757863757135	0.998043617620652	pseudobulk	DESeq2	LRT	0.229326259575793
334	KC_G1	CG5500	-0.101896545994669	0.741698651242446	0.998043617620652	pseudobulk	DESeq2	LRT	0.129772510739447
334	KC_G1	CG5504	0.131356076211546	0.668890350758737	0.998043617620652	pseudobulk	DESeq2	LRT	0.174645069023389
334	KC_G1	CG5509	2.07738706174348	0.431998797123676	0.998043617620652	pseudobulk	DESeq2	LRT	0.364517462451933
334	KC_G1	CG5510	0.0774529455065236	0.831777250330481	0.998043617620652	pseudobulk	DESeq2	LRT	0.0799929620560203
334	KC_G1	CG5515	0.0309497574113356	0.902980485410906	0.998043617620652	pseudobulk	DESeq2	LRT	0.0443216352567472
334	KC_G1	CG5516	-0.0504530559862598	0.847527425974649	0.998043617620652	pseudobulk	DESeq2	LRT	0.0718462391346386
334	KC_G1	CG5521	-0.231151206888932	0.407561751215904	0.998043617620652	pseudobulk	DESeq2	LRT	0.389806580345965
334	KC_G1	CG5522	-0.120567109387921	0.653304380249406	0.998043617620652	pseudobulk	DESeq2	LRT	0.184884429959936
335	KC_G1	CG5532	0.317607787602124	0.101793119322353	0.998043617620652	pseudobulk	DESeq2	LRT	0.99228157702211
335	KC_G1	CG5535	0.157688973255609	0.742838873929206	0.998043617620652	pseudobulk	DESeq2	LRT	0.129105377022744
335	KC_G1	CG5541	-1.4435316870235	0.406502272210352	0.998043617620652	pseudobulk	DESeq2	LRT	0.390937022503715
335	KC_G1	CG5543	0.17324636993234	0.799257041283519	0.998043617620652	pseudobulk	DESeq2	LRT	0.0973135289979603
335	KC_G1	CG5555	0.159490639196388	0.587399204474704	0.998043617620652	pseudobulk	DESeq2	LRT	0.23106664598564
335	KC_G1	CG5567	-0.14004525500385	0.684920891450699	0.998043617620652	pseudobulk	DESeq2	LRT	0.164359586741799
335	KC_G1	CG5569	0.0415609635189998	0.908484967278499	0.998043617620652	pseudobulk	DESeq2	LRT	0.0416822545772019
335	KC_G1	CG5577	-0.261535813389697	0.888716661211162	0.998043617620652	pseudobulk	DESeq2	LRT	0.051236677821781
335	KC_G1	CG5589	1.42133401847614	0.139725575271612	0.998043617620652	pseudobulk	DESeq2	LRT	0.854724093651421

335	KC_G1	CG5590	1.24826614839891	0.0536934114886322	0.998043617620652	pseudobulk	DESeq2	LRT	1.27007900163148
336	KC_G1	CG5599	1.2719105042186	0.421250476028924	0.998043617620652	pseudobulk	DESeq2	LRT	0.375459595351186
336	KC_G1	CG5608	-0.73647191644449	0.268338767380426	0.998043617620652	pseudobulk	DESeq2	LRT	0.571316579496747
336	KC_G1	CG5611	-0.728118248324612	0.255290835577506	0.998043617620652	pseudobulk	DESeq2	LRT	0.592964775205761
336	KC_G1	CG5612	1.13582588427274	0.704684208645453	0.998043617620652	pseudobulk	DESeq2	LRT	0.152005460551806
336	KC_G1	CG5618	-0.438880129145979	0.590719957008185	0.998043617620652	pseudobulk	DESeq2	LRT	0.228618356597576
336	KC_G1	CG5639	1.13582588427274	0.704684208645453	0.998043617620652	pseudobulk	DESeq2	LRT	0.152005460551806
336	KC_G1	CG5641	-0.127999602218888	0.570504222027459	0.998043617620652	pseudobulk	DESeq2	LRT	0.243741137229214
336	KC_G1	CG5644	1.07455184868711	0.56139687154546	0.998043617620652	pseudobulk	DESeq2	LRT	0.250730011855354
336	KC_G1	CG5645	0.44485853062771	0.604788345988697	0.998043617620652	pseudobulk	DESeq2	LRT	0.218396586094094
336	KC_G1	CG5646	0.2477734841762	0.551435403158753	0.998043617620652	pseudobulk	DESeq2	LRT	0.258505354837221
337	KC_G1	CG5664	-1.07868038394563	0.333535579184724	0.998043617620652	pseudobulk	DESeq2	LRT	0.476857831854116
337	KC_G1	CG5674	-0.0710529867062795	0.844286336521457	0.998043617620652	pseudobulk	DESeq2	LRT	0.0735102390595414
337	KC_G1	CG5676	0.170176196966453	0.237522175343125	0.998043617620652	pseudobulk	DESeq2	LRT	0.624295837914583
337	KC_G1	CG5687	1.86075207015273	0.642542236374991	0.998043617620652	pseudobulk	DESeq2	LRT	0.192098319480391
337	KC_G1	CG5697	0.172845155749872	0.90730697492154	0.998043617620652	pseudobulk	DESeq2	LRT	0.0422457504714555
337	KC_G1	CG5707	1.13582588427274	0.633424328955532	0.998043617620652	pseudobulk	DESeq2	LRT	0.198305259991224
337	KC_G1	CG5708	-0.0666306005607734	0.703380827076348	0.998043617620652	pseudobulk	DESeq2	LRT	0.152809473961113
337	KC_G1	CG5721	0.595253072521205	0.390627657705075	0.998043617620652	pseudobulk	DESeq2	LRT	0.408237010501681
337	KC_G1	CG5726	-0.13881560868843	0.670327136912781	0.998043617620652	pseudobulk	DESeq2	LRT	0.173713198668457
337	KC_G1	CG5727	-0.0418887297784786	0.872707700141018	0.998043617620652	pseudobulk	DESeq2	LRT	0.0591311921162385
338	KC_G1	CG5728	0.889103468955063	0.15217039487365	0.998043617620652	pseudobulk	DESeq2	LRT	0.817669832411982
338	KC_G1	CG5731	1.13582588427274	0.633424328955532	0.998043617620652	pseudobulk	DESeq2	LRT	0.198305259991224
338	KC_G1	CG5742	-0.23092890253157	0.429835111274353	0.998043617620652	pseudobulk	DESeq2	LRT	0.366698111855531
338	KC_G1	CG5745	0.0391792413286733	0.941909157610742	0.998043617620652	pseudobulk	DESeq2	LRT	0.0259909807005478
338	KC_G1	CG5746	-0.114013680328164	0.44201865922663	0.998043617620652	pseudobulk	DESeq2	LRT	0.354559397103129
338	KC_G1	CG5757	1.12508065046033	0.900770125530981	0.998043617620652	pseudobulk	DESeq2	LRT	0.0453860258366365
338	KC_G1	CG5758	0.0248040078147456	0.882788854135134	0.998043617620652	pseudobulk	DESeq2	LRT	0.0541431587661202
338	KC_G1	CG5776	-0.18572774504768	0.872158344054852	0.998043617620652	pseudobulk	DESeq2	LRT	0.0594046598996235
338	KC_G1	CG5787	-0.00663585912688296	0.971269081086904	0.998043617620652	pseudobulk	DESeq2	LRT	0.0126604361659573
338	KC_G1	CG5789	0.0689606023414312	0.958769899960873	0.998043617620652	pseudobulk	DESeq2	LRT	0.0182856088541682
339	KC_G1	CG5793	0.894674592980583	0.208731721999084	0.998043617620652	pseudobulk	DESeq2	LRT	0.680411544023917
339	KC_G1	CG5800	-0.250014944492868	0.780394556760678	0.998043617620652	pseudobulk	DESeq2	LRT	0.107685768463892
339	KC_G1	CG5805	-0.0866887100377287	0.770099217375773	0.998043617620652	pseudobulk	DESeq2	LRT	0.113453317966582
339	KC_G1	CG5808	0.45205642582722	0.435903375295135	0.998043617620652	pseudobulk	DESeq2	LRT	0.360609768132174
339	KC_G1	CG5810	-1.32234376952914	0.542789226792531	0.998043617620652	pseudobulk	DESeq2	LRT	0.265368780745144
339	KC_G1	CG5815	-0.217551163726719	0.690872470577112	0.998043617620652	pseudobulk	DESeq2	LRT	0.160602112446591
339	KC_G1	CG5823	-0.247051154207687	0.220806070170776	0.998043617620652	pseudobulk	DESeq2	LRT	0.655988991606628
339	KC_G1	CG5828	-0.255971642490482	0.541713160699473	0.998043617620652	pseudobulk	DESeq2	LRT	0.266230613277982
339	KC_G1	CG5830	0.0307351618872077	0.789132413417127	0.998043617620652	pseudobulk	DESeq2	LRT	0.102850117713977
339	KC_G1	CG5835	-0.522583198971382	0.590926870753872	0.998043617620652	pseudobulk	DESeq2	LRT	0.228466261239151
340	KC_G1	CG5846	0.689221748404605	0.284641148719683	0.998043617620652	pseudobulk	DESeq2	LRT	0.545702316584444
340	KC_G1	CG5850	0.584856308547682	0.328720000772135	0.998043617620652	pseudobulk	DESeq2	LRT	0.483173870726064
340	KC_G1	CG5854	0.284677351255641	0.703089506483673	0.998043617620652	pseudobulk	DESeq2	LRT	0.152989383803012
340	KC_G1	CG5862	0.0438774170279235	0.913717463399597	0.998043617620652	pseudobulk	DESeq2	LRT	0.0391880745677064
340	KC_G1	CG5877	-0.377382101832394	0.494249433629601	0.998043617620652	pseudobulk	DESeq2	LRT	0.306053819680815
340	KC_G1	CG5880	-1.04972748902737	0.157894765723148	0.998043617620652	pseudobulk	DESeq2	LRT	0.801632266794949
340	KC_G1	CG5885	-0.0921296242010146	0.741623346344992	0.998043617620652	pseudobulk	DESeq2	LRT	0.129816607033449
340	KC_G1	CG5886	0.311647790363993	0.440348868530374	0.998043617620652	pseudobulk	DESeq2	LRT	0.356203115232304
340	KC_G1	CG5888	-0.0987998282484869	0.851568594412938	0.998043617620652	pseudobulk	DESeq2	LRT	0.0697803635848953
340	KC_G1	CG5890	0.0867102826795723	0.523745741561609	0.998043617620652	pseudobulk	DESeq2	LRT	0.280879495145101
341	KC_G1	CG5895	-0.55946729406634	0.36275787092581	0.998043617620652	pseudobulk	DESeq2	LRT	0.440383155699152
341	KC_G1	CG5897	1.79207265922759	0.449242408626613	0.998043617620652	pseudobulk	DESeq2	LRT	0.347519252937804
341	KC_G1	CG5902	0.155828666824955	0.554651104360354	0.998043617620652	pseudobulk	DESeq2	LRT	0.255980117954691
341	KC_G1	CG5903	0.0824893330718708	0.569003695669191	0.998043617620652	pseudobulk	DESeq2	LRT	0.244884912860598
341	KC_G1	CG5910	0.145756391869411	0.792351225499248	0.998043617620652	pseudobulk	DESeq2	LRT	0.101082266031626

341	KC_G1	CG5913	0.0425877034866143	0.928976057080832	0.998043617620652	pseudobulk	DESeq2	LRT	0.0319954791297964
341	KC_G1	CG5916	-0.165943781416816	0.685521447959411	0.998043617620652	pseudobulk	DESeq2	LRT	0.163978952858855
341	KC_G1	CG5934	0.116167030728276	0.546436985171659	0.998043617620652	pseudobulk	DESeq2	LRT	0.26245991344006
341	KC_G1	CG5937	-0.0839749748215015	0.741452314405784	0.998043617620652	pseudobulk	DESeq2	LRT	0.129916774853233
341	KC_G1	CG5938	0.126608693845338	0.559149092516026	0.998043617620652	pseudobulk	DESeq2	LRT	0.252472375613219
342	KC_G1	CG5946	0.0533497805688402	0.782186966541586	0.998043617620652	pseudobulk	DESeq2	LRT	0.106689424904251
342	KC_G1	CG5953	0.630147809623973	0.720764674673621	0.998043617620652	pseudobulk	DESeq2	LRT	0.142206506671425
342	KC_G1	CG5955	0.680052858717164	0.653669420671648	0.998043617620652	pseudobulk	DESeq2	LRT	0.184641831301781
342	KC_G1	CG5961	0.372974932011766	0.310860875428401	0.998043617620652	pseudobulk	DESeq2	LRT	0.507433934292536
342	KC_G1	CG5969	0.301253655189572	0.314528293294226	0.998043617620652	pseudobulk	DESeq2	LRT	0.502340281637231
342	KC_G1	CG5973	1.31239830911385	0.17191624624886	0.998043617620652	pseudobulk	DESeq2	LRT	0.764683080131809
342	KC_G1	CG5986	0.124576851305384	0.616661431133797	0.998043617620652	pseudobulk	DESeq2	LRT	0.209953213515601
342	KC_G1	CG5989	-0.0955654530952821	0.802625161821765	0.998043617620652	pseudobulk	DESeq2	LRT	0.0954872295148912
342	KC_G1	CG6005	0.00327191923230387	0.970052724563497	0.998043617620652	pseudobulk	DESeq2	LRT	0.0132046602031394
342	KC_G1	CG6006	0.0966444447758755	0.937272283594084	0.998043617620652	pseudobulk	DESeq2	LRT	0.0281342254575942
343	KC_G1	CG6013	-0.208556695093598	0.440225500220885	0.998043617620652	pseudobulk	DESeq2	LRT	0.356324804389528
343	KC_G1	CG6023	2.04147257822512	0.408147672536479	0.998043617620652	pseudobulk	DESeq2	LRT	0.389182675723292
343	KC_G1	CG6024	-0.00661594845150717	0.962970793349224	0.998043617620652	pseudobulk	DESeq2	LRT	0.0163868847130778
343	KC_G1	CG6028	0.0249451844327351	0.949134403261615	0.998043617620652	pseudobulk	DESeq2	LRT	0.0226722844516783
343	KC_G1	CG6036	-0.794610277746993	0.879485534599449	0.998043617620652	pseudobulk	DESeq2	LRT	0.0557712992369932
343	KC_G1	CG6040	0.373938772800577	0.58969466494613	0.998043617620652	pseudobulk	DESeq2	LRT	0.229372801330109
343	KC_G1	CG6041	-0.273597618291752	0.575702149473566	0.998043617620652	pseudobulk	DESeq2	LRT	0.239802149035806
343	KC_G1	CG6044	0.256932230133816	0.0749135350937518	0.998043617620652	pseudobulk	DESeq2	LRT	1.12543970852924
343	KC_G1	CG6051	-0.279015174440478	0.278611798510857	0.998043617620652	pseudobulk	DESeq2	LRT	0.555000496240615
343	KC_G1	CG6066	-0.190265128549718	0.699867921437923	0.998043617620652	pseudobulk	DESeq2	LRT	0.1549839119899
344	KC_G1	CG6073	-0.931761390893072	0.651700646380486	0.998043617620652	pseudobulk	DESeq2	LRT	0.18595184825507
344	KC_G1	CG6083	1.65759895375003	0.676417887400967	0.998043617620652	pseudobulk	DESeq2	LRT	0.169784916306674
344	KC_G1	CG6084	-0.141238218583767	0.811758311485944	0.998043617620652	pseudobulk	DESeq2	LRT	0.0905732559954595
344	KC_G1	CG6094	0.180953680310661	0.629262917286702	0.998043617620652	pseudobulk	DESeq2	LRT	0.201167860644508
344	KC_G1	CG6115	0.266239727271503	0.107205853577283	0.998043617620652	pseudobulk	DESeq2	LRT	0.969781500959395
344	KC_G1	CG6123	0.173105107343345	0.973555957903825	0.998043617620652	pseudobulk	DESeq2	LRT	0.0116390811132178
344	KC_G1	CG6125	0.173105107343345	0.973555957903825	0.998043617620652	pseudobulk	DESeq2	LRT	0.0116390811132178
344	KC_G1	CG6126	-1.84079135412168	0.114894657387203	0.998043617620652	pseudobulk	DESeq2	LRT	0.939700165578053
344	KC_G1	CG6136	0.455571298536756	0.412118694493624	0.998043617620652	pseudobulk	DESeq2	LRT	0.384977684598743
344	KC_G1	CG6142	1.13582588427274	0.704684208645453	0.998043617620652	pseudobulk	DESeq2	LRT	0.152005460551806
345	KC_G1	CG6144	-0.0113940194509122	0.920214524400557	0.998043617620652	pseudobulk	DESeq2	LRT	0.0361109162383237
345	KC_G1	CG6153	0.295427490927356	0.725114077812092	0.998043617620652	pseudobulk	DESeq2	LRT	0.139593663129812
345	KC_G1	CG6154	-0.275878494365019	0.0772442222134042	0.998043617620652	pseudobulk	DESeq2	LRT	1.11213399545884
345	KC_G1	CG6163	-0.0562081335204214	0.930298663580466	0.998043617620652	pseudobulk	DESeq2	LRT	0.0313776029278292
345	KC_G1	CG6171	0.127478090778528	0.690550412380235	0.998043617620652	pseudobulk	DESeq2	LRT	0.16080461104715
345	KC_G1	CG6175	-0.88271904440604	0.661655916427707	0.998043617620652	pseudobulk	DESeq2	LRT	0.1793677997526
345	KC_G1	CG6178	0.266316621577999	0.583707371815532	0.998043617620652	pseudobulk	DESeq2	LRT	0.233804821819889
345	KC_G1	CG6179	0.205522551103368	0.624148976745469	0.998043617620652	pseudobulk	DESeq2	LRT	0.204711737150347
345	KC_G1	CG6180	-0.0872025534173491	0.642696372533094	0.998043617620652	pseudobulk	DESeq2	LRT	0.191994151300595
345	KC_G1	CG6184	0.134130073309053	0.699815150329773	0.998043617620652	pseudobulk	DESeq2	LRT	0.155016659690499
346	KC_G1	CG6191	-0.2162194526274	0.441123673395184	0.998043617620652	pseudobulk	DESeq2	LRT	0.355439634697188
346	KC_G1	CG6195	0.0613086977632211	0.877293200834208	0.998043617620652	pseudobulk	DESeq2	LRT	0.0568552364816599
346	KC_G1	CG6201	-0.446638329289717	0.463587419438956	0.998043617620652	pseudobulk	DESeq2	LRT	0.333868358175169
346	KC_G1	CG6204	0.340548836360458	0.649836190510036	0.998043617620652	pseudobulk	DESeq2	LRT	0.187196105700893
346	KC_G1	CG6218	0.358662979062118	0.3355316925792	0.998043617620652	pseudobulk	DESeq2	LRT	0.474266452358746
346	KC_G1	CG6227	-0.0193990096252542	0.937030599739004	0.998043617620652	pseudobulk	DESeq2	LRT	0.0282462265286503
346	KC_G1	CG6230	-0.322846415270637	0.585794442463887	0.998043617620652	pseudobulk	DESeq2	LRT	0.232254752863904
346	KC_G1	CG6231	0.217551468520664	0.52386231688546	0.998043617620652	pseudobulk	DESeq2	LRT	0.280782840638341
346	KC_G1	CG6236	-0.523183122475957	0.470317005163235	0.998043617620652	pseudobulk	DESeq2	LRT	0.327609318266377
346	KC_G1	CG6254	-0.344674282636424	0.381798807154246	0.998043617620652	pseudobulk	DESeq2	LRT	0.418165432797087
347	KC_G1	CG6276	-0.0038232085012919	0.960581645927054	0.998043617620652	pseudobulk	DESeq2	LRT	0.0174657157906992

347	KC_G1	CG6282	-0.0450567720068002	0.785567580296641	0.998043617620652	pseudobulk	DESeq2	LRT	0.104816447813773
347	KC_G1	CG6287	-0.765570986635886	0.244794509780108	0.998043617620652	pseudobulk	DESeq2	LRT	0.611198326718231
347	KC_G1	CG6293	-0.23019596793113	0.912068659233978	0.998043617620652	pseudobulk	DESeq2	LRT	0.0399724673688649
347	KC_G1	CG6294	1.69981218657454	0.566400560818652	0.998043617620652	pseudobulk	DESeq2	LRT	0.246876325303589
347	KC_G1	CG6310	-0.65817001371651	0.770857516266391	0.998043617620652	pseudobulk	DESeq2	LRT	0.113025888638787
347	KC_G1	CG6325	0.024973768559414	0.965520245677776	0.998043617620652	pseudobulk	DESeq2	LRT	0.0152386152103706
347	KC_G1	CG6329	-0.0175956549637991	0.885775974448549	0.998043617620652	pseudobulk	DESeq2	LRT	0.0526761035792857
347	KC_G1	CG6330	0.0416649765723423	0.895249546774808	0.998043617620652	pseudobulk	DESeq2	LRT	0.0480558901800636
347	KC_G1	CG6340	0.108498720134352	0.489630121169093	0.998043617620652	pseudobulk	DESeq2	LRT	0.310131873021107
348	KC_G1	CG6345	0.130334943367314	0.876849083329593	0.998043617620652	pseudobulk	DESeq2	LRT	0.0570751477025838
348	KC_G1	CG6356	-0.439882715337993	0.365266424622812	0.998043617620652	pseudobulk	DESeq2	LRT	0.437390246406185
348	KC_G1	CG6357	0.157367486193761	0.708857048065712	0.998043617620652	pseudobulk	DESeq2	LRT	0.149441338153799
348	KC_G1	CG6362	1.12508065046033	0.900770125530981	0.998043617620652	pseudobulk	DESeq2	LRT	0.0453860258366365
348	KC_G1	CG6379	0.143384740254689	0.656154827169276	0.998043617620652	pseudobulk	DESeq2	LRT	0.182993671826242
348	KC_G1	CG6388	-0.241581713103165	0.714349787635859	0.998043617620652	pseudobulk	DESeq2	LRT	0.146089080042058
348	KC_G1	CG6405	-0.794610277746993	0.879485534599449	0.998043617620652	pseudobulk	DESeq2	LRT	0.0557712992369932
348	KC_G1	CG6418	0.0103537171562366	0.959540332933499	0.998043617620652	pseudobulk	DESeq2	LRT	0.017936765584774
348	KC_G1	CG6420	0.471014157067393	0.200339494587734	0.998043617620652	pseudobulk	DESeq2	LRT	0.698233426189594
348	KC_G1	CG6424	-0.10807558253191	0.934918303107865	0.998043617620652	pseudobulk	DESeq2	LRT	0.0292263378543401
349	KC_G1	CG6425	0.949135017209972	0.666764854931464	0.998043617620652	pseudobulk	DESeq2	LRT	0.176027299833216
349	KC_G1	CG6426	1.12508065046033	0.900770125530981	0.998043617620652	pseudobulk	DESeq2	LRT	0.0453860258366365
349	KC_G1	CG6428	0.456431646154627	0.339086437230803	0.998043617620652	pseudobulk	DESeq2	LRT	0.469689580772249
349	KC_G1	CG6435	-0.794610277746993	0.879485534599449	0.998043617620652	pseudobulk	DESeq2	LRT	0.0557712992369932
349	KC_G1	CG6443	-0.328968035007442	0.261932722781504	0.998043617620652	pseudobulk	DESeq2	LRT	0.581810242561001
349	KC_G1	CG6454	0.0403254302834216	0.872810890664253	0.998043617620652	pseudobulk	DESeq2	LRT	0.0590798433937282
349	KC_G1	CG6465	-0.169667562787439	0.783787748475374	0.998043617620652	pseudobulk	DESeq2	LRT	0.105801529338555
349	KC_G1	CG6472	0.00914014173009732	0.973729404153932	0.998043617620652	pseudobulk	DESeq2	LRT	0.0115617152057909
349	KC_G1	CG6479	0.107425452044339	0.918970442780321	0.998043617620652	pseudobulk	DESeq2	LRT	0.0366984567791096
349	KC_G1	CG6481	0.162283725687404	0.891946194041148	0.998043617620652	pseudobulk	DESeq2	LRT	0.0496613433097801
350	KC_G1	CG6495	0.0684486835199483	0.762136692806331	0.998043617620652	pseudobulk	DESeq2	LRT	0.117967128913028
350	KC_G1	CG6511	0.730462028151964	0.249567004746069	0.998043617620652	pseudobulk	DESeq2	LRT	0.602812833268186
350	KC_G1	CG6512	-0.151918978947937	0.635920028319919	0.998043617620652	pseudobulk	DESeq2	LRT	0.196597496683557
350	KC_G1	CG6523	0.0744912503272873	0.721150032183361	0.998043617620652	pseudobulk	DESeq2	LRT	0.141974372768873
350	KC_G1	CG6550	-0.438042023646733	0.504186984134401	0.998043617620652	pseudobulk	DESeq2	LRT	0.297408370066892
350	KC_G1	CG6565	0.234411658128337	0.64457640606342	0.998043617620652	pseudobulk	DESeq2	LRT	0.190725595354859
350	KC_G1	CG6567	0.278396437107975	0.643168456349734	0.998043617620652	pseudobulk	DESeq2	LRT	0.191675263341038
350	KC_G1	CG6574	0.163802850097141	0.776059112975652	0.998043617620652	pseudobulk	DESeq2	LRT	0.110105196961701
350	KC_G1	CG6583	0.102330070484251	0.769782590977203	0.998043617620652	pseudobulk	DESeq2	LRT	0.113631914919824
350	KC_G1	CG6607	0.0980116878276473	0.762813473700739	0.998043617620652	pseudobulk	DESeq2	LRT	0.117581644542913
351	KC_G1	CG6610	-0.389670669293323	0.486959328877276	0.998043617620652	pseudobulk	DESeq2	LRT	0.312507309795168
351	KC_G1	CG6617	0.0996437882518405	0.637207668476675	0.998043617620652	pseudobulk	DESeq2	LRT	0.19571900632184
351	KC_G1	CG6621	-0.487117601583575	0.198043699250389	0.998043617620652	pseudobulk	DESeq2	LRT	0.703238970095418
351	KC_G1	CG6628	1.6685533946513	0.467165782963826	0.998043617620652	pseudobulk	DESeq2	LRT	0.330528974145221
351	KC_G1	CG6638	3.00294902032064	0.0317225618525267	0.998043617620652	pseudobulk	DESeq2	LRT	1.49863174715869
351	KC_G1	CG6650	0.491904282768415	0.54825072361999	0.998043617620652	pseudobulk	DESeq2	LRT	0.26102078643756
351	KC_G1	CG6654	0.181420854966083	0.809123495475675	0.998043617620652	pseudobulk	DESeq2	LRT	0.091985187521944
351	KC_G1	CG6659	0.216399744868924	0.641046992556158	0.998043617620652	pseudobulk	DESeq2	LRT	0.193110132946431
351	KC_G1	CG6664	0.176864213990262	0.514889767845438	0.998043617620652	pseudobulk	DESeq2	LRT	0.288285738610556
351	KC_G1	CG6665	-0.309613032333511	0.574650903465006	0.998043617620652	pseudobulk	DESeq2	LRT	0.240595906163785
352	KC_G1	CG6674	0.501677304759766	0.156561149462445	0.998043617620652	pseudobulk	DESeq2	LRT	0.805315998774657
352	KC_G1	CG6678	2.50145379121547	0.286990765027193	0.998043617620652	pseudobulk	DESeq2	LRT	0.542132078047399
352	KC_G1	CG6683	-0.227405209660026	0.894013055641612	0.998043617620652	pseudobulk	DESeq2	LRT	0.0486561389762197
352	KC_G1	CG6685	-0.525123716284249	0.416000908391697	0.998043617620652	pseudobulk	DESeq2	LRT	0.380905721034146
352	KC_G1	CG6686	0.0987616030580088	0.639228344083829	0.998043617620652	pseudobulk	DESeq2	LRT	0.194343976174846
352	KC_G1	CG6689	-0.264225316036163	0.543470454156598	0.998043617620652	pseudobulk	DESeq2	LRT	0.264824061413091
352	KC_G1	CG6693	0.217923311914193	0.4811662929706	0.998043617620652	pseudobulk	DESeq2	LRT	0.317704803796035

352	KC_G1	CG6695	-0.755926270748831	0.141537275635718	0.998043617620652	pseudobulk	DESeq2	LRT	0.849129168118647
352	KC_G1	CG6700	0.00984038756134084	0.936823265588027	0.998043617620652	pseudobulk	DESeq2	LRT	0.028342332291979
352	KC_G1	CG6701	1.75162781140343	0.300412726727111	0.998043617620652	pseudobulk	DESeq2	LRT	0.522281672765476
353	KC_G1	CG6707	-0.010231779380795	0.95115132568236	0.998043617620652	pseudobulk	DESeq2	LRT	0.0217503824520608
353	KC_G1	CG6712	0.565703129293127	0.325062845990401	0.998043617620652	pseudobulk	DESeq2	LRT	0.488032666626474
353	KC_G1	CG6723	-0.071227241176518	0.764578460858503	0.998043617620652	pseudobulk	DESeq2	LRT	0.116577940769067
353	KC_G1	CG6724	0.264210769543313	0.66115015669521	0.998043617620652	pseudobulk	DESeq2	LRT	0.179699894798363
353	KC_G1	CG6729	-0.0153765918193811	0.953358594337386	0.998043617620652	pseudobulk	DESeq2	LRT	0.0207437140004221
353	KC_G1	CG6738	-0.794610277746993	0.821479263487599	0.998043617620652	pseudobulk	DESeq2	LRT	0.085403394939296
353	KC_G1	CG6752	-0.412452877678771	0.374761889154086	0.998043617620652	pseudobulk	DESeq2	LRT	0.426244580462044
353	KC_G1	CG6758	-0.134182047210375	0.75245612158982	0.998043617620652	pseudobulk	DESeq2	LRT	0.123518820261722
353	KC_G1	CG6762	-0.168910319544364	0.675778204557103	0.998043617620652	pseudobulk	DESeq2	LRT	0.170195819357843
353	KC_G1	CG6765	-0.273367747470776	0.126487195672352	0.998043617620652	pseudobulk	DESeq2	LRT	0.897953435992474
354	KC_G1	CG6766	-0.16672206633221	0.601860012541176	0.998043617620652	pseudobulk	DESeq2	LRT	0.220504510155203
354	KC_G1	CG6767	0.101141441294368	0.48136319939123	0.998043617620652	pseudobulk	DESeq2	LRT	0.317527114958857
354	KC_G1	CG6769	0.212939378332696	0.606854685998903	0.998043617620652	pseudobulk	DESeq2	LRT	0.216915290183536
354	KC_G1	CG6770	-0.0739585384201379	0.542499506821317	0.998043617620652	pseudobulk	DESeq2	LRT	0.265600652290268
354	KC_G1	CG6785	0.173105107343345	0.973555957903825	0.998043617620652	pseudobulk	DESeq2	LRT	0.0116390811132178
354	KC_G1	CG6791	0.0325577392782076	0.897126637931636	0.998043617620652	pseudobulk	DESeq2	LRT	0.0471462478444577
354	KC_G1	CG6800	1.12508065046033	0.900770125530981	0.998043617620652	pseudobulk	DESeq2	LRT	0.0453860258366365
354	KC_G1	CG6805	0.366527817464349	0.553339924581985	0.998043617620652	pseudobulk	DESeq2	LRT	0.257007993440084
354	KC_G1	CG6808	0.520814978847402	0.470568526905988	0.998043617620652	pseudobulk	DESeq2	LRT	0.32737712316116
354	KC_G1	CG6813	0.57284086327499	0.32641085786277	0.998043617620652	pseudobulk	DESeq2	LRT	0.486235403130628
355	KC_G1	CG6833	0.0139571240374648	0.932280146383486	0.998043617620652	pseudobulk	DESeq2	LRT	0.0304535643127899
355	KC_G1	CG6834	0.265915083649717	0.865595237253845	0.998043617620652	pseudobulk	DESeq2	LRT	0.0626851418401678
355	KC_G1	CG6836	1.19274501688866	0.168811844966072	0.998043617620652	pseudobulk	DESeq2	LRT	0.772597083640912
355	KC_G1	CG6841	-0.076302923081059	0.864736888312414	0.998043617620652	pseudobulk	DESeq2	LRT	0.0631160143112735
355	KC_G1	CG6843	0.0790517116656326	0.799430352260624	0.998043617620652	pseudobulk	DESeq2	LRT	0.0972193667475929
355	KC_G1	CG6847	0.332737559759639	0.845408557106103	0.998043617620652	pseudobulk	DESeq2	LRT	0.0729333606172984
355	KC_G1	CG6865	0.1729159612032	0.899814506689544	0.998043617620652	pseudobulk	DESeq2	LRT	0.0458470094762598
355	KC_G1	CG6867	0.140112330555651	0.606317467490681	0.998043617620652	pseudobulk	DESeq2	LRT	0.217299919930066
355	KC_G1	CG6878	0.175455006432548	0.407261558293409	0.998043617620652	pseudobulk	DESeq2	LRT	0.390126581342801
355	KC_G1	CG6891	1.2609445879621	0.626159485520488	0.998043617620652	pseudobulk	DESeq2	LRT	0.203315036023015
356	KC_G1	CG6903	-0.265171558624648	0.571522609440085	0.998043617620652	pseudobulk	DESeq2	LRT	0.242966584234855
356	KC_G1	CG6907	0.345108286340418	0.583328108776549	0.998043617620652	pseudobulk	DESeq2	LRT	0.234087095758527
356	KC_G1	CG6908	-0.357698834169739	0.66473034804818	0.998043617620652	pseudobulk	DESeq2	LRT	0.177354493199693
356	KC_G1	CG6910	3.41759447620195	0.0959031285514964	0.998043617620652	pseudobulk	DESeq2	LRT	1.0181672250453
356	KC_G1	CG6912	0.126502039697208	0.967151103131291	0.998043617620652	pseudobulk	DESeq2	LRT	0.0145056684924722
356	KC_G1	CG6923	-0.279101837480214	0.489411047823375	0.998043617620652	pseudobulk	DESeq2	LRT	0.310326231235611
356	KC_G1	CG6928	0.113846903319156	0.939520003465777	0.998043617620652	pseudobulk	DESeq2	LRT	0.0270939688340005
356	KC_G1	CG6937	-0.258607170814181	0.576597682540046	0.998043617620652	pseudobulk	DESeq2	LRT	0.239127107459591
356	KC_G1	CG6951	0.186508224338217	0.845899508961721	0.998043617620652	pseudobulk	DESeq2	LRT	0.072681227144939
356	KC_G1	CG6959	0.111778274533814	0.657153348675896	0.998043617620652	pseudobulk	DESeq2	LRT	0.182333274713175
357	KC_G1	CG6961	-0.297299602296098	0.638581449575025	0.998043617620652	pseudobulk	DESeq2	LRT	0.194783701619027
357	KC_G1	CG6962	-0.163414561997344	0.934124159466925	0.998043617620652	pseudobulk	DESeq2	LRT	0.0295953955175657
357	KC_G1	CG6966	0.158512221926611	0.442373850731019	0.998043617620652	pseudobulk	DESeq2	LRT	0.354210552632078
357	KC_G1	CG6974	-0.794610277746993	0.879485534599449	0.998043617620652	pseudobulk	DESeq2	LRT	0.0557712992369932
357	KC_G1	CG6983	0.232866410770052	0.483100435730054	0.998043617620652	pseudobulk	DESeq2	LRT	0.315962570803539
357	KC_G1	CG6984	-1.20768673048298	0.0994317501488627	0.998043617620652	pseudobulk	DESeq2	LRT	1.00247491627969
357	KC_G1	CG6999	-0.794610277746993	0.879485534599449	0.998043617620652	pseudobulk	DESeq2	LRT	0.0557712992369932
357	KC_G1	CG7006	0.705686327845721	0.304438261358191	0.998043617620652	pseudobulk	DESeq2	LRT	0.516500766972071
357	KC_G1	CG7009	-0.163489543573567	0.811965190555256	0.998043617620652	pseudobulk	DESeq2	LRT	0.0904625888300254
357	KC_G1	CG7011	-0.343626903544103	0.511910109469482	0.998043617620652	pseudobulk	DESeq2	LRT	0.290806293689728
358	KC_G1	CG7028	-0.17766658883055	0.531773623142215	0.998043617620652	pseudobulk	DESeq2	LRT	0.27427320820017
358	KC_G1	CG7029	0.0611638946581152	0.705710744520147	0.998043617620652	pseudobulk	DESeq2	LRT	0.151373270336465
358	KC_G1	CG7044	0.83185093992037	0.196632983368231	0.998043617620652	pseudobulk	DESeq2	LRT	0.706343631502108

358	KC_G1	CG7049	0.495702358087403	0.368282134428701	0.998043617620652	pseudobulk	DESeq2	LRT	0.433819348501109
358	KC_G1	CG7054	1.13582588427274	0.633424328955532	0.998043617620652	pseudobulk	DESeq2	LRT	0.198305259991224
358	KC_G1	CG7058	0.379787845563079	0.233019135959239	0.998043617620652	pseudobulk	DESeq2	LRT	0.632608412449296
358	KC_G1	CG7059	-0.380389862783807	0.451818114413936	0.998043617620652	pseudobulk	DESeq2	LRT	0.34503636121488
358	KC_G1	CG7065	-0.0938232168339148	0.723162900545789	0.998043617620652	pseudobulk	DESeq2	LRT	0.140763861987238
358	KC_G1	CG7069	-0.920326080227842	0.209639970652994	0.998043617620652	pseudobulk	DESeq2	LRT	0.678525909765301
358	KC_G1	CG7071	1.13582588427274	0.633424328955532	0.998043617620652	pseudobulk	DESeq2	LRT	0.198305259991224
359	KC_G1	CG7079	-1.73907471612892	0.507597351592657	0.998043617620652	pseudobulk	DESeq2	LRT	0.294480652518419
359	KC_G1	CG7083	-0.312079419787524	0.466331685211042	0.998043617620652	pseudobulk	DESeq2	LRT	0.331305075120647
359	KC_G1	CG7084	-0.0564765426729619	0.856936733929794	0.998043617620652	pseudobulk	DESeq2	LRT	0.0670512400597805
359	KC_G1	CG7099	0.191710775819906	0.79209949771666	0.998043617620652	pseudobulk	DESeq2	LRT	0.101220262103372
359	KC_G1	CG7101	-0.297712987737331	0.71622087201608	0.998043617620652	pseudobulk	DESeq2	LRT	0.144953026986041
359	KC_G1	CG7102	-1.67593316621313	0.495877122847873	0.998043617620652	pseudobulk	DESeq2	LRT	0.304625927300966
359	KC_G1	CG7120	-0.0435306613440758	0.867802960732321	0.998043617620652	pseudobulk	DESeq2	LRT	0.0615788724972932
359	KC_G1	CG7130	0.736557199601235	0.72773060799759	0.998043617620652	pseudobulk	DESeq2	LRT	0.138029358478431
359	KC_G1	CG7131	-0.0942152128166348	0.937123272289403	0.998043617620652	pseudobulk	DESeq2	LRT	0.028203276824364
359	KC_G1	CG7133	-0.0488421260552897	0.887400276989734	0.998043617620652	pseudobulk	DESeq2	LRT	0.0518804400604213
360	KC_G1	CG7137	1.04729726481344	0.322389174683618	0.998043617620652	pseudobulk	DESeq2	LRT	0.491619549540958
360	KC_G1	CG7139	-0.00792574527527063	0.95157595446517	0.998043617620652	pseudobulk	DESeq2	LRT	0.0215565407573343
360	KC_G1	CG7148	-0.106537548621265	0.777452199172166	0.998043617620652	pseudobulk	DESeq2	LRT	0.109326303618087
360	KC_G1	CG7149	-0.0545470191152329	0.938711844546908	0.998043617620652	pseudobulk	DESeq2	LRT	0.0274677022269223
360	KC_G1	CG7156	-0.410053590872786	0.533206738738841	0.998043617620652	pseudobulk	DESeq2	LRT	0.273104370551126
360	KC_G1	CG7166	-0.131398557757751	0.420469302414035	0.998043617620652	pseudobulk	DESeq2	LRT	0.376265705642154
360	KC_G1	CG7168	-0.0893212398632705	0.852086197290826	0.998043617620652	pseudobulk	DESeq2	LRT	0.0695164696532194
360	KC_G1	CG7182	0.00885599084829825	0.914314265713849	0.998043617620652	pseudobulk	DESeq2	LRT	0.0389045040399663
360	KC_G1	CG7183	-0.0568456669882095	0.909111176784321	0.998043617620652	pseudobulk	DESeq2	LRT	0.0413830029040041
360	KC_G1	CG7191	-0.794610277746993	0.821479263487599	0.998043617620652	pseudobulk	DESeq2	LRT	0.085403394939296
361	KC_G1	CG7194	1.12508065046033	0.900770125530981	0.998043617620652	pseudobulk	DESeq2	LRT	0.0453860258366365
361	KC_G1	CG7203	0.676667972197729	0.426383448455757	0.998043617620652	pseudobulk	DESeq2	LRT	0.370199662292572
361	KC_G1	CG7206	0.0296282655970051	0.937080235521601	0.998043617620652	pseudobulk	DESeq2	LRT	0.0282232219698092
361	KC_G1	CG7213	-1.60891734856068	0.545249891924755	0.998043617620652	pseudobulk	DESeq2	LRT	0.263404411831769
361	KC_G1	CG7214	-1.24117042129705	0.493813606390446	0.998043617620652	pseudobulk	DESeq2	LRT	0.306436947820695
361	KC_G1	CG7218	0.51279592413058	0.365874452637654	0.998043617620652	pseudobulk	DESeq2	LRT	0.436667914279025
361	KC_G1	CG7220	0.157462068064989	0.428974537595848	0.998043617620652	pseudobulk	DESeq2	LRT	0.367568485227397
361	KC_G1	CG7222	0.0390119454562671	0.883081202203534	0.998043617620652	pseudobulk	DESeq2	LRT	0.0539993597888335
361	KC_G1	CG7227	-0.794610277746993	0.815039924133852	0.998043617620652	pseudobulk	DESeq2	LRT	0.0888211171417967
361	KC_G1	CG7231	-0.179297104419809	0.643482976089408	0.998043617620652	pseudobulk	DESeq2	LRT	0.191462938251792
362	KC_G1	CG7239	-0.370932736241216	0.353372719007109	0.998043617620652	pseudobulk	DESeq2	LRT	0.451766981829833
362	KC_G1	CG7246	-0.371228450039906	0.731212671560618	0.998043617620652	pseudobulk	DESeq2	LRT	0.135956291096477
362	KC_G1	CG7265	-0.55934344315874	0.37240067392002	0.998043617620652	pseudobulk	DESeq2	LRT	0.42898954176298
362	KC_G1	CG7272	-0.0968203309470832	0.852439245691023	0.998043617620652	pseudobulk	DESeq2	LRT	0.069336563891326
362	KC_G1	CG7275	-0.117381331939408	0.857341567818291	0.998043617620652	pseudobulk	DESeq2	LRT	0.0668461191911902
362	KC_G1	CG7289	-0.401624669693807	0.324829553518819	0.998043617620652	pseudobulk	DESeq2	LRT	0.488344464818008
362	KC_G1	CG7322	-0.794610277746993	0.879485534599449	0.998043617620652	pseudobulk	DESeq2	LRT	0.0557712992369932
362	KC_G1	CG7324	0.0364166445340859	0.892096297676319	0.998043617620652	pseudobulk	DESeq2	LRT	0.0495882630093674
362	KC_G1	CG7326	0.196520730318006	0.645300681034205	0.998043617620652	pseudobulk	DESeq2	LRT	0.190237876549095
362	KC_G1	CG7328	-1.23554018065503	0.4288549272787	0.998043617620652	pseudobulk	DESeq2	LRT	0.367689595781836
363	KC_G1	CG7332	-0.00168322736956552	0.965763479389921	0.998043617620652	pseudobulk	DESeq2	LRT	0.0151292215897596
363	KC_G1	CG7339	-0.0556622401095349	0.90737428286145	0.998043617620652	pseudobulk	DESeq2	LRT	0.0422135338308974
363	KC_G1	CG7341	-0.971064177850534	0.627910581449623	0.998043617620652	pseudobulk	DESeq2	LRT	0.202102198218191
363	KC_G1	CG7352	-2.5287247349577	0.0348652142134781	0.998043617620652	pseudobulk	DESeq2	LRT	1.45760766212561
363	KC_G1	CG7366	-0.794610277746993	0.821479263487599	0.998043617620652	pseudobulk	DESeq2	LRT	0.085403394939296
363	KC_G1	CG7367	-0.346928387643943	0.768328648390971	0.998043617620652	pseudobulk	DESeq2	LRT	0.114452973129552
363	KC_G1	CG7368	0.315740625760291	0.30389370186789	0.998043617620652	pseudobulk	DESeq2	LRT	0.517278300487234
363	KC_G1	CG7369	0.0609857774381587	0.863881132438459	0.998043617620652	pseudobulk	DESeq2	LRT	0.0635460110830465
363	KC_G1	CG7376	0.591387589357357	0.559861726186905	0.998043617620652	pseudobulk	DESeq2	LRT	0.25191922115296

363	KC_G1	CG7377	1.13582588427274	0.633424328955532	0.998043617620652	pseudobulk	DESeq2	LRT	0.198305259991224
364	KC_G1	CG7378	0.232230380479877	0.118902641251405	0.998043617620652	pseudobulk	DESeq2	LRT	0.924808498045986
364	KC_G1	CG7379	-0.147622279221754	0.607949505634731	0.998043617620652	pseudobulk	DESeq2	LRT	0.21613249035699
364	KC_G1	CG7381	0.218009027936945	0.446968217004153	0.998043617620652	pseudobulk	DESeq2	LRT	0.349723357562608
364	KC_G1	CG7382	0.237075708922458	0.327904670209738	0.998043617620652	pseudobulk	DESeq2	LRT	0.484252397811041
364	KC_G1	CG7386	1.29327629737686	0.437177235691708	0.998043617620652	pseudobulk	DESeq2	LRT	0.359342460333655
364	KC_G1	CG7394	0.00766394988034466	0.954303982452614	0.998043617620652	pseudobulk	DESeq2	LRT	0.0203132637936535
364	KC_G1	CG7407	0.166574130742559	0.437210901921905	0.998043617620652	pseudobulk	DESeq2	LRT	0.359309017386801
364	KC_G1	CG7409	2.23045770445974	0.357070320422792	0.998043617620652	pseudobulk	DESeq2	LRT	0.447246246733247
364	KC_G1	CG7414	-0.131930064144468	0.636859584914956	0.998043617620652	pseudobulk	DESeq2	LRT	0.195956310551196
364	KC_G1	CG7420	0.931896205467505	0.303457218565176	0.998043617620652	pseudobulk	DESeq2	LRT	0.517902527161038
365	KC_G1	CG7430	0.124280714309984	0.425620719949437	0.998043617620652	pseudobulk	DESeq2	LRT	0.37097723799527
365	KC_G1	CG7453	-0.218428741143345	0.665834917725423	0.998043617620652	pseudobulk	DESeq2	LRT	0.176633433303961
365	KC_G1	CG7457	-0.757449795571951	0.820028655901106	0.998043617620652	pseudobulk	DESeq2	LRT	0.0861709709305825
365	KC_G1	CG7461	0.330544298704162	0.51496177558787	0.998043617620652	pseudobulk	DESeq2	LRT	0.288225006429412
365	KC_G1	CG7483	-0.296623846071809	0.359571742910599	0.998043617620652	pseudobulk	DESeq2	LRT	0.444214444803616
365	KC_G1	CG7484	-0.157454173268316	0.645731841154731	0.998043617620652	pseudobulk	DESeq2	LRT	0.189947797926126
365	KC_G1	CG7488	0.136816132072842	0.765248007243731	0.998043617620652	pseudobulk	DESeq2	LRT	0.116197792660622
365	KC_G1	CG7492	0.101201566216922	0.668406562641989	0.998043617620652	pseudobulk	DESeq2	LRT	0.174959294707415
365	KC_G1	CG7497	-0.794610277746993	0.815039924133852	0.998043617620652	pseudobulk	DESeq2	LRT	0.0888211171417967
365	KC_G1	CG7504	0.38600835019256	0.52614876025634	0.998043617620652	pseudobulk	DESeq2	LRT	0.278891448575415
366	KC_G1	CG7506	-0.022352972989799	0.926388157747128	0.998043617620652	pseudobulk	DESeq2	LRT	0.0332070052767728
366	KC_G1	CG7509	-0.524674912768771	0.766109503826225	0.998043617620652	pseudobulk	DESeq2	LRT	0.115709150069037
366	KC_G1	CG7518	-0.0350939886178588	0.880400843336749	0.998043617620652	pseudobulk	DESeq2	LRT	0.0553195501074209
366	KC_G1	CG7519	-0.199362644792881	0.584124410877297	0.998043617620652	pseudobulk	DESeq2	LRT	0.233494643978104
366	KC_G1	CG7523	0.590831814199054	0.210763843490574	0.998043617620652	pseudobulk	DESeq2	LRT	0.676203890230547
366	KC_G1	CG7530	0.616025221144949	0.576720197098271	0.998043617620652	pseudobulk	DESeq2	LRT	0.239034839061209
366	KC_G1	CG7536	0.0845686735456315	0.923832400010032	0.998043617620652	pseudobulk	DESeq2	LRT	0.0344068105471241
366	KC_G1	CG7544	-0.426791115996392	0.692142009777341	0.998043617620652	pseudobulk	DESeq2	LRT	0.159804790318856
366	KC_G1	CG7546	-0.172744338846237	0.455798087520416	0.998043617620652	pseudobulk	DESeq2	LRT	0.341227501404596
366	KC_G1	CG7550	-0.166006689784861	0.728423354918133	0.998043617620652	pseudobulk	DESeq2	LRT	0.137616138179882
367	KC_G1	CG7556	-0.327503001672882	0.488804917693409	0.998043617620652	pseudobulk	DESeq2	LRT	0.310864433460331
367	KC_G1	CG7564	-0.10657079670863	0.697506095232208	0.998043617620652	pseudobulk	DESeq2	LRT	0.156451992908633
367	KC_G1	CG7565	0.131672908826606	0.75379826941915	0.998043617620652	pseudobulk	DESeq2	LRT	0.122744863944754
367	KC_G1	CG7568	0.764815221789058	0.808325187140535	0.998043617620652	pseudobulk	DESeq2	LRT	0.0924138885208533
367	KC_G1	CG7582	0.0210924974934566	0.910438055843073	0.998043617620652	pseudobulk	DESeq2	LRT	0.0407495972833333
367	KC_G1	CG7600	0.237569648742654	0.543952446143404	0.998043617620652	pseudobulk	DESeq2	LRT	0.26443906589048
367	KC_G1	CG7601	0.168079421524465	0.79423970908006	0.998043617620652	pseudobulk	DESeq2	LRT	0.100048403593919
367	KC_G1	CG7607	0.119396408578921	0.629399063256225	0.998043617620652	pseudobulk	DESeq2	LRT	0.201073907789108
367	KC_G1	CG7611	0.174219240180881	0.544845713320083	0.998043617620652	pseudobulk	DESeq2	LRT	0.26372646164999
367	KC_G1	CG7627	-1.35311537024356	0.412971878866092	0.998043617620652	pseudobulk	DESeq2	LRT	0.38407952042371
368	KC_G1	CG7630	-0.108627276222363	0.383222658409056	0.998043617620652	pseudobulk	DESeq2	LRT	0.416548820754496
368	KC_G1	CG7632	-0.899877435500109	0.119222552468968	0.998043617620652	pseudobulk	DESeq2	LRT	0.923641584474989
368	KC_G1	CG7637	-0.834966365525318	0.0983355446432903	0.998043617620652	pseudobulk	DESeq2	LRT	1.00728947248136
368	KC_G1	CG7638	0.0258211294018273	0.931931204247065	0.998043617620652	pseudobulk	DESeq2	LRT	0.030616146357009
368	KC_G1	CG7639	-0.056477115045059	0.827429621143759	0.998043617620652	pseudobulk	DESeq2	LRT	0.0822689358589079
368	KC_G1	CG7646	-0.0267838261567743	0.78811567728714	0.998043617620652	pseudobulk	DESeq2	LRT	0.103410033372865
368	KC_G1	CG7650	0.21763614117873	0.689793283198303	0.998043617620652	pseudobulk	DESeq2	LRT	0.16128103885253
368	KC_G1	CG7655	-0.171019947513347	0.777751777546101	0.998043617620652	pseudobulk	DESeq2	LRT	0.109158987641206
368	KC_G1	CG7656	0.17729890271928	0.445731574851053	0.998043617620652	pseudobulk	DESeq2	LRT	0.350926600156873
368	KC_G1	CG7668	-2.13994406550903	0.0495619921322482	0.998043617620652	pseudobulk	DESeq2	LRT	1.30485124558381
369	KC_G1	CG7685	0.0824878125436563	0.863378727266949	0.998043617620652	pseudobulk	DESeq2	LRT	0.0637986561017957
369	KC_G1	CG7692	-0.504248294667445	0.50336358996611	0.998043617620652	pseudobulk	DESeq2	LRT	0.298118201677373
369	KC_G1	CG7694	0.0117224598786276	0.959584121117368	0.998043617620652	pseudobulk	DESeq2	LRT	0.0179169472070862
369	KC_G1	CG7702	-0.385763176122313	0.744824350328121	0.998043617620652	pseudobulk	DESeq2	LRT	0.127946133530114
369	KC_G1	CG7706	0.104783732973093	0.769333338310633	0.998043617620652	pseudobulk	DESeq2	LRT	0.113885447426245

369	KC_G1	CG7718	0.146195945734307	0.831659382120538	0.998043617620652	pseudobulk	DESeq2	LRT	0.0800545087506851
369	KC_G1	CG7720	2.23045770445974	0.357070320422792	0.998043617620652	pseudobulk	DESeq2	LRT	0.447246246733247
369	KC_G1	CG7724	-0.794610277746993	0.821479263487599	0.998043617620652	pseudobulk	DESeq2	LRT	0.085403394939296
369	KC_G1	CG7728	0.46493519303332	0.536856347832144	0.998043617620652	pseudobulk	DESeq2	LRT	0.270141907391469
369	KC_G1	CG7737	-0.557769209960009	0.0573608668212323	0.998043617620652	pseudobulk	DESeq2	LRT	1.24138429435501
370	KC_G1	CG7739	-0.129299209480841	0.589540247962334	0.998043617620652	pseudobulk	DESeq2	LRT	0.229486540235943
370	KC_G1	CG7741	-0.0374200012676902	0.932543424541426	0.998043617620652	pseudobulk	DESeq2	LRT	0.0303309358199487
370	KC_G1	CG7744	0.165705524727995	0.868954323947896	0.998043617620652	pseudobulk	DESeq2	LRT	0.0610030513749596
370	KC_G1	CG7745	0.170862779284561	0.7249190054061	0.998043617620652	pseudobulk	DESeq2	LRT	0.139710514077539
370	KC_G1	CG7747	0.0245470877087937	0.945287352791356	0.998043617620652	pseudobulk	DESeq2	LRT	0.024436152589432
370	KC_G1	CG7766	0.0101241261721271	0.953455054762775	0.998043617620652	pseudobulk	DESeq2	LRT	0.020699774488531
370	KC_G1	CG7772	0.208681072859148	0.586891842863559	0.998043617620652	pseudobulk	DESeq2	LRT	0.231441926649254
370	KC_G1	CG7781	-0.0161037245904655	0.894526869705186	0.998043617620652	pseudobulk	DESeq2	LRT	0.0484066096081462
370	KC_G1	CG7785	-0.195478090619463	0.542214475511747	0.998043617620652	pseudobulk	DESeq2	LRT	0.265828892231453
370	KC_G1	CG7789	-0.121309417792452	0.718286561670402	0.998043617620652	pseudobulk	DESeq2	LRT	0.143702258649514
371	KC_G1	CG7791	-0.12953188466741	0.791234513856863	0.998043617620652	pseudobulk	DESeq2	LRT	0.101694776958571
371	KC_G1	CG7806	0.392889587957272	0.648961509841229	0.998043617620652	pseudobulk	DESeq2	LRT	0.187781060609908
371	KC_G1	CG7810	0.034738591751675	0.933436765488579	0.998043617620652	pseudobulk	DESeq2	LRT	0.0299150974573323
371	KC_G1	CG7824	1.12508065046033	0.900770125530981	0.998043617620652	pseudobulk	DESeq2	LRT	0.0453860258366365
371	KC_G1	CG7834	0.208116460416361	0.489765589344807	0.998043617620652	pseudobulk	DESeq2	LRT	0.310011731425775
371	KC_G1	CG7839	0.0469026184551711	0.910241498269273	0.998043617620652	pseudobulk	DESeq2	LRT	0.0408433687213839
371	KC_G1	CG7840	-0.149190331987136	0.792623641077045	0.998043617620652	pseudobulk	DESeq2	LRT	0.100932978385643
371	KC_G1	CG7841	0.486497735485993	0.283606502196998	0.998043617620652	pseudobulk	DESeq2	LRT	0.547283816380702
371	KC_G1	CG7849	0.176279570978046	0.811293337328202	0.998043617620652	pseudobulk	DESeq2	LRT	0.0908220906109284
371	KC_G1	CG7857	0.048246857333485	0.891143219933209	0.998043617620652	pseudobulk	DESeq2	LRT	0.0500524927889143
372	KC_G1	CG7860	-0.752595479487864	0.785589861204421	0.998043617620652	pseudobulk	DESeq2	LRT	0.104804130174502
372	KC_G1	CG7872	0.451809005535714	0.352049062302551	0.998043617620652	pseudobulk	DESeq2	LRT	0.45339680810576
372	KC_G1	CG7878	0.112311378953092	0.754421066314434	0.998043617620652	pseudobulk	DESeq2	LRT	0.122386192942555
372	KC_G1	CG7879	-0.0529300535315181	0.817917136784972	0.998043617620652	pseudobulk	DESeq2	LRT	0.087290692489926
372	KC_G1	CG7881	-0.794610277746993	0.815039924133852	0.998043617620652	pseudobulk	DESeq2	LRT	0.0888211171417967
372	KC_G1	CG7882	1.12508065046033	0.900770125530981	0.998043617620652	pseudobulk	DESeq2	LRT	0.0453860258366365
372	KC_G1	CG7888	-0.19630020713848	0.398173783498383	0.998043617620652	pseudobulk	DESeq2	LRT	0.399927338122788
372	KC_G1	CG7889	0.510929684726677	0.539707057115449	0.998043617620652	pseudobulk	DESeq2	LRT	0.267841903138678
372	KC_G1	CG7900	0.173105107343345	0.973555957903825	0.998043617620652	pseudobulk	DESeq2	LRT	0.0116390811132178
372	KC_G1	CG7903	-0.172583436378872	0.466934887755278	0.998043617620652	pseudobulk	DESeq2	LRT	0.330743675879533
373	KC_G1	CG7910	-1.72984073695683	0.480047293856853	0.998043617620652	pseudobulk	DESeq2	LRT	0.318715974188453
373	KC_G1	CG7914	1.05248876619625	0.430329210551367	0.998043617620652	pseudobulk	DESeq2	LRT	0.366199173204679
373	KC_G1	CG7920	0.116087912243334	0.458874430367292	0.998043617620652	pseudobulk	DESeq2	LRT	0.338306141607533
373	KC_G1	CG7943	-0.00965071956513394	0.943536386253822	0.998043617620652	pseudobulk	DESeq2	LRT	0.0252413471741432
373	KC_G1	CG7945	0.00232471026210334	0.955387707398198	0.998043617620652	pseudobulk	DESeq2	LRT	0.0198203509147913
373	KC_G1	CG7946	-0.166451923158212	0.643309088375301	0.998043617620652	pseudobulk	DESeq2	LRT	0.191580313043341
373	KC_G1	CG7956	-0.343723652535294	0.406523995513756	0.998043617620652	pseudobulk	DESeq2	LRT	0.390913814616901
373	KC_G1	CG7963	-0.794610277746993	0.821479263487599	0.998043617620652	pseudobulk	DESeq2	LRT	0.085403394939296
373	KC_G1	CG7966	-0.955380993395623	0.583258276219124	0.998043617620652	pseudobulk	DESeq2	LRT	0.234139090012486
373	KC_G1	CG7970	-0.335757784735235	0.532224609090106	0.998043617620652	pseudobulk	DESeq2	LRT	0.273905048339785
374	KC_G1	CG7971	-0.041748303410316	0.67808514810574	0.998043617620652	pseudobulk	DESeq2	LRT	0.168715767739728
374	KC_G1	CG7974	0.413155087173283	0.45370995629018	0.998043617620652	pseudobulk	DESeq2	LRT	0.343221690395781
374	KC_G1	CG7985	0.232857736251717	0.465306227683254	0.998043617620652	pseudobulk	DESeq2	LRT	0.332261134807432
374	KC_G1	CG7987	-0.315297655669176	0.312396071209584	0.998043617620652	pseudobulk	DESeq2	LRT	0.505294436583237
374	KC_G1	CG7988	-0.756318288818244	0.215979712376525	0.998043617620652	pseudobulk	DESeq2	LRT	0.665587041519097
374	KC_G1	CG7990	-0.0746834743410244	0.578069856724789	0.998043617620652	pseudobulk	DESeq2	LRT	0.238019676186897
374	KC_G1	CG7991	0.10203878542178	0.745609275533839	0.998043617620652	pseudobulk	DESeq2	LRT	0.127488697907151
374	KC_G1	CG7997	-1.71758122468821	0.412995430020467	0.998043617620652	pseudobulk	DESeq2	LRT	0.384054753980099
374	KC_G1	CG8001	0.4414630275122	0.419942362999827	0.998043617620652	pseudobulk	DESeq2	LRT	0.376810312337407
374	KC_G1	CG8003	0.0527300958789679	0.908046707629789	0.998043617620652	pseudobulk	DESeq2	LRT	0.0418918118928243
375	KC_G1	CG8004	0.0641037512565707	0.806936306384567	0.998043617620652	pseudobulk	DESeq2	LRT	0.0931607439364116

375	KC_G1	CG8005	0.432273709330202	0.619819917930581	0.998043617620652	pseudobulk	DESeq2	LRT	0.207734471807041
375	KC_G1	CG8006	1.12508065046033	0.900770125530981	0.998043617620652	pseudobulk	DESeq2	LRT	0.0453860258366365
375	KC_G1	CG8009	-0.144646933354322	0.583722442827779	0.998043617620652	pseudobulk	DESeq2	LRT	0.23379360871333
375	KC_G1	CG8010	-0.533273252208724	0.357639924397858	0.998043617620652	pseudobulk	DESeq2	LRT	0.446554005598021
375	KC_G1	CG8027	0.343327699507429	0.545936546785578	0.998043617620652	pseudobulk	DESeq2	LRT	0.262857831621806
375	KC_G1	CG8028	-0.794610277746993	0.821479263487599	0.998043617620652	pseudobulk	DESeq2	LRT	0.085403394939296
375	KC_G1	CG8031	0.105352111991169	0.785685433884028	0.998043617620652	pseudobulk	DESeq2	LRT	0.104751298328224
375	KC_G1	CG8032	-0.240991283180758	0.542490477612715	0.998043617620652	pseudobulk	DESeq2	LRT	0.265607880625143
375	KC_G1	CG8034	-0.105336870061195	0.66293336272189	0.998043617620652	pseudobulk	DESeq2	LRT	0.178530124169346
376	KC_G1	CG8036	0.50715444847355	0.423894817061695	0.998043617620652	pseudobulk	DESeq2	LRT	0.372741893493743
376	KC_G1	CG8038	1.13863728409464	0.111767575708134	0.998043617620652	pseudobulk	DESeq2	LRT	0.95168416902045
376	KC_G1	CG8042	-0.160988627665959	0.650768911390132	0.998043617620652	pseudobulk	DESeq2	LRT	0.186573202407291
376	KC_G1	CG8043	0.460186216237483	0.370723199176922	0.998043617620652	pseudobulk	DESeq2	LRT	0.430950235769009
376	KC_G1	CG8060	-0.116260298275168	0.810709911062897	0.998043617620652	pseudobulk	DESeq2	LRT	0.0911345176283195
376	KC_G1	CG8064	-0.358849378898476	0.57806551065373	0.998043617620652	pseudobulk	DESeq2	LRT	0.238022941331675
376	KC_G1	CG8066	-0.89870847191934	0.574706015570166	0.998043617620652	pseudobulk	DESeq2	LRT	0.240554256989749
376	KC_G1	CG8067	-0.238232806811151	0.720801184942886	0.998043617620652	pseudobulk	DESeq2	LRT	0.142184508080972
376	KC_G1	CG8078	-0.145283874587509	0.760222165835865	0.998043617620652	pseudobulk	DESeq2	LRT	0.11905947180229
376	KC_G1	CG8079	-0.131112093243692	0.748774100790839	0.998043617620652	pseudobulk	DESeq2	LRT	0.125649185741836
377	KC_G1	CG8080	0.726302906077929	0.288783084309017	0.998043617620652	pseudobulk	DESeq2	LRT	0.539428249490237
377	KC_G1	CG8086	1.13582588427274	0.633424328955532	0.998043617620652	pseudobulk	DESeq2	LRT	0.198305259991224
377	KC_G1	CG8089	2.23045770445974	0.357070320422792	0.998043617620652	pseudobulk	DESeq2	LRT	0.447246246733247
377	KC_G1	CG8097	0.527858557625462	0.585697159417796	0.998043617620652	pseudobulk	DESeq2	LRT	0.232326882258822
377	KC_G1	CG8108	0.0632196151508144	0.609591261052868	0.998043617620652	pseudobulk	DESeq2	LRT	0.214961267560386
377	KC_G1	CG8111	-0.502799552298642	0.206866426114847	0.998043617620652	pseudobulk	DESeq2	LRT	0.684309988484673
377	KC_G1	CG8112	0.0631175696968907	0.939080148497766	0.998043617620652	pseudobulk	DESeq2	LRT	0.0272973400396678
377	KC_G1	CG8128	0.442228791120765	0.555515528610897	0.998043617620652	pseudobulk	DESeq2	LRT	0.255303796496715
377	KC_G1	CG8132	-0.0912430277098084	0.813573661612603	0.998043617620652	pseudobulk	DESeq2	LRT	0.0896031195758009
377	KC_G1	CG8134	0.0765948444830355	0.785739712690697	0.998043617620652	pseudobulk	DESeq2	LRT	0.104721296280361
378	KC_G1	CG8136	-0.647335375037142	0.762664938290985	0.998043617620652	pseudobulk	DESeq2	LRT	0.117666218809609
378	KC_G1	CG8142	2.79271636980726	0.0452970504481082	0.998043617620652	pseudobulk	DESeq2	LRT	1.34393007648199
378	KC_G1	CG8149	-0.018006915856339	0.934450374829804	0.998043617620652	pseudobulk	DESeq2	LRT	0.0294437574310464
378	KC_G1	CG8152	-1.10628277074181	0.13230652412859	0.998043617620652	pseudobulk	DESeq2	LRT	0.878418739915397
378	KC_G1	CG8155	0.177842356020794	0.691530315220928	0.998043617620652	pseudobulk	DESeq2	LRT	0.160188776589257
378	KC_G1	CG8157	-2.19332442993981	0.381369935212232	0.998043617620652	pseudobulk	DESeq2	LRT	0.418653546964052
378	KC_G1	CG8159	-0.0904936911764536	0.90150169670324	0.998043617620652	pseudobulk	DESeq2	LRT	0.0450334515614783
378	KC_G1	CG8160	-0.65817001371651	0.770857516266391	0.998043617620652	pseudobulk	DESeq2	LRT	0.113025888638787
378	KC_G1	CG8173	-2.3243999702458	0.347033693779396	0.998043617620652	pseudobulk	DESeq2	LRT	0.459628357162795
378	KC_G1	CG8176	0.0258706929675995	0.902354217016375	0.998043617620652	pseudobulk	DESeq2	LRT	0.0446229477152951
379	KC_G1	CG8177	-0.0159223005622877	0.909390405210692	0.998043617620652	pseudobulk	DESeq2	LRT	0.0412496322576245
379	KC_G1	CG8180	-0.971064177850534	0.627910581449623	0.998043617620652	pseudobulk	DESeq2	LRT	0.202102198218191
379	KC_G1	CG8187	-0.0157173957498633	0.959193700455737	0.998043617620652	pseudobulk	DESeq2	LRT	0.0180936821467972
379	KC_G1	CG8188	-0.00887847856414913	0.953795406255803	0.998043617620652	pseudobulk	DESeq2	LRT	0.0205447735802397
379	KC_G1	CG8195	0.218585287217094	0.461956034382716	0.998043617620652	pseudobulk	DESeq2	LRT	0.33539935546891
379	KC_G1	CG8199	-0.683822448120418	0.751359293327437	0.998043617620652	pseudobulk	DESeq2	LRT	0.124152337569353
379	KC_G1	CG8202	0.811762701213526	0.317651803522534	0.998043617620652	pseudobulk	DESeq2	LRT	0.498048674540396
379	KC_G1	CG8204	-0.584516186613412	0.44441816061281	0.998043617620652	pseudobulk	DESeq2	LRT	0.352208202447682
379	KC_G1	CG8206	0.0816043980815755	0.772276750201965	0.998043617620652	pseudobulk	DESeq2	LRT	0.112227039628781
379	KC_G1	CG8207	0.352316983466468	0.59618986741746	0.998043617620652	pseudobulk	DESeq2	LRT	0.224615409319958
380	KC_G1	CG8209	0.154758918949467	0.568782272389312	0.998043617620652	pseudobulk	DESeq2	LRT	0.245053948008736
380	KC_G1	CG8223	0.256039703347995	0.341161342083862	0.998043617620652	pseudobulk	DESeq2	LRT	0.467040185801184
380	KC_G1	CG8229	0.363605119539789	0.330351161280467	0.998043617620652	pseudobulk	DESeq2	LRT	0.481024162181493
380	KC_G1	CG8230	0.0532343747036127	0.903155841470029	0.998043617620652	pseudobulk	DESeq2	LRT	0.0442373047796381
380	KC_G1	CG8236	-2.23422618188688	0.336998892702854	0.998043617620652	pseudobulk	DESeq2	LRT	0.472371526113321
380	KC_G1	CG8237	0.302894522182286	0.552733813607028	0.998043617620652	pseudobulk	DESeq2	LRT	0.25748396654948
380	KC_G1	CG8243	0.0681843035585978	0.770352024449559	0.998043617620652	pseudobulk	DESeq2	LRT	0.113310771789026

380	KC_G1	CG8245	0.0598444711730184	0.705636712092005	0.998043617620652	pseudobulk	DESeq2	LRT	0.151418832292032
380	KC_G1	CG8248	0.117166019825876	0.623064614852899	0.998043617620652	pseudobulk	DESeq2	LRT	0.20546691253997
380	KC_G1	CG8270	0.167264599553241	0.80046673377443	0.998043617620652	pseudobulk	DESeq2	LRT	0.096656712012514
381	KC_G1	CG8272	0.239611047495357	0.734129455145441	0.998043617620652	pseudobulk	DESeq2	LRT	0.134227350577101
381	KC_G1	CG8281	-0.0340727346124575	0.924488018238923	0.998043617620652	pseudobulk	DESeq2	LRT	0.0340987131110167
381	KC_G1	CG8289	-0.00204616366676146	0.954006618169188	0.998043617620652	pseudobulk	DESeq2	LRT	0.0204486124820801
381	KC_G1	CG8292	1.13582588427274	0.633424328955532	0.998043617620652	pseudobulk	DESeq2	LRT	0.198305259991224
381	KC_G1	CG8298	0.191957386362295	0.736683502019611	0.998043617620652	pseudobulk	DESeq2	LRT	0.132719056033638
381	KC_G1	CG8300	-0.467281020858579	0.206441498548264	0.998043617620652	pseudobulk	DESeq2	LRT	0.685202997069244
381	KC_G1	CG8301	-0.397940111544823	0.142993758364248	0.998043617620652	pseudobulk	DESeq2	LRT	0.8446829189484
381	KC_G1	CG8306	0.172834960772825	0.908610676445368	0.998043617620652	pseudobulk	DESeq2	LRT	0.0416221644022337
381	KC_G1	CG8311	-0.573172529322342	0.20394407180884	0.998043617620652	pseudobulk	DESeq2	LRT	0.690488914118041
381	KC_G1	CG8312	-0.464544775860082	0.782064954368218	0.998043617620652	pseudobulk	DESeq2	LRT	0.106757175134272
382	KC_G1	CG8314	0.166000898517175	0.587206212501191	0.998043617620652	pseudobulk	DESeq2	LRT	0.231209358336003
382	KC_G1	CG8319	-0.752143630974366	0.25203376508462	0.998043617620652	pseudobulk	DESeq2	LRT	0.598541272680294
382	KC_G1	CG8320	0.359123291001394	0.392632522821886	0.998043617620652	pseudobulk	DESeq2	LRT	0.40601372945053
382	KC_G1	CG8321	-0.0350946033578653	0.91585547945946	0.998043617620652	pseudobulk	DESeq2	LRT	0.0381730519053864
382	KC_G1	CG8323	-0.180202152655524	0.771350720569878	0.998043617620652	pseudobulk	DESeq2	LRT	0.112748110437839
382	KC_G1	CG8326	0.685771901904342	0.299397980538314	0.998043617620652	pseudobulk	DESeq2	LRT	0.523751133331726
382	KC_G1	CG8336	0.373391590569797	0.52194409207595	0.998043617620652	pseudobulk	DESeq2	LRT	0.282376013862227
382	KC_G1	CG8353	1.14077974240158	0.0875271189796949	0.998043617620652	pseudobulk	DESeq2	LRT	1.05785736642355
382	KC_G1	CG8360	0.204560314765579	0.457560270698991	0.998043617620652	pseudobulk	DESeq2	LRT	0.339551691744
382	KC_G1	CG8366	-0.286531588589287	0.520026861631027	0.998043617620652	pseudobulk	DESeq2	LRT	0.283974222602068
383	KC_G1	CG8369	-0.0924512312577638	0.402138583529166	0.998043617620652	pseudobulk	DESeq2	LRT	0.395624256143745
383	KC_G1	CG8370	-0.0309083069784681	0.917996490701789	0.998043617620652	pseudobulk	DESeq2	LRT	0.0371589790076482
383	KC_G1	CG8372	0.097301822092739	0.65290172625893	0.998043617620652	pseudobulk	DESeq2	LRT	0.185152183128212
383	KC_G1	CG8379	0.0219202922692452	0.949072435856765	0.998043617620652	pseudobulk	DESeq2	LRT	0.0227006397409323
383	KC_G1	CG8388	0.336747217896824	0.600218863794096	0.998043617620652	pseudobulk	DESeq2	LRT	0.221690359606025
383	KC_G1	CG8389	-0.0126048734403851	0.967459264822188	0.998043617620652	pseudobulk	DESeq2	LRT	0.014367312030376
383	KC_G1	CG8397	-0.0192601182450138	0.922118385263504	0.998043617620652	pseudobulk	DESeq2	LRT	0.0352133188963004
383	KC_G1	CG8399	-1.72343437992478	0.512120560562093	0.998043617620652	pseudobulk	DESeq2	LRT	0.290627787806665
383	KC_G1	CG8401	-2.01544921444574	0.386064837560981	0.998043617620652	pseudobulk	DESeq2	LRT	0.413339751726489
383	KC_G1	CG8405	-0.13348508421219	0.648263881767924	0.998043617620652	pseudobulk	DESeq2	LRT	0.188248174580734
384	KC_G1	CG8407	1.60419461962953	0.365238904223767	0.998043617620652	pseudobulk	DESeq2	LRT	0.437422968843594
384	KC_G1	CG8412	0.10687622937671	0.788797862070378	0.998043617620652	pseudobulk	DESeq2	LRT	0.103034275161541
384	KC_G1	CG8414	-0.187867112386651	0.734673737311414	0.998043617620652	pseudobulk	DESeq2	LRT	0.13390548478417
384	KC_G1	CG8420	-1.60891734856068	0.545249891924755	0.998043617620652	pseudobulk	DESeq2	LRT	0.263404411831769
384	KC_G1	CG8435	0.168865040515224	0.733744300461853	0.998043617620652	pseudobulk	DESeq2	LRT	0.134455259219737
384	KC_G1	CG8441	0.0273438239378999	0.950391288423612	0.998043617620652	pseudobulk	DESeq2	LRT	0.0220975532203035
384	KC_G1	CG8460	0.817563417454186	0.394960299454955	0.998043617620652	pseudobulk	DESeq2	LRT	0.403446556510661
384	KC_G1	CG8461	0.0261882764048074	0.949977899697802	0.998043617620652	pseudobulk	DESeq2	LRT	0.0222864980279276
384	KC_G1	CG8475	0.139920394686873	0.718338652075157	0.998043617620652	pseudobulk	DESeq2	LRT	0.143670764596453
384	KC_G1	CG8485	-0.0966630116892715	0.564928044383469	0.998043617620652	pseudobulk	DESeq2	LRT	0.248006865308506
385	KC_G1	CG8490	-0.34308216588665	0.370072878743108	0.998043617620652	pseudobulk	DESeq2	LRT	0.431712741556618
385	KC_G1	CG8500	0.0778385242029306	0.729526975809147	0.998043617620652	pseudobulk	DESeq2	LRT	0.136958644512883
385	KC_G1	CG8507	-0.164315472816325	0.705027426439672	0.998043617620652	pseudobulk	DESeq2	LRT	0.151793988087005
385	KC_G1	CG8516	-0.297671521369952	0.590598514903638	0.998043617620652	pseudobulk	DESeq2	LRT	0.228707649429806
385	KC_G1	CG8520	0.336397018140899	0.469137802437495	0.998043617620652	pseudobulk	DESeq2	LRT	0.328699570829128
385	KC_G1	CG8531	-0.494849260626887	0.308257470464515	0.998043617620652	pseudobulk	DESeq2	LRT	0.511086389696698
385	KC_G1	CG8545	-1.05114976547828	0.285193728791205	0.998043617620652	pseudobulk	DESeq2	LRT	0.544860028544291
385	KC_G1	CG8547	-1.73628797603996	0.457283161399782	0.998043617620652	pseudobulk	DESeq2	LRT	0.339814790409823
385	KC_G1	CG8549	0.0493048953923942	0.903327458159059	0.998043617620652	pseudobulk	DESeq2	LRT	0.0441547884549231
385	KC_G1	CG8569	-0.137957581196873	0.808526163389734	0.998043617620652	pseudobulk	DESeq2	LRT	0.0923059220384162
386	KC_G1	CG8578	0.173845759113773	0.54631935537906	0.998043617620652	pseudobulk	DESeq2	LRT	0.2625534127283
386	KC_G1	CG8607	0.333826256537693	0.748296311446576	0.998043617620652	pseudobulk	DESeq2	LRT	0.125926395525791
386	KC_G1	CG8611	-0.341718490643611	0.573267093302903	0.998043617620652	pseudobulk	DESeq2	LRT	0.241642986896406

386	KC_G1	CG8613	0.200048387720915	0.459640921653855	0.998043617620652	pseudobulk	DESeq2	LRT	0.337581313192375
386	KC_G1	CG8617	-0.0252213677662385	0.900544703214302	0.998043617620652	pseudobulk	DESeq2	LRT	0.0454947238467571
386	KC_G1	CG8620	-0.455449419090036	0.834875862426107	0.998043617620652	pseudobulk	DESeq2	LRT	0.0783780949011207
386	KC_G1	CG8630	-2.98257659561824	0.142657801786736	0.998043617620652	pseudobulk	DESeq2	LRT	0.845704472305002
386	KC_G1	CG8635	0.0690224778588165	0.763500877375306	0.998043617620652	pseudobulk	DESeq2	LRT	0.117190459538752
386	KC_G1	CG8641	-0.192189769144202	0.629527975462743	0.998043617620652	pseudobulk	DESeq2	LRT	0.200984965605146
386	KC_G1	CG8646	-1.66808346769027	0.498191247612582	0.998043617620652	pseudobulk	DESeq2	LRT	0.302603906560549
387	KC_G1	CG8654	2.18876425250967	0.402105238436886	0.998043617620652	pseudobulk	DESeq2	LRT	0.395660269077107
387	KC_G1	CG8668	-0.193525106524629	0.415195248107715	0.998043617620652	pseudobulk	DESeq2	LRT	0.381747625613374
387	KC_G1	CG8671	-0.171620955352313	0.29035034122112	0.998043617620652	pseudobulk	DESeq2	LRT	0.537077659245389
387	KC_G1	CG8673	0.0234880544568819	0.970946622040714	0.998043617620652	pseudobulk	DESeq2	LRT	0.0128046448503595
387	KC_G1	CG8675	-0.170403320795456	0.727796790049106	0.998043617620652	pseudobulk	DESeq2	LRT	0.137989864203253
387	KC_G1	CG8677	-0.0799832398132891	0.556994644498337	0.998043617620652	pseudobulk	DESeq2	LRT	0.254148980546202
387	KC_G1	CG8679	-1.1288547182254	0.259972201394639	0.998043617620652	pseudobulk	DESeq2	LRT	0.585073088284397
387	KC_G1	CG8712	-0.15674608052272	0.784218464485688	0.998043617620652	pseudobulk	DESeq2	LRT	0.105562936417551
387	KC_G1	CG8726	-0.0303806719326713	0.818708995540861	0.998043617620652	pseudobulk	DESeq2	LRT	0.0868704377864576
387	KC_G1	CG8728	0.0507744629381143	0.87284115304131	0.998043617620652	pseudobulk	DESeq2	LRT	0.0590647856582375
388	KC_G1	CG8736	1.34823946227952	0.488653680999865	0.998043617620652	pseudobulk	DESeq2	LRT	0.310998825366197
388	KC_G1	CG8740	0.223010873659658	0.862033475640735	0.998043617620652	pseudobulk	DESeq2	LRT	0.0644758687416903
388	KC_G1	CG8745	0.0625687297763759	0.831494757998675	0.998043617620652	pseudobulk	DESeq2	LRT	0.080140484363194
388	KC_G1	CG8757	0.4383721837851	0.658761071597598	0.998043617620652	pseudobulk	DESeq2	LRT	0.181272072662378
388	KC_G1	CG8765	0.0254927056750382	0.952764941528453	0.998043617620652	pseudobulk	DESeq2	LRT	0.0210142317738727
388	KC_G1	CG8768	0.304360865224485	0.494263174880377	0.998043617620652	pseudobulk	DESeq2	LRT	0.306041745480981
388	KC_G1	CG8777	-0.631725612098876	0.425308489732545	0.998043617620652	pseudobulk	DESeq2	LRT	0.371295948094353
388	KC_G1	CG8778	0.0475025306977919	0.898307687890346	0.998043617620652	pseudobulk	DESeq2	LRT	0.0465748835271721
388	KC_G1	CG8785	-0.794610277746993	0.879485534599449	0.998043617620652	pseudobulk	DESeq2	LRT	0.0557712992369932
388	KC_G1	CG8814	-0.234007328770704	0.460381955210075	0.998043617620652	pseudobulk	DESeq2	LRT	0.336881706993312
389	KC_G1	CG8818	-0.092132582530593	0.627614588551661	0.998043617620652	pseudobulk	DESeq2	LRT	0.202306970034352
389	KC_G1	CG8833	-0.434493234580379	0.338746469991144	0.998043617620652	pseudobulk	DESeq2	LRT	0.470125221789434
389	KC_G1	CG8837	1.7806006469093	0.255376547133645	0.998043617620652	pseudobulk	DESeq2	LRT	0.592818989289229
389	KC_G1	CG8839	0.174250406602385	0.748995779832799	0.998043617620652	pseudobulk	DESeq2	LRT	0.125520629297184
389	KC_G1	CG8841	0.0362496235981195	0.941291047057357	0.998043617620652	pseudobulk	DESeq2	LRT	0.0262760720164
389	KC_G1	CG8850	0.665708931789394	0.758505174577836	0.998043617620652	pseudobulk	DESeq2	LRT	0.120041452078391
389	KC_G1	CG8858	-0.228730032928782	0.644546421499504	0.998043617620652	pseudobulk	DESeq2	LRT	0.190745798442377
389	KC_G1	CG8860	0.0304958782516302	0.891048479502541	0.998043617620652	pseudobulk	DESeq2	LRT	0.0500986665493507
389	KC_G1	CG8861	0.280278498260658	0.691069634084423	0.998043617620652	pseudobulk	DESeq2	LRT	0.160478189709813
389	KC_G1	CG8878	-0.112388350856673	0.564928742664844	0.998043617620652	pseudobulk	DESeq2	LRT	0.248006328497555
390	KC_G1	CG8888	1.11667571124907	0.561494942151568	0.998043617620652	pseudobulk	DESeq2	LRT	0.250654151433793
390	KC_G1	CG8891	0.727929714076359	0.0861833930645352	0.998043617620652	pseudobulk	DESeq2	LRT	1.0645764116146
390	KC_G1	CG8892	0.454741103011102	0.230359781849093	0.998043617620652	pseudobulk	DESeq2	LRT	0.637593341419175
390	KC_G1	CG8908	-1.71430442789954	0.393403271836839	0.998043617620652	pseudobulk	DESeq2	LRT	0.405162032482834
390	KC_G1	CG8910	-0.285907826791733	0.0253506424346878	0.998043617620652	pseudobulk	DESeq2	LRT	1.59601103032258
390	KC_G1	CG8915	-0.344966381365637	0.605458213412682	0.998043617620652	pseudobulk	DESeq2	LRT	0.217915824957791
390	KC_G1	CG8924	0.259288279735374	0.388440339936632	0.998043617620652	pseudobulk	DESeq2	LRT	0.41067567448457
390	KC_G1	CG8927	-0.794610277746993	0.879485534599449	0.998043617620652	pseudobulk	DESeq2	LRT	0.0557712992369932
390	KC_G1	CG8929	0.0329945974883247	0.845673031673099	0.998043617620652	pseudobulk	DESeq2	LRT	0.0727975187433772
390	KC_G1	CG8939	1.14730457912117	0.297973244817134	0.998043617620652	pseudobulk	DESeq2	LRT	0.525822729715463
391	KC_G1	CG8944	0.0302247071556751	0.91586578201503	0.998043617620652	pseudobulk	DESeq2	LRT	0.0381681665081071
391	KC_G1	CG8950	-0.28074032961616	0.813084129378099	0.998043617620652	pseudobulk	DESeq2	LRT	0.0898645158642577
391	KC_G1	CG8963	-0.111421831290247	0.620877020576293	0.998043617620652	pseudobulk	DESeq2	LRT	0.206994413630573
391	KC_G1	CG8974	0.0564504319552555	0.758070671982305	0.998043617620652	pseudobulk	DESeq2	LRT	0.120290304894033
391	KC_G1	CG8993	-0.0456508936659645	0.776307713681738	0.998043617620652	pseudobulk	DESeq2	LRT	0.109966098503889
391	KC_G1	CG9003	0.197965382942165	0.305050295975279	0.998043617620652	pseudobulk	DESeq2	LRT	0.515628549296842
391	KC_G1	CG9004	0.558260677558799	0.209823934720773	0.998043617620652	pseudobulk	DESeq2	LRT	0.678144973128783
391	KC_G1	CG9005	-0.112787808705348	0.467212840389008	0.998043617620652	pseudobulk	DESeq2	LRT	0.330485230036364
391	KC_G1	CG9008	0.777030258285493	0.70316795941598	0.998043617620652	pseudobulk	DESeq2	LRT	0.152940926566052

391	KC_G1	CG9018	-0.10510776429544	0.745375836568379	0.998043617620652	pseudobulk	DESeq2	LRT	0.127624690217961
392	KC_G1	CG9034	-0.0143836892977947	0.922631023927612	0.998043617620652	pseudobulk	DESeq2	LRT	0.0349719461140092
392	KC_G1	CG9044	0.150292315407378	0.670379201272123	0.998043617620652	pseudobulk	DESeq2	LRT	0.173679468293157
392	KC_G1	CG9062	-1.00764407854557	0.109409620056622	0.998043617620652	pseudobulk	DESeq2	LRT	0.960944490124851
392	KC_G1	CG9065	-0.0721457648455493	0.655575434406045	0.998043617620652	pseudobulk	DESeq2	LRT	0.183377328594193
392	KC_G1	CG9067	-0.0522735673235856	0.89293803215252	0.998043617620652	pseudobulk	DESeq2	LRT	0.0491786791047572
392	KC_G1	CG9072	-1.72984073695683	0.480047293856853	0.998043617620652	pseudobulk	DESeq2	LRT	0.318715974188453
392	KC_G1	CG9084	-0.794610277746993	0.815039924133852	0.998043617620652	pseudobulk	DESeq2	LRT	0.0888211171417967
392	KC_G1	CG9098	-0.464290338072693	0.268172648537864	0.998043617620652	pseudobulk	DESeq2	LRT	0.571585518784261
392	KC_G1	CG9107	-0.448010426014062	0.331900421677959	0.998043617620652	pseudobulk	DESeq2	LRT	0.478992195821905
392	KC_G1	CG9117	1.06667464613488	0.669131470313168	0.998043617620652	pseudobulk	DESeq2	LRT	0.174488544082685
393	KC_G1	CG9119	-1.70832308423201	0.223125671547179	0.998043617620652	pseudobulk	DESeq2	LRT	0.651450459434286
393	KC_G1	CG9121	0.262199846745384	0.486867350959205	0.998043617620652	pseudobulk	DESeq2	LRT	0.312589348012483
393	KC_G1	CG9123	-0.359905508736202	0.161810809558745	0.998043617620652	pseudobulk	DESeq2	LRT	0.790992469281809
393	KC_G1	CG9125	-0.0439650669247371	0.924844252282607	0.998043617620652	pseudobulk	DESeq2	LRT	0.03393139814678
393	KC_G1	CG9129	-0.794610277746993	0.879485534599449	0.998043617620652	pseudobulk	DESeq2	LRT	0.0557712992369932
393	KC_G1	CG9132	0.0799770767495486	0.625745020531677	0.998043617620652	pseudobulk	DESeq2	LRT	0.203602597675344
393	KC_G1	CG9135	0.0447685044842976	0.89109008940426	0.998043617620652	pseudobulk	DESeq2	LRT	0.0500783864757048
393	KC_G1	CG9143	0.427062728078362	0.52719807406174	0.998043617620652	pseudobulk	DESeq2	LRT	0.278026184957838
393	KC_G1	CG9147	0.419949786177214	0.464633317052312	0.998043617620652	pseudobulk	DESeq2	LRT	0.332889651847046
393	KC_G1	CG9149	0.244843508479744	0.495705261498164	0.998043617620652	pseudobulk	DESeq2	LRT	0.304776471396458
394	KC_G1	CG9154	-0.670247306072936	0.142729434549407	0.998043617620652	pseudobulk	DESeq2	LRT	0.845486454746794
394	KC_G1	CG9164	0.0829369092498513	0.835854101673366	0.998043617620652	pseudobulk	DESeq2	LRT	0.0778695220440528
394	KC_G1	CG9168	1.13582588427274	0.704684208645453	0.998043617620652	pseudobulk	DESeq2	LRT	0.152005460551806
394	KC_G1	CG9170	-0.00531206315416094	0.971773762955468	0.998043617620652	pseudobulk	DESeq2	LRT	0.0124348306871744
394	KC_G1	CG9171	-0.0203708324512654	0.885861963535727	0.998043617620652	pseudobulk	DESeq2	LRT	0.0526339453196394
394	KC_G1	CG9175	-0.248780662547339	0.478895221382551	0.998043617620652	pseudobulk	DESeq2	LRT	0.319759496508328
394	KC_G1	CG9205	-0.0848853312439707	0.70534810329051	0.998043617620652	pseudobulk	DESeq2	LRT	0.151596497154615
394	KC_G1	CG9213	-0.287927964449908	0.662487191779864	0.998043617620652	pseudobulk	DESeq2	LRT	0.178822513758097
394	KC_G1	CG9215	0.305476186267151	0.507180433703969	0.998043617620652	pseudobulk	DESeq2	LRT	0.294837509263364
394	KC_G1	CG9220	0.660223260418914	0.760341410401239	0.998043617620652	pseudobulk	DESeq2	LRT	0.118991355930445
395	KC_G1	CG9231	0.041411040123867	0.846002014598574	0.998043617620652	pseudobulk	DESeq2	LRT	0.0726286027671202
395	KC_G1	CG9240	0.0995315835412411	0.802427421720748	0.998043617620652	pseudobulk	DESeq2	LRT	0.0955942383892891
395	KC_G1	CG9246	0.546235709815949	0.368409446388925	0.998043617620652	pseudobulk	DESeq2	LRT	0.433669242584887
395	KC_G1	CG9248	0.277031880165207	0.79471870460022	0.998043617620652	pseudobulk	DESeq2	LRT	0.0997865652528114
395	KC_G1	CG9253	0.428668404343327	0.251159384034272	0.998043617620652	pseudobulk	DESeq2	LRT	0.600050590714997
395	KC_G1	CG9257	-0.0362966334106496	0.906977486149624	0.998043617620652	pseudobulk	DESeq2	LRT	0.0424034932732437
395	KC_G1	CG9259	1.12508065046033	0.900770125530981	0.998043617620652	pseudobulk	DESeq2	LRT	0.0453860258366365
395	KC_G1	CG9263	-0.30659909266699	0.819137275569851	0.998043617620652	pseudobulk	DESeq2	LRT	0.0866433106612572
395	KC_G1	CG9265	-0.202774142690916	0.35183272776355	0.998043617620652	pseudobulk	DESeq2	LRT	0.453663764577195
395	KC_G1	CG9272	0.578607081540458	0.719391896764007	0.998043617620652	pseudobulk	DESeq2	LRT	0.143034458386423
396	KC_G1	CG9281	0.133759889452776	0.538554232197202	0.998043617620652	pseudobulk	DESeq2	LRT	0.268770556882118
396	KC_G1	CG9286	-0.121361851420092	0.845370847428358	0.998043617620652	pseudobulk	DESeq2	LRT	0.072952732872387
396	KC_G1	CG9289	1.24545707098284	0.613638418851987	0.998043617620652	pseudobulk	DESeq2	LRT	0.212087457773066
396	KC_G1	CG9300	0.0359538122207968	0.943648356329654	0.998043617620652	pseudobulk	DESeq2	LRT	0.0251898122236614
396	KC_G1	CG9302	0.625872156026696	0.560529564249618	0.998043617620652	pseudobulk	DESeq2	LRT	0.251401476286024
396	KC_G1	CG9304	-0.0635339564693385	0.908812280519224	0.998043617620652	pseudobulk	DESeq2	LRT	0.0415258130968552
396	KC_G1	CG9328	-0.0901745095254588	0.571608809075779	0.998043617620652	pseudobulk	DESeq2	LRT	0.242901086907795
396	KC_G1	CG9330	-0.0799883199859659	0.806644584331531	0.998043617620652	pseudobulk	DESeq2	LRT	0.0933177776246421
396	KC_G1	CG9331	1.69817635634112	0.0893094739017211	0.998043617620652	pseudobulk	DESeq2	LRT	1.04910246893898
396	KC_G1	CG9336	1.80291676816611	0.443552628154353	0.998043617620652	pseudobulk	DESeq2	LRT	0.353054843112933
397	KC_G1	CG9338	1.13582588427274	0.633424328955532	0.998043617620652	pseudobulk	DESeq2	LRT	0.198305259991224
397	KC_G1	CG9344	0.0501219031999065	0.852993436581291	0.998043617620652	pseudobulk	DESeq2	LRT	0.0690543105294222
397	KC_G1	CG9346	-0.0312226375386978	0.905878847802247	0.998043617620652	pseudobulk	DESeq2	LRT	0.0429298809646996
397	KC_G1	CG9356	0.128040623415726	0.821872355485999	0.998043617620652	pseudobulk	DESeq2	LRT	0.0851956272442146
397	KC_G1	CG9360	-0.788151846119435	0.526816684730579	0.998043617620652	pseudobulk	DESeq2	LRT	0.278340479018385

397	KC_G1	CG9368	-0.0435774909613666	0.761696780196415	0.998043617620652	pseudobulk	DESeq2	LRT	0.118217880228967
397	KC_G1	CG9372	1.12508065046033	0.900770125530981	0.998043617620652	pseudobulk	DESeq2	LRT	0.0453860258366365
397	KC_G1	CG9377	0.370968012757542	0.699449609000128	0.998043617620652	pseudobulk	DESeq2	LRT	0.155243568265056
397	KC_G1	CG9380	0.747394379064102	0.747279275622384	0.998043617620652	pseudobulk	DESeq2	LRT	0.126517061913032
397	KC_G1	CG9384	0.408167057795842	0.27324544711922	0.998043617620652	pseudobulk	DESeq2	LRT	0.563447065636164
398	KC_G1	CG9386	-0.245264783574285	0.677597432227586	0.998043617620652	pseudobulk	DESeq2	LRT	0.169028248444031
398	KC_G1	CG9389	1.69981218657454	0.566400560818652	0.998043617620652	pseudobulk	DESeq2	LRT	0.246876325303589
398	KC_G1	CG9391	0.201816508758249	0.288401812763443	0.998043617620652	pseudobulk	DESeq2	LRT	0.540002014165209
398	KC_G1	CG9393	0.394658735818272	0.416958931326702	0.998043617620652	pseudobulk	DESeq2	LRT	0.379906719070712
398	KC_G1	CG9394	-0.0862093519020588	0.658430963644188	0.998043617620652	pseudobulk	DESeq2	LRT	0.181489754005362
398	KC_G1	CG9395	-0.104591302506492	0.76633208854181	0.998043617620652	pseudobulk	DESeq2	LRT	0.115582988896049
398	KC_G1	CG9396	-0.464390851238486	0.829772603746012	0.998043617620652	pseudobulk	DESeq2	LRT	0.0810409081890728
398	KC_G1	CG9399	0.0924059043491161	0.802440359144338	0.998043617620652	pseudobulk	DESeq2	LRT	0.0955872363773576
398	KC_G1	CG9410	0.160725408226694	0.475692862357585	0.998043617620652	pseudobulk	DESeq2	LRT	0.322673365002151
398	KC_G1	CG9422	-0.375435858647802	0.182503769154252	0.998043617620652	pseudobulk	DESeq2	LRT	0.738728161859617
399	KC_G1	CG9425	-0.112739157718239	0.40985600009152	0.998043617620652	pseudobulk	DESeq2	LRT	0.387368702671874
399	KC_G1	CG9426	-0.0913439154666784	0.72589120611445	0.998043617620652	pseudobulk	DESeq2	LRT	0.139128464871123
399	KC_G1	CG9436	0.169296618821234	0.813869184881652	0.998043617620652	pseudobulk	DESeq2	LRT	0.0894453946803194
399	KC_G1	CG9445	1.12508065046033	0.900770125530981	0.998043617620652	pseudobulk	DESeq2	LRT	0.0453860258366365
399	KC_G1	CG9451	0.172834960772825	0.908610676445368	0.998043617620652	pseudobulk	DESeq2	LRT	0.0416221644022337
399	KC_G1	CG9467	0.0408519412573445	0.907844937774245	0.998043617620652	pseudobulk	DESeq2	LRT	0.0419883237474383
399	KC_G1	CG9471	0.239581174411601	0.575256215548565	0.998043617620652	pseudobulk	DESeq2	LRT	0.240138680153695
399	KC_G1	CG9485	-0.442585573743524	0.615982132670005	0.998043617620652	pseudobulk	DESeq2	LRT	0.210431884905967
399	KC_G1	CG9503	-0.794610277746993	0.821479263487599	0.998043617620652	pseudobulk	DESeq2	LRT	0.085403394939296
399	KC_G1	CG9512	-0.861978399584815	0.657286761709498	0.998043617620652	pseudobulk	DESeq2	LRT	0.182245114675988
400	KC_G1	CG9514	0.173116475207952	0.97305562682953	0.998043617620652	pseudobulk	DESeq2	LRT	0.011862331638572
400	KC_G1	CG9515	0.441522504918689	0.533259592346957	0.998043617620652	pseudobulk	DESeq2	LRT	0.273061323659343
400	KC_G1	CG9518	0.730757173260101	0.679347744797991	0.998043617620652	pseudobulk	DESeq2	LRT	0.167907861968013
400	KC_G1	CG9522	-0.767324608732104	0.724630599467818	0.998043617620652	pseudobulk	DESeq2	LRT	0.139883330666603
400	KC_G1	CG9527	0.0792119148662025	0.913334412045076	0.998043617620652	pseudobulk	DESeq2	LRT	0.0393701789668501
400	KC_G1	CG9536	0.500595446304149	0.378956298737686	0.998043617620652	pseudobulk	DESeq2	LRT	0.421410870009171
400	KC_G1	CG9547	0.129865608374264	0.947640458270124	0.998043617620652	pseudobulk	DESeq2	LRT	0.0233564059184837
400	KC_G1	CG9570	1.12508065046033	0.900770125530981	0.998043617620652	pseudobulk	DESeq2	LRT	0.0453860258366365
400	KC_G1	CG9572	-0.65817001371651	0.770857516266391	0.998043617620652	pseudobulk	DESeq2	LRT	0.113025888638787
400	KC_G1	CG9577	-1.08018490478502	0.0609352959242534	0.998043617620652	pseudobulk	DESeq2	LRT	1.21513107543322
401	KC_G1	CG9578	-0.335751212772228	0.375448493274349	0.998043617620652	pseudobulk	DESeq2	LRT	0.425449634215172
401	KC_G1	CG9581	0.2660483643935	0.606370104597776	0.998043617620652	pseudobulk	DESeq2	LRT	0.217262218537399
401	KC_G1	CG9586	-0.169434117121121	0.58387085016003	0.998043617620652	pseudobulk	DESeq2	LRT	0.233683206424785
401	KC_G1	CG9588	-0.216178147536884	0.58698311481274	0.998043617620652	pseudobulk	DESeq2	LRT	0.23137439151069
401	KC_G1	CG9590	-0.0549313960103343	0.889698246133906	0.998043617620652	pseudobulk	DESeq2	LRT	0.0507572655575274
401	KC_G1	CG9593	-2.23422618188688	0.336998892702854	0.998043617620652	pseudobulk	DESeq2	LRT	0.472371526113321
401	KC_G1	CG9596	1.17437439077929	0.170635289145018	0.998043617620652	pseudobulk	DESeq2	LRT	0.767931147282989
401	KC_G1	CG9601	0.124081310677001	0.705566750487299	0.998043617620652	pseudobulk	DESeq2	LRT	0.151461893324264
401	KC_G1	CG9609	0.0297763817710733	0.898570020211565	0.998043617620652	pseudobulk	DESeq2	LRT	0.0464480752532132
401	KC_G1	CG9616	1.12508065046033	0.900770125530981	0.998043617620652	pseudobulk	DESeq2	LRT	0.0453860258366365
402	KC_G1	CG9624	1.12508065046033	0.900770125530981	0.998043617620652	pseudobulk	DESeq2	LRT	0.0453860258366365
402	KC_G1	CG9628	-0.794610277746993	0.815039924133852	0.998043617620652	pseudobulk	DESeq2	LRT	0.0888211171417967
402	KC_G1	CG9630	-0.663008699443227	0.547638269965503	0.998043617620652	pseudobulk	DESeq2	LRT	0.261506210183603
402	KC_G1	CG9636	-0.126244325761963	0.600775196943119	0.998043617620652	pseudobulk	DESeq2	LRT	0.221288005519688
402	KC_G1	CG9641	-0.167095769846973	0.622710377587369	0.998043617620652	pseudobulk	DESeq2	LRT	0.205713896617808
402	KC_G1	CG9643	0.339238744754827	0.462196267730011	0.998043617620652	pseudobulk	DESeq2	LRT	0.335173565803698
402	KC_G1	CG9646	-0.186384228213342	0.265720836529604	0.998043617620652	pseudobulk	DESeq2	LRT	0.575574389003259
402	KC_G1	CG9650	-0.512586510723346	0.784137594652279	0.998043617620652	pseudobulk	DESeq2	LRT	0.10560772385243
402	KC_G1	CG9657	-0.145985309850775	0.570323652688054	0.998043617620652	pseudobulk	DESeq2	LRT	0.243878616808908
402	KC_G1	CG9662	-0.0472572577699909	0.86772948547403	0.998043617620652	pseudobulk	DESeq2	LRT	0.0616156449578957
403	KC_G1	CG9663	-0.117946640308169	0.843823830336422	0.998043617620652	pseudobulk	DESeq2	LRT	0.0737482139180267

403	KC_G1	CG9667	0.169216000503194	0.717670150553226	0.998043617620652	pseudobulk	DESeq2	LRT	0.14407511663463
403	KC_G1	CG9674	-0.183348917410388	0.254023073869942	0.998043617620652	pseudobulk	DESeq2	LRT	0.595126832989266
403	KC_G1	CG9676	1.12508065046033	0.900770125530981	0.998043617620652	pseudobulk	DESeq2	LRT	0.0453860258366365
403	KC_G1	CG9686	-0.107723022096191	0.447877619232982	0.998043617620652	pseudobulk	DESeq2	LRT	0.348840639020966
403	KC_G1	CG9689	-0.24731949914203	0.686574987759256	0.998043617620652	pseudobulk	DESeq2	LRT	0.163312022168642
403	KC_G1	CG9691	-0.140590727951069	0.807500532351159	0.998043617620652	pseudobulk	DESeq2	LRT	0.0928571826846678
403	KC_G1	CG9705	-0.101948928080046	0.706614833433023	0.998043617620652	pseudobulk	DESeq2	LRT	0.150817249988504
403	KC_G1	CG9706	-0.28837309359066	0.768337238207736	0.998043617620652	pseudobulk	DESeq2	LRT	0.11444811779951
403	KC_G1	CG9723	-0.0132579189182772	0.969186322101855	0.998043617620652	pseudobulk	DESeq2	LRT	0.0135927235869509
404	KC_G1	CG9727	-0.270513186416958	0.511282025326946	0.998043617620652	pseudobulk	DESeq2	LRT	0.291339475097714
404	KC_G1	CG9743	-0.86183262160441	0.672975120964107	0.998043617620652	pseudobulk	DESeq2	LRT	0.172000990794925
404	KC_G1	CG9752	1.3847234673349	0.451108629472005	0.998043617620652	pseudobulk	DESeq2	LRT	0.345718864996665
404	KC_G1	CG9760	0.00961823492852598	0.944950595169236	0.998043617620652	pseudobulk	DESeq2	LRT	0.024590897105836
404	KC_G1	CG9766	0.557053324091746	0.594568747377518	0.998043617620652	pseudobulk	DESeq2	LRT	0.225797922575079
404	KC_G1	CG9773	-0.267100003146482	0.580868541488588	0.998043617620652	pseudobulk	DESeq2	LRT	0.235922143282537
404	KC_G1	CG9775	-0.0414423843489283	0.795083348879086	0.998043617620652	pseudobulk	DESeq2	LRT	0.0995873417072141
404	KC_G1	CG9776	-0.151728216125136	0.505464665472421	0.998043617620652	pseudobulk	DESeq2	LRT	0.296309198384954
404	KC_G1	CG9777	-1.73288829970739	0.458469392065673	0.998043617620652	pseudobulk	DESeq2	LRT	0.338689653016193
404	KC_G1	CG9782	-0.980377785265078	0.52657631243277	0.998043617620652	pseudobulk	DESeq2	LRT	0.278538681141683
405	KC_G1	CG9784	0.0247084025750976	0.932011899174738	0.998043617620652	pseudobulk	DESeq2	LRT	0.0305785428896465
405	KC_G1	CG9795	-0.026277189386445	0.916814981512647	0.998043617620652	pseudobulk	DESeq2	LRT	0.037718298588822
405	KC_G1	CG9799	0.385696145852286	0.589567787577491	0.998043617620652	pseudobulk	DESeq2	LRT	0.229466253201103
405	KC_G1	CG9804	0.107886417969005	0.828051575510491	0.998043617620652	pseudobulk	DESeq2	LRT	0.0819426121740217
405	KC_G1	CG9812	-0.414623651988844	0.853296900411484	0.998043617620652	pseudobulk	DESeq2	LRT	0.0688998319328524
405	KC_G1	CG9813	0.0319806984126427	0.785574889874742	0.998043617620652	pseudobulk	DESeq2	LRT	0.104812406793312
405	KC_G1	CG9837	0.300186387385776	0.340242723021166	0.998043617620652	pseudobulk	DESeq2	LRT	0.468211154546518
405	KC_G1	CG9839	0.614842128029125	0.293385426804648	0.998043617620652	pseudobulk	DESeq2	LRT	0.532561462461081
405	KC_G1	CG9849	0.243407143135163	0.536308415581538	0.998043617620652	pseudobulk	DESeq2	LRT	0.270585388172214
405	KC_G1	CG9853	-0.15831030707817	0.575618408191436	0.998043617620652	pseudobulk	DESeq2	LRT	0.239865325840143
406	KC_G1	CG9855	-0.209039911859274	0.528838080349255	0.998043617620652	pseudobulk	DESeq2	LRT	0.276677279902707
406	KC_G1	CG9864	-0.794610277746993	0.879485534599449	0.998043617620652	pseudobulk	DESeq2	LRT	0.0557712992369932
406	KC_G1	CG9886	-0.622156388040849	0.445698654243988	0.998043617620652	pseudobulk	DESeq2	LRT	0.350958677233753
406	KC_G1	CG9890	0.431298165390024	0.534466380858715	0.998043617620652	pseudobulk	DESeq2	LRT	0.272079607698874
406	KC_G1	CG9896	0.647232669512344	0.629499756923923	0.998043617620652	pseudobulk	DESeq2	LRT	0.201004433255276
406	KC_G1	CG9902	0.629762443248671	0.372335936198021	0.998043617620652	pseudobulk	DESeq2	LRT	0.429065045599166
406	KC_G1	CG9911	-0.324795429456313	0.287690043023341	0.998043617620652	pseudobulk	DESeq2	LRT	0.541075168818225
406	KC_G1	CG9915	0.24505011225728	0.461497121840238	0.998043617620652	pseudobulk	DESeq2	LRT	0.335831003138178
406	KC_G1	CG9917	0.196947548815143	0.483109703730382	0.998043617620652	pseudobulk	DESeq2	LRT	0.315954239196911
406	KC_G1	CG9919	0.018551911038889	0.91216911061704	0.998043617620652	pseudobulk	DESeq2	LRT	0.0399246386459558
407	KC_G1	CG9921	0.272398012091606	0.285188511328155	0.998043617620652	pseudobulk	DESeq2	LRT	0.544867973796625
407	KC_G1	CG9922	-0.517621424650103	0.0533323206060813	0.998043617620652	pseudobulk	DESeq2	LRT	1.27300951880186
407	KC_G1	CG9932	-0.00832602090836742	0.955861774477433	0.998043617620652	pseudobulk	DESeq2	LRT	0.019604905759422
407	KC_G1	CG9934	-0.301775173762891	0.375047834301773	0.998043617620652	pseudobulk	DESeq2	LRT	0.425913338009719
407	KC_G1	CG9941	0.0542550606452141	0.859018024391515	0.998043617620652	pseudobulk	DESeq2	LRT	0.0659977234660225
407	KC_G1	CG9945	-0.0438966508968673	0.919707424198617	0.998043617620652	pseudobulk	DESeq2	LRT	0.0363503077247055
407	KC_G1	CG9948	0.558208333830142	0.253853070298521	0.998043617620652	pseudobulk	DESeq2	LRT	0.595417579528858
407	KC_G1	CG9951	0.210109328118656	0.7042011335881	0.998043617620652	pseudobulk	DESeq2	LRT	0.152303280159529
407	KC_G1	CG9953	-0.131878645313132	0.733129304153342	0.998043617620652	pseudobulk	DESeq2	LRT	0.134819420818918
407	KC_G1	CG9967	1.40833198431338	0.231559231545735	0.998043617620652	pseudobulk	DESeq2	LRT	0.635337900355884
408	KC_G1	CG9986	0.409690850138652	0.457641442626075	0.998043617620652	pseudobulk	DESeq2	LRT	0.339474654038329
408	KC_G1	CG9992	-0.381364505297525	0.323417924237002	0.998043617620652	pseudobulk	DESeq2	LRT	0.4902359146043
408	KC_G1	Charon	-0.378296868659845	0.557150920522523	0.998043617620652	pseudobulk	DESeq2	LRT	0.254027147605277
408	KC_G1	chas	1.12508065046033	0.900770125530981	0.998043617620652	pseudobulk	DESeq2	LRT	0.0453860258366365
408	KC_G1	ChAT	-0.0986120168892051	0.641123007206277	0.998043617620652	pseudobulk	DESeq2	LRT	0.193058637835588
408	KC_G1	chb	-0.109870731478289	0.646502859181606	0.998043617620652	pseudobulk	DESeq2	LRT	0.189429550096738
408	KC_G1	Chc	-0.0432790621412661	0.80125084992769	0.998043617620652	pseudobulk	DESeq2	LRT	0.096231496794549

408	KC_G1	Chchd3	0.205273859150527	0.300964312679141	0.998043617620652	pseudobulk	DESeq2	LRT	0.521484998510425
408	KC_G1	Chd1	-0.232213427752824	0.27602636412063	0.998043617620652	pseudobulk	DESeq2	LRT	0.559049435161993
408	KC_G1	Chd64	0.0873476709069203	0.776395657443855	0.998043617620652	pseudobulk	DESeq2	LRT	0.109916902383939
409	KC_G1	CheB42c	0.0279776291156683	0.936493196103831	0.998043617620652	pseudobulk	DESeq2	LRT	0.0284953735533413
409	KC_G1	CheB53a	1.09309732960812	0.339852990356124	0.998043617620652	pseudobulk	DESeq2	LRT	0.468708904380972
409	KC_G1	CheB74a	1.13582588427274	0.704684208645453	0.998043617620652	pseudobulk	DESeq2	LRT	0.152005460551806
409	KC_G1	cher	-0.725964118517062	0.682641307086038	0.998043617620652	pseudobulk	DESeq2	LRT	0.165807435810156
409	KC_G1	CHES-1-like	0.0426376627955006	0.778957535434017	0.998043617620652	pseudobulk	DESeq2	LRT	0.108486217076288
409	KC_G1	Chi	-0.0559008816889512	0.80891605428755	0.998043617620652	pseudobulk	DESeq2	LRT	0.0920965452002271
409	KC_G1	chic	-0.113241872609137	0.381920732661379	0.998043617620652	pseudobulk	DESeq2	LRT	0.418026765203447
409	KC_G1	chico	0.00932176273064048	0.933586258123152	0.998043617620652	pseudobulk	DESeq2	LRT	0.0298455494918304
409	KC_G1	chif	-0.141475818896969	0.534130576715524	0.998043617620652	pseudobulk	DESeq2	LRT	0.272352559797708
409	KC_G1	chinmo	-0.19321441101838	0.135083029258395	0.998043617620652	pseudobulk	DESeq2	LRT	0.869399208804661
410	KC_G1	CHKov1	-0.0529835607621915	0.947851946606671	0.998043617620652	pseudobulk	DESeq2	LRT	0.0232594936674039
410	KC_G1	CHKov2	-1.60891734856068	0.545249891924755	0.998043617620652	pseudobulk	DESeq2	LRT	0.263404411831769
410	KC_G1	chm	-0.0272972906218406	0.942031764328279	0.998043617620652	pseudobulk	DESeq2	LRT	0.0259344530033937
410	KC_G1	Chmp1	0.246628253693647	0.236953308358224	0.998043617620652	pseudobulk	DESeq2	LRT	0.625337223273124
410	KC_G1	CHMP2B	-0.0447702154389791	0.909190221698938	0.998043617620652	pseudobulk	DESeq2	LRT	0.0413452437401046
410	KC_G1	chn	-0.126982463877833	0.267738268308419	0.998043617620652	pseudobulk	DESeq2	LRT	0.572289549891219
410	KC_G1	CHOp24	-0.121760035892445	0.632318290039137	0.998043617620652	pseudobulk	DESeq2	LRT	0.199064255877452
410	KC_G1	CHORD	0.420734842084571	0.30537718171808	0.998043617620652	pseudobulk	DESeq2	LRT	0.515163417260459
410	KC_G1	Chrac-14	0.158719215274239	0.778042076574	0.998043617620652	pseudobulk	DESeq2	LRT	0.108996915696972
410	KC_G1	Chrac-16	-0.21930390183861	0.715034308614973	0.998043617620652	pseudobulk	DESeq2	LRT	0.145673119479068
411	KC_G1	chrb	-0.0305843230633526	0.823161461597476	0.998043617620652	pseudobulk	DESeq2	LRT	0.0845149703731068
411	KC_G1	ChT	-0.205906617903722	0.269569664168525	0.998043617620652	pseudobulk	DESeq2	LRT	0.569328982409297
411	KC_G1	Cht11	1.27658683355474	0.16334427610638	0.998043617620652	pseudobulk	DESeq2	LRT	0.786896079425265
411	KC_G1	Cht2	-2.09327344068102	0.405011885302763	0.998043617620652	pseudobulk	DESeq2	LRT	0.392532231981212
411	KC_G1	Cht5	-0.288885753612645	0.795182966717474	0.998043617620652	pseudobulk	DESeq2	LRT	0.0995329313519882
411	KC_G1	Cht8	-1.66808346769027	0.498191247612582	0.998043617620652	pseudobulk	DESeq2	LRT	0.302603906560549
411	KC_G1	ci	-1.36262208775125	0.554321947234893	0.998043617620652	pseudobulk	DESeq2	LRT	0.256237926072317
411	KC_G1	CIA30	-0.294758186127765	0.569147695094169	0.998043617620652	pseudobulk	DESeq2	LRT	0.244775018594323
411	KC_G1	Ciao1	-0.0672423022984429	0.885572458938511	0.998043617620652	pseudobulk	DESeq2	LRT	0.0527758983590302
411	KC_G1	CIAPIN1	-0.0355736225044751	0.917933100977359	0.998043617620652	pseudobulk	DESeq2	LRT	0.0371889690548318
412	KC_G1	cib	0.0768429825704753	0.765413609185775	0.998043617620652	pseudobulk	DESeq2	LRT	0.116103820212197
412	KC_G1	cic	0.00715802909467844	0.95766632283079	0.998043617620652	pseudobulk	DESeq2	LRT	0.0187857846546734
412	KC_G1	cin	-0.164133178250061	0.629713032936783	0.998043617620652	pseudobulk	DESeq2	LRT	0.200857318173315
412	KC_G1	cindr	-0.0634270402103309	0.768190558279091	0.998043617620652	pseudobulk	DESeq2	LRT	0.114531034989757
412	KC_G1	Cip4	-0.0506739015883606	0.688184718521461	0.998043617620652	pseudobulk	DESeq2	LRT	0.162294975329575
412	KC_G1	Cirl	-0.122047902866135	0.376510883891225	0.998043617620652	pseudobulk	DESeq2	LRT	0.424222465026149
412	KC_G1	Cisd2	0.090800663418018	0.546222449913029	0.998043617620652	pseudobulk	DESeq2	LRT	0.262630454190823
412	KC_G1	ck	-3.27644942312625	0.0905582584116004	0.998043617620652	pseudobulk	DESeq2	LRT	1.04307193829256
412	KC_G1	Cka	-0.0116219685345675	0.923306466330407	0.998043617620652	pseudobulk	DESeq2	LRT	0.0346541228691625
412	KC_G1	CkIalpha	-0.0422324832488753	0.719606190350512	0.998043617620652	pseudobulk	DESeq2	LRT	0.142905109329398
413	KC_G1	CkIIalpha	-0.0583130747569272	0.668801897228298	0.998043617620652	pseudobulk	DESeq2	LRT	0.174702503579488
413	KC_G1	CkIIalpha-i1	-0.337308519017717	0.607647680841913	0.998043617620652	pseudobulk	DESeq2	LRT	0.216348155292997
413	KC_G1	CkIIalpha-i3	0.0219029470816176	0.942174181572388	0.998043617620652	pseudobulk	DESeq2	LRT	0.025868800919558
413	KC_G1	CkIIbeta	-0.0103149522443515	0.945552645124733	0.998043617620652	pseudobulk	DESeq2	LRT	0.0243142861201805
413	KC_G1	ckn	-0.0683748355594252	0.686124376663606	0.998043617620652	pseudobulk	DESeq2	LRT	0.163597150761874
413	KC_G1	Cks30A	-0.711691946027098	0.741255142397939	0.998043617620652	pseudobulk	DESeq2	LRT	0.130032280734188
413	KC_G1	Cks85A	0.408259343346452	0.106779768164526	0.998043617620652	pseudobulk	DESeq2	LRT	0.971511026397909
413	KC_G1	cl	0.0561733990212976	0.826552760475134	0.998043617620652	pseudobulk	DESeq2	LRT	0.0827294193437276
413	KC_G1	Clamp	0.00567556973327459	0.944101199845935	0.998043617620652	pseudobulk	DESeq2	LRT	0.0249814504275582
413	KC_G1	Clbn	-0.0560885147760759	0.854123675997812	0.998043617620652	pseudobulk	DESeq2	LRT	0.0684792394813624
414	KC_G1	Clc	0.057687527483734	0.717503864953653	0.998043617620652	pseudobulk	DESeq2	LRT	0.144175755188362
414	KC_G1	ClC-a	-0.0731710642349294	0.917568626372704	0.998043617620652	pseudobulk	DESeq2	LRT	0.0373614443070394
414	KC_G1	ClC-b	0.277099744430536	0.683633087172658	0.998043617620652	pseudobulk	DESeq2	LRT	0.165176926014547

414	KC_G1	ClC-c	-0.149110243763405	0.563041884999099	0.998043617620652	pseudobulk	DESeq2	LRT	0.249459296538763
414	KC_G1	Clic	-0.140908520193457	0.509374603397113	0.998043617620652	pseudobulk	DESeq2	LRT	0.292962712060757
414	KC_G1	CLIP-190	0.00670107037647375	0.958496120040972	0.998043617620652	pseudobulk	DESeq2	LRT	0.0184096407913504
414	KC_G1	Clk	1.07786256089755	0.612614778068263	0.998043617620652	pseudobulk	DESeq2	LRT	0.212812530944254
414	KC_G1	clos	-0.794610277746993	0.879485534599449	0.998043617620652	pseudobulk	DESeq2	LRT	0.0557712992369932
414	KC_G1	Clp	0.185833348381079	0.718382811958203	0.998043617620652	pseudobulk	DESeq2	LRT	0.143644067155071
414	KC_G1	ClpP	0.0866504309569014	0.736926214445543	0.998043617620652	pseudobulk	DESeq2	LRT	0.132575994180765
415	KC_G1	CLS	0.180046830699845	0.680350188666266	0.998043617620652	pseudobulk	DESeq2	LRT	0.167267490443466
415	KC_G1	clt	1.39833140020692	0.126448602337744	0.998043617620652	pseudobulk	DESeq2	LRT	0.898085966638754
415	KC_G1	clu	0.186372906675901	0.457674488714085	0.998043617620652	pseudobulk	DESeq2	LRT	0.33944329495632
415	KC_G1	cm	0.0987557376001126	0.802142137942947	0.998043617620652	pseudobulk	DESeq2	LRT	0.0957486688046426
415	KC_G1	cmb	0.490324896935188	0.495796374088134	0.998043617620652	pseudobulk	DESeq2	LRT	0.304696653686135
415	KC_G1	cmpy	-0.199348356553009	0.114325048053047	0.998043617620652	pseudobulk	DESeq2	LRT	0.941858607404804
415	KC_G1	cN-IIIB	0.293095482872366	0.574874281469922	0.998043617620652	pseudobulk	DESeq2	LRT	0.240427120238246
415	KC_G1	Cnb	1.13582588427274	0.704684208645453	0.998043617620652	pseudobulk	DESeq2	LRT	0.152005460551806
415	KC_G1	CNBP	-0.0601383343731994	0.680075048920276	0.998043617620652	pseudobulk	DESeq2	LRT	0.167443158568055
415	KC_G1	cnc	0.481088177962876	0.0549319285156322	0.998043617620652	pseudobulk	DESeq2	LRT	1.26017515377319
416	KC_G1	Cndp2	0.0912759878512589	0.830387308020698	0.998043617620652	pseudobulk	DESeq2	LRT	0.0807192973794077
416	KC_G1	CngA	0.245760947937641	0.700074463411315	0.998043617620652	pseudobulk	DESeq2	LRT	0.154855763801878
416	KC_G1	CngB	0.159168014444066	0.559941467113446	0.998043617620652	pseudobulk	DESeq2	LRT	0.251857369133527
416	KC_G1	Cngl	0.0157570355696451	0.865057646664268	0.998043617620652	pseudobulk	DESeq2	LRT	0.0629549505768072
416	KC_G1	cni	0.246517759684528	0.316618854441877	0.998043617620652	pseudobulk	DESeq2	LRT	0.499463226756495
416	KC_G1	cnir	0.0665530081954131	0.857555336952925	0.998043617620652	pseudobulk	DESeq2	LRT	0.0667378459115595
416	KC_G1	cnk	-0.411077000113557	0.357527388889865	0.998043617620652	pseudobulk	DESeq2	LRT	0.446690682847204
416	KC_G1	CNMaR	-2.23422618188688	0.336998892702854	0.998043617620652	pseudobulk	DESeq2	LRT	0.472371526113321
416	KC_G1	cnn	0.301135150308418	0.391875708493367	0.998043617620652	pseudobulk	DESeq2	LRT	0.406851656639929
416	KC_G1	cno	-0.112894865925477	0.506003968848885	0.998043617620652	pseudobulk	DESeq2	LRT	0.295846076752275
417	KC_G1	Cnot4	0.0392836069885578	0.843360954998843	0.998043617620652	pseudobulk	DESeq2	LRT	0.0739865093456648
417	KC_G1	CNPYb	-0.0580075481903933	0.867602671906506	0.998043617620652	pseudobulk	DESeq2	LRT	0.0616791191838184
417	KC_G1	Cnx14D	-0.244421538327994	0.814148350350011	0.998043617620652	pseudobulk	DESeq2	LRT	0.089296452766598
417	KC_G1	Cnx99A	0.302762786162758	0.24738768451919	0.998043617620652	pseudobulk	DESeq2	LRT	0.606621924264241
417	KC_G1	Cog1	0.695511931081279	0.189547328704553	0.998043617620652	pseudobulk	DESeq2	LRT	0.722282331717878
417	KC_G1	Cog2	0.191869008908638	0.764750545511866	0.998043617620652	pseudobulk	DESeq2	LRT	0.116480204552229
417	KC_G1	Cog3	-0.321137626829118	0.675767400477157	0.998043617620652	pseudobulk	DESeq2	LRT	0.170202762745155
417	KC_G1	Cog4	-0.362067783883608	0.544428126943891	0.998043617620652	pseudobulk	DESeq2	LRT	0.264059445810775
417	KC_G1	Cog6	0.156442747084458	0.726497969862187	0.998043617620652	pseudobulk	DESeq2	LRT	0.13876559496387
417	KC_G1	Cog7	-0.21079649603549	0.677115818244497	0.998043617620652	pseudobulk	DESeq2	LRT	0.169337040439683
418	KC_G1	Cog8	-0.0913828138276936	0.881396173123326	0.998043617620652	pseudobulk	DESeq2	LRT	0.054828839456949
418	KC_G1	coil	0.0159526034363009	0.954590028413924	0.998043617620652	pseudobulk	DESeq2	LRT	0.0201831065583221
418	KC_G1	Col4a1	-0.557459575305809	0.371099728232219	0.998043617620652	pseudobulk	DESeq2	LRT	0.430509363698349
418	KC_G1	cold	0.252524346709528	0.719021797629807	0.998043617620652	pseudobulk	DESeq2	LRT	0.143257943487981
418	KC_G1	colt	0.126681227707164	0.776506900568508	0.998043617620652	pseudobulk	DESeq2	LRT	0.109854680482095
418	KC_G1	comm	-0.281628997052641	0.106670401480219	0.998043617620652	pseudobulk	DESeq2	LRT	0.971956070332373
418	KC_G1	comt	-0.133757394157782	0.412355796939982	0.998043617620652	pseudobulk	DESeq2	LRT	0.384727895701675
418	KC_G1	Con	-0.00800876195266648	0.946851113258976	0.998043617620652	pseudobulk	DESeq2	LRT	0.0237183058684737
418	KC_G1	Cont	-0.362842632775048	0.568433788502462	0.998043617620652	pseudobulk	DESeq2	LRT	0.245320114903547
418	KC_G1	conu	-0.079057340368544	0.640590922729375	0.998043617620652	pseudobulk	DESeq2	LRT	0.193419219647336
419	KC_G1	conv	-0.794610277746993	0.821479263487599	0.998043617620652	pseudobulk	DESeq2	LRT	0.085403394939296
419	KC_G1	Coprox	-0.283018387929392	0.745168953648679	0.998043617620652	pseudobulk	DESeq2	LRT	0.127745247629711
419	KC_G1	Coq3	0.450761541743364	0.420521867073204	0.998043617620652	pseudobulk	DESeq2	LRT	0.376211416031803
419	KC_G1	Coq4	0.173105107343345	0.973555957903825	0.998043617620652	pseudobulk	DESeq2	LRT	0.0116390811132178
419	KC_G1	Coq5	-0.286475179739766	0.716434582893138	0.998043617620652	pseudobulk	DESeq2	LRT	0.144823458561199
419	KC_G1	Coq6	-0.051548315899369	0.885288543742716	0.998043617620652	pseudobulk	DESeq2	LRT	0.0529151558204932
419	KC_G1	Coq7	0.177622651011792	0.504559026212778	0.998043617620652	pseudobulk	DESeq2	LRT	0.297088020192209
419	KC_G1	Coq8	-0.207606453075204	0.718486477655262	0.998043617620652	pseudobulk	DESeq2	LRT	0.14358140113444
419	KC_G1	Coq9	0.389683802326743	0.370749848197408	0.998043617620652	pseudobulk	DESeq2	LRT	0.430919018120326

419	KC_G1	CoRest	-0.172145550330041	0.38601701079517	0.998043617620652	pseudobulk	DESeq2	LRT	0.413393556645099
420	KC_G1	Corin	0.02657966095253	0.872909203001366	0.998043617620652	pseudobulk	DESeq2	LRT	0.0590309277563228
420	KC_G1	corn	-0.211669464310006	0.15362540922627	0.998043617620652	pseudobulk	DESeq2	LRT	0.813536947233403
420	KC_G1	coro	0.341252059771843	0.05989088620183	0.998043617620652	pseudobulk	DESeq2	LRT	1.222639260639
420	KC_G1	Cortactin	-0.135620366535251	0.739729695736647	0.998043617620652	pseudobulk	DESeq2	LRT	0.130926946614172
420	KC_G1	corto	-0.200072516753551	0.0957734640709222	0.998043617620652	pseudobulk	DESeq2	LRT	1.01875480411515
420	KC_G1	cos	0.0697163649140702	0.755667069720637	0.998043617620652	pseudobulk	DESeq2	LRT	0.121669502951237
420	KC_G1	Cow	-0.00840286590491794	0.943710637030121	0.998043617620652	pseudobulk	DESeq2	LRT	0.0251611497756094
420	KC_G1	COX4	-0.0397311125856023	0.718086000739752	0.998043617620652	pseudobulk	DESeq2	LRT	0.143823539866257
420	KC_G1	COX5A	0.00591644458280384	0.96030808474539	0.998043617620652	pseudobulk	DESeq2	LRT	0.0175894148355383
420	KC_G1	COX5B	0.00681458683776358	0.961932077888801	0.998043617620652	pseudobulk	DESeq2	LRT	0.0168555924523157
421	KC_G1	COX5BL	-1.24029575767303	0.590792835712426	0.998043617620652	pseudobulk	DESeq2	LRT	0.22856477982831
421	KC_G1	COX6B	-0.0802866224832092	0.498564556745856	0.998043617620652	pseudobulk	DESeq2	LRT	0.302278598990738
421	KC_G1	COX7A	-0.121124026658861	0.345985360865985	0.998043617620652	pseudobulk	DESeq2	LRT	0.460942276437346
421	KC_G1	COX7C	0.0508859338155282	0.686449508877828	0.998043617620652	pseudobulk	DESeq2	LRT	0.163391401358284
421	KC_G1	Cp1	0.129801372026347	0.534989130839885	0.998043617620652	pseudobulk	DESeq2	LRT	0.271655041276364
421	KC_G1	Cp110	-0.177751587736203	0.394454613570137	0.998043617620652	pseudobulk	DESeq2	LRT	0.404002960033749
421	KC_G1	Cp18	0.763164787219597	0.794486984020146	0.998043617620652	pseudobulk	DESeq2	LRT	0.0999132133899323
421	KC_G1	Cp190	-0.0972412570842388	0.623468367425399	0.998043617620652	pseudobulk	DESeq2	LRT	0.205185576188801
421	KC_G1	Cp7Fa	0.989324252065154	0.475255048280916	0.998043617620652	pseudobulk	DESeq2	LRT	0.323073261250735
421	KC_G1	Cp7Fb	0.173195945230598	0.976215465741119	0.998043617620652	pseudobulk	DESeq2	LRT	0.0104543162934892
422	KC_G1	Cp7Fc	1.13582588427274	0.704684208645453	0.998043617620652	pseudobulk	DESeq2	LRT	0.152005460551806
422	KC_G1	cpa	-0.171010706823623	0.66741949541241	0.998043617620652	pseudobulk	DESeq2	LRT	0.17560111169625
422	KC_G1	cpb	-0.156860216049757	0.615492706981385	0.998043617620652	pseudobulk	DESeq2	LRT	0.21077708868962
422	KC_G1	Cpes	-0.128651079068567	0.792520402504212	0.998043617620652	pseudobulk	DESeq2	LRT	0.100989548566437
422	KC_G1	cpo	0.0709835186894464	0.427706541622601	0.998043617620652	pseudobulk	DESeq2	LRT	0.368854107309539
422	KC_G1	Cpr	-1.24390661586413	0.120347180282224	0.998043617620652	pseudobulk	DESeq2	LRT	0.919564080729347
422	KC_G1	Cpr47Eg	-0.794610277746993	0.815039924133852	0.998043617620652	pseudobulk	DESeq2	LRT	0.0888211171417967
422	KC_G1	Cpr51A	0.400329425834043	0.865512925764825	0.998043617620652	pseudobulk	DESeq2	LRT	0.0627264418886215
422	KC_G1	Cpr64Ac	-0.794610277746993	0.879485534599449	0.998043617620652	pseudobulk	DESeq2	LRT	0.0557712992369932
422	KC_G1	Cpr64Ad	1.06667464613488	0.669131470313168	0.998043617620652	pseudobulk	DESeq2	LRT	0.174488544082685
423	KC_G1	Cpr65Ea	-0.228311249709788	0.83269369013844	0.998043617620652	pseudobulk	DESeq2	LRT	0.0795147262609284
423	KC_G1	Cpr65Eb	-0.794610277746993	0.815039924133852	0.998043617620652	pseudobulk	DESeq2	LRT	0.0888211171417967
423	KC_G1	Cpr67B	1.88542683250862	0.272972020122331	0.998043617620652	pseudobulk	DESeq2	LRT	0.563881866253108
423	KC_G1	Cpr73D	1.13582588427274	0.633424328955532	0.998043617620652	pseudobulk	DESeq2	LRT	0.198305259991224
423	KC_G1	Cpsf100	-0.646734411962421	0.272472433880555	0.998043617620652	pseudobulk	DESeq2	LRT	0.564677428870414
423	KC_G1	Cpsf160	0.764163334845867	0.282787447338461	0.998043617620652	pseudobulk	DESeq2	LRT	0.548539872359694
423	KC_G1	Cpsf5	-0.121706566662133	0.501097422175448	0.998043617620652	pseudobulk	DESeq2	LRT	0.300077831418086
423	KC_G1	Cpsf6	-0.160078914362189	0.370958144051005	0.998043617620652	pseudobulk	DESeq2	LRT	0.430675089935261
423	KC_G1	Cpsf73	-0.247458000315962	0.724014081924158	0.998043617620652	pseudobulk	DESeq2	LRT	0.140252986782851
423	KC_G1	CPT2	1.13582588427274	0.704684208645453	0.998043617620652	pseudobulk	DESeq2	LRT	0.152005460551806
424	KC_G1	cpx	-0.0139810226484432	0.896813759073458	0.998043617620652	pseudobulk	DESeq2	LRT	0.0472977373401127
424	KC_G1	CR12460	0.173195945230598	0.976215465741119	0.998043617620652	pseudobulk	DESeq2	LRT	0.0104543162934892
424	KC_G1	CR12628	-0.794610277746993	0.879485534599449	0.998043617620652	pseudobulk	DESeq2	LRT	0.0557712992369932
424	KC_G1	CR12842	0.0633971358049626	0.837895469551944	0.998043617620652	pseudobulk	DESeq2	LRT	0.0768101577759079
424	KC_G1	CR14033	-0.794610277746993	0.821479263487599	0.998043617620652	pseudobulk	DESeq2	LRT	0.085403394939296
424	KC_G1	CR14578	0.173195945230598	0.976215465741119	0.998043617620652	pseudobulk	DESeq2	LRT	0.0104543162934892
424	KC_G1	CR14798	0.896791238125609	0.213182633900817	0.998043617620652	pseudobulk	DESeq2	LRT	0.671248176325714
424	KC_G1	CR15345	-0.446743815663938	0.592641875692454	0.998043617620652	pseudobulk	DESeq2	LRT	0.227207664803952
424	KC_G1	CR18166	-0.272935390754724	0.437764297577567	0.998043617620652	pseudobulk	DESeq2	LRT	0.358759660761042
424	KC_G1	CR18217	-1.27845746407632	0.556113633048063	0.998043617620652	pseudobulk	DESeq2	LRT	0.254836458124014
425	KC_G1	CR18228	-1.36976482397271	0.357462015334623	0.998043617620652	pseudobulk	DESeq2	LRT	0.446770100455956
425	KC_G1	CR18275	-0.0286187421241508	0.945951311780326	0.998043617620652	pseudobulk	DESeq2	LRT	0.0241312162084578
425	KC_G1	CR31032	0.431456263585784	0.510130122757925	0.998043617620652	pseudobulk	DESeq2	LRT	0.292319030984998
425	KC_G1	CR31953	1.12508065046033	0.900770125530981	0.998043617620652	pseudobulk	DESeq2	LRT	0.0453860258366365
425	KC_G1	CR32009	0.173105107343345	0.973555957903825	0.998043617620652	pseudobulk	DESeq2	LRT	0.0116390811132178

425	KC_G1	CR32010	-2.27643791964046	0.298906309922724	0.998043617620652	pseudobulk	DESeq2	LRT	0.52446491689215
425	KC_G1	CR32011	0.173105107343345	0.973555957903825	0.998043617620652	pseudobulk	DESeq2	LRT	0.0116390811132178
425	KC_G1	CR32733	-2.31444314558634	0.21499390294467	0.998043617620652	pseudobulk	DESeq2	LRT	0.667573856154309
425	KC_G1	CR32745	-0.133444433257376	0.771211529543167	0.998043617620652	pseudobulk	DESeq2	LRT	0.112826486393157
425	KC_G1	CR33294	1.29691620864451	0.42272282053264	0.998043617620652	pseudobulk	DESeq2	LRT	0.373944306313686
426	KC_G1	CR40450	0.173195945230598	0.976215465741119	0.998043617620652	pseudobulk	DESeq2	LRT	0.0104543162934892
426	KC_G1	CR41320	-0.669691047955192	0.416004339744354	0.998043617620652	pseudobulk	DESeq2	LRT	0.380902138803076
426	KC_G1	CR41440	-0.71088171110674	0.784645801042283	0.998043617620652	pseudobulk	DESeq2	LRT	0.105326344996467
426	KC_G1	CR41507	-0.794610277746993	0.821479263487599	0.998043617620652	pseudobulk	DESeq2	LRT	0.085403394939296
426	KC_G1	CR41597	0.00992554853666142	0.936002339936546	0.998043617620652	pseudobulk	DESeq2	LRT	0.0287230655564889
426	KC_G1	CR42875	-0.794610277746993	0.879485534599449	0.998043617620652	pseudobulk	DESeq2	LRT	0.0557712992369932
426	KC_G1	CR43086	1.13582588427274	0.704684208645453	0.998043617620652	pseudobulk	DESeq2	LRT	0.152005460551806
426	KC_G1	CR43087	-1.73907471612892	0.507597351592657	0.998043617620652	pseudobulk	DESeq2	LRT	0.294480652518419
426	KC_G1	CR43170	1.34983175981687	0.498849471288393	0.998043617620652	pseudobulk	DESeq2	LRT	0.302030483737643
426	KC_G1	CR43212	-0.191789512291141	0.918149124617736	0.998043617620652	pseudobulk	DESeq2	LRT	0.0370867755102277
427	KC_G1	CR43275	1.13582588427274	0.704684208645453	0.998043617620652	pseudobulk	DESeq2	LRT	0.152005460551806
427	KC_G1	CR43281	0.0572225258796679	0.854253721581657	0.998043617620652	pseudobulk	DESeq2	LRT	0.0684131205082957
427	KC_G1	CR43361	2.16327421405881	0.307865902526682	0.998043617620652	pseudobulk	DESeq2	LRT	0.511638408415809
427	KC_G1	CR43670	0.0785784696450389	0.824357454383607	0.998043617620652	pseudobulk	DESeq2	LRT	0.0838844305309053
427	KC_G1	CR43671	0.929140337457024	0.579338403865936	0.998043617620652	pseudobulk	DESeq2	LRT	0.237067681522965
427	KC_G1	CR43975	-1.72984073695683	0.480047293856853	0.998043617620652	pseudobulk	DESeq2	LRT	0.318715974188453
427	KC_G1	CR44003	0.495089355409986	0.29197194101272	0.998043617620652	pseudobulk	DESeq2	LRT	0.534658882965505
427	KC_G1	CR44383	1.12689409771332	0.593137690901603	0.998043617620652	pseudobulk	DESeq2	LRT	0.226844477871081
427	KC_G1	CR45470	0.396985550026124	0.783958656942991	0.998043617620652	pseudobulk	DESeq2	LRT	0.105706839783619
427	KC_G1	CR4575	1.13582588427274	0.633424328955532	0.998043617620652	pseudobulk	DESeq2	LRT	0.198305259991224
428	KC_G1	CR46405	1.90250740646012	0.460774913678782	0.998043617620652	pseudobulk	DESeq2	LRT	0.336511173568024
428	KC_G1	CR46418	1.13582588427274	0.704684208645453	0.998043617620652	pseudobulk	DESeq2	LRT	0.152005460551806
428	KC_G1	CR46425	-0.794610277746993	0.879485534599449	0.998043617620652	pseudobulk	DESeq2	LRT	0.0557712992369932
428	KC_G1	CR6900	0.300831832790326	0.832978671966532	0.998043617620652	pseudobulk	DESeq2	LRT	0.0793661183601116
428	KC_G1	CR7448	1.11154651156394	0.675883747053588	0.998043617620652	pseudobulk	DESeq2	LRT	0.170127996891038
428	KC_G1	Crag	0.0750899863334394	0.815938499834724	0.998043617620652	pseudobulk	DESeq2	LRT	0.0883425743208606
428	KC_G1	CRAT	0.209475739130332	0.764478933502605	0.998043617620652	pseudobulk	DESeq2	LRT	0.116634477798593
428	KC_G1	crb	-1.05103333137896	0.36325578092143	0.998043617620652	pseudobulk	DESeq2	LRT	0.439787465530147
428	KC_G1	crc	-0.0164328321788819	0.934618110315957	0.998043617620652	pseudobulk	DESeq2	LRT	0.0293658078033789
428	KC_G1	CrebA	-0.629978101324826	0.306436119802784	0.998043617620652	pseudobulk	DESeq2	LRT	0.513660045480348
429	KC_G1	CrebB	0.00800792589198229	0.965504702129189	0.998043617620652	pseudobulk	DESeq2	LRT	0.0152456068107518
429	KC_G1	CREG	0.233937869089527	0.872252936385156	0.998043617620652	pseudobulk	DESeq2	LRT	0.0593575598520801
429	KC_G1	Crg-1	0.864750312591438	0.0805701553800589	0.998043617620652	pseudobulk	DESeq2	LRT	1.09382579881546
429	KC_G1	CRIF	-0.130455794560038	0.64701555476744	0.998043617620652	pseudobulk	DESeq2	LRT	0.189085278421773
429	KC_G1	crim	0.0248445340990691	0.95155831648491	0.998043617620652	pseudobulk	DESeq2	LRT	0.0215645907174619
429	KC_G1	Crk	-0.127201951265753	0.418525412378301	0.998043617620652	pseudobulk	DESeq2	LRT	0.378278167012176
429	KC_G1	crm	-0.774213714406026	0.234123661131688	0.998043617620652	pseudobulk	DESeq2	LRT	0.630554693182248
429	KC_G1	CRMP	0.0386802532754577	0.823077877464682	0.998043617620652	pseudobulk	DESeq2	LRT	0.0845590710416027
429	KC_G1	crok	-1.11846266848916	0.394361124304184	0.998043617620652	pseudobulk	DESeq2	LRT	0.404105903904062
429	KC_G1	crol	0.0671587381092325	0.587712247893364	0.998043617620652	pseudobulk	DESeq2	LRT	0.230835258516371
430	KC_G1	CROT	0.825441251989776	0.381854201958167	0.998043617620652	pseudobulk	DESeq2	LRT	0.418102426019339
430	KC_G1	crp	-0.207108795227758	0.15667280799945	0.998043617620652	pseudobulk	DESeq2	LRT	0.805006372775448
430	KC_G1	crq	0.0551729260491969	0.844775167483476	0.998043617620652	pseudobulk	DESeq2	LRT	0.0732588608891049
430	KC_G1	Crtc	0.271160572975611	0.483061449122512	0.998043617620652	pseudobulk	DESeq2	LRT	0.315997620144231
430	KC_G1	cry	-0.148575860353028	0.86232815051558	0.998043617620652	pseudobulk	DESeq2	LRT	0.0643274362187147
430	KC_G1	Csas	-0.548436644749858	0.280963809339559	0.998043617620652	pseudobulk	DESeq2	LRT	0.551349617519668
430	KC_G1	Cse1	-0.0419882030661636	0.922027883347614	0.998043617620652	pseudobulk	DESeq2	LRT	0.0352559451059163
430	KC_G1	Csk	-0.121199150769225	0.440497063041206	0.998043617620652	pseudobulk	DESeq2	LRT	0.356056982846144
430	KC_G1	Csl4	-0.127723584595738	0.808629035599091	0.998043617620652	pseudobulk	DESeq2	LRT	0.0922506684264522
430	KC_G1	CSN1b	-0.123183228420901	0.805307408792422	0.998043617620652	pseudobulk	DESeq2	LRT	0.0940383053992338
431	KC_G1	CSN3	0.0533052834046746	0.908817694851193	0.998043617620652	pseudobulk	DESeq2	LRT	0.0415232257556151

431	KC_G1	CSN5	-0.234112205221921	0.471541870153574	0.998043617620652	pseudobulk	DESeq2	LRT	0.326479738410609
431	KC_G1	CSN6	0.0350965530516479	0.893727984110656	0.998043617620652	pseudobulk	DESeq2	LRT	0.0487946433693487
431	KC_G1	CSN7	-0.102265325272989	0.631628055728984	0.998043617620652	pseudobulk	DESeq2	LRT	0.199538587692124
431	KC_G1	CSN8	-0.0717524480071692	0.764989178978264	0.998043617620652	pseudobulk	DESeq2	LRT	0.116344708040195
431	KC_G1	Csp	-0.02468689578762	0.875644951882999	0.998043617620652	pseudobulk	DESeq2	LRT	0.0576719517025167
431	KC_G1	CstF50	0.0264801101658656	0.961461487418234	0.998043617620652	pseudobulk	DESeq2	LRT	0.0170681073035327
431	KC_G1	CstF64	-0.870890895491663	0.105495345501146	0.998043617620652	pseudobulk	DESeq2	LRT	0.976766701198142
431	KC_G1	csul	0.196777102394628	0.790142364562099	0.998043617620652	pseudobulk	DESeq2	LRT	0.102294652287489
431	KC_G1	csw	-0.137137087832015	0.289201627462603	0.998043617620652	pseudobulk	DESeq2	LRT	0.5387992674078
432	KC_G1	ct	0.079540734966363	0.88178883666056	0.998043617620652	pseudobulk	DESeq2	LRT	0.0546354035902215
432	KC_G1	cta	-0.0466229038683654	0.81246292531068	0.998043617620652	pseudobulk	DESeq2	LRT	0.0901964478264494
432	KC_G1	CtBP	-0.0332380927565057	0.769728543319535	0.998043617620652	pseudobulk	DESeq2	LRT	0.113662408495312
432	KC_G1	CTCF	-0.631283463801767	0.217332364129353	0.998043617620652	pseudobulk	DESeq2	LRT	0.662875595740253
432	KC_G1	CtIP	0.0702440000219355	0.87859268869552	0.998043617620652	pseudobulk	DESeq2	LRT	0.0562124150843596
432	KC_G1	Ctl1	-0.044930791463304	0.911343424576914	0.998043617620652	pseudobulk	DESeq2	LRT	0.0403179355480582
432	KC_G1	Ctl2	0.278737225299593	0.350665492393439	0.998043617620652	pseudobulk	DESeq2	LRT	0.455106969221059
432	KC_G1	ctp	-0.037907801490377	0.77294549451503	0.998043617620652	pseudobulk	DESeq2	LRT	0.111851129970356
432	KC_G1	CTPsyn	-0.19773476590345	0.531344527110037	0.998043617620652	pseudobulk	DESeq2	LRT	0.274623788329117
432	KC_G1	Ctr1A	-0.348099584684014	0.212270272491107	0.998043617620652	pseudobulk	DESeq2	LRT	0.673110822588873
433	KC_G1	Ctr1B	0.108965432571815	0.945978168508996	0.998043617620652	pseudobulk	DESeq2	LRT	0.0241188862255642
433	KC_G1	Ctr9	0.393958077075902	0.0808481680264889	0.998043617620652	pseudobulk	DESeq2	LRT	1.09232981651207
433	KC_G1	ctrip	-0.207974524544243	0.207023447599846	0.998043617620652	pseudobulk	DESeq2	LRT	0.683980463303809
433	KC_G1	CtsB1	-0.0503088561375512	0.842059554023913	0.998043617620652	pseudobulk	DESeq2	LRT	0.0746571922659875
433	KC_G1	cu	0.14719435842814	0.838266104039973	0.998043617620652	pseudobulk	DESeq2	LRT	0.0766180945307966
433	KC_G1	Cubn	-0.944972423315806	0.467795197402586	0.998043617620652	pseudobulk	DESeq2	LRT	0.329944241167871
433	KC_G1	cue	-0.148182027552742	0.599294483814009	0.998043617620652	pseudobulk	DESeq2	LRT	0.222359719734122
433	KC_G1	Cul1	0.0305577344425918	0.862971378025462	0.998043617620652	pseudobulk	DESeq2	LRT	0.0640036081917875
433	KC_G1	Cul2	-0.182252085871149	0.441198738175721	0.998043617620652	pseudobulk	DESeq2	LRT	0.355365738308241
433	KC_G1	Cul3	-0.289711492083902	0.212704607705169	0.998043617620652	pseudobulk	DESeq2	LRT	0.672223102108297
434	KC_G1	Cul4	-0.056986179842456	0.872687770795963	0.998043617620652	pseudobulk	DESeq2	LRT	0.0591411098738239
434	KC_G1	Cul5	-0.267372559125196	0.567110059609333	0.998043617620652	pseudobulk	DESeq2	LRT	0.246332648968498
434	KC_G1	Culd	2.37279544779784	0.320199125517434	0.998043617620652	pseudobulk	DESeq2	LRT	0.494579858498766
434	KC_G1	cutlet	-0.794610277746993	0.879485534599449	0.998043617620652	pseudobulk	DESeq2	LRT	0.0557712992369932
434	KC_G1	cv	-0.794610277746993	0.879485534599449	0.998043617620652	pseudobulk	DESeq2	LRT	0.0557712992369932
434	KC_G1	cv-2	-0.27398841537092	0.849208903412199	0.998043617620652	pseudobulk	DESeq2	LRT	0.0709854611823008
434	KC_G1	cv-c	-0.0988616441685211	0.449705887157273	0.998043617620652	pseudobulk	DESeq2	LRT	0.347071426990248
434	KC_G1	cv-d	1.90250740646012	0.460774913678782	0.998043617620652	pseudobulk	DESeq2	LRT	0.336511173568024
434	KC_G1	Cwc25	-0.619757951394328	0.0765319074411419	0.998043617620652	pseudobulk	DESeq2	LRT	1.11615746240765
434	KC_G1	cwo	-0.516124203728686	0.0207184639574127	0.998043617620652	pseudobulk	DESeq2	LRT	1.68364244581618
435	KC_G1	cyc	0.16395625095493	0.693052609985837	0.998043617620652	pseudobulk	DESeq2	LRT	0.159233796615741
435	KC_G1	CycC	-0.129312300656851	0.725091467188663	0.998043617620652	pseudobulk	DESeq2	LRT	0.139607205581118
435	KC_G1	CycD	0.448828861383911	0.741765961266935	0.998043617620652	pseudobulk	DESeq2	LRT	0.129733099792956
435	KC_G1	CycE	2.02093374629628	0.211007585685658	0.998043617620652	pseudobulk	DESeq2	LRT	0.675701931611288
435	KC_G1	CycG	-0.00576695403247126	0.955980347069865	0.998043617620652	pseudobulk	DESeq2	LRT	0.0195510358063712
435	KC_G1	CycH	-0.299687141892491	0.716885478473439	0.998043617620652	pseudobulk	DESeq2	LRT	0.144550216772185
435	KC_G1	CycJ	-0.781515823731114	0.644615176883988	0.998043617620652	pseudobulk	DESeq2	LRT	0.190699473630734
435	KC_G1	CycT	-0.162489821006184	0.248598569877887	0.998043617620652	pseudobulk	DESeq2	LRT	0.604501374068987
435	KC_G1	CycY	-0.195385040857733	0.382723349872231	0.998043617620652	pseudobulk	DESeq2	LRT	0.417115040750899
435	KC_G1	CYLD	-0.113536368837617	0.716458065484558	0.998043617620652	pseudobulk	DESeq2	LRT	0.144809223915098
436	KC_G1	Cyp1	-0.0821930290163516	0.436294442294209	0.998043617620652	pseudobulk	DESeq2	LRT	0.360220319207381
436	KC_G1	Cyp12a4	-1.3120605000424	0.377688102564812	0.998043617620652	pseudobulk	DESeq2	LRT	0.422866695532104
436	KC_G1	Cyp12b2	-0.220728140714359	0.661800998041221	0.998043617620652	pseudobulk	DESeq2	LRT	0.179272582229855
436	KC_G1	Cyp12e1	3.05294074758062	0.111188494490705	0.998043617620652	pseudobulk	DESeq2	LRT	0.953940150143498
436	KC_G1	Cyp18a1	-1.73907471612892	0.507597351592657	0.998043617620652	pseudobulk	DESeq2	LRT	0.294480652518419
436	KC_G1	Cyp28a5	-0.382023571421525	0.603091083980993	0.998043617620652	pseudobulk	DESeq2	LRT	0.219617092033902
436	KC_G1	Cyp28c1	1.98003016758422	0.223064603952322	0.998043617620652	pseudobulk	DESeq2	LRT	0.651569338401808

436	KC_G1	Cyp28d1	-0.0176053395478718	0.93174528748795	0.998043617620652	pseudobulk	DESeq2	LRT	0.0307027951109136
436	KC_G1	Cyp28d2	-0.704123705228411	0.78667415397905	0.998043617620652	pseudobulk	DESeq2	LRT	0.104205118254245
436	KC_G1	Cyp303a1	-0.794610277746993	0.879485534599449	0.998043617620652	pseudobulk	DESeq2	LRT	0.0557712992369932
437	KC_G1	Cyp305a1	-2.66786719284931	0.228087652304378	0.998043617620652	pseudobulk	DESeq2	LRT	0.64189822494353
437	KC_G1	Cyp317a1	1.13582588427274	0.633424328955532	0.998043617620652	pseudobulk	DESeq2	LRT	0.198305259991224
437	KC_G1	cyp33	-0.191020203357651	0.658619595483346	0.998043617620652	pseudobulk	DESeq2	LRT	0.181365352148729
437	KC_G1	Cyp49a1	-1.94776694850945	0.153556837875797	0.998043617620652	pseudobulk	DESeq2	LRT	0.813730839685866
437	KC_G1	Cyp4ac3	-1.67593316621313	0.495877122847873	0.998043617620652	pseudobulk	DESeq2	LRT	0.304625927300966
437	KC_G1	Cyp4ae1	-0.104512018727332	0.88967472146253	0.998043617620652	pseudobulk	DESeq2	LRT	0.0507687489678678
437	KC_G1	Cyp4d14	1.13582588427274	0.633424328955532	0.998043617620652	pseudobulk	DESeq2	LRT	0.198305259991224
437	KC_G1	Cyp4d2	1.76911534427852	0.454897600650965	0.998043617620652	pseudobulk	DESeq2	LRT	0.342086353842267
437	KC_G1	Cyp4g1	2.63732022130093	0.22943645835378	0.998043617620652	pseudobulk	DESeq2	LRT	0.639337569853304
437	KC_G1	Cyp4g15	0.322369884021613	0.61269544813999	0.998043617620652	pseudobulk	DESeq2	LRT	0.21275534613292
438	KC_G1	Cyp4p3	-0.794610277746993	0.821479263487599	0.998043617620652	pseudobulk	DESeq2	LRT	0.085403394939296
438	KC_G1	Cyp4s3	1.03479139487875	0.378407942156206	0.998043617620652	pseudobulk	DESeq2	LRT	0.422039757052439
438	KC_G1	Cyp6a13	0.0558547895336278	0.950565350737723	0.998043617620652	pseudobulk	DESeq2	LRT	0.0220180203144185
438	KC_G1	Cyp6a15Psi	1.13582588427274	0.704684208645453	0.998043617620652	pseudobulk	DESeq2	LRT	0.152005460551806
438	KC_G1	Cyp6a17	-1.50763406412345	0.166918889067177	0.998043617620652	pseudobulk	DESeq2	LRT	0.77749451440845
438	KC_G1	Cyp6a19	-0.794610277746993	0.879485534599449	0.998043617620652	pseudobulk	DESeq2	LRT	0.0557712992369932
438	KC_G1	Cyp6a2	-0.794610277746993	0.821479263487599	0.998043617620652	pseudobulk	DESeq2	LRT	0.085403394939296
438	KC_G1	Cyp6a20	0.105688403756531	0.936019838550303	0.998043617620652	pseudobulk	DESeq2	LRT	0.0287149464738379
438	KC_G1	Cyp6a23	1.13582588427274	0.704684208645453	0.998043617620652	pseudobulk	DESeq2	LRT	0.152005460551806
438	KC_G1	Cyp6a9	0.940568543778285	0.571268210709151	0.998043617620652	pseudobulk	DESeq2	LRT	0.243159942397914
439	KC_G1	Cyp6g1	-0.136523184444227	0.931168876013455	0.998043617620652	pseudobulk	DESeq2	LRT	0.0309715485892779
439	KC_G1	Cyp6t1	1.15793661454007	0.402167408337566	0.998043617620652	pseudobulk	DESeq2	LRT	0.395593127554969
439	KC_G1	Cyp6t2Psi	-1.74720907763611	0.407846032164752	0.998043617620652	pseudobulk	DESeq2	LRT	0.389503758482075
439	KC_G1	Cyp6u1	0.36739364685422	0.611826995068915	0.998043617620652	pseudobulk	DESeq2	LRT	0.213371364960321
439	KC_G1	Cyp6v1	0.172845155749872	0.90730697492154	0.998043617620652	pseudobulk	DESeq2	LRT	0.0422457504714555
439	KC_G1	Cyp6w1	-0.604006427184727	0.449259719414869	0.998043617620652	pseudobulk	DESeq2	LRT	0.347502518464766
439	KC_G1	Cyp9c1	-0.752107099832492	0.841251974639758	0.998043617620652	pseudobulk	DESeq2	LRT	0.0750739033633379
439	KC_G1	Cyp9f2	0.628102248633374	0.373400785618102	0.998043617620652	pseudobulk	DESeq2	LRT	0.427824772650338
439	KC_G1	Cyp9f3	-0.140467275673349	0.898055452977052	0.998043617620652	pseudobulk	DESeq2	LRT	0.0466968457621063
439	KC_G1	cype	-0.120361337724682	0.278871849690894	0.998043617620652	pseudobulk	DESeq2	LRT	0.554595322739801
440	KC_G1	Cypl	0.447689267072431	0.278702288762067	0.998043617620652	pseudobulk	DESeq2	LRT	0.554859464754028
440	KC_G1	cyr	-0.794610277746993	0.815039924133852	0.998043617620652	pseudobulk	DESeq2	LRT	0.0888211171417967
440	KC_G1	Cys	-0.0702363819085799	0.849674795989198	0.998043617620652	pseudobulk	DESeq2	LRT	0.0707472640879455
440	KC_G1	CysRS	0.0230995733569928	0.967838385404094	0.998043617620652	pseudobulk	DESeq2	LRT	0.014197157347674
440	KC_G1	CysRS-m	0.236351377673133	0.686795219340068	0.998043617620652	pseudobulk	DESeq2	LRT	0.163172736547601
440	KC_G1	Cyt-b5	0.131718560662304	0.458450190071598	0.998043617620652	pseudobulk	DESeq2	LRT	0.33870784287762
440	KC_G1	Cyt-c-d	-0.641125686021162	0.707504209839923	0.998043617620652	pseudobulk	DESeq2	LRT	0.150270971627264
440	KC_G1	Cyt-c-p	-0.0822123901944765	0.532347132493547	0.998043617620652	pseudobulk	DESeq2	LRT	0.273805080932846
440	KC_G1	Cyt-c1	0.0171339260124505	0.902275310982261	0.998043617620652	pseudobulk	DESeq2	LRT	0.0446609260977772
440	KC_G1	D	-1.68732172404213	0.425379511022056	0.998043617620652	pseudobulk	DESeq2	LRT	0.371223432309067
441	KC_G1	D1	0.0987482939331767	0.478470945195425	0.998043617620652	pseudobulk	DESeq2	LRT	0.320144429307016
441	KC_G1	D12	-0.389043772020096	0.515077292306619	0.998043617620652	pseudobulk	DESeq2	LRT	0.288127596000441
441	KC_G1	D19A	-0.457966357238983	0.224995322970319	0.998043617620652	pseudobulk	DESeq2	LRT	0.647826509574387
441	KC_G1	D19B	0.0328603281074	0.923882229749043	0.998043617620652	pseudobulk	DESeq2	LRT	0.0343833861715805
441	KC_G1	D2hgdh	0.248585902305283	0.775637992103234	0.998043617620652	pseudobulk	DESeq2	LRT	0.110340926570266
441	KC_G1	d4	0.0669475576945083	0.659507942645858	0.998043617620652	pseudobulk	DESeq2	LRT	0.180779969751435
441	KC_G1	da	-0.130348269654087	0.430618905582642	0.998043617620652	pseudobulk	DESeq2	LRT	0.365906907141468
441	KC_G1	DAAM	-0.287277395475089	0.338457967068782	0.998043617620652	pseudobulk	DESeq2	LRT	0.470495258451683
441	KC_G1	Dab	-0.118533669186678	0.404720021613237	0.998043617620652	pseudobulk	DESeq2	LRT	0.392845310406889
441	KC_G1	dac	-0.202273604157164	0.0953449839562075	0.998043617620652	pseudobulk	DESeq2	LRT	1.02070214999545
442	KC_G1	Dad	-0.184963395358134	0.477174588462911	0.998043617620652	pseudobulk	DESeq2	LRT	0.321322692378452
442	KC_G1	Dad1	0.0655471711250265	0.764147544019267	0.998043617620652	pseudobulk	DESeq2	LRT	0.116822778378683
442	KC_G1	daed	0.231575328235844	0.693222615661448	0.998043617620652	pseudobulk	DESeq2	LRT	0.159127277324601

442	KC_G1	Dak1	0.0239224747100578	0.911370425311356	0.998043617620652	pseudobulk	DESeq2	LRT	0.0403050687231564
442	KC_G1	dally	0.0993314387206689	0.797601623680713	0.998043617620652	pseudobulk	DESeq2	LRT	0.0982139706005426
442	KC_G1	dan	0.413194291449855	0.823382923314524	0.998043617620652	pseudobulk	DESeq2	LRT	0.0843981443669232
442	KC_G1	danr	-0.229117006093756	0.767788736792015	0.998043617620652	pseudobulk	DESeq2	LRT	0.114758263128949
442	KC_G1	dany	1.12508065046033	0.900770125530981	0.998043617620652	pseudobulk	DESeq2	LRT	0.0453860258366365
442	KC_G1	dao	-0.0525359221841326	0.823427470821918	0.998043617620652	pseudobulk	DESeq2	LRT	0.0843746483570399
442	KC_G1	dap	-0.794610277746993	0.815039924133852	0.998043617620652	pseudobulk	DESeq2	LRT	0.0888211171417967
443	KC_G1	Dap160	-0.112667227482285	0.486194238130615	0.998043617620652	pseudobulk	DESeq2	LRT	0.313190192269538
443	KC_G1	dare	0.117368080001209	0.830846291732875	0.998043617620652	pseudobulk	DESeq2	LRT	0.0804793141541904
443	KC_G1	Dark	-0.0958878289223253	0.773873810841461	0.998043617620652	pseudobulk	DESeq2	LRT	0.111329850328919
443	KC_G1	DAT	-1.66628396221779	0.391636561643864	0.998043617620652	pseudobulk	DESeq2	LRT	0.40711677095731
443	KC_G1	dati	-0.195868437136344	0.221699873867244	0.998043617620652	pseudobulk	DESeq2	LRT	0.654234553970652
443	KC_G1	daw	1.06585281705208	0.683859663547483	0.998043617620652	pseudobulk	DESeq2	LRT	0.1650330117303
443	KC_G1	Daxx	-0.292295028028378	0.27931026280695	0.998043617620652	pseudobulk	DESeq2	LRT	0.553913106552694
443	KC_G1	dbe	-0.0783584139905745	0.875117318390625	0.998043617620652	pseudobulk	DESeq2	LRT	0.0579337214756418
443	KC_G1	dbo	-0.173600777278282	0.391532073621985	0.998043617620652	pseudobulk	DESeq2	LRT	0.407232655505166
443	KC_G1	Dbp21E2	0.23507988071183	0.820729006223934	0.998043617620652	pseudobulk	DESeq2	LRT	0.0858002174596747
444	KC_G1	Dbp45A	-0.0471174209684259	0.896683357278187	0.998043617620652	pseudobulk	DESeq2	LRT	0.0473608908068226
444	KC_G1	Dbp73D	0.479342423612837	0.578236285930591	0.998043617620652	pseudobulk	DESeq2	LRT	0.237894658627031
444	KC_G1	Dbp80	0.0217818624430608	0.864836312324696	0.998043617620652	pseudobulk	DESeq2	LRT	0.0630660837273183
444	KC_G1	dbr	-0.184587320789327	0.681789507225568	0.998043617620652	pseudobulk	DESeq2	LRT	0.166349686869377
444	KC_G1	DCAF12	0.415452694412249	0.413303981399229	0.998043617620652	pseudobulk	DESeq2	LRT	0.383730411095288
444	KC_G1	Dci	0.413468797093092	0.472649892209402	0.998043617620652	pseudobulk	DESeq2	LRT	0.325460436813728
444	KC_G1	dco	0.200929909300624	0.215676185094168	0.998043617620652	pseudobulk	DESeq2	LRT	0.666197806928919
444	KC_G1	Dcp-1	-0.145346077831491	0.705115813363969	0.998043617620652	pseudobulk	DESeq2	LRT	0.151739545456694
444	KC_G1	DCP1	-0.212194344505922	0.460970539485881	0.998043617620652	pseudobulk	DESeq2	LRT	0.336326829377283
444	KC_G1	DCP2	-0.0780022581040023	0.566986533607715	0.998043617620652	pseudobulk	DESeq2	LRT	0.246427255832511
445	KC_G1	Dcr-1	-0.739306402603735	0.407650521590726	0.998043617620652	pseudobulk	DESeq2	LRT	0.389711997657799
445	KC_G1	Dcr-2	0.321403833618947	0.491513834655817	0.998043617620652	pseudobulk	DESeq2	LRT	0.308464253558964
445	KC_G1	DCTN1-p150	-0.451098513558675	0.114021272308466	0.998043617620652	pseudobulk	DESeq2	LRT	0.943014117221878
445	KC_G1	DCTN2-p50	-0.362248909744893	0.246473251786859	0.998043617620652	pseudobulk	DESeq2	LRT	0.608230205115692
445	KC_G1	DCTN3-p24	0.29168759379467	0.353395912063731	0.998043617620652	pseudobulk	DESeq2	LRT	0.451738478537326
445	KC_G1	DCTN4-p62	-0.244567376362208	0.568200794406212	0.998043617620652	pseudobulk	DESeq2	LRT	0.245498163421965
445	KC_G1	DCTN5-p25	-0.111142270936793	0.759988864766204	0.998043617620652	pseudobulk	DESeq2	LRT	0.119192770885025
445	KC_G1	DCTN6-p27	-0.0209605066607955	0.938054896020584	0.998043617620652	pseudobulk	DESeq2	LRT	0.0277717454789289
445	KC_G1	Dd	0.0177368297627344	0.913334791988941	0.998043617620652	pseudobulk	DESeq2	LRT	0.0393699983019289
445	KC_G1	Ddr	0.173116475207952	0.97305562682953	0.998043617620652	pseudobulk	DESeq2	LRT	0.011862331638572
446	KC_G1	Ddx1	0.0668984404185529	0.896806242992972	0.998043617620652	pseudobulk	DESeq2	LRT	0.047301377121423
446	KC_G1	Deaf1	-0.111829441677744	0.497167583002301	0.998043617620652	pseudobulk	DESeq2	LRT	0.303497196567388
446	KC_G1	Debcl	2.18731290743882	0.327254619360621	0.998043617620652	pseudobulk	DESeq2	LRT	0.48511421445278
446	KC_G1	DEF8	0.220223997316011	0.668410519489072	0.998043617620652	pseudobulk	DESeq2	LRT	0.174956723769511
446	KC_G1	defl	-0.0291557886743456	0.965228313172125	0.998043617620652	pseudobulk	DESeq2	LRT	0.0153699473578711
446	KC_G1	Dek	-0.0487377985632935	0.71812338147268	0.998043617620652	pseudobulk	DESeq2	LRT	0.143800932792045
446	KC_G1	del	-0.100533699034279	0.889844784719778	0.998043617620652	pseudobulk	DESeq2	LRT	0.0506857405668182
446	KC_G1	deltaCOP	-0.00578142471551597	0.930750809774086	0.998043617620652	pseudobulk	DESeq2	LRT	0.031166577263463
446	KC_G1	Den1	-0.269043167805338	0.190398434038569	0.998043617620652	pseudobulk	DESeq2	LRT	0.720336627859134
446	KC_G1	DENR	0.0787942321558613	0.727508660202175	0.998043617620652	pseudobulk	DESeq2	LRT	0.138161832506137
447	KC_G1	Der-1	-0.00928623533547628	0.972792722894688	0.998043617620652	pseudobulk	DESeq2	LRT	0.0119796868568072
447	KC_G1	Der-2	0.121827922006003	0.702559820652342	0.998043617620652	pseudobulk	DESeq2	LRT	0.153316691105248
447	KC_G1	Dera	2.27552427769476	0.294492323893065	0.998043617620652	pseudobulk	DESeq2	LRT	0.530926020857631
447	KC_G1	Desat1	-0.0723353706168199	0.527904434973027	0.998043617620652	pseudobulk	DESeq2	LRT	0.277444689436392
447	KC_G1	Desat2	1.13582588427274	0.704684208645453	0.998043617620652	pseudobulk	DESeq2	LRT	0.152005460551806
447	KC_G1	Det	1.42248086069383	0.388666685355129	0.998043617620652	pseudobulk	DESeq2	LRT	0.410422683414259
447	KC_G1	Df31	-0.122249924998435	0.336336306917359	0.998043617620652	pseudobulk	DESeq2	LRT	0.473226248823411
447	KC_G1	Dg	-0.0985602217421915	0.60099773418022	0.998043617620652	pseudobulk	DESeq2	LRT	0.221127165326516
447	KC_G1	Dgk	0.210403070507647	0.427734244990924	0.998043617620652	pseudobulk	DESeq2	LRT	0.368825978138741

447	KC_G1	Dgkepsilon	0.319426851782314	0.263063280657627	0.998043617620652	pseudobulk	DESeq2	LRT	0.579939768108486
448	KC_G1	Dgp-1	-0.246174888118564	0.521579409157405	0.998043617620652	pseudobulk	DESeq2	LRT	0.282679561957558
448	KC_G1	dgrn	-0.575387673639577	0.214556066994643	0.998043617620652	pseudobulk	DESeq2	LRT	0.668459200427315
448	KC_G1	dgt1	0.19378122857149	0.653096404750103	0.998043617620652	pseudobulk	DESeq2	LRT	0.185022706993322
448	KC_G1	dgt2	0.763164787219597	0.794486984020146	0.998043617620652	pseudobulk	DESeq2	LRT	0.0999132133899323
448	KC_G1	dgt5	1.13582588427274	0.704684208645453	0.998043617620652	pseudobulk	DESeq2	LRT	0.152005460551806
448	KC_G1	dgt6	1.13582588427274	0.704684208645453	0.998043617620652	pseudobulk	DESeq2	LRT	0.152005460551806
448	KC_G1	Dh31	-0.794610277746993	0.821479263487599	0.998043617620652	pseudobulk	DESeq2	LRT	0.085403394939296
448	KC_G1	Dh44	1.69981218657454	0.566400560818652	0.998043617620652	pseudobulk	DESeq2	LRT	0.246876325303589
448	KC_G1	Dh44-R1	0.607627059851831	0.729635445868723	0.998043617620652	pseudobulk	DESeq2	LRT	0.136894076034813
448	KC_G1	Dh44-R2	-0.453707293226567	0.740193762077386	0.998043617620652	pseudobulk	DESeq2	LRT	0.130654579206842
449	KC_G1	Dhap-at	0.00527702757892935	0.929396248644016	0.998043617620652	pseudobulk	DESeq2	LRT	0.0317990848122736
449	KC_G1	Dhc36C	-1.73907471612892	0.507597351592657	0.998043617620652	pseudobulk	DESeq2	LRT	0.294480652518419
449	KC_G1	Dhc64C	-0.178880116938297	0.340644779428715	0.998043617620652	pseudobulk	DESeq2	LRT	0.467698262478804
449	KC_G1	Dhc98D	-1.00048251095297	0.492741987519929	0.998043617620652	pseudobulk	DESeq2	LRT	0.307380429055974
449	KC_G1	Dhfr	-2.67240485668008	0.203373709471669	0.998043617620652	pseudobulk	DESeq2	LRT	0.691705189905663
449	KC_G1	Dhit	-0.0828271694697394	0.454664547697685	0.998043617620652	pseudobulk	DESeq2	LRT	0.342308908428558
449	KC_G1	Dhod	-0.242842391719256	0.613774360796933	0.998043617620652	pseudobulk	DESeq2	LRT	0.211991257313417
449	KC_G1	DhpD	0.410417751416254	0.680444745820509	0.998043617620652	pseudobulk	DESeq2	LRT	0.16720713505951
449	KC_G1	Dhpr	0.250485483602923	0.181936189393469	0.998043617620652	pseudobulk	DESeq2	LRT	0.740080905715826
449	KC_G1	Dhx15	0.00458198823526414	0.963169602794614	0.998043617620652	pseudobulk	DESeq2	LRT	0.0162972320111638
450	KC_G1	dia	0.194650399133573	0.702904013535879	0.998043617620652	pseudobulk	DESeq2	LRT	0.153103976883249
450	KC_G1	Diap1	-0.0638668865965877	0.652742855121953	0.998043617620652	pseudobulk	DESeq2	LRT	0.185257873236297
450	KC_G1	Diap2	0.40884663688177	0.111151846044612	0.998043617620652	pseudobulk	DESeq2	LRT	0.954083320006549
450	KC_G1	Dic1	0.0793795335672191	0.684596056968977	0.998043617620652	pseudobulk	DESeq2	LRT	0.164565606573466
450	KC_G1	Dif	-0.118995212054329	0.341657276993294	0.998043617620652	pseudobulk	DESeq2	LRT	0.466409324760186
450	KC_G1	dikar	-0.141970446235696	0.309544026617627	0.998043617620652	pseudobulk	DESeq2	LRT	0.509277572306974
450	KC_G1	Dim1	0.448916484581875	0.235705594015373	0.998043617620652	pseudobulk	DESeq2	LRT	0.627630110215689
450	KC_G1	dind	-0.394979498737108	0.345049388497598	0.998043617620652	pseudobulk	DESeq2	LRT	0.462118737921376
450	KC_G1	DIP-alpha	-0.130837548866322	0.880230231498645	0.998043617620652	pseudobulk	DESeq2	LRT	0.0554037196772154
450	KC_G1	Dip-B	0.563405032031189	0.40056158910584	0.998043617620652	pseudobulk	DESeq2	LRT	0.397330698674934
451	KC_G1	Dip-C	-0.0656453048989443	0.843048158782431	0.998043617620652	pseudobulk	DESeq2	LRT	0.0741476157713781
451	KC_G1	DIP-delta	-0.794610277746993	0.879485534599449	0.998043617620652	pseudobulk	DESeq2	LRT	0.0557712992369932
451	KC_G1	DIP-epsilon	-0.794610277746993	0.815039924133852	0.998043617620652	pseudobulk	DESeq2	LRT	0.0888211171417967
451	KC_G1	DIP-eta	1.12508065046033	0.900770125530981	0.998043617620652	pseudobulk	DESeq2	LRT	0.0453860258366365
451	KC_G1	DIP-gamma	0.276761360372124	0.298595205231597	0.998043617620652	pseudobulk	DESeq2	LRT	0.524917170349031
451	KC_G1	DIP-iota	-0.794610277746993	0.879485534599449	0.998043617620652	pseudobulk	DESeq2	LRT	0.0557712992369932
451	KC_G1	DIP-kappa	1.48628506564816	0.152836781256613	0.998043617620652	pseudobulk	DESeq2	LRT	0.815772117131307
451	KC_G1	DIP-lambda	-0.0225937895962645	0.944247625346435	0.998043617620652	pseudobulk	DESeq2	LRT	0.0249140986902562
451	KC_G1	DIP-zeta	2.38466886841527	0.34619303318143	0.998043617620652	pseudobulk	DESeq2	LRT	0.460681676152437
451	KC_G1	DIP1	-0.0585665002251138	0.715553727104112	0.998043617620652	pseudobulk	DESeq2	LRT	0.145357751828966
452	KC_G1	DIP2	-0.136087824251707	0.46741466126867	0.998043617620652	pseudobulk	DESeq2	LRT	0.330297669333139
452	KC_G1	Dis3	-0.235915558209601	0.780292050380552	0.998043617620652	pseudobulk	DESeq2	LRT	0.107742817656356
452	KC_G1	Dis3l2	0.125257268462835	0.753190051572955	0.998043617620652	pseudobulk	DESeq2	LRT	0.123095424947862
452	KC_G1	disp	0.0722281681548179	0.846794267755623	0.998043617620652	pseudobulk	DESeq2	LRT	0.0722220905327954
452	KC_G1	DJ-1alpha	0.0920890622382135	0.899382127427308	0.998043617620652	pseudobulk	DESeq2	LRT	0.0460557470069252
452	KC_G1	dj-1beta	0.338364560709406	0.118043143044906	0.998043617620652	pseudobulk	DESeq2	LRT	0.927959235389576
452	KC_G1	dl	-1.29273764381934	0.501511065295134	0.998043617620652	pseudobulk	DESeq2	LRT	0.29971948030338
452	KC_G1	Dlc90F	-0.181173441529274	0.381276761156329	0.998043617620652	pseudobulk	DESeq2	LRT	0.418759664196917
452	KC_G1	dlg1	-0.0422502964424541	0.695553242690575	0.998043617620652	pseudobulk	DESeq2	LRT	0.157669620349585
452	KC_G1	Dlg5	-0.125344025042197	0.673951433341113	0.998043617620652	pseudobulk	DESeq2	LRT	0.171371398704919
453	KC_G1	Dlic	-0.0374732841649658	0.829667326576156	0.998043617620652	pseudobulk	DESeq2	LRT	0.0810960126772371
453	KC_G1	Dlip1	-0.07242406787003	0.914684205025602	0.998043617620652	pseudobulk	DESeq2	LRT	0.0387288203458628
453	KC_G1	Dlip2	-0.113314979417841	0.764853618749746	0.998043617620652	pseudobulk	DESeq2	LRT	0.11642167419574
453	KC_G1	Dlip3	0.0588214360255493	0.841164394581979	0.998043617620652	pseudobulk	DESeq2	LRT	0.0751191187292225
453	KC_G1	Dll	0.491101241374254	0.781910745324933	0.998043617620652	pseudobulk	DESeq2	LRT	0.106842818583577

453	KC_G1	dlp	-0.145923095289256	0.328281757948612	0.998043617620652	pseudobulk	DESeq2	LRT	0.483753249595881
453	KC_G1	dlt	-0.62861542298888	0.513318915845484	0.998043617620652	pseudobulk	DESeq2	LRT	0.289612731655953
453	KC_G1	DMAP1	0.0752337724145533	0.81543494874999	0.998043617620652	pseudobulk	DESeq2	LRT	0.0886106790452466
453	KC_G1	dmpd	-0.0833433503530417	0.759334861155248	0.998043617620652	pseudobulk	DESeq2	LRT	0.119566661152227
453	KC_G1	dmrt99B	1.12508065046033	0.900770125530981	0.998043617620652	pseudobulk	DESeq2	LRT	0.0453860258366365
454	KC_G1	dmt	1.97637197407483	0.0864278174886119	0.998043617620652	pseudobulk	DESeq2	LRT	1.06334645384732
454	KC_G1	Dmtn	0.069222264005166	0.760704976777805	0.998043617620652	pseudobulk	DESeq2	LRT	0.118783742440829
454	KC_G1	Dnah3	1.12508065046033	0.900770125530981	0.998043617620652	pseudobulk	DESeq2	LRT	0.0453860258366365
454	KC_G1	DnaJ-1	-0.379785555172606	0.0754580742748979	0.998043617620652	pseudobulk	DESeq2	LRT	1.12229428239049
454	KC_G1	DnaJ-H	0.168332334809455	0.526805422892825	0.998043617620652	pseudobulk	DESeq2	LRT	0.278349763094635
454	KC_G1	DNAlig1	0.813904900580572	0.524164175470526	0.998043617620652	pseudobulk	DESeq2	LRT	0.280532664669716
454	KC_G1	DNAlig3	-0.0551613656937807	0.900788622249214	0.998043617620652	pseudobulk	DESeq2	LRT	0.0453771079785074
454	KC_G1	DNAlig4	-0.177705145972825	0.851880591981889	0.998043617620652	pseudobulk	DESeq2	LRT	0.0696212759817427
454	KC_G1	DNApol-alpha50	-0.794610277746993	0.815039924133852	0.998043617620652	pseudobulk	DESeq2	LRT	0.0888211171417967
454	KC_G1	DNApol-delta	-0.358610478357155	0.744520429054811	0.998043617620652	pseudobulk	DESeq2	LRT	0.128123381049414
455	KC_G1	DNApol-eta	0.500894172080459	0.484850305206264	0.998043617620652	pseudobulk	DESeq2	LRT	0.314392326671638
455	KC_G1	DNApol-iota	-1.07864547936019	0.052321225712195	0.998043617620652	pseudobulk	DESeq2	LRT	1.28132209049022
455	KC_G1	DNApol-theta	0.141794404464921	0.919015926285883	0.998043617620652	pseudobulk	DESeq2	LRT	0.0366769623482262
455	KC_G1	DNApol-zeta	0.563407947186984	0.627130869506566	0.998043617620652	pseudobulk	DESeq2	LRT	0.202641821252397
455	KC_G1	DNaseII	-0.547577064822933	0.223764575097393	0.998043617620652	pseudobulk	DESeq2	LRT	0.650208666947402
455	KC_G1	dnc	-0.251025290832832	0.0165519767508045	0.998043617620652	pseudobulk	DESeq2	LRT	1.7811501323595
455	KC_G1	dnd	0.24445214668132	0.829123570049649	0.998043617620652	pseudobulk	DESeq2	LRT	0.0813807387001491
455	KC_G1	dnr1	-0.758968034998576	0.47097452634335	0.998043617620652	pseudobulk	DESeq2	LRT	0.327002581974493
455	KC_G1	dnt	-0.870227057217724	0.0551770152281629	0.998043617620652	pseudobulk	DESeq2	LRT	1.25824179615474
455	KC_G1	Dnz1	-0.116923613622774	0.623058137083894	0.998043617620652	pseudobulk	DESeq2	LRT	0.205471427760208
456	KC_G1	Doa	-0.0409594562545821	0.860801587349353	0.998043617620652	pseudobulk	DESeq2	LRT	0.0650969408221688
456	KC_G1	dock	-0.167726393498798	0.223278765228244	0.998043617620652	pseudobulk	DESeq2	LRT	0.651152578239432
456	KC_G1	dod	-0.0730404088507876	0.701321232674591	0.998043617620652	pseudobulk	DESeq2	LRT	0.154083012528821
456	KC_G1	Dok	2.04147257822512	0.408147672536479	0.998043617620652	pseudobulk	DESeq2	LRT	0.389182675723292
456	KC_G1	dom	0.132443008357699	0.535214957753196	0.998043617620652	pseudobulk	DESeq2	LRT	0.271471757760972
456	KC_G1	dome	0.11755463412918	0.737857804559995	0.998043617620652	pseudobulk	DESeq2	LRT	0.132027324687876
456	KC_G1	dop	-0.0933272750374959	0.724098423219474	0.998043617620652	pseudobulk	DESeq2	LRT	0.140202398228352
456	KC_G1	Dop1R1	-0.072670599275772	0.553624750587198	0.998043617620652	pseudobulk	DESeq2	LRT	0.256784502360611
456	KC_G1	Dop1R2	-0.0882697569569048	0.499895695576904	0.998043617620652	pseudobulk	DESeq2	LRT	0.301120602785811
456	KC_G1	Dop2R	-0.0613985036900256	0.546763089260994	0.998043617620652	pseudobulk	DESeq2	LRT	0.262200811352459
457	KC_G1	DopEcR	0.0504306573182642	0.691960839527562	0.998043617620652	pseudobulk	DESeq2	LRT	0.159918483084177
457	KC_G1	dor	0.254004537323268	0.636255588493718	0.998043617620652	pseudobulk	DESeq2	LRT	0.196368390051924
457	KC_G1	DOR	0.0813692729486713	0.567298768206715	0.998043617620652	pseudobulk	DESeq2	LRT	0.246188159431256
457	KC_G1	dos	-0.485577516700591	0.249988216576305	0.998043617620652	pseudobulk	DESeq2	LRT	0.602080461713944
457	KC_G1	Dp	0.198079977555105	0.345364161317589	0.998043617620652	pseudobulk	DESeq2	LRT	0.461722731474853
457	KC_G1	Dp1	0.0612815024670034	0.853673169257525	0.998043617620652	pseudobulk	DESeq2	LRT	0.0687083681349824
457	KC_G1	dpa	1.12508065046033	0.900770125530981	0.998043617620652	pseudobulk	DESeq2	LRT	0.0453860258366365
457	KC_G1	Dpck	0.364575658996383	0.328803479466415	0.998043617620652	pseudobulk	DESeq2	LRT	0.483063595313652
457	KC_G1	DPCoAC	-0.492467383076921	0.44941563898613	0.998043617620652	pseudobulk	DESeq2	LRT	0.347351818861253
457	KC_G1	Dph1	-0.39281639395816	0.830357322847986	0.998043617620652	pseudobulk	DESeq2	LRT	0.0807349799772098
458	KC_G1	Dph5	-0.234961526603385	0.515590533069175	0.998043617620652	pseudobulk	DESeq2	LRT	0.287695065472171
458	KC_G1	Dpit47	0.339027498404645	0.579177937620867	0.998043617620652	pseudobulk	DESeq2	LRT	0.237187989891031
458	KC_G1	dpn	-0.833495705181951	0.590440938773247	0.998043617620652	pseudobulk	DESeq2	LRT	0.228823537928029
458	KC_G1	DppIII	-0.0491993467887953	0.876289023199616	0.998043617620652	pseudobulk	DESeq2	LRT	0.0573526284456599
458	KC_G1	dpr10	-0.216233466753989	0.160225108283319	0.998043617620652	pseudobulk	DESeq2	LRT	0.795269426238936
458	KC_G1	dpr11	-1.08952227041994	0.308636522430039	0.998043617620652	pseudobulk	DESeq2	LRT	0.510552683096876
458	KC_G1	dpr12	-0.123900428578245	0.299112482360244	0.998043617620652	pseudobulk	DESeq2	LRT	0.524165462906293
458	KC_G1	dpr13	2.04147257822512	0.408147672536479	0.998043617620652	pseudobulk	DESeq2	LRT	0.389182675723292
458	KC_G1	dpr14	0.0633240277837964	0.6387056838655	0.998043617620652	pseudobulk	DESeq2	LRT	0.194699219022164
458	KC_G1	dpr15	-0.979410789304664	0.32351300763227	0.998043617620652	pseudobulk	DESeq2	LRT	0.490108252771643
459	KC_G1	dpr16	-0.631074066544808	0.426316182418929	0.998043617620652	pseudobulk	DESeq2	LRT	0.370268181774971

459	KC_G1	dpr17	-0.412817233091792	0.552567623936617	0.998043617620652	pseudobulk	DESeq2	LRT	0.25761456487298
459	KC_G1	dpr18	-0.835846789879216	0.668046316875268	0.998043617620652	pseudobulk	DESeq2	LRT	0.175193426048375
459	KC_G1	dpr19	-0.116500566897845	0.48434062261843	0.998043617620652	pseudobulk	DESeq2	LRT	0.314849104277513
459	KC_G1	dpr2	1.31388825333297	0.494798724636074	0.998043617620652	pseudobulk	DESeq2	LRT	0.305571428453243
459	KC_G1	dpr20	0.618741750737151	0.482216291475559	0.998043617620652	pseudobulk	DESeq2	LRT	0.316758121253426
459	KC_G1	dpr21	0.0180098655584032	0.868053379175832	0.998043617620652	pseudobulk	DESeq2	LRT	0.0614535679470976
459	KC_G1	dpr3	-0.723888430348189	0.658313366042614	0.998043617620652	pseudobulk	DESeq2	LRT	0.181567327135442
459	KC_G1	dpr4	0.245632487904688	0.190118465522701	0.998043617620652	pseudobulk	DESeq2	LRT	0.720975699625085
459	KC_G1	dpr5	-0.027819537694904	0.884410024951876	0.998043617620652	pseudobulk	DESeq2	LRT	0.0533463432568183
460	KC_G1	dpr6	0.339326521248876	0.0639026140439975	0.998043617620652	pseudobulk	DESeq2	LRT	1.19448137593138
460	KC_G1	dpr7	-0.0472678269279471	0.746551201780312	0.998043617620652	pseudobulk	DESeq2	LRT	0.126940401082838
460	KC_G1	dpr8	-0.0494068154970612	0.616676647446971	0.998043617620652	pseudobulk	DESeq2	LRT	0.209942497295999
460	KC_G1	dpr9	-0.20866458330546	0.0679248200378275	0.998043617620652	pseudobulk	DESeq2	LRT	1.16797150354212
460	KC_G1	Dpy-30L1	0.0272633486367655	0.899196657615159	0.998043617620652	pseudobulk	DESeq2	LRT	0.0461453160788296
460	KC_G1	Drak	-0.0268634648835166	0.877295333986919	0.998043617620652	pseudobulk	DESeq2	LRT	0.0568541804888354
460	KC_G1	Drat	-0.0580223692796005	0.69526113455852	0.998043617620652	pseudobulk	DESeq2	LRT	0.157852047212308
460	KC_G1	drd	1.90250740646012	0.460774913678782	0.998043617620652	pseudobulk	DESeq2	LRT	0.336511173568024
460	KC_G1	dre4	0.0808302124498443	0.815031264787529	0.998043617620652	pseudobulk	DESeq2	LRT	0.0888257313038656
460	KC_G1	Dredd	-0.651522973901016	0.8143389309148	0.998043617620652	pseudobulk	DESeq2	LRT	0.0891948024940098
461	KC_G1	Dref	-0.126963641144183	0.749709749540228	0.998043617620652	pseudobulk	DESeq2	LRT	0.125106841369453
461	KC_G1	Drep1	-0.191729063626919	0.574633055763255	0.998043617620652	pseudobulk	DESeq2	LRT	0.240609394837882
461	KC_G1	Drep2	-0.110620942487542	0.426495530309976	0.998043617620652	pseudobulk	DESeq2	LRT	0.370085515895954
461	KC_G1	Drep3	-0.232719903075739	0.29233302753716	0.998043617620652	pseudobulk	DESeq2	LRT	0.534122115648924
461	KC_G1	Drep4	0.290285700425709	0.44477358816071	0.998043617620652	pseudobulk	DESeq2	LRT	0.351861010222377
461	KC_G1	Drgx	-0.528415462266719	0.655159199994471	0.998043617620652	pseudobulk	DESeq2	LRT	0.183653156072093
461	KC_G1	Drice	0.662616826694913	0.298862753486646	0.998043617620652	pseudobulk	DESeq2	LRT	0.524528206617335
461	KC_G1	drk	-0.260065426521098	0.1983454575869	0.998043617620652	pseudobulk	DESeq2	LRT	0.702577741081098
461	KC_G1	drl	0.0369956396727704	0.902714781249087	0.998043617620652	pseudobulk	DESeq2	LRT	0.0444494462492885
461	KC_G1	Drl-2	0.119721897555883	0.657305524238407	0.998043617620652	pseudobulk	DESeq2	LRT	0.182232717733058
462	KC_G1	Dro	1.24545707098284	0.613638418851987	0.998043617620652	pseudobulk	DESeq2	LRT	0.212087457773066
462	KC_G1	Droj2	-0.0109099415619473	0.938300237133522	0.998043617620652	pseudobulk	DESeq2	LRT	0.0276581739164273
462	KC_G1	Dronc	-0.102136900053008	0.878585867309881	0.998043617620652	pseudobulk	DESeq2	LRT	0.056215786955854
462	KC_G1	drongo	-0.199535585236034	0.397037615209424	0.998043617620652	pseudobulk	DESeq2	LRT	0.401168346375356
462	KC_G1	drosha	-0.594862630836326	0.365397794125932	0.998043617620652	pseudobulk	DESeq2	LRT	0.437234078772827
462	KC_G1	Drp1	0.0592323245397708	0.799054017182515	0.998043617620652	pseudobulk	DESeq2	LRT	0.0974238607719492
462	KC_G1	drpr	-0.228157142525671	0.405867769208271	0.998043617620652	pseudobulk	DESeq2	LRT	0.391615435528666
462	KC_G1	Drs	0.173195945230598	0.976215465741119	0.998043617620652	pseudobulk	DESeq2	LRT	0.0104543162934892
462	KC_G1	Drsl2	-0.794610277746993	0.815039924133852	0.998043617620652	pseudobulk	DESeq2	LRT	0.0888211171417967
462	KC_G1	ds	-1.73907471612892	0.507597351592657	0.998043617620652	pseudobulk	DESeq2	LRT	0.294480652518419
463	KC_G1	Dscam1	-0.156401327481826	0.284228740117765	0.998043617620652	pseudobulk	DESeq2	LRT	0.546332009999387
463	KC_G1	Dscam4	0.0808674017676304	0.571521399281408	0.998043617620652	pseudobulk	DESeq2	LRT	0.242967503823658
463	KC_G1	dsd	0.0804104204896724	0.68040133677631	0.998043617620652	pseudobulk	DESeq2	LRT	0.167234841805493
463	KC_G1	dsh	-0.0297968273797286	0.912404895114981	0.998043617620652	pseudobulk	DESeq2	LRT	0.0398123933693897
463	KC_G1	Dsor1	-0.118409175221028	0.593109169417875	0.998043617620652	pseudobulk	DESeq2	LRT	0.226865361758602
463	KC_G1	Dsp1	-0.0713939602282949	0.524505804572159	0.998043617620652	pseudobulk	DESeq2	LRT	0.280249701217402
463	KC_G1	dsx	-2.31256857938916	0.316871197308952	0.998043617620652	pseudobulk	DESeq2	LRT	0.499117235149486
463	KC_G1	dsx-c73A	-1.66808346769027	0.498191247612582	0.998043617620652	pseudobulk	DESeq2	LRT	0.302603906560549
463	KC_G1	Dtd	-0.328602272293921	0.60400788300875	0.998043617620652	pseudobulk	DESeq2	LRT	0.218957393291351
463	KC_G1	dtn	-0.145066209178549	0.278169154009969	0.998043617620652	pseudobulk	DESeq2	LRT	0.55569103029646
464	KC_G1	dtr	0.639186812859026	0.77748208633511	0.998043617620652	pseudobulk	DESeq2	LRT	0.109309608597507
464	KC_G1	Duba	0.291689227590525	0.267143376081179	0.998043617620652	pseudobulk	DESeq2	LRT	0.573255589839888
464	KC_G1	DUBAI	-0.262303712249878	0.444570698747614	0.998043617620652	pseudobulk	DESeq2	LRT	0.352059164650015
464	KC_G1	dunk	0.0526046663654203	0.930989632125525	0.998043617620652	pseudobulk	DESeq2	LRT	0.0310551554695116
464	KC_G1	dup	0.357056964567674	0.623342677680587	0.998043617620652	pseudobulk	DESeq2	LRT	0.205273137753166
464	KC_G1	dUTPase	0.22363650969347	0.851295525542033	0.998043617620652	pseudobulk	DESeq2	LRT	0.0699196492782634
464	KC_G1	dve	-0.626425489033532	0.615230163923718	0.998043617620652	pseudobulk	DESeq2	LRT	0.210962379798823

464	KC_G1	dwg	-0.836174685469797	0.136173584072446	0.998043617620652	pseudobulk	DESeq2	LRT	0.865907131808238
464	KC_G1	dx	-0.794610277746993	0.821479263487599	0.998043617620652	pseudobulk	DESeq2	LRT	0.085403394939296
464	KC_G1	Dyb	0.0574747095939855	0.729980092568634	0.998043617620652	pseudobulk	DESeq2	LRT	0.136688983448694
465	KC_G1	Dyrk2	-0.00855090587788392	0.951061560529018	0.998043617620652	pseudobulk	DESeq2	LRT	0.0217913710407466
465	KC_G1	Dyrk3	-0.136450529329945	0.295600807798441	0.998043617620652	pseudobulk	DESeq2	LRT	0.529294383464201
465	KC_G1	Dys	-0.183118913157046	0.240222351401261	0.998043617620652	pseudobulk	DESeq2	LRT	0.619386586281073
465	KC_G1	Dysb	0.58308279367979	0.587531644174504	0.998043617620652	pseudobulk	DESeq2	LRT	0.230968737532359
465	KC_G1	dysc	-0.0638926663755288	0.534095754880539	0.998043617620652	pseudobulk	DESeq2	LRT	0.272380873892469
465	KC_G1	dyw	0.1729159612032	0.899814506689544	0.998043617620652	pseudobulk	DESeq2	LRT	0.0458470094762598
465	KC_G1	e	-0.866606292568381	0.155149109254572	0.998043617620652	pseudobulk	DESeq2	LRT	0.809250713453547
465	KC_G1	E(bx)	-0.104887225091025	0.460979100734259	0.998043617620652	pseudobulk	DESeq2	LRT	0.336318763637536
465	KC_G1	E(Pc)	-0.12806708105004	0.367192455668644	0.998043617620652	pseudobulk	DESeq2	LRT	0.435106250387834
465	KC_G1	e(r)	0.0551757135394664	0.735997934023746	0.998043617620652	pseudobulk	DESeq2	LRT	0.133123404743135
466	KC_G1	E(spl)m3-HLH	-1.28709035773722	0.371964235694939	0.998043617620652	pseudobulk	DESeq2	LRT	0.429498815459566
466	KC_G1	E(spl)mbeta-HLH	0.539213322894882	0.561374042519817	0.998043617620652	pseudobulk	DESeq2	LRT	0.250747672661742
466	KC_G1	E(var)3-9	0.202067728209456	0.652627921455235	0.998043617620652	pseudobulk	DESeq2	LRT	0.185334349680025
466	KC_G1	e(y)2	0.267480764678111	0.528648070911059	0.998043617620652	pseudobulk	DESeq2	LRT	0.276833348237612
466	KC_G1	e(y)2b	1.13582588427274	0.633424328955532	0.998043617620652	pseudobulk	DESeq2	LRT	0.198305259991224
466	KC_G1	e(y)3	-0.216858981077211	0.232764897316561	0.998043617620652	pseudobulk	DESeq2	LRT	0.633082513932726
466	KC_G1	E(z)	0.165738447196522	0.602712832414824	0.998043617620652	pseudobulk	DESeq2	LRT	0.219889561829552
466	KC_G1	E23	2.32560081242934	0.127444438221726	0.998043617620652	pseudobulk	DESeq2	LRT	0.894679112743316
466	KC_G1	E2f1	-0.325613213392899	0.222561979235581	0.998043617620652	pseudobulk	DESeq2	LRT	0.652549025169835
466	KC_G1	E2f2	0.219162924965174	0.532787163998211	0.998043617620652	pseudobulk	DESeq2	LRT	0.273446246810538
467	KC_G1	Eaat1	0.00274765163111724	0.955692996736288	0.998043617620652	pseudobulk	DESeq2	LRT	0.0196815964647494
467	KC_G1	Eaat2	-0.0533653775175956	0.77374791759819	0.998043617620652	pseudobulk	DESeq2	LRT	0.111400506794872
467	KC_G1	EAChm	1.13582588427274	0.704684208645453	0.998043617620652	pseudobulk	DESeq2	LRT	0.152005460551806
467	KC_G1	Eaf	-0.322078030106626	0.184649571934102	0.998043617620652	pseudobulk	DESeq2	LRT	0.733651694819787
467	KC_G1	Eaf6	0.0359716308711627	0.92818917834816	0.998043617620652	pseudobulk	DESeq2	LRT	0.032363499277338
467	KC_G1	eag	0.29301677106338	0.137183097482384	0.998043617620652	pseudobulk	DESeq2	LRT	0.862699395351858
467	KC_G1	ear	0.302162973302648	0.191017645999637	0.998043617620652	pseudobulk	DESeq2	LRT	0.718926511253405
467	KC_G1	eas	0.0837422438817174	0.685062681769622	0.998043617620652	pseudobulk	DESeq2	LRT	0.16426968967366
467	KC_G1	east	-0.10212108334604	0.494659978301696	0.998043617620652	pseudobulk	DESeq2	LRT	0.305693225891824
467	KC_G1	Eato	0.288246813336878	0.548740405195189	0.998043617620652	pseudobulk	DESeq2	LRT	0.260633060384837
468	KC_G1	Eb1	-0.0155385226683765	0.914124902413605	0.998043617620652	pseudobulk	DESeq2	LRT	0.0389944599303217
468	KC_G1	ebd1	0.0750173073944201	0.865047435899512	0.998043617620652	pseudobulk	DESeq2	LRT	0.0629600768305066
468	KC_G1	ebi	-0.0765391773234769	0.843256016203275	0.998043617620652	pseudobulk	DESeq2	LRT	0.074040551657215
468	KC_G1	ebo	0.230228739890007	0.578893919616228	0.998043617620652	pseudobulk	DESeq2	LRT	0.237401011999941
468	KC_G1	EbpIII	-0.50233911270044	0.619647975754016	0.998043617620652	pseudobulk	DESeq2	LRT	0.207854964704595
468	KC_G1	eca	-0.0645068019451925	0.804968342818358	0.998043617620652	pseudobulk	DESeq2	LRT	0.0942211988987677
468	KC_G1	ecd	0.00259679441275933	0.967447284120588	0.998043617620652	pseudobulk	DESeq2	LRT	0.0143726902256142
468	KC_G1	Echs1	0.203116890482904	0.684170629254839	0.998043617620652	pseudobulk	DESeq2	LRT	0.164835573568583
468	KC_G1	eco	-0.292657163532917	0.851097861291722	0.998043617620652	pseudobulk	DESeq2	LRT	0.0700205008137889
468	KC_G1	EcR	-0.0647721845672807	0.667272964899101	0.998043617620652	pseudobulk	DESeq2	LRT	0.175696470583047
469	KC_G1	ECSIT	0.110343908524324	0.832106119800949	0.998043617620652	pseudobulk	DESeq2	LRT	0.0798212839195423
469	KC_G1	Ect3	-0.403197816758646	0.537433344749652	0.998043617620652	pseudobulk	DESeq2	LRT	0.269675391509999
469	KC_G1	Ect4	0.405511161366612	0.280680181082562	0.998043617620652	pseudobulk	DESeq2	LRT	0.551788251955349
469	KC_G1	ed	-0.289373845816902	0.083596742292174	0.998043617620652	pseudobulk	DESeq2	LRT	1.07781064638992
469	KC_G1	Edc3	-0.11727934701348	0.586022984684515	0.998043617620652	pseudobulk	DESeq2	LRT	0.232085349978678
469	KC_G1	Edem1	0.232567291318655	0.753832818928737	0.998043617620652	pseudobulk	DESeq2	LRT	0.122724958993887
469	KC_G1	Edem2	0.205290650550213	0.612140500854662	0.998043617620652	pseudobulk	DESeq2	LRT	0.213148885464378
469	KC_G1	Edg78E	0.173105107343345	0.973555957903825	0.998043617620652	pseudobulk	DESeq2	LRT	0.0116390811132178
469	KC_G1	EDTP	-0.0280126304882987	0.936286297418968	0.998043617620652	pseudobulk	DESeq2	LRT	0.0285913324826499
469	KC_G1	eEF1alpha1	0.201976105435345	0.365253827900113	0.998043617620652	pseudobulk	DESeq2	LRT	0.437405223916088
470	KC_G1	eEF1alpha2	-0.105271952699599	0.418548145426572	0.998043617620652	pseudobulk	DESeq2	LRT	0.378254578075861
470	KC_G1	eEF1delta	-0.0358094693479202	0.86741071044245	0.998043617620652	pseudobulk	DESeq2	LRT	0.0617752196586225
470	KC_G1	eEF1gamma	-0.244882267947298	0.1892776346286	0.998043617620652	pseudobulk	DESeq2	LRT	0.722900699988787

470	KC_G1	eEF2	-0.221610243698414	0.040045962025259	0.998043617620652	pseudobulk	DESeq2	LRT	1.3974412688072
470	KC_G1	eEF5	-0.0679500741620827	0.549625226158657	0.998043617620652	pseudobulk	DESeq2	LRT	0.259933342669601
470	KC_G1	eEFSec	-0.361907261134579	0.73487640481804	0.998043617620652	pseudobulk	DESeq2	LRT	0.133785696588009
470	KC_G1	Efa6	-0.161903747165657	0.57137772031959	0.998043617620652	pseudobulk	DESeq2	LRT	0.243076698029063
470	KC_G1	Efhc1.1	-0.794610277746993	0.815039924133852	0.998043617620652	pseudobulk	DESeq2	LRT	0.0888211171417967
470	KC_G1	Efr	-0.393811025985031	0.491421293592539	0.998043617620652	pseudobulk	DESeq2	LRT	0.308546029196226
470	KC_G1	Egfr	1.13582588427274	0.704684208645453	0.998043617620652	pseudobulk	DESeq2	LRT	0.152005460551806
471	KC_G1	egg	-0.36613783432743	0.274503000856931	0.998043617620652	pseudobulk	DESeq2	LRT	0.561452903496285
471	KC_G1	egh	0.0391831353974492	0.773128057474756	0.998043617620652	pseudobulk	DESeq2	LRT	0.111748565527319
471	KC_G1	egl	0.303882296075956	0.31476756660959	0.998043617620652	pseudobulk	DESeq2	LRT	0.502010023352828
471	KC_G1	Egm	0.505363793602114	0.383424643168762	0.998043617620652	pseudobulk	DESeq2	LRT	0.41631997792568
471	KC_G1	egr	-0.593242590523872	0.741700559108196	0.998043617620652	pseudobulk	DESeq2	LRT	0.12977139360852
471	KC_G1	Ehbp1	-0.339364757323807	0.0774178008000132	0.998043617620652	pseudobulk	DESeq2	LRT	1.11115916980322
471	KC_G1	eIF1A	-0.0051024047882737	0.962765137448615	0.998043617620652	pseudobulk	DESeq2	LRT	0.0164796442879668
471	KC_G1	eIF2alpha	0.158913822639869	0.468674337088101	0.998043617620652	pseudobulk	DESeq2	LRT	0.329128826224102
471	KC_G1	eIF2Balpha	0.072182118567779	0.867489105106068	0.998043617620652	pseudobulk	DESeq2	LRT	0.061735970856429
471	KC_G1	eIF2Bbeta	-0.292455011202537	0.439352771667336	0.998043617620652	pseudobulk	DESeq2	LRT	0.357186629497083
472	KC_G1	eIF2Bdelta	0.0539580203730102	0.882107019827098	0.998043617620652	pseudobulk	DESeq2	LRT	0.0544787217833532
472	KC_G1	eIF2Bepsilon	-0.429556923143773	0.435269735655646	0.998043617620652	pseudobulk	DESeq2	LRT	0.361241528327738
472	KC_G1	eIF2beta	-0.0433694318360193	0.789439831163424	0.998043617620652	pseudobulk	DESeq2	LRT	0.102680965072402
472	KC_G1	eIF2Bgamma	-0.438109661861965	0.453730584575278	0.998043617620652	pseudobulk	DESeq2	LRT	0.343201945299575
472	KC_G1	eIF2D	-0.168698420685068	0.653674474986557	0.998043617620652	pseudobulk	DESeq2	LRT	0.184638473254856
472	KC_G1	eIF2gamma	0.275370763743194	0.402450352091411	0.998043617620652	pseudobulk	DESeq2	LRT	0.395287688321354
472	KC_G1	eIF3a	-0.131901916779966	0.412929993626298	0.998043617620652	pseudobulk	DESeq2	LRT	0.384123570520065
472	KC_G1	eIF3b	0.00880185773833641	0.937394182958934	0.998043617620652	pseudobulk	DESeq2	LRT	0.0280777458410644
472	KC_G1	eIF3c	-0.106357262779694	0.530519075162857	0.998043617620652	pseudobulk	DESeq2	LRT	0.275298996137691
472	KC_G1	eIF3d1	0.155368387327641	0.379771210392537	0.998043617620652	pseudobulk	DESeq2	LRT	0.420477961246089
473	KC_G1	eIF3e	-0.0721579320583179	0.676797877540977	0.998043617620652	pseudobulk	DESeq2	LRT	0.16954101192779
473	KC_G1	eIF3f1	-0.0723095637963695	0.677158036601985	0.998043617620652	pseudobulk	DESeq2	LRT	0.169309962903434
473	KC_G1	eIF3g1	-0.0668989072200398	0.673815901004166	0.998043617620652	pseudobulk	DESeq2	LRT	0.171458744561661
473	KC_G1	eIF3g2	1.13582588427274	0.633424328955532	0.998043617620652	pseudobulk	DESeq2	LRT	0.198305259991224
473	KC_G1	eIF3h	-0.137285724816298	0.357449746804126	0.998043617620652	pseudobulk	DESeq2	LRT	0.446785006225811
473	KC_G1	eIF3i	0.0259179658615178	0.860469735035188	0.998043617620652	pseudobulk	DESeq2	LRT	0.0652644003374238
473	KC_G1	eIF3j	0.200953210638041	0.335740579918796	0.998043617620652	pseudobulk	DESeq2	LRT	0.473996163757322
473	KC_G1	eIF3k	0.408101597100299	0.0269511100659839	0.998043617620652	pseudobulk	DESeq2	LRT	1.56942334232871
473	KC_G1	eIF3l	-0.0516010011221605	0.775330568910948	0.998043617620652	pseudobulk	DESeq2	LRT	0.11051309279094
473	KC_G1	eIF3m	-0.0475382741126425	0.792260502357549	0.998043617620652	pseudobulk	DESeq2	LRT	0.101131995008348
474	KC_G1	eIF4A	-0.109683934345797	0.21585726131223	0.998043617620652	pseudobulk	DESeq2	LRT	0.665833337349198
474	KC_G1	eIF4B	-0.0709188539539777	0.607671308705136	0.998043617620652	pseudobulk	DESeq2	LRT	0.216331268449779
474	KC_G1	eIF4E1	-0.0151371612954077	0.925123747529771	0.998043617620652	pseudobulk	DESeq2	LRT	0.0338001707477148
474	KC_G1	eIF4EHP	0.00711329553971483	0.956337329564124	0.998043617620652	pseudobulk	DESeq2	LRT	0.0193888916907907
474	KC_G1	eIF4G1	-0.155077711867305	0.30094726717919	0.998043617620652	pseudobulk	DESeq2	LRT	0.521509596032119
474	KC_G1	eIF4G2	-0.281109206120058	0.0563665852355549	0.998043617620652	pseudobulk	DESeq2	LRT	1.24897827453365
474	KC_G1	eIF4H1	-0.0390763544622012	0.809972183479752	0.998043617620652	pseudobulk	DESeq2	LRT	0.0915298956505922
474	KC_G1	eIF5	-0.275680671722986	0.161338327898171	0.998043617620652	pseudobulk	DESeq2	LRT	0.792262448374589
474	KC_G1	eIF5B	-0.102504819677238	0.562390129945806	0.998043617620652	pseudobulk	DESeq2	LRT	0.249962309872455
474	KC_G1	eIF6	-0.315755347152206	0.633666051603964	0.998043617620652	pseudobulk	DESeq2	LRT	0.198139559398355
475	KC_G1	Eip55E	0.344378649252427	0.522816721693664	0.998043617620652	pseudobulk	DESeq2	LRT	0.281650530458078
475	KC_G1	Eip63E	-0.116598599473559	0.439918035437463	0.998043617620652	pseudobulk	DESeq2	LRT	0.356628232771018
475	KC_G1	Eip63F-1	1.6676684059637	0.0613578850701618	0.998043617620652	pseudobulk	DESeq2	LRT	1.21212961839069
475	KC_G1	Eip71CD	0.20481781364237	0.497039163725042	0.998043617620652	pseudobulk	DESeq2	LRT	0.303609390100529
475	KC_G1	Eip74EF	-0.266573983220353	0.420657537508837	0.998043617620652	pseudobulk	DESeq2	LRT	0.376071324817627
475	KC_G1	Eip75B	-0.126607938367421	0.354793562821861	0.998043617620652	pseudobulk	DESeq2	LRT	0.450024268367668
475	KC_G1	Eip78C	-0.064501492594698	0.893532406363027	0.998043617620652	pseudobulk	DESeq2	LRT	0.0488896920118078
475	KC_G1	Eip93F	-0.0854136899764421	0.547044900002952	0.998043617620652	pseudobulk	DESeq2	LRT	0.261977026457702
475	KC_G1	Ekar	-0.752595479487864	0.785589861204421	0.998043617620652	pseudobulk	DESeq2	LRT	0.104804130174502

475	KC_G1	Elal	1.08490994459604	0.308473370408381	0.998043617620652	pseudobulk	DESeq2	LRT	0.51078232136776
476	KC_G1	elav	-0.0839249140993458	0.435735927225434	0.998043617620652	pseudobulk	DESeq2	LRT	0.360776630212306
476	KC_G1	elB	0.193963765881825	0.403304500040493	0.998043617620652	pseudobulk	DESeq2	LRT	0.394366932141027
476	KC_G1	Elba2	-0.794610277746993	0.821479263487599	0.998043617620652	pseudobulk	DESeq2	LRT	0.085403394939296
476	KC_G1	elgi	0.120717001389309	0.761120448910913	0.998043617620652	pseudobulk	DESeq2	LRT	0.11854660977603
476	KC_G1	Elk	-0.209891489557926	0.121934217921303	0.998043617620652	pseudobulk	DESeq2	LRT	0.91387440292087
476	KC_G1	elm	0.105558062714205	0.59845865070146	0.998043617620652	pseudobulk	DESeq2	LRT	0.222965850925992
476	KC_G1	EloA	-0.125105546900919	0.626718065478016	0.998043617620652	pseudobulk	DESeq2	LRT	0.202927786352629
476	KC_G1	EloB	-0.0638469864634115	0.786164069347124	0.998043617620652	pseudobulk	DESeq2	LRT	0.104486808952538
476	KC_G1	EloC	-0.0727180098799889	0.671498254543363	0.998043617620652	pseudobulk	DESeq2	LRT	0.17295511187564
476	KC_G1	Elp1	0.719422451055001	0.371665102405027	0.998043617620652	pseudobulk	DESeq2	LRT	0.429848215187396
477	KC_G1	Elp2	-0.308055535781501	0.754044595555829	0.998043617620652	pseudobulk	DESeq2	LRT	0.122602968412606
477	KC_G1	Elp3	-0.660558913550332	0.338754663491732	0.998043617620652	pseudobulk	DESeq2	LRT	0.470114717328505
477	KC_G1	Elys	0.107135076104885	0.799202288435925	0.998043617620652	pseudobulk	DESeq2	LRT	0.0973432812214242
477	KC_G1	ema	-0.0333062706030395	0.920874589726525	0.998043617620652	pseudobulk	DESeq2	LRT	0.0357995106264537
477	KC_G1	emb	0.0485090236088103	0.787062221020434	0.998043617620652	pseudobulk	DESeq2	LRT	0.103990933234727
477	KC_G1	emc	-0.138028420048889	0.249060041047799	0.998043617620652	pseudobulk	DESeq2	LRT	0.603695944661385
477	KC_G1	EMC1	0.252634728110016	0.427946723791786	0.998043617620652	pseudobulk	DESeq2	LRT	0.368610294078913
477	KC_G1	EMC3	-0.151827379545955	0.556408181283318	0.998043617620652	pseudobulk	DESeq2	LRT	0.25460649287827
477	KC_G1	EMC8-9	0.182909157138939	0.421747763848122	0.998043617620652	pseudobulk	DESeq2	LRT	0.374947211408655
477	KC_G1	EMRE	0.27743435123935	0.180728199999726	0.998043617620652	pseudobulk	DESeq2	LRT	0.742974076831819
478	KC_G1	ena	0.0935999153632891	0.680178011058483	0.998043617620652	pseudobulk	DESeq2	LRT	0.167377412142085
478	KC_G1	enc	-0.0181353632734801	0.895158346929931	0.998043617620652	pseudobulk	DESeq2	LRT	0.0481001343878268
478	KC_G1	EndoA	-0.134442914888153	0.275997930111372	0.998043617620652	pseudobulk	DESeq2	LRT	0.559094174980362
478	KC_G1	EndoB	0.00712829027926937	0.923941846641591	0.998043617620652	pseudobulk	DESeq2	LRT	0.0343553626299442
478	KC_G1	EndoG	0.179514912808929	0.588911587109229	0.998043617620652	pseudobulk	DESeq2	LRT	0.229949900650456
478	KC_G1	EndoGI	0.563652346543769	0.350096108822267	0.998043617620652	pseudobulk	DESeq2	LRT	0.455812716216998
478	KC_G1	endos	0.0573253788499108	0.721866817683696	0.998043617620652	pseudobulk	DESeq2	LRT	0.141542921101299
478	KC_G1	EndoU	0.129865608374264	0.947640458270124	0.998043617620652	pseudobulk	DESeq2	LRT	0.0233564059184837
478	KC_G1	Eno	-0.0399872883953518	0.80013427129488	0.998043617620652	pseudobulk	DESeq2	LRT	0.0968371275213478
478	KC_G1	enok	-0.169221709481854	0.527826484989926	0.998043617620652	pseudobulk	DESeq2	LRT	0.277508821777213
479	KC_G1	ens	-0.0736901963852931	0.640738050899758	0.998043617620652	pseudobulk	DESeq2	LRT	0.193319484209949
479	KC_G1	Ent1	-0.0361301837931715	0.944739711971824	0.998043617620652	pseudobulk	DESeq2	LRT	0.0246878287660096
479	KC_G1	Ent2	0.277076998622587	0.712406390096952	0.998043617620652	pseudobulk	DESeq2	LRT	0.147272193684652
479	KC_G1	Ent3	-0.043296744630001	0.834565973826419	0.998043617620652	pseudobulk	DESeq2	LRT	0.0785393259275298
479	KC_G1	Eogt	0.12060304920718	0.848303193350106	0.998043617620652	pseudobulk	DESeq2	LRT	0.071448898138358
479	KC_G1	Epac	-0.0840983398768814	0.768926486373178	0.998043617620652	pseudobulk	DESeq2	LRT	0.114115179169444
479	KC_G1	Epg5	-0.336725458573944	0.486402600990812	0.998043617620652	pseudobulk	DESeq2	LRT	0.313004111381422
479	KC_G1	Eph	-0.0254747746691383	0.849356659635656	0.998043617620652	pseudobulk	DESeq2	LRT	0.0709099036482593
479	KC_G1	Ephrin	0.0560948233243349	0.741491578972691	0.998043617620652	pseudobulk	DESeq2	LRT	0.129893776835814
479	KC_G1	Eps-15	-0.0380903258753903	0.820993637298122	0.998043617620652	pseudobulk	DESeq2	LRT	0.0856602086503806
480	KC_G1	epsilonCOP	0.152128312519276	0.608400752966324	0.998043617620652	pseudobulk	DESeq2	LRT	0.215810257129037
480	KC_G1	Ercc1	-0.649842062786484	0.49771714193954	0.998043617620652	pseudobulk	DESeq2	LRT	0.303017401406773
480	KC_G1	eRF1	-0.2373559384203	0.190792555049713	0.998043617620652	pseudobulk	DESeq2	LRT	0.719438575983655
480	KC_G1	eRF3	0.115861217150648	0.728557907551783	0.998043617620652	pseudobulk	DESeq2	LRT	0.137535923742002
480	KC_G1	ergic53	0.358314880223799	0.341879561225589	0.998043617620652	pseudobulk	DESeq2	LRT	0.466126862093932
480	KC_G1	erm	-0.794610277746993	0.879485534599449	0.998043617620652	pseudobulk	DESeq2	LRT	0.0557712992369932
480	KC_G1	Ero1L	-0.0415157810138147	0.916197201306616	0.998043617620652	pseudobulk	DESeq2	LRT	0.0380110391905284
480	KC_G1	ERp60	-0.0564121573041507	0.843829668170387	0.998043617620652	pseudobulk	DESeq2	LRT	0.0737452093450147
480	KC_G1	ERR	-0.386165136909498	0.276761494204166	0.998043617620652	pseudobulk	DESeq2	LRT	0.557894333355173
480	KC_G1	Es2	0.226372909749638	0.602191782641831	0.998043617620652	pseudobulk	DESeq2	LRT	0.2202651750565
481	KC_G1	esc	-0.942163205545466	0.530785481786521	0.998043617620652	pseudobulk	DESeq2	LRT	0.275080964606077
481	KC_G1	escl	-1.26590483973602	0.488684990522889	0.998043617620652	pseudobulk	DESeq2	LRT	0.310970999693313
481	KC_G1	esn	1.12508065046033	0.900770125530981	0.998043617620652	pseudobulk	DESeq2	LRT	0.0453860258366365
481	KC_G1	Est-Q	-0.194483284482112	0.899393095895953	0.998043617620652	pseudobulk	DESeq2	LRT	0.0460504505748355
481	KC_G1	Esyt2	0.252782489021596	0.446796695780434	0.998043617620652	pseudobulk	DESeq2	LRT	0.349890047305826

481	KC_G1	Etf-QO	-0.305133613156425	0.629884527064386	0.998043617620652	pseudobulk	DESeq2	LRT	0.20073905984046
481	KC_G1	ETHR	0.765052904958762	0.810501863366603	0.998043617620652	pseudobulk	DESeq2	LRT	0.0912459823590772
481	KC_G1	Etl1	0.0343955636306886	0.930634703769753	0.998043617620652	pseudobulk	DESeq2	LRT	0.0312207564721182
481	KC_G1	Ets21C	-0.794610277746993	0.821479263487599	0.998043617620652	pseudobulk	DESeq2	LRT	0.085403394939296
481	KC_G1	Ets97D	-0.128033816721765	0.814380181429876	0.998043617620652	pseudobulk	DESeq2	LRT	0.0891728037700539
482	KC_G1	euc	0.246072403300218	0.609623989568736	0.998043617620652	pseudobulk	DESeq2	LRT	0.214937951228281
482	KC_G1	Evi5	-0.0536015689769536	0.720859667301868	0.998043617620652	pseudobulk	DESeq2	LRT	0.142149272934275
482	KC_G1	ewg	-0.0946927070702652	0.6417296928311	0.998043617620652	pseudobulk	DESeq2	LRT	0.192647865439434
482	KC_G1	ex	-1.72343437992478	0.51212056056209 3	0.998043617620652	pseudobulk	DESeq2	LRT	0.290627787806665
482	KC_G1	exd	-0.126922747013066	0.46984608547162	0.998043617620652	pseudobulk	DESeq2	LRT	0.328044387123322
482	KC_G1	Exn	0.104299791741988	0.835230085933496	0.998043617620652	pseudobulk	DESeq2	LRT	0.0781938702810271
482	KC_G1	Exo70	0.0535243852846187	0.906742409509009	0.998043617620652	pseudobulk	DESeq2	LRT	0.0425160712833981
482	KC_G1	Exo84	-0.0731599794585263	0.87676531499814	0.998043617620652	pseudobulk	DESeq2	LRT	0.0571166392918072
482	KC_G1	exp	1.13582588427274	0.704684208645453	0.998043617620652	pseudobulk	DESeq2	LRT	0.152005460551806
482	KC_G1	ey	-0.134858196525632	0.275702789828084	0.998043617620652	pseudobulk	DESeq2	LRT	0.559558839259434
483	KC_G1	eya	-3.53529829470596	0.313322155874592	0.998043617620652	pseudobulk	DESeq2	LRT	0.504008893870725
483	KC_G1	eys	-0.185487002639236	0.139152431806231	0.998043617620652	pseudobulk	DESeq2	LRT	0.856509199600876
483	KC_G1	f-cup	-0.190451161838844	0.676301188874492	0.998043617620652	pseudobulk	DESeq2	LRT	0.169859849124742
483	KC_G1	Faa	-0.794610277746993	0.879485534599449	0.998043617620652	pseudobulk	DESeq2	LRT	0.0557712992369932
483	KC_G1	fabp	-0.111477517023365	0.610210936673243	0.998043617620652	pseudobulk	DESeq2	LRT	0.21452001286163
483	KC_G1	Fadd	-0.133748371995784	0.884381372855223	0.998043617620652	pseudobulk	DESeq2	LRT	0.0533604132568054
483	KC_G1	faf	-0.235034366001847	0.183214705821816	0.998043617620652	pseudobulk	DESeq2	LRT	0.737039670400998
483	KC_G1	Faf2	-0.184191559012549	0.552467163833655	0.998043617620652	pseudobulk	DESeq2	LRT	0.257693529388796
483	KC_G1	Fak	-0.0350689111448717	0.839780769878688	0.998043617620652	pseudobulk	DESeq2	LRT	0.0758340744839715
483	KC_G1	fal	-0.212034433232675	0.67672272549617	0.998043617620652	pseudobulk	DESeq2	LRT	0.169589238926809
484	KC_G1	FAM21	-0.252312721250274	0.531767565280681	0.998043617620652	pseudobulk	DESeq2	LRT	0.274278155626494
484	KC_G1	fan	-0.794610277746993	0.815039924133852	0.998043617620652	pseudobulk	DESeq2	LRT	0.0888211171417967
484	KC_G1	Fancd2	1.13582588427274	0.704684208645453	0.998043617620652	pseudobulk	DESeq2	LRT	0.152005460551806
484	KC_G1	Fancm	0.166244942088426	0.883298372245562	0.998043617620652	pseudobulk	DESeq2	LRT	0.0538925698862252
484	KC_G1	fand	0.788166140645819	0.192634458331487	0.998043617620652	pseudobulk	DESeq2	LRT	0.715266023936977
484	KC_G1	Fas1	0.0771301044330067	0.596313615075707	0.998043617620652	pseudobulk	DESeq2	LRT	0.224525274697951
484	KC_G1	Fas3	-0.43448595517548	0.162274056757396	0.998043617620652	pseudobulk	DESeq2	LRT	0.789750906592147
484	KC_G1	FASN1	-0.265648732744872	0.177307407013357	0.998043617620652	pseudobulk	DESeq2	LRT	0.751273121376882
484	KC_G1	fat-spondin	0.447675383662238	0.75232315093263	0.998043617620652	pseudobulk	DESeq2	LRT	0.123595573608711
484	KC_G1	Fatp1	0.0511679558935937	0.876768173294822	0.998043617620652	pseudobulk	DESeq2	LRT	0.0571152234734209
485	KC_G1	Fatp3	-0.616680624547631	0.689887645626378	0.998043617620652	pseudobulk	DESeq2	LRT	0.161221632244785
485	KC_G1	fax	-0.129696473478393	0.192321549854846	0.998043617620652	pseudobulk	DESeq2	LRT	0.715972049829729
485	KC_G1	fbl	-0.21784545800233	0.655328591735487	0.998043617620652	pseudobulk	DESeq2	LRT	0.183540883528576
485	KC_G1	Fbl6	-0.0573026501475961	0.823755738959713	0.998043617620652	pseudobulk	DESeq2	LRT	0.0842015467349862
485	KC_G1	fbp	0.173195945230598	0.976215465741119	0.998043617620652	pseudobulk	DESeq2	LRT	0.0104543162934892
485	KC_G1	Fbw5	0.893046301156573	0.373399264872655	0.998043617620652	pseudobulk	DESeq2	LRT	0.427826541400502
485	KC_G1	Fbxl4	0.0566549896864248	0.919284586668663	0.998043617620652	pseudobulk	DESeq2	LRT	0.0365500214898024
485	KC_G1	FBXO11	-0.0686365459224472	0.743095060586878	0.998043617620652	pseudobulk	DESeq2	LRT	0.128955625475542
485	KC_G1	Fcp1	-0.327448576915996	0.318191513737807	0.998043617620652	pseudobulk	DESeq2	LRT	0.497311407297826
485	KC_G1	Fcp3C	1.12508065046033	0.900770125530981	0.998043617620652	pseudobulk	DESeq2	LRT	0.0453860258366365
486	KC_G1	fd102C	1.13582588427274	0.704684208645453	0.998043617620652	pseudobulk	DESeq2	LRT	0.152005460551806
486	KC_G1	fd3F	-0.794610277746993	0.815039924133852	0.998043617620652	pseudobulk	DESeq2	LRT	0.0888211171417967
486	KC_G1	fd96Ca	1.13582588427274	0.633424328955532	0.998043617620652	pseudobulk	DESeq2	LRT	0.198305259991224
486	KC_G1	Fdh	0.217825785268553	0.841830681721033	0.998043617620652	pseudobulk	DESeq2	LRT	0.0747752498171312
486	KC_G1	fdl	-0.110781711491395	0.66099767361483	0.998043617620652	pseudobulk	DESeq2	LRT	0.179800069013634
486	KC_G1	Fdx1	0.331442310356715	0.382629802374103	0.998043617620652	pseudobulk	DESeq2	LRT	0.4172212065446
486	KC_G1	Fdx2	0.59068291263763	0.0809193237925127	0.998043617620652	pseudobulk	DESeq2	LRT	1.09194775509559
486	KC_G1	FeCH	0.902157986165909	0.0745264860260861	0.998043617620652	pseudobulk	DESeq2	LRT	1.12768935556377
486	KC_G1	Fem-1	0.266494667151084	0.36908790653654	0.998043617620652	pseudobulk	DESeq2	LRT	0.432870184580804
486	KC_G1	Fen1	0.814730332011429	0.458592944834809	0.998043617620652	pseudobulk	DESeq2	LRT	0.338572630903246
487	KC_G1	fend	0.0538940038511175	0.659373557905313	0.998043617620652	pseudobulk	DESeq2	LRT	0.180868472852658

487	KC_G1	FER	-0.481044106248732	0.525154279932989	0.998043617620652	pseudobulk	DESeq2	LRT	0.279713090726357
487	KC_G1	Fer1	0.121483991571076	0.960019907175548	0.998043617620652	pseudobulk	DESeq2	LRT	0.0177197612449608
487	KC_G1	Fer1HCH	-0.0794902379596191	0.557366489966528	0.998043617620652	pseudobulk	DESeq2	LRT	0.253859145526018
487	KC_G1	Fer2LCH	-0.030083149222742	0.789276593533779	0.998043617620652	pseudobulk	DESeq2	LRT	0.102770776264504
487	KC_G1	fest	0.0256098738591729	0.934746655914375	0.998043617620652	pseudobulk	DESeq2	LRT	0.0293060798731616
487	KC_G1	Fgop2	-0.10174557807693	0.813355174107821	0.998043617620652	pseudobulk	DESeq2	LRT	0.0897197662491881
487	KC_G1	fh	0.434681208248014	0.402190377278804	0.998043617620652	pseudobulk	DESeq2	LRT	0.395568324452131
487	KC_G1	Fhos	-1.67562001284769	0.424675803229702	0.998043617620652	pseudobulk	DESeq2	LRT	0.371942483118544
487	KC_G1	Fib	0.0612123916125023	0.947743081086377	0.998043617620652	pseudobulk	DESeq2	LRT	0.0233093774179882
488	KC_G1	Fibp	0.065435064423849	0.768885481583999	0.998043617620652	pseudobulk	DESeq2	LRT	0.114138339548017
488	KC_G1	Fic	0.0619372640897455	0.906251955540332	0.998043617620652	pseudobulk	DESeq2	LRT	0.0427510432849949
488	KC_G1	fid	0.0606130289458977	0.856771019792495	0.998043617620652	pseudobulk	DESeq2	LRT	0.0671352319019953
488	KC_G1	fidipidine	-0.688659254769565	0.268214620766906	0.998043617620652	pseudobulk	DESeq2	LRT	0.571517551818801
488	KC_G1	Fie	0.070896197016386	0.80979885749217	0.998043617620652	pseudobulk	DESeq2	LRT	0.0916228402932121
488	KC_G1	Fife	-0.0383578237958266	0.786825103982807	0.998043617620652	pseudobulk	DESeq2	LRT	0.104121792185237
488	KC_G1	FIG4	-0.643766604047088	0.434824636475084	0.998043617620652	pseudobulk	DESeq2	LRT	0.36168585745127
488	KC_G1	Fili	2.02093374629628	0.211007585685658	0.998043617620652	pseudobulk	DESeq2	LRT	0.675701931611288
488	KC_G1	Fim	-0.0603715662078676	0.685538258268582	0.998043617620652	pseudobulk	DESeq2	LRT	0.16396830325041
488	KC_G1	Fip1	-0.00912895591104438	0.969244960748688	0.998043617620652	pseudobulk	DESeq2	LRT	0.0135664482776538
489	KC_G1	fipi	-0.200688446423141	0.20856020196183	0.998043617620652	pseudobulk	DESeq2	LRT	0.680768561285135
489	KC_G1	Fis1	0.0112624326024007	0.937499125880787	0.998043617620652	pseudobulk	DESeq2	LRT	0.0280291285339263
489	KC_G1	Fit1	0.0265963338419953	0.925711397638636	0.998043617620652	pseudobulk	DESeq2	LRT	0.0335243890728183
489	KC_G1	Fitm	0.14203440607093	0.821689483917815	0.998043617620652	pseudobulk	DESeq2	LRT	0.0852922711445327
489	KC_G1	Fkbp12	-0.0806463406586674	0.765379675544439	0.998043617620652	pseudobulk	DESeq2	LRT	0.116123074533905
489	KC_G1	Fkbp14	0.453218564110749	0.604107401986967	0.998043617620652	pseudobulk	DESeq2	LRT	0.218885842929033
489	KC_G1	Fkbp39	0.134026828611739	0.674014195066689	0.998043617620652	pseudobulk	DESeq2	LRT	0.171330956915399
489	KC_G1	Fkbp59	-0.184195819777337	0.517162024417858	0.998043617620652	pseudobulk	DESeq2	LRT	0.286373373179285
489	KC_G1	fl(2)d	-0.0172817936323477	0.911774760241445	0.998043617620652	pseudobulk	DESeq2	LRT	0.0401124341121016
489	KC_G1	Flacc	-0.0418947399145422	0.880670514820352	0.998043617620652	pseudobulk	DESeq2	LRT	0.0551865437557721
490	KC_G1	FLASH	-0.882792088409424	0.191886634884202	0.998043617620652	pseudobulk	DESeq2	LRT	0.71695527330089
490	KC_G1	flfl	-0.138339261132697	0.441597648206401	0.998043617620652	pseudobulk	DESeq2	LRT	0.354973248172205
490	KC_G1	fliI	0.0742752906926271	0.874720881830934	0.998043617620652	pseudobulk	DESeq2	LRT	0.0581305056277105
490	KC_G1	fln	-0.794610277746993	0.879485534599449	0.998043617620652	pseudobulk	DESeq2	LRT	0.0557712992369932
490	KC_G1	Flo1	0.00652203748183875	0.96513070518764	0.998043617620652	pseudobulk	DESeq2	LRT	0.0154138672802113
490	KC_G1	Flo2	-0.251464381160519	0.157523932969204	0.998043617620652	pseudobulk	DESeq2	LRT	0.802653453513541
490	KC_G1	flr	-0.156321851770391	0.770130346433562	0.998043617620652	pseudobulk	DESeq2	LRT	0.113435763209364
490	KC_G1	flw	-0.0363953433981779	0.821105469583701	0.998043617620652	pseudobulk	DESeq2	LRT	0.0856010549191631
490	KC_G1	flz	0.173195945230598	0.976215465741119	0.998043617620652	pseudobulk	DESeq2	LRT	0.0104543162934892
490	KC_G1	Fmo-2	-0.425614289477036	0.845154747277466	0.998043617620652	pseudobulk	DESeq2	LRT	0.0730637647359404
491	KC_G1	FMRFaR	3.28579684404318	0.126723705412286	0.998043617620652	pseudobulk	DESeq2	LRT	0.897142136760093
491	KC_G1	fmt	-0.160966891087137	0.359218019119347	0.998043617620652	pseudobulk	DESeq2	LRT	0.444641886348209
491	KC_G1	fne	-0.0466546068702419	0.613300833111265	0.998043617620652	pseudobulk	DESeq2	LRT	0.212326445364065
491	KC_G1	fng	0.072548324066887	0.926635140678524	0.998043617620652	pseudobulk	DESeq2	LRT	0.0330912341205698
491	KC_G1	Fnta	-0.195975561046873	0.616196990292765	0.998043617620652	pseudobulk	DESeq2	LRT	0.210280427249907
491	KC_G1	fog	0.344996342777788	0.519984607664087	0.998043617620652	pseudobulk	DESeq2	LRT	0.284009511952681
491	KC_G1	foi	-0.184257753705389	0.611656509762043	0.998043617620652	pseudobulk	DESeq2	LRT	0.213492397778838
491	KC_G1	fok	-0.166537290806702	0.316529503587588	0.998043617620652	pseudobulk	DESeq2	LRT	0.499585803346125
491	KC_G1	fon	-0.794610277746993	0.821479263487599	0.998043617620652	pseudobulk	DESeq2	LRT	0.085403394939296
491	KC_G1	for	-1.74278423686085	0.385072726505398	0.998043617620652	pseudobulk	DESeq2	LRT	0.414457240005315
492	KC_G1	form3	0.155916667524066	0.846443154433124	0.998043617620652	pseudobulk	DESeq2	LRT	0.0724022029950779
492	KC_G1	FoxK	-0.128365378347699	0.410044148782207	0.998043617620652	pseudobulk	DESeq2	LRT	0.387169380986936
492	KC_G1	foxo	-0.794610277746993	0.815039924133852	0.998043617620652	pseudobulk	DESeq2	LRT	0.0888211171417967
492	KC_G1	FoxP	-0.362436087250033	0.0730513842536644	0.998043617620652	pseudobulk	DESeq2	LRT	1.13637155018783
492	KC_G1	Fpgs	0.714374395293509	0.687995291928297	0.998043617620652	pseudobulk	DESeq2	LRT	0.162414533707159
492	KC_G1	Fpps	-0.332663852048825	0.662362515236016	0.998043617620652	pseudobulk	DESeq2	LRT	0.178904253346956
492	KC_G1	fra	-3.48571529295288	0.0663180080507057	0.998043617620652	pseudobulk	DESeq2	LRT	1.17836852687242

492	KC_G1	fray	-0.211817143553776	0.606868221230123	0.998043617620652	pseudobulk	DESeq2	LRT	0.216905603827292
492	KC_G1	fred	0.362581007161159	0.636587382770277	0.998043617620652	pseudobulk	DESeq2	LRT	0.196141973392597
492	KC_G1	FRG1	-0.248566769951847	0.496486700462437	0.998043617620652	pseudobulk	DESeq2	LRT	0.304092380588812
493	KC_G1	Frl	0.327662088714472	0.117532880604825	0.998043617620652	pseudobulk	DESeq2	LRT	0.929840619625376
493	KC_G1	frm	-0.794610277746993	0.821479263487599	0.998043617620652	pseudobulk	DESeq2	LRT	0.085403394939296
493	KC_G1	Frq1	-0.140746221098482	0.339996083260202	0.998043617620652	pseudobulk	DESeq2	LRT	0.468526085982095
493	KC_G1	Frq2	0.496525854072214	0.340249353245215	0.998043617620652	pseudobulk	DESeq2	LRT	0.468202691642045
493	KC_G1	frtz	0.424870434575735	0.686037344953951	0.998043617620652	pseudobulk	DESeq2	LRT	0.163652242506104
493	KC_G1	fru	-0.0230218433071378	0.878866439735688	0.998043617620652	pseudobulk	DESeq2	LRT	0.0560771191162222
493	KC_G1	fry	0.249548942088539	0.453460579800208	0.998043617620652	pseudobulk	DESeq2	LRT	0.343460461017028
493	KC_G1	fs(1)h	-0.130037822875461	0.189776663717961	0.998043617620652	pseudobulk	DESeq2	LRT	0.721757192549488
493	KC_G1	fs(1)K10	-0.684611790281564	0.163630650576654	0.998043617620652	pseudobulk	DESeq2	LRT	0.786135342904818
493	KC_G1	Fs(2)Ket	-0.0824085106376205	0.46206811013509	0.998043617620652	pseudobulk	DESeq2	LRT	0.335294003500915
494	KC_G1	fs(2)ltoPP43	0.0712301109399845	0.83791020071224	0.998043617620652	pseudobulk	DESeq2	LRT	0.0768025224495185
494	KC_G1	fsd	-0.557566491335852	0.314341845595553	0.998043617620652	pseudobulk	DESeq2	LRT	0.502597801287353
494	KC_G1	Fsn	0.00665295594010163	0.972066775668915	0.998043617620652	pseudobulk	DESeq2	LRT	0.0123039003942694
494	KC_G1	ftz-f1	-0.050463296161	0.819070420671009	0.998043617620652	pseudobulk	DESeq2	LRT	0.0866787575874774
494	KC_G1	fu	-0.0130605171347933	0.969651141501976	0.998043617620652	pseudobulk	DESeq2	LRT	0.0133844869418363
494	KC_G1	FucT6	-0.0149378050060208	0.964248168442543	0.998043617620652	pseudobulk	DESeq2	LRT	0.0158111773891204
494	KC_G1	FucTA	0.233973383122057	0.399662821182913	0.998043617620652	pseudobulk	DESeq2	LRT	0.398306250304072
494	KC_G1	FucTB	-0.228820499631298	0.842233436361539	0.998043617620652	pseudobulk	DESeq2	LRT	0.0745675212403037
494	KC_G1	FucTC	-0.794610277746993	0.879485534599449	0.998043617620652	pseudobulk	DESeq2	LRT	0.0557712992369932
494	KC_G1	Fum1	-0.00599561311077754	0.971698483509608	0.998043617620652	pseudobulk	DESeq2	LRT	0.0124684750554062
495	KC_G1	Fum2	1.13582588427274	0.704684208645453	0.998043617620652	pseudobulk	DESeq2	LRT	0.152005460551806
495	KC_G1	Fur1	0.263348215684397	0.263367424747376	0.998043617620652	pseudobulk	DESeq2	LRT	0.579437942841753
495	KC_G1	Fur2	-0.0335888218986555	0.889148807178891	0.998043617620652	pseudobulk	DESeq2	LRT	0.0510255497964104
495	KC_G1	fus	1.13582588427274	0.704684208645453	0.998043617620652	pseudobulk	DESeq2	LRT	0.152005460551806
495	KC_G1	fusl	1.13582588427274	0.704684208645453	0.998043617620652	pseudobulk	DESeq2	LRT	0.152005460551806
495	KC_G1	futsch	0.0214539971099259	0.880736210237169	0.998043617620652	pseudobulk	DESeq2	LRT	0.0551541478796607
495	KC_G1	fwd	-0.131979548074894	0.43837614224456	0.998043617620652	pseudobulk	DESeq2	LRT	0.35815308949969
495	KC_G1	fwe	0.147209637788067	0.191915613355711	0.998043617620652	pseudobulk	DESeq2	LRT	0.716889691661636
495	KC_G1	fws	0.141608951176687	0.771312900643658	0.998043617620652	pseudobulk	DESeq2	LRT	0.112769404756368
495	KC_G1	fy	0.0122871503711493	0.972467777611102	0.998043617620652	pseudobulk	DESeq2	LRT	0.012124779963728
496	KC_G1	fz2	-0.0910357423130299	0.662410346483474	0.998043617620652	pseudobulk	DESeq2	LRT	0.178872892730442
496	KC_G1	fz3	1.12508065046033	0.900770125530981	0.998043617620652	pseudobulk	DESeq2	LRT	0.0453860258366365
496	KC_G1	fz4	0.415406385812468	0.500817232593065	0.998043617620652	pseudobulk	DESeq2	LRT	0.300320735925189
496	KC_G1	fzo	1.06585281705208	0.683859663547483	0.998043617620652	pseudobulk	DESeq2	LRT	0.1650330117303
496	KC_G1	fzr	-0.116030405965397	0.639116372891495	0.998043617620652	pseudobulk	DESeq2	LRT	0.194420056547285
496	KC_G1	fzy	1.13582588427274	0.633424328955532	0.998043617620652	pseudobulk	DESeq2	LRT	0.198305259991224
496	KC_G1	g	-0.00089980336149460	0.973176027343777	0.998043617620652	pseudobulk	DESeq2	LRT	0.0118085977689062
496	KC_G1	G6P	0.403892446078499	0.507465409082809	0.998043617620652	pseudobulk	DESeq2	LRT	0.29459355569347
496	KC_G1	G9a	0.0621436454518005	0.687065480471026	0.998043617620652	pseudobulk	DESeq2	LRT	0.163001870724918
496	KC_G1	GAA1	0.362201269637387	0.393085570090762	0.998043617620652	pseudobulk	DESeq2	LRT	0.405512898548215
497	KC_G1	GABA-B-R1	-0.14545329447758	0.250356524155718	0.998043617620652	pseudobulk	DESeq2	LRT	0.601441086638115
497	KC_G1	GABA-B-R2	0.0239069501686625	0.888519559809109	0.998043617620652	pseudobulk	DESeq2	LRT	0.0513330072242829
497	KC_G1	Gabat	0.122010636244931	0.444154986331442	0.998043617620652	pseudobulk	DESeq2	LRT	0.352465457895728
497	KC_G1	GABPI	-0.16531962197747	0.575245475108386	0.998043617620652	pseudobulk	DESeq2	LRT	0.240146788814361
497	KC_G1	Gad1	-0.473785012655387	0.776866150462736	0.998043617620652	pseudobulk	DESeq2	LRT	0.109653801177068
497	KC_G1	Gadd45	0.764815221789058	0.808325187140535	0.998043617620652	pseudobulk	DESeq2	LRT	0.0924138885208533
497	KC_G1	Gal	-1.31047986807593	0.611671045333228	0.998043617620652	pseudobulk	DESeq2	LRT	0.213482077209615
497	KC_G1	Gale	-0.173474533821702	0.712799238139174	0.998043617620652	pseudobulk	DESeq2	LRT	0.147032773157671
497	KC_G1	galectin	-0.142506347991123	0.477446737171956	0.998043617620652	pseudobulk	DESeq2	LRT	0.321075070242589
497	KC_G1	Galk	0.219375510190088	0.74750978836551	0.998043617620652	pseudobulk	DESeq2	LRT	0.126383116039928
498	KC_G1	galla-1	0.491762433797231	0.136918498314415	0.998043617620652	pseudobulk	DESeq2	LRT	0.863537872734128
498	KC_G1	galla-2	0.263366925331748	0.379151613571579	0.998043617620652	pseudobulk	DESeq2	LRT	0.421187091428067
498	KC_G1	Galphai	0.0881589035836021	0.896825229339388	0.998043617620652	pseudobulk	DESeq2	LRT	0.0472921827405173

498	KC_G1	Galphao	-0.0282775699652481	0.814446359294433	0.998043617620652	pseudobulk	DESeq2	LRT	0.0891375137244923
498	KC_G1	Galphaq	-0.178457024081108	0.169670667026827	0.998043617620652	pseudobulk	DESeq2	LRT	0.770393232812007
498	KC_G1	Galphas	-0.0297851100786921	0.812668488483893	0.998043617620652	pseudobulk	DESeq2	LRT	0.0900865798471253
498	KC_G1	Galt	-0.0910654237852307	0.852477204178649	0.998043617620652	pseudobulk	DESeq2	LRT	0.0693172255105847
498	KC_G1	GalT1	0.043716266250954	0.909202131776272	0.998043617620652	pseudobulk	DESeq2	LRT	0.0413395546699151
498	KC_G1	gammaCOP	-0.634685690425654	0.150888037254244	0.998043617620652	pseudobulk	DESeq2	LRT	0.821345190710921
498	KC_G1	gammaSnap2	-0.077534803370097	0.789195878071149	0.998043617620652	pseudobulk	DESeq2	LRT	0.102815191711318
499	KC_G1	gammaTub23C	-0.0875977553252277	0.840000987925017	0.998043617620652	pseudobulk	DESeq2	LRT	0.075720203164153
499	KC_G1	GAPcenA	0.0405680814438766	0.863335792032425	0.998043617620652	pseudobulk	DESeq2	LRT	0.063820253806834
499	KC_G1	Gapdh1	0.13175397233194	0.42448804706366	0.998043617620652	pseudobulk	DESeq2	LRT	0.372134534318452
499	KC_G1	GAPsec	-0.437110566131606	0.408504588058151	0.998043617620652	pseudobulk	DESeq2	LRT	0.38880306139069
499	KC_G1	Gapvd1	-0.292383927976504	0.397139944724972	0.998043617620652	pseudobulk	DESeq2	LRT	0.401056428974447
499	KC_G1	garz	0.00356344613220773	0.975900996109729	0.998043617620652	pseudobulk	DESeq2	LRT	0.0105942387105057
499	KC_G1	gas	-0.794610277746993	0.879485534599449	0.998043617620652	pseudobulk	DESeq2	LRT	0.0557712992369932
499	KC_G1	Gas41	0.200721965183985	0.602380897360056	0.998043617620652	pseudobulk	DESeq2	LRT	0.220128808890124
499	KC_G1	Gas8	-0.765625368254185	0.836625948066609	0.998043617620652	pseudobulk	DESeq2	LRT	0.0774686698445758
499	KC_G1	Gasz	0.234839401543992	0.582498260115589	0.998043617620652	pseudobulk	DESeq2	LRT	0.234705367509372
500	KC_G1	Gat	-0.0537134260903878	0.8318171252003	0.998043617620652	pseudobulk	DESeq2	LRT	0.0799721427551294
500	KC_G1	GatA	-0.0594787428508725	0.903326840701995	0.998043617620652	pseudobulk	DESeq2	LRT	0.0441550853110461
500	KC_G1	GATAd	0.0823594504829053	0.721370611591729	0.998043617620652	pseudobulk	DESeq2	LRT	0.141841554675717
500	KC_G1	GatB	0.365887700209341	0.5074313802083	0.998043617620652	pseudobulk	DESeq2	LRT	0.294622678955237
500	KC_G1	gb	0.31198182704087	0.624663985576354	0.998043617620652	pseudobulk	DESeq2	LRT	0.204353532178405
500	KC_G1	Gba1a	-0.0320680392280564	0.95371756350833	0.998043617620652	pseudobulk	DESeq2	LRT	0.0205802193950135
500	KC_G1	Gba1b	-0.794610277746993	0.879485534599449	0.998043617620652	pseudobulk	DESeq2	LRT	0.0557712992369932
500	KC_G1	gbb	0.955401970680431	0.430936470761591	0.998043617620652	pseudobulk	DESeq2	LRT	0.365586749401421
500	KC_G1	Gbeta13F	-0.0254358883345412	0.809618460603489	0.998043617620652	pseudobulk	DESeq2	LRT	0.0917195977784572
500	KC_G1	Gbeta5	0.103909504069374	0.545439333257091	0.998043617620652	pseudobulk	DESeq2	LRT	0.263253546994737
501	KC_G1	Gbp1	0.765052904958762	0.810501863366603	0.998043617620652	pseudobulk	DESeq2	LRT	0.0912459823590772
501	KC_G1	Gbs-70E	-0.794610277746993	0.821479263487599	0.998043617620652	pseudobulk	DESeq2	LRT	0.085403394939296
501	KC_G1	Gbs-76A	0.162846111697999	0.650028084557616	0.998043617620652	pseudobulk	DESeq2	LRT	0.187067879195745
501	KC_G1	GC	0.0987146900435731	0.769218918111868	0.998043617620652	pseudobulk	DESeq2	LRT	0.113950043296585
501	KC_G1	GC1	0.0672227527926414	0.770842220453753	0.998043617620652	pseudobulk	DESeq2	LRT	0.113034506253315
501	KC_G1	GCC185	0.246072238690769	0.534496321698941	0.998043617620652	pseudobulk	DESeq2	LRT	0.272055279176157
501	KC_G1	GCC88	-0.135083038225737	0.78795088432972	0.998043617620652	pseudobulk	DESeq2	LRT	0.103500852725382
501	KC_G1	gce	-0.0274758456115246	0.864421296669059	0.998043617620652	pseudobulk	DESeq2	LRT	0.0632745419832427
501	KC_G1	GckIII	0.200915381421123	0.472722581809903	0.998043617620652	pseudobulk	DESeq2	LRT	0.325393651089965
501	KC_G1	gcl	-0.159035608926027	0.571742243267556	0.998043617620652	pseudobulk	DESeq2	LRT	0.242799718682647
502	KC_G1	Gclc	-0.0853107114950567	0.756102486095124	0.998043617620652	pseudobulk	DESeq2	LRT	0.121419333957859
502	KC_G1	Gclm	0.02064486889871	0.949107939997287	0.998043617620652	pseudobulk	DESeq2	LRT	0.0226843933899414
502	KC_G1	Gcn2	-0.310902767086089	0.59764267867718	0.998043617620652	pseudobulk	DESeq2	LRT	0.223558396379795
502	KC_G1	Gcn5	-0.180845644341133	0.742711675376579	0.998043617620652	pseudobulk	DESeq2	LRT	0.129179748951283
502	KC_G1	GCS1	0.548180199536125	0.325784601718274	0.998043617620652	pseudobulk	DESeq2	LRT	0.487069446567787
502	KC_G1	GCS2alpha	-0.239988523932528	0.24382453033794	0.998043617620652	pseudobulk	DESeq2	LRT	0.612922603662311
502	KC_G1	GCS2beta	0.0361266779839145	0.924699909347374	0.998043617620652	pseudobulk	DESeq2	LRT	0.0339991849471184
502	KC_G1	Gdap1	2.66264011801741	0.281871598697827	0.998043617620652	pseudobulk	DESeq2	LRT	0.549948681312517
502	KC_G1	Gdap2	-0.0076625988385592	0.94177624036764	0.998043617620652	pseudobulk	DESeq2	LRT	0.0260522703674814
502	KC_G1	Gdh	-0.0600865213973014	0.652407514433294	0.998043617620652	pseudobulk	DESeq2	LRT	0.185481045396753
503	KC_G1	Gdi	-0.00565759042259363	0.965942586997926	0.998043617620652	pseudobulk	DESeq2	LRT	0.0150486861010538
503	KC_G1	gdl	0.616235831075479	0.340045448099151	0.998043617620652	pseudobulk	DESeq2	LRT	0.468463034311852
503	KC_G1	Gdn1	0.420670711671861	0.512789313547844	0.998043617620652	pseudobulk	DESeq2	LRT	0.290061034026606
503	KC_G1	Ge-1	-0.213860474635637	0.507111034099005	0.998043617620652	pseudobulk	DESeq2	LRT	0.294896939647008
503	KC_G1	GEFmeso	-0.012477857020195	0.950531684133411	0.998043617620652	pseudobulk	DESeq2	LRT	0.0220334021915246
503	KC_G1	gek	0.0734387334254661	0.858602716394026	0.998043617620652	pseudobulk	DESeq2	LRT	0.0662077418604585
503	KC_G1	geko	-0.794610277746993	0.815039924133852	0.998043617620652	pseudobulk	DESeq2	LRT	0.0888211171417967
503	KC_G1	Gel	-0.385497650928642	0.546711218457338	0.998043617620652	pseudobulk	DESeq2	LRT	0.262242014339412
503	KC_G1	Gem2	-0.0588025542851723	0.945763324019226	0.998043617620652	pseudobulk	DESeq2	LRT	0.024217531603005

503	KC_G1	Gem3	0.245490791689901	0.713075201238349	0.998043617620652	pseudobulk	DESeq2	LRT	0.146864666837272
504	KC_G1	geminin	0.149217237388225	0.774368343893824	0.998043617620652	pseudobulk	DESeq2	LRT	0.111052409254979
504	KC_G1	Gen	0.299243349670864	0.891861473457015	0.998043617620652	pseudobulk	DESeq2	LRT	0.0497025962799188
504	KC_G1	Gfat1	-0.299540469042341	0.870848058508045	0.998043617620652	pseudobulk	DESeq2	LRT	0.0600576120506324
504	KC_G1	Gfat2	0.037387820864135	0.914558495382726	0.998043617620652	pseudobulk	DESeq2	LRT	0.038788511719016
504	KC_G1	Gfrl	0.0988409128933569	0.375875835565238	0.998043617620652	pseudobulk	DESeq2	LRT	0.424955593461512
504	KC_G1	gfzf	-0.291490764111882	0.672046757851924	0.998043617620652	pseudobulk	DESeq2	LRT	0.172600509727963
504	KC_G1	Gga	0.0386576739367334	0.909772192691695	0.998043617620652	pseudobulk	DESeq2	LRT	0.041067341571916
504	KC_G1	Ggamma1	-0.102962120964805	0.359627728122362	0.998043617620652	pseudobulk	DESeq2	LRT	0.444146830547586
504	KC_G1	Ggamma30A	-0.245975302705388	0.0988478158072955	0.998043617620652	pseudobulk	DESeq2	LRT	1.00503292264084
504	KC_G1	Ggt-1	1.10637961057774	0.292596679485622	0.998043617620652	pseudobulk	DESeq2	LRT	0.53373060674541
505	KC_G1	ghi	0.268317453020441	0.139202035536649	0.998043617620652	pseudobulk	DESeq2	LRT	0.856354414036729
505	KC_G1	gig	-0.44922192965265	0.525433318530516	0.998043617620652	pseudobulk	DESeq2	LRT	0.279482391399293
505	KC_G1	GIIIspla2	0.193244124810025	0.716189192689972	0.998043617620652	pseudobulk	DESeq2	LRT	0.144972236787565
505	KC_G1	GILT1	0.814587722132563	0.0165990037365402	0.998043617620652	pseudobulk	DESeq2	LRT	1.77991797730375
505	KC_G1	GILT2	-0.334165059404598	0.622181509974618	0.998043617620652	pseudobulk	DESeq2	LRT	0.206082899423574
505	KC_G1	GILT3	-0.221167287014265	0.651000409695415	0.998043617620652	pseudobulk	DESeq2	LRT	0.18641873811629
505	KC_G1	Gip	-0.776957416996528	0.494016760177329	0.998043617620652	pseudobulk	DESeq2	LRT	0.306258316807009
505	KC_G1	Girdin	0.0776545161247487	0.836204501696825	0.998043617620652	pseudobulk	DESeq2	LRT	0.0776874987602179
505	KC_G1	gish	0.0289858841401829	0.786799109584127	0.998043617620652	pseudobulk	DESeq2	LRT	0.104136140240905
505	KC_G1	Git	0.173674464511605	0.722481282431956	0.998043617620652	pseudobulk	DESeq2	LRT	0.141173399840362
506	KC_G1	Gk1	-0.332168692746323	0.466399200071814	0.998043617620652	pseudobulk	DESeq2	LRT	0.331242203113311
506	KC_G1	Gk2	-0.570058840045551	0.436373892659644	0.998043617620652	pseudobulk	DESeq2	LRT	0.360141240240019
506	KC_G1	gkt	-0.640304511824468	0.224089834225961	0.998043617620652	pseudobulk	DESeq2	LRT	0.649577844668459
506	KC_G1	GLaz	0.0377419054115094	0.959092752731591	0.998043617620652	pseudobulk	DESeq2	LRT	0.0181393906899866
506	KC_G1	GlcAT-I	0.302323799784603	0.349615892277052	0.998043617620652	pseudobulk	DESeq2	LRT	0.456408834128717
506	KC_G1	GlcAT-P	0.13675753538267	0.463978831076333	0.998043617620652	pseudobulk	DESeq2	LRT	0.333501833575421
506	KC_G1	GlcAT-S	0.081069199189423	0.781890474391478	0.998043617620652	pseudobulk	DESeq2	LRT	0.10685407775697
506	KC_G1	GlcT	-0.53258435563125	0.415867985519217	0.998043617620652	pseudobulk	DESeq2	LRT	0.381044511343413
506	KC_G1	Gld	-0.794610277746993	0.879485534599449	0.998043617620652	pseudobulk	DESeq2	LRT	0.0557712992369932
506	KC_G1	Gld2	0.1729159612032	0.899814506689544	0.998043617620652	pseudobulk	DESeq2	LRT	0.0458470094762598
507	KC_G1	Gle1	0.580491674454054	0.178743158410862	0.998043617620652	pseudobulk	DESeq2	LRT	0.747770572303237
507	KC_G1	glec	-0.0077961293254861	0.937957512698926	0.998043617620652	pseudobulk	DESeq2	LRT	0.027816833708694
507	KC_G1	Glg1	-0.114407867556591	0.794606620398272	0.998043617620652	pseudobulk	DESeq2	LRT	0.0998478208682977
507	KC_G1	Gli	-2.0362634091035	0.379377865813874	0.998043617620652	pseudobulk	DESeq2	LRT	0.420928010915229
507	KC_G1	GlnRS	-0.141079025151785	0.75028257452221	0.998043617620652	pseudobulk	DESeq2	LRT	0.124775140017554
507	KC_G1	glo	-0.0580046255324676	0.689058285614569	0.998043617620652	pseudobulk	DESeq2	LRT	0.161744040719128
507	KC_G1	Glo1	0.410008275045113	0.400189291665036	0.998043617620652	pseudobulk	DESeq2	LRT	0.397734536471934
507	KC_G1	glob1	-0.228202206825728	0.780083091205039	0.998043617620652	pseudobulk	DESeq2	LRT	0.107859135604511
507	KC_G1	Glos	0.874209999598728	0.438677120269748	0.998043617620652	pseudobulk	DESeq2	LRT	0.357855016152879
507	KC_G1	GLS	-0.0279659126757985	0.914230336554097	0.998043617620652	pseudobulk	DESeq2	LRT	0.038944371780607
508	KC_G1	glu	-0.0316639758177821	0.934849429153258	0.998043617620652	pseudobulk	DESeq2	LRT	0.0292583328213405
508	KC_G1	GluClalpha	-0.187876086612184	0.681746955193441	0.998043617620652	pseudobulk	DESeq2	LRT	0.166376793020568
508	KC_G1	GluProRS	-0.0223825615994081	0.955498274917639	0.998043617620652	pseudobulk	DESeq2	LRT	0.0197700926952338
508	KC_G1	GluRIA	-0.15427311991917	0.248150002806436	0.998043617620652	pseudobulk	DESeq2	LRT	0.605285715555033
508	KC_G1	GluRIB	0.0514812017813465	0.620662872422213	0.998043617620652	pseudobulk	DESeq2	LRT	0.207144233000976
508	KC_G1	GluRIIE	-0.794610277746993	0.815039924133852	0.998043617620652	pseudobulk	DESeq2	LRT	0.0888211171417967
508	KC_G1	GluRS-m	-0.815364173896266	0.602630419923059	0.998043617620652	pseudobulk	DESeq2	LRT	0.219948949544047
508	KC_G1	Glut1	0.0975053947898268	0.56194510615558	0.998043617620652	pseudobulk	DESeq2	LRT	0.250306106598375
508	KC_G1	Glut4EF	-0.0150661838835475	0.904250245854415	0.998043617620652	pseudobulk	DESeq2	LRT	0.0437113644870491
508	KC_G1	GlyRS	0.257257364804373	0.486826310131052	0.998043617620652	pseudobulk	DESeq2	LRT	0.31262595871649
509	KC_G1	GlyS	0.421964827958866	0.378680454490504	0.998043617620652	pseudobulk	DESeq2	LRT	0.421727110316149
509	KC_G1	GlyT	0.455528613078507	0.00382387696415375	0.998043617620652	pseudobulk	DESeq2	LRT	2.41749608989194
509	KC_G1	GM130	0.634758612695567	0.0741424473478972	0.998043617620652	pseudobulk	DESeq2	LRT	1.12993308256131
509	KC_G1	Gmap	0.114541060056024	0.774945420301729	0.998043617620652	pseudobulk	DESeq2	LRT	0.110728883940776
509	KC_G1	Gmd	0.473591238324865	0.540348319772603	0.998043617620652	pseudobulk	DESeq2	LRT	0.267326194644211

509	KC_G1	Gmer	-0.197661598235452	0.799122107817445	0.998043617620652	pseudobulk	DESeq2	LRT	0.0973868543537275
509	KC_G1	GMF	-0.098677131845775	0.811893967605202	0.998043617620652	pseudobulk	DESeq2	LRT	0.0905006854026963
509	KC_G1	GNBP1	0.173116475207952	0.97305562682953	0.998043617620652	pseudobulk	DESeq2	LRT	0.011862331638572
509	KC_G1	GNBP3	-0.794610277746993	0.815039924133852	0.998043617620652	pseudobulk	DESeq2	LRT	0.0888211171417967
509	KC_G1	Gnf1	0.213003517635187	0.521875666557658	0.998043617620652	pseudobulk	DESeq2	LRT	0.28243295247603
510	KC_G1	Gnpnat	-0.229201876287566	0.611905761385339	0.998043617620652	pseudobulk	DESeq2	LRT	0.213315457693886
510	KC_G1	gny	0.0775687619480349	0.921354091298612	0.998043617620652	pseudobulk	DESeq2	LRT	0.0355734313197217
510	KC_G1	goe	-1.27245096663137	0.354128077051167	0.998043617620652	pseudobulk	DESeq2	LRT	0.45083963883074
510	KC_G1	gogo	-0.44012786403399	0.396270594068363	0.998043617620652	pseudobulk	DESeq2	LRT	0.402008154028853
510	KC_G1	gol	-0.208719422597575	0.206117217647285	0.998043617620652	pseudobulk	DESeq2	LRT	0.685885728662867
510	KC_G1	Golgin104	0.239754322557094	0.56675578611266	0.998043617620652	pseudobulk	DESeq2	LRT	0.246604037387642
510	KC_G1	Golgin245	0.105820867441347	0.700449062926123	0.998043617620652	pseudobulk	DESeq2	LRT	0.154623441383833
510	KC_G1	Golgin84	-0.070775451481062	0.873074241079896	0.998043617620652	pseudobulk	DESeq2	LRT	0.0589488248849974
510	KC_G1	gom	0.0256306537950771	0.954028568164332	0.998043617620652	pseudobulk	DESeq2	LRT	0.0204386202535952
510	KC_G1	Gorab	-0.513061359197937	0.555863274483933	0.998043617620652	pseudobulk	DESeq2	LRT	0.255032018560566
511	KC_G1	Gos28	0.143435869362071	0.636401113149835	0.998043617620652	pseudobulk	DESeq2	LRT	0.196269069386223
511	KC_G1	Got1	-0.226339262987914	0.458621713780565	0.998043617620652	pseudobulk	DESeq2	LRT	0.33854538712933
511	KC_G1	Got2	0.22345129016808	0.403341098496703	0.998043617620652	pseudobulk	DESeq2	LRT	0.394327523241709
511	KC_G1	Gp150	1.06667464613488	0.669131470313168	0.998043617620652	pseudobulk	DESeq2	LRT	0.174488544082685
511	KC_G1	Gp210	-0.466882003969687	0.387914981237929	0.998043617620652	pseudobulk	DESeq2	LRT	0.411263447666437
511	KC_G1	Gp93	-0.479811594586871	0.168374404754547	0.998043617620652	pseudobulk	DESeq2	LRT	0.773723926605394
511	KC_G1	Gpat4	-0.211518172963499	0.434453932153472	0.998043617620652	pseudobulk	DESeq2	LRT	0.362056267712982
511	KC_G1	Gpb5	0.218483520231522	0.502401069263636	0.998043617620652	pseudobulk	DESeq2	LRT	0.298949444957957
511	KC_G1	Gpdh1	0.130148246100438	0.437422021854448	0.998043617620652	pseudobulk	DESeq2	LRT	0.359099356348412
511	KC_G1	Gpdh3	0.334990990480067	0.615847634718145	0.998043617620652	pseudobulk	DESeq2	LRT	0.210526722228621
512	KC_G1	GPHR	-0.932260050555882	0.258726758583252	0.998043617620652	pseudobulk	DESeq2	LRT	0.5871586524428
512	KC_G1	Gpo1	-0.0305912758062083	0.917841847604688	0.998043617620652	pseudobulk	DESeq2	LRT	0.0372321451928103
512	KC_G1	gpp	-0.0623265320123284	0.623576084717312	0.998043617620652	pseudobulk	DESeq2	LRT	0.205110549151041
512	KC_G1	Gprk1	0.0169696813452039	0.875854650866646	0.998043617620652	pseudobulk	DESeq2	LRT	0.0575679595443098
512	KC_G1	Gprk2	-0.261394915991684	0.063387986368621	0.998043617620652	pseudobulk	DESeq2	LRT	1.19799304414042
512	KC_G1	gprs	-0.283789059918025	0.0382200333392844	0.998043617620652	pseudobulk	DESeq2	LRT	1.41770893844634
512	KC_G1	Gr32a	-1.66808346769027	0.498191247612582	0.998043617620652	pseudobulk	DESeq2	LRT	0.302603906560549
512	KC_G1	Gr33a	-0.794610277746993	0.821479263487599	0.998043617620652	pseudobulk	DESeq2	LRT	0.085403394939296
512	KC_G1	Gr39a	0.173195945230598	0.976215465741119	0.998043617620652	pseudobulk	DESeq2	LRT	0.0104543162934892
512	KC_G1	Gr43a	1.12508065046033	0.900770125530981	0.998043617620652	pseudobulk	DESeq2	LRT	0.0453860258366365
513	KC_G1	Gr47b	-0.794610277746993	0.815039924133852	0.998043617620652	pseudobulk	DESeq2	LRT	0.0888211171417967
513	KC_G1	Gr77a	-1.14969695804883	0.256943066032051	0.998043617620652	pseudobulk	DESeq2	LRT	0.590163097864587
513	KC_G1	Graf	-0.0307620683596217	0.856182487651229	0.998043617620652	pseudobulk	DESeq2	LRT	0.067433659466224
513	KC_G1	GramD1B	-0.193462197332081	0.29223389293422	0.998043617620652	pseudobulk	DESeq2	LRT	0.534269416530749
513	KC_G1	Grasp65	0.296068368826461	0.469801695190573	0.998043617620652	pseudobulk	DESeq2	LRT	0.328085420486077
513	KC_G1	grass	-0.323009177088943	0.862831841642416	0.998043617620652	pseudobulk	DESeq2	LRT	0.0640738362231444
513	KC_G1	grau	0.117364354559788	0.799266450410858	0.998043617620652	pseudobulk	DESeq2	LRT	0.0973084163648282
513	KC_G1	grh	-1.60891734856068	0.545249891924755	0.998043617620652	pseudobulk	DESeq2	LRT	0.263404411831769
513	KC_G1	Grik	0.043330515215642	0.920991035881463	0.998043617620652	pseudobulk	DESeq2	LRT	0.0357445968239564
513	KC_G1	grim	-1.67593316621313	0.495877122847873	0.998043617620652	pseudobulk	DESeq2	LRT	0.304625927300966
514	KC_G1	Grip128	0.139835753460434	0.884544090557708	0.998043617620652	pseudobulk	DESeq2	LRT	0.05328051458164
514	KC_G1	Grip163	-0.301171660058028	0.481090849617506	0.998043617620652	pseudobulk	DESeq2	LRT	0.317772903331101
514	KC_G1	Grip71	0.604591135102487	0.51385585523224	0.998043617620652	pseudobulk	DESeq2	LRT	0.289158690453637
514	KC_G1	Grip75	0.0490178365019074	0.919645762992334	0.998043617620652	pseudobulk	DESeq2	LRT	0.0363794257014296
514	KC_G1	Grip84	0.264557349728027	0.608039651207601	0.998043617620652	pseudobulk	DESeq2	LRT	0.216068098787844
514	KC_G1	Grip91	-0.39329467162888	0.453950375658882	0.998043617620652	pseudobulk	DESeq2	LRT	0.342991620172513
514	KC_G1	grk	-0.794610277746993	0.879485534599449	0.998043617620652	pseudobulk	DESeq2	LRT	0.0557712992369932
514	KC_G1	gro	-0.174310134370647	0.216787321038368	0.998043617620652	pseudobulk	DESeq2	LRT	0.663966121414521
514	KC_G1	grp	-0.142255277950964	0.333360262421116	0.998043617620652	pseudobulk	DESeq2	LRT	0.477086170674135
514	KC_G1	grsm	0.175247715919415	0.72474521678581	0.998043617620652	pseudobulk	DESeq2	LRT	0.139814642244622
515	KC_G1	Grx1	-0.131428960052918	0.524083384418146	0.998043617620652	pseudobulk	DESeq2	LRT	0.280599608986399

515	KC_G1	gry	-0.588076447510599	0.217290634792833	0.998043617620652	pseudobulk	DESeq2	LRT	0.662958991329923
515	KC_G1	Gs1	0.0928867788851349	0.626324496579884	0.998043617620652	pseudobulk	DESeq2	LRT	0.203200601995934
515	KC_G1	Gs1l	-0.0102674724710491	0.942562157969149	0.998043617620652	pseudobulk	DESeq2	LRT	0.025690000302127
515	KC_G1	Gs2	-0.206559615013672	0.258810567417997	0.998043617620652	pseudobulk	DESeq2	LRT	0.587017995091324
515	KC_G1	GstD1	0.0534969986561013	0.734075336385355	0.998043617620652	pseudobulk	DESeq2	LRT	0.134259367199059
515	KC_G1	GstD10	1.10221512528253	0.490304820317606	0.998043617620652	pseudobulk	DESeq2	LRT	0.30953383706782
515	KC_G1	GstD11	-0.340266887491361	0.410692746512525	0.998043617620652	pseudobulk	DESeq2	LRT	0.386482967410295
515	KC_G1	GstD3	0.102985916219267	0.812573078245849	0.998043617620652	pseudobulk	DESeq2	LRT	0.0901375705931108
515	KC_G1	GstD6	-1.28588412734293	0.498027003720065	0.998043617620652	pseudobulk	DESeq2	LRT	0.302747108548181
516	KC_G1	GstD7	-0.718676691072112	0.739205546127982	0.998043617620652	pseudobulk	DESeq2	LRT	0.131234783341328
516	KC_G1	GstD8	1.54235971072306	0.529343359605328	0.998043617620652	pseudobulk	DESeq2	LRT	0.276262530595783
516	KC_G1	GstD9	0.365209743518153	0.362337522598244	0.998043617620652	pseudobulk	DESeq2	LRT	0.440886689389256
516	KC_G1	GstE1	-0.12858175280697	0.894252315322512	0.998043617620652	pseudobulk	DESeq2	LRT	0.0485399267389717
516	KC_G1	GstE11	0.425650441423024	0.840749189965507	0.998043617620652	pseudobulk	DESeq2	LRT	0.0753335424417301
516	KC_G1	GstE12	-0.291394608708216	0.433285287988747	0.998043617620652	pseudobulk	DESeq2	LRT	0.363226056993267
516	KC_G1	GstE13	1.291396989734	0.30316219981931	0.998043617620652	pseudobulk	DESeq2	LRT	0.518324950247429
516	KC_G1	GstE2	-0.268982901505309	0.514056750178749	0.998043617620652	pseudobulk	DESeq2	LRT	0.288988933672053
516	KC_G1	GstE3	-0.0580164858581658	0.953987606720781	0.998043617620652	pseudobulk	DESeq2	LRT	0.0204572671908087
516	KC_G1	GstE6	0.206943506810418	0.875878711834172	0.998043617620652	pseudobulk	DESeq2	LRT	0.0575560290237766
517	KC_G1	GstE7	-0.794610277746993	0.879485534599449	0.998043617620652	pseudobulk	DESeq2	LRT	0.0557712992369932
517	KC_G1	GstE8	-0.149372637832219	0.921010617785056	0.998043617620652	pseudobulk	DESeq2	LRT	0.035735363050839
517	KC_G1	GstO1	0.416431064791215	0.848864097724124	0.998043617620652	pseudobulk	DESeq2	LRT	0.0711618342941965
517	KC_G1	GstO2	-0.322724723011653	0.32668540982445	0.998043617620652	pseudobulk	DESeq2	LRT	0.485870261221611
517	KC_G1	GstO3	-1.7689014815933	0.397834883217982	0.998043617620652	pseudobulk	DESeq2	LRT	0.400297139449685
517	KC_G1	GstS1	-0.44883264029243	0.810013528581648	0.998043617620652	pseudobulk	DESeq2	LRT	0.0915077276160505
517	KC_G1	GstT1	0.0440196467220027	0.930880344343172	0.998043617620652	pseudobulk	DESeq2	LRT	0.0311061397825621
517	KC_G1	GstT2	-0.411062089322353	0.814122275450454	0.998043617620652	pseudobulk	DESeq2	LRT	0.0893103622295772
517	KC_G1	GstT3	2.10447822489395	0.214284492130699	0.998043617620652	pseudobulk	DESeq2	LRT	0.669009257916476
517	KC_G1	GstZ1	-0.794610277746993	0.821479263487599	0.998043617620652	pseudobulk	DESeq2	LRT	0.085403394939296
518	KC_G1	GstZ2	-0.272350786602014	0.680532688341936	0.998043617620652	pseudobulk	DESeq2	LRT	0.167151009291551
518	KC_G1	gt	-0.0980866384992532	0.923551444367409	0.998043617620652	pseudobulk	DESeq2	LRT	0.0345389081472661
518	KC_G1	Gtp-bp	0.311948335976366	0.54567687808584	0.998043617620652	pseudobulk	DESeq2	LRT	0.263064448145526
518	KC_G1	Gug	-0.0745219196473991	0.565475847426579	0.998043617620652	pseudobulk	DESeq2	LRT	0.247585939904516
518	KC_G1	gukh	-0.587199115572713	0.719693269653897	0.998043617620652	pseudobulk	DESeq2	LRT	0.142852558679523
518	KC_G1	gus	-0.0595374471746672	0.665096476642899	0.998043617620652	pseudobulk	DESeq2	LRT	0.177115352840334
518	KC_G1	GV1	-0.794610277746993	0.879485534599449	0.998043617620652	pseudobulk	DESeq2	LRT	0.0557712992369932
518	KC_G1	gw	-0.0385221638647621	0.719735624666048	0.998043617620652	pseudobulk	DESeq2	LRT	0.142827000559721
518	KC_G1	GXIVsPLA2	-0.519867143266413	0.183719583612816	0.998043617620652	pseudobulk	DESeq2	LRT	0.735844547550114
518	KC_G1	Gyc32E	-1.66808346769027	0.498191247612582	0.998043617620652	pseudobulk	DESeq2	LRT	0.302603906560549
519	KC_G1	Gyc88E	0.923237516940646	0.242919978774413	0.998043617620652	pseudobulk	DESeq2	LRT	0.614536765496765
519	KC_G1	Gyc89Da	-0.462941228701907	0.524372307778474	0.998043617620652	pseudobulk	DESeq2	LRT	0.280360251567304
519	KC_G1	Gyc89Db	-0.794610277746993	0.815039924133852	0.998043617620652	pseudobulk	DESeq2	LRT	0.0888211171417967
519	KC_G1	Gycalpha99B	0.0492518496368817	0.737753424108705	0.998043617620652	pseudobulk	DESeq2	LRT	0.132088766147806
519	KC_G1	Gycbeta100B	0.18282130025912	0.304009594076805	0.998043617620652	pseudobulk	DESeq2	LRT	0.517112710506812
519	KC_G1	Gyf	-0.234173748623051	0.0927499579907703	0.998043617620652	pseudobulk	DESeq2	LRT	1.03268627841783
519	KC_G1	Gyg	-1.63879769624238	0.231450966007564	0.998043617620652	pseudobulk	DESeq2	LRT	0.635541002261258
519	KC_G1	gzl	-0.142455279310463	0.445765345981474	0.998043617620652	pseudobulk	DESeq2	LRT	0.350893696811379
519	KC_G1	h	1.66849878552847	0.468694210005612	0.998043617620652	pseudobulk	DESeq2	LRT	0.329110411485433
519	KC_G1	H	0.146363811378133	0.522427660416055	0.998043617620652	pseudobulk	DESeq2	LRT	0.281973837000898
520	KC_G1	h-cup	1.12508065046033	0.900770125530981	0.998043617620652	pseudobulk	DESeq2	LRT	0.0453860258366365
520	KC_G1	Hacd1	-0.127511578476819	0.753616125124861	0.998043617620652	pseudobulk	DESeq2	LRT	0.122849817516368
520	KC_G1	Hacd2	0.179871449337841	0.60305287730324	0.998043617620652	pseudobulk	DESeq2	LRT	0.219644606078425
520	KC_G1	Hacl	0.118404108235	0.773298737461661	0.998043617620652	pseudobulk	DESeq2	LRT	0.111652698879085
520	KC_G1	Had1	-0.936804819187673	0.623872142997351	0.998043617620652	pseudobulk	DESeq2	LRT	0.204904405959043
520	KC_G1	haf	0.463566531153641	0.608801091179131	0.998043617620652	pseudobulk	DESeq2	LRT	0.215524577832999
520	KC_G1	Hakai	-0.0240474450767659	0.952233617338062	0.998043617620652	pseudobulk	DESeq2	LRT	0.0212564904033064

520	KC_G1	ham	1.12508065046033	0.900770125530981	0.998043617620652	pseudobulk	DESeq2	LRT	0.0453860258366365
520	KC_G1	hang	0.112378557127246	0.510630321073618	0.998043617620652	pseudobulk	DESeq2	LRT	0.291893400491452
520	KC_G1	Hasp	-0.115342162970508	0.397194315915093	0.998043617620652	pseudobulk	DESeq2	LRT	0.400996975142349
521	KC_G1	Haspin	-0.0853589290523767	0.640222683449268	0.998043617620652	pseudobulk	DESeq2	LRT	0.193668942621866
521	KC_G1	Hat1	0.25785201771001	0.501831825718691	0.998043617620652	pseudobulk	DESeq2	LRT	0.299441799586334
521	KC_G1	hay	-0.553212572337144	0.406917534104864	0.998043617620652	pseudobulk	DESeq2	LRT	0.390493595964064
521	KC_G1	hb	1.13582588427274	0.633424328955532	0.998043617620652	pseudobulk	DESeq2	LRT	0.198305259991224
521	KC_G1	HBS1	0.27043265510244	0.557908239581587	0.998043617620652	pseudobulk	DESeq2	LRT	0.253437224575664
521	KC_G1	Hcf	-0.0149424478317441	0.931066688244332	0.998043617620652	pseudobulk	DESeq2	LRT	0.0310192112858177
521	KC_G1	Hcs	0.214487913046759	0.616992298726657	0.998043617620652	pseudobulk	DESeq2	LRT	0.209720256779384
521	KC_G1	hd	1.77059796461121	0.322230660296093	0.998043617620652	pseudobulk	DESeq2	LRT	0.491833138765044
521	KC_G1	HDAC1	-0.265661454030523	0.334420183479898	0.998043617620652	pseudobulk	DESeq2	LRT	0.475707519183311
521	KC_G1	HDAC11	0.286253925912706	0.594484854280744	0.998043617620652	pseudobulk	DESeq2	LRT	0.225859205445772
522	KC_G1	HDAC4	0.0944093090023039	0.530755618150404	0.998043617620652	pseudobulk	DESeq2	LRT	0.275105400046927
522	KC_G1	HDAC6	-0.268320310679985	0.35301799600898	0.998043617620652	pseudobulk	DESeq2	LRT	0.452203154758966
522	KC_G1	hdc	-0.771673938818691	0.0118017411904437	0.998043617620652	pseudobulk	DESeq2	LRT	1.92805391357387
522	KC_G1	Hdc	-0.794610277746993	0.879485534599449	0.998043617620652	pseudobulk	DESeq2	LRT	0.0557712992369932
522	KC_G1	hdly	-0.811589868987547	0.465593915927647	0.998043617620652	pseudobulk	DESeq2	LRT	0.331992703393207
522	KC_G1	hdm	-1.26205935493385	0.492267772487709	0.998043617620652	pseudobulk	DESeq2	LRT	0.307798595445795
522	KC_G1	HEATR2	0.337799855986802	0.656235675542259	0.998043617620652	pseudobulk	DESeq2	LRT	0.18294016335871
522	KC_G1	hebe	1.13582588427274	0.633424328955532	0.998043617620652	pseudobulk	DESeq2	LRT	0.198305259991224
522	KC_G1	hec	-0.415318554004845	0.524751895416109	0.998043617620652	pseudobulk	DESeq2	LRT	0.280045984060911
522	KC_G1	heix	-0.0886588589488638	0.798854299440127	0.998043617620652	pseudobulk	DESeq2	LRT	0.0975324230880805
523	KC_G1	Hel25E	-0.1276197848669	0.552311757088068	0.998043617620652	pseudobulk	DESeq2	LRT	0.257815711829511
523	KC_G1	Hel89B	-0.190257923944822	0.448266248797134	0.998043617620652	pseudobulk	DESeq2	LRT	0.348463959133244
523	KC_G1	Hem	-0.0874574681850793	0.837835553670747	0.998043617620652	pseudobulk	DESeq2	LRT	0.0768412142357247
523	KC_G1	HemK1	0.0398967186275321	0.884476885887594	0.998043617620652	pseudobulk	DESeq2	LRT	0.0533135120619315
523	KC_G1	Hen1	-1.37938314115665	0.159368833244161	0.998043617620652	pseudobulk	DESeq2	LRT	0.797596606864149
523	KC_G1	hep	-0.146584787044423	0.434195415715873	0.998043617620652	pseudobulk	DESeq2	LRT	0.362314766187472
523	KC_G1	heph	0.235080962323272	0.158420364424638	0.998043617620652	pseudobulk	DESeq2	LRT	0.800188992009282
523	KC_G1	her	0.150694879813088	0.678325414679631	0.998043617620652	pseudobulk	DESeq2	LRT	0.168561911007584
523	KC_G1	HERC2	0.0147795342664665	0.925345375565469	0.998043617620652	pseudobulk	DESeq2	LRT	0.0336961410914533
523	KC_G1	Herc4	-0.050090578800544	0.864968566053867	0.998043617620652	pseudobulk	DESeq2	LRT	0.0629996750061735
524	KC_G1	Herp	0.040731981782481	0.904707085318407	0.998043617620652	pseudobulk	DESeq2	LRT	0.0434920084424078
524	KC_G1	Hers	0.0382119265598017	0.792138090432344	0.998043617620652	pseudobulk	DESeq2	LRT	0.10119910289898
524	KC_G1	Hex-A	0.201272486041483	0.213316700454974	0.998043617620652	pseudobulk	DESeq2	LRT	0.670975142527507
524	KC_G1	Hexim	0.209918018218298	0.624079779039783	0.998043617620652	pseudobulk	DESeq2	LRT	0.204759888871947
524	KC_G1	Hexo1	-0.481244503982543	0.435192223565079	0.998043617620652	pseudobulk	DESeq2	LRT	0.361318873633748
524	KC_G1	Hexo2	0.0452684614958783	0.957849996259916	0.998043617620652	pseudobulk	DESeq2	LRT	0.0187024981212296
524	KC_G1	Hey	-0.905292084769011	0.42051868749127	0.998043617620652	pseudobulk	DESeq2	LRT	0.376214699761419
524	KC_G1	Hf	1.01366954950999	0.560147007721114	0.998043617620652	pseudobulk	DESeq2	LRT	0.251697979666132
524	KC_G1	hfp	0.0322779975020334	0.808822942319061	0.998043617620652	pseudobulk	DESeq2	LRT	0.0921465384491299
524	KC_G1	hfw	0.0944310886653461	0.94861180912836	0.998043617620652	pseudobulk	DESeq2	LRT	0.0229114731826085
525	KC_G1	hgo	1.12508065046033	0.900770125530981	0.998043617620652	pseudobulk	DESeq2	LRT	0.0453860258366365
525	KC_G1	hid	1.24545707098284	0.613638418851987	0.998043617620652	pseudobulk	DESeq2	LRT	0.212087457773066
525	KC_G1	hig	-0.23076913197992	0.851827800548946	0.998043617620652	pseudobulk	DESeq2	LRT	0.0696481902449193
525	KC_G1	Hil	-0.794610277746993	0.821479263487599	0.998043617620652	pseudobulk	DESeq2	LRT	0.085403394939296
525	KC_G1	HINT1	0.0906507500719682	0.622676521759517	0.998043617620652	pseudobulk	DESeq2	LRT	0.205737509198329
525	KC_G1	HIP	-0.528992971768017	0.235615616907258	0.998043617620652	pseudobulk	DESeq2	LRT	0.627795927332004
525	KC_G1	HIP-R	-0.0708038839578425	0.793117499906012	0.998043617620652	pseudobulk	DESeq2	LRT	0.100662467435715
525	KC_G1	Hip1	-0.301229478046077	0.483661945418646	0.998043617620652	pseudobulk	DESeq2	LRT	0.315458081610604
525	KC_G1	Hip14	0.413514568886228	0.227632512450952	0.998043617620652	pseudobulk	DESeq2	LRT	0.642765708139775
525	KC_G1	HipHop	-0.310435645600001	0.777136127170405	0.998043617620652	pseudobulk	DESeq2	LRT	0.109502901275247
526	KC_G1	Hipk	-0.144248825873537	0.248208886791163	0.998043617620652	pseudobulk	DESeq2	LRT	0.605182673218952
526	KC_G1	HIPP1	-0.0848783481188403	0.701912401656495	0.998043617620652	pseudobulk	DESeq2	LRT	0.15371708423916
526	KC_G1	Hira	0.0833939258152102	0.91526966215107	0.998043617620652	pseudobulk	DESeq2	LRT	0.0384509326764335

526	KC_G1	His2Av	-0.0771798376342064	0.625773330047976	0.998043617620652	pseudobulk	DESeq2	LRT	0.203582950074156
526	KC_G1	His2B:CG33868	-0.794610277746993	0.879485534599449	0.998043617620652	pseudobulk	DESeq2	LRT	0.0557712992369932
526	KC_G1	His3.3A	-0.035334820028542	0.79718520292088	0.998043617620652	pseudobulk	DESeq2	LRT	0.0984407711223916
526	KC_G1	His3.3B	-0.0623239872112424	0.513575196300579	0.998043617620652	pseudobulk	DESeq2	LRT	0.289395959179684
526	KC_G1	His4r	-0.0244334664529834	0.826118898332712	0.998043617620652	pseudobulk	DESeq2	LRT	0.0829574427840581
526	KC_G1	HisCl1	-0.419806131594975	0.569437442096684	0.998043617620652	pseudobulk	DESeq2	LRT	0.244553980179282
526	KC_G1	HisRS	-0.234396312119959	0.597062698277643	0.998043617620652	pseudobulk	DESeq2	LRT	0.223980060685953
527	KC_G1	hiw	-0.197679334380449	0.234251379740169	0.998043617620652	pseudobulk	DESeq2	LRT	0.630317842439002
527	KC_G1	Hk	-0.114209532625171	0.41650937260648	0.998043617620652	pseudobulk	DESeq2	LRT	0.380375221326878
527	KC_G1	hkb	2.07738706174348	0.431998797123676	0.998043617620652	pseudobulk	DESeq2	LRT	0.364517462451933
527	KC_G1	Hlc	-0.0426153517724507	0.874471370551732	0.998043617620652	pseudobulk	DESeq2	LRT	0.0582544043841996
527	KC_G1	HLH3B	-1.72984073695683	0.480047293856853	0.998043617620652	pseudobulk	DESeq2	LRT	0.318715974188453
527	KC_G1	Hmg-2	0.11882728310302	0.784156823470291	0.998043617620652	pseudobulk	DESeq2	LRT	0.105597074105214
527	KC_G1	Hmgcl	1.90250740646012	0.460774913678782	0.998043617620652	pseudobulk	DESeq2	LRT	0.336511173568024
527	KC_G1	Hmgcr	-0.062090874274654	0.866472103077076	0.998043617620652	pseudobulk	DESeq2	LRT	0.0622454152636484
527	KC_G1	HmgD	-0.12249138145006	0.524804220513433	0.998043617620652	pseudobulk	DESeq2	LRT	0.280002680990772
527	KC_G1	Hmgs	0.313294427596674	0.426646735591607	0.998043617620652	pseudobulk	DESeq2	LRT	0.369931572942336
528	KC_G1	HmgZ	-0.076474545522893	0.610929879486608	0.998043617620652	pseudobulk	DESeq2	LRT	0.214008633782997
528	KC_G1	Hmr	-0.157206472315674	0.797825216022922	0.998043617620652	pseudobulk	DESeq2	LRT	0.0980922415201031
528	KC_G1	Hmt-1	0.268760176562809	0.539439668506511	0.998043617620652	pseudobulk	DESeq2	LRT	0.268057120211584
528	KC_G1	Hmt4-20	-0.0512764237490878	0.773766690722012	0.998043617620652	pseudobulk	DESeq2	LRT	0.111389969816364
528	KC_G1	Hmu	-0.0156424499349156	0.953084150590708	0.998043617620652	pseudobulk	DESeq2	LRT	0.0208687525371584
528	KC_G1	Hnf4	0.0653956413154282	0.959537569642328	0.998043617620652	pseudobulk	DESeq2	LRT	0.0179380162709559
528	KC_G1	hng1	-0.243341434713583	0.551844844583602	0.998043617620652	pseudobulk	DESeq2	LRT	0.258183010337713
528	KC_G1	hng2	0.583933255385257	0.519501334197175	0.998043617620652	pseudobulk	DESeq2	LRT	0.284413332738706
528	KC_G1	hng3	-0.96241646348977	0.149384508337256	0.998043617620652	pseudobulk	DESeq2	LRT	0.825694437945574
528	KC_G1	HnRNP-K	-0.0967902381100341	0.535373345123695	0.998043617620652	pseudobulk	DESeq2	LRT	0.271343255027797
529	KC_G1	Ho	0.0654202045353129	0.844300603134334	0.998043617620652	pseudobulk	DESeq2	LRT	0.0735029004841889
529	KC_G1	hob	0.463328401907968	0.309093736605818	0.998043617620652	pseudobulk	DESeq2	LRT	0.509909795276687
529	KC_G1	hoe1	-0.0165437236366426	0.914091132740762	0.998043617620652	pseudobulk	DESeq2	LRT	0.0390105039670119
529	KC_G1	hoe2	0.106205384060575	0.686826419213858	0.998043617620652	pseudobulk	DESeq2	LRT	0.163153007777108
529	KC_G1	hog	-3.31304495092316	0.10740080824123	0.998043617620652	pseudobulk	DESeq2	LRT	0.968992450355406
529	KC_G1	hoip	0.476910640288866	0.215597832766979	0.998043617620652	pseudobulk	DESeq2	LRT	0.666355609079327
529	KC_G1	holn1	-0.234016013006075	0.579984380322418	0.998043617620652	pseudobulk	DESeq2	LRT	0.236583702352798
529	KC_G1	homer	-0.107467076857477	0.741288217626137	0.998043617620652	pseudobulk	DESeq2	LRT	0.130012902698464
529	KC_G1	hook	-0.00623283492868577	0.973165689991927	0.998043617620652	pseudobulk	DESeq2	LRT	0.0118132109926026
529	KC_G1	hop	0.433771222711587	0.321022430635797	0.998043617620652	pseudobulk	DESeq2	LRT	0.493464621299138
530	KC_G1	how	0.0651787272338549	0.972889136226367	0.998043617620652	pseudobulk	DESeq2	LRT	0.0119366461327872
530	KC_G1	HP1b	-0.0779096188895459	0.745513771661503	0.998043617620652	pseudobulk	DESeq2	LRT	0.12754432953982
530	KC_G1	HP1c	-0.157208440606249	0.529716749462579	0.998043617620652	pseudobulk	DESeq2	LRT	0.275956294604203
530	KC_G1	HP4	0.271316446685519	0.375131524055961	0.998043617620652	pseudobulk	DESeq2	LRT	0.425816438519799
530	KC_G1	HP5	0.267323959052649	0.335359311663517	0.998043617620652	pseudobulk	DESeq2	LRT	0.474489630435782
530	KC_G1	Hpd	-0.704123705228411	0.78667415397905	0.998043617620652	pseudobulk	DESeq2	LRT	0.104205118254245
530	KC_G1	Hph	0.477617642733417	0.177870459695833	0.998043617620652	pseudobulk	DESeq2	LRT	0.749896172525155
530	KC_G1	hpo	-0.0507146861500426	0.887554180419085	0.998043617620652	pseudobulk	DESeq2	LRT	0.0518051261164477
530	KC_G1	hppy	-0.283930728342383	0.134781571662133	0.998043617620652	pseudobulk	DESeq2	LRT	0.870369483709887
530	KC_G1	Hpr1	0.218270331333293	0.700558268939851	0.998043617620652	pseudobulk	DESeq2	LRT	0.154555736428733
531	KC_G1	hpRNA:CR18854	0.0512684866911902	0.709365793791743	0.998043617620652	pseudobulk	DESeq2	LRT	0.149129757400108
531	KC_G1	hpRNA:CR33940	-2.52390306080032	0.179198923484501	0.998043617620652	pseudobulk	DESeq2	LRT	0.746664603636757
531	KC_G1	hpRNA:CR46342	-0.0866253300928367	0.840124744598327	0.998043617620652	pseudobulk	DESeq2	LRT	0.0756562236186057
531	KC_G1	HPS1	-0.0754479425593791	0.834390757675321	0.998043617620652	pseudobulk	DESeq2	LRT	0.0786305151194433
531	KC_G1	HPS4	-0.221234456936306	0.508759531340039	0.998043617620652	pseudobulk	DESeq2	LRT	0.293487441413684
531	KC_G1	Hr3	-0.20949540253328	0.204562038244517	0.998043617620652	pseudobulk	DESeq2	LRT	0.68917495767464
531	KC_G1	Hr38	0.27535086951148	0.123383458630592	0.998043617620652	pseudobulk	DESeq2	LRT	0.908743059970398
531	KC_G1	Hr39	-0.101086054769992	0.602678049311739	0.998043617620652	pseudobulk	DESeq2	LRT	0.219914626080437
531	KC_G1	Hr4	-1.31004124147473	0.225777326369437	0.998043617620652	pseudobulk	DESeq2	LRT	0.646319674134101

531	KC_G1	Hr51	0.195027521803491	0.230501035666891	0.998043617620652	pseudobulk	DESeq2	LRT	0.637327118936349
532	KC_G1	Hr78	-0.00596156905891444	0.974061879512477	0.998043617620652	pseudobulk	DESeq2	LRT	0.0114134526930687
532	KC_G1	Hr96	0.104668431997841	0.814035659682531	0.998043617620652	pseudobulk	DESeq2	LRT	0.0893565699705813
532	KC_G1	Hrb27C	-0.201383134243013	0.0330025565538773	0.998043617620652	pseudobulk	DESeq2	LRT	1.48145241605436
532	KC_G1	Hrb87F	0.194030850088946	0.380965338837971	0.998043617620652	pseudobulk	DESeq2	LRT	0.419114535705407
532	KC_G1	Hrb98DE	-0.0529549288871209	0.680849967963537	0.998043617620652	pseudobulk	DESeq2	LRT	0.166948578636601
532	KC_G1	Hrd3	-0.251632263771153	0.517908886825383	0.998043617620652	pseudobulk	DESeq2	LRT	0.285746636836687
532	KC_G1	hrg	-0.0970752737983627	0.564890409568437	0.998043617620652	pseudobulk	DESeq2	LRT	0.248035798439214
532	KC_G1	hrm	-0.0628959178703491	0.724902597076272	0.998043617620652	pseudobulk	DESeq2	LRT	0.139720344317466
532	KC_G1	Hrs	-0.157323153439769	0.732875974649696	0.998043617620652	pseudobulk	DESeq2	LRT	0.134969515239753
532	KC_G1	Hs2st	-0.139973696738182	0.735316912458882	0.998043617620652	pseudobulk	DESeq2	LRT	0.133525445039957
533	KC_G1	Hs3st-A	-0.161270782608321	0.265281223312755	0.998043617620652	pseudobulk	DESeq2	LRT	0.576293488427943
533	KC_G1	Hs3st-B	0.363123682136113	0.374510870521146	0.998043617620652	pseudobulk	DESeq2	LRT	0.426535571986324
533	KC_G1	Hs6st	-0.0982513276660171	0.777646816702005	0.998043617620652	pseudobulk	DESeq2	LRT	0.109217601446882
533	KC_G1	Hsc20	-0.767518898891	0.171402830348002	0.998043617620652	pseudobulk	DESeq2	LRT	0.765982010917078
533	KC_G1	Hsc70-2	5.26697188768257	0.191162545309942	0.998043617620652	pseudobulk	DESeq2	LRT	0.718597195525677
533	KC_G1	Hsc70-3	-0.230166149416597	0.234321585070514	0.998043617620652	pseudobulk	DESeq2	LRT	0.630187703527511
533	KC_G1	Hsc70-4	-0.0757947648570847	0.570153057599175	0.998043617620652	pseudobulk	DESeq2	LRT	0.244008542313961
533	KC_G1	Hsc70-5	-0.107027492851116	0.603389872238265	0.998043617620652	pseudobulk	DESeq2	LRT	0.219401983633931
533	KC_G1	Hsc70Cb	0.0148260039573334	0.930929011546159	0.998043617620652	pseudobulk	DESeq2	LRT	0.0310834350973109
533	KC_G1	Hsepi	0.247922507497775	0.583583801282304	0.998043617620652	pseudobulk	DESeq2	LRT	0.233896771458752
534	KC_G1	Hsf	-0.213668701251243	0.351557680215477	0.998043617620652	pseudobulk	DESeq2	LRT	0.454003409921011
534	KC_G1	Hsl	-0.0541627271016173	0.931454640067608	0.998043617620652	pseudobulk	DESeq2	LRT	0.0308382895041784
534	KC_G1	Hsp26	-1.71572821461986	0.00571812041969235	0.998043617620652	pseudobulk	DESeq2	LRT	2.24274670293794
534	KC_G1	Hsp60A	0.0536409969396445	0.694592891615803	0.998043617620652	pseudobulk	DESeq2	LRT	0.158269665522562
534	KC_G1	Hsp60D	1.22022446978015	0.459450377644848	0.998043617620652	pseudobulk	DESeq2	LRT	0.337761387169619
534	KC_G1	Hsp67Ba	-0.584012766488194	0.66651048526244	0.998043617620652	pseudobulk	DESeq2	LRT	0.176193014058373
534	KC_G1	Hsp67Bc	-2.4414149157025	0.013566386666886	0.998043617620652	pseudobulk	DESeq2	LRT	1.86753580890583
534	KC_G1	Hsp70Aa	-1.80611282456541	0.218298892913147	0.998043617620652	pseudobulk	DESeq2	LRT	0.660948466777946
534	KC_G1	Hsp70Ba	0.172845155749872	0.90730697492154	0.998043617620652	pseudobulk	DESeq2	LRT	0.0422457504714555
534	KC_G1	Hsp70Bb	0.22866917516379	0.537148342338602	0.998043617620652	pseudobulk	DESeq2	LRT	0.269905760180594
535	KC_G1	Hsp70Bc	-0.949915708124251	0.312267183144048	0.998043617620652	pseudobulk	DESeq2	LRT	0.505473654349129
535	KC_G1	Hsp83	-0.227099611527917	0.0301911606441299	0.998043617620652	pseudobulk	DESeq2	LRT	1.52012019099638
535	KC_G1	HSPBAP1	0.473612895228291	0.486601748541913	0.998043617620652	pseudobulk	DESeq2	LRT	0.312826334820038
535	KC_G1	HSPC300	0.16547062132001	0.624631217952712	0.998043617620652	pseudobulk	DESeq2	LRT	0.20437631430083
535	KC_G1	hth	-0.0399061364539518	0.787809822817669	0.998043617620652	pseudobulk	DESeq2	LRT	0.103578608486366
535	KC_G1	htk	-0.25529487640207	0.213808559895417	0.998043617620652	pseudobulk	DESeq2	LRT	0.669974911657979
535	KC_G1	htl	-0.449098581485316	0.634069005458115	0.998043617620652	pseudobulk	DESeq2	LRT	0.19786347546388
535	KC_G1	HtrA2	-0.581099241710993	0.423127360878335	0.998043617620652	pseudobulk	DESeq2	LRT	0.373528890777698
535	KC_G1	hts	-0.0899259256706677	0.508392150225706	0.998043617620652	pseudobulk	DESeq2	LRT	0.293801163743479
535	KC_G1	htt	-0.067433818132031	0.852000288861254	0.998043617620652	pseudobulk	DESeq2	LRT	0.0695602579905447
536	KC_G1	Hug	1.13582588427274	0.704684208645453	0.998043617620652	pseudobulk	DESeq2	LRT	0.152005460551806
536	KC_G1	Hus1-like	0.417303845199765	0.790423200749783	0.998043617620652	pseudobulk	DESeq2	LRT	0.102140320685447
536	KC_G1	HUWE1	-0.179446102465738	0.356506239962527	0.998043617620652	pseudobulk	DESeq2	LRT	0.447932864248087
536	KC_G1	hwt	0.0542520594675442	0.711387676560635	0.998043617620652	pseudobulk	DESeq2	LRT	0.147893662411513
536	KC_G1	Hyccin	-0.0717802201007349	0.859528939998073	0.998043617620652	pseudobulk	DESeq2	LRT	0.0657394962080176
536	KC_G1	hyd	-0.297485054106923	0.209586315741722	0.998043617620652	pseudobulk	DESeq2	LRT	0.678637076611352
536	KC_G1	Hydr1	0.0145256314797857	0.954178649201027	0.998043617620652	pseudobulk	DESeq2	LRT	0.0203703054860836
536	KC_G1	Hydr2	-0.0294825201529106	0.921405643236449	0.998043617620652	pseudobulk	DESeq2	LRT	0.035549132197356
536	KC_G1	hyx	-0.172645672718517	0.666971034643417	0.998043617620652	pseudobulk	DESeq2	LRT	0.175893026304859
536	KC_G1	I-2	-0.0837741095881399	0.704190631604205	0.998043617620652	pseudobulk	DESeq2	LRT	0.152309756984752
537	KC_G1	I-3	0.990418046679697	0.24567234897815	0.998043617620652	pseudobulk	DESeq2	LRT	0.609643721672083
537	KC_G1	IA-2	-0.0563569813542039	0.589336710970123	0.998043617620652	pseudobulk	DESeq2	LRT	0.229636504984662
537	KC_G1	Ibf1	-0.413046262040367	0.347936423119955	0.998043617620652	pseudobulk	DESeq2	LRT	0.458500105509317
537	KC_G1	Ibf2	0.242374156748202	0.544439217828973	0.998043617620652	pseudobulk	DESeq2	LRT	0.264050598617012
537	KC_G1	IBIN	1.69981218657454	0.566400560818652	0.998043617620652	pseudobulk	DESeq2	LRT	0.246876325303589

537	KC_G1	ICA69	-0.0128081775399464	0.953474214966553	0.998043617620652	pseudobulk	DESeq2	LRT	0.0206910471897212
537	KC_G1	Ice1	-0.458445386010734	0.467630641663511	0.998043617620652	pseudobulk	DESeq2	LRT	0.33009703927799
537	KC_G1	Ice2	0.259859305121756	0.767723858276052	0.998043617620652	pseudobulk	DESeq2	LRT	0.114794962771249
537	KC_G1	icln	-0.0446221341196637	0.92468071983484	0.998043617620652	pseudobulk	DESeq2	LRT	0.0340081975854828
537	KC_G1	ics	-0.0415999083343842	0.907195839515812	0.998043617620652	pseudobulk	DESeq2	LRT	0.0422989501613407
538	KC_G1	ida	-0.300659285014806	0.797666620736804	0.998043617620652	pseudobulk	DESeq2	LRT	0.0981785811130194
538	KC_G1	Ide	0.148643511699327	0.6149932081866	0.998043617620652	pseudobulk	DESeq2	LRT	0.211129680425184
538	KC_G1	Idgf1	-0.794610277746993	0.821479263487599	0.998043617620652	pseudobulk	DESeq2	LRT	0.085403394939296
538	KC_G1	Idgf2	-0.85964384870952	0.629203512053989	0.998043617620652	pseudobulk	DESeq2	LRT	0.201208861920635
538	KC_G1	Idgf3	-0.701733521711777	0.224346198714713	0.998043617620652	pseudobulk	DESeq2	LRT	0.649081284683215
538	KC_G1	Idgf4	0.523247592243661	0.570646944091895	0.998043617620652	pseudobulk	DESeq2	LRT	0.243632504109968
538	KC_G1	Idgf6	0.562077451288907	0.794187709521711	0.998043617620652	pseudobulk	DESeq2	LRT	0.100076838158817
538	KC_G1	Idh	-0.0819923656789634	0.805950563078344	0.998043617620652	pseudobulk	DESeq2	LRT	0.0936915969551495
538	KC_G1	Idh3b	-0.119042358059834	0.463292747303064	0.998043617620652	pseudobulk	DESeq2	LRT	0.334144498482173
538	KC_G1	Idi	0.144952961910221	0.745764880401206	0.998043617620652	pseudobulk	DESeq2	LRT	0.127398072311648
539	KC_G1	Ids	0.779324925349875	0.588005163230038	0.998043617620652	pseudobulk	DESeq2	LRT	0.230618860399254
539	KC_G1	if	0.0467071786550197	0.91943412597561	0.998043617620652	pseudobulk	DESeq2	LRT	0.0364793808904202
539	KC_G1	ifc	0.0708468834055744	0.856562761322161	0.998043617620652	pseudobulk	DESeq2	LRT	0.0672408102848159
539	KC_G1	IFT43	2.85111933021711	0.0495439877678682	0.998043617620652	pseudobulk	DESeq2	LRT	1.30500904022334
539	KC_G1	IFT54	1.13582588427274	0.704684208645453	0.998043617620652	pseudobulk	DESeq2	LRT	0.152005460551806
539	KC_G1	igl	0.0375974535177121	0.771399650764078	0.998043617620652	pseudobulk	DESeq2	LRT	0.112720562088162
539	KC_G1	Ih	0.0326492024909202	0.760526618455907	0.998043617620652	pseudobulk	DESeq2	LRT	0.11888558102425
539	KC_G1	IKKepsilon	0.216860573413775	0.782104619181959	0.998043617620652	pseudobulk	DESeq2	LRT	0.1067351491212
539	KC_G1	IleRS	0.063697794815419	0.878525712088375	0.998043617620652	pseudobulk	DESeq2	LRT	0.0562455233494611
539	KC_G1	IleRS-m	-0.44622724822281	0.623897842402104	0.998043617620652	pseudobulk	DESeq2	LRT	0.204886516268147
540	KC_G1	Ilk	-0.287828145301181	0.566514462277109	0.998043617620652	pseudobulk	DESeq2	LRT	0.24678899876268
540	KC_G1	Ilp1	-0.794610277746993	0.821479263487599	0.998043617620652	pseudobulk	DESeq2	LRT	0.085403394939296
540	KC_G1	Ilp3	1.13582588427274	0.704684208645453	0.998043617620652	pseudobulk	DESeq2	LRT	0.152005460551806
540	KC_G1	Ilp5	-0.794610277746993	0.879485534599449	0.998043617620652	pseudobulk	DESeq2	LRT	0.0557712992369932
540	KC_G1	Ilp6	-0.0762409283719008	0.934280270862082	0.998043617620652	pseudobulk	DESeq2	LRT	0.0295228220248722
540	KC_G1	imd	0.351128165176349	0.51178300687679	0.998043617620652	pseudobulk	DESeq2	LRT	0.290914138421794
540	KC_G1	Iml1	0.0702256547519556	0.862014235105813	0.998043617620652	pseudobulk	DESeq2	LRT	0.0644855622763805
540	KC_G1	Imp	-0.0141053118838415	0.876607048030721	0.998043617620652	pseudobulk	DESeq2	LRT	0.0571950418858639
540	KC_G1	Impbeta11	0.00325433614961916	0.970742408241297	0.998043617620652	pseudobulk	DESeq2	LRT	0.012895997198917
540	KC_G1	ImpE1	1.23357253155843	0.311022036814766	0.998043617620652	pseudobulk	DESeq2	LRT	0.50720883885742
541	KC_G1	ImpE3	-0.776296732928036	0.736588270584829	0.998043617620652	pseudobulk	DESeq2	LRT	0.132775201121675
541	KC_G1	ImpL2	0.12089709174338	0.880358064975126	0.998043617620652	pseudobulk	DESeq2	LRT	0.0553406528334259
541	KC_G1	inaE	0.0476264482840851	0.764735581124386	0.998043617620652	pseudobulk	DESeq2	LRT	0.116488702767146
541	KC_G1	inc	-0.023238630712432	0.917038278784757	0.998043617620652	pseudobulk	DESeq2	LRT	0.0376125357388928
541	KC_G1	ind	-0.794610277746993	0.879485534599449	0.998043617620652	pseudobulk	DESeq2	LRT	0.0557712992369932
541	KC_G1	Indy	-0.796199114069944	0.532924154509807	0.998043617620652	pseudobulk	DESeq2	LRT	0.273334595141734
541	KC_G1	ine	-0.392603294095548	0.652118518145487	0.998043617620652	pseudobulk	DESeq2	LRT	0.185673466999701
541	KC_G1	Ing3	0.384127400072551	0.348208231154582	0.998043617620652	pseudobulk	DESeq2	LRT	0.458160966984445
541	KC_G1	Ing5	0.251716601232176	0.450544093753285	0.998043617620652	pseudobulk	DESeq2	LRT	0.346262699165186
541	KC_G1	Ino80	-0.105248214103423	0.840919501782186	0.998043617620652	pseudobulk	DESeq2	LRT	0.0752455756706263
542	KC_G1	Inos	-0.146415938548574	0.494977655174886	0.998043617620652	pseudobulk	DESeq2	LRT	0.305414406022557
542	KC_G1	INPP5E	0.414116407696116	0.364343964207763	0.998043617620652	pseudobulk	DESeq2	LRT	0.438488420685572
542	KC_G1	InR	-0.15002230769363	0.614351158327849	0.998043617620652	pseudobulk	DESeq2	LRT	0.211583318549227
542	KC_G1	insv	0.141957418053871	0.932413273299005	0.998043617620652	pseudobulk	DESeq2	LRT	0.0303915527410317
542	KC_G1	IntS1	-0.242793719912088	0.666735954089317	0.998043617620652	pseudobulk	DESeq2	LRT	0.17604612468309
542	KC_G1	IntS10	-0.254583309798939	0.788656009583821	0.998043617620652	pseudobulk	DESeq2	LRT	0.10311238299522
542	KC_G1	IntS11	-0.872063763704955	0.202837833400299	0.998043617620652	pseudobulk	DESeq2	LRT	0.692851036988885
542	KC_G1	IntS12	-0.57667607182573	0.475743623418908	0.998043617620652	pseudobulk	DESeq2	LRT	0.322627024023687
542	KC_G1	IntS14	0.727473910915701	0.388738501935725	0.998043617620652	pseudobulk	DESeq2	LRT	0.410342443289133
542	KC_G1	IntS2	-0.565058141105682	0.386321352274543	0.998043617620652	pseudobulk	DESeq2	LRT	0.413051287405949
543	KC_G1	IntS3	-0.0106900727162868	0.959459258628277	0.998043617620652	pseudobulk	DESeq2	LRT	0.0179734619171316

543	KC_G1	IntS4	-0.35781231513921	0.593174763114806	0.998043617620652	pseudobulk	DESeq2	LRT	0.226817334503283
543	KC_G1	IntS6	-0.123847885368298	0.623405405013082	0.998043617620652	pseudobulk	DESeq2	LRT	0.205229436648239
543	KC_G1	IntS8	-0.575739817164087	0.415710734812976	0.998043617620652	pseudobulk	DESeq2	LRT	0.381208760651723
543	KC_G1	IntS9	0.328797050646225	0.733059184534567	0.998043617620652	pseudobulk	DESeq2	LRT	0.134860960585142
543	KC_G1	Invadolysin	0.0293917679090387	0.927412449657532	0.998043617620652	pseudobulk	DESeq2	LRT	0.0327270783978627
543	KC_G1	Inx2	-0.15559042248718	0.670217281597541	0.998043617620652	pseudobulk	DESeq2	LRT	0.173784378044037
543	KC_G1	Inx3	-1.00503328236423	0.0825946398088528	0.998043617620652	pseudobulk	DESeq2	LRT	1.08304813642021
543	KC_G1	Inx7	1.14275767135013	0.613878323120671	0.998043617620652	pseudobulk	DESeq2	LRT	0.211917701878358
543	KC_G1	Ip259	-0.0351559879052689	0.793231307298916	0.998043617620652	pseudobulk	DESeq2	LRT	0.100600153368848
544	KC_G1	IP3K2	0.6926125679762	0.69577237828425	0.998043617620652	pseudobulk	DESeq2	LRT	0.157532816457708
544	KC_G1	IPIP	-0.0461292372662958	0.879107777548652	0.998043617620652	pseudobulk	DESeq2	LRT	0.0559578776847234
544	KC_G1	Ipk2	0.619829839162314	0.294551798998371	0.998043617620652	pseudobulk	DESeq2	LRT	0.530838320432047
544	KC_G1	iPLA2-VIA	0.129189497990598	0.72806377010163	0.998043617620652	pseudobulk	DESeq2	LRT	0.137830579766181
544	KC_G1	Ipod	-0.794610277746993	0.821479263487599	0.998043617620652	pseudobulk	DESeq2	LRT	0.085403394939296
544	KC_G1	Ipp	0.10608874469459	0.526938001413751	0.998043617620652	pseudobulk	DESeq2	LRT	0.278240480096454
544	KC_G1	Ir25a	-1.66808346769027	0.498191247612582	0.998043617620652	pseudobulk	DESeq2	LRT	0.302603906560549
544	KC_G1	Ir47a	1.14482122992599	0.210558020762873	0.998043617620652	pseudobulk	DESeq2	LRT	0.676628210398028
544	KC_G1	Ir52c	1.13582588427274	0.704684208645453	0.998043617620652	pseudobulk	DESeq2	LRT	0.152005460551806
544	KC_G1	Ir56a	0.191862616849124	0.813700448779928	0.998043617620652	pseudobulk	DESeq2	LRT	0.0895354444780347
545	KC_G1	Ir62a	-2.31679508011858	0.210450581917964	0.998043617620652	pseudobulk	DESeq2	LRT	0.67684986904444
545	KC_G1	Ir68a	-0.0120378302036855	0.948175729029377	0.998043617620652	pseudobulk	DESeq2	LRT	0.0231111657472031
545	KC_G1	Ir75d	1.13582588427274	0.704684208645453	0.998043617620652	pseudobulk	DESeq2	LRT	0.152005460551806
545	KC_G1	Ir76a	0.880406818340079	0.355604033008363	0.998043617620652	pseudobulk	DESeq2	LRT	0.449033322214351
545	KC_G1	Ir85a	-0.794610277746993	0.879485534599449	0.998043617620652	pseudobulk	DESeq2	LRT	0.0557712992369932
545	KC_G1	Ir93a	-0.765625368254185	0.836625948066609	0.998043617620652	pseudobulk	DESeq2	LRT	0.0774686698445758
545	KC_G1	Irbp	0.762683499481874	0.747823257359372	0.998043617620652	pseudobulk	DESeq2	LRT	0.126201032380818
545	KC_G1	Irbp18	-0.407859091871254	0.297347232822039	0.998043617620652	pseudobulk	DESeq2	LRT	0.526736098784716
545	KC_G1	Irc	0.57121397949792	0.467140134669891	0.998043617620652	pseudobulk	DESeq2	LRT	0.330552818396485
545	KC_G1	Ire1	-0.261450948339763	0.494465909088642	0.998043617620652	pseudobulk	DESeq2	LRT	0.30586364543193
546	KC_G1	Irk2	-0.0837514699642596	0.761115362791484	0.998043617620652	pseudobulk	DESeq2	LRT	0.118549511919818
546	KC_G1	Irk3	1.04860663650527	0.695005286771939	0.998043617620652	pseudobulk	DESeq2	LRT	0.15801189180248
546	KC_G1	Irp-1A	0.0869408407310068	0.746915856470531	0.998043617620652	pseudobulk	DESeq2	LRT	0.126728320713158
546	KC_G1	Irp-1B	0.43444662453411	0.389189822430279	0.998043617620652	pseudobulk	DESeq2	LRT	0.40983852534141
546	KC_G1	Iru	0.221607245301774	0.461846490211458	0.998043617620652	pseudobulk	DESeq2	LRT	0.335502352436462
546	KC_G1	IscU	0.12951941008188	0.454297496035162	0.998043617620652	pseudobulk	DESeq2	LRT	0.342659656892415
546	KC_G1	isopeptidase-T-3	-0.314874776157746	0.55065201237557	0.998043617620652	pseudobulk	DESeq2	LRT	0.259122769294216
546	KC_G1	isoQC	0.169500597614686	0.770433845688087	0.998043617620652	pseudobulk	DESeq2	LRT	0.113264646609509
546	KC_G1	Iswi	-0.148713989070348	0.597179360139785	0.998043617620652	pseudobulk	DESeq2	LRT	0.223895210880537
546	KC_G1	Itgbn	0.949247102466247	0.731817859237848	0.998043617620652	pseudobulk	DESeq2	LRT	0.135596996224368
547	KC_G1	ITP	0.97255465224963	0.572312935353716	0.998043617620652	pseudobulk	DESeq2	LRT	0.242366438104797
547	KC_G1	Itpr	-0.170323268255827	0.566351378231346	0.998043617620652	pseudobulk	DESeq2	LRT	0.246914038284781
547	KC_G1	ix	0.137552900431922	0.722885762865026	0.998043617620652	pseudobulk	DESeq2	LRT	0.140930328538471
547	KC_G1	Jafrac1	-0.0295201896015221	0.853858514129849	0.998043617620652	pseudobulk	DESeq2	LRT	0.0686140867153187
547	KC_G1	Jafrac2	0.185171615659378	0.810448803438598	0.998043617620652	pseudobulk	DESeq2	LRT	0.0912744146049475
547	KC_G1	jagn	-0.0855096088977954	0.79836196949216	0.998043617620652	pseudobulk	DESeq2	LRT	0.0978001591368659
547	KC_G1	janA	0.0211058078820192	0.955266809657869	0.998043617620652	pseudobulk	DESeq2	LRT	0.0198753113705942
547	KC_G1	jar	0.0956883502322707	0.708077606151092	0.998043617620652	pseudobulk	DESeq2	LRT	0.149919140507804
547	KC_G1	Jarid2	-0.267986478051288	0.105733625200427	0.998043617620652	pseudobulk	DESeq2	LRT	0.975786877245977
547	KC_G1	jbug	-0.70885790669773	0.737685902243277	0.998043617620652	pseudobulk	DESeq2	LRT	0.132128516170813
548	KC_G1	jdp	-0.130987546448383	0.235819180226639	0.998043617620652	pseudobulk	DESeq2	LRT	0.627420874695428
548	KC_G1	jeb	0.118057057571498	0.480087946130779	0.998043617620652	pseudobulk	DESeq2	LRT	0.318679197997937
548	KC_G1	jef	-0.180576116365705	0.26473940126322	0.998043617620652	pseudobulk	DESeq2	LRT	0.577181417679539
548	KC_G1	jet	0.0459610423727029	0.918837557301697	0.998043617620652	pseudobulk	DESeq2	LRT	0.0367612614139678
548	KC_G1	JHDM2	0.446899503736839	0.477212656657299	0.998043617620652	pseudobulk	DESeq2	LRT	0.321288046469567
548	KC_G1	Jhe	-0.794610277746993	0.821479263487599	0.998043617620652	pseudobulk	DESeq2	LRT	0.085403394939296
548	KC_G1	Jheh1	3.1403106328029	0.147253744000083	0.998043617620652	pseudobulk	DESeq2	LRT	0.831933654246089

548	KC_G1	Jheh2	-0.915513521343951	0.628202000295396	0.998043617620652	pseudobulk	DESeq2	LRT	0.201900685072392
548	KC_G1	Jheh3	-0.0399551737313366	0.93684715214744	0.998043617620652	pseudobulk	DESeq2	LRT	0.0283312590521527
548	KC_G1	Jhl-21	0.188851464250296	0.872806287354339	0.998043617620652	pseudobulk	DESeq2	LRT	0.0590821339212496
549	KC_G1	Jhl-26	0.108835214069397	0.935420444000122	0.998043617620652	pseudobulk	DESeq2	LRT	0.0289931426385301
549	KC_G1	JIL-1	-0.103110785636315	0.527455899219013	0.998043617620652	pseudobulk	DESeq2	LRT	0.277813846033097
549	KC_G1	jim	-0.143518970329095	0.164586896926634	0.998043617620652	pseudobulk	DESeq2	LRT	0.783604742750555
549	KC_G1	jing	-0.217541727752304	0.215981653785202	0.998043617620652	pseudobulk	DESeq2	LRT	0.665583137729815
549	KC_G1	JMJD4	0.0761841564249506	0.902532326569387	0.998043617620652	pseudobulk	DESeq2	LRT	0.0445372337444383
549	KC_G1	JMJD5	-0.377260146551117	0.417230131329229	0.998043617620652	pseudobulk	DESeq2	LRT	0.379624335438483
549	KC_G1	JMJD7	0.0370719679620743	0.939114019077631	0.998043617620652	pseudobulk	DESeq2	LRT	0.0272816762641224
549	KC_G1	jnj	-0.363559947503754	0.487047456331365	0.998043617620652	pseudobulk	DESeq2	LRT	0.312428720471765
549	KC_G1	Jon44E	-0.794610277746993	0.815039924133852	0.998043617620652	pseudobulk	DESeq2	LRT	0.0888211171417967
549	KC_G1	jp	-0.0511120643086084	0.819539209804107	0.998043617620652	pseudobulk	DESeq2	LRT	0.0864302633330209
550	KC_G1	Jra	-0.089323656259549	0.811331510461357	0.998043617620652	pseudobulk	DESeq2	LRT	0.0908016565825554
550	KC_G1	jtb	1.69981218657454	0.566400560818652	0.998043617620652	pseudobulk	DESeq2	LRT	0.246876325303589
550	KC_G1	JTBR	-0.269429625897009	0.489350753814514	0.998043617620652	pseudobulk	DESeq2	LRT	0.310379738340873
550	KC_G1	jub	-0.0277774748171461	0.951745757737031	0.998043617620652	pseudobulk	DESeq2	LRT	0.0214790503113408
550	KC_G1	jumu	0.041384394420024	0.909127101578125	0.998043617620652	pseudobulk	DESeq2	LRT	0.0413753954851558
550	KC_G1	Jupiter	0.0421409328618123	0.740344714105995	0.998043617620652	pseudobulk	DESeq2	LRT	0.130566020031463
550	KC_G1	jus	-0.135685940255156	0.227388303286272	0.998043617620652	pseudobulk	DESeq2	LRT	0.643231878913357
550	KC_G1	jv	1.89294495797411	0.417209912563316	0.998043617620652	pseudobulk	DESeq2	LRT	0.379645381644337
550	KC_G1	jvl	0.0192981350932553	0.887920397119003	0.998043617620652	pseudobulk	DESeq2	LRT	0.0516259673763308
550	KC_G1	Jwa	0.0637567907380727	0.77224375788837	0.998043617620652	pseudobulk	DESeq2	LRT	0.112245593451365
551	KC_G1	KaiR1D	-0.264955159500794	0.441881038597454	0.998043617620652	pseudobulk	DESeq2	LRT	0.354694633896347
551	KC_G1	Kank	0.136536327467343	0.540750518365946	0.998043617620652	pseudobulk	DESeq2	LRT	0.267003055571974
551	KC_G1	Kap-alpha1	-0.0566043804365982	0.843068778778405	0.998043617620652	pseudobulk	DESeq2	LRT	0.0741369935538211
551	KC_G1	Kap-alpha3	-0.101735833077885	0.555761262930269	0.998043617620652	pseudobulk	DESeq2	LRT	0.25511172722744
551	KC_G1	Kap3	-0.179024191950628	0.412018856804352	0.998043617620652	pseudobulk	DESeq2	LRT	0.385082907222516
551	KC_G1	kappaB-Ras	-0.794610277746993	0.879485534599449	0.998043617620652	pseudobulk	DESeq2	LRT	0.0557712992369932
551	KC_G1	kar	0.178176515508843	0.940951159325412	0.998043617620652	pseudobulk	DESeq2	LRT	0.0264329183218905
551	KC_G1	Karl	-1.27845746407632	0.556113633048063	0.998043617620652	pseudobulk	DESeq2	LRT	0.254836458124014
551	KC_G1	karr	0.172845155749872	0.90730697492154	0.998043617620652	pseudobulk	DESeq2	LRT	0.0422457504714555
551	KC_G1	Karybeta3	-0.286601028794945	0.266617802491456	0.998043617620652	pseudobulk	DESeq2	LRT	0.574110855426006
552	KC_G1	kat-60L1	-0.257309530838713	0.479207420686617	0.998043617620652	pseudobulk	DESeq2	LRT	0.319476465371882
552	KC_G1	Kat60	0.0937016844737058	0.788897716837116	0.998043617620652	pseudobulk	DESeq2	LRT	0.102979300837186
552	KC_G1	kay	-0.125795172888945	0.686071813553661	0.998043617620652	pseudobulk	DESeq2	LRT	0.163630422780518
552	KC_G1	Kaz-m1	1.12508065046033	0.900770125530981	0.998043617620652	pseudobulk	DESeq2	LRT	0.0453860258366365
552	KC_G1	Kaz1-ORFB	-0.400223903015648	0.618851928299532	0.998043617620652	pseudobulk	DESeq2	LRT	0.208413251488943
552	KC_G1	kcc	-0.153129152674848	0.402501577734378	0.998043617620652	pseudobulk	DESeq2	LRT	0.395232412935886
552	KC_G1	Kcmf1	-0.0369852222408071	0.873103882344189	0.998043617620652	pseudobulk	DESeq2	LRT	0.0589340806417295
552	KC_G1	KCNQ	0.260016793851047	0.338630499612095	0.998043617620652	pseudobulk	DESeq2	LRT	0.470273928634921
552	KC_G1	KdelR	0.0267784118159303	0.904010830852545	0.998043617620652	pseudobulk	DESeq2	LRT	0.0438263662599057
552	KC_G1	Kdm2	-0.163059109613592	0.48998267996618	0.998043617620652	pseudobulk	DESeq2	LRT	0.309819271253236
553	KC_G1	Kdm4A	0.016846894817581	0.963803965936365	0.998043617620652	pseudobulk	DESeq2	LRT	0.0160112909620409
553	KC_G1	Kdm4B	-0.156516810706424	0.345861992061914	0.998043617620652	pseudobulk	DESeq2	LRT	0.461097161445398
553	KC_G1	kdn	-0.0754518334616207	0.646867955334958	0.998043617620652	pseudobulk	DESeq2	LRT	0.189184362487435
553	KC_G1	Keap1	0.032056046194166	0.950223159496625	0.998043617620652	pseudobulk	DESeq2	LRT	0.0221743888620709
553	KC_G1	Kebab	0.847403418802011	0.716263869720618	0.998043617620652	pseudobulk	DESeq2	LRT	0.144926955272638
553	KC_G1	kek1	-0.0291817819666716	0.792087210813555	0.998043617620652	pseudobulk	DESeq2	LRT	0.101226998852506
553	KC_G1	kek2	-0.71088171110674	0.784645801042283	0.998043617620652	pseudobulk	DESeq2	LRT	0.105326344996467
553	KC_G1	kek4	0.204831644364406	0.495125886219139	0.998043617620652	pseudobulk	DESeq2	LRT	0.305284367249125
553	KC_G1	kek5	-0.304600091833261	0.831162196280645	0.998043617620652	pseudobulk	DESeq2	LRT	0.0803142180014972
553	KC_G1	kek6	-0.19899247186192	0.346006849325809	0.998043617620652	pseudobulk	DESeq2	LRT	0.460915304111959
554	KC_G1	kel	0.0138585190537536	0.935591323817486	0.998043617620652	pseudobulk	DESeq2	LRT	0.0289138142631208
554	KC_G1	ken	0.17172214391994	0.93725366732732	0.998043617620652	pseudobulk	DESeq2	LRT	0.0281428515765614
554	KC_G1	kermit	2.63732022130093	0.22943645835378	0.998043617620652	pseudobulk	DESeq2	LRT	0.639337569853304

554	KC_G1	key	0.0510974815089595	0.878368378708935	0.998043617620652	pseudobulk	DESeq2	LRT	0.0563233072284673
554	KC_G1	KFase	1.05099079238442	0.422852154264809	0.998043617620652	pseudobulk	DESeq2	LRT	0.373811452508129
554	KC_G1	Khc	-0.0378232531770655	0.847729669442447	0.998043617620652	pseudobulk	DESeq2	LRT	0.0717426168313082
554	KC_G1	Khc-73	-0.0985977111168893	0.475222757573501	0.998043617620652	pseudobulk	DESeq2	LRT	0.323102769937768
554	KC_G1	kibra	-0.382475980578845	0.637140773269998	0.998043617620652	pseudobulk	DESeq2	LRT	0.195764601730758
554	KC_G1	Kif19A	2.04147257822512	0.408147672536479	0.998043617620652	pseudobulk	DESeq2	LRT	0.389182675723292
554	KC_G1	Kif3C	0.248591258312063	0.66764261746644	0.998043617620652	pseudobulk	DESeq2	LRT	0.175455948895472
555	KC_G1	kin17	-0.260812033548195	0.56120573255153	0.998043617620652	pseudobulk	DESeq2	LRT	0.250877901430596
555	KC_G1	kirre	0.259643335920668	0.3216768108928	0.998043617620652	pseudobulk	DESeq2	LRT	0.492580245483747
555	KC_G1	kis	-0.0563291759805765	0.635965705343861	0.998043617620652	pseudobulk	DESeq2	LRT	0.196566303190413
555	KC_G1	kl-5	2.04147257822512	0.408147672536479	0.998043617620652	pseudobulk	DESeq2	LRT	0.389182675723292
555	KC_G1	klar	-0.0627528913192017	0.651858020006168	0.998043617620652	pseudobulk	DESeq2	LRT	0.185846986848636
555	KC_G1	Klc	-0.0764886900832093	0.749206283528674	0.998043617620652	pseudobulk	DESeq2	LRT	0.125398588891052
555	KC_G1	klg	0.250308964678167	0.128975269088157	0.998043617620652	pseudobulk	DESeq2	LRT	0.88949355736138
555	KC_G1	KLHL18	0.118022559868054	0.693269349214687	0.998043617620652	pseudobulk	DESeq2	LRT	0.15909800037989
555	KC_G1	Klp10A	0.0747724748272964	0.73503298768636	0.998043617620652	pseudobulk	DESeq2	LRT	0.133693169693119
555	KC_G1	Klp31E	-0.551696463556055	0.343630326995716	0.998043617620652	pseudobulk	DESeq2	LRT	0.463908514614717
556	KC_G1	Klp64D	-0.019796122576777	0.931625911819528	0.998043617620652	pseudobulk	DESeq2	LRT	0.0307584407001118
556	KC_G1	Klp68D	-0.144086872820207	0.518711748463815	0.998043617620652	pseudobulk	DESeq2	LRT	0.285073915424145
556	KC_G1	Klp98A	0.378830523507263	0.113900208364285	0.998043617620652	pseudobulk	DESeq2	LRT	0.943475481439984
556	KC_G1	klu	0.172834960772825	0.908610676445368	0.998043617620652	pseudobulk	DESeq2	LRT	0.0416221644022337
556	KC_G1	Kmn1	0.183712784481643	0.770388371752134	0.998043617620652	pseudobulk	DESeq2	LRT	0.11329028107822
556	KC_G1	Kmn2	1.90250740646012	0.460774913678782	0.998043617620652	pseudobulk	DESeq2	LRT	0.336511173568024
556	KC_G1	kmr	-0.168936044498305	0.306980463618158	0.998043617620652	pseudobulk	DESeq2	LRT	0.512889262349539
556	KC_G1	knk	2.04147257822512	0.408147672536479	0.998043617620652	pseudobulk	DESeq2	LRT	0.389182675723292
556	KC_G1	ko	-0.411062089322353	0.814122275450454	0.998043617620652	pseudobulk	DESeq2	LRT	0.0893103622295772
556	KC_G1	koi	-0.095483090208534	0.790608621591094	0.998043617620652	pseudobulk	DESeq2	LRT	0.102038453983661
557	KC_G1	koko	-0.124893958697055	0.835397793660137	0.998043617620652	pseudobulk	DESeq2	LRT	0.0781066760693332
557	KC_G1	kon	-2.73425569144472	0.216926985303784	0.998043617620652	pseudobulk	DESeq2	LRT	0.663686419218864
557	KC_G1	Kr-h1	-0.192972507945313	0.348197851831545	0.998043617620652	pseudobulk	DESeq2	LRT	0.458173912542594
557	KC_G1	Kr-h2	-0.0736936246801365	0.787033859963246	0.998043617620652	pseudobulk	DESeq2	LRT	0.104006582915566
557	KC_G1	kra	0.0529568195246074	0.657387010127749	0.998043617620652	pseudobulk	DESeq2	LRT	0.182178881765766
557	KC_G1	kraken	0.0584709089039359	0.84776681770271	0.998043617620652	pseudobulk	DESeq2	LRT	0.071723586081826
557	KC_G1	kri	-0.139623577839726	0.624390466533172	0.998043617620652	pseudobulk	DESeq2	LRT	0.204543736557162
557	KC_G1	Krn	-0.122175173624694	0.851508607698861	0.998043617620652	pseudobulk	DESeq2	LRT	0.0698109574991224
557	KC_G1	KrT95D	-0.097819862785927	0.557882480265336	0.998043617620652	pseudobulk	DESeq2	LRT	0.25345727695414
557	KC_G1	krz	-0.0575526927729805	0.770103241532914	0.998043617620652	pseudobulk	DESeq2	LRT	0.113451048564621
558	KC_G1	ksh	-0.016574217303172	0.95841146491799	0.998043617620652	pseudobulk	DESeq2	LRT	0.0184479997118203
558	KC_G1	ksr	-0.115675532196364	0.775812447668287	0.998043617620652	pseudobulk	DESeq2	LRT	0.110243256560813
558	KC_G1	kst	-0.794610277746993	0.879485534599449	0.998043617620652	pseudobulk	DESeq2	LRT	0.0557712992369932
558	KC_G1	Ktl	-0.0822997755958016	0.504821557115244	0.998043617620652	pseudobulk	DESeq2	LRT	0.296862107931315
558	KC_G1	kto	0.088339614731213	0.848262688363793	0.998043617620652	pseudobulk	DESeq2	LRT	0.0714696354315432
558	KC_G1	ktub	-0.729213989376275	0.285149484269377	0.998043617620652	pseudobulk	DESeq2	LRT	0.544927409557277
558	KC_G1	Ku80	0.071726378609606	0.915926370335082	0.998043617620652	pseudobulk	DESeq2	LRT	0.0381394370771591
558	KC_G1	Kua	1.23311342636175	0.526335393467589	0.998043617620652	pseudobulk	DESeq2	LRT	0.278737424831974
558	KC_G1	kud	0.232927432785415	0.370416592950612	0.998043617620652	pseudobulk	DESeq2	LRT	0.431309567169683
558	KC_G1	kug	2.37279544779784	0.320199125517434	0.998043617620652	pseudobulk	DESeq2	LRT	0.494579858498766
559	KC_G1	kuk	0.372030200137557	0.17976350603065	0.998043617620652	pseudobulk	DESeq2	LRT	0.745298470212226
559	KC_G1	Kul	0.0612356850170904	0.815711045353582	0.998043617620652	pseudobulk	DESeq2	LRT	0.088463656972433
559	KC_G1	kune	0.0944931688758468	0.776220440282804	0.998043617620652	pseudobulk	DESeq2	LRT	0.110014925129696
559	KC_G1	kuz	-0.179473252000084	0.0698037096097438	0.998043617620652	pseudobulk	DESeq2	LRT	1.15612149685775
559	KC_G1	Kyat	0.152308435920551	0.761991650133958	0.998043617620652	pseudobulk	DESeq2	LRT	0.118049787611338
559	KC_G1	kz	-0.378789719101994	0.575079639996359	0.998043617620652	pseudobulk	DESeq2	LRT	0.240272007804388
559	KC_G1	l(1)10Bb	-0.114715722392398	0.645276205971973	0.998043617620652	pseudobulk	DESeq2	LRT	0.190254348848545
559	KC_G1	l(1)G0004	-0.555280358123429	0.306643667124665	0.998043617620652	pseudobulk	DESeq2	LRT	0.513366000033108
559	KC_G1	l(1)G0007	0.606571953611349	0.111940511760539	0.998043617620652	pseudobulk	DESeq2	LRT	0.951012711952515

559	KC_G1	l(1)G0020	1.69981218657454	0.566400560818652	0.998043617620652	pseudobulk	DESeq2	LRT	0.246876325303589
560	KC_G1	l(1)G0193	-0.176053092366407	0.396030012729081	0.998043617620652	pseudobulk	DESeq2	LRT	0.402271900264598
560	KC_G1	l(1)G0196	-0.105085218565581	0.632296278523743	0.998043617620652	pseudobulk	DESeq2	LRT	0.199079374285721
560	KC_G1	l(1)G0289	-0.226603916725941	0.168893009332692	0.998043617620652	pseudobulk	DESeq2	LRT	0.772388325982973
560	KC_G1	l(1)G0320	-0.0760511270427061	0.680115217704782	0.998043617620652	pseudobulk	DESeq2	LRT	0.167417507625111
560	KC_G1	l(1)G0469	0.0200661568064264	0.885274157873546	0.998043617620652	pseudobulk	DESeq2	LRT	0.0529222131289891
560	KC_G1	l(2)01289	1.13582588427274	0.633424328955532	0.998043617620652	pseudobulk	DESeq2	LRT	0.198305259991224
560	KC_G1	l(2)05287	-0.199089552519909	0.785925707900474	0.998043617620652	pseudobulk	DESeq2	LRT	0.104618505072277
560	KC_G1	l(2)05714	0.17140329244265	0.693218214747905	0.998043617620652	pseudobulk	DESeq2	LRT	0.159130034445461
560	KC_G1	l(2)09851	0.24660229264039	0.625938904154575	0.998043617620652	pseudobulk	DESeq2	LRT	0.203468054782257
560	KC_G1	l(2)10685	-0.122756845259847	0.867443380076005	0.998043617620652	pseudobulk	DESeq2	LRT	0.0617588629613559
561	KC_G1	l(2)34Fd	-0.546349030412128	0.529009027972119	0.998043617620652	pseudobulk	DESeq2	LRT	0.276536916310716
561	KC_G1	l(2)35Be	-0.246588476222236	0.520986006632962	0.998043617620652	pseudobulk	DESeq2	LRT	0.283173941429271
561	KC_G1	l(2)37Bb	0.185080923716325	0.706074790498615	0.998043617620652	pseudobulk	DESeq2	LRT	0.151149294159928
561	KC_G1	l(2)37Cb	0.302703030712623	0.621503632816885	0.998043617620652	pseudobulk	DESeq2	LRT	0.206556328474121
561	KC_G1	l(2)37Cc	-0.093384483790728	0.66773148584091	0.998043617620652	pseudobulk	DESeq2	LRT	0.175398144802059
561	KC_G1	l(2)37Cd	-0.184716447992253	0.892221110690518	0.998043617620652	pseudobulk	DESeq2	LRT	0.0495275052056596
561	KC_G1	l(2)37Cg	-0.654291200119665	0.304111587550301	0.998043617620652	pseudobulk	DESeq2	LRT	0.51696703163726
561	KC_G1	l(2)41Ab	0.460226474341439	0.394748629421443	0.998043617620652	pseudobulk	DESeq2	LRT	0.403679369199941
561	KC_G1	l(2)dtl	0.633210062258421	0.712133823703895	0.998043617620652	pseudobulk	DESeq2	LRT	0.1474383863702
561	KC_G1	l(2)efl	0.0831851935237755	0.896229174861066	0.998043617620652	pseudobulk	DESeq2	LRT	0.0475809226468779
562	KC_G1	l(2)gd1	-0.040717407086466	0.945613025237903	0.998043617620652	pseudobulk	DESeq2	LRT	0.0242865542822073
562	KC_G1	l(2)gl	2.04147257822512	0.408147672536479	0.998043617620652	pseudobulk	DESeq2	LRT	0.389182675723292
562	KC_G1	l(2)k01209	0.077649811500369	0.861401702614299	0.998043617620652	pseudobulk	DESeq2	LRT	0.0647942741623852
562	KC_G1	l(2)k05819	0.604878580241287	0.157032263268783	0.998043617620652	pseudobulk	DESeq2	LRT	0.804011109883124
562	KC_G1	l(2)k09022	-0.87100820852986	0.405378263419245	0.998043617620652	pseudobulk	DESeq2	LRT	0.392139542101233
562	KC_G1	l(2)k09848	0.428701272292406	0.586733512210138	0.998043617620652	pseudobulk	DESeq2	LRT	0.231559105660796
562	KC_G1	l(2)k09913	0.0350092384078951	0.845280281687154	0.998043617620652	pseudobulk	DESeq2	LRT	0.0729992619297603
562	KC_G1	l(2)k10201	0.287239208700978	0.668566309376977	0.998043617620652	pseudobulk	DESeq2	LRT	0.1748555123087
562	KC_G1	l(2)k14505	0.0640303469304343	0.800466688361169	0.998043617620652	pseudobulk	DESeq2	LRT	0.0966567366515504
562	KC_G1	l(2)SH0834	-0.165059064403591	0.414780206217806	0.998043617620652	pseudobulk	DESeq2	LRT	0.382181976814079
563	KC_G1	l(3)02640	-0.0341330464342621	0.898315861396666	0.998043617620652	pseudobulk	DESeq2	LRT	0.046570931994093
563	KC_G1	l(3)04053	-0.27612777788047	0.437615768460317	0.998043617620652	pseudobulk	DESeq2	LRT	0.358907037607159
563	KC_G1	l(3)05822	-0.237713983188897	0.401308818755549	0.998043617620652	pseudobulk	DESeq2	LRT	0.396521296546275
563	KC_G1	l(3)07882	0.605156542097123	0.21094782068265	0.998043617620652	pseudobulk	DESeq2	LRT	0.675824956986952
563	KC_G1	l(3)72Ab	0.200542633818039	0.575805129300233	0.998043617620652	pseudobulk	DESeq2	LRT	0.239724470724235
563	KC_G1	l(3)72Dn	0.942123988234404	0.247955937417938	0.998043617620652	pseudobulk	DESeq2	LRT	0.605625487869043
563	KC_G1	l(3)72Dp	0.118598840617802	0.878342947934182	0.998043617620652	pseudobulk	DESeq2	LRT	0.0563358812296465
563	KC_G1	l(3)72Dr	0.19478960837647	0.896496195445196	0.998043617620652	pseudobulk	DESeq2	LRT	0.0474515491588686
563	KC_G1	l(3)73Ah	0.0911177472221162	0.789815559881402	0.998043617620652	pseudobulk	DESeq2	LRT	0.10247431463079
563	KC_G1	l(3)76BDm	-0.653509471268369	0.240484729075504	0.998043617620652	pseudobulk	DESeq2	LRT	0.618912496374591
564	KC_G1	l(3)80Fg	-0.0375444071908065	0.744618567704194	0.998043617620652	pseudobulk	DESeq2	LRT	0.128066138476299
564	KC_G1	l(3)80Fj	0.0219294849973939	0.849018274122112	0.998043617620652	pseudobulk	DESeq2	LRT	0.0710829619763254
564	KC_G1	l(3)87Df	0.157962611395582	0.552599682286534	0.998043617620652	pseudobulk	DESeq2	LRT	0.257589369114123
564	KC_G1	l(3)L1231	-0.0739997042624964	0.550579545368449	0.998043617620652	pseudobulk	DESeq2	LRT	0.259179927157405
564	KC_G1	l(3)mbt	0.169750214758199	0.612654025917844	0.998043617620652	pseudobulk	DESeq2	LRT	0.212784708274731
564	KC_G1	l(3)neo38	0.0243793487789172	0.845850630268846	0.998043617620652	pseudobulk	DESeq2	LRT	0.0727063227499125
564	KC_G1	l(3)neo43	-0.0960594422473281	0.755568508514325	0.998043617620652	pseudobulk	DESeq2	LRT	0.121726151416517
564	KC_G1	l(3)psg2	0.0133119276553634	0.94986511305183	0.998043617620652	pseudobulk	DESeq2	LRT	0.0223380629390353
564	KC_G1	L2HGDH	0.365818488179114	0.325308236238557	0.998043617620652	pseudobulk	DESeq2	LRT	0.487704940998271
564	KC_G1	La	0.138803614759141	0.671188456982905	0.998043617620652	pseudobulk	DESeq2	LRT	0.173155521058581
565	KC_G1	Lac	-0.164051117858296	0.866531069753257	0.998043617620652	pseudobulk	DESeq2	LRT	0.0622158609010336
565	KC_G1	lace	-0.276261743988025	0.402511234480107	0.998043617620652	pseudobulk	DESeq2	LRT	0.395221993545487
565	KC_G1	Lam	-0.150162340789378	0.517683261251386	0.998043617620652	pseudobulk	DESeq2	LRT	0.285935877250484
565	KC_G1	lama	0.0611694082171272	0.975321175268176	0.998043617620652	pseudobulk	DESeq2	LRT	0.0108523466831239
565	KC_G1	LamC	0.825707223018978	0.318185261386754	0.998043617620652	pseudobulk	DESeq2	LRT	0.49731994111511

565	KC_G1	Lamp1	-0.0816476531819948	0.561216384383195	0.998043617620652	pseudobulk	DESeq2	LRT	0.250869658485906
565	KC_G1	LanB1	1.12508065046033	0.900770125530981	0.998043617620652	pseudobulk	DESeq2	LRT	0.0453860258366365
565	KC_G1	lap	-0.0761358085754575	0.534922423170806	0.998043617620652	pseudobulk	DESeq2	LRT	0.271709196729701
565	KC_G1	Lap1	-0.376961190784866	0.372778803624451	0.998043617620652	pseudobulk	DESeq2	LRT	0.428548789801549
565	KC_G1	Lapsyn	-0.408289272051397	0.720351948938445	0.998043617620652	pseudobulk	DESeq2	LRT	0.142455264490591
566	KC_G1	Lar	-0.0343553247373797	0.764512819738511	0.998043617620652	pseudobulk	DESeq2	LRT	0.116615227720575
566	KC_G1	lark	0.131664459343734	0.442120423853978	0.998043617620652	pseudobulk	DESeq2	LRT	0.354459422297917
566	KC_G1	larp	-0.0924258193008385	0.492044813682914	0.998043617620652	pseudobulk	DESeq2	LRT	0.3079953414422
566	KC_G1	Larp4B	0.03642839783415	0.75139295768482	0.998043617620652	pseudobulk	DESeq2	LRT	0.124132879611941
566	KC_G1	Larp7	-0.170799530545777	0.683608551273404	0.998043617620652	pseudobulk	DESeq2	LRT	0.165192513318731
566	KC_G1	Las	-0.303937844783216	0.534834575658563	0.998043617620652	pseudobulk	DESeq2	LRT	0.271780524498426
566	KC_G1	lawc	-0.0590283332848736	0.748800879437947	0.998043617620652	pseudobulk	DESeq2	LRT	0.12563365420742
566	KC_G1	lbk	-0.297984925321534	0.228945266520431	0.998043617620652	pseudobulk	DESeq2	LRT	0.640268331150514
566	KC_G1	lbm	-3.31620141218876	0.034219843707052	0.998043617620652	pseudobulk	DESeq2	LRT	1.4657219783519
566	KC_G1	LBR	0.113132616032968	0.874614678339248	0.998043617620652	pseudobulk	DESeq2	LRT	0.0581832383240611
567	KC_G1	Lcch3	0.0481603502441304	0.79846329939687	0.998043617620652	pseudobulk	DESeq2	LRT	0.0977450409979135
567	KC_G1	ldbr	-0.598412421903087	0.64110515663732	0.998043617620652	pseudobulk	DESeq2	LRT	0.193070729916881
567	KC_G1	Ldh	0.434282453356282	0.590770365452005	0.998043617620652	pseudobulk	DESeq2	LRT	0.228581298132365
567	KC_G1	lds	-0.794610277746993	0.815039924133852	0.998043617620652	pseudobulk	DESeq2	LRT	0.0888211171417967
567	KC_G1	Ldsdh1	0.143266455699269	0.870892408495902	0.998043617620652	pseudobulk	DESeq2	LRT	0.0600354951447351
567	KC_G1	Leash	-0.259106890113038	0.652736135918581	0.998043617620652	pseudobulk	DESeq2	LRT	0.185262343799159
567	KC_G1	lectin-24Db	-0.794610277746993	0.821479263487599	0.998043617620652	pseudobulk	DESeq2	LRT	0.085403394939296
567	KC_G1	lectin-46Ca	-1.67593316621313	0.495877122847873	0.998043617620652	pseudobulk	DESeq2	LRT	0.304625927300966
567	KC_G1	lectin-46Cb	1.5681698674963	0.509898658126755	0.998043617620652	pseudobulk	DESeq2	LRT	0.292516130940769
567	KC_G1	Lerp	0.0957514300304275	0.704522218359486	0.998043617620652	pseudobulk	DESeq2	LRT	0.152105306090798
568	KC_G1	Letm1	-0.0299606674266654	0.924921335646708	0.998043617620652	pseudobulk	DESeq2	LRT	0.0338952023391454
568	KC_G1	LeuRS	-0.143932101966235	0.794681310815122	0.998043617620652	pseudobulk	DESeq2	LRT	0.0998070005294832
568	KC_G1	LeuRS-m	0.080370655508677	0.88822502866513	0.998043617620652	pseudobulk	DESeq2	LRT	0.0514769933127509
568	KC_G1	levy	-0.0247355534951379	0.826915814793396	0.998043617620652	pseudobulk	DESeq2	LRT	0.0825387020940081
568	KC_G1	Lgr1	1.12392859879163	0.334926225088247	0.998043617620652	pseudobulk	DESeq2	LRT	0.475050845397727
568	KC_G1	Lgr3	-0.317873560178461	0.142431093240928	0.998043617620652	pseudobulk	DESeq2	LRT	0.846395192237741
568	KC_G1	lgs	-0.0626094436869502	0.690614956162509	0.998043617620652	pseudobulk	DESeq2	LRT	0.160764020674496
568	KC_G1	Lhr	0.26515247969308	0.642254355144811	0.998043617620652	pseudobulk	DESeq2	LRT	0.192292942088558
568	KC_G1	lic	0.308047217555534	0.337171905728373	0.998043617620652	pseudobulk	DESeq2	LRT	0.472148619383736
568	KC_G1	lid	-0.0390371875743158	0.80792658665171	0.998043617620652	pseudobulk	DESeq2	LRT	0.0926281001914575
569	KC_G1	lig	-0.174191699457413	0.319072641917868	0.998043617620652	pseudobulk	DESeq2	LRT	0.496110431699513
569	KC_G1	lili	-0.0642968135952821	0.872223670754381	0.998043617620652	pseudobulk	DESeq2	LRT	0.0593721314455363
569	KC_G1	lilli	0.0369552954263365	0.780069368093901	0.998043617620652	pseudobulk	DESeq2	LRT	0.107866775718663
569	KC_G1	Lim1	0.983615621582388	0.630810344880065	0.998043617620652	pseudobulk	DESeq2	LRT	0.200101193137223
569	KC_G1	LIMK1	-0.0318599507405441	0.898946547568707	0.998043617620652	pseudobulk	DESeq2	LRT	0.04626613116567
569	KC_G1	lin	0.349374322170178	0.508220133058351	0.998043617620652	pseudobulk	DESeq2	LRT	0.293948134439223
569	KC_G1	lin-28	-0.158044750338727	0.9319696184822	0.998043617620652	pseudobulk	DESeq2	LRT	0.0305982450930102
569	KC_G1	lin-52	0.527179615835053	0.136380082345933	0.998043617620652	pseudobulk	DESeq2	LRT	0.865249051665448
569	KC_G1	link	-0.17623541249224	0.860366425478791	0.998043617620652	pseudobulk	DESeq2	LRT	0.0653165456506785
569	KC_G1	Lint-1	-0.288473595787613	0.432215453466721	0.998043617620652	pseudobulk	DESeq2	LRT	0.364299709374706
570	KC_G1	Lip4	2.04147257822512	0.408147672536479	0.998043617620652	pseudobulk	DESeq2	LRT	0.389182675723292
570	KC_G1	Liprin-alpha	-0.265234711789183	0.198254223678373	0.998043617620652	pseudobulk	DESeq2	LRT	0.702777551547958
570	KC_G1	Liprin-beta	-0.0838213768995345	0.863023289201649	0.998043617620652	pseudobulk	DESeq2	LRT	0.0639774844292959
570	KC_G1	Liprin-gamma	-0.0538267812984452	0.687975777231811	0.998043617620652	pseudobulk	DESeq2	LRT	0.162426852461805
570	KC_G1	List	0.283048539751481	0.484390380126026	0.998043617620652	pseudobulk	DESeq2	LRT	0.314804490425081
570	KC_G1	Lk6	-0.0934348493556166	0.412687839062949	0.998043617620652	pseudobulk	DESeq2	LRT	0.384378328567873
570	KC_G1	Lkb1	0.314797101844723	0.415083168530185	0.998043617620652	pseudobulk	DESeq2	LRT	0.381864876745947
570	KC_G1	LKRSDH	0.215620560252525	0.888894521282258	0.998043617620652	pseudobulk	DESeq2	LRT	0.0511497705740695
570	KC_G1	LManI	1.12508065046033	0.900770125530981	0.998043617620652	pseudobulk	DESeq2	LRT	0.0453860258366365
570	KC_G1	LManII	1.79280049005751	0.438378263525642	0.998043617620652	pseudobulk	DESeq2	LRT	0.358150987974651
571	KC_G1	Lmpt	0.420042227580506	0.643118463688712	0.998043617620652	pseudobulk	DESeq2	LRT	0.191709021807349

571	KC_G1	lms	0.481247568780457	0.809503883669727	0.998043617620652	pseudobulk	DESeq2	LRT	0.0917810633376787
571	KC_G1	lncRNA:acal	-0.794610277746993	0.879485534599449	0.998043617620652	pseudobulk	DESeq2	LRT	0.0557712992369932
571	KC_G1	lncRNA:alphagamma-ele	-0.114577737456103	0.670441514990167	0.998043617620652	pseudobulk	DESeq2	LRT	0.173639101220087
571	KC_G1	lncRNA:cherub	0.103543789152	0.883785244550016	0.998043617620652	pseudobulk	DESeq2	LRT	0.0536532535816015
571	KC_G1	lncRNA:CR13130	0.425806370366354	0.820769217095803	0.998043617620652	pseudobulk	DESeq2	LRT	0.0857789401174023
571	KC_G1	lncRNA:CR30055	-0.279669585478935	0.155023157788764	0.998043617620652	pseudobulk	DESeq2	LRT	0.809603420870725
571	KC_G1	lncRNA:CR30121	1.12508065046033	0.900770125530981	0.998043617620652	pseudobulk	DESeq2	LRT	0.0453860258366365
571	KC_G1	lncRNA:CR31044	-1.24029575767303	0.590792835712426	0.998043617620652	pseudobulk	DESeq2	LRT	0.22856477982831
571	KC_G1	lncRNA:CR31386	0.277898324400098	0.730819779435391	0.998043617620652	pseudobulk	DESeq2	LRT	0.136189707100288
572	KC_G1	lncRNA:CR31451	-0.0417350801724972	0.674294616382026	0.998043617620652	pseudobulk	DESeq2	LRT	0.171150307748708
572	KC_G1	lncRNA:CR31526	1.12508065046033	0.900770125530981	0.998043617620652	pseudobulk	DESeq2	LRT	0.0453860258366365
572	KC_G1	lncRNA:CR32218	-0.0334008838544882	0.93171076127056	0.998043617620652	pseudobulk	DESeq2	LRT	0.0307188883755818
572	KC_G1	lncRNA:CR32582	-0.92823935520201	0.527548635206689	0.998043617620652	pseudobulk	DESeq2	LRT	0.277737496166322
572	KC_G1	lncRNA:CR32636	-1.67593316621313	0.495877122847873	0.998043617620652	pseudobulk	DESeq2	LRT	0.304625927300966
572	KC_G1	lncRNA:CR32773	-0.192147123873615	0.854452898575191	0.998043617620652	pseudobulk	DESeq2	LRT	0.0683118726187108
572	KC_G1	lncRNA:CR33938	-0.794610277746993	0.879485534599449	0.998043617620652	pseudobulk	DESeq2	LRT	0.0557712992369932
572	KC_G1	lncRNA:CR34044	0.228319291893143	0.924568785563363	0.998043617620652	pseudobulk	DESeq2	LRT	0.034060772899258
572	KC_G1	lncRNA:CR34046	-0.794610277746993	0.821479263487599	0.998043617620652	pseudobulk	DESeq2	LRT	0.085403394939296
572	KC_G1	lncRNA:CR34335	-0.215651264649889	0.0889706451888008	0.998043617620652	pseudobulk	DESeq2	LRT	1.0507532600452
573	KC_G1	lncRNA:CR40469	0.492015747103888	0.0923804658144533	0.998043617620652	pseudobulk	DESeq2	LRT	1.03441985222006
573	KC_G1	lncRNA:CR42491	0.542602929224614	0.655385216048846	0.998043617620652	pseudobulk	DESeq2	LRT	0.183503359506623
573	KC_G1	lncRNA:CR42549	-0.177159840566507	0.692203038821905	0.998043617620652	pseudobulk	DESeq2	LRT	0.159766498453206
573	KC_G1	lncRNA:CR42736	1.90250740646012	0.460774913678782	0.998043617620652	pseudobulk	DESeq2	LRT	0.336511173568024
573	KC_G1	lncRNA:CR42746	1.12508065046033	0.900770125530981	0.998043617620652	pseudobulk	DESeq2	LRT	0.0453860258366365
573	KC_G1	lncRNA:CR42755	0.173195945230598	0.976215465741119	0.998043617620652	pseudobulk	DESeq2	LRT	0.0104543162934892
573	KC_G1	lncRNA:CR42765	1.12508065046033	0.900770125530981	0.998043617620652	pseudobulk	DESeq2	LRT	0.0453860258366365
573	KC_G1	lncRNA:CR42861	0.148824036626064	0.440144047618896	0.998043617620652	pseudobulk	DESeq2	LRT	0.356405167041254
573	KC_G1	lncRNA:CR42862	-0.0436921473258199	0.673765559470805	0.998043617620652	pseudobulk	DESeq2	LRT	0.171491192396246
573	KC_G1	lncRNA:CR43130	-0.794610277746993	0.821479263487599	0.998043617620652	pseudobulk	DESeq2	LRT	0.085403394939296
574	KC_G1	lncRNA:CR43146	-2.67240485668008	0.203373709471669	0.998043617620652	pseudobulk	DESeq2	LRT	0.691705189905663
574	KC_G1	lncRNA:CR43148	-0.52105934144612	0.379570719892826	0.998043617620652	pseudobulk	DESeq2	LRT	0.420707296453981
574	KC_G1	lncRNA:CR43166	1.79207265922759	0.449242408626613	0.998043617620652	pseudobulk	DESeq2	LRT	0.347519252937804
574	KC_G1	lncRNA:CR43174	-0.794610277746993	0.879485534599449	0.998043617620652	pseudobulk	DESeq2	LRT	0.0557712992369932
574	KC_G1	lncRNA:CR43241	0.591772478231005	0.159505469372554	0.998043617620652	pseudobulk	DESeq2	LRT	0.797224420584261
574	KC_G1	lncRNA:CR43242	2.08291528148028	0.0160216378569384	0.998043617620652	pseudobulk	DESeq2	LRT	1.79529308900888
574	KC_G1	lncRNA:CR43253	1.12508065046033	0.900770125530981	0.998043617620652	pseudobulk	DESeq2	LRT	0.0453860258366365
574	KC_G1	lncRNA:CR43256	-1.00128329705775	0.537148986912056	0.998043617620652	pseudobulk	DESeq2	LRT	0.269905239031213
574	KC_G1	lncRNA:CR43264	0.143209121279157	0.84948263119497	0.998043617620652	pseudobulk	DESeq2	LRT	0.0708454964338954
574	KC_G1	lncRNA:CR43285	-1.72343437992478	0.512120560562093	0.998043617620652	pseudobulk	DESeq2	LRT	0.290627787806665
575	KC_G1	lncRNA:CR43287	-0.64277910557112	0.764034577741626	0.998043617620652	pseudobulk	DESeq2	LRT	0.116886986211521
575	KC_G1	lncRNA:CR43309	0.172834960772825	0.908610676445368	0.998043617620652	pseudobulk	DESeq2	LRT	0.0416221644022337
575	KC_G1	lncRNA:CR43334	1.07467244916653	0.260243255168948	0.998043617620652	pseudobulk	DESeq2	LRT	0.584620517459564
575	KC_G1	lncRNA:CR43363	1.12508065046033	0.900770125530981	0.998043617620652	pseudobulk	DESeq2	LRT	0.0453860258366365
575	KC_G1	lncRNA:CR43399	1.13582588427274	0.633424328955532	0.998043617620652	pseudobulk	DESeq2	LRT	0.198305259991224
575	KC_G1	lncRNA:CR43404	1.13582588427274	0.704684208645453	0.998043617620652	pseudobulk	DESeq2	LRT	0.152005460551806
575	KC_G1	lncRNA:CR43406	1.12508065046033	0.900770125530981	0.998043617620652	pseudobulk	DESeq2	LRT	0.0453860258366365
575	KC_G1	lncRNA:CR43410	1.12508065046033	0.900770125530981	0.998043617620652	pseudobulk	DESeq2	LRT	0.0453860258366365
575	KC_G1	lncRNA:CR43411	0.830738961435447	0.687963126928521	0.998043617620652	pseudobulk	DESeq2	LRT	0.162434838218871
575	KC_G1	lncRNA:CR43432	2.07738706174348	0.431998797123676	0.998043617620652	pseudobulk	DESeq2	LRT	0.364517462451933
576	KC_G1	lncRNA:CR43455	1.13582588427274	0.704684208645453	0.998043617620652	pseudobulk	DESeq2	LRT	0.152005460551806
576	KC_G1	lncRNA:CR43459	0.130975340083098	0.673208757786528	0.998043617620652	pseudobulk	DESeq2	LRT	0.171850242913648
576	KC_G1	lncRNA:CR43461	-1.04361183419037	0.435538266876581	0.998043617620652	pseudobulk	DESeq2	LRT	0.360973681383686
576	KC_G1	lncRNA:CR43492	0.658316683882339	0.452498749067882	0.998043617620652	pseudobulk	DESeq2	LRT	0.344382617063567
576	KC_G1	lncRNA:CR43493	0.416431064791215	0.848864097724124	0.998043617620652	pseudobulk	DESeq2	LRT	0.0711618342941965
576	KC_G1	lncRNA:CR43496	-1.40915284876445	0.255975438428895	0.998043617620652	pseudobulk	DESeq2	LRT	0.591801704479336
576	KC_G1	lncRNA:CR43604	0.0781071480641268	0.970967397587672	0.998043617620652	pseudobulk	DESeq2	LRT	0.0127953522603565

576	KC_G1	lncRNA:CR43605	1.13582588427274	0.704684208645453	0.998043617620652	pseudobulk	DESeq2	LRT	0.152005460551806
576	KC_G1	lncRNA:CR43606	1.12508065046033	0.900770125530981	0.998043617620652	pseudobulk	DESeq2	LRT	0.0453860258366365
576	KC_G1	lncRNA:CR43611	-0.794610277746993	0.879485534599449	0.998043617620652	pseudobulk	DESeq2	LRT	0.0557712992369932
577	KC_G1	lncRNA:CR43641	-2.27643791964046	0.298906309922724	0.998043617620652	pseudobulk	DESeq2	LRT	0.52446491689215
577	KC_G1	lncRNA:CR43651	0.0623344047142108	0.82324267560701	0.998043617620652	pseudobulk	DESeq2	LRT	0.0844721245184042
577	KC_G1	lncRNA:CR43653	1.38669271937	0.601428581131124	0.998043617620652	pseudobulk	DESeq2	LRT	0.220815937172328
577	KC_G1	lncRNA:CR43654	-0.794610277746993	0.815039924133852	0.998043617620652	pseudobulk	DESeq2	LRT	0.0888211171417967
577	KC_G1	lncRNA:CR43656	1.12508065046033	0.900770125530981	0.998043617620652	pseudobulk	DESeq2	LRT	0.0453860258366365
577	KC_G1	lncRNA:CR43685	0.51311725105534	0.141152757713092	0.998043617620652	pseudobulk	DESeq2	LRT	0.850310632586994
577	KC_G1	lncRNA:CR43700	1.18241880387395	0.451989020389883	0.998043617620652	pseudobulk	DESeq2	LRT	0.344872114839067
577	KC_G1	lncRNA:CR43704	1.12508065046033	0.900770125530981	0.998043617620652	pseudobulk	DESeq2	LRT	0.0453860258366365
577	KC_G1	lncRNA:CR43765	1.90250740646012	0.460774913678782	0.998043617620652	pseudobulk	DESeq2	LRT	0.336511173568024
577	KC_G1	lncRNA:CR43785	-0.142702768502676	0.925857932937563	0.998043617620652	pseudobulk	DESeq2	LRT	0.0334556479559611
578	KC_G1	lncRNA:CR43802	1.13582588427274	0.704684208645453	0.998043617620652	pseudobulk	DESeq2	LRT	0.152005460551806
578	KC_G1	lncRNA:CR43818	-0.794610277746993	0.815039924133852	0.998043617620652	pseudobulk	DESeq2	LRT	0.0888211171417967
578	KC_G1	lncRNA:CR43827	1.2609445879621	0.626159485520488	0.998043617620652	pseudobulk	DESeq2	LRT	0.203315036023015
578	KC_G1	lncRNA:CR43828	-1.67593316621313	0.495877122847873	0.998043617620652	pseudobulk	DESeq2	LRT	0.304625927300966
578	KC_G1	lncRNA:CR43832	1.13582588427274	0.704684208645453	0.998043617620652	pseudobulk	DESeq2	LRT	0.152005460551806
578	KC_G1	lncRNA:CR43836	0.0106127858529861	0.955037825711813	0.998043617620652	pseudobulk	DESeq2	LRT	0.0199794271884085
578	KC_G1	lncRNA:CR43837	-2.41166857926136	0.110779093045704	0.998043617620652	pseudobulk	DESeq2	LRT	0.955542194766681
578	KC_G1	lncRNA:CR43838	1.12508065046033	0.900770125530981	0.998043617620652	pseudobulk	DESeq2	LRT	0.0453860258366365
578	KC_G1	lncRNA:CR43849	-0.794610277746993	0.821479263487599	0.998043617620652	pseudobulk	DESeq2	LRT	0.085403394939296
578	KC_G1	lncRNA:CR43855	0.646720739111718	0.705868581629832	0.998043617620652	pseudobulk	DESeq2	LRT	0.151276148219711
579	KC_G1	lncRNA:CR43856	0.173105107343345	0.973555957903825	0.998043617620652	pseudobulk	DESeq2	LRT	0.0116390811132178
579	KC_G1	lncRNA:CR43870	-0.765625368254185	0.836625948066609	0.998043617620652	pseudobulk	DESeq2	LRT	0.0774686698445758
579	KC_G1	lncRNA:CR43887	1.24406349205447	0.626072434068117	0.998043617620652	pseudobulk	DESeq2	LRT	0.203375417754029
579	KC_G1	lncRNA:CR43890	-0.794610277746993	0.879485534599449	0.998043617620652	pseudobulk	DESeq2	LRT	0.0557712992369932
579	KC_G1	lncRNA:CR43900	-0.1607036254114	0.862072275228704	0.998043617620652	pseudobulk	DESeq2	LRT	0.0644563218583863
579	KC_G1	lncRNA:CR43905	1.13582588427274	0.704684208645453	0.998043617620652	pseudobulk	DESeq2	LRT	0.152005460551806
579	KC_G1	lncRNA:CR43910	-0.133268490736772	0.928873864264751	0.998043617620652	pseudobulk	DESeq2	LRT	0.0320432566985322
579	KC_G1	lncRNA:CR43937	-0.794610277746993	0.821479263487599	0.998043617620652	pseudobulk	DESeq2	LRT	0.085403394939296
579	KC_G1	lncRNA:CR43943	0.0466400198729007	0.973462604518624	0.998043617620652	pseudobulk	DESeq2	LRT	0.011680727208302
579	KC_G1	lncRNA:CR43958	-0.0651975577770557	0.929301249447299	0.998043617620652	pseudobulk	DESeq2	LRT	0.0318434789398791
580	KC_G1	lncRNA:CR43963	-0.340042308334467	0.84227681899998	0.998043617620652	pseudobulk	DESeq2	LRT	0.0745451517231942
580	KC_G1	lncRNA:CR43974	1.12508065046033	0.900770125530981	0.998043617620652	pseudobulk	DESeq2	LRT	0.0453860258366365
580	KC_G1	lncRNA:CR43992	0.173195945230598	0.976215465741119	0.998043617620652	pseudobulk	DESeq2	LRT	0.0104543162934892
580	KC_G1	lncRNA:CR43995	-0.369845134518329	0.628399540678399	0.998043617620652	pseudobulk	DESeq2	LRT	0.201764141074632
580	KC_G1	lncRNA:CR44017	-0.794610277746993	0.821479263487599	0.998043617620652	pseudobulk	DESeq2	LRT	0.085403394939296
580	KC_G1	lncRNA:CR44023	0.506233083697581	0.698534539594452	0.998043617620652	pseudobulk	DESeq2	LRT	0.155812114983701
580	KC_G1	lncRNA:CR44024	-0.0984341402968669	0.414858808038544	0.998043617620652	pseudobulk	DESeq2	LRT	0.382099684789404
580	KC_G1	lncRNA:CR44042	-0.794610277746993	0.879485534599449	0.998043617620652	pseudobulk	DESeq2	LRT	0.0557712992369932
580	KC_G1	lncRNA:CR44043	-0.690259696519426	0.643030386274126	0.998043617620652	pseudobulk	DESeq2	LRT	0.191768504090751
580	KC_G1	lncRNA:CR44048	1.13582588427274	0.633424328955532	0.998043617620652	pseudobulk	DESeq2	LRT	0.198305259991224
581	KC_G1	lncRNA:CR44050	-0.221805622979812	0.81135561509345	0.998043617620652	pseudobulk	DESeq2	LRT	0.0907887538996632
581	KC_G1	lncRNA:CR44051	-0.486532099301731	0.814737268792241	0.998043617620652	pseudobulk	DESeq2	LRT	0.0889824171610406
581	KC_G1	lncRNA:CR44056	1.13582588427274	0.633424328955532	0.998043617620652	pseudobulk	DESeq2	LRT	0.198305259991224
581	KC_G1	lncRNA:CR44060	-0.794610277746993	0.821479263487599	0.998043617620652	pseudobulk	DESeq2	LRT	0.085403394939296
581	KC_G1	lncRNA:CR44073	1.13582588427274	0.704684208645453	0.998043617620652	pseudobulk	DESeq2	LRT	0.152005460551806
581	KC_G1	lncRNA:CR44074	-1.31483872207159	0.633038743121339	0.998043617620652	pseudobulk	DESeq2	LRT	0.198569709557826
581	KC_G1	lncRNA:CR44092	-0.0530598021217566	0.892505233007536	0.998043617620652	pseudobulk	DESeq2	LRT	0.0493892288184202
581	KC_G1	lncRNA:CR44109	-0.794610277746993	0.879485534599449	0.998043617620652	pseudobulk	DESeq2	LRT	0.0557712992369932
581	KC_G1	lncRNA:CR44125	-2.31763425292248	0.315578515403755	0.998043617620652	pseudobulk	DESeq2	LRT	0.50089257123956
581	KC_G1	lncRNA:CR44126	-0.794610277746993	0.821479263487599	0.998043617620652	pseudobulk	DESeq2	LRT	0.085403394939296
582	KC_G1	lncRNA:CR44127	0.172834960772825	0.908610676445368	0.998043617620652	pseudobulk	DESeq2	LRT	0.0416221644022337
582	KC_G1	lncRNA:CR44129	1.69981218657454	0.566400560818652	0.998043617620652	pseudobulk	DESeq2	LRT	0.246876325303589
582	KC_G1	lncRNA:CR44138	-0.108862988675086	0.935601167166634	0.998043617620652	pseudobulk	DESeq2	LRT	0.0289092450782411

582	KC_G1	lncRNA:CR44157	-0.739609749351626	0.677113896978351	0.998043617620652	pseudobulk	DESeq2	LRT	0.169338272719961
582	KC_G1	lncRNA:CR44161	0.1729159612032	0.899814506689544	0.998043617620652	pseudobulk	DESeq2	LRT	0.0458470094762598
582	KC_G1	lncRNA:CR44170	1.13582588427274	0.633424328955532	0.998043617620652	pseudobulk	DESeq2	LRT	0.198305259991224
582	KC_G1	lncRNA:CR44171	1.45999539932625	0.418540392615003	0.998043617620652	pseudobulk	DESeq2	LRT	0.378262622632991
582	KC_G1	lncRNA:CR44178	-0.823722390931642	0.517938671841393	0.998043617620652	pseudobulk	DESeq2	LRT	0.285721661215531
582	KC_G1	lncRNA:CR44187	1.13582588427274	0.633424328955532	0.998043617620652	pseudobulk	DESeq2	LRT	0.198305259991224
582	KC_G1	lncRNA:CR44206	0.710801355262282	0.42972205366925	0.998043617620652	pseudobulk	DESeq2	LRT	0.366812357414334
583	KC_G1	lncRNA:CR44218	1.13582588427274	0.704684208645453	0.998043617620652	pseudobulk	DESeq2	LRT	0.152005460551806
583	KC_G1	lncRNA:CR44219	1.13582588427274	0.704684208645453	0.998043617620652	pseudobulk	DESeq2	LRT	0.152005460551806
583	KC_G1	lncRNA:CR44221	1.12508065046033	0.900770125530981	0.998043617620652	pseudobulk	DESeq2	LRT	0.0453860258366365
583	KC_G1	lncRNA:CR44222	0.173105107343345	0.973555957903825	0.998043617620652	pseudobulk	DESeq2	LRT	0.0116390811132178
583	KC_G1	lncRNA:CR44260	-0.794610277746993	0.821479263487599	0.998043617620652	pseudobulk	DESeq2	LRT	0.085403394939296
583	KC_G1	lncRNA:CR44272	0.296175279079998	0.885816336538646	0.998043617620652	pseudobulk	DESeq2	LRT	0.0526563145647348
583	KC_G1	lncRNA:CR44276	1.43067171916479	0.566959027189548	0.998043617620652	pseudobulk	DESeq2	LRT	0.246448325425342
583	KC_G1	lncRNA:CR44279	1.12508065046033	0.900770125530981	0.998043617620652	pseudobulk	DESeq2	LRT	0.0453860258366365
583	KC_G1	lncRNA:CR44294	0.324541429746258	0.803280193312258	0.998043617620652	pseudobulk	DESeq2	LRT	0.0951329414160805
583	KC_G1	lncRNA:CR44317	1.06667464613488	0.669131470313168	0.998043617620652	pseudobulk	DESeq2	LRT	0.174488544082685
584	KC_G1	lncRNA:CR44320	0.172845155749872	0.90730697492154	0.998043617620652	pseudobulk	DESeq2	LRT	0.0422457504714555
584	KC_G1	lncRNA:CR44331	-0.042698288924232	0.945035891943232	0.998043617620652	pseudobulk	DESeq2	LRT	0.0245516969120269
584	KC_G1	lncRNA:CR44332	0.131796077129131	0.825817208349695	0.998043617620652	pseudobulk	DESeq2	LRT	0.0831160715540167
584	KC_G1	lncRNA:CR44347	-0.0559329745985775	0.917635891518619	0.998043617620652	pseudobulk	DESeq2	LRT	0.037329608202209
584	KC_G1	lncRNA:CR44348	0.763164787219597	0.794486984020146	0.998043617620652	pseudobulk	DESeq2	LRT	0.0999132133899323
584	KC_G1	lncRNA:CR44357	1.29684295133038	0.121668479369279	0.998043617620652	pseudobulk	DESeq2	LRT	0.914821919790223
584	KC_G1	lncRNA:CR44360	-0.341896175419091	0.879866942952392	0.998043617620652	pseudobulk	DESeq2	LRT	0.0555829986573842
584	KC_G1	lncRNA:CR44366	-0.794610277746993	0.879485534599449	0.998043617620652	pseudobulk	DESeq2	LRT	0.0557712992369932
584	KC_G1	lncRNA:CR44371	-0.794610277746993	0.821479263487599	0.998043617620652	pseudobulk	DESeq2	LRT	0.085403394939296
584	KC_G1	lncRNA:CR44384	-0.508697718760178	0.65547553123216	0.998043617620652	pseudobulk	DESeq2	LRT	0.183443515795704
585	KC_G1	lncRNA:CR44392	-1.39542401216867	0.462167035373762	0.998043617620652	pseudobulk	DESeq2	LRT	0.335201034334682
585	KC_G1	lncRNA:CR44409	-0.718676691072112	0.739205546127982	0.998043617620652	pseudobulk	DESeq2	LRT	0.131234783341328
585	KC_G1	lncRNA:CR44410	1.13582588427274	0.704684208645453	0.998043617620652	pseudobulk	DESeq2	LRT	0.152005460551806
585	KC_G1	lncRNA:CR44417	-0.289409253515507	0.170895880717672	0.998043617620652	pseudobulk	DESeq2	LRT	0.767268405407672
585	KC_G1	lncRNA:CR44441	0.228570564635066	0.925908451678216	0.998043617620652	pseudobulk	DESeq2	LRT	0.0334319516511941
585	KC_G1	lncRNA:CR44444	0.172834960772825	0.908610676445368	0.998043617620652	pseudobulk	DESeq2	LRT	0.0416221644022337
585	KC_G1	lncRNA:CR44445	-0.0234291604783265	0.931230317769559	0.998043617620652	pseudobulk	DESeq2	LRT	0.0309428932765682
585	KC_G1	lncRNA:CR44457	0.173195945230598	0.976215465741119	0.998043617620652	pseudobulk	DESeq2	LRT	0.0104543162934892
585	KC_G1	lncRNA:CR44458	1.13582588427274	0.704684208645453	0.998043617620652	pseudobulk	DESeq2	LRT	0.152005460551806
585	KC_G1	lncRNA:CR44464	1.13582588427274	0.633424328955532	0.998043617620652	pseudobulk	DESeq2	LRT	0.198305259991224
586	KC_G1	lncRNA:CR44471	-1.22190253579857	0.521632143130424	0.998043617620652	pseudobulk	DESeq2	LRT	0.282635655094256
586	KC_G1	lncRNA:CR44472	-1.2833596927783	0.554636446631459	0.998043617620652	pseudobulk	DESeq2	LRT	0.255991595178557
586	KC_G1	lncRNA:CR44473	-2.31763425292248	0.315578515403755	0.998043617620652	pseudobulk	DESeq2	LRT	0.50089257123956
586	KC_G1	lncRNA:CR44474	-2.52653385939235	0.162672845093281	0.998043617620652	pseudobulk	DESeq2	LRT	0.78868493760038
586	KC_G1	lncRNA:CR44503	-0.794610277746993	0.821479263487599	0.998043617620652	pseudobulk	DESeq2	LRT	0.085403394939296
586	KC_G1	lncRNA:CR44504	-0.794610277746993	0.821479263487599	0.998043617620652	pseudobulk	DESeq2	LRT	0.085403394939296
586	KC_G1	lncRNA:CR44506	0.132810618652188	0.777362873928525	0.998043617620652	pseudobulk	DESeq2	LRT	0.109376204678504
586	KC_G1	lncRNA:CR44514	-0.65817001371651	0.770857516266391	0.998043617620652	pseudobulk	DESeq2	LRT	0.113025888638787
586	KC_G1	lncRNA:CR44524	1.69981218657454	0.566400560818652	0.998043617620652	pseudobulk	DESeq2	LRT	0.246876325303589
586	KC_G1	lncRNA:CR44526	1.12508065046033	0.900770125530981	0.998043617620652	pseudobulk	DESeq2	LRT	0.0453860258366365
587	KC_G1	lncRNA:CR44544	0.173105107343345	0.973555957903825	0.998043617620652	pseudobulk	DESeq2	LRT	0.0116390811132178
587	KC_G1	lncRNA:CR44546	-1.73907471612892	0.507597351592657	0.998043617620652	pseudobulk	DESeq2	LRT	0.294480652518419
587	KC_G1	lncRNA:CR44564	1.13582588427274	0.633424328955532	0.998043617620652	pseudobulk	DESeq2	LRT	0.198305259991224
587	KC_G1	lncRNA:CR44565	-0.794610277746993	0.879485534599449	0.998043617620652	pseudobulk	DESeq2	LRT	0.0557712992369932
587	KC_G1	lncRNA:CR44567	2.07738706174348	0.431998797123676	0.998043617620652	pseudobulk	DESeq2	LRT	0.364517462451933
587	KC_G1	lncRNA:CR44589	0.410432688826314	0.851008243997148	0.998043617620652	pseudobulk	DESeq2	LRT	0.0700662327413482
587	KC_G1	lncRNA:CR44595	1.13582588427274	0.704684208645453	0.998043617620652	pseudobulk	DESeq2	LRT	0.152005460551806
587	KC_G1	lncRNA:CR44596	-0.794610277746993	0.815039924133852	0.998043617620652	pseudobulk	DESeq2	LRT	0.0888211171417967
587	KC_G1	lncRNA:CR44597	-0.794610277746993	0.815039924133852	0.998043617620652	pseudobulk	DESeq2	LRT	0.0888211171417967

587	KC_G1	lncRNA:CR44606	0.949176147629133	0.729784453976284	0.998043617620652	pseudobulk	DESeq2	LRT	0.136805392306293
588	KC_G1	lncRNA:CR44608	0.177622136115678	0.935661853927379	0.998043617620652	pseudobulk	DESeq2	LRT	0.0288810759485907
588	KC_G1	lncRNA:CR44619	1.12508065046033	0.900770125530981	0.998043617620652	pseudobulk	DESeq2	LRT	0.0453860258366365
588	KC_G1	lncRNA:CR44628	0.801380794885844	0.538772574038544	0.998043617620652	pseudobulk	DESeq2	LRT	0.268594519940285
588	KC_G1	lncRNA:CR44642	1.13582588427274	0.633424328955532	0.998043617620652	pseudobulk	DESeq2	LRT	0.198305259991224
588	KC_G1	lncRNA:CR44657	-2.01544921444574	0.386064837560981	0.998043617620652	pseudobulk	DESeq2	LRT	0.413339751726489
588	KC_G1	lncRNA:CR44661	-0.794610277746993	0.821479263487599	0.998043617620652	pseudobulk	DESeq2	LRT	0.085403394939296
588	KC_G1	lncRNA:CR44662	-1.04114317517866	0.373060508735541	0.998043617620652	pseudobulk	DESeq2	LRT	0.428220721867717
588	KC_G1	lncRNA:CR44663	-0.183293308708977	0.824918431195727	0.998043617620652	pseudobulk	DESeq2	LRT	0.0835889928233577
588	KC_G1	lncRNA:CR44679	2.38466886841527	0.34619303318143	0.998043617620652	pseudobulk	DESeq2	LRT	0.460681676152437
588	KC_G1	lncRNA:CR44686	-0.794610277746993	0.879485534599449	0.998043617620652	pseudobulk	DESeq2	LRT	0.0557712992369932
589	KC_G1	lncRNA:CR44692	-0.794610277746993	0.815039924133852	0.998043617620652	pseudobulk	DESeq2	LRT	0.0888211171417967
589	KC_G1	lncRNA:CR44702	0.173195945230598	0.976215465741119	0.998043617620652	pseudobulk	DESeq2	LRT	0.0104543162934892
589	KC_G1	lncRNA:CR44708	-1.49152468382924	0.124771679948816	0.998043617620652	pseudobulk	DESeq2	LRT	0.903883977455365
589	KC_G1	lncRNA:CR44713	1.90250740646012	0.460774913678782	0.998043617620652	pseudobulk	DESeq2	LRT	0.336511173568024
589	KC_G1	lncRNA:CR44714	-0.794610277746993	0.879485534599449	0.998043617620652	pseudobulk	DESeq2	LRT	0.0557712992369932
589	KC_G1	lncRNA:CR44716	-1.13818012732623	0.489879468119492	0.998043617620652	pseudobulk	DESeq2	LRT	0.309910762358872
589	KC_G1	lncRNA:CR44717	1.24545707098284	0.613638418851987	0.998043617620652	pseudobulk	DESeq2	LRT	0.212087457773066
589	KC_G1	lncRNA:CR44718	-0.216367205190702	0.814211563635031	0.998043617620652	pseudobulk	DESeq2	LRT	0.0892627339546267
589	KC_G1	lncRNA:CR44727	-1.08931899821065	0.586916235035588	0.998043617620652	pseudobulk	DESeq2	LRT	0.231423877044868
589	KC_G1	lncRNA:CR44728	-0.794610277746993	0.815039924133852	0.998043617620652	pseudobulk	DESeq2	LRT	0.0888211171417967
590	KC_G1	lncRNA:CR44732	1.12508065046033	0.900770125530981	0.998043617620652	pseudobulk	DESeq2	LRT	0.0453860258366365
590	KC_G1	lncRNA:CR44741	1.11121460044925	0.691240116375203	0.998043617620652	pseudobulk	DESeq2	LRT	0.16037106535726
590	KC_G1	lncRNA:CR44749	-0.638137141652138	0.570669885981764	0.998043617620652	pseudobulk	DESeq2	LRT	0.243615044389909
590	KC_G1	lncRNA:CR44761	1.13582588427274	0.704684208645453	0.998043617620652	pseudobulk	DESeq2	LRT	0.152005460551806
590	KC_G1	lncRNA:CR44766	1.24728680252724	0.542457192173983	0.998043617620652	pseudobulk	DESeq2	LRT	0.2656345283295
590	KC_G1	lncRNA:CR44779	0.210922363524271	0.902763848327324	0.998043617620652	pseudobulk	DESeq2	LRT	0.0444258408064128
590	KC_G1	lncRNA:CR44785	-0.510007090755602	0.749432549716208	0.998043617620652	pseudobulk	DESeq2	LRT	0.125267448345039
590	KC_G1	lncRNA:CR44805	1.06595574389305	0.681978466270766	0.998043617620652	pseudobulk	DESeq2	LRT	0.166229338140333
590	KC_G1	lncRNA:CR44813	-0.794610277746993	0.815039924133852	0.998043617620652	pseudobulk	DESeq2	LRT	0.0888211171417967
590	KC_G1	lncRNA:CR44816	-0.335835559911393	0.756915878789205	0.998043617620652	pseudobulk	DESeq2	LRT	0.12095238391827
591	KC_G1	lncRNA:CR44828	0.675827160429678	0.656312557011257	0.998043617620652	pseudobulk	DESeq2	LRT	0.182889286450789
591	KC_G1	lncRNA:CR44830	-0.794610277746993	0.879485534599449	0.998043617620652	pseudobulk	DESeq2	LRT	0.0557712992369932
591	KC_G1	lncRNA:CR44831	2.26259189257468	0.268414136743927	0.998043617620652	pseudobulk	DESeq2	LRT	0.571194614627486
591	KC_G1	lncRNA:CR44832	-0.486532099301731	0.814737268792241	0.998043617620652	pseudobulk	DESeq2	LRT	0.0889824171610406
591	KC_G1	lncRNA:CR44833	-0.794610277746993	0.821479263487599	0.998043617620652	pseudobulk	DESeq2	LRT	0.085403394939296
591	KC_G1	lncRNA:CR44844	-0.794610277746993	0.815039924133852	0.998043617620652	pseudobulk	DESeq2	LRT	0.0888211171417967
591	KC_G1	lncRNA:CR44845	-1.73907471612892	0.507597351592657	0.998043617620652	pseudobulk	DESeq2	LRT	0.294480652518419
591	KC_G1	lncRNA:CR44851	1.13582588427274	0.704684208645453	0.998043617620652	pseudobulk	DESeq2	LRT	0.152005460551806
591	KC_G1	lncRNA:CR44854	-0.718676691072112	0.739205546127982	0.998043617620652	pseudobulk	DESeq2	LRT	0.131234783341328
591	KC_G1	lncRNA:CR44886	-0.106767813222394	0.747130996021789	0.998043617620652	pseudobulk	DESeq2	LRT	0.126603245759263
592	KC_G1	lncRNA:CR44900	1.30081682196589	0.248441777532933	0.998043617620652	pseudobulk	DESeq2	LRT	0.604775372157197
592	KC_G1	lncRNA:CR44901	-0.5425457669829	0.619429139440849	0.998043617620652	pseudobulk	DESeq2	LRT	0.208008368238679
592	KC_G1	lncRNA:CR44908	1.13582588427274	0.704684208645453	0.998043617620652	pseudobulk	DESeq2	LRT	0.152005460551806
592	KC_G1	lncRNA:CR44914	0.173195945230598	0.976215465741119	0.998043617620652	pseudobulk	DESeq2	LRT	0.0104543162934892
592	KC_G1	lncRNA:CR44915	0.172834960772825	0.908610676445368	0.998043617620652	pseudobulk	DESeq2	LRT	0.0416221644022337
592	KC_G1	lncRNA:CR44922	-0.422186092323197	0.224113548571576	0.998043617620652	pseudobulk	DESeq2	LRT	0.649531887811162
592	KC_G1	lncRNA:CR44923	-0.794610277746993	0.815039924133852	0.998043617620652	pseudobulk	DESeq2	LRT	0.0888211171417967
592	KC_G1	lncRNA:CR44938	0.765052904958762	0.810501863366603	0.998043617620652	pseudobulk	DESeq2	LRT	0.0912459823590772
592	KC_G1	lncRNA:CR44947	0.531219532446616	0.662118890566078	0.998043617620652	pseudobulk	DESeq2	LRT	0.179064021314312
592	KC_G1	lncRNA:CR44953	-0.794610277746993	0.815039924133852	0.998043617620652	pseudobulk	DESeq2	LRT	0.0888211171417967
593	KC_G1	lncRNA:CR44957	0.173195945230598	0.976215465741119	0.998043617620652	pseudobulk	DESeq2	LRT	0.0104543162934892
593	KC_G1	lncRNA:CR44965	2.37279544779784	0.320199125517434	0.998043617620652	pseudobulk	DESeq2	LRT	0.494579858498766
593	KC_G1	lncRNA:CR44973	-2.73026776509574	0.214564985109251	0.998043617620652	pseudobulk	DESeq2	LRT	0.668441149166746
593	KC_G1	lncRNA:CR44977	2.50145379121547	0.286990765027193	0.998043617620652	pseudobulk	DESeq2	LRT	0.542132078047399
593	KC_G1	lncRNA:CR44978	0.522269609915347	0.712879035261765	0.998043617620652	pseudobulk	DESeq2	LRT	0.146984157069882

593	KC_G1	lncRNA:CR44979	0.173105107343345	0.973555957903825	0.998043617620652	pseudobulk	DESeq2	LRT	0.0116390811132178
593	KC_G1	lncRNA:CR44985	-3.02561526164544	0.135534071676351	0.998043617620652	pseudobulk	DESeq2	LRT	0.867951514524196
593	KC_G1	lncRNA:CR44986	1.3098112086877	0.136528129174901	0.998043617620652	pseudobulk	DESeq2	LRT	0.864777860807299
593	KC_G1	lncRNA:CR44993	1.24545707098284	0.613638418851987	0.998043617620652	pseudobulk	DESeq2	LRT	0.212087457773066
593	KC_G1	lncRNA:CR44997	-0.308356898052274	0.0670130909637269	0.998043617620652	pseudobulk	DESeq2	LRT	1.17384034986693
594	KC_G1	lncRNA:CR44999	-0.411822761736907	0.310151496895926	0.998043617620652	pseudobulk	DESeq2	LRT	0.508426118442036
594	KC_G1	lncRNA:CR45010	-0.794610277746993	0.815039924133852	0.998043617620652	pseudobulk	DESeq2	LRT	0.0888211171417967
594	KC_G1	lncRNA:CR45013	-2.19332442993981	0.381369935212232	0.998043617620652	pseudobulk	DESeq2	LRT	0.418653546964052
594	KC_G1	lncRNA:CR45014	2.52420870383484	0.282654181793565	0.998043617620652	pseudobulk	DESeq2	LRT	0.548744584884793
594	KC_G1	lncRNA:CR45018	-0.90788303004483	0.515213655586398	0.998043617620652	pseudobulk	DESeq2	LRT	0.288012634648195
594	KC_G1	lncRNA:CR45025	2.84706876674335	0.20790991633284	0.998043617620652	pseudobulk	DESeq2	LRT	0.682124796354298
594	KC_G1	lncRNA:CR45030	-1.59634756047548	0.279931654650329	0.998043617620652	pseudobulk	DESeq2	LRT	0.552947988769796
594	KC_G1	lncRNA:CR45035	-0.00872935586404877	0.974559251680104	0.998043617620652	pseudobulk	DESeq2	LRT	0.0111917513175823
594	KC_G1	lncRNA:CR45036	-0.794610277746993	0.821479263487599	0.998043617620652	pseudobulk	DESeq2	LRT	0.085403394939296
594	KC_G1	lncRNA:CR45043	-0.794610277746993	0.815039924133852	0.998043617620652	pseudobulk	DESeq2	LRT	0.0888211171417967
595	KC_G1	lncRNA:CR45048	-1.27845746407632	0.556113633048063	0.998043617620652	pseudobulk	DESeq2	LRT	0.254836458124014
595	KC_G1	lncRNA:CR45054	0.124297522260102	0.356044016586549	0.998043617620652	pseudobulk	DESeq2	LRT	0.448496308265982
595	KC_G1	lncRNA:CR45055	-0.120688771676719	0.937008693034803	0.998043617620652	pseudobulk	DESeq2	LRT	0.0282563799558364
595	KC_G1	lncRNA:CR45082	-0.794610277746993	0.821479263487599	0.998043617620652	pseudobulk	DESeq2	LRT	0.085403394939296
595	KC_G1	lncRNA:CR45102	-0.147112676693748	0.482785955447837	0.998043617620652	pseudobulk	DESeq2	LRT	0.316245372295117
595	KC_G1	lncRNA:CR45117	-0.794610277746993	0.821479263487599	0.998043617620652	pseudobulk	DESeq2	LRT	0.085403394939296
595	KC_G1	lncRNA:CR45155	-2.70723887115547	0.222713116467202	0.998043617620652	pseudobulk	DESeq2	LRT	0.652254204871744
595	KC_G1	lncRNA:CR45156	-1.60891734856068	0.545249891924755	0.998043617620652	pseudobulk	DESeq2	LRT	0.263404411831769
595	KC_G1	lncRNA:CR45169	-0.374081852389163	0.868267388341527	0.998043617620652	pseudobulk	DESeq2	LRT	0.0613465105393336
595	KC_G1	lncRNA:CR45170	-0.0504308465056773	0.953939081123331	0.998043617620652	pseudobulk	DESeq2	LRT	0.0204793586048471
596	KC_G1	lncRNA:CR45177	-2.54467069327032	0.226913622827011	0.998043617620652	pseudobulk	DESeq2	LRT	0.644139430333658
596	KC_G1	lncRNA:CR45178	-0.794610277746993	0.879485534599449	0.998043617620652	pseudobulk	DESeq2	LRT	0.0557712992369932
596	KC_G1	lncRNA:CR45179	-1.66808346769027	0.498191247612582	0.998043617620652	pseudobulk	DESeq2	LRT	0.302603906560549
596	KC_G1	lncRNA:CR45180	1.43665189530809	0.453797653603825	0.998043617620652	pseudobulk	DESeq2	LRT	0.343137753998782
596	KC_G1	lncRNA:CR45183	0.173116475207952	0.97305562682953	0.998043617620652	pseudobulk	DESeq2	LRT	0.011862331638572
596	KC_G1	lncRNA:CR45187	-1.70024284854914	0.238055042603883	0.998043617620652	pseudobulk	DESeq2	LRT	0.623322614643804
596	KC_G1	lncRNA:CR45188	-0.518444081909213	0.795762981353597	0.998043617620652	pseudobulk	DESeq2	LRT	0.0992162679639495
596	KC_G1	lncRNA:CR45196	1.69981218657454	0.566400560818652	0.998043617620652	pseudobulk	DESeq2	LRT	0.246876325303589
596	KC_G1	lncRNA:CR45199	-0.139046923079547	0.930850499186999	0.998043617620652	pseudobulk	DESeq2	LRT	0.0311200640151481
596	KC_G1	lncRNA:CR45200	2.52420870383484	0.282654181793565	0.998043617620652	pseudobulk	DESeq2	LRT	0.548744584884793
597	KC_G1	lncRNA:CR45203	-1.60891734856068	0.545249891924755	0.998043617620652	pseudobulk	DESeq2	LRT	0.263404411831769
597	KC_G1	lncRNA:CR45217	1.13582588427274	0.633424328955532	0.998043617620652	pseudobulk	DESeq2	LRT	0.198305259991224
597	KC_G1	lncRNA:CR45223	-0.794610277746993	0.815039924133852	0.998043617620652	pseudobulk	DESeq2	LRT	0.0888211171417967
597	KC_G1	lncRNA:CR45224	0.46925474263489	0.830177751593747	0.998043617620652	pseudobulk	DESeq2	LRT	0.0808289097066435
597	KC_G1	lncRNA:CR45225	-1.31047986807593	0.611671045333228	0.998043617620652	pseudobulk	DESeq2	LRT	0.213482077209615
597	KC_G1	lncRNA:CR45233	-1.60891734856068	0.545249891924755	0.998043617620652	pseudobulk	DESeq2	LRT	0.263404411831769
597	KC_G1	lncRNA:CR45238	1.12508065046033	0.900770125530981	0.998043617620652	pseudobulk	DESeq2	LRT	0.0453860258366365
597	KC_G1	lncRNA:CR45240	0.126382127902694	0.951792398662627	0.998043617620652	pseudobulk	DESeq2	LRT	0.0214577679466525
597	KC_G1	lncRNA:CR45242	0.173105107343345	0.973555957903825	0.998043617620652	pseudobulk	DESeq2	LRT	0.0116390811132178
597	KC_G1	lncRNA:CR45251	0.603088533052036	0.791040718043767	0.998043617620652	pseudobulk	DESeq2	LRT	0.101801161045192
598	KC_G1	lncRNA:CR45262	-0.794610277746993	0.815039924133852	0.998043617620652	pseudobulk	DESeq2	LRT	0.0888211171417967
598	KC_G1	lncRNA:CR45266	-1.42445394601728	0.418237957223043	0.998043617620652	pseudobulk	DESeq2	LRT	0.378576555295339
598	KC_G1	lncRNA:CR45279	-0.794610277746993	0.879485534599449	0.998043617620652	pseudobulk	DESeq2	LRT	0.0557712992369932
598	KC_G1	lncRNA:CR45283	1.12508065046033	0.900770125530981	0.998043617620652	pseudobulk	DESeq2	LRT	0.0453860258366365
598	KC_G1	lncRNA:CR45284	-1.60891734856068	0.545249891924755	0.998043617620652	pseudobulk	DESeq2	LRT	0.263404411831769
598	KC_G1	lncRNA:CR45290	2.83301634290227	0.134305128615741	0.998043617620652	pseudobulk	DESeq2	LRT	0.871907402915879
598	KC_G1	lncRNA:CR45306	-0.794610277746993	0.815039924133852	0.998043617620652	pseudobulk	DESeq2	LRT	0.0888211171417967
598	KC_G1	lncRNA:CR45308	-0.427208156903352	0.445309353135501	0.998043617620652	pseudobulk	DESeq2	LRT	0.351338182990319
598	KC_G1	lncRNA:CR45312	1.98658207734826	0.416578288758803	0.998043617620652	pseudobulk	DESeq2	LRT	0.380303368372803
598	KC_G1	lncRNA:CR45332	-0.394854741810359	0.857115676329067	0.998043617620652	pseudobulk	DESeq2	LRT	0.0669605617393276
599	KC_G1	lncRNA:CR45333	0.255317222375299	0.88228563803942	0.998043617620652	pseudobulk	DESeq2	LRT	0.0543907902178957

599	KC_G1	lncRNA:CR45339	-0.794610277746993	0.879485534599449	0.998043617620652	pseudobulk	DESeq2	LRT	0.0557712992369932
599	KC_G1	lncRNA:CR45340	-0.794610277746993	0.879485534599449	0.998043617620652	pseudobulk	DESeq2	LRT	0.0557712992369932
599	KC_G1	lncRNA:CR45358	0.759469876908125	0.223619664330313	0.998043617620652	pseudobulk	DESeq2	LRT	0.650490008770228
599	KC_G1	lncRNA:CR45364	-2.28172252880459	0.324719824317488	0.998043617620652	pseudobulk	DESeq2	LRT	0.488491196656175
599	KC_G1	lncRNA:CR45380	-0.794610277746993	0.821479263487599	0.998043617620652	pseudobulk	DESeq2	LRT	0.085403394939296
599	KC_G1	lncRNA:CR45387	1.11201871582686	0.425379383597473	0.998043617620652	pseudobulk	DESeq2	LRT	0.371223562404194
599	KC_G1	lncRNA:CR45388	0.0781468596477246	0.815201644836554	0.998043617620652	pseudobulk	DESeq2	LRT	0.0887349527225351
599	KC_G1	lncRNA:CR45401	-3.43347082273389	0.0951998328779775	0.998043617620652	pseudobulk	DESeq2	LRT	1.02136381401296
599	KC_G1	lncRNA:CR45402	1.12508065046033	0.900770125530981	0.998043617620652	pseudobulk	DESeq2	LRT	0.0453860258366365
600	KC_G1	lncRNA:CR45403	1.12508065046033	0.900770125530981	0.998043617620652	pseudobulk	DESeq2	LRT	0.0453860258366365
600	KC_G1	lncRNA:CR45405	2.43658683107279	0.288749127391824	0.998043617620652	pseudobulk	DESeq2	LRT	0.539479319548244
600	KC_G1	lncRNA:CR45409	-0.283884330642636	0.465856799383946	0.998043617620652	pseudobulk	DESeq2	LRT	0.331747561410687
600	KC_G1	lncRNA:CR45418	0.0287187511538292	0.973581032562605	0.998043617620652	pseudobulk	DESeq2	LRT	0.0116278956794269
600	KC_G1	lncRNA:CR45444	1.52715265276022	0.29234683398325	0.998043617620652	pseudobulk	DESeq2	LRT	0.534101605062714
600	KC_G1	lncRNA:CR45449	-0.794610277746993	0.815039924133852	0.998043617620652	pseudobulk	DESeq2	LRT	0.0888211171417967
600	KC_G1	lncRNA:CR45450	-0.794610277746993	0.815039924133852	0.998043617620652	pseudobulk	DESeq2	LRT	0.0888211171417967
600	KC_G1	lncRNA:CR45456	-1.67593316621313	0.495877122847873	0.998043617620652	pseudobulk	DESeq2	LRT	0.304625927300966
600	KC_G1	lncRNA:CR45464	1.12508065046033	0.900770125530981	0.998043617620652	pseudobulk	DESeq2	LRT	0.0453860258366365
600	KC_G1	lncRNA:CR45471	2.07738706174348	0.431998797123676	0.998043617620652	pseudobulk	DESeq2	LRT	0.364517462451933
601	KC_G1	lncRNA:CR45473	0.826071642033711	0.0770669269678752	0.998043617620652	pseudobulk	DESeq2	LRT	1.1131319580959
601	KC_G1	lncRNA:CR45480	-0.794610277746993	0.879485534599449	0.998043617620652	pseudobulk	DESeq2	LRT	0.0557712992369932
601	KC_G1	lncRNA:CR45504	1.06411570142544	0.444352212547236	0.998043617620652	pseudobulk	DESeq2	LRT	0.352272653023194
601	KC_G1	lncRNA:CR45505	-1.60891734856068	0.545249891924755	0.998043617620652	pseudobulk	DESeq2	LRT	0.263404411831769
601	KC_G1	lncRNA:CR45517	1.12508065046033	0.900770125530981	0.998043617620652	pseudobulk	DESeq2	LRT	0.0453860258366365
601	KC_G1	lncRNA:CR45522	-2.02477450664369	0.298464370325329	0.998043617620652	pseudobulk	DESeq2	LRT	0.52510750605779
601	KC_G1	lncRNA:CR45527	0.326079055963941	0.678958215357321	0.998043617620652	pseudobulk	DESeq2	LRT	0.168156952373594
601	KC_G1	lncRNA:CR45530	-0.769849674437473	0.725455892474086	0.998043617620652	pseudobulk	DESeq2	LRT	0.13938898741292
601	KC_G1	lncRNA:CR45531	-0.794610277746993	0.821479263487599	0.998043617620652	pseudobulk	DESeq2	LRT	0.085403394939296
601	KC_G1	lncRNA:CR45539	2.86622177009429	0.203230025119761	0.998043617620652	pseudobulk	DESeq2	LRT	0.692012129161051
602	KC_G1	lncRNA:CR45541	-0.794610277746993	0.815039924133852	0.998043617620652	pseudobulk	DESeq2	LRT	0.0888211171417967
602	KC_G1	lncRNA:CR45556	-0.42958161660918	0.754089638184325	0.998043617620652	pseudobulk	DESeq2	LRT	0.122577026736362
602	KC_G1	lncRNA:CR45563	1.13582588427274	0.633424328955532	0.998043617620652	pseudobulk	DESeq2	LRT	0.198305259991224
602	KC_G1	lncRNA:CR45566	-0.794610277746993	0.821479263487599	0.998043617620652	pseudobulk	DESeq2	LRT	0.085403394939296
602	KC_G1	lncRNA:CR45574	-0.794610277746993	0.815039924133852	0.998043617620652	pseudobulk	DESeq2	LRT	0.0888211171417967
602	KC_G1	lncRNA:CR45597	-1.72984073695683	0.480047293856853	0.998043617620652	pseudobulk	DESeq2	LRT	0.318715974188453
602	KC_G1	lncRNA:CR45616	1.13582588427274	0.633424328955532	0.998043617620652	pseudobulk	DESeq2	LRT	0.198305259991224
602	KC_G1	lncRNA:CR45625	-1.26071441347596	0.342095363434176	0.998043617620652	pseudobulk	DESeq2	LRT	0.465852811955241
602	KC_G1	lncRNA:CR45626	-1.66808346769027	0.498191247612582	0.998043617620652	pseudobulk	DESeq2	LRT	0.302603906560549
602	KC_G1	lncRNA:CR45635	-0.794610277746993	0.815039924133852	0.998043617620652	pseudobulk	DESeq2	LRT	0.0888211171417967
603	KC_G1	lncRNA:CR45640	-0.391253826066496	0.670263744731373	0.998043617620652	pseudobulk	DESeq2	LRT	0.173754271414779
603	KC_G1	lncRNA:CR45641	0.730757173260101	0.679347744797991	0.998043617620652	pseudobulk	DESeq2	LRT	0.167907861968013
603	KC_G1	lncRNA:CR45643	-1.07148960690765	0.350737438326822	0.998043617620652	pseudobulk	DESeq2	LRT	0.455017874292581
603	KC_G1	lncRNA:CR45660	0.0534317753438379	0.885003748766966	0.998043617620652	pseudobulk	DESeq2	LRT	0.0530548896802998
603	KC_G1	lncRNA:CR45668	-0.291164338266869	0.260454931619228	0.998043617620652	pseudobulk	DESeq2	LRT	0.584267414945661
603	KC_G1	lncRNA:CR45669	0.464306926412256	0.531707540223669	0.998043617620652	pseudobulk	DESeq2	LRT	0.274327180847543
603	KC_G1	lncRNA:CR45673	1.13582588427274	0.633424328955532	0.998043617620652	pseudobulk	DESeq2	LRT	0.198305259991224
603	KC_G1	lncRNA:CR45674	0.949176147629133	0.729784453976284	0.998043617620652	pseudobulk	DESeq2	LRT	0.136805392306293
603	KC_G1	lncRNA:CR45678	-1.60891734856068	0.545249891924755	0.998043617620652	pseudobulk	DESeq2	LRT	0.263404411831769
603	KC_G1	lncRNA:CR45684	-0.672980545926867	0.490226704727394	0.998043617620652	pseudobulk	DESeq2	LRT	0.309603034577507
604	KC_G1	lncRNA:CR45706	0.496363633982644	0.752296834797086	0.998043617620652	pseudobulk	DESeq2	LRT	0.123610765421344
604	KC_G1	lncRNA:CR45708	-0.794610277746993	0.815039924133852	0.998043617620652	pseudobulk	DESeq2	LRT	0.0888211171417967
604	KC_G1	lncRNA:CR45709	-0.794610277746993	0.821479263487599	0.998043617620652	pseudobulk	DESeq2	LRT	0.085403394939296
604	KC_G1	lncRNA:CR45720	-0.794610277746993	0.821479263487599	0.998043617620652	pseudobulk	DESeq2	LRT	0.085403394939296
604	KC_G1	lncRNA:CR45736	0.840167866502407	0.558431421986135	0.998043617620652	pseudobulk	DESeq2	LRT	0.253030152685662
604	KC_G1	lncRNA:CR45740	-0.794610277746993	0.879485534599449	0.998043617620652	pseudobulk	DESeq2	LRT	0.0557712992369932
604	KC_G1	lncRNA:CR45745	-0.794610277746993	0.815039924133852	0.998043617620652	pseudobulk	DESeq2	LRT	0.0888211171417967

604	KC_G1	lncRNA:CR45758	0.305743171191195	0.305674947405906	0.998043617620652	pseudobulk	DESeq2	LRT	0.514740153845784
604	KC_G1	lncRNA:CR45761	-0.794610277746993	0.821479263487599	0.998043617620652	pseudobulk	DESeq2	LRT	0.085403394939296
604	KC_G1	lncRNA:CR45805	0.69451681481409	0.666050512560935	0.998043617620652	pseudobulk	DESeq2	LRT	0.176492833150107
605	KC_G1	lncRNA:CR45808	0.489134379042483	0.718576237703757	0.998043617620652	pseudobulk	DESeq2	LRT	0.143527148396002
605	KC_G1	lncRNA:CR45809	-0.794610277746993	0.879485534599449	0.998043617620652	pseudobulk	DESeq2	LRT	0.0557712992369932
605	KC_G1	lncRNA:CR45810	1.12508065046033	0.900770125530981	0.998043617620652	pseudobulk	DESeq2	LRT	0.0453860258366365
605	KC_G1	lncRNA:CR45819	0.173195945230598	0.976215465741119	0.998043617620652	pseudobulk	DESeq2	LRT	0.0104543162934892
605	KC_G1	lncRNA:CR45820	-0.794610277746993	0.821479263487599	0.998043617620652	pseudobulk	DESeq2	LRT	0.085403394939296
605	KC_G1	lncRNA:CR45821	-1.47492726446814	0.187274960002018	0.998043617620652	pseudobulk	DESeq2	LRT	0.727520287013085
605	KC_G1	lncRNA:CR45825	0.0402257138156654	0.955036107182284	0.998043617620652	pseudobulk	DESeq2	LRT	0.0199802086742748
605	KC_G1	lncRNA:CR45881	1.13582588427274	0.633424328955532	0.998043617620652	pseudobulk	DESeq2	LRT	0.198305259991224
605	KC_G1	lncRNA:CR45883	0.880548665281164	0.621648416418504	0.998043617620652	pseudobulk	DESeq2	LRT	0.206455168331118
605	KC_G1	lncRNA:CR45898	-0.794610277746993	0.821479263487599	0.998043617620652	pseudobulk	DESeq2	LRT	0.085403394939296
606	KC_G1	lncRNA:CR45910	-1.20286539254962	0.38766032631347	0.998043617620652	pseudobulk	DESeq2	LRT	0.411548643008217
606	KC_G1	lncRNA:CR45911	0.0838976321318009	0.838129729427426	0.998043617620652	pseudobulk	DESeq2	LRT	0.0766887541454878
606	KC_G1	lncRNA:CR45918	0.759237566123917	0.625267036210959	0.998043617620652	pseudobulk	DESeq2	LRT	0.203934466520103
606	KC_G1	lncRNA:CR45922	-1.7392816361115	0.38609804243622	0.998043617620652	pseudobulk	DESeq2	LRT	0.413302400295928
606	KC_G1	lncRNA:CR45938	3.51433627222488	0.0724261714418414	0.998043617620652	pseudobulk	DESeq2	LRT	1.14010447166285
606	KC_G1	lncRNA:CR45940	1.13582588427274	0.704684208645453	0.998043617620652	pseudobulk	DESeq2	LRT	0.152005460551806
606	KC_G1	lncRNA:CR45941	-0.704797641189757	0.838804762683723	0.998043617620652	pseudobulk	DESeq2	LRT	0.0763391123013672
606	KC_G1	lncRNA:CR45950	-1.66808346769027	0.498191247612582	0.998043617620652	pseudobulk	DESeq2	LRT	0.302603906560549
606	KC_G1	lncRNA:CR45953	-0.794610277746993	0.815039924133852	0.998043617620652	pseudobulk	DESeq2	LRT	0.0888211171417967
606	KC_G1	lncRNA:CR45954	-1.73634999673762	0.424993395565854	0.998043617620652	pseudobulk	DESeq2	LRT	0.371617818871082
607	KC_G1	lncRNA:CR45959	-0.0488165746185401	0.947773425099207	0.998043617620652	pseudobulk	DESeq2	LRT	0.0232954727779719
607	KC_G1	lncRNA:CR45961	1.52554480922443	0.250194031868432	0.998043617620652	pseudobulk	DESeq2	LRT	0.601723054185048
607	KC_G1	lncRNA:CR45962	0.167315374939485	0.901778771259059	0.998043617620652	pseudobulk	DESeq2	LRT	0.0448999926195634
607	KC_G1	lncRNA:CR45965	1.13582588427274	0.633424328955532	0.998043617620652	pseudobulk	DESeq2	LRT	0.198305259991224
607	KC_G1	lncRNA:CR45973	1.12508065046033	0.900770125530981	0.998043617620652	pseudobulk	DESeq2	LRT	0.0453860258366365
607	KC_G1	lncRNA:CR45979	0.150694077076766	0.867164110786489	0.998043617620652	pseudobulk	DESeq2	LRT	0.061898704525616
607	KC_G1	lncRNA:CR45981	1.42944759940171	0.552522613276143	0.998043617620652	pseudobulk	DESeq2	LRT	0.25764994276489
607	KC_G1	lncRNA:CR45993	1.13582588427274	0.704684208645453	0.998043617620652	pseudobulk	DESeq2	LRT	0.152005460551806
607	KC_G1	lncRNA:CR45994	-2.05526326317471	0.184897881327805	0.998043617620652	pseudobulk	DESeq2	LRT	0.733068065222194
607	KC_G1	lncRNA:CR45996	2.4186435506362	0.235816992353809	0.998043617620652	pseudobulk	DESeq2	LRT	0.627424903992385
608	KC_G1	lncRNA:CR46003	-0.23367628392681	0.67340329483056	0.998043617620652	pseudobulk	DESeq2	LRT	0.171724763024808
608	KC_G1	lncRNA:CR46004	0.104296924186726	0.604972508457081	0.998043617620652	pseudobulk	DESeq2	LRT	0.218264360383339
608	KC_G1	lncRNA:CR46006	-0.0201581335082522	0.951674067860847	0.998043617620652	pseudobulk	DESeq2	LRT	0.0215117646050881
608	KC_G1	lncRNA:CR46009	0.173105107343345	0.973555957903825	0.998043617620652	pseudobulk	DESeq2	LRT	0.0116390811132178
608	KC_G1	lncRNA:CR46024	-0.794610277746993	0.879485534599449	0.998043617620652	pseudobulk	DESeq2	LRT	0.0557712992369932
608	KC_G1	lncRNA:CR46035	-1.71016868383871	0.415184478138195	0.998043617620652	pseudobulk	DESeq2	LRT	0.381758891154151
608	KC_G1	lncRNA:CR46036	-0.087837150536357	0.937636323616979	0.998043617620652	pseudobulk	DESeq2	LRT	0.0279655766237108
608	KC_G1	lncRNA:CR46039	-1.60891734856068	0.545249891924755	0.998043617620652	pseudobulk	DESeq2	LRT	0.263404411831769
608	KC_G1	lncRNA:CR46064	-0.0155795578875177	0.946763455269049	0.998043617620652	pseudobulk	DESeq2	LRT	0.0237585140311907
608	KC_G1	lncRNA:CR46073	1.13582588427274	0.704684208645453	0.998043617620652	pseudobulk	DESeq2	LRT	0.152005460551806
609	KC_G1	lncRNA:CR46077	1.12508065046033	0.900770125530981	0.998043617620652	pseudobulk	DESeq2	LRT	0.0453860258366365
609	KC_G1	lncRNA:CR46078	-0.794610277746993	0.821479263487599	0.998043617620652	pseudobulk	DESeq2	LRT	0.085403394939296
609	KC_G1	lncRNA:CR46079	0.172834960772825	0.908610676445368	0.998043617620652	pseudobulk	DESeq2	LRT	0.0416221644022337
609	KC_G1	lncRNA:CR46080	0.0721553293344594	0.973275465908855	0.998043617620652	pseudobulk	DESeq2	LRT	0.0117642240762772
609	KC_G1	lncRNA:CR46084	-0.835846789879216	0.668046316875268	0.998043617620652	pseudobulk	DESeq2	LRT	0.175193426048375
609	KC_G1	lncRNA:CR46088	-1.32234376952914	0.542789226792531	0.998043617620652	pseudobulk	DESeq2	LRT	0.265368780745144
609	KC_G1	lncRNA:CR46094	1.12508065046033	0.900770125530981	0.998043617620652	pseudobulk	DESeq2	LRT	0.0453860258366365
609	KC_G1	lncRNA:CR46095	-1.11565309267186	0.59352253515558	0.998043617620652	pseudobulk	DESeq2	LRT	0.226562786889814
609	KC_G1	lncRNA:CR46110	-0.794610277746993	0.821479263487599	0.998043617620652	pseudobulk	DESeq2	LRT	0.085403394939296
609	KC_G1	lncRNA:CR46125	-1.72984073695683	0.480047293856853	0.998043617620652	pseudobulk	DESeq2	LRT	0.318715974188453
610	KC_G1	lncRNA:CR46127	-1.75798523386955	0.479693425296976	0.998043617620652	pseudobulk	DESeq2	LRT	0.319036233956057
610	KC_G1	lncRNA:CR46194	-1.52001993126824	0.293776823982742	0.998043617620652	pseudobulk	DESeq2	LRT	0.531982468632281
610	KC_G1	lncRNA:CR46204	1.13582588427274	0.704684208645453	0.998043617620652	pseudobulk	DESeq2	LRT	0.152005460551806

610	KC_G1	lncRNA:CR46208	0.0798854628074558	0.950989306590081	0.998043617620652	pseudobulk	DESeq2	LRT	0.0218243664643007
610	KC_G1	lncRNA:CR46230	-0.794610277746993	0.815039924133852	0.998043617620652	pseudobulk	DESeq2	LRT	0.0888211171417967
610	KC_G1	lncRNA:CR46231	-2.3243999702458	0.347033693779396	0.998043617620652	pseudobulk	DESeq2	LRT	0.459628357162795
610	KC_G1	lncRNA:CR46248	-0.794610277746993	0.879485534599449	0.998043617620652	pseudobulk	DESeq2	LRT	0.0557712992369932
610	KC_G1	lncRNA:CR46265	-1.72984073695683	0.480047293856853	0.998043617620652	pseudobulk	DESeq2	LRT	0.318715974188453
610	KC_G1	lncRNA:CR46268	0.1948790595899	0.689075994016177	0.998043617620652	pseudobulk	DESeq2	LRT	0.161732879744155
610	KC_G1	lncRNA:CR46271	1.06585281705208	0.683859663547483	0.998043617620652	pseudobulk	DESeq2	LRT	0.1650330117303
611	KC_G1	lncRNA:CR46322	-0.504126279533823	0.607173285200406	0.998043617620652	pseudobulk	DESeq2	LRT	0.216687345059353
611	KC_G1	lncRNA:CR46348	-1.90496033312483	0.229613153696898	0.998043617620652	pseudobulk	DESeq2	LRT	0.639003236420623
611	KC_G1	lncRNA:CR46350	2.38466886841527	0.34619303318143	0.998043617620652	pseudobulk	DESeq2	LRT	0.460681676152437
611	KC_G1	lncRNA:CR46352	1.13582588427274	0.704684208645453	0.998043617620652	pseudobulk	DESeq2	LRT	0.152005460551806
611	KC_G1	lncRNA:CR46391	-0.794610277746993	0.815039924133852	0.998043617620652	pseudobulk	DESeq2	LRT	0.0888211171417967
611	KC_G1	lncRNA:CR46393	0.948682853426738	0.716182290134517	0.998043617620652	pseudobulk	DESeq2	LRT	0.144976422491876
611	KC_G1	lncRNA:CR46431	0.173195945230598	0.976215465741119	0.998043617620652	pseudobulk	DESeq2	LRT	0.0104543162934892
611	KC_G1	lncRNA:CR9162	2.07738706174348	0.431998797123676	0.998043617620652	pseudobulk	DESeq2	LRT	0.364517462451933
611	KC_G1	lncRNA:flam	0.373747765547784	0.836188499316185	0.998043617620652	pseudobulk	DESeq2	LRT	0.0776958098995318
611	KC_G1	lncRNA:Hsromega	-0.356229645144835	0.154934531962142	0.998043617620652	pseudobulk	DESeq2	LRT	0.809851775467355
612	KC_G1	lncRNA:let7C	-0.0436456347604505	0.827080912695841	0.998043617620652	pseudobulk	DESeq2	LRT	0.0824520016706613
612	KC_G1	lncRNA:noe	0.0327084465508789	0.727984267231698	0.998043617620652	pseudobulk	DESeq2	LRT	0.137878006302746
612	KC_G1	lncRNA:roX1	-0.0982950490959809	0.882725858408188	0.998043617620652	pseudobulk	DESeq2	LRT	0.0541741510840129
612	KC_G1	lncRNA:roX2	2.04147257822512	0.408147672536479	0.998043617620652	pseudobulk	DESeq2	LRT	0.389182675723292
612	KC_G1	lncRNA:sphinx	1.12508065046033	0.900770125530981	0.998043617620652	pseudobulk	DESeq2	LRT	0.0453860258366365
612	KC_G1	Lnk	0.141618838715111	0.591426276413372	0.998043617620652	pseudobulk	DESeq2	LRT	0.228099384159202
612	KC_G1	Lnpk	-0.0421349974357446	0.906046037501115	0.998043617620652	pseudobulk	DESeq2	LRT	0.0428497346358484
612	KC_G1	lobo	-1.67593316621313	0.495877122847873	0.998043617620652	pseudobulk	DESeq2	LRT	0.304625927300966
612	KC_G1	loco	0.228570564635066	0.925908451678216	0.998043617620652	pseudobulk	DESeq2	LRT	0.0334319516511941
612	KC_G1	loh	2.02532906892505	0.22895961242719	0.998043617620652	pseudobulk	DESeq2	LRT	0.640241118738366
613	KC_G1	loj	0.0721618625107759	0.852761263406636	0.998043617620652	pseudobulk	DESeq2	LRT	0.0691725356519702
613	KC_G1	lok	-0.794610277746993	0.821479263487599	0.998043617620652	pseudobulk	DESeq2	LRT	0.085403394939296
613	KC_G1	lola	-0.149604405398998	0.122716966993294	0.998043617620652	pseudobulk	DESeq2	LRT	0.911095387051824
613	KC_G1	lolal	-0.0701780719243533	0.580004144780162	0.998043617620652	pseudobulk	DESeq2	LRT	0.236568902904785
613	KC_G1	Lon	-0.359101369810503	0.37445746506252	0.998043617620652	pseudobulk	DESeq2	LRT	0.426597507037305
613	KC_G1	loqs	-0.0314082933128741	0.834240828016322	0.998043617620652	pseudobulk	DESeq2	LRT	0.0787085594574627
613	KC_G1	lost	-0.109999134057978	0.624847097388085	0.998043617620652	pseudobulk	DESeq2	LRT	0.204226243271473
613	KC_G1	lovit	0.2586706452275	0.671422262875661	0.998043617620652	pseudobulk	DESeq2	LRT	0.173004262605799
613	KC_G1	Loxl1	-0.382484970133439	0.785868342928481	0.998043617620652	pseudobulk	DESeq2	LRT	0.104650205524112
613	KC_G1	LPCAT	-0.102095515326442	0.653505239100438	0.998043617620652	pseudobulk	DESeq2	LRT	0.184750926364554
614	KC_G1	LpR1	-0.731557740128382	0.713134171087021	0.998043617620652	pseudobulk	DESeq2	LRT	0.146828753064178
614	KC_G1	LpR2	0.00612428421134756	0.932886184033773	0.998043617620652	pseudobulk	DESeq2	LRT	0.0301713387414667
614	KC_G1	Lpt	-0.447722786489309	0.347797359276322	0.998043617620652	pseudobulk	DESeq2	LRT	0.458673719791833
614	KC_G1	lqf	-0.17277805687974	0.268868880747138	0.998043617620652	pseudobulk	DESeq2	LRT	0.570459460706733
614	KC_G1	lqfR	-0.0178665150406234	0.945101475825	0.998043617620652	pseudobulk	DESeq2	LRT	0.0245215586602251
614	KC_G1	Lrch	0.0797505584079016	0.734884097496417	0.998043617620652	pseudobulk	DESeq2	LRT	0.133781150421794
614	KC_G1	LRP1	-0.225221703005467	0.558153083792057	0.998043617620652	pseudobulk	DESeq2	LRT	0.253246671442046
614	KC_G1	Lrp4	-0.105183810258065	0.520576725513877	0.998043617620652	pseudobulk	DESeq2	LRT	0.283515252682504
614	KC_G1	Lrpprc2	-0.0479895771250189	0.94844179219988	0.998043617620652	pseudobulk	DESeq2	LRT	0.0229893174923473
614	KC_G1	LRR	-0.0415929660886627	0.867564644515036	0.998043617620652	pseudobulk	DESeq2	LRT	0.0616981549115238
615	KC_G1	Lrr47	-1.18126915954966	0.175481021323415	0.998043617620652	pseudobulk	DESeq2	LRT	0.755769846606774
615	KC_G1	Lrrk	0.285689578872176	0.53428468348901	0.998043617620652	pseudobulk	DESeq2	LRT	0.27222727569834
615	KC_G1	Lrt	1.5684833933741	0.521894780878679	0.998043617620652	pseudobulk	DESeq2	LRT	0.282417046212031
615	KC_G1	Lsd-2	-0.572823650606733	0.416951273189076	0.998043617620652	pseudobulk	DESeq2	LRT	0.379914695677532
615	KC_G1	LSm1	-0.233491939727401	0.581181225191948	0.998043617620652	pseudobulk	DESeq2	LRT	0.235688423842765
615	KC_G1	Lsm10	0.178176515508843	0.940951159325412	0.998043617620652	pseudobulk	DESeq2	LRT	0.0264329183218905
615	KC_G1	Lsm11	-0.612655222947186	0.431342602557772	0.998043617620652	pseudobulk	DESeq2	LRT	0.365177645628623
615	KC_G1	Lsm12a	-0.00538967114171997	0.965790208227347	0.998043617620652	pseudobulk	DESeq2	LRT	0.0151172020568034
615	KC_G1	LSm3	0.201436791329535	0.59925337078012	0.998043617620652	pseudobulk	DESeq2	LRT	0.222389514395537

615	KC_G1	LSm7	-0.385900479798968	0.30405550315234	0.998043617620652	pseudobulk	DESeq2	LRT	0.517047131810377
616	KC_G1	lsn	-0.35108465715326	0.330954639024203	0.998043617620652	pseudobulk	DESeq2	LRT	0.480231526984018
616	KC_G1	Lst8	0.199169329018349	0.58784259316209	0.998043617620652	pseudobulk	DESeq2	LRT	0.230738949557903
616	KC_G1	lt	-0.0188940596293257	0.941868249015128	0.998043617620652	pseudobulk	DESeq2	LRT	0.0260098432023252
616	KC_G1	Ltn1	-0.151822489680507	0.752088502012676	0.998043617620652	pseudobulk	DESeq2	LRT	0.123731050799077
616	KC_G1	LTV1	-0.020777151736259	0.93744112610878	0.998043617620652	pseudobulk	DESeq2	LRT	0.028055997636465
616	KC_G1	luna	-0.442696465478769	0.746473411292009	0.998043617620652	pseudobulk	DESeq2	LRT	0.126985656840244
616	KC_G1	lute	-0.137191911369065	0.441858649679658	0.998043617620652	pseudobulk	DESeq2	LRT	0.354716638981297
616	KC_G1	lva	0.12568452654269	0.695160590880957	0.998043617620652	pseudobulk	DESeq2	LRT	0.157914856306316
616	KC_G1	lwr	-0.041610263273526	0.759251700447697	0.998043617620652	pseudobulk	DESeq2	LRT	0.119614226746905
616	KC_G1	Lztr1	0.10123259721206	0.718958602556803	0.998043617620652	pseudobulk	DESeq2	LRT	0.14329611545681
617	KC_G1	m-cup	-0.0601330133178596	0.857370716012317	0.998043617620652	pseudobulk	DESeq2	LRT	0.066831354148747
617	KC_G1	M1BP	-0.0591640924265815	0.838951409960897	0.998043617620652	pseudobulk	DESeq2	LRT	0.0762631917302336
617	KC_G1	M6	-0.501598052888507	0.160381267448264	0.998043617620652	pseudobulk	DESeq2	LRT	0.794846358738531
617	KC_G1	mAChR-A	0.24898496218049	0.0712585159375025	0.998043617620652	pseudobulk	DESeq2	LRT	1.14716322670666
617	KC_G1	mAChR-B	0.0163751079593791	0.915224651910848	0.998043617620652	pseudobulk	DESeq2	LRT	0.0384722905127418
617	KC_G1	mAcon1	-0.16109103540402	0.359463741558527	0.998043617620652	pseudobulk	DESeq2	LRT	0.444344909551488
617	KC_G1	Mad	-0.0229091576477312	0.905029790105331	0.998043617620652	pseudobulk	DESeq2	LRT	0.0433371252528861
617	KC_G1	Mad1	-0.754871368071505	0.0832372023193671	0.998043617620652	pseudobulk	DESeq2	LRT	1.07968252526098
617	KC_G1	Madm	0.10043673468982	0.748741844695829	0.998043617620652	pseudobulk	DESeq2	LRT	0.125667894917007
617	KC_G1	mael	-0.270803293383352	0.876556106260612	0.998043617620652	pseudobulk	DESeq2	LRT	0.0572202805290658
618	KC_G1	maf-S	-0.0913083108194086	0.598572660842031	0.998043617620652	pseudobulk	DESeq2	LRT	0.222883122972925
618	KC_G1	Maf1	-0.111690795750226	0.437123091611282	0.998043617620652	pseudobulk	DESeq2	LRT	0.359396250718155
618	KC_G1	MAGE	0.866966299923626	0.691235567110852	0.998043617620652	pseudobulk	DESeq2	LRT	0.160373923592567
618	KC_G1	Magi	-0.901785468644521	0.00758618718110317	0.998043617620652	pseudobulk	DESeq2	LRT	2.11997644574642
618	KC_G1	mago	0.169492675484683	0.443125457659758	0.998043617620652	pseudobulk	DESeq2	LRT	0.353473298933496
618	KC_G1	MagR	0.173620656454771	0.347189270869898	0.998043617620652	pseudobulk	DESeq2	LRT	0.459433704215579
618	KC_G1	magu	1.24386239920316	0.627869490249816	0.998043617620652	pseudobulk	DESeq2	LRT	0.202130619884913
618	KC_G1	mahe	-0.219802887831428	0.0915815152060544	0.998043617620652	pseudobulk	DESeq2	LRT	1.03819217539464
618	KC_G1	mahj	0.463738831014736	0.459115900648085	0.998043617620652	pseudobulk	DESeq2	LRT	0.338077665970333
618	KC_G1	mal	1.52851469353885	0.0965345638733963	0.998043617620652	pseudobulk	DESeq2	LRT	1.01531716114463
619	KC_G1	Mal-A5	-1.22856335835256	0.349166601129869	0.998043617620652	pseudobulk	DESeq2	LRT	0.45696730459619
619	KC_G1	mam	0.330592497213447	0.0414758315526706	0.998043617620652	pseudobulk	DESeq2	LRT	1.38220489800725
619	KC_G1	mamo	-0.243723318493473	0.00635843169880088	0.998043617620652	pseudobulk	DESeq2	LRT	2.196649989469
619	KC_G1	MAN1	0.158705988372679	0.715555454084871	0.998043617620652	pseudobulk	DESeq2	LRT	0.145356703665453
619	KC_G1	Manf	0.095455152224063	0.709060139282954	0.998043617620652	pseudobulk	DESeq2	LRT	0.149316928355595
619	KC_G1	Map205	-0.215133770990369	0.362631302741576	0.998043617620652	pseudobulk	DESeq2	LRT	0.440534709834643
619	KC_G1	Map60	0.02416779960835	0.913749194734627	0.998043617620652	pseudobulk	DESeq2	LRT	0.0391729927672836
619	KC_G1	MAPk-Ak2	-0.00420454201261015	0.968811834433046	0.998043617620652	pseudobulk	DESeq2	LRT	0.0137605647477694
619	KC_G1	Mapmodulin	-0.0300754076006397	0.806793057883428	0.998043617620652	pseudobulk	DESeq2	LRT	0.0932378473654336
619	KC_G1	Marc	-0.650158397859419	0.654228825636088	0.998043617620652	pseudobulk	DESeq2	LRT	0.184270324566137
620	KC_G1	Marcal1	1.03405156229777	0.279231238884375	0.998043617620652	pseudobulk	DESeq2	LRT	0.554035996807537
620	KC_G1	Marf	-0.00215715918643668	0.973601149162855	0.998043617620652	pseudobulk	DESeq2	LRT	0.0116189221703575
620	KC_G1	Marf1	0.173116475207952	0.97305562682953	0.998043617620652	pseudobulk	DESeq2	LRT	0.011862331638572
620	KC_G1	mars	-0.300631190805425	0.880118789916364	0.998043617620652	pseudobulk	DESeq2	LRT	0.0554587070276954
620	KC_G1	mask	-0.213129508675809	0.0960161218832478	0.998043617620652	pseudobulk	DESeq2	LRT	1.01765583928248
620	KC_G1	Mat1	-0.419769638826331	0.322769250004243	0.998043617620652	pseudobulk	DESeq2	LRT	0.491107846904103
620	KC_G1	Mat89Ba	-0.454580147695657	0.481346666731242	0.998043617620652	pseudobulk	DESeq2	LRT	0.317542031276343
620	KC_G1	mats	-0.159972037123313	0.603965753740417	0.998043617620652	pseudobulk	DESeq2	LRT	0.218987686185525
620	KC_G1	Mau2	-0.127044901576324	0.834958687523164	0.998043617620652	pseudobulk	DESeq2	LRT	0.0783350122105083
620	KC_G1	mav	0.0729807627318175	0.919867230457595	0.998043617620652	pseudobulk	DESeq2	LRT	0.0362748522639046
621	KC_G1	Max	0.172786941088846	0.507793794879609	0.998043617620652	pseudobulk	DESeq2	LRT	0.294312610405996
621	KC_G1	mbc	0.19294017102612	0.74292620468815	0.998043617620652	pseudobulk	DESeq2	LRT	0.129054322832147
621	KC_G1	MBD-like	-0.097723421837902	0.68969723472706	0.998043617620652	pseudobulk	DESeq2	LRT	0.161341515268397
621	KC_G1	MBD-R2	0.261137444094295	0.486777105110954	0.998043617620652	pseudobulk	DESeq2	LRT	0.312669856402949
621	KC_G1	mbf1	-0.0434141387113143	0.781569189137954	0.998043617620652	pseudobulk	DESeq2	LRT	0.107032569620805

621	KC_G1	mbl	-1.22800134405817	0.107969607744967	0.998043617620652	pseudobulk	DESeq2	LRT	0.966698476422239
621	KC_G1	mbm	0.090788642828008	0.905322191213511	0.998043617620652	pseudobulk	DESeq2	LRT	0.0431968340938192
621	KC_G1	mbo	-0.150073642780638	0.840604309474764	0.998043617620652	pseudobulk	DESeq2	LRT	0.0754083878538834
621	KC_G1	Mbs	-0.118637155302519	0.474514430199242	0.998043617620652	pseudobulk	DESeq2	LRT	0.323750575943512
621	KC_G1	mbt	0.135565738713119	0.594367801967598	0.998043617620652	pseudobulk	DESeq2	LRT	0.225944725166977
622	KC_G1	Mccc2	-1.31047986807593	0.611671045333228	0.998043617620652	pseudobulk	DESeq2	LRT	0.213482077209615
622	KC_G1	Mcm3	1.12508065046033	0.900770125530981	0.998043617620652	pseudobulk	DESeq2	LRT	0.0453860258366365
622	KC_G1	Mcm5	1.12508065046033	0.900770125530981	0.998043617620652	pseudobulk	DESeq2	LRT	0.0453860258366365
622	KC_G1	Mco1	1.13582588427274	0.704684208645453	0.998043617620652	pseudobulk	DESeq2	LRT	0.152005460551806
622	KC_G1	MCPH1	0.825496035528663	0.699446040114278	0.998043617620652	pseudobulk	DESeq2	LRT	0.155245784223669
622	KC_G1	Mcr	-0.125134513703598	0.777054760330479	0.998043617620652	pseudobulk	DESeq2	LRT	0.109548374672249
622	KC_G1	Mct1	2.07738706174348	0.431998797123676	0.998043617620652	pseudobulk	DESeq2	LRT	0.364517462451933
622	KC_G1	Mctp	-0.211932710684631	0.525348883982679	0.998043617620652	pseudobulk	DESeq2	LRT	0.27955218599269
622	KC_G1	MCTS1	0.0307820338781412	0.892791477173386	0.998043617620652	pseudobulk	DESeq2	LRT	0.0492499642781764
622	KC_G1	MCU	-0.186725087912491	0.680930604060547	0.998043617620652	pseudobulk	DESeq2	LRT	0.1668971462506
623	KC_G1	Mdh1	-0.0596941116608117	0.765341968851679	0.998043617620652	pseudobulk	DESeq2	LRT	0.116144470727863
623	KC_G1	Mdh2	0.0165595533987153	0.898902245375104	0.998043617620652	pseudobulk	DESeq2	LRT	0.0462875347434318
623	KC_G1	mdlc	-0.507589010038046	0.500259015545987	0.998043617620652	pseudobulk	DESeq2	LRT	0.300805075871977
623	KC_G1	Mdr49	0.411058658572132	0.888736347846225	0.998043617620652	pseudobulk	DESeq2	LRT	0.051227057542728
623	KC_G1	Mdr65	-1.22556023088413	0.51681529142114	0.998043617620652	pseudobulk	DESeq2	LRT	0.286664645009925
623	KC_G1	mdy	-0.0521110517591673	0.794928575795606	0.998043617620652	pseudobulk	DESeq2	LRT	0.099671890879798
623	KC_G1	me31B	0.121891968072476	0.564364128987147	0.998043617620652	pseudobulk	DESeq2	LRT	0.248440597819921
623	KC_G1	Med	-0.0268518853084551	0.911393976997944	0.998043617620652	pseudobulk	DESeq2	LRT	0.0402938458053593
623	KC_G1	MED10	0.0617481752551491	0.81155866101844	0.998043617620652	pseudobulk	DESeq2	LRT	0.0906800830640507
623	KC_G1	MED11	-0.148211269724533	0.635180002393082	0.998043617620652	pseudobulk	DESeq2	LRT	0.197103183419512
624	KC_G1	MED14	-0.274288737363711	0.442509253462881	0.998043617620652	pseudobulk	DESeq2	LRT	0.354077643190226
624	KC_G1	MED15	0.180524108100332	0.72479660308874	0.998043617620652	pseudobulk	DESeq2	LRT	0.139783850738629
624	KC_G1	MED16	0.968837464421114	0.256068219950258	0.998043617620652	pseudobulk	DESeq2	LRT	0.591644317496676
624	KC_G1	MED17	0.0972118057497021	0.845198045192939	0.998043617620652	pseudobulk	DESeq2	LRT	0.0730415160718083
624	KC_G1	MED18	-0.209392832676049	0.560741393313861	0.998043617620652	pseudobulk	DESeq2	LRT	0.251237383576441
624	KC_G1	MED19	-0.294735134988287	0.19449937785144	0.998043617620652	pseudobulk	DESeq2	LRT	0.711081783521402
624	KC_G1	MED20	0.0658482457583519	0.910302100992721	0.998043617620652	pseudobulk	DESeq2	LRT	0.040814454908606
624	KC_G1	MED21	-0.123677804300705	0.451454388441541	0.998043617620652	pseudobulk	DESeq2	LRT	0.34538612098455
624	KC_G1	MED22	-0.115121015841567	0.651917098514914	0.998043617620652	pseudobulk	DESeq2	LRT	0.185807628112608
624	KC_G1	MED23	3.13177453673002	0.0848837394184004	0.998043617620652	pseudobulk	DESeq2	LRT	1.07117549653126
625	KC_G1	MED24	-0.172901223888877	0.650362572333253	0.998043617620652	pseudobulk	DESeq2	LRT	0.186844459875316
625	KC_G1	MED25	-0.245606728367567	0.667676949948603	0.998043617620652	pseudobulk	DESeq2	LRT	0.175433616552084
625	KC_G1	MED26	-0.12209139968519	0.336710614779748	0.998043617620652	pseudobulk	DESeq2	LRT	0.47274319229143
625	KC_G1	MED27	-0.0521903928202243	0.906601371477066	0.998043617620652	pseudobulk	DESeq2	LRT	0.0425836282902681
625	KC_G1	MED28	-0.0849212541803242	0.78654316107955	0.998043617620652	pseudobulk	DESeq2	LRT	0.104277440740158
625	KC_G1	MED30	0.659740779652058	0.254255103027521	0.998043617620652	pseudobulk	DESeq2	LRT	0.594730321809532
625	KC_G1	MED31	0.0345210197700769	0.891115636935359	0.998043617620652	pseudobulk	DESeq2	LRT	0.0500659354420006
625	KC_G1	MED4	-0.362974148057199	0.335384156918671	0.998043617620652	pseudobulk	DESeq2	LRT	0.474457456712965
625	KC_G1	MED6	0.23080675666001	0.487403025722222	0.998043617620652	pseudobulk	DESeq2	LRT	0.312111779110289
625	KC_G1	MED7	0.312941245962785	0.291011327169886	0.998043617620652	pseudobulk	DESeq2	LRT	0.536090106438019
626	KC_G1	MED8	0.194778208620264	0.575106037557191	0.998043617620652	pseudobulk	DESeq2	LRT	0.24025207308388
626	KC_G1	Mef2	-0.086380485332297	0.410098103775349	0.998043617620652	pseudobulk	DESeq2	LRT	0.387112238812506
626	KC_G1	mEFG1	0.804617097541669	0.126745913877465	0.998043617620652	pseudobulk	DESeq2	LRT	0.897066032855172
626	KC_G1	mEFTs	0.311392273419282	0.213320626636495	0.998043617620652	pseudobulk	DESeq2	LRT	0.670967149232682
626	KC_G1	mEFTu1	0.189412960302751	0.422581938504515	0.998043617620652	pseudobulk	DESeq2	LRT	0.374089068986145
626	KC_G1	mEFTu2	-0.040002544196597	0.938459041525671	0.998043617620652	pseudobulk	DESeq2	LRT	0.02758467714765
626	KC_G1	Megf8	0.111387188451765	0.764252855458914	0.998043617620652	pseudobulk	DESeq2	LRT	0.116762929955183
626	KC_G1	mei-41	-1.07453185815843	0.306373014471006	0.998043617620652	pseudobulk	DESeq2	LRT	0.513749490287658
626	KC_G1	mei-9	-0.405482832811601	0.62910060246094	0.998043617620652	pseudobulk	DESeq2	LRT	0.201279898910787
626	KC_G1	mei-P26	-0.0109774889466871	0.903595282295679	0.998043617620652	pseudobulk	DESeq2	LRT	0.0440260452132754
627	KC_G1	mei-S332	-0.44345127483287	0.646013786342359	0.998043617620652	pseudobulk	DESeq2	LRT	0.189758213787385

627	KC_G1	mei-W68	-0.898063730818328	0.430310413673861	0.998043617620652	pseudobulk	DESeq2	LRT	0.366218143700458
627	KC_G1	Meics	0.213650978253795	0.656477912594852	0.998043617620652	pseudobulk	DESeq2	LRT	0.182779881302672
627	KC_G1	meigo	0.0370064985989199	0.947001245282766	0.998043617620652	pseudobulk	DESeq2	LRT	0.0236494499100547
627	KC_G1	Mekk1	0.19928658721828	0.671900164663454	0.998043617620652	pseudobulk	DESeq2	LRT	0.172695252477134
627	KC_G1	melt	2.38466886841527	0.34619303318143	0.998043617620652	pseudobulk	DESeq2	LRT	0.460681676152437
627	KC_G1	Meltrin	0.618330509715188	0.426782111095925	0.998043617620652	pseudobulk	DESeq2	LRT	0.369793792657004
627	KC_G1	Membrin	0.0492975315693394	0.913736233301546	0.998043617620652	pseudobulk	DESeq2	LRT	0.0391791532310316
627	KC_G1	Men	-0.165948125321084	0.444424818715186	0.998043617620652	pseudobulk	DESeq2	LRT	0.352201696063115
627	KC_G1	Men-b	-0.0902696368901823	0.583019178018936	0.998043617620652	pseudobulk	DESeq2	LRT	0.234317159184709
628	KC_G1	meng	0.148666533398974	0.360216764510872	0.998043617620652	pseudobulk	DESeq2	LRT	0.443436078953638
628	KC_G1	MEP-1	-0.565212742567452	0.37933106918285	0.998043617620652	pseudobulk	DESeq2	LRT	0.420981584868858
628	KC_G1	Mer	-0.119904278099225	0.82590943695005	0.998043617620652	pseudobulk	DESeq2	LRT	0.0830675715526836
628	KC_G1	Mes2	-0.537464128795103	0.643757074636488	0.998043617620652	pseudobulk	DESeq2	LRT	0.191277985199365
628	KC_G1	Mes4	-0.393296165521088	0.236397261066516	0.998043617620652	pseudobulk	DESeq2	LRT	0.626357559561464
628	KC_G1	Mesh1	0.0944619859688371	0.942964501597669	0.998043617620652	pseudobulk	DESeq2	LRT	0.0255046562026834
628	KC_G1	MESK2	0.0455187634644245	0.7125746773733	0.998043617620652	pseudobulk	DESeq2	LRT	0.147169615148697
628	KC_G1	MESR3	-0.193365988364178	0.459157551592523	0.998043617620652	pseudobulk	DESeq2	LRT	0.338038268609332
628	KC_G1	MESR4	-0.0558392259220656	0.794367077015467	0.998043617620652	pseudobulk	DESeq2	LRT	0.0999787637156037
628	KC_G1	MESR6	-0.730127782236543	0.212945284743917	0.998043617620652	pseudobulk	DESeq2	LRT	0.671731972084102
629	KC_G1	Met	-0.145759115103354	0.468346905128444	0.998043617620652	pseudobulk	DESeq2	LRT	0.329432345275023
629	KC_G1	metl	0.165199666278277	0.634196756823294	0.998043617620652	pseudobulk	DESeq2	LRT	0.197775983212981
629	KC_G1	metro	0.0787118223447954	0.646801774486207	0.998043617620652	pseudobulk	DESeq2	LRT	0.189228797286801
629	KC_G1	MetRS	-0.163453250163982	0.705823072619502	0.998043617620652	pseudobulk	DESeq2	LRT	0.151304149110713
629	KC_G1	MetRS-m	-0.404608596899273	0.626574183908111	0.998043617620652	pseudobulk	DESeq2	LRT	0.203027502874713
629	KC_G1	Mettl14	0.317595253076299	0.599100075680994	0.998043617620652	pseudobulk	DESeq2	LRT	0.222500625547615
629	KC_G1	Mettl3	0.31878946173363	0.548041904968844	0.998043617620652	pseudobulk	DESeq2	LRT	0.261186232754798
629	KC_G1	mew	0.402154288384183	0.696839475823573	0.998043617620652	pseudobulk	DESeq2	LRT	0.156867254603822
629	KC_G1	Mf	1.90250740646012	0.460774913678782	0.998043617620652	pseudobulk	DESeq2	LRT	0.336511173568024
629	KC_G1	mfas	0.0759629547021026	0.868450766985195	0.998043617620652	pseudobulk	DESeq2	LRT	0.0612547969436699
630	KC_G1	Mfe2	0.516366859765519	0.31716089179355	0.998043617620652	pseudobulk	DESeq2	LRT	0.498720369668965
630	KC_G1	mfrn	0.28461814970711	0.373322573379842	0.998043617620652	pseudobulk	DESeq2	LRT	0.427915749163148
630	KC_G1	MFS10	-0.34594900542584	0.576483704060681	0.998043617620652	pseudobulk	DESeq2	LRT	0.239212964755322
630	KC_G1	MFS14	1.7689881492081	0.456439984610994	0.998043617620652	pseudobulk	DESeq2	LRT	0.340616317945116
630	KC_G1	MFS15	0.0555054089773146	0.935044694528297	0.998043617620652	pseudobulk	DESeq2	LRT	0.0291676296375396
630	KC_G1	MFS16	-0.357307054148907	0.538968365565339	0.998043617620652	pseudobulk	DESeq2	LRT	0.268436724727167
630	KC_G1	MFS17	-0.0217070576187356	0.876637283669001	0.998043617620652	pseudobulk	DESeq2	LRT	0.0571800626035974
630	KC_G1	MFS18	0.0695741661467676	0.84743451379679	0.998043617620652	pseudobulk	DESeq2	LRT	0.0718938522936592
630	KC_G1	MFS9	-0.161277558929997	0.813072988113735	0.998043617620652	pseudobulk	DESeq2	LRT	0.0898704668139939
630	KC_G1	Mgat1	-0.147398587811452	0.687048819559156	0.998043617620652	pseudobulk	DESeq2	LRT	0.163012402224502
631	KC_G1	Mgat2	-0.0129249615282436	0.941308403007328	0.998043617620652	pseudobulk	DESeq2	LRT	0.0262680643721818
631	KC_G1	mge	-0.114239343274242	0.450839442258338	0.998043617620652	pseudobulk	DESeq2	LRT	0.345978096162249
631	KC_G1	mgl	-0.160928647787196	0.177810656503011	0.998043617620652	pseudobulk	DESeq2	LRT	0.750042214559465
631	KC_G1	mGluR	-0.123223648356818	0.298190523693281	0.998043617620652	pseudobulk	DESeq2	LRT	0.52550616226837
631	KC_G1	mgr	-0.0137169400257755	0.951589806501131	0.998043617620652	pseudobulk	DESeq2	LRT	0.0215502188037722
631	KC_G1	Mgstl	-0.0879729072953507	0.83030115171121	0.998043617620652	pseudobulk	DESeq2	LRT	0.0807643596662034
631	KC_G1	mh	-0.0221352112334005	0.963417071617604	0.998043617620652	pseudobulk	DESeq2	LRT	0.0161856623150934
631	KC_G1	Mhcl	-0.0543198627338227	0.815866037548114	0.998043617620652	pseudobulk	DESeq2	LRT	0.0883811450816598
631	KC_G1	mi	0.0473405360263665	0.889409261292366	0.998043617620652	pseudobulk	DESeq2	LRT	0.0508983526167572
631	KC_G1	Mi-2	-0.244548722020495	0.0896200581414195	0.998043617620652	pseudobulk	DESeq2	LRT	1.04759477867237
632	KC_G1	mib1	-0.377083066289663	0.285787102255547	0.998043617620652	pseudobulk	DESeq2	LRT	0.543957375074933
632	KC_G1	mib2	0.124481978168727	0.596628727167837	0.998043617620652	pseudobulk	DESeq2	LRT	0.224295839559085
632	KC_G1	Mical	-0.272536432768636	0.0817194580332314	0.998043617620652	pseudobulk	DESeq2	LRT	1.0876745222887
632	KC_G1	MICAL-like	0.155976192989332	0.711688915117569	0.998043617620652	pseudobulk	DESeq2	LRT	0.14770979845685
632	KC_G1	Mics1	-1.18758623933229	0.520801314224088	0.998043617620652	pseudobulk	DESeq2	LRT	0.283327928511391
632	KC_G1	MICU1	0.199706723140711	0.524925785728182	0.998043617620652	pseudobulk	DESeq2	LRT	0.279902093026479
632	KC_G1	MICU3	-0.147168517283123	0.552368299082428	0.998043617620652	pseudobulk	DESeq2	LRT	0.257771253932347

632	KC_G1	mid	1.13582588427274	0.633424328955532	0.998043617620652	pseudobulk	DESeq2	LRT	0.198305259991224
632	KC_G1	Mid1	-0.0464193833635366	0.683340258121808	0.998043617620652	pseudobulk	DESeq2	LRT	0.165362992616808
632	KC_G1	mIF2	-0.310565728721388	0.759737779872807	0.998043617620652	pseudobulk	DESeq2	LRT	0.119336276672817
633	KC_G1	mIF3	0.2203045185427	0.679660049311174	0.998043617620652	pseudobulk	DESeq2	LRT	0.167708257330388
633	KC_G1	Miga	-0.445122874721781	0.172155154093467	0.998043617620652	pseudobulk	DESeq2	LRT	0.76407997057276
633	KC_G1	milt	-0.0438141699124871	0.792367326389369	0.998043617620652	pseudobulk	DESeq2	LRT	0.101073441085739
633	KC_G1	mim	-0.263072468390182	0.115760764108792	0.998043617620652	pseudobulk	DESeq2	LRT	0.936438615206775
633	KC_G1	mino	-0.172649276627931	0.915034031068773	0.998043617620652	pseudobulk	DESeq2	LRT	0.038562753769021
633	KC_G1	mio	-0.602459626878086	0.399607946176257	0.998043617620652	pseudobulk	DESeq2	LRT	0.398365884444581
633	KC_G1	mip120	-0.034486656251889	0.941624585236885	0.998043617620652	pseudobulk	DESeq2	LRT	0.0261222108556515
633	KC_G1	mip130	0.397643695994148	0.441587427123977	0.998043617620652	pseudobulk	DESeq2	LRT	0.354983300334136
633	KC_G1	mip40	0.580238552570738	0.165761543999088	0.998043617620652	pseudobulk	DESeq2	LRT	0.78051621664552
633	KC_G1	miple1	0.0446319160584443	0.663410953432808	0.998043617620652	pseudobulk	DESeq2	LRT	0.178217362216363
634	KC_G1	miple2	-0.0100571098870573	0.960111073765	0.998043617620652	pseudobulk	DESeq2	LRT	0.0176785211971143
634	KC_G1	Mipp1	-2.28172252880459	0.324719824317488	0.998043617620652	pseudobulk	DESeq2	LRT	0.488491196656175
634	KC_G1	Mipp2	1.69583482309045	0.375639127722993	0.998043617620652	pseudobulk	DESeq2	LRT	0.425229176605247
634	KC_G1	mira	1.24386239920316	0.627869490249816	0.998043617620652	pseudobulk	DESeq2	LRT	0.202130619884913
634	KC_G1	Miro	-0.186605041280829	0.545737644141901	0.998043617620652	pseudobulk	DESeq2	LRT	0.263016088219349
634	KC_G1	mirr	-1.66808346769027	0.498191247612582	0.998043617620652	pseudobulk	DESeq2	LRT	0.302603906560549
634	KC_G1	Mis12	-0.173762041095858	0.775488679790754	0.998043617620652	pseudobulk	DESeq2	LRT	0.110424537425221
634	KC_G1	Mitf	-0.638888336562564	0.142865065751243	0.998043617620652	pseudobulk	DESeq2	LRT	0.845073954597825
634	KC_G1	Mitofilin	-0.0555610794479453	0.831112547029515	0.998043617620652	pseudobulk	DESeq2	LRT	0.0803401612408923
634	KC_G1	mkg-p	1.13582588427274	0.704684208645453	0.998043617620652	pseudobulk	DESeq2	LRT	0.152005460551806
635	KC_G1	Mkk4	-0.122001722130902	0.453293333740529	0.998043617620652	pseudobulk	DESeq2	LRT	0.343620667751352
635	KC_G1	MKP-4	0.796045150802942	0.0687100751737504	0.998043617620652	pseudobulk	DESeq2	LRT	1.16297957630952
635	KC_G1	Mkrn1	-0.0433806721375082	0.852576726850512	0.998043617620652	pseudobulk	DESeq2	LRT	0.069266526648247
635	KC_G1	Mlc-c	0.188270550638865	0.499093665727556	0.998043617620652	pseudobulk	DESeq2	LRT	0.301817941969233
635	KC_G1	mld	-0.277137064225152	0.0489272292581404	0.998043617620652	pseudobulk	DESeq2	LRT	1.3104493775769
635	KC_G1	mldr	1.55727421337762	0.295297451594503	0.998043617620652	pseudobulk	DESeq2	LRT	0.529740300963201
635	KC_G1	mle	0.0502123278372901	0.800975863446121	0.998043617620652	pseudobulk	DESeq2	LRT	0.0963805707198792
635	KC_G1	Mlf	0.00345432881340073	0.974728531018847	0.998043617620652	pseudobulk	DESeq2	LRT	0.0111163216313619
635	KC_G1	Mlh1	-0.752595479487864	0.785589861204421	0.998043617620652	pseudobulk	DESeq2	LRT	0.104804130174502
635	KC_G1	Mlp84B	-0.401380895651674	0.516334763492702	0.998043617620652	pseudobulk	DESeq2	LRT	0.287068634037032
636	KC_G1	mlt	-0.188396861775761	0.39291109847013	0.998043617620652	pseudobulk	DESeq2	LRT	0.405705703597816
636	KC_G1	mmd	-0.131973077877461	0.192817197702742	0.998043617620652	pseudobulk	DESeq2	LRT	0.714854233222027
636	KC_G1	MME1	0.358132151406752	0.53390348696123	0.998043617620652	pseudobulk	DESeq2	LRT	0.272537242725017
636	KC_G1	Mmp1	-2.64287806331783	0.244986690217598	0.998043617620652	pseudobulk	DESeq2	LRT	0.610857509603096
636	KC_G1	Mmp2	1.57374684956626	0.0569985183614433	0.998043617620652	pseudobulk	DESeq2	LRT	1.24413643337685
636	KC_G1	Mms19	0.456301790812996	0.549474482432395	0.998043617620652	pseudobulk	DESeq2	LRT	0.260052471386306
636	KC_G1	mmy	-0.0999603439020818	0.739057259921239	0.998043617620652	pseudobulk	DESeq2	LRT	0.131321912478696
636	KC_G1	mnd	0.151534803522502	0.823792070437262	0.998043617620652	pseudobulk	DESeq2	LRT	0.0841823927412365
636	KC_G1	Mnn1	0.0861614519247383	0.536316930448495	0.998043617620652	pseudobulk	DESeq2	LRT	0.270578493015862
636	KC_G1	Mnt	-0.0542630764835528	0.69449006818794	0.998043617620652	pseudobulk	DESeq2	LRT	0.158333960670808
637	KC_G1	Mo25	0.098832931368775	0.624796954193635	0.998043617620652	pseudobulk	DESeq2	LRT	0.204261096256431
637	KC_G1	Mob2	-0.116770607635016	0.416422449257707	0.998043617620652	pseudobulk	DESeq2	LRT	0.380465865794688
637	KC_G1	Mob3	0.0717292379215338	0.813104703767362	0.998043617620652	pseudobulk	DESeq2	LRT	0.0898535265577962
637	KC_G1	Mob4	0.0647444965225729	0.690068491111243	0.998043617620652	pseudobulk	DESeq2	LRT	0.161107802254813
637	KC_G1	Moca-cyp	-0.180717133571037	0.37768416270551	0.998043617620652	pseudobulk	DESeq2	LRT	0.42287122590536
637	KC_G1	Mocs1	-1.35105769263999	0.533988334747655	0.998043617620652	pseudobulk	DESeq2	LRT	0.272468230256199
637	KC_G1	mod	0.0224304757207507	0.918493778285765	0.998043617620652	pseudobulk	DESeq2	LRT	0.036923781182138
637	KC_G1	modSP	1.13582588427274	0.633424328955532	0.998043617620652	pseudobulk	DESeq2	LRT	0.198305259991224
637	KC_G1	Moe	-0.0888243291701238	0.59814240359275	0.998043617620652	pseudobulk	DESeq2	LRT	0.223195408432958
637	KC_G1	mof	-0.593176642232675	0.273394477759828	0.998043617620652	pseudobulk	DESeq2	LRT	0.563210261906658
638	KC_G1	mol	0.343588087965571	0.874039307776167	0.998043617620652	pseudobulk	DESeq2	LRT	0.0584690355962459
638	KC_G1	Mon1	0.717809182304191	0.353210899227376	0.998043617620652	pseudobulk	DESeq2	LRT	0.451965903618609
638	KC_G1	mon2	-0.236634186306485	0.483534924071602	0.998043617620652	pseudobulk	DESeq2	LRT	0.31557215284504

638	KC_G1	Mondo	0.0562215039992741	0.864555627447295	0.998043617620652	pseudobulk	DESeq2	LRT	0.0632070580104475
638	KC_G1	moody	0.612162064178887	0.436547688352149	0.998043617620652	pseudobulk	DESeq2	LRT	0.359968307154453
638	KC_G1	mop	-0.0611255464617168	0.844064175701688	0.998043617620652	pseudobulk	DESeq2	LRT	0.0736245319364992
638	KC_G1	mor	-0.102048790366932	0.67367828262322	0.998043617620652	pseudobulk	DESeq2	LRT	0.17154745277931
638	KC_G1	morgue	0.343155520319054	0.290182048471297	0.998043617620652	pseudobulk	DESeq2	LRT	0.537329457821791
638	KC_G1	Mos	1.11121460044925	0.691240116375203	0.998043617620652	pseudobulk	DESeq2	LRT	0.16037106535726
638	KC_G1	Mp	-0.156849113288231	0.2577567553145	0.998043617620652	pseudobulk	DESeq2	LRT	0.588789943862619
639	KC_G1	MP1	-1.30561205223248	0.636331582167641	0.998043617620652	pseudobulk	DESeq2	LRT	0.196316521489919
639	KC_G1	Mpc1	-0.15051003039526	0.606080435203453	0.998043617620652	pseudobulk	DESeq2	LRT	0.217469735162275
639	KC_G1	Mpcp1	1.12508065046033	0.900770125530981	0.998043617620652	pseudobulk	DESeq2	LRT	0.0453860258366365
639	KC_G1	Mpcp2	-0.00439888632187991	0.966600585604188	0.998043617620652	pseudobulk	DESeq2	LRT	0.014752946084589
639	KC_G1	Mpi	-0.0538291296008852	0.899088497359284	0.998043617620652	pseudobulk	DESeq2	LRT	0.0461975585190389
639	KC_G1	Mpp6	0.246195002637658	0.619391124142144	0.998043617620652	pseudobulk	DESeq2	LRT	0.208035022362681
639	KC_G1	Mppe	-1.05008433927172	0.101417549024394	0.998043617620652	pseudobulk	DESeq2	LRT	0.993886889331855
639	KC_G1	Mps1	0.577804435163831	0.707989301574907	0.998043617620652	pseudobulk	DESeq2	LRT	0.149973304883801
639	KC_G1	mr	-0.0241394848453206	0.961162532205599	0.998043617620652	pseudobulk	DESeq2	LRT	0.0172031670956761
639	KC_G1	mre11	-0.260703533213292	0.839883853785095	0.998043617620652	pseudobulk	DESeq2	LRT	0.0757807676855175
640	KC_G1	mRF1	0.362339324282995	0.676532510414999	0.998043617620652	pseudobulk	DESeq2	LRT	0.169711328771325
640	KC_G1	MRG15	0.0139082755247176	0.91758209520054	0.998043617620652	pseudobulk	DESeq2	LRT	0.0373550694218181
640	KC_G1	MrgBP	0.329604007839235	0.154314466568385	0.998043617620652	pseudobulk	DESeq2	LRT	0.811593358082561
640	KC_G1	mri	0.0411617160739671	0.889763865489966	0.998043617620652	pseudobulk	DESeq2	LRT	0.0507252355142127
640	KC_G1	mrj	0.097423281737029	0.581404309361338	0.998043617620652	pseudobulk	DESeq2	LRT	0.235521753568492
640	KC_G1	mrn	0.0516403291008404	0.939603988403125	0.998043617620652	pseudobulk	DESeq2	LRT	0.0270551484101912
640	KC_G1	mRNA-cap	0.133983976403074	0.64739923130352	0.998043617620652	pseudobulk	DESeq2	LRT	0.188827820597391
640	KC_G1	MRP	0.163752116455333	0.596853572513047	0.998043617620652	pseudobulk	DESeq2	LRT	0.224132202287066
640	KC_G1	Mrp4	0.175579629065889	0.920099974205621	0.998043617620652	pseudobulk	DESeq2	LRT	0.0361649814730035
640	KC_G1	mRpL1	-0.0256734177012376	0.919509125767636	0.998043617620652	pseudobulk	DESeq2	LRT	0.0364439562020208
641	KC_G1	mRpL10	0.064415571748939	0.840202485909057	0.998043617620652	pseudobulk	DESeq2	LRT	0.0756160378480282
641	KC_G1	mRpL11	0.202487193925453	0.558485036230792	0.998043617620652	pseudobulk	DESeq2	LRT	0.252988458661991
641	KC_G1	mRpL12	0.00197815997411689	0.967577076889358	0.998043617620652	pseudobulk	DESeq2	LRT	0.014314429167523
641	KC_G1	mRpL13	-0.125922269296941	0.624052175355035	0.998043617620652	pseudobulk	DESeq2	LRT	0.204779098584391
641	KC_G1	mRpL14	0.135557799639913	0.639145236032873	0.998043617620652	pseudobulk	DESeq2	LRT	0.194400443812388
641	KC_G1	mRpL15	-0.317960911068822	0.379571772956956	0.998043617620652	pseudobulk	DESeq2	LRT	0.420706091568351
641	KC_G1	mRpL16	0.224652342831193	0.442813205428869	0.998043617620652	pseudobulk	DESeq2	LRT	0.353779436213879
641	KC_G1	mRpL17	-0.0819461683224553	0.755021993425906	0.998043617620652	pseudobulk	DESeq2	LRT	0.122040397398109
641	KC_G1	mRpL18	0.0598189224681287	0.823983962764687	0.998043617620652	pseudobulk	DESeq2	LRT	0.0840812409128115
641	KC_G1	mRpL19	0.0180781113870401	0.942023821107051	0.998043617620652	pseudobulk	DESeq2	LRT	0.0259381149942338
642	KC_G1	mRpL2	0.232119354905295	0.412674179886259	0.998043617620652	pseudobulk	DESeq2	LRT	0.384392703120925
642	KC_G1	mRpL20	0.0559982692690233	0.843915720270919	0.998043617620652	pseudobulk	DESeq2	LRT	0.0737009231005311
642	KC_G1	mRpL21	-0.319417731067618	0.274878391068451	0.998043617620652	pseudobulk	DESeq2	LRT	0.560859399874966
642	KC_G1	mRpL22	-0.170175534260638	0.422135576540175	0.998043617620652	pseudobulk	DESeq2	LRT	0.374548045040589
642	KC_G1	mRpL23	-0.0175462131563403	0.932013770177195	0.998043617620652	pseudobulk	DESeq2	LRT	0.0305776710496756
642	KC_G1	mRpL24	-0.020420532101863	0.923705264329154	0.998043617620652	pseudobulk	DESeq2	LRT	0.0344665812633715
642	KC_G1	mRpL27	0.0110153636956581	0.941465816010332	0.998043617620652	pseudobulk	DESeq2	LRT	0.0261954443011686
642	KC_G1	mRpL28	0.0532724936665269	0.812177687724169	0.998043617620652	pseudobulk	DESeq2	LRT	0.0903489456898318
642	KC_G1	mRpL3	0.0671051784634657	0.87659293395671	0.998043617620652	pseudobulk	DESeq2	LRT	0.0572020344303803
642	KC_G1	mRpL30	0.183910981989703	0.434760357568161	0.998043617620652	pseudobulk	DESeq2	LRT	0.36175006273269
643	KC_G1	mRpL32	-0.125068846586857	0.70532651244825	0.998043617620652	pseudobulk	DESeq2	LRT	0.151609791196415
643	KC_G1	mRpL33	0.0309713902084938	0.89092038413293	0.998043617620652	pseudobulk	DESeq2	LRT	0.0501611043544217
643	KC_G1	mRpL34	-0.171536995556204	0.526163076960294	0.998043617620652	pseudobulk	DESeq2	LRT	0.278879631421399
643	KC_G1	mRpL35	-0.0710268860241583	0.79055865017171	0.998043617620652	pseudobulk	DESeq2	LRT	0.102065904984053
643	KC_G1	mRpL36	-0.0605608199134727	0.776417884243062	0.998043617620652	pseudobulk	DESeq2	LRT	0.109904469498744
643	KC_G1	mRpL37	-0.182389239415968	0.599361756757034	0.998043617620652	pseudobulk	DESeq2	LRT	0.222310971365775
643	KC_G1	mRpL38	0.330250462980072	0.332005129531849	0.998043617620652	pseudobulk	DESeq2	LRT	0.47885520632558
643	KC_G1	mRpL39	0.019601753752193	0.945946129475279	0.998043617620652	pseudobulk	DESeq2	LRT	0.0241335954563352
643	KC_G1	mRpL4	-0.169030123779193	0.532625845891246	0.998043617620652	pseudobulk	DESeq2	LRT	0.273577763064931

643	KC_G1	mRpL40	0.133463970720124	0.644689871008882	0.998043617620652	pseudobulk	DESeq2	LRT	0.190649153116809
644	KC_G1	mRpL41	0.154618699337444	0.538167077952444	0.998043617620652	pseudobulk	DESeq2	LRT	0.269082873463586
644	KC_G1	mRpL42	-0.152613024993289	0.590415426990068	0.998043617620652	pseudobulk	DESeq2	LRT	0.228842303337576
644	KC_G1	mRpL43	-0.0290119050660551	0.925956236577393	0.998043617620652	pseudobulk	DESeq2	LRT	0.0334095388710732
644	KC_G1	mRpL44	0.0896615981821261	0.78719771459338	0.998043617620652	pseudobulk	DESeq2	LRT	0.103916175426467
644	KC_G1	mRpL45	0.198949499486109	0.781907508041563	0.998043617620652	pseudobulk	DESeq2	LRT	0.106844616662479
644	KC_G1	mRpL46	0.104176931397724	0.822225080208736	0.998043617620652	pseudobulk	DESeq2	LRT	0.0850092801339518
644	KC_G1	mRpL47	0.288489535800767	0.22957878631484	0.998043617620652	pseudobulk	DESeq2	LRT	0.639068244375305
644	KC_G1	mRpL48	0.0098366676911606	0.952717353145274	0.998043617620652	pseudobulk	DESeq2	LRT	0.0210359243104861
644	KC_G1	mRpL49	-0.16005941354944	0.503593041771446	0.998043617620652	pseudobulk	DESeq2	LRT	0.297920279241581
644	KC_G1	mRpL50	0.337280568182885	0.163288846366645	0.998043617620652	pseudobulk	DESeq2	LRT	0.787043479236704
645	KC_G1	mRpL51	0.397436184357332	0.331151932469407	0.998043617620652	pseudobulk	DESeq2	LRT	0.479972706241882
645	KC_G1	mRpL52	-0.0565182617546242	0.810501617320647	0.998043617620652	pseudobulk	DESeq2	LRT	0.0912461141988943
645	KC_G1	mRpL54	0.0916712564959616	0.697815816725666	0.998043617620652	pseudobulk	DESeq2	LRT	0.156259191037478
645	KC_G1	mRpL55	-0.0706620153060356	0.679046134892876	0.998043617620652	pseudobulk	DESeq2	LRT	0.168100718431208
645	KC_G1	mRpL9	0.017883510618647	0.947073253877762	0.998043617620652	pseudobulk	DESeq2	LRT	0.0236164280458303
645	KC_G1	mRpS10	-0.220474480856665	0.325231019811317	0.998043617620652	pseudobulk	DESeq2	LRT	0.487808039060712
645	KC_G1	mRpS11	-0.0808734275891468	0.734483209116323	0.998043617620652	pseudobulk	DESeq2	LRT	0.134018128085405
645	KC_G1	mRpS14	-0.207492471617378	0.410865896254085	0.998043617620652	pseudobulk	DESeq2	LRT	0.386299905667647
645	KC_G1	mRpS16	0.0603882493924316	0.830696823069853	0.998043617620652	pseudobulk	DESeq2	LRT	0.0805574504543137
645	KC_G1	mRpS17	-0.0183552925628841	0.948212888863167	0.998043617620652	pseudobulk	DESeq2	LRT	0.023094145701192
646	KC_G1	mRpS18A	0.0855838750479874	0.678181834522202	0.998043617620652	pseudobulk	DESeq2	LRT	0.168653847221492
646	KC_G1	mRpS18B	0.173824943623421	0.683043314441718	0.998043617620652	pseudobulk	DESeq2	LRT	0.165551755137551
646	KC_G1	mRpS18C	-0.00528281941447364	0.970433947467809	0.998043617620652	pseudobulk	DESeq2	LRT	0.0130340194992023
646	KC_G1	mRpS2	0.278027065131465	0.403418680538414	0.998043617620652	pseudobulk	DESeq2	LRT	0.394243995397141
646	KC_G1	mRpS21	0.150101071387197	0.625151960701512	0.998043617620652	pseudobulk	DESeq2	LRT	0.204014402380034
646	KC_G1	mRpS22	0.095269695977383	0.79258569572445	0.998043617620652	pseudobulk	DESeq2	LRT	0.100953769907452
646	KC_G1	mRpS23	0.241745128020747	0.396412457186513	0.998043617620652	pseudobulk	DESeq2	LRT	0.401852706350344
646	KC_G1	mRpS24	-0.15735415900215	0.539178905739102	0.998043617620652	pseudobulk	DESeq2	LRT	0.268267107014856
646	KC_G1	mRpS25	-0.0679350064882861	0.806535135764352	0.998043617620652	pseudobulk	DESeq2	LRT	0.0933767083293353
646	KC_G1	mRpS26	0.0618879162038213	0.812648000380801	0.998043617620652	pseudobulk	DESeq2	LRT	0.0900975289394144
647	KC_G1	mRpS28	0.0576146338432613	0.794174908668787	0.998043617620652	pseudobulk	DESeq2	LRT	0.100083838247745
647	KC_G1	mRpS29	-0.241065227096189	0.479691393283979	0.998043617620652	pseudobulk	DESeq2	LRT	0.31903807366003
647	KC_G1	mRpS30	0.0126081099483553	0.946294971292168	0.998043617620652	pseudobulk	DESeq2	LRT	0.02397346778784
647	KC_G1	mRpS31	-0.13813691438758	0.687660638760496	0.998043617620652	pseudobulk	DESeq2	LRT	0.162625833675053
647	KC_G1	mRpS33	-0.0216427718073088	0.913185740801207	0.998043617620652	pseudobulk	DESeq2	LRT	0.0394408785437172
647	KC_G1	mRpS34	0.0982197659776604	0.734614873794502	0.998043617620652	pseudobulk	DESeq2	LRT	0.133940282713455
647	KC_G1	mRpS35	-0.159426718047316	0.610070177256225	0.998043617620652	pseudobulk	DESeq2	LRT	0.214620204592306
647	KC_G1	mRpS5	0.0788780228943539	0.591394540050256	0.998043617620652	pseudobulk	DESeq2	LRT	0.228122689341506
647	KC_G1	mRpS6	0.634507095021256	0.0465120650529265	0.998043617620652	pseudobulk	DESeq2	LRT	1.3324343781582
647	KC_G1	mRpS7	0.142766176046011	0.557199972756066	0.998043617620652	pseudobulk	DESeq2	LRT	0.253988913482623
648	KC_G1	mRpS9	0.0406207836715442	0.860351874805997	0.998043617620652	pseudobulk	DESeq2	LRT	0.0653238905797136
648	KC_G1	mRRF1	-0.0839351274105051	0.865512977669982	0.998043617620652	pseudobulk	DESeq2	LRT	0.0627264158438082
648	KC_G1	mRRF2	-0.636174990507257	0.447954698677445	0.998043617620652	pseudobulk	DESeq2	LRT	0.348765903656297
648	KC_G1	mrt	-0.27977779612067	0.260140715139829	0.998043617620652	pseudobulk	DESeq2	LRT	0.584791670192691
648	KC_G1	Mrtf	-0.173016444811722	0.379025904602333	0.998043617620652	pseudobulk	DESeq2	LRT	0.421331107073113
648	KC_G1	mrva	-0.113980055870366	0.636640185382024	0.998043617620652	pseudobulk	DESeq2	LRT	0.196105951736298
648	KC_G1	Ms	-0.129416207962863	0.886055498049692	0.998043617620652	pseudobulk	DESeq2	LRT	0.0525390752439962
648	KC_G1	ms(3)76Ba	0.173195945230598	0.976215465741119	0.998043617620652	pseudobulk	DESeq2	LRT	0.0104543162934892
648	KC_G1	MSBP	-0.00599723389625866	0.970990130827191	0.998043617620652	pseudobulk	DESeq2	LRT	0.0127851842517005
648	KC_G1	msd1	1.13582588427274	0.704684208645453	0.998043617620652	pseudobulk	DESeq2	LRT	0.152005460551806
649	KC_G1	msd5	0.681405831386831	0.333182722548511	0.998043617620652	pseudobulk	DESeq2	LRT	0.477317527362458
649	KC_G1	Msh6	0.957051818803407	0.285000537787932	0.998043617620652	pseudobulk	DESeq2	LRT	0.545154320489346
649	KC_G1	msi	-0.0689654515354244	0.925545954819386	0.998043617620652	pseudobulk	DESeq2	LRT	0.033602012968347
649	KC_G1	msk	-0.176121869701674	0.605505958898716	0.998043617620652	pseudobulk	DESeq2	LRT	0.217881578525724
649	KC_G1	msl-1	-0.24807906095737	0.409362688325127	0.998043617620652	pseudobulk	DESeq2	LRT	0.387891743971902

649	KC_G1	msl-2	0.331600888460399	0.549322418255569	0.998043617620652	pseudobulk	DESeq2	LRT	0.26017267673685
649	KC_G1	msl-3	0.242999403393044	0.630518092150476	0.998043617620652	pseudobulk	DESeq2	LRT	0.200302447221927
649	KC_G1	msn	-0.0757830443067735	0.592034442342247	0.998043617620652	pseudobulk	DESeq2	LRT	0.227653026918854
649	KC_G1	Msp300	-0.0512114310524174	0.726822213619618	0.998043617620652	pseudobulk	DESeq2	LRT	0.138571807966922
649	KC_G1	mspo	-0.192160644432299	0.0751640256618696	0.998043617620652	pseudobulk	DESeq2	LRT	1.12398996784883
650	KC_G1	msps	-0.0694622971122571	0.676541232882855	0.998043617620652	pseudobulk	DESeq2	LRT	0.169705729490905
650	KC_G1	Msr-110	0.018554542386496	0.946415367376921	0.998043617620652	pseudobulk	DESeq2	LRT	0.0239182164857753
650	KC_G1	MsR1	0.235158925806966	0.659993352842592	0.998043617620652	pseudobulk	DESeq2	LRT	0.180460438455586
650	KC_G1	MsR2	1.13582588427274	0.704684208645453	0.998043617620652	pseudobulk	DESeq2	LRT	0.152005460551806
650	KC_G1	mst	1.90250740646012	0.460774913678782	0.998043617620652	pseudobulk	DESeq2	LRT	0.336511173568024
650	KC_G1	Mst27D	1.13582588427274	0.704684208645453	0.998043617620652	pseudobulk	DESeq2	LRT	0.152005460551806
650	KC_G1	Mst36Fb	1.13582588427274	0.704684208645453	0.998043617620652	pseudobulk	DESeq2	LRT	0.152005460551806
650	KC_G1	Mst77F	0.262556186562495	0.65439190844076	0.998043617620652	pseudobulk	DESeq2	LRT	0.184162079356987
650	KC_G1	Mst85C	-0.0572283380635683	0.87164277189895	0.998043617620652	pseudobulk	DESeq2	LRT	0.0596614668792804
650	KC_G1	Mst87F	1.11121460044925	0.691240116375203	0.998043617620652	pseudobulk	DESeq2	LRT	0.16037106535726
651	KC_G1	mt:ATPase6	0.405905472375465	0.0096082423070996	0.998043617620652	pseudobulk	DESeq2	LRT	2.01735605313748
651	KC_G1	mt:ATPase8	0.649570001037317	0.329178256416401	0.998043617620652	pseudobulk	DESeq2	LRT	0.482568859456868
651	KC_G1	mt:CoI	-0.198021934302591	0.220744115739884	0.998043617620652	pseudobulk	DESeq2	LRT	0.656110864356322
651	KC_G1	mt:CoII	0.0519847791605345	0.741311920467804	0.998043617620652	pseudobulk	DESeq2	LRT	0.129999016266003
651	KC_G1	mt:CoIII	-0.0743943218304317	0.614363063047678	0.998043617620652	pseudobulk	DESeq2	LRT	0.21157490299736
651	KC_G1	mt:Cyt-b	-0.211091675238897	0.431317639212562	0.998043617620652	pseudobulk	DESeq2	LRT	0.365202780537021
651	KC_G1	mt:lrRNA	0.0344244091090911	0.746980988672744	0.998043617620652	pseudobulk	DESeq2	LRT	0.126690451222871
651	KC_G1	mt:ND2	-0.34731194639019	0.102835923368753	0.998043617620652	pseudobulk	DESeq2	LRT	0.987855148030221
651	KC_G1	mt:ND3	-0.287661872454476	0.171534584140149	0.998043617620652	pseudobulk	DESeq2	LRT	0.7656483060176
651	KC_G1	mt:ND4	-0.375579330168625	0.0293467570694239	0.998043617620652	pseudobulk	DESeq2	LRT	1.53243988299107
652	KC_G1	mt:ND4L	-0.169147855466172	0.951137033692631	0.998043617620652	pseudobulk	DESeq2	LRT	0.0217569082053667
652	KC_G1	mt:ND5	-0.379431908180862	0.0142849350112353	0.998043617620652	pseudobulk	DESeq2	LRT	1.84512173108285
652	KC_G1	mt:ND6	-0.336070171302815	0.133901165875034	0.998043617620652	pseudobulk	DESeq2	LRT	0.873215641577314
652	KC_G1	mt:srRNA	-0.118089264689118	0.363409080370714	0.998043617620652	pseudobulk	DESeq2	LRT	0.439604225333911
652	KC_G1	mt:tRNA:Ala-TGC	-2.50708586646449	0.206802189652552	0.998043617620652	pseudobulk	DESeq2	LRT	0.684444867178742
652	KC_G1	mt:tRNA:Arg-TCG	0.222317754595892	0.927814330005406	0.998043617620652	pseudobulk	DESeq2	LRT	0.0325389241278725
652	KC_G1	mt:tRNA:Gly-TCC	1.12508065046033	0.900770125530981	0.998043617620652	pseudobulk	DESeq2	LRT	0.0453860258366365
652	KC_G1	mt:tRNA:Lys-CTT	-0.794610277746993	0.815039924133852	0.998043617620652	pseudobulk	DESeq2	LRT	0.0888211171417967
652	KC_G1	mt:tRNA:Met-CAT	-0.794610277746993	0.821479263487599	0.998043617620652	pseudobulk	DESeq2	LRT	0.085403394939296
652	KC_G1	mt:tRNA:Ser-GCT	-1.71127439730422	0.490925983916286	0.998043617620652	pseudobulk	DESeq2	LRT	0.308983980789077
653	KC_G1	mt:tRNA:Trp-TCA	1.06585281705208	0.683859663547483	0.998043617620652	pseudobulk	DESeq2	LRT	0.1650330117303
653	KC_G1	mt:tRNA:Val-TAC	-1.70407762731104	0.493422538534865	0.998043617620652	pseudobulk	DESeq2	LRT	0.306781016707324
653	KC_G1	Mt2	-2.70281465192265	0.197132217389703	0.998043617620652	pseudobulk	DESeq2	LRT	0.705242393015524
653	KC_G1	MTA1-like	-0.30532707504146	0.216978855305206	0.998043617620652	pseudobulk	DESeq2	LRT	0.663582586297559
653	KC_G1	Mtap	-0.357114235631411	0.325251309776621	0.998043617620652	pseudobulk	DESeq2	LRT	0.487780945872867
653	KC_G1	Mtch	0.00888154067301359	0.94547787664367	0.998043617620652	pseudobulk	DESeq2	LRT	0.0243486288115216
653	KC_G1	mtd	0.0854286309309179	0.449050552937914	0.998043617620652	pseudobulk	DESeq2	LRT	0.347704764506425
653	KC_G1	mtDNA-helicase	-0.794610277746993	0.815039924133852	0.998043617620652	pseudobulk	DESeq2	LRT	0.0888211171417967
653	KC_G1	mTerf3	-0.160235137738506	0.628226988412096	0.998043617620652	pseudobulk	DESeq2	LRT	0.201883410397428
653	KC_G1	mTerf5	0.370920868346307	0.549833346575136	0.998043617620652	pseudobulk	DESeq2	LRT	0.259768924379055
654	KC_G1	MTF-1	-0.0612117301085071	0.875092366816805	0.998043617620652	pseudobulk	DESeq2	LRT	0.0579461043700082
654	KC_G1	mtg	1.76911534427852	0.454897600650965	0.998043617620652	pseudobulk	DESeq2	LRT	0.342086353842267
654	KC_G1	mtgo	0.172734465715371	0.232613998273765	0.998043617620652	pseudobulk	DESeq2	LRT	0.633364153794475
654	KC_G1	mth	1.12508065046033	0.900770125530981	0.998043617620652	pseudobulk	DESeq2	LRT	0.0453860258366365
654	KC_G1	mthl1	0.174619737100021	0.923414878852843	0.998043617620652	pseudobulk	DESeq2	LRT	0.0346031320031716
654	KC_G1	mthl14	0.855976205877101	0.680248096882705	0.998043617620652	pseudobulk	DESeq2	LRT	0.167332664564002
654	KC_G1	mthl5	-1.67593316621313	0.495877122847873	0.998043617620652	pseudobulk	DESeq2	LRT	0.304625927300966
654	KC_G1	Mtk	-0.794610277746993	0.879485534599449	0.998043617620652	pseudobulk	DESeq2	LRT	0.0557712992369932
654	KC_G1	Mtl	0.227206862165522	0.62623599114155	0.998043617620652	pseudobulk	DESeq2	LRT	0.20326197615539
654	KC_G1	mtm	0.237376225708194	0.599949058433199	0.998043617620652	pseudobulk	DESeq2	LRT	0.221885623917343
655	KC_G1	MtnA	0.583979556270869	0.411248661824498	0.998043617620652	pseudobulk	DESeq2	LRT	0.385895502204721

655	KC_G1	Mtor	-0.230446235457818	0.388707533420398	0.998043617620652	pseudobulk	DESeq2	LRT	0.410377042360232
655	KC_G1	Mtp	1.23649614330592	0.320002357133248	0.998043617620652	pseudobulk	DESeq2	LRT	0.494846822660742
655	KC_G1	Mtpalpha	0.68709360162209	0.182378485373131	0.998043617620652	pseudobulk	DESeq2	LRT	0.739026395365225
655	KC_G1	MTPAP	0.706761039499917	0.403058068326023	0.998043617620652	pseudobulk	DESeq2	LRT	0.394632380814591
655	KC_G1	Mtpbeta	-0.183972240936726	0.659029577581106	0.998043617620652	pseudobulk	DESeq2	LRT	0.181095093612356
655	KC_G1	Mtr3	0.540912303106088	0.384335132366495	0.998043617620652	pseudobulk	DESeq2	LRT	0.415289914532475
655	KC_G1	Mtr4	0.391720416074802	0.508710232633768	0.998043617620652	pseudobulk	DESeq2	LRT	0.293529526509228
655	KC_G1	mtrm	1.06585281705208	0.683859663547483	0.998043617620652	pseudobulk	DESeq2	LRT	0.1650330117303
655	KC_G1	mtRNApol	-0.316386775192594	0.510323041741834	0.998043617620652	pseudobulk	DESeq2	LRT	0.292154822270519
656	KC_G1	mts	-0.0457230595992343	0.666753769286365	0.998043617620652	pseudobulk	DESeq2	LRT	0.176034520481518
656	KC_G1	mtSSB	0.147340131092927	0.736038811834999	0.998043617620652	pseudobulk	DESeq2	LRT	0.133099284410658
656	KC_G1	mtt	-0.226479383380612	0.115002635631046	0.998043617620652	pseudobulk	DESeq2	LRT	0.939292206368968
656	KC_G1	mTTF	-0.184037984096999	0.762173302438778	0.998043617620652	pseudobulk	DESeq2	LRT	0.117946267852733
656	KC_G1	mtTFB2	0.214598182304239	0.809199461134599	0.998043617620652	pseudobulk	DESeq2	LRT	0.091944415108287
656	KC_G1	mu2	1.58866591819823	0.0757478002550273	0.998043617620652	pseudobulk	DESeq2	LRT	1.12062997471971
656	KC_G1	mub	-0.113778188049475	0.214174802775563	0.998043617620652	pseudobulk	DESeq2	LRT	0.669231624350188
656	KC_G1	muc	0.0163529398962192	0.933160375382958	0.998043617620652	pseudobulk	DESeq2	LRT	0.0300437108533061
656	KC_G1	Muc12Ea	-0.55595906156778	0.790614830449785	0.998043617620652	pseudobulk	DESeq2	LRT	0.102035043367578
656	KC_G1	Muc14A	-3.27644942312625	0.0905582584116004	0.998043617620652	pseudobulk	DESeq2	LRT	1.04307193829256
657	KC_G1	Muc68Ca	-0.794610277746993	0.815039924133852	0.998043617620652	pseudobulk	DESeq2	LRT	0.0888211171417967
657	KC_G1	Muc91C	-0.525809424813515	0.801332005230554	0.998043617620652	pseudobulk	DESeq2	LRT	0.0961875111745507
657	KC_G1	Muc96D	-0.794610277746993	0.879485534599449	0.998043617620652	pseudobulk	DESeq2	LRT	0.0557712992369932
657	KC_G1	Mul1	0.14883341323743	0.794237177279822	0.998043617620652	pseudobulk	DESeq2	LRT	0.100049787997912
657	KC_G1	Mulk	-0.0540211854312123	0.915955474750092	0.998043617620652	pseudobulk	DESeq2	LRT	0.0381256371840353
657	KC_G1	Mur2B	-0.689527053921232	0.480164247089866	0.998043617620652	pseudobulk	DESeq2	LRT	0.318610180533985
657	KC_G1	mura	-0.183816000778201	0.128091032708832	0.998043617620652	pseudobulk	DESeq2	LRT	0.892481272920327
657	KC_G1	mus101	-0.43820374275106	0.732657251631068	0.998043617620652	pseudobulk	DESeq2	LRT	0.135099147511553
657	KC_G1	mus201	-0.436468245532766	0.431413472663255	0.998043617620652	pseudobulk	DESeq2	LRT	0.365106296387056
657	KC_G1	mus304	-0.191128388247172	0.872090392166693	0.998043617620652	pseudobulk	DESeq2	LRT	0.0594384981125623
658	KC_G1	mus81	0.438611375820504	0.752254908033481	0.998043617620652	pseudobulk	DESeq2	LRT	0.123634970055274
658	KC_G1	muskelin	0.847981234747265	0.401090139859693	0.998043617620652	pseudobulk	DESeq2	LRT	0.396758014301477
658	KC_G1	mute	-0.145912669560476	0.78424445074162	0.998043617620652	pseudobulk	DESeq2	LRT	0.105548545656446
658	KC_G1	mv	-0.41706654361699	0.652771572872282	0.998043617620652	pseudobulk	DESeq2	LRT	0.185238766652355
658	KC_G1	Mvb12	0.0669578622769104	0.863854485206677	0.998043617620652	pseudobulk	DESeq2	LRT	0.0635594075143106
658	KC_G1	Mvd	0.130436769520859	0.890935698492627	0.998043617620652	pseudobulk	DESeq2	LRT	0.050153639170252
658	KC_G1	Mvl	-0.0210818467605343	0.95835384312856	0.998043617620652	pseudobulk	DESeq2	LRT	0.0184741112290619
658	KC_G1	mxc	-0.0849546903907684	0.850111814321434	0.998043617620652	pseudobulk	DESeq2	LRT	0.07052394822793
658	KC_G1	mxt	0.152319394038952	0.598585534785922	0.998043617620652	pseudobulk	DESeq2	LRT	0.222873782381493
658	KC_G1	Myb	-1.2833596927783	0.554636446631459	0.998043617620652	pseudobulk	DESeq2	LRT	0.255991595178557
659	KC_G1	Mybbp1A	-0.963631380082264	0.257823364718304	0.998043617620652	pseudobulk	DESeq2	LRT	0.588677728144626
659	KC_G1	Myc	-0.536803443383876	0.021703177053143	0.998043617620652	pseudobulk	DESeq2	LRT	1.66347668662827
659	KC_G1	Myd88	-1.46101529683674	0.404718176012336	0.998043617620652	pseudobulk	DESeq2	LRT	0.392847290877514
659	KC_G1	myo	-0.421145644510514	0.451063226670361	0.998043617620652	pseudobulk	DESeq2	LRT	0.345762577701954
659	KC_G1	Myo10A	0.763164787219597	0.794486984020146	0.998043617620652	pseudobulk	DESeq2	LRT	0.0999132133899323
659	KC_G1	Myo28B1	-0.330711929721525	0.690500393872429	0.998043617620652	pseudobulk	DESeq2	LRT	0.160836069356786
659	KC_G1	Myo31DF	-0.0559442230061323	0.910313577058356	0.998043617620652	pseudobulk	DESeq2	LRT	0.0408089798464723
659	KC_G1	Myo81F	1.36662147236043	0.0565130435323022	0.998043617620652	pseudobulk	DESeq2	LRT	1.24785130297124
659	KC_G1	Myo95E	-0.567083906753696	0.0695981582040562	0.998043617620652	pseudobulk	DESeq2	LRT	1.15740225309632
659	KC_G1	MYPT-75D	0.126637778139189	0.566655211668135	0.998043617620652	pseudobulk	DESeq2	LRT	0.246681112564216
660	KC_G1	mys	0.137483512841925	0.509873175087235	0.998043617620652	pseudobulk	DESeq2	LRT	0.292537836077324
660	KC_G1	Mys45A	-0.29339577564456	0.526359583529185	0.998043617620652	pseudobulk	DESeq2	LRT	0.278717465374581
660	KC_G1	Myt1	0.0312146697649754	0.955077761875356	0.998043617620652	pseudobulk	DESeq2	LRT	0.0199612669727958
660	KC_G1	N	-0.588259215211707	0.42032076488426	0.998043617620652	pseudobulk	DESeq2	LRT	0.37641915424417
660	KC_G1	na	-0.115844085353877	0.582896368226809	0.998043617620652	pseudobulk	DESeq2	LRT	0.234408650571737
660	KC_G1	Naa15-16	0.0637927065307616	0.844247114452114	0.998043617620652	pseudobulk	DESeq2	LRT	0.0735304150628985
660	KC_G1	Naa20A	0.378457777028882	0.130814873763882	0.998043617620652	pseudobulk	DESeq2	LRT	0.883342873547419

660	KC_G1	Naa30A	0.133970727716611	0.545778470496747	0.998043617620652	pseudobulk	DESeq2	LRT	0.262983600085832
660	KC_G1	Naa35	-0.713571793182105	0.115832981521496	0.998043617620652	pseudobulk	DESeq2	LRT	0.936167764845373
660	KC_G1	Naa40	-0.130305387943327	0.871441271068382	0.998043617620652	pseudobulk	DESeq2	LRT	0.0597618759210106
661	KC_G1	Naa60	-0.0656151882332782	0.833875011495435	0.998043617620652	pseudobulk	DESeq2	LRT	0.0788990403523672
661	KC_G1	Naa80	-0.116794813967579	0.650337324172614	0.998043617620652	pseudobulk	DESeq2	LRT	0.186861320239301
661	KC_G1	Naam	0.401394071414729	0.717752290814686	0.998043617620652	pseudobulk	DESeq2	LRT	0.144025412712906
661	KC_G1	Nab2	-0.198140738950015	0.39264853752769	0.998043617620652	pseudobulk	DESeq2	LRT	0.405996015796879
661	KC_G1	nac	0.220889400074788	0.814169096261373	0.998043617620652	pseudobulk	DESeq2	LRT	0.0892853863313084
661	KC_G1	Nacalpha	-0.220909190769656	0.0442698628031174	0.998043617620652	pseudobulk	DESeq2	LRT	1.35389182394257
661	KC_G1	nAChRalpha2	0.0306022201606966	0.841852588100792	0.998043617620652	pseudobulk	DESeq2	LRT	0.0747639486181241
661	KC_G1	nAChRalpha3	0.791931205742411	0.340748477086617	0.998043617620652	pseudobulk	DESeq2	LRT	0.4675660764892
661	KC_G1	nAChRalpha4	-0.0317982112228419	0.790005313681217	0.998043617620652	pseudobulk	DESeq2	LRT	0.102369987577065
661	KC_G1	nAChRalpha5	-0.0902199192340235	0.432338649185069	0.998043617620652	pseudobulk	DESeq2	LRT	0.364175938702932
662	KC_G1	nAChRalpha6	-0.268675249960643	0.0069360211192569	0.998043617620652	pseudobulk	DESeq2	LRT	2.1588895931612
662	KC_G1	nAChRalpha7	0.134976318557934	0.341812512762745	0.998043617620652	pseudobulk	DESeq2	LRT	0.466212043065408
662	KC_G1	nAChRbeta1	0.0625139286042957	0.515935400976257	0.998043617620652	pseudobulk	DESeq2	LRT	0.287404671930587
662	KC_G1	nAChRbeta2	-0.0979168897891301	0.630917363673029	0.998043617620652	pseudobulk	DESeq2	LRT	0.200027520076006
662	KC_G1	NaCP60E	-0.310209300812341	0.0383071413652616	0.998043617620652	pseudobulk	DESeq2	LRT	1.41672025562888
662	KC_G1	Nadsyn	0.10823970633868	0.539730011598171	0.998043617620652	pseudobulk	DESeq2	LRT	0.267823432390412
662	KC_G1	nahoda	0.172530702244785	0.946040718765468	0.998043617620652	pseudobulk	DESeq2	LRT	0.0240901706229663
662	KC_G1	Nak	-0.286901756178415	0.238233511350882	0.998043617620652	pseudobulk	DESeq2	LRT	0.622997148096276
662	KC_G1	NANS	-0.0225643646157204	0.96139798747385	0.998043617620652	pseudobulk	DESeq2	LRT	0.0170967913293986
662	KC_G1	Nap1	0.0278843806840913	0.869744133464997	0.998043617620652	pseudobulk	DESeq2	LRT	0.0606084919421122
663	KC_G1	NaPi-III	-0.124027448871921	0.384439132335269	0.998043617620652	pseudobulk	DESeq2	LRT	0.415172411606308
663	KC_G1	Naprt	0.136728712443149	0.740425788956078	0.998043617620652	pseudobulk	DESeq2	LRT	0.13051846322239
663	KC_G1	NAT1	0.0545843010416486	0.680914224596497	0.998043617620652	pseudobulk	DESeq2	LRT	0.166907593124876
663	KC_G1	natalisin	1.13582588427274	0.704684208645453	0.998043617620652	pseudobulk	DESeq2	LRT	0.152005460551806
663	KC_G1	Naus	0.0449710995374459	0.849932595462315	0.998043617620652	pseudobulk	DESeq2	LRT	0.0706155149674914
663	KC_G1	Naxd	0.497239634494379	0.405072508769331	0.998043617620652	pseudobulk	DESeq2	LRT	0.392467230267203
663	KC_G1	Naxe	-0.525346591362653	0.479346072279869	0.998043617620652	pseudobulk	DESeq2	LRT	0.319350826849424
663	KC_G1	naz	-0.337225655218998	0.722022421378251	0.998043617620652	pseudobulk	DESeq2	LRT	0.141449315824187
663	KC_G1	Nbr	-0.567317050113026	0.187406480607419	0.998043617620652	pseudobulk	DESeq2	LRT	0.727215395073759
663	KC_G1	nbs	-0.603943741406191	0.253721063056055	0.998043617620652	pseudobulk	DESeq2	LRT	0.595643477637134
664	KC_G1	NC2alpha	0.0621082170560285	0.852912704299495	0.998043617620652	pseudobulk	DESeq2	LRT	0.0690954166415127
664	KC_G1	Nc73EF	-0.00619853731235176	0.967416235439633	0.998043617620652	pseudobulk	DESeq2	LRT	0.0143866284395234
664	KC_G1	Nca	-0.00872124328888932	0.948672638737059	0.998043617620652	pseudobulk	DESeq2	LRT	0.0228836249983962
664	KC_G1	Ncc69	-0.994969031467467	0.565611147086461	0.998043617620652	pseudobulk	DESeq2	LRT	0.247482040034292
664	KC_G1	ncd	0.78480577599884	0.596788997132032	0.998043617620652	pseudobulk	DESeq2	LRT	0.224179192453774
664	KC_G1	Nckx30C	0.083992679960237	0.477930260935956	0.998043617620652	pseudobulk	DESeq2	LRT	0.320635470543371
664	KC_G1	nclb	-0.203321603966621	0.778491049042811	0.998043617620652	pseudobulk	DESeq2	LRT	0.10874637651097
664	KC_G1	ncm	0.190688402246157	0.456814547704646	0.998043617620652	pseudobulk	DESeq2	LRT	0.340260074010786
664	KC_G1	Ncoa6	-0.166384811852865	0.241559416380752	0.998043617620652	pseudobulk	DESeq2	LRT	0.616976028338952
664	KC_G1	Nct	0.0428057748373746	0.93295007969324	0.998043617620652	pseudobulk	DESeq2	LRT	0.0301415938674127
665	KC_G1	ND-13A	0.00645677369292187	0.954900781410156	0.998043617620652	pseudobulk	DESeq2	LRT	0.0200417512692622
665	KC_G1	ND-13B	0.0937580199854177	0.513953642814817	0.998043617620652	pseudobulk	DESeq2	LRT	0.289076051389256
665	KC_G1	ND-15	-0.128207939969849	0.335786278094977	0.998043617620652	pseudobulk	DESeq2	LRT	0.473937055277765
665	KC_G1	ND-19	-0.0267726118394989	0.863618436417678	0.998043617620652	pseudobulk	DESeq2	LRT	0.0636780949749092
665	KC_G1	ND-20	0.10955856561594	0.456224193219254	0.998043617620652	pseudobulk	DESeq2	LRT	0.340821688145236
665	KC_G1	ND-23	-0.111383633755946	0.500846615544276	0.998043617620652	pseudobulk	DESeq2	LRT	0.300295256611744
665	KC_G1	ND-24	0.14517184586458	0.494027200566681	0.998043617620652	pseudobulk	DESeq2	LRT	0.306249138665824
665	KC_G1	ND-30	-0.0224332096693766	0.886918567316056	0.998043617620652	pseudobulk	DESeq2	LRT	0.0521162532108811
665	KC_G1	ND-39	-0.0538848397366449	0.78679223964728	0.998043617620652	pseudobulk	DESeq2	LRT	0.104139932299962
665	KC_G1	ND-42	0.0497616338669948	0.775777990726994	0.998043617620652	pseudobulk	DESeq2	LRT	0.110262545748345
666	KC_G1	ND-49	0.129680180012232	0.369742525758645	0.998043617620652	pseudobulk	DESeq2	LRT	0.432100596383363
666	KC_G1	ND-51	0.027351616891107	0.859871836347876	0.998043617620652	pseudobulk	DESeq2	LRT	0.0655662754015514
666	KC_G1	ND-51L1	-1.31481767438999	0.589948159507212	0.998043617620652	pseudobulk	DESeq2	LRT	0.229186149424154

666	KC_G1	ND-51L2	-1.67593316621313	0.495877122847873	0.998043617620652	pseudobulk	DESeq2	LRT	0.304625927300966
666	KC_G1	ND-75	-0.0806310509077555	0.668807306354372	0.998043617620652	pseudobulk	DESeq2	LRT	0.174698991113217
666	KC_G1	ND-ACP	-0.0893637909470489	0.480600037350763	0.998043617620652	pseudobulk	DESeq2	LRT	0.31821619978008
666	KC_G1	ND-AGGG	-0.00880577021050106	0.964607620663224	0.998043617620652	pseudobulk	DESeq2	LRT	0.0156493113557564
666	KC_G1	ND-ASHI	0.0641951758661591	0.697653443691868	0.998043617620652	pseudobulk	DESeq2	LRT	0.156360257703591
666	KC_G1	ND-B12	-0.0097913173786775	0.933225593641257	0.998043617620652	pseudobulk	DESeq2	LRT	0.0300133592216713
666	KC_G1	ND-B14	-0.0785301304533911	0.582282576630531	0.998043617620652	pseudobulk	DESeq2	LRT	0.234866204888412
667	KC_G1	ND-B14.5A	0.0453851652552781	0.788521002036866	0.998043617620652	pseudobulk	DESeq2	LRT	0.103186734868686
667	KC_G1	ND-B14.5B	-0.127641533690116	0.416884780490434	0.998043617620652	pseudobulk	DESeq2	LRT	0.379983959682227
667	KC_G1	ND-B14.7	-0.125715676340933	0.437182136333723	0.998043617620652	pseudobulk	DESeq2	LRT	0.35933759203384
667	KC_G1	ND-B15	-0.0831090893652329	0.566334595047265	0.998043617620652	pseudobulk	DESeq2	LRT	0.246926908302433
667	KC_G1	ND-B16.6	-0.148843937561209	0.235472002401387	0.998043617620652	pseudobulk	DESeq2	LRT	0.628060723037928
667	KC_G1	ND-B17	0.0573372214722581	0.71353088253655	0.998043617620652	pseudobulk	DESeq2	LRT	0.146587225317435
667	KC_G1	ND-B17.2	0.0729565750765226	0.622987304968447	0.998043617620652	pseudobulk	DESeq2	LRT	0.205520803161578
667	KC_G1	ND-B18	-0.0226909716737289	0.892563619783488	0.998043617620652	pseudobulk	DESeq2	LRT	0.0493608186486033
667	KC_G1	ND-B22	0.0807646959280035	0.558061806858056	0.998043617620652	pseudobulk	DESeq2	LRT	0.253317699107386
667	KC_G1	ND-B8	1.13582588427274	0.704684208645453	0.998043617620652	pseudobulk	DESeq2	LRT	0.152005460551806
668	KC_G1	ND-MNLL	-0.247257075254351	0.762776256451738	0.998043617620652	pseudobulk	DESeq2	LRT	0.117602834048265
668	KC_G1	ND-MWFE	0.0501307421803165	0.776595983128481	0.998043617620652	pseudobulk	DESeq2	LRT	0.10980486013255
668	KC_G1	ND-PDSW	0.0531965391724238	0.694372214695655	0.998043617620652	pseudobulk	DESeq2	LRT	0.15840766577768
668	KC_G1	ND-SGDH	-0.0485596416071484	0.725047846702328	0.998043617620652	pseudobulk	DESeq2	LRT	0.139633332914316
668	KC_G1	Ndae1	-0.140202054873028	0.324406151020714	0.998043617620652	pseudobulk	DESeq2	LRT	0.488910919784469
668	KC_G1	Ndc1	-1.81106524013563	0.224263762105032	0.998043617620652	pseudobulk	DESeq2	LRT	0.6492408966768
668	KC_G1	Ndf	0.100956974644708	0.597921029067482	0.998043617620652	pseudobulk	DESeq2	LRT	0.223356172040022
668	KC_G1	Ndfip	-0.0389591318111658	0.832266283881204	0.998043617620652	pseudobulk	DESeq2	LRT	0.0797376988080437
668	KC_G1	Ndg	-0.794610277746993	0.815039924133852	0.998043617620652	pseudobulk	DESeq2	LRT	0.0888211171417967
668	KC_G1	neb	-1.73907471612892	0.507597351592657	0.998043617620652	pseudobulk	DESeq2	LRT	0.294480652518419
669	KC_G1	Neb-cGP	-0.013107826081171	0.92373976310584	0.998043617620652	pseudobulk	DESeq2	LRT	0.0344503614266495
669	KC_G1	nec	0.904363188455548	0.47969290585193	0.998043617620652	pseudobulk	DESeq2	LRT	0.319036704240258
669	KC_G1	Nedd4	0.33206360724634	0.144619948958246	0.998043617620652	pseudobulk	DESeq2	LRT	0.839771796080775
669	KC_G1	Nedd8	0.107061064999774	0.645005559064607	0.998043617620652	pseudobulk	DESeq2	LRT	0.190436542324919
669	KC_G1	nej	-0.235337994795247	0.0776805727206057	0.998043617620652	pseudobulk	DESeq2	LRT	1.1096875811371
669	KC_G1	Nelf-A	-0.101137967725078	0.689283961383795	0.998043617620652	pseudobulk	DESeq2	LRT	0.16160182677718
669	KC_G1	NELF-B	-1.25395966281858	0.0630396972559412	0.998043617620652	pseudobulk	DESeq2	LRT	1.20038588118458
669	KC_G1	Nelf-E	-0.15238405433689	0.63744839863159	0.998043617620652	pseudobulk	DESeq2	LRT	0.195554965555318
669	KC_G1	nemy	0.327895865548423	0.735139762155252	0.998043617620652	pseudobulk	DESeq2	LRT	0.133630086544918
669	KC_G1	Neos	0.153662390556197	0.666735953806086	0.998043617620652	pseudobulk	DESeq2	LRT	0.17604612486758
670	KC_G1	Nep1	-0.0339984577133421	0.778627761559363	0.998043617620652	pseudobulk	DESeq2	LRT	0.108670115802675
670	KC_G1	Nep2	1.12508065046033	0.900770125530981	0.998043617620652	pseudobulk	DESeq2	LRT	0.0453860258366365
670	KC_G1	Nep3	-0.223351129518183	0.302520326015079	0.998043617620652	pseudobulk	DESeq2	LRT	0.519245440252415
670	KC_G1	Nep4	-0.497643634749085	0.500861565184204	0.998043617620652	pseudobulk	DESeq2	LRT	0.300282293662549
670	KC_G1	Nepl11	-0.636005980820086	0.767232986536363	0.998043617620652	pseudobulk	DESeq2	LRT	0.115072733310345
670	KC_G1	Nepl16	-0.0670580318063762	0.821708857996072	0.998043617620652	pseudobulk	DESeq2	LRT	0.0852820313200603
670	KC_G1	Nepl17	1.12508065046033	0.900770125530981	0.998043617620652	pseudobulk	DESeq2	LRT	0.0453860258366365
670	KC_G1	Nepl18	-0.794610277746993	0.821479263487599	0.998043617620652	pseudobulk	DESeq2	LRT	0.085403394939296
670	KC_G1	Nepl20	-0.794610277746993	0.879485534599449	0.998043617620652	pseudobulk	DESeq2	LRT	0.0557712992369932
670	KC_G1	Nepl3	-0.479558819091213	0.74479461320578	0.998043617620652	pseudobulk	DESeq2	LRT	0.127963473089368
671	KC_G1	Nepl5	-0.0886978631046777	0.745740826787914	0.998043617620652	pseudobulk	DESeq2	LRT	0.127412080104542
671	KC_G1	nero	-0.329201540322022	0.436515215625792	0.998043617620652	pseudobulk	DESeq2	LRT	0.360000613476967
671	KC_G1	nes	0.0769756178652397	0.824117280673768	0.998043617620652	pseudobulk	DESeq2	LRT	0.0840109791705673
671	KC_G1	net	0.38549429370966	0.808565123498174	0.998043617620652	pseudobulk	DESeq2	LRT	0.0922849953779979
671	KC_G1	NetA	-2.31256857938916	0.316871197308952	0.998043617620652	pseudobulk	DESeq2	LRT	0.499117235149486
671	KC_G1	NetB	-0.0841047019599079	0.564218953075828	0.998043617620652	pseudobulk	DESeq2	LRT	0.248552329244113
671	KC_G1	Neto	-0.553531401622242	0.740683526379698	0.998043617620652	pseudobulk	DESeq2	LRT	0.130367314430293
671	KC_G1	neur	-0.432728111293617	0.173751721057555	0.998043617620652	pseudobulk	DESeq2	LRT	0.760070884912827
671	KC_G1	Neurl4	-0.536399132329327	0.254791395422693	0.998043617620652	pseudobulk	DESeq2	LRT	0.593815242677123

671	KC_G1	Nf-YA	0.086352271411372	0.818012934067171	0.998043617620652	pseudobulk	DESeq2	LRT	0.0872398293976352
672	KC_G1	Nf-YB	0.100704654733084	0.76295976978064	0.998043617620652	pseudobulk	DESeq2	LRT	0.117498361419826
672	KC_G1	Nf-YC	-0.334183414011619	0.345005740040711	0.998043617620652	pseudobulk	DESeq2	LRT	0.462173679282468
672	KC_G1	Nf1	0.0947237727354951	0.621385387818226	0.998043617620652	pseudobulk	DESeq2	LRT	0.206638963608445
672	KC_G1	NFAT	0.200498505461289	0.135963354801475	0.998043617620652	pseudobulk	DESeq2	LRT	0.86657812804166
672	KC_G1	NfI	0.0289058954940874	0.827602711712963	0.998043617620652	pseudobulk	DESeq2	LRT	0.0821780949999629
672	KC_G1	Nfs1	-0.232465426619365	0.456234077519426	0.998043617620652	pseudobulk	DESeq2	LRT	0.340812279063944
672	KC_G1	ng3	-0.794610277746993	0.815039924133852	0.998043617620652	pseudobulk	DESeq2	LRT	0.0888211171417967
672	KC_G1	Nha1	0.332532667600005	0.843004403387413	0.998043617620652	pseudobulk	DESeq2	LRT	0.07417015685585
672	KC_G1	Nhe1	0.130706303455538	0.829349691804516	0.998043617620652	pseudobulk	DESeq2	LRT	0.0812623123941676
672	KC_G1	Nhe2	-0.168664536599301	0.488053565112907	0.998043617620652	pseudobulk	DESeq2	LRT	0.31153251046384
673	KC_G1	Nhe3	-0.0486131692621694	0.798856312133592	0.998043617620652	pseudobulk	DESeq2	LRT	0.0975313288953535
673	KC_G1	NHP2	0.821124345525163	0.0810764844729967	0.998043617620652	pseudobulk	DESeq2	LRT	1.09110509085075
673	KC_G1	NijA	-1.67593316621313	0.495877122847873	0.998043617620652	pseudobulk	DESeq2	LRT	0.304625927300966
673	KC_G1	NijB	-0.794610277746993	0.821479263487599	0.998043617620652	pseudobulk	DESeq2	LRT	0.085403394939296
673	KC_G1	NimA	-0.794610277746993	0.815039924133852	0.998043617620652	pseudobulk	DESeq2	LRT	0.0888211171417967
673	KC_G1	NimB3	1.13582588427274	0.704684208645453	0.998043617620652	pseudobulk	DESeq2	LRT	0.152005460551806
673	KC_G1	NimC4	1.12508065046033	0.900770125530981	0.998043617620652	pseudobulk	DESeq2	LRT	0.0453860258366365
673	KC_G1	ninaA	-0.794610277746993	0.879485534599449	0.998043617620652	pseudobulk	DESeq2	LRT	0.0557712992369932
673	KC_G1	ninaB	-0.743539102981812	0.121240211640496	0.998043617620652	pseudobulk	DESeq2	LRT	0.916353314186472
673	KC_G1	ninaG	0.198564725165423	0.889422506255578	0.998043617620652	pseudobulk	DESeq2	LRT	0.050891885209843
674	KC_G1	NiPp1	0.115150911665894	0.741328800209569	0.998043617620652	pseudobulk	DESeq2	LRT	0.129989127452613
674	KC_G1	Nipped-A	0.0645358531156414	0.693996924280946	0.998043617620652	pseudobulk	DESeq2	LRT	0.158642454286893
674	KC_G1	Nipped-B	-0.105896296275938	0.37067572931815	0.998043617620652	pseudobulk	DESeq2	LRT	0.431005849277357
674	KC_G1	Nipsnap	0.172176342459104	0.425903039899103	0.998043617620652	pseudobulk	DESeq2	LRT	0.37068926012185
674	KC_G1	NitFhit	-0.42455362209349	0.590004827971243	0.998043617620652	pseudobulk	DESeq2	LRT	0.229144434539736
674	KC_G1	nito	0.139763886372739	0.712092053987341	0.998043617620652	pseudobulk	DESeq2	LRT	0.147463860359544
674	KC_G1	NK7.1	-0.191731147700835	0.234216491853998	0.998043617620652	pseudobulk	DESeq2	LRT	0.630382528268686
674	KC_G1	NKAIN	0.208066427143979	0.478035206955582	0.998043617620652	pseudobulk	DESeq2	LRT	0.320540116727914
674	KC_G1	NKCC	3.81492523872396	0.0160439186250714	0.998043617620652	pseudobulk	DESeq2	LRT	1.79468954943111
674	KC_G1	nkd	0.221490980243074	0.566978834940141	0.998043617620652	pseudobulk	DESeq2	LRT	0.246433152818324
675	KC_G1	NLaz	0.00365900336080904	0.944277874512887	0.998043617620652	pseudobulk	DESeq2	LRT	0.024900186200248
675	KC_G1	Nle	-0.063794549899806	0.904684655205621	0.998043617620652	pseudobulk	DESeq2	LRT	0.0435027758997734
675	KC_G1	Nlg2	0.00645725120456191	0.958006462760148	0.998043617620652	pseudobulk	DESeq2	LRT	0.0186315611389889
675	KC_G1	Nlg3	0.0048453760051787	0.968072197075315	0.998043617620652	pseudobulk	DESeq2	LRT	0.0140922525860298
675	KC_G1	Nlg4	-0.294919316539187	0.0882929691397932	0.998043617620652	pseudobulk	DESeq2	LRT	1.05407387836312
675	KC_G1	Nlp	0.1185708191733	0.540938399046929	0.998043617620652	pseudobulk	DESeq2	LRT	0.266852188640615
675	KC_G1	nmd	-0.0240560273101672	0.946678581025505	0.998043617620652	pseudobulk	DESeq2	LRT	0.0237974488545915
675	KC_G1	Nmd3	-0.233830245573797	0.705049235558609	0.998043617620652	pseudobulk	DESeq2	LRT	0.151780553952159
675	KC_G1	Nmda1	0.115641955552422	0.636204985672356	0.998043617620652	pseudobulk	DESeq2	LRT	0.196402931831125
675	KC_G1	Nmdar1	-0.160485723850397	0.592255344743486	0.998043617620652	pseudobulk	DESeq2	LRT	0.227491011344532
676	KC_G1	Nmdar2	-0.0868637511822516	0.662356314328052	0.998043617620652	pseudobulk	DESeq2	LRT	0.178908319145753
676	KC_G1	Nmdmc	0.209823315495265	0.80229367285451	0.998043617620652	pseudobulk	DESeq2	LRT	0.0956666327697192
676	KC_G1	nmdyn-D6	0.411212293701226	0.677871935473924	0.998043617620652	pseudobulk	DESeq2	LRT	0.16885234591223
676	KC_G1	Nmnat	-0.045970651543095	0.904711247083951	0.998043617620652	pseudobulk	DESeq2	LRT	0.0434900106381654
676	KC_G1	nmo	-0.114078204754484	0.295990889829846	0.998043617620652	pseudobulk	DESeq2	LRT	0.52872165568942
676	KC_G1	Nmt	0.037567166502663	0.939569927980887	0.998043617620652	pseudobulk	DESeq2	LRT	0.0270708917677398
676	KC_G1	Nna1	0.320036901313671	0.755284557129908	0.998043617620652	pseudobulk	DESeq2	LRT	0.121889394982205
676	KC_G1	Nnf1a	-0.843507811008217	0.540987981342569	0.998043617620652	pseudobulk	DESeq2	LRT	0.266812383127408
676	KC_G1	Nnp-1	0.422895579616122	0.144420575765624	0.998043617620652	pseudobulk	DESeq2	LRT	0.840370927923692
676	KC_G1	NO66	0.158326000232128	0.825443310239947	0.998043617620652	pseudobulk	DESeq2	LRT	0.0833127478347183
677	KC_G1	Noa36	-0.0672982914430065	0.839883921043077	0.998043617620652	pseudobulk	DESeq2	LRT	0.0757807329071741
677	KC_G1	noc	-0.125129242134797	0.706388710303682	0.998043617620652	pseudobulk	DESeq2	LRT	0.150956250387445
677	KC_G1	nocte	-0.0982896514272791	0.420588109919391	0.998043617620652	pseudobulk	DESeq2	LRT	0.376143009037686
677	KC_G1	noi	-0.19467144809887	0.632946803014195	0.998043617620652	pseudobulk	DESeq2	LRT	0.198632789395334
677	KC_G1	nolo	0.346543185661291	0.406086345683241	0.998043617620652	pseudobulk	DESeq2	LRT	0.391381613056422

677	KC_G1	nom	-0.332086284814294	0.682590202570135	0.998043617620652	pseudobulk	DESeq2	LRT	0.165839949576664
677	KC_G1	Non1	-0.112200109671848	0.552214342500151	0.998043617620652	pseudobulk	DESeq2	LRT	0.257892317747858
677	KC_G1	Non2	-0.194838780561229	0.323760375718925	0.998043617620652	pseudobulk	DESeq2	LRT	0.489776304624146
677	KC_G1	Non3	-0.0282074753306132	0.917560622402648	0.998043617620652	pseudobulk	DESeq2	LRT	0.0373652326832871
677	KC_G1	nonA	-0.0701767749714357	0.631210496416701	0.998043617620652	pseudobulk	DESeq2	LRT	0.199825787843708
678	KC_G1	nonA-l	-0.0931483808094422	0.792160112705074	0.998043617620652	pseudobulk	DESeq2	LRT	0.101187029223068
678	KC_G1	nonC	-0.0710362809391272	0.8892928735556	0.998043617620652	pseudobulk	DESeq2	LRT	0.0509551879360842
678	KC_G1	Nop17l	0.0640952896286628	0.810335314118799	0.998043617620652	pseudobulk	DESeq2	LRT	0.0913352342846202
678	KC_G1	nop5	0.58799610461613	0.639801249027944	0.998043617620652	pseudobulk	DESeq2	LRT	0.193954916416003
678	KC_G1	Nop56	-0.125476496440043	0.72704456491168	0.998043617620652	pseudobulk	DESeq2	LRT	0.138438967817652
678	KC_G1	Nop60B	0.386451405280915	0.523679332451981	0.998043617620652	pseudobulk	DESeq2	LRT	0.280934565642483
678	KC_G1	nopo	-2.02105752212082	0.354058454366442	0.998043617620652	pseudobulk	DESeq2	LRT	0.4509250308752
678	KC_G1	Nopp140	0.155483706564643	0.767630572674779	0.998043617620652	pseudobulk	DESeq2	LRT	0.114847736803234
678	KC_G1	nord	-1.66808346769027	0.498191247612582	0.998043617620652	pseudobulk	DESeq2	LRT	0.302603906560549
678	KC_G1	norpA	-0.042208867862553	0.945397815573338	0.998043617620652	pseudobulk	DESeq2	LRT	0.0243854055083974
679	KC_G1	Nost	0.0156374816800736	0.967745719899034	0.998043617620652	pseudobulk	DESeq2	LRT	0.0142387407822876
679	KC_G1	not	-0.157154596065453	0.631697672276547	0.998043617620652	pseudobulk	DESeq2	LRT	0.199490723421151
679	KC_G1	Not1	-0.24140941544634	0.0756188434666096	0.998043617620652	pseudobulk	DESeq2	LRT	1.12136996913046
679	KC_G1	Not10	-0.0194135634082093	0.969354280392013	0.998043617620652	pseudobulk	DESeq2	LRT	0.0135174676354436
679	KC_G1	Not11	-0.198619031457214	0.500718915909015	0.998043617620652	pseudobulk	DESeq2	LRT	0.300406001731247
679	KC_G1	Not3	-0.0975694782992755	0.520039116873634	0.998043617620652	pseudobulk	DESeq2	LRT	0.283963987897062
679	KC_G1	NP15.6	0.0275482154097317	0.85637726944098	0.998043617620652	pseudobulk	DESeq2	LRT	0.0673348685697563
679	KC_G1	Npc1a	-0.275464827819118	0.344454745140684	0.998043617620652	pseudobulk	DESeq2	LRT	0.462867828115432
679	KC_G1	Npc2a	0.122696324581981	0.746290414661494	0.998043617620652	pseudobulk	DESeq2	LRT	0.127092136413042
679	KC_G1	Npc2b	-0.504623460885858	0.623224616310177	0.998043617620652	pseudobulk	DESeq2	LRT	0.205355401104845
680	KC_G1	Npc2f	-1.73907471612892	0.507597351592657	0.998043617620652	pseudobulk	DESeq2	LRT	0.294480652518419
680	KC_G1	Nph	0.340306707268302	0.265159915192444	0.998043617620652	pseudobulk	DESeq2	LRT	0.576492128557281
680	KC_G1	Npl4	-0.14063617827555	0.745088740044516	0.998043617620652	pseudobulk	DESeq2	LRT	0.127791999712713
680	KC_G1	Nplp1	0.985376447334228	0.242780201992748	0.998043617620652	pseudobulk	DESeq2	LRT	0.614786731583065
680	KC_G1	Nplp3	-0.13012277986102	0.894189403923206	0.998043617620652	pseudobulk	DESeq2	LRT	0.048570480794253
680	KC_G1	Nprl2	0.86464243552641	0.210906905404272	0.998043617620652	pseudobulk	DESeq2	LRT	0.675909200583537
680	KC_G1	Nprl3	-0.327767776024569	0.523221268461437	0.998043617620652	pseudobulk	DESeq2	LRT	0.281314610655301
680	KC_G1	Nrd1	-0.13855255291921	0.763122588163235	0.998043617620652	pseudobulk	DESeq2	LRT	0.117405691289655
680	KC_G1	Nrg	-0.171669408864649	0.255012689474092	0.998043617620652	pseudobulk	DESeq2	LRT	0.593438208462277
680	KC_G1	Nrk	0.529729419352036	0.570091813170153	0.998043617620652	pseudobulk	DESeq2	LRT	0.244055195657079
681	KC_G1	nrm	-0.173038680625403	0.243656405859011	0.998043617620652	pseudobulk	DESeq2	LRT	0.613222166295206
681	KC_G1	Nrt	-0.409697381209999	0.127970781833846	0.998043617620652	pseudobulk	DESeq2	LRT	0.892889176733575
681	KC_G1	nrv1	-0.472213577572761	0.713895986744516	0.998043617620652	pseudobulk	DESeq2	LRT	0.146365059475235
681	KC_G1	nrv2	0.00624395072184011	0.961507597998217	0.998043617620652	pseudobulk	DESeq2	LRT	0.0170472795423383
681	KC_G1	nrv3	-0.0146578747349569	0.905124323439735	0.998043617620652	pseudobulk	DESeq2	LRT	0.0432917641367055
681	KC_G1	Nrx-1	-0.138479426425091	0.245438925339405	0.998043617620652	pseudobulk	DESeq2	LRT	0.610056559296417
681	KC_G1	Nrx-IV	-0.334945958624087	0.32514307009508	0.998043617620652	pseudobulk	DESeq2	LRT	0.487925497850268
681	KC_G1	Ns1	-0.00345897194518074	0.967747722928657	0.998043617620652	pseudobulk	DESeq2	LRT	0.0142378418851968
681	KC_G1	Ns2	-0.26782099771629	0.42479971405865	0.998043617620652	pseudobulk	DESeq2	LRT	0.371815784258961
681	KC_G1	Ns3	0.402548126968077	0.464994268326609	0.998043617620652	pseudobulk	DESeq2	LRT	0.332552400334707
682	KC_G1	Ns4	0.215272179901352	0.663905022692901	0.998043617620652	pseudobulk	DESeq2	LRT	0.177894045738439
682	KC_G1	NSD	0.135026768459656	0.859825407550654	0.998043617620652	pseudobulk	DESeq2	LRT	0.065589725774007
682	KC_G1	Nse1	1.13582588427274	0.704684208645453	0.998043617620652	pseudobulk	DESeq2	LRT	0.152005460551806
682	KC_G1	Nse4	-0.0721839325651668	0.895403224848467	0.998043617620652	pseudobulk	DESeq2	LRT	0.0479813458121091
682	KC_G1	Nsf2	0.324030777822812	0.350233562201444	0.998043617620652	pseudobulk	DESeq2	LRT	0.455642238662406
682	KC_G1	nsl1	-0.182943443616927	0.240913776140552	0.998043617620652	pseudobulk	DESeq2	LRT	0.618138365087748
682	KC_G1	nSMase	0.347051294209861	0.396688896830236	0.998043617620652	pseudobulk	DESeq2	LRT	0.401549955091373
682	KC_G1	nst	-0.119493537320826	0.746667747603478	0.998043617620652	pseudobulk	DESeq2	LRT	0.126872607665116
682	KC_G1	Nsun2	0.446495354634895	0.589546056346781	0.998043617620652	pseudobulk	DESeq2	LRT	0.229482261415461
682	KC_G1	Nsun5	-0.190911496083221	0.695222911399027	0.998043617620652	pseudobulk	DESeq2	LRT	0.157875923944013
683	KC_G1	nSyb	-0.0561120291375376	0.562405044485576	0.998043617620652	pseudobulk	DESeq2	LRT	0.249950792571403

683	KC_G1	NT5E-2	-0.280801067112539	0.883392759535737	0.998043617620652	pseudobulk	DESeq2	LRT	0.053846164627732
683	KC_G1	Ntan1	-0.261677666099405	0.406406541509233	0.998043617620652	pseudobulk	DESeq2	LRT	0.391039310275074
683	KC_G1	ntc	0.67057408137533	0.328636463942283	0.998043617620652	pseudobulk	DESeq2	LRT	0.483284250972956
683	KC_G1	Ntf-2	0.0506332957474467	0.76370578386754	0.998043617620652	pseudobulk	DESeq2	LRT	0.117073920288792
683	KC_G1	Ntl	-0.552829843018635	0.697551482935962	0.998043617620652	pseudobulk	DESeq2	LRT	0.156423733674603
683	KC_G1	Ntmt	0.404530795676633	0.662886881249478	0.998043617620652	pseudobulk	DESeq2	LRT	0.178560575728793
683	KC_G1	NTPase	0.322766457215791	0.438434899126944	0.998043617620652	pseudobulk	DESeq2	LRT	0.358094883595276
683	KC_G1	Nuak1	0.169812236224463	0.618625242163347	0.998043617620652	pseudobulk	DESeq2	LRT	0.208572363174427
683	KC_G1	nub	-0.794610277746993	0.821479263487599	0.998043617620652	pseudobulk	DESeq2	LRT	0.085403394939296
684	KC_G1	NUCB1	-0.01253384886818	0.944962685602824	0.998043617620652	pseudobulk	DESeq2	LRT	0.0245853404396709
684	KC_G1	nudC	0.159019754779577	0.688582035582661	0.998043617620652	pseudobulk	DESeq2	LRT	0.162044311792674
684	KC_G1	nudE	0.00611215593859335	0.950562346927905	0.998043617620652	pseudobulk	DESeq2	LRT	0.0220193926977995
684	KC_G1	nuf	-0.641449096647741	0.0284769230110215	0.998043617620652	pseudobulk	DESeq2	LRT	1.545506938895
684	KC_G1	Nuf2	0.173116475207952	0.97305562682953	0.998043617620652	pseudobulk	DESeq2	LRT	0.011862331638572
684	KC_G1	Nufip	0.943081660912536	0.144585295536432	0.998043617620652	pseudobulk	DESeq2	LRT	0.839875872962031
684	KC_G1	Nulp1	0.440645006560775	0.122753273430313	0.998043617620652	pseudobulk	DESeq2	LRT	0.910966917830115
684	KC_G1	numb	-0.266540821646836	0.154236025935592	0.998043617620652	pseudobulk	DESeq2	LRT	0.811814173382103
684	KC_G1	Nup107	0.270263084022325	0.625778772949614	0.998043617620652	pseudobulk	DESeq2	LRT	0.203579172649083
684	KC_G1	Nup133	0.351797599726013	0.750523218937431	0.998043617620652	pseudobulk	DESeq2	LRT	0.124635867444137
685	KC_G1	Nup153	0.00205544724884429	0.967668582434964	0.998043617620652	pseudobulk	DESeq2	LRT	0.0142733590807789
685	KC_G1	Nup154	0.10070546675747	0.889728666366066	0.998043617620652	pseudobulk	DESeq2	LRT	0.050742416575674
685	KC_G1	Nup160	-0.201016636605183	0.814324214694114	0.998043617620652	pseudobulk	DESeq2	LRT	0.0892026508614956
685	KC_G1	Nup188	-0.159408612285356	0.84973023197277	0.998043617620652	pseudobulk	DESeq2	LRT	0.0707189300046913
685	KC_G1	Nup205	0.134870586867507	0.900043455629917	0.998043617620652	pseudobulk	DESeq2	LRT	0.0457365215777038
685	KC_G1	Nup214	0.905761503420846	0.191845371463221	0.998043617620652	pseudobulk	DESeq2	LRT	0.717048674298508
685	KC_G1	Nup35	-0.597876990167528	0.216020979764254	0.998043617620652	pseudobulk	DESeq2	LRT	0.665504068509312
685	KC_G1	Nup358	-0.28685864596313	0.282436524896721	0.998043617620652	pseudobulk	DESeq2	LRT	0.549079140710267
685	KC_G1	Nup37	-0.351998988652686	0.617373374540566	0.998043617620652	pseudobulk	DESeq2	LRT	0.209452104266706
685	KC_G1	Nup43	-0.116957020655787	0.835019638500427	0.998043617620652	pseudobulk	DESeq2	LRT	0.0783033103941539
686	KC_G1	Nup44A	-0.141401171461328	0.730249535126905	0.998043617620652	pseudobulk	DESeq2	LRT	0.136528710823956
686	KC_G1	Nup50	-0.222970436814011	0.632564037840548	0.998043617620652	pseudobulk	DESeq2	LRT	0.198895501973499
686	KC_G1	Nup54	0.675487317995563	0.413594509500435	0.998043617620652	pseudobulk	DESeq2	LRT	0.38342523516917
686	KC_G1	Nup58	-0.0543447419900678	0.903427565800184	0.998043617620652	pseudobulk	DESeq2	LRT	0.0441066621781395
686	KC_G1	Nup62	0.107053406063203	0.784055850330578	0.998043617620652	pseudobulk	DESeq2	LRT	0.105653000292001
686	KC_G1	Nup75	-0.451746412781683	0.640033598046446	0.998043617620652	pseudobulk	DESeq2	LRT	0.193797227479887
686	KC_G1	Nup93-1	0.70443072082768	0.358848628376401	0.998043617620652	pseudobulk	DESeq2	LRT	0.445088709428039
686	KC_G1	Nup93-2	0.0281844950955373	0.939401412678827	0.998043617620652	pseudobulk	DESeq2	LRT	0.0271487910570086
686	KC_G1	Nup98-96	-0.00750111820372709	0.958490253245711	0.998043617620652	pseudobulk	DESeq2	LRT	0.0184122990437449
686	KC_G1	Nurf-38	0.262754403609225	0.215361459544127	0.998043617620652	pseudobulk	DESeq2	LRT	0.666832014152753
687	KC_G1	nvd	0.172834960772825	0.908610676445368	0.998043617620652	pseudobulk	DESeq2	LRT	0.0416221644022337
687	KC_G1	nvy	-0.0479381947366717	0.939627854878895	0.998043617620652	pseudobulk	DESeq2	LRT	0.0270441172234144
687	KC_G1	nwk	-0.118582270402932	0.428726952695562	0.998043617620652	pseudobulk	DESeq2	LRT	0.367819212922755
687	KC_G1	nxf2	-0.794610277746993	0.821479263487599	0.998043617620652	pseudobulk	DESeq2	LRT	0.085403394939296
687	KC_G1	Nxf3	0.0732469139170124	0.867235329405386	0.998043617620652	pseudobulk	DESeq2	LRT	0.0618630381704542
687	KC_G1	Nxt1	0.190488811615727	0.477768088274404	0.998043617620652	pseudobulk	DESeq2	LRT	0.320782861595709
687	KC_G1	O-fut1	-0.0113071491762835	0.963692709822407	0.998043617620652	pseudobulk	DESeq2	LRT	0.0160614263703466
687	KC_G1	O-fut2	-0.997053291448509	0.515823986256437	0.998043617620652	pseudobulk	DESeq2	LRT	0.287498466663754
687	KC_G1	oaf	-0.202497121807339	0.512147296204288	0.998043617620652	pseudobulk	DESeq2	LRT	0.290605115725722
687	KC_G1	Oamb	0.0230267519718888	0.888021839747258	0.998043617620652	pseudobulk	DESeq2	LRT	0.0515763531796364
688	KC_G1	Oat	-1.74005979220898	0.48579817606524	0.998043617620652	pseudobulk	DESeq2	LRT	0.313544120091384
688	KC_G1	Oatp30B	0.0250355684376519	0.920543107365874	0.998043617620652	pseudobulk	DESeq2	LRT	0.0359558694600428
688	KC_G1	Oatp33Ea	0.510053926575127	0.743568590955233	0.998043617620652	pseudobulk	DESeq2	LRT	0.128678963524829
688	KC_G1	Oatp58Dc	-0.448521937116971	0.571647953575817	0.998043617620652	pseudobulk	DESeq2	LRT	0.242871346886262
688	KC_G1	Oatp74D	-0.384078432777025	0.765692854032222	0.998043617620652	pseudobulk	DESeq2	LRT	0.115945406019364
688	KC_G1	obe	-0.544689349616086	0.44316209957558	0.998043617620652	pseudobulk	DESeq2	LRT	0.353437388733184
688	KC_G1	Obp18a	1.69981218657454	0.566400560818652	0.998043617620652	pseudobulk	DESeq2	LRT	0.246876325303589

688	KC_G1	Obp19a	1.13582588427274	0.633424328955532	0.998043617620652	pseudobulk	DESeq2	LRT	0.198305259991224
688	KC_G1	Obp44a	-0.103018655066788	0.339948657201125	0.998043617620652	pseudobulk	DESeq2	LRT	0.46858666995183
688	KC_G1	Obp49a	1.13582588427274	0.704684208645453	0.998043617620652	pseudobulk	DESeq2	LRT	0.152005460551806
689	KC_G1	Obp50e	1.13582588427274	0.704684208645453	0.998043617620652	pseudobulk	DESeq2	LRT	0.152005460551806
689	KC_G1	Obp56a	-3.27393176540752	0.093620372181894	0.998043617620652	pseudobulk	DESeq2	LRT	1.02862963669478
689	KC_G1	Obp69a	-0.794610277746993	0.879485534599449	0.998043617620652	pseudobulk	DESeq2	LRT	0.0557712992369932
689	KC_G1	Obp73a	-0.794610277746993	0.879485534599449	0.998043617620652	pseudobulk	DESeq2	LRT	0.0557712992369932
689	KC_G1	Obp83g	-0.794610277746993	0.821479263487599	0.998043617620652	pseudobulk	DESeq2	LRT	0.085403394939296
689	KC_G1	Obp85a	-1.01536159427276	0.537796284932125	0.998043617620652	pseudobulk	DESeq2	LRT	0.26938220218533
689	KC_G1	Obp8a	1.12508065046033	0.900770125530981	0.998043617620652	pseudobulk	DESeq2	LRT	0.0453860258366365
689	KC_G1	Obp99a	-0.49318008997193	0.662055854100169	0.998043617620652	pseudobulk	DESeq2	LRT	0.179105369919447
689	KC_G1	obst-E	-2.66786719284931	0.228087652304378	0.998043617620652	pseudobulk	DESeq2	LRT	0.64189822494353
689	KC_G1	ocm	-0.0207617507809697	0.942022685550155	0.998043617620652	pseudobulk	DESeq2	LRT	0.0259386385121957
690	KC_G1	Ocrl	-0.149073899973892	0.570176357503442	0.998043617620652	pseudobulk	DESeq2	LRT	0.243990794775891
690	KC_G1	Oct-TyrR	0.26544584174294	0.544302612463652	0.998043617620652	pseudobulk	DESeq2	LRT	0.264159581215123
690	KC_G1	Octalpha2R	-0.280362917612333	0.0876819812944492	0.998043617620652	pseudobulk	DESeq2	LRT	1.05708964527024
690	KC_G1	Octbeta1R	0.241305389387275	0.0378121066808633	0.998043617620652	pseudobulk	DESeq2	LRT	1.42236912548363
690	KC_G1	Oda	0.0190725453113227	0.851168540053761	0.998043617620652	pseudobulk	DESeq2	LRT	0.0699844366626849
690	KC_G1	Odc1	0.999663307776749	0.620079451707173	0.998043617620652	pseudobulk	DESeq2	LRT	0.207552660135146
690	KC_G1	Odj	-0.0524931767772223	0.886570494149776	0.998043617620652	pseudobulk	DESeq2	LRT	0.0522867265006683
690	KC_G1	OdsH	-1.72343437992478	0.512120560562093	0.998043617620652	pseudobulk	DESeq2	LRT	0.290627787806665
690	KC_G1	Oga	-0.284038022111574	0.635452589173933	0.998043617620652	pseudobulk	DESeq2	LRT	0.196916846413261
690	KC_G1	Ogg1	-0.22888814724952	0.759972932303626	0.998043617620652	pseudobulk	DESeq2	LRT	0.11920187556199
691	KC_G1	ogre	-0.0981447248654086	0.765029162914942	0.998043617620652	pseudobulk	DESeq2	LRT	0.116322009223334
691	KC_G1	ohgt	0.150807206195611	0.630858024048615	0.998043617620652	pseudobulk	DESeq2	LRT	0.200068368663973
691	KC_G1	okr	-1.39864796843056	0.34842914444729	0.998043617620652	pseudobulk	DESeq2	LRT	0.457885525469022
691	KC_G1	olf186-F	-0.1569167963043	0.560007460995911	0.998043617620652	pseudobulk	DESeq2	LRT	0.25180618683707
691	KC_G1	olf186-M	-0.757449795571951	0.820028655901106	0.998043617620652	pseudobulk	DESeq2	LRT	0.0861709709305825
691	KC_G1	olf413	-0.115093476105321	0.361161994504524	0.998043617620652	pseudobulk	DESeq2	LRT	0.442297957266027
691	KC_G1	Oli	-0.178048566977317	0.359046382411568	0.998043617620652	pseudobulk	DESeq2	LRT	0.444849444668907
691	KC_G1	omd	-0.576800790329685	0.326026602043083	0.998043617620652	pseudobulk	DESeq2	LRT	0.486746962357923
691	KC_G1	ome	0.223741781259723	0.652017938967836	0.998043617620652	pseudobulk	DESeq2	LRT	0.185740455360768
691	KC_G1	onecut	-0.103485302953298	0.486313797198859	0.998043617620652	pseudobulk	DESeq2	LRT	0.313083408897113
692	KC_G1	opa	-0.794610277746993	0.879485534599449	0.998043617620652	pseudobulk	DESeq2	LRT	0.0557712992369932
692	KC_G1	Opa1	-0.0637512675824037	0.695316462976391	0.998043617620652	pseudobulk	DESeq2	LRT	0.157817487721878
692	KC_G1	Opbp	0.0615623148903281	0.901943954085668	0.998043617620652	pseudobulk	DESeq2	LRT	0.0448204482535763
692	KC_G1	opm	0.129853378894344	0.781730385409655	0.998043617620652	pseudobulk	DESeq2	LRT	0.106943006938525
692	KC_G1	or	0.0372080581272715	0.910518427894292	0.998043617620652	pseudobulk	DESeq2	LRT	0.0407112601361492
692	KC_G1	Or13a	1.13582588427274	0.704684208645453	0.998043617620652	pseudobulk	DESeq2	LRT	0.152005460551806
692	KC_G1	Or2a	-1.72984073695683	0.480047293856853	0.998043617620652	pseudobulk	DESeq2	LRT	0.318715974188453
692	KC_G1	Or35a	0.177622136115678	0.935661853927379	0.998043617620652	pseudobulk	DESeq2	LRT	0.0288810759485907
692	KC_G1	Or43b	-0.0659011480477128	0.913331636796549	0.998043617620652	pseudobulk	DESeq2	LRT	0.0393714986115898
692	KC_G1	Or45a	-2.23422618188688	0.336998892702854	0.998043617620652	pseudobulk	DESeq2	LRT	0.472371526113321
693	KC_G1	Or56a	-0.794610277746993	0.821479263487599	0.998043617620652	pseudobulk	DESeq2	LRT	0.085403394939296
693	KC_G1	Or63a	1.79280049005751	0.438378263525642	0.998043617620652	pseudobulk	DESeq2	LRT	0.358150987974651
693	KC_G1	Or67d	1.12508065046033	0.900770125530981	0.998043617620652	pseudobulk	DESeq2	LRT	0.0453860258366365
693	KC_G1	Or71a	-1.66808346769027	0.498191247612582	0.998043617620652	pseudobulk	DESeq2	LRT	0.302603906560549
693	KC_G1	Or98a	1.12508065046033	0.900770125530981	0.998043617620652	pseudobulk	DESeq2	LRT	0.0453860258366365
693	KC_G1	Or9a	-0.794610277746993	0.879485534599449	0.998043617620652	pseudobulk	DESeq2	LRT	0.0557712992369932
693	KC_G1	orb	-0.505374635316686	0.20326040592316	0.998043617620652	pseudobulk	DESeq2	LRT	0.691947211444476
693	KC_G1	orb2	-0.111989953943095	0.429637489354876	0.998043617620652	pseudobulk	DESeq2	LRT	0.366897829939811
693	KC_G1	Orc1	1.13582588427274	0.633424328955532	0.998043617620652	pseudobulk	DESeq2	LRT	0.198305259991224
693	KC_G1	Orc4	0.38653658691394	0.684586488169804	0.998043617620652	pseudobulk	DESeq2	LRT	0.164571676876793
694	KC_G1	Orc6	-0.794610277746993	0.815039924133852	0.998043617620652	pseudobulk	DESeq2	LRT	0.0888211171417967
694	KC_G1	Orct	0.278606965928751	0.81696180165722	0.998043617620652	pseudobulk	DESeq2	LRT	0.0877982491191284
694	KC_G1	Orct2	-0.284863936404597	0.893509866109171	0.998043617620652	pseudobulk	DESeq2	LRT	0.0489006476652036

694	KC_G1	Ork1	-0.273615692082939	0.758200210476101	0.998043617620652	pseudobulk	DESeq2	LRT	0.120216099349578
694	KC_G1	ORMDL	0.254081028069353	0.467556101535042	0.998043617620652	pseudobulk	DESeq2	LRT	0.330166271154465
694	KC_G1	ORY	-1.66808346769027	0.498191247612582	0.998043617620652	pseudobulk	DESeq2	LRT	0.302603906560549
694	KC_G1	OS9	1.12508065046033	0.900770125530981	0.998043617620652	pseudobulk	DESeq2	LRT	0.0453860258366365
694	KC_G1	osa	-0.0924690681429888	0.464526759934742	0.998043617620652	pseudobulk	DESeq2	LRT	0.332989262602001
694	KC_G1	Osbp	-0.405193553117914	0.375854558936352	0.998043617620652	pseudobulk	DESeq2	LRT	0.424980177601222
694	KC_G1	Oscillin	0.0853600966663969	0.898979521160775	0.998043617620652	pseudobulk	DESeq2	LRT	0.0462502014238035
695	KC_G1	Oseg1	-0.303619550308009	0.81021396382032	0.998043617620652	pseudobulk	DESeq2	LRT	0.0914002761390365
695	KC_G1	Oseg4	-0.674877813922711	0.75415793290657	0.998043617620652	pseudobulk	DESeq2	LRT	0.122537696295389
695	KC_G1	Oseg6	0.930467025409575	0.566030965130532	0.998043617620652	pseudobulk	DESeq2	LRT	0.247159809770255
695	KC_G1	Osi2	-0.794610277746993	0.821479263487599	0.998043617620652	pseudobulk	DESeq2	LRT	0.085403394939296
695	KC_G1	Osi24	-0.966816426564127	0.626346344839242	0.998043617620652	pseudobulk	DESeq2	LRT	0.20318545263959
695	KC_G1	osp	-0.106621672311867	0.781012414641424	0.998043617620652	pseudobulk	DESeq2	LRT	0.107342062707317
695	KC_G1	Ost48	-0.0675803839754792	0.811379375941548	0.998043617620652	pseudobulk	DESeq2	LRT	0.0907760356119922
695	KC_G1	OstDelta	-0.641195025622463	0.199918578499647	0.998043617620652	pseudobulk	DESeq2	LRT	0.699146844876574
695	KC_G1	Ostgamma	0.0841180467472002	0.808124677953668	0.998043617620652	pseudobulk	DESeq2	LRT	0.0925216308464949
695	KC_G1	otk	-0.813824759361713	0.232631154128088	0.998043617620652	pseudobulk	DESeq2	LRT	0.633332124689691
696	KC_G1	otk2	-1.31275600133313	0.545710131686654	0.998043617620652	pseudobulk	DESeq2	LRT	0.263037983004907
696	KC_G1	OtopLa	-0.0680839146331529	0.580655268739404	0.998043617620652	pseudobulk	DESeq2	LRT	0.236081628913974
696	KC_G1	OtopLc	-0.18752715173916	0.366894666779926	0.998043617620652	pseudobulk	DESeq2	LRT	0.435458601149157
696	KC_G1	ouib	-0.794610277746993	0.821479263487599	0.998043617620652	pseudobulk	DESeq2	LRT	0.085403394939296
696	KC_G1	out	2.12862821144174	0.00782599425994217	0.998043617620652	pseudobulk	DESeq2	LRT	2.10646047497308
696	KC_G1	ova	-0.212355790213097	0.29055751845824	0.998043617620652	pseudobulk	DESeq2	LRT	0.536767882287915
696	KC_G1	ox	0.00403228783549976	0.974266331472959	0.998043617620652	pseudobulk	DESeq2	LRT	0.0113223054643041
696	KC_G1	OXA1L	-0.290087199519811	0.510882280882746	0.998043617620652	pseudobulk	DESeq2	LRT	0.291679159851101
696	KC_G1	oxt	0.241072460447723	0.562996759338871	0.998043617620652	pseudobulk	DESeq2	LRT	0.249494104980442
696	KC_G1	oys	-0.220329377975284	0.50235128448759	0.998043617620652	pseudobulk	DESeq2	LRT	0.298992482933348
697	KC_G1	p	0.207820135274165	0.428440082983801	0.998043617620652	pseudobulk	DESeq2	LRT	0.368109905243642
697	KC_G1	p115	-0.217547066907362	0.739435858019427	0.998043617620652	pseudobulk	DESeq2	LRT	0.131099492683161
697	KC_G1	p120ctn	0.0678414968682059	0.536401864760293	0.998043617620652	pseudobulk	DESeq2	LRT	0.270509721026074
697	KC_G1	p130CAS	-0.353310308839928	0.132014932332264	0.998043617620652	pseudobulk	DESeq2	LRT	0.879376942561374
697	KC_G1	p23	0.103175625211298	0.435386214089268	0.998043617620652	pseudobulk	DESeq2	LRT	0.361125326419741
697	KC_G1	p24-1	-0.0726843949723067	0.779906368682899	0.998043617620652	pseudobulk	DESeq2	LRT	0.107957533213591
697	KC_G1	P32	-0.0190053270801709	0.938485398510894	0.998043617620652	pseudobulk	DESeq2	LRT	0.0275724799903995
697	KC_G1	p38a	-2.64357131381291	0.236669501362524	0.998043617620652	pseudobulk	DESeq2	LRT	0.625857704231308
697	KC_G1	p38b	0.087786539959637	0.769425135041645	0.998043617620652	pseudobulk	DESeq2	LRT	0.113833630569379
697	KC_G1	p47	0.103373672778118	0.686537089949159	0.998043617620652	pseudobulk	DESeq2	LRT	0.163335995171389
698	KC_G1	p53	0.41772187499381	0.71951341275312	0.998043617620652	pseudobulk	DESeq2	LRT	0.142961105785231
698	KC_G1	P58IPK	-0.255619125228266	0.581423642304791	0.998043617620652	pseudobulk	DESeq2	LRT	0.235507312583221
698	KC_G1	P5CDh1	0.167761552173154	0.312542558408393	0.998043617620652	pseudobulk	DESeq2	LRT	0.505090837124786
698	KC_G1	P5cr	0.053674395971831	0.845471316765939	0.998043617620652	pseudobulk	DESeq2	LRT	0.0729011215749083
698	KC_G1	P5cr-2	2.76740599570183	0.225374198521499	0.998043617620652	pseudobulk	DESeq2	LRT	0.647095804710182
698	KC_G1	Pa1	0.00172163440155636	0.974148491725125	0.998043617620652	pseudobulk	DESeq2	LRT	0.0113748375551981
698	KC_G1	pAbp	-0.0409100129778888	0.655645619930064	0.998043617620652	pseudobulk	DESeq2	LRT	0.183330835768717
698	KC_G1	Pabp2	-0.148474325546015	0.482180392166649	0.998043617620652	pseudobulk	DESeq2	LRT	0.31679045415551
698	KC_G1	pad	-0.251388573492096	0.667769652536909	0.998043617620652	pseudobulk	DESeq2	LRT	0.175373321782455
698	KC_G1	Paf-AHalpha	-0.0106250209098209	0.959784262574301	0.998043617620652	pseudobulk	DESeq2	LRT	0.0178263754008611
699	KC_G1	Paics	0.110418073342572	0.834859579527058	0.998043617620652	pseudobulk	DESeq2	LRT	0.0783865651929151
699	KC_G1	pain	3.63769416840192	0.0572856794485612	0.998043617620652	pseudobulk	DESeq2	LRT	1.2419539315016
699	KC_G1	Paip2	0.0771740811442556	0.659092234208924	0.998043617620652	pseudobulk	DESeq2	LRT	0.18105380542868
699	KC_G1	Pak	-0.0506889141035577	0.802511929420759	0.998043617620652	pseudobulk	DESeq2	LRT	0.0955485030438371
699	KC_G1	Pak3	0.0874897817788142	0.786857873990664	0.998043617620652	pseudobulk	DESeq2	LRT	0.104103704890928
699	KC_G1	Pal1	-0.424792412933116	0.359732564076006	0.998043617620652	pseudobulk	DESeq2	LRT	0.444020246756434
699	KC_G1	Pal2	0.166531041369835	0.270586666842327	0.998043617620652	pseudobulk	DESeq2	LRT	0.567693607071231
699	KC_G1	pall	-0.012937713493197	0.955791090189602	0.998043617620652	pseudobulk	DESeq2	LRT	0.0196370222558255
699	KC_G1	pan	-0.0296111392924067	0.752093630672109	0.998043617620652	pseudobulk	DESeq2	LRT	0.123728089258126

699	KC_G1	PAN2	-0.551353928419938	0.287883612382971	0.998043617620652	pseudobulk	DESeq2	LRT	0.540783056393925
700	KC_G1	Panx	-0.17492880579078	0.665719388209304	0.998043617620652	pseudobulk	DESeq2	LRT	0.176708794603534
700	KC_G1	papi	0.429243291554972	0.44295528022996	0.998043617620652	pseudobulk	DESeq2	LRT	0.353640116959109
700	KC_G1	PAPLA1	-0.0265651668416737	0.888727608298433	0.998043617620652	pseudobulk	DESeq2	LRT	0.0512313282761705
700	KC_G1	Papss	-0.481664291405523	0.272153971717866	0.998043617620652	pseudobulk	DESeq2	LRT	0.565185323362095
700	KC_G1	Papst2	-0.0872152337738965	0.829333799522061	0.998043617620652	pseudobulk	DESeq2	LRT	0.0812706345733068
700	KC_G1	par-1	-0.0962430068205924	0.487638565566309	0.998043617620652	pseudobulk	DESeq2	LRT	0.311901954919282
700	KC_G1	par-6	-0.212809343226769	0.441139495214687	0.998043617620652	pseudobulk	DESeq2	LRT	0.355424058100206
700	KC_G1	para	-0.0776994755517274	0.413380756313625	0.998043617620652	pseudobulk	DESeq2	LRT	0.383649744499559
700	KC_G1	park	0.599665790247294	0.498611258934085	0.998043617620652	pseudobulk	DESeq2	LRT	0.302237919097912
700	KC_G1	Parp	0.120231989205904	0.476199751933024	0.998043617620652	pseudobulk	DESeq2	LRT	0.322210835168322
701	KC_G1	parvin	0.045240445362076	0.907840374536105	0.998043617620652	pseudobulk	DESeq2	LRT	0.041990506712879
701	KC_G1	pasha	-0.306269350208852	0.570258172259064	0.998043617620652	pseudobulk	DESeq2	LRT	0.243928482216882
701	KC_G1	pasi1	-0.102990498371366	0.863439712870896	0.998043617620652	pseudobulk	DESeq2	LRT	0.0637679803681285
701	KC_G1	pasi2	-0.101656695183474	0.846707681018206	0.998043617620652	pseudobulk	DESeq2	LRT	0.0722665004519292
701	KC_G1	Pask	0.119285102265941	0.780351858237771	0.998043617620652	pseudobulk	DESeq2	LRT	0.107709531110675
701	KC_G1	Past1	-0.19505846968536	0.337702729701835	0.998043617620652	pseudobulk	DESeq2	LRT	0.47146542888294
701	KC_G1	Pat1	0.0663788933060854	0.784117470318101	0.998043617620652	pseudobulk	DESeq2	LRT	0.105618869854737
701	KC_G1	path	-0.0385649143290734	0.875327742492425	0.998043617620652	pseudobulk	DESeq2	LRT	0.0578293068570358
701	KC_G1	Patj	-0.0275345584588645	0.944218138604991	0.998043617620652	pseudobulk	DESeq2	LRT	0.0249276609473478
701	KC_G1	Patr-1	-0.0899292020355007	0.788167753826973	0.998043617620652	pseudobulk	DESeq2	LRT	0.103381337323096
702	KC_G1	Patronin	-0.214682510005805	0.245036821531	0.998043617620652	pseudobulk	DESeq2	LRT	0.610768649569506
702	KC_G1	Patsas	0.377596376989812	0.389997810778624	0.998043617620652	pseudobulk	DESeq2	LRT	0.408937830843838
702	KC_G1	Pax	-0.233907138393923	0.482025895775475	0.998043617620652	pseudobulk	DESeq2	LRT	0.316929629623611
702	KC_G1	Pbgs	-0.479144285222452	0.350669715020828	0.998043617620652	pseudobulk	DESeq2	LRT	0.455101739585487
702	KC_G1	pbl	-1.73288829970739	0.458469392065673	0.998043617620652	pseudobulk	DESeq2	LRT	0.338689653016193
702	KC_G1	Pbp45	-2.58872325909597	0.082877026095475	0.998043617620652	pseudobulk	DESeq2	LRT	1.0815658412559
702	KC_G1	Pbp95	0.112531638245939	0.913635388633854	0.998043617620652	pseudobulk	DESeq2	LRT	0.039227086866672
702	KC_G1	Pc	0.111802460372387	0.617035648841333	0.998043617620652	pseudobulk	DESeq2	LRT	0.209689744155033
702	KC_G1	PCB	-0.264064912525893	0.356095914538493	0.998043617620652	pseudobulk	DESeq2	LRT	0.448433008924922
702	KC_G1	Pcd	0.159069260577189	0.366450482303938	0.998043617620652	pseudobulk	DESeq2	LRT	0.435984702373662
703	KC_G1	Pcf11	0.0892832653833942	0.572916511000504	0.998043617620652	pseudobulk	DESeq2	LRT	0.241908661546717
703	KC_G1	pch2	0.290339519084345	0.807480142371572	0.998043617620652	pseudobulk	DESeq2	LRT	0.0928681490767247
703	KC_G1	PCID2	-0.0927814912134973	0.860347339370423	0.998043617620652	pseudobulk	DESeq2	LRT	0.0653261800148765
703	KC_G1	pck	0.153999020825333	0.699036571257335	0.998043617620652	pseudobulk	DESeq2	LRT	0.155500102824011
703	KC_G1	Pcl	-0.0345500867569658	0.883497088941044	0.998043617620652	pseudobulk	DESeq2	LRT	0.0537948771237409
703	KC_G1	pcm	-0.0864759399805487	0.85545575648322	0.998043617620652	pseudobulk	DESeq2	LRT	0.0678024469201624
703	KC_G1	Pcmt	0.139092646860962	0.525047867529499	0.998043617620652	pseudobulk	DESeq2	LRT	0.279801101058294
703	KC_G1	PCNA	-1.72343437992478	0.512120560562093	0.998043617620652	pseudobulk	DESeq2	LRT	0.290627787806665
703	KC_G1	PCNA2	-0.259542932086844	0.633980653558279	0.998043617620652	pseudobulk	DESeq2	LRT	0.197923994769813
703	KC_G1	pcs	0.0489831912483214	0.765674676864108	0.998043617620652	pseudobulk	DESeq2	LRT	0.115955716077073
704	KC_G1	pcx	0.0206606718129578	0.947413858198132	0.998043617620652	pseudobulk	DESeq2	LRT	0.0234602669640259
704	KC_G1	Pcyt1	0.09279221584749	0.688658919702508	0.998043617620652	pseudobulk	DESeq2	LRT	0.161995823038543
704	KC_G1	Pcyt2	0.440393476541841	0.423996929854794	0.998043617620652	pseudobulk	DESeq2	LRT	0.37263728810526
704	KC_G1	PDCD-5	0.19730011164608	0.388749853439296	0.998043617620652	pseudobulk	DESeq2	LRT	0.41032976169634
704	KC_G1	Pdcd4	-0.0487572760995762	0.708172199836721	0.998043617620652	pseudobulk	DESeq2	LRT	0.149861126002765
704	KC_G1	Pde11	-0.752479231340634	0.607531954999017	0.998043617620652	pseudobulk	DESeq2	LRT	0.216430874083953
704	KC_G1	Pde1c	-0.129805785593071	0.184389355194889	0.998043617620652	pseudobulk	DESeq2	LRT	0.734264154397143
704	KC_G1	Pde6	0.0752428275807584	0.802074297967291	0.998043617620652	pseudobulk	DESeq2	LRT	0.0957854001663688
704	KC_G1	Pde8	0.226817665176368	0.23950600638796	0.998043617620652	pseudobulk	DESeq2	LRT	0.620683590773685
704	KC_G1	Pde9	0.0746864822074829	0.565622102951163	0.998043617620652	pseudobulk	DESeq2	LRT	0.247473627849476
705	KC_G1	Pdf	1.12508065046033	0.900770125530981	0.998043617620652	pseudobulk	DESeq2	LRT	0.0453860258366365
705	KC_G1	pdgy	-0.320493118280498	0.670721081015116	0.998043617620652	pseudobulk	DESeq2	LRT	0.173458043406392
705	KC_G1	Pdh	1.12508065046033	0.900770125530981	0.998043617620652	pseudobulk	DESeq2	LRT	0.0453860258366365
705	KC_G1	Pdha	-0.110394458016388	0.561079249878765	0.998043617620652	pseudobulk	DESeq2	LRT	0.25097579230133
705	KC_G1	Pdhb	0.0434682676966805	0.782827301146326	0.998043617620652	pseudobulk	DESeq2	LRT	0.106334036705344

705	KC_G1	Pdi	0.0174778973244484	0.927680327476088	0.998043617620652	pseudobulk	DESeq2	LRT	0.0326016530168085
705	KC_G1	Pdk	-0.335116294605401	0.258395987096305	0.998043617620652	pseudobulk	DESeq2	LRT	0.587714235241442
705	KC_G1	Pdk1	-0.15995313726167	0.415052403968181	0.998043617620652	pseudobulk	DESeq2	LRT	0.381897066378497
705	KC_G1	pdm3	-0.227714616306521	0.844615016181732	0.998043617620652	pseudobulk	DESeq2	LRT	0.0733412016384454
705	KC_G1	Pdp	0.263637433350613	0.52730814292682	0.998043617620652	pseudobulk	DESeq2	LRT	0.277935522048246
706	KC_G1	Pdp1	-0.0689963539019989	0.5447041923049	0.998043617620652	pseudobulk	DESeq2	LRT	0.263839282175228
706	KC_G1	Pdrg1	-0.326589244148876	0.704052908067036	0.998043617620652	pseudobulk	DESeq2	LRT	0.152394703332066
706	KC_G1	pds5	-0.417454252433062	0.191029987011619	0.998043617620652	pseudobulk	DESeq2	LRT	0.718898453844101
706	KC_G1	Pdss2	0.136618388272435	0.619478808322348	0.998043617620652	pseudobulk	DESeq2	LRT	0.20797354576263
706	KC_G1	Pdxk	-0.260324964406206	0.605627537736857	0.998043617620652	pseudobulk	DESeq2	LRT	0.217794385794543
706	KC_G1	PDZ-GEF	-0.247101669511364	0.191528926380766	0.998043617620652	pseudobulk	DESeq2	LRT	0.717765625774493
706	KC_G1	pea	-0.209920600721629	0.618809177483416	0.998043617620652	pseudobulk	DESeq2	LRT	0.20844325395643
706	KC_G1	Pect	0.330587728400096	0.0639694600382489	0.998043617620652	pseudobulk	DESeq2	LRT	1.1940273151164
706	KC_G1	PEK	0.0717974913021226	0.868754573053714	0.998043617620652	pseudobulk	DESeq2	LRT	0.0611028963048325
706	KC_G1	pelo	-0.228832032074544	0.48542888545101	0.998043617620652	pseudobulk	DESeq2	LRT	0.313874384537387
707	KC_G1	pen-2	-0.503118619122706	0.178152862159186	0.998043617620652	pseudobulk	DESeq2	LRT	0.749207195990733
707	KC_G1	peng	0.198236344268476	0.693808768311824	0.998043617620652	pseudobulk	DESeq2	LRT	0.158760215872775
707	KC_G1	Pep	-0.110207843932778	0.439839995422192	0.998043617620652	pseudobulk	DESeq2	LRT	0.356705282020697
707	KC_G1	per	0.891158582353255	0.241204333630479	0.998043617620652	pseudobulk	DESeq2	LRT	0.61761489365089
707	KC_G1	pes	-0.744247091260973	0.363905217948643	0.998043617620652	pseudobulk	DESeq2	LRT	0.439011717125895
707	KC_G1	Pex1	-0.2936357271366	0.814367059457141	0.998043617620652	pseudobulk	DESeq2	LRT	0.0891798015413563
707	KC_G1	Pex10	0.362990339617412	0.559650669089778	0.998043617620652	pseudobulk	DESeq2	LRT	0.252082972682861
707	KC_G1	Pex11	0.343091197101798	0.493273827390133	0.998043617620652	pseudobulk	DESeq2	LRT	0.306911927152065
707	KC_G1	Pex12	1.59189023847191	0.0742930630495159	0.998043617620652	pseudobulk	DESeq2	LRT	1.12905173563328
707	KC_G1	Pex13	0.248266619494039	0.297837373059707	0.998043617620652	pseudobulk	DESeq2	LRT	0.526020807271482
708	KC_G1	Pex14	0.06110394971544	0.843541560405609	0.998043617620652	pseudobulk	DESeq2	LRT	0.0738935153153117
708	KC_G1	Pex16	-0.56416947825478	0.313310274683176	0.998043617620652	pseudobulk	DESeq2	LRT	0.504025362650887
708	KC_G1	Pex19	0.0321317428313215	0.861232001993919	0.998043617620652	pseudobulk	DESeq2	LRT	0.0648798408653331
708	KC_G1	Pex2	0.151084434182609	0.774938134915275	0.998043617620652	pseudobulk	DESeq2	LRT	0.110732966832198
708	KC_G1	Pex23	0.0539453210775479	0.863847438173026	0.998043617620652	pseudobulk	DESeq2	LRT	0.0635629503567268
708	KC_G1	Pex3	-0.251778911270027	0.54480926242924	0.998043617620652	pseudobulk	DESeq2	LRT	0.263755517490805
708	KC_G1	Pex5	-0.352608847567956	0.507487979263028	0.998043617620652	pseudobulk	DESeq2	LRT	0.294574240314382
708	KC_G1	Pex6	-0.316627632351343	0.591042981054301	0.998043617620652	pseudobulk	DESeq2	LRT	0.228380935775302
708	KC_G1	Pez	-2.27643791964046	0.298906309922724	0.998043617620652	pseudobulk	DESeq2	LRT	0.52446491689215
708	KC_G1	Pfas	1.1112567833086	0.689254921431598	0.998043617620652	pseudobulk	DESeq2	LRT	0.161620124253084
709	KC_G1	Pfdn1	0.151336293513968	0.568884081778905	0.998043617620652	pseudobulk	DESeq2	LRT	0.244976218266511
709	KC_G1	Pfdn2	-0.040786993957314	0.845152498829092	0.998043617620652	pseudobulk	DESeq2	LRT	0.0730649201338409
709	KC_G1	Pfdn4	-0.0277695459526013	0.873666833249509	0.998043617620652	pseudobulk	DESeq2	LRT	0.058654150965734
709	KC_G1	Pfdn5	-0.0193122473313454	0.927512742993033	0.998043617620652	pseudobulk	DESeq2	LRT	0.0326801149489918
709	KC_G1	Pfdn6	0.11228693952041	0.503202085118181	0.998043617620652	pseudobulk	DESeq2	LRT	0.298257567973273
709	KC_G1	Pfk	-0.0355333910874092	0.852327488892067	0.998043617620652	pseudobulk	DESeq2	LRT	0.0693935046565192
709	KC_G1	Pfrx	0.0149757367782159	0.930741606798898	0.998043617620652	pseudobulk	DESeq2	LRT	0.0311708714537653
709	KC_G1	Pgam5	-0.10171130687793	0.761085934455396	0.998043617620652	pseudobulk	DESeq2	LRT	0.118566304132516
709	KC_G1	Pgant1	0.127325327336283	0.775041259856937	0.998043617620652	pseudobulk	DESeq2	LRT	0.11067517691147
709	KC_G1	Pgant2	-0.146347128211287	0.328040278435722	0.998043617620652	pseudobulk	DESeq2	LRT	0.484072828152928
710	KC_G1	Pgant3	-0.0484214042988729	0.933611361582193	0.998043617620652	pseudobulk	DESeq2	LRT	0.0298338717844212
710	KC_G1	Pgant35A	-0.414761699073369	0.577967955681815	0.998043617620652	pseudobulk	DESeq2	LRT	0.238096239531631
710	KC_G1	Pgant5	-0.235122725358036	0.31164975708563	0.998043617620652	pseudobulk	DESeq2	LRT	0.506333207281245
710	KC_G1	Pgant6	-0.348877517373298	0.627265295932404	0.998043617620652	pseudobulk	DESeq2	LRT	0.202548739566092
710	KC_G1	Pgant7	0.00747489892033488	0.958133976970836	0.998043617620652	pseudobulk	DESeq2	LRT	0.0185737587806657
710	KC_G1	PGAP1	-0.0427859561619623	0.930318750207775	0.998043617620652	pseudobulk	DESeq2	LRT	0.0313682259206741
710	KC_G1	PGAP2	-0.297224648235003	0.331135752704722	0.998043617620652	pseudobulk	DESeq2	LRT	0.479993925970649
710	KC_G1	PGAP3	0.0401794403594666	0.928411700476457	0.998043617620652	pseudobulk	DESeq2	LRT	0.0322593949068343
710	KC_G1	PGAP5	-0.149301565974471	0.746653959248015	0.998043617620652	pseudobulk	DESeq2	LRT	0.126880627647234
710	KC_G1	Pgd	-3.02853303434734	0.132089272249034	0.998043617620652	pseudobulk	DESeq2	LRT	0.879132452576506
711	KC_G1	Pgi	-0.187632935942249	0.697130845536796	0.998043617620652	pseudobulk	DESeq2	LRT	0.156685700866915

711	KC_G1	Pgk	-0.0882804043345521	0.640553516750193	0.998043617620652	pseudobulk	DESeq2	LRT	0.193444580113889
711	KC_G1	Pglym78	0.055998413487406	0.728765930930266	0.998043617620652	pseudobulk	DESeq2	LRT	0.137411938388012
711	KC_G1	Pgm1	0.0517116656809222	0.901672726510034	0.998043617620652	pseudobulk	DESeq2	LRT	0.0449510665180811
711	KC_G1	Pgm2a	-0.688464672931893	0.0779331379607859	0.998043617620652	pseudobulk	DESeq2	LRT	1.10827783663626
711	KC_G1	PGRP-LA	-1.02203883678781	0.35927141404699	0.998043617620652	pseudobulk	DESeq2	LRT	0.44457733669331
711	KC_G1	PGRP-LC	-0.794610277746993	0.879485534599449	0.998043617620652	pseudobulk	DESeq2	LRT	0.0557712992369932
711	KC_G1	PGRP-LD	1.12508065046033	0.900770125530981	0.998043617620652	pseudobulk	DESeq2	LRT	0.0453860258366365
711	KC_G1	PGRP-LE	-0.206979236105756	0.671758988175798	0.998043617620652	pseudobulk	DESeq2	LRT	0.172786513969544
711	KC_G1	PGRP-SA	-0.794610277746993	0.815039924133852	0.998043617620652	pseudobulk	DESeq2	LRT	0.0888211171417967
712	KC_G1	ph-d	0.124968435063771	0.71749383258377	0.998043617620652	pseudobulk	DESeq2	LRT	0.144181827675988
712	KC_G1	ph-p	0.026382588822348	0.926761630189404	0.998043617620652	pseudobulk	DESeq2	LRT	0.0330319551819826
712	KC_G1	PH4alphaEFB	-0.320549120244609	0.268755763699542	0.998043617620652	pseudobulk	DESeq2	LRT	0.570642213156095
712	KC_G1	PH4alphaMP	1.24386239920316	0.627869490249816	0.998043617620652	pseudobulk	DESeq2	LRT	0.202130619884913
712	KC_G1	PH4alphaNE1	-0.604665000608147	0.36844491562057	0.998043617620652	pseudobulk	DESeq2	LRT	0.433627432174616
712	KC_G1	Phax	0.0335626047677211	0.934432844781459	0.998043617620652	pseudobulk	DESeq2	LRT	0.0294519047600867
712	KC_G1	Phb2	-0.0810943466026469	0.688668408099614	0.998043617620652	pseudobulk	DESeq2	LRT	0.161989839336017
712	KC_G1	pHCl-1	-0.0356862174150621	0.779397233776839	0.998043617620652	pseudobulk	DESeq2	LRT	0.108241139931045
712	KC_G1	PheRS-m	0.0233761487912311	0.944105625360315	0.998043617620652	pseudobulk	DESeq2	LRT	0.0249794146585426
712	KC_G1	Phf5a	-0.0241006664345662	0.93472960511394	0.998043617620652	pseudobulk	DESeq2	LRT	0.0293140019513347
713	KC_G1	PHGPx	0.0211967109935149	0.912801858305058	0.998043617620652	pseudobulk	DESeq2	LRT	0.0396234844412758
713	KC_G1	Phk-3	1.13582588427274	0.704684208645453	0.998043617620652	pseudobulk	DESeq2	LRT	0.152005460551806
713	KC_G1	PhKgamma	-0.0291644498625412	0.868880839779706	0.998043617620652	pseudobulk	DESeq2	LRT	0.061039779563498
713	KC_G1	Phlpp	-1.72984073695683	0.480047293856853	0.998043617620652	pseudobulk	DESeq2	LRT	0.318715974188453
713	KC_G1	Phm	-0.0449462335360014	0.801250240170998	0.998043617620652	pseudobulk	DESeq2	LRT	0.0962318272953745
713	KC_G1	pho	0.0966013862058677	0.59341327985669	0.998043617620652	pseudobulk	DESeq2	LRT	0.226642738936017
713	KC_G1	phr	0.866908566235863	0.55507680590413	0.998043617620652	pseudobulk	DESeq2	LRT	0.255646919449439
713	KC_G1	phr6-4	-0.71088171110674	0.784645801042283	0.998043617620652	pseudobulk	DESeq2	LRT	0.105326344996467
713	KC_G1	phtf	0.231643953383331	0.407000754615013	0.998043617620652	pseudobulk	DESeq2	LRT	0.390404785554062
713	KC_G1	phyl	0.00937937041767067	0.939545511428679	0.998043617620652	pseudobulk	DESeq2	LRT	0.0270821779012783
714	KC_G1	PI31	0.0029694253408361	0.960065304174777	0.998043617620652	pseudobulk	DESeq2	LRT	0.0176992250040199
714	KC_G1	Pi3K21B	-0.22522233681445	0.371598049652202	0.998043617620652	pseudobulk	DESeq2	LRT	0.429926574085041
714	KC_G1	Pi3K59F	0.377970098615467	0.586698320706133	0.998043617620652	pseudobulk	DESeq2	LRT	0.231585154853557
714	KC_G1	Pi3K68D	0.0701726252215513	0.791263871712525	0.998043617620652	pseudobulk	DESeq2	LRT	0.101678663255288
714	KC_G1	Pi4KIIalpha	-0.564305888551152	0.150303370592425	0.998043617620652	pseudobulk	DESeq2	LRT	0.823031280136462
714	KC_G1	PI4KIIIalpha	0.062094364794133	0.805802159192992	0.998043617620652	pseudobulk	DESeq2	LRT	0.0937715732291679
714	KC_G1	pic	-0.498764797713899	0.175184667163173	0.998043617620652	pseudobulk	DESeq2	LRT	0.756503907630419
714	KC_G1	PICK1	-0.0289201103767804	0.923415425781605	0.998043617620652	pseudobulk	DESeq2	LRT	0.0346028747752686
714	KC_G1	pico	-0.194458511547045	0.327987049899288	0.998043617620652	pseudobulk	DESeq2	LRT	0.484143303447799
714	KC_G1	Picot	-0.162524452796199	0.641272675489304	0.998043617620652	pseudobulk	DESeq2	LRT	0.192957264896476
715	KC_G1	pie	1.13582588427274	0.633424328955532	0.998043617620652	pseudobulk	DESeq2	LRT	0.198305259991224
715	KC_G1	Piezo	-0.411818624361387	0.188187612717676	0.998043617620652	pseudobulk	DESeq2	LRT	0.725408967016163
715	KC_G1	Pif1A	-0.619015293963197	0.785650117817309	0.998043617620652	pseudobulk	DESeq2	LRT	0.104770820031127
715	KC_G1	PIG-A	-0.145380591082036	0.796970043031103	0.998043617620652	pseudobulk	DESeq2	LRT	0.0985580028081534
715	KC_G1	PIG-B	-0.018681434638796	0.941764831801924	0.998043617620652	pseudobulk	DESeq2	LRT	0.0260575313912096
715	KC_G1	PIG-C	0.298016580972839	0.751464728669579	0.998043617620652	pseudobulk	DESeq2	LRT	0.124091398980809
715	KC_G1	PIG-F	0.240200246940378	0.44342190187067	0.998043617620652	pseudobulk	DESeq2	LRT	0.353182859650348
715	KC_G1	PIG-G	0.125295024299817	0.887210796944977	0.998043617620652	pseudobulk	DESeq2	LRT	0.0519731816637959
715	KC_G1	PIG-H	-0.0903152702206791	0.861881848239689	0.998043617620652	pseudobulk	DESeq2	LRT	0.0645522657002151
715	KC_G1	PIG-K	0.42461245500292	0.231194267603249	0.998043617620652	pseudobulk	DESeq2	LRT	0.636022938327377
716	KC_G1	PIG-L	-0.257878734447256	0.536910123868578	0.998043617620652	pseudobulk	DESeq2	LRT	0.270098406990745
716	KC_G1	PIG-N	1.51643689583361	0.396657895687765	0.998043617620652	pseudobulk	DESeq2	LRT	0.401583896427599
716	KC_G1	PIG-O	-0.38745831326309	0.425490215763297	0.998043617620652	pseudobulk	DESeq2	LRT	0.371110422157221
716	KC_G1	PIG-Q	0.561791570958894	0.539412306269534	0.998043617620652	pseudobulk	DESeq2	LRT	0.268079149681449
716	KC_G1	PIG-S	0.0889234508655432	0.888637161840983	0.998043617620652	pseudobulk	DESeq2	LRT	0.0512755289918281
716	KC_G1	PIG-T	0.0368652772402786	0.89073163806013	0.998043617620652	pseudobulk	DESeq2	LRT	0.0502531216251163
716	KC_G1	PIG-U	-0.111020271046852	0.848080191394377	0.998043617620652	pseudobulk	DESeq2	LRT	0.0715630804868244

716	KC_G1	PIG-V	0.360724049858719	0.604006560723503	0.998043617620652	pseudobulk	DESeq2	LRT	0.218958344043537
716	KC_G1	PIG-Wa	1.03649448874304	0.607834051197003	0.998043617620652	pseudobulk	DESeq2	LRT	0.216214974159449
716	KC_G1	PIG-X	0.423916994078464	0.553718867123199	0.998043617620652	pseudobulk	DESeq2	LRT	0.256710678315959
717	KC_G1	pigeon	0.102444315139612	0.812940003813768	0.998043617620652	pseudobulk	DESeq2	LRT	0.0899415048045526
717	KC_G1	pigs	-0.0071152374390709	0.953312590701436	0.998043617620652	pseudobulk	DESeq2	LRT	0.0207646710751408
717	KC_G1	Pih1D1	-0.0272486543996259	0.903424528891309	0.998043617620652	pseudobulk	DESeq2	LRT	0.044108122079335
717	KC_G1	Pink1	-0.0400026847613582	0.872543537529246	0.998043617620652	pseudobulk	DESeq2	LRT	0.0592128937246929
717	KC_G1	Pino	-0.184153590145716	0.473153510635925	0.998043617620652	pseudobulk	DESeq2	LRT	0.324997933245572
717	KC_G1	pio	-0.794610277746993	0.879485534599449	0.998043617620652	pseudobulk	DESeq2	LRT	0.0557712992369932
717	KC_G1	PIP4K	-0.0114634315886813	0.927266043888302	0.998043617620652	pseudobulk	DESeq2	LRT	0.0327956436160919
717	KC_G1	PIP5K59B	-0.0715711547216396	0.633363137237382	0.998043617620652	pseudobulk	DESeq2	LRT	0.198347216872328
717	KC_G1	pirk	-1.27257469338748	0.647928580095599	0.998043617620652	pseudobulk	DESeq2	LRT	0.188472862925399
717	KC_G1	Pis	0.137602778236537	0.401280573877984	0.998043617620652	pseudobulk	DESeq2	LRT	0.396551864093233
718	KC_G1	Pisd	0.161336492782029	0.702169191295359	0.998043617620652	pseudobulk	DESeq2	LRT	0.153558229760942
718	KC_G1	pit	0.289652748892659	0.599303332556123	0.998043617620652	pseudobulk	DESeq2	LRT	0.222353307308169
718	KC_G1	pita	0.696911696715529	0.244289505313693	0.998043617620652	pseudobulk	DESeq2	LRT	0.61209518993304
718	KC_G1	Pits	-0.00522842527847661	0.958515082957136	0.998043617620652	pseudobulk	DESeq2	LRT	0.0184010487812067
718	KC_G1	Pitslre	0.0199092073437419	0.866833330300761	0.998043617620652	pseudobulk	DESeq2	LRT	0.0620643981281688
718	KC_G1	piwi	-0.776296732928036	0.736588270584829	0.998043617620652	pseudobulk	DESeq2	LRT	0.132775201121675
718	KC_G1	pix	-0.166269287399025	0.443846734399143	0.998043617620652	pseudobulk	DESeq2	LRT	0.352766971092122
718	KC_G1	pk	0.766315774919769	0.530965576217316	0.998043617620652	pseudobulk	DESeq2	LRT	0.274933634367645
718	KC_G1	PK2-R2	-1.42584159700357	0.401048183634649	0.998043617620652	pseudobulk	DESeq2	LRT	0.396803446258787
718	KC_G1	Pka-C1	-0.120140258287103	0.281619849962241	0.998043617620652	pseudobulk	DESeq2	LRT	0.550336737224371
719	KC_G1	Pka-C3	-0.275355534508649	0.104586509767517	0.998043617620652	pseudobulk	DESeq2	LRT	0.980524329917684
719	KC_G1	Pka-R1	-0.0136430788856299	0.900796388135168	0.998043617620652	pseudobulk	DESeq2	LRT	0.045373363851644
719	KC_G1	Pka-R2	-0.0841731244109749	0.480907306638147	0.998043617620652	pseudobulk	DESeq2	LRT	0.317938624447252
719	KC_G1	pkaap	-0.617306435507006	0.296414050786228	0.998043617620652	pseudobulk	DESeq2	LRT	0.528101213532475
719	KC_G1	Pkc53E	-0.0525040371034198	0.615707041596034	0.998043617620652	pseudobulk	DESeq2	LRT	0.210625879527222
719	KC_G1	Pkc98E	-0.0713992270028844	0.560443951974591	0.998043617620652	pseudobulk	DESeq2	LRT	0.251467813158682
719	KC_G1	PKD	-0.746975463085029	0.0935062789582347	0.998043617620652	pseudobulk	DESeq2	LRT	1.02915922521994
719	KC_G1	Pkg21D	-0.460172020241812	0.831468030455414	0.998043617620652	pseudobulk	DESeq2	LRT	0.0801544445367282
719	KC_G1	Pkn	-0.016986103737657	0.920503038243445	0.998043617620652	pseudobulk	DESeq2	LRT	0.0359747737105529
719	KC_G1	Plap	0.0992118846553562	0.874647914342896	0.998043617620652	pseudobulk	DESeq2	LRT	0.058166735126603
720	KC_G1	Plc21C	-0.0945866199304843	0.407536717307053	0.998043617620652	pseudobulk	DESeq2	LRT	0.389833257094597
720	KC_G1	PLCXD	0.116916603086997	0.739150496014905	0.998043617620652	pseudobulk	DESeq2	LRT	0.131267127322977
720	KC_G1	Pld	0.764815221789058	0.808325187140535	0.998043617620652	pseudobulk	DESeq2	LRT	0.0924138885208533
720	KC_G1	Pldn	0.417598402436883	0.22136867333151	0.998043617620652	pseudobulk	DESeq2	LRT	0.654883837666249
720	KC_G1	ple	-0.794610277746993	0.815039924133852	0.998043617620652	pseudobulk	DESeq2	LRT	0.0888211171417967
720	KC_G1	Plekhm1	0.225875050284706	0.640664392583524	0.998043617620652	pseudobulk	DESeq2	LRT	0.193369412943088
720	KC_G1	PlexA	-0.0785123073556237	0.517542026389149	0.998043617620652	pseudobulk	DESeq2	LRT	0.286054378068489
720	KC_G1	Pli	-0.102484952716829	0.486049179654481	0.998043617620652	pseudobulk	DESeq2	LRT	0.313319785527879
720	KC_G1	pll	-0.0572251021667741	0.9477382143067	0.998043617620652	pseudobulk	DESeq2	LRT	0.0233116075803895
720	KC_G1	Plod	0.0144189287326259	0.926164161879563	0.998043617620652	pseudobulk	DESeq2	LRT	0.0333120281349085
721	KC_G1	Plp	-0.074491827702535	0.85211916717459	0.998043617620652	pseudobulk	DESeq2	LRT	0.0694996657645708
721	KC_G1	plum	-0.0689762828627162	0.86932129235989	0.998043617620652	pseudobulk	DESeq2	LRT	0.060819683033099
721	KC_G1	plx	-0.220453952736729	0.211010294447751	0.998043617620652	pseudobulk	DESeq2	LRT	0.675696356490018
721	KC_G1	Plzf	-0.274199715486177	0.779649505834608	0.998043617620652	pseudobulk	DESeq2	LRT	0.108100592042647
721	KC_G1	PMCA	0.0387382990829162	0.728874227616655	0.998043617620652	pseudobulk	DESeq2	LRT	0.137347405789733
721	KC_G1	Pmm2	0.119497857090166	0.802933189816739	0.998043617620652	pseudobulk	DESeq2	LRT	0.0953205898409192
721	KC_G1	PMP34	-0.0590067605443471	0.781443465798588	0.998043617620652	pseudobulk	DESeq2	LRT	0.107102435917125
721	KC_G1	Pmp70	0.651398388687958	0.200726129935506	0.998043617620652	pseudobulk	DESeq2	LRT	0.697396088657804
721	KC_G1	Pms2	-0.0800778147257421	0.951115307052722	0.998043617620652	pseudobulk	DESeq2	LRT	0.0217668288238051
721	KC_G1	pn	0.125950069120782	0.82383020047475	0.998043617620652	pseudobulk	DESeq2	LRT	0.084162291453252
722	KC_G1	Pngl	0.0189875190498987	0.964367384417087	0.998043617620652	pseudobulk	DESeq2	LRT	0.0157574861909046
722	KC_G1	Pnn	-0.0868930501078635	0.70643277916396	0.998043617620652	pseudobulk	DESeq2	LRT	0.150929157279091
722	KC_G1	PNPase	-0.687367771560989	0.599501495401692	0.998043617620652	pseudobulk	DESeq2	LRT	0.222209729255886

722	KC_G1	pns	-0.1652988114739	0.59639601715974	0.998043617620652	pseudobulk	DESeq2	LRT	0.224465265506305
722	KC_G1	pnt	-0.773789098152905	0.487348442156775	0.998043617620652	pseudobulk	DESeq2	LRT	0.312160417849665
722	KC_G1	pnut	-0.0391631154886294	0.841528285336053	0.998043617620652	pseudobulk	DESeq2	LRT	0.0749312819948511
722	KC_G1	PNUTS	0.0259332539632912	0.863010093741886	0.998043617620652	pseudobulk	DESeq2	LRT	0.0639841247589885
722	KC_G1	pod1	0.0304121896103484	0.864838472582552	0.998043617620652	pseudobulk	DESeq2	LRT	0.0630649989129078
722	KC_G1	poe	-0.202001579035864	0.342791508181317	0.998043617620652	pseudobulk	DESeq2	LRT	0.464969945195965
722	KC_G1	Pof	-0.367151811453762	0.448884732499272	0.998043617620652	pseudobulk	DESeq2	LRT	0.347865165603198
723	KC_G1	Pol31	0.697417729493668	0.465701616946383	0.998043617620652	pseudobulk	DESeq2	LRT	0.331892254165889
723	KC_G1	Pol32	0.724095705993171	0.184602042098495	0.998043617620652	pseudobulk	DESeq2	LRT	0.733763499045738
723	KC_G1	POLDIP2	-0.162411520428281	0.554224879075653	0.998043617620652	pseudobulk	DESeq2	LRT	0.256313982698946
723	KC_G1	poly	0.0791299252517108	0.73833993819833	0.998043617620652	pseudobulk	DESeq2	LRT	0.131743639135394
723	KC_G1	polybromo	-0.220015943484267	0.586148232531956	0.998043617620652	pseudobulk	DESeq2	LRT	0.231992540245045
723	KC_G1	Pomp	0.00519087343396204	0.974766565432358	0.998043617620652	pseudobulk	DESeq2	LRT	0.0110993755657451
723	KC_G1	pont	0.125988798416236	0.797238557808452	0.998043617620652	pseudobulk	DESeq2	LRT	0.0984117051565743
723	KC_G1	Pop2	-0.0209284145501221	0.910741140021988	0.998043617620652	pseudobulk	DESeq2	LRT	0.0406050450196473
723	KC_G1	por	0.698652146744374	0.417909906664184	0.998043617620652	pseudobulk	DESeq2	LRT	0.378917333669502
723	KC_G1	porin	-0.188552728800941	0.176503142803956	0.998043617620652	pseudobulk	DESeq2	LRT	0.753247557186843
724	KC_G1	POSH	-0.0167060774408904	0.972111996467346	0.998043617620652	pseudobulk	DESeq2	LRT	0.0122836973722935
724	KC_G1	Pp1-87B	-0.144657819484239	0.234899315359775	0.998043617620652	pseudobulk	DESeq2	LRT	0.629118249020202
724	KC_G1	Pp1alpha-96A	-0.0170931254604603	0.896708686807456	0.998043617620652	pseudobulk	DESeq2	LRT	0.04734862302095
724	KC_G1	Pp2A-29B	-0.208357185628607	0.184010026650443	0.998043617620652	pseudobulk	DESeq2	LRT	0.735158511771338
724	KC_G1	Pp2B-14D	-0.013975945074787	0.919521715726593	0.998043617620652	pseudobulk	DESeq2	LRT	0.0364380098637876
724	KC_G1	Pp2C1	-0.0908069724570851	0.659540445445835	0.998043617620652	pseudobulk	DESeq2	LRT	0.18075856676606
724	KC_G1	Pp4-19C	-0.120119202705547	0.707558608822491	0.998043617620652	pseudobulk	DESeq2	LRT	0.150237580631966
724	KC_G1	Ppa	-0.109685525572056	0.787448347923734	0.998043617620652	pseudobulk	DESeq2	LRT	0.103777923820771
724	KC_G1	ppan	0.105892244759425	0.872251201363942	0.998043617620652	pseudobulk	DESeq2	LRT	0.0593584237194945
724	KC_G1	Ppat-Dpck	0.630797449924123	0.383720287666489	0.998043617620652	pseudobulk	DESeq2	LRT	0.415985238621115
725	KC_G1	Ppcs	-0.259368834342579	0.121951467098352	0.998043617620652	pseudobulk	DESeq2	LRT	0.913812970677139
725	KC_G1	PpD3	-0.198609348729631	0.590059288649891	0.998043617620652	pseudobulk	DESeq2	LRT	0.229104348629316
725	KC_G1	PpD6	-0.794610277746993	0.879485534599449	0.998043617620652	pseudobulk	DESeq2	LRT	0.0557712992369932
725	KC_G1	ppk21	2.63732022130093	0.22943645835378	0.998043617620652	pseudobulk	DESeq2	LRT	0.639337569853304
725	KC_G1	ppk25	-0.794610277746993	0.821479263487599	0.998043617620652	pseudobulk	DESeq2	LRT	0.085403394939296
725	KC_G1	ppk28	-1.7689014815933	0.397834883217982	0.998043617620652	pseudobulk	DESeq2	LRT	0.400297139449685
725	KC_G1	ppk29	2.07738706174348	0.431998797123676	0.998043617620652	pseudobulk	DESeq2	LRT	0.364517462451933
725	KC_G1	ppk8	-0.380335434673006	0.699255178391615	0.998043617620652	pseudobulk	DESeq2	LRT	0.155364308741095
725	KC_G1	ppl	-0.0314389360539004	0.927262892145156	0.998043617620652	pseudobulk	DESeq2	LRT	0.032797119769556
725	KC_G1	Ppm1	1.12508065046033	0.900770125530981	0.998043617620652	pseudobulk	DESeq2	LRT	0.0453860258366365
726	KC_G1	Ppn	1.1112567833086	0.689254921431598	0.998043617620652	pseudobulk	DESeq2	LRT	0.161620124253084
726	KC_G1	PPO1	1.90250740646012	0.460774913678782	0.998043617620652	pseudobulk	DESeq2	LRT	0.336511173568024
726	KC_G1	PPO2	-1.67593316621313	0.495877122847873	0.998043617620652	pseudobulk	DESeq2	LRT	0.304625927300966
726	KC_G1	Ppox	0.32171467688495	0.750008148740644	0.998043617620652	pseudobulk	DESeq2	LRT	0.124934018029805
726	KC_G1	PPP1R15	0.0328898250077178	0.828851549103794	0.998043617620652	pseudobulk	DESeq2	LRT	0.0815232465043281
726	KC_G1	PPP4R2r	-0.310134014504518	0.141981752027469	0.998043617620652	pseudobulk	DESeq2	LRT	0.847767469018548
726	KC_G1	pps	0.229389560395391	0.544309314964828	0.998043617620652	pseudobulk	DESeq2	LRT	0.264154233378667
726	KC_G1	Ppt1	0.506884443083301	0.823561077413028	0.998043617620652	pseudobulk	DESeq2	LRT	0.0843041869012458
726	KC_G1	Ppt2	0.0243786844822966	0.950447433962582	0.998043617620652	pseudobulk	DESeq2	LRT	0.0220718974953039
726	KC_G1	PpV	-0.0246691411498209	0.934490236652071	0.998043617620652	pseudobulk	DESeq2	LRT	0.0294252316744272
727	KC_G1	PQBP1	-0.173864709646873	0.700388792648023	0.998043617620652	pseudobulk	DESeq2	LRT	0.154660811946191
727	KC_G1	pr	-0.383871650726839	0.337483073907027	0.998043617620652	pseudobulk	DESeq2	LRT	0.47174800384959
727	KC_G1	PR-Set7	0.0655681004896406	0.787250371867647	0.998043617620652	pseudobulk	DESeq2	LRT	0.103887125546892
727	KC_G1	prage	-0.046837135191262	0.908101137886929	0.998043617620652	pseudobulk	DESeq2	LRT	0.0418657801351238
727	KC_G1	PRAS40	-0.187285379043111	0.711427273450338	0.998043617620652	pseudobulk	DESeq2	LRT	0.147869489611385
727	KC_G1	Prat	1.13582588427274	0.633424328955532	0.998043617620652	pseudobulk	DESeq2	LRT	0.198305259991224
727	KC_G1	PrBP	-0.0846134806044996	0.714582224286247	0.998043617620652	pseudobulk	DESeq2	LRT	0.145947791366734
727	KC_G1	prd1	-0.0189241605013288	0.911934259526367	0.998043617620652	pseudobulk	DESeq2	LRT	0.0400364684194006
727	KC_G1	pre-lola-G	-1.52640725960363	0.249738464174148	0.998043617620652	pseudobulk	DESeq2	LRT	0.602514563407161

727	KC_G1	pre-mod(mdg4)-AB	-1.72984073695683	0.480047293856853	0.998043617620652	pseudobulk	DESeq2	LRT	0.318715974188453
728	KC_G1	pre-mod(mdg4)-B	-0.355934189672209	0.842102986012508	0.998043617620652	pseudobulk	DESeq2	LRT	0.0746347926786073
728	KC_G1	pre-mod(mdg4)-I	-0.794610277746993	0.821479263487599	0.998043617620652	pseudobulk	DESeq2	LRT	0.085403394939296
728	KC_G1	pre-rRNA:CR45845	-0.0766027638944479	0.852241307442197	0.998043617620652	pseudobulk	DESeq2	LRT	0.0694374197255186
728	KC_G1	prel	-0.038673682995161	0.866429408451932	0.998043617620652	pseudobulk	DESeq2	LRT	0.0622668152566516
728	KC_G1	Prestin	-0.0281081919016414	0.921879268612057	0.998043617620652	pseudobulk	DESeq2	LRT	0.0353259514071044
728	KC_G1	prg	-0.140466877827058	0.766766381344127	0.998043617620652	pseudobulk	DESeq2	LRT	0.115336936895703
728	KC_G1	prim	0.604834824860058	0.120604052449811	0.998043617620652	pseudobulk	DESeq2	LRT	0.918638099102813
728	KC_G1	Prip	-0.794610277746993	0.879485534599449	0.998043617620652	pseudobulk	DESeq2	LRT	0.0557712992369932
728	KC_G1	PRL-1	-0.0609843085007048	0.741049117206605	0.998043617620652	pseudobulk	DESeq2	LRT	0.130153005752315
728	KC_G1	Proc	-0.053055203690748	0.877184870363348	0.998043617620652	pseudobulk	DESeq2	LRT	0.0569088676173787
729	KC_G1	Proc-R	0.980636021447039	0.338053381947157	0.998043617620652	pseudobulk	DESeq2	LRT	0.471014714945607
729	KC_G1	prod	-0.26623323459324	0.403690946395892	0.998043617620652	pseudobulk	DESeq2	LRT	0.393950990432133
729	KC_G1	prominin-like	-0.136511643633325	0.389581948886135	0.998043617620652	pseudobulk	DESeq2	LRT	0.409401174177305
729	KC_G1	ProRS-m	0.350500717651748	0.571611578117718	0.998043617620652	pseudobulk	DESeq2	LRT	0.242898983062119
729	KC_G1	pros	0.0768985676977329	0.474993129304778	0.998043617620652	pseudobulk	DESeq2	LRT	0.323312672325875
729	KC_G1	Prosalpha1	-0.215143972586456	0.815582001177049	0.998043617620652	pseudobulk	DESeq2	LRT	0.0885323670966208
729	KC_G1	Prosalpha2	0.0973937454846453	0.665269395171366	0.998043617620652	pseudobulk	DESeq2	LRT	0.177002455231119
729	KC_G1	Prosalpha3	0.265129333476328	0.278802762701181	0.998043617620652	pseudobulk	DESeq2	LRT	0.554702927059203
729	KC_G1	Prosalpha4	-0.163914679350248	0.54443633839522	0.998043617620652	pseudobulk	DESeq2	LRT	0.264052895522628
729	KC_G1	Prosalpha5	0.0651262740313038	0.783030858778822	0.998043617620652	pseudobulk	DESeq2	LRT	0.106221122318456
730	KC_G1	Prosalpha6	0.123345066755921	0.650753696775959	0.998043617620652	pseudobulk	DESeq2	LRT	0.186583356088739
730	KC_G1	Prosalpha7	-0.0846750080231172	0.769875878259242	0.998043617620652	pseudobulk	DESeq2	LRT	0.113579287466814
730	KC_G1	Prosap	-0.101098434361722	0.446831162419337	0.998043617620652	pseudobulk	DESeq2	LRT	0.349856546400599
730	KC_G1	Prosbeta1	-0.0834826172190679	0.706560571486909	0.998043617620652	pseudobulk	DESeq2	LRT	0.150850601352679
730	KC_G1	Prosbeta2	0.0407967271498618	0.868952314006413	0.998043617620652	pseudobulk	DESeq2	LRT	0.0610040559243134
730	KC_G1	Prosbeta2R1	-0.794610277746993	0.879485534599449	0.998043617620652	pseudobulk	DESeq2	LRT	0.0557712992369932
730	KC_G1	Prosbeta3	0.0756629881442396	0.787160067414127	0.998043617620652	pseudobulk	DESeq2	LRT	0.103936945752499
730	KC_G1	Prosbeta4	-0.0468672478249494	0.831041785482982	0.998043617620652	pseudobulk	DESeq2	LRT	0.0803771389734744
730	KC_G1	Prosbeta5	0.0147030272654473	0.941958612665413	0.998043617620652	pseudobulk	DESeq2	LRT	0.0259681786146589
730	KC_G1	Prosbeta5R2	1.13582588427274	0.704684208645453	0.998043617620652	pseudobulk	DESeq2	LRT	0.152005460551806
731	KC_G1	Prosbeta6	-0.101062434277415	0.761376505783153	0.998043617620652	pseudobulk	DESeq2	LRT	0.118400528580435
731	KC_G1	Prosbeta7	-0.109578418403632	0.736539822503089	0.998043617620652	pseudobulk	DESeq2	LRT	0.132803767184144
731	KC_G1	ProtB	-0.794610277746993	0.821479263487599	0.998043617620652	pseudobulk	DESeq2	LRT	0.085403394939296
731	KC_G1	Prp18	-0.269568128877508	0.56647304877457	0.998043617620652	pseudobulk	DESeq2	LRT	0.246820747842809
731	KC_G1	Prp19	-0.191860303598678	0.678673978200917	0.998043617620652	pseudobulk	DESeq2	LRT	0.168338802260431
731	KC_G1	Prp3	0.642403093976243	0.102484708167133	0.998043617620652	pseudobulk	DESeq2	LRT	0.98934093123334
731	KC_G1	Prp31	0.0968293880777019	0.780247392709107	0.998043617620652	pseudobulk	DESeq2	LRT	0.107767673907666
731	KC_G1	Prp38	-0.108681617067413	0.796403074720898	0.998043617620652	pseudobulk	DESeq2	LRT	0.0988670719347566
731	KC_G1	Prp8	-0.0464356339417386	0.889494628679763	0.998043617620652	pseudobulk	DESeq2	LRT	0.0508566701115711
731	KC_G1	Prpk	0.418717952677143	0.404328082624469	0.998043617620652	pseudobulk	DESeq2	LRT	0.393266093676731
732	KC_G1	prt	-0.0297068931688548	0.791238802706552	0.998043617620652	pseudobulk	DESeq2	LRT	0.101692422892017
732	KC_G1	prtp	-0.128458934971517	0.554026589853775	0.998043617620652	pseudobulk	DESeq2	LRT	0.256469391319129
732	KC_G1	Prx2540-1	-1.11566560098809	0.409995403925208	0.998043617620652	pseudobulk	DESeq2	LRT	0.387221011721993
732	KC_G1	Prx2540-2	-1.35574846224606	0.532546703611297	0.998043617620652	pseudobulk	DESeq2	LRT	0.273642299192052
732	KC_G1	Prx3	-0.130225984147368	0.539602630821059	0.998043617620652	pseudobulk	DESeq2	LRT	0.267925941601536
732	KC_G1	Prx6005	0.200088644712319	0.797623848535198	0.998043617620652	pseudobulk	DESeq2	LRT	0.0982018693247886
732	KC_G1	PRY	1.69981218657454	0.566400560818652	0.998043617620652	pseudobulk	DESeq2	LRT	0.246876325303589
732	KC_G1	ps	-0.0623357375706875	0.533379802507846	0.998043617620652	pseudobulk	DESeq2	LRT	0.272963433763253
732	KC_G1	Psa	-0.0177822378858575	0.921842656503315	0.998043617620652	pseudobulk	DESeq2	LRT	0.0353431996011876
732	KC_G1	Psc	0.191177971930507	0.582493643219997	0.998043617620652	pseudobulk	DESeq2	LRT	0.234708809751801
733	KC_G1	psd	1.12508065046033	0.900770125530981	0.998043617620652	pseudobulk	DESeq2	LRT	0.0453860258366365
733	KC_G1	Psf3	1.15921177587288	0.201314439465528	0.998043617620652	pseudobulk	DESeq2	LRT	0.696125073818255
733	KC_G1	PsGEF	0.153239208426921	0.482060502299725	0.998043617620652	pseudobulk	DESeq2	LRT	0.316898451043441
733	KC_G1	Psi	-0.0717262395280906	0.643180883392193	0.998043617620652	pseudobulk	DESeq2	LRT	0.191666872158644
733	KC_G1	psidin	0.174847271473601	0.702993722350348	0.998043617620652	pseudobulk	DESeq2	LRT	0.153048553160512

733	KC_G1	PSMG1	-0.0450655494941229	0.906261542034104	0.998043617620652	pseudobulk	DESeq2	LRT	0.0427464492653074
733	KC_G1	Psn	-0.748470588226494	0.088599463404326	0.998043617620652	pseudobulk	DESeq2	LRT	1.05256890837532
733	KC_G1	psq	-0.0704697275328681	0.558215373328383	0.998043617620652	pseudobulk	DESeq2	LRT	0.253198207152748
733	KC_G1	PSR	0.149542313124236	0.745713882411712	0.998043617620652	pseudobulk	DESeq2	LRT	0.12742777189033
733	KC_G1	pst	1.0166688969795	0.424692669878205	0.998043617620652	pseudobulk	DESeq2	LRT	0.371925234792037
734	KC_G1	ptc	-3.96925766903619	0.0279676538121379	0.998043617620652	pseudobulk	DESeq2	LRT	1.55334396477541
734	KC_G1	Pten	0.051759032377628	0.846332294081187	0.998043617620652	pseudobulk	DESeq2	LRT	0.0724590871367038
734	KC_G1	Ptip	-0.141989084961253	0.727588535631162	0.998043617620652	pseudobulk	DESeq2	LRT	0.13811415258732
734	KC_G1	PTP-ER	-0.0585404581346546	0.802453789815577	0.998043617620652	pseudobulk	DESeq2	LRT	0.0955799675286159
734	KC_G1	Ptp10D	-0.32813880391413	0.75578086517038	0.998043617620652	pseudobulk	DESeq2	LRT	0.121604107730083
734	KC_G1	Ptp36E	0.088189810908183	0.6054954334592	0.998043617620652	pseudobulk	DESeq2	LRT	0.217889127881704
734	KC_G1	Ptp4E	-0.400052965017177	0.149953147741077	0.998043617620652	pseudobulk	DESeq2	LRT	0.824044413317379
734	KC_G1	Ptp61F	-0.346890711940254	0.309680214962541	0.998043617620652	pseudobulk	DESeq2	LRT	0.509086540226259
734	KC_G1	Ptp69D	0.332821585364505	0.439292700453376	0.998043617620652	pseudobulk	DESeq2	LRT	0.357246013168
734	KC_G1	Ptp99A	0.0135253581101271	0.914823581653295	0.998043617620652	pseudobulk	DESeq2	LRT	0.0386626489954359
735	KC_G1	Ptpa	0.29879017485074	0.408898095034488	0.998043617620652	pseudobulk	DESeq2	LRT	0.388384912722595
735	KC_G1	Ptpmeg	-0.119173509865387	0.609589196718141	0.998043617620652	pseudobulk	DESeq2	LRT	0.214962738268312
735	KC_G1	Ptpmeg2	-0.214657052270145	0.113880535217825	0.998043617620652	pseudobulk	DESeq2	LRT	0.943550500415128
735	KC_G1	PTPMT1	-0.0319601043292204	0.941859755027016	0.998043617620652	pseudobulk	DESeq2	LRT	0.0260137597891768
735	KC_G1	Ptr	1.49494828808535	0.426113511221201	0.998043617620652	pseudobulk	DESeq2	LRT	0.370474694957534
735	KC_G1	Pu	0.0297995556013223	0.931403827671193	0.998043617620652	pseudobulk	DESeq2	LRT	0.0308619816351299
735	KC_G1	puc	-0.09361284448787	0.644639431776059	0.998043617620652	pseudobulk	DESeq2	LRT	0.190683132768788
735	KC_G1	puf	0.0927371626662784	0.692932779305371	0.998043617620652	pseudobulk	DESeq2	LRT	0.159308893804818
735	KC_G1	pum	-0.107525920480273	0.259022303630528	0.998043617620652	pseudobulk	DESeq2	LRT	0.586662838518664
735	KC_G1	Pur-alpha	0.161158056968711	0.238781487716232	0.998043617620652	pseudobulk	DESeq2	LRT	0.621999346279414
736	KC_G1	Pura	-2.95536489399173	0.171903558814414	0.998043617620652	pseudobulk	DESeq2	LRT	0.7647151322864
736	KC_G1	Pus1	-0.431166072121234	0.511374073820986	0.998043617620652	pseudobulk	DESeq2	LRT	0.291261294068587
736	KC_G1	Pus7	0.946103688382722	0.104472869087363	0.998043617620652	pseudobulk	DESeq2	LRT	0.980996478313435
736	KC_G1	put	-0.062280313285033	0.779532923860286	0.998043617620652	pseudobulk	DESeq2	LRT	0.108165537500301
736	KC_G1	Pvf1	-0.794610277746993	0.815039924133852	0.998043617620652	pseudobulk	DESeq2	LRT	0.0888211171417967
736	KC_G1	Pvf3	-0.252382951029794	0.789679021950088	0.998043617620652	pseudobulk	DESeq2	LRT	0.102549398991281
736	KC_G1	Pvr	-0.794610277746993	0.821479263487599	0.998043617620652	pseudobulk	DESeq2	LRT	0.085403394939296
736	KC_G1	PVRAP	-0.209824771966673	0.168802470637756	0.998043617620652	pseudobulk	DESeq2	LRT	0.772621201214558
736	KC_G1	px	-0.0729999085341314	0.578455028461933	0.998043617620652	pseudobulk	DESeq2	LRT	0.23773039928788
736	KC_G1	pxb	-0.0952688633966547	0.80714340846023	0.998043617620652	pseudobulk	DESeq2	LRT	0.0930492955509106
737	KC_G1	Pxn	0.0528506619624022	0.718996967112345	0.998043617620652	pseudobulk	DESeq2	LRT	0.143272941563085
737	KC_G1	pyd	0.215796657483988	0.128091352319491	0.998043617620652	pseudobulk	DESeq2	LRT	0.892480189277161
737	KC_G1	pyd3	0.879101348401791	0.572183658264483	0.998043617620652	pseudobulk	DESeq2	LRT	0.242464549933249
737	KC_G1	pygo	-0.143006592650368	0.667058948250749	0.998043617620652	pseudobulk	DESeq2	LRT	0.175835785620476
737	KC_G1	PyK	0.0678764117279901	0.646461270273535	0.998043617620652	pseudobulk	DESeq2	LRT	0.189457488742332
737	KC_G1	Pym	0.26433349270951	0.535715754241666	0.998043617620652	pseudobulk	DESeq2	LRT	0.271065581777939
737	KC_G1	Pyroxd1	-0.0257941912911927	0.911509805881667	0.998043617620652	pseudobulk	DESeq2	LRT	0.0402386549109343
737	KC_G1	pzg	-0.0130064941686903	0.955136199501054	0.998043617620652	pseudobulk	DESeq2	LRT	0.0199346949354251
737	KC_G1	Pzl	0.415774738963452	0.722622168082056	0.998043617620652	pseudobulk	DESeq2	LRT	0.14108871959053
737	KC_G1	QIL1	0.174624931637588	0.387312048887483	0.998043617620652	pseudobulk	DESeq2	LRT	0.411938992340142
738	KC_G1	qin	-2.75206882815525	0.120012181316925	0.998043617620652	pseudobulk	DESeq2	LRT	0.92077467053379
738	KC_G1	qjt	-1.24380500878551	0.492833919729808	0.998043617620652	pseudobulk	DESeq2	LRT	0.307299409113661
738	KC_G1	qkr54B	-0.298529848584479	0.128885454029614	0.998043617620652	pseudobulk	DESeq2	LRT	0.889796094214786
738	KC_G1	qkr58E-1	-0.0509973908773018	0.757096765607782	0.998043617620652	pseudobulk	DESeq2	LRT	0.120848609160745
738	KC_G1	qkr58E-2	0.142618623958469	0.585427931789735	0.998043617620652	pseudobulk	DESeq2	LRT	0.232526560459179
738	KC_G1	qkr58E-3	-0.0514562103224356	0.719995373289228	0.998043617620652	pseudobulk	DESeq2	LRT	0.142670294348473
738	KC_G1	qless	-0.0624911318777077	0.854007438018138	0.998043617620652	pseudobulk	DESeq2	LRT	0.0685383467862098
738	KC_G1	qm	-0.109520461516202	0.563005178759584	0.998043617620652	pseudobulk	DESeq2	LRT	0.249487610306085
738	KC_G1	Qsox1	-0.148495893356579	0.744162198885224	0.998043617620652	pseudobulk	DESeq2	LRT	0.128332394557042
738	KC_G1	qtc	0.111976899081824	0.601438057090103	0.998043617620652	pseudobulk	DESeq2	LRT	0.220809094590508
739	KC_G1	Qtzl	0.0199410985416462	0.904172219556685	0.998043617620652	pseudobulk	DESeq2	LRT	0.0437488406755135

739	KC_G1	qvr	-0.0214330876648373	0.877809421439595	0.998043617620652	pseudobulk	DESeq2	LRT	0.0565997622273527
739	KC_G1	r	-1.72984073695683	0.480047293856853	0.998043617620652	pseudobulk	DESeq2	LRT	0.318715974188453
739	KC_G1	r-cup	1.06667464613488	0.669131470313168	0.998043617620652	pseudobulk	DESeq2	LRT	0.174488544082685
739	KC_G1	r-l	0.600384389815853	0.732462615918579	0.998043617620652	pseudobulk	DESeq2	LRT	0.135214536318005
739	KC_G1	r2d2	-0.0756809250383143	0.789380525411537	0.998043617620652	pseudobulk	DESeq2	LRT	0.102713592167302
739	KC_G1	Rab1	-0.106950524787373	0.433230104676078	0.998043617620652	pseudobulk	DESeq2	LRT	0.363281372359666
739	KC_G1	Rab10	-0.0957670396698197	0.74798050584824	0.998043617620652	pseudobulk	DESeq2	LRT	0.126109720735622
739	KC_G1	Rab11	-0.0199926157290069	0.887455433066628	0.998043617620652	pseudobulk	DESeq2	LRT	0.051853447466364
739	KC_G1	Rab14	-0.097731532639131	0.490438527621641	0.998043617620652	pseudobulk	DESeq2	LRT	0.309415420063382
740	KC_G1	Rab18	-0.0445507751687733	0.863647987491684	0.998043617620652	pseudobulk	DESeq2	LRT	0.0636632346520328
740	KC_G1	Rab19	0.335877492178015	0.494207245854168	0.998043617620652	pseudobulk	DESeq2	LRT	0.30609089144828
740	KC_G1	Rab2	0.0922988862278173	0.437082738549246	0.998043617620652	pseudobulk	DESeq2	LRT	0.359436344507461
740	KC_G1	Rab26	0.00732905668856872	0.951103093838788	0.998043617620652	pseudobulk	DESeq2	LRT	0.0217724056086955
740	KC_G1	Rab3	-0.023150873872988	0.849894143746354	0.998043617620652	pseudobulk	DESeq2	LRT	0.0706351632853803
740	KC_G1	Rab3-GAP	0.415809539302014	0.621783661239083	0.998043617620652	pseudobulk	DESeq2	LRT	0.206360694204915
740	KC_G1	Rab3-GEF	-0.115849139880591	0.436311081667026	0.998043617620652	pseudobulk	DESeq2	LRT	0.360203756424774
740	KC_G1	Rab30	-0.197894763151239	0.491478509174506	0.998043617620652	pseudobulk	DESeq2	LRT	0.308495467762628
740	KC_G1	Rab32	1.12508065046033	0.900770125530981	0.998043617620652	pseudobulk	DESeq2	LRT	0.0453860258366365
740	KC_G1	Rab35	-0.106478910865254	0.486978234071498	0.998043617620652	pseudobulk	DESeq2	LRT	0.312490449532573
741	KC_G1	Rab39	0.126424267381962	0.655588597363637	0.998043617620652	pseudobulk	DESeq2	LRT	0.1833686087091
741	KC_G1	Rab4	-0.227997217409734	0.197474688651398	0.998043617620652	pseudobulk	DESeq2	LRT	0.704488562232381
741	KC_G1	Rab40	-0.144651736735727	0.628411924503956	0.998043617620652	pseudobulk	DESeq2	LRT	0.201755582548149
741	KC_G1	Rab5	0.0977497616822818	0.44971910117095	0.998043617620652	pseudobulk	DESeq2	LRT	0.347058666007845
741	KC_G1	Rab6	0.0682917277371052	0.778044994537039	0.998043617620652	pseudobulk	DESeq2	LRT	0.108995286925331
741	KC_G1	Rab7	0.0560322254235404	0.791213295835898	0.998043617620652	pseudobulk	DESeq2	LRT	0.101706423307179
741	KC_G1	Rab8	0.0606073091121541	0.766987240606846	0.998043617620652	pseudobulk	DESeq2	LRT	0.115211860796953
741	KC_G1	Rab9	0.354858749422438	0.470358069866513	0.998043617620652	pseudobulk	DESeq2	LRT	0.327571400446464
741	KC_G1	Rabex-5	0.501167993399288	0.27683722517293	0.998043617620652	pseudobulk	DESeq2	LRT	0.557775512480422
741	KC_G1	RabX1	-0.58170497582931	0.194202142862887	0.998043617620652	pseudobulk	DESeq2	LRT	0.711745982314764
742	KC_G1	RabX4	-0.0662661082588147	0.705607575336423	0.998043617620652	pseudobulk	DESeq2	LRT	0.151436765306584
742	KC_G1	RabX6	-0.0676635642242632	0.904905069062127	0.998043617620652	pseudobulk	DESeq2	LRT	0.0433969789660209
742	KC_G1	Rac1	-0.121083302795159	0.412348400557896	0.998043617620652	pseudobulk	DESeq2	LRT	0.384735685665485
742	KC_G1	Rac2	0.92092825848129	0.251962832261784	0.998043617620652	pseudobulk	DESeq2	LRT	0.598663518480799
742	KC_G1	Rack1	-0.0369875543315362	0.718953838015863	0.998043617620652	pseudobulk	DESeq2	LRT	0.143298993537256
742	KC_G1	rad	-0.0100877064844551	0.909630673939534	0.998043617620652	pseudobulk	DESeq2	LRT	0.0411349030937764
742	KC_G1	Rad17	0.628299949375863	0.244334397224887	0.998043617620652	pseudobulk	DESeq2	LRT	0.612015389050327
742	KC_G1	Rad23	-0.113149948559174	0.496960871508626	0.998043617620652	pseudobulk	DESeq2	LRT	0.30367780433882
742	KC_G1	rad50	0.0828915377844879	0.891778260817457	0.998043617620652	pseudobulk	DESeq2	LRT	0.0497431188019469
742	KC_G1	Rad51D	-0.794610277746993	0.879485534599449	0.998043617620652	pseudobulk	DESeq2	LRT	0.0557712992369932
743	KC_G1	Rad60	-0.022917812759495	0.939583864611681	0.998043617620652	pseudobulk	DESeq2	LRT	0.0270644499300636
743	KC_G1	Rad9	0.351916373909209	0.703675677508613	0.998043617620652	pseudobulk	DESeq2	LRT	0.152627460065898
743	KC_G1	Rae1	-0.0678833396999309	0.902740561660616	0.998043617620652	pseudobulk	DESeq2	LRT	0.0444370435160799
743	KC_G1	Raf	0.0256101775925633	0.924591737879991	0.998043617620652	pseudobulk	DESeq2	LRT	0.0340499917211694
743	KC_G1	RAF2	0.133146483230354	0.741749679351003	0.998043617620652	pseudobulk	DESeq2	LRT	0.129742632751087
743	KC_G1	RagA-B	-0.208291554091035	0.725258195883374	0.998043617620652	pseudobulk	DESeq2	LRT	0.139507354691018
743	KC_G1	RagC-D	0.104229451749856	0.746070647279304	0.998043617620652	pseudobulk	DESeq2	LRT	0.127220046158895
743	KC_G1	Rala	-0.0480058378897814	0.830010827698846	0.998043617620652	pseudobulk	DESeq2	LRT	0.0809162421068317
743	KC_G1	Ran	-0.15797542236342	0.372588261011951	0.998043617620652	pseudobulk	DESeq2	LRT	0.428770832365286
743	KC_G1	Ranbp16	0.513667096324366	0.0715810947272282	0.998043617620652	pseudobulk	DESeq2	LRT	1.14520166400191
744	KC_G1	Ranbp21	0.576452829516527	0.363587064289647	0.998043617620652	pseudobulk	DESeq2	LRT	0.439391576515972
744	KC_G1	RanBP3	0.263992605767222	0.527695383585557	0.998043617620652	pseudobulk	DESeq2	LRT	0.277616705125678
744	KC_G1	Ranbp9	-0.850699033232888	0.545280679038673	0.998043617620652	pseudobulk	DESeq2	LRT	0.263379890421645
744	KC_G1	RanBPM	-0.329298106825688	0.128836040543601	0.998043617620652	pseudobulk	DESeq2	LRT	0.889962630611803
744	KC_G1	RanGAP	0.560580513283846	0.569269358011628	0.998043617620652	pseudobulk	DESeq2	LRT	0.244682192272437
744	KC_G1	ranshi	-0.665853666077599	0.528386883281666	0.998043617620652	pseudobulk	DESeq2	LRT	0.277047971884721
744	KC_G1	Rap1	-0.0428822355883801	0.687860171594753	0.998043617620652	pseudobulk	DESeq2	LRT	0.162499836294525

744	KC_G1	Rap2l	-0.0535971405270965	0.782636609781194	0.998043617620652	pseudobulk	DESeq2	LRT	0.106439840751614
744	KC_G1	RapGAP1	-0.00357001950299438	0.963724096286632	0.998043617620652	pseudobulk	DESeq2	LRT	0.016047282083364
744	KC_G1	raptor	0.117371772357582	0.631858329481423	0.998043617620652	pseudobulk	DESeq2	LRT	0.199380285042742
745	KC_G1	ras	0.0754017707701173	0.780936380453388	0.998043617620652	pseudobulk	DESeq2	LRT	0.107384344795475
745	KC_G1	Ras64B	-0.196014138880427	0.668415178343532	0.998043617620652	pseudobulk	DESeq2	LRT	0.174953696725989
745	KC_G1	Ras85D	-0.00424203403490537	0.971766631091388	0.998043617620652	pseudobulk	DESeq2	LRT	0.0124380179934172
745	KC_G1	RasGAP1	0.206010094038231	0.388278485125987	0.998043617620652	pseudobulk	DESeq2	LRT	0.410856673450687
745	KC_G1	raskol	-0.2401518478744	0.153389359789766	0.998043617620652	pseudobulk	DESeq2	LRT	0.814204765190558
745	KC_G1	Rassf	0.285041279753035	0.556246426813283	0.998043617620652	pseudobulk	DESeq2	LRT	0.25473276580024
745	KC_G1	RASSF8	0.147149406402331	0.470162552958498	0.998043617620652	pseudobulk	DESeq2	LRT	0.327751964090539
745	KC_G1	Rat1	-0.415848426678602	0.305857802952323	0.998043617620652	pseudobulk	DESeq2	LRT	0.514480435434581
745	KC_G1	rau	-0.794610277746993	0.821479263487599	0.998043617620652	pseudobulk	DESeq2	LRT	0.085403394939296
745	KC_G1	Ravus	-0.170552736597543	0.664438487202764	0.998043617620652	pseudobulk	DESeq2	LRT	0.177545219243403
746	KC_G1	raw	0.0932925606952839	0.694322853761409	0.998043617620652	pseudobulk	DESeq2	LRT	0.158438539627326
746	KC_G1	rb	-0.0534143653588523	0.879023315301728	0.998043617620652	pseudobulk	DESeq2	LRT	0.0559996055034802
746	KC_G1	Rb97D	0.196892692572018	0.349273362727596	0.998043617620652	pseudobulk	DESeq2	LRT	0.456834534492724
746	KC_G1	Rbbp5	-0.00433558993230799	0.930425751943375	0.998043617620652	pseudobulk	DESeq2	LRT	0.0313182778882358
746	KC_G1	Rbcn-3A	-0.0688461362862892	0.731319194338551	0.998043617620652	pseudobulk	DESeq2	LRT	0.135893027853038
746	KC_G1	Rbcn-3B	-0.406768959720111	0.0752417400185775	0.998043617620652	pseudobulk	DESeq2	LRT	1.12354116963776
746	KC_G1	Rbf	0.381514841148753	0.375346975199487	0.998043617620652	pseudobulk	DESeq2	LRT	0.425567079622998
746	KC_G1	Rbf2	-0.794610277746993	0.815039924133852	0.998043617620652	pseudobulk	DESeq2	LRT	0.0888211171417967
746	KC_G1	Rbfox1	-0.0451937296326732	0.661078088243463	0.998043617620652	pseudobulk	DESeq2	LRT	0.179747237503474
746	KC_G1	Rbm13	0.126234972651469	0.933375088027233	0.998043617620652	pseudobulk	DESeq2	LRT	0.029943794705081
747	KC_G1	Rbp	-0.0953991003973672	0.414301775234065	0.998043617620652	pseudobulk	DESeq2	LRT	0.382683205797272
747	KC_G1	Rbp1	0.123086385682082	0.516325103181064	0.998043617620652	pseudobulk	DESeq2	LRT	0.287076759500552
747	KC_G1	Rbp1-like	-0.0355476849482718	0.73382604283042	0.998043617620652	pseudobulk	DESeq2	LRT	0.134406879583551
747	KC_G1	Rbp6	0.0485484956411786	0.627186491511894	0.998043617620652	pseudobulk	DESeq2	LRT	0.202603304158155
747	KC_G1	Rbp9	-0.224036647816811	0.126695397391341	0.998043617620652	pseudobulk	DESeq2	LRT	0.89723916194253
747	KC_G1	Rbpn-5	-0.154896449971074	0.656326237304507	0.998043617620652	pseudobulk	DESeq2	LRT	0.182880234035311
747	KC_G1	Rbsn-5	-0.0331753962415824	0.93352004664801	0.998043617620652	pseudobulk	DESeq2	LRT	0.0298763514640921
747	KC_G1	Rca1	-0.539516268280558	0.218768719542977	0.998043617620652	pseudobulk	DESeq2	LRT	0.660014775122176
747	KC_G1	Rcc1	0.0324088374537574	0.92021552067318	0.998043617620652	pseudobulk	DESeq2	LRT	0.0361104460485396
747	KC_G1	Rcd-1	0.43185266890572	0.0415672764263823	0.998043617620652	pseudobulk	DESeq2	LRT	1.38124843043108
748	KC_G1	Rcd1	-0.126035847124341	0.632309680998225	0.998043617620652	pseudobulk	DESeq2	LRT	0.199070168855891
748	KC_G1	Rcd2	-0.794610277746993	0.815039924133852	0.998043617620652	pseudobulk	DESeq2	LRT	0.0888211171417967
748	KC_G1	Rcd4	0.260820583796782	0.473319940059262	0.998043617620652	pseudobulk	DESeq2	LRT	0.324845199154884
748	KC_G1	Rcd5	0.24661868637146	0.391726990382647	0.998043617620652	pseudobulk	DESeq2	LRT	0.407016504095694
748	KC_G1	Rchy1	0.823330094954898	0.567905367737824	0.998043617620652	pseudobulk	DESeq2	LRT	0.24572402642501
748	KC_G1	rdgA	-0.0577430050585519	0.923768831772209	0.998043617620652	pseudobulk	DESeq2	LRT	0.0344366950638119
748	KC_G1	rdgB	0.0683176932191183	0.864307757073465	0.998043617620652	pseudobulk	DESeq2	LRT	0.0633315892340054
748	KC_G1	rdgBbeta	0.0121113632825033	0.950328837623666	0.998043617620652	pseudobulk	DESeq2	LRT	0.0221260919172377
748	KC_G1	rdgC	-0.206347507659223	0.196999990531633	0.998043617620652	pseudobulk	DESeq2	LRT	0.705533794711806
748	KC_G1	Rdh	0.0272839943304464	0.86770006215213	0.998043617620652	pseudobulk	DESeq2	LRT	0.0616303714402922
749	KC_G1	rdhB	0.173105107343345	0.973555957903825	0.998043617620652	pseudobulk	DESeq2	LRT	0.0116390811132178
749	KC_G1	Rdl	-0.0681622672956256	0.532151391466921	0.998043617620652	pseudobulk	DESeq2	LRT	0.27396479792031
749	KC_G1	rdo	-0.81630081790348	0.281523514498806	0.998043617620652	pseudobulk	DESeq2	LRT	0.550485324469573
749	KC_G1	rdog	-0.573929500370849	0.396600626391651	0.998043617620652	pseudobulk	DESeq2	LRT	0.401646604204789
749	KC_G1	rdx	-0.130288961948792	0.304470549966003	0.998043617620652	pseudobulk	DESeq2	LRT	0.516454708304653
749	KC_G1	rec	-0.617005528374926	0.429132138817762	0.998043617620652	pseudobulk	DESeq2	LRT	0.367408958810299
749	KC_G1	Reck	-0.794610277746993	0.821479263487599	0.998043617620652	pseudobulk	DESeq2	LRT	0.085403394939296
749	KC_G1	RecQ5	-0.359351849049364	0.594467365532398	0.998043617620652	pseudobulk	DESeq2	LRT	0.225871981849695
749	KC_G1	red	0.14011653452013	0.650959806000217	0.998043617620652	pseudobulk	DESeq2	LRT	0.18644582644136
749	KC_G1	ReepA	-0.464788347433322	0.0351098871619861	0.998043617620652	pseudobulk	DESeq2	LRT	1.454570566291
750	KC_G1	ReepB	0.117886412793292	0.501347892753343	0.998043617620652	pseudobulk	DESeq2	LRT	0.29986080612902
750	KC_G1	ref(2)P	0.110656751031449	0.623099062911212	0.998043617620652	pseudobulk	DESeq2	LRT	0.205442901887264
750	KC_G1	Ref1	-0.216201217437362	0.180799575459499	0.998043617620652	pseudobulk	DESeq2	LRT	0.742802593638384

750	KC_G1	REG	0.0427822933695546	0.834306928948304	0.998043617620652	pseudobulk	DESeq2	LRT	0.0786741495702975
750	KC_G1	Reg-5	-0.794610277746993	0.815039924133852	0.998043617620652	pseudobulk	DESeq2	LRT	0.0888211171417967
750	KC_G1	regucalcin	0.0682307830555064	0.876752945129376	0.998043617620652	pseudobulk	DESeq2	LRT	0.0571227665912694
750	KC_G1	Rel	0.0537308412667674	0.94050006966298	0.998043617620652	pseudobulk	DESeq2	LRT	0.026641167945344
750	KC_G1	rempA	0.977212865865193	0.6319571448318	0.998043617620652	pseudobulk	DESeq2	LRT	0.19931237170831
750	KC_G1	Rep	-0.438979416242989	0.456638330388317	0.998043617620652	pseudobulk	DESeq2	LRT	0.340427636478171
750	KC_G1	Reph	0.00806489815280687	0.946463106630205	0.998043617620652	pseudobulk	DESeq2	LRT	0.0238963102783133
751	KC_G1	repo	0.916746623983069	0.389970677213161	0.998043617620652	pseudobulk	DESeq2	LRT	0.408968047340892
751	KC_G1	Reps	0.00208536192237772	0.976186769447	0.998043617620652	pseudobulk	DESeq2	LRT	0.0104670827633927
751	KC_G1	rept	0.910929815160619	0.131216077515704	0.998043617620652	pseudobulk	DESeq2	LRT	0.882012948888936
751	KC_G1	REPTOR	-0.0969793841212337	0.483723586100479	0.998043617620652	pseudobulk	DESeq2	LRT	0.315402736131176
751	KC_G1	REPTOR-BP	-0.0682183480590544	0.744599099285899	0.998043617620652	pseudobulk	DESeq2	LRT	0.128077493467345
751	KC_G1	retm	-0.258498128610814	0.260571855511125	0.998043617620652	pseudobulk	DESeq2	LRT	0.584072494441917
751	KC_G1	retn	-0.794610277746993	0.879485534599449	0.998043617620652	pseudobulk	DESeq2	LRT	0.0557712992369932
751	KC_G1	Rev1	0.0995449377472826	0.914291780944057	0.998043617620652	pseudobulk	DESeq2	LRT	0.0389151843190407
751	KC_G1	Rev7	-0.241212660414825	0.784596115317364	0.998043617620652	pseudobulk	DESeq2	LRT	0.105353846474657
751	KC_G1	Rexo5	0.677278962103739	0.207548912394521	0.998043617620652	pseudobulk	DESeq2	LRT	0.68287953808549
752	KC_G1	RfC3	0.501993546023412	0.563735879880447	0.998043617620652	pseudobulk	DESeq2	LRT	0.248924322926496
752	KC_G1	RfC38	-0.275556626369628	0.629212403192956	0.998043617620652	pseudobulk	DESeq2	LRT	0.201202725042446
752	KC_G1	RfC4	0.517446808431942	0.404030294669318	0.998043617620652	pseudobulk	DESeq2	LRT	0.393586069754625
752	KC_G1	RFeSP	0.0232622178205235	0.839960023660483	0.998043617620652	pseudobulk	DESeq2	LRT	0.0757413828866856
752	KC_G1	Rfx	-0.698933275872664	0.30440221918318	0.998043617620652	pseudobulk	DESeq2	LRT	0.516552185753316
752	KC_G1	rg	-0.569886917542595	0.245383718637519	0.998043617620652	pseudobulk	DESeq2	LRT	0.610154256362305
752	KC_G1	Rga	-0.13578162199522	0.469004487229783	0.998043617620652	pseudobulk	DESeq2	LRT	0.328823002125405
752	KC_G1	Rgk1	-0.153832165622062	0.113936678534189	0.998043617620652	pseudobulk	DESeq2	LRT	0.943336445185632
752	KC_G1	Rgk2	0.502937143734166	0.09601084032889	0.998043617620652	pseudobulk	DESeq2	LRT	1.01767972915594
752	KC_G1	Rgk3	-0.166741818307257	0.292479564069418	0.998043617620652	pseudobulk	DESeq2	LRT	0.533904473246547
753	KC_G1	Rgl	-0.0179417759376351	0.903014696875369	0.998043617620652	pseudobulk	DESeq2	LRT	0.0443051813366238
753	KC_G1	rgn	-0.119793309367815	0.40042298607238	0.998043617620652	pseudobulk	DESeq2	LRT	0.397481000029224
753	KC_G1	rgr	-0.794610277746993	0.815039924133852	0.998043617620652	pseudobulk	DESeq2	LRT	0.0888211171417967
753	KC_G1	Rh4	-0.857010988773076	0.630208042193331	0.998043617620652	pseudobulk	DESeq2	LRT	0.20051605902
753	KC_G1	Rh5	-2.19332442993981	0.381369935212232	0.998043617620652	pseudobulk	DESeq2	LRT	0.418653546964052
753	KC_G1	Rh50	-0.187473439822482	0.451992506400244	0.998043617620652	pseudobulk	DESeq2	LRT	0.344868765312514
753	KC_G1	Rh7	0.88736756871806	0.0883855102451179	0.998043617620652	pseudobulk	DESeq2	LRT	1.05361892657491
753	KC_G1	rha	1.13582588427274	0.704684208645453	0.998043617620652	pseudobulk	DESeq2	LRT	0.152005460551806
753	KC_G1	Rhau	-0.433914605075261	0.528772153999918	0.998043617620652	pseudobulk	DESeq2	LRT	0.276731423572721
753	KC_G1	Rheb	0.10355064414145	0.641525401153813	0.998043617620652	pseudobulk	DESeq2	LRT	0.192786143088687
754	KC_G1	rho	-1.06794783455796	0.111327384361845	0.998043617620652	pseudobulk	DESeq2	LRT	0.953397994565452
754	KC_G1	rho-4	-0.110215631830808	0.866281750788812	0.998043617620652	pseudobulk	DESeq2	LRT	0.0623408344122585
754	KC_G1	rho-5	-0.0270711351766633	0.907935953081522	0.998043617620652	pseudobulk	DESeq2	LRT	0.041944786065165
754	KC_G1	rho-7	0.055542016708493	0.876605779892492	0.998043617620652	pseudobulk	DESeq2	LRT	0.0571956701557762
754	KC_G1	RhoBTB	-0.320973622060648	0.213124969377664	0.998043617620652	pseudobulk	DESeq2	LRT	0.671365666063955
754	KC_G1	RhoGAP100F	-0.108513064419523	0.407264268902701	0.998043617620652	pseudobulk	DESeq2	LRT	0.390123690820196
754	KC_G1	RhoGAP102A	-0.118932458246347	0.70985150026833	0.998043617620652	pseudobulk	DESeq2	LRT	0.148832495449137
754	KC_G1	RhoGAP15B	-0.776296732928036	0.736588270584829	0.998043617620652	pseudobulk	DESeq2	LRT	0.132775201121675
754	KC_G1	RhoGAP16F	0.199390646752094	0.798737359735161	0.998043617620652	pseudobulk	DESeq2	LRT	0.0975960016231603
754	KC_G1	RhoGAP18B	0.283267866218719	0.775664820360537	0.998043617620652	pseudobulk	DESeq2	LRT	0.110325905177907
755	KC_G1	RhoGAP19D	-0.0256561189524359	0.854185304546693	0.998043617620652	pseudobulk	DESeq2	LRT	0.0684479044722708
755	KC_G1	RhoGAP1A	0.152166071664028	0.531028796025522	0.998043617620652	pseudobulk	DESeq2	LRT	0.274881927852807
755	KC_G1	RhoGAP54D	-1.72984073695683	0.480047293856853	0.998043617620652	pseudobulk	DESeq2	LRT	0.318715974188453
755	KC_G1	RhoGAP5A	0.730763067353811	0.212739680021491	0.998043617620652	pseudobulk	DESeq2	LRT	0.672151498310008
755	KC_G1	RhoGAP68F	-0.310094514950169	0.205718237792431	0.998043617620652	pseudobulk	DESeq2	LRT	0.686727204554627
755	KC_G1	RhoGAP71E	0.073266715074249	0.585746280119185	0.998043617620652	pseudobulk	DESeq2	LRT	0.232290460782798
755	KC_G1	RhoGAP92B	0.0734023092858215	0.825443442520555	0.998043617620652	pseudobulk	DESeq2	LRT	0.0833126782372873
755	KC_G1	RhoGAP93B	0.00713308724404342	0.959369417883027	0.998043617620652	pseudobulk	DESeq2	LRT	0.0180141297895875
755	KC_G1	RhoGAPp190	-0.129737094359295	0.264138454049189	0.998043617620652	pseudobulk	DESeq2	LRT	0.57816836832861

755	KC_G1	RhoGDI	-0.0278528776011229	0.821937772917123	0.998043617620652	pseudobulk	DESeq2	LRT	0.085161060685849
756	KC_G1	RhoGEF2	-1.09315705876164	0.0897236627639618	0.998043617620652	pseudobulk	DESeq2	LRT	1.04709300564516
756	KC_G1	RhoGEF3	0.074883483619769	0.710737708403151	0.998043617620652	pseudobulk	DESeq2	LRT	0.14829064232179
756	KC_G1	RhoGEF64C	-1.72984073695683	0.480047293856853	0.998043617620652	pseudobulk	DESeq2	LRT	0.318715974188453
756	KC_G1	RhoU	-1.66526761788406	0.13057647708133	0.998043617620652	pseudobulk	DESeq2	LRT	0.884135052723267
756	KC_G1	Rhp	-0.265764833943883	0.67004304216726	0.998043617620652	pseudobulk	DESeq2	LRT	0.173897298231534
756	KC_G1	Ric	0.0151622317950541	0.878495871361301	0.998043617620652	pseudobulk	DESeq2	LRT	0.0562602752036525
756	KC_G1	RIC-3	-0.0099163404913499	0.943839662273381	0.998043617620652	pseudobulk	DESeq2	LRT	0.0251017765750308
756	KC_G1	ric8a	0.0368886770405178	0.919824564505121	0.998043617620652	pseudobulk	DESeq2	LRT	0.0362949964943444
756	KC_G1	Rich	0.0109152593853965	0.942376881918193	0.998043617620652	pseudobulk	DESeq2	LRT	0.0257753763966486
756	KC_G1	rictor	-0.207563571456291	0.595220059800551	0.998043617620652	pseudobulk	DESeq2	LRT	0.225322440846108
757	KC_G1	Rif1	1.86162656703847	0.194427718537778	0.998043617620652	pseudobulk	DESeq2	LRT	0.711241819914554
757	KC_G1	Rift	-0.0866478775119144	0.84609979187202	0.998043617620652	pseudobulk	DESeq2	LRT	0.0725784117795593
757	KC_G1	rig	-1.23302523133249	0.00972668990862019	0.998043617620652	pseudobulk	DESeq2	LRT	2.01203492941308
757	KC_G1	Rilpl	-0.00391035569542128	0.944230343369239	0.998043617620652	pseudobulk	DESeq2	LRT	0.024922047384874
757	KC_G1	Rim	0.0874163510197215	0.421695292571147	0.998043617620652	pseudobulk	DESeq2	LRT	0.375001247033004
757	KC_G1	Rim2	-0.0410008169930311	0.874543027230198	0.998043617620652	pseudobulk	DESeq2	LRT	0.0582188185135443
757	KC_G1	rin	-0.304407992552097	0.015499611396153	0.998043617620652	pseudobulk	DESeq2	LRT	1.80967919025694
757	KC_G1	ringer	0.154771973508626	0.226246512465142	0.998043617620652	pseudobulk	DESeq2	LRT	0.645418106614772
757	KC_G1	Rint1	0.243801897763115	0.742184858935557	0.998043617620652	pseudobulk	DESeq2	LRT	0.129487909792964
757	KC_G1	RIOK1	-0.456412578287252	0.565653469459569	0.998043617620652	pseudobulk	DESeq2	LRT	0.247449544766888
758	KC_G1	RIOK2	0.26407818461865	0.651740370450013	0.998043617620652	pseudobulk	DESeq2	LRT	0.185925376870516
758	KC_G1	Rip11	0.231768880425955	0.498870564564912	0.998043617620652	pseudobulk	DESeq2	LRT	0.302012120482883
758	KC_G1	Ripalpha	-0.0723389354717192	0.815760891164534	0.998043617620652	pseudobulk	DESeq2	LRT	0.0884371192672299
758	KC_G1	rk	-0.410204446226527	0.854948780430877	0.998043617620652	pseudobulk	DESeq2	LRT	0.0680599028654498
758	KC_G1	rl	-0.0847535220181235	0.501949277915242	0.998043617620652	pseudobulk	DESeq2	LRT	0.299340166190572
758	KC_G1	Rlip	0.0980560959801692	0.654887544660186	0.998043617620652	pseudobulk	DESeq2	LRT	0.183833269361687
758	KC_G1	RluA-2	-0.25814083730797	0.654068265519033	0.998043617620652	pseudobulk	DESeq2	LRT	0.184376921720059
758	KC_G1	Rm62	-0.181710676026427	0.189259339225476	0.998043617620652	pseudobulk	DESeq2	LRT	0.722942680525304
758	KC_G1	Rme-8	0.460658262189197	0.231677812770024	0.998043617620652	pseudobulk	DESeq2	LRT	0.635115555562338
758	KC_G1	rn	-2.15503262085468	0.16871366603772	0.998043617620652	pseudobulk	DESeq2	LRT	0.772849737538973
759	KC_G1	RN-tre	-0.181718143239168	0.472356467212791	0.998043617620652	pseudobulk	DESeq2	LRT	0.325730134175456
759	KC_G1	RNASEK	0.0769314333377049	0.491601790300037	0.998043617620652	pseudobulk	DESeq2	LRT	0.308386544182631
759	KC_G1	RNaseMRP:RNA	0.52942967196676	0.3760397698749	0.998043617620652	pseudobulk	DESeq2	LRT	0.424766221764227
759	KC_G1	RNaseP:RNA	2.78724684285116	0.139381364315635	0.998043617620652	pseudobulk	DESeq2	LRT	0.855795288762513
759	KC_G1	RNaseX25	-0.0676355741957204	0.880400058173087	0.998043617620652	pseudobulk	DESeq2	LRT	0.0553199374223586
759	KC_G1	RNaseZ	0.481981168847419	0.664525423828694	0.998043617620652	pseudobulk	DESeq2	LRT	0.177488398893871
759	KC_G1	Rnb	0.538775161422891	0.392396623912057	0.998043617620652	pseudobulk	DESeq2	LRT	0.40627473784324
759	KC_G1	Rnf146	0.0423110911171268	0.893189790839462	0.998043617620652	pseudobulk	DESeq2	LRT	0.0490562495587561
759	KC_G1	rngo	0.220960597132893	0.694397179986853	0.998043617620652	pseudobulk	DESeq2	LRT	0.158392051539277
759	KC_G1	rnh1	0.0501659404765286	0.926392239060586	0.998043617620652	pseudobulk	DESeq2	LRT	0.0332050919448775
760	KC_G1	Rnmt	0.353000794340593	0.554077639114609	0.998043617620652	pseudobulk	DESeq2	LRT	0.256429376288554
760	KC_G1	rno	-0.111433420875575	0.526649438950875	0.998043617620652	pseudobulk	DESeq2	LRT	0.278478374140449
760	KC_G1	Rnp4F	0.299452380931079	0.369856181199666	0.998043617620652	pseudobulk	DESeq2	LRT	0.431967118780447
760	KC_G1	RnpS1	-0.0972589109966523	0.676081941625898	0.998043617620652	pseudobulk	DESeq2	LRT	0.170000664059391
760	KC_G1	RnrL	-1.67593316621313	0.495877122847873	0.998043617620652	pseudobulk	DESeq2	LRT	0.304625927300966
760	KC_G1	RnrS	-0.794610277746993	0.879485534599449	0.998043617620652	pseudobulk	DESeq2	LRT	0.0557712992369932
760	KC_G1	ro	1.13582588427274	0.633424328955532	0.998043617620652	pseudobulk	DESeq2	LRT	0.198305259991224
760	KC_G1	robl	-0.112269494775199	0.47750517952112	0.998043617620652	pseudobulk	DESeq2	LRT	0.321021913242033
760	KC_G1	robls54B	-0.794610277746993	0.879485534599449	0.998043617620652	pseudobulk	DESeq2	LRT	0.0557712992369932
760	KC_G1	robo1	-0.23152109510383	0.193687862305363	0.998043617620652	pseudobulk	DESeq2	LRT	0.712897594040661
761	KC_G1	Roc1a	-0.0863387071649593	0.640574101590895	0.998043617620652	pseudobulk	DESeq2	LRT	0.193430623841929
761	KC_G1	Roc1b	0.177622136115678	0.935661853927379	0.998043617620652	pseudobulk	DESeq2	LRT	0.0288810759485907
761	KC_G1	Roc2	0.026481953101729	0.889335664164384	0.998043617620652	pseudobulk	DESeq2	LRT	0.0509342912453117
761	KC_G1	rod	-1.60891734856068	0.545249891924755	0.998043617620652	pseudobulk	DESeq2	LRT	0.263404411831769
761	KC_G1	Roe1	-0.0287303003116369	0.915685628960295	0.998043617620652	pseudobulk	DESeq2	LRT	0.038253601705124

761	KC_G1	rogdi	0.221374918245084	0.465047187766265	0.998043617620652	pseudobulk	DESeq2	LRT	0.332502977547148
761	KC_G1	roh	0.018106833396458	0.879551419227978	0.998043617620652	pseudobulk	DESeq2	LRT	0.055738766287002
761	KC_G1	Rok	-0.108306618020524	0.677539619103556	0.998043617620652	pseudobulk	DESeq2	LRT	0.169065304357657
761	KC_G1	rols	-0.718676691072112	0.739205546127982	0.998043617620652	pseudobulk	DESeq2	LRT	0.131234783341328
761	KC_G1	Rop	-0.08773148864454	0.535565170239038	0.998043617620652	pseudobulk	DESeq2	LRT	0.271187674499541
762	KC_G1	roq	-0.0700869627482078	0.753992309927571	0.998043617620652	pseudobulk	DESeq2	LRT	0.122633083537458
762	KC_G1	Ror	0.00495013526874438	0.955602828844918	0.998043617620652	pseudobulk	DESeq2	LRT	0.0197225732901801
762	KC_G1	row	-0.208940934744515	0.404873799485098	0.998043617620652	pseudobulk	DESeq2	LRT	0.39268032673174
762	KC_G1	Rox8	-0.278530876432939	0.0957337545310279	0.998043617620652	pseudobulk	DESeq2	LRT	1.01893490838931
762	KC_G1	RpA-70	0.843515980496434	0.150187332113874	0.998043617620652	pseudobulk	DESeq2	LRT	0.823366697325576
762	KC_G1	RPA2	0.194850076648254	0.773557699723747	0.998043617620652	pseudobulk	DESeq2	LRT	0.111507286700175
762	KC_G1	RPA3	0.642134986013494	0.189220460829316	0.998043617620652	pseudobulk	DESeq2	LRT	0.723031904166695
762	KC_G1	Rpb10	-0.016738168943719	0.939545586086945	0.998043617620652	pseudobulk	DESeq2	LRT	0.0270821433913247
762	KC_G1	Rpb11	0.174143451656038	0.645422488796308	0.998043617620652	pseudobulk	DESeq2	LRT	0.190155906316704
762	KC_G1	Rpb12	0.111794575796043	0.677815325911732	0.998043617620652	pseudobulk	DESeq2	LRT	0.168888615664403
763	KC_G1	Rpb4	2.50145379121547	0.286990765027193	0.998043617620652	pseudobulk	DESeq2	LRT	0.542132078047399
763	KC_G1	Rpb5	-0.177382944889624	0.655282552067643	0.998043617620652	pseudobulk	DESeq2	LRT	0.183571395666056
763	KC_G1	Rpb7	-0.0122166527492714	0.970959545424027	0.998043617620652	pseudobulk	DESeq2	LRT	0.0127988643918037
763	KC_G1	Rpb8	-0.40725279493997	0.264477161926273	0.998043617620652	pseudobulk	DESeq2	LRT	0.577611824111301
763	KC_G1	Rpe	0.303648634521122	0.817650257752191	0.998043617620652	pseudobulk	DESeq2	LRT	0.0874324220116669
763	KC_G1	Rph	0.475348995533121	0.185502870897205	0.998043617620652	pseudobulk	DESeq2	LRT	0.731649364727284
763	KC_G1	Rpi	0.471951711175174	0.724240136813817	0.998043617620652	pseudobulk	DESeq2	LRT	0.140117410596415
763	KC_G1	RpI1	-0.285474395346443	0.708646676539648	0.998043617620652	pseudobulk	DESeq2	LRT	0.149570245319268
763	KC_G1	RpI12	-1.07827276367577	0.484524495131448	0.998043617620652	pseudobulk	DESeq2	LRT	0.314684262304625
763	KC_G1	RpI135	0.378523952086633	0.731641724873394	0.998043617620652	pseudobulk	DESeq2	LRT	0.13570153506337
764	KC_G1	RpII140	0.0228169441721413	0.933301073961545	0.998043617620652	pseudobulk	DESeq2	LRT	0.0299782344231025
764	KC_G1	RpII15	0.192772431517794	0.592125188199063	0.998043617620652	pseudobulk	DESeq2	LRT	0.227586464229279
764	KC_G1	RpII18	0.00317509534561698	0.972794071421992	0.998043617620652	pseudobulk	DESeq2	LRT	0.0119790848194491
764	KC_G1	RpII215	-0.280617150021811	0.159731936987284	0.998043617620652	pseudobulk	DESeq2	LRT	0.796608241841127
764	KC_G1	RpII33	0.3286604578671	0.327811120766624	0.998043617620652	pseudobulk	DESeq2	LRT	0.484376317374255
764	KC_G1	RpIII128	-0.425992341297856	0.576275306884644	0.998043617620652	pseudobulk	DESeq2	LRT	0.239369989326327
764	KC_G1	RpIIIC160	-0.418680270990006	0.592672054051365	0.998043617620652	pseudobulk	DESeq2	LRT	0.227185550333816
764	KC_G1	RpIIIC53	-0.468485143305296	0.472879990983951	0.998043617620652	pseudobulk	DESeq2	LRT	0.325249061939537
764	KC_G1	RpL10	-0.119695452762742	0.232221014550541	0.998043617620652	pseudobulk	DESeq2	LRT	0.634098481882624
764	KC_G1	RpL10Ab	-0.100254077345677	0.487816583031797	0.998043617620652	pseudobulk	DESeq2	LRT	0.311743440191233
765	KC_G1	RpL11	-0.0884735260407471	0.592255980654088	0.998043617620652	pseudobulk	DESeq2	LRT	0.227490545038369
765	KC_G1	RpL12	-0.0748639479365851	0.543103372192908	0.998043617620652	pseudobulk	DESeq2	LRT	0.265117500613329
765	KC_G1	RpL13	-0.123467289800881	0.277427128277472	0.998043617620652	pseudobulk	DESeq2	LRT	0.556851073597432
765	KC_G1	RpL13A	-0.0623541347212207	0.620095484478027	0.998043617620652	pseudobulk	DESeq2	LRT	0.207541431164606
765	KC_G1	RpL14	-0.127199508159591	0.177105212619465	0.998043617620652	pseudobulk	DESeq2	LRT	0.751768656322319
765	KC_G1	RpL15	-0.0649968731451909	0.479254578179796	0.998043617620652	pseudobulk	DESeq2	LRT	0.31943372974103
765	KC_G1	RpL17	-0.187879744551678	0.0503634158596514	0.998043617620652	pseudobulk	DESeq2	LRT	1.29788482187855
765	KC_G1	RpL18	-0.0792791198513381	0.450900322983926	0.998043617620652	pseudobulk	DESeq2	LRT	0.345919453604304
765	KC_G1	RpL18A	-0.085308077244795	0.470707965566444	0.998043617620652	pseudobulk	DESeq2	LRT	0.327248452272792
765	KC_G1	RpL19	-0.149374928343737	0.131243062272935	0.998043617620652	pseudobulk	DESeq2	LRT	0.881923644845558
766	KC_G1	RpL21	-0.108582040866804	0.335587676883215	0.998043617620652	pseudobulk	DESeq2	LRT	0.474193995280409
766	KC_G1	RpL22	-0.215755480215576	0.309862718352144	0.998043617620652	pseudobulk	DESeq2	LRT	0.508830673479779
766	KC_G1	RpL23	0.0126943265579183	0.920266722799076	0.998043617620652	pseudobulk	DESeq2	LRT	0.03608628194609
766	KC_G1	RpL23A	-0.182762576957248	0.2147636121502	0.998043617620652	pseudobulk	DESeq2	LRT	0.668039300165556
766	KC_G1	RpL24	-0.0868980137670263	0.360425561156893	0.998043617620652	pseudobulk	DESeq2	LRT	0.443184416705905
766	KC_G1	RpL24-like	-0.0896935314247625	0.560627838037096	0.998043617620652	pseudobulk	DESeq2	LRT	0.251325341099939
766	KC_G1	RpL26	-0.0490399876232908	0.647288593879134	0.998043617620652	pseudobulk	DESeq2	LRT	0.188902045784474
766	KC_G1	RpL27	-0.0600506899666172	0.692424346020686	0.998043617620652	pseudobulk	DESeq2	LRT	0.159627670495436
766	KC_G1	RpL27A	-0.111016327263018	0.339638017465258	0.998043617620652	pseudobulk	DESeq2	LRT	0.468983702847184
766	KC_G1	RpL28	-0.0895167159345339	0.49502186016438	0.998043617620652	pseudobulk	DESeq2	LRT	0.305375622199485
767	KC_G1	RpL29	-0.134073257664213	0.246775495878537	0.998043617620652	pseudobulk	DESeq2	LRT	0.607697966733929

767	KC_G1	RpL3	-0.270967443347443	0.0638585834748672	0.998043617620652	pseudobulk	DESeq2	LRT	1.19478071930537
767	KC_G1	RpL30	0.13933110365036	0.400780743872488	0.998043617620652	pseudobulk	DESeq2	LRT	0.397093152986172
767	KC_G1	RpL31	-0.144672862588461	0.268036838769897	0.998043617620652	pseudobulk	DESeq2	LRT	0.571805512780914
767	KC_G1	RpL32	-0.185364082783114	0.166180604267033	0.998043617620652	pseudobulk	DESeq2	LRT	0.779419666179777
767	KC_G1	RpL34a	0.0293473397145951	0.862579237210403	0.998043617620652	pseudobulk	DESeq2	LRT	0.0642009997878811
767	KC_G1	RpL34b	-0.143903596929316	0.16952747185641	0.998043617620652	pseudobulk	DESeq2	LRT	0.770759914517551
767	KC_G1	RpL35	-0.106230876532449	0.411715697070239	0.998043617620652	pseudobulk	DESeq2	LRT	0.385402574778006
767	KC_G1	RpL35A	-0.0708955201730002	0.59935030443843	0.998043617620652	pseudobulk	DESeq2	LRT	0.222319269736895
767	KC_G1	RpL36	-0.0690009157739741	0.449060525605251	0.998043617620652	pseudobulk	DESeq2	LRT	0.347695119654028
768	KC_G1	RpL36A	-0.172072449484759	0.0975733410144627	0.998043617620652	pseudobulk	DESeq2	LRT	1.01066882405275
768	KC_G1	RpL37a	-0.103796485694016	0.306859119552242	0.998043617620652	pseudobulk	DESeq2	LRT	0.513060965381961
768	KC_G1	RpL37A	-0.189530154561193	0.125167239820736	0.998043617620652	pseudobulk	DESeq2	LRT	0.902509324694419
768	KC_G1	RpL38	-0.0242391278588642	0.833151024736232	0.998043617620652	pseudobulk	DESeq2	LRT	0.0792762671831753
768	KC_G1	RpL39	-0.296698948232915	0.01561583621703	0.998043617620652	pseudobulk	DESeq2	LRT	1.80643475464748
768	KC_G1	RpL4	-0.165401917492644	0.221492122420942	0.998043617620652	pseudobulk	DESeq2	LRT	0.65464171526463
768	KC_G1	RpL40	-0.0145628447843957	0.88434426344934	0.998043617620652	pseudobulk	DESeq2	LRT	0.0533786370105332
768	KC_G1	RpL41	-0.283424623350664	0.00589757435137855	0.998043617620652	pseudobulk	DESeq2	LRT	2.22932657520881
768	KC_G1	RpL5	-0.0975468832933827	0.423119685777556	0.998043617620652	pseudobulk	DESeq2	LRT	0.373536768510163
768	KC_G1	RpL6	0.00344121516961885	0.972065561062581	0.998043617620652	pseudobulk	DESeq2	LRT	0.0123044430495389
769	KC_G1	RpL7	-0.143171671675849	0.207889835786003	0.998043617620652	pseudobulk	DESeq2	LRT	0.682166743809279
769	KC_G1	RpL7-like	1.13582588427274	0.633424328955532	0.998043617620652	pseudobulk	DESeq2	LRT	0.198305259991224
769	KC_G1	RpL7A	-0.118483886165105	0.297117365326095	0.998043617620652	pseudobulk	DESeq2	LRT	0.527071964672589
769	KC_G1	RpL8	-0.158894536817061	0.121029872603905	0.998043617620652	pseudobulk	DESeq2	LRT	0.917107423850134
769	KC_G1	RpL9	-0.103433601647001	0.388651274255418	0.998043617620652	pseudobulk	DESeq2	LRT	0.410439904052179
769	KC_G1	RpLP0	-0.313539897837066	0.00604272691779493	0.998043617620652	pseudobulk	DESeq2	LRT	2.21876703189329
769	KC_G1	RpLP0-like	0.351105131724574	0.470944616846184	0.998043617620652	pseudobulk	DESeq2	LRT	0.32703016296098
769	KC_G1	RpLP1	-0.0583071091631605	0.592120700902737	0.998043617620652	pseudobulk	DESeq2	LRT	0.22758975545123
769	KC_G1	RpLP2	-0.187102338388843	0.0912853562481459	0.998043617620652	pseudobulk	DESeq2	LRT	1.0395988852335
769	KC_G1	Rpn1	-0.226881392572486	0.488126280682094	0.998043617620652	pseudobulk	DESeq2	LRT	0.311467809331891
770	KC_G1	Rpn10	0.0714238234170131	0.788478989817451	0.998043617620652	pseudobulk	DESeq2	LRT	0.103209874596223
770	KC_G1	Rpn11	0.175052750879142	0.525678702229914	0.998043617620652	pseudobulk	DESeq2	LRT	0.279279617981373
770	KC_G1	Rpn12	-0.0244525995609359	0.924709331962326	0.998043617620652	pseudobulk	DESeq2	LRT	0.0339947595451172
770	KC_G1	Rpn13	-0.196413917650145	0.436277465571058	0.998043617620652	pseudobulk	DESeq2	LRT	0.36023721843854
770	KC_G1	Rpn13R	-0.766096012895124	0.424515240406899	0.998043617620652	pseudobulk	DESeq2	LRT	0.372106713651345
770	KC_G1	Rpn2	0.0746506401302245	0.782340026143546	0.998043617620652	pseudobulk	DESeq2	LRT	0.106604449776233
770	KC_G1	Rpn3	0.0732237175696687	0.808574618797335	0.998043617620652	pseudobulk	DESeq2	LRT	0.0922798953165477
770	KC_G1	Rpn5	0.0643677356310834	0.744200028615638	0.998043617620652	pseudobulk	DESeq2	LRT	0.128310317615192
770	KC_G1	Rpn6	0.0579748973083813	0.834511187811242	0.998043617620652	pseudobulk	DESeq2	LRT	0.0785678366095193
770	KC_G1	Rpn7	-0.317807392877076	0.264552457007755	0.998043617620652	pseudobulk	DESeq2	LRT	0.577488200641261
771	KC_G1	Rpn8	-0.0221054474918079	0.914770385456767	0.998043617620652	pseudobulk	DESeq2	LRT	0.0386879035765422
771	KC_G1	Rpn9	-0.0594713701891105	0.808479480713545	0.998043617620652	pseudobulk	DESeq2	LRT	0.0923309980531461
771	KC_G1	Rpp30	0.430324381807229	0.504524753747607	0.998043617620652	pseudobulk	DESeq2	LRT	0.297117520899298
771	KC_G1	rpr	1.90250740646012	0.460774913678782	0.998043617620652	pseudobulk	DESeq2	LRT	0.336511173568024
771	KC_G1	RpS10a	-0.794610277746993	0.815039924133852	0.998043617620652	pseudobulk	DESeq2	LRT	0.0888211171417967
771	KC_G1	RpS10b	-0.127011180320995	0.277648109363363	0.998043617620652	pseudobulk	DESeq2	LRT	0.556505279496851
771	KC_G1	RpS11	-0.0510785949042283	0.706517752381136	0.998043617620652	pseudobulk	DESeq2	LRT	0.150876921339404
771	KC_G1	RpS12	-0.100161158580735	0.472949947485752	0.998043617620652	pseudobulk	DESeq2	LRT	0.325184818418529
771	KC_G1	RpS13	-0.123550815979262	0.272502893521214	0.998043617620652	pseudobulk	DESeq2	LRT	0.564628881887798
771	KC_G1	RpS14a	-0.0740889168328409	0.509518859649406	0.998043617620652	pseudobulk	DESeq2	LRT	0.292839736112748
772	KC_G1	RpS14b	-0.0624319503415441	0.653000428676757	0.998043617620652	pseudobulk	DESeq2	LRT	0.185086533622493
772	KC_G1	RpS15	-0.114996998275117	0.346176429169957	0.998043617620652	pseudobulk	DESeq2	LRT	0.460702506159979
772	KC_G1	RpS15Aa	-0.116668233877636	0.318633564216787	0.998043617620652	pseudobulk	DESeq2	LRT	0.496708478420731
772	KC_G1	RpS15Ab	-0.237007801923045	0.561605358997519	0.998043617620652	pseudobulk	DESeq2	LRT	0.250568756697434
772	KC_G1	RpS16	-0.0920405037799053	0.520114482135412	0.998043617620652	pseudobulk	DESeq2	LRT	0.283901053504668
772	KC_G1	RpS17	-0.174302003389067	0.109923322997882	0.998043617620652	pseudobulk	DESeq2	LRT	0.958910151301357
772	KC_G1	RpS18	-0.108907106814786	0.366401087302235	0.998043617620652	pseudobulk	DESeq2	LRT	0.436043246225092

772	KC_G1	RpS19a	-0.188290925734513	0.17290621128018	0.998043617620652	pseudobulk	DESeq2	LRT	0.762189405359647
772	KC_G1	RpS2	-0.12281752275628	0.308936005650028	0.998043617620652	pseudobulk	DESeq2	LRT	0.510131472909747
772	KC_G1	RpS20	-0.133055712261791	0.181692808059224	0.998043617620652	pseudobulk	DESeq2	LRT	0.740662263016729
773	KC_G1	RpS21	-0.174208703192194	0.18024429395893	0.998043617620652	pseudobulk	DESeq2	LRT	0.744138474965676
773	KC_G1	RpS23	-0.097894278609879	0.383604730235238	0.998043617620652	pseudobulk	DESeq2	LRT	0.416116046177089
773	KC_G1	RpS24	-0.137507072908383	0.253811559418574	0.998043617620652	pseudobulk	DESeq2	LRT	0.595488602582602
773	KC_G1	RpS25	-0.164379866288528	0.106800374459092	0.998043617620652	pseudobulk	DESeq2	LRT	0.971427224599296
773	KC_G1	RpS26	-0.0705462981633188	0.433162938976819	0.998043617620652	pseudobulk	DESeq2	LRT	0.363348708294167
773	KC_G1	RpS27	-0.128918084280162	0.240101852212832	0.998043617620652	pseudobulk	DESeq2	LRT	0.619604489660967
773	KC_G1	RpS27A	-0.0487349169638508	0.600256983489179	0.998043617620652	pseudobulk	DESeq2	LRT	0.2216627785876
773	KC_G1	RpS28b	-0.121429075068716	0.292900292880804	0.998043617620652	pseudobulk	DESeq2	LRT	0.533280194052599
773	KC_G1	RpS29	-0.138879667343494	0.26020727428538	0.998043617620652	pseudobulk	DESeq2	LRT	0.584680566583651
773	KC_G1	RpS3	-0.095498587863766	0.322653244781186	0.998043617620652	pseudobulk	DESeq2	LRT	0.491263963027092
774	KC_G1	RpS30	-0.128623852132654	0.218351030526344	0.998043617620652	pseudobulk	DESeq2	LRT	0.660844754047892
774	KC_G1	RpS3A	-0.0909393692542915	0.425243439709067	0.998043617620652	pseudobulk	DESeq2	LRT	0.37136237758636
774	KC_G1	RpS4	-0.1045130676288	0.289049942568423	0.998043617620652	pseudobulk	DESeq2	LRT	0.539027112579194
774	KC_G1	RpS5a	-0.0794988528363279	0.610861432550363	0.998043617620652	pseudobulk	DESeq2	LRT	0.214057293691531
774	KC_G1	RpS6	-0.160550812903291	0.14060422069796	0.998043617620652	pseudobulk	DESeq2	LRT	0.852001642343781
774	KC_G1	RpS7	-0.0719207977160199	0.798621121440969	0.998043617620652	pseudobulk	DESeq2	LRT	0.0976592080360446
774	KC_G1	RpS8	-0.0800113245000102	0.419907985515371	0.998043617620652	pseudobulk	DESeq2	LRT	0.376845866175864
774	KC_G1	RpS9	-0.282409211709314	0.00796820732266179	0.998043617620652	pseudobulk	DESeq2	LRT	2.09863937464502
774	KC_G1	Rpt1	-0.162261455092968	0.438620143864132	0.998043617620652	pseudobulk	DESeq2	LRT	0.357911426994686
774	KC_G1	Rpt2	-0.104608577204959	0.669613358609392	0.998043617620652	pseudobulk	DESeq2	LRT	0.174175890868329
775	KC_G1	Rpt3	0.140767812638734	0.496201214580392	0.998043617620652	pseudobulk	DESeq2	LRT	0.304342177014804
775	KC_G1	Rpt4	0.0621304646628247	0.791079033961333	0.998043617620652	pseudobulk	DESeq2	LRT	0.101780125480027
775	KC_G1	Rpt5	0.0515031316072181	0.866847189234957	0.998043617620652	pseudobulk	DESeq2	LRT	0.0620574546820391
775	KC_G1	Rpt6	0.0265967569091146	0.90337635802388	0.998043617620652	pseudobulk	DESeq2	LRT	0.0441312794090324
775	KC_G1	Rrp1	1.20273941560079	0.00881016787480077	0.998043617620652	pseudobulk	DESeq2	LRT	2.05501581617308
775	KC_G1	Rrp4	-1.50520854765008	0.129481719215309	0.998043617620652	pseudobulk	DESeq2	LRT	0.887791542813139
775	KC_G1	Rrp40	0.14421614102708	0.862761240266	0.998043617620652	pseudobulk	DESeq2	LRT	0.0641093739041085
775	KC_G1	Rrp42	0.335578090355673	0.618013540880925	0.998043617620652	pseudobulk	DESeq2	LRT	0.209002009271312
775	KC_G1	Rrp45	-0.776296732928036	0.736588270584829	0.998043617620652	pseudobulk	DESeq2	LRT	0.132775201121675
775	KC_G1	Rrp46	0.957292107657299	0.2360546016448	0.998043617620652	pseudobulk	DESeq2	LRT	0.626987529020207
776	KC_G1	Rrp47	0.143599197141787	0.593800445361782	0.998043617620652	pseudobulk	DESeq2	LRT	0.226359481010617
776	KC_G1	Rrp6	0.369132666245244	0.471508195195064	0.998043617620652	pseudobulk	DESeq2	LRT	0.326510754470526
776	KC_G1	Rs1	0.605554451516051	0.282319017042187	0.998043617620652	pseudobulk	DESeq2	LRT	0.549259866740384
776	KC_G1	RSG7	-0.0315093120842758	0.779169454073896	0.998043617620652	pseudobulk	DESeq2	LRT	0.108368081523668
776	KC_G1	rst	1.12508065046033	0.900770125530981	0.998043617620652	pseudobulk	DESeq2	LRT	0.0453860258366365
776	KC_G1	rswl	0.024114816714816	0.950225633120145	0.998043617620652	pseudobulk	DESeq2	LRT	0.0221732583069632
776	KC_G1	rt	-0.0274295672039124	0.91064972755021	0.998043617620652	pseudobulk	DESeq2	LRT	0.0406486380043386
776	KC_G1	Rtc1	0.566565192183208	0.41157799396893	0.998043617620652	pseudobulk	DESeq2	LRT	0.385547853912895
776	KC_G1	Rtca	0.231067162584944	0.372616795720393	0.998043617620652	pseudobulk	DESeq2	LRT	0.428737573153441
776	KC_G1	RtcB	0.0240465131917465	0.921507558428451	0.998043617620652	pseudobulk	DESeq2	LRT	0.0355010982416457
777	KC_G1	rtet	0.17952093930434	0.862529151299258	0.998043617620652	pseudobulk	DESeq2	LRT	0.0642262179538613
777	KC_G1	Rtf1	0.0964770540431692	0.747031807663218	0.998043617620652	pseudobulk	DESeq2	LRT	0.126660906084836
777	KC_G1	RtGEF	0.149231520014992	0.736376810656765	0.998043617620652	pseudobulk	DESeq2	LRT	0.132899896379834
777	KC_G1	Rtnl1	0.0428446329737965	0.7259408037405	0.998043617620652	pseudobulk	DESeq2	LRT	0.139098792050616
777	KC_G1	rtp	-0.711691946027098	0.741255142397939	0.998043617620652	pseudobulk	DESeq2	LRT	0.130032280734188
777	KC_G1	rtv	-0.794610277746993	0.815039924133852	0.998043617620652	pseudobulk	DESeq2	LRT	0.0888211171417967
777	KC_G1	ru	-0.76872985355345	0.507418021821954	0.998043617620652	pseudobulk	DESeq2	LRT	0.294634112126436
777	KC_G1	Rubicon	-0.313774007493843	0.464971250049021	0.998043617620652	pseudobulk	DESeq2	LRT	0.33257389943493
777	KC_G1	rudhira	-0.0887356607223603	0.467211427683287	0.998043617620652	pseudobulk	DESeq2	LRT	0.330486543209235
777	KC_G1	rumi	0.598396706281001	0.510322196170036	0.998043617620652	pseudobulk	DESeq2	LRT	0.292155541868578
778	KC_G1	rump	0.0533845992119472	0.772865437807877	0.998043617620652	pseudobulk	DESeq2	LRT	0.111896113720258
778	KC_G1	rush	0.552592575076804	0.276889018878174	0.998043617620652	pseudobulk	DESeq2	LRT	0.557694267566742
778	KC_G1	rut	-0.065420445072921	0.491710470675203	0.998043617620652	pseudobulk	DESeq2	LRT	0.308290543574704

778	KC_G1	rux	-0.0363388152917795	0.914737082957651	0.998043617620652	pseudobulk	DESeq2	LRT	0.0387037144894254
778	KC_G1	RYa-R	-0.765625368254185	0.836625948066609	0.998043617620652	pseudobulk	DESeq2	LRT	0.0774686698445758
778	KC_G1	RYBP	0.0590425681922662	0.870186508687786	0.998043617620652	pseudobulk	DESeq2	LRT	0.0603876542630999
778	KC_G1	RyR	0.112886783963404	0.750711112171573	0.998043617620652	pseudobulk	DESeq2	LRT	0.124527155574831
778	KC_G1	S	0.00910364807112389	0.963888827676211	0.998043617620652	pseudobulk	DESeq2	LRT	0.0159730535566973
778	KC_G1	s-cup	-0.272127313287725	0.348357796057238	0.998043617620652	pseudobulk	DESeq2	LRT	0.457974465747318
778	KC_G1	S-Lap1	1.9128994548756	0.395667867821107	0.998043617620652	pseudobulk	DESeq2	LRT	0.402669217347329
779	KC_G1	S1P	-0.114317036014326	0.87865519480867	0.998043617620652	pseudobulk	DESeq2	LRT	0.0561815189765396
779	KC_G1	S2P	0.381219033156271	0.580191486686506	0.998043617620652	pseudobulk	DESeq2	LRT	0.236428648012153
779	KC_G1	S6k	-0.319878822412822	0.16496061435652	0.998043617620652	pseudobulk	DESeq2	LRT	0.782619734631182
779	KC_G1	S6kII	-0.2318705094129	0.352624530107014	0.998043617620652	pseudobulk	DESeq2	LRT	0.452687479559206
779	KC_G1	S6KL	0.0435735217098324	0.872005506622074	0.998043617620652	pseudobulk	DESeq2	LRT	0.0594807725352887
779	KC_G1	SA	0.364158191121916	0.353859906704186	0.998043617620652	pseudobulk	DESeq2	LRT	0.451168641328369
779	KC_G1	Sac1	-0.0588812449852386	0.852963087269978	0.998043617620652	pseudobulk	DESeq2	LRT	0.0690697629026215
779	KC_G1	Saf-B	0.0394374353560255	0.814294105637608	0.998043617620652	pseudobulk	DESeq2	LRT	0.089218708886797
779	KC_G1	Saf6	0.13191777238962	0.813120830029655	0.998043617620652	pseudobulk	DESeq2	LRT	0.089844913303875
779	KC_G1	sage	0.623537271411544	0.56893512160495	0.998043617620652	pseudobulk	DESeq2	LRT	0.24493725545984
780	KC_G1	SAK	-0.396459764132828	0.639910106655057	0.998043617620652	pseudobulk	DESeq2	LRT	0.193881030587496
780	KC_G1	salm	1.13582588427274	0.704684208645453	0.998043617620652	pseudobulk	DESeq2	LRT	0.152005460551806
780	KC_G1	sals	0.282777219597046	0.105967201566697	0.998043617620652	pseudobulk	DESeq2	LRT	0.974828534572628
780	KC_G1	Sam-S	-0.0808414640112195	0.56774953726673	0.998043617620652	pseudobulk	DESeq2	LRT	0.245843211071393
780	KC_G1	SamDC	0.185696146588674	0.531971880310925	0.998043617620652	pseudobulk	DESeq2	LRT	0.274111323623293
780	KC_G1	Samtor	-0.755843323827747	0.229596281777976	0.998043617620652	pseudobulk	DESeq2	LRT	0.639035149446464
780	KC_G1	Samuel	-0.794610277746993	0.821479263487599	0.998043617620652	pseudobulk	DESeq2	LRT	0.085403394939296
780	KC_G1	san	0.0996884388852048	0.692570249913175	0.998043617620652	pseudobulk	DESeq2	LRT	0.159536167964211
780	KC_G1	sand	1.13582588427274	0.704684208645453	0.998043617620652	pseudobulk	DESeq2	LRT	0.152005460551806
780	KC_G1	sano	-0.259242581009848	0.818789806119208	0.998043617620652	pseudobulk	DESeq2	LRT	0.0868275729144363
781	KC_G1	santa-maria	0.255680306802332	0.760378372417957	0.998043617620652	pseudobulk	DESeq2	LRT	0.118970244348831
781	KC_G1	Sap-r	0.0128456316949201	0.949663835012179	0.998043617620652	pseudobulk	DESeq2	LRT	0.0224301004332417
781	KC_G1	Sap130	0.324417938442463	0.337377563936203	0.998043617620652	pseudobulk	DESeq2	LRT	0.471883801956394
781	KC_G1	Sap30	0.346199321692574	0.295770870222876	0.998043617620652	pseudobulk	DESeq2	LRT	0.529044600874699
781	KC_G1	Sap47	-0.0817584275400676	0.543799895111534	0.998043617620652	pseudobulk	DESeq2	LRT	0.264560880515503
781	KC_G1	Sar1	-0.0509117645954234	0.746259464410908	0.998043617620652	pseudobulk	DESeq2	LRT	0.127110147902284
781	KC_G1	Sara	-0.217835352972485	0.441176005398656	0.998043617620652	pseudobulk	DESeq2	LRT	0.355388115919821
781	KC_G1	Sas-4	0.506884443083301	0.823561077413028	0.998043617620652	pseudobulk	DESeq2	LRT	0.0843041869012458
781	KC_G1	Sas-6	-0.0784030421371325	0.952092824531386	0.998043617620652	pseudobulk	DESeq2	LRT	0.0213207079006827
781	KC_G1	Sas10	0.594415291192484	0.388651970437038	0.998043617620652	pseudobulk	DESeq2	LRT	0.410439126111682
782	KC_G1	sau	0.117705285590821	0.541261752241765	0.998043617620652	pseudobulk	DESeq2	LRT	0.266592660840263
782	KC_G1	sav	-1.75826727288977	0.444072765119988	0.998043617620652	pseudobulk	DESeq2	LRT	0.352545861189756
782	KC_G1	sax	-0.35599462240122	0.323651358812222	0.998043617620652	pseudobulk	DESeq2	LRT	0.489922565307843
782	KC_G1	sba	-0.0582137934744607	0.763942413063005	0.998043617620652	pseudobulk	DESeq2	LRT	0.116939377853072
782	KC_G1	Sbat	-1.26710492585102	0.0631698881366977	0.998043617620652	pseudobulk	DESeq2	LRT	1.19948989216781
782	KC_G1	sbb	-0.056485080946933	0.574007865304947	0.998043617620652	pseudobulk	DESeq2	LRT	0.241082156669738
782	KC_G1	Sbf	-0.0587132608809753	0.83864649671205	0.998043617620652	pseudobulk	DESeq2	LRT	0.0764210628653428
782	KC_G1	sbm	-0.0981588811179199	0.587166337697017	0.998043617620652	pseudobulk	DESeq2	LRT	0.231238850522998
782	KC_G1	sbr	-0.00736097996817532	0.959998631996718	0.998043617620652	pseudobulk	DESeq2	LRT	0.0177293858319942
782	KC_G1	Sc2	-0.134470849054774	0.659909107259119	0.998043617620652	pseudobulk	DESeq2	LRT	0.180515877994768
783	KC_G1	SC35	-0.204848246015789	0.27315319569733	0.998043617620652	pseudobulk	DESeq2	LRT	0.563593714184369
783	KC_G1	sca	-0.0555610563917148	0.778841890046452	0.998043617620652	pseudobulk	DESeq2	LRT	0.108550697975367
783	KC_G1	scaf	0.555543903626307	0.759514315344191	0.998043617620652	pseudobulk	DESeq2	LRT	0.119464036131151
783	KC_G1	scaf6	-0.208889571524557	0.279436023159718	0.998043617620652	pseudobulk	DESeq2	LRT	0.553717608060646
783	KC_G1	SCaMC	0.05415285220664	0.810389509650101	0.998043617620652	pseudobulk	DESeq2	LRT	0.0913061894771958
783	KC_G1	Scamp	0.150898764783824	0.382818977881029	0.998043617620652	pseudobulk	DESeq2	LRT	0.417006540632102
783	KC_G1	SCAP	-0.179507578887927	0.403386654448485	0.998043617620652	pseudobulk	DESeq2	LRT	0.394278473984569
783	KC_G1	SCAR	0.279103523481598	0.18873962021103	0.998043617620652	pseudobulk	DESeq2	LRT	0.724136923190307
783	KC_G1	scaRNA:MeU2-G25	2.60856600434519	0.174399014900371	0.998043617620652	pseudobulk	DESeq2	LRT	0.758455972525822

783	KC_G1	scaRNA:MeU5-C46	1.12508065046033	0.900770125530981	0.998043617620652	pseudobulk	DESeq2	LRT	0.0453860258366365
784	KC_G1	scat	0.252264692956776	0.648849826399457	0.998043617620652	pseudobulk	DESeq2	LRT	0.18785580722561
784	KC_G1	scb	2.26259189257468	0.268414136743927	0.998043617620652	pseudobulk	DESeq2	LRT	0.571194614627486
784	KC_G1	Sccpdh1	0.51243884061745	0.273954204797271	0.998043617620652	pseudobulk	DESeq2	LRT	0.562322029391571
784	KC_G1	Sccpdh2	-1.5316118979248	0.385179847058233	0.998043617620652	pseudobulk	DESeq2	LRT	0.414336443614907
784	KC_G1	SCCRO	-0.03111631007464	0.892888956574117	0.998043617620652	pseudobulk	DESeq2	LRT	0.0492025484415257
784	KC_G1	SCCRO3	0.128085437047254	0.72156058901569	0.998043617620652	pseudobulk	DESeq2	LRT	0.141727195588666
784	KC_G1	SCCRO4	-0.422162395475841	0.102974063233886	0.998043617620652	pseudobulk	DESeq2	LRT	0.987272150177126
784	KC_G1	Sce	-0.223870905930801	0.474234038320572	0.998043617620652	pseudobulk	DESeq2	LRT	0.324007277594715
784	KC_G1	sced	0.257846244433834	0.347547037129265	0.998043617620652	pseudobulk	DESeq2	LRT	0.458986409539311
784	KC_G1	scf	0.154605247777285	0.583430883174369	0.998043617620652	pseudobulk	DESeq2	LRT	0.234010585777841
785	KC_G1	Scgalpha	-0.0555557590703454	0.821959700718705	0.998043617620652	pseudobulk	DESeq2	LRT	0.0851494746552351
785	KC_G1	Scgbeta	-0.0164881439412459	0.920376016405994	0.998043617620652	pseudobulk	DESeq2	LRT	0.0360347069071793
785	KC_G1	Scgdelta	1.69981218657454	0.566400560818652	0.998043617620652	pseudobulk	DESeq2	LRT	0.246876325303589
785	KC_G1	Schip1	-0.0590580798186302	0.698606584499946	0.998043617620652	pseudobulk	DESeq2	LRT	0.15576732537098
785	KC_G1	schlank	0.165775925899201	0.386179390090581	0.998043617620652	pseudobulk	DESeq2	LRT	0.41321090768965
785	KC_G1	Scm	-0.088813327873756	0.708106680217528	0.998043617620652	pseudobulk	DESeq2	LRT	0.149901308497178
785	KC_G1	scny	-0.173108556462575	0.498342802615518	0.998043617620652	pseudobulk	DESeq2	LRT	0.302471809715544
785	KC_G1	SCOT	1.10605959195112	0.291587026616923	0.998043617620652	pseudobulk	DESeq2	LRT	0.535231802692955
785	KC_G1	Scox	0.312294050366739	0.296831015893994	0.998043617620652	pseudobulk	DESeq2	LRT	0.527490721560644
785	KC_G1	Scp1	0.228319291893143	0.924568785563363	0.998043617620652	pseudobulk	DESeq2	LRT	0.034060772899258
786	KC_G1	Scp2	1.12438553390531	0.461541081520658	0.998043617620652	pseudobulk	DESeq2	LRT	0.335789636601934
786	KC_G1	ScpX	0.304619621512987	0.617525208238862	0.998043617620652	pseudobulk	DESeq2	LRT	0.209345309200959
786	KC_G1	scra	1.13582588427274	0.704684208645453	0.998043617620652	pseudobulk	DESeq2	LRT	0.152005460551806
786	KC_G1	scramb1	0.202302500293669	0.486459111521803	0.998043617620652	pseudobulk	DESeq2	LRT	0.312953657732171
786	KC_G1	scramb2	0.304540005313514	0.355520470766341	0.998043617620652	pseudobulk	DESeq2	LRT	0.449135387663032
786	KC_G1	scrib	0.262045784098933	0.0290125080476244	0.998043617620652	pseudobulk	DESeq2	LRT	1.53741472606939
786	KC_G1	scro	-0.122999314358769	0.91077086934659	0.998043617620652	pseudobulk	DESeq2	LRT	0.0405908685755062
786	KC_G1	scrt	-0.117083224178814	0.363662618602746	0.998043617620652	pseudobulk	DESeq2	LRT	0.439301338397887
786	KC_G1	Scsalpha1	-0.0986203936882033	0.449657842348401	0.998043617620652	pseudobulk	DESeq2	LRT	0.347117827783859
786	KC_G1	ScsbetaA	-0.0193953297971915	0.896940832671875	0.998043617620652	pseudobulk	DESeq2	LRT	0.0472362045496916
787	KC_G1	ScsbetaG	0.228912991969076	0.726354780417433	0.998043617620652	pseudobulk	DESeq2	LRT	0.138851200867032
787	KC_G1	scu	0.783108870491612	0.0345277398235879	0.998043617620652	pseudobulk	DESeq2	LRT	1.4618318495182
787	KC_G1	scyl	-0.203383776588526	0.0662300078815394	0.998043617620652	pseudobulk	DESeq2	LRT	1.17894519327096
787	KC_G1	sd	-0.164541258360486	0.205142549361747	0.998043617620652	pseudobulk	DESeq2	LRT	0.687944251691657
787	KC_G1	Sdc	0.284349901380735	0.0088013160392267	0.998043617620652	pseudobulk	DESeq2	LRT	2.05545238400428
787	KC_G1	SdhA	0.198643014190251	0.381556468205956	0.998043617620652	pseudobulk	DESeq2	LRT	0.418441179817971
787	KC_G1	Sdhaf3	0.0240554245844522	0.928582398273981	0.998043617620652	pseudobulk	DESeq2	LRT	0.0321795528526281
787	KC_G1	SdhAL	1.12508065046033	0.900770125530981	0.998043617620652	pseudobulk	DESeq2	LRT	0.0453860258366365
787	KC_G1	SdhB	0.0921229729514133	0.558408665935084	0.998043617620652	pseudobulk	DESeq2	LRT	0.253047850523534
787	KC_G1	SdhC	0.0391980056965078	0.774456142804293	0.998043617620652	pseudobulk	DESeq2	LRT	0.11100317116157
788	KC_G1	SdhD	0.00665023647205734	0.957569412127513	0.998043617620652	pseudobulk	DESeq2	LRT	0.0188297351542786
788	KC_G1	Sdic2	-0.794610277746993	0.821479263487599	0.998043617620652	pseudobulk	DESeq2	LRT	0.085403394939296
788	KC_G1	sdk	1.69981218657454	0.566400560818652	0.998043617620652	pseudobulk	DESeq2	LRT	0.246876325303589
788	KC_G1	Sdr	-2.73975578120602	0.247094641820741	0.998043617620652	pseudobulk	DESeq2	LRT	0.60713667205205
788	KC_G1	sds22	0.526303156067102	0.186026617415983	0.998043617620652	pseudobulk	DESeq2	LRT	0.730424910782977
788	KC_G1	sdt	-0.146703657459398	0.249665073651151	0.998043617620652	pseudobulk	DESeq2	LRT	0.602642208075156
788	KC_G1	Sec10	-0.0324619607221608	0.931304870507916	0.998043617620652	pseudobulk	DESeq2	LRT	0.0309081257799857
788	KC_G1	Sec13	0.174943025658063	0.469473269191186	0.998043617620652	pseudobulk	DESeq2	LRT	0.328389130497456
788	KC_G1	Sec15	0.344736558632876	0.540910815513623	0.998043617620652	pseudobulk	DESeq2	LRT	0.266874334751455
788	KC_G1	Sec16	-0.179790934747835	0.332517763507126	0.998043617620652	pseudobulk	DESeq2	LRT	0.478185149196789
789	KC_G1	Sec22	0.0852433424489461	0.771879364756554	0.998043617620652	pseudobulk	DESeq2	LRT	0.112450569242489
789	KC_G1	Sec23	0.040178490678665	0.895454783707574	0.998043617620652	pseudobulk	DESeq2	LRT	0.0479563391081684
789	KC_G1	Sec24AB	-0.206877284664864	0.458397819850098	0.998043617620652	pseudobulk	DESeq2	LRT	0.338757456549662
789	KC_G1	Sec24CD	0.16142383952598	0.579652254172309	0.998043617620652	pseudobulk	DESeq2	LRT	0.236832470896222
789	KC_G1	Sec3	0.00199705115217438	0.974933359634228	0.998043617620652	pseudobulk	DESeq2	LRT	0.0110250689499101

789	KC_G1	Sec31	-0.0981261657692898	0.82749665403116	0.998043617620652	pseudobulk	DESeq2	LRT	0.0822337536111458
789	KC_G1	Sec5	-0.0628601008314931	0.910091500937627	0.998043617620652	pseudobulk	DESeq2	LRT	0.0409149413551933
789	KC_G1	Sec6	0.237622172423465	0.64582841604745	0.998043617620652	pseudobulk	DESeq2	LRT	0.189882850212161
789	KC_G1	Sec61alpha	0.018097848758936	0.953762118161625	0.998043617620652	pseudobulk	DESeq2	LRT	0.0205599310110703
789	KC_G1	Sec61beta	0.0948363590508876	0.673128925911623	0.998043617620652	pseudobulk	DESeq2	LRT	0.171901746400792
790	KC_G1	Sec61gamma	-0.296101056693543	0.0959841546828173	0.998043617620652	pseudobulk	DESeq2	LRT	1.01780045552081
790	KC_G1	Sec63	0.151058787006316	0.588139524926623	0.998043617620652	pseudobulk	DESeq2	LRT	0.23051963358771
790	KC_G1	Sec71	-0.192987494864246	0.677510005045605	0.998043617620652	pseudobulk	DESeq2	LRT	0.169084287015897
790	KC_G1	Sec8	0.0199260616613255	0.954806464540408	0.998043617620652	pseudobulk	DESeq2	LRT	0.0200846492539685
790	KC_G1	Secp43	0.0712843435273985	0.869393554772623	0.998043617620652	pseudobulk	DESeq2	LRT	0.0607835837645285
790	KC_G1	SecS	0.299741735579241	0.315057783735343	0.998043617620652	pseudobulk	DESeq2	LRT	0.501609786350236
790	KC_G1	sei	-0.0354132879551622	0.901558539785745	0.998043617620652	pseudobulk	DESeq2	LRT	0.0450060685194977
790	KC_G1	Seipin	-2.23957353771189	0.33560557159327	0.998043617620652	pseudobulk	DESeq2	LRT	0.474170837795283
790	KC_G1	sel	0.78396115405255	0.473165450665336	0.998043617620652	pseudobulk	DESeq2	LRT	0.324986973962077
790	KC_G1	SelG	0.13387347166446	0.633156796891611	0.998043617620652	pseudobulk	DESeq2	LRT	0.19848872664559
791	KC_G1	SelR	-0.153047864368843	0.332224237443463	0.998043617620652	pseudobulk	DESeq2	LRT	0.478568686743225
791	KC_G1	SelT	0.123864941053725	0.61017311838365	0.998043617620652	pseudobulk	DESeq2	LRT	0.214546929428464
791	KC_G1	Sem1	-0.209849077968784	0.369151233253592	0.998043617620652	pseudobulk	DESeq2	LRT	0.432795676356144
791	KC_G1	Sema1a	-0.255421228928049	0.0427140355231458	0.998043617620652	pseudobulk	DESeq2	LRT	1.36942939549801
791	KC_G1	Sema1b	-0.248450010792368	0.324469515856898	0.998043617620652	pseudobulk	DESeq2	LRT	0.488826099224249
791	KC_G1	Sema2a	-0.0501074571220494	0.803726701100139	0.998043617620652	pseudobulk	DESeq2	LRT	0.0948916034677891
791	KC_G1	Sema5c	-2.31623874979045	0.266794518586219	0.998043617620652	pseudobulk	DESeq2	LRT	0.573823097439944
791	KC_G1	senju	0.88434874232861	0.251745632351544	0.998043617620652	pseudobulk	DESeq2	LRT	0.599038055479719
791	KC_G1	Sep1	0.0746860432625547	0.81597070200521	0.998043617620652	pseudobulk	DESeq2	LRT	0.0883254346112084
791	KC_G1	Sep4	0.0506110528267495	0.800274730565775	0.998043617620652	pseudobulk	DESeq2	LRT	0.0967608961500324
792	KC_G1	Sep5	1.13582588427274	0.633424328955532	0.998043617620652	pseudobulk	DESeq2	LRT	0.198305259991224
792	KC_G1	SERCA	-0.0592433744725148	0.761892046297111	0.998043617620652	pseudobulk	DESeq2	LRT	0.118106560181163
792	KC_G1	serp	-0.794610277746993	0.815039924133852	0.998043617620652	pseudobulk	DESeq2	LRT	0.0888211171417967
792	KC_G1	SerRS	0.119465862595633	0.808998574845345	0.998043617620652	pseudobulk	DESeq2	LRT	0.0920522434524343
792	KC_G1	SerRS-m	-0.615371183526692	0.36219660870566	0.998043617620652	pseudobulk	DESeq2	LRT	0.441055620359104
792	KC_G1	sesB	-0.0611579982110496	0.605511899048068	0.998043617620652	pseudobulk	DESeq2	LRT	0.217877318020285
792	KC_G1	Sesn	0.173195945230598	0.976215465741119	0.998043617620652	pseudobulk	DESeq2	LRT	0.0104543162934892
792	KC_G1	Set	0.176828102071628	0.486869587212005	0.998043617620652	pseudobulk	DESeq2	LRT	0.312587353239124
792	KC_G1	Set1	-0.118589882435975	0.452141974950247	0.998043617620652	pseudobulk	DESeq2	LRT	0.344725173029696
792	KC_G1	Set2	-0.335137520198916	0.342515705259825	0.998043617620652	pseudobulk	DESeq2	LRT	0.465319510153154
793	KC_G1	Setd3	0.0413487798396554	0.937323089328548	0.998043617620652	pseudobulk	DESeq2	LRT	0.0281106847506762
793	KC_G1	sev	-1.31729413969264	0.525615640677063	0.998043617620652	pseudobulk	DESeq2	LRT	0.279331720010916
793	KC_G1	SF1	-0.0449595931412401	0.868293958319139	0.998043617620652	pseudobulk	DESeq2	LRT	0.0613332208335777
793	KC_G1	SF2	-0.0557358395212653	0.716872572468734	0.998043617620652	pseudobulk	DESeq2	LRT	0.144558035394892
793	KC_G1	Sf3a1	-0.111824065043078	0.733080929144138	0.998043617620652	pseudobulk	DESeq2	LRT	0.134848078373053
793	KC_G1	Sf3a2	-0.0485244638714115	0.891942797004804	0.998043617620652	pseudobulk	DESeq2	LRT	0.0496629973523204
793	KC_G1	Sf3b1	-0.125826794963296	0.715404970138409	0.998043617620652	pseudobulk	DESeq2	LRT	0.1454480469992
793	KC_G1	Sf3b2	-0.0891179687389385	0.695951948053697	0.998043617620652	pseudobulk	DESeq2	LRT	0.15742074518189
793	KC_G1	Sf3b3	-0.681853230937937	0.391418981347733	0.998043617620652	pseudobulk	DESeq2	LRT	0.407358117628714
793	KC_G1	Sf3b5	0.252541587309149	0.281680756073292	0.998043617620652	pseudobulk	DESeq2	LRT	0.550242822224943
794	KC_G1	Sf3b6	-0.166868563050303	0.377631120253049	0.998043617620652	pseudobulk	DESeq2	LRT	0.422932223067653
794	KC_G1	sff	-0.0190036196221776	0.884271257285549	0.998043617620652	pseudobulk	DESeq2	LRT	0.0534144912408346
794	KC_G1	sfl	-0.0339868199521144	0.893014357594886	0.998043617620652	pseudobulk	DESeq2	LRT	0.0491415586096544
794	KC_G1	Sfmbt	-0.134042806500245	0.640241683195173	0.998043617620652	pseudobulk	DESeq2	LRT	0.193656054352548
794	KC_G1	Sfp36F	-0.794610277746993	0.879485534599449	0.998043617620652	pseudobulk	DESeq2	LRT	0.0557712992369932
794	KC_G1	Sfp53D	-1.72343437992478	0.512120560562093	0.998043617620652	pseudobulk	DESeq2	LRT	0.290627787806665
794	KC_G1	Sfp78E	-0.794610277746993	0.815039924133852	0.998043617620652	pseudobulk	DESeq2	LRT	0.0888211171417967
794	KC_G1	Sfxn2	-0.100770139973496	0.734957468803958	0.998043617620652	pseudobulk	DESeq2	LRT	0.133737792341913
794	KC_G1	Sgf11	-0.0707567092823006	0.864698485682251	0.998043617620652	pseudobulk	DESeq2	LRT	0.0631353015892187
794	KC_G1	Sgf29	-0.0303277038903867	0.936106494926139	0.998043617620652	pseudobulk	DESeq2	LRT	0.0286747415098877
795	KC_G1	sgg	-0.0720691181612291	0.504923437007306	0.998043617620652	pseudobulk	DESeq2	LRT	0.296774470210213

795	KC_G1	sgl	-0.206776715122913	0.649670459209341	0.998043617620652	pseudobulk	DESeq2	LRT	0.187306880339524
795	KC_G1	sgll	0.260599445177193	0.591911757225791	0.998043617620652	pseudobulk	DESeq2	LRT	0.227743033490604
795	KC_G1	Sgp	-0.794610277746993	0.821479263487599	0.998043617620652	pseudobulk	DESeq2	LRT	0.085403394939296
795	KC_G1	Sgs4	-1.08678070850198	0.370907279143842	0.998043617620652	pseudobulk	DESeq2	LRT	0.430734643454935
795	KC_G1	Sgsh	0.173116475207952	0.97305562682953	0.998043617620652	pseudobulk	DESeq2	LRT	0.011862331638572
795	KC_G1	Sgt	-0.0172899967535299	0.945893832597673	0.998043617620652	pseudobulk	DESeq2	LRT	0.0241576062033604
795	KC_G1	Sgt1	0.00591481163607784	0.940803433324885	0.998043617620652	pseudobulk	DESeq2	LRT	0.026501106370688
795	KC_G1	Sh	-0.225787390664529	0.024136009140173	0.998043617620652	pseudobulk	DESeq2	LRT	1.61733453836812
795	KC_G1	Sh3beta	-0.0234632133078363	0.893648170043498	0.998043617620652	pseudobulk	DESeq2	LRT	0.0488334296191121
796	KC_G1	SH3PX1	-0.269789716963131	0.522536703327915	0.998043617620652	pseudobulk	DESeq2	LRT	0.28188319901031
796	KC_G1	Shab	-0.0485384476832191	0.672559459658937	0.998043617620652	pseudobulk	DESeq2	LRT	0.172269314507326
796	KC_G1	shakB	-0.173890757963625	0.108827670406669	0.998043617620652	pseudobulk	DESeq2	LRT	0.963260667348586
796	KC_G1	Shal	0.0239236103022245	0.864562683579281	0.998043617620652	pseudobulk	DESeq2	LRT	0.0632035134997388
796	KC_G1	shams	0.0881584283115404	0.894991194904312	0.998043617620652	pseudobulk	DESeq2	LRT	0.0481812373357983
796	KC_G1	Shark	-1.44549953086359	0.334075313211798	0.998043617620652	pseudobulk	DESeq2	LRT	0.476155615747721
796	KC_G1	Shaw	0.131910122739194	0.44295938006688	0.998043617620652	pseudobulk	DESeq2	LRT	0.353636097302069
796	KC_G1	Shawl	-0.161753714373724	0.217400027551823	0.998043617620652	pseudobulk	DESeq2	LRT	0.662740405210148
796	KC_G1	Shc	0.118179475621313	0.724934775602398	0.998043617620652	pseudobulk	DESeq2	LRT	0.139701066353471
796	KC_G1	shep	-0.114609034158004	0.386873298481505	0.998043617620652	pseudobulk	DESeq2	LRT	0.412431243714561
797	KC_G1	shf	-0.794610277746993	0.879485534599449	0.998043617620652	pseudobulk	DESeq2	LRT	0.0557712992369932
797	KC_G1	shg	0.36797330944789	0.865994775535327	0.998043617620652	pseudobulk	DESeq2	LRT	0.0624847280323331
797	KC_G1	shi	-0.0255740602193535	0.800863375600999	0.998043617620652	pseudobulk	DESeq2	LRT	0.0964415666666726
797	KC_G1	Shmt	1.28818576032116	0.043787528123608	0.998043617620652	pseudobulk	DESeq2	LRT	1.35864957073794
797	KC_G1	shn	0.109592692799869	0.734338452113767	0.998043617620652	pseudobulk	DESeq2	LRT	0.134103730264744
797	KC_G1	shop	0.421920517780495	0.412828518686479	0.998043617620652	pseudobulk	DESeq2	LRT	0.384230308763703
797	KC_G1	shot	-0.232004648082047	0.0557474945774562	0.998043617620652	pseudobulk	DESeq2	LRT	1.25377464604906
797	KC_G1	shrb	0.0521498362420595	0.804426658863256	0.998043617620652	pseudobulk	DESeq2	LRT	0.0945135452292507
797	KC_G1	Shrm	-0.794610277746993	0.815039924133852	0.998043617620652	pseudobulk	DESeq2	LRT	0.0888211171417967
797	KC_G1	shtd	0.100922027738727	0.89509949743187	0.998043617620652	pseudobulk	DESeq2	LRT	0.0481286867132479
798	KC_G1	shv	-0.495850118961131	0.463364834096666	0.998043617620652	pseudobulk	DESeq2	LRT	0.334076928977815
798	KC_G1	SiaT	1.12508065046033	0.900770125530981	0.998043617620652	pseudobulk	DESeq2	LRT	0.0453860258366365
798	KC_G1	sick	-0.153443468070288	0.39369871078297	0.998043617620652	pseudobulk	DESeq2	LRT	0.404836007360542
798	KC_G1	side	0.674390140972484	0.367938856930939	0.998043617620652	pseudobulk	DESeq2	LRT	0.434224345195289
798	KC_G1	side-II	0.0594686734145949	0.619856786833908	0.998043617620652	pseudobulk	DESeq2	LRT	0.207708639327957
798	KC_G1	side-III	-0.0725010189519345	0.54889713636081	0.998043617620652	pseudobulk	DESeq2	LRT	0.260509034960674
798	KC_G1	side-IV	-0.0369023329497541	0.860417365693949	0.998043617620652	pseudobulk	DESeq2	LRT	0.0652908328859837
798	KC_G1	side-V	0.447606057322383	0.686229438507168	0.998043617620652	pseudobulk	DESeq2	LRT	0.163530655112571
798	KC_G1	side-VI	-0.0187628957963311	0.882207656526805	0.998043617620652	pseudobulk	DESeq2	LRT	0.0544291773749854
798	KC_G1	side-VII	-0.357908773882494	0.0793062385706209	0.998043617620652	pseudobulk	DESeq2	LRT	1.10069264786238
799	KC_G1	SIDL	-0.265497968224105	0.590929416833432	0.998043617620652	pseudobulk	DESeq2	LRT	0.228464390033125
799	KC_G1	Sidpn	0.0953507438900313	0.764266152876597	0.998043617620652	pseudobulk	DESeq2	LRT	0.116755373627664
799	KC_G1	sif	-0.239852086124272	0.131296462642796	0.998043617620652	pseudobulk	DESeq2	LRT	0.881746974408682
799	KC_G1	SIFa	-1.66808346769027	0.498191247612582	0.998043617620652	pseudobulk	DESeq2	LRT	0.302603906560549
799	KC_G1	SIFaR	0.135329679763582	0.719191061688915	0.998043617620652	pseudobulk	DESeq2	LRT	0.143155718776641
799	KC_G1	sigmar	0.0244980171097553	0.926109853352574	0.998043617620652	pseudobulk	DESeq2	LRT	0.033337495094337
799	KC_G1	Sik2	-0.0798124100504557	0.834052619242471	0.998043617620652	pseudobulk	DESeq2	LRT	0.078806549451262
799	KC_G1	Sik3	0.201418823371573	0.383606020842498	0.998043617620652	pseudobulk	DESeq2	LRT	0.416114585030694
799	KC_G1	sima	0.0219184530941658	0.893010171440323	0.998043617620652	pseudobulk	DESeq2	LRT	0.049143594442643
799	KC_G1	simj	-0.0976753274813893	0.549705268801268	0.998043617620652	pseudobulk	DESeq2	LRT	0.259870100399467
800	KC_G1	Sin	0.43472878353064	0.568016915434622	0.998043617620652	pseudobulk	DESeq2	LRT	0.245638730890163
800	KC_G1	Sin1	-0.320286183165982	0.446998884426564	0.998043617620652	pseudobulk	DESeq2	LRT	0.349693560733817
800	KC_G1	Sin3A	-0.0204373076770112	0.875524824281735	0.998043617620652	pseudobulk	DESeq2	LRT	0.057731535593405
800	KC_G1	sina	0.0867932363653275	0.55307804113324	0.998043617620652	pseudobulk	DESeq2	LRT	0.25721358398966
800	KC_G1	sinu	0.512215373032986	0.234065019157933	0.998043617620652	pseudobulk	DESeq2	LRT	0.630663486439391
800	KC_G1	sip1	-0.136278582645873	0.843630289972232	0.998043617620652	pseudobulk	DESeq2	LRT	0.0738478356043883
800	KC_G1	sip3	0.0872244787420828	0.847900207365963	0.998043617620652	pseudobulk	DESeq2	LRT	0.0716552585222797

800	KC_G1	Sirt1	-0.0825077409335748	0.637479230261871	0.998043617620652	pseudobulk	DESeq2	LRT	0.195533960430638
800	KC_G1	Sirt2	0.0171081494189987	0.956741679054652	0.998043617620652	pseudobulk	DESeq2	LRT	0.019205306220122
800	KC_G1	Sirt4	0.0980020744945556	0.762733030019946	0.998043617620652	pseudobulk	DESeq2	LRT	0.11762744615777
801	KC_G1	Sirt6	-0.0695277338571478	0.925783324588144	0.998043617620652	pseudobulk	DESeq2	LRT	0.0334906460900136
801	KC_G1	Sirt7	-0.169499790964949	0.763783490246666	0.998043617620652	pseudobulk	DESeq2	LRT	0.117029733462391
801	KC_G1	Sirup	0.0425450379478434	0.843567180778817	0.998043617620652	pseudobulk	DESeq2	LRT	0.0738803249542666
801	KC_G1	sisRNA:1	0.265915083649717	0.865595237253845	0.998043617620652	pseudobulk	DESeq2	LRT	0.0626851418401678
801	KC_G1	sisRNA:CR46357	-0.794610277746993	0.879485534599449	0.998043617620652	pseudobulk	DESeq2	LRT	0.0557712992369932
801	KC_G1	sisRNA:CR46364	1.42944759940171	0.552522613276143	0.998043617620652	pseudobulk	DESeq2	LRT	0.25764994276489
801	KC_G1	siz	-0.25845003552345	0.0690379619345539	0.998043617620652	pseudobulk	DESeq2	LRT	1.16091203788441
801	KC_G1	SK	-0.328245339934822	0.0608286125898813	0.998043617620652	pseudobulk	DESeq2	LRT	1.21589208903486
801	KC_G1	Sk1	1.80955778343681	0.306633857547153	0.998043617620652	pseudobulk	DESeq2	LRT	0.513379893401812
801	KC_G1	Sk2	-0.526051467170073	0.339255318322479	0.998043617620652	pseudobulk	DESeq2	LRT	0.469473335414721
802	KC_G1	skd	-0.00889406595796089	0.938683106824123	0.998043617620652	pseudobulk	DESeq2	LRT	0.0274809979209581
802	KC_G1	Skeletor	-0.794610277746993	0.821479263487599	0.998043617620652	pseudobulk	DESeq2	LRT	0.085403394939296
802	KC_G1	Ski6	0.717366274932133	0.239193841517682	0.998043617620652	pseudobulk	DESeq2	LRT	0.621250006244322
802	KC_G1	SKIP	0.419423333472738	0.561207497301111	0.998043617620652	pseudobulk	DESeq2	LRT	0.250876535764219
802	KC_G1	skl	1.15577256681319	0.576039154373569	0.998043617620652	pseudobulk	DESeq2	LRT	0.239547995829467
802	KC_G1	Skp2	-0.794610277746993	0.821479263487599	0.998043617620652	pseudobulk	DESeq2	LRT	0.085403394939296
802	KC_G1	SkpA	-0.0145569280794283	0.908678113536204	0.998043617620652	pseudobulk	DESeq2	LRT	0.0415899322588334
802	KC_G1	sky	-0.229456081726751	0.189740448169943	0.998043617620652	pseudobulk	DESeq2	LRT	0.721840077943681
802	KC_G1	sl	0.520525872096381	0.367548262752671	0.998043617620652	pseudobulk	DESeq2	LRT	0.434685625638394
802	KC_G1	Slbp	-0.260326588678293	0.403993629356658	0.998043617620652	pseudobulk	DESeq2	LRT	0.39362548329797
803	KC_G1	SLC22A	-0.284060731273072	0.584617183221172	0.998043617620652	pseudobulk	DESeq2	LRT	0.233128423893971
803	KC_G1	Slc25A46a	-0.351874075739237	0.342550501714783	0.998043617620652	pseudobulk	DESeq2	LRT	0.465275392057013
803	KC_G1	Slc45-1	-0.794610277746993	0.815039924133852	0.998043617620652	pseudobulk	DESeq2	LRT	0.0888211171417967
803	KC_G1	SLC5A11	-1.2524604568689	0.366564763895376	0.998043617620652	pseudobulk	DESeq2	LRT	0.43584928400357
803	KC_G1	Sld5	1.163150625825	0.55539228501963	0.998043617620652	pseudobulk	DESeq2	LRT	0.255400157348948
803	KC_G1	sle	0.463987480644299	0.341459541639152	0.998043617620652	pseudobulk	DESeq2	LRT	0.466660746986186
803	KC_G1	slgA	0.0315274202605752	0.823563256184672	0.998043617620652	pseudobulk	DESeq2	LRT	0.0843030379551912
803	KC_G1	Slh	-0.0368227862657022	0.932462147952741	0.998043617620652	pseudobulk	DESeq2	LRT	0.0303687887620706
803	KC_G1	sli	0.987654982484343	0.640647112714077	0.998043617620652	pseudobulk	DESeq2	LRT	0.193381126803329
803	KC_G1	slif	-0.534809962592458	0.654689724481653	0.998043617620652	pseudobulk	DESeq2	LRT	0.183964475384613
804	KC_G1	Slik	-0.124642807925676	0.541383965974412	0.998043617620652	pseudobulk	DESeq2	LRT	0.266494610759735
804	KC_G1	slim	-0.148291887305017	0.612784761423833	0.998043617620652	pseudobulk	DESeq2	LRT	0.212692043166295
804	KC_G1	Slimp	0.876123286965939	0.569438989206183	0.998043617620652	pseudobulk	DESeq2	LRT	0.244552800242461
804	KC_G1	Slip1	-0.0315776029136417	0.838180491283219	0.998043617620652	pseudobulk	DESeq2	LRT	0.0766624516227252
804	KC_G1	SLIRP2	-0.172542186933171	0.798806303279418	0.998043617620652	pseudobulk	DESeq2	LRT	0.0975585168250351
804	KC_G1	sll	-0.286981231501401	0.648134613810998	0.998043617620652	pseudobulk	DESeq2	LRT	0.188334784308773
804	KC_G1	Slmap	-0.0644599675429005	0.726747758443757	0.998043617620652	pseudobulk	DESeq2	LRT	0.138616299078702
804	KC_G1	slmb	0.040693954153297	0.836769527189327	0.998043617620652	pseudobulk	DESeq2	LRT	0.0773941439816959
804	KC_G1	slmo	-0.276997003498871	0.547638710550467	0.998043617620652	pseudobulk	DESeq2	LRT	0.261505860785922
804	KC_G1	Sln	0.0533702247464516	0.846580448216691	0.998043617620652	pseudobulk	DESeq2	LRT	0.0723317657812789
805	KC_G1	slo	-0.180690135895455	0.0606197149736761	0.998043617620652	pseudobulk	DESeq2	LRT	1.21738610996197
805	KC_G1	SLO2	-0.062266733639687	0.579608412334547	0.998043617620652	pseudobulk	DESeq2	LRT	0.2368653198814
805	KC_G1	Slob	-0.137077069614979	0.194285954379316	0.998043617620652	pseudobulk	DESeq2	LRT	0.71155859495372
805	KC_G1	slpr	0.0725802748458026	0.758427591958658	0.998043617620652	pseudobulk	DESeq2	LRT	0.120085875541545
805	KC_G1	sls	-2.27131838799805	0.327405917019026	0.998043617620652	pseudobulk	DESeq2	LRT	0.484913476098507
805	KC_G1	Slu7	0.0216956906406735	0.955326625310282	0.998043617620652	pseudobulk	DESeq2	LRT	0.0198481181359611
805	KC_G1	slv	-0.222985744472093	0.66116462922546	0.998043617620652	pseudobulk	DESeq2	LRT	0.179690388226867
805	KC_G1	slx1	1.13582588427274	0.704684208645453	0.998043617620652	pseudobulk	DESeq2	LRT	0.152005460551806
805	KC_G1	sm	-0.100754445770076	0.347440389338642	0.998043617620652	pseudobulk	DESeq2	LRT	0.459119696978981
805	KC_G1	smal	0.820154422759637	0.57263913319579	0.998043617620652	pseudobulk	DESeq2	LRT	0.242118976345399
806	KC_G1	smash	-0.0786318019315392	0.630217044154219	0.998043617620652	pseudobulk	DESeq2	LRT	0.200509855554229
806	KC_G1	SmB	0.0285133419065546	0.87854872703745	0.998043617620652	pseudobulk	DESeq2	LRT	0.0562341461816388
806	KC_G1	SMC1	0.113601911309332	0.60452888298303	0.998043617620652	pseudobulk	DESeq2	LRT	0.218582944727955

806	KC_G1	SMC2	-0.609645264464077	0.367399129696612	0.998043617620652	pseudobulk	DESeq2	LRT	0.434861876795607
806	KC_G1	SMC3	-0.449744376465185	0.142294700804549	0.998043617620652	pseudobulk	DESeq2	LRT	0.846811273170781
806	KC_G1	SMC5	0.415674679622022	0.47065130025684	0.998043617620652	pseudobulk	DESeq2	LRT	0.327300737159549
806	KC_G1	SmD1	-0.170017889964407	0.606000110366871	0.998043617620652	pseudobulk	DESeq2	LRT	0.217527296738469
806	KC_G1	SmD2	-0.069127677651956	0.734066616853663	0.998043617620652	pseudobulk	DESeq2	LRT	0.134264525888912
806	KC_G1	SmD3	-0.0127882007859391	0.952052840473727	0.998043617620652	pseudobulk	DESeq2	LRT	0.0213389468998595
806	KC_G1	SmE	-0.00724909690036726	0.966594139056307	0.998043617620652	pseudobulk	DESeq2	LRT	0.0147558425338047
807	KC_G1	SmF	-0.0903798548073355	0.810634583219727	0.998043617620652	pseudobulk	DESeq2	LRT	0.091174872366947
807	KC_G1	smg	0.0787772672869276	0.766463524715699	0.998043617620652	pseudobulk	DESeq2	LRT	0.115508507987194
807	KC_G1	Smg5	-0.0595907487494404	0.714384904183379	0.998043617620652	pseudobulk	DESeq2	LRT	0.146067731189956
807	KC_G1	Smg6	-0.609598759286312	0.119192887851693	0.998043617620652	pseudobulk	DESeq2	LRT	0.923749657841708
807	KC_G1	smid	-0.123090672545176	0.792322109587941	0.998043617620652	pseudobulk	DESeq2	LRT	0.101098225004794
807	KC_G1	Smn	0.444850217281358	0.174530662611961	0.998043617620652	pseudobulk	DESeq2	LRT	0.758128262496572
807	KC_G1	smo	-0.0727491010560564	0.895525075488944	0.998043617620652	pseudobulk	DESeq2	LRT	0.0479222490176171
807	KC_G1	smog	0.0238379980836169	0.859630655609869	0.998043617620652	pseudobulk	DESeq2	LRT	0.0656881053667128
807	KC_G1	Smox	0.0235074782783505	0.835152316746803	0.998043617620652	pseudobulk	DESeq2	LRT	0.078234309797886
807	KC_G1	smp-30	0.699291096014708	0.448078772854712	0.998043617620652	pseudobulk	DESeq2	LRT	0.348645629732185
808	KC_G1	Smr	-0.125146486959738	0.283668724516786	0.998043617620652	pseudobulk	DESeq2	LRT	0.547188544072056
808	KC_G1	Sms	0.0286467195045449	0.918633503788819	0.998043617620652	pseudobulk	DESeq2	LRT	0.0368577193303908
808	KC_G1	SMSr	-0.340460007570555	0.410432189914212	0.998043617620652	pseudobulk	DESeq2	LRT	0.386758585143111
808	KC_G1	smt3	0.0484824631494165	0.657665678942162	0.998043617620652	pseudobulk	DESeq2	LRT	0.181994821718294
808	KC_G1	Smu1	0.390039033386584	0.390819801244003	0.998043617620652	pseudobulk	DESeq2	LRT	0.408023440467212
808	KC_G1	Smurf	-0.0292974795281802	0.881633220213632	0.998043617620652	pseudobulk	DESeq2	LRT	0.0547120538329765
808	KC_G1	Smyd3	-0.101694659910158	0.866766804521118	0.998043617620652	pseudobulk	DESeq2	LRT	0.0620977296657441
808	KC_G1	Smyd4-1	0.1729159612032	0.899814506689544	0.998043617620652	pseudobulk	DESeq2	LRT	0.0458470094762598
808	KC_G1	Smyd4-4	0.418453303755629	0.304927948861689	0.998043617620652	pseudobulk	DESeq2	LRT	0.515802767566005
808	KC_G1	Smyd5	0.545357018920913	0.453226635187818	0.998043617620652	pseudobulk	DESeq2	LRT	0.343684575474157
809	KC_G1	SmydA-1	0.557364465696897	0.114105670102747	0.998043617620652	pseudobulk	DESeq2	LRT	0.942692774222003
809	KC_G1	SmydA-3	-0.538349058549581	0.722406183657356	0.998043617620652	pseudobulk	DESeq2	LRT	0.141218545201785
809	KC_G1	SmydA-4	0.812030379720217	0.377468462690116	0.998043617620652	pseudobulk	DESeq2	LRT	0.423119327615741
809	KC_G1	SmydA-7	-0.718676691072112	0.739205546127982	0.998043617620652	pseudobulk	DESeq2	LRT	0.131234783341328
809	KC_G1	sn	0.418597820642074	0.375670889418939	0.998043617620652	pseudobulk	DESeq2	LRT	0.425192456931667
809	KC_G1	snama	-0.154587020434008	0.516452405405176	0.998043617620652	pseudobulk	DESeq2	LRT	0.286969695484758
809	KC_G1	Snap24	-0.059522892681548	0.65608808913852	0.998043617620652	pseudobulk	DESeq2	LRT	0.183037846510906
809	KC_G1	Snap25	0.026658527974936	0.782305186908652	0.998043617620652	pseudobulk	DESeq2	LRT	0.10662379024696
809	KC_G1	Snap29	-0.156912316765562	0.572913572827015	0.998043617620652	pseudobulk	DESeq2	LRT	0.241910888809819
809	KC_G1	snf	0.136265964061458	0.493110833672819	0.998043617620652	pseudobulk	DESeq2	LRT	0.307055455889793
810	KC_G1	SNF4Agamma	0.0146309962881522	0.938958576545441	0.998043617620652	pseudobulk	DESeq2	LRT	0.0273535668122245
810	KC_G1	sni	-0.121670225784581	0.810414935448901	0.998043617620652	pseudobulk	DESeq2	LRT	0.0912925637937877
810	KC_G1	Snm1	0.777030258285493	0.70316795941598	0.998043617620652	pseudobulk	DESeq2	LRT	0.152940926566052
810	KC_G1	snmRNA:CR43701	0.172845155749872	0.90730697492154	0.998043617620652	pseudobulk	DESeq2	LRT	0.0422457504714555
810	KC_G1	sno	-0.0445635897683464	0.792834726375507	0.998043617620652	pseudobulk	DESeq2	LRT	0.100817335890379
810	KC_G1	Snoo	-0.164536057215681	0.338882785138198	0.998043617620652	pseudobulk	DESeq2	LRT	0.46995049227026
810	KC_G1	snoRNA:2R:9445205	2.07738706174348	0.431998797123676	0.998043617620652	pseudobulk	DESeq2	LRT	0.364517462451933
810	KC_G1	snoRNA:2R:9445410	-4.29653025243735	0.251224612781474	0.998043617620652	pseudobulk	DESeq2	LRT	0.599937814490794
810	KC_G1	snoRNA:Dek-a	0.608376062239557	0.663686665843393	0.998043617620652	pseudobulk	DESeq2	LRT	0.178036907698936
810	KC_G1	snoRNA:gcl-a	1.12508065046033	0.900770125530981	0.998043617620652	pseudobulk	DESeq2	LRT	0.0453860258366365
811	KC_G1	snoRNA:lola-a	-0.794610277746993	0.821479263487599	0.998043617620652	pseudobulk	DESeq2	LRT	0.085403394939296
811	KC_G1	snoRNA:lola-c	0.948682853426738	0.716182290134517	0.998043617620652	pseudobulk	DESeq2	LRT	0.144976422491876
811	KC_G1	snoRNA:Or-CD2	-0.794610277746993	0.879485534599449	0.998043617620652	pseudobulk	DESeq2	LRT	0.0557712992369932
811	KC_G1	snoRNA:Pi4KIIalpha-a	1.12508065046033	0.900770125530981	0.998043617620652	pseudobulk	DESeq2	LRT	0.0453860258366365
811	KC_G1	snoRNA:Psi18S-1086	-0.794610277746993	0.821479263487599	0.998043617620652	pseudobulk	DESeq2	LRT	0.085403394939296
811	KC_G1	snoRNA:Psi18S-1377a	-0.794610277746993	0.815039924133852	0.998043617620652	pseudobulk	DESeq2	LRT	0.0888211171417967
811	KC_G1	snoRNA:Psi18S-1377b	1.12508065046033	0.900770125530981	0.998043617620652	pseudobulk	DESeq2	LRT	0.0453860258366365
811	KC_G1	snoRNA:Psi18S-1854b	1.13582588427274	0.633424328955532	0.998043617620652	pseudobulk	DESeq2	LRT	0.198305259991224
811	KC_G1	snoRNA:Psi18S-525a	-0.794610277746993	0.821479263487599	0.998043617620652	pseudobulk	DESeq2	LRT	0.085403394939296

811	KC_G1	snoRNA:Psi18S-525f	1.12508065046033	0.900770125530981	0.998043617620652	pseudobulk	DESeq2	LRT	0.0453860258366365
812	KC_G1	snoRNA:Psi18S-525g	-0.794610277746993	0.815039924133852	0.998043617620652	pseudobulk	DESeq2	LRT	0.0888211171417967
812	KC_G1	snoRNA:Psi18S-841d	-0.794610277746993	0.815039924133852	0.998043617620652	pseudobulk	DESeq2	LRT	0.0888211171417967
812	KC_G1	snoRNA:Psi28S-1135d	1.13582588427274	0.633424328955532	0.998043617620652	pseudobulk	DESeq2	LRT	0.198305259991224
812	KC_G1	snoRNA:Psi28S-1153	0.1729159612032	0.899814506689544	0.998043617620652	pseudobulk	DESeq2	LRT	0.0458470094762598
812	KC_G1	snoRNA:Psi28S-1175b	0.172834960772825	0.908610676445368	0.998043617620652	pseudobulk	DESeq2	LRT	0.0416221644022337
812	KC_G1	snoRNA:Psi28S-1175c	1.12508065046033	0.900770125530981	0.998043617620652	pseudobulk	DESeq2	LRT	0.0453860258366365
812	KC_G1	snoRNA:Psi28S-2566	0.172845155749872	0.90730697492154	0.998043617620652	pseudobulk	DESeq2	LRT	0.0422457504714555
812	KC_G1	snoRNA:Psi28S-2622	0.543454260015587	0.760285377265059	0.998043617620652	pseudobulk	DESeq2	LRT	0.119023362313675
812	KC_G1	snoRNA:Psi28S-2626	-1.66808346769027	0.498191247612582	0.998043617620652	pseudobulk	DESeq2	LRT	0.302603906560549
812	KC_G1	snoRNA:Psi28S-2876	-0.711364822669339	0.836431126230485	0.998043617620652	pseudobulk	DESeq2	LRT	0.0775698140913142
813	KC_G1	snoRNA:Psi28S-3308	1.13582588427274	0.633424328955532	0.998043617620652	pseudobulk	DESeq2	LRT	0.198305259991224
813	KC_G1	snoRNA:Psi28S-3342	-1.36262208775125	0.554321947234893	0.998043617620652	pseudobulk	DESeq2	LRT	0.256237926072317
813	KC_G1	snoRNA:Psi28S-3385a	-0.794610277746993	0.815039924133852	0.998043617620652	pseudobulk	DESeq2	LRT	0.0888211171417967
813	KC_G1	snoRNA:Psi28S-3385b	-0.794610277746993	0.879485534599449	0.998043617620652	pseudobulk	DESeq2	LRT	0.0557712992369932
813	KC_G1	snoRNA:Psi28S-3405c	1.13582588427274	0.633424328955532	0.998043617620652	pseudobulk	DESeq2	LRT	0.198305259991224
813	KC_G1	snoRNA:SC35-b	0.173116475207952	0.97305562682953	0.998043617620652	pseudobulk	DESeq2	LRT	0.011862331638572
813	KC_G1	snoRNA:Tudor-SN-a	-2.31679508011858	0.210450581917964	0.998043617620652	pseudobulk	DESeq2	LRT	0.67684986904444
813	KC_G1	Snp	-0.377368590755313	0.517823882773389	0.998043617620652	pseudobulk	DESeq2	LRT	0.285817923160508
813	KC_G1	sNPF	-0.0603197000218166	0.508947320711844	0.998043617620652	pseudobulk	DESeq2	LRT	0.293327167581006
813	KC_G1	sNPF-R	-1.72343437992478	0.512120560562093	0.998043617620652	pseudobulk	DESeq2	LRT	0.290627787806665
814	KC_G1	Snr1	0.230998076763601	0.416397705266818	0.998043617620652	pseudobulk	DESeq2	LRT	0.380491672515739
814	KC_G1	snRNA:7SK	-0.128722298547221	0.400356283295524	0.998043617620652	pseudobulk	DESeq2	LRT	0.397553351172895
814	KC_G1	snRNA:U11	2.63732022130093	0.22943645835378	0.998043617620652	pseudobulk	DESeq2	LRT	0.639337569853304
814	KC_G1	snRNA:U12	0.6813990701437	0.582272948059992	0.998043617620652	pseudobulk	DESeq2	LRT	0.234873386400955
814	KC_G1	snRNA:U2:34ABa	0.173195945230598	0.976215465741119	0.998043617620652	pseudobulk	DESeq2	LRT	0.0104543162934892
814	KC_G1	snRNA:U5:14B	1.12508065046033	0.900770125530981	0.998043617620652	pseudobulk	DESeq2	LRT	0.0453860258366365
814	KC_G1	snRNA:U6:96Ab	-0.794610277746993	0.815039924133852	0.998043617620652	pseudobulk	DESeq2	LRT	0.0888211171417967
814	KC_G1	snRNP-U1-70K	-0.156841364741586	0.490982783918381	0.998043617620652	pseudobulk	DESeq2	LRT	0.30893373594288
814	KC_G1	snRNP-U1-C	-0.263098576101354	0.373630922391806	0.998043617620652	pseudobulk	DESeq2	LRT	0.427557187933403
814	KC_G1	sns	0.957098833493547	0.492021750526364	0.998043617620652	pseudobulk	DESeq2	LRT	0.308015698198523
815	KC_G1	snsl	-1.30936440818423	0.372326952041248	0.998043617620652	pseudobulk	DESeq2	LRT	0.429075524890595
815	KC_G1	snu	-1.60891734856068	0.545249891924755	0.998043617620652	pseudobulk	DESeq2	LRT	0.263404411831769
815	KC_G1	Snx1	-0.0525159331864817	0.875626031051478	0.998043617620652	pseudobulk	DESeq2	LRT	0.0576813359871909
815	KC_G1	Snx16	0.186988314913983	0.645926902639637	0.998043617620652	pseudobulk	DESeq2	LRT	0.189816626860118
815	KC_G1	Snx17	0.103301963724672	0.717784111477874	0.998043617620652	pseudobulk	DESeq2	LRT	0.144006159229102
815	KC_G1	Snx21	-0.0549650829585134	0.89430751666554	0.998043617620652	pseudobulk	DESeq2	LRT	0.0485131189819709
815	KC_G1	Snx27	-0.057492158554541	0.701851385085992	0.998043617620652	pseudobulk	DESeq2	LRT	0.153754838682096
815	KC_G1	Snx3	0.155084189301055	0.52865021231699	0.998043617620652	pseudobulk	DESeq2	LRT	0.276831589035324
815	KC_G1	Snx6	-0.132733999786298	0.590747257174679	0.998043617620652	pseudobulk	DESeq2	LRT	0.228598286109757
815	KC_G1	snz	0.125317319834524	0.6956403612468	0.998043617620652	pseudobulk	DESeq2	LRT	0.157615228051946
816	KC_G1	Sobp	1.06595574389305	0.681978466270766	0.998043617620652	pseudobulk	DESeq2	LRT	0.166229338140333
816	KC_G1	Socs16D	0.0345757949793633	0.880468468713394	0.998043617620652	pseudobulk	DESeq2	LRT	0.0552861923474474
816	KC_G1	Socs36E	-0.010815264285815	0.946035483706518	0.998043617620652	pseudobulk	DESeq2	LRT	0.0240925738636089
816	KC_G1	Socs44A	-0.04636476509831	0.875629393239983	0.998043617620652	pseudobulk	DESeq2	LRT	0.0576796684064433
816	KC_G1	Sod1	-0.00917965862488865	0.939348762033886	0.998043617620652	pseudobulk	DESeq2	LRT	0.0271731326484353
816	KC_G1	Sod2	0.300994120466241	0.052496355753932	0.998043617620652	pseudobulk	DESeq2	LRT	1.2798708438491
816	KC_G1	Sod3	0.0446755003380855	0.828286609014335	0.998043617620652	pseudobulk	DESeq2	LRT	0.0818193598630861
816	KC_G1	Sodh-2	0.0856757944483831	0.942247052808922	0.998043617620652	pseudobulk	DESeq2	LRT	0.0258352122766851
816	KC_G1	sog	1.66385764610404	0.257567996747379	0.998043617620652	pseudobulk	DESeq2	LRT	0.589108099772852
816	KC_G1	sol	-0.104922986458889	0.602092807324807	0.998043617620652	pseudobulk	DESeq2	LRT	0.220336560897494
817	KC_G1	Son	-0.231682230399428	0.424524659289966	0.998043617620652	pseudobulk	DESeq2	LRT	0.372097077899377
817	KC_G1	sona	-1.36262208775125	0.554321947234893	0.998043617620652	pseudobulk	DESeq2	LRT	0.256237926072317
817	KC_G1	Sos	0.171972936534834	0.59680864066426	0.998043617620652	pseudobulk	DESeq2	LRT	0.224164897724324
817	KC_G1	soti	0.562077451288907	0.794187709521711	0.998043617620652	pseudobulk	DESeq2	LRT	0.100076838158817
817	KC_G1	sotv	0.0111137936276892	0.963095111041459	0.998043617620652	pseudobulk	DESeq2	LRT	0.0163308217427583

817	KC_G1	sov	0.00985903724820962	0.957367891292255	0.998043617620652	pseudobulk	DESeq2	LRT	0.0189211422065181
817	KC_G1	sowah	0.244290629386077	0.445491286541501	0.998043617620652	pseudobulk	DESeq2	LRT	0.351160786001858
817	KC_G1	sowi	1.13582588427274	0.704684208645453	0.998043617620652	pseudobulk	DESeq2	LRT	0.152005460551806
817	KC_G1	Sox100B	-0.794610277746993	0.879485534599449	0.998043617620652	pseudobulk	DESeq2	LRT	0.0557712992369932
817	KC_G1	Sox102F	-2.55498106343824	0.188771323840336	0.998043617620652	pseudobulk	DESeq2	LRT	0.724063978490222
818	KC_G1	Sox14	0.660333798080835	0.729870210504599	0.998043617620652	pseudobulk	DESeq2	LRT	0.136754361623511
818	KC_G1	Sox21a	-0.794610277746993	0.821479263487599	0.998043617620652	pseudobulk	DESeq2	LRT	0.085403394939296
818	KC_G1	SoxN	-1.60891734856068	0.545249891924755	0.998043617620652	pseudobulk	DESeq2	LRT	0.263404411831769
818	KC_G1	SP1029	-1.31481767438999	0.589948159507212	0.998043617620652	pseudobulk	DESeq2	LRT	0.229186149424154
818	KC_G1	SP1173	2.18844257130017	0.203572224866984	0.998043617620652	pseudobulk	DESeq2	LRT	0.691281476874902
818	KC_G1	SP2353	0.154112552061864	0.575458572407501	0.998043617620652	pseudobulk	DESeq2	LRT	0.239985936017551
818	KC_G1	Sp7	-0.271491815162026	0.833685922906465	0.998043617620652	pseudobulk	DESeq2	LRT	0.0789975316631653
818	KC_G1	spab	-0.317331985392112	0.154035715199383	0.998043617620652	pseudobulk	DESeq2	LRT	0.812378570620233
818	KC_G1	spag	0.195975816243888	0.671530242816368	0.998043617620652	pseudobulk	DESeq2	LRT	0.172934423810597
818	KC_G1	SPARC	0.134618351242275	0.610395873616182	0.998043617620652	pseudobulk	DESeq2	LRT	0.214388410950587
819	KC_G1	spartin	0.286277929994668	0.679498265109616	0.998043617620652	pseudobulk	DESeq2	LRT	0.167811647767811
819	KC_G1	spas	0.135828861908224	0.681227918799862	0.998043617620652	pseudobulk	DESeq2	LRT	0.166707561630959
819	KC_G1	Spase12	0.0507217867876146	0.838867768570549	0.998043617620652	pseudobulk	DESeq2	LRT	0.0763064919794881
819	KC_G1	Spase22-23	-0.161938972407204	0.595492657359573	0.998043617620652	pseudobulk	DESeq2	LRT	0.225123589156653
819	KC_G1	Spase25	-0.0717293468912034	0.774538751825701	0.998043617620652	pseudobulk	DESeq2	LRT	0.110956848681992
819	KC_G1	Spat	1.12508065046033	0.900770125530981	0.998043617620652	pseudobulk	DESeq2	LRT	0.0453860258366365
819	KC_G1	spdo	1.20573119267355	0.526009093077135	0.998043617620652	pseudobulk	DESeq2	LRT	0.279006748167384
819	KC_G1	SpdS	0.137020286862441	0.687720576186991	0.998043617620652	pseudobulk	DESeq2	LRT	0.162587981633278
819	KC_G1	SPE	-0.794610277746993	0.821479263487599	0.998043617620652	pseudobulk	DESeq2	LRT	0.085403394939296
819	KC_G1	Spec2	0.0658669448092059	0.855288693927063	0.998043617620652	pseudobulk	DESeq2	LRT	0.0678872688776263
820	KC_G1	spel1	-0.23144352430696	0.79236471696519	0.998043617620652	pseudobulk	DESeq2	LRT	0.101074871306736
820	KC_G1	spen	-0.162223722071668	0.0969066701632994	0.998043617620652	pseudobulk	DESeq2	LRT	1.01364632908528
820	KC_G1	Spf45	-0.122518173069428	0.568192646433972	0.998043617620652	pseudobulk	DESeq2	LRT	0.245504391228736
820	KC_G1	spg	-0.517461722887922	0.10959745917155	0.998043617620652	pseudobulk	DESeq2	LRT	0.960199514104992
820	KC_G1	Spg7	0.0574159208656719	0.887784186614451	0.998043617620652	pseudobulk	DESeq2	LRT	0.0516925949800011
820	KC_G1	spi	-0.0991320429440443	0.660621613427487	0.998043617620652	pseudobulk	DESeq2	LRT	0.18004722168496
820	KC_G1	spict	-0.246046056330199	0.653788550289603	0.998043617620652	pseudobulk	DESeq2	LRT	0.184562689429535
820	KC_G1	spidey	-0.0546463363137728	0.750243939653704	0.998043617620652	pseudobulk	DESeq2	LRT	0.124797504047842
820	KC_G1	spin	-0.133819078586404	0.74849673592282	0.998043617620652	pseudobulk	DESeq2	LRT	0.1258100892071
820	KC_G1	spir	0.0502940189329438	0.767588558634924	0.998043617620652	pseudobulk	DESeq2	LRT	0.114871507306485
821	KC_G1	spirit	1.12508065046033	0.900770125530981	0.998043617620652	pseudobulk	DESeq2	LRT	0.0453860258366365
821	KC_G1	sPLA2	-0.718676691072112	0.739205546127982	0.998043617620652	pseudobulk	DESeq2	LRT	0.131234783341328
821	KC_G1	Sply	0.592563857672794	0.182976865889879	0.998043617620652	pseudobulk	DESeq2	LRT	0.737603815468882
821	KC_G1	Spn	-0.0546722693022864	0.661282070124742	0.998043617620652	pseudobulk	DESeq2	LRT	0.179613252513696
821	KC_G1	spn-A	-1.67593316621313	0.495877122847873	0.998043617620652	pseudobulk	DESeq2	LRT	0.304625927300966
821	KC_G1	spn-B	-2.31763425292248	0.315578515403755	0.998043617620652	pseudobulk	DESeq2	LRT	0.50089257123956
821	KC_G1	spn-F	-0.157693113325197	0.685935143210657	0.998043617620652	pseudobulk	DESeq2	LRT	0.163716945921979
821	KC_G1	Spn27A	-0.649003342031703	0.673358054134622	0.998043617620652	pseudobulk	DESeq2	LRT	0.17175394085149
821	KC_G1	Spn31A	-0.711364822669339	0.836431126230485	0.998043617620652	pseudobulk	DESeq2	LRT	0.0775698140913142
821	KC_G1	Spn42Da	0.0695739505334072	0.879795715827008	0.998043617620652	pseudobulk	DESeq2	LRT	0.055618157154814
822	KC_G1	Spn42Dc	-0.794610277746993	0.821479263487599	0.998043617620652	pseudobulk	DESeq2	LRT	0.085403394939296
822	KC_G1	Spn42Dd	-1.67593316621313	0.495877122847873	0.998043617620652	pseudobulk	DESeq2	LRT	0.304625927300966
822	KC_G1	Spn43Ab	-0.0268522587984855	0.908680360231602	0.998043617620652	pseudobulk	DESeq2	LRT	0.0415888584724251
822	KC_G1	Spn43Ad	1.12508065046033	0.900770125530981	0.998043617620652	pseudobulk	DESeq2	LRT	0.0453860258366365
822	KC_G1	Spn77Ba	-0.86183262160441	0.672975120964107	0.998043617620652	pseudobulk	DESeq2	LRT	0.172000990794925
822	KC_G1	Spn77Bc	1.13582588427274	0.633424328955532	0.998043617620652	pseudobulk	DESeq2	LRT	0.198305259991224
822	KC_G1	Spn88Ea	0.0160228970110423	0.960033355180798	0.998043617620652	pseudobulk	DESeq2	LRT	0.0177136776694816
822	KC_G1	SPoCk	0.0235479844320391	0.859380751979539	0.998043617620652	pseudobulk	DESeq2	LRT	0.0658143776740925
822	KC_G1	spok	1.13582588427274	0.704684208645453	0.998043617620652	pseudobulk	DESeq2	LRT	0.152005460551806
822	KC_G1	spoon	0.0644731862738429	0.803989784120738	0.998043617620652	pseudobulk	DESeq2	LRT	0.094749469570179
823	KC_G1	Spp	0.106047996379314	0.755401544576867	0.998043617620652	pseudobulk	DESeq2	LRT	0.121822131501445

823	KC_G1	SppL	0.180380850756668	0.445196120185965	0.998043617620652	pseudobulk	DESeq2	LRT	0.351448629128813
823	KC_G1	Spps	0.199343102778732	0.699189864665796	0.998043617620652	pseudobulk	DESeq2	LRT	0.155404875785009
823	KC_G1	SPR	-0.431356981065851	0.153307044899343	0.998043617620652	pseudobulk	DESeq2	LRT	0.814437887607391
823	KC_G1	Spred	-0.0460602523727446	0.648378502914214	0.998043617620652	pseudobulk	DESeq2	LRT	0.188171392683026
823	KC_G1	spri	-0.380070528982535	0.0315356379904787	0.998043617620652	pseudobulk	DESeq2	LRT	1.50119837846671
823	KC_G1	sprt	-0.71088171110674	0.784645801042283	0.998043617620652	pseudobulk	DESeq2	LRT	0.105326344996467
823	KC_G1	Sps2	0.416899517485575	0.595809285777716	0.998043617620652	pseudobulk	DESeq2	LRT	0.22489273252313
823	KC_G1	Spt	0.69448447990701	0.405864796319715	0.998043617620652	pseudobulk	DESeq2	LRT	0.39161861664804
823	KC_G1	Spt-I	0.00775862034279168	0.943337421746107	0.998043617620652	pseudobulk	DESeq2	LRT	0.025332936964355
824	KC_G1	Spt20	-0.0344460343295026	0.895304657174605	0.998043617620652	pseudobulk	DESeq2	LRT	0.0480291563984423
824	KC_G1	Spt3	-0.178050999679569	0.735101718970725	0.998043617620652	pseudobulk	DESeq2	LRT	0.133652561689757
824	KC_G1	spt4	-0.147133343555815	0.635366273904128	0.998043617620652	pseudobulk	DESeq2	LRT	0.196975841831927
824	KC_G1	Spt5	0.126904783391658	0.613720724519172	0.998043617620652	pseudobulk	DESeq2	LRT	0.212029210930918
824	KC_G1	Spt6	-0.0243218691622456	0.909131001327681	0.998043617620652	pseudobulk	DESeq2	LRT	0.0413735325596336
824	KC_G1	Spt7	0.282703044477787	0.685733825969629	0.998043617620652	pseudobulk	DESeq2	LRT	0.163844427070103
824	KC_G1	Sptr	0.162948174413626	0.575807323381553	0.998043617620652	pseudobulk	DESeq2	LRT	0.239722815866461
824	KC_G1	Spx	0.442783689890719	0.332595849481058	0.998043617620652	pseudobulk	DESeq2	LRT	0.47808317471578
824	KC_G1	spz	0.493160138245581	0.318000701614728	0.998043617620652	pseudobulk	DESeq2	LRT	0.497571921817238
824	KC_G1	spz5	1.06667464613488	0.669131470313168	0.998043617620652	pseudobulk	DESeq2	LRT	0.174488544082685
825	KC_G1	sqd	-0.222402761419638	0.0749031496738385	0.998043617620652	pseudobulk	DESeq2	LRT	1.1254999198545
825	KC_G1	sqh	0.0165692742052845	0.917991368960778	0.998043617620652	pseudobulk	DESeq2	LRT	0.0371614020561963
825	KC_G1	squ	-0.160808133137973	0.849195415651945	0.998043617620652	pseudobulk	DESeq2	LRT	0.0709923590213913
825	KC_G1	sqz	0.227938693800064	0.447249944877493	0.998043617620652	pseudobulk	DESeq2	LRT	0.349449704286363
825	KC_G1	sr	0.179586849341942	0.732336253240336	0.998043617620652	pseudobulk	DESeq2	LRT	0.135289466214758
825	KC_G1	sra	-0.0432229761279528	0.799582604371041	0.998043617620652	pseudobulk	DESeq2	LRT	0.0971366629129081
825	KC_G1	Sra-1	0.464868173856926	0.32250248967328	0.998043617620652	pseudobulk	DESeq2	LRT	0.491466928324237
825	KC_G1	Sras	-0.530114099514837	0.301301613138885	0.998043617620652	pseudobulk	DESeq2	LRT	0.520998543150935
825	KC_G1	Src42A	0.00630283327942325	0.956167528652487	0.998043617620652	pseudobulk	DESeq2	LRT	0.0194660089841906
825	KC_G1	Src64B	-0.0264676150856948	0.86726969720522	0.998043617620652	pseudobulk	DESeq2	LRT	0.0618458277898604
826	KC_G1	SREBP	-0.0777968288251241	0.811039619929945	0.998043617620652	pseudobulk	DESeq2	LRT	0.0909579296400637
826	KC_G1	srl	-0.0536132104136351	0.929383195421273	0.998043617620652	pseudobulk	DESeq2	LRT	0.0318051844521492
826	KC_G1	Srlp	0.248795077050351	0.388678986986566	0.998043617620652	pseudobulk	DESeq2	LRT	0.41040893784175
826	KC_G1	SRm160	-0.170141359013659	0.385813384320741	0.998043617620652	pseudobulk	DESeq2	LRT	0.413622710243676
826	KC_G1	Srp14	-0.109152125891694	0.676484348307515	0.998043617620652	pseudobulk	DESeq2	LRT	0.169742247140732
826	KC_G1	Srp19	-0.0366680390959576	0.89756648842655	0.998043617620652	pseudobulk	DESeq2	LRT	0.0469333706190168
826	KC_G1	Srp54	-0.13919853410059	0.503388668646843	0.998043617620652	pseudobulk	DESeq2	LRT	0.298096564710454
826	KC_G1	Srp54k	0.136264769112479	0.652107880225296	0.998043617620652	pseudobulk	DESeq2	LRT	0.185680551643968
826	KC_G1	Srp68	-0.265941704025294	0.569379683277201	0.998043617620652	pseudobulk	DESeq2	LRT	0.244598033497494
826	KC_G1	Srp72	-0.0330480146601506	0.930328080565684	0.998043617620652	pseudobulk	DESeq2	LRT	0.0313638703139173
827	KC_G1	Srp9	0.0620275775924763	0.797580061169732	0.998043617620652	pseudobulk	DESeq2	LRT	0.0982257115572306
827	KC_G1	SRPK	0.00338007506693556	0.969723441695083	0.998043617620652	pseudobulk	DESeq2	LRT	0.0133521058070171
827	KC_G1	Srpk79D	-0.14128176569574	0.599236610219013	0.998043617620652	pseudobulk	DESeq2	LRT	0.222401661379356
827	KC_G1	SrpRbeta	0.337878294217159	0.405279050154502	0.998043617620652	pseudobulk	DESeq2	LRT	0.392245845400798
827	KC_G1	Sry-delta	0.0798611772260355	0.85926863725147	0.998043617620652	pseudobulk	DESeq2	LRT	0.065871039385451
827	KC_G1	Ssadh	0.0997842493012775	0.709632175068283	0.998043617620652	pseudobulk	DESeq2	LRT	0.14896670160668
827	KC_G1	Ssb-c31a	-0.0885570626496052	0.674758796872104	0.998043617620652	pseudobulk	DESeq2	LRT	0.170851444810558
827	KC_G1	Ssdp	-0.138748753957221	0.226887959714505	0.998043617620652	pseudobulk	DESeq2	LRT	0.644188550251218
827	KC_G1	ssh	0.169275667352736	0.524184043701468	0.998043617620652	pseudobulk	DESeq2	LRT	0.280516203225119
827	KC_G1	Ssk	0.14841936635658	0.860458601841878	0.998043617620652	pseudobulk	DESeq2	LRT	0.0652700194957778
828	KC_G1	Ssl1	0.368987129624368	0.47416097830369	0.998043617620652	pseudobulk	DESeq2	LRT	0.32407418971822
828	KC_G1	ssp	-0.176935855059723	0.741288230629441	0.998043617620652	pseudobulk	DESeq2	LRT	0.130012895080289
828	KC_G1	ssp2	-2.23422618188688	0.336998892702854	0.998043617620652	pseudobulk	DESeq2	LRT	0.472371526113321
828	KC_G1	ssp3	-0.0232833724304632	0.924310784508417	0.998043617620652	pseudobulk	DESeq2	LRT	0.0341819797501847
828	KC_G1	ssp7	0.280546867130476	0.740666724556009	0.998043617620652	pseudobulk	DESeq2	LRT	0.130377166171041
828	KC_G1	SsRbeta	0.138927891351694	0.619761311815234	0.998043617620652	pseudobulk	DESeq2	LRT	0.207775537792729
828	KC_G1	Ssrp	-0.146290705541544	0.77928330202004	0.998043617620652	pseudobulk	DESeq2	LRT	0.108304629443159

828	KC_G1	sstn	0.190364732038919	0.823603998797309	0.998043617620652	pseudobulk	DESeq2	LRT	0.0842815534437711
828	KC_G1	Ssu72	0.123487116427132	0.820444702852947	0.998043617620652	pseudobulk	DESeq2	LRT	0.0859506846354016
828	KC_G1	ssx	0.0564601297232191	0.746698941612754	0.998043617620652	pseudobulk	DESeq2	LRT	0.126854464249709
829	KC_G1	St3	0.123818941797702	0.598555289119363	0.998043617620652	pseudobulk	DESeq2	LRT	0.222895727211756
829	KC_G1	St4	-4.40328353291134	0.243707085848407	0.998043617620652	pseudobulk	DESeq2	LRT	0.613131843404013
829	KC_G1	stac	0.926311272074596	0.529658880241282	0.998043617620652	pseudobulk	DESeq2	LRT	0.27600374195489
829	KC_G1	Stacl	-0.0379715067898219	0.743042597393908	0.998043617620652	pseudobulk	DESeq2	LRT	0.128986288146792
829	KC_G1	stai	0.0148449992513418	0.882560172638378	0.998043617620652	pseudobulk	DESeq2	LRT	0.054255674887526
829	KC_G1	Stam	0.0847308418699944	0.793798770078919	0.998043617620652	pseudobulk	DESeq2	LRT	0.100289578329274
829	KC_G1	Start1	0.312755980594704	0.51279626435011	0.998043617620652	pseudobulk	DESeq2	LRT	0.290055147252973
829	KC_G1	stas	0.0886121196278588	0.686032318866122	0.998043617620652	pseudobulk	DESeq2	LRT	0.163655424271968
829	KC_G1	Stat92E	-0.139934816345681	0.400852342267611	0.998043617620652	pseudobulk	DESeq2	LRT	0.397015574381857
829	KC_G1	stau	-0.0608376693289497	0.728104517379435	0.998043617620652	pseudobulk	DESeq2	LRT	0.137806274446445
830	KC_G1	stc	-0.289967575780356	0.540579087641216	0.998043617620652	pseudobulk	DESeq2	LRT	0.267140759039981
830	KC_G1	stck	-0.1619515109892	0.513101763479097	0.998043617620652	pseudobulk	DESeq2	LRT	0.289796492715645
830	KC_G1	ste14	-1.23741704929648	0.311874601596664	0.998043617620652	pseudobulk	DESeq2	LRT	0.50601999182045
830	KC_G1	ste24a	-0.229067228447697	0.59314095276341	0.998043617620652	pseudobulk	DESeq2	LRT	0.226842089547577
830	KC_G1	ste24b	-1.22749171231044	0.519584846383208	0.998043617620652	pseudobulk	DESeq2	LRT	0.284343523549975
830	KC_G1	step	0.0807991163909236	0.717107021309665	0.998043617620652	pseudobulk	DESeq2	LRT	0.144416025230655
830	KC_G1	stet	-1.3255619052647	0.196656128848142	0.998043617620652	pseudobulk	DESeq2	LRT	0.706292514123539
830	KC_G1	Stim	-0.176868592879141	0.580505986095142	0.998043617620652	pseudobulk	DESeq2	LRT	0.236193297519405
830	KC_G1	Sting	0.228319291893143	0.924568785563363	0.998043617620652	pseudobulk	DESeq2	LRT	0.034060772899258
830	KC_G1	Stip1	0.100916949398287	0.783111805536542	0.998043617620652	pseudobulk	DESeq2	LRT	0.106176228923956
831	KC_G1	stj	-0.0944533435899711	0.517962974431337	0.998043617620652	pseudobulk	DESeq2	LRT	0.285701283835596
831	KC_G1	stl	-1.35335158257345	0.648211282942961	0.998043617620652	pseudobulk	DESeq2	LRT	0.188283413789429
831	KC_G1	Stlk	-0.103045093652587	0.565056934443706	0.998043617620652	pseudobulk	DESeq2	LRT	0.247907790996503
831	KC_G1	stmA	-0.111106652977016	0.536812601197737	0.998043617620652	pseudobulk	DESeq2	LRT	0.27017729804322
831	KC_G1	Stoml2	0.0517292977436561	0.829996445497824	0.998043617620652	pseudobulk	DESeq2	LRT	0.080923767508247
831	KC_G1	stops	0.328051165567269	0.699433833086589	0.998043617620652	pseudobulk	DESeq2	LRT	0.155253363780524
831	KC_G1	strat	-0.217465918819028	0.627539583346692	0.998043617620652	pseudobulk	DESeq2	LRT	0.202358874967468
831	KC_G1	Strip	-0.227931584784368	0.452399754048066	0.998043617620652	pseudobulk	DESeq2	LRT	0.344477639855329
831	KC_G1	Strn-Mlck	-0.794610277746993	0.815039924133852	0.998043617620652	pseudobulk	DESeq2	LRT	0.0888211171417967
831	KC_G1	Strump	-0.239882521105742	0.68408902216281	0.998043617620652	pseudobulk	DESeq2	LRT	0.164887378808558
832	KC_G1	Stt3A	0.0695543959802234	0.855435016084256	0.998043617620652	pseudobulk	DESeq2	LRT	0.067812976453628
832	KC_G1	Stt3B	-0.0542378837885931	0.902968460327843	0.998043617620652	pseudobulk	DESeq2	LRT	0.0443274188390915
832	KC_G1	STUB1	-0.19456533892567	0.329156924525101	0.998043617620652	pseudobulk	DESeq2	LRT	0.482597004155653
832	KC_G1	stumps	-0.0766826143505645	0.941856063975779	0.998043617620652	pseudobulk	DESeq2	LRT	0.0260154617477936
832	KC_G1	sturkopf	0.364004914115178	0.221505360127873	0.998043617620652	pseudobulk	DESeq2	LRT	0.654615759979098
832	KC_G1	stv	-0.27465030631151	0.101313637396545	0.998043617620652	pseudobulk	DESeq2	LRT	0.994332092177349
832	KC_G1	stw	0.8855571736987	0.256267191955182	0.998043617620652	pseudobulk	DESeq2	LRT	0.591306989854946
832	KC_G1	stwl	-1.09256594818858	0.0722507163303233	0.998043617620652	pseudobulk	DESeq2	LRT	1.14115784273384
832	KC_G1	stx	-0.150933313020142	0.286421446811647	0.998043617620652	pseudobulk	DESeq2	LRT	0.542994465822191
832	KC_G1	sty	0.281657896629674	0.664799947246363	0.998043617620652	pseudobulk	DESeq2	LRT	0.177309023686362
833	KC_G1	Su(dx)	-0.147276967874127	0.605109131696658	0.998043617620652	pseudobulk	DESeq2	LRT	0.218166293083859
833	KC_G1	su(f)	0.153214484979133	0.679084416801995	0.998043617620652	pseudobulk	DESeq2	LRT	0.168076235332921
833	KC_G1	Su(fu)	-0.167849896414325	0.760909953136161	0.998043617620652	pseudobulk	DESeq2	LRT	0.118666735042777
833	KC_G1	Su(H)	-0.59279863243791	0.123668295297882	0.998043617620652	pseudobulk	DESeq2	LRT	0.907741625690392
833	KC_G1	su(Hw)	-0.207725060866249	0.527466677175351	0.998043617620652	pseudobulk	DESeq2	LRT	0.277804971814138
833	KC_G1	Su(P)	0.182199924443679	0.431158544631889	0.998043617620652	pseudobulk	DESeq2	LRT	0.365363002698664
833	KC_G1	su(r)	1.13582588427274	0.633424328955532	0.998043617620652	pseudobulk	DESeq2	LRT	0.198305259991224
833	KC_G1	su(sable)	-0.0793821232400105	0.774850467258949	0.998043617620652	pseudobulk	DESeq2	LRT	0.110782100732616
833	KC_G1	Su(Tpl)	-0.213883083851075	0.189028193570741	0.998043617620652	pseudobulk	DESeq2	LRT	0.723473415937695
833	KC_G1	Su(var)2-10	-0.258388238834317	0.255899952729109	0.998043617620652	pseudobulk	DESeq2	LRT	0.591929794337469
834	KC_G1	Su(var)2-HP2	-0.545658832439391	0.0198790936952812	0.998043617620652	pseudobulk	DESeq2	LRT	1.70160341934064
834	KC_G1	Su(var)205	0.0612318948552392	0.766654514230733	0.998043617620652	pseudobulk	DESeq2	LRT	0.115400302759935
834	KC_G1	Su(var)3-3	0.215416704976389	0.512246920078253	0.998043617620652	pseudobulk	DESeq2	LRT	0.290520644145978

834	KC_G1	Su(var)3-7	-0.078509033976267	0.84902546796914	0.998043617620652	pseudobulk	DESeq2	LRT	0.0710792821558468
834	KC_G1	Su(var)3-9	2.95840825636686	0.160769763152811	0.998043617620652	pseudobulk	DESeq2	LRT	0.793795628042056
834	KC_G1	su(w[a])	-0.0893756547541374	0.748430069746224	0.998043617620652	pseudobulk	DESeq2	LRT	0.12584877213072
834	KC_G1	Su(z)12	0.298313165688377	0.400728747325473	0.998043617620652	pseudobulk	DESeq2	LRT	0.397149501198454
834	KC_G1	Su(z)2	-0.0118546048093096	0.946608232887464	0.998043617620652	pseudobulk	DESeq2	LRT	0.0238297226843705
834	KC_G1	subdued	0.274446639984006	0.536624067940745	0.998043617620652	pseudobulk	DESeq2	LRT	0.270329852835436
834	KC_G1	sud1	0.112147034242137	0.892171114570616	0.998043617620652	pseudobulk	DESeq2	LRT	0.0495518418270461
835	KC_G1	sug	-0.0386222860498856	0.966666281578012	0.998043617620652	pseudobulk	DESeq2	LRT	0.01472342982958
835	KC_G1	Sugb	0.192787872086831	0.78246853989632	0.998043617620652	pseudobulk	DESeq2	LRT	0.106533114770769
835	KC_G1	Sulf1	-0.409344286133919	0.805586088867645	0.998043617620652	pseudobulk	DESeq2	LRT	0.0938880419333141
835	KC_G1	sun	-0.0842521740092327	0.552005322679864	0.998043617620652	pseudobulk	DESeq2	LRT	0.258056734590821
835	KC_G1	Sur-8	-0.131287228837818	0.484270097244217	0.998043617620652	pseudobulk	DESeq2	LRT	0.314912346982308
835	KC_G1	Surf1	0.119065577505311	0.815132309231211	0.998043617620652	pseudobulk	DESeq2	LRT	0.088771892480482
835	KC_G1	Surf4	0.421673764198118	0.125468698524327	0.998043617620652	pseudobulk	DESeq2	LRT	0.901464606881885
835	KC_G1	Surf6	-0.0529263202771557	0.900048211183885	0.998043617620652	pseudobulk	DESeq2	LRT	0.045734226904733
835	KC_G1	sut1	1.12508065046033	0.900770125530981	0.998043617620652	pseudobulk	DESeq2	LRT	0.0453860258366365
835	KC_G1	sut2	-0.0450936767131922	0.898745297830386	0.998043617620652	pseudobulk	DESeq2	LRT	0.0463633688000578
836	KC_G1	sut3	0.1729159612032	0.899814506689544	0.998043617620652	pseudobulk	DESeq2	LRT	0.0458470094762598
836	KC_G1	Suv3	-0.429791568737585	0.3792462136668	0.998043617620652	pseudobulk	DESeq2	LRT	0.421078746445843
836	KC_G1	svr	-0.0553599087096486	0.690660087874065	0.998043617620652	pseudobulk	DESeq2	LRT	0.160735640442218
836	KC_G1	sw	-0.230041518006443	0.358341041008135	0.998043617620652	pseudobulk	DESeq2	LRT	0.445703448985339
836	KC_G1	swi2	-0.794610277746993	0.821479263487599	0.998043617620652	pseudobulk	DESeq2	LRT	0.085403394939296
836	KC_G1	SWIP	0.101959893384695	0.89286354858913	0.998043617620652	pseudobulk	DESeq2	LRT	0.0492149068704176
836	KC_G1	Swip-1	-0.0732599867159891	0.759695626407663	0.998043617620652	pseudobulk	DESeq2	LRT	0.119360373835923
836	KC_G1	swm	-0.0784464647880166	0.638871953854068	0.998043617620652	pseudobulk	DESeq2	LRT	0.194586176750889
836	KC_G1	sws	0.0296889236632277	0.890397576936419	0.998043617620652	pseudobulk	DESeq2	LRT	0.0504160305293393
836	KC_G1	sxc	-0.00954750535064602	0.94947221720346	0.998043617620652	pseudobulk	DESeq2	LRT	0.0225177387645201
837	KC_G1	sxe2	-0.794610277746993	0.821479263487599	0.998043617620652	pseudobulk	DESeq2	LRT	0.085403394939296
837	KC_G1	Sxl	-0.25252468194384	0.0331778499766519	0.998043617620652	pseudobulk	DESeq2	LRT	1.47915176097381
837	KC_G1	Syb	-0.299928484977523	0.691867541922181	0.998043617620652	pseudobulk	DESeq2	LRT	0.159977043286868
837	KC_G1	syd	-0.144237788753188	0.440006065229161	0.998043617620652	pseudobulk	DESeq2	LRT	0.356541336974264
837	KC_G1	Sym	-0.195803268775008	0.795079333440237	0.998043617620652	pseudobulk	DESeq2	LRT	0.0995895350462403
837	KC_G1	Syn	-0.185366564337854	0.218998697666551	0.998043617620652	pseudobulk	DESeq2	LRT	0.65955846779875
837	KC_G1	Syn1	-0.0680833187201957	0.682935431181869	0.998043617620652	pseudobulk	DESeq2	LRT	0.165620355186695
837	KC_G1	Syn2	-0.253418478339558	0.25683729979273	0.998043617620652	pseudobulk	DESeq2	LRT	0.590341904599188
837	KC_G1	Synd	0.0822297546806472	0.696507138255452	0.998043617620652	pseudobulk	DESeq2	LRT	0.157074428286583
837	KC_G1	Syngr	0.101553571563482	0.531867493068083	0.998043617620652	pseudobulk	DESeq2	LRT	0.274196552286242
838	KC_G1	Synj	-0.39311671586554	0.0336338398767254	0.998043617620652	pseudobulk	DESeq2	LRT	1.47322354772923
838	KC_G1	Syp	-0.0373125056039317	0.801428777018739	0.998043617620652	pseudobulk	DESeq2	LRT	0.0961350673487107
838	KC_G1	Syt1	-0.0711392089149438	0.501519513814162	0.998043617620652	pseudobulk	DESeq2	LRT	0.299712164185067
838	KC_G1	Syt12	-0.228505608394749	0.674484670189019	0.998043617620652	pseudobulk	DESeq2	LRT	0.171027916604346
838	KC_G1	Syt4	-0.0270761583848092	0.818421757202368	0.998043617620652	pseudobulk	DESeq2	LRT	0.0870228337102034
838	KC_G1	Syt7	-0.0206063247328559	0.842641357665643	0.998043617620652	pseudobulk	DESeq2	LRT	0.0743572290862369
838	KC_G1	Sytalpha	-0.0433991395581629	0.712569777976235	0.998043617620652	pseudobulk	DESeq2	LRT	0.147172601205447
838	KC_G1	Sytbeta	-0.139939834507951	0.397229855005261	0.998043617620652	pseudobulk	DESeq2	LRT	0.400958118241144
838	KC_G1	Syx13	0.0131610864543311	0.933692161795787	0.998043617620652	pseudobulk	DESeq2	LRT	0.0297962870142079
838	KC_G1	Syx16	-0.0421522535929765	0.870752568720616	0.998043617620652	pseudobulk	DESeq2	LRT	0.0601052356988461
839	KC_G1	Syx17	-0.114190681551363	0.707861664355178	0.998043617620652	pseudobulk	DESeq2	LRT	0.150051607108679
839	KC_G1	Syx18	-0.0541924851409319	0.897724819805313	0.998043617620652	pseudobulk	DESeq2	LRT	0.0468567675140258
839	KC_G1	Syx1A	0.0423416087900529	0.712825618030874	0.998043617620652	pseudobulk	DESeq2	LRT	0.147016700708667
839	KC_G1	Syx4	0.547121279492053	0.758762468274186	0.998043617620652	pseudobulk	DESeq2	LRT	0.119894159365532
839	KC_G1	Syx5	0.104371911512046	0.731975954952038	0.998043617620652	pseudobulk	DESeq2	LRT	0.135503185066148
839	KC_G1	Syx6	-0.00903882174948767	0.965432691796344	0.998043617620652	pseudobulk	DESeq2	LRT	0.01527799904709
839	KC_G1	Syx7	-0.00336786453232369	0.953303793058102	0.998043617620652	pseudobulk	DESeq2	LRT	0.0207686789793908
839	KC_G1	Syx8	0.204620914230669	0.564842009341147	0.998043617620652	pseudobulk	DESeq2	LRT	0.248073010705226
839	KC_G1	t-cup	0.186152091849065	0.932459292672547	0.998043617620652	pseudobulk	DESeq2	LRT	0.0303701186115809

839	KC_G1	T48	-1.66808346769027	0.498191247612582	0.998043617620652	pseudobulk	DESeq2	LRT	0.302603906560549
840	KC_G1	Tab2	0.0936493870350509	0.957782618298444	0.998043617620652	pseudobulk	DESeq2	LRT	0.0187330487358505
840	KC_G1	tacc	-0.00244062168058195	0.962153056219207	0.998043617620652	pseudobulk	DESeq2	LRT	0.0167558362947152
840	KC_G1	Tace	-0.0759005265344107	0.759466778500903	0.998043617620652	pseudobulk	DESeq2	LRT	0.119491218811458
840	KC_G1	tadr	-0.235308487242798	0.595441075808399	0.998043617620652	pseudobulk	DESeq2	LRT	0.225161209357396
840	KC_G1	Taf1	0.343288402447555	0.346780177795557	0.998043617620652	pseudobulk	DESeq2	LRT	0.459945735071383
840	KC_G1	Taf10	-0.0455839512994931	0.860999337585763	0.998043617620652	pseudobulk	DESeq2	LRT	0.0649971826728941
840	KC_G1	Taf10b	0.137212317665563	0.458265226305905	0.998043617620652	pseudobulk	DESeq2	LRT	0.338883096271307
840	KC_G1	Taf11	0.356019698735531	0.33413520013063	0.998043617620652	pseudobulk	DESeq2	LRT	0.476077770344403
840	KC_G1	Taf12	-0.122197434479777	0.543038116618644	0.998043617620652	pseudobulk	DESeq2	LRT	0.265169685593172
840	KC_G1	Taf12L	1.12508065046033	0.900770125530981	0.998043617620652	pseudobulk	DESeq2	LRT	0.0453860258366365
841	KC_G1	Taf13	0.123305465712131	0.717891905720487	0.998043617620652	pseudobulk	DESeq2	LRT	0.143940943338783
841	KC_G1	TAF1B	-0.0452674530353937	0.914245525178282	0.998043617620652	pseudobulk	DESeq2	LRT	0.0389371566613817
841	KC_G1	TAF1C-like	0.702305377763643	0.526007911047768	0.998043617620652	pseudobulk	DESeq2	LRT	0.279007724099958
841	KC_G1	Taf2	0.243946176982955	0.652407864672363	0.998043617620652	pseudobulk	DESeq2	LRT	0.185480812249754
841	KC_G1	Taf4	-0.0258045545706008	0.895613026742805	0.998043617620652	pseudobulk	DESeq2	LRT	0.0478795982089372
841	KC_G1	Taf5	0.184863739119518	0.611086595157711	0.998043617620652	pseudobulk	DESeq2	LRT	0.213897242892657
841	KC_G1	Taf6	-0.0380380251255364	0.884709883362636	0.998043617620652	pseudobulk	DESeq2	LRT	0.0531991210659645
841	KC_G1	Taf7	-0.0370277557684675	0.93215506404276	0.998043617620652	pseudobulk	DESeq2	LRT	0.0305118367270189
841	KC_G1	Taf8	-0.300673300590017	0.618345489068603	0.998043617620652	pseudobulk	DESeq2	LRT	0.208768803113298
841	KC_G1	tai	-0.147174241471116	0.296141584648893	0.998043617620652	pseudobulk	DESeq2	LRT	0.528500604041023
842	KC_G1	Tailor	-0.195391154013723	0.578429755326887	0.998043617620652	pseudobulk	DESeq2	LRT	0.237749374354795
842	KC_G1	Tak1	-0.0815367992063166	0.841961367810196	0.998043617620652	pseudobulk	DESeq2	LRT	0.0747078350226324
842	KC_G1	Taldo	-0.385174829271671	0.454259191364216	0.998043617620652	pseudobulk	DESeq2	LRT	0.342696276527631
842	KC_G1	tam	-0.355934189672209	0.842102986012508	0.998043617620652	pseudobulk	DESeq2	LRT	0.0746347926786073
842	KC_G1	tamo	-0.323795499002864	0.268726932714335	0.998043617620652	pseudobulk	DESeq2	LRT	0.57068880493618
842	KC_G1	Tango1	-0.186936696242437	0.472122386433059	0.998043617620652	pseudobulk	DESeq2	LRT	0.325945406317646
842	KC_G1	Tango10	-0.0129958300081178	0.942071236379007	0.998043617620652	pseudobulk	DESeq2	LRT	0.0259162560217887
842	KC_G1	Tango11	0.31455089728409	0.115908087073087	0.998043617620652	pseudobulk	DESeq2	LRT	0.935886261631839
842	KC_G1	Tango14	0.157587814560779	0.60740023474597	0.998043617620652	pseudobulk	DESeq2	LRT	0.216525044573072
842	KC_G1	Tango2	1.24137469996061	0.181634078885325	0.998043617620652	pseudobulk	DESeq2	LRT	0.740802664166712
843	KC_G1	Tango4	-0.0388502175626178	0.931337981371789	0.998043617620652	pseudobulk	DESeq2	LRT	0.0308926854979604
843	KC_G1	Tango5	0.0809227784598407	0.728719165366571	0.998043617620652	pseudobulk	DESeq2	LRT	0.13743980834883
843	KC_G1	Tango6	0.342686634139974	0.619317699598372	0.998043617620652	pseudobulk	DESeq2	LRT	0.208086508028436
843	KC_G1	Tango8	1.13582588427274	0.704684208645453	0.998043617620652	pseudobulk	DESeq2	LRT	0.152005460551806
843	KC_G1	Tango9	-0.0553462638145199	0.856962148436895	0.998043617620652	pseudobulk	DESeq2	LRT	0.0670383602098559
843	KC_G1	tank	-0.473797915712573	0.531405806063083	0.998043617620652	pseudobulk	DESeq2	LRT	0.274573704861037
843	KC_G1	tant	-0.111324486300888	0.770942045840153	0.998043617620652	pseudobulk	DESeq2	LRT	0.11297826801626
843	KC_G1	Tao	-0.00417919900496264	0.966634165194578	0.998043617620652	pseudobulk	DESeq2	LRT	0.0147378590075471
843	KC_G1	Tap42	0.231221193144079	0.602124351658257	0.998043617620652	pseudobulk	DESeq2	LRT	0.220313808306976
843	KC_G1	tapas	0.0419431632342308	0.860889457042146	0.998043617620652	pseudobulk	DESeq2	LRT	0.0650526107748424
844	KC_G1	Tapdelta	-0.0519469183009461	0.826198303981923	0.998043617620652	pseudobulk	DESeq2	LRT	0.0829157008772834
844	KC_G1	tara	-0.294960586989673	0.0529761724115438	0.998043617620652	pseudobulk	DESeq2	LRT	1.27591942317347
844	KC_G1	Task6	0.334548212372839	0.037327275774133	0.998043617620652	pseudobulk	DESeq2	LRT	1.42797370467411
844	KC_G1	Task7	-0.119373885367613	0.552267670432806	0.998043617620652	pseudobulk	DESeq2	LRT	0.257850379487877
844	KC_G1	Tasp1	-0.0752815709779462	0.90391366096336	0.998043617620652	pseudobulk	DESeq2	LRT	0.0438730500090469
844	KC_G1	Tat	-0.108297994383238	0.53951709833842	0.998043617620652	pseudobulk	DESeq2	LRT	0.267994787132402
844	KC_G1	tau	-0.189465354955791	0.157826053808559	0.998043617620652	pseudobulk	DESeq2	LRT	0.801821302195671
844	KC_G1	tay	-0.0384548598707788	0.788265998633796	0.998043617620652	pseudobulk	DESeq2	LRT	0.103327206056917
844	KC_G1	Taz	0.685853437604324	0.090815339327061	0.998043617620652	pseudobulk	DESeq2	LRT	1.04184078999811
844	KC_G1	Tbc1d15-17	0.196876936503427	0.700608647272655	0.998043617620652	pseudobulk	DESeq2	LRT	0.154524506699141
845	KC_G1	TBC1D16	0.105482108194155	0.672570925908084	0.998043617620652	pseudobulk	DESeq2	LRT	0.172261910423996
845	KC_G1	TBC1D5	0.107646801359966	0.848403536091993	0.998043617620652	pseudobulk	DESeq2	LRT	0.0713975300399175
845	KC_G1	TBC1d7	0.168986020853459	0.716726135180668	0.998043617620652	pseudobulk	DESeq2	LRT	0.144646758837559
845	KC_G1	TBCB	-0.00109872108392505	0.965986014376119	0.998043617620652	pseudobulk	DESeq2	LRT	0.0150291612897307
845	KC_G1	TBCC	0.125152505920761	0.642800003750584	0.998043617620652	pseudobulk	DESeq2	LRT	0.191924129374689

845	KC_G1	TBCD	0.138969780861888	0.836976379958235	0.998043617620652	pseudobulk	DESeq2	LRT	0.077286797918979
845	KC_G1	Tbce	0.0292949629093419	0.914399555115889	0.998043617620652	pseudobulk	DESeq2	LRT	0.038863993910633
845	KC_G1	Tbh	-1.76164463706579	0.438917041178301	0.998043617620652	pseudobulk	DESeq2	LRT	0.35761755712261
845	KC_G1	Tbp	0.134570314139202	0.758914028982194	0.998043617620652	pseudobulk	DESeq2	LRT	0.119807418904705
845	KC_G1	TBPH	0.0182679324196586	0.90742226345325	0.998043617620652	pseudobulk	DESeq2	LRT	0.0421905695969129
846	KC_G1	tbrd-1	-0.718676691072112	0.739205546127982	0.998043617620652	pseudobulk	DESeq2	LRT	0.131234783341328
846	KC_G1	Tcs3	0.284210466280601	0.609425280687568	0.998043617620652	pseudobulk	DESeq2	LRT	0.215079533973895
846	KC_G1	Tctp	-0.357011817390057	0.0128082212788646	0.998043617620652	pseudobulk	DESeq2	LRT	1.8925111780157
846	KC_G1	Tdc1	1.13582588427274	0.633424328955532	0.998043617620652	pseudobulk	DESeq2	LRT	0.198305259991224
846	KC_G1	Tdc2	1.12508065046033	0.900770125530981	0.998043617620652	pseudobulk	DESeq2	LRT	0.0453860258366365
846	KC_G1	Tdrd3	-0.30614918615677	0.389761837851314	0.998043617620652	pseudobulk	DESeq2	LRT	0.409200685541482
846	KC_G1	tea	0.197998072196195	0.666670241801942	0.998043617620652	pseudobulk	DESeq2	LRT	0.176088930069643
846	KC_G1	tef	0.424032955202735	0.491083692645643	0.998043617620652	pseudobulk	DESeq2	LRT	0.308844487191139
846	KC_G1	tefu	0.140013293872111	0.902505270256321	0.998043617620652	pseudobulk	DESeq2	LRT	0.0445502533153179
846	KC_G1	Teh1	0.473793603521749	0.68112817629928	0.998043617620652	pseudobulk	DESeq2	LRT	0.166771153841318
847	KC_G1	Teh2	-0.115283688530464	0.468146430616838	0.998043617620652	pseudobulk	DESeq2	LRT	0.329618283545291
847	KC_G1	Teh3	-1.40762380271461	0.252046385685861	0.998043617620652	pseudobulk	DESeq2	LRT	0.598519525910579
847	KC_G1	Teh4	0.138602271111199	0.742964752644849	0.998043617620652	pseudobulk	DESeq2	LRT	0.129031789328185
847	KC_G1	Tehao	0.642124263472541	0.578809007959458	0.998043617620652	pseudobulk	DESeq2	LRT	0.237464718609248
847	KC_G1	Tektin-C	-1.37709505804248	0.394075634671621	0.998043617620652	pseudobulk	DESeq2	LRT	0.404420416326448
847	KC_G1	temp	0.294266069072678	0.431428971203899	0.998043617620652	pseudobulk	DESeq2	LRT	0.365090694626322
847	KC_G1	Ten-a	-0.0921400378668506	0.387371590424557	0.998043617620652	pseudobulk	DESeq2	LRT	0.411872233318178
847	KC_G1	Ten-m	0.31042051041104	0.31542430589081	0.998043617620652	pseudobulk	DESeq2	LRT	0.501104843957216
847	KC_G1	teq	0.516913364029147	0.674083105230223	0.998043617620652	pseudobulk	DESeq2	LRT	0.171286557592309
847	KC_G1	TER94	-0.0843316555212367	0.716257238537931	0.998043617620652	pseudobulk	DESeq2	LRT	0.144930975996707
848	KC_G1	Tes	0.477654722176716	0.371411924266683	0.998043617620652	pseudobulk	DESeq2	LRT	0.430144157246509
848	KC_G1	Tet	-0.0683624158877684	0.539276155556346	0.998043617620652	pseudobulk	DESeq2	LRT	0.268188781898445
848	KC_G1	tex	0.05928970938651	0.930325632055859	0.998043617620652	pseudobulk	DESeq2	LRT	0.0313650133254284
848	KC_G1	tey	-0.794610277746993	0.879485534599449	0.998043617620652	pseudobulk	DESeq2	LRT	0.0557712992369932
848	KC_G1	TFAM	0.0744402927907044	0.779788593131349	0.998043617620652	pseudobulk	DESeq2	LRT	0.1080231220287
848	KC_G1	Tfb1	0.205797460937684	0.667190128300754	0.998043617620652	pseudobulk	DESeq2	LRT	0.175750388114062
848	KC_G1	Tfb4	0.112615670850724	0.856284318900018	0.998043617620652	pseudobulk	DESeq2	LRT	0.0673820091226177
848	KC_G1	TfIIA-L	-0.119497142665666	0.670330557310826	0.998043617620652	pseudobulk	DESeq2	LRT	0.173710982651642
848	KC_G1	TfIIA-S	0.0210750151891118	0.886889429034083	0.998043617620652	pseudobulk	DESeq2	LRT	0.0521305214914394
848	KC_G1	TfIIB	0.0177687749259892	0.952038490253915	0.998043617620652	pseudobulk	DESeq2	LRT	0.0213454930367781
849	KC_G1	TfIIEalpha	0.219034065477519	0.647575340938473	0.998043617620652	pseudobulk	DESeq2	LRT	0.188709697130287
849	KC_G1	TfIIEbeta	-0.497209992792473	0.307044567314281	0.998043617620652	pseudobulk	DESeq2	LRT	0.512798582392877
849	KC_G1	TfIIFalpha	0.10261028777492	0.673564403511441	0.998043617620652	pseudobulk	DESeq2	LRT	0.171620872468987
849	KC_G1	TfIIFbeta	0.369322863823472	0.332434176675346	0.998043617620652	pseudobulk	DESeq2	LRT	0.478294333929698
849	KC_G1	TfIIS	0.0365945981832471	0.837170438315363	0.998043617620652	pseudobulk	DESeq2	LRT	0.0771861156214344
849	KC_G1	Tg	-0.566062869281458	0.798174612094218	0.998043617620652	pseudobulk	DESeq2	LRT	0.0979020901359823
849	KC_G1	Tgi	-0.152100792003776	0.646328957406842	0.998043617620652	pseudobulk	DESeq2	LRT	0.189546385992419
849	KC_G1	tgo	0.0574826680899806	0.908113162968194	0.998043617620652	pseudobulk	DESeq2	LRT	0.0418600292428032
849	KC_G1	Tgt	-0.382417055811407	0.614332162199951	0.998043617620652	pseudobulk	DESeq2	LRT	0.211596747417979
849	KC_G1	TH1	-0.415494473316154	0.343802237651235	0.998043617620652	pseudobulk	DESeq2	LRT	0.463691301015979
850	KC_G1	THADA	-0.0205841423843653	0.941161123088262	0.998043617620652	pseudobulk	DESeq2	LRT	0.0263360206980498
850	KC_G1	Thd1	-0.10248928856372	0.399888891211316	0.998043617620652	pseudobulk	DESeq2	LRT	0.39806066026417
850	KC_G1	THG	-0.266075009362414	0.778073116907865	0.998043617620652	pseudobulk	DESeq2	LRT	0.108979589671532
850	KC_G1	tho2	-0.245280507375623	0.364931753494565	0.998043617620652	pseudobulk	DESeq2	LRT	0.43778834609688
850	KC_G1	thoc5	0.0383351357115194	0.94228249132913	0.998043617620652	pseudobulk	DESeq2	LRT	0.0258188784878911
850	KC_G1	thoc6	-0.192493084770478	0.655516834210798	0.998043617620652	pseudobulk	DESeq2	LRT	0.183416150791625
850	KC_G1	thoc7	-0.110181742060342	0.583878266161839	0.998043617620652	pseudobulk	DESeq2	LRT	0.233677690293592
850	KC_G1	Thor	0.295139818810763	0.873246841757395	0.998043617620652	pseudobulk	DESeq2	LRT	0.0588629763857822
850	KC_G1	ThrRS	-0.148547533868573	0.599074968899943	0.998043617620652	pseudobulk	DESeq2	LRT	0.222518826121095
850	KC_G1	ths	-0.245510378998854	0.276500241378885	0.998043617620652	pseudobulk	DESeq2	LRT	0.558304485229186
851	KC_G1	Tie	-3.5532467158306	0.31170690255513	0.998043617620652	pseudobulk	DESeq2	LRT	0.506253580435986

851	KC_G1	Tif-IA	-0.0840635709357442	0.736196494266778	0.998043617620652	pseudobulk	DESeq2	LRT	0.133006254974408
851	KC_G1	Tig	-3.27393176540752	0.093620372181894	0.998043617620652	pseudobulk	DESeq2	LRT	1.02862963669478
851	KC_G1	tim	0.889397033965463	0.595477973237312	0.998043617620652	pseudobulk	DESeq2	LRT	0.225134298460654
851	KC_G1	Tim10	0.172845155749872	0.90730697492154	0.998043617620652	pseudobulk	DESeq2	LRT	0.0422457504714555
851	KC_G1	Tim17b1	1.06595574389305	0.681978466270766	0.998043617620652	pseudobulk	DESeq2	LRT	0.166229338140333
851	KC_G1	Tim23	-0.0270561636754788	0.878656143379421	0.998043617620652	pseudobulk	DESeq2	LRT	0.0561810501250232
851	KC_G1	Tim8	-0.0725431892025506	0.611427813457073	0.998043617620652	pseudobulk	DESeq2	LRT	0.213654809384566
851	KC_G1	Tim9a	-0.027215856271809	0.90259437953273	0.998043617620652	pseudobulk	DESeq2	LRT	0.0445073751649988
851	KC_G1	timeout	-0.571760862530955	0.607263151303947	0.998043617620652	pseudobulk	DESeq2	LRT	0.21662307104422
852	KC_G1	Timp	-0.219288938305418	0.765367396961816	0.998043617620652	pseudobulk	DESeq2	LRT	0.116130041747201
852	KC_G1	tin	1.13582588427274	0.704684208645453	0.998043617620652	pseudobulk	DESeq2	LRT	0.152005460551806
852	KC_G1	Tina-1	0.215612306255151	0.32024194873252	0.998043617620652	pseudobulk	DESeq2	LRT	0.494521780131265
852	KC_G1	tinc	-0.206382518785253	0.389881433128368	0.998043617620652	pseudobulk	DESeq2	LRT	0.409067446222356
852	KC_G1	tio	1.13582588427274	0.633424328955532	0.998043617620652	pseudobulk	DESeq2	LRT	0.198305259991224
852	KC_G1	Tip60	0.0894577045143193	0.85143934321597	0.998043617620652	pseudobulk	DESeq2	LRT	0.0698462858633416
852	KC_G1	tipE	-0.113660166391059	0.635861723235749	0.998043617620652	pseudobulk	DESeq2	LRT	0.196637317315216
852	KC_G1	Tis11	-1.07832719318413	0.585615590450897	0.998043617620652	pseudobulk	DESeq2	LRT	0.232387369865193
852	KC_G1	Tk	-1.60891734856068	0.545249891924755	0.998043617620652	pseudobulk	DESeq2	LRT	0.263404411831769
852	KC_G1	tko	0.0736178123230492	0.716915505294883	0.998043617620652	pseudobulk	DESeq2	LRT	0.144532026684422
853	KC_G1	TkR86C	-0.577957609530933	0.0431663748833358	0.998043617620652	pseudobulk	DESeq2	LRT	1.36485442190823
853	KC_G1	TkR99D	-0.79461027774699 3	0.815039924133852	0.998043617620652	pseudobulk	DESeq2	LRT	0.0888211171417967
853	KC_G1	tkv	-0.241649313358346	0.391623267460156	0.998043617620652	pseudobulk	DESeq2	LRT	0.407131513423122
853	KC_G1	Tl	-0.156486647547419	0.357868263326574	0.998043617620652	pseudobulk	DESeq2	LRT	0.446276814248396
853	KC_G1	tld	1.90250740646012	0.460774913678782	0.998043617620652	pseudobulk	DESeq2	LRT	0.336511173568024
853	KC_G1	Tlk	-0.0371637754074312	0.773708075814119	0.998043617620652	pseudobulk	DESeq2	LRT	0.111422870037573
853	KC_G1	Tm1	-0.0428369101376766	0.742877474470758	0.998043617620652	pseudobulk	DESeq2	LRT	0.129082810128039
853	KC_G1	Tm2	0.765327129627148	0.50375242974034	0.998043617620652	pseudobulk	DESeq2	LRT	0.297782846120755
853	KC_G1	TM4SF	-0.0210958933906492	0.861497212282914	0.998043617620652	pseudobulk	DESeq2	LRT	0.0647461235463143
853	KC_G1	TM9SF2	0.0188569259338099	0.937770824476396	0.998043617620652	pseudobulk	DESeq2	LRT	0.027903282970607
854	KC_G1	TM9SF3	-0.166510672668502	0.666519126524021	0.998043617620652	pseudobulk	DESeq2	LRT	0.17618738349745
854	KC_G1	TM9SF4	0.328567348211907	0.27067528666392	0.998043617620652	pseudobulk	DESeq2	LRT	0.567551394600542
854	KC_G1	Tmc	0.379501417534186	0.819889808017155	0.998043617620652	pseudobulk	DESeq2	LRT	0.0862445122331468
854	KC_G1	Tmem18	0.147052974372177	0.8015494352081	0.998043617620652	pseudobulk	DESeq2	LRT	0.0960696875630904
854	KC_G1	TMEM216	-0.134042070641123	0.646888143414022	0.998043617620652	pseudobulk	DESeq2	LRT	0.189170808817857
854	KC_G1	Tmem63	-0.60600618309551	0.206804740974276	0.998043617620652	pseudobulk	DESeq2	LRT	0.684439509314237
854	KC_G1	tmod	0.0961298795221474	0.57380546241659	0.998043617620652	pseudobulk	DESeq2	LRT	0.241235321746885
854	KC_G1	TMS1	0.074353847746767	0.643961489191936	0.998043617620652	pseudobulk	DESeq2	LRT	0.191140103962438
854	KC_G1	Tmtc3	-0.397658787824801	0.19084586388341	0.998043617620652	pseudobulk	DESeq2	LRT	0.719317247881906
854	KC_G1	tn	1.12508065046033	0.900770125530981	0.998043617620652	pseudobulk	DESeq2	LRT	0.0453860258366365
855	KC_G1	tna	-0.115931953631918	0.3324708275027	0.998043617620652	pseudobulk	DESeq2	LRT	0.478246455655379
855	KC_G1	tnc	1.99001068938097	0.0534689915996017	0.998043617620652	pseudobulk	DESeq2	LRT	1.27189800641506
855	KC_G1	Tnks	-0.0303523371982513	0.925141200518098	0.998043617620652	pseudobulk	DESeq2	LRT	0.0337919776113409
855	KC_G1	Tnpo	-0.127379706065663	0.436008404252933	0.998043617620652	pseudobulk	DESeq2	LRT	0.360505139434404
855	KC_G1	Tnpo-SR	-0.704490838867314	0.227795429328386	0.998043617620652	pseudobulk	DESeq2	LRT	0.642454994205009
855	KC_G1	Tob	-0.0324696648563972	0.765259183630181	0.998043617620652	pseudobulk	DESeq2	LRT	0.116191449870478
855	KC_G1	toc	-0.0603788925553508	0.616071583032651	0.998043617620652	pseudobulk	DESeq2	LRT	0.210368823046773
855	KC_G1	tok	-0.33596088239938	0.231579317483046	0.998043617620652	pseudobulk	DESeq2	LRT	0.635300230368094
855	KC_G1	Toll-6	-0.228357926006417	0.289083014403634	0.998043617620652	pseudobulk	DESeq2	LRT	0.538977425338981
855	KC_G1	Toll-7	0.0967781802690043	0.698768202727304	0.998043617620652	pseudobulk	DESeq2	LRT	0.155666865701504
856	KC_G1	Tollo	2.23045770445974	0.357070320422792	0.998043617620652	pseudobulk	DESeq2	LRT	0.447246246733247
856	KC_G1	Tom20	0.0424334942008	0.772647989802528	0.998043617620652	pseudobulk	DESeq2	LRT	0.112018320965772
856	KC_G1	Tom40	0.0590449137366411	0.731171051537104	0.998043617620652	pseudobulk	DESeq2	LRT	0.135981011484523
856	KC_G1	Tom7	0.00243765518558365	0.963085690896149	0.998043617620652	pseudobulk	DESeq2	LRT	0.0163350696483782
856	KC_G1	Tom70	0.0435079109757149	0.891761368795616	0.998043617620652	pseudobulk	DESeq2	LRT	0.0497513452654939
856	KC_G1	tomboy20	1.12508065046033	0.900770125530981	0.998043617620652	pseudobulk	DESeq2	LRT	0.0453860258366365
856	KC_G1	tomboy40	-0.561162396149956	0.740999159491454	0.998043617620652	pseudobulk	DESeq2	LRT	0.130182284636637

856	KC_G1	Tomosyn	-0.0887391233990783	0.38271340392375	0.998043617620652	pseudobulk	DESeq2	LRT	0.417126327040776
856	KC_G1	Top1	0.00577074100108439	0.967245676820416	0.998043617620652	pseudobulk	DESeq2	LRT	0.0144632027152557
856	KC_G1	Top2	-0.217421893233482	0.474932069136708	0.998043617620652	pseudobulk	DESeq2	LRT	0.323368504288512
857	KC_G1	Top3alpha	0.377227081924756	0.637263834338879	0.998043617620652	pseudobulk	DESeq2	LRT	0.195680727672238
857	KC_G1	Top3beta	-0.297625400812135	0.384905135180442	0.998043617620652	pseudobulk	DESeq2	LRT	0.414646294762378
857	KC_G1	Topors	0.0801558324402737	0.874225673042469	0.998043617620652	pseudobulk	DESeq2	LRT	0.0583764439033754
857	KC_G1	Tor	-0.23489838697416	0.584431273746771	0.998043617620652	pseudobulk	DESeq2	LRT	0.23326655240193
857	KC_G1	TORIP	-0.420094829933129	0.27576536565122	0.998043617620652	pseudobulk	DESeq2	LRT	0.559460279317087
857	KC_G1	Torsin	0.364318878217333	0.239846043309273	0.998043617620652	pseudobulk	DESeq2	LRT	0.620067441605519
857	KC_G1	tos	-1.01536159427276	0.537796284932125	0.998043617620652	pseudobulk	DESeq2	LRT	0.26938220218533
857	KC_G1	TotA	0.727008253840136	0.383115544209406	0.998043617620652	pseudobulk	DESeq2	LRT	0.416670226958664
857	KC_G1	TotB	-1.23495981023028	0.688295289311108	0.998043617620652	pseudobulk	DESeq2	LRT	0.162225202744963
857	KC_G1	TotC	-0.794610277746993	0.879485534599449	0.998043617620652	pseudobulk	DESeq2	LRT	0.0557712992369932
858	KC_G1	tou	-0.184180789492682	0.156269962832843	0.998043617620652	pseudobulk	DESeq2	LRT	0.806124491139976
858	KC_G1	tow	0.020276602416501	0.858402046769309	0.998043617620652	pseudobulk	DESeq2	LRT	0.0663092555114005
858	KC_G1	toy	-0.148236435187964	0.129862693173181	0.998043617620652	pseudobulk	DESeq2	LRT	0.886515594701189
858	KC_G1	Tpc1	0.120132763843688	0.816723434221915	0.998043617620652	pseudobulk	DESeq2	LRT	0.0879249830336907
858	KC_G1	Tpi	-0.136691254678038	0.731175820030689	0.998043617620652	pseudobulk	DESeq2	LRT	0.135978179146326
858	KC_G1	TpnC25D	-0.794610277746993	0.821479263487599	0.998043617620652	pseudobulk	DESeq2	LRT	0.085403394939296
858	KC_G1	TpnC73F	-0.794610277746993	0.879485534599449	0.998043617620652	pseudobulk	DESeq2	LRT	0.0557712992369932
858	KC_G1	TppII	-0.334111266165654	0.313116230017068	0.998043617620652	pseudobulk	DESeq2	LRT	0.504294420648264
858	KC_G1	Tpr2	0.00610619768745627	0.970719515004051	0.998043617620652	pseudobulk	DESeq2	LRT	0.0129062393844479
858	KC_G1	Tpst	-0.151338905170592	0.698724372924839	0.998043617620652	pseudobulk	DESeq2	LRT	0.155694107408102
859	KC_G1	tra	-0.720310796761098	0.0183247722809603	0.998043617620652	pseudobulk	DESeq2	LRT	1.73696141356197
859	KC_G1	tra2	-0.128791672698651	0.727818792728168	0.998043617620652	pseudobulk	DESeq2	LRT	0.137976734854793
859	KC_G1	Traf6	-0.026939781856463	0.954417861849389	0.998043617620652	pseudobulk	DESeq2	LRT	0.0202614414772395
859	KC_G1	tral	-0.101365787088745	0.451783580953547	0.998043617620652	pseudobulk	DESeq2	LRT	0.345069556573792
859	KC_G1	TRAM	-0.488583440675295	0.33040736209699	0.998043617620652	pseudobulk	DESeq2	LRT	0.480950284346581
859	KC_G1	Trap1	0.0128108371211569	0.970184086928409	0.998043617620652	pseudobulk	DESeq2	LRT	0.0131458529998531
859	KC_G1	Trax	0.484421244564355	0.356011186364728	0.998043617620652	pseudobulk	DESeq2	LRT	0.448536355678395
859	KC_G1	trbd	0.134052703219995	0.7032521807753	0.998043617620652	pseudobulk	DESeq2	LRT	0.152888912419483
859	KC_G1	trbl	-0.794610277746993	0.879485534599449	0.998043617620652	pseudobulk	DESeq2	LRT	0.0557712992369932
859	KC_G1	trc	0.0532965616742012	0.835779024017678	0.998043617620652	pseudobulk	DESeq2	LRT	0.077908532770869
860	KC_G1	Trc8	-0.0529927621257388	0.878702743746355	0.998043617620652	pseudobulk	DESeq2	LRT	0.056158017513566
860	KC_G1	Tre1	0.159541552346245	0.91821407427774	0.998043617620652	pseudobulk	DESeq2	LRT	0.0370560547040494
860	KC_G1	Treh	0.379766907498836	0.217858492657315	0.998043617620652	pseudobulk	DESeq2	LRT	0.661825505536271
860	KC_G1	trem	0.11963338152835	0.867441714235478	0.998043617620652	pseudobulk	DESeq2	LRT	0.0617596969824111
860	KC_G1	Tret1-1	-2.48098175888638	0.0994192010309972	0.998043617620652	pseudobulk	DESeq2	LRT	1.00252973133204
860	KC_G1	Trf	0.192970379031131	0.762759881507608	0.998043617620652	pseudobulk	DESeq2	LRT	0.117612157390663
860	KC_G1	Trf2	-0.244085275723803	0.0920663545378088	0.998043617620652	pseudobulk	DESeq2	LRT	1.03589905284566
860	KC_G1	Trf4-1	-0.0829826986057943	0.647929855016875	0.998043617620652	pseudobulk	DESeq2	LRT	0.18847200837021
860	KC_G1	Trf4-2	1.12508065046033	0.900770125530981	0.998043617620652	pseudobulk	DESeq2	LRT	0.0453860258366365
860	KC_G1	trio	-0.069428671718576	0.524426101725484	0.998043617620652	pseudobulk	DESeq2	LRT	0.280315700747709
861	KC_G1	Trissin	1.13582588427274	0.704684208645453	0.998043617620652	pseudobulk	DESeq2	LRT	0.152005460551806
861	KC_G1	TrissinR	-1.67593316621313	0.495877122847873	0.998043617620652	pseudobulk	DESeq2	LRT	0.304625927300966
861	KC_G1	trk	1.12508065046033	0.900770125530981	0.998043617620652	pseudobulk	DESeq2	LRT	0.0453860258366365
861	KC_G1	Trl	-0.131804179720845	0.796791318219472	0.998043617620652	pseudobulk	DESeq2	LRT	0.0986554065998164
861	KC_G1	trn	0.0906034379428689	0.762279977558549	0.998043617620652	pseudobulk	DESeq2	LRT	0.117885487484379
861	KC_G1	tRNA:Ala-CGC-1-2	1.13582588427274	0.633424328955532	0.998043617620652	pseudobulk	DESeq2	LRT	0.198305259991224
861	KC_G1	tRNA:Gln-CTG-1-1	1.13582588427274	0.704684208645453	0.998043617620652	pseudobulk	DESeq2	LRT	0.152005460551806
861	KC_G1	tRNA:Glu-CTC-2-2	1.12508065046033	0.900770125530981	0.998043617620652	pseudobulk	DESeq2	LRT	0.0453860258366365
861	KC_G1	tRNA:Lys-CTT-2-1Psi	3.04510097614695	0.164711640221344	0.998043617620652	pseudobulk	DESeq2	LRT	0.783275708023541
861	KC_G1	tRNA:Trp-CCA-2-2	-0.794610277746993	0.821479263487599	0.998043617620652	pseudobulk	DESeq2	LRT	0.085403394939296
862	KC_G1	trol	0.0491304627833906	0.904159457026821	0.998043617620652	pseudobulk	DESeq2	LRT	0.0437549708521476
862	KC_G1	Trp1	-0.0640296981939939	0.731584825155761	0.998043617620652	pseudobulk	DESeq2	LRT	0.135735311423431
862	KC_G1	Trpm	-0.271700765256009	0.0370098140822823	0.998043617620652	pseudobulk	DESeq2	LRT	1.43168309656503

862	KC_G1	TrpRS	0.160426609976255	0.80187198819535	0.998043617620652	pseudobulk	DESeq2	LRT	0.0958949574736341
862	KC_G1	TrpRS-m	0.203770980206899	0.767121612467682	0.998043617620652	pseudobulk	DESeq2	LRT	0.115135781504774
862	KC_G1	trr	-0.119569427892116	0.715038007722237	0.998043617620652	pseudobulk	DESeq2	LRT	0.145670872736922
862	KC_G1	Trs20	0.00948617100501378	0.940886084374749	0.998043617620652	pseudobulk	DESeq2	LRT	0.0264629545985019
862	KC_G1	Trs23	-0.382786981370849	0.305960453637212	0.998043617620652	pseudobulk	DESeq2	LRT	0.514334703835535
862	KC_G1	Trs31	0.155660699218631	0.574614217995241	0.998043617620652	pseudobulk	DESeq2	LRT	0.240623632224192
862	KC_G1	Trs33	0.524963255361211	0.293042428711544	0.998043617620652	pseudobulk	DESeq2	LRT	0.533069494931654
863	KC_G1	trsn	-0.00845559468376667	0.954582354786678	0.998043617620652	pseudobulk	DESeq2	LRT	0.0201865977192023
863	KC_G1	trus	0.0245393775993181	0.942455089034297	0.998043617620652	pseudobulk	DESeq2	LRT	0.0257393361346916
863	KC_G1	trv	-0.0343811599751267	0.734055401575393	0.998043617620652	pseudobulk	DESeq2	LRT	0.134271161214048
863	KC_G1	trx	-0.140727149775129	0.336921784436801	0.998043617620652	pseudobulk	DESeq2	LRT	0.472470907823182
863	KC_G1	Trx-2	0.0309108537461669	0.812817912724024	0.998043617620652	pseudobulk	DESeq2	LRT	0.0900067340568606
863	KC_G1	Trxr-1	-0.0845621549245189	0.69118038130988	0.998043617620652	pseudobulk	DESeq2	LRT	0.160408597510907
863	KC_G1	Try29F	-0.799744840544538	0.403655420254928	0.998043617620652	pseudobulk	DESeq2	LRT	0.393989211467444
863	KC_G1	Tsc1	-0.00907424328552556	0.973159576134724	0.998043617620652	pseudobulk	DESeq2	LRT	0.0118159394311554
863	KC_G1	Tsen15	-0.152478690309214	0.917713637820275	0.998043617620652	pseudobulk	DESeq2	LRT	0.0372928143498358
863	KC_G1	Tsen2	0.202554469544764	0.792474418203811	0.998043617620652	pseudobulk	DESeq2	LRT	0.101014748305468
864	KC_G1	Tsen34	0.050015204752925	0.896470078109521	0.998043617620652	pseudobulk	DESeq2	LRT	0.0474642015047983
864	KC_G1	Tsen54	0.91988444782621	0.390374905449111	0.998043617620652	pseudobulk	DESeq2	LRT	0.408518107950178
864	KC_G1	Tsf1	0.207382193865068	0.575382659859108	0.998043617620652	pseudobulk	DESeq2	LRT	0.240043230455737
864	KC_G1	Tsf2	0.0987286186620603	0.850531484235538	0.998043617620652	pseudobulk	DESeq2	LRT	0.0703096054180408
864	KC_G1	TSG101	-0.219662100809261	0.405412599642102	0.998043617620652	pseudobulk	DESeq2	LRT	0.392102758183691
864	KC_G1	tsh	1.12508065046033	0.900770125530981	0.998043617620652	pseudobulk	DESeq2	LRT	0.0453860258366365
864	KC_G1	tsl	-2.72659795038729	0.21856759243411	0.998043617620652	pseudobulk	DESeq2	LRT	0.660414231546479
864	KC_G1	Tsp26A	0.363608937246108	0.285173732427347	0.998043617620652	pseudobulk	DESeq2	LRT	0.544890480177934
864	KC_G1	Tsp39D	-0.0335374319944598	0.848049442181136	0.998043617620652	pseudobulk	DESeq2	LRT	0.071578827176665
864	KC_G1	Tsp3A	-0.0354148649770726	0.927898257230463	0.998043617620652	pseudobulk	DESeq2	LRT	0.0324996409640904
865	KC_G1	Tsp42Ea	0.173108424760601	0.83305213418604	0.998043617620652	pseudobulk	DESeq2	LRT	0.0793278186642552
865	KC_G1	Tsp42Ee	-0.229778516277648	0.315909071098701	0.998043617620652	pseudobulk	DESeq2	LRT	0.50043790346633
865	KC_G1	Tsp42Ef	-0.244421538327994	0.814148350350011	0.998043617620652	pseudobulk	DESeq2	LRT	0.089296452766598
865	KC_G1	Tsp42Ei	1.13582588427274	0.704684208645453	0.998043617620652	pseudobulk	DESeq2	LRT	0.152005460551806
865	KC_G1	Tsp42Ej	-0.082460206670368	0.561158148983337	0.998043617620652	pseudobulk	DESeq2	LRT	0.250914725996048
865	KC_G1	Tsp42Ek	0.278781495982864	0.596579616728809	0.998043617620652	pseudobulk	DESeq2	LRT	0.224331589213024
865	KC_G1	Tsp42En	0.733933466938299	0.315093278157046	0.998043617620652	pseudobulk	DESeq2	LRT	0.501560861473544
865	KC_G1	Tsp42Ep	0.693814139898402	0.747913497313991	0.998043617620652	pseudobulk	DESeq2	LRT	0.126148629156436
865	KC_G1	Tsp5D	-0.115258146149043	0.731534401727018	0.998043617620652	pseudobulk	DESeq2	LRT	0.135765245575711
865	KC_G1	Tsp66E	0.973794929407998	0.204622203837849	0.998043617620652	pseudobulk	DESeq2	LRT	0.689047242172989
866	KC_G1	Tsp86D	0.371811976720643	0.38988071184999	0.998043617620652	pseudobulk	DESeq2	LRT	0.409068249665358
866	KC_G1	Tsp96F	0.0177186849404754	0.908367132458858	0.998043617620652	pseudobulk	DESeq2	LRT	0.0417385882898087
866	KC_G1	Tsp97E	-0.0257026460823943	0.847445126157682	0.998043617620652	pseudobulk	DESeq2	LRT	0.0718884136894459
866	KC_G1	Tspo	0.0544841490395785	0.887249104593817	0.998043617620652	pseudobulk	DESeq2	LRT	0.051954430267392
866	KC_G1	tsr	0.0171133133646527	0.905270353292207	0.998043617620652	pseudobulk	DESeq2	LRT	0.0432217021109225
866	KC_G1	Tsr1	0.0798211636714719	0.897528141201201	0.998043617620652	pseudobulk	DESeq2	LRT	0.0469519256167235
866	KC_G1	tst	0.316731742233484	0.588984980572139	0.998043617620652	pseudobulk	DESeq2	LRT	0.229895779810197
866	KC_G1	tsu	-0.326413131801534	0.181838388609157	0.998043617620652	pseudobulk	DESeq2	LRT	0.740314425838145
866	KC_G1	Ttc19	-0.172277300035087	0.680687201243701	0.998043617620652	pseudobulk	DESeq2	LRT	0.167052415226887
866	KC_G1	Ttc7	0.160325303551786	0.629760069157498	0.998043617620652	pseudobulk	DESeq2	LRT	0.200824879892447
867	KC_G1	Ttd14	-0.142907431979313	0.404510519026608	0.998043617620652	pseudobulk	DESeq2	LRT	0.393070180366142
867	KC_G1	tth	0.285124600758246	0.277929227919252	0.998043617620652	pseudobulk	DESeq2	LRT	0.556065779035584
867	KC_G1	ttk	0.344638463335585	0.882705185027019	0.998043617620652	pseudobulk	DESeq2	LRT	0.0541843223511353
867	KC_G1	TTLL12	0.341077318009061	0.498604804831623	0.998043617620652	pseudobulk	DESeq2	LRT	0.302243540710292
867	KC_G1	TTLL15	0.427023914994283	0.885426509517172	0.998043617620652	pseudobulk	DESeq2	LRT	0.0528474794605408
867	KC_G1	TTLL3A	0.325847557781978	0.407352207435193	0.998043617620652	pseudobulk	DESeq2	LRT	0.390029925912008
867	KC_G1	TTLL3B	0.173195945230598	0.976215465741119	0.998043617620652	pseudobulk	DESeq2	LRT	0.0104543162934892
867	KC_G1	TTLL4A	-0.145254210933798	0.621146389850137	0.998043617620652	pseudobulk	DESeq2	LRT	0.206806034587027
867	KC_G1	TTLL5	-0.108938738095346	0.769389268970945	0.998043617620652	pseudobulk	DESeq2	LRT	0.11385387529357

867	KC_G1	TTLL6A	-0.794610277746993	0.821479263487599	0.998043617620652	pseudobulk	DESeq2	LRT	0.085403394939296
868	KC_G1	TTLL6B	-0.794610277746993	0.879485534599449	0.998043617620652	pseudobulk	DESeq2	LRT	0.0557712992369932
868	KC_G1	ttm2	2.04147257822512	0.408147672536479	0.998043617620652	pseudobulk	DESeq2	LRT	0.389182675723292
868	KC_G1	ttm50	0.176638834191395	0.525594572254074	0.998043617620652	pseudobulk	DESeq2	LRT	0.279349128327036
868	KC_G1	ttv	-0.0109129957642266	0.938045763809708	0.998043617620652	pseudobulk	DESeq2	LRT	0.0277759734703965
868	KC_G1	tty	-0.0880275531205001	0.536927054515655	0.998043617620652	pseudobulk	DESeq2	LRT	0.270084712388343
868	KC_G1	tub	0.0188382195616951	0.927095302185242	0.998043617620652	pseudobulk	DESeq2	LRT	0.0328756195925503
868	KC_G1	tud	-0.0135826826921001	0.945570224358427	0.998043617620652	pseudobulk	DESeq2	LRT	0.0243062120131905
868	KC_G1	Tudor-SN	-0.260608280938752	0.367194295195947	0.998043617620652	pseudobulk	DESeq2	LRT	0.435104074704363
868	KC_G1	tun	-0.184314140485619	0.388116312641977	0.998043617620652	pseudobulk	DESeq2	LRT	0.411038103352455
868	KC_G1	Tusp	-0.0452079090198865	0.857844156044031	0.998043617620652	pseudobulk	DESeq2	LRT	0.0665916029643826
869	KC_G1	tut	-0.280963499300447	0.480840207838641	0.998043617620652	pseudobulk	DESeq2	LRT	0.317999223799944
869	KC_G1	tutl	-0.0579576785336146	0.631678216652247	0.998043617620652	pseudobulk	DESeq2	LRT	0.199504099440672
869	KC_G1	TwdlG	0.173195945230598	0.976215465741119	0.998043617620652	pseudobulk	DESeq2	LRT	0.0104543162934892
869	KC_G1	TwdlU	-2.27643791964046	0.298906309922724	0.998043617620652	pseudobulk	DESeq2	LRT	0.52446491689215
869	KC_G1	TwdlW	-0.794610277746993	0.815039924133852	0.998043617620652	pseudobulk	DESeq2	LRT	0.0888211171417967
869	KC_G1	twe	1.13582588427274	0.704684208645453	0.998043617620652	pseudobulk	DESeq2	LRT	0.152005460551806
869	KC_G1	tweek	-0.0928374082325573	0.692481067367858	0.998043617620652	pseudobulk	DESeq2	LRT	0.159592095837617
869	KC_G1	twf	0.412445237595357	0.242839841934741	0.998043617620652	pseudobulk	DESeq2	LRT	0.614680058482716
869	KC_G1	twi	-1.60891734856068	0.545249891924755	0.998043617620652	pseudobulk	DESeq2	LRT	0.263404411831769
869	KC_G1	twin	-0.0726777482043544	0.708272361357633	0.998043617620652	pseudobulk	DESeq2	LRT	0.149799705178971
870	KC_G1	twr	-0.0724793922133111	0.761975333144019	0.998043617620652	pseudobulk	DESeq2	LRT	0.118059087522386
870	KC_G1	tws	-0.239737937012085	0.176261022165815	0.998043617620652	pseudobulk	DESeq2	LRT	0.753843715663544
870	KC_G1	twz	0.936398828030704	0.248197213791437	0.998043617620652	pseudobulk	DESeq2	LRT	0.605203098106411
870	KC_G1	tx	1.13582588427274	0.633424328955532	0.998043617620652	pseudobulk	DESeq2	LRT	0.198305259991224
870	KC_G1	Txl	0.10498964081834	0.712685142593125	0.998043617620652	pseudobulk	DESeq2	LRT	0.147102294882124
870	KC_G1	tyf	-0.210772904855865	0.145842054754223	0.998043617620652	pseudobulk	DESeq2	LRT	0.83611722557816
870	KC_G1	tyn	-0.650945705693917	0.142869348826804	0.998043617620652	pseudobulk	DESeq2	LRT	0.845060934702514
870	KC_G1	TyrR	0.510087119817468	0.527214767842665	0.998043617620652	pseudobulk	DESeq2	LRT	0.278012433196383
870	KC_G1	TyrRII	1.54235971072306	0.529343359605328	0.998043617620652	pseudobulk	DESeq2	LRT	0.276262530595783
870	KC_G1	TyrRS	0.148408521439186	0.598022730587726	0.998043617620652	pseudobulk	DESeq2	LRT	0.223282308350709
871	KC_G1	TyrRS-m	0.254982221910819	0.656881847633641	0.998043617620652	pseudobulk	DESeq2	LRT	0.182512739321873
871	KC_G1	tzn	0.0887459963569806	0.748938441711647	0.998043617620652	pseudobulk	DESeq2	LRT	0.125553877258641
871	KC_G1	U2A	0.00629569059753538	0.941276484283565	0.998043617620652	pseudobulk	DESeq2	LRT	0.0262827910659896
871	KC_G1	U2af38	0.00814369591754347	0.931959365142533	0.998043617620652	pseudobulk	DESeq2	LRT	0.0306030231386091
871	KC_G1	U2af50	-0.0103936023014441	0.952904375553456	0.998043617620652	pseudobulk	DESeq2	LRT	0.0209506788507985
871	KC_G1	U3-55K	0.851865136118754	0.352053681634361	0.998043617620652	pseudobulk	DESeq2	LRT	0.453391109646744
871	KC_G1	U4-U6-60K	-0.226803932714778	0.637298416168473	0.998043617620652	pseudobulk	DESeq2	LRT	0.195657160839367
871	KC_G1	Uba1	0.135157492258801	0.516985311354165	0.998043617620652	pseudobulk	DESeq2	LRT	0.286521795955294
871	KC_G1	Uba2	-0.422490916262853	0.104313970767213	0.998043617620652	pseudobulk	DESeq2	LRT	0.98165752263018
871	KC_G1	Uba3	0.459847331030359	0.335817366715366	0.998043617620652	pseudobulk	DESeq2	LRT	0.473896848181217
872	KC_G1	Uba4	-0.628629200121936	0.581601838287601	0.998043617620652	pseudobulk	DESeq2	LRT	0.235374229458338
872	KC_G1	Uba5	0.760113222287725	0.231514627821012	0.998043617620652	pseudobulk	DESeq2	LRT	0.635421563690308
872	KC_G1	Ubc10	0.0540800455607519	0.826079504174625	0.998043617620652	pseudobulk	DESeq2	LRT	0.0829781529668712
872	KC_G1	Ubc2	-0.0129684487472491	0.935127065358876	0.998043617620652	pseudobulk	DESeq2	LRT	0.0291293730474437
872	KC_G1	Ubc4	-0.24970621990066	0.2748760670564	0.998043617620652	pseudobulk	DESeq2	LRT	0.56086307171644
872	KC_G1	Ubc6	-0.113517048846808	0.438686856741709	0.998043617620652	pseudobulk	DESeq2	LRT	0.357845377060878
872	KC_G1	Ubc7	-0.557980249569817	0.0991133297381158	0.998043617620652	pseudobulk	DESeq2	LRT	1.00386793338144
872	KC_G1	UbcE2H	-0.117076399798247	0.506490437239842	0.998043617620652	pseudobulk	DESeq2	LRT	0.295428749895357
872	KC_G1	UbcE2M	-0.113819746201826	0.598164832046633	0.998043617620652	pseudobulk	DESeq2	LRT	0.223179124064745
872	KC_G1	Ube3a	-0.21361391157069	0.587316697406607	0.998043617620652	pseudobulk	DESeq2	LRT	0.231127651994657
873	KC_G1	Ubi-p5E	-0.213230410557204	0.0765705385414888	0.998043617620652	pseudobulk	DESeq2	LRT	1.11593829838374
873	KC_G1	Ubi-p63E	-0.0437710708115094	0.80817436289793	0.998043617620652	pseudobulk	DESeq2	LRT	0.0924949304691659
873	KC_G1	ubl	-0.00991714371161871	0.974284883690407	0.998043617620652	pseudobulk	DESeq2	LRT	0.0113140356014418
873	KC_G1	UBL3	0.0717320475861419	0.630620237981519	0.998043617620652	pseudobulk	DESeq2	LRT	0.200232095905488
873	KC_G1	Ublcp1	-0.0312743240223002	0.927323267152238	0.998043617620652	pseudobulk	DESeq2	LRT	0.032768843345408

873	KC_G1	Ubqn	-0.0270439586036135	0.897710129803663	0.998043617620652	pseudobulk	DESeq2	LRT	0.0468638741893828
873	KC_G1	Ubr1	-0.0558793465262157	0.742548465611723	0.998043617620652	pseudobulk	DESeq2	LRT	0.12927519499282
873	KC_G1	Ubr3	-0.033903752603813	0.822017024782634	0.998043617620652	pseudobulk	DESeq2	LRT	0.0851191877001116
873	KC_G1	Uch	0.127489747612467	0.694357475155709	0.998043617620652	pseudobulk	DESeq2	LRT	0.158416884707629
873	KC_G1	Uch-L5	-0.143115794374256	0.637141504255859	0.998043617620652	pseudobulk	DESeq2	LRT	0.195764103468902
874	KC_G1	Uck	0.0876668203021167	0.706323181690451	0.998043617620652	pseudobulk	DESeq2	LRT	0.150996539869418
874	KC_G1	Ucp4A	0.0679128002877804	0.780571446279865	0.998043617620652	pseudobulk	DESeq2	LRT	0.107587339488158
874	KC_G1	udd	-0.348698798483951	0.595618190744812	0.998043617620652	pseudobulk	DESeq2	LRT	0.225032046951672
874	KC_G1	udt	0.647238018104412	0.387261632721578	0.998043617620652	pseudobulk	DESeq2	LRT	0.411995527859492
874	KC_G1	Uev1A	0.1082496192087	0.536634585823031	0.998043617620652	pseudobulk	DESeq2	LRT	0.270321340706096
874	KC_G1	uex	-0.0721870382646728	0.566957204831078	0.998043617620652	pseudobulk	DESeq2	LRT	0.246449721366603
874	KC_G1	Ufc1	-0.0916708086648027	0.812763273535288	0.998043617620652	pseudobulk	DESeq2	LRT	0.0900359291513577
874	KC_G1	Ufd1-like	0.0207092170553075	0.917820782833992	0.998043617620652	pseudobulk	DESeq2	LRT	0.0372421125076364
874	KC_G1	Ufd4	-0.162689691402782	0.394466113855341	0.998043617620652	pseudobulk	DESeq2	LRT	0.403990298405701
874	KC_G1	Ufl1	-0.162085492458412	0.790527317053559	0.998043617620652	pseudobulk	DESeq2	LRT	0.102083118216727
875	KC_G1	Ufm1	-0.045045351111765	0.8895433106357	0.998043617620652	pseudobulk	DESeq2	LRT	0.0508329018665333
875	KC_G1	Ugalt	-0.248612614077022	0.715198535676805	0.998043617620652	pseudobulk	DESeq2	LRT	0.145573383401364
875	KC_G1	Uggt	0.231467070616799	0.622776135096431	0.998043617620652	pseudobulk	DESeq2	LRT	0.205668038034732
875	KC_G1	UGP	-0.45751398867724	0.371192018100884	0.998043617620652	pseudobulk	DESeq2	LRT	0.43040137117088
875	KC_G1	Ugt301D1	-0.704797641189757	0.838804762683723	0.998043617620652	pseudobulk	DESeq2	LRT	0.0763391123013672
875	KC_G1	Ugt302C1	1.13582588427274	0.633424328955532	0.998043617620652	pseudobulk	DESeq2	LRT	0.198305259991224
875	KC_G1	Ugt302K1	0.965935117414534	0.651883564872895	0.998043617620652	pseudobulk	DESeq2	LRT	0.185829968146785
875	KC_G1	Ugt303A1	4.84310938856991	0.193064723930093	0.998043617620652	pseudobulk	DESeq2	LRT	0.714297071648073
875	KC_G1	Ugt304A1	1.69981218657454	0.566400560818652	0.998043617620652	pseudobulk	DESeq2	LRT	0.246876325303589
875	KC_G1	Ugt316A1	0.00031106364579024	0.974339411034868	0.998043617620652	pseudobulk	DESeq2	LRT	0.0112897303262972
876	KC_G1	Ugt317A1	1.54956406646152	0.423960268859398	0.998043617620652	pseudobulk	DESeq2	LRT	0.372674841104481
876	KC_G1	Ugt35A1	3.64752844916685	0.0772828199905454	0.998043617620652	pseudobulk	DESeq2	LRT	1.11191703923247
876	KC_G1	Ugt35B1	0.13806508763381	0.698838238369429	0.998043617620652	pseudobulk	DESeq2	LRT	0.155623339724458
876	KC_G1	Ugt35C1	-0.270803293383352	0.876556106260612	0.998043617620652	pseudobulk	DESeq2	LRT	0.0572202805290658
876	KC_G1	Ugt35E1	0.173195945230598	0.976215465741119	0.998043617620652	pseudobulk	DESeq2	LRT	0.0104543162934892
876	KC_G1	Ugt35E2	0.52665625992831	0.521502359919146	0.998043617620652	pseudobulk	DESeq2	LRT	0.282743721949734
876	KC_G1	Ugt36D1	-1.66808346769027	0.498191247612582	0.998043617620652	pseudobulk	DESeq2	LRT	0.302603906560549
876	KC_G1	Ugt37A2	-0.794610277746993	0.815039924133852	0.998043617620652	pseudobulk	DESeq2	LRT	0.0888211171417967
876	KC_G1	Ugt37D1	0.256843183134888	0.864135082995499	0.998043617620652	pseudobulk	DESeq2	LRT	0.0634183625975666
876	KC_G1	Ugt49B1	-1.31486389469209	0.16031262488121	0.998043617620652	pseudobulk	DESeq2	LRT	0.795032274898855
877	KC_G1	Ugt49C1	0.172834960772825	0.908610676445368	0.998043617620652	pseudobulk	DESeq2	LRT	0.0416221644022337
877	KC_G1	Uhg1	-0.0281798512350680	0.939575394108323	0.998043617620652	pseudobulk	DESeq2	LRT	0.0270683651840266
877	KC_G1	Uhg2	0.298224243653339	0.587068226266018	0.998043617620652	pseudobulk	DESeq2	LRT	0.231311424188432
877	KC_G1	Uhg3	0.120207299819671	0.745052034763604	0.998043617620652	pseudobulk	DESeq2	LRT	0.127813394873801
877	KC_G1	Uhg4	-0.075945763926609	0.905276212623245	0.998043617620652	pseudobulk	DESeq2	LRT	0.0432188911640126
877	KC_G1	Uhg5	-0.428983340491456	0.226301840223029	0.998043617620652	pseudobulk	DESeq2	LRT	0.645311914470117
877	KC_G1	Uhg8	-1.74005979220898	0.48579817606524	0.998043617620652	pseudobulk	DESeq2	LRT	0.313544120091384
877	KC_G1	Ulp1	0.192960467526311	0.40622219718872	0.998043617620652	pseudobulk	DESeq2	LRT	0.391236349140991
877	KC_G1	unc-104	-0.153641649009704	0.164077405352566	0.998043617620652	pseudobulk	DESeq2	LRT	0.784951220326018
877	KC_G1	Unc-115a	0.117770758670807	0.663106511599043	0.998043617620652	pseudobulk	DESeq2	LRT	0.178416707365253
878	KC_G1	Unc-115b	2.38466886841527	0.34619303318143	0.998043617620652	pseudobulk	DESeq2	LRT	0.460681676152437
878	KC_G1	unc-119	0.0878681354822206	0.584443314467134	0.998043617620652	pseudobulk	DESeq2	LRT	0.233257604960579
878	KC_G1	unc-13	-0.0715238229713063	0.455463820732864	0.998043617620652	pseudobulk	DESeq2	LRT	0.341546115024925
878	KC_G1	unc-13-4A	-0.231212924545275	0.0649698260062731	0.998043617620652	pseudobulk	DESeq2	LRT	1.187288296304
878	KC_G1	unc-45	0.274089542551529	0.596799889617552	0.998043617620652	pseudobulk	DESeq2	LRT	0.22417126586128
878	KC_G1	unc-5	0.41982223599858	0.155687962917006	0.998043617620652	pseudobulk	DESeq2	LRT	0.80774496380186
878	KC_G1	Unc-76	0.101253911696691	0.448961390045984	0.998043617620652	pseudobulk	DESeq2	LRT	0.347791006014426
878	KC_G1	unc79	0.0401489729943972	0.799928699240612	0.998043617620652	pseudobulk	DESeq2	LRT	0.0969487216410008
878	KC_G1	unc80	-0.157568259189231	0.349566655260653	0.998043617620652	pseudobulk	DESeq2	LRT	0.456470000885919
878	KC_G1	und	0.115287450701202	0.586136273329583	0.998043617620652	pseudobulk	DESeq2	LRT	0.232001401260562
879	KC_G1	unk	-0.139533555539285	0.432830828232022	0.998043617620652	pseudobulk	DESeq2	LRT	0.363681814319588

879	KC_G1	Unr	-0.0897307386445873	0.360890882727358	0.998043617620652	pseudobulk	DESeq2	LRT	0.44262408948722
879	KC_G1	Updo	-0.171501094753712	0.512667527558596	0.998043617620652	pseudobulk	DESeq2	LRT	0.290164189968879
879	KC_G1	Upf1	-0.0581583237119105	0.857502999491721	0.998043617620652	pseudobulk	DESeq2	LRT	0.0667643521476704
879	KC_G1	Upf2	0.209157639709664	0.696186341219974	0.998043617620652	pseudobulk	DESeq2	LRT	0.157274501578857
879	KC_G1	Upf3	0.157870742747839	0.74694423051677	0.998043617620652	pseudobulk	DESeq2	LRT	0.126711822927563
879	KC_G1	upSET	-0.0815840103559049	0.528027287430109	0.998043617620652	pseudobulk	DESeq2	LRT	0.277343633386446
879	KC_G1	UQCR-11	0.114236859564575	0.325434911078367	0.998043617620652	pseudobulk	DESeq2	LRT	0.487535859895082
879	KC_G1	UQCR-11L	-2.27131838799805	0.327405917019026	0.998043617620652	pseudobulk	DESeq2	LRT	0.484913476098507
879	KC_G1	UQCR-14	-0.0225276375462773	0.866344446399293	0.998043617620652	pseudobulk	DESeq2	LRT	0.0623094042541235
880	KC_G1	UQCR-6.4	-0.0112910644210148	0.929879972125585	0.998043617620652	pseudobulk	DESeq2	LRT	0.0315731060779622
880	KC_G1	UQCR-C1	-0.104741109503041	0.455741042191339	0.998043617620652	pseudobulk	DESeq2	LRT	0.341281858855332
880	KC_G1	UQCR-C2	0.0361258248202963	0.765912331169246	0.998043617620652	pseudobulk	DESeq2	LRT	0.115820938289577
880	KC_G1	UQCR-Q	0.0178598562416901	0.890625967321525	0.998043617620652	pseudobulk	DESeq2	LRT	0.0503046466177287
880	KC_G1	uri	-0.333408444643735	0.717865054502203	0.998043617620652	pseudobulk	DESeq2	LRT	0.143957187503023
880	KC_G1	Urm1	-0.164523392068038	0.825381812905233	0.998043617620652	pseudobulk	DESeq2	LRT	0.0833451049302771
880	KC_G1	Uros1	0.0297756866627536	0.915135228662648	0.998043617620652	pseudobulk	DESeq2	LRT	0.0385147259088516
880	KC_G1	Uros2	1.90250740646012	0.460774913678782	0.998043617620652	pseudobulk	DESeq2	LRT	0.336511173568024
880	KC_G1	Use1	0.151233095884241	0.719820842341617	0.998043617620652	pseudobulk	DESeq2	LRT	0.142775582547087
880	KC_G1	Usf	-0.171468344376668	0.391897958022255	0.998043617620652	pseudobulk	DESeq2	LRT	0.406826999400125
881	KC_G1	ush	0.208594480859484	0.854663553836208	0.998043617620652	pseudobulk	DESeq2	LRT	0.0682048156445907
881	KC_G1	usp	-0.0370046650082386	0.863978931878944	0.998043617620652	pseudobulk	DESeq2	LRT	0.0634968476603153
881	KC_G1	Usp1	0.10777577411943	0.639921341198603	0.998043617620652	pseudobulk	DESeq2	LRT	0.193873405989308
881	KC_G1	Usp12-46	0.0208128594679883	0.958219028197938	0.998043617620652	pseudobulk	DESeq2	LRT	0.0185352092247809
881	KC_G1	Usp14	-0.34221974487227	0.276108221410435	0.998043617620652	pseudobulk	DESeq2	LRT	0.558920661596785
881	KC_G1	Usp15-31	-0.495545670056752	0.162736585692512	0.998043617620652	pseudobulk	DESeq2	LRT	0.788514799993695
881	KC_G1	Usp16-45	0.114062919418598	0.777705157248218	0.998043617620652	pseudobulk	DESeq2	LRT	0.109185021069285
881	KC_G1	Usp2	-0.0948730389024958	0.515852302282018	0.998043617620652	pseudobulk	DESeq2	LRT	0.287474626833762
881	KC_G1	Usp20-33	0.0107320715522059	0.954500243190699	0.998043617620652	pseudobulk	DESeq2	LRT	0.0202239566193294
881	KC_G1	Usp30	-0.199509628781928	0.481504894823277	0.998043617620652	pseudobulk	DESeq2	LRT	0.317399293619542
882	KC_G1	Usp32	0.134074006869282	0.772106484496173	0.998043617620652	pseudobulk	DESeq2	LRT	0.112322800130606
882	KC_G1	Usp39	-0.273152424397316	0.562711104395227	0.998043617620652	pseudobulk	DESeq2	LRT	0.249714514506594
882	KC_G1	Usp47	-0.152318774597963	0.601341358286577	0.998043617620652	pseudobulk	DESeq2	LRT	0.220878925777061
882	KC_G1	Usp5	0.324051503346611	0.356574745231157	0.998043617620652	pseudobulk	DESeq2	LRT	0.447849419419641
882	KC_G1	Usp7	-0.0877180405536961	0.604869551332902	0.998043617620652	pseudobulk	DESeq2	LRT	0.218338276991957
882	KC_G1	Usp8	-0.349288726116931	0.393705483898836	0.998043617620652	pseudobulk	DESeq2	LRT	0.404828535907213
882	KC_G1	Utx	0.0487170562200446	0.858595870957903	0.998043617620652	pseudobulk	DESeq2	LRT	0.0662112044013157
882	KC_G1	Uvrag	0.577724177153388	0.274955689669048	0.998043617620652	pseudobulk	DESeq2	LRT	0.560737289016089
882	KC_G1	Uxs	0.275180471773154	0.6701986304611	0.998043617620652	pseudobulk	DESeq2	LRT	0.173796463972838
882	KC_G1	Uxt	0.5950872414663	0.180391996259263	0.998043617620652	pseudobulk	DESeq2	LRT	0.74378273540576
883	KC_G1	uzip	-0.0234287076918304	0.908804209850063	0.998043617620652	pseudobulk	DESeq2	LRT	0.0415296698478262
883	KC_G1	VAChT	-0.158541424088525	0.260015622312687	0.998043617620652	pseudobulk	DESeq2	LRT	0.585000557873908
883	KC_G1	Vajk2	-0.794610277746993	0.815039924133852	0.998043617620652	pseudobulk	DESeq2	LRT	0.0888211171417967
883	KC_G1	Vajk3	-0.794610277746993	0.879485534599449	0.998043617620652	pseudobulk	DESeq2	LRT	0.0557712992369932
883	KC_G1	ValRS	-0.349825466541419	0.455536726487003	0.998043617620652	pseudobulk	DESeq2	LRT	0.341476603394256
883	KC_G1	ValRS-m	-2.10799043634561	0.17921494884931	0.998043617620652	pseudobulk	DESeq2	LRT	0.746625767374834
883	KC_G1	Vamp7	-0.251342567046903	0.392969062022071	0.998043617620652	pseudobulk	DESeq2	LRT	0.405641639756862
883	KC_G1	Vang	0.0976883844930068	0.786226109662011	0.998043617620652	pseudobulk	DESeq2	LRT	0.104452537857762
883	KC_G1	vap	-0.0447425563887505	0.856243143648199	0.998043617620652	pseudobulk	DESeq2	LRT	0.0674028930909632
883	KC_G1	Vap33	-0.0401134019518882	0.70641459273168	0.998043617620652	pseudobulk	DESeq2	LRT	0.150940337916636
884	KC_G1	vari	0.23915382906757	0.763971023629669	0.998043617620652	pseudobulk	DESeq2	LRT	0.116923113304401
884	KC_G1	Vav	0.0286582132748531	0.929050353183504	0.998043617620652	pseudobulk	DESeq2	LRT	0.0319607472365827
884	KC_G1	veli	0.0552018374342765	0.733666804524942	0.998043617620652	pseudobulk	DESeq2	LRT	0.134501130561022
884	KC_G1	velo	-0.0192823915545804	0.935030087568753	0.998043617620652	pseudobulk	DESeq2	LRT	0.0291744140955602
884	KC_G1	VepD	-0.474534434408118	0.268153181368163	0.998043617620652	pseudobulk	DESeq2	LRT	0.571617046202528
884	KC_G1	ver	0.786528278421459	0.541890726945837	0.998043617620652	pseudobulk	DESeq2	LRT	0.266088280747548
884	KC_G1	vers	0.0918481864080705	0.791765469360091	0.998043617620652	pseudobulk	DESeq2	LRT	0.10140344271185

884	KC_G1	VGAT	-0.0444339350381213	0.825545729607914	0.998043617620652	pseudobulk	DESeq2	LRT	0.0832588647799774
884	KC_G1	VGlut	-0.757449795571951	0.820028655901106	0.998043617620652	pseudobulk	DESeq2	LRT	0.0861709709305825
884	KC_G1	Vha100-1	-0.201283299815802	0.157916369899953	0.998043617620652	pseudobulk	DESeq2	LRT	0.801572847897204
885	KC_G1	Vha100-2	-2.59533401427988	0.480645361956175	0.998043617620652	pseudobulk	DESeq2	LRT	0.318175244107255
885	KC_G1	Vha13	-0.119446784531651	0.373906395690093	0.998043617620652	pseudobulk	DESeq2	LRT	0.427237106150515
885	KC_G1	Vha14-1	-0.0391754697588798	0.763794219547461	0.998043617620652	pseudobulk	DESeq2	LRT	0.117023632723817
885	KC_G1	Vha16-1	-0.03380339578794	0.733060841759138	0.998043617620652	pseudobulk	DESeq2	LRT	0.134859978778095
885	KC_G1	Vha26	-0.0393956655295317	0.737712219659696	0.998043617620652	pseudobulk	DESeq2	LRT	0.132113022713745
885	KC_G1	Vha36-1	0.00591082408740272	0.961987224467292	0.998043617620652	pseudobulk	DESeq2	LRT	0.0168306955091729
885	KC_G1	Vha36-3	2.97392976749169	0.209628783309326	0.998043617620652	pseudobulk	DESeq2	LRT	0.678549086315318
885	KC_G1	Vha44	-0.00521760924590219	0.964937603747049	0.998043617620652	pseudobulk	DESeq2	LRT	0.0155007687541001
885	KC_G1	Vha55	-0.112516902613449	0.472177991289863	0.998043617620652	pseudobulk	DESeq2	LRT	0.325894259705668
885	KC_G1	Vha68-1	-0.0330898508938675	0.819785075110042	0.998043617620652	pseudobulk	DESeq2	LRT	0.0862999926508354
886	KC_G1	Vha68-2	-0.0377377325830112	0.9699011244768	0.998043617620652	pseudobulk	DESeq2	LRT	0.0132725371593619
886	KC_G1	VhaAC39-1	-0.0399940774223219	0.780266914496119	0.998043617620652	pseudobulk	DESeq2	LRT	0.107756807997193
886	KC_G1	VhaAC45	-0.0372729053492679	0.874689014703592	0.998043617620652	pseudobulk	DESeq2	LRT	0.0581463277830417
886	KC_G1	VhaM9.7-a	0.0404750776428899	0.744355351670039	0.998043617620652	pseudobulk	DESeq2	LRT	0.128219684849254
886	KC_G1	VhaM9.7-b	-0.0621416526929995	0.729812717349066	0.998043617620652	pseudobulk	DESeq2	LRT	0.136788573108391
886	KC_G1	VhaM9.7-c	0.24244263697621	0.695397329631088	0.998043617620652	pseudobulk	DESeq2	LRT	0.157766981367327
886	KC_G1	VhaPPA1-1	0.056937875162867	0.713650545561918	0.998043617620652	pseudobulk	DESeq2	LRT	0.146514397867885
886	KC_G1	VhaSFD	-0.111648887479895	0.518816201198603	0.998043617620652	pseudobulk	DESeq2	LRT	0.284986470557708
886	KC_G1	Vhl	0.15344189814365	0.624484468394224	0.998043617620652	pseudobulk	DESeq2	LRT	0.204478358530141
886	KC_G1	viaf	0.176899079969761	0.43281587844328	0.998043617620652	pseudobulk	DESeq2	LRT	0.36369681492135
887	KC_G1	vib	-0.154625367794243	0.364075130450902	0.998043617620652	pseudobulk	DESeq2	LRT	0.438808986204272
887	KC_G1	Victoria	0.487109334592596	0.72002714109208	0.998043617620652	pseudobulk	DESeq2	LRT	0.142651132729338
887	KC_G1	vig	-0.0952603755452511	0.410695975604101	0.998043617620652	pseudobulk	DESeq2	LRT	0.386479552762357
887	KC_G1	vig2	0.0500760880976105	0.786938926196664	0.998043617620652	pseudobulk	DESeq2	LRT	0.104058971637132
887	KC_G1	vih	0.0234583411304225	0.905950032044253	0.998043617620652	pseudobulk	DESeq2	LRT	0.0428957553098307
887	KC_G1	vimar	-0.0575594075775779	0.847685096622291	0.998043617620652	pseudobulk	DESeq2	LRT	0.071765452222657
887	KC_G1	Vinc	-0.13891125771222	0.869652612080077	0.998043617620652	pseudobulk	DESeq2	LRT	0.0606541942611324
887	KC_G1	vir	0.327787041917756	0.641372954410033	0.998043617620652	pseudobulk	DESeq2	LRT	0.19288935746983
887	KC_G1	vir-1	-0.44192263771079	0.229215706204661	0.998043617620652	pseudobulk	DESeq2	LRT	0.639755627174482
887	KC_G1	vis	-0.794610277746993	0.879485534599449	0.998043617620652	pseudobulk	DESeq2	LRT	0.0557712992369932
888	KC_G1	vito	0.422893744004948	0.377232158567796	0.998043617620652	pseudobulk	DESeq2	LRT	0.423391291297333
888	KC_G1	vkg	-0.323697787232406	0.609343948379535	0.998043617620652	pseudobulk	DESeq2	LRT	0.215137497650347
888	KC_G1	Vkor	1.12508065046033	0.900770125530981	0.998043617620652	pseudobulk	DESeq2	LRT	0.0453860258366365
888	KC_G1	vlc	-0.0885499012897732	0.69372198806721	0.998043617620652	pseudobulk	DESeq2	LRT	0.158814539975141
888	KC_G1	Vlet	-0.0266354959983465	0.935887856784682	0.998043617620652	pseudobulk	DESeq2	LRT	0.0287761876907624
888	KC_G1	vls	-0.498453168234282	0.393151546560227	0.998043617620652	pseudobulk	DESeq2	LRT	0.405440011588074
888	KC_G1	Vm26Ab	-0.794610277746993	0.879485534599449	0.998043617620652	pseudobulk	DESeq2	LRT	0.0557712992369932
888	KC_G1	Vm32E	-0.754130632248373	0.654966161309093	0.998043617620652	pseudobulk	DESeq2	LRT	0.183781137162967
888	KC_G1	Vmat	0.277241755568701	0.834745491408907	0.998043617620652	pseudobulk	DESeq2	LRT	0.0784459179597499
888	KC_G1	vnc	1.12508065046033	0.900770125530981	0.998043617620652	pseudobulk	DESeq2	LRT	0.0453860258366365
889	KC_G1	vnd	-0.280801067112539	0.883392759535737	0.998043617620652	pseudobulk	DESeq2	LRT	0.053846164627732
889	KC_G1	Vps11	0.154542788875472	0.451436345473701	0.998043617620652	pseudobulk	DESeq2	LRT	0.345403478481037
889	KC_G1	Vps13	-0.336013545178457	0.408314819723751	0.998043617620652	pseudobulk	DESeq2	LRT	0.38900485713934
889	KC_G1	Vps13B	0.0340755836366138	0.959140917391855	0.998043617620652	pseudobulk	DESeq2	LRT	0.0181175814114764
889	KC_G1	Vps13D	0.371581126021383	0.632819276517388	0.998043617620652	pseudobulk	DESeq2	LRT	0.198720300120961
889	KC_G1	Vps15	0.171217728375716	0.776955549383981	0.998043617620652	pseudobulk	DESeq2	LRT	0.109603827028514
889	KC_G1	Vps16A	0.135059392739785	0.755359283482768	0.998043617620652	pseudobulk	DESeq2	LRT	0.121846428874836
889	KC_G1	Vps16B	-0.084857431772751	0.883390232607335	0.998043617620652	pseudobulk	DESeq2	LRT	0.0538474069207198
889	KC_G1	Vps2	0.0307692812940976	0.909382409268175	0.998043617620652	pseudobulk	DESeq2	LRT	0.0412534508694767
889	KC_G1	Vps20	0.014891424536205	0.965415130177499	0.998043617620652	pseudobulk	DESeq2	LRT	0.0152858991146883
890	KC_G1	Vps24	0.118319044405285	0.505184834025322	0.998043617620652	pseudobulk	DESeq2	LRT	0.296549695721576
890	KC_G1	Vps25	0.19784529461193	0.539097032787765	0.998043617620652	pseudobulk	DESeq2	LRT	0.268333058539471
890	KC_G1	Vps26	-0.0228631134670611	0.945367934706313	0.998043617620652	pseudobulk	DESeq2	LRT	0.0243991323232212

890	KC_G1	Vps28	0.0253063135492488	0.921149029448353	0.998043617620652	pseudobulk	DESeq2	LRT	0.0356701011482625
890	KC_G1	Vps29	0.0807496072372778	0.762760162867194	0.998043617620652	pseudobulk	DESeq2	LRT	0.117611997192289
890	KC_G1	Vps33B	0.364624411460514	0.641866413501565	0.998043617620652	pseudobulk	DESeq2	LRT	0.192555348747187
890	KC_G1	Vps35	-0.124893449154929	0.73351351119041	0.998043617620652	pseudobulk	DESeq2	LRT	0.134591882118727
890	KC_G1	Vps36	0.470225830951333	0.417856188205201	0.998043617620652	pseudobulk	DESeq2	LRT	0.378973161802488
890	KC_G1	Vps37B	-0.0907447050719505	0.766846019750549	0.998043617620652	pseudobulk	DESeq2	LRT	0.11529183225197
890	KC_G1	Vps39	1.04239686887247	0.133406138615976	0.998043617620652	pseudobulk	DESeq2	LRT	0.874824186118814
891	KC_G1	Vps4	-0.0154384380156526	0.940932659745407	0.998043617620652	pseudobulk	DESeq2	LRT	0.0264414568569824
891	KC_G1	Vps45	-0.21643287292895	0.613978593146402	0.998043617620652	pseudobulk	DESeq2	LRT	0.211846770618543
891	KC_G1	Vps50	0.412078205947939	0.342643098096236	0.998043617620652	pseudobulk	DESeq2	LRT	0.465158011807795
891	KC_G1	Vps51	0.41447227006145	0.529919187634299	0.998043617620652	pseudobulk	DESeq2	LRT	0.275790355003824
891	KC_G1	Vps52	-0.106726389245365	0.843173595115881	0.998043617620652	pseudobulk	DESeq2	LRT	0.0740830023153817
891	KC_G1	Vps53	0.251357323690016	0.618303567770941	0.998043617620652	pseudobulk	DESeq2	LRT	0.208798247502804
891	KC_G1	Vps60	0.14593892639781	0.519248452635156	0.998043617620652	pseudobulk	DESeq2	LRT	0.284624788992218
891	KC_G1	Vps8	-0.0672467285699744	0.88949798946633	0.998043617620652	pseudobulk	DESeq2	LRT	0.0508550292154302
891	KC_G1	Vrp1	0.0881358638423292	0.598041929282934	0.998043617620652	pseudobulk	DESeq2	LRT	0.223268366148972
891	KC_G1	vrs	1.69981218657454	0.566400560818652	0.998043617620652	pseudobulk	DESeq2	LRT	0.246876325303589
892	KC_G1	Vsp37A	0.054625861070535	0.81767822670558	0.998043617620652	pseudobulk	DESeq2	LRT	0.0874175665716277
892	KC_G1	Vsx1	-0.0659776385319925	0.775236051545967	0.998043617620652	pseudobulk	DESeq2	LRT	0.11056603907466
892	KC_G1	Vsx2	-1.43301945927599	0.112630040762615	0.998043617620652	pseudobulk	DESeq2	LRT	0.948345758708557
892	KC_G1	Vta1	0.296813341656116	0.350431265737661	0.998043617620652	pseudobulk	DESeq2	LRT	0.455397152699227
892	KC_G1	vtd	-0.105566531504158	0.547650037306855	0.998043617620652	pseudobulk	DESeq2	LRT	0.261496878409709
892	KC_G1	Vti1a	-0.0885109705061858	0.723831637672784	0.998043617620652	pseudobulk	DESeq2	LRT	0.140362438406009
892	KC_G1	Vti1b	0.110649844888441	0.659286368913961	0.998043617620652	pseudobulk	DESeq2	LRT	0.180925903426095
892	KC_G1	vvl	0.444085607660512	0.664248257001326	0.998043617620652	pseudobulk	DESeq2	LRT	0.177669576512264
892	KC_G1	w	0.239600306545768	0.414504683579145	0.998043617620652	pseudobulk	DESeq2	LRT	0.382470557897576
892	KC_G1	wac	0.267227104503393	0.62005016701928	0.998043617620652	pseudobulk	DESeq2	LRT	0.207573171182002
893	KC_G1	wake	-0.102640357415311	0.534427529426528	0.998043617620652	pseudobulk	DESeq2	LRT	0.272111178582491
893	KC_G1	wal	-0.0642931201443599	0.852247715600699	0.998043617620652	pseudobulk	DESeq2	LRT	0.0694341541980336
893	KC_G1	wap	-0.174572638697463	0.386615390123834	0.998043617620652	pseudobulk	DESeq2	LRT	0.412720861862504
893	KC_G1	wapl	-0.203007085640788	0.426909920146792	0.998043617620652	pseudobulk	DESeq2	LRT	0.369663753336238
893	KC_G1	wash	0.428585025428335	0.464659704370127	0.998043617620652	pseudobulk	DESeq2	LRT	0.332864988223715
893	KC_G1	WASp	-0.0291151997287261	0.89016850727208	0.998043617620652	pseudobulk	DESeq2	LRT	0.0505277744209307
893	KC_G1	wat	1.12508065046033	0.900770125530981	0.998043617620652	pseudobulk	DESeq2	LRT	0.0453860258366365
893	KC_G1	Wbp2	-0.0157890357978932	0.953493479216936	0.998043617620652	pseudobulk	DESeq2	LRT	0.0206822726754332
893	KC_G1	wcd	-0.322527221774642	0.680894170186565	0.998043617620652	pseudobulk	DESeq2	LRT	0.16692038423353
893	KC_G1	wcy	-0.191475125310198	0.253473224726993	0.998043617620652	pseudobulk	DESeq2	LRT	0.596067909969575
894	KC_G1	wda	1.39194301121435	0.109056383628316	0.998043617620652	pseudobulk	DESeq2	LRT	0.962348907862355
894	KC_G1	wdb	-0.149438420328788	0.176339373580728	0.998043617620652	pseudobulk	DESeq2	LRT	0.753650706307248
894	KC_G1	wde	-0.170563602300804	0.470268765043437	0.998043617620652	pseudobulk	DESeq2	LRT	0.32765386586324
894	KC_G1	Wdfy2	0.238152815952189	0.457233213974869	0.998043617620652	pseudobulk	DESeq2	LRT	0.339862229453302
894	KC_G1	wdn	0.0903555572885963	0.871594303379872	0.998043617620652	pseudobulk	DESeq2	LRT	0.0596856169053278
894	KC_G1	wdp	0.939030717735832	0.470528651905813	0.998043617620652	pseudobulk	DESeq2	LRT	0.327413925933375
894	KC_G1	Wdr24	-1.03427559110835	0.249218805280131	0.998043617620652	pseudobulk	DESeq2	LRT	0.603419190258236
894	KC_G1	Wdr33	-0.113206677257896	0.740485254534911	0.998043617620652	pseudobulk	DESeq2	LRT	0.130483585269554
894	KC_G1	Wdr59	0.336940080771167	0.794502185772582	0.998043617620652	pseudobulk	DESeq2	LRT	0.0999049036577781
894	KC_G1	Wdr62	0.04560993161188	0.786817771455632	0.998043617620652	pseudobulk	DESeq2	LRT	0.104125839451794
895	KC_G1	WDR79	0.16996472852145	0.87159651018405	0.998043617620652	pseudobulk	DESeq2	LRT	0.0596845173092654
895	KC_G1	Wdr81	-0.427927937565972	0.440864891433552	0.998043617620652	pseudobulk	DESeq2	LRT	0.355694485124189
895	KC_G1	Wdr82	0.0901583889948516	0.763008180939713	0.998043617620652	pseudobulk	DESeq2	LRT	0.117470805534075
895	KC_G1	Wdr92	0.0136141290581831	0.955618681459829	0.998043617620652	pseudobulk	DESeq2	LRT	0.0197153687844308
895	KC_G1	wds	-0.0349493253223574	0.896932165109932	0.998043617620652	pseudobulk	DESeq2	LRT	0.0472404013622061
895	KC_G1	WDY	-2.06523701187827	0.319262499860211	0.998043617620652	pseudobulk	DESeq2	LRT	0.495852090105138
895	KC_G1	wech	-0.260651876427729	0.17755024941268	0.998043617620652	pseudobulk	DESeq2	LRT	0.750678713289291
895	KC_G1	Wee1	-0.328546395141845	0.43502788196343	0.998043617620652	pseudobulk	DESeq2	LRT	0.361482907195231
895	KC_G1	wek	-0.256822938556908	0.765417115364841	0.998043617620652	pseudobulk	DESeq2	LRT	0.116101830816249

895	KC_G1	wfs1	0.0567753431492649	0.927646734092069	0.998043617620652	pseudobulk	DESeq2	LRT	0.0326173800782108
896	KC_G1	wge	-0.0649736538999292	0.699369353630844	0.998043617620652	pseudobulk	DESeq2	LRT	0.155293402396552
896	KC_G1	wgn	-2.01296149677607	0.124583062073511	0.998043617620652	pseudobulk	DESeq2	LRT	0.904540998993376
896	KC_G1	whd	-0.355010168947574	0.475582815395459	0.998043617620652	pseudobulk	DESeq2	LRT	0.322773846470841
896	KC_G1	wisp	-1.31481767438999	0.589948159507212	0.998043617620652	pseudobulk	DESeq2	LRT	0.229186149424154
896	KC_G1	wit	0.0391777348744861	0.891130526676305	0.998043617620652	pseudobulk	DESeq2	LRT	0.0500586788323783
896	KC_G1	wkd	0.0616147525504137	0.834208583844169	0.998043617620652	pseudobulk	DESeq2	LRT	0.0787253456613979
896	KC_G1	wls	0.643295469879006	0.288991541224624	0.998043617620652	pseudobulk	DESeq2	LRT	0.539114868846331
896	KC_G1	wmd	-0.124900244573476	0.515399121467488	0.998043617620652	pseudobulk	DESeq2	LRT	0.287856326068675
896	KC_G1	wnd	-0.0710358220503653	0.686635849483775	0.998043617620652	pseudobulk	DESeq2	LRT	0.163273525660432
896	KC_G1	Wnk	-0.0386944170308258	0.850861597324192	0.998043617620652	pseudobulk	DESeq2	LRT	0.0701410772911083
897	KC_G1	Wnt5	-0.18580676375736	0.62030267326865	0.998043617620652	pseudobulk	DESeq2	LRT	0.207396347186925
897	KC_G1	woc	-0.343958049050635	0.20501981162548	0.998043617620652	pseudobulk	DESeq2	LRT	0.688204169852088
897	KC_G1	wol	0.283593623597296	0.533552746128319	0.998043617620652	pseudobulk	DESeq2	LRT	0.272822640494165
897	KC_G1	wrapper	1.2839143003249	0.151178591460372	0.998043617620652	pseudobulk	DESeq2	LRT	0.820509705322355
897	KC_G1	wrd	-0.0775734024597756	0.58566621841107	0.998043617620652	pseudobulk	DESeq2	LRT	0.232349825623354
897	KC_G1	wry	-0.0996977667788728	0.37568582962352	0.998043617620652	pseudobulk	DESeq2	LRT	0.42517518564562
897	KC_G1	Wsck	2.07738706174348	0.431998797123676	0.998043617620652	pseudobulk	DESeq2	LRT	0.364517462451933
897	KC_G1	wtrw	0.355414913940488	0.610526554110961	0.998043617620652	pseudobulk	DESeq2	LRT	0.214295442197286
897	KC_G1	wts	0.0918206184895635	0.619674367043727	0.998043617620652	pseudobulk	DESeq2	LRT	0.207836468158381
897	KC_G1	wuho	-0.0371100672263752	0.884032109235714	0.998043617620652	pseudobulk	DESeq2	LRT	0.0535319605405044
898	KC_G1	wun	-0.253430945545074	0.427533537564421	0.998043617620652	pseudobulk	DESeq2	LRT	0.369029811677629
898	KC_G1	wun2	-0.12528472243799	0.779703546812247	0.998043617620652	pseudobulk	DESeq2	LRT	0.108070490202201
898	KC_G1	wus	-1.31275600133313	0.545710131686654	0.998043617620652	pseudobulk	DESeq2	LRT	0.263037983004907
898	KC_G1	Wwox	0.511728027617707	0.326517478745125	0.998043617620652	pseudobulk	DESeq2	LRT	0.486093565631234
898	KC_G1	X11L	-0.140434676042432	0.446970063241539	0.998043617620652	pseudobulk	DESeq2	LRT	0.349721563678786
898	KC_G1	X11Lbeta	-0.359770843946819	0.0329799784610044	0.998043617620652	pseudobulk	DESeq2	LRT	1.48174963232613
898	KC_G1	x16	-0.364461143142369	0.0564111517409405	0.998043617620652	pseudobulk	DESeq2	LRT	1.24863503323634
898	KC_G1	Xbp1	-0.196313662233468	0.280582796938782	0.998043617620652	pseudobulk	DESeq2	LRT	0.551938959906343
898	KC_G1	Xe7	-0.113889698081754	0.389047318425055	0.998043617620652	pseudobulk	DESeq2	LRT	0.409997573787085
898	KC_G1	xit	-2.34812502759229	0.279659715382945	0.998043617620652	pseudobulk	DESeq2	LRT	0.553370088676474
899	KC_G1	xmas	0.241301834978182	0.777319417571363	0.998043617620652	pseudobulk	DESeq2	LRT	0.109400483408927
899	KC_G1	XNP	-0.0730663256299895	0.660432689301646	0.998043617620652	pseudobulk	DESeq2	LRT	0.18017143869215
899	KC_G1	Xpac	0.395937143094533	0.481977933768823	0.998043617620652	pseudobulk	DESeq2	LRT	0.316972844461993
899	KC_G1	Xpc	-0.178934425043343	0.377992611530753	0.998043617620652	pseudobulk	DESeq2	LRT	0.422516689059028
899	KC_G1	Xpd	0.396788777233581	0.71155659873379	0.998043617620652	pseudobulk	DESeq2	LRT	0.147790549493754
899	KC_G1	XRCC1	-0.832037784394787	0.691090238521595	0.998043617620652	pseudobulk	DESeq2	LRT	0.160465241289671
899	KC_G1	Xrp1	0.0408968615032372	0.819759343834548	0.998043617620652	pseudobulk	DESeq2	LRT	0.0863136244266279
899	KC_G1	Xxylt	0.209806865936662	0.313619840416151	0.998043617620652	pseudobulk	DESeq2	LRT	0.503596470507072
899	KC_G1	y	-0.794610277746993	0.879485534599449	0.998043617620652	pseudobulk	DESeq2	LRT	0.0557712992369932
899	KC_G1	yata	0.244117319964362	0.553335730511277	0.998043617620652	pseudobulk	DESeq2	LRT	0.257011285211719
900	KC_G1	yellow-b	-0.491270237326388	0.706073078679256	0.998043617620652	pseudobulk	DESeq2	LRT	0.151150347071905
900	KC_G1	yellow-c	-1.63882223825269	0.36415089042169	0.998043617620652	pseudobulk	DESeq2	LRT	0.438718623771626
900	KC_G1	yellow-g	1.13582588427274	0.633424328955532	0.998043617620652	pseudobulk	DESeq2	LRT	0.198305259991224
900	KC_G1	yem	-0.224733088592468	0.693857547338626	0.998043617620652	pseudobulk	DESeq2	LRT	0.158729683370915
900	KC_G1	Yeti	0.0894381334698145	0.509880702215097	0.998043617620652	pseudobulk	DESeq2	LRT	0.292531424745791
900	KC_G1	Yif1	-0.0181528208978736	0.955429152991986	0.998043617620652	pseudobulk	DESeq2	LRT	0.0198015112310341
900	KC_G1	yin	0.0961465739681288	0.957451802323201	0.998043617620652	pseudobulk	DESeq2	LRT	0.0188830789905862
900	KC_G1	Yip1d1	-0.0140061353553046	0.946956369797672	0.998043617620652	pseudobulk	DESeq2	LRT	0.0236700302814396
900	KC_G1	yip2	0.324348720894916	0.784340282499644	0.998043617620652	pseudobulk	DESeq2	LRT	0.10549547972654
900	KC_G1	Yippee	-0.186943314402287	0.36871976378876	0.998043617620652	pseudobulk	DESeq2	LRT	0.433303583103515
901	KC_G1	yki	-0.0936057009020158	0.796565560609219	0.998043617620652	pseudobulk	DESeq2	LRT	0.0987784741766279
901	KC_G1	Ykt6	0.224185873313303	0.357916391724703	0.998043617620652	pseudobulk	DESeq2	LRT	0.44621841149384
901	KC_G1	yl	0.25609124879822	0.843281542713157	0.998043617620652	pseudobulk	DESeq2	LRT	0.0740274051698137
901	KC_G1	YL-1	0.0375285931300387	0.874627664338853	0.998043617620652	pseudobulk	DESeq2	LRT	0.0581767901060722
901	KC_G1	YME1L	-0.152027627760063	0.622290450320823	0.998043617620652	pseudobulk	DESeq2	LRT	0.206006863654439

901	KC_G1	Yp1	-1.60891734856068	0.545249891924755	0.998043617620652	pseudobulk	DESeq2	LRT	0.263404411831769
901	KC_G1	Yp3	-0.494057164586944	0.702698669806958	0.998043617620652	pseudobulk	DESeq2	LRT	0.153230868571853
901	KC_G1	yps	-0.128597443562962	0.453836768472089	0.998043617620652	pseudobulk	DESeq2	LRT	0.343100321810127
901	KC_G1	yrt	-0.0875452791637148	0.7176553285109	0.998043617620652	pseudobulk	DESeq2	LRT	0.144084086211617
901	KC_G1	Ythdc1	-0.0577269342152301	0.688453305358978	0.998043617620652	pseudobulk	DESeq2	LRT	0.162125510617861
902	KC_G1	Ythdf	-0.203283473799011	0.316289119250794	0.998043617620652	pseudobulk	DESeq2	LRT	0.499915748111526
902	KC_G1	ytr	-0.174631746064008	0.407154239832836	0.998043617620652	pseudobulk	DESeq2	LRT	0.390241038394372
902	KC_G1	z	0.0149250840481238	0.94953844526024	0.998043617620652	pseudobulk	DESeq2	LRT	0.0224874466969852
902	KC_G1	ZAP3	-0.131256960197985	0.498893270713503	0.998043617620652	pseudobulk	DESeq2	LRT	0.301992353971627
902	KC_G1	Zasp52	-0.752595479487864	0.785589861204421	0.998043617620652	pseudobulk	DESeq2	LRT	0.104804130174502
902	KC_G1	ZC3H3	-0.190842793984724	0.620174853173265	0.998043617620652	pseudobulk	DESeq2	LRT	0.20748584749808
902	KC_G1	Zcchc7	-0.266662074054536	0.27455222238651	0.998043617620652	pseudobulk	DESeq2	LRT	0.561375036506447
902	KC_G1	zda	0.300310852251717	0.185786465515169	0.998043617620652	pseudobulk	DESeq2	LRT	0.730985927404681
902	KC_G1	Zdhhc8	0.058953559170399	0.684183762045096	0.998043617620652	pseudobulk	DESeq2	LRT	0.164827237280299
902	KC_G1	zetaCOP	0.166713802693408	0.410130116768469	0.998043617620652	pseudobulk	DESeq2	LRT	0.387078338329884
903	KC_G1	zf30C	-0.185505889778808	0.552987794648509	0.998043617620652	pseudobulk	DESeq2	LRT	0.25728445418409
903	KC_G1	zfh1	-0.439182223008672	0.813628398089446	0.998043617620652	pseudobulk	DESeq2	LRT	0.0895739016311467
903	KC_G1	zfh2	-0.211960138572568	0.120338710729837	0.998043617620652	pseudobulk	DESeq2	LRT	0.919594645710551
903	KC_G1	Zfrp8	-0.339184867452106	0.436932406533467	0.998043617620652	pseudobulk	DESeq2	LRT	0.359585743212775
903	KC_G1	Zif	-0.0360541391043466	0.924881204654281	0.998043617620652	pseudobulk	DESeq2	LRT	0.0339140461542959
903	KC_G1	zip	0.00406501974847402	0.952906077722432	0.998043617620652	pseudobulk	DESeq2	LRT	0.0209499030731318
903	KC_G1	Zip102B	0.158043600565003	0.463907735486091	0.998043617620652	pseudobulk	DESeq2	LRT	0.333568385724579
903	KC_G1	Zip48C	-0.131152217316903	0.817218455869307	0.998043617620652	pseudobulk	DESeq2	LRT	0.0876618339244524
903	KC_G1	Zip71B	-0.446738448430009	0.733847502672901	0.998043617620652	pseudobulk	DESeq2	LRT	0.134394179359851
903	KC_G1	Zip88E	1.00070826990715	0.157977599844947	0.998043617620652	pseudobulk	DESeq2	LRT	0.801404488700019
904	KC_G1	Zip89B	1.12508065046033	0.900770125530981	0.998043617620652	pseudobulk	DESeq2	LRT	0.0453860258366365
904	KC_G1	Zip99C	-0.114801806474161	0.778339810601517	0.998043617620652	pseudobulk	DESeq2	LRT	0.108830755646065
904	KC_G1	ZIPIC	0.337840500015943	0.631681993466585	0.998043617620652	pseudobulk	DESeq2	LRT	0.199501502794659
904	KC_G1	Zir	0.240488808178153	0.531348557287769	0.998043617620652	pseudobulk	DESeq2	LRT	0.274620494275593
904	KC_G1	Ziz	-0.0237216946831259	0.908226561507027	0.998043617620652	pseudobulk	DESeq2	LRT	0.041805801105774
904	KC_G1	zld	-2.23422618188688	0.336998892702854	0.998043617620652	pseudobulk	DESeq2	LRT	0.472371526113321
904	KC_G1	Zn72D	-0.101080928440421	0.655848350467648	0.998043617620652	pseudobulk	DESeq2	LRT	0.183196569418319
904	KC_G1	ZnT33D	0.0951399138004301	0.859393133963345	0.998043617620652	pseudobulk	DESeq2	LRT	0.0658081203911704
904	KC_G1	ZnT41F	1.12508065046033	0.900770125530981	0.998043617620652	pseudobulk	DESeq2	LRT	0.0453860258366365
904	KC_G1	ZnT49B	-0.328959465073532	0.275654720216408	0.998043617620652	pseudobulk	DESeq2	LRT	0.559634566412037
905	KC_G1	ZnT63C	0.0973599138852203	0.726410105573063	0.998043617620652	pseudobulk	DESeq2	LRT	0.138818122686187
905	KC_G1	ZnT77C	0.173105107343345	0.973555957903825	0.998043617620652	pseudobulk	DESeq2	LRT	0.0116390811132178
905	KC_G1	ZnT86D	-0.228426035427458	0.765272681670173	0.998043617620652	pseudobulk	DESeq2	LRT	0.116183789625804
905	KC_G1	zormin	2.86622177009429	0.203230025119761	0.998043617620652	pseudobulk	DESeq2	LRT	0.692012129161051
905	KC_G1	Zpr1	0.342573567087466	0.653425317144776	0.998043617620652	pseudobulk	DESeq2	LRT	0.184804042674681
905	KC_G1	Zw	-0.794610277746993	0.815039924133852	0.998043617620652	pseudobulk	DESeq2	LRT	0.0888211171417967
905	KC_G1	Zw10	0.365341022328497	0.509165993973058	0.998043617620652	pseudobulk	DESeq2	LRT	0.293140609580485
905	KC_G1	zyd	-0.0909653020903254	0.844650958867041	0.998043617620652	pseudobulk	DESeq2	LRT	0.0733227205820251
905	KC_G1	Zyx	-0.0824387635214529	0.628277562922954	0.998043617620652	pseudobulk	DESeq2	LRT	0.201848449551518
905	KC_G1	CG34219	0.00441341807117164	0.976436919507507	0.998159533876185	pseudobulk	DESeq2	LRT	0.0103558080798636
906	KC_G1	Arpc4	-0.00592222599853471	0.976775088450509	0.998348238340216	pseudobulk	DESeq2	LRT	0.0102054251139938
906	KC_G1	pins	-0.00859072882096398	0.976945796678094	0.998348238340216	pseudobulk	DESeq2	LRT	0.0101295313238333
906	KC_G1	Sil1	-0.00836389106076241	0.976851375565747	0.998348238340216	pseudobulk	DESeq2	LRT	0.0101715076033386
906	KC_G1	CG12868	-0.0127607144507255	0.977156665442452	0.998453253902698	pseudobulk	DESeq2	LRT	0.010035801191563
906	KC_G1	CG11070	-0.00543368155907835	0.978130610442288	0.998454279182689	pseudobulk	DESeq2	LRT	0.00960314970417829
906	KC_G1	CG12948	0.0131417610790588	0.978025728588701	0.998454279182689	pseudobulk	DESeq2	LRT	0.00964972022556207
906	KC_G1	CG13390	-0.00652853812311531	0.977483993201716	0.998454279182689	pseudobulk	DESeq2	LRT	0.00989034566722178
906	KC_G1	CG13605	0.00087271076631603	0.97806397073997	0.998454279182689	pseudobulk	DESeq2	LRT	0.00963273904593619
906	KC_G1	CG17221	0.0105091773047455	0.977969646521863	0.998454279182689	pseudobulk	DESeq2	LRT	0.00967462430506864
906	KC_G1	CG9684	-0.00139858122220927	0.97782892723448	0.998454279182689	pseudobulk	DESeq2	LRT	0.00973711909459667
907	KC_G1	Chro	0.00537879804676261	0.97741009362372	0.998454279182689	pseudobulk	DESeq2	LRT	0.00992318036570539

907	KC_G1	MED1	0.0079087035046904	0.977876796187519	0.998454279182689	pseudobulk	DESeq2	LRT	0.00971585902268039
907	KC_G1	sta	0.00230218754674023	0.97785863808835	0.998454279182689	pseudobulk	DESeq2	LRT	0.0097239234695715
907	KC_G1	CG6145	-0.0074185730365096	0.978718895033717	0.998944382200399	pseudobulk	DESeq2	LRT	0.00934202716120492
907	KC_G1	28SrRNA-Psi:CR45848	-0.0183690589502723	0.979568773853548	0.999284271253931	pseudobulk	DESeq2	LRT	0.00896506752368483
907	KC_G1	BI-1	-0.00220619208157272	0.979924020368076	0.999284271253931	pseudobulk	DESeq2	LRT	0.00880759656679197
907	KC_G1	CG12325	-0.0134843034961465	0.979809134829782	0.999284271253931	pseudobulk	DESeq2	LRT	0.00885851590267861
907	KC_G1	CG14971	-0.00936801839073968	0.979745770651985	0.999284271253931	pseudobulk	DESeq2	LRT	0.00888660260002
907	KC_G1	CG5149	0.00948537079826056	0.979900820056345	0.999284271253931	pseudobulk	DESeq2	LRT	0.00881787888096193
907	KC_G1	Gapdh2	0.00875775928370647	0.97968961100718	0.999284271253931	pseudobulk	DESeq2	LRT	0.0089114973468227
908	KC_G1	Mkp3	0.00273545201938848	0.979941347572289	0.999284271253931	pseudobulk	DESeq2	LRT	0.00879991735647516
908	KC_G1	Rcp	-0.00887284866011632	0.980025652773723	0.999284271253931	pseudobulk	DESeq2	LRT	0.00876255623376776
908	KC_G1	SNRPG	0.00437825590515441	0.979949958536269	0.999284271253931	pseudobulk	DESeq2	LRT	0.00879610113041306
908	KC_G1	asRNA:CR44107	0.0142965740858526	0.981853620982279	0.999382856886966	pseudobulk	DESeq2	LRT	0.00795325390163776
908	KC_G1	BORCS5	-0.00659379135854572	0.981050614503202	0.999382856886966	pseudobulk	DESeq2	LRT	0.00830858585919906
908	KC_G1	bves	-0.00143338510019871	0.981173242283682	0.999382856886966	pseudobulk	DESeq2	LRT	0.00825430401130915
908	KC_G1	CG5861	-0.00441613709099554	0.980510090943367	0.999382856886966	pseudobulk	DESeq2	LRT	0.00854793242101058
908	KC_G1	crn	0.00198694955107775	0.98088355514812	0.999382856886966	pseudobulk	DESeq2	LRT	0.00838254650207896
908	KC_G1	e(y)1	0.00117310128425943	0.981172074540907	0.999382856886966	pseudobulk	DESeq2	LRT	0.00825482088694687
908	KC_G1	ebd2	-0.00026243592805979	0.981833067404463	0.999382856886966	pseudobulk	DESeq2	LRT	0.00796234527602228
909	KC_G1	kat80	0.00016087128643637	0.980338839661223	0.999382856886966	pseudobulk	DESeq2	LRT	0.00862379087790895
909	KC_G1	Lis-1	0.00190878211136859	0.981032710104243	0.999382856886966	pseudobulk	DESeq2	LRT	0.00831651190553174
909	KC_G1	lncRNA:CR44268	0.00600365410811582	0.981853334807544	0.999382856886966	pseudobulk	DESeq2	LRT	0.00795338048275305
909	KC_G1	rhea	0.00265586325285576	0.980767972104535	0.999382856886966	pseudobulk	DESeq2	LRT	0.0084337248866097
909	KC_G1	Rlb1	0.0016493382393551	0.981679855719799	0.999382856886966	pseudobulk	DESeq2	LRT	0.00803012072945988
909	KC_G1	Svil	0.00398934840990462	0.981533728898225	0.999382856886966	pseudobulk	DESeq2	LRT	0.00809477194508112
909	KC_G1	Tim17b	-0.00065310703340597	0.981486259731601	0.999382856886966	pseudobulk	DESeq2	LRT	0.00811577590555944
909	KC_G1	Usp10	0.00672829840325275	0.980898558546301	0.999382856886966	pseudobulk	DESeq2	LRT	0.00837590367156841
909	KC_G1	vsg	0.00406667839929027	0.981449507541504	0.999382856886966	pseudobulk	DESeq2	LRT	0.00813203856032862
909	KC_G1	CG33056	0.0150068423069974	0.982167315117061	0.999481855709694	pseudobulk	DESeq2	LRT	0.00781452255677692
910	KC_G1	sicily	0.00247999394703222	0.98214888574771	0.999481855709694	pseudobulk	DESeq2	LRT	0.00782267172686584
910	KC_G1	CG7154	-0.00514590052599857	0.982375724785625	0.999583804574202	pseudobulk	DESeq2	LRT	0.00772237780093941
910	KC_G1	CG2617	-0.00037827086456928	0.983320062479506	0.999883749538778	pseudobulk	DESeq2	LRT	0.0073050999253762
910	KC_G1	cora	0.00216109408683395	0.983014452542752	0.999883749538778	pseudobulk	DESeq2	LRT	0.00744009700669864
910	KC_G1	lncRNA:CR43878	-0.0104390838176132	0.98322755437653	0.999883749538778	pseudobulk	DESeq2	LRT	0.00734595910248352
910	KC_G1	Lpin	-0.00238241314139909	0.983258408208576	0.999883749538778	pseudobulk	DESeq2	LRT	0.00733233108859554
910	KC_G1	Sps1	0.00067216790360348	0.982976124279554	0.999883749538778	pseudobulk	DESeq2	LRT	0.0074570307126954
910	KC_G1	Syt14	0.00107424157146318	0.983200845424789	0.999883749538778	pseudobulk	DESeq2	LRT	0.00735775668469967
910	KC_AB1	128up	-0.329200152244302	0.723083269993867	1	pseudobulk	DESeq2	LRT	0.140811686638435
910	KC_AB1	14-3-3epsilon	-0.0531088911848018	0.713328237149763	1	pseudobulk	DESeq2	LRT	0.146710584060625
911	KC_AB1	14-3-3zeta	-0.138247693518334	0.57828679954864	1	pseudobulk	DESeq2	LRT	0.237856721147236
911	KC_AB1	140up	0.131991332541927	0.848241380133929	1	pseudobulk	DESeq2	LRT	0.0714805449799745
911	KC_AB1	18SrRNA-Psi:CR41602	-0.284695663406991	0.792593223367249	1	pseudobulk	DESeq2	LRT	0.100949645182237
911	KC_AB1	18w	0.0337989143642107	0.921135431781516	1	pseudobulk	DESeq2	LRT	0.0356765120927159
911	KC_AB1	26-29-p	-0.223647671046346	0.715480140652152	1	pseudobulk	DESeq2	LRT	0.145402416306555
911	KC_AB1	28SrRNA-Psi:CR40596	-0.318339795761335	0.603198350143659	1	pseudobulk	DESeq2	LRT	0.219539855010439
911	KC_AB1	28SrRNA-Psi:CR40741	1.74550908011916	0.381854164771694	1	pseudobulk	DESeq2	LRT	0.418102468312655
911	KC_AB1	28SrRNA-Psi:CR41609	0.709686956006578	0.915714550115026	1	pseudobulk	DESeq2	LRT	0.0382398850985033
911	KC_AB1	28SrRNA-Psi:CR45848	-1.53448908949263	0.360596596867549	1	pseudobulk	DESeq2	LRT	0.44297837625094
911	KC_AB1	28SrRNA-Psi:CR45855	2.7166725478541	0.398265714122996	1	pseudobulk	DESeq2	LRT	0.39982707950105
912	KC_AB1	28SrRNA-Psi:CR45859	1.08028432644311	0.159455328138108	1	pseudobulk	DESeq2	LRT	0.797360964396448
912	KC_AB1	28SrRNA-Psi:CR45860	-0.205210074260242	0.846121047555605	1	pseudobulk	DESeq2	LRT	0.0725675015887905
912	KC_AB1	2mit	-0.409964114051124	0.163668151890344	1	pseudobulk	DESeq2	LRT	0.78603582152834
912	KC_AB1	312	-0.837944359393996	0.20197140862885	1	pseudobulk	DESeq2	LRT	0.694710105571044
912	KC_AB1	4E-T	-0.138229624464365	0.669656095539681	1	pseudobulk	DESeq2	LRT	0.174148173648398
912	KC_AB1	5-HT1A	-0.257838713276677	0.225078554534284	1	pseudobulk	DESeq2	LRT	0.647665882569937
912	KC_AB1	5-HT1B	0.032606561231349	0.917897727476353	1	pseudobulk	DESeq2	LRT	0.0372057053641436

912	KC_AB1	5-HT2A	-0.760959112540794	1	1	pseudobulk	DESeq2	LRT	0
912	KC_AB1	5-HT2B	-0.0108141376872241	0.956450322674242	1	pseudobulk	DESeq2	LRT	0.0193375819866349
912	KC_AB1	5-HT7	-0.760959112540794	1	1	pseudobulk	DESeq2	LRT	0
913	KC_AB1	5.8SrRNA-Psi:CR45854	-0.760959112540794	0.804334723897681	1	pseudobulk	DESeq2	LRT	0.0945631819854715
913	KC_AB1	5PtaseI	0.136696179747959	0.686955041895494	1	pseudobulk	DESeq2	LRT	0.16307168462255
913	KC_AB1	7B2	0.0980249200303084	0.417629815230665	1	pseudobulk	DESeq2	LRT	0.379208503952314
913	KC_AB1	a	-0.33621024909827	0.218528626217939	1	pseudobulk	DESeq2	LRT	0.660491664441053
913	KC_AB1	A16	0.234352667109427	0.752048307434494	1	pseudobulk	DESeq2	LRT	0.123754261830681
913	KC_AB1	a6	0.00108349407582741	0.97360197874785	1	pseudobulk	DESeq2	LRT	0.0116185521173517
913	KC_AB1	AANAT1	0.950584022303911	0.0477041951849018	1	pseudobulk	DESeq2	LRT	1.32144342671379
913	KC_AB1	AANATL2	0.719075238377752	0.512161513952683	1	pseudobulk	DESeq2	LRT	0.290593059420803
913	KC_AB1	AANATL7	0.525046934748695	0.696668447610074	1	pseudobulk	DESeq2	LRT	0.156973858389127
913	KC_AB1	Aasdh	0.548238141063047	0.665575425647947	1	pseudobulk	DESeq2	LRT	0.17680272142664
914	KC_AB1	Aatf	-0.341008130448017	0.598408677003051	1	pseudobulk	DESeq2	LRT	0.223002117771898
914	KC_AB1	aay	-0.0671201224059978	0.874605124855111	1	pseudobulk	DESeq2	LRT	0.0581879821823644
914	KC_AB1	ab	-0.581265889649191	0.484428572361677	1	pseudobulk	DESeq2	LRT	0.314770249399633
914	KC_AB1	ABCA	-0.0030259422938123	0.927770434066244	1	pseudobulk	DESeq2	LRT	0.0325594715741855
914	KC_AB1	ABCB7	0.324757230887004	0.770530251701818	1	pseudobulk	DESeq2	LRT	0.113210305823879
914	KC_AB1	ABCD	0.0658259466472664	0.764419703708695	1	pseudobulk	DESeq2	LRT	0.116668127083177
914	KC_AB1	Abi	-0.17014870317562	0.757286996319361	1	pseudobulk	DESeq2	LRT	0.120739500557182
914	KC_AB1	Abl	-0.072138381669245	0.760681202198008	1	pseudobulk	DESeq2	LRT	0.118797315810903
914	KC_AB1	abo	2.01414189802547	0.43447424094886	1	pseudobulk	DESeq2	LRT	0.362035966845671
914	KC_AB1	Abp1	-0.49352180191063	0.301015653790407	1	pseudobulk	DESeq2	LRT	0.52141091909711
915	KC_AB1	abs	0.0725383401232157	0.92912762278498	1	pseudobulk	DESeq2	LRT	0.0319246282404554
915	KC_AB1	Ac13E	2.4138856564567	0.409611385497059	1	pseudobulk	DESeq2	LRT	0.387627980279722
915	KC_AB1	Ac3	-0.395010706404157	0.343989520788547	1	pseudobulk	DESeq2	LRT	0.463454787466345
915	KC_AB1	Ac76E	-0.0443191998461451	0.968394902602301	1	pseudobulk	DESeq2	LRT	0.0139475052459171
915	KC_AB1	Ac78C	-0.358447605138646	0.523149188150099	1	pseudobulk	DESeq2	LRT	0.281374444304693
915	KC_AB1	Acbp1	-0.0909467690186207	0.746589314109817	1	pseudobulk	DESeq2	LRT	0.126918230397328
915	KC_AB1	Acbp2	0.369216784119794	0.578892760053324	1	pseudobulk	DESeq2	LRT	0.237401881921455
915	KC_AB1	ACC	-0.420379378585987	0.382690589334953	1	pseudobulk	DESeq2	LRT	0.417152217289877
915	KC_AB1	AcCoAS	-0.142543462297516	0.604221185480031	1	pseudobulk	DESeq2	LRT	0.218804051363679
915	KC_AB1	Ace	-0.16589427269719	0.71982206359068	1	pseudobulk	DESeq2	LRT	0.142774845723078
916	KC_AB1	Acer	1.35968990391017	0.292236855881415	1	pseudobulk	DESeq2	LRT	0.534265013259517
916	KC_AB1	Acf	-0.486220942785522	0.297132728489822	1	pseudobulk	DESeq2	LRT	0.527049509018641
916	KC_AB1	achi	0.102699684624887	0.883986715583655	1	pseudobulk	DESeq2	LRT	0.0535542614485378
916	KC_AB1	AchI	1.25804452752548	0.727552935095488	1	pseudobulk	DESeq2	LRT	0.138135402914536
916	KC_AB1	acj6	-0.760959112540794	0.805265236978025	1	pseudobulk	DESeq2	LRT	0.0940610488455931
916	KC_AB1	Ack	0.279303468025868	0.723603366164882	1	pseudobulk	DESeq2	LRT	0.140499421492388
916	KC_AB1	Ack-like	-0.263603852396468	0.410211323179419	1	pseudobulk	DESeq2	LRT	0.386992355848521
916	KC_AB1	Acn	0.231137321596907	0.437289132520362	1	pseudobulk	DESeq2	LRT	0.359231315577816
916	KC_AB1	Acox57D-p	-1.77199356422654	0.54193209410651	1	pseudobulk	DESeq2	LRT	0.266055128595237
916	KC_AB1	Acp53C14b	0.152115475746305	1	1	pseudobulk	DESeq2	LRT	0
917	KC_AB1	Acp95EF	-0.760959112540794	0.804334723897681	1	pseudobulk	DESeq2	LRT	0.0945631819854715
917	KC_AB1	Acph-1	0.336100637353695	0.790427599994554	1	pseudobulk	DESeq2	LRT	0.102137903546869
917	KC_AB1	Acsl	0.117838141996241	0.717263880139662	1	pseudobulk	DESeq2	LRT	0.144321038746325
917	KC_AB1	Act42A	0.490486474668098	0.708030698589507	1	pseudobulk	DESeq2	LRT	0.149947911887924
917	KC_AB1	Act5C	-0.121107797013061	0.708422118061636	1	pseudobulk	DESeq2	LRT	0.149707887908045
917	KC_AB1	Act79B	0.382562896925494	0.667656361203507	1	pseudobulk	DESeq2	LRT	0.175447008831465
917	KC_AB1	Actbeta	-0.114456336690653	0.682464355451602	1	pseudobulk	DESeq2	LRT	0.165920026536191
917	KC_AB1	Actn	-0.635442421491505	0.477600514289208	1	pseudobulk	DESeq2	LRT	0.320935214221977
917	KC_AB1	ACXC	0.293544814793053	0.855484935116898	1	pseudobulk	DESeq2	LRT	0.0677876338764847
917	KC_AB1	Acyp2	-0.0831343981340643	0.858392846575204	1	pseudobulk	DESeq2	LRT	0.0663139102241423
918	KC_AB1	Ada1-2	-0.00190788348617797	0.988674410401985	1	pseudobulk	DESeq2	LRT	0.00494670642748723
918	KC_AB1	Ada2a	0.943798956650056	0.807634491832869	1	pseudobulk	DESeq2	LRT	0.0927851418175362
918	KC_AB1	Ada2b	0.042585293966118	0.923581338355194	1	pseudobulk	DESeq2	LRT	0.0345248509077869

918	KC_AB1	Ada3	0.0225318947417405	0.957427310013086	1	pseudobulk	DESeq2	LRT	0.0188941886998771
918	KC_AB1	AdamTS-A	1.25804452752548	0.727552935095488	1	pseudobulk	DESeq2	LRT	0.138135402914536
918	KC_AB1	AdamTS-B	3.37429492540794	0.281278028870551	1	pseudobulk	DESeq2	LRT	0.550864190027974
918	KC_AB1	Adar	-0.152012575364126	0.658675949884197	1	pseudobulk	DESeq2	LRT	0.181328193584849
918	KC_AB1	Adat1	0.402513268746747	0.741939521106922	1	pseudobulk	DESeq2	LRT	0.129631494613188
918	KC_AB1	Adck1	1.05495364216678	0.607662541024221	1	pseudobulk	DESeq2	LRT	0.216337534638187
918	KC_AB1	Adck5	0.550276305763905	0.63718483783673	1	pseudobulk	DESeq2	LRT	0.195734567023741
919	KC_AB1	ADD1	0.0716264119812338	0.886172147487879	1	pseudobulk	DESeq2	LRT	0.052481904025025
919	KC_AB1	AdenoK	0.255924672984571	0.746496981963079	1	pseudobulk	DESeq2	LRT	0.126971943758688
919	KC_AB1	Adf1	-0.0112666740905104	0.933903637185372	1	pseudobulk	DESeq2	LRT	0.0296979331892553
919	KC_AB1	Adgf-B	-0.760959112540794	1	1	pseudobulk	DESeq2	LRT	0
919	KC_AB1	Adgf-C	-0.718853741766346	0.977252643133665	1	pseudobulk	DESeq2	LRT	0.0099931462763106
919	KC_AB1	Adgf-D	0.18023660397398	0.719037619992349	1	pseudobulk	DESeq2	LRT	0.143248386769171
919	KC_AB1	Adh	1.25804452752548	0.727552935095488	1	pseudobulk	DESeq2	LRT	0.138135402914536
919	KC_AB1	Adi1	0.726279426592153	0.36904857871689	1	pseudobulk	DESeq2	LRT	0.432916462892246
919	KC_AB1	AdipoR	0.012145128706102	0.967577378815998	1	pseudobulk	DESeq2	LRT	0.0143142936485485
919	KC_AB1	Adk1	-0.0877689370487378	0.790415016629944	1	pseudobulk	DESeq2	LRT	0.10214481743666
920	KC_AB1	Adk2	0.264445611100235	0.391154268159739	1	pseudobulk	DESeq2	LRT	0.407651926499104
920	KC_AB1	Adk3	-0.872378506213532	0.473934537949025	1	pseudobulk	DESeq2	LRT	0.324281640964841
920	KC_AB1	AdoR	0.253119741765009	0.89124707280026	1	pseudobulk	DESeq2	LRT	0.0500018835286135
920	KC_AB1	adp	1.33570531297198	0.0679638865716143	1	pseudobulk	DESeq2	LRT	1.16772179360019
920	KC_AB1	ADPS	-0.185631286777625	0.775956046567212	1	pseudobulk	DESeq2	LRT	0.110162878321636
920	KC_AB1	AdSL	0.900711984999322	0.297296942478372	1	pseudobulk	DESeq2	LRT	0.526809557231656
920	KC_AB1	AdSS	0.631888385614293	0.201029562880276	1	pseudobulk	DESeq2	LRT	0.696740071675569
920	KC_AB1	Aduk	0.0518789325475165	0.92694647721882	1	pseudobulk	DESeq2	LRT	0.0329453417126701
920	KC_AB1	Ady43A	1.05183519070701	0.173298079924602	1	pseudobulk	DESeq2	LRT	0.761206249073559
920	KC_AB1	Aef1	-0.176710330813469	0.624072537770614	1	pseudobulk	DESeq2	LRT	0.204764928069807
921	KC_AB1	aft	0.0997197654571035	0.945443855083898	1	pseudobulk	DESeq2	LRT	0.0243642565082458
921	KC_AB1	Afti	0.144685453515729	0.755707518973779	1	pseudobulk	DESeq2	LRT	0.121646256712254
921	KC_AB1	Ag5r	3.42238926897485	0.134493898221636	1	pseudobulk	DESeq2	LRT	0.871297418477818
921	KC_AB1	AGBE	0.615600820058616	0.646373335736699	1	pseudobulk	DESeq2	LRT	0.189516567427259
921	KC_AB1	ago	-0.197451232957978	0.758936347246476	1	pseudobulk	DESeq2	LRT	0.119794647291407
921	KC_AB1	AGO1	-0.174726390093698	0.229926291693583	1	pseudobulk	DESeq2	LRT	0.638411365030921
921	KC_AB1	AGO2	-0.00466282529323832	0.958895643302225	1	pseudobulk	DESeq2	LRT	0.0182286545645578
921	KC_AB1	AGO3	0.586700138454857	0.772610332244894	1	pseudobulk	DESeq2	LRT	0.112039488260913
921	KC_AB1	Agpat1	-0.0657984710477683	0.928821345064246	1	pseudobulk	DESeq2	LRT	0.0320678127170488
921	KC_AB1	Agpat2	0.0984102316122483	0.951586353860821	1	pseudobulk	DESeq2	LRT	0.0215517945513734
922	KC_AB1	Agpat3	-0.015141096320643	0.936405571949932	1	pseudobulk	DESeq2	LRT	0.0285360107593632
922	KC_AB1	agt	1.49161085205854	0.443354850394934	1	pseudobulk	DESeq2	LRT	0.353248535900648
922	KC_AB1	Ahcy	0.325081213629183	0.681122581618542	1	pseudobulk	DESeq2	LRT	0.166774721083191
922	KC_AB1	AhcyL1	-0.372593460966376	0.589273827797147	1	pseudobulk	DESeq2	LRT	0.229682847375394
922	KC_AB1	AhcyL2	-0.760959112540794	1	1	pseudobulk	DESeq2	LRT	0
922	KC_AB1	AIF	-0.20056949090594	0.830793162786786	1	pseudobulk	DESeq2	LRT	0.0805070862536802
922	KC_AB1	AIMP1	0.134716032147234	0.841354286856474	1	pseudobulk	DESeq2	LRT	0.075021088118057
922	KC_AB1	AIMP2	-0.0308773073862005	0.948971446469178	1	pseudobulk	DESeq2	LRT	0.0227468548302774
922	KC_AB1	AIMP3	-0.0830698802589158	0.800716913408465	1	pseudobulk	DESeq2	LRT	0.0965209978667678
922	KC_AB1	Ak6	0.904767089471667	0.267499527284703	1	pseudobulk	DESeq2	LRT	0.572676981111263
923	KC_AB1	Akap200	0.65368330602772	0.331626628904152	1	pseudobulk	DESeq2	LRT	0.479350603809924
923	KC_AB1	Akh	-0.760959112540794	0.805265236978025	1	pseudobulk	DESeq2	LRT	0.0940610488455931
923	KC_AB1	akirin	0.200154389884127	0.500965061528713	1	pseudobulk	DESeq2	LRT	0.300192561786236
923	KC_AB1	Akt1	0.0518017135231888	0.918113254177861	1	pseudobulk	DESeq2	LRT	0.0371037429483049
923	KC_AB1	Aladin	-0.020895280284949	0.926322455867559	1	pseudobulk	DESeq2	LRT	0.0332378076735959
923	KC_AB1	AlaRS	0.0854489896219347	0.933862249018105	1	pseudobulk	DESeq2	LRT	0.0297171804116143
923	KC_AB1	AlaRS-m	1.21490537884565	0.325407037313189	1	pseudobulk	DESeq2	LRT	0.487573059164257
923	KC_AB1	Alas	-0.133724681532817	0.641662780047498	1	pseudobulk	DESeq2	LRT	0.192693151434681
923	KC_AB1	alc	0.318719631935487	0.410333686348995	1	pseudobulk	DESeq2	LRT	0.386862828158725

923	KC_AB1	Ald1	-0.225372802425958	0.711509112663633	1	pseudobulk	DESeq2	LRT	0.147819533310986
924	KC_AB1	Aldh	1.53044130215465	0.0549845150954434	1	pseudobulk	DESeq2	LRT	1.25975960060524
924	KC_AB1	Aldh-III	0.12067850944564	0.853634962050361	1	pseudobulk	DESeq2	LRT	0.0687278059610419
924	KC_AB1	Aldh7A1	3.4290155526378	0.191656243008451	1	pseudobulk	DESeq2	LRT	0.717477029475316
924	KC_AB1	Alg-2	0.253917232881257	0.374741393386305	1	pseudobulk	DESeq2	LRT	0.4262683327232
924	KC_AB1	Alg1	1.07358028627203	0.35697050006378	1	pseudobulk	DESeq2	LRT	0.447367672372059
924	KC_AB1	Alg10	-0.157321701043241	0.894918741576472	1	pseudobulk	DESeq2	LRT	0.0482163967366731
924	KC_AB1	Alg11	0.14273596415999	0.781932419810908	1	pseudobulk	DESeq2	LRT	0.10683078015176
924	KC_AB1	Alg14	-0.212479395525805	0.828413945688222	1	pseudobulk	DESeq2	LRT	0.0817525987157381
924	KC_AB1	Alg2	-0.54957072630758	0.487468889086707	1	pseudobulk	DESeq2	LRT	0.31205309633286
924	KC_AB1	Alg3	0.571006949102751	0.527773127711484	1	pseudobulk	DESeq2	LRT	0.277552726248828
925	KC_AB1	Alg9	-0.70089073339255	0.71820853506906	1	pseudobulk	DESeq2	LRT	0.143749438096927
925	KC_AB1	Alh	-0.300333357121121	0.459173468099148	1	pseudobulk	DESeq2	LRT	0.33802321423166
925	KC_AB1	alien	-0.516645335020642	0.361259796967122	1	pseudobulk	DESeq2	LRT	0.442180366488496
925	KC_AB1	ALiX	-0.0418809221044174	0.950216810484254	1	pseudobulk	DESeq2	LRT	0.0221772906543736
925	KC_AB1	Alk	-0.445305793228856	0.0136837878200293	1	pseudobulk	DESeq2	LRT	1.86379366857824
925	KC_AB1	AlkB	2.09987857572813	0.503115176013642	1	pseudobulk	DESeq2	LRT	0.29833258237694
925	KC_AB1	Alp12	0.709713058804466	0.916223368658127	1	pseudobulk	DESeq2	LRT	0.037998635557264
925	KC_AB1	Alp4	-0.760959112540794	1	1	pseudobulk	DESeq2	LRT	0
925	KC_AB1	Alp6	-0.385011853448768	0.890534840916548	1	pseudobulk	DESeq2	LRT	0.0503490847107839
925	KC_AB1	alph	-0.474598560535547	0.146702447140283	1	pseudobulk	DESeq2	LRT	0.833562641639625
926	KC_AB1	alpha-Cat	0.00996125094810687	0.96221060565818	1	pseudobulk	DESeq2	LRT	0.0167298605352626
926	KC_AB1	alpha-Catr	-0.465746405219058	0.263935864541715	1	pseudobulk	DESeq2	LRT	0.578501592295034
926	KC_AB1	alpha-Est1	-0.760959112540794	1	1	pseudobulk	DESeq2	LRT	0
926	KC_AB1	alpha-Est10	1.90188165576851	0.466613294362074	1	pseudobulk	DESeq2	LRT	0.331042891804934
926	KC_AB1	alpha-Est2	-0.760959112540794	1	1	pseudobulk	DESeq2	LRT	0
926	KC_AB1	alpha-Est3	1.25804452752548	0.727552935095488	1	pseudobulk	DESeq2	LRT	0.138135402914536
926	KC_AB1	alpha-Est4	-0.760959112540794	1	1	pseudobulk	DESeq2	LRT	0
926	KC_AB1	alpha-Est7	6.09973870457195	0.152300562657299	1	pseudobulk	DESeq2	LRT	0.817298492208878
926	KC_AB1	alpha-Est8	0.497035326776464	0.170651606551785	1	pseudobulk	DESeq2	LRT	0.767889618822255
926	KC_AB1	alpha-Est9	0.0921737304980925	0.819717378355858	1	pseudobulk	DESeq2	LRT	0.086335857588688
927	KC_AB1	alpha-Man-Ia	-0.200597350038868	0.52192160033335	1	pseudobulk	DESeq2	LRT	0.282394728989608
927	KC_AB1	alpha-Man-Ib	-0.0487817225588033	0.916627107486732	1	pseudobulk	DESeq2	LRT	0.0378073034758845
927	KC_AB1	alpha-Man-IIa	0.228890306042004	0.778461746055804	1	pseudobulk	DESeq2	LRT	0.108762723988688
927	KC_AB1	alpha-Man-IIb	0.173210024383695	0.775407392952842	1	pseudobulk	DESeq2	LRT	0.110470062622956
927	KC_AB1	alpha-PheRS	0.0126159811338817	0.987546113125417	1	pseudobulk	DESeq2	LRT	0.00544261599685891
927	KC_AB1	alpha-Spec	-0.16999588959593	0.476790236780396	1	pseudobulk	DESeq2	LRT	0.321672646212056
927	KC_AB1	alpha4GT1	0.265862516674041	0.606063643579953	1	pseudobulk	DESeq2	LRT	0.217481767575841
927	KC_AB1	alpha4GT2	1.67020383217772	0.598754457572587	1	pseudobulk	DESeq2	LRT	0.222751240354882
927	KC_AB1	alphaCOP	0.153387007970604	0.825995809425619	1	pseudobulk	DESeq2	LRT	0.083022156006429
927	KC_AB1	alphaKap4	1.11388934932283	1	1	pseudobulk	DESeq2	LRT	0
928	KC_AB1	alphaSnap	-0.00697615004040755	0.962158822318393	1	pseudobulk	DESeq2	LRT	0.0167532336136357
928	KC_AB1	alphaTub67C	0.472135228513213	0.737510945185932	1	pseudobulk	DESeq2	LRT	0.132231530063588
928	KC_AB1	alphaTub84B	-0.09738799860993	0.549835696847334	1	pseudobulk	DESeq2	LRT	0.25976706798371
928	KC_AB1	alphaTub84D	-0.137134562809198	0.597309427732596	1	pseudobulk	DESeq2	LRT	0.223800630439718
928	KC_AB1	Alr	-0.0495650394756373	0.948652051019355	1	pseudobulk	DESeq2	LRT	0.0228930499874268
928	KC_AB1	alrm	-0.220843388034127	0.540238460556359	1	pseudobulk	DESeq2	LRT	0.267414500835688
928	KC_AB1	Als2	-0.577590041229445	0.512410100363005	1	pseudobulk	DESeq2	LRT	0.290382318254663
928	KC_AB1	alt	-0.0385743987541704	0.879085901135292	1	pseudobulk	DESeq2	LRT	0.0559686851466335
928	KC_AB1	Amacr	-0.227098346492693	0.698616994146073	1	pseudobulk	DESeq2	LRT	0.155760854177764
928	KC_AB1	amn	0.0725089541868366	0.931191553990277	1	pseudobulk	DESeq2	LRT	0.0309609717749983
929	KC_AB1	Amnionless	-0.737210620319495	0.602775548075391	1	pseudobulk	DESeq2	LRT	0.219844373396749
929	KC_AB1	amon	-0.0188860938242942	0.907844529171138	1	pseudobulk	DESeq2	LRT	0.0419885192148657
929	KC_AB1	AMPdeam	0.799560111150704	0.228638635249483	1	pseudobulk	DESeq2	LRT	0.640850380861483
929	KC_AB1	Amph	0.157689831125822	0.655567241509771	1	pseudobulk	DESeq2	LRT	0.183382756118914
929	KC_AB1	AMPKalpha	0.397530449237552	0.345554321070607	1	pseudobulk	DESeq2	LRT	0.46148367191687

929	KC_AB1	Amt	1.25804452752548	0.727552935095488	1	pseudobulk	DESeq2	LRT	0.138135402914536
929	KC_AB1	Amun	-0.0851756402357738	0.780893442494941	1	pseudobulk	DESeq2	LRT	0.107408224117215
929	KC_AB1	amx	-0.637597541610401	0.421286198836506	1	pseudobulk	DESeq2	LRT	0.375422767949641
929	KC_AB1	Amyrel	1.11388934932283	1	1	pseudobulk	DESeq2	LRT	0
929	KC_AB1	ana	-2.06414433794571	0.306333893243809	1	pseudobulk	DESeq2	LRT	0.513804949538749
930	KC_AB1	ana2	0.943805700130147	0.808046339172447	1	pseudobulk	DESeq2	LRT	0.0925637329508755
930	KC_AB1	ana3	2.4138856564567	0.409611385497059	1	pseudobulk	DESeq2	LRT	0.387627980279722
930	KC_AB1	Ance	3.27763741706176	0.159341631577409	1	pseudobulk	DESeq2	LRT	0.797670740192683
930	KC_AB1	Ance-5	4.31851526800926	0.128069104667069	1	pseudobulk	DESeq2	LRT	0.892555626624673
930	KC_AB1	anchor	0.017071548447306	0.931065846682826	1	pseudobulk	DESeq2	LRT	0.0310196038309364
930	KC_AB1	angel	0.16130915394907	0.85023471331661	1	pseudobulk	DESeq2	LRT	0.0704611676656601
930	KC_AB1	Ank	-0.01400294922307	0.946460433256686	1	pseudobulk	DESeq2	LRT	0.0238975369854072
930	KC_AB1	Ank2	-0.333308765421564	0.0241814771045326	1	pseudobulk	DESeq2	LRT	1.61651717416364
930	KC_AB1	Ankle2	-0.461498746476539	0.614065612123908	1	pseudobulk	DESeq2	LRT	0.211785222570593
930	KC_AB1	anne	0.0162001618060477	0.926245825582413	1	pseudobulk	DESeq2	LRT	0.0332737362926248
931	KC_AB1	Ant2	-1.13555266551754	0.522308841542567	1	pseudobulk	DESeq2	LRT	0.28207262244819
931	KC_AB1	antr	-0.760959112540794	0.804334723897681	1	pseudobulk	DESeq2	LRT	0.0945631819854715
931	KC_AB1	AnxB10	-0.32076332237265	0.669195142785923	1	pseudobulk	DESeq2	LRT	0.174447219934579
931	KC_AB1	AnxB11	0.116489544259549	0.77920035224661	1	pseudobulk	DESeq2	LRT	0.108350859801509
931	KC_AB1	AnxB9	1.66794340412386	0.0403879352777277	1	pseudobulk	DESeq2	LRT	1.39374834837519
931	KC_AB1	aop	0.930909357264679	0.180338854426236	1	pseudobulk	DESeq2	LRT	0.743910693438543
931	KC_AB1	aos	-0.570723996670538	0.824991814167186	1	pseudobulk	DESeq2	LRT	0.0835503606375391
931	KC_AB1	Aos1	-0.0737230555293915	0.891488058066309	1	pseudobulk	DESeq2	LRT	0.0498844700440298
931	KC_AB1	ap	-2.00963963748391	0.498268873088307	1	pseudobulk	DESeq2	LRT	0.302536242405947
931	KC_AB1	AP-1-2beta	-0.0764999380909439	0.826841867687441	1	pseudobulk	DESeq2	LRT	0.0825775406982736
932	KC_AB1	AP-1gamma	-0.019314236590761	0.939403382256325	1	pseudobulk	DESeq2	LRT	0.0271478805029785
932	KC_AB1	AP-1mu	-0.114566865517892	0.747435040453578	1	pseudobulk	DESeq2	LRT	0.126426545877721
932	KC_AB1	AP-1sigma	-0.0253119062215483	0.909940863242742	1	pseudobulk	DESeq2	LRT	0.0409868314172513
932	KC_AB1	AP-2alpha	-0.183067003957192	0.440333428467106	1	pseudobulk	DESeq2	LRT	0.356218343276063
932	KC_AB1	AP-2mu	0.0227643567901285	0.918402832843299	1	pseudobulk	DESeq2	LRT	0.0369667853486326
932	KC_AB1	AP-2sigma	-0.0452061565775551	0.835991273625177	1	pseudobulk	DESeq2	LRT	0.0777982558578882
932	KC_AB1	Apc	-0.221663738805498	0.518594006333866	1	pseudobulk	DESeq2	LRT	0.285172506909457
932	KC_AB1	APC10	0.738868524327376	0.216341774546252	1	pseudobulk	DESeq2	LRT	0.664859612307563
932	KC_AB1	APC4	0.717428582039926	0.615365846912163	1	pseudobulk	DESeq2	LRT	0.210866610963415
932	KC_AB1	ApepP	0.466848276876482	0.752264927181003	1	pseudobulk	DESeq2	LRT	0.123629185803873
933	KC_AB1	Apf	0.528177741430467	0.419851474933901	1	pseudobulk	DESeq2	LRT	0.376904316803441
933	KC_AB1	aph-1	0.289354901714321	0.57309505295007	1	pseudobulk	DESeq2	LRT	0.241773340426842
933	KC_AB1	aPKC	-0.177810545847877	0.484836205170986	1	pseudobulk	DESeq2	LRT	0.314404956665869
933	KC_AB1	Apl	1.07495868254215	0.265447218516774	1	pseudobulk	DESeq2	LRT	0.576021821044789
933	KC_AB1	Aplip1	-0.133047659528493	0.519236746264149	1	pseudobulk	DESeq2	LRT	0.284634580200291
933	KC_AB1	apolpp	-1.37955363234889	0.170561992655382	1	pseudobulk	DESeq2	LRT	0.76811773881334
933	KC_AB1	Apoltp	-0.553640768412315	0.518909930657158	1	pseudobulk	DESeq2	LRT	0.284908017899529
933	KC_AB1	app	3.3598694717628	0.0868045308369516	1	pseudobulk	DESeq2	LRT	1.06145760585885
933	KC_AB1	APP-BP1	0.542524363443524	0.650735371275138	1	pseudobulk	DESeq2	LRT	0.186595586178227
933	KC_AB1	Appl	-0.302280576259828	0.0799276211481211	1	pseudobulk	DESeq2	LRT	1.09730311256019
934	KC_AB1	Aprt	0.114805563222061	0.922354180659191	1	pseudobulk	DESeq2	LRT	0.0351022794136267
934	KC_AB1	Aps	0.155696104634745	0.556562682361404	1	pseudobulk	DESeq2	LRT	0.254485916557211
934	KC_AB1	apt	2.98306427813109	0.227187865000518	1	pseudobulk	DESeq2	LRT	0.643614869721849
934	KC_AB1	aqz	-0.0148652277319437	0.891085826704225	1	pseudobulk	DESeq2	LRT	0.0500804640114703
934	KC_AB1	aralar1	0.293725665047423	0.545917982740779	1	pseudobulk	DESeq2	LRT	0.262872599637143
934	KC_AB1	Arc1	3.15757718410008	0.0424201669661576	1	pseudobulk	DESeq2	LRT	1.37242762642912
934	KC_AB1	Arc2	0.368604002143308	0.901433387014827	1	pseudobulk	DESeq2	LRT	0.045066360700604
934	KC_AB1	Arc42	-0.719116901473915	0.574570952428012	1	pseudobulk	DESeq2	LRT	0.240656333650552
934	KC_AB1	Archease	0.247614096290572	0.715393002996649	1	pseudobulk	DESeq2	LRT	0.14545531184543
934	KC_AB1	Arf102F	0.0460477489228077	0.863808879444643	1	pseudobulk	DESeq2	LRT	0.0635823359750328
935	KC_AB1	Arf51F	0.214856749610403	0.5445505436292	1	pseudobulk	DESeq2	LRT	0.263961804061523

935	KC_AB1	Arf79F	0.0228102936174451	0.889198465160119	1	pseudobulk	DESeq2	LRT	0.0510012956053752
935	KC_AB1	ArfGAP1	0.741425202485737	0.19379480761291	1	pseudobulk	DESeq2	LRT	0.712657863277361
935	KC_AB1	ArfGAP3	-0.16513303496515	0.800126884272699	1	pseudobulk	DESeq2	LRT	0.0968411370456182
935	KC_AB1	Arfip	0.0113860897739546	0.972521256302375	1	pseudobulk	DESeq2	LRT	0.0121008975663261
935	KC_AB1	Arfrp1	-0.626657204390229	0.585728711988324	1	pseudobulk	DESeq2	LRT	0.232303486655894
935	KC_AB1	arg	-0.456309318863165	0.629960294315136	1	pseudobulk	DESeq2	LRT	0.200686822773814
935	KC_AB1	Argk	0.171157433042176	0.360742118057699	1	pseudobulk	DESeq2	LRT	0.442803149136599
935	KC_AB1	Argl	2.09987857572813	0.503115176013642	1	pseudobulk	DESeq2	LRT	0.29833258237694
935	KC_AB1	ArgRS	-0.0363675812969883	0.973579100134921	1	pseudobulk	DESeq2	LRT	0.0116287576965419
936	KC_AB1	ArgRS-m	-0.831738361393379	0.670126551277974	1	pseudobulk	DESeq2	LRT	0.173843174415868
936	KC_AB1	ari-1	-0.165275983672382	0.538964257740668	1	pseudobulk	DESeq2	LRT	0.26844003477744
936	KC_AB1	ari-2	-0.0516685664588453	0.897928645819525	1	pseudobulk	DESeq2	LRT	0.046758173308278
936	KC_AB1	Arl1	-0.00347085133685413	0.971264210775172	1	pseudobulk	DESeq2	LRT	0.0126626138887476
936	KC_AB1	Arl2	0.0545345589017893	0.929767283162011	1	pseudobulk	DESeq2	LRT	0.0316257399255613
936	KC_AB1	Arl4	0.00311329272443188	0.986926074581242	1	pseudobulk	DESeq2	LRT	0.00571537681752832
936	KC_AB1	Arl5	0.448442970620591	0.431818391791591	1	pseudobulk	DESeq2	LRT	0.364698864355889
936	KC_AB1	Arl6IP1	0.0219905995334842	0.917727551455967	1	pseudobulk	DESeq2	LRT	0.0372862299762918
936	KC_AB1	Arl8	0.12411716888055	0.672875451818005	1	pseudobulk	DESeq2	LRT	0.172065315558941
936	KC_AB1	arm	-0.109756196741099	0.627758366495424	1	pseudobulk	DESeq2	LRT	0.20220749048508
937	KC_AB1	armi	-0.240249417813258	0.85878040775026	1	pseudobulk	DESeq2	LRT	0.0661178721340302
937	KC_AB1	Arp1	0.154234878476804	0.633221509194968	1	pseudobulk	DESeq2	LRT	0.198444341494409
937	KC_AB1	Arp10	-1.07793519424401	0.194811229463358	1	pseudobulk	DESeq2	LRT	0.710386012788977
937	KC_AB1	Arp2	-0.368361443993083	0.373603101104673	1	pseudobulk	DESeq2	LRT	0.427589527551533
937	KC_AB1	Arp3	0.142491177854825	0.790390643668217	1	pseudobulk	DESeq2	LRT	0.102158209396282
937	KC_AB1	Arp5	0.0206231440970071	0.9099740102179	1	pseudobulk	DESeq2	LRT	0.0409710113933432
937	KC_AB1	Arp6	0.295928468547158	0.687015313601368	1	pseudobulk	DESeq2	LRT	0.163033582389575
937	KC_AB1	Arp8	-0.822118288651597	0.476243549613567	1	pseudobulk	DESeq2	LRT	0.322170893492083
937	KC_AB1	Arpc1	-0.0252010980297523	0.911838405286956	1	pseudobulk	DESeq2	LRT	0.0400821199066555
937	KC_AB1	Arpc2	0.431062026024805	0.43349747923575	1	pseudobulk	DESeq2	LRT	0.363013423579205
938	KC_AB1	Arpc3A	-0.0241006990264451	0.934994286822546	1	pseudobulk	DESeq2	LRT	0.0291910428269722
938	KC_AB1	Arpc3B	1.25804452752548	0.727552935095488	1	pseudobulk	DESeq2	LRT	0.138135402914536
938	KC_AB1	Arpc4	0.0542915963224809	0.877009741222242	1	pseudobulk	DESeq2	LRT	0.0569955827621561
938	KC_AB1	Arpc5	-0.0142206699134019	0.953414891786782	1	pseudobulk	DESeq2	LRT	0.0207180689284692
938	KC_AB1	arr	-0.0412459827974958	0.900320316198926	1	pseudobulk	DESeq2	LRT	0.0456029496627313
938	KC_AB1	Ars2	-0.113028547978073	0.733909514863709	1	pseudobulk	DESeq2	LRT	0.134357481797553
938	KC_AB1	Art1	0.0981067627217187	0.904565869528205	1	pseudobulk	DESeq2	LRT	0.0435598027886425
938	KC_AB1	Art2	0.296275065198765	1	1	pseudobulk	DESeq2	LRT	0
938	KC_AB1	Art3	0.227024542126378	0.890442681950368	1	pseudobulk	DESeq2	LRT	0.0503940309607974
938	KC_AB1	Art4	-1.63380232881218	0.187786226399952	1	pseudobulk	DESeq2	LRT	0.726336265200183
939	KC_AB1	Art7	0.710774764320166	0.757586131680434	1	pseudobulk	DESeq2	LRT	0.1205679840969
939	KC_AB1	Art8	0.70842991589991	0.874943022847655	1	pseudobulk	DESeq2	LRT	0.0580202277417387
939	KC_AB1	Arts	-0.26980074633335	0.853670134705622	1	pseudobulk	DESeq2	LRT	0.0687099119242642
939	KC_AB1	aru	1.57240873769761	0.656008201898012	1	pseudobulk	DESeq2	LRT	0.183090730720712
939	KC_AB1	Arv1	0.554335107868482	0.503502478551762	1	pseudobulk	DESeq2	LRT	0.297998387238061
939	KC_AB1	arx	-0.576303105040742	1	1	pseudobulk	DESeq2	LRT	0
939	KC_AB1	Asap	-0.0871747307767884	0.849708386366184	1	pseudobulk	DESeq2	LRT	0.0707300953698797
939	KC_AB1	Asator	-0.111910338033861	0.53013375234092	1	pseudobulk	DESeq2	LRT	0.27561454440823
939	KC_AB1	Asciz	0.275673852691292	0.769311440136032	1	pseudobulk	DESeq2	LRT	0.113897809287185
939	KC_AB1	asf1	0.379219202446681	0.524715098059527	1	pseudobulk	DESeq2	LRT	0.280076439309182
940	KC_AB1	ash1	-0.327573658698829	0.596055208584762	1	pseudobulk	DESeq2	LRT	0.224713512620036
940	KC_AB1	ash2	-0.233257946324948	0.856191935842256	1	pseudobulk	DESeq2	LRT	0.0674288669428475
940	KC_AB1	Ask1	-0.381095050555968	0.696384827910162	1	pseudobulk	DESeq2	LRT	0.15715069939657
940	KC_AB1	asl	-0.69028093239528	0.701664338880608	1	pseudobulk	DESeq2	LRT	0.153870595326575
940	KC_AB1	AsnRS	0.139559088847048	0.804800755729858	1	pseudobulk	DESeq2	LRT	0.0943116244735937
940	KC_AB1	AsnRS-m	-0.498389868786758	0.720495935187853	1	pseudobulk	DESeq2	LRT	0.14236846499637
940	KC_AB1	AsnS	-0.570723996670538	0.824991814167186	1	pseudobulk	DESeq2	LRT	0.0835503606375391

940	KC_AB1	Asph	-1.24690460300918	0.561814127849183	1	pseudobulk	DESeq2	LRT	0.250407343863891
940	KC_AB1	ASPP	2.09987857572813	0.503115176013642	1	pseudobulk	DESeq2	LRT	0.29833258237694
940	KC_AB1	AspRS	0.0836613082898708	0.921597872445958	1	pseudobulk	DESeq2	LRT	0.0354585365098897
941	KC_AB1	AspRS-m	-0.462520392257636	0.398828208528507	1	pseudobulk	DESeq2	LRT	0.399214132268551
941	KC_AB1	asrij	0.471293847788211	0.426327675790077	1	pseudobulk	DESeq2	LRT	0.370256473468812
941	KC_AB1	asRNA:ASTR	1.90071181822722	0.545896518579078	1	pseudobulk	DESeq2	LRT	0.262889675371117
941	KC_AB1	asRNA:CR17025	-0.760959112540794	0.804334723897681	1	pseudobulk	DESeq2	LRT	0.0945631819854715
941	KC_AB1	asRNA:CR30267	-0.760959112540794	1	1	pseudobulk	DESeq2	LRT	0
941	KC_AB1	asRNA:CR31144	1.11388934932283	1	1	pseudobulk	DESeq2	LRT	0
941	KC_AB1	asRNA:CR31429	1.11388934932283	1	1	pseudobulk	DESeq2	LRT	0
941	KC_AB1	asRNA:CR31912	1.11388934932283	1	1	pseudobulk	DESeq2	LRT	0
941	KC_AB1	asRNA:CR42254	0.656653620108979	0.506727707230325	1	pseudobulk	DESeq2	LRT	0.295225348386938
941	KC_AB1	asRNA:CR42547	-1.39725211774206	0.311554381589485	1	pseudobulk	DESeq2	LRT	0.506466136603877
942	KC_AB1	asRNA:CR42860	0.152115475746306	1	1	pseudobulk	DESeq2	LRT	0
942	KC_AB1	asRNA:CR43152	-1.61102155603974	0.57025980967112	1	pseudobulk	DESeq2	LRT	0.243927235206257
942	KC_AB1	asRNA:CR43259	-0.513175087458894	0.670759956665034	1	pseudobulk	DESeq2	LRT	0.17343287200289
942	KC_AB1	asRNA:CR43425	0.489755363651781	0.761990030473202	1	pseudobulk	DESeq2	LRT	0.118050710732315
942	KC_AB1	asRNA:CR43426	0.943022244937108	0.768808261856119	1	pseudobulk	DESeq2	LRT	0.114181958248924
942	KC_AB1	asRNA:CR43460	0.296275065198765	1	1	pseudobulk	DESeq2	LRT	0
942	KC_AB1	asRNA:CR43466	-0.760959112540794	1	1	pseudobulk	DESeq2	LRT	0
942	KC_AB1	asRNA:CR43629	-1.06209051173125	0.473515806314813	1	pseudobulk	DESeq2	LRT	0.324665519341324
942	KC_AB1	asRNA:CR43730	2.27122133462827	0.453795586678749	1	pseudobulk	DESeq2	LRT	0.343139732096712
942	KC_AB1	asRNA:CR43767	-0.0136402186529002	0.935946539207552	1	pseudobulk	DESeq2	LRT	0.0287489572343713
943	KC_AB1	asRNA:CR43786	0.152115475746305	1	1	pseudobulk	DESeq2	LRT	0
943	KC_AB1	asRNA:CR43809	1.11388934932283	1	1	pseudobulk	DESeq2	LRT	0
943	KC_AB1	asRNA:CR43833	1.25804452752548	0.573436424985144	1	pseudobulk	DESeq2	LRT	0.241514723898045
943	KC_AB1	asRNA:CR43842	0.427923703255051	0.828404471014199	1	pseudobulk	DESeq2	LRT	0.0817575658245109
943	KC_AB1	asRNA:CR43865	1.25804452752548	0.727552935095488	1	pseudobulk	DESeq2	LRT	0.138135402914536
943	KC_AB1	asRNA:CR43875	1.25804452752548	0.573436424985144	1	pseudobulk	DESeq2	LRT	0.241514723898045
943	KC_AB1	asRNA:CR43885	1.11388934932283	1	1	pseudobulk	DESeq2	LRT	0
943	KC_AB1	asRNA:CR43906	0.149421800989093	0.872376023187452	1	pseudobulk	DESeq2	LRT	0.0592962792928785
943	KC_AB1	asRNA:CR43933	0.209525692748117	0.829620020888538	1	pseudobulk	DESeq2	LRT	0.0811207758373159
943	KC_AB1	asRNA:CR43944	0.738275688199472	0.649939847706789	1	pseudobulk	DESeq2	LRT	0.187126835692321
944	KC_AB1	asRNA:CR43957	-0.468770266124086	0.782951884436652	1	pseudobulk	DESeq2	LRT	0.106264926276315
944	KC_AB1	asRNA:CR43959	1.66157861402315	0.376434202576341	1	pseudobulk	DESeq2	LRT	0.424310923726877
944	KC_AB1	asRNA:CR44018	0.495051573971122	0.492930953177013	1	pseudobulk	DESeq2	LRT	0.307213909838985
944	KC_AB1	asRNA:CR44027	0.709713058804466	0.916223368658127	1	pseudobulk	DESeq2	LRT	0.037998635557264
944	KC_AB1	asRNA:CR44029	0.0706237169050683	0.935921793605685	1	pseudobulk	DESeq2	LRT	0.0287604397496257
944	KC_AB1	asRNA:CR44030	-0.353449998949848	0.485121435304544	1	pseudobulk	DESeq2	LRT	0.314149535456781
944	KC_AB1	asRNA:CR44031	-0.78812043421859	0.560674342792397	1	pseudobulk	DESeq2	LRT	0.251289317342978
944	KC_AB1	asRNA:CR44046	-0.331817662839821	0.876863542488445	1	pseudobulk	DESeq2	LRT	0.0570679862864997
944	KC_AB1	asRNA:CR44065	1.25804452752548	0.727552935095488	1	pseudobulk	DESeq2	LRT	0.138135402914536
944	KC_AB1	asRNA:CR44067	1.25804452752548	0.727552935095488	1	pseudobulk	DESeq2	LRT	0.138135402914536
945	KC_AB1	asRNA:CR44069	-0.760959112540794	0.805265236978025	1	pseudobulk	DESeq2	LRT	0.0940610488455931
945	KC_AB1	asRNA:CR44071	1.25804452752548	0.727552935095488	1	pseudobulk	DESeq2	LRT	0.138135402914536
945	KC_AB1	asRNA:CR44093	1.06432145904834	0.547425335337415	1	pseudobulk	DESeq2	LRT	0.261675106921112
945	KC_AB1	asRNA:CR44095	1.25804452752548	0.727552935095488	1	pseudobulk	DESeq2	LRT	0.138135402914536
945	KC_AB1	asRNA:CR44106	0.709686956006578	0.915714550115026	1	pseudobulk	DESeq2	LRT	0.0382398850985033
945	KC_AB1	asRNA:CR44107	-0.2083746467376	0.78068525916412	1	pseudobulk	DESeq2	LRT	0.107524020871153
945	KC_AB1	asRNA:CR44114	1.25804452752548	0.727552935095488	1	pseudobulk	DESeq2	LRT	0.138135402914536
945	KC_AB1	asRNA:CR44116	0.152115475746305	1	1	pseudobulk	DESeq2	LRT	0
945	KC_AB1	asRNA:CR44119	0.152115475746306	1	1	pseudobulk	DESeq2	LRT	0
945	KC_AB1	asRNA:CR44124	-0.760959112540794	0.804334723897681	1	pseudobulk	DESeq2	LRT	0.0945631819854715
946	KC_AB1	asRNA:CR44137	0.23315489977832	0.900752424241	1	pseudobulk	DESeq2	LRT	0.0453945603649028
946	KC_AB1	asRNA:CR44179	-0.575069369497329	1	1	pseudobulk	DESeq2	LRT	0
946	KC_AB1	asRNA:CR44228	2.73062989528267	0.270641314998426	1	pseudobulk	DESeq2	LRT	0.567605905054642

946	KC_AB1	asRNA:CR44340	0.155633894665599	0.979438786698484	1	pseudobulk	DESeq2	LRT	0.0090227015067096
946	KC_AB1	asRNA:CR44351	1.25804452752548	0.573436424985144	1	pseudobulk	DESeq2	LRT	0.241514723898045
946	KC_AB1	asRNA:CR44368	-0.760959112540794	1	1	pseudobulk	DESeq2	LRT	0
946	KC_AB1	asRNA:CR44429	1.67020383217772	0.598754457572587	1	pseudobulk	DESeq2	LRT	0.222751240354882
946	KC_AB1	asRNA:CR44512	1.25804452752548	0.727552935095488	1	pseudobulk	DESeq2	LRT	0.138135402914536
946	KC_AB1	asRNA:CR44599	-0.760959112540794	1	1	pseudobulk	DESeq2	LRT	0
946	KC_AB1	asRNA:CR44690	0.296275065198765	1	1	pseudobulk	DESeq2	LRT	0
947	KC_AB1	asRNA:CR44806	0.418029487522826	0.855473542493551	1	pseudobulk	DESeq2	LRT	0.0677934174808431
947	KC_AB1	asRNA:CR44840	-0.190875068635993	0.901399347552063	1	pseudobulk	DESeq2	LRT	0.0450827606145437
947	KC_AB1	asRNA:CR44841	-1.43550613534661	0.602448798560473	1	pseudobulk	DESeq2	LRT	0.220079857379737
947	KC_AB1	asRNA:CR44843	-0.275986048128865	0.89150058311238	1	pseudobulk	DESeq2	LRT	0.0498783684253554
947	KC_AB1	asRNA:CR44892	-0.104488679882155	0.93395511494691	1	pseudobulk	DESeq2	LRT	0.0296739950753514
947	KC_AB1	asRNA:CR44894	-0.557774656731068	0.51603637461587	1	pseudobulk	DESeq2	LRT	0.287319684539112
947	KC_AB1	asRNA:CR44907	-1.77067014871429	0.542156052888816	1	pseudobulk	DESeq2	LRT	0.265875689192671
947	KC_AB1	asRNA:CR44911	1.25804452752548	0.727552935095488	1	pseudobulk	DESeq2	LRT	0.138135402914536
947	KC_AB1	asRNA:CR44960	2.27122133462827	0.453795586678749	1	pseudobulk	DESeq2	LRT	0.343139732096712
947	KC_AB1	asRNA:CR44961	-0.760959112540794	0.805265236978025	1	pseudobulk	DESeq2	LRT	0.0940610488455931
948	KC_AB1	asRNA:CR44981	-2.69171538695884	0.2586956059267	1	pseudobulk	DESeq2	LRT	0.587210947928196
948	KC_AB1	asRNA:CR44987	-1.44267557892052	0.345124311355298	1	pseudobulk	DESeq2	LRT	0.462024446922452
948	KC_AB1	asRNA:CR44989	1.39934341023393	0.63869615168108	1	pseudobulk	DESeq2	LRT	0.194705700577112
948	KC_AB1	asRNA:CR44995	-0.760959112540794	0.805265236978025	1	pseudobulk	DESeq2	LRT	0.0940610488455931
948	KC_AB1	asRNA:CR44998	-1.8475170584281	0.444320346123096	1	pseudobulk	DESeq2	LRT	0.352303799280682
948	KC_AB1	asRNA:CR45000	-0.884107465927591	0.759545559416804	1	pseudobulk	DESeq2	LRT	0.11944617096526
948	KC_AB1	asRNA:CR45007	-0.760959112540794	0.805265236978025	1	pseudobulk	DESeq2	LRT	0.0940610488455931
948	KC_AB1	asRNA:CR45009	-0.732263793092199	0.659022837150594	1	pseudobulk	DESeq2	LRT	0.181099535517314
948	KC_AB1	asRNA:CR45016	-2.24978065768543	0.364534962203659	1	pseudobulk	DESeq2	LRT	0.438260812575185
948	KC_AB1	asRNA:CR45041	-1.61102155603974	0.57025980967112	1	pseudobulk	DESeq2	LRT	0.243927235206257
949	KC_AB1	asRNA:CR45044	-0.760959112540794	1	1	pseudobulk	DESeq2	LRT	0
949	KC_AB1	asRNA:CR45104	0.293544814793053	0.855484935116898	1	pseudobulk	DESeq2	LRT	0.0677876338764847
949	KC_AB1	asRNA:CR45124	1.90071181822722	0.545896518579078	1	pseudobulk	DESeq2	LRT	0.262889675371117
949	KC_AB1	asRNA:CR45126	-0.408644529962761	0.764555977372665	1	pseudobulk	DESeq2	LRT	0.116590711986025
949	KC_AB1	asRNA:CR45129	0.601081948650873	0.631780284171064	1	pseudobulk	DESeq2	LRT	0.199433931152167
949	KC_AB1	asRNA:CR45131	-1.64360588109794	0.294936945628774	1	pseudobulk	DESeq2	LRT	0.530270821623518
949	KC_AB1	asRNA:CR45132	1.11388934932283	1	1	pseudobulk	DESeq2	LRT	0
949	KC_AB1	asRNA:CR45134	-0.90916630048409	0.455683720215623	1	pseudobulk	DESeq2	LRT	0.341336486771546
949	KC_AB1	asRNA:CR45135	-1.61102155603974	0.57025980967112	1	pseudobulk	DESeq2	LRT	0.243927235206257
949	KC_AB1	asRNA:CR45139	-0.760959112540794	1	1	pseudobulk	DESeq2	LRT	0
950	KC_AB1	asRNA:CR45145	-0.830147482649859	0.68141410227595	1	pseudobulk	DESeq2	LRT	0.166588882688818
950	KC_AB1	asRNA:CR45151	-0.760959112540794	0.805265236978025	1	pseudobulk	DESeq2	LRT	0.0940610488455931
950	KC_AB1	asRNA:CR45161	0.100131380823274	0.926758135890657	1	pseudobulk	DESeq2	LRT	0.0330335926661805
950	KC_AB1	asRNA:CR45162	-0.676570628329761	0.796496269754596	1	pseudobulk	DESeq2	LRT	0.0988162537972989
950	KC_AB1	asRNA:CR45164	0.558410721770849	0.6908895313452	1	pseudobulk	DESeq2	LRT	0.160591387882697
950	KC_AB1	asRNA:CR45182	-0.739275375046187	0.658607044318237	1	pseudobulk	DESeq2	LRT	0.181373628479595
950	KC_AB1	asRNA:CR45192	1.25804452752548	0.727552935095488	1	pseudobulk	DESeq2	LRT	0.138135402914536
950	KC_AB1	asRNA:CR45198	0.770280458153283	0.637938370812944	1	pseudobulk	DESeq2	LRT	0.195221275054185
950	KC_AB1	asRNA:CR45208	1.11388934932283	1	1	pseudobulk	DESeq2	LRT	0
950	KC_AB1	asRNA:CR45214	1.13658746815362	0.691887308070876	1	pseudobulk	DESeq2	LRT	0.159964635988989
951	KC_AB1	asRNA:CR45281	-0.22473168090823	0.887334128738235	1	pseudobulk	DESeq2	LRT	0.0519128142799486
951	KC_AB1	asRNA:CR45467	-0.760959112540794	1	1	pseudobulk	DESeq2	LRT	0
951	KC_AB1	asRNA:CR45469	-0.81795170937462	0.613123217509235	1	pseudobulk	DESeq2	LRT	0.21245223786848
951	KC_AB1	asRNA:CR45479	1.25804452752548	0.727552935095488	1	pseudobulk	DESeq2	LRT	0.138135402914536
951	KC_AB1	asRNA:CR45501	-0.760959112540794	0.804334723897681	1	pseudobulk	DESeq2	LRT	0.0945631819854715
951	KC_AB1	asRNA:CR45520	0.498362126058204	0.839254472147245	1	pseudobulk	DESeq2	LRT	0.0761063358425998
951	KC_AB1	asRNA:CR45528	0.124481543023846	0.91941190661028	1	pseudobulk	DESeq2	LRT	0.0364898763289548
951	KC_AB1	asRNA:CR45535	-0.760959112540794	0.805265236978025	1	pseudobulk	DESeq2	LRT	0.0940610488455931
951	KC_AB1	asRNA:CR45600	-0.627997274260802	0.869037682892539	1	pseudobulk	DESeq2	LRT	0.0609613914253611

951	KC_AB1	asRNA:CR45620	1.18583548841699	0.284381302016234	1	pseudobulk	DESeq2	LRT	0.546098961733144
952	KC_AB1	asRNA:CR45714	1.25804452752548	0.573436424985144	1	pseudobulk	DESeq2	LRT	0.241514723898045
952	KC_AB1	asRNA:CR45789	1.25804452752548	0.727552935095488	1	pseudobulk	DESeq2	LRT	0.138135402914536
952	KC_AB1	asRNA:CR45798	1.25804452752548	0.727552935095488	1	pseudobulk	DESeq2	LRT	0.138135402914536
952	KC_AB1	asRNA:CR45804	1.11388934932283	1	1	pseudobulk	DESeq2	LRT	0
952	KC_AB1	asRNA:CR45807	-1.61102155603974	0.57025980967112	1	pseudobulk	DESeq2	LRT	0.243927235206257
952	KC_AB1	asRNA:CR45815	-1.15624160463964	0.66675496312278	1	pseudobulk	DESeq2	LRT	0.176033742868961
952	KC_AB1	asRNA:CR45822	0.0477408310492424	0.94735643147763	1	pseudobulk	DESeq2	LRT	0.0234865921686919
952	KC_AB1	asRNA:CR45835	-0.352686279529387	0.760621245388427	1	pseudobulk	DESeq2	LRT	0.118831548203822
952	KC_AB1	asRNA:CR45865	-0.760959112540794	1	1	pseudobulk	DESeq2	LRT	0
952	KC_AB1	asRNA:CR45873	3.22729981873597	0.166473509642973	1	pseudobulk	DESeq2	LRT	0.778654864447011
953	KC_AB1	asRNA:CR45874	0.650467818135431	0.4653663367885	1	pseudobulk	DESeq2	LRT	0.332205035479368
953	KC_AB1	asRNA:CR45875	-0.760959112540794	0.805265236978025	1	pseudobulk	DESeq2	LRT	0.0940610488455931
953	KC_AB1	asRNA:CR45882	1.25804452752548	0.727552935095488	1	pseudobulk	DESeq2	LRT	0.138135402914536
953	KC_AB1	asRNA:CR45889	1.90071181822722	0.545896518579078	1	pseudobulk	DESeq2	LRT	0.262889675371117
953	KC_AB1	asRNA:CR45891	0.296275065198765	1	1	pseudobulk	DESeq2	LRT	0
953	KC_AB1	asRNA:CR45894	1.25804452752548	0.573436424985144	1	pseudobulk	DESeq2	LRT	0.241514723898045
953	KC_AB1	asRNA:CR45907	-0.113670996893063	0.916970535977506	1	pseudobulk	DESeq2	LRT	0.0376446188206688
953	KC_AB1	asRNA:CR45912	-0.760959112540794	1	1	pseudobulk	DESeq2	LRT	0
953	KC_AB1	asRNA:CR45925	3.41769529955874	0.139189414962003	1	pseudobulk	DESeq2	LRT	0.856393790575925
953	KC_AB1	asRNA:CR45928	0.152115475746305	1	1	pseudobulk	DESeq2	LRT	0
954	KC_AB1	asRNA:CR45943	-0.760959112540794	0.804334723897681	1	pseudobulk	DESeq2	LRT	0.0945631819854715
954	KC_AB1	asRNA:CR45951	0.295216749156716	0.845806418600799	1	pseudobulk	DESeq2	LRT	0.0727290234308643
954	KC_AB1	asRNA:CR45960	-0.760959112540794	0.804334723897681	1	pseudobulk	DESeq2	LRT	0.0945631819854715
954	KC_AB1	asRNA:CR46029	0.296275065198765	1	1	pseudobulk	DESeq2	LRT	0
954	KC_AB1	asRNA:CR46033	-0.760959112540794	1	1	pseudobulk	DESeq2	LRT	0
954	KC_AB1	asRNA:CR46055	-0.760959112540794	0.804334723897681	1	pseudobulk	DESeq2	LRT	0.0945631819854715
954	KC_AB1	asRNA:CR46067	0.205714513942468	0.928254565221226	1	pseudobulk	DESeq2	LRT	0.0323329062057501
954	KC_AB1	asRNA:CR46068	1.11388934932283	1	1	pseudobulk	DESeq2	LRT	0
954	KC_AB1	asRNA:CR46092	1.25804452752548	0.727552935095488	1	pseudobulk	DESeq2	LRT	0.138135402914536
954	KC_AB1	asRNA:CR46104	0.743223296152397	0.756560927764596	1	pseudobulk	DESeq2	LRT	0.121156091399604
955	KC_AB1	asRNA:CR46105	1.11388934932283	1	1	pseudobulk	DESeq2	LRT	0
955	KC_AB1	asRNA:CR46135	-0.135383796145683	0.920030501246283	1	pseudobulk	DESeq2	LRT	0.0361977744986053
955	KC_AB1	asRNA:CR46246	-1.61102155603974	0.57025980967112	1	pseudobulk	DESeq2	LRT	0.243927235206257
955	KC_AB1	asRNA:CR46262	0.152115475746305	1	1	pseudobulk	DESeq2	LRT	0
955	KC_AB1	asRNA:CR46263	-2.24978065768543	0.364534962203659	1	pseudobulk	DESeq2	LRT	0.438260812575185
955	KC_AB1	asRNA:CR46354	-0.0640932783892343	0.920707956625074	1	pseudobulk	DESeq2	LRT	0.0358781037221619
955	KC_AB1	ast	0.0795114741086723	0.946175003802741	1	pseudobulk	DESeq2	LRT	0.0240285293945457
955	KC_AB1	AstA-R1	-1.77199356422654	0.54193209410651	1	pseudobulk	DESeq2	LRT	0.266055128595237
955	KC_AB1	AstA-R2	2.09987857572813	0.503115176013642	1	pseudobulk	DESeq2	LRT	0.29833258237694
955	KC_AB1	AstC	1.25804452752548	0.727552935095488	1	pseudobulk	DESeq2	LRT	0.138135402914536
956	KC_AB1	AstC-R2	-1.77199356422654	0.54193209410651	1	pseudobulk	DESeq2	LRT	0.266055128595237
956	KC_AB1	asun	-1.61102155603974	0.57025980967112	1	pseudobulk	DESeq2	LRT	0.243927235206257
956	KC_AB1	Asx	-0.31275089035552	0.495508213314165	1	pseudobulk	DESeq2	LRT	0.30494914245528
956	KC_AB1	Atac1	0.708565633201413	0.559079650309287	1	pseudobulk	DESeq2	LRT	0.252526315144696
956	KC_AB1	Atac2	-1.13433975450635	0.373302317383353	1	pseudobulk	DESeq2	LRT	0.427939314055268
956	KC_AB1	Atac3	0.340573467623691	0.613499968777596	1	pseudobulk	DESeq2	LRT	0.212185455039207
956	KC_AB1	ATbp	-0.194673849679119	0.841561807383859	1	pseudobulk	DESeq2	LRT	0.0749139823381669
956	KC_AB1	Ate1	-0.26926664604251	0.588086851405514	1	pseudobulk	DESeq2	LRT	0.230558530555225
956	KC_AB1	Atet	-0.00498041493674247	0.976260588405278	1	pseudobulk	DESeq2	LRT	0.0104342427832249
956	KC_AB1	Atf-2	0.165817113704092	0.842238918968199	1	pseudobulk	DESeq2	LRT	0.0745646941641439
957	KC_AB1	Atf3	-0.388276176950892	0.775573837792042	1	pseudobulk	DESeq2	LRT	0.110376849276398
957	KC_AB1	Atf6	-0.0880534274861788	0.706465610637161	1	pseudobulk	DESeq2	LRT	0.150908973906034
957	KC_AB1	Atg1	0.0484117947108374	0.83976963803593	1	pseudobulk	DESeq2	LRT	0.075839831379222
957	KC_AB1	Atg10	-0.7138557713115	0.813479660285309	1	pseudobulk	DESeq2	LRT	0.0896533014072523
957	KC_AB1	Atg101	-0.0521613476580301	0.9243337917862	1	pseudobulk	DESeq2	LRT	0.0341711697395281

957	KC_AB1	Atg12	0.0823484458104194	0.813855851842444	1	pseudobulk	DESeq2	LRT	0.089452509475785
957	KC_AB1	Atg13	0.0371374948126186	0.941605725397776	1	pseudobulk	DESeq2	LRT	0.0261309094455255
957	KC_AB1	Atg14	0.0135988702813704	0.954287909161714	1	pseudobulk	DESeq2	LRT	0.0203205786539697
957	KC_AB1	Atg16	-0.389372434352379	0.641601381259216	1	pseudobulk	DESeq2	LRT	0.192734709760349
957	KC_AB1	Atg17	0.203123259662484	0.487237067416476	1	pseudobulk	DESeq2	LRT	0.312259679402131
958	KC_AB1	Atg18a	0.715031889488773	0.190881289115333	1	pseudobulk	DESeq2	LRT	0.719236640659352
958	KC_AB1	Atg18b	0.0187672473601127	0.940803929672519	1	pseudobulk	DESeq2	LRT	0.0265008772463314
958	KC_AB1	Atg2	-0.660051803555408	0.495372841719108	1	pseudobulk	DESeq2	LRT	0.305067806823333
958	KC_AB1	Atg3	0.287012481346224	0.558992712372906	1	pseudobulk	DESeq2	LRT	0.25259385400421
958	KC_AB1	Atg4a	0.619855808611894	0.32016977015583	1	pseudobulk	DESeq2	LRT	0.494619675771759
958	KC_AB1	Atg4b	-0.0835582316429647	0.917349793563808	1	pseudobulk	DESeq2	LRT	0.0374650324347519
958	KC_AB1	Atg5	0.390965541430573	0.525098839653265	1	pseudobulk	DESeq2	LRT	0.279758941402032
958	KC_AB1	Atg6	-0.845774272695658	0.166762279420637	1	pseudobulk	DESeq2	LRT	0.77790217727182
958	KC_AB1	Atg7	0.415838784236863	0.701114750969401	1	pseudobulk	DESeq2	LRT	0.15421089553649
958	KC_AB1	Atg8a	0.00843619902364581	0.943383404686741	1	pseudobulk	DESeq2	LRT	0.0253117678151058
959	KC_AB1	Atg9	0.48698895538185	0.598681057415111	1	pseudobulk	DESeq2	LRT	0.222804482943737
959	KC_AB1	atilla	-3.46937979852651	0.174958762313963	1	pseudobulk	DESeq2	LRT	0.757064302227605
959	KC_AB1	atl	0.147981060001559	0.798931043835069	1	pseudobulk	DESeq2	LRT	0.0974907032570583
959	KC_AB1	atms	-0.141378636675141	0.837977189925176	1	pseudobulk	DESeq2	LRT	0.0767678028786175
959	KC_AB1	Atox1	0.0361189193967198	0.873506839568863	1	pseudobulk	DESeq2	LRT	0.0587336901368182
959	KC_AB1	ATP6AP2	-0.0116934119957685	0.910993035839066	1	pseudobulk	DESeq2	LRT	0.0404849430140765
959	KC_AB1	ATP7	0.390533552941413	0.721729713095934	1	pseudobulk	DESeq2	LRT	0.141625414741063
959	KC_AB1	ATP8A	-0.0773662699680455	0.796246653634991	1	pseudobulk	DESeq2	LRT	0.0989523798508307
959	KC_AB1	Atpalpha	-0.205253961239498	0.27547522908215	1	pseudobulk	DESeq2	LRT	0.559917447108373
959	KC_AB1	ATPCL	-0.116029623842138	0.668880373473521	1	pseudobulk	DESeq2	LRT	0.174651547083994
960	KC_AB1	ATPsynB	-0.0638949695297167	0.707188834706367	1	pseudobulk	DESeq2	LRT	0.150464604702693
960	KC_AB1	ATPsynbeta	0.0478962051265879	0.775755724260046	1	pseudobulk	DESeq2	LRT	0.110275011096192
960	KC_AB1	ATPsynC	0.0193390590656116	0.869699610001202	1	pseudobulk	DESeq2	LRT	0.0606307246756725
960	KC_AB1	ATPsynCF6	0.0553024709300691	0.750434221913137	1	pseudobulk	DESeq2	LRT	0.124687369126397
960	KC_AB1	ATPsynCF6L	-2.24978065768543	0.364534962203659	1	pseudobulk	DESeq2	LRT	0.438260812575185
960	KC_AB1	ATPsynD	-0.105623359961619	0.594005728466318	1	pseudobulk	DESeq2	LRT	0.226209366753966
960	KC_AB1	ATPsyndelta	-0.00264469714027784	0.960171329567103	1	pseudobulk	DESeq2	LRT	0.0176512660784725
960	KC_AB1	ATPsynE	-0.0528328614673306	0.757446525270342	1	pseudobulk	DESeq2	LRT	0.120648022363317
960	KC_AB1	ATPsynepsilonL	1.25804452752548	0.573436424985144	1	pseudobulk	DESeq2	LRT	0.241514723898045
960	KC_AB1	ATPsynF	0.0347490109767446	0.806902495135008	1	pseudobulk	DESeq2	LRT	0.0931789415892356
961	KC_AB1	ATPsynG	-0.173312362148863	0.319731804474156	1	pseudobulk	DESeq2	LRT	0.495214161286581
961	KC_AB1	ATPsyngamma	-0.0658685145646014	0.733222264992039	1	pseudobulk	DESeq2	LRT	0.134764355744235
961	KC_AB1	ATPsynO	0.0206594803198822	0.918005923628733	1	pseudobulk	DESeq2	LRT	0.0371545164120783
961	KC_AB1	AttA	-2.51931741568571	0.36419964883203	1	pseudobulk	DESeq2	LRT	0.438660477295588
961	KC_AB1	Atu	0.0994986144563871	0.92500465809888	1	pseudobulk	DESeq2	LRT	0.0338560802538905
961	KC_AB1	Atx2	-0.282486035116568	0.24255664353243	1	pseudobulk	DESeq2	LRT	0.615186825717524
961	KC_AB1	Atxn7	0.314461816581923	0.504949434246744	1	pseudobulk	DESeq2	LRT	0.296752110053887
961	KC_AB1	aux	-0.343234917411036	0.478920060744811	1	pseudobulk	DESeq2	LRT	0.319736971083714
961	KC_AB1	ave	0.0449364163495398	0.928332263998384	1	pseudobulk	DESeq2	LRT	0.0322965554683011
961	KC_AB1	Aven	-0.154628011334995	0.88635721675453	1	pseudobulk	DESeq2	LRT	0.0523912149078911
962	KC_AB1	awd	-0.0611312583528499	0.762786097595294	1	pseudobulk	DESeq2	LRT	0.117597230928431
962	KC_AB1	axed	-0.441858677235222	0.258897863719562	1	pseudobulk	DESeq2	LRT	0.586871533105621
962	KC_AB1	Axn	0.0342429156166257	0.951103186558647	1	pseudobulk	DESeq2	LRT	0.0217723632707815
962	KC_AB1	axo	-0.390379327575227	0.516390321773491	1	pseudobulk	DESeq2	LRT	0.287021905909404
962	KC_AB1	Axud1	0.14571606534243	0.769421232669428	1	pseudobulk	DESeq2	LRT	0.113835833230744
962	KC_AB1	az2	0.269149561601759	0.781291561637847	1	pseudobulk	DESeq2	LRT	0.107186866275231
962	KC_AB1	azot	1.25804452752548	0.573436424985144	1	pseudobulk	DESeq2	LRT	0.241514723898045
962	KC_AB1	B4	-0.160197325441966	0.508189674178133	1	pseudobulk	DESeq2	LRT	0.293974163553688
962	KC_AB1	B52	0.00748767347549808	0.96673033508776	1	pseudobulk	DESeq2	LRT	0.014694653441331
962	KC_AB1	b6	1.11388934932283	1	1	pseudobulk	DESeq2	LRT	0
963	KC_AB1	bab2	-0.760959112540794	0.805265236978025	1	pseudobulk	DESeq2	LRT	0.0940610488455931

963	KC_AB1	babo	-0.0460954928163339	0.887988477928967	1	pseudobulk	DESeq2	LRT	0.0515926693614146
963	KC_AB1	babos	-0.406341748776776	0.911427038729199	1	pseudobulk	DESeq2	LRT	0.0402780916228869
963	KC_AB1	Bacc	-0.0535581731067337	0.60487065987538	1	pseudobulk	DESeq2	LRT	0.218337481062589
963	KC_AB1	baf	0.215388780457573	0.813055035801266	1	pseudobulk	DESeq2	LRT	0.0898800559608854
963	KC_AB1	bai	0.150014676284851	0.701686494950061	1	pseudobulk	DESeq2	LRT	0.153856882064755
963	KC_AB1	Balat	-2.89873240646708	0.213780650480074	1	pseudobulk	DESeq2	LRT	0.670031605815827
963	KC_AB1	Baldspot	0.063807041660133	0.792894071072034	1	pseudobulk	DESeq2	LRT	0.100784829607734
963	KC_AB1	ball	0.0906978998908236	0.931623014025735	1	pseudobulk	DESeq2	LRT	0.0307597915618634
963	KC_AB1	Bap111	-0.697395265161334	0.36002000714221	1	pseudobulk	DESeq2	LRT	0.443673363815984
964	KC_AB1	Bap170	-0.183304733434017	0.886577452015809	1	pseudobulk	DESeq2	LRT	0.0522833181411679
964	KC_AB1	Bap55	-0.0965488883825223	0.850368893376835	1	pseudobulk	DESeq2	LRT	0.0703926347522535
964	KC_AB1	Bap60	-0.284934193106141	0.501360964364032	1	pseudobulk	DESeq2	LRT	0.299849482945123
964	KC_AB1	barc	0.176224298726491	0.617194403747381	1	pseudobulk	DESeq2	LRT	0.209578020445329
964	KC_AB1	bark	2.58743201691345	0.417636627932844	1	pseudobulk	DESeq2	LRT	0.379201419460921
964	KC_AB1	barr	0.296275065198765	1	1	pseudobulk	DESeq2	LRT	0
964	KC_AB1	baz	4.10969727974049	0.0485790756676427	1	pseudobulk	DESeq2	LRT	1.31355075293684
964	KC_AB1	bbc	0.0571134167407379	0.848138197096808	1	pseudobulk	DESeq2	LRT	0.071533377287442
964	KC_AB1	bbg	1.63195036548663	0.633334503478132	1	pseudobulk	DESeq2	LRT	0.198366851366371
964	KC_AB1	BBS5	-0.760959112540794	0.804334723897681	1	pseudobulk	DESeq2	LRT	0.0945631819854715
965	KC_AB1	bbx	-1.76291464024628	0.328726760540759	1	pseudobulk	DESeq2	LRT	0.483164940025102
965	KC_AB1	bc10	0.156463935190915	0.740256749849432	1	pseudobulk	DESeq2	LRT	0.130617623913
965	KC_AB1	BCAS2	-0.327917215233004	0.648908055990375	1	pseudobulk	DESeq2	LRT	0.187816834177741
965	KC_AB1	bchs	-0.0889431806054475	0.769132677118541	1	pseudobulk	DESeq2	LRT	0.113998736960207
965	KC_AB1	BCL7-like	0.380803124986182	0.667372169843052	1	pseudobulk	DESeq2	LRT	0.175631907863425
965	KC_AB1	bcn92	-0.0916701358296921	0.812981418027022	1	pseudobulk	DESeq2	LRT	0.089919380778076
965	KC_AB1	Bdbt	-0.398302885783464	0.478747947105924	1	pseudobulk	DESeq2	LRT	0.319893075294821
965	KC_AB1	bdg	0.947105872752402	0.296728575398845	1	pseudobulk	DESeq2	LRT	0.527640628470956
965	KC_AB1	bdl	0.450615988127258	0.426800480797732	1	pseudobulk	DESeq2	LRT	0.369775100007445
965	KC_AB1	Bdp1	-0.0318607596223879	0.941836993035024	1	pseudobulk	DESeq2	LRT	0.0260242555417763
966	KC_AB1	be	3.16938821910393	0.134030613785328	1	pseudobulk	DESeq2	LRT	0.872795993579006
966	KC_AB1	BEAF-32	5.29283192513551E-05	0.917366149281484	1	pseudobulk	DESeq2	LRT	0.0374572893310174
966	KC_AB1	beag	0.576193557088465	0.233711011377657	1	pseudobulk	DESeq2	LRT	0.63132082518631
966	KC_AB1	beat-Ia	-0.438584120588001	0.828770538443449	1	pseudobulk	DESeq2	LRT	0.0815656958455328
966	KC_AB1	beat-Ib	0.54108872683334	0.659924996080123	1	pseudobulk	DESeq2	LRT	0.180505421487744
966	KC_AB1	beat-Ic	2.97629800173664	0.151394995253965	1	pseudobulk	DESeq2	LRT	0.81988848130564
966	KC_AB1	beat-IIa	-0.760959112540794	1	1	pseudobulk	DESeq2	LRT	0
966	KC_AB1	beat-IIIb	0.0400962965897458	0.860069604926192	1	pseudobulk	DESeq2	LRT	0.0654664001376923
966	KC_AB1	beat-IIIc	-0.462749674967007	0.842212253597878	1	pseudobulk	DESeq2	LRT	0.0745784441895268
966	KC_AB1	beat-IV	-0.468839607248572	0.300019897394466	1	pseudobulk	DESeq2	LRT	0.52284994180678
967	KC_AB1	beat-Va	-0.760959112540794	0.804334723897681	1	pseudobulk	DESeq2	LRT	0.0945631819854715
967	KC_AB1	beat-Vb	1.11388934932283	1	1	pseudobulk	DESeq2	LRT	0
967	KC_AB1	beat-VI	0.63825163975652	0.791548424121757	1	pseudobulk	DESeq2	LRT	0.101522511394706
967	KC_AB1	beat-VII	-0.234491397077252	0.326576671281251	1	pseudobulk	DESeq2	LRT	0.486014841937631
967	KC_AB1	beg	0.740290355897972	0.522957747094894	1	pseudobulk	DESeq2	LRT	0.281533398981337
967	KC_AB1	bel	-0.105708334390217	0.624343651280022	1	pseudobulk	DESeq2	LRT	0.204576300104247
967	KC_AB1	Bem46	0.171249033134658	0.800825138891507	1	pseudobulk	DESeq2	LRT	0.0964623022738664
967	KC_AB1	ben	0.148945303787375	0.438702213045005	1	pseudobulk	DESeq2	LRT	0.357830174779441
967	KC_AB1	Best1	-0.689523811371961	0.579994052106235	1	pseudobulk	DESeq2	LRT	0.236576460145142
967	KC_AB1	Best2	-2.1900801774161	0.437871127685654	1	pseudobulk	DESeq2	LRT	0.35865369034154
968	KC_AB1	Bet1	-0.0977971517115374	0.825820499730594	1	pseudobulk	DESeq2	LRT	0.0831143406313629
968	KC_AB1	Bet3	0.341493526307086	0.427284529926111	1	pseudobulk	DESeq2	LRT	0.369282830771261
968	KC_AB1	Bet5	-0.164662281902846	0.728627857429055	1	pseudobulk	DESeq2	LRT	0.137494228514856
968	KC_AB1	beta-Man	1.13698605850655	0.780204112083573	1	pseudobulk	DESeq2	LRT	0.10779176505922
968	KC_AB1	beta-PheRS	0.0480416554182562	0.951688681670479	1	pseudobulk	DESeq2	LRT	0.0215050956746549
968	KC_AB1	beta-Spec	-0.150111764251724	0.516908231742167	1	pseudobulk	DESeq2	LRT	0.286586551655614
968	KC_AB1	beta'COP	0.0349196463643654	0.941241918831233	1	pseudobulk	DESeq2	LRT	0.0262987394733879

968	KC_AB1	beta3GalTII	1.58131881636881	0.489254654443304	1	pseudobulk	DESeq2	LRT	0.310465034061524
968	KC_AB1	beta4GalNAcTA	-0.0753406160783801	0.87992807412521	1	pseudobulk	DESeq2	LRT	0.0555528259034213
968	KC_AB1	beta4GalNAcTB	-0.097730079453183	0.892592268225492	1	pseudobulk	DESeq2	LRT	0.0493468794062527
969	KC_AB1	beta4GalT7	-0.28834565597777	0.776452432817831	1	pseudobulk	DESeq2	LRT	0.109885144954747
969	KC_AB1	betaCOP	0.0608749933818489	0.911185259080175	1	pseudobulk	DESeq2	LRT	0.0403933147761049
969	KC_AB1	betaggt-I	-0.1941513602632	0.78262161107423	1	pseudobulk	DESeq2	LRT	0.106448163794526
969	KC_AB1	betaggt-II	-0.0766044515730832	0.906703437885617	1	pseudobulk	DESeq2	LRT	0.0425347375821649
969	KC_AB1	betaGlu	0.943022244937108	0.768808261856119	1	pseudobulk	DESeq2	LRT	0.114181958248924
969	KC_AB1	betaTub56D	0.072764982868565	0.763722309491977	1	pseudobulk	DESeq2	LRT	0.117064522808761
969	KC_AB1	betaTub60D	4.28228179718745	0.0862498656767637	1	pseudobulk	DESeq2	LRT	1.06424157261278
969	KC_AB1	betaTub85D	0.943798956650056	0.807634491832869	1	pseudobulk	DESeq2	LRT	0.0927851418175362
969	KC_AB1	betaTub97EF	0.294301698737335	0.847711943127507	1	pseudobulk	DESeq2	LRT	0.0717516981711856
969	KC_AB1	Bgb	0.194380280650324	0.549887301006689	1	pseudobulk	DESeq2	LRT	0.259726309716926
970	KC_AB1	BHD	0.97875952485993	0.365761434906424	1	pseudobulk	DESeq2	LRT	0.436802087515347
970	KC_AB1	BI-1	0.116034736779443	0.610236760583284	1	pseudobulk	DESeq2	LRT	0.214501634062343
970	KC_AB1	bib	1.13988912687578	0.653854063433791	1	pseudobulk	DESeq2	LRT	0.184519172956856
970	KC_AB1	bic	-0.0187091256263616	0.899592716269834	1	pseudobulk	DESeq2	LRT	0.0459540695732496
970	KC_AB1	BicD	0.0484276063227584	0.825890235751885	1	pseudobulk	DESeq2	LRT	0.0830776683877258
970	KC_AB1	bif	-0.38867704115585	0.238020171267454	1	pseudobulk	DESeq2	LRT	0.623386236645427
970	KC_AB1	bigmax	0.516602848969255	0.565196482590882	1	pseudobulk	DESeq2	LRT	0.247800549576706
970	KC_AB1	Bili	0.0133349541335071	0.962644820810211	1	pseudobulk	DESeq2	LRT	0.0165339214064412
970	KC_AB1	Bin1	-0.0326578209496662	0.929840193943365	1	pseudobulk	DESeq2	LRT	0.0315916846204291
970	KC_AB1	bin3	-0.137571327394759	0.646510920081157	1	pseudobulk	DESeq2	LRT	0.189424135144089
971	KC_AB1	bip1	-2.89873240646708	0.213780650480074	1	pseudobulk	DESeq2	LRT	0.670031605815827
971	KC_AB1	bip2	0.0167109204600286	0.927056366505099	1	pseudobulk	DESeq2	LRT	0.0328938592558133
971	KC_AB1	Bka	0.452155728578977	0.534468021890459	1	pseudobulk	DESeq2	LRT	0.272078274238132
971	KC_AB1	Blimp-1	-0.0768402809011623	0.785455312162359	1	pseudobulk	DESeq2	LRT	0.104878518750522
971	KC_AB1	Blm	0.214659056804751	0.829297792519012	1	pseudobulk	DESeq2	LRT	0.0812894906505836
971	KC_AB1	Blos1	0.023045677849124	0.952984489428205	1	pseudobulk	DESeq2	LRT	0.020914167788284
971	KC_AB1	Blos2	-0.728887181423058	0.258540596064201	1	pseudobulk	DESeq2	LRT	0.587471254262827
971	KC_AB1	Blos3	0.534665204687787	0.214520306855857	1	pseudobulk	DESeq2	LRT	0.668531590481531
971	KC_AB1	Blos4	0.709896458038611	0.323738191144498	1	pseudobulk	DESeq2	LRT	0.489806064190433
971	KC_AB1	blot	0.783501343733593	0.77504585330222	1	pseudobulk	DESeq2	LRT	0.11067260298137
972	KC_AB1	blow	1.35383628429856	0.5799218302356	1	pseudobulk	DESeq2	LRT	0.236630542618548
972	KC_AB1	blp	-0.0986998834581187	0.872057381740445	1	pseudobulk	DESeq2	LRT	0.0594549373686628
972	KC_AB1	blw	-0.083659363208823	0.606962112051166	1	pseudobulk	DESeq2	LRT	0.216838417723199
972	KC_AB1	bma	-0.396632502142492	0.113841358383405	1	pseudobulk	DESeq2	LRT	0.94369993078307
972	KC_AB1	Bmcp	0.593503526368383	0.251806651883657	1	pseudobulk	DESeq2	LRT	0.598932801479113
972	KC_AB1	bmm	-0.0482713976289112	0.928806656058408	1	pseudobulk	DESeq2	LRT	0.0320746809965714
972	KC_AB1	bnb	0.193302527845237	0.637315608452285	1	pseudobulk	DESeq2	LRT	0.195645445113905
972	KC_AB1	boca	0.208840682604977	0.626902994931337	1	pseudobulk	DESeq2	LRT	0.202799655381895
972	KC_AB1	bocks	0.20145432184923	0.830465141411606	1	pseudobulk	DESeq2	LRT	0.0806785922436616
972	KC_AB1	BOD1	-0.26806346955761	0.66930707076667	1	pseudobulk	DESeq2	LRT	0.174374586951188
973	KC_AB1	boi	1.90071181822722	0.545896518579078	1	pseudobulk	DESeq2	LRT	0.262889675371117
973	KC_AB1	boil	1.25804452752548	0.727552935095488	1	pseudobulk	DESeq2	LRT	0.138135402914536
973	KC_AB1	bol	-0.108091011894535	0.450341589151854	1	pseudobulk	DESeq2	LRT	0.34645794398728
973	KC_AB1	Bom065	-4.3174157809514	0.11831908209128	1	pseudobulk	DESeq2	LRT	0.926945208215341
973	KC_AB1	Bom067	-5.64882520034584	0.173082460076891	1	pseudobulk	DESeq2	LRT	0.761746940661747
973	KC_AB1	Bom107	-3.73432699611557	0.200340531487601	1	pseudobulk	DESeq2	LRT	0.698231178411511
973	KC_AB1	Bom23	-4.21820615809929	0.130345278170634	1	pseudobulk	DESeq2	LRT	0.884904696767083
973	KC_AB1	Bom836	-0.760959112540794	1	1	pseudobulk	DESeq2	LRT	0
973	KC_AB1	bon	-0.422941351205125	0.265958402428503	1	pseudobulk	DESeq2	LRT	0.575186284453424
973	KC_AB1	bond	5.28597690611768	0.118965415964152	1	pseudobulk	DESeq2	LRT	0.924579272547603
974	KC_AB1	bonsai	-0.379089152130117	0.411975783428259	1	pseudobulk	DESeq2	LRT	0.385128311715841
974	KC_AB1	bor	0.15453018969056	0.83039395229492	1	pseudobulk	DESeq2	LRT	0.0807158224225307
974	KC_AB1	BORCS5	-0.0815595244204696	0.853599421688421	1	pseudobulk	DESeq2	LRT	0.0687458878169661

974	KC_AB1	BORCS6	0.21669069561532	0.699347522978753	1	pseudobulk	DESeq2	LRT	0.155306959009615
974	KC_AB1	borr	1.11388934932283	1	1	pseudobulk	DESeq2	LRT	0
974	KC_AB1	botv	-0.170414805675017	0.848838097713161	1	pseudobulk	DESeq2	LRT	0.0711751365816314
974	KC_AB1	bou	1.91236374149585	0.3661640675514	1	pseudobulk	DESeq2	LRT	0.436324276172531
974	KC_AB1	br	-0.451765040474321	0.640007457921409	1	pseudobulk	DESeq2	LRT	0.193814965211046
974	KC_AB1	Br140	1.43364069553254	0.213121093083747	1	pseudobulk	DESeq2	LRT	0.671373565036903
974	KC_AB1	brat	-0.385049328110262	0.0594718514267276	1	pseudobulk	DESeq2	LRT	1.22568854120653
975	KC_AB1	Brca2	0.0611374507774082	0.936370779454951	1	pseudobulk	DESeq2	LRT	0.0285521474310724
975	KC_AB1	Brd8	-0.00609329144286741	0.963438415915014	1	pseudobulk	DESeq2	LRT	0.016176040721107
975	KC_AB1	Bre1	-0.261608167014292	0.647448358111552	1	pseudobulk	DESeq2	LRT	0.188794866137226
975	KC_AB1	Brf	-0.865169124641758	0.30889322662598	1	pseudobulk	DESeq2	LRT	0.510191614752004
975	KC_AB1	brk	-0.0602873115688322	0.968831634546913	1	pseudobulk	DESeq2	LRT	0.013751688935133
975	KC_AB1	brm	-0.104604803760893	0.826160210165575	1	pseudobulk	DESeq2	LRT	0.0829357255076476
975	KC_AB1	Brms1	0.0569104076515074	0.90076911977807	1	pseudobulk	DESeq2	LRT	0.0453865107474603
975	KC_AB1	brn	-2.24978065768543	0.364534962203659	1	pseudobulk	DESeq2	LRT	0.438260812575185
975	KC_AB1	brp	-0.133682988755817	0.291444849967385	1	pseudobulk	DESeq2	LRT	0.53544361456141
975	KC_AB1	bru1	-0.424979887463108	0.0899094423042965	1	pseudobulk	DESeq2	LRT	1.04619469619431
976	KC_AB1	bru2	-0.0454643050395634	0.773107885701597	1	pseudobulk	DESeq2	LRT	0.111759896902472
976	KC_AB1	bru3	-0.207286084328437	0.119005408630673	1	pseudobulk	DESeq2	LRT	0.92443330007741
976	KC_AB1	Bruce	-0.0844431668318798	0.82017144025588	1	pseudobulk	DESeq2	LRT	0.0860953576469566
976	KC_AB1	brun	0.646783405266232	0.407326349904167	1	pseudobulk	DESeq2	LRT	0.390057494535099
976	KC_AB1	BRWD3	-0.0496070930084397	0.925687183068443	1	pseudobulk	DESeq2	LRT	0.033535749408001
976	KC_AB1	brwl	0.399288712519298	0.54422508858977	1	pseudobulk	DESeq2	LRT	0.264221441268233
976	KC_AB1	bs	0.438047232560727	0.601044710347169	1	pseudobulk	DESeq2	LRT	0.221093220618173
976	KC_AB1	bsf	0.0975482051672158	0.90546582573346	1	pseudobulk	DESeq2	LRT	0.0431279362670479
976	KC_AB1	Bsg	-0.0959933235670491	0.517585496444488	1	pseudobulk	DESeq2	LRT	0.286017901779807
976	KC_AB1	Bsg25D	0.0290589030465512	0.953784702890631	1	pseudobulk	DESeq2	LRT	0.0205496472024898
977	KC_AB1	bsk	0.0349404258406634	0.857576344806222	1	pseudobulk	DESeq2	LRT	0.0667272069677852
977	KC_AB1	bt	-1.36962193441694	0.0417926508510959	1	pseudobulk	DESeq2	LRT	1.37890008127343
977	KC_AB1	BTBD9	0.75448236401742	0.517293902162817	1	pseudobulk	DESeq2	LRT	0.286262641004505
977	KC_AB1	BtbVII	-0.156181375364605	0.473038425935071	1	pseudobulk	DESeq2	LRT	0.32510357914858
977	KC_AB1	Btk29A	2.39871981814587	0.0937137881771156	1	pseudobulk	DESeq2	LRT	1.02819650635132
977	KC_AB1	btl	1.45283908506523	0.572202451205467	1	pseudobulk	DESeq2	LRT	0.242450286092574
977	KC_AB1	btsz	-0.127411111844272	0.618272195159974	1	pseudobulk	DESeq2	LRT	0.208820284085174
977	KC_AB1	btz	-0.481686976808811	0.525476875064218	1	pseudobulk	DESeq2	LRT	0.279446391439708
977	KC_AB1	Bub1	-0.0649889220642193	0.925498222562408	1	pseudobulk	DESeq2	LRT	0.0336244109813953
977	KC_AB1	Bub3	0.00466685492476846	0.981615136614887	1	pseudobulk	DESeq2	LRT	0.00805875336007027
978	KC_AB1	BubR1	2.09987857572813	0.503115176013642	1	pseudobulk	DESeq2	LRT	0.29833258237694
978	KC_AB1	bug	0.0442360774074892	0.947229333068167	1	pseudobulk	DESeq2	LRT	0.0235448615160125
978	KC_AB1	Bug22	-0.0686500963335964	0.883073288367471	1	pseudobulk	DESeq2	LRT	0.0540032517873549
978	KC_AB1	BuGZ	-0.395852631699184	0.167594808504723	1	pseudobulk	DESeq2	LRT	0.775739438406648
978	KC_AB1	bun	-0.0969767697178841	0.509945752346363	1	pseudobulk	DESeq2	LRT	0.292476021372073
978	KC_AB1	bur	-0.979581662678061	0.154641911097092	1	pseudobulk	DESeq2	LRT	0.810672791844267
978	KC_AB1	bves	-0.0460129122615414	0.893751491934371	1	pseudobulk	DESeq2	LRT	0.0487832202249061
978	KC_AB1	bwa	0.0748415733167668	0.918010651449198	1	pseudobulk	DESeq2	LRT	0.0371522797586981
978	KC_AB1	Bx	-0.0265680931193357	0.872817558633599	1	pseudobulk	DESeq2	LRT	0.0590765255487496
978	KC_AB1	Bx42	0.0103705384600103	0.977799754386973	1	pseudobulk	DESeq2	LRT	0.00975007616238143
979	KC_AB1	by	2.38099453019352	0.347403959581892	1	pseudobulk	DESeq2	LRT	0.45916523593277
979	KC_AB1	bys	-0.303994922826178	0.751795141293733	1	pseudobulk	DESeq2	LRT	0.123900485374188
979	KC_AB1	c-cup	-0.760959112540794	0.804334723897681	1	pseudobulk	DESeq2	LRT	0.0945631819854715
979	KC_AB1	c(3)G	-0.718853741766346	0.977252643133665	1	pseudobulk	DESeq2	LRT	0.0099931462763106
979	KC_AB1	c11.1	0.368465058087778	0.424299060579969	1	pseudobulk	DESeq2	LRT	0.37232792978273
979	KC_AB1	c12.1	0.254132675973257	0.491278736217316	1	pseudobulk	DESeq2	LRT	0.308672032818837
979	KC_AB1	c12.2	-0.455675119186451	0.576743928776686	1	pseudobulk	DESeq2	LRT	0.239016968480318
979	KC_AB1	C1GalTA	0.0544994776438701	0.894594248050676	1	pseudobulk	DESeq2	LRT	0.0483738985262974
979	KC_AB1	C3G	0.134111235934888	0.741852589155359	1	pseudobulk	DESeq2	LRT	0.129682383233833

979	KC_AB1	C901	0.620144285060225	0.744430887615277	1	pseudobulk	DESeq2	LRT	0.128175615602287
980	KC_AB1	ca	-0.75291560886291	0.309820069468665	1	pseudobulk	DESeq2	LRT	0.50889045301585
980	KC_AB1	Ca-alpha1D	-0.154400533363206	0.471147202559106	1	pseudobulk	DESeq2	LRT	0.326843383165681
980	KC_AB1	Ca-alpha1T	-0.309536880360217	0.0399342456167201	1	pseudobulk	DESeq2	LRT	1.3986545152528
980	KC_AB1	Ca-beta	-0.300939409796874	0.0830284230472913	1	pseudobulk	DESeq2	LRT	1.08077321052145
980	KC_AB1	Ca-Ma2d	-0.0154998409599346	0.952897057646863	1	pseudobulk	DESeq2	LRT	0.0209540140633736
980	KC_AB1	CaBP1	-0.783424009050378	0.336828980970035	1	pseudobulk	DESeq2	LRT	0.472590548603845
980	KC_AB1	cac	-0.206885515894946	0.0909873401722563	1	pseudobulk	DESeq2	LRT	1.04101903049002
980	KC_AB1	cact	0.0123311425648843	0.949599808003456	1	pseudobulk	DESeq2	LRT	0.0224593818620668
980	KC_AB1	cactin	-0.710583049752198	0.399557651863056	1	pseudobulk	DESeq2	LRT	0.398420547815419
980	KC_AB1	Cad86C	-0.760959112540794	0.804334723897681	1	pseudobulk	DESeq2	LRT	0.0945631819854715
981	KC_AB1	Cad87A	0.663335865740543	0.567812269802584	1	pseudobulk	DESeq2	LRT	0.245795227079654
981	KC_AB1	Cad96Ca	0.194200969423553	0.901395091900093	1	pseudobulk	DESeq2	LRT	0.045084810993807
981	KC_AB1	Cad99C	0.479565861540495	0.826521710136558	1	pseudobulk	DESeq2	LRT	0.0827457343869347
981	KC_AB1	CadN	-0.207717659126582	0.490161017250466	1	pseudobulk	DESeq2	LRT	0.309661231366723
981	KC_AB1	Cadps	-0.196692677636124	0.179700883690699	1	pseudobulk	DESeq2	LRT	0.745449787214618
981	KC_AB1	Caf1-105	0.258747451825288	0.846251855402985	1	pseudobulk	DESeq2	LRT	0.0725003661172633
981	KC_AB1	Caf1-55	0.214336331547314	0.648314026696159	1	pseudobulk	DESeq2	LRT	0.188214582051513
981	KC_AB1	cag	1.01915322421846	0.227462023807506	1	pseudobulk	DESeq2	LRT	0.643091101106029
981	KC_AB1	CAH1	0.174023426435876	0.562313580524344	1	pseudobulk	DESeq2	LRT	0.25002142764912
981	KC_AB1	CAH2	0.28536616752409	0.867568636565854	1	pseudobulk	DESeq2	LRT	0.061696156534038
982	KC_AB1	CAH3	0.419993863405523	0.308082326657275	1	pseudobulk	DESeq2	LRT	0.511333214553462
982	KC_AB1	CAH7	0.270096330583755	0.417303095572881	1	pseudobulk	DESeq2	LRT	0.379548393660603
982	KC_AB1	CAH8	0.462241288198732	0.453222844572242	1	pseudobulk	DESeq2	LRT	0.343688207763578
982	KC_AB1	CAHbeta	0.452774699656057	0.52289859863602	1	pseudobulk	DESeq2	LRT	0.281582522074454
982	KC_AB1	cal1	-0.77908083721046	0.275495350571751	1	pseudobulk	DESeq2	LRT	0.559885726169987
982	KC_AB1	CalpA	0.134057053130037	0.864031232327372	1	pseudobulk	DESeq2	LRT	0.0634705586990225
982	KC_AB1	CalpB	0.331440606772381	0.687672367262241	1	pseudobulk	DESeq2	LRT	0.162618426561916
982	KC_AB1	CalpC	1.77006317075202	0.596029081111493	1	pseudobulk	DESeq2	LRT	0.224732549893771
982	KC_AB1	Calr	0.0390292491639298	0.929763613213412	1	pseudobulk	DESeq2	LRT	0.031627454162661
982	KC_AB1	Cals	0.0100554589478136	0.918287921313655	1	pseudobulk	DESeq2	LRT	0.0370211281313617
983	KC_AB1	Calx	-0.107752172356464	0.76141282920764	1	pseudobulk	DESeq2	LRT	0.118379809939374
983	KC_AB1	calypso	-0.260402592550702	0.721558290109561	1	pseudobulk	DESeq2	LRT	0.141728579261571
983	KC_AB1	Cam	-0.208795700445129	0.0240610944973496	1	pseudobulk	DESeq2	LRT	1.61868462124604
983	KC_AB1	CaMKI	-0.0375274713209794	0.831614701233829	1	pseudobulk	DESeq2	LRT	0.0800778418414161
983	KC_AB1	CaMKII	-0.108984367660087	0.390428765227061	1	pseudobulk	DESeq2	LRT	0.408458192749274
983	KC_AB1	Camp	-0.37720232998877	0.64891423563395	1	pseudobulk	DESeq2	LRT	0.187812698349404
983	KC_AB1	Camta	-0.0298310181963514	0.936048849492843	1	pseudobulk	DESeq2	LRT	0.0287014861853975
983	KC_AB1	cana	0.296275065198765	1	1	pseudobulk	DESeq2	LRT	0
983	KC_AB1	CanA-14F	-0.317545584853275	0.193552573995832	1	pseudobulk	DESeq2	LRT	0.713201048758599
983	KC_AB1	CanB	0.0588170169820014	0.733209559285902	1	pseudobulk	DESeq2	LRT	0.134771881519348
984	KC_AB1	CanB2	-0.12865842012841	0.692915301386434	1	pseudobulk	DESeq2	LRT	0.159319848199972
984	KC_AB1	Cand1	-0.11867073553702	0.854239470083334	1	pseudobulk	DESeq2	LRT	0.0684203658951794
984	KC_AB1	CAP	0.0516695591485895	0.963599468113461	1	pseudobulk	DESeq2	LRT	0.0161034483950023
984	KC_AB1	Cap-D3	-0.285977389512892	0.850053433176486	1	pseudobulk	DESeq2	LRT	0.0705537742805976
984	KC_AB1	Cap-G	-0.766136796302483	0.633862628050465	1	pseudobulk	DESeq2	LRT	0.198004853070929
984	KC_AB1	Cap-H2	-0.270700133903167	0.687147946819186	1	pseudobulk	DESeq2	LRT	0.162949746827305
984	KC_AB1	Capa	-1.60958773650785	0.570513631639934	1	pseudobulk	DESeq2	LRT	0.243733974251419
984	KC_AB1	Caper	-0.0667859111829603	0.74931057933431	1	pseudobulk	DESeq2	LRT	0.125338135660169
984	KC_AB1	Capr	-0.475832263713218	0.264733801535828	1	pseudobulk	DESeq2	LRT	0.57719060390726
984	KC_AB1	caps	-0.0430722072307604	0.828096762107781	1	pseudobulk	DESeq2	LRT	0.081918913463685
985	KC_AB1	capt	-0.588798801477647	0.181464014872518	1	pseudobulk	DESeq2	LRT	0.741209484632049
985	KC_AB1	capu	-0.0181727568573144	0.916745616480547	1	pseudobulk	DESeq2	LRT	0.03775115798897
985	KC_AB1	car	-0.0373384440231391	0.903631570597312	1	pseudobulk	DESeq2	LRT	0.0440086043378992
985	KC_AB1	CARPB	-2.0067102530413	0.298385132842808	1	pseudobulk	DESeq2	LRT	0.525222819554535
985	KC_AB1	CarT	1.25804452752548	0.573436424985144	1	pseudobulk	DESeq2	LRT	0.241514723898045

985	KC_AB1	CASK	-0.0848553788610091	0.558327676530098	1	pseudobulk	DESeq2	LRT	0.2531108434618
985	KC_AB1	casp	-0.117349720220215	0.810127076782439	1	pseudobulk	DESeq2	LRT	0.0914468522118402
985	KC_AB1	cass	0.0584011682838815	0.803956183298167	1	pseudobulk	DESeq2	LRT	0.0947676202445519
985	KC_AB1	Cat	3.93648461469108	0.00393761330181932	1	pseudobulk	DESeq2	LRT	2.4047669365301
985	KC_AB1	cathD	0.586365348072809	0.325746671944359	1	pseudobulk	DESeq2	LRT	0.487120012648635
986	KC_AB1	Catsup	-1.0633744177432	0.342534336755046	1	pseudobulk	DESeq2	LRT	0.465295886899173
986	KC_AB1	cav	-1.56760195037366	0.41399484686445	1	pseudobulk	DESeq2	LRT	0.383005064657513
986	KC_AB1	caz	-0.183253053518321	0.585532031043734	1	pseudobulk	DESeq2	LRT	0.232449342222627
986	KC_AB1	cbc	-0.0112818934402018	0.922377204736315	1	pseudobulk	DESeq2	LRT	0.0350914385619714
986	KC_AB1	Cbl	-0.326041226827341	0.361739435832637	1	pseudobulk	DESeq2	LRT	0.441604143074395
986	KC_AB1	Cbp20	0.676477079992643	0.215199378400983	1	pseudobulk	DESeq2	LRT	0.667158987454593
986	KC_AB1	Cbp53E	-1.50800858067884	0.00824770369340629	1	pseudobulk	DESeq2	LRT	2.08366694988629
986	KC_AB1	Cbp80	-0.325636105121401	0.655903402879596	1	pseudobulk	DESeq2	LRT	0.183160115937426
986	KC_AB1	cbs	0.0451827930678255	0.94523617807827	1	pseudobulk	DESeq2	LRT	0.0244596644845752
986	KC_AB1	Cbs	-0.808269655015268	0.312244354639212	1	pseudobulk	DESeq2	LRT	0.505505404903234
987	KC_AB1	cbt	-0.0969094757856477	0.833552553142455	1	pseudobulk	DESeq2	LRT	0.0790670139280218
987	KC_AB1	CCAP-R	-1.43517655682975	0.211752556129488	1	pseudobulk	DESeq2	LRT	0.674171338464876
987	KC_AB1	CCDC53	-0.128954992246769	0.894077245795155	1	pseudobulk	DESeq2	LRT	0.0486249577439683
987	KC_AB1	Ccdc58	0.61299778941965	0.338510504949232	1	pseudobulk	DESeq2	LRT	0.470427849369697
987	KC_AB1	Ccdc85	3.84359757126144	0.13868838448017	1	pseudobulk	DESeq2	LRT	0.857959910717689
987	KC_AB1	Cchl	-0.133860787230027	0.640806871902156	1	pseudobulk	DESeq2	LRT	0.193272839599873
987	KC_AB1	CCKLR-17D1	-0.552817196283089	0.592807422165546	1	pseudobulk	DESeq2	LRT	0.227086367469077
987	KC_AB1	CCKLR-17D3	-0.4089259919482	0.764034409046628	1	pseudobulk	DESeq2	LRT	0.116887082101572
987	KC_AB1	Ccm3	0.0407492332665401	0.897810641163925	1	pseudobulk	DESeq2	LRT	0.0468152514946395
987	KC_AB1	Ccn	0.00266107817178911	0.948054707637217	1	pseudobulk	DESeq2	LRT	0.0231666009114765
988	KC_AB1	Ccp84Aa	-0.760959112540794	1	1	pseudobulk	DESeq2	LRT	0
988	KC_AB1	Ccp84Ab	-2.73719572742657	0.395113780272619	1	pseudobulk	DESeq2	LRT	0.40327782328744
988	KC_AB1	Ccp84Ae	1.25804452752548	0.573436424985144	1	pseudobulk	DESeq2	LRT	0.241514723898045
988	KC_AB1	Ccs	-0.341481607003654	0.694775927528792	1	pseudobulk	DESeq2	LRT	0.158155237321259
988	KC_AB1	CCT1	-0.226641884053234	0.746607653658169	1	pseudobulk	DESeq2	LRT	0.126907562327646
988	KC_AB1	CCT2	-0.19402459130235	0.765043058214917	1	pseudobulk	DESeq2	LRT	0.116314121161554
988	KC_AB1	CCT3	-0.213963892083048	0.719240612253437	1	pseudobulk	DESeq2	LRT	0.14312579794391
988	KC_AB1	CCT4	0.418935341192026	0.379913038169213	1	pseudobulk	DESeq2	LRT	0.420315801713037
988	KC_AB1	CCT5	0.744126099763073	0.358227957870902	1	pseudobulk	DESeq2	LRT	0.445840522687888
988	KC_AB1	CCT6	0.00291563701853602	0.985753611911821	1	pseudobulk	DESeq2	LRT	0.00623162294812085
989	KC_AB1	CCT7	-0.581169297708217	0.494200735094514	1	pseudobulk	DESeq2	LRT	0.306096612945972
989	KC_AB1	CCT8	-0.349582356140431	0.49867362063362	1	pseudobulk	DESeq2	LRT	0.302183604944393
989	KC_AB1	Ccz1	-0.195088299165947	0.792260794133538	1	pseudobulk	DESeq2	LRT	0.10113183506515
989	KC_AB1	CD98hc	0.323432014116451	0.410028736828053	1	pseudobulk	DESeq2	LRT	0.387185704722942
989	KC_AB1	Cda4	1.13953643968657	0.648138242491997	1	pseudobulk	DESeq2	LRT	0.188332352851342
989	KC_AB1	Cda5	-0.493811921275517	0.343849497369839	1	pseudobulk	DESeq2	LRT	0.463631606161758
989	KC_AB1	CDase	0.0153675459254184	0.97614243773384	1	pseudobulk	DESeq2	LRT	0.0104868058903441
989	KC_AB1	cdc14	-0.156925413187463	0.627770936127546	1	pseudobulk	DESeq2	LRT	0.202198794675498
989	KC_AB1	Cdc16	-0.663732915204377	0.694500251717523	1	pseudobulk	DESeq2	LRT	0.158327592518862
989	KC_AB1	Cdc23	-0.451536717373587	0.778136788080441	1	pseudobulk	DESeq2	LRT	0.108944051999097
990	KC_AB1	Cdc27	-0.00152596051478121	0.974604496060283	1	pseudobulk	DESeq2	LRT	0.0111715894561943
990	KC_AB1	Cdc2rk	-0.339039293703916	0.674145981204766	1	pseudobulk	DESeq2	LRT	0.171246050101286
990	KC_AB1	Cdc37	-0.0505426859561101	0.878667403633666	1	pseudobulk	DESeq2	LRT	0.0561754845405958
990	KC_AB1	Cdc42	0.217800522116127	0.573562116126276	1	pseudobulk	DESeq2	LRT	0.241419541616151
990	KC_AB1	CDC45L	0.035279431899523	0.98040948339088	1	pseudobulk	DESeq2	LRT	0.00859249651786932
990	KC_AB1	Cdc5	-0.236936503725735	0.714716625094577	1	pseudobulk	DESeq2	LRT	0.145866115614154
990	KC_AB1	CDC50	0.0128178934950102	0.931995491741381	1	pseudobulk	DESeq2	LRT	0.0305861884148776
990	KC_AB1	Cdc7	0.278112241458485	0.76692518983594	1	pseudobulk	DESeq2	LRT	0.115246997493002
990	KC_AB1	Cdep	-0.0871607214334711	0.677445008787902	1	pseudobulk	DESeq2	LRT	0.169125952629587
990	KC_AB1	CdGAPr	-0.653749002958833	0.332100398136415	1	pseudobulk	DESeq2	LRT	0.478730603750171
991	KC_AB1	cdi	-0.0476416841346672	0.886442642402937	1	pseudobulk	DESeq2	LRT	0.0523493603380669

991	KC_AB1	Cdk12	-0.0692129413558228	0.85019700968972	1	pseudobulk	DESeq2	LRT	0.0704804268654115
991	KC_AB1	Cdk2	0.502887419745495	0.511072890683319	1	pseudobulk	DESeq2	LRT	0.29151715512164
991	KC_AB1	CDK2AP1	-0.0686256467965646	0.90995119990803	1	pseudobulk	DESeq2	LRT	0.0409818979854433
991	KC_AB1	Cdk4	1.25804452752548	0.573436424985144	1	pseudobulk	DESeq2	LRT	0.241514723898045
991	KC_AB1	Cdk5	-0.00312513546908974	0.952409738375807	1	pseudobulk	DESeq2	LRT	0.0211761725896554
991	KC_AB1	Cdk5alpha	-0.0475180065873845	0.80430552721938	1	pseudobulk	DESeq2	LRT	0.0945789467983544
991	KC_AB1	Cdk7	-0.543313663564211	0.566693366420165	1	pseudobulk	DESeq2	LRT	0.246651871078248
991	KC_AB1	Cdk8	1.14299353039912	0.594643517791466	1	pseudobulk	DESeq2	LRT	0.225743310998691
991	KC_AB1	Cdk9	0.0653857936196338	0.897932508511362	1	pseudobulk	DESeq2	LRT	0.046756305072812
992	KC_AB1	cdm	0.313539527468293	0.492384599981362	1	pseudobulk	DESeq2	LRT	0.307695538697068
992	KC_AB1	Cds	-0.306260948668695	0.273323126201509	1	pseudobulk	DESeq2	LRT	0.563323620569356
992	KC_AB1	CecC	3.80522001411205	0.202854565618816	1	pseudobulk	DESeq2	LRT	0.692815213245607
992	KC_AB1	Ced-12	-0.692474687957088	0.532922228897175	1	pseudobulk	DESeq2	LRT	0.273336164379016
992	KC_AB1	ced-6	1.0624128091856	0.197805365795134	1	pseudobulk	DESeq2	LRT	0.703761931628575
992	KC_AB1	Cen	-0.115519880525147	0.807694694744508	1	pseudobulk	DESeq2	LRT	0.0927527697257686
992	KC_AB1	CenB1A	0.578217583761014	0.41651643170693	1	pseudobulk	DESeq2	LRT	0.380367860862516
992	KC_AB1	CenG1A	-0.00032051067758581	0.969952495233214	1	pseudobulk	DESeq2	LRT	0.0132495353866299
992	KC_AB1	Cenp-C	-0.716419614646419	0.812870875922622	1	pseudobulk	DESeq2	LRT	0.0899784363592155
992	KC_AB1	Cep135	-0.0518653414751322	0.981940836459101	1	pseudobulk	DESeq2	LRT	0.0079146783774002
993	KC_AB1	cep290	1.11388934932283	1	1	pseudobulk	DESeq2	LRT	0
993	KC_AB1	Cep97	0.0206605651947879	0.962667887504171	1	pseudobulk	DESeq2	LRT	0.0165235150575298
993	KC_AB1	cer	0.20482283917089	0.703484711165454	1	pseudobulk	DESeq2	LRT	0.152745336649463
993	KC_AB1	Cerk	0.0410784409352867	0.919938557666403	1	pseudobulk	DESeq2	LRT	0.0362411780430597
993	KC_AB1	cert	1.19327823394853	0.10098633295	1	pseudobulk	DESeq2	LRT	0.995737397762158
993	KC_AB1	cerv	-0.507507182625871	0.6537341909138	1	pseudobulk	DESeq2	LRT	0.184598800429705
993	KC_AB1	Cf2	-0.219008367181844	0.509956034175329	1	pseudobulk	DESeq2	LRT	0.292467264957205
993	KC_AB1	Cfp1	-0.31147909864846	0.657685914367081	1	pseudobulk	DESeq2	LRT	0.181981459308689
993	KC_AB1	cg	-0.0642147002266802	0.740197576333039	1	pseudobulk	DESeq2	LRT	0.130652341271318
993	KC_AB1	CG10011	-0.326903702811777	0.371523484308749	1	pseudobulk	DESeq2	LRT	0.430013728919968
994	KC_AB1	CG10019	0.809063967351792	0.359225077251074	1	pseudobulk	DESeq2	LRT	0.444633353152811
994	KC_AB1	CG10031	3.80522001411205	0.202854565618816	1	pseudobulk	DESeq2	LRT	0.692815213245607
994	KC_AB1	CG10038	-0.0465596286229724	0.91548168743477	1	pseudobulk	DESeq2	LRT	0.0383503385556996
994	KC_AB1	CG10050	-0.0457917461860773	0.927637130426549	1	pseudobulk	DESeq2	LRT	0.0326218762300085
994	KC_AB1	CG10053	0.587898962099741	0.567607041285769	1	pseudobulk	DESeq2	LRT	0.24595222566596
994	KC_AB1	CG10055	0.0574873525062341	0.914845013131141	1	pseudobulk	DESeq2	LRT	0.0386524749425017
994	KC_AB1	CG10068	0.145181624827248	0.870677718704213	1	pseudobulk	DESeq2	LRT	0.0601425693197772
994	KC_AB1	CG10075	-0.228005113222332	0.702631372469401	1	pseudobulk	DESeq2	LRT	0.153272462875669
994	KC_AB1	CG10077	-0.399175004059574	0.0252218947815554	1	pseudobulk	DESeq2	LRT	1.59822229039325
994	KC_AB1	CG10082	-0.180473749010781	0.381609626363212	1	pseudobulk	DESeq2	LRT	0.41838067845512
995	KC_AB1	CG10089	0.0362586235859154	0.899445630391848	1	pseudobulk	DESeq2	LRT	0.0460250837185964
995	KC_AB1	CG10098	-0.0579276708063217	0.881775750260556	1	pseudobulk	DESeq2	LRT	0.0546418488901009
995	KC_AB1	CG10103	0.159314719796612	0.742078123431591	1	pseudobulk	DESeq2	LRT	0.129550371285336
995	KC_AB1	CG10116	-1.43550613534661	0.602448798560473	1	pseudobulk	DESeq2	LRT	0.220079857379737
995	KC_AB1	CG10126	-0.459184491799161	0.866831670429805	1	pseudobulk	DESeq2	LRT	0.0620652297453457
995	KC_AB1	CG10132	-0.229845744136717	0.417258358005462	1	pseudobulk	DESeq2	LRT	0.379594955309477
995	KC_AB1	CG10137	0.230735586489542	0.481681282852437	1	pseudobulk	DESeq2	LRT	0.317240229151281
995	KC_AB1	CG10139	-0.412968321812755	0.304486482850789	1	pseudobulk	DESeq2	LRT	0.516431982353322
995	KC_AB1	CG10147	0.0726112275538023	0.936247854693822	1	pseudobulk	DESeq2	LRT	0.0286091644280742
995	KC_AB1	CG10151	-0.0497232244836006	0.907534567862037	1	pseudobulk	DESeq2	LRT	0.0421368237620256
996	KC_AB1	CG10165	-0.215442658093258	0.91428032817234	1	pseudobulk	DESeq2	LRT	0.0389206244934089
996	KC_AB1	CG10166	0.0263531524993638	0.950108097917669	1	pseudobulk	DESeq2	LRT	0.0222269803338848
996	KC_AB1	CG10171	-0.546981231080862	0.669503947069789	1	pseudobulk	DESeq2	LRT	0.174246858255294
996	KC_AB1	CG10177	1.25804452752548	0.727552935095488	1	pseudobulk	DESeq2	LRT	0.138135402914536
996	KC_AB1	CG10184	2.42480980534803	0.407219734637837	1	pseudobulk	DESeq2	LRT	0.390171183430048
996	KC_AB1	CG10188	-0.0880464420084557	0.797238036429894	1	pseudobulk	DESeq2	LRT	0.0984119891768377
996	KC_AB1	CG10189	-0.318343745269223	0.766244942161421	1	pseudobulk	DESeq2	LRT	0.115632379160165

996	KC_AB1	CG10194	-0.853919115510791	0.233060802673538	1	pseudobulk	DESeq2	LRT	0.632530762146645
996	KC_AB1	CG10205	-0.760959112540794	0.804334723897681	1	pseudobulk	DESeq2	LRT	0.0945631819854715
996	KC_AB1	CG10208	2.23566351327757	0.13421503770526	1	pseudobulk	DESeq2	LRT	0.872198822270304
997	KC_AB1	CG10209	0.892400613474924	0.212739367239717	1	pseudobulk	DESeq2	LRT	0.672152136834511
997	KC_AB1	CG10214	-0.220641050766849	0.721724171670939	1	pseudobulk	DESeq2	LRT	0.141628749257424
997	KC_AB1	CG10222	-0.528355933357655	0.82219917838478	1	pseudobulk	DESeq2	LRT	0.0850229615414808
997	KC_AB1	CG10237	-1.69241498201531	0.47867400278784	1	pseudobulk	DESeq2	LRT	0.319960158798347
997	KC_AB1	CG1024	0.456715346884493	0.606142870495065	1	pseudobulk	DESeq2	LRT	0.217424998680935
997	KC_AB1	CG10252	-0.894800917414213	0.550360601453699	1	pseudobulk	DESeq2	LRT	0.259352663405692
997	KC_AB1	CG10254	-0.185773129813764	0.674855575436378	1	pseudobulk	DESeq2	LRT	0.170789159764747
997	KC_AB1	CG10257	0.748785147228638	0.100605293050728	1	pseudobulk	DESeq2	LRT	0.997379169555919
997	KC_AB1	CG10265	-0.172566286720282	0.80818609933966	1	pseudobulk	DESeq2	LRT	0.0924886236186836
997	KC_AB1	CG10268	-0.216128478718779	0.885119152899794	1	pseudobulk	DESeq2	LRT	0.0529982615466265
998	KC_AB1	CG10274	-3.69213946721089	0.077243850771794	1	pseudobulk	DESeq2	LRT	1.11213608384066
998	KC_AB1	CG10283	0.616371785704442	0.404025413561399	1	pseudobulk	DESeq2	LRT	0.393591316517226
998	KC_AB1	CG10286	0.554352390590955	0.640714199157078	1	pseudobulk	DESeq2	LRT	0.193335651305103
998	KC_AB1	CG10311	0.204125434488284	0.882441289270024	1	pseudobulk	DESeq2	LRT	0.0543141795313561
998	KC_AB1	CG10321	0.263793458099004	0.602187633686832	1	pseudobulk	DESeq2	LRT	0.220268167250218
998	KC_AB1	CG10324	-0.017544932475016	0.974783606617164	1	pseudobulk	DESeq2	LRT	0.0110917831553184
998	KC_AB1	CG10333	0.356897947804914	0.467098302120023	1	pseudobulk	DESeq2	LRT	0.330591711349008
998	KC_AB1	CG10336	0.458089484063483	0.831486480898279	1	pseudobulk	DESeq2	LRT	0.0801448075617294
998	KC_AB1	CG10337	1.25804452752548	0.573436424985144	1	pseudobulk	DESeq2	LRT	0.241514723898045
998	KC_AB1	CG10338	-0.897327358347942	0.643463597839386	1	pseudobulk	DESeq2	LRT	0.191476017065201
999	KC_AB1	CG10340	0.393939461668632	0.322313948196276	1	pseudobulk	DESeq2	LRT	0.491720899906217
999	KC_AB1	CG10341	0.0697592241799393	0.944271131318967	1	pseudobulk	DESeq2	LRT	0.0249032875567932
999	KC_AB1	CG10343	-0.111920140124504	0.845756961041648	1	pseudobulk	DESeq2	LRT	0.0727544190445222
999	KC_AB1	CG10345	-2.00963963748391	0.498268873088307	1	pseudobulk	DESeq2	LRT	0.302536242405947
999	KC_AB1	CG10347	-0.605795689883648	0.582168917312033	1	pseudobulk	DESeq2	LRT	0.234950985774957
999	KC_AB1	CG10352	-0.964075444888033	0.581980066698765	1	pseudobulk	DESeq2	LRT	0.235091890042524
999	KC_AB1	CG10353	-0.061109046003101	0.886142299148255	1	pseudobulk	DESeq2	LRT	0.052496532319929
999	KC_AB1	CG10359	1.90071181822722	0.545896518579078	1	pseudobulk	DESeq2	LRT	0.262889675371117
999	KC_AB1	CG10361	-0.760959112540794	1	1	pseudobulk	DESeq2	LRT	0
999	KC_AB1	CG10362	-0.138377736365717	0.812494638278211	1	pseudobulk	DESeq2	LRT	0.0901794962861583
100	KC_AB1	CG10365	0.572483092980604	0.46565772147075	1	pseudobulk	DESeq2	LRT	0.331933191239423
100	KC_AB1	CG10366	0.535976756077629	0.422284073190503	1	pseudobulk	DESeq2	LRT	0.374395298058673
100	KC_AB1	CG10375	0.236872044479714	0.549165489804422	1	pseudobulk	DESeq2	LRT	0.260296762146167
100	KC_AB1	CG10376	-0.402461313198868	0.707196383910055	1	pseudobulk	DESeq2	LRT	0.150459968656641
100	KC_AB1	CG10383	0.123942820441882	0.868917947153104	1	pseudobulk	DESeq2	LRT	0.0610212325054283
100	KC_AB1	CG10384	-2.52011257447171	0.364021130127131	1	pseudobulk	DESeq2	LRT	0.438873406374921
100	KC_AB1	CG10395	0.169619914147643	0.630103499237919	1	pseudobulk	DESeq2	LRT	0.200588108552626
100	KC_AB1	CG10413	-0.264496758668928	0.74175736728377	1	pseudobulk	DESeq2	LRT	0.129738131489316
100	KC_AB1	CG10417	0.00170214347250866	0.962700259117788	1	pseudobulk	DESeq2	LRT	0.0165089112912942
100	KC_AB1	CG10418	-0.177468031303081	0.807469059427562	1	pseudobulk	DESeq2	LRT	0.0928741099594835
100	KC_AB1	CG10425	1.10646413810219	0.387624161625968	1	pseudobulk	DESeq2	LRT	0.411589160068669
100	KC_AB1	CG10428	0.133061504224556	0.84401970543691	1	pseudobulk	DESeq2	LRT	0.0736474137267016
100	KC_AB1	CG10431	-0.633130885014403	0.688923660759773	1	pseudobulk	DESeq2	LRT	0.161828899351572
100	KC_AB1	CG10433	0.22666433045786	0.814359477559163	1	pseudobulk	DESeq2	LRT	0.0891838449168147
100	KC_AB1	CG10435	0.162497200801829	0.802084592305757	1	pseudobulk	DESeq2	LRT	0.0957798261868611
100	KC_AB1	CG10444	-0.520554165130691	0.665433941244627	1	pseudobulk	DESeq2	LRT	0.176895051198913
100	KC_AB1	CG10462	1.63195036548663	0.633334503478132	1	pseudobulk	DESeq2	LRT	0.198366851366371
100	KC_AB1	CG10463	-0.110310177444139	0.921574661662466	1	pseudobulk	DESeq2	LRT	0.035469474514884
100	KC_AB1	CG10465	0.00278262797054879	0.99120300006728	1	pseudobulk	DESeq2	LRT	0.00383739215389224
100	KC_AB1	CG10466	0.124856677502901	0.885992483456649	1	pseudobulk	DESeq2	LRT	0.0525699625453954
100	KC_AB1	CG10467	1.13698282223142	0.780593459054812	1	pseudobulk	DESeq2	LRT	0.107575092189158
100	KC_AB1	CG10470	0.22156665950351	0.668220181633983	1	pseudobulk	DESeq2	LRT	0.175080411906633
100	KC_AB1	CG10472	-0.760959112540794	1	1	pseudobulk	DESeq2	LRT	0

100	KC_AB1	CG10479	-0.396721808097649	1	1	pseudobulk	DESeq2	LRT	0
100	KC_AB1	CG10481	-0.760959112540794	1	1	pseudobulk	DESeq2	LRT	0
100	KC_AB1	CG10483	-0.0343751343546174	0.912130615215621	1	pseudobulk	DESeq2	LRT	0.0399429671477702
100	KC_AB1	CG10492	-0.317522544983634	0.642611782188025	1	pseudobulk	DESeq2	LRT	0.192051315989085
100	KC_AB1	CG10494	0.0289820609110743	0.959600366177541	1	pseudobulk	DESeq2	LRT	0.0179095949800981
100	KC_AB1	CG10495	0.663646672725888	0.538830825648738	1	pseudobulk	DESeq2	LRT	0.268547566946445
100	KC_AB1	CG10505	-0.760959112540794	0.805265236978025	1	pseudobulk	DESeq2	LRT	0.0940610488455931
100	KC_AB1	CG10508	-0.0688112585410496	0.805253560897347	1	pseudobulk	DESeq2	LRT	0.094067346018176
100	KC_AB1	CG10512	0.220915678169206	0.853606078461656	1	pseudobulk	DESeq2	LRT	0.068742500995774
100	KC_AB1	CG10513	0.116692568523245	0.835095084781126	1	pseudobulk	DESeq2	LRT	0.0782640724867774
100	KC_AB1	CG10514	-0.388228815901435	0.738507123983372	1	pseudobulk	DESeq2	LRT	0.13164531092516
100	KC_AB1	CG10516	0.345404732028448	0.740041865396424	1	pseudobulk	DESeq2	LRT	0.130743710814497
100	KC_AB1	CG10527	0.34491273795705	0.705635297926373	1	pseudobulk	DESeq2	LRT	0.15141970266192
100	KC_AB1	CG10543	-0.123967514622978	0.567748713781007	1	pseudobulk	DESeq2	LRT	0.245843840989238
100	KC_AB1	CG10550	1.72969687629436	0.252986105053425	1	pseudobulk	DESeq2	LRT	0.596903331252402
100	KC_AB1	CG10553	7.60144797161059	0.102234057379799	1	pseudobulk	DESeq2	LRT	0.990404402947109
100	KC_AB1	CG10555	0.113911520952346	0.846173521788678	1	pseudobulk	DESeq2	LRT	0.0725405686064528
100	KC_AB1	CG10559	-0.760959112540794	1	1	pseudobulk	DESeq2	LRT	0
100	KC_AB1	CG10560	5.21925596926529	0.0156940008946237	1	pseudobulk	DESeq2	LRT	1.80426632697019
100	KC_AB1	CG10561	0.511586594929196	0.412726845765815	1	pseudobulk	DESeq2	LRT	0.384337281573833
100	KC_AB1	CG10562	4.97143583030972	0.00950247633269421	1	pseudobulk	DESeq2	LRT	2.02216320339738
100	KC_AB1	CG10565	0.114673784912615	0.787997382426391	1	pseudobulk	DESeq2	LRT	0.10347522514961
100	KC_AB1	CG10576	0.767378471558662	0.214299052944907	1	pseudobulk	DESeq2	LRT	0.668979748238199
100	KC_AB1	CG10581	0.555812243594097	0.729894427679723	1	pseudobulk	DESeq2	LRT	0.136739951923139
100	KC_AB1	CG10584	1.25804452752548	0.727552935095488	1	pseudobulk	DESeq2	LRT	0.138135402914536
100	KC_AB1	CG10600	-0.228473408999256	0.579532757723834	1	pseudobulk	DESeq2	LRT	0.236922010784731
100	KC_AB1	CG10602	0.504476626665182	0.560068524399892	1	pseudobulk	DESeq2	LRT	0.251758833800728
100	KC_AB1	CG10616	-0.422319934006771	0.455864457867722	1	pseudobulk	DESeq2	LRT	0.341164266876001
100	KC_AB1	CG10621	1.39934341023393	0.63869615168108	1	pseudobulk	DESeq2	LRT	0.194705700577112
100	KC_AB1	CG10623	-0.0585849722432592	0.969849752036919	1	pseudobulk	DESeq2	LRT	0.013295540904263
100	KC_AB1	CG10628	-0.517920843485017	0.511586202660994	1	pseudobulk	DESeq2	LRT	0.291081176838023
100	KC_AB1	CG10631	-0.170754782580086	0.795692166289282	1	pseudobulk	DESeq2	LRT	0.0992549176132762
100	KC_AB1	CG10638	-0.330946572927199	0.530294812433846	1	pseudobulk	DESeq2	LRT	0.275482621311335
100	KC_AB1	CG10646	0.799202920347441	0.365183684328411	1	pseudobulk	DESeq2	LRT	0.437488634107145
100	KC_AB1	CG10650	0.152115475746305	1	1	pseudobulk	DESeq2	LRT	0
100	KC_AB1	CG10654	0.157446329513415	0.776919010012906	1	pseudobulk	DESeq2	LRT	0.109624251904045
100	KC_AB1	CG10657	6.58024552288296	0.133883681676339	1	pseudobulk	DESeq2	LRT	0.873272353456976
100	KC_AB1	CG10660	0.630267064935367	0.824180501587846	1	pseudobulk	DESeq2	LRT	0.0839776642023209
100	KC_AB1	CG10669	1.19481682048219	0.366100279750992	1	pseudobulk	DESeq2	LRT	0.436399939257595
100	KC_AB1	CG10672	-0.972610376096757	0.188178713124664	1	pseudobulk	DESeq2	LRT	0.725429505751283
100	KC_AB1	CG10674	0.287646704659454	0.516071156199679	1	pseudobulk	DESeq2	LRT	0.287290413461293
100	KC_AB1	CG10681	-0.801330456084413	0.267864778938332	1	pseudobulk	DESeq2	LRT	0.572084387244077
100	KC_AB1	CG10702	0.296275065198765	1	1	pseudobulk	DESeq2	LRT	0
100	KC_AB1	CG10710	1.25804452752548	0.727552935095488	1	pseudobulk	DESeq2	LRT	0.138135402914536
100	KC_AB1	CG10713	0.092816480123693	0.865035580198834	1	pseudobulk	DESeq2	LRT	0.0629660289904153
100	KC_AB1	CG10731	-1.56550428309243	0.081304230532424	1	pseudobulk	DESeq2	LRT	1.08988685601619
100	KC_AB1	CG10737	0.061043612323735	0.894614802096232	1	pseudobulk	DESeq2	LRT	0.0483639203646452
100	KC_AB1	CG10738	0.231693152129735	0.599976002244942	1	pseudobulk	DESeq2	LRT	0.221866120118069
100	KC_AB1	CG1074	-0.150035853931585	0.813707110477164	1	pseudobulk	DESeq2	LRT	0.0895318889601394
100	KC_AB1	CG10747	-0.547098164219775	0.59767802684623	1	pseudobulk	DESeq2	LRT	0.223532710361479
100	KC_AB1	CG10778	-0.221955896830232	0.827277463383147	1	pseudobulk	DESeq2	LRT	0.0823488065219596
100	KC_AB1	CG10793	-0.760959112540794	0.805265236978025	1	pseudobulk	DESeq2	LRT	0.0940610488455931
100	KC_AB1	CG10795	-0.623714742239174	0.511961377206296	1	pseudobulk	DESeq2	LRT	0.29076280132666
100	KC_AB1	CG10802	0.189834428435449	0.795623235861522	1	pseudobulk	DESeq2	LRT	0.099292541964036
100	KC_AB1	CG10803	0.0990093399967334	0.791148336875762	1	pseudobulk	DESeq2	LRT	0.101742080539217
100	KC_AB1	CG10804	-0.0411813170109065	0.878982969442973	1	pseudobulk	DESeq2	LRT	0.0560195394297464

100	KC_AB1	CG10809	-0.213134637404433	0.576690017321129	1	pseudobulk	DESeq2	LRT	0.239057566293059
100	KC_AB1	CG10814	1.11388934932283	1	1	pseudobulk	DESeq2	LRT	0
100	KC_AB1	CG10841	1.11388934932283	1	1	pseudobulk	DESeq2	LRT	0
100	KC_AB1	CG10863	2.188454392137	0.00845494868125733	1	pseudobulk	DESeq2	LRT	2.07288902408213
100	KC_AB1	CG10864	0.152115475746306	1	1	pseudobulk	DESeq2	LRT	0
100	KC_AB1	CG10866	0.140729785218674	0.876190840955392	1	pseudobulk	DESeq2	LRT	0.0574012909242469
100	KC_AB1	CG10869	-0.570723996670538	0.824991814167186	1	pseudobulk	DESeq2	LRT	0.0835503606375391
100	KC_AB1	CG10874	0.0292342116969145	0.968485030931401	1	pseudobulk	DESeq2	LRT	0.0139070874220325
100	KC_AB1	CG10889	0.305198019612103	0.755846714197277	1	pseudobulk	DESeq2	LRT	0.121566270541309
100	KC_AB1	CG10898	0.0640025672299825	0.927076256893062	1	pseudobulk	DESeq2	LRT	0.0328845413832655
100	KC_AB1	CG1090	-0.0858341750690753	0.78006901095699	1	pseudobulk	DESeq2	LRT	0.1078669745505
100	KC_AB1	CG10903	0.78792930942799	0.290467965058817	1	pseudobulk	DESeq2	LRT	0.536901757818736
100	KC_AB1	CG10904	-0.00697199956556241	0.971108931819632	1	pseudobulk	DESeq2	LRT	0.0127320514156691
100	KC_AB1	CG10907	-0.019827413561564	0.950324028658367	1	pseudobulk	DESeq2	LRT	0.0221282895906054
100	KC_AB1	CG10914	-0.133431810505672	0.922243861591481	1	pseudobulk	DESeq2	LRT	0.0351542267336548
100	KC_AB1	CG10916	-0.0216287720297185	0.950337699348434	1	pseudobulk	DESeq2	LRT	0.0221220421819555
100	KC_AB1	CG1092	3.99740073762969	0.17240197409338	1	pseudobulk	DESeq2	LRT	0.763457765582343
100	KC_AB1	CG10927	-0.385011853448768	0.890534840916548	1	pseudobulk	DESeq2	LRT	0.0503490847107839
100	KC_AB1	CG10932	-0.640324673694742	0.572526535795109	1	pseudobulk	DESeq2	LRT	0.242204379586649
100	KC_AB1	CG10936	-3.58279186106554	0.225990959339248	1	pseudobulk	DESeq2	LRT	0.64590893424911
100	KC_AB1	CG10939	-1.19292085827426	0.456915384074668	1	pseudobulk	DESeq2	LRT	0.340164219256048
101	KC_AB1	CG10947	-0.305452566539259	0.44225724552298	1	pseudobulk	DESeq2	LRT	0.354325043291338
101	KC_AB1	CG10948	-0.304100840745277	0.397906844054746	1	pseudobulk	DESeq2	LRT	0.400218590862037
101	KC_AB1	CG10949	-0.626003622368699	0.409833657358857	1	pseudobulk	DESeq2	LRT	0.38739237827988
101	KC_AB1	CG10958	-0.792170470096014	0.516141090440579	1	pseudobulk	DESeq2	LRT	0.287231564992746
101	KC_AB1	CG10959	1.61715260650853	0.146360811362713	1	pseudobulk	DESeq2	LRT	0.834575191633008
101	KC_AB1	CG10960	0.259487413764769	0.644326837941389	1	pseudobulk	DESeq2	LRT	0.190893778746616
101	KC_AB1	CG10973	-0.0697503800424562	0.911841319841525	1	pseudobulk	DESeq2	LRT	0.0400807317517626
101	KC_AB1	CG10979	0.269604865917767	0.666668717234069	1	pseudobulk	DESeq2	LRT	0.176089923232574
101	KC_AB1	CG10984	0.108894066445455	0.825916411740519	1	pseudobulk	DESeq2	LRT	0.0830639039590138
101	KC_AB1	CG11000	-0.0200623436407695	0.909902957907858	1	pseudobulk	DESeq2	LRT	0.0410049231651103
101	KC_AB1	CG11007	0.447738922498412	0.522825226746336	1	pseudobulk	DESeq2	LRT	0.281643465520698
101	KC_AB1	CG1103	1.33642528709509	0.0766584419202964	1	pseudobulk	DESeq2	LRT	1.11544001200958
101	KC_AB1	CG11030	-0.0410414065462782	0.940480447544822	1	pseudobulk	DESeq2	LRT	0.02665022894046
101	KC_AB1	CG11035	-0.0912112056918425	0.875340543250174	1	pseudobulk	DESeq2	LRT	0.0578229557984144
101	KC_AB1	CG1105	-0.404488824326514	0.609274172790068	1	pseudobulk	DESeq2	LRT	0.215187231283851
101	KC_AB1	CG11050	0.0128991037819421	0.959550154431326	1	pseudobulk	DESeq2	LRT	0.0179323203307975
101	KC_AB1	CG11052	2.09987857572813	0.503115176013642	1	pseudobulk	DESeq2	LRT	0.29833258237694
101	KC_AB1	CG11069	1.13698282223142	0.780593459054812	1	pseudobulk	DESeq2	LRT	0.107575092189158
101	KC_AB1	CG11070	-0.517440838029503	0.444806279048598	1	pseudobulk	DESeq2	LRT	0.351829090722514
101	KC_AB1	CG11076	0.211073731656466	0.490054641442148	1	pseudobulk	DESeq2	LRT	0.309755493127045
101	KC_AB1	CG11077	0.617438469738514	0.130395063697368	1	pseudobulk	DESeq2	LRT	0.884738849168303
101	KC_AB1	CG11089	1.06135829763288	0.326875433498326	1	pseudobulk	DESeq2	LRT	0.485617717832074
101	KC_AB1	CG11095	0.904615860968118	0.425891983136295	1	pseudobulk	DESeq2	LRT	0.370700534879147
101	KC_AB1	CG11099	0.489511613815149	0.296347708211303	1	pseudobulk	DESeq2	LRT	0.528198427004459
101	KC_AB1	CG11103	0.21711423933491	0.803446388053707	1	pseudobulk	DESeq2	LRT	0.0950430973066628
101	KC_AB1	CG11109	-1.02709564654544	0.282591122626033	1	pseudobulk	DESeq2	LRT	0.548841485286767
101	KC_AB1	CG11110	-0.191971143883095	0.763278899590935	1	pseudobulk	DESeq2	LRT	0.117316743275268
101	KC_AB1	CG11122	-0.106467384607288	0.654300679322226	1	pseudobulk	DESeq2	LRT	0.184222628798777
101	KC_AB1	CG11123	3.3946049719343	0.0850732437526713	1	pseudobulk	DESeq2	LRT	1.07020700769083
101	KC_AB1	CG11127	-0.257706676310115	0.806299395512547	1	pseudobulk	DESeq2	LRT	0.0935036657936811
101	KC_AB1	CG1113	1.89990005719648	0.501443369585822	1	pseudobulk	DESeq2	LRT	0.299778106841186
101	KC_AB1	CG11131	2.77905602015038	0.396550243710121	1	pseudobulk	DESeq2	LRT	0.401701778879434
101	KC_AB1	CG11134	0.997928153496135	0.615652126063321	1	pseudobulk	DESeq2	LRT	0.210664616418083
101	KC_AB1	CG11137	0.0894140780674594	0.749039080494154	1	pseudobulk	DESeq2	LRT	0.125495522754216
101	KC_AB1	CG11141	-0.284728578145493	0.726960474215676	1	pseudobulk	DESeq2	LRT	0.13848920165359

101	KC_AB1	CG11147	1.04414577861848	0.560882751576686	1	pseudobulk	DESeq2	LRT	0.251127915330556
101	KC_AB1	CG11151	0.0176660228131265	0.934541285867114	1	pseudobulk	DESeq2	LRT	0.0294015077381138
101	KC_AB1	CG11155	-0.0591837017295598	0.713651487075999	1	pseudobulk	DESeq2	LRT	0.146513824906647
101	KC_AB1	CG11159	0.539316144272388	0.575553542948064	1	pseudobulk	DESeq2	LRT	0.239914268341332
101	KC_AB1	CG1116	-0.344346486948198	0.70806140784441	1	pseudobulk	DESeq2	LRT	0.149929075740347
101	KC_AB1	CG11168	-0.760959112540794	1	1	pseudobulk	DESeq2	LRT	0
101	KC_AB1	CG11170	-0.0934947478005799	0.922463513336733	1	pseudobulk	DESeq2	LRT	0.0350508026972458
101	KC_AB1	CG11178	0.00242604108994093	0.963695596001087	1	pseudobulk	DESeq2	LRT	0.016060125696818
101	KC_AB1	CG11180	0.496158712663431	0.644351657560384	1	pseudobulk	DESeq2	LRT	0.190877049944806
101	KC_AB1	CG11191	-0.141113437406226	0.334771797371154	1	pseudobulk	DESeq2	LRT	0.475251135989384
101	KC_AB1	CG11200	1.55093389295942	0.276482676406289	1	pseudobulk	DESeq2	LRT	0.558332075125475
101	KC_AB1	CG11211	-0.760959112540794	0.805265236978025	1	pseudobulk	DESeq2	LRT	0.0940610488455931
101	KC_AB1	CG11241	-1.22498523733724	0.615248217301746	1	pseudobulk	DESeq2	LRT	0.210949636003277
101	KC_AB1	CG11247	-0.151274741727081	0.638088744848795	1	pseudobulk	DESeq2	LRT	0.195118915764963
101	KC_AB1	CG11248	0.142824771890532	0.819184266873954	1	pseudobulk	DESeq2	LRT	0.0866183972806462
101	KC_AB1	CG11257	0.0123713864672923	0.977870415968485	1	pseudobulk	DESeq2	LRT	0.00971869261375252
101	KC_AB1	CG11267	0.334494657090494	0.146878936448572	1	pseudobulk	DESeq2	LRT	0.833040480860894
101	KC_AB1	CG11269	-0.760959112540794	0.805265236978025	1	pseudobulk	DESeq2	LRT	0.0940610488455931
101	KC_AB1	CG11275	3.4290155526378	0.191656243008451	1	pseudobulk	DESeq2	LRT	0.717477029475316
101	KC_AB1	CG11279	0.232554164458403	0.729451051080357	1	pseudobulk	DESeq2	LRT	0.137003845592236
101	KC_AB1	CG1129	0.148647688332832	0.818340629724321	1	pseudobulk	DESeq2	LRT	0.0870658860388212
101	KC_AB1	CG11307	0.124713343532449	0.895737468843567	1	pseudobulk	DESeq2	LRT	0.047819258796971
101	KC_AB1	CG11309	-0.175561436305943	0.864899964867936	1	pseudobulk	DESeq2	LRT	0.0630341205330844
101	KC_AB1	CG11317	0.159877344527052	0.651319732463317	1	pseudobulk	DESeq2	LRT	0.186205764187169
101	KC_AB1	CG11319	-0.110183417120139	0.419149863000185	1	pseudobulk	DESeq2	LRT	0.3776306714551
101	KC_AB1	CG11334	0.339504694459315	0.627010933419385	1	pseudobulk	DESeq2	LRT	0.202724886151249
101	KC_AB1	CG11342	-0.0514521474285079	0.956803617156654	1	pseudobulk	DESeq2	LRT	0.0191771915201654
101	KC_AB1	CG11357	0.00125664786940199	0.956871309008675	1	pseudobulk	DESeq2	LRT	0.0191464671819689
101	KC_AB1	CG11360	-0.302109656574569	0.260149710524676	1	pseudobulk	DESeq2	LRT	0.5847766530184
101	KC_AB1	CG11367	-0.456459010610097	0.611007542484549	1	pseudobulk	DESeq2	LRT	0.213953428645823
101	KC_AB1	CG11374	1.05093696471708	0.640069150462412	1	pseudobulk	DESeq2	LRT	0.193773104075583
101	KC_AB1	CG11377	0.511318431391731	0.517997345439011	1	pseudobulk	DESeq2	LRT	0.285672465861221
101	KC_AB1	CG11378	-0.205858952508648	0.881310275773043	1	pseudobulk	DESeq2	LRT	0.0548711661248875
101	KC_AB1	CG11380	-0.760959112540794	1	1	pseudobulk	DESeq2	LRT	0
101	KC_AB1	CG11391	-0.718853741766346	0.977252643133665	1	pseudobulk	DESeq2	LRT	0.0099931462763106
101	KC_AB1	CG11396	0.545002655162246	0.460197353467276	1	pseudobulk	DESeq2	LRT	0.337055883229008
101	KC_AB1	CG11398	0.0423301552797832	0.947914603907498	1	pseudobulk	DESeq2	LRT	0.0232307857866761
101	KC_AB1	CG11399	0.0653934739534826	0.902899159570736	1	pseudobulk	DESeq2	LRT	0.0443607512230228
101	KC_AB1	CG11403	0.291853629993484	0.902736173524132	1	pseudobulk	DESeq2	LRT	0.0444391545856091
101	KC_AB1	CG11406	0.210025833244221	0.682099310938526	1	pseudobulk	DESeq2	LRT	0.16615238919594
101	KC_AB1	CG11407	-0.532370299087338	0.512307421128458	1	pseudobulk	DESeq2	LRT	0.290469353021189
101	KC_AB1	CG11409	-0.760959112540794	1	1	pseudobulk	DESeq2	LRT	0
101	KC_AB1	CG11413	7.71266597450146	0.00955188481377807	1	pseudobulk	DESeq2	LRT	2.01991092334224
101	KC_AB1	CG11414	-0.444998820157925	0.50589126818572	1	pseudobulk	DESeq2	LRT	0.295942816561951
101	KC_AB1	CG11417	-1.43743706063807	0.396971108835272	1	pseudobulk	DESeq2	LRT	0.401241099609669
101	KC_AB1	CG1142	0.0829560764940202	0.892300251567942	1	pseudobulk	DESeq2	LRT	0.0494889845724997
101	KC_AB1	CG11425	-0.204781890645532	0.933341089913253	1	pseudobulk	DESeq2	LRT	0.0299596141354504
101	KC_AB1	CG11426	1.82002062570305	0.389438666078937	1	pseudobulk	DESeq2	LRT	0.409560931010498
101	KC_AB1	CG1143	1.11388934932283	1	1	pseudobulk	DESeq2	LRT	0
101	KC_AB1	CG11436	-1.40497295344719	0.189546766192824	1	pseudobulk	DESeq2	LRT	0.722283620557474
101	KC_AB1	CG11438	-3.39161266055751	0.175647375301919	1	pseudobulk	DESeq2	LRT	0.75535833548431
101	KC_AB1	CG11444	0.161479140769738	0.6517465497781	1	pseudobulk	DESeq2	LRT	0.185921259225684
101	KC_AB1	CG11447	-0.741005553478096	0.403660099370312	1	pseudobulk	DESeq2	LRT	0.393984177217657
101	KC_AB1	CG11453	-0.760959112540794	1	1	pseudobulk	DESeq2	LRT	0
101	KC_AB1	CG11454	-0.0366514013547321	0.904659509674049	1	pseudobulk	DESeq2	LRT	0.0435148471970081
101	KC_AB1	CG11456	-0.234940183619683	0.712049726209347	1	pseudobulk	DESeq2	LRT	0.147489676216749

101	KC_AB1	CG1146	0.851951216522685	0.701796797121782	1	pseudobulk	DESeq2	LRT	0.153788618160382
101	KC_AB1	CG11474	-0.574326160250485	0.294086175142175	1	pseudobulk	DESeq2	LRT	0.531525390999861
101	KC_AB1	CG11486	-0.345139372251533	0.0605821362363821	1	pseudobulk	DESeq2	LRT	1.21765541671932
101	KC_AB1	CG11504	-0.137069689092473	0.788087040729221	1	pseudobulk	DESeq2	LRT	0.10342581395609
101	KC_AB1	CG11523	-0.157211636042676	0.698873920713613	1	pseudobulk	DESeq2	LRT	0.155601165423285
101	KC_AB1	CG11537	-0.0565752785389157	0.771356139619348	1	pseudobulk	DESeq2	LRT	0.112745059354911
101	KC_AB1	CG11539	0.861468815698364	0.644713991007348	1	pseudobulk	DESeq2	LRT	0.190632905015578
101	KC_AB1	CG11555	0.521093635863112	0.38918082222113	1	pseudobulk	DESeq2	LRT	0.409848568734481
101	KC_AB1	CG11560	-0.0116846879612857	0.961935698885179	1	pseudobulk	DESeq2	LRT	0.0168539576427252
102	KC_AB1	CG11562	0.300454602866195	0.790506007815021	1	pseudobulk	DESeq2	LRT	0.102094825098
102	KC_AB1	CG11563	0.0782990452432692	0.926483887109726	1	pseudobulk	DESeq2	LRT	0.033162129286458
102	KC_AB1	CG11583	-0.288837819875467	0.64550966770841	1	pseudobulk	DESeq2	LRT	0.19009724898087
102	KC_AB1	CG11586	0.0972525713313357	0.817465590344511	1	pseudobulk	DESeq2	LRT	0.0875305190811525
102	KC_AB1	CG11590	0.369578697135892	0.692091688811909	1	pseudobulk	DESeq2	LRT	0.159836366081884
102	KC_AB1	CG11593	-0.10773082442055	0.8869765658672	1	pseudobulk	DESeq2	LRT	0.0520878541806815
102	KC_AB1	CG11594	-2.1900801774161	0.437871127685654	1	pseudobulk	DESeq2	LRT	0.35865369034154
102	KC_AB1	CG11596	0.41870198175886	0.640503154121929	1	pseudobulk	DESeq2	LRT	0.193478727255164
102	KC_AB1	CG11597	1.03091791266704	0.609326680396086	1	pseudobulk	DESeq2	LRT	0.215149805143035
102	KC_AB1	CG11601	2.09987857572813	0.503115176013642	1	pseudobulk	DESeq2	LRT	0.29833258237694
102	KC_AB1	CG1161	-0.136637551942434	0.673782712015374	1	pseudobulk	DESeq2	LRT	0.171480136383463
102	KC_AB1	CG11626	1.94571210489703	0.479499733936668	1	pseudobulk	DESeq2	LRT	0.319211629473233
102	KC_AB1	CG11635	2.09987857572813	0.503115176013642	1	pseudobulk	DESeq2	LRT	0.29833258237694
102	KC_AB1	CG11638	0.371055422160238	0.881976387776155	1	pseudobulk	DESeq2	LRT	0.054543041620775
102	KC_AB1	CG11655	1.65693832904969	0.309290007630012	1	pseudobulk	DESeq2	LRT	0.50963411071537
102	KC_AB1	CG11658	0.0111005431597901	0.92788320424124	1	pseudobulk	DESeq2	LRT	0.0325066864382385
102	KC_AB1	CG11668	3.31338974495522	0.157494751650168	1	pseudobulk	DESeq2	LRT	0.802733914048024
102	KC_AB1	CG11679	0.152115475746305	1	1	pseudobulk	DESeq2	LRT	0
102	KC_AB1	CG11686	1.84094063849367	0.301869369254155	1	pseudobulk	DESeq2	LRT	0.520180952690024
102	KC_AB1	CG11695	0.208899291692333	0.836396631854455	1	pseudobulk	DESeq2	LRT	0.0775877247424211
102	KC_AB1	CG11696	-0.217047539790791	0.774919866702233	1	pseudobulk	DESeq2	LRT	0.110743204910925
102	KC_AB1	CG11699	0.284910485683162	0.429950733022267	1	pseudobulk	DESeq2	LRT	0.366581306286463
102	KC_AB1	CG11700	-1.77067014871429	0.542156052888816	1	pseudobulk	DESeq2	LRT	0.265875689192671
102	KC_AB1	CG11710	1.04023841384252	0.109544900062259	1	pseudobulk	DESeq2	LRT	0.96040783650926
102	KC_AB1	CG1172	0.364616871533953	0.526436490521898	1	pseudobulk	DESeq2	LRT	0.278654014753065
102	KC_AB1	CG11722	0.18314021649462	0.76153859630146	1	pseudobulk	DESeq2	LRT	0.118308080855885
102	KC_AB1	CG11723	-0.230310205892974	0.80238425967107	1	pseudobulk	DESeq2	LRT	0.0956175994356038
102	KC_AB1	CG11737	0.318832377005632	0.827633204400028	1	pseudobulk	DESeq2	LRT	0.0821620938902683
102	KC_AB1	CG11752	0.0129900268340672	0.959306717210169	1	pseudobulk	DESeq2	LRT	0.018042514523984
102	KC_AB1	CG11753	0.359110812187543	0.395705758515831	1	pseudobulk	DESeq2	LRT	0.402627629608999
102	KC_AB1	CG11755	0.304942036117215	0.766449166558359	1	pseudobulk	DESeq2	LRT	0.115516643699735
102	KC_AB1	CG11760	1.3724368174049	0.345877958372562	1	pseudobulk	DESeq2	LRT	0.46107711321598
102	KC_AB1	CG11768	-0.0148621311899659	0.971488824092861	1	pseudobulk	DESeq2	LRT	0.0125621911143465
102	KC_AB1	CG11771	0.0915944534204492	0.878597400189808	1	pseudobulk	DESeq2	LRT	0.0562100861661808
102	KC_AB1	CG11777	0.0506010033994659	0.903470721752984	1	pseudobulk	DESeq2	LRT	0.04408591680216
102	KC_AB1	CG11779	1.07772009115263	0.145082844786755	1	pseudobulk	DESeq2	LRT	0.838383937354926
102	KC_AB1	CG11781	0.318632701440533	0.41499498909433	1	pseudobulk	DESeq2	LRT	0.381957147195908
102	KC_AB1	CG11788	0.84880183353381	0.649902681599463	1	pseudobulk	DESeq2	LRT	0.187151671062708
102	KC_AB1	CG11790	0.0155072538597503	0.963244124071005	1	pseudobulk	DESeq2	LRT	0.0162636315662691
102	KC_AB1	CG11791	-0.189719127917919	0.883150811860196	1	pseudobulk	DESeq2	LRT	0.0539651274914342
102	KC_AB1	CG11807	-0.163614772247526	0.796815100022409	1	pseudobulk	DESeq2	LRT	0.0986424444208649
102	KC_AB1	CG11808	-0.435838499933394	0.690857416101621	1	pseudobulk	DESeq2	LRT	0.160611576055502
102	KC_AB1	CG11811	-0.07906974015829	0.864285496896137	1	pseudobulk	DESeq2	LRT	0.0633427745977778
102	KC_AB1	CG11825	3.51235137991753	0.182155193970943	1	pseudobulk	DESeq2	LRT	0.739558440778038
102	KC_AB1	CG11828	2.4138856564567	0.409611385497059	1	pseudobulk	DESeq2	LRT	0.387627980279722
102	KC_AB1	CG11835	0.943798956650056	0.807634491832869	1	pseudobulk	DESeq2	LRT	0.0927851418175362
102	KC_AB1	CG11837	0.916395010650245	0.47640320950229	1	pseudobulk	DESeq2	LRT	0.322025321361303

102	KC_AB1	CG11839	-0.613836156035403	0.419806541131781	1	pseudobulk	DESeq2	LRT	0.376950798828163
102	KC_AB1	CG11841	-4.99323691487998	0.0566196667811208	1	pseudobulk	DESeq2	LRT	1.24703269087954
102	KC_AB1	CG11843	-0.760959112540794	1	1	pseudobulk	DESeq2	LRT	0
102	KC_AB1	CG11852	-0.248147337867302	0.94697012791198	1	pseudobulk	DESeq2	LRT	0.0236637205612551
102	KC_AB1	CG11857	-0.408507801167826	0.375726021381344	1	pseudobulk	DESeq2	LRT	0.4251287262808
102	KC_AB1	CG11858	0.41710343072151	0.471368102868893	1	pseudobulk	DESeq2	LRT	0.326639809203974
102	KC_AB1	CG11873	-0.292782433130545	0.29148845660867	1	pseudobulk	DESeq2	LRT	0.535378639294064
102	KC_AB1	CG11882	0.100538303815713	0.895833925606362	1	pseudobulk	DESeq2	LRT	0.0477724946657798
102	KC_AB1	CG11883	-0.210761215297297	0.616873912242907	1	pseudobulk	DESeq2	LRT	0.209803595793493
102	KC_AB1	CG11885	0.0597328523670119	0.921658372283553	1	pseudobulk	DESeq2	LRT	0.0354300274561887
102	KC_AB1	CG11892	-0.760959112540794	0.804334723897681	1	pseudobulk	DESeq2	LRT	0.0945631819854715
102	KC_AB1	CG11893	1.63195036548663	0.633334503478132	1	pseudobulk	DESeq2	LRT	0.198366851366371
102	KC_AB1	CG11898	0.296275065198765	1	1	pseudobulk	DESeq2	LRT	0
102	KC_AB1	CG11899	-0.339914322499397	0.784754492885513	1	pseudobulk	DESeq2	LRT	0.105266189193004
102	KC_AB1	CG11902	-0.229445403387411	0.787828089610357	1	pseudobulk	DESeq2	LRT	0.103568538701672
102	KC_AB1	CG11905	2.95597035741046	0.225995086366541	1	pseudobulk	DESeq2	LRT	0.645901003273377
102	KC_AB1	CG11906	-0.0728855710908522	0.901143814203671	1	pseudobulk	DESeq2	LRT	0.0452058941141976
102	KC_AB1	CG11911	-0.398753196791637	0.871522191505789	1	pseudobulk	DESeq2	LRT	0.0597215500045825
102	KC_AB1	CG11920	0.765398525300913	0.579487929709584	1	pseudobulk	DESeq2	LRT	0.236955605628109
102	KC_AB1	CG11927	-0.02650468930659	0.915989566014999	1	pseudobulk	DESeq2	LRT	0.038109473327694
102	KC_AB1	CG11951	-0.760959112540794	1	1	pseudobulk	DESeq2	LRT	0
102	KC_AB1	CG11961	0.323670736863392	0.914596139679247	1	pseudobulk	DESeq2	LRT	0.0387706360184678
102	KC_AB1	CG11964	0.715223456311661	0.51224192311575	1	pseudobulk	DESeq2	LRT	0.290524880704054
102	KC_AB1	CG11970	-0.886111845297661	0.449374062531317	1	pseudobulk	DESeq2	LRT	0.347391998282279
102	KC_AB1	CG11975	-0.00220153556953762	0.996765140606124	1	pseudobulk	DESeq2	LRT	0.00140715879398308
102	KC_AB1	CG11980	-0.071356862320747	0.879464287313703	1	pseudobulk	DESeq2	LRT	0.0557817913827504
102	KC_AB1	CG11983	-0.697357195569961	0.770678219972763	1	pseudobulk	DESeq2	LRT	0.113126914374965
102	KC_AB1	CG11986	0.0353511519075622	0.971660734477814	1	pseudobulk	DESeq2	LRT	0.0124853470737709
102	KC_AB1	CG11999	-0.449378742421508	0.58602391691055	1	pseudobulk	DESeq2	LRT	0.23208465911789
102	KC_AB1	CG12004	-0.0979459560982034	0.738297994703558	1	pseudobulk	DESeq2	LRT	0.131768311166269
102	KC_AB1	CG12007	-0.123133701531611	0.872442291719388	1	pseudobulk	DESeq2	LRT	0.0592632901185774
102	KC_AB1	CG12010	-1.26615411498443	0.406559248641923	1	pseudobulk	DESeq2	LRT	0.390876154908248
102	KC_AB1	CG12012	0.38709747193078	0.520309018054875	1	pseudobulk	DESeq2	LRT	0.283738646797546
102	KC_AB1	CG12024	-0.10629358661036	0.802509335671419	1	pseudobulk	DESeq2	LRT	0.0955499067025304
102	KC_AB1	CG12025	0.206679304464911	0.456022360687661	1	pseudobulk	DESeq2	LRT	0.341013861534753
102	KC_AB1	CG12038	0.474979580942996	0.149317563680359	1	pseudobulk	DESeq2	LRT	0.825889104794543
102	KC_AB1	CG12054	0.0433097399496745	0.816283679284279	1	pseudobulk	DESeq2	LRT	0.0881588866656372
102	KC_AB1	CG12056	0.357502952774197	0.671745786325184	1	pseudobulk	DESeq2	LRT	0.172795049094959
102	KC_AB1	CG12065	2.35061922459225	0.0913648013058769	1	pseudobulk	DESeq2	LRT	1.03922108591677
102	KC_AB1	CG12071	-0.192318652445292	0.488560462271298	1	pseudobulk	DESeq2	LRT	0.311081682086657
102	KC_AB1	CG12075	0.293544814793053	0.855484935116898	1	pseudobulk	DESeq2	LRT	0.0677876338764847
102	KC_AB1	CG1208	5.51721280758424	0.17859004781649	1	pseudobulk	DESeq2	LRT	0.748142746448641
102	KC_AB1	CG12081	0.586787542529054	0.292195978698931	1	pseudobulk	DESeq2	LRT	0.5343257652699
102	KC_AB1	CG12084	-0.413338706456631	0.497844363187769	1	pseudobulk	DESeq2	LRT	0.302906405780479
102	KC_AB1	CG12091	0.0278263837559658	0.903598178931693	1	pseudobulk	DESeq2	LRT	0.0440246530070018
102	KC_AB1	CG12093	0.991301595006691	0.163620579749219	1	pseudobulk	DESeq2	LRT	0.786162072856484
102	KC_AB1	CG12096	-0.255268625168166	0.751373100552651	1	pseudobulk	DESeq2	LRT	0.124144356904604
102	KC_AB1	CG12099	0.0829430174724794	0.823451136891421	1	pseudobulk	DESeq2	LRT	0.0843621665100524
102	KC_AB1	CG12104	-0.345051118852611	0.69740249367015	1	pseudobulk	DESeq2	LRT	0.156516504070381
102	KC_AB1	CG12105	-0.760959112540794	0.804334723897681	1	pseudobulk	DESeq2	LRT	0.0945631819854715
102	KC_AB1	CG12106	-0.640202873858422	0.415142170147276	1	pseudobulk	DESeq2	LRT	0.381803148741331
102	KC_AB1	CG12107	-0.0148505255035931	0.96985917999625	1	pseudobulk	DESeq2	LRT	0.0132913191257832
102	KC_AB1	CG12112	0.942515514173974	0.714955176215666	1	pseudobulk	DESeq2	LRT	0.145721185237255
103	KC_AB1	CG12118	-0.0861943334454995	0.89481144891467	1	pseudobulk	DESeq2	LRT	0.0482684678379741
103	KC_AB1	CG12121	0.246982164698388	0.630774459608054	1	pseudobulk	DESeq2	LRT	0.200125899800722
103	KC_AB1	CG12123	0.325448135482772	0.54673702733954	1	pseudobulk	DESeq2	LRT	0.262221512856749

103	KC_AB1	CG12128	0.100272279377509	0.89670826381465	1	pseudobulk	DESeq2	LRT	0.0473488278851243
103	KC_AB1	CG12129	0.373972523910649	0.553879848784334	1	pseudobulk	DESeq2	LRT	0.256584435035596
103	KC_AB1	CG1213	8.61632463839364	0.0787244139862525	1	pseudobulk	DESeq2	LRT	1.10389056350798
103	KC_AB1	CG12134	-0.181134628200276	0.900938013069757	1	pseudobulk	DESeq2	LRT	0.0453050886078817
103	KC_AB1	CG12155	0.354946120441245	0.604468995464788	1	pseudobulk	DESeq2	LRT	0.218625970144612
103	KC_AB1	CG12158	1.25804452752548	0.727552935095488	1	pseudobulk	DESeq2	LRT	0.138135402914536
103	KC_AB1	CG12159	0.091288391490477	0.842802181886711	1	pseudobulk	DESeq2	LRT	0.0742743487400979
103	KC_AB1	CG12163	0.0797706131608046	0.898441550063056	1	pseudobulk	DESeq2	LRT	0.046510171544194
103	KC_AB1	CG12164	1.25804452752548	0.727552935095488	1	pseudobulk	DESeq2	LRT	0.138135402914536
103	KC_AB1	CG12170	0.469523169697173	0.563283209041691	1	pseudobulk	DESeq2	LRT	0.249273194493791
103	KC_AB1	CG12171	0.228874370479804	0.819200743485959	1	pseudobulk	DESeq2	LRT	0.0866096622133337
103	KC_AB1	CG12173	0.334659768249482	0.585068780712538	1	pseudobulk	DESeq2	LRT	0.232793075237435
103	KC_AB1	CG12177	0.286027570046924	0.65358268695981	1	pseudobulk	DESeq2	LRT	0.184699460541438
103	KC_AB1	CG12179	-1.73533618889279	0.492709206576031	1	pseudobulk	DESeq2	LRT	0.307409322588473
103	KC_AB1	CG1218	-0.554703293556128	0.44045337853484	1	pseudobulk	DESeq2	LRT	0.356100054372485
103	KC_AB1	CG12182	-0.686946213647028	0.7088038181097	1	pseudobulk	DESeq2	LRT	0.149473951702964
103	KC_AB1	CG12194	-0.181684657336304	0.439202305068361	1	pseudobulk	DESeq2	LRT	0.357335389241383
103	KC_AB1	CG12204	0.389099173695241	0.631426291072519	1	pseudobulk	DESeq2	LRT	0.199677339089344
103	KC_AB1	CG12206	-0.573338767577075	0.738934348585005	1	pseudobulk	DESeq2	LRT	0.131394145250392
103	KC_AB1	CG12213	0.245425953781926	0.602694837381729	1	pseudobulk	DESeq2	LRT	0.219902528635341
103	KC_AB1	CG12219	0.401466154258396	0.688773756387866	1	pseudobulk	DESeq2	LRT	0.161923408698805
103	KC_AB1	CG12236	-0.549748682875004	0.357818278478599	1	pseudobulk	DESeq2	LRT	0.446337478079338
103	KC_AB1	CG12237	0.0607385052766334	0.874132975723177	1	pseudobulk	DESeq2	LRT	0.0584224961642453
103	KC_AB1	CG12241	0.415317278376278	0.542150774430412	1	pseudobulk	DESeq2	LRT	0.265879917526017
103	KC_AB1	CG12253	0.889462953627714	0.186618975474268	1	pseudobulk	DESeq2	LRT	0.729044199149107
103	KC_AB1	CG12259	0.130331071493578	0.849583768672538	1	pseudobulk	DESeq2	LRT	0.0707937933945361
103	KC_AB1	CG12267	-0.835903794569968	0.551479602404141	1	pseudobulk	DESeq2	LRT	0.258470546192331
103	KC_AB1	CG12268	0.664026106360037	0.787782864887872	1	pseudobulk	DESeq2	LRT	0.103593469789349
103	KC_AB1	CG1227	-0.549118462108481	0.614298710759963	1	pseudobulk	DESeq2	LRT	0.211620396141322
103	KC_AB1	CG12278	-0.760959112540794	0.804334723897681	1	pseudobulk	DESeq2	LRT	0.0945631819854715
103	KC_AB1	CG12279	-0.231137271347197	0.558650065653321	1	pseudobulk	DESeq2	LRT	0.252860145859078
103	KC_AB1	CG12288	-0.697357195569961	0.770678219972763	1	pseudobulk	DESeq2	LRT	0.113126914374965
103	KC_AB1	CG12290	0.166467630523283	0.929380664278055	1	pseudobulk	DESeq2	LRT	0.0318063672398678
103	KC_AB1	CG12299	0.375527291234767	0.432595340983267	1	pseudobulk	DESeq2	LRT	0.363918162188083
103	KC_AB1	CG12301	-0.115339844861282	0.885436341580179	1	pseudobulk	DESeq2	LRT	0.0528426569405954
103	KC_AB1	CG1231	0.0268544106739412	0.973615507833322	1	pseudobulk	DESeq2	LRT	0.0116125172422478
103	KC_AB1	CG12316	-0.219431604708708	0.77012756944609	1	pseudobulk	DESeq2	LRT	0.113437329220255
103	KC_AB1	CG12320	1.07465566575952	0.425879366006107	1	pseudobulk	DESeq2	LRT	0.370713401125525
103	KC_AB1	CG12321	0.434145391704515	0.529287882750281	1	pseudobulk	DESeq2	LRT	0.276308048413656
103	KC_AB1	CG12325	-0.38782029347039	0.702788491506592	1	pseudobulk	DESeq2	LRT	0.153175358895731
103	KC_AB1	CG1233	-0.388883259757297	0.755836564289598	1	pseudobulk	DESeq2	LRT	0.121572102515453
103	KC_AB1	CG12333	-0.212768110346647	0.629829398536037	1	pseudobulk	DESeq2	LRT	0.200777071670267
103	KC_AB1	CG12338	-0.37207163196066	0.830615681553245	1	pseudobulk	DESeq2	LRT	0.0805998739150963
103	KC_AB1	CG1234	-0.00968664917892965	0.989489535527744	1	pseudobulk	DESeq2	LRT	0.00458879436975078
103	KC_AB1	CG12341	0.38077204194224	0.488602989164673	1	pseudobulk	DESeq2	LRT	0.311043880437179
103	KC_AB1	CG12343	-0.0673528105391455	0.851745001787651	1	pseudobulk	DESeq2	LRT	0.0696904062806739
103	KC_AB1	CG12344	-0.716419614646419	0.812870875922622	1	pseudobulk	DESeq2	LRT	0.0899784363592155
103	KC_AB1	CG12355	-0.125799875677609	0.776366164279165	1	pseudobulk	DESeq2	LRT	0.109933400366439
103	KC_AB1	CG1236	0.554367873805515	0.644714224735985	1	pseudobulk	DESeq2	LRT	0.190632747570509
103	KC_AB1	CG12375	-0.553602135110652	0.318385452221643	1	pseudobulk	DESeq2	LRT	0.497046784414912
103	KC_AB1	CG12379	1.16599462037993	0.307742580413023	1	pseudobulk	DESeq2	LRT	0.51181240898812
103	KC_AB1	CG12384	-0.297397190463665	0.297993569892828	1	pseudobulk	DESeq2	LRT	0.52579310703177
103	KC_AB1	CG1239	0.0561583165629429	0.938160920679725	1	pseudobulk	DESeq2	LRT	0.0277226616538699
103	KC_AB1	CG12391	-0.227792412629985	0.567365541962869	1	pseudobulk	DESeq2	LRT	0.246137043918752
103	KC_AB1	CG12402	-0.395578318545968	0.514322453598143	1	pseudobulk	DESeq2	LRT	0.288764515420479
103	KC_AB1	CG12470	1.13698282223142	0.780593459054812	1	pseudobulk	DESeq2	LRT	0.107575092189158

103	KC_AB1	CG12499	-0.919719573666537	0.548887520100804	1	pseudobulk	DESeq2	LRT	0.260516643536058
103	KC_AB1	CG12502	-0.212559983188017	0.826577388118522	1	pseudobulk	DESeq2	LRT	0.082716479467656
103	KC_AB1	CG12512	2.74179455111232	0.326270680492327	1	pseudobulk	DESeq2	LRT	0.486421951258097
103	KC_AB1	CG12531	-0.387271562055667	0.112561082399695	1	pseudobulk	DESeq2	LRT	0.948611739329934
103	KC_AB1	CG12535	0.432401479180099	0.826492410621546	1	pseudobulk	DESeq2	LRT	0.0827611300421001
103	KC_AB1	CG12539	2.58743201691345	0.417636627932844	1	pseudobulk	DESeq2	LRT	0.379201419460921
103	KC_AB1	CG12541	-0.332365956284298	0.429345854117189	1	pseudobulk	DESeq2	LRT	0.367192726413148
103	KC_AB1	CG12547	-0.156682111136191	0.700593981848868	1	pseudobulk	DESeq2	LRT	0.154533597622171
103	KC_AB1	CG12560	0.404226958181589	0.651088359335101	1	pseudobulk	DESeq2	LRT	0.186360069238765
103	KC_AB1	CG12567	-0.327931710922359	0.441166683053192	1	pseudobulk	DESeq2	LRT	0.355397292951029
103	KC_AB1	CG12576	-0.128530877445618	0.712522050301593	1	pseudobulk	DESeq2	LRT	0.147201691072257
103	KC_AB1	CG12581	2.78044614204691	0.247077463052649	1	pseudobulk	DESeq2	LRT	0.60716686656978
103	KC_AB1	CG12594	0.291853629993484	0.902736173524132	1	pseudobulk	DESeq2	LRT	0.0444391545856091
103	KC_AB1	CG12605	-0.120100334133556	0.550497499603602	1	pseudobulk	DESeq2	LRT	0.259244649282283
103	KC_AB1	CG12607	-0.760959112540794	0.805265236978025	1	pseudobulk	DESeq2	LRT	0.0940610488455931
103	KC_AB1	CG12608	-0.139538859256104	0.906625199761536	1	pseudobulk	DESeq2	LRT	0.0425722138397814
103	KC_AB1	CG12640	-0.534400629357398	0.80033240638072	1	pseudobulk	DESeq2	LRT	0.0967295976663479
103	KC_AB1	CG12643	2.09987857572813	0.503115176013642	1	pseudobulk	DESeq2	LRT	0.29833258237694
103	KC_AB1	CG1265	0.261537279946484	0.542969248204514	1	pseudobulk	DESeq2	LRT	0.265224766568206
103	KC_AB1	CG12655	1.67020383217772	0.598754457572587	1	pseudobulk	DESeq2	LRT	0.222751240354882
103	KC_AB1	CG12659	0.124241991315699	0.890509995997119	1	pseudobulk	DESeq2	LRT	0.050361201206886
103	KC_AB1	CG12672	-0.431575413180694	0.798460994444905	1	pseudobulk	DESeq2	LRT	0.0977462946928114
103	KC_AB1	CG12693	0.291868230578568	0.902153667810848	1	pseudobulk	DESeq2	LRT	0.0447194808636415
103	KC_AB1	CG12698	-0.760959112540794	1	1	pseudobulk	DESeq2	LRT	0
103	KC_AB1	CG12699	-0.760959112540794	0.804334723897681	1	pseudobulk	DESeq2	LRT	0.0945631819854715
103	KC_AB1	CG1271	-0.0935063638857447	0.923643649354769	1	pseudobulk	DESeq2	LRT	0.0344955514738061
103	KC_AB1	CG12713	-1.78357119214903	0.248404537044365	1	pseudobulk	DESeq2	LRT	0.604840476147079
103	KC_AB1	CG12717	0.138415112664847	0.869584383626475	1	pseudobulk	DESeq2	LRT	0.0606882681004037
103	KC_AB1	CG12728	-0.554254498298108	0.469638839984768	1	pseudobulk	DESeq2	LRT	0.32823599333298
103	KC_AB1	CG1273	2.09987857572813	0.503115176013642	1	pseudobulk	DESeq2	LRT	0.29833258237694
103	KC_AB1	CG12730	0.331610571598356	0.449002960180272	1	pseudobulk	DESeq2	LRT	0.347750795776811
103	KC_AB1	CG12744	-0.760959112540794	1	1	pseudobulk	DESeq2	LRT	0
103	KC_AB1	CG12746	0.00170105153595489	0.951950353757108	1	pseudobulk	DESeq2	LRT	0.0213857004091748
103	KC_AB1	CG1275	0.416490766090608	0.360818561447746	1	pseudobulk	DESeq2	LRT	0.442711129314725
103	KC_AB1	CG12768	-0.770970636051058	0.701392221065259	1	pseudobulk	DESeq2	LRT	0.154039055060115
103	KC_AB1	CG12769	0.0780993442800054	0.859144820259155	1	pseudobulk	DESeq2	LRT	0.0659336239007045
103	KC_AB1	CG12773	0.824228820319488	0.362957663815855	1	pseudobulk	DESeq2	LRT	0.440144029078022
103	KC_AB1	CG12795	-0.0481430240381988	0.9279242913093	1	pseudobulk	DESeq2	LRT	0.0324874561172254
103	KC_AB1	CG12811	1.42395346677582	0.522472482557159	1	pseudobulk	DESeq2	LRT	0.281936577921758
103	KC_AB1	CG12814	2.01602929805121	0.267954886089276	1	pseudobulk	DESeq2	LRT	0.571938319298617
103	KC_AB1	CG12817	0.0208612258462811	0.963046597240995	1	pseudobulk	DESeq2	LRT	0.0163526989242256
104	KC_AB1	CG12818	-0.934783680378571	0.325327091680051	1	pseudobulk	DESeq2	LRT	0.487679769250456
104	KC_AB1	CG12822	-0.217558390580249	0.52823677368162	1	pseudobulk	DESeq2	LRT	0.27717136827281
104	KC_AB1	CG12825	0.152115475746305	1	1	pseudobulk	DESeq2	LRT	0
104	KC_AB1	CG12836	0.71591774407153	0.525113032644683	1	pseudobulk	DESeq2	LRT	0.279747202936647
104	KC_AB1	CG12848	-0.084638859827334	0.878190044721181	1	pseudobulk	DESeq2	LRT	0.0564114904235723
104	KC_AB1	CG12851	0.00767414527315833	0.933662904187286	1	pseudobulk	DESeq2	LRT	0.0298098960146149
104	KC_AB1	CG12863	0.106550877879002	0.940594908770438	1	pseudobulk	DESeq2	LRT	0.0265973763223325
104	KC_AB1	CG12868	-0.15446653494958	0.832355627834322	1	pseudobulk	DESeq2	LRT	0.0796910797102232
104	KC_AB1	CG12877	0.323466542688985	0.671304112143947	1	pseudobulk	DESeq2	LRT	0.173080692490404
104	KC_AB1	CG12883	0.720480718783116	0.539925248667964	1	pseudobulk	DESeq2	LRT	0.267666363025553
104	KC_AB1	CG12895	0.245736716750047	0.574963686737911	1	pseudobulk	DESeq2	LRT	0.240359583392721
104	KC_AB1	CG12896	3.43555075139483	0.0355140763050699	1	pseudobulk	DESeq2	LRT	1.44959947652418
104	KC_AB1	CG12909	-0.108735187097257	0.925842729548966	1	pseudobulk	DESeq2	LRT	0.0334627795057931
104	KC_AB1	CG12910	-0.189167559525861	0.815507483203128	1	pseudobulk	DESeq2	LRT	0.0885720494625594
104	KC_AB1	CG12913	0.0731088205040105	0.889491740484669	1	pseudobulk	DESeq2	LRT	0.05085808027099

104	KC_AB1	CG12923	0.70842991589991	0.874943022847655	1	pseudobulk	DESeq2	LRT	0.0580202277417387
104	KC_AB1	CG12926	-0.155637626944815	0.843037827923148	1	pseudobulk	DESeq2	LRT	0.0741529377243689
104	KC_AB1	CG12929	0.644078112004703	0.48712635815899	1	pseudobulk	DESeq2	LRT	0.312358370339346
104	KC_AB1	CG12935	0.462161132144307	0.626512295937204	1	pseudobulk	DESeq2	LRT	0.203070401117915
104	KC_AB1	CG12942	0.351934700050603	0.776778838731447	1	pseudobulk	DESeq2	LRT	0.109702614134221
104	KC_AB1	CG12945	0.155282889545687	0.797730608451432	1	pseudobulk	DESeq2	LRT	0.0981437440067756
104	KC_AB1	CG12947	-6.86931705768883	0.124748742861454	1	pseudobulk	DESeq2	LRT	0.90396382222629
104	KC_AB1	CG12948	0.35200650313017	0.646407680451366	1	pseudobulk	DESeq2	LRT	0.189493492026431
104	KC_AB1	CG12970	-1.77067014871429	0.542156052888816	1	pseudobulk	DESeq2	LRT	0.265875689192671
104	KC_AB1	CG12975	0.388453244733204	0.453817405113257	1	pseudobulk	DESeq2	LRT	0.343118851774816
104	KC_AB1	CG1299	0.0751137719366014	0.960814369320806	1	pseudobulk	DESeq2	LRT	0.0173605105280585
104	KC_AB1	CG12991	-0.299436576984281	0.538453742330415	1	pseudobulk	DESeq2	LRT	0.268851600283451
104	KC_AB1	CG12992	-1.11000032652526	0.814275384621196	1	pseudobulk	DESeq2	LRT	0.0892286936423418
104	KC_AB1	CG12994	0.152115475746305	1	1	pseudobulk	DESeq2	LRT	0
104	KC_AB1	CG13001	-0.125635976473177	0.825327083188732	1	pseudobulk	DESeq2	LRT	0.0833739032410359
104	KC_AB1	CG13005	0.704879510996967	0.591208899747224	1	pseudobulk	DESeq2	LRT	0.228259036923755
104	KC_AB1	CG13008	1.25804452752548	0.727552935095488	1	pseudobulk	DESeq2	LRT	0.138135402914536
104	KC_AB1	CG13012	0.89169359928517	0.634678257415671	1	pseudobulk	DESeq2	LRT	0.197446379338693
104	KC_AB1	CG13014	-0.538218969557373	0.775353846208946	1	pseudobulk	DESeq2	LRT	0.110500054416431
104	KC_AB1	CG13016	0.749910513217183	0.423183635197372	1	pseudobulk	DESeq2	LRT	0.373471135114874
104	KC_AB1	CG13018	0.422586847575944	0.549197790836377	1	pseudobulk	DESeq2	LRT	0.260271218393587
104	KC_AB1	CG13022	1.25804452752548	0.727552935095488	1	pseudobulk	DESeq2	LRT	0.138135402914536
104	KC_AB1	CG13024	-4.7696400627133	0.272214481512222	1	pseudobulk	DESeq2	LRT	0.565088774527415
104	KC_AB1	CG13025	-0.530269268340368	0.675410782151454	1	pseudobulk	DESeq2	LRT	0.170432010636493
104	KC_AB1	CG13038	-0.760959112540794	1	1	pseudobulk	DESeq2	LRT	0
104	KC_AB1	CG13049	1.25804452752548	0.727552935095488	1	pseudobulk	DESeq2	LRT	0.138135402914536
104	KC_AB1	CG13051	1.25804452752548	0.573436424985144	1	pseudobulk	DESeq2	LRT	0.241514723898045
104	KC_AB1	CG13054	-0.273701563821817	0.815461151183453	1	pseudobulk	DESeq2	LRT	0.0885967240517102
104	KC_AB1	CG13055	-0.457762503394937	0.391099068009858	1	pseudobulk	DESeq2	LRT	0.407713218971567
104	KC_AB1	CG13065	-4.93204172539748	0.0608358005647135	1	pseudobulk	DESeq2	LRT	1.21584077250395
104	KC_AB1	CG13067	2.87650224989178	0.470206486530405	1	pseudobulk	DESeq2	LRT	0.327711384047892
104	KC_AB1	CG13068	4.71102227889991	0.0512520887489454	1	pseudobulk	DESeq2	LRT	1.29028843049355
104	KC_AB1	CG13069	2.09987857572813	0.503115176013642	1	pseudobulk	DESeq2	LRT	0.29833258237694
104	KC_AB1	CG1307	0.133722424607861	0.770247273568899	1	pseudobulk	DESeq2	LRT	0.113369830273051
104	KC_AB1	CG13082	7.45178225170975	0.10633960125164	1	pseudobulk	DESeq2	LRT	0.973304972520401
104	KC_AB1	CG13085	-1.43550613534661	0.602448798560473	1	pseudobulk	DESeq2	LRT	0.220079857379737
104	KC_AB1	CG13088	-0.716419614646419	0.812870875922622	1	pseudobulk	DESeq2	LRT	0.0899784363592155
104	KC_AB1	CG1309	0.466826839334583	0.527495107482985	1	pseudobulk	DESeq2	LRT	0.277781564092822
104	KC_AB1	CG13096	0.743326353614495	0.487613143565972	1	pseudobulk	DESeq2	LRT	0.31192459652937
104	KC_AB1	CG13097	0.857250147564235	0.363758829912096	1	pseudobulk	DESeq2	LRT	0.439186455777704
104	KC_AB1	CG13101	2.58743201691345	0.417636627932844	1	pseudobulk	DESeq2	LRT	0.379201419460921
104	KC_AB1	CG13108	0.0982350482629386	0.808753517333218	1	pseudobulk	DESeq2	LRT	0.0921838175379747
104	KC_AB1	CG13116	1.25804452752548	0.727552935095488	1	pseudobulk	DESeq2	LRT	0.138135402914536
104	KC_AB1	CG13117	0.317406094409081	0.922353623686712	1	pseudobulk	DESeq2	LRT	0.0351025416666225
104	KC_AB1	CG13123	0.272003450784741	0.772641487487527	1	pseudobulk	DESeq2	LRT	0.112021975840236
104	KC_AB1	CG13124	3.62318786614656	0.113754069414538	1	pseudobulk	DESeq2	LRT	0.944033058032029
104	KC_AB1	CG13126	-0.880414378885395	0.567422399691676	1	pseudobulk	DESeq2	LRT	0.246093523897102
104	KC_AB1	CG1314	1.25804452752548	0.727552935095488	1	pseudobulk	DESeq2	LRT	0.138135402914536
104	KC_AB1	CG13148	-0.703608099257977	0.349370323969212	1	pseudobulk	DESeq2	LRT	0.45671398740051
104	KC_AB1	CG13151	0.093287088252146	1	1	pseudobulk	DESeq2	LRT	0
104	KC_AB1	CG1316	0.216477800484264	0.419439517110568	1	pseudobulk	DESeq2	LRT	0.377330655285599
104	KC_AB1	CG13168	-1.22498523733724	0.615248217301746	1	pseudobulk	DESeq2	LRT	0.210949636003277
104	KC_AB1	CG1317	-0.0437532410717749	0.916701868299721	1	pseudobulk	DESeq2	LRT	0.0377718835278111
104	KC_AB1	CG13170	1.11388934932283	1	1	pseudobulk	DESeq2	LRT	0
104	KC_AB1	CG13185	0.256441005141838	0.855088195867257	1	pseudobulk	DESeq2	LRT	0.0679890887769996
104	KC_AB1	CG13192	-0.531453119293437	0.529330974924297	1	pseudobulk	DESeq2	LRT	0.276272691602727

104	KC_AB1	CG13197	0.0726737961358805	0.899888699809242	1	pseudobulk	DESeq2	LRT	0.0458112017248686
104	KC_AB1	CG13204	0.105053461859363	0.702633481186712	1	pseudobulk	DESeq2	LRT	0.153271159485366
104	KC_AB1	CG13220	0.131968843426606	0.789770070672576	1	pseudobulk	DESeq2	LRT	0.102499328421752
104	KC_AB1	CG13229	-0.84572271983469	0.729929556961718	1	pseudobulk	DESeq2	LRT	0.136719050152121
104	KC_AB1	CG13248	-0.00223249606649231	0.952550487210917	1	pseudobulk	DESeq2	LRT	0.021111996506908
104	KC_AB1	CG13252	0.152115475746306	1	1	pseudobulk	DESeq2	LRT	0
104	KC_AB1	CG13255	-0.637817183844118	0.691170320238326	1	pseudobulk	DESeq2	LRT	0.160414919304434
104	KC_AB1	CG13284	-0.653500876336709	0.586004876041269	1	pseudobulk	DESeq2	LRT	0.232098770280533
104	KC_AB1	CG13293	-0.417034010388768	0.183142594115791	1	pseudobulk	DESeq2	LRT	0.737210638559227
104	KC_AB1	CG13295	-0.176640038481217	0.851707920920829	1	pseudobulk	DESeq2	LRT	0.0697093137780667
104	KC_AB1	CG13300	0.519723456195346	0.797972646103151	1	pseudobulk	DESeq2	LRT	0.0980119956794342
104	KC_AB1	CG13314	-1.43550613534661	0.602448798560473	1	pseudobulk	DESeq2	LRT	0.220079857379737
104	KC_AB1	CG13315	3.17598958573675	0.0427065003107349	1	pseudobulk	DESeq2	LRT	1.36950601644511
104	KC_AB1	CG13319	0.378250309708948	0.757650446853144	1	pseudobulk	DESeq2	LRT	0.120531116290219
104	KC_AB1	CG13339	-0.16179694372658	0.757226814854419	1	pseudobulk	DESeq2	LRT	0.120774015235038
104	KC_AB1	CG13344	0.0288263669430966	0.946585834252282	1	pseudobulk	DESeq2	LRT	0.023839999077933
104	KC_AB1	CG13360	7.23315917993736	0.112605553807758	1	pseudobulk	DESeq2	LRT	0.948440189159796
104	KC_AB1	CG13364	-0.129482982615389	0.63635499052499	1	pseudobulk	DESeq2	LRT	0.196300545646933
104	KC_AB1	CG13365	0.0601810836305497	0.874739516636085	1	pseudobulk	DESeq2	LRT	0.0581212536400053
104	KC_AB1	CG13366	-0.202241556126214	0.633649716286579	1	pseudobulk	DESeq2	LRT	0.198150755247694
104	KC_AB1	CG13367	-1.40672261935731	0.137554231572193	1	pseudobulk	DESeq2	LRT	0.861526044894904
104	KC_AB1	CG13369	1.00815644124703	0.168837559207081	1	pseudobulk	DESeq2	LRT	0.772530934831762
104	KC_AB1	CG13373	-0.760959112540794	1	1	pseudobulk	DESeq2	LRT	0
104	KC_AB1	CG13375	0.0426392347495894	0.900977657401667	1	pseudobulk	DESeq2	LRT	0.0452859785963845
104	KC_AB1	CG13377	-1.59146823391525	0.486084569581447	1	pseudobulk	DESeq2	LRT	0.313288165084674
104	KC_AB1	CG13384	-0.207867412242104	0.690851645623839	1	pseudobulk	DESeq2	LRT	0.160615203572652
104	KC_AB1	CG13390	-0.751101605776213	0.432701751383442	1	pseudobulk	DESeq2	LRT	0.363811346967614
104	KC_AB1	CG13392	-0.370398379495017	0.735658885462908	1	pseudobulk	DESeq2	LRT	0.133323515148936
104	KC_AB1	CG13397	-1.66790167605961	0.564477250177486	1	pseudobulk	DESeq2	LRT	0.24835355652285
105	KC_AB1	CG13398	0.55865759236893	0.576472345057161	1	pseudobulk	DESeq2	LRT	0.239221522154457
105	KC_AB1	CG13404	0.206403026607288	0.667087223495406	1	pseudobulk	DESeq2	LRT	0.175817377168703
105	KC_AB1	CG13408	1.25804452752548	0.727552935095488	1	pseudobulk	DESeq2	LRT	0.138135402914536
105	KC_AB1	CG13409	0.195609218023172	0.778515694449159	1	pseudobulk	DESeq2	LRT	0.108732627869191
105	KC_AB1	CG13428	-2.35716291794055	0.404761325252261	1	pseudobulk	DESeq2	LRT	0.392800990812383
105	KC_AB1	CG13437	-1.60958773650785	0.570513631639934	1	pseudobulk	DESeq2	LRT	0.243733974251419
105	KC_AB1	CG1344	-0.419032325186387	0.561316164955919	1	pseudobulk	DESeq2	LRT	0.250792450653893
105	KC_AB1	CG13458	3.58032143331778	0.112075923402402	1	pseudobulk	DESeq2	LRT	0.950487674260824
105	KC_AB1	CG13473	-0.760959112540794	1	1	pseudobulk	DESeq2	LRT	0
105	KC_AB1	CG13482	-1.31030390722135	0.538170489756924	1	pseudobulk	DESeq2	LRT	0.269080120186354
105	KC_AB1	CG13484	0.6766028148838	0.3435845102043	1	pseudobulk	DESeq2	LRT	0.463966423666277
105	KC_AB1	CG13502	1.25804452752548	0.573436424985144	1	pseudobulk	DESeq2	LRT	0.241514723898045
105	KC_AB1	CG13506	0.528902848420052	0.230000586422748	1	pseudobulk	DESeq2	LRT	0.63827105667876
105	KC_AB1	CG13526	-1.61102155603974	0.57025980967112	1	pseudobulk	DESeq2	LRT	0.243927235206257
105	KC_AB1	CG13531	-0.681771412045424	0.590481125608576	1	pseudobulk	DESeq2	LRT	0.228793979803304
105	KC_AB1	CG13532	0.119058387821977	0.836000272561616	1	pseudobulk	DESeq2	LRT	0.0777935809676983
105	KC_AB1	CG1354	0.475400586317592	0.484516216903108	1	pseudobulk	DESeq2	LRT	0.314691682403375
105	KC_AB1	CG13551	-0.0452534618124017	0.837084320988666	1	pseudobulk	DESeq2	LRT	0.0772307925496094
105	KC_AB1	CG13578	0.111662612562811	0.853776160945219	1	pseudobulk	DESeq2	LRT	0.0686559756909897
105	KC_AB1	CG13579	1.67020383217772	0.598754457572587	1	pseudobulk	DESeq2	LRT	0.222751240354882
105	KC_AB1	CG1358	-0.0930228816125427	0.820938808084507	1	pseudobulk	DESeq2	LRT	0.0856892135281339
105	KC_AB1	CG13585	-0.0464290901520275	0.852301356453568	1	pseudobulk	DESeq2	LRT	0.0694068203690518
105	KC_AB1	CG1360	-0.486037170095308	0.797951198334821	1	pseudobulk	DESeq2	LRT	0.0980236687269516
105	KC_AB1	CG13601	-0.207279714917377	0.700897777339182	1	pseudobulk	DESeq2	LRT	0.15434531723269
105	KC_AB1	CG13603	0.323848092308837	0.51576082957961	1	pseudobulk	DESeq2	LRT	0.287551644251974
105	KC_AB1	CG13604	0.14655262505158	0.912852444920468	1	pseudobulk	DESeq2	LRT	0.0395994169188565
105	KC_AB1	CG13605	-0.0650759611946568	0.929788975261217	1	pseudobulk	DESeq2	LRT	0.0316156076599268

105	KC_AB1	CG13606	0.00440449811984049	0.996363527352628	1	pseudobulk	DESeq2	LRT	0.00158217852554091
105	KC_AB1	CG13609	1.45044921743621	0.272719867161272	1	pseudobulk	DESeq2	LRT	0.564283223318989
105	KC_AB1	CG13616	1.25804452752548	0.727552935095488	1	pseudobulk	DESeq2	LRT	0.138135402914536
105	KC_AB1	CG13623	0.463154357925148	0.365542829154273	1	pseudobulk	DESeq2	LRT	0.437061731221435
105	KC_AB1	CG13625	-0.359984946486634	0.547432014526318	1	pseudobulk	DESeq2	LRT	0.261669808084904
105	KC_AB1	CG13630	0.240695695752075	0.751007899285474	1	pseudobulk	DESeq2	LRT	0.124355494955818
105	KC_AB1	CG13631	0.723636307836193	0.281059937969719	1	pseudobulk	DESeq2	LRT	0.551201053925199
105	KC_AB1	CG13654	-0.267888439911603	0.658198924615854	1	pseudobulk	DESeq2	LRT	0.181642831603757
105	KC_AB1	CG13658	-1.60958773650785	0.570513631639934	1	pseudobulk	DESeq2	LRT	0.243733974251419
105	KC_AB1	CG13663	0.0999491740871975	0.90433895156337	1	pseudobulk	DESeq2	LRT	0.0436687628835469
105	KC_AB1	CG13667	1.08452285071736	0.638378235735759	1	pseudobulk	DESeq2	LRT	0.194921927830551
105	KC_AB1	CG13671	-1.65950548603614	0.200076594366692	1	pseudobulk	DESeq2	LRT	0.69880371362232
105	KC_AB1	CG13676	-0.967258610721754	0.749177533540542	1	pseudobulk	DESeq2	LRT	0.125415254796089
105	KC_AB1	CG13678	2.77905602015038	0.396550243710121	1	pseudobulk	DESeq2	LRT	0.401701778879434
105	KC_AB1	CG13679	-0.760959112540794	1	1	pseudobulk	DESeq2	LRT	0
105	KC_AB1	CG13685	3.11388989581827	0.250155454630956	1	pseudobulk	DESeq2	LRT	0.601790022901309
105	KC_AB1	CG13689	0.225577500584166	0.803379720694786	1	pseudobulk	DESeq2	LRT	0.0950791351404537
105	KC_AB1	CG13693	1.11388934932283	1	1	pseudobulk	DESeq2	LRT	0
105	KC_AB1	CG13698	1.24321728307373	0.594687101689029	1	pseudobulk	DESeq2	LRT	0.225711480915601
105	KC_AB1	CG13707	-0.739191433798481	0.435824279273954	1	pseudobulk	DESeq2	LRT	0.360688579365141
105	KC_AB1	CG1371	-0.334065005437504	0.60782695271603	1	pseudobulk	DESeq2	LRT	0.216220046019296
105	KC_AB1	CG13716	-0.760959112540794	0.804334723897681	1	pseudobulk	DESeq2	LRT	0.0945631819854715
105	KC_AB1	CG13722	-0.760959112540794	1	1	pseudobulk	DESeq2	LRT	0
105	KC_AB1	CG13737	0.118864039578646	0.891881989640698	1	pseudobulk	DESeq2	LRT	0.0496926059808118
105	KC_AB1	CG13738	1.11388934932283	1	1	pseudobulk	DESeq2	LRT	0
105	KC_AB1	CG13739	-0.195677904113983	0.214958415050273	1	pseudobulk	DESeq2	LRT	0.667645548740212
105	KC_AB1	CG13741	-0.569432576450917	0.825325802164462	1	pseudobulk	DESeq2	LRT	0.0833745773279703
105	KC_AB1	CG13743	-0.807031191187054	0.661699097911657	1	pseudobulk	DESeq2	LRT	0.179339457425706
105	KC_AB1	CG13748	2.99960667015886	0.357405021116046	1	pseudobulk	DESeq2	LRT	0.44683935046587
105	KC_AB1	CG13751	0.327684072873387	0.537685607050606	1	pseudobulk	DESeq2	LRT	0.269471588708228
105	KC_AB1	CG13760	0.0454205678794414	0.95018421550495	1	pseudobulk	DESeq2	LRT	0.0221921883727578
105	KC_AB1	CG13766	-0.298343268665913	0.666082257980053	1	pseudobulk	DESeq2	LRT	0.176472134221808
105	KC_AB1	CG13773	-0.0573068390491313	0.951377074977875	1	pseudobulk	DESeq2	LRT	0.0216473178420799
105	KC_AB1	CG13775	-0.191451000564008	0.90484624429844	1	pseudobulk	DESeq2	LRT	0.0434252118730387
105	KC_AB1	CG13784	-0.402336712756093	0.125811400685124	1	pseudobulk	DESeq2	LRT	0.900280002529081
105	KC_AB1	CG13793	1.25804452752548	0.727552935095488	1	pseudobulk	DESeq2	LRT	0.138135402914536
105	KC_AB1	CG13796	2.6268597268973	0.378437501240742	1	pseudobulk	DESeq2	LRT	0.422005833752979
105	KC_AB1	CG13807	-0.563914215155261	0.445882631764855	1	pseudobulk	DESeq2	LRT	0.350779444168872
105	KC_AB1	CG13827	-2.27165469313822	0.263380052378072	1	pseudobulk	DESeq2	LRT	0.579417120301276
105	KC_AB1	CG13833	-2.00963963748391	0.498268873088307	1	pseudobulk	DESeq2	LRT	0.302536242405947
105	KC_AB1	CG13850	0.246329075092971	0.748872791249954	1	pseudobulk	DESeq2	LRT	0.125591948322122
105	KC_AB1	CG13856	-1.79467717658919	0.371992102892408	1	pseudobulk	DESeq2	LRT	0.429466279759647
105	KC_AB1	CG13868	0.133339670053861	0.478442432652183	1	pseudobulk	DESeq2	LRT	0.320170310103513
105	KC_AB1	CG13875	-2.00963963748391	0.498268873088307	1	pseudobulk	DESeq2	LRT	0.302536242405947
105	KC_AB1	CG13876	0.993152279961008	0.357709512416523	1	pseudobulk	DESeq2	LRT	0.446469510689511
105	KC_AB1	CG13887	-0.053358638458065	0.889092198632077	1	pseudobulk	DESeq2	LRT	0.0510532004685499
105	KC_AB1	CG13893	-1.12405481456887	0.299008132301807	1	pseudobulk	DESeq2	LRT	0.524316999749915
105	KC_AB1	CG13894	2.04480874254212	0.526249918436357	1	pseudobulk	DESeq2	LRT	0.278807958467711
105	KC_AB1	CG13895	-0.353831226871126	0.856766795213782	1	pseudobulk	DESeq2	LRT	0.0671373733326742
105	KC_AB1	CG13901	0.00936326110271288	0.982965619638404	1	pseudobulk	DESeq2	LRT	0.00746167185498724
105	KC_AB1	CG13907	-0.128284952605286	0.807889861180168	1	pseudobulk	DESeq2	LRT	0.0926478421239774
105	KC_AB1	CG13917	0.0914360335389476	0.547901986132504	1	pseudobulk	DESeq2	LRT	0.261297125254607
105	KC_AB1	CG13919	0.794386525759442	0.257336902158878	1	pseudobulk	DESeq2	LRT	0.589497931415688
105	KC_AB1	CG13920	0.20626007359966	0.350968070560155	1	pseudobulk	DESeq2	LRT	0.454732391825582
105	KC_AB1	CG13921	0.132954694985854	0.634960864148217	1	pseudobulk	DESeq2	LRT	0.197253041650155
105	KC_AB1	CG13924	2.4138856564567	0.409611385497059	1	pseudobulk	DESeq2	LRT	0.387627980279722

105	KC_AB1	CG13926	0.651434554212253	0.356151185389798	1	pseudobulk	DESeq2	LRT	0.448365605840284
105	KC_AB1	CG13928	-0.096600124306525	0.629062658078041	1	pseudobulk	DESeq2	LRT	0.201306094296766
105	KC_AB1	CG13933	-0.362257755648691	0.572344075867629	1	pseudobulk	DESeq2	LRT	0.24234280805103
105	KC_AB1	CG13937	0.202789629928417	0.805755276433136	1	pseudobulk	DESeq2	LRT	0.0937968418587032
105	KC_AB1	CG13954	-0.227036984555043	0.363851570211722	1	pseudobulk	DESeq2	LRT	0.43907574652839
105	KC_AB1	CG13955	-0.760959112540794	0.805265236978025	1	pseudobulk	DESeq2	LRT	0.0940610488455931
105	KC_AB1	CG13982	0.0137496827567787	0.974799432820043	1	pseudobulk	DESeq2	LRT	0.0110847321783241
105	KC_AB1	CG13994	-0.621387638969194	0.304061063457623	1	pseudobulk	DESeq2	LRT	0.51703918987939
105	KC_AB1	CG13995	0.0342399911285283	0.962907795077055	1	pseudobulk	DESeq2	LRT	0.0164152975134966
105	KC_AB1	CG13999	-0.0746925371210123	0.869057229029508	1	pseudobulk	DESeq2	LRT	0.0609516235129529
105	KC_AB1	CG14005	-0.317656366090915	0.64754446694423	1	pseudobulk	DESeq2	LRT	0.188730403176562
105	KC_AB1	CG14015	0.998973174442886	0.14454588663611	1	pseudobulk	DESeq2	LRT	0.83999426260043
105	KC_AB1	CG14024	-0.183835813047545	0.654467049911764	1	pseudobulk	DESeq2	LRT	0.184112213745346
105	KC_AB1	CG14043	-0.226172650255969	0.687925683553001	1	pseudobulk	DESeq2	LRT	0.162458475959439
105	KC_AB1	CG14044	2.09987857572813	0.503115176013642	1	pseudobulk	DESeq2	LRT	0.29833258237694
105	KC_AB1	CG14052	0.897272604463685	0.699914436311722	1	pseudobulk	DESeq2	LRT	0.154955048712815
105	KC_AB1	CG14053	-0.760959112540794	1	1	pseudobulk	DESeq2	LRT	0
106	KC_AB1	CG14057	-0.996176787778342	0.40482738749982	1	pseudobulk	DESeq2	LRT	0.39273011415813
106	KC_AB1	CG1407	0.379019230154924	0.64023528406197	1	pseudobulk	DESeq2	LRT	0.193660395091679
106	KC_AB1	CG14073	0.00390269939096327	0.95330126253046	1	pseudobulk	DESeq2	LRT	0.0207698318077524
106	KC_AB1	CG14074	0.202432644860328	0.742716018206688	1	pseudobulk	DESeq2	LRT	0.12917720952488
106	KC_AB1	CG14082	-0.0457888591345113	0.968402248481414	1	pseudobulk	DESeq2	LRT	0.0139442108639923
106	KC_AB1	CG14086	-0.760959112540794	1	1	pseudobulk	DESeq2	LRT	0
106	KC_AB1	CG14096	2.4138856564567	0.409611385497059	1	pseudobulk	DESeq2	LRT	0.387627980279722
106	KC_AB1	CG14100	-0.330887333770101	0.684527449203002	1	pseudobulk	DESeq2	LRT	0.164609132192694
106	KC_AB1	CG14102	-0.91201534681688	0.365234366171958	1	pseudobulk	DESeq2	LRT	0.437428364936619
106	KC_AB1	CG14104	0.877201836726486	0.431031347581513	1	pseudobulk	DESeq2	LRT	0.365491143791461
106	KC_AB1	CG14105	-2.73719572742657	0.395113780272619	1	pseudobulk	DESeq2	LRT	0.40327782328744
106	KC_AB1	CG14107	-0.760959112540794	0.805265236978025	1	pseudobulk	DESeq2	LRT	0.0940610488455931
106	KC_AB1	CG14117	1.39929782761041	0.639042277522582	1	pseudobulk	DESeq2	LRT	0.194470408997475
106	KC_AB1	CG14118	1.11388934932283	1	1	pseudobulk	DESeq2	LRT	0
106	KC_AB1	CG14130	-0.204542496305708	0.819881093554881	1	pseudobulk	DESeq2	LRT	0.0862491282961284
106	KC_AB1	CG14131	1.25804452752548	0.727552935095488	1	pseudobulk	DESeq2	LRT	0.138135402914536
106	KC_AB1	CG14132	-0.243258381349887	0.94370439481472	1	pseudobulk	DESeq2	LRT	0.025164022445009
106	KC_AB1	CG14135	0.147479434394636	0.832416800139097	1	pseudobulk	DESeq2	LRT	0.0796591632825189
106	KC_AB1	CG14137	1.25804452752548	0.573436424985144	1	pseudobulk	DESeq2	LRT	0.241514723898045
106	KC_AB1	CG14141	-0.189448922997618	0.593147919945584	1	pseudobulk	DESeq2	LRT	0.226836988245791
106	KC_AB1	CG14142	-0.570723996670538	0.824991814167186	1	pseudobulk	DESeq2	LRT	0.0835503606375391
106	KC_AB1	CG14147	2.09987857572813	0.503115176013642	1	pseudobulk	DESeq2	LRT	0.29833258237694
106	KC_AB1	CG14153	0.89249181112131	0.550311100069833	1	pseudobulk	DESeq2	LRT	0.259391727148032
106	KC_AB1	CG14154	-0.760959112540794	0.805265236978025	1	pseudobulk	DESeq2	LRT	0.0940610488455931
106	KC_AB1	CG1416	0.213072881878396	0.529793151682943	1	pseudobulk	DESeq2	LRT	0.275893659865762
106	KC_AB1	CG14174	-0.219618509055647	0.885397987441767	1	pseudobulk	DESeq2	LRT	0.0528614695312488
106	KC_AB1	CG1418	0.747129701395273	0.289592020294893	1	pseudobulk	DESeq2	LRT	0.538213409289951
106	KC_AB1	CG14182	-0.0624411029942075	0.919448511796526	1	pseudobulk	DESeq2	LRT	0.0364725858046295
106	KC_AB1	CG14183	-1.36212040035207	0.550436212376225	1	pseudobulk	DESeq2	LRT	0.259293002247405
106	KC_AB1	CG14184	0.193833464273377	0.71021260068154	1	pseudobulk	DESeq2	LRT	0.148611626658573
106	KC_AB1	CG14186	0.00518143611080944	0.911989726042717	1	pseudobulk	DESeq2	LRT	0.0400100541585224
106	KC_AB1	CG14194	0.0806492937236765	0.891343115248469	1	pseudobulk	DESeq2	LRT	0.0499550856660362
106	KC_AB1	CG14196	2.13102056038908	0.437616900286912	1	pseudobulk	DESeq2	LRT	0.358905914372019
106	KC_AB1	CG14200	0.431713288880491	0.38304290656781	1	pseudobulk	DESeq2	LRT	0.416752575791301
106	KC_AB1	CG14207	0.392453359301707	0.252134635840341	1	pseudobulk	DESeq2	LRT	0.598367491010918
106	KC_AB1	CG14210	-0.772943895782742	0.405679752166347	1	pseudobulk	DESeq2	LRT	0.391816667774768
106	KC_AB1	CG14218	-3.22129287111021	0.293514748994408	1	pseudobulk	DESeq2	LRT	0.532370070750446
106	KC_AB1	CG14220	-0.479051166210471	0.693183186027941	1	pseudobulk	DESeq2	LRT	0.159151980152935
106	KC_AB1	CG14223	-1.43550613534661	0.602448798560473	1	pseudobulk	DESeq2	LRT	0.220079857379737

106	KC_AB1	CG14229	0.15457361072086	0.75846544379457	1	pseudobulk	DESeq2	LRT	0.120064201180753
106	KC_AB1	CG14230	-0.112378123305689	0.908147958243081	1	pseudobulk	DESeq2	LRT	0.0418433891289944
106	KC_AB1	CG14231	0.0412069486636499	0.919344259045904	1	pseudobulk	DESeq2	LRT	0.0365218315870632
106	KC_AB1	CG14232	0.608084108468825	0.265395294093014	1	pseudobulk	DESeq2	LRT	0.576106782178274
106	KC_AB1	CG14234	-0.0883904711315211	0.82566848802355	1	pseudobulk	DESeq2	LRT	0.0831942901148045
106	KC_AB1	CG14246	-0.760959112540794	0.804334723897681	1	pseudobulk	DESeq2	LRT	0.0945631819854715
106	KC_AB1	CG14252	-0.0195911292228683	0.969352336113063	1	pseudobulk	DESeq2	LRT	0.0135183387209517
106	KC_AB1	CG14253	2.09987857572813	0.503115176013642	1	pseudobulk	DESeq2	LRT	0.29833258237694
106	KC_AB1	CG14257	1.25804452752548	0.573436424985144	1	pseudobulk	DESeq2	LRT	0.241514723898045
106	KC_AB1	CG14258	-1.43550613534661	0.602448798560473	1	pseudobulk	DESeq2	LRT	0.220079857379737
106	KC_AB1	CG14259	-4.93204172539748	0.0608358005647135	1	pseudobulk	DESeq2	LRT	1.21584077250395
106	KC_AB1	CG14270	0.287590284714851	0.68508939262539	1	pseudobulk	DESeq2	LRT	0.164252756695948
106	KC_AB1	CG14275	-2.41876763198367	0.438413379977707	1	pseudobulk	DESeq2	LRT	0.358116200048746
106	KC_AB1	CG14286	0.343550920689657	0.737403510215215	1	pseudobulk	DESeq2	LRT	0.132294799380472
106	KC_AB1	CG14301	-0.575069369497329	1	1	pseudobulk	DESeq2	LRT	0
106	KC_AB1	CG14304	0.676641301832977	0.79473536735153	1	pseudobulk	DESeq2	LRT	0.0997774595591333
106	KC_AB1	CG14312	0.111371951949021	0.730574598206003	1	pseudobulk	DESeq2	LRT	0.136335432111438
106	KC_AB1	CG14314	0.10161861129121	0.896250156086202	1	pseudobulk	DESeq2	LRT	0.0475707556896996
106	KC_AB1	CG14321	0.252608360669686	0.54827981803959	1	pseudobulk	DESeq2	LRT	0.260997740028163
106	KC_AB1	CG14322	-0.487649411348889	0.620138224405366	1	pseudobulk	DESeq2	LRT	0.207511498556784
106	KC_AB1	CG1434	0.137369078038645	0.884531660567763	1	pseudobulk	DESeq2	LRT	0.0532866175153782
106	KC_AB1	CG14340	1.68373311097749	0.307773224292913	1	pseudobulk	DESeq2	LRT	0.511769165685999
106	KC_AB1	CG14341	0.0928418131767049	0.86061168266366	1	pseudobulk	DESeq2	LRT	0.0651927627364708
106	KC_AB1	CG14352	-0.92717186431982	0.491220903249063	1	pseudobulk	DESeq2	LRT	0.308723160652385
106	KC_AB1	CG14356	-1.43550613534661	0.602448798560473	1	pseudobulk	DESeq2	LRT	0.220079857379737
106	KC_AB1	CG14367	0.152115475746305	1	1	pseudobulk	DESeq2	LRT	0
106	KC_AB1	CG14397	1.11388934932283	1	1	pseudobulk	DESeq2	LRT	0
106	KC_AB1	CG1440	0.108443640470843	0.858100401595516	1	pseudobulk	DESeq2	LRT	0.0664618947761387
106	KC_AB1	CG14401	2.74009504005053	0.0834025961386429	1	pseudobulk	DESeq2	LRT	1.07882043052367
106	KC_AB1	CG14407	-0.109534089744416	0.713536216374949	1	pseudobulk	DESeq2	LRT	0.146583978859598
106	KC_AB1	CG14408	0.0146024300590583	0.948222889465561	1	pseudobulk	DESeq2	LRT	0.0230895653125652
106	KC_AB1	CG14410	1.13988912687578	0.653854063433791	1	pseudobulk	DESeq2	LRT	0.184519172956856
106	KC_AB1	CG14411	0.0253510368049252	0.918981239313529	1	pseudobulk	DESeq2	LRT	0.0366933544961264
106	KC_AB1	CG14414	-0.147915190003234	0.721173518399193	1	pseudobulk	DESeq2	LRT	0.141960229015991
106	KC_AB1	CG14431	-0.209909292079914	0.679705622983782	1	pseudobulk	DESeq2	LRT	0.167679137285731
106	KC_AB1	CG14434	-0.308076769973854	0.634958288906249	1	pseudobulk	DESeq2	LRT	0.197254803043191
106	KC_AB1	CG14435	-0.0381995819999947	0.917009378993542	1	pseudobulk	DESeq2	LRT	0.0376262224277859
106	KC_AB1	CG14439	1.25804452752548	0.727552935095488	1	pseudobulk	DESeq2	LRT	0.138135402914536
106	KC_AB1	CG14440	-0.192662392275351	0.480709037858782	1	pseudobulk	DESeq2	LRT	0.318117712580529
106	KC_AB1	CG14441	0.377509156203833	0.824293756381265	1	pseudobulk	DESeq2	LRT	0.0839179897103834
106	KC_AB1	CG14442	0.158285320119644	0.559954155946027	1	pseudobulk	DESeq2	LRT	0.25184752769856
106	KC_AB1	CG14448	1.67020383217772	0.598754457572587	1	pseudobulk	DESeq2	LRT	0.222751240354882
106	KC_AB1	CG14450	0.0653149831344768	0.916739927681771	1	pseudobulk	DESeq2	LRT	0.037753852980403
106	KC_AB1	CG14457	-0.760959112540794	1	1	pseudobulk	DESeq2	LRT	0
106	KC_AB1	CG14459	-1.43550613534661	0.602448798560473	1	pseudobulk	DESeq2	LRT	0.220079857379737
106	KC_AB1	CG14464	0.0286846621522268	0.894471863914335	1	pseudobulk	DESeq2	LRT	0.0484333158444314
106	KC_AB1	CG14478	0.196355516443564	0.31608242865105	1	pseudobulk	DESeq2	LRT	0.500199646368569
106	KC_AB1	CG14483	0.405186785623699	0.409610804163387	1	pseudobulk	DESeq2	LRT	0.387628596644878
106	KC_AB1	CG14492	1.11388934932283	1	1	pseudobulk	DESeq2	LRT	0
106	KC_AB1	CG14502	-0.853791455458968	0.76171504745363	1	pseudobulk	DESeq2	LRT	0.118207464963883
106	KC_AB1	CG14505	-0.760959112540794	0.804334723897681	1	pseudobulk	DESeq2	LRT	0.0945631819854715
106	KC_AB1	CG14511	-2.60994204730986	0.339314469994084	1	pseudobulk	DESeq2	LRT	0.469397619563291
106	KC_AB1	CG14512	-0.0495618637086571	0.918724638504937	1	pseudobulk	DESeq2	LRT	0.0368146364893908
106	KC_AB1	CG14515	-0.760959112540794	0.805265236978025	1	pseudobulk	DESeq2	LRT	0.0940610488455931
106	KC_AB1	CG14535	0.0168756877116723	0.959323320595527	1	pseudobulk	DESeq2	LRT	0.0180349979538271
106	KC_AB1	CG14543	0.100246835287674	0.889029381870548	1	pseudobulk	DESeq2	LRT	0.0510838856289053

106	KC_AB1	CG14544	0.296275065198765	1	1	pseudobulk	DESeq2	LRT	0
106	KC_AB1	CG14561	-0.967258610721754	0.749177533540542	1	pseudobulk	DESeq2	LRT	0.125415254796089
106	KC_AB1	CG14562	-0.0101422957304741	0.984248043708377	1	pseudobulk	DESeq2	LRT	0.00689543974068036
106	KC_AB1	CG14563	1.12864329517458	0.503674927737232	1	pseudobulk	DESeq2	LRT	0.297849667201407
106	KC_AB1	CG14565	5.33604790728318	0.022416994040812	1	pseudobulk	DESeq2	LRT	1.64942262363273
107	KC_AB1	CG14566	5.08196896214782	0.0555130220361641	1	pseudobulk	DESeq2	LRT	1.25560512975865
107	KC_AB1	CG14570	1.25804452752548	0.727552935095488	1	pseudobulk	DESeq2	LRT	0.138135402914536
107	KC_AB1	CG14572	3.7951235223405	0.206529069530445	1	pseudobulk	DESeq2	LRT	0.685018811571072
107	KC_AB1	CG14589	-0.252676765451564	0.908741057977342	1	pseudobulk	DESeq2	LRT	0.0415598495746707
107	KC_AB1	CG14591	-0.224914106440757	0.639098892554284	1	pseudobulk	DESeq2	LRT	0.194431935006569
107	KC_AB1	CG14609	-0.576303105040742	1	1	pseudobulk	DESeq2	LRT	0
107	KC_AB1	CG1461	-0.898663366816474	0.670306150466251	1	pseudobulk	DESeq2	LRT	0.173726795672216
107	KC_AB1	CG14613	0.197024307579013	0.803842400642104	1	pseudobulk	DESeq2	LRT	0.0948290896104793
107	KC_AB1	CG14615	0.152115475746306	1	1	pseudobulk	DESeq2	LRT	0
107	KC_AB1	CG14618	-0.373625649819147	0.733286107077992	1	pseudobulk	DESeq2	LRT	0.134726543119106
107	KC_AB1	CG14621	0.603607093822533	0.404768448859249	1	pseudobulk	DESeq2	LRT	0.392793347503132
107	KC_AB1	CG14629	4.90190122290401	0.0687021279919401	1	pseudobulk	DESeq2	LRT	1.16302981081754
107	KC_AB1	CG1463	-0.100931329573406	0.896194936094322	1	pseudobulk	DESeq2	LRT	0.0475975143757833
107	KC_AB1	CG14636	-0.760959112540794	1	1	pseudobulk	DESeq2	LRT	0
107	KC_AB1	CG14641	-0.104209841764811	0.775096439504625	1	pseudobulk	DESeq2	LRT	0.11064425808855
107	KC_AB1	CG14644	1.25804452752548	0.727552935095488	1	pseudobulk	DESeq2	LRT	0.138135402914536
107	KC_AB1	CG14646	0.33347330655618	0.638884740353197	1	pseudobulk	DESeq2	LRT	0.19457748478936
107	KC_AB1	CG14647	0.205612219130294	0.747966713518584	1	pseudobulk	DESeq2	LRT	0.126117728949528
107	KC_AB1	CG14650	0.356015943298342	0.595895741870141	1	pseudobulk	DESeq2	LRT	0.224829717929649
107	KC_AB1	CG14657	0.0782688329708911	0.8715180704215	1	pseudobulk	DESeq2	LRT	0.0597236036165447
107	KC_AB1	CG14661	4.15858286796254	0.335785439349295	1	pseudobulk	DESeq2	LRT	0.473938140084085
107	KC_AB1	CG14667	0.658571031981829	0.273875509534271	1	pseudobulk	DESeq2	LRT	0.562446801443227
107	KC_AB1	CG14668	-0.760959112540794	0.804334723897681	1	pseudobulk	DESeq2	LRT	0.0945631819854715
107	KC_AB1	CG14669	0.0647885032991115	0.857127765845747	1	pseudobulk	DESeq2	LRT	0.0669544361094968
107	KC_AB1	CG14671	0.37292252922773	0.603410349047309	1	pseudobulk	DESeq2	LRT	0.219387245543867
107	KC_AB1	CG1468	-0.110161756371095	0.886992309288976	1	pseudobulk	DESeq2	LRT	0.0520801457238986
107	KC_AB1	CG14683	0.813638458404516	0.414469379115706	1	pseudobulk	DESeq2	LRT	0.382507549485992
107	KC_AB1	CG14687	-2.58139793223099	0.198146444243264	1	pseudobulk	DESeq2	LRT	0.703013716715536
107	KC_AB1	CG14688	-0.224267146663162	0.72569898286697	1	pseudobulk	DESeq2	LRT	0.139243485621189
107	KC_AB1	CG14692	2.88322402903533	0.302751069383099	1	pseudobulk	DESeq2	LRT	0.518914314158952
107	KC_AB1	CG14693	0.70842991589991	0.874943022847655	1	pseudobulk	DESeq2	LRT	0.0580202277417387
107	KC_AB1	CG14694	0.70842991589991	0.874943022847655	1	pseudobulk	DESeq2	LRT	0.0580202277417387
107	KC_AB1	CG14696	0.494775748955277	0.827825706404751	1	pseudobulk	DESeq2	LRT	0.0820610916144188
107	KC_AB1	CG14701	-1.04719991015243	0.496188651494385	1	pseudobulk	DESeq2	LRT	0.304353172852459
107	KC_AB1	CG14710	0.291133702397169	0.737965109958448	1	pseudobulk	DESeq2	LRT	0.131964170576295
107	KC_AB1	CG14711	0.291514333344835	0.73263436800012	1	pseudobulk	DESeq2	LRT	0.135112712367196
107	KC_AB1	CG14712	-0.233367691398086	0.885502433279975	1	pseudobulk	DESeq2	LRT	0.0528102410706157
107	KC_AB1	CG14715	-0.245199178419094	0.722196106184175	1	pseudobulk	DESeq2	LRT	0.141344857459019
107	KC_AB1	CG14721	1.05394771467329	0.0795835553800348	1	pseudobulk	DESeq2	LRT	1.09917666273375
107	KC_AB1	CG14722	-0.230803634788678	0.779064096324156	1	pseudobulk	DESeq2	LRT	0.108426809933663
107	KC_AB1	CG14743	1.25804452752548	0.727552935095488	1	pseudobulk	DESeq2	LRT	0.138135402914536
107	KC_AB1	CG14752	-4.21820615809929	0.130345278170634	1	pseudobulk	DESeq2	LRT	0.884904696767083
107	KC_AB1	CG14757	1.09803950274617	0.276122367246561	1	pseudobulk	DESeq2	LRT	0.558898411983312
107	KC_AB1	CG14762	-0.227778830203994	0.201430457605616	1	pseudobulk	DESeq2	LRT	0.6958748606446
107	KC_AB1	CG14764	0.0124954122009817	0.960436919268321	1	pseudobulk	DESeq2	LRT	0.0175311539811777
107	KC_AB1	CG14767	0.0268245288230111	0.895838247168571	1	pseudobulk	DESeq2	LRT	0.0477703996054787
107	KC_AB1	CG14777	0.0100986897301902	0.913990740587332	1	pseudobulk	DESeq2	LRT	0.0390582039731672
107	KC_AB1	CG14778	0.152115475746305	1	1	pseudobulk	DESeq2	LRT	0
107	KC_AB1	CG14787	1.25804452752548	0.727552935095488	1	pseudobulk	DESeq2	LRT	0.138135402914536
107	KC_AB1	CG14795	0.152115475746305	1	1	pseudobulk	DESeq2	LRT	0
107	KC_AB1	CG14797	1.40153657836866	0.531630688489197	1	pseudobulk	DESeq2	LRT	0.274389957268748

107	KC_AB1	CG14803	-0.538218969557373	0.775353846208946	1	pseudobulk	DESeq2	LRT	0.110500054416431
107	KC_AB1	CG14805	-0.100687590633902	0.868554258948305	1	pseudobulk	DESeq2	LRT	0.0612030458112526
107	KC_AB1	CG14806	0.0251657734724564	0.928789172320508	1	pseudobulk	DESeq2	LRT	0.0320828561773908
107	KC_AB1	CG14811	-0.760959112540794	1	1	pseudobulk	DESeq2	LRT	0
107	KC_AB1	CG14812	-0.00919664138791502	0.940238467829582	1	pseudobulk	DESeq2	LRT	0.0267619845619746
107	KC_AB1	CG14814	1.25804452752548	0.573436424985144	1	pseudobulk	DESeq2	LRT	0.241514723898045
107	KC_AB1	CG14817	0.213187859851216	0.530556363874012	1	pseudobulk	DESeq2	LRT	0.27526847185845
107	KC_AB1	CG14818	-0.0394281631860675	0.855685366974244	1	pseudobulk	DESeq2	LRT	0.0676858947919126
107	KC_AB1	CG14830	0.847525847835988	0.691106783674185	1	pseudobulk	DESeq2	LRT	0.16045484412001
107	KC_AB1	CG14834	-0.760959112540794	0.805265236978025	1	pseudobulk	DESeq2	LRT	0.0940610488455931
107	KC_AB1	CG14853	-0.0195553898810119	0.929040430322688	1	pseudobulk	DESeq2	LRT	0.0319653858083204
107	KC_AB1	CG14855	1.90071181822722	0.545896518579078	1	pseudobulk	DESeq2	LRT	0.262889675371117
107	KC_AB1	CG14857	1.13734983340557	0.742793668201421	1	pseudobulk	DESeq2	LRT	0.1291318069744
107	KC_AB1	CG1486	0.306942187499727	0.662129159304085	1	pseudobulk	DESeq2	LRT	0.179057285935313
107	KC_AB1	CG14868	0.256703636403752	0.561865100958861	1	pseudobulk	DESeq2	LRT	0.250367942334155
107	KC_AB1	CG14879	-0.696045889415738	0.771031022267663	1	pseudobulk	DESeq2	LRT	0.112928147852258
107	KC_AB1	CG14881	0.262194941621994	0.793539944980657	1	pseudobulk	DESeq2	LRT	0.100431206970827
107	KC_AB1	CG14882	0.041504444717871	0.918298893800517	1	pseudobulk	DESeq2	LRT	0.0370159388416884
107	KC_AB1	CG14883	-0.0654325364568011	0.915549877970171	1	pseudobulk	DESeq2	LRT	0.0383179909175534
107	KC_AB1	CG14894	-0.146154324877282	0.749431151132111	1	pseudobulk	DESeq2	LRT	0.125268258822144
107	KC_AB1	CG14903	0.0151883771787276	0.929777711326062	1	pseudobulk	DESeq2	LRT	0.0316208689553864
107	KC_AB1	CG14906	-0.348092194126679	0.737791709978765	1	pseudobulk	DESeq2	LRT	0.132066228928548
107	KC_AB1	CG1492	0.0294710737815691	0.964990914698185	1	pseudobulk	DESeq2	LRT	0.0154767754800611
107	KC_AB1	CG14965	0.323457627426421	0.83692834997867	1	pseudobulk	DESeq2	LRT	0.0773117206696276
107	KC_AB1	CG14966	-1.5198053212444	0.184533535940932	1	pseudobulk	DESeq2	LRT	0.733924696439418
107	KC_AB1	CG14968	0.171051251501439	0.921020148144152	1	pseudobulk	DESeq2	LRT	0.035730869116815
107	KC_AB1	CG14969	-0.284996743730678	0.738711080857774	1	pseudobulk	DESeq2	LRT	0.131525386400304
107	KC_AB1	CG14971	-0.575875057345459	0.559991827720888	1	pseudobulk	DESeq2	LRT	0.251818310853836
107	KC_AB1	CG14977	0.500112999748252	0.338357158854221	1	pseudobulk	DESeq2	LRT	0.470624630417413
107	KC_AB1	CG14982	-0.45127366060238	0.144206237112926	1	pseudobulk	DESeq2	LRT	0.841015955392265
107	KC_AB1	CG14984	-0.450632937417343	0.904305002564132	1	pseudobulk	DESeq2	LRT	0.0436850666596476
107	KC_AB1	CG14985	2.25798792028661	0.402405101834573	1	pseudobulk	DESeq2	LRT	0.395336521777783
107	KC_AB1	CG14989	-2.4407905271123	0.120474096388018	1	pseudobulk	DESeq2	LRT	0.91910632242596
107	KC_AB1	CG14995	-0.0368650111358571	0.926207197595933	1	pseudobulk	DESeq2	LRT	0.0332918484079308
107	KC_AB1	CG14997	-1.89022041803328	0.314778881512739	1	pseudobulk	DESeq2	LRT	0.501994412113923
107	KC_AB1	CG15011	-0.974021821636439	0.403537048513156	1	pseudobulk	DESeq2	LRT	0.394116586775373
107	KC_AB1	CG15012	0.109435068398481	0.719753244087502	1	pseudobulk	DESeq2	LRT	0.142816368983919
107	KC_AB1	CG15014	-0.0770499727857483	0.89424235094863	1	pseudobulk	DESeq2	LRT	0.0485447659735238
107	KC_AB1	CG15019	0.0158893588737159	0.904425594256091	1	pseudobulk	DESeq2	LRT	0.0436271560934739
107	KC_AB1	CG15027	-0.76424810863457	0.509767713751264	1	pseudobulk	DESeq2	LRT	0.292627674130959
107	KC_AB1	CG1504	0.943798956650056	0.807634491832869	1	pseudobulk	DESeq2	LRT	0.0927851418175362
107	KC_AB1	CG15040	-2.36175013442542	0.448372544778042	1	pseudobulk	DESeq2	LRT	0.348360988442027
107	KC_AB1	CG15073	0.631049649912437	0.5007551396861	1	pseudobulk	DESeq2	LRT	0.300374584469021
107	KC_AB1	CG15080	-2.73719572742657	0.395113780272619	1	pseudobulk	DESeq2	LRT	0.40327782328744
107	KC_AB1	CG15083	-2.00963963748391	0.498268873088307	1	pseudobulk	DESeq2	LRT	0.302536242405947
107	KC_AB1	CG15084	-0.79240447732575	0.651761600160387	1	pseudobulk	DESeq2	LRT	0.185911230446074
107	KC_AB1	CG15093	-0.385011853448768	0.890534840916548	1	pseudobulk	DESeq2	LRT	0.0503490847107839
107	KC_AB1	CG15096	1.25804452752548	0.573436424985144	1	pseudobulk	DESeq2	LRT	0.241514723898045
107	KC_AB1	CG15097	-0.0700762977935429	0.82763781652006	1	pseudobulk	DESeq2	LRT	0.0821596737206954
108	KC_AB1	CG15098	0.462065446381201	0.446731882113181	1	pseudobulk	DESeq2	LRT	0.349953051934409
108	KC_AB1	CG15099	0.0149885854135768	0.960961680543771	1	pseudobulk	DESeq2	LRT	0.0172939299799391
108	KC_AB1	CG15107	-0.254186238466469	0.827797567863204	1	pseudobulk	DESeq2	LRT	0.0820758539260073
108	KC_AB1	CG15109	-1.43550613534661	0.602448798560473	1	pseudobulk	DESeq2	LRT	0.220079857379737
108	KC_AB1	CG15111	-0.282809983859126	0.585950526475284	1	pseudobulk	DESeq2	LRT	0.232139051193215
108	KC_AB1	CG15117	1.11388934932283	1	1	pseudobulk	DESeq2	LRT	0
108	KC_AB1	CG15118	-0.379404310734451	0.580382845738963	1	pseudobulk	DESeq2	LRT	0.236285432400752

108	KC_AB1	CG1513	-0.342051533605944	0.299094281439791	1	pseudobulk	DESeq2	LRT	0.524191890421993
108	KC_AB1	CG15141	-0.369312756440128	0.479781151862482	1	pseudobulk	DESeq2	LRT	0.318956817232928
108	KC_AB1	CG15160	-0.485511107373798	0.423811826601876	1	pseudobulk	DESeq2	LRT	0.372826928332469
108	KC_AB1	CG15168	0.00486213258622807	0.985469166621914	1	pseudobulk	DESeq2	LRT	0.00635695938600371
108	KC_AB1	CG15199	-0.760959112540794	0.805265236978025	1	pseudobulk	DESeq2	LRT	0.0940610488455931
108	KC_AB1	CG15201	0.00452214400564445	0.910937579745066	1	pseudobulk	DESeq2	LRT	0.0405113812059743
108	KC_AB1	CG15202	-1.77199356422654	0.54193209410651	1	pseudobulk	DESeq2	LRT	0.266055128595237
108	KC_AB1	CG15203	-0.272879066277626	0.831853217320665	1	pseudobulk	DESeq2	LRT	0.0799532993480955
108	KC_AB1	CG15209	-0.204824797244939	0.406866660478816	1	pseudobulk	DESeq2	LRT	0.390547895704076
108	KC_AB1	CG15210	1.78435940631618	0.198498673527931	1	pseudobulk	DESeq2	LRT	0.70224239107429
108	KC_AB1	CG15211	-1.43550613534661	0.602448798560473	1	pseudobulk	DESeq2	LRT	0.220079857379737
108	KC_AB1	CG1523	0.480667498938149	0.559422523549571	1	pseudobulk	DESeq2	LRT	0.252260051985998
108	KC_AB1	CG15236	-1.38204092468574	0.565879008743161	1	pseudobulk	DESeq2	LRT	0.247276415900504
108	KC_AB1	CG15237	0.149988658881275	0.686467648704581	1	pseudobulk	DESeq2	LRT	0.163379925026128
108	KC_AB1	CG15270	-1.14782596152845	0.101538283153039	1	pseudobulk	DESeq2	LRT	0.993370184081549
108	KC_AB1	CG15279	-0.760959112540794	1	1	pseudobulk	DESeq2	LRT	0
108	KC_AB1	CG1529	-0.760959112540794	0.804334723897681	1	pseudobulk	DESeq2	LRT	0.0945631819854715
108	KC_AB1	CG15309	-0.0405672789961384	0.891762319201923	1	pseudobulk	DESeq2	LRT	0.0497508824107336
108	KC_AB1	CG15312	0.226115516655957	0.515553507448036	1	pseudobulk	DESeq2	LRT	0.287726254175834
108	KC_AB1	CG15317	0.0833875415213821	0.900862989944555	1	pseudobulk	DESeq2	LRT	0.0453412547874482
108	KC_AB1	CG1532	0.239965240383154	0.732823859710424	1	pseudobulk	DESeq2	LRT	0.135000399094727
108	KC_AB1	CG15322	1.25804452752548	0.573436424985144	1	pseudobulk	DESeq2	LRT	0.241514723898045
108	KC_AB1	CG15332	0.943805700130147	0.808046339172447	1	pseudobulk	DESeq2	LRT	0.0925637329508755
108	KC_AB1	CG15343	0.798281285674532	0.582417791917613	1	pseudobulk	DESeq2	LRT	0.234765366501307
108	KC_AB1	CG15347	1.25804452752548	0.727552935095488	1	pseudobulk	DESeq2	LRT	0.138135402914536
108	KC_AB1	CG15356	0.2097008391494	0.796195648960786	1	pseudobulk	DESeq2	LRT	0.0989802000720468
108	KC_AB1	CG15365	-1.61102155603974	0.57025980967112	1	pseudobulk	DESeq2	LRT	0.243927235206257
108	KC_AB1	CG1537	-0.120602722483614	0.839364191744367	1	pseudobulk	DESeq2	LRT	0.0760495622385891
108	KC_AB1	CG15373	-0.760959112540794	1	1	pseudobulk	DESeq2	LRT	0
108	KC_AB1	CG15382	0.0952154049763301	0.952334545355926	1	pseudobulk	DESeq2	LRT	0.0212104616161752
108	KC_AB1	CG15385	0.307680976955862	0.577351879262846	1	pseudobulk	DESeq2	LRT	0.238559416232068
108	KC_AB1	CG15387	-0.245911646783014	0.737128827368462	1	pseudobulk	DESeq2	LRT	0.132456604236097
108	KC_AB1	CG15390	-0.0357227995853342	0.930119495513514	1	pseudobulk	DESeq2	LRT	0.0314612526198844
108	KC_AB1	CG15394	1.25804452752548	0.727552935095488	1	pseudobulk	DESeq2	LRT	0.138135402914536
108	KC_AB1	CG1542	0.877854234779683	0.318919360457651	1	pseudobulk	DESeq2	LRT	0.496319115486719
108	KC_AB1	CG15431	-0.196468593119274	0.362908134385747	1	pseudobulk	DESeq2	LRT	0.440203297218977
108	KC_AB1	CG15432	0.13303076761247	0.791434355860413	1	pseudobulk	DESeq2	LRT	0.101585101104682
108	KC_AB1	CG15435	-1.46538517282785	0.434971213096776	1	pseudobulk	DESeq2	LRT	0.361539484210674
108	KC_AB1	CG15436	2.88322402903533	0.302751069383099	1	pseudobulk	DESeq2	LRT	0.518914314158952
108	KC_AB1	CG15439	-0.10584497416081	0.923695604559336	1	pseudobulk	DESeq2	LRT	0.0344711229790318
108	KC_AB1	CG15443	-0.0718608351183907	0.878684303734623	1	pseudobulk	DESeq2	LRT	0.0561671314937258
108	KC_AB1	CG15445	-0.0803478218729439	0.941354380309166	1	pseudobulk	DESeq2	LRT	0.0262468521947683
108	KC_AB1	CG15446	-0.760959112540794	1	1	pseudobulk	DESeq2	LRT	0
108	KC_AB1	CG15449	-0.729530424241359	0.550693294661244	1	pseudobulk	DESeq2	LRT	0.259090211532791
108	KC_AB1	CG1545	-0.0762609038568811	0.870175754693017	1	pseudobulk	DESeq2	LRT	0.0603930214222079
108	KC_AB1	CG15456	0.206616692304912	0.673123574547887	1	pseudobulk	DESeq2	LRT	0.171905199048406
108	KC_AB1	CG15458	-0.760959112540794	0.804334723897681	1	pseudobulk	DESeq2	LRT	0.0945631819854715
108	KC_AB1	CG15459	0.7713768060463	0.0505971787310457	1	pseudobulk	DESeq2	LRT	1.2958736984902
108	KC_AB1	CG15461	1.47902393337271	0.619084561357736	1	pseudobulk	DESeq2	LRT	0.208250026225741
108	KC_AB1	CG15473	1.11388934932283	1	1	pseudobulk	DESeq2	LRT	0
108	KC_AB1	CG15506	-1.43550613534661	0.602448798560473	1	pseudobulk	DESeq2	LRT	0.220079857379737
108	KC_AB1	CG15514	0.127827178163726	0.816609005179367	1	pseudobulk	DESeq2	LRT	0.0879858351916848
108	KC_AB1	CG15515	-3.41337562833488	0.256593506563309	1	pseudobulk	DESeq2	LRT	0.590754338215985
108	KC_AB1	CG1552	-0.0549384182191573	0.777147704956684	1	pseudobulk	DESeq2	LRT	0.109496431197402
108	KC_AB1	CG15522	-0.0961654804704742	0.941969813383146	1	pseudobulk	DESeq2	LRT	0.0259630145013753
108	KC_AB1	CG15525	-0.779841663330548	0.292659028790918	1	pseudobulk	DESeq2	LRT	0.533638072972702

108	KC_AB1	CG15535	0.00093821264021597	0.959121534763277	1	pseudobulk	DESeq2	LRT	0.0181263578629228
108	KC_AB1	CG15536	-1.32993309508204	0.27597162302178	1	pseudobulk	DESeq2	LRT	0.559135572277691
108	KC_AB1	CG15537	-0.760959112540794	1	1	pseudobulk	DESeq2	LRT	0
108	KC_AB1	CG15539	-0.535979953737589	0.799855814031602	1	pseudobulk	DESeq2	LRT	0.0969882940257126
108	KC_AB1	CG15544	-0.760959112540794	1	1	pseudobulk	DESeq2	LRT	0
108	KC_AB1	CG15547	2.4138856564567	0.409611385497059	1	pseudobulk	DESeq2	LRT	0.387627980279722
108	KC_AB1	CG15554	-1.22404819087985	0.615494548704634	1	pseudobulk	DESeq2	LRT	0.210775789163178
108	KC_AB1	CG15561	-0.50924274700815	0.667490709998997	1	pseudobulk	DESeq2	LRT	0.175554774344284
108	KC_AB1	CG15579	-2.35716291794055	0.404761325252261	1	pseudobulk	DESeq2	LRT	0.392800990812383
108	KC_AB1	CG15601	0.134357098658246	0.871473723813106	1	pseudobulk	DESeq2	LRT	0.0597457029601958
108	KC_AB1	CG15602	0.913781661508743	0.225519850982829	1	pseudobulk	DESeq2	LRT	0.646815224105931
108	KC_AB1	CG15611	0.386628173623796	0.856111654016185	1	pseudobulk	DESeq2	LRT	0.0674695909760058
108	KC_AB1	CG15614	-1.43550613534661	0.602448798560473	1	pseudobulk	DESeq2	LRT	0.220079857379737
108	KC_AB1	CG15628	-0.14873244212382	0.427615302758982	1	pseudobulk	DESeq2	LRT	0.368946761403235
108	KC_AB1	CG15629	5.34754619907802	0.00523916340067905	1	pseudobulk	DESeq2	LRT	2.28073805642768
108	KC_AB1	CG15631	1.47306986287108	0.669507364477944	1	pseudobulk	DESeq2	LRT	0.174244641453253
108	KC_AB1	CG15638	1.13661584234725	0.691531818919754	1	pseudobulk	DESeq2	LRT	0.160187832238187
108	KC_AB1	CG15642	0.178576210923891	0.794554910112585	1	pseudobulk	DESeq2	LRT	0.0998760841899404
108	KC_AB1	CG15643	0.494552132362229	0.590276799400937	1	pseudobulk	DESeq2	LRT	0.228944286218348
108	KC_AB1	CG15651	-0.63543303110202	0.223140084865018	1	pseudobulk	DESeq2	LRT	0.651422406085733
108	KC_AB1	CG15653	-0.492150268293339	0.622455412174087	1	pseudobulk	DESeq2	LRT	0.205891752570039
108	KC_AB1	CG15657	-0.760959112540794	0.805265236978025	1	pseudobulk	DESeq2	LRT	0.0940610488455931
108	KC_AB1	CG15673	4.11570063625529	0.0979522044090902	1	pseudobulk	DESeq2	LRT	1.00898578578531
108	KC_AB1	CG15706	0.7030878490581	0.572782422078777	1	pseudobulk	DESeq2	LRT	0.242010318408524
108	KC_AB1	CG15715	-0.0868665393835007	0.845520925693039	1	pseudobulk	DESeq2	LRT	0.0728756396326993
108	KC_AB1	CG15717	0.296275065198765	1	1	pseudobulk	DESeq2	LRT	0
108	KC_AB1	CG1572	0.934338086067662	0.587035184948862	1	pseudobulk	DESeq2	LRT	0.231335867796351
108	KC_AB1	CG15725	0.296275065198765	1	1	pseudobulk	DESeq2	LRT	0
108	KC_AB1	CG15743	-0.291055976515378	0.691187948072102	1	pseudobulk	DESeq2	LRT	0.160403843057251
108	KC_AB1	CG15744	-0.292590009773637	0.818007356267939	1	pseudobulk	DESeq2	LRT	0.0872427907391806
108	KC_AB1	CG15747	0.0653300438907325	0.898082691380021	1	pseudobulk	DESeq2	LRT	0.0466836736259391
108	KC_AB1	CG1575	0.0431942687094826	0.967464167684518	1	pseudobulk	DESeq2	LRT	0.0143651111308262
108	KC_AB1	CG15760	0.39691169684404	0.41215117537392	1	pseudobulk	DESeq2	LRT	0.384943457296247
108	KC_AB1	CG15765	0.124650617063399	0.585082803528096	1	pseudobulk	DESeq2	LRT	0.232782666275895
108	KC_AB1	CG15767	-0.760959112540794	1	1	pseudobulk	DESeq2	LRT	0
108	KC_AB1	CG15771	-0.281174591690853	0.582059357348927	1	pseudobulk	DESeq2	LRT	0.235032724534597
108	KC_AB1	CG15772	0.222610796226353	0.337230351149912	1	pseudobulk	DESeq2	LRT	0.472073345246168
109	KC_AB1	CG1578	-0.137354619367971	0.702192267245303	1	pseudobulk	DESeq2	LRT	0.153543957427184
109	KC_AB1	CG15784	2.58743201691345	0.417636627932844	1	pseudobulk	DESeq2	LRT	0.379201419460921
109	KC_AB1	CG15803	-0.241718298588286	0.547737909268599	1	pseudobulk	DESeq2	LRT	0.261427200249929
109	KC_AB1	CG15812	-0.306489673803103	0.74155886351352	1	pseudobulk	DESeq2	LRT	0.129854369808268
109	KC_AB1	CG15814	0.186457598681325	0.726567458689496	1	pseudobulk	DESeq2	LRT	0.138724057103723
109	KC_AB1	CG1582	-0.0315723563036977	0.96852035172492	1	pseudobulk	DESeq2	LRT	0.0138912489263207
109	KC_AB1	CG15864	0.152115475746305	1	1	pseudobulk	DESeq2	LRT	0
109	KC_AB1	CG15877	-0.409423908286584	0.541573252174416	1	pseudobulk	DESeq2	LRT	0.266342793214921
109	KC_AB1	CG15878	1.25804452752548	0.573436424985144	1	pseudobulk	DESeq2	LRT	0.241514723898045
109	KC_AB1	CG15879	1.25804452752548	0.727552935095488	1	pseudobulk	DESeq2	LRT	0.138135402914536
109	KC_AB1	CG15890	0.152115475746305	1	1	pseudobulk	DESeq2	LRT	0
109	KC_AB1	CG15891	0.388363361387456	0.339806015901837	1	pseudobulk	DESeq2	LRT	0.46876893667991
109	KC_AB1	CG15892	-1.31349914273857	0.746788708402592	1	pseudobulk	DESeq2	LRT	0.126802257294357
109	KC_AB1	CG15894	-0.143819620369484	0.558585891456062	1	pseudobulk	DESeq2	LRT	0.252910037736624
109	KC_AB1	CG15908	0.216746549362493	0.698541022072827	1	pseudobulk	DESeq2	LRT	0.15580808470121
109	KC_AB1	CG15912	0.0464174623636003	0.961833767696579	1	pseudobulk	DESeq2	LRT	0.0168999799479475
109	KC_AB1	CG15914	1.25804452752548	0.727552935095488	1	pseudobulk	DESeq2	LRT	0.138135402914536
109	KC_AB1	CG15916	0.121768495505688	0.652954254001521	1	pseudobulk	DESeq2	LRT	0.185117244346124
109	KC_AB1	CG15922	0.500508586401865	0.389128937726442	1	pseudobulk	DESeq2	LRT	0.409906471515436

109	KC_AB1	CG1598	0.187941145465022	0.676335422861427	1	pseudobulk	DESeq2	LRT	0.169837865937989
109	KC_AB1	CG1602	1.35097129913916	0.335932847448949	1	pseudobulk	DESeq2	LRT	0.473747528851508
109	KC_AB1	CG1603	-0.0916534844585328	0.914011739806327	1	pseudobulk	DESeq2	LRT	0.0390482260382006
109	KC_AB1	CG1607	0.0372172370637245	0.940267662461712	1	pseudobulk	DESeq2	LRT	0.026748499822487
109	KC_AB1	CG1620	-0.414777241425364	0.477225793250779	1	pseudobulk	DESeq2	LRT	0.321276091481668
109	KC_AB1	CG1622	-0.00031460096254854	0.971146584033255	1	pseudobulk	DESeq2	LRT	0.0127152131073451
109	KC_AB1	CG1628	-0.760959112540794	0.805265236978025	1	pseudobulk	DESeq2	LRT	0.0940610488455931
109	KC_AB1	CG1632	3.23211873039625	0.231855719297104	1	pseudobulk	DESeq2	LRT	0.634782186684127
109	KC_AB1	CG1635	-0.383470481271617	0.603105707409475	1	pseudobulk	DESeq2	LRT	0.219606561622377
109	KC_AB1	CG1636	-0.250785949277916	0.429473433566725	1	pseudobulk	DESeq2	LRT	0.367063695656293
109	KC_AB1	CG1637	0.275466408350802	0.624670015517508	1	pseudobulk	DESeq2	LRT	0.204349339912498
109	KC_AB1	CG1640	-0.0450568554509058	0.872774942970493	1	pseudobulk	DESeq2	LRT	0.059097730666572
109	KC_AB1	CG1646	-0.0538596726791426	0.875147697944789	1	pseudobulk	DESeq2	LRT	0.0579186452756214
109	KC_AB1	CG1647	-0.0829527228439185	0.863252787237114	1	pseudobulk	DESeq2	LRT	0.0638620107430676
109	KC_AB1	CG1648	-0.273866642582404	0.571215531139456	1	pseudobulk	DESeq2	LRT	0.243199992764683
109	KC_AB1	CG1662	-0.162651874936917	0.839711048351717	1	pseudobulk	DESeq2	LRT	0.0758701326226562
109	KC_AB1	CG1663	0.278924788989368	0.821969117855878	1	pseudobulk	DESeq2	LRT	0.0851444990009811
109	KC_AB1	CG16700	0.296275065198765	1	1	pseudobulk	DESeq2	LRT	0
109	KC_AB1	CG1671	-3.02999637893047	0.191309629999418	1	pseudobulk	DESeq2	LRT	0.718263168235408
109	KC_AB1	CG16711	-0.323187506953575	0.150020700290511	1	pseudobulk	DESeq2	LRT	0.823848811599788
109	KC_AB1	CG16717	0.60944353366574	0.321482749911437	1	pseudobulk	DESeq2	LRT	0.492842325443534
109	KC_AB1	CG16721	1.09775282232052	0.151718646305516	1	pseudobulk	DESeq2	LRT	0.818961040901142
109	KC_AB1	CG16734	0.254196856837688	0.890998468402047	1	pseudobulk	DESeq2	LRT	0.0501230425008484
109	KC_AB1	CG16739	1.13698605850655	0.780204112083573	1	pseudobulk	DESeq2	LRT	0.10779176505922
109	KC_AB1	CG16743	1.85630024725514	0.233135517815724	1	pseudobulk	DESeq2	LRT	0.632391557383253
109	KC_AB1	CG16749	1.25804452752548	0.573436424985144	1	pseudobulk	DESeq2	LRT	0.241514723898045
109	KC_AB1	CG16753	1.00455945790418	0.311443647656503	1	pseudobulk	DESeq2	LRT	0.506620522763392
109	KC_AB1	CG16758	-0.400746619592429	0.813644283277972	1	pseudobulk	DESeq2	LRT	0.0895654225977311
109	KC_AB1	CG1677	-0.116288527634576	0.622270414679799	1	pseudobulk	DESeq2	LRT	0.206020846688268
109	KC_AB1	CG16779	-0.214152816404445	0.162455821854076	1	pseudobulk	DESeq2	LRT	0.789264720429835
109	KC_AB1	CG16782	1.11388934932283	1	1	pseudobulk	DESeq2	LRT	0
109	KC_AB1	CG16786	-1.43550613534661	0.602448798560473	1	pseudobulk	DESeq2	LRT	0.220079857379737
109	KC_AB1	CG16787	-0.198651590661262	0.784308693423941	1	pseudobulk	DESeq2	LRT	0.105512971162137
109	KC_AB1	CG16789	1.25804452752548	0.573436424985144	1	pseudobulk	DESeq2	LRT	0.241514723898045
109	KC_AB1	CG16790	0.0154463574099955	0.985519122520666	1	pseudobulk	DESeq2	LRT	0.00633494446963829
109	KC_AB1	CG16791	-0.155974483193456	0.382958552774759	1	pseudobulk	DESeq2	LRT	0.416848226750058
109	KC_AB1	CG16798	0.440748176240683	0.795420799786285	1	pseudobulk	DESeq2	LRT	0.0994030566561732
109	KC_AB1	CG16799	0.939729030686247	0.766958937810335	1	pseudobulk	DESeq2	LRT	0.115227887106769
109	KC_AB1	CG16812	-0.448421481433013	0.256496347643053	1	pseudobulk	DESeq2	LRT	0.590918814605815
109	KC_AB1	CG16820	5.59013216133267	0.175005344615962	1	pseudobulk	DESeq2	LRT	0.756948687874984
109	KC_AB1	CG16825	1.11388934932283	1	1	pseudobulk	DESeq2	LRT	0
109	KC_AB1	CG16852	1.11388934932283	1	1	pseudobulk	DESeq2	LRT	0
109	KC_AB1	CG16854	0.358804627383182	0.723456703955741	1	pseudobulk	DESeq2	LRT	0.140587454587444
109	KC_AB1	CG16863	0.781923423666886	0.675743786148264	1	pseudobulk	DESeq2	LRT	0.170217939197758
109	KC_AB1	CG16865	0.362697263808429	0.73555138414842	1	pseudobulk	DESeq2	LRT	0.133386982932879
109	KC_AB1	CG16868	0.404415522636419	0.615321188789138	1	pseudobulk	DESeq2	LRT	0.210898129580005
109	KC_AB1	CG1688	-0.0523872651343934	0.927333445882245	1	pseudobulk	DESeq2	LRT	0.0327640763540355
109	KC_AB1	CG16890	-1.18608266531542	0.498960298359081	1	pseudobulk	DESeq2	LRT	0.301934009265439
109	KC_AB1	CG16896	-0.621428018569091	0.534696796847686	1	pseudobulk	DESeq2	LRT	0.271892417574508
109	KC_AB1	CG16903	-0.527235189987449	0.365485755418649	1	pseudobulk	DESeq2	LRT	0.437129544735206
109	KC_AB1	CG16908	-0.71791931041265	0.786153997290876	1	pseudobulk	DESeq2	LRT	0.104492373015615
109	KC_AB1	CG16935	0.312861577284269	0.538011937920316	1	pseudobulk	DESeq2	LRT	0.269208087687889
109	KC_AB1	CG1695	0.0260299992007518	0.915693633583251	1	pseudobulk	DESeq2	LRT	0.0382498052620273
109	KC_AB1	CG16952	0.107435076333617	0.740041413870406	1	pseudobulk	DESeq2	LRT	0.130743975793179
109	KC_AB1	CG16953	0.0603172828677428	0.886623690910071	1	pseudobulk	DESeq2	LRT	0.0522606683738679
109	KC_AB1	CG16959	-2.00963963748391	0.498268873088307	1	pseudobulk	DESeq2	LRT	0.302536242405947

109	KC_AB1	CG16965	2.09987857572813	0.503115176013642	1	pseudobulk	DESeq2	LRT	0.29833258237694
109	KC_AB1	CG16972	-0.0304381621318107	0.931504232687295	1	pseudobulk	DESeq2	LRT	0.0308151673608043
109	KC_AB1	CG16974	-0.0442943174970867	0.901106867701092	1	pseudobulk	DESeq2	LRT	0.0452237003631939
109	KC_AB1	CG16978	-6.37725053580735	0.142164710192674	1	pseudobulk	DESeq2	LRT	0.847208195945585
109	KC_AB1	CG16979	-0.0836280443755861	0.925503129802101	1	pseudobulk	DESeq2	LRT	0.0336221082417267
109	KC_AB1	CG1698	2.09987857572813	0.503115176013642	1	pseudobulk	DESeq2	LRT	0.29833258237694
109	KC_AB1	CG16985	1.1513697260209	0.563321772543092	1	pseudobulk	DESeq2	LRT	0.249243462837813
109	KC_AB1	CG16986	0.595271261595902	0.454809982093909	1	pseudobulk	DESeq2	LRT	0.34217001205658
109	KC_AB1	CG16989	-0.0938980940182102	0.923408701818215	1	pseudobulk	DESeq2	LRT	0.0346060371556524
109	KC_AB1	CG17002	-0.213971630994849	0.754821827271146	1	pseudobulk	DESeq2	LRT	0.122155549781149
109	KC_AB1	CG17003	-0.760959112540794	0.805265236978025	1	pseudobulk	DESeq2	LRT	0.0940610488455931
109	KC_AB1	CG17010	-0.191864985972428	0.904626820903834	1	pseudobulk	DESeq2	LRT	0.0435305401830064
109	KC_AB1	CG17019	-0.229406670734282	0.636590890817291	1	pseudobulk	DESeq2	LRT	0.196139580129231
109	KC_AB1	CG17026	0.433152216824259	0.67964978098271	1	pseudobulk	DESeq2	LRT	0.167714818716602
109	KC_AB1	CG17027	0.571778004001766	0.784559566227574	1	pseudobulk	DESeq2	LRT	0.105374077823533
109	KC_AB1	CG17029	0.289493448759282	0.694218455562421	1	pseudobulk	DESeq2	LRT	0.158503844939486
109	KC_AB1	CG1703	-0.196871758281089	0.713803464321557	1	pseudobulk	DESeq2	LRT	0.14642134860215
109	KC_AB1	CG17030	-0.760959112540794	0.804334723897681	1	pseudobulk	DESeq2	LRT	0.0945631819854715
109	KC_AB1	CG17032	-3.99653340182271	0.160092881750206	1	pseudobulk	DESeq2	LRT	0.795627977795456
109	KC_AB1	CG17048	-0.760959112540794	1	1	pseudobulk	DESeq2	LRT	0
109	KC_AB1	CG17059	-0.00459127666455647	0.944465249377902	1	pseudobulk	DESeq2	LRT	0.0248140168645021
109	KC_AB1	CG17065	0.0122380743769597	0.973171163979506	1	pseudobulk	DESeq2	LRT	0.0118107681239956
109	KC_AB1	CG17068	-0.261020254038969	0.846892000684762	1	pseudobulk	DESeq2	LRT	0.0721719692409563
109	KC_AB1	CG17075	-0.137454839922535	0.807670456595672	1	pseudobulk	DESeq2	LRT	0.0927658026852613
109	KC_AB1	CG17078	0.317752018969599	0.507528650433154	1	pseudobulk	DESeq2	LRT	0.294539436422011
110	KC_AB1	CG17104	-2.73719572742657	0.395113780272619	1	pseudobulk	DESeq2	LRT	0.40327782328744
110	KC_AB1	CG17107	-0.760959112540794	1	1	pseudobulk	DESeq2	LRT	0
110	KC_AB1	CG17111	1.74218151256907	0.459503365947811	1	pseudobulk	DESeq2	LRT	0.337711302978182
110	KC_AB1	CG17118	-2.10264006323213	0.319081610547617	1	pseudobulk	DESeq2	LRT	0.496098224536762
110	KC_AB1	CG17121	0.675910847019996	0.771613529648262	1	pseudobulk	DESeq2	LRT	0.112600165942529
110	KC_AB1	CG17124	0.101727660780836	0.682735934757316	1	pseudobulk	DESeq2	LRT	0.165747238123718
110	KC_AB1	CG17129	1.11388934932283	1	1	pseudobulk	DESeq2	LRT	0
110	KC_AB1	CG17141	0.112738471368112	0.909803628199428	1	pseudobulk	DESeq2	LRT	0.04105233558273
110	KC_AB1	CG17162	0.786571582083693	0.447605870397396	1	pseudobulk	DESeq2	LRT	0.349104226289181
110	KC_AB1	CG17163	0.0694262327029944	0.879992919836219	1	pseudobulk	DESeq2	LRT	0.0555208220412301
110	KC_AB1	CG17167	-1.43179340439473	0.560037947646132	1	pseudobulk	DESeq2	LRT	0.251782544609954
110	KC_AB1	CG17168	-0.425711940331056	0.28535626396143	1	pseudobulk	DESeq2	LRT	0.544612589650767
110	KC_AB1	CG17186	1.16539528072623	0.384677337528553	1	pseudobulk	DESeq2	LRT	0.414903398502662
110	KC_AB1	CG17187	1.51738599905927	0.158161801580302	1	pseudobulk	DESeq2	LRT	0.800898396729744
110	KC_AB1	CG17189	-4.73107474809667	0.0765809337696049	1	pseudobulk	DESeq2	LRT	1.11587934249977
110	KC_AB1	CG17193	1.25804452752548	0.727552935095488	1	pseudobulk	DESeq2	LRT	0.138135402914536
110	KC_AB1	CG17202	-0.0896842960863976	0.781614515882659	1	pseudobulk	DESeq2	LRT	0.107007383642918
110	KC_AB1	CG17211	-1.76935196357135	0.542380240270211	1	pseudobulk	DESeq2	LRT	0.265696140855463
110	KC_AB1	CG1722	-0.760959112540794	1	1	pseudobulk	DESeq2	LRT	0
110	KC_AB1	CG17221	0.415367632449781	0.763241710511371	1	pseudobulk	DESeq2	LRT	0.117337903830767
110	KC_AB1	CG17224	-0.760959112540794	0.805265236978025	1	pseudobulk	DESeq2	LRT	0.0940610488455931
110	KC_AB1	CG17230	-0.214226366478692	0.738529130166309	1	pseudobulk	DESeq2	LRT	0.131632369924164
110	KC_AB1	CG17233	-0.238845224481969	0.592044649597037	1	pseudobulk	DESeq2	LRT	0.22764553932033
110	KC_AB1	CG17249	0.455956900960104	0.554017851375402	1	pseudobulk	DESeq2	LRT	0.25647624135654
110	KC_AB1	CG17258	1.67020383217772	0.598754457572587	1	pseudobulk	DESeq2	LRT	0.222751240354882
110	KC_AB1	CG17260	-1.60128328867486	0.326094663255326	1	pseudobulk	DESeq2	LRT	0.48665630863878
110	KC_AB1	CG17266	-0.712668323281839	0.557247066821123	1	pseudobulk	DESeq2	LRT	0.253952208835617
110	KC_AB1	CG17270	-0.0495038731317751	0.908747182587287	1	pseudobulk	DESeq2	LRT	0.041556922585386
110	KC_AB1	CG17271	-0.0174671285432254	0.957818313969559	1	pseudobulk	DESeq2	LRT	0.0187168632843466
110	KC_AB1	CG17272	0.00301128215747699	0.938109859477062	1	pseudobulk	DESeq2	LRT	0.0277462996050611
110	KC_AB1	CG17278	2.77905602015038	0.396550243710121	1	pseudobulk	DESeq2	LRT	0.401701778879434

110	KC_AB1	CG17292	2.04425205959347	0.490670545152587	1	pseudobulk	DESeq2	LRT	0.309210011841057
110	KC_AB1	CG17294	0.355497091649322	0.491586649676611	1	pseudobulk	DESeq2	LRT	0.308399920029905
110	KC_AB1	CG17298	-0.760959112540794	1	1	pseudobulk	DESeq2	LRT	0
110	KC_AB1	CG17321	-0.0787829772888177	0.766140462048367	1	pseudobulk	DESeq2	LRT	0.11569160073264
110	KC_AB1	CG17328	-0.599007515880506	0.588513380345536	1	pseudobulk	DESeq2	LRT	0.230243658658825
110	KC_AB1	CG17329	1.25804452752548	0.727552935095488	1	pseudobulk	DESeq2	LRT	0.138135402914536
110	KC_AB1	CG17333	0.88913407318103	0.671008192168902	1	pseudobulk	DESeq2	LRT	0.173272177607415
110	KC_AB1	CG17343	-0.428874470902854	0.49453973830644	1	pseudobulk	DESeq2	LRT	0.305798805312893
110	KC_AB1	CG17350	-0.760959112540794	0.804334723897681	1	pseudobulk	DESeq2	LRT	0.0945631819854715
110	KC_AB1	CG17359	0.629688850140186	0.517431579520283	1	pseudobulk	DESeq2	LRT	0.286147069255201
110	KC_AB1	CG17361	-0.189990502558036	0.829390275012951	1	pseudobulk	DESeq2	LRT	0.0812410612471071
110	KC_AB1	CG17364	-0.0033922187103120	0.981986681873165	1	pseudobulk	DESeq2	LRT	0.0078944022621502
110	KC_AB1	CG1737	0.355068053616025	0.308969867138696	1	pseudobulk	DESeq2	LRT	0.510083873887501
110	KC_AB1	CG17378	0.0186681928868088	0.929791415050796	1	pseudobulk	DESeq2	LRT	0.0316144680618143
110	KC_AB1	CG1738	1.31177435728837	0.136260768763384	1	pseudobulk	DESeq2	LRT	0.865629165163582
110	KC_AB1	CG17385	0.310332191694956	0.312610541228756	1	pseudobulk	DESeq2	LRT	0.504996381658178
110	KC_AB1	CG17454	-0.0099960767344155	0.953414423371671	1	pseudobulk	DESeq2	LRT	0.0207182822984818
110	KC_AB1	CG17486	0.431025475086748	0.231998452103073	1	pseudobulk	DESeq2	LRT	0.63451491271831
110	KC_AB1	CG17493	0.134783044930165	0.613682864296733	1	pseudobulk	DESeq2	LRT	0.212056003235982
110	KC_AB1	CG1750	0.465688986005349	0.60682765138406	1	pseudobulk	DESeq2	LRT	0.216934637888758
110	KC_AB1	CG17508	0.330206333475842	0.351212233937409	1	pseudobulk	DESeq2	LRT	0.454430364522853
110	KC_AB1	CG17528	-0.41873641308779	0.324665548659556	1	pseudobulk	DESeq2	LRT	0.488563793360004
110	KC_AB1	CG17544	-0.272055422857379	0.861799169225079	1	pseudobulk	DESeq2	LRT	0.064593928907593
110	KC_AB1	CG17549	0.759569512462419	0.82124704597288	1	pseudobulk	DESeq2	LRT	0.0855261795873957
110	KC_AB1	CG17556	-0.547992472621797	0.290015755136622	1	pseudobulk	DESeq2	LRT	0.537578408366433
110	KC_AB1	CG17565	0.318396793606053	0.479377443279445	1	pseudobulk	DESeq2	LRT	0.319322405199613
110	KC_AB1	CG17568	0.236104335259898	0.696600145658922	1	pseudobulk	DESeq2	LRT	0.157016439066902
110	KC_AB1	CG17574	1.28834788459536	0.090218952614303	1	pseudobulk	DESeq2	LRT	1.04470221909675
110	KC_AB1	CG17593	-0.333980108792987	0.533003823484349	1	pseudobulk	DESeq2	LRT	0.273269675565926
110	KC_AB1	CG17598	0.26737877199051	0.671633638405841	1	pseudobulk	DESeq2	LRT	0.172867560586119
110	KC_AB1	CG17600	0.0473918932864909	0.906145955463735	1	pseudobulk	DESeq2	LRT	0.0428018436618001
110	KC_AB1	CG17612	-1.19726917963794	0.331854520810698	1	pseudobulk	DESeq2	LRT	0.479052261644761
110	KC_AB1	CG17633	1.25804452752548	0.573436424985144	1	pseudobulk	DESeq2	LRT	0.241514723898045
110	KC_AB1	CG17637	-0.760959112540794	0.804334723897681	1	pseudobulk	DESeq2	LRT	0.0945631819854715
110	KC_AB1	CG1764	0.272549523687846	0.575077980781601	1	pseudobulk	DESeq2	LRT	0.240273260828845
110	KC_AB1	CG17646	1.73370397098747	0.29838861243171	1	pseudobulk	DESeq2	LRT	0.525217755101645
110	KC_AB1	CG17650	1.90071181822722	0.545896518579078	1	pseudobulk	DESeq2	LRT	0.262889675371117
110	KC_AB1	CG17652	-0.287735095862825	0.670283370087329	1	pseudobulk	DESeq2	LRT	0.173741555436798
110	KC_AB1	CG17660	0.212404871824114	0.784163774324087	1	pseudobulk	DESeq2	LRT	0.10559322448741
110	KC_AB1	CG17669	-3.09859732262995	0.319636127445685	1	pseudobulk	DESeq2	LRT	0.49534413967225
110	KC_AB1	CG17683	0.477305788382256	0.248379638687216	1	pseudobulk	DESeq2	LRT	0.604884009011672
110	KC_AB1	CG17684	-0.143961895370963	0.455219477760139	1	pseudobulk	DESeq2	LRT	0.341779163786154
110	KC_AB1	CG17691	0.553432969534224	0.319776824714329	1	pseudobulk	DESeq2	LRT	0.495153014208899
110	KC_AB1	CG17698	-0.138297623982507	0.556136148391944	1	pseudobulk	DESeq2	LRT	0.254818875221738
110	KC_AB1	CG17712	0.557842210879566	0.384002744138903	1	pseudobulk	DESeq2	LRT	0.415665672090477
110	KC_AB1	CG17715	0.348692535711351	0.241222638246252	1	pseudobulk	DESeq2	LRT	0.617581936979349
110	KC_AB1	CG17716	1.71612496767129	0.464351466120566	1	pseudobulk	DESeq2	LRT	0.333153178893667
110	KC_AB1	CG17721	1.23531857104863	0.236971045313355	1	pseudobulk	DESeq2	LRT	0.625304715714975
110	KC_AB1	CG17724	0.0235405009616985	0.954683780513462	1	pseudobulk	DESeq2	LRT	0.0201404557668654
110	KC_AB1	CG17726	1.08150172717967	0.659846871007429	1	pseudobulk	DESeq2	LRT	0.180556838386156
110	KC_AB1	CG17734	0.36025310628656	0.286318082910368	1	pseudobulk	DESeq2	LRT	0.543151222498171
110	KC_AB1	CG17739	0.810312643297938	0.609365858297171	1	pseudobulk	DESeq2	LRT	0.215121882191643
110	KC_AB1	CG17746	-0.661613501477861	0.330851871330231	1	pseudobulk	DESeq2	LRT	0.480366404601783
110	KC_AB1	CG17754	0.111421866919338	0.861338508903811	1	pseudobulk	DESeq2	LRT	0.0648261358204402
110	KC_AB1	CG17760	1.93086056272323	0.489320537915144	1	pseudobulk	DESeq2	LRT	0.310406555513252
110	KC_AB1	CG17765	0.0725733647989583	0.816989211055558	1	pseudobulk	DESeq2	LRT	0.0877836786083124

110	KC_AB1	CG17776	0.102516084960114	0.799707448103467	1	pseudobulk	DESeq2	LRT	0.0970688591469569
110	KC_AB1	CG17777	-2.36175013442542	0.448372544778042	1	pseudobulk	DESeq2	LRT	0.348360988442027
110	KC_AB1	CG17778	0.140056538700172	0.53856389640046	1	pseudobulk	DESeq2	LRT	0.268762763660491
110	KC_AB1	CG17780	1.25804452752548	0.727552935095488	1	pseudobulk	DESeq2	LRT	0.138135402914536
110	KC_AB1	CG17781	1.25804452752548	0.727552935095488	1	pseudobulk	DESeq2	LRT	0.138135402914536
110	KC_AB1	CG17786	-0.313054495292384	0.803573289057949	1	pseudobulk	DESeq2	LRT	0.0949745077221173
110	KC_AB1	CG17802	-0.0759700922063825	0.904110973243732	1	pseudobulk	DESeq2	LRT	0.04377825966903
110	KC_AB1	CG17803	0.240717662902349	0.905733587629501	1	pseudobulk	DESeq2	LRT	0.042999526867284
110	KC_AB1	CG17806	-0.5132710932668	0.666765871009954	1	pseudobulk	DESeq2	LRT	0.176026638015272
110	KC_AB1	CG17807	-0.56957330721045	0.553720368243747	1	pseudobulk	DESeq2	LRT	0.256709500954078
110	KC_AB1	CG17816	-0.0223543301640073	0.88431237525826	1	pseudobulk	DESeq2	LRT	0.0533942973316183
110	KC_AB1	CG17829	-0.674826351489401	0.43244327149094	1	pseudobulk	DESeq2	LRT	0.364070855816116
110	KC_AB1	CG17834	1.63121327894087	0.595535911010076	1	pseudobulk	DESeq2	LRT	0.225092045292395
111	KC_AB1	CG17839	0.75177436220672	0.267579066379502	1	pseudobulk	DESeq2	LRT	0.572547865909757
111	KC_AB1	CG17841	-0.0819015976189702	0.602290540222417	1	pseudobulk	DESeq2	LRT	0.2201939579509
111	KC_AB1	CG1785	0.524991281257573	0.689431294614688	1	pseudobulk	DESeq2	LRT	0.161509007017417
111	KC_AB1	CG17883	0.011068934020606	0.962047945344042	1	pseudobulk	DESeq2	LRT	0.0168032835968218
111	KC_AB1	CG1789	-0.13243307516951	0.825039005866705	1	pseudobulk	DESeq2	LRT	0.0835255185625312
111	KC_AB1	CG17896	0.657485638617616	0.48728108672755	1	pseudobulk	DESeq2	LRT	0.312220444947999
111	KC_AB1	CG17904	0.128129522376374	0.815639100672509	1	pseudobulk	DESeq2	LRT	0.0885019628849785
111	KC_AB1	CG17919	-2.43835914691588	0.435837756451753	1	pseudobulk	DESeq2	LRT	0.360675149701555
111	KC_AB1	CG1792	0.136664852340824	0.878824095365128	1	pseudobulk	DESeq2	LRT	0.0560980442177803
111	KC_AB1	CG17928	-2.13621400928779	0.457001334179545	1	pseudobulk	DESeq2	LRT	0.340082532039628
111	KC_AB1	CG17931	-0.13312157656317	0.758923442369542	1	pseudobulk	DESeq2	LRT	0.119802032053664
111	KC_AB1	CG17977	0.0249273080353783	0.955410414203923	1	pseudobulk	DESeq2	LRT	0.0198100291122764
111	KC_AB1	CG17982	-0.709483972524545	0.583552279038548	1	pseudobulk	DESeq2	LRT	0.233920230482519
111	KC_AB1	CG17985	0.666787558910982	0.374886146229904	1	pseudobulk	DESeq2	LRT	0.426100608463688
111	KC_AB1	CG17996	-0.363727505363799	0.480647020870701	1	pseudobulk	DESeq2	LRT	0.318173745172202
111	KC_AB1	CG18004	-0.0574814389063739	0.909010814983106	1	pseudobulk	DESeq2	LRT	0.0414309497158279
111	KC_AB1	CG18011	1.90071181822722	0.545896518579078	1	pseudobulk	DESeq2	LRT	0.262889675371117
111	KC_AB1	CG18048	-2.16098594158411	0.0735824862722331	1	pseudobulk	DESeq2	LRT	1.13322554191847
111	KC_AB1	CG1806	2.58743201691345	0.417636627932844	1	pseudobulk	DESeq2	LRT	0.379201419460921
111	KC_AB1	CG18067	2.36468246449593	0.410782157831629	1	pseudobulk	DESeq2	LRT	0.386388428082423
111	KC_AB1	CG18081	0.321987667046252	0.575635656008976	1	pseudobulk	DESeq2	LRT	0.239852312844635
111	KC_AB1	CG18094	-1.60958773650785	0.570513631639934	1	pseudobulk	DESeq2	LRT	0.243733974251419
111	KC_AB1	CG1812	-0.779622857796165	0.501329261680729	1	pseudobulk	DESeq2	LRT	0.299876945665048
111	KC_AB1	CG18135	0.335265688767217	0.338900638835311	1	pseudobulk	DESeq2	LRT	0.469927612507004
111	KC_AB1	CG1814	-0.293793521519728	0.433845603088854	1	pseudobulk	DESeq2	LRT	0.362664799650286
111	KC_AB1	CG1815	0.119694137192252	0.829726551126857	1	pseudobulk	DESeq2	LRT	0.0810650123271466
111	KC_AB1	CG18171	-1.77067014871429	0.542156052888816	1	pseudobulk	DESeq2	LRT	0.265875689192671
111	KC_AB1	CG18178	0.0548035754628123	0.938890315611907	1	pseudobulk	DESeq2	LRT	0.0273851405420388
111	KC_AB1	CG18213	1.25804452752548	0.727552935095488	1	pseudobulk	DESeq2	LRT	0.138135402914536
111	KC_AB1	CG1824	0.0972138766070129	0.892447371714093	1	pseudobulk	DESeq2	LRT	0.049417385129574
111	KC_AB1	CG18249	2.09987857572813	0.503115176013642	1	pseudobulk	DESeq2	LRT	0.29833258237694
111	KC_AB1	CG18259	-0.367370107268341	0.681211787958132	1	pseudobulk	DESeq2	LRT	0.166717845440977
111	KC_AB1	CG18262	0.145607428135125	0.874584589221293	1	pseudobulk	DESeq2	LRT	0.0581981794895838
111	KC_AB1	CG18265	0.0273806397156867	0.922837858169023	1	pseudobulk	DESeq2	LRT	0.0348745974445615
111	KC_AB1	CG1827	-1.60958773650785	0.570513631639934	1	pseudobulk	DESeq2	LRT	0.243733974251419
111	KC_AB1	CG18273	1.14800504822344	0.772124825498498	1	pseudobulk	DESeq2	LRT	0.112312483805518
111	KC_AB1	CG18281	0.00635494054748053	0.990437883202087	1	pseudobulk	DESeq2	LRT	0.0041727567014062
111	KC_AB1	CG18302	-0.760959112540794	1	1	pseudobulk	DESeq2	LRT	0
111	KC_AB1	CG18324	-0.132576168497511	0.839780952045934	1	pseudobulk	DESeq2	LRT	0.0758339802757877
111	KC_AB1	CG18327	-0.485382126094376	0.810832531129408	1	pseudobulk	DESeq2	LRT	0.0910688354519273
111	KC_AB1	CG18335	-0.760959112540794	1	1	pseudobulk	DESeq2	LRT	0
111	KC_AB1	CG18343	0.245556270924701	0.973663313394496	1	pseudobulk	DESeq2	LRT	0.0115911934429027
111	KC_AB1	CG1835	1.25804452752548	0.727552935095488	1	pseudobulk	DESeq2	LRT	0.138135402914536

111	KC_AB1	CG18371	1.11388934932283	1	1	pseudobulk	DESeq2	LRT	0
111	KC_AB1	CG1840	0.329478566183371	0.549101004931295	1	pseudobulk	DESeq2	LRT	0.260347761470524
111	KC_AB1	CG18428	0.34325933136953	0.372518469819499	1	pseudobulk	DESeq2	LRT	0.428852189649019
111	KC_AB1	CG18467	-0.352260942629163	0.248111939051064	1	pseudobulk	DESeq2	LRT	0.605352337141566
111	KC_AB1	CG1847	0.532848940978205	0.260854514691041	1	pseudobulk	DESeq2	LRT	0.583601642405515
111	KC_AB1	CG18473	-0.760959112540794	1	1	pseudobulk	DESeq2	LRT	0
111	KC_AB1	CG18476	0.0492852831512267	0.917983376630369	1	pseudobulk	DESeq2	LRT	0.0371651831811843
111	KC_AB1	CG18480	1.11388934932283	1	1	pseudobulk	DESeq2	LRT	0
111	KC_AB1	CG18507	1.53612086133758	0.411154738423893	1	pseudobulk	DESeq2	LRT	0.38599470027411
111	KC_AB1	CG18508	-0.181315527002829	0.631285081780784	1	pseudobulk	DESeq2	LRT	0.199774473585426
111	KC_AB1	CG18528	0.263282778576749	0.788771315966497	1	pseudobulk	DESeq2	LRT	0.103048891099267
111	KC_AB1	CG18549	0.67634054407156	0.358909259490355	1	pseudobulk	DESeq2	LRT	0.445015337170542
111	KC_AB1	CG18568	-0.760959112540794	1	1	pseudobulk	DESeq2	LRT	0
111	KC_AB1	CG18596	-0.565367420403992	0.79202177089376	1	pseudobulk	DESeq2	LRT	0.101262880469753
111	KC_AB1	CG18598	1.25804452752548	0.727552935095488	1	pseudobulk	DESeq2	LRT	0.138135402914536
111	KC_AB1	CG18600	0.518066954478603	0.667334654516312	1	pseudobulk	DESeq2	LRT	0.175656321763312
111	KC_AB1	CG18622	0.683441894478493	0.802755862961851	1	pseudobulk	DESeq2	LRT	0.0954165138615147
111	KC_AB1	CG18628	-0.287723761595917	0.908359580254375	1	pseudobulk	DESeq2	LRT	0.0417421990483341
111	KC_AB1	CG18659	-0.0158467822442237	0.978098352367164	1	pseudobulk	DESeq2	LRT	0.00961747267382103
111	KC_AB1	CG18661	-0.488810412070296	0.501545506336948	1	pseudobulk	DESeq2	LRT	0.299689656353585
111	KC_AB1	CG18675	1.67020383217772	0.598754457572587	1	pseudobulk	DESeq2	LRT	0.222751240354882
111	KC_AB1	CG18731	-0.419519606822291	0.430152398897325	1	pseudobulk	DESeq2	LRT	0.366377650759398
111	KC_AB1	CG18764	-0.976419312497587	0.549831191126641	1	pseudobulk	DESeq2	LRT	0.25977062689715
111	KC_AB1	CG18766	0.0393657967197092	0.893354027358055	1	pseudobulk	DESeq2	LRT	0.0489764003961148
111	KC_AB1	CG18787	-1.07774211660359	0.432286779523818	1	pseudobulk	DESeq2	LRT	0.3642280461403
111	KC_AB1	CG18789	-1.49818075798149	0.686309014577879	1	pseudobulk	DESeq2	LRT	0.163480296671447
111	KC_AB1	CG18809	0.11364690329444	0.859661550610822	1	pseudobulk	DESeq2	LRT	0.0656724971665649
111	KC_AB1	CG18810	1.25804452752548	0.727552935095488	1	pseudobulk	DESeq2	LRT	0.138135402914536
111	KC_AB1	CG18815	0.0862906350794938	0.696263482470426	1	pseudobulk	DESeq2	LRT	0.157226382043254
111	KC_AB1	CG1882	0.0728828978213625	0.877456316501523	1	pseudobulk	DESeq2	LRT	0.0567744953459811
111	KC_AB1	CG18858	1.11388934932283	1	1	pseudobulk	DESeq2	LRT	0
111	KC_AB1	CG18870	-0.502071036216038	0.595896069384964	1	pseudobulk	DESeq2	LRT	0.224829479233798
111	KC_AB1	CG1888	0.306072107774023	0.69109383262561	1	pseudobulk	DESeq2	LRT	0.160462982691018
111	KC_AB1	CG1889	-3.58279186106554	0.225990959339248	1	pseudobulk	DESeq2	LRT	0.64590893424911
111	KC_AB1	CG1890	0.724384597811199	0.397523971742533	1	pseudobulk	DESeq2	LRT	0.400636677116698
111	KC_AB1	CG1896	-0.00017397590638634	0.979804840689471	1	pseudobulk	DESeq2	LRT	0.00886041925860977
111	KC_AB1	CG1902	0.536958274932362	0.282270579270883	1	pseudobulk	DESeq2	LRT	0.549334385495182
111	KC_AB1	CG1907	-0.244201101910543	0.483787879776552	1	pseudobulk	DESeq2	LRT	0.315345016114896
111	KC_AB1	CG1909	-0.190721717018762	0.418703780766117	1	pseudobulk	DESeq2	LRT	0.37809311754572
111	KC_AB1	CG1910	-0.250436041193019	0.424713585681017	1	pseudobulk	DESeq2	LRT	0.37190384663369
111	KC_AB1	CG1924	1.11388934932283	1	1	pseudobulk	DESeq2	LRT	0
111	KC_AB1	CG1927	0.483935631641669	0.398956506697071	1	pseudobulk	DESeq2	LRT	0.399074447498806
111	KC_AB1	CG1943	-0.0373589652419279	0.857874524930077	1	pseudobulk	DESeq2	LRT	0.0665762286028643
111	KC_AB1	CG1951	0.243833972221972	0.719430115106038	1	pseudobulk	DESeq2	LRT	0.143011386714015
111	KC_AB1	CG1965	0.0859120078430704	0.805500256530867	1	pseudobulk	DESeq2	LRT	0.0939343169332521
111	KC_AB1	CG1983	-0.241368986201766	0.726090135392593	1	pseudobulk	DESeq2	LRT	0.139009463495751
111	KC_AB1	CG1998	0.384960081726643	0.205857205273965	1	pseudobulk	DESeq2	LRT	0.686433927520225
111	KC_AB1	CG2003	-0.778260625186364	0.631906915353234	1	pseudobulk	DESeq2	LRT	0.199346891853426
111	KC_AB1	CG2004	-0.553320644723408	0.783050201440138	1	pseudobulk	DESeq2	LRT	0.106210394379414
111	KC_AB1	CG2006	-0.345708066847191	0.794239667786369	1	pseudobulk	DESeq2	LRT	0.100048426173528
111	KC_AB1	CG2016	1.25804452752548	0.573436424985144	1	pseudobulk	DESeq2	LRT	0.241514723898045
111	KC_AB1	CG2017	-0.140286625605346	0.69295732390949	1	pseudobulk	DESeq2	LRT	0.15929351078692
111	KC_AB1	CG2021	-0.396963485018388	0.468236639241178	1	pseudobulk	DESeq2	LRT	0.329534606022377
111	KC_AB1	CG2023	0.246400518221364	0.75954699729301	1	pseudobulk	DESeq2	LRT	0.1194453488143
111	KC_AB1	CG2034	0.296297640570191	0.686545697451581	1	pseudobulk	DESeq2	LRT	0.163330550210839
111	KC_AB1	CG2053	1.90071181822722	0.545896518579078	1	pseudobulk	DESeq2	LRT	0.262889675371117

111	KC_AB1	CG2059	0.0560576491948578	0.889888385646183	1	pseudobulk	DESeq2	LRT	0.0506644613750131
112	KC_AB1	CG2061	0.138337489937166	0.773083130794103	1	pseudobulk	DESeq2	LRT	0.111773803230488
112	KC_AB1	CG2063	-0.212170053649953	0.690274275308843	1	pseudobulk	DESeq2	LRT	0.160978311313407
112	KC_AB1	CG2064	3.58333718222294	0.241420367068608	1	pseudobulk	DESeq2	LRT	0.617226094087932
112	KC_AB1	CG2076	0.108877837742867	0.860710158692054	1	pseudobulk	DESeq2	LRT	0.0651430711625295
112	KC_AB1	CG2082	-1.2711787771144	0.517114470977494	1	pseudobulk	DESeq2	LRT	0.286413308722346
112	KC_AB1	CG2091	0.818296506457921	0.194999294942993	1	pseudobulk	DESeq2	LRT	0.709966958908873
112	KC_AB1	CG2100	3.45705217163	0.0534610115017557	1	pseudobulk	DESeq2	LRT	1.2719628284924
112	KC_AB1	CG2111	-2.24976163616681	0.429894962609849	1	pseudobulk	DESeq2	LRT	0.366637643795677
112	KC_AB1	CG2116	-0.14000104655205	0.867466146225334	1	pseudobulk	DESeq2	LRT	0.0617474650032738
112	KC_AB1	CG2121	-1.43550613534661	0.602448798560473	1	pseudobulk	DESeq2	LRT	0.220079857379737
112	KC_AB1	CG2124	0.501541492532629	0.657088644303167	1	pseudobulk	DESeq2	LRT	0.182376038149748
112	KC_AB1	CG2126	-0.766238176191561	0.471820804698935	1	pseudobulk	DESeq2	LRT	0.32622291303343
112	KC_AB1	CG2129	-0.37711032078547	0.799223615364324	1	pseudobulk	DESeq2	LRT	0.0973316921107897
112	KC_AB1	CG2145	-2.13518333033153	0.457250610250827	1	pseudobulk	DESeq2	LRT	0.33984570623779
112	KC_AB1	CG2147	0.496349257240435	0.439812332441304	1	pseudobulk	DESeq2	LRT	0.356732597085015
112	KC_AB1	CG2162	-0.0734143677103616	0.872665547149299	1	pseudobulk	DESeq2	LRT	0.0591521696483866
112	KC_AB1	CG2182	0.240072653218351	0.553541862903514	1	pseudobulk	DESeq2	LRT	0.256849529002979
112	KC_AB1	CG2186	-0.31960448730569	0.387692570525126	1	pseudobulk	DESeq2	LRT	0.411512521435031
112	KC_AB1	CG2199	-0.0743363629590034	0.903585996933749	1	pseudobulk	DESeq2	LRT	0.0440305080543807
112	KC_AB1	CG2200	0.309280023494127	0.657643061818842	1	pseudobulk	DESeq2	LRT	0.182009757362756
112	KC_AB1	CG2201	-0.479412607238293	0.563269230840053	1	pseudobulk	DESeq2	LRT	0.249283971898594
112	KC_AB1	CG2202	0.593045414502284	0.595023433816722	1	pseudobulk	DESeq2	LRT	0.225465930108617
112	KC_AB1	CG2209	1.78945402998133	0.367358455068288	1	pseudobulk	DESeq2	LRT	0.434909960046515
112	KC_AB1	CG2211	1.29699409341884	0.15158949197502	1	pseudobulk	DESeq2	LRT	0.819330902499691
112	KC_AB1	CG2218	0.524446158933456	0.456957415322267	1	pseudobulk	DESeq2	LRT	0.340124270722895
112	KC_AB1	CG2224	-0.498679987673893	0.559292214153306	1	pseudobulk	DESeq2	LRT	0.252361226396393
112	KC_AB1	CG2225	-0.226971886259584	0.390832393020271	1	pseudobulk	DESeq2	LRT	0.408009448210833
112	KC_AB1	CG2246	-0.323519761130962	0.356970052844343	1	pseudobulk	DESeq2	LRT	0.447368216464794
112	KC_AB1	CG2247	-0.0338658163412472	0.935304842402199	1	pseudobulk	DESeq2	LRT	0.0290468171534426
112	KC_AB1	CG2256	0.366322103092889	0.859845041285473	1	pseudobulk	DESeq2	LRT	0.0655798089638444
112	KC_AB1	CG2258	0.184446007763833	0.591315107014034	1	pseudobulk	DESeq2	LRT	0.228181025433462
112	KC_AB1	CG2260	-0.21151201994571	0.836582055197722	1	pseudobulk	DESeq2	LRT	0.0774914553329544
112	KC_AB1	CG2269	-0.222769734444939	0.259506320859871	1	pseudobulk	DESeq2	LRT	0.585852059468198
112	KC_AB1	CG2277	-0.3749585751734	0.48139161159623	1	pseudobulk	DESeq2	LRT	0.317501481716182
112	KC_AB1	CG2278	-0.53888744935476	0.412851387579867	1	pseudobulk	DESeq2	LRT	0.384206251416218
112	KC_AB1	CG2310	1.60354496085297	0.384532372344667	1	pseudobulk	DESeq2	LRT	0.4150670926926
112	KC_AB1	CG2321	-0.353239788504467	0.856926651726396	1	pseudobulk	DESeq2	LRT	0.0670563497381105
112	KC_AB1	CG2444	3.99740073762969	0.17240197409338	1	pseudobulk	DESeq2	LRT	0.763457765582343
112	KC_AB1	CG2493	-3.22611930138691	0.0731007849258575	1	pseudobulk	DESeq2	LRT	1.13607795974339
112	KC_AB1	CG2519	0.0165143823343137	0.937265155300758	1	pseudobulk	DESeq2	LRT	0.0281375284361242
112	KC_AB1	CG2528	-0.760959112540794	1	1	pseudobulk	DESeq2	LRT	0
112	KC_AB1	CG2540	-0.568336237928263	0.58569232138817	1	pseudobulk	DESeq2	LRT	0.232330469672922
112	KC_AB1	CG2574	1.11388934932283	1	1	pseudobulk	DESeq2	LRT	0
112	KC_AB1	CG2608	0.639702603578432	0.416492064615528	1	pseudobulk	DESeq2	LRT	0.380393268750636
112	KC_AB1	CG2611	0.322833478836048	0.784725322781885	1	pseudobulk	DESeq2	LRT	0.105282332650113
112	KC_AB1	CG2614	0.313058971480077	0.742879352090012	1	pseudobulk	DESeq2	LRT	0.129081712452205
112	KC_AB1	CG2616	-1.77199356422654	0.54193209410651	1	pseudobulk	DESeq2	LRT	0.266055128595237
112	KC_AB1	CG2617	0.102104172385983	0.948998877714171	1	pseudobulk	DESeq2	LRT	0.0227343011689463
112	KC_AB1	CG2656	0.319340104423775	0.695615150232151	1	pseudobulk	DESeq2	LRT	0.157630967798464
112	KC_AB1	CG2662	0.808428525593901	0.339343342220729	1	pseudobulk	DESeq2	LRT	0.469360667071198
112	KC_AB1	CG2680	0.7039057256451	0.392461313286583	1	pseudobulk	DESeq2	LRT	0.40620314720976
112	KC_AB1	CG2685	-0.194698925449632	0.8244567336159	1	pseudobulk	DESeq2	LRT	0.083832130613646
112	KC_AB1	CG2691	3.20684218769807	0.0867005910727372	1	pseudobulk	DESeq2	LRT	1.06197794175386
112	KC_AB1	CG2694	-0.716419614646419	0.812870875922622	1	pseudobulk	DESeq2	LRT	0.0899784363592155
112	KC_AB1	CG2698	0.0338753264465929	0.926770488898792	1	pseudobulk	DESeq2	LRT	0.0330278038769532

112	KC_AB1	CG2701	0.39508731475905	0.514847455850512	1	pseudobulk	DESeq2	LRT	0.288321429009042
112	KC_AB1	CG2712	-0.190763391373104	0.879848197266967	1	pseudobulk	DESeq2	LRT	0.05559225145922
112	KC_AB1	CG2747	0.210310117188517	0.600787246400076	1	pseudobulk	DESeq2	LRT	0.221279295173096
112	KC_AB1	CG2765	0.486516167814119	0.300244355155145	1	pseudobulk	DESeq2	LRT	0.522525148947594
112	KC_AB1	CG2767	0.339553540743216	0.748462511284172	1	pseudobulk	DESeq2	LRT	0.125829947558942
112	KC_AB1	CG2772	0.0839129092915158	0.911882706920417	1	pseudobulk	DESeq2	LRT	0.0400610202387001
112	KC_AB1	CG2790	0.0340169735791343	0.953946923274769	1	pseudobulk	DESeq2	LRT	0.0204757883673302
112	KC_AB1	CG2794	1.49161085205854	0.443354850394934	1	pseudobulk	DESeq2	LRT	0.353248535900648
112	KC_AB1	CG2811	-0.316842119800981	0.576185762500135	1	pseudobulk	DESeq2	LRT	0.239437477301737
112	KC_AB1	CG2812	2.59005369605578	0.365116889791152	1	pseudobulk	DESeq2	LRT	0.437568076736828
112	KC_AB1	CG2816	2.50740851728363	0.324019330850488	1	pseudobulk	DESeq2	LRT	0.489429079203069
112	KC_AB1	CG2818	0.0128866905010662	0.956496140492494	1	pseudobulk	DESeq2	LRT	0.0193167780320794
112	KC_AB1	CG2841	2.5734022108142	0.186690145624425	1	pseudobulk	DESeq2	LRT	0.728878605531823
112	KC_AB1	CG2846	0.852217635572229	0.164414941915624	1	pseudobulk	DESeq2	LRT	0.784058716621825
112	KC_AB1	CG2852	-0.210181488291874	0.390486182858655	1	pseudobulk	DESeq2	LRT	0.408394328789002
112	KC_AB1	CG2861	-0.760959112540794	0.804334723897681	1	pseudobulk	DESeq2	LRT	0.0945631819854715
112	KC_AB1	CG2865	-0.309047597836848	0.155861215530717	1	pseudobulk	DESeq2	LRT	0.807261941107074
112	KC_AB1	CG2875	0.42640186605551	0.843822415785108	1	pseudobulk	DESeq2	LRT	0.0737489419519159
112	KC_AB1	CG2889	-0.665228757538575	0.366172178591344	1	pseudobulk	DESeq2	LRT	0.436314656056337
112	KC_AB1	CG2906	-0.396825829742307	0.728544304465378	1	pseudobulk	DESeq2	LRT	0.137544032638295
112	KC_AB1	CG2909	-0.687338374992581	0.449008273965368	1	pseudobulk	DESeq2	LRT	0.347745656091566
112	KC_AB1	CG2911	1.37600630439252	0.418923330668471	1	pseudobulk	DESeq2	LRT	0.377865452246981
112	KC_AB1	CG2915	0.970851126764727	0.435581744314761	1	pseudobulk	DESeq2	LRT	0.360930330269575
112	KC_AB1	CG2918	-0.396341831999533	0.437370019760005	1	pseudobulk	DESeq2	LRT	0.359150989700378
112	KC_AB1	CG2921	-0.0147404390711681	0.941006131174778	1	pseudobulk	DESeq2	LRT	0.0264075468950606
112	KC_AB1	CG2924	0.00127303774230557	0.963466442685799	1	pseudobulk	DESeq2	LRT	0.0161634071218467
112	KC_AB1	CG2926	-0.27751783770561	0.308284866504123	1	pseudobulk	DESeq2	LRT	0.511047793973024
112	KC_AB1	CG2930	1.77006317075202	0.596029081111493	1	pseudobulk	DESeq2	LRT	0.224732549893771
112	KC_AB1	CG2931	-0.0822380876004754	0.830784910083565	1	pseudobulk	DESeq2	LRT	0.0805114003494523
112	KC_AB1	CG2938	-0.451506014055545	0.574990327485733	1	pseudobulk	DESeq2	LRT	0.240339460971073
112	KC_AB1	CG2972	-0.280136830065852	0.811882828063432	1	pseudobulk	DESeq2	LRT	0.0905066441545822
112	KC_AB1	CG2975	2.99960667015886	0.357405021116046	1	pseudobulk	DESeq2	LRT	0.44683935046587
112	KC_AB1	CG2991	-0.117776388599623	0.766260980326833	1	pseudobulk	DESeq2	LRT	0.115623289098408
112	KC_AB1	CG2993	-0.0410725894671159	0.861413791808895	1	pseudobulk	DESeq2	LRT	0.0647881791736639
112	KC_AB1	CG30001	1.25804452752548	0.727552935095488	1	pseudobulk	DESeq2	LRT	0.138135402914536
112	KC_AB1	CG30008	0.152115475746305	1	1	pseudobulk	DESeq2	LRT	0
112	KC_AB1	CG30010	0.741545889548342	0.44150154821377	1	pseudobulk	DESeq2	LRT	0.355067769142996
112	KC_AB1	CG30015	-0.760959112540794	0.804334723897681	1	pseudobulk	DESeq2	LRT	0.0945631819854715
112	KC_AB1	CG30020	-0.909483399244631	0.252376145693577	1	pseudobulk	DESeq2	LRT	0.597951696508853
112	KC_AB1	CG30022	0.751169558371074	0.552709510896747	1	pseudobulk	DESeq2	LRT	0.257503062115463
112	KC_AB1	CG30054	-0.760959112540794	1	1	pseudobulk	DESeq2	LRT	0
112	KC_AB1	CG30060	-0.101820149591026	0.915990878764734	1	pseudobulk	DESeq2	LRT	0.0381088509193407
112	KC_AB1	CG30062	-0.760959112540794	1	1	pseudobulk	DESeq2	LRT	0
112	KC_AB1	CG30069	-0.537320146289207	0.61586589296589	1	pseudobulk	DESeq2	LRT	0.210513846740827
112	KC_AB1	CG3008	0.374479725068548	0.565915174226715	1	pseudobulk	DESeq2	LRT	0.247248660907265
113	KC_AB1	CG30082	1.11388934932283	1	1	pseudobulk	DESeq2	LRT	0
113	KC_AB1	CG30089	0.126085239973399	0.819346942383729	1	pseudobulk	DESeq2	LRT	0.0865321626382187
113	KC_AB1	CG3009	-0.231764949532385	0.753544607544024	1	pseudobulk	DESeq2	LRT	0.12289103367874
113	KC_AB1	CG30094	-0.453208047809637	0.35666544851915	1	pseudobulk	DESeq2	LRT	0.44773896031197
113	KC_AB1	CG30096	0.67657742278782	0.477283651413924	1	pseudobulk	DESeq2	LRT	0.321223441439999
113	KC_AB1	CG30099	0.278593247633235	0.951871828976203	1	pseudobulk	DESeq2	LRT	0.0214215261069401
113	KC_AB1	CG30105	0.124481543023846	0.91941190661028	1	pseudobulk	DESeq2	LRT	0.0364898763289548
113	KC_AB1	CG30108	2.09987857572813	0.503115176013642	1	pseudobulk	DESeq2	LRT	0.29833258237694
113	KC_AB1	CG30109	-0.226589905719819	0.783590432104894	1	pseudobulk	DESeq2	LRT	0.105910875522397
113	KC_AB1	CG30110	1.25804452752548	0.727552935095488	1	pseudobulk	DESeq2	LRT	0.138135402914536
113	KC_AB1	CG30116	-0.207735650199453	0.125305552940022	1	pseudobulk	DESeq2	LRT	0.902029682734713

113	KC_AB1	CG30122	-0.101069966986098	0.7980613960822	1	pseudobulk	DESeq2	LRT	0.0979636964261712
113	KC_AB1	CG3014	-0.760959112540794	0.804334723897681	1	pseudobulk	DESeq2	LRT	0.0945631819854715
113	KC_AB1	CG30148	2.88322402903533	0.302751069383099	1	pseudobulk	DESeq2	LRT	0.518914314158952
113	KC_AB1	CG30154	-1.42421292828182	0.506005285421213	1	pseudobulk	DESeq2	LRT	0.295844946762416
113	KC_AB1	CG30156	-0.396285069997305	0.774400534135319	1	pseudobulk	DESeq2	LRT	0.111034356149119
113	KC_AB1	CG30157	0.840326773475871	0.732104296481109	1	pseudobulk	DESeq2	LRT	0.135427044406559
113	KC_AB1	CG30158	-0.115324884688005	0.572412707736369	1	pseudobulk	DESeq2	LRT	0.242290733335529
113	KC_AB1	CG30159	2.915829539802	0.119117575297687	1	pseudobulk	DESeq2	LRT	0.924024155463673
113	KC_AB1	CG30172	0.168718685402563	0.423542151632968	1	pseudobulk	DESeq2	LRT	0.373103361459277
113	KC_AB1	CG30183	0.550850173735056	0.79752055926651	1	pseudobulk	DESeq2	LRT	0.0982581124577293
113	KC_AB1	CG30187	1.90071181822722	0.545896518579078	1	pseudobulk	DESeq2	LRT	0.262889675371117
113	KC_AB1	CG30197	0.189851905407949	0.647588800589014	1	pseudobulk	DESeq2	LRT	0.188700670549376
113	KC_AB1	CG3021	-0.191114105941031	0.808929973437033	1	pseudobulk	DESeq2	LRT	0.0920890722890728
113	KC_AB1	CG30284	-0.760959112540794	0.804334723897681	1	pseudobulk	DESeq2	LRT	0.0945631819854715
113	KC_AB1	CG30285	0.26438296751355	0.789609029290101	1	pseudobulk	DESeq2	LRT	0.102587894091655
113	KC_AB1	CG30291	-0.6739473440946	0.409683175732778	1	pseudobulk	DESeq2	LRT	0.38755187064887
113	KC_AB1	CG3032	0.152115475746305	1	1	pseudobulk	DESeq2	LRT	0
113	KC_AB1	CG30325	0.254196856837688	0.890998468402047	1	pseudobulk	DESeq2	LRT	0.0501230425008484
113	KC_AB1	CG30334	-0.760959112540794	0.805265236978025	1	pseudobulk	DESeq2	LRT	0.0940610488455931
113	KC_AB1	CG30338	-2.52011257447171	0.364021130127131	1	pseudobulk	DESeq2	LRT	0.438873406374921
113	KC_AB1	CG30344	0.460437794023063	0.645835543822212	1	pseudobulk	DESeq2	LRT	0.189878057087411
113	KC_AB1	CG30345	2.4138856564567	0.409611385497059	1	pseudobulk	DESeq2	LRT	0.387627980279722
113	KC_AB1	CG30349	0.054241109331408	0.919104069192695	1	pseudobulk	DESeq2	LRT	0.0366353111196115
113	KC_AB1	CG30355	1.25804452752548	0.727552935095488	1	pseudobulk	DESeq2	LRT	0.138135402914536
113	KC_AB1	CG30356	-2.61077565254151	0.274959422178212	1	pseudobulk	DESeq2	LRT	0.560731393531137
113	KC_AB1	CG30357	0.494775748955277	0.827825706404751	1	pseudobulk	DESeq2	LRT	0.0820610916144188
113	KC_AB1	CG3036	2.58743201691345	0.417636627932844	1	pseudobulk	DESeq2	LRT	0.379201419460921
113	KC_AB1	CG30373	-0.294321315112837	0.583081898923588	1	pseudobulk	DESeq2	LRT	0.234270440519655
113	KC_AB1	CG30380	-3.63326965964202	0.149907780941669	1	pseudobulk	DESeq2	LRT	0.824175824574467
113	KC_AB1	CG30389	-0.0488069920682593	0.827835155232331	1	pseudobulk	DESeq2	LRT	0.082056134592394
113	KC_AB1	CG30392	-0.00196460805410648	0.978254821737383	1	pseudobulk	DESeq2	LRT	0.00954800282030261
113	KC_AB1	CG30394	0.108610881258111	0.92085394054634	1	pseudobulk	DESeq2	LRT	0.0358092491138183
113	KC_AB1	CG3040	0.0617121837258547	0.897357696758252	1	pseudobulk	DESeq2	LRT	0.0470344078333644
113	KC_AB1	CG30403	0.328994270041178	0.853003887577764	1	pseudobulk	DESeq2	LRT	0.0690489895244775
113	KC_AB1	CG30419	-0.0180931188327371	0.935293450562273	1	pseudobulk	DESeq2	LRT	0.0290521068120902
113	KC_AB1	CG30423	0.0223312633112923	0.930035053825279	1	pseudobulk	DESeq2	LRT	0.0315006822030671
113	KC_AB1	CG30424	-0.760959112540794	1	1	pseudobulk	DESeq2	LRT	0
113	KC_AB1	CG30427	-0.760959112540794	1	1	pseudobulk	DESeq2	LRT	0
113	KC_AB1	CG30428	-0.666065275003097	0.0779592758103803	1	pseudobulk	DESeq2	LRT	1.10813220384144
113	KC_AB1	CG30432	1.11388934932283	1	1	pseudobulk	DESeq2	LRT	0
113	KC_AB1	CG3045	0.817525591775603	0.749273870186925	1	pseudobulk	DESeq2	LRT	0.125359412512672
113	KC_AB1	CG30456	2.09987857572813	0.503115176013642	1	pseudobulk	DESeq2	LRT	0.29833258237694
113	KC_AB1	CG30460	-0.203156363118671	0.914073048477194	1	pseudobulk	DESeq2	LRT	0.0390190960792063
113	KC_AB1	CG30466	2.09948827516949	0.496374591357787	1	pseudobulk	DESeq2	LRT	0.304190457462447
113	KC_AB1	CG30467	-0.271527773791945	0.658768322674723	1	pseudobulk	DESeq2	LRT	0.181267292347348
113	KC_AB1	CG30480	0.296275065198765	1	1	pseudobulk	DESeq2	LRT	0
113	KC_AB1	CG30484	0.705746498062877	0.729457903182219	1	pseudobulk	DESeq2	LRT	0.136999766064148
113	KC_AB1	CG30491	0.45146499101711	0.709064903051867	1	pseudobulk	DESeq2	LRT	0.149314010589544
113	KC_AB1	CG30495	0.489854451465621	0.856861061698406	1	pseudobulk	DESeq2	LRT	0.0670895923468823
113	KC_AB1	CG30496	0.898739311998799	0.740697588966623	1	pseudobulk	DESeq2	LRT	0.130359069011421
113	KC_AB1	CG3061	0.0324303194623056	0.901092818464507	1	pseudobulk	DESeq2	LRT	0.0452304715395189
113	KC_AB1	CG3065	-0.0981031925492399	0.855245572058208	1	pseudobulk	DESeq2	LRT	0.0679091656499615
113	KC_AB1	CG3071	0.398330555033387	0.741630239337352	1	pseudobulk	DESeq2	LRT	0.129812570517273
113	KC_AB1	CG3078	-0.307502373093055	0.565842188443468	1	pseudobulk	DESeq2	LRT	0.247304675250994
113	KC_AB1	CG3091	-1.8414491861354	0.311487877093495	1	pseudobulk	DESeq2	LRT	0.506558851135957
113	KC_AB1	CG3097	0.291868230578568	0.902153667810848	1	pseudobulk	DESeq2	LRT	0.0447194808636415

113	KC_AB1	CG31030	-0.118670322154276	0.538575014438662	1	pseudobulk	DESeq2	LRT	0.268753798238126
113	KC_AB1	CG31038	-0.185940401056842	0.865472931633117	1	pseudobulk	DESeq2	LRT	0.0627465104878398
113	KC_AB1	CG3104	-0.0845888758675486	0.785304197859216	1	pseudobulk	DESeq2	LRT	0.104962081011678
113	KC_AB1	CG31051	0.0202454687054655	0.939112565295928	1	pseudobulk	DESeq2	LRT	0.027282348567855
113	KC_AB1	CG31064	0.269548593501415	0.403441850224353	1	pseudobulk	DESeq2	LRT	0.394219053126773
113	KC_AB1	CG3107	0.16815126553043	0.566934341671498	1	pseudobulk	DESeq2	LRT	0.24646723511221
113	KC_AB1	CG31075	2.99960667015886	0.357405021116046	1	pseudobulk	DESeq2	LRT	0.44683935046587
113	KC_AB1	CG31098	-0.460779653915884	0.589650403013095	1	pseudobulk	DESeq2	LRT	0.229405400293392
113	KC_AB1	CG31100	-1.65908590696825	0.518650538121198	1	pseudobulk	DESeq2	LRT	0.285125167169863
113	KC_AB1	CG31102	4.07290908771404	0.0961759302950378	1	pseudobulk	DESeq2	LRT	1.01693360413685
113	KC_AB1	CG31103	3.67717523121923	0.149514598696356	1	pseudobulk	DESeq2	LRT	0.825316400491839
113	KC_AB1	CG31109	0.25557157072519	0.550371728277153	1	pseudobulk	DESeq2	LRT	0.259343883218363
113	KC_AB1	CG31111	-1.72274967638748	0.175411010345429	1	pseudobulk	DESeq2	LRT	0.755943149954079
113	KC_AB1	CG31121	4.1240316074106	0.0980837594853901	1	pseudobulk	DESeq2	LRT	1.00840289628744
113	KC_AB1	CG31122	-0.224302073220885	0.612560176086897	1	pseudobulk	DESeq2	LRT	0.212851241071561
113	KC_AB1	CG31125	0.999452692841403	0.279361597124115	1	pseudobulk	DESeq2	LRT	0.553833295084321
113	KC_AB1	CG31126	0.293921200667297	0.554045454756599	1	pseudobulk	DESeq2	LRT	0.256454603610624
113	KC_AB1	CG31139	-0.760959112540794	1	1	pseudobulk	DESeq2	LRT	0
113	KC_AB1	CG31140	-0.122533831165106	0.359452231592313	1	pseudobulk	DESeq2	LRT	0.444358815807575
113	KC_AB1	CG31142	-0.144395013377727	0.858854518255877	1	pseudobulk	DESeq2	LRT	0.0660803952718946
113	KC_AB1	CG31145	2.29873183706224	0.171606644009843	1	pseudobulk	DESeq2	LRT	0.765465901796437
113	KC_AB1	CG31156	-0.111219422109134	0.912846873768688	1	pseudobulk	DESeq2	LRT	0.0396020674324993
113	KC_AB1	CG31161	1.90071181822722	0.545896518579078	1	pseudobulk	DESeq2	LRT	0.262889675371117
113	KC_AB1	CG31174	0.310433205716855	0.80492504377499	1	pseudobulk	DESeq2	LRT	0.0942445601173025
113	KC_AB1	CG31189	-2.00963963748391	0.498268873088307	1	pseudobulk	DESeq2	LRT	0.302536242405947
113	KC_AB1	CG31191	-0.113315450564809	0.739036630244688	1	pseudobulk	DESeq2	LRT	0.131334035327927
113	KC_AB1	CG31195	-0.671530071791384	0.491369351798413	1	pseudobulk	DESeq2	LRT	0.308591935279471
113	KC_AB1	CG31200	0.296275065198765	1	1	pseudobulk	DESeq2	LRT	0
113	KC_AB1	CG31207	-3.99653340182271	0.160092881750206	1	pseudobulk	DESeq2	LRT	0.795627977795456
113	KC_AB1	CG31211	-0.175939938367111	0.599526801597741	1	pseudobulk	DESeq2	LRT	0.222191397175933
113	KC_AB1	CG31213	-0.760959112540794	1	1	pseudobulk	DESeq2	LRT	0
113	KC_AB1	CG31221	-0.385817613198168	0.00560002440070713	1	pseudobulk	DESeq2	LRT	2.25181008065998
113	KC_AB1	CG31223	-0.814009673942439	0.566503764695823	1	pseudobulk	DESeq2	LRT	0.246797199690806
114	KC_AB1	CG31224	-0.0137092096502241	0.922803055293751	1	pseudobulk	DESeq2	LRT	0.0348909762499686
114	KC_AB1	CG31229	-0.249711624045913	0.67271027480985	1	pseudobulk	DESeq2	LRT	0.172171938960363
114	KC_AB1	CG31248	0.512944689199439	0.479534925437216	1	pseudobulk	DESeq2	LRT	0.319179756851413
114	KC_AB1	CG31249	0.157168138893531	0.847304148958319	1	pseudobulk	DESeq2	LRT	0.0719606669965254
114	KC_AB1	CG31251	1.90617938173577	0.291589940267081	1	pseudobulk	DESeq2	LRT	0.535227463076484
114	KC_AB1	CG31278	-0.139538859256104	0.906625199761536	1	pseudobulk	DESeq2	LRT	0.0425722138397814
114	KC_AB1	CG31300	0.709713058804466	0.916223368658127	1	pseudobulk	DESeq2	LRT	0.037998635557264
114	KC_AB1	CG31301	0.518854845124317	0.351239233446789	1	pseudobulk	DESeq2	LRT	0.454396979332593
114	KC_AB1	CG31313	-0.613486300063999	0.82208091225047	1	pseudobulk	DESeq2	LRT	0.0850854354836129
114	KC_AB1	CG31324	0.431282203897532	0.251695085195594	1	pseudobulk	DESeq2	LRT	0.599125264758641
114	KC_AB1	CG31326	-0.760959112540794	1	1	pseudobulk	DESeq2	LRT	0
114	KC_AB1	CG31337	-0.760959112540794	0.804334723897681	1	pseudobulk	DESeq2	LRT	0.0945631819854715
114	KC_AB1	CG31342	-0.124791691332229	0.79957629974451	1	pseudobulk	DESeq2	LRT	0.0971400872936947
114	KC_AB1	CG31344	0.298238146352233	0.523827764754161	1	pseudobulk	DESeq2	LRT	0.280811486132327
114	KC_AB1	CG31345	0.897272604463685	0.699914436311722	1	pseudobulk	DESeq2	LRT	0.154955048712815
114	KC_AB1	CG31357	-0.214033969491375	0.683999782103588	1	pseudobulk	DESeq2	LRT	0.164944036629627
114	KC_AB1	CG31360	-0.00865092388815053	0.950815693345468	1	pseudobulk	DESeq2	LRT	0.0219036587937803
114	KC_AB1	CG31365	-0.741027132908439	0.509492876949946	1	pseudobulk	DESeq2	LRT	0.292861883341475
114	KC_AB1	CG31368	0.64645076616205	0.448150187191804	1	pseudobulk	DESeq2	LRT	0.348576417836618
114	KC_AB1	CG31370	-0.760959112540794	0.804334723897681	1	pseudobulk	DESeq2	LRT	0.0945631819854715
114	KC_AB1	CG31371	1.25804452752548	0.573436424985144	1	pseudobulk	DESeq2	LRT	0.241514723898045
114	KC_AB1	CG31381	0.012232718593166	0.975134693959253	1	pseudobulk	DESeq2	LRT	0.0109353916829806
114	KC_AB1	CG31388	-0.330026039193132	0.507305472097518	1	pseudobulk	DESeq2	LRT	0.294730453099629

114	KC_AB1	CG31415	0.943022244937108	0.768808261856119	1	pseudobulk	DESeq2	LRT	0.114181958248924
114	KC_AB1	CG31431	2.58743201691345	0.417636627932844	1	pseudobulk	DESeq2	LRT	0.379201419460921
114	KC_AB1	CG31436	0.756310590091493	0.722405032634678	1	pseudobulk	DESeq2	LRT	0.141219237171493
114	KC_AB1	CG31441	1.00861802550899	0.415763172006186	1	pseudobulk	DESeq2	LRT	0.381153982784369
114	KC_AB1	CG31445	-0.760959112540794	1	1	pseudobulk	DESeq2	LRT	0
114	KC_AB1	CG31454	-0.760959112540794	1	1	pseudobulk	DESeq2	LRT	0
114	KC_AB1	CG31457	0.229083678438483	0.798390280234822	1	pseudobulk	DESeq2	LRT	0.0977847588775959
114	KC_AB1	CG31460	-0.1763310501063	0.805563412825049	1	pseudobulk	DESeq2	LRT	0.0939002668449827
114	KC_AB1	CG31467	3.59348491827797	0.110146741159828	1	pseudobulk	DESeq2	LRT	0.958028347512222
114	KC_AB1	CG31469	0.291853629993484	0.902736173524132	1	pseudobulk	DESeq2	LRT	0.0444391545856091
114	KC_AB1	CG31473	0.943022244937108	0.768808261856119	1	pseudobulk	DESeq2	LRT	0.114181958248924
114	KC_AB1	CG31475	-0.0666351889256063	0.835831693227841	1	pseudobulk	DESeq2	LRT	0.0778811652179913
114	KC_AB1	CG3149	1.13179822378469	0.330686782611737	1	pseudobulk	DESeq2	LRT	0.48058316327651
114	KC_AB1	CG31510	0.249384353848127	0.595345204919761	1	pseudobulk	DESeq2	LRT	0.225231139954808
114	KC_AB1	CG31522	0.0239890495913311	0.947547370793589	1	pseudobulk	DESeq2	LRT	0.023399069106592
114	KC_AB1	CG31523	-0.138694472453223	0.658565570678831	1	pseudobulk	DESeq2	LRT	0.181400977625089
114	KC_AB1	CG31538	-0.522425698611801	0.845792159978981	1	pseudobulk	DESeq2	LRT	0.0727363448377909
114	KC_AB1	CG31548	-1.84720757905536	0.438680975312332	1	pseudobulk	DESeq2	LRT	0.357851199641171
114	KC_AB1	CG31549	0.989945809056186	0.527327580592124	1	pseudobulk	DESeq2	LRT	0.277919513353309
114	KC_AB1	CG31550	0.113000420442641	0.799587174535415	1	pseudobulk	DESeq2	LRT	0.0971341806284188
114	KC_AB1	CG3156	-0.0267040370154327	0.978165348376113	1	pseudobulk	DESeq2	LRT	0.00958772617594734
114	KC_AB1	CG31612	-2.60267693238046	0.191335433513622	1	pseudobulk	DESeq2	LRT	0.718204595291875
114	KC_AB1	CG3163	0.311942190345548	0.585893863769346	1	pseudobulk	DESeq2	LRT	0.232181050456501
114	KC_AB1	CG31633	-0.530487073306293	0.598104623738681	1	pseudobulk	DESeq2	LRT	0.223222840195428
114	KC_AB1	CG31635	0.125489067145479	0.612784843616011	1	pseudobulk	DESeq2	LRT	0.212691984914835
114	KC_AB1	CG31637	-0.0714777700705623	0.807792784448704	1	pseudobulk	DESeq2	LRT	0.0927000304531156
114	KC_AB1	CG31638	0.158156140363583	0.695211951233426	1	pseudobulk	DESeq2	LRT	0.157882770635749
114	KC_AB1	CG3164	0.0990903291385928	0.880638364022198	1	pseudobulk	DESeq2	LRT	0.0552023989125776
114	KC_AB1	CG31642	0.498362126058204	0.839254472147245	1	pseudobulk	DESeq2	LRT	0.0761063358425998
114	KC_AB1	CG31643	-0.899922622441345	0.66991531885731	1	pseudobulk	DESeq2	LRT	0.173980091145856
114	KC_AB1	CG31648	0.310429800166429	0.637087646686326	1	pseudobulk	DESeq2	LRT	0.19580081593189
114	KC_AB1	CG3165	-0.240325408587205	0.650557894895718	1	pseudobulk	DESeq2	LRT	0.186714048349257
114	KC_AB1	CG31650	-0.0914765269604194	0.787727527391114	1	pseudobulk	DESeq2	LRT	0.103623977705529
114	KC_AB1	CG31659	1.25804452752548	0.727552935095488	1	pseudobulk	DESeq2	LRT	0.138135402914536
114	KC_AB1	CG31663	-0.295053693533136	0.756542165111618	1	pseudobulk	DESeq2	LRT	0.121166862002999
114	KC_AB1	CG31673	1.18205607558444	0.654796763918293	1	pseudobulk	DESeq2	LRT	0.183893475588595
114	KC_AB1	CG31674	-0.716419614646419	0.812870875922622	1	pseudobulk	DESeq2	LRT	0.0899784363592155
114	KC_AB1	CG31676	0.537232314425659	0.333918772245414	1	pseudobulk	DESeq2	LRT	0.476359165092296
114	KC_AB1	CG31678	0.0404565605750378	0.950814353831677	1	pseudobulk	DESeq2	LRT	0.0219042706303978
114	KC_AB1	CG3168	0.856976546560544	0.386861847119595	1	pseudobulk	DESeq2	LRT	0.412444098923004
114	KC_AB1	CG31683	0.943798956650056	0.807634491832869	1	pseudobulk	DESeq2	LRT	0.0927851418175362
114	KC_AB1	CG31687	-0.81985698686445	0.271245996795999	1	pseudobulk	DESeq2	LRT	0.566636662663182
114	KC_AB1	CG31688	0.12964309057032	0.556314381806526	1	pseudobulk	DESeq2	LRT	0.254679712558761
114	KC_AB1	CG31689	-0.760959112540794	0.805265236978025	1	pseudobulk	DESeq2	LRT	0.0940610488455931
114	KC_AB1	CG31690	0.0360099827412559	0.90308420196373	1	pseudobulk	DESeq2	LRT	0.044271754954078
114	KC_AB1	CG31694	-0.222884100823879	0.525571595364773	1	pseudobulk	DESeq2	LRT	0.279368114357111
114	KC_AB1	CG31697	0.291868230578568	0.902153667810848	1	pseudobulk	DESeq2	LRT	0.0447194808636415
114	KC_AB1	CG31704	-2.14017374284611	0.585464980093351	1	pseudobulk	DESeq2	LRT	0.232499077375685
114	KC_AB1	CG31705	-0.348546622449466	0.328365470457969	1	pseudobulk	DESeq2	LRT	0.483642517746254
114	KC_AB1	CG31706	-0.0778452890288027	0.814065198526411	1	pseudobulk	DESeq2	LRT	0.0893408110489699
114	KC_AB1	CG31710	-0.389155174451514	0.772446899339324	1	pseudobulk	DESeq2	LRT	0.112131365775693
114	KC_AB1	CG31712	0.00665967311624768	0.953660633770912	1	pseudobulk	DESeq2	LRT	0.0206061442701742
114	KC_AB1	CG31715	0.150090090503826	0.642442517814273	1	pseudobulk	DESeq2	LRT	0.192165724514745
114	KC_AB1	CG31717	0.194432485557029	0.728803735695442	1	pseudobulk	DESeq2	LRT	0.137389409926718
114	KC_AB1	CG31729	0.303049163875703	0.567408542818099	1	pseudobulk	DESeq2	LRT	0.246104129818223
114	KC_AB1	CG31751	-0.00184063739771072	0.950690359799891	1	pseudobulk	DESeq2	LRT	0.021960909905056

114	KC_AB1	CG31752	-0.760959112540794	1	1	pseudobulk	DESeq2	LRT	0
114	KC_AB1	CG3176	-0.0305366301252753	0.943025802209839	1	pseudobulk	DESeq2	LRT	0.0254764243315455
114	KC_AB1	CG31760	-0.208036609093917	0.485418957728761	1	pseudobulk	DESeq2	LRT	0.31388326657801
114	KC_AB1	CG31777	3.80522001411205	0.202854565618816	1	pseudobulk	DESeq2	LRT	0.692815213245607
114	KC_AB1	CG31778	3.58333718222294	0.241420367068608	1	pseudobulk	DESeq2	LRT	0.617226094087932
114	KC_AB1	CG31788	-1.77067014871429	0.542156052888816	1	pseudobulk	DESeq2	LRT	0.265875689192671
114	KC_AB1	CG31793	-0.538218969557373	0.775353846208946	1	pseudobulk	DESeq2	LRT	0.110500054416431
114	KC_AB1	CG31800	-0.237218841137166	0.67914388202288	1	pseudobulk	DESeq2	LRT	0.168038207238036
114	KC_AB1	CG31806	0.293544814793053	0.855484935116898	1	pseudobulk	DESeq2	LRT	0.0677876338764847
114	KC_AB1	CG31808	0.0603364264185376	0.823133826624821	1	pseudobulk	DESeq2	LRT	0.0845295506446031
114	KC_AB1	CG31815	0.625071353643838	0.733662361365517	1	pseudobulk	DESeq2	LRT	0.134503760699733
114	KC_AB1	CG31817	1.11388934932283	1	1	pseudobulk	DESeq2	LRT	0
114	KC_AB1	CG31826	2.58743201691345	0.417636627932844	1	pseudobulk	DESeq2	LRT	0.379201419460921
114	KC_AB1	CG31832	1.25804452752548	0.727552935095488	1	pseudobulk	DESeq2	LRT	0.138135402914536
114	KC_AB1	CG3184	-1.06740882909207	0.165551893203033	1	pseudobulk	DESeq2	LRT	0.781065848418835
114	KC_AB1	CG31849	0.0644849663738589	0.90984369920205	1	pseudobulk	DESeq2	LRT	0.0410332081231789
114	KC_AB1	CG31855	0.198459206257002	0.603955884089962	1	pseudobulk	DESeq2	LRT	0.218994783226585
114	KC_AB1	CG31861	-0.760959112540794	1	1	pseudobulk	DESeq2	LRT	0
114	KC_AB1	CG31869	1.34709519886762	0.65416280962107	1	pseudobulk	DESeq2	LRT	0.184314149962587
114	KC_AB1	CG31886	0.222510755266233	0.900985513452252	1	pseudobulk	DESeq2	LRT	0.0452821917937752
114	KC_AB1	CG31897	0.709713058804466	0.916223368658127	1	pseudobulk	DESeq2	LRT	0.037998635557264
115	KC_AB1	CG31898	-0.84572271983469	0.729929556961718	1	pseudobulk	DESeq2	LRT	0.136719050152121
115	KC_AB1	CG31904	-5.69750804190157	0.170803210081688	1	pseudobulk	DESeq2	LRT	0.767503971424163
115	KC_AB1	CG31908	-0.0334236025565045	0.954800848795319	1	pseudobulk	DESeq2	LRT	0.0200872035876131
115	KC_AB1	CG31909	-0.760959112540794	0.804334723897681	1	pseudobulk	DESeq2	LRT	0.0945631819854715
115	KC_AB1	CG3191	0.617462696065864	0.741516486410255	1	pseudobulk	DESeq2	LRT	0.129879188698438
115	KC_AB1	CG31915	0.376508731187096	0.753299180488883	1	pseudobulk	DESeq2	LRT	0.12303250503436
115	KC_AB1	CG31921	0.110369418103439	0.95352570733554	1	pseudobulk	DESeq2	LRT	0.0206675937522512
115	KC_AB1	CG31922	0.363136171351042	0.618457236166026	1	pseudobulk	DESeq2	LRT	0.20869032471586
115	KC_AB1	CG31935	0.0880376913570003	0.926072507057229	1	pseudobulk	DESeq2	LRT	0.0333550088017741
115	KC_AB1	CG31937	-0.892336217061449	0.412663389049143	1	pseudobulk	DESeq2	LRT	0.384404059446364
115	KC_AB1	CG31955	-1.30943155764665	0.665865564455012	1	pseudobulk	DESeq2	LRT	0.176613444266292
115	KC_AB1	CG31957	1.02028366838149	0.251007995217872	1	pseudobulk	DESeq2	LRT	0.600312444958394
115	KC_AB1	CG31974	0.449214902360038	0.568496911227471	1	pseudobulk	DESeq2	LRT	0.245271890590159
115	KC_AB1	CG3198	-0.0652409576801447	0.814205024487204	1	pseudobulk	DESeq2	LRT	0.0892662219021679
115	KC_AB1	CG31997	0.202981124226315	0.491128701891074	1	pseudobulk	DESeq2	LRT	0.308804684665641
115	KC_AB1	CG31998	0.0902071782589618	0.714933934476237	1	pseudobulk	DESeq2	LRT	0.145734088573817
115	KC_AB1	CG31999	-0.00309256210444318	0.993346467643741	1	pseudobulk	DESeq2	LRT	0.00289924823989458
115	KC_AB1	CG32017	-0.0361526133888662	0.863792140890195	1	pseudobulk	DESeq2	LRT	0.0635907516471082
115	KC_AB1	CG32022	0.280451997007129	0.764492538381702	1	pseudobulk	DESeq2	LRT	0.116626749042331
115	KC_AB1	CG32032	-0.616343092161894	0.506126839783727	1	pseudobulk	DESeq2	LRT	0.295740631549352
115	KC_AB1	CG32036	3.11480971027333	0.190195286845861	1	pseudobulk	DESeq2	LRT	0.720800249344957
115	KC_AB1	CG32039	0.0503446049636838	0.899219178948717	1	pseudobulk	DESeq2	LRT	0.0461344388493356
115	KC_AB1	CG32052	0.662825325020837	0.500105233724679	1	pseudobulk	DESeq2	LRT	0.300938600429628
115	KC_AB1	CG32055	0.833665146301187	0.716955005728418	1	pseudobulk	DESeq2	LRT	0.144508098693451
115	KC_AB1	CG32066	0.00998575599717344	0.961954633457247	1	pseudobulk	DESeq2	LRT	0.0168454091511316
115	KC_AB1	CG32069	-0.0412495525232614	0.937020301375991	1	pseudobulk	DESeq2	LRT	0.0282509996351081
115	KC_AB1	CG32074	1.11388934932283	1	1	pseudobulk	DESeq2	LRT	0
115	KC_AB1	CG32075	-0.457361177890421	0.775742106521328	1	pseudobulk	DESeq2	LRT	0.110282634837153
115	KC_AB1	CG32085	-0.133160547837538	0.723105105140561	1	pseudobulk	DESeq2	LRT	0.140798572325135
115	KC_AB1	CG32091	1.07738641754216	0.489877663136758	1	pseudobulk	DESeq2	LRT	0.309912362539195
115	KC_AB1	CG32095	0.410395801995819	0.682878072202144	1	pseudobulk	DESeq2	LRT	0.165656832624259
115	KC_AB1	CG32099	0.0618385357221477	0.897869118217563	1	pseudobulk	DESeq2	LRT	0.0467869655359779
115	KC_AB1	CG32100	-0.330892983588205	0.489754229972745	1	pseudobulk	DESeq2	LRT	0.310021804346215
115	KC_AB1	CG32104	0.300838673246413	0.795006233863822	1	pseudobulk	DESeq2	LRT	0.099629465906996
115	KC_AB1	CG32109	-0.294790995614151	0.568383007088671	1	pseudobulk	DESeq2	LRT	0.245358914625531

115	KC_AB1	CG32110	-0.760959112540794	1	1	pseudobulk	DESeq2	LRT	0
115	KC_AB1	CG32112	0.790054050667481	0.422827251298766	1	pseudobulk	DESeq2	LRT	0.373837030098567
115	KC_AB1	CG32115	-0.570723996670538	0.824991814167186	1	pseudobulk	DESeq2	LRT	0.0835503606375391
115	KC_AB1	CG32137	2.18601646603334	0.291851216495891	1	pseudobulk	DESeq2	LRT	0.534838492121334
115	KC_AB1	CG32147	0.122050645074413	0.872245868574005	1	pseudobulk	DESeq2	LRT	0.0593610789274427
115	KC_AB1	CG32163	-0.00485381590429082	0.951755047474565	1	pseudobulk	DESeq2	LRT	0.02147481129895
115	KC_AB1	CG32170	0.431223946605786	0.908522984959789	1	pseudobulk	DESeq2	LRT	0.041664080887669
115	KC_AB1	CG32176	-0.634377852630013	0.252569910554722	1	pseudobulk	DESeq2	LRT	0.597618389562214
115	KC_AB1	CG32181	1.11388934932283	1	1	pseudobulk	DESeq2	LRT	0
115	KC_AB1	CG32187	-0.206733749121875	0.844379091618653	1	pseudobulk	DESeq2	LRT	0.073462529162424
115	KC_AB1	CG32192	0.296275065198765	1	1	pseudobulk	DESeq2	LRT	0
115	KC_AB1	CG32195	-0.195508291748675	0.906294033540402	1	pseudobulk	DESeq2	LRT	0.0427308791144272
115	KC_AB1	CG32196	0.120124769521856	0.919173783850717	1	pseudobulk	DESeq2	LRT	0.036602370842111
115	KC_AB1	CG32202	0.068708938541843	0.900974230527211	1	pseudobulk	DESeq2	LRT	0.0452876304414492
115	KC_AB1	CG32204	-0.0285276382840848	0.917514544561272	1	pseudobulk	DESeq2	LRT	0.0373870425280412
115	KC_AB1	CG32206	-0.453103918624985	0.183540560769258	1	pseudobulk	DESeq2	LRT	0.736267945734966
115	KC_AB1	CG3222	1.67020383217772	0.598754457572587	1	pseudobulk	DESeq2	LRT	0.222751240354882
115	KC_AB1	CG32221	-0.120962788870925	0.924237818584296	1	pseudobulk	DESeq2	LRT	0.0342162647002007
115	KC_AB1	CG32225	-0.136870344239011	0.787707916451187	1	pseudobulk	DESeq2	LRT	0.103634789856581
115	KC_AB1	CG32228	-0.760959112540794	1	1	pseudobulk	DESeq2	LRT	0
115	KC_AB1	CG3223	-0.0490629917354548	0.930256161018642	1	pseudobulk	DESeq2	LRT	0.0313974449962551
115	KC_AB1	CG32232	-0.718853741766346	0.977252643133665	1	pseudobulk	DESeq2	LRT	0.0099931462763106
115	KC_AB1	CG3224	-0.0488686761648928	0.889590512536182	1	pseudobulk	DESeq2	LRT	0.0508098574786937
115	KC_AB1	CG32243	-0.0282130559214287	0.940977520579929	1	pseudobulk	DESeq2	LRT	0.0264207514980405
115	KC_AB1	CG3225	-0.438584120588001	0.828770538443449	1	pseudobulk	DESeq2	LRT	0.0815656958455328
115	KC_AB1	CG3226	0.282586300268508	0.553705064640052	1	pseudobulk	DESeq2	LRT	0.256721504056844
115	KC_AB1	CG32260	1.25804452752548	0.727552935095488	1	pseudobulk	DESeq2	LRT	0.138135402914536
115	KC_AB1	CG32262	-0.661401517721364	0.46242787363789	1	pseudobulk	DESeq2	LRT	0.334955995949372
115	KC_AB1	CG32264	-0.0533220025069624	0.759324274392715	1	pseudobulk	DESeq2	LRT	0.119572716194391
115	KC_AB1	CG32266	-0.760959112540794	1	1	pseudobulk	DESeq2	LRT	0
115	KC_AB1	CG32267	-0.130886316646932	0.620470254521694	1	pseudobulk	DESeq2	LRT	0.207279033837673
115	KC_AB1	CG32276	0.10232128442989	0.720564104625345	1	pseudobulk	DESeq2	LRT	0.142327376339768
115	KC_AB1	CG32278	-0.153693249454597	0.55185545575846	1	pseudobulk	DESeq2	LRT	0.258174659565884
115	KC_AB1	CG32280	0.429147614903084	0.530735953133343	1	pseudobulk	DESeq2	LRT	0.27512149138223
115	KC_AB1	CG32281	-0.402757785381544	0.738218242964504	1	pseudobulk	DESeq2	LRT	0.131815226654692
115	KC_AB1	CG32295	-0.760959112540794	0.804334723897681	1	pseudobulk	DESeq2	LRT	0.0945631819854715
115	KC_AB1	CG32313	1.11388934932283	1	1	pseudobulk	DESeq2	LRT	0
115	KC_AB1	CG32333	-0.281288311764053	0.847349657687754	1	pseudobulk	DESeq2	LRT	0.0719373416542057
115	KC_AB1	CG32344	1.33651186842738	0.226554568032779	1	pseudobulk	DESeq2	LRT	0.644827176691909
115	KC_AB1	CG32354	2.17914818942692	0.381516710961221	1	pseudobulk	DESeq2	LRT	0.418486434591672
115	KC_AB1	CG32365	-0.428877212918458	0.819863073543117	1	pseudobulk	DESeq2	LRT	0.086258673677451
115	KC_AB1	CG32368	-0.156387909775947	0.907737442005518	1	pseudobulk	DESeq2	LRT	0.0420397505730791
115	KC_AB1	CG32369	-0.197761502561936	0.55419789317466	1	pseudobulk	DESeq2	LRT	0.256335129554074
115	KC_AB1	CG32373	-0.334346980933823	0.673987438398242	1	pseudobulk	DESeq2	LRT	0.171348197656321
115	KC_AB1	CG32392	0.217358744610343	0.855263173571899	1	pseudobulk	DESeq2	LRT	0.0679002276772221
115	KC_AB1	CG32396	0.943798956650056	0.807634491832869	1	pseudobulk	DESeq2	LRT	0.0927851418175362
115	KC_AB1	CG32407	0.91747866794934	0.415445402463659	1	pseudobulk	DESeq2	LRT	0.381486042798662
115	KC_AB1	CG32409	-0.339545472179584	0.760748209572162	1	pseudobulk	DESeq2	LRT	0.11875906108461
115	KC_AB1	CG32425	0.198404049207041	0.552993582265507	1	pseudobulk	DESeq2	LRT	0.25727990884512
115	KC_AB1	CG32428	-0.286163114180893	0.416893534489365	1	pseudobulk	DESeq2	LRT	0.379974840199596
115	KC_AB1	CG32432	-0.311940684133857	0.146287505225781	1	pseudobulk	DESeq2	LRT	0.834792766446328
115	KC_AB1	CG32441	0.118921420156893	0.736490159869541	1	pseudobulk	DESeq2	LRT	0.132833051323602
115	KC_AB1	CG32444	5.94141718056109	0.0559903327649451	1	pseudobulk	DESeq2	LRT	1.25188695137356
115	KC_AB1	CG32448	-0.235697291890171	0.654023991958665	1	pseudobulk	DESeq2	LRT	0.184406319895699
115	KC_AB1	CG32452	-0.316884761040416	0.766884502550021	1	pseudobulk	DESeq2	LRT	0.115270038504999
115	KC_AB1	CG32457	-1.22121879113183	0.605446734719958	1	pseudobulk	DESeq2	LRT	0.217924058688964

115	KC_AB1	CG32459	-2.13621400928779	0.457001334179545	1	pseudobulk	DESeq2	LRT	0.340082532039628
115	KC_AB1	CG32473	0.801496098125195	0.347836669759404	1	pseudobulk	DESeq2	LRT	0.458624635574589
115	KC_AB1	CG32485	-0.138770010065857	0.685653312117277	1	pseudobulk	DESeq2	LRT	0.163895421746751
115	KC_AB1	CG32486	-0.0796771616270448	0.649700851350278	1	pseudobulk	DESeq2	LRT	0.187286564146613
115	KC_AB1	CG32500	1.11388934932283	1	1	pseudobulk	DESeq2	LRT	0
115	KC_AB1	CG32506	0.00118103749281875	0.927326793967114	1	pseudobulk	DESeq2	LRT	0.0327671916308649
115	KC_AB1	CG32512	1.58151592924504	0.390033634464958	1	pseudobulk	DESeq2	LRT	0.408897940069172
115	KC_AB1	CG32521	1.0877817538669	0.205332192034689	1	pseudobulk	DESeq2	LRT	0.687542956489073
115	KC_AB1	CG3253	0.0306494074828644	0.941350385058414	1	pseudobulk	DESeq2	LRT	0.0262486954103241
116	KC_AB1	CG32533	0.0932657353674954	0.946195086777147	1	pseudobulk	DESeq2	LRT	0.0240193114038277
116	KC_AB1	CG32537	-0.303368360672905	0.797112045941881	1	pseudobulk	DESeq2	LRT	0.098480627770626
116	KC_AB1	CG32544	0.00577506260228377	0.967370555935173	1	pseudobulk	DESeq2	LRT	0.0144071354605711
116	KC_AB1	CG32547	-0.305624464049066	0.299201003387335	1	pseudobulk	DESeq2	LRT	0.524036954374246
116	KC_AB1	CG32548	-0.760959112540794	0.805265236978025	1	pseudobulk	DESeq2	LRT	0.0940610488455931
116	KC_AB1	CG32549	-0.306465594403407	0.474580545017606	1	pseudobulk	DESeq2	LRT	0.323690069246956
116	KC_AB1	CG32554	-1.14451487677508	0.399476806674976	1	pseudobulk	DESeq2	LRT	0.398508430431283
116	KC_AB1	CG3257	0.154088141414344	0.739382829370352	1	pseudobulk	DESeq2	LRT	0.13113063923261
116	KC_AB1	CG32576	-0.336871369509523	0.662522179302668	1	pseudobulk	DESeq2	LRT	0.178799578240169
116	KC_AB1	CG32579	2.58743201691345	0.417636627932844	1	pseudobulk	DESeq2	LRT	0.379201419460921
116	KC_AB1	CG32581	-1.53380862629999	0.626768306149835	1	pseudobulk	DESeq2	LRT	0.202892972656934
116	KC_AB1	CG32590	0.667875124796416	0.227357484386766	1	pseudobulk	DESeq2	LRT	0.643290744672545
116	KC_AB1	CG32591	1.11388934932283	1	1	pseudobulk	DESeq2	LRT	0
116	KC_AB1	CG3262	0.197998778067058	0.523103660789793	1	pseudobulk	DESeq2	LRT	0.281412240677509
116	KC_AB1	CG32638	-0.287023665706868	0.804288669588488	1	pseudobulk	DESeq2	LRT	0.0945880493751125
116	KC_AB1	CG32641	-2.8708363720268	0.13125667312555	1	pseudobulk	DESeq2	LRT	0.88187860770919
116	KC_AB1	CG32647	-0.48706878241161	0.12921119782358	1	pseudobulk	DESeq2	LRT	0.888699847468982
116	KC_AB1	CG32650	0.291868230578568	0.902153667810848	1	pseudobulk	DESeq2	LRT	0.0447194808636415
116	KC_AB1	CG32683	-0.180663079796793	0.538461541409685	1	pseudobulk	DESeq2	LRT	0.268845309914761
116	KC_AB1	CG32686	-0.760959112540794	0.804334723897681	1	pseudobulk	DESeq2	LRT	0.0945631819854715
116	KC_AB1	CG32694	1.16954034939163	0.51888123967062	1	pseudobulk	DESeq2	LRT	0.284932031087333
116	KC_AB1	CG32695	-0.760959112540794	0.804334723897681	1	pseudobulk	DESeq2	LRT	0.0945631819854715
116	KC_AB1	CG3270	-0.640573819164632	0.678497771070001	1	pseudobulk	DESeq2	LRT	0.168451574700765
116	KC_AB1	CG32700	-0.235426421262434	0.246080660309784	1	pseudobulk	DESeq2	LRT	0.608922516531984
116	KC_AB1	CG32708	-0.395450636086134	0.680624746049647	1	pseudobulk	DESeq2	LRT	0.1670922649413
116	KC_AB1	CG32712	-0.760959112540794	1	1	pseudobulk	DESeq2	LRT	0
116	KC_AB1	CG32755	-1.21296207351619	0.645188741988394	1	pseudobulk	DESeq2	LRT	0.190313219297606
116	KC_AB1	CG32756	0.301805699024739	0.616360651103481	1	pseudobulk	DESeq2	LRT	0.210165094733149
116	KC_AB1	CG32762	1.11388934932283	1	1	pseudobulk	DESeq2	LRT	0
116	KC_AB1	CG32767	-0.134746235049729	0.344645711246607	1	pseudobulk	DESeq2	LRT	0.462627121474224
116	KC_AB1	CG3277	1.11388934932283	1	1	pseudobulk	DESeq2	LRT	0
116	KC_AB1	CG32772	-0.0739725977119902	0.827787198141871	1	pseudobulk	DESeq2	LRT	0.0820812943152235
116	KC_AB1	CG32795	-0.234111717930152	0.546595013001573	1	pseudobulk	DESeq2	LRT	0.262334335021291
116	KC_AB1	CG32801	-0.760959112540794	1	1	pseudobulk	DESeq2	LRT	0
116	KC_AB1	CG32803	-1.5856771266849	0.235620525475588	1	pseudobulk	DESeq2	LRT	0.627786879791088
116	KC_AB1	CG32808	-0.655109288491529	0.675019855045408	1	pseudobulk	DESeq2	LRT	0.170683452635879
116	KC_AB1	CG32809	-0.0276323068038848	0.890505619172064	1	pseudobulk	DESeq2	LRT	0.0503633357541115
116	KC_AB1	CG3281	0.699322671709551	0.60097276290899	1	pseudobulk	DESeq2	LRT	0.221145210503713
116	KC_AB1	CG32813	0.391277400028353	0.536103871171447	1	pseudobulk	DESeq2	LRT	0.270751056750952
116	KC_AB1	CG32815	-0.0562590592532102	0.883002610374228	1	pseudobulk	DESeq2	LRT	0.0540380125385729
116	KC_AB1	CG32816	2.8058186523884	0.144063412552211	1	pseudobulk	DESeq2	LRT	0.841446301935831
116	KC_AB1	CG32817	-1.61102155603974	0.57025980967112	1	pseudobulk	DESeq2	LRT	0.243927235206257
116	KC_AB1	CG32829	0.70842991589991	0.874943022847655	1	pseudobulk	DESeq2	LRT	0.0580202277417387
116	KC_AB1	CG32846	-0.760959112540794	0.804334723897681	1	pseudobulk	DESeq2	LRT	0.0945631819854715
116	KC_AB1	CG32850	0.00156349473729475	0.98273004626931	1	pseudobulk	DESeq2	LRT	0.00756576550136928
116	KC_AB1	CG32856	-0.104158971177614	0.881329264048449	1	pseudobulk	DESeq2	LRT	0.0548618091315332
116	KC_AB1	CG3294	0.282105058968163	0.779261544588076	1	pseudobulk	DESeq2	LRT	0.108316755026409

116	KC_AB1	CG32944	-0.760959112540794	0.805265236978025	1	pseudobulk	DESeq2	LRT	0.0940610488455931
116	KC_AB1	CG3295	0.416089767666734	0.540247930200401	1	pseudobulk	DESeq2	LRT	0.267406888311943
116	KC_AB1	CG32982	0.015059148816058	0.958066023554897	1	pseudobulk	DESeq2	LRT	0.0186045611954167
116	KC_AB1	CG3301	-2.95661566151694	0.246821028878379	1	pseudobulk	DESeq2	LRT	0.607617841653462
116	KC_AB1	CG33051	-0.204932579896622	0.874182614600733	1	pseudobulk	DESeq2	LRT	0.0583978348379544
116	KC_AB1	CG33054	-0.683435076813743	0.639513477807421	1	pseudobulk	DESeq2	LRT	0.194150298291331
116	KC_AB1	CG33056	1.08358785800801	0.659233701656387	1	pseudobulk	DESeq2	LRT	0.180960598535959
116	KC_AB1	CG3308	0.751927574733431	0.415438437540217	1	pseudobulk	DESeq2	LRT	0.381493323787105
116	KC_AB1	CG33080	0.371055422160238	0.881976387776155	1	pseudobulk	DESeq2	LRT	0.054543041620775
116	KC_AB1	CG33082	0.296275065198765	1	1	pseudobulk	DESeq2	LRT	0
116	KC_AB1	CG3309	0.259193769051601	0.799655786108426	1	pseudobulk	DESeq2	LRT	0.0970969159621923
116	KC_AB1	CG33090	-0.355585096838986	0.170112144710758	1	pseudobulk	DESeq2	LRT	0.769264679964228
116	KC_AB1	CG33096	-0.639995317903431	0.614849021526526	1	pseudobulk	DESeq2	LRT	0.211231513765442
116	KC_AB1	CG33098	0.170401532593089	0.901886517204887	1	pseudobulk	DESeq2	LRT	0.0448481055315592
116	KC_AB1	CG33107	0.617210184432208	0.406494283172863	1	pseudobulk	DESeq2	LRT	0.390945557828678
116	KC_AB1	CG33108	0.0794663526207919	0.949105701189911	1	pseudobulk	DESeq2	LRT	0.0226854178285697
116	KC_AB1	CG33111	0.498265597566991	0.667192620537695	1	pseudobulk	DESeq2	LRT	0.175748765843762
116	KC_AB1	CG33116	-0.220315926906396	0.516808782944556	1	pseudobulk	DESeq2	LRT	0.286670114301007
116	KC_AB1	CG33127	1.90071181822722	0.545896518579078	1	pseudobulk	DESeq2	LRT	0.262889675371117
116	KC_AB1	CG33129	0.10302466639485	0.743001121629957	1	pseudobulk	DESeq2	LRT	0.129010530630631
116	KC_AB1	CG33143	1.34746974546903	0.647188649921216	1	pseudobulk	DESeq2	LRT	0.188969107773733
116	KC_AB1	CG33144	0.146948200620227	0.644176624105519	1	pseudobulk	DESeq2	LRT	0.190995038917412
116	KC_AB1	CG33155	-0.484164075788247	0.81120251308976	1	pseudobulk	DESeq2	LRT	0.0908707125727156
116	KC_AB1	CG33156	0.141893455423468	0.789509003045954	1	pseudobulk	DESeq2	LRT	0.102642913216315
116	KC_AB1	CG33158	-0.569299277860249	0.60217328746345	1	pseudobulk	DESeq2	LRT	0.220278513792591
116	KC_AB1	CG33169	-0.631720743928554	0.379034116031686	1	pseudobulk	DESeq2	LRT	0.421321698376282
116	KC_AB1	CG33170	0.209904442481466	0.568466847423089	1	pseudobulk	DESeq2	LRT	0.245294857978965
116	KC_AB1	CG33172	0.0957631127924135	0.94578634399932	1	pseudobulk	DESeq2	LRT	0.0242069609576786
116	KC_AB1	CG33178	-2.5009690989343	0.281691945291831	1	pseudobulk	DESeq2	LRT	0.550225571066492
116	KC_AB1	CG33181	-0.0271677813130518	0.867935078530801	1	pseudobulk	DESeq2	LRT	0.0615127587985049
116	KC_AB1	CG33199	-0.148732223445665	0.665386839392225	1	pseudobulk	DESeq2	LRT	0.176925793241555
116	KC_AB1	CG33203	0.0728335582655864	0.882390019482123	1	pseudobulk	DESeq2	LRT	0.0543394127486836
116	KC_AB1	CG33213	0.761113687519194	0.502848610029198	1	pseudobulk	DESeq2	LRT	0.298562746007924
116	KC_AB1	CG33217	-0.0123342324838705	0.901945812125002	1	pseudobulk	DESeq2	LRT	0.0448195535911141
116	KC_AB1	CG33228	0.985684823487533	0.245304912078717	1	pseudobulk	DESeq2	LRT	0.610293755228909
116	KC_AB1	CG33229	-0.212597970090843	0.31583897715368	1	pseudobulk	DESeq2	LRT	0.500534275473324
116	KC_AB1	CG33230	0.0732877074362207	0.944302383551165	1	pseudobulk	DESeq2	LRT	0.0248889140924983
116	KC_AB1	CG33233	2.13148430488057	0.45484681020722	1	pseudobulk	DESeq2	LRT	0.342134846603996
116	KC_AB1	CG33267	0.609672776282129	0.67817257301287	1	pseudobulk	DESeq2	LRT	0.16865977815278
116	KC_AB1	CG33272	1.11388934932283	1	1	pseudobulk	DESeq2	LRT	0
116	KC_AB1	CG3328	1.57407577798265	0.414193581864721	1	pseudobulk	DESeq2	LRT	0.382796634999137
116	KC_AB1	CG33281	1.25804452752548	0.727552935095488	1	pseudobulk	DESeq2	LRT	0.138135402914536
116	KC_AB1	CG33288	1.25804452752548	0.727552935095488	1	pseudobulk	DESeq2	LRT	0.138135402914536
116	KC_AB1	CG33296	0.960927098283632	0.703352211962905	1	pseudobulk	DESeq2	LRT	0.152827142396451
116	KC_AB1	CG33298	0.0811982592084966	0.843171814552427	1	pseudobulk	DESeq2	LRT	0.0740839194334029
116	KC_AB1	CG33303	-0.16670032812704	0.614736836977648	1	pseudobulk	DESeq2	LRT	0.211310761798966
116	KC_AB1	CG33310	-0.314297949106024	0.700119820816478	1	pseudobulk	DESeq2	LRT	0.154827627033996
116	KC_AB1	CG33331	1.43728569018017	0.23539770553847	1	pseudobulk	DESeq2	LRT	0.62819777461424
116	KC_AB1	CG33332	-2.19587645960801	0.169271706112456	1	pseudobulk	DESeq2	LRT	0.771415628460105
116	KC_AB1	CG3335	-0.667392334448537	0.474264585943299	1	pseudobulk	DESeq2	LRT	0.323979303565758
116	KC_AB1	CG3337	-1.01280143360762	0.155685791077782	1	pseudobulk	DESeq2	LRT	0.807751022230218
116	KC_AB1	CG3342	-0.487987262199727	0.556792236953983	1	pseudobulk	DESeq2	LRT	0.254306828482895
116	KC_AB1	CG3345	-0.760959112540794	0.804334723897681	1	pseudobulk	DESeq2	LRT	0.0945631819854715
117	KC_AB1	CG33458	0.943022244937108	0.768808261856119	1	pseudobulk	DESeq2	LRT	0.114181958248924
117	KC_AB1	CG33460	1.25804452752548	0.727552935095488	1	pseudobulk	DESeq2	LRT	0.138135402914536
117	KC_AB1	CG33467	1.11388934932283	1	1	pseudobulk	DESeq2	LRT	0

117	KC_AB1	CG33475	-0.760959112540794	0.805265236978025	1	pseudobulk	DESeq2	LRT	0.0940610488455931
117	KC_AB1	CG33493	-0.53545933184993	0.750016491128434	1	pseudobulk	DESeq2	LRT	0.124929187371846
117	KC_AB1	CG33494	3.80522001411205	0.202854565618816	1	pseudobulk	DESeq2	LRT	0.692815213245607
117	KC_AB1	CG33506	0.514355949396621	0.479588401187583	1	pseudobulk	DESeq2	LRT	0.319131328828281
117	KC_AB1	CG33521	-0.267908824936174	0.836481364267609	1	pseudobulk	DESeq2	LRT	0.0775437301180819
117	KC_AB1	CG33523	0.418554666370692	0.517452562651556	1	pseudobulk	DESeq2	LRT	0.286129457896104
117	KC_AB1	CG33543	1.76450331337746	0.0956683038791389	1	pseudobulk	DESeq2	LRT	1.01923192565544
117	KC_AB1	CG3356	0.713046515752688	0.34942616274322	1	pseudobulk	DESeq2	LRT	0.456644581008404
117	KC_AB1	CG3358	-0.483569614705714	0.663146094966785	1	pseudobulk	DESeq2	LRT	0.178390783437782
117	KC_AB1	CG33635	-0.0998444466045379	0.871645312595764	1	pseudobulk	DESeq2	LRT	0.0596602009833954
117	KC_AB1	CG33642	-0.0383051347773867	0.983080967019333	1	pseudobulk	DESeq2	LRT	0.00741071199326591
117	KC_AB1	CG3368	-0.413941090377384	0.67011949599327	1	pseudobulk	DESeq2	LRT	0.173847746816915
117	KC_AB1	CG33695	0.571899715037065	0.446671231542931	1	pseudobulk	DESeq2	LRT	0.350012017943418
117	KC_AB1	CG33700	0.23933665343942	0.896765478834469	1	pseudobulk	DESeq2	LRT	0.0473211183437949
117	KC_AB1	CG33757	-0.760959112540794	0.804334723897681	1	pseudobulk	DESeq2	LRT	0.0945631819854715
117	KC_AB1	CG3376	0.296829314647626	0.803645564941873	1	pseudobulk	DESeq2	LRT	0.0949354476805981
117	KC_AB1	CG33774	0.223587296692286	0.683795003294169	1	pseudobulk	DESeq2	LRT	0.165074077054632
117	KC_AB1	CG33777	-1.8856918730571	0.468164907518632	1	pseudobulk	DESeq2	LRT	0.329601143057579
117	KC_AB1	CG33792	1.11388934932283	1	1	pseudobulk	DESeq2	LRT	0
117	KC_AB1	CG3386	-0.192822838130415	0.827647873047545	1	pseudobulk	DESeq2	LRT	0.0821543966927824
117	KC_AB1	CG33914	-0.760959112540794	0.804334723897681	1	pseudobulk	DESeq2	LRT	0.0945631819854715
117	KC_AB1	CG33939	0.342819821862687	0.924402470642007	1	pseudobulk	DESeq2	LRT	0.0341389024564978
117	KC_AB1	CG33941	0.171041848368747	0.792094206068387	1	pseudobulk	DESeq2	LRT	0.101223163432471
117	KC_AB1	CG33947	-0.276574462987903	0.678544377230461	1	pseudobulk	DESeq2	LRT	0.168421743942238
117	KC_AB1	CG33958	2.14204208446331	0.343930330823948	1	pseudobulk	DESeq2	LRT	0.463529522553617
117	KC_AB1	CG33969	-0.287029132047178	0.735670224270797	1	pseudobulk	DESeq2	LRT	0.133316821362122
117	KC_AB1	CG33970	-1.53448070799783	0.0695334914287046	1	pseudobulk	DESeq2	LRT	1.15780596319095
117	KC_AB1	CG33977	0.435132757342656	0.255373033869207	1	pseudobulk	DESeq2	LRT	0.592824964003319
117	KC_AB1	CG33978	-0.390659561835172	0.68061412149361	1	pseudobulk	DESeq2	LRT	0.167099044333469
117	KC_AB1	CG33993	-0.438091648593083	0.707174574584479	1	pseudobulk	DESeq2	LRT	0.150473362129825
117	KC_AB1	CG33998	-1.02958735051477	0.546975774145321	1	pseudobulk	DESeq2	LRT	0.262031908379543
117	KC_AB1	CG34001	0.547096880680561	0.672959041703848	1	pseudobulk	DESeq2	LRT	0.172011367430273
117	KC_AB1	CG34015	0.109132127062697	0.81339410880847	1	pseudobulk	DESeq2	LRT	0.0896989773964408
117	KC_AB1	CG3402	-0.0133157117631663	0.978871473861771	1	pseudobulk	DESeq2	LRT	0.00927432745827812
117	KC_AB1	CG34026	-0.760959112540794	1	1	pseudobulk	DESeq2	LRT	0
117	KC_AB1	CG34027	-0.760959112540794	0.805265236978025	1	pseudobulk	DESeq2	LRT	0.0940610488455931
117	KC_AB1	CG34028	-0.64145515134999	0.782847173450071	1	pseudobulk	DESeq2	LRT	0.106323012150752
117	KC_AB1	CG34031	1.67020383217772	0.598754457572587	1	pseudobulk	DESeq2	LRT	0.222751240354882
117	KC_AB1	CG34039	-0.28956437506048	0.706738479078093	1	pseudobulk	DESeq2	LRT	0.150741262447346
117	KC_AB1	CG34041	-0.0518653414751322	0.981940836459101	1	pseudobulk	DESeq2	LRT	0.0079146783774002
117	KC_AB1	CG34045	-1.38718159242805	0.788203284813311	1	pseudobulk	DESeq2	LRT	0.103361759557548
117	KC_AB1	CG34051	1.25804452752548	0.727552935095488	1	pseudobulk	DESeq2	LRT	0.138135402914536
117	KC_AB1	CG34056	1.71412565764297	0.464865561800447	1	pseudobulk	DESeq2	LRT	0.33267262605614
117	KC_AB1	CG34057	2.09987857572813	0.503115176013642	1	pseudobulk	DESeq2	LRT	0.29833258237694
117	KC_AB1	CG3407	-1.76080866184573	0.3168694952839	1	pseudobulk	DESeq2	LRT	0.499119567901973
117	KC_AB1	CG3408	0.474831245983554	0.732982070008181	1	pseudobulk	DESeq2	LRT	0.134906648812824
117	KC_AB1	CG34107	-0.760959112540794	1	1	pseudobulk	DESeq2	LRT	0
117	KC_AB1	CG34109	1.93087103138744	0.48962757043079	1	pseudobulk	DESeq2	LRT	0.310134135493139
117	KC_AB1	CG34112	1.25804452752548	0.573436424985144	1	pseudobulk	DESeq2	LRT	0.241514723898045
117	KC_AB1	CG34115	-1.43550613534661	0.602448798560473	1	pseudobulk	DESeq2	LRT	0.220079857379737
117	KC_AB1	CG34116	1.19609114399685	0.27609172167342	1	pseudobulk	DESeq2	LRT	0.558946615039304
117	KC_AB1	CG34117	0.447078173223282	0.447467537223809	1	pseudobulk	DESeq2	LRT	0.349238466305519
117	KC_AB1	CG34120	0.48909204473378	0.865165105786238	1	pseudobulk	DESeq2	LRT	0.0629010050328645
117	KC_AB1	CG34125	0.161786271741787	0.657759229084799	1	pseudobulk	DESeq2	LRT	0.18193304957068
117	KC_AB1	CG34126	0.555983786265626	0.321047626114367	1	pseudobulk	DESeq2	LRT	0.493430536989482
117	KC_AB1	CG34132	-0.0425209604961513	0.906974730663874	1	pseudobulk	DESeq2	LRT	0.0424048127040923

117	KC_AB1	CG34133	-0.120298410002271	0.856061001952877	1	pseudobulk	DESeq2	LRT	0.06749528687942
117	KC_AB1	CG34134	0.999497670405064	0.304091976421425	1	pseudobulk	DESeq2	LRT	0.516995038724237
117	KC_AB1	CG34136	-0.459167792686404	0.819293366635313	1	pseudobulk	DESeq2	LRT	0.0865605613682894
117	KC_AB1	CG34141	-0.760959112540794	0.804334723897681	1	pseudobulk	DESeq2	LRT	0.0945631819854715
117	KC_AB1	CG34148	-0.00241743770099407	0.902010518346108	1	pseudobulk	DESeq2	LRT	0.0447883981198774
117	KC_AB1	CG34149	0.919816652527727	0.420922025501851	1	pseudobulk	DESeq2	LRT	0.37579834841771
117	KC_AB1	CG34150	0.00306352167882319	0.988746545201313	1	pseudobulk	DESeq2	LRT	0.00491502096847891
117	KC_AB1	CG34155	0.709686956006578	0.915714550115026	1	pseudobulk	DESeq2	LRT	0.0382398850985033
117	KC_AB1	CG34159	-0.268274145471738	0.403412610870746	1	pseudobulk	DESeq2	LRT	0.394250529658276
117	KC_AB1	CG34163	0.156984933439541	0.890697725918172	1	pseudobulk	DESeq2	LRT	0.0502696565003366
117	KC_AB1	CG34174	-0.119552010804202	0.885305002694777	1	pseudobulk	DESeq2	LRT	0.0529070816556773
117	KC_AB1	CG34180	-2.73719572742657	0.395113780272619	1	pseudobulk	DESeq2	LRT	0.40327782328744
117	KC_AB1	CG34183	-1.01981000585408	0.250343616627491	1	pseudobulk	DESeq2	LRT	0.601463477957105
117	KC_AB1	CG34189	-0.760959112540794	1	1	pseudobulk	DESeq2	LRT	0
117	KC_AB1	CG34190	-0.760959112540794	0.805265236978025	1	pseudobulk	DESeq2	LRT	0.0940610488455931
117	KC_AB1	CG34195	0.819639133051019	0.427616556282465	1	pseudobulk	DESeq2	LRT	0.368945488302085
117	KC_AB1	CG3420	0.026174858745819	0.93134529804754	1	pseudobulk	DESeq2	LRT	0.0308892736544769
117	KC_AB1	CG34200	0.0711596317558561	0.829295648251301	1	pseudobulk	DESeq2	LRT	0.0812906135823545
117	KC_AB1	CG34205	-0.760959112540794	0.805265236978025	1	pseudobulk	DESeq2	LRT	0.0940610488455931
117	KC_AB1	CG34208	2.58743201691345	0.417636627932844	1	pseudobulk	DESeq2	LRT	0.379201419460921
117	KC_AB1	CG34213	-0.366232033321717	0.778854623283006	1	pseudobulk	DESeq2	LRT	0.108543597780528
117	KC_AB1	CG34214	4.58085424419702	0.0980644594377514	1	pseudobulk	DESeq2	LRT	1.00848836129205
117	KC_AB1	CG34217	1.25804452752548	0.573436424985144	1	pseudobulk	DESeq2	LRT	0.241514723898045
117	KC_AB1	CG34219	0.0139750310733068	0.959896526292262	1	pseudobulk	DESeq2	LRT	0.0177755799628206
117	KC_AB1	CG34220	-0.760959112540794	0.804334723897681	1	pseudobulk	DESeq2	LRT	0.0945631819854715
117	KC_AB1	CG34227	-4.52627084654204	0.0554374783094185	1	pseudobulk	DESeq2	LRT	1.25619653274712
117	KC_AB1	CG34228	0.527616267413403	0.425576561926644	1	pseudobulk	DESeq2	LRT	0.371022298255814
117	KC_AB1	CG34229	-0.175126886015218	0.838954351929473	1	pseudobulk	DESeq2	LRT	0.0762616687832988
117	KC_AB1	CG34231	-0.615822159288746	0.634425076250099	1	pseudobulk	DESeq2	LRT	0.19761965945178
117	KC_AB1	CG34232	-3.53300872248455	0.10299931101079	1	pseudobulk	DESeq2	LRT	0.98716568039398
117	KC_AB1	CG34234	1.65806569952475	0.525043668677946	1	pseudobulk	DESeq2	LRT	0.279804574161347
117	KC_AB1	CG34242	0.655300962810282	0.46924138456908	1	pseudobulk	DESeq2	LRT	0.328603692427354
117	KC_AB1	CG34248	4.12174664643008	0.157202596236497	1	pseudobulk	DESeq2	LRT	0.803540285765608
117	KC_AB1	CG34250	0.324065676547478	0.471522866131532	1	pseudobulk	DESeq2	LRT	0.326497241645733
117	KC_AB1	CG34251	1.63195036548663	0.633334503478132	1	pseudobulk	DESeq2	LRT	0.198366851366371
117	KC_AB1	CG34253	1.25804452752548	0.727552935095488	1	pseudobulk	DESeq2	LRT	0.138135402914536
117	KC_AB1	CG34256	0.152115475746306	1	1	pseudobulk	DESeq2	LRT	0
117	KC_AB1	CG34260	1.11388934932283	1	1	pseudobulk	DESeq2	LRT	0
117	KC_AB1	CG34261	1.90071181822722	0.545896518579078	1	pseudobulk	DESeq2	LRT	0.262889675371117
117	KC_AB1	CG34263	-0.760959112540794	0.804334723897681	1	pseudobulk	DESeq2	LRT	0.0945631819854715
117	KC_AB1	CG34279	0.860681888856842	0.743699219954362	1	pseudobulk	DESeq2	LRT	0.128602674031803
118	KC_AB1	CG34286	-1.43262420355084	0.559815650460642	1	pseudobulk	DESeq2	LRT	0.251954964366308
118	KC_AB1	CG34288	1.25804452752548	0.727552935095488	1	pseudobulk	DESeq2	LRT	0.138135402914536
118	KC_AB1	CG34293	0.237971676251475	0.682148885285449	1	pseudobulk	DESeq2	LRT	0.166120826221722
118	KC_AB1	CG34296	3.37429492540794	0.281278028870551	1	pseudobulk	DESeq2	LRT	0.550864190027974
118	KC_AB1	CG34298	1.11388934932283	1	1	pseudobulk	DESeq2	LRT	0
118	KC_AB1	CG34310	0.0343086176960997	1	1	pseudobulk	DESeq2	LRT	0
118	KC_AB1	CG34325	-1.30943155764665	0.665865564455012	1	pseudobulk	DESeq2	LRT	0.176613444266292
118	KC_AB1	CG34330	2.33888469383904	0.218102303520766	1	pseudobulk	DESeq2	LRT	0.661339747529624
118	KC_AB1	CG34331	5.24198564656588	0.0455071079077502	1	pseudobulk	DESeq2	LRT	1.3419207641336
118	KC_AB1	CG34334	-0.696045889415738	0.771031022267663	1	pseudobulk	DESeq2	LRT	0.112928147852258
118	KC_AB1	CG3434	1.13941025380657	0.211115374435117	1	pseudobulk	DESeq2	LRT	0.675480138131466
118	KC_AB1	CG34347	0.0704970160248509	0.88628816897379	1	pseudobulk	DESeq2	LRT	0.0524250480370886
118	KC_AB1	CG34348	0.565447630519144	0.578988804578402	1	pseudobulk	DESeq2	LRT	0.237329833780042
118	KC_AB1	CG34351	0.377162636402593	0.571423439818781	1	pseudobulk	DESeq2	LRT	0.243041948805983
118	KC_AB1	CG34353	-0.0767494780506271	0.884961114296959	1	pseudobulk	DESeq2	LRT	0.0530758120335657

118	KC_AB1	CG34354	-0.399951481738326	0.0854368094822449	1	pseudobulk	DESeq2	LRT	1.06835497813068
118	KC_AB1	CG34355	0.0975732168366572	0.708444695328344	1	pseudobulk	DESeq2	LRT	0.149694047253078
118	KC_AB1	CG34357	-0.34615858783327	0.227994691963965	1	pseudobulk	DESeq2	LRT	0.642075263866188
118	KC_AB1	CG3436	-0.0746467991832734	0.872579849812335	1	pseudobulk	DESeq2	LRT	0.0591948202471525
118	KC_AB1	CG34367	0.045079269859952	0.960619924541133	1	pseudobulk	DESeq2	LRT	0.0174484097556354
118	KC_AB1	CG3437	0.316535792447268	0.759836264431204	1	pseudobulk	DESeq2	LRT	0.119279982870636
118	KC_AB1	CG34370	0.494882982107155	0.232346636119579	1	pseudobulk	DESeq2	LRT	0.633863610791224
118	KC_AB1	CG34376	0.449981351787791	0.680336941125354	1	pseudobulk	DESeq2	LRT	0.167275946956049
118	KC_AB1	CG34377	2.3321795939829	0.218301687810501	1	pseudobulk	DESeq2	LRT	0.660942906508207
118	KC_AB1	CG34384	-0.498622496810971	0.0875119510451717	1	pseudobulk	DESeq2	LRT	1.05793263362282
118	KC_AB1	CG34398	4.97662503037777	0.0190865977036632	1	pseudobulk	DESeq2	LRT	1.71927148020834
118	KC_AB1	CG34401	-0.113052867308739	0.575284005035519	1	pseudobulk	DESeq2	LRT	0.240117700755277
118	KC_AB1	CG34402	0.182228493487422	0.704865404699445	1	pseudobulk	DESeq2	LRT	0.151893804394294
118	KC_AB1	CG34404	0.374175884739235	0.595171685585154	1	pseudobulk	DESeq2	LRT	0.225357737889971
118	KC_AB1	CG34408	-0.346960470201054	0.611081811720634	1	pseudobulk	DESeq2	LRT	0.213900642457423
118	KC_AB1	CG34409	-0.491407404788854	0.731627892084806	1	pseudobulk	DESeq2	LRT	0.135709746132176
118	KC_AB1	CG34417	1.24357339059926	0.493627462036988	1	pseudobulk	DESeq2	LRT	0.306600687144664
118	KC_AB1	CG34423	1.25804452752548	0.727552935095488	1	pseudobulk	DESeq2	LRT	0.138135402914536
118	KC_AB1	CG34425	-0.760959112540794	1	1	pseudobulk	DESeq2	LRT	0
118	KC_AB1	CG34428	1.25804452752548	0.727552935095488	1	pseudobulk	DESeq2	LRT	0.138135402914536
118	KC_AB1	CG34431	1.25804452752548	0.573436424985144	1	pseudobulk	DESeq2	LRT	0.241514723898045
118	KC_AB1	CG34436	1.25804452752548	0.727552935095488	1	pseudobulk	DESeq2	LRT	0.138135402914536
118	KC_AB1	CG34454	-4.63017880774413	0.0415946405657798	1	pseudobulk	DESeq2	LRT	1.38096262424476
118	KC_AB1	CG34457	-2.24976163616681	0.429894962609849	1	pseudobulk	DESeq2	LRT	0.366637643795677
118	KC_AB1	CG34461	-0.760959112540794	1	1	pseudobulk	DESeq2	LRT	0
118	KC_AB1	CG3448	2.09987857572813	0.503115176013642	1	pseudobulk	DESeq2	LRT	0.29833258237694
118	KC_AB1	CG3500	-0.213810451433154	0.745710221129263	1	pseudobulk	DESeq2	LRT	0.127429904180884
118	KC_AB1	CG3505	1.21641357259129	0.57305989573505	1	pseudobulk	DESeq2	LRT	0.241799983568774
118	KC_AB1	CG3511	-0.849431446308255	0.320264041566956	1	pseudobulk	DESeq2	LRT	0.494491820080197
118	KC_AB1	CG3520	-0.760959112540794	1	1	pseudobulk	DESeq2	LRT	0
118	KC_AB1	CG3527	-0.51295051167133	0.482276244752695	1	pseudobulk	DESeq2	LRT	0.316704129384153
118	KC_AB1	CG3529	0.379361267982493	0.363074160512406	1	pseudobulk	DESeq2	LRT	0.440004658127022
118	KC_AB1	CG3530	-0.128054766611105	0.62466249025971	1	pseudobulk	DESeq2	LRT	0.204354571790996
118	KC_AB1	CG3534	1.15229247079122	0.390121553379459	1	pseudobulk	DESeq2	LRT	0.408800055186321
118	KC_AB1	CG3542	-0.842792349684755	0.192101034117541	1	pseudobulk	DESeq2	LRT	0.716470297239644
118	KC_AB1	CG3548	-0.0858485583743094	0.840943724160367	1	pseudobulk	DESeq2	LRT	0.0752330661573423
118	KC_AB1	CG3552	-0.206894470850536	0.827528096221529	1	pseudobulk	DESeq2	LRT	0.0822172521421789
118	KC_AB1	CG3556	-0.282924903887063	0.507944957641647	1	pseudobulk	DESeq2	LRT	0.294183346550021
118	KC_AB1	CG3558	-0.146102640817689	0.632397895243087	1	pseudobulk	DESeq2	LRT	0.199009584163349
118	KC_AB1	CG3566	-0.309637313600694	0.329312332519105	1	pseudobulk	DESeq2	LRT	0.482392004955551
118	KC_AB1	CG3568	1.25413552494658	0.244922681119266	1	pseudobulk	DESeq2	LRT	0.610970995076051
118	KC_AB1	CG3587	0.818321807317723	0.432034317272312	1	pseudobulk	DESeq2	LRT	0.36448175501364
118	KC_AB1	CG3589	0.406809127213965	0.817314604556935	1	pseudobulk	DESeq2	LRT	0.087610740623817
118	KC_AB1	CG3594	0.293585083399965	0.729676460099581	1	pseudobulk	DESeq2	LRT	0.136869664181428
118	KC_AB1	CG3603	0.296275065198765	1	1	pseudobulk	DESeq2	LRT	0
118	KC_AB1	CG3609	2.15341322612959	0.0365953597264732	1	pseudobulk	DESeq2	LRT	1.43657397944023
118	KC_AB1	CG3610	-0.760959112540794	0.804334723897681	1	pseudobulk	DESeq2	LRT	0.0945631819854715
118	KC_AB1	CG3618	0.168070790087838	0.67974967866579	1	pseudobulk	DESeq2	LRT	0.167650989041981
118	KC_AB1	CG3625	0.100665014487092	0.562468220180938	1	pseudobulk	DESeq2	LRT	0.249902010443182
118	KC_AB1	CG3626	-0.657614550250885	0.778711989736868	1	pseudobulk	DESeq2	LRT	0.108623138471343
118	KC_AB1	CG3631	-0.159804738220203	0.865097191770069	1	pseudobulk	DESeq2	LRT	0.0629350977626463
118	KC_AB1	CG3632	0.0119801546609725	0.922702091541122	1	pseudobulk	DESeq2	LRT	0.0349384949477714
118	KC_AB1	CG3634	0.307278215699584	0.688515486609426	1	pseudobulk	DESeq2	LRT	0.162086286818276
118	KC_AB1	CG3638	-0.149618479185974	0.619526463270125	1	pseudobulk	DESeq2	LRT	0.207940137864634
118	KC_AB1	CG3645	0.308376868197591	0.708890812704823	1	pseudobulk	DESeq2	LRT	0.149420652111016
118	KC_AB1	CG3651	0.3533587246197	0.529875584423456	1	pseudobulk	DESeq2	LRT	0.275826091420671

118	KC_AB1	CG3652	-0.143245752772631	0.75704495902534	1	pseudobulk	DESeq2	LRT	0.120878328060269
118	KC_AB1	CG3655	0.267538863089677	0.806685680384206	1	pseudobulk	DESeq2	LRT	0.0932956522244075
118	KC_AB1	CG3662	-0.106113337725628	0.616065606986611	1	pseudobulk	DESeq2	LRT	0.210373035830608
118	KC_AB1	CG3663	0.291853629993484	0.902736173524132	1	pseudobulk	DESeq2	LRT	0.0444391545856091
118	KC_AB1	CG3678	0.809146913619864	0.145459681187539	1	pseudobulk	DESeq2	LRT	0.837257368632069
118	KC_AB1	CG3700	0.0936434187773718	0.926617892480295	1	pseudobulk	DESeq2	LRT	0.0330993180650501
118	KC_AB1	CG3702	-0.102376839852031	0.838289141523676	1	pseudobulk	DESeq2	LRT	0.0766061592819271
118	KC_AB1	CG3703	0.0828649271330989	0.833767843436148	1	pseudobulk	DESeq2	LRT	0.0789548586550823
118	KC_AB1	CG3704	-1.39287721968276	0.263361060374394	1	pseudobulk	DESeq2	LRT	0.579448437858237
118	KC_AB1	CG3709	0.898739311998799	0.740697588966623	1	pseudobulk	DESeq2	LRT	0.130359069011421
118	KC_AB1	CG3713	6.15179475412755	0.0950166965505545	1	pseudobulk	DESeq2	LRT	1.02220007278868
118	KC_AB1	CG3719	0.144930561161798	0.697062607014533	1	pseudobulk	DESeq2	LRT	0.156728213781664
118	KC_AB1	CG3726	4.11275859650143	0.078919693628753	1	pseudobulk	DESeq2	LRT	1.10281460937525
118	KC_AB1	CG3732	-0.0379486625257945	0.912220659518691	1	pseudobulk	DESeq2	LRT	0.0399000962988022
118	KC_AB1	CG3735	0.258885747972015	0.806260241851186	1	pseudobulk	DESeq2	LRT	0.0935247555179741
118	KC_AB1	CG3740	-0.50193960198851	0.742762686738789	1	pseudobulk	DESeq2	LRT	0.129149921505323
118	KC_AB1	CG3744	-0.201671873473432	0.694685754134871	1	pseudobulk	DESeq2	LRT	0.15821160707649
118	KC_AB1	CG3756	-0.404210285980601	0.694613058639474	1	pseudobulk	DESeq2	LRT	0.158257056266036
118	KC_AB1	CG3760	-0.0558188510595429	0.910860032456867	1	pseudobulk	DESeq2	LRT	0.0405483538742294
118	KC_AB1	CG3764	-0.155928280098826	0.779923367305183	1	pseudobulk	DESeq2	LRT	0.107948067555016
118	KC_AB1	CG3770	0.293544814793053	0.855484935116898	1	pseudobulk	DESeq2	LRT	0.0677876338764847
118	KC_AB1	CG3776	-0.438904006860441	0.447799223421921	1	pseudobulk	DESeq2	LRT	0.348916663914351
118	KC_AB1	CG3777	-0.343178129746324	0.751048164300737	1	pseudobulk	DESeq2	LRT	0.124332211039365
118	KC_AB1	CG3781	-0.366742725655172	0.567484551211082	1	pseudobulk	DESeq2	LRT	0.246045956892731
118	KC_AB1	CG3792	-0.309120023394715	0.643789027665586	1	pseudobulk	DESeq2	LRT	0.191256429428748
118	KC_AB1	CG3793	-0.180897124726529	0.678429796215864	1	pseudobulk	DESeq2	LRT	0.168495086388192
118	KC_AB1	CG3803	-0.253626011010283	0.761961797793888	1	pseudobulk	DESeq2	LRT	0.118066802181676
118	KC_AB1	CG3808	-0.724552214263518	0.73364105491175	1	pseudobulk	DESeq2	LRT	0.134516373326676
118	KC_AB1	CG3815	0.516513973185274	0.54797532643314	1	pseudobulk	DESeq2	LRT	0.261238995959385
119	KC_AB1	CG3817	0.047183254602915	0.956239383987962	1	pseudobulk	DESeq2	LRT	0.0194333732773222
119	KC_AB1	CG3819	1.03806863903421	0.409873917125134	1	pseudobulk	DESeq2	LRT	0.387349717714345
119	KC_AB1	CG3823	-1.36249801790501	0.405798306187922	1	pseudobulk	DESeq2	LRT	0.391689770054704
119	KC_AB1	CG3829	-1.21296207351619	0.645188741988394	1	pseudobulk	DESeq2	LRT	0.190313219297606
119	KC_AB1	CG3831	4.0018123891836	0.0629978653172461	1	pseudobulk	DESeq2	LRT	1.20067416636622
119	KC_AB1	CG3842	-2.11277601324589	0.381129799043716	1	pseudobulk	DESeq2	LRT	0.418927094118639
119	KC_AB1	CG3847	-0.0576113248461291	0.921337891432531	1	pseudobulk	DESeq2	LRT	0.0355810674439492
119	KC_AB1	CG3857	2.08060467995205	0.164540474161934	1	pseudobulk	DESeq2	LRT	0.783727255506702
119	KC_AB1	CG3860	-0.00627961114627232	0.979603729054255	1	pseudobulk	DESeq2	LRT	0.00894957031681823
119	KC_AB1	CG3862	0.171301252790533	0.840822581836117	1	pseudobulk	DESeq2	LRT	0.0752956330465116
119	KC_AB1	CG3894	-0.0186327093188297	0.925867996846604	1	pseudobulk	DESeq2	LRT	0.0334509272787891
119	KC_AB1	CG3902	3.2367878119458	0.0140813481403782	1	pseudobulk	DESeq2	LRT	1.85135576409313
119	KC_AB1	CG3907	-0.167781851346288	0.719152505708288	1	pseudobulk	DESeq2	LRT	0.143179002017188
119	KC_AB1	CG3909	1.14020976902537	0.323119265523564	1	pseudobulk	DESeq2	LRT	0.490637147023714
119	KC_AB1	CG3918	0.381473869398585	0.549704505774748	1	pseudobulk	DESeq2	LRT	0.259870703228757
119	KC_AB1	CG3919	-2.41876763198367	0.438413379977707	1	pseudobulk	DESeq2	LRT	0.358116200048746
119	KC_AB1	CG3939	1.40876879934001	0.481366337382109	1	pseudobulk	DESeq2	LRT	0.317524283816629
119	KC_AB1	CG3955	0.129263409667479	0.585153295996731	1	pseudobulk	DESeq2	LRT	0.232730344372319
119	KC_AB1	CG3961	-0.869947673188567	0.504691260248874	1	pseudobulk	DESeq2	LRT	0.296974215889559
119	KC_AB1	CG3995	-0.377315202795931	0.594164466948262	1	pseudobulk	DESeq2	LRT	0.226093324039799
119	KC_AB1	CG40006	-0.444419681814891	0.854527096765258	1	pseudobulk	DESeq2	LRT	0.0682741613937445
119	KC_AB1	CG4004	0.310850011972653	0.553338621895339	1	pseudobulk	DESeq2	LRT	0.257009015868185
119	KC_AB1	CG40045	0.149903265154225	0.58130728028033	1	pseudobulk	DESeq2	LRT	0.235594237914462
119	KC_AB1	CG40160	1.95854191278228	0.349477299785091	1	pseudobulk	DESeq2	LRT	0.456581028496975
119	KC_AB1	CG4017	4.6144452059428	0.0983980161710624	1	pseudobulk	DESeq2	LRT	1.00701365740855
119	KC_AB1	CG40191	0.195126060656828	0.639631821243604	1	pseudobulk	DESeq2	LRT	0.194069938536284
119	KC_AB1	CG40198	-5.40133162876647	0.185215303891571	1	pseudobulk	DESeq2	LRT	0.732323131471225

119	KC_AB1	CG4020	1.11388934932283	1	1	pseudobulk	DESeq2	LRT	0
119	KC_AB1	CG4022	0.148717718293794	0.786855200726842	1	pseudobulk	DESeq2	LRT	0.104105180361578
119	KC_AB1	CG40228	0.00649205585485581	0.929292063983152	1	pseudobulk	DESeq2	LRT	0.0318477716448663
119	KC_AB1	CG4025	0.599545084805005	0.441736567227401	1	pseudobulk	DESeq2	LRT	0.354836648086541
119	KC_AB1	CG4036	0.661253776896304	0.465691226483598	1	pseudobulk	DESeq2	LRT	0.331901943999097
119	KC_AB1	CG4038	0.182050870661587	0.824193079928138	1	pseudobulk	DESeq2	LRT	0.0839710362099269
119	KC_AB1	CG4041	0.910829896215429	0.423430639821055	1	pseudobulk	DESeq2	LRT	0.373217719241131
119	KC_AB1	CG4042	1.45873466875515	0.0589901911986973	1	pseudobulk	DESeq2	LRT	1.22922019619558
119	KC_AB1	CG40439	-1.46862564106327	0.349913418558087	1	pseudobulk	DESeq2	LRT	0.456039402775763
119	KC_AB1	CG4045	-0.136245601431195	0.856046697769877	1	pseudobulk	DESeq2	LRT	0.0675025436983061
119	KC_AB1	CG40470	-0.385011853448768	0.890534840916548	1	pseudobulk	DESeq2	LRT	0.0503490847107839
119	KC_AB1	CG40472	-0.614853728043603	0.576225690401287	1	pseudobulk	DESeq2	LRT	0.239407383072618
119	KC_AB1	CG40485	-0.760959112540794	1	1	pseudobulk	DESeq2	LRT	0
119	KC_AB1	CG4049	-0.77793515211394	0.117149663347395	1	pseudobulk	DESeq2	LRT	0.93125895509586
119	KC_AB1	CG40498	-0.0558027347692185	0.718244872312413	1	pseudobulk	DESeq2	LRT	0.143727465836361
119	KC_AB1	CG4069	-1.85230509384503	0.343759988451396	1	pseudobulk	DESeq2	LRT	0.46374467391426
119	KC_AB1	CG4074	0.463487396988047	0.482288987250149	1	pseudobulk	DESeq2	LRT	0.31669265479198
119	KC_AB1	CG4078	0.638836553066287	0.637121372009369	1	pseudobulk	DESeq2	LRT	0.195777826419987
119	KC_AB1	CG4080	-0.142601592651682	0.687141045359862	1	pseudobulk	DESeq2	LRT	0.162954108742039
119	KC_AB1	CG4101	-0.127401902439732	0.777547479398598	1	pseudobulk	DESeq2	LRT	0.109273082158049
119	KC_AB1	CG41099	0.317189615801396	0.374327035032668	1	pseudobulk	DESeq2	LRT	0.426748805689467
119	KC_AB1	CG41128	0.109336668311677	0.592116463725448	1	pseudobulk	DESeq2	LRT	0.227592863245463
119	KC_AB1	CG4115	4.99697088313761	0.012407510860415	1	pseudobulk	DESeq2	LRT	1.90631533598829
119	KC_AB1	CG4119	-0.443077907370337	0.384452731421119	1	pseudobulk	DESeq2	LRT	0.415157049217337
119	KC_AB1	CG4133	-0.229005355962106	0.566145247586223	1	pseudobulk	DESeq2	LRT	0.247072133948248
119	KC_AB1	CG41378	-0.152021139845419	0.741504806646517	1	pseudobulk	DESeq2	LRT	0.129886029405265
119	KC_AB1	CG41520	-0.325934945069582	0.878443443182604	1	pseudobulk	DESeq2	LRT	0.0562861944468762
119	KC_AB1	CG4168	0.296275065198765	1	1	pseudobulk	DESeq2	LRT	0
119	KC_AB1	CG4186	0.0823847094875262	0.918677062041134	1	pseudobulk	DESeq2	LRT	0.0368371271572685
119	KC_AB1	CG4199	1.48611475147597	0.0547282891000772	1	pseudobulk	DESeq2	LRT	1.26178812843359
119	KC_AB1	CG4210	2.09987857572813	0.503115176013642	1	pseudobulk	DESeq2	LRT	0.29833258237694
119	KC_AB1	CG42231	1.16219436697981	0.572739167454846	1	pseudobulk	DESeq2	LRT	0.242043116122109
119	KC_AB1	CG42232	-0.870910127582936	0.0669433244075905	1	pseudobulk	DESeq2	LRT	1.17429272436512
119	KC_AB1	CG42233	0.315128028820568	0.883237338721326	1	pseudobulk	DESeq2	LRT	0.0539225794950124
119	KC_AB1	CG42235	-0.121719540798344	0.882341583026737	1	pseudobulk	DESeq2	LRT	0.0543632528445627
119	KC_AB1	CG42237	4.68534789162203	0.0338149600254317	1	pseudobulk	DESeq2	LRT	1.4708911216423
119	KC_AB1	CG42238	-0.101614600649198	0.500191490275217	1	pseudobulk	DESeq2	LRT	0.300863701165998
119	KC_AB1	CG42239	1.73658870300863	0.169794959042132	1	pseudobulk	DESeq2	LRT	0.770075207452981
119	KC_AB1	CG42240	0.056514366382602	0.947313645957802	1	pseudobulk	DESeq2	LRT	0.0235062066814151
119	KC_AB1	CG42246	2.4138856564567	0.409611385497059	1	pseudobulk	DESeq2	LRT	0.387627980279722
119	KC_AB1	CG42249	-0.760959112540794	1	1	pseudobulk	DESeq2	LRT	0
119	KC_AB1	CG42258	-0.1892365428765	0.297595574677702	1	pseudobulk	DESeq2	LRT	0.526373531148148
119	KC_AB1	CG42259	0.949977091978715	0.53779387816678	1	pseudobulk	DESeq2	LRT	0.269384145760018
119	KC_AB1	CG42260	-0.2116718860864	0.169990070447499	1	pseudobulk	DESeq2	LRT	0.769576446126456
119	KC_AB1	CG42264	-0.396721808097649	1	1	pseudobulk	DESeq2	LRT	0
119	KC_AB1	CG42268	-0.242813003354012	0.249468121992027	1	pseudobulk	DESeq2	LRT	0.60298494233505
119	KC_AB1	CG42269	-1.43550613534661	0.602448798560473	1	pseudobulk	DESeq2	LRT	0.220079857379737
119	KC_AB1	CG42271	-0.116353208116351	0.854949768522531	1	pseudobulk	DESeq2	LRT	0.0680594009377147
119	KC_AB1	CG4230	-0.0506456292958021	0.828575678289071	1	pseudobulk	DESeq2	LRT	0.0816678189738505
119	KC_AB1	CG42302	1.11388934932283	1	1	pseudobulk	DESeq2	LRT	0
119	KC_AB1	CG42307	-0.686484777040423	0.708974175368212	1	pseudobulk	DESeq2	LRT	0.149369583856912
119	KC_AB1	CG42322	-0.100604032482532	0.68305604980306	1	pseudobulk	DESeq2	LRT	0.165543657780505
119	KC_AB1	CG42324	-0.130593844305611	0.643815002627025	1	pseudobulk	DESeq2	LRT	0.19123890729903
119	KC_AB1	CG42326	3.37429492540794	0.281278028870551	1	pseudobulk	DESeq2	LRT	0.550864190027974
119	KC_AB1	CG42327	-0.325131862337446	0.867719131485936	1	pseudobulk	DESeq2	LRT	0.0616208271106266
119	KC_AB1	CG42336	0.405757853294425	0.431024468496344	1	pseudobulk	DESeq2	LRT	0.365498075010237

119	KC_AB1	CG42337	0.0220360061318315	0.908774891914878	1	pseudobulk	DESeq2	LRT	0.0415436803714508
119	KC_AB1	CG42339	-0.196487829381789	0.460779706185211	1	pseudobulk	DESeq2	LRT	0.336506656507284
119	KC_AB1	CG42340	-0.31443442999509	0.571481937356039	1	pseudobulk	DESeq2	LRT	0.242997491656397
119	KC_AB1	CG42342	-0.521943899989716	0.288641487243771	1	pseudobulk	DESeq2	LRT	0.539641246402222
119	KC_AB1	CG42346	0.267939891077111	0.34772556064697	1	pseudobulk	DESeq2	LRT	0.458763383979031
119	KC_AB1	CG42354	0.719266940197495	0.699065588649202	1	pseudobulk	DESeq2	LRT	0.155482075395787
119	KC_AB1	CG42359	-0.0848899749207392	0.909436755082482	1	pseudobulk	DESeq2	LRT	0.0412274976711184
119	KC_AB1	CG42360	-0.0177871605470405	0.94068362478928	1	pseudobulk	DESeq2	LRT	0.0265564160107351
119	KC_AB1	CG42361	0.12373200721205	0.588769389893096	1	pseudobulk	DESeq2	LRT	0.230054777043664
119	KC_AB1	CG42364	3.32493825860261	0.146251142462174	1	pseudobulk	DESeq2	LRT	0.834900732677714
119	KC_AB1	CG42365	-0.646092448159359	0.530991142612113	1	pseudobulk	DESeq2	LRT	0.274912723262724
119	KC_AB1	CG42369	4.58085424419702	0.0980644594377514	1	pseudobulk	DESeq2	LRT	1.00848836129205
119	KC_AB1	CG42376	-0.200769784715171	0.631985795872338	1	pseudobulk	DESeq2	LRT	0.199292682546074
119	KC_AB1	CG4238	0.0297563227678754	0.933689398290625	1	pseudobulk	DESeq2	LRT	0.0297975724237548
119	KC_AB1	CG42382	0.323007173003343	0.768426951104391	1	pseudobulk	DESeq2	LRT	0.114397411495592
119	KC_AB1	CG4239	-0.344811180200083	0.707532301406322	1	pseudobulk	DESeq2	LRT	0.150253728238589
119	KC_AB1	CG42390	2.95597035741046	0.225995086366541	1	pseudobulk	DESeq2	LRT	0.645901003273377
120	KC_AB1	CG42391	-0.569432576450917	0.825325802164462	1	pseudobulk	DESeq2	LRT	0.0833745773279703
120	KC_AB1	CG42394	0.45787206225416	0.63761157437969	1	pseudobulk	DESeq2	LRT	0.195443807917455
120	KC_AB1	CG42399	0.156817684681086	0.895503552168549	1	pseudobulk	DESeq2	LRT	0.0479326871079255
120	KC_AB1	CG42402	-0.0418709764642109	0.909925106344606	1	pseudobulk	DESeq2	LRT	0.0409943518984592
120	KC_AB1	CG42404	-0.264435903813543	0.464353023505981	1	pseudobulk	DESeq2	LRT	0.333151722318622
120	KC_AB1	CG42445	-1.77067014871429	0.542156052888816	1	pseudobulk	DESeq2	LRT	0.265875689192671
120	KC_AB1	CG42450	-0.194048470963833	0.23098606413269	1	pseudobulk	DESeq2	LRT	0.636414221202412
120	KC_AB1	CG42489	0.137074306501724	1	1	pseudobulk	DESeq2	LRT	0
120	KC_AB1	CG42495	0.569638398106976	0.587372613930005	1	pseudobulk	DESeq2	LRT	0.231086306189697
120	KC_AB1	CG42500	-0.760959112540794	1	1	pseudobulk	DESeq2	LRT	0
120	KC_AB1	CG42514	1.26905628300702	0.535404324629871	1	pseudobulk	DESeq2	LRT	0.271318125201189
120	KC_AB1	CG42518	0.152115475746306	1	1	pseudobulk	DESeq2	LRT	0
120	KC_AB1	CG42524	0.296275065198765	1	1	pseudobulk	DESeq2	LRT	0
120	KC_AB1	CG42526	2.27122133462827	0.453795586678749	1	pseudobulk	DESeq2	LRT	0.343139732096712
120	KC_AB1	CG42540	-0.224203925599166	0.258620882437861	1	pseudobulk	DESeq2	LRT	0.587336410772807
120	KC_AB1	CG42541	-0.0745084939961749	0.624461364102684	1	pseudobulk	DESeq2	LRT	0.204494426587
120	KC_AB1	CG42542	-0.0850234716287794	0.843111880745565	1	pseudobulk	DESeq2	LRT	0.0741147907769181
120	KC_AB1	CG42553	0.817896530538267	0.495321304311856	1	pseudobulk	DESeq2	LRT	0.305112992134443
120	KC_AB1	CG42554	-0.344109580953055	0.673226357183091	1	pseudobulk	DESeq2	LRT	0.171838889494939
120	KC_AB1	CG42556	1.11388934932283	1	1	pseudobulk	DESeq2	LRT	0
120	KC_AB1	CG42562	1.80127577335365	0.482317890987887	1	pseudobulk	DESeq2	LRT	0.316666628160628
120	KC_AB1	CG42588	-0.175234342398536	0.720913424917991	1	pseudobulk	DESeq2	LRT	0.142116886928211
120	KC_AB1	CG4259	1.25804452752548	0.727552935095488	1	pseudobulk	DESeq2	LRT	0.138135402914536
120	KC_AB1	CG42594	0.428396471271059	0.675788788839294	1	pseudobulk	DESeq2	LRT	0.170189017334158
120	KC_AB1	CG42613	-0.127095022312473	0.398406309466556	1	pseudobulk	DESeq2	LRT	0.399673792374817
120	KC_AB1	CG42656	1.11388934932283	1	1	pseudobulk	DESeq2	LRT	0
120	KC_AB1	CG4266	-0.221965756882554	0.638952851671418	1	pseudobulk	DESeq2	LRT	0.194531187247526
120	KC_AB1	CG42663	0.807925200609904	0.638322389996672	1	pseudobulk	DESeq2	LRT	0.194959921853347
120	KC_AB1	CG42668	0.126820767253543	0.550285224518214	1	pseudobulk	DESeq2	LRT	0.25941214809445
120	KC_AB1	CG42671	0.221837403755476	0.677414987915373	1	pseudobulk	DESeq2	LRT	0.169145198749946
120	KC_AB1	CG42672	-0.345055719047591	0.631041334912059	1	pseudobulk	DESeq2	LRT	0.199942192362415
120	KC_AB1	CG42673	0.261147483792215	0.379292673444173	1	pseudobulk	DESeq2	LRT	0.421025546207038
120	KC_AB1	CG42674	-0.513575814835815	0.284790421149286	1	pseudobulk	DESeq2	LRT	0.545474622169902
120	KC_AB1	CG42675	0.943798956650056	0.807634491832869	1	pseudobulk	DESeq2	LRT	0.0927851418175362
120	KC_AB1	CG42676	-0.52171349645397	0.461370277792551	1	pseudobulk	DESeq2	LRT	0.335950386855366
120	KC_AB1	CG4269	-0.760959112540794	1	1	pseudobulk	DESeq2	LRT	0
120	KC_AB1	CG42692	-0.967142463471365	0.620452767258733	1	pseudobulk	DESeq2	LRT	0.207291274116361
120	KC_AB1	CG42697	1.11388934932283	1	1	pseudobulk	DESeq2	LRT	0
120	KC_AB1	CG42699	-0.331268367283011	0.479608894400352	1	pseudobulk	DESeq2	LRT	0.31911277145898

120	KC_AB1	CG42700	0.0294356578749646	0.903923059696164	1	pseudobulk	DESeq2	LRT	0.0438685343160671
120	KC_AB1	CG42709	0.0841507911092239	0.894237842180901	1	pseudobulk	DESeq2	LRT	0.0485469556907509
120	KC_AB1	CG42711	4.69601879312554	0.0866214174418427	1	pseudobulk	DESeq2	LRT	1.06237471390872
120	KC_AB1	CG42724	-0.158586710674105	0.661086997450976	1	pseudobulk	DESeq2	LRT	0.179741384649389
120	KC_AB1	CG42741	3.52278680896166	0.181142264725337	1	pseudobulk	DESeq2	LRT	0.741980206806527
120	KC_AB1	CG42747	-0.488454231205858	0.800108174944632	1	pseudobulk	DESeq2	LRT	0.0968512922511212
120	KC_AB1	CG42748	0.107285257141535	0.585223445716355	1	pseudobulk	DESeq2	LRT	0.232678283125559
120	KC_AB1	CG42749	0.9057111034072	0.728478230736048	1	pseudobulk	DESeq2	LRT	0.137583421810903
120	KC_AB1	CG42750	-0.0322843963689254	0.890788543469573	1	pseudobulk	DESeq2	LRT	0.050225377111665
120	KC_AB1	CG42762	-0.760959112540794	0.805265236978025	1	pseudobulk	DESeq2	LRT	0.0940610488455931
120	KC_AB1	CG42763	-0.843114884391027	0.727032647709573	1	pseudobulk	DESeq2	LRT	0.138446086525063
120	KC_AB1	CG42766	2.4138856564567	0.409611385497059	1	pseudobulk	DESeq2	LRT	0.387627980279722
120	KC_AB1	CG4278	0.220454670209132	0.794574183934431	1	pseudobulk	DESeq2	LRT	0.0998655494706343
120	KC_AB1	CG42784	-0.145318897947147	0.511638195274788	1	pseudobulk	DESeq2	LRT	0.291037041640825
120	KC_AB1	CG42788	-0.241316214585059	0.389583772392471	1	pseudobulk	DESeq2	LRT	0.409399141390908
120	KC_AB1	CG42789	1.67020383217772	0.598754457572587	1	pseudobulk	DESeq2	LRT	0.222751240354882
120	KC_AB1	CG42795	-0.252889213365349	0.225484733178928	1	pseudobulk	DESeq2	LRT	0.646882857425047
120	KC_AB1	CG42797	0.273675721850226	0.618195021218961	1	pseudobulk	DESeq2	LRT	0.208874496952816
120	KC_AB1	CG42806	-0.427522082851143	0.547263744173835	1	pseudobulk	DESeq2	LRT	0.261803322598952
120	KC_AB1	CG42807	4.45565821955857	0.111691140174007	1	pseudobulk	DESeq2	LRT	0.951981275641296
120	KC_AB1	CG42809	-1.41796165839591	0.141862353367642	1	pseudobulk	DESeq2	LRT	0.84813283987658
120	KC_AB1	CG4281	0.0663097004105115	0.901884926425535	1	pseudobulk	DESeq2	LRT	0.0448488715562095
120	KC_AB1	CG42810	-0.760959112540794	1	1	pseudobulk	DESeq2	LRT	0
120	KC_AB1	CG42813	-0.258008641367765	0.528198557728833	1	pseudobulk	DESeq2	LRT	0.277202788989118
120	KC_AB1	CG4282	-1.03867812003717	0.362897508118064	1	pseudobulk	DESeq2	LRT	0.44021601392755
120	KC_AB1	CG42825	1.11388934932283	1	1	pseudobulk	DESeq2	LRT	0
120	KC_AB1	CG42846	-0.760959112540794	1	1	pseudobulk	DESeq2	LRT	0
120	KC_AB1	CG42855	0.581932459321301	0.706580429790591	1	pseudobulk	DESeq2	LRT	0.150838395420359
120	KC_AB1	CG4287	0.0225939607522036	0.945227500894208	1	pseudobulk	DESeq2	LRT	0.0244636512875995
120	KC_AB1	CG4291	-0.268223343717534	0.715864124139755	1	pseudobulk	DESeq2	LRT	0.145169401904764
120	KC_AB1	CG4293	0.0925114528230854	0.928304784448005	1	pseudobulk	DESeq2	LRT	0.0323094112034656
120	KC_AB1	CG4294	-0.060607319379222	0.881852042760065	1	pseudobulk	DESeq2	LRT	0.0546042747356229
120	KC_AB1	CG4301	2.58743201691345	0.417636627932844	1	pseudobulk	DESeq2	LRT	0.379201419460921
120	KC_AB1	CG43049	-2.13621400928779	0.457001334179545	1	pseudobulk	DESeq2	LRT	0.340082532039628
120	KC_AB1	CG43051	-0.377193558252637	0.82538328898947	1	pseudobulk	DESeq2	LRT	0.0833443282537675
120	KC_AB1	CG43055	-1.77067014871429	0.542156052888816	1	pseudobulk	DESeq2	LRT	0.265875689192671
120	KC_AB1	CG43066	0.0347322835650437	0.912642165890851	1	pseudobulk	DESeq2	LRT	0.0396994698273514
120	KC_AB1	CG43074	-0.760959112540794	0.805265236978025	1	pseudobulk	DESeq2	LRT	0.0940610488455931
120	KC_AB1	CG43078	0.124010009662819	0.722666060582945	1	pseudobulk	DESeq2	LRT	0.141062341086145
120	KC_AB1	CG43085	1.13658746815362	0.691887308070876	1	pseudobulk	DESeq2	LRT	0.159964635988989
120	KC_AB1	CG43091	-2.41876763198367	0.438413379977707	1	pseudobulk	DESeq2	LRT	0.358116200048746
120	KC_AB1	CG43092	-2.41876763198367	0.438413379977707	1	pseudobulk	DESeq2	LRT	0.358116200048746
120	KC_AB1	CG43093	-3.22129287111021	0.293514748994408	1	pseudobulk	DESeq2	LRT	0.532370070750446
120	KC_AB1	CG43102	-0.260867681720809	0.263111557364563	1	pseudobulk	DESeq2	LRT	0.579860074797553
120	KC_AB1	CG43103	1.11471421835543	0.626302681986126	1	pseudobulk	DESeq2	LRT	0.203215728536149
120	KC_AB1	CG43107	-1.30943155764665	0.665865564455012	1	pseudobulk	DESeq2	LRT	0.176613444266292
120	KC_AB1	CG43117	0.614013856580899	0.721615659068091	1	pseudobulk	DESeq2	LRT	0.141694051168614
120	KC_AB1	CG4313	-0.704766371521546	0.665171429826296	1	pseudobulk	DESeq2	LRT	0.177066412692524
120	KC_AB1	CG43155	-0.760959112540794	0.805265236978025	1	pseudobulk	DESeq2	LRT	0.0940610488455931
120	KC_AB1	CG43163	0.296275065198765	1	1	pseudobulk	DESeq2	LRT	0
120	KC_AB1	CG43191	-0.760959112540794	1	1	pseudobulk	DESeq2	LRT	0
120	KC_AB1	CG43219	1.5226431882713	0.415383456081278	1	pseudobulk	DESeq2	LRT	0.381550804564736
120	KC_AB1	CG43222	0.316515566929995	0.290920814593709	1	pseudobulk	DESeq2	LRT	0.536225205051319
120	KC_AB1	CG43232	1.25804452752548	0.727552935095488	1	pseudobulk	DESeq2	LRT	0.138135402914536
120	KC_AB1	CG43236	5.74251229139202	0.167654972946523	1	pseudobulk	DESeq2	LRT	0.775583560092586
120	KC_AB1	CG43267	1.25804452752548	0.727552935095488	1	pseudobulk	DESeq2	LRT	0.138135402914536

120	KC_AB1	CG43273	2.4460746975579	0.337865831265829	1	pseudobulk	DESeq2	LRT	0.471255726671495
120	KC_AB1	CG43293	1.25804452752548	0.573436424985144	1	pseudobulk	DESeq2	LRT	0.241514723898045
120	KC_AB1	CG43313	0.296275065198765	1	1	pseudobulk	DESeq2	LRT	0
120	KC_AB1	CG43317	-0.760959112540794	0.805265236978025	1	pseudobulk	DESeq2	LRT	0.0940610488455931
120	KC_AB1	CG4332	0.939481058652844	0.479395500011608	1	pseudobulk	DESeq2	LRT	0.319306046917508
121	KC_AB1	CG43322	0.761614969214348	0.643593923962231	1	pseudobulk	DESeq2	LRT	0.191388064638469
121	KC_AB1	CG43324	0.0903297900073405	0.566810622166089	1	pseudobulk	DESeq2	LRT	0.246562019566264
121	KC_AB1	CG43341	1.09694335164528	0.595638420134218	1	pseudobulk	DESeq2	LRT	0.225017296960592
121	KC_AB1	CG43342	-1.4358639907461	0.442454635269018	1	pseudobulk	DESeq2	LRT	0.354131250756678
121	KC_AB1	CG43347	0.0132811524528475	0.954672631228594	1	pseudobulk	DESeq2	LRT	0.0201455277092914
121	KC_AB1	CG4335	1.13988912687578	0.653854063433791	1	pseudobulk	DESeq2	LRT	0.184519172956856
121	KC_AB1	CG43367	0.0192846904699044	0.969508782046176	1	pseudobulk	DESeq2	LRT	0.013448252622491
121	KC_AB1	CG43373	0.158134295058976	0.521079528215636	1	pseudobulk	DESeq2	LRT	0.283095988736192
121	KC_AB1	CG4338	-0.27865990124743	0.715600315253949	1	pseudobulk	DESeq2	LRT	0.145329476778476
121	KC_AB1	CG43394	-0.00396295911251939	0.933243054546409	1	pseudobulk	DESeq2	LRT	0.0300052335295906
121	KC_AB1	CG43396	0.152115475746305	1	1	pseudobulk	DESeq2	LRT	0
121	KC_AB1	CG4341	0.0483771643825897	0.903026122179155	1	pseudobulk	DESeq2	LRT	0.0442996865035768
121	KC_AB1	CG43428	0.0955131218172105	0.939378068076343	1	pseudobulk	DESeq2	LRT	0.0271595836296821
121	KC_AB1	CG4360	-0.389684147576413	0.597624507458161	1	pseudobulk	DESeq2	LRT	0.223571601226781
121	KC_AB1	CG4364	0.0293759050865391	0.899692525094967	1	pseudobulk	DESeq2	LRT	0.0459058877496581
121	KC_AB1	CG43647	-0.760959112540794	0.805265236978025	1	pseudobulk	DESeq2	LRT	0.0940610488455931
121	KC_AB1	CG43658	0.897242408364078	0.36645858209181	1	pseudobulk	DESeq2	LRT	0.435975103111357
121	KC_AB1	CG43693	0.0493179803763964	0.938549530865104	1	pseudobulk	DESeq2	LRT	0.0275428030492623
121	KC_AB1	CG43707	-0.425388815319096	0.343498030255608	1	pseudobulk	DESeq2	LRT	0.464075749002023
121	KC_AB1	CG43736	-0.0612692582330587	0.778348015410284	1	pseudobulk	DESeq2	LRT	0.108826177588585
121	KC_AB1	CG43737	0.0540415899335757	0.759301856161335	1	pseudobulk	DESeq2	LRT	0.1195855384605
121	KC_AB1	CG43740	0.44731244841668	0.590317024060229	1	pseudobulk	DESeq2	LRT	0.228914692047641
121	KC_AB1	CG43755	1.25804452752548	0.573436424985144	1	pseudobulk	DESeq2	LRT	0.241514723898045
121	KC_AB1	CG43759	0.025883104359201	0.915359785426748	1	pseudobulk	DESeq2	LRT	0.0384081713831511
121	KC_AB1	CG43778	-0.0539673930895625	0.846036533385997	1	pseudobulk	DESeq2	LRT	0.0726108829357138
121	KC_AB1	CG43779	1.66148392095495	0.561456215207444	1	pseudobulk	DESeq2	LRT	0.250684106250671
121	KC_AB1	CG43783	-0.0123927445813111	0.959192933930979	1	pseudobulk	DESeq2	LRT	0.0180940292066306
121	KC_AB1	CG43788	-0.760959112540794	0.804334723897681	1	pseudobulk	DESeq2	LRT	0.0945631819854715
121	KC_AB1	CG43789	-1.11000032652526	0.814275384621196	1	pseudobulk	DESeq2	LRT	0.0892286936423418
121	KC_AB1	CG43795	1.25804452752548	0.727552935095488	1	pseudobulk	DESeq2	LRT	0.138135402914536
121	KC_AB1	CG43799	1.25804452752548	0.727552935095488	1	pseudobulk	DESeq2	LRT	0.138135402914536
121	KC_AB1	CG43800	-0.52368574550482	0.70490908137215	1	pseudobulk	DESeq2	LRT	0.151866894362743
121	KC_AB1	CG43813	-0.760959112540794	0.805265236978025	1	pseudobulk	DESeq2	LRT	0.0940610488455931
121	KC_AB1	CG43843	-2.89515599797543	0.274544979992475	1	pseudobulk	DESeq2	LRT	0.561386492881572
121	KC_AB1	CG43845	-0.62846884635387	0.770813910320913	1	pseudobulk	DESeq2	LRT	0.113050456547587
121	KC_AB1	CG43850	-0.258968868869343	0.599897536613513	1	pseudobulk	DESeq2	LRT	0.22192292142203
121	KC_AB1	CG43861	-0.526376839681175	0.150440150041913	1	pseudobulk	DESeq2	LRT	0.822636242104582
121	KC_AB1	CG43867	-0.111350007594117	0.595835904662062	1	pseudobulk	DESeq2	LRT	0.224873330045479
121	KC_AB1	CG43897	-2.77318649358755	0.304421239040066	1	pseudobulk	DESeq2	LRT	0.516525050731551
121	KC_AB1	CG4390	0.0727912801030364	0.892925400416821	1	pseudobulk	DESeq2	LRT	0.0491848227919068
121	KC_AB1	CG43901	-0.0941061394565032	0.793872871065887	1	pseudobulk	DESeq2	LRT	0.100249038901683
121	KC_AB1	CG43902	-1.63619566506195	0.255253524458564	1	pseudobulk	DESeq2	LRT	0.593028252601136
121	KC_AB1	CG43925	0.254196856837688	0.890998468402047	1	pseudobulk	DESeq2	LRT	0.0501230425008484
121	KC_AB1	CG4393	0.0645990499456189	0.923254977515583	1	pseudobulk	DESeq2	LRT	0.0346783422724626
121	KC_AB1	CG43980	-0.760959112540794	0.805265236978025	1	pseudobulk	DESeq2	LRT	0.0940610488455931
121	KC_AB1	CG44002	-0.731830846713147	0.652210243478249	1	pseudobulk	DESeq2	LRT	0.185612384537814
121	KC_AB1	CG44006	-0.760959112540794	1	1	pseudobulk	DESeq2	LRT	0
121	KC_AB1	CG44008	-1.69030812397019	0.43179876096644	1	pseudobulk	DESeq2	LRT	0.364718608194956
121	KC_AB1	CG4404	-0.135243683131171	0.749083952778085	1	pseudobulk	DESeq2	LRT	0.125469506486026
121	KC_AB1	CG4407	0.327231067704956	0.646815857411338	1	pseudobulk	DESeq2	LRT	0.189219341422065
121	KC_AB1	CG44085	1.25804452752548	0.573436424985144	1	pseudobulk	DESeq2	LRT	0.241514723898045

121	KC_AB1	CG4409	-0.128182564962674	0.692683897314196	1	pseudobulk	DESeq2	LRT	0.159464908203892
121	KC_AB1	CG44098	2.69781908144165	0.281090649306432	1	pseudobulk	DESeq2	LRT	0.551153601287484
121	KC_AB1	CG44102	-0.760959112540794	0.805265236978025	1	pseudobulk	DESeq2	LRT	0.0940610488455931
121	KC_AB1	CG44153	0.412964613102417	0.222144618134382	1	pseudobulk	DESeq2	LRT	0.653364203885423
121	KC_AB1	CG44158	-0.0898816554815313	0.905736266496574	1	pseudobulk	DESeq2	LRT	0.0429982423665398
121	KC_AB1	CG44195	1.11388934932283	1	1	pseudobulk	DESeq2	LRT	0
121	KC_AB1	CG4424	-1.39815810870357	0.267546326039814	1	pseudobulk	DESeq2	LRT	0.57260100840339
121	KC_AB1	CG44242	0.148194696092237	0.56971428082272	1	pseudobulk	DESeq2	LRT	0.24434289410349
121	KC_AB1	CG44243	0.846192514155193	0.393338231228291	1	pseudobulk	DESeq2	LRT	0.40523383949275
121	KC_AB1	CG44245	0.719266940197495	0.699065588649202	1	pseudobulk	DESeq2	LRT	0.155482075395787
121	KC_AB1	CG44247	-0.246422917983444	0.43025020102548	1	pseudobulk	DESeq2	LRT	0.366278918085268
121	KC_AB1	CG44249	0.359026488718433	0.847265072367733	1	pseudobulk	DESeq2	LRT	0.0719806965679971
121	KC_AB1	CG44250	2.09987857572813	0.503115176013642	1	pseudobulk	DESeq2	LRT	0.29833258237694
121	KC_AB1	CG44261	-0.760959112540794	0.805265236978025	1	pseudobulk	DESeq2	LRT	0.0940610488455931
121	KC_AB1	CG44296	-1.30678381920874	0.666522996268782	1	pseudobulk	DESeq2	LRT	0.176184862033548
121	KC_AB1	CG44325	0.981743649296881	0.33266007319668	1	pseudobulk	DESeq2	LRT	0.477999321255968
121	KC_AB1	CG4438	-0.576936076780698	0.707314744464879	1	pseudobulk	DESeq2	LRT	0.150387288654449
121	KC_AB1	CG44422	0.0286799801620792	0.962633647464508	1	pseudobulk	DESeq2	LRT	0.0165389622589343
121	KC_AB1	CG44437	0.293544814793053	0.855484935116898	1	pseudobulk	DESeq2	LRT	0.0677876338764847
121	KC_AB1	CG4452	0.353600823770186	0.541572392251215	1	pseudobulk	DESeq2	LRT	0.266343482798825
121	KC_AB1	CG4455	0.883494273274129	0.71183745910665	1	pseudobulk	DESeq2	LRT	0.147619161801035
121	KC_AB1	CG4459	0.296275065198765	1	1	pseudobulk	DESeq2	LRT	0
121	KC_AB1	CG4462	-0.760959112540794	1	1	pseudobulk	DESeq2	LRT	0
121	KC_AB1	CG4467	-0.216686361880485	0.120372675803737	1	pseudobulk	DESeq2	LRT	0.919472085290933
121	KC_AB1	CG4476	1.11388934932283	1	1	pseudobulk	DESeq2	LRT	0
121	KC_AB1	CG44774	0.0995400833675602	0.639188349469689	1	pseudobulk	DESeq2	LRT	0.194371149537521
121	KC_AB1	CG44815	-1.31431541683768	0.746522178624388	1	pseudobulk	DESeq2	LRT	0.12695728517793
121	KC_AB1	CG44837	0.709686956006578	0.915714550115026	1	pseudobulk	DESeq2	LRT	0.0382398850985033
121	KC_AB1	CG44838	-0.267586635956112	0.301566272647466	1	pseudobulk	DESeq2	LRT	0.520617231841264
121	KC_AB1	CG4496	3.58333718222294	0.241420367068608	1	pseudobulk	DESeq2	LRT	0.617226094087932
121	KC_AB1	CG4497	-0.439700820772335	0.580085172518955	1	pseudobulk	DESeq2	LRT	0.236508235334846
121	KC_AB1	CG45002	-0.0347706756685908	0.946675680640581	1	pseudobulk	DESeq2	LRT	0.0237987794256244
121	KC_AB1	CG4502	-0.201298719492964	0.238781172232444	1	pseudobulk	DESeq2	LRT	0.621999920080004
121	KC_AB1	CG45049	0.0601611463778228	0.782559504856573	1	pseudobulk	DESeq2	LRT	0.10648262931083
121	KC_AB1	CG45050	-0.0656026062393744	0.716401496940228	1	pseudobulk	DESeq2	LRT	0.144843515352038
121	KC_AB1	CG45067	-0.760959112540794	0.804334723897681	1	pseudobulk	DESeq2	LRT	0.0945631819854715
121	KC_AB1	CG45071	-0.33314612541202	0.530575640523563	1	pseudobulk	DESeq2	LRT	0.275252692968555
121	KC_AB1	CG45081	0.296275065198765	1	1	pseudobulk	DESeq2	LRT	0
121	KC_AB1	CG4511	-0.247867203014299	0.732100818653077	1	pseudobulk	DESeq2	LRT	0.135429107507565
121	KC_AB1	CG45218	1.39934341023393	0.63869615168108	1	pseudobulk	DESeq2	LRT	0.194705700577112
121	KC_AB1	CG45263	-0.280764326623998	0.117278082815691	1	pseudobulk	DESeq2	LRT	0.930783142203047
121	KC_AB1	CG4537	-0.811116343061765	0.534701402211675	1	pseudobulk	DESeq2	LRT	0.271888676995612
121	KC_AB1	CG4538	-0.429906377022176	0.483122581832799	1	pseudobulk	DESeq2	LRT	0.315942662500704
121	KC_AB1	CG4552	-0.416022244871399	0.549509181167377	1	pseudobulk	DESeq2	LRT	0.260025047012976
121	KC_AB1	CG4553	-0.606522227936007	0.700918639316185	1	pseudobulk	DESeq2	LRT	0.15433239080183
121	KC_AB1	CG4554	-0.713971960584339	0.760574873905167	1	pseudobulk	DESeq2	LRT	0.118858025893382
121	KC_AB1	CG4557	-0.313871386681804	0.718627309609599	1	pseudobulk	DESeq2	LRT	0.14349628255666
121	KC_AB1	CG4558	0.902473116302646	0.207519550800699	1	pseudobulk	DESeq2	LRT	0.682940981338007
121	KC_AB1	CG4562	-0.890378709757347	0.174154734772882	1	pseudobulk	DESeq2	LRT	0.759064713811067
122	KC_AB1	CG4565	-0.411752043544056	0.835902280270588	1	pseudobulk	DESeq2	LRT	0.0778444900487799
122	KC_AB1	CG45676	2.4460746975579	0.337865831265829	1	pseudobulk	DESeq2	LRT	0.471255726671495
122	KC_AB1	CG4572	1.36653252487245	0.362001249115361	1	pseudobulk	DESeq2	LRT	0.441289930895087
122	KC_AB1	CG4577	-0.169066153540618	0.377605054755594	1	pseudobulk	DESeq2	LRT	0.422962200714624
122	KC_AB1	CG4587	-0.129769920004157	0.466509373103152	1	pseudobulk	DESeq2	LRT	0.331139625988043
122	KC_AB1	CG4592	2.58743201691345	0.417636627932844	1	pseudobulk	DESeq2	LRT	0.379201419460921
122	KC_AB1	CG4593	-0.446129533331396	0.518856810856217	1	pseudobulk	DESeq2	LRT	0.284952478057146

122	KC_AB1	CG4594	1.11388934932283	1	1	pseudobulk	DESeq2	LRT	0
122	KC_AB1	CG4596	0.0691416148133287	0.857024633055984	1	pseudobulk	DESeq2	LRT	0.0670066951753537
122	KC_AB1	CG4598	1.2214138316321	0.183469802157826	1	pseudobulk	DESeq2	LRT	0.736435407354456
122	KC_AB1	CG4603	0.0588643195907516	0.910833817239299	1	pseudobulk	DESeq2	LRT	0.0405608533667585
122	KC_AB1	CG4607	-1.60958773650785	0.570513631639934	1	pseudobulk	DESeq2	LRT	0.243733974251419
122	KC_AB1	CG4610	-0.41108798428629	0.770374239816432	1	pseudobulk	DESeq2	LRT	0.1132982478106
122	KC_AB1	CG4611	0.189735967335765	0.881944901594433	1	pseudobulk	DESeq2	LRT	0.0545585460257189
122	KC_AB1	CG4612	-0.247967907224013	0.158019245346781	1	pseudobulk	DESeq2	LRT	0.801290016596752
122	KC_AB1	CG4615	0.140511508451576	0.809174459152398	1	pseudobulk	DESeq2	LRT	0.0919578337907943
122	KC_AB1	CG4617	-0.0708016183154698	0.903007389015604	1	pseudobulk	DESeq2	LRT	0.0443086959815391
122	KC_AB1	CG4619	0.995874702702384	0.467210584415943	1	pseudobulk	DESeq2	LRT	0.330487327065671
122	KC_AB1	CG4622	0.392338793295052	0.55243025007249	1	pseudobulk	DESeq2	LRT	0.257722548268892
122	KC_AB1	CG4627	-0.524821125766217	0.511709123389671	1	pseudobulk	DESeq2	LRT	0.290976839814147
122	KC_AB1	CG46280	-0.238474209812985	0.578403591472764	1	pseudobulk	DESeq2	LRT	0.237769019046927
122	KC_AB1	CG46281	-0.659105826146651	0.60092448073457	1	pseudobulk	DESeq2	LRT	0.221180103140412
122	KC_AB1	CG4629	0.130661214321197	0.774332416890432	1	pseudobulk	DESeq2	LRT	0.111072558918132
122	KC_AB1	CG4630	1.89988258304539	0.501753467924434	1	pseudobulk	DESeq2	LRT	0.299509617155892
122	KC_AB1	CG46301	0.0640437306982205	0.853283738153222	1	pseudobulk	DESeq2	LRT	0.0689065310550659
122	KC_AB1	CG46304	1.25804452752548	0.727552935095488	1	pseudobulk	DESeq2	LRT	0.138135402914536
122	KC_AB1	CG4631	-1.64386070186712	0.29487016188623	1	pseudobulk	DESeq2	LRT	0.530369171781127
122	KC_AB1	CG46338	0.166570088373513	0.667803136209509	1	pseudobulk	DESeq2	LRT	0.175351545696015
122	KC_AB1	CG46339	-0.564484868110028	0.0359137058234201	1	pseudobulk	DESeq2	LRT	1.44473977906708
122	KC_AB1	CG46385	-0.210036020619654	0.445828011176626	1	pseudobulk	DESeq2	LRT	0.350832648468458
122	KC_AB1	CG46387	0.943805700130147	0.808046339172447	1	pseudobulk	DESeq2	LRT	0.0925637329508755
122	KC_AB1	CG46399	1.11388934932283	1	1	pseudobulk	DESeq2	LRT	0
122	KC_AB1	CG46448	-0.403113036875962	0.813769315244996	1	pseudobulk	DESeq2	LRT	0.0894986900922433
122	KC_AB1	CG4645	1.15844887443264	0.0844434018758742	1	pseudobulk	DESeq2	LRT	1.0734342790811
122	KC_AB1	CG4646	0.0203041214015418	0.957397628399371	1	pseudobulk	DESeq2	LRT	0.0189076526576444
122	KC_AB1	CG4653	-0.760959112540794	0.804334723897681	1	pseudobulk	DESeq2	LRT	0.0945631819854715
122	KC_AB1	CG4658	-0.389644798535301	0.225235112028285	1	pseudobulk	DESeq2	LRT	0.647363906132031
122	KC_AB1	CG4660	0.159154940941033	0.603273252006386	1	pseudobulk	DESeq2	LRT	0.219485930051658
122	KC_AB1	CG4661	-0.357486815143958	0.831537302364907	1	pseudobulk	DESeq2	LRT	0.0801182637651249
122	KC_AB1	CG4676	0.476096751586624	0.522266610239585	1	pseudobulk	DESeq2	LRT	0.282107738766169
122	KC_AB1	CG4678	-0.0826746628625548	0.863945545857913	1	pseudobulk	DESeq2	LRT	0.0635136300659067
122	KC_AB1	CG4679	0.799566010024493	0.345633519226274	1	pseudobulk	DESeq2	LRT	0.461384146664462
122	KC_AB1	CG4686	0.0176882981419403	0.933145302453124	1	pseudobulk	DESeq2	LRT	0.0300507258780483
122	KC_AB1	CG4704	1.25804452752548	0.573436424985144	1	pseudobulk	DESeq2	LRT	0.241514723898045
122	KC_AB1	CG4707	0.13883031301183	0.906600456464526	1	pseudobulk	DESeq2	LRT	0.0425840666142216
122	KC_AB1	CG4709	-1.32513966923614	0.360894115374712	1	pseudobulk	DESeq2	LRT	0.442620199351206
122	KC_AB1	CG4730	0.847525847835988	0.691106783674185	1	pseudobulk	DESeq2	LRT	0.16045484412001
122	KC_AB1	CG4743	0.95910478124215	0.451488214664919	1	pseudobulk	DESeq2	LRT	0.345353581724329
122	KC_AB1	CG4751	-0.380425446824178	0.678467484081369	1	pseudobulk	DESeq2	LRT	0.168470961301841
122	KC_AB1	CG4752	3.88868554264359	0.118962980968666	1	pseudobulk	DESeq2	LRT	0.924588161819474
122	KC_AB1	CG4757	3.37429492540794	0.281278028870551	1	pseudobulk	DESeq2	LRT	0.550864190027974
122	KC_AB1	CG4768	0.22343383190995	0.543667727065298	1	pseudobulk	DESeq2	LRT	0.26466644660736
122	KC_AB1	CG4788	0.569529417287972	0.413687698861638	1	pseudobulk	DESeq2	LRT	0.383327392800255
122	KC_AB1	CG4789	0.226604940943965	0.567226113822534	1	pseudobulk	DESeq2	LRT	0.246243783417435
122	KC_AB1	CG4797	1.67020383217772	0.598754457572587	1	pseudobulk	DESeq2	LRT	0.222751240354882
122	KC_AB1	CG4806	0.741032937234257	0.247521540088366	1	pseudobulk	DESeq2	LRT	0.606387001439641
122	KC_AB1	CG4813	-0.602236383050668	0.597325744266534	1	pseudobulk	DESeq2	LRT	0.22378876710132
122	KC_AB1	CG4820	-0.864209511715103	0.428852054371129	1	pseudobulk	DESeq2	LRT	0.367692505138836
122	KC_AB1	CG4825	-0.0559019589276888	0.924596306379141	1	pseudobulk	DESeq2	LRT	0.0340478458345167
122	KC_AB1	CG4829	-0.402637844669718	0.534935466733949	1	pseudobulk	DESeq2	LRT	0.271698607010167
122	KC_AB1	CG4842	1.25804452752548	0.573436424985144	1	pseudobulk	DESeq2	LRT	0.241514723898045
122	KC_AB1	CG4847	-0.760959112540794	1	1	pseudobulk	DESeq2	LRT	0
122	KC_AB1	CG4849	0.905984500227007	0.487764527744493	1	pseudobulk	DESeq2	LRT	0.311789786566336

122	KC_AB1	CG4853	-0.0917551226156182	0.85876440815404	1	pseudobulk	DESeq2	LRT	0.0661259633771662
122	KC_AB1	CG4854	0.680704633252605	0.635962739698485	1	pseudobulk	DESeq2	LRT	0.196568328404059
122	KC_AB1	CG4858	-0.363841408799401	0.557064693539926	1	pseudobulk	DESeq2	LRT	0.254094366018545
122	KC_AB1	CG4866	-0.0509614323591569	0.92080119592682	1	pseudobulk	DESeq2	LRT	0.0358341253208347
122	KC_AB1	CG4872	1.01690356025801	0.621073425118329	1	pseudobulk	DESeq2	LRT	0.206857053223266
122	KC_AB1	CG4880	2.46450052269655	0.253507764940297	1	pseudobulk	DESeq2	LRT	0.59600873369129
122	KC_AB1	CG4882	-0.439771220594217	0.715925917294073	1	pseudobulk	DESeq2	LRT	0.145131915366683
122	KC_AB1	CG4887	-0.0766319297859533	0.880334925879572	1	pseudobulk	DESeq2	LRT	0.0553520678632667
122	KC_AB1	CG4896	0.152298632922924	0.75132156411562	1	pseudobulk	DESeq2	LRT	0.124174146043748
122	KC_AB1	CG4901	0.520642893835083	0.6843054212264	1	pseudobulk	DESeq2	LRT	0.164750019408359
122	KC_AB1	CG4908	-0.164972642710553	0.834316854587551	1	pseudobulk	DESeq2	LRT	0.0786689828569897
122	KC_AB1	CG4911	-0.0218210750376601	0.93572501062749	1	pseudobulk	DESeq2	LRT	0.0288517622808684
122	KC_AB1	CG4928	-0.546071868931801	0.867512976428594	1	pseudobulk	DESeq2	LRT	0.0617240202267904
122	KC_AB1	CG4935	-0.729029161901678	0.483891256219823	1	pseudobulk	DESeq2	LRT	0.315252225400759
122	KC_AB1	CG4936	-1.06391384484042	0.1715253639034	1	pseudobulk	DESeq2	LRT	0.765671650613586
122	KC_AB1	CG4942	0.44483354384671	0.785668144147546	1	pseudobulk	DESeq2	LRT	0.104760855486145
122	KC_AB1	CG4949	2.28670213262649	0.175824729477126	1	pseudobulk	DESeq2	LRT	0.754920042116926
122	KC_AB1	CG4950	2.09987857572813	0.503115176013642	1	pseudobulk	DESeq2	LRT	0.29833258237694
122	KC_AB1	CG4951	1.55701404371006	0.126235412487242	1	pseudobulk	DESeq2	LRT	0.898818796514863
122	KC_AB1	CG4953	-0.194735910343471	0.818870481168685	1	pseudobulk	DESeq2	LRT	0.0867847841515712
122	KC_AB1	CG4957	0.249723771453582	0.73728279112896	1	pseudobulk	DESeq2	LRT	0.132365902818661
122	KC_AB1	CG4968	0.552493138341005	0.209815072609693	1	pseudobulk	DESeq2	LRT	0.67816331635176
122	KC_AB1	CG4972	-0.475169450975996	0.566493712232233	1	pseudobulk	DESeq2	LRT	0.246804906203054
122	KC_AB1	CG5001	3.01175963014276	0.14696244435794	1	pseudobulk	DESeq2	LRT	0.832793633224182
122	KC_AB1	CG5003	0.440736239328437	0.576876101988355	1	pseudobulk	DESeq2	LRT	0.23891745201007
122	KC_AB1	CG5004	0.0824817194643503	0.884241616294713	1	pseudobulk	DESeq2	LRT	0.0534290491430719
122	KC_AB1	CG5009	0.497156217270963	0.555499326861024	1	pseudobulk	DESeq2	LRT	0.255316462989036
122	KC_AB1	CG5013	0.055826750325224	0.934518686235559	1	pseudobulk	DESeq2	LRT	0.0294120102317999
122	KC_AB1	CG5021	0.492432380876145	0.260799617205367	1	pseudobulk	DESeq2	LRT	0.583693050385358
122	KC_AB1	CG5022	0.0452106778771016	0.933581702265917	1	pseudobulk	DESeq2	LRT	0.0298476688337421
122	KC_AB1	CG5027	-0.0399232959345679	0.932904265269811	1	pseudobulk	DESeq2	LRT	0.0301629213106234
122	KC_AB1	CG5028	0.0315165885373417	0.898770489307974	1	pseudobulk	DESeq2	LRT	0.0463511958669614
122	KC_AB1	CG5033	-1.45627569616489	0.300693732132515	1	pseudobulk	DESeq2	LRT	0.521875624538766
122	KC_AB1	CG5037	0.089171347477008	0.842833098440231	1	pseudobulk	DESeq2	LRT	0.0742584177866447
122	KC_AB1	CG5038	0.245556270924701	0.973663313394496	1	pseudobulk	DESeq2	LRT	0.0115911934429027
122	KC_AB1	CG5039	1.39934341023393	0.63869615168108	1	pseudobulk	DESeq2	LRT	0.194705700577112
122	KC_AB1	CG5044	-0.0246303247633094	0.971432489022313	1	pseudobulk	DESeq2	LRT	0.0125873758813446
123	KC_AB1	CG5050	1.25804452752548	0.727552935095488	1	pseudobulk	DESeq2	LRT	0.138135402914536
123	KC_AB1	CG5056	-0.0780790626366876	0.872936648387402	1	pseudobulk	DESeq2	LRT	0.0590172731948955
123	KC_AB1	CG5059	0.178821127224344	0.769249178577365	1	pseudobulk	DESeq2	LRT	0.113932958830271
123	KC_AB1	CG5062	-0.760959112540794	0.805265236978025	1	pseudobulk	DESeq2	LRT	0.0940610488455931
123	KC_AB1	CG5065	-0.100691266826162	0.644860083899561	1	pseudobulk	DESeq2	LRT	0.1905345045616
123	KC_AB1	CG5068	0.0179794020340706	0.924943326330373	1	pseudobulk	DESeq2	LRT	0.0338848767918063
123	KC_AB1	CG5071	-2.13518333033153	0.457250610250827	1	pseudobulk	DESeq2	LRT	0.33984570623779
123	KC_AB1	CG5078	0.124481543023846	0.91941190661028	1	pseudobulk	DESeq2	LRT	0.0364898763289548
123	KC_AB1	CG5079	-0.760959112540794	1	1	pseudobulk	DESeq2	LRT	0
123	KC_AB1	CG5080	3.81210075880085	0.0933186077838385	1	pseudobulk	DESeq2	LRT	1.03003174905003
123	KC_AB1	CG5087	0.11597031631839	0.857034615102032	1	pseudobulk	DESeq2	LRT	0.0670016368350178
123	KC_AB1	CG5098	-0.325537962445495	0.590459620929334	1	pseudobulk	DESeq2	LRT	0.22880979662317
123	KC_AB1	CG5104	-1.6947626985936	0.385987008094487	1	pseudobulk	DESeq2	LRT	0.413427312965109
123	KC_AB1	CG5110	0.360162389017868	0.38821845834652	1	pseudobulk	DESeq2	LRT	0.410923819366353
123	KC_AB1	CG5112	-0.286971596832756	0.929394666011936	1	pseudobulk	DESeq2	LRT	0.0317998243557841
123	KC_AB1	CG5114	-0.504581808778173	0.640592974692623	1	pseudobulk	DESeq2	LRT	0.19341782850229
123	KC_AB1	CG5116	1.61458718980285	0.542390465593699	1	pseudobulk	DESeq2	LRT	0.265687953315843
123	KC_AB1	CG5126	0.658790525980753	0.59147082675182	1	pseudobulk	DESeq2	LRT	0.228066671311885
123	KC_AB1	CG5131	-3.3933489483767	0.12154019762821	1	pseudobulk	DESeq2	LRT	0.915280061811549

123	KC_AB1	CG5144	-0.760959112540794	0.805265236978025	1	pseudobulk	DESeq2	LRT	0.0940610488455931
123	KC_AB1	CG5149	0.0659091251948274	0.88973628599702	1	pseudobulk	DESeq2	LRT	0.0507386972962655
123	KC_AB1	CG5151	0.232783963152198	0.493093802685094	1	pseudobulk	DESeq2	LRT	0.307070455746249
123	KC_AB1	CG5160	-2.61077565254151	0.274959422178212	1	pseudobulk	DESeq2	LRT	0.560731393531137
123	KC_AB1	CG5174	0.318960452801058	0.308495204575853	1	pseudobulk	DESeq2	LRT	0.510751582497618
123	KC_AB1	CG5180	0.0815327405361911	0.912006954611046	1	pseudobulk	DESeq2	LRT	0.0400018498978063
123	KC_AB1	CG5181	-0.108949519875683	0.845052671099073	1	pseudobulk	DESeq2	LRT	0.0731162211629822
123	KC_AB1	CG5188	-0.623074259447947	0.657290540482466	1	pseudobulk	DESeq2	LRT	0.182242617903458
123	KC_AB1	CG5191	-0.568295245030019	0.610328718673293	1	pseudobulk	DESeq2	LRT	0.214436194080939
123	KC_AB1	CG5196	0.372563690216788	0.566833054631256	1	pseudobulk	DESeq2	LRT	0.246544831986007
123	KC_AB1	CG5199	-0.219733611863415	0.83801566507117	1	pseudobulk	DESeq2	LRT	0.0767478630041142
123	KC_AB1	CG5214	-0.123195942245899	0.687514043692869	1	pseudobulk	DESeq2	LRT	0.162718426172572
123	KC_AB1	CG5217	2.99960667015886	0.357405021116046	1	pseudobulk	DESeq2	LRT	0.44683935046587
123	KC_AB1	CG5220	-0.204840304946014	0.782307545932499	1	pseudobulk	DESeq2	LRT	0.106622480643609
123	KC_AB1	CG5254	2.56935349350891	0.0876804752419828	1	pseudobulk	DESeq2	LRT	1.05709710490885
123	KC_AB1	CG5262	0.650551578161284	0.532021850408584	1	pseudobulk	DESeq2	LRT	0.274070530642916
123	KC_AB1	CG5267	1.90071181822722	0.545896518579078	1	pseudobulk	DESeq2	LRT	0.262889675371117
123	KC_AB1	CG5270	-0.349052028566246	0.752754653840154	1	pseudobulk	DESeq2	LRT	0.123346550819054
123	KC_AB1	CG5273	0.453770181744967	0.373143535484918	1	pseudobulk	DESeq2	LRT	0.428124077901411
123	KC_AB1	CG5274	-0.69135834091982	0.418449629536892	1	pseudobulk	DESeq2	LRT	0.378356812296178
123	KC_AB1	CG5276	0.685009183469792	0.44870794774196	1	pseudobulk	DESeq2	LRT	0.34803623795543
123	KC_AB1	CG5280	-0.760959112540794	0.805265236978025	1	pseudobulk	DESeq2	LRT	0.0940610488455931
123	KC_AB1	CG5281	0.0700062694969692	0.857250356919821	1	pseudobulk	DESeq2	LRT	0.0668923253926314
123	KC_AB1	CG5282	0.0454861365188554	0.887475124291635	1	pseudobulk	DESeq2	LRT	0.0518438112692437
123	KC_AB1	CG5285	0.399324989179401	0.615993580694211	1	pseudobulk	DESeq2	LRT	0.210423813620652
123	KC_AB1	CG5287	0.280724476599986	0.822545628365307	1	pseudobulk	DESeq2	LRT	0.0848400014659653
123	KC_AB1	CG5290	-0.569432576450917	0.825325802164462	1	pseudobulk	DESeq2	LRT	0.0833745773279703
123	KC_AB1	CG5292	-0.500729440837778	0.340713357058155	1	pseudobulk	DESeq2	LRT	0.4676108403595
123	KC_AB1	CG5316	-0.720556468276127	0.171711519634606	1	pseudobulk	DESeq2	LRT	0.765200568287403
123	KC_AB1	CG5321	0.965672978329062	0.671140964934474	1	pseudobulk	DESeq2	LRT	0.17318625201663
123	KC_AB1	CG5323	-0.0902074443630864	0.903637270416215	1	pseudobulk	DESeq2	LRT	0.0440058649558645
123	KC_AB1	CG5335	-2.52011257447171	0.364021130127131	1	pseudobulk	DESeq2	LRT	0.438873406374921
123	KC_AB1	CG5339	0.0838492127147981	0.884566096557659	1	pseudobulk	DESeq2	LRT	0.053269710184698
123	KC_AB1	CG5346	0.632939939812144	0.805000180713481	1	pseudobulk	DESeq2	LRT	0.0942040221378969
123	KC_AB1	CG5348	-2.60570519386268	0.202447166741081	1	pseudobulk	DESeq2	LRT	0.693688296827525
123	KC_AB1	CG5355	-0.0748150371852072	0.879126695765832	1	pseudobulk	DESeq2	LRT	0.0559485318580388
123	KC_AB1	CG5359	0.299494381199507	0.787165846795202	1	pseudobulk	DESeq2	LRT	0.103933757145506
123	KC_AB1	CG5377	1.13698605850655	0.780204112083573	1	pseudobulk	DESeq2	LRT	0.10779176505922
123	KC_AB1	CG5380	0.0314985280431219	0.968479888083717	1	pseudobulk	DESeq2	LRT	0.0139093936180289
123	KC_AB1	CG5381	3.12964491182152	0.252827515563327	1	pseudobulk	DESeq2	LRT	0.597175662956756
123	KC_AB1	CG5382	0.271569789940837	0.692600698122417	1	pseudobulk	DESeq2	LRT	0.159517075029468
123	KC_AB1	CG5390	0.487669929581329	0.759420047887929	1	pseudobulk	DESeq2	LRT	0.119517942128831
123	KC_AB1	CG5397	1.60862935509599	0.40383466995631	1	pseudobulk	DESeq2	LRT	0.393796398804068
123	KC_AB1	CG5399	-0.760959112540794	0.804334723897681	1	pseudobulk	DESeq2	LRT	0.0945631819854715
123	KC_AB1	CG5404	-0.628405612780789	0.527015963025381	1	pseudobulk	DESeq2	LRT	0.278176230045815
123	KC_AB1	CG5412	-0.0531871302812389	0.926591425025534	1	pseudobulk	DESeq2	LRT	0.0331117232148027
123	KC_AB1	CG5435	-1.61102155603974	0.57025980967112	1	pseudobulk	DESeq2	LRT	0.243927235206257
123	KC_AB1	CG5439	-0.526774428189941	0.459522990001907	1	pseudobulk	DESeq2	LRT	0.337692755918345
123	KC_AB1	CG5445	0.0729651534959216	0.886994747780506	1	pseudobulk	DESeq2	LRT	0.0520789517767258
123	KC_AB1	CG5446	0.490801252003677	0.212924023807674	1	pseudobulk	DESeq2	LRT	0.671775335186881
123	KC_AB1	CG5447	-0.334990844193037	0.416947207386576	1	pseudobulk	DESeq2	LRT	0.37991893061891
123	KC_AB1	CG5455	0.00531820136217769	0.987677077411947	1	pseudobulk	DESeq2	LRT	0.00538502547511715
123	KC_AB1	CG5466	0.143989272649724	0.692019321342482	1	pseudobulk	DESeq2	LRT	0.159881779769715
123	KC_AB1	CG5498	-0.388488822750371	0.741834910145444	1	pseudobulk	DESeq2	LRT	0.129692732982625
123	KC_AB1	CG5500	-0.287551253152422	0.508756961601823	1	pseudobulk	DESeq2	LRT	0.293489635035379
123	KC_AB1	CG5504	0.203863699896422	0.700978007874312	1	pseudobulk	DESeq2	LRT	0.154295607152678

123	KC_AB1	CG5509	-0.760959112540794	0.805265236978025	1	pseudobulk	DESeq2	LRT	0.0940610488455931
123	KC_AB1	CG5510	-0.0916402178327588	0.896458106239993	1	pseudobulk	DESeq2	LRT	0.0474700013097424
123	KC_AB1	CG5515	-0.215716478499816	0.640963086967005	1	pseudobulk	DESeq2	LRT	0.193166980759103
123	KC_AB1	CG5516	0.0255618460189615	0.926156653183738	1	pseudobulk	DESeq2	LRT	0.0333155491072329
123	KC_AB1	CG5521	-0.18478256457641	0.703678454524809	1	pseudobulk	DESeq2	LRT	0.15262574615071
123	KC_AB1	CG5522	0.594822968525524	0.247275576668303	1	pseudobulk	DESeq2	LRT	0.606818776651196
123	KC_AB1	CG5532	0.26312198610794	0.490921319126376	1	pseudobulk	DESeq2	LRT	0.308988107484768
123	KC_AB1	CG5535	-0.0865506275086234	0.878118694314878	1	pseudobulk	DESeq2	LRT	0.0564467770301549
123	KC_AB1	CG5541	1.91493096567444	0.491917945257778	1	pseudobulk	DESeq2	LRT	0.308107334008522
123	KC_AB1	CG5543	1.80472472999959	0.249655753654827	1	pseudobulk	DESeq2	LRT	0.602658420589253
123	KC_AB1	CG5554	0.120573312346831	0.827838679269483	1	pseudobulk	DESeq2	LRT	0.0820542858346768
123	KC_AB1	CG5555	-0.0381995324519181	0.906090919686733	1	pseudobulk	DESeq2	LRT	0.0428282218186454
123	KC_AB1	CG5567	0.0610089822767939	0.926558268462306	1	pseudobulk	DESeq2	LRT	0.0331272640127222
123	KC_AB1	CG5569	-0.45994786983904	0.485872616706258	1	pseudobulk	DESeq2	LRT	0.313477576649042
123	KC_AB1	CG5577	0.869549207220843	0.687084778212549	1	pseudobulk	DESeq2	LRT	0.162989672782803
123	KC_AB1	CG5589	1.93735620446146	0.313101918355473	1	pseudobulk	DESeq2	LRT	0.504314271480357
123	KC_AB1	CG5590	1.18376203849985	0.209728520371509	1	pseudobulk	DESeq2	LRT	0.678342507075211
123	KC_AB1	CG5599	-0.385011853448768	0.890534840916548	1	pseudobulk	DESeq2	LRT	0.0503490847107839
123	KC_AB1	CG5608	-0.548525402933867	0.575804148589476	1	pseudobulk	DESeq2	LRT	0.23972521041482
123	KC_AB1	CG5611	0.104578129526764	0.897463958778072	1	pseudobulk	DESeq2	LRT	0.0469829832154867
123	KC_AB1	CG5613	1.25804452752548	0.727552935095488	1	pseudobulk	DESeq2	LRT	0.138135402914536
123	KC_AB1	CG5618	0.00485851413330386	0.916540121498708	1	pseudobulk	DESeq2	LRT	0.0378485190660749
123	KC_AB1	CG5641	-0.195387959134362	0.586091048895416	1	pseudobulk	DESeq2	LRT	0.232034911351725
123	KC_AB1	CG5644	-0.175924288629025	0.720682866061064	1	pseudobulk	DESeq2	LRT	0.142255802990631
123	KC_AB1	CG5645	0.0467600061082849	0.96948009970652	1	pseudobulk	DESeq2	LRT	0.0134611011560321
124	KC_AB1	CG5646	0.734972466145086	0.367265303383173	1	pseudobulk	DESeq2	LRT	0.435020098774203
124	KC_AB1	CG5662	0.296275065198765	1	1	pseudobulk	DESeq2	LRT	0
124	KC_AB1	CG5664	1.17270293184914	0.515302174544892	1	pseudobulk	DESeq2	LRT	0.287938024837868
124	KC_AB1	CG5674	-0.301613365725871	0.697160053729447	1	pseudobulk	DESeq2	LRT	0.156667505299687
124	KC_AB1	CG5676	0.291778581575306	0.229551965687289	1	pseudobulk	DESeq2	LRT	0.639118983954429
124	KC_AB1	CG5687	1.11388934932283	1	1	pseudobulk	DESeq2	LRT	0
124	KC_AB1	CG5697	-0.760959112540794	0.804334723897681	1	pseudobulk	DESeq2	LRT	0.0945631819854715
124	KC_AB1	CG5704	1.11388934932283	1	1	pseudobulk	DESeq2	LRT	0
124	KC_AB1	CG5708	0.489779743757208	0.119603854108576	1	pseudobulk	DESeq2	LRT	0.92225482543859
124	KC_AB1	CG5715	0.161070628177329	0.889471426554504	1	pseudobulk	DESeq2	LRT	0.0508679986638383
124	KC_AB1	CG5721	0.687874238998828	0.422973222863436	1	pseudobulk	DESeq2	LRT	0.373687125605446
124	KC_AB1	CG5726	0.174934875307999	0.755165681786894	1	pseudobulk	DESeq2	LRT	0.121957754606748
124	KC_AB1	CG5727	-0.13954561071408	0.762111854985874	1	pseudobulk	DESeq2	LRT	0.117981282678677
124	KC_AB1	CG5728	-0.875990936861892	0.429682072849974	1	pseudobulk	DESeq2	LRT	0.366852765526585
124	KC_AB1	CG5731	-1.77199356422654	0.54193209410651	1	pseudobulk	DESeq2	LRT	0.266055128595237
124	KC_AB1	CG5742	0.043483605354688	0.926271796118239	1	pseudobulk	DESeq2	LRT	0.0332615595011381
124	KC_AB1	CG5745	0.184745717588664	0.869644488844979	1	pseudobulk	DESeq2	LRT	0.0606582509299742
124	KC_AB1	CG5746	-0.308848306471709	0.185307330100629	1	pseudobulk	DESeq2	LRT	0.732107401192211
124	KC_AB1	CG5757	-1.43550613534661	0.602448798560473	1	pseudobulk	DESeq2	LRT	0.220079857379737
124	KC_AB1	CG5758	0.0832500366064899	0.828044940970423	1	pseudobulk	DESeq2	LRT	0.0819460918554445
124	KC_AB1	CG5768	1.90071181822722	0.545896518579078	1	pseudobulk	DESeq2	LRT	0.262889675371117
124	KC_AB1	CG5776	-0.118435090707104	0.938860818754357	1	pseudobulk	DESeq2	LRT	0.0273987848660748
124	KC_AB1	CG5787	-0.242334711944134	0.73159395483254	1	pseudobulk	DESeq2	LRT	0.135729891760023
124	KC_AB1	CG5789	-1.43504920888332	0.179164887412823	1	pseudobulk	DESeq2	LRT	0.746747099009954
124	KC_AB1	CG5793	0.207741036192086	0.864397233372155	1	pseudobulk	DESeq2	LRT	0.0632866318086127
124	KC_AB1	CG5800	0.452100558121015	0.761846331920954	1	pseudobulk	DESeq2	LRT	0.118132619118312
124	KC_AB1	CG5805	0.532581734227883	0.267977787765304	1	pseudobulk	DESeq2	LRT	0.571901202429217
124	KC_AB1	CG5808	-0.851248147859418	0.40904564626336	1	pseudobulk	DESeq2	LRT	0.388228225454372
124	KC_AB1	CG5810	-0.760959112540794	0.804334723897681	1	pseudobulk	DESeq2	LRT	0.0945631819854715
124	KC_AB1	CG5815	0.604255507692152	0.463833859670634	1	pseudobulk	DESeq2	LRT	0.333637551231946
124	KC_AB1	CG5823	0.00522470955627162	0.934892955843248	1	pseudobulk	DESeq2	LRT	0.0292381124942693

124	KC_AB1	CG5828	0.150987412712499	0.838907651505038	1	pseudobulk	DESeq2	LRT	0.0762858444742655
124	KC_AB1	CG5830	-0.155993837859275	0.178464890281413	1	pseudobulk	DESeq2	LRT	0.748447210682455
124	KC_AB1	CG5835	-0.502491250160929	0.68361232654153	1	pseudobulk	DESeq2	LRT	0.165190114908728
124	KC_AB1	CG5846	0.567308063991037	0.613971843648202	1	pseudobulk	DESeq2	LRT	0.211851544866324
124	KC_AB1	CG5850	0.132742856981614	0.897312601265945	1	pseudobulk	DESeq2	LRT	0.047056233261125
124	KC_AB1	CG5853	2.88322402903533	0.302751069383099	1	pseudobulk	DESeq2	LRT	0.518914314158952
124	KC_AB1	CG5854	-0.0015617828346662	0.956380096981119	1	pseudobulk	DESeq2	LRT	0.0193694704705399
124	KC_AB1	CG5861	0.0511248474720098	0.888776802827114	1	pseudobulk	DESeq2	LRT	0.0512072890532942
124	KC_AB1	CG5862	-0.760037098604068	0.345494219571649	1	pseudobulk	DESeq2	LRT	0.461559214365431
124	KC_AB1	CG5867	7.62551147819056	0.101611869299091	1	pseudobulk	DESeq2	LRT	0.993055559079435
124	KC_AB1	CG5877	1.35060184397851	0.127209538183486	1	pseudobulk	DESeq2	LRT	0.895480324024449
124	KC_AB1	CG5880	-0.0520732055750323	0.965296921805553	1	pseudobulk	DESeq2	LRT	0.0153390787109543
124	KC_AB1	CG5885	-0.134996271174016	0.789454070777899	1	pseudobulk	DESeq2	LRT	0.102673131504886
124	KC_AB1	CG5886	-0.104026137396374	0.890139316074098	1	pseudobulk	DESeq2	LRT	0.0505420164256432
124	KC_AB1	CG5888	-0.754004526339494	0.511640482485792	1	pseudobulk	DESeq2	LRT	0.291035100189021
124	KC_AB1	CG5890	-0.20699745124903	0.41450705958311	1	pseudobulk	DESeq2	LRT	0.382468068462609
124	KC_AB1	CG5895	0.126448156933747	0.862362022429313	1	pseudobulk	DESeq2	LRT	0.0643103776357196
124	KC_AB1	CG5902	-0.11408307354261	0.790801628951102	1	pseudobulk	DESeq2	LRT	0.101932444765869
124	KC_AB1	CG5903	0.236687383353547	0.325291940571089	1	pseudobulk	DESeq2	LRT	0.487726696659318
124	KC_AB1	CG5909	-2.00963963748391	0.498268873088307	1	pseudobulk	DESeq2	LRT	0.302536242405947
124	KC_AB1	CG5910	3.91655691480688	0.0385954753156341	1	pseudobulk	DESeq2	LRT	1.41346360622546
124	KC_AB1	CG5913	-0.510311607069423	0.632304688395041	1	pseudobulk	DESeq2	LRT	0.199073597980319
124	KC_AB1	CG5916	-0.0106874848497096	0.943971343032204	1	pseudobulk	DESeq2	LRT	0.025041189761137
124	KC_AB1	CG5934	-0.161022886441827	0.565169698618052	1	pseudobulk	DESeq2	LRT	0.247821130750841
124	KC_AB1	CG5937	-0.0220713717991712	0.924929713485125	1	pseudobulk	DESeq2	LRT	0.033891268564049
124	KC_AB1	CG5938	0.334274713673896	0.330086238170085	1	pseudobulk	DESeq2	LRT	0.481372581732639
124	KC_AB1	CG5946	-0.0673465205330595	0.819710695492842	1	pseudobulk	DESeq2	LRT	0.0863393982509197
124	KC_AB1	CG5953	1.59155565536523	0.419961120357547	1	pseudobulk	DESeq2	LRT	0.376790914353912
124	KC_AB1	CG5955	-0.0115845992169638	0.935696897983855	1	pseudobulk	DESeq2	LRT	0.0288648102909906
124	KC_AB1	CG5958	2.99960667015886	0.357405021116046	1	pseudobulk	DESeq2	LRT	0.44683935046587
124	KC_AB1	CG5961	0.292175867170122	0.60344218670927	1	pseudobulk	DESeq2	LRT	0.219364331524962
124	KC_AB1	CG5969	0.607555948515552	0.245919154773919	1	pseudobulk	DESeq2	LRT	0.609207642515336
124	KC_AB1	CG5973	0.312450700387557	0.878402024513267	1	pseudobulk	DESeq2	LRT	0.0563066719446128
124	KC_AB1	CG5986	0.257313056494308	0.509968693443079	1	pseudobulk	DESeq2	LRT	0.292456484063321
124	KC_AB1	CG5989	-0.0759899541569865	0.917154090854024	1	pseudobulk	DESeq2	LRT	0.0375576924808793
124	KC_AB1	CG6000	1.25804452752548	0.727552935095488	1	pseudobulk	DESeq2	LRT	0.138135402914536
124	KC_AB1	CG6005	0.0368857918751082	0.928616218612464	1	pseudobulk	DESeq2	LRT	0.0321637354960488
124	KC_AB1	CG6006	-0.216396662612736	0.730241755525708	1	pseudobulk	DESeq2	LRT	0.136533337538121
124	KC_AB1	CG6013	0.0933298181506425	0.829248578372852	1	pseudobulk	DESeq2	LRT	0.0813152643431255
124	KC_AB1	CG6023	-0.760959112540794	1	1	pseudobulk	DESeq2	LRT	0
124	KC_AB1	CG6024	-0.174426256924203	0.358864478303893	1	pseudobulk	DESeq2	LRT	0.445069527568365
124	KC_AB1	CG6028	-0.3946295699428	0.692723186267944	1	pseudobulk	DESeq2	LRT	0.159440275767665
124	KC_AB1	CG6040	-0.778723311093523	0.409017150271828	1	pseudobulk	DESeq2	LRT	0.388258481449232
124	KC_AB1	CG6041	-0.17507498349115	0.815765942431191	1	pseudobulk	DESeq2	LRT	0.0884344300840787
124	KC_AB1	CG6044	0.141132683166637	0.30576182215877	1	pseudobulk	DESeq2	LRT	0.514616742144305
124	KC_AB1	CG6051	0.0635296098828312	0.901312617509502	1	pseudobulk	DESeq2	LRT	0.0451245491860497
124	KC_AB1	CG6052	1.11388934932283	1	1	pseudobulk	DESeq2	LRT	0
124	KC_AB1	CG6066	0.445186351371328	0.602042189374937	1	pseudobulk	DESeq2	LRT	0.220373073574871
124	KC_AB1	CG6073	-0.385011853448768	0.890534840916548	1	pseudobulk	DESeq2	LRT	0.0503490847107839
124	KC_AB1	CG6083	1.7504506057736	0.277135185507566	1	pseudobulk	DESeq2	LRT	0.55730833202471
124	KC_AB1	CG6084	2.38169297104649	0.00868498047299485	1	pseudobulk	DESeq2	LRT	2.06123115366825
124	KC_AB1	CG6094	0.553919111594823	0.451188642923147	1	pseudobulk	DESeq2	LRT	0.345641840713287
124	KC_AB1	CG6115	-0.0032015680209676	0.9633961720942	1	pseudobulk	DESeq2	LRT	0.0161950836201615
124	KC_AB1	CG6126	-2.73719572742657	0.395113780272619	1	pseudobulk	DESeq2	LRT	0.40327782328744
124	KC_AB1	CG6136	-0.79397585053343	0.443496653556754	1	pseudobulk	DESeq2	LRT	0.353109652827337
124	KC_AB1	CG6144	0.0185024144442362	0.935866937261609	1	pseudobulk	DESeq2	LRT	0.028785895408316

124	KC_AB1	CG6145	-0.287709022981928	0.711512967811075	1	pseudobulk	DESeq2	LRT	0.147817180193232
124	KC_AB1	CG6153	-0.347596430695603	0.751466559762996	1	pseudobulk	DESeq2	LRT	0.124090340737132
124	KC_AB1	CG6154	-0.31697891485055	0.228128151283149	1	pseudobulk	DESeq2	LRT	0.641821118964688
124	KC_AB1	CG6163	-0.409858947027979	0.831895553802382	1	pseudobulk	DESeq2	LRT	0.0799311968519545
124	KC_AB1	CG6171	0.209104405464453	0.685010060298327	1	pseudobulk	DESeq2	LRT	0.164303050259448
124	KC_AB1	CG6175	-0.449393077929445	0.79728162558987	1	pseudobulk	DESeq2	LRT	0.0983882446823618
124	KC_AB1	CG6178	-0.569885208355244	0.599192805008182	1	pseudobulk	DESeq2	LRT	0.222433410201788
124	KC_AB1	CG6179	-0.0824746865427683	0.904274619840423	1	pseudobulk	DESeq2	LRT	0.0436996582751782
124	KC_AB1	CG6180	0.106755053695081	0.77269041378279	1	pseudobulk	DESeq2	LRT	0.111994475702508
124	KC_AB1	CG6184	-0.500573652525122	0.323481183327573	1	pseudobulk	DESeq2	LRT	0.490150976864453
124	KC_AB1	CG6191	-0.475476060057685	0.284712609908554	1	pseudobulk	DESeq2	LRT	0.54559329754421
124	KC_AB1	CG6195	-0.279651026781615	0.716025648028643	1	pseudobulk	DESeq2	LRT	0.145071420991288
124	KC_AB1	CG6201	-0.386268293802892	0.767045789770678	1	pseudobulk	DESeq2	LRT	0.115178709517572
125	KC_AB1	CG6204	1.31338049418156	0.305948643895526	1	pseudobulk	DESeq2	LRT	0.51435146745506
125	KC_AB1	CG6218	0.0319817600699813	0.955837982441589	1	pseudobulk	DESeq2	LRT	0.019615715772703
125	KC_AB1	CG6227	-0.14272938096306	0.833231007200729	1	pseudobulk	DESeq2	LRT	0.0792345769316711
125	KC_AB1	CG6230	-1.47054140301306	0.168177438978773	1	pseudobulk	DESeq2	LRT	0.774232265281302
125	KC_AB1	CG6231	-0.765721779569942	0.720047078762894	1	pseudobulk	DESeq2	LRT	0.142639107209684
125	KC_AB1	CG6236	1.34777517093	0.197546531138693	1	pseudobulk	DESeq2	LRT	0.704330592004053
125	KC_AB1	CG6254	-0.185614719776925	0.790596827139732	1	pseudobulk	DESeq2	LRT	0.102044932920535
125	KC_AB1	CG6276	-0.225352046189171	0.644257621532984	1	pseudobulk	DESeq2	LRT	0.190940435066324
125	KC_AB1	CG6282	-0.207043614487577	0.420839075146389	1	pseudobulk	DESeq2	LRT	0.375883942493601
125	KC_AB1	CG6287	0.30357622880776	0.658865208015101	1	pseudobulk	DESeq2	LRT	0.18120342516759
125	KC_AB1	CG6293	-0.760959112540794	0.805265236978025	1	pseudobulk	DESeq2	LRT	0.0940610488455931
125	KC_AB1	CG6310	3.80522001411205	0.202854565618816	1	pseudobulk	DESeq2	LRT	0.692815213245607
125	KC_AB1	CG6325	-0.573760582981819	0.64704692122472	1	pseudobulk	DESeq2	LRT	0.189064224910727
125	KC_AB1	CG6329	-0.00820942379086746	0.964569374147732	1	pseudobulk	DESeq2	LRT	0.0156665313938111
125	KC_AB1	CG6330	0.0273016622515497	0.933678768692809	1	pseudobulk	DESeq2	LRT	0.0298025166824803
125	KC_AB1	CG6340	-0.12701242232514	0.621097928151855	1	pseudobulk	DESeq2	LRT	0.206839919464347
125	KC_AB1	CG6345	1.27179366484067	0.458649426559353	1	pseudobulk	DESeq2	LRT	0.338519145146168
125	KC_AB1	CG6356	0.0486570381277616	0.927614961728325	1	pseudobulk	DESeq2	LRT	0.0326322551357659
125	KC_AB1	CG6357	2.20907213147572	0.00222514749061617	1	pseudobulk	DESeq2	LRT	2.65264119716044
125	KC_AB1	CG6362	-2.00963963748391	0.498268873088307	1	pseudobulk	DESeq2	LRT	0.302536242405947
125	KC_AB1	CG6379	-0.180447559662373	0.767653578215412	1	pseudobulk	DESeq2	LRT	0.114834721389319
125	KC_AB1	CG6388	-0.13816602462961	0.900041758026404	1	pseudobulk	DESeq2	LRT	0.0457373407165229
125	KC_AB1	CG6398	1.23928812133252	0.708326500086237	1	pseudobulk	DESeq2	LRT	0.14976650996508
125	KC_AB1	CG6409	2.99960667015886	0.357405021116046	1	pseudobulk	DESeq2	LRT	0.44683935046587
125	KC_AB1	CG6418	-0.447856574306972	0.361309986138372	1	pseudobulk	DESeq2	LRT	0.442120034931129
125	KC_AB1	CG6420	0.148096362787357	0.836520608302947	1	pseudobulk	DESeq2	LRT	0.0775233554045101
125	KC_AB1	CG6421	-2.41876763198367	0.438413379977707	1	pseudobulk	DESeq2	LRT	0.358116200048746
125	KC_AB1	CG6424	-0.760959112540794	0.805265236978025	1	pseudobulk	DESeq2	LRT	0.0940610488455931
125	KC_AB1	CG6425	-2.79680560112717	0.189271245666978	1	pseudobulk	DESeq2	LRT	0.722915359605666
125	KC_AB1	CG6426	-2.37287777141478	0.396160654274306	1	pseudobulk	DESeq2	LRT	0.402128659741638
125	KC_AB1	CG6428	-0.315580551417905	0.70868705135484	1	pseudobulk	DESeq2	LRT	0.149545502297768
125	KC_AB1	CG6435	-0.760959112540794	1	1	pseudobulk	DESeq2	LRT	0
125	KC_AB1	CG6443	0.305182891679483	0.458678479154893	1	pseudobulk	DESeq2	LRT	0.338491636156517
125	KC_AB1	CG6454	-0.260983491851304	0.572803098445125	1	pseudobulk	DESeq2	LRT	0.241994641478866
125	KC_AB1	CG6465	0.70533223341955	0.484715534864733	1	pseudobulk	DESeq2	LRT	0.314513061156309
125	KC_AB1	CG6470	1.25804452752548	0.573436424985144	1	pseudobulk	DESeq2	LRT	0.241514723898045
125	KC_AB1	CG6472	-1.60958773650785	0.570513631639934	1	pseudobulk	DESeq2	LRT	0.243733974251419
125	KC_AB1	CG6479	-0.770452856372761	0.605481900710816	1	pseudobulk	DESeq2	LRT	0.217898834418371
125	KC_AB1	CG6481	-0.298280314257323	0.850661090003833	1	pseudobulk	DESeq2	LRT	0.0702434317573342
125	KC_AB1	CG6495	-0.0103773000182178	0.96288199625782	1	pseudobulk	DESeq2	LRT	0.0164269335548595
125	KC_AB1	CG6511	-1.1639934974822	0.535383489520649	1	pseudobulk	DESeq2	LRT	0.271335025978069
125	KC_AB1	CG6512	-0.541068397353496	0.354670164335606	1	pseudobulk	DESeq2	LRT	0.450175343835361
125	KC_AB1	CG6523	0.210546152023484	0.558324240917313	1	pseudobulk	DESeq2	LRT	0.253113515857111

125	KC_AB1	CG6550	1.32088399334002	0.284630448295826	1	pseudobulk	DESeq2	LRT	0.545718643185174
125	KC_AB1	CG6553	1.25804452752548	0.727552935095488	1	pseudobulk	DESeq2	LRT	0.138135402914536
125	KC_AB1	CG6565	0.291294995883017	0.745269375135371	1	pseudobulk	DESeq2	LRT	0.127686724580808
125	KC_AB1	CG6567	-0.0612476300036748	0.939441057417248	1	pseudobulk	DESeq2	LRT	0.0271304632925376
125	KC_AB1	CG6574	1.55702380897078	0.208537337305273	1	pseudobulk	DESeq2	LRT	0.680816176019093
125	KC_AB1	CG6583	0.302589044521635	0.612967684942648	1	pseudobulk	DESeq2	LRT	0.212562420458779
125	KC_AB1	CG6607	-0.172708475247497	0.74295314918134	1	pseudobulk	DESeq2	LRT	0.129038572098844
125	KC_AB1	CG6610	-0.362310354899662	0.761070642302137	1	pseudobulk	DESeq2	LRT	0.11857503030153
125	KC_AB1	CG6617	0.092192333184161	0.790991408633952	1	pseudobulk	DESeq2	LRT	0.101828233573284
125	KC_AB1	CG6621	0.0323741238372006	0.956360783919094	1	pseudobulk	DESeq2	LRT	0.0193782406665971
125	KC_AB1	CG6628	-0.259457548523114	0.9024835187052	1	pseudobulk	DESeq2	LRT	0.0445607205034607
125	KC_AB1	CG6638	0.093287088252146	1	1	pseudobulk	DESeq2	LRT	0
125	KC_AB1	CG6650	0.182652742669916	0.880802329203035	1	pseudobulk	DESeq2	LRT	0.0551215455818451
125	KC_AB1	CG6654	-0.281087242965851	0.827378822789937	1	pseudobulk	DESeq2	LRT	0.0822955993012445
125	KC_AB1	CG6656	1.63195036548663	0.633334503478132	1	pseudobulk	DESeq2	LRT	0.198366851366371
125	KC_AB1	CG6659	-0.631041484864315	0.498651246711943	1	pseudobulk	DESeq2	LRT	0.302203090813125
125	KC_AB1	CG6664	-0.00078084392419092	0.958427678529935	1	pseudobulk	DESeq2	LRT	0.0184406527393593
125	KC_AB1	CG6665	0.355118493457009	0.637920953473238	1	pseudobulk	DESeq2	LRT	0.195233132559759
125	KC_AB1	CG6674	0.01765575862355	0.967173083514297	1	pseudobulk	DESeq2	LRT	0.014495798420934
125	KC_AB1	CG6675	-0.760959112540794	1	1	pseudobulk	DESeq2	LRT	0
125	KC_AB1	CG6678	1.11388934932283	1	1	pseudobulk	DESeq2	LRT	0
125	KC_AB1	CG6683	-0.760959112540794	1	1	pseudobulk	DESeq2	LRT	0
125	KC_AB1	CG6685	1.09030244551261	0.213970661472939	1	pseudobulk	DESeq2	LRT	0.669645770734443
125	KC_AB1	CG6686	-0.154068414050568	0.674203171098878	1	pseudobulk	DESeq2	LRT	0.171209209116284
125	KC_AB1	CG6689	-0.0152262972782524	0.942842079224533	1	pseudobulk	DESeq2	LRT	0.0255610430683674
125	KC_AB1	CG6693	-0.278273732952826	0.577425355575362	1	pseudobulk	DESeq2	LRT	0.238504149543123
125	KC_AB1	CG6695	-0.606104589423016	0.562944049350377	1	pseudobulk	DESeq2	LRT	0.24953476725502
125	KC_AB1	CG6700	0.157356514483863	0.397249744653538	1	pseudobulk	DESeq2	LRT	0.400936373278783
125	KC_AB1	CG6701	2.07671723064908	0.301306449502811	1	pseudobulk	DESeq2	LRT	0.520991572098617
125	KC_AB1	CG6707	0.16197405387045	0.563875322790051	1	pseudobulk	DESeq2	LRT	0.248816911282577
125	KC_AB1	CG6712	-0.0809007022256024	0.920924848853674	1	pseudobulk	DESeq2	LRT	0.0357758085217249
125	KC_AB1	CG6723	0.243083560460202	0.46534626599014	1	pseudobulk	DESeq2	LRT	0.332223766582721
125	KC_AB1	CG6724	1.26469182876422	0.297184875100828	1	pseudobulk	DESeq2	LRT	0.526973297291675
125	KC_AB1	CG6729	-0.539706979118566	0.566957516953084	1	pseudobulk	DESeq2	LRT	0.246449482278315
125	KC_AB1	CG6752	0.0939641887548904	0.929711041518087	1	pseudobulk	DESeq2	LRT	0.0316520112031477
125	KC_AB1	CG6753	4.18873486899365	0.081841852281291	1	pseudobulk	DESeq2	LRT	1.08702455003832
125	KC_AB1	CG6758	-0.987610715384711	0.174258488518881	1	pseudobulk	DESeq2	LRT	0.758806057263703
125	KC_AB1	CG6762	0.475643211833436	0.501252789746152	1	pseudobulk	DESeq2	LRT	0.299943197278518
125	KC_AB1	CG6763	-0.570723996670538	0.824991814167186	1	pseudobulk	DESeq2	LRT	0.0835503606375391
125	KC_AB1	CG6765	-0.236562394980021	0.421166049921589	1	pseudobulk	DESeq2	LRT	0.375546644433289
125	KC_AB1	CG6766	0.103173921690959	0.841932271551407	1	pseudobulk	DESeq2	LRT	0.0747228435057597
125	KC_AB1	CG6767	0.133562817806179	0.485432622211018	1	pseudobulk	DESeq2	LRT	0.313871041415348
125	KC_AB1	CG6769	-0.440692583643271	0.53451837334722	1	pseudobulk	DESeq2	LRT	0.272037361912324
125	KC_AB1	CG6770	0.0165485391513913	0.945484212675089	1	pseudobulk	DESeq2	LRT	0.0243457184374871
125	KC_AB1	CG6785	1.25804452752548	0.573436424985144	1	pseudobulk	DESeq2	LRT	0.241514723898045
125	KC_AB1	CG6791	-0.0826103646923336	0.866500889376651	1	pseudobulk	DESeq2	LRT	0.0622309871899099
125	KC_AB1	CG6805	-0.590008911058084	0.614138486089139	1	pseudobulk	DESeq2	LRT	0.211733685922804
125	KC_AB1	CG6808	0.566235631321027	0.719630559594535	1	pseudobulk	DESeq2	LRT	0.142890402328331
125	KC_AB1	CG6813	0.474212119657127	0.525626343963142	1	pseudobulk	DESeq2	LRT	0.279322876418002
125	KC_AB1	CG6830	1.25804452752548	0.573436424985144	1	pseudobulk	DESeq2	LRT	0.241514723898045
125	KC_AB1	CG6833	-0.423376283498701	0.580400431406155	1	pseudobulk	DESeq2	LRT	0.236272273427198
125	KC_AB1	CG6834	1.36214222908153	0.53658260858479	1	pseudobulk	DESeq2	LRT	0.270363407545665
125	KC_AB1	CG6836	2.27122133462827	0.453795586678749	1	pseudobulk	DESeq2	LRT	0.343139732096712
125	KC_AB1	CG6841	-0.257599848877474	0.763900635677203	1	pseudobulk	DESeq2	LRT	0.116963128575558
125	KC_AB1	CG6843	-0.259667721563521	0.644855955192326	1	pseudobulk	DESeq2	LRT	0.190537285134556
125	KC_AB1	CG6847	0.359026488718433	0.847265072367733	1	pseudobulk	DESeq2	LRT	0.0719806965679971

125	KC_AB1	CG6867	0.421166869561208	0.457818159823276	1	pseudobulk	DESeq2	LRT	0.33930698458716
126	KC_AB1	CG6870	-1.43550613534661	0.602448798560473	1	pseudobulk	DESeq2	LRT	0.220079857379737
126	KC_AB1	CG6878	0.123552692959278	0.777788356027733	1	pseudobulk	DESeq2	LRT	0.109138562796627
126	KC_AB1	CG6891	-0.437255301577789	0.907572497431198	1	pseudobulk	DESeq2	LRT	0.042118673204513
126	KC_AB1	CG6903	-0.43726027539763	0.629080827240353	1	pseudobulk	DESeq2	LRT	0.201293550787977
126	KC_AB1	CG6907	2.24650593211836	0.0862675070734304	1	pseudobulk	DESeq2	LRT	1.06415275186239
126	KC_AB1	CG6908	0.609087844155955	0.692126707419092	1	pseudobulk	DESeq2	LRT	0.159814392110279
126	KC_AB1	CG6912	0.242946154045532	0.885105647793542	1	pseudobulk	DESeq2	LRT	0.0530048880418836
126	KC_AB1	CG6923	-0.142785524229663	0.830826304184602	1	pseudobulk	DESeq2	LRT	0.0804897620389824
126	KC_AB1	CG6928	0.371055422160238	0.881976387776155	1	pseudobulk	DESeq2	LRT	0.054543041620775
126	KC_AB1	CG6937	-0.176208840857838	0.819345358219148	1	pseudobulk	DESeq2	LRT	0.0865330023247609
126	KC_AB1	CG6938	-3.02999637893047	0.191309629999418	1	pseudobulk	DESeq2	LRT	0.718263168235408
126	KC_AB1	CG6951	-0.287723761595917	0.908359580254375	1	pseudobulk	DESeq2	LRT	0.0417421990483341
126	KC_AB1	CG6959	1.27428717733693	0.456228518993862	1	pseudobulk	DESeq2	LRT	0.34081757032079
126	KC_AB1	CG6961	-0.0243177393220388	0.918483519375263	1	pseudobulk	DESeq2	LRT	0.0369286319641556
126	KC_AB1	CG6962	-0.197024465433583	0.911474558289662	1	pseudobulk	DESeq2	LRT	0.0402554491687578
126	KC_AB1	CG6966	-0.0626905057775443	0.837577327509619	1	pseudobulk	DESeq2	LRT	0.0769750871443177
126	KC_AB1	CG6983	0.456891775535424	0.28991020407645	1	pseudobulk	DESeq2	LRT	0.537736498352817
126	KC_AB1	CG6984	1.3619355327589	0.236284103231238	1	pseudobulk	DESeq2	LRT	0.626565495935081
126	KC_AB1	CG6985	1.25804452752548	0.727552935095488	1	pseudobulk	DESeq2	LRT	0.138135402914536
126	KC_AB1	CG6999	1.11388934932283	1	1	pseudobulk	DESeq2	LRT	0
126	KC_AB1	CG7006	-0.335168925066409	0.725749248802182	1	pseudobulk	DESeq2	LRT	0.139213405019177
126	KC_AB1	CG7009	0.574954892713762	0.573975254965506	1	pseudobulk	DESeq2	LRT	0.241106830360884
126	KC_AB1	CG7011	0.453759279441529	0.578479509890602	1	pseudobulk	DESeq2	LRT	0.237712019424493
126	KC_AB1	CG7028	-0.0310299860086939	0.901229475741746	1	pseudobulk	DESeq2	LRT	0.0451646126177032
126	KC_AB1	CG7029	0.097839175518764	0.699818669932918	1	pseudobulk	DESeq2	LRT	0.155014475484599
126	KC_AB1	CG7044	0.634459245752475	0.736631801158783	1	pseudobulk	DESeq2	LRT	0.132749536133218
126	KC_AB1	CG7049	0.78782132044083	0.421400496488778	1	pseudobulk	DESeq2	LRT	0.375304957047307
126	KC_AB1	CG7054	-0.396721808097649	1	1	pseudobulk	DESeq2	LRT	0
126	KC_AB1	CG7058	-0.244297812535529	0.653221622799453	1	pseudobulk	DESeq2	LRT	0.184939447776367
126	KC_AB1	CG7059	0.302295088307325	0.65367990454933	1	pseudobulk	DESeq2	LRT	0.184634865925139
126	KC_AB1	CG7065	-0.149658303479624	0.785132049491044	1	pseudobulk	DESeq2	LRT	0.105057294156167
126	KC_AB1	CG7069	-0.654377852934675	0.71437466970849	1	pseudobulk	DESeq2	LRT	0.146073953056882
126	KC_AB1	CG7071	-0.760959112540794	0.805265236978025	1	pseudobulk	DESeq2	LRT	0.0940610488455931
126	KC_AB1	CG7079	-1.43550613534661	0.602448798560473	1	pseudobulk	DESeq2	LRT	0.220079857379737
126	KC_AB1	CG7083	0.0693866727375873	0.910055555618325	1	pseudobulk	DESeq2	LRT	0.0409320947536163
126	KC_AB1	CG7084	0.0378035511276476	0.943523161510738	1	pseudobulk	DESeq2	LRT	0.0252474343513629
126	KC_AB1	CG7099	-0.220744381641908	0.885275129951742	1	pseudobulk	DESeq2	LRT	0.0529217362507688
126	KC_AB1	CG7101	-1.47509336359606	0.390345852680409	1	pseudobulk	DESeq2	LRT	0.408550430536725
126	KC_AB1	CG7102	2.09987857572813	0.503115176013642	1	pseudobulk	DESeq2	LRT	0.29833258237694
126	KC_AB1	CG7110	-0.385011853448768	0.890534840916548	1	pseudobulk	DESeq2	LRT	0.0503490847107839
126	KC_AB1	CG7115	0.28780531950182	0.485058013292039	1	pseudobulk	DESeq2	LRT	0.314206316354668
126	KC_AB1	CG7120	-0.0432245726321772	0.90551873758955	1	pseudobulk	DESeq2	LRT	0.0431025585497563
126	KC_AB1	CG7130	1.11388934932283	1	1	pseudobulk	DESeq2	LRT	0
126	KC_AB1	CG7133	0.0450380703406494	0.93335174729656	1	pseudobulk	DESeq2	LRT	0.0299546551591589
126	KC_AB1	CG7137	-0.0272546240539262	0.979528540661769	1	pseudobulk	DESeq2	LRT	0.00898290538508334
126	KC_AB1	CG7139	0.219942854355032	0.742367740949606	1	pseudobulk	DESeq2	LRT	0.129380908330101
126	KC_AB1	CG7148	0.207365501241996	0.72677399279911	1	pseudobulk	DESeq2	LRT	0.138600622071106
126	KC_AB1	CG7149	-0.648573342794347	0.594917358395331	1	pseudobulk	DESeq2	LRT	0.225543359121219
126	KC_AB1	CG7154	-0.02452391160689	0.908075758527205	1	pseudobulk	DESeq2	LRT	0.0418779178469345
126	KC_AB1	CG7156	-1.05033558300333	0.412152268819952	1	pseudobulk	DESeq2	LRT	0.384942305105069
126	KC_AB1	CG7166	-0.12111950439153	0.657810997040446	1	pseudobulk	DESeq2	LRT	0.181898870418488
126	KC_AB1	CG7168	-0.635235909950149	0.419908977230665	1	pseudobulk	DESeq2	LRT	0.376844840484555
126	KC_AB1	CG7182	-0.113514081031942	0.860325801810445	1	pseudobulk	DESeq2	LRT	0.0653370520895648
126	KC_AB1	CG7183	-0.0409415884780198	0.929975794353489	1	pseudobulk	DESeq2	LRT	0.0315283552261925
126	KC_AB1	CG7194	2.09987857572813	0.503115176013642	1	pseudobulk	DESeq2	LRT	0.29833258237694

126	KC_AB1	CG7206	-0.094462912799286	0.897334273627247	1	pseudobulk	DESeq2	LRT	0.0470457440812699
126	KC_AB1	CG7218	0.875990009462132	0.433583152316392	1	pseudobulk	DESeq2	LRT	0.362927601465465
126	KC_AB1	CG7220	0.271856672463175	0.45241078216793	1	pseudobulk	DESeq2	LRT	0.34446705321556
126	KC_AB1	CG7222	0.394253500187861	0.457398334396961	1	pseudobulk	DESeq2	LRT	0.339705421197407
126	KC_AB1	CG7231	0.0651056560010909	0.87892960005021	1	pseudobulk	DESeq2	LRT	0.056045909379184
126	KC_AB1	CG7239	0.308856092693327	0.633616870378366	1	pseudobulk	DESeq2	LRT	0.198173267948405
126	KC_AB1	CG7246	0.401292905331247	0.811745887893916	1	pseudobulk	DESeq2	LRT	0.0905799027259886
126	KC_AB1	CG7255	-0.760959112540794	1	1	pseudobulk	DESeq2	LRT	0
126	KC_AB1	CG7265	-0.163764785543378	0.852411535146395	1	pseudobulk	DESeq2	LRT	0.0693506818854157
126	KC_AB1	CG7275	-0.422960681515034	0.740694531507248	1	pseudobulk	DESeq2	LRT	0.130360861700496
126	KC_AB1	CG7289	0.220071094930464	0.797737917543873	1	pseudobulk	DESeq2	LRT	0.0981397648640181
126	KC_AB1	CG7324	-0.300157019323071	0.531517677586613	1	pseudobulk	DESeq2	LRT	0.274482286830901
126	KC_AB1	CG7326	0.915007662202113	0.37783087224052	1	pseudobulk	DESeq2	LRT	0.422702559130111
126	KC_AB1	CG7328	-0.716419614646419	0.812870875922622	1	pseudobulk	DESeq2	LRT	0.0899784363592155
126	KC_AB1	CG7332	0.016275496273376	0.952941058467731	1	pseudobulk	DESeq2	LRT	0.020933960614401
126	KC_AB1	CG7339	0.242935547144694	0.739956440191182	1	pseudobulk	DESeq2	LRT	0.130793845595265
126	KC_AB1	CG7341	2.17914818942692	0.381516710961221	1	pseudobulk	DESeq2	LRT	0.418486434591672
126	KC_AB1	CG7352	-0.760959112540794	0.805265236978025	1	pseudobulk	DESeq2	LRT	0.0940610488455931
126	KC_AB1	CG7362	-1.61102155603974	0.57025980967112	1	pseudobulk	DESeq2	LRT	0.243927235206257
126	KC_AB1	CG7365	2.77905602015038	0.396550243710121	1	pseudobulk	DESeq2	LRT	0.401701778879434
126	KC_AB1	CG7367	-0.119679433470562	0.938579946146907	1	pseudobulk	DESeq2	LRT	0.0275287292315365
126	KC_AB1	CG7368	0.110700093960145	0.828559332814988	1	pseudobulk	DESeq2	LRT	0.0816763864703444
126	KC_AB1	CG7369	-0.520814629658122	0.466624418667316	1	pseudobulk	DESeq2	LRT	0.33103253812052
126	KC_AB1	CG7370	1.25804452752548	0.727552935095488	1	pseudobulk	DESeq2	LRT	0.138135402914536
126	KC_AB1	CG7376	0.97008428177413	0.590896237053326	1	pseudobulk	DESeq2	LRT	0.228488775686571
126	KC_AB1	CG7378	-0.0198578847113684	0.890286425567891	1	pseudobulk	DESeq2	LRT	0.050470248375967
126	KC_AB1	CG7379	-0.34098834691496	0.505755889054476	1	pseudobulk	DESeq2	LRT	0.296059051574514
126	KC_AB1	CG7381	1.25804452752548	0.727552935095488	1	pseudobulk	DESeq2	LRT	0.138135402914536
126	KC_AB1	CG7382	-0.296348608985113	0.460778448233393	1	pseudobulk	DESeq2	LRT	0.336507842154723
126	KC_AB1	CG7384	1.25804452752548	0.573436424985144	1	pseudobulk	DESeq2	LRT	0.241514723898045
126	KC_AB1	CG7386	-0.760959112540794	1	1	pseudobulk	DESeq2	LRT	0
126	KC_AB1	CG7394	-0.576407983993865	0.19313866302539	1	pseudobulk	DESeq2	LRT	0.714130779261411
126	KC_AB1	CG7407	-0.192677959176619	0.534287291510499	1	pseudobulk	DESeq2	LRT	0.272225155767502
126	KC_AB1	CG7414	0.0174709378655451	0.938090861584195	1	pseudobulk	DESeq2	LRT	0.0277550946981956
126	KC_AB1	CG7420	0.444920770393282	0.735050727278635	1	pseudobulk	DESeq2	LRT	0.133682688375684
126	KC_AB1	CG7430	0.185528551723725	0.482373903286978	1	pseudobulk	DESeq2	LRT	0.316616195820298
126	KC_AB1	CG7453	0.353992152900822	0.664743430112118	1	pseudobulk	DESeq2	LRT	0.177345946256875
126	KC_AB1	CG7457	0.152115475746305	1	1	pseudobulk	DESeq2	LRT	0
126	KC_AB1	CG7460	0.152115475746305	1	1	pseudobulk	DESeq2	LRT	0
126	KC_AB1	CG7461	-0.46872022853379	0.520340215423296	1	pseudobulk	DESeq2	LRT	0.283712607581789
126	KC_AB1	CG7483	0.0479575604528391	0.901585644731408	1	pseudobulk	DESeq2	LRT	0.0449930118505571
126	KC_AB1	CG7484	-0.0187800671416801	0.970315527598631	1	pseudobulk	DESeq2	LRT	0.0130870187105441
126	KC_AB1	CG7488	-0.654089816098664	0.302008466648455	1	pseudobulk	DESeq2	LRT	0.519980881654895
126	KC_AB1	CG7492	-0.218731404602001	0.648448449055463	1	pseudobulk	DESeq2	LRT	0.18812454414528
126	KC_AB1	CG7497	-0.916882210103909	0.735691134343467	1	pseudobulk	DESeq2	LRT	0.133304477515894
127	KC_AB1	CG7504	-0.356088770115506	0.777360506788111	1	pseudobulk	DESeq2	LRT	0.109377527146582
127	KC_AB1	CG7506	0.435729842205492	0.327488788352907	1	pseudobulk	DESeq2	LRT	0.484803563580024
127	KC_AB1	CG7509	0.709686956006578	0.915714550115026	1	pseudobulk	DESeq2	LRT	0.0382398850985033
127	KC_AB1	CG7518	-0.10394920804815	0.821088343464176	1	pseudobulk	DESeq2	LRT	0.0856101132643441
127	KC_AB1	CG7519	-0.188998915839923	0.751224920112075	1	pseudobulk	DESeq2	LRT	0.124230013809501
127	KC_AB1	CG7523	-0.0304232258656891	0.965432703633775	1	pseudobulk	DESeq2	LRT	0.0152779937220883
127	KC_AB1	CG7530	0.868014633555195	0.610949502767246	1	pseudobulk	DESeq2	LRT	0.213994684316917
127	KC_AB1	CG7536	1.51094059755698	0.406244295079419	1	pseudobulk	DESeq2	LRT	0.391212724801806
127	KC_AB1	CG7544	0.296275065198765	1	1	pseudobulk	DESeq2	LRT	0
127	KC_AB1	CG7546	0.200164209101514	0.648915825794949	1	pseudobulk	DESeq2	LRT	0.187811634114313
127	KC_AB1	CG7550	-1.01829423517932	0.213327419177319	1	pseudobulk	DESeq2	LRT	0.670953320677612

127	KC_AB1	CG7556	0.308830301895931	0.665872460354765	1	pseudobulk	DESeq2	LRT	0.176608946608113
127	KC_AB1	CG7560	-0.760959112540794	0.804334723897681	1	pseudobulk	DESeq2	LRT	0.0945631819854715
127	KC_AB1	CG7564	-0.155625444739891	0.750098821254903	1	pseudobulk	DESeq2	LRT	0.124881517010293
127	KC_AB1	CG7565	0.0345165843679426	0.950388316218232	1	pseudobulk	DESeq2	LRT	0.0220989114129026
127	KC_AB1	CG7568	-0.570723996670538	0.824991814167186	1	pseudobulk	DESeq2	LRT	0.0835503606375391
127	KC_AB1	CG7582	0.15356061917797	0.683996419184818	1	pseudobulk	DESeq2	LRT	0.164946171865183
127	KC_AB1	CG7600	0.621956375549372	0.421138467656945	1	pseudobulk	DESeq2	LRT	0.375575087413441
127	KC_AB1	CG7601	0.0897677539642821	0.916103507575485	1	pseudobulk	DESeq2	LRT	0.0380554540294876
127	KC_AB1	CG7607	-0.354784117927217	0.429084219322055	1	pseudobulk	DESeq2	LRT	0.367457457472403
127	KC_AB1	CG7611	-0.311725681929042	0.539890299416174	1	pseudobulk	DESeq2	LRT	0.267694475729118
127	KC_AB1	CG7630	0.020959739019017	0.892973766201465	1	pseudobulk	DESeq2	LRT	0.0491612996347418
127	KC_AB1	CG7632	-0.703448719535984	0.577145027424715	1	pseudobulk	DESeq2	LRT	0.238715041790375
127	KC_AB1	CG7637	-0.20219693729756	0.810795904460156	1	pseudobulk	DESeq2	LRT	0.0910884537072562
127	KC_AB1	CG7638	0.325793233075846	0.561267659331146	1	pseudobulk	DESeq2	LRT	0.250829981437319
127	KC_AB1	CG7639	-0.0960654865430675	0.836839302928718	1	pseudobulk	DESeq2	LRT	0.0773579309574093
127	KC_AB1	CG7646	-0.0126593099360317	0.907890137454942	1	pseudobulk	DESeq2	LRT	0.0419667016813314
127	KC_AB1	CG7650	-0.0400809375755854	0.909274650142265	1	pseudobulk	DESeq2	LRT	0.0413049165304378
127	KC_AB1	CG7655	0.305596065579278	0.785528698960445	1	pseudobulk	DESeq2	LRT	0.104837943568024
127	KC_AB1	CG7656	0.00939659066319001	0.916045684273928	1	pseudobulk	DESeq2	LRT	0.0380828670162808
127	KC_AB1	CG7668	-1.45051948968537	0.20920739486408	1	pseudobulk	DESeq2	LRT	0.67942296850503
127	KC_AB1	CG7675	-2.99952832076105	0.338679393613493	1	pseudobulk	DESeq2	LRT	0.47021122647966
127	KC_AB1	CG7685	-0.255219253791939	0.774942637432999	1	pseudobulk	DESeq2	LRT	0.110730443517324
127	KC_AB1	CG7692	-0.451292468588751	0.658835748136001	1	pseudobulk	DESeq2	LRT	0.181222844236649
127	KC_AB1	CG7694	0.0407475110367662	0.93484486399004	1	pseudobulk	DESeq2	LRT	0.0292604536228019
127	KC_AB1	CG7695	-1.43550613534661	0.602448798560473	1	pseudobulk	DESeq2	LRT	0.220079857379737
127	KC_AB1	CG7702	0.943798956650056	0.807634491832869	1	pseudobulk	DESeq2	LRT	0.0927851418175362
127	KC_AB1	CG7706	-0.54514485936894	0.343842857651512	1	pseudobulk	DESeq2	LRT	0.463639992449637
127	KC_AB1	CG7718	-0.837446516594411	0.451811626059495	1	pseudobulk	DESeq2	LRT	0.345042597965186
127	KC_AB1	CG7720	-1.43550613534661	0.602448798560473	1	pseudobulk	DESeq2	LRT	0.220079857379737
127	KC_AB1	CG7724	-0.854632178888923	0.755457236781058	1	pseudobulk	DESeq2	LRT	0.121790114191456
127	KC_AB1	CG7728	-1.80903920340208	0.411521856685128	1	pseudobulk	DESeq2	LRT	0.385607093657874
127	KC_AB1	CG7737	0.754468740984315	0.389436490268736	1	pseudobulk	DESeq2	LRT	0.409563357438807
127	KC_AB1	CG7739	0.00072807116230929	0.988475961457841	1	pseudobulk	DESeq2	LRT	0.00503388773943743
127	KC_AB1	CG7741	-0.0857280005422969	0.93038054219399	1	pseudobulk	DESeq2	LRT	0.0313393809388347
127	KC_AB1	CG7744	-2.29825733823672	0.207183770272687	1	pseudobulk	DESeq2	LRT	0.683644268024676
127	KC_AB1	CG7745	0.742189141019244	0.296293001183717	1	pseudobulk	DESeq2	LRT	0.528278606984986
127	KC_AB1	CG7747	0.49234874396613	0.471838386802589	1	pseudobulk	DESeq2	LRT	0.326206729625974
127	KC_AB1	CG7766	-0.0983865127406808	0.800934842036286	1	pseudobulk	DESeq2	LRT	0.096402813372824
127	KC_AB1	CG7772	-0.0892865524527621	0.907252182181087	1	pseudobulk	DESeq2	LRT	0.0422719785332315
127	KC_AB1	CG7781	-0.0999717689609146	0.608120838554202	1	pseudobulk	DESeq2	LRT	0.216010114308034
127	KC_AB1	CG7785	0.0272374395707528	0.955563447814664	1	pseudobulk	DESeq2	LRT	0.0197404712243813
127	KC_AB1	CG7789	0.122088252647975	0.867751756505145	1	pseudobulk	DESeq2	LRT	0.0616044985557497
127	KC_AB1	CG7791	0.466906500067359	0.631552553556338	1	pseudobulk	DESeq2	LRT	0.199590504538774
127	KC_AB1	CG7800	-0.287723761595917	0.908359580254375	1	pseudobulk	DESeq2	LRT	0.0417421990483341
127	KC_AB1	CG7806	0.470767000077022	0.746384267349181	1	pseudobulk	DESeq2	LRT	0.12703752343552
127	KC_AB1	CG7810	-0.0477264225534411	0.943393980731077	1	pseudobulk	DESeq2	LRT	0.0253068990714682
127	KC_AB1	CG7824	2.09987857572813	0.503115176013642	1	pseudobulk	DESeq2	LRT	0.29833258237694
127	KC_AB1	CG7834	0.0920323644265257	0.847129743297622	1	pseudobulk	DESeq2	LRT	0.072050069618448
127	KC_AB1	CG7839	0.412527033454875	0.567372360119908	1	pseudobulk	DESeq2	LRT	0.246131824937394
127	KC_AB1	CG7840	-1.1666790793267	0.398349396946026	1	pseudobulk	DESeq2	LRT	0.399735835968507
127	KC_AB1	CG7841	1.36145419086214	0.20637247237679	1	pseudobulk	DESeq2	LRT	0.685348232878925
127	KC_AB1	CG7849	-0.339902225163692	0.794531425704039	1	pseudobulk	DESeq2	LRT	0.0998889206844884
127	KC_AB1	CG7857	1.45917382732887	0.0361408005358085	1	pseudobulk	DESeq2	LRT	1.44200223184056
127	KC_AB1	CG7860	0.494775748955277	0.827825706404751	1	pseudobulk	DESeq2	LRT	0.0820610916144188
127	KC_AB1	CG7872	0.25115047084483	0.722714096473927	1	pseudobulk	DESeq2	LRT	0.141033474324286
127	KC_AB1	CG7878	0.617787346262563	0.283292211675163	1	pseudobulk	DESeq2	LRT	0.547765365024196

127	KC_AB1	CG7879	-0.148596177077302	0.700840523295352	1	pseudobulk	DESeq2	LRT	0.154380794775545
127	KC_AB1	CG7884	1.25804452752548	0.727552935095488	1	pseudobulk	DESeq2	LRT	0.138135402914536
127	KC_AB1	CG7888	-0.327410666063955	0.421512919374609	1	pseudobulk	DESeq2	LRT	0.375189109705631
127	KC_AB1	CG7889	0.0104265948157896	0.974922664647641	1	pseudobulk	DESeq2	LRT	0.0110298331720897
127	KC_AB1	CG7896	-2.00963963748391	0.498268873088307	1	pseudobulk	DESeq2	LRT	0.302536242405947
127	KC_AB1	CG7900	-0.877833043512066	0.751842013479608	1	pseudobulk	DESeq2	LRT	0.12387340925197
127	KC_AB1	CG7903	-0.01151539012923	0.941330206200462	1	pseudobulk	DESeq2	LRT	0.0262580050794211
127	KC_AB1	CG7914	0.220614585804744	0.864454273705771	1	pseudobulk	DESeq2	LRT	0.0632579742841892
127	KC_AB1	CG7920	0.279178375258364	0.237908895724154	1	pseudobulk	DESeq2	LRT	0.623589318821566
127	KC_AB1	CG7943	0.42985191267958	0.362705432453401	1	pseudobulk	DESeq2	LRT	0.440445939685992
127	KC_AB1	CG7945	-0.123579122480847	0.692439716607371	1	pseudobulk	DESeq2	LRT	0.159618030038786
127	KC_AB1	CG7946	0.779328126695051	0.211304107240873	1	pseudobulk	DESeq2	LRT	0.675092061231118
127	KC_AB1	CG7953	-0.760959112540794	1	1	pseudobulk	DESeq2	LRT	0
127	KC_AB1	CG7956	-0.0544227628127224	0.908101250718715	1	pseudobulk	DESeq2	LRT	0.0418657261739327
127	KC_AB1	CG7966	4.7449443527972	0.0678535993682284	1	pseudobulk	DESeq2	LRT	1.16842710976845
127	KC_AB1	CG7970	0.28727417421595	0.739301570802229	1	pseudobulk	DESeq2	LRT	0.13117837105121
127	KC_AB1	CG7971	-0.0106032556361899	0.945274531202207	1	pseudobulk	DESeq2	LRT	0.0244420432671866
127	KC_AB1	CG7974	1.01258527853401	0.278953312100587	1	pseudobulk	DESeq2	LRT	0.554468477707748
127	KC_AB1	CG7985	-0.129241801494228	0.548186953176295	1	pseudobulk	DESeq2	LRT	0.261071304861767
127	KC_AB1	CG7987	0.0150521491405112	0.974077676764488	1	pseudobulk	DESeq2	LRT	0.0114064093995267
127	KC_AB1	CG7988	0.103326968131049	0.898549439771292	1	pseudobulk	DESeq2	LRT	0.0464580222510003
127	KC_AB1	CG7990	-0.274696622950244	0.229417997948568	1	pseudobulk	DESeq2	LRT	0.639372514494297
127	KC_AB1	CG7991	-3.30270707798607	0.137902667339578	1	pseudobulk	DESeq2	LRT	0.860427333536574
127	KC_AB1	CG7997	-2.35716291794055	0.404761325252261	1	pseudobulk	DESeq2	LRT	0.392800990812383
127	KC_AB1	CG8001	0.825698813969927	0.334531192575106	1	pseudobulk	DESeq2	LRT	0.475563381239485
127	KC_AB1	CG8003	0.273236734298678	0.755011969541984	1	pseudobulk	DESeq2	LRT	0.122046163251833
127	KC_AB1	CG8004	-0.0316281764128094	0.938682865311387	1	pseudobulk	DESeq2	LRT	0.0274811096601183
127	KC_AB1	CG8005	-1.81083883321868	0.24699430896512	1	pseudobulk	DESeq2	LRT	0.607313053272797
127	KC_AB1	CG8006	1.25804452752548	0.573436424985144	1	pseudobulk	DESeq2	LRT	0.241514723898045
127	KC_AB1	CG8008	-2.36175013442542	0.448372544778042	1	pseudobulk	DESeq2	LRT	0.348360988442027
127	KC_AB1	CG8009	0.278444517980005	0.53531362832818	1	pseudobulk	DESeq2	LRT	0.271391699952297
127	KC_AB1	CG8010	-1.17056939481669	0.334513181602447	1	pseudobulk	DESeq2	LRT	0.475586764041414
127	KC_AB1	CG8027	0.9102053909742	0.394752033078954	1	pseudobulk	DESeq2	LRT	0.403675624580726
128	KC_AB1	CG8031	1.11851839779133	0.1054824182666	1	pseudobulk	DESeq2	LRT	0.976819922225197
128	KC_AB1	CG8032	-0.588140235910128	0.312067680294593	1	pseudobulk	DESeq2	LRT	0.505751207292798
128	KC_AB1	CG8034	0.556780593584531	0.704131609388816	1	pseudobulk	DESeq2	LRT	0.152346159196863
128	KC_AB1	CG8036	1.03521963526943	0.386838477205957	1	pseudobulk	DESeq2	LRT	0.412470334984227
128	KC_AB1	CG8038	0.0255909774768194	0.921417428245023	1	pseudobulk	DESeq2	LRT	0.0355435774978603
128	KC_AB1	CG8042	-0.083194377002097	0.879876030168584	1	pseudobulk	DESeq2	LRT	0.0555785133116167
128	KC_AB1	CG8043	-0.668643129789577	0.579915891353366	1	pseudobulk	DESeq2	LRT	0.236634990178297
128	KC_AB1	CG8060	-0.180123390539422	0.841544815543244	1	pseudobulk	DESeq2	LRT	0.0749227511975082
128	KC_AB1	CG8064	0.256531107369856	0.830157228082486	1	pseudobulk	DESeq2	LRT	0.080839646392673
128	KC_AB1	CG8066	-2.17628857351781	0.230899283144635	1	pseudobulk	DESeq2	LRT	0.636577415401632
128	KC_AB1	CG8067	-0.0314782056353535	0.977996108046488	1	pseudobulk	DESeq2	LRT	0.00966287349184355
128	KC_AB1	CG8078	0.152719507639627	0.846855356787389	1	pseudobulk	DESeq2	LRT	0.0721907609956497
128	KC_AB1	CG8079	-0.717050183829282	0.357870612266688	1	pseudobulk	DESeq2	LRT	0.446273963678825
128	KC_AB1	CG8080	0.212102334211717	0.831329660385192	1	pseudobulk	DESeq2	LRT	0.080226724355768
128	KC_AB1	CG8097	3.00052729308995	0.20959030983652	1	pseudobulk	DESeq2	LRT	0.678628800322909
128	KC_AB1	CG8108	-0.084387487074057	0.662832762036256	1	pseudobulk	DESeq2	LRT	0.178596033716912
128	KC_AB1	CG8111	-0.039193268892206	0.945365003375014	1	pseudobulk	DESeq2	LRT	0.0244004789555054
128	KC_AB1	CG8112	0.0385617171861529	0.961057066817037	1	pseudobulk	DESeq2	LRT	0.0172508234990893
128	KC_AB1	CG8128	0.300492807978046	0.764673878321778	1	pseudobulk	DESeq2	LRT	0.116523745294887
128	KC_AB1	CG8132	0.673394487160123	0.246172248919781	1	pseudobulk	DESeq2	LRT	0.608760906808039
128	KC_AB1	CG8134	0.194081444106507	0.682564606657527	1	pseudobulk	DESeq2	LRT	0.165856235148866
128	KC_AB1	CG8142	0.370263142375327	0.863762501301616	1	pseudobulk	DESeq2	LRT	0.0636056539945678
128	KC_AB1	CG8149	0.42262835144957	0.352737879003608	1	pseudobulk	DESeq2	LRT	0.45254790081186

128	KC_AB1	CG8152	0.386383082700322	0.718265125328243	1	pseudobulk	DESeq2	LRT	0.14371521980531
128	KC_AB1	CG8155	-0.678603252068186	0.406750075261766	1	pseudobulk	DESeq2	LRT	0.390672358032735
128	KC_AB1	CG8157	1.13734983340557	0.742793668201421	1	pseudobulk	DESeq2	LRT	0.1291318069744
128	KC_AB1	CG8159	-0.986914887916529	0.443854412450751	1	pseudobulk	DESeq2	LRT	0.352759458348769
128	KC_AB1	CG8160	1.11388934932283	1	1	pseudobulk	DESeq2	LRT	0
128	KC_AB1	CG8173	0.0828732960497558	0.91780347980775	1	pseudobulk	DESeq2	LRT	0.0372503000315981
128	KC_AB1	CG8176	0.228150633154664	0.564695060456064	1	pseudobulk	DESeq2	LRT	0.248186011140501
128	KC_AB1	CG8177	0.139955460103571	0.559226795522529	1	pseudobulk	DESeq2	LRT	0.252412027411211
128	KC_AB1	CG8180	4.5921249058718	0.0212123433254252	1	pseudobulk	DESeq2	LRT	1.6734113523657
128	KC_AB1	CG8187	-0.553111674092548	0.283132981550634	1	pseudobulk	DESeq2	LRT	0.548009537660175
128	KC_AB1	CG8188	-0.0403886526749548	0.874610300716443	1	pseudobulk	DESeq2	LRT	0.0581854120609457
128	KC_AB1	CG8191	-0.760959112540794	1	1	pseudobulk	DESeq2	LRT	0
128	KC_AB1	CG8195	0.374016307844965	0.410942276768058	1	pseudobulk	DESeq2	LRT	0.386219177252117
128	KC_AB1	CG8199	-0.760959112540794	0.805265236978025	1	pseudobulk	DESeq2	LRT	0.0940610488455931
128	KC_AB1	CG8202	1.30959696927673	0.397105374014993	1	pseudobulk	DESeq2	LRT	0.401094235602285
128	KC_AB1	CG8204	0.157074661812188	0.859566933455133	1	pseudobulk	DESeq2	LRT	0.0657202996652218
128	KC_AB1	CG8206	0.272574567232098	0.581129658646462	1	pseudobulk	DESeq2	LRT	0.235726959257827
128	KC_AB1	CG8207	-0.452650942954775	0.645026466348386	1	pseudobulk	DESeq2	LRT	0.190422465282181
128	KC_AB1	CG8209	0.149628870587668	0.770305873497825	1	pseudobulk	DESeq2	LRT	0.113336790678366
128	KC_AB1	CG8219	1.25804452752548	0.573436424985144	1	pseudobulk	DESeq2	LRT	0.241514723898045
128	KC_AB1	CG8223	0.028808791380841	0.951708101910405	1	pseudobulk	DESeq2	LRT	0.0214962335150569
128	KC_AB1	CG8229	-0.0474561683969958	0.925972861122959	1	pseudobulk	DESeq2	LRT	0.033401741652041
128	KC_AB1	CG8230	0.16982555969311	0.818969711021172	1	pseudobulk	DESeq2	LRT	0.0867321599973545
128	KC_AB1	CG8237	0.592688502766677	0.443320767328956	1	pseudobulk	DESeq2	LRT	0.353281923730858
128	KC_AB1	CG8243	0.241524428348886	0.50364416131002	1	pseudobulk	DESeq2	LRT	0.297876196410902
128	KC_AB1	CG8245	0.141111961120265	0.541773206023858	1	pseudobulk	DESeq2	LRT	0.266182477272081
128	KC_AB1	CG8248	-0.283072521358939	0.498796366938262	1	pseudobulk	DESeq2	LRT	0.302076718434095
128	KC_AB1	CG8270	-2.09383583728264	0.110496848969382	1	pseudobulk	DESeq2	LRT	0.95665010654649
128	KC_AB1	CG8272	0.150686746316668	0.904196849730142	1	pseudobulk	DESeq2	LRT	0.0437370104041347
128	KC_AB1	CG8281	0.162911738619242	0.789679733364715	1	pseudobulk	DESeq2	LRT	0.10254900773952
128	KC_AB1	CG8289	-0.307432347310218	0.458107853925553	1	pseudobulk	DESeq2	LRT	0.339032262497459
128	KC_AB1	CG8292	1.11388934932283	1	1	pseudobulk	DESeq2	LRT	0
128	KC_AB1	CG8298	-0.502579979504666	0.748544033513919	1	pseudobulk	DESeq2	LRT	0.125782646958033
128	KC_AB1	CG8300	0.102136058106929	0.856662304763038	1	pseudobulk	DESeq2	LRT	0.0671903426982895
128	KC_AB1	CG8301	-0.455314528600414	0.298634469571312	1	pseudobulk	DESeq2	LRT	0.524860065731436
128	KC_AB1	CG8303	1.13734983340557	0.742793668201421	1	pseudobulk	DESeq2	LRT	0.1291318069744
128	KC_AB1	CG8306	3.26726616948798	0.160845947750703	1	pseudobulk	DESeq2	LRT	0.79358987595988
128	KC_AB1	CG8311	-0.18488853838531	0.815731935872573	1	pseudobulk	DESeq2	LRT	0.0884525347484545
128	KC_AB1	CG8312	0.12206176563451	0.934258554218887	1	pseudobulk	DESeq2	LRT	0.0295329169912476
128	KC_AB1	CG8314	0.327101157508272	0.521482614097583	1	pseudobulk	DESeq2	LRT	0.282760166101012
128	KC_AB1	CG8319	0.443302328374104	0.68326327186321	1	pseudobulk	DESeq2	LRT	0.165411923717297
128	KC_AB1	CG8320	0.265182362842651	0.747113932394331	1	pseudobulk	DESeq2	LRT	0.12661316466761
128	KC_AB1	CG8321	-0.00908164003130161	0.953142311947158	1	pseudobulk	DESeq2	LRT	0.020842250800171
128	KC_AB1	CG8323	0.199829194819027	0.836203998269455	1	pseudobulk	DESeq2	LRT	0.0776877602223295
128	KC_AB1	CG8326	-0.566621973379529	0.588734438131064	1	pseudobulk	DESeq2	LRT	0.230080559307851
128	KC_AB1	CG8336	-0.123444367224865	0.890321408604803	1	pseudobulk	DESeq2	LRT	0.0504531834843034
128	KC_AB1	CG8343	1.25804452752548	0.727552935095488	1	pseudobulk	DESeq2	LRT	0.138135402914536
128	KC_AB1	CG8353	-0.354809234975995	0.822270464282259	1	pseudobulk	DESeq2	LRT	0.0849853091931144
128	KC_AB1	CG8360	-0.00634386304501498	0.93071004378284	1	pseudobulk	DESeq2	LRT	0.0311855993610982
128	KC_AB1	CG8366	-0.69150238267904	0.354767009615221	1	pseudobulk	DESeq2	LRT	0.450056772741708
128	KC_AB1	CG8369	-0.0542328150841312	0.68290038064655	1	pseudobulk	DESeq2	LRT	0.165642645207133
128	KC_AB1	CG8370	-0.200689051495307	0.833101982728466	1	pseudobulk	DESeq2	LRT	0.079301831936265
128	KC_AB1	CG8372	0.313256603051586	0.37950780607867	1	pseudobulk	DESeq2	LRT	0.420779286693334
128	KC_AB1	CG8379	1.0054894802377	0.189001779444031	1	pseudobulk	DESeq2	LRT	0.723534106942705
128	KC_AB1	CG8388	-0.381998896801297	0.713555200808928	1	pseudobulk	DESeq2	LRT	0.146572424119908
128	KC_AB1	CG8389	-0.0750606872097162	0.884330223159501	1	pseudobulk	DESeq2	LRT	0.0533855321406975

128	KC_AB1	CG8397	-0.0436868938853624	0.888906570591492	1	pseudobulk	DESeq2	LRT	0.0511438835841978
128	KC_AB1	CG8401	0.296275065198765	1	1	pseudobulk	DESeq2	LRT	0
128	KC_AB1	CG8405	0.404933119831178	0.412215626308868	1	pseudobulk	DESeq2	LRT	0.384875548968543
128	KC_AB1	CG8407	1.25804452752548	0.727552935095488	1	pseudobulk	DESeq2	LRT	0.138135402914536
128	KC_AB1	CG8412	0.311403627822937	0.661918519125124	1	pseudobulk	DESeq2	LRT	0.179195468071277
128	KC_AB1	CG8414	-0.258852269576678	0.826151666526968	1	pseudobulk	DESeq2	LRT	0.0829402167361554
128	KC_AB1	CG8435	-0.0532965709201217	0.947919759637744	1	pseudobulk	DESeq2	LRT	0.0232284236550057
128	KC_AB1	CG8441	-0.560371517604659	0.428997124690287	1	pseudobulk	DESeq2	LRT	0.367545618620234
128	KC_AB1	CG8460	0.986765630111248	0.518716021514778	1	pseudobulk	DESeq2	LRT	0.285070337801667
128	KC_AB1	CG8461	-0.220080952751388	0.820147953836543	1	pseudobulk	DESeq2	LRT	0.0861077942764994
128	KC_AB1	CG8475	-0.030082793247415	0.959360233230836	1	pseudobulk	DESeq2	LRT	0.0180182875861406
128	KC_AB1	CG8485	-0.0731862084301332	0.793184488600593	1	pseudobulk	DESeq2	LRT	0.100625787382819
128	KC_AB1	CG8490	0.561982454257405	0.368502951546087	1	pseudobulk	DESeq2	LRT	0.433559029283775
128	KC_AB1	CG8500	0.0702979304119323	0.755810251086122	1	pseudobulk	DESeq2	LRT	0.121587222024793
128	KC_AB1	CG8501	4.16690208976446	0.147959240986735	1	pseudobulk	DESeq2	LRT	0.829857905229841
128	KC_AB1	CG8507	0.433751618745526	0.541732817915446	1	pseudobulk	DESeq2	LRT	0.266214854263512
128	KC_AB1	CG8509	-0.760959112540794	1	1	pseudobulk	DESeq2	LRT	0
128	KC_AB1	CG8516	0.443100694673436	0.669547784327311	1	pseudobulk	DESeq2	LRT	0.174218422790698
128	KC_AB1	CG8519	1.1332161709987	0.32951683828293	1	pseudobulk	DESeq2	LRT	0.482122388091164
128	KC_AB1	CG8520	0.761781989355316	0.393374310370476	1	pseudobulk	DESeq2	LRT	0.405194005445255
128	KC_AB1	CG8531	-0.881268034841729	0.262279237213157	1	pseudobulk	DESeq2	LRT	0.581236088062554
129	KC_AB1	CG8545	0.13633722683056	0.926753893606694	1	pseudobulk	DESeq2	LRT	0.0330355806764947
129	KC_AB1	CG8547	1.25804452752548	0.573436424985144	1	pseudobulk	DESeq2	LRT	0.241514723898045
129	KC_AB1	CG8549	0.568208657832426	0.416883153180162	1	pseudobulk	DESeq2	LRT	0.379985654954599
129	KC_AB1	CG8565	1.25804452752548	0.727552935095488	1	pseudobulk	DESeq2	LRT	0.138135402914536
129	KC_AB1	CG8569	0.194259604983429	0.806551183925135	1	pseudobulk	DESeq2	LRT	0.0933680669719722
129	KC_AB1	CG8578	-0.011412539242899	0.978751046933142	1	pseudobulk	DESeq2	LRT	0.00932776038529674
129	KC_AB1	CG8586	0.106531657243297	0.956043605272399	1	pseudobulk	DESeq2	LRT	0.019522299044709
129	KC_AB1	CG8607	1.25804452752548	0.573436424985144	1	pseudobulk	DESeq2	LRT	0.241514723898045
129	KC_AB1	CG8611	-0.794902313862786	0.462615689152375	1	pseudobulk	DESeq2	LRT	0.334779642648926
129	KC_AB1	CG8613	-0.0763629636869244	0.825044469804373	1	pseudobulk	DESeq2	LRT	0.0835226423953389
129	KC_AB1	CG8617	-0.0268044805722852	0.92732193356966	1	pseudobulk	DESeq2	LRT	0.0327694679042776
129	KC_AB1	CG8620	-1.76935196357135	0.542380240270211	1	pseudobulk	DESeq2	LRT	0.265696140855463
129	KC_AB1	CG8630	0.773244287926187	0.836877627343698	1	pseudobulk	DESeq2	LRT	0.0773380421916931
129	KC_AB1	CG8635	0.266088763941611	0.532948872109709	1	pseudobulk	DESeq2	LRT	0.273314452560063
129	KC_AB1	CG8641	0.19129035730113	0.887304185013865	1	pseudobulk	DESeq2	LRT	0.0519274701049267
129	KC_AB1	CG8654	-0.760959112540794	1	1	pseudobulk	DESeq2	LRT	0
129	KC_AB1	CG8665	-0.760959112540794	0.804334723897681	1	pseudobulk	DESeq2	LRT	0.0945631819854715
129	KC_AB1	CG8668	-0.757722436524803	0.0683268580681542	1	pseudobulk	DESeq2	LRT	1.16540854934955
129	KC_AB1	CG8671	-0.0232726139488303	0.904912362000526	1	pseudobulk	DESeq2	LRT	0.0433934788528613
129	KC_AB1	CG8673	1.80346638298797	0.51759809238489	1	pseudobulk	DESeq2	LRT	0.286007332934878
129	KC_AB1	CG8675	0.0670249694169819	0.929082359409139	1	pseudobulk	DESeq2	LRT	0.0319457858432582
129	KC_AB1	CG8677	-0.0740628434592613	0.762294802210654	1	pseudobulk	DESeq2	LRT	0.117877041503649
129	KC_AB1	CG8679	-1.59146823391525	0.486084569581447	1	pseudobulk	DESeq2	LRT	0.313288165084674
129	KC_AB1	CG8712	0.559325689655977	0.631065237766349	1	pseudobulk	DESeq2	LRT	0.199925742281342
129	KC_AB1	CG8726	-0.207358520275909	0.368574522508023	1	pseudobulk	DESeq2	LRT	0.433474688424029
129	KC_AB1	CG8728	-0.224735274644372	0.739134283787039	1	pseudobulk	DESeq2	LRT	0.131276653066863
129	KC_AB1	CG8738	-0.385011853448768	0.890534840916548	1	pseudobulk	DESeq2	LRT	0.0503490847107839
129	KC_AB1	CG8740	2.94103562241097	0.277249645217157	1	pseudobulk	DESeq2	LRT	0.557129000923904
129	KC_AB1	CG8745	-0.686137674013748	0.30842690953121	1	pseudobulk	DESeq2	LRT	0.510847737783586
129	KC_AB1	CG8765	-0.270651583807437	0.721315100409265	1	pseudobulk	DESeq2	LRT	0.141874975953878
129	KC_AB1	CG8768	0.89723701357417	0.388724795936583	1	pseudobulk	DESeq2	LRT	0.410357755754207
129	KC_AB1	CG8777	-0.126536520532211	0.882780744470617	1	pseudobulk	DESeq2	LRT	0.0541471483936632
129	KC_AB1	CG8778	0.574878975082321	0.398091378666212	1	pseudobulk	DESeq2	LRT	0.400017227686523
129	KC_AB1	CG8785	2.09987857572813	0.503115176013642	1	pseudobulk	DESeq2	LRT	0.29833258237694
129	KC_AB1	CG8814	-0.122270751449961	0.839290411847619	1	pseudobulk	DESeq2	LRT	0.0760877382897971

129	KC_AB1	CG8818	0.104563087684561	0.737513509547474	1	pseudobulk	DESeq2	LRT	0.132230020003108
129	KC_AB1	CG8833	0.437265228982838	0.588004697318858	1	pseudobulk	DESeq2	LRT	0.23061920451653
129	KC_AB1	CG8839	0.0249592905677713	0.972752474837972	1	pseudobulk	DESeq2	LRT	0.0119976556081493
129	KC_AB1	CG8841	-0.855295458798765	0.324893709195905	1	pseudobulk	DESeq2	LRT	0.488258697666882
129	KC_AB1	CG8850	1.25804452752548	0.727552935095488	1	pseudobulk	DESeq2	LRT	0.138135402914536
129	KC_AB1	CG8854	-2.00963963748391	0.498268873088307	1	pseudobulk	DESeq2	LRT	0.302536242405947
129	KC_AB1	CG8858	-0.804959006931996	0.426218610134915	1	pseudobulk	DESeq2	LRT	0.370367591453237
129	KC_AB1	CG8860	0.0493359163439872	0.895483014991285	1	pseudobulk	DESeq2	LRT	0.0479426471857101
129	KC_AB1	CG8861	-0.657059716098719	0.650263111317708	1	pseudobulk	DESeq2	LRT	0.186910882322363
129	KC_AB1	CG8878	-0.0200821052114455	0.943351910301367	1	pseudobulk	DESeq2	LRT	0.0253262667622251
129	KC_AB1	CG8888	-1.49339698681285	0.562776365708835	1	pseudobulk	DESeq2	LRT	0.249664149435163
129	KC_AB1	CG8891	0.583457947487164	0.480478513075259	1	pseudobulk	DESeq2	LRT	0.318326029143198
129	KC_AB1	CG8892	-0.390981497018252	0.550271311325444	1	pseudobulk	DESeq2	LRT	0.259423128762229
129	KC_AB1	CG8910	0.278294305372047	0.30739246505743	1	pseudobulk	DESeq2	LRT	0.512306782327772
129	KC_AB1	CG8915	0.207601913729876	0.852056185523562	1	pseudobulk	DESeq2	LRT	0.0695317664324646
129	KC_AB1	CG8916	1.11388934932283	1	1	pseudobulk	DESeq2	LRT	0
129	KC_AB1	CG8924	0.0622014133587821	0.897854123821531	1	pseudobulk	DESeq2	LRT	0.0467942183055627
129	KC_AB1	CG8927	1.94091064698073	0.616174344495411	1	pseudobulk	DESeq2	LRT	0.210296388258659
129	KC_AB1	CG8929	-0.207092969509487	0.463643552371678	1	pseudobulk	DESeq2	LRT	0.33381577532644
129	KC_AB1	CG8939	-0.416612610694332	0.777736209875977	1	pseudobulk	DESeq2	LRT	0.109167680672956
129	KC_AB1	CG8944	0.325510528282467	0.707293581068246	1	pseudobulk	DESeq2	LRT	0.150400283270996
129	KC_AB1	CG8950	-0.248843574470018	0.887931446317941	1	pseudobulk	DESeq2	LRT	0.0516205630897671
129	KC_AB1	CG8963	-0.361443180837202	0.381675627763531	1	pseudobulk	DESeq2	LRT	0.418305571425676
129	KC_AB1	CG8974	-0.0549984397326645	0.839124746804869	1	pseudobulk	DESeq2	LRT	0.0761734708497935
129	KC_AB1	CG8993	0.0441606503832492	0.857631095910494	1	pseudobulk	DESeq2	LRT	0.0666994807558915
129	KC_AB1	CG9003	0.0687126739719847	0.829476059348314	1	pseudobulk	DESeq2	LRT	0.0811961442302949
129	KC_AB1	CG9004	-0.60442630066423	0.566835381135731	1	pseudobulk	DESeq2	LRT	0.246543049475311
129	KC_AB1	CG9005	-0.0422106279404992	0.84111139987294	1	pseudobulk	DESeq2	LRT	0.0751464808416528
129	KC_AB1	CG9008	1.13988912687578	0.653854063433791	1	pseudobulk	DESeq2	LRT	0.184519172956856
129	KC_AB1	CG9016	1.11388934932283	1	1	pseudobulk	DESeq2	LRT	0
129	KC_AB1	CG9018	-0.153101891096822	0.779242153102345	1	pseudobulk	DESeq2	LRT	0.108327562335062
129	KC_AB1	CG9034	0.00824104317832346	0.95675708019434	1	pseudobulk	DESeq2	LRT	0.0191983152252762
129	KC_AB1	CG9044	-0.402015427289495	0.51330744019213	1	pseudobulk	DESeq2	LRT	0.289622440763651
129	KC_AB1	CG9062	-0.14905601255371	0.858422669709028	1	pseudobulk	DESeq2	LRT	0.0662988217975393
129	KC_AB1	CG9065	0.0378955240173494	0.897253234105843	1	pseudobulk	DESeq2	LRT	0.0470849675984671
129	KC_AB1	CG9067	0.036727922806816	0.958566794981411	1	pseudobulk	DESeq2	LRT	0.0183776191645014
129	KC_AB1	CG9072	-0.760959112540794	1	1	pseudobulk	DESeq2	LRT	0
129	KC_AB1	CG9095	4.29764035701693	0.0723138580006471	1	pseudobulk	DESeq2	LRT	1.14077846789917
129	KC_AB1	CG9098	-1.2274657738723	0.537169033458596	1	pseudobulk	DESeq2	LRT	0.269889031345258
129	KC_AB1	CG9106	-0.760959112540794	0.804334723897681	1	pseudobulk	DESeq2	LRT	0.0945631819854715
129	KC_AB1	CG9107	-0.757748357328474	0.452464362326306	1	pseudobulk	DESeq2	LRT	0.344415621662177
129	KC_AB1	CG9117	2.13409626988176	0.404972692061512	1	pseudobulk	DESeq2	LRT	0.392574260950386
129	KC_AB1	CG9119	-0.14993231604172	1	1	pseudobulk	DESeq2	LRT	0
129	KC_AB1	CG9121	-0.108803961701762	0.886308731265505	1	pseudobulk	DESeq2	LRT	0.0524149723229471
129	KC_AB1	CG9123	-0.142083530001089	0.758755715417595	1	pseudobulk	DESeq2	LRT	0.119898024529569
129	KC_AB1	CG9125	-0.125425255172401	0.886993923122915	1	pseudobulk	DESeq2	LRT	0.052079355549588
129	KC_AB1	CG9132	0.0353561541684047	0.894060039318244	1	pseudobulk	DESeq2	LRT	0.0486333158024213
129	KC_AB1	CG9135	-0.0888382881554794	0.877475949908454	1	pseudobulk	DESeq2	LRT	0.0567647779563646
129	KC_AB1	CG9143	-0.169214885172879	0.829808119271818	1	pseudobulk	DESeq2	LRT	0.0810223201244272
129	KC_AB1	CG9147	-0.396367083332471	0.658890729285313	1	pseudobulk	DESeq2	LRT	0.181186603013333
129	KC_AB1	CG9149	0.170002675034452	0.765067572797384	1	pseudobulk	DESeq2	LRT	0.116300205111725
129	KC_AB1	CG9154	0.111673173663607	0.871434708830978	1	pseudobulk	DESeq2	LRT	0.0597651463126232
129	KC_AB1	CG9164	-0.416359569744652	0.672991245979053	1	pseudobulk	DESeq2	LRT	0.171990584881102
129	KC_AB1	CG9170	-0.0444562974921338	0.854289596676983	1	pseudobulk	DESeq2	LRT	0.0683948823333855
129	KC_AB1	CG9171	-0.111965641147858	0.643415214092538	1	pseudobulk	DESeq2	LRT	0.191508674055254
129	KC_AB1	CG9175	0.27454860396043	0.684780940017109	1	pseudobulk	DESeq2	LRT	0.164448336182236

129	KC_AB1	CG9205	-0.232820559789079	0.573661302846927	1	pseudobulk	DESeq2	LRT	0.241344445092053
129	KC_AB1	CG9213	-0.713882473030676	0.610339453290554	1	pseudobulk	DESeq2	LRT	0.214428555665932
129	KC_AB1	CG9215	-0.0551696758688432	0.911122642681618	1	pseudobulk	DESeq2	LRT	0.0404231603980815
129	KC_AB1	CG9220	1.11388934932283	1	1	pseudobulk	DESeq2	LRT	0
129	KC_AB1	CG9231	0.34619006427721	0.367808647125687	1	pseudobulk	DESeq2	LRT	0.434378064780309
129	KC_AB1	CG9240	-0.477292323848196	0.391957044889127	1	pseudobulk	DESeq2	LRT	0.406761525300479
129	KC_AB1	CG9246	0.311902751596414	0.764002358259744	1	pseudobulk	DESeq2	LRT	0.116905300877696
129	KC_AB1	CG9248	-0.722499828593808	0.75996324350504	1	pseudobulk	DESeq2	LRT	0.119207412362586
129	KC_AB1	CG9253	-0.367372507256304	0.635708614952109	1	pseudobulk	DESeq2	LRT	0.196741903087694
130	KC_AB1	CG9257	-0.105315560780876	0.854086893903582	1	pseudobulk	DESeq2	LRT	0.0684979423987203
130	KC_AB1	CG9263	0.943805700130147	0.808046339172447	1	pseudobulk	DESeq2	LRT	0.0925637329508755
130	KC_AB1	CG9265	-0.267617644686843	0.546469338961651	1	pseudobulk	DESeq2	LRT	0.262434200216235
130	KC_AB1	CG9270	1.25804452752548	0.727552935095488	1	pseudobulk	DESeq2	LRT	0.138135402914536
130	KC_AB1	CG9272	-0.273025315354969	0.880667207053856	1	pseudobulk	DESeq2	LRT	0.0551881749531499
130	KC_AB1	CG9281	-0.0919812533489665	0.747512184240407	1	pseudobulk	DESeq2	LRT	0.126381724066744
130	KC_AB1	CG9286	0.682823178061361	0.42749970593603	1	pseudobulk	DESeq2	LRT	0.369064179673594
130	KC_AB1	CG9289	-0.760959112540794	1	1	pseudobulk	DESeq2	LRT	0
130	KC_AB1	CG9300	0.279973078866262	0.864639057586978	1	pseudobulk	DESeq2	LRT	0.0631651503529349
130	KC_AB1	CG9302	0.550793828766587	0.68673759813867	1	pseudobulk	DESeq2	LRT	0.163209174803031
130	KC_AB1	CG9304	-0.21672064602522	0.818563478439529	1	pseudobulk	DESeq2	LRT	0.0869476360235281
130	KC_AB1	CG9328	-0.164656321924146	0.408562458427974	1	pseudobulk	DESeq2	LRT	0.388741541880314
130	KC_AB1	CG9330	0.316488689372523	0.498334136712345	1	pseudobulk	DESeq2	LRT	0.302479361919835
130	KC_AB1	CG9331	-1.20417092131936	0.459206484505431	1	pseudobulk	DESeq2	LRT	0.337991987846488
130	KC_AB1	CG9336	3.7951235223405	0.206529069530445	1	pseudobulk	DESeq2	LRT	0.685018811571072
130	KC_AB1	CG9338	1.25804452752548	0.573436424985144	1	pseudobulk	DESeq2	LRT	0.241514723898045
130	KC_AB1	CG9344	-0.498686718104902	0.360622858407549	1	pseudobulk	DESeq2	LRT	0.442946748590788
130	KC_AB1	CG9346	0.380782101767243	0.46798175279295	1	pseudobulk	DESeq2	LRT	0.329771080293026
130	KC_AB1	CG9356	0.642956716720681	0.631803843434296	1	pseudobulk	DESeq2	LRT	0.199417736491602
130	KC_AB1	CG9360	-0.0864047982602675	0.937687279127165	1	pseudobulk	DESeq2	LRT	0.027941975686924
130	KC_AB1	CG9368	-0.0178778772886782	0.940670567477848	1	pseudobulk	DESeq2	LRT	0.0265624443474795
130	KC_AB1	CG9372	0.492376813758313	0.8196555319725	1	pseudobulk	DESeq2	LRT	0.0863686256585538
130	KC_AB1	CG9377	-0.672462237120784	0.643393982105123	1	pseudobulk	DESeq2	LRT	0.191523005527242
130	KC_AB1	CG9380	-0.760959112540794	1	1	pseudobulk	DESeq2	LRT	0
130	KC_AB1	CG9384	0.324589581102624	0.603832065597995	1	pseudobulk	DESeq2	LRT	0.219083828142162
130	KC_AB1	CG9386	0.122541061795167	0.911348450780606	1	pseudobulk	DESeq2	LRT	0.040315540351637
130	KC_AB1	CG9391	-0.0224243273503721	0.947781856043081	1	pseudobulk	DESeq2	LRT	0.0232916095169654
130	KC_AB1	CG9393	0.843094662360718	0.409274914101646	1	pseudobulk	DESeq2	LRT	0.387984873974589
130	KC_AB1	CG9394	0.162988051805901	0.537962739528886	1	pseudobulk	DESeq2	LRT	0.269247803473733
130	KC_AB1	CG9395	-0.390432197223553	0.53094781208644	1	pseudobulk	DESeq2	LRT	0.27494816448677
130	KC_AB1	CG9396	2.09987857572813	0.503115176013642	1	pseudobulk	DESeq2	LRT	0.29833258237694
130	KC_AB1	CG9399	0.18172754939373	0.790030359459514	1	pseudobulk	DESeq2	LRT	0.102356219225508
130	KC_AB1	CG9410	-0.091035067804866	0.811527004300144	1	pseudobulk	DESeq2	LRT	0.090697024052893
130	KC_AB1	CG9422	-0.28297791994031	0.535178397969619	1	pseudobulk	DESeq2	LRT	0.271501424821569
130	KC_AB1	CG9425	0.121906637777674	0.560910044237673	1	pseudobulk	DESeq2	LRT	0.251106782992909
130	KC_AB1	CG9426	0.332836527082675	0.467257871409744	1	pseudobulk	DESeq2	LRT	0.330443373777868
130	KC_AB1	CG9427	-3.41337562833488	0.256593506563309	1	pseudobulk	DESeq2	LRT	0.590754338215985
130	KC_AB1	CG9436	0.54770880545899	0.549846420422461	1	pseudobulk	DESeq2	LRT	0.259758597918713
130	KC_AB1	CG9449	-0.760959112540794	1	1	pseudobulk	DESeq2	LRT	0
130	KC_AB1	CG9451	1.25804452752548	0.573436424985144	1	pseudobulk	DESeq2	LRT	0.241514723898045
130	KC_AB1	CG9467	-0.0272179562135579	0.939043523055081	1	pseudobulk	DESeq2	LRT	0.0273142784637963
130	KC_AB1	CG9471	0.82989074311655	0.37637151437569	1	pseudobulk	DESeq2	LRT	0.424383253516816
130	KC_AB1	CG9485	-2.16413011538117	0.251573800616462	1	pseudobulk	DESeq2	LRT	0.599334589141537
130	KC_AB1	CG9497	2.88322402903533	0.302751069383099	1	pseudobulk	DESeq2	LRT	0.518914314158952
130	KC_AB1	CG9498	2.58743201691345	0.417636627932844	1	pseudobulk	DESeq2	LRT	0.379201419460921
130	KC_AB1	CG9514	2.09987857572813	0.503115176013642	1	pseudobulk	DESeq2	LRT	0.29833258237694
130	KC_AB1	CG9515	-0.251874263776774	0.851320105400901	1	pseudobulk	DESeq2	LRT	0.0699071098690403

130	KC_AB1	CG9518	0.393853628865458	0.873915328625432	1	pseudobulk	DESeq2	LRT	0.0585306429858249
130	KC_AB1	CG9527	0.383235307248486	0.787995113719793	1	pseudobulk	DESeq2	LRT	0.103476475519468
130	KC_AB1	CG9536	0.479462767895919	0.544510520697378	1	pseudobulk	DESeq2	LRT	0.263993724656275
130	KC_AB1	CG9547	1.39929782761041	0.639042277522582	1	pseudobulk	DESeq2	LRT	0.194470408997475
130	KC_AB1	CG9572	1.2640260768691	0.572041290112849	1	pseudobulk	DESeq2	LRT	0.242572622568898
130	KC_AB1	CG9577	0.809752279480054	0.32372194233658	1	pseudobulk	DESeq2	LRT	0.489827862498168
130	KC_AB1	CG9578	0.233988987524434	0.734553286802884	1	pseudobulk	DESeq2	LRT	0.13397669364496
130	KC_AB1	CG9581	0.165316120114608	0.851178162504656	1	pseudobulk	DESeq2	LRT	0.0699795269961407
130	KC_AB1	CG9586	-0.00860757483450128	0.91612838799059	1	pseudobulk	DESeq2	LRT	0.0380436592048098
130	KC_AB1	CG9588	-0.404073625329996	0.6400420839923	1	pseudobulk	DESeq2	LRT	0.193791469383691
130	KC_AB1	CG9590	0.399644562246205	0.521510198752928	1	pseudobulk	DESeq2	LRT	0.28273719400867
130	KC_AB1	CG9593	-0.760959112540794	0.804334723897681	1	pseudobulk	DESeq2	LRT	0.0945631819854715
130	KC_AB1	CG9596	0.734884454940186	0.537077515319217	1	pseudobulk	DESeq2	LRT	0.269963028926293
130	KC_AB1	CG9601	-0.0990618887162815	0.879415051724226	1	pseudobulk	DESeq2	LRT	0.0558061054381131
130	KC_AB1	CG9609	-0.245631632050741	0.512169700998601	1	pseudobulk	DESeq2	LRT	0.29058611715679
130	KC_AB1	CG9628	0.420904902712445	0.665102156860671	1	pseudobulk	DESeq2	LRT	0.177111643789412
130	KC_AB1	CG9630	0.689052239134538	0.623452341615605	1	pseudobulk	DESeq2	LRT	0.205196739562241
130	KC_AB1	CG9631	-2.00963963748391	0.498268873088307	1	pseudobulk	DESeq2	LRT	0.302536242405947
130	KC_AB1	CG9636	0.0156308144624975	0.961953363987319	1	pseudobulk	DESeq2	LRT	0.0168459822801849
130	KC_AB1	CG9641	-0.0284200145401499	0.958579785523403	1	pseudobulk	DESeq2	LRT	0.0183717336252719
130	KC_AB1	CG9643	-0.819290819227606	0.314889090557505	1	pseudobulk	DESeq2	LRT	0.501842385386792
130	KC_AB1	CG9646	-0.0752747543272278	0.768894629976784	1	pseudobulk	DESeq2	LRT	0.114133172233815
130	KC_AB1	CG9649	-2.00963963748391	0.498268873088307	1	pseudobulk	DESeq2	LRT	0.302536242405947
130	KC_AB1	CG9657	-0.0690242125165676	0.87100168866443	1	pseudobulk	DESeq2	LRT	0.0599810029981546
130	KC_AB1	CG9662	-0.245415138385867	0.599105697162934	1	pseudobulk	DESeq2	LRT	0.222496550490322
130	KC_AB1	CG9663	0.0887382564954287	0.96078348649418	1	pseudobulk	DESeq2	LRT	0.0173744699953319
130	KC_AB1	CG9664	-1.22121879113183	0.605446734719958	1	pseudobulk	DESeq2	LRT	0.217924058688964
130	KC_AB1	CG9667	-0.247712012072528	0.766891518119797	1	pseudobulk	DESeq2	LRT	0.115266065534761
130	KC_AB1	CG9674	-0.0425222595338769	0.852462988983022	1	pseudobulk	DESeq2	LRT	0.0693244675018814
130	KC_AB1	CG9684	1.81448253843153	0.251392535517801	1	pseudobulk	DESeq2	LRT	0.599647621763653
130	KC_AB1	CG9686	0.087925692860895	0.682526918983838	1	pseudobulk	DESeq2	LRT	0.165880215297326
130	KC_AB1	CG9689	-0.395128557665161	0.730152910900134	1	pseudobulk	DESeq2	LRT	0.136586179049485
130	KC_AB1	CG9691	-0.205351818925393	0.806288909842461	1	pseudobulk	DESeq2	LRT	0.0935093136935732
130	KC_AB1	CG9701	-3.41337562833488	0.256593506563309	1	pseudobulk	DESeq2	LRT	0.590754338215985
130	KC_AB1	CG9705	-0.0600492141572709	0.898437921871392	1	pseudobulk	DESeq2	LRT	0.0465119253664656
130	KC_AB1	CG9706	-0.140324461546405	0.91919658269752	1	pseudobulk	DESeq2	LRT	0.0365915988958907
130	KC_AB1	CG9722	1.25804452752548	0.727552935095488	1	pseudobulk	DESeq2	LRT	0.138135402914536
130	KC_AB1	CG9723	-0.670908876772088	0.655489332307642	1	pseudobulk	DESeq2	LRT	0.18343437179636
130	KC_AB1	CG9727	-0.280297292022638	0.713068402291612	1	pseudobulk	DESeq2	LRT	0.146868807717676
130	KC_AB1	CG9743	-0.476900423470843	0.711817320807324	1	pseudobulk	DESeq2	LRT	0.147631448420511
130	KC_AB1	CG9747	-2.77900935124229	0.387147734759486	1	pseudobulk	DESeq2	LRT	0.412123277493911
130	KC_AB1	CG9752	1.47597293528938	0.454525713615767	1	pseudobulk	DESeq2	LRT	0.342441542660822
130	KC_AB1	CG9760	-0.796925297569485	0.682978713483224	1	pseudobulk	DESeq2	LRT	0.165592831839459
130	KC_AB1	CG9766	1.1578251023428	0.523005293265409	1	pseudobulk	DESeq2	LRT	0.281493915675125
130	KC_AB1	CG9773	-0.494629860189985	0.538605843225634	1	pseudobulk	DESeq2	LRT	0.268728939326135
130	KC_AB1	CG9775	-0.0566509631115088	0.819967409722481	1	pseudobulk	DESeq2	LRT	0.0862034086637997
130	KC_AB1	CG9776	-0.156369750430455	0.708482886564901	1	pseudobulk	DESeq2	LRT	0.149670635692022
130	KC_AB1	CG9777	-0.760959112540794	1	1	pseudobulk	DESeq2	LRT	0
130	KC_AB1	CG9782	-0.828859111376547	0.658347641935711	1	pseudobulk	DESeq2	LRT	0.181544715649042
130	KC_AB1	CG9784	-0.0998386766359652	0.841529189933889	1	pseudobulk	DESeq2	LRT	0.0749308151517936
130	KC_AB1	CG9795	0.407031333789608	0.376951899918964	1	pseudobulk	DESeq2	LRT	0.423714063408268
130	KC_AB1	CG9799	-0.250860359535846	0.81077746514412	1	pseudobulk	DESeq2	LRT	0.091098330649435
130	KC_AB1	CG9801	0.174317818593935	0.83773025225922	1	pseudobulk	DESeq2	LRT	0.07689580095725
131	KC_AB1	CG9804	-0.576356170842841	0.459774516670486	1	pseudobulk	DESeq2	LRT	0.337455103480156
131	KC_AB1	CG9812	2.54574003167073	0.323440918906978	1	pseudobulk	DESeq2	LRT	0.490205037826797
131	KC_AB1	CG9813	0.0276751223007706	0.867465598564101	1	pseudobulk	DESeq2	LRT	0.0617477391884135

131	KC_AB1	CG9837	-0.171747711199917	0.764750093218222	1	pseudobulk	DESeq2	LRT	0.116480461405517
131	KC_AB1	CG9839	1.66330087317089	0.141239436620724	1	pseudobulk	DESeq2	LRT	0.850044023429802
131	KC_AB1	CG9849	0.0374591376334013	0.954070603982811	1	pseudobulk	DESeq2	LRT	0.020419485059495
131	KC_AB1	CG9853	0.212272011053025	0.700450256939556	1	pseudobulk	DESeq2	LRT	0.154622701068753
131	KC_AB1	CG9855	0.0743823519099387	0.905080093874285	1	pseudobulk	DESeq2	LRT	0.0433129867743612
131	KC_AB1	CG9886	0.458967701820029	0.728231580306794	1	pseudobulk	DESeq2	LRT	0.137730491488879
131	KC_AB1	CG9890	0.0530936360937484	0.951069329867587	1	pseudobulk	DESeq2	LRT	0.0217878232503335
131	KC_AB1	CG9896	-2.35716291794055	0.404761325252261	1	pseudobulk	DESeq2	LRT	0.392800990812383
131	KC_AB1	CG9902	-0.159185198089599	0.882014136530962	1	pseudobulk	DESeq2	LRT	0.054524454133271
131	KC_AB1	CG9911	-0.0858532014479882	0.868908334546056	1	pseudobulk	DESeq2	LRT	0.0610260370158005
131	KC_AB1	CG9915	0.330963000205779	0.577586025409489	1	pseudobulk	DESeq2	LRT	0.238383322988565
131	KC_AB1	CG9917	0.272430175960852	0.571189277986309	1	pseudobulk	DESeq2	LRT	0.243219953464174
131	KC_AB1	CG9919	-0.253346956077229	0.460508743182336	1	pseudobulk	DESeq2	LRT	0.336762119908399
131	KC_AB1	CG9921	0.70132713332091	0.238486469200133	1	pseudobulk	DESeq2	LRT	0.622536256114079
131	KC_AB1	CG9922	-0.354143715115856	0.452940534544692	1	pseudobulk	DESeq2	LRT	0.343958811705302
131	KC_AB1	CG9928	-5.05193869967269	0.0527455211291201	1	pseudobulk	DESeq2	LRT	1.27781441245666
131	KC_AB1	CG9932	1.01170397847183	0.0355890397173856	1	pseudobulk	DESeq2	LRT	1.4486837302054
131	KC_AB1	CG9934	0.0847073540671214	0.894998977798956	1	pseudobulk	DESeq2	LRT	0.0481774607025533
131	KC_AB1	CG9941	0.241745191997966	0.615537437951179	1	pseudobulk	DESeq2	LRT	0.210745527458662
131	KC_AB1	CG9945	0.103008353327044	0.883273984597563	1	pseudobulk	DESeq2	LRT	0.0539045608142076
131	KC_AB1	CG9948	-0.638361321913574	0.496386763571727	1	pseudobulk	DESeq2	LRT	0.304179807722063
131	KC_AB1	CG9951	0.247273491540892	0.816532935219101	1	pseudobulk	DESeq2	LRT	0.0880262931129332
131	KC_AB1	CG9953	-0.1105135588989	0.861835331853859	1	pseudobulk	DESeq2	LRT	0.0645757055196035
131	KC_AB1	CG9967	0.106063023679513	0.94789355012569	1	pseudobulk	DESeq2	LRT	0.0232404318484292
131	KC_AB1	CG9986	0.0216289321999812	0.943560383896571	1	pseudobulk	DESeq2	LRT	0.0252303015892105
131	KC_AB1	CG9992	0.465531232293316	0.473872139475924	1	pseudobulk	DESeq2	LRT	0.324338824171568
131	KC_AB1	Charon	-0.927743708403157	0.359029543668941	1	pseudobulk	DESeq2	LRT	0.44486981291338
131	KC_AB1	chas	4.38246987091381	0.0653657068115437	1	pseudobulk	DESeq2	LRT	1.18465003835189
131	KC_AB1	ChAT	-0.0646454682420273	0.853484558744481	1	pseudobulk	DESeq2	LRT	0.0688043317404933
131	KC_AB1	chb	0.161774419190738	0.679070782036084	1	pseudobulk	DESeq2	LRT	0.168084955254772
131	KC_AB1	Chc	-0.0736798319724051	0.790018554052774	1	pseudobulk	DESeq2	LRT	0.102362708927137
131	KC_AB1	Chchd2	0.143235570844139	0.390533201966099	1	pseudobulk	DESeq2	LRT	0.408342037797694
131	KC_AB1	Chchd3	-0.0609720008077172	0.846022887155619	1	pseudobulk	DESeq2	LRT	0.0726178879882317
131	KC_AB1	Chd1	-0.104102549191942	0.770137532258845	1	pseudobulk	DESeq2	LRT	0.113431710973631
131	KC_AB1	Chd64	0.705078287117683	0.197052878720633	1	pseudobulk	DESeq2	LRT	0.705417216198765
131	KC_AB1	CheB42c	0.344809363606593	0.805942697163666	1	pseudobulk	DESeq2	LRT	0.0936958356022411
131	KC_AB1	CheB53a	0.295216749156716	0.845806418600799	1	pseudobulk	DESeq2	LRT	0.0727290234308643
131	KC_AB1	CheB53b	0.296275065198765	1	1	pseudobulk	DESeq2	LRT	0
131	KC_AB1	cher	1.16136453850355	0.60284986933682	1	pseudobulk	DESeq2	LRT	0.219790828882339
131	KC_AB1	CHES-1-like	-0.604694184369713	0.101712829133172	1	pseudobulk	DESeq2	LRT	0.992624265657858
131	KC_AB1	Chi	-0.146185289223579	0.716511738989619	1	pseudobulk	DESeq2	LRT	0.144776689932859
131	KC_AB1	chic	0.459501394632384	0.0824372821547463	1	pseudobulk	DESeq2	LRT	1.08387633475616
131	KC_AB1	chico	-0.253474181085845	0.634592419582404	1	pseudobulk	DESeq2	LRT	0.197505119996708
131	KC_AB1	chif	-0.537553155735963	0.238608674214109	1	pseudobulk	DESeq2	LRT	0.622313772338572
131	KC_AB1	chinmo	-0.316329617207493	0.105540673551961	1	pseudobulk	DESeq2	LRT	0.976580138518128
131	KC_AB1	CHKov1	3.92593675984459	0.0130977609944098	1	pseudobulk	DESeq2	LRT	1.88280293876262
131	KC_AB1	CHKov2	1.76844611331216	0.36832537115236	1	pseudobulk	DESeq2	LRT	0.433768364848676
131	KC_AB1	chm	-0.523063277285698	0.472559807744347	1	pseudobulk	DESeq2	LRT	0.325543218838075
131	KC_AB1	Chmp1	0.0694565185321994	0.80979286746236	1	pseudobulk	DESeq2	LRT	0.0916260527533065
131	KC_AB1	CHMP2B	0.266440641126175	0.677772517662343	1	pseudobulk	DESeq2	LRT	0.168916044925566
131	KC_AB1	chn	-0.227382123907699	0.183382094784916	1	pseudobulk	DESeq2	LRT	0.736643070605595
131	KC_AB1	CHOp24	-0.0551905678694893	0.878974190733757	1	pseudobulk	DESeq2	LRT	0.0560238769017361
131	KC_AB1	CHORD	-0.732146043272733	0.323433221140345	1	pseudobulk	DESeq2	LRT	0.49021537398762
131	KC_AB1	Chrac-14	0.252371593101468	0.82476716811161	1	pseudobulk	DESeq2	LRT	0.0836686355418968
131	KC_AB1	Chrac-16	-0.24700425080567	0.822464789481691	1	pseudobulk	DESeq2	LRT	0.0848826855495731
131	KC_AB1	chrb	-0.100126366440668	0.659872823508277	1	pseudobulk	DESeq2	LRT	0.180539757443956

131	KC_AB1	Chro	0.0972982702231199	0.799012681338037	1	pseudobulk	DESeq2	LRT	0.0974463278306551
131	KC_AB1	ChT	-0.272443467097116	0.421321769677	1	pseudobulk	DESeq2	LRT	0.375386100316935
131	KC_AB1	Cht11	1.07156632681045	0.467063984181433	1	pseudobulk	DESeq2	LRT	0.330623620347231
131	KC_AB1	Cht2	0.752127344690506	0.611405608129273	1	pseudobulk	DESeq2	LRT	0.213670582017475
131	KC_AB1	Cht5	3.30291575684105	0.170854577908311	1	pseudobulk	DESeq2	LRT	0.767373380144807
131	KC_AB1	Cht6	-2.41876763198367	0.438413379977707	1	pseudobulk	DESeq2	LRT	0.358116200048746
131	KC_AB1	Cht7	-2.41876763198367	0.438413379977707	1	pseudobulk	DESeq2	LRT	0.358116200048746
131	KC_AB1	ci	2.77905602015038	0.396550243710121	1	pseudobulk	DESeq2	LRT	0.401701778879434
131	KC_AB1	CIA30	0.460671812205392	0.543333823333146	1	pseudobulk	DESeq2	LRT	0.264933258651682
131	KC_AB1	Ciao1	0.43164522063131	0.592656343295792	1	pseudobulk	DESeq2	LRT	0.227197062914578
131	KC_AB1	CIAPIN1	-0.79843771252186	0.269574419287462	1	pseudobulk	DESeq2	LRT	0.569321321667101
131	KC_AB1	cib	1.50291047960229	0.276129839250192	1	pseudobulk	DESeq2	LRT	0.558886659925439
131	KC_AB1	cic	-0.0276304594906477	0.892325313818194	1	pseudobulk	DESeq2	LRT	0.0494767866109774
131	KC_AB1	cid	3.37429492540794	0.281278028870551	1	pseudobulk	DESeq2	LRT	0.550864190027974
131	KC_AB1	cin	0.167814916014967	0.772922819225633	1	pseudobulk	DESeq2	LRT	0.111863870710268
131	KC_AB1	cindr	0.399721839569684	0.303127798480567	1	pseudobulk	DESeq2	LRT	0.518374234621718
131	KC_AB1	Cip4	-0.0629491959838355	0.743596506479966	1	pseudobulk	DESeq2	LRT	0.128662659271356
131	KC_AB1	Cirl	-0.0700169857406288	0.727450577906931	1	pseudobulk	DESeq2	LRT	0.138196506770093
131	KC_AB1	Cisd2	0.0481188709081706	0.833930705965094	1	pseudobulk	DESeq2	LRT	0.0788700348146105
131	KC_AB1	ck	-0.760959112540794	0.805265236978025	1	pseudobulk	DESeq2	LRT	0.0940610488455931
131	KC_AB1	Cka	-0.34973042274807	0.153212908759771	1	pseudobulk	DESeq2	LRT	0.814704642229796
131	KC_AB1	ckd	-1.38718159242805	0.788203284813311	1	pseudobulk	DESeq2	LRT	0.103361759557548
131	KC_AB1	CkIalpha	-0.114550405442792	0.488374987616837	1	pseudobulk	DESeq2	LRT	0.311246586779065
131	KC_AB1	CkIIalpha	0.0451779386364449	0.821618196988754	1	pseudobulk	DESeq2	LRT	0.0853299506616457
131	KC_AB1	CkIIalpha-i1	0.328279183946642	0.699275991529791	1	pseudobulk	DESeq2	LRT	0.155351382277595
131	KC_AB1	CkIIalpha-i3	-0.473303010882613	0.47411907583107	1	pseudobulk	DESeq2	LRT	0.324112570811589
131	KC_AB1	CkIIbeta	-0.101355655457625	0.635151439767523	1	pseudobulk	DESeq2	LRT	0.197122713111149
131	KC_AB1	ckn	0.0525787479200878	0.840440826483056	1	pseudobulk	DESeq2	LRT	0.075492858829575
131	KC_AB1	Cks85A	0.188350464321208	0.347435067954574	1	pseudobulk	DESeq2	LRT	0.459126348668036
131	KC_AB1	cl	0.0298253517022509	0.94348024846779	1	pseudobulk	DESeq2	LRT	0.0252671872544672
131	KC_AB1	Clamp	-0.150142517798889	0.689276662665462	1	pseudobulk	DESeq2	LRT	0.161606425476783
131	KC_AB1	Clbn	0.139748885942913	0.786157883560204	1	pseudobulk	DESeq2	LRT	0.104490226131979
131	KC_AB1	Clc	0.0928490982487669	0.706727607828452	1	pseudobulk	DESeq2	LRT	0.150747942938338
131	KC_AB1	ClC-a	0.515515222542274	0.650861120786363	1	pseudobulk	DESeq2	LRT	0.186511670280589
131	KC_AB1	ClC-b	1.05480565223818	0.303340620886713	1	pseudobulk	DESeq2	LRT	0.518069428641862
131	KC_AB1	ClC-c	-0.238602527325553	0.598127714580122	1	pseudobulk	DESeq2	LRT	0.223206073845447
131	KC_AB1	Clect27	1.66284943962668	0.564594668040363	1	pseudobulk	DESeq2	LRT	0.248263227591123
131	KC_AB1	Clic	0.652218882011227	0.20203983950589	1	pseudobulk	DESeq2	LRT	0.694562985149463
131	KC_AB1	CLIP-190	-0.00113126928952439	0.977679222139741	1	pseudobulk	DESeq2	LRT	0.00980361444180933
131	KC_AB1	Clk	0.0943998277587013	1	1	pseudobulk	DESeq2	LRT	0
131	KC_AB1	Cln3	-0.760959112540794	1	1	pseudobulk	DESeq2	LRT	0
132	KC_AB1	Cln7	-1.31232364397782	0.537553723231048	1	pseudobulk	DESeq2	LRT	0.269578125762371
132	KC_AB1	clos	-0.760959112540794	1	1	pseudobulk	DESeq2	LRT	0
132	KC_AB1	Clp	0.135662780438623	0.873907005641738	1	pseudobulk	DESeq2	LRT	0.0585347791337849
132	KC_AB1	ClpP	-0.249234659760235	0.573231744872625	1	pseudobulk	DESeq2	LRT	0.241669766911714
132	KC_AB1	CLS	0.102997381671626	0.875789132859118	1	pseudobulk	DESeq2	LRT	0.057600448009616
132	KC_AB1	clt	0.25899150418759	0.857099870235462	1	pseudobulk	DESeq2	LRT	0.0669685706495803
132	KC_AB1	clu	-0.206957064957859	0.643081732227341	1	pseudobulk	DESeq2	LRT	0.191733827076453
132	KC_AB1	cm	1.29736542980625	0.0285546100580349	1	pseudobulk	DESeq2	LRT	1.54432376618736
132	KC_AB1	cmb	0.101713782364021	0.923543616651405	1	pseudobulk	DESeq2	LRT	0.0345425890989813
132	KC_AB1	cmpy	0.599247655112026	0.527861378063827	1	pseudobulk	DESeq2	LRT	0.27748011278095
132	KC_AB1	cN-IIIB	-0.960513706223122	0.434066305358132	1	pseudobulk	DESeq2	LRT	0.362443925207099
132	KC_AB1	CNBP	0.0452474275482513	0.824504226185388	1	pseudobulk	DESeq2	LRT	0.0838071139378496
132	KC_AB1	cnc	0.0521035872628214	0.91817766417905	1	pseudobulk	DESeq2	LRT	0.0370732761983717
132	KC_AB1	Cndp2	0.678360997313938	0.589867366092606	1	pseudobulk	DESeq2	LRT	0.229245630136911
132	KC_AB1	CngA	1.11388934932283	1	1	pseudobulk	DESeq2	LRT	0

132	KC_AB1	CngB	0.571248237367883	0.0952484376962445	1	pseudobulk	DESeq2	LRT	1.02114213906929
132	KC_AB1	CngI	0.212544069606034	0.291402109990572	1	pseudobulk	DESeq2	LRT	0.535507307905958
132	KC_AB1	cni	-0.20327213845803	0.59078328023924	1	pseudobulk	DESeq2	LRT	0.228571804156613
132	KC_AB1	cnir	0.432609541566368	0.494831436462406	1	pseudobulk	DESeq2	LRT	0.305542717594919
132	KC_AB1	cnk	0.201098330885464	0.790010268684416	1	pseudobulk	DESeq2	LRT	0.102367263641211
132	KC_AB1	CNMaR	2.04480391452018	0.525926445146098	1	pseudobulk	DESeq2	LRT	0.279074991019079
132	KC_AB1	cnn	-0.497685623339445	0.430547876221553	1	pseudobulk	DESeq2	LRT	0.365978548687091
132	KC_AB1	cno	0.621050736768382	0.708431212781622	1	pseudobulk	DESeq2	LRT	0.149702312473215
132	KC_AB1	Cnot4	-0.209326793180162	0.519389369971934	1	pseudobulk	DESeq2	LRT	0.284506943058224
132	KC_AB1	CNPYb	0.448055513548729	0.456839463629815	1	pseudobulk	DESeq2	LRT	0.340236387038079
132	KC_AB1	Cnx14D	-0.530055384727197	0.784817145589627	1	pseudobulk	DESeq2	LRT	0.105231517664772
132	KC_AB1	Cnx99A	-0.318851001656319	0.459584810314306	1	pseudobulk	DESeq2	LRT	0.337634333564459
132	KC_AB1	Coa7	0.267346431500609	0.497187429199908	1	pseudobulk	DESeq2	LRT	0.30347986051738
132	KC_AB1	Cog1	0.99492212325421	0.303654841217852	1	pseudobulk	DESeq2	LRT	0.517619790459619
132	KC_AB1	Cog2	0.472972838626667	0.665711953511914	1	pseudobulk	DESeq2	LRT	0.176713644794378
132	KC_AB1	Cog3	-0.517410719778163	0.742791087079311	1	pseudobulk	DESeq2	LRT	0.129133316100131
132	KC_AB1	Cog4	-0.386265090248276	0.697812180340184	1	pseudobulk	DESeq2	LRT	0.156261454193781
132	KC_AB1	Cog6	0.0503376336563946	0.928674765995534	1	pseudobulk	DESeq2	LRT	0.0321363549664318
132	KC_AB1	Cog7	0.326793759463629	0.673321052064166	1	pseudobulk	DESeq2	LRT	0.171777806663245
132	KC_AB1	Cog8	0.0952981858979752	0.920545140368746	1	pseudobulk	DESeq2	LRT	0.0359549103295612
132	KC_AB1	coil	0.863075184598245	0.433212064628339	1	pseudobulk	DESeq2	LRT	0.363299457105457
132	KC_AB1	Col4a1	0.465317115047142	0.542747624927046	1	pseudobulk	DESeq2	LRT	0.265402068349517
132	KC_AB1	cold	0.498905294787256	0.594405937165318	1	pseudobulk	DESeq2	LRT	0.225916861318043
132	KC_AB1	colt	-0.695576836199831	0.356533365027312	1	pseudobulk	DESeq2	LRT	0.447899821859192
132	KC_AB1	comm	-0.153471447760792	0.627665418096164	1	pseudobulk	DESeq2	LRT	0.20227179861349
132	KC_AB1	comm2	1.25804452752548	0.727552935095488	1	pseudobulk	DESeq2	LRT	0.138135402914536
132	KC_AB1	comt	-0.0697337272545673	0.799659668518975	1	pseudobulk	DESeq2	LRT	0.097094807423226
132	KC_AB1	Con	-0.19318197577792	0.363666463326359	1	pseudobulk	DESeq2	LRT	0.43929674696248
132	KC_AB1	Cont	-0.175155438635382	0.882741171427455	1	pseudobulk	DESeq2	LRT	0.0541666172590741
132	KC_AB1	conu	0.0197295620515884	0.920395140765451	1	pseudobulk	DESeq2	LRT	0.036025682859024
132	KC_AB1	conv	-2.99952832076105	0.338679393613493	1	pseudobulk	DESeq2	LRT	0.47021122647966
132	KC_AB1	Coop	-0.459184491799161	0.866831670429805	1	pseudobulk	DESeq2	LRT	0.0620652297453457
132	KC_AB1	Coprox	1.25047854069538	0.420939236267639	1	pseudobulk	DESeq2	LRT	0.375780591238584
132	KC_AB1	Coq2	-0.0501343632356184	0.921169980158729	1	pseudobulk	DESeq2	LRT	0.0356602236212311
132	KC_AB1	Coq3	0.266764436811899	0.787344873806957	1	pseudobulk	DESeq2	LRT	0.103834995742825
132	KC_AB1	Coq4	1.11388934932283	1	1	pseudobulk	DESeq2	LRT	0
132	KC_AB1	Coq5	1.71741102154904	0.400050445173363	1	pseudobulk	DESeq2	LRT	0.397885241974284
132	KC_AB1	Coq6	-0.448255090244125	0.43371357811368	1	pseudobulk	DESeq2	LRT	0.362796981338582
132	KC_AB1	Coq7	0.425962449326134	0.313358371327881	1	pseudobulk	DESeq2	LRT	0.503958698688785
132	KC_AB1	Coq8	0.164448725778088	0.865775929114149	1	pseudobulk	DESeq2	LRT	0.0625944929028815
132	KC_AB1	Coq9	0.65483474466478	0.522278978867981	1	pseudobulk	DESeq2	LRT	0.282097453667812
132	KC_AB1	cora	-0.208312244782861	0.304236087140003	1	pseudobulk	DESeq2	LRT	0.51678927313563
132	KC_AB1	CoRest	-0.451806467215841	0.191611781651715	1	pseudobulk	DESeq2	LRT	0.717577790930654
132	KC_AB1	Corin	-0.174488871862372	0.649804098492424	1	pseudobulk	DESeq2	LRT	0.187217553768151
132	KC_AB1	corn	-0.236413557097004	0.348953328825579	1	pseudobulk	DESeq2	LRT	0.457232654384395
132	KC_AB1	coro	-0.0582777230782563	0.878948896431965	1	pseudobulk	DESeq2	LRT	0.0560363748042586
132	KC_AB1	Cortactin	-0.31680730011324	0.724136604955502	1	pseudobulk	DESeq2	LRT	0.140179498468371
132	KC_AB1	corto	-0.159917915545054	0.462223405726466	1	pseudobulk	DESeq2	LRT	0.335148066817789
132	KC_AB1	cos	0.0272569275120081	0.939381008298247	1	pseudobulk	DESeq2	LRT	0.0271582243046206
132	KC_AB1	Cow	-0.205285939937676	0.328998191391991	1	pseudobulk	DESeq2	LRT	0.482806489498771
132	KC_AB1	COX4	0.0434623208411866	0.744286754212829	1	pseudobulk	DESeq2	LRT	0.12825970991242
132	KC_AB1	COX5A	0.107131763970676	0.502690387436383	1	pseudobulk	DESeq2	LRT	0.298699419373577
132	KC_AB1	COX5B	0.0391675466765202	0.830083424296763	1	pseudobulk	DESeq2	LRT	0.080878258357581
132	KC_AB1	COX6B	0.0971881508440322	0.483009736000548	1	pseudobulk	DESeq2	LRT	0.316044115110395
132	KC_AB1	COX7A	0.0833266474405413	0.603457622298629	1	pseudobulk	DESeq2	LRT	0.219353222746531
132	KC_AB1	COX7C	-0.0660177013407649	0.745267197075224	1	pseudobulk	DESeq2	LRT	0.127687993814345

132	KC_AB1	COX8	-0.134922036136678	0.443874386337686	1	pseudobulk	DESeq2	LRT	0.35273991510788
132	KC_AB1	Cp1	0.0884581016575084	0.814942986633062	1	pseudobulk	DESeq2	LRT	0.0888727734155576
132	KC_AB1	Cp110	0.00293378838950875	0.992043367916928	1	pseudobulk	DESeq2	LRT	0.00346934192311315
132	KC_AB1	Cp18	-0.760959112540794	0.804334723897681	1	pseudobulk	DESeq2	LRT	0.0945631819854715
132	KC_AB1	Cp190	-0.125573679996539	0.73144427034248	1	pseudobulk	DESeq2	LRT	0.135818757720143
132	KC_AB1	Cp7Fa	0.943022244937108	0.768808261856119	1	pseudobulk	DESeq2	LRT	0.114181958248924
132	KC_AB1	cpa	-0.375645756463434	0.629238061530255	1	pseudobulk	DESeq2	LRT	0.201185015526469
132	KC_AB1	cpb	-0.188607465407929	0.707192902973998	1	pseudobulk	DESeq2	LRT	0.150462106330244
132	KC_AB1	Cpes	0.769345689179016	0.257897295922027	1	pseudobulk	DESeq2	LRT	0.588553211457638
132	KC_AB1	cpo	-0.223250982694649	0.113185548585948	1	pseudobulk	DESeq2	LRT	0.94620901987945
132	KC_AB1	Cpr	1.64316006335886	0.236069052574921	1	pseudobulk	DESeq2	LRT	0.626960942937702
132	KC_AB1	Cpr49Ae	-3.73432699611557	0.200340531487601	1	pseudobulk	DESeq2	LRT	0.698231178411511
132	KC_AB1	Cpr51A	0.291868230578568	0.902153667810848	1	pseudobulk	DESeq2	LRT	0.0447194808636415
132	KC_AB1	Cpr62Bb	7.71322639702951	0.0993387066969484	1	pseudobulk	DESeq2	LRT	1.00288149843819
132	KC_AB1	Cpr64Aa	-2.73719572742657	0.395113780272619	1	pseudobulk	DESeq2	LRT	0.40327782328744
132	KC_AB1	Cpr64Ad	2.77905602015038	0.396550243710121	1	pseudobulk	DESeq2	LRT	0.401701778879434
132	KC_AB1	Cpr67B	-1.84720757905536	0.438680975312332	1	pseudobulk	DESeq2	LRT	0.357851199641171
132	KC_AB1	Cpr73D	3.12964491182152	0.252827515563327	1	pseudobulk	DESeq2	LRT	0.597175662956756
132	KC_AB1	Cpr76Bb	-5.4886327142356	0.180831626106085	1	pseudobulk	DESeq2	LRT	0.742725612344016
132	KC_AB1	Cpr76Bd	-3.58279186106554	0.225990959339248	1	pseudobulk	DESeq2	LRT	0.64590893424911
132	KC_AB1	Cpr92A	-2.73719572742657	0.395113780272619	1	pseudobulk	DESeq2	LRT	0.40327782328744
132	KC_AB1	Cpr97Ea	-5.96441495846645	0.15886531117893	1	pseudobulk	DESeq2	LRT	0.798970922227619
132	KC_AB1	Cpr97Eb	-3.57686879066961	0.396086972268792	1	pseudobulk	DESeq2	LRT	0.402209441778543
132	KC_AB1	Cpsf100	0.372150318273174	0.696911398435175	1	pseudobulk	DESeq2	LRT	0.1568224322558
132	KC_AB1	Cpsf160	-0.415399642591149	0.753066009673212	1	pseudobulk	DESeq2	LRT	0.123166954237059
132	KC_AB1	Cpsf5	-0.3574475065444	0.296457012764591	1	pseudobulk	DESeq2	LRT	0.528038271853198
132	KC_AB1	Cpsf6	0.240963082295097	0.415658544498392	1	pseudobulk	DESeq2	LRT	0.381263287481681
132	KC_AB1	Cpsf73	-0.165326676288081	0.864496247737024	1	pseudobulk	DESeq2	LRT	0.0632368873994766
132	KC_AB1	cpx	-0.133872607641634	0.504867736472294	1	pseudobulk	DESeq2	LRT	0.296822381967919
133	KC_AB1	CR12460	-0.760959112540794	0.805265236978025	1	pseudobulk	DESeq2	LRT	0.0940610488455931
133	KC_AB1	CR12842	-0.283759648944245	0.678705891399228	1	pseudobulk	DESeq2	LRT	0.168318380967557
133	KC_AB1	CR14578	0.296275065198765	1	1	pseudobulk	DESeq2	LRT	0
133	KC_AB1	CR14798	0.093287088252146	1	1	pseudobulk	DESeq2	LRT	0
133	KC_AB1	CR15345	0.852234742909098	0.575210167979696	1	pseudobulk	DESeq2	LRT	0.240173445541648
133	KC_AB1	CR18166	1.55092930200642	0.0835642006471062	1	pseudobulk	DESeq2	LRT	1.07797973681039
133	KC_AB1	CR18228	-1.48093423670513	0.465491529647032	1	pseudobulk	DESeq2	LRT	0.332088217284191
133	KC_AB1	CR18275	0.620406727884998	0.748661774708428	1	pseudobulk	DESeq2	LRT	0.125714340582394
133	KC_AB1	CR31032	-0.871278937704112	0.503656398016602	1	pseudobulk	DESeq2	LRT	0.297865644775451
133	KC_AB1	CR31953	-0.760959112540794	1	1	pseudobulk	DESeq2	LRT	0
133	KC_AB1	CR32010	-0.396721808097649	1	1	pseudobulk	DESeq2	LRT	0
133	KC_AB1	CR32745	-0.380155245313748	0.710679708437135	1	pseudobulk	DESeq2	LRT	0.148326084500712
133	KC_AB1	CR33294	-0.760959112540794	0.805265236978025	1	pseudobulk	DESeq2	LRT	0.0940610488455931
133	KC_AB1	CR40354	1.25804452752548	0.727552935095488	1	pseudobulk	DESeq2	LRT	0.138135402914536
133	KC_AB1	CR41320	0.0431942687094826	0.967464167684518	1	pseudobulk	DESeq2	LRT	0.0143651111308262
133	KC_AB1	CR41440	-0.197901312682693	0.911320066175192	1	pseudobulk	DESeq2	LRT	0.0403290669774481
133	KC_AB1	CR41571	1.11388934932283	1	1	pseudobulk	DESeq2	LRT	0
133	KC_AB1	CR41597	0.541925787044556	0.423367272238798	1	pseudobulk	DESeq2	LRT	0.373282717491517
133	KC_AB1	CR42875	0.296275065198765	1	1	pseudobulk	DESeq2	LRT	0
133	KC_AB1	CR43170	-1.21296207351619	0.645188741988394	1	pseudobulk	DESeq2	LRT	0.190313219297606
133	KC_AB1	CR43212	0.253119741765009	0.89124707280026	1	pseudobulk	DESeq2	LRT	0.0500018835286135
133	KC_AB1	CR43281	0.503682733849293	0.495149813103903	1	pseudobulk	DESeq2	LRT	0.305263380540007
133	KC_AB1	CR43361	-0.760959112540794	1	1	pseudobulk	DESeq2	LRT	0
133	KC_AB1	CR43670	0.74679171926964	0.313443155718809	1	pseudobulk	DESeq2	LRT	0.503841208887732
133	KC_AB1	CR43671	1.11388934932283	1	1	pseudobulk	DESeq2	LRT	0
133	KC_AB1	CR43972	0.0343086176960997	1	1	pseudobulk	DESeq2	LRT	0
133	KC_AB1	CR44003	1.36460636454741	0.276838807830233	1	pseudobulk	DESeq2	LRT	0.557773029659088

133	KC_AB1	CR44383	0.709713058804466	0.916223368658127	1	pseudobulk	DESeq2	LRT	0.037998635557264
133	KC_AB1	CR45470	-1.42222334914379	0.486292011727057	1	pseudobulk	DESeq2	LRT	0.31310286448766
133	KC_AB1	CR46418	1.11388934932283	1	1	pseudobulk	DESeq2	LRT	0
133	KC_AB1	CR6434	1.11388934932283	1	1	pseudobulk	DESeq2	LRT	0
133	KC_AB1	CR6900	2.36468246449593	0.410782157831629	1	pseudobulk	DESeq2	LRT	0.386388428082423
133	KC_AB1	CR7448	-0.760959112540794	0.804334723897681	1	pseudobulk	DESeq2	LRT	0.0945631819854715
133	KC_AB1	Crag	-0.778490417563708	0.257043403079345	1	pseudobulk	DESeq2	LRT	0.58999353765574
133	KC_AB1	CRAT	-0.317675416070531	0.730719868784556	1	pseudobulk	DESeq2	LRT	0.136249083723373
133	KC_AB1	crb	0.0299035695687231	0.876490918381509	1	pseudobulk	DESeq2	LRT	0.0572525794187092
133	KC_AB1	crc	-0.361744290153694	0.369755059292684	1	pseudobulk	DESeq2	LRT	0.432085874916296
133	KC_AB1	CrebA	-0.506928699775509	0.706406244410685	1	pseudobulk	DESeq2	LRT	0.150945470385871
133	KC_AB1	CrebB	-0.200949179936773	0.65000319326026	1	pseudobulk	DESeq2	LRT	0.187084509800369
133	KC_AB1	CREG	0.133877951387114	0.92347787474446	1	pseudobulk	DESeq2	LRT	0.0345735051955846
133	KC_AB1	Crg-1	1.27903693849691	0.23509067024564	1	pseudobulk	DESeq2	LRT	0.628764605844162
133	KC_AB1	CRIF	0.503531592407044	0.365873704769339	1	pseudobulk	DESeq2	LRT	0.436668802002701
133	KC_AB1	crim	0.814977096906329	0.469298774732219	1	pseudobulk	DESeq2	LRT	0.328550579662917
133	KC_AB1	Crk	0.112835599429545	0.638180957838434	1	pseudobulk	DESeq2	LRT	0.195056158508334
133	KC_AB1	crm	0.0524503042102574	0.949856088004952	1	pseudobulk	DESeq2	LRT	0.0223421893635401
133	KC_AB1	CRMP	-0.0260550671070885	0.927108069774781	1	pseudobulk	DESeq2	LRT	0.0328696387020307
133	KC_AB1	crn	0.315105283032408	0.672796915841348	1	pseudobulk	DESeq2	LRT	0.172116008048661
133	KC_AB1	crok	1.26277639574724	0.585985910697965	1	pseudobulk	DESeq2	LRT	0.232112825926769
133	KC_AB1	crol	0.147549398563677	0.472232328065371	1	pseudobulk	DESeq2	LRT	0.325844285322055
133	KC_AB1	CROT	-0.337955973119823	0.768746679343853	1	pseudobulk	DESeq2	LRT	0.114216747178485
133	KC_AB1	crp	-0.119932542461277	0.63885712726312	1	pseudobulk	DESeq2	LRT	0.194596255736692
133	KC_AB1	crq	-0.330049287457982	0.478374408503359	1	pseudobulk	DESeq2	LRT	0.320232061760151
133	KC_AB1	Crtc	0.354514093808277	0.264344701766255	1	pseudobulk	DESeq2	LRT	0.577829389691397
133	KC_AB1	cry	1.85943222322464	0.0831977749296994	1	pseudobulk	DESeq2	LRT	1.07988828847701
133	KC_AB1	Csas	-1.40531133040423	0.131831271460955	1	pseudobulk	DESeq2	LRT	0.879981559301636
133	KC_AB1	Cse1	-0.223399236607419	0.748473948587209	1	pseudobulk	DESeq2	LRT	0.125823311128165
133	KC_AB1	Csk	-0.0467228033466277	0.877781764627635	1	pseudobulk	DESeq2	LRT	0.0566134455961432
133	KC_AB1	Csl4	0.432860011868618	0.630387180377696	1	pseudobulk	DESeq2	LRT	0.200392627288547
133	KC_AB1	CSN1b	0.445044246024841	0.634028077980913	1	pseudobulk	DESeq2	LRT	0.197891508928739
133	KC_AB1	CSN3	-0.0239125702765452	0.936008128950661	1	pseudobulk	DESeq2	LRT	0.028720379527829
133	KC_AB1	CSN4	0.0309651584339231	0.953551866858396	1	pseudobulk	DESeq2	LRT	0.0206556792537744
133	KC_AB1	CSN5	-0.143534536403002	0.783878958505049	1	pseudobulk	DESeq2	LRT	0.10575099307029
133	KC_AB1	CSN6	0.32368142159957	0.565464989747466	1	pseudobulk	DESeq2	LRT	0.247594278855538
133	KC_AB1	CSN7	-0.165330087416181	0.662766951416132	1	pseudobulk	DESeq2	LRT	0.17863915561699
133	KC_AB1	CSN8	0.0366335217299844	0.920429859602816	1	pseudobulk	DESeq2	LRT	0.0360093008569494
133	KC_AB1	Csp	-0.121836452386929	0.545758768141106	1	pseudobulk	DESeq2	LRT	0.262999278202956
133	KC_AB1	CstF50	-0.751107722437315	0.475054122418089	1	pseudobulk	DESeq2	LRT	0.32325690884141
133	KC_AB1	CstF64	-0.317987391129137	0.767558724384102	1	pseudobulk	DESeq2	LRT	0.114888387576735
133	KC_AB1	csul	0.610997734981205	0.704105357412258	1	pseudobulk	DESeq2	LRT	0.152362351199812
133	KC_AB1	csw	-0.0437801868745607	0.835984101935646	1	pseudobulk	DESeq2	LRT	0.0778019815409752
133	KC_AB1	ct	1.53392759713581	0.120597481037473	1	pseudobulk	DESeq2	LRT	0.918661763364614
133	KC_AB1	cta	0.0142375700150929	0.961182181186039	1	pseudobulk	DESeq2	LRT	0.0171942889337926
133	KC_AB1	CtBP	-0.030506053264386	0.844607136474749	1	pseudobulk	DESeq2	LRT	0.0733452533414805
133	KC_AB1	CTCF	-0.0503508207730834	0.945486616980192	1	pseudobulk	DESeq2	LRT	0.0243446140561581
133	KC_AB1	CtIP	0.716116169020808	0.380504056772298	1	pseudobulk	DESeq2	LRT	0.41964070860581
133	KC_AB1	Ctl1	0.277114623362617	0.657907232359531	1	pseudobulk	DESeq2	LRT	0.181835339387021
133	KC_AB1	Ctl2	1.10296182336386	0.287552484349361	1	pseudobulk	DESeq2	LRT	0.541282875902954
133	KC_AB1	ctp	-0.206862457336333	0.228726322825527	1	pseudobulk	DESeq2	LRT	0.640683852008751
133	KC_AB1	CTPsyn	0.0264069547088938	0.914736830087936	1	pseudobulk	DESeq2	LRT	0.0387038345457117
133	KC_AB1	Ctr1A	0.527095176896032	0.449960355295426	1	pseudobulk	DESeq2	LRT	0.346825748968883
133	KC_AB1	Ctr1B	1.33291464456605	0.667248269932866	1	pseudobulk	DESeq2	LRT	0.175712543594533
133	KC_AB1	Ctr9	0.452421967281612	0.275912862270405	1	pseudobulk	DESeq2	LRT	0.559228053479286
133	KC_AB1	ctrip	-0.303397017050643	0.303632983891571	1	pseudobulk	DESeq2	LRT	0.517651052460287

133	KC_AB1	CtsB1	0.277622136397814	0.566078324568041	1	pseudobulk	DESeq2	LRT	0.247123474157474
133	KC_AB1	cu	0.478718472385228	0.812520071585028	1	pseudobulk	DESeq2	LRT	0.0901659018925309
133	KC_AB1	Cubn	0.152115475746305	1	1	pseudobulk	DESeq2	LRT	0
133	KC_AB1	cue	0.189769022420616	0.712125300019226	1	pseudobulk	DESeq2	LRT	0.147443584566449
133	KC_AB1	Cul1	-0.151103666280572	0.65855567365021	1	pseudobulk	DESeq2	LRT	0.181407504320997
133	KC_AB1	Cul2	-0.00205177591348223	0.985160688188776	1	pseudobulk	DESeq2	LRT	0.00649292655616006
133	KC_AB1	Cul3	0.121417463883209	0.760767917669743	1	pseudobulk	DESeq2	LRT	0.118747810309314
133	KC_AB1	Cul4	0.323533983187942	0.662776295351593	1	pseudobulk	DESeq2	LRT	0.178633032814705
133	KC_AB1	Cul5	-0.877088546394373	0.349644366297302	1	pseudobulk	DESeq2	LRT	0.456373465009058
133	KC_AB1	Cul6	0.296275065198765	1	1	pseudobulk	DESeq2	LRT	0
133	KC_AB1	Culd	-0.760959112540794	0.804334723897681	1	pseudobulk	DESeq2	LRT	0.0945631819854715
133	KC_AB1	cutlet	2.09987857572813	0.503115176013642	1	pseudobulk	DESeq2	LRT	0.29833258237694
133	KC_AB1	cv	0.417118300384725	0.823463300155454	1	pseudobulk	DESeq2	LRT	0.0843557515581485
133	KC_AB1	cv-2	-0.141999915912079	0.921515470408231	1	pseudobulk	DESeq2	LRT	0.0354973694448746
133	KC_AB1	cv-c	-0.0911595926143326	0.679223080285181	1	pseudobulk	DESeq2	LRT	0.167987565004697
133	KC_AB1	Cwc25	0.374009859629009	0.499306477797483	1	pseudobulk	DESeq2	LRT	0.301632799549832
133	KC_AB1	cwo	-0.168127481882085	0.676279218500531	1	pseudobulk	DESeq2	LRT	0.169873957878832
134	KC_AB1	cyc	0.273690387749786	0.689967724842962	1	pseudobulk	DESeq2	LRT	0.161171224118454
134	KC_AB1	CycC	0.126276260763978	0.828324079829875	1	pseudobulk	DESeq2	LRT	0.0817997132816242
134	KC_AB1	CycD	1.13661584234725	0.691531818919754	1	pseudobulk	DESeq2	LRT	0.160187832238187
134	KC_AB1	CycE	-2.69245487400416	0.326477446722968	1	pseudobulk	DESeq2	LRT	0.486146814701527
134	KC_AB1	CycG	-0.114602819086913	0.43933802356478	1	pseudobulk	DESeq2	LRT	0.357201208048705
134	KC_AB1	CycH	-0.289127604211337	0.865787150256184	1	pseudobulk	DESeq2	LRT	0.0625888641387615
134	KC_AB1	CycJ	2.13148430488057	0.45484681020722	1	pseudobulk	DESeq2	LRT	0.342134846603996
134	KC_AB1	CycK	-0.0183504479241563	0.963754672506926	1	pseudobulk	DESeq2	LRT	0.0160335033751704
134	KC_AB1	CycT	-0.294793900883599	0.272099518546034	1	pseudobulk	DESeq2	LRT	0.565272226661939
134	KC_AB1	CycY	-0.00463434922137778	0.958611553273293	1	pseudobulk	DESeq2	LRT	0.0183573411562452
134	KC_AB1	CYLD	-0.263471189321571	0.664814281963533	1	pseudobulk	DESeq2	LRT	0.177299659333258
134	KC_AB1	Cyp1	0.0779180576500904	0.689699998981738	1	pseudobulk	DESeq2	LRT	0.161339774652244
134	KC_AB1	Cyp12a4	0.322762400411895	0.82993799611934	1	pseudobulk	DESeq2	LRT	0.080954352140893
134	KC_AB1	Cyp12b2	0.551646434647088	0.479167674778545	1	pseudobulk	DESeq2	LRT	0.319512487653023
134	KC_AB1	Cyp12e1	-0.569432576450917	0.825325802164462	1	pseudobulk	DESeq2	LRT	0.0833745773279703
134	KC_AB1	Cyp18a1	3.37429492540794	0.281278028870551	1	pseudobulk	DESeq2	LRT	0.550864190027974
134	KC_AB1	Cyp28a5	2.18618421964141	0.0157946069666274	1	pseudobulk	DESeq2	LRT	1.80149117661851
134	KC_AB1	Cyp28c1	-0.84572271983469	0.729929556961718	1	pseudobulk	DESeq2	LRT	0.136719050152121
134	KC_AB1	Cyp28d1	-0.297675580267774	0.455120300855801	1	pseudobulk	DESeq2	LRT	0.341873792151229
134	KC_AB1	Cyp301a1	1.25804452752548	0.573436424985144	1	pseudobulk	DESeq2	LRT	0.241514723898045
134	KC_AB1	Cyp303a1	-0.760959112540794	0.804334723897681	1	pseudobulk	DESeq2	LRT	0.0945631819854715
134	KC_AB1	Cyp305a1	0.556210909873717	0.787496141578709	1	pseudobulk	DESeq2	LRT	0.103751565405258
134	KC_AB1	Cyp311a1	0.296275065198765	1	1	pseudobulk	DESeq2	LRT	0
134	KC_AB1	cyp33	0.248464461425503	0.729643582568583	1	pseudobulk	DESeq2	LRT	0.136889232925605
134	KC_AB1	Cyp49a1	0.0491935792632814	0.965528299435152	1	pseudobulk	DESeq2	LRT	0.0152349926164219
134	KC_AB1	Cyp4ac1	-0.760959112540794	0.805265236978025	1	pseudobulk	DESeq2	LRT	0.0940610488455931
134	KC_AB1	Cyp4ae1	-0.331884882767343	0.847006761080179	1	pseudobulk	DESeq2	LRT	0.0721131229773285
134	KC_AB1	Cyp4d2	2.58743201691345	0.417636627932844	1	pseudobulk	DESeq2	LRT	0.379201419460921
134	KC_AB1	Cyp4d20	-1.43550613534661	0.602448798560473	1	pseudobulk	DESeq2	LRT	0.220079857379737
134	KC_AB1	Cyp4d8	1.25804452752548	0.727552935095488	1	pseudobulk	DESeq2	LRT	0.138135402914536
134	KC_AB1	Cyp4e2	3.10235661376378	0.172835810485278	1	pseudobulk	DESeq2	LRT	0.762366269467828
134	KC_AB1	Cyp4e3	-0.760959112540794	0.805265236978025	1	pseudobulk	DESeq2	LRT	0.0940610488455931
134	KC_AB1	Cyp4g15	0.825082424540477	0.413166094817561	1	pseudobulk	DESeq2	LRT	0.383875324707592
134	KC_AB1	Cyp4p1	-0.760959112540794	1	1	pseudobulk	DESeq2	LRT	0
134	KC_AB1	Cyp4s3	0.924677456282491	0.719745633946136	1	pseudobulk	DESeq2	LRT	0.142820960918588
134	KC_AB1	Cyp6a13	0.132853734329562	0.909210999021931	1	pseudobulk	DESeq2	LRT	0.0413353191133254
134	KC_AB1	Cyp6a15Psi	-1.60958773650785	0.570513631639934	1	pseudobulk	DESeq2	LRT	0.243733974251419
134	KC_AB1	Cyp6a16	-1.43550613534661	0.602448798560473	1	pseudobulk	DESeq2	LRT	0.220079857379737
134	KC_AB1	Cyp6a17	-0.690211045665743	0.546037299946508	1	pseudobulk	DESeq2	LRT	0.26277768951569

134	KC_AB1	Cyp6a2	1.25804452752548	0.727552935095488	1	pseudobulk	DESeq2	LRT	0.138135402914536
134	KC_AB1	Cyp6a20	-0.967258610721754	0.749177533540542	1	pseudobulk	DESeq2	LRT	0.125415254796089
134	KC_AB1	Cyp6a23	-0.760959112540794	1	1	pseudobulk	DESeq2	LRT	0
134	KC_AB1	Cyp6a9	-0.569432576450917	0.825325802164462	1	pseudobulk	DESeq2	LRT	0.0833745773279703
134	KC_AB1	Cyp6d4	-0.760959112540794	0.804334723897681	1	pseudobulk	DESeq2	LRT	0.0945631819854715
134	KC_AB1	Cyp6d5	6.69460572134293	0.129766350393835	1	pseudobulk	DESeq2	LRT	0.886837909483749
134	KC_AB1	Cyp6g1	-1.22404819087985	0.615494548704634	1	pseudobulk	DESeq2	LRT	0.210775789163178
134	KC_AB1	Cyp6t1	1.53654127413203	0.423514874625857	1	pseudobulk	DESeq2	LRT	0.373131331839802
134	KC_AB1	Cyp6u1	0.440782523377994	0.699252437058124	1	pseudobulk	DESeq2	LRT	0.155366011336055
134	KC_AB1	Cyp6v1	2.04480874254212	0.526249918436357	1	pseudobulk	DESeq2	LRT	0.278807958467711
134	KC_AB1	Cyp6w1	-0.543633737493513	0.611948787906964	1	pseudobulk	DESeq2	LRT	0.213284921089785
134	KC_AB1	Cyp9b2	-1.43550613534661	0.602448798560473	1	pseudobulk	DESeq2	LRT	0.220079857379737
134	KC_AB1	Cyp9f2	0.565534560896615	0.521484793666077	1	pseudobulk	DESeq2	LRT	0.282758350944444
134	KC_AB1	Cyp9f3	0.425113145054655	0.86166643315149	1	pseudobulk	DESeq2	LRT	0.0646608249852909
134	KC_AB1	cype	-0.0524695340265991	0.759300618991155	1	pseudobulk	DESeq2	LRT	0.119586246079764
134	KC_AB1	Cypl	0.526552857630619	0.489030826478766	1	pseudobulk	DESeq2	LRT	0.310663763887298
134	KC_AB1	cyr	0.943805700130147	0.808046339172447	1	pseudobulk	DESeq2	LRT	0.0925637329508755
134	KC_AB1	Cys	1.57543755764429	0.22919728065387	1	pseudobulk	DESeq2	LRT	0.639790539427269
134	KC_AB1	CysRS	0.216677206129076	0.86387091041294	1	pseudobulk	DESeq2	LRT	0.0635511499793314
134	KC_AB1	CysRS-m	-0.684245870392073	0.484107525135417	1	pseudobulk	DESeq2	LRT	0.31505816648246
134	KC_AB1	cysu	-1.43550613534661	0.602448798560473	1	pseudobulk	DESeq2	LRT	0.220079857379737
134	KC_AB1	Cyt-b5	-0.0248094874800644	0.924765177817973	1	pseudobulk	DESeq2	LRT	0.0339685320444692
134	KC_AB1	Cyt-c-d	1.13698282223142	0.780593459054812	1	pseudobulk	DESeq2	LRT	0.107575092189158
134	KC_AB1	Cyt-c-p	-0.0458116717355733	0.834406146476178	1	pseudobulk	DESeq2	LRT	0.0786225054314112
134	KC_AB1	Cyt-c1	0.0183569707859393	0.927075283393388	1	pseudobulk	DESeq2	LRT	0.0328849974253177
134	KC_AB1	d	2.88322402903533	0.302751069383099	1	pseudobulk	DESeq2	LRT	0.518914314158952
134	KC_AB1	D1	-0.0370809355649707	0.889116049195462	1	pseudobulk	DESeq2	LRT	0.0510415503504248
134	KC_AB1	D12	0.221502682963823	0.877265351424048	1	pseudobulk	DESeq2	LRT	0.0568690232488694
134	KC_AB1	D19A	0.17505349640508	0.796848320993556	1	pseudobulk	DESeq2	LRT	0.0986243381077445
134	KC_AB1	D19B	-0.273882647571027	0.638727859167671	1	pseudobulk	DESeq2	LRT	0.194684140959715
134	KC_AB1	D2hgdh	0.874569144195404	0.495048164827316	1	pseudobulk	DESeq2	LRT	0.305352545104581
134	KC_AB1	d4	-0.0115368133071074	0.947314564646027	1	pseudobulk	DESeq2	LRT	0.0235057855104176
134	KC_AB1	da	-0.0835330703755255	0.748656930852628	1	pseudobulk	DESeq2	LRT	0.125717150484971
134	KC_AB1	DAAM	-0.0745217549771658	0.872570219045215	1	pseudobulk	DESeq2	LRT	0.0591996136332248
134	KC_AB1	Dab	-0.132778274888834	0.671648866238452	1	pseudobulk	DESeq2	LRT	0.172857714014528
134	KC_AB1	dac	-0.232287187560525	0.203136912021083	1	pseudobulk	DESeq2	LRT	0.692211153746766
134	KC_AB1	Dad	0.46853183642212	0.496596168226939	1	pseudobulk	DESeq2	LRT	0.30399663581713
134	KC_AB1	Dad1	0.0199805262011494	0.955577668017229	1	pseudobulk	DESeq2	LRT	0.0197340083259018
134	KC_AB1	daed	-1.1968621763306	0.272097933604918	1	pseudobulk	DESeq2	LRT	0.565274756373088
134	KC_AB1	Dak1	-0.231973242775836	0.459369839455076	1	pseudobulk	DESeq2	LRT	0.337837522393548
134	KC_AB1	dally	0.402818899478777	0.599908610294659	1	pseudobulk	DESeq2	LRT	0.221914904729285
134	KC_AB1	danr	1.11388934932283	1	1	pseudobulk	DESeq2	LRT	0
134	KC_AB1	dao	-0.202236441103799	0.588156193006175	1	pseudobulk	DESeq2	LRT	0.230507325704814
134	KC_AB1	dap	2.78044614204691	0.247077463052649	1	pseudobulk	DESeq2	LRT	0.60716686656978
134	KC_AB1	Dap160	-0.153318085980347	0.546241934785379	1	pseudobulk	DESeq2	LRT	0.262614962294407
134	KC_AB1	dare	0.294871581684691	0.742343859369717	1	pseudobulk	DESeq2	LRT	0.129394879579956
134	KC_AB1	Dark	-0.13328287693309	0.824701619051042	1	pseudobulk	DESeq2	LRT	0.0837031528307779
134	KC_AB1	DAT	-1.01764348127783	0.636757081905014	1	pseudobulk	DESeq2	LRT	0.196026216187409
134	KC_AB1	dati	-0.148958726795418	0.437630616958607	1	pseudobulk	DESeq2	LRT	0.358892302051594
134	KC_AB1	Daxx	-0.261056681138496	0.620529367481725	1	pseudobulk	DESeq2	LRT	0.207237660042042
134	KC_AB1	dbe	1.49652551246773	0.145097971980779	1	pseudobulk	DESeq2	LRT	0.838338657608522
134	KC_AB1	dbo	0.0490957810295764	0.875982352981701	1	pseudobulk	DESeq2	LRT	0.0575046427815379
134	KC_AB1	Dbp21E2	-0.0464886201005713	0.941860752483164	1	pseudobulk	DESeq2	LRT	0.0260132998592679
134	KC_AB1	Dbp45A	0.0767959223601765	0.916370870098585	1	pseudobulk	DESeq2	LRT	0.0379287247603758
134	KC_AB1	Dbp73D	1.08489993238655	0.507721737495604	1	pseudobulk	DESeq2	LRT	0.294374242401388
134	KC_AB1	Dbp80	-0.0401177824217376	0.835604545788924	1	pseudobulk	DESeq2	LRT	0.0779992060712458

134	KC_AB1	dbr	0.664470203619423	0.232833170277659	1	pseudobulk	DESeq2	LRT	0.632955148410641
134	KC_AB1	DCAF12	-0.460836917284998	0.627049502512313	1	pseudobulk	DESeq2	LRT	0.202698172376129
134	KC_AB1	Dci	0.652054544643725	0.571867802776209	1	pseudobulk	DESeq2	LRT	0.24270435435428
134	KC_AB1	dco	0.0762066829090072	0.794199477957626	1	pseudobulk	DESeq2	LRT	0.100070402742039
134	KC_AB1	Dcp-1	-0.890066023856454	0.262783446534311	1	pseudobulk	DESeq2	LRT	0.580401995677061
135	KC_AB1	DCP1	0.14825304991319	0.786012287594084	1	pseudobulk	DESeq2	LRT	0.104570664657037
135	KC_AB1	DCP2	-0.125965193079905	0.610662168365047	1	pseudobulk	DESeq2	LRT	0.214198984505899
135	KC_AB1	Dcr-1	-0.403113036875962	0.813769315244996	1	pseudobulk	DESeq2	LRT	0.0894986900922433
135	KC_AB1	Dcr-2	-0.425725314941269	0.651364608417619	1	pseudobulk	DESeq2	LRT	0.18617584231167
135	KC_AB1	DCTN1-p150	-0.647521415222369	0.163254018261877	1	pseudobulk	DESeq2	LRT	0.787136120389898
135	KC_AB1	DCTN2-p50	0.079829542709255	0.877022496487149	1	pseudobulk	DESeq2	LRT	0.0569892664116975
135	KC_AB1	DCTN3-p24	0.342811438498188	0.501379673072612	1	pseudobulk	DESeq2	LRT	0.299833277181444
135	KC_AB1	DCTN4-p62	-0.187614072126497	0.810012009600117	1	pseudobulk	DESeq2	LRT	0.0915085420295041
135	KC_AB1	DCTN5-p25	0.450560757066179	0.516271490025392	1	pseudobulk	DESeq2	LRT	0.287121857262524
135	KC_AB1	DCTN6-p27	-0.0211080491736426	0.953553735762348	1	pseudobulk	DESeq2	LRT	0.0206548280637068
135	KC_AB1	Dd	0.112000265529319	0.698806543915334	1	pseudobulk	DESeq2	LRT	0.155643036755774
135	KC_AB1	Ddc	0.683367188772378	0.10380675967498	1	pseudobulk	DESeq2	LRT	0.983774365235556
135	KC_AB1	Ddr	-1.61102155603974	0.57025980967112	1	pseudobulk	DESeq2	LRT	0.243927235206257
135	KC_AB1	Ddx1	0.347251135106756	0.794095575146283	1	pseudobulk	DESeq2	LRT	0.100127223944871
135	KC_AB1	Deaf1	0.0153308622750715	0.937659583197612	1	pseudobulk	DESeq2	LRT	0.0279548033825556
135	KC_AB1	Debcl	0.220614585804744	0.864454273705771	1	pseudobulk	DESeq2	LRT	0.0632579742841892
135	KC_AB1	DEF8	0.00905869658001549	0.977007061732056	1	pseudobulk	DESeq2	LRT	0.0101022972226998
135	KC_AB1	defl	0.00693174237228643	0.989180347068661	1	pseudobulk	DESeq2	LRT	0.00472452074222115
135	KC_AB1	Dek	-0.146854358034421	0.565409696203226	1	pseudobulk	DESeq2	LRT	0.247636748072448
135	KC_AB1	del	1.7967066599999	0.378890869950886	1	pseudobulk	DESeq2	LRT	0.421485859696563
135	KC_AB1	deltaCOP	-0.174348737730318	0.724034582082858	1	pseudobulk	DESeq2	LRT	0.140240690088654
135	KC_AB1	Den1	-0.0545733632818732	0.868223648177499	1	pseudobulk	DESeq2	LRT	0.0613683892723411
135	KC_AB1	DENR	0.0224089117166651	0.940972350539701	1	pseudobulk	DESeq2	LRT	0.0264231376614307
135	KC_AB1	Der-1	-0.29035459494353	0.552336682062138	1	pseudobulk	DESeq2	LRT	0.257796113234485
135	KC_AB1	Der-2	0.56570715731196	0.396121051982495	1	pseudobulk	DESeq2	LRT	0.402172076260492
135	KC_AB1	Dera	0.943022244937108	0.768808261856119	1	pseudobulk	DESeq2	LRT	0.114181958248924
135	KC_AB1	Desat1	-0.050697966348382	0.814003028006768	1	pseudobulk	DESeq2	LRT	0.0893739795774024
135	KC_AB1	Det	3.52278680896166	0.181142264725337	1	pseudobulk	DESeq2	LRT	0.741980206806527
135	KC_AB1	Df31	-0.00591070769951625	0.961048513566493	1	pseudobulk	DESeq2	LRT	0.0172546886660726
135	KC_AB1	Dg	0.0415697621989226	0.864001329938773	1	pseudobulk	DESeq2	LRT	0.0634855890203825
135	KC_AB1	Dgk	0.0578172720374426	0.889644734602673	1	pseudobulk	DESeq2	LRT	0.0507833872923374
135	KC_AB1	Dgkepsilon	-0.100486024494221	0.805033071326632	1	pseudobulk	DESeq2	LRT	0.0941862781418874
135	KC_AB1	Dgp-1	0.351768403627754	0.51449210094089	1	pseudobulk	DESeq2	LRT	0.288621288625782
135	KC_AB1	dgrn	-0.184719568835197	0.826660147217336	1	pseudobulk	DESeq2	LRT	0.0826729989402614
135	KC_AB1	dgt1	-0.129213186798251	0.838580451179045	1	pseudobulk	DESeq2	LRT	0.0764552660011669
135	KC_AB1	dgt2	-1.61102155603974	0.57025980967112	1	pseudobulk	DESeq2	LRT	0.243927235206257
135	KC_AB1	dgt6	1.25804452752548	0.573436424985144	1	pseudobulk	DESeq2	LRT	0.241514723898045
135	KC_AB1	Dh31	0.943798956650056	0.807634491832869	1	pseudobulk	DESeq2	LRT	0.0927851418175362
135	KC_AB1	Dh44-R1	0.330196008951434	0.325697694102978	1	pseudobulk	DESeq2	LRT	0.487185316172301
135	KC_AB1	Dh44-R2	0.166214233310139	0.554889512718907	1	pseudobulk	DESeq2	LRT	0.255793483171685
135	KC_AB1	Dhap-at	0.189329121385371	0.788526257977567	1	pseudobulk	DESeq2	LRT	0.103183840058744
135	KC_AB1	Dhc64C	-0.117958002156385	0.753756520604387	1	pseudobulk	DESeq2	LRT	0.122768917836529
135	KC_AB1	Dhfr	0.291868230578568	0.902153667810848	1	pseudobulk	DESeq2	LRT	0.0447194808636415
135	KC_AB1	Dhit	-0.0874246825165794	0.681032819191468	1	pseudobulk	DESeq2	LRT	0.166831958791596
135	KC_AB1	Dhod	-0.29229669238263	0.757514523723415	1	pseudobulk	DESeq2	LRT	0.120609036076524
135	KC_AB1	DhpD	1.91302694696753	0.237211687593868	1	pseudobulk	DESeq2	LRT	0.624863916772886
135	KC_AB1	Dhpr	-0.212850859838749	0.421536235493948	1	pseudobulk	DESeq2	LRT	0.375165087234551
135	KC_AB1	Dhx15	-0.171581919877744	0.824598117080282	1	pseudobulk	DESeq2	LRT	0.0837576612186006
135	KC_AB1	dia	0.519652711630882	0.776521194173146	1	pseudobulk	DESeq2	LRT	0.10984668624987
135	KC_AB1	Diap1	-0.0931937428404386	0.695488854172103	1	pseudobulk	DESeq2	LRT	0.157709825571775
135	KC_AB1	Diap2	0.19565270617767	0.658547405415671	1	pseudobulk	DESeq2	LRT	0.181412956967327

135	KC_AB1	Dic1	0.162712393369774	0.652758210986151	1	pseudobulk	DESeq2	LRT	0.185247656519881
135	KC_AB1	Dic3	-0.760959112540794	0.804334723897681	1	pseudobulk	DESeq2	LRT	0.0945631819854715
135	KC_AB1	didum	-0.246602131349129	0.684223585837187	1	pseudobulk	DESeq2	LRT	0.164801959349447
135	KC_AB1	Dif	-0.206224655531938	0.390255296281588	1	pseudobulk	DESeq2	LRT	0.408651194273847
135	KC_AB1	dikar	-0.134434379537847	0.522668164363673	1	pseudobulk	DESeq2	LRT	0.281773951905783
135	KC_AB1	Dim1	0.188217450595414	0.772931994623836	1	pseudobulk	DESeq2	LRT	0.111858715213421
135	KC_AB1	dind	0.0182427519125676	0.912518899294894	1	pseudobulk	DESeq2	LRT	0.0397581320501322
135	KC_AB1	DIP-alpha	1.36842129972114	0.633412197704719	1	pseudobulk	DESeq2	LRT	0.198313577615757
135	KC_AB1	Dip-B	4.9630175372339	0.0443949778318029	1	pseudobulk	DESeq2	LRT	1.35266615653625
135	KC_AB1	DIP-beta	-0.760959112540794	0.804334723897681	1	pseudobulk	DESeq2	LRT	0.0945631819854715
135	KC_AB1	Dip-C	0.506245599643555	0.392817778521385	1	pseudobulk	DESeq2	LRT	0.405808864726721
135	KC_AB1	DIP-delta	-1.77199356422654	0.54193209410651	1	pseudobulk	DESeq2	LRT	0.266055128595237
135	KC_AB1	DIP-epsilon	1.11388934932283	1	1	pseudobulk	DESeq2	LRT	0
135	KC_AB1	DIP-eta	1.13734983340557	0.742793668201421	1	pseudobulk	DESeq2	LRT	0.1291318069744
135	KC_AB1	DIP-gamma	0.709686956006578	0.915714550115026	1	pseudobulk	DESeq2	LRT	0.0382398850985033
135	KC_AB1	DIP-kappa	-0.125854653216084	0.333645829201699	1	pseudobulk	DESeq2	LRT	0.476714299753743
135	KC_AB1	DIP-lambda	0.943022244937108	0.768808261856119	1	pseudobulk	DESeq2	LRT	0.114181958248924
135	KC_AB1	DIP1	-0.0189764184518468	0.937339479507094	1	pseudobulk	DESeq2	LRT	0.0281030906756479
135	KC_AB1	DIP2	-0.0761408022332006	0.815305851543967	1	pseudobulk	DESeq2	LRT	0.0886794406832847
135	KC_AB1	Dis3	0.156649164773119	0.918205691542813	1	pseudobulk	DESeq2	LRT	0.0370600195661032
135	KC_AB1	Dis3l2	0.646242786561644	0.303433857230738	1	pseudobulk	DESeq2	LRT	0.517935962151421
135	KC_AB1	disp	-0.565928544519901	0.530469398804475	1	pseudobulk	DESeq2	LRT	0.275339664191814
135	KC_AB1	DJ-1alpha	0.444106679859171	0.646580175491052	1	pseudobulk	DESeq2	LRT	0.189377615231469
135	KC_AB1	dj-1beta	0.228534836810456	0.509243811158595	1	pseudobulk	DESeq2	LRT	0.293074240277732
135	KC_AB1	dl	3.1557909146352	0.0469252876665541	1	pseudobulk	DESeq2	LRT	1.32859305633871
135	KC_AB1	Dl	1.25804452752548	0.573436424985144	1	pseudobulk	DESeq2	LRT	0.241514723898045
135	KC_AB1	Dlc90F	0.0384869774221075	0.885616819935987	1	pseudobulk	DESeq2	LRT	0.0527541437824311
135	KC_AB1	dlg1	-0.0659741652104248	0.612407189938791	1	pseudobulk	DESeq2	LRT	0.212959719129211
135	KC_AB1	Dlg5	-0.0862202493820702	0.870721943972209	1	pseudobulk	DESeq2	LRT	0.0601205102941934
135	KC_AB1	Dlic	0.0804832948001747	0.77813579619222	1	pseudobulk	DESeq2	LRT	0.108944605593095
135	KC_AB1	Dlip1	0.169511562869455	0.868946649784161	1	pseudobulk	DESeq2	LRT	0.061006886860426
135	KC_AB1	Dlip2	0.063638282900322	0.905817906890186	1	pseudobulk	DESeq2	LRT	0.0429590981079899
135	KC_AB1	Dlip3	0.380120429075845	0.522295385295012	1	pseudobulk	DESeq2	LRT	0.282083811325108
135	KC_AB1	Dlish	-0.716419614646419	0.812870875922622	1	pseudobulk	DESeq2	LRT	0.0899784363592155
135	KC_AB1	Dll	-2.82259727438982	0.296520083348317	1	pseudobulk	DESeq2	LRT	0.527945886475195
135	KC_AB1	dlp	2.24725108027632	0.16229010425153	1	pseudobulk	DESeq2	LRT	0.789707960765135
135	KC_AB1	dlt	0.238170797775381	0.88516030757908	1	pseudobulk	DESeq2	LRT	0.0529780689719277
135	KC_AB1	DMAP1	0.45692533042384	0.333955491054394	1	pseudobulk	DESeq2	LRT	0.476311411264923
135	KC_AB1	dmpd	0.248275522923523	0.6564311142683	1	pseudobulk	DESeq2	LRT	0.182810841947434
135	KC_AB1	dmrt11E	0.152115475746306	1	1	pseudobulk	DESeq2	LRT	0
135	KC_AB1	dmt	0.137866841408349	0.929465368589904	1	pseudobulk	DESeq2	LRT	0.0317667871836343
135	KC_AB1	Dmtn	-0.365562291049045	0.42432182717457	1	pseudobulk	DESeq2	LRT	0.372304627489439
135	KC_AB1	DnaJ-1	0.243465901144561	0.536296022040925	1	pseudobulk	DESeq2	LRT	0.270595424390806
135	KC_AB1	DnaJ-H	0.288665675733363	0.529103789827373	1	pseudobulk	DESeq2	LRT	0.276459127722865
135	KC_AB1	DNAlig1	-0.396721808097649	1	1	pseudobulk	DESeq2	LRT	0
135	KC_AB1	DNAlig3	0.13492535191351	0.842431056063955	1	pseudobulk	DESeq2	LRT	0.0744656313422807
135	KC_AB1	DNAlig4	1.20057365920444	0.430066798442848	1	pseudobulk	DESeq2	LRT	0.366464084090031
135	KC_AB1	DNApol-alpha180	1.25804452752548	0.573436424985144	1	pseudobulk	DESeq2	LRT	0.241514723898045
135	KC_AB1	DNApol-alpha50	0.296275065198765	1	1	pseudobulk	DESeq2	LRT	0
136	KC_AB1	DNApol-alpha73	1.25804452752548	0.573436424985144	1	pseudobulk	DESeq2	LRT	0.241514723898045
136	KC_AB1	DNApol-delta	0.0254716872453063	0.975369293438105	1	pseudobulk	DESeq2	LRT	0.0108309209817951
136	KC_AB1	DNApol-eta	-0.963578006576247	0.514579502904031	1	pseudobulk	DESeq2	LRT	0.288547516907179
136	KC_AB1	DNApol-iota	-0.0764230737370616	0.921380858841239	1	pseudobulk	DESeq2	LRT	0.0355608142083387
136	KC_AB1	DNApol-theta	-1.77199356422654	0.54193209410651	1	pseudobulk	DESeq2	LRT	0.266055128595237
136	KC_AB1	DNApol-zeta	-1.87044563070574	0.449059757773538	1	pseudobulk	DESeq2	LRT	0.347695862238472
136	KC_AB1	DNaseII	0.156604560395552	0.815389937129734	1	pseudobulk	DESeq2	LRT	0.0886346525551836

136	KC_AB1	dnc	-0.284588052249558	0.097936810018916	1	pseudobulk	DESeq2	LRT	1.00905404585327
136	KC_AB1	dnd	-0.647842733348031	0.704843036613106	1	pseudobulk	DESeq2	LRT	0.1519075864448
136	KC_AB1	dnk	0.0925710254541478	0.895595048518262	1	pseudobulk	DESeq2	LRT	0.0478883161728872
136	KC_AB1	dnr1	-1.1057387065675	0.673959620645904	1	pseudobulk	DESeq2	LRT	0.171366122835592
136	KC_AB1	dnt	0.496927720782221	0.567495763961174	1	pseudobulk	DESeq2	LRT	0.246037375888414
136	KC_AB1	Dnz1	0.130837668355551	0.740633125246201	1	pseudobulk	DESeq2	LRT	0.13039686777953
136	KC_AB1	Doa	-0.212745757138582	0.558838222715485	1	pseudobulk	DESeq2	LRT	0.252713897212982
136	KC_AB1	Doc3	-0.760959112540794	1	1	pseudobulk	DESeq2	LRT	0
136	KC_AB1	dock	0.044332807032006	0.839702678824732	1	pseudobulk	DESeq2	LRT	0.0758744613229959
136	KC_AB1	dod	0.241961635849947	0.474066254997341	1	pseudobulk	DESeq2	LRT	0.324160957547591
136	KC_AB1	Dok	4.45565821955857	0.111691140174007	1	pseudobulk	DESeq2	LRT	0.951981275641296
136	KC_AB1	dom	-0.0638765200486518	0.866286045700562	1	pseudobulk	DESeq2	LRT	0.0623386812422909
136	KC_AB1	dome	-0.169484772454669	0.808733410648055	1	pseudobulk	DESeq2	LRT	0.092194614809056
136	KC_AB1	dop	-0.0837598202689192	0.855466398311191	1	pseudobulk	DESeq2	LRT	0.0677970443515504
136	KC_AB1	Dop1R1	-0.324441126078213	0.0458359374965093	1	pseudobulk	DESeq2	LRT	1.33879388146461
136	KC_AB1	Dop1R2	-0.379879823335678	0.0251989579648183	1	pseudobulk	DESeq2	LRT	1.59861741792823
136	KC_AB1	Dop2R	0.0635227587712985	0.626877331080247	1	pseudobulk	DESeq2	LRT	0.202817434682915
136	KC_AB1	DopEcR	-0.152914917881327	0.552075915505617	1	pseudobulk	DESeq2	LRT	0.258001198686998
136	KC_AB1	dor	0.184229527415129	0.86388029578838	1	pseudobulk	DESeq2	LRT	0.0635464316880059
136	KC_AB1	DOR	-0.221102168722701	0.237119552688633	1	pseudobulk	DESeq2	LRT	0.625032632969667
136	KC_AB1	dos	-0.084821974785683	0.884080863319173	1	pseudobulk	DESeq2	LRT	0.0535080100014262
136	KC_AB1	Dp	-0.226548211739127	0.535263598668719	1	pseudobulk	DESeq2	LRT	0.271432290401082
136	KC_AB1	Dp1	-0.742682791607749	0.264770728965877	1	pseudobulk	DESeq2	LRT	0.577130028866998
136	KC_AB1	dpa	0.763804777867263	0.744585110462139	1	pseudobulk	DESeq2	LRT	0.128085652658032
136	KC_AB1	Dpck	0.0904730608189249	0.877364513779315	1	pseudobulk	DESeq2	LRT	0.056819935216567
136	KC_AB1	DPCoAC	0.467211472552978	0.69847646670758	1	pseudobulk	DESeq2	LRT	0.155848221691834
136	KC_AB1	Dph1	-0.338376517634981	0.859710437232504	1	pseudobulk	DESeq2	LRT	0.0656478007190349
136	KC_AB1	Dph5	-0.222279060843864	0.665026117093409	1	pseudobulk	DESeq2	LRT	0.177161298626482
136	KC_AB1	Dpit47	0.157177832908244	0.894109842959881	1	pseudobulk	DESeq2	LRT	0.0486091240889142
136	KC_AB1	dpn	1.90071181822722	0.545896518579078	1	pseudobulk	DESeq2	LRT	0.262889675371117
136	KC_AB1	dpp	-0.760959112540794	1	1	pseudobulk	DESeq2	LRT	0
136	KC_AB1	DppIII	-0.470271178398105	0.452117717411353	1	pseudobulk	DESeq2	LRT	0.344748473670824
136	KC_AB1	dpr1	0.0888834419427011	0.896519202451729	1	pseudobulk	DESeq2	LRT	0.0474404038937531
136	KC_AB1	dpr10	-0.295622108921804	0.330129509001	1	pseudobulk	DESeq2	LRT	0.48131565402923
136	KC_AB1	dpr12	-0.25517511991471	0.186969482998157	1	pseudobulk	DESeq2	LRT	0.728229272859475
136	KC_AB1	dpr13	1.90071181822722	0.545896518579078	1	pseudobulk	DESeq2	LRT	0.262889675371117
136	KC_AB1	dpr14	-0.0418606566007098	0.860454380670735	1	pseudobulk	DESeq2	LRT	0.0652721500292254
136	KC_AB1	dpr17	-0.385011853448768	0.890534840916548	1	pseudobulk	DESeq2	LRT	0.0503490847107839
136	KC_AB1	dpr19	-0.129519837335967	0.617505054038422	1	pseudobulk	DESeq2	LRT	0.209359483522522
136	KC_AB1	dpr2	-0.167399643098084	0.920613467105548	1	pseudobulk	DESeq2	LRT	0.0359226763605239
136	KC_AB1	dpr20	0.0461767631761319	0.922522824333818	1	pseudobulk	DESeq2	LRT	0.0350228800636384
136	KC_AB1	dpr21	-0.107047210575085	0.524897056022192	1	pseudobulk	DESeq2	LRT	0.279925863042304
136	KC_AB1	dpr3	0.0653579911535846	0.946396025515762	1	pseudobulk	DESeq2	LRT	0.0239270922391682
136	KC_AB1	dpr4	-0.0673649093915467	0.823889691749404	1	pseudobulk	DESeq2	LRT	0.0841309308652821
136	KC_AB1	dpr5	-0.152589528315131	0.77791175073785	1	pseudobulk	DESeq2	LRT	0.109069668230664
136	KC_AB1	dpr6	-0.560531989834697	0.0517717215538328	1	pseudobulk	DESeq2	LRT	1.28590739327778
136	KC_AB1	dpr7	0.0713449960254031	0.760448994939433	1	pseudobulk	DESeq2	LRT	0.118929909762466
136	KC_AB1	dpr8	-0.141839956148563	0.402538944349316	1	pseudobulk	DESeq2	LRT	0.395192096667968
136	KC_AB1	dpr9	-0.0909111153816357	0.623445146995255	1	pseudobulk	DESeq2	LRT	0.205201751335776
136	KC_AB1	dpy	1.55770427203681	0.475647125641621	1	pseudobulk	DESeq2	LRT	0.322715123371671
136	KC_AB1	Dpy-30L1	0.0774433500898661	0.834724102437669	1	pseudobulk	DESeq2	LRT	0.0784570461794158
136	KC_AB1	Drak	-0.384658769965873	0.20313039655367	1	pseudobulk	DESeq2	LRT	0.692225083647032
136	KC_AB1	Drat	-0.0873450469789499	0.682652676015482	1	pseudobulk	DESeq2	LRT	0.16580020298941
136	KC_AB1	dre4	0.736079885183383	0.261090560489978	1	pseudobulk	DESeq2	LRT	0.583208829445615
136	KC_AB1	Dredd	0.494775748955277	0.827825706404751	1	pseudobulk	DESeq2	LRT	0.0820610916144188
136	KC_AB1	Dref	-0.48557963704572	0.544556024535397	1	pseudobulk	DESeq2	LRT	0.263957432905592

136	KC_AB1	Drep1	-1.22347731385815	0.400405476797214	1	pseudobulk	DESeq2	LRT	0.397499990816741
136	KC_AB1	Drep2	0.0533831091683067	0.807420313231396	1	pseudobulk	DESeq2	LRT	0.092900328726383
136	KC_AB1	Drep3	-0.760959112540794	0.804334723897681	1	pseudobulk	DESeq2	LRT	0.0945631819854715
136	KC_AB1	Drep4	-0.353365058879159	0.621721568623483	1	pseudobulk	DESeq2	LRT	0.206404065924948
136	KC_AB1	Drgx	0.296275065198765	1	1	pseudobulk	DESeq2	LRT	0
136	KC_AB1	Drice	-0.0684631209298324	0.931602312225201	1	pseudobulk	DESeq2	LRT	0.0307694422225872
136	KC_AB1	Drip	2.99960667015886	0.357405021116046	1	pseudobulk	DESeq2	LRT	0.44683935046587
136	KC_AB1	drk	-0.297648129020289	0.489078217742646	1	pseudobulk	DESeq2	LRT	0.310621679081864
136	KC_AB1	drl	-0.0609672871935737	0.888075551378934	1	pseudobulk	DESeq2	LRT	0.0515500858528845
136	KC_AB1	Drl-2	1.19128241789501	0.625893738281617	1	pseudobulk	DESeq2	LRT	0.203499393299466
136	KC_AB1	Droj2	0.0581930662462919	0.762168929303678	1	pseudobulk	DESeq2	LRT	0.117948759718934
136	KC_AB1	Dronc	0.541206450200653	0.578337352178548	1	pseudobulk	DESeq2	LRT	0.237818757685733
136	KC_AB1	drongo	-0.192824383725687	0.650832838314428	1	pseudobulk	DESeq2	LRT	0.186530542491545
136	KC_AB1	drosha	-0.00111202739145071	0.981874852504291	1	pseudobulk	DESeq2	LRT	0.00794386285498641
136	KC_AB1	Drp1	-0.121864704775047	0.751615826606701	1	pseudobulk	DESeq2	LRT	0.124004083633259
136	KC_AB1	drpr	-0.0725573200164696	0.876807520636854	1	pseudobulk	DESeq2	LRT	0.0570957337717973
136	KC_AB1	Drs	5.59002623798477	0.00216987581419707	1	pseudobulk	DESeq2	LRT	2.66356512087601
136	KC_AB1	Drsl2	1.25804452752548	0.727552935095488	1	pseudobulk	DESeq2	LRT	0.138135402914536
136	KC_AB1	Drsl6	-0.760959112540794	0.805265236978025	1	pseudobulk	DESeq2	LRT	0.0940610488455931
136	KC_AB1	ds	-0.438584120588001	0.828770538443449	1	pseudobulk	DESeq2	LRT	0.0815656958455328
136	KC_AB1	dsb	2.36511635630727	0.443679267663499	1	pseudobulk	DESeq2	LRT	0.352930864611606
136	KC_AB1	Dscam1	-0.0106779967070511	0.93891366537665	1	pseudobulk	DESeq2	LRT	0.0273743399749029
136	KC_AB1	Dscam2	-0.0379604697491833	0.81039181208821	1	pseudobulk	DESeq2	LRT	0.0913049555832065
136	KC_AB1	Dscam3	1.74758121156501	0.493314355044031	1	pseudobulk	DESeq2	LRT	0.306876246739962
136	KC_AB1	Dscam4	-0.0458864153223919	0.87127238900319	1	pseudobulk	DESeq2	LRT	0.0598460487268079
136	KC_AB1	dsd	0.0261178971109271	0.921987518011493	1	pseudobulk	DESeq2	LRT	0.035274958442494
136	KC_AB1	dsh	-0.191702015932543	0.695580095949112	1	pseudobulk	DESeq2	LRT	0.157652853844633
136	KC_AB1	Dsor1	0.0862260553197566	0.822680015959959	1	pseudobulk	DESeq2	LRT	0.084769052181744
136	KC_AB1	Dsp1	-0.0288574955841014	0.853454573266902	1	pseudobulk	DESeq2	LRT	0.0688195900788921
136	KC_AB1	dsx	-1.59393039059934	0.58799577039461	1	pseudobulk	DESeq2	LRT	0.230625797904964
136	KC_AB1	Dtd	-0.384988031783525	0.743689169544396	1	pseudobulk	DESeq2	LRT	0.128608543161373
136	KC_AB1	Dtg	-1.77199356422654	0.54193209410651	1	pseudobulk	DESeq2	LRT	0.266055128595237
136	KC_AB1	dtn	-0.118723337505537	0.576337237701566	1	pseudobulk	DESeq2	LRT	0.239323319329196
136	KC_AB1	dtr	-0.84572271983469	0.729929556961718	1	pseudobulk	DESeq2	LRT	0.136719050152121
136	KC_AB1	Duba	-0.0925572218792948	0.822450602815833	1	pseudobulk	DESeq2	LRT	0.0848901767442303
136	KC_AB1	DUBAI	0.197224396017449	0.75503142710269	1	pseudobulk	DESeq2	LRT	0.122034971108263
136	KC_AB1	dunk	0.603348748252756	0.419904944913022	1	pseudobulk	DESeq2	LRT	0.376849010963885
137	KC_AB1	dup	-2.56001970754374	0.130098844686787	1	pseudobulk	DESeq2	LRT	0.885726560074704
137	KC_AB1	dUTPase	-2.47819805667031	0.220400880205568	1	pseudobulk	DESeq2	LRT	0.656786675393492
137	KC_AB1	dve	-0.760959112540794	0.805265236978025	1	pseudobulk	DESeq2	LRT	0.0940610488455931
137	KC_AB1	dwg	-0.551411259163765	0.609420086865905	1	pseudobulk	DESeq2	LRT	0.215083235260618
137	KC_AB1	Dyb	-0.0489647803696739	0.802899027209849	1	pseudobulk	DESeq2	LRT	0.0953390682741014
137	KC_AB1	Dyrk2	-0.230909266552034	0.38428464589889	1	pseudobulk	DESeq2	LRT	0.415346967434165
137	KC_AB1	Dyrk3	-0.00391174869364424	0.95645824322432	1	pseudobulk	DESeq2	LRT	0.019333985524939
137	KC_AB1	Dys	-0.398192226547001	0.218722066837104	1	pseudobulk	DESeq2	LRT	0.66010739884562
137	KC_AB1	Dysb	1.4790069735779	0.618734192823006	1	pseudobulk	DESeq2	LRT	0.208495883100692
137	KC_AB1	dysc	-0.191718636724753	0.196322526799972	1	pseudobulk	DESeq2	LRT	0.707029864925635
137	KC_AB1	dyw	-1.2237060175761	0.71867115550687	1	pseudobulk	DESeq2	LRT	0.14346978558097
137	KC_AB1	e	-0.00380455707178423	0.98721003556768	1	pseudobulk	DESeq2	LRT	0.00559043843085644
137	KC_AB1	E(bx)	-0.386248831992303	0.0932531509962571	1	pseudobulk	DESeq2	LRT	1.03033648458987
137	KC_AB1	E(Pc)	-0.315108746696888	0.205178296253732	1	pseudobulk	DESeq2	LRT	0.687868580771501
137	KC_AB1	e(r)	0.0546575142654902	0.860635451827102	1	pseudobulk	DESeq2	LRT	0.0651807681584509
137	KC_AB1	E(spl)m2-BFM	1.25804452752548	0.727552935095488	1	pseudobulk	DESeq2	LRT	0.138135402914536
137	KC_AB1	E(spl)m3-HLH	0.393853628865458	0.873915328625432	1	pseudobulk	DESeq2	LRT	0.0585306429858249
137	KC_AB1	E(spl)mbeta-HLH	0.729858847558949	0.582216722770204	1	pseudobulk	DESeq2	LRT	0.234915324657222
137	KC_AB1	E(var)3-9	0.500293721872576	0.519528940494823	1	pseudobulk	DESeq2	LRT	0.284390254945827

137	KC_AB1	e(y)1	1.30517276478937	0.135232602655473	1	pseudobulk	DESeq2	LRT	0.868918593553815
137	KC_AB1	e(y)2	-0.113709290824191	0.87105556399298	1	pseudobulk	DESeq2	LRT	0.0599541407835268
137	KC_AB1	e(y)2b	0.943798956650056	0.807634491832869	1	pseudobulk	DESeq2	LRT	0.0927851418175362
137	KC_AB1	e(y)3	-0.149764943595299	0.687624055345911	1	pseudobulk	DESeq2	LRT	0.162648938673103
137	KC_AB1	E(z)	0.0606197869306338	0.922110953415805	1	pseudobulk	DESeq2	LRT	0.0352168191230654
137	KC_AB1	E23	0.296275065198765	1	1	pseudobulk	DESeq2	LRT	0
137	KC_AB1	E2f1	0.146471460127068	0.817499988575442	1	pseudobulk	DESeq2	LRT	0.0875122447369395
137	KC_AB1	E2f2	0.465869320473349	0.422077955604697	1	pseudobulk	DESeq2	LRT	0.374607329696441
137	KC_AB1	Eaat1	0.3413443302168	0.470245219949411	1	pseudobulk	DESeq2	LRT	0.327675610365877
137	KC_AB1	Eaat2	-0.195999017430874	0.646622649077269	1	pseudobulk	DESeq2	LRT	0.189349087542266
137	KC_AB1	Eaf	-0.421407472289108	0.475658371516667	1	pseudobulk	DESeq2	LRT	0.322704855331612
137	KC_AB1	Eaf6	-0.0348710487808121	0.96527311739579	1	pseudobulk	DESeq2	LRT	0.0153497886293676
137	KC_AB1	eag	-0.101245862227419	0.683043477498271	1	pseudobulk	DESeq2	LRT	0.165551651462497
137	KC_AB1	ear	0.299192453546536	0.472086427900254	1	pseudobulk	DESeq2	LRT	0.325978485001396
137	KC_AB1	eas	-0.10695600279847	0.777804666961967	1	pseudobulk	DESeq2	LRT	0.109129455339049
137	KC_AB1	east	-0.0633701239836644	0.797598135600056	1	pseudobulk	DESeq2	LRT	0.0982158698663533
137	KC_AB1	Eato	0.439236437815522	0.628940674310657	1	pseudobulk	DESeq2	LRT	0.201390318043808
137	KC_AB1	Eb1	0.0812256523121123	0.707741856322806	1	pseudobulk	DESeq2	LRT	0.150125119171501
137	KC_AB1	ebd1	0.275980239407524	0.742525181868333	1	pseudobulk	DESeq2	LRT	0.12928881317437
137	KC_AB1	ebd2	0.715979393092862	0.248227964774422	1	pseudobulk	DESeq2	LRT	0.605149293493683
137	KC_AB1	ebi	-0.290740639834937	0.669842710610284	1	pseudobulk	DESeq2	LRT	0.174027164364096
137	KC_AB1	ebo	-0.253994132944198	0.757448825551895	1	pseudobulk	DESeq2	LRT	0.120646703460879
137	KC_AB1	EbpIII	0.282069487234871	0.878594408586741	1	pseudobulk	DESeq2	LRT	0.0562115649309891
137	KC_AB1	ec	1.0671889502546	0.731277402076364	1	pseudobulk	DESeq2	LRT	0.135917846930155
137	KC_AB1	eca	-0.0853963499535558	0.84132203344615	1	pseudobulk	DESeq2	LRT	0.0750377371645303
137	KC_AB1	ecd	-0.355317153184679	0.67169527679476	1	pseudobulk	DESeq2	LRT	0.172827705548538
137	KC_AB1	Echs1	-0.255136366788162	0.762625142364567	1	pseudobulk	DESeq2	LRT	0.117688880926761
137	KC_AB1	eco	2.13148430488057	0.45484681020722	1	pseudobulk	DESeq2	LRT	0.342134846603996
137	KC_AB1	EcR	-0.0816582120006107	0.619929975273835	1	pseudobulk	DESeq2	LRT	0.207657363839933
137	KC_AB1	ECSIT	-0.210555219921822	0.838770438740107	1	pseudobulk	DESeq2	LRT	0.0763568840224789
137	KC_AB1	ect	4.23269314069016	0.0125942197250196	1	pseudobulk	DESeq2	LRT	1.89982873405038
137	KC_AB1	Ect3	0.16665717199831	0.849614625619712	1	pseudobulk	DESeq2	LRT	0.0707780200723243
137	KC_AB1	Ect4	0.305743193568094	0.821909752109152	1	pseudobulk	DESeq2	LRT	0.0851758665386986
137	KC_AB1	ed	1.54737454083896	0.143211074275176	1	pseudobulk	DESeq2	LRT	0.844023397456653
137	KC_AB1	Edc3	-0.422587729939476	0.236079184089921	1	pseudobulk	DESeq2	LRT	0.626942304464796
137	KC_AB1	Edem1	1.32166019297021	0.27726670996505	1	pseudobulk	DESeq2	LRT	0.557102270870668
137	KC_AB1	Edem2	-0.532272789631039	0.395994248321238	1	pseudobulk	DESeq2	LRT	0.402311122005015
137	KC_AB1	Edg78E	-0.760959112540794	0.805265236978025	1	pseudobulk	DESeq2	LRT	0.0940610488455931
137	KC_AB1	Edg91	3.12964491182152	0.252827515563327	1	pseudobulk	DESeq2	LRT	0.597175662956756
137	KC_AB1	edl	4.31851526800926	0.128069104667069	1	pseudobulk	DESeq2	LRT	0.892555626624673
137	KC_AB1	EDTP	-0.730134173081534	0.439914984924286	1	pseudobulk	DESeq2	LRT	0.356631244299362
137	KC_AB1	eEF1alpha1	0.303306851639452	0.54355811495955	1	pseudobulk	DESeq2	LRT	0.264754016146268
137	KC_AB1	eEF1alpha2	-0.160451538113295	0.387594394848385	1	pseudobulk	DESeq2	LRT	0.411622512075395
137	KC_AB1	eEF1beta	-0.168575685520193	0.642474948000118	1	pseudobulk	DESeq2	LRT	0.192143802093831
137	KC_AB1	eEF1delta	0.470953633835579	0.274025287323198	1	pseudobulk	DESeq2	LRT	0.562209358208116
137	KC_AB1	eEF1gamma	-0.189189036204121	0.617658233975799	1	pseudobulk	DESeq2	LRT	0.209251764644334
137	KC_AB1	eEF2	-0.251788392910367	0.182432072940934	1	pseudobulk	DESeq2	LRT	0.738898807034063
137	KC_AB1	eEF5	-0.011205352108553	0.933326739760536	1	pseudobulk	DESeq2	LRT	0.029966291480011
137	KC_AB1	eEFSec	0.293544814793053	0.855484935116898	1	pseudobulk	DESeq2	LRT	0.0677876338764847
137	KC_AB1	Efa6	-0.115583749427443	0.820889650821287	1	pseudobulk	DESeq2	LRT	0.0857152195679885
137	KC_AB1	eff	-0.126435010730417	0.370472954737382	1	pseudobulk	DESeq2	LRT	0.431243490888519
137	KC_AB1	Efhc1.2	-0.760959112540794	0.805265236978025	1	pseudobulk	DESeq2	LRT	0.0940610488455931
137	KC_AB1	Efr	0.265954184054188	0.728472818240923	1	pseudobulk	DESeq2	LRT	0.137586648572389
137	KC_AB1	Egfr	0.847525847835988	0.691106783674185	1	pseudobulk	DESeq2	LRT	0.16045484412001
137	KC_AB1	egg	-0.101254694299902	0.869213041625512	1	pseudobulk	DESeq2	LRT	0.0608737661716877
137	KC_AB1	egh	0.0320199050626663	0.859017383121811	1	pseudobulk	DESeq2	LRT	0.0659980476734199

137	KC_AB1	egl	-0.0267281134946077	0.929962951642477	1	pseudobulk	DESeq2	LRT	0.0315343527554525
137	KC_AB1	Eglp2	1.25804452752548	0.727552935095488	1	pseudobulk	DESeq2	LRT	0.138135402914536
137	KC_AB1	Eglp4	0.205634162617708	0.9324006963501	1	pseudobulk	DESeq2	LRT	0.0303974108047287
137	KC_AB1	Egm	0.101697740358445	0.904634584633344	1	pseudobulk	DESeq2	LRT	0.0435268129771143
137	KC_AB1	egr	1.56812162677943	0.515342563766959	1	pseudobulk	DESeq2	LRT	0.287903986307172
137	KC_AB1	Eh	-0.760959112540794	1	1	pseudobulk	DESeq2	LRT	0
137	KC_AB1	Ehbp1	-0.100805288866315	0.76040055406337	1	pseudobulk	DESeq2	LRT	0.118957575359229
137	KC_AB1	eIF1	0.0990797487254778	0.632086912870435	1	pseudobulk	DESeq2	LRT	0.199223201488394
137	KC_AB1	eIF1A	0.12296819569946	0.472629692068084	1	pseudobulk	DESeq2	LRT	0.325478998115708
137	KC_AB1	eIF2alpha	0.0134381437324845	0.974425761683534	1	pseudobulk	DESeq2	LRT	0.0112512427642511
137	KC_AB1	eIF2Balpha	-0.514136144452142	0.478291150875128	1	pseudobulk	DESeq2	LRT	0.320307654172261
137	KC_AB1	eIF2Bbeta	0.615684006658019	0.356953852949423	1	pseudobulk	DESeq2	LRT	0.447387925918183
137	KC_AB1	eIF2Bdelta	0.555162998016652	0.4074917984746	1	pseudobulk	DESeq2	LRT	0.389881127814971
137	KC_AB1	eIF2Bepsilon	-1.04596673402543	0.28259544092311	1	pseudobulk	DESeq2	LRT	0.548834848849481
137	KC_AB1	eIF2beta	-0.176422061371	0.542807462316602	1	pseudobulk	DESeq2	LRT	0.265354190451053
137	KC_AB1	eIF2Bgamma	0.0984765839133569	0.922128558472067	1	pseudobulk	DESeq2	LRT	0.0352085275983051
137	KC_AB1	eIF2D	0.395513614994445	0.536491827367999	1	pseudobulk	DESeq2	LRT	0.270436889459474
137	KC_AB1	eIF2gamma	0.187882970790274	0.762939286621243	1	pseudobulk	DESeq2	LRT	0.117510021068034
137	KC_AB1	eIF3a	-0.161592281269866	0.604416287901746	1	pseudobulk	DESeq2	LRT	0.218663840741786
137	KC_AB1	eIF3b	-0.0987640799617218	0.779778967504903	1	pseudobulk	DESeq2	LRT	0.108028482946118
137	KC_AB1	eIF3c	0.178440278749638	0.566961926639375	1	pseudobulk	DESeq2	LRT	0.246446104432678
137	KC_AB1	eIF3d1	-0.207759025867694	0.501018714796904	1	pseudobulk	DESeq2	LRT	0.300146051415741
137	KC_AB1	eIF3e	0.115055393459595	0.696465319250898	1	pseudobulk	DESeq2	LRT	0.157100504556539
137	KC_AB1	eIF3f1	0.160773183036183	0.610235184393802	1	pseudobulk	DESeq2	LRT	0.214502755809381
137	KC_AB1	eIF3g1	0.222982537815769	0.417738135682924	1	pseudobulk	DESeq2	LRT	0.379095875798682
138	KC_AB1	eIF3g2	-0.760959112540794	1	1	pseudobulk	DESeq2	LRT	0
138	KC_AB1	eIF3h	0.0493318428983701	0.848093363532204	1	pseudobulk	DESeq2	LRT	0.0715563352021134
138	KC_AB1	eIF3i	0.0699452037576403	0.760739455565227	1	pseudobulk	DESeq2	LRT	0.118764058584088
138	KC_AB1	eIF3j	-0.152399368928904	0.64567386846885	1	pseudobulk	DESeq2	LRT	0.189986789882578
138	KC_AB1	eIF3k	0.296844975264698	0.311580204415584	1	pseudobulk	DESeq2	LRT	0.506430142097586
138	KC_AB1	eIF3l	-0.0778561232275472	0.810894202213395	1	pseudobulk	DESeq2	LRT	0.0910358047187822
138	KC_AB1	eIF3m	-0.0333105182265661	0.891854297564059	1	pseudobulk	DESeq2	LRT	0.0497060906154676
138	KC_AB1	eIF4A	0.0612887156485433	0.611767776792992	1	pseudobulk	DESeq2	LRT	0.213413402031863
138	KC_AB1	eIF4B	0.00678028515147659	0.96166473660852	1	pseudobulk	DESeq2	LRT	0.0169763088547172
138	KC_AB1	eIF4E1	-0.101149404659251	0.642785969506117	1	pseudobulk	DESeq2	LRT	0.191933611424208
138	KC_AB1	eIF4EHP	-0.215129228774382	0.389333167161345	1	pseudobulk	DESeq2	LRT	0.40967859730525
138	KC_AB1	eIF4G1	-0.0608253261888288	0.837543883229522	1	pseudobulk	DESeq2	LRT	0.0769924287745306
138	KC_AB1	eIF4G2	-0.308240275905339	0.235975482147361	1	pseudobulk	DESeq2	LRT	0.627133117882656
138	KC_AB1	eIF4H1	-0.442306786541776	0.140757047086966	1	pseudobulk	DESeq2	LRT	0.851529852715561
138	KC_AB1	eIF5	0.372546425330994	0.322302827859113	1	pseudobulk	DESeq2	LRT	0.491735884006062
138	KC_AB1	eIF5B	-0.04491788680983	0.860078920072416	1	pseudobulk	DESeq2	LRT	0.0654616964547898
138	KC_AB1	eIF6	0.335413303838465	0.700074314670396	1	pseudobulk	DESeq2	LRT	0.154855856074015
138	KC_AB1	Eip55E	1.10074266054233	0.318261203079851	1	pseudobulk	DESeq2	LRT	0.497216299849877
138	KC_AB1	Eip63E	0.312842175306344	0.245396878319221	1	pseudobulk	DESeq2	LRT	0.610130966211133
138	KC_AB1	Eip63F-1	-0.297569732291354	0.825189327414947	1	pseudobulk	DESeq2	LRT	0.083446397609659
138	KC_AB1	Eip71CD	0.152909867177597	0.72541449817906	1	pseudobulk	DESeq2	LRT	0.13941376883245
138	KC_AB1	Eip74EF	0.317178114563986	0.565021704048037	1	pseudobulk	DESeq2	LRT	0.247934869406656
138	KC_AB1	Eip75B	-0.186962112239434	0.313247900524497	1	pseudobulk	DESeq2	LRT	0.504111831087783
138	KC_AB1	Eip78C	-0.585760850805192	0.462690841306014	1	pseudobulk	DESeq2	LRT	0.334709097019718
138	KC_AB1	Eip93F	-0.196485719201508	0.21449885533694	1	pseudobulk	DESeq2	LRT	0.668575021065164
138	KC_AB1	Ekar	1.47737206823489	0.580967731351599	1	pseudobulk	DESeq2	LRT	0.235847988926577
138	KC_AB1	Elal	-0.66634233137268	0.653001238607046	1	pseudobulk	DESeq2	LRT	0.185085994958164
138	KC_AB1	elav	-0.0442860736742142	0.794790354788514	1	pseudobulk	DESeq2	LRT	0.0997474119290343
138	KC_AB1	elB	0.22166299065601	0.548204601640592	1	pseudobulk	DESeq2	LRT	0.261057323304494
138	KC_AB1	elg1	1.25804452752548	0.727552935095488	1	pseudobulk	DESeq2	LRT	0.138135402914536
138	KC_AB1	elgi	0.266030127227725	0.623245183837655	1	pseudobulk	DESeq2	LRT	0.20534106884635

138	KC_AB1	Elk	-0.255718288046544	0.0904846376537146	1	pseudobulk	DESeq2	LRT	1.0434251484031
138	KC_AB1	elm	-0.0368775902998677	0.897557552101786	1	pseudobulk	DESeq2	LRT	0.0469376945503394
138	KC_AB1	EloA	0.204074476248895	0.698868992509141	1	pseudobulk	DESeq2	LRT	0.15560422792067
138	KC_AB1	EloB	-0.0280124987667858	0.910263556755869	1	pseudobulk	DESeq2	LRT	0.04083284430237
138	KC_AB1	EloC	0.0565186201074258	0.813608019542853	1	pseudobulk	DESeq2	LRT	0.0895847793256713
138	KC_AB1	ELOVL	-2.73719572742657	0.395113780272619	1	pseudobulk	DESeq2	LRT	0.40327782328744
138	KC_AB1	Elp1	-0.239862590629801	0.831963740104805	1	pseudobulk	DESeq2	LRT	0.0798956013722057
138	KC_AB1	Elp2	-1.92658935381723	0.303299890006664	1	pseudobulk	DESeq2	LRT	0.518127747188537
138	KC_AB1	Elp3	-1.09626951731138	0.492114026757792	1	pseudobulk	DESeq2	LRT	0.307934256065919
138	KC_AB1	Elys	-0.289227546583756	0.719096985348855	1	pseudobulk	DESeq2	LRT	0.14321253192406
138	KC_AB1	ema	-0.751551595304169	0.217395832806048	1	pseudobulk	DESeq2	LRT	0.66274878502748
138	KC_AB1	emb	-0.160846635988615	0.643295734481792	1	pseudobulk	DESeq2	LRT	0.191589328278105
138	KC_AB1	emc	0.00332968436071022	0.96783571827878	1	pseudobulk	DESeq2	LRT	0.0141983541584431
138	KC_AB1	EMC1	-0.192482151331195	0.742552556196517	1	pseudobulk	DESeq2	LRT	0.129272802538195
138	KC_AB1	EMC3	0.130670102573439	0.770638369630761	1	pseudobulk	DESeq2	LRT	0.113149371518104
138	KC_AB1	EMC8-9	0.125879653649262	0.726811151433932	1	pseudobulk	DESeq2	LRT	0.138578417950315
138	KC_AB1	emp	0.625808972515325	0.741557467852634	1	pseudobulk	DESeq2	LRT	0.12985518717884
138	KC_AB1	EMRE	0.173913909448641	0.537896767847633	1	pseudobulk	DESeq2	LRT	0.269301065329894
138	KC_AB1	ena	-0.0387542562392367	0.904423269938956	1	pseudobulk	DESeq2	LRT	0.0436282722045275
138	KC_AB1	enc	-0.158426580004262	0.528961774356424	1	pseudobulk	DESeq2	LRT	0.276575711303006
138	KC_AB1	EndoA	-0.0988826399672798	0.630931635335445	1	pseudobulk	DESeq2	LRT	0.20001769623141
138	KC_AB1	EndoB	-0.160108060047389	0.742859056481704	1	pseudobulk	DESeq2	LRT	0.129093577623954
138	KC_AB1	EndoG	0.00049882960973624	0.968195527424906	1	pseudobulk	DESeq2	LRT	0.0140369279119954
138	KC_AB1	EndoGI	0.939208659913323	0.436224902435388	1	pseudobulk	DESeq2	LRT	0.360289545814688
138	KC_AB1	endos	0.214101775566473	0.409086992243416	1	pseudobulk	DESeq2	LRT	0.388184329562295
138	KC_AB1	EndoU	2.78011544604646	0.271256374274721	1	pseudobulk	DESeq2	LRT	0.566620047503558
138	KC_AB1	Eno	-0.0041063721907819	0.988127204675354	1	pseudobulk	DESeq2	LRT	0.0051871437397586
138	KC_AB1	enok	-0.107790189798549	0.828507827058716	1	pseudobulk	DESeq2	LRT	0.0817033843691427
138	KC_AB1	ens	-0.168682764845908	0.545198448420391	1	pseudobulk	DESeq2	LRT	0.263445388793749
138	KC_AB1	Ent1	0.457473903155997	0.601570873208407	1	pseudobulk	DESeq2	LRT	0.220713199529268
138	KC_AB1	Ent2	0.106550877879002	0.940594908770438	1	pseudobulk	DESeq2	LRT	0.0265973763223325
138	KC_AB1	Ent3	-0.0923431296937537	0.772459128265065	1	pseudobulk	DESeq2	LRT	0.11212449033489
138	KC_AB1	Eogt	0.265019874552321	0.836599147296053	1	pseudobulk	DESeq2	LRT	0.0774825824099517
138	KC_AB1	Epac	-0.760959112540794	1	1	pseudobulk	DESeq2	LRT	0
138	KC_AB1	Epg5	-0.159056560812856	0.81577900012214	1	pseudobulk	DESeq2	LRT	0.088427478534075
138	KC_AB1	Eph	-0.175500521107863	0.456752354316764	1	pseudobulk	DESeq2	LRT	0.340319205411344
138	KC_AB1	Ephrin	-0.0760573409368172	0.76379595305328	1	pseudobulk	DESeq2	LRT	0.117022647051056
138	KC_AB1	Eps-15	-0.0996183818438865	0.723842214740719	1	pseudobulk	DESeq2	LRT	0.140356092277381
138	KC_AB1	epsilonCOP	0.088333732878148	0.853546337523102	1	pseudobulk	DESeq2	LRT	0.0687728968288882
138	KC_AB1	Ercc1	-1.58594280928057	0.44963436139752	1	pseudobulk	DESeq2	LRT	0.34714050705842
138	KC_AB1	eRF1	0.244113325072012	0.405964475762464	1	pseudobulk	DESeq2	LRT	0.391511968036931
138	KC_AB1	eRF3	-0.575805742731545	0.301473239631987	1	pseudobulk	DESeq2	LRT	0.5207512321008
138	KC_AB1	ergic53	-0.0770290812214845	0.907372780355512	1	pseudobulk	DESeq2	LRT	0.0422142529724804
138	KC_AB1	erm	1.25804452752548	0.573436424985144	1	pseudobulk	DESeq2	LRT	0.241514723898045
138	KC_AB1	Ero1L	0.602216192928034	0.402093054852457	1	pseudobulk	DESeq2	LRT	0.395673428178617
138	KC_AB1	ERp60	-0.317492511589524	0.561065765554028	1	pseudobulk	DESeq2	LRT	0.250986229754146
138	KC_AB1	ERR	0.0541449590063581	0.917873595483818	1	pseudobulk	DESeq2	LRT	0.0372171233344154
138	KC_AB1	Es2	-0.360788568246666	0.651031237536762	1	pseudobulk	DESeq2	LRT	0.186398172776858
138	KC_AB1	escl	0.278593247633235	0.951871828976203	1	pseudobulk	DESeq2	LRT	0.0214215261069401
138	KC_AB1	esn	0.34466469438782	0.905972261010375	1	pseudobulk	DESeq2	LRT	0.0428850993149653
138	KC_AB1	Est-6	7.06075026218591	0.117848800603585	1	pseudobulk	DESeq2	LRT	0.928674833113311
138	KC_AB1	Est-Q	1.11388934932283	1	1	pseudobulk	DESeq2	LRT	0
138	KC_AB1	Esyt2	-0.287400078948367	0.598989622596486	1	pseudobulk	DESeq2	LRT	0.222580701630927
138	KC_AB1	Etf-QO	-1.00526052787074	0.36421170908163	1	pseudobulk	DESeq2	LRT	0.438646096136384
138	KC_AB1	ETHR	0.634605556595444	0.839081746941067	1	pseudobulk	DESeq2	LRT	0.0761957262797966
138	KC_AB1	Etl1	-0.190274782890791	0.775081075788563	1	pseudobulk	DESeq2	LRT	0.110652866621497

138	KC_AB1	Ets97D	1.09655471831605	0.208584690301337	1	pseudobulk	DESeq2	LRT	0.68071757108519
138	KC_AB1	Ets98B	0.296275065198765	1	1	pseudobulk	DESeq2	LRT	0
138	KC_AB1	euc	-0.161106485610153	0.858110390373063	1	pseudobulk	DESeq2	LRT	0.0664568393703692
138	KC_AB1	Evi5	-0.0653191046785821	0.80946989185402	1	pseudobulk	DESeq2	LRT	0.0917993001469667
138	KC_AB1	ewg	0.17898791398441	0.605138483379991	1	pseudobulk	DESeq2	LRT	0.218145227520169
138	KC_AB1	ex	2.09987857572813	0.503115176013642	1	pseudobulk	DESeq2	LRT	0.29833258237694
138	KC_AB1	exd	-0.155869964304737	0.579820954674544	1	pseudobulk	DESeq2	LRT	0.236706093335839
138	KC_AB1	Exd2	-0.00362579695724307	0.986532181329215	1	pseudobulk	DESeq2	LRT	0.00588874320880989
138	KC_AB1	Exn	0.205289349298962	0.674324358260945	1	pseudobulk	DESeq2	LRT	0.171131152250913
138	KC_AB1	Exo70	-0.0424410290383744	0.9304424345494	1	pseudobulk	DESeq2	LRT	0.0313104910242408
138	KC_AB1	Exo84	-0.431860119198449	0.612369563870739	1	pseudobulk	DESeq2	LRT	0.212986402839013
138	KC_AB1	exp	-3.87140133208745	0.178628418456448	1	pseudobulk	DESeq2	LRT	0.748049446919988
138	KC_AB1	ey	-0.307115897388563	0.181481020446953	1	pseudobulk	DESeq2	LRT	0.741168787411118
139	KC_AB1	eya	-0.760959112540794	1	1	pseudobulk	DESeq2	LRT	0
139	KC_AB1	eys	-0.0811614196155972	0.747385684748714	1	pseudobulk	DESeq2	LRT	0.126455224782046
139	KC_AB1	f-cup	-0.698231873810775	0.410239096421446	1	pseudobulk	DESeq2	LRT	0.386962953058362
139	KC_AB1	Faa	-3.87140133208745	0.178628418456448	1	pseudobulk	DESeq2	LRT	0.748049446919988
139	KC_AB1	fabp	-0.19141338278095	0.66670405558439	1	pseudobulk	DESeq2	LRT	0.176066903037681
139	KC_AB1	Fadd	-0.729549586412807	0.731890099720189	1	pseudobulk	DESeq2	LRT	0.135554127504983
139	KC_AB1	faf	-0.340321832841009	0.28994599577969	1	pseudobulk	DESeq2	LRT	0.537682884580167
139	KC_AB1	Faf2	-0.0358365580093718	0.945181807069007	1	pseudobulk	DESeq2	LRT	0.0244846462922922
139	KC_AB1	Fak	0.260647621986141	0.376955724078276	1	pseudobulk	DESeq2	LRT	0.423709657533505
139	KC_AB1	fal	0.190792172000023	0.858841458310306	1	pseudobulk	DESeq2	LRT	0.0660869993070359
139	KC_AB1	FAM21	-0.0707699521965246	0.915253177143855	1	pseudobulk	DESeq2	LRT	0.0384587548652793
139	KC_AB1	Fancd2	1.11388934932283	1	1	pseudobulk	DESeq2	LRT	0
139	KC_AB1	Fancm	-0.262488525155374	0.901290248884416	1	pseudobulk	DESeq2	LRT	0.0451353275672721
139	KC_AB1	fand	0.127701946437395	0.882785480838901	1	pseudobulk	DESeq2	LRT	0.05414481828723
139	KC_AB1	Fas1	0.0246778953545921	0.878864461222962	1	pseudobulk	DESeq2	LRT	0.056078096805308
139	KC_AB1	Fas2	-0.118912193202137	0.401357384496838	1	pseudobulk	DESeq2	LRT	0.396468742113581
139	KC_AB1	Fas3	0.757854419538024	0.459568507612206	1	pseudobulk	DESeq2	LRT	0.337649739424231
139	KC_AB1	FASN1	0.171498148870867	0.67286346089385	1	pseudobulk	DESeq2	LRT	0.172073054938615
139	KC_AB1	fat-spondin	1.65160760123991	0.280260887909269	1	pseudobulk	DESeq2	LRT	0.552437506415189
139	KC_AB1	Fatp1	0.162258448354786	0.785203552603288	1	pseudobulk	DESeq2	LRT	0.10501774412996
139	KC_AB1	Fatp3	1.40344192422149	0.480174234999883	1	pseudobulk	DESeq2	LRT	0.318601146856199
139	KC_AB1	fax	0.0720370122687006	0.649519842945421	1	pseudobulk	DESeq2	LRT	0.187407576615489
139	KC_AB1	fbl	0.249245247237533	0.760585860543834	1	pseudobulk	DESeq2	LRT	0.118851752478036
139	KC_AB1	Fbl6	-0.0918790208275763	0.839017010460356	1	pseudobulk	DESeq2	LRT	0.0762292340769274
139	KC_AB1	fbp	-1.61102155603974	0.57025980967112	1	pseudobulk	DESeq2	LRT	0.243927235206257
139	KC_AB1	Fbw5	0.0173153031215059	0.972899175228068	1	pseudobulk	DESeq2	LRT	0.011932164778863
139	KC_AB1	Fbxl4	-0.301681829737599	0.759176725150255	1	pseudobulk	DESeq2	LRT	0.119657114982446
139	KC_AB1	Fbxl7	0.296275065198765	1	1	pseudobulk	DESeq2	LRT	0
139	KC_AB1	FBXO11	-0.163073015395393	0.643618625482059	1	pseudobulk	DESeq2	LRT	0.191371396476305
139	KC_AB1	Fcp1	0.309354630611305	0.60236291304897	1	pseudobulk	DESeq2	LRT	0.220141775110824
139	KC_AB1	Fdh	3.10217251417204	0.0549406660618807	1	pseudobulk	DESeq2	LRT	1.26010607980359
139	KC_AB1	fdl	0.0335275079261811	0.921097452742094	1	pseudobulk	DESeq2	LRT	0.0356944187183003
139	KC_AB1	Fdx1	0.0471685018570138	0.927876679338214	1	pseudobulk	DESeq2	LRT	0.0325097404210346
139	KC_AB1	Fdx2	0.0700677534340871	0.902547707615248	1	pseudobulk	DESeq2	LRT	0.0445298325176591
139	KC_AB1	FeCH	0.437940188379677	0.60699058782467	1	pseudobulk	DESeq2	LRT	0.216818043171037
139	KC_AB1	Fem-1	0.103268511056352	0.866237594366197	1	pseudobulk	DESeq2	LRT	0.0623629719898159
139	KC_AB1	Fen1	-0.81150164565937	0.654904099122705	1	pseudobulk	DESeq2	LRT	0.183822291261838
139	KC_AB1	fend	-0.119078014350678	0.431256641578825	1	pseudobulk	DESeq2	LRT	0.365264203504005
139	KC_AB1	FER	1.29248728958489	0.567005050171457	1	pseudobulk	DESeq2	LRT	0.246413072938083
139	KC_AB1	Fer1	1.25804452752548	0.727552935095488	1	pseudobulk	DESeq2	LRT	0.138135402914536
139	KC_AB1	Fer1HCH	-0.189526284226717	0.498602166500297	1	pseudobulk	DESeq2	LRT	0.302245838754268
139	KC_AB1	Fer2LCH	0.247370438266411	0.165558102113445	1	pseudobulk	DESeq2	LRT	0.781049560807049
139	KC_AB1	fest	0.783783673387297	0.577875487135558	1	pseudobulk	DESeq2	LRT	0.238165727453588

139	KC_AB1	Fgop2	-0.204333076155403	0.767576990009896	1	pseudobulk	DESeq2	LRT	0.114878052776474
139	KC_AB1	fh	0.261406895586606	0.808375686039999	1	pseudobulk	DESeq2	LRT	0.0923867574742262
139	KC_AB1	Fhos	-2.75413151147551	0.0758167152794205	1	pseudobulk	DESeq2	LRT	1.12023503508927
139	KC_AB1	Fib	-0.694849654980486	0.612533038236705	1	pseudobulk	DESeq2	LRT	0.212870481760275
139	KC_AB1	Fibp	0.168219737468911	0.69198257676646	1	pseudobulk	DESeq2	LRT	0.159904840383479
139	KC_AB1	Fic	0.303902850807232	0.735576204125417	1	pseudobulk	DESeq2	LRT	0.133372328623983
139	KC_AB1	fid	0.0198924622716091	0.928305916833486	1	pseudobulk	DESeq2	LRT	0.0323088814329915
139	KC_AB1	fidipidine	-0.408932797763055	0.703401043583461	1	pseudobulk	DESeq2	LRT	0.152796991688394
139	KC_AB1	Fie	-0.223853425529211	0.691011220602621	1	pseudobulk	DESeq2	LRT	0.160514900518367
139	KC_AB1	Fife	-0.110502240774282	0.556290048694104	1	pseudobulk	DESeq2	LRT	0.254698708953463
139	KC_AB1	FIG4	1.36756769743414	0.240481105767308	1	pseudobulk	DESeq2	LRT	0.618919039803009
139	KC_AB1	Fili	3.49689207403346	0.121845565419218	1	pseudobulk	DESeq2	LRT	0.914190272380033
139	KC_AB1	Fim	-0.145383777827983	0.605250587800129	1	pseudobulk	DESeq2	LRT	0.218064780113031
139	KC_AB1	Fip1	0.56685410980358	0.329827370505238	1	pseudobulk	DESeq2	LRT	0.481713307556974
139	KC_AB1	fipi	0.108869622209954	0.731162809981948	1	pseudobulk	DESeq2	LRT	0.135985906757786
139	KC_AB1	firl	4.38871030373311	0.12299216393188	1	pseudobulk	DESeq2	LRT	0.910122557418755
139	KC_AB1	Fis1	-0.00461656035401294	0.95757348975758	1	pseudobulk	DESeq2	LRT	0.018827885796473
139	KC_AB1	Fit1	-0.372794085239466	0.55565096150824	1	pseudobulk	DESeq2	LRT	0.255197929804733
139	KC_AB1	Fitm	-0.717263113911223	0.54304385950197	1	pseudobulk	DESeq2	LRT	0.265165092749181
139	KC_AB1	fj	2.88322402903533	0.302751069383099	1	pseudobulk	DESeq2	LRT	0.518914314158952
139	KC_AB1	Fkbp12	0.0236012197621153	0.934750213444652	1	pseudobulk	DESeq2	LRT	0.0293044270051693
139	KC_AB1	Fkbp14	0.0357672423445405	0.93954685917185	1	pseudobulk	DESeq2	LRT	0.027081554922407
139	KC_AB1	Fkbp39	-0.360943014271337	0.571969715744068	1	pseudobulk	DESeq2	LRT	0.242626965321351
139	KC_AB1	Fkbp59	-0.16681979701395	0.726831180706465	1	pseudobulk	DESeq2	LRT	0.138566449940928
139	KC_AB1	fl(2)d	0.11597329787799	0.66790122555577	1	pseudobulk	DESeq2	LRT	0.175287759632107
139	KC_AB1	Flacc	0.226681319428351	0.594126253207258	1	pseudobulk	DESeq2	LRT	0.226121256626623
139	KC_AB1	FLASH	-0.104800697785399	0.8899442621234	1	pseudobulk	DESeq2	LRT	0.0506371926924634
139	KC_AB1	flfl	0.174741698772841	0.595543832931839	1	pseudobulk	DESeq2	LRT	0.225086268270453
139	KC_AB1	fliI	0.560635861701823	0.51934274530612	1	pseudobulk	DESeq2	LRT	0.284545930656277
139	KC_AB1	fln	2.4138856564567	0.409611385497059	1	pseudobulk	DESeq2	LRT	0.387627980279722
139	KC_AB1	Flo1	0.0313940522919492	0.891215008108986	1	pseudobulk	DESeq2	LRT	0.0500175085661781
139	KC_AB1	Flo2	-0.394876234811117	0.181776941079004	1	pseudobulk	DESeq2	LRT	0.740461209107976
139	KC_AB1	flr	-0.814378439486053	0.483549390551565	1	pseudobulk	DESeq2	LRT	0.315559159743351
139	KC_AB1	flw	-0.0890356306618147	0.748513370199332	1	pseudobulk	DESeq2	LRT	0.125800437736518
139	KC_AB1	Fmo-2	3.84338117373556	0.0131336303620329	1	pseudobulk	DESeq2	LRT	1.88161521082811
139	KC_AB1	Fmr1	-0.043850496072448	0.913215201614148	1	pseudobulk	DESeq2	LRT	0.0394268677444489
139	KC_AB1	FMRFaR	-0.591806728486254	0.624234638956344	1	pseudobulk	DESeq2	LRT	0.204652135878621
139	KC_AB1	fmt	-0.167346943906149	0.560637037939586	1	pseudobulk	DESeq2	LRT	0.251318214386223
139	KC_AB1	fne	-0.0197617283373915	0.899833264991045	1	pseudobulk	DESeq2	LRT	0.0458379558970426
139	KC_AB1	fng	-0.556087273845979	0.630428333610591	1	pseudobulk	DESeq2	LRT	0.200364276396974
139	KC_AB1	Fnta	0.355030335288488	0.605520864751105	1	pseudobulk	DESeq2	LRT	0.21787088754958
139	KC_AB1	fog	0.473084959199542	0.591216425408961	1	pseudobulk	DESeq2	LRT	0.228253508704263
139	KC_AB1	foi	-0.247664480048022	0.6678905986509	1	pseudobulk	DESeq2	LRT	0.17529466970007
139	KC_AB1	fok	-0.0420121612789784	0.831711090732998	1	pseudobulk	DESeq2	LRT	0.0800275072312616
139	KC_AB1	for	2.25902685349739	0.371031433750581	1	pseudobulk	DESeq2	LRT	0.430589295435818
139	KC_AB1	form3	2.56394879315952	0.345952836871008	1	pseudobulk	DESeq2	LRT	0.460983103758642
139	KC_AB1	FoxK	-0.00652808512868829	0.930587743543706	1	pseudobulk	DESeq2	LRT	0.0312426717107561
139	KC_AB1	foxo	1.37756415309218	0.416562217870442	1	pseudobulk	DESeq2	LRT	0.380320123045225
139	KC_AB1	FoxP	-0.231857906628094	0.367991027446979	1	pseudobulk	DESeq2	LRT	0.434162770396513
139	KC_AB1	Fpps	-0.298107369830599	0.838687907752257	1	pseudobulk	DESeq2	LRT	0.0763996186187964
139	KC_AB1	fra	1.123587512929	0.532681643022363	1	pseudobulk	DESeq2	LRT	0.273532269371468
139	KC_AB1	fray	1.0079397458521	0.329539838931771	1	pseudobulk	DESeq2	LRT	0.482092074901961
139	KC_AB1	FRG1	-0.0709297370729561	0.902139503336244	1	pseudobulk	DESeq2	LRT	0.0447262996592319
139	KC_AB1	frj	0.165449461905185	0.73277177112649	1	pseudobulk	DESeq2	LRT	0.135031269525979
139	KC_AB1	Frl	0.282817701588247	0.292817331721889	1	pseudobulk	DESeq2	LRT	0.533403221164152
139	KC_AB1	frm	4.8100079129786	0.0801803238368656	1	pseudobulk	DESeq2	LRT	1.09593219402863

139	KC_AB1	Frq1	-0.159164672332863	0.763920040348569	1	pseudobulk	DESeq2	LRT	0.116952096728915
140	KC_AB1	Frq2	0.958236221785649	0.209594435702704	1	pseudobulk	DESeq2	LRT	0.678620251152472
140	KC_AB1	frtz	0.63825163975652	0.791548424121757	1	pseudobulk	DESeq2	LRT	0.101522511394706
140	KC_AB1	fru	0.41445219485094	0.674114542248083	1	pseudobulk	DESeq2	LRT	0.171266303999059
140	KC_AB1	fry	-0.175475032166914	0.780629526218357	1	pseudobulk	DESeq2	LRT	0.107555026163669
140	KC_AB1	fs(1)h	-0.150339940143689	0.27240973707161	1	pseudobulk	DESeq2	LRT	0.56477737296743
140	KC_AB1	fs(1)K10	-0.323048686841899	0.738554968888542	1	pseudobulk	DESeq2	LRT	0.131617175644443
140	KC_AB1	Fs(2)Ket	-0.266067003806082	0.144013078567344	1	pseudobulk	DESeq2	LRT	0.841598065601222
140	KC_AB1	fs(2)ltoPP43	0.313010979567132	0.680136970550783	1	pseudobulk	DESeq2	LRT	0.167403617346432
140	KC_AB1	fsd	-0.0476732685146433	0.946282291602442	1	pseudobulk	DESeq2	LRT	0.0239792870686567
140	KC_AB1	Fsn	0.125895210638148	0.768081836335878	1	pseudobulk	DESeq2	LRT	0.11459250500021
140	KC_AB1	ft	2.09987857572813	0.503115176013642	1	pseudobulk	DESeq2	LRT	0.29833258237694
140	KC_AB1	ftz-f1	0.0332785024611	0.929030227362947	1	pseudobulk	DESeq2	LRT	0.0319701553676442
140	KC_AB1	fu	-0.599503557323791	0.594757833914045	1	pseudobulk	DESeq2	LRT	0.22565982889854
140	KC_AB1	FucT6	0.433588279469886	0.489178479511373	1	pseudobulk	DESeq2	LRT	0.310532657185712
140	KC_AB1	FucTA	0.426757126749892	0.45278232641453	1	pseudobulk	DESeq2	LRT	0.344110533432957
140	KC_AB1	FucTB	0.69942874119457	0.71363110281359	1	pseudobulk	DESeq2	LRT	0.146526229980099
140	KC_AB1	Fum1	0.170065858448112	0.635509749374273	1	pseudobulk	DESeq2	LRT	0.196877782534094
140	KC_AB1	Fur1	-0.287512816785586	0.187267149778139	1	pseudobulk	DESeq2	LRT	0.727538399460497
140	KC_AB1	Fur2	-0.354545823535346	0.351649843858795	1	pseudobulk	DESeq2	LRT	0.453889571087995
140	KC_AB1	fus	3.58333718222294	0.241420367068608	1	pseudobulk	DESeq2	LRT	0.617226094087932
140	KC_AB1	fuss	-2.24978065768543	0.364534962203659	1	pseudobulk	DESeq2	LRT	0.438260812575185
140	KC_AB1	futsch	0.0366109941859317	0.828170912107381	1	pseudobulk	DESeq2	LRT	0.0818800273148068
140	KC_AB1	fw	2.58743201691345	0.417636627932844	1	pseudobulk	DESeq2	LRT	0.379201419460921
140	KC_AB1	fwd	0.230462889547416	0.455928635988108	1	pseudobulk	DESeq2	LRT	0.34110312975229
140	KC_AB1	fwe	0.125137472212897	0.483032421562005	1	pseudobulk	DESeq2	LRT	0.316023718041625
140	KC_AB1	fws	-0.420537194450958	0.668009587032655	1	pseudobulk	DESeq2	LRT	0.175217304641966
140	KC_AB1	fy	-0.467232592483773	0.73177294677253	1	pseudobulk	DESeq2	LRT	0.135623650171334
140	KC_AB1	fz2	-0.648569830818168	0.15712511184979	1	pseudobulk	DESeq2	LRT	0.803754400152577
140	KC_AB1	fz3	-0.423450042768175	0.658165894640259	1	pseudobulk	DESeq2	LRT	0.181664626072785
140	KC_AB1	fz4	-0.834596787753045	0.54539036611004	1	pseudobulk	DESeq2	LRT	0.263292537795565
140	KC_AB1	fzr	0.310303243514766	0.425895184499128	1	pseudobulk	DESeq2	LRT	0.370697270368325
140	KC_AB1	fzy	1.34516178575742	0.538610711629975	1	pseudobulk	DESeq2	LRT	0.268725013799496
140	KC_AB1	g	0.373861676704474	0.219054690538381	1	pseudobulk	DESeq2	LRT	0.659447442996528
140	KC_AB1	G6P	0.0283752159890608	0.926912567890614	1	pseudobulk	DESeq2	LRT	0.0329612292572653
140	KC_AB1	G9a	-0.22353972523617	0.431105770278235	1	pseudobulk	DESeq2	LRT	0.365416164148605
140	KC_AB1	GAA1	0.0734175928477673	0.92062867508844	1	pseudobulk	DESeq2	LRT	0.0359155021351467
140	KC_AB1	GABA-B-R1	0.0170940468973246	0.913886691613554	1	pseudobulk	DESeq2	LRT	0.0391076469986265
140	KC_AB1	GABA-B-R2	-0.0682795662366145	0.808409263471141	1	pseudobulk	DESeq2	LRT	0.0923687185964294
140	KC_AB1	Gabat	0.140107743897586	0.630533309266335	1	pseudobulk	DESeq2	LRT	0.200291965952978
140	KC_AB1	GABPI	0.107890818248604	0.816401920783784	1	pseudobulk	DESeq2	LRT	0.088095982171527
140	KC_AB1	Gad1	-1.49412986484045	0.562581821332303	1	pseudobulk	DESeq2	LRT	0.249814305271815
140	KC_AB1	Gadd45	2.54366026396482	0.300503109684473	1	pseudobulk	DESeq2	LRT	0.522151029445537
140	KC_AB1	Gagr	1.11388934932283	1	1	pseudobulk	DESeq2	LRT	0
140	KC_AB1	Gal	2.77905602015038	0.396550243710121	1	pseudobulk	DESeq2	LRT	0.401701778879434
140	KC_AB1	Gale	0.164746109566872	0.808392547002334	1	pseudobulk	DESeq2	LRT	0.0923776991283826
140	KC_AB1	galectin	0.258686174126466	0.469052571227481	1	pseudobulk	DESeq2	LRT	0.328778479002428
140	KC_AB1	Galk	0.566702474735465	0.717245118296405	1	pseudobulk	DESeq2	LRT	0.144332398960875
140	KC_AB1	galla-1	0.814821132697793	0.0670273810090145	1	pseudobulk	DESeq2	LRT	1.17374774965825
140	KC_AB1	galla-2	0.577186434020255	0.258455176475275	1	pseudobulk	DESeq2	LRT	0.587614765132452
140	KC_AB1	Galphai	0.647899373901874	0.588843149896654	1	pseudobulk	DESeq2	LRT	0.230000372795255
140	KC_AB1	Galphao	-0.0908821188561175	0.621131461071326	1	pseudobulk	DESeq2	LRT	0.206816472648456
140	KC_AB1	Galphaq	-0.310101716395613	0.200072826969684	1	pseudobulk	DESeq2	LRT	0.698811891366155
140	KC_AB1	Galphas	-0.157430508270244	0.395802426457967	1	pseudobulk	DESeq2	LRT	0.402521547688805
140	KC_AB1	Galt	-0.425019925884143	0.71511507357218	1	pseudobulk	DESeq2	LRT	0.145624067574812
140	KC_AB1	GalT1	-0.591815557038899	0.552158652679018	1	pseudobulk	DESeq2	LRT	0.257936117772202

140	KC_AB1	gammaCOP	0.235992459844781	0.735268144218254	1	pseudobulk	DESeq2	LRT	0.133554249600031
140	KC_AB1	gammaSnap1	0.340693009500591	0.127824538536328	1	pseudobulk	DESeq2	LRT	0.893385766459733
140	KC_AB1	gammaSnap2	-0.131706412300653	0.795423700380346	1	pseudobulk	DESeq2	LRT	0.0994014729539387
140	KC_AB1	gammaTub23C	0.474694136386039	0.573104581450966	1	pseudobulk	DESeq2	LRT	0.241766119738167
140	KC_AB1	GAPcenA	0.32418874902776	0.450598428076789	1	pseudobulk	DESeq2	LRT	0.346210327656057
140	KC_AB1	Gapdh1	0.120434751502971	0.718890623621236	1	pseudobulk	DESeq2	LRT	0.143337180787009
140	KC_AB1	Gapdh2	0.00542525311067956	0.993024882498142	1	pseudobulk	DESeq2	LRT	0.0030398691317596
140	KC_AB1	GAPsec	0.50945679923045	0.543412451419716	1	pseudobulk	DESeq2	LRT	0.26487041464665
140	KC_AB1	Gapvd1	0.295112258507642	0.663419712119143	1	pseudobulk	DESeq2	LRT	0.178211628479206
140	KC_AB1	garz	-0.764974362010578	0.247718811128294	1	pseudobulk	DESeq2	LRT	0.606041012957882
140	KC_AB1	gas	1.67020383217772	0.598754457572587	1	pseudobulk	DESeq2	LRT	0.222751240354882
140	KC_AB1	Gas41	0.169598612821846	0.796394771289158	1	pseudobulk	DESeq2	LRT	0.098871599985312
140	KC_AB1	Gasp	-1.52516013248832	0.322406425888566	1	pseudobulk	DESeq2	LRT	0.49159631084916
140	KC_AB1	Gasz	0.178641017169845	0.798171683795702	1	pseudobulk	DESeq2	LRT	0.0979036834542869
140	KC_AB1	Gat	0.12980336431813	0.754221879180083	1	pseudobulk	DESeq2	LRT	0.122500873323456
140	KC_AB1	GatA	-0.575831156995342	0.571355542152482	1	pseudobulk	DESeq2	LRT	0.243093555603637
140	KC_AB1	GATAd	-0.113670745846245	0.758478871023894	1	pseudobulk	DESeq2	LRT	0.120056512867417
140	KC_AB1	GatB	0.162102198361938	0.865215158014294	1	pseudobulk	DESeq2	LRT	0.0628758806056935
140	KC_AB1	gb	-0.186914857804884	0.878246769222826	1	pseudobulk	DESeq2	LRT	0.0563834391576614
140	KC_AB1	Gba1a	-2.24978065768543	0.364534962203659	1	pseudobulk	DESeq2	LRT	0.438260812575185
140	KC_AB1	gbb	-1.74861749711195	0.37959826562342	1	pseudobulk	DESeq2	LRT	0.420675780522666
140	KC_AB1	Gbeta13F	-0.0631301596809223	0.698887064284341	1	pseudobulk	DESeq2	LRT	0.155592997817664
140	KC_AB1	Gbeta5	0.124813508177882	0.625923322010804	1	pseudobulk	DESeq2	LRT	0.203478866258016
140	KC_AB1	Gbp1	-1.77067014871429	0.542156052888816	1	pseudobulk	DESeq2	LRT	0.265875689192671
140	KC_AB1	Gbs-76A	-0.753284926683968	0.226280102192579	1	pseudobulk	DESeq2	LRT	0.645353633795589
140	KC_AB1	GC	-0.067506888924505	0.819015589533448	1	pseudobulk	DESeq2	LRT	0.086707831592554
140	KC_AB1	GC1	0.340178502198881	0.578429318748912	1	pseudobulk	DESeq2	LRT	0.237749702144773
140	KC_AB1	GC2	-0.760959112540794	0.805265236978025	1	pseudobulk	DESeq2	LRT	0.0940610488455931
140	KC_AB1	GCC185	0.553756961143686	0.417074375427492	1	pseudobulk	DESeq2	LRT	0.37978649189068
140	KC_AB1	GCC88	0.0168352793034492	0.93072537847028	1	pseudobulk	DESeq2	LRT	0.0311784438400877
140	KC_AB1	gce	0.295377740278737	0.308146421784612	1	pseudobulk	DESeq2	LRT	0.511242870958523
140	KC_AB1	GckIII	-0.205319466221241	0.677744535006239	1	pseudobulk	DESeq2	LRT	0.168933975667363
140	KC_AB1	gcl	0.0892404943167015	0.881880545341923	1	pseudobulk	DESeq2	LRT	0.0545902380113423
140	KC_AB1	Gclc	0.458880124086628	0.409879972945871	1	pseudobulk	DESeq2	LRT	0.38734330113112
140	KC_AB1	Gclm	0.735808072428731	0.219213937747829	1	pseudobulk	DESeq2	LRT	0.659131836617691
140	KC_AB1	Gcn2	-0.146613850449463	0.89125931687639	1	pseudobulk	DESeq2	LRT	0.0499959171716567
140	KC_AB1	Gcn5	-0.439497077164453	0.644928717450296	1	pseudobulk	DESeq2	LRT	0.190488284324063
140	KC_AB1	GCS1	0.381646140184293	0.645381760441653	1	pseudobulk	DESeq2	LRT	0.19018331264063
140	KC_AB1	GCS2alpha	-0.302168951331876	0.330689730632305	1	pseudobulk	DESeq2	LRT	0.480579291626884
140	KC_AB1	GCS2beta	-0.211351851965944	0.778421130340203	1	pseudobulk	DESeq2	LRT	0.108785383600962
140	KC_AB1	Gdap2	-0.11427681592821	0.460246574072176	1	pseudobulk	DESeq2	LRT	0.337009435560693
140	KC_AB1	Gdh	0.0788240987681161	0.716790623125127	1	pseudobulk	DESeq2	LRT	0.144607684640088
140	KC_AB1	Gdi	0.00385702577761073	0.983920906202652	1	pseudobulk	DESeq2	LRT	0.00703981150802776
140	KC_AB1	gdl	-0.990001801707088	0.43136219991255	1	pseudobulk	DESeq2	LRT	0.365157914608192
140	KC_AB1	Gdn1	0.159406597190823	0.856378998165703	1	pseudobulk	DESeq2	LRT	0.06733399188272
141	KC_AB1	Ge-1	-0.389976339544557	0.517907506634121	1	pseudobulk	DESeq2	LRT	0.285747794202901
141	KC_AB1	GEFmeso	0.152115475746305	1	1	pseudobulk	DESeq2	LRT	0
141	KC_AB1	gek	0.0427693128113331	0.926669663226098	1	pseudobulk	DESeq2	LRT	0.0330750544269947
141	KC_AB1	geko	-1.61102155603974	0.57025980967112	1	pseudobulk	DESeq2	LRT	0.243927235206257
141	KC_AB1	Gel	0.55420281087534	0.562313775858183	1	pseudobulk	DESeq2	LRT	0.250021276785978
141	KC_AB1	Gem2	-1.21392496296945	0.264511103100341	1	pseudobulk	DESeq2	LRT	0.577556093330102
141	KC_AB1	Gem3	-0.226485185058318	0.860474431675859	1	pseudobulk	DESeq2	LRT	0.0652620298652532
141	KC_AB1	geminin	-0.319649417516712	0.692768985884731	1	pseudobulk	DESeq2	LRT	0.159411563197142
141	KC_AB1	Gfat1	-2.38324878285857	0.390434993490158	1	pseudobulk	DESeq2	LRT	0.408451264779408
141	KC_AB1	Gfat2	0.472350884135763	0.447161072682762	1	pseudobulk	DESeq2	LRT	0.349536010700607
141	KC_AB1	Gfrl	-0.158172004887305	0.377813789980922	1	pseudobulk	DESeq2	LRT	0.422722194631965

141	KC_AB1	gfzf	-1.77073327401384	0.084514517905676	1	pseudobulk	DESeq2	LRT	1.07306868152661
141	KC_AB1	Gga	-0.373166071968207	0.646735646334159	1	pseudobulk	DESeq2	LRT	0.1892732012462
141	KC_AB1	Ggamma1	-0.109250503086164	0.543184404220126	1	pseudobulk	DESeq2	LRT	0.265052707907183
141	KC_AB1	Ggamma30A	-0.180352738014103	0.491600036428926	1	pseudobulk	DESeq2	LRT	0.308388093603157
141	KC_AB1	Ggt-1	0.703352139488717	0.686040494780681	1	pseudobulk	DESeq2	LRT	0.16365024851957
141	KC_AB1	ghi	0.331970832560268	0.23616152779041	1	pseudobulk	DESeq2	LRT	0.626790850283889
141	KC_AB1	gig	-1.56462338535918	0.262330984082195	1	pseudobulk	DESeq2	LRT	0.581150411578035
141	KC_AB1	GIIIspla2	0.43230389276222	0.639954230713255	1	pseudobulk	DESeq2	LRT	0.19385108548403
141	KC_AB1	GILT1	-0.520653313882343	0.560345279311377	1	pseudobulk	DESeq2	LRT	0.251544282475237
141	KC_AB1	GILT2	-0.183155418985521	0.870398328074899	1	pseudobulk	DESeq2	LRT	0.0602819518698646
141	KC_AB1	GILT3	-0.336864443716637	0.683835444603351	1	pseudobulk	DESeq2	LRT	0.165048392576122
141	KC_AB1	Gip	0.106846458001798	0.947606372787514	1	pseudobulk	DESeq2	LRT	0.0233720272473466
141	KC_AB1	Girdin	-0.425694956354204	0.547471433772323	1	pseudobulk	DESeq2	LRT	0.261638536722932
141	KC_AB1	gish	-0.0401702465081508	0.815148382102064	1	pseudobulk	DESeq2	LRT	0.0887633290973087
141	KC_AB1	Git	-0.134619708788315	0.875084179976904	1	pseudobulk	DESeq2	LRT	0.0579501673879929
141	KC_AB1	Gk1	0.317720453942042	0.664032538109522	1	pseudobulk	DESeq2	LRT	0.177810639344041
141	KC_AB1	Gk2	1.85892747469106	0.335073527609369	1	pseudobulk	DESeq2	LRT	0.474859882123885
141	KC_AB1	gkt	0.592204875518287	0.536953589455501	1	pseudobulk	DESeq2	LRT	0.270063250081484
141	KC_AB1	GLaz	0.382105587669069	0.709723081686413	1	pseudobulk	DESeq2	LRT	0.148911070372021
141	KC_AB1	GlcAT-I	-0.133391760751608	0.837069479794738	1	pseudobulk	DESeq2	LRT	0.0772384924976365
141	KC_AB1	GlcAT-P	-0.163128783495107	0.350084350297943	1	pseudobulk	DESeq2	LRT	0.455827302920078
141	KC_AB1	GlcAT-S	-0.0523208729687633	0.916653489001463	1	pseudobulk	DESeq2	LRT	0.037794804193129
141	KC_AB1	GlcT	-0.312838675492648	0.751623333150087	1	pseudobulk	DESeq2	LRT	0.123999746265716
141	KC_AB1	Gld	-2.75941668356407	0.168183568678903	1	pseudobulk	DESeq2	LRT	0.774216436484968
141	KC_AB1	Gld2	-0.760959112540794	0.804334723897681	1	pseudobulk	DESeq2	LRT	0.0945631819854715
141	KC_AB1	Gle1	0.239275389805432	0.737619850837661	1	pseudobulk	DESeq2	LRT	0.132167404056853
141	KC_AB1	glec	-0.0333427821875005	0.884318511570943	1	pseudobulk	DESeq2	LRT	0.053391283738723
141	KC_AB1	Glg1	0.187130746261624	0.798569042319006	1	pseudobulk	DESeq2	LRT	0.0976875298674855
141	KC_AB1	Gli	1.55458657914315	0.53203197753872	1	pseudobulk	DESeq2	LRT	0.274062263849225
141	KC_AB1	GlnRS	-0.00974322876170711	0.890135436416559	1	pseudobulk	DESeq2	LRT	0.05054390929511
141	KC_AB1	glo	-0.183526977930152	0.389169206441911	1	pseudobulk	DESeq2	LRT	0.409861531202862
141	KC_AB1	Glo1	-0.0561315103429655	0.924589729597415	1	pseudobulk	DESeq2	LRT	0.0340509350423701
141	KC_AB1	glob1	0.262037226492968	0.910093627964325	1	pseudobulk	DESeq2	LRT	0.0409139263420023
141	KC_AB1	Glos	-0.760959112540794	1	1	pseudobulk	DESeq2	LRT	0
141	KC_AB1	GLS	0.47488246140688	0.792176344596482	1	pseudobulk	DESeq2	LRT	0.101178130329355
141	KC_AB1	Glt	3.32088770837427	0.291144101679295	1	pseudobulk	DESeq2	LRT	0.535892003886997
141	KC_AB1	glu	-0.229730868132152	0.794843576775709	1	pseudobulk	DESeq2	LRT	0.0997183310005209
141	KC_AB1	GluClalpha	-0.242662486481496	0.129944532874856	1	pseudobulk	DESeq2	LRT	0.886241987746106
141	KC_AB1	GluProRS	-0.264436266578836	0.720810235777711	1	pseudobulk	DESeq2	LRT	0.142179054839461
141	KC_AB1	GluRIA	-0.1485146024737	0.427915934912361	1	pseudobulk	DESeq2	LRT	0.368641540775583
141	KC_AB1	GluRIB	-0.0255936132505572	0.870257653889063	1	pseudobulk	DESeq2	LRT	0.0603521484203846
141	KC_AB1	GluRS-m	1.11471421835543	0.626302681986126	1	pseudobulk	DESeq2	LRT	0.203215728536149
141	KC_AB1	Glut1	-0.0797541114963247	0.748924843628615	1	pseudobulk	DESeq2	LRT	0.125561762587674
141	KC_AB1	Glut4EF	-0.0514428049727209	0.816857850434475	1	pseudobulk	DESeq2	LRT	0.0878535127982358
141	KC_AB1	Glyat	1.25804452752548	0.727552935095488	1	pseudobulk	DESeq2	LRT	0.138135402914536
141	KC_AB1	GlyP	-0.12152813281644	0.684386458504607	1	pseudobulk	DESeq2	LRT	0.164698592139276
141	KC_AB1	GlyRS	-0.213069706107534	0.757043583781713	1	pseudobulk	DESeq2	LRT	0.120879116997906
141	KC_AB1	GlyS	-0.103822620826482	0.910921798083149	1	pseudobulk	DESeq2	LRT	0.0405189052649325
141	KC_AB1	GlyT	-0.0524852011586536	0.840078160974846	1	pseudobulk	DESeq2	LRT	0.0756803052465603
141	KC_AB1	GM130	0.417743263865658	0.42190460716523	1	pseudobulk	DESeq2	LRT	0.374785732130534
141	KC_AB1	Gmap	-0.237011606087416	0.73811143380988	1	pseudobulk	DESeq2	LRT	0.131878067126275
141	KC_AB1	Gmd	-1.09257685278925	0.470078327943705	1	pseudobulk	DESeq2	LRT	0.327829770661182
141	KC_AB1	Gmer	0.070095028419854	0.941910568503293	1	pseudobulk	DESeq2	LRT	0.0259903301681841
141	KC_AB1	GMF	0.314460449706306	0.690458301047398	1	pseudobulk	DESeq2	LRT	0.160862544705703
141	KC_AB1	GNBP1	-1.43550613534661	0.602448798560473	1	pseudobulk	DESeq2	LRT	0.220079857379737
141	KC_AB1	GNBP3	-2.00963963748391	0.498268873088307	1	pseudobulk	DESeq2	LRT	0.302536242405947

141	KC_AB1	Gnf1	-0.195530852663144	0.746160821510349	1	pseudobulk	DESeq2	LRT	0.127167558094115
141	KC_AB1	Gnpnat	-0.1594439810224	0.82201548460395	1	pseudobulk	DESeq2	LRT	0.0851200014201564
141	KC_AB1	gny	0.92713094420774	0.480836256366698	1	pseudobulk	DESeq2	LRT	0.318002792780832
141	KC_AB1	goe	-2.24976163616681	0.429894962609849	1	pseudobulk	DESeq2	LRT	0.366637643795677
141	KC_AB1	gogo	-0.0380340023516267	0.967934070269778	1	pseudobulk	DESeq2	LRT	0.0141542231597427
141	KC_AB1	gol	-0.198775501516883	0.469053226294995	1	pseudobulk	DESeq2	LRT	0.328777872477647
141	KC_AB1	Golgin104	-1.078596872588	0.178264441947943	1	pseudobulk	DESeq2	LRT	0.748935276038622
141	KC_AB1	Golgin245	0.0129748252371836	0.977153235584911	1	pseudobulk	DESeq2	LRT	0.0100373255846015
141	KC_AB1	Golgin84	-0.578117446668251	0.447547570319122	1	pseudobulk	DESeq2	LRT	0.349160796260703
141	KC_AB1	gom	-0.904535042075943	0.169205697293277	1	pseudobulk	DESeq2	LRT	0.771585018002814
141	KC_AB1	Gorab	-0.0816120212783007	0.928708392507219	1	pseudobulk	DESeq2	LRT	0.0321206298228017
141	KC_AB1	Gos28	0.12345028683296	0.793041162140027	1	pseudobulk	DESeq2	LRT	0.100704270404544
141	KC_AB1	Got1	0.402044847231806	0.403962992263778	1	pseudobulk	DESeq2	LRT	0.393658419522588
141	KC_AB1	Got2	-0.209082944034935	0.5789742317115	1	pseudobulk	DESeq2	LRT	0.23734076489871
141	KC_AB1	Gp150	-0.0476678268419955	0.958295777945237	1	pseudobulk	DESeq2	LRT	0.0185004252600021
141	KC_AB1	Gp210	0.0237705674676568	0.961792977446334	1	pseudobulk	DESeq2	LRT	0.0169183982618375
141	KC_AB1	Gp93	-0.21521975596946	0.768751567394661	1	pseudobulk	DESeq2	LRT	0.114213985739898
141	KC_AB1	Gpat4	-0.175860206434094	0.692412298761939	1	pseudobulk	DESeq2	LRT	0.159635226705067
141	KC_AB1	Gpb5	-0.503021829575818	0.526654320845444	1	pseudobulk	DESeq2	LRT	0.278474348369435
141	KC_AB1	Gpdh1	-0.00914368832055287	0.955559795572237	1	pseudobulk	DESeq2	LRT	0.0197421311369357
141	KC_AB1	Gpdh3	0.243359306179557	0.859210827662157	1	pseudobulk	DESeq2	LRT	0.0659002586879355
141	KC_AB1	GPHR	-1.48327740112355	0.27604530884282	1	pseudobulk	DESeq2	LRT	0.559019628929525
141	KC_AB1	Gpo1	-0.290929243494633	0.555494901249494	1	pseudobulk	DESeq2	LRT	0.255319922986609
141	KC_AB1	gpp	-0.126076187258939	0.535883360003138	1	pseudobulk	DESeq2	LRT	0.270929728254045
141	KC_AB1	Gprk1	0.0440353235111058	0.763578541415946	1	pseudobulk	DESeq2	LRT	0.117146284934725
141	KC_AB1	Gprk2	-0.0391440953357502	0.835111250300324	1	pseudobulk	DESeq2	LRT	0.0782556656263346
141	KC_AB1	gprs	-0.330807641017738	0.0824743462528296	1	pseudobulk	DESeq2	LRT	1.0836811182793
141	KC_AB1	Gr2a	-0.760959112540794	1	1	pseudobulk	DESeq2	LRT	0
141	KC_AB1	Gr33a	-0.760959112540794	0.805265236978025	1	pseudobulk	DESeq2	LRT	0.0940610488455931
141	KC_AB1	Gr77a	1.11388934932283	1	1	pseudobulk	DESeq2	LRT	0
141	KC_AB1	Gr98a	-0.760959112540794	0.804334723897681	1	pseudobulk	DESeq2	LRT	0.0945631819854715
141	KC_AB1	Graf	-0.158137988019151	0.583888401291404	1	pseudobulk	DESeq2	LRT	0.233670151748493
141	KC_AB1	GramD1B	-0.339468522347508	0.253334511683918	1	pseudobulk	DESeq2	LRT	0.596305642373214
142	KC_AB1	Grasp65	0.295399184428944	0.662909759769751	1	pseudobulk	DESeq2	LRT	0.178545586981948
142	KC_AB1	grass	0.288790771135495	0.869654775371181	1	pseudobulk	DESeq2	LRT	0.060653113939855
142	KC_AB1	grau	-0.160802275793752	0.818815098198205	1	pseudobulk	DESeq2	LRT	0.0868141579444198
142	KC_AB1	grh	-0.59186849862222	0.799543843626489	1	pseudobulk	DESeq2	LRT	0.0971577163793154
142	KC_AB1	Grik	-1.69921111931132	0.393175053847316	1	pseudobulk	DESeq2	LRT	0.405414045062041
142	KC_AB1	grim	0.152115475746305	1	1	pseudobulk	DESeq2	LRT	0
142	KC_AB1	Grip128	0.639526966217148	0.676188063340523	1	pseudobulk	DESeq2	LRT	0.169932500046292
142	KC_AB1	Grip163	0.184641381580155	0.799970717094258	1	pseudobulk	DESeq2	LRT	0.0969259100544751
142	KC_AB1	Grip71	0.296275065198765	1	1	pseudobulk	DESeq2	LRT	0
142	KC_AB1	Grip75	0.224769158308897	0.783495381064205	1	pseudobulk	DESeq2	LRT	0.105963559481595
142	KC_AB1	Grip84	-0.451445905742044	0.660057887501778	1	pseudobulk	DESeq2	LRT	0.18041797488214
142	KC_AB1	Grip91	-1.07492008032779	0.240187375683582	1	pseudobulk	DESeq2	LRT	0.619449822974082
142	KC_AB1	grnd	2.04480874254212	0.526249918436357	1	pseudobulk	DESeq2	LRT	0.278807958467711
142	KC_AB1	gro	0.123079364118547	0.560655225233631	1	pseudobulk	DESeq2	LRT	0.251304125924714
142	KC_AB1	grp	0.110163051066451	0.619627950054048	1	pseudobulk	DESeq2	LRT	0.207869000402073
142	KC_AB1	grsm	0.421039309590203	0.581292692681995	1	pseudobulk	DESeq2	LRT	0.235605136441228
142	KC_AB1	Grx1	0.0343851381797794	0.919854874500378	1	pseudobulk	DESeq2	LRT	0.0362806858884759
142	KC_AB1	gry	-0.400720616132071	0.680646301846165	1	pseudobulk	DESeq2	LRT	0.167078510790726
142	KC_AB1	Gs1	0.157057054161667	0.560847086023145	1	pseudobulk	DESeq2	LRT	0.251155532235416
142	KC_AB1	Gs1l	-0.0449069710449556	0.885283942249973	1	pseudobulk	DESeq2	LRT	0.0529174131727594
142	KC_AB1	Gs2	-0.0498918795950169	0.87784570613453	1	pseudobulk	DESeq2	LRT	0.0565818108170299
142	KC_AB1	GstD1	1.09049462914192	0.0242864293392559	1	pseudobulk	DESeq2	LRT	1.61463633172628
142	KC_AB1	GstD11	-0.131971950765939	0.912287453915878	1	pseudobulk	DESeq2	LRT	0.0398682976590263

142	KC_AB1	GstD3	0.525452136714529	0.562177887765971	1	pseudobulk	DESeq2	LRT	0.250126240567926
142	KC_AB1	GstD4	1.25804452752548	0.573436424985144	1	pseudobulk	DESeq2	LRT	0.241514723898045
142	KC_AB1	GstD6	1.25804452752548	0.727552935095488	1	pseudobulk	DESeq2	LRT	0.138135402914536
142	KC_AB1	GstD9	0.783115877127557	0.551497261778809	1	pseudobulk	DESeq2	LRT	0.258456639519903
142	KC_AB1	GstE1	1.7026319996257	0.206014953616404	1	pseudobulk	DESeq2	LRT	0.6861012551775
142	KC_AB1	GstE11	0.152115475746305	1	1	pseudobulk	DESeq2	LRT	0
142	KC_AB1	GstE12	0.742935101271294	0.143330066186405	1	pseudobulk	DESeq2	LRT	0.843662698582314
142	KC_AB1	GstE13	-1.38718159242805	0.788203284813311	1	pseudobulk	DESeq2	LRT	0.103361759557548
142	KC_AB1	GstE2	0.495155061233169	0.449292218187617	1	pseudobulk	DESeq2	LRT	0.347471103391049
142	KC_AB1	GstE3	1.61339788797931	0.2470824894146	1	pseudobulk	DESeq2	LRT	0.607158031692538
142	KC_AB1	GstE4	-2.69245487400416	0.326477446722968	1	pseudobulk	DESeq2	LRT	0.486146814701527
142	KC_AB1	GstE5	1.25804452752548	0.727552935095488	1	pseudobulk	DESeq2	LRT	0.138135402914536
142	KC_AB1	GstE6	2.08943175300695	0.285812032003975	1	pseudobulk	DESeq2	LRT	0.543919492399696
142	KC_AB1	GstE7	-0.760959112540794	1	1	pseudobulk	DESeq2	LRT	0
142	KC_AB1	GstE9	-0.760959112540794	1	1	pseudobulk	DESeq2	LRT	0
142	KC_AB1	GstO1	0.152115475746305	1	1	pseudobulk	DESeq2	LRT	0
142	KC_AB1	GstO2	0.374358072814307	0.591872710559178	1	pseudobulk	DESeq2	LRT	0.227771683557088
142	KC_AB1	GstO3	1.68896388261654	0.450838191809837	1	pseudobulk	DESeq2	LRT	0.345979300723311
142	KC_AB1	GstS1	-1.16569815005703	0.560966767727447	1	pseudobulk	DESeq2	LRT	0.251062866052365
142	KC_AB1	GstT1	-0.160161553706093	0.899681220899758	1	pseudobulk	DESeq2	LRT	0.0459113444810486
142	KC_AB1	GstT2	0.152115475746305	1	1	pseudobulk	DESeq2	LRT	0
142	KC_AB1	GstT3	1.39934341023393	0.63869615168108	1	pseudobulk	DESeq2	LRT	0.194705700577112
142	KC_AB1	GstT4	0.012422098459362	0.966978303871404	1	pseudobulk	DESeq2	LRT	0.0145832700893187
142	KC_AB1	GstZ1	0.296275065198765	1	1	pseudobulk	DESeq2	LRT	0
142	KC_AB1	GstZ2	0.0425859030771875	0.936517016000441	1	pseudobulk	DESeq2	LRT	0.0284843273245588
142	KC_AB1	Gtp-bp	0.0537518040725233	0.917149543974123	1	pseudobulk	DESeq2	LRT	0.0375598455430148
142	KC_AB1	Gug	-0.0634564465681614	0.71754309629119	1	pseudobulk	DESeq2	LRT	0.144152009688913
142	KC_AB1	gukh	-0.206096972441685	0.88860351558333	1	pseudobulk	DESeq2	LRT	0.0512919728918835
142	KC_AB1	gus	-0.0617721295424449	0.768531656772989	1	pseudobulk	DESeq2	LRT	0.11433823866695
142	KC_AB1	GV1	2.99960667015886	0.357405021116046	1	pseudobulk	DESeq2	LRT	0.44683935046587
142	KC_AB1	gw	-0.0706158351981429	0.622878177177998	1	pseudobulk	DESeq2	LRT	0.205596884565252
142	KC_AB1	GXIVsPLA2	0.398199713303772	0.503118311752311	1	pseudobulk	DESeq2	LRT	0.298329875581714
142	KC_AB1	Gyc88E	0.237287113611087	0.374863355220966	1	pseudobulk	DESeq2	LRT	0.4261270119742
142	KC_AB1	Gyc89Da	-0.74637898135139	0.655975296223717	1	pseudobulk	DESeq2	LRT	0.18311251567882
142	KC_AB1	Gyc89Db	0.735455941845419	0.135839021574367	1	pseudobulk	DESeq2	LRT	0.86697545523899
142	KC_AB1	Gycalpha99B	-0.0135770858118338	0.951478455418185	1	pseudobulk	DESeq2	LRT	0.0216010411120416
142	KC_AB1	Gycbeta100B	-0.0565759200141151	0.879269126610662	1	pseudobulk	DESeq2	LRT	0.0558781757649336
142	KC_AB1	Gyf	-0.331980259632557	0.170810417422138	1	pseudobulk	DESeq2	LRT	0.767485645994876
142	KC_AB1	Gyg	0.567808204119751	0.771741767317255	1	pseudobulk	DESeq2	LRT	0.112527994729259
142	KC_AB1	gzl	0.184099457300857	0.515056777291697	1	pseudobulk	DESeq2	LRT	0.288144893861061
142	KC_AB1	h	2.84818442498864	0.325704866558735	1	pseudobulk	DESeq2	LRT	0.487175752323016
142	KC_AB1	H	-0.13993621794381	0.780874904084709	1	pseudobulk	DESeq2	LRT	0.107418534390547
142	KC_AB1	h-cup	-0.760959112540794	1	1	pseudobulk	DESeq2	LRT	0
142	KC_AB1	Hacd1	1.24688375143297	0.127677722837945	1	pseudobulk	DESeq2	LRT	0.893884871667955
142	KC_AB1	Hacd2	-0.591181084679704	0.317373148847188	1	pseudobulk	DESeq2	LRT	0.498429819234554
142	KC_AB1	Hacl	-0.0179044520303376	0.976447234380505	1	pseudobulk	DESeq2	LRT	0.0103512203090878
142	KC_AB1	Had1	2.42480980534803	0.407219734637837	1	pseudobulk	DESeq2	LRT	0.390171183430048
142	KC_AB1	Had2	5.44758850151535	0.181766513334031	1	pseudobulk	DESeq2	LRT	0.740486123390143
142	KC_AB1	haf	0.546370224207986	0.528035767978257	1	pseudobulk	DESeq2	LRT	0.277336658319483
142	KC_AB1	Hakai	-0.247306401042268	0.741911356374585	1	pseudobulk	DESeq2	LRT	0.129647981159141
142	KC_AB1	Hand	1.25804452752548	0.573436424985144	1	pseudobulk	DESeq2	LRT	0.241514723898045
142	KC_AB1	hang	-0.353755199096169	0.247011053383083	1	pseudobulk	DESeq2	LRT	0.60728361226312
142	KC_AB1	Hasp	-0.0424172562512678	0.827397285888267	1	pseudobulk	DESeq2	LRT	0.0822859080545777
142	KC_AB1	Haspin	-0.169198823033439	0.570108367687061	1	pseudobulk	DESeq2	LRT	0.244042584651634
142	KC_AB1	Hat1	-0.78994303580482	0.219410598825933	1	pseudobulk	DESeq2	LRT	0.658742397269788
142	KC_AB1	hay	0.493571675846425	0.646606864022485	1	pseudobulk	DESeq2	LRT	0.18935968946887

142	KC_AB1	Hayan	2.58743201691345	0.417636627932844	1	pseudobulk	DESeq2	LRT	0.379201419460921
142	KC_AB1	hbs	-0.760959112540794	1	1	pseudobulk	DESeq2	LRT	0
142	KC_AB1	HBS1	-0.0516514469191043	0.899119503469536	1	pseudobulk	DESeq2	LRT	0.0461825816280645
142	KC_AB1	Hcf	0.0987002721400183	0.760650426322795	1	pseudobulk	DESeq2	LRT	0.118814886986749
142	KC_AB1	Hcs	-0.311885182437529	0.667698622607528	1	pseudobulk	DESeq2	LRT	0.175419519669718
142	KC_AB1	hd	1.74758121156501	0.493314355044031	1	pseudobulk	DESeq2	LRT	0.306876246739962
142	KC_AB1	HDAC1	-0.460516404104093	0.493489337017921	1	pseudobulk	DESeq2	LRT	0.30672222683286
142	KC_AB1	HDAC11	-0.443744494735725	0.667625566078658	1	pseudobulk	DESeq2	LRT	0.175467040785048
142	KC_AB1	HDAC4	-0.0289416908156606	0.904545102100548	1	pseudobulk	DESeq2	LRT	0.0435697736299858
142	KC_AB1	HDAC6	0.290513356853424	0.571784395625843	1	pseudobulk	DESeq2	LRT	0.242767700999824
142	KC_AB1	hdc	-0.729542636560733	0.246775010556396	1	pseudobulk	DESeq2	LRT	0.607698820841968
142	KC_AB1	hdly	1.75836959453326	0.188814743547393	1	pseudobulk	DESeq2	LRT	0.723964096948544
142	KC_AB1	hdm	0.245556270924701	0.973663313394496	1	pseudobulk	DESeq2	LRT	0.0115911934429027
142	KC_AB1	HEATR2	-1.43179340439473	0.560037947646132	1	pseudobulk	DESeq2	LRT	0.251782544609954
142	KC_AB1	hebe	1.25804452752548	0.573436424985144	1	pseudobulk	DESeq2	LRT	0.241514723898045
142	KC_AB1	hec	0.443818561654439	0.632966851259631	1	pseudobulk	DESeq2	LRT	0.198619033571884
142	KC_AB1	heix	-0.712855694422856	0.396432813335155	1	pseudobulk	DESeq2	LRT	0.401830405497053
142	KC_AB1	Hel25E	0.450216770687501	0.290176838192672	1	pseudobulk	DESeq2	LRT	0.537337255738935
142	KC_AB1	Hel89B	0.187589246089583	0.662944232526207	1	pseudobulk	DESeq2	LRT	0.17852300330683
142	KC_AB1	Hem	-0.132752148849192	0.857544616794894	1	pseudobulk	DESeq2	LRT	0.0667432749892855
142	KC_AB1	HemK1	-0.00263337123093593	0.990281629783134	1	pseudobulk	DESeq2	LRT	0.00424127725398892
143	KC_AB1	Hen1	-1.04944860929885	0.521440930358567	1	pseudobulk	DESeq2	LRT	0.282794882006978
143	KC_AB1	hep	0.196216921863637	0.551483116253236	1	pseudobulk	DESeq2	LRT	0.258467779017617
143	KC_AB1	heph	-0.0656276140939384	0.706145078863358	1	pseudobulk	DESeq2	LRT	0.151106063145215
143	KC_AB1	her	-0.406974350128001	0.564999454320976	1	pseudobulk	DESeq2	LRT	0.24795197162393
143	KC_AB1	HERC2	-0.0303975047595984	0.933147612237606	1	pseudobulk	DESeq2	LRT	0.0300496508842496
143	KC_AB1	Herc4	-0.199383840604504	0.670684978828009	1	pseudobulk	DESeq2	LRT	0.173481420340668
143	KC_AB1	Herp	-0.368542860411453	0.535208001638753	1	pseudobulk	DESeq2	LRT	0.271477402262693
143	KC_AB1	Hers	-0.177748028392403	0.46170412158556	1	pseudobulk	DESeq2	LRT	0.33563624852905
143	KC_AB1	Hex-A	0.0710153838349372	0.796667283936037	1	pseudobulk	DESeq2	LRT	0.0987230172737289
143	KC_AB1	Hexim	-0.852781211065831	0.331475537351056	1	pseudobulk	DESeq2	LRT	0.479548516687446
143	KC_AB1	Hexo1	-0.726461863783541	0.535879952885138	1	pseudobulk	DESeq2	LRT	0.27093248948412
143	KC_AB1	Hexo2	0.914918180656396	0.612323972236839	1	pseudobulk	DESeq2	LRT	0.213018737777427
143	KC_AB1	Hey	-0.165722375354793	0.811935144896175	1	pseudobulk	DESeq2	LRT	0.0904786595994698
143	KC_AB1	Hf	0.709713058804466	0.916223368658127	1	pseudobulk	DESeq2	LRT	0.037998635557264
143	KC_AB1	hfp	-0.179153656593475	0.475951639313806	1	pseudobulk	DESeq2	LRT	0.32243717300671
143	KC_AB1	hfw	-0.697357195569961	0.770678219972763	1	pseudobulk	DESeq2	LRT	0.113126914374965
143	KC_AB1	hgo	-2.82194858466035	0.296654834627456	1	pseudobulk	DESeq2	LRT	0.527748569507829
143	KC_AB1	hid	3.16053952073859	0.192216230189951	1	pseudobulk	DESeq2	LRT	0.716209944532498
143	KC_AB1	hig	0.195581174995313	0.898850546967239	1	pseudobulk	DESeq2	LRT	0.0463125129644206
143	KC_AB1	Hil	0.256777459400988	0.881454288986532	1	pseudobulk	DESeq2	LRT	0.0548002046983542
143	KC_AB1	HINT1	0.0753543233982261	0.825505856253976	1	pseudobulk	DESeq2	LRT	0.0832798414444764
143	KC_AB1	HIP	-0.824770780347034	0.381436599974105	1	pseudobulk	DESeq2	LRT	0.418577637445826
143	KC_AB1	HIP-R	0.232247612596294	0.597877942808078	1	pseudobulk	DESeq2	LRT	0.223387468478893
143	KC_AB1	Hip1	0.121326973858901	0.857683601686957	1	pseudobulk	DESeq2	LRT	0.0666728932509571
143	KC_AB1	Hip14	-0.173103354273407	0.768605345165175	1	pseudobulk	DESeq2	LRT	0.114296599617092
143	KC_AB1	HipHop	1.11388934932283	1	1	pseudobulk	DESeq2	LRT	0
143	KC_AB1	Hipk	-0.133825961302589	0.536452016693357	1	pseudobulk	DESeq2	LRT	0.27046911771899
143	KC_AB1	HIPP1	-0.044740240317595	0.911410774856257	1	pseudobulk	DESeq2	LRT	0.0402858414185308
143	KC_AB1	Hira	0.724376203863392	0.67494136527916	1	pseudobulk	DESeq2	LRT	0.170733954341991
143	KC_AB1	His-Psi:CR31614	-0.760959112540794	0.805265236978025	1	pseudobulk	DESeq2	LRT	0.0940610488455931
143	KC_AB1	His1:CG33801	-0.760959112540794	0.804334723897681	1	pseudobulk	DESeq2	LRT	0.0945631819854715
143	KC_AB1	His2Av	-0.0798838614142825	0.730660155208041	1	pseudobulk	DESeq2	LRT	0.136284575212739
143	KC_AB1	His2B:CG33868	1.13988912687578	0.653854063433791	1	pseudobulk	DESeq2	LRT	0.184519172956856
143	KC_AB1	His3.3A	-0.107807713579825	0.651205027168916	1	pseudobulk	DESeq2	LRT	0.186282255441809
143	KC_AB1	His3.3B	-0.135447438772329	0.247018148235762	1	pseudobulk	DESeq2	LRT	0.607271138282399

143	KC_AB1	His4r	0.0859595184234334	0.566590325250704	1	pseudobulk	DESeq2	LRT	0.246730845498789
143	KC_AB1	HisCl1	0.55008748560714	0.797802742076999	1	pseudobulk	DESeq2	LRT	0.0981044753377069
143	KC_AB1	HisRS	0.573521189890432	0.341491688896628	1	pseudobulk	DESeq2	LRT	0.466619861558599
143	KC_AB1	hiw	-0.3230869914964	0.2680846762018	1	pseudobulk	DESeq2	LRT	0.571728009706783
143	KC_AB1	Hk	-0.00258767257651324	0.988187009855316	1	pseudobulk	DESeq2	LRT	0.00516085939745776
143	KC_AB1	hkb	-0.760959112540794	0.805265236978025	1	pseudobulk	DESeq2	LRT	0.0940610488455931
143	KC_AB1	Hlc	-0.306669800538513	0.483834467652149	1	pseudobulk	DESeq2	LRT	0.315303196375274
143	KC_AB1	HLH4C	1.67020383217772	0.598754457572587	1	pseudobulk	DESeq2	LRT	0.222751240354882
143	KC_AB1	Hmg-2	-0.179863088672721	0.807413552743114	1	pseudobulk	DESeq2	LRT	0.0929039650667122
143	KC_AB1	Hmgcr	0.317513666629741	0.603242770714722	1	pseudobulk	DESeq2	LRT	0.219507873990278
143	KC_AB1	HmgD	-0.0370577657735822	0.928764505430312	1	pseudobulk	DESeq2	LRT	0.0320943903736046
143	KC_AB1	Hmgs	0.256588658545168	0.730100203309504	1	pseudobulk	DESeq2	LRT	0.136617530622103
143	KC_AB1	HmgZ	0.494871709638151	0.00300631523751131	1	pseudobulk	DESeq2	LRT	2.52196548192159
143	KC_AB1	Hml	3.32088770837427	0.291144101679295	1	pseudobulk	DESeq2	LRT	0.535892003886997
143	KC_AB1	Hmr	0.0804303750888063	0.938017369972137	1	pseudobulk	DESeq2	LRT	0.0277891193893752
143	KC_AB1	Hmt-1	-0.0176357852137434	0.979979082097236	1	pseudobulk	DESeq2	LRT	0.00878319433474839
143	KC_AB1	Hmt4-20	-0.218814069691021	0.511315498624191	1	pseudobulk	DESeq2	LRT	0.291311043054903
143	KC_AB1	Hmu	-0.83575901317924	0.20898796553692	1	pseudobulk	DESeq2	LRT	0.679878721790323
143	KC_AB1	Hn	-0.760959112540794	1	1	pseudobulk	DESeq2	LRT	0
143	KC_AB1	Hnf4	0.719266940197495	0.699065588649202	1	pseudobulk	DESeq2	LRT	0.155482075395787
143	KC_AB1	hng1	0.608495764301487	0.396764361380086	1	pseudobulk	DESeq2	LRT	0.40146734445908
143	KC_AB1	hng2	0.321465262907466	0.833743527724279	1	pseudobulk	DESeq2	LRT	0.0789675244511562
143	KC_AB1	hng3	0.950877883082144	0.509007340305081	1	pseudobulk	DESeq2	LRT	0.293275954733956
143	KC_AB1	HnRNP-K	-0.099813176289681	0.709532760263784	1	pseudobulk	DESeq2	LRT	0.149027547671868
143	KC_AB1	Ho	0.809300988319799	0.16237220206442	1	pseudobulk	DESeq2	LRT	0.78948831945057
143	KC_AB1	hob	-1.35947077554453	0.202654103918583	1	pseudobulk	DESeq2	LRT	0.693244596999278
143	KC_AB1	hoe1	-0.196504248773356	0.555297067255653	1	pseudobulk	DESeq2	LRT	0.255474620203014
143	KC_AB1	hoe2	-0.0937449676162853	0.834749601369422	1	pseudobulk	DESeq2	LRT	0.078443779668742
143	KC_AB1	hog	-0.760959112540794	1	1	pseudobulk	DESeq2	LRT	0
143	KC_AB1	hoip	-0.106876060368331	0.816613383038416	1	pseudobulk	DESeq2	LRT	0.0879835069355436
143	KC_AB1	holn1	0.44376500361458	0.54708197884633	1	pseudobulk	DESeq2	LRT	0.261947590864094
143	KC_AB1	homer	-0.368946343860684	0.563630262767358	1	pseudobulk	DESeq2	LRT	0.249005696542055
143	KC_AB1	hook	-0.0157243399231482	0.951731783649158	1	pseudobulk	DESeq2	LRT	0.0214854269237472
143	KC_AB1	hop	0.382019069521375	0.604965822700895	1	pseudobulk	DESeq2	LRT	0.218269159945341
143	KC_AB1	how	-0.388422036839266	0.821772538129019	1	pseudobulk	DESeq2	LRT	0.0852483760192765
143	KC_AB1	HP1b	0.255029324886722	0.568675199825501	1	pseudobulk	DESeq2	LRT	0.245135711107401
143	KC_AB1	HP1c	0.315794783707878	0.446947032712987	1	pseudobulk	DESeq2	LRT	0.349743941663306
143	KC_AB1	HP4	0.307917477625368	0.581361708403587	1	pseudobulk	DESeq2	LRT	0.235553576584645
143	KC_AB1	HP5	0.140439275043809	0.758340813613318	1	pseudobulk	DESeq2	LRT	0.120135569821083
143	KC_AB1	Hph	-0.0526702212507914	0.899466115828646	1	pseudobulk	DESeq2	LRT	0.0460151925027768
143	KC_AB1	hpo	0.37820103900749	0.608807747192582	1	pseudobulk	DESeq2	LRT	0.215519829723717
143	KC_AB1	hppy	-0.286438434542119	0.389422335695436	1	pseudobulk	DESeq2	LRT	0.409579142720744
143	KC_AB1	Hpr1	0.547781186176568	0.650157400272074	1	pseudobulk	DESeq2	LRT	0.186981489827378
143	KC_AB1	hpRNA:CR18854	-0.190327411973862	0.468627577622549	1	pseudobulk	DESeq2	LRT	0.329172157785819
143	KC_AB1	hpRNA:CR32207	0.152115475746305	1	1	pseudobulk	DESeq2	LRT	0
143	KC_AB1	hpRNA:CR33940	1.21647593365317	0.460399337944506	1	pseudobulk	DESeq2	LRT	0.336865309558437
143	KC_AB1	hpRNA:CR46342	-0.255424123355249	0.71182922075651	1	pseudobulk	DESeq2	LRT	0.147624188075874
143	KC_AB1	HPS1	0.178392335889419	0.807991037231775	1	pseudobulk	DESeq2	LRT	0.0925934566788441
143	KC_AB1	HPS4	0.0385286749606563	0.950545491622749	1	pseudobulk	DESeq2	LRT	0.0220270936455016
143	KC_AB1	Hr3	-0.0894650219433334	0.781004776498122	1	pseudobulk	DESeq2	LRT	0.107346310040198
143	KC_AB1	Hr38	-0.300790449323881	0.23924907343746	1	pseudobulk	DESeq2	LRT	0.621149735481224
143	KC_AB1	Hr39	-0.322325433863466	0.245893100250956	1	pseudobulk	DESeq2	LRT	0.60925365737343
143	KC_AB1	Hr4	-0.60693742682916	0.630059954460226	1	pseudobulk	DESeq2	LRT	0.200618122526796
143	KC_AB1	Hr51	0.14965336858242	0.534976887936204	1	pseudobulk	DESeq2	LRT	0.27166497995744
143	KC_AB1	Hr78	0.0660683865710783	0.897262798898196	1	pseudobulk	DESeq2	LRT	0.0470803380086912
143	KC_AB1	Hr96	-0.81779675251503	0.250038060702134	1	pseudobulk	DESeq2	LRT	0.601993878148805

143	KC_AB1	Hrb27C	-0.142185476646795	0.266910446414081	1	pseudobulk	DESeq2	LRT	0.573634428343597
143	KC_AB1	Hrb87F	-0.142021238572987	0.710056492961462	1	pseudobulk	DESeq2	LRT	0.148707096907118
143	KC_AB1	Hrb98DE	-0.112893612795341	0.610873704799578	1	pseudobulk	DESeq2	LRT	0.214048568772436
143	KC_AB1	Hrd3	0.0332193445269784	0.916006628353868	1	pseudobulk	DESeq2	LRT	0.0381013837043179
143	KC_AB1	hrg	-0.27077891816276	0.396163337953431	1	pseudobulk	DESeq2	LRT	0.402125717745569
143	KC_AB1	hrm	0.260358743454281	0.40780336446872	1	pseudobulk	DESeq2	LRT	0.38954919551675
143	KC_AB1	Hrs	0.621056226163625	0.402373426417219	1	pseudobulk	DESeq2	LRT	0.39537070872113
143	KC_AB1	Hs2st	0.497645593216758	0.488180573772341	1	pseudobulk	DESeq2	LRT	0.311419506506981
143	KC_AB1	Hs3st-A	-0.176853225912055	0.262355526190104	1	pseudobulk	DESeq2	LRT	0.581109783500741
144	KC_AB1	Hs3st-B	0.659451562848833	0.665323820526716	1	pseudobulk	DESeq2	LRT	0.176966927270749
144	KC_AB1	Hs6st	-0.150807717996567	0.715883845859096	1	pseudobulk	DESeq2	LRT	0.145157437461212
144	KC_AB1	Hsc20	-0.911139957682025	0.373590568800007	1	pseudobulk	DESeq2	LRT	0.427604095958579
144	KC_AB1	Hsc70-2	2.4138856564567	0.409611385497059	1	pseudobulk	DESeq2	LRT	0.387627980279722
144	KC_AB1	Hsc70-3	-0.357867574619793	0.430186480773873	1	pseudobulk	DESeq2	LRT	0.366343242060046
144	KC_AB1	Hsc70-4	0.214724354958386	0.326698676396176	1	pseudobulk	DESeq2	LRT	0.485852625044005
144	KC_AB1	Hsc70-5	-0.0652922523380854	0.848792940039821	1	pseudobulk	DESeq2	LRT	0.0711982413999528
144	KC_AB1	Hsc70Cb	-0.0433786293977162	0.876628200581078	1	pseudobulk	DESeq2	LRT	0.0571845624754071
144	KC_AB1	Hsepi	-0.373312529575833	0.625966057555484	1	pseudobulk	DESeq2	LRT	0.203449215377442
144	KC_AB1	Hsf	0.134026582070015	0.754995472099905	1	pseudobulk	DESeq2	LRT	0.122055652937596
144	KC_AB1	Hsl	0.0696359086412373	0.943510741434889	1	pseudobulk	DESeq2	LRT	0.0252531512284129
144	KC_AB1	Hsp23	-2.61097310396083	0.00142945069015249	1	pseudobulk	DESeq2	LRT	2.84483082132316
144	KC_AB1	Hsp26	-1.40182449609462	0.00452349942192349	1	pseudobulk	DESeq2	LRT	2.34452546084755
144	KC_AB1	Hsp27	-1.79147040116853	0.0693914808176379	1	pseudobulk	DESeq2	LRT	1.15869384454507
144	KC_AB1	Hsp60A	0.238255637658469	0.43416095382195	1	pseudobulk	DESeq2	LRT	0.362349237312231
144	KC_AB1	Hsp60D	0.745451863273431	0.62952825782577	1	pseudobulk	DESeq2	LRT	0.200984770810503
144	KC_AB1	Hsp67Bc	-1.2139739447694	0.779153091164369	1	pseudobulk	DESeq2	LRT	0.108377201999191
144	KC_AB1	Hsp68	-3.05456157809614	0.00284689525568395	1	pseudobulk	DESeq2	LRT	2.54562851132698
144	KC_AB1	Hsp70Aa	-0.760959112540794	1	1	pseudobulk	DESeq2	LRT	0
144	KC_AB1	Hsp70Ab	-0.659176193826906	0.361635699333126	1	pseudobulk	DESeq2	LRT	0.441728704142169
144	KC_AB1	Hsp70Ba	0.296275065198765	1	1	pseudobulk	DESeq2	LRT	0
144	KC_AB1	Hsp70Bb	0.10528635987039	0.846530656331508	1	pseudobulk	DESeq2	LRT	0.0723573096938154
144	KC_AB1	Hsp70Bc	-1.69248851744212	0.0181229337101891	1	pseudobulk	DESeq2	LRT	1.74177149810419
144	KC_AB1	Hsp83	0.145773669447129	0.360128979595936	1	pseudobulk	DESeq2	LRT	0.443541929525121
144	KC_AB1	HSPBAP1	-0.207806667574786	0.837094947085565	1	pseudobulk	DESeq2	LRT	0.0772252795733882
144	KC_AB1	HSPC300	0.248351638854166	0.690289675331512	1	pseudobulk	DESeq2	LRT	0.16096862230934
144	KC_AB1	hth	0.486338669906489	0.287660458976816	1	pseudobulk	DESeq2	LRT	0.541119830943746
144	KC_AB1	htk	0.200249123278005	0.569220345711703	1	pseudobulk	DESeq2	LRT	0.244719585270101
144	KC_AB1	htl	1.27179366484067	0.458649426559353	1	pseudobulk	DESeq2	LRT	0.338519145146168
144	KC_AB1	HtrA2	3.11480971027333	0.190195286845861	1	pseudobulk	DESeq2	LRT	0.720800249344957
144	KC_AB1	hts	0.00138901578128673	0.924628534999765	1	pseudobulk	DESeq2	LRT	0.0340327079110096
144	KC_AB1	htt	0.247852397679731	0.664116973782225	1	pseudobulk	DESeq2	LRT	0.177755419737435
144	KC_AB1	Hug	-0.182009407901012	0.869570481578722	1	pseudobulk	DESeq2	LRT	0.0606952112228907
144	KC_AB1	Hus1-like	0.152115475746306	1	1	pseudobulk	DESeq2	LRT	0
144	KC_AB1	HUWE1	0.0685201201505492	0.823973932188323	1	pseudobulk	DESeq2	LRT	0.0840865277274544
144	KC_AB1	hwt	-0.0153759456259164	0.933876905490647	1	pseudobulk	DESeq2	LRT	0.02971036444362
144	KC_AB1	Hyccin	-0.126233949858979	0.828056442860468	1	pseudobulk	DESeq2	LRT	0.0819400593656145
144	KC_AB1	hyd	-0.167908295949884	0.686711648811107	1	pseudobulk	DESeq2	LRT	0.163225585529231
144	KC_AB1	Hydr1	-0.0675304471364952	0.947851379201739	1	pseudobulk	DESeq2	LRT	0.0232597536456693
144	KC_AB1	Hydr2	-0.235655219351179	0.682754234927371	1	pseudobulk	DESeq2	LRT	0.165735597376608
144	KC_AB1	hyx	-0.964606134286705	0.153799459358133	1	pseudobulk	DESeq2	LRT	0.813045191180831
144	KC_AB1	I-2	0.0818252044393563	0.821920765963448	1	pseudobulk	DESeq2	LRT	0.0851700468923388
144	KC_AB1	I-3	-0.285236855150662	0.908999161438407	1	pseudobulk	DESeq2	LRT	0.0414365174192214
144	KC_AB1	IA-2	-0.218231507297639	0.291444375492607	1	pseudobulk	DESeq2	LRT	0.53544432159722
144	KC_AB1	Ibf1	-1.42191981722392	0.115243386085265	1	pseudobulk	DESeq2	LRT	0.938383989748257
144	KC_AB1	Ibf2	0.723403812737728	0.341719771326736	1	pseudobulk	DESeq2	LRT	0.466329892931719
144	KC_AB1	ICA69	0.357978453579943	0.261662499067077	1	pseudobulk	DESeq2	LRT	0.582258515095117

144	KC_AB1	Ice1	-0.549109388839176	0.716245469296439	1	pseudobulk	DESeq2	LRT	0.144938112201907
144	KC_AB1	Ice2	1.03735658381811	0.430104270137722	1	pseudobulk	DESeq2	LRT	0.366426245685751
144	KC_AB1	icln	-0.110078593131636	0.865899789790963	1	pseudobulk	DESeq2	LRT	0.0625323657875453
144	KC_AB1	ics	-0.0418431206172878	0.913049687249877	1	pseudobulk	DESeq2	LRT	0.0395055879517023
144	KC_AB1	ida	0.142467235535596	0.924530346898152	1	pseudobulk	DESeq2	LRT	0.0340788289351972
144	KC_AB1	Ide	0.0222540189579871	0.960421339871457	1	pseudobulk	DESeq2	LRT	0.0175381987967515
144	KC_AB1	Idgf1	2.99960667015886	0.357405021116046	1	pseudobulk	DESeq2	LRT	0.44683935046587
144	KC_AB1	Idgf2	0.323196446171093	0.836065213949708	1	pseudobulk	DESeq2	LRT	0.0777598458218715
144	KC_AB1	Idgf3	0.169341402605741	0.811956588598694	1	pseudobulk	DESeq2	LRT	0.0904671897687119
144	KC_AB1	Idgf4	2.07851675316354	0.0411370732724201	1	pseudobulk	DESeq2	LRT	1.38576660974514
144	KC_AB1	Idgf6	0.191188498942827	0.921486604582843	1	pseudobulk	DESeq2	LRT	0.0355109736278027
144	KC_AB1	Idh	0.879258260069296	0.123476525291457	1	pseudobulk	DESeq2	LRT	0.908415600342996
144	KC_AB1	Idh3a	-0.329848499655302	0.143620094080001	1	pseudobulk	DESeq2	LRT	0.842784793119141
144	KC_AB1	Idh3b	0.0901214900663168	0.737091339085131	1	pseudobulk	DESeq2	LRT	0.13247869178443
144	KC_AB1	Idi	-0.125209872522577	0.833102087045463	1	pseudobulk	DESeq2	LRT	0.0793017775560159
144	KC_AB1	Ids	0.709713058804466	0.916223368658127	1	pseudobulk	DESeq2	LRT	0.037998635557264
144	KC_AB1	if	0.593020121173868	0.728195768213239	1	pseudobulk	DESeq2	LRT	0.137751849223798
144	KC_AB1	ifc	0.0238369140300835	0.97005914976041	1	pseudobulk	DESeq2	LRT	0.0132017836395744
144	KC_AB1	IFT20	-0.760959112540794	0.805265236978025	1	pseudobulk	DESeq2	LRT	0.0940610488455931
144	KC_AB1	IFT43	1.51908833610022	0.184867668108403	1	pseudobulk	DESeq2	LRT	0.733139036866341
144	KC_AB1	igl	-0.139916867739755	0.51973933897689	1	pseudobulk	DESeq2	LRT	0.284214410261994
144	KC_AB1	Ih	-0.171085117819596	0.322750774906665	1	pseudobulk	DESeq2	LRT	0.491132706343386
144	KC_AB1	ihog	1.25804452752548	0.727552935095488	1	pseudobulk	DESeq2	LRT	0.138135402914536
144	KC_AB1	IKKbeta	-0.760959112540794	0.804334723897681	1	pseudobulk	DESeq2	LRT	0.0945631819854715
144	KC_AB1	IKKepsilon	0.781029026918308	0.487755933853195	1	pseudobulk	DESeq2	LRT	0.311797438439804
144	KC_AB1	IleRS	-0.139044948639415	0.846475371549339	1	pseudobulk	DESeq2	LRT	0.0723856732973025
144	KC_AB1	IleRS-m	-0.0733049160264654	0.948645165671152	1	pseudobulk	DESeq2	LRT	0.0228962021226897
144	KC_AB1	Ilk	0.211201229034391	0.798217574298041	1	pseudobulk	DESeq2	LRT	0.0978787146168404
144	KC_AB1	Ilp6	0.390408741546128	0.768618862050547	1	pseudobulk	DESeq2	LRT	0.114288962073132
144	KC_AB1	Ilp8	1.25804452752548	0.727552935095488	1	pseudobulk	DESeq2	LRT	0.138135402914536
144	KC_AB1	IM14	-1.43550613534661	0.602448798560473	1	pseudobulk	DESeq2	LRT	0.220079857379737
144	KC_AB1	imd	0.346341572617093	0.625757990149888	1	pseudobulk	DESeq2	LRT	0.203593596284898
144	KC_AB1	Iml1	-0.334346493074715	0.661961465883078	1	pseudobulk	DESeq2	LRT	0.179167290985419
144	KC_AB1	Imp	1.68178678514115	0.257335163240769	1	pseudobulk	DESeq2	LRT	0.589500866109796
144	KC_AB1	Impbeta11	0.936133352746756	0.602176825399324	1	pseudobulk	DESeq2	LRT	0.220275962198978
144	KC_AB1	ImpE1	1.70002804781309	0.432453003928593	1	pseudobulk	DESeq2	LRT	0.364061081825666
144	KC_AB1	ImpE3	-0.760959112540794	0.805265236978025	1	pseudobulk	DESeq2	LRT	0.0940610488455931
144	KC_AB1	ImpL2	-0.661215574434626	0.574150527833396	1	pseudobulk	DESeq2	LRT	0.240974231579316
144	KC_AB1	in	-0.760959112540794	1	1	pseudobulk	DESeq2	LRT	0
144	KC_AB1	inaE	-0.00483221875867245	0.946323269224246	1	pseudobulk	DESeq2	LRT	0.023960480873217
144	KC_AB1	inc	0.0668764258738974	0.864042993080124	1	pseudobulk	DESeq2	LRT	0.0634646473441189
144	KC_AB1	Indy	0.927458351763772	0.650688073626843	1	pseudobulk	DESeq2	LRT	0.186627153317656
144	KC_AB1	ine	-0.137765582447334	0.896272740714824	1	pseudobulk	DESeq2	LRT	0.0475598120307879
144	KC_AB1	Ing3	0.697618357738528	0.329789340651488	1	pseudobulk	DESeq2	LRT	0.481763385595842
144	KC_AB1	Ing5	-0.162644892702942	0.749979048580534	1	pseudobulk	DESeq2	LRT	0.124950868892243
144	KC_AB1	Ino80	0.0869319650651909	0.926276771524337	1	pseudobulk	DESeq2	LRT	0.0332592267240643
144	KC_AB1	Inos	0.859244747025971	0.0944364324538049	1	pseudobulk	DESeq2	LRT	1.02486042772304
144	KC_AB1	INPP5E	0.133349724876805	0.855384319815313	1	pseudobulk	DESeq2	LRT	0.0678387151267882
144	KC_AB1	InR	-0.195785547732497	0.749126664238727	1	pseudobulk	DESeq2	LRT	0.125444744477981
144	KC_AB1	insv	-0.716419614646419	0.812870875922622	1	pseudobulk	DESeq2	LRT	0.0899784363592155
144	KC_AB1	IntS1	0.89185349696562	0.372606475561688	1	pseudobulk	DESeq2	LRT	0.428749601731052
144	KC_AB1	IntS10	0.570909447936835	0.696833062134693	1	pseudobulk	DESeq2	LRT	0.156871251855132
144	KC_AB1	IntS11	-0.309220780627619	0.749868467695669	1	pseudobulk	DESeq2	LRT	0.125014908293137
145	KC_AB1	IntS12	-1.35317881049632	0.456576913328023	1	pseudobulk	DESeq2	LRT	0.34048605225825
145	KC_AB1	IntS14	-0.28602311894876	0.813994634381712	1	pseudobulk	DESeq2	LRT	0.0893784578455588
145	KC_AB1	IntS2	0.0904330688970173	0.925559304771182	1	pseudobulk	DESeq2	LRT	0.0335957488076584

145	KC_AB1	IntS3	-0.347705359729978	0.426498608255584	1	pseudobulk	DESeq2	LRT	0.37008238167795
145	KC_AB1	IntS4	0.234311107634923	0.849590826157841	1	pseudobulk	DESeq2	LRT	0.0707901857288701
145	KC_AB1	IntS6	-0.00512056432880625	0.955618107921003	1	pseudobulk	DESeq2	LRT	0.0197156294373739
145	KC_AB1	IntS8	-0.621590918585173	0.619527209032847	1	pseudobulk	DESeq2	LRT	0.207939615077538
145	KC_AB1	IntS9	0.989044191816059	0.576765572004992	1	pseudobulk	DESeq2	LRT	0.239000671198642
145	KC_AB1	Invadolysin	0.509924477010191	0.416794171168571	1	pseudobulk	DESeq2	LRT	0.380078363237865
145	KC_AB1	Inx2	-0.179803874523322	0.731808991379746	1	pseudobulk	DESeq2	LRT	0.135602258852391
145	KC_AB1	Inx3	-0.469305640792447	0.621348995391844	1	pseudobulk	DESeq2	LRT	0.206664399502356
145	KC_AB1	Inx5	1.25804452752548	0.727552935095488	1	pseudobulk	DESeq2	LRT	0.138135402914536
145	KC_AB1	Inx7	-0.760959112540794	1	1	pseudobulk	DESeq2	LRT	0
145	KC_AB1	Ip259	-0.0688972446458372	0.721252057973057	1	pseudobulk	DESeq2	LRT	0.141912934647211
145	KC_AB1	IP3K1	4.96668881692218	0.033454935401041	1	pseudobulk	DESeq2	LRT	1.47553980437366
145	KC_AB1	IP3K2	1.21671269245038	0.638896979472215	1	pseudobulk	DESeq2	LRT	0.194569165086909
145	KC_AB1	IPIP	0.496763336801533	0.294161233852778	1	pseudobulk	DESeq2	LRT	0.531414561496792
145	KC_AB1	Ipk1	1.25804452752548	0.727552935095488	1	pseudobulk	DESeq2	LRT	0.138135402914536
145	KC_AB1	Ipk2	0.191734106385892	0.882731272629374	1	pseudobulk	DESeq2	LRT	0.0541714873360879
145	KC_AB1	iPLA2-VIA	-0.396182538615624	0.500293910175254	1	pseudobulk	DESeq2	LRT	0.300774783531501
145	KC_AB1	Ipod	1.11388934932283	1	1	pseudobulk	DESeq2	LRT	0
145	KC_AB1	Ipp	-0.164040520100226	0.546264859137584	1	pseudobulk	DESeq2	LRT	0.262596736467857
145	KC_AB1	Ir40a	-0.760959112540794	1	1	pseudobulk	DESeq2	LRT	0
145	KC_AB1	Ir41a	-1.61102155603974	0.57025980967112	1	pseudobulk	DESeq2	LRT	0.243927235206257
145	KC_AB1	Ir47a	-0.396051030308863	0.847780548125483	1	pseudobulk	DESeq2	LRT	0.0717165523097658
145	KC_AB1	Ir47b	-0.84572271983469	0.729929556961718	1	pseudobulk	DESeq2	LRT	0.136719050152121
145	KC_AB1	Ir56a	-0.973804716840323	0.283636391632572	1	pseudobulk	DESeq2	LRT	0.54723804827293
145	KC_AB1	Ir68a	0.499178434206606	0.708400871677791	1	pseudobulk	DESeq2	LRT	0.149720913088087
145	KC_AB1	Ir76a	3.51235137991753	0.182155193970943	1	pseudobulk	DESeq2	LRT	0.739558440778038
145	KC_AB1	Ir85a	1.25804452752548	0.727552935095488	1	pseudobulk	DESeq2	LRT	0.138135402914536
145	KC_AB1	Irbp	-0.459184491799161	0.866831670429805	1	pseudobulk	DESeq2	LRT	0.0620652297453457
145	KC_AB1	Irbp18	0.112368711947361	0.83980191126422	1	pseudobulk	DESeq2	LRT	0.0758231413073196
145	KC_AB1	Irc	-0.107272914935357	0.911921554537141	1	pseudobulk	DESeq2	LRT	0.0400425190147065
145	KC_AB1	Ire1	-0.603122455779584	0.355123980702694	1	pseudobulk	DESeq2	LRT	0.449619999804258
145	KC_AB1	Irk1	-0.570723996670538	0.824991814167186	1	pseudobulk	DESeq2	LRT	0.0835503606375391
145	KC_AB1	Irk2	-0.494696230667333	0.249717110854524	1	pseudobulk	DESeq2	LRT	0.602551698357102
145	KC_AB1	Irk3	-0.569432576450917	0.825325802164462	1	pseudobulk	DESeq2	LRT	0.0833745773279703
145	KC_AB1	Irp-1A	0.0511747177625373	0.906455673152722	1	pseudobulk	DESeq2	LRT	0.0426534286074821
145	KC_AB1	Irp-1B	0.281727968646565	0.859384745448132	1	pseudobulk	DESeq2	LRT	0.0658123595496003
145	KC_AB1	IRSp53	3.9691052230278	0.118978671314663	1	pseudobulk	DESeq2	LRT	0.924530885334839
145	KC_AB1	Iru	-0.0259813513343058	0.942795666438881	1	pseudobulk	DESeq2	LRT	0.0255824223780981
145	KC_AB1	IscU	0.124389242345592	0.6214471480852	1	pseudobulk	DESeq2	LRT	0.206595800684407
145	KC_AB1	isopeptidase-T-3	0.181376444156891	0.864263695593432	1	pseudobulk	DESeq2	LRT	0.0633537296640322
145	KC_AB1	isoQC	-1.19301887438313	0.31505934812555	1	pseudobulk	DESeq2	LRT	0.501607629906617
145	KC_AB1	Iswi	-0.512654479704206	0.385600606521632	1	pseudobulk	DESeq2	LRT	0.413862291655123
145	KC_AB1	ITP	-1.61102155603974	0.57025980967112	1	pseudobulk	DESeq2	LRT	0.243927235206257
145	KC_AB1	Itpr	0.146478141666771	0.843470656376388	1	pseudobulk	DESeq2	LRT	0.0739300215460875
145	KC_AB1	ix	0.270562508958491	0.683925160903985	1	pseudobulk	DESeq2	LRT	0.164991418725287
145	KC_AB1	Jabba	2.58743201691345	0.417636627932844	1	pseudobulk	DESeq2	LRT	0.379201419460921
145	KC_AB1	Jafrac1	0.0394979678218041	0.901588764561155	1	pseudobulk	DESeq2	LRT	0.044991509028826
145	KC_AB1	Jafrac2	1.01004852098025	0.531844797138611	1	pseudobulk	DESeq2	LRT	0.274215084960958
145	KC_AB1	jagn	-0.118696781582043	0.807636571427524	1	pseudobulk	DESeq2	LRT	0.0927840235451871
145	KC_AB1	janA	0.315553439335578	0.570322058227228	1	pseudobulk	DESeq2	LRT	0.243879830973012
145	KC_AB1	janB	1.11388934932283	1	1	pseudobulk	DESeq2	LRT	0
145	KC_AB1	jar	0.0728481950703065	0.854788465190489	1	pseudobulk	DESeq2	LRT	0.0681413469875191
145	KC_AB1	Jarid2	-0.0996637460337332	0.720097426900848	1	pseudobulk	DESeq2	LRT	0.14260874092573
145	KC_AB1	jbug	1.55886998782004	0.47712740176907	1	pseudobulk	DESeq2	LRT	0.321365640881087
145	KC_AB1	jdp	-0.20561963436433	0.62367208815777	1	pseudobulk	DESeq2	LRT	0.205043691944234
145	KC_AB1	jeb	-0.368230814101715	0.29678185912059	1	pseudobulk	DESeq2	LRT	0.527562648961094

145	KC_AB1	jef	-0.268868512421791	0.351388714298857	1	pseudobulk	DESeq2	LRT	0.454212191037761
145	KC_AB1	jet	1.03651318142597	0.189148085010053	1	pseudobulk	DESeq2	LRT	0.723198051271987
145	KC_AB1	JHDM2	0.423136522103981	0.707137975312011	1	pseudobulk	DESeq2	LRT	0.150495839286065
145	KC_AB1	Jheh1	2.04480874254212	0.526249918436357	1	pseudobulk	DESeq2	LRT	0.278807958467711
145	KC_AB1	Jheh2	0.296275065198765	1	1	pseudobulk	DESeq2	LRT	0
145	KC_AB1	Jheh3	0.176251722124659	0.895963371648921	1	pseudobulk	DESeq2	LRT	0.0477097445967047
145	KC_AB1	JhI-21	0.883557623728307	0.628142879885958	1	pseudobulk	DESeq2	LRT	0.201941558670098
145	KC_AB1	JhI-26	1.03024943107471	0.481056975383004	1	pseudobulk	DESeq2	LRT	0.317803483648901
145	KC_AB1	jigr1	-0.0856015387898768	0.731693170950413	1	pseudobulk	DESeq2	LRT	0.13567099831141
145	KC_AB1	JIL-1	0.108132845572127	0.660824702487546	1	pseudobulk	DESeq2	LRT	0.179913730891198
145	KC_AB1	jim	-0.140318493486966	0.433813044207957	1	pseudobulk	DESeq2	LRT	0.362697393441315
145	KC_AB1	jing	-0.0157441513972527	0.930581744043145	1	pseudobulk	DESeq2	LRT	0.0312454716169497
145	KC_AB1	JMJD4	-0.437679040595599	0.728111122805216	1	pseudobulk	DESeq2	LRT	0.137802334508035
145	KC_AB1	JMJD5	-0.0287633598217589	0.97158572698257	1	pseudobulk	DESeq2	LRT	0.0125188737951084
145	KC_AB1	JMJD7	0.00569215138886444	0.963716194007152	1	pseudobulk	DESeq2	LRT	0.0160508431964004
145	KC_AB1	jnj	0.197091640219387	0.828675398965353	1	pseudobulk	DESeq2	LRT	0.0816155539428147
145	KC_AB1	jp	-0.448580586178488	0.188539532307041	1	pseudobulk	DESeq2	LRT	0.724597574568286
145	KC_AB1	Jra	0.519312660733262	0.418932785045402	1	pseudobulk	DESeq2	LRT	0.377855651080279
145	KC_AB1	jtb	4.31851526800926	0.128069104667069	1	pseudobulk	DESeq2	LRT	0.892555626624673
145	KC_AB1	JTBR	0.390403374528477	0.595456743108928	1	pseudobulk	DESeq2	LRT	0.225149782311249
145	KC_AB1	jub	0.427002706273038	0.667633223365644	1	pseudobulk	DESeq2	LRT	0.175462059701964
145	KC_AB1	jumu	-1.35490988619187	0.112846073189915	1	pseudobulk	DESeq2	LRT	0.94751354887353
145	KC_AB1	Jupiter	-0.0274020331292226	0.901314814684281	1	pseudobulk	DESeq2	LRT	0.0451234904859931
145	KC_AB1	jus	-0.00066465007008833	0.983853054886264	1	pseudobulk	DESeq2	LRT	0.0070697615458793
145	KC_AB1	jv	1.43133021453264	0.505109318794817	1	pseudobulk	DESeq2	LRT	0.296614619086474
145	KC_AB1	jvl	0.108694540156353	0.670750221019863	1	pseudobulk	DESeq2	LRT	0.173439175551005
145	KC_AB1	Jwa	-0.174353339641267	0.627894560734052	1	pseudobulk	DESeq2	LRT	0.202113279090749
145	KC_AB1	KaiR1D	-0.241788493707108	0.725845795247615	1	pseudobulk	DESeq2	LRT	0.139155634653078
145	KC_AB1	Kank	1.39629359694355	0.212931966691684	1	pseudobulk	DESeq2	LRT	0.671759134636585
145	KC_AB1	Kap-alpha1	-0.232088302232607	0.638743890214141	1	pseudobulk	DESeq2	LRT	0.19467324100044
145	KC_AB1	Kap-alpha3	-0.0344967081487579	0.890530760423115	1	pseudobulk	DESeq2	LRT	0.0503510746832817
145	KC_AB1	Kap3	0.245055744761737	0.519243024827921	1	pseudobulk	DESeq2	LRT	0.284629328782446
145	KC_AB1	kar	0.943022244937108	0.768808261856119	1	pseudobulk	DESeq2	LRT	0.114181958248924
145	KC_AB1	Karl	3.99740073762969	0.17240197409338	1	pseudobulk	DESeq2	LRT	0.763457765582343
145	KC_AB1	Karybeta3	-0.00504565826506461	0.968612084304796	1	pseudobulk	DESeq2	LRT	0.0138501170423673
145	KC_AB1	kat-60L1	0.2206361105714	0.671856418326433	1	pseudobulk	DESeq2	LRT	0.172723529611578
145	KC_AB1	Kat60	-0.0892263319342922	0.886849883938854	1	pseudobulk	DESeq2	LRT	0.0521498864753704
145	KC_AB1	kat80	-0.163470230836305	0.77953844516661	1	pseudobulk	DESeq2	LRT	0.108162461473229
145	KC_AB1	kay	1.16953229790777	0.128590943377418	1	pseudobulk	DESeq2	LRT	0.890789617568229
145	KC_AB1	Kaz1-ORFB	-1.77282884497951	0.463390561057957	1	pseudobulk	DESeq2	LRT	0.334052816731183
145	KC_AB1	kcc	-0.417232326370122	0.0955366468585879	1	pseudobulk	DESeq2	LRT	1.01983000563554
146	KC_AB1	Kcmf1	0.244364504273529	0.51665505725218	1	pseudobulk	DESeq2	LRT	0.286799315184137
146	KC_AB1	KCNQ	0.106847338390274	0.61870464948306	1	pseudobulk	DESeq2	LRT	0.208516620302531
146	KC_AB1	KdelR	0.172993291451126	0.680909315758562	1	pseudobulk	DESeq2	LRT	0.166910724046268
146	KC_AB1	Kdm2	-0.00248740566960619	0.987283043309172	1	pseudobulk	DESeq2	LRT	0.00555832197563061
146	KC_AB1	Kdm4A	-0.440844985591023	0.597557025297513	1	pseudobulk	DESeq2	LRT	0.223620643366781
146	KC_AB1	Kdm4B	-0.0152414455867342	0.929199427019254	1	pseudobulk	DESeq2	LRT	0.0318910666747004
146	KC_AB1	kdn	-0.208294549496387	0.564923197977285	1	pseudobulk	DESeq2	LRT	0.248010591051124
146	KC_AB1	Keap1	0.476747606386216	0.519045244450974	1	pseudobulk	DESeq2	LRT	0.284794783656429
146	KC_AB1	Kebab	-0.655759015510318	0.785501562616293	1	pseudobulk	DESeq2	LRT	0.104852946671165
146	KC_AB1	kek1	0.178549241378093	0.403384765444841	1	pseudobulk	DESeq2	LRT	0.394280507730036
146	KC_AB1	kek3	-0.329980918580231	0.282172010939782	1	pseudobulk	DESeq2	LRT	0.549486066759018
146	KC_AB1	kek4	0.396514332889468	0.476147667364287	1	pseudobulk	DESeq2	LRT	0.322258338926634
146	KC_AB1	kek5	2.44410518519652	0.318957889796972	1	pseudobulk	DESeq2	LRT	0.496266650597989
146	KC_AB1	kek6	0.205485123632582	0.624199772686237	1	pseudobulk	DESeq2	LRT	0.204676393826935
146	KC_AB1	kel	0.078704280164934	0.883665874494944	1	pseudobulk	DESeq2	LRT	0.0537119163143271

146	KC_AB1	ken	0.632201060941383	0.83966373596901	1	pseudobulk	DESeq2	LRT	0.0758946030469265
146	KC_AB1	kermit	2.04425205959347	0.490670545152587	1	pseudobulk	DESeq2	LRT	0.309210011841057
146	KC_AB1	key	0.0913481373262397	0.863184495165791	1	pseudobulk	DESeq2	LRT	0.0638963692107063
146	KC_AB1	KFase	-3.6278247242408	0.079375151269777	1	pseudobulk	DESeq2	LRT	1.10031543403928
146	KC_AB1	Khc	0.226184164738745	0.596920257003481	1	pseudobulk	DESeq2	LRT	0.224083682700629
146	KC_AB1	Khc-73	-0.106948837229731	0.631313948527182	1	pseudobulk	DESeq2	LRT	0.199754615073939
146	KC_AB1	kibra	2.76419632791174	0.0858754021114601	1	pseudobulk	DESeq2	LRT	1.06613121634428
146	KC_AB1	Kif19A	0.152115475746306	1	1	pseudobulk	DESeq2	LRT	0
146	KC_AB1	Kif3C	-0.175167891240356	0.885725091544597	1	pseudobulk	DESeq2	LRT	0.0527010520987533
146	KC_AB1	kin17	-0.0386092336001841	0.941607558372045	1	pseudobulk	DESeq2	LRT	0.0261300640281557
146	KC_AB1	kirre	0.200827465508599	0.305950746446998	1	pseudobulk	DESeq2	LRT	0.514348482890876
146	KC_AB1	kis	-0.17341346937854	0.297094654644993	1	pseudobulk	DESeq2	LRT	0.527105161993264
146	KC_AB1	kkv	-2.38324878285857	0.390434993490158	1	pseudobulk	DESeq2	LRT	0.408451264779408
146	KC_AB1	klar	-0.393149146438151	0.376308480963415	1	pseudobulk	DESeq2	LRT	0.424455993760638
146	KC_AB1	Klc	0.181951807045667	0.667157472105405	1	pseudobulk	DESeq2	LRT	0.175771645551687
146	KC_AB1	klg	0.104643396117619	0.722215374418304	1	pseudobulk	DESeq2	LRT	0.141333270611544
146	KC_AB1	KLHL18	0.437681409229738	0.378335916263892	1	pseudobulk	DESeq2	LRT	0.422122428216091
146	KC_AB1	Klp10A	-0.145218781248617	0.727654277492533	1	pseudobulk	DESeq2	LRT	0.138074913324449
146	KC_AB1	Klp31E	-0.387624902683464	0.645688839178262	1	pseudobulk	DESeq2	LRT	0.18997672037004
146	KC_AB1	Klp54D	-0.273024051754392	0.843392992864782	1	pseudobulk	DESeq2	LRT	0.0739700115412179
146	KC_AB1	Klp64D	-0.144066709170307	0.717388688485234	1	pseudobulk	DESeq2	LRT	0.144245475397959
146	KC_AB1	Klp68D	-0.282404621353199	0.512377295235698	1	pseudobulk	DESeq2	LRT	0.290410123213689
146	KC_AB1	Klp98A	0.291505833086682	0.417958515653789	1	pseudobulk	DESeq2	LRT	0.378866821853487
146	KC_AB1	Kmn1	-0.766255903148525	0.527306256517184	1	pseudobulk	DESeq2	LRT	0.277937075710506
146	KC_AB1	Kmn2	-1.61102155603974	0.57025980967112	1	pseudobulk	DESeq2	LRT	0.243927235206257
146	KC_AB1	kmr	-0.506539373628383	0.0848569504667009	1	pseudobulk	DESeq2	LRT	1.07131257967854
146	KC_AB1	kni	3.32088770837427	0.291144101679295	1	pseudobulk	DESeq2	LRT	0.535892003886997
146	KC_AB1	knrl	1.23813923640809	0.515089765370176	1	pseudobulk	DESeq2	LRT	0.288117079293203
146	KC_AB1	ko	2.66900722365034	0.14741903308453	1	pseudobulk	DESeq2	LRT	0.831446441646858
146	KC_AB1	koi	0.117501219448708	0.804384688089618	1	pseudobulk	DESeq2	LRT	0.0945362050338707
146	KC_AB1	koko	0.85524736181663	0.588395513167185	1	pseudobulk	DESeq2	LRT	0.230330647661303
146	KC_AB1	kon	1.25804452752548	0.727552935095488	1	pseudobulk	DESeq2	LRT	0.138135402914536
146	KC_AB1	Kr-h1	-0.066448103198392	0.828738932368696	1	pseudobulk	DESeq2	LRT	0.0815822584584206
146	KC_AB1	Kr-h2	0.135763525432487	0.746384011617537	1	pseudobulk	DESeq2	LRT	0.127037672236702
146	KC_AB1	kra	0.199169002384459	0.248241686015536	1	pseudobulk	DESeq2	LRT	0.605125287759222
146	KC_AB1	kraken	0.077508934486947	0.876556876322941	1	pseudobulk	DESeq2	LRT	0.0572198989976712
146	KC_AB1	kri	0.268730919917187	0.51661095304733	1	pseudobulk	DESeq2	LRT	0.286836390269577
146	KC_AB1	Krn	0.892793958708771	0.450229879481512	1	pseudobulk	DESeq2	LRT	0.346565686449356
146	KC_AB1	KrT95D	-0.200605163689057	0.308939374844567	1	pseudobulk	DESeq2	LRT	0.510126736606463
146	KC_AB1	krz	0.142436125432936	0.706916100633341	1	pseudobulk	DESeq2	LRT	0.150632126788856
146	KC_AB1	ksh	-0.0381241238175783	0.943166894253149	1	pseudobulk	DESeq2	LRT	0.0254114516529013
146	KC_AB1	ksr	0.278338070521334	0.671622748962359	1	pseudobulk	DESeq2	LRT	0.172874602019549
146	KC_AB1	kst	-2.99952832076105	0.338679393613493	1	pseudobulk	DESeq2	LRT	0.47021122647966
146	KC_AB1	Ktl	-0.12659813522818	0.5644260629044	1	pseudobulk	DESeq2	LRT	0.248392940498436
146	KC_AB1	kto	0.25734868599338	0.763437962957251	1	pseudobulk	DESeq2	LRT	0.117226247985397
146	KC_AB1	ktub	0.26843276775	0.814784035363662	1	pseudobulk	DESeq2	LRT	0.0889574890255697
146	KC_AB1	Ku80	0.348082755086629	0.843373162060664	1	pseudobulk	DESeq2	LRT	0.0739802232813283
146	KC_AB1	Kua	1.11388934932283	1	1	pseudobulk	DESeq2	LRT	0
146	KC_AB1	kud	-0.236907517165257	0.619230062914439	1	pseudobulk	DESeq2	LRT	0.208147967310766
146	KC_AB1	kug	-0.767891757946563	0.0463000778358902	1	pseudobulk	DESeq2	LRT	1.3344182788812
146	KC_AB1	kuk	0.0148289372276362	0.970142307887158	1	pseudobulk	DESeq2	LRT	0.0131645554275742
146	KC_AB1	Kul	-0.123062646623886	0.76898043507167	1	pseudobulk	DESeq2	LRT	0.114084709676468
146	KC_AB1	kune	0.0133825185455463	0.945595315191216	1	pseudobulk	DESeq2	LRT	0.0242946881037292
146	KC_AB1	kuz	0.00216504272340388	0.933530480899731	1	pseudobulk	DESeq2	LRT	0.0298714972430862
146	KC_AB1	Kyat	-0.415793867092819	0.665190671884925	1	pseudobulk	DESeq2	LRT	0.177053849616842
146	KC_AB1	kz	-0.34107018979271	0.817167008319255	1	pseudobulk	DESeq2	LRT	0.0876891755615287

146	KC_AB1	l(1)10Bb	-0.151057636894127	0.73802487606152	1	pseudobulk	DESeq2	LRT	0.131928999485666
146	KC_AB1	l(1)G0004	-0.207609554455808	0.809484859469408	1	pseudobulk	DESeq2	LRT	0.0917912698388124
146	KC_AB1	l(1)G0007	-1.11477901753615	0.106803468700936	1	pseudobulk	DESeq2	LRT	0.97141464231483
146	KC_AB1	l(1)G0020	-0.760959112540794	0.804334723897681	1	pseudobulk	DESeq2	LRT	0.0945631819854715
146	KC_AB1	l(1)G0193	0.0301762558617438	0.953460455525053	1	pseudobulk	DESeq2	LRT	0.0206973144726125
146	KC_AB1	l(1)G0196	-0.0121057206704811	0.968312223014817	1	pseudobulk	DESeq2	LRT	0.013984586008576
146	KC_AB1	l(1)G0289	-0.165367350840454	0.442253391052321	1	pseudobulk	DESeq2	LRT	0.354328828379427
146	KC_AB1	l(1)G0320	-0.178169829827202	0.593967132306538	1	pseudobulk	DESeq2	LRT	0.226237586420886
146	KC_AB1	l(1)G0469	0.17536952699145	0.573669852622776	1	pseudobulk	DESeq2	LRT	0.241337972469995
146	KC_AB1	l(2)05287	-0.436182345135797	0.724035872387712	1	pseudobulk	DESeq2	LRT	0.140239916131452
146	KC_AB1	l(2)05714	0.617872879716436	0.337909504528208	1	pseudobulk	DESeq2	LRT	0.471199592462744
146	KC_AB1	l(2)09851	0.433911886852759	0.62157721508587	1	pseudobulk	DESeq2	LRT	0.206504913679215
146	KC_AB1	l(2)10685	-0.429149437742635	0.785775721530297	1	pseudobulk	DESeq2	LRT	0.104701393910966
146	KC_AB1	l(2)34Fc	-6.44430001085188	0.139638824006022	1	pseudobulk	DESeq2	LRT	0.854993817333125
146	KC_AB1	l(2)34Fd	-0.659191843696584	0.576251253693811	1	pseudobulk	DESeq2	LRT	0.239388116748999
146	KC_AB1	l(2)35Be	-0.118228370637361	0.854886714805138	1	pseudobulk	DESeq2	LRT	0.0680914319322651
146	KC_AB1	l(2)37Bb	0.179578282670576	0.83413199114959	1	pseudobulk	DESeq2	LRT	0.0787652221532026
146	KC_AB1	l(2)37Cb	2.0820540707346	0.121147307307814	1	pseudobulk	DESeq2	LRT	0.916686234306944
146	KC_AB1	l(2)37Cc	0.070960493110898	0.854550475239603	1	pseudobulk	DESeq2	LRT	0.0682622799641704
146	KC_AB1	l(2)37Cd	0.296275065198765	1	1	pseudobulk	DESeq2	LRT	0
146	KC_AB1	l(2)37Cg	-0.0447594370515972	0.892831353215321	1	pseudobulk	DESeq2	LRT	0.0492305671863824
146	KC_AB1	l(2)41Ab	-0.946415152454216	0.568834162729333	1	pseudobulk	DESeq2	LRT	0.245014328875863
146	KC_AB1	l(2)dtl	-1.80072588255208	0.369082379242925	1	pseudobulk	DESeq2	LRT	0.43287668842731
146	KC_AB1	l(2)efl	-1.12375367880335	0.595220395960318	1	pseudobulk	DESeq2	LRT	0.225322195571628
146	KC_AB1	l(2)gd1	0.46157491254255	0.578995350240743	1	pseudobulk	DESeq2	LRT	0.23732492396323
146	KC_AB1	l(2)gl	2.71834409762744	0.198750042297838	1	pseudobulk	DESeq2	LRT	0.701692770245251
146	KC_AB1	l(2)k01209	0.248753351216599	0.619900049388498	1	pseudobulk	DESeq2	LRT	0.20767832904744
146	KC_AB1	l(2)k05819	-0.122553462807514	0.833230034282833	1	pseudobulk	DESeq2	LRT	0.0792350840336834
146	KC_AB1	l(2)k09022	0.573419110879053	0.727008638535867	1	pseudobulk	DESeq2	LRT	0.138460428692311
147	KC_AB1	l(2)k09848	0.417787688032883	0.699306371706949	1	pseudobulk	DESeq2	LRT	0.155332514681881
147	KC_AB1	l(2)k09913	0.0214554798653984	0.919368476865117	1	pseudobulk	DESeq2	LRT	0.0365103913386296
147	KC_AB1	l(2)k10201	0.0999228888511161	0.910068353395984	1	pseudobulk	DESeq2	LRT	0.0409259874724686
147	KC_AB1	l(2)k14505	0.395222418518605	0.330599563356236	1	pseudobulk	DESeq2	LRT	0.480697724364397
147	KC_AB1	l(2)SH0834	-0.263663056611124	0.437783858960247	1	pseudobulk	DESeq2	LRT	0.358740254859736
147	KC_AB1	l(3)02640	-0.0814227566968211	0.866247947182724	1	pseudobulk	DESeq2	LRT	0.062357781561403
147	KC_AB1	l(3)04053	-0.0408093479700576	0.945280573716871	1	pseudobulk	DESeq2	LRT	0.0244392671187755
147	KC_AB1	l(3)05822	0.281974670631612	0.572089493538556	1	pseudobulk	DESeq2	LRT	0.242536028008022
147	KC_AB1	l(3)07882	0.642680893362048	0.518991852015315	1	pseudobulk	DESeq2	LRT	0.284839460364577
147	KC_AB1	l(3)72Ab	0.250909975413813	0.711700572255167	1	pseudobulk	DESeq2	LRT	0.147702684970937
147	KC_AB1	l(3)72Dn	0.872729964365509	0.559490230236604	1	pseudobulk	DESeq2	LRT	0.252207492676967
147	KC_AB1	l(3)72Dp	0.261798924827972	0.831028531620899	1	pseudobulk	DESeq2	LRT	0.0803840653701584
147	KC_AB1	l(3)72Dr	-1.15478402271996	0.586097111234091	1	pseudobulk	DESeq2	LRT	0.232030419171504
147	KC_AB1	l(3)73Ah	-0.161329567209046	0.740857507371086	1	pseudobulk	DESeq2	LRT	0.130265313900923
147	KC_AB1	l(3)76BDm	-1.31395201666731	0.111844415953053	1	pseudobulk	DESeq2	LRT	0.951385694010929
147	KC_AB1	l(3)80Fg	-0.186154658928915	0.216848665095944	1	pseudobulk	DESeq2	LRT	0.663843246991123
147	KC_AB1	l(3)80Fj	-0.132440038530933	0.336043186721304	1	pseudobulk	DESeq2	LRT	0.473604905498546
147	KC_AB1	l(3)87Df	0.271808129422422	0.623578490207767	1	pseudobulk	DESeq2	LRT	0.205108873831475
147	KC_AB1	l(3)L1231	0.0496743638839138	0.806349794404236	1	pseudobulk	DESeq2	LRT	0.0934765204471315
147	KC_AB1	l(3)mbt	0.518360030810253	0.431853514546225	1	pseudobulk	DESeq2	LRT	0.364663541640346
147	KC_AB1	l(3)neo38	-0.214553801526685	0.223327049202993	1	pseudobulk	DESeq2	LRT	0.651058672324183
147	KC_AB1	l(3)neo43	-0.496929565770421	0.399761171915508	1	pseudobulk	DESeq2	LRT	0.398199390412347
147	KC_AB1	l(3)psg2	-0.169730899184909	0.669753212447867	1	pseudobulk	DESeq2	LRT	0.174085194636459
147	KC_AB1	L2HGDH	0.396943697783728	0.612945340307793	1	pseudobulk	DESeq2	LRT	0.212578252171827
147	KC_AB1	La	0.0630220435207405	0.886355452230935	1	pseudobulk	DESeq2	LRT	0.0523920794843944
147	KC_AB1	lab	-0.760959112540794	1	1	pseudobulk	DESeq2	LRT	0
147	KC_AB1	Lac	2.18849803868924	0.25050777940595	1	pseudobulk	DESeq2	LRT	0.601178782768327

147	KC_AB1	lace	0.193406567868613	0.752585141865184	1	pseudobulk	DESeq2	LRT	0.123444360118811
147	KC_AB1	Lam	0.320241319676898	0.475384910365216	1	pseudobulk	DESeq2	LRT	0.322954607734316
147	KC_AB1	lama	0.293544814793053	0.855484935116898	1	pseudobulk	DESeq2	LRT	0.0677876338764847
147	KC_AB1	LamC	1.93087103138744	0.48962757043079	1	pseudobulk	DESeq2	LRT	0.310134135493139
147	KC_AB1	Lamp1	0.0679573093413333	0.752523363926768	1	pseudobulk	DESeq2	LRT	0.123480011791325
147	KC_AB1	LanA	1.25804452752548	0.727552935095488	1	pseudobulk	DESeq2	LRT	0.138135402914536
147	KC_AB1	LanB1	0.939729030686247	0.766958937810335	1	pseudobulk	DESeq2	LRT	0.115227887106769
147	KC_AB1	LanB2	1.63194393720054	0.632980320284965	1	pseudobulk	DESeq2	LRT	0.198609792233049
147	KC_AB1	lap	-0.0661379505738014	0.776910231906883	1	pseudobulk	DESeq2	LRT	0.109629158856372
147	KC_AB1	Lap1	-0.40561576724841	0.629446860159406	1	pseudobulk	DESeq2	LRT	0.201040928484926
147	KC_AB1	Lapsyn	0.362671466789992	0.748256725898995	1	pseudobulk	DESeq2	LRT	0.125949370702016
147	KC_AB1	Lar	0.104177254671753	0.495891030392138	1	pseudobulk	DESeq2	LRT	0.304613747095922
147	KC_AB1	lark	0.226770850979355	0.442317911961189	1	pseudobulk	DESeq2	LRT	0.354265473227292
147	KC_AB1	larp	-0.0543707531110438	0.806279004658542	1	pseudobulk	DESeq2	LRT	0.0935146489934754
147	KC_AB1	Larp4B	-0.232505495758157	0.198648047330216	1	pseudobulk	DESeq2	LRT	0.701915699613805
147	KC_AB1	Larp7	0.306605071169375	0.754150158603575	1	pseudobulk	DESeq2	LRT	0.122542173281106
147	KC_AB1	Las	-0.340633471349068	0.645579261559603	1	pseudobulk	DESeq2	LRT	0.190050429238416
147	KC_AB1	lawc	-0.285153534595804	0.280485292012986	1	pseudobulk	DESeq2	LRT	0.552089907188826
147	KC_AB1	laza	-1.77199356422654	0.54193209410651	1	pseudobulk	DESeq2	LRT	0.266055128595237
147	KC_AB1	lbk	-0.277580772155082	0.479428662966238	1	pseudobulk	DESeq2	LRT	0.319276004937422
147	KC_AB1	LBR	-0.887395765817507	0.533519278311795	1	pseudobulk	DESeq2	LRT	0.272849883056447
147	KC_AB1	Lcch3	0.157796137291602	0.622006614127935	1	pseudobulk	DESeq2	LRT	0.206204997199869
147	KC_AB1	ldbr	2.62156539671496	0.291067440166642	1	pseudobulk	DESeq2	LRT	0.536006373560968
147	KC_AB1	Ldh	2.09948827516949	0.496374591357787	1	pseudobulk	DESeq2	LRT	0.304190457462447
147	KC_AB1	lds	-0.760959112540794	0.805265236978025	1	pseudobulk	DESeq2	LRT	0.0940610488455931
147	KC_AB1	Ldsdh1	0.0567841347233075	0.959297317965683	1	pseudobulk	DESeq2	LRT	0.0180467697428196
147	KC_AB1	Leash	-0.122471990789027	0.87976943945755	1	pseudobulk	DESeq2	LRT	0.0556311281803277
147	KC_AB1	lectin-24Db	4.90190122290401	0.0687021279919401	1	pseudobulk	DESeq2	LRT	1.16302981081754
147	KC_AB1	lectin-28C	5.57288044543161	0.175616368620586	1	pseudobulk	DESeq2	LRT	0.755435007391804
147	KC_AB1	Lerp	-0.106250644878066	0.793460259178098	1	pseudobulk	DESeq2	LRT	0.100474820203234
147	KC_AB1	Letm1	0.775639277463231	0.197080286939469	1	pseudobulk	DESeq2	LRT	0.705356814084679
147	KC_AB1	LeuRS	-0.231538791636931	0.787399442693243	1	pseudobulk	DESeq2	LRT	0.10380489693128
147	KC_AB1	LeuRS-m	0.175823661809132	0.862200243906373	1	pseudobulk	DESeq2	LRT	0.0643918586252796
147	KC_AB1	levy	0.0247240316050284	0.896350557477995	1	pseudobulk	DESeq2	LRT	0.0475221070752629
147	KC_AB1	lft	0.490578286518508	0.757370656515503	1	pseudobulk	DESeq2	LRT	0.120691525146357
147	KC_AB1	Lgr1	-0.0521710417050604	0.858053955606835	1	pseudobulk	DESeq2	LRT	0.0664854022613611
147	KC_AB1	Lgr3	1.67020383217772	0.598754457572587	1	pseudobulk	DESeq2	LRT	0.222751240354882
147	KC_AB1	lgs	-0.00505869204054625	0.980335413988003	1	pseudobulk	DESeq2	LRT	0.00862530846908808
147	KC_AB1	Lhr	-0.54706618918355	0.690654634265278	1	pseudobulk	DESeq2	LRT	0.160739069743542
147	KC_AB1	lic	-0.392222133524325	0.582313884575983	1	pseudobulk	DESeq2	LRT	0.234842854537616
147	KC_AB1	lid	0.166654363937116	0.53769753846379	1	pseudobulk	DESeq2	LRT	0.269461951683639
147	KC_AB1	lig	-0.411523010179099	0.202444462950792	1	pseudobulk	DESeq2	LRT	0.693694097101555
147	KC_AB1	lili	0.099517201770048	0.907754730652648	1	pseudobulk	DESeq2	LRT	0.0420314791366482
147	KC_AB1	lilli	-0.00897513970370252	0.946131408449612	1	pseudobulk	DESeq2	LRT	0.0240485401297856
147	KC_AB1	LIMK1	0.358755109907339	0.449684649739162	1	pseudobulk	DESeq2	LRT	0.347091937087072
147	KC_AB1	lin	0.834583223111115	0.449162647275652	1	pseudobulk	DESeq2	LRT	0.347596367184779
147	KC_AB1	lin-52	0.00323917583627405	0.919097060946692	1	pseudobulk	DESeq2	LRT	0.0366386226642703
147	KC_AB1	link	-2.35716291794055	0.404761325252261	1	pseudobulk	DESeq2	LRT	0.392800990812383
147	KC_AB1	Lint-1	-0.427462879116211	0.509341126044992	1	pseudobulk	DESeq2	LRT	0.292991255900575
147	KC_AB1	Liprin-alpha	0.439903506591287	0.321937690106107	1	pseudobulk	DESeq2	LRT	0.492228176321814
147	KC_AB1	Liprin-beta	-0.128171676718411	0.838490121325395	1	pseudobulk	DESeq2	LRT	0.0765020496698718
147	KC_AB1	Liprin-gamma	0.132679182489316	0.620871902444375	1	pseudobulk	DESeq2	LRT	0.206997993704465
147	KC_AB1	Lis-1	-0.0353622093450445	0.827072994362876	1	pseudobulk	DESeq2	LRT	0.0824561595525826
147	KC_AB1	List	0.0264801058917779	0.970154449064183	1	pseudobulk	DESeq2	LRT	0.0131591203350641
147	KC_AB1	Lk6	-0.0317418850037165	0.867383720339088	1	pseudobulk	DESeq2	LRT	0.0617887332516446
147	KC_AB1	Lkb1	-0.425515250915263	0.491029060823964	1	pseudobulk	DESeq2	LRT	0.308892804044031

147	KC_AB1	LKRSDH	-0.215433646642641	0.893570649868376	1	pseudobulk	DESeq2	LRT	0.0488711044510217
147	KC_AB1	LManI	-0.760959112540794	1	1	pseudobulk	DESeq2	LRT	0
147	KC_AB1	LManII	-0.068399712718329	0.975596996756336	1	pseudobulk	DESeq2	LRT	0.0107295452731888
147	KC_AB1	Lmpt	-0.284477408415921	0.429467233991871	1	pseudobulk	DESeq2	LRT	0.367069964869643
147	KC_AB1	lms	-1.61102155603974	0.57025980967112	1	pseudobulk	DESeq2	LRT	0.243927235206257
147	KC_AB1	Lmx1a	-0.760959112540794	0.805265236978025	1	pseudobulk	DESeq2	LRT	0.0940610488455931
147	KC_AB1	lncRNA:acal	-0.760959112540794	1	1	pseudobulk	DESeq2	LRT	0
147	KC_AB1	lncRNA:alphagamma-ele	0.244130101810533	0.629852721257808	1	pseudobulk	DESeq2	LRT	0.20076098994741
147	KC_AB1	lncRNA:cherub	-0.541365012892463	0.256646992954204	1	pseudobulk	DESeq2	LRT	0.590663819854598
147	KC_AB1	lncRNA:CR13130	-1.04456951810107	0.623763115739014	1	pseudobulk	DESeq2	LRT	0.204980309451609
147	KC_AB1	lncRNA:CR30055	-0.0539170169709579	0.841500244348773	1	pseudobulk	DESeq2	LRT	0.0749457535808389
147	KC_AB1	lncRNA:CR30121	1.11388934932283	1	1	pseudobulk	DESeq2	LRT	0
147	KC_AB1	lncRNA:CR31044	0.0513205106298865	0.915163004381908	1	pseudobulk	DESeq2	LRT	0.0385015446235411
147	KC_AB1	lncRNA:CR31386	-1.43550613534661	0.602448798560473	1	pseudobulk	DESeq2	LRT	0.220079857379737
147	KC_AB1	lncRNA:CR31451	-0.140453908294631	0.391955394628258	1	pseudobulk	DESeq2	LRT	0.406763353818954
147	KC_AB1	lncRNA:CR32218	0.502105900481512	0.502540424873372	1	pseudobulk	DESeq2	LRT	0.298828997403404
147	KC_AB1	lncRNA:CR32582	-1.4049914550416	0.364294121487634	1	pseudobulk	DESeq2	LRT	0.438547836790148
148	KC_AB1	lncRNA:CR32636	-0.760959112540794	0.804334723897681	1	pseudobulk	DESeq2	LRT	0.0945631819854715
148	KC_AB1	lncRNA:CR32657	1.25804452752548	0.727552935095488	1	pseudobulk	DESeq2	LRT	0.138135402914536
148	KC_AB1	lncRNA:CR32773	0.296275065198765	1	1	pseudobulk	DESeq2	LRT	0
148	KC_AB1	lncRNA:CR33938	1.33029054390672	0.628370150742698	1	pseudobulk	DESeq2	LRT	0.201784453287741
148	KC_AB1	lncRNA:CR33963	1.25804452752548	0.727552935095488	1	pseudobulk	DESeq2	LRT	0.138135402914536
148	KC_AB1	lncRNA:CR34046	-0.760959112540794	1	1	pseudobulk	DESeq2	LRT	0
148	KC_AB1	lncRNA:CR34335	-0.0633824583669713	0.693979103133981	1	pseudobulk	DESeq2	LRT	0.158653606678052
148	KC_AB1	lncRNA:CR40469	-0.209543745579842	0.638842160397062	1	pseudobulk	DESeq2	LRT	0.194606430317524
148	KC_AB1	lncRNA:CR42491	0.204416482446379	0.911290842863504	1	pseudobulk	DESeq2	LRT	0.0403429937271972
148	KC_AB1	lncRNA:CR42549	0.913316395789094	0.365754220966653	1	pseudobulk	DESeq2	LRT	0.436810653221932
148	KC_AB1	lncRNA:CR42736	1.11388934932283	1	1	pseudobulk	DESeq2	LRT	0
148	KC_AB1	lncRNA:CR42767	1.90071181822722	0.545896518579078	1	pseudobulk	DESeq2	LRT	0.262889675371117
148	KC_AB1	lncRNA:CR42861	0.145911919528694	0.68056396436252	1	pseudobulk	DESeq2	LRT	0.16713105038102
148	KC_AB1	lncRNA:CR42862	-0.142282087709743	0.411351534061239	1	pseudobulk	DESeq2	LRT	0.385786878731948
148	KC_AB1	lncRNA:CR42874	1.25804452752548	0.727552935095488	1	pseudobulk	DESeq2	LRT	0.138135402914536
148	KC_AB1	lncRNA:CR43144	2.77905602015038	0.396550243710121	1	pseudobulk	DESeq2	LRT	0.401701778879434
148	KC_AB1	lncRNA:CR43146	-0.760959112540794	0.804334723897681	1	pseudobulk	DESeq2	LRT	0.0945631819854715
148	KC_AB1	lncRNA:CR43148	-0.535979953737589	0.799855814031602	1	pseudobulk	DESeq2	LRT	0.0969882940257126
148	KC_AB1	lncRNA:CR43241	1.06550853919877	0.244168041809124	1	pseudobulk	DESeq2	LRT	0.612311179761367
148	KC_AB1	lncRNA:CR43242	0.293282570782599	0.872045801793267	1	pseudobulk	DESeq2	LRT	0.0594607043521855
148	KC_AB1	lncRNA:CR43253	-0.760959112540794	0.805265236978025	1	pseudobulk	DESeq2	LRT	0.0940610488455931
148	KC_AB1	lncRNA:CR43256	3.73367756122872	0.0995449623879823	1	pseudobulk	DESeq2	LRT	1.00198071316002
148	KC_AB1	lncRNA:CR43264	1.15461620943185	0.256161271849059	1	pseudobulk	DESeq2	LRT	0.591486529133172
148	KC_AB1	lncRNA:CR43285	-0.697357195569961	0.770678219972763	1	pseudobulk	DESeq2	LRT	0.113126914374965
148	KC_AB1	lncRNA:CR43287	2.61561465745786	0.313337449404899	1	pseudobulk	DESeq2	LRT	0.503987696092006
148	KC_AB1	lncRNA:CR43301	-0.641369210765349	0.791257849325846	1	pseudobulk	DESeq2	LRT	0.101681968725624
148	KC_AB1	lncRNA:CR43309	0.296275065198765	1	1	pseudobulk	DESeq2	LRT	0
148	KC_AB1	lncRNA:CR43314	2.09987857572813	0.503115176013642	1	pseudobulk	DESeq2	LRT	0.29833258237694
148	KC_AB1	lncRNA:CR43334	-0.651945920336254	0.570298604906698	1	pseudobulk	DESeq2	LRT	0.24389769080673
148	KC_AB1	lncRNA:CR43363	1.11388934932283	1	1	pseudobulk	DESeq2	LRT	0
148	KC_AB1	lncRNA:CR43411	-0.287780930859244	0.929076631507939	1	pseudobulk	DESeq2	LRT	0.0319484633276888
148	KC_AB1	lncRNA:CR43431	-0.760959112540794	0.804334723897681	1	pseudobulk	DESeq2	LRT	0.0945631819854715
148	KC_AB1	lncRNA:CR43432	2.42480980534803	0.407219734637837	1	pseudobulk	DESeq2	LRT	0.390171183430048
148	KC_AB1	lncRNA:CR43437	1.25804452752548	0.727552935095488	1	pseudobulk	DESeq2	LRT	0.138135402914536
148	KC_AB1	lncRNA:CR43452	-3.73432699611557	0.200340531487601	1	pseudobulk	DESeq2	LRT	0.698231178411511
148	KC_AB1	lncRNA:CR43459	-0.234598189998658	0.710680897188008	1	pseudobulk	DESeq2	LRT	0.148325358058819
148	KC_AB1	lncRNA:CR43461	2.09987857572813	0.503115176013642	1	pseudobulk	DESeq2	LRT	0.29833258237694
148	KC_AB1	lncRNA:CR43492	0.871633315497164	0.546079804515516	1	pseudobulk	DESeq2	LRT	0.262743884533221
148	KC_AB1	lncRNA:CR43496	-2.60091049889292	0.198562096894842	1	pseudobulk	DESeq2	LRT	0.702103649498395

148	KC_AB1	lncRNA:CR43604	-1.21568980399399	0.778574693542857	1	pseudobulk	DESeq2	LRT	0.108699716509594
148	KC_AB1	lncRNA:CR43635	0.296275065198765	1	1	pseudobulk	DESeq2	LRT	0
148	KC_AB1	lncRNA:CR43641	-1.61102155603974	0.57025980967112	1	pseudobulk	DESeq2	LRT	0.243927235206257
148	KC_AB1	lncRNA:CR43651	0.0787545670744297	0.848441962769071	1	pseudobulk	DESeq2	LRT	0.0713778600183076
148	KC_AB1	lncRNA:CR43653	0.293544814793053	0.855484935116898	1	pseudobulk	DESeq2	LRT	0.0677876338764847
148	KC_AB1	lncRNA:CR43685	-0.158347530300049	0.783837389163805	1	pseudobulk	DESeq2	LRT	0.105774024450361
148	KC_AB1	lncRNA:CR43686	-0.760959112540794	1	1	pseudobulk	DESeq2	LRT	0
148	KC_AB1	lncRNA:CR43700	0.152115475746305	1	1	pseudobulk	DESeq2	LRT	0
148	KC_AB1	lncRNA:CR43785	-0.760959112540794	0.805265236978025	1	pseudobulk	DESeq2	LRT	0.0940610488455931
148	KC_AB1	lncRNA:CR43818	1.11388934932283	1	1	pseudobulk	DESeq2	LRT	0
148	KC_AB1	lncRNA:CR43828	1.25804452752548	0.727552935095488	1	pseudobulk	DESeq2	LRT	0.138135402914536
148	KC_AB1	lncRNA:CR43836	1.33029054390672	0.628370150742698	1	pseudobulk	DESeq2	LRT	0.201784453287741
148	KC_AB1	lncRNA:CR43837	-1.48008690624249	0.268859655488375	1	pseudobulk	DESeq2	LRT	0.570474362199607
148	KC_AB1	lncRNA:CR43849	-0.760959112540794	0.805265236978025	1	pseudobulk	DESeq2	LRT	0.0940610488455931
148	KC_AB1	lncRNA:CR43855	-0.760959112540794	1	1	pseudobulk	DESeq2	LRT	0
148	KC_AB1	lncRNA:CR43856	0.229133303077424	0.809268887083457	1	pseudobulk	DESeq2	LRT	0.091907156045987
148	KC_AB1	lncRNA:CR43870	-1.60958773650785	0.570513631639934	1	pseudobulk	DESeq2	LRT	0.243733974251419
148	KC_AB1	lncRNA:CR43878	0.186372093533363	0.910273834150017	1	pseudobulk	DESeq2	LRT	0.0408279408979254
148	KC_AB1	lncRNA:CR43884	1.11388934932283	1	1	pseudobulk	DESeq2	LRT	0
148	KC_AB1	lncRNA:CR43890	1.25804452752548	0.573436424985144	1	pseudobulk	DESeq2	LRT	0.241514723898045
148	KC_AB1	lncRNA:CR43893	-0.760959112540794	1	1	pseudobulk	DESeq2	LRT	0
148	KC_AB1	lncRNA:CR43900	-0.345634349144491	0.774022285027069	1	pseudobulk	DESeq2	LRT	0.111246535279692
148	KC_AB1	lncRNA:CR43904	1.11388934932283	1	1	pseudobulk	DESeq2	LRT	0
148	KC_AB1	lncRNA:CR43907	-0.760959112540794	0.805265236978025	1	pseudobulk	DESeq2	LRT	0.0940610488455931
148	KC_AB1	lncRNA:CR43910	1.67020383217772	0.598754457572587	1	pseudobulk	DESeq2	LRT	0.222751240354882
148	KC_AB1	lncRNA:CR43951	-1.43550613534661	0.602448798560473	1	pseudobulk	DESeq2	LRT	0.220079857379737
148	KC_AB1	lncRNA:CR43956	-1.77199356422654	0.54193209410651	1	pseudobulk	DESeq2	LRT	0.266055128595237
148	KC_AB1	lncRNA:CR43958	0.281727968646565	0.859384745448132	1	pseudobulk	DESeq2	LRT	0.0658123595496003
148	KC_AB1	lncRNA:CR43963	-0.696045889415738	0.771031022267663	1	pseudobulk	DESeq2	LRT	0.112928147852258
148	KC_AB1	lncRNA:CR43966	-0.760959112540794	0.805265236978025	1	pseudobulk	DESeq2	LRT	0.0940610488455931
148	KC_AB1	lncRNA:CR43970	-0.760959112540794	0.804334723897681	1	pseudobulk	DESeq2	LRT	0.0945631819854715
148	KC_AB1	lncRNA:CR43973	1.13661584234725	0.691531818919754	1	pseudobulk	DESeq2	LRT	0.160187832238187
148	KC_AB1	lncRNA:CR43995	-0.41986557426529	0.803731316149176	1	pseudobulk	DESeq2	LRT	0.0948891097288435
148	KC_AB1	lncRNA:CR44017	0.296275065198765	1	1	pseudobulk	DESeq2	LRT	0
148	KC_AB1	lncRNA:CR44023	0.554910817793805	0.645639272516625	1	pseudobulk	DESeq2	LRT	0.190010060506371
148	KC_AB1	lncRNA:CR44024	0.0983325033870087	0.638699529955856	1	pseudobulk	DESeq2	LRT	0.194703403456315
148	KC_AB1	lncRNA:CR44042	2.13148430488057	0.45484681020722	1	pseudobulk	DESeq2	LRT	0.342134846603996
148	KC_AB1	lncRNA:CR44048	2.77905602015038	0.396550243710121	1	pseudobulk	DESeq2	LRT	0.401701778879434
148	KC_AB1	lncRNA:CR44050	-0.0831775083988207	0.938803827882818	1	pseudobulk	DESeq2	LRT	0.0274251482756223
148	KC_AB1	lncRNA:CR44051	-0.760959112540794	1	1	pseudobulk	DESeq2	LRT	0
148	KC_AB1	lncRNA:CR44060	1.25804452752548	0.573436424985144	1	pseudobulk	DESeq2	LRT	0.241514723898045
148	KC_AB1	lncRNA:CR44070	3.66097360166198	0.0970577125909502	1	pseudobulk	DESeq2	LRT	1.01296994814163
148	KC_AB1	lncRNA:CR44074	-0.760959112540794	0.805265236978025	1	pseudobulk	DESeq2	LRT	0.0940610488455931
148	KC_AB1	lncRNA:CR44092	-0.777733393415448	0.344238897094967	1	pseudobulk	DESeq2	LRT	0.463140058356878
148	KC_AB1	lncRNA:CR44097	-0.760959112540794	0.805265236978025	1	pseudobulk	DESeq2	LRT	0.0940610488455931
148	KC_AB1	lncRNA:CR44103	1.25804452752548	0.727552935095488	1	pseudobulk	DESeq2	LRT	0.138135402914536
148	KC_AB1	lncRNA:CR44111	-0.760959112540794	1	1	pseudobulk	DESeq2	LRT	0
148	KC_AB1	lncRNA:CR44129	0.943022244937108	0.768808261856119	1	pseudobulk	DESeq2	LRT	0.114181958248924
148	KC_AB1	lncRNA:CR44133	2.58743201691345	0.417636627932844	1	pseudobulk	DESeq2	LRT	0.379201419460921
148	KC_AB1	lncRNA:CR44157	0.152115475746305	1	1	pseudobulk	DESeq2	LRT	0
148	KC_AB1	lncRNA:CR44178	0.181412809615233	0.880171565156353	1	pseudobulk	DESeq2	LRT	0.0554326658743717
148	KC_AB1	lncRNA:CR44186	1.25804452752548	0.727552935095488	1	pseudobulk	DESeq2	LRT	0.138135402914536
148	KC_AB1	lncRNA:CR44187	1.11388934932283	1	1	pseudobulk	DESeq2	LRT	0
148	KC_AB1	lncRNA:CR44206	-0.235486009704009	0.724789867917395	1	pseudobulk	DESeq2	LRT	0.1397878864382
148	KC_AB1	lncRNA:CR44222	-0.760959112540794	0.804334723897681	1	pseudobulk	DESeq2	LRT	0.0945631819854715
148	KC_AB1	lncRNA:CR44260	2.04480874254212	0.526249918436357	1	pseudobulk	DESeq2	LRT	0.278807958467711

148	KC_AB1	lncRNA:CR44265	1.11388934932283	1	1	pseudobulk	DESeq2	LRT	0
148	KC_AB1	lncRNA:CR44268	0.137074306501724	1	1	pseudobulk	DESeq2	LRT	0
148	KC_AB1	lncRNA:CR44272	-0.760959112540794	0.804334723897681	1	pseudobulk	DESeq2	LRT	0.0945631819854715
148	KC_AB1	lncRNA:CR44281	-0.760959112540794	0.804334723897681	1	pseudobulk	DESeq2	LRT	0.0945631819854715
148	KC_AB1	lncRNA:CR44283	1.77006317075202	0.596029081111493	1	pseudobulk	DESeq2	LRT	0.224732549893771
149	KC_AB1	lncRNA:CR44294	0.0365329709774743	0.932958014128651	1	pseudobulk	DESeq2	LRT	0.0301379003505374
149	KC_AB1	lncRNA:CR44317	0.683441894478493	0.802755862961851	1	pseudobulk	DESeq2	LRT	0.0954165138615147
149	KC_AB1	lncRNA:CR44322	2.09987857572813	0.503115176013642	1	pseudobulk	DESeq2	LRT	0.29833258237694
149	KC_AB1	lncRNA:CR44330	2.13148430488057	0.45484681020722	1	pseudobulk	DESeq2	LRT	0.342134846603996
149	KC_AB1	lncRNA:CR44332	-2.51992355268831	0.220571581663641	1	pseudobulk	DESeq2	LRT	0.656450442583478
149	KC_AB1	lncRNA:CR44347	-0.351095630438326	0.663428146048561	1	pseudobulk	DESeq2	LRT	0.178206107410631
149	KC_AB1	lncRNA:CR44348	-0.760959112540794	0.804334723897681	1	pseudobulk	DESeq2	LRT	0.0945631819854715
149	KC_AB1	lncRNA:CR44357	-0.199038057269904	0.83188163520503	1	pseudobulk	DESeq2	LRT	0.0799384631735099
149	KC_AB1	lncRNA:CR44360	2.27122133462827	0.453795586678749	1	pseudobulk	DESeq2	LRT	0.343139732096712
149	KC_AB1	lncRNA:CR44366	1.25804452752548	0.727552935095488	1	pseudobulk	DESeq2	LRT	0.138135402914536
149	KC_AB1	lncRNA:CR44371	1.25804452752548	0.727552935095488	1	pseudobulk	DESeq2	LRT	0.138135402914536
149	KC_AB1	lncRNA:CR44384	0.152115475746305	1	1	pseudobulk	DESeq2	LRT	0
149	KC_AB1	lncRNA:CR44392	-0.696045889415738	0.771031022267663	1	pseudobulk	DESeq2	LRT	0.112928147852258
149	KC_AB1	lncRNA:CR44408	-1.22121879113183	0.605446734719958	1	pseudobulk	DESeq2	LRT	0.217924058688964
149	KC_AB1	lncRNA:CR44409	-1.77067014871429	0.542156052888816	1	pseudobulk	DESeq2	LRT	0.265875689192671
149	KC_AB1	lncRNA:CR44417	-0.151145649757215	0.664598964210181	1	pseudobulk	DESeq2	LRT	0.17744033991279
149	KC_AB1	lncRNA:CR44441	0.152115475746305	1	1	pseudobulk	DESeq2	LRT	0
149	KC_AB1	lncRNA:CR44444	1.11388934932283	1	1	pseudobulk	DESeq2	LRT	0
149	KC_AB1	lncRNA:CR44445	1.65806569952475	0.525043668677946	1	pseudobulk	DESeq2	LRT	0.279804574161347
149	KC_AB1	lncRNA:CR44464	-0.697357195569961	0.770678219972763	1	pseudobulk	DESeq2	LRT	0.113126914374965
149	KC_AB1	lncRNA:CR44471	-0.760959112540794	1	1	pseudobulk	DESeq2	LRT	0
149	KC_AB1	lncRNA:CR44474	1.25804452752548	0.727552935095488	1	pseudobulk	DESeq2	LRT	0.138135402914536
149	KC_AB1	lncRNA:CR44506	0.012232718593166	0.975134693959253	1	pseudobulk	DESeq2	LRT	0.0109353916829806
149	KC_AB1	lncRNA:CR44544	1.25804452752548	0.727552935095488	1	pseudobulk	DESeq2	LRT	0.138135402914536
149	KC_AB1	lncRNA:CR44564	-0.760959112540794	1	1	pseudobulk	DESeq2	LRT	0
149	KC_AB1	lncRNA:CR44567	2.09987857572813	0.503115176013642	1	pseudobulk	DESeq2	LRT	0.29833258237694
149	KC_AB1	lncRNA:CR44589	0.43379013272813	0.832859882065532	1	pseudobulk	DESeq2	LRT	0.079428056893678
149	KC_AB1	lncRNA:CR44595	-1.66454882767785	0.285148105738668	1	pseudobulk	DESeq2	LRT	0.544929509121752
149	KC_AB1	lncRNA:CR44596	0.284830539002579	0.83165374637133	1	pseudobulk	DESeq2	LRT	0.0800574517621246
149	KC_AB1	lncRNA:CR44597	0.544557704566096	0.546580827119933	1	pseudobulk	DESeq2	LRT	0.262345606492708
149	KC_AB1	lncRNA:CR44598	0.13202369622706	0.840404360758233	1	pseudobulk	DESeq2	LRT	0.0755117027578463
149	KC_AB1	lncRNA:CR44605	-0.760959112540794	1	1	pseudobulk	DESeq2	LRT	0
149	KC_AB1	lncRNA:CR44619	-1.60958773650785	0.570513631639934	1	pseudobulk	DESeq2	LRT	0.243733974251419
149	KC_AB1	lncRNA:CR44620	0.152115475746305	1	1	pseudobulk	DESeq2	LRT	0
149	KC_AB1	lncRNA:CR44628	0.296275065198765	1	1	pseudobulk	DESeq2	LRT	0
149	KC_AB1	lncRNA:CR44655	-0.760959112540794	0.804334723897681	1	pseudobulk	DESeq2	LRT	0.0945631819854715
149	KC_AB1	lncRNA:CR44661	-0.760959112540794	0.804334723897681	1	pseudobulk	DESeq2	LRT	0.0945631819854715
149	KC_AB1	lncRNA:CR44662	-1.84720757905536	0.438680975312332	1	pseudobulk	DESeq2	LRT	0.357851199641171
149	KC_AB1	lncRNA:CR44663	0.289188866933129	0.486336246784712	1	pseudobulk	DESeq2	LRT	0.31306336112902
149	KC_AB1	lncRNA:CR44676	1.25804452752548	0.727552935095488	1	pseudobulk	DESeq2	LRT	0.138135402914536
149	KC_AB1	lncRNA:CR44683	1.25804452752548	0.727552935095488	1	pseudobulk	DESeq2	LRT	0.138135402914536
149	KC_AB1	lncRNA:CR44708	-0.631606837090285	0.552684001268926	1	pseudobulk	DESeq2	LRT	0.257523106905808
149	KC_AB1	lncRNA:CR44713	-0.760959112540794	1	1	pseudobulk	DESeq2	LRT	0
149	KC_AB1	lncRNA:CR44716	-2.00963963748391	0.498268873088307	1	pseudobulk	DESeq2	LRT	0.302536242405947
149	KC_AB1	lncRNA:CR44718	-0.262488525155374	0.901290248884416	1	pseudobulk	DESeq2	LRT	0.0451353275672721
149	KC_AB1	lncRNA:CR44728	-0.760959112540794	0.805265236978025	1	pseudobulk	DESeq2	LRT	0.0940610488455931
149	KC_AB1	lncRNA:CR44749	0.293544814793053	0.855484935116898	1	pseudobulk	DESeq2	LRT	0.0677876338764847
149	KC_AB1	lncRNA:CR44759	-0.760959112540794	0.804334723897681	1	pseudobulk	DESeq2	LRT	0.0945631819854715
149	KC_AB1	lncRNA:CR44760	1.11388934932283	1	1	pseudobulk	DESeq2	LRT	0
149	KC_AB1	lncRNA:CR44763	-0.760959112540794	1	1	pseudobulk	DESeq2	LRT	0
149	KC_AB1	lncRNA:CR44766	-2.24976163616681	0.429894962609849	1	pseudobulk	DESeq2	LRT	0.366637643795677

149	KC_AB1	lncRNA:CR44772	1.25804452752548	0.573436424985144	1	pseudobulk	DESeq2	LRT	0.241514723898045
149	KC_AB1	lncRNA:CR44773	1.11388934932283	1	1	pseudobulk	DESeq2	LRT	0
149	KC_AB1	lncRNA:CR44779	-0.760959112540794	1	1	pseudobulk	DESeq2	LRT	0
149	KC_AB1	lncRNA:CR44785	-0.511868633442244	0.830414221287437	1	pseudobulk	DESeq2	LRT	0.0807052219067765
149	KC_AB1	lncRNA:CR44805	1.11388934932283	1	1	pseudobulk	DESeq2	LRT	0
149	KC_AB1	lncRNA:CR44811	2.80815610391224	0.158132717814292	1	pseudobulk	DESeq2	LRT	0.800978264816936
149	KC_AB1	lncRNA:CR44813	-2.10264006323213	0.319081610547617	1	pseudobulk	DESeq2	LRT	0.496098224536762
149	KC_AB1	lncRNA:CR44814	-0.760959112540794	0.805265236978025	1	pseudobulk	DESeq2	LRT	0.0940610488455931
149	KC_AB1	lncRNA:CR44816	-0.489975111905223	0.815559055007702	1	pseudobulk	DESeq2	LRT	0.088544586021147
149	KC_AB1	lncRNA:CR44831	1.11388934932283	1	1	pseudobulk	DESeq2	LRT	0
149	KC_AB1	lncRNA:CR44832	-0.760959112540794	1	1	pseudobulk	DESeq2	LRT	0
149	KC_AB1	lncRNA:CR44833	-0.760959112540794	0.805265236978025	1	pseudobulk	DESeq2	LRT	0.0940610488455931
149	KC_AB1	lncRNA:CR44845	0.296275065198765	1	1	pseudobulk	DESeq2	LRT	0
149	KC_AB1	lncRNA:CR44851	1.25804452752548	0.573436424985144	1	pseudobulk	DESeq2	LRT	0.241514723898045
149	KC_AB1	lncRNA:CR44854	-1.43550613534661	0.602448798560473	1	pseudobulk	DESeq2	LRT	0.220079857379737
149	KC_AB1	lncRNA:CR44855	-0.760959112540794	0.804334723897681	1	pseudobulk	DESeq2	LRT	0.0945631819854715
149	KC_AB1	lncRNA:CR44886	0.0783073588068288	0.877778236815703	1	pseudobulk	DESeq2	LRT	0.0566151910326464
149	KC_AB1	lncRNA:CR44895	1.25804452752548	0.727552935095488	1	pseudobulk	DESeq2	LRT	0.138135402914536
149	KC_AB1	lncRNA:CR44900	0.342632118294865	0.879250049710544	1	pseudobulk	DESeq2	LRT	0.0558875984571225
149	KC_AB1	lncRNA:CR44901	-0.62846884635387	0.770813910320913	1	pseudobulk	DESeq2	LRT	0.113050456547587
149	KC_AB1	lncRNA:CR44914	2.09987857572813	0.503115176013642	1	pseudobulk	DESeq2	LRT	0.29833258237694
149	KC_AB1	lncRNA:CR44922	0.0332941859269835	0.949018740473528	1	pseudobulk	DESeq2	LRT	0.0227252113831595
149	KC_AB1	lncRNA:CR44938	-0.760959112540794	1	1	pseudobulk	DESeq2	LRT	0
149	KC_AB1	lncRNA:CR44947	2.09948827516949	0.496374591357787	1	pseudobulk	DESeq2	LRT	0.304190457462447
149	KC_AB1	lncRNA:CR44951	-0.853791455458968	0.76171504745363	1	pseudobulk	DESeq2	LRT	0.118207464963883
149	KC_AB1	lncRNA:CR44965	-1.77067014871429	0.542156052888816	1	pseudobulk	DESeq2	LRT	0.265875689192671
149	KC_AB1	lncRNA:CR44973	-0.760959112540794	0.804334723897681	1	pseudobulk	DESeq2	LRT	0.0945631819854715
149	KC_AB1	lncRNA:CR44978	0.0329601418282168	0.922790465777888	1	pseudobulk	DESeq2	LRT	0.0348969012352991
149	KC_AB1	lncRNA:CR44979	-0.760959112540794	0.805265236978025	1	pseudobulk	DESeq2	LRT	0.0940610488455931
149	KC_AB1	lncRNA:CR44985	-1.77067014871429	0.542156052888816	1	pseudobulk	DESeq2	LRT	0.265875689192671
149	KC_AB1	lncRNA:CR44986	-0.5458338201468	0.716151064748245	1	pseudobulk	DESeq2	LRT	0.144995358046101
149	KC_AB1	lncRNA:CR44991	1.25804452752548	0.727552935095488	1	pseudobulk	DESeq2	LRT	0.138135402914536
149	KC_AB1	lncRNA:CR44993	-0.718853741766346	0.977252643133665	1	pseudobulk	DESeq2	LRT	0.0099931462763106
149	KC_AB1	lncRNA:CR44994	1.25804452752548	0.573436424985144	1	pseudobulk	DESeq2	LRT	0.241514723898045
149	KC_AB1	lncRNA:CR44997	0.259362063462386	0.485918312165905	1	pseudobulk	DESeq2	LRT	0.313436733942246
149	KC_AB1	lncRNA:CR44999	-0.253872548075375	0.785659774972846	1	pseudobulk	DESeq2	LRT	0.104765481746961
149	KC_AB1	lncRNA:CR45018	-0.474613422728405	0.759263964115592	1	pseudobulk	DESeq2	LRT	0.119607211944992
149	KC_AB1	lncRNA:CR45025	-0.760959112540794	1	1	pseudobulk	DESeq2	LRT	0
149	KC_AB1	lncRNA:CR45030	0.291868230578568	0.902153667810848	1	pseudobulk	DESeq2	LRT	0.0447194808636415
149	KC_AB1	lncRNA:CR45035	-1.72528247055183	0.445024651908338	1	pseudobulk	DESeq2	LRT	0.351615930836547
149	KC_AB1	lncRNA:CR45048	2.09987857572813	0.503115176013642	1	pseudobulk	DESeq2	LRT	0.29833258237694
149	KC_AB1	lncRNA:CR45054	-0.0186598596577536	0.926186108885284	1	pseudobulk	DESeq2	LRT	0.0333017369241912
149	KC_AB1	lncRNA:CR45102	-0.464806978338019	0.242161846343305	1	pseudobulk	DESeq2	LRT	0.615894280795899
149	KC_AB1	lncRNA:CR45112	-0.760959112540794	1	1	pseudobulk	DESeq2	LRT	0
149	KC_AB1	lncRNA:CR45128	-0.570723996670538	0.824991814167186	1	pseudobulk	DESeq2	LRT	0.0835503606375391
149	KC_AB1	lncRNA:CR45156	1.25804452752548	0.727552935095488	1	pseudobulk	DESeq2	LRT	0.138135402914536
149	KC_AB1	lncRNA:CR45165	0.296275065198765	1	1	pseudobulk	DESeq2	LRT	0
149	KC_AB1	lncRNA:CR45169	0.296275065198765	1	1	pseudobulk	DESeq2	LRT	0
149	KC_AB1	lncRNA:CR45170	-1.57992700724272	0.534361421248136	1	pseudobulk	DESeq2	LRT	0.272164903720841
150	KC_AB1	lncRNA:CR45177	-0.760959112540794	0.804334723897681	1	pseudobulk	DESeq2	LRT	0.0945631819854715
150	KC_AB1	lncRNA:CR45179	-2.69245487400416	0.326477446722968	1	pseudobulk	DESeq2	LRT	0.486146814701527
150	KC_AB1	lncRNA:CR45180	1.13658746815362	0.691887308070876	1	pseudobulk	DESeq2	LRT	0.159964635988989
150	KC_AB1	lncRNA:CR45187	-0.239373860046804	0.909220793071153	1	pseudobulk	DESeq2	LRT	0.0413306409048264
150	KC_AB1	lncRNA:CR45188	-0.760959112540794	0.804334723897681	1	pseudobulk	DESeq2	LRT	0.0945631819854715
150	KC_AB1	lncRNA:CR45199	1.25804452752548	0.727552935095488	1	pseudobulk	DESeq2	LRT	0.138135402914536
150	KC_AB1	lncRNA:CR45200	-1.77199356422654	0.54193209410651	1	pseudobulk	DESeq2	LRT	0.266055128595237

150	KC_AB1	lncRNA:CR45217	1.67020383217772	0.598754457572587	1	pseudobulk	DESeq2	LRT	0.222751240354882
150	KC_AB1	lncRNA:CR45226	1.25804452752548	0.727552935095488	1	pseudobulk	DESeq2	LRT	0.138135402914536
150	KC_AB1	lncRNA:CR45240	-1.44259395822269	0.609836273405539	1	pseudobulk	DESeq2	LRT	0.214786747121594
150	KC_AB1	lncRNA:CR45247	-1.43550613534661	0.602448798560473	1	pseudobulk	DESeq2	LRT	0.220079857379737
150	KC_AB1	lncRNA:CR45251	1.13698282223142	0.780593459054812	1	pseudobulk	DESeq2	LRT	0.107575092189158
150	KC_AB1	lncRNA:CR45270	1.25804452752548	0.727552935095488	1	pseudobulk	DESeq2	LRT	0.138135402914536
150	KC_AB1	lncRNA:CR45284	-0.760959112540794	1	1	pseudobulk	DESeq2	LRT	0
150	KC_AB1	lncRNA:CR45290	0.152115475746306	1	1	pseudobulk	DESeq2	LRT	0
150	KC_AB1	lncRNA:CR45308	1.25804452752548	0.727552935095488	1	pseudobulk	DESeq2	LRT	0.138135402914536
150	KC_AB1	lncRNA:CR45312	0.168416881203352	0.748983714131008	1	pseudobulk	DESeq2	LRT	0.125527625478012
150	KC_AB1	lncRNA:CR45356	-0.760959112540794	0.804334723897681	1	pseudobulk	DESeq2	LRT	0.0945631819854715
150	KC_AB1	lncRNA:CR45358	0.296275065198765	1	1	pseudobulk	DESeq2	LRT	0
150	KC_AB1	lncRNA:CR45387	0.943022244937108	0.768808261856119	1	pseudobulk	DESeq2	LRT	0.114181958248924
150	KC_AB1	lncRNA:CR45388	-0.242455885224342	0.66383222254454	1	pseudobulk	DESeq2	LRT	0.177941670677575
150	KC_AB1	lncRNA:CR45393	1.25804452752548	0.727552935095488	1	pseudobulk	DESeq2	LRT	0.138135402914536
150	KC_AB1	lncRNA:CR45403	1.11388934932283	1	1	pseudobulk	DESeq2	LRT	0
150	KC_AB1	lncRNA:CR45409	0.529922793972426	0.517381307458213	1	pseudobulk	DESeq2	LRT	0.286189266022256
150	KC_AB1	lncRNA:CR45418	1.67020383217772	0.598754457572587	1	pseudobulk	DESeq2	LRT	0.222751240354882
150	KC_AB1	lncRNA:CR45422	-0.760959112540794	1	1	pseudobulk	DESeq2	LRT	0
150	KC_AB1	lncRNA:CR45444	1.30305219044131	0.58441618457351	1	pseudobulk	DESeq2	LRT	0.233277765404341
150	KC_AB1	lncRNA:CR45456	-0.760959112540794	0.804334723897681	1	pseudobulk	DESeq2	LRT	0.0945631819854715
150	KC_AB1	lncRNA:CR45471	0.296275065198765	1	1	pseudobulk	DESeq2	LRT	0
150	KC_AB1	lncRNA:CR45473	-0.906640945973523	0.379385178380583	1	pseudobulk	DESeq2	LRT	0.420919639902975
150	KC_AB1	lncRNA:CR45474	-0.760959112540794	0.804334723897681	1	pseudobulk	DESeq2	LRT	0.0945631819854715
150	KC_AB1	lncRNA:CR45522	-2.69104391023971	0.258829318222164	1	pseudobulk	DESeq2	LRT	0.586986531631839
150	KC_AB1	lncRNA:CR45527	-1.73533618889279	0.492709206576031	1	pseudobulk	DESeq2	LRT	0.307409322588473
150	KC_AB1	lncRNA:CR45530	-0.718853741766346	0.977252643133665	1	pseudobulk	DESeq2	LRT	0.0099931462763106
150	KC_AB1	lncRNA:CR45531	1.13661584234725	0.691531818919754	1	pseudobulk	DESeq2	LRT	0.160187832238187
150	KC_AB1	lncRNA:CR45539	0.293544814793053	0.855484935116898	1	pseudobulk	DESeq2	LRT	0.0677876338764847
150	KC_AB1	lncRNA:CR45541	1.25804452752548	0.727552935095488	1	pseudobulk	DESeq2	LRT	0.138135402914536
150	KC_AB1	lncRNA:CR45555	-2.00963963748391	0.498268873088307	1	pseudobulk	DESeq2	LRT	0.302536242405947
150	KC_AB1	lncRNA:CR45556	0.374893549239772	0.848756618381963	1	pseudobulk	DESeq2	LRT	0.0712168261835375
150	KC_AB1	lncRNA:CR45562	1.25804452752548	0.727552935095488	1	pseudobulk	DESeq2	LRT	0.138135402914536
150	KC_AB1	lncRNA:CR45566	1.25804452752548	0.727552935095488	1	pseudobulk	DESeq2	LRT	0.138135402914536
150	KC_AB1	lncRNA:CR45578	-0.760959112540794	0.804334723897681	1	pseudobulk	DESeq2	LRT	0.0945631819854715
150	KC_AB1	lncRNA:CR45585	1.25804452752548	0.573436424985144	1	pseudobulk	DESeq2	LRT	0.241514723898045
150	KC_AB1	lncRNA:CR45596	1.25804452752548	0.727552935095488	1	pseudobulk	DESeq2	LRT	0.138135402914536
150	KC_AB1	lncRNA:CR45618	-0.760959112540794	0.804334723897681	1	pseudobulk	DESeq2	LRT	0.0945631819854715
150	KC_AB1	lncRNA:CR45625	-0.536464614365819	0.838047114315769	1	pseudobulk	DESeq2	LRT	0.0767315650067374
150	KC_AB1	lncRNA:CR45641	-1.20936316999684	0.542145505743777	1	pseudobulk	DESeq2	LRT	0.265884138072697
150	KC_AB1	lncRNA:CR45643	-0.623612063033828	0.736215813351559	1	pseudobulk	DESeq2	LRT	0.132994858476467
150	KC_AB1	lncRNA:CR45660	0.464383425124741	0.519655403568128	1	pseudobulk	DESeq2	LRT	0.284284552400392
150	KC_AB1	lncRNA:CR45667	-0.760959112540794	0.804334723897681	1	pseudobulk	DESeq2	LRT	0.0945631819854715
150	KC_AB1	lncRNA:CR45668	0.095487329789835	0.853945420821423	1	pseudobulk	DESeq2	LRT	0.0685698859779256
150	KC_AB1	lncRNA:CR45669	-2.00963963748391	0.498268873088307	1	pseudobulk	DESeq2	LRT	0.302536242405947
150	KC_AB1	lncRNA:CR45674	1.11388934932283	1	1	pseudobulk	DESeq2	LRT	0
150	KC_AB1	lncRNA:CR45684	-1.86925490517174	0.219579219803991	1	pseudobulk	DESeq2	LRT	0.658408762361654
150	KC_AB1	lncRNA:CR45687	-0.760959112540794	0.805265236978025	1	pseudobulk	DESeq2	LRT	0.0940610488455931
150	KC_AB1	lncRNA:CR45699	1.11388934932283	1	1	pseudobulk	DESeq2	LRT	0
150	KC_AB1	lncRNA:CR45736	-0.84572271983469	0.729929556961718	1	pseudobulk	DESeq2	LRT	0.136719050152121
150	KC_AB1	lncRNA:CR45753	1.25804452752548	0.573436424985144	1	pseudobulk	DESeq2	LRT	0.241514723898045
150	KC_AB1	lncRNA:CR45758	0.241695352988319	0.585311457628926	1	pseudobulk	DESeq2	LRT	0.232612974371528
150	KC_AB1	lncRNA:CR45761	-0.760959112540794	1	1	pseudobulk	DESeq2	LRT	0
150	KC_AB1	lncRNA:CR45805	-1.77199356422654	0.54193209410651	1	pseudobulk	DESeq2	LRT	0.266055128595237
150	KC_AB1	lncRNA:CR45819	1.47737206823489	0.580967731351599	1	pseudobulk	DESeq2	LRT	0.235847988926577
150	KC_AB1	lncRNA:CR45821	-0.570723996670538	0.824991814167186	1	pseudobulk	DESeq2	LRT	0.0835503606375391

150	KC_AB1	lncRNA:CR45825	-0.268625053440567	0.860127453697522	1	pseudobulk	DESeq2	LRT	0.0654371902258892
150	KC_AB1	lncRNA:CR45883	-0.262488525155374	0.901290248884416	1	pseudobulk	DESeq2	LRT	0.0451353275672721
150	KC_AB1	lncRNA:CR45910	-0.325934945069582	0.878443443182604	1	pseudobulk	DESeq2	LRT	0.0562861944468762
150	KC_AB1	lncRNA:CR45911	-0.30642379653085	0.675152960811974	1	pseudobulk	DESeq2	LRT	0.1705978234491
150	KC_AB1	lncRNA:CR45918	0.833665146301187	0.716955005728418	1	pseudobulk	DESeq2	LRT	0.144508098693451
150	KC_AB1	lncRNA:CR45919	-1.00294344476931	0.278438374605384	1	pseudobulk	DESeq2	LRT	0.555270910114427
150	KC_AB1	lncRNA:CR45922	-1.76935196357135	0.542380240270211	1	pseudobulk	DESeq2	LRT	0.265696140855463
150	KC_AB1	lncRNA:CR45938	-1.77199356422654	0.54193209410651	1	pseudobulk	DESeq2	LRT	0.266055128595237
150	KC_AB1	lncRNA:CR45939	-1.60958773650785	0.570513631639934	1	pseudobulk	DESeq2	LRT	0.243733974251419
150	KC_AB1	lncRNA:CR45953	1.11388934932283	1	1	pseudobulk	DESeq2	LRT	0
150	KC_AB1	lncRNA:CR45961	-0.716419614646419	0.812870875922622	1	pseudobulk	DESeq2	LRT	0.0899784363592155
150	KC_AB1	lncRNA:CR45962	0.291853629993484	0.902736173524132	1	pseudobulk	DESeq2	LRT	0.0444391545856091
150	KC_AB1	lncRNA:CR45972	1.11388934932283	1	1	pseudobulk	DESeq2	LRT	0
150	KC_AB1	lncRNA:CR45979	-0.462121716796105	0.476702127114941	1	pseudobulk	DESeq2	LRT	0.321752910182838
150	KC_AB1	lncRNA:CR45981	-1.60958773650785	0.570513631639934	1	pseudobulk	DESeq2	LRT	0.243733974251419
150	KC_AB1	lncRNA:CR45994	0.152115475746306	1	1	pseudobulk	DESeq2	LRT	0
150	KC_AB1	lncRNA:CR46003	-1.10839877042791	0.244273859024571	1	pseudobulk	DESeq2	LRT	0.612123006578849
150	KC_AB1	lncRNA:CR46004	0.296148206947171	0.456957313593304	1	pseudobulk	DESeq2	LRT	0.340124367406591
150	KC_AB1	lncRNA:CR46006	-0.370087311720107	0.0792440625577309	1	pseudobulk	DESeq2	LRT	1.10103326784855
150	KC_AB1	lncRNA:CR46010	-0.760959112540794	1	1	pseudobulk	DESeq2	LRT	0
150	KC_AB1	lncRNA:CR46016	0.296275065198765	1	1	pseudobulk	DESeq2	LRT	0
150	KC_AB1	lncRNA:CR46024	0.70842991589991	0.874943022847655	1	pseudobulk	DESeq2	LRT	0.0580202277417387
150	KC_AB1	lncRNA:CR46036	-0.760959112540794	1	1	pseudobulk	DESeq2	LRT	0
150	KC_AB1	lncRNA:CR46061	1.11388934932283	1	1	pseudobulk	DESeq2	LRT	0
150	KC_AB1	lncRNA:CR46064	-0.0899526427502253	0.831385102408214	1	pseudobulk	DESeq2	LRT	0.0801977618840181
150	KC_AB1	lncRNA:CR46076	-0.760959112540794	0.805265236978025	1	pseudobulk	DESeq2	LRT	0.0940610488455931
150	KC_AB1	lncRNA:CR46079	-0.760959112540794	0.805265236978025	1	pseudobulk	DESeq2	LRT	0.0940610488455931
150	KC_AB1	lncRNA:CR46080	-2.24978065768543	0.364534962203659	1	pseudobulk	DESeq2	LRT	0.438260812575185
150	KC_AB1	lncRNA:CR46084	0.152115475746305	1	1	pseudobulk	DESeq2	LRT	0
150	KC_AB1	lncRNA:CR46095	-1.61102155603974	0.57025980967112	1	pseudobulk	DESeq2	LRT	0.243927235206257
150	KC_AB1	lncRNA:CR46124	-0.760959112540794	1	1	pseudobulk	DESeq2	LRT	0
150	KC_AB1	lncRNA:CR46127	-0.760959112540794	0.804334723897681	1	pseudobulk	DESeq2	LRT	0.0945631819854715
150	KC_AB1	lncRNA:CR46194	-2.35899449797085	0.448742383752913	1	pseudobulk	DESeq2	LRT	0.348002909382209
150	KC_AB1	lncRNA:CR46204	0.494775748955277	0.827825706404751	1	pseudobulk	DESeq2	LRT	0.0820610916144188
150	KC_AB1	lncRNA:CR46208	-0.194713606698067	0.780873506259043	1	pseudobulk	DESeq2	LRT	0.107419311811506
150	KC_AB1	lncRNA:CR46228	1.25804452752548	0.727552935095488	1	pseudobulk	DESeq2	LRT	0.138135402914536
150	KC_AB1	lncRNA:CR46233	-0.760959112540794	0.805265236978025	1	pseudobulk	DESeq2	LRT	0.0940610488455931
151	KC_AB1	lncRNA:CR46254	-0.760959112540794	1	1	pseudobulk	DESeq2	LRT	0
151	KC_AB1	lncRNA:CR46258	-0.760959112540794	0.805265236978025	1	pseudobulk	DESeq2	LRT	0.0940610488455931
151	KC_AB1	lncRNA:CR46268	-1.1315122855643	0.227426379587188	1	pseudobulk	DESeq2	LRT	0.643159162143031
151	KC_AB1	lncRNA:CR46271	-0.814953172532845	0.571809586161946	1	pseudobulk	DESeq2	LRT	0.242748568141482
151	KC_AB1	lncRNA:CR46322	-0.0502041966748531	0.969584455517293	1	pseudobulk	DESeq2	LRT	0.0134143557780147
151	KC_AB1	lncRNA:CR46350	-0.760959112540794	0.804334723897681	1	pseudobulk	DESeq2	LRT	0.0945631819854715
151	KC_AB1	lncRNA:CR46393	4.05098780010447	0.0672652969646423	1	pseudobulk	DESeq2	LRT	1.17220893613028
151	KC_AB1	lncRNA:CR9162	1.11388934932283	1	1	pseudobulk	DESeq2	LRT	0
151	KC_AB1	lncRNA:dntRL	1.25804452752548	0.573436424985144	1	pseudobulk	DESeq2	LRT	0.241514723898045
151	KC_AB1	lncRNA:flam	0.152115475746305	1	1	pseudobulk	DESeq2	LRT	0
151	KC_AB1	lncRNA:Hsromega	-0.209315925669878	0.633254502722638	1	pseudobulk	DESeq2	LRT	0.198421713498499
151	KC_AB1	lncRNA:let7C	-0.0738709068499055	0.767368888842847	1	pseudobulk	DESeq2	LRT	0.114995812223664
151	KC_AB1	lncRNA:noe	-0.328298444853855	0.0583446156256024	1	pseudobulk	DESeq2	LRT	1.23399921694486
151	KC_AB1	lncRNA:roX1	2.58743201691345	0.417636627932844	1	pseudobulk	DESeq2	LRT	0.379201419460921
151	KC_AB1	Lnk	0.102723266732238	0.819931084264891	1	pseudobulk	DESeq2	LRT	0.0862226488129237
151	KC_AB1	Lnpk	-0.254211604309173	0.726429126954586	1	pseudobulk	DESeq2	LRT	0.138806750635035
151	KC_AB1	loco	3.41249799143893	0.117930065112952	1	pseudobulk	DESeq2	LRT	0.928375461672341
151	KC_AB1	loh	-0.760959112540794	1	1	pseudobulk	DESeq2	LRT	0
151	KC_AB1	loj	-0.312174406283774	0.679296919336114	1	pseudobulk	DESeq2	LRT	0.167940354963756

151	KC_AB1	lola	-0.151889338887731	0.211899768689453	1	pseudobulk	DESeq2	LRT	0.67386951736636
151	KC_AB1	lolal	-0.0661591988531456	0.70937588250916	1	pseudobulk	DESeq2	LRT	0.149123580836218
151	KC_AB1	Lon	-0.522658046263833	0.495893305057931	1	pseudobulk	DESeq2	LRT	0.30461175497976
151	KC_AB1	loqs	-0.210155637858102	0.372249036732326	1	pseudobulk	DESeq2	LRT	0.429166417396812
151	KC_AB1	lost	-0.0807711390106785	0.840554923055558	1	pseudobulk	DESeq2	LRT	0.07543390387751
151	KC_AB1	lov	-1.43550613534661	0.602448798560473	1	pseudobulk	DESeq2	LRT	0.220079857379737
151	KC_AB1	lovit	0.174937210311579	0.83822726554914	1	pseudobulk	DESeq2	LRT	0.0766382167007533
151	KC_AB1	Loxl1	-1.22121879113183	0.605446734719958	1	pseudobulk	DESeq2	LRT	0.217924058688964
151	KC_AB1	Loxl2	3.7951235223405	0.206529069530445	1	pseudobulk	DESeq2	LRT	0.685018811571072
151	KC_AB1	LPCAT	-0.0775961946047768	0.863618566669765	1	pseudobulk	DESeq2	LRT	0.0636780294740399
151	KC_AB1	Lpin	-0.11777324654713	0.739760056846246	1	pseudobulk	DESeq2	LRT	0.130909122006273
151	KC_AB1	LpR2	0.791242850419789	0.629977936455981	1	pseudobulk	DESeq2	LRT	0.200674660456279
151	KC_AB1	Lpt	0.257109412248171	0.796362954639492	1	pseudobulk	DESeq2	LRT	0.0988889507664876
151	KC_AB1	lqf	-0.136719259371904	0.575188392221851	1	pseudobulk	DESeq2	LRT	0.240189886959083
151	KC_AB1	lqfR	-0.377421847233307	0.474238492566273	1	pseudobulk	DESeq2	LRT	0.324003198500663
151	KC_AB1	Lrch	0.05747582339969	0.870387076244982	1	pseudobulk	DESeq2	LRT	0.0602875661260873
151	KC_AB1	LRP1	-0.186289310404615	0.793823924245465	1	pseudobulk	DESeq2	LRT	0.100275816475444
151	KC_AB1	Lrp4	-0.235676144030148	0.40602556173826	1	pseudobulk	DESeq2	LRT	0.391446624126206
151	KC_AB1	Lrpprc2	0.124481543023846	0.91941190661028	1	pseudobulk	DESeq2	LRT	0.0364898763289548
151	KC_AB1	LRR	-0.00167152164840225	0.963720814733681	1	pseudobulk	DESeq2	LRT	0.0160487608912202
151	KC_AB1	Lrr47	-1.23989790937637	0.28154955168205	1	pseudobulk	DESeq2	LRT	0.550445159859704
151	KC_AB1	Lrrk	0.192210292818437	0.816218820823993	1	pseudobulk	DESeq2	LRT	0.0881933952454646
151	KC_AB1	Lsd-1	1.25804452752548	0.727552935095488	1	pseudobulk	DESeq2	LRT	0.138135402914536
151	KC_AB1	Lsd-2	-0.485518315752466	0.582588855852699	1	pseudobulk	DESeq2	LRT	0.234637827102735
151	KC_AB1	LSm1	-0.638699112316074	0.409677788028231	1	pseudobulk	DESeq2	LRT	0.387557582051923
151	KC_AB1	Lsm11	-1.08233555646652	0.546572251204739	1	pseudobulk	DESeq2	LRT	0.262352420675863
151	KC_AB1	Lsm12a	-0.0248936774176422	0.948754242627421	1	pseudobulk	DESeq2	LRT	0.0228462690194588
151	KC_AB1	LSm3	-0.563904644922052	0.509283961942119	1	pseudobulk	DESeq2	LRT	0.293040000143691
151	KC_AB1	LSm7	-0.162877729946732	0.80755687688545	1	pseudobulk	DESeq2	LRT	0.0928268802072479
151	KC_AB1	lsn	-0.0971929625642407	0.862518483828826	1	pseudobulk	DESeq2	LRT	0.0642315891951535
151	KC_AB1	Lst	-1.43550613534661	0.602448798560473	1	pseudobulk	DESeq2	LRT	0.220079857379737
151	KC_AB1	Lst8	0.146693844911578	0.85674599341431	1	pseudobulk	DESeq2	LRT	0.0671479178781071
151	KC_AB1	lt	0.39661389468582	0.383489233465238	1	pseudobulk	DESeq2	LRT	0.416246824444831
151	KC_AB1	Ltn1	-0.567566369057597	0.470860389519214	1	pseudobulk	DESeq2	LRT	0.327107842444905
151	KC_AB1	LTV1	-0.0411264311212803	0.920553895337642	1	pseudobulk	DESeq2	LRT	0.035950779932872
151	KC_AB1	LUBEL	1.25804452752548	0.573436424985144	1	pseudobulk	DESeq2	LRT	0.241514723898045
151	KC_AB1	luna	2.95620245049424	0.0355687968465117	1	pseudobulk	DESeq2	LRT	1.44893082505201
151	KC_AB1	lush	1.25804452752548	0.727552935095488	1	pseudobulk	DESeq2	LRT	0.138135402914536
151	KC_AB1	lute	-0.0512473168625524	0.857247674605137	1	pseudobulk	DESeq2	LRT	0.0668936842912066
151	KC_AB1	lva	0.457996952573562	0.430500361098285	1	pseudobulk	DESeq2	LRT	0.366026479929428
151	KC_AB1	lwr	-0.0942553369297285	0.662997049778673	1	pseudobulk	DESeq2	LRT	0.178488404125684
151	KC_AB1	Lztr1	0.241867438697139	0.635838614551097	1	pseudobulk	DESeq2	LRT	0.196653100867255
151	KC_AB1	m-cup	-0.300569900306881	0.613641993098775	1	pseudobulk	DESeq2	LRT	0.212084928154501
151	KC_AB1	M1BP	-0.293085747140553	0.59245838913749	1	pseudobulk	DESeq2	LRT	0.227342146588027
151	KC_AB1	M6	-0.569999614403812	0.322404093707921	1	pseudobulk	DESeq2	LRT	0.49159945240208
151	KC_AB1	mAChR-A	0.0203068963144698	0.915481862542745	1	pseudobulk	DESeq2	LRT	0.0383502554864029
151	KC_AB1	mAChR-B	-0.0473748751044584	0.782040104771042	1	pseudobulk	DESeq2	LRT	0.106770974773713
151	KC_AB1	mAcon1	-0.179090764223939	0.609375196930371	1	pseudobulk	DESeq2	LRT	0.215115226607423
151	KC_AB1	Mad	0.0615744850122392	0.848991428878176	1	pseudobulk	DESeq2	LRT	0.0710966942197716
151	KC_AB1	Mad1	-0.487243748319303	0.530597354272107	1	pseudobulk	DESeq2	LRT	0.2752349198793
151	KC_AB1	Madm	0.261404711719961	0.617001506234746	1	pseudobulk	DESeq2	LRT	0.209713775758266
151	KC_AB1	maf-S	-0.0541125129532672	0.835717482187677	1	pseudobulk	DESeq2	LRT	0.0779405128283663
151	KC_AB1	Maf1	-0.173126430995599	0.439362458018671	1	pseudobulk	DESeq2	LRT	0.357177054770726
151	KC_AB1	MAGE	-0.716419614646419	0.812870875922622	1	pseudobulk	DESeq2	LRT	0.0899784363592155
151	KC_AB1	Magi	-0.273877296404183	0.599230850763627	1	pseudobulk	DESeq2	LRT	0.222405835543068
151	KC_AB1	mago	0.146441120278149	0.715532846044143	1	pseudobulk	DESeq2	LRT	0.145370425456533

151	KC_AB1	MagR	0.0286545311625314	0.919001516316401	1	pseudobulk	DESeq2	LRT	0.0366837720444626
151	KC_AB1	magu	2.98306427813109	0.227187865000518	1	pseudobulk	DESeq2	LRT	0.643614869721849
151	KC_AB1	mahe	-0.281587236158294	0.223032572447443	1	pseudobulk	DESeq2	LRT	0.651631706453327
151	KC_AB1	mahj	-0.406096204211701	0.664214828645115	1	pseudobulk	DESeq2	LRT	0.177691432973014
151	KC_AB1	mal	-0.309967844309286	0.804172072416322	1	pseudobulk	DESeq2	LRT	0.0946510133098153
151	KC_AB1	Mal-A5	1.91515398583233	0.457300554437087	1	pseudobulk	DESeq2	LRT	0.339798272074771
151	KC_AB1	mam	-0.30924664382258	0.390490619128086	1	pseudobulk	DESeq2	LRT	0.408389394846459
151	KC_AB1	mamo	0.819422219520177	0.216403602922386	1	pseudobulk	DESeq2	LRT	0.664735512898715
151	KC_AB1	MAN1	0.0761904112820608	0.917342571925455	1	pseudobulk	DESeq2	LRT	0.0374684513378295
151	KC_AB1	Manf	0.47993759213218	0.292478819933859	1	pseudobulk	DESeq2	LRT	0.533905578193416
151	KC_AB1	Map205	0.0285672852909864	0.900446668602164	1	pseudobulk	DESeq2	LRT	0.0455420043521448
151	KC_AB1	Map60	-0.113793044305073	0.814199028547004	1	pseudobulk	DESeq2	LRT	0.0892694201301976
151	KC_AB1	MAPk-Ak2	0.265532210386069	0.616559306793956	1	pseudobulk	DESeq2	LRT	0.210025142304987
151	KC_AB1	Mapmodulin	-0.00875745495691245	0.96971387316051	1	pseudobulk	DESeq2	LRT	0.0133563911342439
151	KC_AB1	Marc	-0.0495551903366867	0.983354422399057	1	pseudobulk	DESeq2	LRT	0.00728992474150539
151	KC_AB1	Marcal1	0.601816686662251	0.749156877473316	1	pseudobulk	DESeq2	LRT	0.125427229180377
151	KC_AB1	Marf	-0.134800940391906	0.665533017181662	1	pseudobulk	DESeq2	LRT	0.176830394247738
151	KC_AB1	Marf1	-0.678984539928844	0.677921935523786	1	pseudobulk	DESeq2	LRT	0.168820313394272
151	KC_AB1	mars	1.11388934932283	1	1	pseudobulk	DESeq2	LRT	0
151	KC_AB1	mask	-0.19721527677984	0.395399159937936	1	pseudobulk	DESeq2	LRT	0.402964257720137
151	KC_AB1	Mat1	0.553547082574251	0.450843561627627	1	pseudobulk	DESeq2	LRT	0.345974127984202
151	KC_AB1	Mat89Ba	-0.197838201425547	0.85944349242678	1	pseudobulk	DESeq2	LRT	0.0657826724777879
151	KC_AB1	mats	0.513135343442553	0.349848711777927	1	pseudobulk	DESeq2	LRT	0.456119720919392
151	KC_AB1	Mau2	0.241368632133967	0.837379754241858	1	pseudobulk	DESeq2	LRT	0.0770775434774301
151	KC_AB1	mav	1.79472274153272	0.391072031541475	1	pseudobulk	DESeq2	LRT	0.407743242553467
152	KC_AB1	Max	-0.17506957792639	0.688247540886749	1	pseudobulk	DESeq2	LRT	0.162255331669065
152	KC_AB1	mbc	-0.417983088700367	0.536028323195904	1	pseudobulk	DESeq2	LRT	0.270812262018593
152	KC_AB1	MBD-like	0.0208089414149249	0.949165488209547	1	pseudobulk	DESeq2	LRT	0.0226580611759788
152	KC_AB1	MBD-R2	0.00777238071418148	0.988602180773645	1	pseudobulk	DESeq2	LRT	0.00497843585691746
152	KC_AB1	mbf1	-0.00762335908621289	0.971505631573866	1	pseudobulk	DESeq2	LRT	0.0125546775609917
152	KC_AB1	mbl	0.0505874488671715	0.93118414736217	1	pseudobulk	DESeq2	LRT	0.030964426134625
152	KC_AB1	mbm	-0.174668262480694	0.884592265266037	1	pseudobulk	DESeq2	LRT	0.0532568623516343
152	KC_AB1	mbo	0.363859670701465	0.769667448771311	1	pseudobulk	DESeq2	LRT	0.113696880489937
152	KC_AB1	Mbs	-0.251059191252655	0.339163672295225	1	pseudobulk	DESeq2	LRT	0.469590671057494
152	KC_AB1	mbt	-0.17679538473041	0.670347253775397	1	pseudobulk	DESeq2	LRT	0.173700165462947
152	KC_AB1	Mcad	2.3498923087154	0.181433929724408	1	pseudobulk	DESeq2	LRT	0.741281492831797
152	KC_AB1	Mccc1	-0.760959112540794	0.805265236978025	1	pseudobulk	DESeq2	LRT	0.0940610488455931
152	KC_AB1	Mccc2	1.11649075726014	0.617975542504054	1	pseudobulk	DESeq2	LRT	0.209028712557973
152	KC_AB1	Mcm3	1.25804452752548	0.573436424985144	1	pseudobulk	DESeq2	LRT	0.241514723898045
152	KC_AB1	Mcm6	1.11388934932283	1	1	pseudobulk	DESeq2	LRT	0
152	KC_AB1	Mcm7	2.4138856564567	0.409611385497059	1	pseudobulk	DESeq2	LRT	0.387627980279722
152	KC_AB1	MCPH1	1.11388934932283	1	1	pseudobulk	DESeq2	LRT	0
152	KC_AB1	Mcr	0.275122351428377	0.760364032651041	1	pseudobulk	DESeq2	LRT	0.118978434666432
152	KC_AB1	Mct1	0.864095242258922	0.275647402308657	1	pseudobulk	DESeq2	LRT	0.559646095941222
152	KC_AB1	Mctp	-0.449059374420733	0.491818629987447	1	pseudobulk	DESeq2	LRT	0.308195024301036
152	KC_AB1	MCTS1	0.173208762311554	0.644254250585902	1	pseudobulk	DESeq2	LRT	0.19094270742989
152	KC_AB1	MCU	0.193057993760483	0.677201093489215	1	pseudobulk	DESeq2	LRT	0.16928234929915
152	KC_AB1	Mdh1	-0.158212789922659	0.627042740124646	1	pseudobulk	DESeq2	LRT	0.202702856031044
152	KC_AB1	Mdh2	-0.0219830659446492	0.916107668106464	1	pseudobulk	DESeq2	LRT	0.0380534816633431
152	KC_AB1	mdlc	-0.63669870918568	0.635853818171059	1	pseudobulk	DESeq2	LRT	0.196642716519459
152	KC_AB1	mdy	0.166959985905824	0.622805654026665	1	pseudobulk	DESeq2	LRT	0.205647453423386
152	KC_AB1	me31B	-0.203117206020178	0.557604978755505	1	pseudobulk	DESeq2	LRT	0.253673357154686
152	KC_AB1	Mec2	2.99960667015886	0.357405021116046	1	pseudobulk	DESeq2	LRT	0.44683935046587
152	KC_AB1	Med	0.386512925275821	0.388795706627035	1	pseudobulk	DESeq2	LRT	0.410278539523753
152	KC_AB1	MED1	-0.0658308982552018	0.903134064201475	1	pseudobulk	DESeq2	LRT	0.0442477767947169
152	KC_AB1	MED10	0.0715000688540666	0.87097908529009	1	pseudobulk	DESeq2	LRT	0.0599922735252414

152	KC_AB1	MED11	0.0938712152207428	0.86121843062973	1	pseudobulk	DESeq2	LRT	0.0648866845671518
152	KC_AB1	MED14	-0.0457464301508746	0.942300430626862	1	pseudobulk	DESeq2	LRT	0.0258106104112534
152	KC_AB1	MED15	0.0359834612282883	0.937394897664863	1	pseudobulk	DESeq2	LRT	0.0280774147181188
152	KC_AB1	MED16	1.01398814557611	0.496834831641456	1	pseudobulk	DESeq2	LRT	0.303787964644266
152	KC_AB1	MED17	0.820367596544291	0.471387458332823	1	pseudobulk	DESeq2	LRT	0.326621976434746
152	KC_AB1	MED18	0.0490946148257469	0.929211130842886	1	pseudobulk	DESeq2	LRT	0.0318855965098877
152	KC_AB1	MED19	0.174684242550665	0.632310038341169	1	pseudobulk	DESeq2	LRT	0.199069923419153
152	KC_AB1	MED20	0.132987505168801	0.860991827635182	1	pseudobulk	DESeq2	LRT	0.0650009707644505
152	KC_AB1	MED21	0.163491595112954	0.591110936928986	1	pseudobulk	DESeq2	LRT	0.22833100511742
152	KC_AB1	MED22	0.0377415790534379	0.893255136582467	1	pseudobulk	DESeq2	LRT	0.0490244777474009
152	KC_AB1	MED24	-0.166466683090329	0.792072316213956	1	pseudobulk	DESeq2	LRT	0.101235165507829
152	KC_AB1	MED25	0.422049183120056	0.656284899912176	1	pseudobulk	DESeq2	LRT	0.18290758806406
152	KC_AB1	MED26	-0.261757778335085	0.191386138166481	1	pseudobulk	DESeq2	LRT	0.718089520769288
152	KC_AB1	MED27	0.431623924319566	0.634887430977364	1	pseudobulk	DESeq2	LRT	0.197303270675225
152	KC_AB1	MED28	-0.741336082556777	0.253450911467866	1	pseudobulk	DESeq2	LRT	0.596106142614765
152	KC_AB1	MED30	0.27059631723065	0.749589450801287	1	pseudobulk	DESeq2	LRT	0.125176534034669
152	KC_AB1	MED31	0.0527666410134192	0.872924147787886	1	pseudobulk	DESeq2	LRT	0.0590234924093971
152	KC_AB1	MED4	-0.155463804107216	0.841137713899237	1	pseudobulk	DESeq2	LRT	0.0751328942255863
152	KC_AB1	MED6	0.539940422807805	0.278499375046326	1	pseudobulk	DESeq2	LRT	0.555175775047654
152	KC_AB1	MED7	-0.488824417907989	0.36449002730481	1	pseudobulk	DESeq2	LRT	0.438314349776023
152	KC_AB1	MED8	0.115620489853525	0.836617977965008	1	pseudobulk	DESeq2	LRT	0.0774728071626219
152	KC_AB1	Mef2	-0.171688920906426	0.27334406647703	1	pseudobulk	DESeq2	LRT	0.563290348969251
152	KC_AB1	mEFG1	0.124712724271237	0.883219034730538	1	pseudobulk	DESeq2	LRT	0.0539315797990288
152	KC_AB1	mEFTs	0.11041509602707	0.797019680320396	1	pseudobulk	DESeq2	LRT	0.0985309547029773
152	KC_AB1	mEFTu1	0.0214907664079375	0.952308166588876	1	pseudobulk	DESeq2	LRT	0.0212224913295674
152	KC_AB1	mEFTu2	-0.40702799101185	0.664702785358521	1	pseudobulk	DESeq2	LRT	0.17737250136231
152	KC_AB1	Megf8	-0.12033682654416	0.860963700315267	1	pseudobulk	DESeq2	LRT	0.0650151587497181
152	KC_AB1	mei-41	1.94274349948711	0.44537359025864	1	pseudobulk	DESeq2	LRT	0.351275539311402
152	KC_AB1	mei-9	0.376387243849355	0.796238618228104	1	pseudobulk	DESeq2	LRT	0.0989567626014066
152	KC_AB1	mei-P26	-0.161041676744415	0.116192834681996	1	pseudobulk	DESeq2	LRT	0.934820652961339
152	KC_AB1	mei-S332	0.628285493771289	0.713716250945462	1	pseudobulk	DESeq2	LRT	0.146474414472455
152	KC_AB1	mei-W68	-1.09953295457447	0.640554772384257	1	pseudobulk	DESeq2	LRT	0.193443728796401
152	KC_AB1	Meics	0.0525697984703889	0.953067493775602	1	pseudobulk	DESeq2	LRT	0.0208763426603346
152	KC_AB1	meigo	-0.316302381422856	0.769864133698341	1	pseudobulk	DESeq2	LRT	0.113585912738551
152	KC_AB1	Mekk1	0.108501725786403	0.883095471268784	1	pseudobulk	DESeq2	LRT	0.0539923423976596
152	KC_AB1	melt	-0.760959112540794	0.804334723897681	1	pseudobulk	DESeq2	LRT	0.0945631819854715
152	KC_AB1	Meltrin	4.13750281880488	0.0493237339573154	1	pseudobulk	DESeq2	LRT	1.30694405341299
152	KC_AB1	Membrin	-0.175814398539637	0.831371601112916	1	pseudobulk	DESeq2	LRT	0.0802048146753662
152	KC_AB1	Men	0.301601869189734	0.583192492737595	1	pseudobulk	DESeq2	LRT	0.234188075196978
152	KC_AB1	Men-b	-0.0130434770445652	0.944093567953952	1	pseudobulk	DESeq2	LRT	0.0249849611761647
152	KC_AB1	meng	0.27246363080805	0.527281601120289	1	pseudobulk	DESeq2	LRT	0.277957382606219
152	KC_AB1	MEP-1	-0.0557453526203042	0.932403639356055	1	pseudobulk	DESeq2	LRT	0.0303960400107834
152	KC_AB1	Mer	-0.456930717907721	0.587541447319598	1	pseudobulk	DESeq2	LRT	0.230961491256616
152	KC_AB1	Mes2	-0.190333227380668	0.901504509699613	1	pseudobulk	DESeq2	LRT	0.0450320964149478
152	KC_AB1	Mes4	-0.081160194708473	0.863039931678832	1	pseudobulk	DESeq2	LRT	0.0639691096074254
152	KC_AB1	Mesh1	-1.22498523733724	0.615248217301746	1	pseudobulk	DESeq2	LRT	0.210949636003277
152	KC_AB1	MESK2	-0.107576756961068	0.552964888422217	1	pseudobulk	DESeq2	LRT	0.257302444190052
152	KC_AB1	MESK4	-0.760959112540794	1	1	pseudobulk	DESeq2	LRT	0
152	KC_AB1	meso18E	-1.61102155603974	0.57025980967112	1	pseudobulk	DESeq2	LRT	0.243927235206257
152	KC_AB1	MESR3	-0.362828753746935	0.514128641982691	1	pseudobulk	DESeq2	LRT	0.288928201018127
152	KC_AB1	MESR4	-0.237145111493212	0.53541906613332	1	pseudobulk	DESeq2	LRT	0.271306167760479
152	KC_AB1	MESR6	0.0415029760602855	0.919327722855322	1	pseudobulk	DESeq2	LRT	0.0365296432863682
152	KC_AB1	Met	0.181347826117032	0.60006574794085	1	pseudobulk	DESeq2	LRT	0.221801162277104
152	KC_AB1	metl	0.7495032271742	0.268612432146865	1	pseudobulk	DESeq2	LRT	0.570873890819075
152	KC_AB1	metro	-0.0349110014736526	0.866791962139204	1	pseudobulk	DESeq2	LRT	0.0620851245958458
152	KC_AB1	MetRS	0.0495060138781488	0.935709921226834	1	pseudobulk	DESeq2	LRT	0.0288587657233379

152	KC_AB1	MetRS-m	0.263460312358242	0.805008029768889	1	pseudobulk	DESeq2	LRT	0.0941997876235263
152	KC_AB1	Mettl14	0.526328443292733	0.604002669942715	1	pseudobulk	DESeq2	LRT	0.218961141612601
152	KC_AB1	Mettl3	-0.394776863147981	0.701157661149318	1	pseudobulk	DESeq2	LRT	0.15418431631522
152	KC_AB1	mew	-0.154590452495372	0.93172201917594	1	pseudobulk	DESeq2	LRT	0.0307136408064335
152	KC_AB1	mex1	1.25804452752548	0.727552935095488	1	pseudobulk	DESeq2	LRT	0.138135402914536
152	KC_AB1	Mf	2.13148430488057	0.45484681020722	1	pseudobulk	DESeq2	LRT	0.342134846603996
152	KC_AB1	Mfap1	0.845310371469311	0.159203715761796	1	pseudobulk	DESeq2	LRT	0.79804680019124
152	KC_AB1	mfas	0.922063534396428	0.255899412040967	1	pseudobulk	DESeq2	LRT	0.591930711954382
152	KC_AB1	Mfe2	0.475656500719694	0.707439950757872	1	pseudobulk	DESeq2	LRT	0.150310418222463
152	KC_AB1	mfrn	-0.0670551668857513	0.893405986933821	1	pseudobulk	DESeq2	LRT	0.048951141540014
152	KC_AB1	MFS10	0.221472533011977	0.808909214275034	1	pseudobulk	DESeq2	LRT	0.092100217512373
152	KC_AB1	MFS14	5.49294694223383	0.1798461709335	1	pseudobulk	DESeq2	LRT	0.745098804218304
152	KC_AB1	MFS15	-0.390523636515956	0.721304678127307	1	pseudobulk	DESeq2	LRT	0.141881251120226
153	KC_AB1	MFS16	-0.0632972089846754	0.942919164715056	1	pseudobulk	DESeq2	LRT	0.0255255371915813
153	KC_AB1	MFS17	0.144397111830592	0.475554565101905	1	pseudobulk	DESeq2	LRT	0.322799644945084
153	KC_AB1	MFS18	0.606470967654982	0.332391102415464	1	pseudobulk	DESeq2	LRT	0.4783506101093
153	KC_AB1	MFS9	-0.720639610746342	0.498881173178061	1	pseudobulk	DESeq2	LRT	0.302002885195231
153	KC_AB1	Mgat1	-0.0268071326567207	0.958756096721885	1	pseudobulk	DESeq2	LRT	0.0182918613592559
153	KC_AB1	Mgat2	-0.146029759077194	0.713678610202168	1	pseudobulk	DESeq2	LRT	0.14649731937082
153	KC_AB1	mge	-0.0366593388048216	0.871233503389083	1	pseudobulk	DESeq2	LRT	0.0598654320846588
153	KC_AB1	mgl	0.311140790899851	0.598325874858934	1	pseudobulk	DESeq2	LRT	0.223062215500807
153	KC_AB1	mGluR	-0.177026128298287	0.732014532600504	1	pseudobulk	DESeq2	LRT	0.135480296857515
153	KC_AB1	mgr	-0.0368494211180471	0.908892408841624	1	pseudobulk	DESeq2	LRT	0.0414875238318402
153	KC_AB1	Mgstl	0.492281881665627	0.573678437691467	1	pseudobulk	DESeq2	LRT	0.241331473226494
153	KC_AB1	mh	0.152115475746305	1	1	pseudobulk	DESeq2	LRT	0
153	KC_AB1	Mhcl	-0.332250575418558	0.440551998045712	1	pseudobulk	DESeq2	LRT	0.356002824751198
153	KC_AB1	mi	-0.414898945089431	0.626449556824353	1	pseudobulk	DESeq2	LRT	0.203113893663955
153	KC_AB1	Mi-2	-0.37397890766562	0.182795327754391	1	pseudobulk	DESeq2	LRT	0.738034909020229
153	KC_AB1	mib1	-0.603895193488039	0.452721392503223	1	pseudobulk	DESeq2	LRT	0.344168983245869
153	KC_AB1	mib2	-0.0303093487794003	0.928282793261038	1	pseudobulk	DESeq2	LRT	0.0323196995962839
153	KC_AB1	Mical	-0.139242840603435	0.78022136717046	1	pseudobulk	DESeq2	LRT	0.107782160257043
153	KC_AB1	MICAL-like	0.0461334072696451	0.920337723809268	1	pseudobulk	DESeq2	LRT	0.0360527762713553
153	KC_AB1	Mics1	2.09987857572813	0.503115176013642	1	pseudobulk	DESeq2	LRT	0.29833258237694
153	KC_AB1	MICU1	-0.0742279352208894	0.877789885737592	1	pseudobulk	DESeq2	LRT	0.0566094275849833
153	KC_AB1	MICU3	-0.178848749713321	0.624683899878409	1	pseudobulk	DESeq2	LRT	0.204339687081143
153	KC_AB1	Mid1	-0.159228468229463	0.437116219056064	1	pseudobulk	DESeq2	LRT	0.359403078854962
153	KC_AB1	mIF2	0.406920632389411	0.841356682947494	1	pseudobulk	DESeq2	LRT	0.0750198512935201
153	KC_AB1	mIF3	0.76151937013326	0.40905636899779	1	pseudobulk	DESeq2	LRT	0.388216840995448
153	KC_AB1	Miga	0.0342662137540202	0.948391396590153	1	pseudobulk	DESeq2	LRT	0.0230123944137668
153	KC_AB1	milt	-0.296199124489789	0.321399126262732	1	pseudobulk	DESeq2	LRT	0.492955308219189
153	KC_AB1	mim	0.0165128078282213	0.925600805601828	1	pseudobulk	DESeq2	LRT	0.0335762760655159
153	KC_AB1	Mink	-0.760959112540794	1	1	pseudobulk	DESeq2	LRT	0
153	KC_AB1	mino	-1.61102155603974	0.57025980967112	1	pseudobulk	DESeq2	LRT	0.243927235206257
153	KC_AB1	mio	1.10467020738974	0.398581715915521	1	pseudobulk	DESeq2	LRT	0.399482627509902
153	KC_AB1	mip120	-0.224501952054482	0.782404513851239	1	pseudobulk	DESeq2	LRT	0.106568652679772
153	KC_AB1	mip130	0.415324305424115	0.691573786003787	1	pseudobulk	DESeq2	LRT	0.160161476950554
153	KC_AB1	mip40	-0.414605701464988	0.541035038740848	1	pseudobulk	DESeq2	LRT	0.266774608019043
153	KC_AB1	miple1	0.166070833932848	0.251631631645795	1	pseudobulk	DESeq2	LRT	0.599234766304094
153	KC_AB1	miple2	-0.739932195741711	0.468881890109578	1	pseudobulk	DESeq2	LRT	0.328936540938617
153	KC_AB1	Mipp1	2.62091928095693	0.110463655692342	1	pseudobulk	DESeq2	LRT	0.956780588301001
153	KC_AB1	Mipp2	1.11388934932283	1	1	pseudobulk	DESeq2	LRT	0
153	KC_AB1	mira	-1.25079861973268	0.596654950892141	1	pseudobulk	DESeq2	LRT	0.224276751359069
153	KC_AB1	Miro	0.293665486696142	0.562220857671703	1	pseudobulk	DESeq2	LRT	0.250093046662237
153	KC_AB1	mirr	2.88281041865688	0.0868296899654731	1	pseudobulk	DESeq2	LRT	1.06133174966861
153	KC_AB1	Mis12	0.471212549053896	0.611888554721415	1	pseudobulk	DESeq2	LRT	0.213327670136936
153	KC_AB1	Mitf	-0.837944764360508	0.312232641267384	1	pseudobulk	DESeq2	LRT	0.505521697105455

153	KC_AB1	Mitofilin	-0.197807635701922	0.683606557693729	1	pseudobulk	DESeq2	LRT	0.165193779835823
153	KC_AB1	mkg-p	0.152115475746305	1	1	pseudobulk	DESeq2	LRT	0
153	KC_AB1	Mkk4	-0.183689597428936	0.563229519230606	1	pseudobulk	DESeq2	LRT	0.249314591609192
153	KC_AB1	MKP-4	0.243440897778981	0.671937803120341	1	pseudobulk	DESeq2	LRT	0.172670924880311
153	KC_AB1	Mkp3	-0.0810426129508226	0.694705211581666	1	pseudobulk	DESeq2	LRT	0.158199443096755
153	KC_AB1	Mkrn1	0.110236828282454	0.79013100469838	1	pseudobulk	DESeq2	LRT	0.102300896176964
153	KC_AB1	Mlc-c	0.277701253590902	0.499209133667442	1	pseudobulk	DESeq2	LRT	0.301717477281799
153	KC_AB1	mld	-0.0132139616694367	0.94080115877235	1	pseudobulk	DESeq2	LRT	0.0265021563528353
153	KC_AB1	mldr	0.312982910567526	0.86186729206288	1	pseudobulk	DESeq2	LRT	0.0645596004882281
153	KC_AB1	mle	-0.234957849386671	0.550510753329114	1	pseudobulk	DESeq2	LRT	0.259234193375455
153	KC_AB1	Mlf	-0.0330053844924873	0.857786622993977	1	pseudobulk	DESeq2	LRT	0.0666207307772898
153	KC_AB1	Mlh1	1.14797610714282	0.77173698641838	1	pseudobulk	DESeq2	LRT	0.112530685168625
153	KC_AB1	Mlp84B	-0.436385585571934	0.740721173534613	1	pseudobulk	DESeq2	LRT	0.130345240851556
153	KC_AB1	mlt	0.202480478213074	0.544343668332512	1	pseudobulk	DESeq2	LRT	0.264126824317646
153	KC_AB1	mmd	-0.248424138459	0.0616002905742902	1	pseudobulk	DESeq2	LRT	1.21041723922335
153	KC_AB1	MME1	1.4584122026083	0.192132826117021	1	pseudobulk	DESeq2	LRT	0.716398429081195
153	KC_AB1	Mmp1	4.38130104917072	0.0487356287502902	1	pseudobulk	DESeq2	LRT	1.31215342662365
153	KC_AB1	Mmp2	-0.32632383435446	0.326671861217463	1	pseudobulk	DESeq2	LRT	0.485888273067545
153	KC_AB1	Mms19	0.490953653815857	0.687765301950131	1	pseudobulk	DESeq2	LRT	0.162559738301309
153	KC_AB1	mmy	-0.299056364170671	0.536695520681782	1	pseudobulk	DESeq2	LRT	0.270272029366034
153	KC_AB1	mnb	0.0769526389025902	0.727551207211094	1	pseudobulk	DESeq2	LRT	0.138136434333025
153	KC_AB1	mnd	0.25967048557255	0.767802458848299	1	pseudobulk	DESeq2	LRT	0.114750501410411
153	KC_AB1	Mnn1	0.0234284641848394	0.888937899519457	1	pseudobulk	DESeq2	LRT	0.0511285774302914
153	KC_AB1	Mnt	-0.169476270921127	0.412890296259452	1	pseudobulk	DESeq2	LRT	0.384165323786706
153	KC_AB1	Mo25	-0.0225560036463346	0.915317604952775	1	pseudobulk	DESeq2	LRT	0.0384281844637624
153	KC_AB1	Mob2	-0.253203974563401	0.277787998724785	1	pseudobulk	DESeq2	LRT	0.556286521018091
153	KC_AB1	Mob3	-0.0461625820186759	0.895024486432465	1	pseudobulk	DESeq2	LRT	0.0481650829220425
153	KC_AB1	Mob4	-0.0013794373957915€	0.926010065016129	1	pseudobulk	DESeq2	LRT	0.0333842928459175
153	KC_AB1	Moca-cyp	0.132325982488455	0.723141733258458	1	pseudobulk	DESeq2	LRT	0.140776574158478
153	KC_AB1	Mocs1	-1.43550613534661	0.602448798560473	1	pseudobulk	DESeq2	LRT	0.220079857379737
153	KC_AB1	mod	0.0340143333977898	0.908102461199264	1	pseudobulk	DESeq2	LRT	0.0418651472685826
153	KC_AB1	modSP	0.612467920120613	0.808548798728348	1	pseudobulk	DESeq2	LRT	0.0922937637861558
153	KC_AB1	Moe	0.277796576828408	0.366481015675494	1	pseudobulk	DESeq2	LRT	0.435948517614719
153	KC_AB1	mof	-0.552955072952247	0.704901421229566	1	pseudobulk	DESeq2	LRT	0.151871613802198
153	KC_AB1	mol	-0.639069712887817	0.788164848031384	1	pseudobulk	DESeq2	LRT	0.103382938471215
153	KC_AB1	Mon1	-0.358255341551491	0.7496923135401	1	pseudobulk	DESeq2	LRT	0.125116941873953
153	KC_AB1	mon2	-0.262949940661552	0.704057693722636	1	pseudobulk	DESeq2	LRT	0.15239175131421
153	KC_AB1	Mondo	0.263596160511002	0.671534257677973	1	pseudobulk	DESeq2	LRT	0.172931827312454
153	KC_AB1	moody	-0.251163832587679	0.881533195336776	1	pseudobulk	DESeq2	LRT	0.0547613291047094
153	KC_AB1	mop	-0.398363986129319	0.465523527004341	1	pseudobulk	DESeq2	LRT	0.332058365402374
153	KC_AB1	mor	0.132412774205034	0.792947110217907	1	pseudobulk	DESeq2	LRT	0.10075577927324
153	KC_AB1	morgue	0.0561902464372397	0.896917671049748	1	pseudobulk	DESeq2	LRT	0.0472474194416813
153	KC_AB1	Mp	-0.498358747654957	0.149516848641757	1	pseudobulk	DESeq2	LRT	0.825309865133227
153	KC_AB1	MP1	-0.05063995050214	0.956948932013945	1	pseudobulk	DESeq2	LRT	0.0191112379141895
153	KC_AB1	Mpc1	0.102461010288198	0.819803551315614	1	pseudobulk	DESeq2	LRT	0.0862902046906149
153	KC_AB1	Mpcp2	0.0100955479708067	0.94948650959048	1	pseudobulk	DESeq2	LRT	0.0225112013872421
153	KC_AB1	Mpi	0.190693159943453	0.761536147408468	1	pseudobulk	DESeq2	LRT	0.118309477426497
153	KC_AB1	Mpp6	-0.123148050819457	0.865471769079572	1	pseudobulk	DESeq2	LRT	0.0627470938578222
153	KC_AB1	Mppe	-0.0312317909188824	0.971884212541915	1	pseudobulk	DESeq2	LRT	0.012385472573166
153	KC_AB1	Mps1	2.73062989528267	0.270641314998426	1	pseudobulk	DESeq2	LRT	0.567605905054642
153	KC_AB1	mr	0.0886641893409992	0.898111589092084	1	pseudobulk	DESeq2	LRT	0.0466696995062951
153	KC_AB1	mre11	-0.242262269731956	0.880939455895153	1	pseudobulk	DESeq2	LRT	0.0550539382101721
153	KC_AB1	mRF1	-0.239090895972507	0.862268445246935	1	pseudobulk	DESeq2	LRT	0.064357506634935
153	KC_AB1	MRG15	0.432242844563181	0.426843509738801	1	pseudobulk	DESeq2	LRT	0.369731317747179
153	KC_AB1	MrgBP	-0.393458375090875	0.3182261643406	1	pseudobulk	DESeq2	LRT	0.497264115822051
153	KC_AB1	mri	0.277452326494003	0.519730089617571	1	pseudobulk	DESeq2	LRT	0.284222139100578

153	KC_AB1	mrj	0.306035075137882	0.241009385522146	1	pseudobulk	DESeq2	LRT	0.617966044557453
154	KC_AB1	mrn	-0.0311006742651814	0.948392550500158	1	pseudobulk	DESeq2	LRT	0.023011866006988
154	KC_AB1	mRNA-cap	0.334772624729276	0.558648963628591	1	pseudobulk	DESeq2	LRT	0.252861002573792
154	KC_AB1	MRP	0.162161771291951	0.81918232278066	1	pseudobulk	DESeq2	LRT	0.0866194279522861
154	KC_AB1	Mrp4	1.90071181822722	0.545896518579078	1	pseudobulk	DESeq2	LRT	0.262889675371117
154	KC_AB1	mRpL1	-0.286063813088111	0.598120899443178	1	pseudobulk	DESeq2	LRT	0.223211022275621
154	KC_AB1	mRpL10	-0.128213585720026	0.818452313417718	1	pseudobulk	DESeq2	LRT	0.0870066193954263
154	KC_AB1	mRpL11	0.0840793978640012	0.901800698753107	1	pseudobulk	DESeq2	LRT	0.0448894325196887
154	KC_AB1	mRpL12	0.0255593635810662	0.93147779816687	1	pseudobulk	DESeq2	LRT	0.0308274920812395
154	KC_AB1	mRpL13	0.00918321884639937	0.938702576416716	1	pseudobulk	DESeq2	LRT	0.0274719901430636
154	KC_AB1	mRpL14	0.100204591229129	0.857697107635795	1	pseudobulk	DESeq2	LRT	0.066666054466982
154	KC_AB1	mRpL15	0.373188668614449	0.525227766859996	1	pseudobulk	DESeq2	LRT	0.279652322423928
154	KC_AB1	mRpL16	0.0966357384971186	0.877784454303917	1	pseudobulk	DESeq2	LRT	0.0566121148442125
154	KC_AB1	mRpL17	0.149702731885776	0.736518703491983	1	pseudobulk	DESeq2	LRT	0.1328162200091
154	KC_AB1	mRpL18	0.13683906631647	0.784153284272522	1	pseudobulk	DESeq2	LRT	0.105599034245691
154	KC_AB1	mRpL19	0.432182021225434	0.481661001750921	1	pseudobulk	DESeq2	LRT	0.317258515425261
154	KC_AB1	mRpL2	0.199495229105535	0.717885838357695	1	pseudobulk	DESeq2	LRT	0.143944613854212
154	KC_AB1	mRpL20	-0.0343824303537152	0.945901121949274	1	pseudobulk	DESeq2	LRT	0.0241542594081176
154	KC_AB1	mRpL21	-0.0857971288020413	0.858275495677133	1	pseudobulk	DESeq2	LRT	0.0663732866876135
154	KC_AB1	mRpL22	0.170768986633564	0.681342708151608	1	pseudobulk	DESeq2	LRT	0.16663438761535
154	KC_AB1	mRpL23	-0.21633851130585	0.575601637857725	1	pseudobulk	DESeq2	LRT	0.239877978961353
154	KC_AB1	mRpL24	0.408451989496935	0.31078246846086	1	pseudobulk	DESeq2	LRT	0.507543488151226
154	KC_AB1	mRpL27	-0.248740183238035	0.525315312213837	1	pseudobulk	DESeq2	LRT	0.279579939929701
154	KC_AB1	mRpL28	-0.00697945509305284	0.94678026027718	1	pseudobulk	DESeq2	LRT	0.0237508053923671
154	KC_AB1	mRpL3	0.398085058636405	0.694880643500588	1	pseudobulk	DESeq2	LRT	0.158089785799275
154	KC_AB1	mRpL30	0.291547239309478	0.495888946397879	1	pseudobulk	DESeq2	LRT	0.304615572233006
154	KC_AB1	mRpL32	0.0638545837149028	0.90434785252733	1	pseudobulk	DESeq2	LRT	0.0436644883573849
154	KC_AB1	mRpL33	0.361040819506966	0.356791288534717	1	pseudobulk	DESeq2	LRT	0.4475857579604
154	KC_AB1	mRpL34	-0.00418739965296259	0.952875202013615	1	pseudobulk	DESeq2	LRT	0.0209639751496157
154	KC_AB1	mRpL35	0.423352384392819	0.381974712478264	1	pseudobulk	DESeq2	LRT	0.417965387336108
154	KC_AB1	mRpL36	-0.277530950012715	0.516097651492894	1	pseudobulk	DESeq2	LRT	0.287268117186582
154	KC_AB1	mRpL37	0.688541705483551	0.338668168914263	1	pseudobulk	DESeq2	LRT	0.470225620345401
154	KC_AB1	mRpL38	0.120748661469418	0.837297284316831	1	pseudobulk	DESeq2	LRT	0.0771203173765953
154	KC_AB1	mRpL39	-0.296087727576868	0.672986263271538	1	pseudobulk	DESeq2	LRT	0.171993800332124
154	KC_AB1	mRpL4	-0.0288761873539846	0.939337245075524	1	pseudobulk	DESeq2	LRT	0.0271784573822608
154	KC_AB1	mRpL40	0.127630692439609	0.81199468261632	1	pseudobulk	DESeq2	LRT	0.09044681474645
154	KC_AB1	mRpL41	-0.329083986496006	0.521335277401763	1	pseudobulk	DESeq2	LRT	0.282882886501043
154	KC_AB1	mRpL42	-0.022175718524567	0.95825595962841	1	pseudobulk	DESeq2	LRT	0.0185184710815056
154	KC_AB1	mRpL43	0.674000648667841	0.343357210447805	1	pseudobulk	DESeq2	LRT	0.464253828057708
154	KC_AB1	mRpL44	-0.249857795118476	0.709996893423442	1	pseudobulk	DESeq2	LRT	0.14874355152319
154	KC_AB1	mRpL45	0.814188348049271	0.496879601296091	1	pseudobulk	DESeq2	LRT	0.303748832247003
154	KC_AB1	mRpL46	-0.418446726180586	0.643498867557799	1	pseudobulk	DESeq2	LRT	0.191452213039054
154	KC_AB1	mRpL47	0.22561599304611	0.598719926861417	1	pseudobulk	DESeq2	LRT	0.222776287232738
154	KC_AB1	mRpL48	-0.324770329219842	0.381572358414444	1	pseudobulk	DESeq2	LRT	0.41842309367151
154	KC_AB1	mRpL49	0.114149431528026	0.805242681715324	1	pseudobulk	DESeq2	LRT	0.094073213487587
154	KC_AB1	mRpL50	0.0748604324994792	0.86992309091415	1	pseudobulk	DESeq2	LRT	0.0605191412526843
154	KC_AB1	mRpL51	-0.411600663483303	0.573445596214215	1	pseudobulk	DESeq2	LRT	0.24150777808478
154	KC_AB1	mRpL52	0.00647546875753208	0.98048978107545	1	pseudobulk	DESeq2	LRT	0.00855692830488658
154	KC_AB1	mRpL54	-0.204040160033717	0.648421559093971	1	pseudobulk	DESeq2	LRT	0.188142553910056
154	KC_AB1	mRpL55	0.0876213501909481	0.772572342742133	1	pseudobulk	DESeq2	LRT	0.112060843187667
154	KC_AB1	mRpL9	-0.549557093671115	0.338994434814075	1	pseudobulk	DESeq2	LRT	0.469807431441098
154	KC_AB1	mRpS10	-0.274103296394556	0.530591981349848	1	pseudobulk	DESeq2	LRT	0.275239317643979
154	KC_AB1	mRpS11	0.374144963615788	0.442018362940399	1	pseudobulk	DESeq2	LRT	0.354559688211922
154	KC_AB1	mRpS14	0.22034841353307	0.655188682846488	1	pseudobulk	DESeq2	LRT	0.183633612804063
154	KC_AB1	mRpS16	0.261365222814921	0.624010952780505	1	pseudobulk	DESeq2	LRT	0.204807787416312
154	KC_AB1	mRpS17	-0.183153900573013	0.738066804127655	1	pseudobulk	DESeq2	LRT	0.131904327403289

154	KC_AB1	mRpS18A	-0.290552496555912	0.533018990033624	1	pseudobulk	DESeq2	LRT	0.273257317952981
154	KC_AB1	mRpS18B	-0.212392767360191	0.773697310785549	1	pseudobulk	DESeq2	LRT	0.111428912659043
154	KC_AB1	mRpS18C	-0.096582266106502	0.806965524135047	1	pseudobulk	DESeq2	LRT	0.093145019178476
154	KC_AB1	mRpS2	0.00698506994998201	0.93373248184846	1	pseudobulk	DESeq2	LRT	0.0297775330832814
154	KC_AB1	mRpS21	0.0460340171471835	0.87346659543423	1	pseudobulk	DESeq2	LRT	0.0587536993770883
154	KC_AB1	mRpS22	-0.346960470201054	0.611081811720634	1	pseudobulk	DESeq2	LRT	0.213900642457423
154	KC_AB1	mRpS23	-0.308972520311825	0.638012763296041	1	pseudobulk	DESeq2	LRT	0.195170633232446
154	KC_AB1	mRpS24	0.113994689298682	0.787468032550269	1	pseudobulk	DESeq2	LRT	0.103767067467013
154	KC_AB1	mRpS25	0.125894825037604	0.7979372946115	1	pseudobulk	DESeq2	LRT	0.0980312360605631
154	KC_AB1	mRpS26	0.182454696120534	0.717694208846573	1	pseudobulk	DESeq2	LRT	0.144060558123438
154	KC_AB1	mRpS28	0.103206815161346	0.827316198168028	1	pseudobulk	DESeq2	LRT	0.082328472462032
154	KC_AB1	mRpS29	0.671661650008804	0.330875436909312	1	pseudobulk	DESeq2	LRT	0.480335472219713
154	KC_AB1	mRpS30	-0.258570417556497	0.719048584617518	1	pseudobulk	DESeq2	LRT	0.143241764250727
154	KC_AB1	mRpS31	-0.43308011891209	0.468338353224511	1	pseudobulk	DESeq2	LRT	0.329440275462149
154	KC_AB1	mRpS33	-0.16657753884498	0.680014288206473	1	pseudobulk	DESeq2	LRT	0.1674819619643
154	KC_AB1	mRpS34	0.0604125716695613	0.907299699719079	1	pseudobulk	DESeq2	LRT	0.0422492328572835
154	KC_AB1	mRpS35	0.176504542953269	0.775265607892887	1	pseudobulk	DESeq2	LRT	0.110549481648493
154	KC_AB1	mRpS5	0.125042839731733	0.689341881721238	1	pseudobulk	DESeq2	LRT	0.161565334667182
154	KC_AB1	mRpS6	0.072516385156322	0.886152048189559	1	pseudobulk	DESeq2	LRT	0.0524917543835226
154	KC_AB1	mRpS7	-0.00286502737338418	0.958492471701708	1	pseudobulk	DESeq2	LRT	0.0184112938565978
154	KC_AB1	mRpS9	0.064931321596605	0.87040951545265	1	pseudobulk	DESeq2	LRT	0.0602763698448959
154	KC_AB1	mRRF1	0.708089556598832	0.453114512979596	1	pseudobulk	DESeq2	LRT	0.343792027412484
154	KC_AB1	mRRF2	-0.0944883797952445	0.952161656027398	1	pseudobulk	DESeq2	LRT	0.0212893117409602
154	KC_AB1	mrt	-0.0043074628455446	0.927035768846932	1	pseudobulk	DESeq2	LRT	0.0329035086676326
154	KC_AB1	Mrtf	0.233381857073555	0.552093469366752	1	pseudobulk	DESeq2	LRT	0.257987390035658
154	KC_AB1	mrva	0.0657522415705225	0.844747669312955	1	pseudobulk	DESeq2	LRT	0.0732729977843996
154	KC_AB1	Ms	-2.33094705302179	0.202889506755561	1	pseudobulk	DESeq2	LRT	0.69274041366651
154	KC_AB1	ms(3)76Ba	1.90071181822722	0.545896518579078	1	pseudobulk	DESeq2	LRT	0.262889675371117
154	KC_AB1	MSBP	0.029801699067299	0.925948111668753	1	pseudobulk	DESeq2	LRT	0.0334133496542725
154	KC_AB1	msd5	-0.831375169831878	0.360908231219335	1	pseudobulk	DESeq2	LRT	0.442603212890974
154	KC_AB1	Msh6	1.16708691745207	0.43100430943667	1	pseudobulk	DESeq2	LRT	0.365518387483794
154	KC_AB1	msi	0.584372341809321	0.439589693258035	1	pseudobulk	DESeq2	LRT	0.356952498718147
154	KC_AB1	msk	0.0445881956890519	0.912500834190084	1	pseudobulk	DESeq2	LRT	0.0397667298479426
154	KC_AB1	msl-1	0.0640293072236477	0.903445391054661	1	pseudobulk	DESeq2	LRT	0.0440980933303642
154	KC_AB1	msl-2	-0.568666222950716	0.51997462103852	1	pseudobulk	DESeq2	LRT	0.284017852926552
154	KC_AB1	msl-3	-0.653187908793239	0.478165009930878	1	pseudobulk	DESeq2	LRT	0.32042220686917
154	KC_AB1	msn	-0.270475859616751	0.408471945019702	1	pseudobulk	DESeq2	LRT	0.388837766650643
154	KC_AB1	Msp300	0.182165961173143	0.581352277364339	1	pseudobulk	DESeq2	LRT	0.235560621908524
154	KC_AB1	mspo	-0.315414657675784	0.0652796515941764	1	pseudobulk	DESeq2	LRT	1.18522217213303
154	KC_AB1	msps	-0.217083640706253	0.4881003501806	1	pseudobulk	DESeq2	LRT	0.311490880765016
154	KC_AB1	Msr-110	0.164857028740013	0.747775696516156	1	pseudobulk	DESeq2	LRT	0.126228653972445
154	KC_AB1	MsR1	-0.760959112540794	1	1	pseudobulk	DESeq2	LRT	0
154	KC_AB1	mst	1.25804452752548	0.727552935095488	1	pseudobulk	DESeq2	LRT	0.138135402914536
154	KC_AB1	Mst36Fa	1.25804452752548	0.727552935095488	1	pseudobulk	DESeq2	LRT	0.138135402914536
154	KC_AB1	Mst57Db	-0.760959112540794	0.805265236978025	1	pseudobulk	DESeq2	LRT	0.0940610488455931
155	KC_AB1	Mst77F	0.202638422330103	0.837190187882802	1	pseudobulk	DESeq2	LRT	0.0771758703636032
155	KC_AB1	Mst85C	-0.355124178355872	0.590984378285546	1	pseudobulk	DESeq2	LRT	0.228423998837953
155	KC_AB1	mt:ATPase6	-0.171344833169922	0.375294092244576	1	pseudobulk	DESeq2	LRT	0.425628272053145
155	KC_AB1	mt:ATPase8	0.710515854616532	0.68624427388324	1	pseudobulk	DESeq2	LRT	0.163521266339719
155	KC_AB1	mt:CoI	-0.180314618616567	0.347268200795226	1	pseudobulk	DESeq2	LRT	0.459334983033727
155	KC_AB1	mt:CoII	-0.144233250586467	0.424265576983819	1	pseudobulk	DESeq2	LRT	0.372362203520772
155	KC_AB1	mt:CoIII	-0.254169139842496	0.135215597926797	1	pseudobulk	DESeq2	LRT	0.868973207042268
155	KC_AB1	mt:Cyt-b	-0.426552915711195	0.0174921295499519	1	pseudobulk	DESeq2	LRT	1.75715731484988
155	KC_AB1	mt:lrRNA	-0.0546591681645978	0.721373786276392	1	pseudobulk	DESeq2	LRT	0.141839643390484
155	KC_AB1	mt:ND1	-0.678322273588592	0.00466584240447597	1	pseudobulk	DESeq2	LRT	2.331069934214
155	KC_AB1	mt:ND2	-0.518611723815019	0.175314409775589	1	pseudobulk	DESeq2	LRT	0.756182386080601

155	KC_AB1	mt:ND3	-0.757573507419463	0.0508850609450193	1	pseudobulk	DESeq2	LRT	1.29340970099192
155	KC_AB1	mt:ND4	-0.435993147783946	0.0240663235762676	1	pseudobulk	DESeq2	LRT	1.61859024842409
155	KC_AB1	mt:ND4L	-0.775101176624684	0.73176432283082	1	pseudobulk	DESeq2	LRT	0.135628768360498
155	KC_AB1	mt:ND5	-0.542494445841813	0.0278675243272912	1	pseudobulk	DESeq2	LRT	1.55490161107689
155	KC_AB1	mt:ND6	-0.499313564721562	0.157950814548433	1	pseudobulk	DESeq2	LRT	0.801478130107187
155	KC_AB1	mt:srRNA	-0.311184037435026	0.223764629893576	1	pseudobulk	DESeq2	LRT	0.650208560595996
155	KC_AB1	mt:tRNA:Ala-TGC	-0.760959112540794	0.805265236978025	1	pseudobulk	DESeq2	LRT	0.0940610488455931
155	KC_AB1	mt:tRNA:Arg-TCG	-1.61102155603974	0.57025980967112	1	pseudobulk	DESeq2	LRT	0.243927235206257
155	KC_AB1	mt:tRNA:Gly-TCC	1.25804452752548	0.727552935095488	1	pseudobulk	DESeq2	LRT	0.138135402914536
155	KC_AB1	mt:tRNA:Met-CAT	-0.760959112540794	0.804334723897681	1	pseudobulk	DESeq2	LRT	0.0945631819854715
155	KC_AB1	mt:tRNA:Ser-GCT	-1.57853843153518	0.534734952250692	1	pseudobulk	DESeq2	LRT	0.271861427881147
155	KC_AB1	mt:tRNA:Val-TAC	0.152115475746305	1	1	pseudobulk	DESeq2	LRT	0
155	KC_AB1	Mt2	0.118864039578646	0.891881989640698	1	pseudobulk	DESeq2	LRT	0.0496926059808118
155	KC_AB1	MTA1-like	-0.0489827671742584	0.925432122145829	1	pseudobulk	DESeq2	LRT	0.0336554300267808
155	KC_AB1	Mtap	-0.393069932879891	0.631341281414726	1	pseudobulk	DESeq2	LRT	0.199735812598084
155	KC_AB1	Mtch	0.0386891061240878	0.88991319275116	1	pseudobulk	DESeq2	LRT	0.0506523548695071
155	KC_AB1	mtd	-0.0786532762776804	0.570868921555351	1	pseudobulk	DESeq2	LRT	0.243463599600443
155	KC_AB1	mTerf3	-0.137560759611587	0.827661042972767	1	pseudobulk	DESeq2	LRT	0.0821474860481341
155	KC_AB1	mTerf5	0.625998637626791	0.522788390942982	1	pseudobulk	DESeq2	LRT	0.281674064942643
155	KC_AB1	MTF-1	0.697735061229971	0.341958612561429	1	pseudobulk	DESeq2	LRT	0.466026453662781
155	KC_AB1	mtg	2.73062989528267	0.270641314998426	1	pseudobulk	DESeq2	LRT	0.567605905054642
155	KC_AB1	mtgo	-0.187541745590079	0.398349637559483	1	pseudobulk	DESeq2	LRT	0.399735573643357
155	KC_AB1	mth	1.13734983340557	0.742793668201421	1	pseudobulk	DESeq2	LRT	0.1291318069744
155	KC_AB1	mthl1	2.23650969909167	0.226285640930319	1	pseudobulk	DESeq2	LRT	0.645343003546728
155	KC_AB1	mthl11	1.25804452752548	0.727552935095488	1	pseudobulk	DESeq2	LRT	0.138135402914536
155	KC_AB1	mthl14	-0.760959112540794	0.804334723897681	1	pseudobulk	DESeq2	LRT	0.0945631819854715
155	KC_AB1	mthl5	-0.877833043512066	0.751842013479608	1	pseudobulk	DESeq2	LRT	0.12387340925197
155	KC_AB1	mthl8	5.05491456844043	0.0194420169513424	1	pseudobulk	DESeq2	LRT	1.71125868254759
155	KC_AB1	Mtk	4.58085424419702	0.0980644594377514	1	pseudobulk	DESeq2	LRT	1.00848836129205
155	KC_AB1	Mtl	0.497080815625381	0.557723112613714	1	pseudobulk	DESeq2	LRT	0.253581357529939
155	KC_AB1	mtm	-0.306876061283496	0.648741919703005	1	pseudobulk	DESeq2	LRT	0.187928038392428
155	KC_AB1	MtnA	-0.10630005568013	0.80403205597039	1	pseudobulk	DESeq2	LRT	0.0947266360108448
155	KC_AB1	MtnE	2.87962473736619	0.299481422195579	1	pseudobulk	DESeq2	LRT	0.523630113135072
155	KC_AB1	Mtor	0.310109396022035	0.550754867062217	1	pseudobulk	DESeq2	LRT	0.25904165626721
155	KC_AB1	Mtp	0.152115475746305	1	1	pseudobulk	DESeq2	LRT	0
155	KC_AB1	Mtpalpha	0.439572036469201	0.581319906464795	1	pseudobulk	DESeq2	LRT	0.235584804998556
155	KC_AB1	MTPAP	2.62968726173762	0.1295732127555	1	pseudobulk	DESeq2	LRT	0.887484772808919
155	KC_AB1	Mtpbeta	-0.0509287106969726	0.924493715569324	1	pseudobulk	DESeq2	LRT	0.0340960366982544
155	KC_AB1	Mtr3	-0.0357884663608928	0.944310301731804	1	pseudobulk	DESeq2	LRT	0.0248852724541836
155	KC_AB1	Mtr4	1.24675618839797	0.271069578389371	1	pseudobulk	DESeq2	LRT	0.566919219708242
155	KC_AB1	mtrm	-0.119679433470562	0.938579946146907	1	pseudobulk	DESeq2	LRT	0.0275287292315365
155	KC_AB1	mtRNApol	0.728605783716694	0.378824376971116	1	pseudobulk	DESeq2	LRT	0.421562082352478
155	KC_AB1	mts	0.00580093795601655	0.942260102815241	1	pseudobulk	DESeq2	LRT	0.0258291973929352
155	KC_AB1	mtSSB	1.12382430558998	0.33214796860791	1	pseudobulk	DESeq2	LRT	0.478668399326821
155	KC_AB1	mtt	-1.2741071451676	0.292845078674124	1	pseudobulk	DESeq2	LRT	0.533362069988865
155	KC_AB1	mTTF	0.354382388224568	0.746986706495986	1	pseudobulk	DESeq2	LRT	0.126687126895189
155	KC_AB1	mtTFB2	-1.03223842853944	0.319142806820308	1	pseudobulk	DESeq2	LRT	0.496014939714367
155	KC_AB1	mu2	0.907676386249195	0.618771740100981	1	pseudobulk	DESeq2	LRT	0.2084695291654
155	KC_AB1	mub	-0.298740636949086	0.0278366314390435	1	pseudobulk	DESeq2	LRT	1.55538332064009
155	KC_AB1	muc	-0.0481667556218725	0.871825275377477	1	pseudobulk	DESeq2	LRT	0.0595705443595753
155	KC_AB1	Muc11A	-0.760959112540794	1	1	pseudobulk	DESeq2	LRT	0
155	KC_AB1	Muc14A	-0.0445449571661783	0.979507396696476	1	pseudobulk	DESeq2	LRT	0.00899228010586056
155	KC_AB1	Muc4B	-0.760959112540794	0.805265236978025	1	pseudobulk	DESeq2	LRT	0.0940610488455931
155	KC_AB1	Muc96D	1.25804452752548	0.727552935095488	1	pseudobulk	DESeq2	LRT	0.138135402914536
155	KC_AB1	Mul1	0.0922126334884315	0.940099002163529	1	pseudobulk	DESeq2	LRT	0.0268264082840562
155	KC_AB1	Mulk	-0.744304707489993	0.419820466912928	1	pseudobulk	DESeq2	LRT	0.376936392693413

155	KC_AB1	Mur2B	2.46901251270079	0.329256345181941	1	pseudobulk	DESeq2	LRT	0.482465846887208
155	KC_AB1	mura	-0.185414551110875	0.35229182585495	1	pseudobulk	DESeq2	LRT	0.453097433535402
155	KC_AB1	mus101	-0.107090169032304	0.930531645204492	1	pseudobulk	DESeq2	LRT	0.031268852942674
155	KC_AB1	mus201	0.491067035947011	0.64737033542682	1	pseudobulk	DESeq2	LRT	0.188847205235592
155	KC_AB1	mus304	1.16304896680274	0.572464331717016	1	pseudobulk	DESeq2	LRT	0.242251567540127
155	KC_AB1	mus81	0.279815096567509	0.951476823927993	1	pseudobulk	DESeq2	LRT	0.0216017857929023
155	KC_AB1	muskelin	-0.975126591445514	0.650803644869077	1	pseudobulk	DESeq2	LRT	0.186550023433251
155	KC_AB1	mute	0.155675327712776	0.88248357528371	1	pseudobulk	DESeq2	LRT	0.0542933689203463
155	KC_AB1	mv	-1.10870879407232	0.397405020283419	1	pseudobulk	DESeq2	LRT	0.40076665089602
155	KC_AB1	Mvb12	-0.403044399880139	0.597983529683319	1	pseudobulk	DESeq2	LRT	0.22331077766068
155	KC_AB1	Mvd	-1.79630530824108	0.304479808527892	1	pseudobulk	DESeq2	LRT	0.516441502163025
155	KC_AB1	Mvl	-0.471451985809013	0.491711698798491	1	pseudobulk	DESeq2	LRT	0.308289458858123
155	KC_AB1	mwh	-2.25167212244253	0.429440893483957	1	pseudobulk	DESeq2	LRT	0.367096602263969
155	KC_AB1	mxc	-0.163529914697917	0.866144888873842	1	pseudobulk	DESeq2	LRT	0.0624094530460487
155	KC_AB1	mxt	0.171275214805121	0.713375243141935	1	pseudobulk	DESeq2	LRT	0.146681966421002
155	KC_AB1	Myb	2.73062989528267	0.270641314998426	1	pseudobulk	DESeq2	LRT	0.567605905054642
155	KC_AB1	Mybbp1A	2.08288483941956	0.290146334684745	1	pseudobulk	DESeq2	LRT	0.537382911352231
155	KC_AB1	Myc	-0.10405497657373	0.85722482297797	1	pseudobulk	DESeq2	LRT	0.0669052614213103
155	KC_AB1	Myd88	1.39878546914222	0.405257126600298	1	pseudobulk	DESeq2	LRT	0.392269339179089
155	KC_AB1	myo	0.121383771066618	0.863949320294891	1	pseudobulk	DESeq2	LRT	0.0635117327083952
155	KC_AB1	Myo10A	1.4033888882443	0.5352172094323	1	pseudobulk	DESeq2	LRT	0.271469930663769
155	KC_AB1	Myo28B1	0.103454450984845	0.944670564090874	1	pseudobulk	DESeq2	LRT	0.0247196170372236
155	KC_AB1	Myo31DF	1.47597293528938	0.454525713615767	1	pseudobulk	DESeq2	LRT	0.342441542660822
155	KC_AB1	Myo61F	1.25804452752548	0.727552935095488	1	pseudobulk	DESeq2	LRT	0.138135402914536
155	KC_AB1	Myo81F	0.507477196888861	0.322997871126973	1	pseudobulk	DESeq2	LRT	0.490800340086405
155	KC_AB1	Myo95E	-0.27583282604113	0.683526962847373	1	pseudobulk	DESeq2	LRT	0.165244349296787
155	KC_AB1	MYPT-75D	-0.140065853047631	0.65771703628527	1	pseudobulk	DESeq2	LRT	0.181960908834279
155	KC_AB1	mys	-0.192393159255835	0.625367893824416	1	pseudobulk	DESeq2	LRT	0.203864419052173
155	KC_AB1	Mys45A	-0.280050057973279	0.703149607384914	1	pseudobulk	DESeq2	LRT	0.152952261396836
155	KC_AB1	Myt1	-0.011707511154538	0.942057930617393	1	pseudobulk	DESeq2	LRT	0.0259223900161536
155	KC_AB1	N	0.469755684517131	0.656337056586695	1	pseudobulk	DESeq2	LRT	0.182873074919071
155	KC_AB1	na	-0.0773334341209027	0.835007060586675	1	pseudobulk	DESeq2	LRT	0.0783098522279335
155	KC_AB1	Naa15-16	-0.0127167060827346	0.948954943260624	1	pseudobulk	DESeq2	LRT	0.022754407549337
156	KC_AB1	Naa20A	0.440056519120232	0.331485604915544	1	pseudobulk	DESeq2	LRT	0.479535326509546
156	KC_AB1	Naa30A	0.473847032734016	0.270150737209894	1	pseudobulk	DESeq2	LRT	0.568393842984115
156	KC_AB1	Naa35	0.336615687819757	0.732249497914368	1	pseudobulk	DESeq2	LRT	0.135340917431402
156	KC_AB1	Naa40	0.21027178680075	0.867768232961271	1	pseudobulk	DESeq2	LRT	0.0615962524577264
156	KC_AB1	Naa60	0.986939248263644	0.0729869768536713	1	pseudobulk	DESeq2	LRT	1.13675462460763
156	KC_AB1	Naa80	-0.0144150052773779	0.95695460711006	1	pseudobulk	DESeq2	LRT	0.0191086623790318
156	KC_AB1	Naam	0.164245182131989	0.929893792485668	1	pseudobulk	DESeq2	LRT	0.0315666514153184
156	KC_AB1	NAAT1	0.449651266543935	0.918820471207416	1	pseudobulk	DESeq2	LRT	0.0367693373414238
156	KC_AB1	nab	2.09987857572813	0.503115176013642	1	pseudobulk	DESeq2	LRT	0.29833258237694
156	KC_AB1	Nab2	0.275531980642222	0.553100459038816	1	pseudobulk	DESeq2	LRT	0.257195981093405
156	KC_AB1	nac	0.244208194607129	0.887912070705464	1	pseudobulk	DESeq2	LRT	0.0516300399625905
156	KC_AB1	Nacalpha	-0.0375545496002303	0.810130973402311	1	pseudobulk	DESeq2	LRT	0.0914447633093865
156	KC_AB1	Nach	1.25804452752548	0.727552935095488	1	pseudobulk	DESeq2	LRT	0.138135402914536
156	KC_AB1	nAChRalpha1	-0.420101247287039	0.0544591461939949	1	pseudobulk	DESeq2	LRT	1.26392917177577
156	KC_AB1	nAChRalpha2	-0.242537088310609	0.472821840959136	1	pseudobulk	DESeq2	LRT	0.325302470390443
156	KC_AB1	nAChRalpha3	0.36344088310091	0.828089548935084	1	pseudobulk	DESeq2	LRT	0.0819226964210471
156	KC_AB1	nAChRalpha4	0.0349469678509987	0.869948501072795	1	pseudobulk	DESeq2	LRT	0.0605064558433293
156	KC_AB1	nAChRalpha5	-0.116018277935008	0.441928888891155	1	pseudobulk	DESeq2	LRT	0.35464760767977
156	KC_AB1	nAChRalpha6	-0.375805991765893	0.130952830027587	1	pseudobulk	DESeq2	LRT	0.882885111579459
156	KC_AB1	nAChRalpha7	1.05655069009539	0.0662819326566041	1	pseudobulk	DESeq2	LRT	1.17860483685708
156	KC_AB1	nAChRbeta1	-0.00118800118981294	0.989680864942863	1	pseudobulk	DESeq2	LRT	0.00450482655227432
156	KC_AB1	nAChRbeta2	-0.00790706159941386	0.97817029404129	1	pseudobulk	DESeq2	LRT	0.00958553036143656
156	KC_AB1	nAChRbeta3	2.09987857572813	0.503115176013642	1	pseudobulk	DESeq2	LRT	0.29833258237694

156	KC_AB1	NaCP60E	-0.222696470259339	0.267826626389281	1	pseudobulk	DESeq2	LRT	0.572146249134661
156	KC_AB1	Nadsyn	0.307442694335424	0.324342777880887	1	pseudobulk	DESeq2	LRT	0.488995768032502
156	KC_AB1	nahoda	0.914648648633956	0.671609903661192	1	pseudobulk	DESeq2	LRT	0.172882908314671
156	KC_AB1	Nak	0.0316846165650311	0.940840078467017	1	pseudobulk	DESeq2	LRT	0.0264841905384183
[truncated: 658,395 more chars]
