# Supplementary material for: Transcriptional changes in specific subsets of Drosophila neurons following inhibition of the serotonin transporter
Source: Transl Psychiatry. 2023 Jun 24;13:226. doi: 10.1038/s41398-023-02521-3 (PMC10290657; doi:10.1038/s41398-023-02521-3)
Supplement: Supplementary file 5 — Supplemental Table 4 [file 41398_2023_2521_MOESM5_ESM.pdf]

scRNA-seq_DEGs_dSERT-TMKO_v_WT_Day4

	cell_type	gene	avg_logFC	p_val	p_val_adj	de_family	de_method	de_type	minuslogP
1	KC_G2	side	5.85581238651	3.91033779474368E-20	2.92102233267353E-16	pseudobulk	DESeq2	LRT	18
2	KC_G1	CG32581	-6.94715969097114	7.57539973095806E-14	6.56105370698277E-10	pseudobulk	DESeq2	LRT	13.1205944458132
3	KC_G1	LysRS	-2.44985682838237	2.74123925813866E-13	1.18709366073695E-09	pseudobulk	DESeq2	LRT	12.562053057144
4	KC_AB1	CG42260	-1.07662793990313	6.84273701127981E-12	3.83543773644867E-08	pseudobulk	DESeq2	LRT	11.1647701510376
5	KC_AB1	LysRS	-2.40433971738634	9.41327214737678E-12	3.83543773644867E-08	pseudobulk	DESeq2	LRT	11.0262593852304
6	KC_G1	CG42260	-0.937269618542383	5.28135400335707E-11	1.52472690076919E-07	pseudobulk	DESeq2	LRT	10.2772547212538
7	KC_AB1	CG32581	-7.78990108295117	1.09336163125422E-09	2.96993464436354E-06	pseudobulk	DESeq2	LRT	8.96123617063739
8	KC_G2	beat-IIa	7.76064464122013	1.76504663852828E-09	6.59244919490311E-06	pseudobulk	DESeq2	LRT	8.75324381458898
9	KC_G1	Myo81F	1.32851734175737	4.12209044473026E-08	8.9253563354522E-05	pseudobulk	DESeq2	LRT	7.38488248339224
10	KC_G2	CG9650	5.55127941148781	1.87655087724426E-07	0.000467261168433821	pseudobulk	DESeq2	LRT	6.72663965645372
11	KC_G1	CG42392	-0.972062653232242	8.89103045094451E-07	0.00154010429471261	pseudobulk	DESeq2	LRT	6.05104790233408
12	KC_AB1	Cbp53E	-2.05590915268787	1.13021951102463E-06	0.00230253969883492	pseudobulk	DESeq2	LRT	5.94683719973039
13	KC_G1	CG13229	2.78877368799257	2.27759249916993E-06	0.00328770477255179	pseudobulk	DESeq2	LRT	5.64252397612738
14	KC_G2	CG44153	5.9475504408771	1.9116283869663E-06	0.00356996601265956	pseudobulk	DESeq2	LRT	5.7185965289928
15	KC_G1	Hsp26	-1.90068898630491	4.02843547125402E-05	0.0498432565950444	pseudobulk	DESeq2	LRT	4.39486358862978
16	KC_G2	CG44837	6.68583825925281	3.68463465237822E-05	0.0550484417065306	pseudobulk	DESeq2	LRT	4.43360556786523
17	KC_G2	nAChRalpha7	1.36903439520838	5.45730232387008E-05	0.0679434139321825	pseudobulk	DESeq2	LRT	4.26302198648593
18	KC_G2	lncRNA:CR31451	0.875626896927615	7.1743952316947E-05	0.0765610462582277	pseudobulk	DESeq2	LRT	4.1442147020654
19	KC_G2	CG32581	-6.30955209561478	9.58748036483694E-05	0.0833771184275811	pseudobulk	DESeq2	LRT	4.0182955124773
20	KC_G2	LysRS	-1.9769148728944	0.000100454359551303	0.0833771184275811	pseudobulk	DESeq2	LRT	3.99803121085212
21	KC_G1	Ca-alpha1T	1.32830587735023	0.000110789142184044	0.118118999972145	pseudobulk	DESeq2	LRT	3.95550280026297
22	KC_G1	Pgant9	0.67729068572643	0.00012274229300881	0.118118999972145	pseudobulk	DESeq2	LRT	3.91100576770867
23	KC_G1	Sdc	0.527162598749261	0.000195848788899428	0.169624636065795	pseudobulk	DESeq2	LRT	3.70807910972205
24	KC_G2	Tektin-C	3.13517360369772	0.000246949481551031	0.18447126271862	pseudobulk	DESeq2	LRT	3.60739188126499
25	KC_G2	beat-IIb	4.51184535236267	0.000286033165314335	0.194242522263462	pseudobulk	DESeq2	LRT	3.54358360785719
26	KC_G1	CG31549	-6.15687206051412	0.000309138298809396	0.243404255089834	pseudobulk	DESeq2	LRT	3.50984718733003
27	KC_AB1	lncRNA:CR44832	-6.39812971863143	0.000212060392017074	0.345616026909428	pseudobulk	DESeq2	LRT	3.67354044008668
28	KC_G2	CG12994	3.74600163693971	0.00060242996851712	0.375012655401907	pseudobulk	DESeq2	LRT	3.22009343183079
29	KC_G1	out	5.87905712517848	0.000952519611843309	0.687481029847908	pseudobulk	DESeq2	LRT	3.02112607368106
30	KC_AB2	128up	2.4643417141186	0.449082701910884	0.988741847156811	pseudobulk	DESeq2	LRT	0.347673673080982
31	KC_AB2	14-3-3epsilon	-0.246492956213492	0.505851690278564	0.988741847156811	pseudobulk	DESeq2	LRT	0.295976794493892
32	KC_AB2	14-3-3zeta	0.024902236874264	0.910903424124876	0.988741847156811	pseudobulk	DESeq2	LRT	0.0405276653903184
33	KC_AB2	140up	-0.405381063532158	0.89155634938256	0.988741847156811	pseudobulk	DESeq2	LRT	0.0498512027381788
34	KC_AB2	18SrRNA-Psi:CR41602	0.331758848923756	0.946375065527934	0.988741847156811	pseudobulk	DESeq2	LRT	0.0239367107367223
35	KC_AB2	18w	3.51593030737787	0.251255733474702	0.988741847156811	pseudobulk	DESeq2	LRT	0.599884019171395
36	KC_AB2	26-29-p	0.157545400630359	0.936236928475676	0.988741847156811	pseudobulk	DESeq2	LRT	0.0286142327696613
37	KC_AB2	28SrRNA-Psi:CR40596	0.568082799876568	0.825260330228634	0.988741847156811	pseudobulk	DESeq2	LRT	0.0834090306653718
38	KC_AB2	28SrRNA-Psi:CR40741	0.818790875285784	0.940355871560339	0.988741847156811	pseudobulk	DESeq2	LRT	0.0267077593721816
39	KC_AB2	28SrRNA-Psi:CR41609	0.818790875285784	0.940355871560339	0.988741847156811	pseudobulk	DESeq2	LRT	0.0267077593721816
40	KC_AB2	28SrRNA-Psi:CR45848	0.818790875285784	0.940355871560339	0.988741847156811	pseudobulk	DESeq2	LRT	0.0267077593721816
41	KC_AB2	28SrRNA-Psi:CR45859	0.216528273680989	0.905903853023375	0.988741847156811	pseudobulk	DESeq2	LRT	0.0429178931810098
42	KC_AB2	2mit	-0.730002850306806	0.311541018274298	0.988741847156811	pseudobulk	DESeq2	LRT	0.506484764936064
43	KC_AB2	312	0.920073134400379	0.728211051768817	0.988741847156811	pseudobulk	DESeq2	LRT	0.137742734237847
44	KC_AB2	4E-T	-0.450880635090309	0.706830283888098	0.988741847156811	pseudobulk	DESeq2	LRT	0.150684851576509
45	KC_AB2	5-HT1A	-0.195626331490006	0.646790403733848	0.988741847156811	pseudobulk	DESeq2	LRT	0.189236432235571
46	KC_AB2	5-HT1B	0.345796998006939	0.690256197762662	0.988741847156811	pseudobulk	DESeq2	LRT	0.160989685171075
47	KC_AB2	5-HT7	1.47543920254359	0.661929169513603	0.988741847156811	pseudobulk	DESeq2	LRT	0.17918848025087
48	KC_AB2	5PtaseI	-0.29105245827331	0.798176520702044	0.988741847156811	pseudobulk	DESeq2	LRT	0.0979010516453521
49	KC_AB2	7B2	0.409729108257107	0.241846181541427	0.988741847156811	pseudobulk	DESeq2	LRT	0.616460765205768
50	KC_AB2	a	-0.617724918533346	0.450543228455699	0.988741847156811	pseudobulk	DESeq2	LRT	0.346263533255196
51	KC_AB2	A16	0.818790875285784	0.940355871560339	0.988741847156811	pseudobulk	DESeq2	LRT	0.0267077593721816
52	KC_AB2	a6	-0.452344515838269	0.871171367298942	0.988741847156811	pseudobulk	DESeq2	LRT	0.0598964069304489
53	KC_AB2	AANAT1	0.924140746192761	0.675445049022325	0.988741847156811	pseudobulk	DESeq2	LRT	0.170409977326066
54	KC_AB2	AANATL7	-0.488465967391191	0.84251072973523	0.988741847156811	pseudobulk	DESeq2	LRT	0.0744245594950451

55	KC_AB2	Aatf	-0.0144409071146711	0.984632974376923	0.988741847156811	pseudobulk	DESeq2	LRT	0.00672562423038896
56	KC_AB2	ab	-0.910037958008752	0.589414059876659	0.988741847156811	pseudobulk	DESeq2	LRT	0.229579508706622
57	KC_AB2	ABCA	-1.87057166357412	0.515275319421977	0.988741847156811	pseudobulk	DESeq2	LRT	0.28796065881108
58	KC_AB2	ABCB7	2.7785831508444	0.373751317747552	0.988741847156811	pseudobulk	DESeq2	LRT	0.427417267458199
59	KC_AB2	ABCD	0.218128533579459	0.776614487610556	0.988741847156811	pseudobulk	DESeq2	LRT	0.109794512025056
60	KC_AB2	Abi	-0.0207956826263153	0.983307927913115	0.988741847156811	pseudobulk	DESeq2	LRT	0.00731045932761182
61	KC_AB2	Abl	-0.421035449250535	0.491733038867031	0.988741847156811	pseudobulk	DESeq2	LRT	0.308270611080191
62	KC_AB2	Abp1	-1.09785658461028	0.483747798014209	0.988741847156811	pseudobulk	DESeq2	LRT	0.315380998846324
63	KC_AB2	abs	-2.26538931672255	0.547771004842275	0.988741847156811	pseudobulk	DESeq2	LRT	0.2614009599796
64	KC_AB2	Ac3	-0.204204712460618	0.847834308435626	0.988741847156811	pseudobulk	DESeq2	LRT	0.0716890132657921
65	KC_AB2	Ac76E	-1.74699812742254	0.480865720047389	0.988741847156811	pseudobulk	DESeq2	LRT	0.317976181805168
66	KC_AB2	Ac78C	-1.06694190804421	0.261330259670917	0.988741847156811	pseudobulk	DESeq2	LRT	0.582810300003884
67	KC_AB2	Acbp1	-0.120849390925995	0.882509107148089	0.988741847156811	pseudobulk	DESeq2	LRT	0.0542808041681818
68	KC_AB2	ACC	1.03734376381036	0.64157927463489	0.988741847156811	pseudobulk	DESeq2	LRT	0.192749673802887
69	KC_AB2	AcCoAS	0.0385701316694874	0.948244152891792	0.988741847156811	pseudobulk	DESeq2	LRT	0.0230798265842111
70	KC_AB2	Ace	-0.393235302513073	0.830081630163786	0.988741847156811	pseudobulk	DESeq2	LRT	0.0808791970378034
71	KC_AB2	Acer	0.802207095184316	0.732945722088841	0.988741847156811	pseudobulk	DESeq2	LRT	0.134928185618234
72	KC_AB2	Acf	-0.0979346299150915	0.956185972694747	0.988741847156811	pseudobulk	DESeq2	LRT	0.0194576317194512
73	KC_AB2	Ack	-1.02592565225788	0.645171277800408	0.988741847156811	pseudobulk	DESeq2	LRT	0.190324975087242
74	KC_AB2	Ack-like	-0.644777562099464	0.61556619894307	0.988741847156811	pseudobulk	DESeq2	LRT	0.210725235519461
75	KC_AB2	Acn	0.959507227343557	0.299213482547484	0.988741847156811	pseudobulk	DESeq2	LRT	0.524018841074788
76	KC_AB2	Acph-1	-1.14755310091518	0.618375466635947	0.988741847156811	pseudobulk	DESeq2	LRT	0.208747748902126
77	KC_AB2	Acsl	1.15254745147349	0.49318467715754	0.988741847156811	pseudobulk	DESeq2	LRT	0.306990425039208
78	KC_AB2	Act42A	4.0494356576363	0.113095797390465	0.988741847156811	pseudobulk	DESeq2	LRT	0.946553533041316
79	KC_AB2	Act5C	1.07303273380608	0.109821863676261	0.988741847156811	pseudobulk	DESeq2	LRT	0.959311190589775
80	KC_AB2	Act88F	-1.80294541848982	0.660135549041553	0.988741847156811	pseudobulk	DESeq2	LRT	0.180366879372519
81	KC_AB2	Actbeta	0.0718837378930457	0.91752586756011	0.988741847156811	pseudobulk	DESeq2	LRT	0.037381682955705
82	KC_AB2	Actn	0.0601466527378941	0.974810960364079	0.988741847156811	pseudobulk	DESeq2	LRT	0.0110795964355292
83	KC_AB2	ACXB	0.818790875285784	0.940355871560339	0.988741847156811	pseudobulk	DESeq2	LRT	0.0267077593721816
84	KC_AB2	Acyp2	-0.53863548005614	0.761930245810466	0.988741847156811	pseudobulk	DESeq2	LRT	0.118084786201414
85	KC_AB2	Ada1-2	-1.1284597703615	0.729754448571724	0.988741847156811	pseudobulk	DESeq2	LRT	0.136823248879498
86	KC_AB2	Ada2b	1.6341263262903	0.579948075372967	0.988741847156811	pseudobulk	DESeq2	LRT	0.236610888486163
87	KC_AB2	Ada3	-0.850804884520864	0.65925312502858	0.988741847156811	pseudobulk	DESeq2	LRT	0.180947802862626
88	KC_AB2	Adar	1.17080655178149	0.379630184435215	0.988741847156811	pseudobulk	DESeq2	LRT	0.42063926407274
89	KC_AB2	Adat1	0.818790875285784	0.940355871560339	0.988741847156811	pseudobulk	DESeq2	LRT	0.0267077593721816
90	KC_AB2	Adck1	-1.80294541848982	0.660135549041553	0.988741847156811	pseudobulk	DESeq2	LRT	0.180366879372519
91	KC_AB2	Adck5	-0.0582963981256801	0.979058404810457	0.988741847156811	pseudobulk	DESeq2	LRT	0.0091913999942843
92	KC_AB2	ADD1	-3.3468609602753	0.15548942579296	0.988741847156811	pseudobulk	DESeq2	LRT	0.808299140244325
93	KC_AB2	AdenoK	0.269971509165439	0.903083168079264	0.988741847156811	pseudobulk	DESeq2	LRT	0.0442722521508783
94	KC_AB2	Adi1	-0.982834386675151	0.729867072195321	0.988741847156811	pseudobulk	DESeq2	LRT	0.13675622901488
95	KC_AB2	AdipoR	-0.758302676904018	0.52157340753453	0.988741847156811	pseudobulk	DESeq2	LRT	0.282684559253555
96	KC_AB2	Adk1	0.0983699107221355	0.936570459984514	0.988741847156811	pseudobulk	DESeq2	LRT	0.0284595442563062
97	KC_AB2	Adk2	-0.117227253463615	0.891930949536298	0.988741847156811	pseudobulk	DESeq2	LRT	0.049668766023121
98	KC_AB2	adp	2.73593896747858	0.422277110754874	0.988741847156811	pseudobulk	DESeq2	LRT	0.374402458575713
99	KC_AB2	ADPS	-0.110914776539818	0.941315433983623	0.988741847156811	pseudobulk	DESeq2	LRT	0.0262648204801763
100	KC_AB2	AdSL	1.42621218304824	0.66738831402044	0.988741847156811	pseudobulk	DESeq2	LRT	0.175621402117653
101	KC_AB2	AdSS	-0.397210918028763	0.775269226750836	0.988741847156811	pseudobulk	DESeq2	LRT	0.110547454412466
102	KC_AB2	Aduk	0.919353707608246	0.746278333243311	0.988741847156811	pseudobulk	DESeq2	LRT	0.127099167101924
103	KC_AB2	Ady43A	1.10527623683615	0.724717602335893	0.988741847156811	pseudobulk	DESeq2	LRT	0.139831190172672
104	KC_AB2	Aef1	0.33844332870745	0.776027921953448	0.988741847156811	pseudobulk	DESeq2	LRT	0.110122652282859
105	KC_AB2	aft	-2.26538931672255	0.547771004842275	0.988741847156811	pseudobulk	DESeq2	LRT	0.2614009599796
106	KC_AB2	Afti	1.19688341589857	0.605793926756547	0.988741847156811	pseudobulk	DESeq2	LRT	0.217675084897749
107	KC_AB2	Ag5r	-2.51746470924027	0.517377929599049	0.988741847156811	pseudobulk	DESeq2	LRT	0.286192101436607
108	KC_AB2	ago	0.668970670557767	0.818048200024539	0.988741847156811	pseudobulk	DESeq2	LRT	0.0872211066123218
109	KC_AB2	AGO1	0.109820102942373	0.78097809172374	0.988741847156811	pseudobulk	DESeq2	LRT	0.107361148935684
110	KC_AB2	AGO2	-0.209294959031791	0.877893213984396	0.988741847156811	pseudobulk	DESeq2	LRT	0.056558308009394

111	KC_AB2	Agpat1	3.06202454821851	0.30627034058063	0.988741847156811	pseudobulk	DESeq2	LRT	0.513895058517773
112	KC_AB2	Agpat3	0.0563120942996901	0.974316031513895	0.988741847156811	pseudobulk	DESeq2	LRT	0.0113001514574287
113	KC_AB2	Ahcy	-0.957133960806503	0.762632518785665	0.988741847156811	pseudobulk	DESeq2	LRT	0.1176846802739
114	KC_AB2	AhcyL1	-1.04089554658427	0.475918141745959	0.988741847156811	pseudobulk	DESeq2	LRT	0.322467739811473
115	KC_AB2	AIF	3.27725343811621	0.25757966897206	0.988741847156811	pseudobulk	DESeq2	LRT	0.589088419269148
116	KC_AB2	AIMP1	0.699674038439951	0.821690393783994	0.988741847156811	pseudobulk	DESeq2	LRT	0.0852917902455435
117	KC_AB2	AIMP2	1.47543920254359	0.661929169513603	0.988741847156811	pseudobulk	DESeq2	LRT	0.17918848025087
118	KC_AB2	AIMP3	0.600313568100488	0.586970440521155	0.988741847156811	pseudobulk	DESeq2	LRT	0.231383769010971
119	KC_AB2	Ak6	-0.158601889198964	0.945838126788742	0.988741847156811	pseudobulk	DESeq2	LRT	0.0241831835324482
120	KC_AB2	Akap200	-0.597492700100737	0.751042608390007	0.988741847156811	pseudobulk	DESeq2	LRT	0.124335423763143
121	KC_AB2	akirin	0.172314530124523	0.840939053779283	0.988741847156811	pseudobulk	DESeq2	LRT	0.0752354781217906
122	KC_AB2	Akt1	0.219663433380893	0.888117667345339	0.988741847156811	pseudobulk	DESeq2	LRT	0.0515294904225896
123	KC_AB2	Aladin	-0.233272055930639	0.937531793764924	0.988741847156811	pseudobulk	DESeq2	LRT	0.028013995469539
124	KC_AB2	AlaRS	2.06137138167525	0.547946929015274	0.988741847156811	pseudobulk	DESeq2	LRT	0.261261502741816
125	KC_AB2	AlaRS-m	-1.80294541848982	0.660135549041553	0.988741847156811	pseudobulk	DESeq2	LRT	0.180366879372519
126	KC_AB2	Alas	0.123764945436762	0.900273943292135	0.988741847156811	pseudobulk	DESeq2	LRT	0.0456253194969434
127	KC_AB2	alc	0.169249522948909	0.880113768825818	0.988741847156811	pseudobulk	DESeq2	LRT	0.0554611846911225
128	KC_AB2	Ald1	0.643262841447784	0.342914954699364	0.988741847156811	pseudobulk	DESeq2	LRT	0.464813574664028
129	KC_AB2	Aldh-III	0.306444003039066	0.865780107924966	0.988741847156811	pseudobulk	DESeq2	LRT	0.0625923967137429
130	KC_AB2	Alg-2	1.00986845121153	0.416811702357981	0.988741847156811	pseudobulk	DESeq2	LRT	0.380060096334849
131	KC_AB2	Alg1	0.157562008957688	0.950463298513166	0.988741847156811	pseudobulk	DESeq2	LRT	0.0220646484576093
132	KC_AB2	Alg10	0.818790875285784	0.940355871560339	0.988741847156811	pseudobulk	DESeq2	LRT	0.0267077593721816
133	KC_AB2	Alg11	0.110731962284482	0.958092313397532	0.988741847156811	pseudobulk	DESeq2	LRT	0.0185926440867238
134	KC_AB2	Alg2	-1.23536834900699	0.725570392794057	0.988741847156811	pseudobulk	DESeq2	LRT	0.139320447155934
135	KC_AB2	Alg3	2.41462217514131	0.497446973552313	0.988741847156811	pseudobulk	DESeq2	LRT	0.303253207020768
136	KC_AB2	Alh	0.139543759454009	0.92013189547714	0.988741847156811	pseudobulk	DESeq2	LRT	0.0361499146411484
137	KC_AB2	alien	1.84969518086931	0.509872718319655	0.988741847156811	pseudobulk	DESeq2	LRT	0.292538225138248
138	KC_AB2	ALiX	-0.452344515838269	0.871171367298942	0.988741847156811	pseudobulk	DESeq2	LRT	0.0598964069304489
139	KC_AB2	Alk	-0.206502442382825	0.679788649747341	0.988741847156811	pseudobulk	DESeq2	LRT	0.167626090993397
140	KC_AB2	alph	-0.220150389094542	0.781470912274576	0.988741847156811	pseudobulk	DESeq2	LRT	0.107087182550417
141	KC_AB2	alpha-Cat	-0.439578694085643	0.547057518885984	0.988741847156811	pseudobulk	DESeq2	LRT	0.261967008544983
142	KC_AB2	alpha-Catr	-0.768039962015224	0.588674610573031	0.988741847156811	pseudobulk	DESeq2	LRT	0.23012469482451
143	KC_AB2	alpha-Est8	-0.999396203754128	0.454054521843969	0.988741847156811	pseudobulk	DESeq2	LRT	0.342891994908779
144	KC_AB2	alpha-Est9	-0.600987133452689	0.687737091287189	0.988741847156811	pseudobulk	DESeq2	LRT	0.162577552498229
145	KC_AB2	alpha-Man-Ia	0.564220856629931	0.221934251283882	0.988741847156811	pseudobulk	DESeq2	LRT	0.653775667593665
146	KC_AB2	alpha-Man-Ib	0.363533173924407	0.854324450514061	0.988741847156811	pseudobulk	DESeq2	LRT	0.0683771640790577
147	KC_AB2	alpha-Man-IIa	-0.213177136262384	0.93155916913195	0.988741847156811	pseudobulk	DESeq2	LRT	0.030789555140895
148	KC_AB2	alpha-Man-IIb	0.157562008957688	0.950463298513166	0.988741847156811	pseudobulk	DESeq2	LRT	0.0220646484576093
149	KC_AB2	alpha-PheRS	0.699674038439951	0.821690393783994	0.988741847156811	pseudobulk	DESeq2	LRT	0.0852917902455435
150	KC_AB2	alpha-Spec	0.799125660621929	0.359848340631644	0.988741847156811	pseudobulk	DESeq2	LRT	0.443880495633657
151	KC_AB2	alpha4GT1	-0.733592669469711	0.588664030710163	0.988741847156811	pseudobulk	DESeq2	LRT	0.23013250018467
152	KC_AB2	alpha4GT2	0.0601466527378941	0.974810960364079	0.988741847156811	pseudobulk	DESeq2	LRT	0.0110795964355292
153	KC_AB2	alphaCOP	-0.140517900335193	0.940720168609696	0.988741847156811	pseudobulk	DESeq2	LRT	0.0265395448004056
154	KC_AB2	alphaSnap	0.374225164094699	0.631583046372098	0.988741847156811	pseudobulk	DESeq2	LRT	0.199569536304444
155	KC_AB2	alphaTub67C	1.12452187615248	0.704078404906088	0.988741847156811	pseudobulk	DESeq2	LRT	0.152378975911753
156	KC_AB2	alphaTub84B	0.206576249997081	0.631442363001625	0.988741847156811	pseudobulk	DESeq2	LRT	0.19966628497045
157	KC_AB2	alphaTub84D	0.0287512265659429	0.977395204134918	0.988741847156811	pseudobulk	DESeq2	LRT	0.00992979629091551
158	KC_AB2	Alr	-1.80294541848982	0.660135549041553	0.988741847156811	pseudobulk	DESeq2	LRT	0.180366879372519
159	KC_AB2	Als2	-0.0384500034601774	0.953910728566112	0.988741847156811	pseudobulk	DESeq2	LRT	0.0204922667059844
160	KC_AB2	alt	0.586065417622254	0.575557260190713	0.988741847156811	pseudobulk	DESeq2	LRT	0.239911463436772
161	KC_AB2	Amacr	-0.110156823709618	0.935376654353758	0.988741847156811	pseudobulk	DESeq2	LRT	0.0290134736533693
162	KC_AB2	amn	0.712171366772551	0.813205723536451	0.988741847156811	pseudobulk	DESeq2	LRT	0.0897995733547685
163	KC_AB2	amon	0.118208771339738	0.826897813854056	0.988741847156811	pseudobulk	DESeq2	LRT	0.0825481562531579
164	KC_AB2	AMPdeam	0.5738945031678	0.80780652681376	0.988741847156811	pseudobulk	DESeq2	LRT	0.0926926421938231
165	KC_AB2	Amph	1.15102549446658	0.491153502372127	0.988741847156811	pseudobulk	DESeq2	LRT	0.30878275469068
166	KC_AB2	AMPKalpha	-0.700885635722807	0.658007405588736	0.988741847156811	pseudobulk	DESeq2	LRT	0.181769218561995

167	KC_AB2	Amun	-0.476767565185764	0.663923101553353	0.988741847156811	pseudobulk	DESeq2	LRT	0.177882219585354
168	KC_AB2	amx	0.353798809751289	0.901648351639924	0.988741847156811	pseudobulk	DESeq2	LRT	0.0449628069360408
169	KC_AB2	ana2	-1.80294541848982	0.660135549041553	0.988741847156811	pseudobulk	DESeq2	LRT	0.180366879372519
170	KC_AB2	anchor	-0.908909516750057	0.671937973991072	0.988741847156811	pseudobulk	DESeq2	LRT	0.172670814441211
171	KC_AB2	angel	0.157562008957688	0.950463298513166	0.988741847156811	pseudobulk	DESeq2	LRT	0.0220646484576093
172	KC_AB2	Ank	-0.380955353136944	0.639152439679823	0.988741847156811	pseudobulk	DESeq2	LRT	0.194395549014909
173	KC_AB2	Ank2	-0.340471696234889	0.313975592193046	0.988741847156811	pseudobulk	DESeq2	LRT	0.503104111760806
174	KC_AB2	Ankle2	-1.77566073155666	0.606304952090798	0.988741847156811	pseudobulk	DESeq2	LRT	0.217308884582246
175	KC_AB2	anne	0.382900998240852	0.544548349403366	0.988741847156811	pseudobulk	DESeq2	LRT	0.263963554022298
176	KC_AB2	Ant2	-0.934020934207768	0.782359118258182	0.988741847156811	pseudobulk	DESeq2	LRT	0.106593851444836
177	KC_AB2	AnxB10	0.574989404325028	0.815857662655918	0.988741847156811	pseudobulk	DESeq2	LRT	0.088385603151941
178	KC_AB2	AnxB11	0.904610447611892	0.552591228417356	0.988741847156811	pseudobulk	DESeq2	LRT	0.257596013158513
179	KC_AB2	aop	-1.3677082045258	0.601030079295445	0.988741847156811	pseudobulk	DESeq2	LRT	0.221103792647606
180	KC_AB2	aos	0.818790875285784	0.940355871560339	0.988741847156811	pseudobulk	DESeq2	LRT	0.0267077593721816
181	KC_AB2	Aos1	2.34726809501174	0.359332409723481	0.988741847156811	pseudobulk	DESeq2	LRT	0.44450361015608
182	KC_AB2	AP-1-2beta	-0.160387205499185	0.891606130698767	0.988741847156811	pseudobulk	DESeq2	LRT	0.0498269539653631
183	KC_AB2	AP-1gamma	-0.422106373510868	0.573230957222659	0.988741847156811	pseudobulk	DESeq2	LRT	0.241670363655129
184	KC_AB2	AP-1mu	-0.531388669299586	0.694448796923205	0.988741847156811	pseudobulk	DESeq2	LRT	0.158359770132968
185	KC_AB2	AP-1sigma	0.245531237833624	0.7924599914102	0.988741847156811	pseudobulk	DESeq2	LRT	0.101022654597134
186	KC_AB2	AP-2alpha	-0.0192319604686272	0.981884362148966	0.988741847156811	pseudobulk	DESeq2	LRT	0.00793965665070562
187	KC_AB2	AP-2mu	0.346329085391022	0.74644274262538	0.988741847156811	pseudobulk	DESeq2	LRT	0.127003500083016
188	KC_AB2	AP-2sigma	-0.115982876981201	0.879125221958876	0.988741847156811	pseudobulk	DESeq2	LRT	0.0559492599291667
189	KC_AB2	Apc	-0.962331574231161	0.384334086796726	0.988741847156811	pseudobulk	DESeq2	LRT	0.415291096016447
190	KC_AB2	APC10	-2.58401630608882	0.379537283165344	0.988741847156811	pseudobulk	DESeq2	LRT	0.42074555553224
191	KC_AB2	ApepP	0.0601466527378941	0.974810960364079	0.988741847156811	pseudobulk	DESeq2	LRT	0.0110795964355292
192	KC_AB2	Apf	-0.114099770495178	0.955170180595609	0.988741847156811	pseudobulk	DESeq2	LRT	0.0199192442181898
193	KC_AB2	aph-1	-2.11248316060727	0.189632949892833	0.988741847156811	pseudobulk	DESeq2	LRT	0.722086199100574
194	KC_AB2	aPKC	-0.711549106375618	0.394192776612044	0.988741847156811	pseudobulk	DESeq2	LRT	0.404291338215562
195	KC_AB2	Apl	-2.9721553030081	0.387854083811327	0.988741847156811	pseudobulk	DESeq2	LRT	0.411331631401987
196	KC_AB2	Aplip1	-0.145050734402952	0.84179428994632	0.988741847156811	pseudobulk	DESeq2	LRT	0.0747940244815761
197	KC_AB2	apolpp	3.51593030737787	0.251255733474702	0.988741847156811	pseudobulk	DESeq2	LRT	0.599884019171395
198	KC_AB2	Apoltp	0.0927862888886855	0.867230104084102	0.988741847156811	pseudobulk	DESeq2	LRT	0.0618656549169814
199	KC_AB2	app	-1.17604213983969	0.530596092629532	0.988741847156811	pseudobulk	DESeq2	LRT	0.275235952536277
200	KC_AB2	APP-BP1	2.00515076565275	0.576456787459964	0.988741847156811	pseudobulk	DESeq2	LRT	0.239233242872451
201	KC_AB2	Appl	-0.250378583280924	0.301262414533012	0.988741847156811	pseudobulk	DESeq2	LRT	0.52105504748067
202	KC_AB2	Aps	0.0237744608187441	0.975317192553908	0.988741847156811	pseudobulk	DESeq2	LRT	0.0108541201239194
203	KC_AB2	aqz	-0.118623325972662	0.669863798224744	0.988741847156811	pseudobulk	DESeq2	LRT	0.174013492362748
204	KC_AB2	aralar1	1.76062409692731	0.431284494748455	0.988741847156811	pseudobulk	DESeq2	LRT	0.365236155037982
205	KC_AB2	Arc42	0.572861358867068	0.813871944286787	0.988741847156811	pseudobulk	DESeq2	LRT	0.0894439222171593
206	KC_AB2	Archease	-4.04128891635342	0.164265069336396	0.988741847156811	pseudobulk	DESeq2	LRT	0.784454778666552
207	KC_AB2	Arf102F	0.118740164647779	0.915704134867007	0.988741847156811	pseudobulk	DESeq2	LRT	0.0382448247496921
208	KC_AB2	Arf51F	0.170681531202091	0.89141402288473	0.988741847156811	pseudobulk	DESeq2	LRT	0.049920538284892
209	KC_AB2	Arf79F	0.657412195337757	0.297744859415491	0.988741847156811	pseudobulk	DESeq2	LRT	0.526155727903147
210	KC_AB2	ArfGAP1	0.363641268105473	0.858330168669459	0.988741847156811	pseudobulk	DESeq2	LRT	0.06634562258353
211	KC_AB2	ArfGAP3	1.12452187615248	0.704078404906088	0.988741847156811	pseudobulk	DESeq2	LRT	0.152378975911753
212	KC_AB2	Arfip	0.854038804238809	0.628126019355827	0.988741847156811	pseudobulk	DESeq2	LRT	0.201953216102996
213	KC_AB2	Arfrp1	1.10527623683615	0.724717602335893	0.988741847156811	pseudobulk	DESeq2	LRT	0.139831190172672
214	KC_AB2	arg	0.997284248814671	0.663837179223659	0.988741847156811	pseudobulk	DESeq2	LRT	0.177938427914901
215	KC_AB2	Argk	0.525797764524739	0.259496376099038	0.988741847156811	pseudobulk	DESeq2	LRT	0.585868702752634
216	KC_AB2	ArgRS	-0.506613484034373	0.961658398065406	0.988741847156811	pseudobulk	DESeq2	LRT	0.0169791713942958
217	KC_AB2	ArgRS-m	-1.23536834900699	0.725570392794057	0.988741847156811	pseudobulk	DESeq2	LRT	0.139320447155934
218	KC_AB2	ari-1	0.178597987521946	0.822688934663491	0.988741847156811	pseudobulk	DESeq2	LRT	0.0847643440052245
219	KC_AB2	ari-2	0.421489448475535	0.787508785880886	0.988741847156811	pseudobulk	DESeq2	LRT	0.103744592283374
220	KC_AB2	Arl1	-0.294666239043538	0.880736594655136	0.988741847156811	pseudobulk	DESeq2	LRT	0.0551539583216942
221	KC_AB2	Arl2	-0.908909516750057	0.671937973991072	0.988741847156811	pseudobulk	DESeq2	LRT	0.172670814441211
222	KC_AB2	Arl4	-0.0995767203663479	0.886434131269302	0.988741847156811	pseudobulk	DESeq2	LRT	0.0523535302143126

223	KC_AB2	Arl5	0.179268503759514	0.917968545859208	0.988741847156811	pseudobulk	DESeq2	LRT	0.0371721996199037
224	KC_AB2	Arl6IP1	0.388563639100183	0.573753525864029	0.988741847156811	pseudobulk	DESeq2	LRT	0.241274632594401
225	KC_AB2	Arl8	-0.205886035987985	0.844694351656192	0.988741847156811	pseudobulk	DESeq2	LRT	0.0733004098693846
226	KC_AB2	arm	0.283668920286327	0.65571304259973	0.988741847156811	pseudobulk	DESeq2	LRT	0.183286177820814
227	KC_AB2	armi	-1.80294541848982	0.660135549041553	0.988741847156811	pseudobulk	DESeq2	LRT	0.180366879372519
228	KC_AB2	Arp1	0.795756898840838	0.455277075804448	0.988741847156811	pseudobulk	DESeq2	LRT	0.341724216817388
229	KC_AB2	Arp10	-1.02592565225788	0.645171277800408	0.988741847156811	pseudobulk	DESeq2	LRT	0.190324975087242
230	KC_AB2	Arp2	0.800572342462331	0.487841515186095	0.988741847156811	pseudobulk	DESeq2	LRT	0.31172124410214
231	KC_AB2	Arp3	1.13539439938001	0.65602501243538	0.988741847156811	pseudobulk	DESeq2	LRT	0.183079601850592
232	KC_AB2	Arp5	-0.506613484034373	0.961658398065406	0.988741847156811	pseudobulk	DESeq2	LRT	0.0169791713942958
233	KC_AB2	Arp6	-0.213177136262384	0.93155916913195	0.988741847156811	pseudobulk	DESeq2	LRT	0.030789555140895
234	KC_AB2	Arp8	-1.80294541848982	0.660135549041553	0.988741847156811	pseudobulk	DESeq2	LRT	0.180366879372519
235	KC_AB2	Arpc1	1.76265209291734	0.284296829372343	0.988741847156811	pseudobulk	DESeq2	LRT	0.546227983762861
236	KC_AB2	Arpc2	0.760118285990313	0.738173623698606	0.988741847156811	pseudobulk	DESeq2	LRT	0.131841477003816
237	KC_AB2	Arpc3A	1.47543920254359	0.661929169513603	0.988741847156811	pseudobulk	DESeq2	LRT	0.17918848025087
238	KC_AB2	Arpc4	-0.159170689593312	0.92478622720461	0.988741847156811	pseudobulk	DESeq2	LRT	0.033958646801551
239	KC_AB2	Arpc5	-0.340221463096046	0.876039462438654	0.988741847156811	pseudobulk	DESeq2	LRT	0.057476329981081
240	KC_AB2	arr	-1.38195849511675	0.232251008226475	0.988741847156811	pseudobulk	DESeq2	LRT	0.634042392011418
241	KC_AB2	Ars2	0.0828026096697369	0.937747088104014	0.988741847156811	pseudobulk	DESeq2	LRT	0.0279142757480974
242	KC_AB2	Art1	2.7785831508444	0.373751317747552	0.988741847156811	pseudobulk	DESeq2	LRT	0.427417267458199
243	KC_AB2	Art2	2.06137138167525	0.547946929015274	0.988741847156811	pseudobulk	DESeq2	LRT	0.261261502741816
244	KC_AB2	Art4	2.00515076565275	0.576456787459964	0.988741847156811	pseudobulk	DESeq2	LRT	0.239233242872451
245	KC_AB2	Art7	0.818790875285784	0.940355871560339	0.988741847156811	pseudobulk	DESeq2	LRT	0.0267077593721816
246	KC_AB2	Arts	0.818790875285784	0.940355871560339	0.988741847156811	pseudobulk	DESeq2	LRT	0.0267077593721816
247	KC_AB2	Arv1	2.00515076565275	0.576456787459964	0.988741847156811	pseudobulk	DESeq2	LRT	0.239233242872451
248	KC_AB2	Asap	-1.43398637937178	0.362623574612833	0.988741847156811	pseudobulk	DESeq2	LRT	0.44054396529441
249	KC_AB2	Asator	0.102350105837576	0.868453159600649	0.988741847156811	pseudobulk	DESeq2	LRT	0.0612536004472224
250	KC_AB2	Asciz	-0.233272055930639	0.937531793764924	0.988741847156811	pseudobulk	DESeq2	LRT	0.028013995469539
251	KC_AB2	asf1	-1.27482208804161	0.504147634250043	0.988741847156811	pseudobulk	DESeq2	LRT	0.297442266428947
252	KC_AB2	ash1	-0.783372789234193	0.572493612365314	0.988741847156811	pseudobulk	DESeq2	LRT	0.242229354629948
253	KC_AB2	ash2	-1.80294541848982	0.660135549041553	0.988741847156811	pseudobulk	DESeq2	LRT	0.180366879372519
254	KC_AB2	Ask1	-3.4227037205893	0.285801360563088	0.988741847156811	pseudobulk	DESeq2	LRT	0.543935708072568
255	KC_AB2	asl	0.818790875285784	0.940355871560339	0.988741847156811	pseudobulk	DESeq2	LRT	0.0267077593721816
256	KC_AB2	AsnRS	0.683017593519122	0.746591264083729	0.988741847156811	pseudobulk	DESeq2	LRT	0.126917096089901
257	KC_AB2	Asph	1.47543920254359	0.661929169513603	0.988741847156811	pseudobulk	DESeq2	LRT	0.17918848025087
258	KC_AB2	AspRS	2.06137138167525	0.547946929015274	0.988741847156811	pseudobulk	DESeq2	LRT	0.261261502741816
259	KC_AB2	AspRS-m	3.30033526000341	0.259137897354645	0.988741847156811	pseudobulk	DESeq2	LRT	0.586469069420752
260	KC_AB2	asrij	-0.37671184873479	0.876517139842803	0.988741847156811	pseudobulk	DESeq2	LRT	0.0572395870814579
261	KC_AB2	asRNA:CR42254	2.00515076565275	0.576456787459964	0.988741847156811	pseudobulk	DESeq2	LRT	0.239233242872451
262	KC_AB2	asRNA:CR43425	-0.341472371153588	0.849350394863359	0.988741847156811	pseudobulk	DESeq2	LRT	0.070913106974026
263	KC_AB2	asRNA:CR43430	-1.80294541848982	0.660135549041553	0.988741847156811	pseudobulk	DESeq2	LRT	0.180366879372519
264	KC_AB2	asRNA:CR43629	-2.51746470924027	0.517377929599049	0.988741847156811	pseudobulk	DESeq2	LRT	0.286192101436607
265	KC_AB2	asRNA:CR43730	-1.80294541848982	0.660135549041553	0.988741847156811	pseudobulk	DESeq2	LRT	0.180366879372519
266	KC_AB2	asRNA:CR43767	-1.80294541848982	0.660135549041553	0.988741847156811	pseudobulk	DESeq2	LRT	0.180366879372519
267	KC_AB2	asRNA:CR43786	-1.65719613210719	0.599376091584556	0.988741847156811	pseudobulk	DESeq2	LRT	0.222300584546838
268	KC_AB2	asRNA:CR43809	0.818790875285784	0.940355871560339	0.988741847156811	pseudobulk	DESeq2	LRT	0.0267077593721816
269	KC_AB2	asRNA:CR43865	2.51243226103394	0.474458247157471	0.988741847156811	pseudobulk	DESeq2	LRT	0.323801999942597
270	KC_AB2	asRNA:CR43889	-1.23536834900699	0.725570392794057	0.988741847156811	pseudobulk	DESeq2	LRT	0.139320447155934
271	KC_AB2	asRNA:CR43906	1.47543920254359	0.661929169513603	0.988741847156811	pseudobulk	DESeq2	LRT	0.17918848025087
272	KC_AB2	asRNA:CR43933	0.818790875285784	0.940355871560339	0.988741847156811	pseudobulk	DESeq2	LRT	0.0267077593721816
273	KC_AB2	asRNA:CR43936	-1.80294541848982	0.660135549041553	0.988741847156811	pseudobulk	DESeq2	LRT	0.180366879372519
274	KC_AB2	asRNA:CR43944	1.10527623683615	0.724717602335893	0.988741847156811	pseudobulk	DESeq2	LRT	0.139831190172672
275	KC_AB2	asRNA:CR43959	-1.80294541848982	0.660135549041553	0.988741847156811	pseudobulk	DESeq2	LRT	0.180366879372519
276	KC_AB2	asRNA:CR44018	0.684221129527733	0.737351212059311	0.988741847156811	pseudobulk	DESeq2	LRT	0.132325601520231
277	KC_AB2	asRNA:CR44027	-1.80933943727629	0.584587525761637	0.988741847156811	pseudobulk	DESeq2	LRT	0.233150456085471
278	KC_AB2	asRNA:CR44029	1.48741827283584	0.532545672123726	0.988741847156811	pseudobulk	DESeq2	LRT	0.273643140376104

279	KC_AB2	asRNA:CR44030	0.925814870582422	0.543485392432563	0.988741847156811	pseudobulk	DESeq2	LRT	0.264812124201765
280	KC_AB2	asRNA:CR44031	1.21185718506466	0.567710906692152	0.988741847156811	pseudobulk	DESeq2	LRT	0.245872762158739
281	KC_AB2	asRNA:CR44046	-1.80294541848982	0.660135549041553	0.988741847156811	pseudobulk	DESeq2	LRT	0.180366879372519
282	KC_AB2	asRNA:CR44065	-1.80294541848982	0.660135549041553	0.988741847156811	pseudobulk	DESeq2	LRT	0.180366879372519
283	KC_AB2	asRNA:CR44106	-1.80294541848982	0.660135549041553	0.988741847156811	pseudobulk	DESeq2	LRT	0.180366879372519
284	KC_AB2	asRNA:CR44107	-1.80294541848982	0.660135549041553	0.988741847156811	pseudobulk	DESeq2	LRT	0.180366879372519
285	KC_AB2	asRNA:CR44114	1.42621218304824	0.66738831402044	0.988741847156811	pseudobulk	DESeq2	LRT	0.175621402117653
286	KC_AB2	asRNA:CR44137	-2.26538931672255	0.547771004842275	0.988741847156811	pseudobulk	DESeq2	LRT	0.2614009599796
287	KC_AB2	asRNA:CR44179	0.818790875285784	0.940355871560339	0.988741847156811	pseudobulk	DESeq2	LRT	0.0267077593721816
288	KC_AB2	asRNA:CR44382	-1.80294541848982	0.660135549041553	0.988741847156811	pseudobulk	DESeq2	LRT	0.180366879372519
289	KC_AB2	asRNA:CR44843	1.42621218304824	0.66738831402044	0.988741847156811	pseudobulk	DESeq2	LRT	0.175621402117653
290	KC_AB2	asRNA:CR44892	-1.23536834900699	0.725570392794057	0.988741847156811	pseudobulk	DESeq2	LRT	0.139320447155934
291	KC_AB2	asRNA:CR44960	-1.80294541848982	0.660135549041553	0.988741847156811	pseudobulk	DESeq2	LRT	0.180366879372519
292	KC_AB2	asRNA:CR44961	-1.80294541848982	0.660135549041553	0.988741847156811	pseudobulk	DESeq2	LRT	0.180366879372519
293	KC_AB2	asRNA:CR44987	-1.80294541848982	0.660135549041553	0.988741847156811	pseudobulk	DESeq2	LRT	0.180366879372519
294	KC_AB2	asRNA:CR45000	0.818790875285784	0.940355871560339	0.988741847156811	pseudobulk	DESeq2	LRT	0.0267077593721816
295	KC_AB2	asRNA:CR45007	0.818790875285784	0.940355871560339	0.988741847156811	pseudobulk	DESeq2	LRT	0.0267077593721816
296	KC_AB2	asRNA:CR45009	2.12627090373229	0.415798287869119	0.988741847156811	pseudobulk	DESeq2	LRT	0.38111730329345
297	KC_AB2	asRNA:CR45126	-0.957133960806503	0.762632518785665	0.988741847156811	pseudobulk	DESeq2	LRT	0.1176846802739
298	KC_AB2	asRNA:CR45129	1.42621218304824	0.66738831402044	0.988741847156811	pseudobulk	DESeq2	LRT	0.175621402117653
299	KC_AB2	asRNA:CR45134	2.4643417141186	0.449082701910884	0.988741847156811	pseudobulk	DESeq2	LRT	0.347673673080982
300	KC_AB2	asRNA:CR45145	1.42621218304824	0.66738831402044	0.988741847156811	pseudobulk	DESeq2	LRT	0.175621402117653
301	KC_AB2	asRNA:CR45162	0.818790875285784	0.940355871560339	0.988741847156811	pseudobulk	DESeq2	LRT	0.0267077593721816
302	KC_AB2	asRNA:CR45182	0.712171366772551	0.813205723536451	0.988741847156811	pseudobulk	DESeq2	LRT	0.0897995733547685
303	KC_AB2	asRNA:CR45198	-1.28585361841952	0.604452679793172	0.988741847156811	pseudobulk	DESeq2	LRT	0.218637692667744
304	KC_AB2	asRNA:CR45469	1.42621218304824	0.66738831402044	0.988741847156811	pseudobulk	DESeq2	LRT	0.175621402117653
305	KC_AB2	asRNA:CR45620	1.42621218304824	0.66738831402044	0.988741847156811	pseudobulk	DESeq2	LRT	0.175621402117653
306	KC_AB2	asRNA:CR45789	0.157562008957688	0.950463298513166	0.988741847156811	pseudobulk	DESeq2	LRT	0.0220646484576093
307	KC_AB2	asRNA:CR45813	-1.80294541848982	0.660135549041553	0.988741847156811	pseudobulk	DESeq2	LRT	0.180366879372519
308	KC_AB2	asRNA:CR45822	0.818790875285784	0.940355871560339	0.988741847156811	pseudobulk	DESeq2	LRT	0.0267077593721816
309	KC_AB2	asRNA:CR45832	1.42621218304824	0.66738831402044	0.988741847156811	pseudobulk	DESeq2	LRT	0.175621402117653
310	KC_AB2	asRNA:CR45835	-0.457212305199634	0.858276431637992	0.988741847156811	pseudobulk	DESeq2	LRT	0.0663728130839547
311	KC_AB2	asRNA:CR45873	-1.80294541848982	0.660135549041553	0.988741847156811	pseudobulk	DESeq2	LRT	0.180366879372519
312	KC_AB2	asRNA:CR45874	-0.124237841338121	0.946426769540675	0.988741847156811	pseudobulk	DESeq2	LRT	0.0239129842514505
313	KC_AB2	asRNA:CR45907	0.818790875285784	0.940355871560339	0.988741847156811	pseudobulk	DESeq2	LRT	0.0267077593721816
314	KC_AB2	asRNA:CR45928	0.818790875285784	0.940355871560339	0.988741847156811	pseudobulk	DESeq2	LRT	0.0267077593721816
315	KC_AB2	asRNA:CR45951	-1.80294541848982	0.660135549041553	0.988741847156811	pseudobulk	DESeq2	LRT	0.180366879372519
316	KC_AB2	asRNA:CR46067	-1.80294541848982	0.660135549041553	0.988741847156811	pseudobulk	DESeq2	LRT	0.180366879372519
317	KC_AB2	asRNA:CR46068	-1.80294541848982	0.660135549041553	0.988741847156811	pseudobulk	DESeq2	LRT	0.180366879372519
318	KC_AB2	asRNA:CR46135	-2.51746470924027	0.517377929599049	0.988741847156811	pseudobulk	DESeq2	LRT	0.286192101436607
319	KC_AB2	asRNA:CR46262	0.818790875285784	0.940355871560339	0.988741847156811	pseudobulk	DESeq2	LRT	0.0267077593721816
320	KC_AB2	ast	0.818790875285784	0.940355871560339	0.988741847156811	pseudobulk	DESeq2	LRT	0.0267077593721816
321	KC_AB2	AstA-R1	-3.32426296135166	0.343738249391692	0.988741847156811	pseudobulk	DESeq2	LRT	0.463772139158982
322	KC_AB2	Asx	-1.65012765780502	0.330235889010299	0.988741847156811	pseudobulk	DESeq2	LRT	0.481175730733544
323	KC_AB2	Atac1	1.47543920254359	0.661929169513603	0.988741847156811	pseudobulk	DESeq2	LRT	0.17918848025087
324	KC_AB2	Atac3	-1.73627324524809	0.521368726288639	0.988741847156811	pseudobulk	DESeq2	LRT	0.282855023048041
325	KC_AB2	ATbp	-2.26538931672255	0.547771004842275	0.988741847156811	pseudobulk	DESeq2	LRT	0.2614009599796
326	KC_AB2	Ate1	-0.794177064289352	0.743016342902793	0.988741847156811	pseudobulk	DESeq2	LRT	0.129001633676244
327	KC_AB2	Atet	0.320759680284561	0.757535772338276	0.988741847156811	pseudobulk	DESeq2	LRT	0.120596854096551
328	KC_AB2	Atf-2	-1.69633662497937	0.605069927920179	0.988741847156811	pseudobulk	DESeq2	LRT	0.2181944310419
329	KC_AB2	Atf6	0.0810382184367213	0.9127897064833	0.988741847156811	pseudobulk	DESeq2	LRT	0.039629266095
330	KC_AB2	Atg1	-0.0741019478375958	0.921108620417206	0.988741847156811	pseudobulk	DESeq2	LRT	0.0356891532273785
331	KC_AB2	Atg10	0.818790875285784	0.940355871560339	0.988741847156811	pseudobulk	DESeq2	LRT	0.0267077593721816
332	KC_AB2	Atg101	0.922664372932389	0.678259639521954	0.988741847156811	pseudobulk	DESeq2	LRT	0.168604025267887
333	KC_AB2	Atg12	-1.03391514514131	0.420088538166151	0.988741847156811	pseudobulk	DESeq2	LRT	0.376659167733849
334	KC_AB2	Atg13	-0.254611003409661	0.868033631089631	0.988741847156811	pseudobulk	DESeq2	LRT	0.0614634481948212

335	KC_AB2	Atg14	-0.349038222693293	0.86868347896181	0.988741847156811	pseudobulk	DESeq2	LRT	0.0611384380287992
336	KC_AB2	Atg16	1.12452187615248	0.704078404906088	0.988741847156811	pseudobulk	DESeq2	LRT	0.152378975911753
337	KC_AB2	Atg17	0.00931377815392388	0.983407354719355	0.988741847156811	pseudobulk	DESeq2	LRT	0.00726654802665084
338	KC_AB2	Atg18a	-1.34257104959132	0.502867704929748	0.988741847156811	pseudobulk	DESeq2	LRT	0.29854625465779
339	KC_AB2	Atg18b	0.952675537494734	0.547147691789924	0.988741847156811	pseudobulk	DESeq2	LRT	0.261895428562901
340	KC_AB2	Atg2	0.818790875285784	0.940355871560339	0.988741847156811	pseudobulk	DESeq2	LRT	0.0267077593721816
341	KC_AB2	Atg3	1.31413323252528	0.624060193048613	0.988741847156811	pseudobulk	DESeq2	LRT	0.204773518894346
342	KC_AB2	Atg4a	-0.68750020136443	0.784083099060507	0.988741847156811	pseudobulk	DESeq2	LRT	0.105637907276101
343	KC_AB2	Atg4b	-1.80294541848982	0.660135549041553	0.988741847156811	pseudobulk	DESeq2	LRT	0.180366879372519
344	KC_AB2	Atg5	1.10527623683615	0.724717602335893	0.988741847156811	pseudobulk	DESeq2	LRT	0.139831190172672
345	KC_AB2	Atg6	-1.21442392643984	0.584026528107531	0.988741847156811	pseudobulk	DESeq2	LRT	0.233567425577577
346	KC_AB2	Atg7	-2.11027789354592	0.473734933668313	0.988741847156811	pseudobulk	DESeq2	LRT	0.32446458879962
347	KC_AB2	Atg8a	0.17448540423682	0.683049280242128	0.988741847156811	pseudobulk	DESeq2	LRT	0.165547961962465
348	KC_AB2	Atg9	-2.51746470924027	0.517377929599049	0.988741847156811	pseudobulk	DESeq2	LRT	0.286192101436607
349	KC_AB2	atl	0.920819427723561	0.702122570790991	0.988741847156811	pseudobulk	DESeq2	LRT	0.153587065688553
350	KC_AB2	atms	-0.385733633096415	0.822744264387791	0.988741847156811	pseudobulk	DESeq2	LRT	0.0847351366281377
351	KC_AB2	Atox1	1.21620241813299	0.208772334676808	0.988741847156811	pseudobulk	DESeq2	LRT	0.68032705209334
352	KC_AB2	ATP6AP2	0.412273882980407	0.549927970036516	0.988741847156811	pseudobulk	DESeq2	LRT	0.259694190986241
353	KC_AB2	ATP8A	-0.333026962726478	0.762810467512672	0.988741847156811	pseudobulk	DESeq2	LRT	0.117583356066774
354	KC_AB2	ATP8B	2.06137138167525	0.547946929015274	0.988741847156811	pseudobulk	DESeq2	LRT	0.261261502741816
355	KC_AB2	Atpalpha	0.140393720594276	0.585875969429403	0.988741847156811	pseudobulk	DESeq2	LRT	0.232194314858562
356	KC_AB2	ATPCL	0.129808739048127	0.893632292772755	0.988741847156811	pseudobulk	DESeq2	LRT	0.0488411457120422
357	KC_AB2	ATPsynB	0.679175395803818	0.213167351287932	0.988741847156811	pseudobulk	DESeq2	LRT	0.671279311096107
358	KC_AB2	ATPsynbeta	0.901472861572014	0.0625806383414713	0.988741847156811	pseudobulk	DESeq2	LRT	1.20356001123194
359	KC_AB2	ATPsynC	0.0500317572312608	0.879016479498138	0.988741847156811	pseudobulk	DESeq2	LRT	0.0560029828465725
360	KC_AB2	ATPsynCF6	0.769334165175847	0.165814277107199	0.988741847156811	pseudobulk	DESeq2	LRT	0.780378078116706
361	KC_AB2	ATPsynD	0.45348596183062	0.398790934880091	0.988741847156811	pseudobulk	DESeq2	LRT	0.399254722417303
362	KC_AB2	ATPsyndelta	0.671399466252732	0.353338846089903	0.988741847156811	pseudobulk	DESeq2	LRT	0.451808613583907
363	KC_AB2	ATPsynE	0.313836913825525	0.590212107408476	0.988741847156811	pseudobulk	DESeq2	LRT	0.228991885776827
364	KC_AB2	ATPsynF	0.189706285879187	0.64207378011252	0.988741847156811	pseudobulk	DESeq2	LRT	0.192415064680842
365	KC_AB2	ATPsynG	-0.02234536389299	0.954460488945561	0.988741847156811	pseudobulk	DESeq2	LRT	0.0202420450472445
366	KC_AB2	ATPsyngamma	0.249835543366995	0.735349487849963	0.988741847156811	pseudobulk	DESeq2	LRT	0.133506205717441
367	KC_AB2	ATPsynO	0.547778603128194	0.350410590689823	0.988741847156811	pseudobulk	DESeq2	LRT	0.45542277633774
368	KC_AB2	AttA	-1.80294541848982	0.660135549041553	0.988741847156811	pseudobulk	DESeq2	LRT	0.180366879372519
369	KC_AB2	Atu	0.0601466527378941	0.974810960364079	0.988741847156811	pseudobulk	DESeq2	LRT	0.0110795964355292
370	KC_AB2	Atx2	-0.0779020649700147	0.926723023956966	0.988741847156811	pseudobulk	DESeq2	LRT	0.0330500470217826
371	KC_AB2	Atxn7	0.0648918484735626	0.963984972209493	0.988741847156811	pseudobulk	DESeq2	LRT	0.015929736364144
372	KC_AB2	aub	0.818790875285784	0.940355871560339	0.988741847156811	pseudobulk	DESeq2	LRT	0.0267077593721816
373	KC_AB2	aux	-0.121120420743075	0.958079731976279	0.988741847156811	pseudobulk	DESeq2	LRT	0.0185983471673402
374	KC_AB2	ave	-1.65418851609544	0.48054246547914	0.988741847156811	pseudobulk	DESeq2	LRT	0.318268227749117
375	KC_AB2	Aven	1.47543920254359	0.661929169513603	0.988741847156811	pseudobulk	DESeq2	LRT	0.17918848025087
376	KC_AB2	awd	0.480492810787021	0.539270585968537	0.988741847156811	pseudobulk	DESeq2	LRT	0.268193267269656
377	KC_AB2	axed	0.0792619315417132	0.942931463966485	0.988741847156811	pseudobulk	DESeq2	LRT	0.025519872377045
378	KC_AB2	Axn	1.76923267226582	0.433375211084549	0.988741847156811	pseudobulk	DESeq2	LRT	0.363135933803196
379	KC_AB2	Axud1	-0.0582963981256801	0.979058404810457	0.988741847156811	pseudobulk	DESeq2	LRT	0.0091913999942843
380	KC_AB2	az2	1.10527623683615	0.724717602335893	0.988741847156811	pseudobulk	DESeq2	LRT	0.139831190172672
381	KC_AB2	B4	0.0171524655419377	0.978755140830388	0.988741847156811	pseudobulk	DESeq2	LRT	0.0093259438321799
382	KC_AB2	B52	0.117643135915546	0.816766728770805	0.988741847156811	pseudobulk	DESeq2	LRT	0.0879019616723022
383	KC_AB2	babo	0.273499199379563	0.78591979425109	0.988741847156811	pseudobulk	DESeq2	LRT	0.104621772906541
384	KC_AB2	Bacc	0.0470198091092975	0.870199331113712	0.988741847156811	pseudobulk	DESeq2	LRT	0.0603812548674062
385	KC_AB2	bai	-1.43317152240612	0.385489057667563	0.988741847156811	pseudobulk	DESeq2	LRT	0.413987945141021
386	KC_AB2	Baldspot	0.00420740665032072	0.960978560262476	0.988741847156811	pseudobulk	DESeq2	LRT	0.0172863014714827
387	KC_AB2	ball	-1.80294541848982	0.660135549041553	0.988741847156811	pseudobulk	DESeq2	LRT	0.180366879372519
388	KC_AB2	Bap111	-1.02592565225788	0.645171277800408	0.988741847156811	pseudobulk	DESeq2	LRT	0.190324975087242
389	KC_AB2	Bap170	-2.26538931672255	0.547771004842275	0.988741847156811	pseudobulk	DESeq2	LRT	0.2614009599796
390	KC_AB2	Bap55	1.91142393796962	0.32880834661643	0.988741847156811	pseudobulk	DESeq2	LRT	0.48305716666894

391	KC_AB2	Bap60	-0.390322594159798	0.793735695476282	0.988741847156811	pseudobulk	DESeq2	LRT	0.100324088385569
392	KC_AB2	barc	0.453257014782104	0.771126676667268	0.988741847156811	pseudobulk	DESeq2	LRT	0.112874272456765
393	KC_AB2	baz	-1.80294541848982	0.660135549041553	0.988741847156811	pseudobulk	DESeq2	LRT	0.180366879372519
394	KC_AB2	bbc	0.356034289956173	0.735133296498869	0.988741847156811	pseudobulk	DESeq2	LRT	0.133633906242045
395	KC_AB2	BBS5	-2.51746470924027	0.517377929599049	0.988741847156811	pseudobulk	DESeq2	LRT	0.286192101436607
396	KC_AB2	bc10	-0.171659813331075	0.918050040188221	0.988741847156811	pseudobulk	DESeq2	LRT	0.0371336460478548
397	KC_AB2	BCAS2	3.25449350606273	0.266356678091136	0.988741847156811	pseudobulk	DESeq2	LRT	0.574536410117102
398	KC_AB2	bchs	-0.0293105030063403	0.944974455663407	0.988741847156811	pseudobulk	DESeq2	LRT	0.0245799310826597
399	KC_AB2	BCL7-like	-0.0582963981256801	0.979058404810457	0.988741847156811	pseudobulk	DESeq2	LRT	0.0091913999942843
400	KC_AB2	bcn92	0.156751703196075	0.889785281257954	0.988741847156811	pseudobulk	DESeq2	LRT	0.0507147825858625
401	KC_AB2	Bdbt	1.40305140665399	0.649741560632143	0.988741847156811	pseudobulk	DESeq2	LRT	0.187259352757606
402	KC_AB2	bdg	1.47543920254359	0.661929169513603	0.988741847156811	pseudobulk	DESeq2	LRT	0.17918848025087
403	KC_AB2	bdl	1.42621218304824	0.66738831402044	0.988741847156811	pseudobulk	DESeq2	LRT	0.175621402117653
404	KC_AB2	Bdp1	1.47543920254359	0.661929169513603	0.988741847156811	pseudobulk	DESeq2	LRT	0.17918848025087
405	KC_AB2	BEAF-32	0.781221941544258	0.607310102152912	0.988741847156811	pseudobulk	DESeq2	LRT	0.216589494650901
406	KC_AB2	beag	-0.943420647347194	0.5905693093219	0.988741847156811	pseudobulk	DESeq2	LRT	0.228729126179734
407	KC_AB2	beat-Ia	0.818790875285784	0.940355871560339	0.988741847156811	pseudobulk	DESeq2	LRT	0.0267077593721816
408	KC_AB2	beat-Ib	0.469215405870985	0.716791926383286	0.988741847156811	pseudobulk	DESeq2	LRT	0.144606895012972
409	KC_AB2	beat-Ic	0.818790875285784	0.940355871560339	0.988741847156811	pseudobulk	DESeq2	LRT	0.0267077593721816
410	KC_AB2	beat-IIIb	2.80917294583258	0.367996267391162	0.988741847156811	pseudobulk	DESeq2	LRT	0.434156586379859
411	KC_AB2	beat-IV	-0.388354882075501	0.606130992196105	0.988741847156811	pseudobulk	DESeq2	LRT	0.217433509430613
412	KC_AB2	beat-Va	2.51243226103394	0.474458247157471	0.988741847156811	pseudobulk	DESeq2	LRT	0.323801999942597
413	KC_AB2	beat-Vb	2.51243226103394	0.474458247157471	0.988741847156811	pseudobulk	DESeq2	LRT	0.323801999942597
414	KC_AB2	beat-VI	1.42621218304824	0.66738831402044	0.988741847156811	pseudobulk	DESeq2	LRT	0.175621402117653
415	KC_AB2	beat-VII	0.133398514264094	0.834812617258743	0.988741847156811	pseudobulk	DESeq2	LRT	0.078410995681764
416	KC_AB2	beg	3.03619069427769	0.314302467188174	0.988741847156811	pseudobulk	DESeq2	LRT	0.502652209876457
417	KC_AB2	bel	-0.0309861597389175	0.942960066688869	0.988741847156811	pseudobulk	DESeq2	LRT	0.0255066987620221
418	KC_AB2	Bem46	-0.742148798960242	0.80644751100302	0.988741847156811	pseudobulk	DESeq2	LRT	0.0934238941443075
419	KC_AB2	ben	0.475360627610852	0.524340023646802	0.988741847156811	pseudobulk	DESeq2	LRT	0.280386990683118
420	KC_AB2	Best1	-0.139875912273695	0.94970305777199	0.988741847156811	pseudobulk	DESeq2	LRT	0.0224121636899844
421	KC_AB2	Bet1	1.32675001876361	0.448590175967266	0.988741847156811	pseudobulk	DESeq2	LRT	0.348150241608878
422	KC_AB2	Bet3	0.10707090889796	0.936703471398759	0.988741847156811	pseudobulk	DESeq2	LRT	0.0283978702828781
423	KC_AB2	Bet5	0.21996598550629	0.885875319835517	0.988741847156811	pseudobulk	DESeq2	LRT	0.0526273974331645
424	KC_AB2	beta-PheRS	2.80917294583258	0.367996267391162	0.988741847156811	pseudobulk	DESeq2	LRT	0.434156586379859
425	KC_AB2	beta-Spec	-0.242533152032149	0.70840661311801	0.988741847156811	pseudobulk	DESeq2	LRT	0.149717393236827
426	KC_AB2	beta'COP	1.75566251772846	0.434141035354741	0.988741847156811	pseudobulk	DESeq2	LRT	0.362369162362515
427	KC_AB2	beta3GalTII	1.12452187615248	0.704078404906088	0.988741847156811	pseudobulk	DESeq2	LRT	0.152378975911753
428	KC_AB2	beta4GalNAcTA	0.668970670557767	0.818048200024539	0.988741847156811	pseudobulk	DESeq2	LRT	0.0872211066123218
429	KC_AB2	beta4GalNAcTB	0.818790875285784	0.940355871560339	0.988741847156811	pseudobulk	DESeq2	LRT	0.0267077593721816
430	KC_AB2	beta4GalT7	2.00515076565275	0.576456787459964	0.988741847156811	pseudobulk	DESeq2	LRT	0.239233242872451
431	KC_AB2	betaCOP	-2.16923839102081	0.498281874778995	0.988741847156811	pseudobulk	DESeq2	LRT	0.302524910193245
432	KC_AB2	betaggt-I	-0.506613484034373	0.961658398065406	0.988741847156811	pseudobulk	DESeq2	LRT	0.0169791713942958
433	KC_AB2	betaggt-II	2.41462217514131	0.497446973552313	0.988741847156811	pseudobulk	DESeq2	LRT	0.303253207020768
434	KC_AB2	betaGlu	0.818790875285784	0.940355871560339	0.988741847156811	pseudobulk	DESeq2	LRT	0.0267077593721816
435	KC_AB2	betaTub56D	0.295165773178752	0.553154346814062	0.988741847156811	pseudobulk	DESeq2	LRT	0.257153670473237
436	KC_AB2	betaTub85D	0.818790875285784	0.940355871560339	0.988741847156811	pseudobulk	DESeq2	LRT	0.0267077593721816
437	KC_AB2	betaTub97EF	0.818790875285784	0.940355871560339	0.988741847156811	pseudobulk	DESeq2	LRT	0.0267077593721816
438	KC_AB2	Bgb	1.22750758992873	0.400591826017322	0.988741847156811	pseudobulk	DESeq2	LRT	0.397297916629536
439	KC_AB2	BHD	1.42621218304824	0.66738831402044	0.988741847156811	pseudobulk	DESeq2	LRT	0.175621402117653
440	KC_AB2	BI-1	0.649742654607029	0.429095076604895	0.988741847156811	pseudobulk	DESeq2	LRT	0.367446468491315
441	KC_AB2	bic	-0.332183074328657	0.481407221332838	0.988741847156811	pseudobulk	DESeq2	LRT	0.317487399392353
442	KC_AB2	BicD	-0.0546290173082529	0.954373310932803	0.988741847156811	pseudobulk	DESeq2	LRT	0.0202817142210752
443	KC_AB2	bif	0.593603752339375	0.647919821385542	0.988741847156811	pseudobulk	DESeq2	LRT	0.188478733765603
444	KC_AB2	bigmax	-0.568233637794205	0.868826397133536	0.988741847156811	pseudobulk	DESeq2	LRT	0.0610669925949191
445	KC_AB2	Bili	0.792624111005831	0.390658126893882	0.988741847156811	pseudobulk	DESeq2	LRT	0.408203136595783
446	KC_AB2	Bin1	-0.335533259674735	0.868442100281927	0.988741847156811	pseudobulk	DESeq2	LRT	0.0612591310064972

447	KC_AB2	bin3	-0.473064888281349	0.698551061975945	0.988741847156811	pseudobulk	DESeq2	LRT	0.155801842772604
448	KC_AB2	bip2	0.444314564486269	0.446020104707885	0.988741847156811	pseudobulk	DESeq2	LRT	0.350645564680448
449	KC_AB2	Bka	1.12452187615248	0.704078404906088	0.988741847156811	pseudobulk	DESeq2	LRT	0.152378975911753
450	KC_AB2	Blimp-1	0.0858462027294632	0.912965361646774	0.988741847156811	pseudobulk	DESeq2	LRT	0.0395456994986024
451	KC_AB2	Blm	0.157562008957688	0.950463298513166	0.988741847156811	pseudobulk	DESeq2	LRT	0.0220646484576093
452	KC_AB2	Blos1	-0.124237841338121	0.946426769540675	0.988741847156811	pseudobulk	DESeq2	LRT	0.0239129842514505
453	KC_AB2	Blos2	-2.51746470924027	0.517377929599049	0.988741847156811	pseudobulk	DESeq2	LRT	0.286192101436607
454	KC_AB2	Blos3	-1.18743225435356	0.64061820392288	0.988741847156811	pseudobulk	DESeq2	LRT	0.193400724506216
455	KC_AB2	Blos4	-1.99867440840856	0.534899605264936	0.988741847156811	pseudobulk	DESeq2	LRT	0.271727722597268
456	KC_AB2	blp	0.157545400630359	0.936236928475676	0.988741847156811	pseudobulk	DESeq2	LRT	0.0286142327696613
457	KC_AB2	blw	-0.0572486192698373	0.907466008789902	0.988741847156811	pseudobulk	DESeq2	LRT	0.042169633478022
458	KC_AB2	bma	-0.616280516917354	0.15023805975053	0.988741847156811	pseudobulk	DESeq2	LRT	0.823220033737972
459	KC_AB2	Bmcp	-0.448374585768327	0.814227572251762	0.988741847156811	pseudobulk	DESeq2	LRT	0.0892541951597077
460	KC_AB2	bmm	0.818790875285784	0.940355871560339	0.988741847156811	pseudobulk	DESeq2	LRT	0.0267077593721816
461	KC_AB2	bnb	0.0601466527378941	0.974810960364079	0.988741847156811	pseudobulk	DESeq2	LRT	0.0110795964355292
462	KC_AB2	boca	0.424080140117926	0.782904709847825	0.988741847156811	pseudobulk	DESeq2	LRT	0.106291094271129
463	KC_AB2	bocks	-0.853565742783243	0.753557841534365	0.988741847156811	pseudobulk	DESeq2	LRT	0.122883406527736
464	KC_AB2	BOD1	-0.331004884396198	0.894406551228697	0.988741847156811	pseudobulk	DESeq2	LRT	0.0484650283842476
465	KC_AB2	bol	-0.323640012623211	0.461384790829192	0.988741847156811	pseudobulk	DESeq2	LRT	0.335936725739987
466	KC_AB2	bon	-0.3086930839159	0.840681528804389	0.988741847156811	pseudobulk	DESeq2	LRT	0.0753684946625984
467	KC_AB2	bonsai	2.18593775365336	0.405686989635832	0.988741847156811	pseudobulk	DESeq2	LRT	0.391808919877551
468	KC_AB2	bor	1.32665372579835	0.53789476225346	0.988741847156811	pseudobulk	DESeq2	LRT	0.26930268463709
469	KC_AB2	BORCS5	0.942071803158662	0.510987668473292	0.988741847156811	pseudobulk	DESeq2	LRT	0.291589580449678
470	KC_AB2	BORCS6	0.157545400630359	0.936236928475676	0.988741847156811	pseudobulk	DESeq2	LRT	0.0286142327696613
471	KC_AB2	botv	0.699674038439951	0.821690393783994	0.988741847156811	pseudobulk	DESeq2	LRT	0.0852917902455435
472	KC_AB2	br	-0.593356633120117	0.629834220145622	0.988741847156811	pseudobulk	DESeq2	LRT	0.200773746975633
473	KC_AB2	Br140	-0.0582963981256801	0.979058404810457	0.988741847156811	pseudobulk	DESeq2	LRT	0.0091913999942843
474	KC_AB2	brat	-0.190798017321194	0.710416673617966	0.988741847156811	pseudobulk	DESeq2	LRT	0.148486854133596
475	KC_AB2	Brca2	-3.32426296135166	0.343738249391692	0.988741847156811	pseudobulk	DESeq2	LRT	0.463772139158982
476	KC_AB2	Bre1	1.7499346849987	0.376777192038282	0.988741847156811	pseudobulk	DESeq2	LRT	0.423915394816844
477	KC_AB2	Brf	0.818790875285784	0.940355871560339	0.988741847156811	pseudobulk	DESeq2	LRT	0.0267077593721816
478	KC_AB2	brm	0.340046974523705	0.83106200337761	0.988741847156811	pseudobulk	DESeq2	LRT	0.0803665734237993
479	KC_AB2	Brms1	-1.04632530781858	0.703665033675239	0.988741847156811	pseudobulk	DESeq2	LRT	0.152634029275562
480	KC_AB2	brn	-1.80294541848982	0.660135549041553	0.988741847156811	pseudobulk	DESeq2	LRT	0.180366879372519
481	KC_AB2	brp	-0.150137339002218	0.619203132728787	0.988741847156811	pseudobulk	DESeq2	LRT	0.208166855097477
482	KC_AB2	bru1	-0.244404239130787	0.775737292012372	0.988741847156811	pseudobulk	DESeq2	LRT	0.11028533021896
483	KC_AB2	bru2	0.099418050043988	0.931297591307328	0.988741847156811	pseudobulk	DESeq2	LRT	0.0309115202956839
484	KC_AB2	bru3	-0.403301488289531	0.127279296521519	0.988741847156811	pseudobulk	DESeq2	LRT	0.895242233720265
485	KC_AB2	Bruce	1.08707461675391	0.453583984898521	0.988741847156811	pseudobulk	DESeq2	LRT	0.343342287873937
486	KC_AB2	brun	2.80917294583258	0.367996267391162	0.988741847156811	pseudobulk	DESeq2	LRT	0.434156586379859
487	KC_AB2	BRWD3	-0.332464417915409	0.87936231254484	0.988741847156811	pseudobulk	DESeq2	LRT	0.0558321511845224
488	KC_AB2	brwl	-0.452344515838269	0.871171367298942	0.988741847156811	pseudobulk	DESeq2	LRT	0.0598964069304489
489	KC_AB2	bs	-1.29600486439944	0.685894431367319	0.988741847156811	pseudobulk	DESeq2	LRT	0.163742723072789
490	KC_AB2	bsf	-0.506613484034373	0.961658398065406	0.988741847156811	pseudobulk	DESeq2	LRT	0.0169791713942958
491	KC_AB2	Bsg	0.0857920300198129	0.82527156682443	0.988741847156811	pseudobulk	DESeq2	LRT	0.0834031174303011
492	KC_AB2	Bsg25D	-0.327901454811136	0.818586700395622	0.988741847156811	pseudobulk	DESeq2	LRT	0.0869353156298082
493	KC_AB2	bsk	0.274746335797456	0.655316057303629	0.988741847156811	pseudobulk	DESeq2	LRT	0.183549190333313
494	KC_AB2	bt	-3.91108067132102	0.221683018369551	0.988741847156811	pseudobulk	DESeq2	LRT	0.654267573962038
495	KC_AB2	BTBD9	0.792281256713368	0.823478838299676	0.988741847156811	pseudobulk	DESeq2	LRT	0.0843475568193814
496	KC_AB2	BtbVII	-0.120983517173504	0.862079456272147	0.988741847156811	pseudobulk	DESeq2	LRT	0.0644527042097946
497	KC_AB2	btl	2.06137138167525	0.547946929015274	0.988741847156811	pseudobulk	DESeq2	LRT	0.261261502741816
498	KC_AB2	btsz	0.113148473950518	0.893675201805685	0.988741847156811	pseudobulk	DESeq2	LRT	0.0488202929418442
499	KC_AB2	btz	1.4413042803421	0.615311685084066	0.988741847156811	pseudobulk	DESeq2	LRT	0.210904837358999
500	KC_AB2	Bub1	0.157562008957688	0.950463298513166	0.988741847156811	pseudobulk	DESeq2	LRT	0.0220646484576093
501	KC_AB2	Bub3	1.63365892747121	0.39368368529591	0.988741847156811	pseudobulk	DESeq2	LRT	0.404852582499004
502	KC_AB2	bug	-1.19564122961809	0.585865739229133	0.988741847156811	pseudobulk	DESeq2	LRT	0.232201898303775

503	KC_AB2	Bug22	3.79415743120494	0.154946854526743	0.988741847156811	pseudobulk	DESeq2	LRT	0.809817235659252
504	KC_AB2	BuGZ	0.397658516314542	0.623375228727006	0.988741847156811	pseudobulk	DESeq2	LRT	0.205250459423497
505	KC_AB2	bun	0.34338095570515	0.329387484425654	0.988741847156811	pseudobulk	DESeq2	LRT	0.482292906526735
506	KC_AB2	bur	1.32467530657598	0.538468278591255	0.988741847156811	pseudobulk	DESeq2	LRT	0.268839876095909
507	KC_AB2	bves	-0.181717084921255	0.860878063970189	0.988741847156811	pseudobulk	DESeq2	LRT	0.0650583582967608
508	KC_AB2	bwa	0.390461963422779	0.886811854140999	0.988741847156811	pseudobulk	DESeq2	LRT	0.0521685102422415
509	KC_AB2	Bx	0.625498445313879	0.172200243694721	0.988741847156811	pseudobulk	DESeq2	LRT	0.763966238276105
510	KC_AB2	Bx42	1.6309072817361	0.479026786495868	0.988741847156811	pseudobulk	DESeq2	LRT	0.319640200777332
511	KC_AB2	bys	-2.21667329608248	0.477261050443944	0.988741847156811	pseudobulk	DESeq2	LRT	0.321244007216606
512	KC_AB2	c(3)G	0.818790875285784	0.940355871560339	0.988741847156811	pseudobulk	DESeq2	LRT	0.0267077593721816
513	KC_AB2	c11.1	1.09755075627148	0.564545147075846	0.988741847156811	pseudobulk	DESeq2	LRT	0.24830132151245
514	KC_AB2	c12.1	-0.372043213450487	0.827349811348178	0.988741847156811	pseudobulk	DESeq2	LRT	0.0823108277910516
515	KC_AB2	c12.2	0.5738945031678	0.80780652681376	0.988741847156811	pseudobulk	DESeq2	LRT	0.0926926421938231
516	KC_AB2	C1GalTA	-0.125329829850647	0.917586939710787	0.988741847156811	pseudobulk	DESeq2	LRT	0.0373527765059852
517	KC_AB2	C3G	0.96682858298164	0.463363520213763	0.988741847156811	pseudobulk	DESeq2	LRT	0.334078160432732
518	KC_AB2	ca	0.572861358867068	0.813871944286787	0.988741847156811	pseudobulk	DESeq2	LRT	0.0894439222171593
519	KC_AB2	Ca-alpha1D	-0.193690605813389	0.74980026191758	0.988741847156811	pseudobulk	DESeq2	LRT	0.125054412208227
520	KC_AB2	Ca-alpha1T	0.0369354395218551	0.912813487894659	0.988741847156811	pseudobulk	DESeq2	LRT	0.0396179513298228
521	KC_AB2	Ca-beta	0.00023669128333278	0.981928716395879	0.988741847156811	pseudobulk	DESeq2	LRT	0.00792003889288878
522	KC_AB2	Ca-Ma2d	1.15108463671715	0.351616244345157	0.988741847156811	pseudobulk	DESeq2	LRT	0.453931069131548
523	KC_AB2	CaBP1	2.41462217514131	0.497446973552313	0.988741847156811	pseudobulk	DESeq2	LRT	0.303253207020768
524	KC_AB2	cac	0.0695357313664203	0.831504506333162	0.988741847156811	pseudobulk	DESeq2	LRT	0.0801353927820175
525	KC_AB2	cact	0.171347689174491	0.83715265489483	0.988741847156811	pseudobulk	DESeq2	LRT	0.0771953411304638
526	KC_AB2	cactin	2.7785831508444	0.373751317747552	0.988741847156811	pseudobulk	DESeq2	LRT	0.427417267458199
527	KC_AB2	Cad87A	-1.08506241312707	0.67856296251147	0.988741847156811	pseudobulk	DESeq2	LRT	0.168409848810952
528	KC_AB2	Cad96Ca	-1.80294541848982	0.660135549041553	0.988741847156811	pseudobulk	DESeq2	LRT	0.180366879372519
529	KC_AB2	CadN	0.678126213977131	0.609246147012426	0.988741847156811	pseudobulk	DESeq2	LRT	0.215207208694791
530	KC_AB2	CadN2	2.7785831508444	0.373751317747552	0.988741847156811	pseudobulk	DESeq2	LRT	0.427417267458199
531	KC_AB2	Cadps	0.051202928371547	0.902632097846788	0.988741847156811	pseudobulk	DESeq2	LRT	0.0444892269095088
532	KC_AB2	Caf1-105	-0.568233637794205	0.868826397133536	0.988741847156811	pseudobulk	DESeq2	LRT	0.0610669925949191
533	KC_AB2	Caf1-55	1.14703591811676	0.660809111836255	0.988741847156811	pseudobulk	DESeq2	LRT	0.179923977199595
534	KC_AB2	cag	-3.51218082781652	0.306311589270116	0.988741847156811	pseudobulk	DESeq2	LRT	0.513836571391778
535	KC_AB2	CAH2	3.41250446005175	0.270453901523293	0.988741847156811	pseudobulk	DESeq2	LRT	0.567906749116294
536	KC_AB2	CAH3	-0.405328955441183	0.727766263566576	0.988741847156811	pseudobulk	DESeq2	LRT	0.138008080498154
537	KC_AB2	CAH7	-1.80294541848982	0.660135549041553	0.988741847156811	pseudobulk	DESeq2	LRT	0.180366879372519
538	KC_AB2	CAH8	0.738261049346199	0.725130373298079	0.988741847156811	pseudobulk	DESeq2	LRT	0.139583903341193
539	KC_AB2	CAHbeta	0.666943671827768	0.808936122914367	0.988741847156811	pseudobulk	DESeq2	LRT	0.0920857707994393
540	KC_AB2	cal1	1.10527623683615	0.724717602335893	0.988741847156811	pseudobulk	DESeq2	LRT	0.139831190172672
541	KC_AB2	CalpA	-1.2337849808706	0.539269081720012	0.988741847156811	pseudobulk	DESeq2	LRT	0.268194478698133
542	KC_AB2	CalpB	0.157562008957688	0.950463298513166	0.988741847156811	pseudobulk	DESeq2	LRT	0.0220646484576093
543	KC_AB2	Calr	0.396604575378234	0.742284447156411	0.988741847156811	pseudobulk	DESeq2	LRT	0.129429638982423
544	KC_AB2	Cals	0.301146336086121	0.516173393237947	0.988741847156811	pseudobulk	DESeq2	LRT	0.287204385432887
545	KC_AB2	Calx	1.40174012881758	0.384485311499011	0.988741847156811	pseudobulk	DESeq2	LRT	0.415120246907482
546	KC_AB2	calypso	1.13539439938001	0.65602501243538	0.988741847156811	pseudobulk	DESeq2	LRT	0.183079601850592
547	KC_AB2	Cam	-0.165216707846051	0.533028359345462	0.988741847156811	pseudobulk	DESeq2	LRT	0.273249684069865
548	KC_AB2	CaMKI	0.166218994885881	0.776283438534537	0.988741847156811	pseudobulk	DESeq2	LRT	0.109979679107466
549	KC_AB2	CaMKII	-0.23230923234276	0.506656740182954	0.988741847156811	pseudobulk	DESeq2	LRT	0.295286175444624
550	KC_AB2	Camp	0.317508825551005	0.883746467592857	0.988741847156811	pseudobulk	DESeq2	LRT	0.053672309102253
551	KC_AB2	Camta	0.632478144582639	0.683818352791159	0.988741847156811	pseudobulk	DESeq2	LRT	0.165059247486532
552	KC_AB2	CanA-14F	0.168061871244848	0.692108562541423	0.988741847156811	pseudobulk	DESeq2	LRT	0.159825777776311
553	KC_AB2	CanB	0.210472965802838	0.644617687569797	0.988741847156811	pseudobulk	DESeq2	LRT	0.190697782117862
554	KC_AB2	CanB2	-0.479402905382796	0.707153626672184	0.988741847156811	pseudobulk	DESeq2	LRT	0.150486226983555
555	KC_AB2	Cand1	-0.568233637794205	0.868826397133536	0.988741847156811	pseudobulk	DESeq2	LRT	0.0610669925949191
556	KC_AB2	Cap-D3	1.42621218304824	0.66738831402044	0.988741847156811	pseudobulk	DESeq2	LRT	0.175621402117653
557	KC_AB2	Cap-G	2.51243226103394	0.474458247157471	0.988741847156811	pseudobulk	DESeq2	LRT	0.323801999942597
558	KC_AB2	Cap-H2	-0.405381063532158	0.89155634938256	0.988741847156811	pseudobulk	DESeq2	LRT	0.0498512027381788

559	KC_AB2	Caper	0.00325298782972698	0.97654911327967	0.988741847156811	pseudobulk	DESeq2	LRT	0.0103059099900771
560	KC_AB2	Capr	0.132692505409233	0.933426327899036	0.988741847156811	pseudobulk	DESeq2	LRT	0.0299199537186821
561	KC_AB2	caps	0.390374785028037	0.59428971888132	0.988741847156811	pseudobulk	DESeq2	LRT	0.226001782903043
562	KC_AB2	capu	0.210756526288454	0.891203404777959	0.988741847156811	pseudobulk	DESeq2	LRT	0.0500231629766132
563	KC_AB2	car	3.25449350606273	0.266356678091136	0.988741847156811	pseudobulk	DESeq2	LRT	0.574536410117102
564	KC_AB2	CARPB	0.363130219669324	0.869039837990352	0.988741847156811	pseudobulk	DESeq2	LRT	0.0609603144341722
565	KC_AB2	CASK	0.225984104243721	0.660307455884841	0.988741847156811	pseudobulk	DESeq2	LRT	0.1802537988479
566	KC_AB2	casp	0.107243313242643	0.927720828069648	0.988741847156811	pseudobulk	DESeq2	LRT	0.0325826930369169
567	KC_AB2	cass	0.0203382542419031	0.95243884170763	0.988741847156811	pseudobulk	DESeq2	LRT	0.0211629018062989
568	KC_AB2	Cat	1.69894780879763	0.540778829726708	0.988741847156811	pseudobulk	DESeq2	LRT	0.26698031838453
569	KC_AB2	cathD	0.758768486184067	0.75667228241667	0.988741847156811	pseudobulk	DESeq2	LRT	0.121092174336864
570	KC_AB2	Catsup	2.00515076565275	0.576456787459964	0.988741847156811	pseudobulk	DESeq2	LRT	0.239233242872451
571	KC_AB2	caz	-0.330094577451426	0.726775471322457	0.988741847156811	pseudobulk	DESeq2	LRT	0.138599738558647
572	KC_AB2	cbc	-0.506613484034373	0.961658398065406	0.988741847156811	pseudobulk	DESeq2	LRT	0.0169791713942958
573	KC_AB2	Cbl	1.37148473388935	0.290819310035272	0.988741847156811	pseudobulk	DESeq2	LRT	0.536376760249933
574	KC_AB2	Cbp20	2.11513947821417	0.415104446921141	0.988741847156811	pseudobulk	DESeq2	LRT	0.381842614096864
575	KC_AB2	Cbp53E	-0.847876556160616	0.0786403241715408	0.988741847156811	pseudobulk	DESeq2	LRT	1.10435470492133
576	KC_AB2	Cbp80	1.12452187615248	0.704078404906088	0.988741847156811	pseudobulk	DESeq2	LRT	0.152378975911753
577	KC_AB2	cbs	0.578095385250022	0.743958705493656	0.988741847156811	pseudobulk	DESeq2	LRT	0.128451169929867
578	KC_AB2	Cbs	-0.568233637794205	0.868826397133536	0.988741847156811	pseudobulk	DESeq2	LRT	0.0610669925949191
579	KC_AB2	cbt	0.740635261447468	0.680864099739953	0.988741847156811	pseudobulk	DESeq2	LRT	0.166939564479132
580	KC_AB2	CCDC53	-1.80294541848982	0.660135549041553	0.988741847156811	pseudobulk	DESeq2	LRT	0.180366879372519
581	KC_AB2	Ccdc58	0.10598154425916	0.959628155914924	0.988741847156811	pseudobulk	DESeq2	LRT	0.0178970181249073
582	KC_AB2	Cchl	-0.294545289015789	0.830865763757625	0.988741847156811	pseudobulk	DESeq2	LRT	0.080469135985388
583	KC_AB2	CCKLR-17D1	0.818790875285784	0.940355871560339	0.988741847156811	pseudobulk	DESeq2	LRT	0.0267077593721816
584	KC_AB2	Ccm3	-0.508306886400093	0.658018482694835	0.988741847156811	pseudobulk	DESeq2	LRT	0.181761907571995
585	KC_AB2	Ccn	-0.368355348007074	0.595094270366725	0.988741847156811	pseudobulk	DESeq2	LRT	0.225414231150251
586	KC_AB2	Ccs	1.42034337486757	0.643720050244361	0.988741847156811	pseudobulk	DESeq2	LRT	0.191302963490459
587	KC_AB2	CCT1	-0.187188766977065	0.932037508762103	0.988741847156811	pseudobulk	DESeq2	LRT	0.0305666096196055
588	KC_AB2	CCT2	0.0471023025337348	0.971010011322875	0.988741847156811	pseudobulk	DESeq2	LRT	0.0127762923990319
589	KC_AB2	CCT3	0.922664372932389	0.678259639521954	0.988741847156811	pseudobulk	DESeq2	LRT	0.168604025267887
590	KC_AB2	CCT4	-0.742381366866637	0.676610375514192	0.988741847156811	pseudobulk	DESeq2	LRT	0.169661346787454
591	KC_AB2	CCT5	-1.65719613210719	0.599376091584556	0.988741847156811	pseudobulk	DESeq2	LRT	0.222300584546838
592	KC_AB2	CCT6	-0.920201035056088	0.72458149710501	0.988741847156811	pseudobulk	DESeq2	LRT	0.139912760292915
593	KC_AB2	CCT7	-1.20081362769313	0.741163517910401	0.988741847156811	pseudobulk	DESeq2	LRT	0.130085965985812
594	KC_AB2	CCT8	1.4129303440845	0.645460595159151	0.988741847156811	pseudobulk	DESeq2	LRT	0.190130265910817
595	KC_AB2	Ccz1	-0.158601889198964	0.945838126788742	0.988741847156811	pseudobulk	DESeq2	LRT	0.0241831835324482
596	KC_AB2	CD98hc	0.930247921330852	0.444720673333196	0.988741847156811	pseudobulk	DESeq2	LRT	0.351912681421668
597	KC_AB2	Cda5	2.44948324680672	0.324428547937906	0.988741847156811	pseudobulk	DESeq2	LRT	0.48888093724017
598	KC_AB2	cdc14	-0.128716737110856	0.903214390096602	0.988741847156811	pseudobulk	DESeq2	LRT	0.0442091518074421
599	KC_AB2	Cdc16	-1.80294541848982	0.660135549041553	0.988741847156811	pseudobulk	DESeq2	LRT	0.180366879372519
600	KC_AB2	Cdc23	-2.11027789354592	0.473734933668313	0.988741847156811	pseudobulk	DESeq2	LRT	0.32446458879962
601	KC_AB2	Cdc27	0.105902682160767	0.948921138997729	0.988741847156811	pseudobulk	DESeq2	LRT	0.0227698785329056
602	KC_AB2	Cdc2rk	1.13539439938001	0.65602501243538	0.988741847156811	pseudobulk	DESeq2	LRT	0.183079601850592
603	KC_AB2	Cdc37	0.616962881190687	0.574634253628327	0.988741847156811	pseudobulk	DESeq2	LRT	0.240608489519883
604	KC_AB2	Cdc42	-0.145713453028948	0.901043760154535	0.988741847156811	pseudobulk	DESeq2	LRT	0.0452541165324353
605	KC_AB2	CDC45L	0.818790875285784	0.940355871560339	0.988741847156811	pseudobulk	DESeq2	LRT	0.0267077593721816
606	KC_AB2	Cdc5	-0.452344515838269	0.871171367298942	0.988741847156811	pseudobulk	DESeq2	LRT	0.0598964069304489
607	KC_AB2	CDC50	0.330391410498902	0.851537379119407	0.988741847156811	pseudobulk	DESeq2	LRT	0.0697962834747243
608	KC_AB2	Cdc7	-0.689687550646419	0.745991540083112	0.988741847156811	pseudobulk	DESeq2	LRT	0.127266097615876
609	KC_AB2	Cdep	-0.175445391836026	0.800727557644516	0.988741847156811	pseudobulk	DESeq2	LRT	0.0965152246625579
610	KC_AB2	CdGAPr	-0.424202661415976	0.818228840339359	0.988741847156811	pseudobulk	DESeq2	LRT	0.0871252168695471
611	KC_AB2	cdi	1.02425983047713	0.43508495124725	0.988741847156811	pseudobulk	DESeq2	LRT	0.361425937859442
612	KC_AB2	Cdk12	0.444811662881181	0.779564417627056	0.988741847156811	pseudobulk	DESeq2	LRT	0.108147992002967
613	KC_AB2	Cdk2	1.4413042803421	0.615311685084066	0.988741847156811	pseudobulk	DESeq2	LRT	0.210904837358999
614	KC_AB2	CDK2AP1	0.321244353479908	0.883160641080799	0.988741847156811	pseudobulk	DESeq2	LRT	0.053960293942671

615	KC_AB2	Cdk5	-0.842398273321037	0.650199938808377	0.988741847156811	pseudobulk	DESeq2	LRT	0.186953075712217
616	KC_AB2	Cdk5alpha	0.523078832599414	0.412862930024456	0.988741847156811	pseudobulk	DESeq2	LRT	0.384194109638157
617	KC_AB2	Cdk7	-0.506613484034373	0.961658398065406	0.988741847156811	pseudobulk	DESeq2	LRT	0.0169791713942958
618	KC_AB2	Cdk8	2.06137138167525	0.547946929015274	0.988741847156811	pseudobulk	DESeq2	LRT	0.261261502741816
619	KC_AB2	Cdk9	0.868542525530208	0.70796968404673	0.988741847156811	pseudobulk	DESeq2	LRT	0.149985338825918
620	KC_AB2	cdm	0.595228774321507	0.644674315580627	0.988741847156811	pseudobulk	DESeq2	LRT	0.190659632136973
621	KC_AB2	Cds	-0.282388760047645	0.697274381198909	0.988741847156811	pseudobulk	DESeq2	LRT	0.156596291066899
622	KC_AB2	Cen	0.172522961347536	0.933397059180592	0.988741847156811	pseudobulk	DESeq2	LRT	0.0299335717641833
623	KC_AB2	CenB1A	-2.51746470924027	0.517377929599049	0.988741847156811	pseudobulk	DESeq2	LRT	0.286192101436607
624	KC_AB2	CenG1A	-0.852974127770145	0.438666793609434	0.988741847156811	pseudobulk	DESeq2	LRT	0.357865239764213
625	KC_AB2	Cep97	-0.781185819204491	0.6421463640911	0.988741847156811	pseudobulk	DESeq2	LRT	0.19236597212993
626	KC_AB2	cer	1.64209625885217	0.483203675195701	0.988741847156811	pseudobulk	DESeq2	LRT	0.315869771176778
627	KC_AB2	Cerk	0.460210744397841	0.788924068927861	0.988741847156811	pseudobulk	DESeq2	LRT	0.102964794043451
628	KC_AB2	cert	-0.233272055930639	0.937531793764924	0.988741847156811	pseudobulk	DESeq2	LRT	0.028013995469539
629	KC_AB2	cerv	3.25449350606273	0.266356678091136	0.988741847156811	pseudobulk	DESeq2	LRT	0.574536410117102
630	KC_AB2	Cf2	0.295917338969104	0.773878102603078	0.988741847156811	pseudobulk	DESeq2	LRT	0.111327441818392
631	KC_AB2	Cfp1	2.4643417141186	0.449082701910884	0.988741847156811	pseudobulk	DESeq2	LRT	0.347673673080982
632	KC_AB2	cg	-0.401760139419386	0.47432684854778	0.988741847156811	pseudobulk	DESeq2	LRT	0.323922292075646
633	KC_AB2	CG10011	0.164972137049222	0.880298218699372	0.988741847156811	pseudobulk	DESeq2	LRT	0.0553701769461937
634	KC_AB2	CG10019	-0.887548319995794	0.488582509792274	0.988741847156811	pseudobulk	DESeq2	LRT	0.311062083896883
635	KC_AB2	CG10038	-1.69633662497937	0.605069927920179	0.988741847156811	pseudobulk	DESeq2	LRT	0.2181944310419
636	KC_AB2	CG10050	0.818790875285784	0.940355871560339	0.988741847156811	pseudobulk	DESeq2	LRT	0.0267077593721816
637	KC_AB2	CG10053	-0.0582963981256801	0.979058404810457	0.988741847156811	pseudobulk	DESeq2	LRT	0.0091913999942843
638	KC_AB2	CG10055	3.48393309996043	0.216218474953395	0.988741847156811	pseudobulk	DESeq2	LRT	0.665107200172195
639	KC_AB2	CG10068	0.922664372932389	0.678259639521954	0.988741847156811	pseudobulk	DESeq2	LRT	0.168604025267887
640	KC_AB2	CG10075	1.14703591811676	0.660809111836255	0.988741847156811	pseudobulk	DESeq2	LRT	0.179923977199595
641	KC_AB2	CG10077	-0.241777696449333	0.492698938845841	0.988741847156811	pseudobulk	DESeq2	LRT	0.307418373089191
642	KC_AB2	CG10082	0.067008779199463	0.91540395269308	0.988741847156811	pseudobulk	DESeq2	LRT	0.0383872166311499
643	KC_AB2	CG10089	-0.0373653752584733	0.962683152418628	0.988741847156811	pseudobulk	DESeq2	LRT	0.0165166285542605
644	KC_AB2	CG10098	-0.106269097517594	0.938540389308507	0.988741847156811	pseudobulk	DESeq2	LRT	0.0275470331370631
645	KC_AB2	CG10103	-0.345036337248882	0.833079212119776	0.988741847156811	pseudobulk	DESeq2	LRT	0.0793137023735677
646	KC_AB2	CG10132	0.887372949159358	0.383114186119329	0.988741847156811	pseudobulk	DESeq2	LRT	0.416671766473519
647	KC_AB2	CG10137	0.234988598610617	0.862939759617794	0.988741847156811	pseudobulk	DESeq2	LRT	0.0640195205984908
648	KC_AB2	CG10139	1.23913594857638	0.441771833901474	0.988741847156811	pseudobulk	DESeq2	LRT	0.354801976946044
649	KC_AB2	CG10147	0.269971509165439	0.903083168079264	0.988741847156811	pseudobulk	DESeq2	LRT	0.0442722521508783
650	KC_AB2	CG10151	0.680485379488395	0.740381497778529	0.988741847156811	pseudobulk	DESeq2	LRT	0.130544442854155
651	KC_AB2	CG10165	1.42621218304824	0.66738831402044	0.988741847156811	pseudobulk	DESeq2	LRT	0.175621402117653
652	KC_AB2	CG10166	-0.488465967391191	0.84251072973523	0.988741847156811	pseudobulk	DESeq2	LRT	0.0744245594950451
653	KC_AB2	CG10171	-0.568233637794205	0.868826397133536	0.988741847156811	pseudobulk	DESeq2	LRT	0.0610669925949191
654	KC_AB2	CG10188	-0.464887843860708	0.688226702105887	0.988741847156811	pseudobulk	DESeq2	LRT	0.162268481450232
655	KC_AB2	CG10189	0.818790875285784	0.940355871560339	0.988741847156811	pseudobulk	DESeq2	LRT	0.0267077593721816
656	KC_AB2	CG10194	-0.421426144018401	0.837702312916935	0.988741847156811	pseudobulk	DESeq2	LRT	0.0769102854580717
657	KC_AB2	CG10209	1.31617713253562	0.59447159965162	0.988741847156811	pseudobulk	DESeq2	LRT	0.225868888579699
658	KC_AB2	CG10214	0.508232850315684	0.818116998464947	0.988741847156811	pseudobulk	DESeq2	LRT	0.0871845836706556
659	KC_AB2	CG10222	1.42621218304824	0.66738831402044	0.988741847156811	pseudobulk	DESeq2	LRT	0.175621402117653
660	KC_AB2	CG10232	1.42621218304824	0.66738831402044	0.988741847156811	pseudobulk	DESeq2	LRT	0.175621402117653
661	KC_AB2	CG1024	-0.331004884396198	0.894406551228697	0.988741847156811	pseudobulk	DESeq2	LRT	0.0484650283842476
662	KC_AB2	CG10252	0.818790875285784	0.940355871560339	0.988741847156811	pseudobulk	DESeq2	LRT	0.0267077593721816
663	KC_AB2	CG10254	1.99386207130934	0.349788165749185	0.988741847156811	pseudobulk	DESeq2	LRT	0.456194887929862
664	KC_AB2	CG10257	-0.275695871128137	0.867470937595485	0.988741847156811	pseudobulk	DESeq2	LRT	0.0617450662239331
665	KC_AB2	CG10265	2.11513947821417	0.415104446921141	0.988741847156811	pseudobulk	DESeq2	LRT	0.381842614096864
666	KC_AB2	CG10268	-0.378852672109618	0.920549594954422	0.988741847156811	pseudobulk	DESeq2	LRT	0.0359528087516892
667	KC_AB2	CG10283	0.177638023285271	0.920343194892319	0.988741847156811	pseudobulk	DESeq2	LRT	0.0360501945515639
668	KC_AB2	CG10286	-1.23536834900699	0.725570392794057	0.988741847156811	pseudobulk	DESeq2	LRT	0.139320447155934
669	KC_AB2	CG10321	-1.79349865679954	0.372423177328072	0.988741847156811	pseudobulk	DESeq2	LRT	0.428963299032091
670	KC_AB2	CG10324	0.666943671827768	0.808936122914367	0.988741847156811	pseudobulk	DESeq2	LRT	0.0920857707994393

671	KC_AB2	CG10333	0.244330481508234	0.890890414664351	0.988741847156811	pseudobulk	DESeq2	LRT	0.0501757137336436
672	KC_AB2	CG10338	1.42621218304824	0.66738831402044	0.988741847156811	pseudobulk	DESeq2	LRT	0.175621402117653
673	KC_AB2	CG10340	1.18229736072854	0.45274905475825	0.988741847156811	pseudobulk	DESeq2	LRT	0.344142447725566
674	KC_AB2	CG10343	2.73593896747858	0.422277110754874	0.988741847156811	pseudobulk	DESeq2	LRT	0.374402458575713
675	KC_AB2	CG10347	1.12452187615248	0.704078404906088	0.988741847156811	pseudobulk	DESeq2	LRT	0.152378975911753
676	KC_AB2	CG10353	-0.996962628187757	0.539879850345917	0.988741847156811	pseudobulk	DESeq2	LRT	0.26770288117273
677	KC_AB2	CG10362	-1.5707624441993	0.507036228886573	0.988741847156811	pseudobulk	DESeq2	LRT	0.294961008233931
678	KC_AB2	CG10365	-3.91108067132102	0.221683018369551	0.988741847156811	pseudobulk	DESeq2	LRT	0.654267573962038
679	KC_AB2	CG10366	-0.463188358016548	0.832830924973029	0.988741847156811	pseudobulk	DESeq2	LRT	0.0794431568215084
680	KC_AB2	CG10375	-0.938999680670882	0.457388532106753	0.988741847156811	pseudobulk	DESeq2	LRT	0.339714728459466
681	KC_AB2	CG10376	0.712171366772551	0.813205723536451	0.988741847156811	pseudobulk	DESeq2	LRT	0.0897995733547685
682	KC_AB2	CG10383	2.7785831508444	0.373751317747552	0.988741847156811	pseudobulk	DESeq2	LRT	0.427417267458199
683	KC_AB2	CG10384	1.42621218304824	0.66738831402044	0.988741847156811	pseudobulk	DESeq2	LRT	0.175621402117653
684	KC_AB2	CG10395	-0.883669024954103	0.485283099155049	0.988741847156811	pseudobulk	DESeq2	LRT	0.314004833497229
685	KC_AB2	CG10413	1.12452187615248	0.704078404906088	0.988741847156811	pseudobulk	DESeq2	LRT	0.152378975911753
686	KC_AB2	CG10417	0.616962881190687	0.574634253628327	0.988741847156811	pseudobulk	DESeq2	LRT	0.240608489519883
687	KC_AB2	CG10418	-0.50879048068994	0.817628596116567	0.988741847156811	pseudobulk	DESeq2	LRT	0.0874439277298491
688	KC_AB2	CG10428	-2.45158172483764	0.378823136429564	0.988741847156811	pseudobulk	DESeq2	LRT	0.421563504545096
689	KC_AB2	CG10433	-1.80294541848982	0.660135549041553	0.988741847156811	pseudobulk	DESeq2	LRT	0.180366879372519
690	KC_AB2	CG10435	-0.00340877878248056	0.980029417943268	0.988741847156811	pseudobulk	DESeq2	LRT	0.0087608877170192
691	KC_AB2	CG10444	-2.51746470924027	0.517377929599049	0.988741847156811	pseudobulk	DESeq2	LRT	0.286192101436607
692	KC_AB2	CG10463	-1.80294541848982	0.660135549041553	0.988741847156811	pseudobulk	DESeq2	LRT	0.180366879372519
693	KC_AB2	CG10465	0.366200411612712	0.749205993869354	0.988741847156811	pseudobulk	DESeq2	LRT	0.125398756798705
694	KC_AB2	CG10466	-0.920201035056088	0.72458149710501	0.988741847156811	pseudobulk	DESeq2	LRT	0.139912760292915
695	KC_AB2	CG10469	0.818790875285784	0.940355871560339	0.988741847156811	pseudobulk	DESeq2	LRT	0.0267077593721816
696	KC_AB2	CG10470	0.335362974997744	0.846331023922517	0.988741847156811	pseudobulk	DESeq2	LRT	0.0724597389177114
697	KC_AB2	CG10479	0.0601466527378941	0.974810960364079	0.988741847156811	pseudobulk	DESeq2	LRT	0.0110795964355292
698	KC_AB2	CG10483	0.892530381217315	0.606077298715004	0.988741847156811	pseudobulk	DESeq2	LRT	0.217471982657939
699	KC_AB2	CG10492	-0.50879048068994	0.817628596116567	0.988741847156811	pseudobulk	DESeq2	LRT	0.0874439277298491
700	KC_AB2	CG10494	1.4413042803421	0.615311685084066	0.988741847156811	pseudobulk	DESeq2	LRT	0.210904837358999
701	KC_AB2	CG10508	0.235343167113255	0.774695485109369	0.988741847156811	pseudobulk	DESeq2	LRT	0.110868975074713
702	KC_AB2	CG10513	2.51243226103394	0.474458247157471	0.988741847156811	pseudobulk	DESeq2	LRT	0.323801999942597
703	KC_AB2	CG10514	1.42621218304824	0.66738831402044	0.988741847156811	pseudobulk	DESeq2	LRT	0.175621402117653
704	KC_AB2	CG10516	-0.934020934207768	0.782359118258182	0.988741847156811	pseudobulk	DESeq2	LRT	0.106593851444836
705	KC_AB2	CG10543	0.108498207000731	0.888671781130135	0.988741847156811	pseudobulk	DESeq2	LRT	0.0512586101929803
706	KC_AB2	CG10555	0.0349875728287032	0.980612927422154	0.988741847156811	pseudobulk	DESeq2	LRT	0.00850238574709158
707	KC_AB2	CG10561	-0.588639054357632	0.8432408255636	0.988741847156811	pseudobulk	DESeq2	LRT	0.0740483752247712
708	KC_AB2	CG10565	1.72140536509799	0.359434023678424	0.988741847156811	pseudobulk	DESeq2	LRT	0.444380815383927
709	KC_AB2	CG10576	-0.507140190294263	0.811190366321596	0.988741847156811	pseudobulk	DESeq2	LRT	0.0908772156515369
710	KC_AB2	CG10584	0.818790875285784	0.940355871560339	0.988741847156811	pseudobulk	DESeq2	LRT	0.0267077593721816
711	KC_AB2	CG10600	-0.252599375136763	0.858731708968153	0.988741847156811	pseudobulk	DESeq2	LRT	0.0661425003298305
712	KC_AB2	CG10602	-1.32980601192291	0.583869450786927	0.988741847156811	pseudobulk	DESeq2	LRT	0.233684247306934
713	KC_AB2	CG10616	-0.0229973822346953	0.971360658954591	0.988741847156811	pseudobulk	DESeq2	LRT	0.0126194898530182
714	KC_AB2	CG10628	2.4643417141186	0.449082701910884	0.988741847156811	pseudobulk	DESeq2	LRT	0.347673673080982
715	KC_AB2	CG10631	3.63001914094513	0.185579857467938	0.988741847156811	pseudobulk	DESeq2	LRT	0.731469163166964
716	KC_AB2	CG10638	-1.54768271596609	0.394513952614918	0.988741847156811	pseudobulk	DESeq2	LRT	0.403937632666189
717	KC_AB2	CG10654	1.4413042803421	0.615311685084066	0.988741847156811	pseudobulk	DESeq2	LRT	0.210904837358999
718	KC_AB2	CG10669	-0.506613484034373	0.961658398065406	0.988741847156811	pseudobulk	DESeq2	LRT	0.0169791713942958
719	KC_AB2	CG10672	-2.11027789354592	0.473734933668313	0.988741847156811	pseudobulk	DESeq2	LRT	0.32446458879962
720	KC_AB2	CG10674	-0.144853288068314	0.931823095060018	0.988741847156811	pseudobulk	DESeq2	LRT	0.0306665298474161
721	KC_AB2	CG10681	0.0601466527378941	0.974810960364079	0.988741847156811	pseudobulk	DESeq2	LRT	0.0110795964355292
722	KC_AB2	CG10713	-0.729234089517982	0.868684292414125	0.988741847156811	pseudobulk	DESeq2	LRT	0.0611380313470854
723	KC_AB2	CG10731	0.518112575170181	0.804900582036886	0.988741847156811	pseudobulk	DESeq2	LRT	0.0942577585627823
724	KC_AB2	CG10737	-1.15218677467413	0.555163608827013	0.988741847156811	pseudobulk	DESeq2	LRT	0.255579009778709
725	KC_AB2	CG10738	-0.0323203545809474	0.954448156132187	0.988741847156811	pseudobulk	DESeq2	LRT	0.020247656706879
726	KC_AB2	CG1074	-0.568233637794205	0.868826397133536	0.988741847156811	pseudobulk	DESeq2	LRT	0.0610669925949191

727	KC_AB2	CG10747	-1.80294541848982	0.660135549041553	0.988741847156811	pseudobulk	DESeq2	LRT	0.180366879372519
728	KC_AB2	CG10778	-1.80294541848982	0.660135549041553	0.988741847156811	pseudobulk	DESeq2	LRT	0.180366879372519
729	KC_AB2	CG10795	-1.29600486439944	0.685894431367319	0.988741847156811	pseudobulk	DESeq2	LRT	0.163742723072789
730	KC_AB2	CG10802	0.818790875285784	0.940355871560339	0.988741847156811	pseudobulk	DESeq2	LRT	0.0267077593721816
731	KC_AB2	CG10803	-0.274911090244434	0.825905725535048	0.988741847156811	pseudobulk	DESeq2	LRT	0.0830695231597301
732	KC_AB2	CG10804	0.0786070046177643	0.92484897646186	0.988741847156811	pseudobulk	DESeq2	LRT	0.0339291797411018
733	KC_AB2	CG10809	0.281219297776739	0.83967129765854	0.988741847156811	pseudobulk	DESeq2	LRT	0.0758906919750188
734	KC_AB2	CG10863	1.42621218304824	0.66738831402044	0.988741847156811	pseudobulk	DESeq2	LRT	0.175621402117653
735	KC_AB2	CG10866	-2.9721553030081	0.387854083811327	0.988741847156811	pseudobulk	DESeq2	LRT	0.411331631401987
736	KC_AB2	CG10874	1.40305140665399	0.649741560632143	0.988741847156811	pseudobulk	DESeq2	LRT	0.187259352757606
737	KC_AB2	CG10889	-0.506613484034373	0.961658398065406	0.988741847156811	pseudobulk	DESeq2	LRT	0.0169791713942958
738	KC_AB2	CG10898	0.157562008957688	0.950463298513166	0.988741847156811	pseudobulk	DESeq2	LRT	0.0220646484576093
739	KC_AB2	CG1090	0.35162338202339	0.731650838802986	0.988741847156811	pseudobulk	DESeq2	LRT	0.135696125168687
740	KC_AB2	CG10903	3.06202454821851	0.30627034058063	0.988741847156811	pseudobulk	DESeq2	LRT	0.513895058517773
741	KC_AB2	CG10904	0.351722119397094	0.929615296941389	0.988741847156811	pseudobulk	DESeq2	LRT	0.0316967385224016
742	KC_AB2	CG10907	2.4643417141186	0.449082701910884	0.988741847156811	pseudobulk	DESeq2	LRT	0.347673673080982
743	KC_AB2	CG10914	1.4413042803421	0.615311685084066	0.988741847156811	pseudobulk	DESeq2	LRT	0.210904837358999
744	KC_AB2	CG10916	-1.20081362769313	0.741163517910401	0.988741847156811	pseudobulk	DESeq2	LRT	0.130085965985812
745	KC_AB2	CG10932	0.818790875285784	0.940355871560339	0.988741847156811	pseudobulk	DESeq2	LRT	0.0267077593721816
746	KC_AB2	CG10947	0.311971974219864	0.850384078638143	0.988741847156811	pseudobulk	DESeq2	LRT	0.0703848795105447
747	KC_AB2	CG10948	-0.153387422245114	0.918842205331607	0.988741847156811	pseudobulk	DESeq2	LRT	0.0367590644982888
748	KC_AB2	CG10949	-0.568233637794205	0.868826397133536	0.988741847156811	pseudobulk	DESeq2	LRT	0.0610669925949191
749	KC_AB2	CG10959	2.06137138167525	0.547946929015274	0.988741847156811	pseudobulk	DESeq2	LRT	0.261261502741816
750	KC_AB2	CG10960	0.385189473162274	0.752939868354927	0.988741847156811	pseudobulk	DESeq2	LRT	0.123239706248719
751	KC_AB2	CG10973	-0.568233637794205	0.868826397133536	0.988741847156811	pseudobulk	DESeq2	LRT	0.0610669925949191
752	KC_AB2	CG10979	1.12919034796087	0.7301237214754	0.988741847156811	pseudobulk	DESeq2	LRT	0.136603541247596
753	KC_AB2	CG10984	-0.0895902818285133	0.954518965493979	0.988741847156811	pseudobulk	DESeq2	LRT	0.0202154381162507
754	KC_AB2	CG11000	-0.385892692916572	0.726292803445357	0.988741847156811	pseudobulk	DESeq2	LRT	0.138888259073431
755	KC_AB2	CG11007	0.157562008957688	0.950463298513166	0.988741847156811	pseudobulk	DESeq2	LRT	0.0220646484576093
756	KC_AB2	CG1103	-0.177983666163382	0.915073671553535	0.988741847156811	pseudobulk	DESeq2	LRT	0.0385439399650358
757	KC_AB2	CG11030	-1.12561613168135	0.232030942874755	0.988741847156811	pseudobulk	DESeq2	LRT	0.634454095179463
758	KC_AB2	CG11035	0.27226851987049	0.911402843231596	0.988741847156811	pseudobulk	DESeq2	LRT	0.0402896209172008
759	KC_AB2	CG1105	1.47543920254359	0.661929169513603	0.988741847156811	pseudobulk	DESeq2	LRT	0.17918848025087
760	KC_AB2	CG11050	1.02040418550165	0.437745405989792	0.988741847156811	pseudobulk	DESeq2	LRT	0.358778403014368
761	KC_AB2	CG11070	-1.80933943727629	0.584587525761637	0.988741847156811	pseudobulk	DESeq2	LRT	0.233150456085471
762	KC_AB2	CG11076	-0.142837436653266	0.902967443807358	0.988741847156811	pseudobulk	DESeq2	LRT	0.0443279077481788
763	KC_AB2	CG11077	0.430971618573129	0.663878665957655	0.988741847156811	pseudobulk	DESeq2	LRT	0.177911287379194
764	KC_AB2	CG11089	-0.506613484034373	0.961658398065406	0.988741847156811	pseudobulk	DESeq2	LRT	0.0169791713942958
765	KC_AB2	CG11095	2.41462217514131	0.497446973552313	0.988741847156811	pseudobulk	DESeq2	LRT	0.303253207020768
766	KC_AB2	CG11099	0.741345218380303	0.489188526027102	0.988741847156811	pseudobulk	DESeq2	LRT	0.310523737943099
767	KC_AB2	CG11103	3.03619069427769	0.314302467188174	0.988741847156811	pseudobulk	DESeq2	LRT	0.502652209876457
768	KC_AB2	CG11109	2.06137138167525	0.547946929015274	0.988741847156811	pseudobulk	DESeq2	LRT	0.261261502741816
769	KC_AB2	CG11110	-0.378852672109618	0.920549594954422	0.988741847156811	pseudobulk	DESeq2	LRT	0.0359528087516892
770	KC_AB2	CG11122	-0.415458143101044	0.674933719994709	0.988741847156811	pseudobulk	DESeq2	LRT	0.17073887376733
771	KC_AB2	CG11123	-1.80294541848982	0.660135549041553	0.988741847156811	pseudobulk	DESeq2	LRT	0.180366879372519
772	KC_AB2	CG1113	-2.51746470924027	0.517377929599049	0.988741847156811	pseudobulk	DESeq2	LRT	0.286192101436607
773	KC_AB2	CG11131	2.00515076565275	0.576456787459964	0.988741847156811	pseudobulk	DESeq2	LRT	0.239233242872451
774	KC_AB2	CG11134	1.47543920254359	0.661929169513603	0.988741847156811	pseudobulk	DESeq2	LRT	0.17918848025087
775	KC_AB2	CG11137	0.368165264923951	0.70128564345444	0.988741847156811	pseudobulk	DESeq2	LRT	0.15410505177901
776	KC_AB2	CG11141	1.12919034796087	0.7301237214754	0.988741847156811	pseudobulk	DESeq2	LRT	0.136603541247596
777	KC_AB2	CG11151	0.0987933044661516	0.918250747088431	0.988741847156811	pseudobulk	DESeq2	LRT	0.0370387096407126
778	KC_AB2	CG11155	-0.255176664395215	0.576878239190451	0.988741847156811	pseudobulk	DESeq2	LRT	0.238915843045243
779	KC_AB2	CG1116	3.27725343811621	0.25757966897206	0.988741847156811	pseudobulk	DESeq2	LRT	0.589088419269148
780	KC_AB2	CG11170	-1.80294541848982	0.660135549041553	0.988741847156811	pseudobulk	DESeq2	LRT	0.180366879372519
781	KC_AB2	CG11178	-0.452344515838269	0.871171367298942	0.988741847156811	pseudobulk	DESeq2	LRT	0.0598964069304489
782	KC_AB2	CG11180	0.818790875285784	0.940355871560339	0.988741847156811	pseudobulk	DESeq2	LRT	0.0267077593721816

783	KC_AB2	CG11191	0.164168593619679	0.747449774141823	0.988741847156811	pseudobulk	DESeq2	LRT	0.126417985004748
784	KC_AB2	CG11200	-0.934020934207768	0.782359118258182	0.988741847156811	pseudobulk	DESeq2	LRT	0.106593851444836
785	KC_AB2	CG11247	-0.504672781004287	0.694888940360225	0.988741847156811	pseudobulk	DESeq2	LRT	0.158084600363647
786	KC_AB2	CG11248	-1.39136845792757	0.465860886257086	0.988741847156811	pseudobulk	DESeq2	LRT	0.33174375144447
787	KC_AB2	CG11257	0.157562008957688	0.950463298513166	0.988741847156811	pseudobulk	DESeq2	LRT	0.0220646484576093
788	KC_AB2	CG11267	1.16451095191646	0.226548735824728	0.988741847156811	pseudobulk	DESeq2	LRT	0.644838356905129
789	KC_AB2	CG11279	2.99842299859728	0.361638898709429	0.988741847156811	pseudobulk	DESeq2	LRT	0.441724861973572
790	KC_AB2	CG1129	0.25858261909978	0.755452448766453	0.988741847156811	pseudobulk	DESeq2	LRT	0.121792866716432
791	KC_AB2	CG11307	-0.506613484034373	0.961658398065406	0.988741847156811	pseudobulk	DESeq2	LRT	0.0169791713942958
792	KC_AB2	CG11309	0.818790875285784	0.940355871560339	0.988741847156811	pseudobulk	DESeq2	LRT	0.0267077593721816
793	KC_AB2	CG11317	2.35604698204764	0.229762088337996	0.988741847156811	pseudobulk	DESeq2	LRT	0.638721630059894
794	KC_AB2	CG11319	0.0597744124257082	0.871811895378637	0.988741847156811	pseudobulk	DESeq2	LRT	0.0595772095761257
795	KC_AB2	CG11334	-0.742148798960242	0.80644751100302	0.988741847156811	pseudobulk	DESeq2	LRT	0.0934238941443075
796	KC_AB2	CG11357	0.495080965515537	0.728381716125761	0.988741847156811	pseudobulk	DESeq2	LRT	0.137640964422081
797	KC_AB2	CG11360	-0.513022670992384	0.539139584597753	0.988741847156811	pseudobulk	DESeq2	LRT	0.268298780318931
798	KC_AB2	CG11367	2.00515076565275	0.576456787459964	0.988741847156811	pseudobulk	DESeq2	LRT	0.239233242872451
799	KC_AB2	CG11374	0.818790875285784	0.940355871560339	0.988741847156811	pseudobulk	DESeq2	LRT	0.0267077593721816
800	KC_AB2	CG11377	-0.729234089517982	0.868684292414125	0.988741847156811	pseudobulk	DESeq2	LRT	0.0611380313470854
801	KC_AB2	CG11396	0.0601466527378941	0.974810960364079	0.988741847156811	pseudobulk	DESeq2	LRT	0.0110795964355292
802	KC_AB2	CG11398	-1.80294541848982	0.660135549041553	0.988741847156811	pseudobulk	DESeq2	LRT	0.180366879372519
803	KC_AB2	CG11399	0.602197121971709	0.777679873261571	0.988741847156811	pseudobulk	DESeq2	LRT	0.109199140654683
804	KC_AB2	CG11406	2.51243226103394	0.474458247157471	0.988741847156811	pseudobulk	DESeq2	LRT	0.323801999942597
805	KC_AB2	CG11414	-2.64843360157809	0.321943703667444	0.988741847156811	pseudobulk	DESeq2	LRT	0.492220064093287
806	KC_AB2	CG11417	-1.65719613210719	0.599376091584556	0.988741847156811	pseudobulk	DESeq2	LRT	0.222300584546838
807	KC_AB2	CG1142	2.55216854135116	0.29732771327113	0.988741847156811	pseudobulk	DESeq2	LRT	0.526764609261937
808	KC_AB2	CG11444	-0.644964795483367	0.684317848487906	0.988741847156811	pseudobulk	DESeq2	LRT	0.164742132517617
809	KC_AB2	CG11447	-0.405381063532158	0.89155634938256	0.988741847156811	pseudobulk	DESeq2	LRT	0.0498512027381788
810	KC_AB2	CG11454	2.18699318931231	0.234444038955972	0.988741847156811	pseudobulk	DESeq2	LRT	0.62996080528572
811	KC_AB2	CG11456	0.158485844312962	0.932199411153928	0.988741847156811	pseudobulk	DESeq2	LRT	0.0304911757358515
812	KC_AB2	CG1146	0.0601466527378941	0.974810960364079	0.988741847156811	pseudobulk	DESeq2	LRT	0.0110795964355292
813	KC_AB2	CG11474	2.4643417141186	0.449082701910884	0.988741847156811	pseudobulk	DESeq2	LRT	0.347673673080982
814	KC_AB2	CG11486	-0.0994080140647224	0.818480779283334	0.988741847156811	pseudobulk	DESeq2	LRT	0.0869915148460516
815	KC_AB2	CG11504	-0.110156823709618	0.935376654353758	0.988741847156811	pseudobulk	DESeq2	LRT	0.0290134736533693
816	KC_AB2	CG11523	-0.889062944005037	0.640569392963698	0.988741847156811	pseudobulk	DESeq2	LRT	0.193433816194413
817	KC_AB2	CG11537	-0.0570385225418247	0.944531760296982	0.988741847156811	pseudobulk	DESeq2	LRT	0.0247834341531407
818	KC_AB2	CG11539	-1.80294541848982	0.660135549041553	0.988741847156811	pseudobulk	DESeq2	LRT	0.180366879372519
819	KC_AB2	CG11555	0.362555753552128	0.897034383127386	0.988741847156811	pseudobulk	DESeq2	LRT	0.0471909102264623
820	KC_AB2	CG11560	-3.09794865339079	0.39855598532175	0.988741847156811	pseudobulk	DESeq2	LRT	0.399510664459796
821	KC_AB2	CG11562	2.43796147164888	0.320874079934739	0.988741847156811	pseudobulk	DESeq2	LRT	0.493665363602429
822	KC_AB2	CG11563	0.666943671827768	0.808936122914367	0.988741847156811	pseudobulk	DESeq2	LRT	0.0920857707994393
823	KC_AB2	CG11583	0.315515278988381	0.901389738439351	0.988741847156811	pseudobulk	DESeq2	LRT	0.0450873903126698
824	KC_AB2	CG11586	1.69922424137453	0.307926208465612	0.988741847156811	pseudobulk	DESeq2	LRT	0.511553345498895
825	KC_AB2	CG11590	-0.506613484034373	0.961658398065406	0.988741847156811	pseudobulk	DESeq2	LRT	0.0169791713942958
826	KC_AB2	CG11593	-3.51218082781652	0.306311589270116	0.988741847156811	pseudobulk	DESeq2	LRT	0.513836571391778
827	KC_AB2	CG11596	0.818790875285784	0.940355871560339	0.988741847156811	pseudobulk	DESeq2	LRT	0.0267077593721816
828	KC_AB2	CG11601	0.712171366772551	0.813205723536451	0.988741847156811	pseudobulk	DESeq2	LRT	0.0897995733547685
829	KC_AB2	CG1161	-0.818911012405941	0.472486049391436	0.988741847156811	pseudobulk	DESeq2	LRT	0.325611009930933
830	KC_AB2	CG11626	-1.23536834900699	0.725570392794057	0.988741847156811	pseudobulk	DESeq2	LRT	0.139320447155934
831	KC_AB2	CG11638	-3.76093835843942	0.0787180363352186	0.988741847156811	pseudobulk	DESeq2	LRT	1.10392574815669
832	KC_AB2	CG11658	0.47541206690141	0.737281054715613	0.988741847156811	pseudobulk	DESeq2	LRT	0.132366925649564
833	KC_AB2	CG11695	-0.378852672109618	0.920549594954422	0.988741847156811	pseudobulk	DESeq2	LRT	0.0359528087516892
834	KC_AB2	CG11696	-0.568233637794205	0.868826397133536	0.988741847156811	pseudobulk	DESeq2	LRT	0.0610669925949191
835	KC_AB2	CG11699	-0.159455945029067	0.908776420260247	0.988741847156811	pseudobulk	DESeq2	LRT	0.0415429499910144
836	KC_AB2	CG11710	2.28068239784515	0.366179304841476	0.988741847156811	pseudobulk	DESeq2	LRT	0.436306204128051
837	KC_AB2	CG1172	-0.603364947088068	0.775557174031654	0.988741847156811	pseudobulk	DESeq2	LRT	0.110386180505197
838	KC_AB2	CG11722	2.43796147164888	0.320874079934739	0.988741847156811	pseudobulk	DESeq2	LRT	0.493665363602429

839	KC_AB2	CG11723	-0.934020934207768	0.782359118258182	0.988741847156811	pseudobulk	DESeq2	LRT	0.106593851444836
840	KC_AB2	CG11737	-0.729234089517982	0.868684292414125	0.988741847156811	pseudobulk	DESeq2	LRT	0.0611380313470854
841	KC_AB2	CG11752	1.1272557671306	0.24951346618778	0.988741847156811	pseudobulk	DESeq2	LRT	0.60290601062886
842	KC_AB2	CG11753	-0.0696062406270821	0.956337869997271	0.988741847156811	pseudobulk	DESeq2	LRT	0.0193886462679046
843	KC_AB2	CG11755	1.47543920254359	0.661929169513603	0.988741847156811	pseudobulk	DESeq2	LRT	0.17918848025087
844	KC_AB2	CG11760	-1.80294541848982	0.660135549041553	0.988741847156811	pseudobulk	DESeq2	LRT	0.180366879372519
845	KC_AB2	CG11768	-0.256985834574047	0.742735753439019	0.988741847156811	pseudobulk	DESeq2	LRT	0.129165669732523
846	KC_AB2	CG11771	2.41462217514131	0.497446973552313	0.988741847156811	pseudobulk	DESeq2	LRT	0.303253207020768
847	KC_AB2	CG11777	-3.6809086040586	0.231552724242678	0.988741847156811	pseudobulk	DESeq2	LRT	0.635350105118793
848	KC_AB2	CG11779	-0.197565662601209	0.921418496992636	0.988741847156811	pseudobulk	DESeq2	LRT	0.0355430737620862
849	KC_AB2	CG11781	-0.979033212163068	0.464563376897005	0.988741847156811	pseudobulk	DESeq2	LRT	0.332955030089927
850	KC_AB2	CG11788	-0.506613484034373	0.961658398065406	0.988741847156811	pseudobulk	DESeq2	LRT	0.0169791713942958
851	KC_AB2	CG11790	-1.7473988760673	0.438033495305606	0.988741847156811	pseudobulk	DESeq2	LRT	0.358492678833342
852	KC_AB2	CG11807	-0.522044679843893	0.796310928443557	0.988741847156811	pseudobulk	DESeq2	LRT	0.0989173240449814
853	KC_AB2	CG11808	2.06137138167525	0.547946929015274	0.988741847156811	pseudobulk	DESeq2	LRT	0.261261502741816
854	KC_AB2	CG11811	1.13539439938001	0.65602501243538	0.988741847156811	pseudobulk	DESeq2	LRT	0.183079601850592
855	KC_AB2	CG11835	0.0651758372004298	0.942953353421588	0.988741847156811	pseudobulk	DESeq2	LRT	0.0255097906695348
856	KC_AB2	CG11837	0.699674038439951	0.821690393783994	0.988741847156811	pseudobulk	DESeq2	LRT	0.0852917902455435
857	KC_AB2	CG11857	0.448047619542509	0.802856870845756	0.988741847156811	pseudobulk	DESeq2	LRT	0.0953618715860232
858	KC_AB2	CG11858	2.80496381579643	0.22093644234667	0.988741847156811	pseudobulk	DESeq2	LRT	0.655732643545827
859	KC_AB2	CG11873	-0.235336557473206	0.784322291938167	0.988741847156811	pseudobulk	DESeq2	LRT	0.105505441335497
860	KC_AB2	CG11882	-1.80294541848982	0.660135549041553	0.988741847156811	pseudobulk	DESeq2	LRT	0.180366879372519
861	KC_AB2	CG11883	-0.254747577233665	0.838531138328601	0.988741847156811	pseudobulk	DESeq2	LRT	0.0764808055048887
862	KC_AB2	CG11885	0.684221129527733	0.737351212059311	0.988741847156811	pseudobulk	DESeq2	LRT	0.132325601520231
863	KC_AB2	CG11898	0.818790875285784	0.940355871560339	0.988741847156811	pseudobulk	DESeq2	LRT	0.0267077593721816
864	KC_AB2	CG11902	-0.0582963981256801	0.979058404810457	0.988741847156811	pseudobulk	DESeq2	LRT	0.0091913999942843
865	KC_AB2	CG11906	-2.51746470924027	0.517377929599049	0.988741847156811	pseudobulk	DESeq2	LRT	0.286192101436607
866	KC_AB2	CG11920	-2.9721553030081	0.387854083811327	0.988741847156811	pseudobulk	DESeq2	LRT	0.411331631401987
867	KC_AB2	CG11927	0.921695369118063	0.730077514579258	0.988741847156811	pseudobulk	DESeq2	LRT	0.136631027048226
868	KC_AB2	CG11964	-0.957133960806503	0.762632518785665	0.988741847156811	pseudobulk	DESeq2	LRT	0.1176846802739
869	KC_AB2	CG11970	-0.233272055930639	0.937531793764924	0.988741847156811	pseudobulk	DESeq2	LRT	0.028013995469539
870	KC_AB2	CG11975	-0.97670612739315	0.676315355917219	0.988741847156811	pseudobulk	DESeq2	LRT	0.169850751692797
871	KC_AB2	CG11980	-0.853565742783243	0.753557841534365	0.988741847156811	pseudobulk	DESeq2	LRT	0.122883406527736
872	KC_AB2	CG11983	1.47543920254359	0.661929169513603	0.988741847156811	pseudobulk	DESeq2	LRT	0.17918848025087
873	KC_AB2	CG11986	0.818790875285784	0.940355871560339	0.988741847156811	pseudobulk	DESeq2	LRT	0.0267077593721816
874	KC_AB2	CG11999	0.10598154425916	0.959628155914924	0.988741847156811	pseudobulk	DESeq2	LRT	0.0178970181249073
875	KC_AB2	CG12004	0.0437717737832368	0.955006127202783	0.988741847156811	pseudobulk	DESeq2	LRT	0.0199938420269116
876	KC_AB2	CG12007	0.699674038439951	0.821690393783994	0.988741847156811	pseudobulk	DESeq2	LRT	0.0852917902455435
877	KC_AB2	CG12012	1.06980162817022	0.618453116986936	0.988741847156811	pseudobulk	DESeq2	LRT	0.208693217305034
878	KC_AB2	CG12024	-0.568771421109962	0.693127386152723	0.988741847156811	pseudobulk	DESeq2	LRT	0.15918694140678
879	KC_AB2	CG12025	0.32140381670586	0.812225745916471	0.988741847156811	pseudobulk	DESeq2	LRT	0.0903232483694482
880	KC_AB2	CG12038	-0.798328910986825	0.405535982867978	0.988741847156811	pseudobulk	DESeq2	LRT	0.391970605157651
881	KC_AB2	CG12054	-0.236652430358731	0.728981887636926	0.988741847156811	pseudobulk	DESeq2	LRT	0.137283262075794
882	KC_AB2	CG12056	-1.21442392643984	0.584026528107531	0.988741847156811	pseudobulk	DESeq2	LRT	0.233567425577577
883	KC_AB2	CG12071	0.160174373950745	0.848497006329143	0.988741847156811	pseudobulk	DESeq2	LRT	0.0713496856232972
884	KC_AB2	CG12081	-0.568233637794205	0.868826397133536	0.988741847156811	pseudobulk	DESeq2	LRT	0.0610669925949191
885	KC_AB2	CG12084	-1.13714576649625	0.404898945130333	0.988741847156811	pseudobulk	DESeq2	LRT	0.392653354682987
886	KC_AB2	CG12091	0.778379984266684	0.491139621104818	0.988741847156811	pseudobulk	DESeq2	LRT	0.30879502914858
887	KC_AB2	CG12093	1.69894780879763	0.540778829726708	0.988741847156811	pseudobulk	DESeq2	LRT	0.26698031838453
888	KC_AB2	CG12096	0.351722119397094	0.929615296941389	0.988741847156811	pseudobulk	DESeq2	LRT	0.0316967385224016
889	KC_AB2	CG12099	-0.930485540236149	0.461027020138395	0.988741847156811	pseudobulk	DESeq2	LRT	0.336273620482105
890	KC_AB2	CG12104	-0.0323203545809474	0.954448156132187	0.988741847156811	pseudobulk	DESeq2	LRT	0.020247656706879
891	KC_AB2	CG12106	0.157562008957688	0.950463298513166	0.988741847156811	pseudobulk	DESeq2	LRT	0.0220646484576093
892	KC_AB2	CG12107	-0.201557921614869	0.922776242739966	0.988741847156811	pseudobulk	DESeq2	LRT	0.034903595099961
893	KC_AB2	CG12118	-0.50879048068994	0.817628596116567	0.988741847156811	pseudobulk	DESeq2	LRT	0.0874439277298491
894	KC_AB2	CG12121	-0.424744147075721	0.775702617077487	0.988741847156811	pseudobulk	DESeq2	LRT	0.110304743323903

895	KC_AB2	CG12123	3.91297093245125	0.13608758477008	0.988741847156811	pseudobulk	DESeq2	LRT	0.866181493546022
896	KC_AB2	CG12128	-1.16822656624734	0.643917245153277	0.988741847156811	pseudobulk	DESeq2	LRT	0.191169943638088
897	KC_AB2	CG12129	0.362555753552128	0.897034383127386	0.988741847156811	pseudobulk	DESeq2	LRT	0.0471909102264623
898	KC_AB2	CG12155	-2.45158172483764	0.378823136429564	0.988741847156811	pseudobulk	DESeq2	LRT	0.421563504545096
899	KC_AB2	CG12159	1.32467530657598	0.538468278591255	0.988741847156811	pseudobulk	DESeq2	LRT	0.268839876095909
900	KC_AB2	CG12163	-0.37671184873479	0.876517139842803	0.988741847156811	pseudobulk	DESeq2	LRT	0.0572395870814579
901	KC_AB2	CG12170	1.76062409692731	0.431284494748455	0.988741847156811	pseudobulk	DESeq2	LRT	0.365236155037982
902	KC_AB2	CG12173	0.998893024789477	0.660591418406266	0.988741847156811	pseudobulk	DESeq2	LRT	0.180067072429364
903	KC_AB2	CG1218	-0.934020934207768	0.782359118258182	0.988741847156811	pseudobulk	DESeq2	LRT	0.106593851444836
904	KC_AB2	CG12182	1.42621218304824	0.66738831402044	0.988741847156811	pseudobulk	DESeq2	LRT	0.175621402117653
905	KC_AB2	CG12194	2.74444227974632	0.293466309349946	0.988741847156811	pseudobulk	DESeq2	LRT	0.532441749623364
906	KC_AB2	CG12204	-0.488465967391191	0.84251072973523	0.988741847156811	pseudobulk	DESeq2	LRT	0.0744245594950451
907	KC_AB2	CG12206	-3.6809086040586	0.231552724242678	0.988741847156811	pseudobulk	DESeq2	LRT	0.635350105118793
908	KC_AB2	CG12213	-0.710955203713478	0.683108711094811	0.988741847156811	pseudobulk	DESeq2	LRT	0.165510176447824
909	KC_AB2	CG12219	-0.840006590521399	0.717017354938613	0.988741847156811	pseudobulk	DESeq2	LRT	0.144470332389583
910	KC_AB2	CG12236	2.80496381579643	0.22093644234667	0.988741847156811	pseudobulk	DESeq2	LRT	0.655732643545827
911	KC_AB2	CG12237	2.19621398363889	0.40208091169913	0.988741847156811	pseudobulk	DESeq2	LRT	0.395686544008532
912	KC_AB2	CG12241	-0.111480487015859	0.943154382753945	0.988741847156811	pseudobulk	DESeq2	LRT	0.0254172127871586
913	KC_AB2	CG12253	0.740635261447468	0.680864099739953	0.988741847156811	pseudobulk	DESeq2	LRT	0.166939564479132
914	KC_AB2	CG12259	0.818790875285784	0.940355871560339	0.988741847156811	pseudobulk	DESeq2	LRT	0.0267077593721816
915	KC_AB2	CG12267	2.06137138167525	0.547946929015274	0.988741847156811	pseudobulk	DESeq2	LRT	0.261261502741816
916	KC_AB2	CG1227	0.499142633167307	0.837505285988482	0.988741847156811	pseudobulk	DESeq2	LRT	0.0770124431945389
917	KC_AB2	CG12279	1.10577060612178	0.488790451261765	0.988741847156811	pseudobulk	DESeq2	LRT	0.310877286817939
918	KC_AB2	CG12288	-0.506613484034373	0.961658398065406	0.988741847156811	pseudobulk	DESeq2	LRT	0.0169791713942958
919	KC_AB2	CG12299	-0.981133689484444	0.501456175383379	0.988741847156811	pseudobulk	DESeq2	LRT	0.299767016025075
920	KC_AB2	CG12301	3.22031971259211	0.311877201824258	0.988741847156811	pseudobulk	DESeq2	LRT	0.506016370942756
921	KC_AB2	CG1231	1.69894780879763	0.540778829726708	0.988741847156811	pseudobulk	DESeq2	LRT	0.26698031838453
922	KC_AB2	CG12310	-2.26538931672255	0.547771004842275	0.988741847156811	pseudobulk	DESeq2	LRT	0.2614009599796
923	KC_AB2	CG12316	-1.61658171076968	0.417711229110782	0.988741847156811	pseudobulk	DESeq2	LRT	0.379123849668325
924	KC_AB2	CG12321	0.0556967317943303	0.951374442369035	0.988741847156811	pseudobulk	DESeq2	LRT	0.0216485196043508
925	KC_AB2	CG1233	-0.957133960806503	0.762632518785665	0.988741847156811	pseudobulk	DESeq2	LRT	0.1176846802739
926	KC_AB2	CG12333	1.48122604986829	0.532335836746603	0.988741847156811	pseudobulk	DESeq2	LRT	0.273814296221732
927	KC_AB2	CG12338	2.80917294583258	0.367996267391162	0.988741847156811	pseudobulk	DESeq2	LRT	0.434156586379859
928	KC_AB2	CG1234	-0.689687550646419	0.745991540083112	0.988741847156811	pseudobulk	DESeq2	LRT	0.127266097615876
929	KC_AB2	CG12341	0.712171366772551	0.813205723536451	0.988741847156811	pseudobulk	DESeq2	LRT	0.0897995733547685
930	KC_AB2	CG12343	1.34246377808695	0.417644008796031	0.988741847156811	pseudobulk	DESeq2	LRT	0.379193744272457
931	KC_AB2	CG12355	-1.80933943727629	0.584587525761637	0.988741847156811	pseudobulk	DESeq2	LRT	0.233150456085471
932	KC_AB2	CG1236	2.51243226103394	0.474458247157471	0.988741847156811	pseudobulk	DESeq2	LRT	0.323801999942597
933	KC_AB2	CG12375	-0.92978684073213	0.72380920826628	0.988741847156811	pseudobulk	DESeq2	LRT	0.140375896118957
934	KC_AB2	CG12379	0.0601466527378941	0.974810960364079	0.988741847156811	pseudobulk	DESeq2	LRT	0.0110795964355292
935	KC_AB2	CG12384	0.114032467760383	0.901493653014474	0.988741847156811	pseudobulk	DESeq2	LRT	0.0450373265904909
936	KC_AB2	CG1239	2.00515076565275	0.576456787459964	0.988741847156811	pseudobulk	DESeq2	LRT	0.239233242872451
937	KC_AB2	CG12391	-0.421375613136114	0.762002825894075	0.988741847156811	pseudobulk	DESeq2	LRT	0.118043418072568
938	KC_AB2	CG12402	-0.640993867785619	0.699564320220935	0.988741847156811	pseudobulk	DESeq2	LRT	0.155172348887922
939	KC_AB2	CG12470	0.818790875285784	0.940355871560339	0.988741847156811	pseudobulk	DESeq2	LRT	0.0267077593721816
940	KC_AB2	CG12499	-1.80294541848982	0.660135549041553	0.988741847156811	pseudobulk	DESeq2	LRT	0.180366879372519
941	KC_AB2	CG12502	-1.23536834900699	0.725570392794057	0.988741847156811	pseudobulk	DESeq2	LRT	0.139320447155934
942	KC_AB2	CG12531	0.69235640356798	0.346758944625326	0.988741847156811	pseudobulk	DESeq2	LRT	0.459972327511145
943	KC_AB2	CG12535	-3.51218082781652	0.306311589270116	0.988741847156811	pseudobulk	DESeq2	LRT	0.513836571391778
944	KC_AB2	CG12541	0.818790875285784	0.940355871560339	0.988741847156811	pseudobulk	DESeq2	LRT	0.0267077593721816
945	KC_AB2	CG12547	0.49344366383116	0.635258191045576	0.988741847156811	pseudobulk	DESeq2	LRT	0.197049726430351
946	KC_AB2	CG12567	0.374424193653406	0.745477697460335	0.988741847156811	pseudobulk	DESeq2	LRT	0.127565344853233
947	KC_AB2	CG12576	0.206486728815455	0.883569745626498	0.988741847156811	pseudobulk	DESeq2	LRT	0.0537591632537657
948	KC_AB2	CG12605	0.153863690119233	0.78292924591317	0.988741847156811	pseudobulk	DESeq2	LRT	0.106277483788679
949	KC_AB2	CG12608	1.47543920254359	0.661929169513603	0.988741847156811	pseudobulk	DESeq2	LRT	0.17918848025087
950	KC_AB2	CG12640	-0.378852672109618	0.920549594954422	0.988741847156811	pseudobulk	DESeq2	LRT	0.0359528087516892

951	KC_AB2	CG1265	-0.130184569162235	0.907645436992454	0.988741847156811	pseudobulk	DESeq2	LRT	0.0420837713358365
952	KC_AB2	CG12659	2.51243226103394	0.474458247157471	0.988741847156811	pseudobulk	DESeq2	LRT	0.323801999942597
953	KC_AB2	CG12672	0.157562008957688	0.950463298513166	0.988741847156811	pseudobulk	DESeq2	LRT	0.0220646484576093
954	KC_AB2	CG1271	-1.80294541848982	0.660135549041553	0.988741847156811	pseudobulk	DESeq2	LRT	0.180366879372519
955	KC_AB2	CG12713	0.818790875285784	0.940355871560339	0.988741847156811	pseudobulk	DESeq2	LRT	0.0267077593721816
956	KC_AB2	CG12717	1.12452187615248	0.704078404906088	0.988741847156811	pseudobulk	DESeq2	LRT	0.152378975911753
957	KC_AB2	CG12728	1.13267300966507	0.729184666160225	0.988741847156811	pseudobulk	DESeq2	LRT	0.137162472589771
958	KC_AB2	CG12730	0.157562008957688	0.950463298513166	0.988741847156811	pseudobulk	DESeq2	LRT	0.0220646484576093
959	KC_AB2	CG12744	-2.26538931672255	0.547771004842275	0.988741847156811	pseudobulk	DESeq2	LRT	0.2614009599796
960	KC_AB2	CG12746	-0.568233637794205	0.868826397133536	0.988741847156811	pseudobulk	DESeq2	LRT	0.0610669925949191
961	KC_AB2	CG1275	-0.0260627328834289	0.938542904403274	0.988741847156811	pseudobulk	DESeq2	LRT	0.0275458693189391
962	KC_AB2	CG12768	1.42621218304824	0.66738831402044	0.988741847156811	pseudobulk	DESeq2	LRT	0.175621402117653
963	KC_AB2	CG12769	-0.59051039257323	0.80673877371878	0.988741847156811	pseudobulk	DESeq2	LRT	0.0932670693649112
964	KC_AB2	CG12773	-0.197565662601209	0.921418496992636	0.988741847156811	pseudobulk	DESeq2	LRT	0.0355430737620862
965	KC_AB2	CG12795	1.92721521352877	0.471759180353374	0.988741847156811	pseudobulk	DESeq2	LRT	0.326279639784082
966	KC_AB2	CG12814	0.0601466527378941	0.974810960364079	0.988741847156811	pseudobulk	DESeq2	LRT	0.0110795964355292
967	KC_AB2	CG12817	-0.300859819000612	0.875340908758419	0.988741847156811	pseudobulk	DESeq2	LRT	0.0578227744539286
968	KC_AB2	CG12818	-1.16822656624734	0.643917245153277	0.988741847156811	pseudobulk	DESeq2	LRT	0.191169943638088
969	KC_AB2	CG12822	0.253591425676655	0.835526722763412	0.988741847156811	pseudobulk	DESeq2	LRT	0.0780396554497065
970	KC_AB2	CG12848	-0.272159080840168	0.881917593135503	0.988741847156811	pseudobulk	DESeq2	LRT	0.0545719936873807
971	KC_AB2	CG12851	0.320348949796381	0.849242092899403	0.988741847156811	pseudobulk	DESeq2	LRT	0.0709684880565728
972	KC_AB2	CG12863	1.47543920254359	0.661929169513603	0.988741847156811	pseudobulk	DESeq2	LRT	0.17918848025087
973	KC_AB2	CG12866	1.42621218304824	0.66738831402044	0.988741847156811	pseudobulk	DESeq2	LRT	0.175621402117653
974	KC_AB2	CG12868	0.666943671827768	0.808936122914367	0.988741847156811	pseudobulk	DESeq2	LRT	0.0920857707994393
975	KC_AB2	CG12877	1.40305140665399	0.649741560632143	0.988741847156811	pseudobulk	DESeq2	LRT	0.187259352757606
976	KC_AB2	CG12883	-2.51746470924027	0.517377929599049	0.988741847156811	pseudobulk	DESeq2	LRT	0.286192101436607
977	KC_AB2	CG12895	-0.204752272798203	0.872124742996086	0.988741847156811	pseudobulk	DESeq2	LRT	0.0594213919937639
978	KC_AB2	CG12910	0.818790875285784	0.940355871560339	0.988741847156811	pseudobulk	DESeq2	LRT	0.0267077593721816
979	KC_AB2	CG12913	-3.90741406860124	0.060562762485885	0.988741847156811	pseudobulk	DESeq2	LRT	1.21779432331951
980	KC_AB2	CG12923	0.818790875285784	0.940355871560339	0.988741847156811	pseudobulk	DESeq2	LRT	0.0267077593721816
981	KC_AB2	CG12929	0.818790875285784	0.940355871560339	0.988741847156811	pseudobulk	DESeq2	LRT	0.0267077593721816
982	KC_AB2	CG12935	-1.65719613210719	0.599376091584556	0.988741847156811	pseudobulk	DESeq2	LRT	0.222300584546838
983	KC_AB2	CG12942	-0.934020934207768	0.782359118258182	0.988741847156811	pseudobulk	DESeq2	LRT	0.106593851444836
984	KC_AB2	CG12945	-1.11186627337669	0.445225152468656	0.988741847156811	pseudobulk	DESeq2	LRT	0.351420308690922
985	KC_AB2	CG12947	-4.67920794546437	0.0978619891848834	0.988741847156811	pseudobulk	DESeq2	LRT	1.00938596082993
986	KC_AB2	CG12948	1.47543920254359	0.661929169513603	0.988741847156811	pseudobulk	DESeq2	LRT	0.17918848025087
987	KC_AB2	CG12975	0.95933273603229	0.581899282803658	0.988741847156811	pseudobulk	DESeq2	LRT	0.235152178078295
988	KC_AB2	CG12991	1.27993084523152	0.29072464139306	0.988741847156811	pseudobulk	DESeq2	LRT	0.536518156507891
989	KC_AB2	CG12992	0.818790875285784	0.940355871560339	0.988741847156811	pseudobulk	DESeq2	LRT	0.0267077593721816
990	KC_AB2	CG13001	0.157562008957688	0.950463298513166	0.988741847156811	pseudobulk	DESeq2	LRT	0.0220646484576093
991	KC_AB2	CG13005	-4.15506802606233	0.145547043709333	0.988741847156811	pseudobulk	DESeq2	LRT	0.836996611343429
992	KC_AB2	CG13014	-2.51746470924027	0.517377929599049	0.988741847156811	pseudobulk	DESeq2	LRT	0.286192101436607
993	KC_AB2	CG13016	-2.21667329608248	0.477261050443944	0.988741847156811	pseudobulk	DESeq2	LRT	0.321244007216606
994	KC_AB2	CG13018	0.998893024789477	0.660591418406266	0.988741847156811	pseudobulk	DESeq2	LRT	0.180067072429364
995	KC_AB2	CG13025	0.699674038439951	0.821690393783994	0.988741847156811	pseudobulk	DESeq2	LRT	0.0852917902455435
996	KC_AB2	CG13054	0.0601466527378941	0.974810960364079	0.988741847156811	pseudobulk	DESeq2	LRT	0.0110795964355292
997	KC_AB2	CG13055	0.641786731973042	0.435949981071089	0.988741847156811	pseudobulk	DESeq2	LRT	0.360563336857481
998	KC_AB2	CG1307	-0.341472371153588	0.849350394863359	0.988741847156811	pseudobulk	DESeq2	LRT	0.070913106974026
999	KC_AB2	CG1309	-1.1284597703615	0.729754448571724	0.988741847156811	pseudobulk	DESeq2	LRT	0.136823248879498
100	KC_AB2	CG13096	-0.92978684073213	0.72380920826628	0.988741847156811	pseudobulk	DESeq2	LRT	0.140375896118957
100	KC_AB2	CG13097	-0.589613642418545	0.823284157979841	0.988741847156811	pseudobulk	DESeq2	LRT	0.0844502414063249
100	KC_AB2	CG13108	0.488726063825165	0.742262466611102	0.988741847156811	pseudobulk	DESeq2	LRT	0.129442499511719
100	KC_AB2	CG13123	-0.729234089517982	0.868684292414125	0.988741847156811	pseudobulk	DESeq2	LRT	0.0611380313470854
100	KC_AB2	CG13126	-1.80294541848982	0.660135549041553	0.988741847156811	pseudobulk	DESeq2	LRT	0.180366879372519
100	KC_AB2	CG13148	0.667839000613305	0.81413342949896	0.988741847156811	pseudobulk	DESeq2	LRT	0.0893044121300904
100	KC_AB2	CG13151	-0.506613484034373	0.961658398065406	0.988741847156811	pseudobulk	DESeq2	LRT	0.0169791713942958

100	KC_AB2	CG1316	4.01250839576074E-05	0.954675594279229	0.988741847156811	pseudobulk	DESeq2	LRT	0.0201441797765016
100	KC_AB2	CG13168	0.818790875285784	0.940355871560339	0.988741847156811	pseudobulk	DESeq2	LRT	0.0267077593721816
100	KC_AB2	CG1317	-0.587425801135323	0.771690223849521	0.988741847156811	pseudobulk	DESeq2	LRT	0.112557001570832
101	KC_AB2	CG13185	1.47543920254359	0.661929169513603	0.988741847156811	pseudobulk	DESeq2	LRT	0.17918848025087
101	KC_AB2	CG13192	-1.40296545307325	0.628223627794903	0.988741847156811	pseudobulk	DESeq2	LRT	0.201885733604539
101	KC_AB2	CG13197	2.10427674742456	0.422430831487357	0.988741847156811	pseudobulk	DESeq2	LRT	0.374244391951096
101	KC_AB2	CG13204	-0.111753468143923	0.920896716851484	0.988741847156811	pseudobulk	DESeq2	LRT	0.0357890753588
101	KC_AB2	CG13220	-0.502584436694424	0.711741493934019	0.988741847156811	pseudobulk	DESeq2	LRT	0.14767771442982
101	KC_AB2	CG13248	0.222311450660881	0.845040182604262	0.988741847156811	pseudobulk	DESeq2	LRT	0.073122639371747
101	KC_AB2	CG13258	-3.6809086040586	0.231552724242678	0.988741847156811	pseudobulk	DESeq2	LRT	0.635350105118793
101	KC_AB2	CG13284	-1.80294541848982	0.660135549041553	0.988741847156811	pseudobulk	DESeq2	LRT	0.180366879372519
101	KC_AB2	CG13293	-0.425205991693087	0.661996040428286	0.988741847156811	pseudobulk	DESeq2	LRT	0.179144608181582
101	KC_AB2	CG13295	0.818790875285784	0.940355871560339	0.988741847156811	pseudobulk	DESeq2	LRT	0.0267077593721816
102	KC_AB2	CG13300	-2.08434100393431	0.328662713614262	0.988741847156811	pseudobulk	DESeq2	LRT	0.483249563305339
102	KC_AB2	CG13319	-0.378852672109618	0.920549594954422	0.988741847156811	pseudobulk	DESeq2	LRT	0.0359528087516892
102	KC_AB2	CG13325	-0.647769774460182	0.665042795114112	0.988741847156811	pseudobulk	DESeq2	LRT	0.177150407202295
102	KC_AB2	CG13339	0.333937454051123	0.859086179292022	0.988741847156811	pseudobulk	DESeq2	LRT	0.0659632677011664
102	KC_AB2	CG13344	0.832064699200183	0.64462948842046	0.988741847156811	pseudobulk	DESeq2	LRT	0.190689831672951
102	KC_AB2	CG13364	-0.485362355909409	0.600300485211078	0.988741847156811	pseudobulk	DESeq2	LRT	0.221631305612295
102	KC_AB2	CG13365	1.31733964585313	0.473926627605351	0.988741847156811	pseudobulk	DESeq2	LRT	0.324288889745005
102	KC_AB2	CG13366	-0.663273929909526	0.684336515129322	0.988741847156811	pseudobulk	DESeq2	LRT	0.164730286109088
102	KC_AB2	CG13369	-0.634227699706429	0.828294391400015	0.988741847156811	pseudobulk	DESeq2	LRT	0.0818152793537044
102	KC_AB2	CG13375	-0.0587253851504102	0.943155278179364	0.988741847156811	pseudobulk	DESeq2	LRT	0.025416800470638
103	KC_AB2	CG13376	-1.80294541848982	0.660135549041553	0.988741847156811	pseudobulk	DESeq2	LRT	0.180366879372519
103	KC_AB2	CG13377	1.42621218304824	0.66738831402044	0.988741847156811	pseudobulk	DESeq2	LRT	0.175621402117653
103	KC_AB2	CG13384	0.716574918408453	0.656497189950173	0.988741847156811	pseudobulk	DESeq2	LRT	0.1827671285109
103	KC_AB2	CG13390	0.818790875285784	0.940355871560339	0.988741847156811	pseudobulk	DESeq2	LRT	0.0267077593721816
103	KC_AB2	CG13392	3.25449350606273	0.266356678091136	0.988741847156811	pseudobulk	DESeq2	LRT	0.574536410117102
103	KC_AB2	CG13398	-1.1284597703615	0.729754448571724	0.988741847156811	pseudobulk	DESeq2	LRT	0.136823248879498
103	KC_AB2	CG13404	0.657088368271084	0.670791547292943	0.988741847156811	pseudobulk	DESeq2	LRT	0.173412418616877
103	KC_AB2	CG13409	0.760546103616116	0.738305064567484	0.988741847156811	pseudobulk	DESeq2	LRT	0.131764152427911
103	KC_AB2	CG13437	-1.80294541848982	0.660135549041553	0.988741847156811	pseudobulk	DESeq2	LRT	0.180366879372519
103	KC_AB2	CG1344	0.997366942311027	0.663723613191462	0.988741847156811	pseudobulk	DESeq2	LRT	0.178012731256061
104	KC_AB2	CG13461	-1.80294541848982	0.660135549041553	0.988741847156811	pseudobulk	DESeq2	LRT	0.180366879372519
104	KC_AB2	CG13482	-2.51746470924027	0.517377929599049	0.988741847156811	pseudobulk	DESeq2	LRT	0.286192101436607
104	KC_AB2	CG13484	-1.11199598795122	0.588851785677079	0.988741847156811	pseudobulk	DESeq2	LRT	0.229994003621588
104	KC_AB2	CG13502	0.0601466527378941	0.974810960364079	0.988741847156811	pseudobulk	DESeq2	LRT	0.0110795964355292
104	KC_AB2	CG13506	-0.390322594159798	0.793735695476282	0.988741847156811	pseudobulk	DESeq2	LRT	0.100324088385569
104	KC_AB2	CG13526	-2.26538931672255	0.547771004842275	0.988741847156811	pseudobulk	DESeq2	LRT	0.2614009599796
104	KC_AB2	CG13531	1.47543920254359	0.661929169513603	0.988741847156811	pseudobulk	DESeq2	LRT	0.17918848025087
104	KC_AB2	CG13532	-0.756918557499763	0.681297390272846	0.988741847156811	pseudobulk	DESeq2	LRT	0.166663274633585
104	KC_AB2	CG1354	-0.0229973822346953	0.971360658954591	0.988741847156811	pseudobulk	DESeq2	LRT	0.0126194898530182
104	KC_AB2	CG13551	0.448116787295899	0.574068571394522	0.988741847156811	pseudobulk	DESeq2	LRT	0.24103622885709
105	KC_AB2	CG13558	-2.51746470924027	0.517377929599049	0.988741847156811	pseudobulk	DESeq2	LRT	0.286192101436607
105	KC_AB2	CG13578	-0.934020934207768	0.782359118258182	0.988741847156811	pseudobulk	DESeq2	LRT	0.106593851444836
105	KC_AB2	CG1358	1.55777494142873	0.214012971444748	0.988741847156811	pseudobulk	DESeq2	LRT	0.669559903023938
105	KC_AB2	CG13585	0.982783325145869	0.458703411003891	0.988741847156811	pseudobulk	DESeq2	LRT	0.338468030361456
105	KC_AB2	CG1360	-2.51746470924027	0.517377929599049	0.988741847156811	pseudobulk	DESeq2	LRT	0.286192101436607
105	KC_AB2	CG13601	-0.779301790094566	0.664769009124738	0.988741847156811	pseudobulk	DESeq2	LRT	0.177329235132244
105	KC_AB2	CG13603	2.46476144614939	0.0920658978505903	0.988741847156811	pseudobulk	DESeq2	LRT	1.03590120713136
105	KC_AB2	CG13604	-0.934020934207768	0.782359118258182	0.988741847156811	pseudobulk	DESeq2	LRT	0.106593851444836
105	KC_AB2	CG13605	-2.9721553030081	0.387854083811327	0.988741847156811	pseudobulk	DESeq2	LRT	0.411331631401987
105	KC_AB2	CG13623	1.6341263262903	0.579948075372967	0.988741847156811	pseudobulk	DESeq2	LRT	0.236610888486163
106	KC_AB2	CG13625	0.928039350209165	0.67716747857253	0.988741847156811	pseudobulk	DESeq2	LRT	0.169303907349344
106	KC_AB2	CG13630	2.7785831508444	0.373751317747552	0.988741847156811	pseudobulk	DESeq2	LRT	0.427417267458199
106	KC_AB2	CG13654	1.94106108959906	0.476420175276185	0.988741847156811	pseudobulk	DESeq2	LRT	0.322009855447888

106	KC_AB2	CG13663	-0.957133960806503	0.762632518785665	0.988741847156811	pseudobulk	DESeq2	LRT	0.1176846802739
106	KC_AB2	CG13671	0.699674038439951	0.821690393783994	0.988741847156811	pseudobulk	DESeq2	LRT	0.0852917902455435
106	KC_AB2	CG1368	1.42621218304824	0.66738831402044	0.988741847156811	pseudobulk	DESeq2	LRT	0.175621402117653
106	KC_AB2	CG13685	-1.80294541848982	0.660135549041553	0.988741847156811	pseudobulk	DESeq2	LRT	0.180366879372519
106	KC_AB2	CG13689	-0.010237273074013	0.943681123757042	0.988741847156811	pseudobulk	DESeq2	LRT	0.0251747319601966
106	KC_AB2	CG13698	-1.80294541848982	0.660135549041553	0.988741847156811	pseudobulk	DESeq2	LRT	0.180366879372519
106	KC_AB2	CG13707	1.42621218304824	0.66738831402044	0.988741847156811	pseudobulk	DESeq2	LRT	0.175621402117653
107	KC_AB2	CG1371	-1.2879653519079	0.633474066910355	0.988741847156811	pseudobulk	DESeq2	LRT	0.19827115951497
107	KC_AB2	CG13737	-1.96071351315253	0.529174679439165	0.988741847156811	pseudobulk	DESeq2	LRT	0.276400944611017
107	KC_AB2	CG13739	-0.119395263246552	0.710308335825174	0.988741847156811	pseudobulk	DESeq2	LRT	0.148553088634108
107	KC_AB2	CG13743	0.818790875285784	0.940355871560339	0.988741847156811	pseudobulk	DESeq2	LRT	0.0267077593721816
107	KC_AB2	CG13751	-0.345036337248882	0.833079212119776	0.988741847156811	pseudobulk	DESeq2	LRT	0.0793137023735677
107	KC_AB2	CG13760	1.1669370038017	0.601782723498991	0.988741847156811	pseudobulk	DESeq2	LRT	0.220560284520093
107	KC_AB2	CG13766	0.27226851987049	0.911402843231596	0.988741847156811	pseudobulk	DESeq2	LRT	0.0402896209172008
107	KC_AB2	CG13773	-1.80294541848982	0.660135549041553	0.988741847156811	pseudobulk	DESeq2	LRT	0.180366879372519
107	KC_AB2	CG13784	0.110954351522016	0.866939426231984	0.988741847156811	pseudobulk	DESeq2	LRT	0.0620112459750199
107	KC_AB2	CG13807	1.15128682504377	0.719737694898673	0.988741847156811	pseudobulk	DESeq2	LRT	0.142825751365876
108	KC_AB2	CG13827	0.997284248814671	0.663837179223659	0.988741847156811	pseudobulk	DESeq2	LRT	0.177938427914901
108	KC_AB2	CG13850	0.792281256713368	0.823478838299676	0.988741847156811	pseudobulk	DESeq2	LRT	0.0843475568193814
108	KC_AB2	CG13868	-0.120702379010257	0.864390437539793	0.988741847156811	pseudobulk	DESeq2	LRT	0.0632900462157659
108	KC_AB2	CG13876	2.06137138167525	0.547946929015274	0.988741847156811	pseudobulk	DESeq2	LRT	0.261261502741816
108	KC_AB2	CG13887	-0.33220627028631	0.821829403472076	0.988741847156811	pseudobulk	DESeq2	LRT	0.0852183245766667
108	KC_AB2	CG13893	-1.5707624441993	0.507036228886573	0.988741847156811	pseudobulk	DESeq2	LRT	0.294961008233931
108	KC_AB2	CG13894	1.42621218304824	0.66738831402044	0.988741847156811	pseudobulk	DESeq2	LRT	0.175621402117653
108	KC_AB2	CG13895	-1.20081362769313	0.741163517910401	0.988741847156811	pseudobulk	DESeq2	LRT	0.130085965985812
108	KC_AB2	CG13896	-1.80294541848982	0.660135549041553	0.988741847156811	pseudobulk	DESeq2	LRT	0.180366879372519
108	KC_AB2	CG13898	1.42621218304824	0.66738831402044	0.988741847156811	pseudobulk	DESeq2	LRT	0.175621402117653
109	KC_AB2	CG13901	1.67796394544623	0.574362267760116	0.988741847156811	pseudobulk	DESeq2	LRT	0.240814098434665
109	KC_AB2	CG13907	-0.418154869600558	0.847540414580397	0.988741847156811	pseudobulk	DESeq2	LRT	0.07183958349565
109	KC_AB2	CG13917	-0.0684288534919126	0.886316082136135	0.988741847156811	pseudobulk	DESeq2	LRT	0.052411370384705
109	KC_AB2	CG13919	-0.631319870539175	0.790908149987646	0.988741847156811	pseudobulk	DESeq2	LRT	0.101873949207374
109	KC_AB2	CG13920	-0.0997356822031418	0.912945914670962	0.988741847156811	pseudobulk	DESeq2	LRT	0.039554950456625
109	KC_AB2	CG13921	1.02151402731699	0.464112348651509	0.988741847156811	pseudobulk	DESeq2	LRT	0.333376876140629
109	KC_AB2	CG13924	1.42621218304824	0.66738831402044	0.988741847156811	pseudobulk	DESeq2	LRT	0.175621402117653
109	KC_AB2	CG13926	0.572861358867068	0.813871944286787	0.988741847156811	pseudobulk	DESeq2	LRT	0.0894439222171593
109	KC_AB2	CG13928	-0.135515278745632	0.799512691318539	0.988741847156811	pseudobulk	DESeq2	LRT	0.0971746379516888
109	KC_AB2	CG13933	-0.957133960806503	0.762632518785665	0.988741847156811	pseudobulk	DESeq2	LRT	0.1176846802739
110	KC_AB2	CG13937	-3.09794865339079	0.39855598532175	0.988741847156811	pseudobulk	DESeq2	LRT	0.399510664459796
110	KC_AB2	CG13954	-0.836810983077869	0.346432890940169	0.988741847156811	pseudobulk	DESeq2	LRT	0.46038088202505
110	KC_AB2	CG13970	1.42621218304824	0.66738831402044	0.988741847156811	pseudobulk	DESeq2	LRT	0.175621402117653
110	KC_AB2	CG13982	-1.23536834900699	0.725570392794057	0.988741847156811	pseudobulk	DESeq2	LRT	0.139320447155934
110	KC_AB2	CG13994	-0.158601889198964	0.945838126788742	0.988741847156811	pseudobulk	DESeq2	LRT	0.0241831835324482
110	KC_AB2	CG13995	3.48393309996043	0.216218474953395	0.988741847156811	pseudobulk	DESeq2	LRT	0.665107200172195
110	KC_AB2	CG13999	-1.43045205048613	0.309919392977523	0.988741847156811	pseudobulk	DESeq2	LRT	0.508751247253417
110	KC_AB2	CG14005	1.0821173077982	0.758638591992449	0.988741847156811	pseudobulk	DESeq2	LRT	0.119965068483926
110	KC_AB2	CG14015	0.606398620649635	0.698113396405247	0.988741847156811	pseudobulk	DESeq2	LRT	0.156074028045672
110	KC_AB2	CG14024	-1.10956356922831	0.345093821863002	0.988741847156811	pseudobulk	DESeq2	LRT	0.462062815715248
111	KC_AB2	CG14043	-0.934020934207768	0.782359118258182	0.988741847156811	pseudobulk	DESeq2	LRT	0.106593851444836
111	KC_AB2	CG14044	1.42621218304824	0.66738831402044	0.988741847156811	pseudobulk	DESeq2	LRT	0.175621402117653
111	KC_AB2	CG14057	0.818790875285784	0.940355871560339	0.988741847156811	pseudobulk	DESeq2	LRT	0.0267077593721816
111	KC_AB2	CG1407	-0.233272055930639	0.937531793764924	0.988741847156811	pseudobulk	DESeq2	LRT	0.028013995469539
111	KC_AB2	CG14073	0.163148460302646	0.823427233429793	0.988741847156811	pseudobulk	DESeq2	LRT	0.0843747735630945
111	KC_AB2	CG14074	-1.80294541848982	0.660135549041553	0.988741847156811	pseudobulk	DESeq2	LRT	0.180366879372519
111	KC_AB2	CG14075	0.818790875285784	0.940355871560339	0.988741847156811	pseudobulk	DESeq2	LRT	0.0267077593721816
111	KC_AB2	CG14082	-0.311374193836261	0.855497389932041	0.988741847156811	pseudobulk	DESeq2	LRT	0.0677813111256177
111	KC_AB2	CG14086	0.699674038439951	0.821690393783994	0.988741847156811	pseudobulk	DESeq2	LRT	0.0852917902455435

111	KC_AB2	CG14100	-1.80294541848982	0.660135549041553	0.988741847156811	pseudobulk	DESeq2	LRT	0.180366879372519
112	KC_AB2	CG14102	-0.729234089517982	0.868684292414125	0.988741847156811	pseudobulk	DESeq2	LRT	0.0611380313470854
112	KC_AB2	CG14104	-0.452344515838269	0.871171367298942	0.988741847156811	pseudobulk	DESeq2	LRT	0.0598964069304489
112	KC_AB2	CG14130	-0.934020934207768	0.782359118258182	0.988741847156811	pseudobulk	DESeq2	LRT	0.106593851444836
112	KC_AB2	CG14135	1.4413042803421	0.615311685084066	0.988741847156811	pseudobulk	DESeq2	LRT	0.210904837358999
112	KC_AB2	CG1416	-0.12058928718113	0.933784783540129	0.988741847156811	pseudobulk	DESeq2	LRT	0.0297532073792971
112	KC_AB2	CG14174	2.06137138167525	0.547946929015274	0.988741847156811	pseudobulk	DESeq2	LRT	0.261261502741816
112	KC_AB2	CG1418	-0.331004884396198	0.894406551228697	0.988741847156811	pseudobulk	DESeq2	LRT	0.0484650283842476
112	KC_AB2	CG14182	0.699674038439951	0.821690393783994	0.988741847156811	pseudobulk	DESeq2	LRT	0.0852917902455435
112	KC_AB2	CG14183	-2.26538931672255	0.547771004842275	0.988741847156811	pseudobulk	DESeq2	LRT	0.2614009599796
112	KC_AB2	CG14184	-0.170889539133738	0.904714490512976	0.988741847156811	pseudobulk	DESeq2	LRT	0.043488453676419
113	KC_AB2	CG14185	0.818790875285784	0.940355871560339	0.988741847156811	pseudobulk	DESeq2	LRT	0.0267077593721816
113	KC_AB2	CG14186	-0.20869210875768	0.752187156570734	0.988741847156811	pseudobulk	DESeq2	LRT	0.123674086332496
113	KC_AB2	CG14194	-0.68750020136443	0.784083099060507	0.988741847156811	pseudobulk	DESeq2	LRT	0.105637907276101
113	KC_AB2	CG14200	-0.932950386820262	0.493177811782866	0.988741847156811	pseudobulk	DESeq2	LRT	0.306996470675312
113	KC_AB2	CG14207	0.2932813851259	0.829789165526879	0.988741847156811	pseudobulk	DESeq2	LRT	0.0810320039651007
113	KC_AB2	CG14210	-0.934020934207768	0.782359118258182	0.988741847156811	pseudobulk	DESeq2	LRT	0.106593851444836
113	KC_AB2	CG14220	-0.957133960806503	0.762632518785665	0.988741847156811	pseudobulk	DESeq2	LRT	0.1176846802739
113	KC_AB2	CG14223	1.42621218304824	0.66738831402044	0.988741847156811	pseudobulk	DESeq2	LRT	0.175621402117653
113	KC_AB2	CG14229	0.738241660464042	0.684578738484932	0.988741847156811	pseudobulk	DESeq2	LRT	0.164576593223353
113	KC_AB2	CG14230	0.818790875285784	0.940355871560339	0.988741847156811	pseudobulk	DESeq2	LRT	0.0267077593721816
114	KC_AB2	CG14231	-0.0582963981256801	0.979058404810457	0.988741847156811	pseudobulk	DESeq2	LRT	0.0091913999942843
114	KC_AB2	CG14232	-0.283552121523166	0.899867754837197	0.988741847156811	pseudobulk	DESeq2	LRT	0.0458213100767619
114	KC_AB2	CG14234	-0.981506968104824	0.5378526929464	0.988741847156811	pseudobulk	DESeq2	LRT	0.269336652587159
114	KC_AB2	CG14252	2.00515076565275	0.576456787459964	0.988741847156811	pseudobulk	DESeq2	LRT	0.239233242872451
114	KC_AB2	CG14270	-0.540579920873242	0.762304662430088	0.988741847156811	pseudobulk	DESeq2	LRT	0.117871423977224
114	KC_AB2	CG14286	2.51243226103394	0.474458247157471	0.988741847156811	pseudobulk	DESeq2	LRT	0.323801999942597
114	KC_AB2	CG14304	-1.80294541848982	0.660135549041553	0.988741847156811	pseudobulk	DESeq2	LRT	0.180366879372519
114	KC_AB2	CG14314	0.712171366772551	0.813205723536451	0.988741847156811	pseudobulk	DESeq2	LRT	0.0897995733547685
114	KC_AB2	CG14321	-0.756557080291009	0.54688797069225	0.988741847156811	pseudobulk	DESeq2	LRT	0.26210162922992
114	KC_AB2	CG14322	0.798955445836505	0.738225347953373	0.988741847156811	pseudobulk	DESeq2	LRT	0.131811046803217
115	KC_AB2	CG14341	0.143727500031408	0.907671353519454	0.988741847156811	pseudobulk	DESeq2	LRT	0.0420713708504496
115	KC_AB2	CG14352	-1.80294541848982	0.660135549041553	0.988741847156811	pseudobulk	DESeq2	LRT	0.180366879372519
115	KC_AB2	CG1440	-0.341472371153588	0.849350394863359	0.988741847156811	pseudobulk	DESeq2	LRT	0.070913106974026
115	KC_AB2	CG14407	0.321649514439517	0.802922233284407	0.988741847156811	pseudobulk	DESeq2	LRT	0.0953265161048456
115	KC_AB2	CG14408	-0.0474644638304076	0.94356095851622	0.988741847156811	pseudobulk	DESeq2	LRT	0.0252300371079215
115	KC_AB2	CG14410	-2.26538931672255	0.547771004842275	0.988741847156811	pseudobulk	DESeq2	LRT	0.2614009599796
115	KC_AB2	CG14411	-1.69687973060914	0.291958167239635	0.988741847156811	pseudobulk	DESeq2	LRT	0.534679371286153
115	KC_AB2	CG14414	3.09521016443865	0.156658299384717	0.988741847156811	pseudobulk	DESeq2	LRT	0.805046592282382
115	KC_AB2	CG14431	0.15525444512595	0.914443567249513	0.988741847156811	pseudobulk	DESeq2	LRT	0.0388430908309321
115	KC_AB2	CG14434	0.353798809751289	0.901648351639924	0.988741847156811	pseudobulk	DESeq2	LRT	0.0449628069360408
116	KC_AB2	CG14435	-1.00225733656146	0.493880149979032	0.988741847156811	pseudobulk	DESeq2	LRT	0.306378428642689
116	KC_AB2	CG14440	1.19089747429732	0.41736614637499	0.988741847156811	pseudobulk	DESeq2	LRT	0.379482780567149
116	KC_AB2	CG14441	1.85196256914229	0.547781346847132	0.988741847156811	pseudobulk	DESeq2	LRT	0.261392760507189
116	KC_AB2	CG14442	1.19214930600409	0.264128623730449	0.988741847156811	pseudobulk	DESeq2	LRT	0.578184531566352
116	KC_AB2	CG14450	-2.47381228748408	0.383327499819241	0.988741847156811	pseudobulk	DESeq2	LRT	0.4164300234502
116	KC_AB2	CG14464	-0.417825132809892	0.544979410748227	0.988741847156811	pseudobulk	DESeq2	LRT	0.263619905002879
116	KC_AB2	CG14478	-0.397125372456277	0.589647813371295	0.988741847156811	pseudobulk	DESeq2	LRT	0.229407307643252
116	KC_AB2	CG14483	1.39329988803621	0.386720653063554	0.988741847156811	pseudobulk	DESeq2	LRT	0.412602633533295
116	KC_AB2	CG14535	0.855986901384231	0.635269510950706	0.988741847156811	pseudobulk	DESeq2	LRT	0.197041987642246
116	KC_AB2	CG14543	0.575200883059258	0.804213580533116	0.988741847156811	pseudobulk	DESeq2	LRT	0.0946285973599483
117	KC_AB2	CG14561	-2.51746470924027	0.517377929599049	0.988741847156811	pseudobulk	DESeq2	LRT	0.286192101436607
117	KC_AB2	CG14562	-0.128589801976955	0.926918300944105	0.988741847156811	pseudobulk	DESeq2	LRT	0.0329585431076954
117	KC_AB2	CG14563	-2.39889516092361	0.434257656394078	0.988741847156811	pseudobulk	DESeq2	LRT	0.362252515770014
117	KC_AB2	CG14589	-1.80294541848982	0.660135549041553	0.988741847156811	pseudobulk	DESeq2	LRT	0.180366879372519
117	KC_AB2	CG14591	-0.487923111280115	0.783394690329388	0.988741847156811	pseudobulk	DESeq2	LRT	0.106019376327165

117	KC_AB2	CG1461	1.42621218304824	0.66738831402044	0.988741847156811	pseudobulk	DESeq2	LRT	0.175621402117653
117	KC_AB2	CG14613	-0.588639054357632	0.8432408255636	0.988741847156811	pseudobulk	DESeq2	LRT	0.0740483752247712
117	KC_AB2	CG14618	-2.9721553030081	0.387854083811327	0.988741847156811	pseudobulk	DESeq2	LRT	0.411331631401987
117	KC_AB2	CG14621	-1.1284597703615	0.729754448571724	0.988741847156811	pseudobulk	DESeq2	LRT	0.136823248879498
117	KC_AB2	CG1463	1.1669370038017	0.601782723498991	0.988741847156811	pseudobulk	DESeq2	LRT	0.220560284520093
118	KC_AB2	CG14641	-0.254611003409661	0.868033631089631	0.988741847156811	pseudobulk	DESeq2	LRT	0.0614634481948212
118	KC_AB2	CG14646	0.157562008957688	0.950463298513166	0.988741847156811	pseudobulk	DESeq2	LRT	0.0220646484576093
118	KC_AB2	CG14647	0.320539134359348	0.848791832994679	0.988741847156811	pseudobulk	DESeq2	LRT	0.0711988078324561
118	KC_AB2	CG14650	-1.14755310091518	0.618375466635947	0.988741847156811	pseudobulk	DESeq2	LRT	0.208747748902126
118	KC_AB2	CG14657	0.876762974009119	0.510411459369096	0.988741847156811	pseudobulk	DESeq2	LRT	0.292079583728711
118	KC_AB2	CG14667	3.06202454821851	0.30627034058063	0.988741847156811	pseudobulk	DESeq2	LRT	0.513895058517773
118	KC_AB2	CG14669	0.699674038439951	0.821690393783994	0.988741847156811	pseudobulk	DESeq2	LRT	0.0852917902455435
118	KC_AB2	CG14671	2.4643417141186	0.449082701910884	0.988741847156811	pseudobulk	DESeq2	LRT	0.347673673080982
118	KC_AB2	CG14683	-1.40296545307325	0.628223627794903	0.988741847156811	pseudobulk	DESeq2	LRT	0.201885733604539
118	KC_AB2	CG14694	0.818790875285784	0.940355871560339	0.988741847156811	pseudobulk	DESeq2	LRT	0.0267077593721816
119	KC_AB2	CG14710	-0.378852672109618	0.920549594954422	0.988741847156811	pseudobulk	DESeq2	LRT	0.0359528087516892
119	KC_AB2	CG14711	0.353798809751289	0.901648351639924	0.988741847156811	pseudobulk	DESeq2	LRT	0.0449628069360408
119	KC_AB2	CG14715	0.398226076509848	0.750746245643468	0.988741847156811	pseudobulk	DESeq2	LRT	0.124506830957689
119	KC_AB2	CG14721	-1.8055859507431	0.413762197354307	0.988741847156811	pseudobulk	DESeq2	LRT	0.38324919039909
119	KC_AB2	CG14722	-0.92978684073213	0.72380920826628	0.988741847156811	pseudobulk	DESeq2	LRT	0.140375896118957
119	KC_AB2	CG14757	-0.853565742783243	0.753557841534365	0.988741847156811	pseudobulk	DESeq2	LRT	0.122883406527736
119	KC_AB2	CG14760	-2.26538931672255	0.547771004842275	0.988741847156811	pseudobulk	DESeq2	LRT	0.2614009599796
119	KC_AB2	CG14762	0.155184557183198	0.750424085535723	0.988741847156811	pseudobulk	DESeq2	LRT	0.124693235333428
119	KC_AB2	CG14764	0.377057232108292	0.707349695257209	0.988741847156811	pseudobulk	DESeq2	LRT	0.150365829238599
119	KC_AB2	CG14767	0.0233304237498781	0.97383535155308	0.988741847156811	pseudobulk	DESeq2	LRT	0.0115144640212205
120	KC_AB2	CG14777	1.49923757263635	0.39257603171533	0.988741847156811	pseudobulk	DESeq2	LRT	0.406076219283976
120	KC_AB2	CG14805	0.107243313242643	0.927720828069648	0.988741847156811	pseudobulk	DESeq2	LRT	0.0325826930369169
120	KC_AB2	CG14806	-0.466144968607287	0.806083667837512	0.988741847156811	pseudobulk	DESeq2	LRT	0.0936198780531005
120	KC_AB2	CG14811	2.06137138167525	0.547946929015274	0.988741847156811	pseudobulk	DESeq2	LRT	0.261261502741816
120	KC_AB2	CG14812	0.539331511638967	0.733549957788951	0.988741847156811	pseudobulk	DESeq2	LRT	0.134570303561331
120	KC_AB2	CG14817	0.444303551450947	0.713607263778834	0.988741847156811	pseudobulk	DESeq2	LRT	0.146540737943859
120	KC_AB2	CG14818	-0.164525850877151	0.850253872752632	0.988741847156811	pseudobulk	DESeq2	LRT	0.070451381257907
120	KC_AB2	CG14834	2.06137138167525	0.547946929015274	0.988741847156811	pseudobulk	DESeq2	LRT	0.261261502741816
120	KC_AB2	CG14853	-0.414572843910669	0.699115343720076	0.988741847156811	pseudobulk	DESeq2	LRT	0.155451166159027
120	KC_AB2	CG1486	0.0960904300109501	0.963271183317648	0.988741847156811	pseudobulk	DESeq2	LRT	0.016251431630502
121	KC_AB2	CG14868	1.1114511822041	0.470185741718989	0.988741847156811	pseudobulk	DESeq2	LRT	0.32773054489771
121	KC_AB2	CG14882	0.351722119397094	0.929615296941389	0.988741847156811	pseudobulk	DESeq2	LRT	0.0316967385224016
121	KC_AB2	CG14883	0.684221129527733	0.737351212059311	0.988741847156811	pseudobulk	DESeq2	LRT	0.132325601520231
121	KC_AB2	CG14894	-1.39136845792757	0.465860886257086	0.988741847156811	pseudobulk	DESeq2	LRT	0.33174375144447
121	KC_AB2	CG14903	-1.05085274221298	0.468804498326943	0.988741847156811	pseudobulk	DESeq2	LRT	0.329008229777516
121	KC_AB2	CG14906	0.818790875285784	0.940355871560339	0.988741847156811	pseudobulk	DESeq2	LRT	0.0267077593721816
121	KC_AB2	CG1492	0.924140746192761	0.675445049022325	0.988741847156811	pseudobulk	DESeq2	LRT	0.170409977326066
121	KC_AB2	CG14965	-1.23536834900699	0.725570392794057	0.988741847156811	pseudobulk	DESeq2	LRT	0.139320447155934
121	KC_AB2	CG14966	-0.506613484034373	0.961658398065406	0.988741847156811	pseudobulk	DESeq2	LRT	0.0169791713942958
121	KC_AB2	CG14969	1.87434911411483	0.392658868335322	0.988741847156811	pseudobulk	DESeq2	LRT	0.405984589410945
122	KC_AB2	CG14971	1.31617713253562	0.59447159965162	0.988741847156811	pseudobulk	DESeq2	LRT	0.225868888579699
122	KC_AB2	CG14977	0.786780068809377	0.754069556893097	0.988741847156811	pseudobulk	DESeq2	LRT	0.122588592085696
122	KC_AB2	CG14982	0.146127880542071	0.9044000470846	0.988741847156811	pseudobulk	DESeq2	LRT	0.04363942371657
122	KC_AB2	CG14984	-1.80294541848982	0.660135549041553	0.988741847156811	pseudobulk	DESeq2	LRT	0.180366879372519
122	KC_AB2	CG14995	-0.644335753748423	0.695988736857784	0.988741847156811	pseudobulk	DESeq2	LRT	0.157397788493019
122	KC_AB2	CG14997	0.818790875285784	0.940355871560339	0.988741847156811	pseudobulk	DESeq2	LRT	0.0267077593721816
122	KC_AB2	CG15011	2.06137138167525	0.547946929015274	0.988741847156811	pseudobulk	DESeq2	LRT	0.261261502741816
122	KC_AB2	CG15012	-0.122449269164875	0.899480235557521	0.988741847156811	pseudobulk	DESeq2	LRT	0.0460083750450669
122	KC_AB2	CG15019	0.157562008957688	0.950463298513166	0.988741847156811	pseudobulk	DESeq2	LRT	0.0220646484576093
122	KC_AB2	CG15027	-1.28585361841952	0.604452679793172	0.988741847156811	pseudobulk	DESeq2	LRT	0.218637692667744
123	KC_AB2	CG15040	0.818790875285784	0.940355871560339	0.988741847156811	pseudobulk	DESeq2	LRT	0.0267077593721816

123	KC_AB2	CG15073	0.712171366772551	0.813205723536451	0.988741847156811	pseudobulk	DESeq2	LRT	0.0897995733547685
123	KC_AB2	CG15097	0.0125844123046364	0.956212472524364	0.988741847156811	pseudobulk	DESeq2	LRT	0.0194455958073709
123	KC_AB2	CG15098	0.321417721695761	0.830003645411771	0.988741847156811	pseudobulk	DESeq2	LRT	0.0809200001796612
123	KC_AB2	CG15099	0.492553384038554	0.738565073853147	0.988741847156811	pseudobulk	DESeq2	LRT	0.131611233635343
123	KC_AB2	CG15107	1.42621218304824	0.66738831402044	0.988741847156811	pseudobulk	DESeq2	LRT	0.175621402117653
123	KC_AB2	CG15111	-0.409028640883814	0.818518332365151	0.988741847156811	pseudobulk	DESeq2	LRT	0.086971589244262
123	KC_AB2	CG15118	-0.0144409071146711	0.984632974376923	0.988741847156811	pseudobulk	DESeq2	LRT	0.00672562423038896
123	KC_AB2	CG1513	-0.31580086986392	0.738932830966709	0.988741847156811	pseudobulk	DESeq2	LRT	0.131395037202335
123	KC_AB2	CG15141	-0.672679070920009	0.691861968213447	0.988741847156811	pseudobulk	DESeq2	LRT	0.159980541992256
124	KC_AB2	CG15160	-0.0493731122220437	0.968729445650825	0.988741847156811	pseudobulk	DESeq2	LRT	0.0137974991799579
124	KC_AB2	CG15168	-0.192755586629418	0.894393915993559	0.988741847156811	pseudobulk	DESeq2	LRT	0.0484711636832913
124	KC_AB2	CG15202	-2.51746470924027	0.517377929599049	0.988741847156811	pseudobulk	DESeq2	LRT	0.286192101436607
124	KC_AB2	CG15209	1.42621218304824	0.66738831402044	0.988741847156811	pseudobulk	DESeq2	LRT	0.175621402117653
124	KC_AB2	CG1523	1.47543920254359	0.661929169513603	0.988741847156811	pseudobulk	DESeq2	LRT	0.17918848025087
124	KC_AB2	CG15236	-1.80294541848982	0.660135549041553	0.988741847156811	pseudobulk	DESeq2	LRT	0.180366879372519
124	KC_AB2	CG15237	1.53519137830027	0.43238982969318	0.988741847156811	pseudobulk	DESeq2	LRT	0.364124529705027
124	KC_AB2	CG15270	0.488478040875569	0.718031020812756	0.988741847156811	pseudobulk	DESeq2	LRT	0.143856792698112
124	KC_AB2	CG15309	-1.3460975324975	0.494732871429856	0.988741847156811	pseudobulk	DESeq2	LRT	0.30562923294222
124	KC_AB2	CG15312	2.51243226103394	0.474458247157471	0.988741847156811	pseudobulk	DESeq2	LRT	0.323801999942597
125	KC_AB2	CG15317	1.7098779479	0.537865353861561	0.988741847156811	pseudobulk	DESeq2	LRT	0.269326429526188
125	KC_AB2	CG1532	-1.40296545307325	0.628223627794903	0.988741847156811	pseudobulk	DESeq2	LRT	0.201885733604539
125	KC_AB2	CG15332	-2.26538931672255	0.547771004842275	0.988741847156811	pseudobulk	DESeq2	LRT	0.2614009599796
125	KC_AB2	CG15343	0.699674038439951	0.821690393783994	0.988741847156811	pseudobulk	DESeq2	LRT	0.0852917902455435
125	KC_AB2	CG15356	-1.1284597703615	0.729754448571724	0.988741847156811	pseudobulk	DESeq2	LRT	0.136823248879498
125	KC_AB2	CG15385	0.578095385250022	0.743958705493656	0.988741847156811	pseudobulk	DESeq2	LRT	0.128451169929867
125	KC_AB2	CG15387	0.668970670557767	0.818048200024539	0.988741847156811	pseudobulk	DESeq2	LRT	0.0872211066123218
125	KC_AB2	CG15390	-1.80294541848982	0.660135549041553	0.988741847156811	pseudobulk	DESeq2	LRT	0.180366879372519
125	KC_AB2	CG1542	2.00515076565275	0.576456787459964	0.988741847156811	pseudobulk	DESeq2	LRT	0.239233242872451
125	KC_AB2	CG15431	-0.185241231458904	0.81201262483097	0.988741847156811	pseudobulk	DESeq2	LRT	0.0904372184782802
126	KC_AB2	CG15432	-0.215812554377026	0.909171219175963	0.988741847156811	pseudobulk	DESeq2	LRT	0.0413543208033163
126	KC_AB2	CG15435	0.818790875285784	0.940355871560339	0.988741847156811	pseudobulk	DESeq2	LRT	0.0267077593721816
126	KC_AB2	CG15439	0.818790875285784	0.940355871560339	0.988741847156811	pseudobulk	DESeq2	LRT	0.0267077593721816
126	KC_AB2	CG15443	0.104637746735554	0.951122131714938	0.988741847156811	pseudobulk	DESeq2	LRT	0.0217637125849166
126	KC_AB2	CG15445	1.42621218304824	0.66738831402044	0.988741847156811	pseudobulk	DESeq2	LRT	0.175621402117653
126	KC_AB2	CG15449	-1.54219110932009	0.464324107836603	0.988741847156811	pseudobulk	DESeq2	LRT	0.333178767058352
126	KC_AB2	CG1545	2.51243226103394	0.474458247157471	0.988741847156811	pseudobulk	DESeq2	LRT	0.323801999942597
126	KC_AB2	CG15456	0.360597065274579	0.876727378089377	0.988741847156811	pseudobulk	DESeq2	LRT	0.0571354312607773
126	KC_AB2	CG15459	1.47543920254359	0.661929169513603	0.988741847156811	pseudobulk	DESeq2	LRT	0.17918848025087
126	KC_AB2	CG15514	-1.52496782475103	0.617219242690575	0.988741847156811	pseudobulk	DESeq2	LRT	0.209560542647675
127	KC_AB2	CG15525	2.06137138167525	0.547946929015274	0.988741847156811	pseudobulk	DESeq2	LRT	0.261261502741816
127	KC_AB2	CG15535	-0.373657285159983	0.842403106308872	0.988741847156811	pseudobulk	DESeq2	LRT	0.0744800403858235
127	KC_AB2	CG15547	1.42621218304824	0.66738831402044	0.988741847156811	pseudobulk	DESeq2	LRT	0.175621402117653
127	KC_AB2	CG15561	-2.51746470924027	0.517377929599049	0.988741847156811	pseudobulk	DESeq2	LRT	0.286192101436607
127	KC_AB2	CG15570	0.818790875285784	0.940355871560339	0.988741847156811	pseudobulk	DESeq2	LRT	0.0267077593721816
127	KC_AB2	CG15579	0.818790875285784	0.940355871560339	0.988741847156811	pseudobulk	DESeq2	LRT	0.0267077593721816
127	KC_AB2	CG15601	0.157562008957688	0.950463298513166	0.988741847156811	pseudobulk	DESeq2	LRT	0.0220646484576093
127	KC_AB2	CG15602	3.30033526000341	0.259137897354645	0.988741847156811	pseudobulk	DESeq2	LRT	0.586469069420752
127	KC_AB2	CG15628	-0.155523789488696	0.82909245137209	0.988741847156811	pseudobulk	DESeq2	LRT	0.0813970389526796
127	KC_AB2	CG15642	0.699674038439951	0.821690393783994	0.988741847156811	pseudobulk	DESeq2	LRT	0.0852917902455435
128	KC_AB2	CG15643	-0.568233637794205	0.868826397133536	0.988741847156811	pseudobulk	DESeq2	LRT	0.0610669925949191
128	KC_AB2	CG15651	0.836206984333835	0.674113903735976	0.988741847156811	pseudobulk	DESeq2	LRT	0.171266715357068
128	KC_AB2	CG15653	0.818790875285784	0.940355871560339	0.988741847156811	pseudobulk	DESeq2	LRT	0.0267077593721816
128	KC_AB2	CG15715	0.308265991867546	0.838215202900907	0.988741847156811	pseudobulk	DESeq2	LRT	0.076644466532362
128	KC_AB2	CG1572	-1.80294541848982	0.660135549041553	0.988741847156811	pseudobulk	DESeq2	LRT	0.180366879372519
128	KC_AB2	CG15743	0.920073134400379	0.728211051768817	0.988741847156811	pseudobulk	DESeq2	LRT	0.137742734237847
128	KC_AB2	CG15747	0.921695369118063	0.730077514579258	0.988741847156811	pseudobulk	DESeq2	LRT	0.136631027048226

128	KC_AB2	CG1575	1.15128682504377	0.719737694898673	0.988741847156811	pseudobulk	DESeq2	LRT	0.142825751365876
128	KC_AB2	CG15760	-0.689440897149644	0.651277754439355	0.988741847156811	pseudobulk	DESeq2	LRT	0.186233755680101
128	KC_AB2	CG15765	0.221848141288485	0.622384880894376	0.988741847156811	pseudobulk	DESeq2	LRT	0.205940965863959
129	KC_AB2	CG15771	0.0563120942996901	0.974316031513895	0.988741847156811	pseudobulk	DESeq2	LRT	0.0113001514574287
129	KC_AB2	CG15772	0.609881048911365	0.482937220574309	0.988741847156811	pseudobulk	DESeq2	LRT	0.316109321692006
129	KC_AB2	CG15773	-1.80294541848982	0.660135549041553	0.988741847156811	pseudobulk	DESeq2	LRT	0.180366879372519
129	KC_AB2	CG1578	0.595388708057048	0.527390541579897	0.988741847156811	pseudobulk	DESeq2	LRT	0.277867663273136
129	KC_AB2	CG15784	-1.80294541848982	0.660135549041553	0.988741847156811	pseudobulk	DESeq2	LRT	0.180366879372519
129	KC_AB2	CG15803	-0.563227798333749	0.715783191409581	0.988741847156811	pseudobulk	DESeq2	LRT	0.145218504274846
129	KC_AB2	CG15812	-3.4227037205893	0.285801360563088	0.988741847156811	pseudobulk	DESeq2	LRT	0.543935708072568
129	KC_AB2	CG15814	-0.385219464369328	0.846043783148359	0.988741847156811	pseudobulk	DESeq2	LRT	0.0726071614435858
129	KC_AB2	CG1582	-1.65719613210719	0.599376091584556	0.988741847156811	pseudobulk	DESeq2	LRT	0.222300584546838
129	KC_AB2	CG15864	0.818790875285784	0.940355871560339	0.988741847156811	pseudobulk	DESeq2	LRT	0.0267077593721816
130	KC_AB2	CG15877	-0.0323203545809474	0.954448156132187	0.988741847156811	pseudobulk	DESeq2	LRT	0.020247656706879
130	KC_AB2	CG15891	0.761842366086347	0.601544884836795	0.988741847156811	pseudobulk	DESeq2	LRT	0.220731961824269
130	KC_AB2	CG15894	-1.17973909372166	0.311635359886974	0.988741847156811	pseudobulk	DESeq2	LRT	0.506353270728172
130	KC_AB2	CG15908	2.00515076565275	0.576456787459964	0.988741847156811	pseudobulk	DESeq2	LRT	0.239233242872451
130	KC_AB2	CG15912	-0.00340877878248056	0.980029417943268	0.988741847156811	pseudobulk	DESeq2	LRT	0.0087608877170192
130	KC_AB2	CG15916	0.54459627071407	0.620482829077201	0.988741847156811	pseudobulk	DESeq2	LRT	0.207270232441164
130	KC_AB2	CG15922	-0.912369387131612	0.601134165098738	0.988741847156811	pseudobulk	DESeq2	LRT	0.221028588464254
130	KC_AB2	CG1598	0.612459217348831	0.742982699573887	0.988741847156811	pseudobulk	DESeq2	LRT	0.129021298711929
130	KC_AB2	CG1602	0.157562008957688	0.950463298513166	0.988741847156811	pseudobulk	DESeq2	LRT	0.0220646484576093
130	KC_AB2	CG1603	1.47543920254359	0.661929169513603	0.988741847156811	pseudobulk	DESeq2	LRT	0.17918848025087
131	KC_AB2	CG1607	0.444303551450947	0.713607263778834	0.988741847156811	pseudobulk	DESeq2	LRT	0.146540737943859
131	KC_AB2	CG1620	0.221609725778056	0.892612653855332	0.988741847156811	pseudobulk	DESeq2	LRT	0.0493369608064992
131	KC_AB2	CG1622	0.0629483694588955	0.970479626220075	0.988741847156811	pseudobulk	DESeq2	LRT	0.0130135775482347
131	KC_AB2	CG1635	-0.0323203545809474	0.954448156132187	0.988741847156811	pseudobulk	DESeq2	LRT	0.020247656706879
131	KC_AB2	CG1636	0.517245766982953	0.674650202430427	0.988741847156811	pseudobulk	DESeq2	LRT	0.170921344991914
131	KC_AB2	CG1637	-1.14529445241152	0.392246360811031	0.988741847156811	pseudobulk	DESeq2	LRT	0.40644107702597
131	KC_AB2	CG1640	0.362935267374622	0.717523186291266	0.988741847156811	pseudobulk	DESeq2	LRT	0.144164060425812
131	KC_AB2	CG1646	-0.366150598907428	0.779385651923885	0.988741847156811	pseudobulk	DESeq2	LRT	0.108247593600603
131	KC_AB2	CG1647	1.31950274499659	0.590522087411301	0.988741847156811	pseudobulk	DESeq2	LRT	0.228763853746569
131	KC_AB2	CG1648	-1.96992303489536	0.292467459495872	0.988741847156811	pseudobulk	DESeq2	LRT	0.533922447351162
132	KC_AB2	CG1662	-1.69633662497937	0.605069927920179	0.988741847156811	pseudobulk	DESeq2	LRT	0.2181944310419
132	KC_AB2	CG16711	0.0519125756900899	0.932966359049352	0.988741847156811	pseudobulk	DESeq2	LRT	0.0301340157847269
132	KC_AB2	CG16717	0.997284248814671	0.663837179223659	0.988741847156811	pseudobulk	DESeq2	LRT	0.177938427914901
132	KC_AB2	CG16721	-1.52496782475103	0.617219242690575	0.988741847156811	pseudobulk	DESeq2	LRT	0.209560542647675
132	KC_AB2	CG16734	0.818790875285784	0.940355871560339	0.988741847156811	pseudobulk	DESeq2	LRT	0.0267077593721816
132	KC_AB2	CG1674	2.83920224641092	0.235465469366797	0.988741847156811	pseudobulk	DESeq2	LRT	0.628072772454433
132	KC_AB2	CG16753	2.06137138167525	0.547946929015274	0.988741847156811	pseudobulk	DESeq2	LRT	0.261261502741816
132	KC_AB2	CG1677	-0.408454607484236	0.592636193002976	0.988741847156811	pseudobulk	DESeq2	LRT	0.227211829161242
132	KC_AB2	CG16779	-0.503582155108194	0.335157255037348	0.988741847156811	pseudobulk	DESeq2	LRT	0.474751375140661
132	KC_AB2	CG16787	-1.1284597703615	0.729754448571724	0.988741847156811	pseudobulk	DESeq2	LRT	0.136823248879498
133	KC_AB2	CG16790	0.818790875285784	0.940355871560339	0.988741847156811	pseudobulk	DESeq2	LRT	0.0267077593721816
133	KC_AB2	CG16791	-0.292613947078114	0.57213537101514	0.988741847156811	pseudobulk	DESeq2	LRT	0.242501202100017
133	KC_AB2	CG16798	0.818790875285784	0.940355871560339	0.988741847156811	pseudobulk	DESeq2	LRT	0.0267077593721816
133	KC_AB2	CG16812	0.189249264265911	0.917387558178736	0.988741847156811	pseudobulk	DESeq2	LRT	0.0374471541658424
133	KC_AB2	CG16820	-2.26538931672255	0.547771004842275	0.988741847156811	pseudobulk	DESeq2	LRT	0.2614009599796
133	KC_AB2	CG16825	-2.26538931672255	0.547771004842275	0.988741847156811	pseudobulk	DESeq2	LRT	0.2614009599796
133	KC_AB2	CG16854	1.94106108959906	0.476420175276185	0.988741847156811	pseudobulk	DESeq2	LRT	0.322009855447888
133	KC_AB2	CG16863	2.06137138167525	0.547946929015274	0.988741847156811	pseudobulk	DESeq2	LRT	0.261261502741816
133	KC_AB2	CG16865	1.12452187615248	0.704078404906088	0.988741847156811	pseudobulk	DESeq2	LRT	0.152378975911753
133	KC_AB2	CG16868	-0.0582963981256801	0.979058404810457	0.988741847156811	pseudobulk	DESeq2	LRT	0.0091913999942843
134	KC_AB2	CG1688	0.594979745961009	0.662663064178255	0.988741847156811	pseudobulk	DESeq2	LRT	0.178707235641905
134	KC_AB2	CG16890	1.42621218304824	0.66738831402044	0.988741847156811	pseudobulk	DESeq2	LRT	0.175621402117653
134	KC_AB2	CG16896	0.818790875285784	0.940355871560339	0.988741847156811	pseudobulk	DESeq2	LRT	0.0267077593721816

134	KC_AB2	CG16903	-1.80860363537534	0.42681091640277	0.988741847156811	pseudobulk	DESeq2	LRT	0.369764481298046
134	KC_AB2	CG16908	2.00515076565275	0.576456787459964	0.988741847156811	pseudobulk	DESeq2	LRT	0.239233242872451
134	KC_AB2	CG16935	0.82812664292794	0.740482099422493	0.988741847156811	pseudobulk	DESeq2	LRT	0.130485435746419
134	KC_AB2	CG1695	0.237781028391615	0.861998859872273	0.988741847156811	pseudobulk	DESeq2	LRT	0.06449330859699
134	KC_AB2	CG16952	0.32140381670586	0.812225745916471	0.988741847156811	pseudobulk	DESeq2	LRT	0.0903232483694482
134	KC_AB2	CG16953	0.422676311221553	0.783086876104452	0.988741847156811	pseudobulk	DESeq2	LRT	0.10619005439185
134	KC_AB2	CG16972	3.4439122560021	0.227129406551316	0.988741847156811	pseudobulk	DESeq2	LRT	0.643726633828736
135	KC_AB2	CG16974	0.688639960934559	0.608191846334261	0.988741847156811	pseudobulk	DESeq2	LRT	0.215959406480236
135	KC_AB2	CG16979	-2.26538931672255	0.547771004842275	0.988741847156811	pseudobulk	DESeq2	LRT	0.2614009599796
135	KC_AB2	CG16985	2.06137138167525	0.547946929015274	0.988741847156811	pseudobulk	DESeq2	LRT	0.261261502741816
135	KC_AB2	CG16986	0.581445489707027	0.811994604428781	0.988741847156811	pseudobulk	DESeq2	LRT	0.0904468565649727
135	KC_AB2	CG16989	-0.506613484034373	0.961658398065406	0.988741847156811	pseudobulk	DESeq2	LRT	0.0169791713942958
135	KC_AB2	CG17002	-0.233272055930639	0.937531793764924	0.988741847156811	pseudobulk	DESeq2	LRT	0.028013995469539
135	KC_AB2	CG17019	0.526465234361098	0.799453536031485	0.988741847156811	pseudobulk	DESeq2	LRT	0.097206772232345
135	KC_AB2	CG17026	-0.568233637794205	0.868826397133536	0.988741847156811	pseudobulk	DESeq2	LRT	0.0610669925949191
135	KC_AB2	CG17027	1.42621218304824	0.66738831402044	0.988741847156811	pseudobulk	DESeq2	LRT	0.175621402117653
135	KC_AB2	CG17029	0.818790875285784	0.940355871560339	0.988741847156811	pseudobulk	DESeq2	LRT	0.0267077593721816
136	KC_AB2	CG1703	-0.840006590521399	0.717017354938613	0.988741847156811	pseudobulk	DESeq2	LRT	0.144470332389583
136	KC_AB2	CG17059	-0.167266173108623	0.849422580649277	0.988741847156811	pseudobulk	DESeq2	LRT	0.0708761981121985
136	KC_AB2	CG17065	0.952276662934622	0.548401675120402	0.988741847156811	pseudobulk	DESeq2	LRT	0.260901227306237
136	KC_AB2	CG17075	-0.837864416490872	0.544634075637226	0.988741847156811	pseudobulk	DESeq2	LRT	0.263895190028277
136	KC_AB2	CG17078	0.11213458976865	0.938112983749562	0.988741847156811	pseudobulk	DESeq2	LRT	0.0277448532370976
136	KC_AB2	CG17124	-0.0776615483235532	0.927655300145524	0.988741847156811	pseudobulk	DESeq2	LRT	0.0326133697449296
136	KC_AB2	CG17141	-2.9721553030081	0.387854083811327	0.988741847156811	pseudobulk	DESeq2	LRT	0.411331631401987
136	KC_AB2	CG17162	-0.452344515838269	0.871171367298942	0.988741847156811	pseudobulk	DESeq2	LRT	0.0598964069304489
136	KC_AB2	CG17163	0.00864923670303397	0.952235192455287	0.988741847156811	pseudobulk	DESeq2	LRT	0.0212557720248108
136	KC_AB2	CG17167	-3.32426296135166	0.343738249391692	0.988741847156811	pseudobulk	DESeq2	LRT	0.463772139158982
137	KC_AB2	CG17168	0.85433262872888	0.629329850990851	0.988741847156811	pseudobulk	DESeq2	LRT	0.201121667882043
137	KC_AB2	CG17186	0.0601466527378941	0.974810960364079	0.988741847156811	pseudobulk	DESeq2	LRT	0.0110795964355292
137	KC_AB2	CG17187	-0.233272055930639	0.937531793764924	0.988741847156811	pseudobulk	DESeq2	LRT	0.028013995469539
137	KC_AB2	CG17193	-2.26538931672255	0.547771004842275	0.988741847156811	pseudobulk	DESeq2	LRT	0.2614009599796
137	KC_AB2	CG17202	0.657999813811193	0.574907576262388	0.988741847156811	pseudobulk	DESeq2	LRT	0.240401968085165
137	KC_AB2	CG17221	0.818790875285784	0.940355871560339	0.988741847156811	pseudobulk	DESeq2	LRT	0.0267077593721816
137	KC_AB2	CG17230	1.31413323252528	0.624060193048613	0.988741847156811	pseudobulk	DESeq2	LRT	0.204773518894346
137	KC_AB2	CG17233	1.16920056293863	0.463335514141164	0.988741847156811	pseudobulk	DESeq2	LRT	0.334104410342018
137	KC_AB2	CG17249	-1.1284597703615	0.729754448571724	0.988741847156811	pseudobulk	DESeq2	LRT	0.136823248879498
137	KC_AB2	CG17260	-0.506613484034373	0.961658398065406	0.988741847156811	pseudobulk	DESeq2	LRT	0.0169791713942958
138	KC_AB2	CG17264	1.42621218304824	0.66738831402044	0.988741847156811	pseudobulk	DESeq2	LRT	0.175621402117653
138	KC_AB2	CG17266	0.818790875285784	0.940355871560339	0.988741847156811	pseudobulk	DESeq2	LRT	0.0267077593721816
138	KC_AB2	CG17270	0.338116808202516	0.828937719305608	0.988741847156811	pseudobulk	DESeq2	LRT	0.0814780981324584
138	KC_AB2	CG17271	0.575200883059258	0.804213580533116	0.988741847156811	pseudobulk	DESeq2	LRT	0.0946285973599483
138	KC_AB2	CG17272	0.170923326936401	0.841945749886111	0.988741847156811	pseudobulk	DESeq2	LRT	0.0747158910230823
138	KC_AB2	CG17294	1.06040655315449	0.536866964313559	0.988741847156811	pseudobulk	DESeq2	LRT	0.270133319183978
138	KC_AB2	CG17321	-0.40433761563393	0.687574317204423	0.988741847156811	pseudobulk	DESeq2	LRT	0.162680353777758
138	KC_AB2	CG17328	-0.506613484034373	0.961658398065406	0.988741847156811	pseudobulk	DESeq2	LRT	0.0169791713942958
138	KC_AB2	CG17343	3.21186121212301	0.139665627418953	0.988741847156811	pseudobulk	DESeq2	LRT	0.854910463313463
138	KC_AB2	CG17359	0.712171366772551	0.813205723536451	0.988741847156811	pseudobulk	DESeq2	LRT	0.0897995733547685
139	KC_AB2	CG17361	0.712171366772551	0.813205723536451	0.988741847156811	pseudobulk	DESeq2	LRT	0.0897995733547685
139	KC_AB2	CG17364	0.907325252206858	0.526656583165861	0.988741847156811	pseudobulk	DESeq2	LRT	0.278472482798178
139	KC_AB2	CG1737	-0.374849006164263	0.728021380994767	0.988741847156811	pseudobulk	DESeq2	LRT	0.137855865863212
139	KC_AB2	CG17378	0.414910419720053	0.750347298666893	0.988741847156811	pseudobulk	DESeq2	LRT	0.124737676630478
139	KC_AB2	CG1738	1.47543920254359	0.661929169513603	0.988741847156811	pseudobulk	DESeq2	LRT	0.17918848025087
139	KC_AB2	CG17385	-0.0305520497669745	0.961951774828401	0.988741847156811	pseudobulk	DESeq2	LRT	0.0168466997406617
139	KC_AB2	CG17454	0.0624746615810756	0.942071380537067	0.988741847156811	pseudobulk	DESeq2	LRT	0.0259161895649828
139	KC_AB2	CG17486	0.12932610628054	0.910174751995068	0.988741847156811	pseudobulk	DESeq2	LRT	0.040875215875664
139	KC_AB2	CG17493	0.979168346553267	0.383079581120958	0.988741847156811	pseudobulk	DESeq2	LRT	0.416710996131843

139	KC_AB2	CG1750	0.297713224699148	0.901701424115727	0.988741847156811	pseudobulk	DESeq2	LRT	0.0449372444148942
140	KC_AB2	CG17508	0.146719198679819	0.900352927347767	0.988741847156811	pseudobulk	DESeq2	LRT	0.0455872190552577
140	KC_AB2	CG17528	0.403500580654007	0.787216114333264	0.988741847156811	pseudobulk	DESeq2	LRT	0.103906024466975
140	KC_AB2	CG17544	-1.80933943727629	0.584587525761637	0.988741847156811	pseudobulk	DESeq2	LRT	0.233150456085471
140	KC_AB2	CG17556	1.94106108959906	0.476420175276185	0.988741847156811	pseudobulk	DESeq2	LRT	0.322009855447888
140	KC_AB2	CG17565	1.99386207130934	0.349788165749185	0.988741847156811	pseudobulk	DESeq2	LRT	0.456194887929862
140	KC_AB2	CG17568	0.370787695871894	0.854629936390885	0.988741847156811	pseudobulk	DESeq2	LRT	0.0682218985751522
140	KC_AB2	CG17574	-0.451045789314127	0.823968225471349	0.988741847156811	pseudobulk	DESeq2	LRT	0.0840895355947879
140	KC_AB2	CG17593	1.01437549054343	0.585186909806483	0.988741847156811	pseudobulk	DESeq2	LRT	0.232705397280827
140	KC_AB2	CG17598	-0.587425801135323	0.771690223849521	0.988741847156811	pseudobulk	DESeq2	LRT	0.112557001570832
140	KC_AB2	CG17600	0.362555753552128	0.897034383127386	0.988741847156811	pseudobulk	DESeq2	LRT	0.0471909102264623
141	KC_AB2	CG17612	2.41462217514131	0.497446973552313	0.988741847156811	pseudobulk	DESeq2	LRT	0.303253207020768
141	KC_AB2	CG17637	1.42621218304824	0.66738831402044	0.988741847156811	pseudobulk	DESeq2	LRT	0.175621402117653
141	KC_AB2	CG1764	1.43050469631063	0.476736087104661	0.988741847156811	pseudobulk	DESeq2	LRT	0.321721972391742
141	KC_AB2	CG17646	-1.80294541848982	0.660135549041553	0.988741847156811	pseudobulk	DESeq2	LRT	0.180366879372519
141	KC_AB2	CG17650	1.42621218304824	0.66738831402044	0.988741847156811	pseudobulk	DESeq2	LRT	0.175621402117653
141	KC_AB2	CG17652	1.49446960201383	0.748153945305307	0.988741847156811	pseudobulk	DESeq2	LRT	0.126009029518429
141	KC_AB2	CG17660	-0.0144409071146711	0.984632974376923	0.988741847156811	pseudobulk	DESeq2	LRT	0.00672562423038896
141	KC_AB2	CG17683	0.961828407721153	0.584174465909025	0.988741847156811	pseudobulk	DESeq2	LRT	0.233457429829368
141	KC_AB2	CG17684	-0.208040576453672	0.641017866282808	0.988741847156811	pseudobulk	DESeq2	LRT	0.193129865769945
141	KC_AB2	CG17691	0.599954338021429	0.688598493387841	0.988741847156811	pseudobulk	DESeq2	LRT	0.162033931840557
142	KC_AB2	CG17698	-0.288536832743132	0.702916159147216	0.988741847156811	pseudobulk	DESeq2	LRT	0.153096472691624
142	KC_AB2	CG17712	0.046530156636047	0.970525086348867	0.988741847156811	pseudobulk	DESeq2	LRT	0.0129932343899138
142	KC_AB2	CG17715	0.336235193760295	0.74901993784284	0.988741847156811	pseudobulk	DESeq2	LRT	0.125506621846753
142	KC_AB2	CG17716	-1.24930044123812	0.502479568210586	0.988741847156811	pseudobulk	DESeq2	LRT	0.298881592800609
142	KC_AB2	CG17724	-0.980809355788834	0.370000214930819	0.988741847156811	pseudobulk	DESeq2	LRT	0.431798023653973
142	KC_AB2	CG17726	-2.26538931672255	0.547771004842275	0.988741847156811	pseudobulk	DESeq2	LRT	0.2614009599796
142	KC_AB2	CG17734	0.957153962054099	0.421559188709695	0.988741847156811	pseudobulk	DESeq2	LRT	0.375141439958911
142	KC_AB2	CG17746	-0.0323203545809474	0.954448156132187	0.988741847156811	pseudobulk	DESeq2	LRT	0.020247656706879
142	KC_AB2	CG17754	0.833854176015168	0.642251910343238	0.988741847156811	pseudobulk	DESeq2	LRT	0.192294595274473
142	KC_AB2	CG17760	0.818790875285784	0.940355871560339	0.988741847156811	pseudobulk	DESeq2	LRT	0.0267077593721816
143	KC_AB2	CG17765	-0.133624712817158	0.89106854152885	0.988741847156811	pseudobulk	DESeq2	LRT	0.0500888884851501
143	KC_AB2	CG17776	1.7602724179441	0.290392949246603	0.988741847156811	pseudobulk	DESeq2	LRT	0.537013932532781
143	KC_AB2	CG17778	0.519511523577052	0.383881163953735	0.988741847156811	pseudobulk	DESeq2	LRT	0.415803197055937
143	KC_AB2	CG17802	-0.729234089517982	0.868684292414125	0.988741847156811	pseudobulk	DESeq2	LRT	0.0611380313470854
143	KC_AB2	CG17803	-1.80294541848982	0.660135549041553	0.988741847156811	pseudobulk	DESeq2	LRT	0.180366879372519
143	KC_AB2	CG17806	-0.233272055930639	0.937531793764924	0.988741847156811	pseudobulk	DESeq2	LRT	0.028013995469539
143	KC_AB2	CG17807	2.00515076565275	0.576456787459964	0.988741847156811	pseudobulk	DESeq2	LRT	0.239233242872451
143	KC_AB2	CG17816	-0.23005675632875	0.481627153433989	0.988741847156811	pseudobulk	DESeq2	LRT	0.31728903617275
143	KC_AB2	CG17829	-1.1284597703615	0.729754448571724	0.988741847156811	pseudobulk	DESeq2	LRT	0.136823248879498
143	KC_AB2	CG17839	-0.167308964337992	0.816554724384833	0.988741847156811	pseudobulk	DESeq2	LRT	0.0880147041276294
144	KC_AB2	CG17841	0.113354314827069	0.825231291970819	0.988741847156811	pseudobulk	DESeq2	LRT	0.0834243123607949
144	KC_AB2	CG1785	0.712171366772551	0.813205723536451	0.988741847156811	pseudobulk	DESeq2	LRT	0.0897995733547685
144	KC_AB2	CG17883	0.798937477954143	0.484439231042713	0.988741847156811	pseudobulk	DESeq2	LRT	0.314760693903326
144	KC_AB2	CG1789	0.356576043500675	0.884817522565587	0.988741847156811	pseudobulk	DESeq2	LRT	0.0531462853633029
144	KC_AB2	CG17904	-0.644945279262214	0.70964919440659	0.988741847156811	pseudobulk	DESeq2	LRT	0.148956285906352
144	KC_AB2	CG1792	3.06202454821851	0.30627034058063	0.988741847156811	pseudobulk	DESeq2	LRT	0.513895058517773
144	KC_AB2	CG17931	-0.220402264164655	0.864409831698392	0.988741847156811	pseudobulk	DESeq2	LRT	0.0632803021450237
144	KC_AB2	CG17977	-0.0323203545809474	0.954448156132187	0.988741847156811	pseudobulk	DESeq2	LRT	0.020247656706879
144	KC_AB2	CG17982	0.50351383114442	0.831654424165643	0.988741847156811	pseudobulk	DESeq2	LRT	0.0800570978140888
144	KC_AB2	CG17985	-0.934020934207768	0.782359118258182	0.988741847156811	pseudobulk	DESeq2	LRT	0.106593851444836
145	KC_AB2	CG17996	0.804708302235815	0.733338290589079	0.988741847156811	pseudobulk	DESeq2	LRT	0.13469563812263
145	KC_AB2	CG18004	-1.68886373956583	0.337098268609638	0.988741847156811	pseudobulk	DESeq2	LRT	0.472243478078884
145	KC_AB2	CG18011	1.42621218304824	0.66738831402044	0.988741847156811	pseudobulk	DESeq2	LRT	0.175621402117653
145	KC_AB2	CG18048	0.818790875285784	0.940355871560339	0.988741847156811	pseudobulk	DESeq2	LRT	0.0267077593721816
145	KC_AB2	CG18081	0.157562008957688	0.950463298513166	0.988741847156811	pseudobulk	DESeq2	LRT	0.0220646484576093

145	KC_AB2	CG1812	-2.51746470924027	0.517377929599049	0.988741847156811	pseudobulk	DESeq2	LRT	0.286192101436607
145	KC_AB2	CG18135	-1.43539591527129	0.311283755210163	0.988741847156811	pseudobulk	DESeq2	LRT	0.506843543007402
145	KC_AB2	CG1814	0.444811662881181	0.779564417627056	0.988741847156811	pseudobulk	DESeq2	LRT	0.108147992002967
145	KC_AB2	CG1815	-2.21667329608248	0.477261050443944	0.988741847156811	pseudobulk	DESeq2	LRT	0.321244007216606
145	KC_AB2	CG18178	-2.45158172483764	0.378823136429564	0.988741847156811	pseudobulk	DESeq2	LRT	0.421563504545096
146	KC_AB2	CG18213	2.51243226103394	0.474458247157471	0.988741847156811	pseudobulk	DESeq2	LRT	0.323801999942597
146	KC_AB2	CG18223	1.42621218304824	0.66738831402044	0.988741847156811	pseudobulk	DESeq2	LRT	0.175621402117653
146	KC_AB2	CG1824	-0.0034087787824805€	0.980029417943268	0.988741847156811	pseudobulk	DESeq2	LRT	0.0087608877170192
146	KC_AB2	CG18259	0.818790875285784	0.940355871560339	0.988741847156811	pseudobulk	DESeq2	LRT	0.0267077593721816
146	KC_AB2	CG18265	1.2869577273174	0.322487488553055	0.988741847156811	pseudobulk	DESeq2	LRT	0.49148712989002
146	KC_AB2	CG1827	-2.9721553030081	0.387854083811327	0.988741847156811	pseudobulk	DESeq2	LRT	0.411331631401987
146	KC_AB2	CG18273	0.351722119397094	0.929615296941389	0.988741847156811	pseudobulk	DESeq2	LRT	0.0316967385224016
146	KC_AB2	CG18281	-2.26538931672255	0.547771004842275	0.988741847156811	pseudobulk	DESeq2	LRT	0.2614009599796
146	KC_AB2	CG18324	0.364417163554467	0.847077934404736	0.988741847156811	pseudobulk	DESeq2	LRT	0.0720766310803004
146	KC_AB2	CG18327	0.818790875285784	0.940355871560339	0.988741847156811	pseudobulk	DESeq2	LRT	0.0267077593721816
147	KC_AB2	CG18335	-2.26538931672255	0.547771004842275	0.988741847156811	pseudobulk	DESeq2	LRT	0.2614009599796
147	KC_AB2	CG1840	3.09926389624515	0.339712049344771	0.988741847156811	pseudobulk	DESeq2	LRT	0.468889048740397
147	KC_AB2	CG18428	-0.416522381580336	0.732928671633917	0.988741847156811	pseudobulk	DESeq2	LRT	0.134938288691893
147	KC_AB2	CG18467	-0.678242971759928	0.496645115409142	0.988741847156811	pseudobulk	DESeq2	LRT	0.303953831532808
147	KC_AB2	CG1847	-0.192181231709668	0.899340686783902	0.988741847156811	pseudobulk	DESeq2	LRT	0.0460757583645606
147	KC_AB2	CG18476	-0.718003415132797	0.760638816665156	0.988741847156811	pseudobulk	DESeq2	LRT	0.118821515588473
147	KC_AB2	CG18507	1.47543920254359	0.661929169513603	0.988741847156811	pseudobulk	DESeq2	LRT	0.17918848025087
147	KC_AB2	CG18508	0.402862698183919	0.757393647968245	0.988741847156811	pseudobulk	DESeq2	LRT	0.12067834149656
147	KC_AB2	CG18528	-1.99867440840856	0.534899605264936	0.988741847156811	pseudobulk	DESeq2	LRT	0.271727722597268
147	KC_AB2	CG18549	1.23898279432937	0.452928554082101	0.988741847156811	pseudobulk	DESeq2	LRT	0.34397029912409
148	KC_AB2	CG18596	-2.16923839102081	0.498281874778995	0.988741847156811	pseudobulk	DESeq2	LRT	0.302524910193245
148	KC_AB2	CG18600	-2.26538931672255	0.547771004842275	0.988741847156811	pseudobulk	DESeq2	LRT	0.2614009599796
148	KC_AB2	CG18628	0.818790875285784	0.940355871560339	0.988741847156811	pseudobulk	DESeq2	LRT	0.0267077593721816
148	KC_AB2	CG18659	1.10527623683615	0.724717602335893	0.988741847156811	pseudobulk	DESeq2	LRT	0.139831190172672
148	KC_AB2	CG18661	0.575200883059258	0.804213580533116	0.988741847156811	pseudobulk	DESeq2	LRT	0.0946285973599483
148	KC_AB2	CG18675	-2.26538931672255	0.547771004842275	0.988741847156811	pseudobulk	DESeq2	LRT	0.2614009599796
148	KC_AB2	CG18731	-1.65719613210719	0.599376091584556	0.988741847156811	pseudobulk	DESeq2	LRT	0.222300584546838
148	KC_AB2	CG18764	2.00515076565275	0.576456787459964	0.988741847156811	pseudobulk	DESeq2	LRT	0.239233242872451
148	KC_AB2	CG18766	-1.08897477461755	0.407479314565392	0.988741847156811	pseudobulk	DESeq2	LRT	0.389894433054372
148	KC_AB2	CG18809	0.804708302235815	0.733338290589079	0.988741847156811	pseudobulk	DESeq2	LRT	0.13469563812263
149	KC_AB2	CG18815	0.0148518679165913	0.974321844241302	0.988741847156811	pseudobulk	DESeq2	LRT	0.0112975604830163
149	KC_AB2	CG1882	1.4107145821539	0.42448312778373	0.988741847156811	pseudobulk	DESeq2	LRT	0.37213956727173
149	KC_AB2	CG18870	0.351722119397094	0.929615296941389	0.988741847156811	pseudobulk	DESeq2	LRT	0.0316967385224016
149	KC_AB2	CG1888	0.086855330069921	0.93457542246533	0.988741847156811	pseudobulk	DESeq2	LRT	0.0293856442704431
149	KC_AB2	CG1890	0.818790875285784	0.940355871560339	0.988741847156811	pseudobulk	DESeq2	LRT	0.0267077593721816
149	KC_AB2	CG1896	-0.233095226066582	0.89156790931789	0.988741847156811	pseudobulk	DESeq2	LRT	0.0498455717047824
149	KC_AB2	CG1902	0.89966407823676	0.632116859490606	0.988741847156811	pseudobulk	DESeq2	LRT	0.199202626242253
149	KC_AB2	CG1907	0.322884219716945	0.836061399620774	0.988741847156811	pseudobulk	DESeq2	LRT	0.0777618271814104
149	KC_AB2	CG1909	-0.477191969921639	0.470548535454695	0.988741847156811	pseudobulk	DESeq2	LRT	0.327395573951885
149	KC_AB2	CG1910	0.282238512673597	0.814503739886007	0.988741847156811	pseudobulk	DESeq2	LRT	0.0891069172386799
150	KC_AB2	CG1927	-0.296576489727943	0.896291428147039	0.988741847156811	pseudobulk	DESeq2	LRT	0.047550757014702
150	KC_AB2	CG1943	0.275917495803887	0.733989674624795	0.988741847156811	pseudobulk	DESeq2	LRT	0.13431004946406
150	KC_AB2	CG1951	0.362555753552128	0.897034383127386	0.988741847156811	pseudobulk	DESeq2	LRT	0.0471909102264623
150	KC_AB2	CG1965	0.228464658616335	0.82873572340351	0.988741847156811	pseudobulk	DESeq2	LRT	0.0815839400960471
150	KC_AB2	CG1983	3.18706398733836	0.133825224129958	0.988741847156811	pseudobulk	DESeq2	LRT	0.873462020589236
150	KC_AB2	CG1998	0.911560374855594	0.349576491308863	0.988741847156811	pseudobulk	DESeq2	LRT	0.456457780952183
150	KC_AB2	CG2004	-0.506613484034373	0.961658398065406	0.988741847156811	pseudobulk	DESeq2	LRT	0.0169791713942958
150	KC_AB2	CG2017	-0.726502369596528	0.602740449747285	0.988741847156811	pseudobulk	DESeq2	LRT	0.219869662169776
150	KC_AB2	CG2021	1.92721521352877	0.471759180353374	0.988741847156811	pseudobulk	DESeq2	LRT	0.326279639784082
150	KC_AB2	CG2023	-2.51746470924027	0.517377929599049	0.988741847156811	pseudobulk	DESeq2	LRT	0.286192101436607
151	KC_AB2	CG2034	0.818790875285784	0.940355871560339	0.988741847156811	pseudobulk	DESeq2	LRT	0.0267077593721816

151	KC_AB2	CG2059	1.22851089263308	0.490964350708434	0.988741847156811	pseudobulk	DESeq2	LRT	0.308950041181895
151	KC_AB2	CG2061	0.922664372932389	0.678259639521954	0.988741847156811	pseudobulk	DESeq2	LRT	0.168604025267887
151	KC_AB2	CG2063	-0.233095226066582	0.89156790931789	0.988741847156811	pseudobulk	DESeq2	LRT	0.0498455717047824
151	KC_AB2	CG2064	-1.80294541848982	0.660135549041553	0.988741847156811	pseudobulk	DESeq2	LRT	0.180366879372519
151	KC_AB2	CG2076	0.935939496831483	0.567502290103793	0.988741847156811	pseudobulk	DESeq2	LRT	0.246032381575485
151	KC_AB2	CG2091	2.56240783746857	0.286713435654989	0.988741847156811	pseudobulk	DESeq2	LRT	0.542551955138264
151	KC_AB2	CG2100	-1.80294541848982	0.660135549041553	0.988741847156811	pseudobulk	DESeq2	LRT	0.180366879372519
151	KC_AB2	CG2111	-1.80294541848982	0.660135549041553	0.988741847156811	pseudobulk	DESeq2	LRT	0.180366879372519
151	KC_AB2	CG2116	-1.65719613210719	0.599376091584556	0.988741847156811	pseudobulk	DESeq2	LRT	0.222300584546838
152	KC_AB2	CG2126	0.157562008957688	0.950463298513166	0.988741847156811	pseudobulk	DESeq2	LRT	0.0220646484576093
152	KC_AB2	CG2129	2.06137138167525	0.547946929015274	0.988741847156811	pseudobulk	DESeq2	LRT	0.261261502741816
152	KC_AB2	CG2147	0.00266298162891975	0.968632759616684	0.988741847156811	pseudobulk	DESeq2	LRT	0.0138408469969281
152	KC_AB2	CG2162	0.194804214247169	0.909397535999427	0.988741847156811	pseudobulk	DESeq2	LRT	0.04124622684446
152	KC_AB2	CG2182	-0.811488116327892	0.485265791022993	0.988741847156811	pseudobulk	DESeq2	LRT	0.314020323342864
152	KC_AB2	CG2186	-0.468006698176309	0.651120919797009	0.988741847156811	pseudobulk	DESeq2	LRT	0.18633835102624
152	KC_AB2	CG2199	2.99842299859728	0.361638898709429	0.988741847156811	pseudobulk	DESeq2	LRT	0.441724861973572
152	KC_AB2	CG2200	-0.0493731122220437	0.968729445650825	0.988741847156811	pseudobulk	DESeq2	LRT	0.0137974991799579
152	KC_AB2	CG2201	1.42034337486757	0.643720050244361	0.988741847156811	pseudobulk	DESeq2	LRT	0.191302963490459
152	KC_AB2	CG2202	-1.1284597703615	0.729754448571724	0.988741847156811	pseudobulk	DESeq2	LRT	0.136823248879498
153	KC_AB2	CG2209	1.47543920254359	0.661929169513603	0.988741847156811	pseudobulk	DESeq2	LRT	0.17918848025087
153	KC_AB2	CG2211	-0.92978684073213	0.72380920826628	0.988741847156811	pseudobulk	DESeq2	LRT	0.140375896118957
153	KC_AB2	CG2218	-1.70869593138706	0.348955663219931	0.988741847156811	pseudobulk	DESeq2	LRT	0.457229749092738
153	KC_AB2	CG2224	-0.233272055930639	0.937531793764924	0.988741847156811	pseudobulk	DESeq2	LRT	0.028013995469539
153	KC_AB2	CG2225	-0.331814631479556	0.696165626954629	0.988741847156811	pseudobulk	DESeq2	LRT	0.157287423729781
153	KC_AB2	CG2246	0.715925243533926	0.602223717518651	0.988741847156811	pseudobulk	DESeq2	LRT	0.220242144564474
153	KC_AB2	CG2247	0.699674038439951	0.821690393783994	0.988741847156811	pseudobulk	DESeq2	LRT	0.0852917902455435
153	KC_AB2	CG2258	1.82764623910507	0.355683754395659	0.988741847156811	pseudobulk	DESeq2	LRT	0.448935970455049
153	KC_AB2	CG2269	0.296031349209421	0.450533151587241	0.988741847156811	pseudobulk	DESeq2	LRT	0.346273246812145
153	KC_AB2	CG2277	2.58110170599353	0.284090348510032	0.988741847156811	pseudobulk	DESeq2	LRT	0.546543520448255
154	KC_AB2	CG2278	-1.27790246201552	0.547658074219306	0.988741847156811	pseudobulk	DESeq2	LRT	0.261490505067465
154	KC_AB2	CG2321	-0.0582963981256801	0.979058404810457	0.988741847156811	pseudobulk	DESeq2	LRT	0.0091913999942843
154	KC_AB2	CG2519	-1.14505507470656	0.445742661070484	0.988741847156811	pseudobulk	DESeq2	LRT	0.350915798534727
154	KC_AB2	CG2540	2.51243226103394	0.474458247157471	0.988741847156811	pseudobulk	DESeq2	LRT	0.323801999942597
154	KC_AB2	CG2608	-1.74699812742254	0.480865720047389	0.988741847156811	pseudobulk	DESeq2	LRT	0.317976181805168
154	KC_AB2	CG2611	2.4643417141186	0.449082701910884	0.988741847156811	pseudobulk	DESeq2	LRT	0.347673673080982
154	KC_AB2	CG2656	-0.634227699706429	0.828294391400015	0.988741847156811	pseudobulk	DESeq2	LRT	0.0818152793537044
154	KC_AB2	CG2662	1.12919034796087	0.7301237214754	0.988741847156811	pseudobulk	DESeq2	LRT	0.136603541247596
154	KC_AB2	CG2680	-0.197565662601209	0.921418496992636	0.988741847156811	pseudobulk	DESeq2	LRT	0.0355430737620862
154	KC_AB2	CG2685	-0.506613484034373	0.961658398065406	0.988741847156811	pseudobulk	DESeq2	LRT	0.0169791713942958
155	KC_AB2	CG2698	-0.864674604872878	0.696981345538496	0.988741847156811	pseudobulk	DESeq2	LRT	0.15677884548608
155	KC_AB2	CG2701	0.284990316553174	0.896610940373611	0.988741847156811	pseudobulk	DESeq2	LRT	0.0473959662120276
155	KC_AB2	CG2712	0.0601466527378941	0.974810960364079	0.988741847156811	pseudobulk	DESeq2	LRT	0.0110795964355292
155	KC_AB2	CG2747	-0.199298282717524	0.882352677272768	0.988741847156811	pseudobulk	DESeq2	LRT	0.0543577922161234
155	KC_AB2	CG2765	-0.730701309720345	0.603567125202526	0.988741847156811	pseudobulk	DESeq2	LRT	0.219274423190608
155	KC_AB2	CG2767	-0.452344515838269	0.871171367298942	0.988741847156811	pseudobulk	DESeq2	LRT	0.0598964069304489
155	KC_AB2	CG2772	-0.368614094711936	0.789733908398645	0.988741847156811	pseudobulk	DESeq2	LRT	0.102519214507805
155	KC_AB2	CG2790	-0.586551448989482	0.787602284627903	0.988741847156811	pseudobulk	DESeq2	LRT	0.103693032757571
155	KC_AB2	CG2794	0.818790875285784	0.940355871560339	0.988741847156811	pseudobulk	DESeq2	LRT	0.0267077593721816
155	KC_AB2	CG2811	-0.244067456186467	0.906772274454269	0.988741847156811	pseudobulk	DESeq2	LRT	0.042501767367312
156	KC_AB2	CG2818	-2.47358677193831	0.276432863915668	0.988741847156811	pseudobulk	DESeq2	LRT	0.558410326823203
156	KC_AB2	CG2841	-2.26538931672255	0.547771004842275	0.988741847156811	pseudobulk	DESeq2	LRT	0.2614009599796
156	KC_AB2	CG2846	-0.385733633096415	0.822744264387791	0.988741847156811	pseudobulk	DESeq2	LRT	0.0847351366281377
156	KC_AB2	CG2852	0.498188609680012	0.532510908939026	0.988741847156811	pseudobulk	DESeq2	LRT	0.273671490906137
156	KC_AB2	CG2865	0.819218154415023	0.293564461660678	0.988741847156811	pseudobulk	DESeq2	LRT	0.532296520414132
156	KC_AB2	CG2875	0.818790875285784	0.940355871560339	0.988741847156811	pseudobulk	DESeq2	LRT	0.0267077593721816
156	KC_AB2	CG2889	-1.23536834900699	0.725570392794057	0.988741847156811	pseudobulk	DESeq2	LRT	0.139320447155934

156	KC_AB2	CG2898	-2.26538931672255	0.547771004842275	0.988741847156811	pseudobulk	DESeq2	LRT	0.2614009599796
156	KC_AB2	CG2909	0.792281256713368	0.823478838299676	0.988741847156811	pseudobulk	DESeq2	LRT	0.0843475568193814
156	KC_AB2	CG2915	-3.09794865339079	0.39855598532175	0.988741847156811	pseudobulk	DESeq2	LRT	0.399510664459796
157	KC_AB2	CG2918	-1.39387680343993	0.390001281887193	0.988741847156811	pseudobulk	DESeq2	LRT	0.408933965497554
157	KC_AB2	CG2921	0.312988786626857	0.865053929025086	0.988741847156811	pseudobulk	DESeq2	LRT	0.0629568169884365
157	KC_AB2	CG2924	-0.410123150276276	0.620430428398306	0.988741847156811	pseudobulk	DESeq2	LRT	0.207306910791675
157	KC_AB2	CG2926	0.0312286266787755	0.950009595013864	0.988741847156811	pseudobulk	DESeq2	LRT	0.0222720083526981
157	KC_AB2	CG2931	4.45442851630551	0.0624556041443036	0.988741847156811	pseudobulk	DESeq2	LRT	1.20442858627705
157	KC_AB2	CG2938	-2.14254423290279	0.31091199741747	0.988741847156811	pseudobulk	DESeq2	LRT	0.507362519154383
157	KC_AB2	CG2972	0.331758848923756	0.946375065527934	0.988741847156811	pseudobulk	DESeq2	LRT	0.0239367107367223
157	KC_AB2	CG2991	-0.828164839089803	0.604188505091408	0.988741847156811	pseudobulk	DESeq2	LRT	0.218827541596436
157	KC_AB2	CG2993	0.25129962274683	0.774046814475837	0.988741847156811	pseudobulk	DESeq2	LRT	0.111232772322666
157	KC_AB2	CG30008	0.818790875285784	0.940355871560339	0.988741847156811	pseudobulk	DESeq2	LRT	0.0267077593721816
158	KC_AB2	CG30010	2.4001665144902	0.374323583152955	0.988741847156811	pseudobulk	DESeq2	LRT	0.426752810581136
158	KC_AB2	CG30015	2.51243226103394	0.474458247157471	0.988741847156811	pseudobulk	DESeq2	LRT	0.323801999942597
158	KC_AB2	CG30020	2.4643417141186	0.449082701910884	0.988741847156811	pseudobulk	DESeq2	LRT	0.347673673080982
158	KC_AB2	CG30022	2.73593896747858	0.422277110754874	0.988741847156811	pseudobulk	DESeq2	LRT	0.374402458575713
158	KC_AB2	CG30060	-0.934020934207768	0.782359118258182	0.988741847156811	pseudobulk	DESeq2	LRT	0.106593851444836
158	KC_AB2	CG30069	0.818790875285784	0.940355871560339	0.988741847156811	pseudobulk	DESeq2	LRT	0.0267077593721816
158	KC_AB2	CG3008	0.699674038439951	0.821690393783994	0.988741847156811	pseudobulk	DESeq2	LRT	0.0852917902455435
158	KC_AB2	CG30089	-0.526561575376116	0.640859104457154	0.988741847156811	pseudobulk	DESeq2	LRT	0.193237441437103
158	KC_AB2	CG30094	1.14703591811676	0.660809111836255	0.988741847156811	pseudobulk	DESeq2	LRT	0.179923977199595
158	KC_AB2	CG30096	-2.26538931672255	0.547771004842275	0.988741847156811	pseudobulk	DESeq2	LRT	0.2614009599796
159	KC_AB2	CG30109	-1.52496782475103	0.617219242690575	0.988741847156811	pseudobulk	DESeq2	LRT	0.209560542647675
159	KC_AB2	CG30116	0.30146838891536	0.54658332859467	0.988741847156811	pseudobulk	DESeq2	LRT	0.262343618910753
159	KC_AB2	CG30122	-1.27541452362739	0.468690171183204	0.988741847156811	pseudobulk	DESeq2	LRT	0.329114153895314
159	KC_AB2	CG30158	-0.127443302992818	0.816143130850548	0.988741847156811	pseudobulk	DESeq2	LRT	0.0882336703055301
159	KC_AB2	CG30172	0.0922458046010496	0.866299560561549	0.988741847156811	pseudobulk	DESeq2	LRT	0.062331905900825
159	KC_AB2	CG3021	1.42621218304824	0.66738831402044	0.988741847156811	pseudobulk	DESeq2	LRT	0.175621402117653
159	KC_AB2	CG30291	-0.0582963981256801	0.979058404810457	0.988741847156811	pseudobulk	DESeq2	LRT	0.0091913999942843
159	KC_AB2	CG3032	-1.80294541848982	0.660135549041553	0.988741847156811	pseudobulk	DESeq2	LRT	0.180366879372519
159	KC_AB2	CG30338	0.818790875285784	0.940355871560339	0.988741847156811	pseudobulk	DESeq2	LRT	0.0267077593721816
159	KC_AB2	CG30344	0.0601466527378941	0.974810960364079	0.988741847156811	pseudobulk	DESeq2	LRT	0.0110795964355292
160	KC_AB2	CG30345	0.818790875285784	0.940355871560339	0.988741847156811	pseudobulk	DESeq2	LRT	0.0267077593721816
160	KC_AB2	CG30349	-0.688968519449025	0.70663956002083	0.988741847156811	pseudobulk	DESeq2	LRT	0.150802052978409
160	KC_AB2	CG30373	3.0880282443908	0.160034807134603	0.988741847156811	pseudobulk	DESeq2	LRT	0.795785549203662
160	KC_AB2	CG3038	1.47543920254359	0.661929169513603	0.988741847156811	pseudobulk	DESeq2	LRT	0.17918848025087
160	KC_AB2	CG30389	0.00131025045932149	0.959962107301429	0.988741847156811	pseudobulk	DESeq2	LRT	0.0177459095798964
160	KC_AB2	CG30392	0.194804214247169	0.909397535999427	0.988741847156811	pseudobulk	DESeq2	LRT	0.04124622684446
160	KC_AB2	CG30394	-2.9721553030081	0.387854083811327	0.988741847156811	pseudobulk	DESeq2	LRT	0.411331631401987
160	KC_AB2	CG3040	-2.08434100393431	0.328662713614262	0.988741847156811	pseudobulk	DESeq2	LRT	0.483249563305339
160	KC_AB2	CG30403	-0.506613484034373	0.961658398065406	0.988741847156811	pseudobulk	DESeq2	LRT	0.0169791713942958
160	KC_AB2	CG30414	0.818790875285784	0.940355871560339	0.988741847156811	pseudobulk	DESeq2	LRT	0.0267077593721816
161	KC_AB2	CG30419	0.44498699490159	0.650895224790592	0.988741847156811	pseudobulk	DESeq2	LRT	0.186488914592243
161	KC_AB2	CG30423	0.930636963272417	0.445890838241667	0.988741847156811	pseudobulk	DESeq2	LRT	0.350771451045696
161	KC_AB2	CG30424	1.4129303440845	0.645460595159151	0.988741847156811	pseudobulk	DESeq2	LRT	0.190130265910817
161	KC_AB2	CG30428	-0.170965113517415	0.87472183660936	0.988741847156811	pseudobulk	DESeq2	LRT	0.0581300315853228
161	KC_AB2	CG30460	-1.80294541848982	0.660135549041553	0.988741847156811	pseudobulk	DESeq2	LRT	0.180366879372519
161	KC_AB2	CG30466	0.818790875285784	0.940355871560339	0.988741847156811	pseudobulk	DESeq2	LRT	0.0267077593721816
161	KC_AB2	CG30467	-1.40296545307325	0.628223627794903	0.988741847156811	pseudobulk	DESeq2	LRT	0.201885733604539
161	KC_AB2	CG30480	-1.80294541848982	0.660135549041553	0.988741847156811	pseudobulk	DESeq2	LRT	0.180366879372519
161	KC_AB2	CG30491	1.4413042803421	0.615311685084066	0.988741847156811	pseudobulk	DESeq2	LRT	0.210904837358999
161	KC_AB2	CG30495	2.00515076565275	0.576456787459964	0.988741847156811	pseudobulk	DESeq2	LRT	0.239233242872451
162	KC_AB2	CG30496	0.0601466527378941	0.974810960364079	0.988741847156811	pseudobulk	DESeq2	LRT	0.0110795964355292
162	KC_AB2	CG3061	-0.29003852570804	0.84572624639156	0.988741847156811	pseudobulk	DESeq2	LRT	0.0727701912413608
162	KC_AB2	CG3065	-0.0384500034601774	0.953910728566112	0.988741847156811	pseudobulk	DESeq2	LRT	0.0204922667059844

162	KC_AB2	CG3071	1.47543920254359	0.661929169513603	0.988741847156811	pseudobulk	DESeq2	LRT	0.17918848025087
162	KC_AB2	CG3078	0.60008651668205	0.68870606200875	0.988741847156811	pseudobulk	DESeq2	LRT	0.161966094326481
162	KC_AB2	CG3097	1.42621218304824	0.66738831402044	0.988741847156811	pseudobulk	DESeq2	LRT	0.175621402117653
162	KC_AB2	CG31029	0.818790875285784	0.940355871560339	0.988741847156811	pseudobulk	DESeq2	LRT	0.0267077593721816
162	KC_AB2	CG31030	0.500210340735127	0.400667826728825	0.988741847156811	pseudobulk	DESeq2	LRT	0.397215529628986
162	KC_AB2	CG31038	1.12919034796087	0.7301237214754	0.988741847156811	pseudobulk	DESeq2	LRT	0.136603541247596
162	KC_AB2	CG3104	0.148659413571328	0.898247616231334	0.988741847156811	pseudobulk	DESeq2	LRT	0.046603926652065
163	KC_AB2	CG31051	2.09183456778624	0.271601620762659	0.988741847156811	pseudobulk	DESeq2	LRT	0.566067642763484
163	KC_AB2	CG31064	0.100212229603488	0.921903517124104	0.988741847156811	pseudobulk	DESeq2	LRT	0.0353145281580556
163	KC_AB2	CG3107	-0.36933023329585	0.709093523188085	0.988741847156811	pseudobulk	DESeq2	LRT	0.149296481423546
163	KC_AB2	CG3108	-1.80294541848982	0.660135549041553	0.988741847156811	pseudobulk	DESeq2	LRT	0.180366879372519
163	KC_AB2	CG31098	0.818790875285784	0.940355871560339	0.988741847156811	pseudobulk	DESeq2	LRT	0.0267077593721816
163	KC_AB2	CG31109	0.317224567334262	0.824573068980285	0.988741847156811	pseudobulk	DESeq2	LRT	0.083770853604817
163	KC_AB2	CG31111	-1.23536834900699	0.725570392794057	0.988741847156811	pseudobulk	DESeq2	LRT	0.139320447155934
163	KC_AB2	CG31122	-0.65464074967228	0.674323305705541	0.988741847156811	pseudobulk	DESeq2	LRT	0.171131830143317
163	KC_AB2	CG31125	1.12452187615248	0.704078404906088	0.988741847156811	pseudobulk	DESeq2	LRT	0.152378975911753
163	KC_AB2	CG31126	0.61702365764228	0.739069534444918	0.988741847156811	pseudobulk	DESeq2	LRT	0.131314699622637
164	KC_AB2	CG31140	0.0864138587293446	0.820467451210545	0.988741847156811	pseudobulk	DESeq2	LRT	0.0859386431788825
164	KC_AB2	CG31142	0.818790875285784	0.940355871560339	0.988741847156811	pseudobulk	DESeq2	LRT	0.0267077593721816
164	KC_AB2	CG31145	0.818790875285784	0.940355871560339	0.988741847156811	pseudobulk	DESeq2	LRT	0.0267077593721816
164	KC_AB2	CG31156	0.104637746735554	0.951122131714938	0.988741847156811	pseudobulk	DESeq2	LRT	0.0217637125849166
164	KC_AB2	CG31157	0.818790875285784	0.940355871560339	0.988741847156811	pseudobulk	DESeq2	LRT	0.0267077593721816
164	KC_AB2	CG31174	0.818790875285784	0.940355871560339	0.988741847156811	pseudobulk	DESeq2	LRT	0.0267077593721816
164	KC_AB2	CG31191	0.327666188211622	0.755739427190058	0.988741847156811	pseudobulk	DESeq2	LRT	0.121627919896272
164	KC_AB2	CG31211	0.112140827038324	0.909855917697253	0.988741847156811	pseudobulk	DESeq2	LRT	0.0410273759242881
164	KC_AB2	CG31221	-0.129485678438078	0.847418692373046	0.988741847156811	pseudobulk	DESeq2	LRT	0.0719019605557827
164	KC_AB2	CG31224	-0.304345638121601	0.830407045896163	0.988741847156811	pseudobulk	DESeq2	LRT	0.0807089745475902
165	KC_AB2	CG31229	1.1327121871749	0.657275461456076	0.988741847156811	pseudobulk	DESeq2	LRT	0.182252581249451
165	KC_AB2	CG31249	-0.488465967391191	0.84251072973523	0.988741847156811	pseudobulk	DESeq2	LRT	0.0744245594950451
165	KC_AB2	CG31251	-2.51746470924027	0.517377929599049	0.988741847156811	pseudobulk	DESeq2	LRT	0.286192101436607
165	KC_AB2	CG31301	0.668970670557767	0.818048200024539	0.988741847156811	pseudobulk	DESeq2	LRT	0.0872211066123218
165	KC_AB2	CG31324	2.06137138167525	0.547946929015274	0.988741847156811	pseudobulk	DESeq2	LRT	0.261261502741816
165	KC_AB2	CG31337	-1.80294541848982	0.660135549041553	0.988741847156811	pseudobulk	DESeq2	LRT	0.180366879372519
165	KC_AB2	CG31342	0.442646110217664	0.78163626900832	0.988741847156811	pseudobulk	DESeq2	LRT	0.106995296954678
165	KC_AB2	CG31344	-0.75820992101805	0.527939958314418	0.988741847156811	pseudobulk	DESeq2	LRT	0.277415466207574
165	KC_AB2	CG31345	2.06137138167525	0.547946929015274	0.988741847156811	pseudobulk	DESeq2	LRT	0.261261502741816
165	KC_AB2	CG31357	-0.0582963981256801	0.979058404810457	0.988741847156811	pseudobulk	DESeq2	LRT	0.0091913999942843
166	KC_AB2	CG31360	1.5475546809088	0.433799017213538	0.988741847156811	pseudobulk	DESeq2	LRT	0.362711436229665
166	KC_AB2	CG31365	1.10527623683615	0.724717602335893	0.988741847156811	pseudobulk	DESeq2	LRT	0.139831190172672
166	KC_AB2	CG31368	-0.742148798960242	0.80644751100302	0.988741847156811	pseudobulk	DESeq2	LRT	0.0934238941443075
166	KC_AB2	CG31388	-0.50879048068994	0.817628596116567	0.988741847156811	pseudobulk	DESeq2	LRT	0.0874439277298491
166	KC_AB2	CG31457	0.818790875285784	0.940355871560339	0.988741847156811	pseudobulk	DESeq2	LRT	0.0267077593721816
166	KC_AB2	CG31460	-2.08434100393431	0.328662713614262	0.988741847156811	pseudobulk	DESeq2	LRT	0.483249563305339
166	KC_AB2	CG31467	-3.09794865339079	0.39855598532175	0.988741847156811	pseudobulk	DESeq2	LRT	0.399510664459796
166	KC_AB2	CG31475	1.24780475844232	0.427738907699139	0.988741847156811	pseudobulk	DESeq2	LRT	0.368821243943537
166	KC_AB2	CG3149	-0.233272055930639	0.937531793764924	0.988741847156811	pseudobulk	DESeq2	LRT	0.028013995469539
166	KC_AB2	CG31510	1.7098779479	0.537865353861561	0.988741847156811	pseudobulk	DESeq2	LRT	0.269326429526188
167	KC_AB2	CG31522	0.229478723420001	0.874269636581332	0.988741847156811	pseudobulk	DESeq2	LRT	0.0583546044140361
167	KC_AB2	CG31523	0.470427817298873	0.780796750129473	0.988741847156811	pseudobulk	DESeq2	LRT	0.107462002979395
167	KC_AB2	CG31550	-0.0895902818285133	0.954518965493979	0.988741847156811	pseudobulk	DESeq2	LRT	0.0202154381162507
167	KC_AB2	CG3156	3.81052043979741	0.156359277889969	0.988741847156811	pseudobulk	DESeq2	LRT	0.805876343926194
167	KC_AB2	CG3163	0.0471023025337348	0.971010011322875	0.988741847156811	pseudobulk	DESeq2	LRT	0.0127762923990319
167	KC_AB2	CG31635	-0.119956399855374	0.888984751835069	0.988741847156811	pseudobulk	DESeq2	LRT	0.0511056881306655
167	KC_AB2	CG31637	-0.433071644233688	0.504367627917241	0.988741847156811	pseudobulk	DESeq2	LRT	0.297252795747011
167	KC_AB2	CG31638	0.960074702290367	0.580130744127381	0.988741847156811	pseudobulk	DESeq2	LRT	0.236474118412647
167	KC_AB2	CG3164	-0.906925799940864	0.671074658720917	0.988741847156811	pseudobulk	DESeq2	LRT	0.173229160804428

167	KC_AB2	CG31643	-1.65719613210719	0.599376091584556	0.988741847156811	pseudobulk	DESeq2	LRT	0.222300584546838
168	KC_AB2	CG31648	0.921695369118063	0.730077514579258	0.988741847156811	pseudobulk	DESeq2	LRT	0.136631027048226
168	KC_AB2	CG3165	1.1327121871749	0.657275461456076	0.988741847156811	pseudobulk	DESeq2	LRT	0.182252581249451
168	KC_AB2	CG31650	0.481728581442314	0.602087231176794	0.988741847156811	pseudobulk	DESeq2	LRT	0.220340583037431
168	KC_AB2	CG31678	0.0932175381512402	0.953765948892824	0.988741847156811	pseudobulk	DESeq2	LRT	0.0205581866955345
168	KC_AB2	CG31687	1.13539439938001	0.65602501243538	0.988741847156811	pseudobulk	DESeq2	LRT	0.183079601850592
168	KC_AB2	CG31688	0.27724578192011	0.752543026915629	0.988741847156811	pseudobulk	DESeq2	LRT	0.123468664082529
168	KC_AB2	CG31690	0.818790875285784	0.940355871560339	0.988741847156811	pseudobulk	DESeq2	LRT	0.0267077593721816
168	KC_AB2	CG31694	0.740546916924993	0.56821917680261	0.988741847156811	pseudobulk	DESeq2	LRT	0.245484113381447
168	KC_AB2	CG31705	1.42621218304824	0.66738831402044	0.988741847156811	pseudobulk	DESeq2	LRT	0.175621402117653
168	KC_AB2	CG31706	1.42621218304824	0.66738831402044	0.988741847156811	pseudobulk	DESeq2	LRT	0.175621402117653
169	KC_AB2	CG31712	0.251621340840358	0.682539031750274	0.988741847156811	pseudobulk	DESeq2	LRT	0.165872507966232
169	KC_AB2	CG31715	1.06053646111741	0.387371266349129	0.988741847156811	pseudobulk	DESeq2	LRT	0.411872596649492
169	KC_AB2	CG31717	-0.896907752083019	0.564045210284217	0.988741847156811	pseudobulk	DESeq2	LRT	0.248686084332176
169	KC_AB2	CG31729	0.526209091021246	0.639773831927187	0.988741847156811	pseudobulk	DESeq2	LRT	0.193973527431094
169	KC_AB2	CG3176	1.42621218304824	0.66738831402044	0.988741847156811	pseudobulk	DESeq2	LRT	0.175621402117653
169	KC_AB2	CG31760	-0.0185036786246888	0.966023204052803	0.988741847156811	pseudobulk	DESeq2	LRT	0.015012441626889
169	KC_AB2	CG31793	-0.729234089517982	0.868684292414125	0.988741847156811	pseudobulk	DESeq2	LRT	0.0611380313470854
169	KC_AB2	CG31800	1.4413042803421	0.615311685084066	0.988741847156811	pseudobulk	DESeq2	LRT	0.210904837358999
169	KC_AB2	CG31808	1.41843039367426	0.307284135392203	0.988741847156811	pseudobulk	DESeq2	LRT	0.512459861121684
169	KC_AB2	CG31810	1.42621218304824	0.66738831402044	0.988741847156811	pseudobulk	DESeq2	LRT	0.175621402117653
170	KC_AB2	CG31815	3.48393309996043	0.216218474953395	0.988741847156811	pseudobulk	DESeq2	LRT	0.665107200172195
170	KC_AB2	CG3184	-0.588639054357632	0.8432408255636	0.988741847156811	pseudobulk	DESeq2	LRT	0.0740483752247712
170	KC_AB2	CG31849	1.47543920254359	0.661929169513603	0.988741847156811	pseudobulk	DESeq2	LRT	0.17918848025087
170	KC_AB2	CG31855	-1.61162288355715	0.33826460401739	0.988741847156811	pseudobulk	DESeq2	LRT	0.470743444376305
170	KC_AB2	CG31897	-2.51746470924027	0.517377929599049	0.988741847156811	pseudobulk	DESeq2	LRT	0.286192101436607
170	KC_AB2	CG31908	-0.602547753666317	0.699576538455223	0.988741847156811	pseudobulk	DESeq2	LRT	0.155164763787827
170	KC_AB2	CG3191	2.06137138167525	0.547946929015274	0.988741847156811	pseudobulk	DESeq2	LRT	0.261261502741816
170	KC_AB2	CG31915	-0.934020934207768	0.782359118258182	0.988741847156811	pseudobulk	DESeq2	LRT	0.106593851444836
170	KC_AB2	CG31921	-0.378852672109618	0.920549594954422	0.988741847156811	pseudobulk	DESeq2	LRT	0.0359528087516892
170	KC_AB2	CG31922	0.0601466527378941	0.974810960364079	0.988741847156811	pseudobulk	DESeq2	LRT	0.0110795964355292
171	KC_AB2	CG31935	-1.99867440840856	0.534899605264936	0.988741847156811	pseudobulk	DESeq2	LRT	0.271727722597268
171	KC_AB2	CG31957	0.712171366772551	0.813205723536451	0.988741847156811	pseudobulk	DESeq2	LRT	0.0897995733547685
171	KC_AB2	CG3198	0.852139004241774	0.317345226145699	0.988741847156811	pseudobulk	DESeq2	LRT	0.498468030433921
171	KC_AB2	CG31997	-0.690674465469211	0.439964832276193	0.988741847156811	pseudobulk	DESeq2	LRT	0.356582036601969
171	KC_AB2	CG31998	0.261206392747338	0.75882761307931	0.988741847156811	pseudobulk	DESeq2	LRT	0.119856873895952
171	KC_AB2	CG31999	3.30033526000341	0.259137897354645	0.988741847156811	pseudobulk	DESeq2	LRT	0.586469069420752
171	KC_AB2	CG32017	-0.124279448493401	0.864333651948864	0.988741847156811	pseudobulk	DESeq2	LRT	0.0633185778582077
171	KC_AB2	CG32022	0.362555753552128	0.897034383127386	0.988741847156811	pseudobulk	DESeq2	LRT	0.0471909102264623
171	KC_AB2	CG32039	-0.661803720718587	0.618104248994811	0.988741847156811	pseudobulk	DESeq2	LRT	0.208938270954919
171	KC_AB2	CG32055	-1.80294541848982	0.660135549041553	0.988741847156811	pseudobulk	DESeq2	LRT	0.180366879372519
172	KC_AB2	CG32066	-0.48937091395036	0.280097354828968	0.988741847156811	pseudobulk	DESeq2	LRT	0.552690992528118
172	KC_AB2	CG32069	3.89657444468943	0.144963977997646	0.988741847156811	pseudobulk	DESeq2	LRT	0.838739901905657
172	KC_AB2	CG32075	1.42621218304824	0.66738831402044	0.988741847156811	pseudobulk	DESeq2	LRT	0.175621402117653
172	KC_AB2	CG32085	0.836441433719504	0.367120184345236	0.988741847156811	pseudobulk	DESeq2	LRT	0.435191737237295
172	KC_AB2	CG32095	0.331758848923756	0.946375065527934	0.988741847156811	pseudobulk	DESeq2	LRT	0.0239367107367223
172	KC_AB2	CG32099	0.818790875285784	0.940355871560339	0.988741847156811	pseudobulk	DESeq2	LRT	0.0267077593721816
172	KC_AB2	CG32100	-0.300859819000612	0.875340908758419	0.988741847156811	pseudobulk	DESeq2	LRT	0.0578227744539286
172	KC_AB2	CG32104	-1.80294541848982	0.660135549041553	0.988741847156811	pseudobulk	DESeq2	LRT	0.180366879372519
172	KC_AB2	CG32109	-1.58605076109552	0.272464042934721	0.988741847156811	pseudobulk	DESeq2	LRT	0.564690803425538
172	KC_AB2	CG32112	0.362555753552128	0.897034383127386	0.988741847156811	pseudobulk	DESeq2	LRT	0.0471909102264623
173	KC_AB2	CG32137	-0.568233637794205	0.868826397133536	0.988741847156811	pseudobulk	DESeq2	LRT	0.0610669925949191
173	KC_AB2	CG32147	1.32714918892411	0.592713712963247	0.988741847156811	pseudobulk	DESeq2	LRT	0.227155024852469
173	KC_AB2	CG32163	-1.80933943727629	0.584587525761637	0.988741847156811	pseudobulk	DESeq2	LRT	0.233150456085471
173	KC_AB2	CG32176	2.08838438366837	0.316299392055167	0.988741847156811	pseudobulk	DESeq2	LRT	0.499901642821362
173	KC_AB2	CG32187	-1.80294541848982	0.660135549041553	0.988741847156811	pseudobulk	DESeq2	LRT	0.180366879372519

173	KC_AB2	CG32192	0.818790875285784	0.940355871560339	0.988741847156811	pseudobulk	DESeq2	LRT	0.0267077593721816
173	KC_AB2	CG32202	1.92721521352877	0.471759180353374	0.988741847156811	pseudobulk	DESeq2	LRT	0.326279639784082
173	KC_AB2	CG32204	0.294070102641522	0.51546878414257	0.988741847156811	pseudobulk	DESeq2	LRT	0.28779762967263
173	KC_AB2	CG32206	0.264169417639439	0.730639021104943	0.988741847156811	pseudobulk	DESeq2	LRT	0.136297137218527
173	KC_AB2	CG32225	2.49991005509522	0.199169931457441	0.988741847156811	pseudobulk	DESeq2	LRT	0.700776226091972
174	KC_AB2	CG3223	-1.31090837363605	0.544429329058634	0.988741847156811	pseudobulk	DESeq2	LRT	0.264058486875707
174	KC_AB2	CG3224	-0.673956917661111	0.634573235370489	0.988741847156811	pseudobulk	DESeq2	LRT	0.197518249247949
174	KC_AB2	CG3226	0.618536349682234	0.738912583449676	0.988741847156811	pseudobulk	DESeq2	LRT	0.131406937479366
174	KC_AB2	CG32262	1.67796394544623	0.574362267760116	0.988741847156811	pseudobulk	DESeq2	LRT	0.240814098434665
174	KC_AB2	CG32264	0.196126678240343	0.607725552934532	0.988741847156811	pseudobulk	DESeq2	LRT	0.216292502561433
174	KC_AB2	CG32267	-0.0375043270549451	0.969417081654388	0.988741847156811	pseudobulk	DESeq2	LRT	0.0134893320416861
174	KC_AB2	CG32276	0.649845493772328	0.498699274131082	0.988741847156811	pseudobulk	DESeq2	LRT	0.302161263907354
174	KC_AB2	CG32278	-0.365810098316308	0.689278196189521	0.988741847156811	pseudobulk	DESeq2	LRT	0.161605459246048
174	KC_AB2	CG32280	2.73593896747858	0.422277110754874	0.988741847156811	pseudobulk	DESeq2	LRT	0.374402458575713
174	KC_AB2	CG32281	-0.568233637794205	0.868826397133536	0.988741847156811	pseudobulk	DESeq2	LRT	0.0610669925949191
175	KC_AB2	CG32333	1.98341701527074	0.35546446228043	0.988741847156811	pseudobulk	DESeq2	LRT	0.449203811550207
175	KC_AB2	CG32344	1.47543920254359	0.661929169513603	0.988741847156811	pseudobulk	DESeq2	LRT	0.17918848025087
175	KC_AB2	CG32368	-0.506613484034373	0.961658398065406	0.988741847156811	pseudobulk	DESeq2	LRT	0.0169791713942958
175	KC_AB2	CG32369	1.42371955497174	0.290301682369639	0.988741847156811	pseudobulk	DESeq2	LRT	0.537150447317712
175	KC_AB2	CG32373	-1.54768271596609	0.394513952614918	0.988741847156811	pseudobulk	DESeq2	LRT	0.403937632666189
175	KC_AB2	CG32392	-2.64843360157809	0.321943703667444	0.988741847156811	pseudobulk	DESeq2	LRT	0.492220064093287
175	KC_AB2	CG32425	0.0346467216493583	0.963408326643192	0.988741847156811	pseudobulk	DESeq2	LRT	0.0161896044409707
175	KC_AB2	CG32428	0.333093728995657	0.800006919360402	0.988741847156811	pseudobulk	DESeq2	LRT	0.0969062567242496
175	KC_AB2	CG32432	0.449049286598989	0.493496542017611	0.988741847156811	pseudobulk	DESeq2	LRT	0.306715886130984
175	KC_AB2	CG32441	0.148305680020782	0.895984215533869	0.988741847156811	pseudobulk	DESeq2	LRT	0.0476996411938156
176	KC_AB2	CG32448	-0.164903967600826	0.915882238866045	0.988741847156811	pseudobulk	DESeq2	LRT	0.0381603629024758
176	KC_AB2	CG32452	-3.76508657848594	0.215111404675873	0.988741847156811	pseudobulk	DESeq2	LRT	0.667336563780367
176	KC_AB2	CG32459	-1.80294541848982	0.660135549041553	0.988741847156811	pseudobulk	DESeq2	LRT	0.180366879372519
176	KC_AB2	CG32473	-1.80294541848982	0.660135549041553	0.988741847156811	pseudobulk	DESeq2	LRT	0.180366879372519
176	KC_AB2	CG32485	0.377313835397995	0.677433071195836	0.988741847156811	pseudobulk	DESeq2	LRT	0.169133605613923
176	KC_AB2	CG32486	-0.424475053671571	0.372527018265303	0.988741847156811	pseudobulk	DESeq2	LRT	0.428842223699554
176	KC_AB2	CG32500	0.818790875285784	0.940355871560339	0.988741847156811	pseudobulk	DESeq2	LRT	0.0267077593721816
176	KC_AB2	CG32506	0.818790875285784	0.940355871560339	0.988741847156811	pseudobulk	DESeq2	LRT	0.0267077593721816
176	KC_AB2	CG32521	4.20307340969562	0.10486203552108	0.988741847156811	pseudobulk	DESeq2	LRT	0.979381716267607
176	KC_AB2	CG3253	-0.933299237375886	0.70112757174831	0.988741847156811	pseudobulk	DESeq2	LRT	0.154202953979679
177	KC_AB2	CG32533	-2.51746470924027	0.517377929599049	0.988741847156811	pseudobulk	DESeq2	LRT	0.286192101436607
177	KC_AB2	CG32537	2.06137138167525	0.547946929015274	0.988741847156811	pseudobulk	DESeq2	LRT	0.261261502741816
177	KC_AB2	CG32544	0.53160046168607	0.684450075406634	0.988741847156811	pseudobulk	DESeq2	LRT	0.164658224324944
177	KC_AB2	CG32547	0.362874457852021	0.879075667294383	0.988741847156811	pseudobulk	DESeq2	LRT	0.0559737409966935
177	KC_AB2	CG32549	-1.42697349706048	0.364426532313976	0.988741847156811	pseudobulk	DESeq2	LRT	0.438390011451845
177	KC_AB2	CG3257	0.0984074645742802	0.937362148994738	0.988741847156811	pseudobulk	DESeq2	LRT	0.0280925874219649
177	KC_AB2	CG32576	1.92721521352877	0.471759180353374	0.988741847156811	pseudobulk	DESeq2	LRT	0.326279639784082
177	KC_AB2	CG32581	-3.55444432281812	0.114209528418996	0.988741847156811	pseudobulk	DESeq2	LRT	0.942297661701681
177	KC_AB2	CG32590	1.4129303440845	0.645460595159151	0.988741847156811	pseudobulk	DESeq2	LRT	0.190130265910817
177	KC_AB2	CG3262	-0.799603656018799	0.415076209620838	0.988741847156811	pseudobulk	DESeq2	LRT	0.38187215779585
178	KC_AB2	CG32638	0.712171366772551	0.813205723536451	0.988741847156811	pseudobulk	DESeq2	LRT	0.0897995733547685
178	KC_AB2	CG32641	0.818790875285784	0.940355871560339	0.988741847156811	pseudobulk	DESeq2	LRT	0.0267077593721816
178	KC_AB2	CG32647	-0.0722082424153435	0.936868231504319	0.988741847156811	pseudobulk	DESeq2	LRT	0.0283214873990461
178	KC_AB2	CG32683	-0.134673841207209	0.801496499864599	0.988741847156811	pseudobulk	DESeq2	LRT	0.0960983698697988
178	KC_AB2	CG3270	-1.80294541848982	0.660135549041553	0.988741847156811	pseudobulk	DESeq2	LRT	0.180366879372519
178	KC_AB2	CG32700	0.087459956691163	0.784104571893929	0.988741847156811	pseudobulk	DESeq2	LRT	0.105626013887014
178	KC_AB2	CG32708	-2.51746470924027	0.517377929599049	0.988741847156811	pseudobulk	DESeq2	LRT	0.286192101436607
178	KC_AB2	CG32756	2.0846618955163	0.461498386685809	0.988741847156811	pseudobulk	DESeq2	LRT	0.335829812849717
178	KC_AB2	CG32767	-0.0760527102396271	0.862147037784931	0.988741847156811	pseudobulk	DESeq2	LRT	0.0644186596359558
178	KC_AB2	CG32772	0.101345049595506	0.936142805998073	0.988741847156811	pseudobulk	DESeq2	LRT	0.0286578957851573
179	KC_AB2	CG32795	-0.349402820837206	0.750588239310201	0.988741847156811	pseudobulk	DESeq2	LRT	0.124598244669402

179	KC_AB2	CG32797	-1.80294541848982	0.660135549041553	0.988741847156811	pseudobulk	DESeq2	LRT	0.180366879372519
179	KC_AB2	CG32803	-1.73627324524809	0.521368726288639	0.988741847156811	pseudobulk	DESeq2	LRT	0.282855023048041
179	KC_AB2	CG32808	1.45259040912931	0.616176917291092	0.988741847156811	pseudobulk	DESeq2	LRT	0.210294574894236
179	KC_AB2	CG32809	0.241425587772833	0.721632827739789	0.988741847156811	pseudobulk	DESeq2	LRT	0.14168371856207
179	KC_AB2	CG3281	-0.568233637794205	0.868826397133536	0.988741847156811	pseudobulk	DESeq2	LRT	0.0610669925949191
179	KC_AB2	CG32813	-0.187188766977065	0.932037508762103	0.988741847156811	pseudobulk	DESeq2	LRT	0.0305666096196055
179	KC_AB2	CG32815	-0.948052007136795	0.338473752056299	0.988741847156811	pseudobulk	DESeq2	LRT	0.47047500431738
179	KC_AB2	CG32845	-2.26538931672255	0.547771004842275	0.988741847156811	pseudobulk	DESeq2	LRT	0.2614009599796
179	KC_AB2	CG32850	-0.187309877408166	0.785397873418423	0.988741847156811	pseudobulk	DESeq2	LRT	0.104910278980923
180	KC_AB2	CG32856	1.12919034796087	0.7301237214754	0.988741847156811	pseudobulk	DESeq2	LRT	0.136603541247596
180	KC_AB2	CG3294	1.47543920254359	0.661929169513603	0.988741847156811	pseudobulk	DESeq2	LRT	0.17918848025087
180	KC_AB2	CG3295	-0.58621066677634	0.754406236055079	0.988741847156811	pseudobulk	DESeq2	LRT	0.122394730301062
180	KC_AB2	CG32982	0.400616899598601	0.662314542488302	0.988741847156811	pseudobulk	DESeq2	LRT	0.178935709013077
180	KC_AB2	CG3308	0.780352552100014	0.834299274204729	0.988741847156811	pseudobulk	DESeq2	LRT	0.0786781342287373
180	KC_AB2	CG3309	-0.634227699706429	0.828294391400015	0.988741847156811	pseudobulk	DESeq2	LRT	0.0818152793537044
180	KC_AB2	CG33090	0.468129051143149	0.580244103853311	0.988741847156811	pseudobulk	DESeq2	LRT	0.236389263930178
180	KC_AB2	CG33096	-3.4227037205893	0.285801360563088	0.988741847156811	pseudobulk	DESeq2	LRT	0.543935708072568
180	KC_AB2	CG33098	1.40305140665399	0.649741560632143	0.988741847156811	pseudobulk	DESeq2	LRT	0.187259352757606
180	KC_AB2	CG33107	0.474661093301217	0.815951154483998	0.988741847156811	pseudobulk	DESeq2	LRT	0.0883358387620737
181	KC_AB2	CG33108	0.712171366772551	0.813205723536451	0.988741847156811	pseudobulk	DESeq2	LRT	0.0897995733547685
181	KC_AB2	CG33111	-1.80294541848982	0.660135549041553	0.988741847156811	pseudobulk	DESeq2	LRT	0.180366879372519
181	KC_AB2	CG33116	0.453574201119133	0.747905894637242	0.988741847156811	pseudobulk	DESeq2	LRT	0.126153043861283
181	KC_AB2	CG33129	0.669361488431062	0.539013439962126	0.988741847156811	pseudobulk	DESeq2	LRT	0.268400405817681
181	KC_AB2	CG33144	-0.152862130754624	0.896599404013288	0.988741847156811	pseudobulk	DESeq2	LRT	0.0474015541539504
181	KC_AB2	CG33155	1.42621218304824	0.66738831402044	0.988741847156811	pseudobulk	DESeq2	LRT	0.175621402117653
181	KC_AB2	CG33156	0.684221129527733	0.737351212059311	0.988741847156811	pseudobulk	DESeq2	LRT	0.132325601520231
181	KC_AB2	CG33158	3.22031971259211	0.311877201824258	0.988741847156811	pseudobulk	DESeq2	LRT	0.506016370942756
181	KC_AB2	CG33169	0.336669057553448	0.835432882951706	0.988741847156811	pseudobulk	DESeq2	LRT	0.0780884347407321
181	KC_AB2	CG33170	0.12918411531674	0.893944347226286	0.988741847156811	pseudobulk	DESeq2	LRT	0.0486895174958681
182	KC_AB2	CG33172	0.818790875285784	0.940355871560339	0.988741847156811	pseudobulk	DESeq2	LRT	0.0267077593721816
182	KC_AB2	CG33181	0.0297113793303201	0.925301876480088	0.988741847156811	pseudobulk	DESeq2	LRT	0.0337165570975091
182	KC_AB2	CG33199	0.464874625167151	0.622359575684813	0.988741847156811	pseudobulk	DESeq2	LRT	0.205958623966905
182	KC_AB2	CG33203	4.27743921536416	0.0818345553383005	0.988741847156811	pseudobulk	DESeq2	LRT	1.08706327305434
182	KC_AB2	CG33213	-2.51746470924027	0.517377929599049	0.988741847156811	pseudobulk	DESeq2	LRT	0.286192101436607
182	KC_AB2	CG33217	1.17122272564433	0.596070420011995	0.988741847156811	pseudobulk	DESeq2	LRT	0.224702429494649
182	KC_AB2	CG33223	1.47543920254359	0.661929169513603	0.988741847156811	pseudobulk	DESeq2	LRT	0.17918848025087
182	KC_AB2	CG33228	-1.73627324524809	0.521368726288639	0.988741847156811	pseudobulk	DESeq2	LRT	0.282855023048041
182	KC_AB2	CG33229	-0.244801138154588	0.728395523318554	0.988741847156811	pseudobulk	DESeq2	LRT	0.137632732020478
182	KC_AB2	CG33230	-1.80294541848982	0.660135549041553	0.988741847156811	pseudobulk	DESeq2	LRT	0.180366879372519
183	KC_AB2	CG33284	-1.80294541848982	0.660135549041553	0.988741847156811	pseudobulk	DESeq2	LRT	0.180366879372519
183	KC_AB2	CG33298	0.440409503894039	0.785162242480352	0.988741847156811	pseudobulk	DESeq2	LRT	0.10504059327568
183	KC_AB2	CG33299	1.42621218304824	0.66738831402044	0.988741847156811	pseudobulk	DESeq2	LRT	0.175621402117653
183	KC_AB2	CG33303	0.185088875960897	0.860772997144181	0.988741847156811	pseudobulk	DESeq2	LRT	0.0651113654871883
183	KC_AB2	CG33310	-0.337072010867851	0.906965066354256	0.988741847156811	pseudobulk	DESeq2	LRT	0.0424094403729327
183	KC_AB2	CG33331	-0.405381063532158	0.89155634938256	0.988741847156811	pseudobulk	DESeq2	LRT	0.0498512027381788
183	KC_AB2	CG33332	0.818790875285784	0.940355871560339	0.988741847156811	pseudobulk	DESeq2	LRT	0.0267077593721816
183	KC_AB2	CG3335	0.792281256713368	0.823478838299676	0.988741847156811	pseudobulk	DESeq2	LRT	0.0843475568193814
183	KC_AB2	CG3337	-0.0362223591928502	0.956694233985992	0.988741847156811	pseudobulk	DESeq2	LRT	0.0192268435304406
183	KC_AB2	CG3342	-0.010237273074013	0.943681123757042	0.988741847156811	pseudobulk	DESeq2	LRT	0.0251747319601966
184	KC_AB2	CG33506	-0.0323203545809474	0.954448156132187	0.988741847156811	pseudobulk	DESeq2	LRT	0.020247656706879
184	KC_AB2	CG33514	0.818790875285784	0.940355871560339	0.988741847156811	pseudobulk	DESeq2	LRT	0.0267077593721816
184	KC_AB2	CG33521	1.42621218304824	0.66738831402044	0.988741847156811	pseudobulk	DESeq2	LRT	0.175621402117653
184	KC_AB2	CG33523	2.41462217514131	0.497446973552313	0.988741847156811	pseudobulk	DESeq2	LRT	0.303253207020768
184	KC_AB2	CG33543	0.154165322676124	0.852490746999489	0.988741847156811	pseudobulk	DESeq2	LRT	0.0693103261757832
184	KC_AB2	CG3356	2.43796147164888	0.320874079934739	0.988741847156811	pseudobulk	DESeq2	LRT	0.493665363602429
184	KC_AB2	CG3358	-2.9721553030081	0.387854083811327	0.988741847156811	pseudobulk	DESeq2	LRT	0.411331631401987

184	KC_AB2	CG33635	-2.15400439962555	0.378907849258803	0.988741847156811	pseudobulk	DESeq2	LRT	0.421466398012216
184	KC_AB2	CG33668	1.42621218304824	0.66738831402044	0.988741847156811	pseudobulk	DESeq2	LRT	0.175621402117653
184	KC_AB2	CG3368	2.7785831508444	0.373751317747552	0.988741847156811	pseudobulk	DESeq2	LRT	0.427417267458199
185	KC_AB2	CG33695	2.7785831508444	0.373751317747552	0.988741847156811	pseudobulk	DESeq2	LRT	0.427417267458199
185	KC_AB2	CG33700	0.712171366772551	0.813205723536451	0.988741847156811	pseudobulk	DESeq2	LRT	0.0897995733547685
185	KC_AB2	CG33774	1.11901260988891	0.403887501935785	0.988741847156811	pseudobulk	DESeq2	LRT	0.393739585612638
185	KC_AB2	CG3386	1.42621218304824	0.66738831402044	0.988741847156811	pseudobulk	DESeq2	LRT	0.175621402117653
185	KC_AB2	CG33947	-0.331004884396198	0.894406551228697	0.988741847156811	pseudobulk	DESeq2	LRT	0.0484650283842476
185	KC_AB2	CG33969	-1.20081362769313	0.741163517910401	0.988741847156811	pseudobulk	DESeq2	LRT	0.130085965985812
185	KC_AB2	CG33970	-1.77566073155666	0.606304952090798	0.988741847156811	pseudobulk	DESeq2	LRT	0.217308884582246
185	KC_AB2	CG33977	-0.790585170260446	0.514449479333457	0.988741847156811	pseudobulk	DESeq2	LRT	0.288657267984151
185	KC_AB2	CG33993	2.43796147164888	0.320874079934739	0.988741847156811	pseudobulk	DESeq2	LRT	0.493665363602429
185	KC_AB2	CG34008	1.47543920254359	0.661929169513603	0.988741847156811	pseudobulk	DESeq2	LRT	0.17918848025087
186	KC_AB2	CG34015	0.148520905817266	0.928829608110371	0.988741847156811	pseudobulk	DESeq2	LRT	0.0320639491329567
186	KC_AB2	CG3402	0.760546103616116	0.738305064567484	0.988741847156811	pseudobulk	DESeq2	LRT	0.131764152427911
186	KC_AB2	CG34026	-2.26538931672255	0.547771004842275	0.988741847156811	pseudobulk	DESeq2	LRT	0.2614009599796
186	KC_AB2	CG34028	-3.60847516454743	0.105967665366411	0.988741847156811	pseudobulk	DESeq2	LRT	0.974826633746592
186	KC_AB2	CG34039	0.818790875285784	0.940355871560339	0.988741847156811	pseudobulk	DESeq2	LRT	0.0267077593721816
186	KC_AB2	CG34045	0.0601466527378941	0.974810960364079	0.988741847156811	pseudobulk	DESeq2	LRT	0.0110795964355292
186	KC_AB2	CG34109	0.818790875285784	0.940355871560339	0.988741847156811	pseudobulk	DESeq2	LRT	0.0267077593721816
186	KC_AB2	CG34116	0.818790875285784	0.940355871560339	0.988741847156811	pseudobulk	DESeq2	LRT	0.0267077593721816
186	KC_AB2	CG34117	0.189166420521999	0.910050831216522	0.988741847156811	pseudobulk	DESeq2	LRT	0.0409343493269188
186	KC_AB2	CG34125	0.0288789541405111	0.981461080552591	0.988741847156811	pseudobulk	DESeq2	LRT	0.00812691749685834
187	KC_AB2	CG34126	0.540892505893564	0.731144256485185	0.988741847156811	pseudobulk	DESeq2	LRT	0.135996927262917
187	KC_AB2	CG34132	1.2117839627016	0.517105339425622	0.988741847156811	pseudobulk	DESeq2	LRT	0.286420977851384
187	KC_AB2	CG34133	-0.187188766977065	0.932037508762103	0.988741847156811	pseudobulk	DESeq2	LRT	0.0305666096196055
187	KC_AB2	CG34134	0.328504829056507	0.864082951513786	0.988741847156811	pseudobulk	DESeq2	LRT	0.0634445634754604
187	KC_AB2	CG34148	1.47543920254359	0.661929169513603	0.988741847156811	pseudobulk	DESeq2	LRT	0.17918848025087
187	KC_AB2	CG34149	-3.4227037205893	0.285801360563088	0.988741847156811	pseudobulk	DESeq2	LRT	0.543935708072568
187	KC_AB2	CG34150	0.475522879236569	0.801582640633501	0.988741847156811	pseudobulk	DESeq2	LRT	0.0960516966149455
187	KC_AB2	CG34155	0.818790875285784	0.940355871560339	0.988741847156811	pseudobulk	DESeq2	LRT	0.0267077593721816
187	KC_AB2	CG34159	0.341323392397052	0.821092715537383	0.988741847156811	pseudobulk	DESeq2	LRT	0.0856078007699234
187	KC_AB2	CG34163	0.818790875285784	0.940355871560339	0.988741847156811	pseudobulk	DESeq2	LRT	0.0267077593721816
188	KC_AB2	CG34174	1.42621218304824	0.66738831402044	0.988741847156811	pseudobulk	DESeq2	LRT	0.175621402117653
188	KC_AB2	CG34183	-2.35595135045048	0.406121109235175	0.988741847156811	pseudobulk	DESeq2	LRT	0.39134443630141
188	KC_AB2	CG34190	0.818790875285784	0.940355871560339	0.988741847156811	pseudobulk	DESeq2	LRT	0.0267077593721816
188	KC_AB2	CG34195	-1.80294541848982	0.660135549041553	0.988741847156811	pseudobulk	DESeq2	LRT	0.180366879372519
188	KC_AB2	CG3420	0.406721093523586	0.798559328613053	0.988741847156811	pseudobulk	DESeq2	LRT	0.0976928126099016
188	KC_AB2	CG34200	0.561866381453155	0.543745963773668	0.988741847156811	pseudobulk	DESeq2	LRT	0.264603953793315
188	KC_AB2	CG34213	1.10527623683615	0.724717602335893	0.988741847156811	pseudobulk	DESeq2	LRT	0.139831190172672
188	KC_AB2	CG34219	0.0607732375614882	0.934718414584717	0.988741847156811	pseudobulk	DESeq2	LRT	0.0293192013310873
188	KC_AB2	CG34228	1.99386207130934	0.349788165749185	0.988741847156811	pseudobulk	DESeq2	LRT	0.456194887929862
188	KC_AB2	CG34229	-0.568233637794205	0.868826397133536	0.988741847156811	pseudobulk	DESeq2	LRT	0.0610669925949191
189	KC_AB2	CG34231	2.06137138167525	0.547946929015274	0.988741847156811	pseudobulk	DESeq2	LRT	0.261261502741816
189	KC_AB2	CG34232	0.818790875285784	0.940355871560339	0.988741847156811	pseudobulk	DESeq2	LRT	0.0267077593721816
189	KC_AB2	CG34242	0.353798809751289	0.901648351639924	0.988741847156811	pseudobulk	DESeq2	LRT	0.0449628069360408
189	KC_AB2	CG34250	0.307241901491808	0.784239664327952	0.988741847156811	pseudobulk	DESeq2	LRT	0.10555119625778
189	KC_AB2	CG34251	2.51243226103394	0.474458247157471	0.988741847156811	pseudobulk	DESeq2	LRT	0.323801999942597
189	KC_AB2	CG34293	-0.0323203545809474	0.954448156132187	0.988741847156811	pseudobulk	DESeq2	LRT	0.020247656706879
189	KC_AB2	CG34309	0.818790875285784	0.940355871560339	0.988741847156811	pseudobulk	DESeq2	LRT	0.0267077593721816
189	KC_AB2	CG34310	-2.51746470924027	0.517377929599049	0.988741847156811	pseudobulk	DESeq2	LRT	0.286192101436607
189	KC_AB2	CG3434	2.00515076565275	0.576456787459964	0.988741847156811	pseudobulk	DESeq2	LRT	0.239233242872451
189	KC_AB2	CG34347	2.51243226103394	0.474458247157471	0.988741847156811	pseudobulk	DESeq2	LRT	0.323801999942597
190	KC_AB2	CG34348	2.06137138167525	0.547946929015274	0.988741847156811	pseudobulk	DESeq2	LRT	0.261261502741816
190	KC_AB2	CG34351	0.818790875285784	0.940355871560339	0.988741847156811	pseudobulk	DESeq2	LRT	0.0267077593721816
190	KC_AB2	CG34353	-0.855352062129536	0.542853392709198	0.988741847156811	pseudobulk	DESeq2	LRT	0.265317443586733

190	KC_AB2	CG34354	1.30223383003068	0.0899989482150303	0.988741847156811	pseudobulk	DESeq2	LRT	1.04576256597265
190	KC_AB2	CG34355	-0.026107411119639	0.948175218111817	0.988741847156811	pseudobulk	DESeq2	LRT	0.0231113997636729
190	KC_AB2	CG34357	-0.233636188199809	0.753984884946845	0.988741847156811	pseudobulk	DESeq2	LRT	0.122637360297303
190	KC_AB2	CG3436	0.32742703490313	0.878511928385878	0.988741847156811	pseudobulk	DESeq2	LRT	0.0562523373024427
190	KC_AB2	CG3437	1.0821173077982	0.758638591992449	0.988741847156811	pseudobulk	DESeq2	LRT	0.119965068483926
190	KC_AB2	CG34370	2.87543539509656	0.0297347607392309	0.988741847156811	pseudobulk	DESeq2	LRT	1.52673555170144
190	KC_AB2	CG34376	2.06137138167525	0.547946929015274	0.988741847156811	pseudobulk	DESeq2	LRT	0.261261502741816
191	KC_AB2	CG34384	-0.504028934407792	0.668671699583778	0.988741847156811	pseudobulk	DESeq2	LRT	0.174787057201908
191	KC_AB2	CG34401	-0.18853697944133	0.773762143232318	0.988741847156811	pseudobulk	DESeq2	LRT	0.111392522207812
191	KC_AB2	CG34402	-0.0334784491582317	0.954213663769481	0.988741847156811	pseudobulk	DESeq2	LRT	0.0203543688950962
191	KC_AB2	CG34404	-0.0979799684794967	0.948221375263194	0.988741847156811	pseudobulk	DESeq2	LRT	0.0230902588312143
191	KC_AB2	CG34408	0.330391410498902	0.851537379119407	0.988741847156811	pseudobulk	DESeq2	LRT	0.0697962834747243
191	KC_AB2	CG34409	-0.934020934207768	0.782359118258182	0.988741847156811	pseudobulk	DESeq2	LRT	0.106593851444836
191	KC_AB2	CG34417	0.818790875285784	0.940355871560339	0.988741847156811	pseudobulk	DESeq2	LRT	0.0267077593721816
191	KC_AB2	CG3448	0.818790875285784	0.940355871560339	0.988741847156811	pseudobulk	DESeq2	LRT	0.0267077593721816
191	KC_AB2	CG3500	-0.509471600872311	0.777256408280785	0.988741847156811	pseudobulk	DESeq2	LRT	0.109435688622808
191	KC_AB2	CG3511	1.85714160457615	0.510506350526785	0.988741847156811	pseudobulk	DESeq2	LRT	0.29199885106662
192	KC_AB2	CG3527	1.91626857490363	0.477198801200827	0.988741847156811	pseudobulk	DESeq2	LRT	0.321300656017034
192	KC_AB2	CG3529	0.61702365764228	0.739069534444918	0.988741847156811	pseudobulk	DESeq2	LRT	0.131314699622637
192	KC_AB2	CG3530	-0.282596147775992	0.743833922517149	0.988741847156811	pseudobulk	DESeq2	LRT	0.128524019542426
192	KC_AB2	CG3534	-0.506613484034373	0.961658398065406	0.988741847156811	pseudobulk	DESeq2	LRT	0.0169791713942958
192	KC_AB2	CG3542	-0.36342523203408	0.870060346655059	0.988741847156811	pseudobulk	DESeq2	LRT	0.0604506240361143
192	KC_AB2	CG3548	-0.244067456186467	0.906772274454269	0.988741847156811	pseudobulk	DESeq2	LRT	0.042501767367312
192	KC_AB2	CG3552	0.699674038439951	0.821690393783994	0.988741847156811	pseudobulk	DESeq2	LRT	0.0852917902455435
192	KC_AB2	CG3556	0.982754671718272	0.513272831853577	0.988741847156811	pseudobulk	DESeq2	LRT	0.289651722858537
192	KC_AB2	CG3558	-0.0121203900611543	0.976613779210563	0.988741847156811	pseudobulk	DESeq2	LRT	0.0102771524736688
192	KC_AB2	CG3566	0.573559175483023	0.61038792550442	0.988741847156811	pseudobulk	DESeq2	LRT	0.214394066040509
193	KC_AB2	CG3568	2.11513947821417	0.415104446921141	0.988741847156811	pseudobulk	DESeq2	LRT	0.381842614096864
193	KC_AB2	CG3587	-4.65112611560589	0.102291582254303	0.988741847156811	pseudobulk	DESeq2	LRT	0.990160103638011
193	KC_AB2	CG3589	1.47543920254359	0.661929169513603	0.988741847156811	pseudobulk	DESeq2	LRT	0.17918848025087
193	KC_AB2	CG3594	-0.957133960806503	0.762632518785665	0.988741847156811	pseudobulk	DESeq2	LRT	0.1176846802739
193	KC_AB2	CG3609	0.818790875285784	0.940355871560339	0.988741847156811	pseudobulk	DESeq2	LRT	0.0267077593721816
193	KC_AB2	CG3618	-0.393953564514768	0.770355756752861	0.988741847156811	pseudobulk	DESeq2	LRT	0.113308667666819
193	KC_AB2	CG3625	0.269152326269824	0.651578547787976	0.988741847156811	pseudobulk	DESeq2	LRT	0.186033222599673
193	KC_AB2	CG3631	-1.80294541848982	0.660135549041553	0.988741847156811	pseudobulk	DESeq2	LRT	0.180366879372519
193	KC_AB2	CG3632	-1.64471537088186	0.426376803926012	0.988741847156811	pseudobulk	DESeq2	LRT	0.37020643015633
193	KC_AB2	CG3634	0.0885531792216718	0.964858407830046	0.988741847156811	pseudobulk	DESeq2	LRT	0.0155364143348316
194	KC_AB2	CG3638	0.00890249398801547	0.959974250020664	0.988741847156811	pseudobulk	DESeq2	LRT	0.0177404161520133
194	KC_AB2	CG3645	-2.26538931672255	0.547771004842275	0.988741847156811	pseudobulk	DESeq2	LRT	0.2614009599796
194	KC_AB2	CG3651	0.353798809751289	0.901648351639924	0.988741847156811	pseudobulk	DESeq2	LRT	0.0449628069360408
194	KC_AB2	CG3652	0.341961843926254	0.81565882734411	0.988741847156811	pseudobulk	DESeq2	LRT	0.0884914593656937
194	KC_AB2	CG3662	0.0137071281065708	0.952920036712468	0.988741847156811	pseudobulk	DESeq2	LRT	0.0209435411996116
194	KC_AB2	CG3678	1.42034337486757	0.643720050244361	0.988741847156811	pseudobulk	DESeq2	LRT	0.191302963490459
194	KC_AB2	CG3700	2.00515076565275	0.576456787459964	0.988741847156811	pseudobulk	DESeq2	LRT	0.239233242872451
194	KC_AB2	CG3702	1.48741827283584	0.532545672123726	0.988741847156811	pseudobulk	DESeq2	LRT	0.273643140376104
194	KC_AB2	CG3703	-0.131982659698664	0.936247161971421	0.988741847156811	pseudobulk	DESeq2	LRT	0.0286094857592535
194	KC_AB2	CG3704	0.666943671827768	0.808936122914367	0.988741847156811	pseudobulk	DESeq2	LRT	0.0920857707994393
195	KC_AB2	CG3713	0.818790875285784	0.940355871560339	0.988741847156811	pseudobulk	DESeq2	LRT	0.0267077593721816
195	KC_AB2	CG3719	-0.0829658698601685	0.952770697057585	0.988741847156811	pseudobulk	DESeq2	LRT	0.0210116082652991
195	KC_AB2	CG3726	-0.44760236997836	0.505209839068298	0.988741847156811	pseudobulk	DESeq2	LRT	0.296528200057603
195	KC_AB2	CG3732	0.0424374425989578	0.963862935393893	0.988741847156811	pseudobulk	DESeq2	LRT	0.0159847198672157
195	KC_AB2	CG3740	0.792281256713368	0.823478838299676	0.988741847156811	pseudobulk	DESeq2	LRT	0.0843475568193814
195	KC_AB2	CG3744	-0.244067456186467	0.906772274454269	0.988741847156811	pseudobulk	DESeq2	LRT	0.042501767367312
195	KC_AB2	CG3756	2.00515076565275	0.576456787459964	0.988741847156811	pseudobulk	DESeq2	LRT	0.239233242872451
195	KC_AB2	CG3760	2.73593896747858	0.422277110754874	0.988741847156811	pseudobulk	DESeq2	LRT	0.374402458575713
195	KC_AB2	CG3764	-0.55246575220467	0.787729781446674	0.988741847156811	pseudobulk	DESeq2	LRT	0.103622734988401

195	KC_AB2	CG3776	0.834352593082403	0.675685094045283	0.988741847156811	pseudobulk	DESeq2	LRT	0.170255661725145
196	KC_AB2	CG3777	1.5974064630256	0.358322459033174	0.988741847156811	pseudobulk	DESeq2	LRT	0.445725970152356
196	KC_AB2	CG3781	-0.934020934207768	0.782359118258182	0.988741847156811	pseudobulk	DESeq2	LRT	0.106593851444836
196	KC_AB2	CG3792	-1.37054505049545	0.574189269529552	0.988741847156811	pseudobulk	DESeq2	LRT	0.240944927875125
196	KC_AB2	CG3793	-1.27734305393251	0.477549169380556	0.988741847156811	pseudobulk	DESeq2	LRT	0.320981905983287
196	KC_AB2	CG3803	-0.522044679843893	0.796310928443557	0.988741847156811	pseudobulk	DESeq2	LRT	0.0989173240449814
196	KC_AB2	CG3815	-1.80294541848982	0.660135549041553	0.988741847156811	pseudobulk	DESeq2	LRT	0.180366879372519
196	KC_AB2	CG3817	-0.405381063532158	0.89155634938256	0.988741847156811	pseudobulk	DESeq2	LRT	0.0498512027381788
196	KC_AB2	CG3847	-0.506613484034373	0.961658398065406	0.988741847156811	pseudobulk	DESeq2	LRT	0.0169791713942958
196	KC_AB2	CG3857	3.66684585317293	0.187794911241363	0.988741847156811	pseudobulk	DESeq2	LRT	0.726316180172881
196	KC_AB2	CG3862	-2.51746470924027	0.517377929599049	0.988741847156811	pseudobulk	DESeq2	LRT	0.286192101436607
197	KC_AB2	CG3894	0.157562008957688	0.950463298513166	0.988741847156811	pseudobulk	DESeq2	LRT	0.0220646484576093
197	KC_AB2	CG3902	0.818790875285784	0.940355871560339	0.988741847156811	pseudobulk	DESeq2	LRT	0.0267077593721816
197	KC_AB2	CG3907	0.970295642204646	0.623136780158323	0.988741847156811	pseudobulk	DESeq2	LRT	0.205416614098497
197	KC_AB2	CG3918	0.513202572024843	0.807055953632252	0.988741847156811	pseudobulk	DESeq2	LRT	0.093096354359303
197	KC_AB2	CG3919	3.30033526000341	0.259137897354645	0.988741847156811	pseudobulk	DESeq2	LRT	0.586469069420752
197	KC_AB2	CG3955	-0.129503796659415	0.856401692170073	0.988741847156811	pseudobulk	DESeq2	LRT	0.0673224832512576
197	KC_AB2	CG3961	-2.23744817905509	0.154034972682415	0.988741847156811	pseudobulk	DESeq2	LRT	0.812380664107439
197	KC_AB2	CG3995	0.353798809751289	0.901648351639924	0.988741847156811	pseudobulk	DESeq2	LRT	0.0449628069360408
197	KC_AB2	CG4000	0.818790875285784	0.940355871560339	0.988741847156811	pseudobulk	DESeq2	LRT	0.0267077593721816
197	KC_AB2	CG4004	0.187367232873995	0.898430173710008	0.988741847156811	pseudobulk	DESeq2	LRT	0.0465156707540569
198	KC_AB2	CG40045	0.629136156474971	0.383374961041686	0.988741847156811	pseudobulk	DESeq2	LRT	0.416376255142178
198	KC_AB2	CG40191	-0.0161083203658194	0.983465619685469	0.988741847156811	pseudobulk	DESeq2	LRT	0.00724081768858799
198	KC_AB2	CG40198	1.47543920254359	0.661929169513603	0.988741847156811	pseudobulk	DESeq2	LRT	0.17918848025087
198	KC_AB2	CG4022	0.80430127541587	0.689337728291919	0.988741847156811	pseudobulk	DESeq2	LRT	0.161567951390344
198	KC_AB2	CG40228	-0.130546401233639	0.86680252697929	0.988741847156811	pseudobulk	DESeq2	LRT	0.0620798312567399
198	KC_AB2	CG4025	-0.405381063532158	0.89155634938256	0.988741847156811	pseudobulk	DESeq2	LRT	0.0498512027381788
198	KC_AB2	CG4036	-1.37054505049545	0.574189269529552	0.988741847156811	pseudobulk	DESeq2	LRT	0.240944927875125
198	KC_AB2	CG4038	0.818790875285784	0.940355871560339	0.988741847156811	pseudobulk	DESeq2	LRT	0.0267077593721816
198	KC_AB2	CG4041	0.699674038439951	0.821690393783994	0.988741847156811	pseudobulk	DESeq2	LRT	0.0852917902455435
198	KC_AB2	CG4042	1.47543920254359	0.661929169513603	0.988741847156811	pseudobulk	DESeq2	LRT	0.17918848025087
199	KC_AB2	CG40439	-1.80294541848982	0.660135549041553	0.988741847156811	pseudobulk	DESeq2	LRT	0.180366879372519
199	KC_AB2	CG40472	-1.10721699134304	0.425888692746304	0.988741847156811	pseudobulk	DESeq2	LRT	0.370703890198789
199	KC_AB2	CG4049	-0.751812832182869	0.678670348965535	0.988741847156811	pseudobulk	DESeq2	LRT	0.168341124673201
199	KC_AB2	CG40498	-0.215717692124402	0.654946513466413	0.988741847156811	pseudobulk	DESeq2	LRT	0.183794165435495
199	KC_AB2	CG4074	-2.45158172483764	0.378823136429564	0.988741847156811	pseudobulk	DESeq2	LRT	0.421563504545096
199	KC_AB2	CG4080	-0.204085973835288	0.853980814467739	0.988741847156811	pseudobulk	DESeq2	LRT	0.0685518860608635
199	KC_AB2	CG4101	0.0885531792216718	0.964858407830046	0.988741847156811	pseudobulk	DESeq2	LRT	0.0155364143348316
199	KC_AB2	CG41099	-0.436372966674237	0.767023026259517	0.988741847156811	pseudobulk	DESeq2	LRT	0.115191598205724
199	KC_AB2	CG41128	-0.0268624142814152	0.926645498000867	0.988741847156811	pseudobulk	DESeq2	LRT	0.0330863798876522
199	KC_AB2	CG4119	-2.45158172483764	0.378823136429564	0.988741847156811	pseudobulk	DESeq2	LRT	0.421563504545096
200	KC_AB2	CG4133	0.0563120942996901	0.974316031513895	0.988741847156811	pseudobulk	DESeq2	LRT	0.0113001514574287
200	KC_AB2	CG41378	-0.734322144038947	0.515446654157592	0.988741847156811	pseudobulk	DESeq2	LRT	0.287816275101741
200	KC_AB2	CG4168	0.919353707608246	0.746278333243311	0.988741847156811	pseudobulk	DESeq2	LRT	0.127099167101924
200	KC_AB2	CG4186	0.699674038439951	0.821690393783994	0.988741847156811	pseudobulk	DESeq2	LRT	0.0852917902455435
200	KC_AB2	CG4199	1.83411718865815	0.518907236503101	0.988741847156811	pseudobulk	DESeq2	LRT	0.284910272740318
200	KC_AB2	CG42231	-3.32426296135166	0.343738249391692	0.988741847156811	pseudobulk	DESeq2	LRT	0.463772139158982
200	KC_AB2	CG42232	0.046530156636047	0.970525086348867	0.988741847156811	pseudobulk	DESeq2	LRT	0.0129932343899138
200	KC_AB2	CG42233	0.818790875285784	0.940355871560339	0.988741847156811	pseudobulk	DESeq2	LRT	0.0267077593721816
200	KC_AB2	CG42238	-0.660470277765608	0.185214948307109	0.988741847156811	pseudobulk	DESeq2	LRT	0.732323965249615
200	KC_AB2	CG42239	0.818790875285784	0.940355871560339	0.988741847156811	pseudobulk	DESeq2	LRT	0.0267077593721816
201	KC_AB2	CG42240	0.331758848923756	0.946375065527934	0.988741847156811	pseudobulk	DESeq2	LRT	0.0239367107367223
201	KC_AB2	CG42258	0.40446683321269	0.463642368229579	0.988741847156811	pseudobulk	DESeq2	LRT	0.333816884512653
201	KC_AB2	CG42259	-0.506613484034373	0.961658398065406	0.988741847156811	pseudobulk	DESeq2	LRT	0.0169791713942958
201	KC_AB2	CG42260	-1.0831603311245	0.0191989683358603	0.988741847156811	pseudobulk	DESeq2	LRT	1.71672210765482
201	KC_AB2	CG42268	0.600937345278347	0.373056737443392	0.988741847156811	pseudobulk	DESeq2	LRT	0.428225112200144

201	KC_AB2	CG42271	-0.97670612739315	0.676315355917219	0.988741847156811	pseudobulk	DESeq2	LRT	0.169850751692797
201	KC_AB2	CG4230	-0.195830310422444	0.799559882403526	0.988741847156811	pseudobulk	DESeq2	LRT	0.097149004558749
201	KC_AB2	CG42307	-2.45158172483764	0.378823136429564	0.988741847156811	pseudobulk	DESeq2	LRT	0.421563504545096
201	KC_AB2	CG42322	0.482983878760558	0.441475566825003	0.988741847156811	pseudobulk	DESeq2	LRT	0.355093327163889
201	KC_AB2	CG42324	0.226321448170741	0.768685124980615	0.988741847156811	pseudobulk	DESeq2	LRT	0.11425152299128
202	KC_AB2	CG42336	-0.300213970456187	0.862708793342715	0.988741847156811	pseudobulk	DESeq2	LRT	0.0641357753007584
202	KC_AB2	CG42337	0.13503365739577	0.861109857422621	0.988741847156811	pseudobulk	DESeq2	LRT	0.0649414392212089
202	KC_AB2	CG42339	1.50848728064736	0.0934387694438696	0.988741847156811	pseudobulk	DESeq2	LRT	1.02947288970146
202	KC_AB2	CG42340	-0.519803281006321	0.529571320183149	0.988741847156811	pseudobulk	DESeq2	LRT	0.276075542872488
202	KC_AB2	CG42342	-0.160356280801779	0.90801875697963	0.988741847156811	pseudobulk	DESeq2	LRT	0.0419051801478428
202	KC_AB2	CG42346	-0.570010944603223	0.680916939869031	0.988741847156811	pseudobulk	DESeq2	LRT	0.166905861298039
202	KC_AB2	CG42359	0.331758848923756	0.946375065527934	0.988741847156811	pseudobulk	DESeq2	LRT	0.0239367107367223
202	KC_AB2	CG42360	-0.246893968726718	0.883832168964877	0.988741847156811	pseudobulk	DESeq2	LRT	0.0536301954081643
202	KC_AB2	CG42361	0.355232416325357	0.621102135583408	0.988741847156811	pseudobulk	DESeq2	LRT	0.206836977483647
202	KC_AB2	CG42365	1.32714918892411	0.592713712963247	0.988741847156811	pseudobulk	DESeq2	LRT	0.227155024852469
203	KC_AB2	CG42376	-0.597492700100737	0.751042608390007	0.988741847156811	pseudobulk	DESeq2	LRT	0.124335423763143
203	KC_AB2	CG4238	-0.286814311689005	0.828931551850599	0.988741847156811	pseudobulk	DESeq2	LRT	0.081481329378401
203	KC_AB2	CG42382	-2.9721553030081	0.387854083811327	0.988741847156811	pseudobulk	DESeq2	LRT	0.411331631401987
203	KC_AB2	CG4239	0.10598154425916	0.959628155914924	0.988741847156811	pseudobulk	DESeq2	LRT	0.0178970181249073
203	KC_AB2	CG42392	-0.480975271687743	0.544217282458827	0.988741847156811	pseudobulk	DESeq2	LRT	0.264227670646442
203	KC_AB2	CG42394	1.47543920254359	0.661929169513603	0.988741847156811	pseudobulk	DESeq2	LRT	0.17918848025087
203	KC_AB2	CG42399	0.818790875285784	0.940355871560339	0.988741847156811	pseudobulk	DESeq2	LRT	0.0267077593721816
203	KC_AB2	CG42402	-0.39593607582776	0.325306264861622	0.988741847156811	pseudobulk	DESeq2	LRT	0.487707572842852
203	KC_AB2	CG42404	0.0573274603352775	0.961049101590826	0.988741847156811	pseudobulk	DESeq2	LRT	0.0172544229400021
203	KC_AB2	CG42450	0.102328221592373	0.859104153846093	0.988741847156811	pseudobulk	DESeq2	LRT	0.0659541811063958
204	KC_AB2	CG42495	-0.568233637794205	0.868826397133536	0.988741847156811	pseudobulk	DESeq2	LRT	0.0610669925949191
204	KC_AB2	CG42540	0.265839167850869	0.484590741951115	0.988741847156811	pseudobulk	DESeq2	LRT	0.314624887264034
204	KC_AB2	CG42541	-0.279066470491531	0.495356281935275	0.988741847156811	pseudobulk	DESeq2	LRT	0.305082325065644
204	KC_AB2	CG42542	-2.50949001284745	0.18699309685154	0.988741847156811	pseudobulk	DESeq2	LRT	0.728174425841155
204	KC_AB2	CG42554	0.176741474148333	0.939387133560172	0.988741847156811	pseudobulk	DESeq2	LRT	0.0271553924837109
204	KC_AB2	CG42556	2.06137138167525	0.547946929015274	0.988741847156811	pseudobulk	DESeq2	LRT	0.261261502741816
204	KC_AB2	CG42561	-1.80294541848982	0.660135549041553	0.988741847156811	pseudobulk	DESeq2	LRT	0.180366879372519
204	KC_AB2	CG42588	-0.300859819000612	0.875340908758419	0.988741847156811	pseudobulk	DESeq2	LRT	0.0578227744539286
204	KC_AB2	CG42594	-0.527838885643666	0.506293445107351	0.988741847156811	pseudobulk	DESeq2	LRT	0.295597695311431
204	KC_AB2	CG42613	-0.0249837990357665	0.952116683602843	0.988741847156811	pseudobulk	DESeq2	LRT	0.0213098247882591
205	KC_AB2	CG4266	0.316148142040676	0.850903064246337	0.988741847156811	pseudobulk	DESeq2	LRT	0.0701199123722693
205	KC_AB2	CG42663	1.42621218304824	0.66738831402044	0.988741847156811	pseudobulk	DESeq2	LRT	0.175621402117653
205	KC_AB2	CG42668	0.519965384343846	0.514928088137605	0.988741847156811	pseudobulk	DESeq2	LRT	0.288253417765964
205	KC_AB2	CG42671	0.313746566266179	0.803982886470425	0.988741847156811	pseudobulk	DESeq2	LRT	0.0947531955184164
205	KC_AB2	CG42672	1.4413042803421	0.615311685084066	0.988741847156811	pseudobulk	DESeq2	LRT	0.210904837358999
205	KC_AB2	CG42673	0.311524215039786	0.754820201334597	0.988741847156811	pseudobulk	DESeq2	LRT	0.12215648528143
205	KC_AB2	CG42674	-0.618001500136109	0.807592904791208	0.988741847156811	pseudobulk	DESeq2	LRT	0.092807505260298
205	KC_AB2	CG42676	1.7098779479	0.537865353861561	0.988741847156811	pseudobulk	DESeq2	LRT	0.269326429526188
205	KC_AB2	CG42692	-2.51746470924027	0.517377929599049	0.988741847156811	pseudobulk	DESeq2	LRT	0.286192101436607
205	KC_AB2	CG42699	0.475522879236569	0.801582640633501	0.988741847156811	pseudobulk	DESeq2	LRT	0.0960516966149455
206	KC_AB2	CG42700	0.191437025632171	0.854987653806422	0.988741847156811	pseudobulk	DESeq2	LRT	0.0680401565261624
206	KC_AB2	CG42709	-0.514347453932055	0.785590925586934	0.988741847156811	pseudobulk	DESeq2	LRT	0.104803541756598
206	KC_AB2	CG42724	-0.403073109136834	0.589780208194824	0.988741847156811	pseudobulk	DESeq2	LRT	0.229309805565157
206	KC_AB2	CG42747	-0.839863902534007	0.348551741280305	0.988741847156811	pseudobulk	DESeq2	LRT	0.457732743291729
206	KC_AB2	CG42748	0.242876843021913	0.736747609713226	0.988741847156811	pseudobulk	DESeq2	LRT	0.132681264490647
206	KC_AB2	CG42750	0.318752828652245	0.614165175491469	0.988741847156811	pseudobulk	DESeq2	LRT	0.211714812641674
206	KC_AB2	CG4278	2.73593896747858	0.422277110754874	0.988741847156811	pseudobulk	DESeq2	LRT	0.374402458575713
206	KC_AB2	CG42784	-0.516934976535402	0.484169720825232	0.988741847156811	pseudobulk	DESeq2	LRT	0.31500237410474
206	KC_AB2	CG42788	0.379923722992013	0.737582534142996	0.988741847156811	pseudobulk	DESeq2	LRT	0.132189375868646
206	KC_AB2	CG42789	-2.26538931672255	0.547771004842275	0.988741847156811	pseudobulk	DESeq2	LRT	0.2614009599796
207	KC_AB2	CG42795	-0.184503536301899	0.709003233284338	0.988741847156811	pseudobulk	DESeq2	LRT	0.149351784288944

207	KC_AB2	CG42797	-0.300859819000612	0.875340908758419	0.988741847156811	pseudobulk	DESeq2	LRT	0.0578227744539286
207	KC_AB2	CG42806	0.37733433210913	0.907416158791808	0.988741847156811	pseudobulk	DESeq2	LRT	0.0421934913124174
207	KC_AB2	CG42809	-1.2165671016221	0.546334558879998	0.988741847156811	pseudobulk	DESeq2	LRT	0.262541326931549
207	KC_AB2	CG4281	-0.957133960806503	0.762632518785665	0.988741847156811	pseudobulk	DESeq2	LRT	0.1176846802739
207	KC_AB2	CG42813	-0.179180647297476	0.908469806707817	0.988741847156811	pseudobulk	DESeq2	LRT	0.0416895020357343
207	KC_AB2	CG4282	1.42034337486757	0.643720050244361	0.988741847156811	pseudobulk	DESeq2	LRT	0.191302963490459
207	KC_AB2	CG42855	-0.934020934207768	0.782359118258182	0.988741847156811	pseudobulk	DESeq2	LRT	0.106593851444836
207	KC_AB2	CG4287	0.666943671827768	0.808936122914367	0.988741847156811	pseudobulk	DESeq2	LRT	0.0920857707994393
207	KC_AB2	CG4291	-2.35595135045048	0.406121109235175	0.988741847156811	pseudobulk	DESeq2	LRT	0.39134443630141
208	KC_AB2	CG4293	1.4413042803421	0.615311685084066	0.988741847156811	pseudobulk	DESeq2	LRT	0.210904837358999
208	KC_AB2	CG4294	-0.941573457310943	0.5203719084311	0.988741847156811	pseudobulk	DESeq2	LRT	0.283686156273895
208	KC_AB2	CG4297	0.611930124744066	0.527182299846205	0.988741847156811	pseudobulk	DESeq2	LRT	0.27803917961315
208	KC_AB2	CG43060	2.4643417141186	0.449082701910884	0.988741847156811	pseudobulk	DESeq2	LRT	0.347673673080982
208	KC_AB2	CG43068	0.818790875285784	0.940355871560339	0.988741847156811	pseudobulk	DESeq2	LRT	0.0267077593721816
208	KC_AB2	CG43078	2.67145324364694	0.110898932131831	0.988741847156811	pseudobulk	DESeq2	LRT	0.955072635739803
208	KC_AB2	CG43095	-1.80294541848982	0.660135549041553	0.988741847156811	pseudobulk	DESeq2	LRT	0.180366879372519
208	KC_AB2	CG43102	-0.271923826998065	0.739174372928204	0.988741847156811	pseudobulk	DESeq2	LRT	0.131253098455697
208	KC_AB2	CG43110	0.818790875285784	0.940355871560339	0.988741847156811	pseudobulk	DESeq2	LRT	0.0267077593721816
208	KC_AB2	CG4313	-0.506613484034373	0.961658398065406	0.988741847156811	pseudobulk	DESeq2	LRT	0.0169791713942958
209	KC_AB2	CG43135	-1.80294541848982	0.660135549041553	0.988741847156811	pseudobulk	DESeq2	LRT	0.180366879372519
209	KC_AB2	CG43163	0.818790875285784	0.940355871560339	0.988741847156811	pseudobulk	DESeq2	LRT	0.0267077593721816
209	KC_AB2	CG43222	-0.31350139879378	0.707534241646364	0.988741847156811	pseudobulk	DESeq2	LRT	0.150252537290293
209	KC_AB2	CG43290	0.818790875285784	0.940355871560339	0.988741847156811	pseudobulk	DESeq2	LRT	0.0267077593721816
209	KC_AB2	CG43322	-0.934020934207768	0.782359118258182	0.988741847156811	pseudobulk	DESeq2	LRT	0.106593851444836
209	KC_AB2	CG43324	0.161847456171582	0.722284635167735	0.988741847156811	pseudobulk	DESeq2	LRT	0.141291623590272
209	KC_AB2	CG43342	0.818790875285784	0.940355871560339	0.988741847156811	pseudobulk	DESeq2	LRT	0.0267077593721816
209	KC_AB2	CG43347	0.200151209901366	0.813693499455038	0.988741847156811	pseudobulk	DESeq2	LRT	0.0895391535411729
209	KC_AB2	CG43366	1.42621218304824	0.66738831402044	0.988741847156811	pseudobulk	DESeq2	LRT	0.175621402117653
209	KC_AB2	CG43367	-0.0375927612005486	0.955878636256887	0.988741847156811	pseudobulk	DESeq2	LRT	0.019597244700702
210	KC_AB2	CG43373	0.985911093532207	0.432943602355928	0.988741847156811	pseudobulk	DESeq2	LRT	0.36356867357932
210	KC_AB2	CG4338	0.667839000613305	0.81413342949896	0.988741847156811	pseudobulk	DESeq2	LRT	0.0893044121300904
210	KC_AB2	CG4341	0.0557672462994941	0.93768168726417	0.988741847156811	pseudobulk	DESeq2	LRT	0.0279445655935353
210	KC_AB2	CG4360	-0.895932805887824	0.726253597795029	0.988741847156811	pseudobulk	DESeq2	LRT	0.138911703140466
210	KC_AB2	CG4364	0.693809979578311	0.690053159116761	0.988741847156811	pseudobulk	DESeq2	LRT	0.161117451550002
210	KC_AB2	CG43647	-1.80294541848982	0.660135549041553	0.988741847156811	pseudobulk	DESeq2	LRT	0.180366879372519
210	KC_AB2	CG43658	3.4822663299396	0.0830592633811486	0.988741847156811	pseudobulk	DESeq2	LRT	1.08061192479032
210	KC_AB2	CG43680	2.06137138167525	0.547946929015274	0.988741847156811	pseudobulk	DESeq2	LRT	0.261261502741816
210	KC_AB2	CG43707	-0.672181874222351	0.581038883278097	0.988741847156811	pseudobulk	DESeq2	LRT	0.235794803533819
210	KC_AB2	CG43736	-0.770018992171093	0.285214342078625	0.988741847156811	pseudobulk	DESeq2	LRT	0.544828639658189
211	KC_AB2	CG43737	-0.0685804796116159	0.862174527271691	0.988741847156811	pseudobulk	DESeq2	LRT	0.0644048124131951
211	KC_AB2	CG43740	1.42621218304824	0.66738831402044	0.988741847156811	pseudobulk	DESeq2	LRT	0.175621402117653
211	KC_AB2	CG43759	-0.0713889842459936	0.872285563980519	0.988741847156811	pseudobulk	DESeq2	LRT	0.0593413148857208
211	KC_AB2	CG43778	-1.0553281277172	0.301534105753834	0.988741847156811	pseudobulk	DESeq2	LRT	0.520663558804131
211	KC_AB2	CG43783	1.64209625885217	0.483203675195701	0.988741847156811	pseudobulk	DESeq2	LRT	0.315869771176778
211	KC_AB2	CG43789	0.818790875285784	0.940355871560339	0.988741847156811	pseudobulk	DESeq2	LRT	0.0267077593721816
211	KC_AB2	CG43795	-0.223362784232142	0.725987666436249	0.988741847156811	pseudobulk	DESeq2	LRT	0.13907075732219
211	KC_AB2	CG43800	1.4129303440845	0.645460595159151	0.988741847156811	pseudobulk	DESeq2	LRT	0.190130265910817
211	KC_AB2	CG43861	0.143727500031408	0.907671353519454	0.988741847156811	pseudobulk	DESeq2	LRT	0.0420713708504496
211	KC_AB2	CG43867	-0.384512414473993	0.675124643698035	0.988741847156811	pseudobulk	DESeq2	LRT	0.170616038912777
212	KC_AB2	CG4390	0.61702365764228	0.739069534444918	0.988741847156811	pseudobulk	DESeq2	LRT	0.131314699622637
212	KC_AB2	CG43901	0.725307151571678	0.513887930668818	0.988741847156811	pseudobulk	DESeq2	LRT	0.289131582169829
212	KC_AB2	CG43902	2.00515076565275	0.576456787459964	0.988741847156811	pseudobulk	DESeq2	LRT	0.239233242872451
212	KC_AB2	CG4393	0.32742703490313	0.878511928385878	0.988741847156811	pseudobulk	DESeq2	LRT	0.0562523373024427
212	KC_AB2	CG4404	-0.233095226066582	0.89156790931789	0.988741847156811	pseudobulk	DESeq2	LRT	0.0498455717047824
212	KC_AB2	CG4407	1.12919034796087	0.7301237214754	0.988741847156811	pseudobulk	DESeq2	LRT	0.136603541247596
212	KC_AB2	CG4409	1.42621218304824	0.66738831402044	0.988741847156811	pseudobulk	DESeq2	LRT	0.175621402117653

212	KC_AB2	CG44098	0.818790875285784	0.940355871560339	0.988741847156811	pseudobulk	DESeq2	LRT	0.0267077593721816
212	KC_AB2	CG44153	-0.538739295554273	0.616195967662835	0.988741847156811	pseudobulk	DESeq2	LRT	0.21028114799816
212	KC_AB2	CG4424	-1.80294541848982	0.660135549041553	0.988741847156811	pseudobulk	DESeq2	LRT	0.180366879372519
213	KC_AB2	CG44242	0.0185316062243429	0.947759931698958	0.988741847156811	pseudobulk	DESeq2	LRT	0.0233016558496115
213	KC_AB2	CG44243	-2.51746470924027	0.517377929599049	0.988741847156811	pseudobulk	DESeq2	LRT	0.286192101436607
213	KC_AB2	CG44245	-1.80294541848982	0.660135549041553	0.988741847156811	pseudobulk	DESeq2	LRT	0.180366879372519
213	KC_AB2	CG44247	-0.162435103588496	0.784686485298068	0.988741847156811	pseudobulk	DESeq2	LRT	0.105303827206009
213	KC_AB2	CG44314	0.818790875285784	0.940355871560339	0.988741847156811	pseudobulk	DESeq2	LRT	0.0267077593721816
213	KC_AB2	CG44385	0.818790875285784	0.940355871560339	0.988741847156811	pseudobulk	DESeq2	LRT	0.0267077593721816
213	KC_AB2	CG44422	3.00331245130986	0.227562950606189	0.988741847156811	pseudobulk	DESeq2	LRT	0.642898443757851
213	KC_AB2	CG4452	-2.77227577437562	0.189386736997104	0.988741847156811	pseudobulk	DESeq2	LRT	0.722650438483001
213	KC_AB2	CG4455	1.42621218304824	0.66738831402044	0.988741847156811	pseudobulk	DESeq2	LRT	0.175621402117653
213	KC_AB2	CG4467	-0.14518891923841	0.724494497222934	0.988741847156811	pseudobulk	DESeq2	LRT	0.139964908791551
214	KC_AB2	CG44774	0.888163875742781	0.228944764825938	0.988741847156811	pseudobulk	DESeq2	LRT	0.640269282833822
214	KC_AB2	CG44815	-2.26538931672255	0.547771004842275	0.988741847156811	pseudobulk	DESeq2	LRT	0.2614009599796
214	KC_AB2	CG44837	-0.360179130062888	0.720527203814486	0.988741847156811	pseudobulk	DESeq2	LRT	0.142349617565488
214	KC_AB2	CG44838	0.0291019590104613	0.96778805362332	0.988741847156811	pseudobulk	DESeq2	LRT	0.0142197431258165
214	KC_AB2	CG4497	-0.853565742783243	0.753557841534365	0.988741847156811	pseudobulk	DESeq2	LRT	0.122883406527736
214	KC_AB2	CG45002	0.818790875285784	0.940355871560339	0.988741847156811	pseudobulk	DESeq2	LRT	0.0267077593721816
214	KC_AB2	CG4502	0.418114005313677	0.50570954572761	0.988741847156811	pseudobulk	DESeq2	LRT	0.296098848586755
214	KC_AB2	CG45049	0.433492217001479	0.546305660877751	0.988741847156811	pseudobulk	DESeq2	LRT	0.26256429925618
214	KC_AB2	CG45050	-0.123827691111931	0.807957043487584	0.988741847156811	pseudobulk	DESeq2	LRT	0.0926117286712422
214	KC_AB2	CG45071	2.7785831508444	0.373751317747552	0.988741847156811	pseudobulk	DESeq2	LRT	0.427417267458199
215	KC_AB2	CG4511	0.198943728147656	0.908294890660497	0.988741847156811	pseudobulk	DESeq2	LRT	0.0417731287976286
215	KC_AB2	CG45218	0.818790875285784	0.940355871560339	0.988741847156811	pseudobulk	DESeq2	LRT	0.0267077593721816
215	KC_AB2	CG45263	0.112464111847285	0.728459657013199	0.988741847156811	pseudobulk	DESeq2	LRT	0.137594494987611
215	KC_AB2	CG4537	-1.23536834900699	0.725570392794057	0.988741847156811	pseudobulk	DESeq2	LRT	0.139320447155934
215	KC_AB2	CG4538	2.82301744158985	0.222767949441939	0.988741847156811	pseudobulk	DESeq2	LRT	0.65214729277021
215	KC_AB2	CG4552	-0.194388201900128	0.928057462354013	0.988741847156811	pseudobulk	DESeq2	LRT	0.0324251328213705
215	KC_AB2	CG4553	1.47543920254359	0.661929169513603	0.988741847156811	pseudobulk	DESeq2	LRT	0.17918848025087
215	KC_AB2	CG4557	-0.568233637794205	0.868826397133536	0.988741847156811	pseudobulk	DESeq2	LRT	0.0610669925949191
215	KC_AB2	CG4558	-1.65719613210719	0.599376091584556	0.988741847156811	pseudobulk	DESeq2	LRT	0.222300584546838
215	KC_AB2	CG45603	0.818790875285784	0.940355871560339	0.988741847156811	pseudobulk	DESeq2	LRT	0.0267077593721816
216	KC_AB2	CG4562	-1.96571807528696	0.246828312188414	0.988741847156811	pseudobulk	DESeq2	LRT	0.607605026478416
216	KC_AB2	CG4565	1.42621218304824	0.66738831402044	0.988741847156811	pseudobulk	DESeq2	LRT	0.175621402117653
216	KC_AB2	CG4572	-1.23536834900699	0.725570392794057	0.988741847156811	pseudobulk	DESeq2	LRT	0.139320447155934
216	KC_AB2	CG4577	0.599385167422956	0.20331697841043	0.988741847156811	pseudobulk	DESeq2	LRT	0.691826353176631
216	KC_AB2	CG4587	0.13728013565709	0.828352003725612	0.988741847156811	pseudobulk	DESeq2	LRT	0.0817850728893918
216	KC_AB2	CG4593	0.27226851987049	0.911402843231596	0.988741847156811	pseudobulk	DESeq2	LRT	0.0402896209172008
216	KC_AB2	CG4596	-0.202209755684413	0.867758037868502	0.988741847156811	pseudobulk	DESeq2	LRT	0.0616013548553182
216	KC_AB2	CG4598	1.42621218304824	0.66738831402044	0.988741847156811	pseudobulk	DESeq2	LRT	0.175621402117653
216	KC_AB2	CG4603	-0.587425801135323	0.771690223849521	0.988741847156811	pseudobulk	DESeq2	LRT	0.112557001570832
216	KC_AB2	CG4610	-1.80294541848982	0.660135549041553	0.988741847156811	pseudobulk	DESeq2	LRT	0.180366879372519
217	KC_AB2	CG4611	-1.80294541848982	0.660135549041553	0.988741847156811	pseudobulk	DESeq2	LRT	0.180366879372519
217	KC_AB2	CG4612	0.0607454187674758	0.91810291213834	0.988741847156811	pseudobulk	DESeq2	LRT	0.0371086350637082
217	KC_AB2	CG4615	0.421489448475535	0.787508785880886	0.988741847156811	pseudobulk	DESeq2	LRT	0.103744592283374
217	KC_AB2	CG4617	-0.840006590521399	0.717017354938613	0.988741847156811	pseudobulk	DESeq2	LRT	0.144470332389583
217	KC_AB2	CG4622	1.7098779479	0.537865353861561	0.988741847156811	pseudobulk	DESeq2	LRT	0.269326429526188
217	KC_AB2	CG4627	1.40305140665399	0.649741560632143	0.988741847156811	pseudobulk	DESeq2	LRT	0.187259352757606
217	KC_AB2	CG46280	0.0277692474095899	0.95479666811421	0.988741847156811	pseudobulk	DESeq2	LRT	0.020089105189097
217	KC_AB2	CG46281	-1.65719613210719	0.599376091584556	0.988741847156811	pseudobulk	DESeq2	LRT	0.222300584546838
217	KC_AB2	CG4629	-0.43853146534373	0.771089970022033	0.988741847156811	pseudobulk	DESeq2	LRT	0.112894945938033
217	KC_AB2	CG46301	-0.203305173466848	0.768973665357665	0.988741847156811	pseudobulk	DESeq2	LRT	0.1140885330018
218	KC_AB2	CG4631	-0.506613484034373	0.961658398065406	0.988741847156811	pseudobulk	DESeq2	LRT	0.0169791713942958
218	KC_AB2	CG46338	0.419671460681067	0.785238231243751	0.988741847156811	pseudobulk	DESeq2	LRT	0.104998563868265
218	KC_AB2	CG46339	-0.305902062373932	0.4848800242607	0.988741847156811	pseudobulk	DESeq2	LRT	0.314365707268365

218	KC_AB2	CG46385	0.196035363156796	0.720822305334986	0.988741847156811	pseudobulk	DESeq2	LRT	0.142171782886347
218	KC_AB2	CG4645	-0.787390638757929	0.708636903053768	0.988741847156811	pseudobulk	DESeq2	LRT	0.149576235046444
218	KC_AB2	CG4646	0.676700101239319	0.68351484567994	0.988741847156811	pseudobulk	DESeq2	LRT	0.165252048284366
218	KC_AB2	CG4658	1.61629386556623	0.232286905400776	0.988741847156811	pseudobulk	DESeq2	LRT	0.63397527178597
218	KC_AB2	CG4660	-0.819831984811786	0.296838974471567	0.988741847156811	pseudobulk	DESeq2	LRT	0.52747907749448
218	KC_AB2	CG4661	1.47543920254359	0.661929169513603	0.988741847156811	pseudobulk	DESeq2	LRT	0.17918848025087
218	KC_AB2	CG4676	2.00515076565275	0.576456787459964	0.988741847156811	pseudobulk	DESeq2	LRT	0.239233242872451
219	KC_AB2	CG4678	1.42621218304824	0.66738831402044	0.988741847156811	pseudobulk	DESeq2	LRT	0.175621402117653
219	KC_AB2	CG4679	-0.233272055930639	0.937531793764924	0.988741847156811	pseudobulk	DESeq2	LRT	0.028013995469539
219	KC_AB2	CG4686	1.79057114677109	0.227338470802418	0.988741847156811	pseudobulk	DESeq2	LRT	0.643327065623606
219	KC_AB2	CG4707	1.47543920254359	0.661929169513603	0.988741847156811	pseudobulk	DESeq2	LRT	0.17918848025087
219	KC_AB2	CG4730	0.818790875285784	0.940355871560339	0.988741847156811	pseudobulk	DESeq2	LRT	0.0267077593721816
219	KC_AB2	CG4751	-1.1284597703615	0.729754448571724	0.988741847156811	pseudobulk	DESeq2	LRT	0.136823248879498
219	KC_AB2	CG4763	-1.80294541848982	0.660135549041553	0.988741847156811	pseudobulk	DESeq2	LRT	0.180366879372519
219	KC_AB2	CG4768	1.12713376258683	0.534020775613435	0.988741847156811	pseudobulk	DESeq2	LRT	0.272441846793991
219	KC_AB2	CG4788	0.699674038439951	0.821690393783994	0.988741847156811	pseudobulk	DESeq2	LRT	0.0852917902455435
219	KC_AB2	CG4789	0.113455905075472	0.923572674051179	0.988741847156811	pseudobulk	DESeq2	LRT	0.0345289251315908
220	KC_AB2	CG4806	0.581445489707027	0.811994604428781	0.988741847156811	pseudobulk	DESeq2	LRT	0.0904468565649727
220	KC_AB2	CG4813	-0.0362223591928502	0.956694233985992	0.988741847156811	pseudobulk	DESeq2	LRT	0.0192268435304406
220	KC_AB2	CG4825	-0.568233637794205	0.868826397133536	0.988741847156811	pseudobulk	DESeq2	LRT	0.0610669925949191
220	KC_AB2	CG4829	-1.54186453418413	0.241871941357003	0.988741847156811	pseudobulk	DESeq2	LRT	0.616414509565384
220	KC_AB2	CG4849	0.818790875285784	0.940355871560339	0.988741847156811	pseudobulk	DESeq2	LRT	0.0267077593721816
220	KC_AB2	CG4853	3.03619069427769	0.314302467188174	0.988741847156811	pseudobulk	DESeq2	LRT	0.502652209876457
220	KC_AB2	CG4858	-0.0582963981256801	0.979058404810457	0.988741847156811	pseudobulk	DESeq2	LRT	0.0091913999942843
220	KC_AB2	CG4866	0.5738945031678	0.80780652681376	0.988741847156811	pseudobulk	DESeq2	LRT	0.0926926421938231
220	KC_AB2	CG4880	0.0601466527378941	0.974810960364079	0.988741847156811	pseudobulk	DESeq2	LRT	0.0110795964355292
220	KC_AB2	CG4882	-0.840006590521399	0.717017354938613	0.988741847156811	pseudobulk	DESeq2	LRT	0.144470332389583
221	KC_AB2	CG4887	0.459041972900521	0.711814196767333	0.988741847156811	pseudobulk	DESeq2	LRT	0.147633354466159
221	KC_AB2	CG4896	0.0536606246612516	0.930818900320667	0.988741847156811	pseudobulk	DESeq2	LRT	0.0311348069263085
221	KC_AB2	CG4901	2.51243226103394	0.474458247157471	0.988741847156811	pseudobulk	DESeq2	LRT	0.323801999942597
221	KC_AB2	CG4908	-0.337072010867851	0.906965066354256	0.988741847156811	pseudobulk	DESeq2	LRT	0.0424094403729327
221	KC_AB2	CG4911	1.12256745044744	0.447112548303483	0.988741847156811	pseudobulk	DESeq2	LRT	0.349583141399053
221	KC_AB2	CG4935	1.69894780879763	0.540778829726708	0.988741847156811	pseudobulk	DESeq2	LRT	0.26698031838453
221	KC_AB2	CG4936	1.12452187615248	0.704078404906088	0.988741847156811	pseudobulk	DESeq2	LRT	0.152378975911753
221	KC_AB2	CG4942	-0.934020934207768	0.782359118258182	0.988741847156811	pseudobulk	DESeq2	LRT	0.106593851444836
221	KC_AB2	CG4951	1.14703591811676	0.660809111836255	0.988741847156811	pseudobulk	DESeq2	LRT	0.179923977199595
221	KC_AB2	CG4953	3.25449350606273	0.266356678091136	0.988741847156811	pseudobulk	DESeq2	LRT	0.574536410117102
222	KC_AB2	CG4957	-0.568233637794205	0.868826397133536	0.988741847156811	pseudobulk	DESeq2	LRT	0.0610669925949191
222	KC_AB2	CG4968	-0.862332580780999	0.666590188569964	0.988741847156811	pseudobulk	DESeq2	LRT	0.176141082936639
222	KC_AB2	CG4972	-3.4227037205893	0.285801360563088	0.988741847156811	pseudobulk	DESeq2	LRT	0.543935708072568
222	KC_AB2	CG5003	-0.957133960806503	0.762632518785665	0.988741847156811	pseudobulk	DESeq2	LRT	0.1176846802739
222	KC_AB2	CG5009	-0.568233637794205	0.868826397133536	0.988741847156811	pseudobulk	DESeq2	LRT	0.0610669925949191
222	KC_AB2	CG5013	0.818790875285784	0.940355871560339	0.988741847156811	pseudobulk	DESeq2	LRT	0.0267077593721816
222	KC_AB2	CG5021	0.366371937423912	0.832374013979327	0.988741847156811	pseudobulk	DESeq2	LRT	0.0796814865593369
222	KC_AB2	CG5022	1.46633849533333	0.484714236800387	0.988741847156811	pseudobulk	DESeq2	LRT	0.314514224195034
222	KC_AB2	CG5027	1.76192653942113	0.294103317637791	0.988741847156811	pseudobulk	DESeq2	LRT	0.531500076398544
222	KC_AB2	CG5028	0.140466524264904	0.881658307772732	0.988741847156811	pseudobulk	DESeq2	LRT	0.0546996958214857
223	KC_AB2	CG5033	-1.99867440840856	0.534899605264936	0.988741847156811	pseudobulk	DESeq2	LRT	0.271727722597268
223	KC_AB2	CG5037	1.32467530657598	0.538468278591255	0.988741847156811	pseudobulk	DESeq2	LRT	0.268839876095909
223	KC_AB2	CG5039	0.818790875285784	0.940355871560339	0.988741847156811	pseudobulk	DESeq2	LRT	0.0267077593721816
223	KC_AB2	CG5044	0.798955445836505	0.738225347953373	0.988741847156811	pseudobulk	DESeq2	LRT	0.131811046803217
223	KC_AB2	CG5048	1.45259040912931	0.616176917291092	0.988741847156811	pseudobulk	DESeq2	LRT	0.210294574894236
223	KC_AB2	CG5050	1.42621218304824	0.66738831402044	0.988741847156811	pseudobulk	DESeq2	LRT	0.175621402117653
223	KC_AB2	CG5056	1.33007272203764	0.517901761928373	0.988741847156811	pseudobulk	DESeq2	LRT	0.285752611487477
223	KC_AB2	CG5059	-0.341523381068643	0.791376846133208	0.988741847156811	pseudobulk	DESeq2	LRT	0.101616660342019
223	KC_AB2	CG5065	0.944044596327862	0.229754031761479	0.988741847156811	pseudobulk	DESeq2	LRT	0.638736858804096

223	KC_AB2	CG5068	0.104637746735554	0.951122131714938	0.988741847156811	pseudobulk	DESeq2	LRT	0.0217637125849166
224	KC_AB2	CG5087	-0.689687550646419	0.745991540083112	0.988741847156811	pseudobulk	DESeq2	LRT	0.127266097615876
224	KC_AB2	CG5098	0.330391410498902	0.851537379119407	0.988741847156811	pseudobulk	DESeq2	LRT	0.0697962834747243
224	KC_AB2	CG5104	0.818790875285784	0.940355871560339	0.988741847156811	pseudobulk	DESeq2	LRT	0.0267077593721816
224	KC_AB2	CG5110	-0.0182232118236305	0.978188153261768	0.988741847156811	pseudobulk	DESeq2	LRT	0.00957760117962873
224	KC_AB2	CG5114	-0.934020934207768	0.782359118258182	0.988741847156811	pseudobulk	DESeq2	LRT	0.106593851444836
224	KC_AB2	CG5126	1.12452187615248	0.704078404906088	0.988741847156811	pseudobulk	DESeq2	LRT	0.152378975911753
224	KC_AB2	CG5144	0.818790875285784	0.940355871560339	0.988741847156811	pseudobulk	DESeq2	LRT	0.0267077593721816
224	KC_AB2	CG5149	-0.088232111136206	0.957691663474435	0.988741847156811	pseudobulk	DESeq2	LRT	0.0187742930152181
224	KC_AB2	CG5151	3.34051319803522	0.114757256234294	0.988741847156811	pseudobulk	DESeq2	LRT	0.940219843979113
224	KC_AB2	CG5160	1.42621218304824	0.66738831402044	0.988741847156811	pseudobulk	DESeq2	LRT	0.175621402117653
225	KC_AB2	CG5171	-1.80294541848982	0.660135549041553	0.988741847156811	pseudobulk	DESeq2	LRT	0.180366879372519
225	KC_AB2	CG5174	0.430998199178423	0.692390158501277	0.988741847156811	pseudobulk	DESeq2	LRT	0.159649113729868
225	KC_AB2	CG5180	-1.48046351229221	0.534490457994632	0.988741847156811	pseudobulk	DESeq2	LRT	0.272060043639989
225	KC_AB2	CG5181	0.315515278988381	0.901389738439351	0.988741847156811	pseudobulk	DESeq2	LRT	0.0450873903126698
225	KC_AB2	CG5188	-3.4227037205893	0.285801360563088	0.988741847156811	pseudobulk	DESeq2	LRT	0.543935708072568
225	KC_AB2	CG5191	-0.506613484034373	0.961658398065406	0.988741847156811	pseudobulk	DESeq2	LRT	0.0169791713942958
225	KC_AB2	CG5196	-1.12199977650382	0.349776063427903	0.988741847156811	pseudobulk	DESeq2	LRT	0.456209914345258
225	KC_AB2	CG5199	0.712171366772551	0.813205723536451	0.988741847156811	pseudobulk	DESeq2	LRT	0.0897995733547685
225	KC_AB2	CG5214	0.323454365629786	0.742710981686644	0.988741847156811	pseudobulk	DESeq2	LRT	0.129180154580899
225	KC_AB2	CG5217	3.09926389624515	0.339712049344771	0.988741847156811	pseudobulk	DESeq2	LRT	0.468889048740397
226	KC_AB2	CG5220	0.712171366772551	0.813205723536451	0.988741847156811	pseudobulk	DESeq2	LRT	0.0897995733547685
226	KC_AB2	CG5262	-0.934020934207768	0.782359118258182	0.988741847156811	pseudobulk	DESeq2	LRT	0.106593851444836
226	KC_AB2	CG5270	-3.83346815451011	0.239050858312983	0.988741847156811	pseudobulk	DESeq2	LRT	0.621509692629463
226	KC_AB2	CG5273	-0.518819613096587	0.759405963298412	0.988741847156811	pseudobulk	DESeq2	LRT	0.119525996849335
226	KC_AB2	CG5274	0.353798809751289	0.901648351639924	0.988741847156811	pseudobulk	DESeq2	LRT	0.0449628069360408
226	KC_AB2	CG5276	-1.80294541848982	0.660135549041553	0.988741847156811	pseudobulk	DESeq2	LRT	0.180366879372519
226	KC_AB2	CG5281	0.157692007916382	0.894844829644515	0.988741847156811	pseudobulk	DESeq2	LRT	0.0482522668870443
226	KC_AB2	CG5282	0.668843710792558	0.553533717792641	0.988741847156811	pseudobulk	DESeq2	LRT	0.25685591949116
226	KC_AB2	CG5285	-3.4227037205893	0.285801360563088	0.988741847156811	pseudobulk	DESeq2	LRT	0.543935708072568
226	KC_AB2	CG5287	-0.506613484034373	0.961658398065406	0.988741847156811	pseudobulk	DESeq2	LRT	0.0169791713942958
227	KC_AB2	CG5290	0.818790875285784	0.940355871560339	0.988741847156811	pseudobulk	DESeq2	LRT	0.0267077593721816
227	KC_AB2	CG5292	-0.114099770495178	0.955170180595609	0.988741847156811	pseudobulk	DESeq2	LRT	0.0199192442181898
227	KC_AB2	CG5316	-0.00340877878248056	0.980029417943268	0.988741847156811	pseudobulk	DESeq2	LRT	0.0087608877170192
227	KC_AB2	CG5323	-1.18743225435356	0.64061820392288	0.988741847156811	pseudobulk	DESeq2	LRT	0.193400724506216
227	KC_AB2	CG5339	-0.957133960806503	0.762632518785665	0.988741847156811	pseudobulk	DESeq2	LRT	0.1176846802739
227	KC_AB2	CG5348	0.351722119397094	0.929615296941389	0.988741847156811	pseudobulk	DESeq2	LRT	0.0316967385224016
227	KC_AB2	CG5355	1.69894780879763	0.540778829726708	0.988741847156811	pseudobulk	DESeq2	LRT	0.26698031838453
227	KC_AB2	CG5359	-0.405381063532158	0.89155634938256	0.988741847156811	pseudobulk	DESeq2	LRT	0.0498512027381788
227	KC_AB2	CG5380	-0.506613484034373	0.961658398065406	0.988741847156811	pseudobulk	DESeq2	LRT	0.0169791713942958
227	KC_AB2	CG5382	0.699674038439951	0.821690393783994	0.988741847156811	pseudobulk	DESeq2	LRT	0.0852917902455435
228	KC_AB2	CG5412	-1.40296545307325	0.628223627794903	0.988741847156811	pseudobulk	DESeq2	LRT	0.201885733604539
228	KC_AB2	CG5439	-0.957133960806503	0.762632518785665	0.988741847156811	pseudobulk	DESeq2	LRT	0.1176846802739
228	KC_AB2	CG5445	0.163898935093501	0.910262514890877	0.988741847156811	pseudobulk	DESeq2	LRT	0.0408333413852996
228	KC_AB2	CG5446	0.717813259931394	0.639514985815729	0.988741847156811	pseudobulk	DESeq2	LRT	0.194149274202023
228	KC_AB2	CG5447	-1.88553080214318	0.332579342074537	0.988741847156811	pseudobulk	DESeq2	LRT	0.4781047301666
228	KC_AB2	CG5455	0.320348949796381	0.849242092899403	0.988741847156811	pseudobulk	DESeq2	LRT	0.0709684880565728
228	KC_AB2	CG5466	0.513202572024843	0.807055953632252	0.988741847156811	pseudobulk	DESeq2	LRT	0.093096354359303
228	KC_AB2	CG5498	-0.934020934207768	0.782359118258182	0.988741847156811	pseudobulk	DESeq2	LRT	0.106593851444836
228	KC_AB2	CG5500	2.09385235217438	0.314672322125116	0.988741847156811	pseudobulk	DESeq2	LRT	0.502141454979881
228	KC_AB2	CG5504	0.639524207038909	0.688561882327919	0.988741847156811	pseudobulk	DESeq2	LRT	0.162057022805973
229	KC_AB2	CG5510	1.17156071944349	0.597348257338218	0.988741847156811	pseudobulk	DESeq2	LRT	0.223772398949354
229	KC_AB2	CG5515	1.09755075627148	0.564545147075846	0.988741847156811	pseudobulk	DESeq2	LRT	0.24830132151245
229	KC_AB2	CG5516	-0.58621066677634	0.754406236055079	0.988741847156811	pseudobulk	DESeq2	LRT	0.122394730301062
229	KC_AB2	CG5521	-0.680955529585605	0.616245856432056	0.988741847156811	pseudobulk	DESeq2	LRT	0.210245987852228
229	KC_AB2	CG5522	0.0766005673852471	0.954816405916504	0.988741847156811	pseudobulk	DESeq2	LRT	0.020080127434663

229	KC_AB2	CG5532	0.717905322993618	0.491275279492022	0.988741847156811	pseudobulk	DESeq2	LRT	0.308675088603449
229	KC_AB2	CG5535	-0.50879048068994	0.817628596116567	0.988741847156811	pseudobulk	DESeq2	LRT	0.0874439277298491
229	KC_AB2	CG5541	0.818790875285784	0.940355871560339	0.988741847156811	pseudobulk	DESeq2	LRT	0.0267077593721816
229	KC_AB2	CG5543	-2.11027789354592	0.473734933668313	0.988741847156811	pseudobulk	DESeq2	LRT	0.32446458879962
229	KC_AB2	CG5554	0.475522879236569	0.801582640633501	0.988741847156811	pseudobulk	DESeq2	LRT	0.0960516966149455
230	KC_AB2	CG5555	-1.06875327715096	0.441789827432436	0.988741847156811	pseudobulk	DESeq2	LRT	0.354784288330637
230	KC_AB2	CG5567	3.64730466917968	0.182487106943786	0.988741847156811	pseudobulk	DESeq2	LRT	0.738767813842945
230	KC_AB2	CG5569	-1.64271834309286	0.357867998682071	0.988741847156811	pseudobulk	DESeq2	LRT	0.446277135410403
230	KC_AB2	CG5590	2.06137138167525	0.547946929015274	0.988741847156811	pseudobulk	DESeq2	LRT	0.261261502741816
230	KC_AB2	CG5608	1.74111111089874	0.563806300883242	0.988741847156811	pseudobulk	DESeq2	LRT	0.248870074928531
230	KC_AB2	CG5611	0.818790875285784	0.940355871560339	0.988741847156811	pseudobulk	DESeq2	LRT	0.0267077593721816
230	KC_AB2	CG5618	1.42621218304824	0.66738831402044	0.988741847156811	pseudobulk	DESeq2	LRT	0.175621402117653
230	KC_AB2	CG5641	0.873917537747718	0.565461162678869	0.988741847156811	pseudobulk	DESeq2	LRT	0.247597218171726
230	KC_AB2	CG5644	0.518112575170181	0.804900582036886	0.988741847156811	pseudobulk	DESeq2	LRT	0.0942577585627823
230	KC_AB2	CG5645	-0.934020934207768	0.782359118258182	0.988741847156811	pseudobulk	DESeq2	LRT	0.106593851444836
231	KC_AB2	CG5646	-0.794177064289352	0.743016342902793	0.988741847156811	pseudobulk	DESeq2	LRT	0.129001633676244
231	KC_AB2	CG5664	0.818790875285784	0.940355871560339	0.988741847156811	pseudobulk	DESeq2	LRT	0.0267077593721816
231	KC_AB2	CG5674	-0.0290481433840362	0.943004399476661	0.988741847156811	pseudobulk	DESeq2	LRT	0.0254862811078676
231	KC_AB2	CG5676	-0.360008716443124	0.741924866753069	0.988741847156811	pseudobulk	DESeq2	LRT	0.129640072627549
231	KC_AB2	CG5697	-1.80294541848982	0.660135549041553	0.988741847156811	pseudobulk	DESeq2	LRT	0.180366879372519
231	KC_AB2	CG5708	0.435469677673563	0.692600063631559	0.988741847156811	pseudobulk	DESeq2	LRT	0.159517472886423
231	KC_AB2	CG5721	-2.9721553030081	0.387854083811327	0.988741847156811	pseudobulk	DESeq2	LRT	0.411331631401987
231	KC_AB2	CG5726	-0.131822678323325	0.930740981183606	0.988741847156811	pseudobulk	DESeq2	LRT	0.0311711633729812
231	KC_AB2	CG5727	0.321244353479908	0.883160641080799	0.988741847156811	pseudobulk	DESeq2	LRT	0.053960293942671
231	KC_AB2	CG5728	-1.08868399753678	0.65578805130173	0.988741847156811	pseudobulk	DESeq2	LRT	0.183236500598949
232	KC_AB2	CG5742	-0.547605794825219	0.713155219555199	0.988741847156811	pseudobulk	DESeq2	LRT	0.146815934861023
232	KC_AB2	CG5745	-2.51746470924027	0.517377929599049	0.988741847156811	pseudobulk	DESeq2	LRT	0.286192101436607
232	KC_AB2	CG5746	-0.151599073611388	0.863812866870754	0.988741847156811	pseudobulk	DESeq2	LRT	0.0635803312339363
232	KC_AB2	CG5757	-1.80294541848982	0.660135549041553	0.988741847156811	pseudobulk	DESeq2	LRT	0.180366879372519
232	KC_AB2	CG5758	-0.192181231709668	0.899340686783902	0.988741847156811	pseudobulk	DESeq2	LRT	0.0460757583645606
232	KC_AB2	CG5768	-4.32774965800022	0.148534134463525	0.988741847156811	pseudobulk	DESeq2	LRT	0.828173730147234
232	KC_AB2	CG5776	0.818790875285784	0.940355871560339	0.988741847156811	pseudobulk	DESeq2	LRT	0.0267077593721816
232	KC_AB2	CG5787	0.712171366772551	0.813205723536451	0.988741847156811	pseudobulk	DESeq2	LRT	0.0897995733547685
232	KC_AB2	CG5805	0.321244353479908	0.883160641080799	0.988741847156811	pseudobulk	DESeq2	LRT	0.053960293942671
232	KC_AB2	CG5808	3.22031971259211	0.311877201824258	0.988741847156811	pseudobulk	DESeq2	LRT	0.506016370942756
233	KC_AB2	CG5815	1.42621218304824	0.66738831402044	0.988741847156811	pseudobulk	DESeq2	LRT	0.175621402117653
233	KC_AB2	CG5823	0.744200558493458	0.552934807395364	0.988741847156811	pseudobulk	DESeq2	LRT	0.257326070245877
233	KC_AB2	CG5828	1.94106108959906	0.476420175276185	0.988741847156811	pseudobulk	DESeq2	LRT	0.322009855447888
233	KC_AB2	CG5830	0.0245589768682133	0.922810581016884	0.988741847156811	pseudobulk	DESeq2	LRT	0.0348874344685636
233	KC_AB2	CG5846	-2.51746470924027	0.517377929599049	0.988741847156811	pseudobulk	DESeq2	LRT	0.286192101436607
233	KC_AB2	CG5854	-2.51746470924027	0.517377929599049	0.988741847156811	pseudobulk	DESeq2	LRT	0.286192101436607
233	KC_AB2	CG5861	1.66791810945549	0.27131009099814	0.988741847156811	pseudobulk	DESeq2	LRT	0.566534052964138
233	KC_AB2	CG5862	1.67796394544623	0.574362267760116	0.988741847156811	pseudobulk	DESeq2	LRT	0.240814098434665
233	KC_AB2	CG5877	2.00515076565275	0.576456787459964	0.988741847156811	pseudobulk	DESeq2	LRT	0.239233242872451
233	KC_AB2	CG5880	0.818790875285784	0.940355871560339	0.988741847156811	pseudobulk	DESeq2	LRT	0.0267077593721816
234	KC_AB2	CG5885	2.01599493208962	0.288210614579416	0.988741847156811	pseudobulk	DESeq2	LRT	0.540290028489488
234	KC_AB2	CG5886	-0.568233637794205	0.868826397133536	0.988741847156811	pseudobulk	DESeq2	LRT	0.0610669925949191
234	KC_AB2	CG5890	-0.0164548285423313	0.937357825943607	0.988741847156811	pseudobulk	DESeq2	LRT	0.0280945903634992
234	KC_AB2	CG5902	-0.457212305199634	0.858276431637992	0.988741847156811	pseudobulk	DESeq2	LRT	0.0663728130839547
234	KC_AB2	CG5903	0.273557203397178	0.739578995084338	0.988741847156811	pseudobulk	DESeq2	LRT	0.13101543181317
234	KC_AB2	CG5913	0.0601466527378941	0.974810960364079	0.988741847156811	pseudobulk	DESeq2	LRT	0.0110795964355292
234	KC_AB2	CG5916	1.33092447354815	0.414370568376215	0.988741847156811	pseudobulk	DESeq2	LRT	0.382611098933015
234	KC_AB2	CG5934	-0.745305729478517	0.557763186272734	0.988741847156811	pseudobulk	DESeq2	LRT	0.253550153616441
234	KC_AB2	CG5937	0.317224567334262	0.824573068980285	0.988741847156811	pseudobulk	DESeq2	LRT	0.083770853604817
234	KC_AB2	CG5938	0.504880555119022	0.633939613272586	0.988741847156811	pseudobulk	DESeq2	LRT	0.197952109423526
235	KC_AB2	CG5946	-0.435732808999485	0.72344254586074	0.988741847156811	pseudobulk	DESeq2	LRT	0.140595953842017

235	KC_AB2	CG5953	-0.506613484034373	0.961658398065406	0.988741847156811	pseudobulk	DESeq2	LRT	0.0169791713942958
235	KC_AB2	CG5961	-0.321985981938315	0.836410605907815	0.988741847156811	pseudobulk	DESeq2	LRT	0.0775804688504965
235	KC_AB2	CG5969	0.325673225559482	0.833945800669666	0.988741847156811	pseudobulk	DESeq2	LRT	0.0788621738646229
235	KC_AB2	CG5986	1.32447065690247	0.462398042244304	0.988741847156811	pseudobulk	DESeq2	LRT	0.334984013351142
235	KC_AB2	CG5989	-0.194388201900128	0.928057462354013	0.988741847156811	pseudobulk	DESeq2	LRT	0.0324251328213705
235	KC_AB2	CG6005	0.880193121489963	0.699473947829638	0.988741847156811	pseudobulk	DESeq2	LRT	0.155228456332311
235	KC_AB2	CG6013	-0.244957054328445	0.882873582432541	0.988741847156811	pseudobulk	DESeq2	LRT	0.0541014780560468
235	KC_AB2	CG6024	0.57366536475622	0.503223069762573	0.988741847156811	pseudobulk	DESeq2	LRT	0.29823945730658
235	KC_AB2	CG6028	3.48393309996043	0.216218474953395	0.988741847156811	pseudobulk	DESeq2	LRT	0.665107200172195
236	KC_AB2	CG6041	1.09347879643988	0.573203382935229	0.988741847156811	pseudobulk	DESeq2	LRT	0.241691255145302
236	KC_AB2	CG6044	-0.331391091547996	0.415006273408776	0.988741847156811	pseudobulk	DESeq2	LRT	0.381945338260867
236	KC_AB2	CG6051	-0.357196493016583	0.842020761741307	0.988741847156811	pseudobulk	DESeq2	LRT	0.0746771999510382
236	KC_AB2	CG6066	-2.72566785950637	0.305305375662423	0.988741847156811	pseudobulk	DESeq2	LRT	0.515265548796741
236	KC_AB2	CG6083	-0.233272055930639	0.937531793764924	0.988741847156811	pseudobulk	DESeq2	LRT	0.028013995469539
236	KC_AB2	CG6084	0.786780068809377	0.754069556893097	0.988741847156811	pseudobulk	DESeq2	LRT	0.122588592085696
236	KC_AB2	CG6094	0.818790875285784	0.940355871560339	0.988741847156811	pseudobulk	DESeq2	LRT	0.0267077593721816
236	KC_AB2	CG6115	-0.0613369256062313	0.946497324825413	0.988741847156811	pseudobulk	DESeq2	LRT	0.0238806091858718
236	KC_AB2	CG6136	-2.9721553030081	0.387854083811327	0.988741847156811	pseudobulk	DESeq2	LRT	0.411331631401987
236	KC_AB2	CG6144	-0.123268804573364	0.929272035250629	0.988741847156811	pseudobulk	DESeq2	LRT	0.0318571319548154
237	KC_AB2	CG6145	-0.233272055930639	0.937531793764924	0.988741847156811	pseudobulk	DESeq2	LRT	0.028013995469539
237	KC_AB2	CG6153	-0.0582963981256801	0.979058404810457	0.988741847156811	pseudobulk	DESeq2	LRT	0.0091913999942843
237	KC_AB2	CG6154	3.66684585317293	0.187794911241363	0.988741847156811	pseudobulk	DESeq2	LRT	0.726316180172881
237	KC_AB2	CG6171	-1.63785643245857	0.555083135854033	0.988741847156811	pseudobulk	DESeq2	LRT	0.255641966897631
237	KC_AB2	CG6175	0.818790875285784	0.940355871560339	0.988741847156811	pseudobulk	DESeq2	LRT	0.0267077593721816
237	KC_AB2	CG6178	0.818790875285784	0.940355871560339	0.988741847156811	pseudobulk	DESeq2	LRT	0.0267077593721816
237	KC_AB2	CG6179	-0.568233637794205	0.868826397133536	0.988741847156811	pseudobulk	DESeq2	LRT	0.0610669925949191
237	KC_AB2	CG6180	-0.407159925347506	0.777044977319523	0.988741847156811	pseudobulk	DESeq2	LRT	0.109553842413568
237	KC_AB2	CG6184	1.19378206033478	0.4111656103304	0.988741847156811	pseudobulk	DESeq2	LRT	0.385983216649727
237	KC_AB2	CG6191	-0.094347335329863	0.917902265641662	0.988741847156811	pseudobulk	DESeq2	LRT	0.0372035581801809
238	KC_AB2	CG6195	3.03619069427769	0.314302467188174	0.988741847156811	pseudobulk	DESeq2	LRT	0.502652209876457
238	KC_AB2	CG6201	-0.0323203545809474	0.954448156132187	0.988741847156811	pseudobulk	DESeq2	LRT	0.020247656706879
238	KC_AB2	CG6204	-1.23536834900699	0.725570392794057	0.988741847156811	pseudobulk	DESeq2	LRT	0.139320447155934
238	KC_AB2	CG6218	-0.233272055930639	0.937531793764924	0.988741847156811	pseudobulk	DESeq2	LRT	0.028013995469539
238	KC_AB2	CG6227	0.818790875285784	0.940355871560339	0.988741847156811	pseudobulk	DESeq2	LRT	0.0267077593721816
238	KC_AB2	CG6230	1.42621218304824	0.66738831402044	0.988741847156811	pseudobulk	DESeq2	LRT	0.175621402117653
238	KC_AB2	CG6236	-2.45158172483764	0.378823136429564	0.988741847156811	pseudobulk	DESeq2	LRT	0.421563504545096
238	KC_AB2	CG6254	-0.588639054357632	0.8432408255636	0.988741847156811	pseudobulk	DESeq2	LRT	0.0740483752247712
238	KC_AB2	CG6276	-0.669068297387362	0.767301512613651	0.988741847156811	pseudobulk	DESeq2	LRT	0.115033945655498
238	KC_AB2	CG6282	-0.217963318638521	0.796760594396976	0.988741847156811	pseudobulk	DESeq2	LRT	0.0986721530722332
239	KC_AB2	CG6287	-2.26538931672255	0.547771004842275	0.988741847156811	pseudobulk	DESeq2	LRT	0.2614009599796
239	KC_AB2	CG6310	1.47543920254359	0.661929169513603	0.988741847156811	pseudobulk	DESeq2	LRT	0.17918848025087
239	KC_AB2	CG6329	-0.0509099959109793	0.884398998714633	0.988741847156811	pseudobulk	DESeq2	LRT	0.0533517577859467
239	KC_AB2	CG6330	-2.04390370956279	0.335138268785348	0.988741847156811	pseudobulk	DESeq2	LRT	0.474775978093194
239	KC_AB2	CG6340	0.33884090569448	0.707884376085509	0.988741847156811	pseudobulk	DESeq2	LRT	0.150037673000617
239	KC_AB2	CG6379	-1.4214940075732	0.494339217113562	0.988741847156811	pseudobulk	DESeq2	LRT	0.305974934551539
239	KC_AB2	CG6388	2.41462217514131	0.497446973552313	0.988741847156811	pseudobulk	DESeq2	LRT	0.303253207020768
239	KC_AB2	CG6418	0.475522879236569	0.801582640633501	0.988741847156811	pseudobulk	DESeq2	LRT	0.0960516966149455
239	KC_AB2	CG6420	1.13267300966507	0.729184666160225	0.988741847156811	pseudobulk	DESeq2	LRT	0.137162472589771
239	KC_AB2	CG6424	-1.80294541848982	0.660135549041553	0.988741847156811	pseudobulk	DESeq2	LRT	0.180366879372519
240	KC_AB2	CG6425	-1.80294541848982	0.660135549041553	0.988741847156811	pseudobulk	DESeq2	LRT	0.180366879372519
240	KC_AB2	CG6428	1.7098779479	0.537865353861561	0.988741847156811	pseudobulk	DESeq2	LRT	0.269326429526188
240	KC_AB2	CG6435	-1.80294541848982	0.660135549041553	0.988741847156811	pseudobulk	DESeq2	LRT	0.180366879372519
240	KC_AB2	CG6443	-0.0229973822346953	0.971360658954591	0.988741847156811	pseudobulk	DESeq2	LRT	0.0126194898530182
240	KC_AB2	CG6454	3.10246270279262	0.151201785676372	0.988741847156811	pseudobulk	DESeq2	LRT	0.820443079834711
240	KC_AB2	CG6472	-2.51746470924027	0.517377929599049	0.988741847156811	pseudobulk	DESeq2	LRT	0.286192101436607
240	KC_AB2	CG6481	1.42621218304824	0.66738831402044	0.988741847156811	pseudobulk	DESeq2	LRT	0.175621402117653

240	KC_AB2	CG6495	-0.855747273710406	0.498453319360163	0.988741847156811	pseudobulk	DESeq2	LRT	0.302375507550045
240	KC_AB2	CG6511	-1.80294541848982	0.660135549041553	0.988741847156811	pseudobulk	DESeq2	LRT	0.180366879372519
240	KC_AB2	CG6512	0.10598154425916	0.959628155914924	0.988741847156811	pseudobulk	DESeq2	LRT	0.0178970181249073
241	KC_AB2	CG6523	0.032036803734416	0.974359377332712	0.988741847156811	pseudobulk	DESeq2	LRT	0.0112808307949485
241	KC_AB2	CG6565	2.7785831508444	0.373751317747552	0.988741847156811	pseudobulk	DESeq2	LRT	0.427417267458199
241	KC_AB2	CG6567	-3.76508657848594	0.215111404675873	0.988741847156811	pseudobulk	DESeq2	LRT	0.667336563780367
241	KC_AB2	CG6574	1.12452187615248	0.704078404906088	0.988741847156811	pseudobulk	DESeq2	LRT	0.152378975911753
241	KC_AB2	CG6583	0.474661093301217	0.815951154483998	0.988741847156811	pseudobulk	DESeq2	LRT	0.0883358387620737
241	KC_AB2	CG6607	1.31413323252528	0.624060193048613	0.988741847156811	pseudobulk	DESeq2	LRT	0.204773518894346
241	KC_AB2	CG6610	1.92721521352877	0.471759180353374	0.988741847156811	pseudobulk	DESeq2	LRT	0.326279639784082
241	KC_AB2	CG6617	0.24619866569863	0.866451184773514	0.988741847156811	pseudobulk	DESeq2	LRT	0.0622559000945371
241	KC_AB2	CG6621	-1.73655864349591	0.445098074963394	0.988741847156811	pseudobulk	DESeq2	LRT	0.351544284024786
241	KC_AB2	CG6638	-0.378852672109618	0.920549594954422	0.988741847156811	pseudobulk	DESeq2	LRT	0.0359528087516892
242	KC_AB2	CG6650	-2.51746470924027	0.517377929599049	0.988741847156811	pseudobulk	DESeq2	LRT	0.286192101436607
242	KC_AB2	CG6659	-2.26538931672255	0.547771004842275	0.988741847156811	pseudobulk	DESeq2	LRT	0.2614009599796
242	KC_AB2	CG6664	1.47562839863574	0.571908161489355	0.988741847156811	pseudobulk	DESeq2	LRT	0.242673705753556
242	KC_AB2	CG6665	0.0601466527378941	0.974810960364079	0.988741847156811	pseudobulk	DESeq2	LRT	0.0110795964355292
242	KC_AB2	CG6674	0.10598154425916	0.959628155914924	0.988741847156811	pseudobulk	DESeq2	LRT	0.0178970181249073
242	KC_AB2	CG6685	0.297713224699148	0.901701424115727	0.988741847156811	pseudobulk	DESeq2	LRT	0.0449372444148942
242	KC_AB2	CG6686	0.283887863858921	0.855130150415141	0.988741847156811	pseudobulk	DESeq2	LRT	0.0679677808209954
242	KC_AB2	CG6689	-1.87057166357412	0.515275319421977	0.988741847156811	pseudobulk	DESeq2	LRT	0.28796065881108
242	KC_AB2	CG6693	0.0912892557890945	0.950837602536886	0.988741847156811	pseudobulk	DESeq2	LRT	0.0218936516689727
242	KC_AB2	CG6700	-0.140365054912596	0.754626289851023	0.988741847156811	pseudobulk	DESeq2	LRT	0.122268068816519
243	KC_AB2	CG6707	0.23485568689033	0.785614753103643	0.988741847156811	pseudobulk	DESeq2	LRT	0.104790369504022
243	KC_AB2	CG6712	0.818790875285784	0.940355871560339	0.988741847156811	pseudobulk	DESeq2	LRT	0.0267077593721816
243	KC_AB2	CG6723	1.42621218304824	0.66738831402044	0.988741847156811	pseudobulk	DESeq2	LRT	0.175621402117653
243	KC_AB2	CG6724	2.06137138167525	0.547946929015274	0.988741847156811	pseudobulk	DESeq2	LRT	0.261261502741816
243	KC_AB2	CG6729	2.51243226103394	0.474458247157471	0.988741847156811	pseudobulk	DESeq2	LRT	0.323801999942597
243	KC_AB2	CG6758	-0.522044679843893	0.796310928443557	0.988741847156811	pseudobulk	DESeq2	LRT	0.0989173240449814
243	KC_AB2	CG6762	-0.158601889198964	0.945838126788742	0.988741847156811	pseudobulk	DESeq2	LRT	0.0241831835324482
243	KC_AB2	CG6765	-0.914833674559355	0.361622867230591	0.988741847156811	pseudobulk	DESeq2	LRT	0.441744114706468
243	KC_AB2	CG6766	0.508232850315684	0.818116998464947	0.988741847156811	pseudobulk	DESeq2	LRT	0.0871845836706556
243	KC_AB2	CG6767	-0.185478134298361	0.646850395029479	0.988741847156811	pseudobulk	DESeq2	LRT	0.189196152292794
244	KC_AB2	CG6769	-0.68750020136443	0.784083099060507	0.988741847156811	pseudobulk	DESeq2	LRT	0.105637907276101
244	KC_AB2	CG6770	-0.117267206043637	0.83765940578076	0.988741847156811	pseudobulk	DESeq2	LRT	0.0769325306034292
244	KC_AB2	CG6791	-0.472580216151724	0.802774194135708	0.988741847156811	pseudobulk	DESeq2	LRT	0.095406596728393
244	KC_AB2	CG6805	2.7785831508444	0.373751317747552	0.988741847156811	pseudobulk	DESeq2	LRT	0.427417267458199
244	KC_AB2	CG6808	0.818790875285784	0.940355871560339	0.988741847156811	pseudobulk	DESeq2	LRT	0.0267077593721816
244	KC_AB2	CG6813	-0.506613484034373	0.961658398065406	0.988741847156811	pseudobulk	DESeq2	LRT	0.0169791713942958
244	KC_AB2	CG6833	2.10427674742456	0.422430831487357	0.988741847156811	pseudobulk	DESeq2	LRT	0.374244391951096
244	KC_AB2	CG6841	-1.63785643245857	0.555083135854033	0.988741847156811	pseudobulk	DESeq2	LRT	0.255641966897631
244	KC_AB2	CG6843	0.922664372932389	0.678259639521954	0.988741847156811	pseudobulk	DESeq2	LRT	0.168604025267887
244	KC_AB2	CG6867	0.924208186908538	0.641079039286817	0.988741847156811	pseudobulk	DESeq2	LRT	0.193088422571717
245	KC_AB2	CG6878	-1.39096835157591	0.461763948360687	0.988741847156811	pseudobulk	DESeq2	LRT	0.335579977090568
245	KC_AB2	CG6903	-1.1284597703615	0.729754448571724	0.988741847156811	pseudobulk	DESeq2	LRT	0.136823248879498
245	KC_AB2	CG6923	-0.378852672109618	0.920549594954422	0.988741847156811	pseudobulk	DESeq2	LRT	0.0359528087516892
245	KC_AB2	CG6937	3.06202454821851	0.30627034058063	0.988741847156811	pseudobulk	DESeq2	LRT	0.513895058517773
245	KC_AB2	CG6961	2.41462217514131	0.497446973552313	0.988741847156811	pseudobulk	DESeq2	LRT	0.303253207020768
245	KC_AB2	CG6962	-1.80294541848982	0.660135549041553	0.988741847156811	pseudobulk	DESeq2	LRT	0.180366879372519
245	KC_AB2	CG6966	0.0482587431604983	0.96365703719964	0.988741847156811	pseudobulk	DESeq2	LRT	0.0160775027708529
245	KC_AB2	CG6983	0.24619866569863	0.866451184773514	0.988741847156811	pseudobulk	DESeq2	LRT	0.0622559000945371
245	KC_AB2	CG6984	1.47543920254359	0.661929169513603	0.988741847156811	pseudobulk	DESeq2	LRT	0.17918848025087
245	KC_AB2	CG7006	-1.80294541848982	0.660135549041553	0.988741847156811	pseudobulk	DESeq2	LRT	0.180366879372519
246	KC_AB2	CG7009	-0.357010642654066	0.890177512296031	0.988741847156811	pseudobulk	DESeq2	LRT	0.0505233810814692
246	KC_AB2	CG7011	1.47543920254359	0.661929169513603	0.988741847156811	pseudobulk	DESeq2	LRT	0.17918848025087
246	KC_AB2	CG7028	-0.828298321971602	0.660886958829763	0.988741847156811	pseudobulk	DESeq2	LRT	0.179872817904349

246	KC_AB2	CG7029	0.132804515223556	0.900760068254448	0.988741847156811	pseudobulk	DESeq2	LRT	0.045390874847457
246	KC_AB2	CG7044	-0.506613484034373	0.961658398065406	0.988741847156811	pseudobulk	DESeq2	LRT	0.0169791713942958
246	KC_AB2	CG7049	3.03619069427769	0.314302467188174	0.988741847156811	pseudobulk	DESeq2	LRT	0.502652209876457
246	KC_AB2	CG7058	-0.650352536437416	0.738896630494989	0.988741847156811	pseudobulk	DESeq2	LRT	0.131416313899713
246	KC_AB2	CG7059	0.818790875285784	0.940355871560339	0.988741847156811	pseudobulk	DESeq2	LRT	0.0267077593721816
246	KC_AB2	CG7065	1.54148253642247	0.322362904046297	0.988741847156811	pseudobulk	DESeq2	LRT	0.49165494048643
246	KC_AB2	CG7069	0.818790875285784	0.940355871560339	0.988741847156811	pseudobulk	DESeq2	LRT	0.0267077593721816
247	KC_AB2	CG7083	-0.00340877878248056	0.980029417943268	0.988741847156811	pseudobulk	DESeq2	LRT	0.0087608877170192
247	KC_AB2	CG7099	0.818790875285784	0.940355871560339	0.988741847156811	pseudobulk	DESeq2	LRT	0.0267077593721816
247	KC_AB2	CG7115	0.38419063455275	0.792537746005855	0.988741847156811	pseudobulk	DESeq2	LRT	0.100980044578122
247	KC_AB2	CG7120	0.444811662881181	0.779564417627056	0.988741847156811	pseudobulk	DESeq2	LRT	0.108147992002967
247	KC_AB2	CG7133	-0.405381063532158	0.89155634938256	0.988741847156811	pseudobulk	DESeq2	LRT	0.0498512027381788
247	KC_AB2	CG7137	-3.09794865339079	0.39855598532175	0.988741847156811	pseudobulk	DESeq2	LRT	0.399510664459796
247	KC_AB2	CG7139	0.331758848923756	0.946375065527934	0.988741847156811	pseudobulk	DESeq2	LRT	0.0239367107367223
247	KC_AB2	CG7148	-0.457212305199634	0.858276431637992	0.988741847156811	pseudobulk	DESeq2	LRT	0.0663728130839547
247	KC_AB2	CG7149	-0.488465967391191	0.84251072973523	0.988741847156811	pseudobulk	DESeq2	LRT	0.0744245594950451
247	KC_AB2	CG7154	0.158152772761942	0.908138927325862	0.988741847156811	pseudobulk	DESeq2	LRT	0.0418477079153554
248	KC_AB2	CG7156	0.818790875285784	0.940355871560339	0.988741847156811	pseudobulk	DESeq2	LRT	0.0267077593721816
248	KC_AB2	CG7166	-0.276944268599992	0.745198281388683	0.988741847156811	pseudobulk	DESeq2	LRT	0.127728155364996
248	KC_AB2	CG7168	2.7785831508444	0.373751317747552	0.988741847156811	pseudobulk	DESeq2	LRT	0.427417267458199
248	KC_AB2	CG7182	1.4413042803421	0.615311685084066	0.988741847156811	pseudobulk	DESeq2	LRT	0.210904837358999
248	KC_AB2	CG7183	-0.729234089517982	0.868684292414125	0.988741847156811	pseudobulk	DESeq2	LRT	0.0611380313470854
248	KC_AB2	CG7206	0.100241700199253	0.948596554657455	0.988741847156811	pseudobulk	DESeq2	LRT	0.0229184570570896
248	KC_AB2	CG7218	-2.51746470924027	0.517377929599049	0.988741847156811	pseudobulk	DESeq2	LRT	0.286192101436607
248	KC_AB2	CG7220	0.263173099036933	0.831581687734519	0.988741847156811	pseudobulk	DESeq2	LRT	0.080095082836738
248	KC_AB2	CG7222	-1.45753859454489	0.461481102811837	0.988741847156811	pseudobulk	DESeq2	LRT	0.335846078197274
248	KC_AB2	CG7231	-0.837864416490872	0.544634075637226	0.988741847156811	pseudobulk	DESeq2	LRT	0.263895190028277
249	KC_AB2	CG7239	-1.29600486439944	0.685894431367319	0.988741847156811	pseudobulk	DESeq2	LRT	0.163742723072789
249	KC_AB2	CG7265	3.06202454821851	0.30627034058063	0.988741847156811	pseudobulk	DESeq2	LRT	0.513895058517773
249	KC_AB2	CG7272	-0.0582963981256801	0.979058404810457	0.988741847156811	pseudobulk	DESeq2	LRT	0.0091913999942843
249	KC_AB2	CG7275	0.818790875285784	0.940355871560339	0.988741847156811	pseudobulk	DESeq2	LRT	0.0267077593721816
249	KC_AB2	CG7289	3.06202454821851	0.30627034058063	0.988741847156811	pseudobulk	DESeq2	LRT	0.513895058517773
249	KC_AB2	CG7324	-0.0361964822545474	0.980570086618169	0.988741847156811	pseudobulk	DESeq2	LRT	0.00852135952428676
249	KC_AB2	CG7326	-0.742148798960242	0.80644751100302	0.988741847156811	pseudobulk	DESeq2	LRT	0.0934238941443075
249	KC_AB2	CG7332	0.249791958635574	0.855016136390457	0.988741847156811	pseudobulk	DESeq2	LRT	0.0680256889218813
249	KC_AB2	CG7339	-0.233272055930639	0.937531793764924	0.988741847156811	pseudobulk	DESeq2	LRT	0.028013995469539
249	KC_AB2	CG7368	-0.0860313693258353	0.916951427846865	0.988741847156811	pseudobulk	DESeq2	LRT	0.0376536688848057
250	KC_AB2	CG7369	-2.51746470924027	0.517377929599049	0.988741847156811	pseudobulk	DESeq2	LRT	0.286192101436607
250	KC_AB2	CG7378	0.0912618473955628	0.852801246411394	0.988741847156811	pseudobulk	DESeq2	LRT	0.0691521735740754
250	KC_AB2	CG7379	-0.385733633096415	0.822744264387791	0.988741847156811	pseudobulk	DESeq2	LRT	0.0847351366281377
250	KC_AB2	CG7381	2.42863809728538	0.323713128875623	0.988741847156811	pseudobulk	DESeq2	LRT	0.489839686502244
250	KC_AB2	CG7382	0.0350487159584134	0.959546876645532	0.988741847156811	pseudobulk	DESeq2	LRT	0.0179338038662933
250	KC_AB2	CG7394	-0.415168986466252	0.791831859553787	0.988741847156811	pseudobulk	DESeq2	LRT	0.101367028284691
250	KC_AB2	CG7407	0.249543471998869	0.839196704258384	0.988741847156811	pseudobulk	DESeq2	LRT	0.0761362303974446
250	KC_AB2	CG7414	0.32742703490313	0.878511928385878	0.988741847156811	pseudobulk	DESeq2	LRT	0.0562523373024427
250	KC_AB2	CG7420	-3.09794865339079	0.39855598532175	0.988741847156811	pseudobulk	DESeq2	LRT	0.399510664459796
250	KC_AB2	CG7430	0.204413618835932	0.862339699403994	0.988741847156811	pseudobulk	DESeq2	LRT	0.0643216198889085
251	KC_AB2	CG7453	-2.51746470924027	0.517377929599049	0.988741847156811	pseudobulk	DESeq2	LRT	0.286192101436607
251	KC_AB2	CG7461	0.351722119397094	0.929615296941389	0.988741847156811	pseudobulk	DESeq2	LRT	0.0316967385224016
251	KC_AB2	CG7483	-2.11027789354592	0.473734933668313	0.988741847156811	pseudobulk	DESeq2	LRT	0.32446458879962
251	KC_AB2	CG7484	-0.950691022789824	0.615632481305353	0.988741847156811	pseudobulk	DESeq2	LRT	0.210678474481334
251	KC_AB2	CG7488	1.12452187615248	0.704078404906088	0.988741847156811	pseudobulk	DESeq2	LRT	0.152378975911753
251	KC_AB2	CG7492	0.33164150909617	0.809558444183357	0.988741847156811	pseudobulk	DESeq2	LRT	0.0917517929018211
251	KC_AB2	CG7504	-0.627361101364054	0.786684305786895	0.988741847156811	pseudobulk	DESeq2	LRT	0.104199513842738
251	KC_AB2	CG7506	0.774944809983748	0.637419511817172	0.988741847156811	pseudobulk	DESeq2	LRT	0.195574646628158
251	KC_AB2	CG7518	0.172522961347536	0.933397059180592	0.988741847156811	pseudobulk	DESeq2	LRT	0.0299335717641833

251	KC_AB2	CG7519	-0.382714549144618	0.863954068453144	0.988741847156811	pseudobulk	DESeq2	LRT	0.063509345886375
252	KC_AB2	CG7523	1.44852261274149	0.476489857350802	0.988741847156811	pseudobulk	DESeq2	LRT	0.321946339398079
252	KC_AB2	CG7546	-1.04975342963815	0.540658091788825	0.988741847156811	pseudobulk	DESeq2	LRT	0.267077292732924
252	KC_AB2	CG7550	-0.853565742783243	0.753557841534365	0.988741847156811	pseudobulk	DESeq2	LRT	0.122883406527736
252	KC_AB2	CG7556	0.998893024789477	0.660591418406266	0.988741847156811	pseudobulk	DESeq2	LRT	0.180067072429364
252	KC_AB2	CG7560	0.818790875285784	0.940355871560339	0.988741847156811	pseudobulk	DESeq2	LRT	0.0267077593721816
252	KC_AB2	CG7564	-0.0427483548288203	0.956155951102391	0.988741847156811	pseudobulk	DESeq2	LRT	0.0194712675779106
252	KC_AB2	CG7565	0.572861358867068	0.813871944286787	0.988741847156811	pseudobulk	DESeq2	LRT	0.0894439222171593
252	KC_AB2	CG7568	1.42621218304824	0.66738831402044	0.988741847156811	pseudobulk	DESeq2	LRT	0.175621402117653
252	KC_AB2	CG7582	-0.140436485485279	0.910585395366499	0.988741847156811	pseudobulk	DESeq2	LRT	0.0406793195040027
252	KC_AB2	CG7600	-2.51746470924027	0.517377929599049	0.988741847156811	pseudobulk	DESeq2	LRT	0.286192101436607
253	KC_AB2	CG7601	-0.378852672109618	0.920549594954422	0.988741847156811	pseudobulk	DESeq2	LRT	0.0359528087516892
253	KC_AB2	CG7611	1.39969057416033	0.384008395907805	0.988741847156811	pseudobulk	DESeq2	LRT	0.415659280172654
253	KC_AB2	CG7630	0.329427133653063	0.438799954389449	0.988741847156811	pseudobulk	DESeq2	LRT	0.357733426239559
253	KC_AB2	CG7632	-1.65719613210719	0.599376091584556	0.988741847156811	pseudobulk	DESeq2	LRT	0.222300584546838
253	KC_AB2	CG7637	-0.349038222693293	0.86868347896181	0.988741847156811	pseudobulk	DESeq2	LRT	0.0611384380287992
253	KC_AB2	CG7638	-1.27790246201552	0.547658074219306	0.988741847156811	pseudobulk	DESeq2	LRT	0.261490505067465
253	KC_AB2	CG7639	1.44456584843033	0.488153456050951	0.988741847156811	pseudobulk	DESeq2	LRT	0.311443631604346
253	KC_AB2	CG7646	0.185631187769723	0.622920662942401	0.988741847156811	pseudobulk	DESeq2	LRT	0.205567262876015
253	KC_AB2	CG7650	0.157562008957688	0.950463298513166	0.988741847156811	pseudobulk	DESeq2	LRT	0.0220646484576093
253	KC_AB2	CG7656	1.05886396715538	0.533819336832516	0.988741847156811	pseudobulk	DESeq2	LRT	0.272605698574819
254	KC_AB2	CG7668	1.42621218304824	0.66738831402044	0.988741847156811	pseudobulk	DESeq2	LRT	0.175621402117653
254	KC_AB2	CG7685	1.47543920254359	0.661929169513603	0.988741847156811	pseudobulk	DESeq2	LRT	0.17918848025087
254	KC_AB2	CG7694	0.470427817298873	0.780796750129473	0.988741847156811	pseudobulk	DESeq2	LRT	0.107462002979395
254	KC_AB2	CG7706	0.310390049490412	0.904941705285892	0.988741847156811	pseudobulk	DESeq2	LRT	0.04337939636174
254	KC_AB2	CG7718	0.0601466527378941	0.974810960364079	0.988741847156811	pseudobulk	DESeq2	LRT	0.0110795964355292
254	KC_AB2	CG7737	-1.80294541848982	0.660135549041553	0.988741847156811	pseudobulk	DESeq2	LRT	0.180366879372519
254	KC_AB2	CG7739	0.589477194229043	0.616183890745962	0.988741847156811	pseudobulk	DESeq2	LRT	0.210289659884038
254	KC_AB2	CG7741	-0.506613484034373	0.961658398065406	0.988741847156811	pseudobulk	DESeq2	LRT	0.0169791713942958
254	KC_AB2	CG7745	-1.48046351229221	0.534490457994632	0.988741847156811	pseudobulk	DESeq2	LRT	0.272060043639989
254	KC_AB2	CG7747	3.25449350606273	0.266356678091136	0.988741847156811	pseudobulk	DESeq2	LRT	0.574536410117102
255	KC_AB2	CG7766	0.593062167968284	0.647146248594642	0.988741847156811	pseudobulk	DESeq2	LRT	0.188997562020287
255	KC_AB2	CG7772	2.06137138167525	0.547946929015274	0.988741847156811	pseudobulk	DESeq2	LRT	0.261261502741816
255	KC_AB2	CG7781	0.489189339136293	0.276122389794744	0.988741847156811	pseudobulk	DESeq2	LRT	0.558898376518779
255	KC_AB2	CG7785	-0.40843269200372	0.838644455540079	0.988741847156811	pseudobulk	DESeq2	LRT	0.0764221198909265
255	KC_AB2	CG7789	-3.76508657848594	0.215111404675873	0.988741847156811	pseudobulk	DESeq2	LRT	0.667336563780367
255	KC_AB2	CG7791	0.157562008957688	0.950463298513166	0.988741847156811	pseudobulk	DESeq2	LRT	0.0220646484576093
255	KC_AB2	CG7810	-1.80294541848982	0.660135549041553	0.988741847156811	pseudobulk	DESeq2	LRT	0.180366879372519
255	KC_AB2	CG7834	-0.657952642813821	0.752317290102074	0.988741847156811	pseudobulk	DESeq2	LRT	0.123598956910529
255	KC_AB2	CG7839	0.362555753552128	0.897034383127386	0.988741847156811	pseudobulk	DESeq2	LRT	0.0471909102264623
255	KC_AB2	CG7840	0.157562008957688	0.950463298513166	0.988741847156811	pseudobulk	DESeq2	LRT	0.0220646484576093
256	KC_AB2	CG7841	-2.5242248176406	0.26191093325055	0.988741847156811	pseudobulk	DESeq2	LRT	0.581846371940267
256	KC_AB2	CG7849	-0.729234089517982	0.868684292414125	0.988741847156811	pseudobulk	DESeq2	LRT	0.0611380313470854
256	KC_AB2	CG7857	0.507344003284788	0.810757234451112	0.988741847156811	pseudobulk	DESeq2	LRT	0.09110916739359
256	KC_AB2	CG7872	1.31950274499659	0.590522087411301	0.988741847156811	pseudobulk	DESeq2	LRT	0.228763853746569
256	KC_AB2	CG7878	-0.659744005757546	0.719120576365438	0.988741847156811	pseudobulk	DESeq2	LRT	0.143198284499195
256	KC_AB2	CG7879	0.613806846667423	0.503996152895127	0.988741847156811	pseudobulk	DESeq2	LRT	0.297572778599701
256	KC_AB2	CG7881	0.818790875285784	0.940355871560339	0.988741847156811	pseudobulk	DESeq2	LRT	0.0267077593721816
256	KC_AB2	CG7882	-2.26538931672255	0.547771004842275	0.988741847156811	pseudobulk	DESeq2	LRT	0.2614009599796
256	KC_AB2	CG7889	0.712171366772551	0.813205723536451	0.988741847156811	pseudobulk	DESeq2	LRT	0.0897995733547685
256	KC_AB2	CG7903	0.760546103616116	0.738305064567484	0.988741847156811	pseudobulk	DESeq2	LRT	0.131764152427911
257	KC_AB2	CG7914	-1.80294541848982	0.660135549041553	0.988741847156811	pseudobulk	DESeq2	LRT	0.180366879372519
257	KC_AB2	CG7920	-0.105882057795448	0.913350165264191	0.988741847156811	pseudobulk	DESeq2	LRT	0.0393626883073033
257	KC_AB2	CG7943	-0.213177136262384	0.93155916913195	0.988741847156811	pseudobulk	DESeq2	LRT	0.030789555140895
257	KC_AB2	CG7945	-0.0220546936417288	0.951721659840408	0.988741847156811	pseudobulk	DESeq2	LRT	0.02149004664722
257	KC_AB2	CG7946	0.104637746735554	0.951122131714938	0.988741847156811	pseudobulk	DESeq2	LRT	0.0217637125849166

257	KC_AB2	CG7956	0.204159020535727	0.899850754138013	0.988741847156811	pseudobulk	DESeq2	LRT	0.0458295150374908
257	KC_AB2	CG7970	-0.794177064289352	0.743016342902793	0.988741847156811	pseudobulk	DESeq2	LRT	0.129001633676244
257	KC_AB2	CG7971	0.00107164342603481	0.959386140683393	0.988741847156811	pseudobulk	DESeq2	LRT	0.0180065596539456
257	KC_AB2	CG7974	1.15128682504377	0.719737694898673	0.988741847156811	pseudobulk	DESeq2	LRT	0.142825751365876
257	KC_AB2	CG7985	3.13082827395258	0.16144057530431	0.988741847156811	pseudobulk	DESeq2	LRT	0.791987303466915
258	KC_AB2	CG7987	-1.40296545307325	0.628223627794903	0.988741847156811	pseudobulk	DESeq2	LRT	0.201885733604539
258	KC_AB2	CG7988	0.818790875285784	0.940355871560339	0.988741847156811	pseudobulk	DESeq2	LRT	0.0267077593721816
258	KC_AB2	CG7990	0.625901997247817	0.54499068722555	0.988741847156811	pseudobulk	DESeq2	LRT	0.26361091886301
258	KC_AB2	CG7991	-0.378852672109618	0.920549594954422	0.988741847156811	pseudobulk	DESeq2	LRT	0.0359528087516892
258	KC_AB2	CG8001	-0.506613484034373	0.961658398065406	0.988741847156811	pseudobulk	DESeq2	LRT	0.0169791713942958
258	KC_AB2	CG8003	1.47543920254359	0.661929169513603	0.988741847156811	pseudobulk	DESeq2	LRT	0.17918848025087
258	KC_AB2	CG8004	0.191875519051305	0.915781167268606	0.988741847156811	pseudobulk	DESeq2	LRT	0.0382082918362987
258	KC_AB2	CG8009	0.48020439743879	0.797798185903029	0.988741847156811	pseudobulk	DESeq2	LRT	0.0981069555583927
258	KC_AB2	CG8010	-0.934020934207768	0.782359118258182	0.988741847156811	pseudobulk	DESeq2	LRT	0.106593851444836
258	KC_AB2	CG8031	0.818790875285784	0.940355871560339	0.988741847156811	pseudobulk	DESeq2	LRT	0.0267077593721816
259	KC_AB2	CG8032	1.10527623683615	0.724717602335893	0.988741847156811	pseudobulk	DESeq2	LRT	0.139831190172672
259	KC_AB2	CG8034	1.42621218304824	0.66738831402044	0.988741847156811	pseudobulk	DESeq2	LRT	0.175621402117653
259	KC_AB2	CG8036	-0.233272055930639	0.937531793764924	0.988741847156811	pseudobulk	DESeq2	LRT	0.028013995469539
259	KC_AB2	CG8038	1.94106108959906	0.476420175276185	0.988741847156811	pseudobulk	DESeq2	LRT	0.322009855447888
259	KC_AB2	CG8042	-1.27482208804161	0.504147634250043	0.988741847156811	pseudobulk	DESeq2	LRT	0.297442266428947
259	KC_AB2	CG8043	1.47543920254359	0.661929169513603	0.988741847156811	pseudobulk	DESeq2	LRT	0.17918848025087
259	KC_AB2	CG8060	-3.51218082781652	0.306311589270116	0.988741847156811	pseudobulk	DESeq2	LRT	0.513836571391778
259	KC_AB2	CG8066	2.41462217514131	0.497446973552313	0.988741847156811	pseudobulk	DESeq2	LRT	0.303253207020768
259	KC_AB2	CG8067	0.331758848923756	0.946375065527934	0.988741847156811	pseudobulk	DESeq2	LRT	0.0239367107367223
259	KC_AB2	CG8078	-0.957133960806503	0.762632518785665	0.988741847156811	pseudobulk	DESeq2	LRT	0.1176846802739
260	KC_AB2	CG8079	-0.810783464999901	0.650359881924785	0.988741847156811	pseudobulk	DESeq2	LRT	0.186846256460819
260	KC_AB2	CG8080	2.80917294583258	0.367996267391162	0.988741847156811	pseudobulk	DESeq2	LRT	0.434156586379859
260	KC_AB2	CG8097	-2.51746470924027	0.517377929599049	0.988741847156811	pseudobulk	DESeq2	LRT	0.286192101436607
260	KC_AB2	CG8108	-0.169332201164316	0.784389001133875	0.988741847156811	pseudobulk	DESeq2	LRT	0.105468504729337
260	KC_AB2	CG8111	2.73593896747858	0.422277110754874	0.988741847156811	pseudobulk	DESeq2	LRT	0.374402458575713
260	KC_AB2	CG8128	-3.09794865339079	0.39855598532175	0.988741847156811	pseudobulk	DESeq2	LRT	0.399510664459796
260	KC_AB2	CG8132	2.80496381579643	0.22093644234667	0.988741847156811	pseudobulk	DESeq2	LRT	0.655732643545827
260	KC_AB2	CG8134	0.528102210160941	0.803006295301726	0.988741847156811	pseudobulk	DESeq2	LRT	0.0952810499839783
260	KC_AB2	CG8149	-1.60581231992925	0.486998362385013	0.988741847156811	pseudobulk	DESeq2	LRT	0.312472499172118
260	KC_AB2	CG8155	2.06137138167525	0.547946929015274	0.988741847156811	pseudobulk	DESeq2	LRT	0.261261502741816
261	KC_AB2	CG8173	0.613750852128688	0.739100932111639	0.988741847156811	pseudobulk	DESeq2	LRT	0.131296250013865
261	KC_AB2	CG8176	0.767467537116339	0.648051188696615	0.988741847156811	pseudobulk	DESeq2	LRT	0.18839068843426
261	KC_AB2	CG8177	0.830254658702368	0.383865802781374	0.988741847156811	pseudobulk	DESeq2	LRT	0.415820575886936
261	KC_AB2	CG8187	0.691967660738031	0.689608980590363	0.988741847156811	pseudobulk	DESeq2	LRT	0.161397091447492
261	KC_AB2	CG8188	0.408244312333748	0.680154026452055	0.988741847156811	pseudobulk	DESeq2	LRT	0.167392726612283
261	KC_AB2	CG8195	1.32377927188868	0.350814137985353	0.988741847156811	pseudobulk	DESeq2	LRT	0.454922912668315
261	KC_AB2	CG8202	0.818790875285784	0.940355871560339	0.988741847156811	pseudobulk	DESeq2	LRT	0.0267077593721816
261	KC_AB2	CG8204	-2.26538931672255	0.547771004842275	0.988741847156811	pseudobulk	DESeq2	LRT	0.2614009599796
261	KC_AB2	CG8206	-0.289656389218279	0.857518756894482	0.988741847156811	pseudobulk	DESeq2	LRT	0.0667563716622416
261	KC_AB2	CG8209	-0.97670612739315	0.676315355917219	0.988741847156811	pseudobulk	DESeq2	LRT	0.169850751692797
262	KC_AB2	CG8223	0.779675161849222	0.644733464660634	0.988741847156811	pseudobulk	DESeq2	LRT	0.190619787303604
262	KC_AB2	CG8229	0.172522961347536	0.933397059180592	0.988741847156811	pseudobulk	DESeq2	LRT	0.0299335717641833
262	KC_AB2	CG8230	1.69894780879763	0.540778829726708	0.988741847156811	pseudobulk	DESeq2	LRT	0.26698031838453
262	KC_AB2	CG8237	1.10527623683615	0.724717602335893	0.988741847156811	pseudobulk	DESeq2	LRT	0.139831190172672
262	KC_AB2	CG8243	1.81656312154912	0.330476819996472	0.988741847156811	pseudobulk	DESeq2	LRT	0.480858996997305
262	KC_AB2	CG8245	-0.112136876124128	0.882150326111154	0.988741847156811	pseudobulk	DESeq2	LRT	0.0544574009933401
262	KC_AB2	CG8248	1.24864182465334	0.46524685826473	0.988741847156811	pseudobulk	DESeq2	LRT	0.332316550906272
262	KC_AB2	CG8270	0.818790875285784	0.940355871560339	0.988741847156811	pseudobulk	DESeq2	LRT	0.0267077593721816
262	KC_AB2	CG8272	0.818790875285784	0.940355871560339	0.988741847156811	pseudobulk	DESeq2	LRT	0.0267077593721816
262	KC_AB2	CG8281	1.69894780879763	0.540778829726708	0.988741847156811	pseudobulk	DESeq2	LRT	0.26698031838453
263	KC_AB2	CG8289	0.328504829056507	0.864082951513786	0.988741847156811	pseudobulk	DESeq2	LRT	0.0634445634754604

263	KC_AB2	CG8298	1.47543920254359	0.661929169513603	0.988741847156811	pseudobulk	DESeq2	LRT	0.17918848025087
263	KC_AB2	CG8300	-0.609556974174627	0.603964384869391	0.988741847156811	pseudobulk	DESeq2	LRT	0.218988670502604
263	KC_AB2	CG8301	0.572178477920992	0.692232729700003	0.988741847156811	pseudobulk	DESeq2	LRT	0.159747870525763
263	KC_AB2	CG8311	-0.862332580780999	0.666590188569964	0.988741847156811	pseudobulk	DESeq2	LRT	0.176141082936639
263	KC_AB2	CG8314	1.14471031909633	0.632897194908847	0.988741847156811	pseudobulk	DESeq2	LRT	0.198666829176629
263	KC_AB2	CG8319	-1.69633662497937	0.605069927920179	0.988741847156811	pseudobulk	DESeq2	LRT	0.2181944310419
263	KC_AB2	CG8320	-2.51746470924027	0.517377929599049	0.988741847156811	pseudobulk	DESeq2	LRT	0.286192101436607
263	KC_AB2	CG8321	-1.59087154787651	0.436900982678057	0.988741847156811	pseudobulk	DESeq2	LRT	0.359616978473819
263	KC_AB2	CG8323	-1.80294541848982	0.660135549041553	0.988741847156811	pseudobulk	DESeq2	LRT	0.180366879372519
264	KC_AB2	CG8326	-3.4227037205893	0.285801360563088	0.988741847156811	pseudobulk	DESeq2	LRT	0.543935708072568
264	KC_AB2	CG8336	2.43796147164888	0.320874079934739	0.988741847156811	pseudobulk	DESeq2	LRT	0.493665363602429
264	KC_AB2	CG8353	0.667839000613305	0.81413342949896	0.988741847156811	pseudobulk	DESeq2	LRT	0.0893044121300904
264	KC_AB2	CG8360	-0.68750020136443	0.784083099060507	0.988741847156811	pseudobulk	DESeq2	LRT	0.105637907276101
264	KC_AB2	CG8366	1.7098779479	0.537865353861561	0.988741847156811	pseudobulk	DESeq2	LRT	0.269326429526188
264	KC_AB2	CG8369	-1.65719613210719	0.599376091584556	0.988741847156811	pseudobulk	DESeq2	LRT	0.222300584546838
264	KC_AB2	CG8370	-2.9721553030081	0.387854083811327	0.988741847156811	pseudobulk	DESeq2	LRT	0.411331631401987
264	KC_AB2	CG8372	0.222008486804317	0.861032622987513	0.988741847156811	pseudobulk	DESeq2	LRT	0.0649803935927369
264	KC_AB2	CG8379	2.80917294583258	0.367996267391162	0.988741847156811	pseudobulk	DESeq2	LRT	0.434156586379859
264	KC_AB2	CG8389	0.104637746735554	0.951122131714938	0.988741847156811	pseudobulk	DESeq2	LRT	0.0217637125849166
265	KC_AB2	CG8397	0.167165629946856	0.902635884466227	0.988741847156811	pseudobulk	DESeq2	LRT	0.0444874050105513
265	KC_AB2	CG8405	0.768649457246436	0.690270902888434	0.988741847156811	pseudobulk	DESeq2	LRT	0.160980433118035
265	KC_AB2	CG8412	3.25449350606273	0.266356678091136	0.988741847156811	pseudobulk	DESeq2	LRT	0.574536410117102
265	KC_AB2	CG8414	0.712171366772551	0.813205723536451	0.988741847156811	pseudobulk	DESeq2	LRT	0.0897995733547685
265	KC_AB2	CG8435	0.699674038439951	0.821690393783994	0.988741847156811	pseudobulk	DESeq2	LRT	0.0852917902455435
265	KC_AB2	CG8441	-0.568233637794205	0.868826397133536	0.988741847156811	pseudobulk	DESeq2	LRT	0.0610669925949191
265	KC_AB2	CG8475	-0.718003415132797	0.760638816665156	0.988741847156811	pseudobulk	DESeq2	LRT	0.118821515588473
265	KC_AB2	CG8485	1.23525746788534	0.322899359177664	0.988741847156811	pseudobulk	DESeq2	LRT	0.490932816877793
265	KC_AB2	CG8490	-0.186211564389806	0.913968884328821	0.988741847156811	pseudobulk	DESeq2	LRT	0.0390685893803034
265	KC_AB2	CG8500	0.138015239609351	0.830043256551629	0.988741847156811	pseudobulk	DESeq2	LRT	0.0808992743803539
266	KC_AB2	CG8507	-1.226194541513	0.455567054772564	0.988741847156811	pseudobulk	DESeq2	LRT	0.341447690316864
266	KC_AB2	CG8509	-1.80294541848982	0.660135549041553	0.988741847156811	pseudobulk	DESeq2	LRT	0.180366879372519
266	KC_AB2	CG8516	-0.506613484034373	0.961658398065406	0.988741847156811	pseudobulk	DESeq2	LRT	0.0169791713942958
266	KC_AB2	CG8519	-1.80933943727629	0.584587525761637	0.988741847156811	pseudobulk	DESeq2	LRT	0.233150456085471
266	KC_AB2	CG8520	-3.4227037205893	0.285801360563088	0.988741847156811	pseudobulk	DESeq2	LRT	0.543935708072568
266	KC_AB2	CG8531	2.06137138167525	0.547946929015274	0.988741847156811	pseudobulk	DESeq2	LRT	0.261261502741816
266	KC_AB2	CG8549	-3.09794865339079	0.39855598532175	0.988741847156811	pseudobulk	DESeq2	LRT	0.399510664459796
266	KC_AB2	CG8569	-2.26538931672255	0.547771004842275	0.988741847156811	pseudobulk	DESeq2	LRT	0.2614009599796
266	KC_AB2	CG8578	0.747259712542553	0.545701441935715	0.988741847156811	pseudobulk	DESeq2	LRT	0.263044898656112
266	KC_AB2	CG8611	1.47543920254359	0.661929169513603	0.988741847156811	pseudobulk	DESeq2	LRT	0.17918848025087
267	KC_AB2	CG8613	-0.978528018992097	0.433541261762505	0.988741847156811	pseudobulk	DESeq2	LRT	0.3629695627714
267	KC_AB2	CG8617	0.247806629114718	0.846700811892383	0.988741847156811	pseudobulk	DESeq2	LRT	0.0722700237877964
267	KC_AB2	CG8635	-1.66893953471286	0.358983023020408	0.988741847156811	pseudobulk	DESeq2	LRT	0.444926089535595
267	KC_AB2	CG8641	2.47759074909051	0.372167924980709	0.988741847156811	pseudobulk	DESeq2	LRT	0.429261058943407
267	KC_AB2	CG8668	-0.395875282423059	0.723234498161534	0.988741847156811	pseudobulk	DESeq2	LRT	0.140720866266299
267	KC_AB2	CG8671	-0.196978849876294	0.825580628056752	0.988741847156811	pseudobulk	DESeq2	LRT	0.0832405061562546
267	KC_AB2	CG8673	0.0601466527378941	0.974810960364079	0.988741847156811	pseudobulk	DESeq2	LRT	0.0110795964355292
267	KC_AB2	CG8675	0.0972124918502767	0.969738987055936	0.988741847156811	pseudobulk	DESeq2	LRT	0.0133451438114273
267	KC_AB2	CG8677	0.0453652090560972	0.941863031820831	0.988741847156811	pseudobulk	DESeq2	LRT	0.026012248851918
267	KC_AB2	CG8712	-0.233272055930639	0.937531793764924	0.988741847156811	pseudobulk	DESeq2	LRT	0.028013995469539
268	KC_AB2	CG8726	-0.0622783686004377	0.925447812242618	0.988741847156811	pseudobulk	DESeq2	LRT	0.0336480669101036
268	KC_AB2	CG8728	0.315515278988381	0.901389738439351	0.988741847156811	pseudobulk	DESeq2	LRT	0.0450873903126698
268	KC_AB2	CG8745	-1.92150817035771	0.457631232406761	0.988741847156811	pseudobulk	DESeq2	LRT	0.339484343482859
268	KC_AB2	CG8757	-1.80294541848982	0.660135549041553	0.988741847156811	pseudobulk	DESeq2	LRT	0.180366879372519
268	KC_AB2	CG8765	1.0821173077982	0.758638591992449	0.988741847156811	pseudobulk	DESeq2	LRT	0.119965068483926
268	KC_AB2	CG8768	0.666943671827768	0.808936122914367	0.988741847156811	pseudobulk	DESeq2	LRT	0.0920857707994393
268	KC_AB2	CG8777	-0.506613484034373	0.961658398065406	0.988741847156811	pseudobulk	DESeq2	LRT	0.0169791713942958

268	KC_AB2	CG8778	1.32714918892411	0.592713712963247	0.988741847156811	pseudobulk	DESeq2	LRT	0.227155024852469
268	KC_AB2	CG8814	0.00266298162891975	0.968632759616684	0.988741847156811	pseudobulk	DESeq2	LRT	0.0138408469969281
268	KC_AB2	CG8818	0.48761912772513	0.718899900147014	0.988741847156811	pseudobulk	DESeq2	LRT	0.143331576710629
269	KC_AB2	CG8833	-0.568233637794205	0.868826397133536	0.988741847156811	pseudobulk	DESeq2	LRT	0.0610669925949191
269	KC_AB2	CG8839	1.69894780879763	0.540778829726708	0.988741847156811	pseudobulk	DESeq2	LRT	0.26698031838453
269	KC_AB2	CG8850	-1.80294541848982	0.660135549041553	0.988741847156811	pseudobulk	DESeq2	LRT	0.180366879372519
269	KC_AB2	CG8860	0.816815757636112	0.564493052070233	0.988741847156811	pseudobulk	DESeq2	LRT	0.248341399117451
269	KC_AB2	CG8878	1.32043022106922	0.348718341355784	0.988741847156811	pseudobulk	DESeq2	LRT	0.457525209664103
269	KC_AB2	CG8888	0.818790875285784	0.940355871560339	0.988741847156811	pseudobulk	DESeq2	LRT	0.0267077593721816
269	KC_AB2	CG8891	-0.908909516750057	0.671937973991072	0.988741847156811	pseudobulk	DESeq2	LRT	0.172670814441211
269	KC_AB2	CG8892	1.6341263262903	0.579948075372967	0.988741847156811	pseudobulk	DESeq2	LRT	0.236610888486163
269	KC_AB2	CG8910	-0.354136367087136	0.550669604833278	0.988741847156811	pseudobulk	DESeq2	LRT	0.259108894496542
269	KC_AB2	CG8915	1.47543920254359	0.661929169513603	0.988741847156811	pseudobulk	DESeq2	LRT	0.17918848025087
27	KC_AB2	CG8924	-0.752984140847189	0.699848618405734	0.988741847156811	pseudobulk	DESeq2	LRT	0.15499589041571
27	KC_AB2	CG8929	0.653880280753836	0.4744658572049	0.988741847156811	pseudobulk	DESeq2	LRT	0.323795034155576
27	KC_AB2	CG8939	0.818790875285784	0.940355871560339	0.988741847156811	pseudobulk	DESeq2	LRT	0.0267077593721816
27	KC_AB2	CG8944	2.41462217514131	0.497446973552313	0.988741847156811	pseudobulk	DESeq2	LRT	0.303253207020768
27	KC_AB2	CG8963	-0.190371847884524	0.906978045889847	0.988741847156811	pseudobulk	DESeq2	LRT	0.0424032252489335
27	KC_AB2	CG8974	0.145583695493984	0.888891033083402	0.988741847156811	pseudobulk	DESeq2	LRT	0.0511514748353189
27	KC_AB2	CG8993	0.464788083613466	0.618284401486032	0.988741847156811	pseudobulk	DESeq2	LRT	0.208811710049718
27	KC_AB2	CG9003	-0.121513111208203	0.903950352540449	0.988741847156811	pseudobulk	DESeq2	LRT	0.0438554215266553
27	KC_AB2	CG9004	2.4643417141186	0.449082701910884	0.988741847156811	pseudobulk	DESeq2	LRT	0.347673673080982
27	KC_AB2	CG9005	0.0499324222838045	0.944692067327935	0.988741847156811	pseudobulk	DESeq2	LRT	0.0247097314418978
271	KC_AB2	CG9008	0.183857755803646	0.831189480771979	0.988741847156811	pseudobulk	DESeq2	LRT	0.080299961687154
271	KC_AB2	CG9018	0.451878341743173	0.8022111819877	0.988741847156811	pseudobulk	DESeq2	LRT	0.0957112886997858
271	KC_AB2	CG9034	0.325267076890603	0.679694045287307	0.988741847156811	pseudobulk	DESeq2	LRT	0.167686534859542
271	KC_AB2	CG9044	0.961828407721153	0.584174465909025	0.988741847156811	pseudobulk	DESeq2	LRT	0.233457429829368
271	KC_AB2	CG9062	-0.233272055930639	0.937531793764924	0.988741847156811	pseudobulk	DESeq2	LRT	0.028013995469539
271	KC_AB2	CG9065	-0.0780716419649062	0.925549019146697	0.988741847156811	pseudobulk	DESeq2	LRT	0.0336005750945907
271	KC_AB2	CG9067	-0.506613484034373	0.961658398065406	0.988741847156811	pseudobulk	DESeq2	LRT	0.0169791713942958
271	KC_AB2	CG9098	-2.26538931672255	0.547771004842275	0.988741847156811	pseudobulk	DESeq2	LRT	0.2614009599796
271	KC_AB2	CG9107	-1.65719613210719	0.599376091584556	0.988741847156811	pseudobulk	DESeq2	LRT	0.222300584546838
271	KC_AB2	CG9121	0.157562008957688	0.950463298513166	0.988741847156811	pseudobulk	DESeq2	LRT	0.0220646484576093
272	KC_AB2	CG9123	-0.502584436694424	0.711741493934019	0.988741847156811	pseudobulk	DESeq2	LRT	0.14767771442982
272	KC_AB2	CG9125	0.818790875285784	0.940355871560339	0.988741847156811	pseudobulk	DESeq2	LRT	0.0267077593721816
272	KC_AB2	CG9132	-0.0823065709591569	0.935198379313832	0.988741847156811	pseudobulk	DESeq2	LRT	0.0290962544720392
272	KC_AB2	CG9135	1.47543920254359	0.661929169513603	0.988741847156811	pseudobulk	DESeq2	LRT	0.17918848025087
272	KC_AB2	CG9143	0.351722119397094	0.929615296941389	0.988741847156811	pseudobulk	DESeq2	LRT	0.0316967385224016
272	KC_AB2	CG9147	0.0601466527378941	0.974810960364079	0.988741847156811	pseudobulk	DESeq2	LRT	0.0110795964355292
272	KC_AB2	CG9149	1.91626857490363	0.477198801200827	0.988741847156811	pseudobulk	DESeq2	LRT	0.321300656017034
272	KC_AB2	CG9154	2.30572175897729	0.372975818883078	0.988741847156811	pseudobulk	DESeq2	LRT	0.428319323864415
272	KC_AB2	CG9164	0.581445489707027	0.811994604428781	0.988741847156811	pseudobulk	DESeq2	LRT	0.0904468565649727
272	KC_AB2	CG9170	0.289751549838805	0.763861179352717	0.988741847156811	pseudobulk	DESeq2	LRT	0.116985560951909
273	KC_AB2	CG9171	-0.233338598315786	0.712155127972572	0.988741847156811	pseudobulk	DESeq2	LRT	0.147425394166172
273	KC_AB2	CG9175	2.30572175897729	0.372975818883078	0.988741847156811	pseudobulk	DESeq2	LRT	0.428319323864415
273	KC_AB2	CG9205	0.693809979578311	0.690053159116761	0.988741847156811	pseudobulk	DESeq2	LRT	0.161117451550002
273	KC_AB2	CG9213	-4.21515119874888	0.130841174999422	0.988741847156811	pseudobulk	DESeq2	LRT	0.883255564413504
273	KC_AB2	CG9215	2.7785831508444	0.373751317747552	0.988741847156811	pseudobulk	DESeq2	LRT	0.427417267458199
273	KC_AB2	CG9231	1.53430585779125	0.273550042862986	0.988741847156811	pseudobulk	DESeq2	LRT	0.562963212836222
273	KC_AB2	CG9240	0.666943671827768	0.808936122914367	0.988741847156811	pseudobulk	DESeq2	LRT	0.0920857707994393
273	KC_AB2	CG9246	-0.378852672109618	0.920549594954422	0.988741847156811	pseudobulk	DESeq2	LRT	0.0359528087516892
273	KC_AB2	CG9253	-0.405381063532158	0.89155634938256	0.988741847156811	pseudobulk	DESeq2	LRT	0.0498512027381788
273	KC_AB2	CG9257	0.804708302235815	0.733338290589079	0.988741847156811	pseudobulk	DESeq2	LRT	0.13469563812263
274	KC_AB2	CG9265	-0.126113705570819	0.910733020209729	0.988741847156811	pseudobulk	DESeq2	LRT	0.0406089170368683
274	KC_AB2	CG9281	0.403235505047614	0.759975897062216	0.988741847156811	pseudobulk	DESeq2	LRT	0.119200181322985
274	KC_AB2	CG9286	-2.26538931672255	0.547771004842275	0.988741847156811	pseudobulk	DESeq2	LRT	0.2614009599796

274	KC_AB2	CG9302	-0.506613484034373	0.961658398065406	0.988741847156811	pseudobulk	DESeq2	LRT	0.0169791713942958
274	KC_AB2	CG9304	-0.378852672109618	0.920549594954422	0.988741847156811	pseudobulk	DESeq2	LRT	0.0359528087516892
274	KC_AB2	CG9328	-0.0865597003766327	0.894964145077352	0.988741847156811	pseudobulk	DESeq2	LRT	0.0481943634628289
274	KC_AB2	CG9330	0.475213242523281	0.812738132485219	0.988741847156811	pseudobulk	DESeq2	LRT	0.0900493633068432
274	KC_AB2	CG9331	1.47543920254359	0.661929169513603	0.988741847156811	pseudobulk	DESeq2	LRT	0.17918848025087
274	KC_AB2	CG9344	-0.217915120660429	0.892149991826569	0.988741847156811	pseudobulk	DESeq2	LRT	0.0495621241592452
274	KC_AB2	CG9346	1.91626857490363	0.477198801200827	0.988741847156811	pseudobulk	DESeq2	LRT	0.321300656017034
275	KC_AB2	CG9356	-0.0582963981256801	0.979058404810457	0.988741847156811	pseudobulk	DESeq2	LRT	0.0091913999942843
275	KC_AB2	CG9368	0.206464292795832	0.81147350068598	0.988741847156811	pseudobulk	DESeq2	LRT	0.0907256578386866
275	KC_AB2	CG9384	0.191875519051305	0.915781167268606	0.988741847156811	pseudobulk	DESeq2	LRT	0.0382082918362987
275	KC_AB2	CG9386	-1.80294541848982	0.660135549041553	0.988741847156811	pseudobulk	DESeq2	LRT	0.180366879372519
275	KC_AB2	CG9391	0.751627904349286	0.521620034545433	0.988741847156811	pseudobulk	DESeq2	LRT	0.282645736437498
275	KC_AB2	CG9393	0.310390049490412	0.904941705285892	0.988741847156811	pseudobulk	DESeq2	LRT	0.04337939636174
275	KC_AB2	CG9395	-2.95479271295451	0.247561026589529	0.988741847156811	pseudobulk	DESeq2	LRT	0.606317725037055
275	KC_AB2	CG9399	1.85714160457615	0.510506350526785	0.988741847156811	pseudobulk	DESeq2	LRT	0.29199885106662
275	KC_AB2	CG9410	-0.104804761051873	0.946661076019174	0.988741847156811	pseudobulk	DESeq2	LRT	0.0238054794555745
275	KC_AB2	CG9422	-0.246577964598332	0.865188932983435	0.988741847156811	pseudobulk	DESeq2	LRT	0.0628890444513524
276	KC_AB2	CG9425	-0.172098537409086	0.82384942507754	0.988741847156811	pseudobulk	DESeq2	LRT	0.0841521570329687
276	KC_AB2	CG9426	0.363641268105473	0.858330168669459	0.988741847156811	pseudobulk	DESeq2	LRT	0.06634562258353
276	KC_AB2	CG9436	3.09926389624515	0.339712049344771	0.988741847156811	pseudobulk	DESeq2	LRT	0.468889048740397
276	KC_AB2	CG9467	0.581445489707027	0.811994604428781	0.988741847156811	pseudobulk	DESeq2	LRT	0.0904468565649727
276	KC_AB2	CG9471	0.818790875285784	0.940355871560339	0.988741847156811	pseudobulk	DESeq2	LRT	0.0267077593721816
276	KC_AB2	CG9485	-0.506613484034373	0.961658398065406	0.988741847156811	pseudobulk	DESeq2	LRT	0.0169791713942958
276	KC_AB2	CG9515	0.818790875285784	0.940355871560339	0.988741847156811	pseudobulk	DESeq2	LRT	0.0267077593721816
276	KC_AB2	CG9518	-3.4227037205893	0.285801360563088	0.988741847156811	pseudobulk	DESeq2	LRT	0.543935708072568
276	KC_AB2	CG9527	2.00515076565275	0.576456787459964	0.988741847156811	pseudobulk	DESeq2	LRT	0.239233242872451
276	KC_AB2	CG9536	0.297713224699148	0.901701424115727	0.988741847156811	pseudobulk	DESeq2	LRT	0.0449372444148942
277	KC_AB2	CG9577	-1.80294541848982	0.660135549041553	0.988741847156811	pseudobulk	DESeq2	LRT	0.180366879372519
277	KC_AB2	CG9578	-1.40296545307325	0.628223627794903	0.988741847156811	pseudobulk	DESeq2	LRT	0.201885733604539
277	KC_AB2	CG9581	1.4413042803421	0.615311685084066	0.988741847156811	pseudobulk	DESeq2	LRT	0.210904837358999
277	KC_AB2	CG9586	-0.933299237375886	0.70112757174831	0.988741847156811	pseudobulk	DESeq2	LRT	0.154202953979679
277	KC_AB2	CG9588	-2.64843360157809	0.321943703667444	0.988741847156811	pseudobulk	DESeq2	LRT	0.492220064093287
277	KC_AB2	CG9590	-1.37054505049545	0.574189269529552	0.988741847156811	pseudobulk	DESeq2	LRT	0.240944927875125
277	KC_AB2	CG9596	1.47543920254359	0.661929169513603	0.988741847156811	pseudobulk	DESeq2	LRT	0.17918848025087
277	KC_AB2	CG9601	1.12452187615248	0.704078404906088	0.988741847156811	pseudobulk	DESeq2	LRT	0.152378975911753
277	KC_AB2	CG9609	1.40398209932659	0.389272992959481	0.988741847156811	pseudobulk	DESeq2	LRT	0.409745725784866
277	KC_AB2	CG9616	0.818790875285784	0.940355871560339	0.988741847156811	pseudobulk	DESeq2	LRT	0.0267077593721816
278	KC_AB2	CG9630	1.42621218304824	0.66738831402044	0.988741847156811	pseudobulk	DESeq2	LRT	0.175621402117653
278	KC_AB2	CG9636	1.09844784868719	0.562810824123525	0.988741847156811	pseudobulk	DESeq2	LRT	0.249637558693024
278	KC_AB2	CG9641	0.507344003284788	0.810757234451112	0.988741847156811	pseudobulk	DESeq2	LRT	0.09110916739359
278	KC_AB2	CG9643	0.818790875285784	0.940355871560339	0.988741847156811	pseudobulk	DESeq2	LRT	0.0267077593721816
278	KC_AB2	CG9646	-0.825309818798736	0.290344727513739	0.988741847156811	pseudobulk	DESeq2	LRT	0.537086056086588
278	KC_AB2	CG9650	-2.51746470924027	0.517377929599049	0.988741847156811	pseudobulk	DESeq2	LRT	0.286192101436607
278	KC_AB2	CG9657	1.42621218304824	0.66738831402044	0.988741847156811	pseudobulk	DESeq2	LRT	0.175621402117653
278	KC_AB2	CG9662	0.362874457852021	0.879075667294383	0.988741847156811	pseudobulk	DESeq2	LRT	0.0559737409966935
278	KC_AB2	CG9667	-0.488465967391191	0.84251072973523	0.988741847156811	pseudobulk	DESeq2	LRT	0.0744245594950451
278	KC_AB2	CG9674	0.477154182776544	0.45950186266697	0.988741847156811	pseudobulk	DESeq2	LRT	0.337712723789628
279	KC_AB2	CG9705	-0.40843269200372	0.838644455540079	0.988741847156811	pseudobulk	DESeq2	LRT	0.0764221198909265
279	KC_AB2	CG9723	-0.0323203545809474	0.954448156132187	0.988741847156811	pseudobulk	DESeq2	LRT	0.020247656706879
279	KC_AB2	CG9727	2.03652262118693	0.452598323593784	0.988741847156811	pseudobulk	DESeq2	LRT	0.344287058987824
279	KC_AB2	CG9743	-1.80294541848982	0.660135549041553	0.988741847156811	pseudobulk	DESeq2	LRT	0.180366879372519
279	KC_AB2	CG9760	0.157562008957688	0.950463298513166	0.988741847156811	pseudobulk	DESeq2	LRT	0.0220646484576093
279	KC_AB2	CG9766	-1.80294541848982	0.660135549041553	0.988741847156811	pseudobulk	DESeq2	LRT	0.180366879372519
279	KC_AB2	CG9773	0.32742703490313	0.878511928385878	0.988741847156811	pseudobulk	DESeq2	LRT	0.0562523373024427
279	KC_AB2	CG9775	-0.234440600061512	0.774633289110377	0.988741847156811	pseudobulk	DESeq2	LRT	0.110903843566956
279	KC_AB2	CG9776	-0.331904876487713	0.763936593751846	0.988741847156811	pseudobulk	DESeq2	LRT	0.11694268609242

279	KC_AB2	CG9782	-1.80294541848982	0.660135549041553	0.988741847156811	pseudobulk	DESeq2	LRT	0.180366879372519
280	KC_AB2	CG9784	-0.717118992470045	0.653956742771855	0.988741847156811	pseudobulk	DESeq2	LRT	0.184450977970538
280	KC_AB2	CG9795	-0.302067522850666	0.813582259571057	0.988741847156811	pseudobulk	DESeq2	LRT	0.0895985299161822
280	KC_AB2	CG9799	2.80917294583258	0.367996267391162	0.988741847156811	pseudobulk	DESeq2	LRT	0.434156586379859
280	KC_AB2	CG9801	0.666943671827768	0.808936122914367	0.988741847156811	pseudobulk	DESeq2	LRT	0.0920857707994393
280	KC_AB2	CG9804	-0.568233637794205	0.868826397133536	0.988741847156811	pseudobulk	DESeq2	LRT	0.0610669925949191
280	KC_AB2	CG9813	0.561734498182309	0.209742374517763	0.988741847156811	pseudobulk	DESeq2	LRT	0.678313819605615
280	KC_AB2	CG9837	-0.596583188746717	0.671040548971783	0.988741847156811	pseudobulk	DESeq2	LRT	0.17325123592249
280	KC_AB2	CG9839	-0.233272055930639	0.937531793764924	0.988741847156811	pseudobulk	DESeq2	LRT	0.028013995469539
280	KC_AB2	CG9849	0.884804879663301	0.704032768176767	0.988741847156811	pseudobulk	DESeq2	LRT	0.15240712678521
280	KC_AB2	CG9853	1.20672691757874	0.527673436745763	0.988741847156811	pseudobulk	DESeq2	LRT	0.277634767799571
281	KC_AB2	CG9855	0.315515278988381	0.901389738439351	0.988741847156811	pseudobulk	DESeq2	LRT	0.0450873903126698
281	KC_AB2	CG9864	-1.80294541848982	0.660135549041553	0.988741847156811	pseudobulk	DESeq2	LRT	0.180366879372519
281	KC_AB2	CG9886	-1.80294541848982	0.660135549041553	0.988741847156811	pseudobulk	DESeq2	LRT	0.180366879372519
281	KC_AB2	CG9890	0.0601466527378941	0.974810960364079	0.988741847156811	pseudobulk	DESeq2	LRT	0.0110795964355292
281	KC_AB2	CG9902	1.47543920254359	0.661929169513603	0.988741847156811	pseudobulk	DESeq2	LRT	0.17918848025087
281	KC_AB2	CG9911	0.455966658845175	0.791510891453656	0.988741847156811	pseudobulk	DESeq2	LRT	0.101543104723699
281	KC_AB2	CG9915	-0.0265683699975436	0.949703263057826	0.988741847156811	pseudobulk	DESeq2	LRT	0.0224120698138033
281	KC_AB2	CG9917	0.481592370266986	0.722751409016093	0.988741847156811	pseudobulk	DESeq2	LRT	0.141011052992043
281	KC_AB2	CG9919	0.830936672127112	0.535413480381358	0.988741847156811	pseudobulk	DESeq2	LRT	0.271310698555256
281	KC_AB2	CG9921	1.32072338097357	0.519646064326127	0.988741847156811	pseudobulk	DESeq2	LRT	0.284292357606853
282	KC_AB2	CG9922	-1.48046351229221	0.534490457994632	0.988741847156811	pseudobulk	DESeq2	LRT	0.272060043639989
282	KC_AB2	CG9932	0.229908221754888	0.860103171165043	0.988741847156811	pseudobulk	DESeq2	LRT	0.0654494511049955
282	KC_AB2	CG9934	-0.50879048068994	0.817628596116567	0.988741847156811	pseudobulk	DESeq2	LRT	0.0874439277298491
282	KC_AB2	CG9941	0.362555753552128	0.897034383127386	0.988741847156811	pseudobulk	DESeq2	LRT	0.0471909102264623
282	KC_AB2	CG9945	1.42034337486757	0.643720050244361	0.988741847156811	pseudobulk	DESeq2	LRT	0.191302963490459
282	KC_AB2	CG9948	0.699674038439951	0.821690393783994	0.988741847156811	pseudobulk	DESeq2	LRT	0.0852917902455435
282	KC_AB2	CG9951	1.42621218304824	0.66738831402044	0.988741847156811	pseudobulk	DESeq2	LRT	0.175621402117653
282	KC_AB2	CG9953	0.0742413024401475	0.943070327868629	0.988741847156811	pseudobulk	DESeq2	LRT	0.0254559192813408
282	KC_AB2	CG9967	0.818790875285784	0.940355871560339	0.988741847156811	pseudobulk	DESeq2	LRT	0.0267077593721816
282	KC_AB2	CG9986	-0.588639054357632	0.8432408255636	0.988741847156811	pseudobulk	DESeq2	LRT	0.0740483752247712
283	KC_AB2	CG9992	0.630543148119245	0.748402447226467	0.988741847156811	pseudobulk	DESeq2	LRT	0.125864801055495
283	KC_AB2	Charon	0.712171366772551	0.813205723536451	0.988741847156811	pseudobulk	DESeq2	LRT	0.0897995733547685
283	KC_AB2	ChAT	-0.357811422665239	0.719961090183378	0.988741847156811	pseudobulk	DESeq2	LRT	0.142690974089942
283	KC_AB2	chb	-0.745050649235211	0.610776378802451	0.988741847156811	pseudobulk	DESeq2	LRT	0.2141177672183
283	KC_AB2	Chc	-0.893079614170889	0.342288245586094	0.988741847156811	pseudobulk	DESeq2	LRT	0.465608014577428
283	KC_AB2	Chchd2	-0.00531908052595913	0.970495551088504	0.988741847156811	pseudobulk	DESeq2	LRT	0.0130064511485106
283	KC_AB2	Chchd3	-0.025899326732957	0.97159641150457	0.988741847156811	pseudobulk	DESeq2	LRT	0.0125140978877405
283	KC_AB2	Chd1	0.392141861029183	0.769123241326674	0.988741847156811	pseudobulk	DESeq2	LRT	0.114004064958352
283	KC_AB2	Chd64	1.45259040912931	0.616176917291092	0.988741847156811	pseudobulk	DESeq2	LRT	0.210294574894236
283	KC_AB2	CheB42c	0.353798809751289	0.901648351639924	0.988741847156811	pseudobulk	DESeq2	LRT	0.0449628069360408
284	KC_AB2	CHES-1-like	0.790076644042166	0.413573089679328	0.988741847156811	pseudobulk	DESeq2	LRT	0.383447727612349
284	KC_AB2	Chi	-1.22754603072947	0.488648268115683	0.988741847156811	pseudobulk	DESeq2	LRT	0.311003636132682
284	KC_AB2	chic	1.30993299556956	0.220921408374436	0.988741847156811	pseudobulk	DESeq2	LRT	0.655762196811314
284	KC_AB2	chico	-4.87269593419189	0.0485583253200299	0.988741847156811	pseudobulk	DESeq2	LRT	1.31373629962661
284	KC_AB2	chif	0.761437443812382	0.602701175671061	0.988741847156811	pseudobulk	DESeq2	LRT	0.219897961366075
284	KC_AB2	chinmo	-0.20556234902979	0.603755068086808	0.988741847156811	pseudobulk	DESeq2	LRT	0.21913921063729
284	KC_AB2	chm	0.353798809751289	0.901648351639924	0.988741847156811	pseudobulk	DESeq2	LRT	0.0449628069360408
284	KC_AB2	Chmp1	-0.258430114643636	0.8328142689009	0.988741847156811	pseudobulk	DESeq2	LRT	0.0794518425130863
284	KC_AB2	CHMP2B	-0.723394771468922	0.738748113587962	0.988741847156811	pseudobulk	DESeq2	LRT	0.131503615091243
284	KC_AB2	chn	-0.0543789453254522	0.893961700570565	0.988741847156811	pseudobulk	DESeq2	LRT	0.048681087006277
285	KC_AB2	CHOp24	-0.11454387205324	0.926484615561739	0.988741847156811	pseudobulk	DESeq2	LRT	0.0331617878206546
285	KC_AB2	CHORD	1.42621218304824	0.66738831402044	0.988741847156811	pseudobulk	DESeq2	LRT	0.175621402117653
285	KC_AB2	Chrac-16	0.297713224699148	0.901701424115727	0.988741847156811	pseudobulk	DESeq2	LRT	0.0449372444148942
285	KC_AB2	chrb	-0.498393307242753	0.117474891667332	0.988741847156811	pseudobulk	DESeq2	LRT	0.9300549468013
285	KC_AB2	ChT	0.354959680977432	0.633772023423832	0.988741847156811	pseudobulk	DESeq2	LRT	0.198066935768657

285	KC_AB2	Cht11	2.00515076565275	0.576456787459964	0.988741847156811	pseudobulk	DESeq2	LRT	0.239233242872451
285	KC_AB2	CIA30	0.699674038439951	0.821690393783994	0.988741847156811	pseudobulk	DESeq2	LRT	0.0852917902455435
285	KC_AB2	Ciao1	-2.35595135045048	0.406121109235175	0.988741847156811	pseudobulk	DESeq2	LRT	0.39134443630141
285	KC_AB2	CIAPIN1	-0.522044679843893	0.796310928443557	0.988741847156811	pseudobulk	DESeq2	LRT	0.0989173240449814
285	KC_AB2	cib	1.7098779479	0.537865353861561	0.988741847156811	pseudobulk	DESeq2	LRT	0.269326429526188
286	KC_AB2	cic	-0.611682304364197	0.263546501469653	0.988741847156811	pseudobulk	DESeq2	LRT	0.579142744588501
286	KC_AB2	cin	-1.28602143946788	0.560977040332514	0.988741847156811	pseudobulk	DESeq2	LRT	0.251054913184025
286	KC_AB2	cindr	-0.952097453718798	0.566656117549707	0.988741847156811	pseudobulk	DESeq2	LRT	0.246680418281264
286	KC_AB2	Cip4	0.214094667679718	0.710604591269585	0.988741847156811	pseudobulk	DESeq2	LRT	0.148371990828391
286	KC_AB2	Cirl	-0.262182999957069	0.693886130491975	0.988741847156811	pseudobulk	DESeq2	LRT	0.158711793171197
286	KC_AB2	Cisd2	0.987569343842076	0.293856179228235	0.988741847156811	pseudobulk	DESeq2	LRT	0.531865172478903
286	KC_AB2	Cka	0.582403985029061	0.49766934223144	0.988741847156811	pseudobulk	DESeq2	LRT	0.303059112138857
286	KC_AB2	CkIalpha	0.162767958213601	0.788921647528634	0.988741847156811	pseudobulk	DESeq2	LRT	0.102966127000548
286	KC_AB2	CkIIalpha	-0.365071902512423	0.596029954876721	0.988741847156811	pseudobulk	DESeq2	LRT	0.224731913228294
286	KC_AB2	CkIIalpha-i3	0.928039350209165	0.67716747857253	0.988741847156811	pseudobulk	DESeq2	LRT	0.169303907349344
287	KC_AB2	CkIIbeta	0.0568811440263765	0.939050309409052	0.988741847156811	pseudobulk	DESeq2	LRT	0.0273111398814414
287	KC_AB2	ckn	-0.143250731196291	0.854823086846284	0.988741847156811	pseudobulk	DESeq2	LRT	0.068123757033766
287	KC_AB2	Cks85A	0.336299849038696	0.783533250599507	0.988741847156811	pseudobulk	DESeq2	LRT	0.105942568760833
287	KC_AB2	cl	-0.698854156894611	0.642847409297697	0.988741847156811	pseudobulk	DESeq2	LRT	0.191892101981606
287	KC_AB2	Clamp	-0.254611003409661	0.868033631089631	0.988741847156811	pseudobulk	DESeq2	LRT	0.0614634481948212
287	KC_AB2	Clbn	0.574989404325028	0.815857662655918	0.988741847156811	pseudobulk	DESeq2	LRT	0.088385603151941
287	KC_AB2	Clc	0.911058663384462	0.305073282470912	0.988741847156811	pseudobulk	DESeq2	LRT	0.515595825079751
287	KC_AB2	ClC-b	1.42621218304824	0.66738831402044	0.988741847156811	pseudobulk	DESeq2	LRT	0.175621402117653
287	KC_AB2	ClC-c	0.167690682683747	0.923775866926924	0.988741847156811	pseudobulk	DESeq2	LRT	0.0344333876159605
287	KC_AB2	Clic	-0.589372447467994	0.699327975248179	0.988741847156811	pseudobulk	DESeq2	LRT	0.155319098310737
288	KC_AB2	CLIP-190	-0.586438247760328	0.287559622418248	0.988741847156811	pseudobulk	DESeq2	LRT	0.541272095312898
288	KC_AB2	Clp	2.06137138167525	0.547946929015274	0.988741847156811	pseudobulk	DESeq2	LRT	0.261261502741816
288	KC_AB2	ClpP	0.0349875728287032	0.980612927422154	0.988741847156811	pseudobulk	DESeq2	LRT	0.00850238574709158
288	KC_AB2	CLS	-0.452344515838269	0.871171367298942	0.988741847156811	pseudobulk	DESeq2	LRT	0.0598964069304489
288	KC_AB2	clu	1.53981939596024	0.431292874271972	0.988741847156811	pseudobulk	DESeq2	LRT	0.365227717114718
288	KC_AB2	cm	0.157562008957688	0.950463298513166	0.988741847156811	pseudobulk	DESeq2	LRT	0.0220646484576093
288	KC_AB2	cmb	2.06137138167525	0.547946929015274	0.988741847156811	pseudobulk	DESeq2	LRT	0.261261502741816
288	KC_AB2	cmpy	0.645136588148733	0.619884323211221	0.988741847156811	pseudobulk	DESeq2	LRT	0.207689346756914
288	KC_AB2	cN-IIIB	-1.23536834900699	0.725570392794057	0.988741847156811	pseudobulk	DESeq2	LRT	0.139320447155934
288	KC_AB2	CNBP	0.32070887417884	0.708200064440167	0.988741847156811	pseudobulk	DESeq2	LRT	0.149844038060811
289	KC_AB2	cnc	1.20464899815983	0.565310923070149	0.988741847156811	pseudobulk	DESeq2	LRT	0.247712622919043
289	KC_AB2	Cndp2	0.6751894878334	0.741835922118177	0.988741847156811	pseudobulk	DESeq2	LRT	0.129692140541008
289	KC_AB2	CngA	-2.41703282302934	0.0195164412792332	0.988741847156811	pseudobulk	DESeq2	LRT	1.70959937077245
289	KC_AB2	CngB	0.0165713603373103	0.973218323345593	0.988741847156811	pseudobulk	DESeq2	LRT	0.0117897229502529
289	KC_AB2	Cngl	-0.195076378466192	0.573618537287087	0.988741847156811	pseudobulk	DESeq2	LRT	0.241376822278531
289	KC_AB2	cni	-0.122143459844207	0.927345423163375	0.988741847156811	pseudobulk	DESeq2	LRT	0.0327584671165663
289	KC_AB2	cnir	-0.546382620888903	0.808721752151108	0.988741847156811	pseudobulk	DESeq2	LRT	0.0922008755339383
289	KC_AB2	cnk	1.12452187615248	0.704078404906088	0.988741847156811	pseudobulk	DESeq2	LRT	0.152378975911753
289	KC_AB2	CNMaR	-1.53965400560324	0.502647226796967	0.988741847156811	pseudobulk	DESeq2	LRT	0.298736709190012
289	KC_AB2	cnn	0.527025244051618	0.797900569756	0.988741847156811	pseudobulk	DESeq2	LRT	0.0980512248106127
290	KC_AB2	cno	-1.06476372447571	0.465427374021298	0.988741847156811	pseudobulk	DESeq2	LRT	0.332148077351859
290	KC_AB2	Cnot4	1.84304108949564	0.207156676644774	0.988741847156811	pseudobulk	DESeq2	LRT	0.683701064860232
290	KC_AB2	CNPYb	1.48741827283584	0.532545672123726	0.988741847156811	pseudobulk	DESeq2	LRT	0.273643140376104
290	KC_AB2	Cnx14D	1.42621218304824	0.66738831402044	0.988741847156811	pseudobulk	DESeq2	LRT	0.175621402117653
290	KC_AB2	Cnx99A	0.156661704037738	0.907366188761799	0.988741847156811	pseudobulk	DESeq2	LRT	0.0422174079086175
290	KC_AB2	Coa7	-0.0885198033477002	0.959687900795474	0.988741847156811	pseudobulk	DESeq2	LRT	0.0178699805019348
290	KC_AB2	Cog1	1.15128682504377	0.719737694898673	0.988741847156811	pseudobulk	DESeq2	LRT	0.142825751365876
290	KC_AB2	Cog2	-2.51746470924027	0.517377929599049	0.988741847156811	pseudobulk	DESeq2	LRT	0.286192101436607
290	KC_AB2	Cog3	0.331758848923756	0.946375065527934	0.988741847156811	pseudobulk	DESeq2	LRT	0.0239367107367223
290	KC_AB2	Cog6	0.157562008957688	0.950463298513166	0.988741847156811	pseudobulk	DESeq2	LRT	0.0220646484576093
291	KC_AB2	Cog7	-1.23536834900699	0.725570392794057	0.988741847156811	pseudobulk	DESeq2	LRT	0.139320447155934

291	KC_AB2	Cog8	0.792281256713368	0.823478838299676	0.988741847156811	pseudobulk	DESeq2	LRT	0.0843475568193814
291	KC_AB2	coil	-0.957133960806503	0.762632518785665	0.988741847156811	pseudobulk	DESeq2	LRT	0.1176846802739
291	KC_AB2	cold	0.818790875285784	0.940355871560339	0.988741847156811	pseudobulk	DESeq2	LRT	0.0267077593721816
291	KC_AB2	colt	-1.23536834900699	0.725570392794057	0.988741847156811	pseudobulk	DESeq2	LRT	0.139320447155934
291	KC_AB2	comm	-0.444344849420398	0.566572265482469	0.988741847156811	pseudobulk	DESeq2	LRT	0.246744688627311
291	KC_AB2	comt	0.953319682696094	0.40764877888496	0.988741847156811	pseudobulk	DESeq2	LRT	0.38971385427045
291	KC_AB2	Con	0.117047974984837	0.819797326255505	0.988741847156811	pseudobulk	DESeq2	LRT	0.086293502455689
291	KC_AB2	Cont	2.7785831508444	0.373751317747552	0.988741847156811	pseudobulk	DESeq2	LRT	0.427417267458199
291	KC_AB2	conu	-0.622214862417408	0.558602402006735	0.988741847156811	pseudobulk	DESeq2	LRT	0.252897201151971
292	KC_AB2	Coprox	-3.4227037205893	0.285801360563088	0.988741847156811	pseudobulk	DESeq2	LRT	0.543935708072568
292	KC_AB2	Coq2	2.7785831508444	0.373751317747552	0.988741847156811	pseudobulk	DESeq2	LRT	0.427417267458199
292	KC_AB2	Coq3	-3.32426296135166	0.343738249391692	0.988741847156811	pseudobulk	DESeq2	LRT	0.463772139158982
292	KC_AB2	Coq5	-1.80294541848982	0.660135549041553	0.988741847156811	pseudobulk	DESeq2	LRT	0.180366879372519
292	KC_AB2	Coq6	0.780352552100014	0.834299274204729	0.988741847156811	pseudobulk	DESeq2	LRT	0.0786781342287373
292	KC_AB2	Coq7	0.415355054823098	0.774384816568148	0.988741847156811	pseudobulk	DESeq2	LRT	0.111043170866649
292	KC_AB2	Coq8	-0.506613484034373	0.961658398065406	0.988741847156811	pseudobulk	DESeq2	LRT	0.0169791713942958
292	KC_AB2	Coq9	3.30033526000341	0.259137897354645	0.988741847156811	pseudobulk	DESeq2	LRT	0.586469069420752
292	KC_AB2	cora	-0.207467400539479	0.773577596282158	0.988741847156811	pseudobulk	DESeq2	LRT	0.111496116422372
292	KC_AB2	CoRest	-0.850877093653734	0.40194300090521	0.988741847156811	pseudobulk	DESeq2	LRT	0.395835529371884
293	KC_AB2	Corin	-2.21667329608248	0.477261050443944	0.988741847156811	pseudobulk	DESeq2	LRT	0.321244007216606
293	KC_AB2	corn	0.42551420096434	0.50068048262055	0.988741847156811	pseudobulk	DESeq2	LRT	0.300439337811005
293	KC_AB2	coro	-0.48857675941716	0.754640406822353	0.988741847156811	pseudobulk	DESeq2	LRT	0.122259944443585
293	KC_AB2	Cortactin	-2.51746470924027	0.517377929599049	0.988741847156811	pseudobulk	DESeq2	LRT	0.286192101436607
293	KC_AB2	corto	0.0560053916259138	0.937810406717111	0.988741847156811	pseudobulk	DESeq2	LRT	0.027884952280962
293	KC_AB2	cos	-1.06622077822007	0.55903750929651	0.988741847156811	pseudobulk	DESeq2	LRT	0.25255905162345
293	KC_AB2	Cow	3.39951966540076	0.0191894765930624	0.988741847156811	pseudobulk	DESeq2	LRT	1.716936870801
293	KC_AB2	COX4	0.405541044744481	0.391997454854282	0.988741847156811	pseudobulk	DESeq2	LRT	0.406716752740587
293	KC_AB2	COX5A	0.531942778387767	0.303756164723757	0.988741847156811	pseudobulk	DESeq2	LRT	0.517474899308555
293	KC_AB2	COX5B	0.285959789796583	0.674850418922182	0.988741847156811	pseudobulk	DESeq2	LRT	0.170792478184715
294	KC_AB2	COX6B	0.441322183110304	0.326202339210778	0.988741847156811	pseudobulk	DESeq2	LRT	0.486512928940469
294	KC_AB2	COX7A	-0.135992050906857	0.738940668195434	0.988741847156811	pseudobulk	DESeq2	LRT	0.131390431036529
294	KC_AB2	COX7C	0.120516645296021	0.839974525268478	0.988741847156811	pseudobulk	DESeq2	LRT	0.07573388501323
294	KC_AB2	COX8	0.350222408116544	0.439966410893329	0.988741847156811	pseudobulk	DESeq2	LRT	0.356580478333146
294	KC_AB2	Cp1	-0.0222133506047509	0.970854555360521	0.988741847156811	pseudobulk	DESeq2	LRT	0.0128458272862036
294	KC_AB2	Cp110	0.527736632909124	0.597470286109144	0.988741847156811	pseudobulk	DESeq2	LRT	0.223683688538352
294	KC_AB2	Cp190	-0.0546881335187679	0.954869152501645	0.988741847156811	pseudobulk	DESeq2	LRT	0.0200561365207969
294	KC_AB2	Cp7Fa	1.4413042803421	0.615311685084066	0.988741847156811	pseudobulk	DESeq2	LRT	0.210904837358999
294	KC_AB2	Cp7Fb	-2.45158172483764	0.378823136429564	0.988741847156811	pseudobulk	DESeq2	LRT	0.421563504545096
294	KC_AB2	Cp7Fc	-1.80294541848982	0.660135549041553	0.988741847156811	pseudobulk	DESeq2	LRT	0.180366879372519
295	KC_AB2	cpa	2.59152681662288	0.284258236371736	0.988741847156811	pseudobulk	DESeq2	LRT	0.546286942791596
295	KC_AB2	cpb	0.673376052367894	0.739921353685403	0.988741847156811	pseudobulk	DESeq2	LRT	0.130814439019823
295	KC_AB2	Cpes	-0.773021474708252	0.784024111360353	0.988741847156811	pseudobulk	DESeq2	LRT	0.105670581104165
295	KC_AB2	cpo	-0.186328084663767	0.576449139239161	0.988741847156811	pseudobulk	DESeq2	LRT	0.239239004973268
295	KC_AB2	Cpr11B	-0.934020934207768	0.782359118258182	0.988741847156811	pseudobulk	DESeq2	LRT	0.106593851444836
295	KC_AB2	Cpr67B	0.818790875285784	0.940355871560339	0.988741847156811	pseudobulk	DESeq2	LRT	0.0267077593721816
295	KC_AB2	Cpsf100	0.818790875285784	0.940355871560339	0.988741847156811	pseudobulk	DESeq2	LRT	0.0267077593721816
295	KC_AB2	Cpsf160	1.47543920254359	0.661929169513603	0.988741847156811	pseudobulk	DESeq2	LRT	0.17918848025087
295	KC_AB2	Cpsf5	0.491571858115809	0.624018734785835	0.988741847156811	pseudobulk	DESeq2	LRT	0.204802371388141
295	KC_AB2	Cpsf6	-0.0322135567609851	0.976706020525706	0.988741847156811	pseudobulk	DESeq2	LRT	0.0102361352329639
296	KC_AB2	Cpsf73	0.818790875285784	0.940355871560339	0.988741847156811	pseudobulk	DESeq2	LRT	0.0267077593721816
296	KC_AB2	cpx	-0.0399706912133739	0.87511093056703	0.988741847156811	pseudobulk	DESeq2	LRT	0.0579368915725023
296	KC_AB2	CR12842	0.139543759454009	0.92013189547714	0.988741847156811	pseudobulk	DESeq2	LRT	0.0361499146411484
296	KC_AB2	CR18166	-1.5707624441993	0.507036228886573	0.988741847156811	pseudobulk	DESeq2	LRT	0.294961008233931
296	KC_AB2	CR18228	1.42621218304824	0.66738831402044	0.988741847156811	pseudobulk	DESeq2	LRT	0.175621402117653
296	KC_AB2	CR18275	-0.400052159425825	0.85291397307595	0.988741847156811	pseudobulk	DESeq2	LRT	0.0690947705939153
296	KC_AB2	CR31032	0.818790875285784	0.940355871560339	0.988741847156811	pseudobulk	DESeq2	LRT	0.0267077593721816

296	KC_AB2	CR32745	-0.97670612739315	0.676315355917219	0.988741847156811	pseudobulk	DESeq2	LRT	0.169850751692797
296	KC_AB2	CR41320	1.47543920254359	0.661929169513603	0.988741847156811	pseudobulk	DESeq2	LRT	0.17918848025087
296	KC_AB2	CR41597	0.973099679487076	0.48594448794672	0.988741847156811	pseudobulk	DESeq2	LRT	0.313413339699419
297	KC_AB2	CR43170	-2.26538931672255	0.547771004842275	0.988741847156811	pseudobulk	DESeq2	LRT	0.2614009599796
297	KC_AB2	CR43212	-1.80294541848982	0.660135549041553	0.988741847156811	pseudobulk	DESeq2	LRT	0.180366879372519
297	KC_AB2	CR43281	0.126434706187641	0.917509849637377	0.988741847156811	pseudobulk	DESeq2	LRT	0.0373892648195234
297	KC_AB2	CR43361	-1.80294541848982	0.660135549041553	0.988741847156811	pseudobulk	DESeq2	LRT	0.180366879372519
297	KC_AB2	CR43670	-0.233272055930639	0.937531793764924	0.988741847156811	pseudobulk	DESeq2	LRT	0.028013995469539
297	KC_AB2	CR43671	-0.378852672109618	0.920549594954422	0.988741847156811	pseudobulk	DESeq2	LRT	0.0359528087516892
297	KC_AB2	CR44003	-1.80294541848982	0.660135549041553	0.988741847156811	pseudobulk	DESeq2	LRT	0.180366879372519
297	KC_AB2	CR44383	-1.80294541848982	0.660135549041553	0.988741847156811	pseudobulk	DESeq2	LRT	0.180366879372519
297	KC_AB2	CR6900	-2.26538931672255	0.547771004842275	0.988741847156811	pseudobulk	DESeq2	LRT	0.2614009599796
297	KC_AB2	Crag	-2.36879023282761	0.4113734544154	0.988741847156811	pseudobulk	DESeq2	LRT	0.385763736396444
298	KC_AB2	CRAT	-2.51746470924027	0.517377929599049	0.988741847156811	pseudobulk	DESeq2	LRT	0.286192101436607
298	KC_AB2	crb	0.0667888239154857	0.88616506465143	0.988741847156811	pseudobulk	DESeq2	LRT	0.0524853751892761
298	KC_AB2	crc	0.251354063541595	0.836642486292507	0.988741847156811	pseudobulk	DESeq2	LRT	0.0774600848977725
298	KC_AB2	CrebA	1.84204627701866	0.0985536859461058	0.988741847156811	pseudobulk	DESeq2	LRT	1.0063271282986
298	KC_AB2	CrebB	-0.551742342733104	0.715456638523198	0.988741847156811	pseudobulk	DESeq2	LRT	0.14541668226864
298	KC_AB2	CREG	-1.80294541848982	0.660135549041553	0.988741847156811	pseudobulk	DESeq2	LRT	0.180366879372519
298	KC_AB2	Crg-1	2.73593896747858	0.422277110754874	0.988741847156811	pseudobulk	DESeq2	LRT	0.374402458575713
298	KC_AB2	CRIF	-1.52426695172354	0.373014920630827	0.988741847156811	pseudobulk	DESeq2	LRT	0.428273796025002
298	KC_AB2	crim	-0.568233637794205	0.868826397133536	0.988741847156811	pseudobulk	DESeq2	LRT	0.0610669925949191
298	KC_AB2	Crk	0.595806094570246	0.386852463871831	0.988741847156811	pseudobulk	DESeq2	LRT	0.412454632766488
299	KC_AB2	crm	1.47543920254359	0.661929169513603	0.988741847156811	pseudobulk	DESeq2	LRT	0.17918848025087
299	KC_AB2	CRMP	0.0395508767643342	0.934213142180049	0.988741847156811	pseudobulk	DESeq2	LRT	0.0295540275041027
299	KC_AB2	crn	1.69894780879763	0.540778829726708	0.988741847156811	pseudobulk	DESeq2	LRT	0.26698031838453
299	KC_AB2	crol	1.09729988177332	0.215824352152756	0.988741847156811	pseudobulk	DESeq2	LRT	0.665899554051255
299	KC_AB2	crp	-0.157084573777931	0.845518036322644	0.988741847156811	pseudobulk	DESeq2	LRT	0.0728771237352504
299	KC_AB2	crq	1.83519154909184	0.267542882731453	0.988741847156811	pseudobulk	DESeq2	LRT	0.572606597788473
299	KC_AB2	Crtc	0.712015159578031	0.348942197433799	0.988741847156811	pseudobulk	DESeq2	LRT	0.457246508326265
299	KC_AB2	Cse1	0.666943671827768	0.808936122914367	0.988741847156811	pseudobulk	DESeq2	LRT	0.0920857707994393
299	KC_AB2	Csk	0.289751549838805	0.763861179352717	0.988741847156811	pseudobulk	DESeq2	LRT	0.116985560951909
299	KC_AB2	Csl4	-1.23536834900699	0.725570392794057	0.988741847156811	pseudobulk	DESeq2	LRT	0.139320447155934
300	KC_AB2	CSN1b	0.157562008957688	0.950463298513166	0.988741847156811	pseudobulk	DESeq2	LRT	0.0220646484576093
300	KC_AB2	CSN3	0.792281256713368	0.823478838299676	0.988741847156811	pseudobulk	DESeq2	LRT	0.0843475568193814
300	KC_AB2	CSN4	0.998893024789477	0.660591418406266	0.988741847156811	pseudobulk	DESeq2	LRT	0.180067072429364
300	KC_AB2	CSN5	0.48020439743879	0.797798185903029	0.988741847156811	pseudobulk	DESeq2	LRT	0.0981069555583927
300	KC_AB2	CSN6	-0.21718335023406	0.885874716609737	0.988741847156811	pseudobulk	DESeq2	LRT	0.0526276931606903
300	KC_AB2	CSN7	0.139543759454009	0.92013189547714	0.988741847156811	pseudobulk	DESeq2	LRT	0.0361499146411484
300	KC_AB2	CSN8	-0.701378075788637	0.705378733592591	0.988741847156811	pseudobulk	DESeq2	LRT	0.151577637980993
300	KC_AB2	Csp	-0.717391973313207	0.416973145752702	0.988741847156811	pseudobulk	DESeq2	LRT	0.379891913915842
300	KC_AB2	CstF50	2.00515076565275	0.576456787459964	0.988741847156811	pseudobulk	DESeq2	LRT	0.239233242872451
300	KC_AB2	CstF64	-3.4227037205893	0.285801360563088	0.988741847156811	pseudobulk	DESeq2	LRT	0.543935708072568
301	KC_AB2	csul	-0.934020934207768	0.782359118258182	0.988741847156811	pseudobulk	DESeq2	LRT	0.106593851444836
301	KC_AB2	csw	0.0515410549039086	0.928124506593374	0.988741847156811	pseudobulk	DESeq2	LRT	0.0323937598808965
301	KC_AB2	cta	-0.175259590848606	0.873183320861962	0.988741847156811	pseudobulk	DESeq2	LRT	0.0588945685730619
301	KC_AB2	CtBP	-0.355103366182449	0.489496496116491	0.988741847156811	pseudobulk	DESeq2	LRT	0.310250412589586
301	KC_AB2	CTCF	-1.16822656624734	0.643917245153277	0.988741847156811	pseudobulk	DESeq2	LRT	0.191169943638088
301	KC_AB2	CtIP	0.0601466527378941	0.974810960364079	0.988741847156811	pseudobulk	DESeq2	LRT	0.0110795964355292
301	KC_AB2	Ctl1	0.0419836735898602	0.932469150056142	0.988741847156811	pseudobulk	DESeq2	LRT	0.0303655275428804
301	KC_AB2	Ctl2	-0.58621066677634	0.754406236055079	0.988741847156811	pseudobulk	DESeq2	LRT	0.122394730301062
301	KC_AB2	ctp	0.0892509790575021	0.754430124647381	0.988741847156811	pseudobulk	DESeq2	LRT	0.122380978400474
301	KC_AB2	CTPsyn	0.275159791831755	0.871229280995972	0.988741847156811	pseudobulk	DESeq2	LRT	0.0598675368779949
302	KC_AB2	Ctr1A	-1.59087154787651	0.436900982678057	0.988741847156811	pseudobulk	DESeq2	LRT	0.359616978473819
302	KC_AB2	Ctr9	2.00447746529068	0.298715182307654	0.988741847156811	pseudobulk	DESeq2	LRT	0.524742703660107
302	KC_AB2	ctrip	-0.121090669659444	0.893066308443463	0.988741847156811	pseudobulk	DESeq2	LRT	0.0491162943902913

302	KC_AB2	CtsB1	0.314117147437151	0.759846610894073	0.988741847156811	pseudobulk	DESeq2	LRT	0.119274069252994
302	KC_AB2	Cubn	0.818790875285784	0.940355871560339	0.988741847156811	pseudobulk	DESeq2	LRT	0.0267077593721816
302	KC_AB2	cue	-0.982137735218619	0.64553975034157	0.988741847156811	pseudobulk	DESeq2	LRT	0.190077010062976
302	KC_AB2	Cul1	-0.124463160497499	0.904027612692689	0.988741847156811	pseudobulk	DESeq2	LRT	0.0438183041961846
302	KC_AB2	Cul2	-0.515929001573633	0.735264703248323	0.988741847156811	pseudobulk	DESeq2	LRT	0.133556282052766
302	KC_AB2	Cul3	0.748122053893993	0.60211661192911	0.988741847156811	pseudobulk	DESeq2	LRT	0.220319390780521
302	KC_AB2	Cul4	-0.689687550646419	0.745991540083112	0.988741847156811	pseudobulk	DESeq2	LRT	0.127266097615876
303	KC_AB2	Cul5	2.41462217514131	0.497446973552313	0.988741847156811	pseudobulk	DESeq2	LRT	0.303253207020768
303	KC_AB2	cv-c	-0.309701298157466	0.708629343750908	0.988741847156811	pseudobulk	DESeq2	LRT	0.149580867857713
303	KC_AB2	Cwc25	3.48393309996043	0.216218474953395	0.988741847156811	pseudobulk	DESeq2	LRT	0.665107200172195
303	KC_AB2	cwo	0.192361721665456	0.878108184854825	0.988741847156811	pseudobulk	DESeq2	LRT	0.0564519747646424
303	KC_AB2	cyc	2.4643417141186	0.449082701910884	0.988741847156811	pseudobulk	DESeq2	LRT	0.347673673080982
303	KC_AB2	CycC	1.45259040912931	0.616176917291092	0.988741847156811	pseudobulk	DESeq2	LRT	0.210294574894236
303	KC_AB2	CycG	0.0176184805584437	0.955539291004942	0.988741847156811	pseudobulk	DESeq2	LRT	0.019751450402999
303	KC_AB2	CycH	2.41462217514131	0.497446973552313	0.988741847156811	pseudobulk	DESeq2	LRT	0.303253207020768
303	KC_AB2	CycJ	-0.506613484034373	0.961658398065406	0.988741847156811	pseudobulk	DESeq2	LRT	0.0169791713942958
303	KC_AB2	CycK	-1.12429747517098	0.477122570541496	0.988741847156811	pseudobulk	DESeq2	LRT	0.321370038419546
304	KC_AB2	CycT	-0.229782470332908	0.789619939295508	0.988741847156811	pseudobulk	DESeq2	LRT	0.102581893498656
304	KC_AB2	CycY	0.832064699200183	0.64462948842046	0.988741847156811	pseudobulk	DESeq2	LRT	0.190689831672951
304	KC_AB2	CYLD	2.78701273797561	0.237560157073908	0.988741847156811	pseudobulk	DESeq2	LRT	0.624226396240996
304	KC_AB2	Cyp1	0.570896233352606	0.277649447157801	0.988741847156811	pseudobulk	DESeq2	LRT	0.556503186936797
304	KC_AB2	Cyp12b2	-0.506613484034373	0.961658398065406	0.988741847156811	pseudobulk	DESeq2	LRT	0.0169791713942958
304	KC_AB2	cyp33	0.167075029051464	0.925389167234494	0.988741847156811	pseudobulk	DESeq2	LRT	0.0336755887326197
304	KC_AB2	Cyp49a1	0.712171366772551	0.813205723536451	0.988741847156811	pseudobulk	DESeq2	LRT	0.0897995733547685
304	KC_AB2	Cyp4s3	0.818790875285784	0.940355871560339	0.988741847156811	pseudobulk	DESeq2	LRT	0.0267077593721816
304	KC_AB2	Cyp6d5	-3.32426296135166	0.343738249391692	0.988741847156811	pseudobulk	DESeq2	LRT	0.463772139158982
304	KC_AB2	Cyp6u1	-0.233272055930639	0.937531793764924	0.988741847156811	pseudobulk	DESeq2	LRT	0.028013995469539
305	KC_AB2	cype	0.605138547910427	0.175839840914579	0.988741847156811	pseudobulk	DESeq2	LRT	0.754882717834324
305	KC_AB2	Cypl	2.7785831508444	0.373751317747552	0.988741847156811	pseudobulk	DESeq2	LRT	0.427417267458199
305	KC_AB2	CysRS	-2.51746470924027	0.517377929599049	0.988741847156811	pseudobulk	DESeq2	LRT	0.286192101436607
305	KC_AB2	CysRS-m	0.699674038439951	0.821690393783994	0.988741847156811	pseudobulk	DESeq2	LRT	0.0852917902455435
305	KC_AB2	Cyt-b5	0.816004132751143	0.494421497413991	0.988741847156811	pseudobulk	DESeq2	LRT	0.305902654413376
305	KC_AB2	Cyt-c-p	0.573975544740248	0.235930187205297	0.988741847156811	pseudobulk	DESeq2	LRT	0.62721648769461
305	KC_AB2	Cyt-c1	1.1069586619716	0.18567387686001	0.988741847156811	pseudobulk	DESeq2	LRT	0.73124919444872
305	KC_AB2	D1	0.611210702682004	0.502129847781185	0.988741847156811	pseudobulk	DESeq2	LRT	0.299183962371141
305	KC_AB2	D12	1.12452187615248	0.704078404906088	0.988741847156811	pseudobulk	DESeq2	LRT	0.152378975911753
305	KC_AB2	D19A	-0.506613484034373	0.961658398065406	0.988741847156811	pseudobulk	DESeq2	LRT	0.0169791713942958
306	KC_AB2	D19B	3.46443801158151	0.218071151261552	0.988741847156811	pseudobulk	DESeq2	LRT	0.6614017836492
306	KC_AB2	d4	0.227877899415833	0.75641543133898	0.988741847156811	pseudobulk	DESeq2	LRT	0.121239619862684
306	KC_AB2	da	0.128616919074783	0.876466746386176	0.988741847156811	pseudobulk	DESeq2	LRT	0.057264556620899
306	KC_AB2	DAAM	1.32424837115872	0.473251147665869	0.988741847156811	pseudobulk	DESeq2	LRT	0.324908324169554
306	KC_AB2	Dab	0.0891444753400672	0.919781951142912	0.988741847156811	pseudobulk	DESeq2	LRT	0.036315116827588
306	KC_AB2	dac	-0.216075562953738	0.649517318911305	0.988741847156811	pseudobulk	DESeq2	LRT	0.187409264287129
306	KC_AB2	Dad	0.684221129527733	0.737351212059311	0.988741847156811	pseudobulk	DESeq2	LRT	0.132325601520231
306	KC_AB2	Dad1	-0.652351539498718	0.624190662883811	0.988741847156811	pseudobulk	DESeq2	LRT	0.204682732127538
306	KC_AB2	daed	2.06137138167525	0.547946929015274	0.988741847156811	pseudobulk	DESeq2	LRT	0.261261502741816
306	KC_AB2	Dak1	0.402157457982861	0.709785839524081	0.988741847156811	pseudobulk	DESeq2	LRT	0.148872669228913
307	KC_AB2	dally	-2.26538931672255	0.547771004842275	0.988741847156811	pseudobulk	DESeq2	LRT	0.2614009599796
307	KC_AB2	danr	0.818790875285784	0.940355871560339	0.988741847156811	pseudobulk	DESeq2	LRT	0.0267077593721816
307	KC_AB2	dao	-1.39194310832104	0.299190148266157	0.988741847156811	pseudobulk	DESeq2	LRT	0.524052711021769
307	KC_AB2	Dap160	0.635591770412003	0.495268672969312	0.988741847156811	pseudobulk	DESeq2	LRT	0.305159141403439
307	KC_AB2	dare	-0.378852672109618	0.920549594954422	0.988741847156811	pseudobulk	DESeq2	LRT	0.0359528087516892
307	KC_AB2	Dark	-2.51746470924027	0.517377929599049	0.988741847156811	pseudobulk	DESeq2	LRT	0.286192101436607
307	KC_AB2	DAT	3.51593030737787	0.251255733474702	0.988741847156811	pseudobulk	DESeq2	LRT	0.599884019171395
307	KC_AB2	dati	-0.130545602883712	0.75867308589426	0.988741847156811	pseudobulk	DESeq2	LRT	0.119945322361311
307	KC_AB2	Daxx	0.225897631647062	0.875606914029851	0.988741847156811	pseudobulk	DESeq2	LRT	0.057690817783461

307	KC_AB2	dbe	1.47543920254359	0.661929169513603	0.988741847156811	pseudobulk	DESeq2	LRT	0.17918848025087
308	KC_AB2	dbo	0.414910419720053	0.750347298666893	0.988741847156811	pseudobulk	DESeq2	LRT	0.124737676630478
308	KC_AB2	Dbp45A	-0.233272055930639	0.937531793764924	0.988741847156811	pseudobulk	DESeq2	LRT	0.028013995469539
308	KC_AB2	Dbp73D	1.42621218304824	0.66738831402044	0.988741847156811	pseudobulk	DESeq2	LRT	0.175621402117653
308	KC_AB2	Dbp80	-0.0696941353944217	0.907316007792485	0.988741847156811	pseudobulk	DESeq2	LRT	0.0422414267898479
308	KC_AB2	dbr	0.0601466527378941	0.974810960364079	0.988741847156811	pseudobulk	DESeq2	LRT	0.0110795964355292
308	KC_AB2	DCAF12	-0.0582963981256801	0.979058404810457	0.988741847156811	pseudobulk	DESeq2	LRT	0.0091913999942843
308	KC_AB2	Dci	1.42621218304824	0.66738831402044	0.988741847156811	pseudobulk	DESeq2	LRT	0.175621402117653
308	KC_AB2	dco	-0.614740949034752	0.485275974940895	0.988741847156811	pseudobulk	DESeq2	LRT	0.314011209218147
308	KC_AB2	Dcp-1	-0.940357095412495	0.643440277414923	0.988741847156811	pseudobulk	DESeq2	LRT	0.191491757062346
308	KC_AB2	DCP1	1.4129303440845	0.645460595159151	0.988741847156811	pseudobulk	DESeq2	LRT	0.190130265910817
309	KC_AB2	DCP2	0.425180485623515	0.642515012981714	0.988741847156811	pseudobulk	DESeq2	LRT	0.192116720169523
309	KC_AB2	Dcr-1	0.818790875285784	0.940355871560339	0.988741847156811	pseudobulk	DESeq2	LRT	0.0267077593721816
309	KC_AB2	Dcr-2	1.47543920254359	0.661929169513603	0.988741847156811	pseudobulk	DESeq2	LRT	0.17918848025087
309	KC_AB2	DCTN1-p150	-0.272159080840168	0.881917593135503	0.988741847156811	pseudobulk	DESeq2	LRT	0.0545719936873807
309	KC_AB2	DCTN2-p50	-0.0477778988959478	0.974735162549842	0.988741847156811	pseudobulk	DESeq2	LRT	0.0111133669343064
309	KC_AB2	DCTN3-p24	0.74211824391331	0.690246317519422	0.988741847156811	pseudobulk	DESeq2	LRT	0.160995901653945
309	KC_AB2	DCTN4-p62	0.668970670557767	0.818048200024539	0.988741847156811	pseudobulk	DESeq2	LRT	0.0872211066123218
309	KC_AB2	DCTN5-p25	-0.213177136262384	0.93155916913195	0.988741847156811	pseudobulk	DESeq2	LRT	0.030789555140895
309	KC_AB2	DCTN6-p27	-0.244067456186467	0.906772274454269	0.988741847156811	pseudobulk	DESeq2	LRT	0.042501767367312
309	KC_AB2	Dd	-0.405237239001491	0.673222706839465	0.988741847156811	pseudobulk	DESeq2	LRT	0.171841244317169
310	KC_AB2	Ddc	1.39373648318353	0.196960492608681	0.988741847156811	pseudobulk	DESeq2	LRT	0.705620878217774
310	KC_AB2	Ddr	3.51593030737787	0.251255733474702	0.988741847156811	pseudobulk	DESeq2	LRT	0.599884019171395
310	KC_AB2	Ddx1	-0.568233637794205	0.868826397133536	0.988741847156811	pseudobulk	DESeq2	LRT	0.0610669925949191
310	KC_AB2	Deaf1	0.401631666228995	0.665354026938417	0.988741847156811	pseudobulk	DESeq2	LRT	0.176947210285234
310	KC_AB2	DEF8	2.06137138167525	0.547946929015274	0.988741847156811	pseudobulk	DESeq2	LRT	0.261261502741816
310	KC_AB2	defl	-1.80294541848982	0.660135549041553	0.988741847156811	pseudobulk	DESeq2	LRT	0.180366879372519
310	KC_AB2	Dek	-0.135853646406395	0.86696263889407	0.988741847156811	pseudobulk	DESeq2	LRT	0.0619996177161264
310	KC_AB2	del	-0.934020934207768	0.782359118258182	0.988741847156811	pseudobulk	DESeq2	LRT	0.106593851444836
310	KC_AB2	deltaCOP	1.64020553681259	0.395043317498507	0.988741847156811	pseudobulk	DESeq2	LRT	0.403355280274522
310	KC_AB2	Den1	-0.0164752064620401	0.975553826317318	0.988741847156811	pseudobulk	DESeq2	LRT	0.0107487633502623
311	KC_AB2	DENR	-0.555005900594584	0.702275031306502	0.988741847156811	pseudobulk	DESeq2	LRT	0.153492772219586
311	KC_AB2	Der-1	-0.0299947124123637	0.983450084395904	0.988741847156811	pseudobulk	DESeq2	LRT	0.00724767806447394
311	KC_AB2	Der-2	-0.50879048068994	0.817628596116567	0.988741847156811	pseudobulk	DESeq2	LRT	0.0874439277298491
311	KC_AB2	Dera	1.42621218304824	0.66738831402044	0.988741847156811	pseudobulk	DESeq2	LRT	0.175621402117653
311	KC_AB2	Desat1	-0.311709384151921	0.633349516905187	0.988741847156811	pseudobulk	DESeq2	LRT	0.198356556378166
311	KC_AB2	Det	0.699674038439951	0.821690393783994	0.988741847156811	pseudobulk	DESeq2	LRT	0.0852917902455435
311	KC_AB2	Df31	0.762714533051965	0.298114046936876	0.988741847156811	pseudobulk	DESeq2	LRT	0.525617559816263
311	KC_AB2	Dg	-0.706670169198358	0.394759114951329	0.988741847156811	pseudobulk	DESeq2	LRT	0.403667833384612
311	KC_AB2	Dgk	-2.43220920834584	0.11236884223464	0.988741847156811	pseudobulk	DESeq2	LRT	0.949354093761853
311	KC_AB2	Dgkepsilon	-1.02592565225788	0.645171277800408	0.988741847156811	pseudobulk	DESeq2	LRT	0.190324975087242
312	KC_AB2	Dgp-1	0.360597065274579	0.876727378089377	0.988741847156811	pseudobulk	DESeq2	LRT	0.0571354312607773
312	KC_AB2	dgrn	0.581445489707027	0.811994604428781	0.988741847156811	pseudobulk	DESeq2	LRT	0.0904468565649727
312	KC_AB2	dgt1	1.4413042803421	0.615311685084066	0.988741847156811	pseudobulk	DESeq2	LRT	0.210904837358999
312	KC_AB2	Dh44-R1	0.125857599958302	0.931359657231176	0.988741847156811	pseudobulk	DESeq2	LRT	0.0308825778928101
312	KC_AB2	Dh44-R2	3.83932053948463	0.189970154032558	0.988741847156811	pseudobulk	DESeq2	LRT	0.721314625137331
312	KC_AB2	Dhap-at	-0.457212305199634	0.858276431637992	0.988741847156811	pseudobulk	DESeq2	LRT	0.0663728130839547
312	KC_AB2	Dhc64C	-0.378908515743588	0.741384881684407	0.988741847156811	pseudobulk	DESeq2	LRT	0.129956274346355
312	KC_AB2	Dhit	0.497145699338316	0.39515182583154	0.988741847156811	pseudobulk	DESeq2	LRT	0.403236007026716
312	KC_AB2	Dhod	2.41462217514131	0.497446973552313	0.988741847156811	pseudobulk	DESeq2	LRT	0.303253207020768
312	KC_AB2	DhpD	2.4643417141186	0.449082701910884	0.988741847156811	pseudobulk	DESeq2	LRT	0.347673673080982
313	KC_AB2	Dhpr	0.267935034546862	0.816553283560044	0.988741847156811	pseudobulk	DESeq2	LRT	0.0880154704483531
313	KC_AB2	Dhx15	2.11513947821417	0.415104446921141	0.988741847156811	pseudobulk	DESeq2	LRT	0.381842614096864
313	KC_AB2	dia	0.0601466527378941	0.974810960364079	0.988741847156811	pseudobulk	DESeq2	LRT	0.0110795964355292
313	KC_AB2	Diap1	-0.140083867255482	0.844240097214764	0.988741847156811	pseudobulk	DESeq2	LRT	0.0735340248592155
313	KC_AB2	Diap2	1.07837905313259	0.605666852796606	0.988741847156811	pseudobulk	DESeq2	LRT	0.217766193946807

313	KC_AB2	dib	-1.80294541848982	0.660135549041553	0.988741847156811	pseudobulk	DESeq2	LRT	0.180366879372519
313	KC_AB2	Dic1	0.161420254574937	0.906571048596072	0.988741847156811	pseudobulk	DESeq2	LRT	0.0425981542776991
313	KC_AB2	didum	-1.53965400560324	0.502647226796967	0.988741847156811	pseudobulk	DESeq2	LRT	0.298736709190012
313	KC_AB2	Dif	0.173174151212747	0.816278529886284	0.988741847156811	pseudobulk	DESeq2	LRT	0.0881616263531316
313	KC_AB2	dikar	-0.277435134138013	0.700967513692148	0.988741847156811	pseudobulk	DESeq2	LRT	0.154302108925159
314	KC_AB2	Dim1	-0.358098920152354	0.844906190835001	0.988741847156811	pseudobulk	DESeq2	LRT	0.0731915076944904
314	KC_AB2	dind	-0.158601889198964	0.945838126788742	0.988741847156811	pseudobulk	DESeq2	LRT	0.0241831835324482
314	KC_AB2	DIP-alpha	-1.1284597703615	0.729754448571724	0.988741847156811	pseudobulk	DESeq2	LRT	0.136823248879498
314	KC_AB2	Dip-B	-2.51746470924027	0.517377929599049	0.988741847156811	pseudobulk	DESeq2	LRT	0.286192101436607
314	KC_AB2	Dip-C	-1.59599518682648	0.371906361547588	0.988741847156811	pseudobulk	DESeq2	LRT	0.429566392866129
314	KC_AB2	DIP-gamma	-1.80294541848982	0.660135549041553	0.988741847156811	pseudobulk	DESeq2	LRT	0.180366879372519
314	KC_AB2	DIP-kappa	-0.324676993248769	0.402334015209248	0.988741847156811	pseudobulk	DESeq2	LRT	0.395413248579158
314	KC_AB2	DIP1	-0.0190634720764426	0.981963000791083	0.988741847156811	pseudobulk	DESeq2	LRT	0.00790487560916621
314	KC_AB2	DIP2	0.714968398960555	0.574828565548121	0.988741847156811	pseudobulk	DESeq2	LRT	0.240461658158431
314	KC_AB2	Dis3	-0.934020934207768	0.782359118258182	0.988741847156811	pseudobulk	DESeq2	LRT	0.106593851444836
315	KC_AB2	Dis3l2	0.351722119397094	0.929615296941389	0.988741847156811	pseudobulk	DESeq2	LRT	0.0316967385224016
315	KC_AB2	disp	0.167075029051464	0.925389167234494	0.988741847156811	pseudobulk	DESeq2	LRT	0.0336755887326197
315	KC_AB2	DJ-1alpha	0.331758848923756	0.946375065527934	0.988741847156811	pseudobulk	DESeq2	LRT	0.0239367107367223
315	KC_AB2	dj-1beta	0.419671460681067	0.785238231243751	0.988741847156811	pseudobulk	DESeq2	LRT	0.104998563868265
315	KC_AB2	dl	-0.043251987344817	0.978652029676919	0.988741847156811	pseudobulk	DESeq2	LRT	0.00937169885514152
315	KC_AB2	Dlc90F	0.841907944116255	0.545061882267876	0.988741847156811	pseudobulk	DESeq2	LRT	0.263554188362243
315	KC_AB2	dlg1	0.107140778700388	0.758622389387629	0.988741847156811	pseudobulk	DESeq2	LRT	0.11997434401655
315	KC_AB2	Dlg5	0.478685616198768	0.78501345906638	0.988741847156811	pseudobulk	DESeq2	LRT	0.105122897206146
315	KC_AB2	Dlic	0.32108157896894	0.751197878568373	0.988741847156811	pseudobulk	DESeq2	LRT	0.124245647216054
315	KC_AB2	Dlip1	1.47543920254359	0.661929169513603	0.988741847156811	pseudobulk	DESeq2	LRT	0.17918848025087
316	KC_AB2	Dlip2	1.47543920254359	0.661929169513603	0.988741847156811	pseudobulk	DESeq2	LRT	0.17918848025087
316	KC_AB2	Dlip3	-0.217915120660429	0.892149991826569	0.988741847156811	pseudobulk	DESeq2	LRT	0.0495621241592452
316	KC_AB2	dlp	0.818790875285784	0.940355871560339	0.988741847156811	pseudobulk	DESeq2	LRT	0.0267077593721816
316	KC_AB2	dlt	-0.934020934207768	0.782359118258182	0.988741847156811	pseudobulk	DESeq2	LRT	0.106593851444836
316	KC_AB2	DMAP1	-0.940357095412495	0.643440277414923	0.988741847156811	pseudobulk	DESeq2	LRT	0.191491757062346
316	KC_AB2	dmpd	0.43338653856073	0.77568785694311	0.988741847156811	pseudobulk	DESeq2	LRT	0.110313007194308
316	KC_AB2	dmt	2.06137138167525	0.547946929015274	0.988741847156811	pseudobulk	DESeq2	LRT	0.261261502741816
316	KC_AB2	Dmtn	-0.300213970456187	0.862708793342715	0.988741847156811	pseudobulk	DESeq2	LRT	0.0641357753007584
316	KC_AB2	Dnah3	0.818790875285784	0.940355871560339	0.988741847156811	pseudobulk	DESeq2	LRT	0.0267077593721816
316	KC_AB2	DnaJ-1	-0.107868598362687	0.919257030793011	0.988741847156811	pseudobulk	DESeq2	LRT	0.0365630398132679
317	KC_AB2	DnaJ-H	0.9844378692756	0.565111304018534	0.988741847156811	pseudobulk	DESeq2	LRT	0.247866005348055
317	KC_AB2	DNAlig1	2.00515076565275	0.576456787459964	0.988741847156811	pseudobulk	DESeq2	LRT	0.239233242872451
317	KC_AB2	DNAlig3	2.7785831508444	0.373751317747552	0.988741847156811	pseudobulk	DESeq2	LRT	0.427417267458199
317	KC_AB2	DNAlig4	1.12452187615248	0.704078404906088	0.988741847156811	pseudobulk	DESeq2	LRT	0.152378975911753
317	KC_AB2	DNApol-eta	-1.99867440840856	0.534899605264936	0.988741847156811	pseudobulk	DESeq2	LRT	0.271727722597268
317	KC_AB2	DNApol-iota	-1.80294541848982	0.660135549041553	0.988741847156811	pseudobulk	DESeq2	LRT	0.180366879372519
317	KC_AB2	DNApol-zeta	0.818790875285784	0.940355871560339	0.988741847156811	pseudobulk	DESeq2	LRT	0.0267077593721816
317	KC_AB2	dnc	-0.0641996929376991	0.799124017874134	0.988741847156811	pseudobulk	DESeq2	LRT	0.0973858163070001
317	KC_AB2	dnk	0.50351383114442	0.831654424165643	0.988741847156811	pseudobulk	DESeq2	LRT	0.0800570978140888
317	KC_AB2	dnt	-0.568233637794205	0.868826397133536	0.988741847156811	pseudobulk	DESeq2	LRT	0.0610669925949191
318	KC_AB2	Dnz1	0.324683740976312	0.825117200039295	0.988741847156811	pseudobulk	DESeq2	LRT	0.0834843596739648
318	KC_AB2	Doa	-0.747479132876222	0.578153580773182	0.988741847156811	pseudobulk	DESeq2	LRT	0.237956780225608
318	KC_AB2	dock	0.21439346789347	0.782079265209087	0.988741847156811	pseudobulk	DESeq2	LRT	0.106749228144291
318	KC_AB2	dod	0.490765851098085	0.671779767498567	0.988741847156811	pseudobulk	DESeq2	LRT	0.17277308027412
318	KC_AB2	dom	-0.663273929909526	0.684336515129322	0.988741847156811	pseudobulk	DESeq2	LRT	0.164730286109088
318	KC_AB2	dome	2.06137138167525	0.547946929015274	0.988741847156811	pseudobulk	DESeq2	LRT	0.261261502741816
318	KC_AB2	dop	1.56645644620297	0.359832388723451	0.988741847156811	pseudobulk	DESeq2	LRT	0.443899748131112
318	KC_AB2	Dop1R1	0.262485438472934	0.536990936007962	0.988741847156811	pseudobulk	DESeq2	LRT	0.27003304479383
318	KC_AB2	Dop1R2	0.273708396936535	0.528816952585898	0.988741847156811	pseudobulk	DESeq2	LRT	0.276694630873971
318	KC_AB2	Dop2R	-0.19924413772457	0.511152691706842	0.988741847156811	pseudobulk	DESeq2	LRT	0.291449347888415
319	KC_AB2	DopEcR	-0.11393827238923	0.8133740764923	0.988741847156811	pseudobulk	DESeq2	LRT	0.0897096733572439

319	KC_AB2	dor	1.47543920254359	0.661929169513603	0.988741847156811	pseudobulk	DESeq2	LRT	0.17918848025087
319	KC_AB2	DOR	-0.0825538371398614	0.866709199770221	0.988741847156811	pseudobulk	DESeq2	LRT	0.0621265935500938
319	KC_AB2	dos	-1.47735078907169	0.488752680123704	0.988741847156811	pseudobulk	DESeq2	LRT	0.310910848092764
319	KC_AB2	Dp	0.380229863992492	0.74050885190232	0.988741847156811	pseudobulk	DESeq2	LRT	0.130469745637905
319	KC_AB2	Dp1	-0.840006590521399	0.717017354938613	0.988741847156811	pseudobulk	DESeq2	LRT	0.144470332389583
319	KC_AB2	Dpck	3.46443801158151	0.218071151261552	0.988741847156811	pseudobulk	DESeq2	LRT	0.6614017836492
319	KC_AB2	DPCoAC	-0.546382620888903	0.808721752151108	0.988741847156811	pseudobulk	DESeq2	LRT	0.0922008755339383
319	KC_AB2	Dph5	-1.92150817035771	0.457631232406761	0.988741847156811	pseudobulk	DESeq2	LRT	0.339484343482859
319	KC_AB2	Dpit47	2.51243226103394	0.474458247157471	0.988741847156811	pseudobulk	DESeq2	LRT	0.323801999942597
320	KC_AB2	DppIII	-0.0323203545809474	0.954448156132187	0.988741847156811	pseudobulk	DESeq2	LRT	0.020247656706879
320	KC_AB2	dpr10	-0.268079324716051	0.616007492517946	0.988741847156811	pseudobulk	DESeq2	LRT	0.210414005466402
320	KC_AB2	dpr12	0.208064068057484	0.66219261003716	0.988741847156811	pseudobulk	DESeq2	LRT	0.179015670213354
320	KC_AB2	dpr14	-0.170728015271172	0.826843780109403	0.988741847156811	pseudobulk	DESeq2	LRT	0.0825765362095349
320	KC_AB2	dpr16	0.758768486184067	0.75667228241667	0.988741847156811	pseudobulk	DESeq2	LRT	0.121092174336864
320	KC_AB2	dpr17	-0.42149359962671	0.511386738315364	0.988741847156811	pseudobulk	DESeq2	LRT	0.291250538631031
320	KC_AB2	dpr19	0.288287282673113	0.730827991413258	0.988741847156811	pseudobulk	DESeq2	LRT	0.136184827105608
320	KC_AB2	dpr2	-0.934020934207768	0.782359118258182	0.988741847156811	pseudobulk	DESeq2	LRT	0.106593851444836
320	KC_AB2	dpr20	1.12747777954303	0.644223852171502	0.988741847156811	pseudobulk	DESeq2	LRT	0.190963199611491
320	KC_AB2	dpr21	-0.0699103610039475	0.864419331519212	0.988741847156811	pseudobulk	DESeq2	LRT	0.0632755292966138
321	KC_AB2	dpr3	0.362555753552128	0.897034383127386	0.988741847156811	pseudobulk	DESeq2	LRT	0.0471909102264623
321	KC_AB2	dpr4	0.186422558697241	0.851580293239807	0.988741847156811	pseudobulk	DESeq2	LRT	0.0697743972997503
321	KC_AB2	dpr5	-1.76792120507835	0.407315406400152	0.988741847156811	pseudobulk	DESeq2	LRT	0.390069162739853
321	KC_AB2	dpr6	0.11624531439252	0.8866896371269	0.988741847156811	pseudobulk	DESeq2	LRT	0.0522283671695271
321	KC_AB2	dpr7	0.0462781679543212	0.939512567772046	0.988741847156811	pseudobulk	DESeq2	LRT	0.0270974060077954
321	KC_AB2	dpr8	0.449039367224693	0.357497767322651	0.988741847156811	pseudobulk	DESeq2	LRT	0.446726666149558
321	KC_AB2	dpr9	0.252623033100268	0.620352692310133	0.988741847156811	pseudobulk	DESeq2	LRT	0.207361328608325
321	KC_AB2	dpy	0.818790875285784	0.940355871560339	0.988741847156811	pseudobulk	DESeq2	LRT	0.0267077593721816
321	KC_AB2	Dpy-30L1	-0.121120420743075	0.958079731976279	0.988741847156811	pseudobulk	DESeq2	LRT	0.0185983471673402
321	KC_AB2	Drak	-0.598618278464639	0.526721248796981	0.988741847156811	pseudobulk	DESeq2	LRT	0.278419161139135
322	KC_AB2	Drat	-0.500130976642994	0.497527643361199	0.988741847156811	pseudobulk	DESeq2	LRT	0.303182784213319
322	KC_AB2	dre4	-0.0384500034601774	0.953910728566112	0.988741847156811	pseudobulk	DESeq2	LRT	0.0204922667059844
322	KC_AB2	Dref	0.770523665597912	0.687741087577941	0.988741847156811	pseudobulk	DESeq2	LRT	0.162575028914724
322	KC_AB2	Drep1	1.42621218304824	0.66738831402044	0.988741847156811	pseudobulk	DESeq2	LRT	0.175621402117653
322	KC_AB2	Drep2	-0.273402220988145	0.736344006051383	0.988741847156811	pseudobulk	DESeq2	LRT	0.132919244051082
322	KC_AB2	Drep3	2.41462217514131	0.497446973552313	0.988741847156811	pseudobulk	DESeq2	LRT	0.303253207020768
322	KC_AB2	Drep4	-0.773021474708252	0.784024111360353	0.988741847156811	pseudobulk	DESeq2	LRT	0.105670581104165
322	KC_AB2	Drice	-1.80294541848982	0.660135549041553	0.988741847156811	pseudobulk	DESeq2	LRT	0.180366879372519
322	KC_AB2	drk	0.400543661483313	0.788405698590208	0.988741847156811	pseudobulk	DESeq2	LRT	0.10325024530414
322	KC_AB2	drl	-3.09794865339079	0.39855598532175	0.988741847156811	pseudobulk	DESeq2	LRT	0.399510664459796
323	KC_AB2	Drl-2	0.432043919174875	0.569658272405676	0.988741847156811	pseudobulk	DESeq2	LRT	0.244385591545013
323	KC_AB2	Droj2	0.400055669405211	0.686679568950352	0.988741847156811	pseudobulk	DESeq2	LRT	0.163245874149588
323	KC_AB2	Dronc	2.00515076565275	0.576456787459964	0.988741847156811	pseudobulk	DESeq2	LRT	0.239233242872451
323	KC_AB2	drongo	0.10598154425916	0.959628155914924	0.988741847156811	pseudobulk	DESeq2	LRT	0.0178970181249073
323	KC_AB2	drosha	0.919353707608246	0.746278333243311	0.988741847156811	pseudobulk	DESeq2	LRT	0.127099167101924
323	KC_AB2	Drp1	-0.209502985988326	0.858793067078383	0.988741847156811	pseudobulk	DESeq2	LRT	0.0661114702229279
323	KC_AB2	drpr	0.48020439743879	0.797798185903029	0.988741847156811	pseudobulk	DESeq2	LRT	0.0981069555583927
323	KC_AB2	Drsl2	0.818790875285784	0.940355871560339	0.988741847156811	pseudobulk	DESeq2	LRT	0.0267077593721816
323	KC_AB2	Dscam1	-0.262353583035908	0.717716569207403	0.988741847156811	pseudobulk	DESeq2	LRT	0.144047027527916
323	KC_AB2	Dscam2	1.12458695628651	0.121417869149319	0.988741847156811	pseudobulk	DESeq2	LRT	0.915717393146589
324	KC_AB2	Dscam3	0.461844695326946	0.426615484652826	0.988741847156811	pseudobulk	DESeq2	LRT	0.369963385226886
324	KC_AB2	Dscam4	-0.690120076831834	0.417794460797977	0.988741847156811	pseudobulk	DESeq2	LRT	0.379037322280124
324	KC_AB2	dsd	0.0213420325014771	0.978751765734874	0.988741847156811	pseudobulk	DESeq2	LRT	0.00932744143645669
324	KC_AB2	dsh	0.469453808210009	0.668927859074603	0.988741847156811	pseudobulk	DESeq2	LRT	0.174620716455169
324	KC_AB2	Dsor1	-0.475200187713831	0.69490666946013	0.988741847156811	pseudobulk	DESeq2	LRT	0.158073520100903
324	KC_AB2	Dsp1	0.184531982577093	0.699563947264299	0.988741847156811	pseudobulk	DESeq2	LRT	0.155172580422104
324	KC_AB2	dsx-c73A	0.818790875285784	0.940355871560339	0.988741847156811	pseudobulk	DESeq2	LRT	0.0267077593721816

324	KC_AB2	Dtd	0.0601466527378941	0.974810960364079	0.988741847156811	pseudobulk	DESeq2	LRT	0.0110795964355292
324	KC_AB2	dtn	-0.204290807905859	0.747391746426606	0.988741847156811	pseudobulk	DESeq2	LRT	0.126451702447276
324	KC_AB2	Duba	1.70483820960947	0.399419083138225	0.988741847156811	pseudobulk	DESeq2	LRT	0.398571189581381
325	KC_AB2	DUBAI	0.752634025970037	0.844139830246018	0.988741847156811	pseudobulk	DESeq2	LRT	0.0735856073147832
325	KC_AB2	dunk	1.42621218304824	0.66738831402044	0.988741847156811	pseudobulk	DESeq2	LRT	0.175621402117653
325	KC_AB2	dup	-1.23536834900699	0.725570392794057	0.988741847156811	pseudobulk	DESeq2	LRT	0.139320447155934
325	KC_AB2	dUTPase	-1.80294541848982	0.660135549041553	0.988741847156811	pseudobulk	DESeq2	LRT	0.180366879372519
325	KC_AB2	dwg	-0.233272055930639	0.937531793764924	0.988741847156811	pseudobulk	DESeq2	LRT	0.028013995469539
325	KC_AB2	Dyb	0.345229835980783	0.558095530311867	0.988741847156811	pseudobulk	DESeq2	LRT	0.253291455656902
325	KC_AB2	Dyrk2	-0.487368007148894	0.551260652774039	0.988741847156811	pseudobulk	DESeq2	LRT	0.258643004968495
325	KC_AB2	Dyrk3	-0.471070330330991	0.526172566258127	0.988741847156811	pseudobulk	DESeq2	LRT	0.278871799035006
325	KC_AB2	Dys	-1.06224769127054	0.275864639769362	0.988741847156811	pseudobulk	DESeq2	LRT	0.559303963664397
325	KC_AB2	dysc	-0.200228905665383	0.454704432921878	0.988741847156811	pseudobulk	DESeq2	LRT	0.342270811829363
326	KC_AB2	E(bx)	-0.519317947509438	0.557694113539089	0.988741847156811	pseudobulk	DESeq2	LRT	0.253603939462148
326	KC_AB2	E(Pc)	0.114206729944084	0.867839505170354	0.988741847156811	pseudobulk	DESeq2	LRT	0.0615605841134858
326	KC_AB2	e(r)	0.496507304952545	0.600930692582553	0.988741847156811	pseudobulk	DESeq2	LRT	0.221175613795337
326	KC_AB2	E(var)3-9	-0.0144409071146711	0.984632974376923	0.988741847156811	pseudobulk	DESeq2	LRT	0.00672562423038896
326	KC_AB2	e(y)1	-0.773021474708252	0.784024111360353	0.988741847156811	pseudobulk	DESeq2	LRT	0.105670581104165
326	KC_AB2	e(y)2	1.31950274499659	0.590522087411301	0.988741847156811	pseudobulk	DESeq2	LRT	0.228763853746569
326	KC_AB2	e(y)3	-0.204752272798203	0.872124742996086	0.988741847156811	pseudobulk	DESeq2	LRT	0.0594213919937639
326	KC_AB2	E(z)	-0.194388201900128	0.928057462354013	0.988741847156811	pseudobulk	DESeq2	LRT	0.0324251328213705
326	KC_AB2	E2f1	-2.89052650748519	0.257350434966322	0.988741847156811	pseudobulk	DESeq2	LRT	0.589475093381118
326	KC_AB2	E2f2	-0.957133960806503	0.762632518785665	0.988741847156811	pseudobulk	DESeq2	LRT	0.1176846802739
327	KC_AB2	Eaat2	-0.541526614003567	0.617819566759176	0.988741847156811	pseudobulk	DESeq2	LRT	0.20913834141985
327	KC_AB2	Eaf	0.364529320645641	0.851733046289313	0.988741847156811	pseudobulk	DESeq2	LRT	0.0696965022875613
327	KC_AB2	Eaf6	-2.9721553030081	0.387854083811327	0.988741847156811	pseudobulk	DESeq2	LRT	0.411331631401987
327	KC_AB2	eag	0.181096898679986	0.855015742087039	0.988741847156811	pseudobulk	DESeq2	LRT	0.0680258892032921
327	KC_AB2	ear	-0.0384500034601774	0.953910728566112	0.988741847156811	pseudobulk	DESeq2	LRT	0.0204922667059844
327	KC_AB2	eas	0.631077867486174	0.560469958606855	0.988741847156811	pseudobulk	DESeq2	LRT	0.251447660786978
327	KC_AB2	east	-0.396368872493801	0.657040328214817	0.988741847156811	pseudobulk	DESeq2	LRT	0.182407973235277
327	KC_AB2	Eato	0.363641268105473	0.858330168669459	0.988741847156811	pseudobulk	DESeq2	LRT	0.06634562258353
327	KC_AB2	Eb1	0.30836222164997	0.680847206517148	0.988741847156811	pseudobulk	DESeq2	LRT	0.166950340086872
327	KC_AB2	ebd1	0.356576043500675	0.884817522565587	0.988741847156811	pseudobulk	DESeq2	LRT	0.0531462853633029
328	KC_AB2	ebd2	-0.407893625579103	0.793321221522551	0.988741847156811	pseudobulk	DESeq2	LRT	0.100550928082573
328	KC_AB2	ebi	-1.63785643245857	0.555083135854033	0.988741847156811	pseudobulk	DESeq2	LRT	0.255641966897631
328	KC_AB2	ebo	-0.586551448989482	0.787602284627903	0.988741847156811	pseudobulk	DESeq2	LRT	0.103693032757571
328	KC_AB2	eca	-0.38262537075071	0.800000775716775	0.988741847156811	pseudobulk	DESeq2	LRT	0.0969095918963668
328	KC_AB2	ecd	2.7785831508444	0.373751317747552	0.988741847156811	pseudobulk	DESeq2	LRT	0.427417267458199
328	KC_AB2	Echs1	0.921695369118063	0.730077514579258	0.988741847156811	pseudobulk	DESeq2	LRT	0.136631027048226
328	KC_AB2	EcR	-0.454033218355476	0.477023350619146	0.988741847156811	pseudobulk	DESeq2	LRT	0.321460361427739
328	KC_AB2	ECSIT	1.47543920254359	0.661929169513603	0.988741847156811	pseudobulk	DESeq2	LRT	0.17918848025087
328	KC_AB2	ed	4.26616734477238	0.0836038712203354	0.988741847156811	pseudobulk	DESeq2	LRT	1.07777361238509
328	KC_AB2	Edc3	0.678200423833269	0.572210441104195	0.988741847156811	pseudobulk	DESeq2	LRT	0.242444221902014
329	KC_AB2	Edem1	0.315515278988381	0.901389738439351	0.988741847156811	pseudobulk	DESeq2	LRT	0.0450873903126698
329	KC_AB2	Edem2	-0.234160738284965	0.896763493077035	0.988741847156811	pseudobulk	DESeq2	LRT	0.047322080027168
329	KC_AB2	EDTP	-0.378852672109618	0.920549594954422	0.988741847156811	pseudobulk	DESeq2	LRT	0.0359528087516892
329	KC_AB2	eEF1alpha1	0.103129660203454	0.930668481606612	0.988741847156811	pseudobulk	DESeq2	LRT	0.0312049938298198
329	KC_AB2	eEF1alpha2	-0.249055880105305	0.449869101969787	0.988741847156811	pseudobulk	DESeq2	LRT	0.346913834140125
329	KC_AB2	eEF1beta	-0.988652125543833	0.322083730966575	0.988741847156811	pseudobulk	DESeq2	LRT	0.492031211628264
329	KC_AB2	eEF1delta	1.23585998362146	0.500078349285153	0.988741847156811	pseudobulk	DESeq2	LRT	0.300961947670948
329	KC_AB2	eEF1gamma	-0.296558141162117	0.820112678521132	0.988741847156811	pseudobulk	DESeq2	LRT	0.0861264740820505
329	KC_AB2	eEF2	-0.0954703749231238	0.869262812862697	0.988741847156811	pseudobulk	DESeq2	LRT	0.0608488991324162
329	KC_AB2	eEF5	-0.14310707874531	0.81507596689452	0.988741847156811	pseudobulk	DESeq2	LRT	0.0888019121611456
330	KC_AB2	Efa6	-0.696382966583408	0.600035814030131	0.988741847156811	pseudobulk	DESeq2	LRT	0.221822827330565
330	KC_AB2	eff	0.056487603279074	0.893259769541823	0.988741847156811	pseudobulk	DESeq2	LRT	0.0490222252403812
330	KC_AB2	Efr	-0.0144409071146711	0.984632974376923	0.988741847156811	pseudobulk	DESeq2	LRT	0.00672562423038896

330	KC_AB2	egg	2.73593896747858	0.422277110754874	0.988741847156811	pseudobulk	DESeq2	LRT	0.374402458575713
330	KC_AB2	egh	0.148628181119261	0.825221216693865	0.988741847156811	pseudobulk	DESeq2	LRT	0.0834296147092866
330	KC_AB2	egl	-2.39529240704571	0.218624420449521	0.988741847156811	pseudobulk	DESeq2	LRT	0.660301328781003
330	KC_AB2	Egm	1.47543920254359	0.661929169513603	0.988741847156811	pseudobulk	DESeq2	LRT	0.17918848025087
330	KC_AB2	Ehbp1	0.587362986980796	0.580969083565195	0.988741847156811	pseudobulk	DESeq2	LRT	0.235846978098974
330	KC_AB2	eIF1	-0.103394151356043	0.892920463431251	0.988741847156811	pseudobulk	DESeq2	LRT	0.0491872240132369
330	KC_AB2	eIF1A	0.294785613275599	0.563417712669758	0.988741847156811	pseudobulk	DESeq2	LRT	0.249169503829095
331	KC_AB2	eIF2alpha	0.24619866569863	0.866451184773514	0.988741847156811	pseudobulk	DESeq2	LRT	0.0622559000945371
331	KC_AB2	eIF2Balpha	1.64755990459092	0.573059482818602	0.988741847156811	pseudobulk	DESeq2	LRT	0.241800296498366
331	KC_AB2	eIF2Bbeta	-1.73627324524809	0.521368726288639	0.988741847156811	pseudobulk	DESeq2	LRT	0.282855023048041
331	KC_AB2	eIF2Bdelta	1.4413042803421	0.615311685084066	0.988741847156811	pseudobulk	DESeq2	LRT	0.210904837358999
331	KC_AB2	eIF2Bepsilon	-0.0323203545809474	0.954448156132187	0.988741847156811	pseudobulk	DESeq2	LRT	0.020247656706879
331	KC_AB2	eIF2beta	0.77723881052738	0.436144751236571	0.988741847156811	pseudobulk	DESeq2	LRT	0.360369349653154
331	KC_AB2	eIF2Bgamma	-1.80294541848982	0.660135549041553	0.988741847156811	pseudobulk	DESeq2	LRT	0.180366879372519
331	KC_AB2	eIF2D	-0.669068297387362	0.767301512613651	0.988741847156811	pseudobulk	DESeq2	LRT	0.115033945655498
331	KC_AB2	eIF2gamma	-0.213177136262384	0.93155916913195	0.988741847156811	pseudobulk	DESeq2	LRT	0.030789555140895
331	KC_AB2	eIF3a	-0.50725960786936	0.576618880809073	0.988741847156811	pseudobulk	DESeq2	LRT	0.239111141176141
332	KC_AB2	eIF3b	-0.740713701809251	0.501258141315197	0.988741847156811	pseudobulk	DESeq2	LRT	0.299938560607069
332	KC_AB2	eIF3c	0.951810075807036	0.373460654258345	0.988741847156811	pseudobulk	DESeq2	LRT	0.427755146294593
332	KC_AB2	eIF3d1	-0.915036479129383	0.424258757743385	0.988741847156811	pseudobulk	DESeq2	LRT	0.372369184012361
332	KC_AB2	eIF3e	0.852684294570646	0.366143821237363	0.988741847156811	pseudobulk	DESeq2	LRT	0.436348290286271
332	KC_AB2	eIF3f1	-0.281760079355484	0.741686732565007	0.988741847156811	pseudobulk	DESeq2	LRT	0.129779489661984
332	KC_AB2	eIF3g1	0.239168812790081	0.814824101434252	0.988741847156811	pseudobulk	DESeq2	LRT	0.0889361336175064
332	KC_AB2	eIF3h	0.205160220218902	0.818045497657992	0.988741847156811	pseudobulk	DESeq2	LRT	0.0872225412769516
332	KC_AB2	eIF3i	-0.76618299737825	0.283894123967934	0.988741847156811	pseudobulk	DESeq2	LRT	0.546843596395281
332	KC_AB2	eIF3j	-0.925849100597597	0.468877589497074	0.988741847156811	pseudobulk	DESeq2	LRT	0.328940524331629
332	KC_AB2	eIF3k	0.556563436426384	0.640519236442799	0.988741847156811	pseudobulk	DESeq2	LRT	0.193467822741297
333	KC_AB2	eIF3l	-0.35659152143169	0.606188121379427	0.988741847156811	pseudobulk	DESeq2	LRT	0.217392578146411
333	KC_AB2	eIF3m	0.0487479791713632	0.918097871640419	0.988741847156811	pseudobulk	DESeq2	LRT	0.0371110194003808
333	KC_AB2	eIF4A	0.118119083739377	0.740511039644163	0.988741847156811	pseudobulk	DESeq2	LRT	0.130468462570452
333	KC_AB2	eIF4B	0.276738665645725	0.727506738711515	0.988741847156811	pseudobulk	DESeq2	LRT	0.138162979563124
333	KC_AB2	eIF4E1	0.288775839263135	0.757352209061727	0.988741847156811	pseudobulk	DESeq2	LRT	0.120702103487205
333	KC_AB2	eIF4E5	0.818790875285784	0.940355887156033	0.988741847156811	pseudobulk	DESeq2	LRT	0.0267077593721816
333	KC_AB2	eIF4EHP	0.201238395156761	0.689347498039282	0.988741847156811	pseudobulk	DESeq2	LRT	0.161561796327116
333	KC_AB2	eIF4G1	-0.181144943621152	0.831332023536838	0.988741847156811	pseudobulk	DESeq2	LRT	0.080225489824739
333	KC_AB2	eIF4G2	-0.0257372494826747	0.950241373022639	0.988741847156811	pseudobulk	DESeq2	LRT	0.0221660645458748
333	KC_AB2	eIF4H1	-0.273354445932037	0.756456141030883	0.988741847156811	pseudobulk	DESeq2	LRT	0.121216247099416
334	KC_AB2	eIF5	0.225171972524541	0.797942541387846	0.988741847156811	pseudobulk	DESeq2	LRT	0.0980283803994243
334	KC_AB2	eIF5B	-0.414370821200611	0.647650284780611	0.988741847156811	pseudobulk	DESeq2	LRT	0.188659439172935
334	KC_AB2	eIF6	-0.452344515838269	0.871171367298942	0.988741847156811	pseudobulk	DESeq2	LRT	0.0598964069304489
334	KC_AB2	Eip55E	-0.0582963981256801	0.979058404810457	0.988741847156811	pseudobulk	DESeq2	LRT	0.0091913999942843
334	KC_AB2	Eip63E	-0.260089175697055	0.747521429501094	0.988741847156811	pseudobulk	DESeq2	LRT	0.126376352728383
334	KC_AB2	Eip71CD	1.14703591811676	0.660809111836255	0.988741847156811	pseudobulk	DESeq2	LRT	0.179923977199595
334	KC_AB2	Eip75B	0.0230609638432377	0.954533133058859	0.988741847156811	pseudobulk	DESeq2	LRT	0.0202089920949462
334	KC_AB2	Eip78C	0.801371043861579	0.637329902962428	0.988741847156811	pseudobulk	DESeq2	LRT	0.195635704324295
334	KC_AB2	Eip93F	0.100193182071793	0.711596614916488	0.988741847156811	pseudobulk	DESeq2	LRT	0.147766126533288
334	KC_AB2	Ekar	1.42621218304824	0.66738831402044	0.988741847156811	pseudobulk	DESeq2	LRT	0.175621402117653
335	KC_AB2	elav	-0.248213532465856	0.628356026078182	0.988741847156811	pseudobulk	DESeq2	LRT	0.201794215579594
335	KC_AB2	elB	1.62636111143805	0.40185851414217	0.988741847156811	pseudobulk	DESeq2	LRT	0.395926825878373
335	KC_AB2	elgi	3.89657444468943	0.144963977997646	0.988741847156811	pseudobulk	DESeq2	LRT	0.838739901905657
335	KC_AB2	Elk	0.648182045642908	0.246165394587369	0.988741847156811	pseudobulk	DESeq2	LRT	0.608772999317247
335	KC_AB2	elm	-0.0561396270891976	0.966784179485183	0.988741847156811	pseudobulk	DESeq2	LRT	0.0146704650274223
335	KC_AB2	EloA	-0.330624682013443	0.829450751309215	0.988741847156811	pseudobulk	DESeq2	LRT	0.0812093951364332
335	KC_AB2	EloB	2.18204160808338	0.285627389905139	0.988741847156811	pseudobulk	DESeq2	LRT	0.544200148736546
335	KC_AB2	EloC	0.864198082535668	0.348362424649569	0.988741847156811	pseudobulk	DESeq2	LRT	0.457968695361878
335	KC_AB2	Elp1	2.06137138167525	0.547946929015274	0.988741847156811	pseudobulk	DESeq2	LRT	0.261261502741816

335	KC_AB2	Elp2	0.818790875285784	0.940355871560339	0.988741847156811	pseudobulk	DESeq2	LRT	0.0267077593721816
336	KC_AB2	Elp3	-2.26538931672255	0.547771004842275	0.988741847156811	pseudobulk	DESeq2	LRT	0.2614009599796
336	KC_AB2	Elys	0.818790875285784	0.940355871560339	0.988741847156811	pseudobulk	DESeq2	LRT	0.0267077593721816
336	KC_AB2	ema	-0.0384805229678164	0.985071128575604	0.988741847156811	pseudobulk	DESeq2	LRT	0.006532409469285
336	KC_AB2	emb	-0.0940725841649018	0.921590757148646	0.988741847156811	pseudobulk	DESeq2	LRT	0.0354618895409469
336	KC_AB2	emc	-0.475043920680493	0.40448707170807	0.988741847156811	pseudobulk	DESeq2	LRT	0.393095354831866
336	KC_AB2	EMC1	-0.75852709693596	0.63425596262606	0.988741847156811	pseudobulk	DESeq2	LRT	0.19773544129437
336	KC_AB2	EMC3	1.35316200662963	0.684467033464499	0.988741847156811	pseudobulk	DESeq2	LRT	0.164647464300239
336	KC_AB2	EMC8-9	0.697217550937474	0.650732865863005	0.988741847156811	pseudobulk	DESeq2	LRT	0.186597258269229
336	KC_AB2	EMRE	0.378474228908169	0.710389218553149	0.988741847156811	pseudobulk	DESeq2	LRT	0.148503638387121
336	KC_AB2	ena	0.0278441094049211	0.938174176084614	0.988741847156811	pseudobulk	DESeq2	LRT	0.0277165254906564
337	KC_AB2	enc	0.0772159339617869	0.877738262685721	0.988741847156811	pseudobulk	DESeq2	LRT	0.0566349693076763
337	KC_AB2	EndoA	0.321795996029919	0.609683419614825	0.988741847156811	pseudobulk	DESeq2	LRT	0.214895615487677
337	KC_AB2	EndoB	-0.0260627328834289	0.938542904403274	0.988741847156811	pseudobulk	DESeq2	LRT	0.0275458693189391
337	KC_AB2	EndoG	-3.6809086040586	0.231552724242678	0.988741847156811	pseudobulk	DESeq2	LRT	0.635350105118793
337	KC_AB2	EndoGI	-0.934020934207768	0.782359118258182	0.988741847156811	pseudobulk	DESeq2	LRT	0.106593851444836
337	KC_AB2	endos	0.235653239649011	0.804215530997118	0.988741847156811	pseudobulk	DESeq2	LRT	0.0946275440642236
337	KC_AB2	Eno	0.346506200011084	0.493446849671276	0.988741847156811	pseudobulk	DESeq2	LRT	0.306759619362304
337	KC_AB2	enok	0.178902983564581	0.90382885537382	0.988741847156811	pseudobulk	DESeq2	LRT	0.043913797625774
337	KC_AB2	ens	0.126456980586585	0.892064608528359	0.988741847156811	pseudobulk	DESeq2	LRT	0.0496036903422372
337	KC_AB2	Ent1	3.30033526000341	0.259137897354645	0.988741847156811	pseudobulk	DESeq2	LRT	0.586469069420752
338	KC_AB2	Ent2	0.712171366772551	0.813205723536451	0.988741847156811	pseudobulk	DESeq2	LRT	0.0897995733547685
338	KC_AB2	Ent3	0.0798435430463032	0.942161133009753	0.988741847156811	pseudobulk	DESeq2	LRT	0.0258748156863621
338	KC_AB2	Eogt	1.15128682504377	0.719737694898673	0.988741847156811	pseudobulk	DESeq2	LRT	0.142825751365876
338	KC_AB2	Epac	0.712171366772551	0.813205723536451	0.988741847156811	pseudobulk	DESeq2	LRT	0.0897995733547685
338	KC_AB2	Epg5	-0.957133960806503	0.762632518785665	0.988741847156811	pseudobulk	DESeq2	LRT	0.1176846802739
338	KC_AB2	Eph	0.323597627453646	0.694605891821291	0.988741847156811	pseudobulk	DESeq2	LRT	0.158261537214973
338	KC_AB2	Ephrin	-0.384447914387812	0.70403740910949	0.988741847156811	pseudobulk	DESeq2	LRT	0.152404263957057
338	KC_AB2	Eps-15	0.0237744608187441	0.975317192553908	0.988741847156811	pseudobulk	DESeq2	LRT	0.0108541201239194
338	KC_AB2	epsilonCOP	1.92721521352877	0.471759180353374	0.988741847156811	pseudobulk	DESeq2	LRT	0.326279639784082
338	KC_AB2	Ercc1	-2.51746470924027	0.517377929599049	0.988741847156811	pseudobulk	DESeq2	LRT	0.286192101436607
339	KC_AB2	eRF1	-0.973758241907998	0.395496783589036	0.988741847156811	pseudobulk	DESeq2	LRT	0.402857044088462
339	KC_AB2	eRF3	-0.00340877878248056	0.980029417943268	0.988741847156811	pseudobulk	DESeq2	LRT	0.0087608877170192
339	KC_AB2	ergic53	0.667839000613305	0.81413342949896	0.988741847156811	pseudobulk	DESeq2	LRT	0.0893044121300904
339	KC_AB2	Ero1L	0.568082799876568	0.825260330228634	0.988741847156811	pseudobulk	DESeq2	LRT	0.0834090306653718
339	KC_AB2	ERp60	-0.0340728771768707	0.969851936619519	0.988741847156811	pseudobulk	DESeq2	LRT	0.0132945626588201
339	KC_AB2	ERR	2.44948324680672	0.324428547937906	0.988741847156811	pseudobulk	DESeq2	LRT	0.48888093724017
339	KC_AB2	Es2	2.06137138167525	0.547946929015274	0.988741847156811	pseudobulk	DESeq2	LRT	0.261261502741816
339	KC_AB2	esn	0.818790875285784	0.940355871560339	0.988741847156811	pseudobulk	DESeq2	LRT	0.0267077593721816
339	KC_AB2	Esyt2	-1.81351761718572	0.117127845628514	0.988741847156811	pseudobulk	DESeq2	LRT	0.931339844763728
339	KC_AB2	Etf-QO	-0.568233637794205	0.868826397133536	0.988741847156811	pseudobulk	DESeq2	LRT	0.0610669925949191
340	KC_AB2	Etl1	-1.40296545307325	0.628223627794903	0.988741847156811	pseudobulk	DESeq2	LRT	0.201885733604539
340	KC_AB2	Ets97D	2.00515076565275	0.576456787459964	0.988741847156811	pseudobulk	DESeq2	LRT	0.239233242872451
340	KC_AB2	euc	1.42621218304824	0.66738831402044	0.988741847156811	pseudobulk	DESeq2	LRT	0.175621402117653
340	KC_AB2	Evi5	1.06436898357337	0.25324052288003	0.988741847156811	pseudobulk	DESeq2	LRT	0.596466798437262
340	KC_AB2	ewg	-0.530830919923514	0.648122549336304	0.988741847156811	pseudobulk	DESeq2	LRT	0.18834286840649
340	KC_AB2	exd	0.0985606357208893	0.932691774030451	0.988741847156811	pseudobulk	DESeq2	LRT	0.0302618535244163
340	KC_AB2	Exd2	-2.26538931672255	0.547771004842275	0.988741847156811	pseudobulk	DESeq2	LRT	0.2614009599796
340	KC_AB2	Exn	0.226504010747893	0.885319833187759	0.988741847156811	pseudobulk	DESeq2	LRT	0.0528998064823762
340	KC_AB2	Exo70	-0.55246575220467	0.787729781446674	0.988741847156811	pseudobulk	DESeq2	LRT	0.103622734988401
340	KC_AB2	Exo84	0.572861358867068	0.813871944286787	0.988741847156811	pseudobulk	DESeq2	LRT	0.0894439222171593
341	KC_AB2	ey	-0.085981207268162	0.8707172790259	0.988741847156811	pseudobulk	DESeq2	LRT	0.0601228370597947
341	KC_AB2	eys	0.535198450325649	0.432188213787418	0.988741847156811	pseudobulk	DESeq2	LRT	0.364327080943944
341	KC_AB2	f-cup	-1.73627324524809	0.521368726288639	0.988741847156811	pseudobulk	DESeq2	LRT	0.282855023048041
341	KC_AB2	fabp	0.819355979274046	0.601805319652072	0.988741847156811	pseudobulk	DESeq2	LRT	0.220543977637265
341	KC_AB2	Fadd	0.818790875285784	0.940355871560339	0.988741847156811	pseudobulk	DESeq2	LRT	0.0267077593721816

341	KC_AB2	faf	0.168397995468545	0.877150195248086	0.988741847156811	pseudobulk	DESeq2	LRT	0.0569260356162578
341	KC_AB2	Faf2	0.0307911778072702	0.947644582406897	0.988741847156811	pseudobulk	DESeq2	LRT	0.0233545158704913
341	KC_AB2	Fak	0.380980655566846	0.738187105452057	0.988741847156811	pseudobulk	DESeq2	LRT	0.131833545268685
341	KC_AB2	fal	-0.213177136262384	0.93155916913195	0.988741847156811	pseudobulk	DESeq2	LRT	0.030789555140895
341	KC_AB2	FAM21	2.73593896747858	0.422277110754874	0.988741847156811	pseudobulk	DESeq2	LRT	0.374402458575713
342	KC_AB2	fand	0.331758848923756	0.946375065527934	0.988741847156811	pseudobulk	DESeq2	LRT	0.0239367107367223
342	KC_AB2	Fas1	0.359395615557153	0.367087835440107	0.988741847156811	pseudobulk	DESeq2	LRT	0.435230006911893
342	KC_AB2	Fas2	-0.119460790677462	0.748211768895004	0.988741847156811	pseudobulk	DESeq2	LRT	0.125975464908249
342	KC_AB2	Fas3	0.829641805680855	0.450737339509337	0.988741847156811	pseudobulk	DESeq2	LRT	0.346076463071346
342	KC_AB2	FASN1	0.875505057383933	0.557000670320507	0.988741847156811	pseudobulk	DESeq2	LRT	0.254144282175782
342	KC_AB2	Fatp1	0.684221129527733	0.737351212059311	0.988741847156811	pseudobulk	DESeq2	LRT	0.132325601520231
342	KC_AB2	fax	-0.196411776745129	0.65002164597135	0.988741847156811	pseudobulk	DESeq2	LRT	0.187072180942703
342	KC_AB2	fbl	-4.21515119874888	0.130841174999422	0.988741847156811	pseudobulk	DESeq2	LRT	0.883255564413504
342	KC_AB2	Fbl6	0.157805303999508	0.909477145538044	0.988741847156811	pseudobulk	DESeq2	LRT	0.0412082099500571
342	KC_AB2	fbp	-2.51746470924027	0.517377929599049	0.988741847156811	pseudobulk	DESeq2	LRT	0.286192101436607
343	KC_AB2	Fbw5	0.818790875285784	0.940355871560339	0.988741847156811	pseudobulk	DESeq2	LRT	0.0267077593721816
343	KC_AB2	Fbxl4	1.47543920254359	0.661929169513603	0.988741847156811	pseudobulk	DESeq2	LRT	0.17918848025087
343	KC_AB2	FBXO11	0.0554292544151258	0.956516388264291	0.988741847156811	pseudobulk	DESeq2	LRT	0.0193075846834416
343	KC_AB2	Fcp1	-0.850804884520864	0.65925312502858	0.988741847156811	pseudobulk	DESeq2	LRT	0.180947802862626
343	KC_AB2	fdl	0.632478144582639	0.683818352791159	0.988741847156811	pseudobulk	DESeq2	LRT	0.165059247486532
343	KC_AB2	Fdx1	3.46443801158151	0.218071151261552	0.988741847156811	pseudobulk	DESeq2	LRT	0.6614017836492
343	KC_AB2	Fdx2	-0.349038222693293	0.86868347896181	0.988741847156811	pseudobulk	DESeq2	LRT	0.0611384380287992
343	KC_AB2	FeCH	-0.0582963981256801	0.979058404810457	0.988741847156811	pseudobulk	DESeq2	LRT	0.0091913999942843
343	KC_AB2	Fem-1	-0.409028640883814	0.818518332365151	0.988741847156811	pseudobulk	DESeq2	LRT	0.086971589244262
343	KC_AB2	fend	-0.0588128788011435	0.90014070799501	0.988741847156811	pseudobulk	DESeq2	LRT	0.0456895973058359
344	KC_AB2	FER	-0.506613484034373	0.961658398065406	0.988741847156811	pseudobulk	DESeq2	LRT	0.0169791713942958
344	KC_AB2	Fer1HCH	0.0104012228744593	0.974910544187924	0.988741847156811	pseudobulk	DESeq2	LRT	0.0110352324531664
344	KC_AB2	Fer2LCH	-0.347580152928789	0.421316664918797	0.988741847156811	pseudobulk	DESeq2	LRT	0.375391362285125
344	KC_AB2	fest	0.148520905817266	0.928829608110371	0.988741847156811	pseudobulk	DESeq2	LRT	0.0320639491329567
344	KC_AB2	Fgop2	1.17156071944349	0.597348257338218	0.988741847156811	pseudobulk	DESeq2	LRT	0.223772398949354
344	KC_AB2	fh	2.7785831508444	0.373751317747552	0.988741847156811	pseudobulk	DESeq2	LRT	0.427417267458199
344	KC_AB2	Fibp	-0.468109176374994	0.793482995826418	0.988741847156811	pseudobulk	DESeq2	LRT	0.100462375648746
344	KC_AB2	Fic	2.06137138167525	0.547946929015274	0.988741847156811	pseudobulk	DESeq2	LRT	0.261261502741816
344	KC_AB2	fid	-0.313445555076898	0.85625978514027	0.988741847156811	pseudobulk	DESeq2	LRT	0.0673944524535131
344	KC_AB2	fidipidine	-2.51746470924027	0.517377929599049	0.988741847156811	pseudobulk	DESeq2	LRT	0.286192101436607
345	KC_AB2	Fie	1.06972865429115	0.466761604053797	0.988741847156811	pseudobulk	DESeq2	LRT	0.330904876348596
345	KC_AB2	Fife	0.615973653170691	0.426844008550001	0.988741847156811	pseudobulk	DESeq2	LRT	0.369730810229036
345	KC_AB2	Fili	3.09926389624515	0.339712049344771	0.988741847156811	pseudobulk	DESeq2	LRT	0.468889048740397
345	KC_AB2	Fim	0.357138713579772	0.757976537663805	0.988741847156811	pseudobulk	DESeq2	LRT	0.120344237271434
345	KC_AB2	fipi	-0.213526059908176	0.853192452565958	0.988741847156811	pseudobulk	DESeq2	LRT	0.0689529950222515
345	KC_AB2	Fis1	-0.212075570998583	0.75584202090959	0.988741847156811	pseudobulk	DESeq2	LRT	0.121568967219398
345	KC_AB2	Fit1	-1.75175850638956	0.237101805679948	0.988741847156811	pseudobulk	DESeq2	LRT	0.62506513858254
345	KC_AB2	Fitm	2.41462217514131	0.497446973552313	0.988741847156811	pseudobulk	DESeq2	LRT	0.303253207020768
345	KC_AB2	Fkbp12	0.511357878546927	0.273796218242813	0.988741847156811	pseudobulk	DESeq2	LRT	0.562572554768941
345	KC_AB2	Fkbp14	0.818790875285784	0.940355871560339	0.988741847156811	pseudobulk	DESeq2	LRT	0.0267077593721816
346	KC_AB2	Fkbp39	0.568082799876568	0.825260330228634	0.988741847156811	pseudobulk	DESeq2	LRT	0.0834090306653718
346	KC_AB2	Fkbp59	0.997366942311027	0.663723613191462	0.988741847156811	pseudobulk	DESeq2	LRT	0.178012731256061
346	KC_AB2	fl(2)d	0.822056291484769	0.428050301631008	0.988741847156811	pseudobulk	DESeq2	LRT	0.368505192582283
346	KC_AB2	Flacc	-0.544182215481797	0.744990859502554	0.988741847156811	pseudobulk	DESeq2	LRT	0.127849055697285
346	KC_AB2	FLASH	0.712171366772551	0.813205723536451	0.988741847156811	pseudobulk	DESeq2	LRT	0.0897995733547685
346	KC_AB2	flfl	-0.619482231223099	0.570992537755858	0.988741847156811	pseudobulk	DESeq2	LRT	0.24336956746804
346	KC_AB2	flil	0.666943671827768	0.808936122914367	0.988741847156811	pseudobulk	DESeq2	LRT	0.0920857707994393
346	KC_AB2	Flo1	0.433974940944578	0.629020736883279	0.988741847156811	pseudobulk	DESeq2	LRT	0.201335036962474
346	KC_AB2	Flo2	-0.238437221799648	0.796433013802662	0.988741847156811	pseudobulk	DESeq2	LRT	0.0988507458631681
346	KC_AB2	flr	-1.83172320633983	0.524697733495197	0.988741847156811	pseudobulk	DESeq2	LRT	0.280090811792999
347	KC_AB2	flw	0.87595614699083	0.400933752018645	0.988741847156811	pseudobulk	DESeq2	LRT	0.39692738176784

347	KC_AB2	Fmr1	-0.391113651195192	0.774011029964072	0.988741847156811	pseudobulk	DESeq2	LRT	0.111252850404445
347	KC_AB2	fmt	0.518163077419865	0.636104216276365	0.988741847156811	pseudobulk	DESeq2	LRT	0.196471725788833
347	KC_AB2	fne	-0.114441716560568	0.755052119706765	0.988741847156811	pseudobulk	DESeq2	LRT	0.122023068874395
347	KC_AB2	Fnta	2.7785831508444	0.373751317747552	0.988741847156811	pseudobulk	DESeq2	LRT	0.427417267458199
347	KC_AB2	fog	-2.26538931672255	0.547771004842275	0.988741847156811	pseudobulk	DESeq2	LRT	0.2614009599796
347	KC_AB2	foi	-0.450294942462673	0.78910063697519	0.988741847156811	pseudobulk	DESeq2	LRT	0.102867606048018
347	KC_AB2	fok	-1.20499905131099	0.0274363190657336	0.988741847156811	pseudobulk	DESeq2	LRT	1.56167415523469
347	KC_AB2	form3	-1.23536834900699	0.725570392794057	0.988741847156811	pseudobulk	DESeq2	LRT	0.139320447155934
347	KC_AB2	FoxK	0.572563863052134	0.58445290392247	0.988741847156811	pseudobulk	DESeq2	LRT	0.233250479182479
348	KC_AB2	FoxP	0.278041306552392	0.571726312802977	0.988741847156811	pseudobulk	DESeq2	LRT	0.242811819606323
348	KC_AB2	fra	1.47543920254359	0.661929169513603	0.988741847156811	pseudobulk	DESeq2	LRT	0.17918848025087
348	KC_AB2	fray	-1.23536834900699	0.725570392794057	0.988741847156811	pseudobulk	DESeq2	LRT	0.139320447155934
348	KC_AB2	FRG1	-0.0034087787824805	0.980029417943268	0.988741847156811	pseudobulk	DESeq2	LRT	0.0087608877170192
348	KC_AB2	frj	-0.990576791433587	0.536423540579161	0.988741847156811	pseudobulk	DESeq2	LRT	0.270492171686769
348	KC_AB2	Frl	0.239168812790081	0.814824101434252	0.988741847156811	pseudobulk	DESeq2	LRT	0.0889361336175064
348	KC_AB2	Frq1	0.189822470203192	0.699668840395856	0.988741847156811	pseudobulk	DESeq2	LRT	0.155107466870289
348	KC_AB2	Frq2	0.145260802214693	0.881362101575346	0.988741847156811	pseudobulk	DESeq2	LRT	0.0548456280155302
348	KC_AB2	fru	2.03852607663652	0.187087703568458	0.988741847156811	pseudobulk	DESeq2	LRT	0.727954755780899
348	KC_AB2	fry	0.219036624197398	0.897526678470329	0.988741847156811	pseudobulk	DESeq2	LRT	0.0469526334011757
349	KC_AB2	fs(1)K10	0.818790875285784	0.940355871560339	0.988741847156811	pseudobulk	DESeq2	LRT	0.0267077593721816
349	KC_AB2	Fs(2)Ket	-0.0271311609619475	0.929755751607669	0.988741847156811	pseudobulk	DESeq2	LRT	0.0316311263502787
349	KC_AB2	fs(2)ltoPP43	0.928039350209165	0.67716747857253	0.988741847156811	pseudobulk	DESeq2	LRT	0.169303907349344
349	KC_AB2	Fsn	-1.40296545307325	0.628223627794903	0.988741847156811	pseudobulk	DESeq2	LRT	0.201885733604539
349	KC_AB2	ftz-f1	-0.601098532458938	0.288373511839451	0.988741847156811	pseudobulk	DESeq2	LRT	0.540044633655358
349	KC_AB2	fu	0.818790875285784	0.940355871560339	0.988741847156811	pseudobulk	DESeq2	LRT	0.0267077593721816
349	KC_AB2	FucT6	0.226504010747893	0.885319833187759	0.988741847156811	pseudobulk	DESeq2	LRT	0.0528998064823762
349	KC_AB2	FucTA	2.34011937438372	0.357833688438857	0.988741847156811	pseudobulk	DESeq2	LRT	0.446318774963809
349	KC_AB2	FucTB	-0.506613484034373	0.961658398065406	0.988741847156811	pseudobulk	DESeq2	LRT	0.0169791713942958
349	KC_AB2	Fum1	0.0182825326159166	0.97104529804109	0.988741847156811	pseudobulk	DESeq2	LRT	0.0127605103284255
350	KC_AB2	Fur1	0.130691985917548	0.904348090302381	0.988741847156811	pseudobulk	DESeq2	LRT	0.0436643741708152
350	KC_AB2	Fur2	-2.02131623479709	0.177155023904376	0.988741847156811	pseudobulk	DESeq2	LRT	0.751646527084626
350	KC_AB2	fuss	2.00515076565275	0.576456787459964	0.988741847156811	pseudobulk	DESeq2	LRT	0.239233242872451
350	KC_AB2	futsch	-0.181043798975918	0.613969309992659	0.988741847156811	pseudobulk	DESeq2	LRT	0.211853337057453
350	KC_AB2	fwd	0.355901461055476	0.755521726342369	0.988741847156811	pseudobulk	DESeq2	LRT	0.121753042251584
350	KC_AB2	fwe	0.239056890788375	0.662975495929167	0.988741847156811	pseudobulk	DESeq2	LRT	0.178502523147885
350	KC_AB2	fws	-1.80294541848982	0.660135549041553	0.988741847156811	pseudobulk	DESeq2	LRT	0.180366879372519
350	KC_AB2	fz	-0.596089946306731	0.223463327862866	0.988741847156811	pseudobulk	DESeq2	LRT	0.650793737917098
350	KC_AB2	fz2	0.718019899564923	0.642887859706877	0.988741847156811	pseudobulk	DESeq2	LRT	0.191864775377246
350	KC_AB2	fz3	0.572861358867068	0.813871944286787	0.988741847156811	pseudobulk	DESeq2	LRT	0.0894439222171593
351	KC_AB2	fz4	2.06137138167525	0.547946929015274	0.988741847156811	pseudobulk	DESeq2	LRT	0.261261502741816
351	KC_AB2	fzr	0.880454992600614	0.54826962515464	0.988741847156811	pseudobulk	DESeq2	LRT	0.261005813925271
351	KC_AB2	g	0.644770321751384	0.330563255252942	0.988741847156811	pseudobulk	DESeq2	LRT	0.480745423383401
351	KC_AB2	G6P	0.818790875285784	0.940355871560339	0.988741847156811	pseudobulk	DESeq2	LRT	0.0267077593721816
351	KC_AB2	G9a	0.840499589548677	0.471261407859071	0.988741847156811	pseudobulk	DESeq2	LRT	0.32673812366089
351	KC_AB2	GAA1	-0.8330424863479	0.701126211347028	0.988741847156811	pseudobulk	DESeq2	LRT	0.15420379664422
351	KC_AB2	GABA-B-R1	0.219270652702647	0.798061918620791	0.988741847156811	pseudobulk	DESeq2	LRT	0.0979634120676578
351	KC_AB2	GABA-B-R2	0.186204595970438	0.867680637480482	0.988741847156811	pseudobulk	DESeq2	LRT	0.0616400938345908
351	KC_AB2	Gabat	0.775182625592722	0.436104728820087	0.988741847156811	pseudobulk	DESeq2	LRT	0.360409204110028
351	KC_AB2	GABPI	0.80430127541587	0.689337728291919	0.988741847156811	pseudobulk	DESeq2	LRT	0.161567951390344
352	KC_AB2	Gale	0.297713224699148	0.901701424115727	0.988741847156811	pseudobulk	DESeq2	LRT	0.0449372444148942
352	KC_AB2	galectin	0.361297473630756	0.86408302051291	0.988741847156811	pseudobulk	DESeq2	LRT	0.0634445287959917
352	KC_AB2	Galk	2.00515076565275	0.576456787459964	0.988741847156811	pseudobulk	DESeq2	LRT	0.239233242872451
352	KC_AB2	galla-1	0.432993905702896	0.77248797019866	0.988741847156811	pseudobulk	DESeq2	LRT	0.112108275031956
352	KC_AB2	galla-2	-0.234160738284965	0.896763493077035	0.988741847156811	pseudobulk	DESeq2	LRT	0.047322080027168
352	KC_AB2	Galphao	-0.0125814360844891	0.964169895576558	0.988741847156811	pseudobulk	DESeq2	LRT	0.0158464326840512
352	KC_AB2	Galphaq	-0.438831408897175	0.556898461363722	0.988741847156811	pseudobulk	DESeq2	LRT	0.254223982006396

352	KC_AB2	Galphas	-0.0334342421144298	0.949704185663437	0.988741847156811	pseudobulk	DESeq2	LRT	0.0224116479111454
352	KC_AB2	Galt	-1.99867440840856	0.534899605264936	0.988741847156811	pseudobulk	DESeq2	LRT	0.271727722597268
352	KC_AB2	GalT1	1.45259040912931	0.616176917291092	0.988741847156811	pseudobulk	DESeq2	LRT	0.210294574894236
353	KC_AB2	gammaCOP	-1.26918136841212	0.54413768650788	0.988741847156811	pseudobulk	DESeq2	LRT	0.264291194190369
353	KC_AB2	gammaSnap1	0.622272672451592	0.422580963899162	0.988741847156811	pseudobulk	DESeq2	LRT	0.374090070605421
353	KC_AB2	gammaSnap2	0.630543148119245	0.748402447226467	0.988741847156811	pseudobulk	DESeq2	LRT	0.125864801055495
353	KC_AB2	gammaTub23C	-0.934020934207768	0.782359118258182	0.988741847156811	pseudobulk	DESeq2	LRT	0.106593851444836
353	KC_AB2	GAPcenA	-0.0244563360567977	0.955338917724215	0.988741847156811	pseudobulk	DESeq2	LRT	0.0198425300019622
353	KC_AB2	Gapdh1	0.143467187426069	0.90336274906372	0.988741847156811	pseudobulk	DESeq2	LRT	0.0441378219101368
353	KC_AB2	Gapdh2	0.306604027478726	0.666206068505266	0.988741847156811	pseudobulk	DESeq2	LRT	0.176391415616102
353	KC_AB2	GAPsec	-0.50879048068994	0.817628596116567	0.988741847156811	pseudobulk	DESeq2	LRT	0.0874439277298491
353	KC_AB2	Gapvd1	0.861054960758556	0.567907291927136	0.988741847156811	pseudobulk	DESeq2	LRT	0.245722554941499
353	KC_AB2	garz	0.666943671827768	0.808936122914367	0.988741847156811	pseudobulk	DESeq2	LRT	0.0920857707994393
354	KC_AB2	Gas41	0.792281256713368	0.823478838299676	0.988741847156811	pseudobulk	DESeq2	LRT	0.0843475568193814
354	KC_AB2	Gas8	1.42621218304824	0.66738831402044	0.988741847156811	pseudobulk	DESeq2	LRT	0.175621402117653
354	KC_AB2	Gasz	-0.618001500136109	0.807592904791208	0.988741847156811	pseudobulk	DESeq2	LRT	0.092807505260298
354	KC_AB2	Gat	-2.51746470924027	0.517377929599049	0.988741847156811	pseudobulk	DESeq2	LRT	0.286192101436607
354	KC_AB2	GatA	-0.934020934207768	0.782359118258182	0.988741847156811	pseudobulk	DESeq2	LRT	0.106593851444836
354	KC_AB2	GATAd	0.0678697882829055	0.946868218762626	0.988741847156811	pseudobulk	DESeq2	LRT	0.0237104601167619
354	KC_AB2	GatB	1.12452187615248	0.704078404906088	0.988741847156811	pseudobulk	DESeq2	LRT	0.152378975911753
354	KC_AB2	gb	-0.506613484034373	0.961658398065406	0.988741847156811	pseudobulk	DESeq2	LRT	0.0169791713942958
354	KC_AB2	Gba1a	-1.80294541848982	0.660135549041553	0.988741847156811	pseudobulk	DESeq2	LRT	0.180366879372519
354	KC_AB2	Gbeta13F	0.0360526856427797	0.904559434641697	0.988741847156811	pseudobulk	DESeq2	LRT	0.0435628922769113
355	KC_AB2	Gbeta5	1.52113550630848	0.162954853583948	0.988741847156811	pseudobulk	DESeq2	LRT	0.787932699615318
355	KC_AB2	Gbs-76A	-0.0885198033477002	0.959687900795474	0.988741847156811	pseudobulk	DESeq2	LRT	0.0178699805019348
355	KC_AB2	GC	0.0232200085868279	0.946273123445589	0.988741847156811	pseudobulk	DESeq2	LRT	0.0239834947974229
355	KC_AB2	GC1	1.92721521352877	0.471759180353374	0.988741847156811	pseudobulk	DESeq2	LRT	0.326279639784082
355	KC_AB2	GCC185	0.0885531792216718	0.964858407830046	0.988741847156811	pseudobulk	DESeq2	LRT	0.0155364143348316
355	KC_AB2	GCC88	2.41462217514131	0.497446973552313	0.988741847156811	pseudobulk	DESeq2	LRT	0.303253207020768
355	KC_AB2	gce	0.413893838466125	0.582226979605498	0.988741847156811	pseudobulk	DESeq2	LRT	0.234907673815946
355	KC_AB2	GckIII	1.64209625885217	0.483203675195701	0.988741847156811	pseudobulk	DESeq2	LRT	0.315869771176778
355	KC_AB2	gcl	-0.0681140289272513	0.944219428467735	0.988741847156811	pseudobulk	DESeq2	LRT	0.0249270676735407
355	KC_AB2	Gclc	0.950064444524007	0.521271724636783	0.988741847156811	pseudobulk	DESeq2	LRT	0.282935831887216
356	KC_AB2	Gclm	0.41260719752318	0.800096267863296	0.988741847156811	pseudobulk	DESeq2	LRT	0.0968577553999308
356	KC_AB2	Gcn2	1.74111111089874	0.563806300883242	0.988741847156811	pseudobulk	DESeq2	LRT	0.248870074928531
356	KC_AB2	Gcn5	2.4643417141186	0.449082701910884	0.988741847156811	pseudobulk	DESeq2	LRT	0.347673673080982
356	KC_AB2	GCS1	0.998893024789477	0.660591418406266	0.988741847156811	pseudobulk	DESeq2	LRT	0.180067072429364
356	KC_AB2	GCS2alpha	-0.223533551847974	0.843262497789343	0.988741847156811	pseudobulk	DESeq2	LRT	0.0740372135176805
356	KC_AB2	GCS2beta	0.157562008957688	0.950463298513166	0.988741847156811	pseudobulk	DESeq2	LRT	0.0220646484576093
356	KC_AB2	Gdap2	0.350794979129823	0.411389881763251	0.988741847156811	pseudobulk	DESeq2	LRT	0.385746394091041
356	KC_AB2	Gdh	0.233807124954093	0.726076259629931	0.988741847156811	pseudobulk	DESeq2	LRT	0.139017763050371
356	KC_AB2	Gdi	0.158789793237827	0.826330145462804	0.988741847156811	pseudobulk	DESeq2	LRT	0.0828464033982917
356	KC_AB2	Gdn1	-0.378852672109618	0.920549594954422	0.988741847156811	pseudobulk	DESeq2	LRT	0.0359528087516892
357	KC_AB2	Ge-1	0.760546103616116	0.738305064567484	0.988741847156811	pseudobulk	DESeq2	LRT	0.131764152427911
357	KC_AB2	GEFmeso	1.42621218304824	0.66738831402044	0.988741847156811	pseudobulk	DESeq2	LRT	0.175621402117653
357	KC_AB2	gek	-0.689687550646419	0.745991540083112	0.988741847156811	pseudobulk	DESeq2	LRT	0.127266097615876
357	KC_AB2	Gel	0.0601466527378941	0.974810960364079	0.988741847156811	pseudobulk	DESeq2	LRT	0.0110795964355292
357	KC_AB2	geminin	-1.52496782475103	0.617219242690575	0.988741847156811	pseudobulk	DESeq2	LRT	0.209560542647675
357	KC_AB2	Gen	1.42621218304824	0.66738831402044	0.988741847156811	pseudobulk	DESeq2	LRT	0.175621402117653
357	KC_AB2	Gfat2	-0.158601889198964	0.945838126788742	0.988741847156811	pseudobulk	DESeq2	LRT	0.0241831835324482
357	KC_AB2	Gfrl	0.0509421711070138	0.875452744166557	0.988741847156811	pseudobulk	DESeq2	LRT	0.0577672916154789
357	KC_AB2	gfzf	1.35316200662963	0.684467033464499	0.988741847156811	pseudobulk	DESeq2	LRT	0.164647464300239
357	KC_AB2	Gga	-3.4227037205893	0.285801360563088	0.988741847156811	pseudobulk	DESeq2	LRT	0.543935708072568
358	KC_AB2	Ggamma1	0.206204540304434	0.605664655504813	0.988741847156811	pseudobulk	DESeq2	LRT	0.21776776952161
358	KC_AB2	Ggamma30A	-0.0827136058818619	0.778230820082627	0.988741847156811	pseudobulk	DESeq2	LRT	0.10889157393478
358	KC_AB2	ghi	-0.285953769659895	0.805421247896835	0.988741847156811	pseudobulk	DESeq2	LRT	0.0939769174121901

358	KC_AB2	gig	1.42621218304824	0.66738831402044	0.988741847156811	pseudobulk	DESeq2	LRT	0.175621402117653
358	KC_AB2	GIIIspla2	2.00515076565275	0.576456787459964	0.988741847156811	pseudobulk	DESeq2	LRT	0.239233242872451
358	KC_AB2	GILT1	-2.01288303445131	0.360334600843078	0.988741847156811	pseudobulk	DESeq2	LRT	0.44329403309331
358	KC_AB2	Girdin	1.12452187615248	0.704078404906088	0.988741847156811	pseudobulk	DESeq2	LRT	0.152378975911753
358	KC_AB2	gish	-0.0541163673899606	0.895650396129903	0.988741847156811	pseudobulk	DESeq2	LRT	0.0478614776819347
358	KC_AB2	Git	2.00515076565275	0.576456787459964	0.988741847156811	pseudobulk	DESeq2	LRT	0.239233242872451
358	KC_AB2	Gk1	0.331758848923756	0.946375065527934	0.988741847156811	pseudobulk	DESeq2	LRT	0.0239367107367223
359	KC_AB2	Gk2	-0.378852672109618	0.920549594954422	0.988741847156811	pseudobulk	DESeq2	LRT	0.0359528087516892
359	KC_AB2	gkt	-1.1284597703615	0.729754448571724	0.988741847156811	pseudobulk	DESeq2	LRT	0.136823248879498
359	KC_AB2	GlcAT-I	0.321802230037868	0.828000394066036	0.988741847156811	pseudobulk	DESeq2	LRT	0.0819694565234964
359	KC_AB2	GlcAT-P	-0.182504956546188	0.670652488518002	0.988741847156811	pseudobulk	DESeq2	LRT	0.17350245958351
359	KC_AB2	GlcAT-S	0.170681531202091	0.89141402288473	0.988741847156811	pseudobulk	DESeq2	LRT	0.049920538284892
359	KC_AB2	GlcT	0.818790875285784	0.940355871560339	0.988741847156811	pseudobulk	DESeq2	LRT	0.0267077593721816
359	KC_AB2	Gle1	3.09926389624515	0.339712049344771	0.988741847156811	pseudobulk	DESeq2	LRT	0.468889048740397
359	KC_AB2	glec	0.600370099908307	0.573544841785534	0.988741847156811	pseudobulk	DESeq2	LRT	0.241432621740566
359	KC_AB2	Glg1	1.4129303440845	0.645460595159151	0.988741847156811	pseudobulk	DESeq2	LRT	0.190130265910817
359	KC_AB2	GlnRS	1.12452187615248	0.704078404906088	0.988741847156811	pseudobulk	DESeq2	LRT	0.152378975911753
360	KC_AB2	glo	-0.0191245218790105	0.965710345977459	0.988741847156811	pseudobulk	DESeq2	LRT	0.0151531158279729
360	KC_AB2	Glo1	-0.732725114995457	0.722759478418736	0.988741847156811	pseudobulk	DESeq2	LRT	0.141006204191428
360	KC_AB2	glob1	0.818790875285784	0.940355871560339	0.988741847156811	pseudobulk	DESeq2	LRT	0.0267077593721816
360	KC_AB2	GLS	0.189249264265911	0.917387558178736	0.988741847156811	pseudobulk	DESeq2	LRT	0.0374471541658424
360	KC_AB2	glu	-2.53258623675656	0.392718648892655	0.988741847156811	pseudobulk	DESeq2	LRT	0.405918475050257
360	KC_AB2	GluClalpha	-0.102632653212166	0.870315225089692	0.988741847156811	pseudobulk	DESeq2	LRT	0.0603234189657771
360	KC_AB2	GluRIA	0.542986845697558	0.445176303039339	0.988741847156811	pseudobulk	DESeq2	LRT	0.351467961437864
360	KC_AB2	GluRIB	0.450697610856773	0.325113924695513	0.988741847156811	pseudobulk	DESeq2	LRT	0.48796442918454
360	KC_AB2	Glut1	-0.791730508617457	0.34644160570364	0.988741847156811	pseudobulk	DESeq2	LRT	0.46036995717876
360	KC_AB2	Glut4EF	0.241652318176394	0.55070115922242	0.988741847156811	pseudobulk	DESeq2	LRT	0.259084009330647
361	KC_AB2	GlyP	0.387626856268115	0.752774921273493	0.988741847156811	pseudobulk	DESeq2	LRT	0.12333485787777
361	KC_AB2	GlyRS	0.575200883059258	0.804213580533116	0.988741847156811	pseudobulk	DESeq2	LRT	0.0946285973599483
361	KC_AB2	GlyS	1.15128682504377	0.719737694898673	0.988741847156811	pseudobulk	DESeq2	LRT	0.142825751365876
361	KC_AB2	GlyT	-0.327276434056388	0.72449988353581	0.988741847156811	pseudobulk	DESeq2	LRT	0.139961680006138
361	KC_AB2	GM130	2.90072538444148	0.201184744653021	0.988741847156811	pseudobulk	DESeq2	LRT	0.696404953855657
361	KC_AB2	Gmap	0.699674038439951	0.821690393783994	0.988741847156811	pseudobulk	DESeq2	LRT	0.0852917902455435
361	KC_AB2	Gmer	-2.26538931672255	0.547771004842275	0.988741847156811	pseudobulk	DESeq2	LRT	0.2614009599796
361	KC_AB2	GMF	-0.194388201900128	0.928057462354013	0.988741847156811	pseudobulk	DESeq2	LRT	0.0324251328213705
361	KC_AB2	GNBP3	0.818790875285784	0.940355871560339	0.988741847156811	pseudobulk	DESeq2	LRT	0.0267077593721816
361	KC_AB2	Gnf1	0.572861358867068	0.813871944286787	0.988741847156811	pseudobulk	DESeq2	LRT	0.0894439222171593
362	KC_AB2	Gnpnat	1.06980162817022	0.618453116986936	0.988741847156811	pseudobulk	DESeq2	LRT	0.208693217305034
362	KC_AB2	gny	1.47543920254359	0.661929169513603	0.988741847156811	pseudobulk	DESeq2	LRT	0.17918848025087
362	KC_AB2	gogo	-2.09209031988317	0.408698600207438	0.988741847156811	pseudobulk	DESeq2	LRT	0.388596849744308
362	KC_AB2	gol	-0.940729884717284	0.405295949052607	0.988741847156811	pseudobulk	DESeq2	LRT	0.392227737025372
362	KC_AB2	Golgin104	0.148158061181916	0.939300423739034	0.988741847156811	pseudobulk	DESeq2	LRT	0.0271954817436067
362	KC_AB2	Golgin245	3.81052043979741	0.156359277889969	0.988741847156811	pseudobulk	DESeq2	LRT	0.805876343926194
362	KC_AB2	Golgin84	-0.957133960806503	0.762632518785665	0.988741847156811	pseudobulk	DESeq2	LRT	0.1176846802739
362	KC_AB2	gom	0.297713224699148	0.901701424115727	0.988741847156811	pseudobulk	DESeq2	LRT	0.0449372444148942
362	KC_AB2	Gorab	-1.99867440840856	0.534899605264936	0.988741847156811	pseudobulk	DESeq2	LRT	0.271727722597268
362	KC_AB2	Gos28	-0.487386868153729	0.832337069706575	0.988741847156811	pseudobulk	DESeq2	LRT	0.0797007628096776
363	KC_AB2	Got1	-0.568233637794205	0.868826397133536	0.988741847156811	pseudobulk	DESeq2	LRT	0.0610669925949191
363	KC_AB2	Got2	2.47120421534988	0.318754600362815	0.988741847156811	pseudobulk	DESeq2	LRT	0.496543538643422
363	KC_AB2	Gp210	-0.213177136262384	0.93155916913195	0.988741847156811	pseudobulk	DESeq2	LRT	0.030789555140895
363	KC_AB2	Gp93	0.513202572024843	0.807055953632252	0.988741847156811	pseudobulk	DESeq2	LRT	0.093096354359303
363	KC_AB2	Gpa2	1.42621218304824	0.66738831402044	0.988741847156811	pseudobulk	DESeq2	LRT	0.175621402117653
363	KC_AB2	Gpat4	0.0885531792216718	0.964858407830046	0.988741847156811	pseudobulk	DESeq2	LRT	0.0155364143348316
363	KC_AB2	Gpb5	0.029375960626152	0.946152502853269	0.988741847156811	pseudobulk	DESeq2	LRT	0.0240388574568496
363	KC_AB2	Gpdh1	0.0593609254655896	0.93757213511186	0.988741847156811	pseudobulk	DESeq2	LRT	0.0279953084793413
363	KC_AB2	Gpdh3	-1.80294541848982	0.660135549041553	0.988741847156811	pseudobulk	DESeq2	LRT	0.180366879372519

363	KC_AB2	GPHR	0.818790875285784	0.940355871560339	0.988741847156811	pseudobulk	DESeq2	LRT	0.0267077593721816
364	KC_AB2	Gpo1	-0.215812554377026	0.909171219175963	0.988741847156811	pseudobulk	DESeq2	LRT	0.0413543208033163
364	KC_AB2	gpp	-0.623562168302718	0.297960846148511	0.988741847156811	pseudobulk	DESeq2	LRT	0.52584080108761
364	KC_AB2	Gprk1	0.169578649526312	0.733059715889408	0.988741847156811	pseudobulk	DESeq2	LRT	0.134860645788743
364	KC_AB2	Gprk2	-0.0644324271693383	0.908502386005499	0.988741847156811	pseudobulk	DESeq2	LRT	0.0416739277641346
364	KC_AB2	gprs	-0.193669160805829	0.769416685996017	0.988741847156811	pseudobulk	DESeq2	LRT	0.11383839957662
364	KC_AB2	Graf	-0.0832562497656724	0.899781902325897	0.988741847156811	pseudobulk	DESeq2	LRT	0.0458627462216192
364	KC_AB2	GramD1B	0.594901446393324	0.641807363494129	0.988741847156811	pseudobulk	DESeq2	LRT	0.192595304525459
364	KC_AB2	Grasp65	0.157562008957688	0.950463298513166	0.988741847156811	pseudobulk	DESeq2	LRT	0.0220646484576093
364	KC_AB2	grau	-1.33788900664848	0.722041597401238	0.988741847156811	pseudobulk	DESeq2	LRT	0.141437781652764
364	KC_AB2	Grik	3.03619069427769	0.314302467188174	0.988741847156811	pseudobulk	DESeq2	LRT	0.502652209876457
365	KC_AB2	Grip128	0.818790875285784	0.940355871560339	0.988741847156811	pseudobulk	DESeq2	LRT	0.0267077593721816
365	KC_AB2	Grip163	2.00515076565275	0.576456787459964	0.988741847156811	pseudobulk	DESeq2	LRT	0.239233242872451
365	KC_AB2	Grip71	1.47543920254359	0.661929169513603	0.988741847156811	pseudobulk	DESeq2	LRT	0.17918848025087
365	KC_AB2	Grip75	0.11213458976865	0.938112983749562	0.988741847156811	pseudobulk	DESeq2	LRT	0.0277448532370976
365	KC_AB2	Grip91	2.06137138167525	0.547946929015274	0.988741847156811	pseudobulk	DESeq2	LRT	0.261261502741816
365	KC_AB2	gro	0.0743879879760201	0.903432800647592	0.988741847156811	pseudobulk	DESeq2	LRT	0.0441041456966392
365	KC_AB2	grp	-0.0399702336628495	0.95788975899235	0.988741847156811	pseudobulk	DESeq2	LRT	0.0186844698527901
365	KC_AB2	grsm	0.320539134359348	0.848791832994679	0.988741847156811	pseudobulk	DESeq2	LRT	0.0711988078324561
365	KC_AB2	Grx1	0.0401806130337701	0.933305788272293	0.988741847156811	pseudobulk	DESeq2	LRT	0.0299760407108805
365	KC_AB2	gry	-0.506613484034373	0.961658398065406	0.988741847156811	pseudobulk	DESeq2	LRT	0.0169791713942958
366	KC_AB2	Gs1	0.235112809488509	0.817829086127029	0.988741847156811	pseudobulk	DESeq2	LRT	0.0873374478048695
366	KC_AB2	Gs1l	-0.297555621265812	0.770273406883377	0.988741847156811	pseudobulk	DESeq2	LRT	0.113355095574041
366	KC_AB2	Gs2	2.06137138167525	0.547946929015274	0.988741847156811	pseudobulk	DESeq2	LRT	0.261261502741816
366	KC_AB2	GstD1	-0.217963318638521	0.796760594396976	0.988741847156811	pseudobulk	DESeq2	LRT	0.0986721530722332
366	KC_AB2	GstD11	-0.506613484034373	0.961658398065406	0.988741847156811	pseudobulk	DESeq2	LRT	0.0169791713942958
366	KC_AB2	GstD9	0.0601466527378941	0.974810960364079	0.988741847156811	pseudobulk	DESeq2	LRT	0.0110795964355292
366	KC_AB2	GstO2	1.69894780879763	0.540778829726708	0.988741847156811	pseudobulk	DESeq2	LRT	0.26698031838453
366	KC_AB2	GstO3	0.818790875285784	0.940355871560339	0.988741847156811	pseudobulk	DESeq2	LRT	0.0267077593721816
366	KC_AB2	GstT1	-0.0582963981256801	0.979058404810457	0.988741847156811	pseudobulk	DESeq2	LRT	0.0091913999942843
366	KC_AB2	GstT2	2.06137138167525	0.547946929015274	0.988741847156811	pseudobulk	DESeq2	LRT	0.261261502741816
367	KC_AB2	GstZ2	0.157562008957688	0.950463298513166	0.988741847156811	pseudobulk	DESeq2	LRT	0.0220646484576093
367	KC_AB2	Gtp-bp	-0.506613484034373	0.961658398065406	0.988741847156811	pseudobulk	DESeq2	LRT	0.0169791713942958
367	KC_AB2	Gug	0.14718065034379	0.773392766406596	0.988741847156811	pseudobulk	DESeq2	LRT	0.111599894228807
367	KC_AB2	gukh	0.699674038439951	0.821690393783994	0.988741847156811	pseudobulk	DESeq2	LRT	0.0852917902455435
367	KC_AB2	gus	-0.344819260829251	0.575347037336824	0.988741847156811	pseudobulk	DESeq2	LRT	0.240070118898613
367	KC_AB2	gw	0.126221181802482	0.792472486170074	0.988741847156811	pseudobulk	DESeq2	LRT	0.101015807106351
367	KC_AB2	GXIVsPLA2	0.362555753552128	0.897034383127386	0.988741847156811	pseudobulk	DESeq2	LRT	0.0471909102264623
367	KC_AB2	Gyc88E	0.646616639464795	0.643967348261282	0.988741847156811	pseudobulk	DESeq2	LRT	0.19113615256169
367	KC_AB2	Gyc89Da	0.157562008957688	0.950463298513166	0.988741847156811	pseudobulk	DESeq2	LRT	0.0220646484576093
367	KC_AB2	Gyc89Db	2.2511916182477	0.415234008889419	0.988741847156811	pseudobulk	DESeq2	LRT	0.381707083704527
368	KC_AB2	Gycalpha99B	-0.610590644230464	0.4561922879852	0.988741847156811	pseudobulk	DESeq2	LRT	0.340852060825661
368	KC_AB2	Gycbeta100B	0.0539453817982923	0.95376265654701	0.988741847156811	pseudobulk	DESeq2	LRT	0.0205596858579748
368	KC_AB2	Gyf	-0.0651213759951502	0.936131771267733	0.988741847156811	pseudobulk	DESeq2	LRT	0.0286630150369529
368	KC_AB2	gzl	-0.579449608485573	0.592124135183874	0.988741847156811	pseudobulk	DESeq2	LRT	0.227587236564423
368	KC_AB2	H	-1.31939971332678	0.402856619184178	0.988741847156811	pseudobulk	DESeq2	LRT	0.394849496233543
368	KC_AB2	h-cup	-0.506613484034373	0.961658398065406	0.988741847156811	pseudobulk	DESeq2	LRT	0.0169791713942958
368	KC_AB2	Hacd1	-0.787390638757929	0.708636903053768	0.988741847156811	pseudobulk	DESeq2	LRT	0.149576235046444
368	KC_AB2	Hacd2	0.559944376141277	0.735204772894766	0.988741847156811	pseudobulk	DESeq2	LRT	0.133591682205281
368	KC_AB2	Hacl	-0.00340877878248056	0.980029417943268	0.988741847156811	pseudobulk	DESeq2	LRT	0.0087608877170192
368	KC_AB2	haf	0.818790875285784	0.940355871560339	0.988741847156811	pseudobulk	DESeq2	LRT	0.0267077593721816
369	KC_AB2	Hakai	1.47543920254359	0.661929169513603	0.988741847156811	pseudobulk	DESeq2	LRT	0.17918848025087
369	KC_AB2	hang	0.300215092025959	0.754586778758957	0.988741847156811	pseudobulk	DESeq2	LRT	0.12229080841455
369	KC_AB2	Hasp	-0.0966959029535496	0.876098706387946	0.988741847156811	pseudobulk	DESeq2	LRT	0.0574469609270518
369	KC_AB2	Haspin	0.103592608324421	0.925119455101218	0.988741847156811	pseudobulk	DESeq2	LRT	0.0338021858104177
369	KC_AB2	Hat1	0.568082799876568	0.825260330228634	0.988741847156811	pseudobulk	DESeq2	LRT	0.0834090306653718

369	KC_AB2	HBS1	-0.934020934207768	0.782359118258182	0.988741847156811	pseudobulk	DESeq2	LRT	0.106593851444836
369	KC_AB2	Hcf	0.334694952499929	0.755180617408417	0.988741847156811	pseudobulk	DESeq2	LRT	0.121949165241494
369	KC_AB2	Hcs	-0.121120420743075	0.958079731976279	0.988741847156811	pseudobulk	DESeq2	LRT	0.0185983471673402
369	KC_AB2	HDAC1	0.920073134400379	0.728211051768817	0.988741847156811	pseudobulk	DESeq2	LRT	0.137742734237847
369	KC_AB2	HDAC11	0.666943671827768	0.808936122914367	0.988741847156811	pseudobulk	DESeq2	LRT	0.0920857707994393
370	KC_AB2	HDAC4	0.156616454711371	0.74970734241014	0.988741847156811	pseudobulk	DESeq2	LRT	0.125108235782416
370	KC_AB2	HDAC6	0.139543759454009	0.92013189547714	0.988741847156811	pseudobulk	DESeq2	LRT	0.0361499146411484
370	KC_AB2	hdc	0.765873557313455	0.651584971610722	0.988741847156811	pseudobulk	DESeq2	LRT	0.186028940971573
370	KC_AB2	hec	-1.80294541848982	0.660135549041553	0.988741847156811	pseudobulk	DESeq2	LRT	0.180366879372519
370	KC_AB2	heix	1.45259040912931	0.616176917291092	0.988741847156811	pseudobulk	DESeq2	LRT	0.210294574894236
370	KC_AB2	Hel25E	0.266200357171911	0.812297104966933	0.988741847156811	pseudobulk	DESeq2	LRT	0.0902850945917375
370	KC_AB2	Hel89B	1.42259303455845	0.424485315934917	0.988741847156811	pseudobulk	DESeq2	LRT	0.372137328550154
370	KC_AB2	Hem	2.00515076565275	0.576456787459964	0.988741847156811	pseudobulk	DESeq2	LRT	0.239233242872451
370	KC_AB2	HemK1	-0.0323203545809474	0.954448156132187	0.988741847156811	pseudobulk	DESeq2	LRT	0.020247656706879
370	KC_AB2	Hen1	2.7785831508444	0.373751317747552	0.988741847156811	pseudobulk	DESeq2	LRT	0.427417267458199
371	KC_AB2	hep	-0.164903967600826	0.915882238866045	0.988741847156811	pseudobulk	DESeq2	LRT	0.0381603629024758
371	KC_AB2	heph	1.10994537305939	0.2955386005317	0.988741847156811	pseudobulk	DESeq2	LRT	0.52938578753031
371	KC_AB2	her	-2.3519140413083	0.314643478010964	0.988741847156811	pseudobulk	DESeq2	LRT	0.502181265960687
371	KC_AB2	HERC2	0.962797039441956	0.473582535877996	0.988741847156811	pseudobulk	DESeq2	LRT	0.324604321301316
371	KC_AB2	Herc4	2.58110170599353	0.284090348510032	0.988741847156811	pseudobulk	DESeq2	LRT	0.546543520448255
371	KC_AB2	Herp	-0.883283128441371	0.63972157328483	0.988741847156811	pseudobulk	DESeq2	LRT	0.194009003353754
371	KC_AB2	Hers	-0.0480887926879544	0.940058940129536	0.988741847156811	pseudobulk	DESeq2	LRT	0.0268449160061022
371	KC_AB2	Hesr	-1.80294541848982	0.660135549041553	0.988741847156811	pseudobulk	DESeq2	LRT	0.180366879372519
371	KC_AB2	Hex-A	0.998437227971566	0.262512059951412	0.988741847156811	pseudobulk	DESeq2	LRT	0.580850740067396
371	KC_AB2	Hexim	-0.233272055930639	0.937531793764924	0.988741847156811	pseudobulk	DESeq2	LRT	0.028013995469539
372	KC_AB2	Hexo1	0.699674038439951	0.821690393783994	0.988741847156811	pseudobulk	DESeq2	LRT	0.0852917902455435
372	KC_AB2	Hey	0.362555753552128	0.897034383127386	0.988741847156811	pseudobulk	DESeq2	LRT	0.0471909102264623
372	KC_AB2	Hf	0.818790875285784	0.940355871560339	0.988741847156811	pseudobulk	DESeq2	LRT	0.0267077593721816
372	KC_AB2	hfp	-0.0145020189918432	0.981189018376986	0.988741847156811	pseudobulk	DESeq2	LRT	0.00824732113112938
372	KC_AB2	hfw	1.42621218304824	0.66738831402044	0.988741847156811	pseudobulk	DESeq2	LRT	0.175621402117653
372	KC_AB2	hgo	-2.26538931672255	0.547771004842275	0.988741847156811	pseudobulk	DESeq2	LRT	0.2614009599796
372	KC_AB2	HINT1	-0.815512053431733	0.451542879548649	0.988741847156811	pseudobulk	DESeq2	LRT	0.345301001790949
372	KC_AB2	HIP	-0.558226010179434	0.754006724696345	0.988741847156811	pseudobulk	DESeq2	LRT	0.122624780807293
372	KC_AB2	HIP-R	0.153977168019861	0.895869830354104	0.988741847156811	pseudobulk	DESeq2	LRT	0.0477550886255582
372	KC_AB2	Hip1	-3.4227037205893	0.285801360563088	0.988741847156811	pseudobulk	DESeq2	LRT	0.543935708072568
373	KC_AB2	Hip14	-0.300859819000612	0.875340908758419	0.988741847156811	pseudobulk	DESeq2	LRT	0.0578227744539286
373	KC_AB2	HipHop	0.818790875285784	0.940355871560339	0.988741847156811	pseudobulk	DESeq2	LRT	0.0267077593721816
373	KC_AB2	Hipk	-0.327945554385118	0.575772078630828	0.988741847156811	pseudobulk	DESeq2	LRT	0.239749399531875
373	KC_AB2	HIPP1	0.43324628136146	0.792959056514803	0.988741847156811	pseudobulk	DESeq2	LRT	0.100749236375646
373	KC_AB2	Hira	-1.80294541848982	0.660135549041553	0.988741847156811	pseudobulk	DESeq2	LRT	0.180366879372519
373	KC_AB2	His2Av	0.642375732928921	0.474660311309576	0.988741847156811	pseudobulk	DESeq2	LRT	0.323617080267827
373	KC_AB2	His2B:CG33868	2.06137138167525	0.547946929015274	0.988741847156811	pseudobulk	DESeq2	LRT	0.261261502741816
373	KC_AB2	His3.3A	0.37865609111053	0.562444706988934	0.988741847156811	pseudobulk	DESeq2	LRT	0.249920165892006
373	KC_AB2	His3.3B	0.20625850912223	0.499675862497874	0.988741847156811	pseudobulk	DESeq2	LRT	0.301311629218947
373	KC_AB2	His4r	0.393346523884927	0.296878496964457	0.988741847156811	pseudobulk	DESeq2	LRT	0.527421257398477
374	KC_AB2	HisRS	-0.957133960806503	0.762632518785665	0.988741847156811	pseudobulk	DESeq2	LRT	0.1176846802739
374	KC_AB2	hiw	-0.57782908547015	0.602700562640007	0.988741847156811	pseudobulk	DESeq2	LRT	0.219898403104286
374	KC_AB2	Hk	-0.334343490913536	0.635309544471822	0.988741847156811	pseudobulk	DESeq2	LRT	0.197014620061703
374	KC_AB2	Hlc	-0.495174913670935	0.758889783099913	0.988741847156811	pseudobulk	DESeq2	LRT	0.119821294021897
374	KC_AB2	Hmg-2	1.10527623683615	0.724717602335893	0.988741847156811	pseudobulk	DESeq2	LRT	0.139831190172672
374	KC_AB2	Hmgcr	-0.522044679843893	0.796310928443557	0.988741847156811	pseudobulk	DESeq2	LRT	0.0989173240449814
374	KC_AB2	HmgD	0.310974079447882	0.852647373225612	0.988741847156811	pseudobulk	DESeq2	LRT	0.0692305415470647
374	KC_AB2	Hmgs	-0.506613484034373	0.961658398065406	0.988741847156811	pseudobulk	DESeq2	LRT	0.0169791713942958
374	KC_AB2	HmgZ	0.146753876958497	0.737678906833256	0.988741847156811	pseudobulk	DESeq2	LRT	0.132132634566415
374	KC_AB2	Hmr	1.12452187615248	0.704078404906088	0.988741847156811	pseudobulk	DESeq2	LRT	0.152378975911753
375	KC_AB2	Hmt-1	1.91626857490363	0.477198801200827	0.988741847156811	pseudobulk	DESeq2	LRT	0.321300656017034

375	KC_AB2	Hmt4-20	-0.288745161276031	0.767766540173715	0.988741847156811	pseudobulk	DESeq2	LRT	0.114770818676756
375	KC_AB2	Hmu	0.475213242523281	0.812738132485219	0.988741847156811	pseudobulk	DESeq2	LRT	0.0900493633068432
375	KC_AB2	hng1	2.4643417141186	0.449082701910884	0.988741847156811	pseudobulk	DESeq2	LRT	0.347673673080982
375	KC_AB2	HnRNP-K	0.165343380305577	0.839946911245435	0.988741847156811	pseudobulk	DESeq2	LRT	0.0757481626068973
375	KC_AB2	Ho	0.998893024789477	0.660591418406266	0.988741847156811	pseudobulk	DESeq2	LRT	0.180067072429364
375	KC_AB2	hob	0.818790875285784	0.940355871560339	0.988741847156811	pseudobulk	DESeq2	LRT	0.0267077593721816
375	KC_AB2	hoe1	1.32121414808556	0.313533554186836	0.988741847156811	pseudobulk	DESeq2	LRT	0.503715974391814
375	KC_AB2	hoe2	0.933701246465306	0.496120101708681	0.988741847156811	pseudobulk	DESeq2	LRT	0.30441317593843
375	KC_AB2	hoip	-0.194774157311496	0.848206982272431	0.988741847156811	pseudobulk	DESeq2	LRT	0.071498156835155
376	KC_AB2	holn1	-0.506613484034373	0.961658398065406	0.988741847156811	pseudobulk	DESeq2	LRT	0.0169791713942958
376	KC_AB2	homer	2.99842299859728	0.361638898709429	0.988741847156811	pseudobulk	DESeq2	LRT	0.441724861973572
376	KC_AB2	hook	-0.4667955180638	0.694711159804506	0.988741847156811	pseudobulk	DESeq2	LRT	0.158195724585285
376	KC_AB2	hop	0.760546103616116	0.738305064567484	0.988741847156811	pseudobulk	DESeq2	LRT	0.131764152427911
376	KC_AB2	HP1b	0.0336799954194084	0.966730614244894	0.988741847156811	pseudobulk	DESeq2	LRT	0.0146945280326403
376	KC_AB2	HP1c	1.96488579948226	0.273023622640308	0.988741847156811	pseudobulk	DESeq2	LRT	0.563799775162552
376	KC_AB2	HP4	0.998893024789477	0.660591418406266	0.988741847156811	pseudobulk	DESeq2	LRT	0.180067072429364
376	KC_AB2	HP5	0.847832741663511	0.608530738759056	0.988741847156811	pseudobulk	DESeq2	LRT	0.215717479329945
376	KC_AB2	Hph	-0.147926357299875	0.943541611067299	0.988741847156811	pseudobulk	DESeq2	LRT	0.0252389422839741
376	KC_AB2	hpo	-0.506613484034373	0.961658398065406	0.988741847156811	pseudobulk	DESeq2	LRT	0.0169791713942958
377	KC_AB2	hppy	-0.50172977155203	0.552581412414892	0.988741847156811	pseudobulk	DESeq2	LRT	0.257603727854927
377	KC_AB2	Hpr1	0.818790875285784	0.940355871560339	0.988741847156811	pseudobulk	DESeq2	LRT	0.0267077593721816
377	KC_AB2	hpRNA:CR18854	0.226251151999351	0.807084586039322	0.988741847156811	pseudobulk	DESeq2	LRT	0.0930809469073623
377	KC_AB2	hpRNA:CR32207	1.47543920254359	0.661929169513603	0.988741847156811	pseudobulk	DESeq2	LRT	0.17918848025087
377	KC_AB2	hpRNA:CR46342	0.780352552100014	0.834299274204729	0.988741847156811	pseudobulk	DESeq2	LRT	0.0786781342287373
377	KC_AB2	HPS1	-1.40296545307325	0.628223627794903	0.988741847156811	pseudobulk	DESeq2	LRT	0.201885733604539
377	KC_AB2	HPS4	0.299229629739512	0.851026027418758	0.988741847156811	pseudobulk	DESeq2	LRT	0.070057157434224
377	KC_AB2	Hr3	-1.10708700962228	0.23849494870589	0.988741847156811	pseudobulk	DESeq2	LRT	0.622520814830952
377	KC_AB2	Hr38	-0.392686915406407	0.347917532955788	0.988741847156811	pseudobulk	DESeq2	LRT	0.458523684865273
377	KC_AB2	Hr39	-0.68289396627029	0.381075056795852	0.988741847156811	pseudobulk	DESeq2	LRT	0.418989476970265
378	KC_AB2	Hr51	1.34186854531998	0.110215149026858	0.988741847156811	pseudobulk	DESeq2	LRT	0.95775870778369
378	KC_AB2	Hr78	0.956149878169744	0.552793572893123	0.988741847156811	pseudobulk	DESeq2	LRT	0.257437014970467
378	KC_AB2	Hr96	-1.23536834900699	0.725570392794057	0.988741847156811	pseudobulk	DESeq2	LRT	0.139320447155934
378	KC_AB2	Hrb27C	-0.0801580330506406	0.820832677753546	0.988741847156811	pseudobulk	DESeq2	LRT	0.0857453624106806
378	KC_AB2	Hrb87F	0.931366871264525	0.546787326434422	0.988741847156811	pseudobulk	DESeq2	LRT	0.262181560167462
378	KC_AB2	Hrb98DE	0.023702002714266	0.954525667995345	0.988741847156811	pseudobulk	DESeq2	LRT	0.0202123885706222
378	KC_AB2	Hrd3	0.575200883059258	0.804213580533116	0.988741847156811	pseudobulk	DESeq2	LRT	0.0946285973599483
378	KC_AB2	hrg	-0.42382980072996	0.702744341354088	0.988741847156811	pseudobulk	DESeq2	LRT	0.153202642737368
378	KC_AB2	hrm	0.256656488129454	0.827490303151939	0.988741847156811	pseudobulk	DESeq2	LRT	0.0822370867513728
378	KC_AB2	Hrs	0.310390049490412	0.904941705285892	0.988741847156811	pseudobulk	DESeq2	LRT	0.04337939636174
379	KC_AB2	Hs2st	-0.957133960806503	0.762632518785665	0.988741847156811	pseudobulk	DESeq2	LRT	0.1176846802739
379	KC_AB2	Hs3st-A	-0.253991480803428	0.516131617783199	0.988741847156811	pseudobulk	DESeq2	LRT	0.287239535605156
379	KC_AB2	Hs3st-B	0.818790875285784	0.940355871560339	0.988741847156811	pseudobulk	DESeq2	LRT	0.0267077593721816
379	KC_AB2	Hs6st	0.641767316647791	0.649691903566914	0.988741847156811	pseudobulk	DESeq2	LRT	0.187292545360415
379	KC_AB2	Hsc70-2	0.818790875285784	0.940355871560339	0.988741847156811	pseudobulk	DESeq2	LRT	0.0267077593721816
379	KC_AB2	Hsc70-3	0.679674663105468	0.518654456437911	0.988741847156811	pseudobulk	DESeq2	LRT	0.285121886161219
379	KC_AB2	Hsc70-4	0.0681747572706948	0.89691034892731	0.988741847156811	pseudobulk	DESeq2	LRT	0.0472509648845389
379	KC_AB2	Hsc70-5	0.64510927968964	0.645092522575761	0.988741847156811	pseudobulk	DESeq2	LRT	0.190377992089684
379	KC_AB2	Hsc70Cb	-0.694673942051206	0.452443529104136	0.988741847156811	pseudobulk	DESeq2	LRT	0.344435618732632
379	KC_AB2	Hsepi	3.27725343811621	0.25757966897206	0.988741847156811	pseudobulk	DESeq2	LRT	0.589088419269148
380	KC_AB2	Hsf	0.572891340160817	0.683926553260069	0.988741847156811	pseudobulk	DESeq2	LRT	0.164990534575993
380	KC_AB2	Hsl	-0.203334765675412	0.946119398140357	0.988741847156811	pseudobulk	DESeq2	LRT	0.0240540531527077
380	KC_AB2	Hsp23	-2.18306924400367	0.621837827414998	0.988741847156811	pseudobulk	DESeq2	LRT	0.206322862643567
380	KC_AB2	Hsp26	-0.739922499234443	0.660373498325336	0.988741847156811	pseudobulk	DESeq2	LRT	0.180210363879885
380	KC_AB2	Hsp27	-3.36413440902233	0.189817425253469	0.988741847156811	pseudobulk	DESeq2	LRT	0.721663921813577
380	KC_AB2	Hsp60A	0.109532976672173	0.895030393368243	0.988741847156811	pseudobulk	DESeq2	LRT	0.0481622166975013
380	KC_AB2	Hsp60D	-0.506613484034373	0.961658398065406	0.988741847156811	pseudobulk	DESeq2	LRT	0.0169791713942958

380	KC_AB2	Hsp68	-2.17804862092214	0.275519887419499	0.988741847156811	pseudobulk	DESeq2	LRT	0.559847047683975
380	KC_AB2	Hsp70Aa	2.7785831508444	0.373751317747552	0.988741847156811	pseudobulk	DESeq2	LRT	0.427417267458199
380	KC_AB2	Hsp70Ab	0.208999414555304	0.864824600856976	0.988741847156811	pseudobulk	DESeq2	LRT	0.0630719649098871
381	KC_AB2	Hsp70Bb	0.316148142040676	0.850903064246337	0.988741847156811	pseudobulk	DESeq2	LRT	0.0701199123722693
381	KC_AB2	Hsp70Bc	0.0803262651121452	0.924190567181922	0.988741847156811	pseudobulk	DESeq2	LRT	0.0342384684528321
381	KC_AB2	Hsp83	-0.442952709232552	0.353733692001715	0.988741847156811	pseudobulk	DESeq2	LRT	0.451323573056129
381	KC_AB2	HSPBAP1	-1.65719613210719	0.599376091584556	0.988741847156811	pseudobulk	DESeq2	LRT	0.222300584546838
381	KC_AB2	HSPC300	0.836206984333835	0.674113903735976	0.988741847156811	pseudobulk	DESeq2	LRT	0.171266715357068
381	KC_AB2	hth	-0.157707372567732	0.849991001711422	0.988741847156811	pseudobulk	DESeq2	LRT	0.0705856718477794
381	KC_AB2	htk	-0.240359842327581	0.866674312366755	0.988741847156811	pseudobulk	DESeq2	LRT	0.0621440754364842
381	KC_AB2	hts	-0.217818607036873	0.77130426531852	0.988741847156811	pseudobulk	DESeq2	LRT	0.112774266979011
381	KC_AB2	htt	-0.0144409071146711	0.984632974376923	0.988741847156811	pseudobulk	DESeq2	LRT	0.00672562423038896
381	KC_AB2	Hug	0.818790875285784	0.940355871560339	0.988741847156811	pseudobulk	DESeq2	LRT	0.0267077593721816
382	KC_AB2	Hus1-like	1.47543920254359	0.661929169513603	0.988741847156811	pseudobulk	DESeq2	LRT	0.17918848025087
382	KC_AB2	HUWE1	-0.301642491090265	0.719216879576314	0.988741847156811	pseudobulk	DESeq2	LRT	0.143140128531744
382	KC_AB2	hwt	-0.257132145548924	0.697577551386537	0.988741847156811	pseudobulk	DESeq2	LRT	0.156407503800578
382	KC_AB2	Hyccin	0.10598154425916	0.959628155914924	0.988741847156811	pseudobulk	DESeq2	LRT	0.0178970181249073
382	KC_AB2	hyd	-0.608203374906063	0.809656234342825	0.988741847156811	pseudobulk	DESeq2	LRT	0.0916993357109913
382	KC_AB2	Hydr2	0.61702365764228	0.739069534444918	0.988741847156811	pseudobulk	DESeq2	LRT	0.131314699622637
382	KC_AB2	hyx	-1.12033987853799	0.612256743736902	0.988741847156811	pseudobulk	DESeq2	LRT	0.213066422615508
382	KC_AB2	I-2	0.779379171321013	0.610545544693969	0.988741847156811	pseudobulk	DESeq2	LRT	0.214281933567587
382	KC_AB2	I-3	-1.80294541848982	0.660135549041553	0.988741847156811	pseudobulk	DESeq2	LRT	0.180366879372519
382	KC_AB2	IA-2	-0.0198361758750251	0.943267921169237	0.988741847156811	pseudobulk	DESeq2	LRT	0.0253649348775919
383	KC_AB2	Ibf1	-1.06622077822007	0.55903750929651	0.988741847156811	pseudobulk	DESeq2	LRT	0.25255905162345
383	KC_AB2	Ibf2	2.41462217514131	0.497446973552313	0.988741847156811	pseudobulk	DESeq2	LRT	0.303253207020768
383	KC_AB2	ICA69	-0.033623501357773	0.970256417661369	0.988741847156811	pseudobulk	DESeq2	LRT	0.0131134759822254
383	KC_AB2	Ice1	-1.80294541848982	0.660135549041553	0.988741847156811	pseudobulk	DESeq2	LRT	0.180366879372519
383	KC_AB2	Ice2	1.42621218304824	0.66738831402044	0.988741847156811	pseudobulk	DESeq2	LRT	0.175621402117653
383	KC_AB2	icln	-3.51218082781652	0.306311589270116	0.988741847156811	pseudobulk	DESeq2	LRT	0.513836571391778
383	KC_AB2	ics	4.25413101604405	0.0876238203774895	0.988741847156811	pseudobulk	DESeq2	LRT	1.05737781561011
383	KC_AB2	Ide	0.801371043861579	0.637329902962428	0.988741847156811	pseudobulk	DESeq2	LRT	0.195635704324295
383	KC_AB2	Idh	-0.180909185132649	0.910622741137549	0.988741847156811	pseudobulk	DESeq2	LRT	0.0406615081819837
383	KC_AB2	Idh3a	1.02685841401898	0.293699788770688	0.988741847156811	pseudobulk	DESeq2	LRT	0.532096365822306
384	KC_AB2	Idh3b	0.532680334117092	0.613352251763877	0.988741847156811	pseudobulk	DESeq2	LRT	0.212290035987388
384	KC_AB2	Idi	1.45259040912931	0.616176917291092	0.988741847156811	pseudobulk	DESeq2	LRT	0.210294574894236
384	KC_AB2	Ids	0.818790875285784	0.940355871560339	0.988741847156811	pseudobulk	DESeq2	LRT	0.0267077593721816
384	KC_AB2	ifc	-2.2040094716458	0.214748633575424	0.988741847156811	pseudobulk	DESeq2	LRT	0.668069590861289
384	KC_AB2	IFT43	0.818790875285784	0.940355871560339	0.988741847156811	pseudobulk	DESeq2	LRT	0.0267077593721816
384	KC_AB2	igl	0.474309226831302	0.272005174676476	0.988741847156811	pseudobulk	DESeq2	LRT	0.565422833789103
384	KC_AB2	Ih	0.115291161973885	0.813724648410489	0.988741847156811	pseudobulk	DESeq2	LRT	0.0895225286565497
384	KC_AB2	IKKepsilon	1.45259040912931	0.616176917291092	0.988741847156811	pseudobulk	DESeq2	LRT	0.210294574894236
384	KC_AB2	IleRS	0.297713224699148	0.901701424115727	0.988741847156811	pseudobulk	DESeq2	LRT	0.0449372444148942
384	KC_AB2	Ilk	0.353798809751289	0.901648351639924	0.988741847156811	pseudobulk	DESeq2	LRT	0.0449628069360408
385	KC_AB2	imd	-0.187188766977065	0.932037508762103	0.988741847156811	pseudobulk	DESeq2	LRT	0.0305666096196055
385	KC_AB2	Iml1	0.666943671827768	0.808936122914367	0.988741847156811	pseudobulk	DESeq2	LRT	0.0920857707994393
385	KC_AB2	Imp	4.72403438814677	0.319817434688489	0.988741847156811	pseudobulk	DESeq2	LRT	0.495097864596618
385	KC_AB2	inaE	-0.231167202575143	0.874125629151683	0.988741847156811	pseudobulk	DESeq2	LRT	0.058426146168257
385	KC_AB2	inc	1.82157231932995	0.355156761598304	0.988741847156811	pseudobulk	DESeq2	LRT	0.449579912663393
385	KC_AB2	Indy	0.818790875285784	0.940355871560339	0.988741847156811	pseudobulk	DESeq2	LRT	0.0267077593721816
385	KC_AB2	Ing3	0.317508825551005	0.883746467592857	0.988741847156811	pseudobulk	DESeq2	LRT	0.053672309102253
385	KC_AB2	Ing5	2.01044021410856	0.465232850276855	0.988741847156811	pseudobulk	DESeq2	LRT	0.332329627154538
385	KC_AB2	Inos	-0.302075029379803	0.819505653511644	0.988741847156811	pseudobulk	DESeq2	LRT	0.0864480460221689
385	KC_AB2	INPP5E	2.99842299859728	0.361638898709429	0.988741847156811	pseudobulk	DESeq2	LRT	0.441724861973572
386	KC_AB2	InR	0.920073134400379	0.728211051768817	0.988741847156811	pseudobulk	DESeq2	LRT	0.137742734237847
386	KC_AB2	IntS1	0.315515278988381	0.901389738439351	0.988741847156811	pseudobulk	DESeq2	LRT	0.0450873903126698
386	KC_AB2	IntS10	1.42621218304824	0.66738831402044	0.988741847156811	pseudobulk	DESeq2	LRT	0.175621402117653

386	KC_AB2	IntS11	-1.65719613210719	0.599376091584556	0.988741847156811	pseudobulk	DESeq2	LRT	0.222300584546838
386	KC_AB2	IntS12	-1.80294541848982	0.660135549041553	0.988741847156811	pseudobulk	DESeq2	LRT	0.180366879372519
386	KC_AB2	IntS14	-0.506613484034373	0.961658398065406	0.988741847156811	pseudobulk	DESeq2	LRT	0.0169791713942958
386	KC_AB2	IntS2	0.818790875285784	0.940355871560339	0.988741847156811	pseudobulk	DESeq2	LRT	0.0267077593721816
386	KC_AB2	IntS3	-0.706431215842201	0.620887238249173	0.988741847156811	pseudobulk	DESeq2	LRT	0.206987266575216
386	KC_AB2	IntS4	-1.23536834900699	0.725570392794057	0.988741847156811	pseudobulk	DESeq2	LRT	0.139320447155934
386	KC_AB2	IntS6	0.215964559351794	0.913537469997374	0.988741847156811	pseudobulk	DESeq2	LRT	0.0392736347598703
387	KC_AB2	IntS8	0.780352552100014	0.834299274204729	0.988741847156811	pseudobulk	DESeq2	LRT	0.0786781342287373
387	KC_AB2	Invadolysin	-0.378852672109618	0.920549594954422	0.988741847156811	pseudobulk	DESeq2	LRT	0.0359528087516892
387	KC_AB2	Inx3	1.15128682504377	0.719737694898673	0.988741847156811	pseudobulk	DESeq2	LRT	0.142825751365876
387	KC_AB2	Ip259	-0.207713707928777	0.689930750470675	0.988741847156811	pseudobulk	DESeq2	LRT	0.161194497955112
387	KC_AB2	IPIP	0.613750852128688	0.739100932111639	0.988741847156811	pseudobulk	DESeq2	LRT	0.131296250013865
387	KC_AB2	Ipk2	-2.35595135045048	0.406121109235175	0.988741847156811	pseudobulk	DESeq2	LRT	0.39134443630141
387	KC_AB2	iPLA2-VIA	0.770523665597912	0.687741087577941	0.988741847156811	pseudobulk	DESeq2	LRT	0.162575028914724
387	KC_AB2	Ipp	0.46102693002637	0.613221641458433	0.988741847156811	pseudobulk	DESeq2	LRT	0.212382526681119
387	KC_AB2	Ir41a	0.818790875285784	0.940355871560339	0.988741847156811	pseudobulk	DESeq2	LRT	0.0267077593721816
387	KC_AB2	Ir56a	1.64209625885217	0.483203675195701	0.988741847156811	pseudobulk	DESeq2	LRT	0.315869771176778
388	KC_AB2	Ir68a	-0.0323203545809474	0.954448156132187	0.988741847156811	pseudobulk	DESeq2	LRT	0.020247656706879
388	KC_AB2	Ir76a	0.818790875285784	0.940355871560339	0.988741847156811	pseudobulk	DESeq2	LRT	0.0267077593721816
388	KC_AB2	Irbp	-1.80294541848982	0.660135549041553	0.988741847156811	pseudobulk	DESeq2	LRT	0.180366879372519
388	KC_AB2	Irbp18	1.09755075627148	0.564545147075846	0.988741847156811	pseudobulk	DESeq2	LRT	0.24830132151245
388	KC_AB2	Ire1	-0.0582963981256801	0.979058404810457	0.988741847156811	pseudobulk	DESeq2	LRT	0.0091913999942843
388	KC_AB2	Irk2	-1.80294541848982	0.660135549041553	0.988741847156811	pseudobulk	DESeq2	LRT	0.180366879372519
388	KC_AB2	Irp-1A	0.361297473630756	0.86408302051291	0.988741847156811	pseudobulk	DESeq2	LRT	0.0634445287959917
388	KC_AB2	Irp-1B	-0.147926357299875	0.943541611067299	0.988741847156811	pseudobulk	DESeq2	LRT	0.0252389422839741
388	KC_AB2	IRSp53	1.12452187615248	0.704078404906088	0.988741847156811	pseudobulk	DESeq2	LRT	0.152378975911753
388	KC_AB2	Iru	-0.309344821003396	0.844574704127334	0.988741847156811	pseudobulk	DESeq2	LRT	0.0733619302784221
389	KC_AB2	IscU	0.485401078930153	0.621377088733374	0.988741847156811	pseudobulk	DESeq2	LRT	0.206644763987559
389	KC_AB2	isopeptidase-T-3	0.5738945031678	0.80780652681376	0.988741847156811	pseudobulk	DESeq2	LRT	0.0926926421938231
389	KC_AB2	isoQC	1.47543920254359	0.661929169513603	0.988741847156811	pseudobulk	DESeq2	LRT	0.17918848025087
389	KC_AB2	Iswi	0.897597620560572	0.634952810743471	0.988741847156811	pseudobulk	DESeq2	LRT	0.197258549976131
389	KC_AB2	Itgbn	-0.934020934207768	0.782359118258182	0.988741847156811	pseudobulk	DESeq2	LRT	0.106593851444836
389	KC_AB2	Itpr	-0.708848427368349	0.719084259593817	0.988741847156811	pseudobulk	DESeq2	LRT	0.143220217638515
389	KC_AB2	ix	1.89586497481491	0.514180746077939	0.988741847156811	pseudobulk	DESeq2	LRT	0.288884189903704
389	KC_AB2	Jafrac1	0.239606247839856	0.799809596089532	0.988741847156811	pseudobulk	DESeq2	LRT	0.0970133895201635
389	KC_AB2	Jafrac2	1.42621218304824	0.66738831402044	0.988741847156811	pseudobulk	DESeq2	LRT	0.175621402117653
389	KC_AB2	jagn	-1.44468641100173	0.432997359740356	0.988741847156811	pseudobulk	DESeq2	LRT	0.363514751807581
390	KC_AB2	janA	-0.145132327794126	0.926520871738359	0.988741847156811	pseudobulk	DESeq2	LRT	0.0331447928818367
390	KC_AB2	janB	1.42621218304824	0.66738831402044	0.988741847156811	pseudobulk	DESeq2	LRT	0.175621402117653
390	KC_AB2	jar	-0.456817829054119	0.768213892126133	0.988741847156811	pseudobulk	DESeq2	LRT	0.114517843461953
390	KC_AB2	Jarid2	0.468868849300992	0.708747372732595	0.988741847156811	pseudobulk	DESeq2	LRT	0.149508537989479
390	KC_AB2	jbug	1.42621218304824	0.66738831402044	0.988741847156811	pseudobulk	DESeq2	LRT	0.175621402117653
390	KC_AB2	jdp	0.330396230710937	0.220000206168276	0.988741847156811	pseudobulk	DESeq2	LRT	0.657576912188236
390	KC_AB2	jeb	0.747439978793343	0.133780727328108	0.988741847156811	pseudobulk	DESeq2	LRT	0.873606447240275
390	KC_AB2	jef	-0.253935737899019	0.74098766190846	0.988741847156811	pseudobulk	DESeq2	LRT	0.130189023342782
390	KC_AB2	jet	0.712171366772551	0.813205723536451	0.988741847156811	pseudobulk	DESeq2	LRT	0.0897995733547685
390	KC_AB2	JHDM2	2.12627090373229	0.415798287869119	0.988741847156811	pseudobulk	DESeq2	LRT	0.38111730329345
391	KC_AB2	JhI-21	2.06137138167525	0.547946929015274	0.988741847156811	pseudobulk	DESeq2	LRT	0.261261502741816
391	KC_AB2	jigr1	-0.202209755684413	0.867758037868502	0.988741847156811	pseudobulk	DESeq2	LRT	0.0616013548553182
391	KC_AB2	JIL-1	-0.114159447470253	0.901176151412792	0.988741847156811	pseudobulk	DESeq2	LRT	0.0451903098985748
391	KC_AB2	jim	0.026968477579474	0.928112994510227	0.988741847156811	pseudobulk	DESeq2	LRT	0.0323991467284126
391	KC_AB2	jing	-0.423065934068027	0.769303809334768	0.988741847156811	pseudobulk	DESeq2	LRT	0.113902117076131
391	KC_AB2	JMJD4	1.47543920254359	0.661929169513603	0.988741847156811	pseudobulk	DESeq2	LRT	0.17918848025087
391	KC_AB2	JMJD5	1.42621218304824	0.66738831402044	0.988741847156811	pseudobulk	DESeq2	LRT	0.175621402117653
391	KC_AB2	JMJD7	-0.457212305199634	0.858276431637992	0.988741847156811	pseudobulk	DESeq2	LRT	0.0663728130839547
391	KC_AB2	jnj	1.42621218304824	0.66738831402044	0.988741847156811	pseudobulk	DESeq2	LRT	0.175621402117653

391	KC_AB2	jp	0.394779682290181	0.744651127765063	0.988741847156811	pseudobulk	DESeq2	LRT	0.128047148423911
392	KC_AB2	Jra	1.31999723788073	0.469291549914029	0.988741847156811	pseudobulk	DESeq2	LRT	0.328557265644203
392	KC_AB2	JTBR	0.157562008957688	0.950463298513166	0.988741847156811	pseudobulk	DESeq2	LRT	0.0220646484576093
392	KC_AB2	jub	-1.21589183877631	0.541005006441934	0.988741847156811	pseudobulk	DESeq2	LRT	0.266798715928467
392	KC_AB2	jumu	-1.23536834900699	0.725570392794057	0.988741847156811	pseudobulk	DESeq2	LRT	0.139320447155934
392	KC_AB2	Jupiter	-0.0288826581258828	0.951942141809603	0.988741847156811	pseudobulk	DESeq2	LRT	0.0213894468428577
392	KC_AB2	jus	0.600552091652545	0.126443564050518	0.988741847156811	pseudobulk	DESeq2	LRT	0.898103271250216
392	KC_AB2	jv	1.74111111089874	0.563806300883242	0.988741847156811	pseudobulk	DESeq2	LRT	0.248870074928531
392	KC_AB2	jvl	0.573199160444146	0.454630909527421	0.988741847156811	pseudobulk	DESeq2	LRT	0.342341040716513
392	KC_AB2	Jwa	0.675181270552491	0.64677866894966	0.988741847156811	pseudobulk	DESeq2	LRT	0.18924431175608
392	KC_AB2	KaiR1D	0.474661093301217	0.815951154483998	0.988741847156811	pseudobulk	DESeq2	LRT	0.0883358387620737
393	KC_AB2	Kank	0.564133973942315	0.758437522118305	0.988741847156811	pseudobulk	DESeq2	LRT	0.120080189322646
393	KC_AB2	Kap-alpha1	0.836206984333835	0.674113903735976	0.988741847156811	pseudobulk	DESeq2	LRT	0.171266715357068
393	KC_AB2	Kap-alpha3	0.507429417106814	0.63048597945996	0.988741847156811	pseudobulk	DESeq2	LRT	0.200324566681503
393	KC_AB2	Kap3	0.529204231013716	0.692002332680952	0.988741847156811	pseudobulk	DESeq2	LRT	0.159892441571049
393	KC_AB2	Karybeta3	0.338871409245135	0.825304127768648	0.988741847156811	pseudobulk	DESeq2	LRT	0.0833859827560807
393	KC_AB2	kat-60L1	0.855065484155306	0.500945099118024	0.988741847156811	pseudobulk	DESeq2	LRT	0.300209867858476
393	KC_AB2	Kat60	0.685691472970664	0.738333928211375	0.988741847156811	pseudobulk	DESeq2	LRT	0.13174717424796
393	KC_AB2	kat80	-0.0148827856665692	0.974851841948725	0.988741847156811	pseudobulk	DESeq2	LRT	0.01106138339212
393	KC_AB2	kay	0.179268503759514	0.917968545859208	0.988741847156811	pseudobulk	DESeq2	LRT	0.0371721996199037
393	KC_AB2	Kaz1-ORFB	-1.80294541848982	0.660135549041553	0.988741847156811	pseudobulk	DESeq2	LRT	0.180366879372519
394	KC_AB2	kcc	-0.321382343995502	0.67726492314182	0.988741847156811	pseudobulk	DESeq2	LRT	0.16924141676103
394	KC_AB2	Kcmf1	0.178553035525572	0.895963172375559	0.988741847156811	pseudobulk	DESeq2	LRT	0.0477098411891928
394	KC_AB2	KCNQ	-0.00695196900751267	0.95032150996818	0.988741847156811	pseudobulk	DESeq2	LRT	0.0221294406240073
394	KC_AB2	KdelR	0.683107107991495	0.647576000014666	0.988741847156811	pseudobulk	DESeq2	LRT	0.188709255123018
394	KC_AB2	Kdm2	-0.94851584115099	0.584922323445749	0.988741847156811	pseudobulk	DESeq2	LRT	0.232901803556663
394	KC_AB2	Kdm4A	0.792281256713368	0.823478838299676	0.988741847156811	pseudobulk	DESeq2	LRT	0.0843475568193814
394	KC_AB2	Kdm4B	-0.308401396569453	0.66794076516544	0.988741847156811	pseudobulk	DESeq2	LRT	0.175262050252673
394	KC_AB2	kdn	-0.526561575376116	0.640859104457154	0.988741847156811	pseudobulk	DESeq2	LRT	0.193237441437103
394	KC_AB2	Keap1	1.94106108959906	0.476420175276185	0.988741847156811	pseudobulk	DESeq2	LRT	0.322009855447888
394	KC_AB2	Kebab	-1.80294541848982	0.660135549041553	0.988741847156811	pseudobulk	DESeq2	LRT	0.180366879372519
395	KC_AB2	kek1	-0.0302978684699669	0.926443179434996	0.988741847156811	pseudobulk	DESeq2	LRT	0.033181211654921
395	KC_AB2	kek2	-0.529500498072998	0.604890413942762	0.988741847156811	pseudobulk	DESeq2	LRT	0.218323297960412
395	KC_AB2	kek3	0.0679962090268417	0.925954027237178	0.988741847156811	pseudobulk	DESeq2	LRT	0.033410575102993
395	KC_AB2	kek4	-1.70869593138706	0.348955663219931	0.988741847156811	pseudobulk	DESeq2	LRT	0.457229749092738
395	KC_AB2	kek5	3.09926389624515	0.339712049344771	0.988741847156811	pseudobulk	DESeq2	LRT	0.468889048740397
395	KC_AB2	kek6	1.83816620003647	0.268454878464454	0.988741847156811	pseudobulk	DESeq2	LRT	0.571128699464045
395	KC_AB2	kel	-1.20081362769313	0.741163517910401	0.988741847156811	pseudobulk	DESeq2	LRT	0.130085965985812
395	KC_AB2	key	0.997366942311027	0.663723613191462	0.988741847156811	pseudobulk	DESeq2	LRT	0.178012731256061
395	KC_AB2	Khc	-0.244337923538221	0.885759363485059	0.988741847156811	pseudobulk	DESeq2	LRT	0.0526842479833275
395	KC_AB2	Khc-73	-0.369365003413062	0.672093890075231	0.988741847156811	pseudobulk	DESeq2	LRT	0.172570052700208
396	KC_AB2	Kif3C	0.712171366772551	0.813205723536451	0.988741847156811	pseudobulk	DESeq2	LRT	0.0897995733547685
396	KC_AB2	kin17	-2.15400439962555	0.378907849258803	0.988741847156811	pseudobulk	DESeq2	LRT	0.421466398012216
396	KC_AB2	kirre	1.88601646191997	0.29868041924867	0.988741847156811	pseudobulk	DESeq2	LRT	0.524793247737327
396	KC_AB2	kis	-0.435215917892862	0.424032851901743	0.988741847156811	pseudobulk	DESeq2	LRT	0.372600495183329
396	KC_AB2	kl-5	-2.26538931672255	0.547771004842275	0.988741847156811	pseudobulk	DESeq2	LRT	0.2614009599796
396	KC_AB2	klar	0.733005456743295	0.517734323676971	0.988741847156811	pseudobulk	DESeq2	LRT	0.285893042108402
396	KC_AB2	Klc	-0.229286408750297	0.852472697089433	0.988741847156811	pseudobulk	DESeq2	LRT	0.0693195216530984
396	KC_AB2	klg	2.42255448296272	0.172209969200981	0.988741847156811	pseudobulk	DESeq2	LRT	0.763941710933155
396	KC_AB2	KLHL18	-1.27482208804161	0.504147634250043	0.988741847156811	pseudobulk	DESeq2	LRT	0.297442266428947
396	KC_AB2	Klp10A	0.616215559710414	0.641745755013331	0.988741847156811	pseudobulk	DESeq2	LRT	0.192636995395722
397	KC_AB2	Klp31E	-0.68750020136443	0.784083099060507	0.988741847156811	pseudobulk	DESeq2	LRT	0.105637907276101
397	KC_AB2	Klp64D	-0.287387311603126	0.823593053147202	0.988741847156811	pseudobulk	DESeq2	LRT	0.0842873252309858
397	KC_AB2	Klp68D	0.629166655703089	0.607915994788094	0.988741847156811	pseudobulk	DESeq2	LRT	0.216156429807163
397	KC_AB2	Klp98A	1.86535356424771	0.118681454095391	0.988741847156811	pseudobulk	DESeq2	LRT	0.925617141309225
397	KC_AB2	Kmn1	0.806799704531645	0.753617870565155	0.988741847156811	pseudobulk	DESeq2	LRT	0.122848811653853

397	KC_AB2	kmr	0.292644121715659	0.727359325919929	0.988741847156811	pseudobulk	DESeq2	LRT	0.138250988437238
397	KC_AB2	ko	-2.468611364	0.221979886610129	0.988741847156811	pseudobulk	DESeq2	LRT	0.653686374783427
397	KC_AB2	koi	0.0701895314896461	0.95462191199686	0.988741847156811	pseudobulk	DESeq2	LRT	0.0201686012393047
397	KC_AB2	koko	-1.80294541848982	0.660135549041553	0.988741847156811	pseudobulk	DESeq2	LRT	0.180366879372519
397	KC_AB2	Kr-h1	-0.0084533446145052	0.983736164328038	0.988741847156811	pseudobulk	DESeq2	LRT	0.00712136268699972
398	KC_AB2	Kr-h2	-0.0265683699975436	0.949703263057826	0.988741847156811	pseudobulk	DESeq2	LRT	0.0224120698138033
398	KC_AB2	kra	0.231877443048908	0.696329110623298	0.988741847156811	pseudobulk	DESeq2	LRT	0.157185448399326
398	KC_AB2	kraken	-0.296576489727943	0.896291428147039	0.988741847156811	pseudobulk	DESeq2	LRT	0.047550757014702
398	KC_AB2	kri	0.944666102106262	0.639482450338318	0.988741847156811	pseudobulk	DESeq2	LRT	0.194171369599417
398	KC_AB2	Krn	0.297713224699148	0.901701424115727	0.988741847156811	pseudobulk	DESeq2	LRT	0.0449372444148942
398	KC_AB2	KrT95D	0.103439624861095	0.905714663141941	0.988741847156811	pseudobulk	DESeq2	LRT	0.0430086011541333
398	KC_AB2	krz	-0.0224714967855894	0.97090477072336	0.988741847156811	pseudobulk	DESeq2	LRT	0.0128233649195244
398	KC_AB2	ksh	1.0388618481634	0.585240842327867	0.988741847156811	pseudobulk	DESeq2	LRT	0.232665373287195
398	KC_AB2	ksr	-1.18743225435356	0.64061820392288	0.988741847156811	pseudobulk	DESeq2	LRT	0.193400724506216
398	KC_AB2	Ktl	-0.054193979402969	0.92764917940507	0.988741847156811	pseudobulk	DESeq2	LRT	0.0326162352625129
399	KC_AB2	kto	-0.506613484034373	0.961658398065406	0.988741847156811	pseudobulk	DESeq2	LRT	0.0169791713942958
399	KC_AB2	ktub	-1.80294541848982	0.660135549041553	0.988741847156811	pseudobulk	DESeq2	LRT	0.180366879372519
399	KC_AB2	Ku80	1.42621218304824	0.66738831402044	0.988741847156811	pseudobulk	DESeq2	LRT	0.175621402117653
399	KC_AB2	Kua	2.06137138167525	0.547946929015274	0.988741847156811	pseudobulk	DESeq2	LRT	0.261261502741816
399	KC_AB2	kud	-1.33031509309313	0.407491680773676	0.988741847156811	pseudobulk	DESeq2	LRT	0.389881253257666
399	KC_AB2	kuk	0.92733860596403	0.602246695479295	0.988741847156811	pseudobulk	DESeq2	LRT	0.220225574291918
399	KC_AB2	Kul	1.53178284040979	0.30790769446616	0.988741847156811	pseudobulk	DESeq2	LRT	0.511579458149608
399	KC_AB2	kune	-0.268451881258776	0.899861448944124	0.988741847156811	pseudobulk	DESeq2	LRT	0.0458243534396852
399	KC_AB2	kuz	-0.170114127690618	0.790764262949465	0.988741847156811	pseudobulk	DESeq2	LRT	0.101952966008022
399	KC_AB2	Kyat	-0.378852672109618	0.920549594954422	0.988741847156811	pseudobulk	DESeq2	LRT	0.0359528087516892
400	KC_AB2	kz	0.818790875285784	0.940355871560339	0.988741847156811	pseudobulk	DESeq2	LRT	0.0267077593721816
400	KC_AB2	l(1)10Bb	2.18696931202408	0.283189555820048	0.988741847156811	pseudobulk	DESeq2	LRT	0.547922767696119
400	KC_AB2	l(1)G0004	-0.568233637794205	0.868826397133536	0.988741847156811	pseudobulk	DESeq2	LRT	0.0610669925949191
400	KC_AB2	l(1)G0007	0.693809979578311	0.690053159116761	0.988741847156811	pseudobulk	DESeq2	LRT	0.161117451550002
400	KC_AB2	l(1)G0193	1.87434911411483	0.392658868335322	0.988741847156811	pseudobulk	DESeq2	LRT	0.405984589410945
400	KC_AB2	l(1)G0196	-0.642027776738386	0.543117906395131	0.988741847156811	pseudobulk	DESeq2	LRT	0.265105878443981
400	KC_AB2	l(1)G0289	-0.0921697126884656	0.88486034505953	0.988741847156811	pseudobulk	DESeq2	LRT	0.05312526733153
400	KC_AB2	l(1)G0320	0.455804215077338	0.660671378800001	0.988741847156811	pseudobulk	DESeq2	LRT	0.18001450702317
400	KC_AB2	l(1)G0469	0.456679322303278	0.614417962656069	0.988741847156811	pseudobulk	DESeq2	LRT	0.21153609608791
400	KC_AB2	l(2)05714	1.12452187615248	0.704078404906088	0.988741847156811	pseudobulk	DESeq2	LRT	0.152378975911753
401	KC_AB2	l(2)09851	0.668970670557767	0.818048200024539	0.988741847156811	pseudobulk	DESeq2	LRT	0.0872211066123218
401	KC_AB2	l(2)10685	0.818790875285784	0.940355871560339	0.988741847156811	pseudobulk	DESeq2	LRT	0.0267077593721816
401	KC_AB2	l(2)34Fd	-1.20081362769313	0.741163517910401	0.988741847156811	pseudobulk	DESeq2	LRT	0.130085965985812
401	KC_AB2	l(2)35Be	-1.52496782475103	0.617219242690575	0.988741847156811	pseudobulk	DESeq2	LRT	0.209560542647675
401	KC_AB2	l(2)37Bb	-0.92978684073213	0.72380920826628	0.988741847156811	pseudobulk	DESeq2	LRT	0.140375896118957
401	KC_AB2	l(2)37Cc	-0.462960135835943	0.762716996537802	0.988741847156811	pseudobulk	DESeq2	LRT	0.117636575598525
401	KC_AB2	l(2)37Cg	2.7785831508444	0.373751317747552	0.988741847156811	pseudobulk	DESeq2	LRT	0.427417267458199
401	KC_AB2	l(2)41Ab	2.51100223967711	0.18508466541614	0.988741847156811	pseudobulk	DESeq2	LRT	0.732629561803714
401	KC_AB2	l(2)dtl	-1.80294541848982	0.660135549041553	0.988741847156811	pseudobulk	DESeq2	LRT	0.180366879372519
401	KC_AB2	l(2)efl	1.13539439938001	0.65602501243538	0.988741847156811	pseudobulk	DESeq2	LRT	0.183079601850592
402	KC_AB2	l(2)gd1	-3.32426296135166	0.343738249391692	0.988741847156811	pseudobulk	DESeq2	LRT	0.463772139158982
402	KC_AB2	l(2)k01209	-1.2879653519079	0.633474066910355	0.988741847156811	pseudobulk	DESeq2	LRT	0.19827115951497
402	KC_AB2	l(2)k05819	0.310974079447882	0.852647373225612	0.988741847156811	pseudobulk	DESeq2	LRT	0.0692305415470647
402	KC_AB2	l(2)k09848	-1.23536834900699	0.725570392794057	0.988741847156811	pseudobulk	DESeq2	LRT	0.139320447155934
402	KC_AB2	l(2)k09913	0.0701431644945393	0.914015547468735	0.988741847156811	pseudobulk	DESeq2	LRT	0.0390464168236618
402	KC_AB2	l(2)k10201	-2.51746470924027	0.517377929599049	0.988741847156811	pseudobulk	DESeq2	LRT	0.286192101436607
402	KC_AB2	l(2)k14505	-0.0169659372998418	0.972349840248525	0.988741847156811	pseudobulk	DESeq2	LRT	0.0121774528163082
402	KC_AB2	l(2)SH0834	0.405123229601684	0.768541212613176	0.988741847156811	pseudobulk	DESeq2	LRT	0.114332838729516
402	KC_AB2	l(3)02640	2.18204160808338	0.285627389905139	0.988741847156811	pseudobulk	DESeq2	LRT	0.544200148736546
402	KC_AB2	l(3)04053	2.00515076565275	0.576456787459964	0.988741847156811	pseudobulk	DESeq2	LRT	0.239233242872451
403	KC_AB2	l(3)05822	-0.299889380661219	0.841793251907184	0.988741847156811	pseudobulk	DESeq2	LRT	0.0747945600220898

403	KC_AB2	l(3)07882	-2.9721553030081	0.387854083811327	0.988741847156811	pseudobulk	DESeq2	LRT	0.411331631401987
403	KC_AB2	l(3)72Ab	0.572861358867068	0.813871944286787	0.988741847156811	pseudobulk	DESeq2	LRT	0.0894439222171593
403	KC_AB2	l(3)72Dn	2.06137138167525	0.547946929015274	0.988741847156811	pseudobulk	DESeq2	LRT	0.261261502741816
403	KC_AB2	l(3)72Dp	-2.51746470924027	0.517377929599049	0.988741847156811	pseudobulk	DESeq2	LRT	0.286192101436607
403	KC_AB2	l(3)72Dr	-3.09794865339079	0.39855598532175	0.988741847156811	pseudobulk	DESeq2	LRT	0.399510664459796
403	KC_AB2	l(3)73Ah	-0.379503436459267	0.855911584421984	0.988741847156811	pseudobulk	DESeq2	LRT	0.0675710955808535
403	KC_AB2	l(3)76BDm	-1.20081362769313	0.741163517910401	0.988741847156811	pseudobulk	DESeq2	LRT	0.130085965985812
403	KC_AB2	l(3)80Fg	-0.284689419362781	0.406373472779176	0.988741847156811	pseudobulk	DESeq2	LRT	0.391074649645489
403	KC_AB2	l(3)80Fj	0.299056577645415	0.5212992666012	0.988741847156811	pseudobulk	DESeq2	LRT	0.28291288606718
404	KC_AB2	l(3)87Df	1.15102549446658	0.491153502372127	0.988741847156811	pseudobulk	DESeq2	LRT	0.30878275469068
404	KC_AB2	l(3)L1231	-0.274035897332517	0.727428285124457	0.988741847156811	pseudobulk	DESeq2	LRT	0.138209815963953
404	KC_AB2	l(3)mbt	0.921695369118063	0.730077514579258	0.988741847156811	pseudobulk	DESeq2	LRT	0.136631027048226
404	KC_AB2	l(3)neo38	-0.540784935797677	0.32868220161674	0.988741847156811	pseudobulk	DESeq2	LRT	0.483223812647668
404	KC_AB2	l(3)neo43	-0.864674604872878	0.696981345538496	0.988741847156811	pseudobulk	DESeq2	LRT	0.15677884548608
404	KC_AB2	l(3)psg2	0.429408125669229	0.795100748264289	0.988741847156811	pseudobulk	DESeq2	LRT	0.0995778378302778
404	KC_AB2	L2HGDH	0.680485379488395	0.740381497778529	0.988741847156811	pseudobulk	DESeq2	LRT	0.130544442854155
404	KC_AB2	La	-0.233272055930639	0.937531793764924	0.988741847156811	pseudobulk	DESeq2	LRT	0.028013995469539
404	KC_AB2	Lac	1.42621218304824	0.66738831402044	0.988741847156811	pseudobulk	DESeq2	LRT	0.175621402117653
404	KC_AB2	lace	0.818790875285784	0.940355871560339	0.988741847156811	pseudobulk	DESeq2	LRT	0.0267077593721816
405	KC_AB2	Lam	-0.452344515838269	0.871171367298942	0.988741847156811	pseudobulk	DESeq2	LRT	0.0598964069304489
405	KC_AB2	Lamp1	-0.106597646238688	0.887271275908896	0.988741847156811	pseudobulk	DESeq2	LRT	0.0519435778929684
405	KC_AB2	lap	-0.239294619517873	0.674965386231301	0.988741847156811	pseudobulk	DESeq2	LRT	0.170718498212219
405	KC_AB2	Lap1	-0.982834386675151	0.729867072195321	0.988741847156811	pseudobulk	DESeq2	LRT	0.13675622901488
405	KC_AB2	Lar	-0.335331602040137	0.496143030237009	0.988741847156811	pseudobulk	DESeq2	LRT	0.30439310518701
405	KC_AB2	lark	0.0607833096403573	0.938493046374102	0.988741847156811	pseudobulk	DESeq2	LRT	0.0275689408716647
405	KC_AB2	larp	-0.399427369819652	0.591337645484957	0.988741847156811	pseudobulk	DESeq2	LRT	0.228164472250741
405	KC_AB2	Larp4B	-0.738436109920065	0.155288061787504	0.988741847156811	pseudobulk	DESeq2	LRT	0.808861930614647
405	KC_AB2	Larp7	0.818790875285784	0.940355871560339	0.988741847156811	pseudobulk	DESeq2	LRT	0.0267077593721816
405	KC_AB2	Las	-0.506613484034373	0.961658398065406	0.988741847156811	pseudobulk	DESeq2	LRT	0.0169791713942958
406	KC_AB2	lawc	-0.509928591824577	0.641094232599673	0.988741847156811	pseudobulk	DESeq2	LRT	0.193078130090849
406	KC_AB2	lbk	-1.00729128970201	0.175998261655228	0.988741847156811	pseudobulk	DESeq2	LRT	0.754491621715797
406	KC_AB2	lbm	-2.19197918823644	0.30500585771122	0.988741847156811	pseudobulk	DESeq2	LRT	0.515691819842622
406	KC_AB2	LBR	0.712171366772551	0.813205723536451	0.988741847156811	pseudobulk	DESeq2	LRT	0.0897995733547685
406	KC_AB2	Lcch3	0.00500644563434207	0.98429507563362	0.988741847156811	pseudobulk	DESeq2	LRT	0.00687468763684353
406	KC_AB2	Ldh	0.818790875285784	0.940355871560339	0.988741847156811	pseudobulk	DESeq2	LRT	0.0267077593721816
406	KC_AB2	Leash	2.06137138167525	0.547946929015274	0.988741847156811	pseudobulk	DESeq2	LRT	0.261261502741816
406	KC_AB2	Lerp	-0.209294959031791	0.877893213984396	0.988741847156811	pseudobulk	DESeq2	LRT	0.056558308009394
406	KC_AB2	Letm1	-0.0384500034601774	0.953910728566112	0.988741847156811	pseudobulk	DESeq2	LRT	0.0204922667059844
406	KC_AB2	LeuRS	2.06137138167525	0.547946929015274	0.988741847156811	pseudobulk	DESeq2	LRT	0.261261502741816
407	KC_AB2	LeuRS-m	0.0601466527378941	0.974810960364079	0.988741847156811	pseudobulk	DESeq2	LRT	0.0110795964355292
407	KC_AB2	levy	0.537800227390284	0.310964428497128	0.988741847156811	pseudobulk	DESeq2	LRT	0.507289287471429
407	KC_AB2	Lgr1	2.12820410041287	0.0918857755118608	0.988741847156811	pseudobulk	DESeq2	LRT	1.03675171489081
407	KC_AB2	lgs	0.865127527500553	0.381554543267517	0.988741847156811	pseudobulk	DESeq2	LRT	0.418443370823289
407	KC_AB2	Lhr	-0.934020934207768	0.782359118258182	0.988741847156811	pseudobulk	DESeq2	LRT	0.106593851444836
407	KC_AB2	lic	-1.08129079499132	0.586874037065494	0.988741847156811	pseudobulk	DESeq2	LRT	0.231455102973389
407	KC_AB2	lid	0.352438831185159	0.671373207411986	0.988741847156811	pseudobulk	DESeq2	LRT	0.173035994195103
407	KC_AB2	lig	-0.243211909245335	0.800403699424586	0.988741847156811	pseudobulk	DESeq2	LRT	0.0966909127445083
407	KC_AB2	lili	-1.16822656624734	0.643917245153277	0.988741847156811	pseudobulk	DESeq2	LRT	0.191169943638088
407	KC_AB2	lilli	-0.167194753967143	0.760128389619193	0.988741847156811	pseudobulk	DESeq2	LRT	0.119113046937761
408	KC_AB2	LIMK1	0.00660524288075413	0.98064423596828	0.988741847156811	pseudobulk	DESeq2	LRT	0.00848852001946118
408	KC_AB2	lin	2.42863809728538	0.323713128875623	0.988741847156811	pseudobulk	DESeq2	LRT	0.489839686502244
408	KC_AB2	lin-52	-2.11027789354592	0.473734933668313	0.988741847156811	pseudobulk	DESeq2	LRT	0.32446458879962
408	KC_AB2	Lint-1	-0.448374585768327	0.814227572251762	0.988741847156811	pseudobulk	DESeq2	LRT	0.0892541951597077
408	KC_AB2	Liprin-alpha	-1.1465808389022	0.451198674656923	0.988741847156811	pseudobulk	DESeq2	LRT	0.345632184711838
408	KC_AB2	Liprin-beta	-1.23536834900699	0.725570392794057	0.988741847156811	pseudobulk	DESeq2	LRT	0.139320447155934
408	KC_AB2	Liprin-gamma	0.155975984309163	0.783550239645276	0.988741847156811	pseudobulk	DESeq2	LRT	0.105933152225113

408	KC_AB2	Lis-1	-0.0348431775456426	0.931566239220666	0.988741847156811	pseudobulk	DESeq2	LRT	0.0307862590659213
408	KC_AB2	Lkb1	-1.1284597703615	0.729754448571724	0.988741847156811	pseudobulk	DESeq2	LRT	0.136823248879498
408	KC_AB2	Lkr	1.42621218304824	0.66738831402044	0.988741847156811	pseudobulk	DESeq2	LRT	0.175621402117653
409	KC_AB2	Lmpt	0.599103181024285	0.554868924147382	0.988741847156811	pseudobulk	DESeq2	LRT	0.25580959749478
409	KC_AB2	lms	0.818790875285784	0.940355871560339	0.988741847156811	pseudobulk	DESeq2	LRT	0.0267077593721816
409	KC_AB2	lncRNA:alphagamma-ele	-0.678474919575052	0.472208995255285	0.988741847156811	pseudobulk	DESeq2	LRT	0.325865744168506
409	KC_AB2	lncRNA:cherub	1.80887083242013	0.327815018695064	0.988741847156811	pseudobulk	DESeq2	LRT	0.484371153306776
409	KC_AB2	lncRNA:CR30055	-1.11139857492744	0.380226739681421	0.988741847156811	pseudobulk	DESeq2	LRT	0.419957344367636
409	KC_AB2	lncRNA:CR30121	-2.26538931672255	0.547771004842275	0.988741847156811	pseudobulk	DESeq2	LRT	0.2614009599796
409	KC_AB2	lncRNA:CR31451	0.220847718993809	0.486064506653187	0.988741847156811	pseudobulk	DESeq2	LRT	0.313306090769629
409	KC_AB2	lncRNA:CR32218	1.32467530657598	0.538468278591255	0.988741847156811	pseudobulk	DESeq2	LRT	0.268839876095909
409	KC_AB2	lncRNA:CR32582	-2.51746470924027	0.517377929599049	0.988741847156811	pseudobulk	DESeq2	LRT	0.286192101436607
409	KC_AB2	lncRNA:CR32773	2.06137138167525	0.547946929015274	0.988741847156811	pseudobulk	DESeq2	LRT	0.261261502741816
410	KC_AB2	lncRNA:CR34335	0.525513595027544	0.214500271361339	0.988741847156811	pseudobulk	DESeq2	LRT	0.668572154058942
410	KC_AB2	lncRNA:CR40469	0.699674038439951	0.821690393783994	0.988741847156811	pseudobulk	DESeq2	LRT	0.0852917902455435
410	KC_AB2	lncRNA:CR42549	-0.213177136262384	0.93155916913195	0.988741847156811	pseudobulk	DESeq2	LRT	0.030789555140895
410	KC_AB2	lncRNA:CR42715	1.42621218304824	0.66738831402044	0.988741847156811	pseudobulk	DESeq2	LRT	0.175621402117653
410	KC_AB2	lncRNA:CR42861	-0.0745083593765503	0.948776116597123	0.988741847156811	pseudobulk	DESeq2	LRT	0.0228362562738991
410	KC_AB2	lncRNA:CR42862	-0.0172903945668188	0.957548683873015	0.988741847156811	pseudobulk	DESeq2	LRT	0.0188391363150395
410	KC_AB2	lncRNA:CR43148	0.321244353479908	0.883160641080799	0.988741847156811	pseudobulk	DESeq2	LRT	0.053960293942671
410	KC_AB2	lncRNA:CR43166	0.818790875285784	0.940355871560339	0.988741847156811	pseudobulk	DESeq2	LRT	0.0267077593721816
410	KC_AB2	lncRNA:CR43196	0.818790875285784	0.940355871560339	0.988741847156811	pseudobulk	DESeq2	LRT	0.0267077593721816
410	KC_AB2	lncRNA:CR43241	0.284990316553174	0.896610940373611	0.988741847156811	pseudobulk	DESeq2	LRT	0.0473959662120276
411	KC_AB2	lncRNA:CR43242	-3.32426296135166	0.343738249391692	0.988741847156811	pseudobulk	DESeq2	LRT	0.463772139158982
411	KC_AB2	lncRNA:CR43256	-0.934020934207768	0.782359118258182	0.988741847156811	pseudobulk	DESeq2	LRT	0.106593851444836
411	KC_AB2	lncRNA:CR43264	4.37399608415456	0.0702592894412528	0.988741847156811	pseudobulk	DESeq2	LRT	1.15329624670601
411	KC_AB2	lncRNA:CR43287	-1.80294541848982	0.660135549041553	0.988741847156811	pseudobulk	DESeq2	LRT	0.180366879372519
411	KC_AB2	lncRNA:CR43334	0.300414301642887	0.881846961452027	0.988741847156811	pseudobulk	DESeq2	LRT	0.0546067771853455
411	KC_AB2	lncRNA:CR43459	-0.257785287384048	0.890752234185173	0.988741847156811	pseudobulk	DESeq2	LRT	0.050243079677957
411	KC_AB2	lncRNA:CR43496	-1.63785643245857	0.555083135854033	0.988741847156811	pseudobulk	DESeq2	LRT	0.255641966897631
411	KC_AB2	lncRNA:CR43604	0.818790875285784	0.940355871560339	0.988741847156811	pseudobulk	DESeq2	LRT	0.0267077593721816
411	KC_AB2	lncRNA:CR43651	-0.11350464060454	0.932722166081082	0.988741847156811	pseudobulk	DESeq2	LRT	0.0302477021346365
411	KC_AB2	lncRNA:CR43685	-0.110541354292284	0.948079934556759	0.988741847156811	pseudobulk	DESeq2	LRT	0.023155044862967
412	KC_AB2	lncRNA:CR43785	1.42621218304824	0.66738831402044	0.988741847156811	pseudobulk	DESeq2	LRT	0.175621402117653
412	KC_AB2	lncRNA:CR43836	-1.65239501904088	0.365358459891242	0.988741847156811	pseudobulk	DESeq2	LRT	0.437280832094468
412	KC_AB2	lncRNA:CR43856	-0.934020934207768	0.782359118258182	0.988741847156811	pseudobulk	DESeq2	LRT	0.106593851444836
412	KC_AB2	lncRNA:CR43878	1.12919034796087	0.7301237214754	0.988741847156811	pseudobulk	DESeq2	LRT	0.136603541247596
412	KC_AB2	lncRNA:CR43910	-1.80294541848982	0.660135549041553	0.988741847156811	pseudobulk	DESeq2	LRT	0.180366879372519
412	KC_AB2	lncRNA:CR43943	-1.80294541848982	0.660135549041553	0.988741847156811	pseudobulk	DESeq2	LRT	0.180366879372519
412	KC_AB2	lncRNA:CR43956	0.818790875285784	0.940355871560339	0.988741847156811	pseudobulk	DESeq2	LRT	0.0267077593721816
412	KC_AB2	lncRNA:CR43958	-1.80294541848982	0.660135549041553	0.988741847156811	pseudobulk	DESeq2	LRT	0.180366879372519
412	KC_AB2	lncRNA:CR43963	1.42621218304824	0.66738831402044	0.988741847156811	pseudobulk	DESeq2	LRT	0.175621402117653
412	KC_AB2	lncRNA:CR43966	0.818790875285784	0.940355871560339	0.988741847156811	pseudobulk	DESeq2	LRT	0.0267077593721816
413	KC_AB2	lncRNA:CR43995	0.269971509165439	0.903083168079264	0.988741847156811	pseudobulk	DESeq2	LRT	0.0442722521508783
413	KC_AB2	lncRNA:CR44023	0.104637746735554	0.951122131714938	0.988741847156811	pseudobulk	DESeq2	LRT	0.0217637125849166
413	KC_AB2	lncRNA:CR44024	-0.0276968352714998	0.962285219251326	0.988741847156811	pseudobulk	DESeq2	LRT	0.0166961849394258
413	KC_AB2	lncRNA:CR44043	2.4643417141186	0.449082701910884	0.988741847156811	pseudobulk	DESeq2	LRT	0.347673673080982
413	KC_AB2	lncRNA:CR44050	1.47543920254359	0.661929169513603	0.988741847156811	pseudobulk	DESeq2	LRT	0.17918848025087
413	KC_AB2	lncRNA:CR44070	-1.80294541848982	0.660135549041553	0.988741847156811	pseudobulk	DESeq2	LRT	0.180366879372519
413	KC_AB2	lncRNA:CR44092	0.110731962284482	0.958092313397532	0.988741847156811	pseudobulk	DESeq2	LRT	0.0185926440867238
413	KC_AB2	lncRNA:CR44126	-2.26538931672255	0.547771004842275	0.988741847156811	pseudobulk	DESeq2	LRT	0.2614009599796
413	KC_AB2	lncRNA:CR44149	-1.80294541848982	0.660135549041553	0.988741847156811	pseudobulk	DESeq2	LRT	0.180366879372519
413	KC_AB2	lncRNA:CR44178	1.42621218304824	0.66738831402044	0.988741847156811	pseudobulk	DESeq2	LRT	0.175621402117653
414	KC_AB2	lncRNA:CR44187	-0.627361101364054	0.786684305786895	0.988741847156811	pseudobulk	DESeq2	LRT	0.104199513842738
414	KC_AB2	lncRNA:CR44206	-1.99867440840856	0.534899605264936	0.988741847156811	pseudobulk	DESeq2	LRT	0.271727722597268
414	KC_AB2	lncRNA:CR44272	-2.51746470924027	0.517377929599049	0.988741847156811	pseudobulk	DESeq2	LRT	0.286192101436607

414	KC_AB2	lncRNA:CR44330	0.818790875285784	0.940355871560339	0.988741847156811	pseudobulk	DESeq2	LRT	0.0267077593721816
414	KC_AB2	lncRNA:CR44332	-2.26538931672255	0.547771004842275	0.988741847156811	pseudobulk	DESeq2	LRT	0.2614009599796
414	KC_AB2	lncRNA:CR44347	-0.416302618095149	0.849281950028502	0.988741847156811	pseudobulk	DESeq2	LRT	0.070948105970953
414	KC_AB2	lncRNA:CR44348	1.42621218304824	0.66738831402044	0.988741847156811	pseudobulk	DESeq2	LRT	0.175621402117653
414	KC_AB2	lncRNA:CR44357	-3.19757568445434	0.226213705653814	0.988741847156811	pseudobulk	DESeq2	LRT	0.645481085929121
414	KC_AB2	lncRNA:CR44360	0.818790875285784	0.940355871560339	0.988741847156811	pseudobulk	DESeq2	LRT	0.0267077593721816
414	KC_AB2	lncRNA:CR44363	-1.80294541848982	0.660135549041553	0.988741847156811	pseudobulk	DESeq2	LRT	0.180366879372519
415	KC_AB2	lncRNA:CR44411	-1.80294541848982	0.660135549041553	0.988741847156811	pseudobulk	DESeq2	LRT	0.180366879372519
415	KC_AB2	lncRNA:CR44417	-0.862877827457123	0.513632719123631	0.988741847156811	pseudobulk	DESeq2	LRT	0.289347318891216
415	KC_AB2	lncRNA:CR44445	-1.80294541848982	0.660135549041553	0.988741847156811	pseudobulk	DESeq2	LRT	0.180366879372519
415	KC_AB2	lncRNA:CR44472	1.42621218304824	0.66738831402044	0.988741847156811	pseudobulk	DESeq2	LRT	0.175621402117653
415	KC_AB2	lncRNA:CR44506	-0.00340877878248056	0.980029417943268	0.988741847156811	pseudobulk	DESeq2	LRT	0.0087608877170192
415	KC_AB2	lncRNA:CR44564	-1.80294541848982	0.660135549041553	0.988741847156811	pseudobulk	DESeq2	LRT	0.180366879372519
415	KC_AB2	lncRNA:CR44589	-1.52496782475103	0.617219242690575	0.988741847156811	pseudobulk	DESeq2	LRT	0.209560542647675
415	KC_AB2	lncRNA:CR44595	0.818790875285784	0.940355871560339	0.988741847156811	pseudobulk	DESeq2	LRT	0.0267077593721816
415	KC_AB2	lncRNA:CR44597	0.699674038439951	0.821690393783994	0.988741847156811	pseudobulk	DESeq2	LRT	0.0852917902455435
415	KC_AB2	lncRNA:CR44598	-0.197729422006198	0.906021765866329	0.988741847156811	pseudobulk	DESeq2	LRT	0.0428613688992414
416	KC_AB2	lncRNA:CR44628	0.818790875285784	0.940355871560339	0.988741847156811	pseudobulk	DESeq2	LRT	0.0267077593721816
416	KC_AB2	lncRNA:CR44663	2.80917294583258	0.367996267391162	0.988741847156811	pseudobulk	DESeq2	LRT	0.434156586379859
416	KC_AB2	lncRNA:CR44679	0.818790875285784	0.940355871560339	0.988741847156811	pseudobulk	DESeq2	LRT	0.0267077593721816
416	KC_AB2	lncRNA:CR44814	0.818790875285784	0.940355871560339	0.988741847156811	pseudobulk	DESeq2	LRT	0.0267077593721816
416	KC_AB2	lncRNA:CR44816	-0.506613484034373	0.961658398065406	0.988741847156811	pseudobulk	DESeq2	LRT	0.0169791713942958
416	KC_AB2	lncRNA:CR44832	-3.6809086040586	0.231552724242678	0.988741847156811	pseudobulk	DESeq2	LRT	0.635350105118793
416	KC_AB2	lncRNA:CR44833	0.818790875285784	0.940355871560339	0.988741847156811	pseudobulk	DESeq2	LRT	0.0267077593721816
416	KC_AB2	lncRNA:CR44886	1.05931975243218	0.418579755662151	0.988741847156811	pseudobulk	DESeq2	LRT	0.378221779859788
416	KC_AB2	lncRNA:CR44900	1.42621218304824	0.66738831402044	0.988741847156811	pseudobulk	DESeq2	LRT	0.175621402117653
416	KC_AB2	lncRNA:CR44922	-0.163522282077002	0.90229247489512	0.988741847156811	pseudobulk	DESeq2	LRT	0.0446526646263037
417	KC_AB2	lncRNA:CR44923	2.51243226103394	0.474458247157471	0.988741847156811	pseudobulk	DESeq2	LRT	0.323801999942597
417	KC_AB2	lncRNA:CR44938	0.818790875285784	0.940355871560339	0.988741847156811	pseudobulk	DESeq2	LRT	0.0267077593721816
417	KC_AB2	lncRNA:CR44977	1.42621218304824	0.66738831402044	0.988741847156811	pseudobulk	DESeq2	LRT	0.175621402117653
417	KC_AB2	lncRNA:CR44986	-2.26538931672255	0.547771004842275	0.988741847156811	pseudobulk	DESeq2	LRT	0.2614009599796
417	KC_AB2	lncRNA:CR44993	-0.729234089517982	0.868684292414125	0.988741847156811	pseudobulk	DESeq2	LRT	0.0611380313470854
417	KC_AB2	lncRNA:CR44994	0.818790875285784	0.940355871560339	0.988741847156811	pseudobulk	DESeq2	LRT	0.0267077593721816
417	KC_AB2	lncRNA:CR44997	0.32742703490313	0.878511928385878	0.988741847156811	pseudobulk	DESeq2	LRT	0.0562523373024427
417	KC_AB2	lncRNA:CR44999	-1.78822362243861	0.217672446660993	0.988741847156811	pseudobulk	DESeq2	LRT	0.662196541195063
417	KC_AB2	lncRNA:CR45025	0.818790875285784	0.940355871560339	0.988741847156811	pseudobulk	DESeq2	LRT	0.0267077593721816
417	KC_AB2	lncRNA:CR45035	1.47543920254359	0.661929169513603	0.988741847156811	pseudobulk	DESeq2	LRT	0.17918848025087
418	KC_AB2	lncRNA:CR45054	-0.468874156366393	0.480221747116447	0.988741847156811	pseudobulk	DESeq2	LRT	0.318558176559679
418	KC_AB2	lncRNA:CR45102	-0.32351155741363	0.808244514436759	0.988741847156811	pseudobulk	DESeq2	LRT	0.0924572342672017
418	KC_AB2	lncRNA:CR45125	0.818790875285784	0.940355871560339	0.988741847156811	pseudobulk	DESeq2	LRT	0.0267077593721816
418	KC_AB2	lncRNA:CR45159	1.42621218304824	0.66738831402044	0.988741847156811	pseudobulk	DESeq2	LRT	0.175621402117653
418	KC_AB2	lncRNA:CR45169	-2.26538931672255	0.547771004842275	0.988741847156811	pseudobulk	DESeq2	LRT	0.2614009599796
418	KC_AB2	lncRNA:CR45170	-3.4227037205893	0.285801360563088	0.988741847156811	pseudobulk	DESeq2	LRT	0.543935708072568
418	KC_AB2	lncRNA:CR45188	-1.80294541848982	0.660135549041553	0.988741847156811	pseudobulk	DESeq2	LRT	0.180366879372519
418	KC_AB2	lncRNA:CR45240	3.09926389624515	0.339712049344771	0.988741847156811	pseudobulk	DESeq2	LRT	0.468889048740397
418	KC_AB2	lncRNA:CR45290	-2.9721553030081	0.387854083811327	0.988741847156811	pseudobulk	DESeq2	LRT	0.411331631401987
418	KC_AB2	lncRNA:CR45308	-1.80294541848982	0.660135549041553	0.988741847156811	pseudobulk	DESeq2	LRT	0.180366879372519
419	KC_AB2	lncRNA:CR45388	1.13091714558663	0.68188196534655	0.988741847156811	pseudobulk	DESeq2	LRT	0.166290795776296
419	KC_AB2	lncRNA:CR45409	2.81309903729385	0.219655468837245	0.988741847156811	pseudobulk	DESeq2	LRT	0.658257979485977
419	KC_AB2	lncRNA:CR45473	2.7785831508444	0.373751317747552	0.988741847156811	pseudobulk	DESeq2	LRT	0.427417267458199
419	KC_AB2	lncRNA:CR45522	-2.9721553030081	0.387854083811327	0.988741847156811	pseudobulk	DESeq2	LRT	0.411331631401987
419	KC_AB2	lncRNA:CR45527	-2.51746470924027	0.517377929599049	0.988741847156811	pseudobulk	DESeq2	LRT	0.286192101436607
419	KC_AB2	lncRNA:CR45660	0.0471023025337348	0.971010011322875	0.988741847156811	pseudobulk	DESeq2	LRT	0.0127762923990319
419	KC_AB2	lncRNA:CR45668	-0.634227699706429	0.828294391400015	0.988741847156811	pseudobulk	DESeq2	LRT	0.0818152793537044
419	KC_AB2	lncRNA:CR45669	-2.51746470924027	0.517377929599049	0.988741847156811	pseudobulk	DESeq2	LRT	0.286192101436607
419	KC_AB2	lncRNA:CR45684	0.341938532160155	0.831023958828318	0.988741847156811	pseudobulk	DESeq2	LRT	0.0803864551124753

419	KC_AB2	lncRNA:CR45736	0.818790875285784	0.940355871560339	0.988741847156811	pseudobulk	DESeq2	LRT	0.0267077593721816
420	KC_AB2	lncRNA:CR45758	0.826276136237366	0.506769691221217	0.988741847156811	pseudobulk	DESeq2	LRT	0.295189367208079
420	KC_AB2	lncRNA:CR45819	0.818790875285784	0.940355871560339	0.988741847156811	pseudobulk	DESeq2	LRT	0.0267077593721816
420	KC_AB2	lncRNA:CR45821	0.818790875285784	0.940355871560339	0.988741847156811	pseudobulk	DESeq2	LRT	0.0267077593721816
420	KC_AB2	lncRNA:CR45825	-1.20081362769313	0.741163517910401	0.988741847156811	pseudobulk	DESeq2	LRT	0.130085965985812
420	KC_AB2	lncRNA:CR45878	-1.80294541848982	0.660135549041553	0.988741847156811	pseudobulk	DESeq2	LRT	0.180366879372519
420	KC_AB2	lncRNA:CR45880	-1.80294541848982	0.660135549041553	0.988741847156811	pseudobulk	DESeq2	LRT	0.180366879372519
420	KC_AB2	lncRNA:CR45883	-1.80294541848982	0.660135549041553	0.988741847156811	pseudobulk	DESeq2	LRT	0.180366879372519
420	KC_AB2	lncRNA:CR45911	-1.2879653519079	0.633474066910355	0.988741847156811	pseudobulk	DESeq2	LRT	0.19827115951497
420	KC_AB2	lncRNA:CR45918	-1.69633662497937	0.605069927920179	0.988741847156811	pseudobulk	DESeq2	LRT	0.2181944310419
420	KC_AB2	lncRNA:CR45919	2.06137138167525	0.547946929015274	0.988741847156811	pseudobulk	DESeq2	LRT	0.261261502741816
421	KC_AB2	lncRNA:CR45959	1.42621218304824	0.66738831402044	0.988741847156811	pseudobulk	DESeq2	LRT	0.175621402117653
421	KC_AB2	lncRNA:CR45971	1.42621218304824	0.66738831402044	0.988741847156811	pseudobulk	DESeq2	LRT	0.175621402117653
421	KC_AB2	lncRNA:CR45972	1.42621218304824	0.66738831402044	0.988741847156811	pseudobulk	DESeq2	LRT	0.175621402117653
421	KC_AB2	lncRNA:CR45979	-0.0144409071146711	0.984632974376923	0.988741847156811	pseudobulk	DESeq2	LRT	0.00672562423038896
421	KC_AB2	lncRNA:CR45982	0.818790875285784	0.940355871560339	0.988741847156811	pseudobulk	DESeq2	LRT	0.0267077593721816
421	KC_AB2	lncRNA:CR45994	-1.23536834900699	0.725570392794057	0.988741847156811	pseudobulk	DESeq2	LRT	0.139320447155934
421	KC_AB2	lncRNA:CR45996	2.00515076565275	0.576456787459964	0.988741847156811	pseudobulk	DESeq2	LRT	0.239233242872451
421	KC_AB2	lncRNA:CR46003	-1.1284597703615	0.729754448571724	0.988741847156811	pseudobulk	DESeq2	LRT	0.136823248879498
421	KC_AB2	lncRNA:CR46004	2.01044021410856	0.465232850276855	0.988741847156811	pseudobulk	DESeq2	LRT	0.332329627154538
421	KC_AB2	lncRNA:CR46006	-0.0780974884201545	0.866153446903191	0.988741847156811	pseudobulk	DESeq2	LRT	0.0624051619781127
422	KC_AB2	lncRNA:CR46064	0.0523160437812123	0.94762594060204	0.988741847156811	pseudobulk	DESeq2	LRT	0.0233630592767058
422	KC_AB2	lncRNA:CR46084	0.157562008957688	0.950463298513166	0.988741847156811	pseudobulk	DESeq2	LRT	0.0220646484576093
422	KC_AB2	lncRNA:CR46208	0.746317762218178	0.547958156869278	0.988741847156811	pseudobulk	DESeq2	LRT	0.261252603805036
422	KC_AB2	lncRNA:CR46233	0.818790875285784	0.940355871560339	0.988741847156811	pseudobulk	DESeq2	LRT	0.0267077593721816
422	KC_AB2	lncRNA:CR46268	-1.14755310091518	0.618375466635947	0.988741847156811	pseudobulk	DESeq2	LRT	0.208747748902126
422	KC_AB2	lncRNA:CR46322	-0.405381063532158	0.89155634938256	0.988741847156811	pseudobulk	DESeq2	LRT	0.0498512027381788
422	KC_AB2	lncRNA:flam	2.00515076565275	0.576456787459964	0.988741847156811	pseudobulk	DESeq2	LRT	0.239233242872451
422	KC_AB2	lncRNA:Hsromega	-0.868091613239444	0.000438183189635751	0.988741847156811	pseudobulk	DESeq2	LRT	3.35834428763444
422	KC_AB2	lncRNA:let7C	0.770981480810769	0.472613722390003	0.988741847156811	pseudobulk	DESeq2	LRT	0.325493672733828
422	KC_AB2	lncRNA:noe	0.510583137353684	0.0128224681261969	0.988741847156811	pseudobulk	DESeq2	LRT	1.89202837182033
423	KC_AB2	lncRNA:roX1	1.42621218304824	0.66738831402044	0.988741847156811	pseudobulk	DESeq2	LRT	0.175621402117653
423	KC_AB2	lncRNA:roX2	0.818790875285784	0.940355871560339	0.988741847156811	pseudobulk	DESeq2	LRT	0.0267077593721816
423	KC_AB2	Lnk	0.738616472640367	0.628202413171786	0.988741847156811	pseudobulk	DESeq2	LRT	0.201900399638919
423	KC_AB2	Lnpk	-0.957133960806503	0.762632518785665	0.988741847156811	pseudobulk	DESeq2	LRT	0.1176846802739
423	KC_AB2	loh	-0.0582963981256801	0.979058404810457	0.988741847156811	pseudobulk	DESeq2	LRT	0.0091913999942843
423	KC_AB2	loj	0.362555753552128	0.897034383127386	0.988741847156811	pseudobulk	DESeq2	LRT	0.0471909102264623
423	KC_AB2	lola	-0.183096461887837	0.568459168543233	0.988741847156811	pseudobulk	DESeq2	LRT	0.245300724491124
423	KC_AB2	lolal	0.251773870302674	0.701274701159621	0.988741847156811	pseudobulk	DESeq2	LRT	0.154111828212235
423	KC_AB2	Lon	-1.40296545307325	0.628223627794903	0.988741847156811	pseudobulk	DESeq2	LRT	0.201885733604539
423	KC_AB2	loqs	-0.230447530535158	0.757550269532342	0.988741847156811	pseudobulk	DESeq2	LRT	0.120588542949577
424	KC_AB2	lost	0.572178477920992	0.692232729700003	0.988741847156811	pseudobulk	DESeq2	LRT	0.159747870525763
424	KC_AB2	lovit	3.63001914094513	0.185579857467938	0.988741847156811	pseudobulk	DESeq2	LRT	0.731469163166964
424	KC_AB2	LPCAT	1.03695592081708	0.452765726625953	0.988741847156811	pseudobulk	DESeq2	LRT	0.344126455716829
424	KC_AB2	Lpin	-1.00975279379859	0.447054107721507	0.988741847156811	pseudobulk	DESeq2	LRT	0.349639910284667
424	KC_AB2	Lpt	2.4643417141186	0.449082701910884	0.988741847156811	pseudobulk	DESeq2	LRT	0.347673673080982
424	KC_AB2	lqf	-0.422923672391685	0.550206533793439	0.988741847156811	pseudobulk	DESeq2	LRT	0.25947425659703
424	KC_AB2	lqfR	0.310390049490412	0.904941705285892	0.988741847156811	pseudobulk	DESeq2	LRT	0.04337939636174
424	KC_AB2	Lrch	0.57339563734307	0.699544809061774	0.988741847156811	pseudobulk	DESeq2	LRT	0.155184461722557
424	KC_AB2	LRP1	-0.290252040608313	0.872221172896038	0.988741847156811	pseudobulk	DESeq2	LRT	0.0593733751719846
424	KC_AB2	Lrp4	-0.0145559267672141	0.964113519520913	0.988741847156811	pseudobulk	DESeq2	LRT	0.0158718270941193
425	KC_AB2	Lrpprc2	2.06137138167525	0.547946929015274	0.988741847156811	pseudobulk	DESeq2	LRT	0.261261502741816
425	KC_AB2	LRR	0.847832741663511	0.608530738759056	0.988741847156811	pseudobulk	DESeq2	LRT	0.215717479329945
425	KC_AB2	Lrr47	1.42621218304824	0.66738831402044	0.988741847156811	pseudobulk	DESeq2	LRT	0.175621402117653
425	KC_AB2	Lrrk	-0.568233637794205	0.868826397133536	0.988741847156811	pseudobulk	DESeq2	LRT	0.0610669925949191
425	KC_AB2	Lsd-2	-0.378852672109618	0.920549594954422	0.988741847156811	pseudobulk	DESeq2	LRT	0.0359528087516892

425	KC_AB2	LSm1	0.818790875285784	0.940355871560339	0.988741847156811	pseudobulk	DESeq2	LRT	0.0267077593721816
425	KC_AB2	Lsm12a	0.570871964820517	0.688301172834114	0.988741847156811	pseudobulk	DESeq2	LRT	0.162221490427453
425	KC_AB2	LSm3	-0.321040059436753	0.870444986106655	0.988741847156811	pseudobulk	DESeq2	LRT	0.0602586719737732
425	KC_AB2	LSm7	-1.73627324524809	0.521368726288639	0.988741847156811	pseudobulk	DESeq2	LRT	0.282855023048041
425	KC_AB2	lsn	1.7098779479	0.537865353861561	0.988741847156811	pseudobulk	DESeq2	LRT	0.269326429526188
426	KC_AB2	Lst8	0.818790875285784	0.940355871560339	0.988741847156811	pseudobulk	DESeq2	LRT	0.0267077593721816
426	KC_AB2	lt	-1.86305953694915	0.283921667450936	0.988741847156811	pseudobulk	DESeq2	LRT	0.54680146307363
426	KC_AB2	Ltn1	-1.02592565225788	0.645171277800408	0.988741847156811	pseudobulk	DESeq2	LRT	0.190324975087242
426	KC_AB2	LTV1	0.739459049639609	0.685859718010821	0.988741847156811	pseudobulk	DESeq2	LRT	0.163764703424277
426	KC_AB2	luna	0.818790875285784	0.940355871560339	0.988741847156811	pseudobulk	DESeq2	LRT	0.0267077593721816
426	KC_AB2	lute	0.55034594750535	0.688537219270625	0.988741847156811	pseudobulk	DESeq2	LRT	0.162072578737538
426	KC_AB2	lva	1.2117839627016	0.517105339425622	0.988741847156811	pseudobulk	DESeq2	LRT	0.286420977851384
426	KC_AB2	lwr	0.0283911800001658	0.973133033155818	0.988741847156811	pseudobulk	DESeq2	LRT	0.0118277849976625
426	KC_AB2	LysRS	-2.71963753322567	0.00477210923850293	0.988741847156811	pseudobulk	DESeq2	LRT	2.32128962343924
426	KC_AB2	Lztr1	-0.022992191949664	0.973602074482358	0.988741847156811	pseudobulk	DESeq2	LRT	0.0116185094130768
427	KC_AB2	m-cup	-1.73627324524809	0.521368726288639	0.988741847156811	pseudobulk	DESeq2	LRT	0.282855023048041
427	KC_AB2	M1BP	0.899175816777536	0.633651248744323	0.988741847156811	pseudobulk	DESeq2	LRT	0.198149704924274
427	KC_AB2	M6	1.67796394544623	0.574362267760116	0.988741847156811	pseudobulk	DESeq2	LRT	0.240814098434665
427	KC_AB2	mAChR-A	0.0326339196522644	0.966341524526128	0.988741847156811	pseudobulk	DESeq2	LRT	0.0148693580523982
427	KC_AB2	mAChR-B	0.842427831324256	0.333973170683432	0.988741847156811	pseudobulk	DESeq2	LRT	0.476288420290662
427	KC_AB2	mAcon1	0.588641544875505	0.74069336233354	0.988741847156811	pseudobulk	DESeq2	LRT	0.130361547227483
427	KC_AB2	Mad	0.0992692229982227	0.933336453414587	0.988741847156811	pseudobulk	DESeq2	LRT	0.0299617715576502
427	KC_AB2	Mad1	-1.1284597703615	0.729754448571724	0.988741847156811	pseudobulk	DESeq2	LRT	0.136823248879498
427	KC_AB2	Madm	-0.00340877878248056	0.980029417943268	0.988741847156811	pseudobulk	DESeq2	LRT	0.0087608877170192
427	KC_AB2	mael	-1.80294541848982	0.660135549041553	0.988741847156811	pseudobulk	DESeq2	LRT	0.180366879372519
428	KC_AB2	maf-S	0.914853256705245	0.455569054470451	0.988741847156811	pseudobulk	DESeq2	LRT	0.34144578399848
428	KC_AB2	Maf1	-0.254228555769709	0.711598168017387	0.988741847156811	pseudobulk	DESeq2	LRT	0.147765178661418
428	KC_AB2	Magi	-1.64271834309286	0.357867998682071	0.988741847156811	pseudobulk	DESeq2	LRT	0.446277135410403
428	KC_AB2	mago	0.323743113177247	0.801220043826253	0.988741847156811	pseudobulk	DESeq2	LRT	0.0962481946577263
428	KC_AB2	MagR	0.604071214267024	0.581069399159887	0.988741847156811	pseudobulk	DESeq2	LRT	0.235771995196119
428	KC_AB2	mahe	0.162941265670117	0.810565682162115	0.988741847156811	pseudobulk	DESeq2	LRT	0.0912117874227548
428	KC_AB2	mahj	-1.37054505049545	0.574189269529552	0.988741847156811	pseudobulk	DESeq2	LRT	0.240944927875125
428	KC_AB2	mal	2.06137138167525	0.547946929015274	0.988741847156811	pseudobulk	DESeq2	LRT	0.261261502741816
428	KC_AB2	mam	-0.285147688836435	0.708885493360072	0.988741847156811	pseudobulk	DESeq2	LRT	0.149423910963718
428	KC_AB2	mamo	5.99963851467141	0.122296253296214	0.988741847156811	pseudobulk	DESeq2	LRT	0.912586847932679
429	KC_AB2	MAN1	-0.244067456186467	0.906772274454269	0.988741847156811	pseudobulk	DESeq2	LRT	0.042501767367312
429	KC_AB2	Manf	1.03300974416562	0.509646295460471	0.988741847156811	pseudobulk	DESeq2	LRT	0.292731128259441
429	KC_AB2	Map205	-0.669805852571175	0.647931160730873	0.988741847156811	pseudobulk	DESeq2	LRT	0.188471133176995
429	KC_AB2	Map60	0.518112575170181	0.804900582036886	0.988741847156811	pseudobulk	DESeq2	LRT	0.0942577585627823
429	KC_AB2	MAPk-Ak2	-0.906529149177788	0.58238998031075	0.988741847156811	pseudobulk	DESeq2	LRT	0.234786105420953
429	KC_AB2	Mapmodulin	-0.230914178947952	0.670381102257266	0.988741847156811	pseudobulk	DESeq2	LRT	0.173678236771973
429	KC_AB2	Marf	0.258054590434572	0.791400255946833	0.988741847156811	pseudobulk	DESeq2	LRT	0.101603813614748
429	KC_AB2	mars	-2.26538931672255	0.547771004842275	0.988741847156811	pseudobulk	DESeq2	LRT	0.2614009599796
429	KC_AB2	mask	-0.498549055038189	0.517432831422461	0.988741847156811	pseudobulk	DESeq2	LRT	0.286146018500722
429	KC_AB2	Mat1	-3.4227037205893	0.285801360563088	0.988741847156811	pseudobulk	DESeq2	LRT	0.543935708072568
430	KC_AB2	mats	-1.54768271596609	0.394513952614918	0.988741847156811	pseudobulk	DESeq2	LRT	0.403937632666189
430	KC_AB2	Mau2	-0.506613484034373	0.961658398065406	0.988741847156811	pseudobulk	DESeq2	LRT	0.0169791713942958
430	KC_AB2	Max	0.731084242513263	0.689241505462626	0.988741847156811	pseudobulk	DESeq2	LRT	0.161628577639223
430	KC_AB2	mbc	-1.48149817242366	0.342629818077936	0.988741847156811	pseudobulk	DESeq2	LRT	0.465174844338402
430	KC_AB2	MBD-like	0.125117747819201	0.919455556622137	0.988741847156811	pseudobulk	DESeq2	LRT	0.0364692582478075
430	KC_AB2	MBD-R2	0.752634025970037	0.844139830246018	0.988741847156811	pseudobulk	DESeq2	LRT	0.0735856073147832
430	KC_AB2	mbf1	0.111497864234101	0.896638200657682	0.988741847156811	pseudobulk	DESeq2	LRT	0.047382762256507
430	KC_AB2	mbo	-0.934020934207768	0.782359118258182	0.988741847156811	pseudobulk	DESeq2	LRT	0.106593851444836
430	KC_AB2	Mbs	-0.318695680945018	0.701230325458373	0.988741847156811	pseudobulk	DESeq2	LRT	0.154139310640922
430	KC_AB2	mbt	0.771676584147554	0.569787294192586	0.988741847156811	pseudobulk	DESeq2	LRT	0.244287239413095
431	KC_AB2	Mcad	0.818790875285784	0.940355871560339	0.988741847156811	pseudobulk	DESeq2	LRT	0.0267077593721816

431	KC_AB2	Mccc1	-1.80294541848982	0.660135549041553	0.988741847156811	pseudobulk	DESeq2	LRT	0.180366879372519
431	KC_AB2	Mcr	1.0821173077982	0.758638591992449	0.988741847156811	pseudobulk	DESeq2	LRT	0.119965068483926
431	KC_AB2	Mctp	3.03619069427769	0.314302467188174	0.988741847156811	pseudobulk	DESeq2	LRT	0.502652209876457
431	KC_AB2	MCTS1	-0.369466933652223	0.83718326075838	0.988741847156811	pseudobulk	DESeq2	LRT	0.0771794638414072
431	KC_AB2	MCU	1.05874540552977	0.480484632780241	0.988741847156811	pseudobulk	DESeq2	LRT	0.318320497705045
431	KC_AB2	Mdh1	0.228233040356986	0.863275058584094	0.988741847156811	pseudobulk	DESeq2	LRT	0.0638508063792085
431	KC_AB2	Mdh2	0.475816033563272	0.534058003446199	0.988741847156811	pseudobulk	DESeq2	LRT	0.272411572168811
431	KC_AB2	mdy	1.05471097764646	0.481231578734858	0.988741847156811	pseudobulk	DESeq2	LRT	0.317645881705618
431	KC_AB2	me31B	0.371559856488034	0.730502142083302	0.988741847156811	pseudobulk	DESeq2	LRT	0.13637850622716
432	KC_AB2	Med	0.48020439743879	0.797798185903029	0.988741847156811	pseudobulk	DESeq2	LRT	0.0981069555583927
432	KC_AB2	MED1	-2.21667329608248	0.477261050443944	0.988741847156811	pseudobulk	DESeq2	LRT	0.321244007216606
432	KC_AB2	MED10	0.269588858012571	0.844077441836281	0.988741847156811	pseudobulk	DESeq2	LRT	0.0736177061951155
432	KC_AB2	MED11	-0.982137735218619	0.64553975034157	0.988741847156811	pseudobulk	DESeq2	LRT	0.190077010062976
432	KC_AB2	MED14	-0.0144409071146711	0.984632974376923	0.988741847156811	pseudobulk	DESeq2	LRT	0.00672562423038896
432	KC_AB2	MED15	1.47543920254359	0.661929169513603	0.988741847156811	pseudobulk	DESeq2	LRT	0.17918848025087
432	KC_AB2	MED16	-2.51746470924027	0.517377929599049	0.988741847156811	pseudobulk	DESeq2	LRT	0.286192101436607
432	KC_AB2	MED17	-1.23536834900699	0.725570392794057	0.988741847156811	pseudobulk	DESeq2	LRT	0.139320447155934
432	KC_AB2	MED18	-0.59051039257323	0.80673877371878	0.988741847156811	pseudobulk	DESeq2	LRT	0.0932670693649112
432	KC_AB2	MED19	-0.0084533446145052	0.983736164328038	0.988741847156811	pseudobulk	DESeq2	LRT	0.00712136268699972
433	KC_AB2	MED20	0.818790875285784	0.940355871560339	0.988741847156811	pseudobulk	DESeq2	LRT	0.0267077593721816
433	KC_AB2	MED21	-0.0276087293668204	0.93870279200093	0.988741847156811	pseudobulk	DESeq2	LRT	0.0274718904021809
433	KC_AB2	MED22	1.5962800986319	0.438718098189139	0.988741847156811	pseudobulk	DESeq2	LRT	0.357814449521309
433	KC_AB2	MED23	0.818790875285784	0.940355871560339	0.988741847156811	pseudobulk	DESeq2	LRT	0.0267077593721816
433	KC_AB2	MED24	-0.588639054357632	0.8432408255636	0.988741847156811	pseudobulk	DESeq2	LRT	0.0740483752247712
433	KC_AB2	MED25	-0.934020934207768	0.782359118258182	0.988741847156811	pseudobulk	DESeq2	LRT	0.106593851444836
433	KC_AB2	MED26	-0.303214642615459	0.657551830946799	0.988741847156811	pseudobulk	DESeq2	LRT	0.182070008607022
433	KC_AB2	MED28	0.353798809751289	0.901648351639924	0.988741847156811	pseudobulk	DESeq2	LRT	0.0449628069360408
433	KC_AB2	MED30	-1.29600486439944	0.685894431367319	0.988741847156811	pseudobulk	DESeq2	LRT	0.163742723072789
433	KC_AB2	MED31	0.769722434695234	0.64745137208058	0.988741847156811	pseudobulk	DESeq2	LRT	0.188792844436106
434	KC_AB2	MED4	0.148520905817266	0.928829608110371	0.988741847156811	pseudobulk	DESeq2	LRT	0.0320639491329567
434	KC_AB2	MED6	-0.771921015954341	0.687452956728941	0.988741847156811	pseudobulk	DESeq2	LRT	0.162757015798882
434	KC_AB2	MED7	1.65216744322178	0.578100610800606	0.988741847156811	pseudobulk	DESeq2	LRT	0.237996571764493
434	KC_AB2	MED8	-0.742148798960242	0.80644751100302	0.988741847156811	pseudobulk	DESeq2	LRT	0.0934238941443075
434	KC_AB2	Mef2	-0.102297640958193	0.769181841597005	0.988741847156811	pseudobulk	DESeq2	LRT	0.113970976887153
434	KC_AB2	mEFG1	0.699674038439951	0.821690393783994	0.988741847156811	pseudobulk	DESeq2	LRT	0.0852917902455435
434	KC_AB2	mEFTs	-0.416943685854669	0.762912824407242	0.988741847156811	pseudobulk	DESeq2	LRT	0.117525084642593
434	KC_AB2	mEFTu1	1.41620968097021	0.411480320407709	0.988741847156811	pseudobulk	DESeq2	LRT	0.385650930664869
434	KC_AB2	mEFTu2	0.310390049490412	0.904941705285892	0.988741847156811	pseudobulk	DESeq2	LRT	0.04337939636174
434	KC_AB2	Megf8	-0.0384805229678164	0.985071128575604	0.988741847156811	pseudobulk	DESeq2	LRT	0.006532409469285
435	KC_AB2	mei-9	1.45259040912931	0.616176917291092	0.988741847156811	pseudobulk	DESeq2	LRT	0.210294574894236
435	KC_AB2	mei-P26	-0.180335281813347	0.544711982599698	0.988741847156811	pseudobulk	DESeq2	LRT	0.263833070991438
435	KC_AB2	mei-S332	-1.80294541848982	0.660135549041553	0.988741847156811	pseudobulk	DESeq2	LRT	0.180366879372519
435	KC_AB2	mei-W68	1.42621218304824	0.66738831402044	0.988741847156811	pseudobulk	DESeq2	LRT	0.175621402117653
435	KC_AB2	Meics	3.46443801158151	0.218071151261552	0.988741847156811	pseudobulk	DESeq2	LRT	0.6614017836492
435	KC_AB2	meigo	-0.0582963981256801	0.979058404810457	0.988741847156811	pseudobulk	DESeq2	LRT	0.0091913999942843
435	KC_AB2	Mekk1	-1.80933943727629	0.584587525761637	0.988741847156811	pseudobulk	DESeq2	LRT	0.233150456085471
435	KC_AB2	Membrin	-1.40296545307325	0.628223627794903	0.988741847156811	pseudobulk	DESeq2	LRT	0.201885733604539
435	KC_AB2	Men	-1.35613101588867	0.53174050073743	0.988741847156811	pseudobulk	DESeq2	LRT	0.27430025979696
435	KC_AB2	Men-b	-0.0578363734723887	0.929620417428513	0.988741847156811	pseudobulk	DESeq2	LRT	0.0316943463574003
436	KC_AB2	meng	2.95265617055103	0.0693160403652321	0.988741847156811	pseudobulk	DESeq2	LRT	1.15916625404592
436	KC_AB2	MEP-1	-0.0362223591928502	0.956694233985992	0.988741847156811	pseudobulk	DESeq2	LRT	0.0192268435304406
436	KC_AB2	Mer	0.356576043500675	0.884817522565587	0.988741847156811	pseudobulk	DESeq2	LRT	0.0531462853633029
436	KC_AB2	Mes4	0.418786747473112	0.814422565982762	0.988741847156811	pseudobulk	DESeq2	LRT	0.0891502014291997
436	KC_AB2	Mesh1	0.818790875285784	0.940355871560339	0.988741847156811	pseudobulk	DESeq2	LRT	0.0267077593721816
436	KC_AB2	MESK2	0.498756355613012	0.40580181026828	0.988741847156811	pseudobulk	DESeq2	LRT	0.391686019925218
436	KC_AB2	MESR3	-1.80294541848982	0.660135549041553	0.988741847156811	pseudobulk	DESeq2	LRT	0.180366879372519

436	KC_AB2	MESR4	0.060968150680055	0.947578193572861	0.988741847156811	pseudobulk	DESeq2	LRT	0.0233849421661848
436	KC_AB2	MESR6	-3.4227037205893	0.285801360563088	0.988741847156811	pseudobulk	DESeq2	LRT	0.543935708072568
436	KC_AB2	Met	-0.146550045083784	0.890216434817828	0.988741847156811	pseudobulk	DESeq2	LRT	0.0505043922094074
437	KC_AB2	metl	-0.957133960806503	0.762632518785665	0.988741847156811	pseudobulk	DESeq2	LRT	0.1176846802739
437	KC_AB2	metro	0.44799842808605	0.568484549998889	0.988741847156811	pseudobulk	DESeq2	LRT	0.245281333863503
437	KC_AB2	MetRS	1.84969518086931	0.509872718319655	0.988741847156811	pseudobulk	DESeq2	LRT	0.292538225138248
437	KC_AB2	MetRS-m	-2.11027789354592	0.473734933668313	0.988741847156811	pseudobulk	DESeq2	LRT	0.32446458879962
437	KC_AB2	Mettl14	1.42621218304824	0.66738831402044	0.988741847156811	pseudobulk	DESeq2	LRT	0.175621402117653
437	KC_AB2	mew	-1.00791327801769	0.419684052903416	0.988741847156811	pseudobulk	DESeq2	LRT	0.377077532736637
437	KC_AB2	Mf	1.47543920254359	0.661929169513603	0.988741847156811	pseudobulk	DESeq2	LRT	0.17918848025087
437	KC_AB2	Mfap1	3.77845897374758	0.158617058409698	0.988741847156811	pseudobulk	DESeq2	LRT	0.799650108476029
437	KC_AB2	Mfe2	-1.65719613210719	0.599376091584556	0.988741847156811	pseudobulk	DESeq2	LRT	0.222300584546838
437	KC_AB2	mfrn	0.972726581225976	0.61661584324292	0.988741847156811	pseudobulk	DESeq2	LRT	0.20998532076329
438	KC_AB2	MFS10	1.12919034796087	0.7301237214754	0.988741847156811	pseudobulk	DESeq2	LRT	0.136603541247596
438	KC_AB2	MFS15	-1.73627324524809	0.521368726288639	0.988741847156811	pseudobulk	DESeq2	LRT	0.282855023048041
438	KC_AB2	MFS16	-1.80294541848982	0.660135549041553	0.988741847156811	pseudobulk	DESeq2	LRT	0.180366879372519
438	KC_AB2	MFS17	0.372358478672921	0.405492265391427	0.988741847156811	pseudobulk	DESeq2	LRT	0.392017425373563
438	KC_AB2	MFS18	-0.244067456186467	0.906772274454269	0.988741847156811	pseudobulk	DESeq2	LRT	0.042501767367312
438	KC_AB2	MFS9	0.818790875285784	0.940355871560339	0.988741847156811	pseudobulk	DESeq2	LRT	0.0267077593721816
438	KC_AB2	Mgat1	-0.68750020136443	0.784083099060507	0.988741847156811	pseudobulk	DESeq2	LRT	0.105637907276101
438	KC_AB2	Mgat2	-0.12058928718113	0.933784783540129	0.988741847156811	pseudobulk	DESeq2	LRT	0.0297532073792971
438	KC_AB2	mge	0.0279692205823342	0.966230207491814	0.988741847156811	pseudobulk	DESeq2	LRT	0.0149193891824173
438	KC_AB2	mgl	-0.101232729823268	0.864951533207512	0.988741847156811	pseudobulk	DESeq2	LRT	0.0630082271597512
439	KC_AB2	mGluR	3.95947413000916	0.139135602718294	0.988741847156811	pseudobulk	DESeq2	LRT	0.856561726329509
439	KC_AB2	mgr	-1.7340172576784	0.242153677587896	0.988741847156811	pseudobulk	DESeq2	LRT	0.61590893093707
439	KC_AB2	Mgstl	-0.452344515838269	0.871171367298942	0.988741847156811	pseudobulk	DESeq2	LRT	0.0598964069304489
439	KC_AB2	Mhcl	-0.0717881058078627	0.958172962302896	0.988741847156811	pseudobulk	DESeq2	LRT	0.0185560882141772
439	KC_AB2	mi	-0.506613484034373	0.961658398065406	0.988741847156811	pseudobulk	DESeq2	LRT	0.0169791713942958
439	KC_AB2	Mi-2	0.27000397029062	0.728690309631866	0.988741847156811	pseudobulk	DESeq2	LRT	0.137457005830485
439	KC_AB2	mib1	-1.99867440840856	0.534899605264936	0.988741847156811	pseudobulk	DESeq2	LRT	0.271727722597268
439	KC_AB2	mib2	0.0336799954194084	0.966730614244894	0.988741847156811	pseudobulk	DESeq2	LRT	0.0146945280326403
439	KC_AB2	Mical	1.31864757175893	0.41576973861634	0.988741847156811	pseudobulk	DESeq2	LRT	0.381147123542797
439	KC_AB2	MICAL-like	1.4413042803421	0.615311685084066	0.988741847156811	pseudobulk	DESeq2	LRT	0.210904837358999
440	KC_AB2	MICU1	-0.150676854040664	0.925449362849159	0.988741847156811	pseudobulk	DESeq2	LRT	0.0336473392415183
440	KC_AB2	MICU3	0.329343227560874	0.812041007439155	0.988741847156811	pseudobulk	DESeq2	LRT	0.0904220386715629
440	KC_AB2	Mid1	0.0983882017958305	0.879473039364149	0.988741847156811	pseudobulk	DESeq2	LRT	0.055777469492304
440	KC_AB2	mIF2	-0.378852672109618	0.920549594954422	0.988741847156811	pseudobulk	DESeq2	LRT	0.0359528087516892
440	KC_AB2	mIF3	-0.506613484034373	0.961658398065406	0.988741847156811	pseudobulk	DESeq2	LRT	0.0169791713942958
440	KC_AB2	Miga	-0.0738214758854805	0.959452700110465	0.988741847156811	pseudobulk	DESeq2	LRT	0.0179764306078861
440	KC_AB2	milt	-0.361221523353386	0.683261459786104	0.988741847156811	pseudobulk	DESeq2	LRT	0.165413075507776
440	KC_AB2	mim	-0.462787570257328	0.666013962791509	0.988741847156811	pseudobulk	DESeq2	LRT	0.176516665875135
440	KC_AB2	mino	1.42621218304824	0.66738831402044	0.988741847156811	pseudobulk	DESeq2	LRT	0.175621402117653
440	KC_AB2	mio	2.51243226103394	0.474458247157471	0.988741847156811	pseudobulk	DESeq2	LRT	0.323801999942597
441	KC_AB2	mip120	-0.0582963981256801	0.979058404810457	0.988741847156811	pseudobulk	DESeq2	LRT	0.0091913999942843
441	KC_AB2	mip130	0.818790875285784	0.940355871560339	0.988741847156811	pseudobulk	DESeq2	LRT	0.0267077593721816
441	KC_AB2	mip40	0.27226851987049	0.911402843231596	0.988741847156811	pseudobulk	DESeq2	LRT	0.0402896209172008
441	KC_AB2	miple1	0.0149601785685774	0.93991125745785	0.988741847156811	pseudobulk	DESeq2	LRT	0.0269131487574516
441	KC_AB2	miple2	0.818790875285784	0.940355871560339	0.988741847156811	pseudobulk	DESeq2	LRT	0.0267077593721816
441	KC_AB2	Miro	1.75566251772846	0.434141035354741	0.988741847156811	pseudobulk	DESeq2	LRT	0.362369162362515
441	KC_AB2	Mis12	2.10427674742456	0.422430831487357	0.988741847156811	pseudobulk	DESeq2	LRT	0.374244391951096
441	KC_AB2	Mitf	-1.54130609826036	0.519800700764531	0.988741847156811	pseudobulk	DESeq2	LRT	0.284163139344502
441	KC_AB2	Mitofilin	1.70483820960947	0.399419083138225	0.988741847156811	pseudobulk	DESeq2	LRT	0.398571189581381
441	KC_AB2	Mkk4	-0.426228249949764	0.696509537453821	0.988741847156811	pseudobulk	DESeq2	LRT	0.157072932312227
442	KC_AB2	MKP-4	-0.010237273074013	0.943681123757042	0.988741847156811	pseudobulk	DESeq2	LRT	0.0251747319601966
442	KC_AB2	Mkp3	-0.547111290989277	0.193023131568612	0.988741847156811	pseudobulk	DESeq2	LRT	0.714390642750573
442	KC_AB2	Mkrn1	-0.140436485485279	0.910585395366499	0.988741847156811	pseudobulk	DESeq2	LRT	0.0406793195040027

442	KC_AB2	Mlc-c	2.68449022421397	0.254379849598147	0.988741847156811	pseudobulk	DESeq2	LRT	0.594517293789866
442	KC_AB2	mld	0.0369226790908019	0.966521596226316	0.988741847156811	pseudobulk	DESeq2	LRT	0.0147884375307895
442	KC_AB2	mle	-0.100146980692724	0.92401128062036	0.988741847156811	pseudobulk	DESeq2	LRT	0.034322726743887
442	KC_AB2	Mlf	0.383914077010211	0.578362472764116	0.988741847156811	pseudobulk	DESeq2	LRT	0.237799894137109
442	KC_AB2	Mlh1	0.818790875285784	0.940355871560339	0.988741847156811	pseudobulk	DESeq2	LRT	0.0267077593721816
442	KC_AB2	Mlp84B	-1.80294541848982	0.660135549041553	0.988741847156811	pseudobulk	DESeq2	LRT	0.180366879372519
442	KC_AB2	mlt	0.866419439444091	0.590034420594869	0.988741847156811	pseudobulk	DESeq2	LRT	0.229122652360602
443	KC_AB2	mmd	-0.368717440442016	0.295915944233595	0.988741847156811	pseudobulk	DESeq2	LRT	0.528831634007613
443	KC_AB2	MME1	-3.32426296135166	0.343738249391692	0.988741847156811	pseudobulk	DESeq2	LRT	0.463772139158982
443	KC_AB2	Mmp2	-0.00834159338679707	0.968248343822115	0.988741847156811	pseudobulk	DESeq2	LRT	0.0140132371970633
443	KC_AB2	Mms19	2.4643417141186	0.449082701910884	0.988741847156811	pseudobulk	DESeq2	LRT	0.347673673080982
443	KC_AB2	mmy	-0.0886264621524277	0.941489029714451	0.988741847156811	pseudobulk	DESeq2	LRT	0.0261847360426809
443	KC_AB2	mnb	0.347244807773816	0.744240924337992	0.988741847156811	pseudobulk	DESeq2	LRT	0.128286452662376
443	KC_AB2	Mnn1	0.205573483357609	0.702121836238634	0.988741847156811	pseudobulk	DESeq2	LRT	0.153587520042558
443	KC_AB2	Mnt	0.335991752769598	0.665840316569879	0.988741847156811	pseudobulk	DESeq2	LRT	0.176629911892291
443	KC_AB2	Mo25	0.21517757608798	0.892303003534357	0.988741847156811	pseudobulk	DESeq2	LRT	0.0494876451556573
443	KC_AB2	Mob2	-0.0280419861580262	0.962945695953258	0.988741847156811	pseudobulk	DESeq2	LRT	0.0163982036468314
444	KC_AB2	Mob3	0.575200883059258	0.804213580533116	0.988741847156811	pseudobulk	DESeq2	LRT	0.0946285973599483
444	KC_AB2	Mob4	-0.279428637709916	0.796159653415563	0.988741847156811	pseudobulk	DESeq2	LRT	0.0989998347184638
444	KC_AB2	Moca-cyp	0.20593975827165	0.865452598028576	0.988741847156811	pseudobulk	DESeq2	LRT	0.0627567140143329
444	KC_AB2	mod	1.1327121871749	0.657275461456076	0.988741847156811	pseudobulk	DESeq2	LRT	0.182252581249451
444	KC_AB2	Moe	0.27033441949988	0.761079127932568	0.988741847156811	pseudobulk	DESeq2	LRT	0.118570188120368
444	KC_AB2	mof	0.712171366772551	0.813205723536451	0.988741847156811	pseudobulk	DESeq2	LRT	0.0897995733547685
444	KC_AB2	mon2	0.10598154425916	0.959628155914924	0.988741847156811	pseudobulk	DESeq2	LRT	0.0178970181249073
444	KC_AB2	Mondo	-0.124237841338121	0.946426769540675	0.988741847156811	pseudobulk	DESeq2	LRT	0.0239129842514505
444	KC_AB2	mop	0.360597065274579	0.876727378089377	0.988741847156811	pseudobulk	DESeq2	LRT	0.0571354312607773
444	KC_AB2	mor	-0.669068297387362	0.767301512613651	0.988741847156811	pseudobulk	DESeq2	LRT	0.115033945655498
445	KC_AB2	morgue	0.0885531792216718	0.964858407830046	0.988741847156811	pseudobulk	DESeq2	LRT	0.0155364143348316
445	KC_AB2	Mp	-0.0223065973244343	0.975107896958091	0.988741847156811	pseudobulk	DESeq2	LRT	0.0109473263928376
445	KC_AB2	MP1	-0.506613484034373	0.961658398065406	0.988741847156811	pseudobulk	DESeq2	LRT	0.0169791713942958
445	KC_AB2	Mpc1	0.320539134359348	0.848791832994679	0.988741847156811	pseudobulk	DESeq2	LRT	0.0711988078324561
445	KC_AB2	Mpcp2	0.474750757805472	0.323745014554161	0.988741847156811	pseudobulk	DESeq2	LRT	0.489796910689189
445	KC_AB2	Mpi	1.13539439938001	0.65602501243538	0.988741847156811	pseudobulk	DESeq2	LRT	0.183079601850592
445	KC_AB2	Mpp6	2.00515076565275	0.576456787459964	0.988741847156811	pseudobulk	DESeq2	LRT	0.239233242872451
445	KC_AB2	Mppe	-0.233272055930639	0.937531793764924	0.988741847156811	pseudobulk	DESeq2	LRT	0.028013995469539
445	KC_AB2	mr	-1.80294541848982	0.660135549041553	0.988741847156811	pseudobulk	DESeq2	LRT	0.180366879372519
445	KC_AB2	mre11	-2.51746470924027	0.517377929599049	0.988741847156811	pseudobulk	DESeq2	LRT	0.286192101436607
446	KC_AB2	MRG15	0.158485844312962	0.932199411153928	0.988741847156811	pseudobulk	DESeq2	LRT	0.0304911757358515
446	KC_AB2	MrgBP	-0.586634344042284	0.73878287657376	0.988741847156811	pseudobulk	DESeq2	LRT	0.131483179143693
446	KC_AB2	mri	0.527025244051618	0.797900569756	0.988741847156811	pseudobulk	DESeq2	LRT	0.0980512248106127
446	KC_AB2	mrj	0.184679091523329	0.843524158399153	0.988741847156811	pseudobulk	DESeq2	LRT	0.0739024747711218
446	KC_AB2	mrn	3.06202454821851	0.30627034058063	0.988741847156811	pseudobulk	DESeq2	LRT	0.513895058517773
446	KC_AB2	mRNA-cap	-1.21589183877631	0.541005006441934	0.988741847156811	pseudobulk	DESeq2	LRT	0.266798715928467
446	KC_AB2	MRP	1.91626857490363	0.477198801200827	0.988741847156811	pseudobulk	DESeq2	LRT	0.321300656017034
446	KC_AB2	mRpL1	0.74211824391331	0.690246317519422	0.988741847156811	pseudobulk	DESeq2	LRT	0.160995901653945
446	KC_AB2	mRpL10	2.81309903729385	0.219655468837245	0.988741847156811	pseudobulk	DESeq2	LRT	0.658257979485977
446	KC_AB2	mRpL11	1.12919034796087	0.7301237214754	0.988741847156811	pseudobulk	DESeq2	LRT	0.136603541247596
447	KC_AB2	mRpL12	0.295735754774966	0.846751555773671	0.988741847156811	pseudobulk	DESeq2	LRT	0.0722439967343508
447	KC_AB2	mRpL13	1.19116218256916	0.585499821265137	0.988741847156811	pseudobulk	DESeq2	LRT	0.232473233168179
447	KC_AB2	mRpL14	0.11213458976865	0.938112983749562	0.988741847156811	pseudobulk	DESeq2	LRT	0.0277448532370976
447	KC_AB2	mRpL15	-1.20081362769313	0.741163517910401	0.988741847156811	pseudobulk	DESeq2	LRT	0.130085965985812
447	KC_AB2	mRpL16	2.02386655318765	0.293915536984342	0.988741847156811	pseudobulk	DESeq2	LRT	0.531777455614304
447	KC_AB2	mRpL17	2.26998970133409	0.374936237995469	0.988741847156811	pseudobulk	DESeq2	LRT	0.426042582515506
447	KC_AB2	mRpL18	0.835224734200581	0.677981786722778	0.988741847156811	pseudobulk	DESeq2	LRT	0.16878197284686
447	KC_AB2	mRpL19	1.35316200662963	0.684467033464499	0.988741847156811	pseudobulk	DESeq2	LRT	0.164647464300239
447	KC_AB2	mRpL2	-0.114099770495178	0.955170180595609	0.988741847156811	pseudobulk	DESeq2	LRT	0.0199192442181898

447	KC_AB2	mRpL20	0.764118474408575	0.696745253983381	0.988741847156811	pseudobulk	DESeq2	LRT	0.156925980886418
448	KC_AB2	mRpL21	3.91297093245125	0.13608758477008	0.988741847156811	pseudobulk	DESeq2	LRT	0.866181493546022
448	KC_AB2	mRpL22	0.24619866569863	0.866451184773514	0.988741847156811	pseudobulk	DESeq2	LRT	0.0622559000945371
448	KC_AB2	mRpL23	0.972726581225976	0.61661584324292	0.988741847156811	pseudobulk	DESeq2	LRT	0.20998532076329
448	KC_AB2	mRpL24	0.61702365764228	0.739069534444918	0.988741847156811	pseudobulk	DESeq2	LRT	0.131314699622637
448	KC_AB2	mRpL27	-0.332119834137093	0.814444373366488	0.988741847156811	pseudobulk	DESeq2	LRT	0.0891385726998192
448	KC_AB2	mRpL28	-0.769414524029196	0.61954429556239	0.988741847156811	pseudobulk	DESeq2	LRT	0.207927637422547
448	KC_AB2	mRpL3	1.13091714558663	0.68188196534655	0.988741847156811	pseudobulk	DESeq2	LRT	0.166290795776296
448	KC_AB2	mRpL30	-0.595873616431378	0.740791062049533	0.988741847156811	pseudobulk	DESeq2	LRT	0.130304266237008
448	KC_AB2	mRpL32	1.83411718865815	0.518907236503101	0.988741847156811	pseudobulk	DESeq2	LRT	0.284910272740318
448	KC_AB2	mRpL33	0.440495658050252	0.722409641534501	0.988741847156811	pseudobulk	DESeq2	LRT	0.14121646640816
449	KC_AB2	mRpL34	0.479184308705444	0.72408973547763	0.988741847156811	pseudobulk	DESeq2	LRT	0.14020760893018
449	KC_AB2	mRpL35	0.760118285990313	0.738173623698606	0.988741847156811	pseudobulk	DESeq2	LRT	0.131841477003816
449	KC_AB2	mRpL36	-0.0286126903153794	0.937098297657482	0.988741847156811	pseudobulk	DESeq2	LRT	0.0282148510640435
449	KC_AB2	mRpL37	1.17156071944349	0.597348257338218	0.988741847156811	pseudobulk	DESeq2	LRT	0.223772398949354
449	KC_AB2	mRpL38	1.47543920254359	0.661929169513603	0.988741847156811	pseudobulk	DESeq2	LRT	0.17918848025087
449	KC_AB2	mRpL39	0.327387326334235	0.866634433974796	0.988741847156811	pseudobulk	DESeq2	LRT	0.06216405914177
449	KC_AB2	mRpL4	0.740635261447468	0.680864099739953	0.988741847156811	pseudobulk	DESeq2	LRT	0.166939564479132
449	KC_AB2	mRpL40	-1.95379733800296	0.37159236682117	0.988741847156811	pseudobulk	DESeq2	LRT	0.429933215780369
449	KC_AB2	mRpL41	0.293731438615369	0.870005790715194	0.988741847156811	pseudobulk	DESeq2	LRT	0.060477856729329
449	KC_AB2	mRpL42	1.86863978865009	0.240736769990432	0.988741847156811	pseudobulk	DESeq2	LRT	0.618457570778838
450	KC_AB2	mRpL43	1.45259040912931	0.616176917291092	0.988741847156811	pseudobulk	DESeq2	LRT	0.210294574894236
450	KC_AB2	mRpL44	0.353798809751289	0.901648351639924	0.988741847156811	pseudobulk	DESeq2	LRT	0.0449628069360408
450	KC_AB2	mRpL45	-0.506613484034373	0.961658398065406	0.988741847156811	pseudobulk	DESeq2	LRT	0.0169791713942958
450	KC_AB2	mRpL46	0.668970670557767	0.818048200024539	0.988741847156811	pseudobulk	DESeq2	LRT	0.0872211066123218
450	KC_AB2	mRpL47	0.0471023025337348	0.971010011322875	0.988741847156811	pseudobulk	DESeq2	LRT	0.0127762923990319
450	KC_AB2	mRpL48	1.56267010177454	0.361437394674886	0.988741847156811	pseudobulk	DESeq2	LRT	0.441966916908436
450	KC_AB2	mRpL49	-0.296576489727943	0.896291428147039	0.988741847156811	pseudobulk	DESeq2	LRT	0.047550757014702
450	KC_AB2	mRpL50	2.69033465208286	0.261035207647621	0.988741847156811	pseudobulk	DESeq2	LRT	0.583300912371034
450	KC_AB2	mRpL51	1.47543920254359	0.661929169513603	0.988741847156811	pseudobulk	DESeq2	LRT	0.17918848025087
450	KC_AB2	mRpL52	2.34726809501174	0.359332409723481	0.988741847156811	pseudobulk	DESeq2	LRT	0.44450361015608
451	KC_AB2	mRpL54	1.06578634547012	0.651954183166929	0.988741847156811	pseudobulk	DESeq2	LRT	0.185782923741917
451	KC_AB2	mRpL55	-0.0220465280775703	0.949755814158781	0.988741847156811	pseudobulk	DESeq2	LRT	0.0223880391269177
451	KC_AB2	mRpL9	0.10598154425916	0.959628155914924	0.988741847156811	pseudobulk	DESeq2	LRT	0.0178970181249073
451	KC_AB2	mRpS10	-0.935464943137344	0.567095154771498	0.988741847156811	pseudobulk	DESeq2	LRT	0.246344063285314
451	KC_AB2	mRpS11	1.10869771177333	0.517540048950725	0.988741847156811	pseudobulk	DESeq2	LRT	0.286056037435637
451	KC_AB2	mRpS14	3.64730466917968	0.182487106943786	0.988741847156811	pseudobulk	DESeq2	LRT	0.738767813842945
451	KC_AB2	mRpS16	1.17122272564433	0.596070420011995	0.988741847156811	pseudobulk	DESeq2	LRT	0.224702429494649
451	KC_AB2	mRpS17	-0.779301790094566	0.664769009124738	0.988741847156811	pseudobulk	DESeq2	LRT	0.177329235132244
451	KC_AB2	mRpS18A	1.76062409692731	0.431284494748455	0.988741847156811	pseudobulk	DESeq2	LRT	0.365236155037982
451	KC_AB2	mRpS18B	3.06202454821851	0.30627034058063	0.988741847156811	pseudobulk	DESeq2	LRT	0.513895058517773
452	KC_AB2	mRpS18C	-0.466144968607287	0.806083667837512	0.988741847156811	pseudobulk	DESeq2	LRT	0.0936198780531005
452	KC_AB2	mRpS2	1.05886396715538	0.533819336832516	0.988741847156811	pseudobulk	DESeq2	LRT	0.272605698574819
452	KC_AB2	mRpS21	-0.634227699706429	0.828294391400015	0.988741847156811	pseudobulk	DESeq2	LRT	0.0818152793537044
452	KC_AB2	mRpS22	-1.63785643245857	0.555083135854033	0.988741847156811	pseudobulk	DESeq2	LRT	0.255641966897631
452	KC_AB2	mRpS23	0.998893024789477	0.660591418406266	0.988741847156811	pseudobulk	DESeq2	LRT	0.180067072429364
452	KC_AB2	mRpS24	-0.380369563278455	0.792274214425817	0.988741847156811	pseudobulk	DESeq2	LRT	0.101124478510891
452	KC_AB2	mRpS25	0.283530900391561	0.863357349909691	0.988741847156811	pseudobulk	DESeq2	LRT	0.0638094094165818
452	KC_AB2	mRpS26	-0.235620053326589	0.908937007481336	0.988741847156811	pseudobulk	DESeq2	LRT	0.0414662138640759
452	KC_AB2	mRpS28	0.334496878588925	0.842710517627863	0.988741847156811	pseudobulk	DESeq2	LRT	0.0743215857402773
452	KC_AB2	mRpS29	1.12452187615248	0.704078404906088	0.988741847156811	pseudobulk	DESeq2	LRT	0.152378975911753
453	KC_AB2	mRpS30	-1.23536834900699	0.725570392794057	0.988741847156811	pseudobulk	DESeq2	LRT	0.139320447155934
453	KC_AB2	mRpS31	-1.07213314691332	0.608918916799374	0.988741847156811	pseudobulk	DESeq2	LRT	0.215440533854871
453	KC_AB2	mRpS33	0.242948347113092	0.872297973775964	0.988741847156811	pseudobulk	DESeq2	LRT	0.0593351363272662
453	KC_AB2	mRpS34	1.32328954264249	0.534544163991607	0.988741847156811	pseudobulk	DESeq2	LRT	0.272016407601135
453	KC_AB2	mRpS35	-1.63785643245857	0.555083135854033	0.988741847156811	pseudobulk	DESeq2	LRT	0.255641966897631

453	KC_AB2	mRpS5	-0.33869336758244	0.673572558437099	0.988741847156811	pseudobulk	DESeq2	LRT	0.171615614445024
453	KC_AB2	mRpS6	2.71138816265562	0.251252315493245	0.988741847156811	pseudobulk	DESeq2	LRT	0.599889927178198
453	KC_AB2	mRpS7	-0.99658637023493	0.632968656238047	0.988741847156811	pseudobulk	DESeq2	LRT	0.198617795132546
453	KC_AB2	mRpS9	-1.32153899439768	0.423612735330985	0.988741847156811	pseudobulk	DESeq2	LRT	0.373030991905364
453	KC_AB2	mRRF1	-0.233272055930639	0.937531793764924	0.988741847156811	pseudobulk	DESeq2	LRT	0.028013995469539
454	KC_AB2	mRRF2	-0.506613484034373	0.961658398065406	0.988741847156811	pseudobulk	DESeq2	LRT	0.0169791713942958
454	KC_AB2	mrt	-0.671911135432045	0.732135319378852	0.988741847156811	pseudobulk	DESeq2	LRT	0.135408641580739
454	KC_AB2	Mrtf	-0.646922107096011	0.651025708006607	0.988741847156811	pseudobulk	DESeq2	LRT	0.186401861470263
454	KC_AB2	mrva	0.668149459031813	0.524107787855779	0.988741847156811	pseudobulk	DESeq2	LRT	0.28057938695327
454	KC_AB2	ms(3)76Ba	1.47543920254359	0.661929169513603	0.988741847156811	pseudobulk	DESeq2	LRT	0.17918848025087
454	KC_AB2	MSBP	0.121052877416246	0.904969508878568	0.988741847156811	pseudobulk	DESeq2	LRT	0.0433660532244827
454	KC_AB2	msi	1.89958882329279	0.395530224447978	0.988741847156811	pseudobulk	DESeq2	LRT	0.402820324278975
454	KC_AB2	msk	-1.29600486439944	0.685894431367319	0.988741847156811	pseudobulk	DESeq2	LRT	0.163742723072789
454	KC_AB2	msl-1	-0.708848427368349	0.719084259593817	0.988741847156811	pseudobulk	DESeq2	LRT	0.143220217638515
454	KC_AB2	msl-2	-2.51746470924027	0.517377929599049	0.988741847156811	pseudobulk	DESeq2	LRT	0.286192101436607
455	KC_AB2	msl-3	0.818790875285784	0.940355871560339	0.988741847156811	pseudobulk	DESeq2	LRT	0.0267077593721816
455	KC_AB2	msn	1.20928694600539	0.167602695888594	0.988741847156811	pseudobulk	DESeq2	LRT	0.775719000024813
455	KC_AB2	Msp300	-0.0466090909003843	0.893540361368331	0.988741847156811	pseudobulk	DESeq2	LRT	0.0488858255605142
455	KC_AB2	mspo	0.139721566122608	0.787623275295276	0.988741847156811	pseudobulk	DESeq2	LRT	0.103681458375788
455	KC_AB2	msps	0.213943057246354	0.850051805612277	0.988741847156811	pseudobulk	DESeq2	LRT	0.0705546058081277
455	KC_AB2	Mst77F	-0.010237273074013	0.943681123757042	0.988741847156811	pseudobulk	DESeq2	LRT	0.0251747319601966
455	KC_AB2	Mst85C	-4.60398629012217	0.0773725664619272	0.988741847156811	pseudobulk	DESeq2	LRT	1.1114129972775
455	KC_AB2	mt:ATPase6	0.532011473131827	0.244996914499277	0.988741847156811	pseudobulk	DESeq2	LRT	0.610839385122716
455	KC_AB2	mt:ATPase8	1.47543920254359	0.661929169513603	0.988741847156811	pseudobulk	DESeq2	LRT	0.17918848025087
455	KC_AB2	mt:CoI	-0.31153250326757	0.500610573783679	0.988741847156811	pseudobulk	DESeq2	LRT	0.300499981560782
456	KC_AB2	mt:CoII	0.00888360079745207	0.972615671126866	0.988741847156811	pseudobulk	DESeq2	LRT	0.0120587372055411
456	KC_AB2	mt:CoIII	-0.0665430010735307	0.876605577468707	0.988741847156811	pseudobulk	DESeq2	LRT	0.057195770442068
456	KC_AB2	mt:Cyt-b	-0.715104495790061	0.171877076466016	0.988741847156811	pseudobulk	DESeq2	LRT	0.764782042034184
456	KC_AB2	mt:lrRNA	-0.00023300853323417	0.978356344166177	0.988741847156811	pseudobulk	DESeq2	LRT	0.00950293445779654
456	KC_AB2	mt:ND1	0.453661801473286	0.52486681443942	0.988741847156811	pseudobulk	DESeq2	LRT	0.279950885340928
456	KC_AB2	mt:ND2	-0.722955552693502	0.450908724098391	0.988741847156811	pseudobulk	DESeq2	LRT	0.345911361963029
456	KC_AB2	mt:ND3	-0.350947300583545	0.694107374230315	0.988741847156811	pseudobulk	DESeq2	LRT	0.15857334160755
456	KC_AB2	mt:ND4	-0.793456551935532	0.295720853370865	0.988741847156811	pseudobulk	DESeq2	LRT	0.529118049215617
456	KC_AB2	mt:ND4L	-2.26538931672255	0.547771004842275	0.988741847156811	pseudobulk	DESeq2	LRT	0.2614009599796
456	KC_AB2	mt:ND5	-0.474620082361734	0.529672580796608	0.988741847156811	pseudobulk	DESeq2	LRT	0.275992508312171
457	KC_AB2	mt:ND6	-1.69072092962661	0.118340785989639	0.988741847156811	pseudobulk	DESeq2	LRT	0.926865550575068
457	KC_AB2	mt:srRNA	0.0614788058399932	0.926932644218196	0.988741847156811	pseudobulk	DESeq2	LRT	0.032951822821141
457	KC_AB2	MTA1-like	-0.409028640883814	0.818518332365151	0.988741847156811	pseudobulk	DESeq2	LRT	0.086971589244262
457	KC_AB2	Mtap	1.69894780879763	0.540778829726708	0.988741847156811	pseudobulk	DESeq2	LRT	0.26698031838453
457	KC_AB2	Mtch	0.126434706187641	0.917509849637377	0.988741847156811	pseudobulk	DESeq2	LRT	0.0373892648195234
457	KC_AB2	mtd	-0.0415779816543713	0.886782609233214	0.988741847156811	pseudobulk	DESeq2	LRT	0.0521828324588904
457	KC_AB2	mTerf3	3.11844405469765	0.153619112391481	0.988741847156811	pseudobulk	DESeq2	LRT	0.813554748563682
457	KC_AB2	mTerf5	0.331758848923756	0.946375065527934	0.988741847156811	pseudobulk	DESeq2	LRT	0.0239367107367223
457	KC_AB2	MTF-1	-0.588639054357632	0.8432408255636	0.988741847156811	pseudobulk	DESeq2	LRT	0.0740483752247712
457	KC_AB2	mtg	-2.51746470924027	0.517377929599049	0.988741847156811	pseudobulk	DESeq2	LRT	0.286192101436607
458	KC_AB2	mtgo	-0.162810779236635	0.759529664294273	0.988741847156811	pseudobulk	DESeq2	LRT	0.119455259605371
458	KC_AB2	mthl5	0.818790875285784	0.940355871560339	0.988741847156811	pseudobulk	DESeq2	LRT	0.0267077593721816
458	KC_AB2	mthl8	1.47543920254359	0.661929169513603	0.988741847156811	pseudobulk	DESeq2	LRT	0.17918848025087
458	KC_AB2	Mtl	-0.457212305199634	0.858276431637992	0.988741847156811	pseudobulk	DESeq2	LRT	0.0663728130839547
458	KC_AB2	mtm	1.4413042803421	0.615311685084066	0.988741847156811	pseudobulk	DESeq2	LRT	0.210904837358999
458	KC_AB2	MtnA	-1.67648953751065	0.306242994739901	0.988741847156811	pseudobulk	DESeq2	LRT	0.513933836931416
458	KC_AB2	Mtor	-0.451045789314127	0.823968225471349	0.988741847156811	pseudobulk	DESeq2	LRT	0.0840895355947879
458	KC_AB2	Mtpalpha	-0.957133960806503	0.762632518785665	0.988741847156811	pseudobulk	DESeq2	LRT	0.1176846802739
458	KC_AB2	MTPAP	0.351722119397094	0.929615296941389	0.988741847156811	pseudobulk	DESeq2	LRT	0.0316967385224016
458	KC_AB2	Mtr3	-1.65719613210719	0.599376091584556	0.988741847156811	pseudobulk	DESeq2	LRT	0.222300584546838
459	KC_AB2	Mtr4	-0.506613484034373	0.961658398065406	0.988741847156811	pseudobulk	DESeq2	LRT	0.0169791713942958

459	KC_AB2	mtRNApol	1.4413042803421	0.615311685084066	0.988741847156811	pseudobulk	DESeq2	LRT	0.210904837358999
459	KC_AB2	mts	0.646136328773692	0.15433235101095	0.988741847156811	pseudobulk	DESeq2	LRT	0.811543027968762
459	KC_AB2	mtSSB	0.804708302235815	0.733338290589079	0.988741847156811	pseudobulk	DESeq2	LRT	0.13469563812263
459	KC_AB2	mtt	0.164972137049222	0.880298218699372	0.988741847156811	pseudobulk	DESeq2	LRT	0.0553701769461937
459	KC_AB2	mtTFB2	2.7785831508444	0.373751317747552	0.988741847156811	pseudobulk	DESeq2	LRT	0.427417267458199
459	KC_AB2	mu2	0.818790875285784	0.940355871560339	0.988741847156811	pseudobulk	DESeq2	LRT	0.0267077593721816
459	KC_AB2	mub	-0.209146858761196	0.36056118479213	0.988741847156811	pseudobulk	DESeq2	LRT	0.443021027857428
459	KC_AB2	muc	0.0230749675120008	0.982497473708522	0.988741847156811	pseudobulk	DESeq2	LRT	0.00766855765061132
459	KC_AB2	Mul1	-1.80294541848982	0.660135549041553	0.988741847156811	pseudobulk	DESeq2	LRT	0.180366879372519
460	KC_AB2	Mulk	-0.0323203545809474	0.954448156132187	0.988741847156811	pseudobulk	DESeq2	LRT	0.020247656706879
460	KC_AB2	Mur2B	-1.92150817035771	0.457631232406761	0.988741847156811	pseudobulk	DESeq2	LRT	0.339484343482859
460	KC_AB2	mura	0.111740943484903	0.83467659258308	0.988741847156811	pseudobulk	DESeq2	LRT	0.0784817655505664
460	KC_AB2	mus101	-0.506613484034373	0.961658398065406	0.988741847156811	pseudobulk	DESeq2	LRT	0.0169791713942958
460	KC_AB2	muskelin	-2.26538931672255	0.547771004842275	0.988741847156811	pseudobulk	DESeq2	LRT	0.2614009599796
460	KC_AB2	mute	0.818790875285784	0.940355871560339	0.988741847156811	pseudobulk	DESeq2	LRT	0.0267077593721816
460	KC_AB2	mv	-2.26538931672255	0.547771004842275	0.988741847156811	pseudobulk	DESeq2	LRT	0.2614009599796
460	KC_AB2	Mvb12	-0.215812554377026	0.909171219175963	0.988741847156811	pseudobulk	DESeq2	LRT	0.0413543208033163
460	KC_AB2	Mvd	0.818790875285784	0.940355871560339	0.988741847156811	pseudobulk	DESeq2	LRT	0.0267077593721816
460	KC_AB2	Mvl	1.4413042803421	0.615311685084066	0.988741847156811	pseudobulk	DESeq2	LRT	0.210904837358999
461	KC_AB2	mxc	1.13539439938001	0.65602501243538	0.988741847156811	pseudobulk	DESeq2	LRT	0.183079601850592
461	KC_AB2	mxt	0.111161386130984	0.934678618050305	0.988741847156811	pseudobulk	DESeq2	LRT	0.0293376922294836
461	KC_AB2	Myb	-0.378852672109618	0.920549594954422	0.988741847156811	pseudobulk	DESeq2	LRT	0.0359528087516892
461	KC_AB2	Mybbp1A	0.818790875285784	0.940355871560339	0.988741847156811	pseudobulk	DESeq2	LRT	0.0267077593721816
461	KC_AB2	Myc	-1.38109913371034	0.444830808749517	0.988741847156811	pseudobulk	DESeq2	LRT	0.351805141375301
461	KC_AB2	myo	-1.80294541848982	0.660135549041553	0.988741847156811	pseudobulk	DESeq2	LRT	0.180366879372519
461	KC_AB2	Myo28B1	-1.80933943727629	0.584587525761637	0.988741847156811	pseudobulk	DESeq2	LRT	0.233150456085471
461	KC_AB2	Myo31DF	2.00515076565275	0.576456787459964	0.988741847156811	pseudobulk	DESeq2	LRT	0.239233242872451
461	KC_AB2	Myo81F	0.616962881190687	0.574634253628327	0.988741847156811	pseudobulk	DESeq2	LRT	0.240608489519883
461	KC_AB2	Myo95E	-0.0493731122220437	0.968729445650825	0.988741847156811	pseudobulk	DESeq2	LRT	0.0137974991799579
462	KC_AB2	MYPT-75D	-0.607320970366458	0.51175733776882	0.988741847156811	pseudobulk	DESeq2	LRT	0.2909359215435
462	KC_AB2	mys	-0.432252713608746	0.706890156381369	0.988741847156811	pseudobulk	DESeq2	LRT	0.150648065953666
462	KC_AB2	Mys45A	3.30033526000341	0.259137897354645	0.988741847156811	pseudobulk	DESeq2	LRT	0.586469069420752
462	KC_AB2	na	0.689240063478862	0.607296849735211	0.988741847156811	pseudobulk	DESeq2	LRT	0.216598971711559
462	KC_AB2	Naa15-16	-1.1284597703615	0.729754448571724	0.988741847156811	pseudobulk	DESeq2	LRT	0.136823248879498
462	KC_AB2	Naa20A	-1.17948993174423	0.426452665360198	0.988741847156811	pseudobulk	DESeq2	LRT	0.370129166873272
462	KC_AB2	Naa30A	-0.544182215481797	0.744990859502554	0.988741847156811	pseudobulk	DESeq2	LRT	0.127849055697285
462	KC_AB2	Naa35	2.41462217514131	0.497446973552313	0.988741847156811	pseudobulk	DESeq2	LRT	0.303253207020768
462	KC_AB2	Naa40	-2.26538931672255	0.547771004842275	0.988741847156811	pseudobulk	DESeq2	LRT	0.2614009599796
462	KC_AB2	Naa80	0.260978420129786	0.850764398268782	0.988741847156811	pseudobulk	DESeq2	LRT	0.0701906922048037
463	KC_AB2	Naam	-1.29600486439944	0.685894431367319	0.988741847156811	pseudobulk	DESeq2	LRT	0.163742723072789
463	KC_AB2	Nab2	-0.377876018271479	0.786431802962154	0.988741847156811	pseudobulk	DESeq2	LRT	0.104338932139685
463	KC_AB2	nac	-1.80294541848982	0.660135549041553	0.988741847156811	pseudobulk	DESeq2	LRT	0.180366879372519
463	KC_AB2	Nacalpha	0.317622098935104	0.506672965980172	0.988741847156811	pseudobulk	DESeq2	LRT	0.295272267287873
463	KC_AB2	Nach	-1.80294541848982	0.660135549041553	0.988741847156811	pseudobulk	DESeq2	LRT	0.180366879372519
463	KC_AB2	nAChRalpha1	-0.0877562162255955	0.867139003174487	0.988741847156811	pseudobulk	DESeq2	LRT	0.0619112791410439
463	KC_AB2	nAChRalpha2	0.533975350473754	0.608716698365518	0.988741847156811	pseudobulk	DESeq2	LRT	0.215584784482359
463	KC_AB2	nAChRalpha3	1.42621218304824	0.66738831402044	0.988741847156811	pseudobulk	DESeq2	LRT	0.175621402117653
463	KC_AB2	nAChRalpha4	-0.154441480707216	0.787396500093585	0.988741847156811	pseudobulk	DESeq2	LRT	0.103806519941295
463	KC_AB2	nAChRalpha5	-0.100087985351474	0.777446738331006	0.988741847156811	pseudobulk	DESeq2	LRT	0.109329354122689
464	KC_AB2	nAChRalpha6	0.82478338959006	0.0346249912869387	0.988741847156811	pseudobulk	DESeq2	LRT	1.4606103272137
464	KC_AB2	nAChRalpha7	2.30464778997969	0.0890405710541847	0.988741847156811	pseudobulk	DESeq2	LRT	1.05041206335342
464	KC_AB2	nAChRbeta1	0.112437890617991	0.721677873939319	0.988741847156811	pseudobulk	DESeq2	LRT	0.14165660961613
464	KC_AB2	nAChRbeta2	-0.179646322707095	0.873889508816825	0.988741847156811	pseudobulk	DESeq2	LRT	0.0585434743960173
464	KC_AB2	NaCP60E	-0.144021053145068	0.809373352772861	0.988741847156811	pseudobulk	DESeq2	LRT	0.0918510981087823
464	KC_AB2	Nadsyn	-0.147108306384959	0.878191871437464	0.988741847156811	pseudobulk	DESeq2	LRT	0.0564105870519372
464	KC_AB2	Nak	-0.79911720984961	0.528461581935601	0.988741847156811	pseudobulk	DESeq2	LRT	0.276986579519909

464	KC_AB2	Nap1	0.31826508416362	0.728863170955846	0.988741847156811	pseudobulk	DESeq2	LRT	0.13735399387176
464	KC_AB2	NaPi-III	0.165998373695658	0.814183696387187	0.988741847156811	pseudobulk	DESeq2	LRT	0.089277598394806
464	KC_AB2	Naprt	-2.3674447802368	0.243624491687148	0.988741847156811	pseudobulk	DESeq2	LRT	0.613279054011611
465	KC_AB2	NAT1	-0.710506928726038	0.278971046137172	0.988741847156811	pseudobulk	DESeq2	LRT	0.55444086896789
465	KC_AB2	Naus	0.364417163554467	0.847077934404736	0.988741847156811	pseudobulk	DESeq2	LRT	0.0720766310803004
465	KC_AB2	Naxd	-3.32426296135166	0.343738249391692	0.988741847156811	pseudobulk	DESeq2	LRT	0.463772139158982
465	KC_AB2	Naxe	0.818790875285784	0.940355871560339	0.988741847156811	pseudobulk	DESeq2	LRT	0.0267077593721816
465	KC_AB2	Nbr	-1.21589183877631	0.541005006441934	0.988741847156811	pseudobulk	DESeq2	LRT	0.266798715928467
465	KC_AB2	nbs	-0.0144409071146711	0.984632974376923	0.988741847156811	pseudobulk	DESeq2	LRT	0.00672562423038896
465	KC_AB2	NC2alpha	-2.51746470924027	0.517377929599049	0.988741847156811	pseudobulk	DESeq2	LRT	0.286192101436607
465	KC_AB2	NC2beta	-1.95379733800296	0.37159236682117	0.988741847156811	pseudobulk	DESeq2	LRT	0.429933215780369
465	KC_AB2	Nc73EF	-0.113527029790247	0.87449132561454	0.988741847156811	pseudobulk	DESeq2	LRT	0.0582444940828731
465	KC_AB2	Nca	0.740258418867227	0.247123631084625	0.988741847156811	pseudobulk	DESeq2	LRT	0.607085723400235
466	KC_AB2	Nckx30C	0.0821394766494197	0.838281959176518	0.988741847156811	pseudobulk	DESeq2	LRT	0.0766098802738211
466	KC_AB2	ncm	2.70180605057798	0.249545627897494	0.988741847156811	pseudobulk	DESeq2	LRT	0.602850034680352
466	KC_AB2	Ncoa6	0.203238026781325	0.794358953741666	0.988741847156811	pseudobulk	DESeq2	LRT	0.0999832048752728
466	KC_AB2	Nct	3.30033526000341	0.259137897354645	0.988741847156811	pseudobulk	DESeq2	LRT	0.586469069420752
466	KC_AB2	ND-13A	0.762251062431613	0.491188103630237	0.988741847156811	pseudobulk	DESeq2	LRT	0.308752160169633
466	KC_AB2	ND-13B	1.1458499753706	0.27547111020181	0.988741847156811	pseudobulk	DESeq2	LRT	0.55992394068815
466	KC_AB2	ND-15	-0.108562598496823	0.879189922789387	0.988741847156811	pseudobulk	DESeq2	LRT	0.0559172984076364
466	KC_AB2	ND-18	0.224668023051545	0.801497641360635	0.988741847156811	pseudobulk	DESeq2	LRT	0.09609775134548
466	KC_AB2	ND-19	-0.534676334507656	0.624613489926596	0.988741847156811	pseudobulk	DESeq2	LRT	0.204388640442969
466	KC_AB2	ND-20	0.782626169159865	0.426392544504393	0.988741847156811	pseudobulk	DESeq2	LRT	0.370190397576487
467	KC_AB2	ND-23	-0.258965541281536	0.810886041947055	0.988741847156811	pseudobulk	DESeq2	LRT	0.0910401751736022
467	KC_AB2	ND-24	0.373758234289767	0.823771489964601	0.988741847156811	pseudobulk	DESeq2	LRT	0.0841932426846645
467	KC_AB2	ND-30	0.540309959080098	0.569645968096918	0.988741847156811	pseudobulk	DESeq2	LRT	0.24439497217085
467	KC_AB2	ND-39	-0.288268913379647	0.746480349090023	0.988741847156811	pseudobulk	DESeq2	LRT	0.126981620482958
467	KC_AB2	ND-42	-0.39295170230343	0.711656311814504	0.988741847156811	pseudobulk	DESeq2	LRT	0.147729694451648
467	KC_AB2	ND-49	-0.387690625503361	0.602678880855544	0.988741847156811	pseudobulk	DESeq2	LRT	0.219914026863928
467	KC_AB2	ND-51	0.215085370254383	0.789466826716495	0.988741847156811	pseudobulk	DESeq2	LRT	0.1026661142645
467	KC_AB2	ND-75	0.387810490232537	0.750933825843026	0.988741847156811	pseudobulk	DESeq2	LRT	0.12439833241988
467	KC_AB2	ND-ACP	-0.526002758479082	0.467569263335178	0.988741847156811	pseudobulk	DESeq2	LRT	0.330154045847814
467	KC_AB2	ND-AGGG	0.208687035921869	0.752076985931246	0.988741847156811	pseudobulk	DESeq2	LRT	0.123737700826163
468	KC_AB2	ND-ASHI	0.842847060415305	0.457885227387636	0.988741847156811	pseudobulk	DESeq2	LRT	0.339243367757572
468	KC_AB2	ND-B12	0.818345855324201	0.221824352288879	0.988741847156811	pseudobulk	DESeq2	LRT	0.653990777915755
468	KC_AB2	ND-B14	0.412701487812743	0.642132465438018	0.988741847156811	pseudobulk	DESeq2	LRT	0.192375372126568
468	KC_AB2	ND-B14.5A	1.63221696668098	0.112096969416977	0.988741847156811	pseudobulk	DESeq2	LRT	0.950406128558241
468	KC_AB2	ND-B14.5B	-0.24019890528004	0.814154506737533	0.988741847156811	pseudobulk	DESeq2	LRT	0.089293168752058
468	KC_AB2	ND-B14.7	-0.630982225390447	0.530257126397974	0.988741847156811	pseudobulk	DESeq2	LRT	0.275513486065491
468	KC_AB2	ND-B15	0.236478826774551	0.775334255108376	0.988741847156811	pseudobulk	DESeq2	LRT	0.110511028005337
468	KC_AB2	ND-B16.6	-0.0232099648494359	0.969248350032045	0.988741847156811	pseudobulk	DESeq2	LRT	0.0135649296270079
468	KC_AB2	ND-B17	0.754953069926051	0.466226350772311	0.988741847156811	pseudobulk	DESeq2	LRT	0.331403184115315
468	KC_AB2	ND-B17.2	0.993422787784725	0.261291549609685	0.988741847156811	pseudobulk	DESeq2	LRT	0.582874635497427
469	KC_AB2	ND-B18	0.412244560388137	0.671440953066184	0.988741847156811	pseudobulk	DESeq2	LRT	0.172992173442117
469	KC_AB2	ND-B22	0.918583277899327	0.22488142786946	0.988741847156811	pseudobulk	DESeq2	LRT	0.648046409868337
469	KC_AB2	ND-MLRQ	0.160864550092764	0.740385238951792	0.988741847156811	pseudobulk	DESeq2	LRT	0.130542248354692
469	KC_AB2	ND-MNLL	-0.506613484034373	0.961658398065406	0.988741847156811	pseudobulk	DESeq2	LRT	0.0169791713942958
469	KC_AB2	ND-MWFE	0.844890208489603	0.391128940896045	0.988741847156811	pseudobulk	DESeq2	LRT	0.40768004800482
469	KC_AB2	ND-PDSW	0.622288218178923	0.386446677145464	0.988741847156811	pseudobulk	DESeq2	LRT	0.41291042262301
469	KC_AB2	ND-SGDH	0.0380500307014163	0.955578757999748	0.988741847156811	pseudobulk	DESeq2	LRT	0.0197335129468876
469	KC_AB2	Ndae1	0.339020050246102	0.615036530435404	0.988741847156811	pseudobulk	DESeq2	LRT	0.211099088297179
469	KC_AB2	Ndc1	0.818790875285784	0.940355871560339	0.988741847156811	pseudobulk	DESeq2	LRT	0.0267077593721816
469	KC_AB2	Ndf	-0.480889460134267	0.655226398850821	0.988741847156811	pseudobulk	DESeq2	LRT	0.183608613316809
470	KC_AB2	Ndfip	-0.244740731439142	0.831029123470398	0.988741847156811	pseudobulk	DESeq2	LRT	0.0803837560704528
470	KC_AB2	Neb-cGP	0.177192330997497	0.728565033865859	0.988741847156811	pseudobulk	DESeq2	LRT	0.137531675756067
470	KC_AB2	Nedd4	-0.368553108134416	0.712004515202298	0.988741847156811	pseudobulk	DESeq2	LRT	0.147517252260299

470	KC_AB2	Nedd8	-0.275695871128137	0.867470937595485	0.988741847156811	pseudobulk	DESeq2	LRT	0.0617450662239331
470	KC_AB2	nej	-0.1667245313416	0.793973143067576	0.988741847156811	pseudobulk	DESeq2	LRT	0.100194187767892
470	KC_AB2	Nelf-A	-0.21718335023406	0.885874716609737	0.988741847156811	pseudobulk	DESeq2	LRT	0.0526276931606903
470	KC_AB2	NELF-B	1.47543920254359	0.661929169513603	0.988741847156811	pseudobulk	DESeq2	LRT	0.17918848025087
470	KC_AB2	Nelf-E	0.417187211196709	0.787956800081191	0.988741847156811	pseudobulk	DESeq2	LRT	0.103497592155939
470	KC_AB2	nemy	0.699674038439951	0.821690393783994	0.988741847156811	pseudobulk	DESeq2	LRT	0.0852917902455435
470	KC_AB2	Neos	3.03619069427769	0.314302467188174	0.988741847156811	pseudobulk	DESeq2	LRT	0.502652209876457
471	KC_AB2	Nep1	-0.151480378940673	0.720868275182824	0.988741847156811	pseudobulk	DESeq2	LRT	0.142144086997192
471	KC_AB2	Nep3	-0.472844194314684	0.704208454632192	0.988741847156811	pseudobulk	DESeq2	LRT	0.152298765153273
471	KC_AB2	Nepl11	-1.80294541848982	0.660135549041553	0.988741847156811	pseudobulk	DESeq2	LRT	0.180366879372519
471	KC_AB2	Nepl16	0.329343227560874	0.812041007439155	0.988741847156811	pseudobulk	DESeq2	LRT	0.0904220386715629
471	KC_AB2	Nepl5	1.42621218304824	0.66738831402044	0.988741847156811	pseudobulk	DESeq2	LRT	0.175621402117653
471	KC_AB2	nero	0.310390049490412	0.904941705285892	0.988741847156811	pseudobulk	DESeq2	LRT	0.04337939636174
471	KC_AB2	nes	-1.29600486439944	0.685894431367319	0.988741847156811	pseudobulk	DESeq2	LRT	0.163742723072789
471	KC_AB2	net	-3.32426296135166	0.343738249391692	0.988741847156811	pseudobulk	DESeq2	LRT	0.463772139158982
471	KC_AB2	NetB	0.149114350996803	0.806979558759488	0.988741847156811	pseudobulk	DESeq2	LRT	0.0931374660590854
471	KC_AB2	neur	-0.394337917977515	0.675243736555729	0.988741847156811	pseudobulk	DESeq2	LRT	0.17053943556243
472	KC_AB2	Neurl4	-2.21667329608248	0.477261050443944	0.988741847156811	pseudobulk	DESeq2	LRT	0.321244007216606
472	KC_AB2	Nf-YA	-1.37054505049545	0.574189269529552	0.988741847156811	pseudobulk	DESeq2	LRT	0.240944927875125
472	KC_AB2	Nf-YB	-0.0732658797326201	0.938469407099271	0.988741847156811	pseudobulk	DESeq2	LRT	0.0275798802557713
472	KC_AB2	Nf-YC	3.03619069427769	0.314302467188174	0.988741847156811	pseudobulk	DESeq2	LRT	0.502652209876457
472	KC_AB2	Nf1	0.104011191821811	0.933863515299783	0.988741847156811	pseudobulk	DESeq2	LRT	0.0297165915252199
472	KC_AB2	NFAT	-0.259607468460984	0.691312160607098	0.988741847156811	pseudobulk	DESeq2	LRT	0.160325803542695
472	KC_AB2	NfI	-0.591659439050435	0.449513307436165	0.988741847156811	pseudobulk	DESeq2	LRT	0.347257446843883
472	KC_AB2	Nfs1	0.760118285990313	0.738173623698606	0.988741847156811	pseudobulk	DESeq2	LRT	0.131841477003816
472	KC_AB2	Nhe1	0.157562008957688	0.950463298513166	0.988741847156811	pseudobulk	DESeq2	LRT	0.0220646484576093
472	KC_AB2	Nhe2	0.299229629739512	0.851026027418758	0.988741847156811	pseudobulk	DESeq2	LRT	0.070057157434224
473	KC_AB2	Nhe3	-0.0898336046579577	0.919665556026907	0.988741847156811	pseudobulk	DESeq2	LRT	0.0363700787185597
473	KC_AB2	NHP2	0.315515278988381	0.901389738439351	0.988741847156811	pseudobulk	DESeq2	LRT	0.0450873903126698
473	KC_AB2	NiPp1	0.353798809751289	0.901648351639924	0.988741847156811	pseudobulk	DESeq2	LRT	0.0449628069360408
473	KC_AB2	Nipped-A	-0.244587024793194	0.800056127891727	0.988741847156811	pseudobulk	DESeq2	LRT	0.0968795440348209
473	KC_AB2	Nipped-B	-0.164744301095736	0.776286172949677	0.988741847156811	pseudobulk	DESeq2	LRT	0.109978149332057
473	KC_AB2	Nipsnap	-1.57622768308261	0.407870247575519	0.988741847156811	pseudobulk	DESeq2	LRT	0.389477973489087
473	KC_AB2	nito	1.12452187615248	0.704078404906088	0.988741847156811	pseudobulk	DESeq2	LRT	0.152378975911753
473	KC_AB2	NK7.1	0.494813342918632	0.699451317351274	0.988741847156811	pseudobulk	DESeq2	LRT	0.155242507535934
473	KC_AB2	NKAIN	1.05672255099273	0.476703557865673	0.988741847156811	pseudobulk	DESeq2	LRT	0.32175160671432
473	KC_AB2	nkd	-0.924485846631166	0.610549321913483	0.988741847156811	pseudobulk	DESeq2	LRT	0.214279246756535
474	KC_AB2	Nle	2.4643417141186	0.449082701910884	0.988741847156811	pseudobulk	DESeq2	LRT	0.347673673080982
474	KC_AB2	Nlg2	0.152894371946455	0.819812016062897	0.988741847156811	pseudobulk	DESeq2	LRT	0.0862857204772311
474	KC_AB2	Nlg3	-0.117249554305846	0.78722691500474	0.988741847156811	pseudobulk	DESeq2	LRT	0.103900065950934
474	KC_AB2	Nlp	0.652762974296675	0.636338569111308	0.988741847156811	pseudobulk	DESeq2	LRT	0.196311752947062
474	KC_AB2	nmd	1.84969518086931	0.509872718319655	0.988741847156811	pseudobulk	DESeq2	LRT	0.292538225138248
474	KC_AB2	Nmd3	-0.0323203545809474	0.954448156132187	0.988741847156811	pseudobulk	DESeq2	LRT	0.020247656706879
474	KC_AB2	Nmda1	0.011720498762202	0.969799729540815	0.988741847156811	pseudobulk	DESeq2	LRT	0.0133179413371659
474	KC_AB2	Nmdar1	1.1669370038017	0.601782723498991	0.988741847156811	pseudobulk	DESeq2	LRT	0.220560284520093
474	KC_AB2	Nmdar2	0.463905213589484	0.776979723284953	0.988741847156811	pseudobulk	DESeq2	LRT	0.109590314764543
474	KC_AB2	Nmnat	-0.742148798960242	0.80644751100302	0.988741847156811	pseudobulk	DESeq2	LRT	0.0934238941443075
475	KC_AB2	nmo	-0.113562489285821	0.853774171888106	0.988741847156811	pseudobulk	DESeq2	LRT	0.0686569874755467
475	KC_AB2	Nmt	2.00515076565275	0.576456787459964	0.988741847156811	pseudobulk	DESeq2	LRT	0.239233242872451
475	KC_AB2	Nna1	0.0601466527378941	0.974810960364079	0.988741847156811	pseudobulk	DESeq2	LRT	0.0110795964355292
475	KC_AB2	Nnf1a	-1.23536834900699	0.725570392794057	0.988741847156811	pseudobulk	DESeq2	LRT	0.139320447155934
475	KC_AB2	Nnp-1	-0.957133960806503	0.762632518785665	0.988741847156811	pseudobulk	DESeq2	LRT	0.1176846802739
475	KC_AB2	NO66	-0.729234089517982	0.868684292414125	0.988741847156811	pseudobulk	DESeq2	LRT	0.0611380313470854
475	KC_AB2	Noa36	-0.124237841338121	0.946426769540675	0.988741847156811	pseudobulk	DESeq2	LRT	0.0239129842514505
475	KC_AB2	noc	2.80917294583258	0.367996267391162	0.988741847156811	pseudobulk	DESeq2	LRT	0.434156586379859
475	KC_AB2	nocte	-0.439656776524136	0.483803593582615	0.988741847156811	pseudobulk	DESeq2	LRT	0.315330910121919

475	KC_AB2	noi	0.818790875285784	0.940355871560339	0.988741847156811	pseudobulk	DESeq2	LRT	0.0267077593721816
476	KC_AB2	nom	-2.51746470924027	0.517377929599049	0.988741847156811	pseudobulk	DESeq2	LRT	0.286192101436607
476	KC_AB2	Non1	-0.591560617752682	0.449665471757954	0.988741847156811	pseudobulk	DESeq2	LRT	0.347110459109174
476	KC_AB2	Non2	-0.153935021515702	0.910160719119377	0.988741847156811	pseudobulk	DESeq2	LRT	0.0408819117848242
476	KC_AB2	Non3	1.12452187615248	0.704078404906088	0.988741847156811	pseudobulk	DESeq2	LRT	0.152378975911753
476	KC_AB2	nonA	1.06719198324779	0.256536547014358	0.988741847156811	pseudobulk	DESeq2	LRT	0.590850755168102
476	KC_AB2	nonA-l	-0.0148827856665692	0.974851841948725	0.988741847156811	pseudobulk	DESeq2	LRT	0.01106138339212
476	KC_AB2	nonC	-2.72566785950637	0.305305375662423	0.988741847156811	pseudobulk	DESeq2	LRT	0.515265548796741
476	KC_AB2	Nop17l	-1.24332780259389	0.399670683419777	0.988741847156811	pseudobulk	DESeq2	LRT	0.39829770687116
476	KC_AB2	nop5	2.06137138167525	0.547946929015274	0.988741847156811	pseudobulk	DESeq2	LRT	0.261261502741816
476	KC_AB2	Nop56	0.369237445508587	0.839866280826322	0.988741847156811	pseudobulk	DESeq2	LRT	0.0757898545596527
477	KC_AB2	Nop60B	-0.213177136262384	0.93155916913195	0.988741847156811	pseudobulk	DESeq2	LRT	0.030789555140895
477	KC_AB2	Nopp140	1.4413042803421	0.615311685084066	0.988741847156811	pseudobulk	DESeq2	LRT	0.210904837358999
477	KC_AB2	norpA	2.29323494570297	0.0851144464096204	0.988741847156811	pseudobulk	DESeq2	LRT	1.06999672119025
477	KC_AB2	not	0.712171366772551	0.813205723536451	0.988741847156811	pseudobulk	DESeq2	LRT	0.0897995733547685
477	KC_AB2	Not1	-0.0400450291076333	0.952509050163218	0.988741847156811	pseudobulk	DESeq2	LRT	0.0211308892300085
477	KC_AB2	Not10	-1.80294541848982	0.660135549041553	0.988741847156811	pseudobulk	DESeq2	LRT	0.180366879372519
477	KC_AB2	Not11	0.998893024789477	0.660591418406266	0.988741847156811	pseudobulk	DESeq2	LRT	0.180067072429364
477	KC_AB2	Not3	0.196729940332691	0.837159137172715	0.988741847156811	pseudobulk	DESeq2	LRT	0.0771919782950244
477	KC_AB2	NP15.6	0.499344950050475	0.653815636504896	0.988741847156811	pseudobulk	DESeq2	LRT	0.184544697144166
477	KC_AB2	Npc1a	-0.779301790094566	0.664769009124738	0.988741847156811	pseudobulk	DESeq2	LRT	0.177329235132244
478	KC_AB2	Npc2a	2.00515076565275	0.576456787459964	0.988741847156811	pseudobulk	DESeq2	LRT	0.239233242872451
478	KC_AB2	Nph	1.56645644620297	0.359832388723451	0.988741847156811	pseudobulk	DESeq2	LRT	0.443899748131112
478	KC_AB2	Npl4	-0.213177136262384	0.93155916913195	0.988741847156811	pseudobulk	DESeq2	LRT	0.030789555140895
478	KC_AB2	Nplp1	2.28068239784515	0.366179304841476	0.988741847156811	pseudobulk	DESeq2	LRT	0.436306204128051
478	KC_AB2	Nprl2	0.818790875285784	0.940355871560339	0.988741847156811	pseudobulk	DESeq2	LRT	0.0267077593721816
478	KC_AB2	Nprl3	-1.65719613210719	0.599376091584556	0.988741847156811	pseudobulk	DESeq2	LRT	0.222300584546838
478	KC_AB2	Nrd1	-0.718003415132797	0.760638816665156	0.988741847156811	pseudobulk	DESeq2	LRT	0.118821515588473
478	KC_AB2	Nrg	0.0522378144858557	0.914875715391906	0.988741847156811	pseudobulk	DESeq2	LRT	0.0386379002347597
478	KC_AB2	Nrk	0.818790875285784	0.940355871560339	0.988741847156811	pseudobulk	DESeq2	LRT	0.0267077593721816
478	KC_AB2	nrm	0.246253370684419	0.735461737261131	0.988741847156811	pseudobulk	DESeq2	LRT	0.133439916721097
479	KC_AB2	Nrt	-0.158583969269452	0.875476454264215	0.988741847156811	pseudobulk	DESeq2	LRT	0.0577555296726338
479	KC_AB2	nrv2	3.06202454821851	0.30627034058063	0.988741847156811	pseudobulk	DESeq2	LRT	0.513895058517773
479	KC_AB2	nrv3	-0.0477862834393494	0.85288633598119	0.988741847156811	pseudobulk	DESeq2	LRT	0.0691088433287925
479	KC_AB2	Nrx-1	0.598883948880699	0.248749762816395	0.988741847156811	pseudobulk	DESeq2	LRT	0.60423732468306
479	KC_AB2	Nrx-IV	-1.09902991128308	0.514235566520712	0.988741847156811	pseudobulk	DESeq2	LRT	0.288837889168248
479	KC_AB2	Ns1	-1.65719613210719	0.599376091584556	0.988741847156811	pseudobulk	DESeq2	LRT	0.222300584546838
479	KC_AB2	Ns2	-0.0323203545809474	0.954448156132187	0.988741847156811	pseudobulk	DESeq2	LRT	0.020247656706879
479	KC_AB2	Ns3	-2.9721553030081	0.387854083811327	0.988741847156811	pseudobulk	DESeq2	LRT	0.411331631401987
479	KC_AB2	Ns4	-0.506613484034373	0.961658398065406	0.988741847156811	pseudobulk	DESeq2	LRT	0.0169791713942958
479	KC_AB2	NSD	0.818790875285784	0.940355871560339	0.988741847156811	pseudobulk	DESeq2	LRT	0.0267077593721816
480	KC_AB2	Nsf2	1.17454914662604	0.606736084931753	0.988741847156811	pseudobulk	DESeq2	LRT	0.217000175122362
480	KC_AB2	nsl1	0.478369466391115	0.666690375932245	0.988741847156811	pseudobulk	DESeq2	LRT	0.176075814125505
480	KC_AB2	nSMase	0.920073134400379	0.728211051768817	0.988741847156811	pseudobulk	DESeq2	LRT	0.137742734237847
480	KC_AB2	nst	-0.588639054357632	0.8432408255636	0.988741847156811	pseudobulk	DESeq2	LRT	0.0740483752247712
480	KC_AB2	Nsun2	-0.934020934207768	0.782359118258182	0.988741847156811	pseudobulk	DESeq2	LRT	0.106593851444836
480	KC_AB2	Nsun5	-0.506613484034373	0.961658398065406	0.988741847156811	pseudobulk	DESeq2	LRT	0.0169791713942958
480	KC_AB2	nSyb	0.00682282848498425	0.958559354671585	0.988741847156811	pseudobulk	DESeq2	LRT	0.0183809901325534
480	KC_AB2	Ntan1	-2.15400439962555	0.378907849258803	0.988741847156811	pseudobulk	DESeq2	LRT	0.421466398012216
480	KC_AB2	Ntf-2	-0.166609570985185	0.859397492906787	0.988741847156811	pseudobulk	DESeq2	LRT	0.065805917603868
480	KC_AB2	Ntf-2r	0.818790875285784	0.940355871560339	0.988741847156811	pseudobulk	DESeq2	LRT	0.0267077593721816
481	KC_AB2	Ntl	0.818790875285784	0.940355871560339	0.988741847156811	pseudobulk	DESeq2	LRT	0.0267077593721816
481	KC_AB2	Ntmt	-3.0394446557563	0.222238357844669	0.988741847156811	pseudobulk	DESeq2	LRT	0.653180980667086
481	KC_AB2	NTPase	0.157545400630359	0.936236928475676	0.988741847156811	pseudobulk	DESeq2	LRT	0.0286142327696613
481	KC_AB2	Nuak1	0.856267380946811	0.618575863460794	0.988741847156811	pseudobulk	DESeq2	LRT	0.208607029968623
481	KC_AB2	NUCB1	-0.0210765408824023	0.954441213131496	0.988741847156811	pseudobulk	DESeq2	LRT	0.0202508159333242

481	KC_AB2	nudC	-0.147926357299875	0.943541611067299	0.988741847156811	pseudobulk	DESeq2	LRT	0.0252389422839741
481	KC_AB2	nudE	0.165793268209952	0.899221224362588	0.988741847156811	pseudobulk	DESeq2	LRT	0.0461334509801176
481	KC_AB2	Nufip	1.47543920254359	0.661929169513603	0.988741847156811	pseudobulk	DESeq2	LRT	0.17918848025087
481	KC_AB2	Nulp1	-0.360747892984485	0.818289253550995	0.988741847156811	pseudobulk	DESeq2	LRT	0.0870931522995831
481	KC_AB2	numb	0.611130734068103	0.611743340788225	0.988741847156811	pseudobulk	DESeq2	LRT	0.213430749519563
482	KC_AB2	Nup107	-1.80294541848982	0.660135549041553	0.988741847156811	pseudobulk	DESeq2	LRT	0.180366879372519
482	KC_AB2	Nup133	-2.51746470924027	0.517377929599049	0.988741847156811	pseudobulk	DESeq2	LRT	0.286192101436607
482	KC_AB2	Nup153	1.93930864874617	0.219703429827876	0.988741847156811	pseudobulk	DESeq2	LRT	0.658163163181022
482	KC_AB2	Nup154	-1.80294541848982	0.660135549041553	0.988741847156811	pseudobulk	DESeq2	LRT	0.180366879372519
482	KC_AB2	Nup160	-1.99867440840856	0.534899605264936	0.988741847156811	pseudobulk	DESeq2	LRT	0.271727722597268
482	KC_AB2	Nup188	-2.26538931672255	0.547771004842275	0.988741847156811	pseudobulk	DESeq2	LRT	0.2614009599796
482	KC_AB2	Nup214	0.353798809751289	0.901648351639924	0.988741847156811	pseudobulk	DESeq2	LRT	0.0449628069360408
482	KC_AB2	Nup35	2.73593896747858	0.422277110754874	0.988741847156811	pseudobulk	DESeq2	LRT	0.374402458575713
482	KC_AB2	Nup358	0.0500444276466639	0.960141847756744	0.988741847156811	pseudobulk	DESeq2	LRT	0.0176646011820455
482	KC_AB2	Nup43	-0.92978684073213	0.72380920826628	0.988741847156811	pseudobulk	DESeq2	LRT	0.140375896118957
483	KC_AB2	Nup44A	1.35316200662963	0.684467033464499	0.988741847156811	pseudobulk	DESeq2	LRT	0.164647464300239
483	KC_AB2	Nup50	1.7098779479	0.537865353861561	0.988741847156811	pseudobulk	DESeq2	LRT	0.269326429526188
483	KC_AB2	Nup54	-1.80933943727629	0.584587525761637	0.988741847156811	pseudobulk	DESeq2	LRT	0.233150456085471
483	KC_AB2	Nup58	1.47543920254359	0.661929169513603	0.988741847156811	pseudobulk	DESeq2	LRT	0.17918848025087
483	KC_AB2	Nup62	2.06137138167525	0.547946929015274	0.988741847156811	pseudobulk	DESeq2	LRT	0.261261502741816
483	KC_AB2	Nup75	-2.26538931672255	0.547771004842275	0.988741847156811	pseudobulk	DESeq2	LRT	0.2614009599796
483	KC_AB2	Nup93-1	0.157562008957688	0.950463298513166	0.988741847156811	pseudobulk	DESeq2	LRT	0.0220646484576093
483	KC_AB2	Nup93-2	-1.65719613210719	0.599376091584556	0.988741847156811	pseudobulk	DESeq2	LRT	0.222300584546838
483	KC_AB2	Nup98-96	0.668970670557767	0.818048200024539	0.988741847156811	pseudobulk	DESeq2	LRT	0.0872211066123218
483	KC_AB2	Nurf-38	-0.609390826095259	0.648557404852734	0.988741847156811	pseudobulk	DESeq2	LRT	0.188051577779716
484	KC_AB2	nwk	0.639621000608741	0.502653041057663	0.988741847156811	pseudobulk	DESeq2	LRT	0.298731685613639
484	KC_AB2	Nxt1	0.30871173965274	0.81537911145609	0.988741847156811	pseudobulk	DESeq2	LRT	0.0886404185835866
484	KC_AB2	O-fut1	2.41462217514131	0.497446973552313	0.988741847156811	pseudobulk	DESeq2	LRT	0.303253207020768
484	KC_AB2	oaf	2.27759571504346	0.215580164727236	0.988741847156811	pseudobulk	DESeq2	LRT	0.666391200562267
484	KC_AB2	Oamb	-0.466513643080198	0.445408382363965	0.988741847156811	pseudobulk	DESeq2	LRT	0.351241614019179
484	KC_AB2	Oatp30B	0.630543148119245	0.748402447226467	0.988741847156811	pseudobulk	DESeq2	LRT	0.125864801055495
484	KC_AB2	Oatp33Ea	1.42621218304824	0.66738831402044	0.988741847156811	pseudobulk	DESeq2	LRT	0.175621402117653
484	KC_AB2	obe	-1.99867440840856	0.534899605264936	0.988741847156811	pseudobulk	DESeq2	LRT	0.271727722597268
484	KC_AB2	Obp56a	-1.80294541848982	0.660135549041553	0.988741847156811	pseudobulk	DESeq2	LRT	0.180366879372519
484	KC_AB2	obst-E	1.47543920254359	0.661929169513603	0.988741847156811	pseudobulk	DESeq2	LRT	0.17918848025087
485	KC_AB2	obst-J	1.42621218304824	0.66738831402044	0.988741847156811	pseudobulk	DESeq2	LRT	0.175621402117653
485	KC_AB2	ocm	-0.853565742783243	0.753557841534365	0.988741847156811	pseudobulk	DESeq2	LRT	0.122883406527736
485	KC_AB2	Ocrl	-0.358098920152354	0.844906190835001	0.988741847156811	pseudobulk	DESeq2	LRT	0.0731915076944904
485	KC_AB2	Octalpha2R	-0.250522378538222	0.778877254268392	0.988741847156811	pseudobulk	DESeq2	LRT	0.108530978776943
485	KC_AB2	Octbeta1R	0.16931867697681	0.72440054446981	0.988741847156811	pseudobulk	DESeq2	LRT	0.140021231935913
485	KC_AB2	Octbeta3R	0.203431429120817	0.742417818243958	0.988741847156811	pseudobulk	DESeq2	LRT	0.129351613470006
485	KC_AB2	Oda	0.00465608256080009	0.942761508432705	0.988741847156811	pseudobulk	DESeq2	LRT	0.0255981573913817
485	KC_AB2	Odj	-1.40296545307325	0.628223627794903	0.988741847156811	pseudobulk	DESeq2	LRT	0.201885733604539
485	KC_AB2	OdsH	-1.80294541848982	0.660135549041553	0.988741847156811	pseudobulk	DESeq2	LRT	0.180366879372519
485	KC_AB2	Oga	-1.80294541848982	0.660135549041553	0.988741847156811	pseudobulk	DESeq2	LRT	0.180366879372519
486	KC_AB2	Ogg1	2.73593896747858	0.422277110754874	0.988741847156811	pseudobulk	DESeq2	LRT	0.374402458575713
486	KC_AB2	ohgt	-1.06622077822007	0.55903750929651	0.988741847156811	pseudobulk	DESeq2	LRT	0.25255905162345
486	KC_AB2	okr	0.818790875285784	0.940355871560339	0.988741847156811	pseudobulk	DESeq2	LRT	0.0267077593721816
486	KC_AB2	olf186-F	-0.333924372251237	0.839017298925653	0.988741847156811	pseudobulk	DESeq2	LRT	0.0762290847606876
486	KC_AB2	olf413	-0.0780216331906279	0.857352495783564	0.988741847156811	pseudobulk	DESeq2	LRT	0.0668405835622784
486	KC_AB2	Oli	0.366657016002069	0.836524773995217	0.988741847156811	pseudobulk	DESeq2	LRT	0.0775211927170117
486	KC_AB2	omd	0.157562008957688	0.950463298513166	0.988741847156811	pseudobulk	DESeq2	LRT	0.0220646484576093
486	KC_AB2	ome	0.461242866101852	0.61635929443413	0.988741847156811	pseudobulk	DESeq2	LRT	0.210166050658321
486	KC_AB2	onecut	-0.281548895555689	0.715603329595706	0.988741847156811	pseudobulk	DESeq2	LRT	0.145327647392426
486	KC_AB2	Opa1	0.678201690449887	0.572760646225092	0.988741847156811	pseudobulk	DESeq2	LRT	0.242026829587121
487	KC_AB2	Opbp	0.712171366772551	0.813205723536451	0.988741847156811	pseudobulk	DESeq2	LRT	0.0897995733547685

487	KC_AB2	opm	-1.1284597703615	0.729754448571724	0.988741847156811	pseudobulk	DESeq2	LRT	0.136823248879498
487	KC_AB2	or	-0.507140190294263	0.811190366321596	0.988741847156811	pseudobulk	DESeq2	LRT	0.0908772156515369
487	KC_AB2	Or43b	0.818790875285784	0.940355871560339	0.988741847156811	pseudobulk	DESeq2	LRT	0.0267077593721816
487	KC_AB2	Or45a	-1.80294541848982	0.660135549041553	0.988741847156811	pseudobulk	DESeq2	LRT	0.180366879372519
487	KC_AB2	Or92a	-1.80294541848982	0.660135549041553	0.988741847156811	pseudobulk	DESeq2	LRT	0.180366879372519
487	KC_AB2	orb	-2.30947243982793	0.260675862596932	0.988741847156811	pseudobulk	DESeq2	LRT	0.583899180670076
487	KC_AB2	orb2	-0.0243802002100301	0.938572934987986	0.988741847156811	pseudobulk	DESeq2	LRT	0.0275319734080359
487	KC_AB2	Orc4	0.699674038439951	0.821690393783994	0.988741847156811	pseudobulk	DESeq2	LRT	0.0852917902455435
487	KC_AB2	ORMDL	-0.0384805229678164	0.985071128575604	0.988741847156811	pseudobulk	DESeq2	LRT	0.006532409469285
488	KC_AB2	ORY	-1.80294541848982	0.660135549041553	0.988741847156811	pseudobulk	DESeq2	LRT	0.180366879372519
488	KC_AB2	osa	0.458960177088376	0.51463568653392	0.988741847156811	pseudobulk	DESeq2	LRT	0.288500101671359
488	KC_AB2	Osbp	0.699674038439951	0.821690393783994	0.988741847156811	pseudobulk	DESeq2	LRT	0.0852917902455435
488	KC_AB2	Oscillin	-1.80294541848982	0.660135549041553	0.988741847156811	pseudobulk	DESeq2	LRT	0.180366879372519
488	KC_AB2	Oseg6	2.06137138167525	0.547946929015274	0.988741847156811	pseudobulk	DESeq2	LRT	0.261261502741816
488	KC_AB2	osp	0.086855330069921	0.93457542246533	0.988741847156811	pseudobulk	DESeq2	LRT	0.0293856442704431
488	KC_AB2	Ost48	2.90072538444148	0.201184744653021	0.988741847156811	pseudobulk	DESeq2	LRT	0.696404953855657
488	KC_AB2	OstDelta	-0.934020934207768	0.782359118258182	0.988741847156811	pseudobulk	DESeq2	LRT	0.106593851444836
488	KC_AB2	Ostgamma	0.569063444495555	0.738733567410712	0.988741847156811	pseudobulk	DESeq2	LRT	0.131512166567168
488	KC_AB2	otk	-0.213177136262384	0.93155916913195	0.988741847156811	pseudobulk	DESeq2	LRT	0.030789555140895
489	KC_AB2	otk2	1.12452187615248	0.704078404906088	0.988741847156811	pseudobulk	DESeq2	LRT	0.152378975911753
489	KC_AB2	OtopLa	-1.06547752640016	0.00913253694583506	0.988741847156811	pseudobulk	DESeq2	LRT	2.03940856216671
489	KC_AB2	OtopLc	-0.0298017824566077	0.933113881958521	0.988741847156811	pseudobulk	DESeq2	LRT	0.0300653495139718
489	KC_AB2	out	1.47543920254359	0.661929169513603	0.988741847156811	pseudobulk	DESeq2	LRT	0.17918848025087
489	KC_AB2	ova	0.57642073069163	0.743498489158833	0.988741847156811	pseudobulk	DESeq2	LRT	0.12871990965803
489	KC_AB2	ox	0.472983442616538	0.493572813536385	0.988741847156811	pseudobulk	DESeq2	LRT	0.306648769672329
489	KC_AB2	OXA1L	0.351722119397094	0.929615296941389	0.988741847156811	pseudobulk	DESeq2	LRT	0.0316967385224016
489	KC_AB2	oxt	1.17122272564433	0.596070420011995	0.988741847156811	pseudobulk	DESeq2	LRT	0.224702429494649
489	KC_AB2	oys	-1.80294541848982	0.660135549041553	0.988741847156811	pseudobulk	DESeq2	LRT	0.180366879372519
489	KC_AB2	p	0.919353707608246	0.746278333243311	0.988741847156811	pseudobulk	DESeq2	LRT	0.127099167101924
490	KC_AB2	p115	-0.0323203545809474	0.954448156132187	0.988741847156811	pseudobulk	DESeq2	LRT	0.020247656706879
490	KC_AB2	p120ctn	-0.151443195393283	0.78601579204226	0.988741847156811	pseudobulk	DESeq2	LRT	0.104568728352559
490	KC_AB2	p130CAS	3.77845897374758	0.158617058409698	0.988741847156811	pseudobulk	DESeq2	LRT	0.799650108476029
490	KC_AB2	p23	0.369368110115516	0.703701999734737	0.988741847156811	pseudobulk	DESeq2	LRT	0.152611214820938
490	KC_AB2	p24-1	0.407127130538907	0.766081148388107	0.988741847156811	pseudobulk	DESeq2	LRT	0.115725224583381
490	KC_AB2	P32	-1.39509543152453	0.47084431190984	0.988741847156811	pseudobulk	DESeq2	LRT	0.32712267175822
490	KC_AB2	p38b	-0.602547753666317	0.699576538455223	0.988741847156811	pseudobulk	DESeq2	LRT	0.155164763787827
490	KC_AB2	p47	-1.59087154787651	0.436900982678057	0.988741847156811	pseudobulk	DESeq2	LRT	0.359616978473819
490	KC_AB2	p53	-2.9721553030081	0.387854083811327	0.988741847156811	pseudobulk	DESeq2	LRT	0.411331631401987
490	KC_AB2	P58IPK	-0.506613484034373	0.961658398065406	0.988741847156811	pseudobulk	DESeq2	LRT	0.0169791713942958
491	KC_AB2	P5CDh1	-0.342711813400469	0.717306693924989	0.988741847156811	pseudobulk	DESeq2	LRT	0.144295116298091
491	KC_AB2	P5cr	-0.742148798960242	0.80644751100302	0.988741847156811	pseudobulk	DESeq2	LRT	0.0934238941443075
491	KC_AB2	Pa1	-0.481648742923465	0.682798403839045	0.988741847156811	pseudobulk	DESeq2	LRT	0.165707502795595
491	KC_AB2	pAbp	-0.0447453734548286	0.891582218049415	0.988741847156811	pseudobulk	DESeq2	LRT	0.0498386017889586
491	KC_AB2	Pabp2	-0.0157450521181259	0.976901073549458	0.988741847156811	pseudobulk	DESeq2	LRT	0.0101494131357394
491	KC_AB2	Paf-AHalpha	0.104738716333549	0.945063852997661	0.988741847156811	pseudobulk	DESeq2	LRT	0.0245388475037562
491	KC_AB2	Paics	2.7785831508444	0.373751317747552	0.988741847156811	pseudobulk	DESeq2	LRT	0.427417267458199
491	KC_AB2	Paip2	-0.556008553680362	0.547040646550856	0.988741847156811	pseudobulk	DESeq2	LRT	0.261980403251738
491	KC_AB2	Pak	0.539111688928056	0.577951925641418	0.988741847156811	pseudobulk	DESeq2	LRT	0.238108284930628
491	KC_AB2	Pak3	1.31617713253562	0.59447159965162	0.988741847156811	pseudobulk	DESeq2	LRT	0.225868888579699
492	KC_AB2	Pal1	-2.51746470924027	0.517377929599049	0.988741847156811	pseudobulk	DESeq2	LRT	0.286192101436607
492	KC_AB2	Pal2	0.134288480661402	0.854118642702492	0.988741847156811	pseudobulk	DESeq2	LRT	0.0684817987580637
492	KC_AB2	pall	1.14703591811676	0.660809111836255	0.988741847156811	pseudobulk	DESeq2	LRT	0.179923977199595
492	KC_AB2	pan	-0.237125211899446	0.410155357263522	0.988741847156811	pseudobulk	DESeq2	LRT	0.387051611518128
492	KC_AB2	PAN2	0.331758848923756	0.946375065527934	0.988741847156811	pseudobulk	DESeq2	LRT	0.0239367107367223
492	KC_AB2	Panx	-1.1284597703615	0.729754448571724	0.988741847156811	pseudobulk	DESeq2	LRT	0.136823248879498
492	KC_AB2	papi	-0.934020934207768	0.782359118258182	0.988741847156811	pseudobulk	DESeq2	LRT	0.106593851444836

492	KC_AB2	PAPLA1	0.621544340195364	0.646803130050567	0.988741847156811	pseudobulk	DESeq2	LRT	0.189227887095259
492	KC_AB2	Papss	-1.08506241312707	0.67856296251147	0.988741847156811	pseudobulk	DESeq2	LRT	0.168409848810952
492	KC_AB2	Papst2	2.80917294583258	0.367996267391162	0.988741847156811	pseudobulk	DESeq2	LRT	0.434156586379859
493	KC_AB2	par-1	-0.882273751381853	0.193845978042421	0.988741847156811	pseudobulk	DESeq2	LRT	0.712543205397568
493	KC_AB2	par-6	-0.625498673989172	0.653868064108824	0.988741847156811	pseudobulk	DESeq2	LRT	0.184509873709284
493	KC_AB2	para	0.217294716780537	0.405861712204521	0.988741847156811	pseudobulk	DESeq2	LRT	0.391621916809354
493	KC_AB2	Parp	-0.185837450965286	0.780654085277236	0.988741847156811	pseudobulk	DESeq2	LRT	0.10754136322162
493	KC_AB2	parvin	0.834352593082403	0.675685094045283	0.988741847156811	pseudobulk	DESeq2	LRT	0.170255661725145
493	KC_AB2	pasha	-1.83172320633983	0.524697733495197	0.988741847156811	pseudobulk	DESeq2	LRT	0.280090811792999
493	KC_AB2	pasi2	2.4643417141186	0.449082701910884	0.988741847156811	pseudobulk	DESeq2	LRT	0.347673673080982
493	KC_AB2	Pask	2.06137138167525	0.547946929015274	0.988741847156811	pseudobulk	DESeq2	LRT	0.261261502741816
493	KC_AB2	Past1	0.866462110987865	0.477694513035217	0.988741847156811	pseudobulk	DESeq2	LRT	0.320849747144976
493	KC_AB2	Pat1	0.155512745384915	0.889802708252214	0.988741847156811	pseudobulk	DESeq2	LRT	0.0507062767435124
494	KC_AB2	path	-0.452344515838269	0.871171367298942	0.988741847156811	pseudobulk	DESeq2	LRT	0.0598964069304489
494	KC_AB2	Patj	-0.187188766977065	0.932037508762103	0.988741847156811	pseudobulk	DESeq2	LRT	0.0305666096196055
494	KC_AB2	Patr-1	-1.28585361841952	0.604452679793172	0.988741847156811	pseudobulk	DESeq2	LRT	0.218637692667744
494	KC_AB2	Patronin	0.504683657845979	0.67160887751512	0.988741847156811	pseudobulk	DESeq2	LRT	0.172883571869384
494	KC_AB2	Patsas	-0.934020934207768	0.782359118258182	0.988741847156811	pseudobulk	DESeq2	LRT	0.106593851444836
494	KC_AB2	Pax	1.4413042803421	0.615311685084066	0.988741847156811	pseudobulk	DESeq2	LRT	0.210904837358999
494	KC_AB2	Pbp95	2.06137138167525	0.547946929015274	0.988741847156811	pseudobulk	DESeq2	LRT	0.261261502741816
494	KC_AB2	Pc	-0.814046802218109	0.506852505804588	0.988741847156811	pseudobulk	DESeq2	LRT	0.295118402075775
494	KC_AB2	PCB	1.88145593350457	0.299839307201757	0.988741847156811	pseudobulk	DESeq2	LRT	0.523111434256836
494	KC_AB2	Pcd	0.450150729692223	0.465255615377958	0.988741847156811	pseudobulk	DESeq2	LRT	0.332308376471362
495	KC_AB2	Pcf11	0.167586562565834	0.83604242666991	0.988741847156811	pseudobulk	DESeq2	LRT	0.0777716828467546
495	KC_AB2	PCID2	-2.9721553030081	0.387854083811327	0.988741847156811	pseudobulk	DESeq2	LRT	0.411331631401987
495	KC_AB2	pck	0.683084472732921	0.745076201728678	0.988741847156811	pseudobulk	DESeq2	LRT	0.127799308059901
495	KC_AB2	Pcl	0.0556967317943303	0.951374442369035	0.988741847156811	pseudobulk	DESeq2	LRT	0.0216485196043508
495	KC_AB2	pcm	-3.09794865339079	0.39855598532175	0.988741847156811	pseudobulk	DESeq2	LRT	0.399510664459796
495	KC_AB2	Pcmt	-0.739808368223237	0.591979617810057	0.988741847156811	pseudobulk	DESeq2	LRT	0.227693246022025
495	KC_AB2	PCNA2	-0.957133960806503	0.762632518785665	0.988741847156811	pseudobulk	DESeq2	LRT	0.1176846802739
495	KC_AB2	pcs	-0.0344106674396631	0.970322150694496	0.988741847156811	pseudobulk	DESeq2	LRT	0.0130840543509222
495	KC_AB2	pcx	-0.235620053326589	0.908937007481336	0.988741847156811	pseudobulk	DESeq2	LRT	0.0414662138640759
495	KC_AB2	Pcyt1	0.261659287484284	0.792713778293956	0.988741847156811	pseudobulk	DESeq2	LRT	0.100883593194223
496	KC_AB2	Pcyt2	-1.23536834900699	0.725570392794057	0.988741847156811	pseudobulk	DESeq2	LRT	0.139320447155934
496	KC_AB2	PDCD-5	-0.885835550404817	0.538926723019376	0.988741847156811	pseudobulk	DESeq2	LRT	0.268470281105936
496	KC_AB2	Pdcd4	0.885452756098897	0.31036325253143	0.988741847156811	pseudobulk	DESeq2	LRT	0.508129705479521
496	KC_AB2	Pde11	0.157562008957688	0.950463298513166	0.988741847156811	pseudobulk	DESeq2	LRT	0.0220646484576093
496	KC_AB2	Pde1c	0.121590269631884	0.732931400419935	0.988741847156811	pseudobulk	DESeq2	LRT	0.134936671761693
496	KC_AB2	Pde6	0.802776349286921	0.419771939535699	0.988741847156811	pseudobulk	DESeq2	LRT	0.376986596034971
496	KC_AB2	Pde8	-0.255019541186223	0.702692250707688	0.988741847156811	pseudobulk	DESeq2	LRT	0.153234835837261
496	KC_AB2	Pde9	-0.0299353914384577	0.919332392978903	0.988741847156811	pseudobulk	DESeq2	LRT	0.0365274371049392
496	KC_AB2	pdgy	1.42621218304824	0.66738831402044	0.988741847156811	pseudobulk	DESeq2	LRT	0.175621402117653
496	KC_AB2	Pdha	-0.390544434647309	0.706643119272642	0.988741847156811	pseudobulk	DESeq2	LRT	0.150799865498941
497	KC_AB2	Pdhb	-0.451869845142519	0.67266982981767	0.988741847156811	pseudobulk	DESeq2	LRT	0.172198050595089
497	KC_AB2	Pdi	0.123764945436762	0.900273943292135	0.988741847156811	pseudobulk	DESeq2	LRT	0.0456253194969434
497	KC_AB2	Pdk	0.853627489729184	0.606283183762972	0.988741847156811	pseudobulk	DESeq2	LRT	0.217324477452366
497	KC_AB2	Pdk1	-0.559431832608895	0.586391947152377	0.988741847156811	pseudobulk	DESeq2	LRT	0.231812002438041
497	KC_AB2	pdm3	0.818790875285784	0.940355871560339	0.988741847156811	pseudobulk	DESeq2	LRT	0.0267077593721816
497	KC_AB2	Pdp	0.924140746192761	0.675445049022325	0.988741847156811	pseudobulk	DESeq2	LRT	0.170409977326066
497	KC_AB2	Pdp1	-0.0273317378094312	0.943542674568975	0.988741847156811	pseudobulk	DESeq2	LRT	0.025238452774403
497	KC_AB2	pds5	0.575200883059258	0.804213580533116	0.988741847156811	pseudobulk	DESeq2	LRT	0.0946285973599483
497	KC_AB2	Pdss2	0.922664372932389	0.678259639521954	0.988741847156811	pseudobulk	DESeq2	LRT	0.168604025267887
497	KC_AB2	Pdxk	1.47543920254359	0.661929169513603	0.988741847156811	pseudobulk	DESeq2	LRT	0.17918848025087
498	KC_AB2	PDZ-GEF	0.100096176606989	0.909081329041193	0.988741847156811	pseudobulk	DESeq2	LRT	0.0413972618013405
498	KC_AB2	pea	2.73593896747858	0.422277110754874	0.988741847156811	pseudobulk	DESeq2	LRT	0.374402458575713
498	KC_AB2	Pect	1.2230628388663	0.216065558037011	0.988741847156811	pseudobulk	DESeq2	LRT	0.665414456377268

498	KC_AB2	PEK	0.818790875285784	0.940355871560339	0.988741847156811	pseudobulk	DESeq2	LRT	0.0267077593721816
498	KC_AB2	pelo	-0.452344515838269	0.871171367298942	0.988741847156811	pseudobulk	DESeq2	LRT	0.0598964069304489
498	KC_AB2	pen-2	3.61120017668169	0.194690754078012	0.988741847156811	pseudobulk	DESeq2	LRT	0.710654672764742
498	KC_AB2	peng	-1.87057166357412	0.515275319421977	0.988741847156811	pseudobulk	DESeq2	LRT	0.28796065881108
498	KC_AB2	Pep	0.196642890365306	0.732523820395188	0.988741847156811	pseudobulk	DESeq2	LRT	0.135178248246231
498	KC_AB2	per	-0.957133960806503	0.762632518785665	0.988741847156811	pseudobulk	DESeq2	LRT	0.1176846802739
498	KC_AB2	pes	0.157562008957688	0.950463298513166	0.988741847156811	pseudobulk	DESeq2	LRT	0.0220646484576093
499	KC_AB2	Pex1	3.06202454821851	0.30627034058063	0.988741847156811	pseudobulk	DESeq2	LRT	0.513895058517773
499	KC_AB2	Pex10	2.7785831508444	0.373751317747552	0.988741847156811	pseudobulk	DESeq2	LRT	0.427417267458199
499	KC_AB2	Pex11	-1.37054505049545	0.574189269529552	0.988741847156811	pseudobulk	DESeq2	LRT	0.240944927875125
499	KC_AB2	Pex12	-1.23536834900699	0.725570392794057	0.988741847156811	pseudobulk	DESeq2	LRT	0.139320447155934
499	KC_AB2	Pex13	-0.20662287788209	0.896484308289624	0.988741847156811	pseudobulk	DESeq2	LRT	0.0474573077558637
499	KC_AB2	Pex14	0.920073134400379	0.728211051768817	0.988741847156811	pseudobulk	DESeq2	LRT	0.137742734237847
499	KC_AB2	Pex16	0.353798809751289	0.901648351639924	0.988741847156811	pseudobulk	DESeq2	LRT	0.0449628069360408
499	KC_AB2	Pex19	-0.226858097285807	0.861876476725134	0.988741847156811	pseudobulk	DESeq2	LRT	0.0645549723663376
499	KC_AB2	Pex2	-1.52496782475103	0.617219242690575	0.988741847156811	pseudobulk	DESeq2	LRT	0.209560542647675
499	KC_AB2	Pex23	-0.488465967391191	0.84251072973523	0.988741847156811	pseudobulk	DESeq2	LRT	0.0744245594950451
500	KC_AB2	Pex3	0.752634025970037	0.844139830246018	0.988741847156811	pseudobulk	DESeq2	LRT	0.0735856073147832
500	KC_AB2	Pex5	1.12452187615248	0.704078404906088	0.988741847156811	pseudobulk	DESeq2	LRT	0.152378975911753
500	KC_AB2	Pex6	-2.51746470924027	0.517377929599049	0.988741847156811	pseudobulk	DESeq2	LRT	0.286192101436607
500	KC_AB2	Pfdn1	0.998893024789477	0.660591418406266	0.988741847156811	pseudobulk	DESeq2	LRT	0.180067072429364
500	KC_AB2	Pfdn2	-0.714805448721718	0.713685201670511	0.988741847156811	pseudobulk	DESeq2	LRT	0.146493308286463
500	KC_AB2	Pfdn4	0.210009840773532	0.863792183456565	0.988741847156811	pseudobulk	DESeq2	LRT	0.0635907302457334
500	KC_AB2	Pfdn5	1.42805793569811	0.496621984925921	0.988741847156811	pseudobulk	DESeq2	LRT	0.303974058602093
500	KC_AB2	Pfdn6	0.801083655688617	0.567824539907119	0.988741847156811	pseudobulk	DESeq2	LRT	0.245785842320994
500	KC_AB2	Pfk	-0.79712441066902	0.508837068952191	0.988741847156811	pseudobulk	DESeq2	LRT	0.29342125770758
500	KC_AB2	Pfrx	0.792918518047366	0.392645891795546	0.988741847156811	pseudobulk	DESeq2	LRT	0.405998942156287
501	KC_AB2	Pgam5	2.80917294583258	0.367996267391162	0.988741847156811	pseudobulk	DESeq2	LRT	0.434156586379859
501	KC_AB2	Pgant1	-0.405381063532158	0.89155634938256	0.988741847156811	pseudobulk	DESeq2	LRT	0.0498512027381788
501	KC_AB2	Pgant2	-0.0156475668208201	0.9727135173651	0.988741847156811	pseudobulk	DESeq2	LRT	0.0120150488862299
501	KC_AB2	Pgant3	-0.506613484034373	0.961658398065406	0.988741847156811	pseudobulk	DESeq2	LRT	0.0169791713942958
501	KC_AB2	Pgant35A	-3.32426296135166	0.343738249391692	0.988741847156811	pseudobulk	DESeq2	LRT	0.463772139158982
501	KC_AB2	Pgant5	-0.958583890310803	0.407311985752355	0.988741847156811	pseudobulk	DESeq2	LRT	0.390072809974103
501	KC_AB2	Pgant7	-0.0323203545809474	0.954448156132187	0.988741847156811	pseudobulk	DESeq2	LRT	0.020247656706879
501	KC_AB2	Pgant9	0.462677580056202	0.291141382505963	0.988741847156811	pseudobulk	DESeq2	LRT	0.535896060048469
501	KC_AB2	PGAP2	2.55216854135116	0.29732771327113	0.988741847156811	pseudobulk	DESeq2	LRT	0.526764609261937
501	KC_AB2	PGAP3	-0.197565662601209	0.921418496992636	0.988741847156811	pseudobulk	DESeq2	LRT	0.0355430737620862
502	KC_AB2	PGAP5	2.7785831508444	0.373751317747552	0.988741847156811	pseudobulk	DESeq2	LRT	0.427417267458199
502	KC_AB2	Pgi	1.49704873313519	0.299071521721802	0.988741847156811	pseudobulk	DESeq2	LRT	0.524224939519366
502	KC_AB2	Pgk	0.214033115776878	0.811930294947106	0.988741847156811	pseudobulk	DESeq2	LRT	0.0904812537874689
502	KC_AB2	Pglym78	0.174552491434442	0.816372495512161	0.988741847156811	pseudobulk	DESeq2	LRT	0.0881116355685525
502	KC_AB2	Pgm1	-0.568233637794205	0.868826397133536	0.988741847156811	pseudobulk	DESeq2	LRT	0.0610669925949191
502	KC_AB2	Pgm2a	-0.400995799654333	0.83163350315977	0.988741847156811	pseudobulk	DESeq2	LRT	0.080068023015021
502	KC_AB2	PGRP-LE	0.572861358867068	0.813871944286787	0.988741847156811	pseudobulk	DESeq2	LRT	0.0894439222171593
502	KC_AB2	ph-d	-0.853565742783243	0.753557841534365	0.988741847156811	pseudobulk	DESeq2	LRT	0.122883406527736
502	KC_AB2	ph-p	2.59981310546494	0.282723565933047	0.988741847156811	pseudobulk	DESeq2	LRT	0.548637990137543
502	KC_AB2	Phax	-0.0323203545809474	0.954448156132187	0.988741847156811	pseudobulk	DESeq2	LRT	0.020247656706879
503	KC_AB2	Phb2	0.641767316647791	0.649691903566914	0.988741847156811	pseudobulk	DESeq2	LRT	0.187292545360415
503	KC_AB2	pHCl-1	0.179424936991763	0.656264161363526	0.988741847156811	pseudobulk	DESeq2	LRT	0.182921311950667
503	KC_AB2	PheRS-m	1.17156071944349	0.597348257338218	0.988741847156811	pseudobulk	DESeq2	LRT	0.223772398949354
503	KC_AB2	Phf5a	2.26998970133409	0.374936237995469	0.988741847156811	pseudobulk	DESeq2	LRT	0.426042582515506
503	KC_AB2	PHGPx	0.0742413024401475	0.943070327868629	0.988741847156811	pseudobulk	DESeq2	LRT	0.0254559192813408
503	KC_AB2	PhKgamma	-0.559583513075519	0.659759241063322	0.988741847156811	pseudobulk	DESeq2	LRT	0.180614518023864
503	KC_AB2	Phlpp	-2.26538931672255	0.547771004842275	0.988741847156811	pseudobulk	DESeq2	LRT	0.2614009599796
503	KC_AB2	Phm	-0.0369711264421468	0.961347051055966	0.988741847156811	pseudobulk	DESeq2	LRT	0.0171198015619325
503	KC_AB2	pho	-0.273819178740373	0.784353555628604	0.988741847156811	pseudobulk	DESeq2	LRT	0.10548813036808

503	KC_AB2	phol	2.70180605057798	0.249545627897494	0.988741847156811	pseudobulk	DESeq2	LRT	0.602850034680352
504	KC_AB2	phr	-1.80294541848982	0.660135549041553	0.988741847156811	pseudobulk	DESeq2	LRT	0.180366879372519
504	KC_AB2	phtf	-0.448374585768327	0.814227572251762	0.988741847156811	pseudobulk	DESeq2	LRT	0.0892541951597077
504	KC_AB2	phyl	0.393508672607934	0.559535595209561	0.988741847156811	pseudobulk	DESeq2	LRT	0.252172280339611
504	KC_AB2	PI31	1.10541528051857	0.364023014516956	0.988741847156811	pseudobulk	DESeq2	LRT	0.438871158214259
504	KC_AB2	Pi3K21B	0.197019222279105	0.906036510922107	0.988741847156811	pseudobulk	DESeq2	LRT	0.0428543010290278
504	KC_AB2	Pi3K59F	1.42621218304824	0.66738831402044	0.988741847156811	pseudobulk	DESeq2	LRT	0.175621402117653
504	KC_AB2	Pi3K68D	-1.38702429620387	0.328699507583205	0.988741847156811	pseudobulk	DESeq2	LRT	0.483200946523965
504	KC_AB2	Pi3K92E	-1.77566073155666	0.606304952090798	0.988741847156811	pseudobulk	DESeq2	LRT	0.217308884582246
504	KC_AB2	Pi4KIIalpha	0.353798809751289	0.901648351639924	0.988741847156811	pseudobulk	DESeq2	LRT	0.0449628069360408
504	KC_AB2	PI4KIIIalpha	0.175405668582614	0.875945301917673	0.988741847156811	pseudobulk	DESeq2	LRT	0.0575230123444743
505	KC_AB2	pic	-0.568233637794205	0.868826397133536	0.988741847156811	pseudobulk	DESeq2	LRT	0.0610669925949191
505	KC_AB2	PICK1	0.821782310181673	0.594817370620634	0.988741847156811	pseudobulk	DESeq2	LRT	0.225616357139772
505	KC_AB2	pico	0.57259795170203	0.639470473018481	0.988741847156811	pseudobulk	DESeq2	LRT	0.19417950388461
505	KC_AB2	Picot	-0.272159080840168	0.881917593135503	0.988741847156811	pseudobulk	DESeq2	LRT	0.0545719936873807
505	KC_AB2	Piezo	-1.77566073155666	0.606304952090798	0.988741847156811	pseudobulk	DESeq2	LRT	0.217308884582246
505	KC_AB2	Pif1A	0.818790875285784	0.940355871560339	0.988741847156811	pseudobulk	DESeq2	LRT	0.0267077593721816
505	KC_AB2	PIG-A	1.47543920254359	0.661929169513603	0.988741847156811	pseudobulk	DESeq2	LRT	0.17918848025087
505	KC_AB2	PIG-B	-0.337041059841174	0.846336073904806	0.988741847156811	pseudobulk	DESeq2	LRT	0.0724571475287197
505	KC_AB2	PIG-F	1.50749703173234	0.386226840419617	0.988741847156811	pseudobulk	DESeq2	LRT	0.413157548679128
505	KC_AB2	PIG-G	-2.26538931672255	0.547771004842275	0.988741847156811	pseudobulk	DESeq2	LRT	0.2614009599796
506	KC_AB2	PIG-H	1.32714918892411	0.592713712963247	0.988741847156811	pseudobulk	DESeq2	LRT	0.227155024852469
506	KC_AB2	PIG-K	1.89266378342788	0.300347618662404	0.988741847156811	pseudobulk	DESeq2	LRT	0.522375807052326
506	KC_AB2	PIG-L	-0.158601889198964	0.945838126788742	0.988741847156811	pseudobulk	DESeq2	LRT	0.0241831835324482
506	KC_AB2	PIG-O	0.712171366772551	0.813205723536451	0.988741847156811	pseudobulk	DESeq2	LRT	0.0897995733547685
506	KC_AB2	PIG-Q	0.818790875285784	0.940355871560339	0.988741847156811	pseudobulk	DESeq2	LRT	0.0267077593721816
506	KC_AB2	PIG-S	-1.80933943727629	0.584587525761637	0.988741847156811	pseudobulk	DESeq2	LRT	0.233150456085471
506	KC_AB2	PIG-T	0.368009530205336	0.821408243890115	0.988741847156811	pseudobulk	DESeq2	LRT	0.0854409427557778
506	KC_AB2	PIG-U	0.802207095184316	0.732945722088841	0.988741847156811	pseudobulk	DESeq2	LRT	0.134928185618234
506	KC_AB2	PIG-X	-0.773021474708252	0.784024111360353	0.988741847156811	pseudobulk	DESeq2	LRT	0.105670581104165
506	KC_AB2	pigeon	-0.0582963981256801	0.979058404810457	0.988741847156811	pseudobulk	DESeq2	LRT	0.0091913999942843
507	KC_AB2	pigs	-0.745125090833299	0.395264887129199	0.988741847156811	pseudobulk	DESeq2	LRT	0.403111763960445
507	KC_AB2	Pih1D1	0.283992347156198	0.862569424275045	0.988741847156811	pseudobulk	DESeq2	LRT	0.0642059404678031
507	KC_AB2	Pink1	-0.0295761453730973	0.975489763368292	0.988741847156811	pseudobulk	DESeq2	LRT	0.0107772836615597
507	KC_AB2	Pino	-0.43023994148358	0.756784218585753	0.988741847156811	pseudobulk	DESeq2	LRT	0.121027932965388
507	KC_AB2	pins	-1.28801548279985	0.516234052853976	0.988741847156811	pseudobulk	DESeq2	LRT	0.287153351053687
507	KC_AB2	PIP4K	-0.0525480902179507	0.919641070331815	0.988741847156811	pseudobulk	DESeq2	LRT	0.0363816417740203
507	KC_AB2	PIP5K59B	-0.44149265806376	0.61081306957923	0.988741847156811	pseudobulk	DESeq2	LRT	0.21409167890865
507	KC_AB2	Pis	0.489813180275169	0.600978797777312	0.988741847156811	pseudobulk	DESeq2	LRT	0.221140849415835
507	KC_AB2	Pisd	0.0490715323267471	0.977258570740954	0.988741847156811	pseudobulk	DESeq2	LRT	0.00999051203495298
507	KC_AB2	pit	1.45259040912931	0.616176917291092	0.988741847156811	pseudobulk	DESeq2	LRT	0.210294574894236
508	KC_AB2	pita	-1.40296545307325	0.628223627794903	0.988741847156811	pseudobulk	DESeq2	LRT	0.201885733604539
508	KC_AB2	Pits	-0.100740541848267	0.85288043812143	0.988741847156811	pseudobulk	DESeq2	LRT	0.06911184656228
508	KC_AB2	Pitslre	0.259990695730617	0.523948780977506	0.988741847156811	pseudobulk	DESeq2	LRT	0.28071116573774
508	KC_AB2	pix	1.53519137830027	0.43238982969318	0.988741847156811	pseudobulk	DESeq2	LRT	0.364124529705027
508	KC_AB2	Pka-C1	0.021990292354396	0.924507200963188	0.988741847156811	pseudobulk	DESeq2	LRT	0.0340897017829072
508	KC_AB2	Pka-C3	3.48228051815311	0.136832429065204	0.988741847156811	pseudobulk	DESeq2	LRT	0.863810963324784
508	KC_AB2	Pka-R1	-0.497407117634035	0.241173356522253	0.988741847156811	pseudobulk	DESeq2	LRT	0.617670672296797
508	KC_AB2	Pka-R2	-0.306229579582902	0.228947735566665	0.988741847156811	pseudobulk	DESeq2	LRT	0.640263647553473
508	KC_AB2	Pkc53E	-0.189656324621836	0.566151030407194	0.988741847156811	pseudobulk	DESeq2	LRT	0.247067697923355
508	KC_AB2	Pkc98E	0.254334139909206	0.59146939407166	0.988741847156811	pseudobulk	DESeq2	LRT	0.228067723275589
509	KC_AB2	PKD	2.4643417141186	0.449082701910884	0.988741847156811	pseudobulk	DESeq2	LRT	0.347673673080982
509	KC_AB2	Pkn	-0.0546881335187679	0.954869152501645	0.988741847156811	pseudobulk	DESeq2	LRT	0.0200561365207969
509	KC_AB2	Plap	-3.32426296135166	0.343738249391692	0.988741847156811	pseudobulk	DESeq2	LRT	0.463772139158982
509	KC_AB2	Plc21C	-0.462364054921526	0.384828074826489	0.988741847156811	pseudobulk	DESeq2	LRT	0.414733251869522
509	KC_AB2	Pldn	0.299229629739512	0.851026027418758	0.988741847156811	pseudobulk	DESeq2	LRT	0.070057157434224

509	KC_AB2	PlexA	0.00982263387072123	0.98077554985457	0.988741847156811	pseudobulk	DESeq2	LRT	0.00843036939131909
509	KC_AB2	PlexB	0.0169005097566288	0.983558193834493	0.988741847156811	pseudobulk	DESeq2	LRT	0.00719993923877838
509	KC_AB2	plh	-1.80294541848982	0.660135549041553	0.988741847156811	pseudobulk	DESeq2	LRT	0.180366879372519
509	KC_AB2	Pli	0.639933817385819	0.428172301152957	0.988741847156811	pseudobulk	DESeq2	LRT	0.36838143102924
509	KC_AB2	pll	1.47543920254359	0.661929169513603	0.988741847156811	pseudobulk	DESeq2	LRT	0.17918848025087
510	KC_AB2	Plod	0.780352552100014	0.834299274204729	0.988741847156811	pseudobulk	DESeq2	LRT	0.0786781342287373
510	KC_AB2	Plp	0.362555753552128	0.897034383127386	0.988741847156811	pseudobulk	DESeq2	LRT	0.0471909102264623
510	KC_AB2	plum	-0.555828083223045	0.769829010190795	0.988741847156811	pseudobulk	DESeq2	LRT	0.113605727005537
510	KC_AB2	plx	-0.778462962198401	0.334022847621189	0.988741847156811	pseudobulk	DESeq2	LRT	0.476223825833826
510	KC_AB2	Plzf	-1.80294541848982	0.660135549041553	0.988741847156811	pseudobulk	DESeq2	LRT	0.180366879372519
510	KC_AB2	PMCA	-0.0283638365428802	0.943897499791728	0.988741847156811	pseudobulk	DESeq2	LRT	0.0250751642737232
510	KC_AB2	Pmm2	-0.114099770495178	0.955170180595609	0.988741847156811	pseudobulk	DESeq2	LRT	0.0199192442181898
510	KC_AB2	PMP34	0.646616639464795	0.643967348261282	0.988741847156811	pseudobulk	DESeq2	LRT	0.19113615256169
510	KC_AB2	Pmp70	-1.08506241312707	0.67856296251147	0.988741847156811	pseudobulk	DESeq2	LRT	0.168409848810952
510	KC_AB2	pn	0.792281256713368	0.823478838299676	0.988741847156811	pseudobulk	DESeq2	LRT	0.0843475568193814
511	KC_AB2	Pngl	0.158485844312962	0.932199411153928	0.988741847156811	pseudobulk	DESeq2	LRT	0.0304911757358515
511	KC_AB2	Pnn	0.299229629739512	0.851026027418758	0.988741847156811	pseudobulk	DESeq2	LRT	0.070057157434224
511	KC_AB2	pns	0.43324628136146	0.792959056514803	0.988741847156811	pseudobulk	DESeq2	LRT	0.100749236375646
511	KC_AB2	pnut	-0.0946379886006171	0.921191284930397	0.988741847156811	pseudobulk	DESeq2	LRT	0.0356501793970614
511	KC_AB2	PNUTS	-0.0687013702155662	0.908656293142618	0.988741847156811	pseudobulk	DESeq2	LRT	0.0416003612437167
511	KC_AB2	pod1	-0.600945882938392	0.698364000134572	0.988741847156811	pseudobulk	DESeq2	LRT	0.155918156109287
511	KC_AB2	poe	0.49621143204106	0.730451555337321	0.988741847156811	pseudobulk	DESeq2	LRT	0.136408581847879
511	KC_AB2	Pof	0.576714922882476	0.824743989322041	0.988741847156811	pseudobulk	DESeq2	LRT	0.0836808408796565
511	KC_AB2	Pol31	0.818790875285784	0.940355871560339	0.988741847156811	pseudobulk	DESeq2	LRT	0.0267077593721816
511	KC_AB2	Pol32	-0.934020934207768	0.782359118258182	0.988741847156811	pseudobulk	DESeq2	LRT	0.106593851444836
512	KC_AB2	POLDIP2	1.76062409692731	0.431284494748455	0.988741847156811	pseudobulk	DESeq2	LRT	0.365236155037982
512	KC_AB2	poly	-0.516865450960998	0.716509776063777	0.988741847156811	pseudobulk	DESeq2	LRT	0.144777879709639
512	KC_AB2	polybromo	-0.0144409071146711	0.984632974376923	0.988741847156811	pseudobulk	DESeq2	LRT	0.00672562423038896
512	KC_AB2	Pomp	0.379357275703035	0.762907306203372	0.988741847156811	pseudobulk	DESeq2	LRT	0.117528225937485
512	KC_AB2	pont	3.77845897374758	0.158617058409698	0.988741847156811	pseudobulk	DESeq2	LRT	0.799650108476029
512	KC_AB2	Pop2	-0.128049386895759	0.920526989119912	0.988741847156811	pseudobulk	DESeq2	LRT	0.0359634738041611
512	KC_AB2	por	0.157562008957688	0.950463298513166	0.988741847156811	pseudobulk	DESeq2	LRT	0.0220646484576093
512	KC_AB2	porin	0.235300284311751	0.589815267564712	0.988741847156811	pseudobulk	DESeq2	LRT	0.229283989781359
512	KC_AB2	POSH	0.818790875285784	0.940355871560339	0.988741847156811	pseudobulk	DESeq2	LRT	0.0267077593721816
512	KC_AB2	Pp1-87B	0.571647926787083	0.412650638313364	0.988741847156811	pseudobulk	DESeq2	LRT	0.384417478760724
513	KC_AB2	Pp1alpha-96A	-0.244240602107271	0.732941046962808	0.988741847156811	pseudobulk	DESeq2	LRT	0.134930955793231
513	KC_AB2	Pp2A-29B	0.127592177196488	0.87397647561548	0.988741847156811	pseudobulk	DESeq2	LRT	0.0585002568940663
513	KC_AB2	Pp2B-14D	-0.434957444315601	0.567676671040945	0.988741847156811	pseudobulk	DESeq2	LRT	0.245898952958602
513	KC_AB2	Pp2C1	-0.0696062406270821	0.956337869997271	0.988741847156811	pseudobulk	DESeq2	LRT	0.0193886462679046
513	KC_AB2	Pp4-19C	0.321601000162439	0.868811750025189	0.988741847156811	pseudobulk	DESeq2	LRT	0.0610743142094242
513	KC_AB2	Ppat-Dpck	-2.51746470924027	0.517377929599049	0.988741847156811	pseudobulk	DESeq2	LRT	0.286192101436607
513	KC_AB2	Ppcs	0.47597472089045	0.427607228667352	0.988741847156811	pseudobulk	DESeq2	LRT	0.36895496168635
513	KC_AB2	PpD3	0.351722119397094	0.929615296941389	0.988741847156811	pseudobulk	DESeq2	LRT	0.0316967385224016
513	KC_AB2	ppk8	0.818790875285784	0.940355871560339	0.988741847156811	pseudobulk	DESeq2	LRT	0.0267077593721816
513	KC_AB2	ppl	-0.957133960806503	0.762632518785665	0.988741847156811	pseudobulk	DESeq2	LRT	0.1176846802739
514	KC_AB2	PPP1R15	0.686073464838417	0.452908812531509	0.988741847156811	pseudobulk	DESeq2	LRT	0.343989228894055
514	KC_AB2	PPP4R2r	0.167165629946856	0.902635884466227	0.988741847156811	pseudobulk	DESeq2	LRT	0.0444874050105513
514	KC_AB2	pps	-2.09209031988317	0.408698600207438	0.988741847156811	pseudobulk	DESeq2	LRT	0.388596849744308
514	KC_AB2	Ppt2	2.7785831508444	0.373751317747552	0.988741847156811	pseudobulk	DESeq2	LRT	0.427417267458199
514	KC_AB2	PpV	1.13539439938001	0.65602501243538	0.988741847156811	pseudobulk	DESeq2	LRT	0.183079601850592
514	KC_AB2	PQBP1	0.667839000613305	0.81413342949896	0.988741847156811	pseudobulk	DESeq2	LRT	0.0893044121300904
514	KC_AB2	pr	-0.908909516750057	0.671937973991072	0.988741847156811	pseudobulk	DESeq2	LRT	0.172670814441211
514	KC_AB2	PR-Set7	1.6341263262903	0.579948075372967	0.988741847156811	pseudobulk	DESeq2	LRT	0.236610888486163
514	KC_AB2	prage	-1.53632809488426	0.249843402254125	0.988741847156811	pseudobulk	DESeq2	LRT	0.602332114712328
514	KC_AB2	PRAS40	0.102529639938564	0.940184605358105	0.988741847156811	pseudobulk	DESeq2	LRT	0.0267868642547249
515	KC_AB2	PrBP	0.387626856268115	0.752774921273493	0.988741847156811	pseudobulk	DESeq2	LRT	0.12333485787777

515	KC_AB2	prd1	0.421373029613324	0.702961212147893	0.988741847156811	pseudobulk	DESeq2	LRT	0.153068637732482
515	KC_AB2	pre-mod(mdg4)-B	2.51243226103394	0.474458247157471	0.988741847156811	pseudobulk	DESeq2	LRT	0.323801999942597
515	KC_AB2	pre-mod(mdg4)-L	-1.80294541848982	0.660135549041553	0.988741847156811	pseudobulk	DESeq2	LRT	0.180366879372519
515	KC_AB2	pre-rRNA:CR45845	0.31938212317522	0.805080575958998	0.988741847156811	pseudobulk	DESeq2	LRT	0.0941606513797738
515	KC_AB2	prel	1.57977661945776	0.30773565028047	0.988741847156811	pseudobulk	DESeq2	LRT	0.511822189084885
515	KC_AB2	Prestin	-0.405381063532158	0.89155634938256	0.988741847156811	pseudobulk	DESeq2	LRT	0.0498512027381788
515	KC_AB2	prg	-2.26538931672255	0.547771004842275	0.988741847156811	pseudobulk	DESeq2	LRT	0.2614009599796
515	KC_AB2	prim	-3.09794865339079	0.39855598532175	0.988741847156811	pseudobulk	DESeq2	LRT	0.399510664459796
515	KC_AB2	PRL-1	-1.14765616172798	0.495130140235091	0.988741847156811	pseudobulk	DESeq2	LRT	0.305280635899647
516	KC_AB2	prod	0.513202572024843	0.807055953632252	0.988741847156811	pseudobulk	DESeq2	LRT	0.093096354359303
516	KC_AB2	prominin-like	-0.0441630166117905	0.955524232756763	0.988741847156811	pseudobulk	DESeq2	LRT	0.0197582944602581
516	KC_AB2	ProRS-m	-0.729234089517982	0.868684292414125	0.988741847156811	pseudobulk	DESeq2	LRT	0.0611380313470854
516	KC_AB2	pros	0.0884823068522782	0.719454836128903	0.988741847156811	pseudobulk	DESeq2	LRT	0.142996463764395
516	KC_AB2	Prosalpha2	-0.663273929909526	0.684336515129322	0.988741847156811	pseudobulk	DESeq2	LRT	0.164730286109088
516	KC_AB2	Prosalpha3	0.732912549131166	0.64451234525678	0.988741847156811	pseudobulk	DESeq2	LRT	0.190768759574273
516	KC_AB2	Prosalpha4	2.68449022421397	0.254379849598147	0.988741847156811	pseudobulk	DESeq2	LRT	0.594517293789866
516	KC_AB2	Prosalpha5	1.88145593350457	0.299839307201757	0.988741847156811	pseudobulk	DESeq2	LRT	0.523111434256836
516	KC_AB2	Prosalpha6	-0.75755554055223	0.732589794692809	0.988741847156811	pseudobulk	DESeq2	LRT	0.1351391355479
516	KC_AB2	Prosalpha7	0.317508825551005	0.883746467592857	0.988741847156811	pseudobulk	DESeq2	LRT	0.053672309102253
517	KC_AB2	Prosap	-0.19699937306426	0.634985220977314	0.988741847156811	pseudobulk	DESeq2	LRT	0.197236382617778
517	KC_AB2	Prosbeta1	0.194804214247169	0.909397535999427	0.988741847156811	pseudobulk	DESeq2	LRT	0.04124622684446
517	KC_AB2	Prosbeta2	-1.40296545307325	0.628223627794903	0.988741847156811	pseudobulk	DESeq2	LRT	0.201885733604539
517	KC_AB2	Prosbeta3	0.189249264265911	0.917387558178736	0.988741847156811	pseudobulk	DESeq2	LRT	0.0374471541658424
517	KC_AB2	Prosbeta4	-0.600987133452689	0.687737091287189	0.988741847156811	pseudobulk	DESeq2	LRT	0.162577552498229
517	KC_AB2	Prosbeta5	0.444811662881181	0.779564417627056	0.988741847156811	pseudobulk	DESeq2	LRT	0.108147992002967
517	KC_AB2	Prosbeta6	-0.333924372251237	0.839017298925653	0.988741847156811	pseudobulk	DESeq2	LRT	0.0762290847606876
517	KC_AB2	Prosbeta7	0.920073134400379	0.728211051768817	0.988741847156811	pseudobulk	DESeq2	LRT	0.137742734237847
517	KC_AB2	Prp18	-2.26538931672255	0.547771004842275	0.988741847156811	pseudobulk	DESeq2	LRT	0.2614009599796
517	KC_AB2	Prp19	2.06137138167525	0.547946929015274	0.988741847156811	pseudobulk	DESeq2	LRT	0.261261502741816
518	KC_AB2	Prp3	0.804708302235815	0.733338290589079	0.988741847156811	pseudobulk	DESeq2	LRT	0.13469563812263
518	KC_AB2	Prp31	1.91626857490363	0.477198801200827	0.988741847156811	pseudobulk	DESeq2	LRT	0.321300656017034
518	KC_AB2	Prp38	2.00515076565275	0.576456787459964	0.988741847156811	pseudobulk	DESeq2	LRT	0.239233242872451
518	KC_AB2	Prp8	-1.80933943727629	0.584587525761637	0.988741847156811	pseudobulk	DESeq2	LRT	0.233150456085471
518	KC_AB2	Prpk	1.42621218304824	0.66738831402044	0.988741847156811	pseudobulk	DESeq2	LRT	0.175621402117653
518	KC_AB2	prt	0.0832928328870661	0.830500234140013	0.988741847156811	pseudobulk	DESeq2	LRT	0.0806602407734521
518	KC_AB2	prtp	0.193852168735538	0.860365519649092	0.988741847156811	pseudobulk	DESeq2	LRT	0.0653170028942847
518	KC_AB2	Prx3	1.00489005324154	0.596692795516291	0.988741847156811	pseudobulk	DESeq2	LRT	0.224249205806662
518	KC_AB2	ps	-0.205366012821024	0.490791097212877	0.988741847156811	pseudobulk	DESeq2	LRT	0.309103323830911
518	KC_AB2	Psa	-0.487923111280115	0.783394690329388	0.988741847156811	pseudobulk	DESeq2	LRT	0.106019376327165
519	KC_AB2	Psc	0.666943671827768	0.808936122914367	0.988741847156811	pseudobulk	DESeq2	LRT	0.0920857707994393
519	KC_AB2	Psf3	-2.9721553030081	0.387854083811327	0.988741847156811	pseudobulk	DESeq2	LRT	0.411331631401987
519	KC_AB2	PsGEF	1.4233011112329	0.430228718890376	0.988741847156811	pseudobulk	DESeq2	LRT	0.366300602690251
519	KC_AB2	Psi	0.065363258845503	0.933026544246434	0.988741847156811	pseudobulk	DESeq2	LRT	0.0301060005667187
519	KC_AB2	psidin	-0.634227699706429	0.828294391400015	0.988741847156811	pseudobulk	DESeq2	LRT	0.0818152793537044
519	KC_AB2	PSMG1	2.00515076565275	0.576456787459964	0.988741847156811	pseudobulk	DESeq2	LRT	0.239233242872451
519	KC_AB2	Psn	0.362555753552128	0.897034383127386	0.988741847156811	pseudobulk	DESeq2	LRT	0.0471909102264623
519	KC_AB2	psq	0.409346832093472	0.463195970700105	0.988741847156811	pseudobulk	DESeq2	LRT	0.334235227152644
519	KC_AB2	PSR	2.80917294583258	0.367996267391162	0.988741847156811	pseudobulk	DESeq2	LRT	0.434156586379859
519	KC_AB2	Pten	-0.732725114995457	0.722759478418736	0.988741847156811	pseudobulk	DESeq2	LRT	0.141006204191428
520	KC_AB2	Ptip	-4.50981472838827	0.120912272850901	0.988741847156811	pseudobulk	DESeq2	LRT	0.917529615095309
520	KC_AB2	PTP-ER	0.405544140866722	0.787221473910918	0.988741847156811	pseudobulk	DESeq2	LRT	0.103903067684162
520	KC_AB2	Ptp10D	-2.51746470924027	0.517377929599049	0.988741847156811	pseudobulk	DESeq2	LRT	0.286192101436607
520	KC_AB2	Ptp36E	0.429050452978174	0.716006373116733	0.988741847156811	pseudobulk	DESeq2	LRT	0.14508311205372
520	KC_AB2	Ptp4E	0.310974079447882	0.852647373225612	0.988741847156811	pseudobulk	DESeq2	LRT	0.0692305415470647
520	KC_AB2	Ptp61F	0.518112575170181	0.804900582036886	0.988741847156811	pseudobulk	DESeq2	LRT	0.0942577585627823
520	KC_AB2	Ptp69D	-0.742148798960242	0.80644751100302	0.988741847156811	pseudobulk	DESeq2	LRT	0.0934238941443075

520	KC_AB2	Ptp99A	-0.168739742522672	0.70559807641503	0.988741847156811	pseudobulk	DESeq2	LRT	0.15144261183808
520	KC_AB2	Ptpa	-0.235620053326589	0.908937007481336	0.988741847156811	pseudobulk	DESeq2	LRT	0.0414662138640759
520	KC_AB2	Ptpmeg	-2.28854450052849	0.257498855448542	0.988741847156811	pseudobulk	DESeq2	LRT	0.589224697005265
521	KC_AB2	Ptpmeg2	-0.611469957658269	0.251243624780864	0.988741847156811	pseudobulk	DESeq2	LRT	0.599904949502276
521	KC_AB2	PTPMT1	-1.77083291708398	0.389176699865528	0.988741847156811	pseudobulk	DESeq2	LRT	0.409853168975988
521	KC_AB2	Pu	-1.40296545307325	0.628223627794903	0.988741847156811	pseudobulk	DESeq2	LRT	0.201885733604539
521	KC_AB2	puc	1.64971861411541	0.153092390460548	0.988741847156811	pseudobulk	DESeq2	LRT	0.815046395605627
521	KC_AB2	puf	-0.531490168490222	0.777932593987025	0.988741847156811	pseudobulk	DESeq2	LRT	0.109058031966236
521	KC_AB2	pum	0.29494123690724	0.321017934024704	0.988741847156811	pseudobulk	DESeq2	LRT	0.493470704572067
521	KC_AB2	Pur-alpha	0.166009218424147	0.757623706677219	0.988741847156811	pseudobulk	DESeq2	LRT	0.120546444355867
521	KC_AB2	Pus1	-0.378852672109618	0.920549594954422	0.988741847156811	pseudobulk	DESeq2	LRT	0.0359528087516892
521	KC_AB2	Pus7	-0.506613484034373	0.961658398065406	0.988741847156811	pseudobulk	DESeq2	LRT	0.0169791713942958
521	KC_AB2	put	-1.0372813493891	0.502890660088475	0.988741847156811	pseudobulk	DESeq2	LRT	0.298526430216623
522	KC_AB2	Pvf1	-0.506613484034373	0.961658398065406	0.988741847156811	pseudobulk	DESeq2	LRT	0.0169791713942958
522	KC_AB2	Pvf3	0.321244353479908	0.883160641080799	0.988741847156811	pseudobulk	DESeq2	LRT	0.053960293942671
522	KC_AB2	PVRAP	-0.0709049897174368	0.920619761416672	0.988741847156811	pseudobulk	DESeq2	LRT	0.0359197070630485
522	KC_AB2	px	-0.252251945818227	0.774007823316667	0.988741847156811	pseudobulk	DESeq2	LRT	0.111254649645159
522	KC_AB2	pxb	-0.495774118063222	0.586647371723876	0.988741847156811	pseudobulk	DESeq2	LRT	0.231622870698102
522	KC_AB2	Pxn	-0.00805983872314296	0.98514696693688	0.988741847156811	pseudobulk	DESeq2	LRT	0.00649897542265854
522	KC_AB2	pyd	2.37856478194333	0.0324913796789984	0.988741847156811	pseudobulk	DESeq2	LRT	1.48823184684972
522	KC_AB2	pygo	0.685691472970664	0.738333928211375	0.988741847156811	pseudobulk	DESeq2	LRT	0.13174717424796
522	KC_AB2	PyK	0.0764778564077434	0.893069184286714	0.988741847156811	pseudobulk	DESeq2	LRT	0.0491148958817686
522	KC_AB2	Pym	0.331758848923756	0.946375065527934	0.988741847156811	pseudobulk	DESeq2	LRT	0.0239367107367223
523	KC_AB2	Pyroxd1	-1.80294541848982	0.660135549041553	0.988741847156811	pseudobulk	DESeq2	LRT	0.180366879372519
523	KC_AB2	pzg	-1.40296545307325	0.628223627794903	0.988741847156811	pseudobulk	DESeq2	LRT	0.201885733604539
523	KC_AB2	Pzl	0.921695369118063	0.730077514579258	0.988741847156811	pseudobulk	DESeq2	LRT	0.136631027048226
523	KC_AB2	QIL1	1.3902798530695	0.40224297533855	0.988741847156811	pseudobulk	DESeq2	LRT	0.395511531562914
523	KC_AB2	qkr54B	-0.160571420196022	0.897478118257074	0.988741847156811	pseudobulk	DESeq2	LRT	0.0469761313134937
523	KC_AB2	qkr58E-1	0.0384281714520565	0.942252280053409	0.988741847156811	pseudobulk	DESeq2	LRT	0.0258328029752892
523	KC_AB2	qkr58E-2	-2.18835334603931	0.281596442673783	0.988741847156811	pseudobulk	DESeq2	LRT	0.550372835811548
523	KC_AB2	qkr58E-3	0.287789955629087	0.636142525811119	0.988741847156811	pseudobulk	DESeq2	LRT	0.196445571083142
523	KC_AB2	qless	0.922664372932389	0.678259639521954	0.988741847156811	pseudobulk	DESeq2	LRT	0.168604025267887
523	KC_AB2	qm	-0.0666123868776222	0.873431827895442	0.988741847156811	pseudobulk	DESeq2	LRT	0.0587709864156995
524	KC_AB2	Qsox1	-3.91108067132102	0.221683018369551	0.988741847156811	pseudobulk	DESeq2	LRT	0.654267573962038
524	KC_AB2	Qsox3	1.47543920254359	0.661929169513603	0.988741847156811	pseudobulk	DESeq2	LRT	0.17918848025087
524	KC_AB2	qtc	0.640837811683353	0.547686469255933	0.988741847156811	pseudobulk	DESeq2	LRT	0.261467988302677
524	KC_AB2	Qtzl	-0.00418280065493296	0.98378080203307	0.988741847156811	pseudobulk	DESeq2	LRT	0.00710165672327501
524	KC_AB2	qvr	0.112485782665613	0.847709096082216	0.988741847156811	pseudobulk	DESeq2	LRT	0.0717531567540759
524	KC_AB2	r2d2	0.997366942311027	0.663723613191462	0.988741847156811	pseudobulk	DESeq2	LRT	0.178012731256061
524	KC_AB2	Rab1	0.431572128533593	0.621380839284037	0.988741847156811	pseudobulk	DESeq2	LRT	0.2066421426509
524	KC_AB2	Rab10	2.34011937438372	0.357833688438857	0.988741847156811	pseudobulk	DESeq2	LRT	0.446318774963809
524	KC_AB2	Rab11	0.436190351820769	0.651627585029194	0.988741847156811	pseudobulk	DESeq2	LRT	0.186000539200259
524	KC_AB2	Rab14	0.117700558745492	0.888937738564818	0.988741847156811	pseudobulk	DESeq2	LRT	0.0511286560653884
525	KC_AB2	Rab18	-0.153630173095315	0.915895323748328	0.988741847156811	pseudobulk	DESeq2	LRT	0.0381541583367161
525	KC_AB2	Rab19	0.818790875285784	0.940355871560339	0.988741847156811	pseudobulk	DESeq2	LRT	0.0267077593721816
525	KC_AB2	Rab2	0.114663039695888	0.833860475928545	0.988741847156811	pseudobulk	DESeq2	LRT	0.078906610757314
525	KC_AB2	Rab21	-0.778776753336558	0.59370665539221	0.988741847156811	pseudobulk	DESeq2	LRT	0.226428082648961
525	KC_AB2	Rab23	0.102946774028289	0.927193086181459	0.988741847156811	pseudobulk	DESeq2	LRT	0.0328298154444129
525	KC_AB2	Rab26	-0.0271192106335428	0.920489423162114	0.988741847156811	pseudobulk	DESeq2	LRT	0.0359811973704178
525	KC_AB2	Rab27	0.114907601909538	0.819339203078554	0.988741847156811	pseudobulk	DESeq2	LRT	0.0865362648728539
525	KC_AB2	Rab3	0.412107734534392	0.508154119051871	0.988741847156811	pseudobulk	DESeq2	LRT	0.294004549718779
525	KC_AB2	Rab3-GAP	-1.80294541848982	0.660135549041553	0.988741847156811	pseudobulk	DESeq2	LRT	0.180366879372519
525	KC_AB2	Rab3-GEF	-0.569095701564549	0.397939339971369	0.988741847156811	pseudobulk	DESeq2	LRT	0.400183124719043
526	KC_AB2	Rab30	1.21185718506466	0.567710906692152	0.988741847156811	pseudobulk	DESeq2	LRT	0.245872762158739
526	KC_AB2	Rab35	0.377057232108292	0.707349695257209	0.988741847156811	pseudobulk	DESeq2	LRT	0.150365829238599
526	KC_AB2	Rab39	0.317508825551005	0.883746467592857	0.988741847156811	pseudobulk	DESeq2	LRT	0.053672309102253

526	KC_AB2	Rab4	0.559228737481376	0.601738870624396	0.988741847156811	pseudobulk	DESeq2	LRT	0.220591933410406
526	KC_AB2	Rab40	-1.08506241312707	0.67856296251147	0.988741847156811	pseudobulk	DESeq2	LRT	0.168409848810952
526	KC_AB2	Rab5	-0.243392792857476	0.719016269531248	0.988741847156811	pseudobulk	DESeq2	LRT	0.143261282513219
526	KC_AB2	Rab6	1.53981939596024	0.431292874271972	0.988741847156811	pseudobulk	DESeq2	LRT	0.365227717114718
526	KC_AB2	Rab7	0.387810490232537	0.750933825843026	0.988741847156811	pseudobulk	DESeq2	LRT	0.12439833241988
526	KC_AB2	Rab8	-0.393953564514768	0.770355756752861	0.988741847156811	pseudobulk	DESeq2	LRT	0.113308667666819
526	KC_AB2	Rab9	0.818790875285784	0.940355871560339	0.988741847156811	pseudobulk	DESeq2	LRT	0.0267077593721816
527	KC_AB2	Rabex-5	1.42621218304824	0.66738831402044	0.988741847156811	pseudobulk	DESeq2	LRT	0.175621402117653
527	KC_AB2	RabX1	0.928039350209165	0.67716747857253	0.988741847156811	pseudobulk	DESeq2	LRT	0.169303907349344
527	KC_AB2	RabX4	-0.63382886236539	0.641240826156846	0.988741847156811	pseudobulk	DESeq2	LRT	0.192978835023143
527	KC_AB2	RabX6	2.73593896747858	0.422277110754874	0.988741847156811	pseudobulk	DESeq2	LRT	0.374402458575713
527	KC_AB2	Rac1	0.00295835444183368	0.976833562314153	0.988741847156811	pseudobulk	DESeq2	LRT	0.0101794271984812
527	KC_AB2	Rack1	0.359443780452712	0.477011517969939	0.988741847156811	pseudobulk	DESeq2	LRT	0.321471134313356
527	KC_AB2	rad	0.0100924115317236	0.96994281596865	0.988741847156811	pseudobulk	DESeq2	LRT	0.0132538692815206
527	KC_AB2	Rad23	0.163248453410678	0.869480649630726	0.988741847156811	pseudobulk	DESeq2	LRT	0.0607400788160828
527	KC_AB2	rad50	0.818790875285784	0.940355871560339	0.988741847156811	pseudobulk	DESeq2	LRT	0.0267077593721816
527	KC_AB2	Rad60	-0.0144409071146711	0.984632974376923	0.988741847156811	pseudobulk	DESeq2	LRT	0.00672562423038896
528	KC_AB2	Rad9	-1.80294541848982	0.660135549041553	0.988741847156811	pseudobulk	DESeq2	LRT	0.180366879372519
528	KC_AB2	Rae1	3.06202454821851	0.30627034058063	0.988741847156811	pseudobulk	DESeq2	LRT	0.513895058517773
528	KC_AB2	Raf	-0.0384805229678164	0.985071128575604	0.988741847156811	pseudobulk	DESeq2	LRT	0.006532409469285
528	KC_AB2	RAF2	-1.80294541848982	0.660135549041553	0.988741847156811	pseudobulk	DESeq2	LRT	0.180366879372519
528	KC_AB2	RagA-B	-0.934020934207768	0.782359118258182	0.988741847156811	pseudobulk	DESeq2	LRT	0.106593851444836
528	KC_AB2	RagC-D	2.28068239784515	0.366179304841476	0.988741847156811	pseudobulk	DESeq2	LRT	0.436306204128051
528	KC_AB2	Rala	0.713780192757491	0.612761158965162	0.988741847156811	pseudobulk	DESeq2	LRT	0.212708771087119
528	KC_AB2	Ran	0.143043117315356	0.868204645919667	0.988741847156811	pseudobulk	DESeq2	LRT	0.06137789450299
528	KC_AB2	Ranbp16	-0.417865920819128	0.808720489563031	0.988741847156811	pseudobulk	DESeq2	LRT	0.0922015535612835
528	KC_AB2	Ranbp21	1.47543920254359	0.661929169513603	0.988741847156811	pseudobulk	DESeq2	LRT	0.17918848025087
529	KC_AB2	RanBP3	-0.405381063532158	0.89155634938256	0.988741847156811	pseudobulk	DESeq2	LRT	0.0498512027381788
529	KC_AB2	RanBPM	-0.594135042870286	0.639909285028113	0.988741847156811	pseudobulk	DESeq2	LRT	0.193881588210002
529	KC_AB2	ranshi	-0.506613484034373	0.961658398065406	0.988741847156811	pseudobulk	DESeq2	LRT	0.0169791713942958
529	KC_AB2	Rap1	0.364883579738484	0.476320376494157	0.988741847156811	pseudobulk	DESeq2	LRT	0.322100839421254
529	KC_AB2	Rap2l	-0.349402820837206	0.750588239310201	0.988741847156811	pseudobulk	DESeq2	LRT	0.124598244669402
529	KC_AB2	RapGAP1	-0.66186606850117	0.675702365632786	0.988741847156811	pseudobulk	DESeq2	LRT	0.170244560608151
529	KC_AB2	raptor	1.51342944320976	0.389648379825243	0.988741847156811	pseudobulk	DESeq2	LRT	0.409327125236218
529	KC_AB2	ras	-0.223716175604984	0.886080944903013	0.988741847156811	pseudobulk	DESeq2	LRT	0.0525266028093553
529	KC_AB2	Ras64B	1.64755990459092	0.573059482818602	0.988741847156811	pseudobulk	DESeq2	LRT	0.241800296498366
529	KC_AB2	Ras85D	1.15102549446658	0.491153502372127	0.988741847156811	pseudobulk	DESeq2	LRT	0.30878275469068
530	KC_AB2	RasGAP1	0.181724337371644	0.891229382321343	0.988741847156811	pseudobulk	DESeq2	LRT	0.0500105039817521
530	KC_AB2	raskol	-0.397548309212435	0.59498054059009	0.988741847156811	pseudobulk	DESeq2	LRT	0.225497238057239
530	KC_AB2	Rassf	-0.506613484034373	0.961658398065406	0.988741847156811	pseudobulk	DESeq2	LRT	0.0169791713942958
530	KC_AB2	RASSF8	0.31709673144345	0.825618921651911	0.988741847156811	pseudobulk	DESeq2	LRT	0.083220362380082
530	KC_AB2	Rat1	3.30033526000341	0.259137897354645	0.988741847156811	pseudobulk	DESeq2	LRT	0.586469069420752
530	KC_AB2	Ravus	3.41250446005175	0.270453901523293	0.988741847156811	pseudobulk	DESeq2	LRT	0.567906749116294
530	KC_AB2	raw	-0.433345196398904	0.645202334998363	0.988741847156811	pseudobulk	DESeq2	LRT	0.190304069561022
530	KC_AB2	rb	-0.401790037290404	0.801973866892007	0.988741847156811	pseudobulk	DESeq2	LRT	0.0958397833985189
530	KC_AB2	Rb97D	0.974871526006433	0.401758729644404	0.988741847156811	pseudobulk	DESeq2	LRT	0.396034677862181
530	KC_AB2	Rbbp5	-2.06919847526573	0.497641705310376	0.988741847156811	pseudobulk	DESeq2	LRT	0.303083230352651
531	KC_AB2	Rbcn-3A	-0.830408318689899	0.532054076756867	0.988741847156811	pseudobulk	DESeq2	LRT	0.274044224765904
531	KC_AB2	Rbcn-3B	-0.201771347089686	0.864005518767894	0.988741847156811	pseudobulk	DESeq2	LRT	0.0634834834901011
531	KC_AB2	Rbf	0.317508825551005	0.883746467592857	0.988741847156811	pseudobulk	DESeq2	LRT	0.053672309102253
531	KC_AB2	Rbfox1	0.23137244797338	0.473947855369507	0.988741847156811	pseudobulk	DESeq2	LRT	0.324269437589676
531	KC_AB2	Rbp1	0.361318996264378	0.69381993094676	0.988741847156811	pseudobulk	DESeq2	LRT	0.158753228599076
531	KC_AB2	Rbp1-like	0.0793453511338701	0.813614129311878	0.988741847156811	pseudobulk	DESeq2	LRT	0.0895815180143928
531	KC_AB2	Rbp6	6.41822309506568	0.228405495281841	0.988741847156811	pseudobulk	DESeq2	LRT	0.64129345146879
531	KC_AB2	Rbp9	0.313101261076357	0.712534931026472	0.988741847156811	pseudobulk	DESeq2	LRT	0.147193840119192
531	KC_AB2	Rbpn-5	-1.19564122961809	0.585865739229133	0.988741847156811	pseudobulk	DESeq2	LRT	0.232201898303775

531	KC_AB2	Rbsn-5	1.4413042803421	0.615311685084066	0.988741847156811	pseudobulk	DESeq2	LRT	0.210904837358999
532	KC_AB2	Rca1	2.7785831508444	0.373751317747552	0.988741847156811	pseudobulk	DESeq2	LRT	0.427417267458199
532	KC_AB2	Rcc1	-1.00727152105576	0.576116916781016	0.988741847156811	pseudobulk	DESeq2	LRT	0.239489372201384
532	KC_AB2	Rcd-1	0.814353701250753	0.537943810346474	0.988741847156811	pseudobulk	DESeq2	LRT	0.269263085171703
532	KC_AB2	Rcd1	-0.498853514795498	0.762821910206645	0.988741847156811	pseudobulk	DESeq2	LRT	0.117576841392698
532	KC_AB2	Rcd4	-0.0582963981256801	0.979058404810457	0.988741847156811	pseudobulk	DESeq2	LRT	0.0091913999942843
532	KC_AB2	Rcd5	-1.21442392643984	0.584026528107531	0.988741847156811	pseudobulk	DESeq2	LRT	0.233567425577577
532	KC_AB2	Rcp	-0.933299237375886	0.70112757174831	0.988741847156811	pseudobulk	DESeq2	LRT	0.154202953979679
532	KC_AB2	rdgA	-0.289313674457293	0.568944389264333	0.988741847156811	pseudobulk	DESeq2	LRT	0.244930181084581
532	KC_AB2	rdgB	0.333646667890861	0.485218476947528	0.988741847156811	pseudobulk	DESeq2	LRT	0.314062669710724
532	KC_AB2	rdgBbeta	1.15643052116892	0.392891159400947	0.988741847156811	pseudobulk	DESeq2	LRT	0.405727743309806
533	KC_AB2	rdgC	-1.33572246196862	0.479260031408656	0.988741847156811	pseudobulk	DESeq2	LRT	0.319428788121614
533	KC_AB2	Rdh	0.166484391730625	0.797786130199298	0.988741847156811	pseudobulk	DESeq2	LRT	0.0981135183273459
533	KC_AB2	Rdl	-0.160200729036098	0.475339288540233	0.988741847156811	pseudobulk	DESeq2	LRT	0.322996288185666
533	KC_AB2	rdog	0.96158523435066	0.743767989760743	0.988741847156811	pseudobulk	DESeq2	LRT	0.128562516713028
533	KC_AB2	rdx	0.535865124770847	0.441194636161841	0.988741847156811	pseudobulk	DESeq2	LRT	0.35536977614906
533	KC_AB2	red	0.924140746192761	0.675445049022325	0.988741847156811	pseudobulk	DESeq2	LRT	0.170409977326066
533	KC_AB2	ReepA	-1.02773720444812	0.469395739888933	0.988741847156811	pseudobulk	DESeq2	LRT	0.328460856260912
533	KC_AB2	ReepB	0.398395823592206	0.676469750088357	0.988741847156811	pseudobulk	DESeq2	LRT	0.169751619115781
533	KC_AB2	ref(2)P	-0.35816879836274	0.738025807430493	0.988741847156811	pseudobulk	DESeq2	LRT	0.131928451417215
533	KC_AB2	Ref1	-0.31806380044257	0.756800579216224	0.988741847156811	pseudobulk	DESeq2	LRT	0.121018544219486
534	KC_AB2	REG	0.0728471578244417	0.935122783687239	0.988741847156811	pseudobulk	DESeq2	LRT	0.0291313615586215
534	KC_AB2	regucalcin	0.818790875285784	0.940355871560339	0.988741847156811	pseudobulk	DESeq2	LRT	0.0267077593721816
534	KC_AB2	Rep	-1.80933943727629	0.584587525761637	0.988741847156811	pseudobulk	DESeq2	LRT	0.233150456085471
534	KC_AB2	Reph	-0.215898191792183	0.745166173469222	0.988741847156811	pseudobulk	DESeq2	LRT	0.127746867958689
534	KC_AB2	Reps	-0.246893968726718	0.883832168964877	0.988741847156811	pseudobulk	DESeq2	LRT	0.0536301954081643
534	KC_AB2	rept	1.47543920254359	0.661929169513603	0.988741847156811	pseudobulk	DESeq2	LRT	0.17918848025087
534	KC_AB2	REPTOR	-0.606608323676834	0.513012630806144	0.988741847156811	pseudobulk	DESeq2	LRT	0.289871942058007
534	KC_AB2	REPTOR-BP	0.191062258256834	0.81883150902371	0.988741847156811	pseudobulk	DESeq2	LRT	0.0868054538309328
534	KC_AB2	retm	1.03379725121881	0.507318132290641	0.988741847156811	pseudobulk	DESeq2	LRT	0.29471961508642
534	KC_AB2	retn	3.4439122560021	0.227129406551316	0.988741847156811	pseudobulk	DESeq2	LRT	0.643726633828736
535	KC_AB2	Rev1	2.80917294583258	0.367996267391162	0.988741847156811	pseudobulk	DESeq2	LRT	0.434156586379859
535	KC_AB2	Rev7	-1.80294541848982	0.660135549041553	0.988741847156811	pseudobulk	DESeq2	LRT	0.180366879372519
535	KC_AB2	Rexo5	-1.28585361841952	0.604452679793172	0.988741847156811	pseudobulk	DESeq2	LRT	0.218637692667744
535	KC_AB2	RfC3	-0.506613484034373	0.961658398065406	0.988741847156811	pseudobulk	DESeq2	LRT	0.0169791713942958
535	KC_AB2	RfC38	0.712171366772551	0.813205723536451	0.988741847156811	pseudobulk	DESeq2	LRT	0.0897995733547685
535	KC_AB2	RfC4	0.353798809751289	0.901648351639924	0.988741847156811	pseudobulk	DESeq2	LRT	0.0449628069360408
535	KC_AB2	RFeSP	0.726125737030048	0.311498518696735	0.988741847156811	pseudobulk	DESeq2	LRT	0.506544014248355
535	KC_AB2	rg	-1.65719613210719	0.599376091584556	0.988741847156811	pseudobulk	DESeq2	LRT	0.222300584546838
535	KC_AB2	Rga	0.284871170148432	0.783154898843832	0.988741847156811	pseudobulk	DESeq2	LRT	0.106152331096701
535	KC_AB2	Rgk1	-0.469373993892207	0.148082427155697	0.988741847156811	pseudobulk	DESeq2	LRT	0.829496475861803
536	KC_AB2	Rgk2	-0.759099626774255	0.432111686713932	0.988741847156811	pseudobulk	DESeq2	LRT	0.364403987781024
536	KC_AB2	Rgk3	-0.576677307669258	0.260695978970925	0.988741847156811	pseudobulk	DESeq2	LRT	0.583865667428551
536	KC_AB2	Rgl	-0.0538044450628395	0.947637419287506	0.988741847156811	pseudobulk	DESeq2	LRT	0.0233577986571391
536	KC_AB2	rgn	-0.0311832380517303	0.956875118121839	0.988741847156811	pseudobulk	DESeq2	LRT	0.0191447383460009
536	KC_AB2	Rh7	2.06137138167525	0.547946929015274	0.988741847156811	pseudobulk	DESeq2	LRT	0.261261502741816
536	KC_AB2	rhea	-0.946233664102057	0.407311813486863	0.988741847156811	pseudobulk	DESeq2	LRT	0.390072993651409
536	KC_AB2	Rheb	-0.121120420743075	0.958079731976279	0.988741847156811	pseudobulk	DESeq2	LRT	0.0185983471673402
536	KC_AB2	rho	0.800670795147795	0.640193342527983	0.988741847156811	pseudobulk	DESeq2	LRT	0.193688846465541
536	KC_AB2	rho-4	1.47543920254359	0.661929169513603	0.988741847156811	pseudobulk	DESeq2	LRT	0.17918848025087
536	KC_AB2	rho-5	0.527971538873239	0.636557145528627	0.988741847156811	pseudobulk	DESeq2	LRT	0.196162602420814
537	KC_AB2	rho-7	-0.506613484034373	0.961658398065406	0.988741847156811	pseudobulk	DESeq2	LRT	0.0169791713942958
537	KC_AB2	Rho1	0.16901165128362	0.846680102810139	0.988741847156811	pseudobulk	DESeq2	LRT	0.0722806461351566
537	KC_AB2	RhoBTB	-0.287387311603126	0.823593053147202	0.988741847156811	pseudobulk	DESeq2	LRT	0.0842873252309858
537	KC_AB2	RhoGAP100F	-0.241662367881248	0.726590757130385	0.988741847156811	pseudobulk	DESeq2	LRT	0.138710131043635
537	KC_AB2	RhoGAP102A	1.08299336710066	0.62245466963101	0.988741847156811	pseudobulk	DESeq2	LRT	0.20589227065141

537	KC_AB2	RhoGAP16F	-0.331004884396198	0.894406551228697	0.988741847156811	pseudobulk	DESeq2	LRT	0.0484650283842476
537	KC_AB2	RhoGAP19D	0.175933859366015	0.811091860942729	0.988741847156811	pseudobulk	DESeq2	LRT	0.0909299565887339
537	KC_AB2	RhoGAP1A	0.972726581225976	0.61661584324292	0.988741847156811	pseudobulk	DESeq2	LRT	0.20998532076329
537	KC_AB2	RhoGAP5A	0.157562008957688	0.950463298513166	0.988741847156811	pseudobulk	DESeq2	LRT	0.0220646484576093
537	KC_AB2	RhoGAP68F	0.877544775091925	0.56010090332143	0.988741847156811	pseudobulk	DESeq2	LRT	0.251733726907771
538	KC_AB2	RhoGAP71E	-0.0218918597500868	0.970708531126026	0.988741847156811	pseudobulk	DESeq2	LRT	0.0129111535378474
538	KC_AB2	RhoGAP92B	1.48741827283584	0.532545672123726	0.988741847156811	pseudobulk	DESeq2	LRT	0.273643140376104
538	KC_AB2	RhoGAP93B	-0.102371542943037	0.89142806433609	0.988741847156811	pseudobulk	DESeq2	LRT	0.0499136973819458
538	KC_AB2	RhoGAPp190	0.27302641250699	0.620768348629958	0.988741847156811	pseudobulk	DESeq2	LRT	0.207070434735991
538	KC_AB2	RhoGDI	0.745329750333927	0.234782735314289	0.988741847156811	pseudobulk	DESeq2	LRT	0.629333841978979
538	KC_AB2	RhoGEF2	-2.51746470924027	0.517377929599049	0.988741847156811	pseudobulk	DESeq2	LRT	0.286192101436607
538	KC_AB2	RhoGEF3	-0.0963030662898338	0.907605298163269	0.988741847156811	pseudobulk	DESeq2	LRT	0.0421029775773661
538	KC_AB2	RhoU	-0.233272055930639	0.937531793764924	0.988741847156811	pseudobulk	DESeq2	LRT	0.028013995469539
538	KC_AB2	Rhp	1.85196256914229	0.547781346847132	0.988741847156811	pseudobulk	DESeq2	LRT	0.261392760507189
538	KC_AB2	Ric	0.122497925037846	0.767546408467322	0.988741847156811	pseudobulk	DESeq2	LRT	0.114895356134892
539	KC_AB2	RIC-3	-0.483900374810182	0.637220252122566	0.988741847156811	pseudobulk	DESeq2	LRT	0.195710429912168
539	KC_AB2	ric8a	2.28068239784515	0.366179304841476	0.988741847156811	pseudobulk	DESeq2	LRT	0.436306204128051
539	KC_AB2	Rich	0.818790875285784	0.940355871560339	0.988741847156811	pseudobulk	DESeq2	LRT	0.0267077593721816
539	KC_AB2	rictor	1.32714918892411	0.592713712963247	0.988741847156811	pseudobulk	DESeq2	LRT	0.227155024852469
539	KC_AB2	Rif1	-1.23536834900699	0.725570392794057	0.988741847156811	pseudobulk	DESeq2	LRT	0.139320447155934
539	KC_AB2	Rift	0.00266298162891975	0.968632759616684	0.988741847156811	pseudobulk	DESeq2	LRT	0.0138408469969281
539	KC_AB2	rig	-1.80294541848982	0.660135549041553	0.988741847156811	pseudobulk	DESeq2	LRT	0.180366879372519
539	KC_AB2	Rilpl	1.1669370038017	0.601782723498991	0.988741847156811	pseudobulk	DESeq2	LRT	0.220560284520093
539	KC_AB2	Rim	0.0515468238756395	0.867377024606995	0.988741847156811	pseudobulk	DESeq2	LRT	0.0617920857825439
539	KC_AB2	Rim2	0.572383115294938	0.737977345694019	0.988741847156811	pseudobulk	DESeq2	LRT	0.131956969872202
540	KC_AB2	rin	0.0765897459553081	0.906530665296926	0.988741847156811	pseudobulk	DESeq2	LRT	0.0426175004002567
540	KC_AB2	ringer	0.0851951012680757	0.891118757047173	0.988741847156811	pseudobulk	DESeq2	LRT	0.0500644148256907
540	KC_AB2	Rint1	2.51243226103394	0.474458247157471	0.988741847156811	pseudobulk	DESeq2	LRT	0.323801999942597
540	KC_AB2	RIOK1	-0.296576489727943	0.896291428147039	0.988741847156811	pseudobulk	DESeq2	LRT	0.047550757014702
540	KC_AB2	RIOK2	0.760546103616116	0.738305064567484	0.988741847156811	pseudobulk	DESeq2	LRT	0.131764152427911
540	KC_AB2	Rip11	-0.448374585768327	0.814227572251762	0.988741847156811	pseudobulk	DESeq2	LRT	0.0892541951597077
540	KC_AB2	Ripalpha	0.575200883059258	0.804213580533116	0.988741847156811	pseudobulk	DESeq2	LRT	0.0946285973599483
540	KC_AB2	rk	1.14335059045916	0.427971878246465	0.988741847156811	pseudobulk	DESeq2	LRT	0.368584767256994
540	KC_AB2	rl	-0.184836693412434	0.740562238520315	0.988741847156811	pseudobulk	DESeq2	LRT	0.130438436521465
540	KC_AB2	Rlb1	-1.80294541848982	0.660135549041553	0.988741847156811	pseudobulk	DESeq2	LRT	0.180366879372519
541	KC_AB2	Rlip	-0.28891603285919	0.742658026465738	0.988741847156811	pseudobulk	DESeq2	LRT	0.129211120839264
541	KC_AB2	RluA-2	1.64209625885217	0.483203675195701	0.988741847156811	pseudobulk	DESeq2	LRT	0.315869771176778
541	KC_AB2	Rm62	0.0590996498086188	0.9062071164567	0.988741847156811	pseudobulk	DESeq2	LRT	0.0427725316230213
541	KC_AB2	Rme-8	0.919353707608246	0.746278333243311	0.988741847156811	pseudobulk	DESeq2	LRT	0.127099167101924
541	KC_AB2	rn	0.01640586526624	0.94220885598526	0.988741847156811	pseudobulk	DESeq2	LRT	0.0258528180690701
541	KC_AB2	RN-tre	0.329343227560874	0.812041007439155	0.988741847156811	pseudobulk	DESeq2	LRT	0.0904220386715629
541	KC_AB2	RNASEK	0.196618587066385	0.680745228607194	0.988741847156811	pseudobulk	DESeq2	LRT	0.167015393978932
541	KC_AB2	RNaseMRP:RNA	2.00515076565275	0.576456787459964	0.988741847156811	pseudobulk	DESeq2	LRT	0.239233242872451
541	KC_AB2	RNaseP:RNA	0.818790875285784	0.940355871560339	0.988741847156811	pseudobulk	DESeq2	LRT	0.0267077593721816
541	KC_AB2	RNaseX25	0.834352593082403	0.675685094045283	0.988741847156811	pseudobulk	DESeq2	LRT	0.170255661725145
542	KC_AB2	RNaseZ	-0.506613484034373	0.961658398065406	0.988741847156811	pseudobulk	DESeq2	LRT	0.0169791713942958
542	KC_AB2	Rnf146	-1.40296545307325	0.628223627794903	0.988741847156811	pseudobulk	DESeq2	LRT	0.201885733604539
542	KC_AB2	rngo	-0.0582963981256801	0.979058404810457	0.988741847156811	pseudobulk	DESeq2	LRT	0.0091913999942843
542	KC_AB2	rnh1	0.351722119397094	0.929615296941389	0.988741847156811	pseudobulk	DESeq2	LRT	0.0316967385224016
542	KC_AB2	Rnmt	-2.9721553030081	0.387854083811327	0.988741847156811	pseudobulk	DESeq2	LRT	0.411331631401987
542	KC_AB2	rno	-0.0277687440024355	0.952356987833531	0.988741847156811	pseudobulk	DESeq2	LRT	0.0212002272616722
542	KC_AB2	Rnp4F	-1.18550161118176	0.488418921384383	0.988741847156811	pseudobulk	DESeq2	LRT	0.311207519801576
542	KC_AB2	RnpS1	1.1114511822041	0.470185741718989	0.988741847156811	pseudobulk	DESeq2	LRT	0.32773054489771
542	KC_AB2	robl	-0.189869899717698	0.844764422521601	0.988741847156811	pseudobulk	DESeq2	LRT	0.0732643848527676
542	KC_AB2	robo1	0.53270467085558	0.676578379675744	0.988741847156811	pseudobulk	DESeq2	LRT	0.169681884374736
543	KC_AB2	robo2	-1.80294541848982	0.660135549041553	0.988741847156811	pseudobulk	DESeq2	LRT	0.180366879372519

543	KC_AB2	Roc1a	-0.0561623711991846	0.938040382370393	0.988741847156811	pseudobulk	DESeq2	LRT	0.0277784649651544
543	KC_AB2	Roc2	-0.0612237599720558	0.953024964971941	0.988741847156811	pseudobulk	DESeq2	LRT	0.020895722648614
543	KC_AB2	Roe1	-0.34786399421262	0.816361540357585	0.988741847156811	pseudobulk	DESeq2	LRT	0.0881174635393994
543	KC_AB2	rogdi	-0.0295761453730973	0.975489763368292	0.988741847156811	pseudobulk	DESeq2	LRT	0.0107772836615597
543	KC_AB2	roh	0.531517984139812	0.357686338400915	0.988741847156811	pseudobulk	DESeq2	LRT	0.446497647131996
543	KC_AB2	Rok	-0.42076958521121	0.784050289861592	0.988741847156811	pseudobulk	DESeq2	LRT	0.105656080288865
543	KC_AB2	Rop	0.0117083433540684	0.94711531462556	0.988741847156811	pseudobulk	DESeq2	LRT	0.0235971408946138
543	KC_AB2	roq	0.572804916048311	0.73434626003257	0.988741847156811	pseudobulk	DESeq2	LRT	0.134099112614647
543	KC_AB2	Ror	1.40305140665399	0.649741560632143	0.988741847156811	pseudobulk	DESeq2	LRT	0.187259352757606
544	KC_AB2	row	0.997362153285053	0.543624053434592	0.988741847156811	pseudobulk	DESeq2	LRT	0.264701335525258
544	KC_AB2	Rox8	0.340858519285158	0.728347730572308	0.988741847156811	pseudobulk	DESeq2	LRT	0.13766122863568
544	KC_AB2	RpA-70	1.67796394544623	0.574362267760116	0.988741847156811	pseudobulk	DESeq2	LRT	0.240814098434665
544	KC_AB2	RPA2	0.0601466527378941	0.974810960364079	0.988741847156811	pseudobulk	DESeq2	LRT	0.0110795964355292
544	KC_AB2	RPA3	-0.0323203545809474	0.954448156132187	0.988741847156811	pseudobulk	DESeq2	LRT	0.020247656706879
544	KC_AB2	Rpb10	0.121160718490023	0.927926435766036	0.988741847156811	pseudobulk	DESeq2	LRT	0.032486452452746
544	KC_AB2	Rpb11	-1.3460975324975	0.494732871429856	0.988741847156811	pseudobulk	DESeq2	LRT	0.30562923294222
544	KC_AB2	Rpb12	0.0111292401420289	0.98080996596634	0.988741847156811	pseudobulk	DESeq2	LRT	0.00841512995637057
544	KC_AB2	Rpb5	-2.64843360157809	0.321943703667444	0.988741847156811	pseudobulk	DESeq2	LRT	0.492220064093287
544	KC_AB2	Rpb7	-2.41084114734092	0.305622632322907	0.988741847156811	pseudobulk	DESeq2	LRT	0.514814488024998
545	KC_AB2	Rpb8	-0.0299086096223997	0.954925398411684	0.988741847156811	pseudobulk	DESeq2	LRT	0.0200305554567448
545	KC_AB2	Rph	-1.60581231992925	0.486998362385013	0.988741847156811	pseudobulk	DESeq2	LRT	0.312472499172118
545	KC_AB2	RpI12	-2.26538931672255	0.547771004842275	0.988741847156811	pseudobulk	DESeq2	LRT	0.2614009599796
545	KC_AB2	RpII140	1.31950274499659	0.590522087411301	0.988741847156811	pseudobulk	DESeq2	LRT	0.228763853746569
545	KC_AB2	RpII15	-0.233272055930639	0.937531793764924	0.988741847156811	pseudobulk	DESeq2	LRT	0.028013995469539
545	KC_AB2	RpII18	-0.203229921684894	0.878271712262333	0.988741847156811	pseudobulk	DESeq2	LRT	0.056371104958466
545	KC_AB2	RpII215	-0.256981645817585	0.839788440609583	0.988741847156811	pseudobulk	DESeq2	LRT	0.0758301075666461
545	KC_AB2	RpII33	1.47543920254359	0.661929169513603	0.988741847156811	pseudobulk	DESeq2	LRT	0.17918848025087
545	KC_AB2	RpIIIC53	1.89586497481491	0.514180746077939	0.988741847156811	pseudobulk	DESeq2	LRT	0.288884189903704
545	KC_AB2	RpL10	-0.138534927345285	0.760390576489494	0.988741847156811	pseudobulk	DESeq2	LRT	0.118963273979099
546	KC_AB2	RpL10Ab	0.370786330546959	0.446895168010778	0.988741847156811	pseudobulk	DESeq2	LRT	0.349794341060385
546	KC_AB2	RpL11	0.46570214673628	0.507571896605429	0.988741847156811	pseudobulk	DESeq2	LRT	0.29450243206013
546	KC_AB2	RpL12	0.0878282493303123	0.853722877045192	0.988741847156811	pseudobulk	DESeq2	LRT	0.0686830807179899
546	KC_AB2	RpL13	-0.251406638484815	0.590428507135667	0.988741847156811	pseudobulk	DESeq2	LRT	0.228832682023714
546	KC_AB2	RpL13A	-0.173544369454388	0.738139736361047	0.988741847156811	pseudobulk	DESeq2	LRT	0.131861414618835
546	KC_AB2	RpL14	0.0631721359793903	0.868396507788884	0.988741847156811	pseudobulk	DESeq2	LRT	0.06128193170664
546	KC_AB2	RpL15	0.108338652086731	0.750978606793093	0.988741847156811	pseudobulk	DESeq2	LRT	0.124372434612403
546	KC_AB2	RpL17	0.146434791865208	0.775269513819618	0.988741847156811	pseudobulk	DESeq2	LRT	0.110547293600763
546	KC_AB2	RpL18	-0.00651390973899303	0.961787897212926	0.988741847156811	pseudobulk	DESeq2	LRT	0.0169206922307213
546	KC_AB2	RpL18A	0.126885782375657	0.774953976589318	0.988741847156811	pseudobulk	DESeq2	LRT	0.110724088857311
547	KC_AB2	RpL19	0.208734407088622	0.639887002267579	0.988741847156811	pseudobulk	DESeq2	LRT	0.193896711366771
547	KC_AB2	RpL21	0.0747987949544468	0.840580384251345	0.988741847156811	pseudobulk	DESeq2	LRT	0.0754207488901796
547	KC_AB2	RpL22	-0.565856135493767	0.50224479562201	0.988741847156811	pseudobulk	DESeq2	LRT	0.29908455481735
547	KC_AB2	RpL23	0.356616993200576	0.454488219746961	0.988741847156811	pseudobulk	DESeq2	LRT	0.342477369131109
547	KC_AB2	RpL23A	-0.157291692094727	0.778167203440757	0.988741847156811	pseudobulk	DESeq2	LRT	0.108927076879552
547	KC_AB2	RpL24	-0.0246760151714607	0.951549174003537	0.988741847156811	pseudobulk	DESeq2	LRT	0.0215687633973878
547	KC_AB2	RpL24-like	0.135750541620777	0.828556427223537	0.988741847156811	pseudobulk	DESeq2	LRT	0.0816779094566849
547	KC_AB2	RpL26	0.130073014112557	0.770646860455375	0.988741847156811	pseudobulk	DESeq2	LRT	0.113144586526715
547	KC_AB2	RpL27	0.0737573628606265	0.877008710935677	0.988741847156811	pseudobulk	DESeq2	LRT	0.0569960929594911
547	KC_AB2	RpL27A	0.0806973758664444	0.832391982477549	0.988741847156811	pseudobulk	DESeq2	LRT	0.0796721115244758
548	KC_AB2	RpL28	-0.214118291248533	0.623761360733939	0.988741847156811	pseudobulk	DESeq2	LRT	0.204981531373959
548	KC_AB2	RpL29	-0.228745402369745	0.61463119119454	0.988741847156811	pseudobulk	DESeq2	LRT	0.211385404021151
548	KC_AB2	RpL3	-0.26342086145082	0.666426639554449	0.988741847156811	pseudobulk	DESeq2	LRT	0.176247650886596
548	KC_AB2	RpL30	0.424718432423446	0.648584761641781	0.988741847156811	pseudobulk	DESeq2	LRT	0.188033259197783
548	KC_AB2	RpL31	-0.102526731433437	0.81656155132559	0.988741847156811	pseudobulk	DESeq2	LRT	0.0880110731519995
548	KC_AB2	RpL32	0.272620704190344	0.553259897910877	0.988741847156811	pseudobulk	DESeq2	LRT	0.257070807731252
548	KC_AB2	RpL34a	0.607364021926361	0.610290605225967	0.988741847156811	pseudobulk	DESeq2	LRT	0.214463315493064

548	KC_AB2	RpL34b	0.123820233869728	0.80539149498905	0.988741847156811	pseudobulk	DESeq2	LRT	0.0939929608954943
548	KC_AB2	RpL35A	-0.138139798546257	0.804057300796257	0.988741847156811	pseudobulk	DESeq2	LRT	0.0947130003400091
548	KC_AB2	RpL36	0.101643722018002	0.782288819572043	0.988741847156811	pseudobulk	DESeq2	LRT	0.106632876622012
549	KC_AB2	RpL36A	0.136645906402219	0.746773253392326	0.988741847156811	pseudobulk	DESeq2	LRT	0.12681124523841
549	KC_AB2	RpL37a	0.0475583239286335	0.903514446952064	0.988741847156811	pseudobulk	DESeq2	LRT	0.0440648987960212
549	KC_AB2	RpL37A	0.449449126141243	0.346783603471946	0.988741847156811	pseudobulk	DESeq2	LRT	0.459941444904012
549	KC_AB2	RpL38	-0.0403140999428091	0.905431219433948	0.988741847156811	pseudobulk	DESeq2	LRT	0.043144535029478
549	KC_AB2	RpL39	-0.361981276166052	0.503171518121286	0.988741847156811	pseudobulk	DESeq2	LRT	0.29828394998099
549	KC_AB2	RpL4	0.0142024382755356	0.93052776997439	0.988741847156811	pseudobulk	DESeq2	LRT	0.0312706615803088
549	KC_AB2	RpL40	-0.129829738648453	0.781514652961401	0.988741847156811	pseudobulk	DESeq2	LRT	0.107062874790512
549	KC_AB2	RpL41	-0.255062261221302	0.434785833854611	0.988741847156811	pseudobulk	DESeq2	LRT	0.361724614491441
549	KC_AB2	RpL5	-0.409867819798997	0.366764625683764	0.988741847156811	pseudobulk	DESeq2	LRT	0.435612558528707
549	KC_AB2	RpL6	0.0457799108504445	0.907181575076328	0.988741847156811	pseudobulk	DESeq2	LRT	0.042305778914064
550	KC_AB2	RpL7	-0.00553582806003975	0.932629887874218	0.988741847156811	pseudobulk	DESeq2	LRT	0.0302906708771734
550	KC_AB2	RpL7A	0.107187476627238	0.825614762391745	0.988741847156811	pseudobulk	DESeq2	LRT	0.0832225502518006
550	KC_AB2	RpL8	0.209517301466379	0.614406171987699	0.988741847156811	pseudobulk	DESeq2	LRT	0.211544430270267
550	KC_AB2	RpL9	-0.041930551395772	0.904039315785069	0.988741847156811	pseudobulk	DESeq2	LRT	0.0438126820719573
550	KC_AB2	RpLP0	0.117858228978864	0.817847462050772	0.988741847156811	pseudobulk	DESeq2	LRT	0.0873276896869935
550	KC_AB2	RpLP0-like	-0.506613484034373	0.961658398065406	0.988741847156811	pseudobulk	DESeq2	LRT	0.0169791713942958
550	KC_AB2	RpLP1	-0.0768320868854945	0.862496509237434	0.988741847156811	pseudobulk	DESeq2	LRT	0.0642426539613407
550	KC_AB2	RpLP2	0.0579524784569482	0.893329049420723	0.988741847156811	pseudobulk	DESeq2	LRT	0.0489885433222937
550	KC_AB2	Rpn1	0.11213458976865	0.938112983749562	0.988741847156811	pseudobulk	DESeq2	LRT	0.0277448532370976
550	KC_AB2	Rpn10	-0.217915120660429	0.892149991826569	0.988741847156811	pseudobulk	DESeq2	LRT	0.0495621241592452
551	KC_AB2	Rpn11	1.35316200662963	0.684467033464499	0.988741847156811	pseudobulk	DESeq2	LRT	0.164647464300239
551	KC_AB2	Rpn12	-0.600987133452689	0.687737091287189	0.988741847156811	pseudobulk	DESeq2	LRT	0.162577552498229
551	KC_AB2	Rpn13	-0.78830405600437	0.624658564378423	0.988741847156811	pseudobulk	DESeq2	LRT	0.204357301255249
551	KC_AB2	Rpn13R	0.818790875285784	0.940355871560339	0.988741847156811	pseudobulk	DESeq2	LRT	0.0267077593721816
551	KC_AB2	Rpn2	-0.339749823279958	0.807568597460787	0.988741847156811	pseudobulk	DESeq2	LRT	0.092820577066997
551	KC_AB2	Rpn3	-1.14834607806119	0.546930865791775	0.988741847156811	pseudobulk	DESeq2	LRT	0.26206756672448
551	KC_AB2	Rpn5	-0.809643441341291	0.611273019854505	0.988741847156811	pseudobulk	DESeq2	LRT	0.213764772518568
551	KC_AB2	Rpn6	0.833854176015168	0.642251910343238	0.988741847156811	pseudobulk	DESeq2	LRT	0.192294595274473
551	KC_AB2	Rpn7	0.924140746192761	0.675445049022325	0.988741847156811	pseudobulk	DESeq2	LRT	0.170409977326066
551	KC_AB2	Rpn8	0.607898459197294	0.69691767241217	0.988741847156811	pseudobulk	DESeq2	LRT	0.156818522517303
552	KC_AB2	Rpn9	-0.555828083223045	0.769829010190795	0.988741847156811	pseudobulk	DESeq2	LRT	0.113605727005537
552	KC_AB2	Rpp30	-1.80294541848982	0.660135549041553	0.988741847156811	pseudobulk	DESeq2	LRT	0.180366879372519
552	KC_AB2	rpr	-1.80294541848982	0.660135549041553	0.988741847156811	pseudobulk	DESeq2	LRT	0.180366879372519
552	KC_AB2	RpS10b	-0.0961645348822374	0.811304659443325	0.988741847156811	pseudobulk	DESeq2	LRT	0.0908160297971826
552	KC_AB2	RpS11	-0.021186866703666	0.928816502058384	0.988741847156811	pseudobulk	DESeq2	LRT	0.032070077195806
552	KC_AB2	RpS12	-0.0846673075170059	0.870238147521224	0.988741847156811	pseudobulk	DESeq2	LRT	0.0603618830119967
552	KC_AB2	RpS13	-0.0277772672529554	0.923072038821987	0.988741847156811	pseudobulk	DESeq2	LRT	0.0347644042373389
552	KC_AB2	RpS14a	-0.0930612032346743	0.829132358921908	0.988741847156811	pseudobulk	DESeq2	LRT	0.0813761351179671
552	KC_AB2	RpS14b	-0.732987772874374	0.41185544298868	0.988741847156811	pseudobulk	DESeq2	LRT	0.385255190104412
552	KC_AB2	RpS15	0.38626760799332	0.413664471347995	0.988741847156811	pseudobulk	DESeq2	LRT	0.383351778017021
553	KC_AB2	RpS15Aa	0.415997593062672	0.421426737145421	0.988741847156811	pseudobulk	DESeq2	LRT	0.3752779143227
553	KC_AB2	RpS15Ab	0.331758848923756	0.946375065527934	0.988741847156811	pseudobulk	DESeq2	LRT	0.0239367107367223
553	KC_AB2	RpS16	-0.185164114275597	0.706662315982385	0.988741847156811	pseudobulk	DESeq2	LRT	0.150788067589013
553	KC_AB2	RpS17	0.0147400983532106	0.936860059257613	0.988741847156811	pseudobulk	DESeq2	LRT	0.0283252757408954
553	KC_AB2	RpS18	0.1235960996517	0.739196524621785	0.988741847156811	pseudobulk	DESeq2	LRT	0.131240083645538
553	KC_AB2	RpS19a	0.150644215646082	0.801004814188077	0.988741847156811	pseudobulk	DESeq2	LRT	0.0963648737172186
553	KC_AB2	RpS2	0.150827429129534	0.742922345260851	0.988741847156811	pseudobulk	DESeq2	LRT	0.129056578954386
553	KC_AB2	RpS20	-0.0391849553087487	0.912230626235095	0.988741847156811	pseudobulk	DESeq2	LRT	0.039895351321536
553	KC_AB2	RpS21	-0.0314627041657357	0.917883436142702	0.988741847156811	pseudobulk	DESeq2	LRT	0.0372124672238541
553	KC_AB2	RpS23	0.0240465640826523	0.917458712909129	0.988741847156811	pseudobulk	DESeq2	LRT	0.0374134705736272
554	KC_AB2	RpS24	-0.176632664247949	0.700024271627725	0.988741847156811	pseudobulk	DESeq2	LRT	0.154886901626824
554	KC_AB2	RpS25	-0.00042669436348796	0.934415284282232	0.988741847156811	pseudobulk	DESeq2	LRT	0.0294600663948342
554	KC_AB2	RpS26	-0.266569521189121	0.518856258898454	0.988741847156811	pseudobulk	DESeq2	LRT	0.284952940058093

554	KC_AB2	RpS27	-0.129806004413115	0.734553323213673	0.988741847156811	pseudobulk	DESeq2	LRT	0.133976672117584
554	KC_AB2	RpS27A	0.0122525240987101	0.928789360941683	0.988741847156811	pseudobulk	DESeq2	LRT	0.0320827679796277
554	KC_AB2	RpS28b	0.0774653328615614	0.834348744854125	0.988741847156811	pseudobulk	DESeq2	LRT	0.078652383045972
554	KC_AB2	RpS29	0.0633233654778361	0.852775053465226	0.988741847156811	pseudobulk	DESeq2	LRT	0.0691655127040659
554	KC_AB2	RpS3	0.261827273298329	0.598061495271676	0.988741847156811	pseudobulk	DESeq2	LRT	0.223254157677079
554	KC_AB2	RpS30	-0.258794066039781	0.466296033879186	0.988741847156811	pseudobulk	DESeq2	LRT	0.331338278458674
554	KC_AB2	RpS3A	-0.17776880949372	0.701113522027277	0.988741847156811	pseudobulk	DESeq2	LRT	0.154211656785986
555	KC_AB2	RpS4	0.0317060651020643	0.930442274631706	0.988741847156811	pseudobulk	DESeq2	LRT	0.0313105656676312
555	KC_AB2	RpS5a	-0.0480970227921346	0.932072378726481	0.988741847156811	pseudobulk	DESeq2	LRT	0.030550361829476
555	KC_AB2	RpS6	0.0280165409677361	0.932375468403891	0.988741847156811	pseudobulk	DESeq2	LRT	0.0304091616604465
555	KC_AB2	RpS7	-0.0834603938495456	0.872454424681658	0.988741847156811	pseudobulk	DESeq2	LRT	0.0592572504733493
555	KC_AB2	RpS8	-0.0519761393440751	0.890534193625901	0.988741847156811	pseudobulk	DESeq2	LRT	0.0503494003804756
555	KC_AB2	RpS9	0.0607403601638433	0.872654485021755	0.988741847156811	pseudobulk	DESeq2	LRT	0.0591576749091545
555	KC_AB2	Rpt1	0.408590043632153	0.766749964275982	0.988741847156811	pseudobulk	DESeq2	LRT	0.115346235579901
555	KC_AB2	Rpt2	-0.0229973822346953	0.971360658954591	0.988741847156811	pseudobulk	DESeq2	LRT	0.0126194898530182
555	KC_AB2	Rpt3	0.0815888660624535	0.944372456947312	0.988741847156811	pseudobulk	DESeq2	LRT	0.0248566878049062
555	KC_AB2	Rpt4	-0.236485694239447	0.849884344958662	0.988741847156811	pseudobulk	DESeq2	LRT	0.0706401704783203
556	KC_AB2	Rpt5	-0.3086930839159	0.840681528804389	0.988741847156811	pseudobulk	DESeq2	LRT	0.0753684946625984
556	KC_AB2	Rpt6	-0.139664928815823	0.900919009635566	0.988741847156811	pseudobulk	DESeq2	LRT	0.0453142492532636
556	KC_AB2	Rrp1	3.92744695471024	0.132139883250188	0.988741847156811	pseudobulk	DESeq2	LRT	0.878966081240594
556	KC_AB2	Rrp42	0.0601466527378941	0.974810960364079	0.988741847156811	pseudobulk	DESeq2	LRT	0.0110795964355292
556	KC_AB2	Rrp45	0.818790875285784	0.940355871560339	0.988741847156811	pseudobulk	DESeq2	LRT	0.0267077593721816
556	KC_AB2	Rrp46	0.157562008957688	0.950463298513166	0.988741847156811	pseudobulk	DESeq2	LRT	0.0220646484576093
556	KC_AB2	Rrp47	-0.663273929909526	0.684336515129322	0.988741847156811	pseudobulk	DESeq2	LRT	0.164730286109088
556	KC_AB2	Rrp6	0.499142633167307	0.837505285988482	0.988741847156811	pseudobulk	DESeq2	LRT	0.0770124431945389
556	KC_AB2	Rs1	1.45259040912931	0.616176917291092	0.988741847156811	pseudobulk	DESeq2	LRT	0.210294574894236
556	KC_AB2	Rsf1	0.405367396120056	0.528706851055304	0.988741847156811	pseudobulk	DESeq2	LRT	0.276785061911446
557	KC_AB2	RSG7	-0.00998838569350015	0.935041011122648	0.988741847156811	pseudobulk	DESeq2	LRT	0.0291693404497692
557	KC_AB2	rswl	0.0885531792216718	0.964858407830046	0.988741847156811	pseudobulk	DESeq2	LRT	0.0155364143348316
557	KC_AB2	rt	0.818790875285784	0.940355871560339	0.988741847156811	pseudobulk	DESeq2	LRT	0.0267077593721816
557	KC_AB2	Rtc1	-2.26538931672255	0.547771004842275	0.988741847156811	pseudobulk	DESeq2	LRT	0.2614009599796
557	KC_AB2	Rtca	3.02031355103516	0.171490739042093	0.988741847156811	pseudobulk	DESeq2	LRT	0.765759328049858
557	KC_AB2	RtcB	-0.568233637794205	0.868826397133536	0.988741847156811	pseudobulk	DESeq2	LRT	0.0610669925949191
557	KC_AB2	Rtf1	-0.0384805229678164	0.985071128575604	0.988741847156811	pseudobulk	DESeq2	LRT	0.006532409469285
557	KC_AB2	RtGEF	-1.1284597703615	0.729754448571724	0.988741847156811	pseudobulk	DESeq2	LRT	0.136823248879498
557	KC_AB2	Rtnl1	-0.16358098565867	0.669776806350046	0.988741847156811	pseudobulk	DESeq2	LRT	0.174069895686261
557	KC_AB2	ru	0.818790875285784	0.940355871560339	0.988741847156811	pseudobulk	DESeq2	LRT	0.0267077593721816
558	KC_AB2	Rubicon	0.29128807176383	0.879400810780693	0.988741847156811	pseudobulk	DESeq2	LRT	0.0558131383098778
558	KC_AB2	rudhira	0.387349143035096	0.501544987649185	0.988741847156811	pseudobulk	DESeq2	LRT	0.299690105491992
558	KC_AB2	rumi	1.4129303440845	0.645460595159151	0.988741847156811	pseudobulk	DESeq2	LRT	0.190130265910817
558	KC_AB2	rump	0.0986724424451138	0.933940879027766	0.988741847156811	pseudobulk	DESeq2	LRT	0.0296806149098062
558	KC_AB2	rush	-2.00182500877706	0.434812851538254	0.988741847156811	pseudobulk	DESeq2	LRT	0.361697628178971
558	KC_AB2	rut	-0.024729330283648	0.925615361579549	0.988741847156811	pseudobulk	DESeq2	LRT	0.0335694464138359
558	KC_AB2	rux	-0.688968519449025	0.70663956002083	0.988741847156811	pseudobulk	DESeq2	LRT	0.150802052978409
558	KC_AB2	RYBP	0.699674038439951	0.821690393783994	0.988741847156811	pseudobulk	DESeq2	LRT	0.0852917902455435
558	KC_AB2	RyR	-1.2782107826776	0.335203110234593	0.988741847156811	pseudobulk	DESeq2	LRT	0.474691960353651
558	KC_AB2	S	0.539595034337559	0.755423503841144	0.988741847156811	pseudobulk	DESeq2	LRT	0.121809506893039
559	KC_AB2	s-cup	0.712171366772551	0.813205723536451	0.988741847156811	pseudobulk	DESeq2	LRT	0.0897995733547685
559	KC_AB2	S1P	0.818790875285784	0.940355871560339	0.988741847156811	pseudobulk	DESeq2	LRT	0.0267077593721816
559	KC_AB2	S2P	-1.20081362769313	0.741163517910401	0.988741847156811	pseudobulk	DESeq2	LRT	0.130085965985812
559	KC_AB2	S6k	0.24619866569863	0.866451184773514	0.988741847156811	pseudobulk	DESeq2	LRT	0.0622559000945371
559	KC_AB2	S6kII	0.0126290187641847	0.96273546980407	0.988741847156811	pseudobulk	DESeq2	LRT	0.0164930272952422
559	KC_AB2	S6KL	0.574989404325028	0.815857662655918	0.988741847156811	pseudobulk	DESeq2	LRT	0.088385603151941
559	KC_AB2	SA	0.157562008957688	0.950463298513166	0.988741847156811	pseudobulk	DESeq2	LRT	0.0220646484576093
559	KC_AB2	Sac1	0.297713224699148	0.901701424115727	0.988741847156811	pseudobulk	DESeq2	LRT	0.0449372444148942
559	KC_AB2	Saf-B	-0.30134054810048	0.741029750318947	0.988741847156811	pseudobulk	DESeq2	LRT	0.130164355934315

559	KC_AB2	Saf6	2.06137138167525	0.547946929015274	0.988741847156811	pseudobulk	DESeq2	LRT	0.261261502741816
560	KC_AB2	sage	-3.09794865339079	0.39855598532175	0.988741847156811	pseudobulk	DESeq2	LRT	0.399510664459796
560	KC_AB2	SAK	-0.337072010867851	0.906965066354256	0.988741847156811	pseudobulk	DESeq2	LRT	0.0424094403729327
560	KC_AB2	sals	-1.88465419320578	0.400636257563581	0.988741847156811	pseudobulk	DESeq2	LRT	0.397249749632452
560	KC_AB2	Sam-S	0.381792557941006	0.636745603922511	0.988741847156811	pseudobulk	DESeq2	LRT	0.196034044713442
560	KC_AB2	SamDC	1.31950274499659	0.590522087411301	0.988741847156811	pseudobulk	DESeq2	LRT	0.228763853746569
560	KC_AB2	Samtor	-0.506613484034373	0.961658398065406	0.988741847156811	pseudobulk	DESeq2	LRT	0.0169791713942958
560	KC_AB2	san	0.167165629946856	0.902635884466227	0.988741847156811	pseudobulk	DESeq2	LRT	0.0444874050105513
560	KC_AB2	sano	0.061704045500587	0.963589002188074	0.988741847156811	pseudobulk	DESeq2	LRT	0.016108165415381
560	KC_AB2	santa-maria	1.42621218304824	0.66738831402044	0.988741847156811	pseudobulk	DESeq2	LRT	0.175621402117653
560	KC_AB2	Sap-r	0.15816514772931	0.891053323592786	0.988741847156811	pseudobulk	DESeq2	LRT	0.0500963055600296
561	KC_AB2	Sap130	-0.088232111136206	0.957691663474435	0.988741847156811	pseudobulk	DESeq2	LRT	0.0187742930152181
561	KC_AB2	Sap30	2.57125803383194	0.282842618467385	0.988741847156811	pseudobulk	DESeq2	LRT	0.548455150848669
561	KC_AB2	Sap47	-0.143634677424837	0.672660585566384	0.988741847156811	pseudobulk	DESeq2	LRT	0.172204018983638
561	KC_AB2	Sar1	0.48757936491321	0.678644970092679	0.988741847156811	pseudobulk	DESeq2	LRT	0.168357365415916
561	KC_AB2	Sara	0.344881888182183	0.808533896365827	0.988741847156811	pseudobulk	DESeq2	LRT	0.0923017683413094
561	KC_AB2	Sas-6	-2.26538931672255	0.547771004842275	0.988741847156811	pseudobulk	DESeq2	LRT	0.2614009599796
561	KC_AB2	Sas10	-1.80294541848982	0.660135549041553	0.988741847156811	pseudobulk	DESeq2	LRT	0.180366879372519
561	KC_AB2	sau	0.572945637735694	0.613235121883156	0.988741847156811	pseudobulk	DESeq2	LRT	0.212372979709313
561	KC_AB2	sax	-0.364162016358913	0.832369175883111	0.988741847156811	pseudobulk	DESeq2	LRT	0.0796840108627881
561	KC_AB2	sba	0.230911957240161	0.831509684184534	0.988741847156811	pseudobulk	DESeq2	LRT	0.0801326884006697
562	KC_AB2	Sbat	3.46443801158151	0.218071151261552	0.988741847156811	pseudobulk	DESeq2	LRT	0.6614017836492
562	KC_AB2	sbb	0.128063229841025	0.782971286294847	0.988741847156811	pseudobulk	DESeq2	LRT	0.106254164420164
562	KC_AB2	Sbf	1.25488503277736	0.451702033471048	0.988741847156811	pseudobulk	DESeq2	LRT	0.345147954328428
562	KC_AB2	sbm	-0.472894928467461	0.662573924352436	0.988741847156811	pseudobulk	DESeq2	LRT	0.178765659810359
562	KC_AB2	Sbp2	2.4643417141186	0.449082701910884	0.988741847156811	pseudobulk	DESeq2	LRT	0.347673673080982
562	KC_AB2	sbr	0.00981849622677228	0.977259783281399	0.988741847156811	pseudobulk	DESeq2	LRT	0.00998997318135465
562	KC_AB2	Sc2	-0.0157607737407134	0.979432010380381	0.988741847156811	pseudobulk	DESeq2	LRT	0.00902570621489607
562	KC_AB2	SC35	0.14285888006002	0.872536856104106	0.988741847156811	pseudobulk	DESeq2	LRT	0.059216219309096
562	KC_AB2	sca	0.906728081066965	0.49744268786112	0.988741847156811	pseudobulk	DESeq2	LRT	0.303256948645812
562	KC_AB2	scaf6	0.853341883758108	0.40710388716046	0.988741847156811	pseudobulk	DESeq2	LRT	0.390294750815691
563	KC_AB2	SCaMC	-0.943352595969729	0.538586166692529	0.988741847156811	pseudobulk	DESeq2	LRT	0.268744805410682
563	KC_AB2	Scamp	-0.396202655351342	0.682508152706857	0.988741847156811	pseudobulk	DESeq2	LRT	0.165892156515114
563	KC_AB2	SCAP	-0.401735364971212	0.677866042706129	0.988741847156811	pseudobulk	DESeq2	LRT	0.168856121267907
563	KC_AB2	SCAR	-0.0887327487996433	0.937469271322228	0.988741847156811	pseudobulk	DESeq2	LRT	0.0280429588150793
563	KC_AB2	scat	-1.80294541848982	0.660135549041553	0.988741847156811	pseudobulk	DESeq2	LRT	0.180366879372519
563	KC_AB2	Sccpdh1	-0.0323203545809474	0.954448156132187	0.988741847156811	pseudobulk	DESeq2	LRT	0.020247656706879
563	KC_AB2	SCCRO	-0.788827489472345	0.690314001057326	0.988741847156811	pseudobulk	DESeq2	LRT	0.160953318093263
563	KC_AB2	SCCRO3	-1.88465419320578	0.400636257563581	0.988741847156811	pseudobulk	DESeq2	LRT	0.397249749632452
563	KC_AB2	SCCRO4	0.197019222279105	0.906036510922107	0.988741847156811	pseudobulk	DESeq2	LRT	0.0428543010290278
563	KC_AB2	Sce	0.0960904300109501	0.963271183317648	0.988741847156811	pseudobulk	DESeq2	LRT	0.016251431630502
564	KC_AB2	sced	-2.45158172483764	0.378823136429564	0.988741847156811	pseudobulk	DESeq2	LRT	0.421563504545096
564	KC_AB2	scf	0.0336799954194084	0.966730614244894	0.988741847156811	pseudobulk	DESeq2	LRT	0.0146945280326403
564	KC_AB2	Scgalpha	0.9844378692756	0.565111304018534	0.988741847156811	pseudobulk	DESeq2	LRT	0.247866005348055
564	KC_AB2	Scgbeta	0.273382020796336	0.758012960802936	0.988741847156811	pseudobulk	DESeq2	LRT	0.120323368567057
564	KC_AB2	Schip1	0.235296808575352	0.800950069560313	0.988741847156811	pseudobulk	DESeq2	LRT	0.0963945565628492
564	KC_AB2	schlank	0.503427650916014	0.63674135228508	0.988741847156811	pseudobulk	DESeq2	LRT	0.19603694456674
564	KC_AB2	Scm	-0.446738973454751	0.810155947697833	0.988741847156811	pseudobulk	DESeq2	LRT	0.0914313753117493
564	KC_AB2	scny	-0.373110296322301	0.847802279612777	0.988741847156811	pseudobulk	DESeq2	LRT	0.0717054200137059
564	KC_AB2	Scox	-1.05053966288872	0.617553465682197	0.988741847156811	pseudobulk	DESeq2	LRT	0.209325436698949
564	KC_AB2	Scp1	-2.26538931672255	0.547771004842275	0.988741847156811	pseudobulk	DESeq2	LRT	0.2614009599796
565	KC_AB2	ScpX	-0.506613484034373	0.961658398065406	0.988741847156811	pseudobulk	DESeq2	LRT	0.0169791713942958
565	KC_AB2	scramb1	2.57125803383194	0.282842618467385	0.988741847156811	pseudobulk	DESeq2	LRT	0.548455150848669
565	KC_AB2	scramb2	-0.840006590521399	0.717017354938613	0.988741847156811	pseudobulk	DESeq2	LRT	0.144470332389583
565	KC_AB2	scrib	0.233800965609364	0.631752177167387	0.988741847156811	pseudobulk	DESeq2	LRT	0.199453252723991
565	KC_AB2	scro	-2.9721553030081	0.387854083811327	0.988741847156811	pseudobulk	DESeq2	LRT	0.411331631401987

565	KC_AB2	scrt	-0.387316946035949	0.49331413442792	0.988741847156811	pseudobulk	DESeq2	LRT	0.306876440961719
565	KC_AB2	ScsbetaA	-0.116678331609373	0.883800289157135	0.988741847156811	pseudobulk	DESeq2	LRT	0.0536458606835152
565	KC_AB2	ScsbetaG	0.818790875285784	0.940355871560339	0.988741847156811	pseudobulk	DESeq2	LRT	0.0267077593721816
565	KC_AB2	scu	-0.588639054357632	0.8432408255636	0.988741847156811	pseudobulk	DESeq2	LRT	0.0740483752247712
565	KC_AB2	scyl	-0.281615332717624	0.566023128053301	0.988741847156811	pseudobulk	DESeq2	LRT	0.247165822909409
566	KC_AB2	sd	-0.108670669299189	0.878734168316089	0.988741847156811	pseudobulk	DESeq2	LRT	0.0561424863532273
566	KC_AB2	Sdc	-0.284870633803525	0.544204847382795	0.988741847156811	pseudobulk	DESeq2	LRT	0.264237594158202
566	KC_AB2	SdhA	0.450908361458216	0.722775881680326	0.988741847156811	pseudobulk	DESeq2	LRT	0.140996347848663
566	KC_AB2	Sdhaf3	1.1327121871749	0.657275461456076	0.988741847156811	pseudobulk	DESeq2	LRT	0.182252581249451
566	KC_AB2	SdhB	0.864169598479159	0.385887882440173	0.988741847156811	pseudobulk	DESeq2	LRT	0.413538858826876
566	KC_AB2	SdhC	-0.276105431556219	0.705993221157952	0.988741847156811	pseudobulk	DESeq2	LRT	0.151199468959286
566	KC_AB2	SdhD	1.0950222008989	0.25895184668117	0.988741847156811	pseudobulk	DESeq2	LRT	0.586780987520842
566	KC_AB2	sds22	0.105902682160767	0.948921138997729	0.988741847156811	pseudobulk	DESeq2	LRT	0.0227698785329056
566	KC_AB2	sdt	-0.0326806577360554	0.922750332363165	0.988741847156811	pseudobulk	DESeq2	LRT	0.0349157897048194
566	KC_AB2	Sec10	1.4129303440845	0.645460595159151	0.988741847156811	pseudobulk	DESeq2	LRT	0.190130265910817
567	KC_AB2	Sec13	0.551429827514599	0.688345219211046	0.988741847156811	pseudobulk	DESeq2	LRT	0.162193699560362
567	KC_AB2	Sec15	-0.506613484034373	0.961658398065406	0.988741847156811	pseudobulk	DESeq2	LRT	0.0169791713942958
567	KC_AB2	Sec16	0.353659730702455	0.739691486939959	0.988741847156811	pseudobulk	DESeq2	LRT	0.130949379535587
567	KC_AB2	Sec22	2.89510379953434	0.0979224548098749	0.988741847156811	pseudobulk	DESeq2	LRT	1.0091177077698
567	KC_AB2	Sec23	0.360597065274579	0.876727378089377	0.988741847156811	pseudobulk	DESeq2	LRT	0.0571354312607773
567	KC_AB2	Sec24AB	-0.382714549144618	0.863954068453144	0.988741847156811	pseudobulk	DESeq2	LRT	0.063509345886375
567	KC_AB2	Sec24CD	-1.15799512945374	0.460399548019562	0.988741847156811	pseudobulk	DESeq2	LRT	0.336865111394779
567	KC_AB2	Sec3	2.4643417141186	0.449082701910884	0.988741847156811	pseudobulk	DESeq2	LRT	0.347673673080982
567	KC_AB2	Sec31	2.4643417141186	0.449082701910884	0.988741847156811	pseudobulk	DESeq2	LRT	0.347673673080982
567	KC_AB2	Sec5	1.12452187615248	0.704078409906088	0.988741847156811	pseudobulk	DESeq2	LRT	0.152378975911753
568	KC_AB2	Sec6	-1.99867440840856	0.534899605264936	0.988741847156811	pseudobulk	DESeq2	LRT	0.271727722597268
568	KC_AB2	Sec61alpha	1.07340560663181	0.618555212422194	0.988741847156811	pseudobulk	DESeq2	LRT	0.208621529050062
568	KC_AB2	Sec61beta	0.906728081066965	0.49744268786112	0.988741847156811	pseudobulk	DESeq2	LRT	0.303256948645812
568	KC_AB2	Sec61gamma	0.626556861342864	0.60807973159509	0.988741847156811	pseudobulk	DESeq2	LRT	0.216039472172116
568	KC_AB2	Sec63	0.510085173370696	0.747317736001631	0.988741847156811	pseudobulk	DESeq2	LRT	0.126494710562999
568	KC_AB2	Sec71	1.47543920254359	0.661929169513603	0.988741847156811	pseudobulk	DESeq2	LRT	0.17918848025087
568	KC_AB2	Sec8	1.69894780879763	0.540778829726708	0.988741847156811	pseudobulk	DESeq2	LRT	0.26698031838453
568	KC_AB2	Secp43	-0.0384805229678164	0.985071128575604	0.988741847156811	pseudobulk	DESeq2	LRT	0.006532409469285
568	KC_AB2	SecS	-0.487923111280115	0.783394690329388	0.988741847156811	pseudobulk	DESeq2	LRT	0.106019376327165
568	KC_AB2	sei	0.765272298362395	0.741528757908517	0.988741847156811	pseudobulk	DESeq2	LRT	0.129872001535403
569	KC_AB2	sel	2.4643417141186	0.449082701910884	0.988741847156811	pseudobulk	DESeq2	LRT	0.347673673080982
569	KC_AB2	SelG	0.475522879236569	0.801582640633501	0.988741847156811	pseudobulk	DESeq2	LRT	0.0960516966149455
569	KC_AB2	SelR	-0.432745491253605	0.54454455005631	0.988741847156811	pseudobulk	DESeq2	LRT	0.263966584131965
569	KC_AB2	SelT	0.938905756580212	0.565739036218207	0.988741847156811	pseudobulk	DESeq2	LRT	0.247383853733943
569	KC_AB2	Sem1	-0.437516661077144	0.789345112385092	0.988741847156811	pseudobulk	DESeq2	LRT	0.102733075833883
569	KC_AB2	Sema1a	0.0478125875761869	0.933300546225807	0.988741847156811	pseudobulk	DESeq2	LRT	0.0299784799952873
569	KC_AB2	Sema1b	-1.15125618006759	0.484195627613811	0.988741847156811	pseudobulk	DESeq2	LRT	0.31497913664515
569	KC_AB2	Sema2a	-0.475994762797899	0.658118766853187	0.988741847156811	pseudobulk	DESeq2	LRT	0.181695724722609
569	KC_AB2	Sema2b	1.45259040912931	0.616176917291092	0.988741847156811	pseudobulk	DESeq2	LRT	0.210294574894236
569	KC_AB2	senju	1.42621218304824	0.66738831402044	0.988741847156811	pseudobulk	DESeq2	LRT	0.175621402117653
570	KC_AB2	Sep1	0.356576043500675	0.884817522565587	0.988741847156811	pseudobulk	DESeq2	LRT	0.0531462853633029
570	KC_AB2	Sep2	-0.0229973822346953	0.971360658954591	0.988741847156811	pseudobulk	DESeq2	LRT	0.0126194898530182
570	KC_AB2	Sep4	0.325884727352926	0.765770333826834	0.988741847156811	pseudobulk	DESeq2	LRT	0.115901462361358
570	KC_AB2	SERCA	-0.325368896347951	0.770108370195498	0.988741847156811	pseudobulk	DESeq2	LRT	0.113448156299893
570	KC_AB2	SerRS	-0.853565742783243	0.753557841534365	0.988741847156811	pseudobulk	DESeq2	LRT	0.122883406527736
570	KC_AB2	SerRS-m	3.06202454821851	0.30627034058063	0.988741847156811	pseudobulk	DESeq2	LRT	0.513895058517773
570	KC_AB2	sesB	-0.149813219755618	0.71264910821993	0.988741847156811	pseudobulk	DESeq2	LRT	0.1471242539832
570	KC_AB2	Sesn	-1.80294541848982	0.660135549041553	0.988741847156811	pseudobulk	DESeq2	LRT	0.180366879372519
570	KC_AB2	Set	-0.448374585768327	0.814227572251762	0.988741847156811	pseudobulk	DESeq2	LRT	0.0892541951597077
570	KC_AB2	Set1	0.8007652250482	0.445750390858351	0.988741847156811	pseudobulk	DESeq2	LRT	0.350908267339258
571	KC_AB2	Set2	-0.194388201900128	0.928057462354013	0.988741847156811	pseudobulk	DESeq2	LRT	0.0324251328213705

571	KC_AB2	Setd3	-0.0144409071146711	0.984632974376923	0.988741847156811	pseudobulk	DESeq2	LRT	0.00672562423038896
571	KC_AB2	SF1	0.780757760356506	0.610007522899248	0.988741847156811	pseudobulk	DESeq2	LRT	0.2146648090327
571	KC_AB2	SF2	-0.231066911307467	0.790910521092435	0.988741847156811	pseudobulk	DESeq2	LRT	0.10187264721525
571	KC_AB2	Sf3a1	0.363641268105473	0.858330168669459	0.988741847156811	pseudobulk	DESeq2	LRT	0.06634562258353
571	KC_AB2	Sf3a2	-0.451045789314127	0.823968225471349	0.988741847156811	pseudobulk	DESeq2	LRT	0.0840895355947879
571	KC_AB2	Sf3b1	1.31950274499659	0.590522087411301	0.988741847156811	pseudobulk	DESeq2	LRT	0.228763853746569
571	KC_AB2	Sf3b2	1.09755075627148	0.564545147075846	0.988741847156811	pseudobulk	DESeq2	LRT	0.24830132151245
571	KC_AB2	Sf3b3	0.699674038439951	0.821690393783994	0.988741847156811	pseudobulk	DESeq2	LRT	0.0852917902455435
571	KC_AB2	Sf3b5	-0.874016633270315	0.834623135226332	0.988741847156811	pseudobulk	DESeq2	LRT	0.0785095810886316
572	KC_AB2	Sf3b6	0.948605040531545	0.5192903465501	0.988741847156811	pseudobulk	DESeq2	LRT	0.284589750732483
572	KC_AB2	sff	-0.013482453244512	0.959979378916202	0.988741847156811	pseudobulk	DESeq2	LRT	0.0177380958344842
572	KC_AB2	sfl	0.843861068130357	0.515383857446488	0.988741847156811	pseudobulk	DESeq2	LRT	0.287869188291331
572	KC_AB2	Sfmbt	-0.840006590521399	0.717017354938613	0.988741847156811	pseudobulk	DESeq2	LRT	0.144470332389583
572	KC_AB2	Sfxn1-3	1.0325721522626	0.627176172308459	0.988741847156811	pseudobulk	DESeq2	LRT	0.202610449736126
572	KC_AB2	Sfxn2	1.87899144193828	0.388696615728932	0.988741847156811	pseudobulk	DESeq2	LRT	0.410389240631014
572	KC_AB2	Sgf11	0.666943671827768	0.808936122914367	0.988741847156811	pseudobulk	DESeq2	LRT	0.0920857707994393
572	KC_AB2	Sgf29	1.64209625885217	0.483203675195701	0.988741847156811	pseudobulk	DESeq2	LRT	0.315869771176778
572	KC_AB2	sgg	0.317427047740387	0.5416529311396	0.988741847156811	pseudobulk	DESeq2	LRT	0.266278902338761
572	KC_AB2	sgl	-1.80294541848982	0.660135549041553	0.988741847156811	pseudobulk	DESeq2	LRT	0.180366879372519
573	KC_AB2	sgll	-1.80294541848982	0.660135549041553	0.988741847156811	pseudobulk	DESeq2	LRT	0.180366879372519
573	KC_AB2	Sgs4	2.4643417141186	0.449082701910884	0.988741847156811	pseudobulk	DESeq2	LRT	0.347673673080982
573	KC_AB2	Sgt	1.1828730956701	0.436095278144291	0.988741847156811	pseudobulk	DESeq2	LRT	0.360418615658563
573	KC_AB2	Sgt1	-0.50879048068994	0.817628596116567	0.988741847156811	pseudobulk	DESeq2	LRT	0.0874439277298491
573	KC_AB2	Sh	-0.158344238026957	0.539789658893508	0.988741847156811	pseudobulk	DESeq2	LRT	0.267775439765618
573	KC_AB2	Sh3beta	1.65183797719551	0.131865839728086	0.988741847156811	pseudobulk	DESeq2	LRT	0.879867695272696
573	KC_AB2	SH3PX1	-0.457212305199634	0.858276431637992	0.988741847156811	pseudobulk	DESeq2	LRT	0.0663728130839547
573	KC_AB2	Shab	-0.169326895119771	0.749330446707857	0.988741847156811	pseudobulk	DESeq2	LRT	0.12532662084034
573	KC_AB2	shakB	-0.272266927379367	0.578642147910851	0.988741847156811	pseudobulk	DESeq2	LRT	0.237589935813438
573	KC_AB2	Shal	-0.319856277438217	0.661883032340985	0.988741847156811	pseudobulk	DESeq2	LRT	0.179218752096769
574	KC_AB2	shams	0.818790875285784	0.940355871560339	0.988741847156811	pseudobulk	DESeq2	LRT	0.0267077593721816
574	KC_AB2	Shark	0.818790875285784	0.940355871560339	0.988741847156811	pseudobulk	DESeq2	LRT	0.0267077593721816
574	KC_AB2	Shaw	-0.382864413882057	0.667862878408255	0.988741847156811	pseudobulk	DESeq2	LRT	0.175312695104647
574	KC_AB2	Shawl	0.152434636789694	0.828517237510465	0.988741847156811	pseudobulk	DESeq2	LRT	0.0816984515442215
574	KC_AB2	Shc	0.11213458976865	0.938112983749562	0.988741847156811	pseudobulk	DESeq2	LRT	0.0277448532370976
574	KC_AB2	shep	0.318798680770766	0.32231109808556	0.988741847156811	pseudobulk	DESeq2	LRT	0.491724740239024
574	KC_AB2	shg	0.818790875285784	0.940355871560339	0.988741847156811	pseudobulk	DESeq2	LRT	0.0267077593721816
574	KC_AB2	shi	0.0258319619154158	0.935353538475701	0.988741847156811	pseudobulk	DESeq2	LRT	0.0290242064659612
574	KC_AB2	Shmt	1.47543920254359	0.661929169513603	0.988741847156811	pseudobulk	DESeq2	LRT	0.17918848025087
574	KC_AB2	shn	0.104637746735554	0.951122131714938	0.988741847156811	pseudobulk	DESeq2	LRT	0.0217637125849166
575	KC_AB2	shop	1.47543920254359	0.661929169513603	0.988741847156811	pseudobulk	DESeq2	LRT	0.17918848025087
575	KC_AB2	shot	-0.197741840055039	0.671111887283685	0.988741847156811	pseudobulk	DESeq2	LRT	0.173205068535836
575	KC_AB2	shrb	-0.984491843965367	0.431268451341265	0.988741847156811	pseudobulk	DESeq2	LRT	0.36525231071456
575	KC_AB2	shv	0.331758848923756	0.946375065527934	0.988741847156811	pseudobulk	DESeq2	LRT	0.0239367107367223
575	KC_AB2	sicily	2.41462217514131	0.497446973552313	0.988741847156811	pseudobulk	DESeq2	LRT	0.303253207020768
575	KC_AB2	sick	-0.051091293768546	0.944336397342974	0.988741847156811	pseudobulk	DESeq2	LRT	0.0248732710778124
575	KC_AB2	side-II	0.483458967642629	0.345647576947851	0.988741847156811	pseudobulk	DESeq2	LRT	0.461366483253893
575	KC_AB2	side-III	0.933280995794211	0.189021862043414	0.988741847156811	pseudobulk	DESeq2	LRT	0.723487962939286
575	KC_AB2	side-IV	-0.0786280431881614	0.950746569695297	0.988741847156811	pseudobulk	DESeq2	LRT	0.0219352328531049
575	KC_AB2	side-V	0.474300517574011	0.81118474384143	0.988741847156811	pseudobulk	DESeq2	LRT	0.0908802258211275
576	KC_AB2	side-VI	0.1313472041639	0.807553153838559	0.988741847156811	pseudobulk	DESeq2	LRT	0.0928288824221927
576	KC_AB2	side-VII	0.592524312040804	0.640550918416189	0.988741847156811	pseudobulk	DESeq2	LRT	0.193446341784662
576	KC_AB2	SIDL	-1.23536834900699	0.725570392794057	0.988741847156811	pseudobulk	DESeq2	LRT	0.139320447155934
576	KC_AB2	Sidpn	-2.26538931672255	0.547771004842275	0.988741847156811	pseudobulk	DESeq2	LRT	0.2614009599796
576	KC_AB2	sif	-0.503580812160565	0.405722121776137	0.988741847156811	pseudobulk	DESeq2	LRT	0.39177131198169
576	KC_AB2	SIFaR	-0.506613484034373	0.961658398065406	0.988741847156811	pseudobulk	DESeq2	LRT	0.0169791713942958
576	KC_AB2	sigmar	-0.159170689593312	0.92478622720461	0.988741847156811	pseudobulk	DESeq2	LRT	0.033958646801551

576	KC_AB2	Sik2	0.317508825551005	0.883746467592857	0.988741847156811	pseudobulk	DESeq2	LRT	0.053672309102253
576	KC_AB2	Sik3	0.773411801951799	0.572026368671613	0.988741847156811	pseudobulk	DESeq2	LRT	0.242583951093992
576	KC_AB2	Sil1	-2.64843360157809	0.321943703667444	0.988741847156811	pseudobulk	DESeq2	LRT	0.492220064093287
577	KC_AB2	sima	-0.263071847608636	0.76134324579809	0.988741847156811	pseudobulk	DESeq2	LRT	0.118419500722838
577	KC_AB2	simj	-0.25824556566031	0.791378564940475	0.988741847156811	pseudobulk	DESeq2	LRT	0.101615717090135
577	KC_AB2	Sin	-0.506613484034373	0.961658398065406	0.988741847156811	pseudobulk	DESeq2	LRT	0.0169791713942958
577	KC_AB2	Sin1	2.4643417141186	0.449082701910884	0.988741847156811	pseudobulk	DESeq2	LRT	0.347673673080982
577	KC_AB2	Sin3A	-0.0263429195970125	0.93794295814278	0.988741847156811	pseudobulk	DESeq2	LRT	0.0278235728332045
577	KC_AB2	sina	-0.0743799866276766	0.904640831805618	0.988741847156811	pseudobulk	DESeq2	LRT	0.0435238138621967
577	KC_AB2	sinu	-1.12033987853799	0.612256743736902	0.988741847156811	pseudobulk	DESeq2	LRT	0.213066422615508
577	KC_AB2	sip1	-1.80294541848982	0.660135549041553	0.988741847156811	pseudobulk	DESeq2	LRT	0.180366879372519
577	KC_AB2	sip3	-1.80294541848982	0.660135549041553	0.988741847156811	pseudobulk	DESeq2	LRT	0.180366879372519
577	KC_AB2	Sirt1	0.664045397997232	0.519959789772553	0.988741847156811	pseudobulk	DESeq2	LRT	0.284030240509659
578	KC_AB2	Sirt2	1.35316200662963	0.684467033464499	0.988741847156811	pseudobulk	DESeq2	LRT	0.164647464300239
578	KC_AB2	Sirt4	3.79415743120494	0.154946854526743	0.988741847156811	pseudobulk	DESeq2	LRT	0.809817235659252
578	KC_AB2	Sirt6	1.42621218304824	0.66738831402044	0.988741847156811	pseudobulk	DESeq2	LRT	0.175621402117653
578	KC_AB2	Sirt7	0.818790875285784	0.940355871560339	0.988741847156811	pseudobulk	DESeq2	LRT	0.0267077593721816
578	KC_AB2	Sirup	-0.924630677931247	0.557647662512588	0.988741847156811	pseudobulk	DESeq2	LRT	0.253640113888557
578	KC_AB2	sisRNA:CR46364	1.42621218304824	0.66738831402044	0.988741847156811	pseudobulk	DESeq2	LRT	0.175621402117653
578	KC_AB2	siz	-0.13321063283771	0.858337965470036	0.988741847156811	pseudobulk	DESeq2	LRT	0.0663416776073288
578	KC_AB2	SK	0.409921929474132	0.395163877318807	0.988741847156811	pseudobulk	DESeq2	LRT	0.40322276195415
578	KC_AB2	Sk2	2.06137138167525	0.547946929015274	0.988741847156811	pseudobulk	DESeq2	LRT	0.261261502741816
578	KC_AB2	skd	0.0602770919303854	0.918819765731291	0.988741847156811	pseudobulk	DESeq2	LRT	0.0367696707955813
579	KC_AB2	Ski6	0.818790875285784	0.940355871560339	0.988741847156811	pseudobulk	DESeq2	LRT	0.0267077593721816
579	KC_AB2	SKIP	0.157562008957688	0.950463298513166	0.988741847156811	pseudobulk	DESeq2	LRT	0.0220646484576093
579	KC_AB2	skl	0.818790875285784	0.940355871560339	0.988741847156811	pseudobulk	DESeq2	LRT	0.0267077593721816
579	KC_AB2	SkpA	-0.42419437295376	0.559435619420308	0.988741847156811	pseudobulk	DESeq2	LRT	0.252249885434621
579	KC_AB2	sky	-0.208148099185337	0.844041645814033	0.988741847156811	pseudobulk	DESeq2	LRT	0.0736361243447031
579	KC_AB2	sl	-3.4227037205893	0.285801360563088	0.988741847156811	pseudobulk	DESeq2	LRT	0.543935708072568
579	KC_AB2	Slbp	1.29518405230266	0.581169015396514	0.988741847156811	pseudobulk	DESeq2	LRT	0.235697547851342
579	KC_AB2	SLC22A	-2.51746470924027	0.517377929599049	0.988741847156811	pseudobulk	DESeq2	LRT	0.286192101436607
579	KC_AB2	Slc25A46a	0.998893024789477	0.660591418406266	0.988741847156811	pseudobulk	DESeq2	LRT	0.180067072429364
579	KC_AB2	sle	1.10527623683615	0.724717602335893	0.988741847156811	pseudobulk	DESeq2	LRT	0.139831190172672
580	KC_AB2	slgA	1.21200182821837	0.197693639776202	0.988741847156811	pseudobulk	DESeq2	LRT	0.704007302636412
580	KC_AB2	Slh	-1.23536834900699	0.725570392794057	0.988741847156811	pseudobulk	DESeq2	LRT	0.139320447155934
580	KC_AB2	Slik	0.0585056918159126	0.958517014634713	0.988741847156811	pseudobulk	DESeq2	LRT	0.0184001735565156
580	KC_AB2	slim	1.43050469631063	0.476736087104661	0.988741847156811	pseudobulk	DESeq2	LRT	0.321721972391742
580	KC_AB2	Slip1	0.0275463519017415	0.971397333752305	0.988741847156811	pseudobulk	DESeq2	LRT	0.0126030928933002
580	KC_AB2	SLIRP2	-2.9721553030081	0.387854083811327	0.988741847156811	pseudobulk	DESeq2	LRT	0.411331631401987
580	KC_AB2	sll	2.41462217514131	0.497446973552313	0.988741847156811	pseudobulk	DESeq2	LRT	0.303253207020768
580	KC_AB2	Slmap	0.262485652016901	0.839859366441491	0.988741847156811	pseudobulk	DESeq2	LRT	0.0757934299997051
580	KC_AB2	slmb	0.646497422309356	0.641983613495159	0.988741847156811	pseudobulk	DESeq2	LRT	0.192476057070483
580	KC_AB2	slmo	0.792281256713368	0.823478838299676	0.988741847156811	pseudobulk	DESeq2	LRT	0.0843475568193814
581	KC_AB2	Sln	0.046530156636047	0.970525086348867	0.988741847156811	pseudobulk	DESeq2	LRT	0.0129932343899138
581	KC_AB2	slo	0.0401470263717051	0.866103722624201	0.988741847156811	pseudobulk	DESeq2	LRT	0.0624300947425633
581	KC_AB2	SLO2	1.56444679815904	0.0640467547548555	0.988741847156811	pseudobulk	DESeq2	LRT	1.19350287103662
581	KC_AB2	Slob	-0.178543375248532	0.687698430469771	0.988741847156811	pseudobulk	DESeq2	LRT	0.16260196684468
581	KC_AB2	slpr	-0.18791684623099	0.895148305484945	0.988741847156811	pseudobulk	DESeq2	LRT	0.0481050061165242
581	KC_AB2	Slu7	-0.488465967391191	0.84251072973523	0.988741847156811	pseudobulk	DESeq2	LRT	0.0744245594950451
581	KC_AB2	slv	0.792281256713368	0.823478838299676	0.988741847156811	pseudobulk	DESeq2	LRT	0.0843475568193814
581	KC_AB2	sm	-0.214448653967286	0.714122114981785	0.988741847156811	pseudobulk	DESeq2	LRT	0.146227517456193
581	KC_AB2	smal	2.4643417141186	0.449082701910884	0.988741847156811	pseudobulk	DESeq2	LRT	0.347673673080982
581	KC_AB2	smash	0.222008486804317	0.861032622987513	0.988741847156811	pseudobulk	DESeq2	LRT	0.0649803935927369
582	KC_AB2	SmB	0.0218104546780889	0.977572066519538	0.988741847156811	pseudobulk	DESeq2	LRT	0.00985121660412526
582	KC_AB2	SMC1	-1.04118960692311	0.546633270199781	0.988741847156811	pseudobulk	DESeq2	LRT	0.262303939009156
582	KC_AB2	SMC2	0.818790875285784	0.940355871560339	0.988741847156811	pseudobulk	DESeq2	LRT	0.0267077593721816

582	KC_AB2	SMC3	0.739194211565721	0.681363203832783	0.988741847156811	pseudobulk	DESeq2	LRT	0.166621323664927
582	KC_AB2	SMC5	-0.331004884396198	0.894406551228697	0.988741847156811	pseudobulk	DESeq2	LRT	0.0484650283842476
582	KC_AB2	SmD1	2.0846618955163	0.461498386685809	0.988741847156811	pseudobulk	DESeq2	LRT	0.335829812849717
582	KC_AB2	SmD2	0.93207702106479	0.499359931265499	0.988741847156811	pseudobulk	DESeq2	LRT	0.301586308457292
582	KC_AB2	SmD3	1.06578634547012	0.651954183166929	0.988741847156811	pseudobulk	DESeq2	LRT	0.185782923741917
582	KC_AB2	SmE	-0.873752018689397	0.57934961351547	0.988741847156811	pseudobulk	DESeq2	LRT	0.237059278417274
582	KC_AB2	SmF	0.972726581225976	0.61661584324292	0.988741847156811	pseudobulk	DESeq2	LRT	0.20998532076329
583	KC_AB2	smg	1.04306579789491	0.585847600161045	0.988741847156811	pseudobulk	DESeq2	LRT	0.232215344761904
583	KC_AB2	Smg5	0.506649538000576	0.596955120775104	0.988741847156811	pseudobulk	DESeq2	LRT	0.224058318003575
583	KC_AB2	Smg6	1.47543920254359	0.661929169513603	0.988741847156811	pseudobulk	DESeq2	LRT	0.17918848025087
583	KC_AB2	smid	0.802207095184316	0.732945722088841	0.988741847156811	pseudobulk	DESeq2	LRT	0.134928185618234
583	KC_AB2	Smn	-2.51746470924027	0.517377929599049	0.988741847156811	pseudobulk	DESeq2	LRT	0.286192101436607
583	KC_AB2	smo	-0.331004884396198	0.894406551228697	0.988741847156811	pseudobulk	DESeq2	LRT	0.0484650283842476
583	KC_AB2	Smox	0.114151653445292	0.842192615242733	0.988741847156811	pseudobulk	DESeq2	LRT	0.0745885710057466
583	KC_AB2	smp-30	0.0601466527378941	0.974810960364079	0.988741847156811	pseudobulk	DESeq2	LRT	0.0110795964355292
583	KC_AB2	Smr	0.153783729652751	0.777848805895307	0.988741847156811	pseudobulk	DESeq2	LRT	0.109104810653679
583	KC_AB2	Sms	0.269815109240075	0.830447388273419	0.988741847156811	pseudobulk	DESeq2	LRT	0.080687876405005
584	KC_AB2	SMSr	0.513202572024843	0.807055953632252	0.988741847156811	pseudobulk	DESeq2	LRT	0.093096354359303
584	KC_AB2	smt3	0.465029389284867	0.412195570319774	0.988741847156811	pseudobulk	DESeq2	LRT	0.384896679699008
584	KC_AB2	Smu1	-0.568233637794205	0.868826397133536	0.988741847156811	pseudobulk	DESeq2	LRT	0.0610669925949191
584	KC_AB2	Smurf	-0.394504591789951	0.78742771750732	0.988741847156811	pseudobulk	DESeq2	LRT	0.10378930208247
584	KC_AB2	Smyd4-1	-1.80294541848982	0.660135549041553	0.988741847156811	pseudobulk	DESeq2	LRT	0.180366879372519
584	KC_AB2	Smyd4-4	-0.729234089517982	0.868684292414125	0.988741847156811	pseudobulk	DESeq2	LRT	0.0611380313470854
584	KC_AB2	Smyd5	-0.506613484034373	0.961658398065406	0.988741847156811	pseudobulk	DESeq2	LRT	0.0169791713942958
584	KC_AB2	SmydA-1	-1.40296545307325	0.628223627794903	0.988741847156811	pseudobulk	DESeq2	LRT	0.201885733604539
584	KC_AB2	SmydA-3	0.818790875285784	0.940355871560339	0.988741847156811	pseudobulk	DESeq2	LRT	0.0267077593721816
584	KC_AB2	SmydA-7	-0.934020934207768	0.782359118258182	0.988741847156811	pseudobulk	DESeq2	LRT	0.106593851444836
585	KC_AB2	sn	-0.0660869289106849	0.958896701534792	0.988741847156811	pseudobulk	DESeq2	LRT	0.018228175279545
585	KC_AB2	snama	1.4775833900109	0.538570614165403	0.988741847156811	pseudobulk	DESeq2	LRT	0.268757346531586
585	KC_AB2	Snap24	-0.0783803242970516	0.906982000548461	0.988741847156811	pseudobulk	DESeq2	LRT	0.0424013316169132
585	KC_AB2	Snap25	0.313872675849748	0.414660288139788	0.988741847156811	pseudobulk	DESeq2	LRT	0.382307554864056
585	KC_AB2	Snap29	-0.626862388405342	0.704279663046672	0.988741847156811	pseudobulk	DESeq2	LRT	0.152254852221227
585	KC_AB2	snf	0.874911712375826	0.500501858041644	0.988741847156811	pseudobulk	DESeq2	LRT	0.300594305925451
585	KC_AB2	SNF4Agamma	0.550470981392675	0.681063982536233	0.988741847156811	pseudobulk	DESeq2	LRT	0.166812086388027
585	KC_AB2	sni	0.818790875285784	0.940355871560339	0.988741847156811	pseudobulk	DESeq2	LRT	0.0267077593721816
585	KC_AB2	Snm1	0.818790875285784	0.940355871560339	0.988741847156811	pseudobulk	DESeq2	LRT	0.0267077593721816
585	KC_AB2	sno	0.695314812006909	0.451383520129528	0.988741847156811	pseudobulk	DESeq2	LRT	0.345454300923872
586	KC_AB2	Snoo	0.103893543332057	0.873953876777459	0.988741847156811	pseudobulk	DESeq2	LRT	0.0585114868044999
586	KC_AB2	snoRNA:Psi18S-1389a	-1.80294541848982	0.660135549041553	0.988741847156811	pseudobulk	DESeq2	LRT	0.180366879372519
586	KC_AB2	snoRNA:Psi28S-1153	1.42621218304824	0.66738831402044	0.988741847156811	pseudobulk	DESeq2	LRT	0.175621402117653
586	KC_AB2	snoRNA:Psi28S-1192b	-1.80294541848982	0.660135549041553	0.988741847156811	pseudobulk	DESeq2	LRT	0.180366879372519
586	KC_AB2	snoRNA:Psi28S-2622	1.42621218304824	0.66738831402044	0.988741847156811	pseudobulk	DESeq2	LRT	0.175621402117653
586	KC_AB2	snoRNA:Psi28S-2626	-1.80294541848982	0.660135549041553	0.988741847156811	pseudobulk	DESeq2	LRT	0.180366879372519
586	KC_AB2	snoRNA:Psi28S-3342	0.818790875285784	0.940355871560339	0.988741847156811	pseudobulk	DESeq2	LRT	0.0267077593721816
586	KC_AB2	snoRNA:Tudor-SN-a	-1.80294541848982	0.660135549041553	0.988741847156811	pseudobulk	DESeq2	LRT	0.180366879372519
586	KC_AB2	Snp	0.575200883059258	0.804213580533116	0.988741847156811	pseudobulk	DESeq2	LRT	0.0946285973599483
586	KC_AB2	sNPF	0.153515992530009	0.576978569921154	0.988741847156811	pseudobulk	DESeq2	LRT	0.238840317065805
587	KC_AB2	sNPF-R	-1.80294541848982	0.660135549041553	0.988741847156811	pseudobulk	DESeq2	LRT	0.180366879372519
587	KC_AB2	Snr1	1.056891727646	0.538230477376054	0.988741847156811	pseudobulk	DESeq2	LRT	0.269031713890201
587	KC_AB2	snRNA:7SK	0.390608789745271	0.67692753145665	0.988741847156811	pseudobulk	DESeq2	LRT	0.169457822264759
587	KC_AB2	snRNA:U12	0.818790875285784	0.940355871560339	0.988741847156811	pseudobulk	DESeq2	LRT	0.0267077593721816
587	KC_AB2	snRNA:U2:14B	-2.26538931672255	0.547771004842275	0.988741847156811	pseudobulk	DESeq2	LRT	0.2614009599796
587	KC_AB2	snRNA:U4:39B	-1.80294541848982	0.660135549041553	0.988741847156811	pseudobulk	DESeq2	LRT	0.180366879372519
587	KC_AB2	snRNA:U4atac	-1.80294541848982	0.660135549041553	0.988741847156811	pseudobulk	DESeq2	LRT	0.180366879372519
587	KC_AB2	snRNP-U1-70K	-0.0140542944791947	0.960562692423774	0.988741847156811	pseudobulk	DESeq2	LRT	0.0174742850602987
587	KC_AB2	snRNP-U1-C	-0.358098920152354	0.844906190835001	0.988741847156811	pseudobulk	DESeq2	LRT	0.0731915076944904

587	KC_AB2	SNRPG	0.586056275133576	0.725643131261145	0.988741847156811	pseudobulk	DESeq2	LRT	0.139276911295247
588	KC_AB2	Snx1	-0.418154869600558	0.847540414580397	0.988741847156811	pseudobulk	DESeq2	LRT	0.07183958349565
588	KC_AB2	Snx16	-1.40296545307325	0.628223627794903	0.988741847156811	pseudobulk	DESeq2	LRT	0.201885733604539
588	KC_AB2	Snx17	1.1669370038017	0.601782723498991	0.988741847156811	pseudobulk	DESeq2	LRT	0.220560284520093
588	KC_AB2	Snx21	-0.382714549144618	0.863954068453144	0.988741847156811	pseudobulk	DESeq2	LRT	0.063509345886375
588	KC_AB2	Snx3	0.0557672462994941	0.93768168726417	0.988741847156811	pseudobulk	DESeq2	LRT	0.0279445655935353
588	KC_AB2	Snx6	1.39720153729184	0.381821978957304	0.988741847156811	pseudobulk	DESeq2	LRT	0.418139075771742
588	KC_AB2	snz	-0.184053861974217	0.929726174796487	0.988741847156811	pseudobulk	DESeq2	LRT	0.0316449420757317
588	KC_AB2	Sobp	1.42621218304824	0.66738831402044	0.988741847156811	pseudobulk	DESeq2	LRT	0.175621402117653
588	KC_AB2	Socs16D	-0.0291311708614138	0.977739902863604	0.988741847156811	pseudobulk	DESeq2	LRT	0.00977666031908129
588	KC_AB2	Socs36E	-0.0239083735188339	0.96203886697272	0.988741847156811	pseudobulk	DESeq2	LRT	0.0168073818386939
589	KC_AB2	Socs44A	0.370787695871894	0.854629936390885	0.988741847156811	pseudobulk	DESeq2	LRT	0.0682218985751522
589	KC_AB2	Sod1	0.0984524088944726	0.863948983225933	0.988741847156811	pseudobulk	DESeq2	LRT	0.0635119021479833
589	KC_AB2	Sod2	0.387952633736696	0.665409459902575	0.988741847156811	pseudobulk	DESeq2	LRT	0.176911029204672
589	KC_AB2	Sod3	1.42621218304824	0.66738831402044	0.988741847156811	pseudobulk	DESeq2	LRT	0.175621402117653
589	KC_AB2	sol	-0.0606679669214317	0.950419719208847	0.988741847156811	pseudobulk	DESeq2	LRT	0.022084561572954
589	KC_AB2	Son	-0.8330424863479	0.701126211347028	0.988741847156811	pseudobulk	DESeq2	LRT	0.15420379664422
589	KC_AB2	Sos	0.338127523453888	0.843168067999016	0.988741847156811	pseudobulk	DESeq2	LRT	0.0740858491837329
589	KC_AB2	sotv	0.572804916048311	0.73434626003257	0.988741847156811	pseudobulk	DESeq2	LRT	0.134099112614647
589	KC_AB2	sov	-0.260156296167586	0.854277965848429	0.988741847156811	pseudobulk	DESeq2	LRT	0.0684007951281407
589	KC_AB2	sowah	-1.58264982430149	0.397409063953286	0.988741847156811	pseudobulk	DESeq2	LRT	0.400762231891513
590	KC_AB2	SP1173	-0.427276807624177	0.498102580616086	0.988741847156811	pseudobulk	DESeq2	LRT	0.302681208228623
590	KC_AB2	SP2353	1.69894780879763	0.540778829726708	0.988741847156811	pseudobulk	DESeq2	LRT	0.26698031838453
590	KC_AB2	spab	-0.0297131143523102	0.965478288113855	0.988741847156811	pseudobulk	DESeq2	LRT	0.0152574882837251
590	KC_AB2	spag	-0.568233637794205	0.868826397133536	0.988741847156811	pseudobulk	DESeq2	LRT	0.0610669925949191
590	KC_AB2	spartin	2.00515076565275	0.576456787459964	0.988741847156811	pseudobulk	DESeq2	LRT	0.239233242872451
590	KC_AB2	spas	0.804708302235815	0.733338290589079	0.988741847156811	pseudobulk	DESeq2	LRT	0.13469563812263
590	KC_AB2	Spase12	1.73504880462037	0.271054122995879	0.988741847156811	pseudobulk	DESeq2	LRT	0.566943982293216
590	KC_AB2	Spase22-23	0.320348949796381	0.849242092899403	0.988741847156811	pseudobulk	DESeq2	LRT	0.0709684880565728
590	KC_AB2	Spase25	-0.589372447467994	0.699327975248179	0.988741847156811	pseudobulk	DESeq2	LRT	0.155319098310737
590	KC_AB2	SpdS	-1.2337849808706	0.539269081720012	0.988741847156811	pseudobulk	DESeq2	LRT	0.268194478698133
591	KC_AB2	Spec2	0.300414301642887	0.881846961452027	0.988741847156811	pseudobulk	DESeq2	LRT	0.0546067771853455
591	KC_AB2	spen	-0.0851394777281511	0.800525585108777	0.988741847156811	pseudobulk	DESeq2	LRT	0.0966247833024635
591	KC_AB2	Spf45	0.801371043861579	0.637329902962428	0.988741847156811	pseudobulk	DESeq2	LRT	0.195635704324295
591	KC_AB2	spg	-1.98612903452902	0.287765957616863	0.988741847156811	pseudobulk	DESeq2	LRT	0.540960583900466
591	KC_AB2	Spg7	-0.158601889198964	0.945838126788742	0.988741847156811	pseudobulk	DESeq2	LRT	0.0241831835324482
591	KC_AB2	spi	-0.13668622971369	0.91203917392455	0.988741847156811	pseudobulk	DESeq2	LRT	0.0399865074458278
591	KC_AB2	spict	-0.506613484034373	0.961658398065406	0.988741847156811	pseudobulk	DESeq2	LRT	0.0169791713942958
591	KC_AB2	spidey	0.151640007554404	0.880349603371991	0.988741847156811	pseudobulk	DESeq2	LRT	0.0553448270954178
591	KC_AB2	spin	0.997284248814671	0.663837179223659	0.988741847156811	pseudobulk	DESeq2	LRT	0.177938427914901
591	KC_AB2	spir	0.689390462272313	0.31337403329805	0.988741847156811	pseudobulk	DESeq2	LRT	0.503936992751223
592	KC_AB2	sPLA2	-2.26538931672255	0.547771004842275	0.988741847156811	pseudobulk	DESeq2	LRT	0.2614009599796
592	KC_AB2	Sply	1.91626857490363	0.477198801200827	0.988741847156811	pseudobulk	DESeq2	LRT	0.321300656017034
592	KC_AB2	Spn	-0.387074762505574	0.443132166731678	0.988741847156811	pseudobulk	DESeq2	LRT	0.353466723615364
592	KC_AB2	spn-A	-1.80294541848982	0.660135549041553	0.988741847156811	pseudobulk	DESeq2	LRT	0.180366879372519
592	KC_AB2	spn-F	-0.983811206789992	0.610594181020465	0.988741847156811	pseudobulk	DESeq2	LRT	0.214247338856132
592	KC_AB2	Spn42Da	2.26998970133409	0.374936237995469	0.988741847156811	pseudobulk	DESeq2	LRT	0.426042582515506
592	KC_AB2	SPoCk	-0.348426913676818	0.648836073525089	0.988741847156811	pseudobulk	DESeq2	LRT	0.187865012530913
592	KC_AB2	spoon	0.841048444940544	0.664765959075749	0.988741847156811	pseudobulk	DESeq2	LRT	0.177331227737896
592	KC_AB2	Spp	0.0912892557890945	0.950837602536886	0.988741847156811	pseudobulk	DESeq2	LRT	0.0218936516689727
592	KC_AB2	SppL	0.772645244709987	0.645928609712238	0.988741847156811	pseudobulk	DESeq2	LRT	0.18981547909676
593	KC_AB2	Spps	2.41462217514131	0.497446973552313	0.988741847156811	pseudobulk	DESeq2	LRT	0.303253207020768
593	KC_AB2	SPR	0.362555753552128	0.897034383127386	0.988741847156811	pseudobulk	DESeq2	LRT	0.0471909102264623
593	KC_AB2	Spred	-0.0215264818280747	0.948792962254257	0.988741847156811	pseudobulk	DESeq2	LRT	0.0228285453810318
593	KC_AB2	spri	-0.268451881258776	0.899861448944124	0.988741847156811	pseudobulk	DESeq2	LRT	0.0458243534396852
593	KC_AB2	Sps1	1.40305140665399	0.649741560632143	0.988741847156811	pseudobulk	DESeq2	LRT	0.187259352757606

593	KC_AB2	Spt	-0.378852672109618	0.920549594954422	0.988741847156811	pseudobulk	DESeq2	LRT	0.0359528087516892
593	KC_AB2	Spt-I	1.40515361337666	0.434775214219175	0.988741847156811	pseudobulk	DESeq2	LRT	0.361735222255163
593	KC_AB2	Spt20	0.921695369118063	0.730077514579258	0.988741847156811	pseudobulk	DESeq2	LRT	0.136631027048226
593	KC_AB2	Spt3	-0.233272055930639	0.937531793764924	0.988741847156811	pseudobulk	DESeq2	LRT	0.028013995469539
593	KC_AB2	spt4	0.525540442231456	0.811763947838672	0.988741847156811	pseudobulk	DESeq2	LRT	0.0905702405309365
594	KC_AB2	Spt5	0.343476917369387	0.818061130055175	0.988741847156811	pseudobulk	DESeq2	LRT	0.0872142422288078
594	KC_AB2	Spt6	-1.03317945058074	0.530784861466785	0.988741847156811	pseudobulk	DESeq2	LRT	0.275081472158762
594	KC_AB2	Spt7	1.42621218304824	0.66738831402044	0.988741847156811	pseudobulk	DESeq2	LRT	0.175621402117653
594	KC_AB2	Sptr	0.725727189706227	0.609272864774982	0.988741847156811	pseudobulk	DESeq2	LRT	0.215188163646275
594	KC_AB2	Spx	2.00515076565275	0.576456787459964	0.988741847156811	pseudobulk	DESeq2	LRT	0.239233242872451
594	KC_AB2	spz	1.42621218304824	0.66738831402044	0.988741847156811	pseudobulk	DESeq2	LRT	0.175621402117653
594	KC_AB2	sqd	0.304034479252221	0.516328071801899	0.988741847156811	pseudobulk	DESeq2	LRT	0.287074262523486
594	KC_AB2	sqh	0.573230942921688	0.573201316517484	0.988741847156811	pseudobulk	DESeq2	LRT	0.241692820794543
594	KC_AB2	squ	-2.26538931672255	0.547771004842275	0.988741847156811	pseudobulk	DESeq2	LRT	0.2614009599796
594	KC_AB2	sqz	-0.934020934207768	0.782359118258182	0.988741847156811	pseudobulk	DESeq2	LRT	0.106593851444836
595	KC_AB2	sr	0.183202733028021	0.792970380950365	0.988741847156811	pseudobulk	DESeq2	LRT	0.100743034157868
595	KC_AB2	sra	0.906084394431434	0.276613271159322	0.988741847156811	pseudobulk	DESeq2	LRT	0.55812698744797
595	KC_AB2	Sra-1	-0.634227699706429	0.828294391400015	0.988741847156811	pseudobulk	DESeq2	LRT	0.0818152793537044
595	KC_AB2	Sras	-2.11027789354592	0.473734933668313	0.988741847156811	pseudobulk	DESeq2	LRT	0.32446458879962
595	KC_AB2	Src42A	-0.115065361347364	0.907953433311489	0.988741847156811	pseudobulk	DESeq2	LRT	0.0419364247988079
595	KC_AB2	Src64B	-0.468321440087857	0.67801523498237	0.988741847156811	pseudobulk	DESeq2	LRT	0.168760547439074
595	KC_AB2	SREBP	0.317508825551005	0.883746467592857	0.988741847156811	pseudobulk	DESeq2	LRT	0.053672309102253
595	KC_AB2	srl	0.818790875285784	0.940355871560339	0.988741847156811	pseudobulk	DESeq2	LRT	0.0267077593721816
595	KC_AB2	Srlp	3.10973530061152	0.150972653486424	0.988741847156811	pseudobulk	DESeq2	LRT	0.821101711749896
595	KC_AB2	SRm160	0.235838008090229	0.860616583759302	0.988741847156811	pseudobulk	DESeq2	LRT	0.0651902894807914
596	KC_AB2	Srp14	-0.0821793982666366	0.94726746716445	0.988741847156811	pseudobulk	DESeq2	LRT	0.0235273777941202
596	KC_AB2	Srp19	-1.1284597703615	0.729754448571724	0.988741847156811	pseudobulk	DESeq2	LRT	0.136823248879498
596	KC_AB2	Srp54	0.817156183996148	0.564025022286417	0.988741847156811	pseudobulk	DESeq2	LRT	0.248701628641018
596	KC_AB2	Srp54k	-0.933299237375886	0.70112757174831	0.988741847156811	pseudobulk	DESeq2	LRT	0.154202953979679
596	KC_AB2	Srp68	-1.80294541848982	0.660135549041553	0.988741847156811	pseudobulk	DESeq2	LRT	0.180366879372519
596	KC_AB2	Srp72	-0.0157607737407134	0.979432010380381	0.988741847156811	pseudobulk	DESeq2	LRT	0.00902570621489607
596	KC_AB2	Srp9	-0.477864718012166	0.75458376081892	0.988741847156811	pseudobulk	DESeq2	LRT	0.122292545361662
596	KC_AB2	SRPK	-0.476171242352823	0.605128869465497	0.988741847156811	pseudobulk	DESeq2	LRT	0.218152127268401
596	KC_AB2	Srpk79D	-0.813347322381823	0.578826803997097	0.988741847156811	pseudobulk	DESeq2	LRT	0.237451366014473
596	KC_AB2	SrpRbeta	0.575200883059258	0.804213580533116	0.988741847156811	pseudobulk	DESeq2	LRT	0.0946285973599483
597	KC_AB2	Sry-delta	-0.588639054357632	0.8432408255636	0.988741847156811	pseudobulk	DESeq2	LRT	0.0740483752247712
597	KC_AB2	Ssadh	-0.663273929909526	0.684336515129322	0.988741847156811	pseudobulk	DESeq2	LRT	0.164730286109088
597	KC_AB2	Ssb-c31a	-0.757401134338791	0.553436109416208	0.988741847156811	pseudobulk	DESeq2	LRT	0.256932508352645
597	KC_AB2	Ssdp	-0.0165562282003392	0.970517145068378	0.988741847156811	pseudobulk	DESeq2	LRT	0.01299678800069
597	KC_AB2	Sse	0.818790875285784	0.940355871560339	0.988741847156811	pseudobulk	DESeq2	LRT	0.0267077593721816
597	KC_AB2	ssh	0.363130219669324	0.869039837990352	0.988741847156811	pseudobulk	DESeq2	LRT	0.0609603144341722
597	KC_AB2	Ssl1	0.157562008957688	0.950463298513166	0.988741847156811	pseudobulk	DESeq2	LRT	0.0220646484576093
597	KC_AB2	ssp	0.157562008957688	0.950463298513166	0.988741847156811	pseudobulk	DESeq2	LRT	0.0220646484576093
597	KC_AB2	ssp2	0.818790875285784	0.940355871560339	0.988741847156811	pseudobulk	DESeq2	LRT	0.0267077593721816
597	KC_AB2	ssp3	0.451878341743173	0.8022111819877	0.988741847156811	pseudobulk	DESeq2	LRT	0.0957112886997858
598	KC_AB2	ssp6	0.818790875285784	0.940355871560339	0.988741847156811	pseudobulk	DESeq2	LRT	0.0267077593721816
598	KC_AB2	SsRbeta	-0.409748433453832	0.770864010582775	0.988741847156811	pseudobulk	DESeq2	LRT	0.113022229812312
598	KC_AB2	Ssrp	-2.26538931672255	0.547771004842275	0.988741847156811	pseudobulk	DESeq2	LRT	0.2614009599796
598	KC_AB2	sstn	-1.80294541848982	0.660135549041553	0.988741847156811	pseudobulk	DESeq2	LRT	0.180366879372519
598	KC_AB2	Ssu72	2.7785831508444	0.373751317747552	0.988741847156811	pseudobulk	DESeq2	LRT	0.427417267458199
598	KC_AB2	ssx	-0.22881819232764	0.762880320539376	0.988741847156811	pseudobulk	DESeq2	LRT	0.117543588134372
598	KC_AB2	St3	0.372213729468475	0.738433039377331	0.988741847156811	pseudobulk	DESeq2	LRT	0.131688880104769
598	KC_AB2	sta	0.16735203903823	0.747682593575037	0.988741847156811	pseudobulk	DESeq2	LRT	0.126282729828518
598	KC_AB2	Stacl	-0.3183351635678	0.34440566134287	0.988741847156811	pseudobulk	DESeq2	LRT	0.462929718223724
598	KC_AB2	stai	0.298652329695411	0.414368420716739	0.988741847156811	pseudobulk	DESeq2	LRT	0.382613349862854
599	KC_AB2	Stam	0.10598154425916	0.959628155914924	0.988741847156811	pseudobulk	DESeq2	LRT	0.0178970181249073

599	KC_AB2	stan	1.42621218304824	0.66738831402044	0.988741847156811	pseudobulk	DESeq2	LRT	0.175621402117653
599	KC_AB2	Start1	0.712171366772551	0.813205723536451	0.988741847156811	pseudobulk	DESeq2	LRT	0.0897995733547685
599	KC_AB2	stas	0.366369789377649	0.766601879605175	0.988741847156811	pseudobulk	DESeq2	LRT	0.115430120248436
599	KC_AB2	Stat92E	-0.448240788523268	0.507598505596245	0.988741847156811	pseudobulk	DESeq2	LRT	0.294479665167285
599	KC_AB2	stau	-0.21029468565071	0.868188879141345	0.988741847156811	pseudobulk	DESeq2	LRT	0.0613857814534123
599	KC_AB2	stc	1.45259040912931	0.616176917291092	0.988741847156811	pseudobulk	DESeq2	LRT	0.210294574894236
599	KC_AB2	stck	0.243724124886986	0.863908585446489	0.988741847156811	pseudobulk	DESeq2	LRT	0.063532209993971
599	KC_AB2	ste14	-1.80294541848982	0.660135549041553	0.988741847156811	pseudobulk	DESeq2	LRT	0.180366879372519
599	KC_AB2	step	0.252942429258202	0.825640947213529	0.988741847156811	pseudobulk	DESeq2	LRT	0.083208776584227
600	KC_AB2	stet	0.157562008957688	0.950463298513166	0.988741847156811	pseudobulk	DESeq2	LRT	0.0220646484576093
600	KC_AB2	Stim	-0.728813744705868	0.691255064977443	0.988741847156811	pseudobulk	DESeq2	LRT	0.160361673504684
600	KC_AB2	Stip1	-0.0323203545809474	0.954448156132187	0.988741847156811	pseudobulk	DESeq2	LRT	0.020247656706879
600	KC_AB2	stj	-0.341366825898176	0.62736403385944	0.988741847156811	pseudobulk	DESeq2	LRT	0.202480382584573
600	KC_AB2	Stlk	-0.784556883954341	0.382514053772933	0.988741847156811	pseudobulk	DESeq2	LRT	0.417352604003407
600	KC_AB2	stmA	-0.288315087326322	0.703147726356827	0.988741847156811	pseudobulk	DESeq2	LRT	0.152953423199676
600	KC_AB2	Stoml2	0.835772402873027	0.561583823515908	0.988741847156811	pseudobulk	DESeq2	LRT	0.250585410598622
600	KC_AB2	stops	-0.957133960806503	0.762632518785665	0.988741847156811	pseudobulk	DESeq2	LRT	0.1176846802739
600	KC_AB2	Strip	-0.568233637794205	0.868826397133536	0.988741847156811	pseudobulk	DESeq2	LRT	0.0610669925949191
600	KC_AB2	Strn-Mlck	0.818790875285784	0.940355871560339	0.988741847156811	pseudobulk	DESeq2	LRT	0.0267077593721816
601	KC_AB2	Strump	1.47543920254359	0.661929169513603	0.988741847156811	pseudobulk	DESeq2	LRT	0.17918848025087
601	KC_AB2	Stt3A	0.508232850315684	0.818116998464947	0.988741847156811	pseudobulk	DESeq2	LRT	0.0871845836706556
601	KC_AB2	Stt3B	-0.659744005757546	0.719120576365438	0.988741847156811	pseudobulk	DESeq2	LRT	0.143198284499195
601	KC_AB2	STUB1	0.0552270667948139	0.965050912194612	0.988741847156811	pseudobulk	DESeq2	LRT	0.0154497744262272
601	KC_AB2	sturkopf	-0.873412790089593	0.682729887711167	0.988741847156811	pseudobulk	DESeq2	LRT	0.165751084721312
601	KC_AB2	stv	0.239727465394146	0.739819780655522	0.988741847156811	pseudobulk	DESeq2	LRT	0.130874061078619
601	KC_AB2	stw	0.157562008957688	0.950463298513166	0.988741847156811	pseudobulk	DESeq2	LRT	0.0220646484576093
601	KC_AB2	stwl	2.00515076565275	0.576456787459964	0.988741847156811	pseudobulk	DESeq2	LRT	0.239233242872451
601	KC_AB2	stx	-0.134257250644375	0.894381064824843	0.988741847156811	pseudobulk	DESeq2	LRT	0.0484774039222474
601	KC_AB2	sty	-0.514789246145311	0.264583764037205	0.988741847156811	pseudobulk	DESeq2	LRT	0.577436809450826
602	KC_AB2	Su(dx)	-1.05294575252525	0.645160213371264	0.988741847156811	pseudobulk	DESeq2	LRT	0.190332423127619
602	KC_AB2	su(f)	1.1327121871749	0.657275461456076	0.988741847156811	pseudobulk	DESeq2	LRT	0.182252581249451
602	KC_AB2	Su(fu)	-0.957133960806503	0.762632518785665	0.988741847156811	pseudobulk	DESeq2	LRT	0.1176846802739
602	KC_AB2	Su(H)	1.91626857490363	0.477198801200827	0.988741847156811	pseudobulk	DESeq2	LRT	0.321300656017034
602	KC_AB2	su(Hw)	1.14703591811676	0.660809111836255	0.988741847156811	pseudobulk	DESeq2	LRT	0.179923977199595
602	KC_AB2	Su(P)	0.983211168940078	0.513070192434908	0.988741847156811	pseudobulk	DESeq2	LRT	0.289823215586416
602	KC_AB2	su(sable)	0.608058389265249	0.698238294085596	0.988741847156811	pseudobulk	DESeq2	LRT	0.155996336481445
602	KC_AB2	Su(Tpl)	-0.203303383046259	0.779847708037916	0.988741847156811	pseudobulk	DESeq2	LRT	0.107990199894594
602	KC_AB2	Su(var)2-10	-0.149066352319491	0.908072435454937	0.988741847156811	pseudobulk	DESeq2	LRT	0.0418795071356874
602	KC_AB2	Su(var)2-HP2	0.805654759462258	0.685063269453114	0.988741847156811	pseudobulk	DESeq2	LRT	0.164269317112732
603	KC_AB2	Su(var)205	0.100673475185603	0.928865504841065	0.988741847156811	pseudobulk	DESeq2	LRT	0.0320471651602236
603	KC_AB2	Su(var)3-3	0.310390049490412	0.904941705285892	0.988741847156811	pseudobulk	DESeq2	LRT	0.04337939636174
603	KC_AB2	Su(var)3-7	0.666943671827768	0.808936122914367	0.988741847156811	pseudobulk	DESeq2	LRT	0.0920857707994393
603	KC_AB2	su(w[a])	0.745939594314228	0.626839506528318	0.988741847156811	pseudobulk	DESeq2	LRT	0.202843639953029
603	KC_AB2	Su(z)12	0.241279285109204	0.928945177902019	0.988741847156811	pseudobulk	DESeq2	LRT	0.0320099153251665
603	KC_AB2	Su(z)2	-0.156893364199718	0.890974226891612	0.988741847156811	pseudobulk	DESeq2	LRT	0.0501348585676638
603	KC_AB2	subdued	-0.568233637794205	0.868826397133536	0.988741847156811	pseudobulk	DESeq2	LRT	0.0610669925949191
603	KC_AB2	sud1	1.12452187615248	0.704078404906088	0.988741847156811	pseudobulk	DESeq2	LRT	0.152378975911753
603	KC_AB2	sug	2.00515076565275	0.576456787459964	0.988741847156811	pseudobulk	DESeq2	LRT	0.239233242872451
603	KC_AB2	sun	0.601245460096697	0.336108656580388	0.988741847156811	pseudobulk	DESeq2	LRT	0.473520302000381
604	KC_AB2	Sur-8	0.325548032832598	0.713852421717042	0.988741847156811	pseudobulk	DESeq2	LRT	0.14639156281564
604	KC_AB2	Surf1	-1.23536834900699	0.725570392794057	0.988741847156811	pseudobulk	DESeq2	LRT	0.139320447155934
604	KC_AB2	Surf4	3.09552462727688	0.154060029930595	0.988741847156811	pseudobulk	DESeq2	LRT	0.812310022100605
604	KC_AB2	Surf6	-0.587425801135323	0.771690223849521	0.988741847156811	pseudobulk	DESeq2	LRT	0.112557001570832
604	KC_AB2	sut2	2.0407202319306	0.462680771122375	0.988741847156811	pseudobulk	DESeq2	LRT	0.334718549276796
604	KC_AB2	Suv3	1.31950274499659	0.590522087411301	0.988741847156811	pseudobulk	DESeq2	LRT	0.228763853746569
604	KC_AB2	sv	1.42621218304824	0.66738831402044	0.988741847156811	pseudobulk	DESeq2	LRT	0.175621402117653

604	KC_AB2	Svil	-0.156751412878358	0.823822503726105	0.988741847156811	pseudobulk	DESeq2	LRT	0.0841663489290255
604	KC_AB2	svr	-0.0198389435329613	0.959813775787338	0.988741847156811	pseudobulk	DESeq2	LRT	0.0178130211201105
604	KC_AB2	sw	-0.341472371153588	0.849350394863359	0.988741847156811	pseudobulk	DESeq2	LRT	0.070913106974026
605	KC_AB2	Swip-1	0.832064699200183	0.64462948842046	0.988741847156811	pseudobulk	DESeq2	LRT	0.190689831672951
605	KC_AB2	swm	0.0969125984762957	0.915732560450358	0.988741847156811	pseudobulk	DESeq2	LRT	0.0382313434494357
605	KC_AB2	sws	0.601576675848729	0.686569785793211	0.988741847156811	pseudobulk	DESeq2	LRT	0.163315312695893
605	KC_AB2	sxc	0.793407239222973	0.349285935976704	0.988741847156811	pseudobulk	DESeq2	LRT	0.456818900908809
605	KC_AB2	Sxl	-0.407749675146486	0.374808096926894	0.988741847156811	pseudobulk	DESeq2	LRT	0.426191035679971
605	KC_AB2	syd	-0.08939915861675	0.933601220234383	0.988741847156811	pseudobulk	DESeq2	LRT	0.0298385893312458
605	KC_AB2	Syn	-0.268239393416362	0.687178776767304	0.988741847156811	pseudobulk	DESeq2	LRT	0.162930261975389
605	KC_AB2	Syn1	0.800046907705718	0.468783664934186	0.988741847156811	pseudobulk	DESeq2	LRT	0.32902752999454
605	KC_AB2	Syn2	0.770355407522861	0.689231851206441	0.988741847156811	pseudobulk	DESeq2	LRT	0.161634660876404
605	KC_AB2	Synd	-0.341472371153588	0.849350394863359	0.988741847156811	pseudobulk	DESeq2	LRT	0.070913106974026
606	KC_AB2	Syngr	-0.246998836869562	0.770979278371727	0.988741847156811	pseudobulk	DESeq2	LRT	0.112957294335231
606	KC_AB2	Synj	0.150266658909438	0.883764416581082	0.988741847156811	pseudobulk	DESeq2	LRT	0.0536634886229997
606	KC_AB2	Syp	-0.737476651182434	0.129160606068066	0.988741847156811	pseudobulk	DESeq2	LRT	0.888869925784026
606	KC_AB2	Syt1	0.0500866531184233	0.881417729976498	0.988741847156811	pseudobulk	DESeq2	LRT	0.054818217777186
606	KC_AB2	Syt12	-0.77701868203408	0.449954408144479	0.988741847156811	pseudobulk	DESeq2	LRT	0.346831489101047
606	KC_AB2	Syt14	-0.40443354135462	0.740373070180136	0.988741847156811	pseudobulk	DESeq2	LRT	0.130549386360063
606	KC_AB2	Syt4	0.543115150543805	0.360139457153169	0.988741847156811	pseudobulk	DESeq2	LRT	0.443529294387836
606	KC_AB2	Syt7	0.0136828891925772	0.957055016198033	0.988741847156811	pseudobulk	DESeq2	LRT	0.0190630961369932
606	KC_AB2	Sytalpha	-0.379028387144983	0.503321362186463	0.988741847156811	pseudobulk	DESeq2	LRT	0.298154636694416
606	KC_AB2	Sytbeta	-0.168112079731616	0.792189951849028	0.988741847156811	pseudobulk	DESeq2	LRT	0.101170670495467
607	KC_AB2	Syx13	1.09347879643988	0.573203382935229	0.988741847156811	pseudobulk	DESeq2	LRT	0.241691255145302
607	KC_AB2	Syx16	0.75376402089636	0.569978636191738	0.988741847156811	pseudobulk	DESeq2	LRT	0.244141422148421
607	KC_AB2	Syx17	0.5738945031678	0.80780652681376	0.988741847156811	pseudobulk	DESeq2	LRT	0.0926926421938231
607	KC_AB2	Syx18	1.4413042803421	0.615311685084066	0.988741847156811	pseudobulk	DESeq2	LRT	0.210904837358999
607	KC_AB2	Syx1A	0.715572981410654	0.11339799995922	0.988741847156811	pseudobulk	DESeq2	LRT	0.945394605181511
607	KC_AB2	Syx5	0.666943671827768	0.808936122914367	0.988741847156811	pseudobulk	DESeq2	LRT	0.0920857707994393
607	KC_AB2	Syx6	-0.535944289431147	0.668915865167044	0.988741847156811	pseudobulk	DESeq2	LRT	0.174628503445197
607	KC_AB2	Syx7	0.598872924576024	0.683432114819113	0.988741847156811	pseudobulk	DESeq2	LRT	0.16530461734143
607	KC_AB2	Syx8	1.31617713253562	0.59447159965162	0.988741847156811	pseudobulk	DESeq2	LRT	0.225868888579699
607	KC_AB2	tacc	0.16541399766479	0.820852896953685	0.988741847156811	pseudobulk	DESeq2	LRT	0.0857346647628669
608	KC_AB2	Tace	0.513202572024843	0.807055953632252	0.988741847156811	pseudobulk	DESeq2	LRT	0.093096354359303
608	KC_AB2	Taf1	-0.0384805229678164	0.985071128575604	0.988741847156811	pseudobulk	DESeq2	LRT	0.006532409469285
608	KC_AB2	Taf10	1.14997463023881	0.4924150857273	0.988741847156811	pseudobulk	DESeq2	LRT	0.30766865040408
608	KC_AB2	Taf10b	0.556489356150423	0.647891481617672	0.988741847156811	pseudobulk	DESeq2	LRT	0.18849773005739
608	KC_AB2	Taf11	-3.46227275985435	0.142395745015585	0.988741847156811	pseudobulk	DESeq2	LRT	0.846502987833018
608	KC_AB2	Taf12	0.138532944989144	0.893477417102256	0.988741847156811	pseudobulk	DESeq2	LRT	0.0489164199370943
608	KC_AB2	Taf13	0.320539134359348	0.848791832994679	0.988741847156811	pseudobulk	DESeq2	LRT	0.0711988078324561
608	KC_AB2	TAF1B	1.42621218304824	0.66738831402044	0.988741847156811	pseudobulk	DESeq2	LRT	0.175621402117653
608	KC_AB2	Taf2	1.42621218304824	0.66738831402044	0.988741847156811	pseudobulk	DESeq2	LRT	0.175621402117653
608	KC_AB2	Taf4	0.230939532034872	0.819306522118525	0.988741847156811	pseudobulk	DESeq2	LRT	0.0865535879102714
609	KC_AB2	Taf5	1.12452187615248	0.704078404906088	0.988741847156811	pseudobulk	DESeq2	LRT	0.152378975911753
609	KC_AB2	Taf6	-1.08752762896699	0.525234556296637	0.988741847156811	pseudobulk	DESeq2	LRT	0.279646708486514
609	KC_AB2	Taf7	1.47543920254359	0.661929169513603	0.988741847156811	pseudobulk	DESeq2	LRT	0.17918848025087
609	KC_AB2	Taf8	2.51243226103394	0.474458247157471	0.988741847156811	pseudobulk	DESeq2	LRT	0.323801999942597
609	KC_AB2	tai	-0.3728123898201	0.490236141982229	0.988741847156811	pseudobulk	DESeq2	LRT	0.309594674143019
609	KC_AB2	Tailor	0.668970670557767	0.818048200024539	0.988741847156811	pseudobulk	DESeq2	LRT	0.0872211066123218
609	KC_AB2	Tak1	-0.934020934207768	0.782359118258182	0.988741847156811	pseudobulk	DESeq2	LRT	0.106593851444836
609	KC_AB2	Taldo	3.09926389624515	0.339712049344771	0.988741847156811	pseudobulk	DESeq2	LRT	0.468889048740397
609	KC_AB2	tamo	2.80917294583258	0.367996267391162	0.988741847156811	pseudobulk	DESeq2	LRT	0.434156586379859
609	KC_AB2	Tango1	0.363641268105473	0.858330168669459	0.988741847156811	pseudobulk	DESeq2	LRT	0.06634562258353
610	KC_AB2	Tango10	-0.332464417915409	0.87936231254484	0.988741847156811	pseudobulk	DESeq2	LRT	0.0558321511845224
610	KC_AB2	Tango11	1.52414543155357	0.248799671964088	0.988741847156811	pseudobulk	DESeq2	LRT	0.604150196586847
610	KC_AB2	Tango4	-0.405381063532158	0.89155634938256	0.988741847156811	pseudobulk	DESeq2	LRT	0.0498512027381788

610	KC_AB2	Tango5	-0.0963677021573955	0.940374582452363	0.988741847156811	pseudobulk	DESeq2	LRT	0.0266991180093106
610	KC_AB2	Tango6	0.699674038439951	0.821690393783994	0.988741847156811	pseudobulk	DESeq2	LRT	0.0852917902455435
610	KC_AB2	Tango9	0.167075029051464	0.925389167234494	0.988741847156811	pseudobulk	DESeq2	LRT	0.0336755887326197
610	KC_AB2	tank	-1.80294541848982	0.660135549041553	0.988741847156811	pseudobulk	DESeq2	LRT	0.180366879372519
610	KC_AB2	tant	0.818790875285784	0.940355871560339	0.988741847156811	pseudobulk	DESeq2	LRT	0.0267077593721816
610	KC_AB2	Tao	0.236871836245088	0.830319292112142	0.988741847156811	pseudobulk	DESeq2	LRT	0.0807548713137575
610	KC_AB2	Tap42	1.10527623683615	0.724717602335893	0.988741847156811	pseudobulk	DESeq2	LRT	0.139831190172672
611	KC_AB2	tapas	-1.34313927715166	0.350716531286915	0.988741847156811	pseudobulk	DESeq2	LRT	0.455043762839686
611	KC_AB2	Tapdelta	1.32139018889898	0.38890393719358	0.988741847156811	pseudobulk	DESeq2	LRT	0.410157660110985
611	KC_AB2	tara	1.03734376381036	0.64157927463489	0.988741847156811	pseudobulk	DESeq2	LRT	0.192749673802887
611	KC_AB2	Task6	-0.102508762418003	0.84809584305395	0.988741847156811	pseudobulk	DESeq2	LRT	0.0715550654821935
611	KC_AB2	Task7	-0.110490738418564	0.909468469457778	0.988741847156811	pseudobulk	DESeq2	LRT	0.0412123529807809
611	KC_AB2	Tasp1	0.818790875285784	0.940355871560339	0.988741847156811	pseudobulk	DESeq2	LRT	0.0267077593721816
611	KC_AB2	Tat	1.38710003515313	0.195528943246674	0.988741847156811	pseudobulk	DESeq2	LRT	0.708788946902685
611	KC_AB2	tau	-0.216164178061638	0.787260586770358	0.988741847156811	pseudobulk	DESeq2	LRT	0.103881490430976
611	KC_AB2	tay	0.0621226932519558	0.922816509747616	0.988741847156811	pseudobulk	DESeq2	LRT	0.0348846442894919
611	KC_AB2	Taz	0.157562008957688	0.950463298513166	0.988741847156811	pseudobulk	DESeq2	LRT	0.0220646484576093
612	KC_AB2	Tbc1d15-17	0.818790875285784	0.940355871560339	0.988741847156811	pseudobulk	DESeq2	LRT	0.0267077593721816
612	KC_AB2	TBC1D16	0.782763495027571	0.564150321480935	0.988741847156811	pseudobulk	DESeq2	LRT	0.248605160039235
612	KC_AB2	TBC1D5	0.818790875285784	0.940355871560339	0.988741847156811	pseudobulk	DESeq2	LRT	0.0267077593721816
612	KC_AB2	TBCB	1.13539439938001	0.65602501243538	0.988741847156811	pseudobulk	DESeq2	LRT	0.183079601850592
612	KC_AB2	TBCC	1.13849377617832	0.544897316370797	0.988741847156811	pseudobulk	DESeq2	LRT	0.263685330998854
612	KC_AB2	TBCD	0.157562008957688	0.950463298513166	0.988741847156811	pseudobulk	DESeq2	LRT	0.0220646484576093
612	KC_AB2	Tbce	1.30889929962086	0.540572983771383	0.988741847156811	pseudobulk	DESeq2	LRT	0.267145662840393
612	KC_AB2	Tbp	1.64755990459092	0.573059482818602	0.988741847156811	pseudobulk	DESeq2	LRT	0.241800296498366
612	KC_AB2	TBPH	0.940151004693715	0.351827363622724	0.988741847156811	pseudobulk	DESeq2	LRT	0.453670386003618
612	KC_AB2	tbrd-1	0.818790875285784	0.940355871560339	0.988741847156811	pseudobulk	DESeq2	LRT	0.0267077593721816
613	KC_AB2	Tcs3	-1.80294541848982	0.660135549041553	0.988741847156811	pseudobulk	DESeq2	LRT	0.180366879372519
613	KC_AB2	Tctp	0.0991618621094575	0.846763399217718	0.988741847156811	pseudobulk	DESeq2	LRT	0.0722379223357976
613	KC_AB2	Tdrd3	-0.742148798960242	0.80644751100302	0.988741847156811	pseudobulk	DESeq2	LRT	0.0934238941443075
613	KC_AB2	tea	2.06137138167525	0.547946929015274	0.988741847156811	pseudobulk	DESeq2	LRT	0.261261502741816
613	KC_AB2	tef	-1.80294541848982	0.660135549041553	0.988741847156811	pseudobulk	DESeq2	LRT	0.180366879372519
613	KC_AB2	tefu	-0.378852672109618	0.920549594954422	0.988741847156811	pseudobulk	DESeq2	LRT	0.0359528087516892
613	KC_AB2	Teh1	-1.40296545307325	0.628223627794903	0.988741847156811	pseudobulk	DESeq2	LRT	0.201885733604539
613	KC_AB2	Teh2	-0.15683810134198	0.853529324057563	0.988741847156811	pseudobulk	DESeq2	LRT	0.0687815535678581
613	KC_AB2	Teh3	-2.51746470924027	0.517377929599049	0.988741847156811	pseudobulk	DESeq2	LRT	0.286192101436607
613	KC_AB2	Teh4	-0.382714549144618	0.863954068453144	0.988741847156811	pseudobulk	DESeq2	LRT	0.063509345886375
614	KC_AB2	Tektin-C	3.83932053948463	0.189970154032558	0.988741847156811	pseudobulk	DESeq2	LRT	0.721314625137331
614	KC_AB2	temp	1.45259040912931	0.616176917291092	0.988741847156811	pseudobulk	DESeq2	LRT	0.210294574894236
614	KC_AB2	Ten-m	1.82735205662592	0.155239510853705	0.988741847156811	pseudobulk	DESeq2	LRT	0.808997734354281
614	KC_AB2	teq	-1.80294541848982	0.660135549041553	0.988741847156811	pseudobulk	DESeq2	LRT	0.180366879372519
614	KC_AB2	TER94	0.602036842358073	0.689862380457653	0.988741847156811	pseudobulk	DESeq2	LRT	0.161237537333613
614	KC_AB2	Tes	0.818790875285784	0.940355871560339	0.988741847156811	pseudobulk	DESeq2	LRT	0.0267077593721816
614	KC_AB2	Tet	1.46939650739252	0.250366425340869	0.988741847156811	pseudobulk	DESeq2	LRT	0.601423911351542
614	KC_AB2	tex	0.818790875285784	0.940355871560339	0.988741847156811	pseudobulk	DESeq2	LRT	0.0267077593721816
614	KC_AB2	TFAM	-0.421426144018401	0.837702312916935	0.988741847156811	pseudobulk	DESeq2	LRT	0.0769102854580717
614	KC_AB2	Tfb1	0.666943671827768	0.808936122914367	0.988741847156811	pseudobulk	DESeq2	LRT	0.0920857707994393
615	KC_AB2	Tfb4	0.37733433210913	0.907416158791808	0.988741847156811	pseudobulk	DESeq2	LRT	0.0421934913124174
615	KC_AB2	TfIIA-L	1.76062409692731	0.431284494748455	0.988741847156811	pseudobulk	DESeq2	LRT	0.365236155037982
615	KC_AB2	TfIIA-S	0.808093551076529	0.41972669302166	0.988741847156811	pseudobulk	DESeq2	LRT	0.377033410432587
615	KC_AB2	TfIIB	-1.19564122961809	0.585865739229133	0.988741847156811	pseudobulk	DESeq2	LRT	0.232201898303775
615	KC_AB2	TfIIEalpha	2.00515076565275	0.576456787459964	0.988741847156811	pseudobulk	DESeq2	LRT	0.239233242872451
615	KC_AB2	TfIIEbeta	1.47543920254359	0.661929169513603	0.988741847156811	pseudobulk	DESeq2	LRT	0.17918848025087
615	KC_AB2	TfIIFalpha	0.100538804910555	0.939639801621475	0.988741847156811	pseudobulk	DESeq2	LRT	0.0270385954933285
615	KC_AB2	TfIIFbeta	0.699674038439951	0.821690393783994	0.988741847156811	pseudobulk	DESeq2	LRT	0.0852917902455435
615	KC_AB2	TfIIS	1.23913594857638	0.441771833901474	0.988741847156811	pseudobulk	DESeq2	LRT	0.354801976946044

615	KC_AB2	Tgi	-1.73627324524809	0.521368726288639	0.988741847156811	pseudobulk	DESeq2	LRT	0.282855023048041
616	KC_AB2	tgo	-0.506613484034373	0.961658398065406	0.988741847156811	pseudobulk	DESeq2	LRT	0.0169791713942958
616	KC_AB2	Tgt	-1.23536834900699	0.725570392794057	0.988741847156811	pseudobulk	DESeq2	LRT	0.139320447155934
616	KC_AB2	TH1	1.4413042803421	0.615311685084066	0.988741847156811	pseudobulk	DESeq2	LRT	0.210904837358999
616	KC_AB2	THADA	0.818790875285784	0.940355871560339	0.988741847156811	pseudobulk	DESeq2	LRT	0.0267077593721816
616	KC_AB2	Thd1	-0.389588844114774	0.565557758655748	0.988741847156811	pseudobulk	DESeq2	LRT	0.247523035328197
616	KC_AB2	Theg	-1.80294541848982	0.660135549041553	0.988741847156811	pseudobulk	DESeq2	LRT	0.180366879372519
616	KC_AB2	tho2	-0.487923111280115	0.783394690329388	0.988741847156811	pseudobulk	DESeq2	LRT	0.106019376327165
616	KC_AB2	thoc5	2.00515076565275	0.576456787459964	0.988741847156811	pseudobulk	DESeq2	LRT	0.239233242872451
616	KC_AB2	thoc6	0.834352593082403	0.675685094045283	0.988741847156811	pseudobulk	DESeq2	LRT	0.170255661725145
616	KC_AB2	thoc7	0.538471591573674	0.740252210768007	0.988741847156811	pseudobulk	DESeq2	LRT	0.130620286913452
617	KC_AB2	ThrRS	0.541098734321378	0.738610285177375	0.988741847156811	pseudobulk	DESeq2	LRT	0.131584649075131
617	KC_AB2	ths	-0.42076958521121	0.784050289861592	0.988741847156811	pseudobulk	DESeq2	LRT	0.105656080288865
617	KC_AB2	Tie	-1.80294541848982	0.660135549041553	0.988741847156811	pseudobulk	DESeq2	LRT	0.180366879372519
617	KC_AB2	Tif-IA	-0.460305896488822	0.736044165460061	0.988741847156811	pseudobulk	DESeq2	LRT	0.133096125553639
617	KC_AB2	Tim17b	0.530418741252958	0.547669915866775	0.988741847156811	pseudobulk	DESeq2	LRT	0.261481114707318
617	KC_AB2	Tim23	-0.518906633246996	0.660482549999843	0.988741847156811	pseudobulk	DESeq2	LRT	0.180138651991752
617	KC_AB2	Tim8	0.410744456354463	0.65563834528724	0.988741847156811	pseudobulk	DESeq2	LRT	0.183335654461418
617	KC_AB2	Tim9a	-0.415086066101405	0.804643838423678	0.988741847156811	pseudobulk	DESeq2	LRT	0.0943963099864128
617	KC_AB2	timeout	1.47543920254359	0.661929169513603	0.988741847156811	pseudobulk	DESeq2	LRT	0.17918848025087
617	KC_AB2	Tina-1	0.129751708593588	0.93205793279696	0.988741847156811	pseudobulk	DESeq2	LRT	0.0305570928905378
618	KC_AB2	tinc	-0.0387118286271094	0.946398809915596	0.988741847156811	pseudobulk	DESeq2	LRT	0.0239258144995413
618	KC_AB2	Tip60	-1.5305745667549	0.670004185239519	0.988741847156811	pseudobulk	DESeq2	LRT	0.17392248443238
618	KC_AB2	tipE	-0.562891240526638	0.668010772958821	0.988741847156811	pseudobulk	DESeq2	LRT	0.17521653363397
618	KC_AB2	tko	0.947270624774323	0.523010143189647	0.988741847156811	pseudobulk	DESeq2	LRT	0.281489888401223
618	KC_AB2	tkv	0.953485198887239	0.548755798091448	0.988741847156811	pseudobulk	DESeq2	LRT	0.260620878019401
618	KC_AB2	Tl	0.0387959805724938	0.91564943413024	0.988741847156811	pseudobulk	DESeq2	LRT	0.0382707686510151
618	KC_AB2	Tlk	-0.500382161994943	0.341884376480961	0.988741847156811	pseudobulk	DESeq2	LRT	0.466120745249444
618	KC_AB2	Tm1	0.487499775306291	0.494301766107416	0.988741847156811	pseudobulk	DESeq2	LRT	0.30600783783108
618	KC_AB2	TM4SF	0.452013519790097	0.376612456225983	0.988741847156811	pseudobulk	DESeq2	LRT	0.424105320058207
618	KC_AB2	TM9SF2	0.802207095184316	0.732945722088841	0.988741847156811	pseudobulk	DESeq2	LRT	0.134928185618234
619	KC_AB2	TM9SF3	0.572861358867068	0.813871944286787	0.988741847156811	pseudobulk	DESeq2	LRT	0.0894439222171593
619	KC_AB2	TM9SF4	0.582030305049867	0.736488992071221	0.988741847156811	pseudobulk	DESeq2	LRT	0.13283373995299
619	KC_AB2	Tmc	1.65216744322178	0.578100610800606	0.988741847156811	pseudobulk	DESeq2	LRT	0.237996571764493
619	KC_AB2	Tmem18	0.699674038439951	0.821690393783994	0.988741847156811	pseudobulk	DESeq2	LRT	0.0852917902455435
619	KC_AB2	TMEM216	-1.44345811118689	0.452828673738114	0.988741847156811	pseudobulk	DESeq2	LRT	0.34406608082163
619	KC_AB2	Tmem63	-0.382714549144618	0.863954068453144	0.988741847156811	pseudobulk	DESeq2	LRT	0.063509345886375
619	KC_AB2	tmod	1.04623600047774	0.336659324574789	0.988741847156811	pseudobulk	DESeq2	LRT	0.472809352218478
619	KC_AB2	TMS1	-0.049708075698151	0.941046785527612	0.988741847156811	pseudobulk	DESeq2	LRT	0.0263887844458784
619	KC_AB2	Tmtc3	-0.673615760041143	0.667898390124049	0.988741847156811	pseudobulk	DESeq2	LRT	0.175289603340303
619	KC_AB2	tn	0.818790875285784	0.940355871560339	0.988741847156811	pseudobulk	DESeq2	LRT	0.0267077593721816
620	KC_AB2	tna	0.234178987909775	0.575338570151177	0.988741847156811	pseudobulk	DESeq2	LRT	0.240076510309055
620	KC_AB2	tnc	-1.80294541848982	0.660135549041553	0.988741847156811	pseudobulk	DESeq2	LRT	0.180366879372519
620	KC_AB2	Tnks	-0.137361950162669	0.926230329403537	0.988741847156811	pseudobulk	DESeq2	LRT	0.0332810021405098
620	KC_AB2	Tnpo	-0.120992867902654	0.875727164843525	0.988741847156811	pseudobulk	DESeq2	LRT	0.0576311783742414
620	KC_AB2	Tnpo-SR	1.47543920254359	0.661929169513603	0.988741847156811	pseudobulk	DESeq2	LRT	0.17918848025087
620	KC_AB2	Tob	0.026282534799284	0.944578855544654	0.988741847156811	pseudobulk	DESeq2	LRT	0.0247617803590003
620	KC_AB2	toc	0.00940064151066716	0.975653552272968	0.988741847156811	pseudobulk	DESeq2	LRT	0.0107043698811166
620	KC_AB2	tok	-1.80294541848982	0.660135549041553	0.988741847156811	pseudobulk	DESeq2	LRT	0.180366879372519
620	KC_AB2	Toll-6	1.29518405230266	0.581169015396514	0.988741847156811	pseudobulk	DESeq2	LRT	0.235697547851342
620	KC_AB2	Toll-7	1.12452187615248	0.704078404906088	0.988741847156811	pseudobulk	DESeq2	LRT	0.152378975911753
621	KC_AB2	Tom20	-0.0524735597302349	0.945962830729012	0.988741847156811	pseudobulk	DESeq2	LRT	0.0241259277910407
621	KC_AB2	Tom40	0.988601462539668	0.444968127510007	0.988741847156811	pseudobulk	DESeq2	LRT	0.351671095855609
621	KC_AB2	Tom7	0.120489658958097	0.862054782893309	0.988741847156811	pseudobulk	DESeq2	LRT	0.0644651342306557
621	KC_AB2	Tom70	-0.00340877878248056	0.980029417943268	0.988741847156811	pseudobulk	DESeq2	LRT	0.0087608877170192
621	KC_AB2	Tomosyn	0.181802634851125	0.659377453052585	0.988741847156811	pseudobulk	DESeq2	LRT	0.18086590733278

621	KC_AB2	Top1	0.147705848059866	0.852383761493385	0.988741847156811	pseudobulk	DESeq2	LRT	0.0693648324925522
621	KC_AB2	Top2	1.45536849314007	0.496856278270181	0.988741847156811	pseudobulk	DESeq2	LRT	0.30376921806916
621	KC_AB2	Top3alpha	2.51243226103394	0.474458247157471	0.988741847156811	pseudobulk	DESeq2	LRT	0.323801999942597
621	KC_AB2	Top3beta	0.699674038439951	0.821690393783994	0.988741847156811	pseudobulk	DESeq2	LRT	0.0852917902455435
621	KC_AB2	Topors	-1.89400523612133	0.268051305520902	0.988741847156811	pseudobulk	DESeq2	LRT	0.571782073239484
622	KC_AB2	Tor	-1.23536834900699	0.725570392794057	0.988741847156811	pseudobulk	DESeq2	LRT	0.139320447155934
622	KC_AB2	TORIP	-1.18743225435356	0.64061820392288	0.988741847156811	pseudobulk	DESeq2	LRT	0.193400724506216
622	KC_AB2	Torsin	-2.13278318874786	0.300019830818357	0.988741847156811	pseudobulk	DESeq2	LRT	0.52285003817919
622	KC_AB2	TotA	1.47543920254359	0.661929169513603	0.988741847156811	pseudobulk	DESeq2	LRT	0.17918848025087
622	KC_AB2	tou	-1.02512116852471	0.286204535295957	0.988741847156811	pseudobulk	DESeq2	LRT	0.543323488541296
622	KC_AB2	tow	-0.0389181587202281	0.939998545170148	0.988741847156811	pseudobulk	DESeq2	LRT	0.0268728185546264
622	KC_AB2	toy	-0.199412504726813	0.585095177683038	0.988741847156811	pseudobulk	DESeq2	LRT	0.232773481301821
622	KC_AB2	Tpc2	-1.80294541848982	0.660135549041553	0.988741847156811	pseudobulk	DESeq2	LRT	0.180366879372519
622	KC_AB2	Tpi	-0.148290017750087	0.854974039377346	0.988741847156811	pseudobulk	DESeq2	LRT	0.0680470720862546
622	KC_AB2	TppII	-0.933299237375886	0.70112757174831	0.988741847156811	pseudobulk	DESeq2	LRT	0.154202953979679
623	KC_AB2	Tpr2	-0.123262046070891	0.896008838167329	0.988741847156811	pseudobulk	DESeq2	LRT	0.0476877064669258
623	KC_AB2	Tpst	1.1669370038017	0.601782723498991	0.988741847156811	pseudobulk	DESeq2	LRT	0.220560284520093
623	KC_AB2	tra	0.581445489707027	0.811994604428781	0.988741847156811	pseudobulk	DESeq2	LRT	0.0904468565649727
623	KC_AB2	tra2	0.0399531707843842	0.971730847635948	0.988741847156811	pseudobulk	DESeq2	LRT	0.0124540103550365
623	KC_AB2	Traf6	-0.506613484034373	0.961658398065406	0.988741847156811	pseudobulk	DESeq2	LRT	0.0169791713942958
623	KC_AB2	tral	0.0358426405719381	0.946096601292998	0.988741847156811	pseudobulk	DESeq2	LRT	0.0240645176504351
623	KC_AB2	TRAM	0.353798809751289	0.901648351639924	0.988741847156811	pseudobulk	DESeq2	LRT	0.0449628069360408
623	KC_AB2	Trap1	-0.405381063532158	0.89155634938256	0.988741847156811	pseudobulk	DESeq2	LRT	0.0498512027381788
623	KC_AB2	Trax	0.921695369118063	0.730077514579258	0.988741847156811	pseudobulk	DESeq2	LRT	0.136631027048226
623	KC_AB2	trbd	0.617577601747753	0.742249230397304	0.988741847156811	pseudobulk	DESeq2	LRT	0.129450244030843
624	KC_AB2	trc	1.53010705085077	0.330992108752279	0.988741847156811	pseudobulk	DESeq2	LRT	0.480182360200698
624	KC_AB2	Trc8	-0.047455149482735	0.975740487907333	0.988741847156811	pseudobulk	DESeq2	LRT	0.0106656737843436
624	KC_AB2	Treh	2.00515076565275	0.576456787459964	0.988741847156811	pseudobulk	DESeq2	LRT	0.239233242872451
624	KC_AB2	trem	0.818790875285784	0.940355871560339	0.988741847156811	pseudobulk	DESeq2	LRT	0.0267077593721816
624	KC_AB2	Trf	-2.9721553030081	0.387854083811327	0.988741847156811	pseudobulk	DESeq2	LRT	0.411331631401987
624	KC_AB2	Trf2	-0.640730906023272	0.48717944944114	0.988741847156811	pseudobulk	DESeq2	LRT	0.312311039715392
624	KC_AB2	Trf4-1	-0.623735960743867	0.589664699033323	0.988741847156811	pseudobulk	DESeq2	LRT	0.229394870990939
624	KC_AB2	trio	0.73660763501669	0.393837844705487	0.988741847156811	pseudobulk	DESeq2	LRT	0.404682553923994
624	KC_AB2	trk	-1.80294541848982	0.660135549041553	0.988741847156811	pseudobulk	DESeq2	LRT	0.180366879372519
624	KC_AB2	Trl	-0.506613484034373	0.961658398065406	0.988741847156811	pseudobulk	DESeq2	LRT	0.0169791713942958
625	KC_AB2	trn	-1.24728087159609	0.373441201656967	0.988741847156811	pseudobulk	DESeq2	LRT	0.427777768162171
625	KC_AB2	tRNA:Cys-GCA-4-1	0.818790875285784	0.940355871560339	0.988741847156811	pseudobulk	DESeq2	LRT	0.0267077593721816
625	KC_AB2	tRNA:Thr-AGT-1-1	-2.26538931672255	0.547771004842275	0.988741847156811	pseudobulk	DESeq2	LRT	0.2614009599796
625	KC_AB2	Trp1	0.57260030201287	0.688930306488789	0.988741847156811	pseudobulk	DESeq2	LRT	0.161824709933087
625	KC_AB2	Trpm	-0.0752212693799757	0.899417726556159	0.988741847156811	pseudobulk	DESeq2	LRT	0.0460385572065276
625	KC_AB2	TrpRS	0.818790875285784	0.940355871560339	0.988741847156811	pseudobulk	DESeq2	LRT	0.0267077593721816
625	KC_AB2	TrpRS-m	-1.80294541848982	0.660135549041553	0.988741847156811	pseudobulk	DESeq2	LRT	0.180366879372519
625	KC_AB2	trr	-0.58621066677634	0.754406236055079	0.988741847156811	pseudobulk	DESeq2	LRT	0.122394730301062
625	KC_AB2	Trs20	0.32140381670586	0.812225745916471	0.988741847156811	pseudobulk	DESeq2	LRT	0.0903232483694482
625	KC_AB2	Trs23	-2.29552462754713	0.335269163364018	0.988741847156811	pseudobulk	DESeq2	LRT	0.474606389299205
626	KC_AB2	Trs31	-0.411829322976732	0.82693929768058	0.988741847156811	pseudobulk	DESeq2	LRT	0.0825263691051666
626	KC_AB2	Trs33	0.0601466527378941	0.974810960364079	0.988741847156811	pseudobulk	DESeq2	LRT	0.0110795964355292
626	KC_AB2	trsn	-0.236009043885908	0.902272246073982	0.988741847156811	pseudobulk	DESeq2	LRT	0.0446624013404197
626	KC_AB2	trus	-1.41593304226044	0.504863266150427	0.988741847156811	pseudobulk	DESeq2	LRT	0.296826227420053
626	KC_AB2	trv	0.22886475811	0.539704682429252	0.988741847156811	pseudobulk	DESeq2	LRT	0.267843814018602
626	KC_AB2	trx	0.103946039617437	0.892688986598918	0.988741847156811	pseudobulk	DESeq2	LRT	0.0492998232286276
626	KC_AB2	Trx-2	0.863876930006289	0.436090888676248	0.988741847156811	pseudobulk	DESeq2	LRT	0.360422987022894
626	KC_AB2	Trxr-1	0.896612917337366	0.481733162526936	0.988741847156811	pseudobulk	DESeq2	LRT	0.317193455809885
626	KC_AB2	Tsc1	-0.934020934207768	0.782359118258182	0.988741847156811	pseudobulk	DESeq2	LRT	0.106593851444836
626	KC_AB2	Tsen2	1.42621218304824	0.66738831402044	0.988741847156811	pseudobulk	DESeq2	LRT	0.175621402117653
627	KC_AB2	Tsen34	-0.0582963981256801	0.979058404810457	0.988741847156811	pseudobulk	DESeq2	LRT	0.0091913999942843

627	KC_AB2	Tsf2	2.80917294583258	0.367996267391162	0.988741847156811	pseudobulk	DESeq2	LRT	0.434156586379859
627	KC_AB2	TSG101	-0.862332580780999	0.666590188569964	0.988741847156811	pseudobulk	DESeq2	LRT	0.176141082936639
627	KC_AB2	Tsp26A	-0.883283128441371	0.63972157328483	0.988741847156811	pseudobulk	DESeq2	LRT	0.194009003353754
627	KC_AB2	Tsp39D	-0.125012379431497	0.855762409774802	0.988741847156811	pseudobulk	DESeq2	LRT	0.0676467942553609
627	KC_AB2	Tsp3A	0.0601466527378941	0.974810960364079	0.988741847156811	pseudobulk	DESeq2	LRT	0.0110795964355292
627	KC_AB2	Tsp42Ee	0.193333972909847	0.875625463031158	0.988741847156811	pseudobulk	DESeq2	LRT	0.0576816177149628
627	KC_AB2	Tsp42Ej	0.191956361438648	0.762705864308469	0.988741847156811	pseudobulk	DESeq2	LRT	0.117642914386142
627	KC_AB2	Tsp42Ek	-2.15400439962555	0.378907849258803	0.988741847156811	pseudobulk	DESeq2	LRT	0.421466398012216
627	KC_AB2	Tsp42En	0.178203781171202	0.844882264967406	0.988741847156811	pseudobulk	DESeq2	LRT	0.0732038061241975
628	KC_AB2	Tsp42Eo	-0.568233637794205	0.868826397133536	0.988741847156811	pseudobulk	DESeq2	LRT	0.0610669925949191
628	KC_AB2	Tsp66E	-0.114088227910669	0.864677579705335	0.988741847156811	pseudobulk	DESeq2	LRT	0.0631458017350175
628	KC_AB2	Tsp86D	-1.2337849808706	0.539269081720012	0.988741847156811	pseudobulk	DESeq2	LRT	0.268194478698133
628	KC_AB2	Tsp96F	0.0479264430774449	0.96220766833674	0.988741847156811	pseudobulk	DESeq2	LRT	0.0167311862995315
628	KC_AB2	Tsp97E	0.0938732479693852	0.892840153156574	0.988741847156811	pseudobulk	DESeq2	LRT	0.0492262867059987
628	KC_AB2	Tspo	2.12627090373229	0.415798287869119	0.988741847156811	pseudobulk	DESeq2	LRT	0.38111730329345
628	KC_AB2	tsr	0.445180583316352	0.574835085472511	0.988741847156811	pseudobulk	DESeq2	LRT	0.240456732253046
628	KC_AB2	Tsr1	0.818790875285784	0.940355871560339	0.988741847156811	pseudobulk	DESeq2	LRT	0.0267077593721816
628	KC_AB2	tst	-0.934020934207768	0.782359118258182	0.988741847156811	pseudobulk	DESeq2	LRT	0.106593851444836
628	KC_AB2	tsu	-0.174924145880005	0.911785725013446	0.988741847156811	pseudobulk	DESeq2	LRT	0.0401072114282213
629	KC_AB2	Ttc19	-1.4911990967693	0.465433041229917	0.988741847156811	pseudobulk	DESeq2	LRT	0.332142789260561
629	KC_AB2	Ttc7	-0.321040059436753	0.870444986106655	0.988741847156811	pseudobulk	DESeq2	LRT	0.0602586719737732
629	KC_AB2	Ttd14	-0.371072049747923	0.672999524542614	0.988741847156811	pseudobulk	DESeq2	LRT	0.171985242594139
629	KC_AB2	tth	-0.417313789773203	0.771921729873694	0.988741847156811	pseudobulk	DESeq2	LRT	0.112426733352402
629	KC_AB2	TTLL12	-0.957133960806503	0.762632518787665	0.988741847156811	pseudobulk	DESeq2	LRT	0.1176846802739
629	KC_AB2	TTLL4A	0.49822346129853	0.793777026118226	0.988741847156811	pseudobulk	DESeq2	LRT	0.10030147480964
629	KC_AB2	TTLL5	-0.296576489727943	0.896291428147039	0.988741847156811	pseudobulk	DESeq2	LRT	0.047550757014702
629	KC_AB2	ttm50	-0.110398938468043	0.9364168551839	0.988741847156811	pseudobulk	DESeq2	LRT	0.0285307777525609
629	KC_AB2	ttv	0.373001863176845	0.746796692412778	0.988741847156811	pseudobulk	DESeq2	LRT	0.126797614223307
629	KC_AB2	tty	-0.337994901578011	0.65724124628141	0.988741847156811	pseudobulk	DESeq2	LRT	0.182275189500028
630	KC_AB2	tub	0.0500444276466639	0.960141847756744	0.988741847156811	pseudobulk	DESeq2	LRT	0.0176646011820455
630	KC_AB2	tud	0.0375965083416778	0.9757586132126	0.988741847156811	pseudobulk	DESeq2	LRT	0.010657606427248
630	KC_AB2	Tudor-SN	1.91780756664311	0.321964775040473	0.988741847156811	pseudobulk	DESeq2	LRT	0.492191640236983
630	KC_AB2	tun	0.23945831241279	0.814784373078597	0.988741847156811	pseudobulk	DESeq2	LRT	0.0889573090174988
630	KC_AB2	Tusp	-1.29316606164068	0.468448242573593	0.988741847156811	pseudobulk	DESeq2	LRT	0.329338386006515
630	KC_AB2	tut	1.42621218304824	0.66738831402044	0.988741847156811	pseudobulk	DESeq2	LRT	0.175621402117653
630	KC_AB2	tutl	-0.0544165921627511	0.900417397663491	0.988741847156811	pseudobulk	DESeq2	LRT	0.0455561222496418
630	KC_AB2	tw	-2.51746470924027	0.517377929599049	0.988741847156811	pseudobulk	DESeq2	LRT	0.286192101436607
630	KC_AB2	tweek	-0.674357272625864	0.695101644019374	0.988741847156811	pseudobulk	DESeq2	LRT	0.157951684317728
630	KC_AB2	twf	-0.457212305199634	0.858276431637992	0.988741847156811	pseudobulk	DESeq2	LRT	0.0663728130839547
631	KC_AB2	twin	0.0420278692617463	0.966048375800203	0.988741847156811	pseudobulk	DESeq2	LRT	0.0150011253266955
631	KC_AB2	twr	1.18200449385783	0.354446766731616	0.988741847156811	pseudobulk	DESeq2	LRT	0.450448980934244
631	KC_AB2	tws	-0.495206102004076	0.646946037109478	0.988741847156811	pseudobulk	DESeq2	LRT	0.18913194307622
631	KC_AB2	Txl	2.28068239784515	0.366179304841476	0.988741847156811	pseudobulk	DESeq2	LRT	0.436306204128051
631	KC_AB2	tyf	0.355829400614185	0.679271649191512	0.988741847156811	pseudobulk	DESeq2	LRT	0.167956511210283
631	KC_AB2	tyn	-1.01910793418747	0.615314659319194	0.988741847156811	pseudobulk	DESeq2	LRT	0.210902738112693
631	KC_AB2	TyrR	-0.161318614227121	0.889091545701168	0.988741847156811	pseudobulk	DESeq2	LRT	0.0510535194055457
631	KC_AB2	TyrRS	-0.043251987344817	0.978652029676919	0.988741847156811	pseudobulk	DESeq2	LRT	0.00937169885514152
631	KC_AB2	tzn	-0.979504571299641	0.522261978516474	0.988741847156811	pseudobulk	DESeq2	LRT	0.282111590325256
631	KC_AB2	U2A	-2.36879023282761	0.4113734544154	0.988741847156811	pseudobulk	DESeq2	LRT	0.385763736396444
632	KC_AB2	U2af38	1.01836685929879	0.557737707431868	0.988741847156811	pseudobulk	DESeq2	LRT	0.253569992811653
632	KC_AB2	U2af50	0.227445254721887	0.844562357603068	0.988741847156811	pseudobulk	DESeq2	LRT	0.0733682791147377
632	KC_AB2	U3-55K	0.818790875285784	0.940355871560339	0.988741847156811	pseudobulk	DESeq2	LRT	0.0267077593721816
632	KC_AB2	U4-U6-60K	0.572861358867068	0.813871944286787	0.988741847156811	pseudobulk	DESeq2	LRT	0.0894439222171593
632	KC_AB2	Uba1	0.0912892557890945	0.950837602536886	0.988741847156811	pseudobulk	DESeq2	LRT	0.0218936516689727
632	KC_AB2	Uba2	-0.0220407025300507	0.955454128336539	0.988741847156811	pseudobulk	DESeq2	LRT	0.0197901587277916
632	KC_AB2	Uba3	2.00515076565275	0.576456787459964	0.988741847156811	pseudobulk	DESeq2	LRT	0.239233242872451

632	KC_AB2	Uba4	-1.80294541848982	0.660135549041553	0.988741847156811	pseudobulk	DESeq2	LRT	0.180366879372519
632	KC_AB2	Uba5	1.42621218304824	0.66738831402044	0.988741847156811	pseudobulk	DESeq2	LRT	0.175621402117653
632	KC_AB2	Ubc10	0.623410352677079	0.741927432229851	0.988741847156811	pseudobulk	DESeq2	LRT	0.129638570898015
633	KC_AB2	Ubc2	0.234945183819853	0.873383975232543	0.988741847156811	pseudobulk	DESeq2	LRT	0.0587947807360958
633	KC_AB2	Ubc4	-0.629447170949724	0.650135809129707	0.988741847156811	pseudobulk	DESeq2	LRT	0.186995912595905
633	KC_AB2	Ubc6	-0.123381745099138	0.904048708775543	0.988741847156811	pseudobulk	DESeq2	LRT	0.0438081697651713
633	KC_AB2	Ubc7	-1.73627324524809	0.521368726288639	0.988741847156811	pseudobulk	DESeq2	LRT	0.282855023048041
633	KC_AB2	UbcE2H	0.653170903663113	0.475763040251454	0.988741847156811	pseudobulk	DESeq2	LRT	0.322609299243495
633	KC_AB2	UbcE2M	-0.308665479596311	0.833807867841278	0.988741847156811	pseudobulk	DESeq2	LRT	0.0789340111719062
633	KC_AB2	Ube3a	0.0885531792216718	0.964858407830046	0.988741847156811	pseudobulk	DESeq2	LRT	0.0155364143348316
633	KC_AB2	Ubi-p5E	-0.0128151773951899	0.97989428067026	0.988741847156811	pseudobulk	DESeq2	LRT	0.00882077716281859
633	KC_AB2	Ubi-p63E	-0.0429319897193177	0.93560689481009	0.988741847156811	pseudobulk	DESeq2	LRT	0.0289065863851777
633	KC_AB2	ubl	2.01599493208962	0.288210614579416	0.988741847156811	pseudobulk	DESeq2	LRT	0.540290028489488
634	KC_AB2	UBL3	0.00035558567498187	0.969197295308531	0.988741847156811	pseudobulk	DESeq2	LRT	0.0135878064972009
634	KC_AB2	Ublcp1	-1.18743225435356	0.64061820392288	0.988741847156811	pseudobulk	DESeq2	LRT	0.193400724506216
634	KC_AB2	Ubqn	-0.0887327487996433	0.937469271322228	0.988741847156811	pseudobulk	DESeq2	LRT	0.0280429588150793
634	KC_AB2	Ubr1	0.325749338297864	0.7107950449778	0.988741847156811	pseudobulk	DESeq2	LRT	0.148255608365751
634	KC_AB2	Ubr3	0.478418028477915	0.627283097908806	0.988741847156811	pseudobulk	DESeq2	LRT	0.202536414333911
634	KC_AB2	Uch	-0.842515666101921	0.635004008404488	0.988741847156811	pseudobulk	DESeq2	LRT	0.197223533255338
634	KC_AB2	Uch-L5	0.363641268105473	0.858330168669459	0.988741847156811	pseudobulk	DESeq2	LRT	0.06634562258353
634	KC_AB2	Uch-L5R	0.818790875285784	0.940355871560339	0.988741847156811	pseudobulk	DESeq2	LRT	0.0267077593721816
634	KC_AB2	Uck	0.100401644177886	0.938906637959083	0.988741847156811	pseudobulk	DESeq2	LRT	0.0273775905188086
634	KC_AB2	Ucp4A	0.0545983678259795	0.958159353457108	0.988741847156811	pseudobulk	DESeq2	LRT	0.0185622565040738
635	KC_AB2	udd	-0.883283128441371	0.63972157328483	0.988741847156811	pseudobulk	DESeq2	LRT	0.194009003353754
635	KC_AB2	udt	2.06137138167525	0.547946929015274	0.988741847156811	pseudobulk	DESeq2	LRT	0.261261502741816
635	KC_AB2	Uev1A	-0.02722977271499	0.975362363565725	0.988741847156811	pseudobulk	DESeq2	LRT	0.0108340065987474
635	KC_AB2	uex	0.127592624966693	0.845672223324167	0.988741847156811	pseudobulk	DESeq2	LRT	0.0727979338703063
635	KC_AB2	Ufc1	-0.114099770495178	0.955170180595609	0.988741847156811	pseudobulk	DESeq2	LRT	0.0199192442181898
635	KC_AB2	Ufd1-like	-0.460305896488822	0.736044165460061	0.988741847156811	pseudobulk	DESeq2	LRT	0.133096125553639
635	KC_AB2	Ufd4	-0.805162593727849	0.59245257238638	0.988741847156811	pseudobulk	DESeq2	LRT	0.227346410508257
635	KC_AB2	Ufl1	-1.52496782475103	0.617219242690575	0.988741847156811	pseudobulk	DESeq2	LRT	0.209560542647675
635	KC_AB2	Ufm1	0.802207095184316	0.732945722088841	0.988741847156811	pseudobulk	DESeq2	LRT	0.134928185618234
635	KC_AB2	Ugalt	2.00515076565275	0.576456787459964	0.988741847156811	pseudobulk	DESeq2	LRT	0.239233242872451
636	KC_AB2	Uggt	-0.568233637794205	0.868826397133536	0.988741847156811	pseudobulk	DESeq2	LRT	0.0610669925949191
636	KC_AB2	Ugt316A1	1.4630491093368	0.486955122389524	0.988741847156811	pseudobulk	DESeq2	LRT	0.312511061365783
636	KC_AB2	Uhg1	0.363641268105473	0.858330168669459	0.988741847156811	pseudobulk	DESeq2	LRT	0.06634562258353
636	KC_AB2	Uhg2	0.760546103616116	0.738305064567484	0.988741847156811	pseudobulk	DESeq2	LRT	0.131764152427911
636	KC_AB2	Uhg3	-1.18743225435356	0.64061820392288	0.988741847156811	pseudobulk	DESeq2	LRT	0.193400724506216
636	KC_AB2	Uhg4	-0.194388201900128	0.928057462354013	0.988741847156811	pseudobulk	DESeq2	LRT	0.0324251328213705
636	KC_AB2	Uhg5	-0.0895902818285133	0.954518965493979	0.988741847156811	pseudobulk	DESeq2	LRT	0.0202154381162507
636	KC_AB2	Ulp1	0.102946774028289	0.927193086181459	0.988741847156811	pseudobulk	DESeq2	LRT	0.0328298154444129
636	KC_AB2	unc-104	-0.505637029951532	0.311827759123521	0.988741847156811	pseudobulk	DESeq2	LRT	0.506085226230078
636	KC_AB2	Unc-115a	0.972726581225976	0.61661584324292	0.988741847156811	pseudobulk	DESeq2	LRT	0.20998532076329
637	KC_AB2	Unc-115b	0.818790875285784	0.940355871560339	0.988741847156811	pseudobulk	DESeq2	LRT	0.0267077593721816
637	KC_AB2	unc-119	0.463070369449867	0.625624031085016	0.988741847156811	pseudobulk	DESeq2	LRT	0.203686577775618
637	KC_AB2	unc-13	-0.121741523802886	0.676209995943117	0.988741847156811	pseudobulk	DESeq2	LRT	0.16991841364885
637	KC_AB2	unc-13-4A	0.489149188382638	0.502485554391892	0.988741847156811	pseudobulk	DESeq2	LRT	0.298876418958352
637	KC_AB2	unc-45	-1.80933943727629	0.584587525761637	0.988741847156811	pseudobulk	DESeq2	LRT	0.233150456085471
637	KC_AB2	unc-5	0.0637197602866855	0.938243537329148	0.988741847156811	pseudobulk	DESeq2	LRT	0.0276844183478195
637	KC_AB2	Unc-76	-0.209124406822051	0.73914309773019	0.988741847156811	pseudobulk	DESeq2	LRT	0.131271474273056
637	KC_AB2	unc79	0.342234102667039	0.638498640671807	0.988741847156811	pseudobulk	DESeq2	LRT	0.194840022988447
637	KC_AB2	unc80	0.0986262249723975	0.901311276640491	0.988741847156811	pseudobulk	DESeq2	LRT	0.0451251952797962
637	KC_AB2	und	-0.30060714052091	0.823783618460654	0.988741847156811	pseudobulk	DESeq2	LRT	0.0841868485569261
638	KC_AB2	unk	-0.603404376004995	0.509608953416975	0.988741847156811	pseudobulk	DESeq2	LRT	0.292762950403028
638	KC_AB2	Unr	0.293285067629023	0.444294339622033	0.988741847156811	pseudobulk	DESeq2	LRT	0.352329219702131
638	KC_AB2	Updo	-0.0312066175844283	0.974804086815535	0.988741847156811	pseudobulk	DESeq2	LRT	0.0110826587264241

638	KC_AB2	Upf1	0.321601000162439	0.868811750025189	0.988741847156811	pseudobulk	DESeq2	LRT	0.0610743142094242
638	KC_AB2	Upf2	-0.457212305199634	0.858276431637992	0.988741847156811	pseudobulk	DESeq2	LRT	0.0663728130839547
638	KC_AB2	Upf3	-1.80933943727629	0.584587525761637	0.988741847156811	pseudobulk	DESeq2	LRT	0.233150456085471
638	KC_AB2	upSET	0.505544624700866	0.514809937261787	0.988741847156811	pseudobulk	DESeq2	LRT	0.288353078596892
638	KC_AB2	UQCR-11	0.440410175626713	0.401353571870294	0.988741847156811	pseudobulk	DESeq2	LRT	0.396472867640102
638	KC_AB2	UQCR-14	0.477751332451075	0.405710891611426	0.988741847156811	pseudobulk	DESeq2	LRT	0.391783333179952
638	KC_AB2	UQCR-6.4	0.458918769690066	0.443472608856496	0.988741847156811	pseudobulk	DESeq2	LRT	0.353133199259183
639	KC_AB2	UQCR-C1	0.11757255939604	0.870771156207028	0.988741847156811	pseudobulk	DESeq2	LRT	0.0600959651472413
639	KC_AB2	UQCR-C2	0.138007392962872	0.821299400096295	0.988741847156811	pseudobulk	DESeq2	LRT	0.0854984943953375
639	KC_AB2	UQCR-Q	0.622353950310605	0.268528298704039	0.988741847156811	pseudobulk	DESeq2	LRT	0.571009939672432
639	KC_AB2	uri	2.06137138167525	0.547946929015274	0.988741847156811	pseudobulk	DESeq2	LRT	0.261261502741816
639	KC_AB2	Urm1	0.0885531792216718	0.964858407830046	0.988741847156811	pseudobulk	DESeq2	LRT	0.0155364143348316
639	KC_AB2	Uros1	2.06137138167525	0.547946929015274	0.988741847156811	pseudobulk	DESeq2	LRT	0.261261502741816
639	KC_AB2	Use1	-1.23536834900699	0.725570392794057	0.988741847156811	pseudobulk	DESeq2	LRT	0.139320447155934
639	KC_AB2	Usf	0.104076277704695	0.934124251647671	0.988741847156811	pseudobulk	DESeq2	LRT	0.0295953526607496
639	KC_AB2	ush	0.818790875285784	0.940355871560339	0.988741847156811	pseudobulk	DESeq2	LRT	0.0267077593721816
639	KC_AB2	usp	-0.209294959031791	0.877893213984396	0.988741847156811	pseudobulk	DESeq2	LRT	0.056558308009394
640	KC_AB2	Usp1	-0.330309511864849	0.785031476083053	0.988741847156811	pseudobulk	DESeq2	LRT	0.10511292973229
640	KC_AB2	Usp10	1.4129303440845	0.645460595159151	0.988741847156811	pseudobulk	DESeq2	LRT	0.190130265910817
640	KC_AB2	Usp14	0.581445489707027	0.811994604428781	0.988741847156811	pseudobulk	DESeq2	LRT	0.0904468565649727
640	KC_AB2	Usp15-31	-0.634227699706429	0.828294391400015	0.988741847156811	pseudobulk	DESeq2	LRT	0.0818152793537044
640	KC_AB2	Usp16-45	0.818790875285784	0.940355871560339	0.988741847156811	pseudobulk	DESeq2	LRT	0.0267077593721816
640	KC_AB2	Usp2	-0.380933170885051	0.622571066537665	0.988741847156811	pseudobulk	DESeq2	LRT	0.205811066652249
640	KC_AB2	Usp20-33	-3.91108067132102	0.221683018369551	0.988741847156811	pseudobulk	DESeq2	LRT	0.654267573962038
640	KC_AB2	Usp30	0.136783607278865	0.925515546186956	0.988741847156811	pseudobulk	DESeq2	LRT	0.0336162818635301
640	KC_AB2	Usp32	-1.39096835157591	0.461763948360687	0.988741847156811	pseudobulk	DESeq2	LRT	0.335579977090568
640	KC_AB2	Usp39	0.666943671827768	0.808936122914367	0.988741847156811	pseudobulk	DESeq2	LRT	0.0920857707994393
641	KC_AB2	Usp47	-0.272159080840168	0.881917593135503	0.988741847156811	pseudobulk	DESeq2	LRT	0.0545719936873807
641	KC_AB2	Usp5	-0.158601889198964	0.945838126788742	0.988741847156811	pseudobulk	DESeq2	LRT	0.0241831835324482
641	KC_AB2	Usp7	-0.207468442863813	0.781141116442133	0.988741847156811	pseudobulk	DESeq2	LRT	0.107270501902475
641	KC_AB2	Usp8	-1.48046351229221	0.534490457994632	0.988741847156811	pseudobulk	DESeq2	LRT	0.272060043639989
641	KC_AB2	Utx	-0.33005096517819	0.848732281696353	0.988741847156811	pseudobulk	DESeq2	LRT	0.07122927903465
641	KC_AB2	Uvrag	-0.382714549144618	0.863954068453144	0.988741847156811	pseudobulk	DESeq2	LRT	0.063509345886375
641	KC_AB2	Uxs	1.47543920254359	0.661929169513603	0.988741847156811	pseudobulk	DESeq2	LRT	0.17918848025087
641	KC_AB2	Uxt	-0.794177064289352	0.743016342902793	0.988741847156811	pseudobulk	DESeq2	LRT	0.129001633676244
641	KC_AB2	uzip	0.870516437313356	0.597259838630625	0.988741847156811	pseudobulk	DESeq2	LRT	0.22383668740848
641	KC_AB2	VAChT	0.303407076978132	0.531745042149846	0.988741847156811	pseudobulk	DESeq2	LRT	0.274296550653257
642	KC_AB2	ValRS	-0.506613484034373	0.961658398065406	0.988741847156811	pseudobulk	DESeq2	LRT	0.0169791713942958
642	KC_AB2	Vamp7	0.194804214247169	0.909397535999427	0.988741847156811	pseudobulk	DESeq2	LRT	0.04124622684446
642	KC_AB2	Vang	-0.812236047217954	0.670110188742971	0.988741847156811	pseudobulk	DESeq2	LRT	0.173853778749341
642	KC_AB2	vanin-like	0.818790875285784	0.940355871560339	0.988741847156811	pseudobulk	DESeq2	LRT	0.0267077593721816
642	KC_AB2	vap	-0.0229973822346953	0.971360658954591	0.988741847156811	pseudobulk	DESeq2	LRT	0.0126194898530182
642	KC_AB2	Vap33	0.120518838745491	0.814689519023103	0.988741847156811	pseudobulk	DESeq2	LRT	0.0890078708499204
642	KC_AB2	vari	1.42621218304824	0.66738831402044	0.988741847156811	pseudobulk	DESeq2	LRT	0.175621402117653
642	KC_AB2	Vav	-0.842515666101921	0.635004008404488	0.988741847156811	pseudobulk	DESeq2	LRT	0.197223533255338
642	KC_AB2	veli	-0.548683120911458	0.568205551827177	0.988741847156811	pseudobulk	DESeq2	LRT	0.245494527184487
642	KC_AB2	velo	-0.116308793952336	0.925127504927551	0.988741847156811	pseudobulk	DESeq2	LRT	0.0337984068606531
643	KC_AB2	VepD	-1.01548768188483	0.25417385294386	0.988741847156811	pseudobulk	DESeq2	LRT	0.594869127685454
643	KC_AB2	vers	3.27725343811621	0.25757966897206	0.988741847156811	pseudobulk	DESeq2	LRT	0.589088419269148
643	KC_AB2	VGAT	-0.0265683699975436	0.949703263057826	0.988741847156811	pseudobulk	DESeq2	LRT	0.0224120698138033
643	KC_AB2	VGlut	0.818790875285784	0.940355871560339	0.988741847156811	pseudobulk	DESeq2	LRT	0.0267077593721816
643	KC_AB2	Vha100-1	-0.330103639755956	0.616721418962119	0.988741847156811	pseudobulk	DESeq2	LRT	0.209910968104389
643	KC_AB2	Vha13	0.187783588562852	0.707033793525968	0.988741847156811	pseudobulk	DESeq2	LRT	0.150559828082719
643	KC_AB2	Vha14-1	0.495983101814607	0.457769490440833	0.988741847156811	pseudobulk	DESeq2	LRT	0.339353155686664
643	KC_AB2	Vha16-1	0.183870375389799	0.581457550364936	0.988741847156811	pseudobulk	DESeq2	LRT	0.235481985690826
643	KC_AB2	Vha26	0.485594344260435	0.456135778979802	0.988741847156811	pseudobulk	DESeq2	LRT	0.340905860659608

643	KC_AB2	Vha36-1	0.0444349154522953	0.921475665487963	0.988741847156811	pseudobulk	DESeq2	LRT	0.0355161292282409
644	KC_AB2	Vha44	0.0591920148643582	0.92581129422129	0.988741847156811	pseudobulk	DESeq2	LRT	0.0334775254455557
644	KC_AB2	Vha55	0.202754285237005	0.776406102159341	0.988741847156811	pseudobulk	DESeq2	LRT	0.109911059935253
644	KC_AB2	Vha68-1	0.732280215804229	0.339650981081478	0.988741847156811	pseudobulk	DESeq2	LRT	0.468967126612319
644	KC_AB2	VhaAC39-1	0.846858371949454	0.340659535110993	0.988741847156811	pseudobulk	DESeq2	LRT	0.467679450587393
644	KC_AB2	VhaAC45	-1.33788900664848	0.722041597401238	0.988741847156811	pseudobulk	DESeq2	LRT	0.141437781652764
644	KC_AB2	VhaM9.7-a	0.346976708397768	0.487417411729078	0.988741847156811	pseudobulk	DESeq2	LRT	0.312098960824676
644	KC_AB2	VhaM9.7-b	0.134877686365273	0.882880893503907	0.988741847156811	pseudobulk	DESeq2	LRT	0.0540978816806791
644	KC_AB2	VhaM9.7-c	-2.9721553030081	0.387854083811327	0.988741847156811	pseudobulk	DESeq2	LRT	0.411331631401987
644	KC_AB2	VhaPPA1-1	0.395078781752148	0.624218137247117	0.988741847156811	pseudobulk	DESeq2	LRT	0.204663616651296
644	KC_AB2	VhaSFD	0.749872667811345	0.447981197611154	0.988741847156811	pseudobulk	DESeq2	LRT	0.348740213557848
645	KC_AB2	Vhl	-1.33572246196862	0.479260031408656	0.988741847156811	pseudobulk	DESeq2	LRT	0.319428788121614
645	KC_AB2	viaf	-0.449611546109821	0.726109844872719	0.988741847156811	pseudobulk	DESeq2	LRT	0.138997674873555
645	KC_AB2	vib	0.713934984175869	0.549038482169359	0.988741847156811	pseudobulk	DESeq2	LRT	0.260397214733703
645	KC_AB2	Victoria	0.818790875285784	0.940355871560339	0.988741847156811	pseudobulk	DESeq2	LRT	0.0267077593721816
645	KC_AB2	vig	0.1088424321575	0.839750276936781	0.988741847156811	pseudobulk	DESeq2	LRT	0.0758498442626054
645	KC_AB2	vig2	0.539629042041861	0.643715700723356	0.988741847156811	pseudobulk	DESeq2	LRT	0.191305897963841
645	KC_AB2	vih	-0.0542211645807959	0.964904866528268	0.988741847156811	pseudobulk	DESeq2	LRT	0.0155155032142331
645	KC_AB2	vimar	-1.27482208804161	0.504147634250043	0.988741847156811	pseudobulk	DESeq2	LRT	0.297442266428947
645	KC_AB2	vir	2.06137138167525	0.547946929015274	0.988741847156811	pseudobulk	DESeq2	LRT	0.261261502741816
645	KC_AB2	vir-1	1.15128682504377	0.719737694898673	0.988741847156811	pseudobulk	DESeq2	LRT	0.142825751365876
646	KC_AB2	vito	0.353798809751289	0.901648351639924	0.988741847156811	pseudobulk	DESeq2	LRT	0.0449628069360408
646	KC_AB2	vlc	0.0831759711005134	0.923552284398709	0.988741847156811	pseudobulk	DESeq2	LRT	0.0345385131278067
646	KC_AB2	Vlet	0.5738945031678	0.80780652681376	0.988741847156811	pseudobulk	DESeq2	LRT	0.0926926421938231
646	KC_AB2	Vps11	0.545941176239014	0.602388031672894	0.988741847156811	pseudobulk	DESeq2	LRT	0.220123665343303
646	KC_AB2	Vps13	-0.010237273074013	0.943681123757042	0.988741847156811	pseudobulk	DESeq2	LRT	0.0251747319601966
646	KC_AB2	Vps13B	0.818790875285784	0.940355871560339	0.988741847156811	pseudobulk	DESeq2	LRT	0.0267077593721816
646	KC_AB2	Vps13D	0.712171366772551	0.813205723536451	0.988741847156811	pseudobulk	DESeq2	LRT	0.0897995733547685
646	KC_AB2	Vps15	-0.506613484034373	0.961658398065406	0.988741847156811	pseudobulk	DESeq2	LRT	0.0169791713942958
646	KC_AB2	Vps16A	2.29067719524526	0.364763866244387	0.988741847156811	pseudobulk	DESeq2	LRT	0.437988189718379
646	KC_AB2	Vps16B	0.157562008957688	0.950463298513166	0.988741847156811	pseudobulk	DESeq2	LRT	0.0220646484576093
647	KC_AB2	Vps2	0.571577592263866	0.685393558115559	0.988741847156811	pseudobulk	DESeq2	LRT	0.164059981738678
647	KC_AB2	Vps20	1.1669370038017	0.601782723498991	0.988741847156811	pseudobulk	DESeq2	LRT	0.220560284520093
647	KC_AB2	Vps24	0.317435678878878	0.767556538310644	0.988741847156811	pseudobulk	DESeq2	LRT	0.114889624486644
647	KC_AB2	Vps25	0.765272298362395	0.741528757908517	0.988741847156811	pseudobulk	DESeq2	LRT	0.129872001535403
647	KC_AB2	Vps26	-0.906925799940864	0.671074658720917	0.988741847156811	pseudobulk	DESeq2	LRT	0.173229160804428
647	KC_AB2	Vps28	0.300414301642887	0.881846961452027	0.988741847156811	pseudobulk	DESeq2	LRT	0.0546067771853455
647	KC_AB2	Vps29	-0.75852709693596	0.63425596262606	0.988741847156811	pseudobulk	DESeq2	LRT	0.19773544129437
647	KC_AB2	Vps35	1.87899144193828	0.388696615728932	0.988741847156811	pseudobulk	DESeq2	LRT	0.410389240631014
647	KC_AB2	Vps36	-1.80294541848982	0.660135549041553	0.988741847156811	pseudobulk	DESeq2	LRT	0.180366879372519
647	KC_AB2	Vps37B	-0.174924145880005	0.911785725013446	0.988741847156811	pseudobulk	DESeq2	LRT	0.0401072114282213
648	KC_AB2	Vps39	-0.957133960806503	0.762632518785665	0.988741847156811	pseudobulk	DESeq2	LRT	0.1176846802739
648	KC_AB2	Vps4	1.12823023143992	0.440729966851103	0.988741847156811	pseudobulk	DESeq2	LRT	0.355827419207317
648	KC_AB2	Vps45	0.780352552100014	0.834299274204729	0.988741847156811	pseudobulk	DESeq2	LRT	0.0786781342287373
648	KC_AB2	Vps50	-1.23536834900699	0.725570392794057	0.988741847156811	pseudobulk	DESeq2	LRT	0.139320447155934
648	KC_AB2	Vps51	2.00515076565275	0.576456787459964	0.988741847156811	pseudobulk	DESeq2	LRT	0.239233242872451
648	KC_AB2	Vps52	1.7098779479	0.537865353861561	0.988741847156811	pseudobulk	DESeq2	LRT	0.269326429526188
648	KC_AB2	Vps53	2.51243226103394	0.474458247157471	0.988741847156811	pseudobulk	DESeq2	LRT	0.323801999942597
648	KC_AB2	Vps60	0.890362606964825	0.536224700197154	0.988741847156811	pseudobulk	DESeq2	LRT	0.270653184921847
648	KC_AB2	Vps8	1.91626857490363	0.477198801200827	0.988741847156811	pseudobulk	DESeq2	LRT	0.321300656017034
648	KC_AB2	Vrp1	-0.385228552068709	0.689469742618927	0.988741847156811	pseudobulk	DESeq2	LRT	0.161484788084173
649	KC_AB2	vrs	0.818790875285784	0.940355871560339	0.988741847156811	pseudobulk	DESeq2	LRT	0.0267077593721816
649	KC_AB2	vsg	0.347870291579992	0.706047650065298	0.988741847156811	pseudobulk	DESeq2	LRT	0.151165988095368
649	KC_AB2	Vsp37A	-0.344521661248047	0.805373000003984	0.988741847156811	pseudobulk	DESeq2	LRT	0.0940029341348875
649	KC_AB2	Vsx1	1.25491583270741	0.403406629988283	0.988741847156811	pseudobulk	DESeq2	LRT	0.394256968434446
649	KC_AB2	Vsx2	0.818790875285784	0.940355871560339	0.988741847156811	pseudobulk	DESeq2	LRT	0.0267077593721816

649	KC_AB2	Vta1	0.677607164055588	0.684117954518064	0.988741847156811	pseudobulk	DESeq2	LRT	0.164869011467283
649	KC_AB2	vtd	-0.545453380331128	0.545026321071166	0.988741847156811	pseudobulk	DESeq2	LRT	0.263582523741819
649	KC_AB2	Vti1a	-0.0220407025300507	0.955454128336539	0.988741847156811	pseudobulk	DESeq2	LRT	0.0197901587277916
649	KC_AB2	Vti1b	0.538471591573674	0.740252210768007	0.988741847156811	pseudobulk	DESeq2	LRT	0.130620286913452
649	KC_AB2	w	0.318736935166122	0.838864137626684	0.988741847156811	pseudobulk	DESeq2	LRT	0.0763083717778976
650	KC_AB2	wac	1.10527623683615	0.724717602335893	0.988741847156811	pseudobulk	DESeq2	LRT	0.139831190172672
650	KC_AB2	wake	0.713570446461972	0.524485337095327	0.988741847156811	pseudobulk	DESeq2	LRT	0.280266648762324
650	KC_AB2	wal	2.41462217514131	0.497446973552313	0.988741847156811	pseudobulk	DESeq2	LRT	0.303253207020768
650	KC_AB2	wap	0.409493204087423	0.781042853093028	0.988741847156811	pseudobulk	DESeq2	LRT	0.107325137247836
650	KC_AB2	wapl	-0.91847055940206	0.560507193370775	0.988741847156811	pseudobulk	DESeq2	LRT	0.251418809436202
650	KC_AB2	wash	-0.0582963981256801	0.979058404810457	0.988741847156811	pseudobulk	DESeq2	LRT	0.0091913999942843
650	KC_AB2	WASp	0.0689101676397734	0.960290025335635	0.988741847156811	pseudobulk	DESeq2	LRT	0.0175975821891999
650	KC_AB2	Wbp2	0.207820301599514	0.886478908997988	0.988741847156811	pseudobulk	DESeq2	LRT	0.0523315926234177
650	KC_AB2	wcy	-0.0112757840402961	0.956147462659906	0.988741847156811	pseudobulk	DESeq2	LRT	0.0194751231206079
650	KC_AB2	wda	2.51243226103394	0.474458247157471	0.988741847156811	pseudobulk	DESeq2	LRT	0.323801999942597
651	KC_AB2	wdb	-0.126917468833762	0.750964496627173	0.988741847156811	pseudobulk	DESeq2	LRT	0.124380594664783
651	KC_AB2	wde	1.59317095140199	0.366075065036893	0.988741847156811	pseudobulk	DESeq2	LRT	0.436429851795017
651	KC_AB2	Wdfy2	-0.487923111280115	0.783394690329388	0.988741847156811	pseudobulk	DESeq2	LRT	0.106019376327165
651	KC_AB2	wdn	-4.4226865976364	0.100197417867286	0.988741847156811	pseudobulk	DESeq2	LRT	0.999143470289518
651	KC_AB2	Wdr24	-1.80294541848982	0.660135549041553	0.988741847156811	pseudobulk	DESeq2	LRT	0.180366879372519
651	KC_AB2	Wdr33	-1.48046351229221	0.534490457994632	0.988741847156811	pseudobulk	DESeq2	LRT	0.272060043639989
651	KC_AB2	Wdr59	-0.934020934207768	0.782359118258182	0.988741847156811	pseudobulk	DESeq2	LRT	0.106593851444836
651	KC_AB2	Wdr62	1.81822696127924	0.19954667084684	0.988741847156811	pseudobulk	DESeq2	LRT	0.699955513406931
651	KC_AB2	Wdr81	-1.23536834900699	0.725570392794057	0.988741847156811	pseudobulk	DESeq2	LRT	0.139320447155934
651	KC_AB2	Wdr82	-0.451045789314127	0.823968225471349	0.988741847156811	pseudobulk	DESeq2	LRT	0.0840895355947879
652	KC_AB2	Wdr92	0.157562008957688	0.950463298513166	0.988741847156811	pseudobulk	DESeq2	LRT	0.0220646484576093
652	KC_AB2	wds	-2.08765824978799	0.252139055416669	0.988741847156811	pseudobulk	DESeq2	LRT	0.598359878487621
652	KC_AB2	WDY	-2.11027789354592	0.473734933668313	0.988741847156811	pseudobulk	DESeq2	LRT	0.32446458879962
652	KC_AB2	wech	-0.137717052573173	0.910233243523681	0.988741847156811	pseudobulk	DESeq2	LRT	0.0408473072439877
652	KC_AB2	Wee1	-0.0144409071146711	0.984632974376923	0.988741847156811	pseudobulk	DESeq2	LRT	0.00672562423038896
652	KC_AB2	wek	-0.506613484034373	0.961658398065406	0.988741847156811	pseudobulk	DESeq2	LRT	0.0169791713942958
652	KC_AB2	wfs1	2.00515076565275	0.576456787459964	0.988741847156811	pseudobulk	DESeq2	LRT	0.239233242872451
652	KC_AB2	wge	0.0761450035124067	0.932110540408034	0.988741847156811	pseudobulk	DESeq2	LRT	0.0305325809480496
652	KC_AB2	whd	-1.1284597703615	0.729754448571724	0.988741847156811	pseudobulk	DESeq2	LRT	0.136823248879498
652	KC_AB2	wit	-1.39751327820725	0.382755011951875	0.988741847156811	pseudobulk	DESeq2	LRT	0.417079113761532
653	KC_AB2	wkd	-0.94851584115099	0.584922323445749	0.988741847156811	pseudobulk	DESeq2	LRT	0.232901803556663
653	KC_AB2	wls	-0.506613484034373	0.961658398065406	0.988741847156811	pseudobulk	DESeq2	LRT	0.0169791713942958
653	KC_AB2	wmd	-0.615999477370307	0.625934574388112	0.988741847156811	pseudobulk	DESeq2	LRT	0.203471058909614
653	KC_AB2	wnd	-0.0921084205185754	0.927409954928414	0.988741847156811	pseudobulk	DESeq2	LRT	0.0327282466467336
653	KC_AB2	Wnk	-0.098595637526155	0.938802299589211	0.988741847156811	pseudobulk	DESeq2	LRT	0.0274258552710571
653	KC_AB2	Wnt5	-1.14305939040284	0.576110433642647	0.988741847156811	pseudobulk	DESeq2	LRT	0.239494259416078
653	KC_AB2	Wnt6	-1.80294541848982	0.660135549041553	0.988741847156811	pseudobulk	DESeq2	LRT	0.180366879372519
653	KC_AB2	woc	0.647516049547349	0.706459800591372	0.988741847156811	pseudobulk	DESeq2	LRT	0.150912545603175
653	KC_AB2	wol	0.321601000162439	0.868811750025189	0.988741847156811	pseudobulk	DESeq2	LRT	0.0610743142094242
653	KC_AB2	wrapper	0.818790875285784	0.940355871560339	0.988741847156811	pseudobulk	DESeq2	LRT	0.0267077593721816
654	KC_AB2	wrd	-0.359948543942498	0.584196716740636	0.988741847156811	pseudobulk	DESeq2	LRT	0.233440888145474
654	KC_AB2	wry	2.42469273075529	0.155101181564376	0.988741847156811	pseudobulk	DESeq2	LRT	0.809384893708419
654	KC_AB2	wtrw	0.818790875285784	0.940355871560339	0.988741847156811	pseudobulk	DESeq2	LRT	0.0267077593721816
654	KC_AB2	wts	-0.419219021606142	0.671185550228392	0.988741847156811	pseudobulk	DESeq2	LRT	0.173157401886783
654	KC_AB2	wuho	-0.205210117329556	0.911317538347789	0.988741847156811	pseudobulk	DESeq2	LRT	0.0403302716288717
654	KC_AB2	wun	-0.767288086811737	0.738313966315624	0.988741847156811	pseudobulk	DESeq2	LRT	0.13175891616857
654	KC_AB2	Wwox	1.45259040912931	0.616176917291092	0.988741847156811	pseudobulk	DESeq2	LRT	0.210294574894236
654	KC_AB2	X11L	-0.512869247294946	0.600618192825471	0.988741847156811	pseudobulk	DESeq2	LRT	0.221401517085341
654	KC_AB2	X11Lbeta	0.325267076890603	0.679694045287307	0.988741847156811	pseudobulk	DESeq2	LRT	0.167686534859542
654	KC_AB2	x16	0.213146834007961	0.891008854060909	0.988741847156811	pseudobulk	DESeq2	LRT	0.0501179803057856
655	KC_AB2	Xbp1	0.198930897450656	0.825276783143617	0.988741847156811	pseudobulk	DESeq2	LRT	0.083400372380586

655	KC_AB2	Xe7	0.188242293566477	0.728606216181374	0.988741847156811	pseudobulk	DESeq2	LRT	0.137507127848765
655	KC_AB2	xit	0.818790875285784	0.940355871560339	0.988741847156811	pseudobulk	DESeq2	LRT	0.0267077593721816
655	KC_AB2	xmas	0.157562008957688	0.950463298513166	0.988741847156811	pseudobulk	DESeq2	LRT	0.0220646484576093
655	KC_AB2	XNP	0.572462130088654	0.649201201487299	0.988741847156811	pseudobulk	DESeq2	LRT	0.187620685089149
655	KC_AB2	Xpac	-3.22628249697182	0.177080137513838	0.988741847156811	pseudobulk	DESeq2	LRT	0.751830149436143
655	KC_AB2	Xpc	0.90738699802277	0.494647387630339	0.988741847156811	pseudobulk	DESeq2	LRT	0.305704280209788
655	KC_AB2	Xpd	1.47543920254359	0.661929169513603	0.988741847156811	pseudobulk	DESeq2	LRT	0.17918848025087
655	KC_AB2	Xrp1	0.216708232038142	0.753185820584323	0.988741847156811	pseudobulk	DESeq2	LRT	0.123097864571398
655	KC_AB2	Xxylt	-0.11350464060454	0.932722166081082	0.988741847156811	pseudobulk	DESeq2	LRT	0.0302477021346365
656	KC_AB2	yata	-3.09794865339079	0.39855598532175	0.988741847156811	pseudobulk	DESeq2	LRT	0.399510664459796
656	KC_AB2	yem	1.7098779479	0.537865353861561	0.988741847156811	pseudobulk	DESeq2	LRT	0.269326429526188
656	KC_AB2	Yeti	-0.400189031404833	0.581662537270792	0.988741847156811	pseudobulk	DESeq2	LRT	0.235328906599606
656	KC_AB2	Yif1	1.92721521352877	0.471759180353374	0.988741847156811	pseudobulk	DESeq2	LRT	0.326279639784082
656	KC_AB2	Yip1d1	-0.114099770495178	0.955170180595609	0.988741847156811	pseudobulk	DESeq2	LRT	0.0199192442181898
656	KC_AB2	yip2	-2.11027789354592	0.473734933668313	0.988741847156811	pseudobulk	DESeq2	LRT	0.32446458879962
656	KC_AB2	Yippee	-0.420573488016776	0.704332856492695	0.988741847156811	pseudobulk	DESeq2	LRT	0.152222051689112
656	KC_AB2	yki	-0.634227699706429	0.828294391400015	0.988741847156811	pseudobulk	DESeq2	LRT	0.0818152793537044
656	KC_AB2	Ykt6	0.637459969522021	0.694477239961861	0.988741847156811	pseudobulk	DESeq2	LRT	0.158341982785895
656	KC_AB2	YL-1	-0.114099770495178	0.955170180595609	0.988741847156811	pseudobulk	DESeq2	LRT	0.0199192442181898
657	KC_AB2	YME1L	0.475522879236569	0.801582640633501	0.988741847156811	pseudobulk	DESeq2	LRT	0.0960516966149455
657	KC_AB2	Yp2	0.818790875285784	0.940355871560339	0.988741847156811	pseudobulk	DESeq2	LRT	0.0267077593721816
657	KC_AB2	Yp3	0.818790875285784	0.940355871560339	0.988741847156811	pseudobulk	DESeq2	LRT	0.0267077593721816
657	KC_AB2	yps	0.0161526179102998	0.956890474136475	0.988741847156811	pseudobulk	DESeq2	LRT	0.0191377688065263
657	KC_AB2	yrt	-0.0391677464905046	0.975854328920695	0.988741847156811	pseudobulk	DESeq2	LRT	0.0106150069927054
657	KC_AB2	Ythdc1	-0.0211210862898794	0.979155082073196	0.988741847156811	pseudobulk	DESeq2	LRT	0.0091485176404978
657	KC_AB2	Ythdf	-0.450252693144999	0.697408964767352	0.988741847156811	pseudobulk	DESeq2	LRT	0.156512474333187
657	KC_AB2	ytr	-0.383190653334554	0.806825465783655	0.988741847156811	pseudobulk	DESeq2	LRT	0.0932204026323272
657	KC_AB2	z	-0.883283128441371	0.63972157328483	0.988741847156811	pseudobulk	DESeq2	LRT	0.194009003353754
657	KC_AB2	ZAP3	-0.686333285688455	0.578711226316624	0.988741847156811	pseudobulk	DESeq2	LRT	0.237538092752969
658	KC_AB2	Zasp52	0.362555753552128	0.897034383127386	0.988741847156811	pseudobulk	DESeq2	LRT	0.0471909102264623
658	KC_AB2	ZC3H3	2.11191162521434	0.263046249242669	0.988741847156811	pseudobulk	DESeq2	LRT	0.579967886396095
658	KC_AB2	Zcchc7	-0.147926357299875	0.943541611067299	0.988741847156811	pseudobulk	DESeq2	LRT	0.0252389422839741
658	KC_AB2	zda	0.607363222622466	0.759039189806662	0.988741847156811	pseudobulk	DESeq2	LRT	0.119735800550637
658	KC_AB2	Zdhhc8	-0.254925072953604	0.606058972849129	0.988741847156811	pseudobulk	DESeq2	LRT	0.217485114551791
658	KC_AB2	zetaCOP	0.772096921612307	0.538119664562819	0.988741847156811	pseudobulk	DESeq2	LRT	0.269121137195141
658	KC_AB2	zf30C	-0.506613484034373	0.961658398065406	0.988741847156811	pseudobulk	DESeq2	LRT	0.0169791713942958
658	KC_AB2	zfh2	-3.09794865339079	0.39855598532175	0.988741847156811	pseudobulk	DESeq2	LRT	0.399510664459796
658	KC_AB2	Zfrp8	-0.506613484034373	0.961658398065406	0.988741847156811	pseudobulk	DESeq2	LRT	0.0169791713942958
658	KC_AB2	Zif	1.76923267226582	0.433375211084549	0.988741847156811	pseudobulk	DESeq2	LRT	0.363135933803196
659	KC_AB2	zip	-0.416214483377362	0.662611900105978	0.988741847156811	pseudobulk	DESeq2	LRT	0.178740768719752
659	KC_AB2	Zip102B	0.428214702684823	0.696308410024718	0.988741847156811	pseudobulk	DESeq2	LRT	0.157198359376666
659	KC_AB2	Zip48C	-1.80294541848982	0.660135549041553	0.988741847156811	pseudobulk	DESeq2	LRT	0.180366879372519
659	KC_AB2	Zip71B	0.818790875285784	0.940355871560339	0.988741847156811	pseudobulk	DESeq2	LRT	0.0267077593721816
659	KC_AB2	Zip88E	0.798955445836505	0.738225347953373	0.988741847156811	pseudobulk	DESeq2	LRT	0.131811046803217
659	KC_AB2	Zip99C	-2.43239194781006	0.303807241077998	0.988741847156811	pseudobulk	DESeq2	LRT	0.517401879180473
659	KC_AB2	ZIPIC	-1.80294541848982	0.660135549041553	0.988741847156811	pseudobulk	DESeq2	LRT	0.180366879372519
659	KC_AB2	Zir	-1.6757158311173	0.467705803824964	0.988741847156811	pseudobulk	DESeq2	LRT	0.330027240838734
659	KC_AB2	Ziz	-1.35237076770213	0.277357171192748	0.988741847156811	pseudobulk	DESeq2	LRT	0.556960600763416
659	KC_AB2	Zn72D	0.880548370541495	0.479447908234134	0.988741847156811	pseudobulk	DESeq2	LRT	0.319258571799723
660	KC_AB2	ZnT49B	0.49822346129853	0.793777026118226	0.988741847156811	pseudobulk	DESeq2	LRT	0.10030147480964
660	KC_AB2	ZnT63C	0.0845670433215681	0.937030397054793	0.988741847156811	pseudobulk	DESeq2	LRT	0.028246320468639
660	KC_AB2	ZnT77C	2.51243226103394	0.474458247157471	0.988741847156811	pseudobulk	DESeq2	LRT	0.323801999942597
660	KC_AB2	ZnT86D	-1.80294541848982	0.660135549041553	0.988741847156811	pseudobulk	DESeq2	LRT	0.180366879372519
660	KC_AB2	Zpr1	1.47543920254359	0.661929169513603	0.988741847156811	pseudobulk	DESeq2	LRT	0.17918848025087
660	KC_AB2	Zw10	0.802207095184316	0.732945722088841	0.988741847156811	pseudobulk	DESeq2	LRT	0.134928185618234
660	KC_AB2	Zyx	0.799363588845852	0.398134319066727	0.988741847156811	pseudobulk	DESeq2	LRT	0.399970384739691

660	KC_AB2	Naa60	0.0206586403639275	0.985434991654523	0.98888056854842	pseudobulk	DESeq2	LRT	0.00637202049356286
660	KC_AB2	RpL35	0.00388021168834873	0.986775776533709	0.989973586957384	pseudobulk	DESeq2	LRT	0.00578152015399425
660	KC_AB2	Ten-a	0.00333101912514595	0.986824148186576	0.989973586957384	pseudobulk	DESeq2	LRT	0.00576023160238682
661	KC_AB2	capt	-0.0194944425626801	0.987665789703758	0.989987649504417	pseudobulk	DESeq2	LRT	0.00538998885586638
661	KC_AB2	CG14312	-0.00906760623344625	0.987482389282728	0.989987649504417	pseudobulk	DESeq2	LRT	0.00547064082102852
661	KC_AB2	CG3860	-0.0199111573035904	0.988037969237252	0.989987649504417	pseudobulk	DESeq2	LRT	0.0052263656215081
661	KC_AB2	CG43066	-0.00906760623344625	0.987482389282728	0.989987649504417	pseudobulk	DESeq2	LRT	0.00547064082102852
661	KC_AB2	fs(1)h	-0.0043360793010885	0.987874043978631	0.989987649504417	pseudobulk	DESeq2	LRT	0.00529842534375379
661	KC_AB2	Lk6	-0.00692637369277255	0.987380692799622	0.989987649504417	pseudobulk	DESeq2	LRT	0.00551536920942349
661	KC_AB2	Rbp	0.00499126422065394	0.987898156999543	0.989987649504417	pseudobulk	DESeq2	LRT	0.00528782477763647
661	KC_AB2	smog	0.00384205717697129	0.987654817192195	0.989987649504417	pseudobulk	DESeq2	LRT	0.00539481369413111
661	KC_AB2	PH4alphaEFB	0.00113849951205831	0.988823641291697	0.990474181512366	pseudobulk	DESeq2	LRT	0.00488115880077864
661	KC_AB2	Tango14	0.00113849951205831	0.988823641291697	0.990474181512366	pseudobulk	DESeq2	LRT	0.00488115880077864
662	KC_AB2	asRNA:CR44894	-0.0198725415743665	0.993168586177113	0.99407215122898	pseudobulk	DESeq2	LRT	0.00297702559118863
662	KC_AB2	Chro	-0.00865113821683175	0.993053580122617	0.99407215122898	pseudobulk	DESeq2	LRT	0.00302731855010983
662	KC_AB2	Eip74EF	-0.0194701504656819	0.99313419414737	0.99407215122898	pseudobulk	DESeq2	LRT	0.00299206485799954
662	KC_AB2	Mtpbeta	-0.0198725415743665	0.993168586177113	0.99407215122898	pseudobulk	DESeq2	LRT	0.00297702559118863
662	KC_AB2	strat	-0.0198725415743665	0.993168586177113	0.99407215122898	pseudobulk	DESeq2	LRT	0.00297702559118863
662	KC_AB2	CG33941	-0.00512692916109577	0.993580969676193	0.994183413799083	pseudobulk	DESeq2	LRT	0.00279673524696999
662	KC_AB2	Fip1	-0.00512692916109577	0.993580969676193	0.994183413799083	pseudobulk	DESeq2	LRT	0.00279673524696999
662	KC_AB1	128up	0.180585403270847	0.831503615391509	0.994766463583882	pseudobulk	DESeq2	LRT	0.0801358581207475
662	KC_AB1	14-3-3epsilon	-0.228633221056701	0.114518831540676	0.994766463583882	pseudobulk	DESeq2	LRT	0.941123091824257
662	KC_AB1	140up	0.109920300218929	0.859061719396129	0.994766463583882	pseudobulk	DESeq2	LRT	0.0659756331067555
663	KC_AB1	18SrRNA-Psi:CR41602	-0.232836268623125	0.834300396158601	0.994766463583882	pseudobulk	DESeq2	LRT	0.0786775501960491
663	KC_AB1	18w	1.58862831782278	0.36842726891665	0.994766463583882	pseudobulk	DESeq2	LRT	0.433648233247851
663	KC_AB1	26-29-p	0.143359051534075	0.826456059336663	0.994766463583882	pseudobulk	DESeq2	LRT	0.0827802318625434
663	KC_AB1	28SrRNA-Psi:CR40596	0.270212953589623	0.751369838658659	0.994766463583882	pseudobulk	DESeq2	LRT	0.124146242287027
663	KC_AB1	28SrRNA-Psi:CR40741	-0.485881628473861	0.752061499239371	0.994766463583882	pseudobulk	DESeq2	LRT	0.123746643865505
663	KC_AB1	28SrRNA-Psi:CR41609	0.597141410293382	0.802139944912821	0.994766463583882	pseudobulk	DESeq2	LRT	0.0957498561530429
663	KC_AB1	28SrRNA-Psi:CR45848	0.179643023247231	0.848500094885823	0.994766463583882	pseudobulk	DESeq2	LRT	0.0713481047801448
663	KC_AB1	28SrRNA-Psi:CR45851	-1.64952313825113	0.817565084244375	0.994766463583882	pseudobulk	DESeq2	LRT	0.0874776642296574
663	KC_AB1	28SrRNA-Psi:CR45855	-0.849377531455818	0.743723328152499	0.994766463583882	pseudobulk	DESeq2	LRT	0.128588595910649
663	KC_AB1	28SrRNA-Psi:CR45859	0.0433038034288563	0.940194119074297	0.994766463583882	pseudobulk	DESeq2	LRT	0.0267824696565612
664	KC_AB1	28SrRNA-Psi:CR45860	0.138973379994452	0.931431561132167	0.994766463583882	pseudobulk	DESeq2	LRT	0.0308490502842029
664	KC_AB1	2mit	-0.380855251333331	0.0661104611515809	0.994766463583882	pseudobulk	DESeq2	LRT	1.17972981342006
664	KC_AB1	312	-0.742378048939341	0.455878495886375	0.994766463583882	pseudobulk	DESeq2	LRT	0.341150893295183
664	KC_AB1	4E-T	-0.0340874075986259	0.854022229724945	0.994766463583882	pseudobulk	DESeq2	LRT	0.0685308247193915
664	KC_AB1	5-HT1A	0.108232494970249	0.524902653547831	0.994766463583882	pseudobulk	DESeq2	LRT	0.279921231731255
664	KC_AB1	5-HT1B	-0.221877106633263	0.700053230072	0.994766463583882	pseudobulk	DESeq2	LRT	0.154868936203425
664	KC_AB1	5-HT2A	1.00206552945986	0.690075213866505	0.994766463583882	pseudobulk	DESeq2	LRT	0.161103571310595
664	KC_AB1	5-HT2B	0.930545135351766	0.436504910444561	0.994766463583882	pseudobulk	DESeq2	LRT	0.360010866352498
664	KC_AB1	5-HT7	2.22023857837763	0.524904842126482	0.994766463583882	pseudobulk	DESeq2	LRT	0.279919420946639
664	KC_AB1	5.8SrRNA-Psi:CR45849	0.949183608371002	0.80156895525114	0.994766463583882	pseudobulk	DESeq2	LRT	0.0960591113673091
665	KC_AB1	5PtaseI	-0.081325916739175	0.870876708439486	0.994766463583882	pseudobulk	DESeq2	LRT	0.0600433244807835
665	KC_AB1	7B2	0.126673765543597	0.42327754728726	0.994766463583882	pseudobulk	DESeq2	LRT	0.373374768019883
665	KC_AB1	a	-0.40040733861881	0.0808325023171186	0.994766463583882	pseudobulk	DESeq2	LRT	1.09241397661781
665	KC_AB1	A16	-0.617285914251587	0.572123732246945	0.994766463583882	pseudobulk	DESeq2	LRT	0.242510036904633
665	KC_AB1	a6	-0.722885399912227	0.480176482001355	0.994766463583882	pseudobulk	DESeq2	LRT	0.318599114556285
665	KC_AB1	AANAT1	0.188051692402645	0.698440930807082	0.994766463583882	pseudobulk	DESeq2	LRT	0.155870317551656
665	KC_AB1	AANATL7	-0.45456966509879	0.684536541335935	0.994766463583882	pseudobulk	DESeq2	LRT	0.164603363779839
665	KC_AB1	Aasdh	0.0822233602948793	0.857848061898428	0.994766463583882	pseudobulk	DESeq2	LRT	0.0665896255805677
665	KC_AB1	Aatf	0.52867572341821	0.589642012992177	0.994766463583882	pseudobulk	DESeq2	LRT	0.229411579829084
665	KC_AB1	aay	0.596808856322697	0.822761281772702	0.994766463583882	pseudobulk	DESeq2	LRT	0.0847261539098712
666	KC_AB1	ab	-0.0859796982194101	0.899826377807229	0.994766463583882	pseudobulk	DESeq2	LRT	0.0458412799321612
666	KC_AB1	ABCB7	0.339668416150084	0.694482484482726	0.994766463583882	pseudobulk	DESeq2	LRT	0.158338703113444
666	KC_AB1	ABCD	-0.045931404899116	0.870904858401501	0.994766463583882	pseudobulk	DESeq2	LRT	0.0600292867008425

666	KC_AB1	Abi	0.297079949781137	0.669317356085015	0.994766463583882	pseudobulk	DESeq2	LRT	0.174367913149467
666	KC_AB1	Abl	-0.135168105923606	0.571744912298836	0.994766463583882	pseudobulk	DESeq2	LRT	0.242797691295542
666	KC_AB1	abo	-0.78410610838983	0.738244895252942	0.994766463583882	pseudobulk	DESeq2	LRT	0.131799547371545
666	KC_AB1	Abp1	0.078314171548223	0.875150537093709	0.994766463583882	pseudobulk	DESeq2	LRT	0.0579172363423499
666	KC_AB1	abs	-0.768062577975913	0.68032889465424	0.994766463583882	pseudobulk	DESeq2	LRT	0.167281083467765
666	KC_AB1	Ac3	0.0606809854080013	0.889722464056016	0.994766463583882	pseudobulk	DESeq2	LRT	0.0507454440586812
666	KC_AB1	Ac76E	0.0946970962658519	0.916629782388576	0.994766463583882	pseudobulk	DESeq2	LRT	0.0378060361192835
667	KC_AB1	Ac78C	-0.26628459433669	0.599307416165452	0.994766463583882	pseudobulk	DESeq2	LRT	0.222350348067231
667	KC_AB1	Acbp1	-0.0334125088696016	0.799849702957934	0.994766463583882	pseudobulk	DESeq2	LRT	0.0969916121433841
667	KC_AB1	ACC	0.181295498358394	0.765010090813631	0.994766463583882	pseudobulk	DESeq2	LRT	0.116332836276742
667	KC_AB1	AcCoAS	-0.244706813137671	0.475590701338896	0.994766463583882	pseudobulk	DESeq2	LRT	0.322766645215425
667	KC_AB1	Ace	0.564554278479158	0.359447643670944	0.994766463583882	pseudobulk	DESeq2	LRT	0.444364359024377
667	KC_AB1	Acer	-0.336314434875304	0.732990524071	0.994766463583882	pseudobulk	DESeq2	LRT	0.13490163977944
667	KC_AB1	Acf	-0.225820334571283	0.73142845604164	0.994766463583882	pseudobulk	DESeq2	LRT	0.135828147550805
667	KC_AB1	achi	1.03617094651386	0.517485475044324	0.994766463583882	pseudobulk	DESeq2	LRT	0.286101835622983
667	KC_AB1	Achl	0.949183608371002	0.80156895525114	0.994766463583882	pseudobulk	DESeq2	LRT	0.0960591113673091
667	KC_AB1	Ack	0.078816712102361	0.879781393233127	0.994766463583882	pseudobulk	DESeq2	LRT	0.0556252272894017
668	KC_AB1	Ack-like	-0.250076177540909	0.544410141230385	0.994766463583882	pseudobulk	DESeq2	LRT	0.264073793389029
668	KC_AB1	Acn	-0.382035482143113	0.245702308020446	0.994766463583882	pseudobulk	DESeq2	LRT	0.609590763927587
668	KC_AB1	Acph-1	0.490115771013445	0.700621520106883	0.994766463583882	pseudobulk	DESeq2	LRT	0.154516527138035
668	KC_AB1	Acsl	0.1550113678126	0.745603631700396	0.994766463583882	pseudobulk	DESeq2	LRT	0.127491985279074
668	KC_AB1	Act42A	0.0110010220157771	0.929620960430592	0.994766463583882	pseudobulk	DESeq2	LRT	0.0316940926810252
668	KC_AB1	Act5C	0.247413897512333	0.362158893365963	0.994766463583882	pseudobulk	DESeq2	LRT	0.441100845566566
668	KC_AB1	Actbeta	-0.186545487171124	0.526515495903126	0.994766463583882	pseudobulk	DESeq2	LRT	0.278588842548416
668	KC_AB1	Actn	-0.271250621609088	0.755335346934147	0.994766463583882	pseudobulk	DESeq2	LRT	0.121860191432501
668	KC_AB1	ACXC	-2.20969975456663	0.610836134188693	0.994766463583882	pseudobulk	DESeq2	LRT	0.214075280039667
668	KC_AB1	Acyp2	-0.558892418547767	0.435777115091238	0.994766463583882	pseudobulk	DESeq2	LRT	0.360735580538956
669	KC_AB1	Ada	-1.64952313825113	0.817565084244375	0.994766463583882	pseudobulk	DESeq2	LRT	0.0874776642296574
669	KC_AB1	Ada1-2	0.254735773073499	0.823152761852656	0.994766463583882	pseudobulk	DESeq2	LRT	0.084519560324546
669	KC_AB1	Ada2a	2.67764418499683	0.378704357417491	0.994766463583882	pseudobulk	DESeq2	LRT	0.421699697806334
669	KC_AB1	Ada2b	0.751606152299477	0.392970222032649	0.994766463583882	pseudobulk	DESeq2	LRT	0.405640357759122
669	KC_AB1	Ada3	0.00115056950562296	0.883953184549431	0.994766463583882	pseudobulk	DESeq2	LRT	0.0535707352473777
669	KC_AB1	Adar	-0.0915493314232254	0.806225955052168	0.994766463583882	pseudobulk	DESeq2	LRT	0.0935432245971468
669	KC_AB1	Adat1	0.233048461606111	0.864499074695876	0.994766463583882	pseudobulk	DESeq2	LRT	0.0632354672306489
669	KC_AB1	Adck1	0.0556507705136479	0.910364932142286	0.994766463583882	pseudobulk	DESeq2	LRT	0.0407844799367282
669	KC_AB1	Adck5	-1.02558554613245	0.565515952145453	0.994766463583882	pseudobulk	DESeq2	LRT	0.247555139932103
669	KC_AB1	ADD1	-0.174044193667625	0.767608840892961	0.994766463583882	pseudobulk	DESeq2	LRT	0.114860031944751
670	KC_AB1	AdenoK	0.159835964233694	0.874618240642596	0.994766463583882	pseudobulk	DESeq2	LRT	0.0581814694473491
670	KC_AB1	Adf1	0.141405444838531	0.745360069629907	0.994766463583882	pseudobulk	DESeq2	LRT	0.127633876948282
670	KC_AB1	Adgf-B	-2.20969975456663	0.610836134188693	0.994766463583882	pseudobulk	DESeq2	LRT	0.214075280039667
670	KC_AB1	Adgf-D	-1.64952313825113	0.817565084244375	0.994766463583882	pseudobulk	DESeq2	LRT	0.0874776642296574
670	KC_AB1	Adi1	0.789488672237544	0.485082977425971	0.994766463583882	pseudobulk	DESeq2	LRT	0.314183965406263
670	KC_AB1	AdipoR	0.276956099371435	0.571909808766285	0.994766463583882	pseudobulk	DESeq2	LRT	0.242672454849479
670	KC_AB1	Adk1	0.348864208880121	0.49213443689982	0.994766463583882	pseudobulk	DESeq2	LRT	0.307916244329276
670	KC_AB1	Adk2	0.124931264346646	0.751712050267959	0.994766463583882	pseudobulk	DESeq2	LRT	0.12394848777049
670	KC_AB1	Adk3	0.651806411443827	0.700247618684875	0.994766463583882	pseudobulk	DESeq2	LRT	0.154748359396614
670	KC_AB1	adp	0.195168059926154	0.862973752606598	0.994766463583882	pseudobulk	DESeq2	LRT	0.0640024131740941
671	KC_AB1	ADPS	-0.141122529942407	0.809456114869216	0.994766463583882	pseudobulk	DESeq2	LRT	0.0918066917985346
671	KC_AB1	AdSL	0.376696648865953	0.7865064166209	0.994766463583882	pseudobulk	DESeq2	LRT	0.104297729885345
671	KC_AB1	AdSS	-0.235360926443567	0.682338628037303	0.994766463583882	pseudobulk	DESeq2	LRT	0.166000042074066
671	KC_AB1	Aduk	-0.0740765511528039	0.835697901784422	0.994766463583882	pseudobulk	DESeq2	LRT	0.077950688229609
671	KC_AB1	Ady43A	-0.530606808431602	0.530544213135583	0.994766463583882	pseudobulk	DESeq2	LRT	0.275278418132659
671	KC_AB1	Aef1	-0.00618768886664219	0.867191404634592	0.994766463583882	pseudobulk	DESeq2	LRT	0.0618850353931726
671	KC_AB1	aft	-2.66944192095839	0.284729472085893	0.994766463583882	pseudobulk	DESeq2	LRT	0.545567577103783
671	KC_AB1	Afti	-0.445766247430343	0.442254986798118	0.994766463583882	pseudobulk	DESeq2	LRT	0.354327261353921
671	KC_AB1	Ag5r	1.4796448321752	0.400364727695328	0.994766463583882	pseudobulk	DESeq2	LRT	0.397544191037997

671	KC_AB1	AGBE	2.09225092496487	0.386054269300348	0.994766463583882	pseudobulk	DESeq2	LRT	0.413351640403342
672	KC_AB1	ago	-0.0527985486063944	0.929091284050854	0.994766463583882	pseudobulk	DESeq2	LRT	0.0319416140881981
672	KC_AB1	AGO2	-0.326499269706985	0.500268226264575	0.994766463583882	pseudobulk	DESeq2	LRT	0.300797079759347
672	KC_AB1	AGO3	0.744277400795692	0.605907389834875	0.994766463583882	pseudobulk	DESeq2	LRT	0.217593750680666
672	KC_AB1	Agpat1	-0.26143131362184	0.772865069649604	0.994766463583882	pseudobulk	DESeq2	LRT	0.111896320598631
672	KC_AB1	Agpat2	-0.535166272824937	0.735950099553911	0.994766463583882	pseudobulk	DESeq2	LRT	0.133151631617763
672	KC_AB1	Agpat3	-0.0894406446964191	0.841816927356402	0.994766463583882	pseudobulk	DESeq2	LRT	0.0747823456543231
672	KC_AB1	agt	0.124698704744493	0.891329405341186	0.994766463583882	pseudobulk	DESeq2	LRT	0.0499617656782218
672	KC_AB1	Ahcy	0.24299114742271	0.811603402710348	0.994766463583882	pseudobulk	DESeq2	LRT	0.0906561408215036
672	KC_AB1	AhcyL1	-0.260494357807535	0.642241888293519	0.994766463583882	pseudobulk	DESeq2	LRT	0.192301372295732
672	KC_AB1	AIF	0.887105353070786	0.380479339226046	0.994766463583882	pseudobulk	DESeq2	LRT	0.419668921294821
673	KC_AB1	AIMP1	-1.06060473488273	0.46503892384712	0.994766463583882	pseudobulk	DESeq2	LRT	0.332510695057255
673	KC_AB1	AIMP2	0.371336127133733	0.66759625765472	0.994766463583882	pseudobulk	DESeq2	LRT	0.175486106511155
673	KC_AB1	AIMP3	0.278726535049856	0.566230422921938	0.994766463583882	pseudobulk	DESeq2	LRT	0.247006800191389
673	KC_AB1	Ak6	-1.09461410731956	0.439908835901812	0.994766463583882	pseudobulk	DESeq2	LRT	0.356637314802266
673	KC_AB1	Akap200	0.22334402545067	0.708747050016786	0.994766463583882	pseudobulk	DESeq2	LRT	0.149508735737976
673	KC_AB1	Akh	2.54275315134261	0.410734813545839	0.994766463583882	pseudobulk	DESeq2	LRT	0.386438485142247
673	KC_AB1	akirin	0.195156569584292	0.569226618722968	0.994766463583882	pseudobulk	DESeq2	LRT	0.244714799216359
673	KC_AB1	Akt1	0.133635014863889	0.811089938847592	0.994766463583882	pseudobulk	DESeq2	LRT	0.0909309857647632
673	KC_AB1	al	0.949183608371002	0.80156895525114	0.994766463583882	pseudobulk	DESeq2	LRT	0.0960591113673091
673	KC_AB1	Aladin	0.464372090648708	0.769322022672416	0.994766463583882	pseudobulk	DESeq2	LRT	0.113891835236487
674	KC_AB1	AlaRS	1.06676047529507	0.403419532607356	0.994766463583882	pseudobulk	DESeq2	LRT	0.394243078115748
674	KC_AB1	AlaRS-m	-0.514015259573243	0.73782094639197	0.994766463583882	pseudobulk	DESeq2	LRT	0.132049019516753
674	KC_AB1	Alas	-0.00495684703919667	0.939048092363726	0.994766463583882	pseudobulk	DESeq2	LRT	0.0273121652276642
674	KC_AB1	alc	0.461837272317204	0.267264563017002	0.994766463583882	pseudobulk	DESeq2	LRT	0.573058621140293
674	KC_AB1	Ald1	0.148855609994396	0.55080394823902	0.994766463583882	pseudobulk	DESeq2	LRT	0.259002955320934
674	KC_AB1	Aldh-III	-0.114572071799641	0.856806226602869	0.994766463583882	pseudobulk	DESeq2	LRT	0.0671173860494023
674	KC_AB1	Aldh7A1	-3.95857014146861	0.177445975557202	0.994766463583882	pseudobulk	DESeq2	LRT	0.750933845925402
674	KC_AB1	Alg-2	-0.0611153574768754	0.843887895005321	0.994766463583882	pseudobulk	DESeq2	LRT	0.0737152427277651
674	KC_AB1	Alg1	0.225870234592611	0.854316558685538	0.994766463583882	pseudobulk	DESeq2	LRT	0.0683811758961231
674	KC_AB1	Alg10	0.436774318627524	0.753023728031073	0.994766463583882	pseudobulk	DESeq2	LRT	0.123191338818418
675	KC_AB1	Alg11	0.0914623221218144	0.827886375189056	0.994766463583882	pseudobulk	DESeq2	LRT	0.0820292646820042
675	KC_AB1	Alg14	0.62506181737221	0.65087233953608	0.994766463583882	pseudobulk	DESeq2	LRT	0.18650418450682
675	KC_AB1	Alg2	0.589735941717492	0.659465606262675	0.994766463583882	pseudobulk	DESeq2	LRT	0.180807849700387
675	KC_AB1	Alg3	0.0312856644723177	0.898318976303112	0.994766463583882	pseudobulk	DESeq2	LRT	0.0465694260824268
675	KC_AB1	Alg9	0.235417202704869	0.842672672282477	0.994766463583882	pseudobulk	DESeq2	LRT	0.074341089939403
675	KC_AB1	Alh	0.143946714108997	0.760630826852651	0.994766463583882	pseudobulk	DESeq2	LRT	0.118826077477284
675	KC_AB1	alien	-0.152707576372581	0.834735921719842	0.994766463583882	pseudobulk	DESeq2	LRT	0.0784508968269862
675	KC_AB1	ALiX	0.255374327926049	0.77937768837198	0.994766463583882	pseudobulk	DESeq2	LRT	0.10825203112689
675	KC_AB1	Alk	-0.346985611001979	0.0598827396035935	0.994766463583882	pseudobulk	DESeq2	LRT	1.22269833913215
675	KC_AB1	AlkB	-0.17805032366327	0.942053914371676	0.994766463583882	pseudobulk	DESeq2	LRT	0.0259242415339998
676	KC_AB1	Alp12	-2.32607225918216	0.391287880902332	0.994766463583882	pseudobulk	DESeq2	LRT	0.407503603003357
676	KC_AB1	alph	0.123285765123153	0.731432769872211	0.994766463583882	pseudobulk	DESeq2	LRT	0.135825586169343
676	KC_AB1	alpha-Cat	0.0480675401901006	0.853428861605728	0.994766463583882	pseudobulk	DESeq2	LRT	0.0688326740802259
676	KC_AB1	alpha-Catr	0.275019126256925	0.632687378776456	0.994766463583882	pseudobulk	DESeq2	LRT	0.198810829018664
676	KC_AB1	alpha-Est8	-0.0799890559508426	0.856733954264854	0.994766463583882	pseudobulk	DESeq2	LRT	0.067154020705494
676	KC_AB1	alpha-Est9	0.688907696275758	0.227070174901417	0.994766463583882	pseudobulk	DESeq2	LRT	0.643839905526471
676	KC_AB1	alpha-Man-Ia	0.167871513159104	0.535432870751551	0.994766463583882	pseudobulk	DESeq2	LRT	0.271294970564459
676	KC_AB1	alpha-Man-IIa	-0.587106875305033	0.596781280595757	0.994766463583882	pseudobulk	DESeq2	LRT	0.224184807957422
676	KC_AB1	alpha-Man-IIb	0.257459990962639	0.717184340137909	0.994766463583882	pseudobulk	DESeq2	LRT	0.144369201912341
676	KC_AB1	alpha-PheRS	-0.111894999250296	0.915793562791506	0.994766463583882	pseudobulk	DESeq2	LRT	0.0382024134988142
677	KC_AB1	alpha-Spec	-0.204528447794364	0.492380425563947	0.994766463583882	pseudobulk	DESeq2	LRT	0.307699220644338
677	KC_AB1	alpha4GT1	0.326450180113141	0.659095773934295	0.994766463583882	pseudobulk	DESeq2	LRT	0.181051473009956
677	KC_AB1	alpha4GT2	1.45682795759908	0.573003993665791	0.994766463583882	pseudobulk	DESeq2	LRT	0.24184235111992
677	KC_AB1	alphaCOP	0.237467919476505	0.824288297721184	0.994766463583882	pseudobulk	DESeq2	LRT	0.0839208657163981
677	KC_AB1	alphaSnap	-0.0146546496004112	0.848798088660422	0.994766463583882	pseudobulk	DESeq2	LRT	0.0711956070581369

677	KC_AB1	alphaTub67C	0.710279390142469	0.624129170388426	0.994766463583882	pseudobulk	DESeq2	LRT	0.204725519001116
677	KC_AB1	alphaTub84B	-0.0229902477458542	0.875744173319918	0.994766463583882	pseudobulk	DESeq2	LRT	0.0576227435374471
677	KC_AB1	alphaTub84D	0.012197406502596	0.944429960968633	0.994766463583882	pseudobulk	DESeq2	LRT	0.0248302438752315
677	KC_AB1	Alr	0.290543819513973	0.789596530609737	0.994766463583882	pseudobulk	DESeq2	LRT	0.102594768570888
677	KC_AB1	Als2	0.810347702661291	0.366998895576613	0.994766463583882	pseudobulk	DESeq2	LRT	0.435335242684435
678	KC_AB1	alt	0.0615200589788837	0.81994917209794	0.994766463583882	pseudobulk	DESeq2	LRT	0.0862130683011119
678	KC_AB1	Amacr	0.174381807395257	0.826622470402448	0.994766463583882	pseudobulk	DESeq2	LRT	0.0826927932968104
678	KC_AB1	amn	0.243064160420582	0.872438103157415	0.994766463583882	pseudobulk	DESeq2	LRT	0.0592653751547291
678	KC_AB1	Amnionless	0.696921508852573	0.723188018198223	0.994766463583882	pseudobulk	DESeq2	LRT	0.14074877787769
678	KC_AB1	amon	0.106117925815692	0.57905425721026	0.994766463583882	pseudobulk	DESeq2	LRT	0.237280741103249
678	KC_AB1	AMPdeam	-0.216805395901866	0.685702400683395	0.994766463583882	pseudobulk	DESeq2	LRT	0.163864330042098
678	KC_AB1	Amph	0.279808743095202	0.610223081496	0.994766463583882	pseudobulk	DESeq2	LRT	0.214511369330839
678	KC_AB1	AMPKalpha	0.555753296689743	0.429261318852802	0.994766463583882	pseudobulk	DESeq2	LRT	0.36727824444776
678	KC_AB1	amx	-0.425954626976384	0.680086773269822	0.994766463583882	pseudobulk	DESeq2	LRT	0.167435671487872
678	KC_AB1	Amyrel	-1.64952313825113	0.817565084244375	0.994766463583882	pseudobulk	DESeq2	LRT	0.0874776642296574
679	KC_AB1	ana1	-2.20969975456663	0.610836134188693	0.994766463583882	pseudobulk	DESeq2	LRT	0.214075280039667
679	KC_AB1	ana2	-0.835152883801839	0.920434724857442	0.994766463583882	pseudobulk	DESeq2	LRT	0.0360070052473159
679	KC_AB1	anchor	-0.262596775021835	0.705240043025049	0.994766463583882	pseudobulk	DESeq2	LRT	0.151663036742683
679	KC_AB1	angel	-1.50142087677551	0.213969234787042	0.994766463583882	pseudobulk	DESeq2	LRT	0.669648666476663
679	KC_AB1	Ank	0.0911446335384828	0.706360471964184	0.994766463583882	pseudobulk	DESeq2	LRT	0.150973611933538
679	KC_AB1	Ank2	-0.0889798427841096	0.588163186157943	0.994766463583882	pseudobulk	DESeq2	LRT	0.230502161992853
679	KC_AB1	Ankle2	0.220531594338488	0.826304637428428	0.994766463583882	pseudobulk	DESeq2	LRT	0.082859809867391
679	KC_AB1	anne	-0.148060097315317	0.541837466616291	0.994766463583882	pseudobulk	DESeq2	LRT	0.26613096795894
679	KC_AB1	Ant2	1.34815820347889	0.526408859243851	0.994766463583882	pseudobulk	DESeq2	LRT	0.278676810335701
679	KC_AB1	AnxB10	0.546994057336545	0.505385489541806	0.994766463583882	pseudobulk	DESeq2	LRT	0.296377231554193
680	KC_AB1	AnxB11	0.0456932439220104	0.900928447966936	0.994766463583882	pseudobulk	DESeq2	LRT	0.0453096994616423
680	KC_AB1	aop	0.233406163052775	0.741093446416521	0.994766463583882	pseudobulk	DESeq2	LRT	0.130127027237519
680	KC_AB1	Aos1	0.49991357729979	0.529807678140963	0.994766463583882	pseudobulk	DESeq2	LRT	0.275881752059761
680	KC_AB1	AP-1-2beta	0.154522739278817	0.737545260252344	0.994766463583882	pseudobulk	DESeq2	LRT	0.132211323587472
680	KC_AB1	AP-1gamma	0.0618307872447242	0.810576178575825	0.994766463583882	pseudobulk	DESeq2	LRT	0.0912061635663058
680	KC_AB1	AP-1mu	-0.023787799371434	0.930829999940402	0.994766463583882	pseudobulk	DESeq2	LRT	0.0311296281800891
680	KC_AB1	AP-1sigma	0.0763583040382953	0.829547993042683	0.994766463583882	pseudobulk	DESeq2	LRT	0.0811584830444884
680	KC_AB1	AP-2alpha	-0.0678428992832755	0.823982728801777	0.994766463583882	pseudobulk	DESeq2	LRT	0.0840818912940181
680	KC_AB1	AP-2sigma	0.121528020472172	0.679026049849974	0.994766463583882	pseudobulk	DESeq2	LRT	0.168113564321728
680	KC_AB1	Apc	-0.188551614840319	0.647137192960589	0.994766463583882	pseudobulk	DESeq2	LRT	0.189003639223739
681	KC_AB1	APC10	-0.278466224413396	0.739405059731785	0.994766463583882	pseudobulk	DESeq2	LRT	0.131117581885612
681	KC_AB1	APC4	1.29169465878791	0.584809831144677	0.994766463583882	pseudobulk	DESeq2	LRT	0.23298533513365
681	KC_AB1	ApepP	-1.22636258381907	0.530619334140568	0.994766463583882	pseudobulk	DESeq2	LRT	0.275216929706905
681	KC_AB1	aph-1	-0.138816094783421	0.798207849837849	0.994766463583882	pseudobulk	DESeq2	LRT	0.0978840055365878
681	KC_AB1	aPKC	-0.245649897472209	0.385252805804797	0.994766463583882	pseudobulk	DESeq2	LRT	0.414254189621604
681	KC_AB1	Apl	-1.53252164312824	0.165682212092829	0.994766463583882	pseudobulk	DESeq2	LRT	0.780724115627788
681	KC_AB1	Aplip1	0.302291376318047	0.291720925940352	0.994766463583882	pseudobulk	DESeq2	LRT	0.535032416629171
681	KC_AB1	Apoltp	0.139684708399015	0.86808940059687	0.994766463583882	pseudobulk	DESeq2	LRT	0.0614355464979729
681	KC_AB1	app	1.1821590793593	0.719511699510136	0.994766463583882	pseudobulk	DESeq2	LRT	0.142962139890846
681	KC_AB1	APP-BP1	0.0899185885057952	0.949843011809794	0.994766463583882	pseudobulk	DESeq2	LRT	0.0223481681202833
682	KC_AB1	Appl	0.00503044453729025	0.956221244190004	0.994766463583882	pseudobulk	DESeq2	LRT	0.0194416118931034
682	KC_AB1	Aprt	-0.280158654560806	0.855726772876627	0.994766463583882	pseudobulk	DESeq2	LRT	0.0676648801520045
682	KC_AB1	Aps	-0.223606563586874	0.559927593068609	0.994766463583882	pseudobulk	DESeq2	LRT	0.251868130072156
682	KC_AB1	apt	2.80395130970481	0.386005157167114	0.994766463583882	pseudobulk	DESeq2	LRT	0.413406892959702
682	KC_AB1	aqz	0.0831596384790693	0.466956228026489	0.994766463583882	pseudobulk	DESeq2	LRT	0.330723827822571
682	KC_AB1	aralar1	0.706610493886114	0.352574437613181	0.994766463583882	pseudobulk	DESeq2	LRT	0.452749178156875
682	KC_AB1	Arc1	-2.79499059556516	0.38564607643653	0.994766463583882	pseudobulk	DESeq2	LRT	0.413811082791365
682	KC_AB1	Arc42	0.442499852341606	0.78480579454661	0.994766463583882	pseudobulk	DESeq2	LRT	0.105237799040025
682	KC_AB1	Archease	-0.377241358994877	0.691715991996555	0.994766463583882	pseudobulk	DESeq2	LRT	0.160072183616428
682	KC_AB1	Arf102F	-0.349085971746137	0.366945496993232	0.994766463583882	pseudobulk	DESeq2	LRT	0.435398437417931
683	KC_AB1	Arf51F	0.178239314340392	0.720703332841334	0.994766463583882	pseudobulk	DESeq2	LRT	0.142243469571953

683	KC_AB1	Arf79F	-0.00970052725707702	0.926705183892993	0.994766463583882	pseudobulk	DESeq2	LRT	0.0330584075736591
683	KC_AB1	ArfGAP1	0.441305731686308	0.581127522692926	0.994766463583882	pseudobulk	DESeq2	LRT	0.235728555518705
683	KC_AB1	ArfGAP3	0.507454021728669	0.62193958690261	0.994766463583882	pseudobulk	DESeq2	LRT	0.206251799150146
683	KC_AB1	Arfip	0.357496847432808	0.55612110127051	0.994766463583882	pseudobulk	DESeq2	LRT	0.254830625887889
683	KC_AB1	Arfrp1	-0.75585279790256	0.549422238125455	0.994766463583882	pseudobulk	DESeq2	LRT	0.260093766284739
683	KC_AB1	arg	0.97244782379049	0.509808131587248	0.994766463583882	pseudobulk	DESeq2	LRT	0.292593241688826
683	KC_AB1	Argk	-0.165479780601784	0.42076154230214	0.994766463583882	pseudobulk	DESeq2	LRT	0.375963961651907
683	KC_AB1	Argl	-2.85724547560282	0.450020141104186	0.994766463583882	pseudobulk	DESeq2	LRT	0.346768048503189
683	KC_AB1	ArgRS	0.280783473609079	0.862060689739496	0.994766463583882	pseudobulk	DESeq2	LRT	0.0644621584314761
684	KC_AB1	ArgRS-m	-0.525050937607539	0.744799300928206	0.994766463583882	pseudobulk	DESeq2	LRT	0.127960739657171
684	KC_AB1	ari-1	0.103330093437846	0.720736000502161	0.994766463583882	pseudobulk	DESeq2	LRT	0.142223784546703
684	KC_AB1	ari-2	0.120032734153479	0.83068852961872	0.994766463583882	pseudobulk	DESeq2	LRT	0.0805617863539376
684	KC_AB1	Arl1	0.210052574855781	0.777438692247811	0.994766463583882	pseudobulk	DESeq2	LRT	0.109333848819808
684	KC_AB1	Arl2	0.488861527675963	0.617455819375221	0.994766463583882	pseudobulk	DESeq2	LRT	0.209394111894997
684	KC_AB1	Arl4	-0.0534243024229424	0.829680131429461	0.994766463583882	pseudobulk	DESeq2	LRT	0.0810893099494811
684	KC_AB1	Arl5	-0.0795101307549168	0.892648113388885	0.994766463583882	pseudobulk	DESeq2	LRT	0.0493197085596145
684	KC_AB1	Arl6IP1	-0.0695992295730406	0.80469810892793	0.994766463583882	pseudobulk	DESeq2	LRT	0.0943670192808865
684	KC_AB1	Arl8	0.202366871127464	0.616265540593993	0.994766463583882	pseudobulk	DESeq2	LRT	0.210232115813509
684	KC_AB1	arm	0.161518155134632	0.563942318822928	0.994766463583882	pseudobulk	DESeq2	LRT	0.248765314275492
685	KC_AB1	armi	1.32710828904082	0.607473293222376	0.994766463583882	pseudobulk	DESeq2	LRT	0.216472810505135
685	KC_AB1	Arp1	0.246265715701858	0.598621264826898	0.994766463583882	pseudobulk	DESeq2	LRT	0.222847859776236
685	KC_AB1	Arp10	1.46864299000817	0.237292391161642	0.994766463583882	pseudobulk	DESeq2	LRT	0.624716187317812
685	KC_AB1	Arp2	0.396308995422786	0.453942804735984	0.994766463583882	pseudobulk	DESeq2	LRT	0.342998863337477
685	KC_AB1	Arp3	-0.816685803304414	0.258180726840259	0.994766463583882	pseudobulk	DESeq2	LRT	0.58807618088964
685	KC_AB1	Arp5	0.387548444328087	0.747734973912615	0.994766463583882	pseudobulk	DESeq2	LRT	0.126252305561645
685	KC_AB1	Arp6	0.218363731778184	0.839419374787492	0.994766463583882	pseudobulk	DESeq2	LRT	0.0760210109808686
685	KC_AB1	Arp8	1.00013562845438	0.441752577312396	0.994766463583882	pseudobulk	DESeq2	LRT	0.354820908014098
685	KC_AB1	Arpc1	0.299022616009186	0.541412605627464	0.994766463583882	pseudobulk	DESeq2	LRT	0.266471636835338
685	KC_AB1	Arpc2	-0.0290688840111205	0.941775430521939	0.994766463583882	pseudobulk	DESeq2	LRT	0.0260526438231446
686	KC_AB1	Arpc3A	-0.271467138388971	0.779883456712824	0.994766463583882	pseudobulk	DESeq2	LRT	0.107970292037905
686	KC_AB1	Arpc4	0.239381820555757	0.680910827483747	0.994766463583882	pseudobulk	DESeq2	LRT	0.166909759845659
686	KC_AB1	Arpc5	0.640843148584935	0.384677132566628	0.994766463583882	pseudobulk	DESeq2	LRT	0.414903629901417
686	KC_AB1	arr	0.190357117552	0.710594015918953	0.994766463583882	pseudobulk	DESeq2	LRT	0.148378454128313
686	KC_AB1	Art1	0.151746214741682	0.864267010072632	0.994766463583882	pseudobulk	DESeq2	LRT	0.0633520641338543
686	KC_AB1	Art2	-3.34100803494108	0.314063158159027	0.994766463583882	pseudobulk	DESeq2	LRT	0.502983006443488
686	KC_AB1	Art3	-0.720688993793705	0.702033725657174	0.994766463583882	pseudobulk	DESeq2	LRT	0.153642023888733
686	KC_AB1	Art4	1.64247040105597	0.257070111814983	0.994766463583882	pseudobulk	DESeq2	LRT	0.589948413549173
686	KC_AB1	Art7	0.209946891738838	0.8829622200177	0.994766463583882	pseudobulk	DESeq2	LRT	0.0540578785162677
686	KC_AB1	Art8	-0.835152883801839	0.920434724857442	0.994766463583882	pseudobulk	DESeq2	LRT	0.0360070052473159
687	KC_AB1	Arts	0.584385001526002	0.808656675386651	0.994766463583882	pseudobulk	DESeq2	LRT	0.0922358240397888
687	KC_AB1	aru	-0.835152883801839	0.920434724857442	0.994766463583882	pseudobulk	DESeq2	LRT	0.0360070052473159
687	KC_AB1	Arv1	0.60745396399637	0.566671771690608	0.994766463583882	pseudobulk	DESeq2	LRT	0.246668420858316
687	KC_AB1	arx	-0.643503469817389	0.769036587373876	0.994766463583882	pseudobulk	DESeq2	LRT	0.114052997888302
687	KC_AB1	Asap	-0.793415932055185	0.193713186865932	0.994766463583882	pseudobulk	DESeq2	LRT	0.712840814034768
687	KC_AB1	Asator	-0.0130411415058866	0.908933977049279	0.994766463583882	pseudobulk	DESeq2	LRT	0.041467661821529
687	KC_AB1	Asciz	1.00714384347961	0.440659126246998	0.994766463583882	pseudobulk	DESeq2	LRT	0.355897231016346
687	KC_AB1	asf1	0.364408023248617	0.616916686790086	0.994766463583882	pseudobulk	DESeq2	LRT	0.20977348249902
687	KC_AB1	ash1	0.531312974295396	0.396241652606938	0.994766463583882	pseudobulk	DESeq2	LRT	0.40203987370777
687	KC_AB1	ash2	0.47865508106661	0.724948634422197	0.994766463583882	pseudobulk	DESeq2	LRT	0.139692763880427
688	KC_AB1	Ask1	-0.375147463127622	0.747419132827644	0.994766463583882	pseudobulk	DESeq2	LRT	0.126435789045766
688	KC_AB1	asl	-1.89157349400865	0.516179879210422	0.994766463583882	pseudobulk	DESeq2	LRT	0.287198928343475
688	KC_AB1	AsnRS	1.01497334165008	0.194076791079816	0.994766463583882	pseudobulk	DESeq2	LRT	0.712026397165106
688	KC_AB1	AsnRS-m	-0.271852016503456	0.882107600545087	0.994766463583882	pseudobulk	DESeq2	LRT	0.0544784358741274
688	KC_AB1	AsnS	-1.64952313825113	0.817565084244375	0.994766463583882	pseudobulk	DESeq2	LRT	0.0874776642296574
688	KC_AB1	Asph	3.13490772141882	0.128535663423906	0.994766463583882	pseudobulk	DESeq2	LRT	0.890976356540434
688	KC_AB1	ASPP	1.1821590793593	0.719511699510136	0.994766463583882	pseudobulk	DESeq2	LRT	0.142962139890846

688	KC_AB1	AspRS	-1.25984172451127	0.377241542158982	0.994766463583882	pseudobulk	DESeq2	LRT	0.423380488424101
688	KC_AB1	AspRS-m	-0.176644983549484	0.839822254003217	0.994766463583882	pseudobulk	DESeq2	LRT	0.0758126214071226
688	KC_AB1	asrij	-0.11795964762979	0.880792051722095	0.994766463583882	pseudobulk	DESeq2	LRT	0.0551266130971695
689	KC_AB1	asRNA:ASTR	-1.64952313825113	0.817565084244375	0.994766463583882	pseudobulk	DESeq2	LRT	0.0874776642296574
689	KC_AB1	asRNA:CR31144	0.217906885051292	0.902008941520292	0.994766463583882	pseudobulk	DESeq2	LRT	0.0447891573209458
689	KC_AB1	asRNA:CR42254	-1.22462960175681	0.228390655439122	0.994766463583882	pseudobulk	DESeq2	LRT	0.641321669140052
689	KC_AB1	asRNA:CR42547	1.08611877493397	0.434259028242099	0.994766463583882	pseudobulk	DESeq2	LRT	0.362251143807826
689	KC_AB1	asRNA:CR42860	0.949183608371002	0.80156895525114	0.994766463583882	pseudobulk	DESeq2	LRT	0.0960591113673091
689	KC_AB1	asRNA:CR43425	0.369214845067095	0.633248298301243	0.994766463583882	pseudobulk	DESeq2	LRT	0.198425968595248
689	KC_AB1	asRNA:CR43426	0.949183608371002	0.80156895525114	0.994766463583882	pseudobulk	DESeq2	LRT	0.0960591113673091
689	KC_AB1	asRNA:CR43460	-1.02319198676748	0.828204539793902	0.994766463583882	pseudobulk	DESeq2	LRT	0.0818623932467813
689	KC_AB1	asRNA:CR43465	0.949183608371002	0.80156895525114	0.994766463583882	pseudobulk	DESeq2	LRT	0.0960591113673091
689	KC_AB1	asRNA:CR43466	-2.57435989741145	0.533617212681275	0.994766463583882	pseudobulk	DESeq2	LRT	0.272770169998685
690	KC_AB1	asRNA:CR43468	0.596808856322697	0.822761281772702	0.994766463583882	pseudobulk	DESeq2	LRT	0.0847261539098712
690	KC_AB1	asRNA:CR43471	-2.20969975456663	0.610836134188693	0.994766463583882	pseudobulk	DESeq2	LRT	0.214075280039667
690	KC_AB1	asRNA:CR43609	-1.64952313825113	0.817565084244375	0.994766463583882	pseudobulk	DESeq2	LRT	0.0874776642296574
690	KC_AB1	asRNA:CR43629	1.06554771508077	0.539714016746269	0.994766463583882	pseudobulk	DESeq2	LRT	0.26783630286028
690	KC_AB1	asRNA:CR43730	1.26433463569552	0.528978780772322	0.994766463583882	pseudobulk	DESeq2	LRT	0.276561748717781
690	KC_AB1	asRNA:CR43767	0.921572355523261	0.540489251608862	0.994766463583882	pseudobulk	DESeq2	LRT	0.26721293818315
690	KC_AB1	asRNA:CR43786	-0.835152883801839	0.920434724857442	0.994766463583882	pseudobulk	DESeq2	LRT	0.0360070052473159
690	KC_AB1	asRNA:CR43806	-1.64952313825113	0.817565084244375	0.994766463583882	pseudobulk	DESeq2	LRT	0.0874776642296574
690	KC_AB1	asRNA:CR43820	0.949183608371002	0.80156895525114	0.994766463583882	pseudobulk	DESeq2	LRT	0.0960591113673091
690	KC_AB1	asRNA:CR43842	-0.283995846067142	0.866480656580972	0.994766463583882	pseudobulk	DESeq2	LRT	0.0622411280844138
691	KC_AB1	asRNA:CR43875	-2.79499059556516	0.38564607643653	0.994766463583882	pseudobulk	DESeq2	LRT	0.413811082791365
691	KC_AB1	asRNA:CR43889	-2.20969975456663	0.610836134188693	0.994766463583882	pseudobulk	DESeq2	LRT	0.214075280039667
691	KC_AB1	asRNA:CR43898	-1.64952313825113	0.817565084244375	0.994766463583882	pseudobulk	DESeq2	LRT	0.0874776642296574
691	KC_AB1	asRNA:CR43906	0.150865497138206	0.867718568240628	0.994766463583882	pseudobulk	DESeq2	LRT	0.0616211090156808
691	KC_AB1	asRNA:CR43933	-0.989583867941823	0.582662397843883	0.994766463583882	pseudobulk	DESeq2	LRT	0.234583008228436
691	KC_AB1	asRNA:CR43944	0.574579387376074	0.69332157464757	0.994766463583882	pseudobulk	DESeq2	LRT	0.159065285298698
691	KC_AB1	asRNA:CR43957	-1.72393135133701	0.425020807862363	0.994766463583882	pseudobulk	DESeq2	LRT	0.371589807552997
691	KC_AB1	asRNA:CR43959	0.491136939565768	0.700586090212329	0.994766463583882	pseudobulk	DESeq2	LRT	0.154538489633227
691	KC_AB1	asRNA:CR43981	-4.62642941169408	0.06337111563309	0.994766463583882	pseudobulk	DESeq2	LRT	1.19810864715865
691	KC_AB1	asRNA:CR44018	-0.475723298102429	0.502110762331955	0.994766463583882	pseudobulk	DESeq2	LRT	0.299200469780228
692	KC_AB1	asRNA:CR44019	-1.64952313825113	0.817565084244375	0.994766463583882	pseudobulk	DESeq2	LRT	0.0874776642296574
692	KC_AB1	asRNA:CR44021	1.80633728195307	0.61161102402172	0.994766463583882	pseudobulk	DESeq2	LRT	0.213524695220716
692	KC_AB1	asRNA:CR44027	-0.482468578584362	0.709187187571662	0.994766463583882	pseudobulk	DESeq2	LRT	0.149239119119048
692	KC_AB1	asRNA:CR44029	0.440069474017581	0.577543583436586	0.994766463583882	pseudobulk	DESeq2	LRT	0.238415236835288
692	KC_AB1	asRNA:CR44030	1.27284532529971	0.36599499739498	0.994766463583882	pseudobulk	DESeq2	LRT	0.436524850721992
692	KC_AB1	asRNA:CR44031	0.0335949479761297	0.934060239284245	0.994766463583882	pseudobulk	DESeq2	LRT	0.0296251144068247
692	KC_AB1	asRNA:CR44046	-0.17805032366327	0.942053914371676	0.994766463583882	pseudobulk	DESeq2	LRT	0.0259242415339998
692	KC_AB1	asRNA:CR44054	-1.64952313825113	0.817565084244375	0.994766463583882	pseudobulk	DESeq2	LRT	0.0874776642296574
692	KC_AB1	asRNA:CR44069	-1.64952313825113	0.817565084244375	0.994766463583882	pseudobulk	DESeq2	LRT	0.0874776642296574
692	KC_AB1	asRNA:CR44093	-0.0175856672650169	0.960737619868475	0.994766463583882	pseudobulk	DESeq2	LRT	0.0173952031763364
693	KC_AB1	asRNA:CR44095	-1.64952313825113	0.817565084244375	0.994766463583882	pseudobulk	DESeq2	LRT	0.0874776642296574
693	KC_AB1	asRNA:CR44119	1.45682795759908	0.573003993665791	0.994766463583882	pseudobulk	DESeq2	LRT	0.24184235111992
693	KC_AB1	asRNA:CR44179	-0.0937128549620675	0.940178389818928	0.994766463583882	pseudobulk	DESeq2	LRT	0.0267897353752199
693	KC_AB1	asRNA:CR44228	-0.835152883801839	0.920434724857442	0.994766463583882	pseudobulk	DESeq2	LRT	0.0360070052473159
693	KC_AB1	asRNA:CR44291	0.949183608371002	0.80156895525114	0.994766463583882	pseudobulk	DESeq2	LRT	0.0960591113673091
693	KC_AB1	asRNA:CR44340	-0.981500021353797	0.667407207223606	0.994766463583882	pseudobulk	DESeq2	LRT	0.175609107779306
693	KC_AB1	asRNA:CR44351	-2.79499059556516	0.38564607643653	0.994766463583882	pseudobulk	DESeq2	LRT	0.413811082791365
693	KC_AB1	asRNA:CR44368	0.806933541094274	0.754600478929312	0.994766463583882	pseudobulk	DESeq2	LRT	0.122282923497213
693	KC_AB1	asRNA:CR44390	0.949183608371002	0.80156895525114	0.994766463583882	pseudobulk	DESeq2	LRT	0.0960591113673091
693	KC_AB1	asRNA:CR44429	0.949183608371002	0.80156895525114	0.994766463583882	pseudobulk	DESeq2	LRT	0.0960591113673091
694	KC_AB1	asRNA:CR44431	0.949183608371002	0.80156895525114	0.994766463583882	pseudobulk	DESeq2	LRT	0.0960591113673091
694	KC_AB1	asRNA:CR44690	-1.02319198676748	0.828204539793902	0.994766463583882	pseudobulk	DESeq2	LRT	0.0818623932467813
694	KC_AB1	asRNA:CR44724	0.949183608371002	0.80156895525114	0.994766463583882	pseudobulk	DESeq2	LRT	0.0960591113673091

694	KC_AB1	asRNA:CR44840	-0.548887546079202	0.814781693583783	0.994766463583882	pseudobulk	DESeq2	LRT	0.0889587372379754
694	KC_AB1	asRNA:CR44843	2.80587074018119	0.385946061723411	0.994766463583882	pseudobulk	DESeq2	LRT	0.413473386345624
694	KC_AB1	asRNA:CR44873	-2.79499059556516	0.38564607643653	0.994766463583882	pseudobulk	DESeq2	LRT	0.413811082791365
694	KC_AB1	asRNA:CR44891	-1.64952313825113	0.817565084244375	0.994766463583882	pseudobulk	DESeq2	LRT	0.0874776642296574
694	KC_AB1	asRNA:CR44892	1.29169465878791	0.584809831144677	0.994766463583882	pseudobulk	DESeq2	LRT	0.23298533513365
694	KC_AB1	asRNA:CR44894	-1.77034956848109	0.0490721634835878	0.994766463583882	pseudobulk	DESeq2	LRT	1.30916479450999
694	KC_AB1	asRNA:CR44907	1.1821590793593	0.719511699510136	0.994766463583882	pseudobulk	DESeq2	LRT	0.142962139890846
695	KC_AB1	asRNA:CR44911	1.80633728195307	0.61161102402172	0.994766463583882	pseudobulk	DESeq2	LRT	0.213524695220716
695	KC_AB1	asRNA:CR44960	-4.02268708669696	0.139249961380398	0.994766463583882	pseudobulk	DESeq2	LRT	0.856204916601505
695	KC_AB1	asRNA:CR44981	0.40411895759739	0.864643228521197	0.994766463583882	pseudobulk	DESeq2	LRT	0.0631630553638974
695	KC_AB1	asRNA:CR44982	-2.79499059556516	0.38564607643653	0.994766463583882	pseudobulk	DESeq2	LRT	0.413811082791365
695	KC_AB1	asRNA:CR44987	0.0538070974577746	0.823068699498313	0.994766463583882	pseudobulk	DESeq2	LRT	0.0845639137940102
695	KC_AB1	asRNA:CR44989	-0.29408219783147	0.915376953506099	0.994766463583882	pseudobulk	DESeq2	LRT	0.0384000260261822
695	KC_AB1	asRNA:CR45000	-0.747760753789965	0.720131677202483	0.994766463583882	pseudobulk	DESeq2	LRT	0.142588084882933
695	KC_AB1	asRNA:CR45007	0.854047063205883	0.719204060467466	0.994766463583882	pseudobulk	DESeq2	LRT	0.143147869337068
695	KC_AB1	asRNA:CR45009	0.819341494982688	0.463997163359446	0.994766463583882	pseudobulk	DESeq2	LRT	0.333484674490605
695	KC_AB1	asRNA:CR45016	0.949183608371002	0.80156895525114	0.994766463583882	pseudobulk	DESeq2	LRT	0.0960591113673091
696	KC_AB1	asRNA:CR45041	-2.20969975456663	0.610836134188693	0.994766463583882	pseudobulk	DESeq2	LRT	0.214075280039667
696	KC_AB1	asRNA:CR45044	2.38929733117871	0.484296857543297	0.994766463583882	pseudobulk	DESeq2	LRT	0.314888348950639
696	KC_AB1	asRNA:CR45053	0.949183608371002	0.80156895525114	0.994766463583882	pseudobulk	DESeq2	LRT	0.0960591113673091
696	KC_AB1	asRNA:CR45056	-1.64952313825113	0.817565084244375	0.994766463583882	pseudobulk	DESeq2	LRT	0.0874776642296574
696	KC_AB1	asRNA:CR45121	2.22023857837763	0.524904842126482	0.994766463583882	pseudobulk	DESeq2	LRT	0.279919420946639
696	KC_AB1	asRNA:CR45124	1.80633728195307	0.61161102402172	0.994766463583882	pseudobulk	DESeq2	LRT	0.213524695220716
696	KC_AB1	asRNA:CR45126	0.4771197147979	0.605323832473037	0.994766463583882	pseudobulk	DESeq2	LRT	0.218012226950203
696	KC_AB1	asRNA:CR45129	1.07578421889948	0.540791135907155	0.994766463583882	pseudobulk	DESeq2	LRT	0.266970435517158
696	KC_AB1	asRNA:CR45133	2.22023857837763	0.524904842126482	0.994766463583882	pseudobulk	DESeq2	LRT	0.279919420946639
696	KC_AB1	asRNA:CR45134	-0.24471288778077	0.786674955291564	0.994766463583882	pseudobulk	DESeq2	LRT	0.104204675878676
697	KC_AB1	asRNA:CR45135	1.1821590793593	0.719511699510136	0.994766463583882	pseudobulk	DESeq2	LRT	0.142962139890846
697	KC_AB1	asRNA:CR45137	1.17930484172975	0.669685881755347	0.994766463583882	pseudobulk	DESeq2	LRT	0.174128856716169
697	KC_AB1	asRNA:CR45139	2.22023857837763	0.524904842126482	0.994766463583882	pseudobulk	DESeq2	LRT	0.279919420946639
697	KC_AB1	asRNA:CR45145	0.134681164264918	0.930208631343071	0.994766463583882	pseudobulk	DESeq2	LRT	0.0314196350166374
697	KC_AB1	asRNA:CR45151	1.1821590793593	0.719511699510136	0.994766463583882	pseudobulk	DESeq2	LRT	0.142962139890846
697	KC_AB1	asRNA:CR45160	0.949183608371002	0.80156895525114	0.994766463583882	pseudobulk	DESeq2	LRT	0.0960591113673091
697	KC_AB1	asRNA:CR45164	1.80939591345248	0.468117841155507	0.994766463583882	pseudobulk	DESeq2	LRT	0.329644806495434
697	KC_AB1	asRNA:CR45182	0.0684327519130825	0.879373721353118	0.994766463583882	pseudobulk	DESeq2	LRT	0.0558265167034121
697	KC_AB1	asRNA:CR45198	-0.126857805158015	0.853645970468236	0.994766463583882	pseudobulk	DESeq2	LRT	0.0687222053653266
697	KC_AB1	asRNA:CR45271	-1.64952313825113	0.817565084244375	0.994766463583882	pseudobulk	DESeq2	LRT	0.0874776642296574
698	KC_AB1	asRNA:CR45275	-1.64952313825113	0.817565084244375	0.994766463583882	pseudobulk	DESeq2	LRT	0.0874776642296574
698	KC_AB1	asRNA:CR45281	-0.648218248148942	0.834000364039372	0.994766463583882	pseudobulk	DESeq2	LRT	0.0788337597936092
698	KC_AB1	asRNA:CR45350	-2.20969975456663	0.610836134188693	0.994766463583882	pseudobulk	DESeq2	LRT	0.214075280039667
698	KC_AB1	asRNA:CR45367	-1.64952313825113	0.817565084244375	0.994766463583882	pseudobulk	DESeq2	LRT	0.0874776642296574
698	KC_AB1	asRNA:CR45384	-2.79499059556516	0.38564607643653	0.994766463583882	pseudobulk	DESeq2	LRT	0.413811082791365
698	KC_AB1	asRNA:CR45469	-1.42386827328458	0.53856681126094	0.994766463583882	pseudobulk	DESeq2	LRT	0.268760413142001
698	KC_AB1	asRNA:CR45479	-2.20969975456663	0.610836134188693	0.994766463583882	pseudobulk	DESeq2	LRT	0.214075280039667
698	KC_AB1	asRNA:CR45520	0.886042894545102	0.648775226723578	0.994766463583882	pseudobulk	DESeq2	LRT	0.187905741877447
698	KC_AB1	asRNA:CR45528	-1.64952313825113	0.817565084244375	0.994766463583882	pseudobulk	DESeq2	LRT	0.0874776642296574
698	KC_AB1	asRNA:CR45549	1.1821590793593	0.719511699510136	0.994766463583882	pseudobulk	DESeq2	LRT	0.142962139890846
699	KC_AB1	asRNA:CR45600	5.59186513772306	0.27354452148699	0.994766463583882	pseudobulk	DESeq2	LRT	0.562971978791083
699	KC_AB1	asRNA:CR45612	0.949183608371002	0.80156895525114	0.994766463583882	pseudobulk	DESeq2	LRT	0.0960591113673091
699	KC_AB1	asRNA:CR45620	-0.606730070108084	0.648681626284305	0.994766463583882	pseudobulk	DESeq2	LRT	0.187968403158639
699	KC_AB1	asRNA:CR45683	-2.3701736114949	0.404790721764187	0.994766463583882	pseudobulk	DESeq2	LRT	0.392769450547692
699	KC_AB1	asRNA:CR45686	1.1821590793593	0.719511699510136	0.994766463583882	pseudobulk	DESeq2	LRT	0.142962139890846
699	KC_AB1	asRNA:CR45789	2.67764418499683	0.378704357417491	0.994766463583882	pseudobulk	DESeq2	LRT	0.421699697806334
699	KC_AB1	asRNA:CR45797	-2.20969975456663	0.610836134188693	0.994766463583882	pseudobulk	DESeq2	LRT	0.214075280039667
699	KC_AB1	asRNA:CR45804	0.178600637834916	0.898376519498662	0.994766463583882	pseudobulk	DESeq2	LRT	0.0465416075763243
699	KC_AB1	asRNA:CR45807	1.1821590793593	0.719511699510136	0.994766463583882	pseudobulk	DESeq2	LRT	0.142962139890846

699	KC_AB1	asRNA:CR45813	2.02670873290438	0.538959414925803	0.994766463583882	pseudobulk	DESeq2	LRT	0.26844393710898
700	KC_AB1	asRNA:CR45818	1.1821590793593	0.719511699510136	0.994766463583882	pseudobulk	DESeq2	LRT	0.142962139890846
700	KC_AB1	asRNA:CR45830	0.949183608371002	0.80156895525114	0.994766463583882	pseudobulk	DESeq2	LRT	0.0960591113673091
700	KC_AB1	asRNA:CR45835	-0.286468722693323	0.805351591403713	0.994766463583882	pseudobulk	DESeq2	LRT	0.0940144787987154
700	KC_AB1	asRNA:CR45873	-0.627447798185778	0.772628937945737	0.994766463583882	pseudobulk	DESeq2	LRT	0.112029029876423
700	KC_AB1	asRNA:CR45874	0.27113225158927	0.761398762938961	0.994766463583882	pseudobulk	DESeq2	LRT	0.118387833128717
700	KC_AB1	asRNA:CR45889	-1.64952313825113	0.817565084244375	0.994766463583882	pseudobulk	DESeq2	LRT	0.0874776642296574
700	KC_AB1	asRNA:CR45896	-1.64952313825113	0.817565084244375	0.994766463583882	pseudobulk	DESeq2	LRT	0.0874776642296574
700	KC_AB1	asRNA:CR45907	0.919544664429766	0.636237482158416	0.994766463583882	pseudobulk	DESeq2	LRT	0.196380749225724
700	KC_AB1	asRNA:CR45912	2.02670873290438	0.538959414925803	0.994766463583882	pseudobulk	DESeq2	LRT	0.26844393710898
700	KC_AB1	asRNA:CR45925	1.1821590793593	0.719511699510136	0.994766463583882	pseudobulk	DESeq2	LRT	0.142962139890846
701	KC_AB1	asRNA:CR45928	-1.02319198676748	0.828204539793902	0.994766463583882	pseudobulk	DESeq2	LRT	0.0818623932467813
701	KC_AB1	asRNA:CR45951	0.971093424651049	0.540872556325323	0.994766463583882	pseudobulk	DESeq2	LRT	0.266905053941267
701	KC_AB1	asRNA:CR45999	0.949183608371002	0.80156895525114	0.994766463583882	pseudobulk	DESeq2	LRT	0.0960591113673091
701	KC_AB1	asRNA:CR46029	-0.235416794582491	0.844788530836776	0.994766463583882	pseudobulk	DESeq2	LRT	0.0732519909136154
701	KC_AB1	asRNA:CR46033	-0.835152883801839	0.920434724857442	0.994766463583882	pseudobulk	DESeq2	LRT	0.0360070052473159
701	KC_AB1	asRNA:CR46050	-0.835152883801839	0.920434724857442	0.994766463583882	pseudobulk	DESeq2	LRT	0.0360070052473159
701	KC_AB1	asRNA:CR46058	-1.64952313825113	0.817565084244375	0.994766463583882	pseudobulk	DESeq2	LRT	0.0874776642296574
701	KC_AB1	asRNA:CR46107	0.949183608371002	0.80156895525114	0.994766463583882	pseudobulk	DESeq2	LRT	0.0960591113673091
701	KC_AB1	asRNA:CR46135	-2.08761707051565	0.300243913107209	0.994766463583882	pseudobulk	DESeq2	LRT	0.522525788357186
701	KC_AB1	asRNA:CR46140	-1.64952313825113	0.817565084244375	0.994766463583882	pseudobulk	DESeq2	LRT	0.0874776642296574
702	KC_AB1	asRNA:CR46246	0.949183608371002	0.80156895525114	0.994766463583882	pseudobulk	DESeq2	LRT	0.0960591113673091
702	KC_AB1	asRNA:CR46256	-1.64952313825113	0.817565084244375	0.994766463583882	pseudobulk	DESeq2	LRT	0.0874776642296574
702	KC_AB1	asRNA:CR46262	-0.356212784647529	0.928203443271547	0.994766463583882	pseudobulk	DESeq2	LRT	0.0323568248514225
702	KC_AB1	asRNA:CR46263	0.949183608371002	0.80156895525114	0.994766463583882	pseudobulk	DESeq2	LRT	0.0960591113673091
702	KC_AB1	asRNA:CR46266	-1.64952313825113	0.817565084244375	0.994766463583882	pseudobulk	DESeq2	LRT	0.0874776642296574
702	KC_AB1	asRNA:CR46354	-1.21457746603801	0.770013447451371	0.994766463583882	pseudobulk	DESeq2	LRT	0.113501690278259
702	KC_AB1	ast	-0.909015896987447	0.61737204435158	0.994766463583882	pseudobulk	DESeq2	LRT	0.209453039996011
702	KC_AB1	AstA-R1	1.06071898122436	0.628579022653205	0.994766463583882	pseudobulk	DESeq2	LRT	0.201640116633702
702	KC_AB1	AstA-R2	0.949183608371002	0.80156895525114	0.994766463583882	pseudobulk	DESeq2	LRT	0.0960591113673091
702	KC_AB1	AstC	-1.64952313825113	0.817565084244375	0.994766463583882	pseudobulk	DESeq2	LRT	0.0874776642296574
703	KC_AB1	AstC-R2	-2.20969975456663	0.610836134188693	0.994766463583882	pseudobulk	DESeq2	LRT	0.214075280039667
703	KC_AB1	asun	0.178600637834916	0.898376519498662	0.994766463583882	pseudobulk	DESeq2	LRT	0.0465416075763243
703	KC_AB1	Asx	-0.782084874082185	0.17406786171154	0.994766463583882	pseudobulk	DESeq2	LRT	0.759281405618723
703	KC_AB1	Atac1	0.0500827443498999	0.957289958771782	0.994766463583882	pseudobulk	DESeq2	LRT	0.0189564964747698
703	KC_AB1	Atac2	0.300827473452712	0.869768734250272	0.994766463583882	pseudobulk	DESeq2	LRT	0.060596208060234
703	KC_AB1	Atac3	-0.720107473192174	0.401349685741834	0.994766463583882	pseudobulk	DESeq2	LRT	0.396477072741128
703	KC_AB1	ATbp	0.309157786862567	0.836236418179691	0.994766463583882	pseudobulk	DESeq2	LRT	0.0776709228056033
703	KC_AB1	Ate1	-0.482878904062354	0.441709960878228	0.994766463583882	pseudobulk	DESeq2	LRT	0.354862806977352
703	KC_AB1	Atet	0.098625840992741	0.721281448784354	0.994766463583882	pseudobulk	DESeq2	LRT	0.141895237634133
703	KC_AB1	Atf-2	-0.272218280507276	0.765247871891623	0.994766463583882	pseudobulk	DESeq2	LRT	0.116197869475822
704	KC_AB1	Atf3	2.60589673069645	0.18159285736869	0.994766463583882	pseudobulk	DESeq2	LRT	0.740901237678201
704	KC_AB1	Atf6	-0.239828094213997	0.384507711686257	0.994766463583882	pseudobulk	DESeq2	LRT	0.415094945565535
704	KC_AB1	Atg1	0.0746654750447652	0.77770354772405	0.994766463583882	pseudobulk	DESeq2	LRT	0.109185919878019
704	KC_AB1	Atg101	0.264215720124774	0.712794418312136	0.994766463583882	pseudobulk	DESeq2	LRT	0.147035709792913
704	KC_AB1	Atg13	0.553827379638862	0.399985783079277	0.994766463583882	pseudobulk	DESeq2	LRT	0.397955444771906
704	KC_AB1	Atg14	0.114781252798674	0.88416733730653	0.994766463583882	pseudobulk	DESeq2	LRT	0.0534655327343165
704	KC_AB1	Atg16	0.288215056586144	0.764456591337516	0.994766463583882	pseudobulk	DESeq2	LRT	0.116647170392695
704	KC_AB1	Atg17	-0.0522326068908121	0.860029688870189	0.994766463583882	pseudobulk	DESeq2	LRT	0.0654865563262645
704	KC_AB1	Atg18a	0.274313925405073	0.671723795726643	0.994766463583882	pseudobulk	DESeq2	LRT	0.172809266604203
704	KC_AB1	Atg18b	-0.366885616857029	0.494481690249194	0.994766463583882	pseudobulk	DESeq2	LRT	0.305849784897861
705	KC_AB1	Atg2	0.44058022730795	0.72393404180013	0.994766463583882	pseudobulk	DESeq2	LRT	0.140301000912172
705	KC_AB1	Atg3	-0.350893866067084	0.575439733776552	0.994766463583882	pseudobulk	DESeq2	LRT	0.24000015363074
705	KC_AB1	Atg4a	-0.50861576435489	0.586697614060145	0.994766463583882	pseudobulk	DESeq2	LRT	0.231585677937785
705	KC_AB1	Atg4b	0.22791341436187	0.828291062978683	0.994766463583882	pseudobulk	DESeq2	LRT	0.0818170245278081
705	KC_AB1	Atg5	-1.0280010311258	0.170375825450792	0.994766463583882	pseudobulk	DESeq2	LRT	0.768592027046599

705	KC_AB1	Atg6	0.83580157546196	0.466770907873617	0.994766463583882	pseudobulk	DESeq2	LRT	0.33089621977251
705	KC_AB1	Atg7	0.704841709348349	0.611084890723616	0.994766463583882	pseudobulk	DESeq2	LRT	0.213898454222377
705	KC_AB1	Atg8a	0.03884394754258	0.826834412865518	0.994766463583882	pseudobulk	DESeq2	LRT	0.0825814563231908
705	KC_AB1	Atg9	-0.323851912944867	0.814846269805775	0.994766463583882	pseudobulk	DESeq2	LRT	0.0889243182202389
705	KC_AB1	atl	-0.360591950674226	0.619246910036137	0.994766463583882	pseudobulk	DESeq2	LRT	0.208136151811327
706	KC_AB1	atms	-0.769071094846486	0.358071152462926	0.994766463583882	pseudobulk	DESeq2	LRT	0.446030665949333
706	KC_AB1	Atox1	0.187529818876897	0.49189634969972	0.994766463583882	pseudobulk	DESeq2	LRT	0.308126400272852
706	KC_AB1	ATP6AP2	-0.177265291475897	0.536812252429167	0.994766463583882	pseudobulk	DESeq2	LRT	0.270177580205588
706	KC_AB1	ATP7	0.0053787865039457	0.899480051130611	0.994766463583882	pseudobulk	DESeq2	LRT	0.0460084640916014
706	KC_AB1	ATP8A	-0.199211676546036	0.55517973830856	0.994766463583882	pseudobulk	DESeq2	LRT	0.255566392159393
706	KC_AB1	ATP8B	0.949183608371002	0.80156895525114	0.994766463583882	pseudobulk	DESeq2	LRT	0.0960591113673091
706	KC_AB1	Atpalpha	0.0200364237187824	0.865392478770212	0.994766463583882	pseudobulk	DESeq2	LRT	0.0627868836263037
706	KC_AB1	ATPCL	0.0763322652563102	0.838241005248203	0.994766463583882	pseudobulk	DESeq2	LRT	0.0766310980745434
706	KC_AB1	ATPsynB	0.0573192694298569	0.796154050873437	0.994766463583882	pseudobulk	DESeq2	LRT	0.0990028908412914
706	KC_AB1	ATPsynbeta	-0.0702405834308234	0.720526321808364	0.994766463583882	pseudobulk	DESeq2	LRT	0.142350149190976
707	KC_AB1	ATPsynC	0.046922335658772	0.755350712092991	0.994766463583882	pseudobulk	DESeq2	LRT	0.121851357030859
707	KC_AB1	ATPsynCF6	0.053581714981137	0.817002660179161	0.994766463583882	pseudobulk	DESeq2	LRT	0.0877765293926157
707	KC_AB1	ATPsynCF6L	0.44976297004525	0.831013436772572	0.994766463583882	pseudobulk	DESeq2	LRT	0.0803919539908534
707	KC_AB1	ATPsynD	-0.089635342328544	0.690320555582359	0.994766463583882	pseudobulk	DESeq2	LRT	0.160949194490764
707	KC_AB1	ATPsyndelta	0.0152019360074848	0.950955547990327	0.994766463583882	pseudobulk	DESeq2	LRT	0.0218397834976139
707	KC_AB1	ATPsynE	0.237465541033639	0.326247550592326	0.994766463583882	pseudobulk	DESeq2	LRT	0.486452740245939
707	KC_AB1	ATPsynF	0.0886138241355003	0.645139595010931	0.994766463583882	pseudobulk	DESeq2	LRT	0.190346302754758
707	KC_AB1	ATPsynG	0.0686860539242123	0.731259504140135	0.994766463583882	pseudobulk	DESeq2	LRT	0.135928476371243
707	KC_AB1	ATPsyngamma	-0.102298203739488	0.721842265493945	0.994766463583882	pseudobulk	DESeq2	LRT	0.141557692609842
707	KC_AB1	ATPsynO	-0.0325614774694	0.88844704413031	0.994766463583882	pseudobulk	DESeq2	LRT	0.0513684532018288
708	KC_AB1	AttB	-1.64952313825113	0.817565084244375	0.994766463583882	pseudobulk	DESeq2	LRT	0.0874776642296574
708	KC_AB1	Atu	0.578517852171463	0.617090838471302	0.994766463583882	pseudobulk	DESeq2	LRT	0.209650901213056
708	KC_AB1	Atx2	-0.321451431028938	0.273772934996133	0.994766463583882	pseudobulk	DESeq2	LRT	0.562609488133432
708	KC_AB1	Atxn7	0.243874063989745	0.654026990117957	0.994766463583882	pseudobulk	DESeq2	LRT	0.184404329019114
708	KC_AB1	aub	-1.64952313825113	0.817565084244375	0.994766463583882	pseudobulk	DESeq2	LRT	0.0874776642296574
708	KC_AB1	aurA	-1.02319198676748	0.828204539793902	0.994766463583882	pseudobulk	DESeq2	LRT	0.0818623932467813
708	KC_AB1	aux	-0.67077238999704	0.277316737255154	0.994766463583882	pseudobulk	DESeq2	LRT	0.557023918095428
708	KC_AB1	ave	0.311610875953933	0.62754133369545	0.994766463583882	pseudobulk	DESeq2	LRT	0.202357663624364
708	KC_AB1	Aven	-0.0876446376047783	0.951298398237017	0.994766463583882	pseudobulk	DESeq2	LRT	0.0216832345024977
708	KC_AB1	awd	0.00825027650014588	0.895265566922614	0.994766463583882	pseudobulk	DESeq2	LRT	0.0480481187161554
709	KC_AB1	axed	0.105622920981131	0.79355658244663	0.994766463583882	pseudobulk	DESeq2	LRT	0.100422101589346
709	KC_AB1	Axn	0.122892948597887	0.8344838955922	0.994766463583882	pseudobulk	DESeq2	LRT	0.0785820401985504
709	KC_AB1	axo	0.206174930707402	0.869576833587812	0.994766463583882	pseudobulk	DESeq2	LRT	0.0606920388148111
709	KC_AB1	Axud1	-0.136136398294117	0.803306966719889	0.994766463583882	pseudobulk	DESeq2	LRT	0.0951184665796241
709	KC_AB1	az2	0.10505681339381	0.885435645035157	0.994766463583882	pseudobulk	DESeq2	LRT	0.0528429985865891
709	KC_AB1	B4	0.0782617820842744	0.772684857751557	0.994766463583882	pseudobulk	DESeq2	LRT	0.111997598508664
709	KC_AB1	B52	0.0692746633930554	0.7002853015176	0.994766463583882	pseudobulk	DESeq2	LRT	0.154724989083667
709	KC_AB1	bab1	-0.0713915988297819	0.864170454131802	0.994766463583882	pseudobulk	DESeq2	LRT	0.063400586239177
709	KC_AB1	babo	0.0806984889737768	0.828624787970346	0.994766463583882	pseudobulk	DESeq2	LRT	0.0816420791022704
709	KC_AB1	babos	0.949183608371002	0.80156895525114	0.994766463583882	pseudobulk	DESeq2	LRT	0.0960591113673091
710	KC_AB1	Bacc	0.0192139816155758	0.871756216402701	0.994766463583882	pseudobulk	DESeq2	LRT	0.0596049470322673
710	KC_AB1	baf	-0.117057088065173	0.898435287836747	0.994766463583882	pseudobulk	DESeq2	LRT	0.046513198630161
710	KC_AB1	bai	-0.0614114958219463	0.902973209682749	0.994766463583882	pseudobulk	DESeq2	LRT	0.0443251345807872
710	KC_AB1	ball	1.31489890371978	0.469805239145257	0.994766463583882	pseudobulk	DESeq2	LRT	0.328082144392836
710	KC_AB1	Bap111	-0.681462436665437	0.430832795340709	0.994766463583882	pseudobulk	DESeq2	LRT	0.36569124526867
710	KC_AB1	Bap170	-0.129539316108106	0.884338368618203	0.994766463583882	pseudobulk	DESeq2	LRT	0.0533815319251868
710	KC_AB1	Bap60	0.411885794374252	0.476271011756423	0.994766463583882	pseudobulk	DESeq2	LRT	0.322145851025352
710	KC_AB1	barc	-0.19125230770867	0.652490104499008	0.994766463583882	pseudobulk	DESeq2	LRT	0.185426070340738
710	KC_AB1	baz	-0.647856137067844	0.762139005512724	0.994766463583882	pseudobulk	DESeq2	LRT	0.117965811046941
710	KC_AB1	bbc	0.293315948739577	0.51859693996194	0.994766463583882	pseudobulk	DESeq2	LRT	0.285170050161277
711	KC_AB1	BBS5	-2.20969975456663	0.610836134188693	0.994766463583882	pseudobulk	DESeq2	LRT	0.214075280039667

711	KC_AB1	BBS9	-1.64952313825113	0.817565084244375	0.994766463583882	pseudobulk	DESeq2	LRT	0.0874776642296574
711	KC_AB1	bbx	0.989440313175187	0.686072261314579	0.994766463583882	pseudobulk	DESeq2	LRT	0.163630139340755
711	KC_AB1	bc10	-0.127561901064385	0.806864390769666	0.994766463583882	pseudobulk	DESeq2	LRT	0.0931994507665549
711	KC_AB1	BCAS2	-0.181305290082486	0.854306803061163	0.994766463583882	pseudobulk	DESeq2	LRT	0.0683861352264572
711	KC_AB1	bcd	1.1821590793593	0.719511699510136	0.994766463583882	pseudobulk	DESeq2	LRT	0.142962139890846
711	KC_AB1	bchs	-0.221309087231381	0.599428885041762	0.994766463583882	pseudobulk	DESeq2	LRT	0.222262333275602
711	KC_AB1	BCL7-like	0.259655625509506	0.790223253874699	0.994766463583882	pseudobulk	DESeq2	LRT	0.102250194496547
711	KC_AB1	bcn92	-0.272727904838113	0.561705110537049	0.994766463583882	pseudobulk	DESeq2	LRT	0.250491624783385
711	KC_AB1	Bdbt	0.86552385425207	0.372361324683604	0.994766463583882	pseudobulk	DESeq2	LRT	0.429035433353288
712	KC_AB1	bdg	-0.987147158338059	0.45217987750788	0.994766463583882	pseudobulk	DESeq2	LRT	0.344688768133375
712	KC_AB1	bdl	0.949183608371002	0.80156895525114	0.994766463583882	pseudobulk	DESeq2	LRT	0.0960591113673091
712	KC_AB1	Bdp1	0.69750158688829	0.750018748510497	0.994766463583882	pseudobulk	DESeq2	LRT	0.124927880244456
712	KC_AB1	be	1.23525719400989	0.501484071198234	0.994766463583882	pseudobulk	DESeq2	LRT	0.299742857061459
712	KC_AB1	BEAF-32	0.0187310697991356	0.885764227138348	0.994766463583882	pseudobulk	DESeq2	LRT	0.0526818633040643
712	KC_AB1	beag	0.036363402175442	0.928586002456503	0.994766463583882	pseudobulk	DESeq2	LRT	0.0321778671933409
712	KC_AB1	beat-Ia	0.193428213414189	0.860316359648965	0.994766463583882	pseudobulk	DESeq2	LRT	0.0653418185409745
712	KC_AB1	beat-Ib	1.24824207461819	0.126172192448632	0.994766463583882	pseudobulk	DESeq2	LRT	0.899036350296537
712	KC_AB1	beat-Ic	1.25204026623933	0.459604466074074	0.994766463583882	pseudobulk	DESeq2	LRT	0.337615759831658
712	KC_AB1	beat-IIIa	-1.64952313825113	0.817565084244375	0.994766463583882	pseudobulk	DESeq2	LRT	0.0874776642296574
713	KC_AB1	beat-IIIb	-0.0420008737491323	0.895021608256808	0.994766463583882	pseudobulk	DESeq2	LRT	0.0481664795070906
713	KC_AB1	beat-IV	-0.790158297816475	0.259559318277844	0.994766463583882	pseudobulk	DESeq2	LRT	0.585763375168852
713	KC_AB1	beat-Va	-2.20969975456663	0.610836134188693	0.994766463583882	pseudobulk	DESeq2	LRT	0.214075280039667
713	KC_AB1	beat-Vb	-1.64952313825113	0.817565084244375	0.994766463583882	pseudobulk	DESeq2	LRT	0.0874776642296574
713	KC_AB1	beat-VI	1.17930484172975	0.669685881755347	0.994766463583882	pseudobulk	DESeq2	LRT	0.174128856716169
713	KC_AB1	beg	0.787373868230252	0.574964265800877	0.994766463583882	pseudobulk	DESeq2	LRT	0.2403591460021
713	KC_AB1	bel	0.0396722944782312	0.845543568009851	0.994766463583882	pseudobulk	DESeq2	LRT	0.0728640097589884
713	KC_AB1	Bem46	-1.59777574752773	0.121399623625758	0.994766463583882	pseudobulk	DESeq2	LRT	0.91578265969824
713	KC_AB1	ben	-0.261718388506682	0.357150560601804	0.994766463583882	pseudobulk	DESeq2	LRT	0.447148663848052
713	KC_AB1	Best1	-0.897448938452856	0.398348566798742	0.994766463583882	pseudobulk	DESeq2	LRT	0.39973674102513
714	KC_AB1	Bet1	-0.217165410492663	0.70678166845677	0.994766463583882	pseudobulk	DESeq2	LRT	0.150714723159774
714	KC_AB1	Bet3	0.70845182304351	0.232354774066394	0.994766463583882	pseudobulk	DESeq2	LRT	0.633848399882376
714	KC_AB1	Bet5	0.245651819638053	0.713248788442257	0.994766463583882	pseudobulk	DESeq2	LRT	0.146758957383174
714	KC_AB1	beta-PheRS	0.115329508020812	0.924742487780173	0.994766463583882	pseudobulk	DESeq2	LRT	0.033979188024322
714	KC_AB1	beta-Spec	-0.0435880386794753	0.821767905214064	0.994766463583882	pseudobulk	DESeq2	LRT	0.0852508244523632
714	KC_AB1	beta'COP	0.102141942626933	0.870568232831477	0.994766463583882	pseudobulk	DESeq2	LRT	0.0601971843618355
714	KC_AB1	beta3GalTII	2.07345913356772	0.326363476291341	0.994766463583882	pseudobulk	DESeq2	LRT	0.486298449589334
714	KC_AB1	beta4GalNAcTA	0.386122894459149	0.574727498834282	0.994766463583882	pseudobulk	DESeq2	LRT	0.240538022796221
714	KC_AB1	beta4GalNAcTB	-0.88447997072047	0.608535812284575	0.994766463583882	pseudobulk	DESeq2	LRT	0.215713858485819
714	KC_AB1	beta4GalT7	2.01197815094411	0.158024496349071	0.994766463583882	pseudobulk	DESeq2	LRT	0.801275585168324
715	KC_AB1	betaCOP	0.253646352856601	0.771657806113206	0.994766463583882	pseudobulk	DESeq2	LRT	0.112575246119342
715	KC_AB1	betaggt-I	-1.409716232016	0.354987864709784	0.994766463583882	pseudobulk	DESeq2	LRT	0.44978649308479
715	KC_AB1	betaggt-II	-1.6073807905348	0.136783843943039	0.994766463583882	pseudobulk	DESeq2	LRT	0.863965195749988
715	KC_AB1	betaGlu	-0.237473683803514	0.877148860518115	0.994766463583882	pseudobulk	DESeq2	LRT	0.0569266964680766
715	KC_AB1	betaNACtes3	1.1821590793593	0.719511699510136	0.994766463583882	pseudobulk	DESeq2	LRT	0.142962139890846
715	KC_AB1	betaNACtes6	-1.64952313825113	0.817565084244375	0.994766463583882	pseudobulk	DESeq2	LRT	0.0874776642296574
715	KC_AB1	betaTub56D	0.0671391902377144	0.736127236596431	0.994766463583882	pseudobulk	DESeq2	LRT	0.133047113140928
715	KC_AB1	betaTub85D	-0.356212784647529	0.928203443271547	0.994766463583882	pseudobulk	DESeq2	LRT	0.0323568248514225
715	KC_AB1	Bgb	-0.0497634452662939	0.922914265796681	0.994766463583882	pseudobulk	DESeq2	LRT	0.0348386409250968
715	KC_AB1	BHD	0.694978102914899	0.701892288551021	0.994766463583882	pseudobulk	DESeq2	LRT	0.153729529005562
716	KC_AB1	bi	0.949183608371002	0.80156895525114	0.994766463583882	pseudobulk	DESeq2	LRT	0.0960591113673091
716	KC_AB1	BI-1	-0.0379027283173516	0.904855434154724	0.994766463583882	pseudobulk	DESeq2	LRT	0.0434208010865302
716	KC_AB1	bib	-0.835152883801839	0.920434724857442	0.994766463583882	pseudobulk	DESeq2	LRT	0.0360070052473159
716	KC_AB1	bic	-0.232016331206537	0.290210727007284	0.994766463583882	pseudobulk	DESeq2	LRT	0.537286538852209
716	KC_AB1	BicD	-0.349750995417165	0.246910868320842	0.994766463583882	pseudobulk	DESeq2	LRT	0.607459793227703
716	KC_AB1	bif	0.165805289155285	0.683180116418972	0.994766463583882	pseudobulk	DESeq2	LRT	0.165464782036825
716	KC_AB1	bigmax	1.02074362672701	0.342959915448666	0.994766463583882	pseudobulk	DESeq2	LRT	0.464756636565835

716	KC_AB1	Bili	0.229173322232218	0.517487573389375	0.994766463583882	pseudobulk	DESeq2	LRT	0.286100074611567
716	KC_AB1	Bin1	0.68307643014517	0.393709459462354	0.994766463583882	pseudobulk	DESeq2	LRT	0.404824150505811
716	KC_AB1	bin3	0.147976723439265	0.712937837985784	0.994766463583882	pseudobulk	DESeq2	LRT	0.146948335220588
717	KC_AB1	bip2	0.00089058314738812	0.918080956108478	0.994766463583882	pseudobulk	DESeq2	LRT	0.0371190211506126
717	KC_AB1	Bka	-0.497898643435852	0.601075833209334	0.994766463583882	pseudobulk	DESeq2	LRT	0.221070732877863
717	KC_AB1	Blimp-1	-0.35537978270288	0.147575916184527	0.994766463583882	pseudobulk	DESeq2	LRT	0.830984511900436
717	KC_AB1	Blm	-0.306571654351356	0.799991596197386	0.994766463583882	pseudobulk	DESeq2	LRT	0.0969145751883964
717	KC_AB1	Blos1	-0.132363676964632	0.839758365260217	0.994766463583882	pseudobulk	DESeq2	LRT	0.0758456612365131
717	KC_AB1	Blos2	-0.23035990116882	0.794579214523759	0.994766463583882	pseudobulk	DESeq2	LRT	0.0998627998843603
717	KC_AB1	Blos3	-0.226261955284549	0.746743505978906	0.994766463583882	pseudobulk	DESeq2	LRT	0.126828545529713
717	KC_AB1	Blos4	0.125400116592342	0.913614159485783	0.994766463583882	pseudobulk	DESeq2	LRT	0.0392371782106514
717	KC_AB1	blot	-0.835152883801839	0.920434724857442	0.994766463583882	pseudobulk	DESeq2	LRT	0.0360070052473159
717	KC_AB1	blow	0.949183608371002	0.80156895525114	0.994766463583882	pseudobulk	DESeq2	LRT	0.0960591113673091
718	KC_AB1	blp	-0.463278861469453	0.637375659047893	0.994766463583882	pseudobulk	DESeq2	LRT	0.195604525962748
718	KC_AB1	blw	0.370372882320071	0.0773639312491817	0.994766463583882	pseudobulk	DESeq2	LRT	1.11146146968453
718	KC_AB1	bma	-0.0934035656681903	0.677941437217493	0.994766463583882	pseudobulk	DESeq2	LRT	0.16880782028009
718	KC_AB1	Bmcp	0.597075399043104	0.426515764220218	0.994766463583882	pseudobulk	DESeq2	LRT	0.370064912474585
718	KC_AB1	bmm	-0.106169214535381	0.865452833967045	0.994766463583882	pseudobulk	DESeq2	LRT	0.0627565956175991
718	KC_AB1	bnb	1.19856662192297	0.608802264240657	0.994766463583882	pseudobulk	DESeq2	LRT	0.215523741018373
718	KC_AB1	boca	0.263465016032282	0.657763567258308	0.994766463583882	pseudobulk	DESeq2	LRT	0.181930185242031
718	KC_AB1	bocks	-1.45196735976243	0.131685226737826	0.994766463583882	pseudobulk	DESeq2	LRT	0.880462944143143
718	KC_AB1	BOD1	0.054752569693181	0.910061615362903	0.994766463583882	pseudobulk	DESeq2	LRT	0.040929202946788
718	KC_AB1	boi	-0.00196656141718931	0.931985985392389	0.994766463583882	pseudobulk	DESeq2	LRT	0.0305906182388444
719	KC_AB1	boil	-1.64952313825113	0.817565084244375	0.994766463583882	pseudobulk	DESeq2	LRT	0.0874776642296574
719	KC_AB1	bol	0.0198901282075345	0.863334673022162	0.994766463583882	pseudobulk	DESeq2	LRT	0.0638208167167724
719	KC_AB1	bon	-0.602283040042831	0.213787577575161	0.994766463583882	pseudobulk	DESeq2	LRT	0.670017533679617
719	KC_AB1	bonsai	-0.133755850940076	0.831677139788561	0.994766463583882	pseudobulk	DESeq2	LRT	0.0800452357537506
719	KC_AB1	bor	0.0561732518697753	0.901199445055537	0.994766463583882	pseudobulk	DESeq2	LRT	0.0451790843798447
719	KC_AB1	BORCS5	-0.687773882215138	0.235560125563179	0.994766463583882	pseudobulk	DESeq2	LRT	0.627898222855591
719	KC_AB1	BORCS6	-0.167507382000589	0.837962326561164	0.994766463583882	pseudobulk	DESeq2	LRT	0.0767755061124271
719	KC_AB1	botv	1.63770650281125	0.256761881233626	0.994766463583882	pseudobulk	DESeq2	LRT	0.590469451003066
719	KC_AB1	br	0.243116616495351	0.800132330884811	0.994766463583882	pseudobulk	DESeq2	LRT	0.0968381807325868
719	KC_AB1	Br140	-0.317569052682345	0.799366589663568	0.994766463583882	pseudobulk	DESeq2	LRT	0.0972540074744318
720	KC_AB1	brat	-0.158259445112816	0.444953717722126	0.994766463583882	pseudobulk	DESeq2	LRT	0.351685160217319
720	KC_AB1	Brca2	0.908271535045557	0.482766483800726	0.994766463583882	pseudobulk	DESeq2	LRT	0.316262888544989
720	KC_AB1	Brd8	-0.33477001592089	0.733219347150302	0.994766463583882	pseudobulk	DESeq2	LRT	0.13476608401293
720	KC_AB1	Bre1	-0.0761431421799816	0.89688004898045	0.994766463583882	pseudobulk	DESeq2	LRT	0.0472656367210475
720	KC_AB1	Brf	0.109738189028991	0.862179069422488	0.994766463583882	pseudobulk	DESeq2	LRT	0.0644025244473965
720	KC_AB1	brk	-0.336079221375189	0.868396536629518	0.994766463583882	pseudobulk	DESeq2	LRT	0.061281917283127
720	KC_AB1	brm	0.265852742312662	0.669999438333998	0.994766463583882	pseudobulk	DESeq2	LRT	0.173925561371633
720	KC_AB1	Brms1	-0.218858564561257	0.757788748907409	0.994766463583882	pseudobulk	DESeq2	LRT	0.120451847099692
720	KC_AB1	brn	0.69750158688829	0.750018748510497	0.994766463583882	pseudobulk	DESeq2	LRT	0.124927880244456
720	KC_AB1	brp	-0.134460858873016	0.278416119823837	0.994766463583882	pseudobulk	DESeq2	LRT	0.555305623415383
721	KC_AB1	bru1	0.217234983815553	0.458541382044887	0.994766463583882	pseudobulk	DESeq2	LRT	0.338621464393613
721	KC_AB1	bru2	-0.0207420576879555	0.906227083606909	0.994766463583882	pseudobulk	DESeq2	LRT	0.0427629625880132
721	KC_AB1	bru3	-0.0157409018161381	0.896479268304234	0.994766463583882	pseudobulk	DESeq2	LRT	0.0474597493423771
721	KC_AB1	Bruce	-0.188280806839671	0.687670813621058	0.994766463583882	pseudobulk	DESeq2	LRT	0.162619407753813
721	KC_AB1	brun	-0.736948954704353	0.468089996004575	0.994766463583882	pseudobulk	DESeq2	LRT	0.329670640492803
721	KC_AB1	BRWD3	0.0426768565077976	0.948124975183785	0.994766463583882	pseudobulk	DESeq2	LRT	0.023134413236414
721	KC_AB1	brwl	0.451013389356975	0.714356598424878	0.994766463583882	pseudobulk	DESeq2	LRT	0.146084939389902
721	KC_AB1	bs	0.478913063357543	0.594997459443561	0.994766463583882	pseudobulk	DESeq2	LRT	0.225484888644556
721	KC_AB1	bsf	0.890293391185717	0.434302920219769	0.994766463583882	pseudobulk	DESeq2	LRT	0.362207250464923
721	KC_AB1	Bsg	-0.0228570490510632	0.88392024834038	0.994766463583882	pseudobulk	DESeq2	LRT	0.0535869174169618
722	KC_AB1	Bsg25D	0.215942458510052	0.716792845156146	0.994766463583882	pseudobulk	DESeq2	LRT	0.144606338341345
722	KC_AB1	bsk	0.0996025822218828	0.686747739587827	0.994766463583882	pseudobulk	DESeq2	LRT	0.163202761373945
722	KC_AB1	bt	-1.50140066042753	0.0289125613045479	0.994766463583882	pseudobulk	DESeq2	LRT	1.53891343336707

722	KC_AB1	BTBD9	0.175564683934576	0.838741551528743	0.994766463583882	pseudobulk	DESeq2	LRT	0.0763718413599726
722	KC_AB1	BtbVII	0.0517199276981426	0.818721431044801	0.994766463583882	pseudobulk	DESeq2	LRT	0.0868638412671144
722	KC_AB1	Btk29A	2.38929733117871	0.484296857543297	0.994766463583882	pseudobulk	DESeq2	LRT	0.314888348950639
722	KC_AB1	btl	-0.835152883801839	0.920434724857442	0.994766463583882	pseudobulk	DESeq2	LRT	0.0360070052473159
722	KC_AB1	btsz	-0.10110200056694	0.701843394965396	0.994766463583882	pseudobulk	DESeq2	LRT	0.153759782869869
722	KC_AB1	btz	0.707011469913861	0.492092250809512	0.994766463583882	pseudobulk	DESeq2	LRT	0.307953473935979
722	KC_AB1	Bub1	-0.279684049759247	0.764120169332102	0.994766463583882	pseudobulk	DESeq2	LRT	0.11683833674647
723	KC_AB1	Bub3	-0.223862671519578	0.703481392039066	0.994766463583882	pseudobulk	DESeq2	LRT	0.152747385708458
723	KC_AB1	BubR1	0.40411895759739	0.864643228521197	0.994766463583882	pseudobulk	DESeq2	LRT	0.0631630553638974
723	KC_AB1	bug	-0.00593719043237892	0.954635232874453	0.994766463583882	pseudobulk	DESeq2	LRT	0.020162541098429
723	KC_AB1	Bug22	-1.00770718537089	0.330736724903604	0.994766463583882	pseudobulk	DESeq2	LRT	0.480517578483814
723	KC_AB1	BuGZ	-0.0528142415546156	0.830962098273719	0.994766463583882	pseudobulk	DESeq2	LRT	0.0804187847429287
723	KC_AB1	bun	-0.00415122790092563	0.948184354398315	0.994766463583882	pseudobulk	DESeq2	LRT	0.0231072150733127
723	KC_AB1	bur	0.401965911417004	0.568997598935359	0.994766463583882	pseudobulk	DESeq2	LRT	0.244889566243461
723	KC_AB1	bves	0.0596818232727586	0.846059573348709	0.994766463583882	pseudobulk	DESeq2	LRT	0.0725990560322302
723	KC_AB1	bw	-3.21143241058256	0.292545415061155	0.994766463583882	pseudobulk	DESeq2	LRT	0.533806704011112
723	KC_AB1	bwa	0.251191756082708	0.849032683948987	0.994766463583882	pseudobulk	DESeq2	LRT	0.0710755910452382
724	KC_AB1	Bx	0.288231998346608	0.0630403244551	0.994766463583882	pseudobulk	DESeq2	LRT	1.20038156029171
724	KC_AB1	Bx42	0.218514589018648	0.716591836593162	0.994766463583882	pseudobulk	DESeq2	LRT	0.144728143620002
724	KC_AB1	bys	-1.85889625777701	0.165346823537919	0.994766463583882	pseudobulk	DESeq2	LRT	0.781604143860072
724	KC_AB1	c(3)G	0.115973679573441	0.948205466729312	0.994766463583882	pseudobulk	DESeq2	LRT	0.0230975451533938
724	KC_AB1	c11.1	-0.309718245307559	0.584571918741092	0.994766463583882	pseudobulk	DESeq2	LRT	0.233162050813519
724	KC_AB1	c12.1	0.461238946943345	0.423338781149094	0.994766463583882	pseudobulk	DESeq2	LRT	0.373311944926462
724	KC_AB1	c12.2	-0.304053171160176	0.727720928901788	0.994766463583882	pseudobulk	DESeq2	LRT	0.138035134799786
724	KC_AB1	C1GalTA	-0.102427609193569	0.825928229548325	0.994766463583882	pseudobulk	DESeq2	LRT	0.0830576898047403
724	KC_AB1	C3G	0.518535243519928	0.172488346347745	0.994766463583882	pseudobulk	DESeq2	LRT	0.763240241389745
724	KC_AB1	C901	-0.747760753789965	0.720131677202483	0.994766463583882	pseudobulk	DESeq2	LRT	0.142588084882933
725	KC_AB1	ca	0.821704410075523	0.265269863407021	0.994766463583882	pseudobulk	DESeq2	LRT	0.576312086238783
725	KC_AB1	Ca-alpha1D	0.0619141626428986	0.742039297077795	0.994766463583882	pseudobulk	DESeq2	LRT	0.12957309465072
725	KC_AB1	Ca-alpha1T	0.0467717707133599	0.734787863091986	0.994766463583882	pseudobulk	DESeq2	LRT	0.133838025795339
725	KC_AB1	Ca-beta	-0.228693154702728	0.261737701035979	0.994766463583882	pseudobulk	DESeq2	LRT	0.582133716517124
725	KC_AB1	Ca-Ma2d	-0.289003671123359	0.572180193454267	0.994766463583882	pseudobulk	DESeq2	LRT	0.242467179775071
725	KC_AB1	CaBP1	0.0779455016253767	0.905879225015968	0.994766463583882	pseudobulk	DESeq2	LRT	0.0429297001217496
725	KC_AB1	cact	-0.36076840129994	0.197219934625919	0.994766463583882	pseudobulk	DESeq2	LRT	0.705049189493799
725	KC_AB1	cactin	0.270549379737223	0.771013601716028	0.994766463583882	pseudobulk	DESeq2	LRT	0.112937960343194
725	KC_AB1	Cad86C	-0.648218248148942	0.834000364039372	0.994766463583882	pseudobulk	DESeq2	LRT	0.0788337597936092
725	KC_AB1	Cad87A	-0.614308893843206	0.357406393577369	0.994766463583882	pseudobulk	DESeq2	LRT	0.446837682746617
726	KC_AB1	Cad96Ca	-0.257056328552413	0.890683115735507	0.994766463583882	pseudobulk	DESeq2	LRT	0.0502767803242406
726	KC_AB1	CadN	0.204104508952552	0.567053341335123	0.994766463583882	pseudobulk	DESeq2	LRT	0.246376086154907
726	KC_AB1	CadN2	1.61316313236924	0.280675657293838	0.994766463583882	pseudobulk	DESeq2	LRT	0.551795251638424
726	KC_AB1	Cadps	-0.0671902964071187	0.696893370089431	0.994766463583882	pseudobulk	DESeq2	LRT	0.156833667130657
726	KC_AB1	Caf1-105	0.475892334225681	0.803946046269915	0.994766463583882	pseudobulk	DESeq2	LRT	0.0947730962683447
726	KC_AB1	Caf1-55	1.1002101744969	0.153196959250888	0.994766463583882	pseudobulk	DESeq2	LRT	0.81474985476651
726	KC_AB1	cag	-0.229976970216705	0.776954692149244	0.994766463583882	pseudobulk	DESeq2	LRT	0.10960430619688
726	KC_AB1	CAH1	1.1821590793593	0.719511699510136	0.994766463583882	pseudobulk	DESeq2	LRT	0.142962139890846
726	KC_AB1	CAH2	-2.32607225918216	0.391287880902332	0.994766463583882	pseudobulk	DESeq2	LRT	0.407503603003357
726	KC_AB1	CAH3	0.00722607041006497	0.854155827695716	0.994766463583882	pseudobulk	DESeq2	LRT	0.0684628916823577
727	KC_AB1	CAH8	0.33722681502809	0.579700464430067	0.994766463583882	pseudobulk	DESeq2	LRT	0.236796351691788
727	KC_AB1	CAHbeta	-0.561865288681391	0.562055441653055	0.994766463583882	pseudobulk	DESeq2	LRT	0.250220843120596
727	KC_AB1	cal1	0.555571129550617	0.522418869687789	0.994766463583882	pseudobulk	DESeq2	LRT	0.281981144800978
727	KC_AB1	CalpA	-0.358580970473246	0.59962172794483	0.994766463583882	pseudobulk	DESeq2	LRT	0.222122638406192
727	KC_AB1	CalpB	0.739982307221385	0.481819043812264	0.994766463583882	pseudobulk	DESeq2	LRT	0.317116038586054
727	KC_AB1	CalpC	1.1821590793593	0.719511699510136	0.994766463583882	pseudobulk	DESeq2	LRT	0.142962139890846
727	KC_AB1	Calr	-0.48655833450079	0.325622633628891	0.994766463583882	pseudobulk	DESeq2	LRT	0.487285415458201
727	KC_AB1	Cals	0.0885026033146484	0.614275627662291	0.994766463583882	pseudobulk	DESeq2	LRT	0.211636715645371
727	KC_AB1	Calx	0.541233159054796	0.180486954235276	0.994766463583882	pseudobulk	DESeq2	LRT	0.743554183832496

727	KC_AB1	calypso	-0.0891184307190675	0.931758117477705	0.994766463583882	pseudobulk	DESeq2	LRT	0.0306968149836559
728	KC_AB1	Cam	-0.0753018018120075	0.540688169878018	0.994766463583882	pseudobulk	DESeq2	LRT	0.267053132575111
728	KC_AB1	CaMKI	0.0639290595539056	0.745938119839217	0.994766463583882	pseudobulk	DESeq2	LRT	0.127297198434874
728	KC_AB1	CaMKII	-0.0444215050783738	0.743490947506873	0.994766463583882	pseudobulk	DESeq2	LRT	0.128724314931868
728	KC_AB1	Camp	0.646517359647679	0.574098746676472	0.994766463583882	pseudobulk	DESeq2	LRT	0.241013401246101
728	KC_AB1	Camta	0.358257083569692	0.463379771120754	0.994766463583882	pseudobulk	DESeq2	LRT	0.334062929290951
728	KC_AB1	can	1.1821590793593	0.719511699510136	0.994766463583882	pseudobulk	DESeq2	LRT	0.142962139890846
728	KC_AB1	CanA-14F	-0.120643849517767	0.409158779149652	0.994766463583882	pseudobulk	DESeq2	LRT	0.388108125911945
728	KC_AB1	CanB	0.226392996834397	0.201660647233187	0.994766463583882	pseudobulk	DESeq2	LRT	0.695378843269311
728	KC_AB1	CanB2	-0.222127108721178	0.554783268676563	0.994766463583882	pseudobulk	DESeq2	LRT	0.255876644987024
728	KC_AB1	Cand1	-0.923508768160507	0.269486466661549	0.994766463583882	pseudobulk	DESeq2	LRT	0.569463039760316
729	KC_AB1	CAP	-1.46061734155032	0.281358430441836	0.994766463583882	pseudobulk	DESeq2	LRT	0.550740067396371
729	KC_AB1	Cap-D3	0.819343340173919	0.649118843931874	0.994766463583882	pseudobulk	DESeq2	LRT	0.187675783109146
729	KC_AB1	Cap-G	-0.583482532595099	0.719903275993293	0.994766463583882	pseudobulk	DESeq2	LRT	0.142725850130121
729	KC_AB1	Cap-H2	-0.367830143178306	0.622157278614876	0.994766463583882	pseudobulk	DESeq2	LRT	0.206099813701137
729	KC_AB1	Caper	0.0369275161116312	0.851719654364555	0.994766463583882	pseudobulk	DESeq2	LRT	0.0697033308176664
729	KC_AB1	Capr	0.128328803402838	0.83748711512042	0.994766463583882	pseudobulk	DESeq2	LRT	0.0770218659330847
729	KC_AB1	caps	0.0465813438943025	0.800074904446272	0.994766463583882	pseudobulk	DESeq2	LRT	0.0968693516769909
729	KC_AB1	capt	-0.266529151549127	0.579745125542676	0.994766463583882	pseudobulk	DESeq2	LRT	0.236762894192895
729	KC_AB1	capu	0.0314511536554277	0.907874771200281	0.994766463583882	pseudobulk	DESeq2	LRT	0.0419740522800517
729	KC_AB1	car	-0.578938496393514	0.487422413470599	0.994766463583882	pseudobulk	DESeq2	LRT	0.312094504238708
730	KC_AB1	CARPB	-0.858415647400736	0.446555217388659	0.994766463583882	pseudobulk	DESeq2	LRT	0.350124832129265
730	KC_AB1	CarT	-1.64952313825113	0.817565084244375	0.994766463583882	pseudobulk	DESeq2	LRT	0.0874776642296574
730	KC_AB1	CASK	0.219622872062119	0.330058909162458	0.994766463583882	pseudobulk	DESeq2	LRT	0.48140853999807
730	KC_AB1	casp	-0.0362626382651212	0.888261226668869	0.994766463583882	pseudobulk	DESeq2	LRT	0.0514592947876599
730	KC_AB1	cass	-0.032004968259983	0.85591762723312	0.994766463583882	pseudobulk	DESeq2	LRT	0.0675680294344018
730	KC_AB1	Cat	-0.0241254203139974	0.855717265753716	0.994766463583882	pseudobulk	DESeq2	LRT	0.0676697051897223
730	KC_AB1	cathD	-0.222084389051785	0.731268179477538	0.994766463583882	pseudobulk	DESeq2	LRT	0.135923324125299
730	KC_AB1	Catsup	0.231810834067005	0.854376073854612	0.994766463583882	pseudobulk	DESeq2	LRT	0.0683509222284516
730	KC_AB1	cav	-0.878551581920065	0.610521464396702	0.994766463583882	pseudobulk	DESeq2	LRT	0.214299062750751
730	KC_AB1	caz	-0.229200890641032	0.515739833910965	0.994766463583882	pseudobulk	DESeq2	LRT	0.287569323936264
731	KC_AB1	cbc	0.565799178003989	0.712636069425347	0.994766463583882	pseudobulk	DESeq2	LRT	0.147132200009199
731	KC_AB1	Cbl	-0.12401434991972	0.746368926654855	0.994766463583882	pseudobulk	DESeq2	LRT	0.127046449732139
731	KC_AB1	Cbp20	-0.330177840743885	0.631986496789053	0.994766463583882	pseudobulk	DESeq2	LRT	0.199292200883203
731	KC_AB1	Cbp80	-0.329747690320764	0.648598854490356	0.994766463583882	pseudobulk	DESeq2	LRT	0.188023822682714
731	KC_AB1	cbs	-0.370871925590033	0.643770918251038	0.994766463583882	pseudobulk	DESeq2	LRT	0.19126864605421
731	KC_AB1	Cbs	0.323763772045841	0.741176596551008	0.994766463583882	pseudobulk	DESeq2	LRT	0.130078302452469
731	KC_AB1	cbt	-0.646291476138187	0.0770960568653747	0.994766463583882	pseudobulk	DESeq2	LRT	1.11296783369225
731	KC_AB1	CCAP-R	-0.286566775158176	0.63000560965925	0.994766463583882	pseudobulk	DESeq2	LRT	0.200655583509575
731	KC_AB1	CCDC53	-0.358757075072528	0.755130447916079	0.994766463583882	pseudobulk	DESeq2	LRT	0.121978018017841
731	KC_AB1	Ccdc58	0.286406906692349	0.738131852267567	0.994766463583882	pseudobulk	DESeq2	LRT	0.131866053356476
732	KC_AB1	Cchl	0.0561992607313278	0.786677130106745	0.994766463583882	pseudobulk	DESeq2	LRT	0.104203475244386
732	KC_AB1	CCKLR-17D1	9.123681531041E-05	0.845445252170248	0.994766463583882	pseudobulk	DESeq2	LRT	0.0729145104200947
732	KC_AB1	CCKLR-17D3	1.32745784723813	0.391152697324798	0.994766463583882	pseudobulk	DESeq2	LRT	0.407653670584168
732	KC_AB1	Ccm3	-0.385456672314754	0.434745136005283	0.994766463583882	pseudobulk	DESeq2	LRT	0.361765268250799
732	KC_AB1	Ccn	0.181948211341559	0.594730992413102	0.994766463583882	pseudobulk	DESeq2	LRT	0.225679429109134
732	KC_AB1	Ccs	-0.593324163943884	0.61510078893245	0.994766463583882	pseudobulk	DESeq2	LRT	0.211053715946842
732	KC_AB1	CCT1	0.700185010644629	0.414564489408329	0.994766463583882	pseudobulk	DESeq2	LRT	0.382407901264109
732	KC_AB1	CCT2	-0.0464872029303842	0.886755439581232	0.994766463583882	pseudobulk	DESeq2	LRT	0.0521961387761019
732	KC_AB1	CCT3	0.204919132896869	0.755708121562537	0.994766463583882	pseudobulk	DESeq2	LRT	0.121645910413109
732	KC_AB1	CCT4	0.557282591275599	0.434916196298165	0.994766463583882	pseudobulk	DESeq2	LRT	0.361594418888019
733	KC_AB1	CCT5	0.671688805587762	0.535998852662638	0.994766463583882	pseudobulk	DESeq2	LRT	0.270836139939354
733	KC_AB1	CCT6	-0.196083391039525	0.795062642098269	0.994766463583882	pseudobulk	DESeq2	LRT	0.0995986524179267
733	KC_AB1	CCT7	0.394898473094263	0.813641325266587	0.994766463583882	pseudobulk	DESeq2	LRT	0.0895670014822426
733	KC_AB1	CCT8	0.633601183247857	0.421919027514441	0.994766463583882	pseudobulk	DESeq2	LRT	0.37477088855936
733	KC_AB1	Ccz1	0.180713308133524	0.825105743740192	0.994766463583882	pseudobulk	DESeq2	LRT	0.0834903896561586

733	KC_AB1	CD98hc	0.0810170420905648	0.819773865001764	0.994766463583882	pseudobulk	DESeq2	LRT	0.086305931428695
733	KC_AB1	Cda4	-1.64952313825113	0.817565084244375	0.994766463583882	pseudobulk	DESeq2	LRT	0.0874776642296574
733	KC_AB1	Cda5	-0.229092192300671	0.557208175368647	0.994766463583882	pseudobulk	DESeq2	LRT	0.253982520224664
733	KC_AB1	cdc14	0.106391970961665	0.76537604136465	0.994766463583882	pseudobulk	DESeq2	LRT	0.116125136658136
733	KC_AB1	Cdc16	-1.13194846419515	0.661925982564074	0.994766463583882	pseudobulk	DESeq2	LRT	0.179190571226749
734	KC_AB1	Cdc23	0.911498702061926	0.666302272230238	0.994766463583882	pseudobulk	DESeq2	LRT	0.176328705694236
734	KC_AB1	Cdc27	0.345208359691101	0.56236012931634	0.994766463583882	pseudobulk	DESeq2	LRT	0.249985477873932
734	KC_AB1	Cdc2rk	-0.768112824641631	0.429174732138638	0.994766463583882	pseudobulk	DESeq2	LRT	0.367365855242418
734	KC_AB1	Cdc37	0.0738974013604094	0.868739172726283	0.994766463583882	pseudobulk	DESeq2	LRT	0.0611105950689165
734	KC_AB1	Cdc42	-0.26172104590121	0.521594312481295	0.994766463583882	pseudobulk	DESeq2	LRT	0.282667152842578
734	KC_AB1	CDC45L	0.10212659033711	0.934458047936592	0.994766463583882	pseudobulk	DESeq2	LRT	0.0294401912981141
734	KC_AB1	Cdc5	0.984708526788619	0.348278621912	0.994766463583882	pseudobulk	DESeq2	LRT	0.458073182649412
734	KC_AB1	CDC50	-0.263656231227534	0.717319202399929	0.994766463583882	pseudobulk	DESeq2	LRT	0.144287543088019
734	KC_AB1	Cdc7	-0.708906562993592	0.147240127546504	0.994766463583882	pseudobulk	DESeq2	LRT	0.83197381501837
734	KC_AB1	Cdep	-0.226835225307573	0.34544734234929	0.994766463583882	pseudobulk	DESeq2	LRT	0.46161814415096
735	KC_AB1	CdGAPr	0.292532568741878	0.721723846855441	0.994766463583882	pseudobulk	DESeq2	LRT	0.141628944713826
735	KC_AB1	cdi	-0.264999850859297	0.545427580044078	0.994766463583882	pseudobulk	DESeq2	LRT	0.263262905341743
735	KC_AB1	Cdk12	-0.278349807977427	0.561623848885984	0.994766463583882	pseudobulk	DESeq2	LRT	0.250554458535535
735	KC_AB1	Cdk2	-0.0807090960428847	0.933481608942962	0.994766463583882	pseudobulk	DESeq2	LRT	0.0298942339155102
735	KC_AB1	CDK2AP1	-0.0790948850180443	0.904643518436616	0.994766463583882	pseudobulk	DESeq2	LRT	0.0435225240826027
735	KC_AB1	Cdk5	0.0864334368077952	0.901308099078929	0.994766463583882	pseudobulk	DESeq2	LRT	0.0451267263822247
735	KC_AB1	Cdk5alpha	0.0713741035121291	0.752762679138092	0.994766463583882	pseudobulk	DESeq2	LRT	0.123341920726084
735	KC_AB1	Cdk7	0.213645468901384	0.825815453008193	0.994766463583882	pseudobulk	DESeq2	LRT	0.0831169946831675
735	KC_AB1	Cdk8	2.22683976195986	0.281030108270102	0.994766463583882	pseudobulk	DESeq2	LRT	0.551247149293865
735	KC_AB1	Cdk9	-0.718716526175906	0.489530175141315	0.994766463583882	pseudobulk	DESeq2	LRT	0.310220532679116
736	KC_AB1	cdm	0.0296096882441901	0.827465865303601	0.994766463583882	pseudobulk	DESeq2	LRT	0.0822499127378078
736	KC_AB1	Cds	0.345445843156801	0.283726750561025	0.994766463583882	pseudobulk	DESeq2	LRT	0.547099715760888
736	KC_AB1	Ced-12	0.352122982566509	0.835944281544217	0.994766463583882	pseudobulk	DESeq2	LRT	0.0778226687623168
736	KC_AB1	ced-6	0.949183608371002	0.80156895525114	0.994766463583882	pseudobulk	DESeq2	LRT	0.0960591113673091
736	KC_AB1	Cen	-0.11560158666694	0.829100380868115	0.994766463583882	pseudobulk	DESeq2	LRT	0.0813928853508778
736	KC_AB1	CenB1A	-0.208299919409689	0.791838073997478	0.994766463583882	pseudobulk	DESeq2	LRT	0.101363619874206
736	KC_AB1	CenG1A	0.201906548544464	0.68048487486761	0.994766463583882	pseudobulk	DESeq2	LRT	0.167181523413676
736	KC_AB1	Cenp-C	2.17073305257299	0.293696003918313	0.994766463583882	pseudobulk	DESeq2	LRT	0.532101962527361
736	KC_AB1	Cep135	2.02670873290438	0.538959414925803	0.994766463583882	pseudobulk	DESeq2	LRT	0.26844393710898
736	KC_AB1	Cep97	-0.242736475828268	0.652404699079232	0.994766463583882	pseudobulk	DESeq2	LRT	0.185482919525031
737	KC_AB1	cer	-0.875298074899105	0.200407110260775	0.994766463583882	pseudobulk	DESeq2	LRT	0.69808687416088
737	KC_AB1	Cerk	-0.123830107255292	0.874589542443469	0.994766463583882	pseudobulk	DESeq2	LRT	0.0581957198636154
737	KC_AB1	cert	0.289819372689915	0.737802594025757	0.994766463583882	pseudobulk	DESeq2	LRT	0.132059822179029
737	KC_AB1	cerv	-0.721914293354382	0.502773574413392	0.994766463583882	pseudobulk	DESeq2	LRT	0.298627556737948
737	KC_AB1	Cf2	-0.397740252753321	0.267869933532003	0.994766463583882	pseudobulk	DESeq2	LRT	0.572076030079475
737	KC_AB1	Cfp1	0.274340432095986	0.803679359163354	0.994766463583882	pseudobulk	DESeq2	LRT	0.0949171854814726
737	KC_AB1	cg	-0.190563883736912	0.415919225610762	0.994766463583882	pseudobulk	DESeq2	LRT	0.380991004175376
737	KC_AB1	CG10011	-0.281591108846521	0.560013982835853	0.994766463583882	pseudobulk	DESeq2	LRT	0.251801129078374
737	KC_AB1	CG10019	-0.789231918673713	0.311636238637267	0.994766463583882	pseudobulk	DESeq2	LRT	0.506352046105057
737	KC_AB1	CG10038	-0.17437526782938	0.858597710512219	0.994766463583882	pseudobulk	DESeq2	LRT	0.0662102739199803
738	KC_AB1	CG10050	-1.03279054540079	0.616964932706771	0.994766463583882	pseudobulk	DESeq2	LRT	0.209739519864242
738	KC_AB1	CG10053	-0.134792610527886	0.897663313171096	0.994766463583882	pseudobulk	DESeq2	LRT	0.0468865237448493
738	KC_AB1	CG10055	-0.092555417821259	0.902004715773597	0.994766463583882	pseudobulk	DESeq2	LRT	0.0447911919158217
738	KC_AB1	CG10068	-0.186004914443213	0.846282247723833	0.994766463583882	pseudobulk	DESeq2	LRT	0.072484769129039
738	KC_AB1	CG10075	0.00599193113353279	0.931941849029971	0.994766463583882	pseudobulk	DESeq2	LRT	0.0306111857504173
738	KC_AB1	CG10077	-0.363788297201651	0.23648621652981	0.994766463583882	pseudobulk	DESeq2	LRT	0.626194166805619
738	KC_AB1	CG10082	0.00535500257829081	0.958727141436186	0.994766463583882	pseudobulk	DESeq2	LRT	0.0183049776364174
738	KC_AB1	CG10089	-0.428096528008419	0.27157974555526	0.994766463583882	pseudobulk	DESeq2	LRT	0.566102622909925
738	KC_AB1	CG10098	0.26737863724985	0.638004521961811	0.994766463583882	pseudobulk	DESeq2	LRT	0.195176243134477
738	KC_AB1	CG10103	0.487138518847389	0.370858417421119	0.994766463583882	pseudobulk	DESeq2	LRT	0.4307918593027
739	KC_AB1	CG10116	-1.64952313825113	0.817565084244375	0.994766463583882	pseudobulk	DESeq2	LRT	0.0874776642296574

739	KC_AB1	CG10132	-0.0358363044246863	0.851012325507327	0.994766463583882	pseudobulk	DESeq2	LRT	0.0700641498319165
739	KC_AB1	CG10137	0.242789042190798	0.559376364310834	0.994766463583882	pseudobulk	DESeq2	LRT	0.252295888100783
739	KC_AB1	CG10139	0.776944056238246	0.166765497939209	0.994766463583882	pseudobulk	DESeq2	LRT	0.77789379545207
739	KC_AB1	CG10147	0.448680542203776	0.715613649275733	0.994766463583882	pseudobulk	DESeq2	LRT	0.14532138449846
739	KC_AB1	CG10151	0.0512232891482382	0.917402186437983	0.994766463583882	pseudobulk	DESeq2	LRT	0.0374402291519112
739	KC_AB1	CG10165	-2.0067226956613	0.355453594452233	0.994766463583882	pseudobulk	DESeq2	LRT	0.449217089698855
739	KC_AB1	CG10166	1.19185762732754	0.388281769581409	0.994766463583882	pseudobulk	DESeq2	LRT	0.410852999760572
739	KC_AB1	CG10171	0.157837167007298	0.861606540806936	0.994766463583882	pseudobulk	DESeq2	LRT	0.0646910127908813
739	KC_AB1	CG10188	-0.264869887960294	0.555560416258577	0.994766463583882	pseudobulk	DESeq2	LRT	0.255268705362228
740	KC_AB1	CG10189	0.393877279003338	0.764461274877752	0.994766463583882	pseudobulk	DESeq2	LRT	0.116644509640636
740	KC_AB1	CG10194	-0.257969555785886	0.786452557296172	0.994766463583882	pseudobulk	DESeq2	LRT	0.104327471039343
740	KC_AB1	CG10209	0.399025248997983	0.6414985607613	0.994766463583882	pseudobulk	DESeq2	LRT	0.192804313652461
740	KC_AB1	CG10214	-0.293598914921238	0.717891613203477	0.994766463583882	pseudobulk	DESeq2	LRT	0.143941120299337
740	KC_AB1	CG10222	2.02670873290438	0.538959414925803	0.994766463583882	pseudobulk	DESeq2	LRT	0.26844393710898
740	KC_AB1	CG1024	0.513714559562998	0.775698051782448	0.994766463583882	pseudobulk	DESeq2	LRT	0.110307299314091
740	KC_AB1	CG10252	-1.28132067551389	0.647201349541384	0.994766463583882	pseudobulk	DESeq2	LRT	0.188960585805912
740	KC_AB1	CG10254	-0.599283164666636	0.34536992566208	0.994766463583882	pseudobulk	DESeq2	LRT	0.461715482888019
740	KC_AB1	CG10257	0.176003616028759	0.689738531038642	0.994766463583882	pseudobulk	DESeq2	LRT	0.161315512230498
740	KC_AB1	CG10265	-0.402641125556042	0.648136878168251	0.994766463583882	pseudobulk	DESeq2	LRT	0.188333267037327
741	KC_AB1	CG10268	0.641037150451937	0.727920539458107	0.994766463583882	pseudobulk	DESeq2	LRT	0.137916026124241
741	KC_AB1	CG10274	0.389275648472954	0.837532199093071	0.994766463583882	pseudobulk	DESeq2	LRT	0.0769984874318538
741	KC_AB1	CG10283	-0.0280071378384702	0.927715435613	0.994766463583882	pseudobulk	DESeq2	LRT	0.0325852174180688
741	KC_AB1	CG10286	0.69750158688829	0.750018748510497	0.994766463583882	pseudobulk	DESeq2	LRT	0.124927880244456
741	KC_AB1	CG10324	0.701125451723029	0.595397772590602	0.994766463583882	pseudobulk	DESeq2	LRT	0.225192794401118
741	KC_AB1	CG10333	0.216188791517303	0.719713170749578	0.994766463583882	pseudobulk	DESeq2	LRT	0.14284054965164
741	KC_AB1	CG10336	0.0212843434763832	0.936637839890193	0.994766463583882	pseudobulk	DESeq2	LRT	0.0284283008315977
741	KC_AB1	CG10338	0.0551104605447623	0.907528926819053	0.994766463583882	pseudobulk	DESeq2	LRT	0.0421395232530871
741	KC_AB1	CG10340	0.394181480430263	0.506903246377786	0.994766463583882	pseudobulk	DESeq2	LRT	0.295074927400698
741	KC_AB1	CG10341	-1.38321716661921	0.272836404022313	0.994766463583882	pseudobulk	DESeq2	LRT	0.564097683094701
742	KC_AB1	CG10343	0.189297132033703	0.839246924015589	0.994766463583882	pseudobulk	DESeq2	LRT	0.0761102418410514
742	KC_AB1	CG10347	1.05270755788331	0.340948004201427	0.994766463583882	pseudobulk	DESeq2	LRT	0.467311847430359
742	KC_AB1	CG10353	0.0479523249958729	0.928515435420962	0.994766463583882	pseudobulk	DESeq2	LRT	0.032210872255455
742	KC_AB1	CG10359	1.80633728195307	0.61161102402172	0.994766463583882	pseudobulk	DESeq2	LRT	0.213524695220716
742	KC_AB1	CG10362	0.380934829173819	0.628211394553258	0.994766463583882	pseudobulk	DESeq2	LRT	0.20189419059475
742	KC_AB1	CG10365	0.448644130219071	0.718858229642214	0.994766463583882	pseudobulk	DESeq2	LRT	0.143356751000968
742	KC_AB1	CG10366	-0.0523006895041167	0.841973970816775	0.994766463583882	pseudobulk	DESeq2	LRT	0.0747013342787055
742	KC_AB1	CG10375	-0.0137118469460505	0.916486805045844	0.994766463583882	pseudobulk	DESeq2	LRT	0.037873783333549
742	KC_AB1	CG10376	0.748656695180982	0.638986291977518	0.994766463583882	pseudobulk	DESeq2	LRT	0.194508458558938
742	KC_AB1	CG10383	0.239623127223831	0.830936921378068	0.994766463583882	pseudobulk	DESeq2	LRT	0.080431943408677
743	KC_AB1	CG10384	-1.60653259065571	0.594621747113632	0.994766463583882	pseudobulk	DESeq2	LRT	0.225759211379423
743	KC_AB1	CG10395	0.0756268361537362	0.82299191419435	0.994766463583882	pseudobulk	DESeq2	LRT	0.0846044316626099
743	KC_AB1	CG10413	-0.766431182101561	0.517619369865955	0.994766463583882	pseudobulk	DESeq2	LRT	0.28598948027502
743	KC_AB1	CG10417	0.173317996278723	0.624623779157242	0.994766463583882	pseudobulk	DESeq2	LRT	0.204381486387926
743	KC_AB1	CG10418	0.400610335089812	0.675914204876544	0.994766463583882	pseudobulk	DESeq2	LRT	0.170108426415399
743	KC_AB1	CG10425	-0.502792276798834	0.78573817439348	0.994766463583882	pseudobulk	DESeq2	LRT	0.104722146529669
743	KC_AB1	CG10428	0.239299028100293	0.793289682834473	0.994766463583882	pseudobulk	DESeq2	LRT	0.100568193913992
743	KC_AB1	CG10431	-1.02319198676748	0.828204539793902	0.994766463583882	pseudobulk	DESeq2	LRT	0.0818623932467813
743	KC_AB1	CG10433	-1.50663180504502	0.668163815680457	0.994766463583882	pseudobulk	DESeq2	LRT	0.175117047219009
743	KC_AB1	CG10435	-0.0230395035217753	0.910358975115498	0.994766463583882	pseudobulk	DESeq2	LRT	0.04078732177766
744	KC_AB1	CG10444	-1.3598691330942	0.354069032425129	0.994766463583882	pseudobulk	DESeq2	LRT	0.450912055831573
744	KC_AB1	CG10463	0.396863759429865	0.803785080857863	0.994766463583882	pseudobulk	DESeq2	LRT	0.0948600590561958
744	KC_AB1	CG10465	-0.258792288725662	0.526473280301228	0.994766463583882	pseudobulk	DESeq2	LRT	0.278623665337374
744	KC_AB1	CG10466	-0.393887149911893	0.6810960557946	0.994766463583882	pseudobulk	DESeq2	LRT	0.166791634695515
744	KC_AB1	CG10467	1.34815820347889	0.526408859243851	0.994766463583882	pseudobulk	DESeq2	LRT	0.278676810335701
744	KC_AB1	CG10469	1.1821590793593	0.719511699510136	0.994766463583882	pseudobulk	DESeq2	LRT	0.142962139890846
744	KC_AB1	CG10470	0.286445085754381	0.683324980382705	0.994766463583882	pseudobulk	DESeq2	LRT	0.165372702438353

744	KC_AB1	CG10472	-2.20969975456663	0.610836134188693	0.994766463583882	pseudobulk	DESeq2	LRT	0.214075280039667
744	KC_AB1	CG10481	1.1821590793593	0.719511699510136	0.994766463583882	pseudobulk	DESeq2	LRT	0.142962139890846
744	KC_AB1	CG10483	0.0662442285611185	0.878916638354003	0.994766463583882	pseudobulk	DESeq2	LRT	0.0560523140271174
745	KC_AB1	CG10492	-0.0919986005324673	0.914564880203784	0.994766463583882	pseudobulk	DESeq2	LRT	0.0387854797829638
745	KC_AB1	CG10494	0.535668448154039	0.610384921668546	0.994766463583882	pseudobulk	DESeq2	LRT	0.214396203292084
745	KC_AB1	CG10495	-0.626185859611983	0.605457935389223	0.994766463583882	pseudobulk	DESeq2	LRT	0.217916024383745
745	KC_AB1	CG10508	0.179023168765213	0.556891878304336	0.994766463583882	pseudobulk	DESeq2	LRT	0.254229115802701
745	KC_AB1	CG10512	1.10204970664493	0.619982503857554	0.994766463583882	pseudobulk	DESeq2	LRT	0.207620566284543
745	KC_AB1	CG10514	-1.64952313825113	0.817565084244375	0.994766463583882	pseudobulk	DESeq2	LRT	0.0874776642296574
745	KC_AB1	CG10516	-0.699469910309698	0.678807554156604	0.994766463583882	pseudobulk	DESeq2	LRT	0.168253333251425
745	KC_AB1	CG10543	-0.459836233583223	0.122830311155047	0.994766463583882	pseudobulk	DESeq2	LRT	0.910694447991348
745	KC_AB1	CG10550	0.949183608371002	0.80156895525114	0.994766463583882	pseudobulk	DESeq2	LRT	0.0960591113673091
745	KC_AB1	CG10555	-0.268884163039519	0.667976125697615	0.994766463583882	pseudobulk	DESeq2	LRT	0.175239059475164
746	KC_AB1	CG10561	0.105919808311088	0.903669661574135	0.994766463583882	pseudobulk	DESeq2	LRT	0.0439902978146117
746	KC_AB1	CG10565	0.107305736373659	0.839578078652056	0.994766463583882	pseudobulk	DESeq2	LRT	0.0759389093520653
746	KC_AB1	CG10576	0.85963952734258	0.353217126004428	0.994766463583882	pseudobulk	DESeq2	LRT	0.451958247481487
746	KC_AB1	CG10581	-2.78645829554714	0.251837285097733	0.994766463583882	pseudobulk	DESeq2	LRT	0.598879971156437
746	KC_AB1	CG10584	0.329367741436991	0.858058210994256	0.994766463583882	pseudobulk	DESeq2	LRT	0.06648324844961
746	KC_AB1	CG10600	0.0629189816840701	0.907200296196922	0.994766463583882	pseudobulk	DESeq2	LRT	0.0422968166558997
746	KC_AB1	CG10602	-0.309976243771578	0.760437779816058	0.994766463583882	pseudobulk	DESeq2	LRT	0.118936314797051
746	KC_AB1	CG10616	0.844499755368768	0.22093075370909	0.994766463583882	pseudobulk	DESeq2	LRT	0.655743825837397
746	KC_AB1	CG10623	0.597141410293382	0.802139944912821	0.994766463583882	pseudobulk	DESeq2	LRT	0.0957498561530429
746	KC_AB1	CG10628	-0.811951718177513	0.527607028156089	0.994766463583882	pseudobulk	DESeq2	LRT	0.277689427930301
747	KC_AB1	CG10631	-0.0187587898331346	0.950583447371493	0.994766463583882	pseudobulk	DESeq2	LRT	0.0220097523995708
747	KC_AB1	CG10638	-0.893067851727655	0.29309461464246	0.994766463583882	pseudobulk	DESeq2	LRT	0.532992161270024
747	KC_AB1	CG10646	-0.357985500219812	0.737157562878838	0.994766463583882	pseudobulk	DESeq2	LRT	0.132439674454102
747	KC_AB1	CG10651	0.949183608371002	0.80156895525114	0.994766463583882	pseudobulk	DESeq2	LRT	0.0960591113673091
747	KC_AB1	CG10654	0.395219111546928	0.62289348110912	0.994766463583882	pseudobulk	DESeq2	LRT	0.205586214210386
747	KC_AB1	CG10669	0.338001565020324	0.77366687787554	0.994766463583882	pseudobulk	DESeq2	LRT	0.111445995702561
747	KC_AB1	CG10672	0.704187075212375	0.527154185294464	0.994766463583882	pseudobulk	DESeq2	LRT	0.278062341089315
747	KC_AB1	CG10674	0.443673533292982	0.515938319369173	0.994766463583882	pseudobulk	DESeq2	LRT	0.287402215347162
747	KC_AB1	CG10681	0.857215294707192	0.546702531755596	0.994766463583882	pseudobulk	DESeq2	LRT	0.26224891490497
747	KC_AB1	CG10713	-0.294239518084037	0.687193366754561	0.994766463583882	pseudobulk	DESeq2	LRT	0.162921041254513
748	KC_AB1	CG10731	0.212804481572611	0.879548480995857	0.994766463583882	pseudobulk	DESeq2	LRT	0.0557402170948801
748	KC_AB1	CG10734	0.949183608371002	0.80156895525114	0.994766463583882	pseudobulk	DESeq2	LRT	0.0960591113673091
748	KC_AB1	CG10737	0.61569392953842	0.299343079623908	0.994766463583882	pseudobulk	DESeq2	LRT	0.523830777658234
748	KC_AB1	CG10738	-0.150976296786208	0.774913997612841	0.994766463583882	pseudobulk	DESeq2	LRT	0.11074649418366
748	KC_AB1	CG1074	0.306087273837667	0.699576273796006	0.994766463583882	pseudobulk	DESeq2	LRT	0.155164928087304
748	KC_AB1	CG10747	0.176468400664913	0.832149270280772	0.994766463583882	pseudobulk	DESeq2	LRT	0.0797987633191649
748	KC_AB1	CG10778	-1.50819280357028	0.237047196594905	0.994766463583882	pseudobulk	DESeq2	LRT	0.625165176436831
748	KC_AB1	CG10793	-0.835152883801839	0.920434724857442	0.994766463583882	pseudobulk	DESeq2	LRT	0.0360070052473159
748	KC_AB1	CG10795	0.628003197167209	0.678980115427516	0.994766463583882	pseudobulk	DESeq2	LRT	0.168142944256401
748	KC_AB1	CG10802	0.596953566659222	0.606436359787754	0.994766463583882	pseudobulk	DESeq2	LRT	0.217214767828191
749	KC_AB1	CG10803	-0.549668844622232	0.269321425565194	0.994766463583882	pseudobulk	DESeq2	LRT	0.569729095401706
749	KC_AB1	CG10804	0.320335258302647	0.371266209957871	0.994766463583882	pseudobulk	DESeq2	LRT	0.430314575407883
749	KC_AB1	CG10809	-0.00215490245473879	0.936346520653725	0.994766463583882	pseudobulk	DESeq2	LRT	0.0285633989568739
749	KC_AB1	CG10863	0.969986341149099	0.640932225194753	0.994766463583882	pseudobulk	DESeq2	LRT	0.193187892135042
749	KC_AB1	CG10864	-1.64952313825113	0.817565084244375	0.994766463583882	pseudobulk	DESeq2	LRT	0.0874776642296574
749	KC_AB1	CG10866	0.564373867639223	0.652184063640896	0.994766463583882	pseudobulk	DESeq2	LRT	0.185629817546896
749	KC_AB1	CG10869	1.1821590793593	0.719511699510136	0.994766463583882	pseudobulk	DESeq2	LRT	0.142962139890846
749	KC_AB1	CG10874	-0.174520167057002	0.780997361980259	0.994766463583882	pseudobulk	DESeq2	LRT	0.10735043306169
749	KC_AB1	CG10889	-0.0538431372555513	0.942386114492196	0.994766463583882	pseudobulk	DESeq2	LRT	0.0257711215848224
749	KC_AB1	CG10898	-0.840757015397742	0.46384276724561	0.994766463583882	pseudobulk	DESeq2	LRT	0.33362921101824
750	KC_AB1	CG1090	-0.0883654828382233	0.778448019708837	0.994766463583882	pseudobulk	DESeq2	LRT	0.108770381820728
750	KC_AB1	CG10903	0.949801583294829	0.383224389866114	0.994766463583882	pseudobulk	DESeq2	LRT	0.416546858551764
750	KC_AB1	CG10904	-1.10361761708623	0.383945888229528	0.994766463583882	pseudobulk	DESeq2	LRT	0.415729979015992

750	KC_AB1	CG10907	0.491547820937883	0.767731576197349	0.994766463583882	pseudobulk	DESeq2	LRT	0.11479059683453
750	KC_AB1	CG10914	0.195456154910949	0.922788246908383	0.994766463583882	pseudobulk	DESeq2	LRT	0.0348979455069694
750	KC_AB1	CG10916	-0.795946324383256	0.478630183145396	0.994766463583882	pseudobulk	DESeq2	LRT	0.319999917590182
750	KC_AB1	CG10927	-2.45516412093746	0.350354315181257	0.994766463583882	pseudobulk	DESeq2	LRT	0.455492529097001
750	KC_AB1	CG10932	0.423247080601528	0.743999051232002	0.994766463583882	pseudobulk	DESeq2	LRT	0.128427618278004
750	KC_AB1	CG10947	0.0585952700200786	0.834112645770491	0.994766463583882	pseudobulk	DESeq2	LRT	0.0787752945265315
750	KC_AB1	CG10948	-0.134210709155939	0.727834879427199	0.994766463583882	pseudobulk	DESeq2	LRT	0.137967135916866
751	KC_AB1	CG10949	1.94950161999548	0.177699581428036	0.994766463583882	pseudobulk	DESeq2	LRT	0.750313595175611
751	KC_AB1	CG10958	-1.02319198676748	0.828204539793902	0.994766463583882	pseudobulk	DESeq2	LRT	0.0818623932467813
751	KC_AB1	CG10959	-0.235416794582491	0.844788530836776	0.994766463583882	pseudobulk	DESeq2	LRT	0.0732519909136154
751	KC_AB1	CG10960	-0.223214086547979	0.647414217750017	0.994766463583882	pseudobulk	DESeq2	LRT	0.188817767363819
751	KC_AB1	CG10973	0.352871610732934	0.74655808774518	0.994766463583882	pseudobulk	DESeq2	LRT	0.126936395298981
751	KC_AB1	CG10979	-0.837030680729309	0.390237698220496	0.994766463583882	pseudobulk	DESeq2	LRT	0.408670778666992
751	KC_AB1	CG10984	-0.62822407890485	0.328055919585072	0.994766463583882	pseudobulk	DESeq2	LRT	0.484052121235635
751	KC_AB1	CG11000	-0.384607108695797	0.21959702579178	0.994766463583882	pseudobulk	DESeq2	LRT	0.658373546239092
751	KC_AB1	CG11007	0.982668293440975	0.246885931332212	0.994766463583882	pseudobulk	DESeq2	LRT	0.607503657410598
751	KC_AB1	CG11029	0.949183608371002	0.80156895525114	0.994766463583882	pseudobulk	DESeq2	LRT	0.0960591113673091
752	KC_AB1	CG1103	0.356203672244682	0.739068912293409	0.994766463583882	pseudobulk	DESeq2	LRT	0.131315065213517
752	KC_AB1	CG11030	-0.64508340950227	0.321595937361616	0.994766463583882	pseudobulk	DESeq2	LRT	0.492689446218912
752	KC_AB1	CG11035	0.724954751128939	0.526575107541206	0.994766463583882	pseudobulk	DESeq2	LRT	0.278539674878667
752	KC_AB1	CG11041	-1.64952313825113	0.817565084244375	0.994766463583882	pseudobulk	DESeq2	LRT	0.0874776642296574
752	KC_AB1	CG11044	-1.64952313825113	0.817565084244375	0.994766463583882	pseudobulk	DESeq2	LRT	0.0874776642296574
752	KC_AB1	CG1105	-0.233714187284861	0.785575562792641	0.994766463583882	pseudobulk	DESeq2	LRT	0.104812034779887
752	KC_AB1	CG11050	0.0651792621673451	0.859550074647602	0.994766463583882	pseudobulk	DESeq2	LRT	0.0657288176276966
752	KC_AB1	CG11052	0.949183608371002	0.80156895525114	0.994766463583882	pseudobulk	DESeq2	LRT	0.0960591113673091
752	KC_AB1	CG11070	1.03701768249268	0.408631852420154	0.994766463583882	pseudobulk	DESeq2	LRT	0.388667783589162
752	KC_AB1	CG11076	0.145715859085614	0.740752673165879	0.994766463583882	pseudobulk	DESeq2	LRT	0.130326772599723
753	KC_AB1	CG11077	-0.0207959538435724	0.945800064940959	0.994766463583882	pseudobulk	DESeq2	LRT	0.0242006605008642
753	KC_AB1	CG11089	0.676120089153066	0.633957105454082	0.994766463583882	pseudobulk	DESeq2	LRT	0.197940126179484
753	KC_AB1	CG11095	0.555134229596722	0.593247795915051	0.994766463583882	pseudobulk	DESeq2	LRT	0.226763866635628
753	KC_AB1	CG11099	0.498654654402709	0.269043587772061	0.994766463583882	pseudobulk	DESeq2	LRT	0.570177354215502
753	KC_AB1	CG11103	0.157743043414351	0.898076774211248	0.994766463583882	pseudobulk	DESeq2	LRT	0.046686535057561
753	KC_AB1	CG11109	0.979674444364178	0.420695446893905	0.994766463583882	pseudobulk	DESeq2	LRT	0.37603218824341
753	KC_AB1	CG11110	0.391880246904931	0.692497629831762	0.994766463583882	pseudobulk	DESeq2	LRT	0.159581708693476
753	KC_AB1	CG11122	-0.103661535827383	0.758738644656804	0.994766463583882	pseudobulk	DESeq2	LRT	0.119907795553975
753	KC_AB1	CG11123	-2.71487103837826	0.315395654019323	0.994766463583882	pseudobulk	DESeq2	LRT	0.501144295307783
753	KC_AB1	CG11127	0.087356802117797	0.960952460588087	0.994766463583882	pseudobulk	DESeq2	LRT	0.0172980968423301
754	KC_AB1	CG1113	-0.643503469817389	0.769036587373876	0.994766463583882	pseudobulk	DESeq2	LRT	0.114052997888302
754	KC_AB1	CG11134	0.209946891738838	0.8829622200177	0.994766463583882	pseudobulk	DESeq2	LRT	0.0540578785162677
754	KC_AB1	CG11137	0.249376253132949	0.526975177060839	0.994766463583882	pseudobulk	DESeq2	LRT	0.278209841560514
754	KC_AB1	CG11141	-0.956776225265878	0.423230026369481	0.994766463583882	pseudobulk	DESeq2	LRT	0.373423528534331
754	KC_AB1	CG11151	0.268651102581419	0.588381339849338	0.994766463583882	pseudobulk	DESeq2	LRT	0.230341109107269
754	KC_AB1	CG11155	0.121295786869834	0.501487904709609	0.994766463583882	pseudobulk	DESeq2	LRT	0.299739537182387
754	KC_AB1	CG1116	-0.619875358718089	0.465512791457232	0.994766463583882	pseudobulk	DESeq2	LRT	0.332068380884645
754	KC_AB1	CG11168	1.17930484172975	0.669685881755347	0.994766463583882	pseudobulk	DESeq2	LRT	0.174128856716169
754	KC_AB1	CG11170	3.00445502450761	0.155116266689596	0.994766463583882	pseudobulk	DESeq2	LRT	0.809342656325038
754	KC_AB1	CG11178	0.631237571671811	0.490563745691128	0.994766463583882	pseudobulk	DESeq2	LRT	0.309304550764937
755	KC_AB1	CG11180	0.180042733083966	0.882000399810078	0.994766463583882	pseudobulk	DESeq2	LRT	0.0545312180027931
755	KC_AB1	CG11191	0.0314523898025174	0.864281933219732	0.994766463583882	pseudobulk	DESeq2	LRT	0.0633445653118352
755	KC_AB1	CG11200	2.48228345696307	0.204115101898692	0.994766463583882	pseudobulk	DESeq2	LRT	0.690124861875632
755	KC_AB1	CG11241	0.209946891738838	0.8829622200177	0.994766463583882	pseudobulk	DESeq2	LRT	0.0540578785162677
755	KC_AB1	CG11247	0.0292058692828505	0.892778131149001	0.994766463583882	pseudobulk	DESeq2	LRT	0.0492564564414827
755	KC_AB1	CG11248	0.316570500246895	0.717183095830713	0.994766463583882	pseudobulk	DESeq2	LRT	0.144369955409298
755	KC_AB1	CG11257	-0.203919210024363	0.84165190436689	0.994766463583882	pseudobulk	DESeq2	LRT	0.0748674895816847
755	KC_AB1	CG11263	1.1821590793593	0.719511699510136	0.994766463583882	pseudobulk	DESeq2	LRT	0.142962139890846
755	KC_AB1	CG11267	0.331146235815285	0.400135559821643	0.994766463583882	pseudobulk	DESeq2	LRT	0.397792851400128

755	KC_AB1	CG11275	-2.79499059556516	0.38564607643653	0.994766463583882	pseudobulk	DESeq2	LRT	0.413811082791365
756	KC_AB1	CG11279	0.478763572639829	0.529704849874514	0.994766463583882	pseudobulk	DESeq2	LRT	0.275966050730445
756	KC_AB1	CG1129	0.299657295663145	0.477044008291267	0.994766463583882	pseudobulk	DESeq2	LRT	0.321441554552252
756	KC_AB1	CG11307	-0.306238156857369	0.817884641254251	0.994766463583882	pseudobulk	DESeq2	LRT	0.0873079471840709
756	KC_AB1	CG11309	-0.081120805124586	0.834857265025168	0.994766463583882	pseudobulk	DESeq2	LRT	0.078387769199933
756	KC_AB1	CG11317	-0.0654049255531153	0.871821901192824	0.994766463583882	pseudobulk	DESeq2	LRT	0.0595722251924817
756	KC_AB1	CG11319	0.105277099265925	0.491592185091108	0.994766463583882	pseudobulk	DESeq2	LRT	0.308395029770094
756	KC_AB1	CG11334	-1.15938616346778	0.317794615945423	0.994766463583882	pseudobulk	DESeq2	LRT	0.497853464853624
756	KC_AB1	CG11342	1.45682795759908	0.573003993665791	0.994766463583882	pseudobulk	DESeq2	LRT	0.24184235111992
756	KC_AB1	CG11357	-0.316298391079282	0.45075716116068	0.994766463583882	pseudobulk	DESeq2	LRT	0.346057364931988
756	KC_AB1	CG11360	-0.112948257128697	0.685287394215173	0.994766463583882	pseudobulk	DESeq2	LRT	0.164127256907459
757	KC_AB1	CG11367	0.3289514164175	0.790191202656507	0.994766463583882	pseudobulk	DESeq2	LRT	0.102267809707727
757	KC_AB1	CG11374	-1.50663180504502	0.668163815680457	0.994766463583882	pseudobulk	DESeq2	LRT	0.175117047219009
757	KC_AB1	CG11377	0.288031046781916	0.826059306423214	0.994766463583882	pseudobulk	DESeq2	LRT	0.0829887716533022
757	KC_AB1	CG11396	-0.431926429157213	0.654221784148639	0.994766463583882	pseudobulk	DESeq2	LRT	0.184274998917802
757	KC_AB1	CG11398	-0.114099908033991	0.918752605478418	0.994766463583882	pseudobulk	DESeq2	LRT	0.0368014162959989
757	KC_AB1	CG11399	-0.753616298449613	0.225514481924803	0.994766463583882	pseudobulk	DESeq2	LRT	0.646825563683669
757	KC_AB1	CG11406	-0.417261698069622	0.569794913187936	0.994766463583882	pseudobulk	DESeq2	LRT	0.244281432218815
757	KC_AB1	CG11413	0.628003197167209	0.678980115427516	0.994766463583882	pseudobulk	DESeq2	LRT	0.168142944256401
757	KC_AB1	CG11414	-0.87157352006628	0.312927677456529	0.994766463583882	pseudobulk	DESeq2	LRT	0.504556023191119
757	KC_AB1	CG11417	-0.0627561689052589	0.891934490418666	0.994766463583882	pseudobulk	DESeq2	LRT	0.0496670419181083
758	KC_AB1	CG1142	-0.59295074122912	0.408625682233895	0.994766463583882	pseudobulk	DESeq2	LRT	0.38867434132092
758	KC_AB1	CG1143	-2.79499059556516	0.38564607643653	0.994766463583882	pseudobulk	DESeq2	LRT	0.413811082791365
758	KC_AB1	CG11436	-0.38643683137782	0.710203122268892	0.994766463583882	pseudobulk	DESeq2	LRT	0.148617422739599
758	KC_AB1	CG11444	0.0990269731573361	0.817260547991042	0.994766463583882	pseudobulk	DESeq2	LRT	0.0876394654802425
758	KC_AB1	CG11447	0.217449228652701	0.804452923642647	0.994766463583882	pseudobulk	DESeq2	LRT	0.0944993656114806
758	KC_AB1	CG11454	0.0970663892339228	0.865144942741678	0.994766463583882	pseudobulk	DESeq2	LRT	0.0629111265703297
758	KC_AB1	CG11456	0.0183223185731951	0.895848616882101	0.994766463583882	pseudobulk	DESeq2	LRT	0.0477653724889047
758	KC_AB1	CG11458	-1.64952313825113	0.817565084244375	0.994766463583882	pseudobulk	DESeq2	LRT	0.0874776642296574
758	KC_AB1	CG1146	2.02670873290438	0.538959414925803	0.994766463583882	pseudobulk	DESeq2	LRT	0.26844393710898
758	KC_AB1	CG11474	-0.797341578326246	0.431151312036181	0.994766463583882	pseudobulk	DESeq2	LRT	0.365370287960178
759	KC_AB1	CG11475	-1.64952313825113	0.817565084244375	0.994766463583882	pseudobulk	DESeq2	LRT	0.0874776642296574
759	KC_AB1	CG11486	-0.0972672777129329	0.578316743953435	0.994766463583882	pseudobulk	DESeq2	LRT	0.237834233424728
759	KC_AB1	CG11504	0.185228907711608	0.775942681030498	0.994766463583882	pseudobulk	DESeq2	LRT	0.110170358937142
759	KC_AB1	CG11523	-0.081994971622975	0.849707847043456	0.994766463583882	pseudobulk	DESeq2	LRT	0.0707303710232246
759	KC_AB1	CG11537	-0.129805197737191	0.596271522913201	0.994766463583882	pseudobulk	DESeq2	LRT	0.224555931450804
759	KC_AB1	CG11539	-0.890489951514268	0.752067066979362	0.994766463583882	pseudobulk	DESeq2	LRT	0.123743428663282
759	KC_AB1	CG11560	0.835177727167096	0.495950262199158	0.994766463583882	pseudobulk	DESeq2	LRT	0.304561875799245
759	KC_AB1	CG11562	-0.867639513548072	0.452850819686036	0.994766463583882	pseudobulk	DESeq2	LRT	0.344044841822512
759	KC_AB1	CG11563	-0.168784416075935	0.834160231819753	0.994766463583882	pseudobulk	DESeq2	LRT	0.0787505187732439
759	KC_AB1	CG11583	-0.133694593675847	0.890185765155659	0.994766463583882	pseudobulk	DESeq2	LRT	0.050519354744326
760	KC_AB1	CG11586	0.204190271081685	0.723378743011786	0.994766463583882	pseudobulk	DESeq2	LRT	0.140634257432739
760	KC_AB1	CG11590	0.48306298809704	0.631467681934527	0.994766463583882	pseudobulk	DESeq2	LRT	0.19964887142165
760	KC_AB1	CG11593	0.404685369074004	0.645381902060591	0.994766463583882	pseudobulk	DESeq2	LRT	0.190183217341506
760	KC_AB1	CG11594	0.949183608371002	0.80156895525114	0.994766463583882	pseudobulk	DESeq2	LRT	0.0960591113673091
760	KC_AB1	CG11596	-0.124428387125022	0.859750925711173	0.994766463583882	pseudobulk	DESeq2	LRT	0.0656273478920639
760	KC_AB1	CG11597	-1.50663180504502	0.668163815680457	0.994766463583882	pseudobulk	DESeq2	LRT	0.175117047219009
760	KC_AB1	CG11601	-0.943532023167133	0.614276262319199	0.994766463583882	pseudobulk	DESeq2	LRT	0.211636266941502
760	KC_AB1	CG1161	0.288158909242118	0.544349412783014	0.994766463583882	pseudobulk	DESeq2	LRT	0.264122241238727
760	KC_AB1	CG11638	0.534021206654981	0.67073539934107	0.994766463583882	pseudobulk	DESeq2	LRT	0.173448772334175
760	KC_AB1	CG11655	0.949183608371002	0.80156895525114	0.994766463583882	pseudobulk	DESeq2	LRT	0.0960591113673091
761	KC_AB1	CG11656	-2.79499059556516	0.38564607643653	0.994766463583882	pseudobulk	DESeq2	LRT	0.413811082791365
761	KC_AB1	CG11658	-0.194832820134894	0.739440443146412	0.994766463583882	pseudobulk	DESeq2	LRT	0.131096799698837
761	KC_AB1	CG11679	-0.835152883801839	0.920434724857442	0.994766463583882	pseudobulk	DESeq2	LRT	0.0360070052473159
761	KC_AB1	CG11695	0.252380788273428	0.848816287332261	0.994766463583882	pseudobulk	DESeq2	LRT	0.071186295658717
761	KC_AB1	CG11696	-0.00336420956509905	0.905063206309395	0.994766463583882	pseudobulk	DESeq2	LRT	0.0433210901949513

761	KC_AB1	CG11699	0.126698327970847	0.808081357691803	0.994766463583882	pseudobulk	DESeq2	LRT	0.0925449122230918
761	KC_AB1	CG11700	-4.07942607047938	0.12890417890847	0.994766463583882	pseudobulk	DESeq2	LRT	0.889733003147214
761	KC_AB1	CG11710	0.691823702207824	0.447283892955942	0.994766463583882	pseudobulk	DESeq2	LRT	0.349416740830929
761	KC_AB1	CG1172	0.00599193113353279	0.931941849029971	0.994766463583882	pseudobulk	DESeq2	LRT	0.0306111857504173
761	KC_AB1	CG11722	-0.00706560148908919	0.898082670844875	0.994766463583882	pseudobulk	DESeq2	LRT	0.0466836835563169
762	KC_AB1	CG11723	0.834567619333679	0.569232767315916	0.994766463583882	pseudobulk	DESeq2	LRT	0.244710108139942
762	KC_AB1	CG11737	1.18284045355602	0.397004066839524	0.994766463583882	pseudobulk	DESeq2	LRT	0.401205044378151
762	KC_AB1	CG11752	-0.450491669447432	0.224066624381966	0.994766463583882	pseudobulk	DESeq2	LRT	0.649622828544016
762	KC_AB1	CG11753	-0.135633061749527	0.779650194084881	0.994766463583882	pseudobulk	DESeq2	LRT	0.108100208661189
762	KC_AB1	CG11755	0.708632460267942	0.638441165890324	0.994766463583882	pseudobulk	DESeq2	LRT	0.194879117987811
762	KC_AB1	CG11760	0.367435071798035	0.704180761660572	0.994766463583882	pseudobulk	DESeq2	LRT	0.152315844103643
762	KC_AB1	CG11768	0.213619851815697	0.568820669810429	0.994766463583882	pseudobulk	DESeq2	LRT	0.245024630594904
762	KC_AB1	CG11771	0.382210989906215	0.648618169376594	0.994766463583882	pseudobulk	DESeq2	LRT	0.188010889845173
762	KC_AB1	CG11777	0.19894273893144	0.826927539410647	0.994766463583882	pseudobulk	DESeq2	LRT	0.0825325443928432
762	KC_AB1	CG11779	-0.0494246678013384	0.949780073241099	0.994766463583882	pseudobulk	DESeq2	LRT	0.0223769463271898
763	KC_AB1	CG11781	-0.12678264456852	0.814357436512699	0.994766463583882	pseudobulk	DESeq2	LRT	0.0891849333996678
763	KC_AB1	CG11788	1.10251292103259	0.639221751231493	0.994766463583882	pseudobulk	DESeq2	LRT	0.194348455410132
763	KC_AB1	CG11790	0.463735534344822	0.662613952037152	0.994766463583882	pseudobulk	DESeq2	LRT	0.178739423828412
763	KC_AB1	CG11791	-2.57435989741145	0.533617212681275	0.994766463583882	pseudobulk	DESeq2	LRT	0.272770169998685
763	KC_AB1	CG11807	-0.246613318312306	0.758962604023263	0.994766463583882	pseudobulk	DESeq2	LRT	0.119779622347536
763	KC_AB1	CG11808	0.209946891738838	0.8829622200177	0.994766463583882	pseudobulk	DESeq2	LRT	0.0540578785162677
763	KC_AB1	CG11811	-0.0554023892405195	0.932563464169676	0.994766463583882	pseudobulk	DESeq2	LRT	0.0303216032717486
763	KC_AB1	CG11828	-1.28132067551389	0.647201349541384	0.994766463583882	pseudobulk	DESeq2	LRT	0.188960585805912
763	KC_AB1	CG11837	-2.81295880623569	0.103755146953526	0.994766463583882	pseudobulk	DESeq2	LRT	0.983990350152533
763	KC_AB1	CG11839	0.0526590720372664	0.866846391535528	0.994766463583882	pseudobulk	DESeq2	LRT	0.062057854333355
764	KC_AB1	CG11852	-1.64952313825113	0.817565084244375	0.994766463583882	pseudobulk	DESeq2	LRT	0.0874776642296574
764	KC_AB1	CG11857	0.141618248716779	0.81893743702239	0.994766463583882	pseudobulk	DESeq2	LRT	0.0867492750329639
764	KC_AB1	CG11858	0.205062466243527	0.758225536650534	0.994766463583882	pseudobulk	DESeq2	LRT	0.120201592845945
764	KC_AB1	CG11873	-0.160728092674694	0.601908438905079	0.994766463583882	pseudobulk	DESeq2	LRT	0.220469567716551
764	KC_AB1	CG11882	0.477114514271121	0.740883202943313	0.994766463583882	pseudobulk	DESeq2	LRT	0.130250251285513
764	KC_AB1	CG11883	-0.0722232739948949	0.854572064174264	0.994766463583882	pseudobulk	DESeq2	LRT	0.0682513083047634
764	KC_AB1	CG11885	-0.215157099318525	0.827698498589796	0.994766463583882	pseudobulk	DESeq2	LRT	0.08212783259233
764	KC_AB1	CG11898	-0.398223642572822	0.840304074335614	0.994766463583882	pseudobulk	DESeq2	LRT	0.0755635307121724
764	KC_AB1	CG11899	1.19856662192297	0.608802264240657	0.994766463583882	pseudobulk	DESeq2	LRT	0.215523741018373
764	KC_AB1	CG11902	0.0934194773052396	0.879799498744361	0.994766463583882	pseudobulk	DESeq2	LRT	0.0556162897933683
765	KC_AB1	CG11906	-0.0768722140518598	0.922380403082776	0.994766463583882	pseudobulk	DESeq2	LRT	0.0350899326468167
765	KC_AB1	CG11920	-0.356212784647529	0.928203443271547	0.994766463583882	pseudobulk	DESeq2	LRT	0.0323568248514225
765	KC_AB1	CG11927	0.221672263440025	0.793404077770106	0.994766463583882	pseudobulk	DESeq2	LRT	0.100505571761409
765	KC_AB1	CG11961	1.1821590793593	0.719511699510136	0.994766463583882	pseudobulk	DESeq2	LRT	0.142962139890846
765	KC_AB1	CG11964	0.480073078474809	0.665327592204209	0.994766463583882	pseudobulk	DESeq2	LRT	0.176964465290539
765	KC_AB1	CG11970	0.388226648920603	0.749409197083901	0.994766463583882	pseudobulk	DESeq2	LRT	0.125280981353939
765	KC_AB1	CG11975	-0.370266989238584	0.591590418611525	0.994766463583882	pseudobulk	DESeq2	LRT	0.227978868444315
765	KC_AB1	CG11980	-0.759578057496218	0.440440944340035	0.994766463583882	pseudobulk	DESeq2	LRT	0.356112314871959
765	KC_AB1	CG11983	-4.2169236809797	0.105189937930809	0.994766463583882	pseudobulk	DESeq2	LRT	0.978025801153034
765	KC_AB1	CG11986	0.513714559562998	0.775698051782448	0.994766463583882	pseudobulk	DESeq2	LRT	0.110307299314091
766	KC_AB1	CG11999	-0.482468578584362	0.709187187571662	0.994766463583882	pseudobulk	DESeq2	LRT	0.149239119119048
766	KC_AB1	CG12004	-0.373916116688419	0.297316453463985	0.994766463583882	pseudobulk	DESeq2	LRT	0.526781056315098
766	KC_AB1	CG12007	0.69181442486348	0.454278356104333	0.994766463583882	pseudobulk	DESeq2	LRT	0.342677954465094
766	KC_AB1	CG12010	-0.513883644356483	0.697477245621635	0.994766463583882	pseudobulk	DESeq2	LRT	0.156469956172153
766	KC_AB1	CG12012	0.0482866912163648	0.9317633682078	0.994766463583882	pseudobulk	DESeq2	LRT	0.0306943676138522
766	KC_AB1	CG12017	0.806933541094274	0.754600478929312	0.994766463583882	pseudobulk	DESeq2	LRT	0.122282923497213
766	KC_AB1	CG12024	-0.167012719857792	0.768361026098828	0.994766463583882	pseudobulk	DESeq2	LRT	0.114434672154288
766	KC_AB1	CG12025	-0.31401705306576	0.390092127882711	0.994766463583882	pseudobulk	DESeq2	LRT	0.408832813727162
766	KC_AB1	CG12038	0.239513500661585	0.577464538519719	0.994766463583882	pseudobulk	DESeq2	LRT	0.23847468017706
766	KC_AB1	CG12054	-0.0356327595472494	0.867523488645215	0.994766463583882	pseudobulk	DESeq2	LRT	0.061718757631147
767	KC_AB1	CG12056	-0.0840070761912168	0.924420430540169	0.994766463583882	pseudobulk	DESeq2	LRT	0.0341304647801118

767	KC_AB1	CG12071	0.0236825169714783	0.880463482099219	0.994766463583882	pseudobulk	DESeq2	LRT	0.0552886520211655
767	KC_AB1	CG12081	-0.450156381642171	0.628900486156597	0.994766463583882	pseudobulk	DESeq2	LRT	0.201418069550279
767	KC_AB1	CG12084	-0.044521798661164	0.923047444486272	0.994766463583882	pseudobulk	DESeq2	LRT	0.0347759757357001
767	KC_AB1	CG12091	0.172414678444939	0.702743989094316	0.994766463583882	pseudobulk	DESeq2	LRT	0.153202860433199
767	KC_AB1	CG12093	0.648730524179276	0.509525016044503	0.994766463583882	pseudobulk	DESeq2	LRT	0.292834488667603
767	KC_AB1	CG12096	-0.111558146737861	0.92127361805634	0.994766463583882	pseudobulk	DESeq2	LRT	0.0356113652820783
767	KC_AB1	CG12099	-0.0287858444445414	0.933242724279429	0.994766463583882	pseudobulk	DESeq2	LRT	0.0300053872228344
767	KC_AB1	CG12104	0.801862047340079	0.550090279554276	0.994766463583882	pseudobulk	DESeq2	LRT	0.259566029242632
767	KC_AB1	CG12106	0.513553586486426	0.586997642272184	0.994766463583882	pseudobulk	DESeq2	LRT	0.231363643130983
768	KC_AB1	CG12107	-0.310630536740601	0.683669546226217	0.994766463583882	pseudobulk	DESeq2	LRT	0.165153765135027
768	KC_AB1	CG12112	3.22147157048093	0.291843394109836	0.994766463583882	pseudobulk	DESeq2	LRT	0.534850132520396
768	KC_AB1	CG12118	1.20118650109295	0.342182841521446	0.994766463583882	pseudobulk	DESeq2	LRT	0.465741771590313
768	KC_AB1	CG12121	0.346262287941008	0.598059688397809	0.994766463583882	pseudobulk	DESeq2	LRT	0.223255469777157
768	KC_AB1	CG12123	0.837542184109659	0.251575715063086	0.994766463583882	pseudobulk	DESeq2	LRT	0.599331284224891
768	KC_AB1	CG12128	-0.0185332235666699	0.902778364661945	0.994766463583882	pseudobulk	DESeq2	LRT	0.0444188574592739
768	KC_AB1	CG12129	0.662200016042879	0.500178800425478	0.994766463583882	pseudobulk	DESeq2	LRT	0.300874719349503
768	KC_AB1	CG12134	0.00502834284390088	0.881785846627974	0.994766463583882	pseudobulk	DESeq2	LRT	0.0546368762294749
768	KC_AB1	CG12147	0.949183608371002	0.80156895525114	0.994766463583882	pseudobulk	DESeq2	LRT	0.0960591113673091
768	KC_AB1	CG12155	0.0362037100820093	0.926615690915921	0.994766463583882	pseudobulk	DESeq2	LRT	0.0331003499125924
769	KC_AB1	CG12158	-3.21143241058256	0.292545415061155	0.994766463583882	pseudobulk	DESeq2	LRT	0.533806704011112
769	KC_AB1	CG12159	-0.29090855333461	0.614927454070909	0.994766463583882	pseudobulk	DESeq2	LRT	0.211176116997087
769	KC_AB1	CG12163	0.152418666623815	0.816449699503223	0.994766463583882	pseudobulk	DESeq2	LRT	0.088070566470611
769	KC_AB1	CG12170	-0.0851981368323784	0.854524706172153	0.994766463583882	pseudobulk	DESeq2	LRT	0.0682753763614735
769	KC_AB1	CG12171	0.806933541094274	0.754600478929312	0.994766463583882	pseudobulk	DESeq2	LRT	0.122282923497213
769	KC_AB1	CG12173	0.468442290284381	0.586131977664259	0.994766463583882	pseudobulk	DESeq2	LRT	0.23200458412207
769	KC_AB1	CG12177	-2.20969975456663	0.610836134188693	0.994766463583882	pseudobulk	DESeq2	LRT	0.214075280039667
769	KC_AB1	CG12179	-1.87227586326617	0.401547731174294	0.994766463583882	pseudobulk	DESeq2	LRT	0.396262823601909
769	KC_AB1	CG1218	-0.804602912459617	0.421731297158738	0.994766463583882	pseudobulk	DESeq2	LRT	0.374964168302231
769	KC_AB1	CG12182	-1.67255867366542	0.393063002702225	0.994766463583882	pseudobulk	DESeq2	LRT	0.405537832492395
770	KC_AB1	CG12194	-0.0995624146424518	0.74836599420009	0.994766463583882	pseudobulk	DESeq2	LRT	0.125885955093447
770	KC_AB1	CG12204	0.688840054315581	0.611254607986344	0.994766463583882	pseudobulk	DESeq2	LRT	0.213777853896137
770	KC_AB1	CG12206	-1.72788865064805	0.316272336056959	0.994766463583882	pseudobulk	DESeq2	LRT	0.499938793614574
770	KC_AB1	CG12213	0.00782647804868089	0.947337107213475	0.994766463583882	pseudobulk	DESeq2	LRT	0.0234954510380746
770	KC_AB1	CG12219	-0.781074387991377	0.447875517807291	0.994766463583882	pseudobulk	DESeq2	LRT	0.348842676719845
770	KC_AB1	CG12236	0.11207756976595	0.8407808508716	0.994766463583882	pseudobulk	DESeq2	LRT	0.075317188101961
770	KC_AB1	CG12237	0.55526209394603	0.446504165347216	0.994766463583882	pseudobulk	DESeq2	LRT	0.350174485310993
770	KC_AB1	CG12241	0.312032307878066	0.699918570003486	0.994766463583882	pseudobulk	DESeq2	LRT	0.154952483778976
770	KC_AB1	CG12253	0.224047146974094	0.774843862142877	0.994766463583882	pseudobulk	DESeq2	LRT	0.110785802837486
770	KC_AB1	CG12259	-0.117192456152474	0.914338129990976	0.994766463583882	pseudobulk	DESeq2	LRT	0.0388931687813945
771	KC_AB1	CG1227	1.09255624499018	0.534555027288718	0.994766463583882	pseudobulk	DESeq2	LRT	0.272007581722247
771	KC_AB1	CG12279	1.04872793259109	0.138200154462347	0.994766463583882	pseudobulk	DESeq2	LRT	0.85949147156307
771	KC_AB1	CG12288	2.17673076324139	0.400454055418109	0.994766463583882	pseudobulk	DESeq2	LRT	0.397447303856867
771	KC_AB1	CG12290	-1.64952313825113	0.817565084244375	0.994766463583882	pseudobulk	DESeq2	LRT	0.0874776642296574
771	KC_AB1	CG12299	-0.69390360021508	0.239138725982827	0.994766463583882	pseudobulk	DESeq2	LRT	0.621350088799376
771	KC_AB1	CG12301	2.07176468007008	0.140548052051348	0.994766463583882	pseudobulk	DESeq2	LRT	0.852175169190039
771	KC_AB1	CG1231	0.978218935568633	0.578530845039478	0.994766463583882	pseudobulk	DESeq2	LRT	0.237673481183578
771	KC_AB1	CG12310	1.1821590793593	0.719511699510136	0.994766463583882	pseudobulk	DESeq2	LRT	0.142962139890846
771	KC_AB1	CG12320	-0.356212784647529	0.928203443271547	0.994766463583882	pseudobulk	DESeq2	LRT	0.0323568248514225
771	KC_AB1	CG12321	0.556852382530406	0.546327517617897	0.994766463583882	pseudobulk	DESeq2	LRT	0.262546924236227
772	KC_AB1	CG12325	-0.0869722546723049	0.918728060542471	0.994766463583882	pseudobulk	DESeq2	LRT	0.036813018845553
772	KC_AB1	CG1233	0.91250407677042	0.489275263328995	0.994766463583882	pseudobulk	DESeq2	LRT	0.310446740649799
772	KC_AB1	CG12333	0.0864400798608833	0.899490562668949	0.994766463583882	pseudobulk	DESeq2	LRT	0.0460033888523932
772	KC_AB1	CG12338	-0.890489951514268	0.752067066979362	0.994766463583882	pseudobulk	DESeq2	LRT	0.123743428663282
772	KC_AB1	CG1234	-0.601439387351816	0.562824041850242	0.994766463583882	pseudobulk	DESeq2	LRT	0.24962735931852
772	KC_AB1	CG12341	0.122615559311543	0.891889967565847	0.994766463583882	pseudobulk	DESeq2	LRT	0.0496887212142019
772	KC_AB1	CG12343	-0.295059040923045	0.6490523664083	0.994766463583882	pseudobulk	DESeq2	LRT	0.187720262329536

772	KC_AB1	CG12355	-0.029955417145092	0.921685563231183	0.994766463583882	pseudobulk	DESeq2	LRT	0.0354172150034643
772	KC_AB1	CG1236	-1.94067945388881	0.245697136107039	0.994766463583882	pseudobulk	DESeq2	LRT	0.609599905710227
772	KC_AB1	CG12375	-0.20378315781085	0.75750651696357	0.994766463583882	pseudobulk	DESeq2	LRT	0.120613626496757
773	KC_AB1	CG12379	1.67933360516431	0.216688718041086	0.994766463583882	pseudobulk	DESeq2	LRT	0.664163699755637
773	KC_AB1	CG12384	-0.284713861021724	0.438637680191195	0.994766463583882	pseudobulk	DESeq2	LRT	0.357894063957576
773	KC_AB1	CG1239	0.239364038579332	0.864004681384457	0.994766463583882	pseudobulk	DESeq2	LRT	0.0634839044031326
773	KC_AB1	CG12391	0.255687402207373	0.644764716013356	0.994766463583882	pseudobulk	DESeq2	LRT	0.190598736804665
773	KC_AB1	CG12402	-0.554098689211639	0.415026873640579	0.994766463583882	pseudobulk	DESeq2	LRT	0.381923781129006
773	KC_AB1	CG12420	1.80633728195307	0.61161102402172	0.994766463583882	pseudobulk	DESeq2	LRT	0.213524695220716
773	KC_AB1	CG1246	-1.64952313825113	0.817565084244375	0.994766463583882	pseudobulk	DESeq2	LRT	0.0874776642296574
773	KC_AB1	CG12470	-1.02319198676748	0.828204539793902	0.994766463583882	pseudobulk	DESeq2	LRT	0.0818623932467813
773	KC_AB1	CG12499	-0.296502187713078	0.869914334351175	0.994766463583882	pseudobulk	DESeq2	LRT	0.0605235128415757
773	KC_AB1	CG12502	0.188707915000012	0.8859007967066	0.994766463583882	pseudobulk	DESeq2	LRT	0.0526149077462058
774	KC_AB1	CG12531	0.0124719246756139	0.872320269391438	0.994766463583882	pseudobulk	DESeq2	LRT	0.0593240360618537
774	KC_AB1	CG12535	0.205511634286069	0.845489900058704	0.994766463583882	pseudobulk	DESeq2	LRT	0.0728915759727697
774	KC_AB1	CG12541	0.385657508888949	0.481576360812229	0.994766463583882	pseudobulk	DESeq2	LRT	0.317334839484398
774	KC_AB1	CG12547	0.446677027742745	0.323837065263015	0.994766463583882	pseudobulk	DESeq2	LRT	0.489673444903381
774	KC_AB1	CG12560	0.207821396572799	0.871477306204092	0.994766463583882	pseudobulk	DESeq2	LRT	0.0597439176976029
774	KC_AB1	CG12567	0.250091845311001	0.571117976527923	0.994766463583882	pseudobulk	DESeq2	LRT	0.243274169752911
774	KC_AB1	CG12576	0.347874417052817	0.489927976949338	0.994766463583882	pseudobulk	DESeq2	LRT	0.30986775979291
774	KC_AB1	CG12605	-0.182516720535183	0.389545059370341	0.994766463583882	pseudobulk	DESeq2	LRT	0.409442299470079
774	KC_AB1	CG12607	0.949183608371002	0.80156895525114	0.994766463583882	pseudobulk	DESeq2	LRT	0.0960591113673091
774	KC_AB1	CG12608	-1.21401623275352	0.293945324884757	0.994766463583882	pseudobulk	DESeq2	LRT	0.5317334427479
775	KC_AB1	CG12617	-2.20969975456663	0.610836134188693	0.994766463583882	pseudobulk	DESeq2	LRT	0.214075280039667
775	KC_AB1	CG12645	-1.64952313825113	0.817565084244375	0.994766463583882	pseudobulk	DESeq2	LRT	0.0874776642296574
775	KC_AB1	CG1265	0.341522800545977	0.490143900087027	0.994766463583882	pseudobulk	DESeq2	LRT	0.309676397851144
775	KC_AB1	CG12655	1.80633728195307	0.61161102402172	0.994766463583882	pseudobulk	DESeq2	LRT	0.213524695220716
775	KC_AB1	CG12659	0.0556507705136479	0.910364932142286	0.994766463583882	pseudobulk	DESeq2	LRT	0.0407844799367282
775	KC_AB1	CG12672	-0.906488689298719	0.45948768502231	0.994766463583882	pseudobulk	DESeq2	LRT	0.337726123882934
775	KC_AB1	CG1271	0.678410316364574	0.676102688830508	0.994766463583882	pseudobulk	DESeq2	LRT	0.16998733689049
775	KC_AB1	CG12713	1.02388942995419	0.598311846205213	0.994766463583882	pseudobulk	DESeq2	LRT	0.223072398310179
775	KC_AB1	CG12715	0.949183608371002	0.80156895525114	0.994766463583882	pseudobulk	DESeq2	LRT	0.0960591113673091
775	KC_AB1	CG12717	0.208680958600259	0.843930449375599	0.994766463583882	pseudobulk	DESeq2	LRT	0.0736933432997158
776	KC_AB1	CG12728	0.549013697426786	0.553120500884363	0.994766463583882	pseudobulk	DESeq2	LRT	0.257180244521338
776	KC_AB1	CG12730	0.909155167252296	0.248617768763809	0.994766463583882	pseudobulk	DESeq2	LRT	0.604467835467895
776	KC_AB1	CG1275	-0.4440153364699	0.270169404106126	0.994766463583882	pseudobulk	DESeq2	LRT	0.568363835107631
776	KC_AB1	CG12768	-0.78410610838983	0.738244895252942	0.994766463583882	pseudobulk	DESeq2	LRT	0.131799547371545
776	KC_AB1	CG12769	0.092819480538948	0.845369928537711	0.994766463583882	pseudobulk	DESeq2	LRT	0.0729532049366363
776	KC_AB1	CG12773	-0.981244292040941	0.41910879543453	0.994766463583882	pseudobulk	DESeq2	LRT	0.377673224948544
776	KC_AB1	CG12795	-0.307562107490048	0.684940659447258	0.994766463583882	pseudobulk	DESeq2	LRT	0.16434705243595
776	KC_AB1	CG12814	-1.64952313825113	0.817565084244375	0.994766463583882	pseudobulk	DESeq2	LRT	0.0874776642296574
776	KC_AB1	CG12817	0.664926085597786	0.390170628427688	0.994766463583882	pseudobulk	DESeq2	LRT	0.408745426873568
776	KC_AB1	CG12818	0.546411703314962	0.676627943713884	0.994766463583882	pseudobulk	DESeq2	LRT	0.169650070470377
777	KC_AB1	CG12822	-0.461878629432023	0.384338901908975	0.994766463583882	pseudobulk	DESeq2	LRT	0.415285655011728
777	KC_AB1	CG12836	-0.20086410634835	0.789329705325207	0.994766463583882	pseudobulk	DESeq2	LRT	0.102741552818523
777	KC_AB1	CG12848	0.835417564267685	0.265443850773647	0.994766463583882	pseudobulk	DESeq2	LRT	0.576027330997173
777	KC_AB1	CG12851	-0.331617950743056	0.562132524851077	0.994766463583882	pseudobulk	DESeq2	LRT	0.250161285807054
777	KC_AB1	CG12863	1.39595377037528	0.546916743048716	0.994766463583882	pseudobulk	DESeq2	LRT	0.262078781137403
777	KC_AB1	CG12868	0.131259856261552	0.865995194418513	0.994766463583882	pseudobulk	DESeq2	LRT	0.0624845179633843
777	KC_AB1	CG12877	-0.418226478821067	0.62723967548046	0.994766463583882	pseudobulk	DESeq2	LRT	0.202566478548653
777	KC_AB1	CG12883	0.542459157000834	0.742353438671798	0.994766463583882	pseudobulk	DESeq2	LRT	0.129389275423419
777	KC_AB1	CG12885	0.806933541094274	0.754600478929312	0.994766463583882	pseudobulk	DESeq2	LRT	0.122282923497213
777	KC_AB1	CG12895	0.0817071361473583	0.818447797311984	0.994766463583882	pseudobulk	DESeq2	LRT	0.0870090157784276
778	KC_AB1	CG12896	-1.64952313825113	0.817565084244375	0.994766463583882	pseudobulk	DESeq2	LRT	0.0874776642296574
778	KC_AB1	CG12909	0.746714342506132	0.723342225081447	0.994766463583882	pseudobulk	DESeq2	LRT	0.140656182235089
778	KC_AB1	CG12910	-0.993217988364291	0.27950666863685	0.994766463583882	pseudobulk	DESeq2	LRT	0.553607825997105

778	KC_AB1	CG12913	-0.0640639991728224	0.941264994671885	0.994766463583882	pseudobulk	DESeq2	LRT	0.0262880922771531
778	KC_AB1	CG12926	1.1821590793593	0.719511699510136	0.994766463583882	pseudobulk	DESeq2	LRT	0.142962139890846
778	KC_AB1	CG12929	0.395440078042145	0.760506659344196	0.994766463583882	pseudobulk	DESeq2	LRT	0.118896978713185
778	KC_AB1	CG12935	0.328189160114535	0.754237590595767	0.994766463583882	pseudobulk	DESeq2	LRT	0.122491826502814
778	KC_AB1	CG12942	1.12700276793276	0.468575134574402	0.994766463583882	pseudobulk	DESeq2	LRT	0.329220761414793
778	KC_AB1	CG12945	-0.107336114434101	0.850317441099928	0.994766463583882	pseudobulk	DESeq2	LRT	0.0704189128959605
778	KC_AB1	CG12947	-5.03066872932344	0.304013904873441	0.994766463583882	pseudobulk	DESeq2	LRT	0.517106552339587
779	KC_AB1	CG12948	0.618654865755989	0.591301679473585	0.994766463583882	pseudobulk	DESeq2	LRT	0.228190887472948
779	KC_AB1	CG12975	-0.382727295330241	0.497556325558697	0.994766463583882	pseudobulk	DESeq2	LRT	0.30315774809477
779	KC_AB1	CG12991	-0.0286232098513662	0.927160173923113	0.994766463583882	pseudobulk	DESeq2	LRT	0.0328452317218781
779	KC_AB1	CG12992	-0.357197804518595	0.787859109265642	0.994766463583882	pseudobulk	DESeq2	LRT	0.103551439286087
779	KC_AB1	CG12994	-1.55427673693219	0.522267206552175	0.994766463583882	pseudobulk	DESeq2	LRT	0.282107242898514
779	KC_AB1	CG13001	0.483998170433412	0.551188957927626	0.994766463583882	pseudobulk	DESeq2	LRT	0.258699491316568
779	KC_AB1	CG13005	-0.927811138542507	0.65055174458984	0.994766463583882	pseudobulk	DESeq2	LRT	0.186718154142994
779	KC_AB1	CG13014	0.217906885051292	0.902008941520292	0.994766463583882	pseudobulk	DESeq2	LRT	0.0447891573209458
779	KC_AB1	CG13016	-1.28349343700037	0.265306417359056	0.994766463583882	pseudobulk	DESeq2	LRT	0.57625224496709
779	KC_AB1	CG13018	0.223387353429062	0.811461662875716	0.994766463583882	pseudobulk	DESeq2	LRT	0.0907319933914443
780	KC_AB1	CG13025	1.82628216844872	0.306752685105949	0.994766463583882	pseudobulk	DESeq2	LRT	0.513211627063572
780	KC_AB1	CG13054	-1.6624765187663	0.0974736889944047	0.994766463583882	pseudobulk	DESeq2	LRT	1.01111259729232
780	KC_AB1	CG13055	-0.18263772111135	0.509031044847041	0.994766463583882	pseudobulk	DESeq2	LRT	0.293255730051033
780	KC_AB1	CG1307	0.0494278778251265	0.888292719140469	0.994766463583882	pseudobulk	DESeq2	LRT	0.0514438975556525
780	KC_AB1	CG13074	0.949183608371002	0.80156895525114	0.994766463583882	pseudobulk	DESeq2	LRT	0.0960591113673091
780	KC_AB1	CG13085	1.1821590793593	0.719511699510136	0.994766463583882	pseudobulk	DESeq2	LRT	0.142962139890846
780	KC_AB1	CG13088	2.02670873290438	0.538959414925803	0.994766463583882	pseudobulk	DESeq2	LRT	0.26844393710898
780	KC_AB1	CG1309	0.416148977039292	0.637778267276585	0.994766463583882	pseudobulk	DESeq2	LRT	0.195330283717494
780	KC_AB1	CG13096	-0.374623798862853	0.776492222458386	0.994766463583882	pseudobulk	DESeq2	LRT	0.109862889916519
780	KC_AB1	CG13097	-1.64861303152014	0.294140614792878	0.994766463583882	pseudobulk	DESeq2	LRT	0.531445004181821
781	KC_AB1	CG13108	-0.327706825563363	0.575432108188697	0.994766463583882	pseudobulk	DESeq2	LRT	0.240005908834066
781	KC_AB1	CG13123	0.370519601293918	0.822121836013648	0.994766463583882	pseudobulk	DESeq2	LRT	0.0850638165384441
781	KC_AB1	CG13124	-0.945063104343641	0.689809728909115	0.994766463583882	pseudobulk	DESeq2	LRT	0.161270684741391
781	KC_AB1	CG13126	0.701125451723029	0.595397772590602	0.994766463583882	pseudobulk	DESeq2	LRT	0.225192794401118
781	KC_AB1	CG13135	-2.79499059556516	0.38564607643653	0.994766463583882	pseudobulk	DESeq2	LRT	0.413811082791365
781	KC_AB1	CG13137	1.80633728195307	0.61161102402172	0.994766463583882	pseudobulk	DESeq2	LRT	0.213524695220716
781	KC_AB1	CG1314	0.949183608371002	0.80156895525114	0.994766463583882	pseudobulk	DESeq2	LRT	0.0960591113673091
781	KC_AB1	CG13148	0.55844128731752	0.592666047107537	0.994766463583882	pseudobulk	DESeq2	LRT	0.227189952086455
781	KC_AB1	CG13151	0.806933541094274	0.754600478929312	0.994766463583882	pseudobulk	DESeq2	LRT	0.122282923497213
781	KC_AB1	CG1316	-0.112864409430075	0.706003961074407	0.994766463583882	pseudobulk	DESeq2	LRT	0.151192862308067
782	KC_AB1	CG1317	-0.331200836921272	0.622095849294284	0.994766463583882	pseudobulk	DESeq2	LRT	0.206142696317838
782	KC_AB1	CG13185	0.207821396572799	0.871477306204092	0.994766463583882	pseudobulk	DESeq2	LRT	0.0597439176976029
782	KC_AB1	CG13192	0.938006920752544	0.509362175105722	0.994766463583882	pseudobulk	DESeq2	LRT	0.292973308592629
782	KC_AB1	CG13197	-0.475670298334195	0.506001023210108	0.994766463583882	pseudobulk	DESeq2	LRT	0.295848604950608
782	KC_AB1	CG13204	-0.0760030017511483	0.843940456864677	0.994766463583882	pseudobulk	DESeq2	LRT	0.0736881933830155
782	KC_AB1	CG13220	-0.256938589739177	0.714486786484707	0.994766463583882	pseudobulk	DESeq2	LRT	0.146005798517296
782	KC_AB1	CG13248	-0.208064712185736	0.694600371009865	0.994766463583882	pseudobulk	DESeq2	LRT	0.158264989053626
782	KC_AB1	CG13250	-1.64952313825113	0.817565084244375	0.994766463583882	pseudobulk	DESeq2	LRT	0.0874776642296574
782	KC_AB1	CG13284	0.0130595435377233	0.946397335223866	0.994766463583882	pseudobulk	DESeq2	LRT	0.0239264912237438
782	KC_AB1	CG13293	0.331552342534428	0.321251351936964	0.994766463583882	pseudobulk	DESeq2	LRT	0.493155035995192
783	KC_AB1	CG13295	-1.70182683137033	0.416861231158718	0.994766463583882	pseudobulk	DESeq2	LRT	0.380008493160473
783	KC_AB1	CG13300	0.475892334225681	0.803946046269915	0.994766463583882	pseudobulk	DESeq2	LRT	0.0947730962683447
783	KC_AB1	CG13319	-0.391985191075348	0.783062587833321	0.994766463583882	pseudobulk	DESeq2	LRT	0.106203524705343
783	KC_AB1	CG13325	1.1821590793593	0.719511699510136	0.994766463583882	pseudobulk	DESeq2	LRT	0.142962139890846
783	KC_AB1	CG13339	-0.831940901464639	0.31486815492318	0.994766463583882	pseudobulk	DESeq2	LRT	0.501871260736877
783	KC_AB1	CG13344	-0.127536367828182	0.834239020398927	0.994766463583882	pseudobulk	DESeq2	LRT	0.0787095004796284
783	KC_AB1	CG13358	-1.64952313825113	0.817565084244375	0.994766463583882	pseudobulk	DESeq2	LRT	0.0874776642296574
783	KC_AB1	CG13364	-0.0562438832581086	0.873068183034628	0.994766463583882	pseudobulk	DESeq2	LRT	0.0589518383569741
783	KC_AB1	CG13365	0.325986468356358	0.599592526634669	0.994766463583882	pseudobulk	DESeq2	LRT	0.222143788868373

783	KC_AB1	CG13366	-0.267152452995003	0.654299249303715	0.994766463583882	pseudobulk	DESeq2	LRT	0.18422357797986
784	KC_AB1	CG13367	0.595449146580039	0.656598515930592	0.994766463583882	pseudobulk	DESeq2	LRT	0.182700103214786
784	KC_AB1	CG13369	-0.201065075972523	0.80802944048564	0.994766463583882	pseudobulk	DESeq2	LRT	0.0925728154537186
784	KC_AB1	CG13375	0.150858250071838	0.761022447086719	0.994766463583882	pseudobulk	DESeq2	LRT	0.118602533108373
784	KC_AB1	CG13376	0.949183608371002	0.80156895525114	0.994766463583882	pseudobulk	DESeq2	LRT	0.0960591113673091
784	KC_AB1	CG13377	-1.21457746603801	0.770013447451371	0.994766463583882	pseudobulk	DESeq2	LRT	0.113501690278259
784	KC_AB1	CG13384	0.195945057824665	0.726509612604906	0.994766463583882	pseudobulk	DESeq2	LRT	0.138758635084268
784	KC_AB1	CG13390	0.716432732607741	0.582478813278281	0.994766463583882	pseudobulk	DESeq2	LRT	0.234719866771764
784	KC_AB1	CG13392	1.40357679012009	0.447970528345062	0.994766463583882	pseudobulk	DESeq2	LRT	0.348750556979833
784	KC_AB1	CG13398	1.26605443375722	0.392391959350221	0.994766463583882	pseudobulk	DESeq2	LRT	0.406279900490885
784	KC_AB1	CG13404	-0.0619853848171717	0.881001651025613	0.994766463583882	pseudobulk	DESeq2	LRT	0.0550232777052717
785	KC_AB1	CG13409	0.531010915867871	0.538558956566895	0.994766463583882	pseudobulk	DESeq2	LRT	0.268766747128956
785	KC_AB1	CG13436	2.02670873290438	0.538959414925803	0.994766463583882	pseudobulk	DESeq2	LRT	0.26844393710898
785	KC_AB1	CG13437	0.949183608371002	0.80156895525114	0.994766463583882	pseudobulk	DESeq2	LRT	0.0960591113673091
785	KC_AB1	CG1344	-0.397637054718499	0.61484368437613	0.994766463583882	pseudobulk	DESeq2	LRT	0.211235283642184
785	KC_AB1	CG13482	-2.85724547560282	0.450020141104186	0.994766463583882	pseudobulk	DESeq2	LRT	0.346768048503189
785	KC_AB1	CG13484	0.384012790321975	0.670862351284366	0.994766463583882	pseudobulk	DESeq2	LRT	0.173366579995109
785	KC_AB1	CG13502	0.949183608371002	0.80156895525114	0.994766463583882	pseudobulk	DESeq2	LRT	0.0960591113673091
785	KC_AB1	CG13506	0.0198095881775078	0.907036841748365	0.994766463583882	pseudobulk	DESeq2	LRT	0.0423750725396079
785	KC_AB1	CG13526	-1.64952313825113	0.817565084244375	0.994766463583882	pseudobulk	DESeq2	LRT	0.0874776642296574
785	KC_AB1	CG13527	-1.64952313825113	0.817565084244375	0.994766463583882	pseudobulk	DESeq2	LRT	0.0874776642296574
786	KC_AB1	CG13531	0.47865508106661	0.724948634422197	0.994766463583882	pseudobulk	DESeq2	LRT	0.139692763880427
786	KC_AB1	CG13532	0.352739169695856	0.597593848437988	0.994766463583882	pseudobulk	DESeq2	LRT	0.223593881746853
786	KC_AB1	CG1354	0.0245308382881326	0.907366496039085	0.994766463583882	pseudobulk	DESeq2	LRT	0.0422172608359045
786	KC_AB1	CG13551	-0.150815655204994	0.625068141183631	0.994766463583882	pseudobulk	DESeq2	LRT	0.204072635892823
786	KC_AB1	CG13578	0.385402972194073	0.594769440345545	0.994766463583882	pseudobulk	DESeq2	LRT	0.225651353919841
786	KC_AB1	CG1358	-0.179959100869852	0.758866350448734	0.994766463583882	pseudobulk	DESeq2	LRT	0.119834704175056
786	KC_AB1	CG13585	-0.078567575686854	0.82928910873221	0.994766463583882	pseudobulk	DESeq2	LRT	0.0812940382816873
786	KC_AB1	CG13594	0.949183608371002	0.80156895525114	0.994766463583882	pseudobulk	DESeq2	LRT	0.0960591113673091
786	KC_AB1	CG1360	0.0638212001481997	0.87295346795195	0.994766463583882	pseudobulk	DESeq2	LRT	0.059008905378384
786	KC_AB1	CG13601	1.00576691606216	0.198861552592485	0.994766463583882	pseudobulk	DESeq2	LRT	0.701449174195575
787	KC_AB1	CG13603	0.416491577367903	0.440680134756924	0.994766463583882	pseudobulk	DESeq2	LRT	0.355876526435625
787	KC_AB1	CG13604	-0.336278648946255	0.843068416031181	0.994766463583882	pseudobulk	DESeq2	LRT	0.0741371804177586
787	KC_AB1	CG13605	0.326846083165676	0.730194151541334	0.994766463583882	pseudobulk	DESeq2	LRT	0.136561649835344
787	KC_AB1	CG13609	-1.19096394013377	0.442698996416221	0.994766463583882	pseudobulk	DESeq2	LRT	0.353891462551063
787	KC_AB1	CG13623	0.0990470535433412	0.882581639313022	0.994766463583882	pseudobulk	DESeq2	LRT	0.0542451115908206
787	KC_AB1	CG13625	0.140767975224652	0.862636632557569	0.994766463583882	pseudobulk	DESeq2	LRT	0.0641721031344164
787	KC_AB1	CG13630	-0.420673485441175	0.672566323460172	0.994766463583882	pseudobulk	DESeq2	LRT	0.172264882340404
787	KC_AB1	CG13658	-1.64952313825113	0.817565084244375	0.994766463583882	pseudobulk	DESeq2	LRT	0.0874776642296574
787	KC_AB1	CG13663	0.194189321002786	0.853447322713856	0.994766463583882	pseudobulk	DESeq2	LRT	0.0688232796584584
787	KC_AB1	CG13667	-1.76643687325323	0.543810819055668	0.994766463583882	pseudobulk	DESeq2	LRT	0.26455215642223
788	KC_AB1	CG13671	-0.283995846067142	0.866480656580972	0.994766463583882	pseudobulk	DESeq2	LRT	0.0622411280844138
788	KC_AB1	CG13679	1.1821590793593	0.719511699510136	0.994766463583882	pseudobulk	DESeq2	LRT	0.142962139890846
788	KC_AB1	CG1368	-1.64952313825113	0.817565084244375	0.994766463583882	pseudobulk	DESeq2	LRT	0.0874776642296574
788	KC_AB1	CG13685	0.949183608371002	0.80156895525114	0.994766463583882	pseudobulk	DESeq2	LRT	0.0960591113673091
788	KC_AB1	CG13689	-1.7842477909154	0.129662761633196	0.994766463583882	pseudobulk	DESeq2	LRT	0.887184732777188
788	KC_AB1	CG1371	0.241680495660871	0.804206431101943	0.994766463583882	pseudobulk	DESeq2	LRT	0.0946324582401721
788	KC_AB1	CG13737	0.0459001822316226	0.860647230539266	0.994766463583882	pseudobulk	DESeq2	LRT	0.0651748244169036
788	KC_AB1	CG13739	-0.282411655606288	0.0440988723656395	0.994766463583882	pseudobulk	DESeq2	LRT	1.35557251555805
788	KC_AB1	CG13741	-2.20969975456663	0.610836134188693	0.994766463583882	pseudobulk	DESeq2	LRT	0.214075280039667
788	KC_AB1	CG13743	-0.248330330520074	0.886602745911857	0.994766463583882	pseudobulk	DESeq2	LRT	0.0522709279740623
789	KC_AB1	CG13751	-0.166828764771886	0.747823020462175	0.994766463583882	pseudobulk	DESeq2	LRT	0.12620116995766
789	KC_AB1	CG13760	0.2492262002991	0.772404271885897	0.994766463583882	pseudobulk	DESeq2	LRT	0.112155332961853
789	KC_AB1	CG13766	-0.115959968467551	0.787077157338916	0.994766463583882	pseudobulk	DESeq2	LRT	0.103982691574825
789	KC_AB1	CG13773	1.00681093575455	0.468341819669546	0.994766463583882	pseudobulk	DESeq2	LRT	0.329437061007537
789	KC_AB1	CG13775	-2.57435989741145	0.533617212681275	0.994766463583882	pseudobulk	DESeq2	LRT	0.272770169998685

789	KC_AB1	CG13784	-0.0971292974143062	0.725145837008506	0.994766463583882	pseudobulk	DESeq2	LRT	0.139574641927318
789	KC_AB1	CG13807	0.244076379320777	0.764334188369379	0.994766463583882	pseudobulk	DESeq2	LRT	0.116716714158199
789	KC_AB1	CG13827	2.64699301431894	0.23725229373035	0.994766463583882	pseudobulk	DESeq2	LRT	0.624789580166765
789	KC_AB1	CG13841	1.80633728195307	0.61161102402172	0.994766463583882	pseudobulk	DESeq2	LRT	0.213524695220716
789	KC_AB1	CG13847	-2.57435989741145	0.533617212681275	0.994766463583882	pseudobulk	DESeq2	LRT	0.272770169998685
790	KC_AB1	CG13850	0.147761474645906	0.881555877378413	0.994766463583882	pseudobulk	DESeq2	LRT	0.0547501547566015
790	KC_AB1	CG13868	-0.295915694731787	0.202159627322036	0.994766463583882	pseudobulk	DESeq2	LRT	0.694305571702173
790	KC_AB1	CG13876	0.430497080411022	0.784250238304693	0.994766463583882	pseudobulk	DESeq2	LRT	0.105545340664138
790	KC_AB1	CG13887	0.329747038377432	0.54094833147839	0.994766463583882	pseudobulk	DESeq2	LRT	0.266844214422882
790	KC_AB1	CG13893	-0.359317276082011	0.932121454008898	0.994766463583882	pseudobulk	DESeq2	LRT	0.0305274960480511
790	KC_AB1	CG13895	-0.506163934170691	0.781042226318591	0.994766463583882	pseudobulk	DESeq2	LRT	0.107325485762373
790	KC_AB1	CG13896	1.45682795759908	0.573003993665791	0.994766463583882	pseudobulk	DESeq2	LRT	0.24184235111992
790	KC_AB1	CG13898	-2.51616293026258	0.362288974826161	0.994766463583882	pseudobulk	DESeq2	LRT	0.440944882208953
790	KC_AB1	CG13901	-0.2150306882027	0.841246640771435	0.994766463583882	pseudobulk	DESeq2	LRT	0.075076656969859
790	KC_AB1	CG13907	0.0695617420854097	0.905812947804246	0.994766463583882	pseudobulk	DESeq2	LRT	0.0429614757487256
791	KC_AB1	CG13917	-0.260934036485914	0.186329229996747	0.994766463583882	pseudobulk	DESeq2	LRT	0.729719010747688
791	KC_AB1	CG13919	0.248075452245275	0.816643301641944	0.994766463583882	pseudobulk	DESeq2	LRT	0.0879675957997844
791	KC_AB1	CG13920	0.18671816694137	0.590331752828158	0.994766463583882	pseudobulk	DESeq2	LRT	0.228903856271985
791	KC_AB1	CG13921	0.408412788768571	0.225991052764012	0.994766463583882	pseudobulk	DESeq2	LRT	0.645908754711613
791	KC_AB1	CG13926	0.0801954384036602	0.912766443017187	0.994766463583882	pseudobulk	DESeq2	LRT	0.0396403347164348
791	KC_AB1	CG13928	-0.00819630460454192	0.904703406189831	0.994766463583882	pseudobulk	DESeq2	LRT	0.0434937745703776
791	KC_AB1	CG13930	-1.64952313825113	0.817565084244375	0.994766463583882	pseudobulk	DESeq2	LRT	0.0874776642296574
791	KC_AB1	CG13933	0.345329747872953	0.650933676149336	0.994766463583882	pseudobulk	DESeq2	LRT	0.186463259588092
791	KC_AB1	CG13937	0.450776450591431	0.611039861580682	0.994766463583882	pseudobulk	DESeq2	LRT	0.213930457351801
791	KC_AB1	CG1394	-1.64952313825113	0.817565084244375	0.994766463583882	pseudobulk	DESeq2	LRT	0.0874776642296574
792	KC_AB1	CG13954	-0.257489378016953	0.377505929322013	0.994766463583882	pseudobulk	DESeq2	LRT	0.423076222706027
792	KC_AB1	CG13962	0.596808856322697	0.822761281772702	0.994766463583882	pseudobulk	DESeq2	LRT	0.0847261539098712
792	KC_AB1	CG13982	-0.937697975730141	0.620877627895693	0.994766463583882	pseudobulk	DESeq2	LRT	0.206993988819658
792	KC_AB1	CG13994	-0.0598153074768417	0.916431409674065	0.994766463583882	pseudobulk	DESeq2	LRT	0.0379000342640059
792	KC_AB1	CG13995	-0.884041689005267	0.497586656257817	0.994766463583882	pseudobulk	DESeq2	LRT	0.303131274602006
792	KC_AB1	CG13999	-0.450464877859995	0.342208956754062	0.994766463583882	pseudobulk	DESeq2	LRT	0.465708627705617
792	KC_AB1	CG14005	-0.176897224128947	0.84129482236813	0.994766463583882	pseudobulk	DESeq2	LRT	0.0750517838810965
792	KC_AB1	CG14024	0.206842372190013	0.654779782120327	0.994766463583882	pseudobulk	DESeq2	LRT	0.183904738925851
792	KC_AB1	CG14043	-0.417131384665412	0.704760456703345	0.994766463583882	pseudobulk	DESeq2	LRT	0.15195847167506
792	KC_AB1	CG14044	-0.438934359445006	0.783821221522672	0.994766463583882	pseudobulk	DESeq2	LRT	0.105782982417717
793	KC_AB1	CG14050	0.949183608371002	0.80156895525114	0.994766463583882	pseudobulk	DESeq2	LRT	0.0960591113673091
793	KC_AB1	CG14052	2.22023857837763	0.524904842126482	0.994766463583882	pseudobulk	DESeq2	LRT	0.279919420946639
793	KC_AB1	CG14053	2.02670873290438	0.538959414925803	0.994766463583882	pseudobulk	DESeq2	LRT	0.26844393710898
793	KC_AB1	CG14054	0.949183608371002	0.80156895525114	0.994766463583882	pseudobulk	DESeq2	LRT	0.0960591113673091
793	KC_AB1	CG14057	0.446619822572333	0.788218561685415	0.994766463583882	pseudobulk	DESeq2	LRT	0.103353342189734
793	KC_AB1	CG1407	0.134752215625706	0.831773596629616	0.994766463583882	pseudobulk	DESeq2	LRT	0.0799948697610083
793	KC_AB1	CG14073	0.177769558046913	0.617277739500678	0.994766463583882	pseudobulk	DESeq2	LRT	0.209519384439559
793	KC_AB1	CG14074	-0.010772370266124	0.952366370125031	0.994766463583882	pseudobulk	DESeq2	LRT	0.0211959487637905
793	KC_AB1	CG14075	-1.64952313825113	0.817565084244375	0.994766463583882	pseudobulk	DESeq2	LRT	0.0874776642296574
793	KC_AB1	CG14082	0.052836682251685	0.942671388066353	0.994766463583882	pseudobulk	DESeq2	LRT	0.0256396744116015
794	KC_AB1	CG14100	0.388241410417166	0.690851043294912	0.994766463583882	pseudobulk	DESeq2	LRT	0.160615582218697
794	KC_AB1	CG14102	-0.729024381689453	0.602472956983519	0.994766463583882	pseudobulk	DESeq2	LRT	0.220062442357105
794	KC_AB1	CG14104	-1.08781805700249	0.355240915470551	0.994766463583882	pseudobulk	DESeq2	LRT	0.449477019414447
794	KC_AB1	CG14115	-1.64952313825113	0.817565084244375	0.994766463583882	pseudobulk	DESeq2	LRT	0.0874776642296574
794	KC_AB1	CG14130	0.501597265419405	0.670101750733099	0.994766463583882	pseudobulk	DESeq2	LRT	0.173859247408465
794	KC_AB1	CG14135	-0.18741882345789	0.802809828996729	0.994766463583882	pseudobulk	DESeq2	LRT	0.0953873189786345
794	KC_AB1	CG14137	0.949183608371002	0.80156895525114	0.994766463583882	pseudobulk	DESeq2	LRT	0.0960591113673091
794	KC_AB1	CG14142	-1.89157349400865	0.516179879210422	0.994766463583882	pseudobulk	DESeq2	LRT	0.287198928343475
794	KC_AB1	CG14153	1.80633728195307	0.61161102402172	0.994766463583882	pseudobulk	DESeq2	LRT	0.213524695220716
794	KC_AB1	CG1416	-0.0708946842212119	0.827193703820714	0.994766463583882	pseudobulk	DESeq2	LRT	0.0823927798670664
795	KC_AB1	CG14174	-0.183835980893786	0.825661259795592	0.994766463583882	pseudobulk	DESeq2	LRT	0.0831980921168219

795	KC_AB1	CG1418	-0.0209961905372902	0.945650356752517	0.994766463583882	pseudobulk	DESeq2	LRT	0.0242694092658605
795	KC_AB1	CG14182	0.325783305173304	0.773808416070257	0.994766463583882	pseudobulk	DESeq2	LRT	0.111366551129383
795	KC_AB1	CG14183	-2.03786985328338	0.468221401672057	0.994766463583882	pseudobulk	DESeq2	LRT	0.329548739260383
795	KC_AB1	CG14184	0.0621805956502849	0.917717151132543	0.994766463583882	pseudobulk	DESeq2	LRT	0.0372911517296593
795	KC_AB1	CG14185	1.17930484172975	0.669685881755347	0.994766463583882	pseudobulk	DESeq2	LRT	0.174128856716169
795	KC_AB1	CG14186	-0.291710037870592	0.197712886414277	0.994766463583882	pseudobulk	DESeq2	LRT	0.703965023573403
795	KC_AB1	CG14190	1.32710828904082	0.607473293222376	0.994766463583882	pseudobulk	DESeq2	LRT	0.216472810505135
795	KC_AB1	CG14194	-0.17224351378577	0.775966468508572	0.994766463583882	pseudobulk	DESeq2	LRT	0.110157045309254
795	KC_AB1	CG14200	0.46265075284243	0.373109890849702	0.994766463583882	pseudobulk	DESeq2	LRT	0.428163238001491
796	KC_AB1	CG14207	0.412535562619188	0.390355219986439	0.994766463583882	pseudobulk	DESeq2	LRT	0.408540008700604
796	KC_AB1	CG14210	-0.766518663766329	0.49479892435644	0.994766463583882	pseudobulk	DESeq2	LRT	0.305571253154822
796	KC_AB1	CG14212	-1.64952313825113	0.817565084244375	0.994766463583882	pseudobulk	DESeq2	LRT	0.0874776642296574
796	KC_AB1	CG14220	-1.56023403232557	0.259270718352209	0.994766463583882	pseudobulk	DESeq2	LRT	0.586246529031423
796	KC_AB1	CG14223	1.1821590793593	0.719511699510136	0.994766463583882	pseudobulk	DESeq2	LRT	0.142962139890846
796	KC_AB1	CG14230	0.395171761365791	0.78362740348029	0.994766463583882	pseudobulk	DESeq2	LRT	0.105890385117092
796	KC_AB1	CG14231	-0.296785582853297	0.873388629927457	0.994766463583882	pseudobulk	DESeq2	LRT	0.0587924661722998
796	KC_AB1	CG14232	0.172737032923137	0.813589612349891	0.994766463583882	pseudobulk	DESeq2	LRT	0.0895946049819707
796	KC_AB1	CG14234	-0.0362503030218479	0.895888451873161	0.994766463583882	pseudobulk	DESeq2	LRT	0.0477460614894185
796	KC_AB1	CG14252	0.343314893283387	0.767874352452666	0.994766463583882	pseudobulk	DESeq2	LRT	0.114709837912136
797	KC_AB1	CG14265	1.1821590793593	0.719511699510136	0.994766463583882	pseudobulk	DESeq2	LRT	0.142962139890846
797	KC_AB1	CG14270	0.452445497863558	0.586537010840464	0.994766463583882	pseudobulk	DESeq2	LRT	0.23170457843908
797	KC_AB1	CG14285	0.949183608371002	0.80156895525114	0.994766463583882	pseudobulk	DESeq2	LRT	0.0960591113673091
797	KC_AB1	CG14286	0.249918213573965	0.860233352807472	0.994766463583882	pseudobulk	DESeq2	LRT	0.0653837230706577
797	KC_AB1	CG14302	0.949183608371002	0.80156895525114	0.994766463583882	pseudobulk	DESeq2	LRT	0.0960591113673091
797	KC_AB1	CG14304	-1.28132067551389	0.647201349541384	0.994766463583882	pseudobulk	DESeq2	LRT	0.188960585805912
797	KC_AB1	CG14312	0.188232968332473	0.639319321871535	0.994766463583882	pseudobulk	DESeq2	LRT	0.194282169873375
797	KC_AB1	CG14314	-0.722346277300025	0.535907392528994	0.994766463583882	pseudobulk	DESeq2	LRT	0.270910252076736
797	KC_AB1	CG14321	-0.306671396599424	0.581083197936561	0.994766463583882	pseudobulk	DESeq2	LRT	0.235761682036588
797	KC_AB1	CG14322	0.09563090553882	0.898001607009681	0.994766463583882	pseudobulk	DESeq2	LRT	0.0467228861447085
798	KC_AB1	CG1434	-0.29408219783147	0.915376953506099	0.994766463583882	pseudobulk	DESeq2	LRT	0.0384000260261822
798	KC_AB1	CG14340	0.825619721188946	0.469631868573349	0.994766463583882	pseudobulk	DESeq2	LRT	0.328242440133637
798	KC_AB1	CG14341	0.681891121783382	0.375218100941654	0.994766463583882	pseudobulk	DESeq2	LRT	0.425716218935139
798	KC_AB1	CG14352	1.28641256445967	0.548214402092837	0.994766463583882	pseudobulk	DESeq2	LRT	0.261049559334121
798	KC_AB1	CG14356	0.949183608371002	0.80156895525114	0.994766463583882	pseudobulk	DESeq2	LRT	0.0960591113673091
798	KC_AB1	CG1440	-0.672168535368103	0.382949460114785	0.994766463583882	pseudobulk	DESeq2	LRT	0.416858538411475
798	KC_AB1	CG14407	0.0935656183636234	0.82756774096598	0.994766463583882	pseudobulk	DESeq2	LRT	0.0821964467079948
798	KC_AB1	CG14408	-0.181600003695323	0.523653068532784	0.994766463583882	pseudobulk	DESeq2	LRT	0.280956347218556
798	KC_AB1	CG14410	1.1821590793593	0.719511699510136	0.994766463583882	pseudobulk	DESeq2	LRT	0.142962139890846
798	KC_AB1	CG14411	-0.0154764892967345	0.908913673727322	0.994766463583882	pseudobulk	DESeq2	LRT	0.0414773629872859
799	KC_AB1	CG14414	0.0322274784344778	0.851438796066129	0.994766463583882	pseudobulk	DESeq2	LRT	0.0698465649486751
799	KC_AB1	CG14431	-0.196590971371658	0.589574558323728	0.994766463583882	pseudobulk	DESeq2	LRT	0.22946126568159
799	KC_AB1	CG14434	-0.733700620273093	0.41439913898808	0.994766463583882	pseudobulk	DESeq2	LRT	0.382581155611072
799	KC_AB1	CG14435	0.0270984721652989	0.924021498899297	0.994766463583882	pseudobulk	DESeq2	LRT	0.034317924077824
799	KC_AB1	CG14439	0.949183608371002	0.80156895525114	0.994766463583882	pseudobulk	DESeq2	LRT	0.0960591113673091
799	KC_AB1	CG14440	0.177550047514854	0.643365223996136	0.994766463583882	pseudobulk	DESeq2	LRT	0.19154241784306
799	KC_AB1	CG14441	-2.00354090274141	0.368550329168343	0.994766463583882	pseudobulk	DESeq2	LRT	0.433503196576757
799	KC_AB1	CG14442	-0.319678594052894	0.3830999841163	0.994766463583882	pseudobulk	DESeq2	LRT	0.416687866023196
799	KC_AB1	CG14450	0.374006845449573	0.672737479732848	0.994766463583882	pseudobulk	DESeq2	LRT	0.172154376111267
799	KC_AB1	CG14459	-3.21143241058256	0.292545415061155	0.994766463583882	pseudobulk	DESeq2	LRT	0.533806704011112
800	KC_AB1	CG14464	0.0349895603569306	0.855796471374842	0.994766463583882	pseudobulk	DESeq2	LRT	0.0676295085340144
800	KC_AB1	CG14478	-0.18087940292874	0.461656953164742	0.994766463583882	pseudobulk	DESeq2	LRT	0.335680619004391
800	KC_AB1	CG14483	0.341664293355289	0.57050650706791	0.994766463583882	pseudobulk	DESeq2	LRT	0.243739397753093
800	KC_AB1	CG14491	0.949183608371002	0.80156895525114	0.994766463583882	pseudobulk	DESeq2	LRT	0.0960591113673091
800	KC_AB1	CG14502	-0.0713915988297819	0.864170454131802	0.994766463583882	pseudobulk	DESeq2	LRT	0.063400586239177
800	KC_AB1	CG14505	-1.02319198676748	0.828204539793902	0.994766463583882	pseudobulk	DESeq2	LRT	0.0818623932467813
800	KC_AB1	CG14511	1.1821590793593	0.719511699510136	0.994766463583882	pseudobulk	DESeq2	LRT	0.142962139890846

800	KC_AB1	CG14512	-1.87227586326617	0.401547731174294	0.994766463583882	pseudobulk	DESeq2	LRT	0.396262823601909
800	KC_AB1	CG14535	-0.241813998693852	0.725131369443316	0.994766463583882	pseudobulk	DESeq2	LRT	0.139583306731123
800	KC_AB1	CG14543	-0.253018658104001	0.809551850076416	0.994766463583882	pseudobulk	DESeq2	LRT	0.0917553303807286
801	KC_AB1	CG14544	0.949183608371002	0.80156895525114	0.994766463583882	pseudobulk	DESeq2	LRT	0.0960591113673091
801	KC_AB1	CG14561	-1.02319198676748	0.828204539793902	0.994766463583882	pseudobulk	DESeq2	LRT	0.0818623932467813
801	KC_AB1	CG14562	0.0666039920717408	0.887805397850909	0.994766463583882	pseudobulk	DESeq2	LRT	0.0516822187950662
801	KC_AB1	CG14563	-4.07488895553346	0.165815616496965	0.994766463583882	pseudobulk	DESeq2	LRT	0.780374570051866
801	KC_AB1	CG14589	-1.23494028045745	0.399351044424667	0.994766463583882	pseudobulk	DESeq2	LRT	0.398645175417621
801	KC_AB1	CG14591	-1.02630534586162	0.184004504143845	0.994766463583882	pseudobulk	DESeq2	LRT	0.735171546007443
801	KC_AB1	CG1461	-1.32806228432949	0.722858830184137	0.994766463583882	pseudobulk	DESeq2	LRT	0.140946509425123
801	KC_AB1	CG14613	0.09563090553882	0.898001607009681	0.994766463583882	pseudobulk	DESeq2	LRT	0.0467228861447085
801	KC_AB1	CG14621	-0.178499321689512	0.841135179021375	0.994766463583882	pseudobulk	DESeq2	LRT	0.0751342030304451
801	KC_AB1	CG1463	0.133558194710007	0.806235751280753	0.994766463583882	pseudobulk	DESeq2	LRT	0.0935379476370789
802	KC_AB1	CG14641	-0.31286555203484	0.51110150523841	0.994766463583882	pseudobulk	DESeq2	LRT	0.291492840007837
802	KC_AB1	CG14646	-0.211600225077898	0.83519068387892	0.994766463583882	pseudobulk	DESeq2	LRT	0.078214358645624
802	KC_AB1	CG14647	0.208517080992539	0.755611502066407	0.994766463583882	pseudobulk	DESeq2	LRT	0.121701439784149
802	KC_AB1	CG14650	0.201626399862517	0.791382879145629	0.994766463583882	pseudobulk	DESeq2	LRT	0.101613349537527
802	KC_AB1	CG14651	0.949183608371002	0.80156895525114	0.994766463583882	pseudobulk	DESeq2	LRT	0.0960591113673091
802	KC_AB1	CG14657	0.181932790079673	0.748620142923295	0.994766463583882	pseudobulk	DESeq2	LRT	0.125738491618143
802	KC_AB1	CG14669	0.139174215264806	0.752126895722161	0.994766463583882	pseudobulk	DESeq2	LRT	0.123708880867566
802	KC_AB1	CG14671	0.464251906825069	0.605281083178372	0.994766463583882	pseudobulk	DESeq2	LRT	0.218042898860661
802	KC_AB1	CG14683	-0.3201594580905	0.805407551446268	0.994766463583882	pseudobulk	DESeq2	LRT	0.0939843027940575
802	KC_AB1	CG14692	-2.5283900847069	0.34947608531242	0.994766463583882	pseudobulk	DESeq2	LRT	0.456582537721455
803	KC_AB1	CG14694	-2.57435989741145	0.533617212681275	0.994766463583882	pseudobulk	DESeq2	LRT	0.272770169998685
803	KC_AB1	CG14701	0.920827557517415	0.400409407244051	0.994766463583882	pseudobulk	DESeq2	LRT	0.39749572773071
803	KC_AB1	CG14710	1.1251734606953	0.394633776558227	0.994766463583882	pseudobulk	DESeq2	LRT	0.403805746389753
803	KC_AB1	CG14711	0.685402888891639	0.537377275497562	0.994766463583882	pseudobulk	DESeq2	LRT	0.269720702872485
803	KC_AB1	CG14712	0.519430482521704	0.787642774112072	0.994766463583882	pseudobulk	DESeq2	LRT	0.103670706885852
803	KC_AB1	CG14715	-0.231389743566286	0.715514963674997	0.994766463583882	pseudobulk	DESeq2	LRT	0.145381279341625
803	KC_AB1	CG14721	-0.0449146850148172	0.956534360073558	0.994766463583882	pseudobulk	DESeq2	LRT	0.0192994248815113
803	KC_AB1	CG14722	-0.160094784926984	0.866082699624827	0.994766463583882	pseudobulk	DESeq2	LRT	0.0624406365324127
803	KC_AB1	CG14757	-0.313829585708233	0.670155724353564	0.994766463583882	pseudobulk	DESeq2	LRT	0.173824268389904
803	KC_AB1	CG14762	-0.198801708550115	0.328180110480871	0.994766463583882	pseudobulk	DESeq2	LRT	0.483887743118856
804	KC_AB1	CG14763	-1.64952313825113	0.817565084244375	0.994766463583882	pseudobulk	DESeq2	LRT	0.0874776642296574
804	KC_AB1	CG14764	0.301657379081337	0.383936535138703	0.994766463583882	pseudobulk	DESeq2	LRT	0.415740558749583
804	KC_AB1	CG14767	-0.418635593435991	0.185692653554763	0.994766463583882	pseudobulk	DESeq2	LRT	0.731205277648155
804	KC_AB1	CG14777	0.298758735725836	0.622166630625534	0.994766463583882	pseudobulk	DESeq2	LRT	0.206093285615334
804	KC_AB1	CG14795	-2.57435989741145	0.533617212681275	0.994766463583882	pseudobulk	DESeq2	LRT	0.272770169998685
804	KC_AB1	CG14797	0.329367741436991	0.858058210994256	0.994766463583882	pseudobulk	DESeq2	LRT	0.06648324844961
804	KC_AB1	CG14803	-1.64952313825113	0.817565084244375	0.994766463583882	pseudobulk	DESeq2	LRT	0.0874776642296574
804	KC_AB1	CG14805	-0.844492860961967	0.392568446519391	0.994766463583882	pseudobulk	DESeq2	LRT	0.40608461062807
804	KC_AB1	CG14806	0.48392964208978	0.377435200607135	0.994766463583882	pseudobulk	DESeq2	LRT	0.423157598827835
804	KC_AB1	CG14810	-1.64952313825113	0.817565084244375	0.994766463583882	pseudobulk	DESeq2	LRT	0.0874776642296574
805	KC_AB1	CG14811	-0.445400199890768	0.799414335150906	0.994766463583882	pseudobulk	DESeq2	LRT	0.0972280682086188
805	KC_AB1	CG14812	-0.154932527333082	0.791082582310327	0.994766463583882	pseudobulk	DESeq2	LRT	0.101778177476268
805	KC_AB1	CG14817	-0.139990811534792	0.724405332663845	0.994766463583882	pseudobulk	DESeq2	LRT	0.140018361314968
805	KC_AB1	CG14818	0.178965828888481	0.569301715208034	0.994766463583882	pseudobulk	DESeq2	LRT	0.244657507731993
805	KC_AB1	CG14830	-1.32806228432949	0.722858830184137	0.994766463583882	pseudobulk	DESeq2	LRT	0.140946509425123
805	KC_AB1	CG14851	1.1821590793593	0.719511699510136	0.994766463583882	pseudobulk	DESeq2	LRT	0.142962139890846
805	KC_AB1	CG14855	0.949183608371002	0.80156895525114	0.994766463583882	pseudobulk	DESeq2	LRT	0.0960591113673091
805	KC_AB1	CG14857	-1.28132067551389	0.647201349541384	0.994766463583882	pseudobulk	DESeq2	LRT	0.188960585805912
805	KC_AB1	CG1486	-0.42100770898643	0.576070621077736	0.994766463583882	pseudobulk	DESeq2	LRT	0.239524272715094
805	KC_AB1	CG14879	-2.57435989741145	0.533617212681275	0.994766463583882	pseudobulk	DESeq2	LRT	0.272770169998685
806	KC_AB1	CG14881	0.909783462033953	0.517149000377203	0.994766463583882	pseudobulk	DESeq2	LRT	0.286384310448352
806	KC_AB1	CG14882	1.49975934979285	0.31265558458276	0.994766463583882	pseudobulk	DESeq2	LRT	0.504933809645025
806	KC_AB1	CG14883	0.20380763690647	0.769753311873592	0.994766463583882	pseudobulk	DESeq2	LRT	0.113648433863112

806	KC_AB1	CG14894	-0.740499805771023	0.257387644079578	0.994766463583882	pseudobulk	DESeq2	LRT	0.589412305282666
806	KC_AB1	CG14903	0.178168646619862	0.691101110715777	0.994766463583882	pseudobulk	DESeq2	LRT	0.160458409046159
806	KC_AB1	CG14906	1.18486022580081	0.528345814397098	0.994766463583882	pseudobulk	DESeq2	LRT	0.27708172874678
806	KC_AB1	CG1492	0.495223744435745	0.69543031795795	0.994766463583882	pseudobulk	DESeq2	LRT	0.157746379751662
806	KC_AB1	CG14965	-1.66185081699538	0.280585023753568	0.994766463583882	pseudobulk	DESeq2	LRT	0.551935513189199
806	KC_AB1	CG14966	0.0173333370322636	0.930447670788901	0.994766463583882	pseudobulk	DESeq2	LRT	0.0313080469573776
806	KC_AB1	CG14969	-0.091808020000748	0.911853162853857	0.994766463583882	pseudobulk	DESeq2	LRT	0.0400750911634365
807	KC_AB1	CG14971	0.28360361599904	0.760384391104353	0.994766463583882	pseudobulk	DESeq2	LRT	0.118966806755075
807	KC_AB1	CG14977	0.421193072718432	0.570657141543245	0.994766463583882	pseudobulk	DESeq2	LRT	0.24362474334418
807	KC_AB1	CG14982	0.346480313935965	0.461040259615924	0.994766463583882	pseudobulk	DESeq2	LRT	0.336261148871495
807	KC_AB1	CG14984	-0.627447798185778	0.772628937945737	0.994766463583882	pseudobulk	DESeq2	LRT	0.112029029876423
807	KC_AB1	CG14985	-4.2169236809797	0.105189937930809	0.994766463583882	pseudobulk	DESeq2	LRT	0.978025801153034
807	KC_AB1	CG14989	2.38929733117871	0.484296857543297	0.994766463583882	pseudobulk	DESeq2	LRT	0.314888348950639
807	KC_AB1	CG14995	0.0314794088953191	0.878740444743609	0.994766463583882	pseudobulk	DESeq2	LRT	0.0561393843820039
807	KC_AB1	CG14997	1.56105609375518	0.376364042628432	0.994766463583882	pseudobulk	DESeq2	LRT	0.424391875239476
807	KC_AB1	CG15011	-0.437773119282954	0.719276925622344	0.994766463583882	pseudobulk	DESeq2	LRT	0.143103871626957
807	KC_AB1	CG15012	-0.0612407075512472	0.888979820606974	0.994766463583882	pseudobulk	DESeq2	LRT	0.0511080971833357
808	KC_AB1	CG15014	-0.406402897342317	0.776524553116885	0.994766463583882	pseudobulk	DESeq2	LRT	0.109844807656489
808	KC_AB1	CG15019	0.518337558007439	0.469981892367706	0.994766463583882	pseudobulk	DESeq2	LRT	0.327918874396789
808	KC_AB1	CG15027	0.570880400828228	0.578416955998652	0.994766463583882	pseudobulk	DESeq2	LRT	0.237758984405237
808	KC_AB1	CG1504	0.53983785550047	0.806887399502234	0.994766463583882	pseudobulk	DESeq2	LRT	0.0931870665007086
808	KC_AB1	CG15040	-0.835152883801839	0.920434724857442	0.994766463583882	pseudobulk	DESeq2	LRT	0.0360070052473159
808	KC_AB1	CG15047	-2.79499059556516	0.38564607643653	0.994766463583882	pseudobulk	DESeq2	LRT	0.413811082791365
808	KC_AB1	CG15073	0.660583441210052	0.569389731492225	0.994766463583882	pseudobulk	DESeq2	LRT	0.244590369286844
808	KC_AB1	CG15084	-0.255006937147076	0.88671163122832	0.994766463583882	pseudobulk	DESeq2	LRT	0.0522175947436219
808	KC_AB1	CG15098	-0.13900390497042	0.862095923403941	0.994766463583882	pseudobulk	DESeq2	LRT	0.0644444085519917
808	KC_AB1	CG15099	-0.170875614379435	0.755650684518699	0.994766463583882	pseudobulk	DESeq2	LRT	0.12167891990246
809	KC_AB1	CG15107	0.311348489636432	0.761414414093696	0.994766463583882	pseudobulk	DESeq2	LRT	0.118378905953356
809	KC_AB1	CG15111	-0.523223933586497	0.520785511010521	0.994766463583882	pseudobulk	DESeq2	LRT	0.283341106958511
809	KC_AB1	CG15117	-1.64952313825113	0.817565084244375	0.994766463583882	pseudobulk	DESeq2	LRT	0.0874776642296574
809	KC_AB1	CG15118	-0.543303596127532	0.49986877265457	0.994766463583882	pseudobulk	DESeq2	LRT	0.301143993248226
809	KC_AB1	CG1513	0.150975084891721	0.652236770027953	0.994766463583882	pseudobulk	DESeq2	LRT	0.185594721368605
809	KC_AB1	CG15141	0.565592335270997	0.463115451472682	0.994766463583882	pseudobulk	DESeq2	LRT	0.33431072888005
809	KC_AB1	CG15160	-0.161154311565791	0.80639320014935	0.994766463583882	pseudobulk	DESeq2	LRT	0.093453143039009
809	KC_AB1	CG15168	0.088345264169525	0.860451140525004	0.994766463583882	pseudobulk	DESeq2	LRT	0.0652737854210516
809	KC_AB1	CG15196	1.1821590793593	0.719511699510136	0.994766463583882	pseudobulk	DESeq2	LRT	0.142962139890846
809	KC_AB1	CG15201	-0.648218248148942	0.834000364039372	0.994766463583882	pseudobulk	DESeq2	LRT	0.0788337597936092
810	KC_AB1	CG15209	-1.28132067551389	0.647201349541384	0.994766463583882	pseudobulk	DESeq2	LRT	0.188960585805912
810	KC_AB1	CG1523	-0.0133237703396228	0.928039121993052	0.994766463583882	pseudobulk	DESeq2	LRT	0.0324337154755588
810	KC_AB1	CG15236	-0.528418652606273	0.661951044924384	0.994766463583882	pseudobulk	DESeq2	LRT	0.179174127940006
810	KC_AB1	CG15237	0.0376675661600145	0.926958257404926	0.994766463583882	pseudobulk	DESeq2	LRT	0.0329398224756497
810	KC_AB1	CG15239	0.949183608371002	0.80156895525114	0.994766463583882	pseudobulk	DESeq2	LRT	0.0960591113673091
810	KC_AB1	CG15256	-2.79499059556516	0.38564607643653	0.994766463583882	pseudobulk	DESeq2	LRT	0.413811082791365
810	KC_AB1	CG15269	-1.64952313825113	0.817565084244375	0.994766463583882	pseudobulk	DESeq2	LRT	0.0874776642296574
810	KC_AB1	CG15270	0.360638310960988	0.644519603134525	0.994766463583882	pseudobulk	DESeq2	LRT	0.190763868995324
810	KC_AB1	CG1529	-3.95857014146861	0.177445975557202	0.994766463583882	pseudobulk	DESeq2	LRT	0.750933845925402
810	KC_AB1	CG15293	-2.20969975456663	0.610836134188693	0.994766463583882	pseudobulk	DESeq2	LRT	0.214075280039667
811	KC_AB1	CG15309	0.473926715647477	0.359903569451251	0.994766463583882	pseudobulk	DESeq2	LRT	0.443813846080363
811	KC_AB1	CG15312	0.651437832448134	0.245243383145856	0.994766463583882	pseudobulk	DESeq2	LRT	0.610402701387397
811	KC_AB1	CG15317	1.26433463569552	0.528978780772322	0.994766463583882	pseudobulk	DESeq2	LRT	0.276561748717781
811	KC_AB1	CG1532	-0.900804202414214	0.326193434700147	0.994766463583882	pseudobulk	DESeq2	LRT	0.48652478425893
811	KC_AB1	CG15332	-1.64952313825113	0.817565084244375	0.994766463583882	pseudobulk	DESeq2	LRT	0.0874776642296574
811	KC_AB1	CG15343	0.0551104605447623	0.907528926819053	0.994766463583882	pseudobulk	DESeq2	LRT	0.0421395232530871
811	KC_AB1	CG15356	-0.190233268832309	0.797143101808394	0.994766463583882	pseudobulk	DESeq2	LRT	0.0984637077795214
811	KC_AB1	CG15365	-2.20969975456663	0.610836134188693	0.994766463583882	pseudobulk	DESeq2	LRT	0.214075280039667
811	KC_AB1	CG1537	-2.79499059556516	0.38564607643653	0.994766463583882	pseudobulk	DESeq2	LRT	0.413811082791365

811	KC_AB1	CG15382	-2.20969975456663	0.610836134188693	0.994766463583882	pseudobulk	DESeq2	LRT	0.214075280039667
812	KC_AB1	CG15385	0.0726318076352171	0.832993019310276	0.994766463583882	pseudobulk	DESeq2	LRT	0.079358638074281
812	KC_AB1	CG15387	0.0939466549969875	0.928743713845331	0.994766463583882	pseudobulk	DESeq2	LRT	0.0321041127215691
812	KC_AB1	CG15390	-0.793793566592106	0.526072810805777	0.994766463583882	pseudobulk	DESeq2	LRT	0.278954143407468
812	KC_AB1	CG15399	0.949183608371002	0.80156895525114	0.994766463583882	pseudobulk	DESeq2	LRT	0.0960591113673091
812	KC_AB1	CG1542	-0.411650495332536	0.745257748210929	0.994766463583882	pseudobulk	DESeq2	LRT	0.127693500048423
812	KC_AB1	CG15431	-0.089951088245069	0.721332015065701	0.994766463583882	pseudobulk	DESeq2	LRT	0.141864791977665
812	KC_AB1	CG15432	0.263084536557237	0.718049792314551	0.994766463583882	pseudobulk	DESeq2	LRT	0.143845439075708
812	KC_AB1	CG15435	1.80939591345248	0.468117841155507	0.994766463583882	pseudobulk	DESeq2	LRT	0.329644806495434
812	KC_AB1	CG15436	-3.21143241058256	0.292545415061155	0.994766463583882	pseudobulk	DESeq2	LRT	0.533806704011112
812	KC_AB1	CG15439	-1.48483364312782	0.358246894519163	0.994766463583882	pseudobulk	DESeq2	LRT	0.445817565617391
813	KC_AB1	CG15443	0.111159299822098	0.872250681310256	0.994766463583882	pseudobulk	DESeq2	LRT	0.0593586826546651
813	KC_AB1	CG15445	0.651806411443827	0.700247618684875	0.994766463583882	pseudobulk	DESeq2	LRT	0.154748359396614
813	KC_AB1	CG15446	1.45682795759908	0.573003993665791	0.994766463583882	pseudobulk	DESeq2	LRT	0.24184235111992
813	KC_AB1	CG15449	-0.336590347790864	0.672654397883786	0.994766463583882	pseudobulk	DESeq2	LRT	0.172208013998101
813	KC_AB1	CG15450	0.949183608371002	0.80156895525114	0.994766463583882	pseudobulk	DESeq2	LRT	0.0960591113673091
813	KC_AB1	CG15456	0.168282001975692	0.772831010210278	0.994766463583882	pseudobulk	DESeq2	LRT	0.111915459971178
813	KC_AB1	CG15459	0.553331303120738	0.235725693114554	0.994766463583882	pseudobulk	DESeq2	LRT	0.627593078613803
813	KC_AB1	CG15514	-0.501941741840406	0.551412723750413	0.994766463583882	pseudobulk	DESeq2	LRT	0.258523216846787
813	KC_AB1	CG1552	-2.66944192095839	0.284729472085893	0.994766463583882	pseudobulk	DESeq2	LRT	0.545567577103783
813	KC_AB1	CG15522	-0.28246076697172	0.779990841548126	0.994766463583882	pseudobulk	DESeq2	LRT	0.107910496653702
814	KC_AB1	CG15525	0.274548288344583	0.791830621490667	0.994766463583882	pseudobulk	DESeq2	LRT	0.101367707323296
814	KC_AB1	CG15535	0.249353714085685	0.68924567310789	0.994766463583882	pseudobulk	DESeq2	LRT	0.161625951593267
814	KC_AB1	CG15536	-0.997712536620101	0.358540977169111	0.994766463583882	pseudobulk	DESeq2	LRT	0.445461202223683
814	KC_AB1	CG15539	1.80633728195307	0.61161102402172	0.994766463583882	pseudobulk	DESeq2	LRT	0.213524695220716
814	KC_AB1	CG15547	0.949183608371002	0.80156895525114	0.994766463583882	pseudobulk	DESeq2	LRT	0.0960591113673091
814	KC_AB1	CG15554	1.1821590793593	0.719511699510136	0.994766463583882	pseudobulk	DESeq2	LRT	0.142962139890846
814	KC_AB1	CG15561	-1.31150738053407	0.344743890205748	0.994766463583882	pseudobulk	DESeq2	LRT	0.462503421956876
814	KC_AB1	CG15579	0.195526510729309	0.835393291548733	0.994766463583882	pseudobulk	DESeq2	LRT	0.0781090165679834
814	KC_AB1	CG15601	0.285707344209802	0.772853973580227	0.994766463583882	pseudobulk	DESeq2	LRT	0.111902555834875
814	KC_AB1	CG15602	-0.555459858615707	0.638466426487005	0.994766463583882	pseudobulk	DESeq2	LRT	0.194861935009474
815	KC_AB1	CG15614	-2.20969975456663	0.610836134188693	0.994766463583882	pseudobulk	DESeq2	LRT	0.214075280039667
815	KC_AB1	CG15628	0.143653843352424	0.53816787122457	0.994766463583882	pseudobulk	DESeq2	LRT	0.269082233302812
815	KC_AB1	CG15631	-3.21143241058256	0.292545415061155	0.994766463583882	pseudobulk	DESeq2	LRT	0.533806704011112
815	KC_AB1	CG15642	-0.143027962583693	0.890517482966978	0.994766463583882	pseudobulk	DESeq2	LRT	0.0503575498879319
815	KC_AB1	CG15643	0.543715369835842	0.671862901610765	0.994766463583882	pseudobulk	DESeq2	LRT	0.172719338774149
815	KC_AB1	CG15651	0.571431537130048	0.479268856933909	0.994766463583882	pseudobulk	DESeq2	LRT	0.319420790706066
815	KC_AB1	CG15653	-1.17855694933581	0.472450577012782	0.994766463583882	pseudobulk	DESeq2	LRT	0.325643616262165
815	KC_AB1	CG15657	-1.64952313825113	0.817565084244375	0.994766463583882	pseudobulk	DESeq2	LRT	0.0874776642296574
815	KC_AB1	CG15674	1.17930484172975	0.669685881755347	0.994766463583882	pseudobulk	DESeq2	LRT	0.174128856716169
815	KC_AB1	CG15706	-2.13955914150295	0.227177081201966	0.994766463583882	pseudobulk	DESeq2	LRT	0.64363548462291
816	KC_AB1	CG15715	0.0401732824372156	0.835909911731294	0.994766463583882	pseudobulk	DESeq2	LRT	0.0778405251282114
816	KC_AB1	CG15719	-2.57435989741145	0.533617212681275	0.994766463583882	pseudobulk	DESeq2	LRT	0.272770169998685
816	KC_AB1	CG1572	0.209946891738838	0.8829622200177	0.994766463583882	pseudobulk	DESeq2	LRT	0.0540578785162677
816	KC_AB1	CG15725	-3.46197803745951	0.239982951034438	0.994766463583882	pseudobulk	DESeq2	LRT	0.619819610516178
816	KC_AB1	CG15743	0.258254127682796	0.74715894590984	0.994766463583882	pseudobulk	DESeq2	LRT	0.126586999270799
816	KC_AB1	CG15744	1.57161582234084	0.52162243080664	0.994766463583882	pseudobulk	DESeq2	LRT	0.282643741344212
816	KC_AB1	CG15747	0.520713841493422	0.588365626369317	0.994766463583882	pseudobulk	DESeq2	LRT	0.230352707654554
816	KC_AB1	CG1575	-0.752935805416951	0.655114189972966	0.994766463583882	pseudobulk	DESeq2	LRT	0.183682993515374
816	KC_AB1	CG15760	0.0467068820823802	0.910435959429397	0.994766463583882	pseudobulk	DESeq2	LRT	0.0407505973095665
816	KC_AB1	CG15765	0.148107526335392	0.45671188929748	0.994766463583882	pseudobulk	DESeq2	LRT	0.340357682526829
817	KC_AB1	CG15771	0.0700921284932173	0.902149413805721	0.994766463583882	pseudobulk	DESeq2	LRT	0.0447215287357217
817	KC_AB1	CG15772	0.121612058303813	0.728831112544031	0.994766463583882	pseudobulk	DESeq2	LRT	0.13737309635643
817	KC_AB1	CG1578	-0.138380177866663	0.735203691666944	0.994766463583882	pseudobulk	DESeq2	LRT	0.133592320900295
817	KC_AB1	CG15803	-0.446390569271649	0.252700607999864	0.994766463583882	pseudobulk	DESeq2	LRT	0.597393713162521
817	KC_AB1	CG15812	-0.165950784372267	0.873991223885494	0.994766463583882	pseudobulk	DESeq2	LRT	0.058492928277769

817	KC_AB1	CG15814	-0.150026246732328	0.799692287526308	0.994766463583882	pseudobulk	DESeq2	LRT	0.0970770924295521
817	KC_AB1	CG1582	0.996571085194043	0.571407718015162	0.994766463583882	pseudobulk	DESeq2	LRT	0.243053897889645
817	KC_AB1	CG15861	1.1821590793593	0.719511699510136	0.994766463583882	pseudobulk	DESeq2	LRT	0.142962139890846
817	KC_AB1	CG15864	-1.21457746603801	0.770013447451371	0.994766463583882	pseudobulk	DESeq2	LRT	0.113501690278259
817	KC_AB1	CG15877	-0.0472521233636037	0.842242078855935	0.994766463583882	pseudobulk	DESeq2	LRT	0.0745630647936626
818	KC_AB1	CG15891	-0.187741377880042	0.716044629218234	0.994766463583882	pseudobulk	DESeq2	LRT	0.145059908391619
818	KC_AB1	CG15894	-0.594253956514975	0.017711793117463	0.994766463583882	pseudobulk	DESeq2	LRT	1.75173746921089
818	KC_AB1	CG15908	0.0644871534385216	0.914630434926408	0.994766463583882	pseudobulk	DESeq2	LRT	0.0387543512817313
818	KC_AB1	CG15912	0.596094018800054	0.670317414574127	0.994766463583882	pseudobulk	DESeq2	LRT	0.173719497665494
818	KC_AB1	CG15914	1.1821590793593	0.719511699510136	0.994766463583882	pseudobulk	DESeq2	LRT	0.142962139890846
818	KC_AB1	CG15916	0.128678146799333	0.744516412266416	0.994766463583882	pseudobulk	DESeq2	LRT	0.128125724133193
818	KC_AB1	CG15922	-0.351608904943565	0.573239267681301	0.994766463583882	pseudobulk	DESeq2	LRT	0.241664067484061
818	KC_AB1	CG15923	0.40411895759739	0.864643228521197	0.994766463583882	pseudobulk	DESeq2	LRT	0.0631630553638974
818	KC_AB1	CG1598	0.511900448422287	0.41123994774615	0.994766463583882	pseudobulk	DESeq2	LRT	0.385904704705759
818	KC_AB1	CG1602	0.0551104605447623	0.907528926819053	0.994766463583882	pseudobulk	DESeq2	LRT	0.0421395232530871
819	KC_AB1	CG1603	0.0139580626656924	0.864364215390348	0.994766463583882	pseudobulk	DESeq2	LRT	0.0633032211735764
819	KC_AB1	CG1607	-0.578199436556155	0.147652193922241	0.994766463583882	pseudobulk	DESeq2	LRT	0.830760095591932
819	KC_AB1	CG1620	-0.294203116135298	0.689181799284486	0.994766463583882	pseudobulk	DESeq2	LRT	0.161666200426982
819	KC_AB1	CG1622	0.187344509140363	0.773552150338064	0.994766463583882	pseudobulk	DESeq2	LRT	0.111510402274133
819	KC_AB1	CG1628	-1.64952313825113	0.817565084244375	0.994766463583882	pseudobulk	DESeq2	LRT	0.0874776642296574
819	KC_AB1	CG1635	-0.525631092898006	0.509977969391117	0.994766463583882	pseudobulk	DESeq2	LRT	0.292448584644275
819	KC_AB1	CG1636	0.333752635604074	0.426811482606597	0.994766463583882	pseudobulk	DESeq2	LRT	0.369763905166964
819	KC_AB1	CG1637	0.323646729661627	0.627786077153486	0.994766463583882	pseudobulk	DESeq2	LRT	0.202188320178606
819	KC_AB1	CG1640	-0.0652512585536957	0.866374670874114	0.994766463583882	pseudobulk	DESeq2	LRT	0.0622942531283539
819	KC_AB1	CG1646	-0.371198110262767	0.359393802795793	0.994766463583882	pseudobulk	DESeq2	LRT	0.444429415915957
820	KC_AB1	CG1647	0.497714886617386	0.52294509356525	0.994766463583882	pseudobulk	DESeq2	LRT	0.281543907334288
820	KC_AB1	CG1648	-2.30418889141531	0.0858619361363975	0.994766463583882	pseudobulk	DESeq2	LRT	1.06619932265983
820	KC_AB1	CG1662	0.897179476381625	0.56780389052509	0.994766463583882	pseudobulk	DESeq2	LRT	0.245801636065552
820	KC_AB1	CG1663	1.31275698734198	0.475724140081379	0.994766463583882	pseudobulk	DESeq2	LRT	0.322644810240526
820	KC_AB1	CG1671	0.208615470155158	0.893577121399079	0.994766463583882	pseudobulk	DESeq2	LRT	0.0488679591598277
820	KC_AB1	CG16711	0.309266216914965	0.266936038164708	0.994766463583882	pseudobulk	DESeq2	LRT	0.573592789571368
820	KC_AB1	CG16717	0.212710162714159	0.755506885153111	0.994766463583882	pseudobulk	DESeq2	LRT	0.121761573455494
820	KC_AB1	CG16721	-0.664763038013954	0.481314640674091	0.994766463583882	pseudobulk	DESeq2	LRT	0.317570927711249
820	KC_AB1	CG16734	2.09225092496487	0.386054269300348	0.994766463583882	pseudobulk	DESeq2	LRT	0.413351640403342
820	KC_AB1	CG16736	2.02670873290438	0.538959414925803	0.994766463583882	pseudobulk	DESeq2	LRT	0.26844393710898
821	KC_AB1	CG16739	1.29169465878791	0.584809831144677	0.994766463583882	pseudobulk	DESeq2	LRT	0.23298533513365
821	KC_AB1	CG16743	-3.21143241058256	0.292545415061155	0.994766463583882	pseudobulk	DESeq2	LRT	0.533806704011112
821	KC_AB1	CG16753	0.358918459628791	0.80536226849843	0.994766463583882	pseudobulk	DESeq2	LRT	0.0940087210990595
821	KC_AB1	CG16758	-1.64952313825113	0.817565084244375	0.994766463583882	pseudobulk	DESeq2	LRT	0.0874776642296574
821	KC_AB1	CG1677	0.133566201019875	0.650130705073293	0.994766463583882	pseudobulk	DESeq2	LRT	0.186999322148503
821	KC_AB1	CG16779	-0.0520629733022278	0.759561053835283	0.994766463583882	pseudobulk	DESeq2	LRT	0.119437311625979
821	KC_AB1	CG16782	-2.20969975456663	0.610836134188693	0.994766463583882	pseudobulk	DESeq2	LRT	0.214075280039667
821	KC_AB1	CG16787	0.535668448154039	0.610384921668546	0.994766463583882	pseudobulk	DESeq2	LRT	0.214396203292084
821	KC_AB1	CG16789	-2.20969975456663	0.610836134188693	0.994766463583882	pseudobulk	DESeq2	LRT	0.214075280039667
821	KC_AB1	CG16790	-0.860286089089135	0.603216243021941	0.994766463583882	pseudobulk	DESeq2	LRT	0.219526972576244
822	KC_AB1	CG16791	0.0731765805663878	0.70936751955811	0.994766463583882	pseudobulk	DESeq2	LRT	0.149128700836759
822	KC_AB1	CG16812	0.550832377715756	0.40031280961145	0.994766463583882	pseudobulk	DESeq2	LRT	0.397600512681406
822	KC_AB1	CG16825	-1.64952313825113	0.817565084244375	0.994766463583882	pseudobulk	DESeq2	LRT	0.0874776642296574
822	KC_AB1	CG16854	-0.746388611130786	0.462598959998668	0.994766463583882	pseudobulk	DESeq2	LRT	0.334795347932341
822	KC_AB1	CG16863	-0.648218248148942	0.834000364039372	0.994766463583882	pseudobulk	DESeq2	LRT	0.0788337597936092
822	KC_AB1	CG16865	0.236289203560025	0.865636944626005	0.994766463583882	pseudobulk	DESeq2	LRT	0.0626642165341611
822	KC_AB1	CG16868	0.41202337022643	0.736849236523088	0.994766463583882	pseudobulk	DESeq2	LRT	0.132621362132795
822	KC_AB1	CG1688	0.132786475050985	0.752240592495295	0.994766463583882	pseudobulk	DESeq2	LRT	0.12364323483139
822	KC_AB1	CG16890	0.475649746043893	0.759920495303806	0.994766463583882	pseudobulk	DESeq2	LRT	0.119231842267898
822	KC_AB1	CG16896	-0.93565799849381	0.534292951808374	0.994766463583882	pseudobulk	DESeq2	LRT	0.272220554828736
823	KC_AB1	CG16903	-0.230745790780838	0.760059829896818	0.994766463583882	pseudobulk	DESeq2	LRT	0.1191522198622

823	KC_AB1	CG16908	-1.50663180504502	0.668163815680457	0.994766463583882	pseudobulk	DESeq2	LRT	0.175117047219009
823	KC_AB1	CG16935	0.337873923209273	0.64113427324955	0.994766463583882	pseudobulk	DESeq2	LRT	0.193051006324344
823	KC_AB1	CG1695	0.158329455042187	0.713044481895219	0.994766463583882	pseudobulk	DESeq2	LRT	0.146883376685217
823	KC_AB1	CG16952	0.103156537904749	0.822189042412939	0.994766463583882	pseudobulk	DESeq2	LRT	0.0850283155042295
823	KC_AB1	CG16953	-0.303358388228699	0.623520510283658	0.994766463583882	pseudobulk	DESeq2	LRT	0.205149256128486
823	KC_AB1	CG16972	-0.548189756359812	0.632213595717961	0.994766463583882	pseudobulk	DESeq2	LRT	0.19913616891751
823	KC_AB1	CG16974	0.407434084948587	0.469411390589173	0.994766463583882	pseudobulk	DESeq2	LRT	0.328446376156251
823	KC_AB1	CG16979	-0.161280462790719	0.893697150630603	0.994766463583882	pseudobulk	DESeq2	LRT	0.0488096267216425
823	KC_AB1	CG1698	1.1821590793593	0.719511699510136	0.994766463583882	pseudobulk	DESeq2	LRT	0.142962139890846
824	KC_AB1	CG16985	2.54275315134261	0.410734813545839	0.994766463583882	pseudobulk	DESeq2	LRT	0.386438485142247
824	KC_AB1	CG16986	-0.356690758758309	0.679885899798148	0.994766463583882	pseudobulk	DESeq2	LRT	0.167563965596527
824	KC_AB1	CG16989	0.593284723012334	0.61807881389025	0.994766463583882	pseudobulk	DESeq2	LRT	0.208956142621164
824	KC_AB1	CG17002	-0.127188631327031	0.873966106587714	0.994766463583882	pseudobulk	DESeq2	LRT	0.0585054094792683
824	KC_AB1	CG17010	-0.17805032366327	0.942053914371676	0.994766463583882	pseudobulk	DESeq2	LRT	0.0259242415339998
824	KC_AB1	CG17019	-0.197424910494686	0.774955324274666	0.994766463583882	pseudobulk	DESeq2	LRT	0.110723333597233
824	KC_AB1	CG17026	-0.221082217345612	0.848858028911001	0.994766463583882	pseudobulk	DESeq2	LRT	0.0711649392216866
824	KC_AB1	CG17029	1.0233103206859	0.557203517140596	0.994766463583882	pseudobulk	DESeq2	LRT	0.253986150916538
824	KC_AB1	CG1703	-0.103706496802672	0.868388652120464	0.994766463583882	pseudobulk	DESeq2	LRT	0.0612858604291152
824	KC_AB1	CG17030	-4.07942607047938	0.12890417890847	0.994766463583882	pseudobulk	DESeq2	LRT	0.889733003147214
825	KC_AB1	CG17032	1.1821590793593	0.719511699510136	0.994766463583882	pseudobulk	DESeq2	LRT	0.142962139890846
825	KC_AB1	CG17059	-0.111593536709864	0.784309829834524	0.994766463583882	pseudobulk	DESeq2	LRT	0.105512341899077
825	KC_AB1	CG17065	0.223023051320607	0.643663843256812	0.994766463583882	pseudobulk	DESeq2	LRT	0.191340885954265
825	KC_AB1	CG17075	-0.189568067131836	0.75500096619167	0.994766463583882	pseudobulk	DESeq2	LRT	0.122052492594067
825	KC_AB1	CG17078	-0.20696773563876	0.77436134519058	0.994766463583882	pseudobulk	DESeq2	LRT	0.111056334405018
825	KC_AB1	CG17111	-1.21457746603801	0.770013447451371	0.994766463583882	pseudobulk	DESeq2	LRT	0.113501690278259
825	KC_AB1	CG17119	1.1821590793593	0.719511699510136	0.994766463583882	pseudobulk	DESeq2	LRT	0.142962139890846
825	KC_AB1	CG17122	-2.20969975456663	0.610836134188693	0.994766463583882	pseudobulk	DESeq2	LRT	0.214075280039667
825	KC_AB1	CG17124	0.0964745521830169	0.714451354508194	0.994766463583882	pseudobulk	DESeq2	LRT	0.146027336065275
825	KC_AB1	CG17141	0.555305428932874	0.734010089415069	0.994766463583882	pseudobulk	DESeq2	LRT	0.13429797040113
826	KC_AB1	CG17162	-0.214359980047456	0.864260306946912	0.994766463583882	pseudobulk	DESeq2	LRT	0.0633554324699897
826	KC_AB1	CG17163	0.542315501789911	0.385409120903563	0.994766463583882	pseudobulk	DESeq2	LRT	0.414078011758366
826	KC_AB1	CG17167	-2.69843228182164	0.130161155753217	0.994766463583882	pseudobulk	DESeq2	LRT	0.885518603778817
826	KC_AB1	CG17168	0.0494587676783366	0.896149124210441	0.994766463583882	pseudobulk	DESeq2	LRT	0.0476197153011748
826	KC_AB1	CG17169	0.949183608371002	0.80156895525114	0.994766463583882	pseudobulk	DESeq2	LRT	0.0960591113673091
826	KC_AB1	CG17186	-1.25978527545468	0.402380742285025	0.994766463583882	pseudobulk	DESeq2	LRT	0.395362812543285
826	KC_AB1	CG17187	-0.202284362695157	0.887661678909256	0.994766463583882	pseudobulk	DESeq2	LRT	0.0517525285679232
826	KC_AB1	CG17202	0.467117742025884	0.310030931317284	0.994766463583882	pseudobulk	DESeq2	LRT	0.508594975100301
826	KC_AB1	CG17211	-3.6748866702475	0.198972138271859	0.994766463583882	pseudobulk	DESeq2	LRT	0.701207732846238
826	KC_AB1	CG17217	-1.64952313825113	0.817565084244375	0.994766463583882	pseudobulk	DESeq2	LRT	0.0874776642296574
827	KC_AB1	CG17219	-2.85724547560282	0.450020141104186	0.994766463583882	pseudobulk	DESeq2	LRT	0.346768048503189
827	KC_AB1	CG17221	0.39509611406087	0.839890017035674	0.994766463583882	pseudobulk	DESeq2	LRT	0.0757775807497476
827	KC_AB1	CG17230	0.130760111646649	0.806807875738471	0.994766463583882	pseudobulk	DESeq2	LRT	0.0932298710281062
827	KC_AB1	CG17233	-0.391891814285214	0.465823069165643	0.994766463583882	pseudobulk	DESeq2	LRT	0.331779007507535
827	KC_AB1	CG17249	0.605675048829576	0.665026024937762	0.994766463583882	pseudobulk	DESeq2	LRT	0.177161358808617
827	KC_AB1	CG17260	0.854047063205883	0.719204060467466	0.994766463583882	pseudobulk	DESeq2	LRT	0.143147869337068
827	KC_AB1	CG17266	-0.739006821616313	0.76979092815726	0.994766463583882	pseudobulk	DESeq2	LRT	0.113627211290866
827	KC_AB1	CG17270	-0.123477762204509	0.822632703282378	0.994766463583882	pseudobulk	DESeq2	LRT	0.0847940293603231
827	KC_AB1	CG17271	0.401311020636304	0.520471901821476	0.994766463583882	pseudobulk	DESeq2	LRT	0.283602711327837
827	KC_AB1	CG17272	0.233473994531388	0.476107221818471	0.994766463583882	pseudobulk	DESeq2	LRT	0.322295230892379
828	KC_AB1	CG17279	-1.64952313825113	0.817565084244375	0.994766463583882	pseudobulk	DESeq2	LRT	0.0874776642296574
828	KC_AB1	CG17294	-0.131879580708344	0.804800539661657	0.994766463583882	pseudobulk	DESeq2	LRT	0.0943117410704523
828	KC_AB1	CG17321	-0.0685318976716936	0.805375227933097	0.994766463583882	pseudobulk	DESeq2	LRT	0.0940017327338203
828	KC_AB1	CG17328	0.893178316517145	0.561798195088671	0.994766463583882	pseudobulk	DESeq2	LRT	0.250419660407295
828	KC_AB1	CG17333	1.80633728195307	0.61161102402172	0.994766463583882	pseudobulk	DESeq2	LRT	0.213524695220716
828	KC_AB1	CG17343	-0.162076489666572	0.821827235791048	0.994766463583882	pseudobulk	DESeq2	LRT	0.0852194700858761
828	KC_AB1	CG17359	1.27269052052074	0.447753170961228	0.994766463583882	pseudobulk	DESeq2	LRT	0.348961329820444

828	KC_AB1	CG17361	1.52018402089637	0.282461270018117	0.994766463583882	pseudobulk	DESeq2	LRT	0.549041092518966
828	KC_AB1	CG17364	-0.135334813076672	0.78019992011536	0.994766463583882	pseudobulk	DESeq2	LRT	0.107794098491621
828	KC_AB1	CG1737	0.153499194610218	0.721491636134213	0.994766463583882	pseudobulk	DESeq2	LRT	0.141768699084434
829	KC_AB1	CG17378	0.0935931404653542	0.821845991808084	0.994766463583882	pseudobulk	DESeq2	LRT	0.0852095585845332
829	KC_AB1	CG1738	0.641833300587602	0.746379423307147	0.994766463583882	pseudobulk	DESeq2	LRT	0.12704034202059
829	KC_AB1	CG17385	0.372120729021485	0.448650048476958	0.994766463583882	pseudobulk	DESeq2	LRT	0.34809228098717
829	KC_AB1	CG17454	0.0181886623915701	0.953348640843999	0.994766463583882	pseudobulk	DESeq2	LRT	0.0207482482542119
829	KC_AB1	CG17470	0.949183608371002	0.80156895525114	0.994766463583882	pseudobulk	DESeq2	LRT	0.0960591113673091
829	KC_AB1	CG17486	-0.214860121026664	0.644209454109445	0.994766463583882	pseudobulk	DESeq2	LRT	0.190972905971722
829	KC_AB1	CG17490	0.949183608371002	0.80156895525114	0.994766463583882	pseudobulk	DESeq2	LRT	0.0960591113673091
829	KC_AB1	CG17493	0.0710793333276803	0.854085235721861	0.994766463583882	pseudobulk	DESeq2	LRT	0.0684987855680458
829	KC_AB1	CG1750	0.881337768677799	0.697697705840746	0.994766463583882	pseudobulk	DESeq2	LRT	0.156332705059186
829	KC_AB1	CG17508	-0.0738189416465981	0.819666197826091	0.994766463583882	pseudobulk	DESeq2	LRT	0.0863629743928199
830	KC_AB1	CG17544	0.454736054816347	0.760196997628283	0.994766463583882	pseudobulk	DESeq2	LRT	0.119073849960545
830	KC_AB1	CG17549	2.02670873290438	0.538959414925803	0.994766463583882	pseudobulk	DESeq2	LRT	0.26844393710898
830	KC_AB1	CG17556	-0.709324923615395	0.357206559561413	0.994766463583882	pseudobulk	DESeq2	LRT	0.447080574545058
830	KC_AB1	CG17565	-0.439316949276503	0.564817283456314	0.994766463583882	pseudobulk	DESeq2	LRT	0.248092022305296
830	KC_AB1	CG17568	1.13724809974279	0.299199679810799	0.994766463583882	pseudobulk	DESeq2	LRT	0.52403887556853
830	KC_AB1	CG17574	-0.392111571278019	0.686145097416025	0.994766463583882	pseudobulk	DESeq2	LRT	0.163584035395531
830	KC_AB1	CG17593	0.735670736195129	0.284981333232529	0.994766463583882	pseudobulk	DESeq2	LRT	0.545183586095381
830	KC_AB1	CG17598	0.403478121108751	0.571642253418229	0.994766463583882	pseudobulk	DESeq2	LRT	0.242875677452502
830	KC_AB1	CG17600	0.206287369862366	0.785570490551986	0.994766463583882	pseudobulk	DESeq2	LRT	0.104814838906369
830	KC_AB1	CG17612	0.750885950240234	0.624053232113199	0.994766463583882	pseudobulk	DESeq2	LRT	0.204778363158943
831	KC_AB1	CG17637	1.06108652407915	0.751249895958721	0.994766463583882	pseudobulk	DESeq2	LRT	0.124215575135036
831	KC_AB1	CG1764	0.128521836844389	0.813096268006814	0.994766463583882	pseudobulk	DESeq2	LRT	0.0898580322791924
831	KC_AB1	CG17646	-0.502709873131976	0.649710969969614	0.994766463583882	pseudobulk	DESeq2	LRT	0.187279800377852
831	KC_AB1	CG17652	-0.782857149878603	0.472630339795083	0.994766463583882	pseudobulk	DESeq2	LRT	0.325478402926549
831	KC_AB1	CG17660	1.0400382696977	0.278473245199587	0.994766463583882	pseudobulk	DESeq2	LRT	0.555216524082159
831	KC_AB1	CG17669	-3.85958892239636	0.166041795813258	0.994766463583882	pseudobulk	DESeq2	LRT	0.779782578186973
831	KC_AB1	CG17683	-0.460409238377302	0.357098430542615	0.994766463583882	pseudobulk	DESeq2	LRT	0.447212058539169
831	KC_AB1	CG17684	-0.0643966932498926	0.694155183116069	0.994766463583882	pseudobulk	DESeq2	LRT	0.158543429203492
831	KC_AB1	CG17691	-0.121941440116093	0.835180555952747	0.994766463583882	pseudobulk	DESeq2	LRT	0.0782196251424902
831	KC_AB1	CG17698	-0.294877538461218	0.25644704158687	0.994766463583882	pseudobulk	DESeq2	LRT	0.591002306658312
832	KC_AB1	CG17712	-0.393144341918669	0.618109614794125	0.994766463583882	pseudobulk	DESeq2	LRT	0.208934500835361
832	KC_AB1	CG17715	0.493877782874385	0.160076834422966	0.994766463583882	pseudobulk	DESeq2	LRT	0.795671512616669
832	KC_AB1	CG17716	2.54275315134261	0.410734813545839	0.994766463583882	pseudobulk	DESeq2	LRT	0.386438485142247
832	KC_AB1	CG17721	0.286343262789313	0.841378666682369	0.994766463583882	pseudobulk	DESeq2	LRT	0.0750085037993935
832	KC_AB1	CG17724	-0.288551020664342	0.504776665986235	0.994766463583882	pseudobulk	DESeq2	LRT	0.296900729175255
832	KC_AB1	CG17726	-2.03736705378311	0.481539256788191	0.994766463583882	pseudobulk	DESeq2	LRT	0.317368301869619
832	KC_AB1	CG17734	0.0302904752361238	0.914100826841296	0.994766463583882	pseudobulk	DESeq2	LRT	0.039005898220502
832	KC_AB1	CG17746	-0.56869584279612	0.593232508361664	0.994766463583882	pseudobulk	DESeq2	LRT	0.226775058224826
832	KC_AB1	CG17754	-1.0506439610207	0.125705623189459	0.994766463583882	pseudobulk	DESeq2	LRT	0.900645294585082
832	KC_AB1	CG17760	-0.0876274309314422	0.917937734184526	0.994766463583882	pseudobulk	DESeq2	LRT	0.0371867769874299
833	KC_AB1	CG17765	0.286071881037189	0.449483533600448	0.994766463583882	pseudobulk	DESeq2	LRT	0.347286213602501
833	KC_AB1	CG17776	0.0262927283162094	0.933483864077034	0.994766463583882	pseudobulk	DESeq2	LRT	0.0298931847345829
833	KC_AB1	CG17778	-0.10881687534595	0.56264940313207	0.994766463583882	pseudobulk	DESeq2	LRT	0.249762137500227
833	KC_AB1	CG17786	-1.08563871313496	0.58745735762205	0.994766463583882	pseudobulk	DESeq2	LRT	0.231023652497186
833	KC_AB1	CG17802	-0.0412609031317249	0.957459292663439	0.994766463583882	pseudobulk	DESeq2	LRT	0.0188796814297893
833	KC_AB1	CG17803	0.283554081956596	0.80686475192066	0.994766463583882	pseudobulk	DESeq2	LRT	0.0931992563771998
833	KC_AB1	CG17806	-0.747142796159516	0.565487201864247	0.994766463583882	pseudobulk	DESeq2	LRT	0.247577219602597
833	KC_AB1	CG17807	0.0401341484424634	0.917448903496856	0.994766463583882	pseudobulk	DESeq2	LRT	0.0374181140484076
833	KC_AB1	CG17816	0.0164466517499289	0.90358963673012	0.994766463583882	pseudobulk	DESeq2	LRT	0.0440287586466862
833	KC_AB1	CG17829	0.30061731945614	0.791789228539443	0.994766463583882	pseudobulk	DESeq2	LRT	0.10139041066376
834	KC_AB1	CG17839	0.503268656241809	0.451644301724244	0.994766463583882	pseudobulk	DESeq2	LRT	0.345203464774698
834	KC_AB1	CG17841	-0.169236249252306	0.396914962392133	0.994766463583882	pseudobulk	DESeq2	LRT	0.401302529306846
834	KC_AB1	CG1785	-0.582438735532103	0.770411347052444	0.994766463583882	pseudobulk	DESeq2	LRT	0.113277329302562

834	KC_AB1	CG17883	-0.166633833628081	0.667676753007511	0.994766463583882	pseudobulk	DESeq2	LRT	0.175433744653619
834	KC_AB1	CG1789	-0.216220119290863	0.759844224632135	0.994766463583882	pseudobulk	DESeq2	LRT	0.119275433136183
834	KC_AB1	CG17896	0.984083019823803	0.702143474713468	0.994766463583882	pseudobulk	DESeq2	LRT	0.153574135862026
834	KC_AB1	CG17904	-0.681420866075745	0.34299671361151	0.994766463583882	pseudobulk	DESeq2	LRT	0.464710041086162
834	KC_AB1	CG1792	-0.0548135331684293	0.95773214554551	0.994766463583882	pseudobulk	DESeq2	LRT	0.0187559355738999
834	KC_AB1	CG17931	-0.00220301790341415	0.949981039582842	0.994766463583882	pseudobulk	DESeq2	LRT	0.0222850625919101
834	KC_AB1	CG17982	1.19529878136551	0.483486253574576	0.994766463583882	pseudobulk	DESeq2	LRT	0.31561586921609
835	KC_AB1	CG17985	0.568408990388771	0.552874673565355	0.994766463583882	pseudobulk	DESeq2	LRT	0.257373304043424
835	KC_AB1	CG17996	-0.00566713415937883	0.939796818502271	0.994766463583882	pseudobulk	DESeq2	LRT	0.0269660295293327
835	KC_AB1	CG18004	-0.0111649788640748	0.889279856168737	0.994766463583882	pseudobulk	DESeq2	LRT	0.0509615451450855
835	KC_AB1	CG1801	-2.79499059556516	0.38564607643653	0.994766463583882	pseudobulk	DESeq2	LRT	0.413811082791365
835	KC_AB1	CG18011	-0.548887546079202	0.814781693583783	0.994766463583882	pseudobulk	DESeq2	LRT	0.0889587372379754
835	KC_AB1	CG18048	0.528936140834362	0.669282619147995	0.994766463583882	pseudobulk	DESeq2	LRT	0.174390453206763
835	KC_AB1	CG18063	0.949183608371002	0.80156895525114	0.994766463583882	pseudobulk	DESeq2	LRT	0.0960591113673091
835	KC_AB1	CG18081	-0.550638727771689	0.59492060300005	0.994766463583882	pseudobulk	DESeq2	LRT	0.225540990539998
835	KC_AB1	CG1812	-0.144669000832402	0.897084746022646	0.994766463583882	pseudobulk	DESeq2	LRT	0.0471665279798717
835	KC_AB1	CG18135	0.129859214583218	0.774919512089999	0.994766463583882	pseudobulk	DESeq2	LRT	0.110743403649116
836	KC_AB1	CG1814	0.199427671187272	0.657259885163669	0.994766463583882	pseudobulk	DESeq2	LRT	0.182262873399994
836	KC_AB1	CG1815	-0.185110422116605	0.772741415898767	0.994766463583882	pseudobulk	DESeq2	LRT	0.111965810656122
836	KC_AB1	CG18171	-0.341391478739861	0.841795132270276	0.994766463583882	pseudobulk	DESeq2	LRT	0.0747935899140484
836	KC_AB1	CG18178	0.894918430295795	0.372973776554218	0.994766463583882	pseudobulk	DESeq2	LRT	0.428321701966526
836	KC_AB1	CG18213	0.949183608371002	0.80156895525114	0.994766463583882	pseudobulk	DESeq2	LRT	0.0960591113673091
836	KC_AB1	CG1824	-0.010772370266124	0.952366370125031	0.994766463583882	pseudobulk	DESeq2	LRT	0.0211959487637905
836	KC_AB1	CG18259	0.153542387541646	0.878854858350053	0.994766463583882	pseudobulk	DESeq2	LRT	0.0560828421303081
836	KC_AB1	CG18262	-2.26306816020352	0.40449476385881	0.994766463583882	pseudobulk	DESeq2	LRT	0.393087095910655
836	KC_AB1	CG18265	-0.11815825400374	0.795515537300665	0.994766463583882	pseudobulk	DESeq2	LRT	0.0993513336817031
836	KC_AB1	CG1827	-2.00354090274141	0.368550329168343	0.994766463583882	pseudobulk	DESeq2	LRT	0.433503196576757
837	KC_AB1	CG18273	-2.57435989741145	0.533617212681275	0.994766463583882	pseudobulk	DESeq2	LRT	0.272770169998685
837	KC_AB1	CG18281	2.64699301431894	0.23725229373035	0.994766463583882	pseudobulk	DESeq2	LRT	0.624789580166765
837	KC_AB1	CG18324	0.233075253987931	0.781807785282148	0.994766463583882	pseudobulk	DESeq2	LRT	0.106900009155444
837	KC_AB1	CG18327	-0.0876446376047783	0.951298398237017	0.994766463583882	pseudobulk	DESeq2	LRT	0.0216832345024977
837	KC_AB1	CG18335	-0.0822426761580336	0.929058352140717	0.994766463583882	pseudobulk	DESeq2	LRT	0.0319570080549424
837	KC_AB1	CG18336	-3.21143241058256	0.292545415061155	0.994766463583882	pseudobulk	DESeq2	LRT	0.533806704011112
837	KC_AB1	CG18371	1.1821590793593	0.719511699510136	0.994766463583882	pseudobulk	DESeq2	LRT	0.142962139890846
837	KC_AB1	CG1840	0.760792888070953	0.305211138091013	0.994766463583882	pseudobulk	DESeq2	LRT	0.515399621689625
837	KC_AB1	CG18420	0.949183608371002	0.80156895525114	0.994766463583882	pseudobulk	DESeq2	LRT	0.0960591113673091
837	KC_AB1	CG18428	-0.271580444217185	0.549006193895642	0.994766463583882	pseudobulk	DESeq2	LRT	0.260422755805783
838	KC_AB1	CG18467	-0.292106383884648	0.48333494474507	0.994766463583882	pseudobulk	DESeq2	LRT	0.315751804568704
838	KC_AB1	CG1847	-0.585739974916409	0.466990514441082	0.994766463583882	pseudobulk	DESeq2	LRT	0.330691940778071
838	KC_AB1	CG18476	-0.503872619281769	0.605390945285014	0.994766463583882	pseudobulk	DESeq2	LRT	0.217964078989227
838	KC_AB1	CG18508	-0.12483479413402	0.81766638767993	0.994766463583882	pseudobulk	DESeq2	LRT	0.087423854693979
838	KC_AB1	CG18528	-0.862128082958893	0.639533020568458	0.994766463583882	pseudobulk	DESeq2	LRT	0.194137026978222
838	KC_AB1	CG18536	-1.64952313825113	0.817565084244375	0.994766463583882	pseudobulk	DESeq2	LRT	0.0874776642296574
838	KC_AB1	CG18537	-2.20969975456663	0.610836134188693	0.994766463583882	pseudobulk	DESeq2	LRT	0.214075280039667
838	KC_AB1	CG18549	0.198543158031602	0.822844178264029	0.994766463583882	pseudobulk	DESeq2	LRT	0.084682399207232
838	KC_AB1	CG18563	-1.64952313825113	0.817565084244375	0.994766463583882	pseudobulk	DESeq2	LRT	0.0874776642296574
838	KC_AB1	CG18596	1.34956689823299	0.418248528631601	0.994766463583882	pseudobulk	DESeq2	LRT	0.378565578179795
839	KC_AB1	CG18598	-1.64952313825113	0.817565084244375	0.994766463583882	pseudobulk	DESeq2	LRT	0.0874776642296574
839	KC_AB1	CG18600	-1.86463546605885	0.172777852669396	0.994766463583882	pseudobulk	DESeq2	LRT	0.762511927828825
839	KC_AB1	CG18628	-0.48167422725367	0.855534332799682	0.994766463583882	pseudobulk	DESeq2	LRT	0.0677625574305792
839	KC_AB1	CG18659	-0.321053085735084	0.633878861264828	0.994766463583882	pseudobulk	DESeq2	LRT	0.197993730936833
839	KC_AB1	CG18661	-0.0436033993737628	0.920286839997701	0.994766463583882	pseudobulk	DESeq2	LRT	0.0360767882931682
839	KC_AB1	CG18675	1.32789395261901	0.50354133529231	0.994766463583882	pseudobulk	DESeq2	LRT	0.297964872771697
839	KC_AB1	CG18731	0.261003039284479	0.759485002606152	0.994766463583882	pseudobulk	DESeq2	LRT	0.119480797640166
839	KC_AB1	CG18754	-2.57435989741145	0.533617212681275	0.994766463583882	pseudobulk	DESeq2	LRT	0.272770169998685
839	KC_AB1	CG18764	-0.364337871477143	0.839054806380232	0.994766463583882	pseudobulk	DESeq2	LRT	0.0762096704810448

839	KC_AB1	CG18766	0.43298355573973	0.41336950304613	0.994766463583882	pseudobulk	DESeq2	LRT	0.383661567252371
840	KC_AB1	CG18787	0.265717584717128	0.835630223492062	0.994766463583882	pseudobulk	DESeq2	LRT	0.0779858606275216
840	KC_AB1	CG18789	-1.60653259065571	0.594621747113632	0.994766463583882	pseudobulk	DESeq2	LRT	0.225759211379423
840	KC_AB1	CG18809	-0.194301171436787	0.813559296325225	0.994766463583882	pseudobulk	DESeq2	LRT	0.0896107879903689
840	KC_AB1	CG18815	0.0719464723754344	0.798320277805942	0.994766463583882	pseudobulk	DESeq2	LRT	0.0978228392528381
840	KC_AB1	CG1882	0.462014517960261	0.486493486777814	0.994766463583882	pseudobulk	DESeq2	LRT	0.312922969733532
840	KC_AB1	CG18858	1.1821590793593	0.719511699510136	0.994766463583882	pseudobulk	DESeq2	LRT	0.142962139890846
840	KC_AB1	CG18870	-0.930782755915602	0.390663659531683	0.994766463583882	pseudobulk	DESeq2	LRT	0.408196986008126
840	KC_AB1	CG1888	0.120405557649058	0.834663374978158	0.994766463583882	pseudobulk	DESeq2	LRT	0.0784886429188593
840	KC_AB1	CG1890	1.42733222800489	0.252859483191278	0.994766463583882	pseudobulk	DESeq2	LRT	0.59712075403297
840	KC_AB1	CG1896	-0.312647001269314	0.628683930060501	0.994766463583882	pseudobulk	DESeq2	LRT	0.201567640612505
841	KC_AB1	CG1902	0.200602981652296	0.755594732329181	0.994766463583882	pseudobulk	DESeq2	LRT	0.121711078447853
841	KC_AB1	CG1907	-0.0470611034894549	0.917446792046214	0.994766463583882	pseudobulk	DESeq2	LRT	0.0374191135508479
841	KC_AB1	CG1909	-0.00614872571550681	0.957724868180809	0.994766463583882	pseudobulk	DESeq2	LRT	0.0187592355898332
841	KC_AB1	CG1910	-0.203418464445795	0.596977492461275	0.994766463583882	pseudobulk	DESeq2	LRT	0.224042042545901
841	KC_AB1	CG1927	-0.15409217012886	0.815361706624813	0.994766463583882	pseudobulk	DESeq2	LRT	0.0886496889987236
841	KC_AB1	CG1943	-0.131213783038263	0.670306434213581	0.994766463583882	pseudobulk	DESeq2	LRT	0.173726611831036
841	KC_AB1	CG1951	-0.368159913495521	0.665976877774783	0.994766463583882	pseudobulk	DESeq2	LRT	0.176540848951534
841	KC_AB1	CG1965	0.210505809061251	0.602453427809825	0.994766463583882	pseudobulk	DESeq2	LRT	0.220076520250127
841	KC_AB1	CG1971	1.1821590793593	0.719511699510136	0.994766463583882	pseudobulk	DESeq2	LRT	0.142962139890846
841	KC_AB1	CG1983	-0.436374231950355	0.559567412148872	0.994766463583882	pseudobulk	DESeq2	LRT	0.25214758570277
842	KC_AB1	CG1998	0.618582300055894	0.109391970822731	0.994766463583882	pseudobulk	DESeq2	LRT	0.961014553280009
842	KC_AB1	CG2003	-0.379314053126175	0.843633212750006	0.994766463583882	pseudobulk	DESeq2	LRT	0.0738463309831402
842	KC_AB1	CG2004	0.597141410293382	0.802139944912821	0.994766463583882	pseudobulk	DESeq2	LRT	0.0957498561530429
842	KC_AB1	CG2006	-0.460790978208488	0.756315108898266	0.994766463583882	pseudobulk	DESeq2	LRT	0.121297223621342
842	KC_AB1	CG2021	-0.0312180697471941	0.958921800303679	0.994766463583882	pseudobulk	DESeq2	LRT	0.018216807929798
842	KC_AB1	CG2023	-0.579658913675809	0.64980613787942	0.994766463583882	pseudobulk	DESeq2	LRT	0.187216190752542
842	KC_AB1	CG2034	0.286343262789313	0.841378666682369	0.994766463583882	pseudobulk	DESeq2	LRT	0.0750085037993935
842	KC_AB1	CG2059	0.0425179877472656	0.947091111006922	0.994766463583882	pseudobulk	DESeq2	LRT	0.0236082394717011
842	KC_AB1	CG2061	0.00716814904038909	0.922796986698075	0.994766463583882	pseudobulk	DESeq2	LRT	0.0348938322941331
842	KC_AB1	CG2063	0.155746713322003	0.83033541779534	0.994766463583882	pseudobulk	DESeq2	LRT	0.0807464369352101
843	KC_AB1	CG2064	0.408451987487849	0.824413195620833	0.994766463583882	pseudobulk	DESeq2	LRT	0.0838550654864088
843	KC_AB1	CG2076	-0.222702554338418	0.75605990985112	0.994766463583882	pseudobulk	DESeq2	LRT	0.121443789833611
843	KC_AB1	CG2082	2.02670873290438	0.538959414925803	0.994766463583882	pseudobulk	DESeq2	LRT	0.26844393710898
843	KC_AB1	CG2091	-0.214183625217408	0.814458181155815	0.994766463583882	pseudobulk	DESeq2	LRT	0.0891312098938665
843	KC_AB1	CG2100	0.144791368864283	0.899543411856493	0.994766463583882	pseudobulk	DESeq2	LRT	0.0459778728133911
843	KC_AB1	CG2104	0.949183608371002	0.80156895525114	0.994766463583882	pseudobulk	DESeq2	LRT	0.0960591113673091
843	KC_AB1	CG2111	1.80633728195307	0.61161102402172	0.994766463583882	pseudobulk	DESeq2	LRT	0.213524695220716
843	KC_AB1	CG2116	0.617650429963499	0.691066078567419	0.994766463583882	pseudobulk	DESeq2	LRT	0.160480424137882
843	KC_AB1	CG2121	2.67764418499683	0.378704357417491	0.994766463583882	pseudobulk	DESeq2	LRT	0.421699697806334
843	KC_AB1	CG2124	0.195526510729309	0.835393291548733	0.994766463583882	pseudobulk	DESeq2	LRT	0.0781090165679834
844	KC_AB1	CG2126	0.0875827259982949	0.900805107127911	0.994766463583882	pseudobulk	DESeq2	LRT	0.0453691602467448
844	KC_AB1	CG2129	-0.16536199357653	0.912961766495686	0.994766463583882	pseudobulk	DESeq2	LRT	0.0395474097029747
844	KC_AB1	CG2147	0.509251197712493	0.626192224074741	0.994766463583882	pseudobulk	DESeq2	LRT	0.203292329664487
844	KC_AB1	CG2162	-0.0820961928430846	0.882676507440636	0.994766463583882	pseudobulk	DESeq2	LRT	0.0541984320675491
844	KC_AB1	CG2182	-0.753785502159183	0.149931303480678	0.994766463583882	pseudobulk	DESeq2	LRT	0.824107683298405
844	KC_AB1	CG2186	-0.33012958915362	0.374886539897559	0.994766463583882	pseudobulk	DESeq2	LRT	0.426100152411624
844	KC_AB1	CG2199	-0.53354759017415	0.540722082042389	0.994766463583882	pseudobulk	DESeq2	LRT	0.267025894316857
844	KC_AB1	CG2200	0.122718001895476	0.899063988386992	0.994766463583882	pseudobulk	DESeq2	LRT	0.0462093974609626
844	KC_AB1	CG2201	1.05951271011724	0.148187490535338	0.994766463583882	pseudobulk	DESeq2	LRT	0.829188456415911
844	KC_AB1	CG2202	1.91594373630906	0.204336794883694	0.994766463583882	pseudobulk	DESeq2	LRT	0.689653423009631
845	KC_AB1	CG2209	1.00918293591045	0.350961151887503	0.994766463583882	pseudobulk	DESeq2	LRT	0.454740953205444
845	KC_AB1	CG2211	-0.659884033871925	0.564313844586318	0.994766463583882	pseudobulk	DESeq2	LRT	0.248479294841272
845	KC_AB1	CG2218	0.251450599441535	0.774649577581123	0.994766463583882	pseudobulk	DESeq2	LRT	0.110894711609047
845	KC_AB1	CG2224	-0.667096458671498	0.647328393340295	0.994766463583882	pseudobulk	DESeq2	LRT	0.188875343390575
845	KC_AB1	CG2225	-0.130717947150833	0.640765821683777	0.994766463583882	pseudobulk	DESeq2	LRT	0.193300661483748

845	KC_AB1	CG2233	0.0462401973101628	0.899563689741424	0.994766463583882	pseudobulk	DESeq2	LRT	0.0459680828753462
845	KC_AB1	CG2246	0.207138263239635	0.621714416733751	0.994766463583882	pseudobulk	DESeq2	LRT	0.206409061801345
845	KC_AB1	CG2247	-0.496455310607818	0.53793324636584	0.994766463583882	pseudobulk	DESeq2	LRT	0.269271613801378
845	KC_AB1	CG2256	0.124698704744493	0.891329405341186	0.994766463583882	pseudobulk	DESeq2	LRT	0.0499617656782218
845	KC_AB1	CG2258	0.131662293455766	0.774315895902241	0.994766463583882	pseudobulk	DESeq2	LRT	0.111081825029679
846	KC_AB1	CG2260	2.12250628594009	0.308188228414228	0.994766463583882	pseudobulk	DESeq2	LRT	0.511183953652572
846	KC_AB1	CG2269	-0.126236755163248	0.487456530700075	0.994766463583882	pseudobulk	DESeq2	LRT	0.312064106773272
846	KC_AB1	CG2277	0.0490758344884723	0.895751983998963	0.994766463583882	pseudobulk	DESeq2	LRT	0.0478122212429597
846	KC_AB1	CG2278	-0.139216215306606	0.853745199108796	0.994766463583882	pseudobulk	DESeq2	LRT	0.0686717254848418
846	KC_AB1	CG2321	-0.390513275370056	0.768235697792098	0.994766463583882	pseudobulk	DESeq2	LRT	0.114505516236295
846	KC_AB1	CG2519	-0.20302913086068	0.666945281391409	0.994766463583882	pseudobulk	DESeq2	LRT	0.175909795715511
846	KC_AB1	CG2528	2.38929733117871	0.484296857543297	0.994766463583882	pseudobulk	DESeq2	LRT	0.314888348950639
846	KC_AB1	CG2540	-0.544666518761678	0.677234170494804	0.994766463583882	pseudobulk	DESeq2	LRT	0.169261137268734
846	KC_AB1	CG2608	0.901220215974966	0.340374906332623	0.994766463583882	pseudobulk	DESeq2	LRT	0.468042465164959
846	KC_AB1	CG2611	0.301066600043355	0.777441146378553	0.994766463583882	pseudobulk	DESeq2	LRT	0.109332477890154
847	KC_AB1	CG2614	-0.158841856211958	0.849375260410304	0.994766463583882	pseudobulk	DESeq2	LRT	0.0709003927728893
847	KC_AB1	CG2617	-0.49335164490778	0.785516041641581	0.994766463583882	pseudobulk	DESeq2	LRT	0.104844941463879
847	KC_AB1	CG2656	-0.618225918837218	0.477817462146877	0.994766463583882	pseudobulk	DESeq2	LRT	0.320737982724581
847	KC_AB1	CG2662	0.551778923865747	0.69197970483606	0.994766463583882	pseudobulk	DESeq2	LRT	0.159906642836539
847	KC_AB1	CG2680	0.212599444766775	0.867049015163162	0.994766463583882	pseudobulk	DESeq2	LRT	0.0619563507208009
847	KC_AB1	CG2681	1.1821590793593	0.719511699510136	0.994766463583882	pseudobulk	DESeq2	LRT	0.142962139890846
847	KC_AB1	CG2685	0.575399281961092	0.646564547540924	0.994766463583882	pseudobulk	DESeq2	LRT	0.189388112328017
847	KC_AB1	CG2691	1.90469791235129	0.446136734974147	0.994766463583882	pseudobulk	DESeq2	LRT	0.350532015405037
847	KC_AB1	CG2694	2.54275315134261	0.410734813545839	0.994766463583882	pseudobulk	DESeq2	LRT	0.386438485142247
847	KC_AB1	CG2698	-0.292911398605474	0.593698775284176	0.994766463583882	pseudobulk	DESeq2	LRT	0.226433846960539
848	KC_AB1	CG2701	-0.350766219496912	0.65401551673566	0.994766463583882	pseudobulk	DESeq2	LRT	0.184411947772471
848	KC_AB1	CG2712	2.25702551253886	0.345690610560104	0.994766463583882	pseudobulk	DESeq2	LRT	0.461312416342215
848	KC_AB1	CG2747	0.171029814372294	0.710154610297429	0.994766463583882	pseudobulk	DESeq2	LRT	0.148647089183534
848	KC_AB1	CG2765	0.234098997920008	0.707147681269349	0.994766463583882	pseudobulk	DESeq2	LRT	0.150489878335116
848	KC_AB1	CG2772	0.680965410383079	0.237438988707324	0.994766463583882	pseudobulk	DESeq2	LRT	0.624447966130531
848	KC_AB1	CG2790	-0.654850140144588	0.355197723296454	0.994766463583882	pseudobulk	DESeq2	LRT	0.449529826572929
848	KC_AB1	CG2794	-1.74683112255917	0.584939641962841	0.994766463583882	pseudobulk	DESeq2	LRT	0.232888945054326
848	KC_AB1	CG2811	0.0132857370085235	0.893712025401144	0.994766463583882	pseudobulk	DESeq2	LRT	0.0488023983479135
848	KC_AB1	CG2812	1.80939591345248	0.468117841155507	0.994766463583882	pseudobulk	DESeq2	LRT	0.329644806495434
848	KC_AB1	CG2818	-0.303170064485423	0.671509899044928	0.994766463583882	pseudobulk	DESeq2	LRT	0.172947580807774
849	KC_AB1	CG2841	-1.64952313825113	0.817565084244375	0.994766463583882	pseudobulk	DESeq2	LRT	0.0874776642296574
849	KC_AB1	CG2846	-0.397417505859348	0.640735619438029	0.994766463583882	pseudobulk	DESeq2	LRT	0.193321132266302
849	KC_AB1	CG2852	0.139124701672522	0.665755977564683	0.994766463583882	pseudobulk	DESeq2	LRT	0.176684925509821
849	KC_AB1	CG2865	-0.0990054517464112	0.683017161619023	0.994766463583882	pseudobulk	DESeq2	LRT	0.165568384015676
849	KC_AB1	CG2875	0.184879239640402	0.916746354121716	0.994766463583882	pseudobulk	DESeq2	LRT	0.0377508085426733
849	KC_AB1	CG2889	-0.678865004748823	0.398194809646648	0.994766463583882	pseudobulk	DESeq2	LRT	0.39990440517378
849	KC_AB1	CG2898	-2.20969975456663	0.610836134188693	0.994766463583882	pseudobulk	DESeq2	LRT	0.214075280039667
849	KC_AB1	CG2906	0.111976988912282	0.911177946410688	0.994766463583882	pseudobulk	DESeq2	LRT	0.040396800197668
849	KC_AB1	CG2909	0.0775272217928546	0.938032236857631	0.994766463583882	pseudobulk	DESeq2	LRT	0.0277822361957651
849	KC_AB1	CG2911	1.56670097165014	0.453537968127182	0.994766463583882	pseudobulk	DESeq2	LRT	0.343386349931565
850	KC_AB1	CG2915	-0.151616337861527	0.869964296759384	0.994766463583882	pseudobulk	DESeq2	LRT	0.0604985704142067
850	KC_AB1	CG2918	-0.132005914777076	0.834191179854144	0.994766463583882	pseudobulk	DESeq2	LRT	0.0787344063877245
850	KC_AB1	CG2921	-0.116074392620576	0.881269656752481	0.994766463583882	pseudobulk	DESeq2	LRT	0.0548911829379329
850	KC_AB1	CG2924	0.228544205339049	0.481229664043887	0.994766463583882	pseudobulk	DESeq2	LRT	0.317647609649947
850	KC_AB1	CG2926	-0.194589580389267	0.58355002064811	0.994766463583882	pseudobulk	DESeq2	LRT	0.233921911237506
850	KC_AB1	CG2931	0.287931437863924	0.624071111177553	0.994766463583882	pseudobulk	DESeq2	LRT	0.204765920842546
850	KC_AB1	CG2938	-0.0577594910851229	0.895612128009874	0.994766463583882	pseudobulk	DESeq2	LRT	0.04788003401652
850	KC_AB1	CG2964	1.1821590793593	0.719511699510136	0.994766463583882	pseudobulk	DESeq2	LRT	0.142962139890846
850	KC_AB1	CG2972	0.670026632288385	0.648488622422812	0.994766463583882	pseudobulk	DESeq2	LRT	0.188097639105477
850	KC_AB1	CG2991	-0.42737108803141	0.370312529595082	0.994766463583882	pseudobulk	DESeq2	LRT	0.431431593265313
851	KC_AB1	CG2993	0.215974954459222	0.459091392230817	0.994766463583882	pseudobulk	DESeq2	LRT	0.338100849995258

851	KC_AB1	CG30001	-0.835152883801839	0.920434724857442	0.994766463583882	pseudobulk	DESeq2	LRT	0.0360070052473159
851	KC_AB1	CG30008	-1.64952313825113	0.817565084244375	0.994766463583882	pseudobulk	DESeq2	LRT	0.0874776642296574
851	KC_AB1	CG30010	-1.6914512220979	0.503726753612354	0.994766463583882	pseudobulk	DESeq2	LRT	0.297804982559676
851	KC_AB1	CG30015	1.1821590793593	0.719511699510136	0.994766463583882	pseudobulk	DESeq2	LRT	0.142962139890846
851	KC_AB1	CG30020	0.102487014382045	0.916161385928346	0.994766463583882	pseudobulk	DESeq2	LRT	0.0380280166765513
851	KC_AB1	CG30022	0.770527850009901	0.394133824393095	0.994766463583882	pseudobulk	DESeq2	LRT	0.404356292571791
851	KC_AB1	CG30054	-1.04501834804609	0.667238444024771	0.994766463583882	pseudobulk	DESeq2	LRT	0.175718939068719
851	KC_AB1	CG30060	-0.407732624188146	0.804420737973046	0.994766463583882	pseudobulk	DESeq2	LRT	0.0945167418157653
851	KC_AB1	CG30069	-0.368301516422592	0.744589348550846	0.994766463583882	pseudobulk	DESeq2	LRT	0.128083180713219
852	KC_AB1	CG3008	0.973947799334459	0.44125546538534	0.994766463583882	pseudobulk	DESeq2	LRT	0.355309902404132
852	KC_AB1	CG30089	0.238189774710377	0.670588565267442	0.994766463583882	pseudobulk	DESeq2	LRT	0.17354385634053
852	KC_AB1	CG3009	0.702046775113123	0.452558166338446	0.994766463583882	pseudobulk	DESeq2	LRT	0.34432559392539
852	KC_AB1	CG30094	0.122703144746986	0.789276228678906	0.994766463583882	pseudobulk	DESeq2	LRT	0.10277097702365
852	KC_AB1	CG30096	0.617650429963499	0.691066078567419	0.994766463583882	pseudobulk	DESeq2	LRT	0.160480424137882
852	KC_AB1	CG30099	0.472964318665191	0.749863557626698	0.994766463583882	pseudobulk	DESeq2	LRT	0.125017752022316
852	KC_AB1	CG30105	1.1821590793593	0.719511699510136	0.994766463583882	pseudobulk	DESeq2	LRT	0.142962139890846
852	KC_AB1	CG30109	-0.701830597349508	0.558164124400653	0.994766463583882	pseudobulk	DESeq2	LRT	0.25323808091714
852	KC_AB1	CG30110	1.32710828904082	0.607473293222376	0.994766463583882	pseudobulk	DESeq2	LRT	0.216472810505135
852	KC_AB1	CG30116	0.0259141423749142	0.876068785622878	0.994766463583882	pseudobulk	DESeq2	LRT	0.0574617933254659
853	KC_AB1	CG30122	-0.228045858899296	0.649213089129883	0.994766463583882	pseudobulk	DESeq2	LRT	0.187612732715836
853	KC_AB1	CG3014	1.1821590793593	0.719511699510136	0.994766463583882	pseudobulk	DESeq2	LRT	0.142962139890846
853	KC_AB1	CG30156	0.340112567480486	0.795364603107022	0.994766463583882	pseudobulk	DESeq2	LRT	0.0994337407543186
853	KC_AB1	CG30157	0.217906885051292	0.902008941520292	0.994766463583882	pseudobulk	DESeq2	LRT	0.0447891573209458
853	KC_AB1	CG30158	0.375395676877865	0.193712109954	0.994766463583882	pseudobulk	DESeq2	LRT	0.712843228419753
853	KC_AB1	CG30172	0.0243070351593997	0.915918853996868	0.994766463583882	pseudobulk	DESeq2	LRT	0.0381430010291429
853	KC_AB1	CG30183	-0.0813546184264884	0.888654438767453	0.994766463583882	pseudobulk	DESeq2	LRT	0.0512670854996878
853	KC_AB1	CG30187	2.67764418499683	0.378704357417491	0.994766463583882	pseudobulk	DESeq2	LRT	0.421699697806334
853	KC_AB1	CG3021	0.265717584717128	0.835630223492062	0.994766463583882	pseudobulk	DESeq2	LRT	0.0779858606275216
853	KC_AB1	CG30259	1.1821590793593	0.719511699510136	0.994766463583882	pseudobulk	DESeq2	LRT	0.142962139890846
854	KC_AB1	CG30270	-2.57435989741145	0.533617212681275	0.994766463583882	pseudobulk	DESeq2	LRT	0.272770169998685
854	KC_AB1	CG30288	0.949183608371002	0.80156895525114	0.994766463583882	pseudobulk	DESeq2	LRT	0.0960591113673091
854	KC_AB1	CG30289	0.949183608371002	0.80156895525114	0.994766463583882	pseudobulk	DESeq2	LRT	0.0960591113673091
854	KC_AB1	CG3032	0.00891646089885312	0.960226720676327	0.994766463583882	pseudobulk	DESeq2	LRT	0.0176262128838119
854	KC_AB1	CG30324	2.54275315134261	0.410734813545839	0.994766463583882	pseudobulk	DESeq2	LRT	0.386438485142247
854	KC_AB1	CG30325	0.806933541094274	0.754600478929312	0.994766463583882	pseudobulk	DESeq2	LRT	0.122282923497213
854	KC_AB1	CG30334	-2.57435989741145	0.533617212681275	0.994766463583882	pseudobulk	DESeq2	LRT	0.272770169998685
854	KC_AB1	CG30338	0.597141410293382	0.802139944912821	0.994766463583882	pseudobulk	DESeq2	LRT	0.0957498561530429
854	KC_AB1	CG30344	-0.539559734574841	0.676430005006863	0.994766463583882	pseudobulk	DESeq2	LRT	0.169777136260736
854	KC_AB1	CG30349	1.46873001528539	0.274753280139054	0.994766463583882	pseudobulk	DESeq2	LRT	0.561057114053562
855	KC_AB1	CG30355	0.949183608371002	0.80156895525114	0.994766463583882	pseudobulk	DESeq2	LRT	0.0960591113673091
855	KC_AB1	CG30357	0.949183608371002	0.80156895525114	0.994766463583882	pseudobulk	DESeq2	LRT	0.0960591113673091
855	KC_AB1	CG3036	2.22023857837763	0.524904842126482	0.994766463583882	pseudobulk	DESeq2	LRT	0.279919420946639
855	KC_AB1	CG30373	-0.158938159180813	0.786406430473157	0.994766463583882	pseudobulk	DESeq2	LRT	0.104352943920084
855	KC_AB1	CG30378	2.02670873290438	0.538959414925803	0.994766463583882	pseudobulk	DESeq2	LRT	0.26844393710898
855	KC_AB1	CG3038	0.949183608371002	0.80156895525114	0.994766463583882	pseudobulk	DESeq2	LRT	0.0960591113673091
855	KC_AB1	CG30380	-1.64952313825113	0.817565084244375	0.994766463583882	pseudobulk	DESeq2	LRT	0.0874776642296574
855	KC_AB1	CG30389	-0.0628679114611637	0.834338483277115	0.994766463583882	pseudobulk	DESeq2	LRT	0.0786577244259454
855	KC_AB1	CG30392	0.00180445057934422	0.894021960866312	0.994766463583882	pseudobulk	DESeq2	LRT	0.048651813009545
855	KC_AB1	CG30394	-0.448773946058956	0.741933225768378	0.994766463583882	pseudobulk	DESeq2	LRT	0.129635179606793
856	KC_AB1	CG3040	0.237575389253231	0.75089814055966	0.994766463583882	pseudobulk	DESeq2	LRT	0.124418971109162
856	KC_AB1	CG30403	1.57630120515932	0.359592692327697	0.994766463583882	pseudobulk	DESeq2	LRT	0.444189142617353
856	KC_AB1	CG30419	0.00887943604643595	0.938501370162074	0.994766463583882	pseudobulk	DESeq2	LRT	0.027565088995332
856	KC_AB1	CG30423	0.117682496143083	0.76522357624775	0.994766463583882	pseudobulk	DESeq2	LRT	0.116211657990573
856	KC_AB1	CG30428	-0.0811319920524052	0.787386959159176	0.994766463583882	pseudobulk	DESeq2	LRT	0.103811782347554
856	KC_AB1	CG3045	1.60312588074698	0.44413726555838	0.994766463583882	pseudobulk	DESeq2	LRT	0.352482785602776
856	KC_AB1	CG30460	-1.92352674994464	0.192756937222168	0.994766463583882	pseudobulk	DESeq2	LRT	0.714989982962649

856	KC_AB1	CG30466	1.11168776257694	0.593310522446876	0.994766463583882	pseudobulk	DESeq2	LRT	0.226717949319565
856	KC_AB1	CG30467	-0.619790843211065	0.568918099252663	0.994766463583882	pseudobulk	DESeq2	LRT	0.244950249602727
856	KC_AB1	CG30480	0.806933541094274	0.754600478929312	0.994766463583882	pseudobulk	DESeq2	LRT	0.122282923497213
857	KC_AB1	CG30485	-1.64952313825113	0.817565084244375	0.994766463583882	pseudobulk	DESeq2	LRT	0.0874776642296574
857	KC_AB1	CG30491	-0.0779013611100221	0.868669992946601	0.994766463583882	pseudobulk	DESeq2	LRT	0.0611451803550822
857	KC_AB1	CG30495	0.329367741436991	0.858058210994256	0.994766463583882	pseudobulk	DESeq2	LRT	0.06648324844961
857	KC_AB1	CG30496	1.1821590793593	0.719511699510136	0.994766463583882	pseudobulk	DESeq2	LRT	0.142962139890846
857	KC_AB1	CG3061	0.290791115084498	0.521423750008931	0.994766463583882	pseudobulk	DESeq2	LRT	0.282809191305557
857	KC_AB1	CG3065	0.366902743081425	0.629731399776004	0.994766463583882	pseudobulk	DESeq2	LRT	0.200844651291447
857	KC_AB1	CG3071	-1.65803656949702	0.174732883507828	0.994766463583882	pseudobulk	DESeq2	LRT	0.757625356138379
857	KC_AB1	CG3078	0.746489201084001	0.282355538694733	0.994766463583882	pseudobulk	DESeq2	LRT	0.549203688709937
857	KC_AB1	CG3097	0.279441413418566	0.89333673383135	0.994766463583882	pseudobulk	DESeq2	LRT	0.0489848075400747
857	KC_AB1	CG31029	0.949183608371002	0.80156895525114	0.994766463583882	pseudobulk	DESeq2	LRT	0.0960591113673091
858	KC_AB1	CG31030	0.0510296403408529	0.839134702626268	0.994766463583882	pseudobulk	DESeq2	LRT	0.0761683181801063
858	KC_AB1	CG31038	-0.870672022345781	0.480502378806094	0.994766463583882	pseudobulk	DESeq2	LRT	0.318304457943817
858	KC_AB1	CG3104	0.0976158184032386	0.806083027772374	0.994766463583882	pseudobulk	DESeq2	LRT	0.0936202229017546
858	KC_AB1	CG31051	-0.0231337959244529	0.849291625652362	0.994766463583882	pseudobulk	DESeq2	LRT	0.0709431582075923
858	KC_AB1	CG31064	-0.309296532012715	0.459459516578818	0.994766463583882	pseudobulk	DESeq2	LRT	0.337752748697841
858	KC_AB1	CG31068	-1.76643687325323	0.543810819055668	0.994766463583882	pseudobulk	DESeq2	LRT	0.26455215642223
858	KC_AB1	CG3107	-0.22362946654818	0.52868512600711	0.994766463583882	pseudobulk	DESeq2	LRT	0.276802907835674
858	KC_AB1	CG3108	-2.79499059556516	0.38564607643653	0.994766463583882	pseudobulk	DESeq2	LRT	0.413811082791365
858	KC_AB1	CG31098	-0.697819747867819	0.632718591226243	0.994766463583882	pseudobulk	DESeq2	LRT	0.198789404440254
858	KC_AB1	CG31104	-1.64952313825113	0.817565084244375	0.994766463583882	pseudobulk	DESeq2	LRT	0.0874776642296574
859	KC_AB1	CG31109	-0.351791612101472	0.59226253362832	0.994766463583882	pseudobulk	DESeq2	LRT	0.227485739844504
859	KC_AB1	CG31111	-0.00181849750312253	0.939313231781171	0.994766463583882	pseudobulk	DESeq2	LRT	0.0271895598639189
859	KC_AB1	CG31115	-2.57435989741145	0.533617212681275	0.994766463583882	pseudobulk	DESeq2	LRT	0.272770169998685
859	KC_AB1	CG31122	-0.357757200579078	0.474705880684762	0.994766463583882	pseudobulk	DESeq2	LRT	0.323575388182371
859	KC_AB1	CG31125	-1.77349715540584	0.167169395359783	0.994766463583882	pseudobulk	DESeq2	LRT	0.776843228350116
859	KC_AB1	CG31126	0.49592736804342	0.495660821891164	0.994766463583882	pseudobulk	DESeq2	LRT	0.304815407318182
859	KC_AB1	CG31140	0.0513109608650507	0.740782868407557	0.994766463583882	pseudobulk	DESeq2	LRT	0.130309069849469
859	KC_AB1	CG31142	-0.288457041494708	0.806005483533209	0.994766463583882	pseudobulk	DESeq2	LRT	0.0936620035297566
859	KC_AB1	CG31145	-1.64952313825113	0.817565084244375	0.994766463583882	pseudobulk	DESeq2	LRT	0.0874776642296574
859	KC_AB1	CG31156	1.20742542775355	0.212070094190174	0.994766463583882	pseudobulk	DESeq2	LRT	0.673520570728655
860	KC_AB1	CG31157	1.80633728195307	0.61161102402172	0.994766463583882	pseudobulk	DESeq2	LRT	0.213524695220716
860	KC_AB1	CG31174	-1.34684885465157	0.273389639209975	0.994766463583882	pseudobulk	DESeq2	LRT	0.563217948141267
860	KC_AB1	CG31191	0.191999401084419	0.531055668549961	0.994766463583882	pseudobulk	DESeq2	LRT	0.274859951090185
860	KC_AB1	CG31195	-0.280158654560806	0.855726772876627	0.994766463583882	pseudobulk	DESeq2	LRT	0.0676648801520045
860	KC_AB1	CG31211	0.148301445394133	0.634950739451245	0.994766463583882	pseudobulk	DESeq2	LRT	0.197259966699132
860	KC_AB1	CG31213	0.596808856322697	0.822761281772702	0.994766463583882	pseudobulk	DESeq2	LRT	0.0847261539098712
860	KC_AB1	CG31221	-0.221664475370794	0.537986871244335	0.994766463583882	pseudobulk	DESeq2	LRT	0.269228322504076
860	KC_AB1	CG31223	0.0730733768153475	0.947920232766504	0.994766463583882	pseudobulk	DESeq2	LRT	0.0232282068886007
860	KC_AB1	CG31224	0.333745454292948	0.488599451299275	0.994766463583882	pseudobulk	DESeq2	LRT	0.311047025078159
860	KC_AB1	CG31229	-0.299940585690935	0.666881492332244	0.994766463583882	pseudobulk	DESeq2	LRT	0.1759513351972
861	KC_AB1	CG31249	-0.202661317500214	0.810639221857383	0.994766463583882	pseudobulk	DESeq2	LRT	0.091172387241077
861	KC_AB1	CG31251	2.66804865204241	0.16130379709819	0.994766463583882	pseudobulk	DESeq2	LRT	0.792355409180278
861	KC_AB1	CG31278	-1.69608958436761	0.458437420803486	0.994766463583882	pseudobulk	DESeq2	LRT	0.338719939502361
861	KC_AB1	CG31301	0.0941509190665895	0.880502971318061	0.994766463583882	pseudobulk	DESeq2	LRT	0.0552691741375214
861	KC_AB1	CG31324	0.751015450017926	0.525114247196763	0.994766463583882	pseudobulk	DESeq2	LRT	0.279746198443094
861	KC_AB1	CG31342	-0.448268261727263	0.408620880931271	0.994766463583882	pseudobulk	DESeq2	LRT	0.388679444258833
861	KC_AB1	CG31344	-1.02011572125615	0.0909046077496316	0.994766463583882	pseudobulk	DESeq2	LRT	1.04141410281189
861	KC_AB1	CG31357	-0.295087747307667	0.695656138698591	0.994766463583882	pseudobulk	DESeq2	LRT	0.157605378159774
861	KC_AB1	CG31360	0.235536390198619	0.752540058923982	0.994766463583882	pseudobulk	DESeq2	LRT	0.123470376921384
861	KC_AB1	CG31365	-0.251357199555348	0.847326580630722	0.994766463583882	pseudobulk	DESeq2	LRT	0.0719491695636336
862	KC_AB1	CG31368	0.698147766237832	0.519125630843771	0.994766463583882	pseudobulk	DESeq2	LRT	0.284727528125163
862	KC_AB1	CG31371	-1.54329720724185	0.472915372414475	0.994766463583882	pseudobulk	DESeq2	LRT	0.325216568737222
862	KC_AB1	CG31381	0.69750158688829	0.750018748510497	0.994766463583882	pseudobulk	DESeq2	LRT	0.124927880244456

862	KC_AB1	CG31388	0.31493961375643	0.673747753868973	0.994766463583882	pseudobulk	DESeq2	LRT	0.171502669647782
862	KC_AB1	CG31415	-0.548887546079202	0.814781693583783	0.994766463583882	pseudobulk	DESeq2	LRT	0.0889587372379754
862	KC_AB1	CG31437	-1.64952313825113	0.817565084244375	0.994766463583882	pseudobulk	DESeq2	LRT	0.0874776642296574
862	KC_AB1	CG31441	1.02996170936706	0.619231122089829	0.994766463583882	pseudobulk	DESeq2	LRT	0.208147224463057
862	KC_AB1	CG31446	1.1821590793593	0.719511699510136	0.994766463583882	pseudobulk	DESeq2	LRT	0.142962139890846
862	KC_AB1	CG31457	-0.593588837429502	0.717888719837047	0.994766463583882	pseudobulk	DESeq2	LRT	0.143942870668869
862	KC_AB1	CG31460	-0.0984344681615454	0.900958321435396	0.994766463583882	pseudobulk	DESeq2	LRT	0.0452952991311345
863	KC_AB1	CG31467	0.949183608371002	0.80156895525114	0.994766463583882	pseudobulk	DESeq2	LRT	0.0960591113673091
863	KC_AB1	CG31469	-1.76643687325323	0.543810819055668	0.994766463583882	pseudobulk	DESeq2	LRT	0.26455215642223
863	KC_AB1	CG31475	-0.392795095091803	0.395117318037946	0.994766463583882	pseudobulk	DESeq2	LRT	0.403273934723796
863	KC_AB1	CG3149	0.546848275227028	0.784144882426977	0.994766463583882	pseudobulk	DESeq2	LRT	0.105603687538331
863	KC_AB1	CG31510	0.187490931741723	0.792107761747117	0.994766463583882	pseudobulk	DESeq2	LRT	0.101215731101757
863	KC_AB1	CG31522	0.22523160050966	0.624473017928831	0.994766463583882	pseudobulk	DESeq2	LRT	0.204486321769867
863	KC_AB1	CG31523	0.035835323449803	0.881865080899541	0.994766463583882	pseudobulk	DESeq2	LRT	0.0545978537603399
863	KC_AB1	CG31538	2.38929733117871	0.484296857543297	0.994766463583882	pseudobulk	DESeq2	LRT	0.314888348950639
863	KC_AB1	CG31549	-4.2169236809797	0.105189937930809	0.994766463583882	pseudobulk	DESeq2	LRT	0.978025801153034
863	KC_AB1	CG31550	-0.182284912379713	0.706592962773105	0.994766463583882	pseudobulk	DESeq2	LRT	0.150830692182843
864	KC_AB1	CG3156	-0.273752191915699	0.778902630837319	0.994766463583882	pseudobulk	DESeq2	LRT	0.108516829276155
864	KC_AB1	CG31612	-2.79499059556516	0.38564607643653	0.994766463583882	pseudobulk	DESeq2	LRT	0.413811082791365
864	KC_AB1	CG31627	-2.79499059556516	0.38564607643653	0.994766463583882	pseudobulk	DESeq2	LRT	0.413811082791365
864	KC_AB1	CG3163	-0.591306548410588	0.547231204660871	0.994766463583882	pseudobulk	DESeq2	LRT	0.261829145890221
864	KC_AB1	CG31633	-0.48247330649414	0.702504053757564	0.994766463583882	pseudobulk	DESeq2	LRT	0.153351165345357
864	KC_AB1	CG31635	-0.228531969642744	0.467032093495821	0.994766463583882	pseudobulk	DESeq2	LRT	0.330653274574717
864	KC_AB1	CG31637	0.273175735342349	0.447678262866415	0.994766463583882	pseudobulk	DESeq2	LRT	0.349033992385269
864	KC_AB1	CG31638	0.523081283448893	0.313719714909187	0.994766463583882	pseudobulk	DESeq2	LRT	0.50345818832414
864	KC_AB1	CG3164	0.0861307169848128	0.935058501013443	0.994766463583882	pseudobulk	DESeq2	LRT	0.0291612170712939
864	KC_AB1	CG31642	1.45682795759908	0.573003993665791	0.994766463583882	pseudobulk	DESeq2	LRT	0.24184235111992
865	KC_AB1	CG31643	-1.16090953734183	0.506143046663762	0.994766463583882	pseudobulk	DESeq2	LRT	0.295726725063221
865	KC_AB1	CG31648	0.292049068461775	0.800437931657679	0.994766463583882	pseudobulk	DESeq2	LRT	0.0966723389272744
865	KC_AB1	CG3165	0.739275718598879	0.457267002876578	0.994766463583882	pseudobulk	DESeq2	LRT	0.33983013687793
865	KC_AB1	CG31650	0.42800102366391	0.249818177398026	0.994766463583882	pseudobulk	DESeq2	LRT	0.602375964454898
865	KC_AB1	CG31663	-2.57435989741145	0.533617212681275	0.994766463583882	pseudobulk	DESeq2	LRT	0.272770169998685
865	KC_AB1	CG31675	-1.64952313825113	0.817565084244375	0.994766463583882	pseudobulk	DESeq2	LRT	0.0874776642296574
865	KC_AB1	CG31678	-0.536608777463881	0.531531999286874	0.994766463583882	pseudobulk	DESeq2	LRT	0.274470584959332
865	KC_AB1	CG31683	0.949183608371002	0.80156895525114	0.994766463583882	pseudobulk	DESeq2	LRT	0.0960591113673091
865	KC_AB1	CG31687	-0.166103994815893	0.789281556812532	0.994766463583882	pseudobulk	DESeq2	LRT	0.102768045260171
865	KC_AB1	CG31688	-0.0136398229405472	0.960946288169822	0.994766463583882	pseudobulk	DESeq2	LRT	0.0173008864244499
866	KC_AB1	CG31690	-0.124802871985165	0.853092789095365	0.994766463583882	pseudobulk	DESeq2	LRT	0.0690037289726112
866	KC_AB1	CG31694	-0.415221934079794	0.44707731038265	0.994766463583882	pseudobulk	DESeq2	LRT	0.349617370448803
866	KC_AB1	CG31705	0.949183608371002	0.80156895525114	0.994766463583882	pseudobulk	DESeq2	LRT	0.0960591113673091
866	KC_AB1	CG31706	0.949183608371002	0.80156895525114	0.994766463583882	pseudobulk	DESeq2	LRT	0.0960591113673091
866	KC_AB1	CG31712	0.0557095604046344	0.783527244821247	0.994766463583882	pseudobulk	DESeq2	LRT	0.105945897638519
866	KC_AB1	CG31715	-0.0735610492270341	0.853367558671763	0.994766463583882	pseudobulk	DESeq2	LRT	0.0688638711528709
866	KC_AB1	CG31717	-0.19382372810314	0.771305031057637	0.994766463583882	pseudobulk	DESeq2	LRT	0.112773835818283
866	KC_AB1	CG31729	-0.494186811600955	0.342738967710901	0.994766463583882	pseudobulk	DESeq2	LRT	0.465036515646808
866	KC_AB1	CG31751	-2.40682050561227	0.195449758131335	0.994766463583882	pseudobulk	DESeq2	LRT	0.708964862669909
866	KC_AB1	CG31752	-0.835152883801839	0.920434724857442	0.994766463583882	pseudobulk	DESeq2	LRT	0.0360070052473159
867	KC_AB1	CG3176	1.00206552945986	0.690075213866505	0.994766463583882	pseudobulk	DESeq2	LRT	0.161103571310595
867	KC_AB1	CG31760	-0.215628538099435	0.506109452202786	0.994766463583882	pseudobulk	DESeq2	LRT	0.295755551643636
867	KC_AB1	CG31776	-1.64952313825113	0.817565084244375	0.994766463583882	pseudobulk	DESeq2	LRT	0.0874776642296574
867	KC_AB1	CG31788	0.949183608371002	0.80156895525114	0.994766463583882	pseudobulk	DESeq2	LRT	0.0960591113673091
867	KC_AB1	CG31793	1.32789395261901	0.50354133529231	0.994766463583882	pseudobulk	DESeq2	LRT	0.297964872771697
867	KC_AB1	CG31800	-0.116135105653099	0.883742126427513	0.994766463583882	pseudobulk	DESeq2	LRT	0.0536744424615972
867	KC_AB1	CG31805	-2.79499059556516	0.38564607643653	0.994766463583882	pseudobulk	DESeq2	LRT	0.413811082791365
867	KC_AB1	CG31806	0.949183608371002	0.80156895525114	0.994766463583882	pseudobulk	DESeq2	LRT	0.0960591113673091
867	KC_AB1	CG31808	0.0502934018227491	0.866546518065734	0.994766463583882	pseudobulk	DESeq2	LRT	0.0622081184699839

867	KC_AB1	CG31815	-1.56758265197595	0.404778638832039	0.994766463583882	pseudobulk	DESeq2	LRT	0.392782414355397
868	KC_AB1	CG31817	-2.57435989741145	0.533617212681275	0.994766463583882	pseudobulk	DESeq2	LRT	0.272770169998685
868	KC_AB1	CG31832	-1.08681444615798	0.701131388297773	0.994766463583882	pseudobulk	DESeq2	LRT	0.154200589927933
868	KC_AB1	CG3184	0.240824995802028	0.807923627478938	0.994766463583882	pseudobulk	DESeq2	LRT	0.0926296908740521
868	KC_AB1	CG31849	0.584385001526002	0.808656675386651	0.994766463583882	pseudobulk	DESeq2	LRT	0.0922358240397888
868	KC_AB1	CG31855	0.660056052484885	0.287170406523927	0.994766463583882	pseudobulk	DESeq2	LRT	0.541860317025708
868	KC_AB1	CG31882	0.949183608371002	0.80156895525114	0.994766463583882	pseudobulk	DESeq2	LRT	0.0960591113673091
868	KC_AB1	CG31883	-2.20969975456663	0.610836134188693	0.994766463583882	pseudobulk	DESeq2	LRT	0.214075280039667
868	KC_AB1	CG31886	1.06071898122436	0.628579022653205	0.994766463583882	pseudobulk	DESeq2	LRT	0.201640116633702
868	KC_AB1	CG31897	2.92183940034187	0.320324374195056	0.994766463583882	pseudobulk	DESeq2	LRT	0.494410013644442
868	KC_AB1	CG31908	0.13849520938936	0.843810904647337	0.994766463583882	pseudobulk	DESeq2	LRT	0.0737548664896146
869	KC_AB1	CG3191	0.601176955527342	0.63471688542155	0.994766463583882	pseudobulk	DESeq2	LRT	0.197419947963246
869	KC_AB1	CG31913	-2.20969975456663	0.610836134188693	0.994766463583882	pseudobulk	DESeq2	LRT	0.214075280039667
869	KC_AB1	CG31915	-0.0411805580936525	0.858506575561409	0.994766463583882	pseudobulk	DESeq2	LRT	0.0662563740975915
869	KC_AB1	CG31921	0.478005084452508	0.815070723509861	0.994766463583882	pseudobulk	DESeq2	LRT	0.0888047059870473
869	KC_AB1	CG31922	0.0971508214061977	0.921438858879437	0.994766463583882	pseudobulk	DESeq2	LRT	0.0355334766491562
869	KC_AB1	CG31935	-0.0843092631143923	0.919316610627249	0.994766463583882	pseudobulk	DESeq2	LRT	0.0365348927837787
869	KC_AB1	CG31937	0.2147019870634	0.90028167280434	0.994766463583882	pseudobulk	DESeq2	LRT	0.0456215907762435
869	KC_AB1	CG31957	0.605020823834196	0.574054776968526	0.994766463583882	pseudobulk	DESeq2	LRT	0.241046664744662
869	KC_AB1	CG3198	0.0340804152255104	0.90151842099688	0.994766463583882	pseudobulk	DESeq2	LRT	0.0450253947812234
869	KC_AB1	CG31997	0.0903242148283129	0.742061463036565	0.994766463583882	pseudobulk	DESeq2	LRT	0.129560121739663
870	KC_AB1	CG31998	0.0996552898408998	0.740897873272259	0.994766463583882	pseudobulk	DESeq2	LRT	0.130241651846619
870	KC_AB1	CG31999	-0.410347151721077	0.310813443190387	0.994766463583882	pseudobulk	DESeq2	LRT	0.507500205517378
870	KC_AB1	CG32017	0.068265216811301	0.775193951533484	0.994766463583882	pseudobulk	DESeq2	LRT	0.110589624535567
870	KC_AB1	CG32022	-0.725298463983326	0.496544110525354	0.994766463583882	pseudobulk	DESeq2	LRT	0.304042164878922
870	KC_AB1	CG32036	2.80395130970481	0.386005157167114	0.994766463583882	pseudobulk	DESeq2	LRT	0.413406892959702
870	KC_AB1	CG32039	-0.100504847287673	0.854632054388595	0.994766463583882	pseudobulk	DESeq2	LRT	0.0682208222805604
870	KC_AB1	CG32052	0.643953881027309	0.407201831863618	0.994766463583882	pseudobulk	DESeq2	LRT	0.390190276922596
870	KC_AB1	CG32055	-2.20969975456663	0.610836134188693	0.994766463583882	pseudobulk	DESeq2	LRT	0.214075280039667
870	KC_AB1	CG32066	-0.0353921453290454	0.827977380375992	0.994766463583882	pseudobulk	DESeq2	LRT	0.0819815276016825
870	KC_AB1	CG32075	0.969986341149099	0.640932225194753	0.994766463583882	pseudobulk	DESeq2	LRT	0.193187892135042
871	KC_AB1	CG32085	-0.324757865344491	0.295547213379344	0.994766463583882	pseudobulk	DESeq2	LRT	0.529373131120303
871	KC_AB1	CG32086	-1.64952313825113	0.817565084244375	0.994766463583882	pseudobulk	DESeq2	LRT	0.0874776642296574
871	KC_AB1	CG32095	-0.370284509002658	0.775254933705214	0.994766463583882	pseudobulk	DESeq2	LRT	0.110555461241385
871	KC_AB1	CG32099	0.887505118989499	0.500969684891544	0.994766463583882	pseudobulk	DESeq2	LRT	0.30018855373886
871	KC_AB1	CG32100	-0.33073739545584	0.598477681347074	0.994766463583882	pseudobulk	DESeq2	LRT	0.222952040827399
871	KC_AB1	CG32104	-1.76733594774124	0.321421088280998	0.994766463583882	pseudobulk	DESeq2	LRT	0.492925632788541
871	KC_AB1	CG32109	0.885794470246143	0.290758548847888	0.994766463583882	pseudobulk	DESeq2	LRT	0.536467507337663
871	KC_AB1	CG32110	0.949183608371002	0.80156895525114	0.994766463583882	pseudobulk	DESeq2	LRT	0.0960591113673091
871	KC_AB1	CG32112	0.863781634854934	0.440177249341938	0.994766463583882	pseudobulk	DESeq2	LRT	0.356372407808544
871	KC_AB1	CG32137	-1.21457746603801	0.770013447451371	0.994766463583882	pseudobulk	DESeq2	LRT	0.113501690278259
872	KC_AB1	CG32147	-0.0414249616448951	0.925993792665326	0.994766463583882	pseudobulk	DESeq2	LRT	0.0333919245710158
872	KC_AB1	CG32163	-1.25251955668738	0.200357421182825	0.994766463583882	pseudobulk	DESeq2	LRT	0.698194566787257
872	KC_AB1	CG32176	-0.0598331956452091	0.877701987019662	0.994766463583882	pseudobulk	DESeq2	LRT	0.056652918447901
872	KC_AB1	CG32187	0.600058992856898	0.743397195411951	0.994766463583882	pseudobulk	DESeq2	LRT	0.128779081684483
872	KC_AB1	CG32191	0.949183608371002	0.80156895525114	0.994766463583882	pseudobulk	DESeq2	LRT	0.0960591113673091
872	KC_AB1	CG32192	0.131756303236789	0.898896171988158	0.994766463583882	pseudobulk	DESeq2	LRT	0.0462904690417512
872	KC_AB1	CG32196	-0.237473683803514	0.877148860518115	0.994766463583882	pseudobulk	DESeq2	LRT	0.0569266964680766
872	KC_AB1	CG32202	0.269608233724417	0.760681684649133	0.994766463583882	pseudobulk	DESeq2	LRT	0.118797040365954
872	KC_AB1	CG32204	0.0952678586734776	0.588724761113654	0.994766463583882	pseudobulk	DESeq2	LRT	0.230087697857136
872	KC_AB1	CG32206	0.233816282191151	0.556631729897793	0.994766463583882	pseudobulk	DESeq2	LRT	0.254432041036905
873	KC_AB1	CG3222	-0.561568325517272	0.770380095220986	0.994766463583882	pseudobulk	DESeq2	LRT	0.113294946869199
873	KC_AB1	CG32221	0.00502834284390088	0.881785846627974	0.994766463583882	pseudobulk	DESeq2	LRT	0.0546368762294749
873	KC_AB1	CG32225	-0.191528078059178	0.770901390399531	0.994766463583882	pseudobulk	DESeq2	LRT	0.113001171033662
873	KC_AB1	CG3223	0.384864374458516	0.70728606411145	0.994766463583882	pseudobulk	DESeq2	LRT	0.150404898879411
873	KC_AB1	CG3224	-0.346422630222373	0.494772468487202	0.994766463583882	pseudobulk	DESeq2	LRT	0.305594474598186

873	KC_AB1	CG32243	-0.467509171684459	0.359076462384107	0.994766463583882	pseudobulk	DESeq2	LRT	0.444813062130203
873	KC_AB1	CG3225	0.329367741436991	0.858058210994256	0.994766463583882	pseudobulk	DESeq2	LRT	0.06648324844961
873	KC_AB1	CG3226	-0.227177242383957	0.672062672406383	0.994766463583882	pseudobulk	DESeq2	LRT	0.172590225441538
873	KC_AB1	CG32262	-0.979658855752947	0.37141044011938	0.994766463583882	pseudobulk	DESeq2	LRT	0.430145892673483
873	KC_AB1	CG32264	-0.107630784576449	0.517992542567874	0.994766463583882	pseudobulk	DESeq2	LRT	0.285676492658117
874	KC_AB1	CG32266	-1.64952313825113	0.817565084244375	0.994766463583882	pseudobulk	DESeq2	LRT	0.0874776642296574
874	KC_AB1	CG32267	-0.0760903836650402	0.816690095681847	0.994766463583882	pseudobulk	DESeq2	LRT	0.0879427112375087
874	KC_AB1	CG32276	0.279838301859921	0.488034969848471	0.994766463583882	pseudobulk	DESeq2	LRT	0.311549057775782
874	KC_AB1	CG32278	0.235818365553598	0.543400585863303	0.994766463583882	pseudobulk	DESeq2	LRT	0.264879897686508
874	KC_AB1	CG32280	-0.313353431730565	0.681091340310071	0.994766463583882	pseudobulk	DESeq2	LRT	0.166794641490199
874	KC_AB1	CG32281	-0.0850866375679959	0.944161605492516	0.994766463583882	pseudobulk	DESeq2	LRT	0.0249536642116559
874	KC_AB1	CG32295	1.1821590793593	0.719511699510136	0.994766463583882	pseudobulk	DESeq2	LRT	0.142962139890846
874	KC_AB1	CG32333	1.64869938577302	0.418869895463942	0.994766463583882	pseudobulk	DESeq2	LRT	0.377920851631398
874	KC_AB1	CG32344	1.3149707025747	0.365540668002764	0.994766463583882	pseudobulk	DESeq2	LRT	0.437064298852024
874	KC_AB1	CG32354	1.1821590793593	0.719511699510136	0.994766463583882	pseudobulk	DESeq2	LRT	0.142962139890846
875	KC_AB1	CG32368	-0.648218248148942	0.834000364039372	0.994766463583882	pseudobulk	DESeq2	LRT	0.0788337597936092
875	KC_AB1	CG32369	-0.310913686472471	0.399862173828835	0.994766463583882	pseudobulk	DESeq2	LRT	0.398089677322837
875	KC_AB1	CG32373	0.0755777693856706	0.899297454011681	0.994766463583882	pseudobulk	DESeq2	LRT	0.0460966361097728
875	KC_AB1	CG32392	-1.11444759506805	0.195961764885748	0.994766463583882	pseudobulk	DESeq2	LRT	0.707828657822107
875	KC_AB1	CG32396	0.329367741436991	0.858058210994256	0.994766463583882	pseudobulk	DESeq2	LRT	0.06648324844961
875	KC_AB1	CG32409	-1.03550855853255	0.623427827695289	0.994766463583882	pseudobulk	DESeq2	LRT	0.205213816199734
875	KC_AB1	CG32425	-0.298778821137794	0.46836426559603	0.994766463583882	pseudobulk	DESeq2	LRT	0.329416247345347
875	KC_AB1	CG32428	0.319484968368681	0.467287039433504	0.994766463583882	pseudobulk	DESeq2	LRT	0.330416264301108
875	KC_AB1	CG32432	-0.208140613261125	0.422784931800746	0.994766463583882	pseudobulk	DESeq2	LRT	0.373880499492338
875	KC_AB1	CG32436	-0.0713915988297819	0.864170454131802	0.994766463583882	pseudobulk	DESeq2	LRT	0.063400586239177
876	KC_AB1	CG32441	0.181443750927308	0.655242209889826	0.994766463583882	pseudobulk	DESeq2	LRT	0.183598133635041
876	KC_AB1	CG32448	-0.198789733151645	0.755267228225458	0.994766463583882	pseudobulk	DESeq2	LRT	0.121899359351057
876	KC_AB1	CG32452	-0.476321779714273	0.695317677387277	0.994766463583882	pseudobulk	DESeq2	LRT	0.157816729201817
876	KC_AB1	CG32457	0.596808856322697	0.822761281772702	0.994766463583882	pseudobulk	DESeq2	LRT	0.0847261539098712
876	KC_AB1	CG32459	0.6144588285492	0.785021674273821	0.994766463583882	pseudobulk	DESeq2	LRT	0.105118352315164
876	KC_AB1	CG3246	1.1821590793593	0.719511699510136	0.994766463583882	pseudobulk	DESeq2	LRT	0.142962139890846
876	KC_AB1	CG32473	0.45173769168661	0.710347081980426	0.994766463583882	pseudobulk	DESeq2	LRT	0.148529399228198
876	KC_AB1	CG32485	0.316459636985441	0.440399464514209	0.994766463583882	pseudobulk	DESeq2	LRT	0.356153217762651
876	KC_AB1	CG32486	-0.255043159000967	0.213216212350677	0.994766463583882	pseudobulk	DESeq2	LRT	0.671179775880247
876	KC_AB1	CG32500	-2.79499059556516	0.38564607643653	0.994766463583882	pseudobulk	DESeq2	LRT	0.413811082791365
877	KC_AB1	CG32506	0.787671347878875	0.429824307118822	0.994766463583882	pseudobulk	DESeq2	LRT	0.366709028237132
877	KC_AB1	CG32521	0.974410468407292	0.486186580189771	0.994766463583882	pseudobulk	DESeq2	LRT	0.313197032802359
877	KC_AB1	CG3253	-0.415467011229896	0.604996711732494	0.994766463583882	pseudobulk	DESeq2	LRT	0.21824698581086
877	KC_AB1	CG32533	2.1636635108575	0.192530064332592	0.994766463583882	pseudobulk	DESeq2	LRT	0.715501444055427
877	KC_AB1	CG32536	-1.64952313825113	0.817565084244375	0.994766463583882	pseudobulk	DESeq2	LRT	0.0874776642296574
877	KC_AB1	CG32537	-0.360177229559183	0.774743485989298	0.994766463583882	pseudobulk	DESeq2	LRT	0.110842066603098
877	KC_AB1	CG32544	0.21637581263816	0.600957219283514	0.994766463583882	pseudobulk	DESeq2	LRT	0.221156443289105
877	KC_AB1	CG32547	0.441276737442987	0.266515488084275	0.994766463583882	pseudobulk	DESeq2	LRT	0.574277547635211
877	KC_AB1	CG32548	-1.64952313825113	0.817565084244375	0.994766463583882	pseudobulk	DESeq2	LRT	0.0874776642296574
877	KC_AB1	CG32549	-0.221548802826748	0.640232179455103	0.994766463583882	pseudobulk	DESeq2	LRT	0.193662501062614
878	KC_AB1	CG32554	0.280783473609079	0.862060689739496	0.994766463583882	pseudobulk	DESeq2	LRT	0.0644621584314761
878	KC_AB1	CG3257	0.226881607504109	0.651793713250822	0.994766463583882	pseudobulk	DESeq2	LRT	0.185889832753422
878	KC_AB1	CG32572	0.949183608371002	0.80156895525114	0.994766463583882	pseudobulk	DESeq2	LRT	0.0960591113673091
878	KC_AB1	CG32576	0.281838034887063	0.782979444956833	0.994766463583882	pseudobulk	DESeq2	LRT	0.106249639039117
878	KC_AB1	CG32590	0.0812747330416153	0.892753017907892	0.994766463583882	pseudobulk	DESeq2	LRT	0.0492686730214543
878	KC_AB1	CG3262	-0.220928936158255	0.538093018689855	0.994766463583882	pseudobulk	DESeq2	LRT	0.269142642527267
878	KC_AB1	CG32638	0.418164275147894	0.779115032024967	0.994766463583882	pseudobulk	DESeq2	LRT	0.108398416415357
878	KC_AB1	CG32641	2.16325088398155	0.257812106654197	0.994766463583882	pseudobulk	DESeq2	LRT	0.588696692375719
878	KC_AB1	CG32642	0.949183608371002	0.80156895525114	0.994766463583882	pseudobulk	DESeq2	LRT	0.0960591113673091
878	KC_AB1	CG32647	-0.293748401437566	0.359808020540297	0.994766463583882	pseudobulk	DESeq2	LRT	0.443929159952198
879	KC_AB1	CG32655	2.22023857837763	0.524904842126482	0.994766463583882	pseudobulk	DESeq2	LRT	0.279919420946639

879	KC_AB1	CG32683	-0.136745066608583	0.627495454411386	0.994766463583882	pseudobulk	DESeq2	LRT	0.202389415872745
879	KC_AB1	CG32700	-0.127735315111673	0.401218859079974	0.994766463583882	pseudobulk	DESeq2	LRT	0.396618661389728
879	KC_AB1	CG32706	0.806933541094274	0.754600478929312	0.994766463583882	pseudobulk	DESeq2	LRT	0.122282923497213
879	KC_AB1	CG32708	-2.12431104022436	0.328743519760125	0.994766463583882	pseudobulk	DESeq2	LRT	0.483142799293539
879	KC_AB1	CG32755	-1.64952313825113	0.817565084244375	0.994766463583882	pseudobulk	DESeq2	LRT	0.0874776642296574
879	KC_AB1	CG32756	-0.193890820340419	0.782566294492241	0.994766463583882	pseudobulk	DESeq2	LRT	0.106478861305336
879	KC_AB1	CG32767	0.0417831817294711	0.786186479172631	0.994766463583882	pseudobulk	DESeq2	LRT	0.104474429443951
879	KC_AB1	CG32772	-0.279018758926249	0.561511655476631	0.994766463583882	pseudobulk	DESeq2	LRT	0.250641224519801
879	KC_AB1	CG32795	0.221000921866761	0.581523232916048	0.994766463583882	pseudobulk	DESeq2	LRT	0.23543292973058
880	KC_AB1	CG32803	0.326582365639225	0.780264504813634	0.994766463583882	pseudobulk	DESeq2	LRT	0.107758149222103
880	KC_AB1	CG32808	-1.21463852839841	0.426781310135024	0.994766463583882	pseudobulk	DESeq2	LRT	0.36979460771747
880	KC_AB1	CG32809	0.242350356462054	0.347047639612572	0.994766463583882	pseudobulk	DESeq2	LRT	0.45961090503405
880	KC_AB1	CG3281	0.613905478359878	0.659335575353829	0.994766463583882	pseudobulk	DESeq2	LRT	0.180893490670624
880	KC_AB1	CG32813	0.677432682517186	0.378904735712651	0.994766463583882	pseudobulk	DESeq2	LRT	0.421469966693813
880	KC_AB1	CG32815	-0.0587633576115121	0.881417008100087	0.994766463583882	pseudobulk	DESeq2	LRT	0.0548185734621919
880	KC_AB1	CG32817	1.1821590793593	0.719511699510136	0.994766463583882	pseudobulk	DESeq2	LRT	0.142962139890846
880	KC_AB1	CG32829	1.17930484172975	0.669685881755347	0.994766463583882	pseudobulk	DESeq2	LRT	0.174128856716169
880	KC_AB1	CG32845	2.02670873290438	0.538959414925803	0.994766463583882	pseudobulk	DESeq2	LRT	0.26844393710898
880	KC_AB1	CG32850	0.0235756953417308	0.926041459678071	0.994766463583882	pseudobulk	DESeq2	LRT	0.0333695691427566
881	KC_AB1	CG32856	0.535366796290342	0.617789838227163	0.994766463583882	pseudobulk	DESeq2	LRT	0.209159239508451
881	KC_AB1	CG3288	-1.64952313825113	0.817565084244375	0.994766463583882	pseudobulk	DESeq2	LRT	0.0874776642296574
881	KC_AB1	CG3294	0.62506181737221	0.65087233953608	0.994766463583882	pseudobulk	DESeq2	LRT	0.18650418450682
881	KC_AB1	CG32944	-2.20969975456663	0.610836134188693	0.994766463583882	pseudobulk	DESeq2	LRT	0.214075280039667
881	KC_AB1	CG3295	-0.101940334403113	0.891580960328494	0.994766463583882	pseudobulk	DESeq2	LRT	0.0498392144319994
881	KC_AB1	CG32982	0.137064743678448	0.727786035880142	0.994766463583882	pseudobulk	DESeq2	LRT	0.137996281531288
881	KC_AB1	CG33051	-1.28289356543793	0.433460930391418	0.994766463583882	pseudobulk	DESeq2	LRT	0.363050041163732
881	KC_AB1	CG33054	-1.76643687325323	0.543810819055668	0.994766463583882	pseudobulk	DESeq2	LRT	0.26455215642223
881	KC_AB1	CG33056	0.858598136557056	0.663400004084503	0.994766463583882	pseudobulk	DESeq2	LRT	0.178224530142619
881	KC_AB1	CG3306	-1.64952313825113	0.817565084244375	0.994766463583882	pseudobulk	DESeq2	LRT	0.0874776642296574
882	KC_AB1	CG3308	-0.758244609296221	0.398539260445273	0.994766463583882	pseudobulk	DESeq2	LRT	0.399528889437563
882	KC_AB1	CG3309	-0.831062844857841	0.466568740979549	0.994766463583882	pseudobulk	DESeq2	LRT	0.331084361287763
882	KC_AB1	CG33090	-0.0972208413876909	0.718571176838912	0.994766463583882	pseudobulk	DESeq2	LRT	0.143530207101954
882	KC_AB1	CG33096	0.508650692587302	0.722748136286243	0.994766463583882	pseudobulk	DESeq2	LRT	0.141013019548891
882	KC_AB1	CG33098	0.128141150800652	0.8795666799127	0.994766463583882	pseudobulk	DESeq2	LRT	0.0557312311122255
882	KC_AB1	CG33107	0.553129876019271	0.519405412869395	0.994766463583882	pseudobulk	DESeq2	LRT	0.284493528778606
882	KC_AB1	CG33108	0.898708898572512	0.615954152972483	0.994766463583882	pseudobulk	DESeq2	LRT	0.210451612270739
882	KC_AB1	CG33111	0.0175877958964353	0.948226500520508	0.994766463583882	pseudobulk	DESeq2	LRT	0.0230879114205651
882	KC_AB1	CG33116	0.0544817032856016	0.831943568948106	0.994766463583882	pseudobulk	DESeq2	LRT	0.0799061310717878
882	KC_AB1	CG33127	1.1821590793593	0.719511699510136	0.994766463583882	pseudobulk	DESeq2	LRT	0.142962139890846
883	KC_AB1	CG33129	0.476771047565184	0.216144722058082	0.994766463583882	pseudobulk	DESeq2	LRT	0.665255364844816
883	KC_AB1	CG33143	-0.835152883801839	0.920434724857442	0.994766463583882	pseudobulk	DESeq2	LRT	0.0360070052473159
883	KC_AB1	CG33144	0.615229998921692	0.159694840427756	0.994766463583882	pseudobulk	DESeq2	LRT	0.796709115232542
883	KC_AB1	CG33155	-0.0529807450373425	0.885277191375424	0.994766463583882	pseudobulk	DESeq2	LRT	0.0529207249675449
883	KC_AB1	CG33156	0.379110767027048	0.630946231757927	0.994766463583882	pseudobulk	DESeq2	LRT	0.200007649069311
883	KC_AB1	CG33158	0.979680678390413	0.391831745975586	0.994766463583882	pseudobulk	DESeq2	LRT	0.406900380634562
883	KC_AB1	CG33169	0.125527707845976	0.836659827505693	0.994766463583882	pseudobulk	DESeq2	LRT	0.0774510833047736
883	KC_AB1	CG33170	0.0617465500705364	0.855675214172094	0.994766463583882	pseudobulk	DESeq2	LRT	0.0676910477748642
883	KC_AB1	CG33172	3.3077188457669	0.230409282515317	0.994766463583882	pseudobulk	DESeq2	LRT	0.637500028443383
883	KC_AB1	CG33181	0.259950017226322	0.124536901226273	0.994766463583882	pseudobulk	DESeq2	LRT	0.904701944757813
884	KC_AB1	CG33199	0.0490995881504113	0.887255276942431	0.994766463583882	pseudobulk	DESeq2	LRT	0.0519514090103341
884	KC_AB1	CG33203	-0.332270888749902	0.623369227811587	0.994766463583882	pseudobulk	DESeq2	LRT	0.205254640175117
884	KC_AB1	CG33213	2.01123047816982	0.344762548401913	0.994766463583882	pseudobulk	DESeq2	LRT	0.462479917748112
884	KC_AB1	CG33217	-0.169919870631949	0.806039620574808	0.994766463583882	pseudobulk	DESeq2	LRT	0.0936436100880849
884	KC_AB1	CG33228	-0.22781016265773	0.861363850775173	0.994766463583882	pseudobulk	DESeq2	LRT	0.0648133584130924
884	KC_AB1	CG33229	0.111195372146861	0.692832016744188	0.994766463583882	pseudobulk	DESeq2	LRT	0.15937205116674
884	KC_AB1	CG33230	-0.278551492753559	0.868752967760857	0.994766463583882	pseudobulk	DESeq2	LRT	0.0611036987989956

884	KC_AB1	CG33233	2.02670873290438	0.538959414925803	0.994766463583882	pseudobulk	DESeq2	LRT	0.26844393710898
884	KC_AB1	CG33234	-1.76643687325323	0.543810819055668	0.994766463583882	pseudobulk	DESeq2	LRT	0.26455215642223
884	KC_AB1	CG33267	2.06285803892769	0.195633124725655	0.994766463583882	pseudobulk	DESeq2	LRT	0.708557608303099
885	KC_AB1	CG33296	1.1821590793593	0.719511699510136	0.994766463583882	pseudobulk	DESeq2	LRT	0.142962139890846
885	KC_AB1	CG33298	0.250212877686345	0.589454389408702	0.994766463583882	pseudobulk	DESeq2	LRT	0.229549793952203
885	KC_AB1	CG33303	-0.230902265427331	0.589135426165035	0.994766463583882	pseudobulk	DESeq2	LRT	0.229784861281321
885	KC_AB1	CG33307	-1.64952313825113	0.817565084244375	0.994766463583882	pseudobulk	DESeq2	LRT	0.0874776642296574
885	KC_AB1	CG33310	0.0067158378322932	0.895164994163819	0.994766463583882	pseudobulk	DESeq2	LRT	0.0480969094318297
885	KC_AB1	CG33322	0.949183608371002	0.80156895525114	0.994766463583882	pseudobulk	DESeq2	LRT	0.0960591113673091
885	KC_AB1	CG33331	-0.0766126098067665	0.868026165321	0.994766463583882	pseudobulk	DESeq2	LRT	0.0614671834833482
885	KC_AB1	CG33332	-1.95577997156375	0.302956686963652	0.994766463583882	pseudobulk	DESeq2	LRT	0.518619457165259
885	KC_AB1	CG3335	-0.792585697017835	0.552961056557077	0.994766463583882	pseudobulk	DESeq2	LRT	0.257305453718661
885	KC_AB1	CG3337	0.319603772966624	0.746367039657276	0.994766463583882	pseudobulk	DESeq2	LRT	0.127047547732931
886	KC_AB1	CG3342	-0.428246777363043	0.71456754763332	0.994766463583882	pseudobulk	DESeq2	LRT	0.1459567113407
886	KC_AB1	CG3345	-3.95857014146861	0.177445975557202	0.994766463583882	pseudobulk	DESeq2	LRT	0.750933845925402
886	KC_AB1	CG33476	-1.64952313825113	0.817565084244375	0.994766463583882	pseudobulk	DESeq2	LRT	0.0874776642296574
886	KC_AB1	CG33477	0.949183608371002	0.80156895525114	0.994766463583882	pseudobulk	DESeq2	LRT	0.0960591113673091
886	KC_AB1	CG33493	0.39509611406087	0.839890017035674	0.994766463583882	pseudobulk	DESeq2	LRT	0.0757775807497476
886	KC_AB1	CG33506	-0.495722336557288	0.649963923821033	0.994766463583882	pseudobulk	DESeq2	LRT	0.187110748157548
886	KC_AB1	CG33521	-1.15527227941764	0.556997014999186	0.994766463583882	pseudobulk	DESeq2	LRT	0.25414713224612
886	KC_AB1	CG33523	-0.770346789746563	0.378488663213753	0.994766463583882	pseudobulk	DESeq2	LRT	0.421947124294542
886	KC_AB1	CG33543	0.633277721969179	0.597377733565909	0.994766463583882	pseudobulk	DESeq2	LRT	0.223750969160208
886	KC_AB1	CG3356	0.568186498903614	0.490195315078455	0.994766463583882	pseudobulk	DESeq2	LRT	0.309630843727144
887	KC_AB1	CG3358	-0.264308911002968	0.800427837755988	0.994766463583882	pseudobulk	DESeq2	LRT	0.0966778156210599
887	KC_AB1	CG33632	-1.64952313825113	0.817565084244375	0.994766463583882	pseudobulk	DESeq2	LRT	0.0874776642296574
887	KC_AB1	CG33635	-0.0482922861938382	0.935472899475181	0.994766463583882	pseudobulk	DESeq2	LRT	0.0289687894348059
887	KC_AB1	CG33642	-1.64952313825113	0.817565084244375	0.994766463583882	pseudobulk	DESeq2	LRT	0.0874776642296574
887	KC_AB1	CG33643	-1.64952313825113	0.817565084244375	0.994766463583882	pseudobulk	DESeq2	LRT	0.0874776642296574
887	KC_AB1	CG33645	0.949183608371002	0.80156895525114	0.994766463583882	pseudobulk	DESeq2	LRT	0.0960591113673091
887	KC_AB1	CG33679	-1.64952313825113	0.817565084244375	0.994766463583882	pseudobulk	DESeq2	LRT	0.0874776642296574
887	KC_AB1	CG3368	-0.579658913675809	0.64980613787942	0.994766463583882	pseudobulk	DESeq2	LRT	0.187216190752542
887	KC_AB1	CG33695	-0.230044029180372	0.806086178777704	0.994766463583882	pseudobulk	DESeq2	LRT	0.0936185252335174
887	KC_AB1	CG33700	-0.326295742109009	0.837898811411484	0.994766463583882	pseudobulk	DESeq2	LRT	0.0768084256406759
888	KC_AB1	CG33702	-2.20969975456663	0.610836134188693	0.994766463583882	pseudobulk	DESeq2	LRT	0.214075280039667
888	KC_AB1	CG33758	1.1821590793593	0.719511699510136	0.994766463583882	pseudobulk	DESeq2	LRT	0.142962139890846
888	KC_AB1	CG33774	0.404584815935288	0.479788458364588	0.994766463583882	pseudobulk	DESeq2	LRT	0.318950203489614
888	KC_AB1	CG3386	-0.416437267072002	0.756149174483499	0.994766463583882	pseudobulk	DESeq2	LRT	0.121392517641534
888	KC_AB1	CG33914	0.949183608371002	0.80156895525114	0.994766463583882	pseudobulk	DESeq2	LRT	0.0960591113673091
888	KC_AB1	CG33941	-0.694708602943261	0.44913801889577	0.994766463583882	pseudobulk	DESeq2	LRT	0.347620180969821
888	KC_AB1	CG33947	-0.715229198952417	0.441019182315165	0.994766463583882	pseudobulk	DESeq2	LRT	0.355542520299852
888	KC_AB1	CG33969	1.30014049639568	0.277284585657651	0.994766463583882	pseudobulk	DESeq2	LRT	0.557074272325824
888	KC_AB1	CG33970	0.636251611667375	0.383022004049849	0.994766463583882	pseudobulk	DESeq2	LRT	0.416776275736527
888	KC_AB1	CG33977	-0.100242201719487	0.807123281404141	0.994766463583882	pseudobulk	DESeq2	LRT	0.0930601253220409
889	KC_AB1	CG33978	-2.42700587839427	0.0201458168485679	0.994766463583882	pseudobulk	DESeq2	LRT	1.69581511866362
889	KC_AB1	CG33993	-0.17060593924229	0.856252464081668	0.994766463583882	pseudobulk	DESeq2	LRT	0.0673981657061963
889	KC_AB1	CG33998	-0.752851781817837	0.667403477692109	0.994766463583882	pseudobulk	DESeq2	LRT	0.17561153466271
889	KC_AB1	CG34001	1.32710828904082	0.607473293222376	0.994766463583882	pseudobulk	DESeq2	LRT	0.216472810505135
889	KC_AB1	CG34015	0.382409824632679	0.636139879536587	0.994766463583882	pseudobulk	DESeq2	LRT	0.196447377698438
889	KC_AB1	CG3402	-0.333216228839979	0.633161253253511	0.994766463583882	pseudobulk	DESeq2	LRT	0.198485669951606
889	KC_AB1	CG34028	-1.07048843143301	0.449633211942428	0.994766463583882	pseudobulk	DESeq2	LRT	0.347141617299728
889	KC_AB1	CG34031	2.86104783705935	0.187735989721046	0.994766463583882	pseudobulk	DESeq2	LRT	0.726452463452278
889	KC_AB1	CG34039	0.529633966672262	0.69336539760355	0.994766463583882	pseudobulk	DESeq2	LRT	0.159037835602624
889	KC_AB1	CG34041	0.329367741436991	0.858058210994256	0.994766463583882	pseudobulk	DESeq2	LRT	0.06648324844961
890	KC_AB1	CG34045	-0.17805032366327	0.942053914371676	0.994766463583882	pseudobulk	DESeq2	LRT	0.0259242415339998
890	KC_AB1	CG34057	-1.64952313825113	0.817565084244375	0.994766463583882	pseudobulk	DESeq2	LRT	0.0874776642296574
890	KC_AB1	CG3407	-0.49050184051698	0.793052437934295	0.994766463583882	pseudobulk	DESeq2	LRT	0.100698095466029

890	KC_AB1	CG3408	2.32845491661673	0.320335798497691	0.994766463583882	pseudobulk	DESeq2	LRT	0.494394524897658
890	KC_AB1	CG34109	-0.0175856672650169	0.960737619868475	0.994766463583882	pseudobulk	DESeq2	LRT	0.0173952031763364
890	KC_AB1	CG34112	2.67764418499683	0.378704357417491	0.994766463583882	pseudobulk	DESeq2	LRT	0.421699697806334
890	KC_AB1	CG34116	-2.06337820213399	0.500651198454719	0.994766463583882	pseudobulk	DESeq2	LRT	0.300464739886804
890	KC_AB1	CG34117	-0.215326382894139	0.686037992154016	0.994766463583882	pseudobulk	DESeq2	LRT	0.163651832797673
890	KC_AB1	CG34125	0.0418875078008009	0.9115034768611	0.994766463583882	pseudobulk	DESeq2	LRT	0.0402416704223782
890	KC_AB1	CG34126	-0.219234871942883	0.676646781612521	0.994766463583882	pseudobulk	DESeq2	LRT	0.169637979510167
891	KC_AB1	CG34132	-0.0506439810532443	0.83123221417451	0.994766463583882	pseudobulk	DESeq2	LRT	0.0802776341622269
891	KC_AB1	CG34133	-0.291259795504723	0.732281073648988	0.994766463583882	pseudobulk	DESeq2	LRT	0.135322190383191
891	KC_AB1	CG34134	0.209349921722947	0.785681625341623	0.994766463583882	pseudobulk	DESeq2	LRT	0.104753403538386
891	KC_AB1	CG34136	-0.835152883801839	0.920434724857442	0.994766463583882	pseudobulk	DESeq2	LRT	0.0360070052473159
891	KC_AB1	CG34141	-1.64952313825113	0.817565084244375	0.994766463583882	pseudobulk	DESeq2	LRT	0.0874776642296574
891	KC_AB1	CG34148	0.591451629598202	0.653995882458773	0.994766463583882	pseudobulk	DESeq2	LRT	0.184424985973995
891	KC_AB1	CG34149	0.541401653315471	0.758301072691094	0.994766463583882	pseudobulk	DESeq2	LRT	0.120158329660947
891	KC_AB1	CG34150	-0.075460426289518	0.863031581240517	0.994766463583882	pseudobulk	DESeq2	LRT	0.0639733116920488
891	KC_AB1	CG34155	0.949183608371002	0.80156895525114	0.994766463583882	pseudobulk	DESeq2	LRT	0.0960591113673091
891	KC_AB1	CG34159	-0.0364895165483126	0.895528076668665	0.994766463583882	pseudobulk	DESeq2	LRT	0.0479207935657893
892	KC_AB1	CG34163	0.62506181737221	0.65087233953608	0.994766463583882	pseudobulk	DESeq2	LRT	0.18650418450682
892	KC_AB1	CG34174	0.812119390043026	0.653364348246366	0.994766463583882	pseudobulk	DESeq2	LRT	0.184844567109283
892	KC_AB1	CG34183	0.296252025933263	0.732279410931194	0.994766463583882	pseudobulk	DESeq2	LRT	0.135323176493592
892	KC_AB1	CG34188	0.949183608371002	0.80156895525114	0.994766463583882	pseudobulk	DESeq2	LRT	0.0960591113673091
892	KC_AB1	CG34195	1.64546047106187	0.132656286333494	0.994766463583882	pseudobulk	DESeq2	LRT	0.877272164790138
892	KC_AB1	CG3420	-0.150948109772912	0.834241525733985	0.994766463583882	pseudobulk	DESeq2	LRT	0.0787081962352447
892	KC_AB1	CG34200	0.123427704351914	0.747003962600176	0.994766463583882	pseudobulk	DESeq2	LRT	0.126677094395008
892	KC_AB1	CG34213	0.300725901683936	0.871293469646105	0.994766463583882	pseudobulk	DESeq2	LRT	0.0598355409955241
892	KC_AB1	CG34214	-1.64952313825113	0.817565084244375	0.994766463583882	pseudobulk	DESeq2	LRT	0.0874776642296574
892	KC_AB1	CG34219	-0.105003749335506	0.750815627895566	0.994766463583882	pseudobulk	DESeq2	LRT	0.124466696308853
893	KC_AB1	CG34221	0.949183608371002	0.80156895525114	0.994766463583882	pseudobulk	DESeq2	LRT	0.0960591113673091
893	KC_AB1	CG34222	-1.64952313825113	0.817565084244375	0.994766463583882	pseudobulk	DESeq2	LRT	0.0874776642296574
893	KC_AB1	CG34228	-0.517349538647577	0.582035812339779	0.994766463583882	pseudobulk	DESeq2	LRT	0.235050292630088
893	KC_AB1	CG34229	-0.180207528019413	0.840958627224353	0.994766463583882	pseudobulk	DESeq2	LRT	0.075225369731391
893	KC_AB1	CG34231	1.64887656706202	0.343058144402985	0.994766463583882	pseudobulk	DESeq2	LRT	0.464632265807652
893	KC_AB1	CG34232	-0.283995846067142	0.866480656580972	0.994766463583882	pseudobulk	DESeq2	LRT	0.0622411280844138
893	KC_AB1	CG34234	0.69750158688829	0.750018748510497	0.994766463583882	pseudobulk	DESeq2	LRT	0.124927880244456
893	KC_AB1	CG34242	0.841170893849574	0.357591628302762	0.994766463583882	pseudobulk	DESeq2	LRT	0.44661265717051
893	KC_AB1	CG34250	-0.0805784402541081	0.855289304527399	0.994766463583882	pseudobulk	DESeq2	LRT	0.0678869588299617
893	KC_AB1	CG34251	3.54390806391042	0.224759461271089	0.994766463583882	pseudobulk	DESeq2	LRT	0.64828201754205
894	KC_AB1	CG34261	-2.79499059556516	0.38564607643653	0.994766463583882	pseudobulk	DESeq2	LRT	0.413811082791365
894	KC_AB1	CG34269	1.1821590793593	0.719511699510136	0.994766463583882	pseudobulk	DESeq2	LRT	0.142962139890846
894	KC_AB1	CG34273	-1.64952313825113	0.817565084244375	0.994766463583882	pseudobulk	DESeq2	LRT	0.0874776642296574
894	KC_AB1	CG34286	0.217906885051292	0.902008941520292	0.994766463583882	pseudobulk	DESeq2	LRT	0.0447891573209458
894	KC_AB1	CG34293	-0.462406001418494	0.555655087975675	0.994766463583882	pseudobulk	DESeq2	LRT	0.255194704586913
894	KC_AB1	CG3430	-0.835152883801839	0.920434724857442	0.994766463583882	pseudobulk	DESeq2	LRT	0.0360070052473159
894	KC_AB1	CG34325	0.949183608371002	0.80156895525114	0.994766463583882	pseudobulk	DESeq2	LRT	0.0960591113673091
894	KC_AB1	CG34330	-0.17805032366327	0.942053914371676	0.994766463583882	pseudobulk	DESeq2	LRT	0.0259242415339998
894	KC_AB1	CG34334	-1.64952313825113	0.817565084244375	0.994766463583882	pseudobulk	DESeq2	LRT	0.0874776642296574
894	KC_AB1	CG3434	-0.282363193791842	0.792295125801106	0.994766463583882	pseudobulk	DESeq2	LRT	0.101113015844453
895	KC_AB1	CG34347	0.939504055401765	0.189795833888141	0.994766463583882	pseudobulk	DESeq2	LRT	0.721713324781482
895	KC_AB1	CG34348	-0.234099045282878	0.824229111320015	0.994766463583882	pseudobulk	DESeq2	LRT	0.0839520504977071
895	KC_AB1	CG34351	1.77931620532018	0.385930959243641	0.994766463583882	pseudobulk	DESeq2	LRT	0.413490381082998
895	KC_AB1	CG34353	0.0220463615327969	0.896416145776536	0.994766463583882	pseudobulk	DESeq2	LRT	0.0474903297825512
895	KC_AB1	CG34354	0.0237446211787613	0.869919013886989	0.994766463583882	pseudobulk	DESeq2	LRT	0.0605211766447395
895	KC_AB1	CG34355	0.136629811961892	0.653627477207262	0.994766463583882	pseudobulk	DESeq2	LRT	0.184669699210242
895	KC_AB1	CG34357	0.25165354215976	0.309527920656184	0.994766463583882	pseudobulk	DESeq2	LRT	0.509300169777957
895	KC_AB1	CG3436	0.0561624493859581	0.842709024595004	0.994766463583882	pseudobulk	DESeq2	LRT	0.07432235518185
895	KC_AB1	CG3437	-1.07190744407775	0.526473837111173	0.994766463583882	pseudobulk	DESeq2	LRT	0.278623206018036

895	KC_AB1	CG34370	0.38288151919322	0.376253802976275	0.994766463583882	pseudobulk	DESeq2	LRT	0.424519101754873
896	KC_AB1	CG34376	-0.559474865077222	0.539355758831859	0.994766463583882	pseudobulk	DESeq2	LRT	0.268124679839349
896	KC_AB1	CG34377	-0.468144946325034	0.77312222961709	0.994766463583882	pseudobulk	DESeq2	LRT	0.111751839261781
896	KC_AB1	CG34384	-0.103596016979315	0.721517197243343	0.994766463583882	pseudobulk	DESeq2	LRT	0.141753313109808
896	KC_AB1	CG34401	0.0344255944073488	0.875509967188803	0.994766463583882	pseudobulk	DESeq2	LRT	0.0577389053538562
896	KC_AB1	CG34402	0.266329962579229	0.415439746504833	0.994766463583882	pseudobulk	DESeq2	LRT	0.381491955412966
896	KC_AB1	CG34404	0.220054586604575	0.714240323132564	0.994766463583882	pseudobulk	DESeq2	LRT	0.146155634933458
896	KC_AB1	CG34408	-0.467748887034749	0.531679386316444	0.994766463583882	pseudobulk	DESeq2	LRT	0.274350177343456
896	KC_AB1	CG34409	0.113215098534601	0.945897917237718	0.994766463583882	pseudobulk	DESeq2	LRT	0.0241557307996513
896	KC_AB1	CG34417	1.16329195923651	0.599946512180099	0.994766463583882	pseudobulk	DESeq2	LRT	0.221887467117198
896	KC_AB1	CG34425	0.949183608371002	0.80156895525114	0.994766463583882	pseudobulk	DESeq2	LRT	0.0960591113673091
897	KC_AB1	CG34431	3.2216075874917	0.25352581096939	0.994766463583882	pseudobulk	DESeq2	LRT	0.595977819403831
897	KC_AB1	CG34454	-1.64952313825113	0.817565084244375	0.994766463583882	pseudobulk	DESeq2	LRT	0.0874776642296574
897	KC_AB1	CG34457	0.949183608371002	0.80156895525114	0.994766463583882	pseudobulk	DESeq2	LRT	0.0960591113673091
897	KC_AB1	CG3448	-2.20969975456663	0.610836134188693	0.994766463583882	pseudobulk	DESeq2	LRT	0.214075280039667
897	KC_AB1	CG3500	-0.855532431115949	0.346549929912533	0.994766463583882	pseudobulk	DESeq2	LRT	0.46023418465011
897	KC_AB1	CG3511	-0.752354840337869	0.400729106389407	0.994766463583882	pseudobulk	DESeq2	LRT	0.397149112058877
897	KC_AB1	CG3520	-3.21143241058256	0.292545415061155	0.994766463583882	pseudobulk	DESeq2	LRT	0.533806704011112
897	KC_AB1	CG3527	0.593284723012334	0.61807881389025	0.994766463583882	pseudobulk	DESeq2	LRT	0.208956142621164
897	KC_AB1	CG3529	0.435308978628834	0.490494612262879	0.994766463583882	pseudobulk	DESeq2	LRT	0.309365758676188
897	KC_AB1	CG3530	0.109177251574179	0.74389762260036	0.994766463583882	pseudobulk	DESeq2	LRT	0.12848682923784
898	KC_AB1	CG3534	0.819007894079427	0.700993735174048	0.994766463583882	pseudobulk	DESeq2	LRT	0.154285863333625
898	KC_AB1	CG3542	0.839264065268158	0.403142358361947	0.994766463583882	pseudobulk	DESeq2	LRT	0.394541567918932
898	KC_AB1	CG3548	-0.201440231202385	0.774608000974737	0.994766463583882	pseudobulk	DESeq2	LRT	0.110918021471763
898	KC_AB1	CG3552	0.251150587069877	0.804745978668943	0.994766463583882	pseudobulk	DESeq2	LRT	0.0943411848147644
898	KC_AB1	CG3556	0.550789644832355	0.223655896155305	0.994766463583882	pseudobulk	DESeq2	LRT	0.650419648200199
898	KC_AB1	CG3558	0.281253930020763	0.420520343127822	0.994766463583882	pseudobulk	DESeq2	LRT	0.376212989891146
898	KC_AB1	CG3566	0.3949053456459	0.377507524872806	0.994766463583882	pseudobulk	DESeq2	LRT	0.42307438713899
898	KC_AB1	CG3568	0.928369888401369	0.417311945101758	0.994766463583882	pseudobulk	DESeq2	LRT	0.37953918390192
898	KC_AB1	CG3587	1.76815648251085	0.422128311747306	0.994766463583882	pseudobulk	DESeq2	LRT	0.374555519147912
898	KC_AB1	CG3589	-0.0876446376047783	0.951298398237017	0.994766463583882	pseudobulk	DESeq2	LRT	0.0216832345024977
899	KC_AB1	CG3594	0.0334820659506235	0.956126514210327	0.994766463583882	pseudobulk	DESeq2	LRT	0.0194846382802211
899	KC_AB1	CG3609	-0.437259370414482	0.77335189263586	0.994766463583882	pseudobulk	DESeq2	LRT	0.111622847277955
899	KC_AB1	CG3618	0.565785220597631	0.325819134542617	0.994766463583882	pseudobulk	DESeq2	LRT	0.48702341425018
899	KC_AB1	CG3625	-0.0266052924434873	0.873596512658685	0.994766463583882	pseudobulk	DESeq2	LRT	0.058689108311628
899	KC_AB1	CG3626	-0.470715481489835	0.744621922714589	0.994766463583882	pseudobulk	DESeq2	LRT	0.128064181690258
899	KC_AB1	CG3631	-0.832502939189491	0.568376435572993	0.994766463583882	pseudobulk	DESeq2	LRT	0.245363935869057
899	KC_AB1	CG3632	0.680918908710485	0.428717684272754	0.994766463583882	pseudobulk	DESeq2	LRT	0.36782860180854
899	KC_AB1	CG3634	-0.548527355401405	0.547711518493686	0.994766463583882	pseudobulk	DESeq2	LRT	0.261448125666711
899	KC_AB1	CG3638	-0.134657713271668	0.656486599148153	0.994766463583882	pseudobulk	DESeq2	LRT	0.182774134731258
899	KC_AB1	CG3645	-0.00049594295957414	0.904598597271527	0.994766463583882	pseudobulk	DESeq2	LRT	0.0435440900343937
900	KC_AB1	CG3651	0.535378382859724	0.455235563487056	0.994766463583882	pseudobulk	DESeq2	LRT	0.341763817740228
900	KC_AB1	CG3652	0.403591664509355	0.499084823252046	0.994766463583882	pseudobulk	DESeq2	LRT	0.301825636461477
900	KC_AB1	CG3662	0.153669713684371	0.447031856528463	0.994766463583882	pseudobulk	DESeq2	LRT	0.349661526931707
900	KC_AB1	CG3678	1.11725180574099	0.1858509333993	0.994766463583882	pseudobulk	DESeq2	LRT	0.730835253405234
900	KC_AB1	CG3700	-0.662584750519014	0.625132088868574	0.994766463583882	pseudobulk	DESeq2	LRT	0.204028207606765
900	KC_AB1	CG3702	-0.102104792211677	0.888858952003305	0.994766463583882	pseudobulk	DESeq2	LRT	0.0511671492959325
900	KC_AB1	CG3703	-0.205458593063448	0.65647305969151	0.994766463583882	pseudobulk	DESeq2	LRT	0.182783091763972
900	KC_AB1	CG3704	2.18625804867272	0.141919132823187	0.994766463583882	pseudobulk	DESeq2	LRT	0.847959051199324
900	KC_AB1	CG3709	0.0236942436874584	0.910119428894173	0.994766463583882	pseudobulk	DESeq2	LRT	0.0409016143750932
900	KC_AB1	CG3713	1.80633728195307	0.61161102402172	0.994766463583882	pseudobulk	DESeq2	LRT	0.213524695220716
901	KC_AB1	CG3719	-0.0570347514619971	0.909223894589487	0.994766463583882	pseudobulk	DESeq2	LRT	0.0413291594494844
901	KC_AB1	CG3726	-1.60653259065571	0.594621747113632	0.994766463583882	pseudobulk	DESeq2	LRT	0.225759211379423
901	KC_AB1	CG3732	0.412910382557018	0.448279621692324	0.994766463583882	pseudobulk	DESeq2	LRT	0.348451003243803
901	KC_AB1	CG3735	0.122731951548588	0.889311740972112	0.994766463583882	pseudobulk	DESeq2	LRT	0.0509459739547277
901	KC_AB1	CG3740	0.80582126571575	0.477505985320416	0.994766463583882	pseudobulk	DESeq2	LRT	0.32102118036225

901	KC_AB1	CG3744	0.583425736559414	0.321923612614628	0.994766463583882	pseudobulk	DESeq2	LRT	0.492247167296558
901	KC_AB1	CG3756	0.188346935071874	0.87177561361097	0.994766463583882	pseudobulk	DESeq2	LRT	0.0595952837724741
901	KC_AB1	CG3760	0.184279987626977	0.804255266407861	0.994766463583882	pseudobulk	DESeq2	LRT	0.0946060865784513
901	KC_AB1	CG3764	0.105919808311088	0.903669661574135	0.994766463583882	pseudobulk	DESeq2	LRT	0.0439902978146117
901	KC_AB1	CG3776	0.0611536548501277	0.911748255478083	0.994766463583882	pseudobulk	DESeq2	LRT	0.0401250589842573
902	KC_AB1	CG3777	0.411156034705056	0.732296335214329	0.994766463583882	pseudobulk	DESeq2	LRT	0.135313139289582
902	KC_AB1	CG3781	1.15763291764847	0.300400700652709	0.994766463583882	pseudobulk	DESeq2	LRT	0.522299058720963
902	KC_AB1	CG3792	-0.306119553565306	0.712090324248647	0.994766463583882	pseudobulk	DESeq2	LRT	0.147464915303042
902	KC_AB1	CG3793	0.620430276895011	0.312541493766185	0.994766463583882	pseudobulk	DESeq2	LRT	0.50509231650419
902	KC_AB1	CG3803	-0.549062748881049	0.646850736109534	0.994766463583882	pseudobulk	DESeq2	LRT	0.189195923292167
902	KC_AB1	CG3808	2.54275315134261	0.410734813545839	0.994766463583882	pseudobulk	DESeq2	LRT	0.386438485142247
902	KC_AB1	CG3815	-0.834023135425377	0.429764065711914	0.994766463583882	pseudobulk	DESeq2	LRT	0.366769900420777
902	KC_AB1	CG3817	-0.212086803287294	0.845414863669783	0.994766463583882	pseudobulk	DESeq2	LRT	0.0729301208871503
902	KC_AB1	CG3819	-0.17805032366327	0.942053914371676	0.994766463583882	pseudobulk	DESeq2	LRT	0.0259242415339998
902	KC_AB1	CG3823	-1.84537628931528	0.391827682668327	0.994766463583882	pseudobulk	DESeq2	LRT	0.40690488430505
903	KC_AB1	CG3842	0.949183608371002	0.80156895525114	0.994766463583882	pseudobulk	DESeq2	LRT	0.0960591113673091
903	KC_AB1	CG3847	-0.535912241891664	0.495425664740489	0.994766463583882	pseudobulk	DESeq2	LRT	0.30502149923087
903	KC_AB1	CG3857	1.14907367453669	0.47719576705586	0.994766463583882	pseudobulk	DESeq2	LRT	0.32130341737478
903	KC_AB1	CG3860	-0.56688507188074	0.307535141337892	0.994766463583882	pseudobulk	DESeq2	LRT	0.512105251214936
903	KC_AB1	CG3862	-0.296318350920996	0.819211109987353	0.994766463583882	pseudobulk	DESeq2	LRT	0.0866041665080351
903	KC_AB1	CG3868	-1.64952313825113	0.817565084244375	0.994766463583882	pseudobulk	DESeq2	LRT	0.0874776642296574
903	KC_AB1	CG3894	0.0600308301817955	0.944339738237278	0.994766463583882	pseudobulk	DESeq2	LRT	0.0248717346238558
903	KC_AB1	CG3902	0.949183608371002	0.80156895525114	0.994766463583882	pseudobulk	DESeq2	LRT	0.0960591113673091
903	KC_AB1	CG3907	0.0871163660183705	0.810037507726224	0.994766463583882	pseudobulk	DESeq2	LRT	0.0914948712183966
903	KC_AB1	CG3909	-0.700012685991242	0.717543027060529	0.994766463583882	pseudobulk	DESeq2	LRT	0.144152051590919
904	KC_AB1	CG3918	-0.700602772026747	0.357292240690284	0.994766463583882	pseudobulk	DESeq2	LRT	0.446976415261271
904	KC_AB1	CG3919	-1.66255151257842	0.356932018620452	0.994766463583882	pseudobulk	DESeq2	LRT	0.447414491869321
904	KC_AB1	CG3955	0.151057137048068	0.653806928678056	0.994766463583882	pseudobulk	DESeq2	LRT	0.184550481322639
904	KC_AB1	CG3961	-0.565673970748789	0.67733869638873	0.994766463583882	pseudobulk	DESeq2	LRT	0.169194112422529
904	KC_AB1	CG3982	-1.64952313825113	0.817565084244375	0.994766463583882	pseudobulk	DESeq2	LRT	0.0874776642296574
904	KC_AB1	CG3995	-0.148278707850144	0.80348911005203	0.994766463583882	pseudobulk	DESeq2	LRT	0.0950200049944277
904	KC_AB1	CG3999	1.1821590793593	0.719511699510136	0.994766463583882	pseudobulk	DESeq2	LRT	0.142962139890846
904	KC_AB1	CG4000	-0.114616759262991	0.92008811516338	0.994766463583882	pseudobulk	DESeq2	LRT	0.0361705790710269
904	KC_AB1	CG40006	0.992630526336814	0.723993771284609	0.994766463583882	pseudobulk	DESeq2	LRT	0.140265170140367
904	KC_AB1	CG4004	0.387010186884471	0.583559695580008	0.994766463583882	pseudobulk	DESeq2	LRT	0.233914710938378
905	KC_AB1	CG40045	0.26285250919756	0.358705480634037	0.994766463583882	pseudobulk	DESeq2	LRT	0.445261987720643
905	KC_AB1	CG40191	-0.341357695181345	0.492788990581111	0.994766463583882	pseudobulk	DESeq2	LRT	0.30733900332594
905	KC_AB1	CG4022	0.519782892347826	0.545890973078996	0.994766463583882	pseudobulk	DESeq2	LRT	0.262894087182212
905	KC_AB1	CG40228	0.122963823282666	0.665716078370574	0.994766463583882	pseudobulk	DESeq2	LRT	0.176710953844042
905	KC_AB1	CG4025	0.32709778429242	0.743480070788539	0.994766463583882	pseudobulk	DESeq2	LRT	0.128730668382871
905	KC_AB1	CG40298	-1.64952313825113	0.817565084244375	0.994766463583882	pseudobulk	DESeq2	LRT	0.0874776642296574
905	KC_AB1	CG4036	-0.358757075072528	0.755130447916079	0.994766463583882	pseudobulk	DESeq2	LRT	0.121978018017841
905	KC_AB1	CG4038	1.12759260057257	0.363238439839995	0.994766463583882	pseudobulk	DESeq2	LRT	0.43980819834746
905	KC_AB1	CG4041	-0.712577741429813	0.527058398894981	0.994766463583882	pseudobulk	DESeq2	LRT	0.278141261613459
905	KC_AB1	CG4042	-0.657962979153648	0.617346778083047	0.994766463583882	pseudobulk	DESeq2	LRT	0.209470814084823
906	KC_AB1	CG40439	0.932211453157225	0.423926581320921	0.994766463583882	pseudobulk	DESeq2	LRT	0.372709351162219
906	KC_AB1	CG4045	-0.0212361570757026	0.894752175470127	0.994766463583882	pseudobulk	DESeq2	LRT	0.0482972370077839
906	KC_AB1	CG40470	-2.85724547560282	0.450020141104186	0.994766463583882	pseudobulk	DESeq2	LRT	0.346768048503189
906	KC_AB1	CG40472	-1.24656414818514	0.215892946636629	0.994766463583882	pseudobulk	DESeq2	LRT	0.665761546117966
906	KC_AB1	CG4049	-0.548189976589977	0.522968135786304	0.994766463583882	pseudobulk	DESeq2	LRT	0.281524771694399
906	KC_AB1	CG40498	0.0389407530304678	0.813877950698921	0.994766463583882	pseudobulk	DESeq2	LRT	0.0894407171158671
906	KC_AB1	CG4069	-0.78410610838983	0.738244895252942	0.994766463583882	pseudobulk	DESeq2	LRT	0.131799547371545
906	KC_AB1	CG4074	0.125400116592342	0.913614159485783	0.994766463583882	pseudobulk	DESeq2	LRT	0.0392371782106514
906	KC_AB1	CG4078	0.794635780147327	0.637089865355295	0.994766463583882	pseudobulk	DESeq2	LRT	0.195799303496328
906	KC_AB1	CG4080	0.0610510061329706	0.883704545635412	0.994766463583882	pseudobulk	DESeq2	LRT	0.0536929110591462
907	KC_AB1	CG4101	0.793345265638146	0.24288238692842	0.994766463583882	pseudobulk	DESeq2	LRT	0.614603977731469

907	KC_AB1	CG41099	0.0813556947300789	0.772988908068204	0.994766463583882	pseudobulk	DESeq2	LRT	0.111826737904876
907	KC_AB1	CG41128	0.203381180464659	0.356791770141207	0.994766463583882	pseudobulk	DESeq2	LRT	0.447585171738408
907	KC_AB1	CG4119	-0.990260746805552	0.162023664823545	0.994766463583882	pseudobulk	DESeq2	LRT	0.790421548718545
907	KC_AB1	CG4133	0.0924713119887776	0.813331894366092	0.994766463583882	pseudobulk	DESeq2	LRT	0.0897321967449725
907	KC_AB1	CG41378	-0.444727493901384	0.260885317303143	0.994766463583882	pseudobulk	DESeq2	LRT	0.583550362423965
907	KC_AB1	CG41520	1.17930484172975	0.669685881755347	0.994766463583882	pseudobulk	DESeq2	LRT	0.174128856716169
907	KC_AB1	CG4168	-1.64952313825113	0.817565084244375	0.994766463583882	pseudobulk	DESeq2	LRT	0.0874776642296574
907	KC_AB1	CG4186	-0.0918902793322549	0.876890556513617	0.994766463583882	pseudobulk	DESeq2	LRT	0.0570546069397898
907	KC_AB1	CG4199	1.34155798429812	0.2718896926267	0.994766463583882	pseudobulk	DESeq2	LRT	0.565607256260228
908	KC_AB1	CG42231	0.478005084452508	0.815070723509861	0.994766463583882	pseudobulk	DESeq2	LRT	0.0888047059870473
908	KC_AB1	CG42232	0.105718419308625	0.831846886506504	0.994766463583882	pseudobulk	DESeq2	LRT	0.0799566045563476
908	KC_AB1	CG42233	-0.927811138542507	0.65055174458984	0.994766463583882	pseudobulk	DESeq2	LRT	0.186718154142994
908	KC_AB1	CG42238	-0.099051654263252	0.611833078821994	0.994766463583882	pseudobulk	DESeq2	LRT	0.213367046538158
908	KC_AB1	CG42239	1.75763353038293	0.303695387390051	0.994766463583882	pseudobulk	DESeq2	LRT	0.517561804216937
908	KC_AB1	CG42240	1.82004586226043	0.141392593666108	0.994766463583882	pseudobulk	DESeq2	LRT	0.849573338871383
908	KC_AB1	CG42258	-0.0106845263094363	0.897956239211916	0.994766463583882	pseudobulk	DESeq2	LRT	0.0467448276220889
908	KC_AB1	CG42259	0.302814609468023	0.806631320373334	0.994766463583882	pseudobulk	DESeq2	LRT	0.0933249189497388
908	KC_AB1	CG42268	-0.037931578732569	0.862215966747652	0.994766463583882	pseudobulk	DESeq2	LRT	0.0643839390253999
908	KC_AB1	CG42271	-0.200728020262471	0.819795418890348	0.994766463583882	pseudobulk	DESeq2	LRT	0.0862945128994901
909	KC_AB1	CG4230	-0.1795284088507	0.520715898764075	0.994766463583882	pseudobulk	DESeq2	LRT	0.283399162020686
909	KC_AB1	CG42307	-0.521620634365461	0.626667185631732	0.994766463583882	pseudobulk	DESeq2	LRT	0.202963045801348
909	KC_AB1	CG42322	0.0740111136760049	0.74678858009008	0.994766463583882	pseudobulk	DESeq2	LRT	0.126802331914421
909	KC_AB1	CG42324	0.0873618470836125	0.73952509921527	0.994766463583882	pseudobulk	DESeq2	LRT	0.131047081618798
909	KC_AB1	CG42327	-0.48167422725367	0.855534332799682	0.994766463583882	pseudobulk	DESeq2	LRT	0.0677625574305792
909	KC_AB1	CG42337	-0.0869200348565723	0.758638661044518	0.994766463583882	pseudobulk	DESeq2	LRT	0.119965028953995
909	KC_AB1	CG42339	0.0183949185006855	0.926649808714948	0.994766463583882	pseudobulk	DESeq2	LRT	0.0330843595735323
909	KC_AB1	CG42340	-0.145332333199916	0.82924153262194	0.994766463583882	pseudobulk	DESeq2	LRT	0.0813189543630081
909	KC_AB1	CG42342	0.148010613131908	0.918322938059544	0.994766463583882	pseudobulk	DESeq2	LRT	0.0370045676504757
909	KC_AB1	CG42346	-0.232579520722589	0.46955695812594	0.994766463583882	pseudobulk	DESeq2	LRT	0.328311719480006
910	KC_AB1	CG42354	-1.56898163967216	0.479395075456954	0.994766463583882	pseudobulk	DESeq2	LRT	0.319306431530683
910	KC_AB1	CG42359	-0.260465148448194	0.850277382984172	0.994766463583882	pseudobulk	DESeq2	LRT	0.0704393728178478
910	KC_AB1	CG42360	0.463751454723646	0.623923061411182	0.994766463583882	pseudobulk	DESeq2	LRT	0.204868961703306
910	KC_AB1	CG42361	0.129962101011269	0.71022385774851	0.994766463583882	pseudobulk	DESeq2	LRT	0.148604743024971
910	KC_AB1	CG42364	1.80633728195307	0.61161102402172	0.994766463583882	pseudobulk	DESeq2	LRT	0.213524695220716
910	KC_AB1	CG42365	0.311817647167105	0.772526563711987	0.994766463583882	pseudobulk	DESeq2	LRT	0.112086578213563
910	KC_AB1	CG42376	-0.0945328469746936	0.861383546357771	0.994766463583882	pseudobulk	DESeq2	LRT	0.0648034281321295
910	KC_AB1	CG4238	-0.256377705211687	0.648737520783845	0.994766463583882	pseudobulk	DESeq2	LRT	0.187930983219541
910	KC_AB1	CG42382	0.381063267506242	0.799390504318056	0.994766463583882	pseudobulk	DESeq2	LRT	0.0972410148784703
910	KC_AB1	CG4239	-0.876842550845305	0.327135511845827	0.994766463583882	pseudobulk	DESeq2	LRT	0.485272308942696
911	KC_AB1	CG42391	-2.20969975456663	0.610836134188693	0.994766463583882	pseudobulk	DESeq2	LRT	0.214075280039667
911	KC_AB1	CG42392	-0.943615579254206	0.00112979008937554	0.994766463583882	pseudobulk	DESeq2	LRT	2.94700223925484
911	KC_AB1	CG42394	0.380494398674726	0.83634888263430 1	0.994766463583882	pseudobulk	DESeq2	LRT	0.0776125482082141
911	KC_AB1	CG42399	0.572668982754792	0.658097527752613	0.994766463583882	pseudobulk	DESeq2	LRT	0.181709740692809
911	KC_AB1	CG42402	0.012670787683832	0.814283590289649	0.994766463583882	pseudobulk	DESeq2	LRT	0.089224317164014
911	KC_AB1	CG42404	0.0469563345234712	0.911853016752631	0.994766463583882	pseudobulk	DESeq2	LRT	0.0400751607480624
911	KC_AB1	CG42445	1.1821590793593	0.719511699510136	0.994766463583882	pseudobulk	DESeq2	LRT	0.142962139890846
911	KC_AB1	CG42450	0.0717621214610846	0.756812449782972	0.994766463583882	pseudobulk	DESeq2	LRT	0.121011732278075
911	KC_AB1	CG42486	1.1821590793593	0.719511699510136	0.994766463583882	pseudobulk	DESeq2	LRT	0.142962139890846
911	KC_AB1	CG42495	-0.449247673996996	0.708134950108127	0.994766463583882	pseudobulk	DESeq2	LRT	0.149883970414142
912	KC_AB1	CG4250	1.1821590793593	0.719511699510136	0.994766463583882	pseudobulk	DESeq2	LRT	0.142962139890846
912	KC_AB1	CG42514	-0.48167422725367	0.855534332799682	0.994766463583882	pseudobulk	DESeq2	LRT	0.0677625574305792
912	KC_AB1	CG42518	0.675821769966658	0.748287716720035	0.994766463583882	pseudobulk	DESeq2	LRT	0.125931383741942
912	KC_AB1	CG42521	-2.79499059556516	0.38564607643653	0.994766463583882	pseudobulk	DESeq2	LRT	0.413811082791365
912	KC_AB1	CG42526	1.45682795759908	0.573003993665791	0.994766463583882	pseudobulk	DESeq2	LRT	0.24184235111992
912	KC_AB1	CG42540	0.0830641309010567	0.597460108207359	0.994766463583882	pseudobulk	DESeq2	LRT	0.223691086804566
912	KC_AB1	CG42541	-0.133753771118854	0.386025834142672	0.994766463583882	pseudobulk	DESeq2	LRT	0.413383629913293

912	KC_AB1	CG42542	-0.129673656486883	0.792312147701715	0.994766463583882	pseudobulk	DESeq2	LRT	0.101103685434793
912	KC_AB1	CG42553	-0.803925612827804	0.675180897915086	0.994766463583882	pseudobulk	DESeq2	LRT	0.170579853182495
912	KC_AB1	CG42554	-0.768063299436135	0.330471858245496	0.994766463583882	pseudobulk	DESeq2	LRT	0.48086551750681
913	KC_AB1	CG42556	-1.02319198676748	0.828204539793902	0.994766463583882	pseudobulk	DESeq2	LRT	0.0818623932467813
913	KC_AB1	CG42561	-3.46197803745951	0.239982951034438	0.994766463583882	pseudobulk	DESeq2	LRT	0.619819610516178
913	KC_AB1	CG42562	-1.50663180504502	0.668163815680457	0.994766463583882	pseudobulk	DESeq2	LRT	0.175117047219009
913	KC_AB1	CG42578	-2.20969975456663	0.610836134188693	0.994766463583882	pseudobulk	DESeq2	LRT	0.214075280039667
913	KC_AB1	CG42588	0.108805951336928	0.845880673216869	0.994766463583882	pseudobulk	DESeq2	LRT	0.0726908977394268
913	KC_AB1	CG42594	0.606300904854108	0.367656894740286	0.994766463583882	pseudobulk	DESeq2	LRT	0.434557285208129
913	KC_AB1	CG42613	-0.0360191273036018	0.848175267991914	0.994766463583882	pseudobulk	DESeq2	LRT	0.0715143953179917
913	KC_AB1	CG4266	-0.636325813601713	0.26417492005864	0.994766463583882	pseudobulk	DESeq2	LRT	0.5781084153254
913	KC_AB1	CG42663	-2.26306816020352	0.40449476385881	0.994766463583882	pseudobulk	DESeq2	LRT	0.393087095910655
913	KC_AB1	CG42668	0.0533820771989768	0.797107540196404	0.994766463583882	pseudobulk	DESeq2	LRT	0.0984830826650639
914	KC_AB1	CG42671	-0.184808215200307	0.719450494716513	0.994766463583882	pseudobulk	DESeq2	LRT	0.142999084439159
914	KC_AB1	CG42672	-0.217893111814699	0.736904529508232	0.994766463583882	pseudobulk	DESeq2	LRT	0.132588774004573
914	KC_AB1	CG42673	0.0159670708967043	0.940402258188997	0.994766463583882	pseudobulk	DESeq2	LRT	0.0266863366740197
914	KC_AB1	CG42674	-0.0718353492627023	0.843438418789447	0.994766463583882	pseudobulk	DESeq2	LRT	0.073946620669687
914	KC_AB1	CG42676	0.340373947787661	0.701393422764277	0.994766463583882	pseudobulk	DESeq2	LRT	0.15403831098171
914	KC_AB1	CG42692	-0.654631659528721	0.683604889040291	0.994766463583882	pseudobulk	DESeq2	LRT	0.165194839930781
914	KC_AB1	CG42699	0.250957759228242	0.690754932698315	0.994766463583882	pseudobulk	DESeq2	LRT	0.16067600509258
914	KC_AB1	CG42700	3.63979474231417E-05	0.912377476572619	0.994766463583882	pseudobulk	DESeq2	LRT	0.0398254444836876
914	KC_AB1	CG42709	-0.329773202114297	0.725047469821185	0.994766463583882	pseudobulk	DESeq2	LRT	0.139633558661409
914	KC_AB1	CG42724	-0.114709678010504	0.696826583917609	0.994766463583882	pseudobulk	DESeq2	LRT	0.156875289360186
915	KC_AB1	CG42733	1.1821590793593	0.719511699510136	0.994766463583882	pseudobulk	DESeq2	LRT	0.142962139890846
915	KC_AB1	CG42747	-1.64952313825113	0.817565084244375	0.994766463583882	pseudobulk	DESeq2	LRT	0.0874776642296574
915	KC_AB1	CG42748	0.187341381490223	0.45381172034839	0.994766463583882	pseudobulk	DESeq2	LRT	0.343124292018951
915	KC_AB1	CG42750	0.0761899584111825	0.734663002577052	0.994766463583882	pseudobulk	DESeq2	LRT	0.133911830553348
915	KC_AB1	CG4278	-0.753156118140222	0.433997589303329	0.994766463583882	pseudobulk	DESeq2	LRT	0.362512682826589
915	KC_AB1	CG42784	0.0433229352924392	0.832868529945328	0.994766463583882	pseudobulk	DESeq2	LRT	0.0794235474833218
915	KC_AB1	CG42788	0.0203822936663081	0.92102139756821	0.994766463583882	pseudobulk	DESeq2	LRT	0.0357302799683506
915	KC_AB1	CG42789	-2.26306816020352	0.40449476385881	0.994766463583882	pseudobulk	DESeq2	LRT	0.393087095910655
915	KC_AB1	CG42795	-0.304330536362117	0.181585391908578	0.994766463583882	pseudobulk	DESeq2	LRT	0.740910092316458
915	KC_AB1	CG42797	0.0771493276444533	0.893888226946094	0.994766463583882	pseudobulk	DESeq2	LRT	0.0487167826082399
916	KC_AB1	CG42806	0.669393080254145	0.527605766414283	0.994766463583882	pseudobulk	DESeq2	LRT	0.277690466521772
916	KC_AB1	CG42809	-0.827768055015405	0.371450516892863	0.994766463583882	pseudobulk	DESeq2	LRT	0.430099032971331
916	KC_AB1	CG4281	1.14279788494456	0.299175550784945	0.994766463583882	pseudobulk	DESeq2	LRT	0.524073900757582
916	KC_AB1	CG42812	-1.64952313825113	0.817565084244375	0.994766463583882	pseudobulk	DESeq2	LRT	0.0874776642296574
916	KC_AB1	CG42813	-0.105112521329786	0.780371437561489	0.994766463583882	pseudobulk	DESeq2	LRT	0.107698634634313
916	KC_AB1	CG4282	-0.935971634505062	0.464576463895599	0.994766463583882	pseudobulk	DESeq2	LRT	0.332942795954601
916	KC_AB1	CG42855	-0.190032389632602	0.85311373472114	0.994766463583882	pseudobulk	DESeq2	LRT	0.0689930660551185
916	KC_AB1	CG4287	0.167538206905468	0.811340825034405	0.994766463583882	pseudobulk	DESeq2	LRT	0.0907966706496747
916	KC_AB1	CG4291	-0.147700889010674	0.895156742853916	0.994766463583882	pseudobulk	DESeq2	LRT	0.0481009126210725
916	KC_AB1	CG4293	-0.914465205529959	0.483999472302982	0.994766463583882	pseudobulk	DESeq2	LRT	0.315155111859777
917	KC_AB1	CG4294	0.03599313539207	0.884511060560233	0.994766463583882	pseudobulk	DESeq2	LRT	0.053296731990841
917	KC_AB1	CG4297	0.104599937419213	0.776469213058644	0.994766463583882	pseudobulk	DESeq2	LRT	0.109875759335054
917	KC_AB1	CG43049	1.1821590793593	0.719511699510136	0.994766463583882	pseudobulk	DESeq2	LRT	0.142962139890846
917	KC_AB1	CG43051	-1.64952313825113	0.817565084244375	0.994766463583882	pseudobulk	DESeq2	LRT	0.0874776642296574
917	KC_AB1	CG43055	1.1821590793593	0.719511699510136	0.994766463583882	pseudobulk	DESeq2	LRT	0.142962139890846
917	KC_AB1	CG43066	-0.121643843262377	0.722008888670681	0.994766463583882	pseudobulk	DESeq2	LRT	0.141457455786502
917	KC_AB1	CG43068	0.949183608371002	0.80156895525114	0.994766463583882	pseudobulk	DESeq2	LRT	0.0960591113673091
917	KC_AB1	CG43078	0.441756518706077	0.264940868556703	0.994766463583882	pseudobulk	DESeq2	LRT	0.576851044271095
917	KC_AB1	CG43102	-0.0602697378472936	0.824934881273902	0.994766463583882	pseudobulk	DESeq2	LRT	0.0835803324435235
917	KC_AB1	CG43110	2.38929733117871	0.484296857543297	0.994766463583882	pseudobulk	DESeq2	LRT	0.314888348950639
918	KC_AB1	CG43111	0.949183608371002	0.80156895525114	0.994766463583882	pseudobulk	DESeq2	LRT	0.0960591113673091
918	KC_AB1	CG43117	-0.484211582768817	0.811014852048671	0.994766463583882	pseudobulk	DESeq2	LRT	0.0909711925166842
918	KC_AB1	CG4313	0.223770973523073	0.840608708929666	0.994766463583882	pseudobulk	DESeq2	LRT	0.075406114901002

918	KC_AB1	CG43163	-0.835152883801839	0.920434724857442	0.994766463583882	pseudobulk	DESeq2	LRT	0.0360070052473159
918	KC_AB1	CG43175	1.1821590793593	0.719511699510136	0.994766463583882	pseudobulk	DESeq2	LRT	0.142962139890846
918	KC_AB1	CG43219	-0.48167422725367	0.855534332799682	0.994766463583882	pseudobulk	DESeq2	LRT	0.0677625574305792
918	KC_AB1	CG43222	-0.303471140956196	0.743783133454191	0.994766463583882	pseudobulk	DESeq2	LRT	0.128553674230376
918	KC_AB1	CG43232	-3.09325714697537	0.37426905025483	0.994766463583882	pseudobulk	DESeq2	LRT	0.426816084879393
918	KC_AB1	CG43236	1.1821590793593	0.719511699510136	0.994766463583882	pseudobulk	DESeq2	LRT	0.142962139890846
918	KC_AB1	CG43245	-0.835152883801839	0.920434724857442	0.994766463583882	pseudobulk	DESeq2	LRT	0.0360070052473159
919	KC_AB1	CG43273	3.3077188457669	0.230409282515317	0.994766463583882	pseudobulk	DESeq2	LRT	0.637500028443383
919	KC_AB1	CG4332	-0.356212784647529	0.928203443271547	0.994766463583882	pseudobulk	DESeq2	LRT	0.0323568248514225
919	KC_AB1	CG43322	1.1344753270176	0.605527010181318	0.994766463583882	pseudobulk	DESeq2	LRT	0.217866479917998
919	KC_AB1	CG43324	-0.00299613600990927	0.943821655934417	0.994766463583882	pseudobulk	DESeq2	LRT	0.0251100620164667
919	KC_AB1	CG43342	-0.0876446376047783	0.951298398237017	0.994766463583882	pseudobulk	DESeq2	LRT	0.0216832345024977
919	KC_AB1	CG43347	0.183173726695681	0.541090771064389	0.994766463583882	pseudobulk	DESeq2	LRT	0.266729873404307
919	KC_AB1	CG43367	0.801669389788771	0.336681425036636	0.994766463583882	pseudobulk	DESeq2	LRT	0.472780843298926
919	KC_AB1	CG43373	0.123953889517356	0.661595752079865	0.994766463583882	pseudobulk	DESeq2	LRT	0.179407291929205
919	KC_AB1	CG43376	0.949183608371002	0.80156895525114	0.994766463583882	pseudobulk	DESeq2	LRT	0.0960591113673091
919	KC_AB1	CG4338	-1.00450377937778	0.32829145106538	0.994766463583882	pseudobulk	DESeq2	LRT	0.483740426449681
920	KC_AB1	CG43394	1.11168776257694	0.593310522446876	0.994766463583882	pseudobulk	DESeq2	LRT	0.226717949319565
920	KC_AB1	CG4341	-0.07870769170716	0.888807555441564	0.994766463583882	pseudobulk	DESeq2	LRT	0.0511922622662967
920	KC_AB1	CG43428	1.10251292103259	0.639221751231493	0.994766463583882	pseudobulk	DESeq2	LRT	0.194348455410132
920	KC_AB1	CG43445	-1.64952313825113	0.817565084244375	0.994766463583882	pseudobulk	DESeq2	LRT	0.0874776642296574
920	KC_AB1	CG4360	-0.0472022391304316	0.953334910826823	0.994766463583882	pseudobulk	DESeq2	LRT	0.0207545029582931
920	KC_AB1	CG4364	0.0267803661889694	0.948859622867309	0.994766463583882	pseudobulk	DESeq2	LRT	0.0227980336460096
920	KC_AB1	CG43647	0.217906885051292	0.902008941520292	0.994766463583882	pseudobulk	DESeq2	LRT	0.0447891573209458
920	KC_AB1	CG43658	3.12489817250917	0.275546095031922	0.994766463583882	pseudobulk	DESeq2	LRT	0.559805739304107
920	KC_AB1	CG43707	-0.529435399633568	0.264832693987398	0.994766463583882	pseudobulk	DESeq2	LRT	0.577028401627166
920	KC_AB1	CG43736	-0.0326684963801037	0.897799547233245	0.994766463583882	pseudobulk	DESeq2	LRT	0.0468206179521349
921	KC_AB1	CG43737	0.0917452071098375	0.615511329757537	0.994766463583882	pseudobulk	DESeq2	LRT	0.210763948571993
921	KC_AB1	CG43740	0.635769616096888	0.35159730673645	0.994766463583882	pseudobulk	DESeq2	LRT	0.453954460316064
921	KC_AB1	CG43755	0.806933541094274	0.754600478929312	0.994766463583882	pseudobulk	DESeq2	LRT	0.122282923497213
921	KC_AB1	CG43759	0.0682973575894538	0.728316948614773	0.994766463583882	pseudobulk	DESeq2	LRT	0.137679583490428
921	KC_AB1	CG43778	0.0729571185321107	0.798628098481218	0.994766463583882	pseudobulk	DESeq2	LRT	0.0976554139004236
921	KC_AB1	CG43783	0.518809945266367	0.534001632814915	0.994766463583882	pseudobulk	DESeq2	LRT	0.272457415028703
921	KC_AB1	CG43795	0.949183608371002	0.80156895525114	0.994766463583882	pseudobulk	DESeq2	LRT	0.0960591113673091
921	KC_AB1	CG43800	0.645846369998674	0.721341252294085	0.994766463583882	pseudobulk	DESeq2	LRT	0.14185923052809
921	KC_AB1	CG43813	-1.64952313825113	0.817565084244375	0.994766463583882	pseudobulk	DESeq2	LRT	0.0874776642296574
921	KC_AB1	CG43841	0.949183608371002	0.80156895525114	0.994766463583882	pseudobulk	DESeq2	LRT	0.0960591113673091
922	KC_AB1	CG43843	2.02670873290438	0.538959414925803	0.994766463583882	pseudobulk	DESeq2	LRT	0.26844393710898
922	KC_AB1	CG43845	-1.08681444615798	0.701131388297773	0.994766463583882	pseudobulk	DESeq2	LRT	0.154200589927933
922	KC_AB1	CG43850	0.0707520479224799	0.900193343034334	0.994766463583882	pseudobulk	DESeq2	LRT	0.0456642030106754
922	KC_AB1	CG43861	0.411766461344308	0.385820434337299	0.994766463583882	pseudobulk	DESeq2	LRT	0.413614774398423
922	KC_AB1	CG43897	-1.64952313825113	0.817565084244375	0.994766463583882	pseudobulk	DESeq2	LRT	0.0874776642296574
922	KC_AB1	CG4390	0.619555480077678	0.406158448436803	0.994766463583882	pseudobulk	DESeq2	LRT	0.391304508645755
922	KC_AB1	CG43901	0.251557035849428	0.545030407176119	0.994766463583882	pseudobulk	DESeq2	LRT	0.263579267814329
922	KC_AB1	CG43902	-1.55427673693219	0.522267206552175	0.994766463583882	pseudobulk	DESeq2	LRT	0.282107242898514
922	KC_AB1	CG43919	-1.64952313825113	0.817565084244375	0.994766463583882	pseudobulk	DESeq2	LRT	0.0874776642296574
922	KC_AB1	CG43925	1.1821590793593	0.719511699510136	0.994766463583882	pseudobulk	DESeq2	LRT	0.142962139890846
923	KC_AB1	CG4393	-0.315424995253158	0.658402286354172	0.994766463583882	pseudobulk	DESeq2	LRT	0.181508669671126
923	KC_AB1	CG44004	1.1821590793593	0.719511699510136	0.994766463583882	pseudobulk	DESeq2	LRT	0.142962139890846
923	KC_AB1	CG44008	0.949183608371002	0.80156895525114	0.994766463583882	pseudobulk	DESeq2	LRT	0.0960591113673091
923	KC_AB1	CG4404	-0.142023310002009	0.788634717390695	0.994766463583882	pseudobulk	DESeq2	LRT	0.103124108267961
923	KC_AB1	CG4407	-0.627683421705811	0.475981833077135	0.994766463583882	pseudobulk	DESeq2	LRT	0.322409622794039
923	KC_AB1	CG4408	-1.64952313825113	0.817565084244375	0.994766463583882	pseudobulk	DESeq2	LRT	0.0874776642296574
923	KC_AB1	CG4409	2.02670873290438	0.538959414925803	0.994766463583882	pseudobulk	DESeq2	LRT	0.26844393710898
923	KC_AB1	CG44098	-0.698785369500767	0.698760305032176	0.994766463583882	pseudobulk	DESeq2	LRT	0.155671774260284
923	KC_AB1	CG44102	0.949183608371002	0.80156895525114	0.994766463583882	pseudobulk	DESeq2	LRT	0.0960591113673091

923	KC_AB1	CG44153	-0.638964597462771	0.138637665929318	0.994766463583882	pseudobulk	DESeq2	LRT	0.858118761910922
924	KC_AB1	CG44158	-0.991039252991293	0.557693955433475	0.994766463583882	pseudobulk	DESeq2	LRT	0.253604062584131
924	KC_AB1	CG44195	2.22023857837763	0.524904842126482	0.994766463583882	pseudobulk	DESeq2	LRT	0.279919420946639
924	KC_AB1	CG4424	0.604255506818112	0.676255203542409	0.994766463583882	pseudobulk	DESeq2	LRT	0.169889380131496
924	KC_AB1	CG44242	0.0637792921370005	0.804214633805356	0.994766463583882	pseudobulk	DESeq2	LRT	0.0946280285682328
924	KC_AB1	CG44243	0.0407692026673759	0.91866574695365	0.994766463583882	pseudobulk	DESeq2	LRT	0.0368424762734297
924	KC_AB1	CG44247	-0.400505288449065	0.0945196841287189	0.994766463583882	pseudobulk	DESeq2	LRT	1.02447773838736
924	KC_AB1	CG44249	-1.21457746603801	0.770013447451371	0.994766463583882	pseudobulk	DESeq2	LRT	0.113501690278259
924	KC_AB1	CG44261	0.949183608371002	0.80156895525114	0.994766463583882	pseudobulk	DESeq2	LRT	0.0960591113673091
924	KC_AB1	CG44296	1.1821590793593	0.719511699510136	0.994766463583882	pseudobulk	DESeq2	LRT	0.142962139890846
924	KC_AB1	CG4438	0.789443942742388	0.72202275028751	0.994766463583882	pseudobulk	DESeq2	LRT	0.141449117986226
925	KC_AB1	CG44385	-0.627447798185778	0.772628937945737	0.994766463583882	pseudobulk	DESeq2	LRT	0.112029029876423
925	KC_AB1	CG44422	3.17361065680552	0.0483595875787294	0.994766463583882	pseudobulk	DESeq2	LRT	1.31551741155498
925	KC_AB1	CG44438	-1.64952313825113	0.817565084244375	0.994766463583882	pseudobulk	DESeq2	LRT	0.0874776642296574
925	KC_AB1	CG4455	-0.151616337861527	0.869964296759384	0.994766463583882	pseudobulk	DESeq2	LRT	0.0604985704142067
925	KC_AB1	CG4467	-0.0972965903017914	0.573774818951228	0.994766463583882	pseudobulk	DESeq2	LRT	0.241258515397268
925	KC_AB1	CG4476	-2.20969975456663	0.610836134188693	0.994766463583882	pseudobulk	DESeq2	LRT	0.214075280039667
925	KC_AB1	CG44774	0.0727395677519632	0.774694197645729	0.994766463583882	pseudobulk	DESeq2	LRT	0.11086969682775
925	KC_AB1	CG44815	1.80633728195307	0.61161102402172	0.994766463583882	pseudobulk	DESeq2	LRT	0.213524695220716
925	KC_AB1	CG44837	-1.15188692395163	0.603405702316466	0.994766463583882	pseudobulk	DESeq2	LRT	0.219390589963361
925	KC_AB1	CG44838	-0.0330887478355111	0.869042598576648	0.994766463583882	pseudobulk	DESeq2	LRT	0.0609589348593369
926	KC_AB1	CG4497	0.619962814805505	0.432295953683684	0.994766463583882	pseudobulk	DESeq2	LRT	0.364218829469985
926	KC_AB1	CG45002	-1.38870517137269	0.20805590549463	0.994766463583882	pseudobulk	DESeq2	LRT	0.681819952606763
926	KC_AB1	CG45012	-1.64952313825113	0.817565084244375	0.994766463583882	pseudobulk	DESeq2	LRT	0.0874776642296574
926	KC_AB1	CG4502	-0.0109880242299053	0.951462481847563	0.994766463583882	pseudobulk	DESeq2	LRT	0.0216083321776148
926	KC_AB1	CG45049	-0.0309911858615486	0.902208236163844	0.994766463583882	pseudobulk	DESeq2	LRT	0.0446932125916857
926	KC_AB1	CG45050	-0.214120953357178	0.290547828535489	0.994766463583882	pseudobulk	DESeq2	LRT	0.536782365995571
926	KC_AB1	CG45066	-2.20969975456663	0.610836134188693	0.994766463583882	pseudobulk	DESeq2	LRT	0.214075280039667
926	KC_AB1	CG45071	0.596913891576544	0.467873800750766	0.994766463583882	pseudobulk	DESeq2	LRT	0.32987127305517
926	KC_AB1	CG45081	0.735981790581923	0.862513997338239	0.994766463583882	pseudobulk	DESeq2	LRT	0.0642338482344774
926	KC_AB1	CG4511	0.572533364081109	0.498567931666738	0.994766463583882	pseudobulk	DESeq2	LRT	0.302275659141655
927	KC_AB1	CG45218	-2.26306816020352	0.40449476385881	0.994766463583882	pseudobulk	DESeq2	LRT	0.393087095910655
927	KC_AB1	CG45263	-0.0298668032098528	0.829300628448023	0.994766463583882	pseudobulk	DESeq2	LRT	0.0812880055070973
927	KC_AB1	CG4537	-0.0957692229228332	0.958527644269769	0.994766463583882	pseudobulk	DESeq2	LRT	0.018395357401787
927	KC_AB1	CG4538	-0.595066385657766	0.392106402767591	0.994766463583882	pseudobulk	DESeq2	LRT	0.406596065978471
927	KC_AB1	CG45428	-1.64952313825113	0.817565084244375	0.994766463583882	pseudobulk	DESeq2	LRT	0.0874776642296574
927	KC_AB1	CG4552	-0.119259894201603	0.883120479403853	0.994766463583882	pseudobulk	DESeq2	LRT	0.0539800439071387
927	KC_AB1	CG4553	-0.849377531455818	0.743723328152499	0.994766463583882	pseudobulk	DESeq2	LRT	0.128588595910649
927	KC_AB1	CG4554	-0.257056328552413	0.890683115735507	0.994766463583882	pseudobulk	DESeq2	LRT	0.0502767803242406
927	KC_AB1	CG4557	0.179613815114444	0.856408523171689	0.994766463583882	pseudobulk	DESeq2	LRT	0.0673190191589941
927	KC_AB1	CG4562	0.0713719139994278	0.857991624494205	0.994766463583882	pseudobulk	DESeq2	LRT	0.0665169516078255
928	KC_AB1	CG4565	1.80633728195307	0.61161102402172	0.994766463583882	pseudobulk	DESeq2	LRT	0.213524695220716
928	KC_AB1	CG4572	0.0697801029220067	0.941980302636401	0.994766463583882	pseudobulk	DESeq2	LRT	0.025958178465889
928	KC_AB1	CG4577	-0.105332953561391	0.530934240665025	0.994766463583882	pseudobulk	DESeq2	LRT	0.274959265518974
928	KC_AB1	CG4587	-0.085320580917837	0.687203137898663	0.994766463583882	pseudobulk	DESeq2	LRT	0.162914866102072
928	KC_AB1	CG4593	0.466399522480077	0.681516099762293	0.994766463583882	pseudobulk	DESeq2	LRT	0.166523880173643
928	KC_AB1	CG4596	0.513328233398401	0.308849581725993	0.994766463583882	pseudobulk	DESeq2	LRT	0.510252982489526
928	KC_AB1	CG4598	-0.275483357530627	0.814751402636802	0.994766463583882	pseudobulk	DESeq2	LRT	0.0889748832016967
928	KC_AB1	CG4603	-0.62566483506072	0.422479498841013	0.994766463583882	pseudobulk	DESeq2	LRT	0.374194360693562
928	KC_AB1	CG4607	0.949183608371002	0.80156895525114	0.994766463583882	pseudobulk	DESeq2	LRT	0.0960591113673091
928	KC_AB1	CG4610	0.478005084452508	0.815070723509861	0.994766463583882	pseudobulk	DESeq2	LRT	0.0880047059870473
929	KC_AB1	CG4611	1.9229621176283	0.320498554408559	0.994766463583882	pseudobulk	DESeq2	LRT	0.49417392500258
929	KC_AB1	CG4612	-0.00773740339395979	0.942763370349227	0.994766463583882	pseudobulk	DESeq2	LRT	0.0255972996778821
929	KC_AB1	CG4615	-0.173088079986125	0.739350087551851	0.994766463583882	pseudobulk	DESeq2	LRT	0.131149871361606
929	KC_AB1	CG4617	-0.303902548192536	0.70705472138272	0.994766463583882	pseudobulk	DESeq2	LRT	0.150546973367317
929	KC_AB1	CG4619	0.696921508852573	0.723188018198223	0.994766463583882	pseudobulk	DESeq2	LRT	0.14074877787769

929	KC_AB1	CG4622	0.774336969289721	0.376852394353629	0.994766463583882	pseudobulk	DESeq2	LRT	0.423828721068149
929	KC_AB1	CG4627	0.102487014382045	0.916161385928346	0.994766463583882	pseudobulk	DESeq2	LRT	0.0380280166765513
929	KC_AB1	CG46280	0.177148050133221	0.759715060097102	0.994766463583882	pseudobulk	DESeq2	LRT	0.119349264339113
929	KC_AB1	CG46281	-0.33399464057665	0.801221707825449	0.994766463583882	pseudobulk	DESeq2	LRT	0.09624729270211
929	KC_AB1	CG4629	-0.207155188203806	0.640725204408167	0.994766463583882	pseudobulk	DESeq2	LRT	0.19332819169397
930	KC_AB1	CG46301	-0.274870901225309	0.491213665620777	0.994766463583882	pseudobulk	DESeq2	LRT	0.308729559576295
930	KC_AB1	CG4631	-2.00005840186615	0.514177117052568	0.994766463583882	pseudobulk	DESeq2	LRT	0.288887255112324
930	KC_AB1	CG46338	-0.674915375519997	0.137456786067811	0.994766463583882	pseudobulk	DESeq2	LRT	0.861833814721535
930	KC_AB1	CG46339	-0.356033363869676	0.136398646156292	0.994766463583882	pseudobulk	DESeq2	LRT	0.865189940308351
930	KC_AB1	CG46385	-0.340466262336144	0.231093980667747	0.994766463583882	pseudobulk	DESeq2	LRT	0.636211366493316
930	KC_AB1	CG46387	-3.21143241058256	0.292545415061155	0.994766463583882	pseudobulk	DESeq2	LRT	0.533806704011112
930	KC_AB1	CG4645	0.451525620774754	0.546761948330545	0.994766463583882	pseudobulk	DESeq2	LRT	0.26220171759563
930	KC_AB1	CG4646	-0.399629360565525	0.580383096865382	0.994766463583882	pseudobulk	DESeq2	LRT	0.23628524448549
930	KC_AB1	CG4658	0.0532803028034771	0.852712234064289	0.994766463583882	pseudobulk	DESeq2	LRT	0.0691975060462567
930	KC_AB1	CG4660	-0.00801986713420632	0.918291086792289	0.994766463583882	pseudobulk	DESeq2	LRT	0.0370196310545711
931	KC_AB1	CG4661	1.00206552945986	0.690075213866505	0.994766463583882	pseudobulk	DESeq2	LRT	0.161103571310595
931	KC_AB1	CG4676	0.104865944831448	0.910466669765977	0.994766463583882	pseudobulk	DESeq2	LRT	0.0407359481685291
931	KC_AB1	CG4678	1.77971970228338	0.0656091605296572	0.994766463583882	pseudobulk	DESeq2	LRT	1.18303551901255
931	KC_AB1	CG4679	-0.516779662780203	0.649343958281209	0.994766463583882	pseudobulk	DESeq2	LRT	0.187525195937104
931	KC_AB1	CG4686	-0.469811479468231	0.429733722036631	0.994766463583882	pseudobulk	DESeq2	LRT	0.366800565050586
931	KC_AB1	CG4702	1.1821590793593	0.719511699510136	0.994766463583882	pseudobulk	DESeq2	LRT	0.142962139890846
931	KC_AB1	CG4707	0.2147019870634	0.90028167280434	0.994766463583882	pseudobulk	DESeq2	LRT	0.0456215907762435
931	KC_AB1	CG4709	-0.813968027733744	0.563252032159626	0.994766463583882	pseudobulk	DESeq2	LRT	0.249297232708224
931	KC_AB1	CG4730	-0.17805032366327	0.942053914371676	0.994766463583882	pseudobulk	DESeq2	LRT	0.0259242415339998
931	KC_AB1	CG4743	-0.484211582768817	0.811014852048671	0.994766463583882	pseudobulk	DESeq2	LRT	0.0909711925166842
932	KC_AB1	CG4751	-0.76033227219371	0.53419718128693	0.994766463583882	pseudobulk	DESeq2	LRT	0.272298407881029
932	KC_AB1	CG4763	-1.64952313825113	0.817565084244375	0.994766463583882	pseudobulk	DESeq2	LRT	0.0874776642296574
932	KC_AB1	CG4768	0.0304676401160729	0.91216515090148	0.994766463583882	pseudobulk	DESeq2	LRT	0.0399265239173711
932	KC_AB1	CG4788	0.661993121129605	0.564969403256765	0.994766463583882	pseudobulk	DESeq2	LRT	0.247975071395712
932	KC_AB1	CG4789	-0.0668265437994585	0.903411662933484	0.994766463583882	pseudobulk	DESeq2	LRT	0.0441143070500732
932	KC_AB1	CG4806	0.20694617009554	0.780730608748397	0.994766463583882	pseudobulk	DESeq2	LRT	0.107498793672405
932	KC_AB1	CG4820	-1.29896771247644	0.32329947741478	0.994766463583882	pseudobulk	DESeq2	LRT	0.490394997386777
932	KC_AB1	CG4825	-1.68309931961457	0.136263045247962	0.994766463583882	pseudobulk	DESeq2	LRT	0.865621909543049
932	KC_AB1	CG4829	-1.58094965174778	0.396883337319333	0.994766463583882	pseudobulk	DESeq2	LRT	0.401337134053717
932	KC_AB1	CG4849	-0.114246412660852	0.897377534076922	0.994766463583882	pseudobulk	DESeq2	LRT	0.0470248072662318
933	KC_AB1	CG4853	-0.434490897857279	0.54810372044274	0.994766463583882	pseudobulk	DESeq2	LRT	0.261137249994451
933	KC_AB1	CG4854	-0.811861194394287	0.593326507472545	0.994766463583882	pseudobulk	DESeq2	LRT	0.226706248676038
933	KC_AB1	CG4858	-0.48856263729682	0.523924823310463	0.994766463583882	pseudobulk	DESeq2	LRT	0.280731024397326
933	KC_AB1	CG4866	0.305142227808619	0.773395307636413	0.994766463583882	pseudobulk	DESeq2	LRT	0.111598467217434
933	KC_AB1	CG4880	-0.437567891750304	0.83748785379873	0.994766463583882	pseudobulk	DESeq2	LRT	0.077021482877911
933	KC_AB1	CG4882	-1.02527065560035	0.306138782908764	0.994766463583882	pseudobulk	DESeq2	LRT	0.514081648722364
933	KC_AB1	CG4887	0.151336506453852	0.72506448600812	0.994766463583882	pseudobulk	DESeq2	LRT	0.139623366295144
933	KC_AB1	CG4896	-0.419421724454706	0.375198974808043	0.994766463583882	pseudobulk	DESeq2	LRT	0.425738356955602
933	KC_AB1	CG4901	-0.613490349353657	0.666983302144621	0.994766463583882	pseudobulk	DESeq2	LRT	0.175885038463097
933	KC_AB1	CG4908	-0.0592283285576927	0.948078789840936	0.994766463583882	pseudobulk	DESeq2	LRT	0.0231555692323234
934	KC_AB1	CG4911	0.300698024433849	0.52899223079772	0.994766463583882	pseudobulk	DESeq2	LRT	0.276550706313503
934	KC_AB1	CG4928	0.949183608371002	0.80156895525114	0.994766463583882	pseudobulk	DESeq2	LRT	0.0960591113673091
934	KC_AB1	CG4935	-0.391985191075348	0.783062587833321	0.994766463583882	pseudobulk	DESeq2	LRT	0.106203524705343
934	KC_AB1	CG4936	-0.270265522536715	0.787878709748015	0.994766463583882	pseudobulk	DESeq2	LRT	0.10354063497431
934	KC_AB1	CG4942	-4.7166963631462	0.0507141158658973	0.994766463583882	pseudobulk	DESeq2	LRT	1.2948711414673
934	KC_AB1	CG4949	2.12250628594009	0.308188228414228	0.994766463583882	pseudobulk	DESeq2	LRT	0.511183953652572
934	KC_AB1	CG4951	1.08591237858894	0.338617922324124	0.994766463583882	pseudobulk	DESeq2	LRT	0.470290059338477
934	KC_AB1	CG4953	-0.747541287559887	0.525861323109755	0.994766463583882	pseudobulk	DESeq2	LRT	0.279128770197586
934	KC_AB1	CG4957	0.0836450248606848	0.84236094327241	0.994766463583882	pseudobulk	DESeq2	LRT	0.0745017777609583
934	KC_AB1	CG4968	0.329610234358076	0.555726966822616	0.994766463583882	pseudobulk	DESeq2	LRT	0.255138528429487
935	KC_AB1	CG4972	-0.0717063621073401	0.904678679004557	0.994766463583882	pseudobulk	DESeq2	LRT	0.0435056447886205

935	KC_AB1	CG4975	1.19856662192297	0.608802264240657	0.994766463583882	pseudobulk	DESeq2	LRT	0.215523741018373
935	KC_AB1	CG5003	0.138445253143608	0.844162309731705	0.994766463583882	pseudobulk	DESeq2	LRT	0.0735740421851072
935	KC_AB1	CG5004	-0.147146663531397	0.864162974025904	0.994766463583882	pseudobulk	DESeq2	LRT	0.0634043454313129
935	KC_AB1	CG5009	0.236795371959263	0.82518825017128	0.994766463583882	pseudobulk	DESeq2	LRT	0.0834469645598971
935	KC_AB1	CG5013	-0.935747278994619	0.575886557130582	0.994766463583882	pseudobulk	DESeq2	LRT	0.239663059049792
935	KC_AB1	CG5021	0.301463749998463	0.60134707041996	0.994766463583882	pseudobulk	DESeq2	LRT	0.220874800439278
935	KC_AB1	CG5022	-0.4183270213525	0.618892643678033	0.994766463583882	pseudobulk	DESeq2	LRT	0.208384679415805
935	KC_AB1	CG5027	0.940842588363453	0.199663787975679	0.994766463583882	pseudobulk	DESeq2	LRT	0.699700693809267
935	KC_AB1	CG5028	-0.116640691034172	0.773329640271868	0.994766463583882	pseudobulk	DESeq2	LRT	0.11163534381158
936	KC_AB1	CG5033	-0.89419351234193	0.548105309335553	0.994766463583882	pseudobulk	DESeq2	LRT	0.261135991024013
936	KC_AB1	CG5037	-0.0196678760136628	0.927761047234153	0.994766463583882	pseudobulk	DESeq2	LRT	0.032563865624538
936	KC_AB1	CG5038	1.32710828904082	0.607473293222376	0.994766463583882	pseudobulk	DESeq2	LRT	0.216472810505135
936	KC_AB1	CG5039	1.66436120996494	0.568567199936166	0.994766463583882	pseudobulk	DESeq2	LRT	0.245218197930149
936	KC_AB1	CG5044	-0.21524034477514	0.794386003728139	0.994766463583882	pseudobulk	DESeq2	LRT	0.0999684162714678
936	KC_AB1	CG5050	-1.64952313825113	0.817565084244375	0.994766463583882	pseudobulk	DESeq2	LRT	0.0874776642296574
936	KC_AB1	CG5056	-0.461220157679299	0.478011194474555	0.994766463583882	pseudobulk	DESeq2	LRT	0.320561932589558
936	KC_AB1	CG5059	-0.354702589547019	0.398261019712722	0.994766463583882	pseudobulk	DESeq2	LRT	0.399832198617311
936	KC_AB1	CG5065	0.0344932347425757	0.914183208351211	0.994766463583882	pseudobulk	DESeq2	LRT	0.0389667600619857
936	KC_AB1	CG5068	0.489032971671211	0.495339064424664	0.994766463583882	pseudobulk	DESeq2	LRT	0.305097420462789
937	KC_AB1	CG5071	0.949183608371002	0.80156895525114	0.994766463583882	pseudobulk	DESeq2	LRT	0.0960591113673091
937	KC_AB1	CG5079	-2.79499059556516	0.38564607643653	0.994766463583882	pseudobulk	DESeq2	LRT	0.413811082791365
937	KC_AB1	CG5087	-0.419856540316325	0.65436726504554	0.994766463583882	pseudobulk	DESeq2	LRT	0.184178434528783
937	KC_AB1	CG5096	-4.07942607047938	0.12890417890847	0.994766463583882	pseudobulk	DESeq2	LRT	0.889733003147214
937	KC_AB1	CG5098	-0.183758146674537	0.737297184430851	0.994766463583882	pseudobulk	DESeq2	LRT	0.132357424564933
937	KC_AB1	CG5104	0.40411895759739	0.864643228521197	0.994766463583882	pseudobulk	DESeq2	LRT	0.0631630553638974
937	KC_AB1	CG5110	-0.377064632826669	0.56962586388981	0.994766463583882	pseudobulk	DESeq2	LRT	0.244410299761625
937	KC_AB1	CG5114	-0.9881577743308	0.564848678589511	0.994766463583882	pseudobulk	DESeq2	LRT	0.248067882898484
937	KC_AB1	CG5116	-1.60653259065571	0.594621747113632	0.994766463583882	pseudobulk	DESeq2	LRT	0.225759211379423
937	KC_AB1	CG5126	1.17285402251974	0.470206749786631	0.994766463583882	pseudobulk	DESeq2	LRT	0.327711140897919
938	KC_AB1	CG5131	-4.63641406512817	0.0608967805548075	0.994766463583882	pseudobulk	DESeq2	LRT	1.21540566671426
938	KC_AB1	CG5149	-0.00626808544845762	0.911829865468639	0.994766463583882	pseudobulk	DESeq2	LRT	0.04008618730864
938	KC_AB1	CG5151	-0.0129468711634064	0.883435965846413	0.994766463583882	pseudobulk	DESeq2	LRT	0.0538249240150522
938	KC_AB1	CG5157	0.949183608371002	0.80156895525114	0.994766463583882	pseudobulk	DESeq2	LRT	0.0960591113673091
938	KC_AB1	CG5160	2.02670873290438	0.538959414925803	0.994766463583882	pseudobulk	DESeq2	LRT	0.26844393710898
938	KC_AB1	CG5174	0.155582894908255	0.68592862615992	0.994766463583882	pseudobulk	DESeq2	LRT	0.163721072161387
938	KC_AB1	CG5177	0.949183608371002	0.80156895525114	0.994766463583882	pseudobulk	DESeq2	LRT	0.0960591113673091
938	KC_AB1	CG5180	-0.3876725532524	0.640816079634874	0.994766463583882	pseudobulk	DESeq2	LRT	0.193266599281681
938	KC_AB1	CG5181	0.569391790993925	0.552063212640855	0.994766463583882	pseudobulk	DESeq2	LRT	0.258011191601713
938	KC_AB1	CG5188	-3.26201275000334	0.140620244371484	0.994766463583882	pseudobulk	DESeq2	LRT	0.851952151678044
939	KC_AB1	CG5191	0.488566031708083	0.756948581006934	0.994766463583882	pseudobulk	DESeq2	LRT	0.120933620821461
939	KC_AB1	CG5196	-0.217160857534294	0.692341169854019	0.994766463583882	pseudobulk	DESeq2	LRT	0.15967984243334
939	KC_AB1	CG5199	-0.170204261305003	0.931920229649395	0.994766463583882	pseudobulk	DESeq2	LRT	0.0306212607208715
939	KC_AB1	CG5214	-0.176746649227503	0.670560504301036	0.994766463583882	pseudobulk	DESeq2	LRT	0.173562029895051
939	KC_AB1	CG5217	5.76452726380439	0.264496457249854	0.994766463583882	pseudobulk	DESeq2	LRT	0.577580140669035
939	KC_AB1	CG5220	0.534661008657919	0.648225398411614	0.994766463583882	pseudobulk	DESeq2	LRT	0.188273956682524
939	KC_AB1	CG5245	-2.79499059556516	0.38564607643653	0.994766463583882	pseudobulk	DESeq2	LRT	0.413811082791365
939	KC_AB1	CG5254	-3.21143241058256	0.292545415061155	0.994766463583882	pseudobulk	DESeq2	LRT	0.533806704011112
939	KC_AB1	CG5262	-0.672815725115348	0.602113564752849	0.994766463583882	pseudobulk	DESeq2	LRT	0.220321588652425
939	KC_AB1	CG5270	-0.655639302645168	0.614613015260702	0.994766463583882	pseudobulk	DESeq2	LRT	0.211398247210044
940	KC_AB1	CG5273	-0.285704024265871	0.608986543599947	0.994766463583882	pseudobulk	DESeq2	LRT	0.215392303598413
940	KC_AB1	CG5274	-0.47222544354889	0.69666602137467	0.994766463583882	pseudobulk	DESeq2	LRT	0.156975370876862
940	KC_AB1	CG5276	0.0871213401343273	0.940955672557668	0.994766463583882	pseudobulk	DESeq2	LRT	0.0264308352518565
940	KC_AB1	CG5281	0.079586986036792	0.856471843117797	0.994766463583882	pseudobulk	DESeq2	LRT	0.0672869100826769
940	KC_AB1	CG5282	0.651653787299378	0.184380053022524	0.994766463583882	pseudobulk	DESeq2	LRT	0.734286064469065
940	KC_AB1	CG5285	0.092564242017207	0.859555839666605	0.994766463583882	pseudobulk	DESeq2	LRT	0.0657259048159609
940	KC_AB1	CG5287	0.131296445082192	0.912473036289518	0.994766463583882	pseudobulk	DESeq2	LRT	0.0397799601467659

940	KC_AB1	CG5290	0.729740153036158	0.752353468463509	0.994766463583882	pseudobulk	DESeq2	LRT	0.123578072524162
940	KC_AB1	CG5292	-0.171807361011059	0.831969684973275	0.994766463583882	pseudobulk	DESeq2	LRT	0.0798924980947149
940	KC_AB1	CG5316	-0.18818925732374	0.749534733745247	0.994766463583882	pseudobulk	DESeq2	LRT	0.125208236965967
941	KC_AB1	CG5323	1.24651046276545	0.334094732888496	0.994766463583882	pseudobulk	DESeq2	LRT	0.476130371100848
941	KC_AB1	CG5335	0.949183608371002	0.80156895525114	0.994766463583882	pseudobulk	DESeq2	LRT	0.0960591113673091
941	KC_AB1	CG5339	0.0946360110034943	0.913570534583484	0.994766463583882	pseudobulk	DESeq2	LRT	0.0392579161846579
941	KC_AB1	CG5346	-1.64952313825113	0.817565084244375	0.994766463583882	pseudobulk	DESeq2	LRT	0.0874776642296574
941	KC_AB1	CG5348	-0.973010073065207	0.575751717314117	0.994766463583882	pseudobulk	DESeq2	LRT	0.239764757976962
941	KC_AB1	CG5355	-0.923281405417497	0.437925824652541	0.994766463583882	pseudobulk	DESeq2	LRT	0.358599443541837
941	KC_AB1	CG5359	-0.262871280601489	0.80729646846138	0.994766463583882	pseudobulk	DESeq2	LRT	0.0929669473445153
941	KC_AB1	CG5367	-2.79499059556516	0.38564607643653	0.994766463583882	pseudobulk	DESeq2	LRT	0.413811082791365
941	KC_AB1	CG5380	0.320312728107376	0.812990699520863	0.994766463583882	pseudobulk	DESeq2	LRT	0.0899144226345564
941	KC_AB1	CG5382	-0.787513072578771	0.451891603196672	0.994766463583882	pseudobulk	DESeq2	LRT	0.344965728417042
942	KC_AB1	CG5404	2.80587074018119	0.385946061723411	0.994766463583882	pseudobulk	DESeq2	LRT	0.413473386345624
942	KC_AB1	CG5412	-0.350056910617872	0.662695166601084	0.994766463583882	pseudobulk	DESeq2	LRT	0.178686196944941
942	KC_AB1	CG5439	0.963061659131036	0.339827715996619	0.994766463583882	pseudobulk	DESeq2	LRT	0.468741203414236
942	KC_AB1	CG5445	-0.196304636629124	0.736687598968895	0.994766463583882	pseudobulk	DESeq2	LRT	0.132716640779986
942	KC_AB1	CG5446	0.210611011775264	0.721241870005708	0.994766463583882	pseudobulk	DESeq2	LRT	0.141919069269916
942	KC_AB1	CG5447	-0.110450851840977	0.875407838966948	0.994766463583882	pseudobulk	DESeq2	LRT	0.0577895687523236
942	KC_AB1	CG5455	0.166600853701067	0.720625103466756	0.994766463583882	pseudobulk	DESeq2	LRT	0.142290613005579
942	KC_AB1	CG5466	0.190914447924117	0.689863111208542	0.994766463583882	pseudobulk	DESeq2	LRT	0.161237077298511
942	KC_AB1	CG5498	0.0616479535884938	0.843951246996812	0.994766463583882	pseudobulk	DESeq2	LRT	0.0736826407817025
942	KC_AB1	CG5500	-0.271770520182134	0.652798206920786	0.994766463583882	pseudobulk	DESeq2	LRT	0.185221047154743
943	KC_AB1	CG5504	0.191938995655594	0.827867324958112	0.994766463583882	pseudobulk	DESeq2	LRT	0.0820392582096381
943	KC_AB1	CG5510	0.543350739785657	0.578064398357804	0.994766463583882	pseudobulk	DESeq2	LRT	0.238023776988601
943	KC_AB1	CG5515	0.0365946855873828	0.953913855774767	0.994766463583882	pseudobulk	DESeq2	LRT	0.0204908429593005
943	KC_AB1	CG5516	0.390484868355206	0.599785448908806	0.994766463583882	pseudobulk	DESeq2	LRT	0.222004074647294
943	KC_AB1	CG5521	-0.131909766726571	0.805205927480189	0.994766463583882	pseudobulk	DESeq2	LRT	0.094093036736112
943	KC_AB1	CG5522	-0.0860202905002042	0.820063257642837	0.994766463583882	pseudobulk	DESeq2	LRT	0.0861526459265064
943	KC_AB1	CG5532	-0.211988444181992	0.653521333987379	0.994766463583882	pseudobulk	DESeq2	LRT	0.184740230452582
943	KC_AB1	CG5541	-0.0627561689052589	0.891934490418666	0.994766463583882	pseudobulk	DESeq2	LRT	0.0496670419181083
943	KC_AB1	CG5543	0.224333375176329	0.888675893031512	0.994766463583882	pseudobulk	DESeq2	LRT	0.0512566007087314
943	KC_AB1	CG5554	0.693282596137273	0.258735965014152	0.994766463583882	pseudobulk	DESeq2	LRT	0.587143198954245
944	KC_AB1	CG5555	0.136972407401388	0.829074716942905	0.994766463583882	pseudobulk	DESeq2	LRT	0.0814063286852013
944	KC_AB1	CG5567	-1.59932275540215	0.151882671067496	0.994766463583882	pseudobulk	DESeq2	LRT	0.818491773794065
944	KC_AB1	CG5569	-0.926640702672519	0.318413571409212	0.994766463583882	pseudobulk	DESeq2	LRT	0.497008430059607
944	KC_AB1	CG5589	-1.21558906437909	0.381807382025214	0.994766463583882	pseudobulk	DESeq2	LRT	0.418155679028202
944	KC_AB1	CG5590	-0.490967350109713	0.745057432024581	0.994766463583882	pseudobulk	DESeq2	LRT	0.127810248795325
944	KC_AB1	CG5608	0.466399522480077	0.681516099762293	0.994766463583882	pseudobulk	DESeq2	LRT	0.166523880173643
944	KC_AB1	CG5613	-0.648218248148942	0.834000364039372	0.994766463583882	pseudobulk	DESeq2	LRT	0.0788337597936092
944	KC_AB1	CG5618	0.309157786862567	0.836236418179691	0.994766463583882	pseudobulk	DESeq2	LRT	0.0776709228056033
944	KC_AB1	CG5641	-0.155219333749645	0.783486717084807	0.994766463583882	pseudobulk	DESeq2	LRT	0.105968361985017
944	KC_AB1	CG5644	-0.477470036085962	0.528810535061645	0.994766463583882	pseudobulk	DESeq2	LRT	0.276699901340942
945	KC_AB1	CG5645	1.11168776257694	0.593310522446876	0.994766463583882	pseudobulk	DESeq2	LRT	0.226717949319565
945	KC_AB1	CG5646	-0.226950955160759	0.825677459836429	0.994766463583882	pseudobulk	DESeq2	LRT	0.0831895710444835
945	KC_AB1	CG5664	-0.255006937147076	0.88671163122832	0.994766463583882	pseudobulk	DESeq2	LRT	0.0522175947436219
945	KC_AB1	CG5674	0.489931202920938	0.549206284785054	0.994766463583882	pseudobulk	DESeq2	LRT	0.260264501603069
945	KC_AB1	CG5676	-0.0428626136403504	0.902482169114781	0.994766463583882	pseudobulk	DESeq2	LRT	0.0445613699558862
945	KC_AB1	CG5708	-0.08179603635942	0.8223361317934	0.994766463583882	pseudobulk	DESeq2	LRT	0.0849506272967725
945	KC_AB1	CG5715	-1.64952313825113	0.817565084244375	0.994766463583882	pseudobulk	DESeq2	LRT	0.0874776642296574
945	KC_AB1	CG5721	0.999926511290955	0.496847057325826	0.994766463583882	pseudobulk	DESeq2	LRT	0.303777278030535
945	KC_AB1	CG5726	0.135124163851429	0.808044738438703	0.994766463583882	pseudobulk	DESeq2	LRT	0.0925645932858423
945	KC_AB1	CG5727	-0.636709303034065	0.317494200952656	0.994766463583882	pseudobulk	DESeq2	LRT	0.498264202709839
946	KC_AB1	CG5728	-1.18608882842302	0.383334637774757	0.994766463583882	pseudobulk	DESeq2	LRT	0.416421936511925
946	KC_AB1	CG5742	0.367260919909362	0.566751974703525	0.994766463583882	pseudobulk	DESeq2	LRT	0.246606958009826
946	KC_AB1	CG5745	-1.09461410731956	0.439908835901812	0.994766463583882	pseudobulk	DESeq2	LRT	0.356637314802266

946	KC_AB1	CG5746	-0.247245240698481	0.367696140181674	0.994766463583882	pseudobulk	DESeq2	LRT	0.434510929028604
946	KC_AB1	CG5758	0.0185718476810963	0.93261961656112	0.994766463583882	pseudobulk	DESeq2	LRT	0.0302954539097809
946	KC_AB1	CG5776	-0.0627561689052589	0.891934490418666	0.994766463583882	pseudobulk	DESeq2	LRT	0.0496670419181083
946	KC_AB1	CG5787	-0.0804965494425137	0.913963685027652	0.994766463583882	pseudobulk	DESeq2	LRT	0.0390710599613803
946	KC_AB1	CG5789	0.819007894079427	0.700993735174048	0.994766463583882	pseudobulk	DESeq2	LRT	0.154285863333625
946	KC_AB1	CG5793	-2.20969975456663	0.610836134188693	0.994766463583882	pseudobulk	DESeq2	LRT	0.214075280039667
946	KC_AB1	CG5800	1.26433463569552	0.528978780772322	0.994766463583882	pseudobulk	DESeq2	LRT	0.276561748717781
947	KC_AB1	CG5805	0.238158942330365	0.745096131455405	0.994766463583882	pseudobulk	DESeq2	LRT	0.127787691456705
947	KC_AB1	CG5808	0.0556507705136479	0.910364932142286	0.994766463583882	pseudobulk	DESeq2	LRT	0.0407844799367282
947	KC_AB1	CG5810	-0.835152883801839	0.920434724857442	0.994766463583882	pseudobulk	DESeq2	LRT	0.0360070052473159
947	KC_AB1	CG5815	-0.608331448605044	0.620660275683701	0.994766463583882	pseudobulk	DESeq2	LRT	0.207146050012458
947	KC_AB1	CG5823	0.200554816445023	0.646535165348804	0.994766463583882	pseudobulk	DESeq2	LRT	0.189407848662004
947	KC_AB1	CG5828	-0.841914684157861	0.533567052518636	0.994766463583882	pseudobulk	DESeq2	LRT	0.272810995716552
947	KC_AB1	CG5830	-0.150076298260383	0.280262843871198	0.994766463583882	pseudobulk	DESeq2	LRT	0.552434475451736
947	KC_AB1	CG5846	0.696058494471157	0.547212332072322	0.994766463583882	pseudobulk	DESeq2	LRT	0.261844123841649
947	KC_AB1	CG5850	0.707218771365051	0.59180376161713	0.994766463583882	pseudobulk	DESeq2	LRT	0.227822278708386
947	KC_AB1	CG5854	0.51986258599767	0.74420743113954	0.994766463583882	pseudobulk	DESeq2	LRT	0.12830599772918
948	KC_AB1	CG5862	0.543055348985514	0.668058414448685	0.994766463583882	pseudobulk	DESeq2	LRT	0.175185561531086
948	KC_AB1	CG5877	0.0796872454937441	0.92013246780261	0.994766463583882	pseudobulk	DESeq2	LRT	0.0361496445084451
948	KC_AB1	CG5880	-1.6729726248308	0.364853270181496	0.994766463583882	pseudobulk	DESeq2	LRT	0.437881756810481
948	KC_AB1	CG5885	0.313122139422371	0.673761312483343	0.994766463583882	pseudobulk	DESeq2	LRT	0.171493929919716
948	KC_AB1	CG5886	-0.481652812062737	0.6190072636756	0.994766463583882	pseudobulk	DESeq2	LRT	0.208304254767083
948	KC_AB1	CG5888	0.0544796448811775	0.868402147670179	0.994766463583882	pseudobulk	DESeq2	LRT	0.0612791111501896
948	KC_AB1	CG5890	-0.210357988272771	0.326956035201574	0.994766463583882	pseudobulk	DESeq2	LRT	0.485510641691491
948	KC_AB1	CG5902	0.229910977053329	0.76269279152032	0.994766463583882	pseudobulk	DESeq2	LRT	0.117650358262842
948	KC_AB1	CG5903	0.222276659937897	0.495222456730072	0.994766463583882	pseudobulk	DESeq2	LRT	0.305199669697109
948	KC_AB1	CG5910	-1.74683112255917	0.584939641962841	0.994766463583882	pseudobulk	DESeq2	LRT	0.232888945054326
949	KC_AB1	CG5913	0.17923522846011	0.90935249359699	0.994766463583882	pseudobulk	DESeq2	LRT	0.0412677379554051
949	KC_AB1	CG5916	-0.0207384681881289	0.920424304709099	0.994766463583882	pseudobulk	DESeq2	LRT	0.0360119218790114
949	KC_AB1	CG5934	-0.0067191872404007	0.949240569065143	0.994766463583882	pseudobulk	DESeq2	LRT	0.0226237089875476
949	KC_AB1	CG5937	0.207943773481415	0.669613710273545	0.994766463583882	pseudobulk	DESeq2	LRT	0.174175662787812
949	KC_AB1	CG5938	-0.133961815301192	0.760891991278162	0.994766463583882	pseudobulk	DESeq2	LRT	0.11867698701524
949	KC_AB1	CG5946	-0.136556058031347	0.774187914333398	0.994766463583882	pseudobulk	DESeq2	LRT	0.111153612633808
949	KC_AB1	CG5953	0.735848705915641	0.730849940572521	0.994766463583882	pseudobulk	DESeq2	LRT	0.136171784015139
949	KC_AB1	CG5961	0.98392689301569	0.306432596156843	0.994766463583882	pseudobulk	DESeq2	LRT	0.513665039372019
949	KC_AB1	CG5968	0.949183608371002	0.80156895525114	0.994766463583882	pseudobulk	DESeq2	LRT	0.0960591113673091
949	KC_AB1	CG5969	-0.448162753082722	0.479483488248773	0.994766463583882	pseudobulk	DESeq2	LRT	0.31922634383412
950	KC_AB1	CG5973	-1.08681444615798	0.701131388297773	0.994766463583882	pseudobulk	DESeq2	LRT	0.154200589927933
950	KC_AB1	CG5986	-0.447006210559621	0.446880996936992	0.994766463583882	pseudobulk	DESeq2	LRT	0.349808112783977
950	KC_AB1	CG5989	-0.341876448006842	0.697859753711147	0.994766463583882	pseudobulk	DESeq2	LRT	0.156231847160783
950	KC_AB1	CG6005	0.255545236863747	0.748365132400536	0.994766463583882	pseudobulk	DESeq2	LRT	0.125886455216393
950	KC_AB1	CG6006	0.0907406717286247	0.903829039999137	0.994766463583882	pseudobulk	DESeq2	LRT	0.0439137089123549
950	KC_AB1	CG6013	-0.223620266415382	0.71601426640182	0.994766463583882	pseudobulk	DESeq2	LRT	0.145078324399042
950	KC_AB1	CG6024	-0.294147101517143	0.252637208296049	0.994766463583882	pseudobulk	DESeq2	LRT	0.597502686371246
950	KC_AB1	CG6028	0.337553115012384	0.773229566703916	0.994766463583882	pseudobulk	DESeq2	LRT	0.111691547800681
950	KC_AB1	CG6040	0.949183608371002	0.80156895525114	0.994766463583882	pseudobulk	DESeq2	LRT	0.0960591113673091
950	KC_AB1	CG6041	0.511020761765907	0.533561281430787	0.994766463583882	pseudobulk	DESeq2	LRT	0.27281569309273
951	KC_AB1	CG6044	0.0379612627602768	0.812956552610167	0.994766463583882	pseudobulk	DESeq2	LRT	0.089932664081039
951	KC_AB1	CG6051	-0.117995409280139	0.832634380625236	0.994766463583882	pseudobulk	DESeq2	LRT	0.0795456604591466
951	KC_AB1	CG6052	0.949183608371002	0.80156895525114	0.994766463583882	pseudobulk	DESeq2	LRT	0.0960591113673091
951	KC_AB1	CG6066	0.0111686508800342	0.917295814967186	0.994766463583882	pseudobulk	DESeq2	LRT	0.03749058789522
951	KC_AB1	CG6083	-0.163300565787796	0.929388701621069	0.994766463583882	pseudobulk	DESeq2	LRT	0.0318026114498443
951	KC_AB1	CG6084	-0.521619058371449	0.728520799982995	0.994766463583882	pseudobulk	DESeq2	LRT	0.137558044183789
951	KC_AB1	CG6094	0.865469283923313	0.383731887333279	0.994766463583882	pseudobulk	DESeq2	LRT	0.415972110320894
951	KC_AB1	CG6115	-0.050504106438929	0.896639650103674	0.994766463583882	pseudobulk	DESeq2	LRT	0.0473820602053488
951	KC_AB1	CG6126	-0.17805032366327	0.942053914371676	0.994766463583882	pseudobulk	DESeq2	LRT	0.0259242415339998

951	KC_AB1	CG6136	-0.278333569327508	0.780755839065052	0.994766463583882	pseudobulk	DESeq2	LRT	0.107484759112815
952	KC_AB1	CG6144	0.056755513394966	0.927622212238575	0.994766463583882	pseudobulk	DESeq2	LRT	0.0326288605761518
952	KC_AB1	CG6145	-0.650779356707895	0.499562307626676	0.994766463583882	pseudobulk	DESeq2	LRT	0.301410336925761
952	KC_AB1	CG6153	-0.474740223751373	0.732868755471168	0.994766463583882	pseudobulk	DESeq2	LRT	0.134973793268981
952	KC_AB1	CG6154	-0.0499303594911506	0.836707487740774	0.994766463583882	pseudobulk	DESeq2	LRT	0.0774263444719793
952	KC_AB1	CG6163	3.46956015450889	0.2028823784209	0.994766463583882	pseudobulk	DESeq2	LRT	0.692755672468422
952	KC_AB1	CG6171	-0.0794247202672377	0.883958668034223	0.994766463583882	pseudobulk	DESeq2	LRT	0.053568041168277
952	KC_AB1	CG6178	-0.775658021735077	0.592070371626213	0.994766463583882	pseudobulk	DESeq2	LRT	0.227626671330083
952	KC_AB1	CG6179	-0.908167604633399	0.245530544823169	0.994766463583882	pseudobulk	DESeq2	LRT	0.609894472490155
952	KC_AB1	CG6180	0.296916638370627	0.608156680835776	0.994766463583882	pseudobulk	DESeq2	LRT	0.21598451800314
952	KC_AB1	CG6184	0.376909134111688	0.521150803612763	0.994766463583882	pseudobulk	DESeq2	LRT	0.283036588216421
953	KC_AB1	CG6191	0.195474955050143	0.679753801654007	0.994766463583882	pseudobulk	DESeq2	LRT	0.167648354858166
953	KC_AB1	CG6195	0.410877204745489	0.643664458489256	0.994766463583882	pseudobulk	DESeq2	LRT	0.191340470843286
953	KC_AB1	CG6204	-0.162131915966366	0.917226267001873	0.994766463583882	pseudobulk	DESeq2	LRT	0.0375235166867285
953	KC_AB1	CG6227	-0.23191627176383	0.792318352878707	0.994766463583882	pseudobulk	DESeq2	LRT	0.101100284169797
953	KC_AB1	CG6230	-0.336693797763883	0.789035438360004	0.994766463583882	pseudobulk	DESeq2	LRT	0.102903490658048
953	KC_AB1	CG6236	1.10720932435937	0.421821605572113	0.994766463583882	pseudobulk	DESeq2	LRT	0.374871179597858
953	KC_AB1	CG6254	0.205511634286069	0.845489900058704	0.994766463583882	pseudobulk	DESeq2	LRT	0.0728915759727697
953	KC_AB1	CG6276	-0.213300189072598	0.73128483608754	0.994766463583882	pseudobulk	DESeq2	LRT	0.135913432006881
953	KC_AB1	CG6282	-0.0511594424000124	0.833670808661292	0.994766463583882	pseudobulk	DESeq2	LRT	0.0790054052431292
953	KC_AB1	CG6293	0.40411895759739	0.864643228521197	0.994766463583882	pseudobulk	DESeq2	LRT	0.0631630553638974
954	KC_AB1	CG6294	1.1821590793593	0.719511699510136	0.994766463583882	pseudobulk	DESeq2	LRT	0.142962139890846
954	KC_AB1	CG6321	-1.50663180504502	0.668163815680457	0.994766463583882	pseudobulk	DESeq2	LRT	0.175117047219009
954	KC_AB1	CG6325	0.134681164264918	0.930208631343071	0.994766463583882	pseudobulk	DESeq2	LRT	0.0314196350166374
954	KC_AB1	CG6329	0.0346362745083735	0.810611619771502	0.994766463583882	pseudobulk	DESeq2	LRT	0.0911871751237173
954	KC_AB1	CG6330	-0.151122219711445	0.752588112814968	0.994766463583882	pseudobulk	DESeq2	LRT	0.123442645675517
954	KC_AB1	CG6340	-0.148713186873547	0.654297487376112	0.994766463583882	pseudobulk	DESeq2	LRT	0.184224747469916
954	KC_AB1	CG6345	-0.500135669249037	0.816878680938803	0.994766463583882	pseudobulk	DESeq2	LRT	0.0878424380956607
954	KC_AB1	CG6347	0.949183608371002	0.80156895525114	0.994766463583882	pseudobulk	DESeq2	LRT	0.0960591113673091
954	KC_AB1	CG6357	-2.03786985328338	0.468221401672057	0.994766463583882	pseudobulk	DESeq2	LRT	0.329548739260383
954	KC_AB1	CG6379	-0.353621194742064	0.667159137950221	0.994766463583882	pseudobulk	DESeq2	LRT	0.17577056115057
955	KC_AB1	CG6388	-0.860405337878537	0.541428394154473	0.994766463583882	pseudobulk	DESeq2	LRT	0.26645897224255
955	KC_AB1	CG6403	-0.341391478739861	0.841795132270276	0.994766463583882	pseudobulk	DESeq2	LRT	0.0747935899140484
955	KC_AB1	CG6405	-1.64952313825113	0.817565084244375	0.994766463583882	pseudobulk	DESeq2	LRT	0.0874776642296574
955	KC_AB1	CG6418	0.707040195188489	0.342694628130781	0.994766463583882	pseudobulk	DESeq2	LRT	0.465092703248698
955	KC_AB1	CG6420	-0.391618259374801	0.6126022862896	0.994766463583882	pseudobulk	DESeq2	LRT	0.212821386698391
955	KC_AB1	CG6425	1.02996170936706	0.619231122089829	0.994766463583882	pseudobulk	DESeq2	LRT	0.208147224463057
955	KC_AB1	CG6428	0.547051934790356	0.653050102927512	0.994766463583882	pseudobulk	DESeq2	LRT	0.185053497759815
955	KC_AB1	CG6443	-0.044375407826136	0.907490101120696	0.994766463583882	pseudobulk	DESeq2	LRT	0.0421581035393492
955	KC_AB1	CG6454	0.707365088205246	0.199666217671052	0.994766463583882	pseudobulk	DESeq2	LRT	0.699695408940721
955	KC_AB1	CG6472	-1.24381441853428	0.402088440665477	0.994766463583882	pseudobulk	DESeq2	LRT	0.395678411919117
956	KC_AB1	CG6479	0.124698704744493	0.891329405341186	0.994766463583882	pseudobulk	DESeq2	LRT	0.0499617656782218
956	KC_AB1	CG6481	-0.283995846067142	0.866480656580972	0.994766463583882	pseudobulk	DESeq2	LRT	0.0622411280844138
956	KC_AB1	CG6495	0.0261704135710794	0.909716926592638	0.994766463583882	pseudobulk	DESeq2	LRT	0.0410937245402006
956	KC_AB1	CG6511	0.641833300587602	0.746379423307147	0.994766463583882	pseudobulk	DESeq2	LRT	0.12704034202059
956	KC_AB1	CG6512	-0.438353849955586	0.538859627022834	0.994766463583882	pseudobulk	DESeq2	LRT	0.268524353828405
956	KC_AB1	CG6523	-0.532992656384548	0.297889385638257	0.994766463583882	pseudobulk	DESeq2	LRT	0.525944971242358
956	KC_AB1	CG6550	1.47117507179207	0.405608662679656	0.994766463583882	pseudobulk	DESeq2	LRT	0.391892778246224
956	KC_AB1	CG6565	0.296158746686587	0.764991442407552	0.994766463583882	pseudobulk	DESeq2	LRT	0.116343423063331
956	KC_AB1	CG6567	0.574579387376074	0.69332157464757	0.994766463583882	pseudobulk	DESeq2	LRT	0.159065285298698
956	KC_AB1	CG6574	0.309157786862567	0.836236418179691	0.994766463583882	pseudobulk	DESeq2	LRT	0.0776709228056033
957	KC_AB1	CG6583	-0.556645835071156	0.382721992495197	0.994766463583882	pseudobulk	DESeq2	LRT	0.417116581034243
957	KC_AB1	CG6607	0.330767534695153	0.672801973831446	0.994766463583882	pseudobulk	DESeq2	LRT	0.172112743097707
957	KC_AB1	CG6610	0.268165013616965	0.808013784558073	0.994766463583882	pseudobulk	DESeq2	LRT	0.092581230182788
957	KC_AB1	CG6617	0.359311779947021	0.485532569324116	0.994766463583882	pseudobulk	DESeq2	LRT	0.313781632484457
957	KC_AB1	CG6621	0.164877051591564	0.801920613987462	0.994766463583882	pseudobulk	DESeq2	LRT	0.0958686225059205

957	KC_AB1	CG6628	-0.0713915988297819	0.864170454131802	0.994766463583882	pseudobulk	DESeq2	LRT	0.063400586239177
957	KC_AB1	CG6638	1.54118100478876	0.611889806163345	0.994766463583882	pseudobulk	DESeq2	LRT	0.213326781913476
957	KC_AB1	CG6650	-0.181260954415712	0.81755037679627	0.994766463583882	pseudobulk	DESeq2	LRT	0.0874854769666871
957	KC_AB1	CG6654	0.385432675694031	0.785526848274965	0.994766463583882	pseudobulk	DESeq2	LRT	0.104838966755897
957	KC_AB1	CG6656	0.806933541094274	0.754600478929312	0.994766463583882	pseudobulk	DESeq2	LRT	0.122282923497213
958	KC_AB1	CG6659	-1.57203219539008	0.173751884330826	0.994766463583882	pseudobulk	DESeq2	LRT	0.760070476809549
958	KC_AB1	CG6660	0.949183608371002	0.80156895525114	0.994766463583882	pseudobulk	DESeq2	LRT	0.0960591113673091
958	KC_AB1	CG6664	-0.280314050391279	0.647946420668221	0.994766463583882	pseudobulk	DESeq2	LRT	0.188460904885867
958	KC_AB1	CG6665	0.206909384322427	0.838007697211646	0.994766463583882	pseudobulk	DESeq2	LRT	0.0767519922990894
958	KC_AB1	CG6674	-0.535690181463401	0.58637609062345	0.994766463583882	pseudobulk	DESeq2	LRT	0.231823746283019
958	KC_AB1	CG6678	-1.64952313825113	0.817565084244375	0.994766463583882	pseudobulk	DESeq2	LRT	0.0874776642296574
958	KC_AB1	CG6685	-1.14674838884359	0.239327399275803	0.994766463583882	pseudobulk	DESeq2	LRT	0.621007578556986
958	KC_AB1	CG6686	-0.00065451374995650	0.954813589184163	0.994766463583882	pseudobulk	DESeq2	LRT	0.020081408616165
958	KC_AB1	CG6689	-0.233707185114798	0.797910812078857	0.994766463583882	pseudobulk	DESeq2	LRT	0.098045649985982
958	KC_AB1	CG6693	-0.448152410197348	0.531732963540347	0.994766463583882	pseudobulk	DESeq2	LRT	0.2743064157815
959	KC_AB1	CG6695	0.651804149095265	0.631731062491537	0.994766463583882	pseudobulk	DESeq2	LRT	0.199467768131748
959	KC_AB1	CG6700	0.173920707008106	0.36612978888326	0.994766463583882	pseudobulk	DESeq2	LRT	0.436364934813322
959	KC_AB1	CG6707	0.185705430622794	0.611178427217913	0.994766463583882	pseudobulk	DESeq2	LRT	0.213831983466341
959	KC_AB1	CG6712	-0.0213847321357524	0.955181648958955	0.994766463583882	pseudobulk	DESeq2	LRT	0.0199140298416121
959	KC_AB1	CG6724	0.427212530942237	0.76871403858854	0.994766463583882	pseudobulk	DESeq2	LRT	0.11423518758448
959	KC_AB1	CG6729	-0.474740223751373	0.732868755471168	0.994766463583882	pseudobulk	DESeq2	LRT	0.134973793268981
959	KC_AB1	CG6733	1.80633728195307	0.61161102402172	0.994766463583882	pseudobulk	DESeq2	LRT	0.213524695220716
959	KC_AB1	CG6749	1.80633728195307	0.61161102402172	0.994766463583882	pseudobulk	DESeq2	LRT	0.213524695220716
959	KC_AB1	CG6752	-0.874780316428275	0.681205769332186	0.994766463583882	pseudobulk	DESeq2	LRT	0.166721682526051
959	KC_AB1	CG6758	-0.314685043316343	0.75948744826486	0.994766463583882	pseudobulk	DESeq2	LRT	0.119479399147283
960	KC_AB1	CG6762	-0.344015423271439	0.71330007008837	0.994766463583882	pseudobulk	DESeq2	LRT	0.14672773330584
960	KC_AB1	CG6765	-0.122273895933151	0.734225070486377	0.994766463583882	pseudobulk	DESeq2	LRT	0.134170790374115
960	KC_AB1	CG6766	0.0618180706260455	0.934069036205201	0.994766463583882	pseudobulk	DESeq2	LRT	0.0296210242677974
960	KC_AB1	CG6767	-0.142852170784502	0.435633481918945	0.994766463583882	pseudobulk	DESeq2	LRT	0.360878748627395
960	KC_AB1	CG6769	-0.543437037821376	0.530939608750971	0.994766463583882	pseudobulk	DESeq2	LRT	0.274954874545213
960	KC_AB1	CG6770	-0.0967804765281836	0.664118154867126	0.994766463583882	pseudobulk	DESeq2	LRT	0.177754647376211
960	KC_AB1	CG6785	1.80633728195307	0.61161102402172	0.994766463583882	pseudobulk	DESeq2	LRT	0.213524695220716
960	KC_AB1	CG6791	0.262871691267003	0.661824441530039	0.994766463583882	pseudobulk	DESeq2	LRT	0.17925719815287
960	KC_AB1	CG6805	-0.911262240521434	0.489372419002349	0.994766463583882	pseudobulk	DESeq2	LRT	0.310360511103204
960	KC_AB1	CG6808	-0.283995846067142	0.866480656580972	0.994766463583882	pseudobulk	DESeq2	LRT	0.0622411280844138
961	KC_AB1	CG6813	0.443767716975362	0.685120154694461	0.994766463583882	pseudobulk	DESeq2	LRT	0.164233256325545
961	KC_AB1	CG6833	-1.28669189388445	0.25923883184132	0.994766463583882	pseudobulk	DESeq2	LRT	0.586299944193165
961	KC_AB1	CG6836	0.992630526336814	0.723993771284609	0.994766463583882	pseudobulk	DESeq2	LRT	0.140265170140367
961	KC_AB1	CG6841	0.405090594232966	0.666441087356724	0.994766463583882	pseudobulk	DESeq2	LRT	0.17623823569756
961	KC_AB1	CG6843	0.0812055593671161	0.870200583867917	0.994766463583882	pseudobulk	DESeq2	LRT	0.0603806296499099
961	KC_AB1	CG6865	-1.64952313825113	0.817565084244375	0.994766463583882	pseudobulk	DESeq2	LRT	0.0874776642296574
961	KC_AB1	CG6867	-0.173253264298498	0.763260806783932	0.994766463583882	pseudobulk	DESeq2	LRT	0.117327037938697
961	KC_AB1	CG6878	0.143426599518619	0.787555743227291	0.994766463583882	pseudobulk	DESeq2	LRT	0.103718697069486
961	KC_AB1	CG6903	0.414119274548239	0.640566655842801	0.994766463583882	pseudobulk	DESeq2	LRT	0.193435671916923
961	KC_AB1	CG6907	-1.19897729889733	0.42375830585098	0.994766463583882	pseudobulk	DESeq2	LRT	0.372881776340728
962	KC_AB1	CG6912	5.17746623910185	0.297016123654443	0.994766463583882	pseudobulk	DESeq2	LRT	0.527219974170719
962	KC_AB1	CG6923	0.864460733252181	0.41243177599077	0.994766463583882	pseudobulk	DESeq2	LRT	0.384647881686737
962	KC_AB1	CG6928	1.34815820347889	0.526408859243851	0.994766463583882	pseudobulk	DESeq2	LRT	0.278676810335701
962	KC_AB1	CG6937	0.313900917523397	0.820412491556931	0.994766463583882	pseudobulk	DESeq2	LRT	0.0859677357086629
962	KC_AB1	CG6938	0.651806411443827	0.700247618684875	0.994766463583882	pseudobulk	DESeq2	LRT	0.154748359396614
962	KC_AB1	CG6951	-0.48167422725367	0.855534332799682	0.994766463583882	pseudobulk	DESeq2	LRT	0.0677625574305792
962	KC_AB1	CG6959	-4.02268708669696	0.139249961380398	0.994766463583882	pseudobulk	DESeq2	LRT	0.856204916601505
962	KC_AB1	CG6961	0.381063267506242	0.799390504318056	0.994766463583882	pseudobulk	DESeq2	LRT	0.0972410148784703
962	KC_AB1	CG6962	-0.835152883801839	0.920434724857442	0.994766463583882	pseudobulk	DESeq2	LRT	0.0360070052473159
962	KC_AB1	CG6966	-0.153001986749532	0.702965621166313	0.994766463583882	pseudobulk	DESeq2	LRT	0.153065913817801
963	KC_AB1	CG6983	-0.118793109278534	0.796675849027555	0.994766463583882	pseudobulk	DESeq2	LRT	0.098718348132592

963	KC_AB1	CG6984	-1.72207085463874	0.464449177681071	0.994766463583882	pseudobulk	DESeq2	LRT	0.333061801713891
963	KC_AB1	CG6999	-0.0627561689052589	0.891934490418666	0.994766463583882	pseudobulk	DESeq2	LRT	0.0496670419181083
963	KC_AB1	CG7006	-1.28458774735446	0.390580717181785	0.994766463583882	pseudobulk	DESeq2	LRT	0.408289201468707
963	KC_AB1	CG7009	0.833441706499796	0.620042047278132	0.994766463583882	pseudobulk	DESeq2	LRT	0.207578858434506
963	KC_AB1	CG7011	-0.0706843479215131	0.955949845227986	0.994766463583882	pseudobulk	DESeq2	LRT	0.0195648927784201
963	KC_AB1	CG7028	-0.0659828986220619	0.877197046484823	0.994766463583882	pseudobulk	DESeq2	LRT	0.0569028392579699
963	KC_AB1	CG7029	0.0223517956321075	0.88536750567727	0.994766463583882	pseudobulk	DESeq2	LRT	0.0528764213271498
963	KC_AB1	CG7044	0.475892334225681	0.803946046269915	0.994766463583882	pseudobulk	DESeq2	LRT	0.0947730962683447
963	KC_AB1	CG7049	0.517530417230426	0.688342551679016	0.994766463583882	pseudobulk	DESeq2	LRT	0.162195382577333
964	KC_AB1	CG7058	0.814677698626753	0.356382777146949	0.994766463583882	pseudobulk	DESeq2	LRT	0.448083292207489
964	KC_AB1	CG7059	-0.416900964575567	0.715879194431464	0.994766463583882	pseudobulk	DESeq2	LRT	0.145160259281998
964	KC_AB1	CG7065	-0.139481097394411	0.816980457925672	0.994766463583882	pseudobulk	DESeq2	LRT	0.0877883316151353
964	KC_AB1	CG7069	-1.74683112255917	0.584939641962841	0.994766463583882	pseudobulk	DESeq2	LRT	0.232888945054326
964	KC_AB1	CG7083	-0.332512504540115	0.698598077574221	0.994766463583882	pseudobulk	DESeq2	LRT	0.155772613802948
964	KC_AB1	CG7099	0.513714559562998	0.775698051782448	0.994766463583882	pseudobulk	DESeq2	LRT	0.110307299314091
964	KC_AB1	CG7101	-0.779567494705216	0.584786387067425	0.994766463583882	pseudobulk	DESeq2	LRT	0.23300274564375
964	KC_AB1	CG7102	1.80633728195307	0.61161102402172	0.994766463583882	pseudobulk	DESeq2	LRT	0.213524695220716
964	KC_AB1	CG7110	-1.60636322925781	0.527082061946688	0.994766463583882	pseudobulk	DESeq2	LRT	0.278121763770116
964	KC_AB1	CG7115	0.223007679564452	0.674303503987313	0.994766463583882	pseudobulk	DESeq2	LRT	0.171144583526088
965	KC_AB1	CG7120	-0.203696237743958	0.635603612881102	0.994766463583882	pseudobulk	DESeq2	LRT	0.196813642852533
965	KC_AB1	CG7130	1.1821590793593	0.719511699510136	0.994766463583882	pseudobulk	DESeq2	LRT	0.142962139890846
965	KC_AB1	CG7133	0.0792507836754811	0.920839785902035	0.994766463583882	pseudobulk	DESeq2	LRT	0.0358159247991691
965	KC_AB1	CG7137	1.70400802738958	0.443122299637011	0.994766463583882	pseudobulk	DESeq2	LRT	0.353476394031547
965	KC_AB1	CG7139	-0.437298985472933	0.52078502607071	0.994766463583882	pseudobulk	DESeq2	LRT	0.283341511360664
965	KC_AB1	CG7148	0.384905358391386	0.624219345136447	0.994766463583882	pseudobulk	DESeq2	LRT	0.204662776273339
965	KC_AB1	CG7154	0.334088608456766	0.562186769536742	0.994766463583882	pseudobulk	DESeq2	LRT	0.250119379263614
965	KC_AB1	CG7156	-0.781432876979296	0.629663167122735	0.994766463583882	pseudobulk	DESeq2	LRT	0.200891710514359
965	KC_AB1	CG7166	-0.0101011274665805	0.958607097706914	0.994766463583882	pseudobulk	DESeq2	LRT	0.0183593597344507
965	KC_AB1	CG7168	0.0757839931471787	0.926942752763998	0.994766463583882	pseudobulk	DESeq2	LRT	0.0329470867038497
966	KC_AB1	CG7182	1.10321068400525	0.331101802862627	0.994766463583882	pseudobulk	DESeq2	LRT	0.480038454490581
966	KC_AB1	CG7183	2.73655040822955	0.141553975604197	0.994766463583882	pseudobulk	DESeq2	LRT	0.849077928780553
966	KC_AB1	CG7206	-0.0183776501347904	0.955972312164308	0.994766463583882	pseudobulk	DESeq2	LRT	0.0195546860171955
966	KC_AB1	CG7218	-0.504856256995261	0.658665667042804	0.994766463583882	pseudobulk	DESeq2	LRT	0.181334973574516
966	KC_AB1	CG7220	-0.211087564843388	0.639782882681253	0.994766463583882	pseudobulk	DESeq2	LRT	0.193967383596274
966	KC_AB1	CG7222	0.555044886791575	0.42318845022765	0.994766463583882	pseudobulk	DESeq2	LRT	0.373466193692264
966	KC_AB1	CG7231	0.253144761659426	0.683099040390243	0.994766463583882	pseudobulk	DESeq2	LRT	0.165516324756711
966	KC_AB1	CG7239	-0.244982420490627	0.769499177089566	0.994766463583882	pseudobulk	DESeq2	LRT	0.113791840271296
966	KC_AB1	CG7246	-1.34847834357702	0.614076432341632	0.994766463583882	pseudobulk	DESeq2	LRT	0.211777570099331
966	KC_AB1	CG7265	-1.83810135661945	0.113624671885185	0.994766463583882	pseudobulk	DESeq2	LRT	0.94452735788359
967	KC_AB1	CG7272	-1.40297575431882	0.402579335798363	0.994766463583882	pseudobulk	DESeq2	LRT	0.395148520999933
967	KC_AB1	CG7275	-0.316331265609368	0.779096233752268	0.994766463583882	pseudobulk	DESeq2	LRT	0.108408895079299
967	KC_AB1	CG7289	-0.0323690841019738	0.938709240165677	0.994766463583882	pseudobulk	DESeq2	LRT	0.027468907144036
967	KC_AB1	CG7324	-0.59828398738052	0.307143765830106	0.994766463583882	pseudobulk	DESeq2	LRT	0.512658295233108
967	KC_AB1	CG7326	0.858863249679495	0.383863061129984	0.994766463583882	pseudobulk	DESeq2	LRT	0.415823677721779
967	KC_AB1	CG7332	-0.063993711751091	0.877464721536696	0.994766463583882	pseudobulk	DESeq2	LRT	0.0567703353179038
967	KC_AB1	CG7339	-0.0616926867627397	0.869536141159153	0.994766463583882	pseudobulk	DESeq2	LRT	0.060712362390659
967	KC_AB1	CG7352	1.1821590793593	0.719511699510136	0.994766463583882	pseudobulk	DESeq2	LRT	0.142962139890846
967	KC_AB1	CG7367	-1.28132067551389	0.647201349541384	0.994766463583882	pseudobulk	DESeq2	LRT	0.188960585805912
967	KC_AB1	CG7368	0.214447675790384	0.722525127904189	0.994766463583882	pseudobulk	DESeq2	LRT	0.141147044460183
968	KC_AB1	CG7369	-0.511442787309368	0.5670163331236	0.994766463583882	pseudobulk	DESeq2	LRT	0.246404430907293
968	KC_AB1	CG7376	-1.02254675626108	0.598976376938724	0.994766463583882	pseudobulk	DESeq2	LRT	0.222590305436173
968	KC_AB1	CG7378	-0.152788927234268	0.381111895889517	0.994766463583882	pseudobulk	DESeq2	LRT	0.418947495104653
968	KC_AB1	CG7379	-0.180164467606467	0.758082806589526	0.994766463583882	pseudobulk	DESeq2	LRT	0.120283353100522
968	KC_AB1	CG7381	-1.64952313825113	0.817565084244375	0.994766463583882	pseudobulk	DESeq2	LRT	0.0874776642296574
968	KC_AB1	CG7382	0.294491338092091	0.595218012460038	0.994766463583882	pseudobulk	DESeq2	LRT	0.225323934663734
968	KC_AB1	CG7386	0.949183608371002	0.80156895525114	0.994766463583882	pseudobulk	DESeq2	LRT	0.0960591113673091

968	KC_AB1	CG7394	0.039249999886415	0.873933695838577	0.994766463583882	pseudobulk	DESeq2	LRT	0.0585215154476844
968	KC_AB1	CG7407	0.235688921145515	0.592256997157566	0.994766463583882	pseudobulk	DESeq2	LRT	0.227489799648728
968	KC_AB1	CG7414	0.499349499383549	0.409802822571832	0.994766463583882	pseudobulk	DESeq2	LRT	0.387425054663106
969	KC_AB1	CG7420	0.151764215534826	0.905029099868869	0.994766463583882	pseudobulk	DESeq2	LRT	0.0433374564751338
969	KC_AB1	CG7430	0.0950794836869836	0.821204221335933	0.994766463583882	pseudobulk	DESeq2	LRT	0.085548826838964
969	KC_AB1	CG7457	1.1821590793593	0.719511699510136	0.994766463583882	pseudobulk	DESeq2	LRT	0.142962139890846
969	KC_AB1	CG7460	-1.64952313825113	0.817565084244375	0.994766463583882	pseudobulk	DESeq2	LRT	0.0874776642296574
969	KC_AB1	CG7461	0.280783473609079	0.862060689739496	0.994766463583882	pseudobulk	DESeq2	LRT	0.0644621584314761
969	KC_AB1	CG7483	0.0992973590394019	0.883178341258599	0.994766463583882	pseudobulk	DESeq2	LRT	0.0539515899627168
969	KC_AB1	CG7484	0.441517612557694	0.61703101949294	0.994766463583882	pseudobulk	DESeq2	LRT	0.20969300248866
969	KC_AB1	CG7488	0.569618865619011	0.625729787074591	0.994766463583882	pseudobulk	DESeq2	LRT	0.203613170491213
969	KC_AB1	CG7492	-0.101457390969057	0.827125723689449	0.994766463583882	pseudobulk	DESeq2	LRT	0.0824284723644315
969	KC_AB1	CG7497	-1.64952313825113	0.817565084244375	0.994766463583882	pseudobulk	DESeq2	LRT	0.0874776642296574
970	KC_AB1	CG7504	-0.662673649541403	0.528740438053054	0.994766463583882	pseudobulk	DESeq2	LRT	0.276757473495567
970	KC_AB1	CG7506	0.487970392446155	0.48163013631947	0.994766463583882	pseudobulk	DESeq2	LRT	0.317286346443394
970	KC_AB1	CG7518	-0.512378628074121	0.394697124841148	0.994766463583882	pseudobulk	DESeq2	LRT	0.403736037197523
970	KC_AB1	CG7519	-0.656078913318665	0.406311252008554	0.994766463583882	pseudobulk	DESeq2	LRT	0.391141150556776
970	KC_AB1	CG7523	-1.40125223445929	0.149724733125524	0.994766463583882	pseudobulk	DESeq2	LRT	0.824706452344377
970	KC_AB1	CG7536	-2.11458293841668	0.293528939879375	0.994766463583882	pseudobulk	DESeq2	LRT	0.532349073938275
970	KC_AB1	CG7544	0.0236942436874584	0.910119428894173	0.994766463583882	pseudobulk	DESeq2	LRT	0.0409016143750932
970	KC_AB1	CG7550	-0.575436763912769	0.663104539309478	0.994766463583882	pseudobulk	DESeq2	LRT	0.178417999097024
970	KC_AB1	CG7556	-0.341365668399102	0.716381387463683	0.994766463583882	pseudobulk	DESeq2	LRT	0.144855706221699
970	KC_AB1	CG7560	1.80939591345248	0.468117841155507	0.994766463583882	pseudobulk	DESeq2	LRT	0.329644806495434
971	KC_AB1	CG7564	-0.292292471493141	0.500539866383024	0.994766463583882	pseudobulk	DESeq2	LRT	0.300561326654892
971	KC_AB1	CG7565	0.855048337518235	0.378352690324072	0.994766463583882	pseudobulk	DESeq2	LRT	0.422103173580265
971	KC_AB1	CG7568	-0.627447798185778	0.772628937945737	0.994766463583882	pseudobulk	DESeq2	LRT	0.112029029876423
971	KC_AB1	CG7582	0.375070043135865	0.490798281025311	0.994766463583882	pseudobulk	DESeq2	LRT	0.309096967017832
971	KC_AB1	CG7600	0.397176479580476	0.68319204410292	0.994766463583882	pseudobulk	DESeq2	LRT	0.165457199728768
971	KC_AB1	CG7601	-2.33697228844215	0.262225832624204	0.994766463583882	pseudobulk	DESeq2	LRT	0.581324526934567
971	KC_AB1	CG7611	0.19843682996269	0.777717555344421	0.994766463583882	pseudobulk	DESeq2	LRT	0.109178097646188
971	KC_AB1	CG7627	-2.20969975456663	0.610836134188693	0.994766463583882	pseudobulk	DESeq2	LRT	0.214075280039667
971	KC_AB1	CG7630	0.0653799139029624	0.734309995871359	0.994766463583882	pseudobulk	DESeq2	LRT	0.134120559873044
971	KC_AB1	CG7632	0.419805627871603	0.73043510901643	0.994766463583882	pseudobulk	DESeq2	LRT	0.136418360219584
972	KC_AB1	CG7637	-0.940372445120932	0.118019233519818	0.994766463583882	pseudobulk	DESeq2	LRT	0.928047210229085
972	KC_AB1	CG7638	-0.132396712969872	0.87081677817022	0.994766463583882	pseudobulk	DESeq2	LRT	0.0600732119270553
972	KC_AB1	CG7639	0.970354646360749	0.16842918695184	0.994766463583882	pseudobulk	DESeq2	LRT	0.773582647790164
972	KC_AB1	CG7646	0.126705548838781	0.387729262389282	0.994766463583882	pseudobulk	DESeq2	LRT	0.411471421034013
972	KC_AB1	CG7650	1.25997470065014	0.2569714073962	0.994766463583882	pseudobulk	DESeq2	LRT	0.590115196905542
972	KC_AB1	CG7655	1.00530764230806	0.505177517558677	0.994766463583882	pseudobulk	DESeq2	LRT	0.296555985546382
972	KC_AB1	CG7656	0.263937243125486	0.649166592340784	0.994766463583882	pseudobulk	DESeq2	LRT	0.187643838099306
972	KC_AB1	CG7668	0.551915197432365	0.629610180993122	0.994766463583882	pseudobulk	DESeq2	LRT	0.2009282579149
972	KC_AB1	CG7685	1.76152171021203	0.334214385144892	0.994766463583882	pseudobulk	DESeq2	LRT	0.475974861281057
972	KC_AB1	CG7692	-0.0751450408454766	0.937038401572958	0.994766463583882	pseudobulk	DESeq2	LRT	0.0282426105535364
973	KC_AB1	CG7694	-0.251131114498766	0.714745949253217	0.994766463583882	pseudobulk	DESeq2	LRT	0.145848297280866
973	KC_AB1	CG7702	-2.03786985328338	0.468221401672057	0.994766463583882	pseudobulk	DESeq2	LRT	0.329548739260383
973	KC_AB1	CG7706	0.702804835505576	0.388751241813529	0.994766463583882	pseudobulk	DESeq2	LRT	0.410328210667609
973	KC_AB1	CG7718	-0.623781400773551	0.780876293833657	0.994766463583882	pseudobulk	DESeq2	LRT	0.107417761462951
973	KC_AB1	CG7724	-1.64952313825113	0.817565084244375	0.994766463583882	pseudobulk	DESeq2	LRT	0.0874776642296574
973	KC_AB1	CG7728	0.475649746043893	0.759920495303806	0.994766463583882	pseudobulk	DESeq2	LRT	0.119231842267898
973	KC_AB1	CG7739	0.510913179283097	0.353406874079034	0.994766463583882	pseudobulk	DESeq2	LRT	0.451725007331919
973	KC_AB1	CG7741	1.08038424636867	0.390662606434371	0.994766463583882	pseudobulk	DESeq2	LRT	0.408198156720981
973	KC_AB1	CG7744	-2.45516412093746	0.350354315181257	0.994766463583882	pseudobulk	DESeq2	LRT	0.455492529097001
973	KC_AB1	CG7745	0.366758029646786	0.72918989382001	0.994766463583882	pseudobulk	DESeq2	LRT	0.137159359063438
974	KC_AB1	CG7747	-0.336314434875304	0.732990524071	0.994766463583882	pseudobulk	DESeq2	LRT	0.13490163977944
974	KC_AB1	CG7766	0.0165314376641943	0.887163537131962	0.994766463583882	pseudobulk	DESeq2	LRT	0.0519963162139512
974	KC_AB1	CG7772	1.15514302919305	0.319448692512326	0.994766463583882	pseudobulk	DESeq2	LRT	0.495598885074004

974	KC_AB1	CG7781	0.0134131365992302	0.944927765088679	0.994766463583882	pseudobulk	DESeq2	LRT	0.0246013898213637
974	KC_AB1	CG7785	-0.624505811028928	0.362831191715281	0.994766463583882	pseudobulk	DESeq2	LRT	0.440295384766474
974	KC_AB1	CG7789	0.0212567318314261	0.870862448146837	0.994766463583882	pseudobulk	DESeq2	LRT	0.0600504359548346
974	KC_AB1	CG7791	-0.607965210763801	0.634106642001654	0.994766463583882	pseudobulk	DESeq2	LRT	0.1978376977363
974	KC_AB1	CG7806	-2.20905774599517	0.253494447678173	0.994766463583882	pseudobulk	DESeq2	LRT	0.596031548634941
974	KC_AB1	CG7810	0.939364783671562	0.478843810897333	0.994766463583882	pseudobulk	DESeq2	LRT	0.319806121505977
974	KC_AB1	CG7834	-0.130667192637795	0.841157273392528	0.994766463583882	pseudobulk	DESeq2	LRT	0.0751227954259474
975	KC_AB1	CG7839	-0.432051346446346	0.6401097693534	0.994766463583882	pseudobulk	DESeq2	LRT	0.193745544552536
975	KC_AB1	CG7840	-0.989583867941823	0.582662397843883	0.994766463583882	pseudobulk	DESeq2	LRT	0.234583008228436
975	KC_AB1	CG7841	0.776541761222719	0.551541933188446	0.994766463583882	pseudobulk	DESeq2	LRT	0.258421462988002
975	KC_AB1	CG7849	2.23036725486622	0.21951548993421	0.994766463583882	pseudobulk	DESeq2	LRT	0.65853482869932
975	KC_AB1	CG7857	-0.104972297059361	0.932023146519923	0.994766463583882	pseudobulk	DESeq2	LRT	0.0305733019367346
975	KC_AB1	CG7872	0.506777756810903	0.600758066016197	0.994766463583882	pseudobulk	DESeq2	LRT	0.221300389474859
975	KC_AB1	CG7878	-0.223267357916067	0.753193345652494	0.994766463583882	pseudobulk	DESeq2	LRT	0.123093525563456
975	KC_AB1	CG7879	0.223273884983401	0.571345001269728	0.994766463583882	pseudobulk	DESeq2	LRT	0.243101567934148
975	KC_AB1	CG7881	1.1821590793593	0.719511699510136	0.994766463583882	pseudobulk	DESeq2	LRT	0.142962139890846
975	KC_AB1	CG7882	-1.64952313825113	0.817565084244375	0.994766463583882	pseudobulk	DESeq2	LRT	0.0874776642296574
976	KC_AB1	CG7888	-1.64952313825113	0.817565084244375	0.994766463583882	pseudobulk	DESeq2	LRT	0.0874776642296574
976	KC_AB1	CG7889	-0.716915414196649	0.655152740527948	0.994766463583882	pseudobulk	DESeq2	LRT	0.183657437969526
976	KC_AB1	CG7900	-3.75446423347832	0.226220543058798	0.994766463583882	pseudobulk	DESeq2	LRT	0.645467959392995
976	KC_AB1	CG7903	-0.320130185798933	0.590917562948864	0.994766463583882	pseudobulk	DESeq2	LRT	0.228473101950565
976	KC_AB1	CG7914	0.992630526336814	0.723993771284609	0.994766463583882	pseudobulk	DESeq2	LRT	0.140265170140367
976	KC_AB1	CG7920	0.123955229202409	0.763124456020138	0.994766463583882	pseudobulk	DESeq2	LRT	0.117404628290124
976	KC_AB1	CG7943	0.307168321904555	0.654592804235889	0.994766463583882	pseudobulk	DESeq2	LRT	0.184028773085184
976	KC_AB1	CG7945	0.031929995150871	0.839233398709468	0.994766463583882	pseudobulk	DESeq2	LRT	0.0761172409887148
976	KC_AB1	CG7946	0.116377677262269	0.883041722275396	0.994766463583882	pseudobulk	DESeq2	LRT	0.0540187762345427
976	KC_AB1	CG7950	1.1821590793593	0.719511699510136	0.994766463583882	pseudobulk	DESeq2	LRT	0.142962139890846
977	KC_AB1	CG7956	0.0244335354834615	0.91092171195646	0.994766463583882	pseudobulk	DESeq2	LRT	0.0405189463270164
977	KC_AB1	CG7970	0.53838719609977	0.587169965010361	0.994766463583882	pseudobulk	DESeq2	LRT	0.231236167608119
977	KC_AB1	CG7971	0.0858694638175973	0.584201067510893	0.994766463583882	pseudobulk	DESeq2	LRT	0.233437653775227
977	KC_AB1	CG7974	0.517975828269768	0.770053709647308	0.994766463583882	pseudobulk	DESeq2	LRT	0.113478982632754
977	KC_AB1	CG7985	0.190763871119014	0.529346042036185	0.994766463583882	pseudobulk	DESeq2	LRT	0.276260329827706
977	KC_AB1	CG7987	0.249263854812366	0.753524331855478	0.994766463583882	pseudobulk	DESeq2	LRT	0.122902719434345
977	KC_AB1	CG7988	0.39894238435642	0.765267492076956	0.994766463583882	pseudobulk	DESeq2	LRT	0.116186734745097
977	KC_AB1	CG7990	0.0577853583585823	0.80230039666977	0.994766463583882	pseudobulk	DESeq2	LRT	0.0956629930755185
977	KC_AB1	CG7991	1.1821590793593	0.719511699510136	0.994766463583882	pseudobulk	DESeq2	LRT	0.142962139890846
977	KC_AB1	CG8001	0.395831746275018	0.767429410406031	0.994766463583882	pseudobulk	DESeq2	LRT	0.114961561233221
978	KC_AB1	CG8003	-0.556378386526742	0.640714482776025	0.994766463583882	pseudobulk	DESeq2	LRT	0.193335459060079
978	KC_AB1	CG8004	0.250093531033916	0.686010172197773	0.994766463583882	pseudobulk	DESeq2	LRT	0.163669444502274
978	KC_AB1	CG8005	-0.0627561689052589	0.891934490418666	0.994766463583882	pseudobulk	DESeq2	LRT	0.0496670419181083
978	KC_AB1	CG8009	0.205034897545033	0.720155017229772	0.994766463583882	pseudobulk	DESeq2	LRT	0.142574009289373
978	KC_AB1	CG8010	-0.0560215070077809	0.958726144558353	0.994766463583882	pseudobulk	DESeq2	LRT	0.0183054292130427
978	KC_AB1	CG8012	2.9088647549091	0.182404758875829	0.994766463583882	pseudobulk	DESeq2	LRT	0.738963835269929
978	KC_AB1	CG8027	0.097819640535638	0.89223956997303	0.994766463583882	pseudobulk	DESeq2	LRT	0.0495185201216824
978	KC_AB1	CG8031	-1.78852296053867	0.14882527611467	0.994766463583882	pseudobulk	DESeq2	LRT	0.827323303031432
978	KC_AB1	CG8032	0.124466798304069	0.808841595811388	0.994766463583882	pseudobulk	DESeq2	LRT	0.0921365226410373
978	KC_AB1	CG8034	0.389275648472954	0.837532199093071	0.994766463583882	pseudobulk	DESeq2	LRT	0.0769984874318538
979	KC_AB1	CG8036	-1.62718364431562	0.251246696629544	0.994766463583882	pseudobulk	DESeq2	LRT	0.599899639601273
979	KC_AB1	CG8038	-0.0713915988297819	0.864170454131802	0.994766463583882	pseudobulk	DESeq2	LRT	0.063400586239177
979	KC_AB1	CG8042	0.078813583167913	0.9112004303607	0.994766463583882	pseudobulk	DESeq2	LRT	0.0403860838112726
979	KC_AB1	CG8043	0.765772002374749	0.553454554439476	0.994766463583882	pseudobulk	DESeq2	LRT	0.256918034345254
979	KC_AB1	CG8060	-0.529428120775926	0.693364812554561	0.994766463583882	pseudobulk	DESeq2	LRT	0.159038202052487
979	KC_AB1	CG8064	0.325501121325807	0.840869281385295	0.994766463583882	pseudobulk	DESeq2	LRT	0.0752715128649677
979	KC_AB1	CG8066	-0.582353494399943	0.802766106531744	0.994766463583882	pseudobulk	DESeq2	LRT	0.0954109720801323
979	KC_AB1	CG8067	0.802876888060307	0.539506607881956	0.994766463583882	pseudobulk	DESeq2	LRT	0.268003231705637
979	KC_AB1	CG8078	1.04459227441778	0.414471626119809	0.994766463583882	pseudobulk	DESeq2	LRT	0.382505195008468

979	KC_AB1	CG8079	0.0117660683531627	0.822369276666607	0.994766463583882	pseudobulk	DESeq2	LRT	0.0849331230854226
980	KC_AB1	CG8080	0.28624314771221	0.796372202718876	0.994766463583882	pseudobulk	DESeq2	LRT	0.098883907379549
980	KC_AB1	CG8097	0.854047063205883	0.719204060467466	0.994766463583882	pseudobulk	DESeq2	LRT	0.143147869337068
980	KC_AB1	CG8108	-0.00932150436398454	0.920852982581854	0.994766463583882	pseudobulk	DESeq2	LRT	0.0358097009106651
980	KC_AB1	CG8111	-0.843420758753941	0.428443454164646	0.994766463583882	pseudobulk	DESeq2	LRT	0.368106488010903
980	KC_AB1	CG8128	0.949183608371002	0.80156895525114	0.994766463583882	pseudobulk	DESeq2	LRT	0.0960591113673091
980	KC_AB1	CG8132	0.64250505258733	0.487378675931608	0.994766463583882	pseudobulk	DESeq2	LRT	0.312133476234237
980	KC_AB1	CG8134	-0.11586251018576	0.865532775916901	0.994766463583882	pseudobulk	DESeq2	LRT	0.0627164816530211
980	KC_AB1	CG8142	-0.0876274309314422	0.917937734184526	0.994766463583882	pseudobulk	DESeq2	LRT	0.0371867769874299
980	KC_AB1	CG8149	0.0753300920288291	0.857783135171306	0.994766463583882	pseudobulk	DESeq2	LRT	0.0666224966537662
980	KC_AB1	CG8152	-0.277919745732209	0.800864745320316	0.994766463583882	pseudobulk	DESeq2	LRT	0.0964408238919987
981	KC_AB1	CG8155	-0.278507658441258	0.748516931571299	0.994766463583882	pseudobulk	DESeq2	LRT	0.125798371399996
981	KC_AB1	CG8159	0.986934735379525	0.609851704889206	0.994766463583882	pseudobulk	DESeq2	LRT	0.214775757740171
981	KC_AB1	CG8160	-3.46197803745951	0.239982951034438	0.994766463583882	pseudobulk	DESeq2	LRT	0.619819610516178
981	KC_AB1	CG8173	0.585263909415425	0.685172618615028	0.994766463583882	pseudobulk	DESeq2	LRT	0.164200000963397
981	KC_AB1	CG8176	-0.0528710837451091	0.79684814817459	0.994766463583882	pseudobulk	DESeq2	LRT	0.0986244322967254
981	KC_AB1	CG8177	0.25018960434776	0.39480597781011	0.994766463583882	pseudobulk	DESeq2	LRT	0.403616280241787
981	KC_AB1	CG8180	0.949183608371002	0.80156895525114	0.994766463583882	pseudobulk	DESeq2	LRT	0.0960591113673091
981	KC_AB1	CG8187	-0.26008073748265	0.73783036231076	0.994766463583882	pseudobulk	DESeq2	LRT	0.132043477175485
981	KC_AB1	CG8188	-0.0157462042711635	0.923167274250312	0.994766463583882	pseudobulk	DESeq2	LRT	0.0347195994054335
981	KC_AB1	CG8195	0.293976546066273	0.619289793557005	0.994766463583882	pseudobulk	DESeq2	LRT	0.208106077488247
982	KC_AB1	CG8199	1.1821590793593	0.719511699510136	0.994766463583882	pseudobulk	DESeq2	LRT	0.142962139890846
982	KC_AB1	CG8202	-1.5496554592572	0.507524021670927	0.994766463583882	pseudobulk	DESeq2	LRT	0.294543397292118
982	KC_AB1	CG8204	0.0950046654641491	0.926035841332843	0.994766463583882	pseudobulk	DESeq2	LRT	0.0333722040396142
982	KC_AB1	CG8206	0.468136948776068	0.490428390666286	0.994766463583882	pseudobulk	DESeq2	LRT	0.309424396660905
982	KC_AB1	CG8207	-0.0821496054323249	0.957377622215522	0.994766463583882	pseudobulk	DESeq2	LRT	0.0189167279527711
982	KC_AB1	CG8209	0.147626989565281	0.835256911677172	0.994766463583882	pseudobulk	DESeq2	LRT	0.0781799219264822
982	KC_AB1	CG8219	-1.64952313825113	0.817565084244375	0.994766463583882	pseudobulk	DESeq2	LRT	0.0874776642296574
982	KC_AB1	CG8223	0.739102885151472	0.311478355441585	0.994766463583882	pseudobulk	DESeq2	LRT	0.506572126978748
982	KC_AB1	CG8229	-0.0327828539565739	0.877400382109004	0.994766463583882	pseudobulk	DESeq2	LRT	0.0568021807952224
982	KC_AB1	CG8230	0.14972191254313	0.869552899230555	0.994766463583882	pseudobulk	DESeq2	LRT	0.0607039925628144
983	KC_AB1	CG8237	-0.115188115175472	0.868945865662506	0.994766463583882	pseudobulk	DESeq2	LRT	0.0610072787600453
983	KC_AB1	CG8243	-0.0925209390545214	0.853616252137937	0.994766463583882	pseudobulk	DESeq2	LRT	0.0687373249019632
983	KC_AB1	CG8245	-0.198488355505056	0.52470651851969	0.994766463583882	pseudobulk	DESeq2	LRT	0.280083540452801
983	KC_AB1	CG8248	0.507230185543395	0.389525693596529	0.994766463583882	pseudobulk	DESeq2	LRT	0.409463890445416
983	KC_AB1	CG8270	1.02241747324668	0.427198002334331	0.994766463583882	pseudobulk	DESeq2	LRT	0.369370786825018
983	KC_AB1	CG8272	-0.059113546855606	0.955083348076834	0.994766463583882	pseudobulk	DESeq2	LRT	0.0199587268137861
983	KC_AB1	CG8281	1.33722467168186	0.332693677315985	0.994766463583882	pseudobulk	DESeq2	LRT	0.477955452611403
983	KC_AB1	CG8289	-0.20602443737692	0.703489499744561	0.994766463583882	pseudobulk	DESeq2	LRT	0.152742380442495
983	KC_AB1	CG8298	-0.497088983767899	0.666498078580041	0.994766463583882	pseudobulk	DESeq2	LRT	0.176201098258057
983	KC_AB1	CG8300	-0.133645645976641	0.864780985216329	0.994766463583882	pseudobulk	DESeq2	LRT	0.0630938682064698
984	KC_AB1	CG8301	-0.0132731607773746	0.943008224668564	0.994766463583882	pseudobulk	DESeq2	LRT	0.0254845194444362
984	KC_AB1	CG8311	-0.433720299910024	0.666276432225428	0.994766463583882	pseudobulk	DESeq2	LRT	0.176345548484032
984	KC_AB1	CG8314	0.229701234272814	0.737865019872812	0.994766463583882	pseudobulk	DESeq2	LRT	0.132023077859827
984	KC_AB1	CG8319	0.196658486623007	0.871325117064304	0.994766463583882	pseudobulk	DESeq2	LRT	0.0598197666898938
984	KC_AB1	CG8320	-0.811828838027059	0.460845055809559	0.994766463583882	pseudobulk	DESeq2	LRT	0.336445067490872
984	KC_AB1	CG8321	-0.345458381077433	0.520243942003953	0.994766463583882	pseudobulk	DESeq2	LRT	0.283792968241895
984	KC_AB1	CG8323	-0.841914684157861	0.533567052518636	0.994766463583882	pseudobulk	DESeq2	LRT	0.272810995716552
984	KC_AB1	CG8326	0.365999213672279	0.809335235757598	0.994766463583882	pseudobulk	DESeq2	LRT	0.0918715514621722
984	KC_AB1	CG8336	1.55851692933824	0.229988041247195	0.994766463583882	pseudobulk	DESeq2	LRT	0.638294745527531
984	KC_AB1	CG8353	0.71642128467878	0.657508806235431	0.994766463583882	pseudobulk	DESeq2	LRT	0.18209842614795
985	KC_AB1	CG8360	0.248401587836167	0.733779060923821	0.994766463583882	pseudobulk	DESeq2	LRT	0.134434685405075
985	KC_AB1	CG8366	0.13270175499534	0.85389283160458	0.994766463583882	pseudobulk	DESeq2	LRT	0.0685966323125475
985	KC_AB1	CG8369	-0.407004751814109	0.784385634855506	0.994766463583882	pseudobulk	DESeq2	LRT	0.105470368548507
985	KC_AB1	CG8370	0.152299701140441	0.913485149634611	0.994766463583882	pseudobulk	DESeq2	LRT	0.0392985085021014
985	KC_AB1	CG8372	-0.117180525826628	0.797401248916752	0.994766463583882	pseudobulk	DESeq2	LRT	0.0983230884664145

985	KC_AB1	CG8388	1.09164688975002	0.600358943660708	0.994766463583882	pseudobulk	DESeq2	LRT	0.221589015215027
985	KC_AB1	CG8389	0.117211791975316	0.867995144517546	0.994766463583882	pseudobulk	DESeq2	LRT	0.0614827042188244
985	KC_AB1	CG8397	-0.0388986735093978	0.918405281080363	0.994766463583882	pseudobulk	DESeq2	LRT	0.0369656276274326
985	KC_AB1	CG8401	2.56230450108566	0.183165247082008	0.994766463583882	pseudobulk	DESeq2	LRT	0.737156923857366
985	KC_AB1	CG8405	-0.472284442421516	0.435257259679763	0.994766463583882	pseudobulk	DESeq2	LRT	0.361253976528682
986	KC_AB1	CG8412	-0.631126217447777	0.433917600217544	0.994766463583882	pseudobulk	DESeq2	LRT	0.362592734009209
986	KC_AB1	CG8414	1.09791586129483	0.408020509999647	0.994766463583882	pseudobulk	DESeq2	LRT	0.389318005645922
986	KC_AB1	CG8435	-0.057125657962392	0.886343118937246	0.994766463583882	pseudobulk	DESeq2	LRT	0.0523981225663713
986	KC_AB1	CG8441	-0.92914149602041	0.372131502922187	0.994766463583882	pseudobulk	DESeq2	LRT	0.42930356307133
986	KC_AB1	CG8460	-0.222850149448178	0.874477855551843	0.994766463583882	pseudobulk	DESeq2	LRT	0.0582511837077826
986	KC_AB1	CG8461	1.57709805573619	0.254077662354211	0.994766463583882	pseudobulk	DESeq2	LRT	0.595033514968272
986	KC_AB1	CG8475	0.00891016188773973	0.943355591440512	0.994766463583882	pseudobulk	DESeq2	LRT	0.0253245720656002
986	KC_AB1	CG8485	0.197129906728403	0.626592456382624	0.994766463583882	pseudobulk	DESeq2	LRT	0.203014837943176
986	KC_AB1	CG8490	-0.590935069498591	0.481749244087182	0.994766463583882	pseudobulk	DESeq2	LRT	0.317178958123518
986	KC_AB1	CG8500	-0.116970717819535	0.596394990556721	0.994766463583882	pseudobulk	DESeq2	LRT	0.224466013077377
987	KC_AB1	CG8507	-0.0163020386956873	0.925877564568879	0.994766463583882	pseudobulk	DESeq2	LRT	0.033446439395491
987	KC_AB1	CG8516	-0.709942174013489	0.581894531589458	0.994766463583882	pseudobulk	DESeq2	LRT	0.235155724112109
987	KC_AB1	CG8519	0.131375113985946	0.919820708602786	0.994766463583882	pseudobulk	DESeq2	LRT	0.036296817059571
987	KC_AB1	CG8520	0.621699397179082	0.571844783886016	0.994766463583882	pseudobulk	DESeq2	LRT	0.242721835978786
987	KC_AB1	CG8531	0.862376901564766	0.481556296015236	0.994766463583882	pseudobulk	DESeq2	LRT	0.317352934666934
987	KC_AB1	CG8545	-0.477409350984238	0.747406977388503	0.994766463583882	pseudobulk	DESeq2	LRT	0.126442852128366
987	KC_AB1	CG8547	-2.79499059556516	0.38564607643653	0.994766463583882	pseudobulk	DESeq2	LRT	0.413811082791365
987	KC_AB1	CG8549	0.247075336347856	0.744433368479619	0.994766463583882	pseudobulk	DESeq2	LRT	0.128174168290096
987	KC_AB1	CG8569	-0.147614626497838	0.90815453477232	0.994766463583882	pseudobulk	DESeq2	LRT	0.0418402441128205
987	KC_AB1	CG8578	0.099252762900135	0.834782426679801	0.994766463583882	pseudobulk	DESeq2	LRT	0.0784267020075451
988	KC_AB1	CG8607	1.1821590793593	0.719511699510136	0.994766463583882	pseudobulk	DESeq2	LRT	0.142962139890846
988	KC_AB1	CG8611	0.877279065609654	0.557431449251523	0.994766463583882	pseudobulk	DESeq2	LRT	0.253808532839968
988	KC_AB1	CG8613	0.202304775716186	0.685433927566122	0.994766463583882	pseudobulk	DESeq2	LRT	0.164034402692729
988	KC_AB1	CG8617	0.127306110255813	0.785578317146777	0.994766463583882	pseudobulk	DESeq2	LRT	0.104810512076328
988	KC_AB1	CG8635	0.227145509452152	0.696895961448435	0.994766463583882	pseudobulk	DESeq2	LRT	0.156832052233928
988	KC_AB1	CG8641	-0.918730336328057	0.579152457917705	0.994766463583882	pseudobulk	DESeq2	LRT	0.237207096174572
988	KC_AB1	CG8646	-2.20969975456663	0.610836134188693	0.994766463583882	pseudobulk	DESeq2	LRT	0.214075280039667
988	KC_AB1	CG8668	-0.0589271529505923	0.830824484174716	0.994766463583882	pseudobulk	DESeq2	LRT	0.080490713406449
988	KC_AB1	CG8671	-0.275126495273125	0.391715743850711	0.994766463583882	pseudobulk	DESeq2	LRT	0.407028972924741
988	KC_AB1	CG8673	-1.64952313825113	0.817565084244375	0.994766463583882	pseudobulk	DESeq2	LRT	0.0874776642296574
989	KC_AB1	CG8675	0.383118414092165	0.699359283179201	0.994766463583882	pseudobulk	DESeq2	LRT	0.155299655992087
989	KC_AB1	CG8677	0.181087062075468	0.534334052570764	0.994766463583882	pseudobulk	DESeq2	LRT	0.272187147785411
989	KC_AB1	CG8679	0.217906885051292	0.902008941520292	0.994766463583882	pseudobulk	DESeq2	LRT	0.0447891573209458
989	KC_AB1	CG8712	-0.336079221375189	0.868396536629518	0.994766463583882	pseudobulk	DESeq2	LRT	0.061281917283127
989	KC_AB1	CG8726	-0.145830040877666	0.600478848328825	0.994766463583882	pseudobulk	DESeq2	LRT	0.221502285872832
989	KC_AB1	CG8728	0.218043966448528	0.794717730060571	0.994766463583882	pseudobulk	DESeq2	LRT	0.0997870978154011
989	KC_AB1	CG8740	-2.20969975456663	0.610836134188693	0.994766463583882	pseudobulk	DESeq2	LRT	0.214075280039667
989	KC_AB1	CG8745	0.854047063205883	0.719204060467466	0.994766463583882	pseudobulk	DESeq2	LRT	0.143147869337068
989	KC_AB1	CG8757	-0.835152883801839	0.920434724857442	0.994766463583882	pseudobulk	DESeq2	LRT	0.0360070052473159
989	KC_AB1	CG8765	0.0395025674733951	0.882236565025252	0.994766463583882	pseudobulk	DESeq2	LRT	0.0544149464900379
990	KC_AB1	CG8768	0.292829964640629	0.78050165504948	0.994766463583882	pseudobulk	DESeq2	LRT	0.10762617168152
990	KC_AB1	CG8777	-2.26306816020352	0.40449476385881	0.994766463583882	pseudobulk	DESeq2	LRT	0.393087095910655
990	KC_AB1	CG8778	-0.95955330432812	0.449621622035742	0.994766463583882	pseudobulk	DESeq2	LRT	0.347152811974095
990	KC_AB1	CG8814	0.425208385067145	0.555482072093873	0.994766463583882	pseudobulk	DESeq2	LRT	0.255329953134178
990	KC_AB1	CG8818	0.0562324682794957	0.90323087148177	0.994766463583882	pseudobulk	DESeq2	LRT	0.0442012271005218
990	KC_AB1	CG8833	1.2737492795601	0.417110231191894	0.994766463583882	pseudobulk	DESeq2	LRT	0.379749157323462
990	KC_AB1	CG8838	1.1821590793593	0.719511699510136	0.994766463583882	pseudobulk	DESeq2	LRT	0.142962139890846
990	KC_AB1	CG8839	-0.932743657785611	0.557416660563585	0.994766463583882	pseudobulk	DESeq2	LRT	0.253820054850025
990	KC_AB1	CG8841	-0.698291033518872	0.594627401193503	0.994766463583882	pseudobulk	DESeq2	LRT	0.225755081823069
990	KC_AB1	CG8858	0.342234930728497	0.763501892898779	0.994766463583882	pseudobulk	DESeq2	LRT	0.11718988188919
991	KC_AB1	CG8860	0.199756364248168	0.669657822279274	0.994766463583882	pseudobulk	DESeq2	LRT	0.174147053801188

991	KC_AB1	CG8861	2.38929733117871	0.484296857543297	0.994766463583882	pseudobulk	DESeq2	LRT	0.314888348950639
991	KC_AB1	CG8870	1.1821590793593	0.719511699510136	0.994766463583882	pseudobulk	DESeq2	LRT	0.142962139890846
991	KC_AB1	CG8878	-0.176472391677357	0.730325952146376	0.994766463583882	pseudobulk	DESeq2	LRT	0.136483266421802
991	KC_AB1	CG8888	-0.835152883801839	0.920434724857442	0.994766463583882	pseudobulk	DESeq2	LRT	0.0360070052473159
991	KC_AB1	CG8891	-0.252948972666318	0.767332733458714	0.994766463583882	pseudobulk	DESeq2	LRT	0.115016274942404
991	KC_AB1	CG8892	0.244551080987866	0.76131262500119	0.994766463583882	pseudobulk	DESeq2	LRT	0.118436968153768
991	KC_AB1	CG8908	-2.20969975456663	0.610836134188693	0.994766463583882	pseudobulk	DESeq2	LRT	0.214075280039667
991	KC_AB1	CG8910	-0.272633649728909	0.342260108599778	0.994766463583882	pseudobulk	DESeq2	LRT	0.465643716183681
991	KC_AB1	CG8915	0.283595977104094	0.852000024768602	0.994766463583882	pseudobulk	DESeq2	LRT	0.0695603926078691
992	KC_AB1	CG8916	2.67764418499683	0.378704357417491	0.994766463583882	pseudobulk	DESeq2	LRT	0.421699697806334
992	KC_AB1	CG8929	-0.115050984966994	0.705116845784372	0.994766463583882	pseudobulk	DESeq2	LRT	0.151738909569444
992	KC_AB1	CG8939	-0.134251336451005	0.913539199375943	0.994766463583882	pseudobulk	DESeq2	LRT	0.0392728126164077
992	KC_AB1	CG8944	-0.111982813744125	0.88971857616731	0.994766463583882	pseudobulk	DESeq2	LRT	0.0507473418328393
992	KC_AB1	CG8950	1.1821590793593	0.719511699510136	0.994766463583882	pseudobulk	DESeq2	LRT	0.142962139890846
992	KC_AB1	CG8963	0.136834193370086	0.768283056445568	0.994766463583882	pseudobulk	DESeq2	LRT	0.114478744546301
992	KC_AB1	CG8974	0.13667027893639	0.712058935639901	0.994766463583882	pseudobulk	DESeq2	LRT	0.147484059222796
992	KC_AB1	CG8993	-0.293935173767048	0.445382416015228	0.994766463583882	pseudobulk	DESeq2	LRT	0.351266933189506
992	KC_AB1	CG9003	-0.423769141403463	0.347869432642702	0.994766463583882	pseudobulk	DESeq2	LRT	0.458583731119708
992	KC_AB1	CG9004	-0.93565799849381	0.534292951808374	0.994766463583882	pseudobulk	DESeq2	LRT	0.272220554828736
993	KC_AB1	CG9005	-0.223437225747956	0.490575708237042	0.994766463583882	pseudobulk	DESeq2	LRT	0.309293960491231
993	KC_AB1	CG9008	2.22023857837763	0.524904842126482	0.994766463583882	pseudobulk	DESeq2	LRT	0.279919420946639
993	KC_AB1	CG9018	0.0778145289182885	0.916208914014414	0.994766463583882	pseudobulk	DESeq2	LRT	0.0380054871850836
993	KC_AB1	CG9034	-0.0876317513650885	0.772435285696337	0.994766463583882	pseudobulk	DESeq2	LRT	0.112137895388258
993	KC_AB1	CG9044	-0.506481204149965	0.506179198053941	0.994766463583882	pseudobulk	DESeq2	LRT	0.295695706581988
993	KC_AB1	CG9062	-0.352115025819036	0.746296592668938	0.994766463583882	pseudobulk	DESeq2	LRT	0.127088541212857
993	KC_AB1	CG9065	0.133867711502489	0.701035565293811	0.994766463583882	pseudobulk	DESeq2	LRT	0.154259948625295
993	KC_AB1	CG9067	1.19029256564107	0.304716416342505	0.994766463583882	pseudobulk	DESeq2	LRT	0.516104147894455
993	KC_AB1	CG9072	1.1821590793593	0.719511699510136	0.994766463583882	pseudobulk	DESeq2	LRT	0.142962139890846
993	KC_AB1	CG9083	-1.64952313825113	0.817565084244375	0.994766463583882	pseudobulk	DESeq2	LRT	0.0874776642296574
994	KC_AB1	CG9098	0.602823085965819	0.693720820939178	0.994766463583882	pseudobulk	DESeq2	LRT	0.158815270639152
994	KC_AB1	CG9101	-1.64952313825113	0.817565084244375	0.994766463583882	pseudobulk	DESeq2	LRT	0.0874776642296574
994	KC_AB1	CG9106	0.949183608371002	0.80156895525114	0.994766463583882	pseudobulk	DESeq2	LRT	0.0960591113673091
994	KC_AB1	CG9107	-0.0810223702009391	0.93176412071797	0.994766463583882	pseudobulk	DESeq2	LRT	0.0306940168693458
994	KC_AB1	CG9117	-2.20969975456663	0.610836134188693	0.994766463583882	pseudobulk	DESeq2	LRT	0.214075280039667
994	KC_AB1	CG9121	-0.0784025292173233	0.903258996148046	0.994766463583882	pseudobulk	DESeq2	LRT	0.044187704315159
994	KC_AB1	CG9123	-0.0364943842984251	0.94000188303254	0.994766463583882	pseudobulk	DESeq2	LRT	0.0268712764111289
994	KC_AB1	CG9125	0.00868240509153601	0.855901732073861	0.994766463583882	pseudobulk	DESeq2	LRT	0.0675760947479731
994	KC_AB1	CG9132	0.394577739057771	0.303822515958763	0.994766463583882	pseudobulk	DESeq2	LRT	0.517380044185222
994	KC_AB1	CG9135	0.390985497167617	0.754067957780671	0.994766463583882	pseudobulk	DESeq2	LRT	0.122589513070281
995	KC_AB1	CG9143	0.519438291079816	0.63974062705667	0.994766463583882	pseudobulk	DESeq2	LRT	0.193996068312816
995	KC_AB1	CG9147	0.553319309502239	0.655288345788071	0.994766463583882	pseudobulk	DESeq2	LRT	0.183567555842029
995	KC_AB1	CG9149	0.973090020385093	0.297001104057791	0.994766463583882	pseudobulk	DESeq2	LRT	0.527241936254117
995	KC_AB1	CG9154	0.16266625150712	0.825756024143639	0.994766463583882	pseudobulk	DESeq2	LRT	0.083148249313451
995	KC_AB1	CG9164	0.0153054756778525	0.915372536456987	0.994766463583882	pseudobulk	DESeq2	LRT	0.0384021216706886
995	KC_AB1	CG9170	0.778785148795807	0.0277385804642608	0.994766463583882	pseudobulk	DESeq2	LRT	1.55691576793155
995	KC_AB1	CG9171	-0.506701480695615	0.0508354269699335	0.994766463583882	pseudobulk	DESeq2	LRT	1.29383352442508
995	KC_AB1	CG9175	0.0412188860033817	0.952956887197573	0.994766463583882	pseudobulk	DESeq2	LRT	0.0209267468703054
995	KC_AB1	CG9205	-0.155995520012607	0.805137752120697	0.994766463583882	pseudobulk	DESeq2	LRT	0.0941298092373141
995	KC_AB1	CG9213	-0.22781016265773	0.861363850775173	0.994766463583882	pseudobulk	DESeq2	LRT	0.0648133584130924
996	KC_AB1	CG9215	-0.34927719661383	0.763814558368357	0.994766463583882	pseudobulk	DESeq2	LRT	0.117012068195239
996	KC_AB1	CG9231	-0.131817537479969	0.769227231127917	0.994766463583882	pseudobulk	DESeq2	LRT	0.113945349863539
996	KC_AB1	CG9240	-0.681110960338701	0.355239255488103	0.994766463583882	pseudobulk	DESeq2	LRT	0.449479048805967
996	KC_AB1	CG9246	-0.229344234824732	0.840165320222248	0.994766463583882	pseudobulk	DESeq2	LRT	0.0756352489429339
996	KC_AB1	CG9248	0.729740153036158	0.752353468463509	0.994766463583882	pseudobulk	DESeq2	LRT	0.123578072524162
996	KC_AB1	CG9253	0.295703440706791	0.755920145011378	0.994766463583882	pseudobulk	DESeq2	LRT	0.121524080706023
996	KC_AB1	CG9254	-3.21143241058256	0.292545415061155	0.994766463583882	pseudobulk	DESeq2	LRT	0.533806704011112

996	KC_AB1	CG9257	-1.07565643089366	0.268106227057821	0.994766463583882	pseudobulk	DESeq2	LRT	0.571693098939746
996	KC_AB1	CG9263	-0.835152883801839	0.920434724857442	0.994766463583882	pseudobulk	DESeq2	LRT	0.0360070052473159
996	KC_AB1	CG9265	-0.0286435484423915	0.901799050915923	0.994766463583882	pseudobulk	DESeq2	LRT	0.0448902260955322
997	KC_AB1	CG9270	2.38929733117871	0.484296857543297	0.994766463583882	pseudobulk	DESeq2	LRT	0.314888348950639
997	KC_AB1	CG9272	0.217906885051292	0.902008941520292	0.994766463583882	pseudobulk	DESeq2	LRT	0.0447891573209458
997	KC_AB1	CG9281	-0.374598124208869	0.353622882969815	0.994766463583882	pseudobulk	DESeq2	LRT	0.45145963951312
997	KC_AB1	CG9286	1.10616549493524	0.430082402033995	0.994766463583882	pseudobulk	DESeq2	LRT	0.366448327396821
997	KC_AB1	CG9289	1.1821590793593	0.719511699510136	0.994766463583882	pseudobulk	DESeq2	LRT	0.142962139890846
997	KC_AB1	CG9300	-0.720688993793705	0.702033725657174	0.994766463583882	pseudobulk	DESeq2	LRT	0.153642023888733
997	KC_AB1	CG9302	-3.54706997395105	0.0888658754382405	0.994766463583882	pseudobulk	DESeq2	LRT	1.05126497641979
997	KC_AB1	CG9304	0.693900228019512	0.634196655303206	0.994766463583882	pseudobulk	DESeq2	LRT	0.197776052733389
997	KC_AB1	CG9328	-0.229681282333826	0.425003820275035	0.994766463583882	pseudobulk	DESeq2	LRT	0.371607166145194
997	KC_AB1	CG9330	0.383215232485139	0.60456553538571	0.994766463583882	pseudobulk	DESeq2	LRT	0.218556614383533
998	KC_AB1	CG9331	-0.835152883801839	0.920434724857442	0.994766463583882	pseudobulk	DESeq2	LRT	0.0360070052473159
998	KC_AB1	CG9344	0.235911411333568	0.787189533344642	0.994766463583882	pseudobulk	DESeq2	LRT	0.103920689018897
998	KC_AB1	CG9346	0.246036817857952	0.720738693420651	0.994766463583882	pseudobulk	DESeq2	LRT	0.142222161875635
998	KC_AB1	CG9356	-0.436750496927036	0.710319699562169	0.994766463583882	pseudobulk	DESeq2	LRT	0.14854614070946
998	KC_AB1	CG9368	0.152971498963255	0.680223107335724	0.994766463583882	pseudobulk	DESeq2	LRT	0.167348619069068
998	KC_AB1	CG9380	-2.00005840186615	0.514177117052568	0.994766463583882	pseudobulk	DESeq2	LRT	0.288887255112324
998	KC_AB1	CG9384	-0.571535636877394	0.361288485973355	0.994766463583882	pseudobulk	DESeq2	LRT	0.442145878891199
998	KC_AB1	CG9386	-2.48367038384519	0.18819830763306	0.994766463583882	pseudobulk	DESeq2	LRT	0.725384286269697
998	KC_AB1	CG9391	-0.152841571995792	0.766929296381936	0.994766463583882	pseudobulk	DESeq2	LRT	0.115244672043903
998	KC_AB1	CG9393	0.926418398444777	0.614807329612462	0.994766463583882	pseudobulk	DESeq2	LRT	0.211260963567752
999	KC_AB1	CG9394	1.1821590793593	0.719511699510136	0.994766463583882	pseudobulk	DESeq2	LRT	0.142962139890846
999	KC_AB1	CG9395	-0.409148706013913	0.55507061433241	0.994766463583882	pseudobulk	DESeq2	LRT	0.255651763788819
999	KC_AB1	CG9399	0.259759697749723	0.821879321299002	0.994766463583882	pseudobulk	DESeq2	LRT	0.0851919463790422
999	KC_AB1	CG9410	-0.0414832809637214	0.935791971140435	0.994766463583882	pseudobulk	DESeq2	LRT	0.028820685265193
999	KC_AB1	CG9422	-0.145490628599698	0.803384043838956	0.994766463583882	pseudobulk	DESeq2	LRT	0.0950767981227803
999	KC_AB1	CG9425	0.052879213416614	0.835651090952283	0.994766463583882	pseudobulk	DESeq2	LRT	0.0779750155079751
999	KC_AB1	CG9426	-0.542622014986542	0.39848681604786	0.994766463583882	pseudobulk	DESeq2	LRT	0.399586042680368
999	KC_AB1	CG9436	0.949183608371002	0.80156895525114	0.994766463583882	pseudobulk	DESeq2	LRT	0.0960591113673091
999	KC_AB1	CG9467	0.135914836388098	0.872780725232483	0.994766463583882	pseudobulk	DESeq2	LRT	0.0590948534114712
999	KC_AB1	CG9471	0.60815136535315	0.57794659231876	0.994766463583882	pseudobulk	DESeq2	LRT	0.238112292605289
100	KC_AB1	CG9485	1.47424991200432	0.518427666092641	0.994766463583882	pseudobulk	DESeq2	LRT	0.285311830226264
100	KC_AB1	CG9503	-1.64952313825113	0.817565084244375	0.994766463583882	pseudobulk	DESeq2	LRT	0.0874776642296574
100	KC_AB1	CG9512	-1.64952313825113	0.817565084244375	0.994766463583882	pseudobulk	DESeq2	LRT	0.0874776642296574
100	KC_AB1	CG9514	-0.646988131279709	0.742219498995513	0.994766463583882	pseudobulk	DESeq2	LRT	0.129467640400983
100	KC_AB1	CG9515	-0.925642226170004	0.657544673459392	0.994766463583882	pseudobulk	DESeq2	LRT	0.182074735951784
100	KC_AB1	CG9518	0.166061709543563	0.875536484819394	0.994766463583882	pseudobulk	DESeq2	LRT	0.0577257515501677
100	KC_AB1	CG9527	-0.159094363498051	0.891520814517808	0.994766463583882	pseudobulk	DESeq2	LRT	0.0498685128086472
100	KC_AB1	CG9536	-1.1090285101074	0.272474944688051	0.994766463583882	pseudobulk	DESeq2	LRT	0.564673426906616
100	KC_AB1	CG9577	0.408270288724494	0.784293047765862	0.994766463583882	pseudobulk	DESeq2	LRT	0.105521634703418
100	KC_AB1	CG9578	1.04312398696209	0.301114201807215	0.994766463583882	pseudobulk	DESeq2	LRT	0.521268760857033
100	KC_AB1	CG9581	-0.760326296539118	0.467622572687302	0.994766463583882	pseudobulk	DESeq2	LRT	0.330104533102739
100	KC_AB1	CG9586	0.210583343430465	0.786666143515511	0.994766463583882	pseudobulk	DESeq2	LRT	0.104209540565321
100	KC_AB1	CG9588	-1.08263241169326	0.283253890797592	0.994766463583882	pseudobulk	DESeq2	LRT	0.547824115920633
100	KC_AB1	CG9590	-0.0738882935491122	0.914039693124075	0.994766463583882	pseudobulk	DESeq2	LRT	0.0390349441672082
100	KC_AB1	CG9596	-0.477586443054731	0.760872601189559	0.994766463583882	pseudobulk	DESeq2	LRT	0.118688054441209
100	KC_AB1	CG9601	-0.343778926351242	0.737074755999418	0.994766463583882	pseudobulk	DESeq2	LRT	0.132488462654405
100	KC_AB1	CG9609	-0.101099180371983	0.841000660700443	0.994766463583882	pseudobulk	DESeq2	LRT	0.0752036630148777
100	KC_AB1	CG9616	1.1821590793593	0.719511699510136	0.994766463583882	pseudobulk	DESeq2	LRT	0.142962139890846
100	KC_AB1	CG9630	1.99910047487298	0.452091988556384	0.994766463583882	pseudobulk	DESeq2	LRT	0.344773188954608
100	KC_AB1	CG9636	-0.463347347418804	0.355454726036761	0.994766463583882	pseudobulk	DESeq2	LRT	0.449215707127003
100	KC_AB1	CG9641	-0.163746409138485	0.782856116365486	0.994766463583882	pseudobulk	DESeq2	LRT	0.106318050982384
100	KC_AB1	CG9646	-0.263631617109078	0.433948728724154	0.994766463583882	pseudobulk	DESeq2	LRT	0.36256157958322
100	KC_AB1	CG9650	0.513714559562998	0.775698051782448	0.994766463583882	pseudobulk	DESeq2	LRT	0.110307299314091

100	KC_AB1	CG9657	-1.64952313825113	0.817565084244375	0.994766463583882	pseudobulk	DESeq2	LRT	0.0874776642296574
100	KC_AB1	CG9662	-0.243203050676319	0.720518287134794	0.994766463583882	pseudobulk	DESeq2	LRT	0.142354992086711
100	KC_AB1	CG9663	-0.627447798185778	0.772628937945737	0.994766463583882	pseudobulk	DESeq2	LRT	0.112029029876423
100	KC_AB1	CG9667	-1.42665377874663	0.239678195233819	0.994766463583882	pseudobulk	DESeq2	LRT	0.620371474186504
100	KC_AB1	CG9674	0.250287148688674	0.334979820291337	0.994766463583882	pseudobulk	DESeq2	LRT	0.474981354754469
100	KC_AB1	CG9686	0.124698704744493	0.891329405341186	0.994766463583882	pseudobulk	DESeq2	LRT	0.0499617656782218
100	KC_AB1	CG9705	0.292356906857153	0.655727473578639	0.994766463583882	pseudobulk	DESeq2	LRT	0.183276619942387
100	KC_AB1	CG9706	0.478005084452508	0.815070723509861	0.994766463583882	pseudobulk	DESeq2	LRT	0.0888047059870473
100	KC_AB1	CG9723	-0.379314053126175	0.843633212750006	0.994766463583882	pseudobulk	DESeq2	LRT	0.0738463309831402
100	KC_AB1	CG9727	0.390160930469258	0.682373266208352	0.994766463583882	pseudobulk	DESeq2	LRT	0.165977996151236
100	KC_AB1	CG9743	3.46956015450889	0.2028823784209	0.994766463583882	pseudobulk	DESeq2	LRT	0.692755672468422
100	KC_AB1	CG9752	2.09225092496487	0.386054269300348	0.994766463583882	pseudobulk	DESeq2	LRT	0.413351640403342
100	KC_AB1	CG9760	-0.309556812373092	0.83169477951301	0.994766463583882	pseudobulk	DESeq2	LRT	0.0800360245425936
100	KC_AB1	CG9766	-1.85542794649627	0.283863219567929	0.994766463583882	pseudobulk	DESeq2	LRT	0.546890875785166
100	KC_AB1	CG9773	0.874654373874532	0.332469308066151	0.994766463583882	pseudobulk	DESeq2	LRT	0.478248440444306
100	KC_AB1	CG9775	-0.00224455514124973	0.906291788617366	0.994766463583882	pseudobulk	DESeq2	LRT	0.0427319548788679
100	KC_AB1	CG9776	0.20954142373226	0.614767332327933	0.994766463583882	pseudobulk	DESeq2	LRT	0.211289218216299
100	KC_AB1	CG9782	1.32710828904082	0.607473293222376	0.994766463583882	pseudobulk	DESeq2	LRT	0.216472810505135
100	KC_AB1	CG9784	0.125760618558311	0.808652796098213	0.994766463583882	pseudobulk	DESeq2	LRT	0.0922379074426156
100	KC_AB1	CG9795	-0.228187219406441	0.669422526680726	0.994766463583882	pseudobulk	DESeq2	LRT	0.174299677325529
100	KC_AB1	CG9799	0.529730398381389	0.671718899469057	0.994766463583882	pseudobulk	DESeq2	LRT	0.172812432228846
100	KC_AB1	CG9801	0.507570605148725	0.683500061706902	0.994766463583882	pseudobulk	DESeq2	LRT	0.165261441887723
100	KC_AB1	CG9804	-0.0436033993737628	0.920286839997701	0.994766463583882	pseudobulk	DESeq2	LRT	0.0360767882931682
100	KC_AB1	CG9813	0.308811269631629	0.119002302189846	0.994766463583882	pseudobulk	DESeq2	LRT	0.924444636769862
100	KC_AB1	CG9837	0.161140446100964	0.768167934425737	0.994766463583882	pseudobulk	DESeq2	LRT	0.114543825513216
100	KC_AB1	CG9839	1.56722973934068	0.177652129338862	0.994766463583882	pseudobulk	DESeq2	LRT	0.750429582688722
100	KC_AB1	CG9849	0.0457154758920991	0.92166600114691	0.994766463583882	pseudobulk	DESeq2	LRT	0.0354264326756857
100	KC_AB1	CG9853	1.11145814403743	0.189210650093158	0.994766463583882	pseudobulk	DESeq2	LRT	0.723054422128114
100	KC_AB1	CG9855	-0.157548030622392	0.826730834223923	0.994766463583882	pseudobulk	DESeq2	LRT	0.0826358643758828
100	KC_AB1	CG9886	-1.28289356543793	0.433460930391418	0.994766463583882	pseudobulk	DESeq2	LRT	0.363050041163732
100	KC_AB1	CG9890	0.314627652481189	0.822736172087847	0.994766463583882	pseudobulk	DESeq2	LRT	0.0847394082574248
100	KC_AB1	CG9902	0.433536955892271	0.747449359558058	0.994766463583882	pseudobulk	DESeq2	LRT	0.126418225892495
100	KC_AB1	CG9911	0.0987282684866057	0.866572611931017	0.994766463583882	pseudobulk	DESeq2	LRT	0.0621950409826853
100	KC_AB1	CG9915	0.165782074046314	0.821040282311585	0.994766463583882	pseudobulk	DESeq2	LRT	0.0856355347727963
100	KC_AB1	CG9917	1.01299835106113	0.156824189737247	0.994766463583882	pseudobulk	DESeq2	LRT	0.804586947651948
100	KC_AB1	CG9919	0.166715566307958	0.69863284639905	0.994766463583882	pseudobulk	DESeq2	LRT	0.155750999754032
100	KC_AB1	CG9921	0.000318363823017542	0.895001352908261	0.994766463583882	pseudobulk	DESeq2	LRT	0.0481763081923022
100	KC_AB1	CG9922	-0.360281632425291	0.637282383690193	0.994766463583882	pseudobulk	DESeq2	LRT	0.195668086497198
100	KC_AB1	CG9932	-0.127854064267603	0.784600164201315	0.994766463583882	pseudobulk	DESeq2	LRT	0.105351605317217
100	KC_AB1	CG9934	0.286384051579236	0.703940115099094	0.994766463583882	pseudobulk	DESeq2	LRT	0.152464285159353
100	KC_AB1	CG9941	0.062841709826032	0.880732841243606	0.994766463583882	pseudobulk	DESeq2	LRT	0.0551558091467864
100	KC_AB1	CG9945	0.205304554736708	0.80319342979502	0.994766463583882	pseudobulk	DESeq2	LRT	0.0951798527581174
100	KC_AB1	CG9948	1.13834558465261	0.330209710662778	0.994766463583882	pseudobulk	DESeq2	LRT	0.481210159343215
100	KC_AB1	CG9951	1.07601805237302	0.538858181621095	0.994766463583882	pseudobulk	DESeq2	LRT	0.268525518753016
100	KC_AB1	CG9953	-0.622056076043519	0.311754275949294	0.994766463583882	pseudobulk	DESeq2	LRT	0.50618758112625
100	KC_AB1	CG9967	0.847270412147379	0.709043889678312	0.994766463583882	pseudobulk	DESeq2	LRT	0.149326881241258
100	KC_AB1	CG9986	0.123743657081107	0.903608111382707	0.994766463583882	pseudobulk	DESeq2	LRT	0.0440198792203122
100	KC_AB1	CG9992	-0.410489407975954	0.587804047593922	0.994766463583882	pseudobulk	DESeq2	LRT	0.230767427719594
100	KC_AB1	Charon	0.593814324176767	0.694846097310903	0.994766463583882	pseudobulk	DESeq2	LRT	0.15811137741022
100	KC_AB1	chas	-1.64952313825113	0.817565084244375	0.994766463583882	pseudobulk	DESeq2	LRT	0.0874776642296574
100	KC_AB1	ChAT	-0.485826035314859	0.278276740205425	0.994766463583882	pseudobulk	DESeq2	LRT	0.555523092705102
100	KC_AB1	chb	-0.0555490114318125	0.872098935241712	0.994766463583882	pseudobulk	DESeq2	LRT	0.0594342437460426
100	KC_AB1	Chc	-0.101250891620903	0.779937773745052	0.994766463583882	pseudobulk	DESeq2	LRT	0.107940045511034
100	KC_AB1	Chchd2	-0.0565729530536253	0.82539695935338	0.994766463583882	pseudobulk	DESeq2	LRT	0.0833371353356088
100	KC_AB1	Chchd3	-0.146988121289604	0.769058571929902	0.994766463583882	pseudobulk	DESeq2	LRT	0.114040582828919
100	KC_AB1	Chd1	-0.191476740303279	0.665439915593687	0.994766463583882	pseudobulk	DESeq2	LRT	0.176891152066299

100	KC_AB1	Chd64	0.0871213401343273	0.940955672557668	0.994766463583882	pseudobulk	DESeq2	LRT	0.0264308352518565
100	KC_AB1	CheB42a	-2.79499059556516	0.38564607643653	0.994766463583882	pseudobulk	DESeq2	LRT	0.413811082791365
100	KC_AB1	CheB42c	0.978218935568633	0.578530845039478	0.994766463583882	pseudobulk	DESeq2	LRT	0.237673481183578
100	KC_AB1	CheB74a	-2.20969975456663	0.610836134188693	0.994766463583882	pseudobulk	DESeq2	LRT	0.214075280039667
100	KC_AB1	cher	0.854047063205883	0.719204060467466	0.994766463583882	pseudobulk	DESeq2	LRT	0.143147869337068
100	KC_AB1	CHES-1-like	0.0925017087612454	0.760507274433169	0.994766463583882	pseudobulk	DESeq2	LRT	0.118896627460983
100	KC_AB1	Chi	-0.430761363369147	0.462639369150402	0.994766463583882	pseudobulk	DESeq2	LRT	0.334757412902902
100	KC_AB1	chic	-0.110190771956882	0.749753121252276	0.994766463583882	pseudobulk	DESeq2	LRT	0.125081717579348
100	KC_AB1	chico	-0.0037323723779622	0.826232728902829	0.994766463583882	pseudobulk	DESeq2	LRT	0.0828976056552809
100	KC_AB1	chif	-0.133530407243501	0.788467269970214	0.994766463583882	pseudobulk	DESeq2	LRT	0.103216329939838
100	KC_AB1	chinmo	-0.131920515664266	0.488115394242083	0.994766463583882	pseudobulk	DESeq2	LRT	0.311477495295819
100	KC_AB1	CHKov1	-2.85724547560282	0.450020141104186	0.994766463583882	pseudobulk	DESeq2	LRT	0.346768048503189
100	KC_AB1	ChLD3	1.1821590793593	0.719511699510136	0.994766463583882	pseudobulk	DESeq2	LRT	0.142962139890846
100	KC_AB1	chm	0.159787458062746	0.841799061362545	0.994766463583882	pseudobulk	DESeq2	LRT	0.0747915628423261
100	KC_AB1	Chmp1	0.0416587977329269	0.925897293961074	0.994766463583882	pseudobulk	DESeq2	LRT	0.033437185175279
100	KC_AB1	CHMP2B	-0.0243914533297685	0.954880848517658	0.994766463583882	pseudobulk	DESeq2	LRT	0.0200508169604227
100	KC_AB1	chn	0.125799571817246	0.518897435929472	0.994766463583882	pseudobulk	DESeq2	LRT	0.284918475314772
100	KC_AB1	CHOp24	0.713189997722789	0.597725007519464	0.994766463583882	pseudobulk	DESeq2	LRT	0.223498573845901
100	KC_AB1	CHORD	-0.0886350945884821	0.897225886082806	0.994766463583882	pseudobulk	DESeq2	LRT	0.0470982049723246
100	KC_AB1	Chrac-14	0.463735534344822	0.662613952037152	0.994766463583882	pseudobulk	DESeq2	LRT	0.178739423828412
100	KC_AB1	Chrac-16	0.461977553433496	0.733514248187333	0.994766463583882	pseudobulk	DESeq2	LRT	0.134591445762025
101	KC_AB1	chrb	-0.0954570481657318	0.549189587367228	0.994766463583882	pseudobulk	DESeq2	LRT	0.260277705579173
101	KC_AB1	Chro	0.285452510843058	0.511401027583906	0.994766463583882	pseudobulk	DESeq2	LRT	0.291238403658976
101	KC_AB1	ChT	-0.559890160133053	0.101524572709393	0.994766463583882	pseudobulk	DESeq2	LRT	0.993428829666966
101	KC_AB1	Cht11	2.25702551253886	0.345690610560104	0.994766463583882	pseudobulk	DESeq2	LRT	0.461312416342215
101	KC_AB1	Cht2	0.802721263912487	0.587102987454726	0.994766463583882	pseudobulk	DESeq2	LRT	0.231285709723168
101	KC_AB1	ci	-2.20969975456663	0.610836134188693	0.994766463583882	pseudobulk	DESeq2	LRT	0.214075280039667
101	KC_AB1	CIA30	-1.32154620690291	0.4256848469537	0.994766463583882	pseudobulk	DESeq2	LRT	0.370911809070033
101	KC_AB1	Ciao1	0.175405382164429	0.816326297183577	0.994766463583882	pseudobulk	DESeq2	LRT	0.0881362128871275
101	KC_AB1	CIAPIN1	-1.11764747269326	0.260211218881585	0.994766463583882	pseudobulk	DESeq2	LRT	0.584673982973008
101	KC_AB1	cib	-0.189202211529974	0.790419493062812	0.994766463583882	pseudobulk	DESeq2	LRT	0.102142357862199
101	KC_AB1	cic	-0.188895624317932	0.458094337024594	0.994766463583882	pseudobulk	DESeq2	LRT	0.339045076951643
101	KC_AB1	cin	0.319788592294525	0.679381408320469	0.994766463583882	pseudobulk	DESeq2	LRT	0.167886342032237
101	KC_AB1	cindr	-0.0257308358029689	0.917605156048952	0.994766463583882	pseudobulk	DESeq2	LRT	0.0373441547871322
101	KC_AB1	Cip4	-0.148820143043288	0.532669670464046	0.994766463583882	pseudobulk	DESeq2	LRT	0.273542030688595
101	KC_AB1	Cirl	-0.245888658224756	0.304598239661874	0.994766463583882	pseudobulk	DESeq2	LRT	0.516272610873068
101	KC_AB1	Cisd2	-0.106231515441957	0.759410641516214	0.994766463583882	pseudobulk	DESeq2	LRT	0.119523321445115
101	KC_AB1	ck	-1.64952313825113	0.817565084244375	0.994766463583882	pseudobulk	DESeq2	LRT	0.0874776642296574
101	KC_AB1	Cka	0.148206897007366	0.609084030322351	0.994766463583882	pseudobulk	DESeq2	LRT	0.215322787190094
101	KC_AB1	CkIalpha	0.135909929643303	0.555428087076958	0.994766463583882	pseudobulk	DESeq2	LRT	0.255372162478969
101	KC_AB1	CkIIalpha	0.0476940410804864	0.840863051821362	0.994766463583882	pseudobulk	DESeq2	LRT	0.0752747303392202
101	KC_AB1	CkIIalpha-i1	0.097819640535638	0.89223956997303	0.994766463583882	pseudobulk	DESeq2	LRT	0.0495185201216824
101	KC_AB1	CkIIalpha-i3	-0.821945313206781	0.381703991201354	0.994766463583882	pseudobulk	DESeq2	LRT	0.418273298925247
101	KC_AB1	CkIIbeta	0.085987561053476	0.742340754104312	0.994766463583882	pseudobulk	DESeq2	LRT	0.129396696261584
101	KC_AB1	ckn	0.108773766091117	0.741516152279642	0.994766463583882	pseudobulk	DESeq2	LRT	0.1298793843935
101	KC_AB1	cl	-0.0164596449094349	0.932373370888885	0.994766463583882	pseudobulk	DESeq2	LRT	0.030410138670512
101	KC_AB1	Clamp	0.24767636371975	0.574608087356977	0.994766463583882	pseudobulk	DESeq2	LRT	0.240628265796575
101	KC_AB1	Clbn	0.229075362651276	0.817418417481978	0.994766463583882	pseudobulk	DESeq2	LRT	0.0875555813042841
101	KC_AB1	Clc	-0.149800611905961	0.668430194418802	0.994766463583882	pseudobulk	DESeq2	LRT	0.174943940326689
101	KC_AB1	ClC-a	1.1821590793593	0.719511699510136	0.994766463583882	pseudobulk	DESeq2	LRT	0.142962139890846
101	KC_AB1	ClC-b	0.654059536312134	0.688397524519674	0.994766463583882	pseudobulk	DESeq2	LRT	0.16216070006641
101	KC_AB1	ClC-c	-0.291781217702307	0.634187271468223	0.994766463583882	pseudobulk	DESeq2	LRT	0.197782478781001
101	KC_AB1	Clic	0.366743767806249	0.494140229685546	0.994766463583882	pseudobulk	DESeq2	LRT	0.306149787237455
101	KC_AB1	CLIP-190	-0.17435770094669	0.494187937413413	0.994766463583882	pseudobulk	DESeq2	LRT	0.306107859457467
101	KC_AB1	Cln7	-2.20969975456663	0.610836134188693	0.994766463583882	pseudobulk	DESeq2	LRT	0.214075280039667
101	KC_AB1	clos	0.949183608371002	0.80156895525114	0.994766463583882	pseudobulk	DESeq2	LRT	0.0960591113673091

101	KC_AB1	Clp	-0.0813546184264884	0.888654438767453	0.994766463583882	pseudobulk	DESeq2	LRT	0.0512670854996878
101	KC_AB1	ClpP	0.115935119351822	0.817104350869315	0.994766463583882	pseudobulk	DESeq2	LRT	0.0877224769892498
101	KC_AB1	CLS	-0.995773839385666	0.295819419123184	0.994766463583882	pseudobulk	DESeq2	LRT	0.528973320057778
101	KC_AB1	clt	0.423699245037111	0.765440172869709	0.994766463583882	pseudobulk	DESeq2	LRT	0.116088748281112
101	KC_AB1	clu	0.284079857936417	0.631300024686987	0.994766463583882	pseudobulk	DESeq2	LRT	0.199764193689561
101	KC_AB1	cm	0.405070735111275	0.668099325047181	0.994766463583882	pseudobulk	DESeq2	LRT	0.175158966995638
101	KC_AB1	cmpy	0.630917086141791	0.601052920776057	0.994766463583882	pseudobulk	DESeq2	LRT	0.221087288081801
101	KC_AB1	cN-IIIB	-0.864920080182367	0.42259792856215	0.994766463583882	pseudobulk	DESeq2	LRT	0.374072636049045
101	KC_AB1	CNBP	0.126201183305222	0.686151814532407	0.994766463583882	pseudobulk	DESeq2	LRT	0.16357978382788
101	KC_AB1	cnc	0.0605110235665934	0.861620814963631	0.994766463583882	pseudobulk	DESeq2	LRT	0.0646838179335504
101	KC_AB1	Cndp2	-0.354871336292288	0.786128083502995	0.994766463583882	pseudobulk	DESeq2	LRT	0.104506688786583
101	KC_AB1	CngA	-4.30725257042072	0.335358709589723	0.994766463583882	pseudobulk	DESeq2	LRT	0.474490410129538
101	KC_AB1	CngB	0.622715232805966	0.156490438003625	0.994766463583882	pseudobulk	DESeq2	LRT	0.805512193894723
101	KC_AB1	Cngl	0.0293149218550946	0.897490711076309	0.994766463583882	pseudobulk	DESeq2	LRT	0.0469700376233793
101	KC_AB1	cni	-0.178564755492682	0.714270855175292	0.994766463583882	pseudobulk	DESeq2	LRT	0.146137070293738
101	KC_AB1	cnir	-0.134417515771676	0.884283138226136	0.994766463583882	pseudobulk	DESeq2	LRT	0.0534086561622528
101	KC_AB1	cnk	0.717957675561536	0.54271555973686	0.994766463583882	pseudobulk	DESeq2	LRT	0.265427726953806
101	KC_AB1	CNMaR	0.469123695720418	0.811531888990343	0.994766463583882	pseudobulk	DESeq2	LRT	0.0906944099838556
101	KC_AB1	cnn	-0.721274741909156	0.305686150426606	0.994766463583882	pseudobulk	DESeq2	LRT	0.514724237196562
101	KC_AB1	cno	2.39871913375783	0.342082927152751	0.994766463583882	pseudobulk	DESeq2	LRT	0.4658686002621
101	KC_AB1	Cnot4	-0.112669327282453	0.772172576548777	0.994766463583882	pseudobulk	DESeq2	LRT	0.112285626261601
101	KC_AB1	CNPYb	-0.267016946846597	0.754377517574611	0.994766463583882	pseudobulk	DESeq2	LRT	0.122411263190639
101	KC_AB1	Cnx14D	0.478005084452508	0.815070723509861	0.994766463583882	pseudobulk	DESeq2	LRT	0.0888047059870473
101	KC_AB1	Cnx99A	0.104240981654125	0.846718133843326	0.994766463583882	pseudobulk	DESeq2	LRT	0.0722611390074186
101	KC_AB1	Coa7	-0.126265226225936	0.776810205310849	0.994766463583882	pseudobulk	DESeq2	LRT	0.109685077537392
101	KC_AB1	Cog1	1.25869713358712	0.315483549912536	0.994766463583882	pseudobulk	DESeq2	LRT	0.501023281010867
101	KC_AB1	Cog2	0.0717925335871559	0.944378479700174	0.994766463583882	pseudobulk	DESeq2	LRT	0.0248539180926242
101	KC_AB1	Cog3	-1.32754262918199	0.499139561833611	0.994766463583882	pseudobulk	DESeq2	LRT	0.301778006561226
101	KC_AB1	Cog4	-0.49050184051698	0.793052437934295	0.994766463583882	pseudobulk	DESeq2	LRT	0.100698095466029
101	KC_AB1	Cog6	0.215650179934063	0.807813637619887	0.994766463583882	pseudobulk	DESeq2	LRT	0.0926888192855298
101	KC_AB1	Cog7	-0.0840559898990188	0.864180255489594	0.994766463583882	pseudobulk	DESeq2	LRT	0.0633956605310083
101	KC_AB1	Cog8	-0.203919210024363	0.84165190436689	0.994766463583882	pseudobulk	DESeq2	LRT	0.0748674895816847
101	KC_AB1	coil	0.757111967132494	0.612617233120852	0.994766463583882	pseudobulk	DESeq2	LRT	0.21281079051341
101	KC_AB1	cold	-0.613309020929571	0.618210854276206	0.994766463583882	pseudobulk	DESeq2	LRT	0.208863374052917
101	KC_AB1	colt	0.475649746043893	0.759920495303806	0.994766463583882	pseudobulk	DESeq2	LRT	0.119231842267898
101	KC_AB1	comm	0.0209217164962367	0.943772143670914	0.994766463583882	pseudobulk	DESeq2	LRT	0.025132845417043
101	KC_AB1	comt	-0.141802259358145	0.667793191407636	0.994766463583882	pseudobulk	DESeq2	LRT	0.175358013177975
101	KC_AB1	Con	0.0661240281934806	0.722592864535456	0.994766463583882	pseudobulk	DESeq2	LRT	0.141106331320692
101	KC_AB1	Cont	-0.640119270719045	0.610998541386736	0.994766463583882	pseudobulk	DESeq2	LRT	0.213959826530683
101	KC_AB1	conu	-0.136091095644562	0.681214821177671	0.994766463583882	pseudobulk	DESeq2	LRT	0.166715911670013
101	KC_AB1	Coop	0.949183608371002	0.80156895525114	0.994766463583882	pseudobulk	DESeq2	LRT	0.0960591113673091
101	KC_AB1	Coprox	-1.6429526227112	0.309589532762348	0.994766463583882	pseudobulk	DESeq2	LRT	0.509213731259172
101	KC_AB1	Coq2	1.09344445092622	0.478500403134131	0.994766463583882	pseudobulk	DESeq2	LRT	0.320117691996114
101	KC_AB1	Coq3	0.333288441391291	0.781331465283699	0.994766463583882	pseudobulk	DESeq2	LRT	0.107164685707165
101	KC_AB1	Coq5	0.115973679573441	0.948205466729312	0.994766463583882	pseudobulk	DESeq2	LRT	0.0230975451533938
101	KC_AB1	Coq6	0.63448683030252	0.463029881347119	0.994766463583882	pseudobulk	DESeq2	LRT	0.334390981151775
101	KC_AB1	Coq7	-0.0985863519073474	0.844455772513941	0.994766463583882	pseudobulk	DESeq2	LRT	0.0734230912156819
101	KC_AB1	Coq8	-0.306571654351356	0.799991596197386	0.994766463583882	pseudobulk	DESeq2	LRT	0.0969145751883964
101	KC_AB1	Coq9	0.828487775258965	0.444277935883962	0.994766463583882	pseudobulk	DESeq2	LRT	0.352345254532666
101	KC_AB1	cora	-0.297308071116019	0.134616343466945	0.994766463583882	pseudobulk	DESeq2	LRT	0.870902210196563
101	KC_AB1	CoRest	-0.0322772697065748	0.930204050689938	0.994766463583882	pseudobulk	DESeq2	LRT	0.0314217736307186
101	KC_AB1	Corin	0.184222100235399	0.709894273848593	0.994766463583882	pseudobulk	DESeq2	LRT	0.148806326919216
101	KC_AB1	corn	-0.0515308829438749	0.850407483654087	0.994766463583882	pseudobulk	DESeq2	LRT	0.0703729266416576
101	KC_AB1	coro	-0.0952797548304897	0.779577226742616	0.994766463583882	pseudobulk	DESeq2	LRT	0.108140856117365
101	KC_AB1	Cortactin	-0.577196243255127	0.461871651054871	0.994766463583882	pseudobulk	DESeq2	LRT	0.335478693237906
101	KC_AB1	corto	0.0226052448869634	0.915992873997228	0.994766463583882	pseudobulk	DESeq2	LRT	0.0381079049300973

101	KC_AB1	cos	-0.141902358054155	0.831990028481103	0.994766463583882	pseudobulk	DESeq2	LRT	0.0798818787592574
101	KC_AB1	Cow	0.142015759637188	0.397536933376094	0.994766463583882	pseudobulk	DESeq2	LRT	0.400622516777795
101	KC_AB1	COX4	-0.0755586708014367	0.708625058056981	0.994766463583882	pseudobulk	DESeq2	LRT	0.149583494419636
101	KC_AB1	COX5A	0.0859331007815409	0.720624384276838	0.994766463583882	pseudobulk	DESeq2	LRT	0.142291046435343
101	KC_AB1	COX5B	0.0444266439516563	0.847342231787913	0.994766463583882	pseudobulk	DESeq2	LRT	0.071941147688027
101	KC_AB1	COX5BL	0.949183608371002	0.80156895525114	0.994766463583882	pseudobulk	DESeq2	LRT	0.0960591113673091
101	KC_AB1	COX6B	0.134918171295404	0.475257558685778	0.994766463583882	pseudobulk	DESeq2	LRT	0.323070967214925
101	KC_AB1	COX7A	0.162158964496159	0.390077085797786	0.994766463583882	pseudobulk	DESeq2	LRT	0.408849560592753
101	KC_AB1	COX7C	-0.156131586413171	0.507444631589213	0.994766463583882	pseudobulk	DESeq2	LRT	0.294611337665184
102	KC_AB1	COX8	-0.153818444797569	0.463290706101427	0.994766463583882	pseudobulk	DESeq2	LRT	0.334146411925812
102	KC_AB1	Cp1	-0.130334641616513	0.755901441462556	0.994766463583882	pseudobulk	DESeq2	LRT	0.121534826482004
102	KC_AB1	Cp110	-0.136247924381912	0.720348628571354	0.994766463583882	pseudobulk	DESeq2	LRT	0.142457266318213
102	KC_AB1	Cp190	-0.229133507079816	0.632678022586735	0.994766463583882	pseudobulk	DESeq2	LRT	0.198817251418965
102	KC_AB1	Cp7Fa	0.2147019870634	0.90028167280434	0.994766463583882	pseudobulk	DESeq2	LRT	0.0456215907762435
102	KC_AB1	Cp7Fb	-3.02774973138291	0.201306382223997	0.994766463583882	pseudobulk	DESeq2	LRT	0.696142456006897
102	KC_AB1	Cp7Fc	-1.64952313825113	0.817565084244375	0.994766463583882	pseudobulk	DESeq2	LRT	0.0874776642296574
102	KC_AB1	cpa	0.210816218283555	0.84256329804951	0.994766463583882	pseudobulk	DESeq2	LRT	0.0743974626097505
102	KC_AB1	cpb	0.589274466591584	0.436716916121276	0.994766463583882	pseudobulk	DESeq2	LRT	0.35979998546396
102	KC_AB1	Cpes	0.389101329135044	0.727344689386608	0.994766463583882	pseudobulk	DESeq2	LRT	0.138259727762336
102	KC_AB1	cpo	0.0369136241205588	0.77572279367551	0.994766463583882	pseudobulk	DESeq2	LRT	0.110293447150558
102	KC_AB1	Cpr	0.881337768677799	0.697697705840746	0.994766463583882	pseudobulk	DESeq2	LRT	0.156332705059186
102	KC_AB1	Cpr51A	-1.64952313825113	0.817565084244375	0.994766463583882	pseudobulk	DESeq2	LRT	0.0874776642296574
102	KC_AB1	Cpr64Ad	0.949183608371002	0.80156895525114	0.994766463583882	pseudobulk	DESeq2	LRT	0.0960591113673091
102	KC_AB1	Cpr67B	-1.36080367513457	0.587855376773158	0.994766463583882	pseudobulk	DESeq2	LRT	0.230729505207589
102	KC_AB1	Cpr73D	-1.08681444615798	0.701131388297773	0.994766463583882	pseudobulk	DESeq2	LRT	0.154200589927933
102	KC_AB1	Cpr92F	0.949183608371002	0.80156895525114	0.994766463583882	pseudobulk	DESeq2	LRT	0.0960591113673091
102	KC_AB1	Cpsf100	-0.50062178940184	0.730933222438512	0.994766463583882	pseudobulk	DESeq2	LRT	0.136122298077968
102	KC_AB1	Cpsf160	0.701822169592534	0.683506037476817	0.994766463583882	pseudobulk	DESeq2	LRT	0.165257644912858
102	KC_AB1	Cpsf5	0.040667591802526	0.906511592219653	0.994766463583882	pseudobulk	DESeq2	LRT	0.0426266378951811
102	KC_AB1	Cpsf6	0.198972215646771	0.592049054701408	0.994766463583882	pseudobulk	DESeq2	LRT	0.227642307967079
102	KC_AB1	Cpsf73	0.431549567083559	0.749733203540373	0.994766463583882	pseudobulk	DESeq2	LRT	0.125093255066858
102	KC_AB1	cpx	0.0176936382448932	0.880755057476277	0.994766463583882	pseudobulk	DESeq2	LRT	0.0551448543315248
102	KC_AB1	CR12842	1.90409869152575	0.234994524341875	0.994766463583882	pseudobulk	DESeq2	LRT	0.628942257199812
102	KC_AB1	CR14798	0.546848275227028	0.784144882426977	0.994766463583882	pseudobulk	DESeq2	LRT	0.105603687538331
102	KC_AB1	CR15345	-1.23323373102833	0.423315008432693	0.994766463583882	pseudobulk	DESeq2	LRT	0.373336333548311
102	KC_AB1	CR18166	0.289289854100641	0.733854615501413	0.994766463583882	pseudobulk	DESeq2	LRT	0.134389969974125
102	KC_AB1	CR18217	-1.64952313825113	0.817565084244375	0.994766463583882	pseudobulk	DESeq2	LRT	0.0874776642296574
102	KC_AB1	CR18228	-1.64952313825113	0.817565084244375	0.994766463583882	pseudobulk	DESeq2	LRT	0.0874776642296574
102	KC_AB1	CR18275	0.440593991678279	0.707094446877444	0.994766463583882	pseudobulk	DESeq2	LRT	0.150522573447505
102	KC_AB1	CR31032	0.436774318627524	0.753023728031073	0.994766463583882	pseudobulk	DESeq2	LRT	0.123191338818418
102	KC_AB1	CR31953	-1.64952313825113	0.817565084244375	0.994766463583882	pseudobulk	DESeq2	LRT	0.0874776642296574
102	KC_AB1	CR32010	-2.57435989741145	0.533617212681275	0.994766463583882	pseudobulk	DESeq2	LRT	0.272770169998685
102	KC_AB1	CR32733	-1.89157349400865	0.516179879210422	0.994766463583882	pseudobulk	DESeq2	LRT	0.287198928343475
102	KC_AB1	CR32745	0.15972320094564	0.857844475714494	0.994766463583882	pseudobulk	DESeq2	LRT	0.0665914411271873
102	KC_AB1	CR33294	-1.64952313825113	0.817565084244375	0.994766463583882	pseudobulk	DESeq2	LRT	0.0874776642296574
102	KC_AB1	CR33319	-1.64952313825113	0.817565084244375	0.994766463583882	pseudobulk	DESeq2	LRT	0.0874776642296574
102	KC_AB1	CR40190	-1.64952313825113	0.817565084244375	0.994766463583882	pseudobulk	DESeq2	LRT	0.0874776642296574
102	KC_AB1	CR41320	-0.467437884468606	0.815276186541128	0.994766463583882	pseudobulk	DESeq2	LRT	0.0886952428284564
102	KC_AB1	CR41440	2.02670873290438	0.538959414925803	0.994766463583882	pseudobulk	DESeq2	LRT	0.26844393710898
102	KC_AB1	CR41597	0.0467914378504958	0.927556137475932	0.994766463583882	pseudobulk	DESeq2	LRT	0.032659796582736
102	KC_AB1	CR42786	-1.64952313825113	0.817565084244375	0.994766463583882	pseudobulk	DESeq2	LRT	0.0874776642296574
102	KC_AB1	CR42875	-2.20969975456663	0.610836134188693	0.994766463583882	pseudobulk	DESeq2	LRT	0.214075280039667
102	KC_AB1	CR43080	-2.20969975456663	0.610836134188693	0.994766463583882	pseudobulk	DESeq2	LRT	0.214075280039667
102	KC_AB1	CR43170	-1.36080367513457	0.587855376773158	0.994766463583882	pseudobulk	DESeq2	LRT	0.230729505207589
102	KC_AB1	CR43212	2.02670873290438	0.538959414925803	0.994766463583882	pseudobulk	DESeq2	LRT	0.26844393710898
102	KC_AB1	CR43281	-0.96411471565984	0.172266803485359	0.994766463583882	pseudobulk	DESeq2	LRT	0.763798404804734

102	KC_AB1	CR43361	0.208615470155158	0.893577121399079	0.994766463583882	pseudobulk	DESeq2	LRT	0.0488679591598277
102	KC_AB1	CR43382	-1.64952313825113	0.817565084244375	0.994766463583882	pseudobulk	DESeq2	LRT	0.0874776642296574
102	KC_AB1	CR43483	-1.64952313825113	0.817565084244375	0.994766463583882	pseudobulk	DESeq2	LRT	0.0874776642296574
102	KC_AB1	CR43670	-0.0184107054716716	0.84712757684154	0.994766463583882	pseudobulk	DESeq2	LRT	0.0720511802878961
102	KC_AB1	CR43972	1.1821590793593	0.719511699510136	0.994766463583882	pseudobulk	DESeq2	LRT	0.142962139890846
102	KC_AB1	CR44003	-1.33819735008926	0.265371695158381	0.994766463583882	pseudobulk	DESeq2	LRT	0.576145401336226
102	KC_AB1	CR44383	-2.85724547560282	0.450020141104186	0.994766463583882	pseudobulk	DESeq2	LRT	0.346768048503189
102	KC_AB1	CR45470	-2.32607225918216	0.391287880902332	0.994766463583882	pseudobulk	DESeq2	LRT	0.407503603003357
102	KC_AB1	CR46418	-1.64952313825113	0.817565084244375	0.994766463583882	pseudobulk	DESeq2	LRT	0.0874776642296574
102	KC_AB1	CR6900	-0.119466828426229	0.885067448591205	0.994766463583882	pseudobulk	DESeq2	LRT	0.0530236316357438
102	KC_AB1	CR7448	-2.79499059556516	0.38564607643653	0.994766463583882	pseudobulk	DESeq2	LRT	0.413811082791365
102	KC_AB1	Crag	-0.182529149063742	0.793830712797362	0.994766463583882	pseudobulk	DESeq2	LRT	0.100272102530912
102	KC_AB1	CRAT	-1.38307071517737	0.314763285421266	0.994766463583882	pseudobulk	DESeq2	LRT	0.502015930280054
102	KC_AB1	crb	0.150013823526172	0.384415312427519	0.994766463583882	pseudobulk	DESeq2	LRT	0.415199321392859
102	KC_AB1	crc	0.316551725854155	0.483886034780384	0.994766463583882	pseudobulk	DESeq2	LRT	0.315256911690378
102	KC_AB1	CrebA	-0.348956612269543	0.689291575730721	0.994766463583882	pseudobulk	DESeq2	LRT	0.161597029261535
102	KC_AB1	CrebB	0.294193781503104	0.59840775500599	0.994766463583882	pseudobulk	DESeq2	LRT	0.223002786910832
102	KC_AB1	CREG	-2.12640346989651	0.44900213027744	0.994766463583882	pseudobulk	DESeq2	LRT	0.347751598494355
102	KC_AB1	Crg-1	0.554313829431432	0.533473597288936	0.994766463583882	pseudobulk	DESeq2	LRT	0.272887069839266
102	KC_AB1	CRIF	-0.448370813884098	0.515035844884917	0.994766463583882	pseudobulk	DESeq2	LRT	0.288162544368526
102	KC_AB1	crim	0.513745436551287	0.672272051037666	0.994766463583882	pseudobulk	DESeq2	LRT	0.17245494367972
102	KC_AB1	Crk	-0.154667490158051	0.519470693769374	0.994766463583882	pseudobulk	DESeq2	LRT	0.284438948382559
102	KC_AB1	crm	-0.243888212302755	0.818811407663963	0.994766463583882	pseudobulk	DESeq2	LRT	0.0868161153854492
102	KC_AB1	CRMP	0.0493850485666035	0.855579437766012	0.994766463583882	pseudobulk	DESeq2	LRT	0.0677396614217629
102	KC_AB1	crn	0.0892513673982007	0.924919212462236	0.994766463583882	pseudobulk	DESeq2	LRT	0.0338961992762072
102	KC_AB1	crol	-0.225528626460063	0.4124707734955	0.994766463583882	pseudobulk	DESeq2	LRT	0.384606818894203
102	KC_AB1	CROT	1.90100515887561	0.379831956883464	0.994766463583882	pseudobulk	DESeq2	LRT	0.420408499013709
102	KC_AB1	crp	-0.0799097458986496	0.75243309217978	0.994766463583882	pseudobulk	DESeq2	LRT	0.123532112330814
102	KC_AB1	crq	-0.567213565677678	0.419885741848612	0.994766463583882	pseudobulk	DESeq2	LRT	0.376868872543781
102	KC_AB1	Crtc	0.510870986885636	0.104048460384971	0.994766463583882	pseudobulk	DESeq2	LRT	0.982764341701832
102	KC_AB1	cry	-2.20969975456663	0.610836134188693	0.994766463583882	pseudobulk	DESeq2	LRT	0.214075280039667
102	KC_AB1	Cse1	0.268246074675702	0.764841692081439	0.994766463583882	pseudobulk	DESeq2	LRT	0.116428446376051
102	KC_AB1	Csk	-0.0325908643071308	0.920357507350653	0.994766463583882	pseudobulk	DESeq2	LRT	0.0360434407955951
102	KC_AB1	Csl4	-0.0632988061789734	0.953193490472487	0.994766463583882	pseudobulk	DESeq2	LRT	0.0208189321895468
102	KC_AB1	CSN1b	0.543715369835842	0.671862901610765	0.994766463583882	pseudobulk	DESeq2	LRT	0.172719338774149
102	KC_AB1	CSN3	0.477704458337075	0.662188828727654	0.994766463583882	pseudobulk	DESeq2	LRT	0.179018150166342
102	KC_AB1	CSN4	0.330992172630199	0.683180648399124	0.994766463583882	pseudobulk	DESeq2	LRT	0.165464443859602
102	KC_AB1	CSN5	-0.148627813039791	0.798323694538401	0.994766463583882	pseudobulk	DESeq2	LRT	0.0978209805190455
102	KC_AB1	CSN6	-0.0612575923223262	0.917396891617345	0.994766463583882	pseudobulk	DESeq2	LRT	0.037442735705804
102	KC_AB1	CSN7	0.230144688182855	0.696051205969803	0.994766463583882	pseudobulk	DESeq2	LRT	0.157358809739863
102	KC_AB1	CSN8	-0.295173497057036	0.620279944137556	0.994766463583882	pseudobulk	DESeq2	LRT	0.207412260897266
102	KC_AB1	Csp	0.122652305085004	0.658445090146586	0.994766463583882	pseudobulk	DESeq2	LRT	0.181480436405543
102	KC_AB1	CstF50	1.58591383223753	0.441074872136218	0.994766463583882	pseudobulk	DESeq2	LRT	0.35548768310445
102	KC_AB1	CstF64	-0.167912757499807	0.858803388235274	0.994766463583882	pseudobulk	DESeq2	LRT	0.0661062508112661
102	KC_AB1	csul	1.00462387452151	0.537284155631748	0.994766463583882	pseudobulk	DESeq2	LRT	0.269795966472366
102	KC_AB1	csw	-0.0466436707926048	0.833099661461639	0.994766463583882	pseudobulk	DESeq2	LRT	0.0793030420099408
102	KC_AB1	ct	-2.57435989741145	0.533617212681275	0.994766463583882	pseudobulk	DESeq2	LRT	0.272770169998685
102	KC_AB1	cta	0.129859315835374	0.747345474627224	0.994766463583882	pseudobulk	DESeq2	LRT	0.126478590902133
102	KC_AB1	CtBP	0.0599469895876392	0.748736359506373	0.994766463583882	pseudobulk	DESeq2	LRT	0.12567107651592
102	KC_AB1	CTCF	-0.139417504866461	0.878843816378714	0.994766463583882	pseudobulk	DESeq2	LRT	0.056088298659679
102	KC_AB1	CtIP	0.00143726467739967	0.960352780858589	0.994766463583882	pseudobulk	DESeq2	LRT	0.0175692017144443
102	KC_AB1	Ctl1	-0.719491354823591	0.155301064836434	0.994766463583882	pseudobulk	DESeq2	LRT	0.808825566480056
102	KC_AB1	Ctl2	-0.560313088202896	0.567921209668176	0.994766463583882	pseudobulk	DESeq2	LRT	0.245711911788724
103	KC_AB1	ctp	-0.043763355669178	0.730117714312159	0.994766463583882	pseudobulk	DESeq2	LRT	0.136607114461975
103	KC_AB1	CTPsyn	0.203093783051529	0.700130708350689	0.994766463583882	pseudobulk	DESeq2	LRT	0.154820873391085
103	KC_AB1	Ctr1A	0.511739679881828	0.420859414312072	0.994766463583882	pseudobulk	DESeq2	LRT	0.375862953534883

103	KC_AB1	Ctr9	0.316574983087653	0.615370619303732	0.994766463583882	pseudobulk	DESeq2	LRT	0.21086324286085
103	KC_AB1	ctrip	-0.0204888946739821	0.950281190675878	0.994766463583882	pseudobulk	DESeq2	LRT	0.0221478668276085
103	KC_AB1	CtsB1	0.441801257983983	0.395659648832463	0.994766463583882	pseudobulk	DESeq2	LRT	0.402678238798851
103	KC_AB1	cu	-1.24802864989897	0.599404151404255	0.994766463583882	pseudobulk	DESeq2	LRT	0.222280253506296
103	KC_AB1	Cubn	-0.835152883801839	0.920434724857442	0.994766463583882	pseudobulk	DESeq2	LRT	0.0360070052473159
103	KC_AB1	cue	-0.214640452756935	0.708369908766418	0.994766463583882	pseudobulk	DESeq2	LRT	0.149739895723444
103	KC_AB1	Cul1	-0.273730238926535	0.491800414266501	0.994766463583882	pseudobulk	DESeq2	LRT	0.308211109770585
103	KC_AB1	Cul2	0.0828922923482624	0.85215658304012	0.994766463583882	pseudobulk	DESeq2	LRT	0.0694805966627119
103	KC_AB1	Cul3	-0.248191730467726	0.601591529377458	0.994766463583882	pseudobulk	DESeq2	LRT	0.220698287394019
103	KC_AB1	Cul4	-0.13285679615772	0.800168295351184	0.994766463583882	pseudobulk	DESeq2	LRT	0.0968186604386661
103	KC_AB1	Cul5	0.423247080601528	0.743999051232002	0.994766463583882	pseudobulk	DESeq2	LRT	0.128427618278004
103	KC_AB1	Culd	0.949183608371002	0.80156895525114	0.994766463583882	pseudobulk	DESeq2	LRT	0.0960591113673091
103	KC_AB1	cv-c	-0.0802053989420487	0.745302761202696	0.994766463583882	pseudobulk	DESeq2	LRT	0.127667269789643
103	KC_AB1	Cwc25	-0.566919807487957	0.510796319673652	0.994766463583882	pseudobulk	DESeq2	LRT	0.291752240522135
103	KC_AB1	cwo	-0.404263327857044	0.376794836815133	0.994766463583882	pseudobulk	DESeq2	LRT	0.423895056935583
103	KC_AB1	cyc	-0.278749994017902	0.676137016995232	0.994766463583882	pseudobulk	DESeq2	LRT	0.169965286757732
103	KC_AB1	CycB	1.80633728195307	0.61161102402172	0.994766463583882	pseudobulk	DESeq2	LRT	0.213524695220716
103	KC_AB1	CycC	1.19775581666753	0.19539297013599	0.994766463583882	pseudobulk	DESeq2	LRT	0.709091065418005
103	KC_AB1	CycG	0.0283119181810046	0.854498563178319	0.994766463583882	pseudobulk	DESeq2	LRT	0.0682886631998298
103	KC_AB1	CycH	-0.525050937607539	0.744799300928206	0.994766463583882	pseudobulk	DESeq2	LRT	0.127960739657171
103	KC_AB1	CycJ	-2.20969975456663	0.610836134188693	0.994766463583882	pseudobulk	DESeq2	LRT	0.214075280039667
103	KC_AB1	CycK	-0.0625325911855844	0.896577631824573	0.994766463583882	pseudobulk	DESeq2	LRT	0.0474121002865736
103	KC_AB1	CycT	-0.175337824497028	0.537298349057922	0.994766463583882	pseudobulk	DESeq2	LRT	0.269784493874142
103	KC_AB1	CycY	-0.0627458576508891	0.910017353470768	0.994766463583882	pseudobulk	DESeq2	LRT	0.0409503258715026
103	KC_AB1	CYLD	-0.5259810253168	0.360278912775817	0.994766463583882	pseudobulk	DESeq2	LRT	0.443361156509428
103	KC_AB1	Cyp1	-0.0577140031720054	0.782298917599109	0.994766463583882	pseudobulk	DESeq2	LRT	0.106627270650127
103	KC_AB1	Cyp12b2	-0.465959464098644	0.655243436081218	0.994766463583882	pseudobulk	DESeq2	LRT	0.183597320916249
103	KC_AB1	Cyp28d1	1.1821590793593	0.719511699510136	0.994766463583882	pseudobulk	DESeq2	LRT	0.142962139890846
103	KC_AB1	Cyp303a1	-1.64952313825113	0.817565084244375	0.994766463583882	pseudobulk	DESeq2	LRT	0.0874776642296574
103	KC_AB1	Cyp313a1	2.02670873290438	0.538959414925803	0.994766463583882	pseudobulk	DESeq2	LRT	0.26844393710898
103	KC_AB1	Cyp49a1	-0.510520998423452	0.708511775830784	0.994766463583882	pseudobulk	DESeq2	LRT	0.14965292715847
103	KC_AB1	Cyp4ac1	2.22023857837763	0.524904842126482	0.994766463583882	pseudobulk	DESeq2	LRT	0.279919420946639
103	KC_AB1	Cyp4ae1	0.0236942436874584	0.910119428894173	0.994766463583882	pseudobulk	DESeq2	LRT	0.0409016143750932
103	KC_AB1	Cyp4d2	-2.79499059556516	0.38564607643653	0.994766463583882	pseudobulk	DESeq2	LRT	0.413811082791365
103	KC_AB1	Cyp4g1	1.1821590793593	0.719511699510136	0.994766463583882	pseudobulk	DESeq2	LRT	0.142962139890846
103	KC_AB1	Cyp6a14	-1.64952313825113	0.817565084244375	0.994766463583882	pseudobulk	DESeq2	LRT	0.0874776642296574
103	KC_AB1	Cyp6a2	2.22023857837763	0.524904842126482	0.994766463583882	pseudobulk	DESeq2	LRT	0.279919420946639
103	KC_AB1	Cyp6g1	0.806933541094274	0.754600478929312	0.994766463583882	pseudobulk	DESeq2	LRT	0.122282923497213
103	KC_AB1	Cyp6t2Psi	0.949183608371002	0.80156895525114	0.994766463583882	pseudobulk	DESeq2	LRT	0.0960591113673091
103	KC_AB1	Cyp6u1	-0.418289200511119	0.734590745307832	0.994766463583882	pseudobulk	DESeq2	LRT	0.133954547386121
103	KC_AB1	Cyp9f2	1.1821590793593	0.719511699510136	0.994766463583882	pseudobulk	DESeq2	LRT	0.142962139890846
103	KC_AB1	Cyp9f3	-0.283995846067142	0.866480656580972	0.994766463583882	pseudobulk	DESeq2	LRT	0.0622411280844138
103	KC_AB1	cype	-0.0375071960003339	0.848033999539277	0.994766463583882	pseudobulk	DESeq2	LRT	0.0715867355778445
103	KC_AB1	Cypl	2.07090257700671	0.0954655433824854	0.994766463583882	pseudobulk	DESeq2	LRT	1.02015335112915
103	KC_AB1	Cys	-2.57435989741145	0.533617212681275	0.994766463583882	pseudobulk	DESeq2	LRT	0.272770169998685
103	KC_AB1	CysRS	0.475649746043893	0.759920495303806	0.994766463583882	pseudobulk	DESeq2	LRT	0.119231842267898
103	KC_AB1	CysRS-m	-0.897294514816777	0.664018850996651	0.994766463583882	pseudobulk	DESeq2	LRT	0.177819591162686
103	KC_AB1	Cyt-b5	0.116624123023339	0.7462220860383	0.994766463583882	pseudobulk	DESeq2	LRT	0.127131901231113
103	KC_AB1	Cyt-c-d	1.1821590793593	0.719511699510136	0.994766463583882	pseudobulk	DESeq2	LRT	0.142962139890846
103	KC_AB1	Cyt-c-p	-0.0547215473566246	0.771836910925661	0.994766463583882	pseudobulk	DESeq2	LRT	0.112474456358001
103	KC_AB1	Cyt-c1	0.0486851553207353	0.878645878872265	0.994766463583882	pseudobulk	DESeq2	LRT	0.0561861236055758
103	KC_AB1	d	1.1821590793593	0.719511699510136	0.994766463583882	pseudobulk	DESeq2	LRT	0.142962139890846
103	KC_AB1	D1	0.342542474810085	0.293617548055429	0.994766463583882	pseudobulk	DESeq2	LRT	0.532217992366658
103	KC_AB1	D12	-0.762295128395995	0.605727091408042	0.994766463583882	pseudobulk	DESeq2	LRT	0.217723001892413
103	KC_AB1	D19A	0.568381837025983	0.56847660539754	0.994766463583882	pseudobulk	DESeq2	LRT	0.245287403194035
103	KC_AB1	D19B	-0.410058773506156	0.671249630871019	0.994766463583882	pseudobulk	DESeq2	LRT	0.173115940116557

103	KC_AB1	d4	0.051492868693414	0.81270785221755	0.994766463583882	pseudobulk	DESeq2	LRT	0.0900655441621704
103	KC_AB1	da	0.0169879996843662	0.855615880668224	0.994766463583882	pseudobulk	DESeq2	LRT	0.0677211632980629
103	KC_AB1	DAAM	-0.141103060284634	0.801917687406475	0.994766463583882	pseudobulk	DESeq2	LRT	0.0958702074512016
103	KC_AB1	Dab	0.110804500696723	0.740876606631041	0.994766463583882	pseudobulk	DESeq2	LRT	0.130254117960803
103	KC_AB1	dac	-0.0514735168476771	0.772313864855243	0.994766463583882	pseudobulk	DESeq2	LRT	0.112206168481485
103	KC_AB1	Dad	-0.804263102433562	0.3472752476366	0.994766463583882	pseudobulk	DESeq2	LRT	0.459326170325665
103	KC_AB1	Dad1	0.336140966474467	0.53340765105641	0.994766463583882	pseudobulk	DESeq2	LRT	0.272940759197795
103	KC_AB1	daed	-0.0490407263388828	0.918034301844095	0.994766463583882	pseudobulk	DESeq2	LRT	0.0371410913224011
103	KC_AB1	Dak1	0.500471482103301	0.235506740224292	0.994766463583882	pseudobulk	DESeq2	LRT	0.627996658809703
103	KC_AB1	dally	-1.02319198676748	0.828204539793902	0.994766463583882	pseudobulk	DESeq2	LRT	0.0818623932467813
103	KC_AB1	Damm	-1.64952313825113	0.817565084244375	0.994766463583882	pseudobulk	DESeq2	LRT	0.0874776642296574
103	KC_AB1	dany	0.949183608371002	0.80156895525114	0.994766463583882	pseudobulk	DESeq2	LRT	0.0960591113673091
103	KC_AB1	dao	0.260162609287766	0.633050182419549	0.994766463583882	pseudobulk	DESeq2	LRT	0.19856186172978
103	KC_AB1	Dap160	0.136243512874429	0.678197615966831	0.994766463583882	pseudobulk	DESeq2	LRT	0.168643741208286
103	KC_AB1	dar1	-2.79499059556516	0.38564607643653	0.994766463583882	pseudobulk	DESeq2	LRT	0.413811082791365
103	KC_AB1	dare	-1.11865416670102	0.343691097286894	0.994766463583882	pseudobulk	DESeq2	LRT	0.463831717351433
103	KC_AB1	Dark	-0.529200334688263	0.483650580414939	0.994766463583882	pseudobulk	DESeq2	LRT	0.315468286706197
103	KC_AB1	DAT	-1.64952313825113	0.817565084244375	0.994766463583882	pseudobulk	DESeq2	LRT	0.0874776642296574
103	KC_AB1	dati	-0.030964611710214	0.833076199269927	0.994766463583882	pseudobulk	DESeq2	LRT	0.0793152730122431
103	KC_AB1	Daxx	-0.026244733426406	0.936028736585072	0.994766463583882	pseudobulk	DESeq2	LRT	0.0287108179833158
103	KC_AB1	dbe	-0.266665488807127	0.829019993243217	0.994766463583882	pseudobulk	DESeq2	LRT	0.0814349955649239
103	KC_AB1	dbo	-0.183159685695243	0.631904757820683	0.994766463583882	pseudobulk	DESeq2	LRT	0.199348374676383
103	KC_AB1	Dbp21E2	1.02966392254836	0.647269520172055	0.994766463583882	pseudobulk	DESeq2	LRT	0.188914843364814
103	KC_AB1	Dbp45A	-0.936780503598291	0.450157075274502	0.994766463583882	pseudobulk	DESeq2	LRT	0.346635919509189
103	KC_AB1	Dbp73D	-0.943532023167133	0.614276262319199	0.994766463583882	pseudobulk	DESeq2	LRT	0.211636266941502
103	KC_AB1	Dbp80	0.0913597809616998	0.673747690837255	0.994766463583882	pseudobulk	DESeq2	LRT	0.171502710277719
103	KC_AB1	dbr	0.0734419174993115	0.89063518632819	0.994766463583882	pseudobulk	DESeq2	LRT	0.0503001511918724
103	KC_AB1	DCAF12	0.433551806610847	0.77643869492305	0.994766463583882	pseudobulk	DESeq2	LRT	0.109892829063169
103	KC_AB1	Dci	-0.0533366771593542	0.954196915753561	0.994766463583882	pseudobulk	DESeq2	LRT	0.0203619915429428
103	KC_AB1	dco	0.0805518526793294	0.787171892073848	0.994766463583882	pseudobulk	DESeq2	LRT	0.103930421862241
103	KC_AB1	Dcp-1	-0.510768524947458	0.546010930131447	0.994766463583882	pseudobulk	DESeq2	LRT	0.262798663433978
103	KC_AB1	DCP1	0.439616261999157	0.643648724951262	0.994766463583882	pseudobulk	DESeq2	LRT	0.191351086735083
103	KC_AB1	DCP2	0.138960887265052	0.649490640756629	0.994766463583882	pseudobulk	DESeq2	LRT	0.187427102785006
103	KC_AB1	Dcr-2	0.62506181737221	0.65087233953608	0.994766463583882	pseudobulk	DESeq2	LRT	0.18650418450682
103	KC_AB1	DCTN1-p150	0.524805425122353	0.441268938341714	0.994766463583882	pseudobulk	DESeq2	LRT	0.355296642191534
103	KC_AB1	DCTN2-p50	0.38407178406732	0.652287705169899	0.994766463583882	pseudobulk	DESeq2	LRT	0.185560807323275
103	KC_AB1	DCTN3-p24	1.11683068356307	0.165444958956064	0.994766463583882	pseudobulk	DESeq2	LRT	0.781346461087492
103	KC_AB1	DCTN4-p62	0.289632408959879	0.801532329368318	0.994766463583882	pseudobulk	DESeq2	LRT	0.0960789559260527
103	KC_AB1	DCTN5-p25	-0.331553613596669	0.653828952697817	0.994766463583882	pseudobulk	DESeq2	LRT	0.184535852005123
103	KC_AB1	DCTN6-p27	1.03011551330837	0.14237300974796	0.994766463583882	pseudobulk	DESeq2	LRT	0.846572333933177
103	KC_AB1	Dd	0.0177725493846112	0.956642478131189	0.994766463583882	pseudobulk	DESeq2	LRT	0.0192503389058341
104	KC_AB1	Ddc	0.134473181869824	0.85868976060491	0.994766463583882	pseudobulk	DESeq2	LRT	0.0661637157891779
104	KC_AB1	Ddr	-1.64952313825113	0.817565084244375	0.994766463583882	pseudobulk	DESeq2	LRT	0.0874776642296574
104	KC_AB1	Ddx1	1.87513041965308	0.212281523311771	0.994766463583882	pseudobulk	DESeq2	LRT	0.673087804576085
104	KC_AB1	Deaf1	0.166205217536337	0.638106883108487	0.994766463583882	pseudobulk	DESeq2	LRT	0.19510657072017
104	KC_AB1	DEF8	1.03099324158227	0.424352022038147	0.994766463583882	pseudobulk	DESeq2	LRT	0.372273724068412
104	KC_AB1	defl	0.217906885051292	0.902008941520292	0.994766463583882	pseudobulk	DESeq2	LRT	0.0447891573209458
104	KC_AB1	Dek	-0.340973762065531	0.245033555014684	0.994766463583882	pseudobulk	DESeq2	LRT	0.610774439064563
104	KC_AB1	del	0.478005084452508	0.815070723509861	0.994766463583882	pseudobulk	DESeq2	LRT	0.0880047059870473
104	KC_AB1	deltaCOP	-0.331011439638478	0.543391962477164	0.994766463583882	pseudobulk	DESeq2	LRT	0.264886789689993
104	KC_AB1	Den1	-0.46496502215171	0.404821319578037	0.994766463583882	pseudobulk	DESeq2	LRT	0.392736623808362
104	KC_AB1	DENR	0.0392139175960726	0.878537834120912	0.994766463583882	pseudobulk	DESeq2	LRT	0.0562395309289232
104	KC_AB1	Der-1	0.214054079552907	0.763886764130954	0.994766463583882	pseudobulk	DESeq2	LRT	0.116971014929369
104	KC_AB1	Der-2	0.12933791510411	0.86403671481271	0.994766463583882	pseudobulk	DESeq2	LRT	0.0634678030051467
104	KC_AB1	Dera	0.949183608371002	0.80156895525114	0.994766463583882	pseudobulk	DESeq2	LRT	0.0960591113673091
104	KC_AB1	Desat1	-0.183311528582728	0.453513935355872	0.994766463583882	pseudobulk	DESeq2	LRT	0.343409363609012

104	KC_AB1	Det	1.33653103630187	0.7897265473711	0.994766463583882	pseudobulk	DESeq2	LRT	0.102523262540324
104	KC_AB1	Df31	0.00176478240753356	0.95678795833198	0.994766463583882	pseudobulk	DESeq2	LRT	0.0191842991404498
104	KC_AB1	Dg	-0.256120196810367	0.41764984214277	0.994766463583882	pseudobulk	DESeq2	LRT	0.379187678406422
104	KC_AB1	Dgk	0.282729110687925	0.633120891217755	0.994766463583882	pseudobulk	DESeq2	LRT	0.198513355739469
104	KC_AB1	Dgkepsilon	0.185710866603829	0.774174349165041	0.994766463583882	pseudobulk	DESeq2	LRT	0.11161222322965
104	KC_AB1	dgo	-1.64952313825113	0.817565084244375	0.994766463583882	pseudobulk	DESeq2	LRT	0.0874776642296574
104	KC_AB1	Dgp-1	0.247446017620634	0.716785580256961	0.994766463583882	pseudobulk	DESeq2	LRT	0.144610740061644
104	KC_AB1	dgrn	-0.853487240431203	0.312388447986628	0.994766463583882	pseudobulk	DESeq2	LRT	0.505305034552817
104	KC_AB1	dgt1	-0.485595720144132	0.665398096792127	0.994766463583882	pseudobulk	DESeq2	LRT	0.176918445658148
104	KC_AB1	dgt2	-0.804068653073624	0.741430092663809	0.994766463583882	pseudobulk	DESeq2	LRT	0.129929791097641
104	KC_AB1	dgt6	-0.835152883801839	0.920434724857442	0.994766463583882	pseudobulk	DESeq2	LRT	0.0360070052473159
104	KC_AB1	Dh44-R1	0.447765531119722	0.335266289277106	0.994766463583882	pseudobulk	DESeq2	LRT	0.474610112294257
104	KC_AB1	Dh44-R2	0.639971679355298	0.00725081960292613	0.994766463583882	pseudobulk	DESeq2	LRT	2.13961289978626
104	KC_AB1	Dhap-at	0.14194578429714	0.88110720181142	0.994766463583882	pseudobulk	DESeq2	LRT	0.0549712489968096
104	KC_AB1	Dhc64C	-0.330278303076118	0.434145597042932	0.994766463583882	pseudobulk	DESeq2	LRT	0.362364599085975
104	KC_AB1	Dhit	0.195298113352075	0.344755827385081	0.994766463583882	pseudobulk	DESeq2	LRT	0.462488384239001
104	KC_AB1	Dhod	-0.255783849941125	0.83458686414983	0.994766463583882	pseudobulk	DESeq2	LRT	0.0785284550809825
104	KC_AB1	DhpD	-0.506163934170691	0.781042226318591	0.994766463583882	pseudobulk	DESeq2	LRT	0.107325485762373
104	KC_AB1	Dhpr	0.057671748881	0.873996912944835	0.994766463583882	pseudobulk	DESeq2	LRT	0.0584901013397287
104	KC_AB1	Dhx15	-0.0509269552408664	0.872027598185169	0.994766463583882	pseudobulk	DESeq2	LRT	0.0594697701708005
104	KC_AB1	dia	-2.26306816020352	0.40449476385881	0.994766463583882	pseudobulk	DESeq2	LRT	0.393087095910655
104	KC_AB1	Diap1	-0.0773401969405695	0.78926907660523	0.994766463583882	pseudobulk	DESeq2	LRT	0.102774912426861
104	KC_AB1	Diap2	-0.251459529995251	0.633284746514443	0.994766463583882	pseudobulk	DESeq2	LRT	0.198400972393314
104	KC_AB1	dib	0.949183608371002	0.80156895525114	0.994766463583882	pseudobulk	DESeq2	LRT	0.0960591113673091
104	KC_AB1	Dic1	-0.301564638196665	0.646261131370581	0.994766463583882	pseudobulk	DESeq2	LRT	0.18959196343195
104	KC_AB1	didum	0.407546444865382	0.50618466654122	0.994766463583882	pseudobulk	DESeq2	LRT	0.295691014723788
104	KC_AB1	Dif	0.0611093014508635	0.828873253241468	0.994766463583882	pseudobulk	DESeq2	LRT	0.0815118743064658
104	KC_AB1	dikar	0.32753919266181	0.18605664971745	0.994766463583882	pseudobulk	DESeq2	LRT	0.730354803557059
104	KC_AB1	Dim1	-0.280603116207208	0.743324761720269	0.994766463583882	pseudobulk	DESeq2	LRT	0.128821399688163
104	KC_AB1	dind	-0.320653051380372	0.71176949769146	0.994766463583882	pseudobulk	DESeq2	LRT	0.147660627273623
104	KC_AB1	DIP-alpha	0.107388526366525	0.848900994921265	0.994766463583882	pseudobulk	DESeq2	LRT	0.0711429574199011
104	KC_AB1	Dip-B	1.47424991200432	0.518427666092641	0.994766463583882	pseudobulk	DESeq2	LRT	0.285311830226264
104	KC_AB1	DIP-beta	-1.64952313825113	0.817565084244375	0.994766463583882	pseudobulk	DESeq2	LRT	0.0874776642296574
104	KC_AB1	Dip-C	-0.909786956081574	0.292226397548151	0.994766463583882	pseudobulk	DESeq2	LRT	0.534280555712871
104	KC_AB1	DIP-kappa	-0.0777003725245307	0.658994986401411	0.994766463583882	pseudobulk	DESeq2	LRT	0.181117889482598
104	KC_AB1	DIP-lambda	-0.0175856672650169	0.960737619868475	0.994766463583882	pseudobulk	DESeq2	LRT	0.0173952031763364
104	KC_AB1	DIP1	-0.123537525363498	0.680105886090362	0.994766463583882	pseudobulk	DESeq2	LRT	0.167423466463183
104	KC_AB1	DIP2	0.234422238861661	0.587785979261923	0.994766463583882	pseudobulk	DESeq2	LRT	0.230780777572358
104	KC_AB1	Dis3	-0.0876274309314422	0.917937734184526	0.994766463583882	pseudobulk	DESeq2	LRT	0.0371867769874299
104	KC_AB1	Dis3l2	-0.431005721912618	0.589945299836246	0.994766463583882	pseudobulk	DESeq2	LRT	0.229188254596126
104	KC_AB1	disp	0.147678092424954	0.878271366440605	0.994766463583882	pseudobulk	DESeq2	LRT	0.0563712759630602
104	KC_AB1	DJ-1alpha	0.696124738815335	0.683158309383576	0.994766463583882	pseudobulk	DESeq2	LRT	0.165478644890948
104	KC_AB1	dj-1beta	0.176313605589008	0.737911334807474	0.994766463583882	pseudobulk	DESeq2	LRT	0.131995818552553
104	KC_AB1	dl	0.689232879559345	0.64702264239009	0.994766463583882	pseudobulk	DESeq2	LRT	0.189080521042256
104	KC_AB1	Dl	-2.79499059556516	0.38564607643653	0.994766463583882	pseudobulk	DESeq2	LRT	0.413811082791365
104	KC_AB1	Dlc90F	-0.192430600973644	0.710444942869033	0.994766463583882	pseudobulk	DESeq2	LRT	0.148469572816957
104	KC_AB1	dlg1	0.0474640569780437	0.764673106999349	0.994766463583882	pseudobulk	DESeq2	LRT	0.116524183365616
104	KC_AB1	Dlg5	-0.377344160217144	0.558642134309275	0.994766463583882	pseudobulk	DESeq2	LRT	0.252866311728586
104	KC_AB1	Dlic	0.114512375748055	0.739565467174824	0.994766463583882	pseudobulk	DESeq2	LRT	0.131023375724808
104	KC_AB1	Dlip1	1.34486185587445	0.354979261041433	0.994766463583882	pseudobulk	DESeq2	LRT	0.449797018996635
104	KC_AB1	Dlip2	-0.0991708779555282	0.90622994419382	0.994766463583882	pseudobulk	DESeq2	LRT	0.0427615917007684
104	KC_AB1	Dlip3	-0.0741349876996741	0.911670076211878	0.994766463583882	pseudobulk	DESeq2	LRT	0.0401622998338667
104	KC_AB1	dlp	1.80633728195307	0.61161102402172	0.994766463583882	pseudobulk	DESeq2	LRT	0.213524695220716
104	KC_AB1	dlt	-0.257056328552413	0.890683115735507	0.994766463583882	pseudobulk	DESeq2	LRT	0.0502767803242406
104	KC_AB1	DMAP1	-0.69044164069589	0.38601412923039	0.994766463583882	pseudobulk	DESeq2	LRT	0.413396798606773
104	KC_AB1	dmpd	0.488573896884441	0.454146440653958	0.994766463583882	pseudobulk	DESeq2	LRT	0.342804085219742

104	KC_AB1	dmrt11E	0.949183608371002	0.80156895525114	0.994766463583882	pseudobulk	DESeq2	LRT	0.0960591113673091
104	KC_AB1	dmt	1.55007561624522	0.49585983061903	0.994766463583882	pseudobulk	DESeq2	LRT	0.304641072283799
104	KC_AB1	Dmtn	-0.303758162571242	0.516023352826958	0.994766463583882	pseudobulk	DESeq2	LRT	0.287330643771256
104	KC_AB1	DnaJ-1	-0.114290739961067	0.815636254221413	0.994766463583882	pseudobulk	DESeq2	LRT	0.0885034785064833
104	KC_AB1	DNAlig1	1.06071898122436	0.628579022653205	0.994766463583882	pseudobulk	DESeq2	LRT	0.201640116633702
104	KC_AB1	DNAlig3	-0.0607283591268674	0.856216896006165	0.994766463583882	pseudobulk	DESeq2	LRT	0.067416206343051
104	KC_AB1	DNAlig4	-1.55868602470456	0.32169593728358	0.994766463583882	pseudobulk	DESeq2	LRT	0.492554423793287
104	KC_AB1	DNApol-delta	-2.2427180161471	0.279084904344673	0.994766463583882	pseudobulk	DESeq2	LRT	0.554263653791237
104	KC_AB1	DNApol-eta	-0.5711947951975	0.76428867169774	0.994766463583882	pseudobulk	DESeq2	LRT	0.116742577488978
104	KC_AB1	DNApol-iota	-1.51769329592495	0.248326907578649	0.994766463583882	pseudobulk	DESeq2	LRT	0.604976219713277
104	KC_AB1	DNApol-theta	1.19856662192297	0.608802264240657	0.994766463583882	pseudobulk	DESeq2	LRT	0.215523741018373
104	KC_AB1	DNApol-zeta	0.729740153036158	0.752353468463509	0.994766463583882	pseudobulk	DESeq2	LRT	0.123578072524162
104	KC_AB1	dnc	0.0514127554204646	0.690687365979189	0.994766463583882	pseudobulk	DESeq2	LRT	0.160718488015433
104	KC_AB1	dnd	0.949183608371002	0.80156895525114	0.994766463583882	pseudobulk	DESeq2	LRT	0.0960591113673091
104	KC_AB1	dnk	0.397926188020889	0.60984890662376	0.994766463583882	pseudobulk	DESeq2	LRT	0.214777750477128
104	KC_AB1	dnr1	-0.189414974595038	0.886657488643929	0.994766463583882	pseudobulk	DESeq2	LRT	0.0522441135606927
104	KC_AB1	dnt	0.868178073072996	0.461476944852132	0.994766463583882	pseudobulk	DESeq2	LRT	0.335849991222272
104	KC_AB1	Dnz1	-0.235430421418346	0.653703261958085	0.994766463583882	pseudobulk	DESeq2	LRT	0.18461934791329
104	KC_AB1	Doa	0.231924875464832	0.599431012657668	0.994766463583882	pseudobulk	DESeq2	LRT	0.222260791791314
104	KC_AB1	Doc3	-1.64952313825113	0.817565084244375	0.994766463583882	pseudobulk	DESeq2	LRT	0.0874776642296574
104	KC_AB1	dock	-0.156174779759541	0.482554624338701	0.994766463583882	pseudobulk	DESeq2	LRT	0.316453518162425
104	KC_AB1	dod	0.0332311666945881	0.929642386741486	0.994766463583882	pseudobulk	DESeq2	LRT	0.0316840829870213
104	KC_AB1	dom	-0.37842469054973	0.467217855391991	0.994766463583882	pseudobulk	DESeq2	LRT	0.330480568399857
104	KC_AB1	dome	0.282244180342504	0.736494522244146	0.994766463583882	pseudobulk	DESeq2	LRT	0.13283047892045
104	KC_AB1	dop	0.122342203291835	0.84607576728496	0.994766463583882	pseudobulk	DESeq2	LRT	0.0725907435326502
104	KC_AB1	Dop1R1	0.0439818772056208	0.800660196928642	0.994766463583882	pseudobulk	DESeq2	LRT	0.0965517609569497
104	KC_AB1	Dop1R2	-0.0464047458965086	0.77448883416941	0.994766463583882	pseudobulk	DESeq2	LRT	0.110984839097244
104	KC_AB1	Dop2R	-0.0278760065155659	0.827326757751488	0.994766463583882	pseudobulk	DESeq2	LRT	0.0823229293098836
104	KC_AB1	DopEcR	-0.356107816478209	0.0698639123342908	0.994766463583882	pseudobulk	DESeq2	LRT	1.15574709780489
105	KC_AB1	dor	-0.193508749283189	0.877179158649257	0.994766463583882	pseudobulk	DESeq2	LRT	0.0569116954978768
105	KC_AB1	DOR	-0.109144678943687	0.560190335260402	0.994766463583882	pseudobulk	DESeq2	LRT	0.251664388156656
105	KC_AB1	dos	1.10131754101191	0.325857885437893	0.994766463583882	pseudobulk	DESeq2	LRT	0.486971765045156
105	KC_AB1	Dp	0.186154716295278	0.659202921967071	0.994766463583882	pseudobulk	DESeq2	LRT	0.180980876263363
105	KC_AB1	Dp1	-0.138927855812447	0.84855172134907	0.994766463583882	pseudobulk	DESeq2	LRT	0.071321681204977
105	KC_AB1	dpa	2.02670873290438	0.538959414925803	0.994766463583882	pseudobulk	DESeq2	LRT	0.26844393710898
105	KC_AB1	Dpck	-0.347954501463515	0.740545267220976	0.994766463583882	pseudobulk	DESeq2	LRT	0.130448389265105
105	KC_AB1	DPCoAC	1.26433463569552	0.528978780772322	0.994766463583882	pseudobulk	DESeq2	LRT	0.276561748717781
105	KC_AB1	Dph1	-0.835152883801839	0.920434724857442	0.994766463583882	pseudobulk	DESeq2	LRT	0.0360070052473159
105	KC_AB1	Dph5	0.0809635048004769	0.894800221912434	0.994766463583882	pseudobulk	DESeq2	LRT	0.0482739168694027
105	KC_AB1	Dpit47	0.22791341436187	0.828291062978683	0.994766463583882	pseudobulk	DESeq2	LRT	0.0818170245278081
105	KC_AB1	DppIII	1.06266737765035	0.250064041720274	0.994766463583882	pseudobulk	DESeq2	LRT	0.601948753712145
105	KC_AB1	dpr1	0.703982768113573	0.518108065638837	0.994766463583882	pseudobulk	DESeq2	LRT	0.28557964678801
105	KC_AB1	dpr10	-0.215363600908375	0.382282026721689	0.994766463583882	pseudobulk	DESeq2	LRT	0.417616120184175
105	KC_AB1	dpr12	-0.0316411955394349	0.878200022671924	0.994766463583882	pseudobulk	DESeq2	LRT	0.0564065560197303
105	KC_AB1	dpr14	0.275912313991578	0.342707121759212	0.994766463583882	pseudobulk	DESeq2	LRT	0.465076870450232
105	KC_AB1	dpr17	-1.64952313825113	0.817565084244375	0.994766463583882	pseudobulk	DESeq2	LRT	0.0874776642296574
105	KC_AB1	dpr19	0.0238857623332517	0.885588479415297	0.994766463583882	pseudobulk	DESeq2	LRT	0.0527680418119317
105	KC_AB1	dpr2	0.475892334225681	0.803946046269915	0.994766463583882	pseudobulk	DESeq2	LRT	0.0947730962683447
105	KC_AB1	dpr20	0.960975307530375	0.132257787463922	0.994766463583882	pseudobulk	DESeq2	LRT	0.878578746869047
105	KC_AB1	dpr21	-0.0224719066167144	0.889199063901157	0.994766463583882	pseudobulk	DESeq2	LRT	0.0510010031736497
105	KC_AB1	dpr3	0.0431580783812038	0.937545822223806	0.994766463583882	pseudobulk	DESeq2	LRT	0.028007497090773
105	KC_AB1	dpr4	-0.0688645935778745	0.820468610565822	0.994766463583882	pseudobulk	DESeq2	LRT	0.0859380295028093
105	KC_AB1	dpr5	0.493481757284275	0.524421642318722	0.994766463583882	pseudobulk	DESeq2	LRT	0.280319393744639
105	KC_AB1	dpr6	0.0119132094728598	0.934941569804311	0.994766463583882	pseudobulk	DESeq2	LRT	0.0292155299879052
105	KC_AB1	dpr7	0.190584166975146	0.510249926947222	0.994766463583882	pseudobulk	DESeq2	LRT	0.29221704879008
105	KC_AB1	dpr8	0.260642297522562	0.146802161767619	0.994766463583882	pseudobulk	DESeq2	LRT	0.833267549073657

105	KC_AB1	dpr9	0.0147093222072498	0.873590750964222	0.994766463583882	pseudobulk	DESeq2	LRT	0.05869197265498
105	KC_AB1	dpy	2.39871913375783	0.342082927152751	0.994766463583882	pseudobulk	DESeq2	LRT	0.4658686002621
105	KC_AB1	Dpy-30L1	-0.276254442814662	0.597505887305651	0.994766463583882	pseudobulk	DESeq2	LRT	0.223657811196961
105	KC_AB1	Drak	0.166016228118068	0.608350296269031	0.994766463583882	pseudobulk	DESeq2	LRT	0.215846276107999
105	KC_AB1	Drat	0.01011360594653	0.948308324873723	0.994766463583882	pseudobulk	DESeq2	LRT	0.0230504369016341
105	KC_AB1	drd	-2.20969975456663	0.610836134188693	0.994766463583882	pseudobulk	DESeq2	LRT	0.214075280039667
105	KC_AB1	dre4	-0.687242899252548	0.352759429414914	0.994766463583882	pseudobulk	DESeq2	LRT	0.452521368535679
105	KC_AB1	Dredd	-2.57435989741145	0.533617212681275	0.994766463583882	pseudobulk	DESeq2	LRT	0.272770169998685
105	KC_AB1	Dref	-0.387225925539747	0.615251762978254	0.994766463583882	pseudobulk	DESeq2	LRT	0.210947133170661
105	KC_AB1	Drep1	-1.16621474548569	0.376106006764966	0.994766463583882	pseudobulk	DESeq2	LRT	0.424689730432833
105	KC_AB1	Drep2	0.0214405595328555	0.89761388296511	0.994766463583882	pseudobulk	DESeq2	LRT	0.0469104390106932
105	KC_AB1	Drep3	1.1821590793593	0.719511699510136	0.994766463583882	pseudobulk	DESeq2	LRT	0.142962139890846
105	KC_AB1	Drep4	-0.315641728875764	0.652237010020278	0.994766463583882	pseudobulk	DESeq2	LRT	0.185594561568778
105	KC_AB1	Drice	-0.925642226170004	0.657544673459392	0.994766463583882	pseudobulk	DESeq2	LRT	0.182074735951784
105	KC_AB1	drk	-0.24363675338939	0.616948170129688	0.994766463583882	pseudobulk	DESeq2	LRT	0.209751319552103
105	KC_AB1	drl	-0.341991342545407	0.618875190280416	0.994766463583882	pseudobulk	DESeq2	LRT	0.208396927131521
105	KC_AB1	Drl-2	0.856602301332277	0.593517168576542	0.994766463583882	pseudobulk	DESeq2	LRT	0.226566713760425
105	KC_AB1	Droj2	0.0981573009377812	0.72406859434245	0.994766463583882	pseudobulk	DESeq2	LRT	0.140220289143609
105	KC_AB1	Dronc	-0.761801639395411	0.575002298922028	0.994766463583882	pseudobulk	DESeq2	LRT	0.240330418950099
105	KC_AB1	drongo	0.152935940848399	0.801931584239272	0.994766463583882	pseudobulk	DESeq2	LRT	0.0958626814100621
105	KC_AB1	drosha	-0.842701189968565	0.514869558767672	0.994766463583882	pseudobulk	DESeq2	LRT	0.288302784711975
105	KC_AB1	Drp1	-0.380667878639089	0.493095752764414	0.994766463583882	pseudobulk	DESeq2	LRT	0.30706873820892
105	KC_AB1	drpr	-0.116980598703715	0.835413309495593	0.994766463583882	pseudobulk	DESeq2	LRT	0.0780986099968633
105	KC_AB1	ds	1.06071898122436	0.628579022653205	0.994766463583882	pseudobulk	DESeq2	LRT	0.201640116633702
105	KC_AB1	Dscam1	-0.244040082786274	0.375103615690976	0.994766463583882	pseudobulk	DESeq2	LRT	0.425848749586758
105	KC_AB1	Dscam2	0.597029268460396	0.00167205056223061	0.994766463583882	pseudobulk	DESeq2	LRT	2.77675059378353
105	KC_AB1	Dscam3	4.61183210745844	0.0361433911903876	0.994766463583882	pseudobulk	DESeq2	LRT	1.44197110174249
105	KC_AB1	Dscam4	-0.341336166666746	0.184071787634398	0.994766463583882	pseudobulk	DESeq2	LRT	0.735012769962102
105	KC_AB1	dsd	-0.263659779254152	0.405356438719772	0.994766463583882	pseudobulk	DESeq2	LRT	0.392162924217547
105	KC_AB1	dsh	0.30803394824421	0.473426575826715	0.994766463583882	pseudobulk	DESeq2	LRT	0.324747366578165
105	KC_AB1	Dsor1	0.404978477432089	0.454727808275279	0.994766463583882	pseudobulk	DESeq2	LRT	0.342248486280439
105	KC_AB1	Dsp1	0.104822145928156	0.564573103372142	0.994766463583882	pseudobulk	DESeq2	LRT	0.248279815766327
105	KC_AB1	dsx-c73A	0.949183608371002	0.80156895525114	0.994766463583882	pseudobulk	DESeq2	LRT	0.0960591113673091
105	KC_AB1	Dtd	0.143454526375248	0.932398724290448	0.994766463583882	pseudobulk	DESeq2	LRT	0.0303983293535177
105	KC_AB1	dtn	0.148090597843791	0.530016350033602	0.994766463583882	pseudobulk	DESeq2	LRT	0.275710733003269
105	KC_AB1	dtr	-1.64952313825113	0.817565084244375	0.994766463583882	pseudobulk	DESeq2	LRT	0.0874776642296574
105	KC_AB1	Duba	0.46280224572423	0.413832127748504	0.994766463583882	pseudobulk	DESeq2	LRT	0.383175796023445
105	KC_AB1	DUBAI	-0.184103017376533	0.831820465529084	0.994766463583882	pseudobulk	DESeq2	LRT	0.0799703987618863
105	KC_AB1	dunk	1.40793671288203	0.350362431344311	0.994766463583882	pseudobulk	DESeq2	LRT	0.455482468527317
105	KC_AB1	dup	-0.254576076293858	0.903238298433106	0.994766463583882	pseudobulk	DESeq2	LRT	0.0441976560636768
105	KC_AB1	dUTPase	3.12553801840555	0.312245092635194	0.994766463583882	pseudobulk	DESeq2	LRT	0.505504378440468
105	KC_AB1	dve	1.1821590793593	0.719511699510136	0.994766463583882	pseudobulk	DESeq2	LRT	0.142962139890846
105	KC_AB1	dwg	-0.0357320543990272	0.95393950423571	0.994766463583882	pseudobulk	DESeq2	LRT	0.0204791659768963
105	KC_AB1	Dyb	-0.0763792305464513	0.701103750941649	0.994766463583882	pseudobulk	DESeq2	LRT	0.154217709383757
105	KC_AB1	Dyrk2	-0.408531082995222	0.173691291536404	0.994766463583882	pseudobulk	DESeq2	LRT	0.76022195548782
105	KC_AB1	Dyrk3	-0.00081808768191586	0.916127415084852	0.994766463583882	pseudobulk	DESeq2	LRT	0.0380441204150764
105	KC_AB1	Dys	0.0704209749829537	0.758532056139197	0.994766463583882	pseudobulk	DESeq2	LRT	0.120026060875686
105	KC_AB1	Dysb	-0.17805032366327	0.942053914371676	0.994766463583882	pseudobulk	DESeq2	LRT	0.0259242415339998
105	KC_AB1	dysc	-0.0762796369586961	0.514279209680966	0.994766463583882	pseudobulk	DESeq2	LRT	0.288801032169941
105	KC_AB1	dyw	-0.835152883801839	0.920434724857442	0.994766463583882	pseudobulk	DESeq2	LRT	0.0360070052473159
105	KC_AB1	E(bx)	-0.0569501048930386	0.831919038305094	0.994766463583882	pseudobulk	DESeq2	LRT	0.079918936844154
105	KC_AB1	E(Pc)	-0.169330522960344	0.494579617906547	0.994766463583882	pseudobulk	DESeq2	LRT	0.305763785291967
105	KC_AB1	e(r)	0.191520243058487	0.619567443737812	0.994766463583882	pseudobulk	DESeq2	LRT	0.207911411081477
105	KC_AB1	E(var)3-9	-0.238875058726736	0.799197101211001	0.994766463583882	pseudobulk	DESeq2	LRT	0.0973461000202494
105	KC_AB1	e(y)1	0.358918459628791	0.80536226849843	0.994766463583882	pseudobulk	DESeq2	LRT	0.0940087210990595
105	KC_AB1	e(y)2	-0.618554699861831	0.601861722419558	0.994766463583882	pseudobulk	DESeq2	LRT	0.2205032763306

105	KC_AB1	e(y)2b	0.596808856322697	0.822761281772702	0.994766463583882	pseudobulk	DESeq2	LRT	0.0847261539098712
105	KC_AB1	e(y)3	0.345413149659599	0.421780223380487	0.994766463583882	pseudobulk	DESeq2	LRT	0.3749137875128
105	KC_AB1	E(z)	-0.485713573511897	0.495611328953805	0.994766463583882	pseudobulk	DESeq2	LRT	0.304858774842605
105	KC_AB1	E2f1	0.447028145778016	0.580756741224756	0.994766463583882	pseudobulk	DESeq2	LRT	0.236005740357308
105	KC_AB1	E2f2	-0.550340271517734	0.493821899679184	0.994766463583882	pseudobulk	DESeq2	LRT	0.306429654179441
105	KC_AB1	Eaat2	-0.458290955989767	0.389562351351943	0.994766463583882	pseudobulk	DESeq2	LRT	0.409423021480707
105	KC_AB1	Eaf	0.120642912809961	0.83121377589269	0.994766463583882	pseudobulk	DESeq2	LRT	0.080287267731195
105	KC_AB1	Eaf6	-0.187175465537356	0.860079040025859	0.994766463583882	pseudobulk	DESeq2	LRT	0.0654616358846333
105	KC_AB1	eag	-0.124578599208428	0.535785145436075	0.994766463583882	pseudobulk	DESeq2	LRT	0.271009331311186
105	KC_AB1	ear	0.19843451980145	0.730730028466034	0.994766463583882	pseudobulk	DESeq2	LRT	0.136243045482019
105	KC_AB1	eas	0.223340064368905	0.638019168535838	0.994766463583882	pseudobulk	DESeq2	LRT	0.195166273215695
105	KC_AB1	east	-0.15498830764878	0.605605713981856	0.994766463583882	pseudobulk	DESeq2	LRT	0.21781003585428
105	KC_AB1	Eato	0.308127057924391	0.765543586419751	0.994766463583882	pseudobulk	DESeq2	LRT	0.11603007759446
105	KC_AB1	Eb1	0.0899174036420653	0.759646431837626	0.994766463583882	pseudobulk	DESeq2	LRT	0.119388497759957
105	KC_AB1	ebd1	-0.451230717201237	0.718954174966302	0.994766463583882	pseudobulk	DESeq2	LRT	0.14329878999751
105	KC_AB1	ebd2	0.305850643188069	0.585356613846617	0.994766463583882	pseudobulk	DESeq2	LRT	0.23257947026109
105	KC_AB1	ebi	-0.118307024683501	0.886548710536826	0.994766463583882	pseudobulk	DESeq2	LRT	0.0522973975293001
106	KC_AB1	ebo	0.0397667720961312	0.912177063813276	0.994766463583882	pseudobulk	DESeq2	LRT	0.0399208520518463
106	KC_AB1	eca	0.17097988217324	0.745690324419114	0.994766463583882	pseudobulk	DESeq2	LRT	0.127441491988669
106	KC_AB1	ecd	-0.303620403973654	0.729470826957328	0.994766463583882	pseudobulk	DESeq2	LRT	0.136992071755167
106	KC_AB1	Echs1	-0.219469276617728	0.822160136016244	0.994766463583882	pseudobulk	DESeq2	LRT	0.0850435846316661
106	KC_AB1	eco	0.596808856322697	0.822761281772702	0.994766463583882	pseudobulk	DESeq2	LRT	0.0847261539098712
106	KC_AB1	EcR	-0.142798690606692	0.470425561849167	0.994766463583882	pseudobulk	DESeq2	LRT	0.327509087721834
106	KC_AB1	ECSIT	0.311348489636432	0.761414414093696	0.994766463583882	pseudobulk	DESeq2	LRT	0.118378905953356
106	KC_AB1	ect	1.1821590793593	0.719511699510136	0.994766463583882	pseudobulk	DESeq2	LRT	0.142962139890846
106	KC_AB1	Ect4	-1.32806228432949	0.722858830184137	0.994766463583882	pseudobulk	DESeq2	LRT	0.140946509425123
106	KC_AB1	ed	0.46344977526387	0.60702657515298	0.994766463583882	pseudobulk	DESeq2	LRT	0.216792295432726
106	KC_AB1	Edc3	-0.31108534240814	0.440103340761418	0.994766463583882	pseudobulk	DESeq2	LRT	0.35644533475734
106	KC_AB1	Edem1	1.07601805237302	0.538858181621095	0.994766463583882	pseudobulk	DESeq2	LRT	0.268525518753016
106	KC_AB1	Edem2	0.316887602557345	0.672367165694128	0.994766463583882	pseudobulk	DESeq2	LRT	0.172393503004773
106	KC_AB1	Edg78E	0.646795838186486	0.85519056880605	0.994766463583882	pseudobulk	DESeq2	LRT	0.0679370972497873
106	KC_AB1	EDTP	-0.42646746248065	0.605906228379623	0.994766463583882	pseudobulk	DESeq2	LRT	0.217594583174375
106	KC_AB1	eEF1alpha1	0.199719229080732	0.679250913795502	0.994766463583882	pseudobulk	DESeq2	LRT	0.167969768653793
106	KC_AB1	eEF1alpha2	-0.175431443224954	0.25867085728663	0.994766463583882	pseudobulk	DESeq2	LRT	0.587252497578548
106	KC_AB1	eEF1beta	-0.491307391761008	0.254566703633587	0.994766463583882	pseudobulk	DESeq2	LRT	0.594198401050958
106	KC_AB1	eEF1delta	-0.579329364261627	0.300515493718248	0.994766463583882	pseudobulk	DESeq2	LRT	0.522133132104248
106	KC_AB1	eEF1gamma	0.176279156444173	0.729379471743353	0.994766463583882	pseudobulk	DESeq2	LRT	0.137046463991862
106	KC_AB1	eEF2	0.124049677061664	0.582997829661402	0.994766463583882	pseudobulk	DESeq2	LRT	0.234333061995413
106	KC_AB1	eEF5	0.0456627983823047	0.811405254343161	0.994766463583882	pseudobulk	DESeq2	LRT	0.090762184301335
106	KC_AB1	eEFSec	2.22023857837763	0.524904842126482	0.994766463583882	pseudobulk	DESeq2	LRT	0.279919420946639
106	KC_AB1	Efa6	-0.530951424062138	0.362203322648804	0.994766463583882	pseudobulk	DESeq2	LRT	0.441047570031286
106	KC_AB1	eff	0.110746379515304	0.575137229336206	0.994766463583882	pseudobulk	DESeq2	LRT	0.240228519079436
106	KC_AB1	Efr	0.357725471970503	0.749465078201219	0.994766463583882	pseudobulk	DESeq2	LRT	0.125248598569999
106	KC_AB1	egg	-0.137551105368642	0.837624867947368	0.994766463583882	pseudobulk	DESeq2	LRT	0.0769504375231043
106	KC_AB1	egh	-0.0789209993466646	0.736648487577734	0.994766463583882	pseudobulk	DESeq2	LRT	0.132739698467257
106	KC_AB1	egl	-0.359090933223968	0.58310650170424	0.994766463583882	pseudobulk	DESeq2	LRT	0.234252116118773
106	KC_AB1	Eglp4	-1.64952313825113	0.817565084244375	0.994766463583882	pseudobulk	DESeq2	LRT	0.0874776642296574
106	KC_AB1	Egm	-0.493477263290571	0.659076115958123	0.994766463583882	pseudobulk	DESeq2	LRT	0.181064426329084
106	KC_AB1	Ehbp1	0.0312474005711411	0.932712998846201	0.994766463583882	pseudobulk	DESeq2	LRT	0.030251970607321
106	KC_AB1	eIF1	0.14570533761826	0.586333912225038	0.994766463583882	pseudobulk	DESeq2	LRT	0.231854986479912
106	KC_AB1	eIF1A	-0.114217928340611	0.605446651504689	0.994766463583882	pseudobulk	DESeq2	LRT	0.217924118380317
106	KC_AB1	eIF2alpha	-0.309031549524474	0.584542717534947	0.994766463583882	pseudobulk	DESeq2	LRT	0.233183745730857
106	KC_AB1	eIF2Balpha	-0.0311929744519815	0.934226724572096	0.994766463583882	pseudobulk	DESeq2	LRT	0.0295477134042785
106	KC_AB1	eIF2Bbeta	-0.220695796864143	0.779874009116636	0.994766463583882	pseudobulk	DESeq2	LRT	0.107975553162126
106	KC_AB1	eIF2Bdelta	-0.431981407764153	0.581245468558953	0.994766463583882	pseudobulk	DESeq2	LRT	0.235640419886959
106	KC_AB1	eIF2Bepsilon	0.679845802233456	0.592307445047853	0.994766463583882	pseudobulk	DESeq2	LRT	0.227452808431352

106	KC_AB1	eIF2beta	0.274801328304381	0.435425275789965	0.994766463583882	pseudobulk	DESeq2	LRT	0.361086364414129
106	KC_AB1	eIF2Bgamma	1.31389883227911	0.348522262207877	0.994766463583882	pseudobulk	DESeq2	LRT	0.457769475685795
106	KC_AB1	eIF2D	-0.250322378945539	0.789332119127266	0.994766463583882	pseudobulk	DESeq2	LRT	0.102740224730521
106	KC_AB1	eIF2gamma	-0.0885143850185449	0.885716155018408	0.994766463583882	pseudobulk	DESeq2	LRT	0.0527054339364448
106	KC_AB1	eIF3a	-0.0380447073387088	0.804781719718708	0.994766463583882	pseudobulk	DESeq2	LRT	0.0943218969942335
106	KC_AB1	eIF3b	0.245368537146572	0.516257425972452	0.994766463583882	pseudobulk	DESeq2	LRT	0.287133688293095
106	KC_AB1	eIF3c	0.212057061087187	0.611002771641733	0.994766463583882	pseudobulk	DESeq2	LRT	0.213956819698558
106	KC_AB1	eIF3d1	-0.643397285599182	0.18511971647018	0.994766463583882	pseudobulk	DESeq2	LRT	0.732547323558328
106	KC_AB1	eIF3e	0.255040498007875	0.530286272573386	0.994766463583882	pseudobulk	DESeq2	LRT	0.275489615240091
106	KC_AB1	eIF3f1	-0.11987967002688	0.742003939260603	0.994766463583882	pseudobulk	DESeq2	LRT	0.129593789067871
106	KC_AB1	eIF3g1	-0.0932967607262528	0.813426496406691	0.994766463583882	pseudobulk	DESeq2	LRT	0.0896816850715878
106	KC_AB1	eIF3h	-0.183169899127902	0.593079783598604	0.994766463583882	pseudobulk	DESeq2	LRT	0.226886879576864
106	KC_AB1	eIF3i	0.153271757088485	0.610908202854961	0.994766463583882	pseudobulk	DESeq2	LRT	0.21402404342139
106	KC_AB1	eIF3j	-0.211471259546208	0.620754597974696	0.994766463583882	pseudobulk	DESeq2	LRT	0.207080054910502
106	KC_AB1	eIF3k	0.344686487234761	0.416400290225334	0.994766463583882	pseudobulk	DESeq2	LRT	0.380488976464054
106	KC_AB1	eIF3l	-0.281754932057047	0.352386022436675	0.994766463583882	pseudobulk	DESeq2	LRT	0.45298132642064
106	KC_AB1	eIF3m	-0.223052038968455	0.446799352031383	0.994766463583882	pseudobulk	DESeq2	LRT	0.349887465389461
106	KC_AB1	eIF4A	-0.0449037962686911	0.775640018181319	0.994766463583882	pseudobulk	DESeq2	LRT	0.110339792132046
106	KC_AB1	eIF4B	0.261047979970305	0.349750173755809	0.994766463583882	pseudobulk	DESeq2	LRT	0.456242061078679
106	KC_AB1	eIF4E1	0.058075951772481	0.827929086625207	0.994766463583882	pseudobulk	DESeq2	LRT	0.0820068595994737
106	KC_AB1	eIF4EHP	0.0525079021583427	0.788014099408158	0.994766463583882	pseudobulk	DESeq2	LRT	0.103466011900824
106	KC_AB1	eIF4G1	-0.141740572036923	0.674847561663516	0.994766463583882	pseudobulk	DESeq2	LRT	0.170794316954114
106	KC_AB1	eIF4G2	-0.00737189775138403	0.902345011103604	0.994766463583882	pseudobulk	DESeq2	LRT	0.0446273784559469
106	KC_AB1	eIF4H1	0.0559924053122764	0.839378035856967	0.994766463583882	pseudobulk	DESeq2	LRT	0.0760423992309393
106	KC_AB1	eIF5	0.506807121757165	0.166540721247278	0.994766463583882	pseudobulk	DESeq2	LRT	0.778479558850085
106	KC_AB1	eIF5B	-0.410763169594701	0.316644261788368	0.994766463583882	pseudobulk	DESeq2	LRT	0.499428377828446
106	KC_AB1	eIF6	0.630457456952196	0.618965083932654	0.994766463583882	pseudobulk	DESeq2	LRT	0.20833384901392
106	KC_AB1	Eip55E	-1.56296734298649	0.35117900667547	0.994766463583882	pseudobulk	DESeq2	LRT	0.454471453920572
106	KC_AB1	Eip63E	0.113335504897748	0.721569612526547	0.994766463583882	pseudobulk	DESeq2	LRT	0.141721764531969
106	KC_AB1	Eip71CD	0.55107851155136	0.531505641043679	0.994766463583882	pseudobulk	DESeq2	LRT	0.274492121806458
106	KC_AB1	Eip74EF	-0.386768651819186	0.531664226378417	0.994766463583882	pseudobulk	DESeq2	LRT	0.274362560692268
106	KC_AB1	Eip75B	-0.24785508121734	0.0940021646949847	0.994766463583882	pseudobulk	DESeq2	LRT	1.02686214529113
106	KC_AB1	Eip78C	-0.7015027501277	0.454513068860231	0.994766463583882	pseudobulk	DESeq2	LRT	0.342453624758221
106	KC_AB1	Eip93F	0.0662326702116725	0.554437011293779	0.994766463583882	pseudobulk	DESeq2	LRT	0.256147786217059
106	KC_AB1	elav	0.0718867957725049	0.697965224750102	0.994766463583882	pseudobulk	DESeq2	LRT	0.156166215020632
106	KC_AB1	elB	0.423637040208068	0.408123226475573	0.994766463583882	pseudobulk	DESeq2	LRT	0.389208688629967
106	KC_AB1	elgi	0.22285141060225	0.80376829195152	0.994766463583882	pseudobulk	DESeq2	LRT	0.0948691303934286
106	KC_AB1	Elk	0.152098451342127	0.375185835701517	0.994766463583882	pseudobulk	DESeq2	LRT	0.425753565796138
106	KC_AB1	elm	0.312933635627735	0.462995258029461	0.994766463583882	pseudobulk	DESeq2	LRT	0.334423456978126
106	KC_AB1	EloA	0.280394352112366	0.626336683160144	0.994766463583882	pseudobulk	DESeq2	LRT	0.203192151882455
106	KC_AB1	EloB	-0.22165898987266	0.683720134956314	0.994766463583882	pseudobulk	DESeq2	LRT	0.165121630321316
106	KC_AB1	EloC	0.286512274738751	0.414368837850502	0.994766463583882	pseudobulk	DESeq2	LRT	0.382612912670271
106	KC_AB1	Elp1	-0.464567019355039	0.710830147581045	0.994766463583882	pseudobulk	DESeq2	LRT	0.148234161268481
106	KC_AB1	Elp2	-1.27285858573635	0.459378181187007	0.994766463583882	pseudobulk	DESeq2	LRT	0.33782963607855
106	KC_AB1	Elp3	-0.0336201792232337	0.910107758858842	0.994766463583882	pseudobulk	DESeq2	LRT	0.040907183165607
106	KC_AB1	Elys	1.9283476086411	0.203682597787108	0.994766463583882	pseudobulk	DESeq2	LRT	0.691046074622216
106	KC_AB1	ema	0.390484868355206	0.599785448908806	0.994766463583882	pseudobulk	DESeq2	LRT	0.222004074647294
106	KC_AB1	emb	0.0471150148285822	0.887643723610252	0.994766463583882	pseudobulk	DESeq2	LRT	0.0517613134082864
106	KC_AB1	emc	-0.0498024178368604	0.78416287081146	0.994766463583882	pseudobulk	DESeq2	LRT	0.105593724881316
106	KC_AB1	EMC1	-0.111370376454852	0.865438221166441	0.994766463583882	pseudobulk	DESeq2	LRT	0.0627639285559129
106	KC_AB1	EMC3	0.174228785671055	0.786198610113744	0.994766463583882	pseudobulk	DESeq2	LRT	0.104467728285537
106	KC_AB1	EMC8-9	0.264994785387831	0.587016097850767	0.994766463583882	pseudobulk	DESeq2	LRT	0.231349988851368
106	KC_AB1	EMRE	-0.158269392381665	0.666287318669885	0.994766463583882	pseudobulk	DESeq2	LRT	0.176338452504192
106	KC_AB1	ena	-0.0134671019664496	0.93656979305679	0.994766463583882	pseudobulk	DESeq2	LRT	0.0284598535156155
106	KC_AB1	enc	-0.154875036863622	0.471896603087896	0.994766463583882	pseudobulk	DESeq2	LRT	0.326153148881869
106	KC_AB1	EndoA	-0.255846519684647	0.265991729234374	0.994766463583882	pseudobulk	DESeq2	LRT	0.575131867142302

106	KC_AB1	EndoB	-0.103371016461104	0.825905203436547	0.994766463583882	pseudobulk	DESeq2	LRT	0.0830697977002288
106	KC_AB1	EndoG	0.106762010381708	0.892771793231278	0.994766463583882	pseudobulk	DESeq2	LRT	0.0492595395506762
106	KC_AB1	EndoGI	1.36313299283604	0.448096172039906	0.994766463583882	pseudobulk	DESeq2	LRT	0.34862876612723
106	KC_AB1	endos	-0.181825713011029	0.617625837150457	0.994766463583882	pseudobulk	DESeq2	LRT	0.209274544445044
106	KC_AB1	Eno	-0.0645952373097655	0.737282177018695	0.994766463583882	pseudobulk	DESeq2	LRT	0.132366264558843
107	KC_AB1	enok	-0.0539187523018235	0.884880540055835	0.994766463583882	pseudobulk	DESeq2	LRT	0.0531153556257756
107	KC_AB1	ens	-0.0417560989767378	0.892239369418879	0.994766463583882	pseudobulk	DESeq2	LRT	0.049518617740723
107	KC_AB1	Ent1	0.175945050470004	0.880942484660225	0.994766463583882	pseudobulk	DESeq2	LRT	0.0550524450613662
107	KC_AB1	Ent2	0.360964907139484	0.733780007671144	0.994766463583882	pseudobulk	DESeq2	LRT	0.134434125063557
107	KC_AB1	Ent3	-0.361732958275508	0.414718338993177	0.994766463583882	pseudobulk	DESeq2	LRT	0.382246759554138
107	KC_AB1	Eo	-1.64952313825113	0.817565084244375	0.994766463583882	pseudobulk	DESeq2	LRT	0.0874776642296574
107	KC_AB1	Eogt	-1.19258227719509	0.47997523657998	0.994766463583882	pseudobulk	DESeq2	LRT	0.318781168653778
107	KC_AB1	Epac	-3.21143241058256	0.292545415061155	0.994766463583882	pseudobulk	DESeq2	LRT	0.533806704011112
107	KC_AB1	Epg5	-0.455493926528657	0.670812679912079	0.994766463583882	pseudobulk	DESeq2	LRT	0.17339873681759
107	KC_AB1	Eph	-0.0200822103798712	0.893451707039043	0.994766463583882	pseudobulk	DESeq2	LRT	0.048928917062395
107	KC_AB1	Ephrin	0.0210207334380034	0.876798344315425	0.994766463583882	pseudobulk	DESeq2	LRT	0.0571002789502096
107	KC_AB1	Eps-15	-0.102626165092751	0.797927949092771	0.994766463583882	pseudobulk	DESeq2	LRT	0.0980363225893044
107	KC_AB1	epsilonCOP	0.286849413853872	0.746191864403378	0.994766463583882	pseudobulk	DESeq2	LRT	0.127149490304545
107	KC_AB1	Ercc1	1.35760970987709	0.457420741813013	0.994766463583882	pseudobulk	DESeq2	LRT	0.339684146133517
107	KC_AB1	eRF1	0.0551493469157326	0.895577998140053	0.994766463583882	pseudobulk	DESeq2	LRT	0.0478965843691785
107	KC_AB1	eRF3	-1.03118064624262	0.220186582247638	0.994766463583882	pseudobulk	DESeq2	LRT	0.65720914963928
107	KC_AB1	ergic53	-0.23014405288163	0.809370477190142	0.994766463583882	pseudobulk	DESeq2	LRT	0.0918526410949965
107	KC_AB1	Ero1L	-1.02403223006933	0.208917092254471	0.994766463583882	pseudobulk	DESeq2	LRT	0.680026027381407
107	KC_AB1	ERp60	0.335454039786283	0.586594111857951	0.994766463583882	pseudobulk	DESeq2	LRT	0.231662300715226
107	KC_AB1	ERR	0.317613836045286	0.690683100203827	0.994766463583882	pseudobulk	DESeq2	LRT	0.160721170283146
107	KC_AB1	Es2	0.396856567510196	0.704439271254455	0.994766463583882	pseudobulk	DESeq2	LRT	0.152156440873845
107	KC_AB1	esc	2.38929733117871	0.484296857543297	0.994766463583882	pseudobulk	DESeq2	LRT	0.314888348950639
107	KC_AB1	escl	-3.21143241058256	0.292545415061155	0.994766463583882	pseudobulk	DESeq2	LRT	0.533806704011112
107	KC_AB1	esn	-0.134650316865125	0.937764244880731	0.994766463583882	pseudobulk	DESeq2	LRT	0.0279063300819481
107	KC_AB1	Est-6	-2.20969975456663	0.610836134188693	0.994766463583882	pseudobulk	DESeq2	LRT	0.214075280039667
107	KC_AB1	Esyt2	-0.828123177839367	0.0757674967520534	0.994766463583882	pseudobulk	DESeq2	LRT	1.12051706097188
107	KC_AB1	Etf-QO	1.76506590371453	0.282399918193823	0.994766463583882	pseudobulk	DESeq2	LRT	0.549135433427516
107	KC_AB1	ETHR	-3.28760177386367	0.140404830791884	0.994766463583882	pseudobulk	DESeq2	LRT	0.852617949541211
107	KC_AB1	Etl1	-0.134704154766894	0.889996265452921	0.994766463583882	pseudobulk	DESeq2	LRT	0.050611815710808
107	KC_AB1	Ets97D	0.861285897944506	0.5339815985864	0.994766463583882	pseudobulk	DESeq2	LRT	0.272473708833022
107	KC_AB1	Ets98B	2.22023857837763	0.524904842126482	0.994766463583882	pseudobulk	DESeq2	LRT	0.279919420946639
107	KC_AB1	Evi5	0.0412295675610194	0.875996062096663	0.994766463583882	pseudobulk	DESeq2	LRT	0.0574978461304737
107	KC_AB1	ewg	0.364883679975879	0.362605128707496	0.994766463583882	pseudobulk	DESeq2	LRT	0.440566057511298
107	KC_AB1	exd	-0.0232337054217678	0.892214248323913	0.994766463583882	pseudobulk	DESeq2	LRT	0.0495308455204929
107	KC_AB1	Exd2	-0.48167422725367	0.855534332799682	0.994766463583882	pseudobulk	DESeq2	LRT	0.0677625574305792
107	KC_AB1	Exn	-0.213527536755599	0.739670070687939	0.994766463583882	pseudobulk	DESeq2	LRT	0.130961953824851
107	KC_AB1	Exo70	0.6586442882962	0.582684673025481	0.994766463583882	pseudobulk	DESeq2	LRT	0.234566405469147
107	KC_AB1	Exo84	0.175060010868176	0.827998227780265	0.994766463583882	pseudobulk	DESeq2	LRT	0.0819705927635198
107	KC_AB1	ey	0.0715039266522195	0.69516193319749	0.994766463583882	pseudobulk	DESeq2	LRT	0.157914017708576
107	KC_AB1	eys	-0.0738041917680781	0.816383706206449	0.994766463583882	pseudobulk	DESeq2	LRT	0.088105671735541
107	KC_AB1	f-cup	0.051336785297812	0.949444476012169	0.994766463583882	pseudobulk	DESeq2	LRT	0.0225304279428742
107	KC_AB1	fabp	-0.140427698789604	0.810973266699084	0.994766463583882	pseudobulk	DESeq2	LRT	0.0909934618386952
107	KC_AB1	Fadd	1.22463728031698	0.574887253091764	0.994766463583882	pseudobulk	DESeq2	LRT	0.240417320808332
107	KC_AB1	faf	-0.0872183822569579	0.77280750989351	0.994766463583882	pseudobulk	DESeq2	LRT	0.111928666238702
107	KC_AB1	Faf2	-0.0231589998862324	0.915384164360725	0.994766463583882	pseudobulk	DESeq2	LRT	0.0383966048978403
107	KC_AB1	Fak	0.302299019799749	0.471991578069919	0.994766463583882	pseudobulk	DESeq2	LRT	0.326065750582846
107	KC_AB1	fal	-0.729024381689453	0.602472956983519	0.994766463583882	pseudobulk	DESeq2	LRT	0.220062442357105
107	KC_AB1	FAM21	-1.00711434915111	0.25722213765376	0.994766463583882	pseudobulk	DESeq2	LRT	0.589691656870927
107	KC_AB1	Fancm	3.38870912535198	0.214276667025744	0.994766463583882	pseudobulk	DESeq2	LRT	0.669025117496047
107	KC_AB1	fand	0.144623388058199	0.885140992614632	0.994766463583882	pseudobulk	DESeq2	LRT	0.0529875457570119
107	KC_AB1	Fas1	0.102364400571275	0.506384857863144	0.994766463583882	pseudobulk	DESeq2	LRT	0.295519289256106

107	KC_AB1	Fas2	0.172510224450693	0.273294296355791	0.994766463583882	pseudobulk	DESeq2	LRT	0.563369431926273
107	KC_AB1	Fas3	1.29038439336772	0.786252121946572	0.994766463583882	pseudobulk	DESeq2	LRT	0.104438169465983
107	KC_AB1	FASN1	0.11856563171458	0.761627864006911	0.994766463583882	pseudobulk	DESeq2	LRT	0.118257175753988
107	KC_AB1	FASN3	-2.57435989741145	0.533617212681275	0.994766463583882	pseudobulk	DESeq2	LRT	0.272770169998685
107	KC_AB1	fat-spondin	1.32710828904082	0.607473293222376	0.994766463583882	pseudobulk	DESeq2	LRT	0.216472810505135
107	KC_AB1	Fatp1	-0.0391379898983419	0.955947042801905	0.994766463583882	pseudobulk	DESeq2	LRT	0.0195661659414141
107	KC_AB1	fax	0.0243632116915273	0.881674342834432	0.994766463583882	pseudobulk	DESeq2	LRT	0.0546917972110777
107	KC_AB1	fbl	0.292420794219599	0.75808542342928	0.994766463583882	pseudobulk	DESeq2	LRT	0.120281853954131
107	KC_AB1	Fbl6	-0.271990760456816	0.622609886937864	0.994766463583882	pseudobulk	DESeq2	LRT	0.205783987077107
107	KC_AB1	fbp	-2.64181948300102	0.294606194330082	0.994766463583882	pseudobulk	DESeq2	LRT	0.530758126009708
107	KC_AB1	Fbw5	-1.34055899623702	0.426436891983479	0.994766463583882	pseudobulk	DESeq2	LRT	0.370145230595502
107	KC_AB1	Fbxl4	-0.360081731541115	0.715615236661111	0.994766463583882	pseudobulk	DESeq2	LRT	0.145320421140723
107	KC_AB1	FBXO11	-0.299892952519213	0.547609906767105	0.994766463583882	pseudobulk	DESeq2	LRT	0.261528703680178
107	KC_AB1	Fcp1	0.633289884870054	0.458501222602157	0.994766463583882	pseudobulk	DESeq2	LRT	0.33865950193799
107	KC_AB1	Fdh	-1.02319198676748	0.828204539793902	0.994766463583882	pseudobulk	DESeq2	LRT	0.0818623932467813
107	KC_AB1	fdl	0.0910551144450977	0.821329688509063	0.994766463583882	pseudobulk	DESeq2	LRT	0.0854824784966503
107	KC_AB1	Fdx1	0.334876607744101	0.762776887162015	0.994766463583882	pseudobulk	DESeq2	LRT	0.117602474947051
107	KC_AB1	Fdx2	-0.172055688206032	0.814184388453835	0.994766463583882	pseudobulk	DESeq2	LRT	0.0892772292390491
107	KC_AB1	FeCH	1.05319368540965	0.359207458530758	0.994766463583882	pseudobulk	DESeq2	LRT	0.444654654284461
107	KC_AB1	Fem-1	0.343385265804775	0.6048254633125	0.994766463583882	pseudobulk	DESeq2	LRT	0.218369933209029
107	KC_AB1	Fen1	-2.20969975456663	0.610836134188693	0.994766463583882	pseudobulk	DESeq2	LRT	0.214075280039667
107	KC_AB1	fend	-0.188949228183682	0.264853769426583	0.994766463583882	pseudobulk	DESeq2	LRT	0.576993841759852
107	KC_AB1	Fer1HCH	-0.0947770210611467	0.671513491525719	0.994766463583882	pseudobulk	DESeq2	LRT	0.172945257401354
107	KC_AB1	Fer2LCH	-0.118713181138915	0.568894379502498	0.994766463583882	pseudobulk	DESeq2	LRT	0.24496835690389
107	KC_AB1	fest	-0.242161866810022	0.835551891379217	0.994766463583882	pseudobulk	DESeq2	LRT	0.0780265733710181
107	KC_AB1	Fgop2	0.587376727163475	0.531710971931122	0.994766463583882	pseudobulk	DESeq2	LRT	0.274324377865287
107	KC_AB1	fh	0.296445471922966	0.835985473040973	0.994766463583882	pseudobulk	DESeq2	LRT	0.0778012692511267
107	KC_AB1	Fhos	-0.874780316428275	0.681205769332186	0.994766463583882	pseudobulk	DESeq2	LRT	0.166721682526051
107	KC_AB1	Fib	0.402369316739516	0.765676178280126	0.994766463583882	pseudobulk	DESeq2	LRT	0.115954864467281
107	KC_AB1	Fibp	0.132997077483568	0.807956227565612	0.994766463583882	pseudobulk	DESeq2	LRT	0.0926121672472678
107	KC_AB1	Fic	-0.533272795926953	0.64213739278252	0.994766463583882	pseudobulk	DESeq2	LRT	0.192372039620532
107	KC_AB1	fid	0.952070976038612	0.227220752919156	0.994766463583882	pseudobulk	DESeq2	LRT	0.643552005414053
107	KC_AB1	fidipidine	1.30206458106543	0.461276364479832	0.994766463583882	pseudobulk	DESeq2	LRT	0.336038797806565
107	KC_AB1	Fie	-0.175544208067704	0.756747602649932	0.994766463583882	pseudobulk	DESeq2	LRT	0.121048946199014
107	KC_AB1	Fife	-0.155596902061578	0.359125548018376	0.994766463583882	pseudobulk	DESeq2	LRT	0.44475369827483
107	KC_AB1	FIG4	-0.654631659528721	0.683604889040291	0.994766463583882	pseudobulk	DESeq2	LRT	0.165194839930781
107	KC_AB1	Fim	-0.168344100687893	0.577922031340475	0.994766463583882	pseudobulk	DESeq2	LRT	0.238130749196501
107	KC_AB1	Fip1	0.149434418704316	0.795287999759526	0.994766463583882	pseudobulk	DESeq2	LRT	0.0994755706436086
107	KC_AB1	fipi	0.271012531716479	0.534120386646875	0.994766463583882	pseudobulk	DESeq2	LRT	0.272360845286299
107	KC_AB1	Fis1	-0.159040131111807	0.512302755583308	0.994766463583882	pseudobulk	DESeq2	LRT	0.290473308126379
107	KC_AB1	Fit1	0.30050740708198	0.685125595720101	0.994766463583882	pseudobulk	DESeq2	LRT	0.164229807298375
107	KC_AB1	Fitm	0.423699245037111	0.765440172869709	0.994766463583882	pseudobulk	DESeq2	LRT	0.116088748281112
107	KC_AB1	fiz	-3.21143241058256	0.292545415061155	0.994766463583882	pseudobulk	DESeq2	LRT	0.533806704011112
107	KC_AB1	Fkbp12	0.120520900283987	0.544384856655342	0.994766463583882	pseudobulk	DESeq2	LRT	0.264093964222734
107	KC_AB1	Fkbp14	-1.64952313825113	0.817565084244375	0.994766463583882	pseudobulk	DESeq2	LRT	0.0874776642296574
107	KC_AB1	Fkbp39	0.237923254970439	0.747555953918305	0.994766463583882	pseudobulk	DESeq2	LRT	0.126356295219582
107	KC_AB1	Fkbp59	0.138934334659647	0.827024447046009	0.994766463583882	pseudobulk	DESeq2	LRT	0.082481652406032
107	KC_AB1	fl(2)d	0.0428216358002503	0.897353820175129	0.994766463583882	pseudobulk	DESeq2	LRT	0.0470362839885495
107	KC_AB1	Flacc	0.288937819438539	0.607399078681753	0.994766463583882	pseudobulk	DESeq2	LRT	0.216525871166081
108	KC_AB1	FLASH	-0.313808595631466	0.749159733319303	0.994766463583882	pseudobulk	DESeq2	LRT	0.125425573618202
108	KC_AB1	flfl	-0.219149283890459	0.538784178953692	0.994766463583882	pseudobulk	DESeq2	LRT	0.268585165536264
108	KC_AB1	flil	-0.290989388163588	0.744105984277256	0.994766463583882	pseudobulk	DESeq2	LRT	0.128365202748913
108	KC_AB1	Flo1	-0.00034420700910662	0.934343445419331	0.994766463583882	pseudobulk	DESeq2	LRT	0.0294934567103023
108	KC_AB1	Flo2	-0.322281710897326	0.306161636591516	0.994766463583882	pseudobulk	DESeq2	LRT	0.514049229249834
108	KC_AB1	flr	0.63683485359238	0.63069318805348	0.994766463583882	pseudobulk	DESeq2	LRT	0.20018185967857
108	KC_AB1	flw	0.0710621273412892	0.77473756319673	0.994766463583882	pseudobulk	DESeq2	LRT	0.11084538672906

108	KC_AB1	Fmr1	0.205118989099747	0.634684388074009	0.994766463583882	pseudobulk	DESeq2	LRT	0.197442184302552
108	KC_AB1	FMRFaR	0.523735058473132	0.547630483496703	0.994766463583882	pseudobulk	DESeq2	LRT	0.261512385143717
108	KC_AB1	fmt	-0.258497636915974	0.428309107245373	0.994766463583882	pseudobulk	DESeq2	LRT	0.368242690991336
108	KC_AB1	fne	-0.0749397123428402	0.576630478874888	0.994766463583882	pseudobulk	DESeq2	LRT	0.239102405899694
108	KC_AB1	fng	0.949183608371002	0.80156895525114	0.994766463583882	pseudobulk	DESeq2	LRT	0.0960591113673091
108	KC_AB1	Fnta	0.193258739405246	0.781488028833807	0.994766463583882	pseudobulk	DESeq2	LRT	0.107077670301531
108	KC_AB1	fog	1.13812620529531	0.339699752370357	0.994766463583882	pseudobulk	DESeq2	LRT	0.468904769715861
108	KC_AB1	foi	-0.297459945106477	0.632578693381559	0.994766463583882	pseudobulk	DESeq2	LRT	0.198885440151329
108	KC_AB1	fok	-0.11040774498758	0.630737237875828	0.994766463583882	pseudobulk	DESeq2	LRT	0.200151528089306
108	KC_AB1	form3	1.80633728195307	0.61161102402172	0.994766463583882	pseudobulk	DESeq2	LRT	0.213524695220716
108	KC_AB1	FoxK	-0.371041262505845	0.211339827637241	0.994766463583882	pseudobulk	DESeq2	LRT	0.675018651110726
108	KC_AB1	FoxP	-0.0111674963517617	0.891191653630568	0.994766463583882	pseudobulk	DESeq2	LRT	0.0500288894943613
108	KC_AB1	Fpgs	-2.45516412093746	0.350354315181257	0.994766463583882	pseudobulk	DESeq2	LRT	0.455492529097001
108	KC_AB1	Fpps	1.62276326474024	0.438522978781363	0.994766463583882	pseudobulk	DESeq2	LRT	0.35800764449526
108	KC_AB1	fra	0.657856549006229	0.681354454667751	0.994766463583882	pseudobulk	DESeq2	LRT	0.166626900336031
108	KC_AB1	fray	0.423943494945989	0.68941729891669	0.994766463583882	pseudobulk	DESeq2	LRT	0.161517823437783
108	KC_AB1	fred	-1.64952313825113	0.817565084244375	0.994766463583882	pseudobulk	DESeq2	LRT	0.0874776642296574
108	KC_AB1	FRG1	0.201409857843024	0.797776206542712	0.994766463583882	pseudobulk	DESeq2	LRT	0.0981189205472358
108	KC_AB1	frj	0.0242275940169364	0.909077552382668	0.994766463583882	pseudobulk	DESeq2	LRT	0.0413990660242542
108	KC_AB1	Frl	-0.379079299748176	0.306908705945238	0.994766463583882	pseudobulk	DESeq2	LRT	0.512990791951527
108	KC_AB1	frma	0.949183608371002	0.80156895525114	0.994766463583882	pseudobulk	DESeq2	LRT	0.0960591113673091
108	KC_AB1	Frq1	-1.4316251896547	0.00451046722165096	0.994766463583882	pseudobulk	DESeq2	LRT	2.3457784689258
108	KC_AB1	Frq2	-0.261990111194344	0.738455491667571	0.994766463583882	pseudobulk	DESeq2	LRT	0.131675675444359
108	KC_AB1	frtz	0.00891646089885312	0.960226720676327	0.994766463583882	pseudobulk	DESeq2	LRT	0.0176262128838119
108	KC_AB1	fru	0.0632790197680773	0.876915428591101	0.994766463583882	pseudobulk	DESeq2	LRT	0.0570422888087113
108	KC_AB1	fry	0.201962323139454	0.750267450180499	0.994766463583882	pseudobulk	DESeq2	LRT	0.124783894698224
108	KC_AB1	Fs	-1.64952313825113	0.817565084244375	0.994766463583882	pseudobulk	DESeq2	LRT	0.0874776642296574
108	KC_AB1	fs(1)h	0.0590419219918274	0.703725040781253	0.994766463583882	pseudobulk	DESeq2	LRT	0.152596995114947
108	KC_AB1	fs(1)K10	0.0485142620298545	0.960760487786471	0.994766463583882	pseudobulk	DESeq2	LRT	0.0173848660226756
108	KC_AB1	Fs(2)Ket	0.168734072374005	0.302579699894325	0.994766463583882	pseudobulk	DESeq2	LRT	0.519160212200509
108	KC_AB1	fs(2)ltoPP43	-0.525817998980722	0.493621478132478	0.994766463583882	pseudobulk	DESeq2	LRT	0.306605951828379
108	KC_AB1	fsd	-0.58120564886084	0.720744071653983	0.994766463583882	pseudobulk	DESeq2	LRT	0.142218921133373
108	KC_AB1	Fsn	0.280438690304955	0.659725178135168	0.994766463583882	pseudobulk	DESeq2	LRT	0.180636940936213
108	KC_AB1	ftz-f1	-0.305296151171217	0.17089062520022	0.994766463583882	pseudobulk	DESeq2	LRT	0.767281761360524
108	KC_AB1	fu	1.24199384524128	0.46412512976611	0.994766463583882	pseudobulk	DESeq2	LRT	0.333364916340097
108	KC_AB1	FucT6	0.211518585293025	0.756966893328905	0.994766463583882	pseudobulk	DESeq2	LRT	0.120923114369128
108	KC_AB1	FucTA	0.0600067379123136	0.909566990655609	0.994766463583882	pseudobulk	DESeq2	LRT	0.0411653091341398
108	KC_AB1	FucTB	-0.124428387125022	0.859750925711173	0.994766463583882	pseudobulk	DESeq2	LRT	0.0656273478920639
108	KC_AB1	FucTC	1.1821590793593	0.719511699510136	0.994766463583882	pseudobulk	DESeq2	LRT	0.142962139890846
108	KC_AB1	Fum1	0.396839909242201	0.453946959831521	0.994766463583882	pseudobulk	DESeq2	LRT	0.342994888108029
108	KC_AB1	Fum2	1.80633728195307	0.61161102402172	0.994766463583882	pseudobulk	DESeq2	LRT	0.213524695220716
108	KC_AB1	Fur1	0.118098406705595	0.66537656613947	0.994766463583882	pseudobulk	DESeq2	LRT	0.176932498591228
108	KC_AB1	Fur2	0.520505628413351	0.373578671434543	0.994766463583882	pseudobulk	DESeq2	LRT	0.427617926721122
108	KC_AB1	fuss	1.82628216844872	0.306752685105949	0.994766463583882	pseudobulk	DESeq2	LRT	0.513211627063572
108	KC_AB1	futsch	-0.13642041749511	0.302412119025118	0.994766463583882	pseudobulk	DESeq2	LRT	0.519400808672575
108	KC_AB1	fwd	0.0235353061493543	0.946175285034013	0.994766463583882	pseudobulk	DESeq2	LRT	0.0240284003093653
108	KC_AB1	fwe	0.05375003331828	0.816240274846292	0.994766463583882	pseudobulk	DESeq2	LRT	0.0881819801190163
108	KC_AB1	fws	0.0399585145231285	0.939467239375386	0.994766463583882	pseudobulk	DESeq2	LRT	0.0271183597960925
108	KC_AB1	fy	1.59417945971126	0.340492614386521	0.994766463583882	pseudobulk	DESeq2	LRT	0.467892303915561
108	KC_AB1	fz	-0.374869210552086	0.0564229696019839	0.994766463583882	pseudobulk	DESeq2	LRT	1.2485440601903
108	KC_AB1	fz2	0.00539999574048975	0.812907577896956	0.994766463583882	pseudobulk	DESeq2	LRT	0.0899588279496026
108	KC_AB1	fz3	0.330312974792486	0.85583656066734	0.994766463583882	pseudobulk	DESeq2	LRT	0.0676091647357904
108	KC_AB1	fz4	1.66002234014917	0.198378529253932	0.994766463583882	pseudobulk	DESeq2	LRT	0.702505333850838
108	KC_AB1	fzr2	-2.79499059556516	0.38564607643653	0.994766463583882	pseudobulk	DESeq2	LRT	0.413811082791365
108	KC_AB1	fzy	0.806933541094274	0.754600478929312	0.994766463583882	pseudobulk	DESeq2	LRT	0.122282923497213
108	KC_AB1	g	0.296599776126521	0.255921487269758	0.994766463583882	pseudobulk	DESeq2	LRT	0.59189324904472

108	KC_AB1	G6P	1.00551886423414	0.482893570735624	0.994766463583882	pseudobulk	DESeq2	LRT	0.316148576775319
108	KC_AB1	G9a	0.262763341675474	0.508702227600633	0.994766463583882	pseudobulk	DESeq2	LRT	0.293536360594427
108	KC_AB1	GAA1	0.491252870950706	0.634537559610235	0.994766463583882	pseudobulk	DESeq2	LRT	0.19754266600633
108	KC_AB1	GABA-B-R1	-0.207376921847896	0.379017983084146	0.994766463583882	pseudobulk	DESeq2	LRT	0.421340183781448
108	KC_AB1	GABA-B-R2	0.122794744806826	0.739635950772503	0.994766463583882	pseudobulk	DESeq2	LRT	0.130981987666104
108	KC_AB1	Gabat	0.0944339647768373	0.797090594443134	0.994766463583882	pseudobulk	DESeq2	LRT	0.098492315453608
108	KC_AB1	GABPI	0.132128143900525	0.815844271805058	0.994766463583882	pseudobulk	DESeq2	LRT	0.0883927313810788
108	KC_AB1	Gale	0.526184102184569	0.631908732684761	0.994766463583882	pseudobulk	DESeq2	LRT	0.199345642846808
108	KC_AB1	galectin	-0.0900210320281324	0.845175928846393	0.994766463583882	pseudobulk	DESeq2	LRT	0.0730528804295338
108	KC_AB1	galla-1	0.667354098974816	0.415819044693893	0.994766463583882	pseudobulk	DESeq2	LRT	0.381095623672043
108	KC_AB1	galla-2	0.6937727719011	0.412321481359454	0.994766463583882	pseudobulk	DESeq2	LRT	0.384764038487871
108	KC_AB1	Galphai	1.1821590793593	0.719511699510136	0.994766463583882	pseudobulk	DESeq2	LRT	0.142962139890846
108	KC_AB1	Galphao	0.0192393780689893	0.895993102042445	0.994766463583882	pseudobulk	DESeq2	LRT	0.0476953338160381
108	KC_AB1	Galphaq	0.0969015669337654	0.717038864484518	0.994766463583882	pseudobulk	DESeq2	LRT	0.144457304340934
108	KC_AB1	Galphas	0.0452573726466513	0.853390323667801	0.994766463583882	pseudobulk	DESeq2	LRT	0.0688522857812575
108	KC_AB1	Galt	0.152814260965494	0.848709196599957	0.994766463583882	pseudobulk	DESeq2	LRT	0.0712410917894434
108	KC_AB1	GalT1	-0.0419263615007874	0.937314592536924	0.994766463583882	pseudobulk	DESeq2	LRT	0.0281146216284548
108	KC_AB1	gammaCOP	0.33899474858927	0.71182438337982	0.994766463583882	pseudobulk	DESeq2	LRT	0.147627139420214
108	KC_AB1	gammaSnap1	0.458355286137577	0.167370967183913	0.994766463583882	pseudobulk	DESeq2	LRT	0.776319874212188
108	KC_AB1	gammaSnap2	0.414048848961226	0.540100559091082	0.994766463583882	pseudobulk	DESeq2	LRT	0.267525373153826
108	KC_AB1	gammaTub23C	0.300905112836808	0.761770040896363	0.994766463583882	pseudobulk	DESeq2	LRT	0.118176111393162
108	KC_AB1	GAPcenA	0.444847001906184	0.373508491668121	0.994766463583882	pseudobulk	DESeq2	LRT	0.427699520106455
108	KC_AB1	Gapdh1	0.0600416156717817	0.882579969210389	0.994766463583882	pseudobulk	DESeq2	LRT	0.0542459334037974
108	KC_AB1	Gapdh2	-0.0141473652191902	0.946469648363784	0.994766463583882	pseudobulk	DESeq2	LRT	0.0238933085459084
108	KC_AB1	GAPsec	0.205511634286069	0.845489900058704	0.994766463583882	pseudobulk	DESeq2	LRT	0.0728915759727697
108	KC_AB1	Gapvd1	-0.54197755628198	0.42060003117794	0.994766463583882	pseudobulk	DESeq2	LRT	0.376130699456624
108	KC_AB1	garz	0.466624627314643	0.610298189938853	0.994766463583882	pseudobulk	DESeq2	LRT	0.214457918099664
108	KC_AB1	Gas41	0.259689270532043	0.766863755127236	0.994766463583882	pseudobulk	DESeq2	LRT	0.115281788140162
108	KC_AB1	Gas8	0.949183608371002	0.80156895525114	0.994766463583882	pseudobulk	DESeq2	LRT	0.0960591113673091
108	KC_AB1	Gasz	0.0295740624463319	0.929070218640702	0.994766463583882	pseudobulk	DESeq2	LRT	0.0319514610163311
108	KC_AB1	GatA	-0.304092593891143	0.801319454331535	0.994766463583882	pseudobulk	DESeq2	LRT	0.0961943133849187
108	KC_AB1	GATAd	-0.162406230935866	0.714234438463227	0.994766463583882	pseudobulk	DESeq2	LRT	0.146159213126772
108	KC_AB1	GatB	-0.405117477040171	0.700148525739994	0.994766463583882	pseudobulk	DESeq2	LRT	0.154809821318515
108	KC_AB1	gb	0.087356802117797	0.960952460588087	0.994766463583882	pseudobulk	DESeq2	LRT	0.0172980968423301
108	KC_AB1	Gba1a	-2.20969975456663	0.610836134188693	0.994766463583882	pseudobulk	DESeq2	LRT	0.214075280039667
108	KC_AB1	gbb	1.10251292103259	0.639221751231493	0.994766463583882	pseudobulk	DESeq2	LRT	0.194348455410132
108	KC_AB1	Gbeta13F	0.0850330223927586	0.542023169422355	0.994766463583882	pseudobulk	DESeq2	LRT	0.26598214863281
109	KC_AB1	Gbeta5	0.118673643922917	0.741221524485775	0.994766463583882	pseudobulk	DESeq2	LRT	0.130051977602374
109	KC_AB1	Gbs-76A	1.53842291025208	0.23479859528084	0.994766463583882	pseudobulk	DESeq2	LRT	0.629304505651018
109	KC_AB1	GC	-0.53186705886107	0.1953152627075	0.994766463583882	pseudobulk	DESeq2	LRT	0.709263817896731
109	KC_AB1	GC1	-0.120890155369706	0.869459490308303	0.994766463583882	pseudobulk	DESeq2	LRT	0.0607506477560465
109	KC_AB1	GC2	0.949183608371002	0.80156895525114	0.994766463583882	pseudobulk	DESeq2	LRT	0.0960591113673091
109	KC_AB1	GCC185	-0.39510402151852	0.72799478108312	0.994766463583882	pseudobulk	DESeq2	LRT	0.137871734086588
109	KC_AB1	GCC88	1.04700356023225	0.264109030264038	0.994766463583882	pseudobulk	DESeq2	LRT	0.578216749392109
109	KC_AB1	gce	0.344911389267349	0.263406547966232	0.994766463583882	pseudobulk	DESeq2	LRT	0.579373433207735
109	KC_AB1	GckIII	0.331289952271735	0.583951789025491	0.994766463583882	pseudobulk	DESeq2	LRT	0.233623006696645
109	KC_AB1	gcl	-0.384897126102517	0.529858191790864	0.994766463583882	pseudobulk	DESeq2	LRT	0.275840346933764
109	KC_AB1	Gclc	-0.125660320042221	0.795089878633151	0.994766463583882	pseudobulk	DESeq2	LRT	0.0995837750062915
109	KC_AB1	Gclm	0.108360928964317	0.882803547119267	0.994766463583882	pseudobulk	DESeq2	LRT	0.0541359305044587
109	KC_AB1	Gcn2	0.603169023602823	0.582188289234911	0.994766463583882	pseudobulk	DESeq2	LRT	0.234936534678397
109	KC_AB1	Gcn5	-0.634603671970339	0.531082734873955	0.994766463583882	pseudobulk	DESeq2	LRT	0.274837816959415
109	KC_AB1	GCS1	0.355051265059293	0.796944328859106	0.994766463583882	pseudobulk	DESeq2	LRT	0.0985720155094666
109	KC_AB1	GCS2alpha	-0.0824933115349995	0.829212307053699	0.994766463583882	pseudobulk	DESeq2	LRT	0.0813342607920625
109	KC_AB1	GCS2beta	0.529511386269915	0.645769346945158	0.994766463583882	pseudobulk	DESeq2	LRT	0.189922573701825
109	KC_AB1	Gdap1	1.1821590793593	0.719511699510136	0.994766463583882	pseudobulk	DESeq2	LRT	0.142962139890846
109	KC_AB1	Gdap2	-0.142707234525574	0.333451359729909	0.994766463583882	pseudobulk	DESeq2	LRT	0.476967507299182

109	KC_AB1	Gdh	-0.348501188235399	0.220926977468125	0.994766463583882	pseudobulk	DESeq2	LRT	0.655751249043998
109	KC_AB1	Gdi	0.0634815919924139	0.7995662171118	0.994766463583882	pseudobulk	DESeq2	LRT	0.0971455637683716
109	KC_AB1	gdl	-0.141608251839736	0.872052832183544	0.994766463583882	pseudobulk	DESeq2	LRT	0.059457203105589
109	KC_AB1	Gdn1	0.5430737767836	0.723065977006754	0.994766463583882	pseudobulk	DESeq2	LRT	0.140822073185874
109	KC_AB1	Ge-1	-0.446191094980955	0.564918760940079	0.994766463583882	pseudobulk	DESeq2	LRT	0.248014002113635
109	KC_AB1	gek	-0.193591230771301	0.821637318779904	0.994766463583882	pseudobulk	DESeq2	LRT	0.0853198433006162
109	KC_AB1	Gel	-2.38885517880981	0.126962133482763	0.994766463583882	pseudobulk	DESeq2	LRT	0.896325788269542
109	KC_AB1	Gem2	0.476553029882346	0.733207148458403	0.994766463583882	pseudobulk	DESeq2	LRT	0.134773309502368
109	KC_AB1	Gem3	-0.144621754554915	0.892052115737041	0.994766463583882	pseudobulk	DESeq2	LRT	0.0496097723998283
109	KC_AB1	geminin	-0.416813612714362	0.650896987300669	0.994766463583882	pseudobulk	DESeq2	LRT	0.186487738600571
109	KC_AB1	Gfat1	0.217906885051292	0.902008941520292	0.994766463583882	pseudobulk	DESeq2	LRT	0.0447891573209458
109	KC_AB1	Gfat2	-0.669973481537783	0.388386063389422	0.994766463583882	pseudobulk	DESeq2	LRT	0.410736362444936
109	KC_AB1	Gfrl	-0.0723894146041463	0.6174704229218	0.994766463583882	pseudobulk	DESeq2	LRT	0.209383840448041
109	KC_AB1	gfzf	-0.370284509002658	0.775254933705214	0.994766463583882	pseudobulk	DESeq2	LRT	0.110555461241385
109	KC_AB1	Gga	1.40357679012009	0.447970528345062	0.994766463583882	pseudobulk	DESeq2	LRT	0.348750556979833
109	KC_AB1	Ggamma1	-0.0231768859895818	0.877129471179501	0.994766463583882	pseudobulk	DESeq2	LRT	0.0569362966354116
109	KC_AB1	Ggamma30A	0.0252735180586929	0.869621175522398	0.994766463583882	pseudobulk	DESeq2	LRT	0.0606698935991303
109	KC_AB1	Ggt-1	-0.835152883801839	0.920434724857442	0.994766463583882	pseudobulk	DESeq2	LRT	0.0360070052473159
109	KC_AB1	ghi	-0.170613141531775	0.694728683290698	0.994766463583882	pseudobulk	DESeq2	LRT	0.158184770021984
109	KC_AB1	gig	0.0152134954449985	0.845725642767136	0.994766463583882	pseudobulk	DESeq2	LRT	0.0727705012126439
109	KC_AB1	GIIIspla2	0.205922444895413	0.864205897628881	0.994766463583882	pseudobulk	DESeq2	LRT	0.0633827742444837
109	KC_AB1	GILT1	-1.56339566464445	0.2199640330425	0.994766463583882	pseudobulk	DESeq2	LRT	0.657648326123974
109	KC_AB1	Gip	-1.64952313825113	0.817565084244375	0.994766463583882	pseudobulk	DESeq2	LRT	0.0874776642296574
109	KC_AB1	Girdin	-0.518482740728574	0.523457204718773	0.994766463583882	pseudobulk	DESeq2	LRT	0.281118818310272
109	KC_AB1	gish	-0.125665837697206	0.456320526503728	0.994766463583882	pseudobulk	DESeq2	LRT	0.340729995073934
109	KC_AB1	Git	1.02944527513828	0.410456086317863	0.994766463583882	pseudobulk	DESeq2	LRT	0.386733300152337
109	KC_AB1	Gk1	-0.331492637676615	0.662625721262295	0.994766463583882	pseudobulk	DESeq2	LRT	0.178731710039519
109	KC_AB1	Gk2	1.45682795759908	0.573003993665791	0.994766463583882	pseudobulk	DESeq2	LRT	0.24184235111992
109	KC_AB1	gkt	1.17958426171951	0.314358588119784	0.994766463583882	pseudobulk	DESeq2	LRT	0.502574670443159
109	KC_AB1	GlcAT-I	0.34452302266208	0.623335418085416	0.994766463583882	pseudobulk	DESeq2	LRT	0.205278195678118
109	KC_AB1	GlcAT-P	0.0490486677431787	0.758169580614067	0.994766463583882	pseudobulk	DESeq2	LRT	0.120233644383966
109	KC_AB1	GlcAT-S	-0.564276761946051	0.464705646075226	0.994766463583882	pseudobulk	DESeq2	LRT	0.332822050903057
109	KC_AB1	GlcT	0.220433615819987	0.855906271422858	0.994766463583882	pseudobulk	DESeq2	LRT	0.0675737914356665
109	KC_AB1	Gld	-2.20969975456663	0.610836134188693	0.994766463583882	pseudobulk	DESeq2	LRT	0.214075280039667
109	KC_AB1	Gle1	-1.30433379309226	0.213325461522593	0.994766463583882	pseudobulk	DESeq2	LRT	0.670957306112534
109	KC_AB1	glec	0.0679002581338311	0.736200931935878	0.994766463583882	pseudobulk	DESeq2	LRT	0.133003637127938
109	KC_AB1	Glg1	-0.465959464098644	0.655243436081218	0.994766463583882	pseudobulk	DESeq2	LRT	0.183597320916249
109	KC_AB1	GlnRS	0.130027161178921	0.821439435009124	0.994766463583882	pseudobulk	DESeq2	LRT	0.0854244517188478
109	KC_AB1	glo	0.0970307953719091	0.661421972545442	0.994766463583882	pseudobulk	DESeq2	LRT	0.179521381849388
109	KC_AB1	Glo1	0.668734954468857	0.451549802700867	0.994766463583882	pseudobulk	DESeq2	LRT	0.345294343145906
109	KC_AB1	Glos	-1.76643687325323	0.543810819055668	0.994766463583882	pseudobulk	DESeq2	LRT	0.26455215642223
109	KC_AB1	GLS	-0.432177182589626	0.810808370932697	0.994766463583882	pseudobulk	DESeq2	LRT	0.0910817762208738
109	KC_AB1	glu	0.247696272506861	0.816409652575967	0.994766463583882	pseudobulk	DESeq2	LRT	0.0880918691743692
109	KC_AB1	GluClalpha	-0.0979259336048786	0.54736189377847	0.994766463583882	pseudobulk	DESeq2	LRT	0.261725440571837
109	KC_AB1	GluProRS	0.675821769966658	0.748287716720035	0.994766463583882	pseudobulk	DESeq2	LRT	0.125931383741942
109	KC_AB1	GluRIA	-0.0368386476914257	0.843464500579354	0.994766463583882	pseudobulk	DESeq2	LRT	0.0739331911150807
109	KC_AB1	GluRIB	0.0809572134004972	0.613016632536098	0.994766463583882	pseudobulk	DESeq2	LRT	0.21252774192424
109	KC_AB1	GluRIIE	-1.64952313825113	0.817565084244375	0.994766463583882	pseudobulk	DESeq2	LRT	0.0874776642296574
109	KC_AB1	GluRS-m	-1.50663180504502	0.668163815680457	0.994766463583882	pseudobulk	DESeq2	LRT	0.175117047219009
109	KC_AB1	Glut1	-0.0286919719209836	0.872993726273101	0.994766463583882	pseudobulk	DESeq2	LRT	0.0589888773193595
109	KC_AB1	Glut4EF	-0.101605103762129	0.520924799173881	0.994766463583882	pseudobulk	DESeq2	LRT	0.2832249670288
109	KC_AB1	GlyP	0.463021519019393	0.272554324772413	0.994766463583882	pseudobulk	DESeq2	LRT	0.564546922387365
109	KC_AB1	GlyRS	0.0554244044541361	0.882679624724941	0.994766463583882	pseudobulk	DESeq2	LRT	0.0541968983040808
109	KC_AB1	GlyS	-0.660662485814339	0.690450451250132	0.994766463583882	pseudobulk	DESeq2	LRT	0.160867482213168
109	KC_AB1	GlyT	-0.0369725170001817	0.907010805983327	0.994766463583882	pseudobulk	DESeq2	LRT	0.0423875387932953
109	KC_AB1	GM130	-0.331821688306419	0.601033799702885	0.994766463583882	pseudobulk	DESeq2	LRT	0.221101104350504

109	KC_AB1	Gmap	0.326323772829261	0.705072899481315	0.994766463583882	pseudobulk	DESeq2	LRT	0.151765977752273
109	KC_AB1	Gmd	1.37841725582716	0.521451667598576	0.994766463583882	pseudobulk	DESeq2	LRT	0.28278593933331
109	KC_AB1	Gmer	-2.00005840186615	0.514177117052568	0.994766463583882	pseudobulk	DESeq2	LRT	0.288887255112324
109	KC_AB1	Gnf1	-0.346818294522983	0.672684109695204	0.994766463583882	pseudobulk	DESeq2	LRT	0.172188831204068
109	KC_AB1	Gnpnat	-0.0153478978403345	0.952765179539307	0.994766463583882	pseudobulk	DESeq2	LRT	0.0210141232824882
109	KC_AB1	gny	-0.356212784647529	0.928203443271547	0.994766463583882	pseudobulk	DESeq2	LRT	0.0323568248514225
109	KC_AB1	gogo	-1.17437683445756	0.417083178765207	0.994766463583882	pseudobulk	DESeq2	LRT	0.379777325178773
109	KC_AB1	gol	-0.380488359527594	0.285751353373311	0.994766463583882	pseudobulk	DESeq2	LRT	0.544011704025703
109	KC_AB1	Golgin104	-0.70187748479703	0.486940817333139	0.994766463583882	pseudobulk	DESeq2	LRT	0.312523819622181
109	KC_AB1	Golgin245	-0.255217350246648	0.698311938280045	0.994766463583882	pseudobulk	DESeq2	LRT	0.1559505332349
109	KC_AB1	Golgin84	0.835433773756853	0.341810200796293	0.994766463583882	pseudobulk	DESeq2	LRT	0.46621498057539
109	KC_AB1	gom	-0.0251785612921815	0.925917813292884	0.994766463583882	pseudobulk	DESeq2	LRT	0.0334275606371245
109	KC_AB1	Gorab	-0.237545514828289	0.854740065533037	0.994766463583882	pseudobulk	DESeq2	LRT	0.0681659382169552
109	KC_AB1	Gos28	-0.178728009267573	0.766598452625234	0.994766463583882	pseudobulk	DESeq2	LRT	0.115432061701808
109	KC_AB1	Got1	-0.297342548149706	0.702469540990022	0.994766463583882	pseudobulk	DESeq2	LRT	0.153372501980519
109	KC_AB1	Got2	0.448446887963334	0.436145972605218	0.994766463583882	pseudobulk	DESeq2	LRT	0.360368133467657
109	KC_AB1	Gp210	-0.787513072578771	0.451891603196672	0.994766463583882	pseudobulk	DESeq2	LRT	0.344965728417042
109	KC_AB1	Gp93	1.14267274814089	0.208253573342209	0.994766463583882	pseudobulk	DESeq2	LRT	0.681407537966845
109	KC_AB1	Gpat4	0.471403195855584	0.408252304262043	0.994766463583882	pseudobulk	DESeq2	LRT	0.389071355334795
109	KC_AB1	Gpb5	0.470496658797308	0.539012758382987	0.994766463583882	pseudobulk	DESeq2	LRT	0.268400954980697
109	KC_AB1	Gpdh1	-0.0504371160511726	0.837616214638178	0.994766463583882	pseudobulk	DESeq2	LRT	0.076954924142338
109	KC_AB1	Gpdh3	0.199781935851346	0.825408767771138	0.994766463583882	pseudobulk	DESeq2	LRT	0.0833309222112076
109	KC_AB1	GPHR	-1.09266285160927	0.465336656261063	0.994766463583882	pseudobulk	DESeq2	LRT	0.332232735163127
109	KC_AB1	Gpo1	0.471637297705831	0.528085404638064	0.994766463583882	pseudobulk	DESeq2	LRT	0.277295835489624
109	KC_AB1	Gpo2	0.949183608371002	0.80156895525114	0.994766463583882	pseudobulk	DESeq2	LRT	0.0960591113673091
110	KC_AB1	Gprk1	-0.0765963641493883	0.670113248007243	0.994766463583882	pseudobulk	DESeq2	LRT	0.17385179606264
110	KC_AB1	Gprk2	0.0180046993858004	0.876005769436333	0.994766463583882	pseudobulk	DESeq2	LRT	0.0574930335281386
110	KC_AB1	gprs	-0.448814155152627	0.060037145692562	0.994766463583882	pseudobulk	DESeq2	LRT	1.22157996332145
110	KC_AB1	Gr2a	1.1821590793593	0.719511699510136	0.994766463583882	pseudobulk	DESeq2	LRT	0.142962139890846
110	KC_AB1	Gr39a	-0.548887546079202	0.814781693583783	0.994766463583882	pseudobulk	DESeq2	LRT	0.0889587372379754
110	KC_AB1	Gr58b	0.949183608371002	0.80156895525114	0.994766463583882	pseudobulk	DESeq2	LRT	0.0960591113673091
110	KC_AB1	Gr77a	0.949183608371002	0.80156895525114	0.994766463583882	pseudobulk	DESeq2	LRT	0.0960591113673091
110	KC_AB1	Gr93b	-1.64952313825113	0.817565084244375	0.994766463583882	pseudobulk	DESeq2	LRT	0.0874776642296574
110	KC_AB1	Graf	-0.0519066391269743	0.859714749206739	0.994766463583882	pseudobulk	DESeq2	LRT	0.0656456224717564
110	KC_AB1	GramD1B	0.036436873654858	0.919712776948017	0.994766463583882	pseudobulk	DESeq2	LRT	0.0363477801135309
110	KC_AB1	Grasp65	-0.389897149608465	0.699034994648653	0.994766463583882	pseudobulk	DESeq2	LRT	0.155501082333884
110	KC_AB1	grass	0.949183608371002	0.80156895525114	0.994766463583882	pseudobulk	DESeq2	LRT	0.0960591113673091
110	KC_AB1	grau	-0.691876036576923	0.471896500991364	0.994766463583882	pseudobulk	DESeq2	LRT	0.326153242843057
110	KC_AB1	Grik	-0.237545514828289	0.854740065533037	0.994766463583882	pseudobulk	DESeq2	LRT	0.0681659382169552
110	KC_AB1	Grip128	-1.60653259065571	0.594621747113632	0.994766463583882	pseudobulk	DESeq2	LRT	0.225759211379423
110	KC_AB1	Grip163	-0.482829422428003	0.645605750658116	0.994766463583882	pseudobulk	DESeq2	LRT	0.190032609841184
110	KC_AB1	Grip71	0.969986341149099	0.640932225194753	0.994766463583882	pseudobulk	DESeq2	LRT	0.193187892135042
110	KC_AB1	Grip75	-0.0419263615007874	0.937314592536924	0.994766463583882	pseudobulk	DESeq2	LRT	0.0281146216284548
110	KC_AB1	Grip84	-0.117273746773661	0.885459360347325	0.994766463583882	pseudobulk	DESeq2	LRT	0.0528313666951664
110	KC_AB1	Grip91	-0.82800657585542	0.56722111013579	0.994766463583882	pseudobulk	DESeq2	LRT	0.246247614487752
110	KC_AB1	gro	0.041922032228353	0.841839393183102	0.994766463583882	pseudobulk	DESeq2	LRT	0.074770755658849
110	KC_AB1	grp	-0.0562749008719734	0.834298907292821	0.994766463583882	pseudobulk	DESeq2	LRT	0.0786783252247707
110	KC_AB1	grsm	-0.293624985455589	0.694199964156309	0.994766463583882	pseudobulk	DESeq2	LRT	0.158515413088573
110	KC_AB1	Grx1	-0.250765621821765	0.580443525678161	0.994766463583882	pseudobulk	DESeq2	LRT	0.236240028603201
110	KC_AB1	gry	-0.123148941429253	0.873256876336373	0.994766463583882	pseudobulk	DESeq2	LRT	0.0588579858870696
110	KC_AB1	Gs1	0.186135538025211	0.6045731222199	0.994766463583882	pseudobulk	DESeq2	LRT	0.218551164354782
110	KC_AB1	Gs1l	0.0839539580890779	0.837239893326561	0.994766463583882	pseudobulk	DESeq2	LRT	0.0771500863070532
110	KC_AB1	Gs2	-0.17805032366327	0.942053914371676	0.994766463583882	pseudobulk	DESeq2	LRT	0.0259242415339998
110	KC_AB1	GstD1	-0.312165750487162	0.756525136051139	0.994766463583882	pseudobulk	DESeq2	LRT	0.121176637677871
110	KC_AB1	GstD11	0.0451380775287877	0.88554115283542	0.994766463583882	pseudobulk	DESeq2	LRT	0.0527912514879692
110	KC_AB1	GstD3	2.22023857837763	0.524904842126482	0.994766463583882	pseudobulk	DESeq2	LRT	0.279919420946639

110	KC_AB1	GstD8	0.949183608371002	0.80156895525114	0.994766463583882	pseudobulk	DESeq2	LRT	0.0960591113673091
110	KC_AB1	GstD9	-0.835152883801839	0.920434724857442	0.994766463583882	pseudobulk	DESeq2	LRT	0.0360070052473159
110	KC_AB1	GstE1	-1.64952313825113	0.817565084244375	0.994766463583882	pseudobulk	DESeq2	LRT	0.0874776642296574
110	KC_AB1	GstE12	0.949183608371002	0.80156895525114	0.994766463583882	pseudobulk	DESeq2	LRT	0.0960591113673091
110	KC_AB1	GstE13	1.1821590793593	0.719511699510136	0.994766463583882	pseudobulk	DESeq2	LRT	0.142962139890846
110	KC_AB1	GstE2	1.1821590793593	0.719511699510136	0.994766463583882	pseudobulk	DESeq2	LRT	0.142962139890846
110	KC_AB1	GstE8	-1.64952313825113	0.817565084244375	0.994766463583882	pseudobulk	DESeq2	LRT	0.0874776642296574
110	KC_AB1	GstO1	0.217906885051292	0.902008941520292	0.994766463583882	pseudobulk	DESeq2	LRT	0.0447891573209458
110	KC_AB1	GstO2	-0.662809696988234	0.472150630664381	0.994766463583882	pseudobulk	DESeq2	LRT	0.325919425878535
110	KC_AB1	GstO3	-3.21143241058256	0.292545415061155	0.994766463583882	pseudobulk	DESeq2	LRT	0.533806704011112
110	KC_AB1	GstS1	-0.835152883801839	0.920434724857442	0.994766463583882	pseudobulk	DESeq2	LRT	0.0360070052473159
110	KC_AB1	GstT1	-0.803925612827804	0.675180897915086	0.994766463583882	pseudobulk	DESeq2	LRT	0.170579853182495
110	KC_AB1	GstT2	0.141089634763157	0.906734058383971	0.994766463583882	pseudobulk	DESeq2	LRT	0.0425200711671416
110	KC_AB1	GstZ2	-0.839415946828871	0.414208886301826	0.994766463583882	pseudobulk	DESeq2	LRT	0.382780588131003
110	KC_AB1	gt	-3.0440420488244	0.18734569390835	0.994766463583882	pseudobulk	DESeq2	LRT	0.727356284600256
110	KC_AB1	Gtp-bp	0.895009372428717	0.46859046023972	0.994766463583882	pseudobulk	DESeq2	LRT	0.329206557197488
110	KC_AB1	Gug	-0.0149635419370983	0.905464482786315	0.994766463583882	pseudobulk	DESeq2	LRT	0.0431285803940272
110	KC_AB1	gukh	-1.32754262918199	0.499139561833611	0.994766463583882	pseudobulk	DESeq2	LRT	0.301778006561226
110	KC_AB1	gus	0.0129207168276821	0.957701428918304	0.994766463583882	pseudobulk	DESeq2	LRT	0.0187698645995547
110	KC_AB1	gw	0.170085238232927	0.360063344901063	0.994766463583882	pseudobulk	DESeq2	LRT	0.443621088341215
110	KC_AB1	GXIVsPLA2	0.131971329363007	0.894112366502519	0.994766463583882	pseudobulk	DESeq2	LRT	0.0486078983344896
110	KC_AB1	Gyc32E	1.1821590793593	0.719511699510136	0.994766463583882	pseudobulk	DESeq2	LRT	0.142962139890846
110	KC_AB1	Gyc76C	1.1821590793593	0.719511699510136	0.994766463583882	pseudobulk	DESeq2	LRT	0.142962139890846
110	KC_AB1	Gyc88E	0.082312487872377	0.757148839195882	0.994766463583882	pseudobulk	DESeq2	LRT	0.120818739142431
110	KC_AB1	Gyc89Da	0.475892334225681	0.803946046269915	0.994766463583882	pseudobulk	DESeq2	LRT	0.0947730962683447
110	KC_AB1	Gyc89Db	0.145757059122065	0.839641871269742	0.994766463583882	pseudobulk	DESeq2	LRT	0.0759059121477838
110	KC_AB1	Gycalpha99B	0.0878045209890323	0.770256339683203	0.994766463583882	pseudobulk	DESeq2	LRT	0.113364718485736
110	KC_AB1	Gycbeta100B	0.742107788931767	0.0339748966988026	0.994766463583882	pseudobulk	DESeq2	LRT	1.46884185507253
110	KC_AB1	Gyf	-0.0727093096519285	0.7983444358805	0.994766463583882	pseudobulk	DESeq2	LRT	0.097809697209446
110	KC_AB1	Gyg	-1.32806228432949	0.722858830184137	0.994766463583882	pseudobulk	DESeq2	LRT	0.140946509425123
110	KC_AB1	gzl	-0.269452978794038	0.464217988366531	0.994766463583882	pseudobulk	DESeq2	LRT	0.33327803471819
110	KC_AB1	H	-0.127068534029641	0.803486534247505	0.994766463583882	pseudobulk	DESeq2	LRT	0.0950213972466323
110	KC_AB1	h-cup	0.949183608371002	0.80156895525114	0.994766463583882	pseudobulk	DESeq2	LRT	0.0960591113673091
110	KC_AB1	Hacd1	-0.268157716424422	0.760248835501535	0.994766463583882	pseudobulk	DESeq2	LRT	0.119044236406645
110	KC_AB1	Hacd2	-0.107430087442589	0.872670606926629	0.994766463583882	pseudobulk	DESeq2	LRT	0.0591496515851824
110	KC_AB1	Hacl	-0.0474718954419185	0.957446134378947	0.994766463583882	pseudobulk	DESeq2	LRT	0.0188856499442294
110	KC_AB1	haf	0.109391035275916	0.882314949344165	0.994766463583882	pseudobulk	DESeq2	LRT	0.0543763623207269
110	KC_AB1	Hakai	-0.266783858174164	0.815144512090292	0.994766463583882	pseudobulk	DESeq2	LRT	0.0887653909657783
110	KC_AB1	hang	-0.318457587000787	0.368081222685534	0.994766463583882	pseudobulk	DESeq2	LRT	0.434056337109326
110	KC_AB1	Hasp	0.212438620221422	0.359965598469079	0.994766463583882	pseudobulk	DESeq2	LRT	0.443739002313113
110	KC_AB1	Haspin	0.166786415356166	0.617583164628633	0.994766463583882	pseudobulk	DESeq2	LRT	0.209304551416719
110	KC_AB1	Hat1	-0.643455256936482	0.388465099781565	0.994766463583882	pseudobulk	DESeq2	LRT	0.410647992701225
110	KC_AB1	hay	-0.775532915871357	0.486218209384934	0.994766463583882	pseudobulk	DESeq2	LRT	0.313168780401552
110	KC_AB1	hbs	-1.64952313825113	0.817565084244375	0.994766463583882	pseudobulk	DESeq2	LRT	0.0874776642296574
110	KC_AB1	HBS1	-0.137304788203967	0.827410571461257	0.994766463583882	pseudobulk	DESeq2	LRT	0.0822789346153193
110	KC_AB1	Hcf	-0.00638634752708181	0.838800728413082	0.994766463583882	pseudobulk	DESeq2	LRT	0.0763412010659632
110	KC_AB1	Hcs	-0.648138194565704	0.415713549393818	0.994766463583882	pseudobulk	DESeq2	LRT	0.381205820259069
110	KC_AB1	HDAC1	0.0838657180303191	0.862002680815284	0.994766463583882	pseudobulk	DESeq2	LRT	0.064491383523925
110	KC_AB1	HDAC11	0.862181354487678	0.457836010519149	0.994766463583882	pseudobulk	DESeq2	LRT	0.339290051428403
110	KC_AB1	HDAC4	-0.0379505671267221	0.81739517002659	0.994766463583882	pseudobulk	DESeq2	LRT	0.0875679328551533
110	KC_AB1	HDAC6	-0.160821426891328	0.763426511285828	0.994766463583882	pseudobulk	DESeq2	LRT	0.117232762509497
110	KC_AB1	hdc	-0.334254638258769	0.226341923432213	0.994766463583882	pseudobulk	DESeq2	LRT	0.645234997839386
110	KC_AB1	Hdc	-1.64952313825113	0.817565084244375	0.994766463583882	pseudobulk	DESeq2	LRT	0.0874776642296574
110	KC_AB1	hdly	1.39363783099471	0.581259211302561	0.994766463583882	pseudobulk	DESeq2	LRT	0.235630151717171
110	KC_AB1	hdm	-2.57435989741145	0.533617212681275	0.994766463583882	pseudobulk	DESeq2	LRT	0.272770169998685
110	KC_AB1	HEATR2	-0.536222806981389	0.726317282473814	0.994766463583882	pseudobulk	DESeq2	LRT	0.138873621825507

110	KC_AB1	hec	-0.237473683803514	0.877148860518115	0.994766463583882	pseudobulk	DESeq2	LRT	0.0569266964680766
110	KC_AB1	heix	0.343737658524127	0.729333664566268	0.994766463583882	pseudobulk	DESeq2	LRT	0.137073739819782
110	KC_AB1	Hel25E	0.149586337028695	0.752192446934833	0.994766463583882	pseudobulk	DESeq2	LRT	0.123671031816295
110	KC_AB1	Hel89B	-0.427391958880249	0.537111626074039	0.994766463583882	pseudobulk	DESeq2	LRT	0.269935446981872
110	KC_AB1	Hem	0.141596294984827	0.90715871773128	0.994766463583882	pseudobulk	DESeq2	LRT	0.0423167215347678
110	KC_AB1	HemK1	0.132596090459081	0.871716145037401	0.994766463583882	pseudobulk	DESeq2	LRT	0.0596249103803766
110	KC_AB1	Hen1	-0.597696727942493	0.699138119961125	0.994766463583882	pseudobulk	DESeq2	LRT	0.155437017657251
110	KC_AB1	hep	0.284213018897912	0.446493983597274	0.994766463583882	pseudobulk	DESeq2	LRT	0.350184388753282
110	KC_AB1	heph	0.236807307531133	0.133004035949081	0.994766463583882	pseudobulk	DESeq2	LRT	0.876135180357567
110	KC_AB1	her	-0.351723507882725	0.725768901498042	0.994766463583882	pseudobulk	DESeq2	LRT	0.139201644839168
110	KC_AB1	HERC2	0.138020881565647	0.759908365844917	0.994766463583882	pseudobulk	DESeq2	LRT	0.119238774307681
110	KC_AB1	Herc4	0.33388498650117	0.588962546451161	0.994766463583882	pseudobulk	DESeq2	LRT	0.229912322168366
110	KC_AB1	Herp	-0.088860298962567	0.845869295429096	0.994766463583882	pseudobulk	DESeq2	LRT	0.0726967393951534
111	KC_AB1	Hers	-0.149498517384971	0.577258492054223	0.994766463583882	pseudobulk	DESeq2	LRT	0.238629669454248
111	KC_AB1	Hex-A	0.0695226967742117	0.790454218289665	0.994766463583882	pseudobulk	DESeq2	LRT	0.102123278571679
111	KC_AB1	Hexim	-0.664802473715852	0.461295304984942	0.994766463583882	pseudobulk	DESeq2	LRT	0.336020965572762
111	KC_AB1	Hexo1	0.197041151763454	0.851237758237255	0.994766463583882	pseudobulk	DESeq2	LRT	0.0699491206806121
111	KC_AB1	Hey	-1.04700181357125	0.262022245596142	0.994766463583882	pseudobulk	DESeq2	LRT	0.581661835666797
111	KC_AB1	Hf	-0.48167422725367	0.855534332799682	0.994766463583882	pseudobulk	DESeq2	LRT	0.0677625574305792
111	KC_AB1	hfp	-0.15582137822684	0.569412526337312	0.994766463583882	pseudobulk	DESeq2	LRT	0.244572983168455
111	KC_AB1	hfw	0.154131236646223	0.930566110411021	0.994766463583882	pseudobulk	DESeq2	LRT	0.0312527677596474
111	KC_AB1	HHEX	0.949183608371002	0.80156895525114	0.994766463583882	pseudobulk	DESeq2	LRT	0.0960591113673091
111	KC_AB1	hig	2.02670873290438	0.538959414925803	0.994766463583882	pseudobulk	DESeq2	LRT	0.26844393710898
111	KC_AB1	Him	-1.64952313825113	0.817565084244375	0.994766463583882	pseudobulk	DESeq2	LRT	0.0874776642296574
111	KC_AB1	HINT1	-0.199163995108722	0.644996691524503	0.994766463583882	pseudobulk	DESeq2	LRT	0.190442513048976
111	KC_AB1	HIP	0.0570275093709044	0.909570938980733	0.994766463583882	pseudobulk	DESeq2	LRT	0.0411634239161072
111	KC_AB1	HIP-R	-0.257452849814615	0.66805421311062	0.994766463583882	pseudobulk	DESeq2	LRT	0.175188292764816
111	KC_AB1	Hip1	0.0933655914520428	0.925249603795792	0.994766463583882	pseudobulk	DESeq2	LRT	0.0337410922034941
111	KC_AB1	Hip14	0.134672120536807	0.798427588877919	0.994766463583882	pseudobulk	DESeq2	LRT	0.0977644648438921
111	KC_AB1	Hipk	-0.14991656788008	0.502189821579184	0.994766463583882	pseudobulk	DESeq2	LRT	0.299132093846894
111	KC_AB1	HIPP1	-0.0962143785388846	0.845594553791231	0.994766463583882	pseudobulk	DESeq2	LRT	0.0728378228458908
111	KC_AB1	Hira	2.25702551253886	0.345690610560104	0.994766463583882	pseudobulk	DESeq2	LRT	0.461312416342215
111	KC_AB1	His2Av	0.0678804733096806	0.800155074712317	0.994766463583882	pseudobulk	DESeq2	LRT	0.0968258360515637
111	KC_AB1	His3.3A	0.134856370032131	0.612654608359502	0.994766463583882	pseudobulk	DESeq2	LRT	0.212784295397206
111	KC_AB1	His3.3B	0.0944471155151381	0.491822603898406	0.994766463583882	pseudobulk	DESeq2	LRT	0.308191515201292
111	KC_AB1	His4r	0.206361888640489	0.198450172354246	0.994766463583882	pseudobulk	DESeq2	LRT	0.702348519571303
111	KC_AB1	HisCl1	0.53983785550047	0.806887399502234	0.994766463583882	pseudobulk	DESeq2	LRT	0.0931870665007086
111	KC_AB1	HisRS	-0.299055218248706	0.74091344855014	0.994766463583882	pseudobulk	DESeq2	LRT	0.130232522132008
111	KC_AB1	hiw	-0.0955210465955141	0.774641428072668	0.994766463583882	pseudobulk	DESeq2	LRT	0.110899280520297
111	KC_AB1	Hk	-0.180023995282087	0.429154994910429	0.994766463583882	pseudobulk	DESeq2	LRT	0.367385828381016
111	KC_AB1	Hlc	0.856504055049228	0.177390706156524	0.994766463583882	pseudobulk	DESeq2	LRT	0.751069137439294
111	KC_AB1	Hmg-2	0.76927424591958	0.565106352578531	0.994766463583882	pseudobulk	DESeq2	LRT	0.247869810602836
111	KC_AB1	Hmgcr	-0.312477229681089	0.679190225894578	0.994766463583882	pseudobulk	DESeq2	LRT	0.168008572573576
111	KC_AB1	HmgD	-0.292623478898885	0.554594209756458	0.994766463583882	pseudobulk	DESeq2	LRT	0.256024668988136
111	KC_AB1	Hmgs	0.783727503912399	0.435123317886685	0.994766463583882	pseudobulk	DESeq2	LRT	0.361387642613585
111	KC_AB1	HmgZ	0.240342078355556	0.192833613307723	0.994766463583882	pseudobulk	DESeq2	LRT	0.714817260880929
111	KC_AB1	Hmr	-0.131352091217192	0.925243881204708	0.994766463583882	pseudobulk	DESeq2	LRT	0.0337437782866934
111	KC_AB1	Hmt-1	1.35679750803729	0.309370781172638	0.994766463583882	pseudobulk	DESeq2	LRT	0.509520706068817
111	KC_AB1	Hmt4-20	-0.205998790018084	0.616913408335055	0.994766463583882	pseudobulk	DESeq2	LRT	0.209775790458495
111	KC_AB1	Hmu	-1.1939443559292	0.141296470419847	0.994766463583882	pseudobulk	DESeq2	LRT	0.849868686674649
111	KC_AB1	hng1	0.416416889173652	0.652015867201434	0.994766463583882	pseudobulk	DESeq2	LRT	0.185741835319956
111	KC_AB1	hng2	-0.720688993793705	0.702033725657174	0.994766463583882	pseudobulk	DESeq2	LRT	0.153642023888733
111	KC_AB1	hng3	-1.21457746603801	0.770013447451371	0.994766463583882	pseudobulk	DESeq2	LRT	0.113501690278259
111	KC_AB1	HnRNP-K	0.101648485354708	0.744615364434532	0.994766463583882	pseudobulk	DESeq2	LRT	0.12806800676886
111	KC_AB1	Ho	0.635047975478013	0.361981350087532	0.994766463583882	pseudobulk	DESeq2	LRT	0.441313804501975
111	KC_AB1	hob	1.01234537391295	0.606064483490932	0.994766463583882	pseudobulk	DESeq2	LRT	0.217481165710913

111	KC_AB1	hoe1	-0.178745209825779	0.667842971259702	0.994766463583882	pseudobulk	DESeq2	LRT	0.175325640417074
111	KC_AB1	hoe2	0.260507485208579	0.57343456312799	0.994766463583882	pseudobulk	DESeq2	LRT	0.24151613398566
111	KC_AB1	hog	-1.64952313825113	0.817565084244375	0.994766463583882	pseudobulk	DESeq2	LRT	0.0874776642296574
111	KC_AB1	hoip	0.201254039132405	0.693621556308909	0.994766463583882	pseudobulk	DESeq2	LRT	0.158877418356911
111	KC_AB1	holn1	0.185800939419852	0.836991118458688	0.994766463583882	pseudobulk	DESeq2	LRT	0.0772791503996282
111	KC_AB1	homer	-0.424241840511207	0.496280062278522	0.994766463583882	pseudobulk	DESeq2	LRT	0.304273171944222
111	KC_AB1	hook	-0.038495252087378	0.878539316675836	0.994766463583882	pseudobulk	DESeq2	LRT	0.0562387980465654
111	KC_AB1	hop	1.12423520937953	0.204483606476571	0.994766463583882	pseudobulk	DESeq2	LRT	0.689341503804061
111	KC_AB1	HP1b	0.383929976829138	0.531283956868454	0.994766463583882	pseudobulk	DESeq2	LRT	0.274673298241777
111	KC_AB1	HP4	0.396094344544461	0.681218349946527	0.994766463583882	pseudobulk	DESeq2	LRT	0.166713661981919
111	KC_AB1	HP5	0.355534194175706	0.5596234268081	0.994766463583882	pseudobulk	DESeq2	LRT	0.252104113478762
111	KC_AB1	Hph	0.260089246026094	0.749103502709628	0.994766463583882	pseudobulk	DESeq2	LRT	0.125458172220264
111	KC_AB1	hpo	1.33295189638571	0.234833845757423	0.994766463583882	pseudobulk	DESeq2	LRT	0.629239309611924
111	KC_AB1	hppy	-0.42276247614501	0.1843823212027	0.994766463583882	pseudobulk	DESeq2	LRT	0.73428072196016
111	KC_AB1	Hpr1	-0.458935030966377	0.68091071153109	0.994766463583882	pseudobulk	DESeq2	LRT	0.166909833801897
111	KC_AB1	hpRNA:CR18854	-0.18185857015319	0.585611729859678	0.994766463583882	pseudobulk	DESeq2	LRT	0.232390232902
111	KC_AB1	hpRNA:CR32207	0.949183608371002	0.80156895525114	0.994766463583882	pseudobulk	DESeq2	LRT	0.0960591113673091
111	KC_AB1	hpRNA:CR46342	0.393007216047432	0.609402754776962	0.994766463583882	pseudobulk	DESeq2	LRT	0.215095586900793
111	KC_AB1	HPS1	-0.157538639793862	0.862502927320356	0.994766463583882	pseudobulk	DESeq2	LRT	0.0642394222640562
111	KC_AB1	HPS4	-0.440882422144248	0.517670465108989	0.994766463583882	pseudobulk	DESeq2	LRT	0.28594661231405
111	KC_AB1	Hr3	-0.221847221573173	0.513262463828872	0.994766463583882	pseudobulk	DESeq2	LRT	0.289660495622487
111	KC_AB1	Hr38	-0.409738444937115	0.261631076277707	0.994766463583882	pseudobulk	DESeq2	LRT	0.582310672219489
111	KC_AB1	Hr39	-0.160876424122736	0.600763443067484	0.994766463583882	pseudobulk	DESeq2	LRT	0.221296502363917
111	KC_AB1	Hr4	1.22459909935685	0.518537437211841	0.994766463583882	pseudobulk	DESeq2	LRT	0.285219883079023
111	KC_AB1	Hr51	0.293793587225579	0.29489757067169	0.994766463583882	pseudobulk	DESeq2	LRT	0.530328805095627
111	KC_AB1	Hr78	0.0840933888210931	0.847922858694329	0.994766463583882	pseudobulk	DESeq2	LRT	0.0716436566669748
111	KC_AB1	Hr96	-0.185341987189016	0.841288555617082	0.994766463583882	pseudobulk	DESeq2	LRT	0.0750550189248217
111	KC_AB1	Hrb27C	0.00080158056063578	0.913846559590165	0.994766463583882	pseudobulk	DESeq2	LRT	0.0391267188367041
111	KC_AB1	Hrb87F	0.268855973492196	0.617948616834962	0.994766463583882	pseudobulk	DESeq2	LRT	0.209047635514404
111	KC_AB1	Hrb98DE	0.158907498333502	0.524261747355328	0.994766463583882	pseudobulk	DESeq2	LRT	0.280451829332936
111	KC_AB1	Hrd3	0.264786871253384	0.741028294415004	0.994766463583882	pseudobulk	DESeq2	LRT	0.130165209195158
111	KC_AB1	hrg	0.141275820112571	0.676225511204508	0.994766463583882	pseudobulk	DESeq2	LRT	0.169908449118383
111	KC_AB1	hrm	0.0449038468868641	0.862806238028034	0.994766463583882	pseudobulk	DESeq2	LRT	0.0640867236397758
111	KC_AB1	Hrs	1.36601186826572	0.243279126234899	0.994766463583882	pseudobulk	DESeq2	LRT	0.613895152679952
111	KC_AB1	Hs2st	-0.43526610590119	0.686623181880165	0.994766463583882	pseudobulk	DESeq2	LRT	0.163281537943943
111	KC_AB1	Hs3st-A	-0.0500278098259391	0.754298145556794	0.994766463583882	pseudobulk	DESeq2	LRT	0.122456959996427
111	KC_AB1	Hs3st-B	0.0839527249744413	0.923173252509127	0.994766463583882	pseudobulk	DESeq2	LRT	0.0347167870046038
111	KC_AB1	Hs6st	0.106277426616061	0.785686552362442	0.994766463583882	pseudobulk	DESeq2	LRT	0.104750680079957
111	KC_AB1	Hsc20	-0.430035809362427	0.753545307930492	0.994766463583882	pseudobulk	DESeq2	LRT	0.122890630021367
111	KC_AB1	Hsc70-1	-1.64952313825113	0.817565084244375	0.994766463583882	pseudobulk	DESeq2	LRT	0.0874776642296574
111	KC_AB1	Hsc70-2	4.24685566446445	0.358830499210615	0.994766463583882	pseudobulk	DESeq2	LRT	0.44511065070057
111	KC_AB1	Hsc70-3	0.260851737990139	0.530652189854661	0.994766463583882	pseudobulk	DESeq2	LRT	0.275190039217503
111	KC_AB1	Hsc70-4	0.0982129071293319	0.693474908376646	0.994766463583882	pseudobulk	DESeq2	LRT	0.158969248146147
111	KC_AB1	Hsc70-5	0.67972899501497	0.227776940876574	0.994766463583882	pseudobulk	DESeq2	LRT	0.642490244071464
111	KC_AB1	Hsc70Cb	0.171610021264347	0.687304093870181	0.994766463583882	pseudobulk	DESeq2	LRT	0.162851069243953
111	KC_AB1	Hsepi	0.123596538059192	0.890531974260843	0.994766463583882	pseudobulk	DESeq2	LRT	0.0503504827187014
111	KC_AB1	Hsf	0.092335265840381	0.859866862404352	0.994766463583882	pseudobulk	DESeq2	LRT	0.0655687875927668
111	KC_AB1	Hsl	-2.87071286854028	0.0902464903691555	0.994766463583882	pseudobulk	DESeq2	LRT	1.04456967854083
111	KC_AB1	Hsp23	-3.40795650067645	0.271605273223615	0.994766463583882	pseudobulk	DESeq2	LRT	0.566061802470747
111	KC_AB1	Hsp26	-1.74713016025828	0.0728544120423454	0.994766463583882	pseudobulk	DESeq2	LRT	1.13754414234803
111	KC_AB1	Hsp27	-3.97553081074123	0.0788692216154595	0.994766463583882	pseudobulk	DESeq2	LRT	1.10309244533828
111	KC_AB1	Hsp60A	0.418360796866032	0.298917381797279	0.994766463583882	pseudobulk	DESeq2	LRT	0.524448830363326
111	KC_AB1	Hsp60D	-0.925642226170004	0.657544673459392	0.994766463583882	pseudobulk	DESeq2	LRT	0.182074735951784
111	KC_AB1	Hsp67Bc	-2.20969975456663	0.610836134188693	0.994766463583882	pseudobulk	DESeq2	LRT	0.214075280039667
111	KC_AB1	Hsp68	-2.90146310912415	0.112705347399995	0.994766463583882	pseudobulk	DESeq2	LRT	0.948055477998111
111	KC_AB1	Hsp70Aa	-4.16874834831721	0.117262996581567	0.994766463583882	pseudobulk	DESeq2	LRT	0.930839011889149

111	KC_AB1	Hsp70Ab	-0.711107348821201	0.271648193167647	0.994766463583882	pseudobulk	DESeq2	LRT	0.565993179273478
112	KC_AB1	Hsp70Ba	1.55007561624522	0.49585983061903	0.994766463583882	pseudobulk	DESeq2	LRT	0.304641072283799
112	KC_AB1	Hsp70Bb	-0.600909238940874	0.231455285299012	0.994766463583882	pseudobulk	DESeq2	LRT	0.635532897620473
112	KC_AB1	Hsp70Bbb	0.806933541094274	0.754600478929312	0.994766463583882	pseudobulk	DESeq2	LRT	0.122282923497213
112	KC_AB1	Hsp70Bc	0.4389989495819	0.808479090069174	0.994766463583882	pseudobulk	DESeq2	LRT	0.0923312078973535
112	KC_AB1	Hsp83	-0.244956288499555	0.22660072639821	0.994766463583882	pseudobulk	DESeq2	LRT	0.644738702282734
112	KC_AB1	HSPBAP1	0.89489462229553	0.681408699787765	0.994766463583882	pseudobulk	DESeq2	LRT	0.166592325940286
112	KC_AB1	HSPC300	0.151393205978689	0.78688685634971	0.994766463583882	pseudobulk	DESeq2	LRT	0.104087708804186
112	KC_AB1	hth	0.152934601321026	0.654747496095091	0.994766463583882	pseudobulk	DESeq2	LRT	0.183926153741469
112	KC_AB1	htk	0.305925784808776	0.473308871916638	0.994766463583882	pseudobulk	DESeq2	LRT	0.324855354842532
112	KC_AB1	htl	-1.64952313825113	0.817565084244375	0.994766463583882	pseudobulk	DESeq2	LRT	0.0874776642296574
112	KC_AB1	HtrA2	-1.35346937897466	0.479502856945209	0.994766463583882	pseudobulk	DESeq2	LRT	0.319208800898233
112	KC_AB1	hts	0.048977036010989	0.870342483426246	0.994766463583882	pseudobulk	DESeq2	LRT	0.0603098170438203
112	KC_AB1	htt	0.043437697379508	0.909311614588769	0.994766463583882	pseudobulk	DESeq2	LRT	0.0412872616570268
112	KC_AB1	Hug	1.73751160151238	0.292874830438191	0.994766463583882	pseudobulk	DESeq2	LRT	0.533317949832554
112	KC_AB1	Hus1-like	0.596808856322697	0.822761281772702	0.994766463583882	pseudobulk	DESeq2	LRT	0.0847261539098712
112	KC_AB1	HUWE1	-0.226030800490554	0.555981911048579	0.994766463583882	pseudobulk	DESeq2	LRT	0.254939338021506
112	KC_AB1	hwt	0.135505029473247	0.615736139817751	0.994766463583882	pseudobulk	DESeq2	LRT	0.210605355320636
112	KC_AB1	Hyccin	0.560937976212545	0.535001252438306	0.994766463583882	pseudobulk	DESeq2	LRT	0.271645201293896
112	KC_AB1	hyd	-0.244015289631149	0.639142230346382	0.994766463583882	pseudobulk	DESeq2	LRT	0.19440248615903
112	KC_AB1	Hydr1	0.30843061333912	0.868412891604321	0.994766463583882	pseudobulk	DESeq2	LRT	0.0612737380607052
112	KC_AB1	Hydr2	-0.217166233352513	0.768260054798802	0.994766463583882	pseudobulk	DESeq2	LRT	0.114491747094935
112	KC_AB1	hyx	0.174750845617111	0.858753149136421	0.994766463583882	pseudobulk	DESeq2	LRT	0.0661316573267887
112	KC_AB1	I-2	-0.055988975457312	0.833533988769167	0.994766463583882	pseudobulk	DESeq2	LRT	0.0790766863771439
112	KC_AB1	I-3	0.644739804935742	0.747875090521859	0.994766463583882	pseudobulk	DESeq2	LRT	0.126170931583437
112	KC_AB1	IA-2	-0.00552161356674143	0.949683806404459	0.994766463583882	pseudobulk	DESeq2	LRT	0.0224209673337769
112	KC_AB1	Ibf1	0.694072616920595	0.382244392911254	0.994766463583882	pseudobulk	DESeq2	LRT	0.417658876473031
112	KC_AB1	Ibf2	0.291613497532675	0.762232386870896	0.994766463583882	pseudobulk	DESeq2	LRT	0.117912602217623
112	KC_AB1	ICA69	-0.1177969186039	0.79252122245252	0.994766463583882	pseudobulk	DESeq2	LRT	0.100989099241927
112	KC_AB1	Ice1	0.44976297004525	0.831013436772572	0.994766463583882	pseudobulk	DESeq2	LRT	0.0803919539908534
112	KC_AB1	Ice2	2.14975120970875	0.206148223372116	0.994766463583882	pseudobulk	DESeq2	LRT	0.685820403685864
112	KC_AB1	icln	0.152299701140441	0.913485149634611	0.994766463583882	pseudobulk	DESeq2	LRT	0.0392985085021014
112	KC_AB1	ics	0.520516757308412	0.365961569217495	0.994766463583882	pseudobulk	DESeq2	LRT	0.436564518849021
112	KC_AB1	ida	2.92183940034187	0.320324374195056	0.994766463583882	pseudobulk	DESeq2	LRT	0.494410013644442
112	KC_AB1	Ide	-0.154653636989407	0.803431537842232	0.994766463583882	pseudobulk	DESeq2	LRT	0.0950511245062342
112	KC_AB1	Idgf3	-1.64952313825113	0.817565084244375	0.994766463583882	pseudobulk	DESeq2	LRT	0.0874776642296574
112	KC_AB1	Idgf5	-2.79499059556516	0.38564607643653	0.994766463583882	pseudobulk	DESeq2	LRT	0.413811082791365
112	KC_AB1	Idgf6	1.47424991200432	0.518427666092641	0.994766463583882	pseudobulk	DESeq2	LRT	0.285311830226264
112	KC_AB1	Idh	-0.286348806572545	0.763302152088461	0.994766463583882	pseudobulk	DESeq2	LRT	0.117303513147257
112	KC_AB1	Idh3a	0.162881323595438	0.669745074662263	0.994766463583882	pseudobulk	DESeq2	LRT	0.174090471530677
112	KC_AB1	Idh3b	0.145899850149639	0.701827533144458	0.994766463583882	pseudobulk	DESeq2	LRT	0.153769598135212
112	KC_AB1	Idi	-0.541468907580694	0.551501653679006	0.994766463583882	pseudobulk	DESeq2	LRT	0.258453180988813
112	KC_AB1	Ids	-3.21143241058256	0.292545415061155	0.994766463583882	pseudobulk	DESeq2	LRT	0.533806704011112
112	KC_AB1	ifc	0.427054797023195	0.634009981679817	0.994766463583882	pseudobulk	DESeq2	LRT	0.197903904650854
112	KC_AB1	IFT43	0.453538483256514	0.771549563983593	0.994766463583882	pseudobulk	DESeq2	LRT	0.112636169830034
112	KC_AB1	igl	-0.0120464477840859	0.934735506107948	0.994766463583882	pseudobulk	DESeq2	LRT	0.0293112602375472
112	KC_AB1	Ih	0.0548340674513513	0.761415405202647	0.994766463583882	pseudobulk	DESeq2	LRT	0.118378340646395
112	KC_AB1	IKKepsilon	-0.415579358246154	0.728255862766451	0.994766463583882	pseudobulk	DESeq2	LRT	0.137716010432709
112	KC_AB1	IleRS	-0.307147054464857	0.746038152614201	0.994766463583882	pseudobulk	DESeq2	LRT	0.127238962009735
112	KC_AB1	IleRS-m	-1.28030881563958	0.433918265864907	0.994766463583882	pseudobulk	DESeq2	LRT	0.362592067784205
112	KC_AB1	Ilk	0.0614614956409816	0.933604765813255	0.994766463583882	pseudobulk	DESeq2	LRT	0.0298369399949103
112	KC_AB1	imd	-1.11368787293548	0.446766644138336	0.994766463583882	pseudobulk	DESeq2	LRT	0.349919259028727
112	KC_AB1	Iml1	0.0791183843967295	0.915622006235528	0.994766463583882	pseudobulk	DESeq2	LRT	0.0382837779548922
112	KC_AB1	Imp	0.586697003207442	0.749024314537001	0.994766463583882	pseudobulk	DESeq2	LRT	0.125504084172555
112	KC_AB1	Impbeta11	0.408623319069178	0.773213167022166	0.994766463583882	pseudobulk	DESeq2	LRT	0.111700758993743
112	KC_AB1	ImpL2	-1.08681444615798	0.701131388297773	0.994766463583882	pseudobulk	DESeq2	LRT	0.154200589927933

112	KC_AB1	inaE	0.10468141820701	0.749095893154975	0.994766463583882	pseudobulk	DESeq2	LRT	0.125462583899517
112	KC_AB1	inc	0.202115183085764	0.744831546294422	0.994766463583882	pseudobulk	DESeq2	LRT	0.127941937704134
112	KC_AB1	Ing3	0.562249771173097	0.594763189239654	0.994766463583882	pseudobulk	DESeq2	LRT	0.225655918436572
112	KC_AB1	Ing5	0.505655865300417	0.486213339235198	0.994766463583882	pseudobulk	DESeq2	LRT	0.313173130484927
112	KC_AB1	Ino80	0.741495366406115	0.651904222871618	0.994766463583882	pseudobulk	DESeq2	LRT	0.185816205700262
112	KC_AB1	Inos	-0.252421321966584	0.597557507950215	0.994766463583882	pseudobulk	DESeq2	LRT	0.223620292582987
112	KC_AB1	INPP5E	-0.480088351554121	0.605760388641987	0.994766463583882	pseudobulk	DESeq2	LRT	0.217699129082818
112	KC_AB1	InR	-0.116345259305076	0.803928862798744	0.994766463583882	pseudobulk	DESeq2	LRT	0.0947823789391142
112	KC_AB1	IntS1	-1.29886349326138	0.294683153254077	0.994766463583882	pseudobulk	DESeq2	LRT	0.530644691628614
112	KC_AB1	IntS10	0.854047063205883	0.719204060467466	0.994766463583882	pseudobulk	DESeq2	LRT	0.143147869337068
112	KC_AB1	IntS11	-0.266231014763455	0.851854448793783	0.994766463583882	pseudobulk	DESeq2	LRT	0.0696346041602012
112	KC_AB1	IntS12	-0.652655655913417	0.673134269875396	0.994766463583882	pseudobulk	DESeq2	LRT	0.171898298554954
112	KC_AB1	IntS14	0.206174930707402	0.869576833587812	0.994766463583882	pseudobulk	DESeq2	LRT	0.0606920388148111
112	KC_AB1	IntS2	0.414386326480507	0.758745451354662	0.994766463583882	pseudobulk	DESeq2	LRT	0.119903899485044
112	KC_AB1	IntS3	-0.0231911596443694	0.894817420174077	0.994766463583882	pseudobulk	DESeq2	LRT	0.0482655697119377
112	KC_AB1	IntS4	0.360808096780607	0.841068473445515	0.994766463583882	pseudobulk	DESeq2	LRT	0.0751686457847223
112	KC_AB1	IntS8	-0.759241975042111	0.621872382431476	0.994766463583882	pseudobulk	DESeq2	LRT	0.206298729927017
112	KC_AB1	IntS9	-1.35346937897466	0.479502856945209	0.994766463583882	pseudobulk	DESeq2	LRT	0.319208800898233
112	KC_AB1	Invadolysin	0.0946970962658519	0.916629782388576	0.994766463583882	pseudobulk	DESeq2	LRT	0.0378060361192835
112	KC_AB1	Inx2	0.39509611406087	0.839890017035674	0.994766463583882	pseudobulk	DESeq2	LRT	0.0757775807497476
112	KC_AB1	Inx3	-1.84153356221341	0.563796952622777	0.994766463583882	pseudobulk	DESeq2	LRT	0.248877275861878
112	KC_AB1	Inx7	-0.0813546184264884	0.888654438767453	0.994766463583882	pseudobulk	DESeq2	LRT	0.0512670854996878
112	KC_AB1	Ip259	0.015482545404384	0.915994228946452	0.994766463583882	pseudobulk	DESeq2	LRT	0.0381072625162173
112	KC_AB1	IP3K1	1.80633728195307	0.61161102402172	0.994766463583882	pseudobulk	DESeq2	LRT	0.213524695220716
112	KC_AB1	IP3K2	0.949183608371002	0.80156895525114	0.994766463583882	pseudobulk	DESeq2	LRT	0.0960591113673091
112	KC_AB1	IPIP	-0.0865007265198023	0.89726804009839	0.994766463583882	pseudobulk	DESeq2	LRT	0.0470778011625438
112	KC_AB1	Ipk2	-0.676525963030744	0.651059679722087	0.994766463583882	pseudobulk	DESeq2	LRT	0.186379199778923
112	KC_AB1	iPLA2-VIA	-0.0851028199673415	0.915032610250427	0.994766463583882	pseudobulk	DESeq2	LRT	0.0385634281199477
112	KC_AB1	Ipod	-1.64952313825113	0.817565084244375	0.994766463583882	pseudobulk	DESeq2	LRT	0.0874776642296574
112	KC_AB1	Ipp	-0.044347483720292	0.890324261989482	0.994766463583882	pseudobulk	DESeq2	LRT	0.0504517916192722
112	KC_AB1	Ir25a	-2.79499059556516	0.38564607643653	0.994766463583882	pseudobulk	DESeq2	LRT	0.413811082791365
112	KC_AB1	Ir40a	0.949183608371002	0.80156895525114	0.994766463583882	pseudobulk	DESeq2	LRT	0.0960591113673091
112	KC_AB1	Ir41a	0.682246656685249	0.775848189487036	0.994766463583882	pseudobulk	DESeq2	LRT	0.110223248996933
112	KC_AB1	Ir47a	2.09225092496487	0.386054269300348	0.994766463583882	pseudobulk	DESeq2	LRT	0.413351640403342
112	KC_AB1	Ir47b	-4.68459955755204	0.323392468066551	0.994766463583882	pseudobulk	DESeq2	LRT	0.490270099195316
112	KC_AB1	Ir51a	-2.79499059556516	0.38564607643653	0.994766463583882	pseudobulk	DESeq2	LRT	0.413811082791365
112	KC_AB1	Ir52b	-1.64952313825113	0.817565084244375	0.994766463583882	pseudobulk	DESeq2	LRT	0.0874776642296574
112	KC_AB1	Ir56a	0.878465045342889	0.422376436154626	0.994766463583882	pseudobulk	DESeq2	LRT	0.374300318536447
112	KC_AB1	Ir62a	0.949183608371002	0.80156895525114	0.994766463583882	pseudobulk	DESeq2	LRT	0.0960591113673091
112	KC_AB1	Ir68a	-0.376985172705412	0.750951845023184	0.994766463583882	pseudobulk	DESeq2	LRT	0.124387911346341
112	KC_AB1	Ir76a	2.13651183194062	0.26871499492646	0.994766463583882	pseudobulk	DESeq2	LRT	0.570708098240757
112	KC_AB1	Ir93a	-2.57435989741145	0.533617212681275	0.994766463583882	pseudobulk	DESeq2	LRT	0.272770169998685
112	KC_AB1	Irbp	-0.33399464057665	0.801221707825449	0.994766463583882	pseudobulk	DESeq2	LRT	0.09624729270211
112	KC_AB1	Irbp18	-0.0311919507248035	0.957106939827696	0.994766463583882	pseudobulk	DESeq2	LRT	0.0190395347598824
112	KC_AB1	Ire1	0.0305212413707843	0.941935249538358	0.994766463583882	pseudobulk	DESeq2	LRT	0.0259789504286806
113	KC_AB1	Irk1	1.1821590793593	0.719511699510136	0.994766463583882	pseudobulk	DESeq2	LRT	0.142962139890846
113	KC_AB1	Irk2	-2.20969975456663	0.610836134188693	0.994766463583882	pseudobulk	DESeq2	LRT	0.214075280039667
113	KC_AB1	Irp-1A	0.0682063538826488	0.899318521719373	0.994766463583882	pseudobulk	DESeq2	LRT	0.0460864620767155
113	KC_AB1	Irp-1B	0.782890272096587	0.62241038932871	0.994766463583882	pseudobulk	DESeq2	LRT	0.20592316667635
113	KC_AB1	IRSp53	0.949183608371002	0.80156895525114	0.994766463583882	pseudobulk	DESeq2	LRT	0.0960591113673091
113	KC_AB1	Iru	0.385188109113314	0.534614256628879	0.994766463583882	pseudobulk	DESeq2	LRT	0.27195946403684
113	KC_AB1	IscU	-0.23511841133025	0.487741297691201	0.994766463583882	pseudobulk	DESeq2	LRT	0.3118104705719
113	KC_AB1	isopeptidase-T-3	-0.281741354115275	0.792332470650017	0.994766463583882	pseudobulk	DESeq2	LRT	0.101092545846519
113	KC_AB1	isoQC	0.617602353429823	0.593817232183209	0.994766463583882	pseudobulk	DESeq2	LRT	0.22634720361857
113	KC_AB1	Iswi	0.274830259589121	0.676546846521493	0.994766463583882	pseudobulk	DESeq2	LRT	0.169702125923154
113	KC_AB1	Itgbn	0.0587775856525997	0.890052531922536	0.994766463583882	pseudobulk	DESeq2	LRT	0.0505843600395771

113	KC_AB1	ITP	-1.36080367513457	0.587855376773158	0.994766463583882	pseudobulk	DESeq2	LRT	0.230729505207589
113	KC_AB1	Itpr	-0.0256361360424631	0.899835841205088	0.994766463583882	pseudobulk	DESeq2	LRT	0.045836712517869
113	KC_AB1	ix	0.735369072590692	0.40464903781389	0.994766463583882	pseudobulk	DESeq2	LRT	0.3929214879481
113	KC_AB1	Jafrac1	0.271230489517811	0.598190899480786	0.994766463583882	pseudobulk	DESeq2	LRT	0.223160198351409
113	KC_AB1	Jafrac2	0.455574030982407	0.793898444101867	0.994766463583882	pseudobulk	DESeq2	LRT	0.100235049193838
113	KC_AB1	jagn	0.141003588051968	0.830556083049914	0.994766463583882	pseudobulk	DESeq2	LRT	0.080631036618062
113	KC_AB1	janA	-0.105850165437588	0.879174898407014	0.994766463583882	pseudobulk	DESeq2	LRT	0.0559247200829112
113	KC_AB1	jar	-0.203060808997961	0.610757395731125	0.994766463583882	pseudobulk	DESeq2	LRT	0.214131265401156
113	KC_AB1	Jarid2	0.184835375441511	0.608565667435645	0.994766463583882	pseudobulk	DESeq2	LRT	0.215692552247046
113	KC_AB1	jbug	-1.08681444615798	0.701131388297773	0.994766463583882	pseudobulk	DESeq2	LRT	0.154200589927933
113	KC_AB1	jdp	0.106508595271538	0.33181046448173	0.994766463583882	pseudobulk	DESeq2	LRT	0.479109921521403
113	KC_AB1	jeb	0.296952756735209	0.505901148816779	0.994766463583882	pseudobulk	DESeq2	LRT	0.295934334380282
113	KC_AB1	jef	0.0809362577047482	0.805606284000889	0.994766463583882	pseudobulk	DESeq2	LRT	0.0938771547977028
113	KC_AB1	jet	0.588179192922397	0.548427268638524	0.994766463583882	pseudobulk	DESeq2	LRT	0.260880959563016
113	KC_AB1	JHDM2	0.0556507705136479	0.910364932142286	0.994766463583882	pseudobulk	DESeq2	LRT	0.0407844799367282
113	KC_AB1	JhI-21	-2.26306816020352	0.40449476385881	0.994766463583882	pseudobulk	DESeq2	LRT	0.393087095910655
113	KC_AB1	jigr1	0.0786103069114407	0.806884356860512	0.994766463583882	pseudobulk	DESeq2	LRT	0.0931887041579608
113	KC_AB1	JIL-1	0.513988553148761	0.152788334184903	0.994766463583882	pseudobulk	DESeq2	LRT	0.815909804088441
113	KC_AB1	jim	0.046300629896682	0.751696618159053	0.994766463583882	pseudobulk	DESeq2	LRT	0.123957403616039
113	KC_AB1	jing	0.184368535617453	0.632975251146284	0.994766463583882	pseudobulk	DESeq2	LRT	0.198613270236554
113	KC_AB1	JMJD4	-0.137995038297896	0.863923751741531	0.994766463583882	pseudobulk	DESeq2	LRT	0.0635245858303229
113	KC_AB1	JMJD5	-0.0831514714410424	0.911983473364721	0.994766463583882	pseudobulk	DESeq2	LRT	0.0400130317280961
113	KC_AB1	JMJD7	-0.130488002146384	0.900775943614201	0.994766463583882	pseudobulk	DESeq2	LRT	0.045383220733308
113	KC_AB1	jnj	0.195168059926154	0.862973752606598	0.994766463583882	pseudobulk	DESeq2	LRT	0.0640024131740941
113	KC_AB1	jp	-0.244253505547717	0.54523348278877	0.994766463583882	pseudobulk	DESeq2	LRT	0.263417481993822
113	KC_AB1	Jra	-0.586841118430635	0.436929535850847	0.994766463583882	pseudobulk	DESeq2	LRT	0.359588596573382
113	KC_AB1	JTBR	0.302752415141855	0.755833498626309	0.994766463583882	pseudobulk	DESeq2	LRT	0.121573864011804
113	KC_AB1	jub	1.49412432961789	0.317245120118075	0.994766463583882	pseudobulk	DESeq2	LRT	0.49860504952354
113	KC_AB1	jumu	0.0088064241494064	0.932465463281092	0.994766463583882	pseudobulk	DESeq2	LRT	0.030367244649795
113	KC_AB1	Jupiter	0.202572760186678	0.306649857262697	0.994766463583882	pseudobulk	DESeq2	LRT	0.513357233128902
113	KC_AB1	jus	0.147143364110879	0.317177104520098	0.994766463583882	pseudobulk	DESeq2	LRT	0.498698169839477
113	KC_AB1	jv	-0.324621418602925	0.866631655762513	0.994766463583882	pseudobulk	DESeq2	LRT	0.0621654513830088
113	KC_AB1	jvl	0.0176105836024662	0.926633450238797	0.994766463583882	pseudobulk	DESeq2	LRT	0.0330920263949859
113	KC_AB1	Jwa	0.153070410081974	0.763015280835455	0.994766463583882	pseudobulk	DESeq2	LRT	0.117466764383175
113	KC_AB1	KaiR1D	0.622885214293614	0.454374450768368	0.994766463583882	pseudobulk	DESeq2	LRT	0.342586096756251
113	KC_AB1	Kank	1.50387178472499	0.216274505908509	0.994766463583882	pseudobulk	DESeq2	LRT	0.664994671480008
113	KC_AB1	Kap-alpha1	0.478003250758157	0.453864088868797	0.994766463583882	pseudobulk	DESeq2	LRT	0.343074178621121
113	KC_AB1	Kap-alpha3	-0.113257225435054	0.735684713252025	0.994766463583882	pseudobulk	DESeq2	LRT	0.133308268042409
113	KC_AB1	Kap3	0.391371296637247	0.478824852110545	0.994766463583882	pseudobulk	DESeq2	LRT	0.319823316798679
113	KC_AB1	kar	0.949183608371002	0.80156895525114	0.994766463583882	pseudobulk	DESeq2	LRT	0.0960591113673091
113	KC_AB1	Karybeta3	-0.522346037693511	0.401020206863131	0.994766463583882	pseudobulk	DESeq2	LRT	0.396833743319838
113	KC_AB1	kat-60L1	-0.239609488126364	0.630394500484365	0.994766463583882	pseudobulk	DESeq2	LRT	0.200387584255353
113	KC_AB1	Kat60	-0.27447314327185	0.735772172984424	0.994766463583882	pseudobulk	DESeq2	LRT	0.133256641277711
113	KC_AB1	kat80	-0.688461898522955	0.284874413201655	0.994766463583882	pseudobulk	DESeq2	LRT	0.545346556391807
113	KC_AB1	kay	-0.457913092327952	0.543636468728136	0.994766463583882	pseudobulk	DESeq2	LRT	0.264691417215216
113	KC_AB1	Kaz1-ORFB	0.575114571237417	0.757941253858868	0.994766463583882	pseudobulk	DESeq2	LRT	0.120364454148462
113	KC_AB1	kcc	0.0960389600655532	0.743588667993633	0.994766463583882	pseudobulk	DESeq2	LRT	0.128667237331196
113	KC_AB1	Kcmf1	-0.28274429430033	0.504132644186311	0.994766463583882	pseudobulk	DESeq2	LRT	0.297455179707332
113	KC_AB1	KCNQ	-0.0338756824393985	0.882148856391419	0.994766463583882	pseudobulk	DESeq2	LRT	0.0544581245567548
113	KC_AB1	KdelR	0.146959372371394	0.788678028835603	0.994766463583882	pseudobulk	DESeq2	LRT	0.103100257675773
113	KC_AB1	Kdm2	-0.0856863300011773	0.861843920476124	0.994766463583882	pseudobulk	DESeq2	LRT	0.0645713775783673
113	KC_AB1	Kdm4A	0.384725650202639	0.711141536939437	0.994766463583882	pseudobulk	DESeq2	LRT	0.148043953989804
113	KC_AB1	Kdm4B	-0.127717224811762	0.66501970943274	0.994766463583882	pseudobulk	DESeq2	LRT	0.177165483161504
113	KC_AB1	kdn	0.16410053740257	0.707984207301726	0.994766463583882	pseudobulk	DESeq2	LRT	0.149976429821829
113	KC_AB1	Keap1	0.130219275495283	0.915869925406145	0.994766463583882	pseudobulk	DESeq2	LRT	0.0381662017575264
113	KC_AB1	Kebab	0.597141410293382	0.802139944912821	0.994766463583882	pseudobulk	DESeq2	LRT	0.0957498561530429

113	KC_AB1	kek1	0.00850047203154228	0.887839575630888	0.994766463583882	pseudobulk	DESeq2	LRT	0.0516655001137171
113	KC_AB1	kek2	1.10251292103259	0.639221751231493	0.994766463583882	pseudobulk	DESeq2	LRT	0.194348455410132
113	KC_AB1	kek3	0.0236659272531523	0.872225648083305	0.994766463583882	pseudobulk	DESeq2	LRT	0.0593711469021259
113	KC_AB1	kek4	0.0261592597930138	0.93121769624147	0.994766463583882	pseudobulk	DESeq2	LRT	0.0309487795724122
113	KC_AB1	kek5	3.680983711627	0.163847950338491	0.994766463583882	pseudobulk	DESeq2	LRT	0.78555898706922
113	KC_AB1	kel	-0.0113269159612402	0.947948299451262	0.994766463583882	pseudobulk	DESeq2	LRT	0.023215348184414
113	KC_AB1	key	0.575853669062856	0.388171538924346	0.994766463583882	pseudobulk	DESeq2	LRT	0.410976310630385
113	KC_AB1	KFase	1.45682795759908	0.573003993665791	0.994766463583882	pseudobulk	DESeq2	LRT	0.24184235111992
113	KC_AB1	Khc	-0.158514390826887	0.757776187939092	0.994766463583882	pseudobulk	DESeq2	LRT	0.120459045945608
113	KC_AB1	Khc-73	-0.00856659377078202	0.907065507077334	0.994766463583882	pseudobulk	DESeq2	LRT	0.0423613476313056
113	KC_AB1	kibra	-3.21143241058256	0.292545415061155	0.994766463583882	pseudobulk	DESeq2	LRT	0.533806704011112
113	KC_AB1	Kif3C	-1.31308249775939	0.126190003612363	0.994766463583882	pseudobulk	DESeq2	LRT	0.898975047215024
113	KC_AB1	kin17	-0.0708276105002205	0.931821212766976	0.994766463583882	pseudobulk	DESeq2	LRT	0.0306674071279982
113	KC_AB1	kirre	0.412565677491153	0.125518641280647	0.994766463583882	pseudobulk	DESeq2	LRT	0.901291770564453
113	KC_AB1	kis	-0.113185202158065	0.546752921398152	0.994766463583882	pseudobulk	DESeq2	LRT	0.262208887771428
113	KC_AB1	kl-3	0.949183608371002	0.80156895525114	0.994766463583882	pseudobulk	DESeq2	LRT	0.0960591113673091
113	KC_AB1	kl-5	0.949183608371002	0.80156895525114	0.994766463583882	pseudobulk	DESeq2	LRT	0.0960591113673091
113	KC_AB1	klar	-0.121291690890284	0.769751044481746	0.994766463583882	pseudobulk	DESeq2	LRT	0.113649713126484
113	KC_AB1	Klc	-0.172479092520342	0.713289088727191	0.994766463583882	pseudobulk	DESeq2	LRT	0.146734419385759
113	KC_AB1	klg	0.224808081565749	0.400984223170832	0.994766463583882	pseudobulk	DESeq2	LRT	0.396872714473685
113	KC_AB1	KLHL18	-0.318796821082065	0.626985012021781	0.994766463583882	pseudobulk	DESeq2	LRT	0.202742840786565
113	KC_AB1	Klp10A	-0.511530459284738	0.399909922459797	0.994766463583882	pseudobulk	DESeq2	LRT	0.398037820132355
113	KC_AB1	Klp31E	0.692987350013221	0.569472291296995	0.994766463583882	pseudobulk	DESeq2	LRT	0.244527402453314
113	KC_AB1	Klp54D	-0.237473683803514	0.877148860518115	0.994766463583882	pseudobulk	DESeq2	LRT	0.0569266964680766
113	KC_AB1	Klp64D	0.13071901240972	0.772192672002105	0.994766463583882	pseudobulk	DESeq2	LRT	0.112274324084865
113	KC_AB1	Klp67A	2.38929733117871	0.484296857543297	0.994766463583882	pseudobulk	DESeq2	LRT	0.314888348950639
113	KC_AB1	Klp68D	-0.0959203360335451	0.863986958738593	0.994766463583882	pseudobulk	DESeq2	LRT	0.0634928128343139
113	KC_AB1	Klp98A	1.01874525545908	0.322765116500562	0.994766463583882	pseudobulk	DESeq2	LRT	0.49111340867721
113	KC_AB1	klu	-2.85724547560282	0.450020141104186	0.994766463583882	pseudobulk	DESeq2	LRT	0.346768048503189
113	KC_AB1	Kmn1	0.304329557009274	0.793071473301212	0.994766463583882	pseudobulk	DESeq2	LRT	0.100687671368952
113	KC_AB1	kmr	-0.359258496376583	0.284254601548692	0.994766463583882	pseudobulk	DESeq2	LRT	0.546292496170466
113	KC_AB1	ko	-0.48167422725367	0.855534332799682	0.994766463583882	pseudobulk	DESeq2	LRT	0.0677625574305792
113	KC_AB1	koi	-0.236965726972717	0.695974477793219	0.994766463583882	pseudobulk	DESeq2	LRT	0.157406686189442
114	KC_AB1	koko	-0.544086788549543	0.664543485961261	0.994766463583882	pseudobulk	DESeq2	LRT	0.177476594713594
114	KC_AB1	Kr-h1	0.188370485484782	0.732964918086695	0.994766463583882	pseudobulk	DESeq2	LRT	0.134916811506177
114	KC_AB1	Kr-h2	-0.186823101184436	0.689529554493611	0.994766463583882	pseudobulk	DESeq2	LRT	0.161447114436046
114	KC_AB1	kra	0.0268603516268624	0.878164813607589	0.994766463583882	pseudobulk	DESeq2	LRT	0.0564239682361896
114	KC_AB1	kraken	0.210058504140436	0.736283985625616	0.994766463583882	pseudobulk	DESeq2	LRT	0.132954645442165
114	KC_AB1	kri	0.0968862657539864	0.857843939982917	0.994766463583882	pseudobulk	DESeq2	LRT	0.0665917123480753
114	KC_AB1	Krn	-0.502004128142127	0.719306100166833	0.994766463583882	pseudobulk	DESeq2	LRT	0.143086256594027
114	KC_AB1	KrT95D	0.336718960127987	0.208370774163508	0.994766463583882	pseudobulk	DESeq2	LRT	0.681163194727608
114	KC_AB1	krz	0.189489350726029	0.672846895552188	0.994766463583882	pseudobulk	DESeq2	LRT	0.172083747041028
114	KC_AB1	ksh	-0.22723256051774	0.709167909610218	0.994766463583882	pseudobulk	DESeq2	LRT	0.149250924783644
114	KC_AB1	ksr	0.284814662288527	0.736148056210715	0.994766463583882	pseudobulk	DESeq2	LRT	0.133034830324469
114	KC_AB1	Ktl	-0.0631637983551259	0.814305864209892	0.994766463583882	pseudobulk	DESeq2	LRT	0.0892124376315438
114	KC_AB1	kto	0.213074256575884	0.833438794283422	0.994766463583882	pseudobulk	DESeq2	LRT	0.0791262881946802
114	KC_AB1	Ku80	-0.263094899609327	0.847557906290387	0.994766463583882	pseudobulk	DESeq2	LRT	0.0718306205310427
114	KC_AB1	kud	0.301078547552848	0.580267435545037	0.994766463583882	pseudobulk	DESeq2	LRT	0.23637180124303
114	KC_AB1	kug	-0.318419791149196	0.354000552869878	0.994766463583882	pseudobulk	DESeq2	LRT	0.450996059702603
114	KC_AB1	kuk	-0.0839589017433113	0.891555429309063	0.994766463583882	pseudobulk	DESeq2	LRT	0.0498516509241505
114	KC_AB1	Kul	-0.0925209390545214	0.853616252137937	0.994766463583882	pseudobulk	DESeq2	LRT	0.0687373249019632
114	KC_AB1	kuz	0.214717721688845	0.409114189287823	0.994766463583882	pseudobulk	DESeq2	LRT	0.388155457625708
114	KC_AB1	Kyat	1.32939657121883	0.310075090317708	0.994766463583882	pseudobulk	DESeq2	LRT	0.508533121128323
114	KC_AB1	kz	-0.110450645050449	0.861952233048675	0.994766463583882	pseudobulk	DESeq2	LRT	0.0645168008785117
114	KC_AB1	l(1)10Bb	-0.303869125404692	0.605941477946858	0.994766463583882	pseudobulk	DESeq2	LRT	0.217569318130816
114	KC_AB1	l(1)G0004	-0.389620648666693	0.738227157071531	0.994766463583882	pseudobulk	DESeq2	LRT	0.131809982508863

114	KC_AB1	l(1)G0007	0.237135476557432	0.743355864218042	0.994766463583882	pseudobulk	DESeq2	LRT	0.128803228141534
114	KC_AB1	l(1)G0020	2.02670873290438	0.538959414925803	0.994766463583882	pseudobulk	DESeq2	LRT	0.26844393710898
114	KC_AB1	l(1)G0193	0.101135908532207	0.907741474642351	0.994766463583882	pseudobulk	DESeq2	LRT	0.0420378212177911
114	KC_AB1	l(1)G0196	-0.37685575191771	0.298507729443048	0.994766463583882	pseudobulk	DESeq2	LRT	0.525044418936611
114	KC_AB1	l(1)G0289	-0.0763694283500961	0.758826537212729	0.994766463583882	pseudobulk	DESeq2	LRT	0.119857489639557
114	KC_AB1	l(1)G0320	-0.216064141465495	0.528963158458498	0.994766463583882	pseudobulk	DESeq2	LRT	0.27657457491256
114	KC_AB1	l(1)G0469	-0.358844904214382	0.243625760548086	0.994766463583882	pseudobulk	DESeq2	LRT	0.613276792096707
114	KC_AB1	l(2)05287	0.225870234592611	0.854316558685538	0.994766463583882	pseudobulk	DESeq2	LRT	0.0683811758961231
114	KC_AB1	l(2)05714	-0.178678259011258	0.884368576555782	0.994766463583882	pseudobulk	DESeq2	LRT	0.0533666972001499
114	KC_AB1	l(2)09851	-0.570349900778484	0.678460420438259	0.994766463583882	pseudobulk	DESeq2	LRT	0.168475482840848
114	KC_AB1	l(2)10685	0.217906885051292	0.902008941520292	0.994766463583882	pseudobulk	DESeq2	LRT	0.0447891573209458
114	KC_AB1	l(2)34Fd	0.0236942436874584	0.910119428894173	0.994766463583882	pseudobulk	DESeq2	LRT	0.0409016143750932
114	KC_AB1	l(2)35Be	-0.646059665156839	0.513823537736598	0.994766463583882	pseudobulk	DESeq2	LRT	0.289186005023
114	KC_AB1	l(2)37Bb	-0.361980392568861	0.736589092775089	0.994766463583882	pseudobulk	DESeq2	LRT	0.132774716356294
114	KC_AB1	l(2)37Cb	0.201201874521541	0.904182091561904	0.994766463583882	pseudobulk	DESeq2	LRT	0.0437440989527594
114	KC_AB1	l(2)37Cc	-0.39402818922359	0.515104365332809	0.994766463583882	pseudobulk	DESeq2	LRT	0.288104769607059
114	KC_AB1	l(2)37Cd	-0.572894902042659	0.792877545406836	0.994766463583882	pseudobulk	DESeq2	LRT	0.100793881359111
114	KC_AB1	l(2)37Cg	-1.29964949805355	0.212483142570471	0.994766463583882	pseudobulk	DESeq2	LRT	0.67267551916203
114	KC_AB1	l(2)41Ab	0.351624531625303	0.710894204316426	0.994766463583882	pseudobulk	DESeq2	LRT	0.148195026415531
114	KC_AB1	l(2)efl	-0.0731715587870181	0.90231973590419	0.994766463583882	pseudobulk	DESeq2	LRT	0.0446395434629419
114	KC_AB1	l(2)gd1	-0.109857757368557	0.88851911130275	0.994766463583882	pseudobulk	DESeq2	LRT	0.051333226447241
114	KC_AB1	l(2)k01209	0.12201439080209	0.822534016133822	0.994766463583882	pseudobulk	DESeq2	LRT	0.0848461326318009
114	KC_AB1	l(2)k05819	0.257169209272517	0.646347873980955	0.994766463583882	pseudobulk	DESeq2	LRT	0.189533675369695
114	KC_AB1	l(2)k09022	0.788455060960304	0.697710180635433	0.994766463583882	pseudobulk	DESeq2	LRT	0.156324939968351
114	KC_AB1	l(2)k09848	0.289091137713428	0.79550333039272	0.994766463583882	pseudobulk	DESeq2	LRT	0.0993579978299034
114	KC_AB1	l(2)k09913	0.0463038647270428	0.853813694039971	0.994766463583882	pseudobulk	DESeq2	LRT	0.0686368839683557
114	KC_AB1	l(2)k10201	-0.262032672895729	0.847553129464493	0.994766463583882	pseudobulk	DESeq2	LRT	0.0718330682162774
114	KC_AB1	l(2)k14505	0.408194790728098	0.509733451526638	0.994766463583882	pseudobulk	DESeq2	LRT	0.292656864671598
114	KC_AB1	l(2)SH0834	-0.124864387171112	0.786233742101974	0.994766463583882	pseudobulk	DESeq2	LRT	0.104448321881684
114	KC_AB1	l(3)02640	-0.296489061261183	0.59654649154824	0.994766463583882	pseudobulk	DESeq2	LRT	0.2243557041545
114	KC_AB1	l(3)04053	0.267978527192979	0.751154934150114	0.994766463583882	pseudobulk	DESeq2	LRT	0.124270475636816
114	KC_AB1	l(3)05822	-0.294701621590472	0.637422500354796	0.994766463583882	pseudobulk	DESeq2	LRT	0.195572610445813
114	KC_AB1	l(3)07882	0.165736800488031	0.854052802276552	0.994766463583882	pseudobulk	DESeq2	LRT	0.0685152779896362
114	KC_AB1	l(3)72Ab	-0.307299030701047	0.737163653421959	0.994766463583882	pseudobulk	DESeq2	LRT	0.132436086241113
114	KC_AB1	l(3)72Dn	0.696124738815335	0.683158309383576	0.994766463583882	pseudobulk	DESeq2	LRT	0.165478644890948
114	KC_AB1	l(3)72Dp	-2.7812351869417	0.120374902907143	0.994766463583882	pseudobulk	DESeq2	LRT	0.919464050163642
114	KC_AB1	l(3)72Dr	-3.0829163216422	0.221862415172695	0.994766463583882	pseudobulk	DESeq2	LRT	0.653916263633792
114	KC_AB1	l(3)73Ah	-0.346051457293913	0.565287283062302	0.994766463583882	pseudobulk	DESeq2	LRT	0.24773078449918
114	KC_AB1	l(3)76BDm	-0.319866993134199	0.750598773405097	0.994766463583882	pseudobulk	DESeq2	LRT	0.124592149626951
114	KC_AB1	l(3)80Fg	-0.00115159970938217	0.955733340644729	0.994766463583882	pseudobulk	DESeq2	LRT	0.0196632634152624
114	KC_AB1	l(3)80Fj	0.0283156699153339	0.875906287370037	0.994766463583882	pseudobulk	DESeq2	LRT	0.0575423562236693
114	KC_AB1	l(3)87Df	-0.274368798973812	0.639664066485433	0.994766463583882	pseudobulk	DESeq2	LRT	0.194048045351555
114	KC_AB1	l(3)mbt	0.197041151763454	0.851237758237255	0.994766463583882	pseudobulk	DESeq2	LRT	0.0699491206806121
114	KC_AB1	l(3)neo43	0.250385399905923	0.647036916715396	0.994766463583882	pseudobulk	DESeq2	LRT	0.189070939936549
114	KC_AB1	l(3)psg2	-0.0710384408299208	0.895368866386832	0.994766463583882	pseudobulk	DESeq2	LRT	0.0479980109035178
114	KC_AB1	L2HGDH	0.0482049638889365	0.903810521567597	0.994766463583882	pseudobulk	DESeq2	LRT	0.0439226072046943
114	KC_AB1	La	0.244161204873732	0.682215402771593	0.994766463583882	pseudobulk	DESeq2	LRT	0.166078479500311
114	KC_AB1	Lac	-0.835152883801839	0.920434724857442	0.994766463583882	pseudobulk	DESeq2	LRT	0.0360070052473159
114	KC_AB1	lace	-0.345226211085554	0.6835498350035	0.994766463583882	pseudobulk	DESeq2	LRT	0.165229817192648
114	KC_AB1	Lam	-0.19409053383077	0.721527047365663	0.994766463583882	pseudobulk	DESeq2	LRT	0.141747384180344
114	KC_AB1	LamC	-0.593588837429502	0.717888719837047	0.994766463583882	pseudobulk	DESeq2	LRT	0.143942870668869
114	KC_AB1	Lamp1	-0.045490497984429	0.869055425759372	0.994766463583882	pseudobulk	DESeq2	LRT	0.0609525246631371
114	KC_AB1	lap	-0.0130797209120961	0.953150696104001	0.994766463583882	pseudobulk	DESeq2	LRT	0.0208384306182399
114	KC_AB1	Lap1	0.600932221349441	0.525601471319202	0.994766463583882	pseudobulk	DESeq2	LRT	0.27934342772355
114	KC_AB1	Lar	0.129771304536967	0.466708922017218	0.994766463583882	pseudobulk	DESeq2	LRT	0.330953896681144
114	KC_AB1	lark	-0.015732454326819	0.912007170144988	0.994766463583882	pseudobulk	DESeq2	LRT	0.0400017472613188

114	KC_AB1	Larp4B	0.112346651546234	0.589512792521543	0.994766463583882	pseudobulk	DESeq2	LRT	0.22950676620717
114	KC_AB1	Las	-0.326410503701217	0.774181998028944	0.994766463583882	pseudobulk	DESeq2	LRT	0.111156931502713
114	KC_AB1	lawc	-0.387830906700066	0.323449878632889	0.994766463583882	pseudobulk	DESeq2	LRT	0.490193007482455
114	KC_AB1	lbk	-0.862782200443248	0.0438225171800982	0.994766463583882	pseudobulk	DESeq2	LRT	1.358302680038
114	KC_AB1	lbm	-1.79554031405525	0.00230246256977653	0.994766463583882	pseudobulk	DESeq2	LRT	2.63780742122737
114	KC_AB1	LBR	0.390543035142486	0.704427322286972	0.994766463583882	pseudobulk	DESeq2	LRT	0.152163807604898
114	KC_AB1	Lcch3	-0.357911516691083	0.463171499306724	0.994766463583882	pseudobulk	DESeq2	LRT	0.334258172239678
114	KC_AB1	ldbr	-1.36080367513457	0.587855376773158	0.994766463583882	pseudobulk	DESeq2	LRT	0.230729505207589
114	KC_AB1	Leash	-0.343475499443363	0.773715728769052	0.994766463583882	pseudobulk	DESeq2	LRT	0.111418574335144
114	KC_AB1	lectin-37Db	0.949183608371002	0.80156895525114	0.994766463583882	pseudobulk	DESeq2	LRT	0.0960591113673091
114	KC_AB1	Lerp	0.222675748176412	0.615829501448783	0.994766463583882	pseudobulk	DESeq2	LRT	0.210539509961042
114	KC_AB1	Letm1	0.141689526337652	0.80473897266406	0.994766463583882	pseudobulk	DESeq2	LRT	0.0943449657376719
114	KC_AB1	LeuRS	1.64360917377131	0.329636648608536	0.994766463583882	pseudobulk	DESeq2	LRT	0.481964509949294
114	KC_AB1	LeuRS-m	-0.502792276798834	0.78573817439348	0.994766463583882	pseudobulk	DESeq2	LRT	0.104722146529669
114	KC_AB1	levy	0.202007407579174	0.364679629164083	0.994766463583882	pseudobulk	DESeq2	LRT	0.438088495498171
114	KC_AB1	Lgr1	0.0749692951454633	0.915321584844678	0.994766463583882	pseudobulk	DESeq2	LRT	0.0384262961123042
114	KC_AB1	Lgr4	-1.64952313825113	0.817565084244375	0.994766463583882	pseudobulk	DESeq2	LRT	0.0874776642296574
114	KC_AB1	lgs	-0.120548131345902	0.689675143051691	0.994766463583882	pseudobulk	DESeq2	LRT	0.16135542636719
114	KC_AB1	Lhr	-0.123148941429253	0.873256876336373	0.994766463583882	pseudobulk	DESeq2	LRT	0.0588579858870696
114	KC_AB1	lic	-0.107777277825002	0.854276260533965	0.994766463583882	pseudobulk	DESeq2	LRT	0.0684016620700852
114	KC_AB1	lid	-0.298521013252948	0.360818541893071	0.994766463583882	pseudobulk	DESeq2	LRT	0.442711152851451
115	KC_AB1	lili	1.26608310608741	0.18570282388353	0.994766463583882	pseudobulk	DESeq2	LRT	0.731181492126079
115	KC_AB1	lilli	0.043471095813003	0.818705029384275	0.994766463583882	pseudobulk	DESeq2	LRT	0.0868725416891889
115	KC_AB1	LIMK1	-0.308256153090408	0.528786756914899	0.994766463583882	pseudobulk	DESeq2	LRT	0.27671942998006
115	KC_AB1	lin	1.08636259809041	0.453259364652031	0.994766463583882	pseudobulk	DESeq2	LRT	0.343653214315326
115	KC_AB1	lin-28	0.949183608371002	0.80156895525114	0.994766463583882	pseudobulk	DESeq2	LRT	0.0960591113673091
115	KC_AB1	lin-52	0.1085294523328	0.844538221419074	0.994766463583882	pseudobulk	DESeq2	LRT	0.0733806907042586
115	KC_AB1	link	-3.21143241058256	0.292545415061155	0.994766463583882	pseudobulk	DESeq2	LRT	0.533806704011112
115	KC_AB1	Lint-1	-0.115983848231057	0.856399995728287	0.994766463583882	pseudobulk	DESeq2	LRT	0.0673233435438548
115	KC_AB1	Liprin-alpha	-0.0413512259451114	0.908478378288721	0.994766463583882	pseudobulk	DESeq2	LRT	0.0416854044061832
115	KC_AB1	Liprin-beta	-0.475938784040097	0.596944565494022	0.994766463583882	pseudobulk	DESeq2	LRT	0.224065997208757
115	KC_AB1	Liprin-gamma	0.129329017870445	0.603193272391244	0.994766463583882	pseudobulk	DESeq2	LRT	0.219543510937441
115	KC_AB1	Lis-1	0.0643649617141525	0.722957206416872	0.994766463583882	pseudobulk	DESeq2	LRT	0.14088740888396
115	KC_AB1	List	-3.21143241058256	0.292545415061155	0.994766463583882	pseudobulk	DESeq2	LRT	0.533806704011112
115	KC_AB1	Lk6	-0.0415012728821859	0.814577546912552	0.994766463583882	pseudobulk	DESeq2	LRT	0.0890675650164968
115	KC_AB1	Lkb1	-0.0398576879810734	0.889686644445898	0.994766463583882	pseudobulk	DESeq2	LRT	0.0507629288056637
115	KC_AB1	LKRSDH	-0.739006821616313	0.76979092815726	0.994766463583882	pseudobulk	DESeq2	LRT	0.113627211290866
115	KC_AB1	Lmpt	-0.203364326052077	0.455721583068378	0.994766463583882	pseudobulk	DESeq2	LRT	0.34130040265404
115	KC_AB1	lms	-0.0627561689052589	0.891934490418666	0.994766463583882	pseudobulk	DESeq2	LRT	0.0496670419181083
115	KC_AB1	lncRNA:acal	1.1821590793593	0.719511699510136	0.994766463583882	pseudobulk	DESeq2	LRT	0.142962139890846
115	KC_AB1	lncRNA:alphagamma-ele	-0.115988279885653	0.717253540388143	0.994766463583882	pseudobulk	DESeq2	LRT	0.144327299384174
115	KC_AB1	lncRNA:cherub	0.201281629016931	0.719802013934812	0.994766463583882	pseudobulk	DESeq2	LRT	0.14278694256842
115	KC_AB1	lncRNA:CR30055	-0.0454759178059218	0.894151510098867	0.994766463583882	pseudobulk	DESeq2	LRT	0.0485888856505999
115	KC_AB1	lncRNA:CR30121	-2.20969975456663	0.610836134188693	0.994766463583882	pseudobulk	DESeq2	LRT	0.214075280039667
115	KC_AB1	lncRNA:CR31044	0.0484485100748806	0.890364545493913	0.994766463583882	pseudobulk	DESeq2	LRT	0.050432142027928
115	KC_AB1	lncRNA:CR31386	0.806933541094274	0.754600478929312	0.994766463583882	pseudobulk	DESeq2	LRT	0.122282923497213
115	KC_AB1	lncRNA:CR31451	-0.0745212625323107	0.694690337678747	0.994766463583882	pseudobulk	DESeq2	LRT	0.158208741606407
115	KC_AB1	lncRNA:CR31526	0.949183608371002	0.80156895525114	0.994766463583882	pseudobulk	DESeq2	LRT	0.0960591113673091
115	KC_AB1	lncRNA:CR32218	-0.338456552082876	0.586630947463675	0.994766463583882	pseudobulk	DESeq2	LRT	0.231635029732252
115	KC_AB1	lncRNA:CR32553	1.1821590793593	0.719511699510136	0.994766463583882	pseudobulk	DESeq2	LRT	0.142962139890846
115	KC_AB1	lncRNA:CR32582	0.603253981482333	0.780782035750319	0.994766463583882	pseudobulk	DESeq2	LRT	0.107470187483089
115	KC_AB1	lncRNA:CR32773	-2.00005840186615	0.514177117052568	0.994766463583882	pseudobulk	DESeq2	LRT	0.288887255112324
115	KC_AB1	lncRNA:CR33938	0.949183608371002	0.80156895525114	0.994766463583882	pseudobulk	DESeq2	LRT	0.0960591113673091
115	KC_AB1	lncRNA:CR34335	0.0259382660466313	0.856492484177488	0.994766463583882	pseudobulk	DESeq2	LRT	0.0672764436670234
115	KC_AB1	lncRNA:CR40469	-0.0660674578084875	0.925714500979406	0.994766463583882	pseudobulk	DESeq2	LRT	0.0335229331530616
115	KC_AB1	lncRNA:CR41257	1.1821590793593	0.719511699510136	0.994766463583882	pseudobulk	DESeq2	LRT	0.142962139890846

115	KC_AB1	lncRNA:CR42491	0.729740153036158	0.752353468463509	0.994766463583882	pseudobulk	DESeq2	LRT	0.123578072524162
115	KC_AB1	lncRNA:CR42549	0.404919255179227	0.710345152925775	0.994766463583882	pseudobulk	DESeq2	LRT	0.148530578621975
115	KC_AB1	lncRNA:CR42736	0.949183608371002	0.80156895525114	0.994766463583882	pseudobulk	DESeq2	LRT	0.0960591113673091
115	KC_AB1	lncRNA:CR42861	0.391191116778717	0.447566066085051	0.994766463583882	pseudobulk	DESeq2	LRT	0.349142848575055
115	KC_AB1	lncRNA:CR42862	-0.3010263397939	0.035148233742841	0.994766463583882	pseudobulk	DESeq2	LRT	1.45409649411531
115	KC_AB1	lncRNA:CR43130	-1.64952313825113	0.817565084244375	0.994766463583882	pseudobulk	DESeq2	LRT	0.0874776642296574
115	KC_AB1	lncRNA:CR43146	1.1821590793593	0.719511699510136	0.994766463583882	pseudobulk	DESeq2	LRT	0.142962139890846
115	KC_AB1	lncRNA:CR43148	0.166337473843927	0.82407849255168	0.994766463583882	pseudobulk	DESeq2	LRT	0.0840314202706638
115	KC_AB1	lncRNA:CR43241	0.281199861760506	0.710066486677315	0.994766463583882	pseudobulk	DESeq2	LRT	0.148700984456702
115	KC_AB1	lncRNA:CR43242	-0.747784688099343	0.585104991460222	0.994766463583882	pseudobulk	DESeq2	LRT	0.232766196959552
115	KC_AB1	lncRNA:CR43256	-0.0349213376805719	0.899314947710806	0.994766463583882	pseudobulk	DESeq2	LRT	0.0460881880228023
115	KC_AB1	lncRNA:CR43264	0.934728130840428	0.2999221696612	0.994766463583882	pseudobulk	DESeq2	LRT	0.522991430853783
115	KC_AB1	lncRNA:CR43301	2.80587074018119	0.385946061723411	0.994766463583882	pseudobulk	DESeq2	LRT	0.413473386345624
115	KC_AB1	lncRNA:CR43309	-1.64952313825113	0.817565084244375	0.994766463583882	pseudobulk	DESeq2	LRT	0.0874776642296574
115	KC_AB1	lncRNA:CR43334	0.51961258492118	0.518872302043974	0.994766463583882	pseudobulk	DESeq2	LRT	0.284939511788246
115	KC_AB1	lncRNA:CR43363	0.949183608371002	0.80156895525114	0.994766463583882	pseudobulk	DESeq2	LRT	0.0960591113673091
115	KC_AB1	lncRNA:CR43399	-1.64952313825113	0.817565084244375	0.994766463583882	pseudobulk	DESeq2	LRT	0.0874776642296574
115	KC_AB1	lncRNA:CR43406	0.949183608371002	0.80156895525114	0.994766463583882	pseudobulk	DESeq2	LRT	0.0960591113673091
115	KC_AB1	lncRNA:CR43408	-1.64952313825113	0.817565084244375	0.994766463583882	pseudobulk	DESeq2	LRT	0.0874776642296574
115	KC_AB1	lncRNA:CR43424	-1.64952313825113	0.817565084244375	0.994766463583882	pseudobulk	DESeq2	LRT	0.0874776642296574
115	KC_AB1	lncRNA:CR43452	-1.64952313825113	0.817565084244375	0.994766463583882	pseudobulk	DESeq2	LRT	0.0874776642296574
115	KC_AB1	lncRNA:CR43459	-0.483884064249894	0.429849180011898	0.994766463583882	pseudobulk	DESeq2	LRT	0.366683897392976
115	KC_AB1	lncRNA:CR43461	-2.20969975456663	0.610836134188693	0.994766463583882	pseudobulk	DESeq2	LRT	0.214075280039667
115	KC_AB1	lncRNA:CR43492	0.833441706499796	0.620042047278132	0.994766463583882	pseudobulk	DESeq2	LRT	0.207578858434506
115	KC_AB1	lncRNA:CR43495	0.949183608371002	0.80156895525114	0.994766463583882	pseudobulk	DESeq2	LRT	0.0960591113673091
115	KC_AB1	lncRNA:CR43496	1.09998363574446	0.471467231246942	0.994766463583882	pseudobulk	DESeq2	LRT	0.326548486984309
115	KC_AB1	lncRNA:CR43604	0.911498702061926	0.666302272230238	0.994766463583882	pseudobulk	DESeq2	LRT	0.176328705694236
115	KC_AB1	lncRNA:CR43607	-2.79499059556516	0.38564607643653	0.994766463583882	pseudobulk	DESeq2	LRT	0.413811082791365
115	KC_AB1	lncRNA:CR43635	3.3077188457669	0.230409282515317	0.994766463583882	pseudobulk	DESeq2	LRT	0.637500028443383
115	KC_AB1	lncRNA:CR43651	0.251265411878081	0.646752320689069	0.994766463583882	pseudobulk	DESeq2	LRT	0.189262004264842
115	KC_AB1	lncRNA:CR43654	-1.64952313825113	0.817565084244375	0.994766463583882	pseudobulk	DESeq2	LRT	0.0874776642296574
115	KC_AB1	lncRNA:CR43656	0.949183608371002	0.80156895525114	0.994766463583882	pseudobulk	DESeq2	LRT	0.0960591113673091
115	KC_AB1	lncRNA:CR43685	-0.17277264740475	0.777399811406635	0.994766463583882	pseudobulk	DESeq2	LRT	0.109355569062442
115	KC_AB1	lncRNA:CR43700	-1.64952313825113	0.817565084244375	0.994766463583882	pseudobulk	DESeq2	LRT	0.0874776642296574
115	KC_AB1	lncRNA:CR43818	-1.64952313825113	0.817565084244375	0.994766463583882	pseudobulk	DESeq2	LRT	0.0874776642296574
115	KC_AB1	lncRNA:CR43828	1.1821590793593	0.719511699510136	0.994766463583882	pseudobulk	DESeq2	LRT	0.142962139890846
115	KC_AB1	lncRNA:CR43832	0.949183608371002	0.80156895525114	0.994766463583882	pseudobulk	DESeq2	LRT	0.0960591113673091
115	KC_AB1	lncRNA:CR43836	2.01123047816982	0.344762548401913	0.994766463583882	pseudobulk	DESeq2	LRT	0.462479917748112
115	KC_AB1	lncRNA:CR43837	0.325299774473276	0.834253692332345	0.994766463583882	pseudobulk	DESeq2	LRT	0.078701862520321
115	KC_AB1	lncRNA:CR43838	0.949183608371002	0.80156895525114	0.994766463583882	pseudobulk	DESeq2	LRT	0.0960591113673091
115	KC_AB1	lncRNA:CR43849	-1.64952313825113	0.817565084244375	0.994766463583882	pseudobulk	DESeq2	LRT	0.0874776642296574
115	KC_AB1	lncRNA:CR43855	-1.64952313825113	0.817565084244375	0.994766463583882	pseudobulk	DESeq2	LRT	0.0874776642296574
115	KC_AB1	lncRNA:CR43856	1.03154945120217	0.533517792213664	0.994766463583882	pseudobulk	DESeq2	LRT	0.272851092769278
115	KC_AB1	lncRNA:CR43878	0.263784927142232	0.765341337463751	0.994766463583882	pseudobulk	DESeq2	LRT	0.116144829010065
115	KC_AB1	lncRNA:CR43887	-1.64952313825113	0.817565084244375	0.994766463583882	pseudobulk	DESeq2	LRT	0.0874776642296574
115	KC_AB1	lncRNA:CR43900	0.408451987487849	0.824413195620833	0.994766463583882	pseudobulk	DESeq2	LRT	0.0838550654864088
115	KC_AB1	lncRNA:CR43910	-1.08681444615798	0.701131388297773	0.994766463583882	pseudobulk	DESeq2	LRT	0.154200589927933
115	KC_AB1	lncRNA:CR43912	-1.64952313825113	0.817565084244375	0.994766463583882	pseudobulk	DESeq2	LRT	0.0874776642296574
115	KC_AB1	lncRNA:CR43937	-1.64952313825113	0.817565084244375	0.994766463583882	pseudobulk	DESeq2	LRT	0.0874776642296574
115	KC_AB1	lncRNA:CR43943	-1.76643687325323	0.543810819055668	0.994766463583882	pseudobulk	DESeq2	LRT	0.26455215642223
115	KC_AB1	lncRNA:CR43956	0.69750158688829	0.750018748510497	0.994766463583882	pseudobulk	DESeq2	LRT	0.124927880244456
115	KC_AB1	lncRNA:CR43958	0.935354820562342	0.628177602332302	0.994766463583882	pseudobulk	DESeq2	LRT	0.20191755242796
115	KC_AB1	lncRNA:CR43963	-2.20969975456663	0.610836134188693	0.994766463583882	pseudobulk	DESeq2	LRT	0.214075280039667
115	KC_AB1	lncRNA:CR43973	2.92183940034187	0.320324374195056	0.994766463583882	pseudobulk	DESeq2	LRT	0.494410013644442
115	KC_AB1	lncRNA:CR43978	2.22023857837763	0.524904842126482	0.994766463583882	pseudobulk	DESeq2	LRT	0.279919420946639
115	KC_AB1	lncRNA:CR43985	1.1821590793593	0.719511699510136	0.994766463583882	pseudobulk	DESeq2	LRT	0.142962139890846

115	KC_AB1	lncRNA:CR43988	-1.64952313825113	0.817565084244375	0.994766463583882	pseudobulk	DESeq2	LRT	0.0874776642296574
115	KC_AB1	lncRNA:CR43992	1.80633728195307	0.61161102402172	0.994766463583882	pseudobulk	DESeq2	LRT	0.213524695220716
115	KC_AB1	lncRNA:CR43995	0.276151574356881	0.773411738208797	0.994766463583882	pseudobulk	DESeq2	LRT	0.111589240847616
115	KC_AB1	lncRNA:CR44017	0.806933541094274	0.754600478929312	0.994766463583882	pseudobulk	DESeq2	LRT	0.122282923497213
115	KC_AB1	lncRNA:CR44023	-0.89419351234193	0.548105309335553	0.994766463583882	pseudobulk	DESeq2	LRT	0.261135991024013
115	KC_AB1	lncRNA:CR44024	-0.0503626013366551	0.813902240800279	0.994766463583882	pseudobulk	DESeq2	LRT	0.0894277558364014
115	KC_AB1	lncRNA:CR44043	1.4112747467628	0.40255604098494	0.994766463583882	pseudobulk	DESeq2	LRT	0.395173651702722
115	KC_AB1	lncRNA:CR44050	-0.702227054342151	0.683650310562093	0.994766463583882	pseudobulk	DESeq2	LRT	0.165165984576943
115	KC_AB1	lncRNA:CR44051	-1.64952313825113	0.817565084244375	0.994766463583882	pseudobulk	DESeq2	LRT	0.0874776642296574
116	KC_AB1	lncRNA:CR44056	-1.64952313825113	0.817565084244375	0.994766463583882	pseudobulk	DESeq2	LRT	0.0874776642296574
116	KC_AB1	lncRNA:CR44074	-1.76643687325323	0.543810819055668	0.994766463583882	pseudobulk	DESeq2	LRT	0.26455215642223
116	KC_AB1	lncRNA:CR44075	0.949183608371002	0.80156895525114	0.994766463583882	pseudobulk	DESeq2	LRT	0.0960591113673091
116	KC_AB1	lncRNA:CR44092	-0.532694279264948	0.617142064396489	0.994766463583882	pseudobulk	DESeq2	LRT	0.209614851069521
116	KC_AB1	lncRNA:CR44097	-1.64952313825113	0.817565084244375	0.994766463583882	pseudobulk	DESeq2	LRT	0.0874776642296574
116	KC_AB1	lncRNA:CR44103	-1.64952313825113	0.817565084244375	0.994766463583882	pseudobulk	DESeq2	LRT	0.0874776642296574
116	KC_AB1	lncRNA:CR44105	0.949183608371002	0.80156895525114	0.994766463583882	pseudobulk	DESeq2	LRT	0.0960591113673091
116	KC_AB1	lncRNA:CR44126	1.1821590793593	0.719511699510136	0.994766463583882	pseudobulk	DESeq2	LRT	0.142962139890846
116	KC_AB1	lncRNA:CR44127	-2.20969975456663	0.610836134188693	0.994766463583882	pseudobulk	DESeq2	LRT	0.214075280039667
116	KC_AB1	lncRNA:CR44138	-1.28132067551389	0.647201349541384	0.994766463583882	pseudobulk	DESeq2	LRT	0.188960585805912
116	KC_AB1	lncRNA:CR44149	0.949183608371002	0.80156895525114	0.994766463583882	pseudobulk	DESeq2	LRT	0.0960591113673091
116	KC_AB1	lncRNA:CR44150	-1.64952313825113	0.817565084244375	0.994766463583882	pseudobulk	DESeq2	LRT	0.0874776642296574
116	KC_AB1	lncRNA:CR44171	0.949183608371002	0.80156895525114	0.994766463583882	pseudobulk	DESeq2	LRT	0.0960591113673091
116	KC_AB1	lncRNA:CR44178	0.986575688715885	0.655962090734864	0.994766463583882	pseudobulk	DESeq2	LRT	0.183121258582249
116	KC_AB1	lncRNA:CR44187	-1.02380338826061	0.503033761543743	0.994766463583882	pseudobulk	DESeq2	LRT	0.298402865918037
116	KC_AB1	lncRNA:CR44188	-1.9788084076398	0.324967565827742	0.994766463583882	pseudobulk	DESeq2	LRT	0.488159982667144
116	KC_AB1	lncRNA:CR44206	-0.286600717542373	0.794566529786165	0.994766463583882	pseudobulk	DESeq2	LRT	0.0998697330578107
116	KC_AB1	lncRNA:CR44208	0.949183608371002	0.80156895525114	0.994766463583882	pseudobulk	DESeq2	LRT	0.0960591113673091
116	KC_AB1	lncRNA:CR44218	1.17930484172975	0.669685881755347	0.994766463583882	pseudobulk	DESeq2	LRT	0.174128856716169
116	KC_AB1	lncRNA:CR44222	2.22023857837763	0.524904842126482	0.994766463583882	pseudobulk	DESeq2	LRT	0.279919420946639
116	KC_AB1	lncRNA:CR44256	-2.79499059556516	0.38564607643653	0.994766463583882	pseudobulk	DESeq2	LRT	0.413811082791365
116	KC_AB1	lncRNA:CR44265	-1.64952313825113	0.817565084244375	0.994766463583882	pseudobulk	DESeq2	LRT	0.0874776642296574
116	KC_AB1	lncRNA:CR44268	1.14573897872306	0.761752264856236	0.994766463583882	pseudobulk	DESeq2	LRT	0.118186245850804
116	KC_AB1	lncRNA:CR44272	-2.20969975456663	0.610836134188693	0.994766463583882	pseudobulk	DESeq2	LRT	0.214075280039667
116	KC_AB1	lncRNA:CR44294	1.45764639006851	0.510259806305237	0.994766463583882	pseudobulk	DESeq2	LRT	0.292208640147749
116	KC_AB1	lncRNA:CR44317	1.16877829733945	0.62091281431072	0.994766463583882	pseudobulk	DESeq2	LRT	0.206969377153521
116	KC_AB1	lncRNA:CR44320	-2.20969975456663	0.610836134188693	0.994766463583882	pseudobulk	DESeq2	LRT	0.214075280039667
116	KC_AB1	lncRNA:CR44322	-1.64952313825113	0.817565084244375	0.994766463583882	pseudobulk	DESeq2	LRT	0.0874776642296574
116	KC_AB1	lncRNA:CR44330	-0.835152883801839	0.920434724857442	0.994766463583882	pseudobulk	DESeq2	LRT	0.0360070052473159
116	KC_AB1	lncRNA:CR44331	-2.57435989741145	0.533617212681275	0.994766463583882	pseudobulk	DESeq2	LRT	0.272770169998685
116	KC_AB1	lncRNA:CR44332	0.729740153036158	0.752353468463509	0.994766463583882	pseudobulk	DESeq2	LRT	0.123578072524162
116	KC_AB1	lncRNA:CR44347	0.0460231183506794	0.955425345062067	0.994766463583882	pseudobulk	DESeq2	LRT	0.0198032421456097
116	KC_AB1	lncRNA:CR44348	-0.437567891750304	0.83748785379873	0.994766463583882	pseudobulk	DESeq2	LRT	0.077021482877911
116	KC_AB1	lncRNA:CR44357	-0.70550979546873	0.381533376429166	0.994766463583882	pseudobulk	DESeq2	LRT	0.418467464091864
116	KC_AB1	lncRNA:CR44360	-0.835152883801839	0.920434724857442	0.994766463583882	pseudobulk	DESeq2	LRT	0.0360070052473159
116	KC_AB1	lncRNA:CR44366	-2.79499059556516	0.38564607643653	0.994766463583882	pseudobulk	DESeq2	LRT	0.413811082791365
116	KC_AB1	lncRNA:CR44371	-2.20969975456663	0.610836134188693	0.994766463583882	pseudobulk	DESeq2	LRT	0.214075280039667
116	KC_AB1	lncRNA:CR44384	-0.739006821616313	0.76979092815726	0.994766463583882	pseudobulk	DESeq2	LRT	0.113627211290866
116	KC_AB1	lncRNA:CR44393	0.949183608371002	0.80156895525114	0.994766463583882	pseudobulk	DESeq2	LRT	0.0960591113673091
116	KC_AB1	lncRNA:CR44408	-1.64952313825113	0.817565084244375	0.994766463583882	pseudobulk	DESeq2	LRT	0.0874776642296574
116	KC_AB1	lncRNA:CR44409	0.949183608371002	0.80156895525114	0.994766463583882	pseudobulk	DESeq2	LRT	0.0960591113673091
116	KC_AB1	lncRNA:CR44410	-1.64952313825113	0.817565084244375	0.994766463583882	pseudobulk	DESeq2	LRT	0.0874776642296574
116	KC_AB1	lncRNA:CR44411	2.22023857837763	0.524904842126482	0.994766463583882	pseudobulk	DESeq2	LRT	0.279919420946639
116	KC_AB1	lncRNA:CR44417	-0.192780952434313	0.651326298259587	0.994766463583882	pseudobulk	DESeq2	LRT	0.186201386191885
116	KC_AB1	lncRNA:CR44440	-1.64952313825113	0.817565084244375	0.994766463583882	pseudobulk	DESeq2	LRT	0.0874776642296574
116	KC_AB1	lncRNA:CR44441	-3.21143241058256	0.292545415061155	0.994766463583882	pseudobulk	DESeq2	LRT	0.533806704011112
116	KC_AB1	lncRNA:CR44445	0.806933541094274	0.754600478929312	0.994766463583882	pseudobulk	DESeq2	LRT	0.122282923497213

116	KC_AB1	lncRNA:CR44458	-1.64952313825113	0.817565084244375	0.994766463583882	pseudobulk	DESeq2	LRT	0.0874776642296574
116	KC_AB1	lncRNA:CR44473	-1.64952313825113	0.817565084244375	0.994766463583882	pseudobulk	DESeq2	LRT	0.0874776642296574
116	KC_AB1	lncRNA:CR44474	-2.20969975456663	0.610836134188693	0.994766463583882	pseudobulk	DESeq2	LRT	0.214075280039667
116	KC_AB1	lncRNA:CR44478	-1.64952313825113	0.817565084244375	0.994766463583882	pseudobulk	DESeq2	LRT	0.0874776642296574
116	KC_AB1	lncRNA:CR44506	1.20457551124537	0.440098421975737	0.994766463583882	pseudobulk	DESeq2	LRT	0.356450188647822
116	KC_AB1	lncRNA:CR44546	-2.20969975456663	0.610836134188693	0.994766463583882	pseudobulk	DESeq2	LRT	0.214075280039667
116	KC_AB1	lncRNA:CR44588	0.949183608371002	0.80156895525114	0.994766463583882	pseudobulk	DESeq2	LRT	0.0960591113673091
116	KC_AB1	lncRNA:CR44589	-1.22412322337131	0.466699816481841	0.994766463583882	pseudobulk	DESeq2	LRT	0.330962369890373
116	KC_AB1	lncRNA:CR44595	1.08611877493397	0.434259028242099	0.994766463583882	pseudobulk	DESeq2	LRT	0.362251143807826
116	KC_AB1	lncRNA:CR44596	0.0551104605447623	0.907528926819053	0.994766463583882	pseudobulk	DESeq2	LRT	0.0421395232530871
116	KC_AB1	lncRNA:CR44597	-0.238351699808084	0.694780060559947	0.994766463583882	pseudobulk	DESeq2	LRT	0.15815265383036
116	KC_AB1	lncRNA:CR44598	-0.404349888528238	0.518598276151765	0.994766463583882	pseudobulk	DESeq2	LRT	0.28516893118221
116	KC_AB1	lncRNA:CR44606	0.949183608371002	0.80156895525114	0.994766463583882	pseudobulk	DESeq2	LRT	0.0960591113673091
116	KC_AB1	lncRNA:CR44619	-4.62642941169408	0.06337111563309	0.994766463583882	pseudobulk	DESeq2	LRT	1.19810864715865
116	KC_AB1	lncRNA:CR44628	1.16481357900673	0.545948709611117	0.994766463583882	pseudobulk	DESeq2	LRT	0.262848156158226
116	KC_AB1	lncRNA:CR44636	0.949183608371002	0.80156895525114	0.994766463583882	pseudobulk	DESeq2	LRT	0.0960591113673091
116	KC_AB1	lncRNA:CR44642	-1.64952313825113	0.817565084244375	0.994766463583882	pseudobulk	DESeq2	LRT	0.0874776642296574
116	KC_AB1	lncRNA:CR44655	-1.64952313825113	0.817565084244375	0.994766463583882	pseudobulk	DESeq2	LRT	0.0874776642296574
116	KC_AB1	lncRNA:CR44662	0.217906885051292	0.902008941520292	0.994766463583882	pseudobulk	DESeq2	LRT	0.0447891573209458
116	KC_AB1	lncRNA:CR44663	0.475603073114276	0.45282411559837	0.994766463583882	pseudobulk	DESeq2	LRT	0.34407045241958
116	KC_AB1	lncRNA:CR44670	-1.64952313825113	0.817565084244375	0.994766463583882	pseudobulk	DESeq2	LRT	0.0874776642296574
116	KC_AB1	lncRNA:CR44679	0.949183608371002	0.80156895525114	0.994766463583882	pseudobulk	DESeq2	LRT	0.0960591113673091
116	KC_AB1	lncRNA:CR44708	-0.0145978093758412	0.928196826203735	0.994766463583882	pseudobulk	DESeq2	LRT	0.0323599209035878
116	KC_AB1	lncRNA:CR44716	0.217906885051292	0.902008941520292	0.994766463583882	pseudobulk	DESeq2	LRT	0.0447891573209458
116	KC_AB1	lncRNA:CR44718	-0.123132359203517	0.905238660116382	0.994766463583882	pseudobulk	DESeq2	LRT	0.043236906864143
116	KC_AB1	lncRNA:CR44719	0.949183608371002	0.80156895525114	0.994766463583882	pseudobulk	DESeq2	LRT	0.0960591113673091
116	KC_AB1	lncRNA:CR44727	-1.64952313825113	0.817565084244375	0.994766463583882	pseudobulk	DESeq2	LRT	0.0874776642296574
116	KC_AB1	lncRNA:CR44728	-1.64952313825113	0.817565084244375	0.994766463583882	pseudobulk	DESeq2	LRT	0.0874776642296574
116	KC_AB1	lncRNA:CR44749	0.949183608371002	0.80156895525114	0.994766463583882	pseudobulk	DESeq2	LRT	0.0960591113673091
116	KC_AB1	lncRNA:CR44761	0.949183608371002	0.80156895525114	0.994766463583882	pseudobulk	DESeq2	LRT	0.0960591113673091
116	KC_AB1	lncRNA:CR44763	0.949183608371002	0.80156895525114	0.994766463583882	pseudobulk	DESeq2	LRT	0.0960591113673091
116	KC_AB1	lncRNA:CR44766	0.949183608371002	0.80156895525114	0.994766463583882	pseudobulk	DESeq2	LRT	0.0960591113673091
116	KC_AB1	lncRNA:CR44779	-1.13194846419515	0.661925982564074	0.994766463583882	pseudobulk	DESeq2	LRT	0.179190571226749
116	KC_AB1	lncRNA:CR44809	-2.20969975456663	0.610836134188693	0.994766463583882	pseudobulk	DESeq2	LRT	0.214075280039667
116	KC_AB1	lncRNA:CR44813	-0.627447798185778	0.772628937945737	0.994766463583882	pseudobulk	DESeq2	LRT	0.112029029876423
116	KC_AB1	lncRNA:CR44816	-0.407624799208101	0.733033534631384	0.994766463583882	pseudobulk	DESeq2	LRT	0.13487615691109
116	KC_AB1	lncRNA:CR44828	0.949183608371002	0.80156895525114	0.994766463583882	pseudobulk	DESeq2	LRT	0.0960591113673091
116	KC_AB1	lncRNA:CR44833	0.596808856322697	0.822761281772702	0.994766463583882	pseudobulk	DESeq2	LRT	0.0847261539098712
116	KC_AB1	lncRNA:CR44851	1.1821590793593	0.719511699510136	0.994766463583882	pseudobulk	DESeq2	LRT	0.142962139890846
116	KC_AB1	lncRNA:CR44886	-0.645041683288466	0.352140962683589	0.994766463583882	pseudobulk	DESeq2	LRT	0.453283452826134
116	KC_AB1	lncRNA:CR44900	1.04577911339294	0.588197883499031	0.994766463583882	pseudobulk	DESeq2	LRT	0.2304765425395
116	KC_AB1	lncRNA:CR44901	1.45682795759908	0.573003993665791	0.994766463583882	pseudobulk	DESeq2	LRT	0.24184235111992
116	KC_AB1	lncRNA:CR44914	1.19856662192297	0.608802264240657	0.994766463583882	pseudobulk	DESeq2	LRT	0.215523741018373
116	KC_AB1	lncRNA:CR44922	0.0146164971128316	0.859222306035331	0.994766463583882	pseudobulk	DESeq2	LRT	0.0658944568978825
116	KC_AB1	lncRNA:CR44938	0.806933541094274	0.754600478929312	0.994766463583882	pseudobulk	DESeq2	LRT	0.122282923497213
116	KC_AB1	lncRNA:CR44953	-1.64952313825113	0.817565084244375	0.994766463583882	pseudobulk	DESeq2	LRT	0.0874776642296574
116	KC_AB1	lncRNA:CR44965	0.949183608371002	0.80156895525114	0.994766463583882	pseudobulk	DESeq2	LRT	0.0960591113673091
116	KC_AB1	lncRNA:CR44978	0.608075812828747	0.791206230175283	0.994766463583882	pseudobulk	DESeq2	LRT	0.101710301643317
116	KC_AB1	lncRNA:CR44979	0.949183608371002	0.80156895525114	0.994766463583882	pseudobulk	DESeq2	LRT	0.0960591113673091
116	KC_AB1	lncRNA:CR44985	-0.0713915988297819	0.864170454131802	0.994766463583882	pseudobulk	DESeq2	LRT	0.063400586239177
116	KC_AB1	lncRNA:CR44986	-0.1751915528619	0.888431419889413	0.994766463583882	pseudobulk	DESeq2	LRT	0.0513760907772097
116	KC_AB1	lncRNA:CR44993	0.0587775856525997	0.890052531922536	0.994766463583882	pseudobulk	DESeq2	LRT	0.0505843600395771
116	KC_AB1	lncRNA:CR44997	0.297646180254642	0.381007648509915	0.994766463583882	pseudobulk	DESeq2	LRT	0.419066306024354
117	KC_AB1	lncRNA:CR44999	-1.09893356072213	0.0613117845547161	0.994766463583882	pseudobulk	DESeq2	LRT	1.21245604301505
117	KC_AB1	lncRNA:CR45004	-0.341391478739861	0.841795132270276	0.994766463583882	pseudobulk	DESeq2	LRT	0.0747935899140484
117	KC_AB1	lncRNA:CR45013	-1.64952313825113	0.817565084244375	0.994766463583882	pseudobulk	DESeq2	LRT	0.0874776642296574

117	KC_AB1	lncRNA:CR45025	-0.648218248148942	0.834000364039372	0.994766463583882	pseudobulk	DESeq2	LRT	0.0788337597936092
117	KC_AB1	lncRNA:CR45029	2.02670873290438	0.538959414925803	0.994766463583882	pseudobulk	DESeq2	LRT	0.26844393710898
117	KC_AB1	lncRNA:CR45030	2.54275315134261	0.410734813545839	0.994766463583882	pseudobulk	DESeq2	LRT	0.386438485142247
117	KC_AB1	lncRNA:CR45048	1.1821590793593	0.719511699510136	0.994766463583882	pseudobulk	DESeq2	LRT	0.142962139890846
117	KC_AB1	lncRNA:CR45054	-0.0591707869400948	0.806735747055771	0.994766463583882	pseudobulk	DESeq2	LRT	0.0932686987219619
117	KC_AB1	lncRNA:CR45102	-0.55841525131377	0.218246953875985	0.994766463583882	pseudobulk	DESeq2	LRT	0.661051809130219
117	KC_AB1	lncRNA:CR45125	0.949183608371002	0.80156895525114	0.994766463583882	pseudobulk	DESeq2	LRT	0.0960591113673091
117	KC_AB1	lncRNA:CR45128	0.217906885051292	0.902008941520292	0.994766463583882	pseudobulk	DESeq2	LRT	0.0447891573209458
117	KC_AB1	lncRNA:CR45155	0.992630526336814	0.723993771284609	0.994766463583882	pseudobulk	DESeq2	LRT	0.140265170140367
117	KC_AB1	lncRNA:CR45156	0.217906885051292	0.902008941520292	0.994766463583882	pseudobulk	DESeq2	LRT	0.0447891573209458
117	KC_AB1	lncRNA:CR45169	1.80633728195307	0.61161102402172	0.994766463583882	pseudobulk	DESeq2	LRT	0.213524695220716
117	KC_AB1	lncRNA:CR45170	-0.599253932718975	0.599554567508431	0.994766463583882	pseudobulk	DESeq2	LRT	0.222171284142554
117	KC_AB1	lncRNA:CR45177	2.92183940034187	0.320324374195056	0.994766463583882	pseudobulk	DESeq2	LRT	0.494410013644442
117	KC_AB1	lncRNA:CR45178	1.80633728195307	0.61161102402172	0.994766463583882	pseudobulk	DESeq2	LRT	0.213524695220716
117	KC_AB1	lncRNA:CR45180	-2.20969975456663	0.610836134188693	0.994766463583882	pseudobulk	DESeq2	LRT	0.214075280039667
117	KC_AB1	lncRNA:CR45196	-1.08681444615798	0.701131388297773	0.994766463583882	pseudobulk	DESeq2	LRT	0.154200589927933
117	KC_AB1	lncRNA:CR45200	0.854047063205883	0.719204060467466	0.994766463583882	pseudobulk	DESeq2	LRT	0.143147869337068
117	KC_AB1	lncRNA:CR45217	-1.64952313825113	0.817565084244375	0.994766463583882	pseudobulk	DESeq2	LRT	0.0874776642296574
117	KC_AB1	lncRNA:CR45224	-1.64952313825113	0.817565084244375	0.994766463583882	pseudobulk	DESeq2	LRT	0.0874776642296574
117	KC_AB1	lncRNA:CR45233	-2.20969975456663	0.610836134188693	0.994766463583882	pseudobulk	DESeq2	LRT	0.214075280039667
117	KC_AB1	lncRNA:CR45239	-1.64952313825113	0.817565084244375	0.994766463583882	pseudobulk	DESeq2	LRT	0.0874776642296574
117	KC_AB1	lncRNA:CR45240	0.233048461606111	0.864499074695876	0.994766463583882	pseudobulk	DESeq2	LRT	0.0632354672306489
117	KC_AB1	lncRNA:CR45247	-1.64952313825113	0.817565084244375	0.994766463583882	pseudobulk	DESeq2	LRT	0.0874776642296574
117	KC_AB1	lncRNA:CR45262	-1.64952313825113	0.817565084244375	0.994766463583882	pseudobulk	DESeq2	LRT	0.0874776642296574
117	KC_AB1	lncRNA:CR45266	-1.64952313825113	0.817565084244375	0.994766463583882	pseudobulk	DESeq2	LRT	0.0874776642296574
117	KC_AB1	lncRNA:CR45306	0.596808856322697	0.822761281772702	0.994766463583882	pseudobulk	DESeq2	LRT	0.0847261539098712
117	KC_AB1	lncRNA:CR45308	1.1821590793593	0.719511699510136	0.994766463583882	pseudobulk	DESeq2	LRT	0.142962139890846
117	KC_AB1	lncRNA:CR45312	0.284378749043483	0.706477837340171	0.994766463583882	pseudobulk	DESeq2	LRT	0.1509014576963
117	KC_AB1	lncRNA:CR45339	-1.64952313825113	0.817565084244375	0.994766463583882	pseudobulk	DESeq2	LRT	0.0874776642296574
117	KC_AB1	lncRNA:CR45352	-1.64952313825113	0.817565084244375	0.994766463583882	pseudobulk	DESeq2	LRT	0.0874776642296574
117	KC_AB1	lncRNA:CR45358	0.40411895759739	0.864643228521197	0.994766463583882	pseudobulk	DESeq2	LRT	0.0631630553638974
117	KC_AB1	lncRNA:CR45387	1.80939591345248	0.468117841155507	0.994766463583882	pseudobulk	DESeq2	LRT	0.329644806495434
117	KC_AB1	lncRNA:CR45388	0.471755571968039	0.387354412399746	0.994766463583882	pseudobulk	DESeq2	LRT	0.411891492569473
117	KC_AB1	lncRNA:CR45403	-1.64952313825113	0.817565084244375	0.994766463583882	pseudobulk	DESeq2	LRT	0.0874776642296574
117	KC_AB1	lncRNA:CR45405	-3.6748866702475	0.198972138271859	0.994766463583882	pseudobulk	DESeq2	LRT	0.701207732846238
117	KC_AB1	lncRNA:CR45409	-0.171751204005691	0.811396736170196	0.994766463583882	pseudobulk	DESeq2	LRT	0.090766743570471
117	KC_AB1	lncRNA:CR45418	-1.14988051517267	0.548065792651115	0.994766463583882	pseudobulk	DESeq2	LRT	0.261167303431303
117	KC_AB1	lncRNA:CR45444	-3.50020903827178	0.46931421925589	0.994766463583882	pseudobulk	DESeq2	LRT	0.328536287358315
117	KC_AB1	lncRNA:CR45456	-1.64952313825113	0.817565084244375	0.994766463583882	pseudobulk	DESeq2	LRT	0.0874776642296574
117	KC_AB1	lncRNA:CR45463	1.1821590793593	0.719511699510136	0.994766463583882	pseudobulk	DESeq2	LRT	0.142962139890846
117	KC_AB1	lncRNA:CR45464	1.1821590793593	0.719511699510136	0.994766463583882	pseudobulk	DESeq2	LRT	0.142962139890846
117	KC_AB1	lncRNA:CR45471	2.22023857837763	0.524904842126482	0.994766463583882	pseudobulk	DESeq2	LRT	0.279919420946639
117	KC_AB1	lncRNA:CR45472	0.949183608371002	0.80156895525114	0.994766463583882	pseudobulk	DESeq2	LRT	0.0960591113673091
117	KC_AB1	lncRNA:CR45473	0.808265210474162	0.353135350943192	0.994766463583882	pseudobulk	DESeq2	LRT	0.452058804807099
117	KC_AB1	lncRNA:CR45475	1.1821590793593	0.719511699510136	0.994766463583882	pseudobulk	DESeq2	LRT	0.142962139890846
117	KC_AB1	lncRNA:CR45503	1.1821590793593	0.719511699510136	0.994766463583882	pseudobulk	DESeq2	LRT	0.142962139890846
117	KC_AB1	lncRNA:CR45519	1.1821590793593	0.719511699510136	0.994766463583882	pseudobulk	DESeq2	LRT	0.142962139890846
117	KC_AB1	lncRNA:CR45522	0.840026963894039	0.672863812242849	0.994766463583882	pseudobulk	DESeq2	LRT	0.172072828163199
117	KC_AB1	lncRNA:CR45527	-1.32806228432949	0.722858830184137	0.994766463583882	pseudobulk	DESeq2	LRT	0.140946509425123
117	KC_AB1	lncRNA:CR45530	-2.57435989741145	0.533617212681275	0.994766463583882	pseudobulk	DESeq2	LRT	0.272770169998685
117	KC_AB1	lncRNA:CR45531	-0.835152883801839	0.920434724857442	0.994766463583882	pseudobulk	DESeq2	LRT	0.0360070052473159
117	KC_AB1	lncRNA:CR45539	0.0236942436874584	0.910119428894173	0.994766463583882	pseudobulk	DESeq2	LRT	0.0409016143750932
117	KC_AB1	lncRNA:CR45540	1.1821590793593	0.719511699510136	0.994766463583882	pseudobulk	DESeq2	LRT	0.142962139890846
117	KC_AB1	lncRNA:CR45556	-2.31317649249275	0.222912522381396	0.994766463583882	pseudobulk	DESeq2	LRT	0.651865533815036
117	KC_AB1	lncRNA:CR45562	-2.79499059556516	0.38564607643653	0.994766463583882	pseudobulk	DESeq2	LRT	0.413811082791365
117	KC_AB1	lncRNA:CR45566	-1.64952313825113	0.817565084244375	0.994766463583882	pseudobulk	DESeq2	LRT	0.0874776642296574

117	KC_AB1	lncRNA:CR45575	0.949183608371002	0.80156895525114	0.994766463583882	pseudobulk	DESeq2	LRT	0.0960591113673091
117	KC_AB1	lncRNA:CR45577	-1.64952313825113	0.817565084244375	0.994766463583882	pseudobulk	DESeq2	LRT	0.0874776642296574
117	KC_AB1	lncRNA:CR45605	0.949183608371002	0.80156895525114	0.994766463583882	pseudobulk	DESeq2	LRT	0.0960591113673091
117	KC_AB1	lncRNA:CR45625	-1.36744229896857	0.477876039484861	0.994766463583882	pseudobulk	DESeq2	LRT	0.320684744286443
117	KC_AB1	lncRNA:CR45626	-2.79499059556516	0.38564607643653	0.994766463583882	pseudobulk	DESeq2	LRT	0.413811082791365
117	KC_AB1	lncRNA:CR45638	-1.64952313825113	0.817565084244375	0.994766463583882	pseudobulk	DESeq2	LRT	0.0874776642296574
117	KC_AB1	lncRNA:CR45641	1.59307955075178	0.558763130036445	0.994766463583882	pseudobulk	DESeq2	LRT	0.25277225851705
117	KC_AB1	lncRNA:CR45643	-1.57928784687645	0.637186187711083	0.994766463583882	pseudobulk	DESeq2	LRT	0.195733646973032
117	KC_AB1	lncRNA:CR45659	1.1821590793593	0.719511699510136	0.994766463583882	pseudobulk	DESeq2	LRT	0.142962139890846
117	KC_AB1	lncRNA:CR45660	0.415717325663807	0.464547225315569	0.994766463583882	pseudobulk	DESeq2	LRT	0.332970129568236
117	KC_AB1	lncRNA:CR45668	0.0546548578430922	0.831527371889816	0.994766463583882	pseudobulk	DESeq2	LRT	0.0801234502748277
117	KC_AB1	lncRNA:CR45670	0.949183608371002	0.80156895525114	0.994766463583882	pseudobulk	DESeq2	LRT	0.0960591113673091
117	KC_AB1	lncRNA:CR45684	0.274926110296238	0.717641148478404	0.994766463583882	pseudobulk	DESeq2	LRT	0.144092667448964
117	KC_AB1	lncRNA:CR45687	-1.28132067551389	0.647201349541384	0.994766463583882	pseudobulk	DESeq2	LRT	0.188960585805912
117	KC_AB1	lncRNA:CR45695	1.1821590793593	0.719511699510136	0.994766463583882	pseudobulk	DESeq2	LRT	0.142962139890846
117	KC_AB1	lncRNA:CR45736	0.389275648472954	0.837532199093071	0.994766463583882	pseudobulk	DESeq2	LRT	0.0769984874318538
117	KC_AB1	lncRNA:CR45740	1.1821590793593	0.719511699510136	0.994766463583882	pseudobulk	DESeq2	LRT	0.142962139890846
117	KC_AB1	lncRNA:CR45743	-1.64952313825113	0.817565084244375	0.994766463583882	pseudobulk	DESeq2	LRT	0.0874776642296574
117	KC_AB1	lncRNA:CR45758	0.145477551247077	0.700612232468328	0.994766463583882	pseudobulk	DESeq2	LRT	0.154522284307625
117	KC_AB1	lncRNA:CR45805	-2.20969975456663	0.610836134188693	0.994766463583882	pseudobulk	DESeq2	LRT	0.214075280039667
117	KC_AB1	lncRNA:CR45809	-1.64952313825113	0.817565084244375	0.994766463583882	pseudobulk	DESeq2	LRT	0.0874776642296574
117	KC_AB1	lncRNA:CR45819	1.32710828904082	0.607473293222376	0.994766463583882	pseudobulk	DESeq2	LRT	0.216472810505135
117	KC_AB1	lncRNA:CR45821	-1.18889040549532	0.611395637693754	0.994766463583882	pseudobulk	DESeq2	LRT	0.213677664288847
117	KC_AB1	lncRNA:CR45825	-1.43638678109459	0.494188861135597	0.994766463583882	pseudobulk	DESeq2	LRT	0.306107047687203
117	KC_AB1	lncRNA:CR45833	0.949183608371002	0.80156895525114	0.994766463583882	pseudobulk	DESeq2	LRT	0.0960591113673091
117	KC_AB1	lncRNA:CR45878	-1.64952313825113	0.817565084244375	0.994766463583882	pseudobulk	DESeq2	LRT	0.0874776642296574
117	KC_AB1	lncRNA:CR45883	0.881337768677799	0.697697705840746	0.994766463583882	pseudobulk	DESeq2	LRT	0.156332705059186
117	KC_AB1	lncRNA:CR45906	0.949183608371002	0.80156895525114	0.994766463583882	pseudobulk	DESeq2	LRT	0.0960591113673091
117	KC_AB1	lncRNA:CR45910	0.44976297004525	0.831013436772572	0.994766463583882	pseudobulk	DESeq2	LRT	0.0803919539908534
117	KC_AB1	lncRNA:CR45911	0.217215841449787	0.837657155469515	0.994766463583882	pseudobulk	DESeq2	LRT	0.0769336973056261
117	KC_AB1	lncRNA:CR45916	1.1821590793593	0.719511699510136	0.994766463583882	pseudobulk	DESeq2	LRT	0.142962139890846
117	KC_AB1	lncRNA:CR45919	0.255645940317457	0.846598932502876	0.994766463583882	pseudobulk	DESeq2	LRT	0.072322283474107
117	KC_AB1	lncRNA:CR45922	-3.46197803745951	0.239982951034438	0.994766463583882	pseudobulk	DESeq2	LRT	0.619819610516178
117	KC_AB1	lncRNA:CR45938	1.80939591345248	0.468117841155507	0.994766463583882	pseudobulk	DESeq2	LRT	0.329644806495434
117	KC_AB1	lncRNA:CR45939	-0.835152883801839	0.920434724857442	0.994766463583882	pseudobulk	DESeq2	LRT	0.0360070052473159
117	KC_AB1	lncRNA:CR45941	0.949183608371002	0.80156895525114	0.994766463583882	pseudobulk	DESeq2	LRT	0.0960591113673091
117	KC_AB1	lncRNA:CR45950	0.949183608371002	0.80156895525114	0.994766463583882	pseudobulk	DESeq2	LRT	0.0960591113673091
117	KC_AB1	lncRNA:CR45954	-1.50663180504502	0.668163815680457	0.994766463583882	pseudobulk	DESeq2	LRT	0.175117047219009
117	KC_AB1	lncRNA:CR45961	-0.991491664151033	0.506831134114466	0.994766463583882	pseudobulk	DESeq2	LRT	0.29513671470651
117	KC_AB1	lncRNA:CR45962	-1.64952313825113	0.817565084244375	0.994766463583882	pseudobulk	DESeq2	LRT	0.0874776642296574
117	KC_AB1	lncRNA:CR45971	2.02670873290438	0.538959414925803	0.994766463583882	pseudobulk	DESeq2	LRT	0.26844393710898
118	KC_AB1	lncRNA:CR45972	-0.341391478739861	0.841795132270276	0.994766463583882	pseudobulk	DESeq2	LRT	0.0747935899140484
118	KC_AB1	lncRNA:CR45979	0.189975581143908	0.797666606285378	0.994766463583882	pseudobulk	DESeq2	LRT	0.0981785889811873
118	KC_AB1	lncRNA:CR45981	2.02670873290438	0.538959414925803	0.994766463583882	pseudobulk	DESeq2	LRT	0.26844393710898
118	KC_AB1	lncRNA:CR45984	-2.20969975456663	0.610836134188693	0.994766463583882	pseudobulk	DESeq2	LRT	0.214075280039667
118	KC_AB1	lncRNA:CR45994	0.217906885051292	0.902008941520292	0.994766463583882	pseudobulk	DESeq2	LRT	0.0447891573209458
118	KC_AB1	lncRNA:CR46003	0.626336038689325	0.27613577953002	0.994766463583882	pseudobulk	DESeq2	LRT	0.558877317208229
118	KC_AB1	lncRNA:CR46004	0.0476767893371444	0.810118872822545	0.994766463583882	pseudobulk	DESeq2	LRT	0.0914512502287124
118	KC_AB1	lncRNA:CR46006	-0.188244748342888	0.357262571434993	0.994766463583882	pseudobulk	DESeq2	LRT	0.447012480217084
118	KC_AB1	lncRNA:CR46009	0.949183608371002	0.80156895525114	0.994766463583882	pseudobulk	DESeq2	LRT	0.0960591113673091
118	KC_AB1	lncRNA:CR46010	0.949183608371002	0.80156895525114	0.994766463583882	pseudobulk	DESeq2	LRT	0.0960591113673091
118	KC_AB1	lncRNA:CR46023	-1.64952313825113	0.817565084244375	0.994766463583882	pseudobulk	DESeq2	LRT	0.0874776642296574
118	KC_AB1	lncRNA:CR46024	-1.64952313825113	0.817565084244375	0.994766463583882	pseudobulk	DESeq2	LRT	0.0874776642296574
118	KC_AB1	lncRNA:CR46035	-2.79499059556516	0.38564607643653	0.994766463583882	pseudobulk	DESeq2	LRT	0.413811082791365
118	KC_AB1	lncRNA:CR46036	0.597141410293382	0.802139944912821	0.994766463583882	pseudobulk	DESeq2	LRT	0.0957498561530429
118	KC_AB1	lncRNA:CR46043	1.80633728195307	0.61161102402172	0.994766463583882	pseudobulk	DESeq2	LRT	0.213524695220716

118	KC_AB1	lncRNA:CR46064	0.384101760035432	0.458781367776829	0.994766463583882	pseudobulk	DESeq2	LRT	0.338394228164017
118	KC_AB1	lncRNA:CR46077	0.949183608371002	0.80156895525114	0.994766463583882	pseudobulk	DESeq2	LRT	0.0960591113673091
118	KC_AB1	lncRNA:CR46080	0.992630526336814	0.723993771284609	0.994766463583882	pseudobulk	DESeq2	LRT	0.140265170140367
118	KC_AB1	lncRNA:CR46084	0.0236942436874584	0.910119428894173	0.994766463583882	pseudobulk	DESeq2	LRT	0.0409016143750932
118	KC_AB1	lncRNA:CR46095	-2.20969975456663	0.610836134188693	0.994766463583882	pseudobulk	DESeq2	LRT	0.214075280039667
118	KC_AB1	lncRNA:CR46125	-2.20969975456663	0.610836134188693	0.994766463583882	pseudobulk	DESeq2	LRT	0.214075280039667
118	KC_AB1	lncRNA:CR46127	0.949183608371002	0.80156895525114	0.994766463583882	pseudobulk	DESeq2	LRT	0.0960591113673091
118	KC_AB1	lncRNA:CR46194	3.12489817250917	0.275546095031922	0.994766463583882	pseudobulk	DESeq2	LRT	0.559805739304107
118	KC_AB1	lncRNA:CR46204	1.1821590793593	0.719511699510136	0.994766463583882	pseudobulk	DESeq2	LRT	0.142962139890846
118	KC_AB1	lncRNA:CR46208	-0.16952114729144	0.748743082404604	0.994766463583882	pseudobulk	DESeq2	LRT	0.125667177006488
118	KC_AB1	lncRNA:CR46221	0.949183608371002	0.80156895525114	0.994766463583882	pseudobulk	DESeq2	LRT	0.0960591113673091
118	KC_AB1	lncRNA:CR46230	-1.64952313825113	0.817565084244375	0.994766463583882	pseudobulk	DESeq2	LRT	0.0874776642296574
118	KC_AB1	lncRNA:CR46250	-1.64952313825113	0.817565084244375	0.994766463583882	pseudobulk	DESeq2	LRT	0.0874776642296574
118	KC_AB1	lncRNA:CR46254	-1.64952313825113	0.817565084244375	0.994766463583882	pseudobulk	DESeq2	LRT	0.0874776642296574
118	KC_AB1	lncRNA:CR46265	-1.64952313825113	0.817565084244375	0.994766463583882	pseudobulk	DESeq2	LRT	0.0874776642296574
118	KC_AB1	lncRNA:CR46268	0.281768793361934	0.753275774018368	0.994766463583882	pseudobulk	DESeq2	LRT	0.123045999618159
118	KC_AB1	lncRNA:CR46271	0.087356802117797	0.960952460588087	0.994766463583882	pseudobulk	DESeq2	LRT	0.0172980968423301
118	KC_AB1	lncRNA:CR46322	0.103234278152915	0.929315018553307	0.994766463583882	pseudobulk	DESeq2	LRT	0.0318370442100611
118	KC_AB1	lncRNA:CR46352	-2.20969975456663	0.610836134188693	0.994766463583882	pseudobulk	DESeq2	LRT	0.214075280039667
118	KC_AB1	lncRNA:CR9162	1.80633728195307	0.61161102402172	0.994766463583882	pseudobulk	DESeq2	LRT	0.213524695220716
118	KC_AB1	lncRNA:flam	-1.21457746603801	0.770013447451371	0.994766463583882	pseudobulk	DESeq2	LRT	0.113501690278259
118	KC_AB1	lncRNA:Hsromega	-0.208922420115711	0.0701676009938042	0.994766463583882	pseudobulk	DESeq2	LRT	1.15386337159664
118	KC_AB1	lncRNA:let7C	-0.300912908054727	0.264069398602393	0.994766463583882	pseudobulk	DESeq2	LRT	0.578281923625106
118	KC_AB1	lncRNA:noe	0.26208340309841	0.0359427867086505	0.994766463583882	pseudobulk	DESeq2	LRT	1.44438825429238
118	KC_AB1	lncRNA:roX1	3.4666683238609	0.0315320624699605	0.994766463583882	pseudobulk	DESeq2	LRT	1.50124762170283
118	KC_AB1	lncRNA:roX2	0.531272492644051	0.909268461892318	0.994766463583882	pseudobulk	DESeq2	LRT	0.0413078722181935
118	KC_AB1	lncRNA:sphinx	0.806933541094274	0.754600478929312	0.994766463583882	pseudobulk	DESeq2	LRT	0.122282923497213
118	KC_AB1	Lnk	-0.163332817972558	0.746146453947408	0.994766463583882	pseudobulk	DESeq2	LRT	0.127175920652425
118	KC_AB1	Lnpk	1.71782699250716	0.0590542353429136	0.994766463583882	pseudobulk	DESeq2	LRT	1.22874894953066
118	KC_AB1	loco	-1.64952313825113	0.817565084244375	0.994766463583882	pseudobulk	DESeq2	LRT	0.0874776642296574
118	KC_AB1	loh	-1.13194846419515	0.661925982564074	0.994766463583882	pseudobulk	DESeq2	LRT	0.179190571226749
118	KC_AB1	loj	-0.333638849882369	0.690059894663351	0.994766463583882	pseudobulk	DESeq2	LRT	0.16111321246146
118	KC_AB1	lola	0.0435975990897748	0.739116703874627	0.994766463583882	pseudobulk	DESeq2	LRT	0.131286982650969
118	KC_AB1	lolal	0.018865441021649	0.929553363159081	0.994766463583882	pseudobulk	DESeq2	LRT	0.0317256734980037
118	KC_AB1	Lon	0.153542387541646	0.878854858350053	0.994766463583882	pseudobulk	DESeq2	LRT	0.0560828421303081
118	KC_AB1	loqs	0.436006495415268	0.193981695473036	0.994766463583882	pseudobulk	DESeq2	LRT	0.712239249090964
118	KC_AB1	lost	0.195189306971027	0.748867847189742	0.994766463583882	pseudobulk	DESeq2	LRT	0.125594815544941
118	KC_AB1	lovit	-0.890230060444725	0.558635438054013	0.994766463583882	pseudobulk	DESeq2	LRT	0.252871517501585
118	KC_AB1	Loxl1	0.00891646089885312	0.960226720676327	0.994766463583882	pseudobulk	DESeq2	LRT	0.0176262128838119
118	KC_AB1	LPCAT	-0.0302090786709198	0.889591603631077	0.994766463583882	pseudobulk	DESeq2	LRT	0.050809324810916
118	KC_AB1	Lpin	-0.121761206479897	0.777653190995042	0.994766463583882	pseudobulk	DESeq2	LRT	0.10921404159312
118	KC_AB1	LpR1	-1.64952313825113	0.817565084244375	0.994766463583882	pseudobulk	DESeq2	LRT	0.0874776642296574
118	KC_AB1	LpR2	-0.9896971674044	0.692304363678608	0.994766463583882	pseudobulk	DESeq2	LRT	0.159702930968995
118	KC_AB1	Lpt	0.302814609468023	0.806631320373334	0.994766463583882	pseudobulk	DESeq2	LRT	0.0933249189497388
118	KC_AB1	lqf	0.0532512073660162	0.841792711449441	0.994766463583882	pseudobulk	DESeq2	LRT	0.0747948388528959
118	KC_AB1	lqfR	0.069365695174017	0.907050025631464	0.994766463583882	pseudobulk	DESeq2	LRT	0.0423687600660623
118	KC_AB1	Lrch	-0.315545865866199	0.53475638298836	0.994766463583882	pseudobulk	DESeq2	LRT	0.27184402287597
118	KC_AB1	LRP1	-1.00729393028558	0.24437350380123	0.994766463583882	pseudobulk	DESeq2	LRT	0.611945884258969
118	KC_AB1	Lrp4	0.138639265086102	0.64105180376088	0.994766463583882	pseudobulk	DESeq2	LRT	0.193106873478958
118	KC_AB1	Lrpprc2	0.694198716856127	0.704969414719965	0.994766463583882	pseudobulk	DESeq2	LRT	0.151829724578085
118	KC_AB1	LRR	0.0435999747007689	0.908676216502922	0.994766463583882	pseudobulk	DESeq2	LRT	0.0415908389296711
118	KC_AB1	Lrr47	-2.03159866077969	0.22951530848695	0.994766463583882	pseudobulk	DESeq2	LRT	0.6391883420671
118	KC_AB1	Lrrk	0.0392170361182535	0.945475664783417	0.994766463583882	pseudobulk	DESeq2	LRT	0.0243496448054964
118	KC_AB1	Lrt	1.1821590793593	0.719511699510136	0.994766463583882	pseudobulk	DESeq2	LRT	0.142962139890846
118	KC_AB1	Lsd-2	0.789443942742388	0.72202275028751	0.994766463583882	pseudobulk	DESeq2	LRT	0.141449117986226
118	KC_AB1	LSm1	0.622759260069492	0.579007502908167	0.994766463583882	pseudobulk	DESeq2	LRT	0.237315808551488

118	KC_AB1	Lsm11	1.20992583284813	0.587067761502213	0.994766463583882	pseudobulk	DESeq2	LRT	0.231311768006113
118	KC_AB1	Lsm12a	0.209583892516363	0.674877803590554	0.994766463583882	pseudobulk	DESeq2	LRT	0.170774855362334
118	KC_AB1	LSm3	0.864774790409014	0.473183494979628	0.994766463583882	pseudobulk	DESeq2	LRT	0.324970412320276
118	KC_AB1	LSm7	-0.358154364191389	0.606450181430945	0.994766463583882	pseudobulk	DESeq2	LRT	0.217204869683286
118	KC_AB1	lsn	0.160006883606011	0.830521530363502	0.994766463583882	pseudobulk	DESeq2	LRT	0.0806491044565843
118	KC_AB1	Lst8	-0.373276858246766	0.729666432250837	0.994766463583882	pseudobulk	DESeq2	LRT	0.136875632675053
118	KC_AB1	lt	-0.381516098554385	0.492293956058608	0.994766463583882	pseudobulk	DESeq2	LRT	0.30777549607065
118	KC_AB1	Ltn1	-0.445737947818481	0.607956693014086	0.994766463583882	pseudobulk	DESeq2	LRT	0.216127356014889
118	KC_AB1	LTV1	-0.0281595747199411	0.912012851468719	0.994766463583882	pseudobulk	DESeq2	LRT	0.0399990418441444
118	KC_AB1	LUBEL	-1.64952313825113	0.817565084244375	0.994766463583882	pseudobulk	DESeq2	LRT	0.0874776642296574
118	KC_AB1	luna	0.519430482521704	0.787642774112072	0.994766463583882	pseudobulk	DESeq2	LRT	0.103670706885852
118	KC_AB1	lute	-0.652665952531615	0.11312219435086	0.994766463583882	pseudobulk	DESeq2	LRT	0.946452178996019
118	KC_AB1	lva	0.427670782485791	0.514196886290179	0.994766463583882	pseudobulk	DESeq2	LRT	0.288870557547472
118	KC_AB1	lwr	0.07374143080196	0.809341116907169	0.994766463583882	pseudobulk	DESeq2	LRT	0.091868395611033
118	KC_AB1	Lztr1	0.190431181697151	0.762392986769026	0.994766463583882	pseudobulk	DESeq2	LRT	0.117821107413698
118	KC_AB1	m-cup	0.625102331211732	0.42816958999782	0.994766463583882	pseudobulk	DESeq2	LRT	0.368384180958275
118	KC_AB1	M1BP	-0.36134405066192	0.607029988769432	0.994766463583882	pseudobulk	DESeq2	LRT	0.216789853182781
118	KC_AB1	M6	0.458577251744448	0.594903494988161	0.994766463583882	pseudobulk	DESeq2	LRT	0.225553479638467
118	KC_AB1	mAChR-A	0.0881463290802803	0.738288677458587	0.994766463583882	pseudobulk	DESeq2	LRT	0.131773791952671
118	KC_AB1	mAChR-B	0.0481147742610195	0.806247716762575	0.994766463583882	pseudobulk	DESeq2	LRT	0.0935315022466836
118	KC_AB1	Mad	-0.227572493618773	0.544565282372189	0.994766463583882	pseudobulk	DESeq2	LRT	0.263950049655613
118	KC_AB1	Mad1	0.932211453157225	0.423926581320921	0.994766463583882	pseudobulk	DESeq2	LRT	0.372709351162219
118	KC_AB1	Madm	0.133306470808152	0.854167719653921	0.994766463583882	pseudobulk	DESeq2	LRT	0.068456845272899
118	KC_AB1	mael	0.0551104605447623	0.907528926819053	0.994766463583882	pseudobulk	DESeq2	LRT	0.0421395232530871
118	KC_AB1	maf-S	-0.0491196564547372	0.905126910057258	0.994766463583882	pseudobulk	DESeq2	LRT	0.043290523034149
118	KC_AB1	Maf1	-0.343029278725714	0.155458528015374	0.994766463583882	pseudobulk	DESeq2	LRT	0.808385448799752
118	KC_AB1	MAGE	-0.29408219783147	0.915376953506099	0.994766463583882	pseudobulk	DESeq2	LRT	0.0384000260261822
118	KC_AB1	Magi	0.0445735422740023	0.869735754779578	0.994766463583882	pseudobulk	DESeq2	LRT	0.0606126757408069
118	KC_AB1	mago	-0.130417892668525	0.795237667013044	0.994766463583882	pseudobulk	DESeq2	LRT	0.0995030574481544
119	KC_AB1	MagR	0.21756232575949	0.643250210796516	0.994766463583882	pseudobulk	DESeq2	LRT	0.19162006279698
119	KC_AB1	mahe	0.0393607861833335	0.862738149318752	0.994766463583882	pseudobulk	DESeq2	LRT	0.0641209975198951
119	KC_AB1	mahj	0.282773765818199	0.801088964239238	0.994766463583882	pseudobulk	DESeq2	LRT	0.0963192510409474
119	KC_AB1	mal	-0.581037478411007	0.779368688262961	0.994766463583882	pseudobulk	DESeq2	LRT	0.108257046307989
119	KC_AB1	mam	0.63840743050667	0.105691841233511	0.994766463583882	pseudobulk	DESeq2	LRT	0.975958536287869
119	KC_AB1	mamo	-0.319688193160969	0.664867070193876	0.994766463583882	pseudobulk	DESeq2	LRT	0.177265176429332
119	KC_AB1	MAN1	0.623574937422903	0.548862915370578	0.994766463583882	pseudobulk	DESeq2	LRT	0.260536111892841
119	KC_AB1	Manf	0.0821527203793472	0.851793787334842	0.994766463583882	pseudobulk	DESeq2	LRT	0.0696655318321636
119	KC_AB1	Map205	-0.31918216940091	0.518471949141052	0.994766463583882	pseudobulk	DESeq2	LRT	0.285274735249478
119	KC_AB1	Map60	0.0370936343384672	0.913416574440635	0.994766463583882	pseudobulk	DESeq2	LRT	0.0393311121478922
119	KC_AB1	MAPk-Ak2	0.435719017798245	0.536131525500264	0.994766463583882	pseudobulk	DESeq2	LRT	0.270728654725341
119	KC_AB1	Mapmodulin	0.0582667618654463	0.820522858652679	0.994766463583882	pseudobulk	DESeq2	LRT	0.0859093155878056
119	KC_AB1	Marc	-1.64952313825113	0.817565084244375	0.994766463583882	pseudobulk	DESeq2	LRT	0.0874776642296574
119	KC_AB1	Marcal1	0.694198716856127	0.704969414719965	0.994766463583882	pseudobulk	DESeq2	LRT	0.151829724578085
119	KC_AB1	Marf	0.157500826517726	0.655221061615691	0.994766463583882	pseudobulk	DESeq2	LRT	0.183612150935577
119	KC_AB1	Marf1	-1.05692259439633	0.554865263022474	0.994766463583882	pseudobulk	DESeq2	LRT	0.255812463057297
119	KC_AB1	mars	-2.20969975456663	0.610836134188693	0.994766463583882	pseudobulk	DESeq2	LRT	0.214075280039667
119	KC_AB1	mask	0.0574706533985569	0.805476800824735	0.994766463583882	pseudobulk	DESeq2	LRT	0.0939469635239611
119	KC_AB1	Mat1	0.102829928491985	0.91756586025848	0.994766463583882	pseudobulk	DESeq2	LRT	0.0373627535387634
119	KC_AB1	Mat89Ba	-0.121474431197956	0.872294656513679	0.994766463583882	pseudobulk	DESeq2	LRT	0.0593367879090517
119	KC_AB1	mats	-0.518668120301808	0.466849155726481	0.994766463583882	pseudobulk	DESeq2	LRT	0.330823422256424
119	KC_AB1	Mau2	-1.0818792844866	0.509837625088707	0.994766463583882	pseudobulk	DESeq2	LRT	0.292568117541826
119	KC_AB1	mav	1.32710828904082	0.607473293222376	0.994766463583882	pseudobulk	DESeq2	LRT	0.216472810505135
119	KC_AB1	Max	-0.218010543220332	0.704282444436034	0.994766463583882	pseudobulk	DESeq2	LRT	0.152253137079174
119	KC_AB1	mbc	-0.806088079577614	0.299135940206935	0.994766463583882	pseudobulk	DESeq2	LRT	0.524131404769252
119	KC_AB1	MBD-like	0.379487756182553	0.547568579774654	0.994766463583882	pseudobulk	DESeq2	LRT	0.26156148022757
119	KC_AB1	MBD-R2	-0.33095317835212	0.700355339789786	0.994766463583882	pseudobulk	DESeq2	LRT	0.154681555765711

119	KC_AB1	mbf1	0.362028219423993	0.282456073178945	0.994766463583882	pseudobulk	DESeq2	LRT	0.549049082921821
119	KC_AB1	mbl	-0.747142796159516	0.565487201864247	0.994766463583882	pseudobulk	DESeq2	LRT	0.247577219602597
119	KC_AB1	mbm	0.215603304854097	0.90258907949938	0.994766463583882	pseudobulk	DESeq2	LRT	0.0445099253492763
119	KC_AB1	mbo	0.502436098844458	0.737522120240099	0.994766463583882	pseudobulk	DESeq2	LRT	0.132224949513668
119	KC_AB1	Mbs	-0.0404848726998966	0.874029802489349	0.994766463583882	pseudobulk	DESeq2	LRT	0.0584737586287503
119	KC_AB1	mbt	0.136560147295089	0.74538473127871	0.994766463583882	pseudobulk	DESeq2	LRT	0.127619507730983
119	KC_AB1	Mcad	-0.0876274309314422	0.917937734184526	0.994766463583882	pseudobulk	DESeq2	LRT	0.0371867769874299
119	KC_AB1	Mccc2	2.92183940034187	0.320324374195056	0.994766463583882	pseudobulk	DESeq2	LRT	0.494410013644442
119	KC_AB1	Mco1	-1.64952313825113	0.817565084244375	0.994766463583882	pseudobulk	DESeq2	LRT	0.0874776642296574
119	KC_AB1	MCPH1	-2.20969975456663	0.610836134188693	0.994766463583882	pseudobulk	DESeq2	LRT	0.214075280039667
119	KC_AB1	Mcr	-0.017136142565629	0.875418657402882	0.994766463583882	pseudobulk	DESeq2	LRT	0.0577842017019179
119	KC_AB1	Mct1	0.699145194041508	0.369371239603438	0.994766463583882	pseudobulk	DESeq2	LRT	0.432536923102019
119	KC_AB1	Mctp	-0.613908997833323	0.366277008400619	0.994766463583882	pseudobulk	DESeq2	LRT	0.436190341608791
119	KC_AB1	MCTS1	-0.111451816744974	0.791585023039261	0.994766463583882	pseudobulk	DESeq2	LRT	0.101502431333929
119	KC_AB1	MCU	0.180257322480729	0.724949740135375	0.994766463583882	pseudobulk	DESeq2	LRT	0.139692101482096
119	KC_AB1	Mdh1	0.156736359344023	0.785775207514575	0.994766463583882	pseudobulk	DESeq2	LRT	0.104701678005089
119	KC_AB1	Mdh2	0.165761202236897	0.606412062792722	0.994766463583882	pseudobulk	DESeq2	LRT	0.217232168272755
119	KC_AB1	mdlc	-0.123132359203517	0.905238660116382	0.994766463583882	pseudobulk	DESeq2	LRT	0.043236906864143
119	KC_AB1	mdy	0.352688939018687	0.483893970947221	0.994766463583882	pseudobulk	DESeq2	LRT	0.315249788928088
119	KC_AB1	me31B	-0.215421724888386	0.619834488592499	0.994766463583882	pseudobulk	DESeq2	LRT	0.207724262577701
119	KC_AB1	Med	0.247880189525111	0.669774821004108	0.994766463583882	pseudobulk	DESeq2	LRT	0.174071183019675
119	KC_AB1	MED1	0.508648566185409	0.499598014790247	0.994766463583882	pseudobulk	DESeq2	LRT	0.301379296013183
119	KC_AB1	MED10	0.371098740280847	0.549842803237774	0.994766463583882	pseudobulk	DESeq2	LRT	0.259761454950165
119	KC_AB1	MED11	-0.794218127112917	0.239886662429998	0.994766463583882	pseudobulk	DESeq2	LRT	0.619993897901611
119	KC_AB1	MED14	-0.349863137475212	0.67299298702089	0.994766463583882	pseudobulk	DESeq2	LRT	0.171989461354029
119	KC_AB1	MED15	0.458217147831435	0.68965933730116	0.994766463583882	pseudobulk	DESeq2	LRT	0.161365379500872
119	KC_AB1	MED16	-3.26634861084024	0.140912144104393	0.994766463583882	pseudobulk	DESeq2	LRT	0.851051576867565
119	KC_AB1	MED17	-0.296875103274503	0.812708680651454	0.994766463583882	pseudobulk	DESeq2	LRT	0.0900651014642336
119	KC_AB1	MED18	-0.329990512351841	0.708458656656857	0.994766463583882	pseudobulk	DESeq2	LRT	0.149685488690597
119	KC_AB1	MED19	-0.00863932602058607	0.885917768001434	0.994766463583882	pseudobulk	DESeq2	LRT	0.0526065880007822
119	KC_AB1	MED20	1.64367646244619	0.361608060724215	0.994766463583882	pseudobulk	DESeq2	LRT	0.441761897087635
119	KC_AB1	MED21	-0.407344191151818	0.219228348694004	0.994766463583882	pseudobulk	DESeq2	LRT	0.659103287389671
119	KC_AB1	MED22	-0.113563369154259	0.823064205375938	0.994766463583882	pseudobulk	DESeq2	LRT	0.0845662851366187
119	KC_AB1	MED24	-0.497372080903573	0.560722004345111	0.994766463583882	pseudobulk	DESeq2	LRT	0.251252400601796
119	KC_AB1	MED25	0.808521164682847	0.595356415133744	0.994766463583882	pseudobulk	DESeq2	LRT	0.225222962366079
119	KC_AB1	MED26	0.0342097300262382	0.849195589477694	0.994766463583882	pseudobulk	DESeq2	LRT	0.0709922701236485
119	KC_AB1	MED27	-0.664325260423037	0.637829291226068	0.994766463583882	pseudobulk	DESeq2	LRT	0.195295540399191
119	KC_AB1	MED28	-0.0688761643542727	0.927490661360236	0.994766463583882	pseudobulk	DESeq2	LRT	0.0326904544785616
119	KC_AB1	MED30	-1.167403425359	0.468878346178673	0.994766463583882	pseudobulk	DESeq2	LRT	0.328939823461328
119	KC_AB1	MED31	0.339515263550364	0.528750140847432	0.994766463583882	pseudobulk	DESeq2	LRT	0.276749503930369
119	KC_AB1	MED4	0.622759260069492	0.579007502908167	0.994766463583882	pseudobulk	DESeq2	LRT	0.237315808551488
119	KC_AB1	MED6	-0.240252143301719	0.729897352908278	0.994766463583882	pseudobulk	DESeq2	LRT	0.136738211386391
119	KC_AB1	MED7	-0.53288260614223	0.456527831942889	0.994766463583882	pseudobulk	DESeq2	LRT	0.340532740822423
119	KC_AB1	MED8	0.238342570225149	0.798316691405116	0.994766463583882	pseudobulk	DESeq2	LRT	0.0978247902963362
119	KC_AB1	Mef2	0.00498484681286897	0.917249857412369	0.994766463583882	pseudobulk	DESeq2	LRT	0.0375123470836263
119	KC_AB1	mEFG1	0.409715022195499	0.720654716173811	0.994766463583882	pseudobulk	DESeq2	LRT	0.142272766873142
119	KC_AB1	mEFTs	0.261216214283339	0.655123447982692	0.994766463583882	pseudobulk	DESeq2	LRT	0.183676856151554
119	KC_AB1	mEFTu1	-0.355983985716229	0.597970711504538	0.994766463583882	pseudobulk	DESeq2	LRT	0.223320087154507
119	KC_AB1	mEFTu2	0.0899116982374453	0.932030989218385	0.994766463583882	pseudobulk	DESeq2	LRT	0.0305696474928
119	KC_AB1	Megf8	0.172656472545795	0.833571892702705	0.994766463583882	pseudobulk	DESeq2	LRT	0.0790569378184423
119	KC_AB1	mei-218	-1.64952313825113	0.817565084244375	0.994766463583882	pseudobulk	DESeq2	LRT	0.0874776642296574
119	KC_AB1	mei-41	-1.64952313825113	0.817565084244375	0.994766463583882	pseudobulk	DESeq2	LRT	0.0874776642296574
119	KC_AB1	mei-9	0.265717584717128	0.835630223492062	0.994766463583882	pseudobulk	DESeq2	LRT	0.0779858606275216
119	KC_AB1	mei-P26	0.024365059954989	0.840060086164982	0.994766463583882	pseudobulk	DESeq2	LRT	0.0756896494659411
119	KC_AB1	mei-S332	-1.2752393488376	0.575318001212773	0.994766463583882	pseudobulk	DESeq2	LRT	0.24009203705556
119	KC_AB1	mei-W68	3.02680884525143	0.293242669533885	0.994766463583882	pseudobulk	DESeq2	LRT	0.532772835549261

119	KC_AB1	Meics	1.29595026443928	0.312615135821411	0.994766463583882	pseudobulk	DESeq2	LRT	0.504989998663013
119	KC_AB1	meigo	-0.313501731982744	0.847735509510735	0.994766463583882	pseudobulk	DESeq2	LRT	0.0717396249569093
119	KC_AB1	Mekk1	0.212901975535535	0.818469733048913	0.994766463583882	pseudobulk	DESeq2	LRT	0.086997376133389
119	KC_AB1	Meltrin	1.83724303791705	0.360409590351551	0.994766463583882	pseudobulk	DESeq2	LRT	0.44320366114097
119	KC_AB1	Membrin	1.03403917101678	0.333556617151903	0.994766463583882	pseudobulk	DESeq2	LRT	0.476830439319442
119	KC_AB1	Men	-0.125248931248561	0.842748780253874	0.994766463583882	pseudobulk	DESeq2	LRT	0.0743018673793963
119	KC_AB1	Men-b	-0.568446615853691	0.164326811460107	0.994766463583882	pseudobulk	DESeq2	LRT	0.784291571566733
119	KC_AB1	meng	-0.420096692652893	0.162631334965922	0.994766463583882	pseudobulk	DESeq2	LRT	0.788795773061207
119	KC_AB1	MEP-1	0.780601895146926	0.514056826637719	0.994766463583882	pseudobulk	DESeq2	LRT	0.288988869076644
119	KC_AB1	Mer	-0.525086037820921	0.661613276530463	0.994766463583882	pseudobulk	DESeq2	LRT	0.179395788422157
119	KC_AB1	Mesh1	-0.835152883801839	0.920434724857442	0.994766463583882	pseudobulk	DESeq2	LRT	0.0360070052473159
119	KC_AB1	MESK2	0.346910017099872	0.0997602066542427	0.994766463583882	pseudobulk	DESeq2	LRT	1.00104265988332
119	KC_AB1	MESR3	0.625732144884864	0.380024095858104	0.994766463583882	pseudobulk	DESeq2	LRT	0.420188865576765
119	KC_AB1	MESR4	-0.124435116571342	0.799858648526629	0.994766463583882	pseudobulk	DESeq2	LRT	0.0969867549941192
119	KC_AB1	MESR6	0.264035888783318	0.845614684934745	0.994766463583882	pseudobulk	DESeq2	LRT	0.07282748368224
119	KC_AB1	Met	-0.0739653655154395	0.844235500910247	0.994766463583882	pseudobulk	DESeq2	LRT	0.0735363892992951
119	KC_AB1	metl	0.0299870550042784	0.915868776975475	0.994766463583882	pseudobulk	DESeq2	LRT	0.0381667463298522
120	KC_AB1	metro	0.0172032378899206	0.943441632022973	0.994766463583882	pseudobulk	DESeq2	LRT	0.0252849631985276
120	KC_AB1	MetRS	-0.152472720452399	0.863285540131596	0.994766463583882	pseudobulk	DESeq2	LRT	0.0638455333777914
120	KC_AB1	MetRS-m	0.946340468446946	0.492510481275863	0.994766463583882	pseudobulk	DESeq2	LRT	0.307584522705792
120	KC_AB1	Mettl14	0.0617404682142172	0.916713335361162	0.994766463583882	pseudobulk	DESeq2	LRT	0.037766450954221
120	KC_AB1	Mettl3	-0.583375864359448	0.630566859745868	0.994766463583882	pseudobulk	DESeq2	LRT	0.200268857894653
120	KC_AB1	mew	-0.723685167858558	0.548197515312647	0.994766463583882	pseudobulk	DESeq2	LRT	0.261062937217629
120	KC_AB1	Mf	0.00891646089885312	0.960226720676327	0.994766463583882	pseudobulk	DESeq2	LRT	0.0176262128838119
120	KC_AB1	Mfap1	0.231044147098365	0.806276315973013	0.994766463583882	pseudobulk	DESeq2	LRT	0.09351609723066
120	KC_AB1	Mfe2	-1.61659307235438	0.237489868182365	0.994766463583882	pseudobulk	DESeq2	LRT	0.624354913560589
120	KC_AB1	mfrn	-0.462270660634008	0.534169564154591	0.994766463583882	pseudobulk	DESeq2	LRT	0.272320860785399
120	KC_AB1	MFS10	-0.226950955160759	0.825677459836429	0.994766463583882	pseudobulk	DESeq2	LRT	0.0831895710444835
120	KC_AB1	MFS15	0.685004330574968	0.631648897608293	0.994766463583882	pseudobulk	DESeq2	LRT	0.199524257480284
120	KC_AB1	MFS16	0.572051602496366	0.601363471543594	0.994766463583882	pseudobulk	DESeq2	LRT	0.220862955664923
120	KC_AB1	MFS17	0.116849745705219	0.478775900564458	0.994766463583882	pseudobulk	DESeq2	LRT	0.319867718155519
120	KC_AB1	MFS18	0.68062550090061	0.47638861953758	0.994766463583882	pseudobulk	DESeq2	LRT	0.322038621939607
120	KC_AB1	MFS9	-0.0876274309314422	0.917937734184526	0.994766463583882	pseudobulk	DESeq2	LRT	0.0371867769874299
120	KC_AB1	Mgat1	-0.595439940544416	0.477232920939503	0.994766463583882	pseudobulk	DESeq2	LRT	0.321269605049441
120	KC_AB1	Mgat2	0.0403081133472983	0.910433512025282	0.994766463583882	pseudobulk	DESeq2	LRT	0.0407517647673319
120	KC_AB1	mge	0.151698358054391	0.68933146058974	0.994766463583882	pseudobulk	DESeq2	LRT	0.161571900167168
120	KC_AB1	mgl	-0.221359900800263	0.46886022854349	0.994766463583882	pseudobulk	DESeq2	LRT	0.328956605087248
120	KC_AB1	mGluR	-0.777272528561556	0.349402466723764	0.994766463583882	pseudobulk	DESeq2	LRT	0.45667403329488
120	KC_AB1	mgr	0.0621584712130673	0.918962610135138	0.994766463583882	pseudobulk	DESeq2	LRT	0.0367021584096738
120	KC_AB1	Mgstl	0.220738941344378	0.802006215051947	0.994766463583882	pseudobulk	DESeq2	LRT	0.0958222661892692
120	KC_AB1	mh	-1.18889040549532	0.611395637693754	0.994766463583882	pseudobulk	DESeq2	LRT	0.213677664288847
120	KC_AB1	Mhcl	0.195798741240441	0.693480987841435	0.994766463583882	pseudobulk	DESeq2	LRT	0.158965440846985
120	KC_AB1	mi	0.311211064395068	0.769571172698664	0.994766463583882	pseudobulk	DESeq2	LRT	0.113751208865747
120	KC_AB1	Mi-2	-0.0480054650468761	0.864304912747719	0.994766463583882	pseudobulk	DESeq2	LRT	0.0633330184436807
120	KC_AB1	mia	-1.64952313825113	0.817565084244375	0.994766463583882	pseudobulk	DESeq2	LRT	0.0874776642296574
120	KC_AB1	mib1	-0.366142865767979	0.644320424640353	0.994766463583882	pseudobulk	DESeq2	LRT	0.190898101514112
120	KC_AB1	mib2	0.104681863589849	0.792304251186762	0.994766463583882	pseudobulk	DESeq2	LRT	0.101108013817199
120	KC_AB1	Mical	-0.209235573325773	0.680211776684831	0.994766463583882	pseudobulk	DESeq2	LRT	0.167355853283976
120	KC_AB1	MICAL-like	0.857234706620012	0.470925698665455	0.994766463583882	pseudobulk	DESeq2	LRT	0.327047609230098
120	KC_AB1	MICU1	-0.263794778523352	0.580367946540274	0.994766463583882	pseudobulk	DESeq2	LRT	0.236296581460168
120	KC_AB1	MICU3	-0.0848927585933798	0.85338505701849	0.994766463583882	pseudobulk	DESeq2	LRT	0.0688549660129503
120	KC_AB1	Mid1	-0.00245524278797101	0.894795141257134	0.994766463583882	pseudobulk	DESeq2	LRT	0.0482763827905895
120	KC_AB1	mIF2	0.645846369998674	0.721341252294085	0.994766463583882	pseudobulk	DESeq2	LRT	0.14185923052809
120	KC_AB1	mIF3	-0.386404584431053	0.709394034186658	0.994766463583882	pseudobulk	DESeq2	LRT	0.149112468148696
120	KC_AB1	Miga	-0.46621658930361	0.498210594048592	0.994766463583882	pseudobulk	DESeq2	LRT	0.30258704177758
120	KC_AB1	milt	0.0162211646918508	0.951064283232413	0.994766463583882	pseudobulk	DESeq2	LRT	0.0217901277422926

120	KC_AB1	mim	-0.220830996376337	0.578651307171837	0.994766463583882	pseudobulk	DESeq2	LRT	0.237583061469705
120	KC_AB1	mino	1.59307955075178	0.558763130036445	0.994766463583882	pseudobulk	DESeq2	LRT	0.25277225851705
120	KC_AB1	mio	-2.6543284975265	0.29870988863229	0.994766463583882	pseudobulk	DESeq2	LRT	0.524750400069619
120	KC_AB1	mip120	-0.275474617440354	0.731210434504284	0.994766463583882	pseudobulk	DESeq2	LRT	0.135957619769715
120	KC_AB1	mip130	-0.503136719012003	0.666236813395659	0.994766463583882	pseudobulk	DESeq2	LRT	0.176371373726985
120	KC_AB1	mip40	0.880438906619327	0.456381020984309	0.994766463583882	pseudobulk	DESeq2	LRT	0.340672424405378
120	KC_AB1	miple1	0.160188457579493	0.346184923328873	0.994766463583882	pseudobulk	DESeq2	LRT	0.460691849972044
120	KC_AB1	miple2	-1.02822158675946	0.428216799251044	0.994766463583882	pseudobulk	DESeq2	LRT	0.368336299024494
120	KC_AB1	Mipp2	0.806933541094274	0.754600478929312	0.994766463583882	pseudobulk	DESeq2	LRT	0.122282923497213
120	KC_AB1	Miro	-0.237957586973467	0.709189110287433	0.994766463583882	pseudobulk	DESeq2	LRT	0.149237941681367
120	KC_AB1	Mis12	0.148951480684317	0.865508980538193	0.994766463583882	pseudobulk	DESeq2	LRT	0.0627284215171288
120	KC_AB1	Mitf	-0.708789274356226	0.239434412731037	0.994766463583882	pseudobulk	DESeq2	LRT	0.620813430432778
120	KC_AB1	Mitofilin	-0.186024454711879	0.772405394300687	0.994766463583882	pseudobulk	DESeq2	LRT	0.112154701869803
120	KC_AB1	Mkk4	-0.271185601873995	0.514128317760438	0.994766463583882	pseudobulk	DESeq2	LRT	0.288928474895068
120	KC_AB1	MKP-4	0.293669449887713	0.7340833647481	0.994766463583882	pseudobulk	DESeq2	LRT	0.134254617475972
120	KC_AB1	Mkp3	-0.307480142420044	0.0662933289074709	0.994766463583882	pseudobulk	DESeq2	LRT	1.17853017241638
120	KC_AB1	Mkrn1	-0.440898503537363	0.424803507597515	0.994766463583882	pseudobulk	DESeq2	LRT	0.371811905947406
120	KC_AB1	Mlc-c	0.412332292381055	0.515396110565923	0.994766463583882	pseudobulk	DESeq2	LRT	0.287858863173804
120	KC_AB1	Mlc1	-1.64952313825113	0.817565084244375	0.994766463583882	pseudobulk	DESeq2	LRT	0.0874776642296574
120	KC_AB1	Mlc2	-1.64952313825113	0.817565084244375	0.994766463583882	pseudobulk	DESeq2	LRT	0.0874776642296574
120	KC_AB1	mld	-0.161604802180027	0.659674870920965	0.994766463583882	pseudobulk	DESeq2	LRT	0.180670059239572
120	KC_AB1	mldr	2.38929733117871	0.484296857543297	0.994766463583882	pseudobulk	DESeq2	LRT	0.314888348950639
120	KC_AB1	mle	-0.0685962794355661	0.87957510056362	0.994766463583882	pseudobulk	DESeq2	LRT	0.0557270733549949
120	KC_AB1	Mlf	0.0931220461719661	0.711703125695819	0.994766463583882	pseudobulk	DESeq2	LRT	0.147701126811173
120	KC_AB1	Mlh1	0.131756303236789	0.898896171988158	0.994766463583882	pseudobulk	DESeq2	LRT	0.0462904690417512
120	KC_AB1	Mlp84B	0.59155389911517	0.73237808349158	0.994766463583882	pseudobulk	DESeq2	LRT	0.13526466049416
120	KC_AB1	mlt	0.674183928174672	0.248248566823	0.994766463583882	pseudobulk	DESeq2	LRT	0.605113250073907
120	KC_AB1	mmd	-0.112905737403639	0.409958784986033	0.994766463583882	pseudobulk	DESeq2	LRT	0.387259802677493
120	KC_AB1	MME1	-1.79038792738658	0.249492868786147	0.994766463583882	pseudobulk	DESeq2	LRT	0.602941863231318
120	KC_AB1	Mmp2	0.25652530340135	0.449799955639588	0.994766463583882	pseudobulk	DESeq2	LRT	0.346980591731608
120	KC_AB1	Mms19	1.0910134037871	0.493041662640795	0.994766463583882	pseudobulk	DESeq2	LRT	0.307116380742039
120	KC_AB1	mmy	0.0596588858950546	0.909762317872738	0.994766463583882	pseudobulk	DESeq2	LRT	0.0410720555021636
120	KC_AB1	mnb	0.287991748281868	0.366653238242261	0.994766463583882	pseudobulk	DESeq2	LRT	0.435744475008928
120	KC_AB1	mnd	0.949183608371002	0.80156895525114	0.994766463583882	pseudobulk	DESeq2	LRT	0.0960591113673091
120	KC_AB1	Mnn1	0.129678957254305	0.564091606954087	0.994766463583882	pseudobulk	DESeq2	LRT	0.248650362037845
120	KC_AB1	Mnt	-0.0118074670520956	0.944816948809176	0.994766463583882	pseudobulk	DESeq2	LRT	0.024652324636415
120	KC_AB1	Mo25	-0.0216414382761835	0.931841972777732	0.994766463583882	pseudobulk	DESeq2	LRT	0.0306577316048824
120	KC_AB1	Mob2	-0.0276954620487747	0.909851746369575	0.994766463583882	pseudobulk	DESeq2	LRT	0.0410293669964021
120	KC_AB1	Mob3	-0.0179962917103589	0.945949816373167	0.994766463583882	pseudobulk	DESeq2	LRT	0.0241319027634449
120	KC_AB1	Mob4	0.132781394984546	0.746467678842401	0.994766463583882	pseudobulk	DESeq2	LRT	0.126988991963444
120	KC_AB1	Moca-cyp	0.116592722655848	0.789459842129154	0.994766463583882	pseudobulk	DESeq2	LRT	0.102669956580676
120	KC_AB1	mod	0.447075246435262	0.479411092241655	0.994766463583882	pseudobulk	DESeq2	LRT	0.319291921817566
120	KC_AB1	Moe	0.0644156344878277	0.814172560058393	0.994766463583882	pseudobulk	DESeq2	LRT	0.0892835386749198
120	KC_AB1	mof	-0.202109242319117	0.878741632898844	0.994766463583882	pseudobulk	DESeq2	LRT	0.0561387971677512
120	KC_AB1	Mon1	0.69750158688829	0.750018748510497	0.994766463583882	pseudobulk	DESeq2	LRT	0.124927880244456
120	KC_AB1	mon2	-0.0484193904228277	0.907261758933581	0.994766463583882	pseudobulk	DESeq2	LRT	0.0422673942413518
120	KC_AB1	Mondo	0.0818088750462293	0.901556681180413	0.994766463583882	pseudobulk	DESeq2	LRT	0.0450069638389209
120	KC_AB1	mop	-0.657727037125695	0.385487476951334	0.994766463583882	pseudobulk	DESeq2	LRT	0.413989725989866
120	KC_AB1	mor	-0.219080842234689	0.719350877000165	0.994766463583882	pseudobulk	DESeq2	LRT	0.143059222586689
120	KC_AB1	Mp	0.522670688970333	0.130020792141128	0.994766463583882	pseudobulk	DESeq2	LRT	0.88598719238458
120	KC_AB1	MP1	-0.574348673714124	0.744554756485959	0.994766463583882	pseudobulk	DESeq2	LRT	0.128103357595833
120	KC_AB1	Mpc1	-0.27447314327185	0.735772172984424	0.994766463583882	pseudobulk	DESeq2	LRT	0.133256641277711
120	KC_AB1	Mpcp2	0.193779006415487	0.312013326457432	0.994766463583882	pseudobulk	DESeq2	LRT	0.505826856355511
120	KC_AB1	Mpp6	1.10244960574289	0.364999851864472	0.994766463583882	pseudobulk	DESeq2	LRT	0.437707311802307
120	KC_AB1	Mppe	0.10810490630346	0.907401708961742	0.994766463583882	pseudobulk	DESeq2	LRT	0.0422004071374968
120	KC_AB1	Mps1	2.02670873290438	0.538959414925803	0.994766463583882	pseudobulk	DESeq2	LRT	0.26844393710898

120	KC_AB1	mr	0.974103902644404	0.32945544942501	0.994766463583882	pseudobulk	DESeq2	LRT	0.482203304520639
120	KC_AB1	mre11	1.39595377037528	0.546916743048716	0.994766463583882	pseudobulk	DESeq2	LRT	0.262078781137403
120	KC_AB1	mRF1	2.39591389251247	0.30161018792818	0.994766463583882	pseudobulk	DESeq2	LRT	0.520553992754974
120	KC_AB1	MRG15	0.0499959620113375	0.931120694856074	0.994766463583882	pseudobulk	DESeq2	LRT	0.0309940207237022
120	KC_AB1	MrgBP	-0.18542041200366	0.689712289123409	0.994766463583882	pseudobulk	DESeq2	LRT	0.161332035790186
121	KC_AB1	mri	0.244405963136964	0.695401283834737	0.994766463583882	pseudobulk	DESeq2	LRT	0.157764511866986
121	KC_AB1	mrj	-0.133763191760124	0.699252539260878	0.994766463583882	pseudobulk	DESeq2	LRT	0.155365947859567
121	KC_AB1	mrn	-1.03279054540079	0.616964932706771	0.994766463583882	pseudobulk	DESeq2	LRT	0.209739519864242
121	KC_AB1	mRNA-cap	-0.169518540777586	0.805536477870019	0.994766463583882	pseudobulk	DESeq2	LRT	0.0939147882317796
121	KC_AB1	MRP	0.417953705585371	0.542335621558796	0.994766463583882	pseudobulk	DESeq2	LRT	0.265731869401251
121	KC_AB1	mRpL1	0.0878867906972697	0.902171814757859	0.994766463583882	pseudobulk	DESeq2	LRT	0.0447107450573509
121	KC_AB1	mRpL10	0.469496715493228	0.582438961982983	0.994766463583882	pseudobulk	DESeq2	LRT	0.234749580796255
121	KC_AB1	mRpL11	0.113428981467109	0.889255800626833	0.994766463583882	pseudobulk	DESeq2	LRT	0.0509732932245451
121	KC_AB1	mRpL12	-0.319546105028557	0.49322015920423	0.994766463583882	pseudobulk	DESeq2	LRT	0.30695918095661
121	KC_AB1	mRpL13	-1.11866879564513	0.129176238337713	0.994766463583882	pseudobulk	DESeq2	LRT	0.88881736643291
121	KC_AB1	mRpL14	0.105562667404763	0.817558870724636	0.994766463583882	pseudobulk	DESeq2	LRT	0.0874809648935954
121	KC_AB1	mRpL15	0.568131686353303	0.522522774030285	0.994766463583882	pseudobulk	DESeq2	LRT	0.281894776183148
121	KC_AB1	mRpL16	0.999835042250151	0.324128053250585	0.994766463583882	pseudobulk	DESeq2	LRT	0.489283379197106
121	KC_AB1	mRpL17	0.0883241565368177	0.880169831646808	0.994766463583882	pseudobulk	DESeq2	LRT	0.0554335212239432
121	KC_AB1	mRpL18	0.0477034621446752	0.914642236445053	0.994766463583882	pseudobulk	DESeq2	LRT	0.038748747596173
121	KC_AB1	mRpL19	-0.250542142514924	0.69566623265957	0.994766463583882	pseudobulk	DESeq2	LRT	0.157599076598548
121	KC_AB1	mRpL2	-0.0518467229219138	0.940124688171028	0.994766463583882	pseudobulk	DESeq2	LRT	0.0268145423647221
121	KC_AB1	mRpL20	-0.241161375798892	0.742203119921706	0.994766463583882	pseudobulk	DESeq2	LRT	0.129477224385063
121	KC_AB1	mRpL21	-0.137667750156826	0.839690333769334	0.994766463583882	pseudobulk	DESeq2	LRT	0.0758808462363522
121	KC_AB1	mRpL22	-0.802682486479233	0.19126754942868	0.994766463583882	pseudobulk	DESeq2	LRT	0.718358706394627
121	KC_AB1	mRpL23	0.149187984049517	0.799608856206028	0.994766463583882	pseudobulk	DESeq2	LRT	0.0971224044237197
121	KC_AB1	mRpL24	-0.498828161996421	0.405786661877398	0.994766463583882	pseudobulk	DESeq2	LRT	0.391702232236743
121	KC_AB1	mRpL27	-0.290076764028994	0.72231167773961	0.994766463583882	pseudobulk	DESeq2	LRT	0.141275363767872
121	KC_AB1	mRpL28	-0.126836652911039	0.811584550866332	0.994766463583882	pseudobulk	DESeq2	LRT	0.0906662286881764
121	KC_AB1	mRpL3	0.838619217768782	0.480247313907071	0.994766463583882	pseudobulk	DESeq2	LRT	0.318535055531546
121	KC_AB1	mRpL32	-0.203150119946828	0.806559035568906	0.994766463583882	pseudobulk	DESeq2	LRT	0.0933638392068482
121	KC_AB1	mRpL33	-0.00473432869400902	0.907616563080101	0.994766463583882	pseudobulk	DESeq2	LRT	0.0420975872817515
121	KC_AB1	mRpL34	0.274760897567606	0.621875289453584	0.994766463583882	pseudobulk	DESeq2	LRT	0.206296699766577
121	KC_AB1	mRpL35	0.358946909967335	0.634476669304472	0.994766463583882	pseudobulk	DESeq2	LRT	0.197584342960319
121	KC_AB1	mRpL36	-0.32698062411337	0.591060637549369	0.994766463583882	pseudobulk	DESeq2	LRT	0.228367962093033
121	KC_AB1	mRpL37	0.310055277307288	0.745583746227075	0.994766463583882	pseudobulk	DESeq2	LRT	0.127503568198111
121	KC_AB1	mRpL38	0.200766267075901	0.77882579578181	0.994766463583882	pseudobulk	DESeq2	LRT	0.108559672483072
121	KC_AB1	mRpL39	-0.155504899483003	0.834008164059189	0.994766463583882	pseudobulk	DESeq2	LRT	0.0788296980571032
121	KC_AB1	mRpL4	0.113476251740828	0.854902406997241	0.994766463583882	pseudobulk	DESeq2	LRT	0.0680834601510053
121	KC_AB1	mRpL40	-0.372460555760559	0.623347289167192	0.994766463583882	pseudobulk	DESeq2	LRT	0.205269924856233
121	KC_AB1	mRpL41	0.598539823067859	0.433725415181219	0.994766463583882	pseudobulk	DESeq2	LRT	0.362785128578552
121	KC_AB1	mRpL42	0.403844379894308	0.536644326710943	0.994766463583882	pseudobulk	DESeq2	LRT	0.270313457547636
121	KC_AB1	mRpL43	-0.0935382439288408	0.896648225170936	0.994766463583882	pseudobulk	DESeq2	LRT	0.0473779068237913
121	KC_AB1	mRpL44	0.354883571128668	0.707359149340444	0.994766463583882	pseudobulk	DESeq2	LRT	0.15036002471395
121	KC_AB1	mRpL45	-1.03387565870085	0.485855201383684	0.994766463583882	pseudobulk	DESeq2	LRT	0.313493143515307
121	KC_AB1	mRpL46	-0.494725135885516	0.694047034638732	0.994766463583882	pseudobulk	DESeq2	LRT	0.158611096991986
121	KC_AB1	mRpL47	-0.0995662648728472	0.880646915355527	0.994766463583882	pseudobulk	DESeq2	LRT	0.0551981817685167
121	KC_AB1	mRpL48	-0.068387362096218	0.916260189459099	0.994766463583882	pseudobulk	DESeq2	LRT	0.0379811826632254
121	KC_AB1	mRpL49	-0.0709794279863658	0.850694436352908	0.994766463583882	pseudobulk	DESeq2	LRT	0.0702264075255699
121	KC_AB1	mRpL50	-1.06791944194785	0.104688172169041	0.994766463583882	pseudobulk	DESeq2	LRT	0.980102382809095
121	KC_AB1	mRpL51	0.942042598242413	0.493406616129545	0.994766463583882	pseudobulk	DESeq2	LRT	0.306795031317121
121	KC_AB1	mRpL52	-0.0815447285440837	0.87945053733307	0.994766463583882	pseudobulk	DESeq2	LRT	0.0557885814110468
121	KC_AB1	mRpL54	-0.138848839906679	0.801281908246074	0.994766463583882	pseudobulk	DESeq2	LRT	0.0962146628718423
121	KC_AB1	mRpL55	0.32654092853962	0.479778584032417	0.994766463583882	pseudobulk	DESeq2	LRT	0.318959141620636
121	KC_AB1	mRpL9	0.462973331337027	0.614237518135291	0.994766463583882	pseudobulk	DESeq2	LRT	0.211663660016859
121	KC_AB1	mRpS10	-0.251154288585498	0.664127091884545	0.994766463583882	pseudobulk	DESeq2	LRT	0.177748803128155

121	KC_AB1	mRpS11	0.657300738088722	0.267689970284256	0.994766463583882	pseudobulk	DESeq2	LRT	0.572367900504525
121	KC_AB1	mRpS14	0.0487083435164557	0.853813560445594	0.994766463583882	pseudobulk	DESeq2	LRT	0.0686369519214764
121	KC_AB1	mRpS16	-0.641877382323047	0.389790496703021	0.994766463583882	pseudobulk	DESeq2	LRT	0.409168753416831
121	KC_AB1	mRpS17	-0.0856426611314282	0.913198640377251	0.994766463583882	pseudobulk	DESeq2	LRT	0.039434743783967
121	KC_AB1	mRpS18A	0.350895348128536	0.542416760257615	0.994766463583882	pseudobulk	DESeq2	LRT	0.265666899570675
121	KC_AB1	mRpS18B	0.45640128275382	0.648330207369383	0.994766463583882	pseudobulk	DESeq2	LRT	0.18820374303043
121	KC_AB1	mRpS18C	-0.259368475614424	0.647441916080723	0.994766463583882	pseudobulk	DESeq2	LRT	0.188799187334932
121	KC_AB1	mRpS2	0.0257400670104793	0.916395138137359	0.994766463583882	pseudobulk	DESeq2	LRT	0.0379172235918915
121	KC_AB1	mRpS21	0.483016937109449	0.420223952204497	0.994766463583882	pseudobulk	DESeq2	LRT	0.376519197018648
121	KC_AB1	mRpS22	-0.0554378939995139	0.914788014548498	0.994766463583882	pseudobulk	DESeq2	LRT	0.0386795341063357
121	KC_AB1	mRpS23	0.652095401144716	0.421894225980261	0.994766463583882	pseudobulk	DESeq2	LRT	0.374796418306364
121	KC_AB1	mRpS24	-0.638304067318771	0.287999683519122	0.994766463583882	pseudobulk	DESeq2	LRT	0.540607989483737
121	KC_AB1	mRpS25	0.0462008703171423	0.939112829338652	0.994766463583882	pseudobulk	DESeq2	LRT	0.027282226460787
121	KC_AB1	mRpS26	-0.0584321756437045	0.924483290475171	0.994766463583882	pseudobulk	DESeq2	LRT	0.034100934066744
121	KC_AB1	mRpS28	0.00504935492447057	0.954350221369855	0.994766463583882	pseudobulk	DESeq2	LRT	0.0202922214208961
121	KC_AB1	mRpS29	-0.274305625768316	0.753396969254547	0.994766463583882	pseudobulk	DESeq2	LRT	0.122976131197622
121	KC_AB1	mRpS30	1.17495938427303	0.378318244408855	0.994766463583882	pseudobulk	DESeq2	LRT	0.422142714337618
121	KC_AB1	mRpS31	0.0512413283848297	0.925557380824461	0.994766463583882	pseudobulk	DESeq2	LRT	0.0335966515702459
121	KC_AB1	mRpS33	-0.617237681070084	0.309202461599612	0.994766463583882	pseudobulk	DESeq2	LRT	0.509757057267062
121	KC_AB1	mRpS34	-0.485720678958188	0.559222535802669	0.994766463583882	pseudobulk	DESeq2	LRT	0.252415335514275
121	KC_AB1	mRpS35	-0.273759330377825	0.755641472634657	0.994766463583882	pseudobulk	DESeq2	LRT	0.121684214273093
121	KC_AB1	mRpS5	-0.17510869546919	0.58259722897724	0.994766463583882	pseudobulk	DESeq2	LRT	0.234631585349882
121	KC_AB1	mRpS6	0.211959398328403	0.734331567586107	0.994766463583882	pseudobulk	DESeq2	LRT	0.134107801856501
121	KC_AB1	mRpS7	-0.325568133582078	0.579512250657991	0.994766463583882	pseudobulk	DESeq2	LRT	0.236937378791102
121	KC_AB1	mRpS9	-0.909358650917312	0.165247356476103	0.994766463583882	pseudobulk	DESeq2	LRT	0.781865479358355
121	KC_AB1	mRRF1	-0.0933153259730406	0.924619366399229	0.994766463583882	pseudobulk	DESeq2	LRT	0.0340370143889207
121	KC_AB1	mRRF2	1.57161582234084	0.52162243080664	0.994766463583882	pseudobulk	DESeq2	LRT	0.282643741344212
121	KC_AB1	mrt	0.390045279115705	0.629202280336841	0.994766463583882	pseudobulk	DESeq2	LRT	0.201209712088305
121	KC_AB1	Mrtf	0.0236348297562339	0.916309077542467	0.994766463583882	pseudobulk	DESeq2	LRT	0.0379580110153923
121	KC_AB1	mrva	0.264436316498212	0.545613811688327	0.994766463583882	pseudobulk	DESeq2	LRT	0.263114644465756
121	KC_AB1	Ms	-3.21143241058256	0.292545415061155	0.994766463583882	pseudobulk	DESeq2	LRT	0.533806704011112
121	KC_AB1	ms(3)76Ba	1.32710828904082	0.607473293222376	0.994766463583882	pseudobulk	DESeq2	LRT	0.216472810505135
121	KC_AB1	MSBP	0.27110286871859	0.562762251279745	0.994766463583882	pseudobulk	DESeq2	LRT	0.24967504167569
121	KC_AB1	msd5	1.49975934979285	0.31265558458276	0.994766463583882	pseudobulk	DESeq2	LRT	0.504933809645025
121	KC_AB1	Msh6	-0.283995846067142	0.866480656580972	0.994766463583882	pseudobulk	DESeq2	LRT	0.0622411280844138
121	KC_AB1	msi	0.180712651309759	0.765112801691498	0.994766463583882	pseudobulk	DESeq2	LRT	0.116274531459822
121	KC_AB1	msk	0.136708243688224	0.877928446905591	0.994766463583882	pseudobulk	DESeq2	LRT	0.0565408786045755
121	KC_AB1	msl-1	0.525574976839765	0.485997442615928	0.994766463583882	pseudobulk	DESeq2	LRT	0.313366016047816
121	KC_AB1	msl-2	-0.819115461778836	0.321055429722816	0.994766463583882	pseudobulk	DESeq2	LRT	0.493419980851934
121	KC_AB1	msl-3	-0.268010088951366	0.837718176906529	0.994766463583882	pseudobulk	DESeq2	LRT	0.076902061083136
121	KC_AB1	msn	0.205256388056708	0.480339445269174	0.994766463583882	pseudobulk	DESeq2	LRT	0.318451747820062
121	KC_AB1	Msp300	0.0184546815996464	0.825502882299455	0.994766463583882	pseudobulk	DESeq2	LRT	0.0832814060298207
121	KC_AB1	mspo	0.0710527467718227	0.706353131909558	0.994766463583882	pseudobulk	DESeq2	LRT	0.150978124872626
121	KC_AB1	msps	-0.0187959631477066	0.958908403800846	0.994766463583882	pseudobulk	DESeq2	LRT	0.0182228752315274
121	KC_AB1	mst	-2.20969975456663	0.610836134188693	0.994766463583882	pseudobulk	DESeq2	LRT	0.214075280039667
121	KC_AB1	Mst27D	0.806933541094274	0.754600478929312	0.994766463583882	pseudobulk	DESeq2	LRT	0.122282923497213
121	KC_AB1	Mst36Fa	2.22023857837763	0.524904842126482	0.994766463583882	pseudobulk	DESeq2	LRT	0.279919420946639
121	KC_AB1	Mst36Fb	1.1821590793593	0.719511699510136	0.994766463583882	pseudobulk	DESeq2	LRT	0.142962139890846
121	KC_AB1	Mst77F	0.109074212069946	0.872350887223992	0.994766463583882	pseudobulk	DESeq2	LRT	0.0593087928961979
122	KC_AB1	Mst85C	0.451320609198601	0.583691711508883	0.994766463583882	pseudobulk	DESeq2	LRT	0.233816473678898
122	KC_AB1	mt:ATPase6	0.239746037772615	0.162948538331963	0.994766463583882	pseudobulk	DESeq2	LRT	0.787949530855234
122	KC_AB1	mt:ATPase8	-1.38643141324562	0.56233606024471	0.994766463583882	pseudobulk	DESeq2	LRT	0.250004066120415
122	KC_AB1	mt:CoI	0.215611985887141	0.290833014329402	0.994766463583882	pseudobulk	DESeq2	LRT	0.536356295449671
122	KC_AB1	mt:CoII	0.662397763447592	0.131372185715049	0.994766463583882	pseudobulk	DESeq2	LRT	0.881496574419579
122	KC_AB1	mt:CoIII	0.375801516548288	0.0547174490818467	0.994766463583882	pseudobulk	DESeq2	LRT	1.26187415755076
122	KC_AB1	mt:Cyt-b	0.209189365711112	0.348976664169327	0.994766463583882	pseudobulk	DESeq2	LRT	0.457203613044636

122	KC_AB1	mt:lrRNA	-0.0312711474377709	0.798257965296549	0.994766463583882	pseudobulk	DESeq2	LRT	0.0978567392249987
122	KC_AB1	mt:ND1	-0.376288067126195	0.26706988453071	0.994766463583882	pseudobulk	DESeq2	LRT	0.573375081351574
122	KC_AB1	mt:ND2	0.0053055800470982	0.946876595730098	0.994766463583882	pseudobulk	DESeq2	LRT	0.0237066179193122
122	KC_AB1	mt:ND3	0.0178608144522299	0.945836171861623	0.994766463583882	pseudobulk	DESeq2	LRT	0.0241840811648375
122	KC_AB1	mt:ND4	-0.226564613220114	0.410743943432851	0.994766463583882	pseudobulk	DESeq2	LRT	0.386428831673995
122	KC_AB1	mt:ND4L	-0.78410610838983	0.738244895252942	0.994766463583882	pseudobulk	DESeq2	LRT	0.131799547371545
122	KC_AB1	mt:ND5	-0.347253858504946	0.256402866579124	0.994766463583882	pseudobulk	DESeq2	LRT	0.591077123722112
122	KC_AB1	mt:ND6	-0.548436202448985	0.192027400585364	0.994766463583882	pseudobulk	DESeq2	LRT	0.716636796952814
122	KC_AB1	mt:srRNA	-0.0845180471256323	0.742308334027045	0.994766463583882	pseudobulk	DESeq2	LRT	0.129415663518931
122	KC_AB1	mt:tRNA:Ala-TGC	-2.57435989741145	0.533617212681275	0.994766463583882	pseudobulk	DESeq2	LRT	0.272770169998685
122	KC_AB1	mt:tRNA:Cys-GCA	0.949183608371002	0.80156895525114	0.994766463583882	pseudobulk	DESeq2	LRT	0.0960591113673091
122	KC_AB1	Mt2	2.02670873290438	0.538959414925803	0.994766463583882	pseudobulk	DESeq2	LRT	0.26844393710898
122	KC_AB1	MTA1-like	0.162413798931541	0.750227429171451	0.994766463583882	pseudobulk	DESeq2	LRT	0.124807061592871
122	KC_AB1	Mtap	-0.365763077795429	0.797574717704792	0.994766463583882	pseudobulk	DESeq2	LRT	0.0982286211649606
122	KC_AB1	Mtch	0.216017512828976	0.646516433626725	0.994766463583882	pseudobulk	DESeq2	LRT	0.189420431429068
122	KC_AB1	mtd	0.10600098065552	0.48359725416468	0.994766463583882	pseudobulk	DESeq2	LRT	0.315516173701491
122	KC_AB1	mTerf3	0.166263589301467	0.821183217234722	0.994766463583882	pseudobulk	DESeq2	LRT	0.0855599350160475
122	KC_AB1	mTerf5	-0.629021289006494	0.670102007381304	0.994766463583882	pseudobulk	DESeq2	LRT	0.173859081074206
122	KC_AB1	MTF-1	0.419145069568253	0.55774148637827	0.994766463583882	pseudobulk	DESeq2	LRT	0.253567050263591
122	KC_AB1	mtg	0.154131236646223	0.930566110411021	0.994766463583882	pseudobulk	DESeq2	LRT	0.0312527677596474
122	KC_AB1	mtgo	-0.20199343160382	0.419806374527674	0.994766463583882	pseudobulk	DESeq2	LRT	0.376950971181977
122	KC_AB1	mthl5	-1.28132067551389	0.647201349541384	0.994766463583882	pseudobulk	DESeq2	LRT	0.188960585805912
122	KC_AB1	mthl8	5.30848986097468	0.289350421992234	0.994766463583882	pseudobulk	DESeq2	LRT	0.538575879919529
122	KC_AB1	Mtl	-0.419264862158829	0.663504926899079	0.994766463583882	pseudobulk	DESeq2	LRT	0.178155847905592
122	KC_AB1	mtm	0.625777177863687	0.589548900613421	0.994766463583882	pseudobulk	DESeq2	LRT	0.229480166165542
122	KC_AB1	MtnA	-1.6193081483015	0.0596598160910239	0.994766463583882	pseudobulk	DESeq2	LRT	1.22431808973991
122	KC_AB1	MtnE	-3.21143241058256	0.292545415061155	0.994766463583882	pseudobulk	DESeq2	LRT	0.533806704011112
122	KC_AB1	Mtor	-0.0290577837383596	0.951647414853196	0.994766463583882	pseudobulk	DESeq2	LRT	0.0215239278200309
122	KC_AB1	Mtpalpha	0.0213805434091974	0.874591392432558	0.994766463583882	pseudobulk	DESeq2	LRT	0.0581948012164513
122	KC_AB1	MTPAP	-2.17599855715197	0.233530714558173	0.994766463583882	pseudobulk	DESeq2	LRT	0.631655991817828
122	KC_AB1	Mtpbeta	0.0328827618284201	0.901529698563744	0.994766463583882	pseudobulk	DESeq2	LRT	0.0450199619976995
122	KC_AB1	Mtr3	-0.141608251839736	0.872052832183544	0.994766463583882	pseudobulk	DESeq2	LRT	0.059457203105589
122	KC_AB1	Mtr4	-0.989583867941823	0.582662397843883	0.994766463583882	pseudobulk	DESeq2	LRT	0.234583008228436
122	KC_AB1	mtrm	-1.64952313825113	0.817565084244375	0.994766463583882	pseudobulk	DESeq2	LRT	0.0874776642296574
122	KC_AB1	mtRNApol	-0.698291033518872	0.594627401193503	0.994766463583882	pseudobulk	DESeq2	LRT	0.225755081823069
122	KC_AB1	mts	0.11562938930896	0.545474136329254	0.994766463583882	pseudobulk	DESeq2	LRT	0.263225836671929
122	KC_AB1	mtSSB	0.793270942940137	0.571451970208732	0.994766463583882	pseudobulk	DESeq2	LRT	0.243020265618444
122	KC_AB1	mtt	-0.722346277300025	0.535907392528994	0.994766463583882	pseudobulk	DESeq2	LRT	0.270910252076736
122	KC_AB1	mTTF	-0.147154317047248	0.897789254164568	0.994766463583882	pseudobulk	DESeq2	LRT	0.0468255970686507
122	KC_AB1	mtTFB2	-0.344213087290732	0.801006449768298	0.994766463583882	pseudobulk	DESeq2	LRT	0.0963639869276172
122	KC_AB1	mu2	0.39509611406087	0.839890017035674	0.994766463583882	pseudobulk	DESeq2	LRT	0.0757775807497476
122	KC_AB1	mub	-0.0624475931464242	0.554385634878371	0.994766463583882	pseudobulk	DESeq2	LRT	0.256188031595894
122	KC_AB1	muc	-0.00414115608264635	0.907266345985071	0.994766463583882	pseudobulk	DESeq2	LRT	0.0422651984846205
122	KC_AB1	Muc14A	1.1821590793593	0.719511699510136	0.994766463583882	pseudobulk	DESeq2	LRT	0.142962139890846
122	KC_AB1	Muc91C	0.949183608371002	0.80156895525114	0.994766463583882	pseudobulk	DESeq2	LRT	0.0960591113673091
122	KC_AB1	Muc96D	0.949183608371002	0.80156895525114	0.994766463583882	pseudobulk	DESeq2	LRT	0.0960591113673091
122	KC_AB1	Mul1	-0.396111237852297	0.820237563250823	0.994766463583882	pseudobulk	DESeq2	LRT	0.0860603458276052
122	KC_AB1	Mulk	1.33415492307137	0.319049418165486	0.994766463583882	pseudobulk	DESeq2	LRT	0.496142043042058
122	KC_AB1	Mur2B	0.128392782174642	0.856790492756748	0.994766463583882	pseudobulk	DESeq2	LRT	0.067125361231045
122	KC_AB1	mura	-0.11638827269302	0.584760167755599	0.994766463583882	pseudobulk	DESeq2	LRT	0.233022217980957
122	KC_AB1	mus101	0.596808856322697	0.822761281772702	0.994766463583882	pseudobulk	DESeq2	LRT	0.0847261539098712
122	KC_AB1	mus201	0.24765763846088	0.832761963650996	0.994766463583882	pseudobulk	DESeq2	LRT	0.0794791194169849
122	KC_AB1	mus304	-2.20969975456663	0.610836134188693	0.994766463583882	pseudobulk	DESeq2	LRT	0.214075280039667
122	KC_AB1	mus81	0.596808856322697	0.822761281772702	0.994766463583882	pseudobulk	DESeq2	LRT	0.0847261539098712
122	KC_AB1	muskelin	-0.874780316428275	0.681205769332186	0.994766463583882	pseudobulk	DESeq2	LRT	0.166721682526051
122	KC_AB1	mute	-0.482042602759363	0.72180943200154	0.994766463583882	pseudobulk	DESeq2	LRT	0.141577447242626

122	KC_AB1	mv	0.00502834284390088	0.881785846627974	0.994766463583882	pseudobulk	DESeq2	LRT	0.0546368762294749
122	KC_AB1	Mvb12	-0.0319048533925738	0.883106741656105	0.994766463583882	pseudobulk	DESeq2	LRT	0.0539867998080439
122	KC_AB1	Mvd	-1.77789784908752	0.354320470691372	0.994766463583882	pseudobulk	DESeq2	LRT	0.450603755791344
122	KC_AB1	Mvl	-1.03257537578435	0.305995404451879	0.994766463583882	pseudobulk	DESeq2	LRT	0.514285095858868
122	KC_AB1	mwh	-1.64952313825113	0.817565084244375	0.994766463583882	pseudobulk	DESeq2	LRT	0.0874776642296574
122	KC_AB1	mxc	-1.03395642252184	0.391390928172853	0.994766463583882	pseudobulk	DESeq2	LRT	0.407389244825514
122	KC_AB1	mxt	0.129434065019313	0.82512395744179	0.994766463583882	pseudobulk	DESeq2	LRT	0.0834808029785056
122	KC_AB1	Myb	-0.647856137067844	0.762139005512724	0.994766463583882	pseudobulk	DESeq2	LRT	0.117965811046941
122	KC_AB1	Mybbp1A	-0.432177182589626	0.810808370932697	0.994766463583882	pseudobulk	DESeq2	LRT	0.0910817762208738
122	KC_AB1	Myc	0.0720407689215499	0.775365369130217	0.994766463583882	pseudobulk	DESeq2	LRT	0.1104936001969
122	KC_AB1	myo	0.0618142061237552	0.931284353621472	0.994766463583882	pseudobulk	DESeq2	LRT	0.0309176935047997
122	KC_AB1	Myo10A	-2.79499059556516	0.38564607643653	0.994766463583882	pseudobulk	DESeq2	LRT	0.413811082791365
122	KC_AB1	Myo28B1	-0.547377887425331	0.700026413180807	0.994766463583882	pseudobulk	DESeq2	LRT	0.154885573011087
122	KC_AB1	Myo31DF	0.986575688715885	0.655962090734864	0.994766463583882	pseudobulk	DESeq2	LRT	0.183121258582249
122	KC_AB1	Myo81F	1.24860959783856	0.181694388519855	0.994766463583882	pseudobulk	DESeq2	LRT	0.740658485308889
122	KC_AB1	Myo95E	0.405461421432652	0.546975409823733	0.994766463583882	pseudobulk	DESeq2	LRT	0.262032197648128
122	KC_AB1	MYPT-75D	-0.150867603431675	0.645393130852216	0.994766463583882	pseudobulk	DESeq2	LRT	0.190175661257577
122	KC_AB1	mys	0.167036201879023	0.702141375285935	0.994766463583882	pseudobulk	DESeq2	LRT	0.153575434415938
122	KC_AB1	Mys45A	-0.280398702414819	0.759826387172161	0.994766463583882	pseudobulk	DESeq2	LRT	0.119285628385572
122	KC_AB1	Myt1	0.149339502694502	0.859454017512158	0.994766463583882	pseudobulk	DESeq2	LRT	0.0657773539681329
122	KC_AB1	Mzt1	0.949183608371002	0.80156895525114	0.994766463583882	pseudobulk	DESeq2	LRT	0.0960591113673091
122	KC_AB1	na	0.0216099819981116	0.921424553305788	0.994766463583882	pseudobulk	DESeq2	LRT	0.0355402192342607
122	KC_AB1	Naa15-16	-0.192466456545752	0.856414938741867	0.994766463583882	pseudobulk	DESeq2	LRT	0.0673157657627296
122	KC_AB1	Naa20A	0.37320861161208	0.565462533568097	0.994766463583882	pseudobulk	DESeq2	LRT	0.247596165280838
122	KC_AB1	Naa35	0.813011069827073	0.484188555111363	0.994766463583882	pseudobulk	DESeq2	LRT	0.314985480302639
122	KC_AB1	Naa60	0.468069425863005	0.471363693903443	0.994766463583882	pseudobulk	DESeq2	LRT	0.326643871418429
122	KC_AB1	Naa80	0.101045312158687	0.835539364633568	0.994766463583882	pseudobulk	DESeq2	LRT	0.0780330844415444
122	KC_AB1	Naam	-0.0916579791897125	0.894579124549956	0.994766463583882	pseudobulk	DESeq2	LRT	0.0483812405234574
122	KC_AB1	Nab2	0.262183346248966	0.658801661626061	0.994766463583882	pseudobulk	DESeq2	LRT	0.18124531412566
122	KC_AB1	nac	2.54275315134261	0.410734813545839	0.994766463583882	pseudobulk	DESeq2	LRT	0.386438485142247
122	KC_AB1	Nacalpha	-0.0328740939355864	0.851878933732451	0.994766463583882	pseudobulk	DESeq2	LRT	0.0696221213693337
122	KC_AB1	nAChRalpha1	-0.0684594222087076	0.726467452576434	0.994766463583882	pseudobulk	DESeq2	LRT	0.138783838328236
122	KC_AB1	nAChRalpha2	-0.0284060506043949	0.888195734058276	0.994766463583882	pseudobulk	DESeq2	LRT	0.0514913170432257
122	KC_AB1	nAChRalpha3	4.0547581424395	0.00396202170469244	0.994766463583882	pseudobulk	DESeq2	LRT	2.40208314964494
122	KC_AB1	nAChRalpha4	0.0765843710197316	0.701152761738043	0.994766463583882	pseudobulk	DESeq2	LRT	0.154187351003184
122	KC_AB1	nAChRalpha5	-0.0639170127619418	0.641720782473522	0.994766463583882	pseudobulk	DESeq2	LRT	0.192653895620111
122	KC_AB1	nAChRalpha6	-0.0271871693529706	0.832317209723283	0.994766463583882	pseudobulk	DESeq2	LRT	0.0797111254192132
123	KC_AB1	nAChRalpha7	0.549790117492084	0.430019008250069	0.994766463583882	pseudobulk	DESeq2	LRT	0.366512346756088
123	KC_AB1	nAChRbeta1	-0.0106651200805297	0.937818215036755	0.994766463583882	pseudobulk	DESeq2	LRT	0.0278813363091272
123	KC_AB1	nAChRbeta2	-0.167485008112433	0.660325526819988	0.994766463583882	pseudobulk	DESeq2	LRT	0.180241913475476
123	KC_AB1	NaCP60E	-0.00980177132704249	0.851148011096707	0.994766463583882	pseudobulk	DESeq2	LRT	0.0699949113452723
123	KC_AB1	Nadsyn	0.366235423010128	0.37281717026479	0.994766463583882	pseudobulk	DESeq2	LRT	0.428504094227037
123	KC_AB1	Nak	0.290943114132391	0.578896078338906	0.994766463583882	pseudobulk	DESeq2	LRT	0.237399392498392
123	KC_AB1	NaPi-III	0.161930352415693	0.546826311679681	0.994766463583882	pseudobulk	DESeq2	LRT	0.26215059662365
123	KC_AB1	Naprt	0.0515701445704788	0.932332148589882	0.994766463583882	pseudobulk	DESeq2	LRT	0.0304293402192867
123	KC_AB1	NAT1	-0.0357086710056069	0.83753720702452	0.994766463583882	pseudobulk	DESeq2	LRT	0.0769958906236426
123	KC_AB1	Naus	-0.0265886890222819	0.855060590982108	0.994766463583882	pseudobulk	DESeq2	LRT	0.068003109369111
123	KC_AB1	Naxd	-0.435784219452019	0.74173284199222	0.994766463583882	pseudobulk	DESeq2	LRT	0.129752491137558
123	KC_AB1	Naxe	-0.552999816355386	0.702085862524176	0.994766463583882	pseudobulk	DESeq2	LRT	0.153609772001011
123	KC_AB1	naz	-0.356212784647529	0.928203443271547	0.994766463583882	pseudobulk	DESeq2	LRT	0.0323568248514225
123	KC_AB1	Nbr	1.45003899530635	0.380474409642142	0.994766463583882	pseudobulk	DESeq2	LRT	0.419674548157403
123	KC_AB1	nbs	0.361477162459739	0.719935988470478	0.994766463583882	pseudobulk	DESeq2	LRT	0.142706116193585
123	KC_AB1	NC2alpha	-0.95342912166881	0.278529606284755	0.994766463583882	pseudobulk	DESeq2	LRT	0.55512863473545
123	KC_AB1	NC2beta	0.375204740236651	0.639522069782465	0.994766463583882	pseudobulk	DESeq2	LRT	0.194144463508463
123	KC_AB1	Nc73EF	-0.0710862369201718	0.806080341771829	0.994766463583882	pseudobulk	DESeq2	LRT	0.0936216700444412
123	KC_AB1	Nca	0.0466479256082427	0.847352542513841	0.994766463583882	pseudobulk	DESeq2	LRT	0.0719358630887673

123	KC_AB1	Nckx30C	0.0116546677049255	0.876261891719897	0.994766463583882	pseudobulk	DESeq2	LRT	0.0573660751898638
123	KC_AB1	nclb	0.597141410293382	0.802139944912821	0.994766463583882	pseudobulk	DESeq2	LRT	0.0957498561530429
123	KC_AB1	ncm	0.395105479351656	0.515232557376398	0.994766463583882	pseudobulk	DESeq2	LRT	0.287996701855211
123	KC_AB1	Ncoa6	0.081978057780511	0.76802534413699	0.994766463583882	pseudobulk	DESeq2	LRT	0.11462444840959
123	KC_AB1	Nct	-0.139072439661287	0.86631359952746	0.994766463583882	pseudobulk	DESeq2	LRT	0.0623248679242385
123	KC_AB1	ND-13A	-0.116691110669832	0.758156339439133	0.994766463583882	pseudobulk	DESeq2	LRT	0.120241229256231
123	KC_AB1	ND-13B	0.0451953005899472	0.88057111135556	0.994766463583882	pseudobulk	DESeq2	LRT	0.0552355664175639
123	KC_AB1	ND-15	-0.199305410689882	0.541595628890233	0.994766463583882	pseudobulk	DESeq2	LRT	0.266324849412484
123	KC_AB1	ND-18	-0.0566865723444791	0.876786695231464	0.994766463583882	pseudobulk	DESeq2	LRT	0.0571060489958812
123	KC_AB1	ND-19	0.372632236368603	0.37878845476041	0.994766463583882	pseudobulk	DESeq2	LRT	0.421603266496267
123	KC_AB1	ND-20	0.30328519760977	0.458021613378158	0.994766463583882	pseudobulk	DESeq2	LRT	0.339114027783475
123	KC_AB1	ND-23	-0.0291463914240659	0.911020480933544	0.994766463583882	pseudobulk	DESeq2	LRT	0.0404718594085178
123	KC_AB1	ND-30	0.127115146571202	0.751729062793855	0.994766463583882	pseudobulk	DESeq2	LRT	0.123938659056806
123	KC_AB1	ND-39	0.229763959440895	0.535748617880063	0.994766463583882	pseudobulk	DESeq2	LRT	0.271038940674069
123	KC_AB1	ND-42	-0.0621239553421563	0.877334886084156	0.994766463583882	pseudobulk	DESeq2	LRT	0.0568346011411365
123	KC_AB1	ND-49	0.261264506941921	0.424237270783305	0.994766463583882	pseudobulk	DESeq2	LRT	0.372391179797572
123	KC_AB1	ND-51	-0.0213498366383925	0.937532384445617	0.994766463583882	pseudobulk	DESeq2	LRT	0.0280137218475815
123	KC_AB1	ND-51L1	2.02670873290438	0.538959414925803	0.994766463583882	pseudobulk	DESeq2	LRT	0.26844393710898
123	KC_AB1	ND-51L2	-1.64952313825113	0.817565084244375	0.994766463583882	pseudobulk	DESeq2	LRT	0.0874776642296574
123	KC_AB1	ND-75	-0.319333429466909	0.466559936957799	0.994766463583882	pseudobulk	DESeq2	LRT	0.331092556380584
123	KC_AB1	ND-ACP	-0.176392001286056	0.57120339852807	0.994766463583882	pseudobulk	DESeq2	LRT	0.243209217272505
123	KC_AB1	ND-AGGG	0.0719781169317252	0.809873844777287	0.994766463583882	pseudobulk	DESeq2	LRT	0.0915826265338311
123	KC_AB1	ND-ASHI	-0.212732042841677	0.565216670393171	0.994766463583882	pseudobulk	DESeq2	LRT	0.247785037635583
123	KC_AB1	ND-B12	0.0313360173765007	0.913216430685187	0.994766463583882	pseudobulk	DESeq2	LRT	0.0394262832399316
123	KC_AB1	ND-B14	-0.0144122553631213	0.936307140670597	0.994766463583882	pseudobulk	DESeq2	LRT	0.028581664490673
123	KC_AB1	ND-B14.5A	-0.254216205236306	0.521326976326631	0.994766463583882	pseudobulk	DESeq2	LRT	0.282889801705101
123	KC_AB1	ND-B14.5B	-0.124616335713038	0.765268601669993	0.994766463583882	pseudobulk	DESeq2	LRT	0.116186105043994
123	KC_AB1	ND-B14.7	0.0326903267700598	0.919083515333495	0.994766463583882	pseudobulk	DESeq2	LRT	0.0366450233249469
123	KC_AB1	ND-B15	0.0717881982454414	0.837853603756286	0.994766463583882	pseudobulk	DESeq2	LRT	0.0768318580224761
123	KC_AB1	ND-B16.6	-0.0506012328333418	0.803524997138025	0.994766463583882	pseudobulk	DESeq2	LRT	0.0950006080727055
123	KC_AB1	ND-B17	0.0698771025657751	0.851157819935617	0.994766463583882	pseudobulk	DESeq2	LRT	0.0699899064577425
123	KC_AB1	ND-B17.2	0.334934762813936	0.363000502545401	0.994766463583882	pseudobulk	DESeq2	LRT	0.440092773717211
123	KC_AB1	ND-B18	-0.17835117405258	0.656948502753181	0.994766463583882	pseudobulk	DESeq2	LRT	0.18246867282482
123	KC_AB1	ND-B22	0.0324773061478707	0.837552379419316	0.994766463583882	pseudobulk	DESeq2	LRT	0.0769880232386494
123	KC_AB1	ND-MLRQ	0.125114267465965	0.549857085124408	0.994766463583882	pseudobulk	DESeq2	LRT	0.2597501745188
123	KC_AB1	ND-MNLL	0.560498429917967	0.620704212676861	0.994766463583882	pseudobulk	DESeq2	LRT	0.20711530707778
123	KC_AB1	ND-MWFE	0.00990734937150899	0.89180948863396	0.994766463583882	pseudobulk	DESeq2	LRT	0.0497279111751903
123	KC_AB1	ND-PDSW	0.0205509881497054	0.934921684991969	0.994766463583882	pseudobulk	DESeq2	LRT	0.0292247668818353
123	KC_AB1	ND-SGDH	0.087469415899646	0.775543108796119	0.994766463583882	pseudobulk	DESeq2	LRT	0.110394056790499
123	KC_AB1	Ndae1	-0.0500028738431626	0.826754852027051	0.994766463583882	pseudobulk	DESeq2	LRT	0.0826232476360425
123	KC_AB1	Ndc1	-2.57435989741145	0.533617212681275	0.994766463583882	pseudobulk	DESeq2	LRT	0.272770169998685
123	KC_AB1	Ndf	0.521960037089123	0.255735406419796	0.994766463583882	pseudobulk	DESeq2	LRT	0.592209139989946
123	KC_AB1	Ndfip	-0.256956770888941	0.52326852791051	0.994766463583882	pseudobulk	DESeq2	LRT	0.281275385202678
123	KC_AB1	Ndg	-1.64952313825113	0.817565084244375	0.994766463583882	pseudobulk	DESeq2	LRT	0.0874776642296574
123	KC_AB1	neb	-3.21143241058256	0.292545415061155	0.994766463583882	pseudobulk	DESeq2	LRT	0.533806704011112
123	KC_AB1	Neb-cGP	-0.0460113326934286	0.818705088618171	0.994766463583882	pseudobulk	DESeq2	LRT	0.0868725102676732
123	KC_AB1	Nedd4	0.042872675552993	0.920847111714078	0.994766463583882	pseudobulk	DESeq2	LRT	0.0358124697496167
123	KC_AB1	Nedd8	0.0327743458224479	0.947565846171935	0.994766463583882	pseudobulk	DESeq2	LRT	0.0233906012696361
123	KC_AB1	nej	0.0135708596957218	0.903932493612892	0.994766463583882	pseudobulk	DESeq2	LRT	0.0438640017659193
123	KC_AB1	Nelf-A	0.28007799505745	0.607226954762701	0.994766463583882	pseudobulk	DESeq2	LRT	0.216648958382541
123	KC_AB1	NELF-B	0.654938316042669	0.62337966492492	0.994766463583882	pseudobulk	DESeq2	LRT	0.205247368813972
123	KC_AB1	Nelf-E	-0.165578607619016	0.757832989297648	0.994766463583882	pseudobulk	DESeq2	LRT	0.120426493336442
123	KC_AB1	nemy	-2.12431104022436	0.328743519760125	0.994766463583882	pseudobulk	DESeq2	LRT	0.483142799293539
123	KC_AB1	Neos	-0.232845012937368	0.78372134785033	0.994766463583882	pseudobulk	DESeq2	LRT	0.105838323287804
123	KC_AB1	Nep1	-0.208406340836456	0.256323499327583	0.994766463583882	pseudobulk	DESeq2	LRT	0.591211576568524
123	KC_AB1	Nep3	-0.0597647471013401	0.907225570827283	0.994766463583882	pseudobulk	DESeq2	LRT	0.0422847173657628

123	KC_AB1	Nepl11	1.00206552945986	0.690075213866505	0.994766463583882	pseudobulk	DESeq2	LRT	0.161103571310595
123	KC_AB1	Nepl16	0.478901176351941	0.400078037520308	0.994766463583882	pseudobulk	DESeq2	LRT	0.397855288774804
123	KC_AB1	Nepl3	0.596808856322697	0.822761281772702	0.994766463583882	pseudobulk	DESeq2	LRT	0.0847261539098712
123	KC_AB1	nero	-0.51577077977574	0.612756113642286	0.994766463583882	pseudobulk	DESeq2	LRT	0.212712346974515
123	KC_AB1	nes	-0.246813187912231	0.796503211879902	0.994766463583882	pseudobulk	DESeq2	LRT	0.0988124685773438
123	KC_AB1	net	-2.21542861135025	0.45379467637256	0.994766463583882	pseudobulk	DESeq2	LRT	0.343140603284892
123	KC_AB1	NetB	0.0387611214194719	0.86951460725142	0.994766463583882	pseudobulk	DESeq2	LRT	0.0607231177493639
123	KC_AB1	Neu2	-1.64952313825113	0.817565084244375	0.994766463583882	pseudobulk	DESeq2	LRT	0.0874776642296574
123	KC_AB1	neur	-0.515304992521756	0.262151476800912	0.994766463583882	pseudobulk	DESeq2	LRT	0.581447691394163
123	KC_AB1	Neurl4	0.408562561446017	0.649539228436078	0.994766463583882	pseudobulk	DESeq2	LRT	0.187394614908347
123	KC_AB1	Nf-YA	-0.0559443190527068	0.947067119102999	0.994766463583882	pseudobulk	DESeq2	LRT	0.0236192412468635
123	KC_AB1	Nf-YB	-0.433811310642744	0.577398620226007	0.994766463583882	pseudobulk	DESeq2	LRT	0.238524258260888
123	KC_AB1	Nf-YC	0.132394492206261	0.796089336136593	0.994766463583882	pseudobulk	DESeq2	LRT	0.0990381935511077
123	KC_AB1	Nf1	-0.464251659014272	0.256601672131109	0.994766463583882	pseudobulk	DESeq2	LRT	0.590740517895044
123	KC_AB1	NFAT	0.0145047438176588	0.886092885920634	0.994766463583882	pseudobulk	DESeq2	LRT	0.0525207502028322
123	KC_AB1	NfI	0.1635936209838	0.534391229382658	0.994766463583882	pseudobulk	DESeq2	LRT	0.272140678268236
123	KC_AB1	Nfs1	0.154681596199988	0.846965722490307	0.994766463583882	pseudobulk	DESeq2	LRT	0.0721341656250724
123	KC_AB1	Nha1	-2.20969975456663	0.610836134188693	0.994766463583882	pseudobulk	DESeq2	LRT	0.214075280039667
123	KC_AB1	Nhe1	-0.437259370414482	0.77335189263586	0.994766463583882	pseudobulk	DESeq2	LRT	0.111622847277955
123	KC_AB1	Nhe2	-0.286616122753554	0.641790903569859	0.994766463583882	pseudobulk	DESeq2	LRT	0.192606442674313
123	KC_AB1	NHP2	-0.0659557474418972	0.935223784402862	0.994766463583882	pseudobulk	DESeq2	LRT	0.029084456825291
123	KC_AB1	NimC2	1.1821590793593	0.719511699510136	0.994766463583882	pseudobulk	DESeq2	LRT	0.142962139890846
123	KC_AB1	NiPp1	0.0458356868383502	0.932017209344618	0.994766463583882	pseudobulk	DESeq2	LRT	0.030576068488934
123	KC_AB1	Nipped-A	-0.277378584750964	0.42540404686517	0.994766463583882	pseudobulk	DESeq2	LRT	0.371198382973834
123	KC_AB1	Nipped-B	-0.0442598226595459	0.834898222336251	0.994766463583882	pseudobulk	DESeq2	LRT	0.078366463644317
124	KC_AB1	Nipsnap	0.0598991495543179	0.841174388677571	0.994766463583882	pseudobulk	DESeq2	LRT	0.0751139587928168
124	KC_AB1	NitFhit	-2.11458293841668	0.293528939879375	0.994766463583882	pseudobulk	DESeq2	LRT	0.532349073938275
124	KC_AB1	nito	-0.421879977660177	0.697862582290467	0.994766463583882	pseudobulk	DESeq2	LRT	0.15623008687314
124	KC_AB1	NK7.1	-0.368564510041951	0.318251136732501	0.994766463583882	pseudobulk	DESeq2	LRT	0.497230036454288
124	KC_AB1	NKAIN	-0.80925573929026	0.234098299344145	0.994766463583882	pseudobulk	DESeq2	LRT	0.630601741314637
124	KC_AB1	nkd	0.371954079961127	0.673401069585505	0.994766463583882	pseudobulk	DESeq2	LRT	0.17172619814289
124	KC_AB1	NLaz	2.22023857837763	0.524904842126482	0.994766463583882	pseudobulk	DESeq2	LRT	0.279919420946639
124	KC_AB1	Nle	0.183615259592078	0.871223383626357	0.994766463583882	pseudobulk	DESeq2	LRT	0.0598704766365706
124	KC_AB1	Nlg2	0.08288706812297	0.721133050928822	0.994766463583882	pseudobulk	DESeq2	LRT	0.141984599423028
124	KC_AB1	Nlg3	0.106190747056286	0.609083324709549	0.994766463583882	pseudobulk	DESeq2	LRT	0.215323290312666
124	KC_AB1	Nlg4	2.3014460663571	0.218767647271671	0.994766463583882	pseudobulk	DESeq2	LRT	0.660016903775
124	KC_AB1	Nlp	0.14738426219865	0.780572736913548	0.994766463583882	pseudobulk	DESeq2	LRT	0.10758662140575
124	KC_AB1	nmd	-0.982888665997249	0.318892017289989	0.994766463583882	pseudobulk	DESeq2	LRT	0.496356352159676
124	KC_AB1	Nmd3	-0.326410503701217	0.774181998028944	0.994766463583882	pseudobulk	DESeq2	LRT	0.111156931502713
124	KC_AB1	Nmda1	0.146798152099406	0.774589686030728	0.994766463583882	pseudobulk	DESeq2	LRT	0.110928290114928
124	KC_AB1	Nmdar1	-0.15825315807999	0.78144357455622	0.994766463583882	pseudobulk	DESeq2	LRT	0.107102375474062
124	KC_AB1	Nmdmc	0.729740153036158	0.752353468463509	0.994766463583882	pseudobulk	DESeq2	LRT	0.123578072524162
124	KC_AB1	nmdyn-D6	1.80939591345248	0.468117841155507	0.994766463583882	pseudobulk	DESeq2	LRT	0.329644806495434
124	KC_AB1	nmo	-0.0287393218927992	0.892333111305711	0.994766463583882	pseudobulk	DESeq2	LRT	0.0494729915925539
124	KC_AB1	Nmt	-0.408497067905854	0.778578758322717	0.994766463583882	pseudobulk	DESeq2	LRT	0.108697449152499
124	KC_AB1	Nna1	0.365999213672279	0.809335235757598	0.994766463583882	pseudobulk	DESeq2	LRT	0.0918715514621722
124	KC_AB1	Nnf1a	0.208615470155158	0.893577121399079	0.994766463583882	pseudobulk	DESeq2	LRT	0.0488679591598277
124	KC_AB1	Nnp-1	-0.396338193970479	0.711003263077872	0.994766463583882	pseudobulk	DESeq2	LRT	0.148128406114872
124	KC_AB1	NO66	-0.255006937147076	0.88671163122832	0.994766463583882	pseudobulk	DESeq2	LRT	0.0522175947436219
124	KC_AB1	Noa36	0.039458336674393	0.960104527524017	0.994766463583882	pseudobulk	DESeq2	LRT	0.0176814823191197
124	KC_AB1	noc	0.379037232717863	0.707381526499106	0.994766463583882	pseudobulk	DESeq2	LRT	0.150346286116206
124	KC_AB1	nocte	-0.259595265208424	0.273724822660775	0.994766463583882	pseudobulk	DESeq2	LRT	0.562685816927547
124	KC_AB1	nod	0.949183608371002	0.80156895525114	0.994766463583882	pseudobulk	DESeq2	LRT	0.0960591113673091
124	KC_AB1	noi	0.360720506091767	0.716790467018288	0.994766463583882	pseudobulk	DESeq2	LRT	0.144607779223281
124	KC_AB1	nol	0.949183608371002	0.80156895525114	0.994766463583882	pseudobulk	DESeq2	LRT	0.0960591113673091
124	KC_AB1	nolo	2.22023857837763	0.524904842126482	0.994766463583882	pseudobulk	DESeq2	LRT	0.279919420946639

124	KC_AB1	nom	-0.0957692229228332	0.958527644269769	0.994766463583882	pseudobulk	DESeq2	LRT	0.018395357401787
124	KC_AB1	Non1	-0.052341786851439	0.863919008112443	0.994766463583882	pseudobulk	DESeq2	LRT	0.0635269704592772
124	KC_AB1	Non2	0.293097335942176	0.586452611823063	0.994766463583882	pseudobulk	DESeq2	LRT	0.231767075202718
124	KC_AB1	Non3	0.770119519815681	0.364855353744652	0.994766463583882	pseudobulk	DESeq2	LRT	0.437879276697319
124	KC_AB1	nonA	0.16720499694411	0.600538794588905	0.994766463583882	pseudobulk	DESeq2	LRT	0.221458932088417
124	KC_AB1	nonA-l	0.0880809947672859	0.842935742212011	0.994766463583882	pseudobulk	DESeq2	LRT	0.0742055307920985
124	KC_AB1	nonC	-0.091434957075701	0.895280738569833	0.994766463583882	pseudobulk	DESeq2	LRT	0.0480407589928704
124	KC_AB1	nop5	0.969986341149099	0.640932225194753	0.994766463583882	pseudobulk	DESeq2	LRT	0.193187892135042
124	KC_AB1	Nop56	-0.0288998809163736	0.95932320116159	0.994766463583882	pseudobulk	DESeq2	LRT	0.0180350520226711
124	KC_AB1	Nop60B	-0.198039756019413	0.827418840761032	0.994766463583882	pseudobulk	DESeq2	LRT	0.0822745942147499
124	KC_AB1	Nopp140	0.652530235949219	0.54952397715094	0.994766463583882	pseudobulk	DESeq2	LRT	0.260013353436731
124	KC_AB1	nord	1.19856662192297	0.608802264240657	0.994766463583882	pseudobulk	DESeq2	LRT	0.215523741018373
124	KC_AB1	norpA	-0.03104579070095	0.891851594951772	0.994766463583882	pseudobulk	DESeq2	LRT	0.0497074066727927
124	KC_AB1	Nos	-2.20969975456663	0.610836134188693	0.994766463583882	pseudobulk	DESeq2	LRT	0.214075280039667
124	KC_AB1	Nost	0.816696443796817	0.683567274556758	0.994766463583882	pseudobulk	DESeq2	LRT	0.165218737085912
124	KC_AB1	not	-0.0270594998045082	0.941035719330395	0.994766463583882	pseudobulk	DESeq2	LRT	0.0263938915422726
124	KC_AB1	Not1	-0.124589364652188	0.643796440411276	0.994766463583882	pseudobulk	DESeq2	LRT	0.19125142888373
124	KC_AB1	Not10	-0.899175258576389	0.418580436119719	0.994766463583882	pseudobulk	DESeq2	LRT	0.378221073856398
124	KC_AB1	Not11	0.771145390646983	0.342192441461985	0.994766463583882	pseudobulk	DESeq2	LRT	0.465729587628364
124	KC_AB1	Not3	-0.152493859049966	0.663229768249452	0.994766463583882	pseudobulk	DESeq2	LRT	0.178335989248592
124	KC_AB1	NP15.6	0.182741854196612	0.680698952480956	0.994766463583882	pseudobulk	DESeq2	LRT	0.167044917725195
124	KC_AB1	Npc1a	0.357444980710496	0.537385931714005	0.994766463583882	pseudobulk	DESeq2	LRT	0.269713707197512
124	KC_AB1	Npc1b	-1.64952313825113	0.817565084244375	0.994766463583882	pseudobulk	DESeq2	LRT	0.0874776642296574
124	KC_AB1	Npc2f	-2.79499059556516	0.38564607643653	0.994766463583882	pseudobulk	DESeq2	LRT	0.413811082791365
124	KC_AB1	Nph	0.494371435126957	0.501074606296672	0.994766463583882	pseudobulk	DESeq2	LRT	0.300097606087449
124	KC_AB1	Npl4	-1.02614674959375	0.351867994138209	0.994766463583882	pseudobulk	DESeq2	LRT	0.453620234731489
124	KC_AB1	Nplp1	1.62881245160202	0.0265453878190204	0.994766463583882	pseudobulk	DESeq2	LRT	1.5760109253829
124	KC_AB1	Nprl2	0.475649746043893	0.759920495303806	0.994766463583882	pseudobulk	DESeq2	LRT	0.119231842267898
124	KC_AB1	Nprl3	-0.833768794268267	0.534488911352943	0.994766463583882	pseudobulk	DESeq2	LRT	0.272061300348903
124	KC_AB1	Nrd1	0.58998597015208	0.681243290826519	0.994766463583882	pseudobulk	DESeq2	LRT	0.166697761810889
124	KC_AB1	Nrg	-0.146958625451083	0.472889807644901	0.994766463583882	pseudobulk	DESeq2	LRT	0.325240046380597
124	KC_AB1	Nrk	0.217906885051292	0.902008941520292	0.994766463583882	pseudobulk	DESeq2	LRT	0.0447891573209458
124	KC_AB1	nrm	0.331185618556971	0.240933678505065	0.994766463583882	pseudobulk	DESeq2	LRT	0.618102488641763
124	KC_AB1	Nrt	0.42619697074529	0.43506658676013	0.994766463583882	pseudobulk	DESeq2	LRT	0.361444269368546
124	KC_AB1	nrv2	-0.300646451743012	0.688060879889804	0.994766463583882	pseudobulk	DESeq2	LRT	0.162373133522534
124	KC_AB1	nrv3	-0.0656715391401914	0.612996079332872	0.994766463583882	pseudobulk	DESeq2	LRT	0.212542303180678
124	KC_AB1	Nrx-1	0.0407220584007917	0.828322180552349	0.994766463583882	pseudobulk	DESeq2	LRT	0.081800709083527
124	KC_AB1	Nrx-IV	0.435038848980511	0.478038118339132	0.994766463583882	pseudobulk	DESeq2	LRT	0.32053747174708
124	KC_AB1	Ns1	0.209768086431507	0.811952079072043	0.994766463583882	pseudobulk	DESeq2	LRT	0.0904696018040429
124	KC_AB1	Ns2	-0.138754333907383	0.850648990714004	0.994766463583882	pseudobulk	DESeq2	LRT	0.0702496089438062
124	KC_AB1	Ns3	0.188006668896169	0.872602146338487	0.994766463583882	pseudobulk	DESeq2	LRT	0.0591837231142514
124	KC_AB1	Ns4	-0.260485920085107	0.7886721533233	0.994766463583882	pseudobulk	DESeq2	LRT	0.103103493105166
124	KC_AB1	NSD	-0.0766126098067665	0.868026165321	0.994766463583882	pseudobulk	DESeq2	LRT	0.0614671834833482
124	KC_AB1	Nse4	0.389275648472954	0.837532199093071	0.994766463583882	pseudobulk	DESeq2	LRT	0.0769984874318538
124	KC_AB1	Nsf2	0.541762642525419	0.464570716131765	0.994766463583882	pseudobulk	DESeq2	LRT	0.33294816910144
124	KC_AB1	nsl1	-0.187763825478636	0.556844202490153	0.994766463583882	pseudobulk	DESeq2	LRT	0.254266297579227
124	KC_AB1	nSMase	-0.157392532494876	0.861838065241481	0.994766463583882	pseudobulk	DESeq2	LRT	0.0645743281181037
124	KC_AB1	nst	0.617421646817878	0.42137416910094	0.994766463583882	pseudobulk	DESeq2	LRT	0.375332090846486
124	KC_AB1	Nsun2	0.124698704744493	0.891329405341186	0.994766463583882	pseudobulk	DESeq2	LRT	0.0499617656782218
124	KC_AB1	Nsun5	-0.0524032630183588	0.867745738945416	0.994766463583882	pseudobulk	DESeq2	LRT	0.0616075102489292
124	KC_AB1	nSyb	-0.0403666224474749	0.7564248868474	0.994766463583882	pseudobulk	DESeq2	LRT	0.121234191034432
124	KC_AB1	NT5E-2	-3.46197803745951	0.239982951034438	0.994766463583882	pseudobulk	DESeq2	LRT	0.619819610516178
124	KC_AB1	Ntan1	-0.0362974210838319	0.950032544046525	0.994766463583882	pseudobulk	DESeq2	LRT	0.0222615173872136
124	KC_AB1	ntc	0.595166718187393	0.669888803300932	0.994766463583882	pseudobulk	DESeq2	LRT	0.173997281061353
124	KC_AB1	Ntf-2	-0.0509875328770942	0.873161619791497	0.994766463583882	pseudobulk	DESeq2	LRT	0.0589053621510495
124	KC_AB1	Ntl	2.80587074018119	0.385946061723411	0.994766463583882	pseudobulk	DESeq2	LRT	0.413473386345624

124	KC_AB1	Ntmt	1.11168776257694	0.593310522446876	0.994766463583882	pseudobulk	DESeq2	LRT	0.226717949319565
124	KC_AB1	NTPase	0.541651225882983	0.514852050501709	0.994766463583882	pseudobulk	DESeq2	LRT	0.288317553253739
124	KC_AB1	Nuak1	-0.675541547019404	0.265015585079145	0.994766463583882	pseudobulk	DESeq2	LRT	0.576728585252449
124	KC_AB1	nub	-1.64952313825113	0.817565084244375	0.994766463583882	pseudobulk	DESeq2	LRT	0.0874776642296574
124	KC_AB1	NUCB1	0.199373193550349	0.687818707188465	0.994766463583882	pseudobulk	DESeq2	LRT	0.162526016478184
124	KC_AB1	nudC	0.38937165229966	0.6129626076499	0.994766463583882	pseudobulk	DESeq2	LRT	0.212566017792554
124	KC_AB1	nudE	0.220758568083543	0.62504000739496	0.994766463583882	pseudobulk	DESeq2	LRT	0.204092183560264
124	KC_AB1	nuf	-2.86399183503704	0.535461900159429	0.994766463583882	pseudobulk	DESeq2	LRT	0.271271425185398
124	KC_AB1	Nufip	-0.0214692856008564	0.895511229011072	0.994766463583882	pseudobulk	DESeq2	LRT	0.0479289640673556
124	KC_AB1	Nulp1	0.21630633757919	0.677310637094217	0.994766463583882	pseudobulk	DESeq2	LRT	0.169212103789702
124	KC_AB1	numb	-0.469006129286077	0.466097888331051	0.994766463583882	pseudobulk	DESeq2	LRT	0.331522864645723
124	KC_AB1	Nup107	0.564373867639223	0.652184063640896	0.994766463583882	pseudobulk	DESeq2	LRT	0.185629817546896
124	KC_AB1	Nup133	0.44976297004525	0.831013436772572	0.994766463583882	pseudobulk	DESeq2	LRT	0.0803919539908534
125	KC_AB1	Nup153	-0.0332503133009137	0.898690207964041	0.994766463583882	pseudobulk	DESeq2	LRT	0.0463899903115308
125	KC_AB1	Nup154	-0.537573952939252	0.794993660143913	0.994766463583882	pseudobulk	DESeq2	LRT	0.09963633470893
125	KC_AB1	Nup160	0.166061709543563	0.875536484819394	0.994766463583882	pseudobulk	DESeq2	LRT	0.0577257515501677
125	KC_AB1	Nup188	-0.790745252180986	0.739109456997044	0.994766463583882	pseudobulk	DESeq2	LRT	0.131291240834227
125	KC_AB1	Nup205	0.30843061333912	0.868412891604321	0.994766463583882	pseudobulk	DESeq2	LRT	0.0612737380607052
125	KC_AB1	Nup214	0.151712632592823	0.89712281616456	0.994766463583882	pseudobulk	DESeq2	LRT	0.0471480979465697
125	KC_AB1	Nup35	1.77005287066664	0.240489362194237	0.994766463583882	pseudobulk	DESeq2	LRT	0.618904129446158
125	KC_AB1	Nup358	0.841309447588279	0.203494788512556	0.994766463583882	pseudobulk	DESeq2	LRT	0.691446708547955
125	KC_AB1	Nup37	-0.723029869770256	0.610723141077256	0.994766463583882	pseudobulk	DESeq2	LRT	0.214155623721589
125	KC_AB1	Nup43	0.245532694227529	0.851579371739034	0.994766463583882	pseudobulk	DESeq2	LRT	0.0697748672529893
125	KC_AB1	Nup44A	0.0169973010502941	0.880707936768717	0.994766463583882	pseudobulk	DESeq2	LRT	0.0551680898617043
125	KC_AB1	Nup50	-0.536447385632354	0.630173466106402	0.994766463583882	pseudobulk	DESeq2	LRT	0.200539887049313
125	KC_AB1	Nup54	3.09002431663951	0.146551983457324	0.994766463583882	pseudobulk	DESeq2	LRT	0.834008299376744
125	KC_AB1	Nup58	1.16403547093092	0.373935050346957	0.994766463583882	pseudobulk	DESeq2	LRT	0.427203824872969
125	KC_AB1	Nup62	-0.133694593675847	0.890185765155659	0.994766463583882	pseudobulk	DESeq2	LRT	0.050519354744326
125	KC_AB1	Nup75	0.222308067172976	0.837607105270583	0.994766463583882	pseudobulk	DESeq2	LRT	0.0769596472715273
125	KC_AB1	Nup93-1	-1.03276852375444	0.60801016269219	0.994766463583882	pseudobulk	DESeq2	LRT	0.216089161575533
125	KC_AB1	Nup93-2	1.71604619433211	0.34896674765592	0.994766463583882	pseudobulk	DESeq2	LRT	0.457215954122666
125	KC_AB1	Nup98-96	0.111259816315197	0.88227494511296	0.994766463583882	pseudobulk	DESeq2	LRT	0.0543960537140901
125	KC_AB1	nvy	0.729740153036158	0.752353468463509	0.994766463583882	pseudobulk	DESeq2	LRT	0.123578072524162
125	KC_AB1	nwk	0.0595904838211971	0.845674064473561	0.994766463583882	pseudobulk	DESeq2	LRT	0.0727969883501277
125	KC_AB1	nxf2	0.949183608371002	0.80156895525114	0.994766463583882	pseudobulk	DESeq2	LRT	0.0960591113673091
125	KC_AB1	Nxf3	0.279195222979199	0.835914054288953	0.994766463583882	pseudobulk	DESeq2	LRT	0.077838372880165
125	KC_AB1	Nxt1	-0.226186984413999	0.714595572812834	0.994766463583882	pseudobulk	DESeq2	LRT	0.145939678742188
125	KC_AB1	O-fut1	-0.54272257299079	0.716487053610636	0.994766463583882	pseudobulk	DESeq2	LRT	0.144791652574744
125	KC_AB1	O-fut2	1.32710828904082	0.607473293222376	0.994766463583882	pseudobulk	DESeq2	LRT	0.216472810505135
125	KC_AB1	oaf	0.39255741121073	0.606006551673473	0.994766463583882	pseudobulk	DESeq2	LRT	0.217522680552764
125	KC_AB1	Oamb	-0.0491558686668464	0.798810528691407	0.994766463583882	pseudobulk	DESeq2	LRT	0.0975562195619194
125	KC_AB1	Oatp30B	0.596787617794487	0.487592017388462	0.994766463583882	pseudobulk	DESeq2	LRT	0.311943413046519
125	KC_AB1	obe	2.36497058321568	0.242487361077422	0.994766463583882	pseudobulk	DESeq2	LRT	0.615310892762252
125	KC_AB1	Obp19a	0.949183608371002	0.80156895525114	0.994766463583882	pseudobulk	DESeq2	LRT	0.0960591113673091
125	KC_AB1	Obp44a	0.89489462229553	0.681408699787765	0.994766463583882	pseudobulk	DESeq2	LRT	0.166592325940286
125	KC_AB1	Obp49a	1.1821590793593	0.719511699510136	0.994766463583882	pseudobulk	DESeq2	LRT	0.142962139890846
125	KC_AB1	Obp56a	1.32710828904082	0.607473293222376	0.994766463583882	pseudobulk	DESeq2	LRT	0.216472810505135
125	KC_AB1	Obp69a	1.1821590793593	0.719511699510136	0.994766463583882	pseudobulk	DESeq2	LRT	0.142962139890846
125	KC_AB1	Obp73a	0.789443942742388	0.72202275028751	0.994766463583882	pseudobulk	DESeq2	LRT	0.141449117986226
125	KC_AB1	Obp85a	1.32710828904082	0.607473293222376	0.994766463583882	pseudobulk	DESeq2	LRT	0.216472810505135
125	KC_AB1	obst-E	1.80939591345248	0.468117841155507	0.994766463583882	pseudobulk	DESeq2	LRT	0.329644806495434
125	KC_AB1	ocm	-0.00045024493673747	0.861714303649633	0.994766463583882	pseudobulk	DESeq2	LRT	0.0646366981133222
125	KC_AB1	Ocrl	0.388680028613153	0.557577551354515	0.994766463583882	pseudobulk	DESeq2	LRT	0.253694719700428
125	KC_AB1	Oct-TyrR	0.949183608371002	0.80156895525114	0.994766463583882	pseudobulk	DESeq2	LRT	0.0960591113673091
125	KC_AB1	Octalpha2R	-0.0600143263491228	0.83261578550691	0.994766463583882	pseudobulk	DESeq2	LRT	0.0795553596111949
125	KC_AB1	Octbeta1R	-0.00186531205616551	0.936876219357653	0.994766463583882	pseudobulk	DESeq2	LRT	0.0283177845668656

125	KC_AB1	Octbeta2R	2.80395130970481	0.386005157167114	0.994766463583882	pseudobulk	DESeq2	LRT	0.413406892959702
125	KC_AB1	Octbeta3R	0.0582704899362421	0.803878304696124	0.994766463583882	pseudobulk	DESeq2	LRT	0.0948096920465997
125	KC_AB1	Oda	0.107502207798981	0.562770876201245	0.994766463583882	pseudobulk	DESeq2	LRT	0.249668385708446
125	KC_AB1	Odc1	-1.64952313825113	0.817565084244375	0.994766463583882	pseudobulk	DESeq2	LRT	0.0874776642296574
125	KC_AB1	Odj	-0.647300708520317	0.392487177296027	0.994766463583882	pseudobulk	DESeq2	LRT	0.406174527251328
125	KC_AB1	OdsH	-2.20969975456663	0.610836134188693	0.994766463583882	pseudobulk	DESeq2	LRT	0.214075280039667
125	KC_AB1	Oga	0.0530374416534935	0.957608660625481	0.994766463583882	pseudobulk	DESeq2	LRT	0.0188119348188055
125	KC_AB1	Ogg1	0.454926457073512	0.745717162400171	0.994766463583882	pseudobulk	DESeq2	LRT	0.127425861670083
125	KC_AB1	ogre	-0.17805032366327	0.942053914371676	0.994766463583882	pseudobulk	DESeq2	LRT	0.0259242415339998
125	KC_AB1	ohgt	-0.25954595738197	0.718116022016158	0.994766463583882	pseudobulk	DESeq2	LRT	0.143805383542177
125	KC_AB1	okr	0.596808856322697	0.822761281772702	0.994766463583882	pseudobulk	DESeq2	LRT	0.0847261539098712
125	KC_AB1	olf186-F	-0.499390912252965	0.366461844809487	0.994766463583882	pseudobulk	DESeq2	LRT	0.435971236442532
125	KC_AB1	Oli	-0.348090441531037	0.501504500715867	0.994766463583882	pseudobulk	DESeq2	LRT	0.29972516508166
125	KC_AB1	omd	0.338062211428192	0.809917075760008	0.994766463583882	pseudobulk	DESeq2	LRT	0.0915594445576766
125	KC_AB1	ome	-0.137690363984372	0.587162594239819	0.994766463583882	pseudobulk	DESeq2	LRT	0.231241619360178
125	KC_AB1	onecut	0.0349430344814138	0.887501350170572	0.994766463583882	pseudobulk	DESeq2	LRT	0.0518309775729438
125	KC_AB1	Opa1	-0.50736553883586	0.205583732098402	0.994766463583882	pseudobulk	DESeq2	LRT	0.687011254168099
125	KC_AB1	Opbp	-0.0489065333590256	0.93491811533056	0.994766463583882	pseudobulk	DESeq2	LRT	0.0292264250819143
125	KC_AB1	opm	-0.257833764191556	0.802377865961044	0.994766463583882	pseudobulk	DESeq2	LRT	0.0956210600768276
125	KC_AB1	Optix	-2.3701736114949	0.404790721764187	0.994766463583882	pseudobulk	DESeq2	LRT	0.392769450547692
125	KC_AB1	or	-0.0389678160333422	0.950241271938886	0.994766463583882	pseudobulk	DESeq2	LRT	0.022166110744788
125	KC_AB1	Or13a	0.949183608371002	0.80156895525114	0.994766463583882	pseudobulk	DESeq2	LRT	0.0960591113673091
125	KC_AB1	Or1a	-1.64952313825113	0.817565084244375	0.994766463583882	pseudobulk	DESeq2	LRT	0.0874776642296574
125	KC_AB1	Or22c	-1.64952313825113	0.817565084244375	0.994766463583882	pseudobulk	DESeq2	LRT	0.0874776642296574
125	KC_AB1	Or2a	-1.64952313825113	0.817565084244375	0.994766463583882	pseudobulk	DESeq2	LRT	0.0874776642296574
125	KC_AB1	Or43b	0.510712924521919	0.711446733003987	0.994766463583882	pseudobulk	DESeq2	LRT	0.147857610588039
125	KC_AB1	Or45a	-0.9896971674044	0.692304363678608	0.994766463583882	pseudobulk	DESeq2	LRT	0.159702930968995
125	KC_AB1	Or63a	-1.76643687325323	0.543810819055668	0.994766463583882	pseudobulk	DESeq2	LRT	0.26455215642223
125	KC_AB1	Or88a	-1.64952313825113	0.817565084244375	0.994766463583882	pseudobulk	DESeq2	LRT	0.0874776642296574
125	KC_AB1	orb	0.162212202964297	0.809236127071964	0.994766463583882	pseudobulk	DESeq2	LRT	0.0919247370755123
125	KC_AB1	orb2	0.235633415818548	0.306803005078597	0.994766463583882	pseudobulk	DESeq2	LRT	0.51314039086823
125	KC_AB1	Orc4	1.34815820347889	0.526408859243851	0.994766463583882	pseudobulk	DESeq2	LRT	0.278676810335701
125	KC_AB1	Orc5	0.217906885051292	0.902008941520292	0.994766463583882	pseudobulk	DESeq2	LRT	0.0447891573209458
125	KC_AB1	Orc6	1.80633728195307	0.61161102402172	0.994766463583882	pseudobulk	DESeq2	LRT	0.213524695220716
125	KC_AB1	Orco	-2.20969975456663	0.610836134188693	0.994766463583882	pseudobulk	DESeq2	LRT	0.214075280039667
125	KC_AB1	Orcokinin	1.80633728195307	0.61161102402172	0.994766463583882	pseudobulk	DESeq2	LRT	0.213524695220716
125	KC_AB1	Ork1	-1.64952313825113	0.817565084244375	0.994766463583882	pseudobulk	DESeq2	LRT	0.0874776642296574
125	KC_AB1	ORMDL	0.236914179035654	0.705551263431111	0.994766463583882	pseudobulk	DESeq2	LRT	0.151471426110292
125	KC_AB1	osa	-0.123855252340919	0.594801885592058	0.994766463583882	pseudobulk	DESeq2	LRT	0.225627663383224
125	KC_AB1	Osbp	0.836616726025013	0.458702420645695	0.994766463583882	pseudobulk	DESeq2	LRT	0.338468968020854
125	KC_AB1	Oscillin	-0.212306191660069	0.848090888647991	0.994766463583882	pseudobulk	DESeq2	LRT	0.0715576025509359
125	KC_AB1	Oseg1	2.12134513261811	0.327739617493912	0.994766463583882	pseudobulk	DESeq2	LRT	0.484471057491497
125	KC_AB1	Oseg4	0.949183608371002	0.80156895525114	0.994766463583882	pseudobulk	DESeq2	LRT	0.0960591113673091
125	KC_AB1	Osi24	1.80633728195307	0.61161102402172	0.994766463583882	pseudobulk	DESeq2	LRT	0.213524695220716
125	KC_AB1	osp	1.09255624499018	0.534555027288718	0.994766463583882	pseudobulk	DESeq2	LRT	0.272007581722247
125	KC_AB1	Ost48	-0.142522674627573	0.831004786504772	0.994766463583882	pseudobulk	DESeq2	LRT	0.0803964747157405
125	KC_AB1	OstDelta	1.01509859858479	0.496285601081083	0.994766463583882	pseudobulk	DESeq2	LRT	0.304268324967387
125	KC_AB1	Ostgamma	-0.02569332748728	0.915802580693671	0.994766463583882	pseudobulk	DESeq2	LRT	0.0381981369827684
125	KC_AB1	otk	0.35067887492192	0.630667219306676	0.994766463583882	pseudobulk	DESeq2	LRT	0.200199742090884
125	KC_AB1	otk2	0.190713923739665	0.848653664295535	0.994766463583882	pseudobulk	DESeq2	LRT	0.0712695092527238
125	KC_AB1	OtopLa	0.040256626574243	0.834405951533806	0.994766463583882	pseudobulk	DESeq2	LRT	0.078622606895676
125	KC_AB1	OtopLc	0.248637482872959	0.475667414476595	0.994766463583882	pseudobulk	DESeq2	LRT	0.322696598838085
125	KC_AB1	out	2.70892393076352	0.223582256563894	0.994766463583882	pseudobulk	DESeq2	LRT	0.650562664927614
125	KC_AB1	ova	-0.22530502421169	0.643143278850144	0.994766463583882	pseudobulk	DESeq2	LRT	0.191692264583959
125	KC_AB1	ovo	0.949183608371002	0.80156895525114	0.994766463583882	pseudobulk	DESeq2	LRT	0.0960591113673091
125	KC_AB1	ox	0.132118998159514	0.583542752729803	0.994766463583882	pseudobulk	DESeq2	LRT	0.2339273202622

125	KC_AB1	OXA1L	1.10252397835024	0.455563154330323	0.994766463583882	pseudobulk	DESeq2	LRT	0.341451408644992
126	KC_AB1	oxt	-0.094156486439272	0.894047253574506	0.994766463583882	pseudobulk	DESeq2	LRT	0.04863952659075
126	KC_AB1	oys	0.279991564552487	0.689403028434446	0.994766463583882	pseudobulk	DESeq2	LRT	0.161526813139444
126	KC_AB1	p	-0.502329040177897	0.480217354119077	0.994766463583882	pseudobulk	DESeq2	LRT	0.318562149439406
126	KC_AB1	p115	-0.522954971746956	0.758936709962299	0.994766463583882	pseudobulk	DESeq2	LRT	0.119794439730597
126	KC_AB1	p120ctn	-0.0505050214310057	0.797486983396769	0.994766463583882	pseudobulk	DESeq2	LRT	0.0982763967787318
126	KC_AB1	p130CAS	-0.172805088443265	0.763788269667543	0.994766463583882	pseudobulk	DESeq2	LRT	0.117027015847195
126	KC_AB1	p23	-0.265288511065753	0.398247220531536	0.994766463583882	pseudobulk	DESeq2	LRT	0.399847246567707
126	KC_AB1	P32	-0.709446911020403	0.392260142452683	0.994766463583882	pseudobulk	DESeq2	LRT	0.406425818284591
126	KC_AB1	p38b	-0.133379809637862	0.863145227521675	0.994766463583882	pseudobulk	DESeq2	LRT	0.0639161264111249
126	KC_AB1	p47	-0.157683897410446	0.820789324587269	0.994766463583882	pseudobulk	DESeq2	LRT	0.0857683007495486
126	KC_AB1	p53	0.949183608371002	0.80156895525114	0.994766463583882	pseudobulk	DESeq2	LRT	0.0960591113673091
126	KC_AB1	P58IPK	0.49012570956059	0.65086593794634	0.994766463583882	pseudobulk	DESeq2	LRT	0.186508455987727
126	KC_AB1	P5CDh1	0.119781812479806	0.750751646527406	0.994766463583882	pseudobulk	DESeq2	LRT	0.124503706645488
126	KC_AB1	P5cr	0.496836080872279	0.548291180837584	0.994766463583882	pseudobulk	DESeq2	LRT	0.2609887396069
126	KC_AB1	P5cr-2	-1.64952313825113	0.817565084244375	0.994766463583882	pseudobulk	DESeq2	LRT	0.0874776642296574
126	KC_AB1	Pa1	-0.259410806056636	0.60142709528567	0.994766463583882	pseudobulk	DESeq2	LRT	0.220817010109829
126	KC_AB1	pAbp	0.118997674203445	0.448448800624633	0.994766463583882	pseudobulk	DESeq2	LRT	0.348287133167201
126	KC_AB1	Pabp2	0.0947284358431591	0.87481819349088	0.994766463583882	pseudobulk	DESeq2	LRT	0.0580821935693185
126	KC_AB1	pad	1.29054725401152	0.507474029696183	0.994766463583882	pseudobulk	DESeq2	LRT	0.294586178140335
126	KC_AB1	Paf-AHalpha	-0.190100548589764	0.706259774647401	0.994766463583882	pseudobulk	DESeq2	LRT	0.151035528487829
126	KC_AB1	Paics	0.370519601293918	0.822121836013648	0.994766463583882	pseudobulk	DESeq2	LRT	0.0850638165384441
126	KC_AB1	Paip2	-0.149793619346921	0.640897238947136	0.994766463583882	pseudobulk	DESeq2	LRT	0.193211599398311
126	KC_AB1	Pak	-0.0621844724846889	0.86201151229732	0.994766463583882	pseudobulk	DESeq2	LRT	0.0644869340664543
126	KC_AB1	Pak3	0.0997751269533826	0.855275033381877	0.994766463583882	pseudobulk	DESeq2	LRT	0.067894205420591
126	KC_AB1	Pal1	1.0347053529509	0.217457900619368	0.994766463583882	pseudobulk	DESeq2	LRT	0.662624809046868
126	KC_AB1	Pal2	-0.0752295015857416	0.728328448035844	0.994766463583882	pseudobulk	DESeq2	LRT	0.137672726454216
126	KC_AB1	pall	0.556847584943023	0.50951300899549	0.994766463583882	pseudobulk	DESeq2	LRT	0.292844723016082
126	KC_AB1	pan	0.0982350389505309	0.434443400144263	0.994766463583882	pseudobulk	DESeq2	LRT	0.362066795984421
126	KC_AB1	PAN2	0.999082548932596	0.384911771163318	0.994766463583882	pseudobulk	DESeq2	LRT	0.414638807343691
126	KC_AB1	Panx	-0.0561526457960697	0.877349456132826	0.994766463583882	pseudobulk	DESeq2	LRT	0.0568273887991944
126	KC_AB1	papi	-0.203915211405038	0.865443435023745	0.994766463583882	pseudobulk	DESeq2	LRT	0.0627613121442725
126	KC_AB1	PAPLA1	0.115939137780266	0.790363402929333	0.994766463583882	pseudobulk	DESeq2	LRT	0.102173177572522
126	KC_AB1	Papss	-1.12654950136113	0.242548647185786	0.994766463583882	pseudobulk	DESeq2	LRT	0.615201143307086
126	KC_AB1	Papst2	-0.479172362493613	0.611474623867461	0.994766463583882	pseudobulk	DESeq2	LRT	0.21362156142902
126	KC_AB1	par-1	0.155889003547217	0.527486282476972	0.994766463583882	pseudobulk	DESeq2	LRT	0.277788829910874
126	KC_AB1	par-6	0.200413894709173	0.699151621983684	0.994766463583882	pseudobulk	DESeq2	LRT	0.155428630477232
126	KC_AB1	para	0.157762160945659	0.201816149519978	0.994766463583882	pseudobulk	DESeq2	LRT	0.695044084051611
126	KC_AB1	Parg	0.71225234369677	0.639838526757513	0.994766463583882	pseudobulk	DESeq2	LRT	0.193929613182139
126	KC_AB1	park	0.280783473609079	0.862060689739496	0.994766463583882	pseudobulk	DESeq2	LRT	0.0644621584314761
126	KC_AB1	Parp	-0.174928372528768	0.482389014566595	0.994766463583882	pseudobulk	DESeq2	LRT	0.316602590933064
126	KC_AB1	parvin	-0.368464524339676	0.614453965856607	0.994766463583882	pseudobulk	DESeq2	LRT	0.21151064837287
126	KC_AB1	pasha	0.373370816333734	0.691374946900514	0.994766463583882	pseudobulk	DESeq2	LRT	0.16028636187778
126	KC_AB1	pasi1	0.245532694227529	0.851579371739034	0.994766463583882	pseudobulk	DESeq2	LRT	0.0697748672529893
126	KC_AB1	pasi2	-0.62302262427717	0.428990834395482	0.994766463583882	pseudobulk	DESeq2	LRT	0.367551986635762
126	KC_AB1	Pask	0.0796393273378819	0.842262884660779	0.994766463583882	pseudobulk	DESeq2	LRT	0.0745523366017824
126	KC_AB1	Past1	0.0778036773608438	0.865646306369292	0.994766463583882	pseudobulk	DESeq2	LRT	0.0626595197250769
126	KC_AB1	Pat1	-0.484507157898159	0.237723135583615	0.994766463583882	pseudobulk	DESeq2	LRT	0.623928550003596
126	KC_AB1	Patj	0.325499522555055	0.744809331723626	0.994766463583882	pseudobulk	DESeq2	LRT	0.127954890712684
126	KC_AB1	Patr-1	-0.104197037815657	0.89351926718492	0.994766463583882	pseudobulk	DESeq2	LRT	0.0488960782541676
126	KC_AB1	Patronin	-0.0995628062123621	0.794366099762322	0.994766463583882	pseudobulk	DESeq2	LRT	0.0999792979974506
126	KC_AB1	Patsas	0.250815197622748	0.791866263745495	0.994766463583882	pseudobulk	DESeq2	LRT	0.101348159094413
126	KC_AB1	Pax	0.182536490575496	0.80989823758166	0.994766463583882	pseudobulk	DESeq2	LRT	0.0915695461005362
126	KC_AB1	pb	0.0462401973101628	0.899563689741424	0.994766463583882	pseudobulk	DESeq2	LRT	0.0459680828753462
126	KC_AB1	Pbgs	-0.522954971746956	0.758936709962299	0.994766463583882	pseudobulk	DESeq2	LRT	0.119794439730597
126	KC_AB1	Pbp45	1.19856662192297	0.608802264240657	0.994766463583882	pseudobulk	DESeq2	LRT	0.215523741018373

126	KC_AB1	Pbp95	-1.13273312364362	0.51815807629079	0.994766463583882	pseudobulk	DESeq2	LRT	0.285537728309122
126	KC_AB1	Pc	-0.17589698737114	0.744167596310723	0.994766463583882	pseudobulk	DESeq2	LRT	0.128329244620626
126	KC_AB1	PCB	-0.008494327098941	0.929137567062471	0.994766463583882	pseudobulk	DESeq2	LRT	0.0319199800935012
126	KC_AB1	Pcd	0.0928454351116358	0.742334517088021	0.994766463583882	pseudobulk	DESeq2	LRT	0.129400345142671
126	KC_AB1	Pcf11	0.0260587682067333	0.925225753588577	0.994766463583882	pseudobulk	DESeq2	LRT	0.0337522871792555
126	KC_AB1	pch2	-0.878551581920065	0.610521464396702	0.994766463583882	pseudobulk	DESeq2	LRT	0.214299062750751
126	KC_AB1	PCID2	0.817773412303538	0.495984517778768	0.994766463583882	pseudobulk	DESeq2	LRT	0.304531879856961
126	KC_AB1	pck	0.0574179976194935	0.938017216279256	0.994766463583882	pseudobulk	DESeq2	LRT	0.0277891905479462
126	KC_AB1	Pcl	-0.777003936885011	0.169026541512776	0.994766463583882	pseudobulk	DESeq2	LRT	0.77204509463196
126	KC_AB1	pcm	-0.323170363764802	0.771351679231675	0.994766463583882	pseudobulk	DESeq2	LRT	0.112747570681722
126	KC_AB1	Pcmt	0.110914965224661	0.833600121213304	0.994766463583882	pseudobulk	DESeq2	LRT	0.0790422308940408
126	KC_AB1	PCNA2	-0.745835241885211	0.479556839804569	0.994766463583882	pseudobulk	DESeq2	LRT	0.319159910390079
126	KC_AB1	pcs	-0.12001606052669	0.751775074840893	0.994766463583882	pseudobulk	DESeq2	LRT	0.123912077449698
126	KC_AB1	pcx	-0.0404920489714367	0.948237968353399	0.994766463583882	pseudobulk	DESeq2	LRT	0.0230826591032917
126	KC_AB1	Pcyt1	-0.213011518760917	0.518320928825178	0.994766463583882	pseudobulk	DESeq2	LRT	0.285401254811286
126	KC_AB1	Pcyt2	-0.177855774869658	0.864788238336197	0.994766463583882	pseudobulk	DESeq2	LRT	0.0630902256926087
126	KC_AB1	PDCD-5	0.0485993712213822	0.931294588667565	0.994766463583882	pseudobulk	DESeq2	LRT	0.0309129205269255
126	KC_AB1	Pdcd4	-0.0431981589462735	0.819993772380618	0.994766463583882	pseudobulk	DESeq2	LRT	0.0861894459467754
126	KC_AB1	Pde11	0.949183608371002	0.80156895525114	0.994766463583882	pseudobulk	DESeq2	LRT	0.0960591113673091
126	KC_AB1	Pde1c	-0.100743289920274	0.544257257734416	0.994766463583882	pseudobulk	DESeq2	LRT	0.264195770880361
126	KC_AB1	Pde6	-0.0617086369994317	0.722582050409206	0.994766463583882	pseudobulk	DESeq2	LRT	0.141112830901204
126	KC_AB1	Pde8	-0.137405418557055	0.547209268818382	0.994766463583882	pseudobulk	DESeq2	LRT	0.261846554996666
126	KC_AB1	Pde9	-0.117773989297492	0.468828350550041	0.994766463583882	pseudobulk	DESeq2	LRT	0.328986133945683
126	KC_AB1	pdgy	-0.835152883801839	0.920434724857442	0.994766463583882	pseudobulk	DESeq2	LRT	0.0360070052473159
126	KC_AB1	Pdha	-0.322775383047652	0.446811832766689	0.994766463583882	pseudobulk	DESeq2	LRT	0.349875334130215
126	KC_AB1	Pdhb	-0.126778877838781	0.754283822558531	0.994766463583882	pseudobulk	DESeq2	LRT	0.122465206680056
126	KC_AB1	Pdi	-0.143681690865871	0.787777147962393	0.994766463583882	pseudobulk	DESeq2	LRT	0.103596621467697
126	KC_AB1	Pdk	0.635677145910794	0.251658228224329	0.994766463583882	pseudobulk	DESeq2	LRT	0.599188865330414
126	KC_AB1	Pdk1	-0.262078444426489	0.399307740680965	0.994766463583882	pseudobulk	DESeq2	LRT	0.398692270816319
126	KC_AB1	pdm3	0.949183608371002	0.80156895525114	0.994766463583882	pseudobulk	DESeq2	LRT	0.0960591113673091
126	KC_AB1	Pdp	-0.35937468640407	0.675713865219438	0.994766463583882	pseudobulk	DESeq2	LRT	0.170237169536602
126	KC_AB1	Pdp1	-0.0345317834366948	0.828052676785295	0.994766463583882	pseudobulk	DESeq2	LRT	0.0819420345804675
126	KC_AB1	Pdrg1	-0.747760753789965	0.720131677202483	0.994766463583882	pseudobulk	DESeq2	LRT	0.142588084882933
126	KC_AB1	pds5	0.699145194041508	0.369371239603438	0.994766463583882	pseudobulk	DESeq2	LRT	0.432536923102019
126	KC_AB1	Pdss2	-0.199468656701034	0.764850857846116	0.994766463583882	pseudobulk	DESeq2	LRT	0.116423241877903
126	KC_AB1	Pdxk	-1.77789784908752	0.354320470691372	0.994766463583882	pseudobulk	DESeq2	LRT	0.450603755791344
126	KC_AB1	PDZ-GEF	-0.17177482308365	0.537126499538708	0.994766463583882	pseudobulk	DESeq2	LRT	0.269923420851951
126	KC_AB1	pea	1.20145523455051	0.251081671159413	0.994766463583882	pseudobulk	DESeq2	LRT	0.600184989417152
126	KC_AB1	PEK	0.440020044112866	0.67483134447979	0.994766463583882	pseudobulk	DESeq2	LRT	0.170804753559995
126	KC_AB1	pelo	-0.0881369989080707	0.900852341259495	0.994766463583882	pseudobulk	DESeq2	LRT	0.0453463884121459
126	KC_AB1	pen-2	-0.21142757519486	0.80614965814586	0.994766463583882	pseudobulk	DESeq2	LRT	0.0935843258457011
126	KC_AB1	peng	-1.02885104131204	0.27026674192134	0.994766463583882	pseudobulk	DESeq2	LRT	0.568207393771145
126	KC_AB1	Pep	0.175436410700873	0.447997099030811	0.994766463583882	pseudobulk	DESeq2	LRT	0.348724798231745
126	KC_AB1	per	0.27113966173194	0.696274340812998	0.994766463583882	pseudobulk	DESeq2	LRT	0.157219609202785
126	KC_AB1	pes	1.61455067784864	0.342588322618789	0.994766463583882	pseudobulk	DESeq2	LRT	0.465227444373
127	KC_AB1	Pex1	-0.382726019862672	0.80381691155456	0.994766463583882	pseudobulk	DESeq2	LRT	0.0948428608989432
127	KC_AB1	Pex10	0.141750428393048	0.880266692630739	0.994766463583882	pseudobulk	DESeq2	LRT	0.0553857305875924
127	KC_AB1	Pex11	0.284952175260303	0.767696399489288	0.994766463583882	pseudobulk	DESeq2	LRT	0.114810496237754
127	KC_AB1	Pex12	2.52085064386908	0.19646857892814	0.994766463583882	pseudobulk	DESeq2	LRT	0.7067068961245
127	KC_AB1	Pex13	0.31213320521618	0.639062508939696	0.994766463583882	pseudobulk	DESeq2	LRT	0.194456659901302
127	KC_AB1	Pex14	0.274010498870805	0.735776457907748	0.994766463583882	pseudobulk	DESeq2	LRT	0.133254112080216
127	KC_AB1	Pex16	-0.45152845621656	0.622157050770132	0.994766463583882	pseudobulk	DESeq2	LRT	0.206099972747308
127	KC_AB1	Pex19	-0.0924255466638914	0.853478755039944	0.994766463583882	pseudobulk	DESeq2	LRT	0.0688072849578645
127	KC_AB1	Pex2	-0.0648109581898329	0.959717552913585	0.994766463583882	pseudobulk	DESeq2	LRT	0.017856562022532
127	KC_AB1	Pex23	0.128932093867734	0.856981827709171	0.994766463583882	pseudobulk	DESeq2	LRT	0.0670283871891776
127	KC_AB1	Pex3	0.63167865108638	0.486927054180672	0.994766463583882	pseudobulk	DESeq2	LRT	0.312536094924294

127	KC_AB1	Pex5	0.900679239362507	0.379151863520703	0.994766463583882	pseudobulk	DESeq2	LRT	0.421186805127058
127	KC_AB1	Pex6	-2.61825188265191	0.0784416287866362	0.994766463583882	pseudobulk	DESeq2	LRT	1.1054533970835
127	KC_AB1	Pfdn1	0.229172762850195	0.715312821532948	0.994766463583882	pseudobulk	DESeq2	LRT	0.14550399043005
127	KC_AB1	Pfdn2	-0.174744743414074	0.749234144268903	0.994766463583882	pseudobulk	DESeq2	LRT	0.12538243907886
127	KC_AB1	Pfdn4	0.392162802295398	0.370782103695539	0.994766463583882	pseudobulk	DESeq2	LRT	0.430881235837789
127	KC_AB1	Pfdn5	0.799547787551979	0.200375761609629	0.994766463583882	pseudobulk	DESeq2	LRT	0.698154813921737
127	KC_AB1	Pfdn6	0.218974218867461	0.607988046052306	0.994766463583882	pseudobulk	DESeq2	LRT	0.216104959517654
127	KC_AB1	Pfk	-0.292444285102923	0.53324159574747	0.994766463583882	pseudobulk	DESeq2	LRT	0.273075980602886
127	KC_AB1	Pfrx	-0.0820877206380247	0.785368689381234	0.994766463583882	pseudobulk	DESeq2	LRT	0.104926416918428
127	KC_AB1	Pgam5	0.28267015084919	0.770036904649889	0.994766463583882	pseudobulk	DESeq2	LRT	0.113488460409684
127	KC_AB1	Pgant1	-0.372082866903301	0.712361172858778	0.994766463583882	pseudobulk	DESeq2	LRT	0.147299759720745
127	KC_AB1	Pgant2	-0.322607203811798	0.368633081007185	0.994766463583882	pseudobulk	DESeq2	LRT	0.433405693929163
127	KC_AB1	Pgant3	-0.437049773881408	0.629291651509181	0.994766463583882	pseudobulk	DESeq2	LRT	0.201148029777296
127	KC_AB1	Pgant35A	0.201519360265386	0.893385042978098	0.994766463583882	pseudobulk	DESeq2	LRT	0.0489613227467115
127	KC_AB1	Pgant5	0.635686524158682	0.259503487948607	0.994766463583882	pseudobulk	DESeq2	LRT	0.58585680048738
127	KC_AB1	Pgant6	0.696124738815335	0.683158309383576	0.994766463583882	pseudobulk	DESeq2	LRT	0.165478644890948
127	KC_AB1	Pgant7	1.57630120515932	0.359592692327697	0.994766463583882	pseudobulk	DESeq2	LRT	0.444189142617353
127	KC_AB1	Pgant9	0.357726238927279	0.111469708249613	0.994766463583882	pseudobulk	DESeq2	LRT	0.952843135551955
127	KC_AB1	PGAP1	0.835018713165695	0.573015421735538	0.994766463583882	pseudobulk	DESeq2	LRT	0.241833689577954
127	KC_AB1	PGAP2	-0.390490145457518	0.544750875381465	0.994766463583882	pseudobulk	DESeq2	LRT	0.263802063196375
127	KC_AB1	PGAP3	0.340714826221888	0.671449383313914	0.994766463583882	pseudobulk	DESeq2	LRT	0.172986720710839
127	KC_AB1	PGAP5	-0.458935030966377	0.68091071153109	0.994766463583882	pseudobulk	DESeq2	LRT	0.166909833801897
127	KC_AB1	Pgi	-0.170629666163624	0.725695344480661	0.994766463583882	pseudobulk	DESeq2	LRT	0.139245663018561
127	KC_AB1	Pgk	0.0317789351251674	0.853233779679644	0.994766463583882	pseudobulk	DESeq2	LRT	0.0689319590851667
127	KC_AB1	Pglym78	-0.178936227873197	0.531142797728489	0.994766463583882	pseudobulk	DESeq2	LRT	0.274788703159156
127	KC_AB1	Pgm1	1.03605116398431	0.401326786708257	0.994766463583882	pseudobulk	DESeq2	LRT	0.39650185214919
127	KC_AB1	Pgm2a	-0.380087175102556	0.680790020222024	0.994766463583882	pseudobulk	DESeq2	LRT	0.166986819248781
127	KC_AB1	PGRP-LB	2.22023857837763	0.524904842126482	0.994766463583882	pseudobulk	DESeq2	LRT	0.279919420946639
127	KC_AB1	PGRP-LE	0.777529257823465	0.4338672659377	0.994766463583882	pseudobulk	DESeq2	LRT	0.362643114929328
127	KC_AB1	ph-d	0.417260068211545	0.613587970139061	0.994766463583882	pseudobulk	DESeq2	LRT	0.212123163650729
127	KC_AB1	ph-p	0.0725877095618282	0.913975312071321	0.994766463583882	pseudobulk	DESeq2	LRT	0.0390655350933026
127	KC_AB1	PH4alphaEFB	0.403145197828963	0.566784304681946	0.994766463583882	pseudobulk	DESeq2	LRT	0.246582184684988
127	KC_AB1	PH4alphaNE1	-2.57435989741145	0.533617212681275	0.994766463583882	pseudobulk	DESeq2	LRT	0.272770169998685
127	KC_AB1	Phax	-0.272903162393116	0.800659938484819	0.994766463583882	pseudobulk	DESeq2	LRT	0.0965519011421927
127	KC_AB1	Phb2	0.247811568174397	0.647467914043776	0.994766463583882	pseudobulk	DESeq2	LRT	0.188781748635135
127	KC_AB1	pHCl-1	-0.0135650851459936	0.913321170716821	0.994766463583882	pseudobulk	DESeq2	LRT	0.0393764753216189
127	KC_AB1	PheRS-m	-0.959613768013954	0.245762771766786	0.994766463583882	pseudobulk	DESeq2	LRT	0.609483903551935
127	KC_AB1	Phf5a	0.591855406833303	0.41261723350743	0.994766463583882	pseudobulk	DESeq2	LRT	0.384452637097525
127	KC_AB1	PHGPx	0.358346315406996	0.503970689496388	0.994766463583882	pseudobulk	DESeq2	LRT	0.297594721015067
127	KC_AB1	PhKgamma	0.0950122604218414	0.825907029126437	0.994766463583882	pseudobulk	DESeq2	LRT	0.0830688376794356
127	KC_AB1	Phm	-0.0727821801034609	0.827278138997444	0.994766463583882	pseudobulk	DESeq2	LRT	0.0823484518459842
127	KC_AB1	pho	-0.322370338930995	0.342494524458885	0.994766463583882	pseudobulk	DESeq2	LRT	0.465346367284841
127	KC_AB1	phol	-0.982593713395423	0.23502436278207	0.994766463583882	pseudobulk	DESeq2	LRT	0.628887116139324
127	KC_AB1	phr	-1.64952313825113	0.817565084244375	0.994766463583882	pseudobulk	DESeq2	LRT	0.0874776642296574
127	KC_AB1	phtf	-0.0440287037766847	0.853892375959708	0.994766463583882	pseudobulk	DESeq2	LRT	0.0685968640560395
127	KC_AB1	phyl	0.0303627541074931	0.900661586191669	0.994766463583882	pseudobulk	DESeq2	LRT	0.0454383598062043
127	KC_AB1	PI31	-0.396521022730443	0.389338582420061	0.994766463583882	pseudobulk	DESeq2	LRT	0.40967255671888
127	KC_AB1	Pi3K21B	-0.184624782692183	0.73733395453336	0.994766463583882	pseudobulk	DESeq2	LRT	0.132335766196002
127	KC_AB1	Pi3K59F	-0.00479207869850181	0.906322781575649	0.994766463583882	pseudobulk	DESeq2	LRT	0.0427171033257767
127	KC_AB1	Pi3K68D	0.343559287932147	0.533019770312211	0.994766463583882	pseudobulk	DESeq2	LRT	0.273256682196201
127	KC_AB1	Pi4KIIalpha	0.242942762672471	0.747877965856039	0.994766463583882	pseudobulk	DESeq2	LRT	0.126169261866952
127	KC_AB1	PI4KIIIalpha	0.0810608577621694	0.844831446345589	0.994766463583882	pseudobulk	DESeq2	LRT	0.0732299291850358
127	KC_AB1	pic	-0.440290784727805	0.642615670490343	0.994766463583882	pseudobulk	DESeq2	LRT	0.192048688176805
127	KC_AB1	PICK1	0.43286281741109	0.457220375582492	0.994766463583882	pseudobulk	DESeq2	LRT	0.339874423933219
127	KC_AB1	pico	0.630190632978053	0.166025832901404	0.994766463583882	pseudobulk	DESeq2	LRT	0.779824332359552
127	KC_AB1	Picot	0.629857653278487	0.527052538889094	0.994766463583882	pseudobulk	DESeq2	LRT	0.278146090266932

127	KC_AB1	Piezo	0.259917101853494	0.875690003055577	0.994766463583882	pseudobulk	DESeq2	LRT	0.0576496082032299
127	KC_AB1	Pif1A	-1.64952313825113	0.817565084244375	0.994766463583882	pseudobulk	DESeq2	LRT	0.0874776642296574
127	KC_AB1	PIG-A	-0.0538431372555513	0.942386114492196	0.994766463583882	pseudobulk	DESeq2	LRT	0.0257711215848224
127	KC_AB1	PIG-B	-0.342872198854419	0.603494874661475	0.994766463583882	pseudobulk	DESeq2	LRT	0.219326413910777
127	KC_AB1	PIG-C	-2.50002631996899	0.341598419302658	0.994766463583882	pseudobulk	DESeq2	LRT	0.466484147613365
127	KC_AB1	PIG-F	0.033844369398576	0.950441162943449	0.994766463583882	pseudobulk	DESeq2	LRT	0.0220747629646528
127	KC_AB1	PIG-G	-0.953147145305224	0.579756746535172	0.994766463583882	pseudobulk	DESeq2	LRT	0.236754188846165
127	KC_AB1	PIG-H	0.0489045257525788	0.869232629681978	0.994766463583882	pseudobulk	DESeq2	LRT	0.0608639792856489
127	KC_AB1	PIG-K	0.132391739880054	0.803468653222431	0.994766463583882	pseudobulk	DESeq2	LRT	0.0950310622709983
127	KC_AB1	PIG-L	-0.502828901945505	0.606090269542419	0.994766463583882	pseudobulk	DESeq2	LRT	0.217462688301417
127	KC_AB1	PIG-N	-1.64952313825113	0.817565084244375	0.994766463583882	pseudobulk	DESeq2	LRT	0.0874776642296574
127	KC_AB1	PIG-O	0.147593431261283	0.896229324366812	0.994766463583882	pseudobulk	DESeq2	LRT	0.0475808501994319
127	KC_AB1	PIG-Q	0.239055006065497	0.838672625396468	0.994766463583882	pseudobulk	DESeq2	LRT	0.0764075322934758
127	KC_AB1	PIG-S	-0.387924780963959	0.682352864731523	0.994766463583882	pseudobulk	DESeq2	LRT	0.165990980805972
127	KC_AB1	PIG-T	0.309395075573876	0.566288502684727	0.994766463583882	pseudobulk	DESeq2	LRT	0.246962255734
127	KC_AB1	PIG-U	-1.7506193918396	0.221689814865153	0.994766463583882	pseudobulk	DESeq2	LRT	0.654254259296298
127	KC_AB1	PIG-V	-0.536671700702827	0.719917995343198	0.994766463583882	pseudobulk	DESeq2	LRT	0.142716970510728
127	KC_AB1	PIG-Wa	-3.21143241058256	0.292545415061155	0.994766463583882	pseudobulk	DESeq2	LRT	0.533806704011112
127	KC_AB1	PIG-X	-0.0838996376011653	0.872276120187817	0.994766463583882	pseudobulk	DESeq2	LRT	0.0593460167969183
127	KC_AB1	Pig1	2.46056380702497	0.287851068109869	0.994766463583882	pseudobulk	DESeq2	LRT	0.540832154698124
127	KC_AB1	pigeon	-0.419033211444223	0.690458913244187	0.994766463583882	pseudobulk	DESeq2	LRT	0.160862159637453
127	KC_AB1	pigs	-0.182398638749037	0.605023840269586	0.994766463583882	pseudobulk	DESeq2	LRT	0.218227512135072
127	KC_AB1	Pih1D1	0.0298691772202344	0.952794829125835	0.994766463583882	pseudobulk	DESeq2	LRT	0.0210006084608481
127	KC_AB1	pim	1.1821590793593	0.719511699510136	0.994766463583882	pseudobulk	DESeq2	LRT	0.142962139890846
127	KC_AB1	Pink1	-0.436031770518007	0.407388354387848	0.994766463583882	pseudobulk	DESeq2	LRT	0.389991389909699
127	KC_AB1	Pino	-0.0429557093754939	0.936545935215054	0.994766463583882	pseudobulk	DESeq2	LRT	0.0284709167178085
127	KC_AB1	pins	0.35704507427356	0.633223432634645	0.994766463583882	pseudobulk	DESeq2	LRT	0.1984430223068
127	KC_AB1	pip	-1.64952313825113	0.817565084244375	0.994766463583882	pseudobulk	DESeq2	LRT	0.0874776642296574
127	KC_AB1	PIP4K	-0.00580285218669384	0.884900150635008	0.994766463583882	pseudobulk	DESeq2	LRT	0.0531057309684751
127	KC_AB1	PIP5K59B	-0.00660419604854042	0.935269189733711	0.994766463583882	pseudobulk	DESeq2	LRT	0.0290633722396623
127	KC_AB1	Pis	0.131006131627222	0.729245086217477	0.994766463583882	pseudobulk	DESeq2	LRT	0.137126488549493
127	KC_AB1	Pisd	-0.140788277006297	0.876963445515196	0.994766463583882	pseudobulk	DESeq2	LRT	0.0570185089622403
127	KC_AB1	pit	1.55735097011084	0.268511327189921	0.994766463583882	pseudobulk	DESeq2	LRT	0.57103738880124
128	KC_AB1	pita	0.0929854754601877	0.898267511644309	0.994766463583882	pseudobulk	DESeq2	LRT	0.04659430750894
128	KC_AB1	Pits	0.0954904870697653	0.650781596739768	0.994766463583882	pseudobulk	DESeq2	LRT	0.186564736846695
128	KC_AB1	Pitslre	-0.0519549589288913	0.756790201639113	0.994766463583882	pseudobulk	DESeq2	LRT	0.121024499494229
128	KC_AB1	piwi	-1.64952313825113	0.817565084244375	0.994766463583882	pseudobulk	DESeq2	LRT	0.0874776642296574
128	KC_AB1	pix	0.112322436055116	0.809654339815448	0.994766463583882	pseudobulk	DESeq2	LRT	0.0917003519246795
128	KC_AB1	pk	2.38929733117871	0.484296857543297	0.994766463583882	pseudobulk	DESeq2	LRT	0.314888348950639
128	KC_AB1	PK2-R2	-2.20969975456663	0.610836134188693	0.994766463583882	pseudobulk	DESeq2	LRT	0.214075280039667
128	KC_AB1	Pka-C1	-0.0465355676814073	0.673650229947465	0.994766463583882	pseudobulk	DESeq2	LRT	0.171565537637118
128	KC_AB1	Pka-C3	1.02996170936706	0.619231122089829	0.994766463583882	pseudobulk	DESeq2	LRT	0.208147224463057
128	KC_AB1	Pka-R1	0.0711412677159562	0.713986192405725	0.994766463583882	pseudobulk	DESeq2	LRT	0.146310186851255
128	KC_AB1	Pka-R2	-0.241682968958322	0.0382787615026799	0.994766463583882	pseudobulk	DESeq2	LRT	1.4170421221296
128	KC_AB1	pkaap	0.151447882878092	0.853904226901008	0.994766463583882	pseudobulk	DESeq2	LRT	0.0685908366422322
128	KC_AB1	Pkc53E	-0.108120059055574	0.413752571918124	0.994766463583882	pseudobulk	DESeq2	LRT	0.383259293599603
128	KC_AB1	Pkc98E	-0.0118422525966666	0.924103517811762	0.994766463583882	pseudobulk	DESeq2	LRT	0.0342793765111857
128	KC_AB1	PKD	-0.294480054404714	0.78250347816745	0.994766463583882	pseudobulk	DESeq2	LRT	0.106513723371835
128	KC_AB1	Pkd2	-1.64952313825113	0.817565084244375	0.994766463583882	pseudobulk	DESeq2	LRT	0.0874776642296574
128	KC_AB1	Pkn	0.257115823634712	0.640510393553774	0.994766463583882	pseudobulk	DESeq2	LRT	0.193473818571242
128	KC_AB1	Plap	0.455524808636317	0.71082824195245	0.994766463583882	pseudobulk	DESeq2	LRT	0.148235325548156
128	KC_AB1	Plc21C	-0.172925437253749	0.310963239933061	0.994766463583882	pseudobulk	DESeq2	LRT	0.507290947429144
128	KC_AB1	PLCXD	-3.21143241058256	0.292545415061155	0.994766463583882	pseudobulk	DESeq2	LRT	0.533806704011112
128	KC_AB1	Pld	0.949183608371002	0.80156895525114	0.994766463583882	pseudobulk	DESeq2	LRT	0.0960591113673091
128	KC_AB1	Pldn	0.313320517262275	0.601052449697153	0.994766463583882	pseudobulk	DESeq2	LRT	0.221087628462891
128	KC_AB1	ple	1.1821590793593	0.719511699510136	0.994766463583882	pseudobulk	DESeq2	LRT	0.142962139890846

128	KC_AB1	Plekhm1	-0.035563162862932	0.929574621491585	0.994766463583882	pseudobulk	DESeq2	LRT	0.0317157415550955
128	KC_AB1	PlexA	0.114144160627631	0.613540048244886	0.994766463583882	pseudobulk	DESeq2	LRT	0.212157083851232
128	KC_AB1	PlexB	0.274491596982404	0.407999120812594	0.994766463583882	pseudobulk	DESeq2	LRT	0.389340772759753
128	KC_AB1	plh	-2.57435989741145	0.533617212681275	0.994766463583882	pseudobulk	DESeq2	LRT	0.272770169998685
128	KC_AB1	Pli	-0.0108998601975742	0.87337813975197	0.994766463583882	pseudobulk	DESeq2	LRT	0.0587976824672363
128	KC_AB1	pll	1.02996170936706	0.619231122089829	0.994766463583882	pseudobulk	DESeq2	LRT	0.208147224463057
128	KC_AB1	Plod	-0.841837210211656	0.601965793791747	0.994766463583882	pseudobulk	DESeq2	LRT	0.220428186465698
128	KC_AB1	Plp	-0.446104368383928	0.59251226930118	0.994766463583882	pseudobulk	DESeq2	LRT	0.227302652179243
128	KC_AB1	plx	-0.260371549084008	0.40827719835479	0.994766463583882	pseudobulk	DESeq2	LRT	0.389044874069683
128	KC_AB1	Plzf	-2.26306816020352	0.40449476385881	0.994766463583882	pseudobulk	DESeq2	LRT	0.393087095910655
128	KC_AB1	PMCA	-0.0205562409073837	0.889914622254416	0.994766463583882	pseudobulk	DESeq2	LRT	0.0506516572454214
128	KC_AB1	Pmm2	-0.440290784727805	0.642615670490343	0.994766463583882	pseudobulk	DESeq2	LRT	0.192048688176805
128	KC_AB1	PMP34	0.22284365233011	0.685859020219235	0.994766463583882	pseudobulk	DESeq2	LRT	0.163765145274383
128	KC_AB1	Pmp70	-0.0374610583477162	0.91826727627285	0.994766463583882	pseudobulk	DESeq2	LRT	0.0370308920930574
128	KC_AB1	Pms2	0.854047063205883	0.719204060467466	0.994766463583882	pseudobulk	DESeq2	LRT	0.143147869337068
128	KC_AB1	pn	-1.46947613556816	0.433536142447172	0.994766463583882	pseudobulk	DESeq2	LRT	0.362974691011138
128	KC_AB1	Pnn	-0.128889363328338	0.782115087270846	0.994766463583882	pseudobulk	DESeq2	LRT	0.10672933634027
128	KC_AB1	PNPase	1.10251292103259	0.639221751231493	0.994766463583882	pseudobulk	DESeq2	LRT	0.194348455410132
128	KC_AB1	pns	-0.385599665404222	0.604808199627981	0.994766463583882	pseudobulk	DESeq2	LRT	0.218382329561998
128	KC_AB1	pnt	-1.64952313825113	0.817565084244375	0.994766463583882	pseudobulk	DESeq2	LRT	0.0874776642296574
128	KC_AB1	pnut	0.0707376311658012	0.840437521503322	0.994766463583882	pseudobulk	DESeq2	LRT	0.0754945666681738
128	KC_AB1	PNUTS	-0.160645529553868	0.476930175403896	0.994766463583882	pseudobulk	DESeq2	LRT	0.3215451988561
128	KC_AB1	pod1	-0.0316868989909329	0.922782834535614	0.994766463583882	pseudobulk	DESeq2	LRT	0.0349004927549742
128	KC_AB1	poe	0.111179952561778	0.801409726297651	0.994766463583882	pseudobulk	DESeq2	LRT	0.0961453910625825
128	KC_AB1	Pof	-0.0366680044621355	0.942927710876703	0.994766463583882	pseudobulk	DESeq2	LRT	0.0255216009750273
128	KC_AB1	Pol31	0.746714342506132	0.723342225081447	0.994766463583882	pseudobulk	DESeq2	LRT	0.140656182235089
128	KC_AB1	Pol32	-1.27641407007208	0.329309040328283	0.994766463583882	pseudobulk	DESeq2	LRT	0.482396346692295
128	KC_AB1	POLDIP2	-0.708954411214443	0.349139430917226	0.994766463583882	pseudobulk	DESeq2	LRT	0.457001100304339
128	KC_AB1	poly	-0.248991981416103	0.609932339280753	0.994766463583882	pseudobulk	DESeq2	LRT	0.214718339262665
128	KC_AB1	polybromo	-0.891258219820146	0.383408376558352	0.994766463583882	pseudobulk	DESeq2	LRT	0.416338403055949
128	KC_AB1	Pomp	0.405413677209124	0.417959155393355	0.994766463583882	pseudobulk	DESeq2	LRT	0.378866157110121
128	KC_AB1	pont	-0.285759800647055	0.783141127606234	0.994766463583882	pseudobulk	DESeq2	LRT	0.106159967932136
128	KC_AB1	Pop2	-0.0650991022375133	0.880962111121313	0.994766463583882	pseudobulk	DESeq2	LRT	0.0550427695502511
128	KC_AB1	por	1.80939591345248	0.468117841155507	0.994766463583882	pseudobulk	DESeq2	LRT	0.329644806495434
128	KC_AB1	porin	0.012214154165728	0.879426653938604	0.994766463583882	pseudobulk	DESeq2	LRT	0.0558003757835906
128	KC_AB1	Porin2	-1.64952313825113	0.817565084244375	0.994766463583882	pseudobulk	DESeq2	LRT	0.0874776642296574
128	KC_AB1	POSH	-1.24408241868343	0.410299533058567	0.994766463583882	pseudobulk	DESeq2	LRT	0.386898977281694
128	KC_AB1	Pp1-87B	0.183528413685833	0.434628839474211	0.994766463583882	pseudobulk	DESeq2	LRT	0.361881459771214
128	KC_AB1	Pp1alpha-96A	-0.00618755427037795	0.944544479845893	0.994766463583882	pseudobulk	DESeq2	LRT	0.0247775857603794
128	KC_AB1	Pp2A-29B	0.289071401274292	0.451775846115069	0.994766463583882	pseudobulk	DESeq2	LRT	0.345076992050792
128	KC_AB1	Pp2B-14D	-0.0229827856262206	0.889000840382133	0.994766463583882	pseudobulk	DESeq2	LRT	0.0510978284863098
128	KC_AB1	Pp2C1	-0.0163563668591145	0.943797625933593	0.994766463583882	pseudobulk	DESeq2	LRT	0.0251211194334562
128	KC_AB1	Pp4-19C	-0.5107739319828	0.48758904997542	0.994766463583882	pseudobulk	DESeq2	LRT	0.311946056106671
128	KC_AB1	Ppa	-0.51517200153518	0.46161243947792	0.994766463583882	pseudobulk	DESeq2	LRT	0.335722496377831
128	KC_AB1	ppan	-0.678846737033219	0.617250082788619	0.994766463583882	pseudobulk	DESeq2	LRT	0.209538843146313
128	KC_AB1	Ppat-Dpck	0.694978102914899	0.701892288551021	0.994766463583882	pseudobulk	DESeq2	LRT	0.153729529005562
128	KC_AB1	Ppcs	0.140497761547525	0.60290602277276	0.994766463583882	pseudobulk	DESeq2	LRT	0.219750377697244
128	KC_AB1	PpD3	0.33317793693356	0.734659455751454	0.994766463583882	pseudobulk	DESeq2	LRT	0.133913927256869
128	KC_AB1	ppk21	-1.64952313825113	0.817565084244375	0.994766463583882	pseudobulk	DESeq2	LRT	0.0874776642296574
128	KC_AB1	ppk27	-1.64952313825113	0.817565084244375	0.994766463583882	pseudobulk	DESeq2	LRT	0.0874776642296574
128	KC_AB1	ppk28	0.949183608371002	0.80156895525114	0.994766463583882	pseudobulk	DESeq2	LRT	0.0960591113673091
128	KC_AB1	ppk29	2.22023857837763	0.524904842126482	0.994766463583882	pseudobulk	DESeq2	LRT	0.279919420946639
128	KC_AB1	ppk8	0.45121766623063	0.728400400720733	0.994766463583882	pseudobulk	DESeq2	LRT	0.137629823955158
128	KC_AB1	ppk9	-3.46197803745951	0.239982951034438	0.994766463583882	pseudobulk	DESeq2	LRT	0.619819610516178
128	KC_AB1	ppl	-0.01654623175958	0.959391208636857	0.994766463583882	pseudobulk	DESeq2	LRT	0.0180042655011356
128	KC_AB1	Ppn	0.949183608371002	0.80156895525114	0.994766463583882	pseudobulk	DESeq2	LRT	0.0960591113673091

128	KC_AB1	PPO2	-2.20969975456663	0.610836134188693	0.994766463583882	pseudobulk	DESeq2	LRT	0.214075280039667
128	KC_AB1	Ppox	-0.679649571882236	0.70757904011027	0.994766463583882	pseudobulk	DESeq2	LRT	0.150225040232736
128	KC_AB1	PPP1R15	0.129712381797171	0.732190565789614	0.994766463583882	pseudobulk	DESeq2	LRT	0.13537587126425
128	KC_AB1	PPP4R2r	-0.115315257745259	0.86780759723752	0.994766463583882	pseudobulk	DESeq2	LRT	0.0615765521511603
128	KC_AB1	Ppr-Y	-2.20969975456663	0.610836134188693	0.994766463583882	pseudobulk	DESeq2	LRT	0.214075280039667
128	KC_AB1	pps	-0.0353001751926636	0.955531837306392	0.994766463583882	pseudobulk	DESeq2	LRT	0.0197548381368221
128	KC_AB1	Ppt1	-2.79499059556516	0.38564607643653	0.994766463583882	pseudobulk	DESeq2	LRT	0.413811082791365
128	KC_AB1	Ppt2	0.0108474956783905	0.911972138188058	0.994766463583882	pseudobulk	DESeq2	LRT	0.040018429671604
128	KC_AB1	PpV	-0.422104641960166	0.637958824401276	0.994766463583882	pseudobulk	DESeq2	LRT	0.195207350922082
128	KC_AB1	PQBP1	0.390985497167617	0.754067957780671	0.994766463583882	pseudobulk	DESeq2	LRT	0.122589513070281
128	KC_AB1	pr	0.315782261166072	0.753467046084511	0.994766463583882	pseudobulk	DESeq2	LRT	0.122935737399277
128	KC_AB1	PR-Set7	0.731488762961974	0.261007487844488	0.994766463583882	pseudobulk	DESeq2	LRT	0.583347033340279
128	KC_AB1	PRAS40	0.365421076687116	0.684867139209893	0.994766463583882	pseudobulk	DESeq2	LRT	0.164393671287818
128	KC_AB1	PrBP	0.00249563065361353	0.880407811215598	0.994766463583882	pseudobulk	DESeq2	LRT	0.0553161129237987
128	KC_AB1	prd1	-0.219126049266535	0.598297025435365	0.994766463583882	pseudobulk	DESeq2	LRT	0.223083156342674
128	KC_AB1	pre-lola-G	0.166061709543563	0.875536484819394	0.994766463583882	pseudobulk	DESeq2	LRT	0.0577257515501677
128	KC_AB1	pre-mod(mdg4)-B	1.3156895206302	0.59850742835191	0.994766463583882	pseudobulk	DESeq2	LRT	0.222930454994874
128	KC_AB1	pre-mod(mdg4)-E	-2.20969975456663	0.610836134188693	0.994766463583882	pseudobulk	DESeq2	LRT	0.214075280039667
128	KC_AB1	pre-mod(mdg4)-L	0.949183608371002	0.80156895525114	0.994766463583882	pseudobulk	DESeq2	LRT	0.0960591113673091
128	KC_AB1	pre-rRNA:CR45845	-0.0295508305314189	0.850510799069338	0.994766463583882	pseudobulk	DESeq2	LRT	0.070320167711085
128	KC_AB1	pre-rRNA:CR45856	2.12231919719301	0.261458487497772	0.994766463583882	pseudobulk	DESeq2	LRT	0.582597255484285
129	KC_AB1	prel	-0.408749095786347	0.465890326225728	0.994766463583882	pseudobulk	DESeq2	LRT	0.331716307174451
129	KC_AB1	Prestin	0.7865441484438	0.486398414644868	0.994766463583882	pseudobulk	DESeq2	LRT	0.313007849261859
129	KC_AB1	prg	-0.810527828107944	0.477502176461364	0.994766463583882	pseudobulk	DESeq2	LRT	0.321024644555614
129	KC_AB1	prim	-2.20969975456663	0.610836134188693	0.994766463583882	pseudobulk	DESeq2	LRT	0.214075280039667
129	KC_AB1	PRL-1	-0.0587585269077856	0.844094403467334	0.994766463583882	pseudobulk	DESeq2	LRT	0.0736089791892824
129	KC_AB1	prod	0.717943590571702	0.393030871678859	0.994766463583882	pseudobulk	DESeq2	LRT	0.405573335443399
129	KC_AB1	prominin-like	0.0358239210326547	0.890800066380295	0.994766463583882	pseudobulk	DESeq2	LRT	0.0502197592754101
129	KC_AB1	ProRS-m	0.47865508106661	0.724948634422197	0.994766463583882	pseudobulk	DESeq2	LRT	0.139692763880427
129	KC_AB1	pros	0.142012707048387	0.207136975955995	0.994766463583882	pseudobulk	DESeq2	LRT	0.683742368415714
129	KC_AB1	Prosalpha1	1.47424991200432	0.518427666092641	0.994766463583882	pseudobulk	DESeq2	LRT	0.285311830226264
129	KC_AB1	Prosalpha2	-0.317148636310302	0.596929069366387	0.994766463583882	pseudobulk	DESeq2	LRT	0.224077271237309
129	KC_AB1	Prosalpha3	-0.142901707273849	0.81886217924293	0.994766463583882	pseudobulk	DESeq2	LRT	0.0867891871663388
129	KC_AB1	Prosalpha4	0.265221073623392	0.709894777381721	0.994766463583882	pseudobulk	DESeq2	LRT	0.148806018871117
129	KC_AB1	Prosalpha5	-0.0810434424406203	0.89904049407493	0.994766463583882	pseudobulk	DESeq2	LRT	0.0462207465790865
129	KC_AB1	Prosalpha6	-0.0658612142329767	0.926128908214864	0.994766463583882	pseudobulk	DESeq2	LRT	0.0333285595059909
129	KC_AB1	Prosalpha7	0.370751616605159	0.581076120584105	0.994766463583882	pseudobulk	DESeq2	LRT	0.235766971595548
129	KC_AB1	Prosap	0.0369296645554367	0.847914946062432	0.994766463583882	pseudobulk	DESeq2	LRT	0.0716477094276344
129	KC_AB1	Prosbeta1	-0.265016577684305	0.626644011017482	0.994766463583882	pseudobulk	DESeq2	LRT	0.202979106628247
129	KC_AB1	Prosbeta2	0.383771411984314	0.610011893546483	0.994766463583882	pseudobulk	DESeq2	LRT	0.214661697364228
129	KC_AB1	Prosbeta3	-0.236296337193226	0.733807176700609	0.994766463583882	pseudobulk	DESeq2	LRT	0.134418045119957
129	KC_AB1	Prosbeta4	0.511984953228884	0.365345627127561	0.994766463583882	pseudobulk	DESeq2	LRT	0.437296086418493
129	KC_AB1	Prosbeta5	-0.00608117276301082	0.936698400272378	0.994766463583882	pseudobulk	DESeq2	LRT	0.0284002214732315
129	KC_AB1	Prosbeta6	0.0995016342742785	0.885367458409366	0.994766463583882	pseudobulk	DESeq2	LRT	0.0528764445132172
129	KC_AB1	Prosbeta7	-0.497955952112731	0.575886306483256	0.994766463583882	pseudobulk	DESeq2	LRT	0.239663248071003
129	KC_AB1	Prp18	-0.732775845037224	0.606903476410082	0.994766463583882	pseudobulk	DESeq2	LRT	0.216880374815693
129	KC_AB1	Prp19	0.357486815364926	0.757743349760693	0.994766463583882	pseudobulk	DESeq2	LRT	0.120477866474571
129	KC_AB1	Prp3	0.139446643833292	0.82110229845183	0.994766463583882	pseudobulk	DESeq2	LRT	0.0856027321796166
129	KC_AB1	Prp31	0.275527142464962	0.71563789546189	0.994766463583882	pseudobulk	DESeq2	LRT	0.145306670125022
129	KC_AB1	Prp38	1.35611051425079	0.268685082784388	0.994766463583882	pseudobulk	DESeq2	LRT	0.570756444635914
129	KC_AB1	Prp8	-0.371617642227204	0.610517478899339	0.994766463583882	pseudobulk	DESeq2	LRT	0.214301897843803
129	KC_AB1	Prpk	0.393100574649199	0.703562803571923	0.994766463583882	pseudobulk	DESeq2	LRT	0.152697129178304
129	KC_AB1	prt	-0.0275574981513784	0.84331996826943	0.994766463583882	pseudobulk	DESeq2	LRT	0.0740076162547111
129	KC_AB1	Prx2540-1	-1.64952313825113	0.817565084244375	0.994766463583882	pseudobulk	DESeq2	LRT	0.0874776642296574
129	KC_AB1	Prx3	-0.612972585245933	0.294315136806513	0.994766463583882	pseudobulk	DESeq2	LRT	0.531187401296614
129	KC_AB1	Prx6005	0.157837167007298	0.861606540806936	0.994766463583882	pseudobulk	DESeq2	LRT	0.0646910127908813

129	KC_AB1	PRY	-0.835152883801839	0.920434724857442	0.994766463583882	pseudobulk	DESeq2	LRT	0.0360070052473159
129	KC_AB1	ps	0.0173301685180258	0.886856127594292	0.994766463583882	pseudobulk	DESeq2	LRT	0.0521468289392445
129	KC_AB1	Psa	-0.102500442256924	0.881534614349977	0.994766463583882	pseudobulk	DESeq2	LRT	0.0547606300169036
129	KC_AB1	Psc	0.0545814295273525	0.928750157079325	0.994766463583882	pseudobulk	DESeq2	LRT	0.0321010997792247
129	KC_AB1	psd	-1.64952313825113	0.817565084244375	0.994766463583882	pseudobulk	DESeq2	LRT	0.0874776642296574
129	KC_AB1	Psf3	-0.504879824932936	0.692877612764512	0.994766463583882	pseudobulk	DESeq2	LRT	0.159343470720145
129	KC_AB1	PsGEF	0.0961603926458096	0.817664548827783	0.994766463583882	pseudobulk	DESeq2	LRT	0.0874248313811105
129	KC_AB1	Psi	-0.054020090980061	0.811428136924972	0.994766463583882	pseudobulk	DESeq2	LRT	0.0907499368592171
129	KC_AB1	psidin	-0.0190623328039083	0.896439967833805	0.994766463583882	pseudobulk	DESeq2	LRT	0.0474787886578154
129	KC_AB1	PSMG1	0.460976867815312	0.673847375942311	0.994766463583882	pseudobulk	DESeq2	LRT	0.171438458497344
129	KC_AB1	Psn	-0.476909458590183	0.532931454329889	0.994766463583882	pseudobulk	DESeq2	LRT	0.273328646359265
129	KC_AB1	psq	0.0492639360998763	0.795439499895396	0.994766463583882	pseudobulk	DESeq2	LRT	0.0993928466406241
129	KC_AB1	PSR	0.561635698520189	0.712293793864732	0.994766463583882	pseudobulk	DESeq2	LRT	0.14734083959765
129	KC_AB1	Pten	-0.155899296808931	0.775601032289308	0.994766463583882	pseudobulk	DESeq2	LRT	0.110361621566963
129	KC_AB1	Ptip	0.314500869590928	0.70919902368928	0.994766463583882	pseudobulk	DESeq2	LRT	0.149231870937237
129	KC_AB1	PTP-ER	-0.160410691899641	0.739532915784956	0.994766463583882	pseudobulk	DESeq2	LRT	0.131042491274068
129	KC_AB1	Ptp10D	1.1821590793593	0.719511699510136	0.994766463583882	pseudobulk	DESeq2	LRT	0.142962139890846
129	KC_AB1	Ptp36E	0.352621900224991	0.340632155948466	0.994766463583882	pseudobulk	DESeq2	LRT	0.467714356691099
129	KC_AB1	Ptp4E	0.894035407380622	0.168843861988658	0.994766463583882	pseudobulk	DESeq2	LRT	0.772514722727168
129	KC_AB1	Ptp61F	-0.702414134821273	0.36562816345327	0.994766463583882	pseudobulk	DESeq2	LRT	0.43696035899602
129	KC_AB1	Ptp69D	0.819839075083587	0.384529383686471	0.994766463583882	pseudobulk	DESeq2	LRT	0.415070468124424
129	KC_AB1	Ptpa	-0.203597703775156	0.771966067350518	0.994766463583882	pseudobulk	DESeq2	LRT	0.112401789154299
129	KC_AB1	Ptpmeg	-0.37192981953015	0.451692365076643	0.994766463583882	pseudobulk	DESeq2	LRT	0.345157250224762
129	KC_AB1	Ptpmeg2	-0.470750683133726	0.0254438895740465	0.994766463583882	pseudobulk	DESeq2	LRT	1.59441649792047
129	KC_AB1	PTPMT1	-1.19553818546818	0.217802064624609	0.994766463583882	pseudobulk	DESeq2	LRT	0.661938007726134
129	KC_AB1	Pu	0.385318137280654	0.735483230572424	0.994766463583882	pseudobulk	DESeq2	LRT	0.133427224979805
129	KC_AB1	puc	-0.671766062117259	0.14517467117291	0.994766463583882	pseudobulk	DESeq2	LRT	0.83810914898985
129	KC_AB1	puf	-0.148067004771709	0.812549647481712	0.994766463583882	pseudobulk	DESeq2	LRT	0.0901500937723279
129	KC_AB1	pum	-0.0241505895082182	0.843612180417986	0.994766463583882	pseudobulk	DESeq2	LRT	0.0738571583657785
129	KC_AB1	Pur-alpha	0.0457455154549278	0.801665617562248	0.994766463583882	pseudobulk	DESeq2	LRT	0.0960067423514643
129	KC_AB1	Pus1	0.923586470469746	0.578911799800899	0.994766463583882	pseudobulk	DESeq2	LRT	0.237387598237323
129	KC_AB1	Pus7	0.209185779762938	0.854790385864033	0.994766463583882	pseudobulk	DESeq2	LRT	0.068140371147275
129	KC_AB1	put	-0.88553424725925	0.0696903460707614	0.994766463583882	pseudobulk	DESeq2	LRT	1.15682737884
129	KC_AB1	Pvf3	0.165850049347175	0.64928429400802	0.994766463583882	pseudobulk	DESeq2	LRT	0.187565102453208
129	KC_AB1	PVRAP	0.120072169137614	0.661878061691138	0.994766463583882	pseudobulk	DESeq2	LRT	0.179222013600389
129	KC_AB1	px	0.496570623373258	0.179649931322782	0.994766463583882	pseudobulk	DESeq2	LRT	0.745572944482947
129	KC_AB1	pxb	0.0731708075182011	0.773113055382149	0.994766463583882	pseudobulk	DESeq2	LRT	0.111756992836595
129	KC_AB1	Pxn	0.373061116175611	0.230513794418067	0.994766463583882	pseudobulk	DESeq2	LRT	0.637303080424476
129	KC_AB1	pyd	0.747623572541386	0.233365680988605	0.994766463583882	pseudobulk	DESeq2	LRT	0.631963011414726
129	KC_AB1	pyd3	-1.64952313825113	0.817565084244375	0.994766463583882	pseudobulk	DESeq2	LRT	0.0874776642296574
129	KC_AB1	pygo	-0.138552681361328	0.778624912509855	0.994766463583882	pseudobulk	DESeq2	LRT	0.108671704917275
129	KC_AB1	PyK	-0.163494498128782	0.53172238773743	0.994766463583882	pseudobulk	DESeq2	LRT	0.274315053685937
129	KC_AB1	Pym	0.639361431495929	0.575907178073145	0.994766463583882	pseudobulk	DESeq2	LRT	0.239647508415518
129	KC_AB1	Pyroxd1	0.410616910826838	0.711880835996428	0.994766463583882	pseudobulk	DESeq2	LRT	0.147592698217694
129	KC_AB1	pzg	0.434932572345315	0.566016938095591	0.994766463583882	pseudobulk	DESeq2	LRT	0.247170572325188
129	KC_AB1	Pzl	0.459285232552808	0.703912077573798	0.994766463583882	pseudobulk	DESeq2	LRT	0.152481583200422
129	KC_AB1	QIL1	-0.549712089861851	0.263017739373262	0.994766463583882	pseudobulk	DESeq2	LRT	0.580014959296895
129	KC_AB1	qjt	0.949183608371002	0.80156895525114	0.994766463583882	pseudobulk	DESeq2	LRT	0.0960591113673091
129	KC_AB1	qkr54B	-0.284602505349153	0.487093888102562	0.994766463583882	pseudobulk	DESeq2	LRT	0.312387319781513
129	KC_AB1	qkr58E-1	0.0618001078473242	0.820519589812151	0.994766463583882	pseudobulk	DESeq2	LRT	0.0859110457556079
129	KC_AB1	qkr58E-2	0.319466871554448	0.599908357906337	0.994766463583882	pseudobulk	DESeq2	LRT	0.221915087441913
129	KC_AB1	qkr58E-3	0.17556304056782	0.482602731783226	0.994766463583882	pseudobulk	DESeq2	LRT	0.316410224086945
129	KC_AB1	qless	-0.195743907485977	0.797238150752332	0.994766463583882	pseudobulk	DESeq2	LRT	0.0984119268998285
129	KC_AB1	qm	-0.138774556856987	0.449147089033187	0.994766463583882	pseudobulk	DESeq2	LRT	0.347611410679418
129	KC_AB1	Qsox1	-0.670217201539292	0.458837436405998	0.994766463583882	pseudobulk	DESeq2	LRT	0.338341155371316
129	KC_AB1	Qsox3	0.949183608371002	0.80156895525114	0.994766463583882	pseudobulk	DESeq2	LRT	0.0960591113673091

129	KC_AB1	qtc	-0.174383192312272	0.608116732930563	0.994766463583882	pseudobulk	DESeq2	LRT	0.216013046382709
129	KC_AB1	Qtzl	0.0666173885215965	0.892621947546966	0.994766463583882	pseudobulk	DESeq2	LRT	0.0493324390489778
129	KC_AB1	qvr	-0.18499236341152	0.403886954377431	0.994766463583882	pseudobulk	DESeq2	LRT	0.393740174394742
129	KC_AB1	r	0.949183608371002	0.80156895525114	0.994766463583882	pseudobulk	DESeq2	LRT	0.0960591113673091
129	KC_AB1	r-l	0.949183608371002	0.80156895525114	0.994766463583882	pseudobulk	DESeq2	LRT	0.0960591113673091
129	KC_AB1	r2d2	0.0362583469451901	0.949834780228413	0.994766463583882	pseudobulk	DESeq2	LRT	0.0223519318431481
129	KC_AB1	Rab1	-0.320607210801572	0.277612836271639	0.994766463583882	pseudobulk	DESeq2	LRT	0.556560456830089
129	KC_AB1	Rab11	-0.120318297586893	0.702324230933287	0.994766463583882	pseudobulk	DESeq2	LRT	0.153462347703488
129	KC_AB1	Rab14	-0.145931956414489	0.650167307475569	0.994766463583882	pseudobulk	DESeq2	LRT	0.186974872028325
130	KC_AB1	Rab18	0.14180356269689	0.770998594037189	0.994766463583882	pseudobulk	DESeq2	LRT	0.112946413910712
130	KC_AB1	Rab19	-1.24586031603935	0.247427449035037	0.994766463583882	pseudobulk	DESeq2	LRT	0.606552122398154
130	KC_AB1	Rab2	-0.157898233865186	0.511375154935592	0.994766463583882	pseudobulk	DESeq2	LRT	0.291260375911732
130	KC_AB1	Rab21	-0.292015541562593	0.572491694563811	0.994766463583882	pseudobulk	DESeq2	LRT	0.242230809479377
130	KC_AB1	Rab23	2.32297872599937	0.365596980899545	0.994766463583882	pseudobulk	DESeq2	LRT	0.436997399327927
130	KC_AB1	Rab26	-0.0200380242449655	0.885606574804514	0.994766463583882	pseudobulk	DESeq2	LRT	0.0527591678850692
130	KC_AB1	Rab27	-0.182185130888453	0.364747563573637	0.994766463583882	pseudobulk	DESeq2	LRT	0.438007600407942
130	KC_AB1	Rab3	-0.187720703014711	0.419163285467426	0.994766463583882	pseudobulk	DESeq2	LRT	0.377616764233276
130	KC_AB1	Rab3-GAP	-1.13492380571364	0.366193755491834	0.994766463583882	pseudobulk	DESeq2	LRT	0.436289065764965
130	KC_AB1	Rab3-GEF	-0.0170329714322229	0.881693684964748	0.994766463583882	pseudobulk	DESeq2	LRT	0.0546822697836406
130	KC_AB1	Rab30	-0.129807474904387	0.821050853351062	0.994766463583882	pseudobulk	DESeq2	LRT	0.0856299431901942
130	KC_AB1	Rab35	-0.0550878053756626	0.856675691292509	0.994766463583882	pseudobulk	DESeq2	LRT	0.0671835563013322
130	KC_AB1	Rab39	-0.330934652401818	0.589930337063015	0.994766463583882	pseudobulk	DESeq2	LRT	0.229199269739833
130	KC_AB1	Rab4	-0.348308109180078	0.377473670338668	0.994766463583882	pseudobulk	DESeq2	LRT	0.423113336022661
130	KC_AB1	Rab40	0.0778035568520684	0.906995466357714	0.994766463583882	pseudobulk	DESeq2	LRT	0.0423948837676321
130	KC_AB1	Rab5	-0.13318404741051	0.610814432746307	0.994766463583882	pseudobulk	DESeq2	LRT	0.214090709683681
130	KC_AB1	Rab6	-0.466490715376734	0.476997201133852	0.994766463583882	pseudobulk	DESeq2	LRT	0.321484169252737
130	KC_AB1	Rab7	0.0508418690767471	0.904027872097623	0.994766463583882	pseudobulk	DESeq2	LRT	0.0438181795781821
130	KC_AB1	Rab8	-0.15237461289551	0.728708147701106	0.994766463583882	pseudobulk	DESeq2	LRT	0.137446374592335
130	KC_AB1	Rab9	1.0233103206859	0.557203517140596	0.994766463583882	pseudobulk	DESeq2	LRT	0.253986150916538
130	KC_AB1	Rabex-5	-0.495722336557288	0.649963923821033	0.994766463583882	pseudobulk	DESeq2	LRT	0.187110748157548
130	KC_AB1	RabX1	-0.746937877520315	0.432772462599537	0.994766463583882	pseudobulk	DESeq2	LRT	0.363740381269201
130	KC_AB1	RabX6	0.372333697898814	0.77212709586564	0.994766463583882	pseudobulk	DESeq2	LRT	0.112311206801542
130	KC_AB1	Rac1	0.161294096847549	0.646312085948912	0.994766463583882	pseudobulk	DESeq2	LRT	0.189557722753106
130	KC_AB1	Rack1	-0.0121800129697508	0.943242100550676	0.994766463583882	pseudobulk	DESeq2	LRT	0.0253768232344326
130	KC_AB1	rad	0.109063486072548	0.421011055381232	0.994766463583882	pseudobulk	DESeq2	LRT	0.375706499822229
130	KC_AB1	Rad17	0.521034936421978	0.709706559812326	0.994766463583882	pseudobulk	DESeq2	LRT	0.148921180571974
130	KC_AB1	Rad23	-0.206735732290321	0.564228089364035	0.994766463583882	pseudobulk	DESeq2	LRT	0.248545296854636
130	KC_AB1	rad50	1.0466358997027	0.491156173962099	0.994766463583882	pseudobulk	DESeq2	LRT	0.308780392387201
130	KC_AB1	Rad60	-0.0681667983360919	0.887887295084049	0.994766463583882	pseudobulk	DESeq2	LRT	0.051642158353749
130	KC_AB1	Rad9	-0.548887546079202	0.814781693583783	0.994766463583882	pseudobulk	DESeq2	LRT	0.0889587372379754
130	KC_AB1	Rae1	0.613254183744571	0.660945102566936	0.994766463583882	pseudobulk	DESeq2	LRT	0.17983461107768
130	KC_AB1	Raf	-0.2185282393638	0.765595825492574	0.994766463583882	pseudobulk	DESeq2	LRT	0.116000443269983
130	KC_AB1	RAF2	-0.466889594475018	0.740811508504711	0.994766463583882	pseudobulk	DESeq2	LRT	0.130292279510155
130	KC_AB1	RagA-B	-1.05458634500605	0.448842576871996	0.994766463583882	pseudobulk	DESeq2	LRT	0.347905952947259
130	KC_AB1	RagC-D	-0.119624050813778	0.865936286293562	0.994766463583882	pseudobulk	DESeq2	LRT	0.0625140612494786
130	KC_AB1	Rala	0.48299073090114	0.327602217695935	0.994766463583882	pseudobulk	DESeq2	LRT	0.484653166954821
130	KC_AB1	Ran	-0.169709580195329	0.673005375190337	0.994766463583882	pseudobulk	DESeq2	LRT	0.171981467118995
130	KC_AB1	Ranbp16	-0.144557326503115	0.758416431708856	0.994766463583882	pseudobulk	DESeq2	LRT	0.120092266224872
130	KC_AB1	Ranbp21	0.575114571237417	0.757941253858868	0.994766463583882	pseudobulk	DESeq2	LRT	0.120364454148462
130	KC_AB1	RanBP3	-0.324365539241911	0.70538815998257	0.994766463583882	pseudobulk	DESeq2	LRT	0.151571834287738
130	KC_AB1	Ranbp9	0.69750158688829	0.750018748510497	0.994766463583882	pseudobulk	DESeq2	LRT	0.124927880244456
130	KC_AB1	RanBPM	0.0610208554471112	0.893293021961483	0.994766463583882	pseudobulk	DESeq2	LRT	0.0490060585282205
130	KC_AB1	RanGAP	-0.356212784647529	0.928203443271547	0.994766463583882	pseudobulk	DESeq2	LRT	0.0323568248514225
130	KC_AB1	ranshi	-1.86252746130758	0.377218729935813	0.994766463583882	pseudobulk	DESeq2	LRT	0.423406751497218
130	KC_AB1	Rap2l	-0.287515923222657	0.51240508720383	0.994766463583882	pseudobulk	DESeq2	LRT	0.290386567191238
130	KC_AB1	RapGAP1	-0.585849952131524	0.636108585284735	0.994766463583882	pseudobulk	DESeq2	LRT	0.196468742897568

130	KC_AB1	raptor	0.232670981170626	0.638693721786616	0.994766463583882	pseudobulk	DESeq2	LRT	0.194707352836582
130	KC_AB1	ras	0.49176491504354	0.471188528000187	0.994766463583882	pseudobulk	DESeq2	LRT	0.326805291834846
130	KC_AB1	Ras64B	0.174750845617111	0.858753149136421	0.994766463583882	pseudobulk	DESeq2	LRT	0.0661316573267887
130	KC_AB1	Ras85D	-0.157102132692026	0.769366824017588	0.994766463583882	pseudobulk	DESeq2	LRT	0.113866544903049
130	KC_AB1	RasGAP1	0.34732873879024	0.504608509409009	0.994766463583882	pseudobulk	DESeq2	LRT	0.297045430080401
130	KC_AB1	raskol	-0.0913850579715411	0.769274122237615	0.994766463583882	pseudobulk	DESeq2	LRT	0.1139188766334
130	KC_AB1	Rassf	-0.266523795249259	0.787391093949676	0.994766463583882	pseudobulk	DESeq2	LRT	0.103809501751003
130	KC_AB1	RASSF8	0.103951286370824	0.819915764169595	0.994766463583882	pseudobulk	DESeq2	LRT	0.0862307635132155
130	KC_AB1	Rat1	0.833441706499796	0.620042047278132	0.994766463583882	pseudobulk	DESeq2	LRT	0.207578858434506
130	KC_AB1	Ravus	0.0421916139963689	0.949494167572222	0.994766463583882	pseudobulk	DESeq2	LRT	0.0225076986457415
130	KC_AB1	raw	-0.488991227206444	0.219145444842585	0.994766463583882	pseudobulk	DESeq2	LRT	0.659267552161698
130	KC_AB1	rb	-0.334566112194722	0.646912729579161	0.994766463583882	pseudobulk	DESeq2	LRT	0.189154302976695
130	KC_AB1	Rb97D	0.3414752384878	0.452160494468006	0.994766463583882	pseudobulk	DESeq2	LRT	0.34470738490096
130	KC_AB1	Rbbp5	1.91201168863931	0.136522487233685	0.994766463583882	pseudobulk	DESeq2	LRT	0.864795808131276
130	KC_AB1	Rbcn-3A	0.187244553274307	0.681658891395694	0.994766463583882	pseudobulk	DESeq2	LRT	0.166432896082713
130	KC_AB1	Rbcn-3B	-0.342671144213612	0.465872495950087	0.994766463583882	pseudobulk	DESeq2	LRT	0.331732928550918
130	KC_AB1	Rbf	0.311958779944412	0.740146205727161	0.994766463583882	pseudobulk	DESeq2	LRT	0.130682482878888
130	KC_AB1	Rbf2	1.80633728195307	0.61161102402172	0.994766463583882	pseudobulk	DESeq2	LRT	0.213524695220716
130	KC_AB1	Rbfox1	-0.0649822381029603	0.614610121333204	0.994766463583882	pseudobulk	DESeq2	LRT	0.211400292106054
130	KC_AB1	Rbm13	0.986575688715885	0.655962090734864	0.994766463583882	pseudobulk	DESeq2	LRT	0.183121258582249
130	KC_AB1	Rbp	0.0654218515042222	0.652918222485851	0.994766463583882	pseudobulk	DESeq2	LRT	0.185141210374531
130	KC_AB1	Rbp1	-0.0339133194788932	0.911759025948991	0.994766463583882	pseudobulk	DESeq2	LRT	0.0401199286991953
130	KC_AB1	Rbp1-like	-0.0887530808709713	0.514681309811564	0.994766463583882	pseudobulk	DESeq2	LRT	0.288461602476629
130	KC_AB1	Rbp6	0.708632460267942	0.638441165890324	0.994766463583882	pseudobulk	DESeq2	LRT	0.194879117987811
130	KC_AB1	Rbp9	-0.0147761014966599	0.816819064558503	0.994766463583882	pseudobulk	DESeq2	LRT	0.0878741343688276
130	KC_AB1	Rbpn-5	0.542076655467575	0.529916457093833	0.994766463583882	pseudobulk	DESeq2	LRT	0.275792592819966
130	KC_AB1	Rbsn-5	0.173955874080729	0.840283367465457	0.994766463583882	pseudobulk	DESeq2	LRT	0.0755742327788707
130	KC_AB1	Rca1	0.570772054671909	0.593312773782636	0.994766463583882	pseudobulk	DESeq2	LRT	0.226716301378385
130	KC_AB1	Rcc1	0.283081330473557	0.696248809364	0.994766463583882	pseudobulk	DESeq2	LRT	0.157235534492666
130	KC_AB1	Rcd-1	-0.0429116208483179	0.911372876968804	0.994766463583882	pseudobulk	DESeq2	LRT	0.0403039004387358
130	KC_AB1	Rcd1	0.111649448312952	0.819367502628188	0.994766463583882	pseudobulk	DESeq2	LRT	0.086521264826531
130	KC_AB1	Rcd2	1.1821590793593	0.719511699510136	0.994766463583882	pseudobulk	DESeq2	LRT	0.142962139890846
130	KC_AB1	Rcd4	-0.605361217931232	0.459251704686052	0.994766463583882	pseudobulk	DESeq2	LRT	0.337949222971263
130	KC_AB1	Rcd5	-0.572755907192249	0.457172986629591	0.994766463583882	pseudobulk	DESeq2	LRT	0.339919439047319
130	KC_AB1	Rchy1	-1.64952313825113	0.817565084244375	0.994766463583882	pseudobulk	DESeq2	LRT	0.0874776642296574
130	KC_AB1	Rcp	-0.255627431645633	0.803048644774244	0.994766463583882	pseudobulk	DESeq2	LRT	0.0952581464807777
130	KC_AB1	rdgA	1.35638125428944	0.00991154256949599	0.994766463583882	pseudobulk	DESeq2	LRT	2.00385874942133
130	KC_AB1	rdgB	-0.0153125840509856	0.927829439154218	0.994766463583882	pseudobulk	DESeq2	LRT	0.0325318518437793
130	KC_AB1	rdgBbeta	0.0751742927141197	0.860263993966738	0.994766463583882	pseudobulk	DESeq2	LRT	0.0653682539548062
130	KC_AB1	rdgC	-0.0975719794757681	0.856197772967964	0.994766463583882	pseudobulk	DESeq2	LRT	0.0674259061314482
130	KC_AB1	Rdh	-0.0437879940972821	0.864227683370896	0.994766463583882	pseudobulk	DESeq2	LRT	0.0633718262651736
130	KC_AB1	Rdl	-0.0144172618355005	0.890740441864485	0.994766463583882	pseudobulk	DESeq2	LRT	0.0502488291709214
130	KC_AB1	rdo	-0.647856137067844	0.762139005512724	0.994766463583882	pseudobulk	DESeq2	LRT	0.117965811046941
130	KC_AB1	rdog	-0.423537318420556	0.770405867494321	0.994766463583882	pseudobulk	DESeq2	LRT	0.113280418237231
130	KC_AB1	rdx	-0.0757519469504642	0.746333078579128	0.994766463583882	pseudobulk	DESeq2	LRT	0.127067309383193
130	KC_AB1	rec	0.307528272827057	0.853070973989708	0.994766463583882	pseudobulk	DESeq2	LRT	0.0690148347998648
130	KC_AB1	RecQ5	0.565415469803353	0.650079306558096	0.994766463583882	pseudobulk	DESeq2	LRT	0.187033658280882
130	KC_AB1	red	-0.105197730250928	0.852955141979696	0.994766463583882	pseudobulk	DESeq2	LRT	0.0690738083435662
130	KC_AB1	ReepA	0.0984289275678541	0.841469209004536	0.994766463583882	pseudobulk	DESeq2	LRT	0.0749617710774377
130	KC_AB1	ReepB	-0.0830385173567298	0.841905782185383	0.994766463583882	pseudobulk	DESeq2	LRT	0.0747365077479577
130	KC_AB1	Reepl1	-1.64952313825113	0.817565084244375	0.994766463583882	pseudobulk	DESeq2	LRT	0.0874776642296574
130	KC_AB1	ref(2)P	0.0325331174051692	0.830571858214922	0.994766463583882	pseudobulk	DESeq2	LRT	0.080622787925178
130	KC_AB1	Ref1	0.116010923053907	0.732894943969323	0.994766463583882	pseudobulk	DESeq2	LRT	0.134958274368843
131	KC_AB1	REG	-0.233148001985776	0.662848375608667	0.994766463583882	pseudobulk	DESeq2	LRT	0.178585803672675
131	KC_AB1	regucalcin	-1.69608958436761	0.458437420803486	0.994766463583882	pseudobulk	DESeq2	LRT	0.338719939502361
131	KC_AB1	Rel	-0.313501731982744	0.847735509510735	0.994766463583882	pseudobulk	DESeq2	LRT	0.0717396249569093

131	KC_AB1	Rep	0.338756512374813	0.737439907685277	0.994766463583882	pseudobulk	DESeq2	LRT	0.13227336358571
131	KC_AB1	Reph	-0.107264092063494	0.622512266577058	0.994766463583882	pseudobulk	DESeq2	LRT	0.205852086394088
131	KC_AB1	Reps	0.215910607856214	0.74777016713496	0.994766463583882	pseudobulk	DESeq2	LRT	0.126231865348037
131	KC_AB1	rept	-0.0367632515634312	0.937038348184011	0.994766463583882	pseudobulk	DESeq2	LRT	0.0282426352980139
131	KC_AB1	REPTOR	-0.303113169165192	0.249719040445592	0.994766463583882	pseudobulk	DESeq2	LRT	0.602548342529732
131	KC_AB1	REPTOR-BP	-0.102768813769936	0.757758137290388	0.994766463583882	pseudobulk	DESeq2	LRT	0.120469391204038
131	KC_AB1	retm	0.244529867204446	0.629907053707913	0.994766463583882	pseudobulk	DESeq2	LRT	0.200723528387274
131	KC_AB1	retn	-0.177662638099235	0.831100779256617	0.994766463583882	pseudobulk	DESeq2	LRT	0.080346310486303
131	KC_AB1	Rev1	1.1609811941338	0.567957038275369	0.994766463583882	pseudobulk	DESeq2	LRT	0.245684514193195
131	KC_AB1	Rev7	0.746548202350824	0.68395571814191	0.994766463583882	pseudobulk	DESeq2	LRT	0.164972015223098
131	KC_AB1	Rexo5	0.0322200586877653	0.868935730625668	0.994766463583882	pseudobulk	DESeq2	LRT	0.0610123442266248
131	KC_AB1	RfC3	-0.464617710384112	0.779488218659824	0.994766463583882	pseudobulk	DESeq2	LRT	0.108190444438827
131	KC_AB1	RfC38	0.437774168891516	0.8147450855668	0.994766463583882	pseudobulk	DESeq2	LRT	0.0889782504610473
131	KC_AB1	RfC4	0.0983090179763487	0.904228590750956	0.994766463583882	pseudobulk	DESeq2	LRT	0.043721765152788
131	KC_AB1	RFeSP	0.109147562602399	0.67164460757058	0.994766463583882	pseudobulk	DESeq2	LRT	0.172860467718008
131	KC_AB1	Rfx	-0.345491814499488	0.748135665390884	0.994766463583882	pseudobulk	DESeq2	LRT	0.126019640921338
131	KC_AB1	rg	0.25185191496266	0.837311556160994	0.994766463583882	pseudobulk	DESeq2	LRT	0.077112914832172
131	KC_AB1	Rga	-0.527863518026126	0.200984741417792	0.994766463583882	pseudobulk	DESeq2	LRT	0.696836912577499
131	KC_AB1	Rgk1	-0.150591070910957	0.278367527229317	0.994766463583882	pseudobulk	DESeq2	LRT	0.555381428428073
131	KC_AB1	Rgk2	0.151729651870779	0.505609087321398	0.994766463583882	pseudobulk	DESeq2	LRT	0.296185129072839
131	KC_AB1	Rgk3	-0.564232804885556	0.0292711466907484	0.994766463583882	pseudobulk	DESeq2	LRT	1.53356026384192
131	KC_AB1	Rgl	-0.24014425975538	0.493735151103664	0.994766463583882	pseudobulk	DESeq2	LRT	0.306505952409965
131	KC_AB1	rgn	-0.161267244461627	0.415155269302832	0.994766463583882	pseudobulk	DESeq2	LRT	0.381789445481015
131	KC_AB1	rgr	-0.285057921432903	0.803684499376314	0.994766463583882	pseudobulk	DESeq2	LRT	0.0949144078078145
131	KC_AB1	Rh5	-0.835152883801839	0.920434724857442	0.994766463583882	pseudobulk	DESeq2	LRT	0.0360070052473159
131	KC_AB1	Rh7	2.67764418499683	0.378704357417491	0.994766463583882	pseudobulk	DESeq2	LRT	0.421699697806334
131	KC_AB1	Rhau	-1.07839387776296	0.528054130523119	0.994766463583882	pseudobulk	DESeq2	LRT	0.277321555908373
131	KC_AB1	rhea	-0.165068653264393	0.651499818637345	0.994766463583882	pseudobulk	DESeq2	LRT	0.186085700849035
131	KC_AB1	Rheb	0.213699328989753	0.705130186377931	0.994766463583882	pseudobulk	DESeq2	LRT	0.151730692929329
131	KC_AB1	rho-4	0.108099221407376	0.864646353744106	0.994766463583882	pseudobulk	DESeq2	LRT	0.0631614856244185
131	KC_AB1	rho-5	-0.341899240802018	0.541696487844806	0.994766463583882	pseudobulk	DESeq2	LRT	0.266243980204869
131	KC_AB1	rho-7	0.0733395698239346	0.933136289907533	0.994766463583882	pseudobulk	DESeq2	LRT	0.0300549204206446
131	KC_AB1	Rho1	0.0086953684163087	0.910249020928674	0.994766463583882	pseudobulk	DESeq2	LRT	0.0408397795244807
131	KC_AB1	RhoBTB	-0.302582236855341	0.494615177634555	0.994766463583882	pseudobulk	DESeq2	LRT	0.3057325611211
131	KC_AB1	RhoGAP100F	-0.146086537398328	0.525093070905936	0.994766463583882	pseudobulk	DESeq2	LRT	0.279763712596911
131	KC_AB1	RhoGAP102A	-0.247531724692478	0.561110727622974	0.994766463583882	pseudobulk	DESeq2	LRT	0.250951428136812
131	KC_AB1	RhoGAP16F	-0.100020773079759	0.902387886558847	0.994766463583882	pseudobulk	DESeq2	LRT	0.0446067431878153
131	KC_AB1	RhoGAP18B	-2.20969975456663	0.610836134188693	0.994766463583882	pseudobulk	DESeq2	LRT	0.214075280039667
131	KC_AB1	RhoGAP19D	0.293893021930184	0.26186904028003	0.994766463583882	pseudobulk	DESeq2	LRT	0.581915843424041
131	KC_AB1	RhoGAP1A	-0.0520182000493539	0.873334202102128	0.994766463583882	pseudobulk	DESeq2	LRT	0.058819531375521
131	KC_AB1	RhoGAP5A	0.00538234121474367	0.859753840920192	0.994766463583882	pseudobulk	DESeq2	LRT	0.065625875306218
131	KC_AB1	RhoGAP68F	-0.201662713970732	0.729174502482888	0.994766463583882	pseudobulk	DESeq2	LRT	0.137168526007993
131	KC_AB1	RhoGAP71E	0.0502175679525461	0.805162523675431	0.994766463583882	pseudobulk	DESeq2	LRT	0.0941164475684481
131	KC_AB1	RhoGAP92B	0.430839546285194	0.591633705551654	0.994766463583882	pseudobulk	DESeq2	LRT	0.227947092081982
131	KC_AB1	RhoGAP93B	-0.0165998688410711	0.948632434613172	0.994766463583882	pseudobulk	DESeq2	LRT	0.0229020305035539
131	KC_AB1	RhoGAPp190	-0.00383434253973627	0.943904722523474	0.994766463583882	pseudobulk	DESeq2	LRT	0.0250718410521437
131	KC_AB1	RhoGDI	0.25889889657863	0.291489755192615	0.994766463583882	pseudobulk	DESeq2	LRT	0.535376704512179
131	KC_AB1	RhoGEF2	-0.513944431263765	0.643107491002432	0.994766463583882	pseudobulk	DESeq2	LRT	0.191716431667313
131	KC_AB1	RhoGEF3	0.0633483199482686	0.864591024789717	0.994766463583882	pseudobulk	DESeq2	LRT	0.0631892771351583
131	KC_AB1	RhoGEF64C	1.1821590793593	0.719511699510136	0.994766463583882	pseudobulk	DESeq2	LRT	0.142962139890846
131	KC_AB1	RhoU	-1.90151065290894	0.121018846931932	0.994766463583882	pseudobulk	DESeq2	LRT	0.917146989343476
131	KC_AB1	Rhp	0.266691964425607	0.800235713438596	0.994766463583882	pseudobulk	DESeq2	LRT	0.0967820705486134
131	KC_AB1	rib	-1.64952313825113	0.817565084244375	0.994766463583882	pseudobulk	DESeq2	LRT	0.0874776642296574
131	KC_AB1	Ric	0.0567053858671341	0.743142534422152	0.994766463583882	pseudobulk	DESeq2	LRT	0.128927880753204
131	KC_AB1	RIC-3	0.585665646528323	0.135961923213216	0.994766463583882	pseudobulk	DESeq2	LRT	0.866582700848468
131	KC_AB1	ric8a	-1.14261984314582	0.270837601896687	0.994766463583882	pseudobulk	DESeq2	LRT	0.567291040272806

131	KC_AB1	Rich	0.235380863421296	0.884419527438558	0.994766463583882	pseudobulk	DESeq2	LRT	0.0533416770327323
131	KC_AB1	rictor	-0.134052656284866	0.895111036690471	0.994766463583882	pseudobulk	DESeq2	LRT	0.0481230880015428
131	KC_AB1	Rif1	0.596808856322697	0.822761281772702	0.994766463583882	pseudobulk	DESeq2	LRT	0.0847261539098712
131	KC_AB1	Rift	-0.270602055661034	0.807870523746295	0.994766463583882	pseudobulk	DESeq2	LRT	0.0926582374039999
131	KC_AB1	rig	0.30061731945614	0.791789228539443	0.994766463583882	pseudobulk	DESeq2	LRT	0.10139041066376
131	KC_AB1	Rilpl	0.34966448024609	0.531212712256491	0.994766463583882	pseudobulk	DESeq2	LRT	0.274731540573798
131	KC_AB1	Rim	-0.0306943415015637	0.793746726914203	0.994766463583882	pseudobulk	DESeq2	LRT	0.100318052548511
131	KC_AB1	Rim2	-0.163561671054452	0.715554403210951	0.994766463583882	pseudobulk	DESeq2	LRT	0.145357341476369
131	KC_AB1	rin	-0.21432299332807	0.389617422742254	0.994766463583882	pseudobulk	DESeq2	LRT	0.409361630767657
131	KC_AB1	ringer	-0.00080808548118995	0.937311498328117	0.994766463583882	pseudobulk	DESeq2	LRT	0.0281160552986858
131	KC_AB1	Rint1	0.303502499879008	0.875810039613479	0.994766463583882	pseudobulk	DESeq2	LRT	0.0575900806970518
131	KC_AB1	RIOK1	1.17653862391274	0.460865504298629	0.994766463583882	pseudobulk	DESeq2	LRT	0.336425797524024
131	KC_AB1	RIOK2	0.0239693664280207	0.959992866220118	0.994766463583882	pseudobulk	DESeq2	LRT	0.0177319942237122
131	KC_AB1	Rip11	-0.0394483678359072	0.951934133076966	0.994766463583882	pseudobulk	DESeq2	LRT	0.0213931005975027
131	KC_AB1	Ripalpha	-0.542825098418351	0.380297299436754	0.994766463583882	pseudobulk	DESeq2	LRT	0.419876758584947
131	KC_AB1	rk	0.328348516004836	0.231416662283572	0.994766463583882	pseudobulk	DESeq2	LRT	0.635605374523023
131	KC_AB1	rl	-0.0469162100444225	0.825565650199293	0.994766463583882	pseudobulk	DESeq2	LRT	0.0832483852895952
131	KC_AB1	Rlb1	0.83015431614211	0.45922483150193	0.994766463583882	pseudobulk	DESeq2	LRT	0.337974636523202
131	KC_AB1	Rlip	0.019797285808491	0.90694099204765	0.994766463583882	pseudobulk	DESeq2	LRT	0.0424209683552853
131	KC_AB1	RluA-2	1.31119132126124	0.173800333250372	0.994766463583882	pseudobulk	DESeq2	LRT	0.759949395156287
131	KC_AB1	Rm62	-0.046602346389079	0.802906268655545	0.994766463583882	pseudobulk	DESeq2	LRT	0.0953351513360827
131	KC_AB1	Rme-8	0.543612089577292	0.575507008207959	0.994766463583882	pseudobulk	DESeq2	LRT	0.239949383402542
131	KC_AB1	rn	1.02388942995419	0.598311846205213	0.994766463583882	pseudobulk	DESeq2	LRT	0.223072398310179
131	KC_AB1	RN-tre	0.255325723776651	0.649163016844344	0.994766463583882	pseudobulk	DESeq2	LRT	0.187646230124202
131	KC_AB1	RNASEK	0.169800419758556	0.398269658681135	0.994766463583882	pseudobulk	DESeq2	LRT	0.399822778123128
131	KC_AB1	RNaseMRP:RNA	0.244551080987866	0.76131262500119	0.994766463583882	pseudobulk	DESeq2	LRT	0.118436968153768
131	KC_AB1	RNaseP:RNA	0.40411895759739	0.864643228521197	0.994766463583882	pseudobulk	DESeq2	LRT	0.0631630553638974
131	KC_AB1	RNaseX25	-0.73502058200215	0.37453345113197	0.994766463583882	pseudobulk	DESeq2	LRT	0.426509387595364
131	KC_AB1	RNaseZ	-0.582353494399943	0.802766106531744	0.994766463583882	pseudobulk	DESeq2	LRT	0.0954109720801323
131	KC_AB1	Rnb	-0.906422515952715	0.461770720149678	0.994766463583882	pseudobulk	DESeq2	LRT	0.335573608189233
131	KC_AB1	Rnf146	0.380559392677029	0.644986118332935	0.994766463583882	pseudobulk	DESeq2	LRT	0.190449632335712
131	KC_AB1	rngo	-1.34647188719569	0.274537866736896	0.994766463583882	pseudobulk	DESeq2	LRT	0.561397745273319
131	KC_AB1	rnh1	-0.177828292865577	0.845041074896038	0.994766463583882	pseudobulk	DESeq2	LRT	0.0731221807933293
131	KC_AB1	Rnmt	0.204332571832929	0.845992358363968	0.994766463583882	pseudobulk	DESeq2	LRT	0.072633559816
131	KC_AB1	rno	0.106223138762153	0.756529008659804	0.994766463583882	pseudobulk	DESeq2	LRT	0.121174414555084
131	KC_AB1	Rnp4F	0.246405332111797	0.715180138791363	0.994766463583882	pseudobulk	DESeq2	LRT	0.145584554800903
131	KC_AB1	RnpS1	0.765221846375828	0.156682130337877	0.994766463583882	pseudobulk	DESeq2	LRT	0.80498053217335
131	KC_AB1	ro	1.80633728195307	0.61161102402172	0.994766463583882	pseudobulk	DESeq2	LRT	0.213524695220716
131	KC_AB1	robl	0.401314623521027	0.276184250298008	0.994766463583882	pseudobulk	DESeq2	LRT	0.558801091158157
131	KC_AB1	robo1	-0.121616293294399	0.780566280471471	0.994766463583882	pseudobulk	DESeq2	LRT	0.107590213651073
131	KC_AB1	robo2	2.67764418499683	0.378704357417491	0.994766463583882	pseudobulk	DESeq2	LRT	0.421699697806334
132	KC_AB1	robo3	0.208615470155158	0.893577121399079	0.994766463583882	pseudobulk	DESeq2	LRT	0.0488679591598277
132	KC_AB1	Roc1a	-0.148845606955439	0.74107118275724	0.994766463583882	pseudobulk	DESeq2	LRT	0.130140074350077
132	KC_AB1	Roc2	0.102013627550191	0.809615110328573	0.994766463583882	pseudobulk	DESeq2	LRT	0.0917213949322895
132	KC_AB1	rod	1.80633728195307	0.61161102402172	0.994766463583882	pseudobulk	DESeq2	LRT	0.213524695220716
132	KC_AB1	Roe1	-0.337275959793466	0.623662382653723	0.994766463583882	pseudobulk	DESeq2	LRT	0.205050450431152
132	KC_AB1	rogdi	-0.1745610146563	0.781042662569127	0.994766463583882	pseudobulk	DESeq2	LRT	0.107325243187591
132	KC_AB1	roh	-0.00559601762624516	0.917183867256826	0.994766463583882	pseudobulk	DESeq2	LRT	0.0375435928681312
132	KC_AB1	Rok	0.407013007062743	0.468813104647917	0.994766463583882	pseudobulk	DESeq2	LRT	0.329000257065193
132	KC_AB1	Rop	0.209047988297043	0.479958464156669	0.994766463583882	pseudobulk	DESeq2	LRT	0.318796345057913
132	KC_AB1	roq	-0.0979892824176091	0.851733082475991	0.994766463583882	pseudobulk	DESeq2	LRT	0.0696964838361527
132	KC_AB1	Ror	0.70784207932579	0.501158286277084	0.994766463583882	pseudobulk	DESeq2	LRT	0.300025084512688
132	KC_AB1	row	-0.0220266327324629	0.926476294838087	0.994766463583882	pseudobulk	DESeq2	LRT	0.0331656882206546
132	KC_AB1	Rox8	0.261484931871221	0.531396580989003	0.994766463583882	pseudobulk	DESeq2	LRT	0.274581244171858
132	KC_AB1	RpA-70	0.160006883606011	0.830521530363502	0.994766463583882	pseudobulk	DESeq2	LRT	0.0806491044565843
132	KC_AB1	RPA2	0.205511634286069	0.845489900058704	0.994766463583882	pseudobulk	DESeq2	LRT	0.0728915759727697

132	KC_AB1	RPA3	-0.0677308467637377	0.864170251872603	0.994766463583882	pseudobulk	DESeq2	LRT	0.0634006878858647
132	KC_AB1	Rpb10	0.181697844393457	0.728261085597115	0.994766463583882	pseudobulk	DESeq2	LRT	0.137712895815279
132	KC_AB1	Rpb11	0.160426187625784	0.76708465856776	0.994766463583882	pseudobulk	DESeq2	LRT	0.115156702909056
132	KC_AB1	Rpb12	-0.088549938335425	0.819628423173957	0.994766463583882	pseudobulk	DESeq2	LRT	0.0863829894929373
132	KC_AB1	Rpb4	1.1821590793593	0.719511699510136	0.994766463583882	pseudobulk	DESeq2	LRT	0.142962139890846
132	KC_AB1	Rpb5	0.58459114477883	0.593733061468598	0.994766463583882	pseudobulk	DESeq2	LRT	0.226408767119754
132	KC_AB1	Rpb7	0.103382404490398	0.832235114390953	0.994766463583882	pseudobulk	DESeq2	LRT	0.0797539640243244
132	KC_AB1	Rpb8	-0.0956969757516636	0.844849791501035	0.994766463583882	pseudobulk	DESeq2	LRT	0.0732204987675088
132	KC_AB1	Rpe	-5.26068106026456	0.0145562992442623	0.994766463583882	pseudobulk	DESeq2	LRT	1.83694902489152
132	KC_AB1	Rph	0.191436526517718	0.760216226905143	0.994766463583882	pseudobulk	DESeq2	LRT	0.119062864566973
132	KC_AB1	RpI12	-3.34100803494108	0.314063158159027	0.994766463583882	pseudobulk	DESeq2	LRT	0.502983006443488
132	KC_AB1	RpI135	0.729740153036158	0.752353468463509	0.994766463583882	pseudobulk	DESeq2	LRT	0.123578072524162
132	KC_AB1	RpII140	1.14286134570665	0.218326260926945	0.994766463583882	pseudobulk	DESeq2	LRT	0.660894022927039
132	KC_AB1	RpII15	-0.468928821781735	0.619471504489291	0.994766463583882	pseudobulk	DESeq2	LRT	0.207978666249482
132	KC_AB1	RpII18	0.388783956882612	0.420465421680626	0.994766463583882	pseudobulk	DESeq2	LRT	0.376269713993948
132	KC_AB1	RpII215	0.0832476202276147	0.855962779096118	0.994766463583882	pseudobulk	DESeq2	LRT	0.0675451198843191
132	KC_AB1	RpII33	0.157918151325261	0.843920222940285	0.994766463583882	pseudobulk	DESeq2	LRT	0.0736986059506122
132	KC_AB1	RpIII128	0.69750158688829	0.750018748510497	0.994766463583882	pseudobulk	DESeq2	LRT	0.124927880244456
132	KC_AB1	RpIIIC160	0.0556507705136479	0.910364932142286	0.994766463583882	pseudobulk	DESeq2	LRT	0.0407844799367282
132	KC_AB1	RpIIIC53	0.0730733768153475	0.947920232766504	0.994766463583882	pseudobulk	DESeq2	LRT	0.0232282068886007
132	KC_AB1	RpL10	-0.161251288594788	0.353523718541304	0.994766463583882	pseudobulk	DESeq2	LRT	0.451581443292686
132	KC_AB1	RpL10Ab	-0.0671355102461259	0.729267336038216	0.994766463583882	pseudobulk	DESeq2	LRT	0.137113238097562
132	KC_AB1	RpL11	0.0347287450760801	0.85487949105964	0.994766463583882	pseudobulk	DESeq2	LRT	0.0680951017121421
132	KC_AB1	RpL12	-0.0992086964194023	0.611115521605556	0.994766463583882	pseudobulk	DESeq2	LRT	0.213876685578101
132	KC_AB1	RpL13	0.138423942954497	0.430866773835038	0.994766463583882	pseudobulk	DESeq2	LRT	0.365656995112058
132	KC_AB1	RpL13A	0.00589035100913675	0.931382948262074	0.994766463583882	pseudobulk	DESeq2	LRT	0.0308717173840446
132	KC_AB1	RpL14	0.0691995092138871	0.666138706051538	0.994766463583882	pseudobulk	DESeq2	LRT	0.176435330888672
132	KC_AB1	RpL15	0.0113044972568667	0.894079321372151	0.994766463583882	pseudobulk	DESeq2	LRT	0.0486239495418313
132	KC_AB1	RpL17	-0.122824297047781	0.552465360137584	0.994766463583882	pseudobulk	DESeq2	LRT	0.257694947276737
132	KC_AB1	RpL18	0.0332028848155398	0.80289361425393	0.994766463583882	pseudobulk	DESeq2	LRT	0.0953419961949619
132	KC_AB1	RpL18A	0.048185482242561	0.77301170854517	0.994766463583882	pseudobulk	DESeq2	LRT	0.111813927921199
132	KC_AB1	RpL19	-0.0603035642849426	0.727675330018743	0.994766463583882	pseudobulk	DESeq2	LRT	0.138062348478772
132	KC_AB1	RpL21	0.045319443052336	0.770141791394848	0.994766463583882	pseudobulk	DESeq2	LRT	0.113429309176457
132	KC_AB1	RpL22	-0.0415868913289548	0.89580339927211	0.994766463583882	pseudobulk	DESeq2	LRT	0.0477872938869561
132	KC_AB1	RpL23	0.169497483146562	0.365351632632883	0.994766463583882	pseudobulk	DESeq2	LRT	0.437288947599269
132	KC_AB1	RpL23A	-0.047841957891135	0.797636293600616	0.994766463583882	pseudobulk	DESeq2	LRT	0.0981950932221391
132	KC_AB1	RpL24	0.138954838545356	0.426532592801198	0.994766463583882	pseudobulk	DESeq2	LRT	0.370047777315016
132	KC_AB1	RpL24-like	0.0623310068535199	0.798990635475461	0.994766463583882	pseudobulk	DESeq2	LRT	0.0974583107800837
132	KC_AB1	RpL26	0.226103137122121	0.246986095364265	0.994766463583882	pseudobulk	DESeq2	LRT	0.607327495633252
132	KC_AB1	RpL27	-0.0737694059025351	0.703895682526406	0.994766463583882	pseudobulk	DESeq2	LRT	0.152491698613641
132	KC_AB1	RpL27A	0.110612756770021	0.493487048584487	0.994766463583882	pseudobulk	DESeq2	LRT	0.306724240769621
132	KC_AB1	RpL28	0.144091108706434	0.437843723722185	0.994766463583882	pseudobulk	DESeq2	LRT	0.358680871312837
132	KC_AB1	RpL29	-0.176204435817963	0.309413017047427	0.994766463583882	pseudobulk	DESeq2	LRT	0.509461419424788
132	KC_AB1	RpL3	-0.0150336063573886	0.846067392030287	0.994766463583882	pseudobulk	DESeq2	LRT	0.0725950426096746
132	KC_AB1	RpL30	-0.18885903722016	0.563772385940405	0.994766463583882	pseudobulk	DESeq2	LRT	0.248896200063219
132	KC_AB1	RpL31	0.00092343570631039	0.864022831958499	0.994766463583882	pseudobulk	DESeq2	LRT	0.0634747810597959
132	KC_AB1	RpL32	0.0891895892410754	0.619330257185336	0.994766463583882	pseudobulk	DESeq2	LRT	0.20807770215152
132	KC_AB1	RpL34a	-0.2204059333011	0.566510918821492	0.994766463583882	pseudobulk	DESeq2	LRT	0.246791715212368
132	KC_AB1	RpL34b	-0.123938365679454	0.554184660452617	0.994766463583882	pseudobulk	DESeq2	LRT	0.256345499436106
132	KC_AB1	RpL35	-0.140733175524479	0.557000888110996	0.994766463583882	pseudobulk	DESeq2	LRT	0.254144112364157
132	KC_AB1	RpL35A	0.140681518495241	0.525120042645005	0.994766463583882	pseudobulk	DESeq2	LRT	0.27974140535794
132	KC_AB1	RpL36	-0.0121730548061629	0.898705986506378	0.994766463583882	pseudobulk	DESeq2	LRT	0.0463823653550629
132	KC_AB1	RpL36A	-0.0779863211554701	0.640979843241869	0.994766463583882	pseudobulk	DESeq2	LRT	0.193155627433554
132	KC_AB1	RpL37a	0.0845051335921952	0.639719525833536	0.994766463583882	pseudobulk	DESeq2	LRT	0.194010393330674
132	KC_AB1	RpL37A	-0.122371579037009	0.510093585625798	0.994766463583882	pseudobulk	DESeq2	LRT	0.292350137642771
132	KC_AB1	RpL38	0.0433927923173955	0.766020615357301	0.994766463583882	pseudobulk	DESeq2	LRT	0.11575954235768

132	KC_AB1	RpL39	-0.197330598385069	0.33583649602517	0.994766463583882	pseudobulk	DESeq2	LRT	0.473872109981518
132	KC_AB1	RpL4	-0.0426419943921612	0.838908121428918	0.994766463583882	pseudobulk	DESeq2	LRT	0.0762856011992278
132	KC_AB1	RpL40	-0.0625853867828382	0.733698101753811	0.994766463583882	pseudobulk	DESeq2	LRT	0.134482604543751
132	KC_AB1	RpL41	-0.236966754468466	0.0870983372894183	0.994766463583882	pseudobulk	DESeq2	LRT	1.05999013561143
132	KC_AB1	RpL5	-0.194855081413886	0.296181933644915	0.994766463583882	pseudobulk	DESeq2	LRT	0.528441435881027
132	KC_AB1	RpL6	0.0271284859434069	0.87706213517102	0.994766463583882	pseudobulk	DESeq2	LRT	0.0569696380980266
132	KC_AB1	RpL7	0.0699361270926752	0.694104688712545	0.994766463583882	pseudobulk	DESeq2	LRT	0.158575021906374
132	KC_AB1	RpL7-like	-1.64952313825113	0.817565084244375	0.994766463583882	pseudobulk	DESeq2	LRT	0.0874776642296574
132	KC_AB1	RpL7A	-0.110093882511657	0.560094063439175	0.994766463583882	pseudobulk	DESeq2	LRT	0.251739030490108
132	KC_AB1	RpL8	0.00417476967402942	0.922968994117012	0.994766463583882	pseudobulk	DESeq2	LRT	0.0348128882591002
132	KC_AB1	RpL9	-0.0785972599697925	0.657842129423287	0.994766463583882	pseudobulk	DESeq2	LRT	0.181878316943097
132	KC_AB1	RpLP0	-0.0498381204151136	0.801314289019573	0.994766463583882	pseudobulk	DESeq2	LRT	0.0961971128598351
132	KC_AB1	RpLP0-like	0.560498429917967	0.620704212676861	0.994766463583882	pseudobulk	DESeq2	LRT	0.20711530707778
132	KC_AB1	RpLP1	-0.0266543125513625	0.826051248423056	0.994766463583882	pseudobulk	DESeq2	LRT	0.0829930081070837
132	KC_AB1	RpLP2	0.122713209409436	0.543347473169234	0.994766463583882	pseudobulk	DESeq2	LRT	0.264922348279872
132	KC_AB1	Rpn1	1.43339366484139	0.15593187230892	0.994766463583882	pseudobulk	DESeq2	LRT	0.807065106407365
132	KC_AB1	Rpn10	-0.277203561099061	0.632829529251952	0.994766463583882	pseudobulk	DESeq2	LRT	0.198713263878323
132	KC_AB1	Rpn11	-0.00622238387926054	0.872311275138165	0.994766463583882	pseudobulk	DESeq2	LRT	0.059328513975345
132	KC_AB1	Rpn12	0.208102747185365	0.760688203325047	0.994766463583882	pseudobulk	DESeq2	LRT	0.118793318687221
132	KC_AB1	Rpn13	0.278917342924449	0.643108374629793	0.994766463583882	pseudobulk	DESeq2	LRT	0.19171583494868
132	KC_AB1	Rpn13R	1.45682795759908	0.573003993665791	0.994766463583882	pseudobulk	DESeq2	LRT	0.24184235111992
132	KC_AB1	Rpn2	-0.273489138011758	0.644279603340778	0.994766463583882	pseudobulk	DESeq2	LRT	0.190925617368172
132	KC_AB1	Rpn3	0.236909878126132	0.719843109072575	0.994766463583882	pseudobulk	DESeq2	LRT	0.142762148414245
132	KC_AB1	Rpn5	-0.0302517348977909	0.944098740342717	0.994766463583882	pseudobulk	DESeq2	LRT	0.0249825818211709
132	KC_AB1	Rpn6	-0.433169064794663	0.493611872077544	0.994766463583882	pseudobulk	DESeq2	LRT	0.306614403440452
132	KC_AB1	Rpn7	-0.0510943780687982	0.924477232803297	0.994766463583882	pseudobulk	DESeq2	LRT	0.0341037797883646
132	KC_AB1	Rpn8	0.180586809011889	0.729444347374147	0.994766463583882	pseudobulk	DESeq2	LRT	0.137007836807258
132	KC_AB1	Rpn9	0.269509321927469	0.647968997093041	0.994766463583882	pseudobulk	DESeq2	LRT	0.188445773008296
132	KC_AB1	Rpp30	-0.38102144946092	0.783286087874966	0.994766463583882	pseudobulk	DESeq2	LRT	0.106079586993445
133	KC_AB1	RpS10b	0.0360920801985761	0.829708475675931	0.994766463583882	pseudobulk	DESeq2	LRT	0.0810744734611696
133	KC_AB1	RpS11	0.050378498501067	0.750172466556933	0.994766463583882	pseudobulk	DESeq2	LRT	0.124838879723857
133	KC_AB1	RpS12	0.0244704532174981	0.85166337655406	0.994766463583882	pseudobulk	DESeq2	LRT	0.0697320279951264
133	KC_AB1	RpS13	-0.0711240114980454	0.715568471633282	0.994766463583882	pseudobulk	DESeq2	LRT	0.145348802952815
133	KC_AB1	RpS14a	0.0594867912327542	0.720843781248657	0.994766463583882	pseudobulk	DESeq2	LRT	0.142158843869623
133	KC_AB1	RpS14b	-0.0308418492930347	0.923805326891397	0.994766463583882	pseudobulk	DESeq2	LRT	0.0344195378321983
133	KC_AB1	RpS15	0.077326355861246	0.659761322800575	0.994766463583882	pseudobulk	DESeq2	LRT	0.180613147697359
133	KC_AB1	RpS15Aa	0.148853625049112	0.480416537655084	0.994766463583882	pseudobulk	DESeq2	LRT	0.318382051042806
133	KC_AB1	RpS15Ab	-0.133740775429898	0.863033586085496	0.994766463583882	pseudobulk	DESeq2	LRT	0.0639723028157587
133	KC_AB1	RpS16	-0.0591761566865639	0.751672638160515	0.994766463583882	pseudobulk	DESeq2	LRT	0.123971258337356
133	KC_AB1	RpS17	-0.0266289393240312	0.85004509580369	0.994766463583882	pseudobulk	DESeq2	LRT	0.0705580338866587
133	KC_AB1	RpS18	-0.115495189548001	0.479086685549315	0.994766463583882	pseudobulk	DESeq2	LRT	0.319585898590428
133	KC_AB1	RpS19a	-0.0663749592560023	0.772688921240642	0.994766463583882	pseudobulk	DESeq2	LRT	0.11199531459404
133	KC_AB1	RpS2	0.0525264251434398	0.75692335172404	0.994766463583882	pseudobulk	DESeq2	LRT	0.120948096204889
133	KC_AB1	RpS20	0.0318164694694553	0.823929020260773	0.994766463583882	pseudobulk	DESeq2	LRT	0.0841102002407932
133	KC_AB1	RpS21	0.0532428736489027	0.752275688922296	0.994766463583882	pseudobulk	DESeq2	LRT	0.123622972924261
133	KC_AB1	RpS23	0.0304017762411203	0.824076016914165	0.994766463583882	pseudobulk	DESeq2	LRT	0.084032724948964
133	KC_AB1	RpS24	0.147115403618622	0.440704886458319	0.994766463583882	pseudobulk	DESeq2	LRT	0.355852134082505
133	KC_AB1	RpS25	-0.0490221307534111	0.741261766219163	0.994766463583882	pseudobulk	DESeq2	LRT	0.130028399916384
133	KC_AB1	RpS26	-0.0235721114108118	0.849794883742187	0.994766463583882	pseudobulk	DESeq2	LRT	0.0706858879431684
133	KC_AB1	RpS27	0.000238869007439356	0.915348946118408	0.994766463583882	pseudobulk	DESeq2	LRT	0.0384133141475007
133	KC_AB1	RpS27A	-0.0885287564531793	0.584754385288831	0.994766463583882	pseudobulk	DESeq2	LRT	0.233026512572081
133	KC_AB1	RpS28b	-0.114727410719527	0.4941999822814	0.994766463583882	pseudobulk	DESeq2	LRT	0.306097274504741
133	KC_AB1	RpS29	-0.0779276488205013	0.607808463522482	0.994766463583882	pseudobulk	DESeq2	LRT	0.216233256813627
133	KC_AB1	RpS3	0.317890756066396	0.109138237878136	0.994766463583882	pseudobulk	DESeq2	LRT	0.962023062518971
133	KC_AB1	RpS30	-0.0510897087783212	0.742670901367694	0.994766463583882	pseudobulk	DESeq2	LRT	0.129203591868718
133	KC_AB1	RpS3A	-0.174485500612335	0.344166584751849	0.994766463583882	pseudobulk	DESeq2	LRT	0.463231297757299

133	KC_AB1	RpS4	0.0416527604453898	0.794726090544681	0.994766463583882	pseudobulk	DESeq2	LRT	0.0997825290321982
133	KC_AB1	RpS5a	0.00047582100226751	0.95136253971927	0.994766463583882	pseudobulk	DESeq2	LRT	0.0216539530984828
133	KC_AB1	RpS6	0.0232056169377441	0.837692581164372	0.994766463583882	pseudobulk	DESeq2	LRT	0.0769153307718094
133	KC_AB1	RpS7	0.148377907467339	0.458952661551709	0.994766463583882	pseudobulk	DESeq2	LRT	0.338232107245099
133	KC_AB1	RpS8	0.0853055883323297	0.614803147162691	0.994766463583882	pseudobulk	DESeq2	LRT	0.211263918023476
133	KC_AB1	RpS9	-0.157307644409645	0.368887442802572	0.994766463583882	pseudobulk	DESeq2	LRT	0.433106128222291
133	KC_AB1	Rpt1	0.109182264863806	0.818463322454978	0.994766463583882	pseudobulk	DESeq2	LRT	0.0870007777209643
133	KC_AB1	Rpt2	-0.0744970942757952	0.89779071058559	0.994766463583882	pseudobulk	DESeq2	LRT	0.0468248925435106
133	KC_AB1	Rpt3	0.229288290311171	0.644624373283621	0.994766463583882	pseudobulk	DESeq2	LRT	0.190693277814583
133	KC_AB1	Rpt4	-0.275604185231395	0.645474489929054	0.994766463583882	pseudobulk	DESeq2	LRT	0.190120916994723
133	KC_AB1	Rpt5	0.452120357011712	0.510592275868535	0.994766463583882	pseudobulk	DESeq2	LRT	0.291925759396724
133	KC_AB1	Rpt6	-0.174614112867898	0.755913490129335	0.994766463583882	pseudobulk	DESeq2	LRT	0.121527904114209
133	KC_AB1	Rrp1	-0.941387677761002	0.558366838309724	0.994766463583882	pseudobulk	DESeq2	LRT	0.253080382588975
133	KC_AB1	Rrp4	1.32710828904082	0.607473293222376	0.994766463583882	pseudobulk	DESeq2	LRT	0.216472810505135
133	KC_AB1	Rrp40	0.478005084452508	0.815070723509861	0.994766463583882	pseudobulk	DESeq2	LRT	0.0888047059870473
133	KC_AB1	Rrp42	-0.287533822320852	0.805229533147958	0.994766463583882	pseudobulk	DESeq2	LRT	0.0940803050104332
133	KC_AB1	Rrp45	0.646795838186486	0.85519056880605	0.994766463583882	pseudobulk	DESeq2	LRT	0.0679370972497873
133	KC_AB1	Rrp46	0.39509611406087	0.839890017035674	0.994766463583882	pseudobulk	DESeq2	LRT	0.0757775807497476
133	KC_AB1	Rrp47	0.105197502712398	0.86210920520485	0.994766463583882	pseudobulk	DESeq2	LRT	0.0644377176858657
133	KC_AB1	Rrp6	0.617995242639145	0.674514491039702	0.994766463583882	pseudobulk	DESeq2	LRT	0.171008715657049
133	KC_AB1	Rs1	-0.121435277351349	0.902488014585122	0.994766463583882	pseudobulk	DESeq2	LRT	0.0445585569947236
133	KC_AB1	RSG7	-0.168456129776248	0.472492083487392	0.994766463583882	pseudobulk	DESeq2	LRT	0.325605463613012
133	KC_AB1	rswl	-0.327841272902287	0.766820594004488	0.994766463583882	pseudobulk	DESeq2	LRT	0.115306232071481
133	KC_AB1	rt	-0.714512617114541	0.562371807948271	0.994766463583882	pseudobulk	DESeq2	LRT	0.249976458897524
133	KC_AB1	Rtc1	-0.0835882191718759	0.822917379789117	0.994766463583882	pseudobulk	DESeq2	LRT	0.084643765398734
133	KC_AB1	Rtca	-0.274984454240223	0.673418193580041	0.994766463583882	pseudobulk	DESeq2	LRT	0.171715154558144
133	KC_AB1	RtcB	0.897179476381625	0.56780389052509	0.994766463583882	pseudobulk	DESeq2	LRT	0.245801636065552
133	KC_AB1	rtet	-0.467437884468606	0.815276186541128	0.994766463583882	pseudobulk	DESeq2	LRT	0.0886952428284564
133	KC_AB1	Rtf1	-0.37337327669852	0.601486948312231	0.994766463583882	pseudobulk	DESeq2	LRT	0.220773791994087
133	KC_AB1	RtGEF	-0.926057967072024	0.406860188789987	0.994766463583882	pseudobulk	DESeq2	LRT	0.390554803719339
133	KC_AB1	Rtnl1	0.0506405152948939	0.76433661998575	0.994766463583882	pseudobulk	DESeq2	LRT	0.116715332516623
133	KC_AB1	rtv	1.1821590793593	0.719511699510136	0.994766463583882	pseudobulk	DESeq2	LRT	0.142962139890846
133	KC_AB1	ru	1.1821590793593	0.719511699510136	0.994766463583882	pseudobulk	DESeq2	LRT	0.142962139890846
133	KC_AB1	Rubicon	-0.350986614302326	0.660635494724235	0.994766463583882	pseudobulk	DESeq2	LRT	0.180038096177831
133	KC_AB1	rudhira	-0.136850744963152	0.52554148304974	0.994766463583882	pseudobulk	DESeq2	LRT	0.279392997716529
133	KC_AB1	rumi	-1.49209352175334	0.304117237221176	0.994766463583882	pseudobulk	DESeq2	LRT	0.516958963552403
133	KC_AB1	rump	0.0464102540550209	0.915370200371726	0.994766463583882	pseudobulk	DESeq2	LRT	0.0384032300175008
133	KC_AB1	rush	-1.21401623275352	0.293945324884757	0.994766463583882	pseudobulk	DESeq2	LRT	0.5317334427479
133	KC_AB1	rut	0.0766326416982401	0.570052139234602	0.994766463583882	pseudobulk	DESeq2	LRT	0.24408542021119
133	KC_AB1	rux	0.60335701921894	0.503302072588343	0.994766463583882	pseudobulk	DESeq2	LRT	0.298171281182778
133	KC_AB1	RYa-R	0.51249784426152	0.886257713466163	0.994766463583882	pseudobulk	DESeq2	LRT	0.052439971948552
133	KC_AB1	RYBP	-0.138125368126983	0.840004157989338	0.994766463583882	pseudobulk	DESeq2	LRT	0.0757185641912658
133	KC_AB1	RyR	-0.110755875716275	0.819326595338417	0.994766463583882	pseudobulk	DESeq2	LRT	0.0865429477144392
133	KC_AB1	S	-0.488527580487576	0.32480040151942	0.994766463583882	pseudobulk	DESeq2	LRT	0.488383442554137
133	KC_AB1	s-cup	-0.454291898309769	0.651757803991801	0.994766463583882	pseudobulk	DESeq2	LRT	0.18591375999042
133	KC_AB1	S-Lap1	1.95659807726805	0.360275532956384	0.994766463583882	pseudobulk	DESeq2	LRT	0.443365230696848
133	KC_AB1	S1P	-0.416437267072002	0.756149174483499	0.994766463583882	pseudobulk	DESeq2	LRT	0.121392517641534
133	KC_AB1	S2P	-0.38846974319923	0.709804595355695	0.994766463583882	pseudobulk	DESeq2	LRT	0.148861193307445
133	KC_AB1	S6k	0.0593899021455125	0.857406064088732	0.994766463583882	pseudobulk	DESeq2	LRT	0.0668134492240789
133	KC_AB1	S6kII	-0.474958515562454	0.333690325597706	0.994766463583882	pseudobulk	DESeq2	LRT	0.476656384296472
133	KC_AB1	S6KL	0.305593700287741	0.729177892163698	0.994766463583882	pseudobulk	DESeq2	LRT	0.137166507127404
133	KC_AB1	SA	0.228099654716539	0.79240989651901	0.994766463583882	pseudobulk	DESeq2	LRT	0.101050109134555
133	KC_AB1	Sac1	0.466113534213697	0.598465405592869	0.994766463583882	pseudobulk	DESeq2	LRT	0.22296094900753
133	KC_AB1	Saf-B	-0.012930838072362	0.890483943169262	0.994766463583882	pseudobulk	DESeq2	LRT	0.0503739071449899
133	KC_AB1	Saf6	0.757111967132494	0.612617233120852	0.994766463583882	pseudobulk	DESeq2	LRT	0.21281079051341
133	KC_AB1	sage	0.854345112468581	0.633619142106123	0.994766463583882	pseudobulk	DESeq2	LRT	0.198171710860665

133	KC_AB1	SAK	-0.225261612367904	0.840497091952346	0.994766463583882	pseudobulk	DESeq2	LRT	0.0754637848434681
133	KC_AB1	sals	0.266602429203314	0.559581380342275	0.994766463583882	pseudobulk	DESeq2	LRT	0.252136744768377
133	KC_AB1	Sam-S	-0.151641476066608	0.661229003639666	0.994766463583882	pseudobulk	DESeq2	LRT	0.179648105124177
133	KC_AB1	SamDC	-0.215815772497137	0.725121148964151	0.994766463583882	pseudobulk	DESeq2	LRT	0.13958942800676
133	KC_AB1	Samtor	-0.059113546855606	0.955083348076834	0.994766463583882	pseudobulk	DESeq2	LRT	0.0199587268137861
133	KC_AB1	san	0.0323745376842194	0.942783610406076	0.994766463583882	pseudobulk	DESeq2	LRT	0.025587975969597
133	KC_AB1	sano	0.700869873716825	0.858452936962073	0.994766463583882	pseudobulk	DESeq2	LRT	0.0662835092135289
133	KC_AB1	Sap-r	0.0147849004149777	0.943981219451118	0.994766463583882	pseudobulk	DESeq2	LRT	0.0250366459242625
133	KC_AB1	Sap130	-0.330425386284875	0.682287888423044	0.994766463583882	pseudobulk	DESeq2	LRT	0.166032337994204
133	KC_AB1	Sap30	-0.748199792947879	0.383032226880945	0.994766463583882	pseudobulk	DESeq2	LRT	0.416764684601185
133	KC_AB1	Sap47	0.0704785891964971	0.617370729351974	0.994766463583882	pseudobulk	DESeq2	LRT	0.209453965042238
133	KC_AB1	Sar1	-0.143735565650485	0.711740753190768	0.994766463583882	pseudobulk	DESeq2	LRT	0.147678166420995
133	KC_AB1	Sara	-0.244585081875953	0.711916834014217	0.994766463583882	pseudobulk	DESeq2	LRT	0.147570737596719
133	KC_AB1	Sas-6	0.858598136557056	0.663400004084503	0.994766463583882	pseudobulk	DESeq2	LRT	0.178224530142619
133	KC_AB1	Sas10	0.256171500829629	0.848335760245047	0.994766463583882	pseudobulk	DESeq2	LRT	0.0714322256191549
133	KC_AB1	sau	-0.25323748702432	0.512422671949579	0.994766463583882	pseudobulk	DESeq2	LRT	0.29037166330524
133	KC_AB1	sax	0.012477577830469	0.90289122111991	0.994766463583882	pseudobulk	DESeq2	LRT	0.0443645696345347
134	KC_AB1	sba	0.17852654274035	0.655245027928356	0.994766463583882	pseudobulk	DESeq2	LRT	0.183596265843355
134	KC_AB1	Sbat	-1.54029849397735	0.195748339873373	0.994766463583882	pseudobulk	DESeq2	LRT	0.708301912472526
134	KC_AB1	sbb	0.288388517758538	0.140245917137398	0.994766463583882	pseudobulk	DESeq2	LRT	0.853109773141382
134	KC_AB1	Sbf	-0.169256285739646	0.756262186127894	0.994766463583882	pseudobulk	DESeq2	LRT	0.121327614223164
134	KC_AB1	sbm	-0.160998227064193	0.679879648946541	0.994766463583882	pseudobulk	DESeq2	LRT	0.167567958505985
134	KC_AB1	Sbp2	0.102846339174642	0.886737502339575	0.994766463583882	pseudobulk	DESeq2	LRT	0.0522049237505309
134	KC_AB1	sbr	-0.0138150750696228	0.893309919236822	0.994766463583882	pseudobulk	DESeq2	LRT	0.0489978436156958
134	KC_AB1	Sc2	0.0284814734048233	0.944962244619239	0.994766463583882	pseudobulk	DESeq2	LRT	0.0245855431109355
134	KC_AB1	SC35	0.0432074687365355	0.911836481557059	0.994766463583882	pseudobulk	DESeq2	LRT	0.0400830361503183
134	KC_AB1	sca	0.0659077473889154	0.85601398375838	0.994766463583882	pseudobulk	DESeq2	LRT	0.067519140673903
134	KC_AB1	scaf	0.53983785550047	0.806887399502234	0.994766463583882	pseudobulk	DESeq2	LRT	0.0931870665007086
134	KC_AB1	scaf6	0.0248703511828422	0.92299501441573	0.994766463583882	pseudobulk	DESeq2	LRT	0.0348006448219592
134	KC_AB1	SCaMC	0.167847459893555	0.715464979283141	0.994766463583882	pseudobulk	DESeq2	LRT	0.145411619313446
134	KC_AB1	Scamp	0.228818386173384	0.562079592692809	0.994766463583882	pseudobulk	DESeq2	LRT	0.250202182260507
134	KC_AB1	SCAP	-0.040300072460715	0.91856554382738	0.994766463583882	pseudobulk	DESeq2	LRT	0.0368898493669211
134	KC_AB1	SCAR	-0.165879072582504	0.689276464347914	0.994766463583882	pseudobulk	DESeq2	LRT	0.161606550431295
134	KC_AB1	scaRNA:MeU2-G25	0.949183608371002	0.80156895525114	0.994766463583882	pseudobulk	DESeq2	LRT	0.0960591113673091
134	KC_AB1	scaRNA:MeU4-A65	0.949183608371002	0.80156895525114	0.994766463583882	pseudobulk	DESeq2	LRT	0.0960591113673091
134	KC_AB1	scat	0.705252918620908	0.595935954645681	0.994766463583882	pseudobulk	DESeq2	LRT	0.224800411465361
134	KC_AB1	scb	0.596808856322697	0.822761281772702	0.994766463583882	pseudobulk	DESeq2	LRT	0.0847261539098712
134	KC_AB1	Sccpdh1	-0.00433667778775492	0.938535119791954	0.994766463583882	pseudobulk	DESeq2	LRT	0.0275494715280071
134	KC_AB1	SCCRO	0.0691190439442516	0.902103230491008	0.994766463583882	pseudobulk	DESeq2	LRT	0.044743761939928
134	KC_AB1	SCCRO3	-0.044466817671108	0.885498555230494	0.994766463583882	pseudobulk	DESeq2	LRT	0.0528121430633318
134	KC_AB1	SCCRO4	-0.259174662539817	0.625325277999148	0.994766463583882	pseudobulk	DESeq2	LRT	0.203894015148449
134	KC_AB1	Sce	-0.137988687454161	0.840116317779646	0.994766463583882	pseudobulk	DESeq2	LRT	0.0756605798040779
134	KC_AB1	sced	-0.295754505400978	0.759005884939491	0.994766463583882	pseudobulk	DESeq2	LRT	0.119754856796431
134	KC_AB1	scf	-0.223146358516813	0.756235835117089	0.994766463583882	pseudobulk	DESeq2	LRT	0.121342746935307
134	KC_AB1	Scgalpha	0.155598708199777	0.787206666613473	0.994766463583882	pseudobulk	DESeq2	LRT	0.103911236653462
134	KC_AB1	Scgbeta	0.396390727104622	0.322260018901817	0.994766463583882	pseudobulk	DESeq2	LRT	0.491793571767778
134	KC_AB1	Scgdelta	-1.28658816324798	0.202126623113134	0.994766463583882	pseudobulk	DESeq2	LRT	0.694376479608748
134	KC_AB1	Schip1	-0.126155923073548	0.702495182665724	0.994766463583882	pseudobulk	DESeq2	LRT	0.153356649570736
134	KC_AB1	schlank	-0.205605189691545	0.653852795311849	0.994766463583882	pseudobulk	DESeq2	LRT	0.184520015253214
134	KC_AB1	Scm	0.0346922891708385	0.94036589046115	0.994766463583882	pseudobulk	DESeq2	LRT	0.026703132262074
134	KC_AB1	scny	-0.335046823739773	0.591193877018203	0.994766463583882	pseudobulk	DESeq2	LRT	0.2282700725682
134	KC_AB1	SCOT	-1.58094965174778	0.396883337319333	0.994766463583882	pseudobulk	DESeq2	LRT	0.401337134053717
134	KC_AB1	Scox	0.63653745137672	0.434742679656409	0.994766463583882	pseudobulk	DESeq2	LRT	0.361767722060369
134	KC_AB1	ScpX	0.614065178265861	0.684407198348364	0.994766463583882	pseudobulk	DESeq2	LRT	0.164685431354004
134	KC_AB1	scra	-1.64952313825113	0.817565084244375	0.994766463583882	pseudobulk	DESeq2	LRT	0.0874776642296574
134	KC_AB1	scramb1	-0.0999680952038485	0.823343829022357	0.994766463583882	pseudobulk	DESeq2	LRT	0.0844187651958996

134	KC_AB1	scramb2	0.202934887596389	0.726847835837374	0.994766463583882	pseudobulk	DESeq2	LRT	0.138556498319742
134	KC_AB1	scrib	0.431092929117558	0.0193693824576473	0.994766463583882	pseudobulk	DESeq2	LRT	1.71288422540951
134	KC_AB1	scrt	-0.0401571305553367	0.872811512375492	0.994766463583882	pseudobulk	DESeq2	LRT	0.0590795340418772
134	KC_AB1	Scsalpha1	-0.116616292728923	0.694713850811084	0.994766463583882	pseudobulk	DESeq2	LRT	0.158194042322049
134	KC_AB1	ScsbetaA	-0.175290616477043	0.589060162047145	0.994766463583882	pseudobulk	DESeq2	LRT	0.229840347469619
134	KC_AB1	ScsbetaG	0.508989497410399	0.705334929266353	0.994766463583882	pseudobulk	DESeq2	LRT	0.151604608694719
134	KC_AB1	scu	0.474281979456428	0.658220348747827	0.994766463583882	pseudobulk	DESeq2	LRT	0.181628695708781
134	KC_AB1	scyl	0.0401665174249777	0.838880662201341	0.994766463583882	pseudobulk	DESeq2	LRT	0.076299816803875
134	KC_AB1	sd	0.242749163293356	0.391245439641392	0.994766463583882	pseudobulk	DESeq2	LRT	0.407550711554432
134	KC_AB1	Sdc	0.826086829513824	0.00142522302686854	0.994766463583882	pseudobulk	DESeq2	LRT	2.84611716950854
134	KC_AB1	SdhA	0.0920618577944539	0.862368796084811	0.994766463583882	pseudobulk	DESeq2	LRT	0.0643069663657712
134	KC_AB1	Sdhaf3	0.257904486767322	0.811216859265235	0.994766463583882	pseudobulk	DESeq2	LRT	0.0908630321111142
134	KC_AB1	SdhB	0.0311803594672391	0.902567784688384	0.994766463583882	pseudobulk	DESeq2	LRT	0.0445201717927771
134	KC_AB1	SdhC	0.0733437489671581	0.802742303092312	0.994766463583882	pseudobulk	DESeq2	LRT	0.0954238498730287
134	KC_AB1	SdhD	-0.0224044314565237	0.928750394813489	0.994766463583882	pseudobulk	DESeq2	LRT	0.0321009886119519
134	KC_AB1	Sdic2	2.22023857837763	0.524904842126482	0.994766463583882	pseudobulk	DESeq2	LRT	0.279919420946639
134	KC_AB1	sdk	1.80633728195307	0.61161102402172	0.994766463583882	pseudobulk	DESeq2	LRT	0.213524695220716
134	KC_AB1	Sdr	2.22023857837763	0.524904842126482	0.994766463583882	pseudobulk	DESeq2	LRT	0.279919420946639
134	KC_AB1	sds22	-0.0761777825496252	0.894546913585524	0.994766463583882	pseudobulk	DESeq2	LRT	0.0483968783754734
134	KC_AB1	sdt	0.161961626962115	0.289622186997216	0.994766463583882	pseudobulk	DESeq2	LRT	0.538168171337555
134	KC_AB1	sea	-1.64952313825113	0.817565084244375	0.994766463583882	pseudobulk	DESeq2	LRT	0.0874776642296574
134	KC_AB1	Sec10	-0.704860146244937	0.516369162766358	0.994766463583882	pseudobulk	DESeq2	LRT	0.287039701417806
134	KC_AB1	Sec13	0.0402164862060926	0.948791784751085	0.994766463583882	pseudobulk	DESeq2	LRT	0.0228290843642115
134	KC_AB1	Sec15	-1.12288491887633	0.363634349508686	0.994766463583882	pseudobulk	DESeq2	LRT	0.439335099329399
134	KC_AB1	Sec16	0.486966280552376	0.35182693398	0.994766463583882	pseudobulk	DESeq2	LRT	0.453670916353436
134	KC_AB1	Sec22	0.145446664307423	0.798825541432175	0.994766463583882	pseudobulk	DESeq2	LRT	0.0975480575648339
134	KC_AB1	Sec23	-0.0632158095029686	0.921621860587645	0.994766463583882	pseudobulk	DESeq2	LRT	0.0354472324668834
134	KC_AB1	Sec24AB	-0.600120827142728	0.405387938838938	0.994766463583882	pseudobulk	DESeq2	LRT	0.392129176643543
134	KC_AB1	Sec24CD	-0.715390579865147	0.337684032304214	0.994766463583882	pseudobulk	DESeq2	LRT	0.471489474887605
134	KC_AB1	Sec3	0.173100165396605	0.920977111550947	0.994766463583882	pseudobulk	DESeq2	LRT	0.0357511629091687
134	KC_AB1	Sec31	-0.364884151067761	0.721225402004336	0.994766463583882	pseudobulk	DESeq2	LRT	0.141928985560238
134	KC_AB1	Sec5	1.03154945120217	0.533517792213664	0.994766463583882	pseudobulk	DESeq2	LRT	0.272851092769278
134	KC_AB1	Sec6	-1.084076980477	0.453039878431191	0.994766463583882	pseudobulk	DESeq2	LRT	0.343863567920177
134	KC_AB1	Sec61alpha	0.259937964686363	0.761084325236938	0.994766463583882	pseudobulk	DESeq2	LRT	0.11856722239339
134	KC_AB1	Sec61beta	0.420761055263054	0.471898353080265	0.994766463583882	pseudobulk	DESeq2	LRT	0.326151538337071
134	KC_AB1	Sec61gamma	-0.154598658681599	0.708496353929845	0.994766463583882	pseudobulk	DESeq2	LRT	0.149662380380844
134	KC_AB1	Sec63	-0.0516991826153754	0.933633654243486	0.994766463583882	pseudobulk	DESeq2	LRT	0.0298235018761287
134	KC_AB1	Sec71	0.180637234636885	0.858707079477097	0.994766463583882	pseudobulk	DESeq2	LRT	0.0661549566131409
134	KC_AB1	Sec8	-0.145247433179025	0.85854449314903	0.994766463583882	pseudobulk	DESeq2	LRT	0.066237193073365
134	KC_AB1	Secp43	-0.224088789098477	0.809753303547181	0.994766463583882	pseudobulk	DESeq2	LRT	0.0916472715247816
134	KC_AB1	SecS	0.198685779314143	0.743741666697963	0.994766463583882	pseudobulk	DESeq2	LRT	0.128577887316995
134	KC_AB1	sei	0.410973395626195	0.533549058778038	0.994766463583882	pseudobulk	DESeq2	LRT	0.272825641887045
134	KC_AB1	sel	-1.43104301918602	0.443250501906941	0.994766463583882	pseudobulk	DESeq2	LRT	0.353350763961017
134	KC_AB1	SelG	0.524606371625833	0.441432609913293	0.994766463583882	pseudobulk	DESeq2	LRT	0.355135587390312
134	KC_AB1	SelR	-0.0938702312456404	0.73758601641283	0.994766463583882	pseudobulk	DESeq2	LRT	0.13218732548522
134	KC_AB1	SelT	-0.0868268011980922	0.883875625500958	0.994766463583882	pseudobulk	DESeq2	LRT	0.0536088424064006
134	KC_AB1	Sem1	0.347291674864466	0.531638627518345	0.994766463583882	pseudobulk	DESeq2	LRT	0.274383471844032
134	KC_AB1	Sema1a	0.0148637072496065	0.90071493415589	0.994766463583882	pseudobulk	DESeq2	LRT	0.0454126364482868
134	KC_AB1	Sema1b	0.315994605365665	0.578120639737884	0.994766463583882	pseudobulk	DESeq2	LRT	0.237981525410804
134	KC_AB1	Sema2a	-0.114687739447406	0.794513211259799	0.994766463583882	pseudobulk	DESeq2	LRT	0.0998988768964671
134	KC_AB1	Sema2b	0.553981902168412	0.202748413441308	0.994766463583882	pseudobulk	DESeq2	LRT	0.693042535569981
134	KC_AB1	senju	1.90100515887561	0.379831956883464	0.994766463583882	pseudobulk	DESeq2	LRT	0.420408499013709
134	KC_AB1	Sep1	-0.0546992708938105	0.879806024971445	0.994766463583882	pseudobulk	DESeq2	LRT	0.0556130682708487
134	KC_AB1	Sep2	-0.262970358240045	0.644518944499743	0.994766463583882	pseudobulk	DESeq2	LRT	0.190764312801217
134	KC_AB1	Sep4	-0.0394414176489261	0.897133296156803	0.994766463583882	pseudobulk	DESeq2	LRT	0.0471430246431871
134	KC_AB1	SERCA	0.143567211804296	0.72415171924921	0.994766463583882	pseudobulk	DESeq2	LRT	0.140170433899879

134	KC_AB1	SerRS	-0.399909979266547	0.73604516043329	0.994766463583882	pseudobulk	DESeq2	LRT	0.133095538481407
134	KC_AB1	SerRS-m	-0.035563162862932	0.929574621491585	0.994766463583882	pseudobulk	DESeq2	LRT	0.0317157415550955
134	KC_AB1	sesB	-0.084327045958566	0.639147332195576	0.994766463583882	pseudobulk	DESeq2	LRT	0.1943990194876
134	KC_AB1	Sesn	0.097819640535638	0.89223956997303	0.994766463583882	pseudobulk	DESeq2	LRT	0.0495185201216824
134	KC_AB1	Set	-0.025805444670482	0.953786341919221	0.994766463583882	pseudobulk	DESeq2	LRT	0.0205489008910233
135	KC_AB1	Set1	-0.084516536081522	0.770092865273605	0.994766463583882	pseudobulk	DESeq2	LRT	0.113456900224862
135	KC_AB1	Set2	0.179965612401022	0.798166225960707	0.994766463583882	pseudobulk	DESeq2	LRT	0.0979066531358397
135	KC_AB1	Setd3	-0.19010249682791	0.836503638318572	0.994766463583882	pseudobulk	DESeq2	LRT	0.0775321657616666
135	KC_AB1	SF1	0.433867660690622	0.462712314054461	0.994766463583882	pseudobulk	DESeq2	LRT	0.33468894256896
135	KC_AB1	SF2	0.0339081779573381	0.916736185850435	0.994766463583882	pseudobulk	DESeq2	LRT	0.0377556256314778
135	KC_AB1	Sf3a1	0.114128299679246	0.883053255754481	0.994766463583882	pseudobulk	DESeq2	LRT	0.0540131039163649
135	KC_AB1	Sf3a2	-0.762659136266111	0.331066452588061	0.994766463583882	pseudobulk	DESeq2	LRT	0.480084824666336
135	KC_AB1	Sf3b1	-0.148300281576646	0.822571480584036	0.994766463583882	pseudobulk	DESeq2	LRT	0.0848263520110069
135	KC_AB1	Sf3b2	-0.485122894353203	0.423927678266436	0.994766463583882	pseudobulk	DESeq2	LRT	0.372708227390347
135	KC_AB1	Sf3b3	0.193428213414189	0.860316359648965	0.994766463583882	pseudobulk	DESeq2	LRT	0.0653418185409745
135	KC_AB1	Sf3b5	-0.0340323172281412	0.935251686122543	0.994766463583882	pseudobulk	DESeq2	LRT	0.0290715001593685
135	KC_AB1	Sf3b6	-0.0514879146706292	0.875333973854672	0.994766463583882	pseudobulk	DESeq2	LRT	0.0578262151732303
135	KC_AB1	sff	-0.251179587642205	0.421019280533579	0.994766463583882	pseudobulk	DESeq2	LRT	0.375698015238896
135	KC_AB1	sfl	-0.111810495209462	0.687991491910047	0.994766463583882	pseudobulk	DESeq2	LRT	0.162416932461241
135	KC_AB1	Sfmbt	-0.548100771452293	0.452493870008261	0.994766463583882	pseudobulk	DESeq2	LRT	0.344387299861251
135	KC_AB1	Sfp33A4	-1.64952313825113	0.817565084244375	0.994766463583882	pseudobulk	DESeq2	LRT	0.0874776642296574
135	KC_AB1	Sfp78E	0.949183608371002	0.80156895525114	0.994766463583882	pseudobulk	DESeq2	LRT	0.0960591113673091
135	KC_AB1	Sfxn1-3	0.122756348292405	0.820984873181609	0.994766463583882	pseudobulk	DESeq2	LRT	0.0856648447737114
135	KC_AB1	Sfxn2	0.0786151859000479	0.890413781211505	0.994766463583882	pseudobulk	DESeq2	LRT	0.0504081269103274
135	KC_AB1	Sgf11	0.292580835917005	0.740248944244588	0.994766463583882	pseudobulk	DESeq2	LRT	0.130622203336268
135	KC_AB1	Sgf29	-0.227507954267356	0.809170236741116	0.994766463583882	pseudobulk	DESeq2	LRT	0.0919601000198925
135	KC_AB1	sgg	0.00639866035505016	0.927752801840521	0.994766463583882	pseudobulk	DESeq2	LRT	0.0325677253951963
135	KC_AB1	sgl	-1.90234634415387	0.102815980896288	0.994766463583882	pseudobulk	DESeq2	LRT	0.987939376822609
135	KC_AB1	sgll	-0.670194014571923	0.533524956560355	0.994766463583882	pseudobulk	DESeq2	LRT	0.272845260882528
135	KC_AB1	Sgs1	-2.79499059556516	0.38564607643653	0.994766463583882	pseudobulk	DESeq2	LRT	0.413811082791365
135	KC_AB1	Sgs4	0.881337768677799	0.697697705840746	0.994766463583882	pseudobulk	DESeq2	LRT	0.156332705059186
135	KC_AB1	Sgt	0.161220391843652	0.768614941554591	0.994766463583882	pseudobulk	DESeq2	LRT	0.114291177285621
135	KC_AB1	Sgt1	-0.0212058566224562	0.959417411213738	0.994766463583882	pseudobulk	DESeq2	LRT	0.0179924043551787
135	KC_AB1	Sh	0.015491767067477	0.890844024407822	0.994766463583882	pseudobulk	DESeq2	LRT	0.0501983288125102
135	KC_AB1	Sh3beta	-0.210423765262467	0.569411713169267	0.994766463583882	pseudobulk	DESeq2	LRT	0.244573603377232
135	KC_AB1	SH3PX1	-0.304389748283173	0.75723811672582	0.994766463583882	pseudobulk	DESeq2	LRT	0.120767533288468
135	KC_AB1	Shab	-0.106147485142423	0.574875594514059	0.994766463583882	pseudobulk	DESeq2	LRT	0.240426128287153
135	KC_AB1	shakB	0.0560373574058309	0.731206125412191	0.994766463583882	pseudobulk	DESeq2	LRT	0.135960179115621
135	KC_AB1	Shal	-0.0661268603221048	0.778651859926859	0.994766463583882	pseudobulk	DESeq2	LRT	0.108656674686664
135	KC_AB1	shams	-0.193367052912822	0.911785806342975	0.994766463583882	pseudobulk	DESeq2	LRT	0.0401071726899928
135	KC_AB1	Shark	1.58149959037531	0.565968974761403	0.994766463583882	pseudobulk	DESeq2	LRT	0.247207375276793
135	KC_AB1	Shaw	0.0584225827412302	0.830205000672404	0.994766463583882	pseudobulk	DESeq2	LRT	0.0808146550120767
135	KC_AB1	Shawl	0.155452197248463	0.524061323260523	0.994766463583882	pseudobulk	DESeq2	LRT	0.28061789088765
135	KC_AB1	Shc	0.00390808915149695	0.904606277105228	0.994766463583882	pseudobulk	DESeq2	LRT	0.0435404029899405
135	KC_AB1	shep	0.219729887957143	0.117865474504901	0.994766463583882	pseudobulk	DESeq2	LRT	0.928613391070041
135	KC_AB1	shg	2.54275315134261	0.410734813545839	0.994766463583882	pseudobulk	DESeq2	LRT	0.386438485142247
135	KC_AB1	shi	0.0228867487768646	0.863014211904634	0.994766463583882	pseudobulk	DESeq2	LRT	0.0639820523717673
135	KC_AB1	Shmt	-0.0942507870169442	0.901080929806495	0.994766463583882	pseudobulk	DESeq2	LRT	0.0452362014848995
135	KC_AB1	shn	-0.241982269586285	0.751912120279442	0.994766463583882	pseudobulk	DESeq2	LRT	0.123832914605072
135	KC_AB1	shop	0.719341889112023	0.55945890814953	0.994766463583882	pseudobulk	DESeq2	LRT	0.252231806578492
135	KC_AB1	shot	-0.113144335435072	0.561757484805472	0.994766463583882	pseudobulk	DESeq2	LRT	0.250451132370233
135	KC_AB1	shrb	0.126501600110336	0.799927107333537	0.994766463583882	pseudobulk	DESeq2	LRT	0.0969495859144631
135	KC_AB1	Shrm	-1.74683112255917	0.584939641962841	0.994766463583882	pseudobulk	DESeq2	LRT	0.232888945054326
135	KC_AB1	shtd	2.02670873290438	0.538959414925803	0.994766463583882	pseudobulk	DESeq2	LRT	0.26844393710898
135	KC_AB1	shv	-0.0821496054323249	0.957377622215522	0.994766463583882	pseudobulk	DESeq2	LRT	0.0189167279527711
135	KC_AB1	sicily	-0.444131386177334	0.636445838773777	0.994766463583882	pseudobulk	DESeq2	LRT	0.196238548683547

135	KC_AB1	sick	0.15601844139253	0.665332354898851	0.994766463583882	pseudobulk	DESeq2	LRT	0.176961356439165
135	KC_AB1	side	-1.64952313825113	0.817565084244375	0.994766463583882	pseudobulk	DESeq2	LRT	0.0874776642296574
135	KC_AB1	side-II	0.119554211102915	0.644656770348411	0.994766463583882	pseudobulk	DESeq2	LRT	0.190671451905563
135	KC_AB1	side-III	-0.0762124135204961	0.713449632569322	0.994766463583882	pseudobulk	DESeq2	LRT	0.146636681371091
135	KC_AB1	side-IV	-0.29570742111507	0.544327557795407	0.994766463583882	pseudobulk	DESeq2	LRT	0.264139678000554
135	KC_AB1	side-V	3.02680884525143	0.293242669533885	0.994766463583882	pseudobulk	DESeq2	LRT	0.532772835549261
135	KC_AB1	side-VI	-0.0040494566455772	0.886883093315911	0.994766463583882	pseudobulk	DESeq2	LRT	0.0521336239946138
135	KC_AB1	side-VII	0.0416598672457149	0.857047861032485	0.994766463583882	pseudobulk	DESeq2	LRT	0.0669949246323279
135	KC_AB1	side-VIII	0.123265284375284	0.880060321591092	0.994766463583882	pseudobulk	DESeq2	LRT	0.0554875591722014
135	KC_AB1	SIDL	0.239623127223831	0.830936921378068	0.994766463583882	pseudobulk	DESeq2	LRT	0.080431943408677
135	KC_AB1	Sidpn	0.339982664370336	0.709866061047446	0.994766463583882	pseudobulk	DESeq2	LRT	0.1488235871054
135	KC_AB1	sif	-0.395992008697707	0.0882040491945403	0.994766463583882	pseudobulk	DESeq2	LRT	1.05451147719834
135	KC_AB1	SIFaR	2.16325088398155	0.257812106654197	0.994766463583882	pseudobulk	DESeq2	LRT	0.588696692375719
135	KC_AB1	Sik2	-0.494757875434432	0.56179596725421	0.994766463583882	pseudobulk	DESeq2	LRT	0.250421382623814
135	KC_AB1	Sik3	0.195727013494369	0.687627932202788	0.994766463583882	pseudobulk	DESeq2	LRT	0.162646490107219
135	KC_AB1	Sil1	0.0867746168095977	0.810183210642082	0.994766463583882	pseudobulk	DESeq2	LRT	0.0914167609068723
135	KC_AB1	sima	0.0408805529223416	0.893511838543893	0.994766463583882	pseudobulk	DESeq2	LRT	0.0488996889555102
135	KC_AB1	simj	0.0903721748680352	0.769814258966063	0.994766463583882	pseudobulk	DESeq2	LRT	0.113614048901434
135	KC_AB1	Sin	0.431549567083559	0.749733203540373	0.994766463583882	pseudobulk	DESeq2	LRT	0.125093255066858
135	KC_AB1	Sin1	0.118269466566299	0.87512589511943	0.994766463583882	pseudobulk	DESeq2	LRT	0.0579294651231942
135	KC_AB1	Sin3A	0.150910240268796	0.59245869748658	0.994766463583882	pseudobulk	DESeq2	LRT	0.227341920556502
135	KC_AB1	sina	0.0202583789169815	0.923641293104013	0.994766463583882	pseudobulk	DESeq2	LRT	0.0344966593772788
135	KC_AB1	sing	0.949183608371002	0.80156895525114	0.994766463583882	pseudobulk	DESeq2	LRT	0.0960591113673091
135	KC_AB1	sinu	-0.218252035065813	0.833574151637176	0.994766463583882	pseudobulk	DESeq2	LRT	0.0790557609056227
135	KC_AB1	sip1	-0.0876274309314422	0.917937734184526	0.994766463583882	pseudobulk	DESeq2	LRT	0.0371867769874299
135	KC_AB1	sip3	0.162326886073605	0.823118626787798	0.994766463583882	pseudobulk	DESeq2	LRT	0.0845375703202388
135	KC_AB1	Sirt1	-0.054987982848337	0.866851874568237	0.994766463583882	pseudobulk	DESeq2	LRT	0.062055107314573
135	KC_AB1	Sirt2	5.67162227262315E-05	0.944321578965334	0.994766463583882	pseudobulk	DESeq2	LRT	0.0248800860117186
135	KC_AB1	Sirt4	-0.218451756832414	0.806968917345498	0.994766463583882	pseudobulk	DESeq2	LRT	0.0931431930168469
135	KC_AB1	Sirt6	-0.862128082958893	0.639533020568458	0.994766463583882	pseudobulk	DESeq2	LRT	0.194137026978222
135	KC_AB1	Sirt7	-0.255783849941125	0.83458686414983	0.994766463583882	pseudobulk	DESeq2	LRT	0.0785284550809825
135	KC_AB1	Sirup	0.242942762672471	0.747877965856039	0.994766463583882	pseudobulk	DESeq2	LRT	0.126169261866952
135	KC_AB1	sisRNA:1	-1.64952313825113	0.817565084244375	0.994766463583882	pseudobulk	DESeq2	LRT	0.0874776642296574
135	KC_AB1	siz	-0.211191597000484	0.461595777932332	0.994766463583882	pseudobulk	DESeq2	LRT	0.335738172185679
135	KC_AB1	SK	0.247700494753786	0.161171829975884	0.994766463583882	pseudobulk	DESeq2	LRT	0.792710862996927
135	KC_AB1	Sk2	0.496776259994135	0.622020381890348	0.994766463583882	pseudobulk	DESeq2	LRT	0.20619538444506
135	KC_AB1	Skadu	-1.64952313825113	0.817565084244375	0.994766463583882	pseudobulk	DESeq2	LRT	0.0874776642296574
135	KC_AB1	skd	-0.0429159704395706	0.835493903010021	0.994766463583882	pseudobulk	DESeq2	LRT	0.0780567150090884
135	KC_AB1	Ski6	1.30483956200775	0.433526362208998	0.994766463583882	pseudobulk	DESeq2	LRT	0.362984488467372
135	KC_AB1	SKIP	1.86018888013104	0.296431693195341	0.994766463583882	pseudobulk	DESeq2	LRT	0.528075365321518
135	KC_AB1	SkpA	0.140436987017315	0.603819706816412	0.994766463583882	pseudobulk	DESeq2	LRT	0.219092717046675
135	KC_AB1	sky	-0.502137087886237	0.160026853306977	0.994766463583882	pseudobulk	DESeq2	LRT	0.795807134440982
135	KC_AB1	Slbp	-0.397610734679982	0.628867470669413	0.994766463583882	pseudobulk	DESeq2	LRT	0.201440869374089
135	KC_AB1	SLC22A	-0.484211582768817	0.811014852048671	0.994766463583882	pseudobulk	DESeq2	LRT	0.0909711925166842
135	KC_AB1	Slc25A46a	1.03308176465576	0.410963057069045	0.994766463583882	pseudobulk	DESeq2	LRT	0.386197216645023
135	KC_AB1	SLC5A11	1.17930484172975	0.669685881755347	0.994766463583882	pseudobulk	DESeq2	LRT	0.174128856716169
135	KC_AB1	Sld5	1.80633728195307	0.61161102402172	0.994766463583882	pseudobulk	DESeq2	LRT	0.213524695220716
135	KC_AB1	sle	0.569315361592199	0.670340775815066	0.994766463583882	pseudobulk	DESeq2	LRT	0.173704362326592
135	KC_AB1	slgA	-0.0543709583241543	0.872907274145783	0.994766463583882	pseudobulk	DESeq2	LRT	0.0590318874119878
136	KC_AB1	Slh	0.468845204245928	0.694689306762149	0.994766463583882	pseudobulk	DESeq2	LRT	0.158209386097481
136	KC_AB1	slif	0.40411895759739	0.864643228521197	0.994766463583882	pseudobulk	DESeq2	LRT	0.0631630553638974
136	KC_AB1	Slik	-0.476787361036291	0.260015646328184	0.994766463583882	pseudobulk	DESeq2	LRT	0.585000517761714
136	KC_AB1	slim	-0.33339033242821	0.588855757340251	0.994766463583882	pseudobulk	DESeq2	LRT	0.229991074420005
136	KC_AB1	Slimp	3.61180882517333	0.168873982898256	0.994766463583882	pseudobulk	DESeq2	LRT	0.772437253650179
136	KC_AB1	Slip1	-0.00077026812085544	0.94643500783639	0.994766463583882	pseudobulk	DESeq2	LRT	0.0239092038947312
136	KC_AB1	SLIRP2	-0.167697376891523	0.827523586618263	0.994766463583882	pseudobulk	DESeq2	LRT	0.0822196188305842

136	KC_AB1	sll	-0.715272341952579	0.623675257950241	0.994766463583882	pseudobulk	DESeq2	LRT	0.205041484662704
136	KC_AB1	Slmap	-0.120731785550192	0.745628432629197	0.994766463583882	pseudobulk	DESeq2	LRT	0.127477539631367
136	KC_AB1	slmb	0.252326961106759	0.610854153016126	0.994766463583882	pseudobulk	DESeq2	LRT	0.21406246913758
136	KC_AB1	slmo	-0.910178052452517	0.352257755897285	0.994766463583882	pseudobulk	DESeq2	LRT	0.453139435957964
136	KC_AB1	Sln	0.157542061007004	0.813110022676128	0.994766463583882	pseudobulk	DESeq2	LRT	0.0898506856380954
136	KC_AB1	slo	-0.0181414735682669	0.872561885736242	0.994766463583882	pseudobulk	DESeq2	LRT	0.0592037612964223
136	KC_AB1	SLO2	-0.0593028614938562	0.726619684759149	0.994766463583882	pseudobulk	DESeq2	LRT	0.138692840898744
136	KC_AB1	Slob	0.114994813278688	0.556044312955083	0.994766463583882	pseudobulk	DESeq2	LRT	0.254890596718291
136	KC_AB1	slow	-2.20969975456663	0.610836134188693	0.994766463583882	pseudobulk	DESeq2	LRT	0.214075280039667
136	KC_AB1	slpr	-0.112373692152438	0.793441164093036	0.994766463583882	pseudobulk	DESeq2	LRT	0.10048527187963
136	KC_AB1	sls	0.596808856322697	0.822761281772702	0.994766463583882	pseudobulk	DESeq2	LRT	0.0847261539098712
136	KC_AB1	Slu7	0.278578232747047	0.727367436926762	0.994766463583882	pseudobulk	DESeq2	LRT	0.138246145513329
136	KC_AB1	slv	0.0871213401343273	0.940955672557668	0.994766463583882	pseudobulk	DESeq2	LRT	0.0264308352518565
136	KC_AB1	slx1	1.45682795759908	0.573003993665791	0.994766463583882	pseudobulk	DESeq2	LRT	0.24184235111992
136	KC_AB1	sm	-0.0830056900292077	0.745093255020472	0.994766463583882	pseudobulk	DESeq2	LRT	0.127789368048728
136	KC_AB1	smash	-0.436608948931595	0.277558652797509	0.994766463583882	pseudobulk	DESeq2	LRT	0.556645229136892
136	KC_AB1	SmB	0.361297352402837	0.536036073917416	0.994766463583882	pseudobulk	DESeq2	LRT	0.27080598236675
136	KC_AB1	SMC1	-0.104511658231562	0.862393399758473	0.994766463583882	pseudobulk	DESeq2	LRT	0.0642945759739454
136	KC_AB1	SMC2	0.99025238934285	0.473935213589507	0.994766463583882	pseudobulk	DESeq2	LRT	0.324281021835614
136	KC_AB1	SMC3	0.268415492138991	0.735733766466113	0.994766463583882	pseudobulk	DESeq2	LRT	0.133279311575571
136	KC_AB1	SMC5	0.143038513181072	0.91078643311207	0.994766463583882	pseudobulk	DESeq2	LRT	0.0405834471702539
136	KC_AB1	SmD1	0.715735493952572	0.491590153629002	0.994766463583882	pseudobulk	DESeq2	LRT	0.308396824458115
136	KC_AB1	SmD3	-0.287842035647804	0.740722265080961	0.994766463583882	pseudobulk	DESeq2	LRT	0.130344600864172
136	KC_AB1	SmE	0.224134183136394	0.676839973256016	0.994766463583882	pseudobulk	DESeq2	LRT	0.16951400036521
136	KC_AB1	SmF	0.0265710550077379	0.932865272249415	0.994766463583882	pseudobulk	DESeq2	LRT	0.0301810740923605
136	KC_AB1	smg	0.635193413241552	0.356910460642088	0.994766463583882	pseudobulk	DESeq2	LRT	0.447440723177541
136	KC_AB1	Smg5	0.0845146816600505	0.790637353733666	0.994766463583882	pseudobulk	DESeq2	LRT	0.102022671226053
136	KC_AB1	Smg6	0.515225524186442	0.608824039958355	0.994766463583882	pseudobulk	DESeq2	LRT	0.215508207395284
136	KC_AB1	smid	-1.18417442487168	0.266061039524852	0.994766463583882	pseudobulk	DESeq2	LRT	0.575018716422555
136	KC_AB1	Smn	0.235260140354015	0.801650642962752	0.994766463583882	pseudobulk	DESeq2	LRT	0.0960148547695694
136	KC_AB1	smo	-0.927296974830984	0.42643456281655	0.994766463583882	pseudobulk	DESeq2	LRT	0.370147602686486
136	KC_AB1	smog	-0.106132651818314	0.653792296772968	0.994766463583882	pseudobulk	DESeq2	LRT	0.184560200746628
136	KC_AB1	Smox	-0.10930468526013	0.625501870345792	0.994766463583882	pseudobulk	DESeq2	LRT	0.20377138736202
136	KC_AB1	smp-30	0.147280766987651	0.885491532343871	0.994766463583882	pseudobulk	DESeq2	LRT	0.05281558746534
136	KC_AB1	Smr	-0.0168042785596081	0.933329003559121	0.994766463583882	pseudobulk	DESeq2	LRT	0.0299652380932394
136	KC_AB1	Sms	-0.0405963926556852	0.915144693565312	0.994766463583882	pseudobulk	DESeq2	LRT	0.0385102341860842
136	KC_AB1	SMSr	0.783951867599183	0.30559285557782	0.994766463583882	pseudobulk	DESeq2	LRT	0.514856803301186
136	KC_AB1	smt3	0.0341992852740923	0.879982274539339	0.994766463583882	pseudobulk	DESeq2	LRT	0.0555260757444733
136	KC_AB1	Smurf	0.258158330417705	0.598391803087086	0.994766463583882	pseudobulk	DESeq2	LRT	0.223014364171719
136	KC_AB1	Smyd3	-1.51775761761922	0.250041490387253	0.994766463583882	pseudobulk	DESeq2	LRT	0.601987921123294
136	KC_AB1	Smyd4-1	-2.00005840186615	0.514177117052568	0.994766463583882	pseudobulk	DESeq2	LRT	0.288887255112324
136	KC_AB1	Smyd4-4	-1.33269804377321	0.153112920337669	0.994766463583882	pseudobulk	DESeq2	LRT	0.814988160088171
136	KC_AB1	Smyd5	-0.90808656036651	0.52494506160691	0.994766463583882	pseudobulk	DESeq2	LRT	0.279886145526293
136	KC_AB1	SmydA-1	1.08180406441536	0.178554773237666	0.994766463583882	pseudobulk	DESeq2	LRT	0.748228535484806
136	KC_AB1	SmydA-3	0.188006668896169	0.872602146338487	0.994766463583882	pseudobulk	DESeq2	LRT	0.0591837231142514
136	KC_AB1	SmydA-4	0.408270288724494	0.784293047765862	0.994766463583882	pseudobulk	DESeq2	LRT	0.105521634703418
136	KC_AB1	SmydA-7	0.0639664593105349	0.947865498209139	0.994766463583882	pseudobulk	DESeq2	LRT	0.023253284528812
136	KC_AB1	sn	1.22776512758308	0.282687379535205	0.994766463583882	pseudobulk	DESeq2	LRT	0.548693579980824
136	KC_AB1	snama	0.264769089465416	0.674338647833451	0.994766463583882	pseudobulk	DESeq2	LRT	0.171121949236579
136	KC_AB1	Snap24	-0.0888512244607552	0.743402324635121	0.994766463583882	pseudobulk	DESeq2	LRT	0.128776085189947
136	KC_AB1	Snap25	0.0969694514809792	0.4988669865834	0.994766463583882	pseudobulk	DESeq2	LRT	0.302015235325308
136	KC_AB1	Snap29	-0.617175881010218	0.388340254523445	0.994766463583882	pseudobulk	DESeq2	LRT	0.410787589079802
136	KC_AB1	snf	-0.404643674709124	0.433313288827315	0.994766463583882	pseudobulk	DESeq2	LRT	0.363197991842901
136	KC_AB1	SNF4Agamma	-0.439803633958202	0.41178151419126	0.994766463583882	pseudobulk	DESeq2	LRT	0.385333153747445
136	KC_AB1	sni	0.504519427853977	0.68772296451421	0.994766463583882	pseudobulk	DESeq2	LRT	0.162586473410946
136	KC_AB1	Snm1	2.67764418499683	0.378704357417491	0.994766463583882	pseudobulk	DESeq2	LRT	0.421699697806334

136	KC_AB1	snmRNA:CR43701	0.806933541094274	0.754600478929312	0.994766463583882	pseudobulk	DESeq2	LRT	0.122282923497213
136	KC_AB1	sno	0.137605882928539	0.656291299529852	0.994766463583882	pseudobulk	DESeq2	LRT	0.182903353157706
136	KC_AB1	Snoo	0.0539186918094909	0.845914552516248	0.994766463583882	pseudobulk	DESeq2	LRT	0.0726735036806786
136	KC_AB1	snoRNA:Dek-a	-1.64952313825113	0.817565084244375	0.994766463583882	pseudobulk	DESeq2	LRT	0.0874776642296574
136	KC_AB1	snoRNA:nop5-x16-a	0.949183608371002	0.80156895525114	0.994766463583882	pseudobulk	DESeq2	LRT	0.0960591113673091
136	KC_AB1	snoRNA:Or-CD14	0.949183608371002	0.80156895525114	0.994766463583882	pseudobulk	DESeq2	LRT	0.0960591113673091
136	KC_AB1	snoRNA:Psi18S-1377b	-2.79499059556516	0.38564607643653	0.994766463583882	pseudobulk	DESeq2	LRT	0.413811082791365
136	KC_AB1	snoRNA:Psi18S-1389a	0.949183608371002	0.80156895525114	0.994766463583882	pseudobulk	DESeq2	LRT	0.0960591113673091
136	KC_AB1	snoRNA:Psi28S-1135d	-1.28132067551389	0.647201349541384	0.994766463583882	pseudobulk	DESeq2	LRT	0.188960585805912
136	KC_AB1	snoRNA:Psi28S-1153	-1.64952313825113	0.817565084244375	0.994766463583882	pseudobulk	DESeq2	LRT	0.0874776642296574
136	KC_AB1	snoRNA:Psi28S-1175c	0.949183608371002	0.80156895525114	0.994766463583882	pseudobulk	DESeq2	LRT	0.0960591113673091
136	KC_AB1	snoRNA:Psi28S-1192b	-1.64952313825113	0.817565084244375	0.994766463583882	pseudobulk	DESeq2	LRT	0.0874776642296574
136	KC_AB1	snoRNA:Psi28S-1837b	0.949183608371002	0.80156895525114	0.994766463583882	pseudobulk	DESeq2	LRT	0.0960591113673091
136	KC_AB1	snoRNA:Psi28S-2622	0.469123695720418	0.811531888990343	0.994766463583882	pseudobulk	DESeq2	LRT	0.0906944099838556
136	KC_AB1	snoRNA:Psi28S-2876	1.1821590793593	0.719511699510136	0.994766463583882	pseudobulk	DESeq2	LRT	0.142962139890846
136	KC_AB1	snoRNA:Psi28S-3091b	-1.64952313825113	0.817565084244375	0.994766463583882	pseudobulk	DESeq2	LRT	0.0874776642296574
136	KC_AB1	snoRNA:Psi28S-3571	-2.20969975456663	0.610836134188693	0.994766463583882	pseudobulk	DESeq2	LRT	0.214075280039667
136	KC_AB1	snoRNA:SC35-b	2.02670873290438	0.538959414925803	0.994766463583882	pseudobulk	DESeq2	LRT	0.26844393710898
136	KC_AB1	snoRNA:Tudor-SN-a	0.949183608371002	0.80156895525114	0.994766463583882	pseudobulk	DESeq2	LRT	0.0960591113673091
136	KC_AB1	Snp	-0.693211348537749	0.433000685243405	0.994766463583882	pseudobulk	DESeq2	LRT	0.363511416355193
136	KC_AB1	sNPF	0.288160121215464	0.0408043574526848	0.994766463583882	pseudobulk	DESeq2	LRT	1.3892934566011
136	KC_AB1	Snr1	0.182469106630482	0.75005346432563	0.994766463583882	pseudobulk	DESeq2	LRT	0.12490777869625
136	KC_AB1	snRNA:7SK	0.199805181412229	0.484207814431528	0.994766463583882	pseudobulk	DESeq2	LRT	0.314968205937024
136	KC_AB1	snRNA:U1:82Eb	2.22023857837763	0.524904842126482	0.994766463583882	pseudobulk	DESeq2	LRT	0.279919420946639
136	KC_AB1	snRNA:U11	-1.64952313825113	0.817565084244375	0.994766463583882	pseudobulk	DESeq2	LRT	0.0874776642296574
136	KC_AB1	snRNA:U12	-1.15188692395163	0.603405702316466	0.994766463583882	pseudobulk	DESeq2	LRT	0.219390589963361
136	KC_AB1	snRNA:U2:14B	-0.78410610838983	0.738244895252942	0.994766463583882	pseudobulk	DESeq2	LRT	0.131799547371545
136	KC_AB1	snRNA:U4:39B	-1.64952313825113	0.817565084244375	0.994766463583882	pseudobulk	DESeq2	LRT	0.0874776642296574
136	KC_AB1	snRNA:U5:38ABa	-1.64952313825113	0.817565084244375	0.994766463583882	pseudobulk	DESeq2	LRT	0.0874776642296574
136	KC_AB1	snRNA:U5:63BC	0.949183608371002	0.80156895525114	0.994766463583882	pseudobulk	DESeq2	LRT	0.0960591113673091
136	KC_AB1	snRNA:U6:96Ab	1.1821590793593	0.719511699510136	0.994766463583882	pseudobulk	DESeq2	LRT	0.142962139890846
136	KC_AB1	snRNP-U1-70K	0.314759549853587	0.489683336880713	0.994766463583882	pseudobulk	DESeq2	LRT	0.310084674057916
136	KC_AB1	snRNP-U1-C	0.620693345805951	0.441663406533709	0.994766463583882	pseudobulk	DESeq2	LRT	0.354908582174337
136	KC_AB1	SNRPG	-0.306328172777603	0.50779432738384	0.994766463583882	pseudobulk	DESeq2	LRT	0.294312154977965
136	KC_AB1	sns	2.83318330719205	0.120398598484359	0.994766463583882	pseudobulk	DESeq2	LRT	0.919378568510517
136	KC_AB1	Snx1	0.0743819617198969	0.879161759473444	0.994766463583882	pseudobulk	DESeq2	LRT	0.055931210496817
136	KC_AB1	Snx16	1.25642584052917	0.191344549437579	0.994766463583882	pseudobulk	DESeq2	LRT	0.718183904397922
137	KC_AB1	Snx17	0.0319008687557541	0.948796232585579	0.994766463583882	pseudobulk	DESeq2	LRT	0.0228270484428632
137	KC_AB1	Snx21	-0.255040701318231	0.80860463536502	0.994766463583882	pseudobulk	DESeq2	LRT	0.0922637733811727
137	KC_AB1	Snx27	-0.127904361661311	0.713810433327976	0.994766463583882	pseudobulk	DESeq2	LRT	0.146417108518698
137	KC_AB1	Snx3	-0.123333800665412	0.792235597064149	0.994766463583882	pseudobulk	DESeq2	LRT	0.101145647590388
137	KC_AB1	Snx6	0.375446241679767	0.535194412691099	0.994766463583882	pseudobulk	DESeq2	LRT	0.271488429152959
137	KC_AB1	snz	0.559919649371256	0.461166121224578	0.994766463583882	pseudobulk	DESeq2	LRT	0.33614260490217
137	KC_AB1	Socs16D	-0.157907515905863	0.746502665046923	0.994766463583882	pseudobulk	DESeq2	LRT	0.126968637486033
137	KC_AB1	Socs36E	0.134325626476446	0.759992912911163	0.994766463583882	pseudobulk	DESeq2	LRT	0.1191904575849
137	KC_AB1	Socs44A	-0.252345191649517	0.752897985706699	0.994766463583882	pseudobulk	DESeq2	LRT	0.123263864763487
137	KC_AB1	Sod1	0.113997226555278	0.659518915757767	0.994766463583882	pseudobulk	DESeq2	LRT	0.180772743875902
137	KC_AB1	Sod2	0.362204172179127	0.430004804999777	0.994766463583882	pseudobulk	DESeq2	LRT	0.366526691459415
137	KC_AB1	Sod3	1.80633728195307	0.61161102402172	0.994766463583882	pseudobulk	DESeq2	LRT	0.213524695220716
137	KC_AB1	sol	0.042949322399353	0.846966704103417	0.994766463583882	pseudobulk	DESeq2	LRT	0.0721336622883979
137	KC_AB1	Son	0.0863018446369049	0.901356130276853	0.994766463583882	pseudobulk	DESeq2	LRT	0.0451035832101545
137	KC_AB1	sona	-2.79499059556516	0.38564607643653	0.994766463583882	pseudobulk	DESeq2	LRT	0.413811082791365
137	KC_AB1	Sos	-0.116739881326613	0.877499829363958	0.994766463583882	pseudobulk	DESeq2	LRT	0.0567529593137607
137	KC_AB1	sosie	0.596808856322697	0.822761281772702	0.994766463583882	pseudobulk	DESeq2	LRT	0.0847261539098712
137	KC_AB1	soti	0.729740153036158	0.752353468463509	0.994766463583882	pseudobulk	DESeq2	LRT	0.123578072524162
137	KC_AB1	sotv	0.384400093499947	0.558517055214363	0.994766463583882	pseudobulk	DESeq2	LRT	0.252963560467097

137	KC_AB1	sov	-0.0906543205279181	0.814709965173779	0.994766463583882	pseudobulk	DESeq2	LRT	0.0889969715578791
137	KC_AB1	sowah	0.646788291801598	0.312834410354187	0.994766463583882	pseudobulk	DESeq2	LRT	0.504685482578254
137	KC_AB1	Sox21b	0.949183608371002	0.80156895525114	0.994766463583882	pseudobulk	DESeq2	LRT	0.0960591113673091
137	KC_AB1	SoxN	0.949183608371002	0.80156895525114	0.994766463583882	pseudobulk	DESeq2	LRT	0.0960591113673091
137	KC_AB1	SP1173	0.0894988238782921	0.674549365402075	0.994766463583882	pseudobulk	DESeq2	LRT	0.170986261948768
137	KC_AB1	SP2353	0.479547418245208	0.414553417766532	0.994766463583882	pseudobulk	DESeq2	LRT	0.382419499983415
137	KC_AB1	spab	-0.342665806993449	0.302265898749385	0.994766463583882	pseudobulk	DESeq2	LRT	0.51961084660438
137	KC_AB1	spag	0.143317708789167	0.865073600924825	0.994766463583882	pseudobulk	DESeq2	LRT	0.0629469409563437
137	KC_AB1	SPARC	-2.20969975456663	0.610836134188693	0.994766463583882	pseudobulk	DESeq2	LRT	0.214075280039667
137	KC_AB1	spartin	0.376288664616786	0.675606970202018	0.994766463583882	pseudobulk	DESeq2	LRT	0.170305878484305
137	KC_AB1	spas	-0.555551924911035	0.432112676935085	0.994766463583882	pseudobulk	DESeq2	LRT	0.364402992558948
137	KC_AB1	Spase12	-0.54042435599816	0.313810950236867	0.994766463583882	pseudobulk	DESeq2	LRT	0.503331906050313
137	KC_AB1	Spase22-23	0.249566171816168	0.69797475917481	0.994766463583882	pseudobulk	DESeq2	LRT	0.156160282461802
137	KC_AB1	Spase25	0.306680249595067	0.589959043323069	0.994766463583882	pseudobulk	DESeq2	LRT	0.22917813730056
137	KC_AB1	SpdS	-0.536123521142269	0.47190186798177	0.994766463583882	pseudobulk	DESeq2	LRT	0.326148303537387
137	KC_AB1	Spec2	-0.633091634849849	0.457487706344002	0.994766463583882	pseudobulk	DESeq2	LRT	0.339620571846052
137	KC_AB1	spel1	0.638800163265667	0.709767226878927	0.994766463583882	pseudobulk	DESeq2	LRT	0.148884057840267
137	KC_AB1	spen	-0.035577141416207	0.78592217057929	0.994766463583882	pseudobulk	DESeq2	LRT	0.104620459764065
137	KC_AB1	Spf45	0.154937111947396	0.721302940101293	0.994766463583882	pseudobulk	DESeq2	LRT	0.141882297579565
137	KC_AB1	spg	0.131259856261552	0.865995194418513	0.994766463583882	pseudobulk	DESeq2	LRT	0.0624845179633843
137	KC_AB1	Spg7	0.669759066232443	0.584943507370748	0.994766463583882	pseudobulk	DESeq2	LRT	0.232886075151763
137	KC_AB1	spi	-0.278985519825026	0.551395226160645	0.994766463583882	pseudobulk	DESeq2	LRT	0.258536998224921
137	KC_AB1	spict	0.954845690117739	0.477651256765233	0.994766463583882	pseudobulk	DESeq2	LRT	0.320889075229045
137	KC_AB1	spidey	-0.185120432578798	0.656417677268226	0.994766463583882	pseudobulk	DESeq2	LRT	0.182819731950644
137	KC_AB1	spin	-0.497493042751891	0.563534477572237	0.994766463583882	pseudobulk	DESeq2	LRT	0.249079508257948
137	KC_AB1	Spindly	1.1821590793593	0.719511699510136	0.994766463583882	pseudobulk	DESeq2	LRT	0.142962139890846
137	KC_AB1	spir	-0.0409168800803106	0.838012975950005	0.994766463583882	pseudobulk	DESeq2	LRT	0.0767492566204934
137	KC_AB1	sPLA2	-4.97250539949279	0.028859085071683	0.994766463583882	pseudobulk	DESeq2	LRT	1.53971744159381
137	KC_AB1	Sply	0.0926898912592504	0.895271876545004	0.994766463583882	pseudobulk	DESeq2	LRT	0.0480450579209773
137	KC_AB1	Spn	-0.301506442015534	0.180378400899781	0.994766463583882	pseudobulk	DESeq2	LRT	0.743815467523761
137	KC_AB1	spn-A	0.949183608371002	0.80156895525114	0.994766463583882	pseudobulk	DESeq2	LRT	0.0960591113673091
137	KC_AB1	spn-B	-3.21143241058256	0.292545415061155	0.994766463583882	pseudobulk	DESeq2	LRT	0.533806704011112
137	KC_AB1	spn-F	0.240362476381514	0.776203412150128	0.994766463583882	pseudobulk	DESeq2	LRT	0.110024452455686
137	KC_AB1	Spn42Da	-0.594559481966011	0.524629160046221	0.994766463583882	pseudobulk	DESeq2	LRT	0.280147574029288
137	KC_AB1	Spn43Ab	-3.95857014146861	0.177445975557202	0.994766463583882	pseudobulk	DESeq2	LRT	0.750933845925402
137	KC_AB1	Spn88Ea	-2.20969975456663	0.610836134188693	0.994766463583882	pseudobulk	DESeq2	LRT	0.214075280039667
137	KC_AB1	SPoCk	-0.322336828161057	0.262995982958153	0.994766463583882	pseudobulk	DESeq2	LRT	0.58005088494154
137	KC_AB1	spok	1.1821590793593	0.719511699510136	0.994766463583882	pseudobulk	DESeq2	LRT	0.142962139890846
137	KC_AB1	spoon	-0.209109208598142	0.704548028495567	0.994766463583882	pseudobulk	DESeq2	LRT	0.152089396025699
137	KC_AB1	Spp	0.556847584943023	0.50951300899549	0.994766463583882	pseudobulk	DESeq2	LRT	0.292844723016082
137	KC_AB1	SppL	0.172384405612246	0.734532125435003	0.994766463583882	pseudobulk	DESeq2	LRT	0.133989205191564
137	KC_AB1	Spps	-1.47534342459056	0.207638284986126	0.994766463583882	pseudobulk	DESeq2	LRT	0.682692566886897
137	KC_AB1	SPR	1.62374189241391	0.0441008571595917	0.994766463583882	pseudobulk	DESeq2	LRT	1.35555296935159
137	KC_AB1	Spred	-0.166834706740528	0.378047939679959	0.994766463583882	pseudobulk	DESeq2	LRT	0.422453124452874
137	KC_AB1	spri	-3.85958892239636	0.166041795813258	0.994766463583882	pseudobulk	DESeq2	LRT	0.779782578186973
137	KC_AB1	sprt	0.916993159321176	0.700096979888278	0.994766463583882	pseudobulk	DESeq2	LRT	0.15484179582425
137	KC_AB1	Sps1	-1.00262466711381	0.27440811492541	0.994766463583882	pseudobulk	DESeq2	LRT	0.561603049615375
137	KC_AB1	Sps2	-0.515861821747466	0.752347659589506	0.994766463583882	pseudobulk	DESeq2	LRT	0.123581425697598
137	KC_AB1	Spt	-1.50663180504502	0.668163815680457	0.994766463583882	pseudobulk	DESeq2	LRT	0.175117047219009
137	KC_AB1	Spt-I	0.112257504475319	0.824522545405648	0.994766463583882	pseudobulk	DESeq2	LRT	0.0837974646872624
137	KC_AB1	Spt20	-0.100303309116115	0.864225657424772	0.994766463583882	pseudobulk	DESeq2	LRT	0.0633728443513452
137	KC_AB1	Spt3	-0.722948597948389	0.57936146535796	0.994766463583882	pseudobulk	DESeq2	LRT	0.237050394080379
137	KC_AB1	spt4	1.11099166822345	0.281901887877468	0.994766463583882	pseudobulk	DESeq2	LRT	0.549902015678517
137	KC_AB1	Spt5	-0.120022918530725	0.810655249532274	0.994766463583882	pseudobulk	DESeq2	LRT	0.0911638006075123
137	KC_AB1	Spt6	0.217474743683927	0.715785381526708	0.994766463583882	pseudobulk	DESeq2	LRT	0.145217175444637
137	KC_AB1	Spt7	-2.08761707051565	0.300243913107209	0.994766463583882	pseudobulk	DESeq2	LRT	0.522525788357186

137	KC_AB1	Sptr	0.412618810232952	0.571571747035401	0.994766463583882	pseudobulk	DESeq2	LRT	0.242929246658316
137	KC_AB1	Spx	-0.0734931455103705	0.882664909409484	0.994766463583882	pseudobulk	DESeq2	LRT	0.0542041385681525
137	KC_AB1	spz	3.80499962290998	0.134213047776818	0.994766463583882	pseudobulk	DESeq2	LRT	0.872205261350181
137	KC_AB1	spz4	2.02670873290438	0.538959414925803	0.994766463583882	pseudobulk	DESeq2	LRT	0.26844393710898
137	KC_AB1	sqa	-1.64952313825113	0.817565084244375	0.994766463583882	pseudobulk	DESeq2	LRT	0.0874776642296574
137	KC_AB1	sqd	0.0904903352123329	0.639964000732427	0.994766463583882	pseudobulk	DESeq2	LRT	0.193844455270771
137	KC_AB1	sqh	0.0901234104819283	0.842909918550875	0.994766463583882	pseudobulk	DESeq2	LRT	0.0742188357746314
137	KC_AB1	squ	-2.00005840186615	0.514177117052568	0.994766463583882	pseudobulk	DESeq2	LRT	0.288887255112324
137	KC_AB1	sqz	0.0775082038820824	0.868123464754239	0.994766463583882	pseudobulk	DESeq2	LRT	0.0614185049521199
137	KC_AB1	sr	-0.0421208727137929	0.913261287776364	0.994766463583882	pseudobulk	DESeq2	LRT	0.0394049512663961
137	KC_AB1	Sr-CII	-0.835152883801839	0.920434724857442	0.994766463583882	pseudobulk	DESeq2	LRT	0.0360070052473159
137	KC_AB1	sra	-0.121972212908309	0.692118509455761	0.994766463583882	pseudobulk	DESeq2	LRT	0.159819536184745
137	KC_AB1	Sra-1	-0.18999932250786	0.83991976969287	0.994766463583882	pseudobulk	DESeq2	LRT	0.0757621963711761
137	KC_AB1	Sras	0.0969379335785026	0.917258812152282	0.994766463583882	pseudobulk	DESeq2	LRT	0.0375081072626919
137	KC_AB1	Src42A	-0.0160293274382752	0.959379084598668	0.994766463583882	pseudobulk	DESeq2	LRT	0.0180097538109164
137	KC_AB1	Src64B	0.420408620689528	0.332307869077192	0.994766463583882	pseudobulk	DESeq2	LRT	0.478459374471097
137	KC_AB1	SREBP	1.35843186412052	0.220302264201083	0.994766463583882	pseudobulk	DESeq2	LRT	0.656981039276908
137	KC_AB1	srl	1.45076656717961	0.419362983759837	0.994766463583882	pseudobulk	DESeq2	LRT	0.377409906389313
137	KC_AB1	Srlp	-0.122588721729708	0.872236752202049	0.994766463583882	pseudobulk	DESeq2	LRT	0.0593656180268732
137	KC_AB1	SRm160	-0.282910630709327	0.639399264600664	0.994766463583882	pseudobulk	DESeq2	LRT	0.194227867563951
137	KC_AB1	Srp14	0.179588947807243	0.729688287361143	0.994766463583882	pseudobulk	DESeq2	LRT	0.13686262479742
137	KC_AB1	Srp19	0.286406906692349	0.738131852267567	0.994766463583882	pseudobulk	DESeq2	LRT	0.131866053356476
137	KC_AB1	Srp54	0.0223253347195809	0.941737203701831	0.994766463583882	pseudobulk	DESeq2	LRT	0.0260702722655922
137	KC_AB1	Srp54k	-0.539532918262969	0.503267885608665	0.994766463583882	pseudobulk	DESeq2	LRT	0.298200781798234
137	KC_AB1	Srp68	-0.702910130620105	0.55984840468256	0.994766463583882	pseudobulk	DESeq2	LRT	0.251929554998602
138	KC_AB1	Srp72	-0.0366979615359374	0.871701134743277	0.994766463583882	pseudobulk	DESeq2	LRT	0.0596323886679738
138	KC_AB1	Srp9	-0.293069229167201	0.610827383944274	0.994766463583882	pseudobulk	DESeq2	LRT	0.214081501364623
138	KC_AB1	SRPK	0.0669640441133607	0.843013424586365	0.994766463583882	pseudobulk	DESeq2	LRT	0.0741655093878815
138	KC_AB1	Srpk79D	0.439440549968378	0.461595616205928	0.994766463583882	pseudobulk	DESeq2	LRT	0.335738324346726
138	KC_AB1	SrpRbeta	1.65762260081243	0.145583931831328	0.994766463583882	pseudobulk	DESeq2	LRT	0.836886555669097
138	KC_AB1	Srr	1.1821590793593	0.719511699510136	0.994766463583882	pseudobulk	DESeq2	LRT	0.142962139890846
138	KC_AB1	Sry-delta	-0.609004467479253	0.53267511188645	0.994766463583882	pseudobulk	DESeq2	LRT	0.273537594228657
138	KC_AB1	Ssadh	-0.122252003968318	0.867830315676062	0.994766463583882	pseudobulk	DESeq2	LRT	0.0615651828529637
138	KC_AB1	Ssb-c31a	0.156198768334985	0.76477977838329	0.994766463583882	pseudobulk	DESeq2	LRT	0.116463603802898
138	KC_AB1	Ssdp	-0.300957899829865	0.21580975139019	0.994766463583882	pseudobulk	DESeq2	LRT	0.665928935560104
138	KC_AB1	ssh	-0.221348549561847	0.740975389718226	0.994766463583882	pseudobulk	DESeq2	LRT	0.130196216159761
138	KC_AB1	Ssl1	1.03078966212968	0.391528351881144	0.994766463583882	pseudobulk	DESeq2	LRT	0.407236783747273
138	KC_AB1	ssp	-0.617611662792199	0.648728213474266	0.994766463583882	pseudobulk	DESeq2	LRT	0.187937214001726
138	KC_AB1	ssp2	-1.02319198676748	0.828204539793902	0.994766463583882	pseudobulk	DESeq2	LRT	0.0818623932467813
138	KC_AB1	ssp3	0.0323949059562154	0.953326651487926	0.994766463583882	pseudobulk	DESeq2	LRT	0.0207582655403664
138	KC_AB1	ssp6	1.80939591345248	0.468117841155507	0.994766463583882	pseudobulk	DESeq2	LRT	0.329644806495434
138	KC_AB1	SsRbeta	0.59942694579313	0.330803259873998	0.994766463583882	pseudobulk	DESeq2	LRT	0.48043021937852
138	KC_AB1	Ssrp	-0.63506514683236	0.58685248371094	0.994766463583882	pseudobulk	DESeq2	LRT	0.231471053031234
138	KC_AB1	sstn	-0.582438735532103	0.770411347052444	0.994766463583882	pseudobulk	DESeq2	LRT	0.113277329302562
138	KC_AB1	Ssu72	0.216066728191716	0.818121058255848	0.994766463583882	pseudobulk	DESeq2	LRT	0.0871824285505266
138	KC_AB1	ssx	0.161132928365325	0.667387243928301	0.994766463583882	pseudobulk	DESeq2	LRT	0.1756220984671
138	KC_AB1	St1	0.208615470155158	0.893577121399079	0.994766463583882	pseudobulk	DESeq2	LRT	0.0488679591598277
138	KC_AB1	St3	-0.529596693808542	0.207370842613012	0.994766463583882	pseudobulk	DESeq2	LRT	0.683252307650292
138	KC_AB1	sta	0.064961207517213	0.720127932360394	0.994766463583882	pseudobulk	DESeq2	LRT	0.142590343315016
138	KC_AB1	Stacl	-0.0438070637811826	0.772215109432026	0.994766463583882	pseudobulk	DESeq2	LRT	0.112261705070482
138	KC_AB1	stai	-0.0090422086000304	0.946715454680156	0.994766463583882	pseudobulk	DESeq2	LRT	0.0237805331736051
138	KC_AB1	Stam	0.368936867142539	0.628866466496612	0.994766463583882	pseudobulk	DESeq2	LRT	0.201441562854154
138	KC_AB1	Start1	0.109236526335941	0.872567412188153	0.994766463583882	pseudobulk	DESeq2	LRT	0.0592010106606129
138	KC_AB1	stas	0.515709694838993	0.273812906763628	0.994766463583882	pseudobulk	DESeq2	LRT	0.562546084309731
138	KC_AB1	Stat92E	-0.15457013062618	0.541638983183819	0.994766463583882	pseudobulk	DESeq2	LRT	0.266290085880611
138	KC_AB1	stau	0.300328212687131	0.517408079489263	0.994766463583882	pseudobulk	DESeq2	LRT	0.286166793922129

138	KC_AB1	stc	-0.730620501214924	0.530799898302928	0.994766463583882	pseudobulk	DESeq2	LRT	0.27506916901499
138	KC_AB1	stck	-0.237827494119397	0.600442362528963	0.994766463583882	pseudobulk	DESeq2	LRT	0.221528674917192
138	KC_AB1	ste14	0.684009707602832	0.690372194095112	0.994766463583882	pseudobulk	DESeq2	LRT	0.160916708883582
138	KC_AB1	ste24a	0.855048337518235	0.378352690324072	0.994766463583882	pseudobulk	DESeq2	LRT	0.422103173580265
138	KC_AB1	ste24b	-1.64952313825113	0.817565084244375	0.994766463583882	pseudobulk	DESeq2	LRT	0.0874776642296574
138	KC_AB1	step	-0.0557230268324947	0.890608295744009	0.994766463583882	pseudobulk	DESeq2	LRT	0.0503132638656236
138	KC_AB1	stet	-4.12219491838083	0.272718961984444	0.994766463583882	pseudobulk	DESeq2	LRT	0.564284664775961
138	KC_AB1	Stim	0.32140288032052	0.586169369631594	0.994766463583882	pseudobulk	DESeq2	LRT	0.231976879428009
138	KC_AB1	Stip1	-0.662059278994238	0.57363801043645	0.994766463583882	pseudobulk	DESeq2	LRT	0.241362079139637
138	KC_AB1	stj	0.0111116817957263	0.883138904157032	0.994766463583882	pseudobulk	DESeq2	LRT	0.0539709832123039
138	KC_AB1	Stlk	-0.189133274374733	0.579082698520898	0.994766463583882	pseudobulk	DESeq2	LRT	0.237259410458009
138	KC_AB1	stmA	0.0515415406661597	0.85974044493663	0.994766463583882	pseudobulk	DESeq2	LRT	0.0656326421815587
138	KC_AB1	Stoml2	0.262208716755974	0.687266479726448	0.994766463583882	pseudobulk	DESeq2	LRT	0.162874837561685
138	KC_AB1	stops	-0.685085496766482	0.503910006376456	0.994766463583882	pseudobulk	DESeq2	LRT	0.297647017570147
138	KC_AB1	strat	-0.182021680517549	0.840755389537643	0.994766463583882	pseudobulk	DESeq2	LRT	0.0753303400240836
138	KC_AB1	Strip	0.778437605525277	0.350545514490421	0.994766463583882	pseudobulk	DESeq2	LRT	0.455255585660983
138	KC_AB1	Strn-Mlck	1.80633728195307	0.61161102402172	0.994766463583882	pseudobulk	DESeq2	LRT	0.213524695220716
138	KC_AB1	Strump	0.0674234022968274	0.891781785648264	0.994766463583882	pseudobulk	DESeq2	LRT	0.0497414022187879
138	KC_AB1	Stt3A	-0.0404157150645551	0.812852589175899	0.994766463583882	pseudobulk	DESeq2	LRT	0.0899882065733567
138	KC_AB1	Stt3B	-0.424545077907149	0.679190206453954	0.994766463583882	pseudobulk	DESeq2	LRT	0.168008585004491
138	KC_AB1	STUB1	-0.270779132615966	0.553427640679841	0.994766463583882	pseudobulk	DESeq2	LRT	0.256939154022399
138	KC_AB1	sturkopf	0.218509711231388	0.766762611839154	0.994766463583882	pseudobulk	DESeq2	LRT	0.115339071938753
138	KC_AB1	stv	-0.350858272986598	0.319041582575054	0.994766463583882	pseudobulk	DESeq2	LRT	0.496152709087131
138	KC_AB1	stw	-0.524315373782565	0.531096363864068	0.994766463583882	pseudobulk	DESeq2	LRT	0.274826671955324
138	KC_AB1	stwl	-0.203159634996468	0.863629878332787	0.994766463583882	pseudobulk	DESeq2	LRT	0.0636723411286881
138	KC_AB1	stx	0.272107780965474	0.42048428271979	0.994766463583882	pseudobulk	DESeq2	LRT	0.376250233054267
138	KC_AB1	sty	-0.68953337194275	0.0337452111315249	0.994766463583882	pseudobulk	DESeq2	LRT	1.47178785029132
138	KC_AB1	Su(dx)	-0.537691350600088	0.353189613560762	0.994766463583882	pseudobulk	DESeq2	LRT	0.451992076440937
138	KC_AB1	su(f)	0.381909923205642	0.591615763655604	0.994766463583882	pseudobulk	DESeq2	LRT	0.227960262705173
138	KC_AB1	Su(fu)	-0.443076351239415	0.687289725932076	0.994766463583882	pseudobulk	DESeq2	LRT	0.162860148167699
138	KC_AB1	Su(H)	0.531290439058193	0.604456583858226	0.994766463583882	pseudobulk	DESeq2	LRT	0.218634887636861
138	KC_AB1	su(Hw)	-0.399207573322805	0.658315298371225	0.994766463583882	pseudobulk	DESeq2	LRT	0.181566052364999
138	KC_AB1	Su(P)	-0.271239169574599	0.639777948557371	0.994766463583882	pseudobulk	DESeq2	LRT	0.193970732968532
138	KC_AB1	su(sable)	0.112131492402274	0.855182123877528	0.994766463583882	pseudobulk	DESeq2	LRT	0.0679413858891852
138	KC_AB1	Su(Tpl)	-0.108538825105694	0.659024195820123	0.994766463583882	pseudobulk	DESeq2	LRT	0.181098640158249
138	KC_AB1	Su(var)2-10	0.498375550105177	0.416934898053664	0.994766463583882	pseudobulk	DESeq2	LRT	0.379931752276373
138	KC_AB1	Su(var)2-HP2	-0.247091465415219	0.62072698345409	0.994766463583882	pseudobulk	DESeq2	LRT	0.207099375106791
138	KC_AB1	Su(var)205	-0.277214214627988	0.503429699874421	0.994766463583882	pseudobulk	DESeq2	LRT	0.29806116679502
138	KC_AB1	Su(var)3-3	0.426135864777146	0.547300700300817	0.994766463583882	pseudobulk	DESeq2	LRT	0.261773996153885
138	KC_AB1	Su(var)3-7	0.103761971531316	0.836152653743656	0.994766463583882	pseudobulk	DESeq2	LRT	0.0777144275533809
138	KC_AB1	su(w[a])	-0.177749792416948	0.683631880107158	0.994766463583882	pseudobulk	DESeq2	LRT	0.165177692832828
138	KC_AB1	Su(z)12	0.352507841916796	0.594321290632316	0.994766463583882	pseudobulk	DESeq2	LRT	0.22597871154137
138	KC_AB1	Su(z)2	0.0481663095888261	0.871621213803484	0.994766463583882	pseudobulk	DESeq2	LRT	0.0596722082953582
138	KC_AB1	subdued	-0.291838638037158	0.760104256154724	0.994766463583882	pseudobulk	DESeq2	LRT	0.119126835656847
138	KC_AB1	sud1	-0.388232531028833	0.798201525582673	0.994766463583882	pseudobulk	DESeq2	LRT	0.0978874464949879
138	KC_AB1	sug	-1.64952313825113	0.817565084244375	0.994766463583882	pseudobulk	DESeq2	LRT	0.0874776642296574
138	KC_AB1	Sugb	1.29169465878791	0.584809831144677	0.994766463583882	pseudobulk	DESeq2	LRT	0.23298533513365
138	KC_AB1	Sulf1	0.949183608371002	0.80156895525114	0.994766463583882	pseudobulk	DESeq2	LRT	0.0960591113673091
138	KC_AB1	sun	0.053294426223922	0.821873727728142	0.994766463583882	pseudobulk	DESeq2	LRT	0.0851949021234714
138	KC_AB1	Sur-8	0.145869661198355	0.659284701709003	0.994766463583882	pseudobulk	DESeq2	LRT	0.180927001672415
138	KC_AB1	Surf1	-0.217635029094979	0.833386270973444	0.994766463583882	pseudobulk	DESeq2	LRT	0.0791536582938921
138	KC_AB1	Surf4	-0.343344186242069	0.545257662488242	0.994766463583882	pseudobulk	DESeq2	LRT	0.263398222580113
138	KC_AB1	Surf6	-0.490560116869612	0.570264497630184	0.994766463583882	pseudobulk	DESeq2	LRT	0.243923664997819
138	KC_AB1	sut2	1.16760653201762	0.269243219445436	0.994766463583882	pseudobulk	DESeq2	LRT	0.569855225056948
138	KC_AB1	Suv3	-0.578189119392459	0.615245779366613	0.994766463583882	pseudobulk	DESeq2	LRT	0.210951356908504
138	KC_AB1	sv	-1.21457746603801	0.770013447451371	0.994766463583882	pseudobulk	DESeq2	LRT	0.113501690278259

138	KC_AB1	Svil	0.222547413225841	0.691251760982186	0.994766463583882	pseudobulk	DESeq2	LRT	0.16036374930913
138	KC_AB1	svr	-0.260599585779528	0.246046313422318	0.994766463583882	pseudobulk	DESeq2	LRT	0.60898313773146
138	KC_AB1	sw	-0.115107467387091	0.792481755355164	0.994766463583882	pseudobulk	DESeq2	LRT	0.10101072739385
138	KC_AB1	SWIP	3.46605577803662	0.204928382770117	0.994766463583882	pseudobulk	DESeq2	LRT	0.688397887246492
138	KC_AB1	Swip-1	-0.0568024632305675	0.888939928406933	0.994766463583882	pseudobulk	DESeq2	LRT	0.051127586209611
138	KC_AB1	swm	-0.160095452946961	0.641354213986033	0.994766463583882	pseudobulk	DESeq2	LRT	0.192902047405668
138	KC_AB1	sws	0.124056754657373	0.742583041554145	0.994766463583882	pseudobulk	DESeq2	LRT	0.12925497302469
138	KC_AB1	sxc	0.107128263220741	0.691212657127814	0.994766463583882	pseudobulk	DESeq2	LRT	0.16038831788096
138	KC_AB1	Sxl	-0.167104352337107	0.330536488920079	0.994766463583882	pseudobulk	DESeq2	LRT	0.480780590453945
138	KC_AB1	Syb	1.1821590793593	0.719511699510136	0.994766463583882	pseudobulk	DESeq2	LRT	0.142962139890846
138	KC_AB1	syd	-0.144079596865968	0.681145174643928	0.994766463583882	pseudobulk	DESeq2	LRT	0.166760315653406
138	KC_AB1	Sym	-0.0349213376805719	0.899314947710806	0.994766463583882	pseudobulk	DESeq2	LRT	0.0460881880228023
138	KC_AB1	Syn	0.111101385695245	0.653792106834201	0.994766463583882	pseudobulk	DESeq2	LRT	0.184560326917235
139	KC_AB1	Syn2	-0.280586305000134	0.578891358866303	0.994766463583882	pseudobulk	DESeq2	LRT	0.237402933115337
139	KC_AB1	Synd	-0.295599201591475	0.62267785714543	0.994766463583882	pseudobulk	DESeq2	LRT	0.205736577815675
139	KC_AB1	Syngr	0.336376389948387	0.247240925682616	0.994766463583882	pseudobulk	DESeq2	LRT	0.606879639056513
139	KC_AB1	Synj	-0.167121174336805	0.70976051833771	0.994766463583882	pseudobulk	DESeq2	LRT	0.14888816270182
139	KC_AB1	Syp	-0.375597995250706	0.0697713138009743	0.994766463583882	pseudobulk	DESeq2	LRT	1.15632309913435
139	KC_AB1	Syt1	-0.027353466649758	0.85206702174534	0.994766463583882	pseudobulk	DESeq2	LRT	0.0695262432269792
139	KC_AB1	Syt12	-0.926584508745924	0.00418998873087663	0.994766463583882	pseudobulk	DESeq2	LRT	2.37778714508255
139	KC_AB1	Syt14	0.295133932027068	0.514814142746462	0.994766463583882	pseudobulk	DESeq2	LRT	0.288349530857822
139	KC_AB1	Syt4	-0.188129305684358	0.417765906829276	0.994766463583882	pseudobulk	DESeq2	LRT	0.379067004949452
139	KC_AB1	Syt7	0.0802600374337658	0.629193641586454	0.994766463583882	pseudobulk	DESeq2	LRT	0.201215674856554
139	KC_AB1	Sytalpha	0.0934889004632395	0.644488686504034	0.994766463583882	pseudobulk	DESeq2	LRT	0.190784701944394
139	KC_AB1	Sytbeta	-0.0999491445320442	0.80063402937012	0.994766463583882	pseudobulk	DESeq2	LRT	0.0965659550083428
139	KC_AB1	Syx13	0.261803201347895	0.690575159418706	0.994766463583882	pseudobulk	DESeq2	LRT	0.160789047650929
139	KC_AB1	Syx16	0.0196851864921876	0.869963933312537	0.994766463583882	pseudobulk	DESeq2	LRT	0.0604987518503801
139	KC_AB1	Syx17	0.532851497801159	0.444739107191042	0.994766463583882	pseudobulk	DESeq2	LRT	0.351894680106894
139	KC_AB1	Syx18	-0.437481318599958	0.618975718142866	0.994766463583882	pseudobulk	DESeq2	LRT	0.208326387625125
139	KC_AB1	Syx1A	-0.0250140836269449	0.876618008180479	0.994766463583882	pseudobulk	DESeq2	LRT	0.0571896119696716
139	KC_AB1	Syx5	0.252588300384675	0.724102723802837	0.994766463583882	pseudobulk	DESeq2	LRT	0.140199818863424
139	KC_AB1	Syx6	-0.373861440316592	0.348059430555085	0.994766463583882	pseudobulk	DESeq2	LRT	0.458346594678515
139	KC_AB1	Syx7	-0.249708690639364	0.627906930969016	0.994766463583882	pseudobulk	DESeq2	LRT	0.202104723081188
139	KC_AB1	Syx8	0.292688564291337	0.69586416590878	0.994766463583882	pseudobulk	DESeq2	LRT	0.157475527276758
139	KC_AB1	Tab2	-2.20969975456663	0.610836134188693	0.994766463583882	pseudobulk	DESeq2	LRT	0.214075280039667
139	KC_AB1	tacc	-0.0306345975493697	0.876896389918338	0.994766463583882	pseudobulk	DESeq2	LRT	0.0570517178596927
139	KC_AB1	Tace	0.00356135959708494	0.894180286938818	0.994766463583882	pseudobulk	DESeq2	LRT	0.0485749088004213
139	KC_AB1	tadr	0.984083019823803	0.702143474713468	0.994766463583882	pseudobulk	DESeq2	LRT	0.153574135862026
139	KC_AB1	Taf1	0.0307319442540752	0.948444263073569	0.994766463583882	pseudobulk	DESeq2	LRT	0.0229881860729818
139	KC_AB1	Taf10	0.515345763981942	0.38081903140244	0.994766463583882	pseudobulk	DESeq2	LRT	0.419281355911166
139	KC_AB1	Taf10b	-0.0284193990607697	0.875956369862496	0.994766463583882	pseudobulk	DESeq2	LRT	0.0575175248819759
139	KC_AB1	Taf11	0.300246856895172	0.710006738649816	0.994766463583882	pseudobulk	DESeq2	LRT	0.148737529387202
139	KC_AB1	Taf12	0.309568100644276	0.412828197355492	0.994766463583882	pseudobulk	DESeq2	LRT	0.384230646803161
139	KC_AB1	Taf13	-0.112434433722364	0.81820650206542	0.994766463583882	pseudobulk	DESeq2	LRT	0.0871370736033347
139	KC_AB1	TAF1B	-0.37194906613358	0.865313209044288	0.994766463583882	pseudobulk	DESeq2	LRT	0.0628266667099524
139	KC_AB1	TAF1C-like	-2.92081757832874	0.216872627338305	0.994766463583882	pseudobulk	DESeq2	LRT	0.663795259171187
139	KC_AB1	Taf2	-0.0821496054323249	0.957377622215522	0.994766463583882	pseudobulk	DESeq2	LRT	0.0189167279527711
139	KC_AB1	Taf4	-0.274494355742039	0.47769864086553	0.994766463583882	pseudobulk	DESeq2	LRT	0.32084599435709
139	KC_AB1	Taf5	-1.11963606217876	0.2470225439286	0.994766463583882	pseudobulk	DESeq2	LRT	0.607263410072258
139	KC_AB1	Taf6	0.174337890401578	0.809480754077779	0.994766463583882	pseudobulk	DESeq2	LRT	0.0917934724167
139	KC_AB1	Taf7	-0.375147463127622	0.747419132827644	0.994766463583882	pseudobulk	DESeq2	LRT	0.126435789045766
139	KC_AB1	Taf8	0.362077002474608	0.721186641660334	0.994766463583882	pseudobulk	DESeq2	LRT	0.141952326191085
139	KC_AB1	tai	0.0271653926680958	0.87992304953692	0.994766463583882	pseudobulk	DESeq2	LRT	0.0555553058302048
139	KC_AB1	Tak1	-0.31957838113757	0.765168724653938	0.994766463583882	pseudobulk	DESeq2	LRT	0.11624278954359
139	KC_AB1	Takl1	-1.64952313825113	0.817565084244375	0.994766463583882	pseudobulk	DESeq2	LRT	0.0874776642296574
139	KC_AB1	Taldo	-0.237473683803514	0.877148860518115	0.994766463583882	pseudobulk	DESeq2	LRT	0.0569266964680766

139	KC_AB1	tam	0.729740153036158	0.752353468463509	0.994766463583882	pseudobulk	DESeq2	LRT	0.123578072524162
139	KC_AB1	tamo	0.0772474286404638	0.905746926014485	0.994766463583882	pseudobulk	DESeq2	LRT	0.0429931312290722
139	KC_AB1	Tango1	0.586244933363419	0.395793885699666	0.994766463583882	pseudobulk	DESeq2	LRT	0.402530919142776
139	KC_AB1	Tango10	0.367987915205135	0.617655758320335	0.994766463583882	pseudobulk	DESeq2	LRT	0.209253505357244
139	KC_AB1	Tango11	-0.00565272136179911	0.887174783911891	0.994766463583882	pseudobulk	DESeq2	LRT	0.051990810595993
139	KC_AB1	Tango14	-0.361998676761988	0.609646255608629	0.994766463583882	pseudobulk	DESeq2	LRT	0.214922089251335
139	KC_AB1	Tango2	1.1821590793593	0.719511699510136	0.994766463583882	pseudobulk	DESeq2	LRT	0.142962139890846
139	KC_AB1	Tango4	0.0550380593595435	0.958373190902215	0.994766463583882	pseudobulk	DESeq2	LRT	0.018465343542076
139	KC_AB1	Tango5	-0.00177565425578907	0.909399075969803	0.994766463583882	pseudobulk	DESeq2	LRT	0.0412454914124362
139	KC_AB1	Tango6	-0.981500021353797	0.667407207223606	0.994766463583882	pseudobulk	DESeq2	LRT	0.175609107779306
139	KC_AB1	Tango8	-1.64952313825113	0.817565084244375	0.994766463583882	pseudobulk	DESeq2	LRT	0.0874776642296574
139	KC_AB1	Tango9	-0.232867760348067	0.753954254797822	0.994766463583882	pseudobulk	DESeq2	LRT	0.122655003588536
139	KC_AB1	tank	0.997097578322961	0.311640569762021	0.994766463583882	pseudobulk	DESeq2	LRT	0.506346010316284
139	KC_AB1	tant	-0.237585636305195	0.777645003118264	0.994766463583882	pseudobulk	DESeq2	LRT	0.109218614284989
139	KC_AB1	Tao	-0.298197767820488	0.487332524383743	0.994766463583882	pseudobulk	DESeq2	LRT	0.3121746030061
139	KC_AB1	Tap42	0.653801143002218	0.551181835277283	0.994766463583882	pseudobulk	DESeq2	LRT	0.258705103453173
139	KC_AB1	tapas	-0.906431214996532	0.0501744637137067	0.994766463583882	pseudobulk	DESeq2	LRT	1.29951726074227
139	KC_AB1	Tapdelta	0.0810730906503988	0.824078335193376	0.994766463583882	pseudobulk	DESeq2	LRT	0.084031503199475
139	KC_AB1	tara	-0.242944815832496	0.50968252210089	0.994766463583882	pseudobulk	DESeq2	LRT	0.292700258868196
139	KC_AB1	Task6	-0.143106727205506	0.414913071497919	0.994766463583882	pseudobulk	DESeq2	LRT	0.382042882862055
139	KC_AB1	Task7	0.15759731918824	0.733179883756789	0.994766463583882	pseudobulk	DESeq2	LRT	0.134789459276204
139	KC_AB1	Tasp1	0.718061526455341	0.662641092639594	0.994766463583882	pseudobulk	DESeq2	LRT	0.178721635533359
139	KC_AB1	Tat	0.108661374108852	0.778042022029873	0.994766463583882	pseudobulk	DESeq2	LRT	0.108996946142902
139	KC_AB1	tau	-0.124854983747231	0.636237098100484	0.994766463583882	pseudobulk	DESeq2	LRT	0.196381011383008
139	KC_AB1	tay	0.074553730683349	0.756039844755732	0.994766463583882	pseudobulk	DESeq2	LRT	0.121455315740126
139	KC_AB1	Taz	0.593284723012334	0.61807881389025	0.994766463583882	pseudobulk	DESeq2	LRT	0.208956142621164
139	KC_AB1	Tbc1d15-17	-0.167440504615799	0.854475412959539	0.994766463583882	pseudobulk	DESeq2	LRT	0.0683004293383574
139	KC_AB1	TBC1D16	-0.322773673422769	0.511562054016971	0.994766463583882	pseudobulk	DESeq2	LRT	0.291101677528472
139	KC_AB1	TBC1D5	-0.88281666035384	0.473572075937262	0.994766463583882	pseudobulk	DESeq2	LRT	0.3246139135991
139	KC_AB1	TBC1d7	-0.768838916112429	0.546990411132265	0.994766463583882	pseudobulk	DESeq2	LRT	0.26202028688201
139	KC_AB1	TBCB	0.385246569714958	0.57850389898402	0.994766463583882	pseudobulk	DESeq2	LRT	0.237693709656849
139	KC_AB1	TBCC	-0.139794748373247	0.828956533385974	0.994766463583882	pseudobulk	DESeq2	LRT	0.0814682412296164
139	KC_AB1	TBCD	-0.324621418602925	0.866631655762513	0.994766463583882	pseudobulk	DESeq2	LRT	0.0621654513830088
139	KC_AB1	Tbce	0.34546080252893	0.662458546143392	0.994766463583882	pseudobulk	DESeq2	LRT	0.178841292854473
139	KC_AB1	Tbp	0.0915853501020483	0.930848972646603	0.994766463583882	pseudobulk	DESeq2	LRT	0.0311207762332912
139	KC_AB1	TBPH	0.175453818065682	0.613632425715992	0.994766463583882	pseudobulk	DESeq2	LRT	0.212091699356582
139	KC_AB1	Tcs3	-0.742458858539035	0.596240052347496	0.994766463583882	pseudobulk	DESeq2	LRT	0.224578853648482
139	KC_AB1	Tctp	0.052335125115466	0.750146514418436	0.994766463583882	pseudobulk	DESeq2	LRT	0.124853904356201
139	KC_AB1	Tdc2	0.949183608371002	0.80156895525114	0.994766463583882	pseudobulk	DESeq2	LRT	0.0960591113673091
139	KC_AB1	Tdrd3	-0.164334478728747	0.849718928536322	0.994766463583882	pseudobulk	DESeq2	LRT	0.0707247071943714
139	KC_AB1	tea	-1.39149857196751	0.236911872367677	0.994766463583882	pseudobulk	DESeq2	LRT	0.62541317492699
139	KC_AB1	tef	-0.778652681115231	0.594422424533581	0.994766463583882	pseudobulk	DESeq2	LRT	0.22590481521738
139	KC_AB1	tefu	-0.835152883801839	0.920434724857442	0.994766463583882	pseudobulk	DESeq2	LRT	0.0360070052473159
139	KC_AB1	Teh1	-0.365250704548884	0.656074294542606	0.994766463583882	pseudobulk	DESeq2	LRT	0.183046977875987
139	KC_AB1	Teh2	0.200122989866542	0.493367381430502	0.994766463583882	pseudobulk	DESeq2	LRT	0.306829566911582
139	KC_AB1	Teh3	0.0270003628820048	0.878754027624612	0.994766463583882	pseudobulk	DESeq2	LRT	0.0561326714502013
139	KC_AB1	Teh4	0.564190116388402	0.526323206357674	0.994766463583882	pseudobulk	DESeq2	LRT	0.278747480883551
139	KC_AB1	Tehao	0.217906885051292	0.902008941520292	0.994766463583882	pseudobulk	DESeq2	LRT	0.0447891573209458
139	KC_AB1	temp	0.557053939519165	0.49284020237341	0.994766463583882	pseudobulk	DESeq2	LRT	0.30729387276572
139	KC_AB1	Ten-a	0.119903265524233	0.295300553031815	0.994766463583882	pseudobulk	DESeq2	LRT	0.529735739697838
139	KC_AB1	Ten-m	-0.126818391598222	0.838655994597118	0.994766463583882	pseudobulk	DESeq2	LRT	0.0764161443976294
139	KC_AB1	TER94	0.596074126896787	0.284935890233198	0.994766463583882	pseudobulk	DESeq2	LRT	0.545252844026462
139	KC_AB1	Tes	1.27424937263938	0.484503487685058	0.994766463583882	pseudobulk	DESeq2	LRT	0.314703092345056
139	KC_AB1	Tet	0.170604869979083	0.314807753686627	0.994766463583882	pseudobulk	DESeq2	LRT	0.501954579547258
139	KC_AB1	tex	0.986575688715885	0.655962090734864	0.994766463583882	pseudobulk	DESeq2	LRT	0.183121258582249
139	KC_AB1	TFAM	0.283335946901596	0.698782573257858	0.994766463583882	pseudobulk	DESeq2	LRT	0.155657934302045

139	KC_AB1	Tfb1	-0.485595720144132	0.665398096792127	0.994766463583882	pseudobulk	DESeq2	LRT	0.176918445658148
140	KC_AB1	Tfb4	-0.162131915966366	0.917226267001873	0.994766463583882	pseudobulk	DESeq2	LRT	0.0375235166867285
140	KC_AB1	TfIIA-L	-0.850591545315322	0.20617183191299	0.994766463583882	pseudobulk	DESeq2	LRT	0.685770670189242
140	KC_AB1	TfIIA-S	-0.135285289995197	0.681620505101166	0.994766463583882	pseudobulk	DESeq2	LRT	0.16645735322032
140	KC_AB1	TfIIB	-0.436500087188552	0.546814828277764	0.994766463583882	pseudobulk	DESeq2	LRT	0.262159716942745
140	KC_AB1	TfIIEalpha	-0.59720340708583	0.519473693621765	0.994766463583882	pseudobulk	DESeq2	LRT	0.284436440415135
140	KC_AB1	TfIIEbeta	1.41161299627246	0.283567078545238	0.994766463583882	pseudobulk	DESeq2	LRT	0.547344191105565
140	KC_AB1	TfIIS	0.419047594114022	0.379542677674298	0.994766463583882	pseudobulk	DESeq2	LRT	0.42073938278209
140	KC_AB1	Tg	2.38929733117871	0.484296857543297	0.994766463583882	pseudobulk	DESeq2	LRT	0.314888348950639
140	KC_AB1	Tgi	0.0672386452883197	0.905605166167103	0.994766463583882	pseudobulk	DESeq2	LRT	0.0430611086475599
140	KC_AB1	tgo	0.0326460829930283	0.909986211069663	0.994766463583882	pseudobulk	DESeq2	LRT	0.0409651884500356
140	KC_AB1	Tgt	0.491495033534201	0.767620227770926	0.994766463583882	pseudobulk	DESeq2	LRT	0.114853589572903
140	KC_AB1	TH1	-0.464521600838446	0.579445806580021	0.994766463583882	pseudobulk	DESeq2	LRT	0.236987175755314
140	KC_AB1	THADA	-2.50002631996899	0.341598419302658	0.994766463583882	pseudobulk	DESeq2	LRT	0.466484147613365
140	KC_AB1	Thd1	-0.104873331855741	0.64269768622508	0.994766463583882	pseudobulk	DESeq2	LRT	0.19199326358966
140	KC_AB1	THG	-0.105860877012275	0.856651644277919	0.994766463583882	pseudobulk	DESeq2	LRT	0.0671957471833973
140	KC_AB1	tho2	0.420555373697688	0.491831780890063	0.994766463583882	pseudobulk	DESeq2	LRT	0.308183411711085
140	KC_AB1	thoc5	-1.40297575431882	0.402579335798363	0.994766463583882	pseudobulk	DESeq2	LRT	0.395148520999933
140	KC_AB1	thoc6	-0.403663386345138	0.648401569915243	0.994766463583882	pseudobulk	DESeq2	LRT	0.188155942304859
140	KC_AB1	thoc7	-0.132312043371294	0.796545967173812	0.994766463583882	pseudobulk	DESeq2	LRT	0.0987891568196579
140	KC_AB1	ThrRS	-0.558116246890175	0.338563262104365	0.994766463583882	pseudobulk	DESeq2	LRT	0.470360169472716
140	KC_AB1	ths	-0.623400458974939	0.550636019735184	0.994766463583882	pseudobulk	DESeq2	LRT	0.259135382734604
140	KC_AB1	Tie	-4.21171921313756	0.142685354071374	0.994766463583882	pseudobulk	DESeq2	LRT	0.845620602725699
140	KC_AB1	Tif-IA	0.203105965741975	0.683370258449372	0.994766463583882	pseudobulk	DESeq2	LRT	0.165343926433355
140	KC_AB1	tim	0.949183608371002	0.80156895525114	0.994766463583882	pseudobulk	DESeq2	LRT	0.0960591113673091
140	KC_AB1	Tim17b	0.164363407455088	0.540786500545219	0.994766463583882	pseudobulk	DESeq2	LRT	0.266974158064745
140	KC_AB1	Tim17b1	-3.34100803494108	0.314063158159027	0.994766463583882	pseudobulk	DESeq2	LRT	0.502983006443488
140	KC_AB1	Tim23	0.0418472510925267	0.913880906287396	0.994766463583882	pseudobulk	DESeq2	LRT	0.0391103962926061
140	KC_AB1	Tim8	0.425973682351076	0.330890650220374	0.994766463583882	pseudobulk	DESeq2	LRT	0.48031550426676
140	KC_AB1	Tim9a	-0.00501271709103898	0.958022024195287	0.994766463583882	pseudobulk	DESeq2	LRT	0.0186245067079539
140	KC_AB1	timeout	-0.580582574734113	0.694218624998318	0.994766463583882	pseudobulk	DESeq2	LRT	0.158503738942497
140	KC_AB1	Timp	-0.271852016503456	0.882107600545087	0.994766463583882	pseudobulk	DESeq2	LRT	0.0544784358741274
140	KC_AB1	Tina-1	0.0594451419728898	0.897251375010557	0.994766463583882	pseudobulk	DESeq2	LRT	0.0470858674510728
140	KC_AB1	tinc	0.228817346763537	0.499889859150794	0.994766463583882	pseudobulk	DESeq2	LRT	0.301125673328472
140	KC_AB1	Tip60	0.547415435260469	0.59181970120186	0.994766463583882	pseudobulk	DESeq2	LRT	0.227810581620739
140	KC_AB1	tipE	-0.207983757085673	0.594707642241739	0.994766463583882	pseudobulk	DESeq2	LRT	0.225696480599274
140	KC_AB1	tko	0.51694936044482	0.336499477662016	0.994766463583882	pseudobulk	DESeq2	LRT	0.473015605581699
140	KC_AB1	TkR86C	0.596808856322697	0.822761281772702	0.994766463583882	pseudobulk	DESeq2	LRT	0.0847261539098712
140	KC_AB1	tkv	0.306501323152476	0.608298092842423	0.994766463583882	pseudobulk	DESeq2	LRT	0.215883545150278
140	KC_AB1	Tl	0.015487370345517	0.872870768396694	0.994766463583882	pseudobulk	DESeq2	LRT	0.059050050368564
140	KC_AB1	Tlk	-0.202647294178553	0.276159950170314	0.994766463583882	pseudobulk	DESeq2	LRT	0.558839304328005
140	KC_AB1	Tm1	0.15405205244654	0.567406848805529	0.994766463583882	pseudobulk	DESeq2	LRT	0.24610542641733
140	KC_AB1	Tm2	0.44976297004525	0.831013436772572	0.994766463583882	pseudobulk	DESeq2	LRT	0.0803919539908534
140	KC_AB1	TM4SF	-0.130831998975655	0.506975883722643	0.994766463583882	pseudobulk	DESeq2	LRT	0.295012699079454
140	KC_AB1	TM9SF2	-0.284320126379728	0.630407839826325	0.994766463583882	pseudobulk	DESeq2	LRT	0.200378394547465
140	KC_AB1	TM9SF3	1.06034276860183	0.281155990678736	0.994766463583882	pseudobulk	DESeq2	LRT	0.551052658407469
140	KC_AB1	TM9SF4	-0.122367361584155	0.858794333847191	0.994766463583882	pseudobulk	DESeq2	LRT	0.0661108296142429
140	KC_AB1	Tmc	0.222646485377116	0.669004390261849	0.994766463583882	pseudobulk	DESeq2	LRT	0.174571032216872
140	KC_AB1	Tmem18	-0.358417306131027	0.807765043785056	0.994766463583882	pseudobulk	DESeq2	LRT	0.0927149449513
140	KC_AB1	Tmem63	0.242977401202156	0.775647966247124	0.994766463583882	pseudobulk	DESeq2	LRT	0.110335341893076
140	KC_AB1	tmod	-0.169860215361044	0.685877658194659	0.994766463583882	pseudobulk	DESeq2	LRT	0.163753343636345
140	KC_AB1	TMS1	0.304240570443281	0.369129349673119	0.994766463583882	pseudobulk	DESeq2	LRT	0.432821422443652
140	KC_AB1	tna	0.091458626851757	0.632024659077471	0.994766463583882	pseudobulk	DESeq2	LRT	0.199265976951231
140	KC_AB1	tnc	2.65338476302536	0.245061773161734	0.994766463583882	pseudobulk	DESeq2	LRT	0.610724428444742
140	KC_AB1	Tnks	0.284183986857514	0.647040695153424	0.994766463583882	pseudobulk	DESeq2	LRT	0.189068403837059
140	KC_AB1	Tnpo	0.085590347510171	0.767829514764576	0.994766463583882	pseudobulk	DESeq2	LRT	0.114735197958353

140	KC_AB1	Tnpo-SR	0.732598344862497	0.611344119428962	0.994766463583882	pseudobulk	DESeq2	LRT	0.213714260953017
140	KC_AB1	Tob	-0.176489585087978	0.266630387378211	0.994766463583882	pseudobulk	DESeq2	LRT	0.574090356352674
140	KC_AB1	toc	-0.0798004141847853	0.699263734855511	0.994766463583882	pseudobulk	DESeq2	LRT	0.15535899451185
140	KC_AB1	tok	0.42250366458368	0.533918872784681	0.994766463583882	pseudobulk	DESeq2	LRT	0.272524727575538
140	KC_AB1	Toll-6	0.192309150866564	0.70786371359007	0.994766463583882	pseudobulk	DESeq2	LRT	0.150050349843082
140	KC_AB1	Toll-7	0.196650280581011	0.861831575084995	0.994766463583882	pseudobulk	DESeq2	LRT	0.0645775986278144
140	KC_AB1	Tom20	-0.0584554437570545	0.828537348062564	0.994766463583882	pseudobulk	DESeq2	LRT	0.0816879100667333
140	KC_AB1	Tom40	0.3920128957464	0.416293772662355	0.994766463583882	pseudobulk	DESeq2	LRT	0.380600085674847
140	KC_AB1	Tom7	0.0904824275856642	0.747596171018965	0.994766463583882	pseudobulk	DESeq2	LRT	0.126332931623855
140	KC_AB1	Tom70	0.197292978383785	0.793361325310676	0.994766463583882	pseudobulk	DESeq2	LRT	0.10052897428479
140	KC_AB1	tomboy40	1.1821590793593	0.719511699510136	0.994766463583882	pseudobulk	DESeq2	LRT	0.142962139890846
140	KC_AB1	Tomosyn	-0.012334245425909	0.923260429695538	0.994766463583882	pseudobulk	DESeq2	LRT	0.0346757776021005
140	KC_AB1	Top1	0.14529603231091	0.584972920814625	0.994766463583882	pseudobulk	DESeq2	LRT	0.232864237529751
140	KC_AB1	Top2	-0.782708544247211	0.401405516364582	0.994766463583882	pseudobulk	DESeq2	LRT	0.396416663462294
140	KC_AB1	Top3alpha	-0.0876274309314422	0.917937734184526	0.994766463583882	pseudobulk	DESeq2	LRT	0.0371867769874299
140	KC_AB1	Top3beta	-0.366256150537405	0.674714126742583	0.994766463583882	pseudobulk	DESeq2	LRT	0.170880196763153
140	KC_AB1	Topors	-0.802328271850364	0.461324660669919	0.994766463583882	pseudobulk	DESeq2	LRT	0.335993329032308
140	KC_AB1	Tor	0.241269142732035	0.815559770293225	0.994766463583882	pseudobulk	DESeq2	LRT	0.08854420512363
140	KC_AB1	TORIP	-0.259610837722904	0.773675862492691	0.994766463583882	pseudobulk	DESeq2	LRT	0.111440952256725
140	KC_AB1	Torsin	0.116436711896678	0.841523144111401	0.994766463583882	pseudobulk	DESeq2	LRT	0.0749339352774036
140	KC_AB1	tos	1.45682795759908	0.573003993665791	0.994766463583882	pseudobulk	DESeq2	LRT	0.24184235111992
140	KC_AB1	TotA	0.911498702061926	0.666302272230238	0.994766463583882	pseudobulk	DESeq2	LRT	0.176328705694236
140	KC_AB1	tou	0.0022358052753584	0.937558120468138	0.994766463583882	pseudobulk	DESeq2	LRT	0.0280018002762883
140	KC_AB1	tow	0.0795065105237033	0.695584365975096	0.994766463583882	pseudobulk	DESeq2	LRT	0.157650187806543
140	KC_AB1	toy	0.0338025394239401	0.814565861768638	0.994766463583882	pseudobulk	DESeq2	LRT	0.089073795030988
140	KC_AB1	Tpc1	-0.701830597349508	0.558164124400653	0.994766463583882	pseudobulk	DESeq2	LRT	0.25323808091714
140	KC_AB1	Tpi	0.224241986434101	0.551377404003361	0.994766463583882	pseudobulk	DESeq2	LRT	0.258551035687137
140	KC_AB1	TppII	-0.263694363733115	0.774661398758273	0.994766463583882	pseudobulk	DESeq2	LRT	0.11088808431203
140	KC_AB1	Tpr2	-0.433216552612495	0.229419241350946	0.994766463583882	pseudobulk	DESeq2	LRT	0.639370160706307
140	KC_AB1	Tpst	0.0012811920042844	0.933446565561157	0.994766463583882	pseudobulk	DESeq2	LRT	0.029910537860734
140	KC_AB1	tra2	-0.409795018872396	0.569224526908077	0.994766463583882	pseudobulk	DESeq2	LRT	0.244716395180609
140	KC_AB1	Traf6	0.192126522269739	0.895209465587023	0.994766463583882	pseudobulk	DESeq2	LRT	0.0480753343991894
140	KC_AB1	tral	-0.0917065191901584	0.703290002240601	0.994766463583882	pseudobulk	DESeq2	LRT	0.152865556342602
140	KC_AB1	TRAM	0.062410820441034	0.876174207328191	0.994766463583882	pseudobulk	DESeq2	LRT	0.0574095356590011
140	KC_AB1	Trap1	-0.233576943044383	0.762468021235285	0.994766463583882	pseudobulk	DESeq2	LRT	0.117778366397298
140	KC_AB1	Trax	-0.388138467952165	0.726050027129496	0.994766463583882	pseudobulk	DESeq2	LRT	0.139033454013741
140	KC_AB1	trbd	0.258699694036345	0.669961101912772	0.994766463583882	pseudobulk	DESeq2	LRT	0.173950411799237
140	KC_AB1	trc	-0.454299053851848	0.450753563493707	0.994766463583882	pseudobulk	DESeq2	LRT	0.346060831217794
140	KC_AB1	Trc8	0.620693345805951	0.441663406533709	0.994766463583882	pseudobulk	DESeq2	LRT	0.354908582174337
140	KC_AB1	Treh	0.725996770139257	0.49096229069436	0.994766463583882	pseudobulk	DESeq2	LRT	0.308951863421372
140	KC_AB1	trem	-0.686281197418189	0.633536894719629	0.994766463583882	pseudobulk	DESeq2	LRT	0.198228088426043
140	KC_AB1	Trf	1.1374547094191	0.481438009632003	0.994766463583882	pseudobulk	DESeq2	LRT	0.317459625066825
140	KC_AB1	Trf2	-0.2001927779553	0.475252651738305	0.994766463583882	pseudobulk	DESeq2	LRT	0.323075451249262
140	KC_AB1	Trf4-1	0.075562908171101	0.79200041853423	0.994766463583882	pseudobulk	DESeq2	LRT	0.101274588906645
140	KC_AB1	Trh	-1.64952313825113	0.817565084244375	0.994766463583882	pseudobulk	DESeq2	LRT	0.0874776642296574
141	KC_AB1	Trim9	0.949183608371002	0.80156895525114	0.994766463583882	pseudobulk	DESeq2	LRT	0.0960591113673091
141	KC_AB1	trio	-0.0301487901472052	0.934554273860211	0.994766463583882	pseudobulk	DESeq2	LRT	0.0293954720769582
141	KC_AB1	TrissinR	0.949183608371002	0.80156895525114	0.994766463583882	pseudobulk	DESeq2	LRT	0.0960591113673091
141	KC_AB1	Trl	-0.139417504866461	0.878843816378714	0.994766463583882	pseudobulk	DESeq2	LRT	0.056088298659679
141	KC_AB1	trn	-0.128135634064389	0.715459302074815	0.994766463583882	pseudobulk	DESeq2	LRT	0.145415065450018
141	KC_AB1	tRNA:Lys-CTT-2-1Psi	2.38929733117871	0.484296857543297	0.994766463583882	pseudobulk	DESeq2	LRT	0.314888348950639
141	KC_AB1	tRNA:Lys-TTT-2-1	1.1821590793593	0.719511699510136	0.994766463583882	pseudobulk	DESeq2	LRT	0.142962139890846
141	KC_AB1	tRNA:Thr-AGT-1-7	0.949183608371002	0.80156895525114	0.994766463583882	pseudobulk	DESeq2	LRT	0.0960591113673091
141	KC_AB1	tRNA:Val-AAC-2-2	-1.64952313825113	0.817565084244375	0.994766463583882	pseudobulk	DESeq2	LRT	0.0874776642296574
141	KC_AB1	trp	1.58149959037531	0.565968974761403	0.994766463583882	pseudobulk	DESeq2	LRT	0.247207375276793
141	KC_AB1	Trp1	-0.15755261063849	0.748382521544373	0.994766463583882	pseudobulk	DESeq2	LRT	0.12587636399067

141	KC_AB1	Trpm	-0.47053037263498	0.277215269046201	0.994766463583882	pseudobulk	DESeq2	LRT	0.557182852415641
141	KC_AB1	TrpRS	0.0334820659506235	0.956126514210327	0.994766463583882	pseudobulk	DESeq2	LRT	0.0194846382802211
141	KC_AB1	TrpRS-m	2.64699301431894	0.23725229373035	0.994766463583882	pseudobulk	DESeq2	LRT	0.624789580166765
141	KC_AB1	trr	-0.20073552680309	0.743731852650585	0.994766463583882	pseudobulk	DESeq2	LRT	0.128583618090103
141	KC_AB1	Trs20	0.365001277475952	0.454124962189174	0.994766463583882	pseudobulk	DESeq2	LRT	0.342824625288951
141	KC_AB1	Trs23	-0.388760266281908	0.641258890855884	0.994766463583882	pseudobulk	DESeq2	LRT	0.192966600479921
141	KC_AB1	Trs31	0.172810335808806	0.763376149133353	0.994766463583882	pseudobulk	DESeq2	LRT	0.117261413238883
141	KC_AB1	Trs33	0.666911753740884	0.618514332618064	0.994766463583882	pseudobulk	DESeq2	LRT	0.208650232162887
141	KC_AB1	trsn	-0.0773394163842039	0.885395874361838	0.994766463583882	pseudobulk	DESeq2	LRT	0.0528625060143455
141	KC_AB1	trus	-6.69532739457689E-0	0.951320539837659	0.994766463583882	pseudobulk	DESeq2	LRT	0.0216731263565233
141	KC_AB1	trv	-0.0668450798411834	0.61235469856169	0.994766463583882	pseudobulk	DESeq2	LRT	0.212996945492393
141	KC_AB1	trx	0.209610434118314	0.478266602406018	0.994766463583882	pseudobulk	DESeq2	LRT	0.320329945068215
141	KC_AB1	Trx-2	0.187829934566456	0.615263550921202	0.994766463583882	pseudobulk	DESeq2	LRT	0.210938812366332
141	KC_AB1	Trxr-1	-0.124715536484819	0.812714993234221	0.994766463583882	pseudobulk	DESeq2	LRT	0.090061728165436
141	KC_AB1	Tsc1	-0.496724515278681	0.619124274005212	0.994766463583882	pseudobulk	DESeq2	LRT	0.208222168269677
141	KC_AB1	Tsen15	-0.0627561689052589	0.891934490418666	0.994766463583882	pseudobulk	DESeq2	LRT	0.0496670419181083
141	KC_AB1	Tsen2	-1.23307819015679	0.379437005780321	0.994766463583882	pseudobulk	DESeq2	LRT	0.42086031545458
141	KC_AB1	Tsen34	0.249128178454185	0.762815383843489	0.994766463583882	pseudobulk	DESeq2	LRT	0.117580557037978
141	KC_AB1	Tsen54	0.694198716856127	0.704969414719965	0.994766463583882	pseudobulk	DESeq2	LRT	0.151829724578085
141	KC_AB1	Tsf2	-0.0147932074866799	0.865163776577981	0.994766463583882	pseudobulk	DESeq2	LRT	0.0629016722676512
141	KC_AB1	TSG101	-0.315097754088565	0.588523412815716	0.994766463583882	pseudobulk	DESeq2	LRT	0.230236255242952
141	KC_AB1	Tsp26A	0.847943622322446	0.317154329905831	0.994766463583882	pseudobulk	DESeq2	LRT	0.498729355080412
141	KC_AB1	Tsp39D	-0.297628350420543	0.28365147097259	0.994766463583882	pseudobulk	DESeq2	LRT	0.54721495990953
141	KC_AB1	Tsp3A	1.86262684648489	0.206750882032249	0.994766463583882	pseudobulk	DESeq2	LRT	0.684552629001914
141	KC_AB1	Tsp42A	-1.64952313825113	0.817565084244375	0.994766463583882	pseudobulk	DESeq2	LRT	0.0874776642296574
141	KC_AB1	Tsp42Ee	0.0523325421200668	0.915545472924958	0.994766463583882	pseudobulk	DESeq2	LRT	0.0383200804721226
141	KC_AB1	Tsp42Ej	0.0829807117519435	0.713201818843951	0.994766463583882	pseudobulk	DESeq2	LRT	0.146787557935152
141	KC_AB1	Tsp42Ek	0.380068550719104	0.765411285782467	0.994766463583882	pseudobulk	DESeq2	LRT	0.116105138509593
141	KC_AB1	Tsp42El	-3.21143241058256	0.292545415061155	0.994766463583882	pseudobulk	DESeq2	LRT	0.533806704011112
141	KC_AB1	Tsp42En	0.18603207050579	0.492593183510476	0.994766463583882	pseudobulk	DESeq2	LRT	0.307511602209195
141	KC_AB1	Tsp42Eo	-0.729024381689453	0.602472956983519	0.994766463583882	pseudobulk	DESeq2	LRT	0.220062442357105
141	KC_AB1	Tsp42Ep	0.949183608371002	0.80156895525114	0.994766463583882	pseudobulk	DESeq2	LRT	0.0960591113673091
141	KC_AB1	Tsp66E	0.0416154387837754	0.872450564525943	0.994766463583882	pseudobulk	DESeq2	LRT	0.0592591720041338
141	KC_AB1	Tsp86D	0.480144758631231	0.655029929509127	0.994766463583882	pseudobulk	DESeq2	LRT	0.183738855849898
141	KC_AB1	Tsp96F	-0.199391984508379	0.603327197681996	0.994766463583882	pseudobulk	DESeq2	LRT	0.219447096468823
141	KC_AB1	Tsp97E	-0.0543481209225234	0.857369221747376	0.994766463583882	pseudobulk	DESeq2	LRT	0.0668321110577151
141	KC_AB1	Tspo	-0.393643246862367	0.609382956965714	0.994766463583882	pseudobulk	DESeq2	LRT	0.215109696157402
141	KC_AB1	tsr	-0.147980786616553	0.630801035987511	0.994766463583882	pseudobulk	DESeq2	LRT	0.200107602084907
141	KC_AB1	Tsr1	0.897179476381625	0.56780389052509	0.994766463583882	pseudobulk	DESeq2	LRT	0.245801636065552
141	KC_AB1	tst	-1.13185434995905	0.62538932952952	0.994766463583882	pseudobulk	DESeq2	LRT	0.203849533016307
141	KC_AB1	tsu	0.121444293008558	0.84654839350099	0.994766463583882	pseudobulk	DESeq2	LRT	0.0723482101129878
141	KC_AB1	Ttc19	-0.433411845893634	0.623645440942525	0.994766463583882	pseudobulk	DESeq2	LRT	0.205062248147079
141	KC_AB1	Ttc7	0.306723404398576	0.657439983577858	0.994766463583882	pseudobulk	DESeq2	LRT	0.182143886930412
141	KC_AB1	tth	-0.426007455569917	0.328813831150564	0.994766463583882	pseudobulk	DESeq2	LRT	0.483049922682983
141	KC_AB1	TTLL12	-0.658427503998106	0.477447638233603	0.994766463583882	pseudobulk	DESeq2	LRT	0.321074250620835
141	KC_AB1	TTLL15	-2.57435989741145	0.533617212681275	0.994766463583882	pseudobulk	DESeq2	LRT	0.272770169998685
141	KC_AB1	TTLL3A	-1.68987254520361	0.228117088060108	0.994766463583882	pseudobulk	DESeq2	LRT	0.641842180868707
141	KC_AB1	TTLL3B	-1.64952313825113	0.817565084244375	0.994766463583882	pseudobulk	DESeq2	LRT	0.0874776642296574
141	KC_AB1	TTLL4A	-0.118310868152535	0.80960222700696	0.994766463583882	pseudobulk	DESeq2	LRT	0.0917283058705017
141	KC_AB1	TTLL5	-0.536472571649912	0.59913373426661	0.994766463583882	pseudobulk	DESeq2	LRT	0.222476226740163
141	KC_AB1	TTLL6B	-1.64952313825113	0.817565084244375	0.994766463583882	pseudobulk	DESeq2	LRT	0.0874776642296574
141	KC_AB1	ttm50	0.0078989419038609	0.942506342460835	0.994766463583882	pseudobulk	DESeq2	LRT	0.0257157185901012
141	KC_AB1	ttv	-0.318668942798449	0.295639521002175	0.994766463583882	pseudobulk	DESeq2	LRT	0.529237510040777
141	KC_AB1	tty	-0.137210934937762	0.5902616485619	0.994766463583882	pseudobulk	DESeq2	LRT	0.228955433548283
141	KC_AB1	tub	0.245739381663409	0.765809474209216	0.994766463583882	pseudobulk	DESeq2	LRT	0.115879265082441
141	KC_AB1	tud	0.22766500936082	0.665164987782249	0.994766463583882	pseudobulk	DESeq2	LRT	0.177070618762725

141	KC_AB1	Tudor-SN	-0.224109428316094	0.759810252160445	0.994766463583882	pseudobulk	DESeq2	LRT	0.119294850783049
141	KC_AB1	tun	-0.221486913521315	0.528181757540855	0.994766463583882	pseudobulk	DESeq2	LRT	0.277216602629583
141	KC_AB1	Tusp	-0.463294677622636	0.44881207079043	0.994766463583882	pseudobulk	DESeq2	LRT	0.347935471254613
141	KC_AB1	tut	3.80499962290998	0.134213047776818	0.994766463583882	pseudobulk	DESeq2	LRT	0.872205261350181
141	KC_AB1	tutl	0.0171421204523568	0.870893172334408	0.994766463583882	pseudobulk	DESeq2	LRT	0.0600351142357972
141	KC_AB1	TwdlM	-2.79499059556516	0.38564607643653	0.994766463583882	pseudobulk	DESeq2	LRT	0.413811082791365
141	KC_AB1	TwdlW	-1.64952313825113	0.817565084244375	0.994766463583882	pseudobulk	DESeq2	LRT	0.0874776642296574
141	KC_AB1	twe	0.949183608371002	0.80156895525114	0.994766463583882	pseudobulk	DESeq2	LRT	0.0960591113673091
141	KC_AB1	tweek	-0.046958262385586	0.912429964304917	0.994766463583882	pseudobulk	DESeq2	LRT	0.039800460880486
141	KC_AB1	twf	0.194033406988599	0.80722004229255	0.994766463583882	pseudobulk	DESeq2	LRT	0.0930080636331379
141	KC_AB1	twin	-0.142713796222665	0.722548281131901	0.994766463583882	pseudobulk	DESeq2	LRT	0.141133127770593
141	KC_AB1	twr	0.517848907587866	0.350458110613267	0.994766463583882	pseudobulk	DESeq2	LRT	0.455363884735003
141	KC_AB1	tws	0.191926623490237	0.569113203408413	0.994766463583882	pseudobulk	DESeq2	LRT	0.24480133865648
141	KC_AB1	twz	-3.95857014146861	0.177445975557202	0.994766463583882	pseudobulk	DESeq2	LRT	0.750933845925402
141	KC_AB1	Txl	0.495622194892297	0.5121998743267	0.994766463583882	pseudobulk	DESeq2	LRT	0.290560532426018
141	KC_AB1	tyf	0.0133928374316825	0.86076616497757	0.994766463583882	pseudobulk	DESeq2	LRT	0.0651148126027975
141	KC_AB1	tyn	0.743136321320853	0.468681409614907	0.994766463583882	pseudobulk	DESeq2	LRT	0.329122272555708
141	KC_AB1	TyrR	0.979680678390413	0.391831745975586	0.994766463583882	pseudobulk	DESeq2	LRT	0.406900380634562
141	KC_AB1	TyrRS	-1.19437067977371	0.126238340789932	0.994766463583882	pseudobulk	DESeq2	LRT	0.898808722234406
141	KC_AB1	TyrRS-m	0.641833300587602	0.746379423307147	0.994766463583882	pseudobulk	DESeq2	LRT	0.12704034202059
141	KC_AB1	tzn	0.255594493607103	0.673342011586669	0.994766463583882	pseudobulk	DESeq2	LRT	0.171764287906828
141	KC_AB1	U2A	-1.02102122337149	0.398779698695405	0.994766463583882	pseudobulk	DESeq2	LRT	0.399266959109081
141	KC_AB1	U2af38	0.26708664178487	0.650090222353993	0.994766463583882	pseudobulk	DESeq2	LRT	0.187026365893515
141	KC_AB1	U2af50	0.37386233951342	0.416281094409803	0.994766463583882	pseudobulk	DESeq2	LRT	0.380613312341522
141	KC_AB1	U3-55K	0.597141410293382	0.802139944912821	0.994766463583882	pseudobulk	DESeq2	LRT	0.0957498561530429
141	KC_AB1	U4-U6-60K	0.621699397179082	0.571844783886016	0.994766463583882	pseudobulk	DESeq2	LRT	0.242721835978786
141	KC_AB1	Uba1	0.266335930330093	0.629886819298968	0.994766463583882	pseudobulk	DESeq2	LRT	0.200737479387256
141	KC_AB1	Uba2	-0.280768988888718	0.67412954099708	0.994766463583882	pseudobulk	DESeq2	LRT	0.171256641247327
141	KC_AB1	Uba3	-0.913775820233965	0.374341299791084	0.994766463583882	pseudobulk	DESeq2	LRT	0.426732256022336
141	KC_AB1	Uba4	-0.119466828426229	0.885067448591205	0.994766463583882	pseudobulk	DESeq2	LRT	0.0530236316357438
141	KC_AB1	Uba5	-0.878608747648701	0.569989373018069	0.994766463583882	pseudobulk	DESeq2	LRT	0.244133241314588
141	KC_AB1	Ubc10	0.0624591462153359	0.874346788111793	0.994766463583882	pseudobulk	DESeq2	LRT	0.0583162809904445
141	KC_AB1	Ubc2	0.135389903148983	0.806183626054018	0.994766463583882	pseudobulk	DESeq2	LRT	0.0935660268063877
142	KC_AB1	Ubc4	-0.471236778151172	0.381007387167226	0.994766463583882	pseudobulk	DESeq2	LRT	0.41906660391792
142	KC_AB1	Ubc6	-0.0920547223955452	0.795837532854112	0.994766463583882	pseudobulk	DESeq2	LRT	0.0991755827479975
142	KC_AB1	Ubc7	-0.320114201999103	0.675710716060922	0.994766463583882	pseudobulk	DESeq2	LRT	0.170239193566925
142	KC_AB1	UbcE2H	-0.118058096016604	0.700668266374044	0.994766463583882	pseudobulk	DESeq2	LRT	0.154487551481349
142	KC_AB1	UbcE2M	0.145267615224374	0.783298904133999	0.994766463583882	pseudobulk	DESeq2	LRT	0.106072481052044
142	KC_AB1	Ube3a	0.599056110745543	0.564450702083178	0.994766463583882	pseudobulk	DESeq2	LRT	0.248373982433719
142	KC_AB1	Ubi-p5E	-0.118190963400195	0.571806015511868	0.994766463583882	pseudobulk	DESeq2	LRT	0.242751280090538
142	KC_AB1	Ubi-p63E	-0.0714994912399489	0.816773611961885	0.994766463583882	pseudobulk	DESeq2	LRT	0.087898301729747
142	KC_AB1	ubl	-0.126320718563719	0.834243291938403	0.994766463583882	pseudobulk	DESeq2	LRT	0.0787072767748759
142	KC_AB1	UBL3	0.0851331392753598	0.717254129547433	0.994766463583882	pseudobulk	DESeq2	LRT	0.144326942650455
142	KC_AB1	Ublcp1	0.308874130535112	0.719377968749604	0.994766463583882	pseudobulk	DESeq2	LRT	0.143042866763529
142	KC_AB1	Ubr1	-0.186166776845038	0.559395578726673	0.994766463583882	pseudobulk	DESeq2	LRT	0.252280970467525
142	KC_AB1	Ubr3	0.22400298668722	0.485836010283622	0.994766463583882	pseudobulk	DESeq2	LRT	0.313510298324901
142	KC_AB1	Uch	0.172730136506832	0.810338441478071	0.994766463583882	pseudobulk	DESeq2	LRT	0.091333558197906
142	KC_AB1	Uch-L5	0.102996588509631	0.8771189662849	0.994766463583882	pseudobulk	DESeq2	LRT	0.0569414979713922
142	KC_AB1	Uck	-0.324617306401707	0.571380555491596	0.994766463583882	pseudobulk	DESeq2	LRT	0.243074543068415
142	KC_AB1	Ucp4A	0.132618141332434	0.796133680571282	0.994766463583882	pseudobulk	DESeq2	LRT	0.0990140027900253
142	KC_AB1	udd	0.413773631531062	0.673109192889571	0.994766463583882	pseudobulk	DESeq2	LRT	0.171914478089888
142	KC_AB1	udt	-1.56928133302804	0.32463984929555	0.994766463583882	pseudobulk	DESeq2	LRT	0.488598171919053
142	KC_AB1	Uev1A	-0.143613062601354	0.731163324835238	0.994766463583882	pseudobulk	DESeq2	LRT	0.135985600946466
142	KC_AB1	uex	0.0896004811096588	0.70578896690832	0.994766463583882	pseudobulk	DESeq2	LRT	0.151325134936501
142	KC_AB1	Ufc1	0.914885114560942	0.27939598530831	0.994766463583882	pseudobulk	DESeq2	LRT	0.55377983863334
142	KC_AB1	Ufd1-like	0.39650339273079	0.49200449390252	0.994766463583882	pseudobulk	DESeq2	LRT	0.308030930427449

142	KC_AB1	Ufd4	-0.10909663660327	0.768271225783041	0.994766463583882	pseudobulk	DESeq2	LRT	0.114485432226017
142	KC_AB1	Ufl1	0.0652668569286296	0.951650244450769	0.994766463583882	pseudobulk	DESeq2	LRT	0.0215226365048171
142	KC_AB1	Ufm1	-0.470234375217398	0.470018628793304	0.994766463583882	pseudobulk	DESeq2	LRT	0.327884928826397
142	KC_AB1	Ugalt	0.491185269389615	0.742709846019877	0.994766463583882	pseudobulk	DESeq2	LRT	0.129180818653734
142	KC_AB1	Uggt	-0.372254356208857	0.749381031826172	0.994766463583882	pseudobulk	DESeq2	LRT	0.125297303872967
142	KC_AB1	UGP	0.0526590720372664	0.866846391535528	0.994766463583882	pseudobulk	DESeq2	LRT	0.062057854333355
142	KC_AB1	Ugt316A1	0.177397715530251	0.803879553410194	0.994766463583882	pseudobulk	DESeq2	LRT	0.0948090174305466
142	KC_AB1	Ugt35E2	-1.64952313825113	0.817565084244375	0.994766463583882	pseudobulk	DESeq2	LRT	0.0874776642296574
142	KC_AB1	Ugt49B1	-2.79499059556516	0.38564607643653	0.994766463583882	pseudobulk	DESeq2	LRT	0.413811082791365
142	KC_AB1	Uhg1	0.447492236500576	0.486501813636852	0.994766463583882	pseudobulk	DESeq2	LRT	0.312915536380195
142	KC_AB1	Uhg2	0.792366317375292	0.34382330966867	0.994766463583882	pseudobulk	DESeq2	LRT	0.463664683445026
142	KC_AB1	Uhg3	-0.365909604932354	0.59890871514359	0.994766463583882	pseudobulk	DESeq2	LRT	0.222639367144158
142	KC_AB1	Uhg4	-0.141927297842106	0.889081800258765	0.994766463583882	pseudobulk	DESeq2	LRT	0.0510582797871691
142	KC_AB1	Uhg5	0.147544943322913	0.753307090097043	0.994766463583882	pseudobulk	DESeq2	LRT	0.123027944985401
142	KC_AB1	Uhg8	1.14691474143735	0.444263448947684	0.994766463583882	pseudobulk	DESeq2	LRT	0.352359416161135
142	KC_AB1	Ulp1	-0.394229606679387	0.558127822378309	0.994766463583882	pseudobulk	DESeq2	LRT	0.253266327592281
142	KC_AB1	unc-104	-0.221292485900631	0.216171302449427	0.994766463583882	pseudobulk	DESeq2	LRT	0.665201960776274
142	KC_AB1	Unc-115a	0.0973759848495486	0.870878042217632	0.994766463583882	pseudobulk	DESeq2	LRT	0.0600426593441
142	KC_AB1	Unc-115b	1.43969401082205	0.471635027324931	0.994766463583882	pseudobulk	DESeq2	LRT	0.326393948256695
142	KC_AB1	unc-119	-0.0831554965990807	0.801521214562621	0.994766463583882	pseudobulk	DESeq2	LRT	0.0960849783060282
142	KC_AB1	unc-13-4A	-0.0489149562140686	0.810265141214163	0.994766463583882	pseudobulk	DESeq2	LRT	0.0913728446717844
142	KC_AB1	unc-45	0.0280558409194442	0.871969609526923	0.994766463583882	pseudobulk	DESeq2	LRT	0.0594986511279343
142	KC_AB1	unc-5	0.0502590873016535	0.871750344656474	0.994766463583882	pseudobulk	DESeq2	LRT	0.0596078722486589
142	KC_AB1	Unc-76	-0.220574195820018	0.341429618491303	0.994766463583882	pseudobulk	DESeq2	LRT	0.466698807212527
142	KC_AB1	unc79	-0.319875335259634	0.183011602112603	0.994766463583882	pseudobulk	DESeq2	LRT	0.737521377079614
142	KC_AB1	unc80	-0.147443030062413	0.617803138714691	0.994766463583882	pseudobulk	DESeq2	LRT	0.209149889619858
142	KC_AB1	und	-0.052988806475674	0.897366395855887	0.994766463583882	pseudobulk	DESeq2	LRT	0.0470301977487995
142	KC_AB1	unk	-0.487895289721984	0.167454647719772	0.994766463583882	pseudobulk	DESeq2	LRT	0.776102794071928
142	KC_AB1	Unr	-0.0739721475860796	0.615542517133902	0.994766463583882	pseudobulk	DESeq2	LRT	0.210741943839223
142	KC_AB1	up	1.1821590793593	0.719511699510136	0.994766463583882	pseudobulk	DESeq2	LRT	0.142962139890846
142	KC_AB1	Updo	-0.0836667891996268	0.801048876784708	0.994766463583882	pseudobulk	DESeq2	LRT	0.0963409842025439
142	KC_AB1	Upf1	-0.849614674456955	0.337264532375937	0.994766463583882	pseudobulk	DESeq2	LRT	0.472029327957905
142	KC_AB1	Upf2	-0.557963522904703	0.619873209311764	0.994766463583882	pseudobulk	DESeq2	LRT	0.207697133287876
142	KC_AB1	Upf3	-0.203915211405038	0.865443435023745	0.994766463583882	pseudobulk	DESeq2	LRT	0.0627613121442725
142	KC_AB1	upSET	0.0192702924007203	0.902238688648543	0.994766463583882	pseudobulk	DESeq2	LRT	0.0446785539770328
142	KC_AB1	UQCR-11	0.0356092593976293	0.862440781204943	0.994766463583882	pseudobulk	DESeq2	LRT	0.064270715708444
142	KC_AB1	UQCR-11L	-1.64952313825113	0.817565084244375	0.994766463583882	pseudobulk	DESeq2	LRT	0.0874776642296574
142	KC_AB1	UQCR-14	0.118748737899008	0.628437629217362	0.994766463583882	pseudobulk	DESeq2	LRT	0.201737818424894
142	KC_AB1	UQCR-6.4	-0.0285995212579196	0.889742672044965	0.994766463583882	pseudobulk	DESeq2	LRT	0.0507355801755253
142	KC_AB1	UQCR-C1	-0.259702277328608	0.450761980507248	0.994766463583882	pseudobulk	DESeq2	LRT	0.346052721623767
142	KC_AB1	UQCR-C2	0.220619270469858	0.430519639811675	0.994766463583882	pseudobulk	DESeq2	LRT	0.366007031742775
142	KC_AB1	UQCR-Q	0.144825274227743	0.543505574220584	0.994766463583882	pseudobulk	DESeq2	LRT	0.264795997408733
142	KC_AB1	uri	-1.74683112255917	0.584939641962841	0.994766463583882	pseudobulk	DESeq2	LRT	0.232888945054326
142	KC_AB1	Urm1	-0.255453090510339	0.7599185014307	0.994766463583882	pseudobulk	DESeq2	LRT	0.11923298176766
142	KC_AB1	Uros1	0.665386920145083	0.657365280485811	0.994766463583882	pseudobulk	DESeq2	LRT	0.182193237418395
142	KC_AB1	Use1	0.32253050235679	0.731185873396593	0.994766463583882	pseudobulk	DESeq2	LRT	0.135972207817404
142	KC_AB1	Usf	0.0520592408443843	0.908446684315538	0.994766463583882	pseudobulk	DESeq2	LRT	0.0417005558485271
142	KC_AB1	ush	-2.12640346989651	0.44900213027744	0.994766463583882	pseudobulk	DESeq2	LRT	0.347751598494355
142	KC_AB1	usp	0.282319791360971	0.586963332597872	0.994766463583882	pseudobulk	DESeq2	LRT	0.231389028135345
142	KC_AB1	Usp1	0.411586138916993	0.423170372191742	0.994766463583882	pseudobulk	DESeq2	LRT	0.373484746559043
142	KC_AB1	Usp10	0.554863749012875	0.345350621156392	0.994766463583882	pseudobulk	DESeq2	LRT	0.461739758524054
142	KC_AB1	Usp12-46	-0.647497366094021	0.656103978584876	0.994766463583882	pseudobulk	DESeq2	LRT	0.183027328692604
142	KC_AB1	Usp14	0.305253110337557	0.698313068977753	0.994766463583882	pseudobulk	DESeq2	LRT	0.15594983003143
142	KC_AB1	Usp15-31	-0.135749930505604	0.860670837714866	0.994766463583882	pseudobulk	DESeq2	LRT	0.0651629120733502
142	KC_AB1	Usp16-45	0.381089760230852	0.681395373059558	0.994766463583882	pseudobulk	DESeq2	LRT	0.166600819787109
142	KC_AB1	Usp2	0.0449611746870495	0.809183270068314	0.994766463583882	pseudobulk	DESeq2	LRT	0.0919531048830952

142	KC_AB1	Usp20-33	0.0757839931471787	0.926942752763998	0.994766463583882	pseudobulk	DESeq2	LRT	0.0329470867038497
142	KC_AB1	Usp30	0.40395912093868	0.573146492597901	0.994766463583882	pseudobulk	DESeq2	LRT	0.241734360937212
142	KC_AB1	Usp32	-0.518347665896845	0.455336495258173	0.994766463583882	pseudobulk	DESeq2	LRT	0.341667539557731
142	KC_AB1	Usp39	0.431549567083559	0.749733203540373	0.994766463583882	pseudobulk	DESeq2	LRT	0.125093255066858
142	KC_AB1	Usp47	-0.462565454755952	0.548169801458443	0.994766463583882	pseudobulk	DESeq2	LRT	0.26108489331532
142	KC_AB1	Usp5	1.26527090625017	0.219306234690367	0.994766463583882	pseudobulk	DESeq2	LRT	0.658949021522961
142	KC_AB1	Usp7	0.0945174404753553	0.736218733567379	0.994766463583882	pseudobulk	DESeq2	LRT	0.132993135841503
142	KC_AB1	Usp8	-0.871539454116811	0.314825502645232	0.994766463583882	pseudobulk	DESeq2	LRT	0.501930094580157
142	KC_AB1	Utx	-0.130969914734172	0.827403997899977	0.994766463583882	pseudobulk	DESeq2	LRT	0.0822823849854613
142	KC_AB1	Uxs	0.504942407037331	0.707053974113553	0.994766463583882	pseudobulk	DESeq2	LRT	0.150547432362976
142	KC_AB1	Uxt	-0.445617314381891	0.621066485771153	0.994766463583882	pseudobulk	DESeq2	LRT	0.20686190568821
142	KC_AB1	uzip	-0.337239754811066	0.508508806247517	0.994766463583882	pseudobulk	DESeq2	LRT	0.293701521656504
142	KC_AB1	VAChT	-0.123424171205765	0.515015913194559	0.994766463583882	pseudobulk	DESeq2	LRT	0.288179351724215
142	KC_AB1	ValRS	0.0323443087568574	0.908897999771351	0.994766463583882	pseudobulk	DESeq2	LRT	0.041484852335811
142	KC_AB1	ValRS-m	2.67764418499683	0.378704357417491	0.994766463583882	pseudobulk	DESeq2	LRT	0.421699697806334
142	KC_AB1	Vamp7	-0.456334170450875	0.510992859029842	0.994766463583882	pseudobulk	DESeq2	LRT	0.29158516895649
142	KC_AB1	Vang	-0.351412060746357	0.634732914575079	0.994766463583882	pseudobulk	DESeq2	LRT	0.197408980419784
142	KC_AB1	vap	-0.0122673925206208	0.940345035389053	0.994766463583882	pseudobulk	DESeq2	LRT	0.0267127639844267
142	KC_AB1	Vap33	0.180049395565954	0.406975118252463	0.994766463583882	pseudobulk	DESeq2	LRT	0.390432141968831
142	KC_AB1	vari	-1.03550855853255	0.623427827695289	0.994766463583882	pseudobulk	DESeq2	LRT	0.205213816199734
142	KC_AB1	Vav	0.879218926713063	0.170685189536277	0.994766463583882	pseudobulk	DESeq2	LRT	0.767804161262069
143	KC_AB1	veli	-0.0489707543643381	0.873548115715848	0.994766463583882	pseudobulk	DESeq2	LRT	0.0587131687412905
143	KC_AB1	velo	0.19114215866351	0.727812795244102	0.994766463583882	pseudobulk	DESeq2	LRT	0.13798031360955
143	KC_AB1	VepD	-0.320566371300488	0.358164804740886	0.994766463583882	pseudobulk	DESeq2	LRT	0.445917092573524
143	KC_AB1	vers	-0.40737759768677	0.618870088370313	0.994766463583882	pseudobulk	DESeq2	LRT	0.208400507401942
143	KC_AB1	vg	1.80633728195307	0.61161102402172	0.994766463583882	pseudobulk	DESeq2	LRT	0.213524695220716
143	KC_AB1	VGAT	0.19869066642166	0.670191668810267	0.994766463583882	pseudobulk	DESeq2	LRT	0.173800975205936
143	KC_AB1	Vha100-1	0.0928870233063869	0.715441063342434	0.994766463583882	pseudobulk	DESeq2	LRT	0.14542613677409
143	KC_AB1	Vha13	0.139395501110379	0.448733008196356	0.994766463583882	pseudobulk	DESeq2	LRT	0.348011983174124
143	KC_AB1	Vha14-1	0.0752559118921596	0.76834519504015	0.994766463583882	pseudobulk	DESeq2	LRT	0.114443620307594
143	KC_AB1	Vha16-1	0.088396146858931	0.509487170919168	0.994766463583882	pseudobulk	DESeq2	LRT	0.292866747220185
143	KC_AB1	Vha26	-0.135522753029572	0.614775327353516	0.994766463583882	pseudobulk	DESeq2	LRT	0.211283570269941
143	KC_AB1	Vha36-1	0.108359207529084	0.673684131926668	0.994766463583882	pseudobulk	DESeq2	LRT	0.171543681974688
143	KC_AB1	Vha44	-0.248896892978899	0.305588205928518	0.994766463583882	pseudobulk	DESeq2	LRT	0.514863411218748
143	KC_AB1	Vha55	-0.115981726973002	0.657856727420649	0.994766463583882	pseudobulk	DESeq2	LRT	0.181868679738248
143	KC_AB1	Vha68-1	0.291169107423978	0.343925122840225	0.994766463583882	pseudobulk	DESeq2	LRT	0.463536098931199
143	KC_AB1	VhaAC39-1	0.0037577207387581	0.950557671616371	0.994766463583882	pseudobulk	DESeq2	LRT	0.0220215287670434
143	KC_AB1	VhaAC45	0.109614640289186	0.775287855431448	0.994766463583882	pseudobulk	DESeq2	LRT	0.11053701902342
143	KC_AB1	VhaM9.7-a	0.0559676026267643	0.776019436843099	0.994766463583882	pseudobulk	DESeq2	LRT	0.110127400896466
143	KC_AB1	VhaM9.7-b	-0.141330629984293	0.700704597520733	0.994766463583882	pseudobulk	DESeq2	LRT	0.154465032968342
143	KC_AB1	VhaM9.7-c	-0.636833106273966	0.6010644353543	0.994766463583882	pseudobulk	DESeq2	LRT	0.221078968232217
143	KC_AB1	VhaPPA1-1	0.431520815713702	0.166173382210849	0.994766463583882	pseudobulk	DESeq2	LRT	0.77943854062857
143	KC_AB1	VhaSFD	0.165921762153817	0.64762504715305	0.994766463583882	pseudobulk	DESeq2	LRT	0.188676363084195
143	KC_AB1	Vhl	0.325393641585061	0.690445093135521	0.994766463583882	pseudobulk	DESeq2	LRT	0.160870852489246
143	KC_AB1	viaf	0.130001732698516	0.746381469569167	0.994766463583882	pseudobulk	DESeq2	LRT	0.127039151367341
143	KC_AB1	vib	-0.0725737200122981	0.836630188041471	0.994766463583882	pseudobulk	DESeq2	LRT	0.0774664688693078
143	KC_AB1	vig	0.0970906497264894	0.636350068615013	0.994766463583882	pseudobulk	DESeq2	LRT	0.196303904725814
143	KC_AB1	vig2	-0.233005403280573	0.621845147198469	0.994766463583882	pseudobulk	DESeq2	LRT	0.206317750502233
143	KC_AB1	vih	-0.27310812722505	0.619560722729859	0.994766463583882	pseudobulk	DESeq2	LRT	0.207916122291739
143	KC_AB1	vimar	0.181871651107799	0.786835300141437	0.994766463583882	pseudobulk	DESeq2	LRT	0.104116164369454
143	KC_AB1	Vinc	-1.06124287291934	0.117073093039202	0.994766463583882	pseudobulk	DESeq2	LRT	0.93154290755226
143	KC_AB1	vir	-1.62718364431562	0.251246696629544	0.994766463583882	pseudobulk	DESeq2	LRT	0.599899639601273
143	KC_AB1	vir-1	1.80939591345248	0.468117841155507	0.994766463583882	pseudobulk	DESeq2	LRT	0.329644806495434
143	KC_AB1	vito	0.124365819606419	0.851456010426308	0.994766463583882	pseudobulk	DESeq2	LRT	0.069837784486597
143	KC_AB1	Vkor	-2.919180786494	0.229509855336647	0.994766463583882	pseudobulk	DESeq2	LRT	0.639198660774101
143	KC_AB1	vlc	-0.351585406851247	0.43192987641058	0.994766463583882	pseudobulk	DESeq2	LRT	0.364586754944165

143	KC_AB1	Vlet	-0.158715272333888	0.812114966508482	0.994766463583882	pseudobulk	DESeq2	LRT	0.0903824858007393
143	KC_AB1	vls	-0.552999816355386	0.702085862524176	0.994766463583882	pseudobulk	DESeq2	LRT	0.153609772001011
143	KC_AB1	vnc	0.949183608371002	0.80156895525114	0.994766463583882	pseudobulk	DESeq2	LRT	0.0960591113673091
143	KC_AB1	vnd	-2.20969975456663	0.610836134188693	0.994766463583882	pseudobulk	DESeq2	LRT	0.214075280039667
143	KC_AB1	Vps11	-0.0280267248021108	0.897182050358274	0.994766463583882	pseudobulk	DESeq2	LRT	0.0471194237965306
143	KC_AB1	Vps13	-0.163838029295056	0.871672341189053	0.994766463583882	pseudobulk	DESeq2	LRT	0.0596467342822527
143	KC_AB1	Vps13B	2.0901407903222	0.265627613563703	0.994766463583882	pseudobulk	DESeq2	LRT	0.575726779473527
143	KC_AB1	Vps13D	-0.273752191915699	0.778902630837319	0.994766463583882	pseudobulk	DESeq2	LRT	0.108516829276155
143	KC_AB1	Vps15	-0.448195634704591	0.611853974542812	0.994766463583882	pseudobulk	DESeq2	LRT	0.213352214484125
143	KC_AB1	Vps16A	0.502195133257193	0.572185901640119	0.994766463583882	pseudobulk	DESeq2	LRT	0.2424628471867
143	KC_AB1	Vps16B	0.10204245100032	0.924992907964844	0.994766463583882	pseudobulk	DESeq2	LRT	0.0338615970377687
143	KC_AB1	Vps2	0.0427700960569864	0.945892182407758	0.994766463583882	pseudobulk	DESeq2	LRT	0.0241583638666148
143	KC_AB1	Vps20	0.311292123431597	0.672004930505722	0.994766463583882	pseudobulk	DESeq2	LRT	0.172627540512287
143	KC_AB1	Vps24	0.229640702757272	0.54000307870064	0.994766463583882	pseudobulk	DESeq2	LRT	0.267603764142054
143	KC_AB1	Vps25	-0.483390228593128	0.582594589239619	0.994766463583882	pseudobulk	DESeq2	LRT	0.234633553134868
143	KC_AB1	Vps26	1.11452825356752	0.179087668074343	0.994766463583882	pseudobulk	DESeq2	LRT	0.746934318515319
143	KC_AB1	Vps28	0.0240417956322772	0.931748926715893	0.994766463583882	pseudobulk	DESeq2	LRT	0.0307010988388221
143	KC_AB1	Vps29	-0.184978362553615	0.809476079353755	0.994766463583882	pseudobulk	DESeq2	LRT	0.0917959804599121
143	KC_AB1	Vps33B	0.651806411443827	0.700247618684875	0.994766463583882	pseudobulk	DESeq2	LRT	0.154748359396614
143	KC_AB1	Vps35	-0.223611100955534	0.754379890857793	0.994766463583882	pseudobulk	DESeq2	LRT	0.122409896895721
143	KC_AB1	Vps36	0.0374906357063049	0.954866464318632	0.994766463583882	pseudobulk	DESeq2	LRT	0.0200573591644324
143	KC_AB1	Vps37B	-0.032391299917942	0.929742516162108	0.994766463583882	pseudobulk	DESeq2	LRT	0.0316373087502008
143	KC_AB1	Vps39	0.945359597186799	0.600116097812217	0.994766463583882	pseudobulk	DESeq2	LRT	0.221764723346804
143	KC_AB1	Vps4	-0.0051035613846535	0.901989289571073	0.994766463583882	pseudobulk	DESeq2	LRT	0.0447986193403179
143	KC_AB1	Vps45	-0.200004605226823	0.840071255212768	0.994766463583882	pseudobulk	DESeq2	LRT	0.0756838753270956
143	KC_AB1	Vps50	-0.245341497325401	0.7718294519399	0.994766463583882	pseudobulk	DESeq2	LRT	0.112478653374188
143	KC_AB1	Vps51	0.0717925335871559	0.944378479700174	0.994766463583882	pseudobulk	DESeq2	LRT	0.0248539180926242
143	KC_AB1	Vps52	0.213074256575884	0.833438794283422	0.994766463583882	pseudobulk	DESeq2	LRT	0.0791262881946802
143	KC_AB1	Vps53	0.653684515051766	0.633388080698312	0.994766463583882	pseudobulk	DESeq2	LRT	0.198330113581185
143	KC_AB1	Vps60	0.21359105859719	0.661528751213999	0.994766463583882	pseudobulk	DESeq2	LRT	0.179451275857068
143	KC_AB1	Vps8	-0.585292622640076	0.638521099995527	0.994766463583882	pseudobulk	DESeq2	LRT	0.194824746857547
143	KC_AB1	Vrp1	0.388101110826748	0.333775822599221	0.994766463583882	pseudobulk	DESeq2	LRT	0.476545125092449
143	KC_AB1	vsg	-0.010930331548155	0.959990746685107	0.994766463583882	pseudobulk	DESeq2	LRT	0.0177329530885205
143	KC_AB1	Vsp37A	-0.12312449856471	0.821786577207366	0.994766463583882	pseudobulk	DESeq2	LRT	0.0852409566399549
143	KC_AB1	Vsx1	0.137724672673484	0.774872708129115	0.994766463583882	pseudobulk	DESeq2	LRT	0.110769635168004
143	KC_AB1	Vsx2	-2.20969975456663	0.610836134188693	0.994766463583882	pseudobulk	DESeq2	LRT	0.214075280039667
143	KC_AB1	Vta1	0.187547281913786	0.732474317674678	0.994766463583882	pseudobulk	DESeq2	LRT	0.135207598124304
143	KC_AB1	vtd	0.0835799339461217	0.802377239318764	0.994766463583882	pseudobulk	DESeq2	LRT	0.0956213992529214
143	KC_AB1	Vti1a	0.184804243241353	0.76498692674761	0.994766463583882	pseudobulk	DESeq2	LRT	0.116345986663229
143	KC_AB1	Vti1b	0.483673275576047	0.405450202044315	0.994766463583882	pseudobulk	DESeq2	LRT	0.392062478827517
143	KC_AB1	vvl	-2.57435989741145	0.533617212681275	0.994766463583882	pseudobulk	DESeq2	LRT	0.272770169998685
143	KC_AB1	w	0.609028574970453	0.380773611555386	0.994766463583882	pseudobulk	DESeq2	LRT	0.41933315680276
143	KC_AB1	wac	0.303757955850157	0.851676637907538	0.994766463583882	pseudobulk	DESeq2	LRT	0.0697252655958528
143	KC_AB1	wake	-0.593327352925642	0.329265297133269	0.994766463583882	pseudobulk	DESeq2	LRT	0.482454039278121
143	KC_AB1	wal	-0.866417203364243	0.398402647789886	0.994766463583882	pseudobulk	DESeq2	LRT	0.399677783911202
143	KC_AB1	wap	0.322203770626992	0.596412260384732	0.994766463583882	pseudobulk	DESeq2	LRT	0.224453437380837
143	KC_AB1	wapl	-0.0240551472191024	0.924999679743212	0.994766463583882	pseudobulk	DESeq2	LRT	0.0338584176239726
143	KC_AB1	wash	-0.327016937545149	0.74291952960321	0.994766463583882	pseudobulk	DESeq2	LRT	0.129058224923041
143	KC_AB1	WASp	-0.00086484152749332	0.917636989050164	0.994766463583882	pseudobulk	DESeq2	LRT	0.0373290887678524
143	KC_AB1	Wbp2	0.087949618424412	0.876066724086759	0.994766463583882	pseudobulk	DESeq2	LRT	0.0574628152940929
143	KC_AB1	wcd	-0.748998551461526	0.620049862224366	0.994766463583882	pseudobulk	DESeq2	LRT	0.207573384665989
143	KC_AB1	wcy	-0.216343563665609	0.561467369660285	0.994766463583882	pseudobulk	DESeq2	LRT	0.250675478206192
143	KC_AB1	wda	2.6407536819221	0.191482018027674	0.994766463583882	pseudobulk	DESeq2	LRT	0.717872004139406
143	KC_AB1	wdb	0.0244586368451875	0.875132912315369	0.994766463583882	pseudobulk	DESeq2	LRT	0.0579259827473741
143	KC_AB1	wde	-0.225967214892904	0.621632951679734	0.994766463583882	pseudobulk	DESeq2	LRT	0.206465972403124
143	KC_AB1	Wdfy2	-0.0771305742763499	0.868494312941043	0.994766463583882	pseudobulk	DESeq2	LRT	0.0612330210469455

143	KC_AB1	wdn	0.191416730989004	0.867823531716541	0.994766463583882	pseudobulk	DESeq2	LRT	0.0615685778112266
143	KC_AB1	Wdr24	0.00891646089885312	0.960226720676327	0.994766463583882	pseudobulk	DESeq2	LRT	0.0176262128838119
143	KC_AB1	Wdr33	0.319788592294525	0.679381408320469	0.994766463583882	pseudobulk	DESeq2	LRT	0.167886342032237
143	KC_AB1	Wdr59	-0.17805032366327	0.942053914371676	0.994766463583882	pseudobulk	DESeq2	LRT	0.0259242415339998
143	KC_AB1	Wdr62	0.283100165686649	0.371181577659324	0.994766463583882	pseudobulk	DESeq2	LRT	0.430413586654254
143	KC_AB1	WDR79	3.80499962290998	0.134213047776818	0.994766463583882	pseudobulk	DESeq2	LRT	0.872205261350181
143	KC_AB1	Wdr81	-0.211325689250036	0.840581276847457	0.994766463583882	pseudobulk	DESeq2	LRT	0.0754202877214833
143	KC_AB1	Wdr82	-0.0969977184088017	0.884360300442126	0.994766463583882	pseudobulk	DESeq2	LRT	0.0533707614414693
143	KC_AB1	Wdr92	0.8210119081552	0.588324383172458	0.994766463583882	pseudobulk	DESeq2	LRT	0.230383151854257
144	KC_AB1	wds	-0.00167072447171023	0.948775442027871	0.994766463583882	pseudobulk	DESeq2	LRT	0.0228365650525487
144	KC_AB1	WDY	0.65123050286007	0.714104934316346	0.994766463583882	pseudobulk	DESeq2	LRT	0.146237966030458
144	KC_AB1	wech	-0.0195560476046288	0.940825328264204	0.994766463583882	pseudobulk	DESeq2	LRT	0.0264909993277662
144	KC_AB1	Wee1	0.108010245219676	0.898026811032234	0.994766463583882	pseudobulk	DESeq2	LRT	0.0467106970637261
144	KC_AB1	wek	-0.327097138188559	0.815573406754661	0.994766463583882	pseudobulk	DESeq2	LRT	0.0885369436197405
144	KC_AB1	wfs1	0.663853768988345	0.615346550881436	0.994766463583882	pseudobulk	DESeq2	LRT	0.210880229351728
144	KC_AB1	wge	0.240230641954611	0.49317361059072	0.994766463583882	pseudobulk	DESeq2	LRT	0.307000170278769
144	KC_AB1	whd	-1.04714963252433	0.454511345499486	0.994766463583882	pseudobulk	DESeq2	LRT	0.342455271460004
144	KC_AB1	wit	-0.296449848759645	0.599261596428673	0.994766463583882	pseudobulk	DESeq2	LRT	0.222383553095304
144	KC_AB1	wkd	-0.251102294653094	0.693867073468077	0.994766463583882	pseudobulk	DESeq2	LRT	0.158723720883268
144	KC_AB1	wls	0.483681883062539	0.74893757554732	0.994766463583882	pseudobulk	DESeq2	LRT	0.125554379530369
144	KC_AB1	wmd	-0.0511090408359284	0.904403844301262	0.994766463583882	pseudobulk	DESeq2	LRT	0.0436376002903263
144	KC_AB1	wnd	-0.0172460033624754	0.892010402328394	0.994766463583882	pseudobulk	DESeq2	LRT	0.049630080996659
144	KC_AB1	Wnk	0.189219712240288	0.695995966262484	0.994766463583882	pseudobulk	DESeq2	LRT	0.157393277393778
144	KC_AB1	Wnt5	-0.06322269800252	0.935542003372282	0.994766463583882	pseudobulk	DESeq2	LRT	0.0289367090476783
144	KC_AB1	Wnt6	1.70400802738958	0.443122299637011	0.994766463583882	pseudobulk	DESeq2	LRT	0.353476394031547
144	KC_AB1	woc	-0.598027068251503	0.338995370149381	0.994766463583882	pseudobulk	DESeq2	LRT	0.469806233160654
144	KC_AB1	wol	-0.953802125116204	0.408307505172674	0.994766463583882	pseudobulk	DESeq2	LRT	0.389012637159732
144	KC_AB1	wrd	-0.0425980050402864	0.855769564226707	0.994766463583882	pseudobulk	DESeq2	LRT	0.0676431634275053
144	KC_AB1	wry	-0.0931611283195694	0.624500419888974	0.994766463583882	pseudobulk	DESeq2	LRT	0.204467265287599
144	KC_AB1	Wsck	0.949183608371002	0.80156895525114	0.994766463583882	pseudobulk	DESeq2	LRT	0.0960591113673091
144	KC_AB1	wtrw	-1.32806228432949	0.722858830184137	0.994766463583882	pseudobulk	DESeq2	LRT	0.140946509425123
144	KC_AB1	wts	0.148357300487803	0.685703887345328	0.994766463583882	pseudobulk	DESeq2	LRT	0.163863388455232
144	KC_AB1	wuho	0.323853356930211	0.668332082488011	0.994766463583882	pseudobulk	DESeq2	LRT	0.175007690585002
144	KC_AB1	wun	-0.751023014001106	0.361188616662123	0.994766463583882	pseudobulk	DESeq2	LRT	0.442265945508944
144	KC_AB1	Wwox	-0.634964558270214	0.581196912388412	0.994766463583882	pseudobulk	DESeq2	LRT	0.235676701559558
144	KC_AB1	X11L	-0.345566821926692	0.327319787910707	0.994766463583882	pseudobulk	DESeq2	LRT	0.485027738900192
144	KC_AB1	X11Lbeta	-0.338208091620109	0.267481147307463	0.994766463583882	pseudobulk	DESeq2	LRT	0.572706822647883
144	KC_AB1	x16	0.067383636113942	0.883161949443619	0.994766463583882	pseudobulk	DESeq2	LRT	0.0539596505553803
144	KC_AB1	Xbp1	0.538253066457704	0.219327587700175	0.994766463583882	pseudobulk	DESeq2	LRT	0.658906737987741
144	KC_AB1	Xe7	0.196722931779753	0.390796290519868	0.994766463583882	pseudobulk	DESeq2	LRT	0.40804956730331
144	KC_AB1	xit	0.124698704744493	0.891329405341186	0.994766463583882	pseudobulk	DESeq2	LRT	0.0499617656782218
144	KC_AB1	xmas	-1.39300279252693	0.541251322968016	0.994766463583882	pseudobulk	DESeq2	LRT	0.266601029101383
144	KC_AB1	XNP	0.125630834781903	0.744919524613747	0.994766463583882	pseudobulk	DESeq2	LRT	0.127890642559058
144	KC_AB1	Xpac	0.441853628060859	0.755189796240372	0.994766463583882	pseudobulk	DESeq2	LRT	0.121943886647538
144	KC_AB1	Xpc	-0.383356859899546	0.395009977757433	0.994766463583882	pseudobulk	DESeq2	LRT	0.403391934170334
144	KC_AB1	Xpd	0.949183608371002	0.80156895525114	0.994766463583882	pseudobulk	DESeq2	LRT	0.0960591113673091
144	KC_AB1	XRCC1	-2.62283777965921	0.304158574375238	0.994766463583882	pseudobulk	DESeq2	LRT	0.516899936059965
144	KC_AB1	Xrp1	-0.0605282693327917	0.837146804341626	0.994766463583882	pseudobulk	DESeq2	LRT	0.0771983762660943
144	KC_AB1	Xxylt	0.398304810746589	0.480498952115697	0.994766463583882	pseudobulk	DESeq2	LRT	0.318307555114826
144	KC_AB1	yata	-1.07175307718381	0.262436009485834	0.994766463583882	pseudobulk	DESeq2	LRT	0.580976574601136
144	KC_AB1	yellow-b	-1.64952313825113	0.817565084244375	0.994766463583882	pseudobulk	DESeq2	LRT	0.0874776642296574
144	KC_AB1	yellow-c	1.00206552945986	0.690075213866505	0.994766463583882	pseudobulk	DESeq2	LRT	0.161103571310595
144	KC_AB1	yellow-f	0.949183608371002	0.80156895525114	0.994766463583882	pseudobulk	DESeq2	LRT	0.0960591113673091
144	KC_AB1	yellow-h	-1.13194846419515	0.661925982564074	0.994766463583882	pseudobulk	DESeq2	LRT	0.179190571226749
144	KC_AB1	yem	0.363809567714451	0.79775377221071	0.994766463583882	pseudobulk	DESeq2	LRT	0.0981311335507192
144	KC_AB1	Yeti	0.174743822194745	0.555375154414035	0.994766463583882	pseudobulk	DESeq2	LRT	0.255413553001752

144	KC_AB1	Yif1	-0.497023336354036	0.596106443729963	0.994766463583882	pseudobulk	DESeq2	LRT	0.224676183553444
144	KC_AB1	Yip1d1	-0.304497105255268	0.687428916828975	0.994766463583882	pseudobulk	DESeq2	LRT	0.16277220313403
144	KC_AB1	yip2	-2.85724547560282	0.450020141104186	0.994766463583882	pseudobulk	DESeq2	LRT	0.346768048503189
144	KC_AB1	Yippee	-0.145006232131391	0.769610119443115	0.994766463583882	pseudobulk	DESeq2	LRT	0.113729230485042
144	KC_AB1	yki	1.07837658243257	0.197586096496525	0.994766463583882	pseudobulk	DESeq2	LRT	0.704243618590892
144	KC_AB1	Ykt6	-0.0697837729029925	0.891471629265668	0.994766463583882	pseudobulk	DESeq2	LRT	0.0498924735199505
144	KC_AB1	YL-1	0.328499937868404	0.544275088752209	0.994766463583882	pseudobulk	DESeq2	LRT	0.264181542708529
144	KC_AB1	YME1L	-0.459855692079665	0.504408310373937	0.994766463583882	pseudobulk	DESeq2	LRT	0.297217766825736
144	KC_AB1	Yp3	0.949183608371002	0.80156895525114	0.994766463583882	pseudobulk	DESeq2	LRT	0.0960591113673091
144	KC_AB1	yps	0.104595256021849	0.718748559193867	0.994766463583882	pseudobulk	DESeq2	LRT	0.143423012889833
144	KC_AB1	yrt	-0.117854836488768	0.803295069727388	0.994766463583882	pseudobulk	DESeq2	LRT	0.0951248985373554
144	KC_AB1	Ythdc1	0.0403203033405859	0.878847593991262	0.994766463583882	pseudobulk	DESeq2	LRT	0.0560864318970894
144	KC_AB1	Ythdf	-0.132987330910916	0.72896415253154	0.994766463583882	pseudobulk	DESeq2	LRT	0.137293827979049
144	KC_AB1	ytr	-0.175829983656219	0.686995812381314	0.994766463583882	pseudobulk	DESeq2	LRT	0.163045910196971
144	KC_AB1	yuri	-1.64952313825113	0.817565084244375	0.994766463583882	pseudobulk	DESeq2	LRT	0.0874776642296574
144	KC_AB1	z	0.354399750564266	0.545045460550794	0.994766463583882	pseudobulk	DESeq2	LRT	0.263567273057359
144	KC_AB1	ZAP3	-0.392958430032464	0.41633877956963	0.994766463583882	pseudobulk	DESeq2	LRT	0.38055313518884
144	KC_AB1	Zasp52	-0.0876446376047783	0.951298398237017	0.994766463583882	pseudobulk	DESeq2	LRT	0.0216832345024977
144	KC_AB1	Zasp66	0.949183608371002	0.80156895525114	0.994766463583882	pseudobulk	DESeq2	LRT	0.0960591113673091
144	KC_AB1	ZC3H3	0.037480503670883	0.945059481004392	0.994766463583882	pseudobulk	DESeq2	LRT	0.024540856613445
144	KC_AB1	Zcchc7	-0.153058206566472	0.815002197160033	0.994766463583882	pseudobulk	DESeq2	LRT	0.088841220446291
144	KC_AB1	zda	0.480542652877339	0.416249144512539	0.994766463583882	pseudobulk	DESeq2	LRT	0.380646646059474
144	KC_AB1	Zdhhc8	-0.0179113315931658	0.933416666721774	0.994766463583882	pseudobulk	DESeq2	LRT	0.0299244487897576
144	KC_AB1	zetaCOP	0.597349269946215	0.242479955236174	0.994766463583882	pseudobulk	DESeq2	LRT	0.615324156814817
144	KC_AB1	zf30C	-0.590517004487605	0.392359721825272	0.994766463583882	pseudobulk	DESeq2	LRT	0.406315582043507
144	KC_AB1	zfh1	0.949183608371002	0.80156895525114	0.994766463583882	pseudobulk	DESeq2	LRT	0.0960591113673091
144	KC_AB1	zfh2	0.271978049387114	0.792202317659012	0.994766463583882	pseudobulk	DESeq2	LRT	0.101163891362365
144	KC_AB1	Zfrp8	0.37651223022131	0.735768374109234	0.994766463583882	pseudobulk	DESeq2	LRT	0.133258883595173
144	KC_AB1	Zif	-0.431943890823217	0.572405232870178	0.994766463583882	pseudobulk	DESeq2	LRT	0.242296404618315
144	KC_AB1	zip	-0.236447931932022	0.62699290803779	0.994766463583882	pseudobulk	DESeq2	LRT	0.202737371477853
144	KC_AB1	Zip102B	-0.118898804908987	0.795952740957414	0.994766463583882	pseudobulk	DESeq2	LRT	0.0991127173762893
144	KC_AB1	Zip48C	0.787234614225033	0.577225777268021	0.994766463583882	pseudobulk	DESeq2	LRT	0.238654282784209
144	KC_AB1	Zip88E	0.0810860743910949	0.927767676489256	0.994766463583882	pseudobulk	DESeq2	LRT	0.0325607624131746
144	KC_AB1	Zip99C	0.0359495648069481	0.939625299730783	0.994766463583882	pseudobulk	DESeq2	LRT	0.0270452982103644
144	KC_AB1	ZIPIC	0.886042894545102	0.648775226723578	0.994766463583882	pseudobulk	DESeq2	LRT	0.187905741877447
144	KC_AB1	Zir	-0.850113112899505	0.16061988314423	0.994766463583882	pseudobulk	DESeq2	LRT	0.794200694391535
144	KC_AB1	Ziz	-0.258047264147392	0.575689720381426	0.994766463583882	pseudobulk	DESeq2	LRT	0.239811525315547
144	KC_AB1	Zn72D	0.261152503735925	0.594861187419657	0.994766463583882	pseudobulk	DESeq2	LRT	0.22558436632358
144	KC_AB1	ZnT33D	0.0236942436874584	0.910119428894173	0.994766463583882	pseudobulk	DESeq2	LRT	0.0409016143750932
144	KC_AB1	ZnT49B	0.289505355928545	0.689954271178008	0.994766463583882	pseudobulk	DESeq2	LRT	0.161179692499229
144	KC_AB1	ZnT63C	0.678922435227978	0.274624107145354	0.994766463583882	pseudobulk	DESeq2	LRT	0.561261342042217
144	KC_AB1	ZnT86D	-0.225261612367904	0.840497091952346	0.994766463583882	pseudobulk	DESeq2	LRT	0.0754637848434681
144	KC_AB1	zormin	0.949183608371002	0.80156895525114	0.994766463583882	pseudobulk	DESeq2	LRT	0.0960591113673091
144	KC_AB1	Zpr1	0.212804481572611	0.879548480995857	0.994766463583882	pseudobulk	DESeq2	LRT	0.0557402170948801
144	KC_AB1	Zw10	1.22657472733779	0.301336468955414	0.994766463583882	pseudobulk	DESeq2	LRT	0.520948305075222
144	KC_AB1	zyd	-2.57435989741145	0.533617212681275	0.994766463583882	pseudobulk	DESeq2	LRT	0.272770169998685
144	KC_AB1	Zyx	0.246047396373856	0.433952146207187	0.994766463583882	pseudobulk	DESeq2	LRT	0.362558159390739
144	KC_AB2	CG32243	-0.00956080697803937	0.994547519373592	0.994999723459394	pseudobulk	DESeq2	LRT	0.0023744615001287
144	KC_AB1	Ars2	-0.00230597760620668	0.961483775014113	0.995190052405692	pseudobulk	DESeq2	LRT	0.0170580400591916
144	KC_AB1	CG8924	0.029374870653165	0.9617168703163	0.995304899188155	pseudobulk	DESeq2	LRT	0.0169527655479346
144	KC_AB1	CG12746	-0.0559561033747208	0.961996951710792	0.995341945085227	pseudobulk	DESeq2	LRT	0.0168263041132055
144	KC_AB1	Nurf-38	0.0203850447071129	0.961890543331235	0.995341945085227	pseudobulk	DESeq2	LRT	0.0168743449391228
144	KC_AB1	alpha-Man-Ib	-0.00962293191637957	0.962745743988871	0.995945736416074	pseudobulk	DESeq2	LRT	0.0164883925870634
145	KC_AB1	prtp	0.0171073039138161	0.962824949255839	0.995945736416074	pseudobulk	DESeq2	LRT	0.0164526645709257
145	KC_AB1	asRNA:CR45822	0.00461038084472217	0.964598377990577	0.996024174984363	pseudobulk	DESeq2	LRT	0.0156534726964649
145	KC_AB1	CG14618	0.0441906805679219	0.965063129228746	0.996024174984363	pseudobulk	DESeq2	LRT	0.0154442765225845

145	KC_AB1	CG14667	0.00436823842756203	0.964043417195023	0.996024174984363	pseudobulk	DESeq2	LRT	0.0159034065291083
145	KC_AB1	CG42237	0.025592605180315	0.965711990005086	0.996024174984363	pseudobulk	DESeq2	LRT	0.0151523764846263
145	KC_AB1	CG5611	0.00603860863649213	0.963653788002836	0.996024174984363	pseudobulk	DESeq2	LRT	0.0160789670995168
145	KC_AB1	CG6985	0.025592605180315	0.965711990005086	0.996024174984363	pseudobulk	DESeq2	LRT	0.0151523764846263
145	KC_AB1	CG7607	0.025592605180315	0.965711990005086	0.996024174984363	pseudobulk	DESeq2	LRT	0.0151523764846263
145	KC_AB1	CG9628	0.025592605180315	0.965711990005086	0.996024174984363	pseudobulk	DESeq2	LRT	0.0151523764846263
145	KC_AB1	CG9684	0.025592605180315	0.965711990005086	0.996024174984363	pseudobulk	DESeq2	LRT	0.0151523764846263
145	KC_AB1	CR43671	0.025592605180315	0.965711990005086	0.996024174984363	pseudobulk	DESeq2	LRT	0.0151523764846263
145	KC_AB1	Galk	0.0441906805679219	0.965063129228746	0.996024174984363	pseudobulk	DESeq2	LRT	0.0154442765225845
145	KC_AB1	kune	-0.0410183498156694	0.96389141959055	0.996024174984363	pseudobulk	DESeq2	LRT	0.0159718857326194
145	KC_AB1	l(3)L1231	-0.00752701166676212	0.964580230680171	0.996024174984363	pseudobulk	DESeq2	LRT	0.0156616432999907
145	KC_AB1	lncRNA:CR44260	0.025592605180315	0.965711990005086	0.996024174984363	pseudobulk	DESeq2	LRT	0.0151523764846263
145	KC_AB1	lncRNA:CR44363	0.025592605180315	0.965711990005086	0.996024174984363	pseudobulk	DESeq2	LRT	0.0151523764846263
145	KC_AB1	lncRNA:CR45035	-0.0769932964184457	0.963154630948346	0.996024174984363	pseudobulk	DESeq2	LRT	0.0163039828894473
145	KC_AB1	lncRNA:CR45290	0.025592605180315	0.965711990005086	0.996024174984363	pseudobulk	DESeq2	LRT	0.0151523764846263
145	KC_AB1	Mtp	0.025592605180315	0.965711990005086	0.996024174984363	pseudobulk	DESeq2	LRT	0.0151523764846263
145	KC_AB1	Nap1	-0.00800084309011487	0.964568814069389	0.996024174984363	pseudobulk	DESeq2	LRT	0.0156667835674865
145	KC_AB1	path	0.039599896299885	0.963656609927451	0.996024174984363	pseudobulk	DESeq2	LRT	0.0160776953310563
145	KC_AB1	qin	0.025592605180315	0.965711990005086	0.996024174984363	pseudobulk	DESeq2	LRT	0.0151523764846263
145	KC_AB1	scro	0.025592605180315	0.965711990005086	0.996024174984363	pseudobulk	DESeq2	LRT	0.0151523764846263
145	KC_AB1	Syn1	-0.0123839086070184	0.96462896400568	0.996024174984363	pseudobulk	DESeq2	LRT	0.0156397020668509
145	KC_AB1	ABCA	0.00466261247578418	0.9664050094001	0.996107423257272	pseudobulk	DESeq2	LRT	0.0148408275338057
145	KC_AB1	CG17528	0.0201722862947644	0.966220532523332	0.996107423257272	pseudobulk	DESeq2	LRT	0.0149237378422173
145	KC_AB1	CG32069	0.0101884551959387	0.966567725902989	0.996107423257272	pseudobulk	DESeq2	LRT	0.014767710227808
145	KC_AB1	CG42336	-0.0051002471719096	0.966309796283795	0.996107423257272	pseudobulk	DESeq2	LRT	0.014883617634992
145	KC_AB1	p24-1	-0.0285849602353448	0.966648362144865	0.996107423257272	pseudobulk	DESeq2	LRT	0.0147314805738911
145	KC_AB1	SmD2	-0.0174002824533641	0.966361957727674	0.996107423257272	pseudobulk	DESeq2	LRT	0.0148601750331087
145	KC_AB1	Tailor	0.0101884551959387	0.966567725902989	0.996107423257272	pseudobulk	DESeq2	LRT	0.014767710227808
145	KC_AB2	Adf1	-0.00100357409435769	0.996086628614907	0.996388518788756	pseudobulk	DESeq2	LRT	0.00170288979578495
145	KC_AB1	CG14868	0.0240112732572243	0.967068851772259	0.996414726652186	pseudobulk	DESeq2	LRT	0.0145426046343907
145	KC_AB1	azot	-0.0505214014622309	0.967608352565547	0.996554272409125	pseudobulk	DESeq2	LRT	0.0143003913875966
145	KC_AB1	CG5599	-0.0505214014622309	0.967608352565547	0.996554272409125	pseudobulk	DESeq2	LRT	0.0143003913875966
145	KC_AB1	HP1c	0.0179295694926819	0.967693454113806	0.996554272409125	pseudobulk	DESeq2	LRT	0.0142621966908248
145	KC_AB1	prage	0.0208838696223727	0.967547039023207	0.996554272409125	pseudobulk	DESeq2	LRT	0.0143279117957551
145	KC_AB2	Snx27	-0.00099114316956731	0.996544196074161	0.996695187619021	pseudobulk	DESeq2	LRT	0.00150343586414511
145	KC_AB2	Scsalpha1	0.00206239594919652	0.99669986886834	0.99669986886834	pseudobulk	DESeq2	LRT	0.00143559887737491
145	KC_G1	128up	1.13488282752664	0.324469944210194	0.997045181597847	pseudobulk	DESeq2	LRT	0.488825525884255
145	KC_G1	14-3-3epsilon	0.0111983663061536	0.931425978003043	0.997045181597847	pseudobulk	DESeq2	LRT	0.0308516535129732
145	KC_G1	14-3-3zeta	-0.0911987935425256	0.427184395694917	0.997045181597847	pseudobulk	DESeq2	LRT	0.369384619713966
145	KC_G1	140up	-0.439874407727673	0.670648499364266	0.997045181597847	pseudobulk	DESeq2	LRT	0.173505042847774
145	KC_G1	18SrRNA-Psi:CR41602	0.119098750855386	0.876701141840289	0.997045181597847	pseudobulk	DESeq2	LRT	0.0571484278079145
145	KC_G1	18w	0.695091824622677	0.0753893512038806	0.997045181597847	pseudobulk	DESeq2	LRT	1.12268999418328
145	KC_G1	26-29-p	0.0978696937084406	0.839324055991442	0.997045181597847	pseudobulk	DESeq2	LRT	0.0760703293298569
145	KC_G1	28SrRNA-Psi:CR40596	1.26000414275446	0.0220003342871085	0.997045181597847	pseudobulk	DESeq2	LRT	1.65757072018036
145	KC_G1	28SrRNA-Psi:CR40741	-0.272650306887422	0.776059591157879	0.997045181597847	pseudobulk	DESeq2	LRT	0.110104929363737
145	KC_G1	28SrRNA-Psi:CR41609	1.47221219840277	0.472374792297434	0.997045181597847	pseudobulk	DESeq2	LRT	0.325713286033591
145	KC_G1	28SrRNA-Psi:CR45848	0.143429430243913	0.883467238796752	0.997045181597847	pseudobulk	DESeq2	LRT	0.0538095505981828
145	KC_G1	28SrRNA-Psi:CR45851	1.36858832791253	0.716834138810678	0.997045181597847	pseudobulk	DESeq2	LRT	0.144581319830245
145	KC_G1	28SrRNA-Psi:CR45855	-1.48889291518014	0.469275581647592	0.997045181597847	pseudobulk	DESeq2	LRT	0.328572043340473
145	KC_G1	28SrRNA-Psi:CR45859	0.349352015451854	0.459423860317032	0.997045181597847	pseudobulk	DESeq2	LRT	0.337786453340097
145	KC_G1	28SrRNA-Psi:CR45860	0.836134253194897	0.379917384909165	0.997045181597847	pseudobulk	DESeq2	LRT	0.420310832801244
145	KC_G1	2mit	-0.141349828110589	0.524106113673553	0.997045181597847	pseudobulk	DESeq2	LRT	0.280580774242833
145	KC_G1	312	-0.0764627240256513	0.912237149658338	0.997045181597847	pseudobulk	DESeq2	LRT	0.0398922456632667
145	KC_G1	4E-T	0.169186370033448	0.606222696317054	0.997045181597847	pseudobulk	DESeq2	LRT	0.217367808151936
145	KC_G1	5-HT1A	-0.340139658781203	0.176393851332751	0.997045181597847	pseudobulk	DESeq2	LRT	0.753516557405998
145	KC_G1	5-HT1B	0.128045699916269	0.550107067815833	0.997045181597847	pseudobulk	DESeq2	LRT	0.259552775167128

145	KC_G1	5-HT2A	0.257463247535115	0.764566458810498	0.997045181597847	pseudobulk	DESeq2	LRT	0.116584758204293
145	KC_G1	5-HT2B	-1.1427746013943	0.716605375446379	0.997045181597847	pseudobulk	DESeq2	LRT	0.144719938399961
145	KC_G1	5-HT7	1.36858832791253	0.716834138810678	0.997045181597847	pseudobulk	DESeq2	LRT	0.144581319830245
145	KC_G1	5.8SrRNA-Psi:CR45849	-0.0403644574426932	0.970076776272712	0.997045181597847	pseudobulk	DESeq2	LRT	0.0131938923398719
145	KC_G1	5.8SrRNA-Psi:CR45854	-1.29145474762545	0.54492644406657	0.997045181597847	pseudobulk	DESeq2	LRT	0.263662116240639
145	KC_G1	5.8SrRNA-Psi:CR45857	1.64950845159325	0.636439517805573	0.997045181597847	pseudobulk	DESeq2	LRT	0.196242861973188
145	KC_G1	5PtaseI	0.244693999382814	0.424153184178871	0.997045181597847	pseudobulk	DESeq2	LRT	0.37247726833903
145	KC_G1	7B2	0.248466523584723	0.0563125024031906	0.997045181597847	pseudobulk	DESeq2	LRT	1.24939517314292
145	KC_G1	a	-0.192640748222763	0.287357655704339	0.997045181597847	pseudobulk	DESeq2	LRT	0.541577228020941
145	KC_G1	A16	0.676618763754435	0.378859865192398	0.997045181597847	pseudobulk	DESeq2	LRT	0.4215213996041
145	KC_G1	a6	-0.417579008496974	0.560568262813564	0.997045181597847	pseudobulk	DESeq2	LRT	0.251371493937686
145	KC_G1	AANAT1	0.141691032000688	0.585881332343208	0.997045181597847	pseudobulk	DESeq2	LRT	0.232190339489483
145	KC_G1	AANATL2	-1.29214429865911	0.777205227288829	0.997045181597847	pseudobulk	DESeq2	LRT	0.10946428710589
145	KC_G1	Aasdh	-0.786412787872399	0.495000884881028	0.997045181597847	pseudobulk	DESeq2	LRT	0.305394024705615
145	KC_G1	Aatf	-0.0112692659301951	0.934817026036611	0.997045181597847	pseudobulk	DESeq2	LRT	0.0292733863029051
145	KC_G1	aay	1.15203722513625	0.688394393399532	0.997045181597847	pseudobulk	DESeq2	LRT	0.162162675424025
145	KC_G1	ab	0.03027210051318	0.839813157485955	0.997045181597847	pseudobulk	DESeq2	LRT	0.0758173254842392
145	KC_G1	ABCA	-0.138225507233865	0.752430812530248	0.997045181597847	pseudobulk	DESeq2	LRT	0.123533428116526
145	KC_G1	ABCB7	0.40942846500662	0.60094760883547	0.997045181597847	pseudobulk	DESeq2	LRT	0.221163388538773
145	KC_G1	ABCD	-0.231122418953094	0.313105898048657	0.997045181597847	pseudobulk	DESeq2	LRT	0.50430875139982
145	KC_G1	Abd-B	-1.29214429865911	0.777205227288829	0.997045181597847	pseudobulk	DESeq2	LRT	0.10946428710589
145	KC_G1	Abi	-0.53017499813611	0.262312622096376	0.997045181597847	pseudobulk	DESeq2	LRT	0.58118081129711
145	KC_G1	Abl	-0.0983076778573097	0.596201266100796	0.997045181597847	pseudobulk	DESeq2	LRT	0.224607106028974
145	KC_G1	abo	0.392497355217198	0.868386177718867	0.997045181597847	pseudobulk	DESeq2	LRT	0.0612870979170629
145	KC_G1	Abp1	0.255344909905933	0.577884539117494	0.997045181597847	pseudobulk	DESeq2	LRT	0.238158924612659
145	KC_G1	abs	-0.174382627473703	0.865408482399168	0.997045181597847	pseudobulk	DESeq2	LRT	0.0627788523299247
145	KC_G1	Ac3	-0.281980465393926	0.350392522965118	0.997045181597847	pseudobulk	DESeq2	LRT	0.455445169825979
145	KC_G1	Ac76E	0.498685724259639	0.465038106197362	0.997045181597847	pseudobulk	DESeq2	LRT	0.332511458651596
145	KC_G1	Acbp1	0.0305311948967763	0.900294037172749	0.997045181597847	pseudobulk	DESeq2	LRT	0.045615626265068
145	KC_G1	Acbp2	2.19919780189638	0.538061297284894	0.997045181597847	pseudobulk	DESeq2	LRT	0.269168245604693
145	KC_G1	ACC	-0.0897316138157014	0.827566546004861	0.997045181597847	pseudobulk	DESeq2	LRT	0.0821970738051725
145	KC_G1	AcCoAS	-0.258990876336941	0.304513165491578	0.997045181597847	pseudobulk	DESeq2	LRT	0.516393926095996
145	KC_G1	Ace	-0.0315698262006094	0.905318270519569	0.997045181597847	pseudobulk	DESeq2	LRT	0.0431987149042622
145	KC_G1	Acer	-0.44793281248538	0.608754528528315	0.997045181597847	pseudobulk	DESeq2	LRT	0.215557795046233
145	KC_G1	Acf	0.405974083944658	0.416783106327222	0.997045181597847	pseudobulk	DESeq2	LRT	0.380089892821675
145	KC_G1	achi	0.217328849931945	0.827841878112108	0.997045181597847	pseudobulk	DESeq2	LRT	0.0820526076852364
145	KC_G1	Achl	-1.29214429865911	0.777205227288829	0.997045181597847	pseudobulk	DESeq2	LRT	0.10946428710589
145	KC_G1	acj6	2.46308127796692	0.441169249831524	0.997045181597847	pseudobulk	DESeq2	LRT	0.355394766163613
145	KC_G1	Ack	-0.308878866767131	0.67423273746478	0.997045181597847	pseudobulk	DESeq2	LRT	0.17119016407383
145	KC_G1	Ack-like	0.139954320172045	0.644741700469782	0.997045181597847	pseudobulk	DESeq2	LRT	0.190614239672189
145	KC_G1	Acn	-0.0567949789314681	0.815781364882055	0.997045181597847	pseudobulk	DESeq2	LRT	0.0884262196138362
146	KC_G1	Acp36DE	1.36858832791253	0.716834138810678	0.997045181597847	pseudobulk	DESeq2	LRT	0.144581319830245
146	KC_G1	Acp53C14c	1.64950845159325	0.636439517805573	0.997045181597847	pseudobulk	DESeq2	LRT	0.196242861973188
146	KC_G1	Acp53Ea	-1.29214429865911	0.777205227288829	0.997045181597847	pseudobulk	DESeq2	LRT	0.10946428710589
146	KC_G1	Acph-1	0.18364401671533	0.834799615675268	0.997045181597847	pseudobulk	DESeq2	LRT	0.0784177595468671
146	KC_G1	Acsl	0.231776155719039	0.499457355099724	0.997045181597847	pseudobulk	DESeq2	LRT	0.301501586988568
146	KC_G1	Act42A	0.00685669069628048	0.967507136584671	0.997045181597847	pseudobulk	DESeq2	LRT	0.0143458228280056
146	KC_G1	Act5C	0.0435333852684307	0.758618679257371	0.997045181597847	pseudobulk	DESeq2	LRT	0.119976467988978
146	KC_G1	Actbeta	-0.178998960244947	0.446017173311387	0.997045181597847	pseudobulk	DESeq2	LRT	0.350648419021527
146	KC_G1	Actn	0.44001432678385	0.521949868361932	0.997045181597847	pseudobulk	DESeq2	LRT	0.282371207609441
146	KC_G1	ACXB	-2.97796175991813	0.349956896818348	0.997045181597847	pseudobulk	DESeq2	LRT	0.455985443154605
146	KC_G1	ACXC	2.19919780189638	0.538061297284894	0.997045181597847	pseudobulk	DESeq2	LRT	0.269168245604693
146	KC_G1	ACXD	1.64950845159325	0.636439517805573	0.997045181597847	pseudobulk	DESeq2	LRT	0.196242861973188
146	KC_G1	Acyp2	-0.279018844591812	0.63806595177037	0.997045181597847	pseudobulk	DESeq2	LRT	0.19513442941423
146	KC_G1	Ada1-2	-0.00656474581546769	0.958380760714589	0.997045181597847	pseudobulk	DESeq2	LRT	0.0184619132345601
146	KC_G1	Ada2a	1.79180563980294	0.408655451028418	0.997045181597847	pseudobulk	DESeq2	LRT	0.388642703680765

146	KC_G1	Ada2b	0.157798825096435	0.780510145706824	0.997045181597847	pseudobulk	DESeq2	LRT	0.107621447251338
146	KC_G1	Ada3	0.310790702696547	0.628033339713501	0.997045181597847	pseudobulk	DESeq2	LRT	0.202017300738484
146	KC_G1	Adar	0.146463385095799	0.628247219646404	0.997045181597847	pseudobulk	DESeq2	LRT	0.201869424732834
146	KC_G1	Adat1	0.205730809143211	0.880242455700119	0.997045181597847	pseudobulk	DESeq2	LRT	0.0553976884524374
146	KC_G1	Adck5	-0.468768272814723	0.672794276847496	0.997045181597847	pseudobulk	DESeq2	LRT	0.172117711538508
146	KC_G1	AdenoK	-0.184224521036929	0.806659502399542	0.997045181597847	pseudobulk	DESeq2	LRT	0.093309745865879
146	KC_G1	Adf1	-0.346350421612812	0.312616245549602	0.997045181597847	pseudobulk	DESeq2	LRT	0.504988456997499
146	KC_G1	Adi1	-0.0291475847597003	0.932768368125347	0.997045181597847	pseudobulk	DESeq2	LRT	0.0302261900524112
146	KC_G1	AdipoR	0.212095639974121	0.512502043173715	0.997045181597847	pseudobulk	DESeq2	LRT	0.290304398882352
146	KC_G1	Adk1	0.276546230063952	0.431550934447095	0.997045181597847	pseudobulk	DESeq2	LRT	0.364967938665214
146	KC_G1	Adk2	-0.0256412159635312	0.92154752799016	0.997045181597847	pseudobulk	DESeq2	LRT	0.035482261517529
146	KC_G1	Adk3	0.718967595863529	0.56096074323549	0.997045181597847	pseudobulk	DESeq2	LRT	0.251067530174758
146	KC_G1	adp	1.13633696445359	0.277465833348288	0.997045181597847	pseudobulk	DESeq2	LRT	0.556790487502936
146	KC_G1	ADPS	0.0130564859549475	0.974923560415173	0.997045181597847	pseudobulk	DESeq2	LRT	0.0110294341386777
146	KC_G1	AdSL	-0.303945820322031	0.726231328455938	0.997045181597847	pseudobulk	DESeq2	LRT	0.138925020250901
146	KC_G1	AdSS	0.401642802144193	0.396516714050339	0.997045181597847	pseudobulk	DESeq2	LRT	0.401738501494315
146	KC_G1	Aduk	-0.12663535770474	0.815195762167216	0.997045181597847	pseudobulk	DESeq2	LRT	0.088738086695665
146	KC_G1	Ady43A	0.179071852127028	0.764342070206953	0.997045181597847	pseudobulk	DESeq2	LRT	0.116712235723283
146	KC_G1	Aef1	0.03715991367727	0.896133359144155	0.997045181597847	pseudobulk	DESeq2	LRT	0.0476273554821824
146	KC_G1	aft	0.133770634408018	0.924931988646301	0.997045181597847	pseudobulk	DESeq2	LRT	0.0338902002788434
146	KC_G1	Afti	-0.395723847894964	0.329756021609388	0.997045181597847	pseudobulk	DESeq2	LRT	0.481807265143684
146	KC_G1	Ag5r	-0.857882572494553	0.362063965423216	0.997045181597847	pseudobulk	DESeq2	LRT	0.4412146963832
146	KC_G1	AGBE	2.047197261705	0.194243531092546	0.997045181597847	pseudobulk	DESeq2	LRT	0.711653435629194
146	KC_G1	ago	0.617335276042277	0.244089850686023	0.997045181597847	pseudobulk	DESeq2	LRT	0.612450278275121
146	KC_G1	AGO1	-0.0371469540515329	0.782702310744519	0.997045181597847	pseudobulk	DESeq2	LRT	0.106403384025891
146	KC_G1	AGO2	-0.222777190949092	0.545429749253294	0.997045181597847	pseudobulk	DESeq2	LRT	0.263261178121204
146	KC_G1	AGO3	0.602194408609344	0.55735812943175	0.997045181597847	pseudobulk	DESeq2	LRT	0.25386566002127
146	KC_G1	Agpat1	0.0470516974716422	0.943568216791135	0.997045181597847	pseudobulk	DESeq2	LRT	0.0252266963416558
146	KC_G1	Agpat2	-1.29214429865911	0.777205227288829	0.997045181597847	pseudobulk	DESeq2	LRT	0.10946428710589
146	KC_G1	Agpat3	0.313990032673849	0.403095280806137	0.997045181597847	pseudobulk	DESeq2	LRT	0.39459228627228
146	KC_G1	agt	0.388648773763985	0.766156826098009	0.997045181597847	pseudobulk	DESeq2	LRT	0.115682324704397
146	KC_G1	Ahcy	0.0295284537821961	0.948055611327026	0.997045181597847	pseudobulk	DESeq2	LRT	0.0231661869403131
146	KC_G1	AhcyL1	0.0942921183901733	0.820953047542747	0.997045181597847	pseudobulk	DESeq2	LRT	0.0856816806103989
146	KC_G1	AIF	0.882100557909339	0.266899945561633	0.997045181597847	pseudobulk	DESeq2	LRT	0.573651514793552
146	KC_G1	AIMP1	-1.11661541934993	0.181896457913114	0.997045181597847	pseudobulk	DESeq2	LRT	0.740175757911595
146	KC_G1	AIMP2	0.337043040759941	0.616965216589093	0.997045181597847	pseudobulk	DESeq2	LRT	0.209739320033602
146	KC_G1	AIMP3	-0.0902704096301289	0.789095883065659	0.997045181597847	pseudobulk	DESeq2	LRT	0.102870222448004
146	KC_G1	Ak6	-1.37464484579625	0.255245556391044	0.997045181597847	pseudobulk	DESeq2	LRT	0.593041809874344
146	KC_G1	Akap200	0.272590095213096	0.46022128321542	0.997045181597847	pseudobulk	DESeq2	LRT	0.337033300988361
146	KC_G1	Akh	-0.938807879001128	0.788738444006119	0.997045181597847	pseudobulk	DESeq2	LRT	0.103066990652583
146	KC_G1	akirin	0.195359882458132	0.464401929683988	0.997045181597847	pseudobulk	DESeq2	LRT	0.333105984348264
146	KC_G1	Akt1	-0.136550851198107	0.730869768838609	0.997045181597847	pseudobulk	DESeq2	LRT	0.136160001583127
146	KC_G1	Aladin	0.195266930133783	0.84001069518878	0.997045181597847	pseudobulk	DESeq2	LRT	0.0757151843763324
146	KC_G1	AlaRS	-0.960070069583366	0.379680073626872	0.997045181597847	pseudobulk	DESeq2	LRT	0.420582194909129
146	KC_G1	AlaRS-m	0.0947662163940839	0.930053826765011	0.997045181597847	pseudobulk	DESeq2	LRT	0.0314919159723565
146	KC_G1	Alas	0.172409354416185	0.632783148754185	0.997045181597847	pseudobulk	DESeq2	LRT	0.198745094776682
146	KC_G1	alc	-0.00452441772537844	0.977669129390283	0.997045181597847	pseudobulk	DESeq2	LRT	0.00980809776100239
146	KC_G1	Ald1	-0.0667961304611033	0.698102381042611	0.997045181597847	pseudobulk	DESeq2	LRT	0.156080880727452
146	KC_G1	Aldh	0.32494873613378	0.903794104744488	0.997045181597847	pseudobulk	DESeq2	LRT	0.0439304958055343
146	KC_G1	Aldh-III	0.168550250309299	0.735290816284674	0.997045181597847	pseudobulk	DESeq2	LRT	0.133540858292909
146	KC_G1	Alg-2	0.18397162811014	0.519230339317807	0.997045181597847	pseudobulk	DESeq2	LRT	0.284639939063332
146	KC_G1	Alg1	-0.161352590260422	0.873565457279235	0.997045181597847	pseudobulk	DESeq2	LRT	0.0587045472693916
146	KC_G1	Alg10	-0.085405738987555	0.929784578037244	0.997045181597847	pseudobulk	DESeq2	LRT	0.0316176615610525
146	KC_G1	Alg11	-0.174592449865515	0.72328247042947	0.997045181597847	pseudobulk	DESeq2	LRT	0.140692060394389
146	KC_G1	Alg14	0.348438929569147	0.777864014867638	0.997045181597847	pseudobulk	DESeq2	LRT	0.109096319147187
146	KC_G1	Alg2	-0.247729924107426	0.778964865234598	0.997045181597847	pseudobulk	DESeq2	LRT	0.108482130490276

146	KC_G1	Alg3	0.0530238779186722	0.949992627793246	0.997045181597847	pseudobulk	DESeq2	LRT	0.0222797649439269
146	KC_G1	Alg9	0.526864799479839	0.707546161438495	0.997045181597847	pseudobulk	DESeq2	LRT	0.150245220815644
146	KC_G1	Alh	0.114651482135219	0.685543637506204	0.997045181597847	pseudobulk	DESeq2	LRT	0.163964895469703
146	KC_G1	alien	-0.00746784872786976	0.960069060967644	0.997045181597847	pseudobulk	DESeq2	LRT	0.0176975255871025
146	KC_G1	ALiX	0.289415705359648	0.65937538041135	0.997045181597847	pseudobulk	DESeq2	LRT	0.180867272466001
146	KC_G1	Alk	-0.208497831332182	0.15072280077691	0.997045181597847	pseudobulk	DESeq2	LRT	0.821821044284109
146	KC_G1	AlkB	-1.71116546183129	0.547140079023179	0.997045181597847	pseudobulk	DESeq2	LRT	0.261901471182909
146	KC_G1	Alp12	2.19919780189638	0.538061297284894	0.997045181597847	pseudobulk	DESeq2	LRT	0.269168245604693
146	KC_G1	Alp5	-1.91128782552124	0.588150992836501	0.997045181597847	pseudobulk	DESeq2	LRT	0.230511165526625
146	KC_G1	Alp6	1.36858832791253	0.716834138810678	0.997045181597847	pseudobulk	DESeq2	LRT	0.144581319830245
146	KC_G1	alph	-0.0125432927729506	0.958550310301991	0.997045181597847	pseudobulk	DESeq2	LRT	0.018385087884371
146	KC_G1	alpha-Cat	-0.0299935432891694	0.878102733455212	0.997045181597847	pseudobulk	DESeq2	LRT	0.0564546709245923
146	KC_G1	alpha-Catr	0.00699600483558946	0.949888184270467	0.997045181597847	pseudobulk	DESeq2	LRT	0.0223275145136588
146	KC_G1	alpha-Est10	-2.93874439765445	0.324809191879395	0.997045181597847	pseudobulk	DESeq2	LRT	0.488371689018258
146	KC_G1	alpha-Est2	-1.29214429865911	0.777205227288829	0.997045181597847	pseudobulk	DESeq2	LRT	0.10946428710589
146	KC_G1	alpha-Est8	0.278172894972208	0.659626339241137	0.997045181597847	pseudobulk	DESeq2	LRT	0.180702011064666
146	KC_G1	alpha-Est9	0.874329189800077	0.0163402545367058	0.997045181597847	pseudobulk	DESeq2	LRT	1.78674118262455
146	KC_G1	alpha-Man-Ia	0.283373964656129	0.177722726006342	0.997045181597847	pseudobulk	DESeq2	LRT	0.75025703393875
146	KC_G1	alpha-Man-Ib	0.0241578713272757	0.952664792681617	0.997045181597847	pseudobulk	DESeq2	LRT	0.0210598845636963
146	KC_G1	alpha-Man-Ic	-1.29214429865911	0.777205227288829	0.997045181597847	pseudobulk	DESeq2	LRT	0.10946428710589
146	KC_G1	alpha-Man-IIa	0.624097651984953	0.555427884900341	0.997045181597847	pseudobulk	DESeq2	LRT	0.255372320562809
146	KC_G1	alpha-Man-IIb	0.26167549275739	0.586464797949755	0.997045181597847	pseudobulk	DESeq2	LRT	0.231758050922689
146	KC_G1	alpha-PheRS	0.152065265085882	0.852821121511632	0.997045181597847	pseudobulk	DESeq2	LRT	0.0691420521702719
146	KC_G1	alpha-Spec	0.0340901587704273	0.869566661661345	0.997045181597847	pseudobulk	DESeq2	LRT	0.0606971190299255
146	KC_G1	alpha4GT2	0.113302218171448	0.941500468680988	0.997045181597847	pseudobulk	DESeq2	LRT	0.0261794594545218
146	KC_G1	alphaCOP	-0.308762390105065	0.653402252604305	0.997045181597847	pseudobulk	DESeq2	LRT	0.184819372627748
146	KC_G1	alphaKap4	-0.0403644574426932	0.970076776272712	0.997045181597847	pseudobulk	DESeq2	LRT	0.0131938923398719
146	KC_G1	alphaSnap	-0.0654045614111029	0.767697442157828	0.997045181597847	pseudobulk	DESeq2	LRT	0.114809906388843
146	KC_G1	alphaTub67C	-0.397887824025521	0.705506959025291	0.997045181597847	pseudobulk	DESeq2	LRT	0.151498698066433
147	KC_G1	alphaTub84B	-0.0527871492600903	0.753665830168239	0.997045181597847	pseudobulk	DESeq2	LRT	0.122821174400202
147	KC_G1	alphaTub84D	0.136608460839771	0.619190211279801	0.997045181597847	pseudobulk	DESeq2	LRT	0.208175917991783
147	KC_G1	Alr	-0.105041904219379	0.903406638167547	0.997045181597847	pseudobulk	DESeq2	LRT	0.044116722598021
147	KC_G1	alrm	1.36858832791253	0.716834138810678	0.997045181597847	pseudobulk	DESeq2	LRT	0.144581319830245
147	KC_G1	Als2	0.458265425434761	0.474851980162108	0.997045181597847	pseudobulk	DESeq2	LRT	0.32344174662152
147	KC_G1	alt	0.181153689086655	0.479165212397553	0.997045181597847	pseudobulk	DESeq2	LRT	0.319514719442188
147	KC_G1	Amacr	0.469360743867586	0.536953128327199	0.997045181597847	pseudobulk	DESeq2	LRT	0.270063623047727
147	KC_G1	amn	-0.419393999901486	0.678781059510804	0.997045181597847	pseudobulk	DESeq2	LRT	0.168270284599361
147	KC_G1	Amnionless	0.287999477713237	0.884201087563109	0.997045181597847	pseudobulk	DESeq2	LRT	0.0534489552496806
147	KC_G1	amon	-0.00773869518450814	0.958958288154597	0.997045181597847	pseudobulk	DESeq2	LRT	0.0182002829422269
147	KC_G1	AMPdeam	0.0749471992589735	0.85647200921539	0.997045181597847	pseudobulk	DESeq2	LRT	0.0672868258589379
147	KC_G1	Amph	0.128982948989273	0.688388010558623	0.997045181597847	pseudobulk	DESeq2	LRT	0.162166702251471
147	KC_G1	AMPKalpha	-0.22596181584096	0.588110364800177	0.997045181597847	pseudobulk	DESeq2	LRT	0.230541166566579
147	KC_G1	Amun	-0.21074658096701	0.422477254923612	0.997045181597847	pseudobulk	DESeq2	LRT	0.374196667370066
147	KC_G1	amx	-0.506854048728115	0.622600938248799	0.997045181597847	pseudobulk	DESeq2	LRT	0.20579022917877
147	KC_G1	ana1	-2.22677228768931	0.246577509926623	0.997045181597847	pseudobulk	DESeq2	LRT	0.608046537473396
147	KC_G1	ana2	-2.93874439765445	0.324809191879395	0.997045181597847	pseudobulk	DESeq2	LRT	0.488371689018258
147	KC_G1	ana3	2.46308127796692	0.441169249831524	0.997045181597847	pseudobulk	DESeq2	LRT	0.355394766163613
147	KC_G1	Ance-5	-1.29214429865911	0.777205227288829	0.997045181597847	pseudobulk	DESeq2	LRT	0.10946428710589
147	KC_G1	angel	0.545647365684172	0.588126374434833	0.997045181597847	pseudobulk	DESeq2	LRT	0.230529344293465
147	KC_G1	Ank	0.0974728504483475	0.632355359300341	0.997045181597847	pseudobulk	DESeq2	LRT	0.199038796383452
147	KC_G1	Ank2	-0.105415355157402	0.410782432867777	0.997045181597847	pseudobulk	DESeq2	LRT	0.386388137303871
147	KC_G1	Ankle2	0.300272855490968	0.643329031487616	0.997045181597847	pseudobulk	DESeq2	LRT	0.191566849764899
147	KC_G1	anne	-0.00703520816743385	0.937791898062685	0.997045181597847	pseudobulk	DESeq2	LRT	0.0278935236145165
147	KC_G1	Ant2	-0.0551033762074761	0.955907732207947	0.997045181597847	pseudobulk	DESeq2	LRT	0.019584025429762
147	KC_G1	antr	-1.29214429865911	0.777205227288829	0.997045181597847	pseudobulk	DESeq2	LRT	0.10946428710589
147	KC_G1	AnxB10	0.0697185668462966	0.908961819576973	0.997045181597847	pseudobulk	DESeq2	LRT	0.0414543586870333

147	KC_G1	AnxB11	0.179391130066255	0.743221736197442	0.997045181597847	pseudobulk	DESeq2	LRT	0.12888159749051
147	KC_G1	AnxB9	1.64950845159325	0.636439517805573	0.997045181597847	pseudobulk	DESeq2	LRT	0.196242861973188
147	KC_G1	aop	-0.324993172294153	0.486538385184801	0.997045181597847	pseudobulk	DESeq2	LRT	0.312882890613872
147	KC_G1	aos	1.19226819192844	0.664018121016277	0.997045181597847	pseudobulk	DESeq2	LRT	0.177820068598864
147	KC_G1	Aos1	-0.192334766805358	0.703185667036793	0.997045181597847	pseudobulk	DESeq2	LRT	0.152929990025111
147	KC_G1	ap	-3.78498433938543	0.0421658887336635	0.997045181597847	pseudobulk	DESeq2	LRT	1.37503874158238
147	KC_G1	AP-1-2beta	0.145939381311382	0.688228185236761	0.997045181597847	pseudobulk	DESeq2	LRT	0.162267545545161
147	KC_G1	AP-1gamma	-0.0477125110774902	0.824013761029363	0.997045181597847	pseudobulk	DESeq2	LRT	0.0840655355247105
147	KC_G1	AP-1mu	0.0738013012632328	0.855709334462135	0.997045181597847	pseudobulk	DESeq2	LRT	0.0676737305054063
147	KC_G1	AP-1sigma	-0.294533246316596	0.266983295955141	0.997045181597847	pseudobulk	DESeq2	LRT	0.573515909801883
147	KC_G1	AP-2alpha	0.0081397902412144	0.954735766267166	0.997045181597847	pseudobulk	DESeq2	LRT	0.0201168076105294
147	KC_G1	AP-2mu	0.0629090702879179	0.809368209306174	0.997045181597847	pseudobulk	DESeq2	LRT	0.0918538580048054
147	KC_G1	AP-2sigma	-0.0241780303303091	0.913531768851762	0.997045181597847	pseudobulk	DESeq2	LRT	0.039276345085267
147	KC_G1	Apc	0.0241183135931134	0.928564521940748	0.997045181597847	pseudobulk	DESeq2	LRT	0.0321879136266684
147	KC_G1	APC10	-0.073385110997451	0.913037762563247	0.997045181597847	pseudobulk	DESeq2	LRT	0.0395112599972581
147	KC_G1	Apc2	-1.29214429865911	0.777205227288829	0.997045181597847	pseudobulk	DESeq2	LRT	0.10946428710589
147	KC_G1	APC4	1.42563515428115	0.37574075310322	0.997045181597847	pseudobulk	DESeq2	LRT	0.42511169850063
147	KC_G1	ApepP	0.657313492186966	0.573999859527016	0.997045181597847	pseudobulk	DESeq2	LRT	0.241088213885388
147	KC_G1	Apf	-0.231372409501597	0.721975079937034	0.997045181597847	pseudobulk	DESeq2	LRT	0.141477792503232
147	KC_G1	aph-1	0.230303558381587	0.591887513035383	0.997045181597847	pseudobulk	DESeq2	LRT	0.227760822178903
147	KC_G1	aPKC	-0.114713958269347	0.574996142833565	0.997045181597847	pseudobulk	DESeq2	LRT	0.240335068617704
147	KC_G1	Apl	-0.879179394233022	0.19783021967521	0.997045181597847	pseudobulk	DESeq2	LRT	0.703707366754324
147	KC_G1	Aplip1	0.0282116152417907	0.893875891437017	0.997045181597847	pseudobulk	DESeq2	LRT	0.0487227758413164
147	KC_G1	apolpp	-3.35254086138237	0.0966851677872241	0.997045181597847	pseudobulk	DESeq2	LRT	1.01464014476114
147	KC_G1	Apoltp	-0.42828032258323	0.641597321862212	0.997045181597847	pseudobulk	DESeq2	LRT	0.192737457540119
147	KC_G1	app	-0.807018732450088	0.653902767241676	0.997045181597847	pseudobulk	DESeq2	LRT	0.184486824748801
147	KC_G1	APP-BP1	-0.438252841176475	0.633178636402515	0.997045181597847	pseudobulk	DESeq2	LRT	0.198473746761391
147	KC_G1	Appl	-0.0482570946871134	0.700215316994147	0.997045181597847	pseudobulk	DESeq2	LRT	0.154768393409203
147	KC_G1	Aprt	1.32652709277313	0.280619897462262	0.997045181597847	pseudobulk	DESeq2	LRT	0.551881538397831
147	KC_G1	Aps	-0.0456596017353235	0.878047951189136	0.997045181597847	pseudobulk	DESeq2	LRT	0.0564817661346772
147	KC_G1	aqz	-0.0263093444050252	0.822505416628873	0.997045181597847	pseudobulk	DESeq2	LRT	0.0848612333119893
147	KC_G1	aralar1	-0.0700899060746046	0.888589634942649	0.997045181597847	pseudobulk	DESeq2	LRT	0.0512987569441982
147	KC_G1	Arc1	-1.29214429865911	0.777205227288829	0.997045181597847	pseudobulk	DESeq2	LRT	0.10946428710589
147	KC_G1	Arc2	-1.9468510462689	0.592905871125718	0.997045181597847	pseudobulk	DESeq2	LRT	0.227014249123956
147	KC_G1	Arc42	-1.7389758146508	0.230172143011939	0.997045181597847	pseudobulk	DESeq2	LRT	0.637947238778629
147	KC_G1	Archease	-0.357593938323708	0.579958080176407	0.997045181597847	pseudobulk	DESeq2	LRT	0.236603396447422
147	KC_G1	Arf102F	0.0188960649159509	0.946816448488834	0.997045181597847	pseudobulk	DESeq2	LRT	0.0237342059331895
147	KC_G1	Arf51F	0.120752783368963	0.735955726481656	0.997045181597847	pseudobulk	DESeq2	LRT	0.133148311101427
147	KC_G1	Arf79F	0.0619882669307115	0.738887789580493	0.997045181597847	pseudobulk	DESeq2	LRT	0.131421510273864
147	KC_G1	ArfGAP1	0.042749679386153	0.942607444878919	0.997045181597847	pseudobulk	DESeq2	LRT	0.0256691344288501
147	KC_G1	ArfGAP3	-0.260106984927197	0.649197792372969	0.997045181597847	pseudobulk	DESeq2	LRT	0.187622965681708
[truncated: 879,427 more chars]
