# Supplementary material for: Transcriptional changes in specific subsets of Drosophila neurons following inhibition of the serotonin transporter
Source: Transl Psychiatry. 2023 Jun 24;13:226. doi: 10.1038/s41398-023-02521-3 (PMC10290657; doi:10.1038/s41398-023-02521-3)
Supplement: Supplementary file 6 — Supplemental Table 5 [file 41398_2023_2521_MOESM6_ESM.pdf]

scRNA-seq_DEGs_CIT_v_VEH_Day4

	cell_type	gene	avg_logFC	p_val	p_val_adj	de_family	de_method	de_type	minuslogP
1	KC_G2	Hsp26	-2.25141749022755	3.20478577863584E-16	2.49364381435655E-12	pseudobulk	DESeq2	LRT	15.4942009952452
2	KC_AB1	Lgr1	-1.15978057081582	5.05910229760803E-09	4.43683271500225E-05	pseudobulk	DESeq2	LRT	8.2959265388485
3	KC_AB1	CG6770	0.824881042938586	2.68137920108384E-07	0.00078385651978351	pseudobulk	DESeq2	LRT	6.57164176367739
4	KC_AB1	mgl	-1.14196281316339	2.62648653851539E-07	0.00078385651978351	pseudobulk	DESeq2	LRT	6.58062482073693
5	KC_AB1	Plc21C	-0.512700264544291	8.3869559563592E-06	0.0183884009343175	pseudobulk	DESeq2	LRT	5.07639563765029
6	KC_G2	Hsp70Bc	-2.71394346231003	7.88396887014504E-06	0.0306725808892993	pseudobulk	DESeq2	LRT	5.10325509920118
7	KC_AB1	CG42342	-1.81721809702619	1.81891244847457E-05	0.0319037243462439	pseudobulk	DESeq2	LRT	4.7401882047625
8	KC_AB1	Ddc	-0.962439921154318	2.51223220915112E-05	0.0367204607904255	pseudobulk	DESeq2	LRT	4.59994022063066
9	KC_AB1	Octalpha2R	-0.909297415771036	3.22386463842291E-05	0.040390418398527	pseudobulk	DESeq2	LRT	4.49162320136451
10	KC_AB1	dpr10	-0.57662950210514	6.40150108353898E-05	0.0701764556282961	pseudobulk	DESeq2	LRT	4.1937181766632
11	KC_AB1	CG17816	-0.431065620392634	0.000162662296712499	0.12968621292442	pseudobulk	DESeq2	LRT	3.78871309996577
12	KC_AB1	CG6044	-0.441798456747824	0.000140478331657339	0.12968621292442	pseudobulk	DESeq2	LRT	3.85239065915661
13	KC_AB1	Tet	0.390411351029462	0.000159827749235879	0.12968621292442	pseudobulk	DESeq2	LRT	3.79634781642513
14	KC_AB1	CG34347	1.58151575369074	0.000222136546173592	0.1623447924952	pseudobulk	DESeq2	LRT	3.6533799849192
15	KC_AB1	CG33978	1.84247931528277	0.000326787428930226	0.191061716781206	pseudobulk	DESeq2	LRT	3.48573465853878
16	KC_AB1	kek1	-0.573262175320552	0.000297497238824034	0.191061716781206	pseudobulk	DESeq2	LRT	3.52651706075577
17	KC_AB1	Rgk2	-0.486879302045636	0.000323214034416963	0.191061716781206	pseudobulk	DESeq2	LRT	3.49050978979871
18	KC_AB1	CG42391	-4.97056628549459	0.000410807164903662	0.22517367726282	pseudobulk	DESeq2	LRT	3.38636199046184
19	KC_AB1	Hsp27	-2.11916084058189	0.000532646390210321	0.274782873067325	pseudobulk	DESeq2	LRT	3.2735610118832
20	KC_AB1	CG33543	-3.9739683923463	0.000570315597089836	0.277870432582103	pseudobulk	DESeq2	LRT	3.24388475109169
21	KC_AB1	dpr20	1.33112607995824	0.000607742188913138	0.280520999829906	pseudobulk	DESeq2	LRT	3.21628061427602
22	KC_AB1	MFS17	0.342953211808	0.000649906616252863	0.284984051226881	pseudobulk	DESeq2	LRT	3.1871490417566
23	KC_AB1	CG30419	-0.727467404665046	0.000736523077789683	0.293604881464342	pseudobulk	DESeq2	LRT	3.13281364067513
24	KC_AB1	CG4393	1.27452241772452	0.000735412383606653	0.293604881464342	pseudobulk	DESeq2	LRT	3.13346906132226
25	KC_AB1	ftz-f1	0.497967256580541	0.000860591418058056	0.328147249407354	pseudobulk	DESeq2	LRT	3.06520298907918
26	KC_AB1	CG1129	0.847560050668904	0.000902771617065753	0.329887795069444	pseudobulk	DESeq2	LRT	3.04442210349934
27	KC_AB1	CG46339	-0.586746632109996	0.000944170329165897	0.331214951471397	pseudobulk	DESeq2	LRT	3.02494965152391
28	KC_AB1	l(3)neo38	0.469340699848409	0.00100099476049595	0.337643232674979	pseudobulk	DESeq2	LRT	2.99956819574111
29	KC_G1	CG43736	0.631000511748803	8.11527059129014E-05	0.358978994605719	pseudobulk	DESeq2	LRT	4.09069699471083
30	KC_G1	nAChRalpha7	-0.577869768927227	4.12423603876539E-05	0.358978994605719	pseudobulk	DESeq2	LRT	4.384656487084
31	KC_AB1	igl	-0.395411614511449	0.00136077978161886	0.417032154161321	pseudobulk	DESeq2	LRT	2.86621215206788
32	KC_AB1	side-II	-0.517755816524351	0.00134169666266565	0.417032154161321	pseudobulk	DESeq2	LRT	2.87234566048692
33	KC_AB1	VepD	-0.677135792747958	0.00137901168422786	0.417032154161321	pseudobulk	DESeq2	LRT	2.86043205407492
34	KC_G1	side-II	-0.461863277603947	0.00020132370377795	0.469530991773869	pseudobulk	DESeq2	LRT	3.69610508843023
35	KC_G1	TotA	2.37059000585876	0.000212289359906802	0.469530991773869	pseudobulk	DESeq2	LRT	3.67307177243581
36	KC_G1	CG41378	-0.835749152968763	0.000275562977385837	0.4875811321865	pseudobulk	DESeq2	LRT	3.55977913145635
37	KC_AB1	porin	-0.462617634886416	0.001985190924983	0.580337480403363	pseudobulk	DESeq2	LRT	2.70219771878542
38	KC_AB1	l(1)G0469	0.665284721315673	0.00218373426885978	0.617785468964524	pseudobulk	DESeq2	LRT	2.66080021055944
39	KC_AB1	CG32428	-0.818021640188788	0.00233632252716101	0.640298392600064	pseudobulk	DESeq2	LRT	2.63146720346997
40	KC_AB1	lncRNA:let7C	0.520405064389386	0.0025684741218687	0.682591456023894	pseudobulk	DESeq2	LRT	2.59032480556209
41	KC_G1	InR	0.793607965327057	0.000479454029520487	0.706954966527958	pseudobulk	DESeq2	LRT	3.31925302703903
42	KC_AB1	5-HT2B	-2.90314452125618	0.0028455619138073	0.724661627637546	pseudobulk	DESeq2	LRT	2.54583196056265
43	KC_AB1	CG31221	-0.371550911644857	0.00289336389076793	0.724661627637546	pseudobulk	DESeq2	LRT	2.53859694284208
44	KC_AB1	Lmpt	-0.50553331492934	0.00297466574628867	0.724661627637546	pseudobulk	DESeq2	LRT	2.52656182748095
45	KC_G1	CG6770	0.413834331405033	0.000673948629049039	0.745302940149606	pseudobulk	DESeq2	LRT	3.17137320579523
46	KC_G1	ics	-1.35534461647696	0.000653582164396613	0.745302940149606	pseudobulk	DESeq2	LRT	3.18469980777586
47	KC_AB1	eys	-0.487434144238663	0.00330868309096933	0.745986428891146	pseudobulk	DESeq2	LRT	2.48034482801522
48	KC_AB1	sif	0.39913825920681	0.00326590955350848	0.745986428891146	pseudobulk	DESeq2	LRT	2.48599584683636
49	KC_AB1	Toll-6	-0.892054804439072	0.00331738548765732	0.745986428891146	pseudobulk	DESeq2	LRT	2.47920405955226
50	KC_AB1	Cad87A	-1.27774522083344	0.00373294384500401	0.779474226682979	pseudobulk	DESeq2	LRT	2.42794854315388

51	KC_AB1	lncRNA:CR43242	2.33401834463734	0.00370762027646251	0.779474226682979	pseudobulk	DESeq2	LRT	2.43090475141038
52	KC_AB1	REPTOR-BP	0.612387022822914	0.00362164910814943	0.779474226682979	pseudobulk	DESeq2	LRT	2.44109362960908
53	KC_AB2	Hsp26	-1.58850748988648	0.000166365731052013	0.804066196398276	pseudobulk	DESeq2	LRT	3.7789361272504
54	KC_AB2	rdgA	-1.20173076114301	0.000228525279635719	0.804066196398276	pseudobulk	DESeq2	LRT	3.64106575095941
55	KC_AB1	CG4629	-0.720189976923238	0.00408927002296274	0.83402088607868	pseudobulk	DESeq2	LRT	2.38835421113536
56	KC_AB1	CG12541	0.788037628716728	0.00430098913407499	0.838214993463058	pseudobulk	DESeq2	LRT	2.36643165463662
57	KC_AB1	Fkbp12	-0.396349414929831	0.00424626543389575	0.838214993463058	pseudobulk	DESeq2	LRT	2.37199286158943
58	KC_AB1	Tl	-0.475187657910673	0.00449638203776021	0.857245010242544	pseudobulk	DESeq2	LRT	2.34713679578367
59	KC_AB1	ebd2	0.886215705829481	0.0048561901128204	0.906144410413509	pseudobulk	DESeq2	LRT	2.31370431959905
60	KC_AB1	DopEcR	-0.410803875331603	0.00543863677466928	0.959913732232306	pseudobulk	DESeq2	LRT	2.26450994505031
61	KC_AB1	lncRNA:CR43264	1.22455153166727	0.0053267790293479	0.959913732232306	pseudobulk	DESeq2	LRT	2.27353531866952
62	KC_AB1	mt:CoII	0.346840883642615	0.00558216651583211	0.959913732232306	pseudobulk	DESeq2	LRT	2.25319721266494
63	KC_AB1	stw	1.47863272561138	0.00549473762933863	0.959913732232306	pseudobulk	DESeq2	LRT	2.26005304006503
64	KC_G2	CG11486	0.773336554887426	0.00037621554230335	0.975777711554122	pseudobulk	DESeq2	LRT	3.42456326674111
65	KC_AB1	128up	-1.02813901797359	0.202502435884502	1	pseudobulk	DESeq2	LRT	0.69356974832667
66	KC_AB1	14-3-3epsilon	0.0445434064438373	0.658317563171332	1	pseudobulk	DESeq2	LRT	0.181564558265591
67	KC_AB1	14-3-3zeta	-0.0648804947395204	0.418074276753163	1	pseudobulk	DESeq2	LRT	0.378746552870129
68	KC_AB1	140up	0.558070780091584	0.378042696089741	1	pseudobulk	DESeq2	LRT	0.422459148234123
69	KC_AB1	18SrRNA-Psi:CR4	0.305752584878383	0.603227875021491	1	pseudobulk	DESeq2	LRT	0.219518598026371
70	KC_AB1	18w	-0.6633432575388	0.572676539101097	1	pseudobulk	DESeq2	LRT	0.24209060830912
71	KC_AB1	26-29-p	-0.401784077379264	0.364923685673476	1	pseudobulk	DESeq2	LRT	0.437797947478173
72	KC_AB1	28SrRNA-Psi:CR4	0.103223406238717	0.832625945301265	1	pseudobulk	DESeq2	LRT	0.079550060269310
73	KC_AB1	28SrRNA-Psi:CR4	-0.381848798175527	0.659741617136701	1	pseudobulk	DESeq2	LRT	0.180626119341187
74	KC_AB1	28SrRNA-Psi:CR4	0.775517076445541	0.619193287112455	1	pseudobulk	DESeq2	LRT	0.208173760635599
75	KC_AB1	28SrRNA-Psi:CR4	-0.360500881231853	0.637263894964337	1	pseudobulk	DESeq2	LRT	0.195680686356069
76	KC_AB1	28SrRNA-Psi:CR4	0.113316967542732	1	1	pseudobulk	DESeq2	LRT	0
77	KC_AB1	28SrRNA-Psi:CR4	-0.82542030717083	1	1	pseudobulk	DESeq2	LRT	0
78	KC_AB1	28SrRNA-Psi:CR4	0.600096879214643	0.692934193703674	1	pseudobulk	DESeq2	LRT	0.159308007333908
79	KC_AB1	28SrRNA-Psi:CR4	0.136047407432427	0.710328972729547	1	pseudobulk	DESeq2	LRT	0.14854047106643
80	KC_AB1	28SrRNA-Psi:CR4	-0.175092143521659	0.830774407002044	1	pseudobulk	DESeq2	LRT	0.080516890891139
81	KC_AB1	2mit	-0.0517470464278889	0.78370019678904	1	pseudobulk	DESeq2	LRT	0.105850044179656
82	KC_AB1	312	-0.388033980994316	0.47303132545863	1	pseudobulk	DESeq2	LRT	0.325110098113451
83	KC_AB1	4E-T	-0.202852492390423	0.444325844503056	1	pseudobulk	DESeq2	LRT	0.352298425002143
84	KC_AB1	5-HT1A	-0.0995206644998566	0.388282593529597	1	pseudobulk	DESeq2	LRT	0.410852078172695
85	KC_AB1	5-HT1B	-0.0859216592621088	0.798555570359081	1	pseudobulk	DESeq2	LRT	0.097694856531679
86	KC_AB1	5-HT2A	0.977851105145882	0.69132261808108	1	pseudobulk	DESeq2	LRT	0.160319234022942
87	KC_AB1	5.8SrRNA-Psi:CR4	-3.03302276430872	0.139789869129298	1	pseudobulk	DESeq2	LRT	0.854524301699563
88	KC_AB1	5.8SrRNA-Psi:CR4	-0.30131615088016	0.807740060504158	1	pseudobulk	DESeq2	LRT	0.092728377407819
89	KC_AB1	5.8SrRNA-Psi:CR4	1.08899820779554	0.878177167143508	1	pseudobulk	DESeq2	LRT	0.056417858865076
90	KC_AB1	5PtaseI	0.0309110230123663	0.899702921662986	1	pseudobulk	DESeq2	LRT	0.045900869206202
91	KC_AB1	7B2	-0.14983748919559	0.161795456426339	1	pseudobulk	DESeq2	LRT	0.791033678500481
92	KC_AB1	a	0.0959995228153245	0.544816148376597	1	pseudobulk	DESeq2	LRT	0.26375002839536
93	KC_AB1	A16	0.0287746773396435	0.948740372977433	1	pseudobulk	DESeq2	LRT	0.022852617930708
94	KC_AB1	a6	0.07268787256618	0.867601749511985	1	pseudobulk	DESeq2	LRT	0.061679580905614
95	KC_AB1	AANAT1	0.0381176321344616	0.88248500206746	1	pseudobulk	DESeq2	LRT	0.054292666761319
96	KC_AB1	AANATL2	0.135795303631797	0.946582799906172	1	pseudobulk	DESeq2	LRT	0.023841391241048
97	KC_AB1	AANATL7	0.0857918826647736	0.875134586218114	1	pseudobulk	DESeq2	LRT	0.057925152055235
98	KC_AB1	Aasdh	-0.628368891320396	0.408933471270363	1	pseudobulk	DESeq2	LRT	0.388347340917658
99	KC_AB1	Aatf	-0.130192338768244	0.798951153479826	1	pseudobulk	DESeq2	LRT	0.097479771903337
100	KC_AB1	aay	2.211521544984	0.400335997833192	1	pseudobulk	DESeq2	LRT	0.39757535679119
101	KC_AB1	ab	-0.371205994179926	0.473045338498785	1	pseudobulk	DESeq2	LRT	0.325097232800847
102	KC_AB1	ABCA	-0.117200790030195	0.725998002059675	1	pseudobulk	DESeq2	LRT	0.139064574472981

103	KC_AB1	ABCB7	-0.65921812643666	0.275495827605799	1	pseudobulk	DESeq2	LRT	0.559884974167911
104	KC_AB1	ABCD	0.233581562833238	0.153468232655522	1	pseudobulk	DESeq2	LRT	0.81398150820121
105	KC_AB1	Abi	0.309079662340219	0.453873124249947	1	pseudobulk	DESeq2	LRT	0.343065532911507
106	KC_AB1	Abl	-0.0944127591685581	0.483351967852067	1	pseudobulk	DESeq2	LRT	0.315736508941261
107	KC_AB1	abo	-0.508514723156182	0.765250990125593	1	pseudobulk	DESeq2	LRT	0.116196099815171
108	KC_AB1	Abp1	0.142172524501041	0.659817886046595	1	pseudobulk	DESeq2	LRT	0.180575915971556
109	KC_AB1	abs	1.4358932882731	0.147575725839658	1	pseudobulk	DESeq2	LRT	0.830985072058095
110	KC_AB1	Ac3	-0.112326133049059	0.646338224009987	1	pseudobulk	DESeq2	LRT	0.189540159432838
111	KC_AB1	Ac76E	1.50057491635846	0.227679205863383	1	pseudobulk	DESeq2	LRT	0.642676632060178
112	KC_AB1	Ac78C	-0.618430389799118	0.0599513853555399	1	pseudobulk	DESeq2	LRT	1.22220077678006
113	KC_AB1	Acbp1	-0.0491483349149471	0.802220220073099	1	pseudobulk	DESeq2	LRT	0.095706395763122
114	KC_AB1	Acbp2	0.113316967542732	1	1	pseudobulk	DESeq2	LRT	0
115	KC_AB1	ACC	0.400688614635769	0.284666421638062	1	pseudobulk	DESeq2	LRT	0.545663757852496
116	KC_AB1	AcCoAS	-0.22512775105272	0.25582135607046	1	pseudobulk	DESeq2	LRT	0.592063203264667
117	KC_AB1	Ace	-0.0157079411000355	0.943609627068369	1	pseudobulk	DESeq2	LRT	0.025207636924486
118	KC_AB1	Acer	0.649340447004886	0.222346542351289	1	pseudobulk	DESeq2	LRT	0.652969619754202
119	KC_AB1	Acf	-0.147803345271764	0.685108354436438	1	pseudobulk	DESeq2	LRT	0.164240736518681
120	KC_AB1	achi	-0.116960616994086	0.867119736173538	1	pseudobulk	DESeq2	LRT	0.061920928857093
121	KC_AB1	Ack	-0.177101799193045	0.726495322750092	1	pseudobulk	DESeq2	LRT	0.138767177388485
122	KC_AB1	Ack-like	0.163726251174424	0.505756430292967	1	pseudobulk	DESeq2	LRT	0.296058586811237
123	KC_AB1	Acn	0.0770130913548289	0.696567348229173	1	pseudobulk	DESeq2	LRT	0.15703688706447
124	KC_AB1	Acp53C14b	0.11400025262841	1	1	pseudobulk	DESeq2	LRT	0
125	KC_AB1	Acp53C14c	0.111831901354254	1	1	pseudobulk	DESeq2	LRT	0
126	KC_AB1	Acp63F	1.08899820779554	0.878177167143508	1	pseudobulk	DESeq2	LRT	0.056417858865076
127	KC_AB1	Acp95EF	-2.3118469151042	0.353435572380865	1	pseudobulk	DESeq2	LRT	0.451689742007392
128	KC_AB1	Acph-1	-0.321505120830407	0.752558711365131	1	pseudobulk	DESeq2	LRT	0.123459612641424
129	KC_AB1	Acsl	0.319698609238392	0.335837808439068	1	pseudobulk	DESeq2	LRT	0.473870412807426
130	KC_AB1	Act42A	-0.0294669205884083	0.930812716258322	1	pseudobulk	DESeq2	LRT	0.031137692249192
131	KC_AB1	Act57B	1.08899820779554	0.878177167143508	1	pseudobulk	DESeq2	LRT	0.056417858865076
132	KC_AB1	Act5C	-0.273263457052959	0.523570233749598	1	pseudobulk	DESeq2	LRT	0.281025052124651
133	KC_AB1	Act87E	-1.44925888589622	0.6315588144614	1	pseudobulk	DESeq2	LRT	0.199586199175701
134	KC_AB1	Actbeta	0.152680227275461	0.393389678995101	1	pseudobulk	DESeq2	LRT	0.405177038453984
135	KC_AB1	Actn	-0.413722314281503	0.393095983302614	1	pseudobulk	DESeq2	LRT	0.405501393825351
136	KC_AB1	ACXC	0.736294445752933	1	1	pseudobulk	DESeq2	LRT	0
137	KC_AB1	ACXD	-0.82542030717083	1	1	pseudobulk	DESeq2	LRT	0
138	KC_AB1	Acyp2	-0.202532272917687	0.649869051976759	1	pseudobulk	DESeq2	LRT	0.18717414448507
139	KC_AB1	Ada1-1	-2.60226475197003	0.286731224506517	1	pseudobulk	DESeq2	LRT	0.542525010602557
140	KC_AB1	Ada1-2	-0.254773661571901	0.683944807193743	1	pseudobulk	DESeq2	LRT	0.164978943452884
141	KC_AB1	Ada2a	0.899120070663023	0.559470205872958	1	pseudobulk	DESeq2	LRT	0.252223036516329
142	KC_AB1	Ada2b	-0.242030280992751	0.628248002440533	1	pseudobulk	DESeq2	LRT	0.201868883603565
143	KC_AB1	Ada3	-0.337520529563587	0.481814998849669	1	pseudobulk	DESeq2	LRT	0.31711968458621
144	KC_AB1	Adar	-0.0305768335205607	0.882072081951795	1	pseudobulk	DESeq2	LRT	0.054495923354826
145	KC_AB1	Adat1	0.473581567897271	0.653262840519536	1	pseudobulk	DESeq2	LRT	0.18491204503428
146	KC_AB1	Adck1	-0.535724275767237	0.592590615335605	1	pseudobulk	DESeq2	LRT	0.227245230581641
147	KC_AB1	Adck5	-0.437196303951055	0.633956153506551	1	pseudobulk	DESeq2	LRT	0.197940778314883
148	KC_AB1	ADD1	0.125065343531384	0.735940377009878	1	pseudobulk	DESeq2	LRT	0.133157369065339
149	KC_AB1	AdenoK	-0.548019153267774	0.337423816751588	1	pseudobulk	DESeq2	LRT	0.471824266386075
150	KC_AB1	Adf1	0.522457897636543	0.0431403701410242	1	pseudobulk	DESeq2	LRT	1.36511613300457
151	KC_AB1	Adgf-B	-0.850996402080018	0.900449987182352	1	pseudobulk	DESeq2	LRT	0.045540403770502
152	KC_AB1	Adgf-D	0.359026773898891	0.856962521129231	1	pseudobulk	DESeq2	LRT	0.067038171335481
153	KC_AB1	Adi1	-0.236708124026235	0.682487766808655	1	pseudobulk	DESeq2	LRT	0.1659051286903
154	KC_AB1	AdipoR	0.0427815150539266	0.875692566031153	1	pseudobulk	DESeq2	LRT	0.057648337108987

155	KC_AB1	Adk1	0.180335352396266	0.483693355386843	1	pseudobulk	DESeq2	LRT	0.315429878579395
156	KC_AB1	Adk2	-0.240983059469206	0.226069949999359	1	pseudobulk	DESeq2	LRT	0.645757161744791
157	KC_AB1	Adk3	0.753491914888755	0.439992211358705	1	pseudobulk	DESeq2	LRT	0.356555011227163
158	KC_AB1	adp	-2.26848750513244	0.0102782338304757	1	pseudobulk	DESeq2	LRT	1.98808150631127
159	KC_AB1	ADPS	-0.450293489552998	0.281959391524828	1	pseudobulk	DESeq2	LRT	0.549813435321125
160	KC_AB1	AdSL	-0.0839986344458565	0.903435817239949	1	pseudobulk	DESeq2	LRT	0.044102695575250
161	KC_AB1	AdSS	-0.399455488497104	0.228468110624352	1	pseudobulk	DESeq2	LRT	0.64117440980145
162	KC_AB1	Aduk	-0.377564364885185	0.399630057940414	1	pseudobulk	DESeq2	LRT	0.398341854012816
163	KC_AB1	Ady43A	-0.74657549043454	0.123840520616776	1	pseudobulk	DESeq2	LRT	0.907137230914109
164	KC_AB1	Aef1	-0.0049876892984731	0.908589947357843	1	pseudobulk	DESeq2	LRT	0.041632072530383
165	KC_AB1	aft	-0.220988788247402	0.876266087348095	1	pseudobulk	DESeq2	LRT	0.057363995750110
166	KC_AB1	Afti	-0.0388722599888741	0.896200150952463	1	pseudobulk	DESeq2	LRT	0.047594987272098
167	KC_AB1	Ag5r	-0.219205332858831	0.780294019042473	1	pseudobulk	DESeq2	LRT	0.107741721941066
168	KC_AB1	AGBE	1.24107984973921	0.189911013316402	1	pseudobulk	DESeq2	LRT	0.721449848934127
169	KC_AB1	ago	-0.29674050205392	0.4812373616838	1	pseudobulk	DESeq2	LRT	0.317640662830058
170	KC_AB1	AGO1	0.0428958338276189	0.650879928672695	1	pseudobulk	DESeq2	LRT	0.186499120685945
171	KC_AB1	AGO2	-0.0666335318800252	0.808734593192839	1	pseudobulk	DESeq2	LRT	0.092193979776186
172	KC_AB1	AGO3	0.0150751716804423	0.982821300267584	1	pseudobulk	DESeq2	LRT	0.007525439810564
173	KC_AB1	Agpat1	0.525377066105318	0.345618647730813	1	pseudobulk	DESeq2	LRT	0.461402833357179
174	KC_AB1	Agpat2	0.462086640409983	0.767092653276588	1	pseudobulk	DESeq2	LRT	0.115152176629203
175	KC_AB1	Agpat3	-0.168134234356957	0.623383925099044	1	pseudobulk	DESeq2	LRT	0.205244400857371
176	KC_AB1	agt	1.04323486363549	0.203523609835759	1	pseudobulk	DESeq2	LRT	0.691385203015527
177	KC_AB1	Ahcy	-0.0815358616180038	0.891604758894396	1	pseudobulk	DESeq2	LRT	0.049827622161225
178	KC_AB1	AhcyL1	0.337321322234073	0.34002743979052	1	pseudobulk	DESeq2	LRT	0.468486034520236
179	KC_AB1	AIF	-0.055224912293259	0.925228224178124	1	pseudobulk	DESeq2	LRT	0.033751127503391
180	KC_AB1	AIMP1	0.29858338130415	0.630511529777074	1	pseudobulk	DESeq2	LRT	0.200306967341847
181	KC_AB1	AIMP2	0.17898228348499	0.724957737528314	1	pseudobulk	DESeq2	LRT	0.139687310523119
182	KC_AB1	AIMP3	0.271348019835163	0.329439712538304	1	pseudobulk	DESeq2	LRT	0.482224049681492
183	KC_AB1	Ak6	-0.708932785977823	0.280668146163226	1	pseudobulk	DESeq2	LRT	0.551806873901136
184	KC_AB1	Akap200	-0.426463490760392	0.161712450771168	1	pseudobulk	DESeq2	LRT	0.791256541051882
185	KC_AB1	Akh	-2.03779390173592	0.320930517906753	1	pseudobulk	DESeq2	LRT	0.493588983034383
186	KC_AB1	akirin	0.40469914362394	0.0371500468773837	1	pseudobulk	DESeq2	LRT	1.43004063389328
187	KC_AB1	Akt1	0.242569916381376	0.433665044626829	1	pseudobulk	DESeq2	LRT	0.362845582549164
188	KC_AB1	al	1.08899820779554	0.670846322504933	1	pseudobulk	DESeq2	LRT	0.173376956629912
189	KC_AB1	Aladin	0.401485094098294	0.658216302048969	1	pseudobulk	DESeq2	LRT	0.181631365733173
190	KC_AB1	AlaRS	0.441286041059998	0.602627518769274	1	pseudobulk	DESeq2	LRT	0.219951040308255
191	KC_AB1	AlaRS-m	-0.387715302756601	0.660950452201	1	pseudobulk	DESeq2	LRT	0.1798310959489
192	KC_AB1	Alas	-0.123336312980424	0.59305653373005	1	pseudobulk	DESeq2	LRT	0.22690390508982
193	KC_AB1	alc	0.24781736149847	0.246977991013046	1	pseudobulk	DESeq2	LRT	0.607341746365688
194	KC_AB1	Ald1	-0.289698794228578	0.140261817626103	1	pseudobulk	DESeq2	LRT	0.853060537461536
195	KC_AB1	Aldh	-0.178088900908889	0.917437930365065	1	pseudobulk	DESeq2	LRT	0.037423308451134
196	KC_AB1	Aldh-III	-0.125080920569736	0.74808312973647	1	pseudobulk	DESeq2	LRT	0.126050139060913
197	KC_AB1	Aldh7A1	-0.82542030717083	1	1	pseudobulk	DESeq2	LRT	0
198	KC_AB1	Alg-2	-0.249166659486284	0.348138307995716	1	pseudobulk	DESeq2	LRT	0.458248185748229
199	KC_AB1	Alg1	0.208807512751513	0.756467708229118	1	pseudobulk	DESeq2	LRT	0.121209606222723
200	KC_AB1	Alg10	0.416705997203199	0.576122840140099	1	pseudobulk	DESeq2	LRT	0.23948490701582
201	KC_AB1	Alg11	0.143091279623538	0.730627265142732	1	pseudobulk	DESeq2	LRT	0.136304125061285
202	KC_AB1	Alg14	-0.926358071018361	0.347404545954774	1	pseudobulk	DESeq2	LRT	0.459164502900561
203	KC_AB1	Alg2	0.206783776993516	0.769392340022558	1	pseudobulk	DESeq2	LRT	0.113852141791218
204	KC_AB1	Alg3	-0.1307161455053	0.830744931000761	1	pseudobulk	DESeq2	LRT	0.080532299998230
205	KC_AB1	Alg9	0.847787571643107	0.582690314631031	1	pseudobulk	DESeq2	LRT	0.234562200611165
206	KC_AB1	Alh	0.0476789519375394	0.853113782807975	1	pseudobulk	DESeq2	LRT	0.068993041575561

207	KC_AB1	alien	-0.333359729220191	0.467145841054647	1	pseudobulk	DESeq2	LRT	0.330547513272625
208	KC_AB1	ALiX	0.188774224103471	0.681157931910915	1	pseudobulk	DESeq2	LRT	0.166752181765096
209	KC_AB1	Alk	0.204177732136899	0.0583190801645932	1	pseudobulk	DESeq2	LRT	1.23418933452876
210	KC_AB1	AlkB	-2.88166845352927	0.192208536768695	1	pseudobulk	DESeq2	LRT	0.716227327441645
211	KC_AB1	Alp12	0.532121747317381	0.74087520745984	1	pseudobulk	DESeq2	LRT	0.130254938141789
212	KC_AB1	Alp5	-0.850932661185203	0.660736244265418	1	pseudobulk	DESeq2	LRT	0.179971869591347
213	KC_AB1	Alp6	0.637884335154205	0.771760557606062	1	pseudobulk	DESeq2	LRT	0.112517420700816
214	KC_AB1	Alp8	0.135879393043758	1	1	pseudobulk	DESeq2	LRT	0
215	KC_AB1	alph	-0.0961378568755329	0.650496975226791	1	pseudobulk	DESeq2	LRT	0.186754718541853
216	KC_AB1	alpha-Cat	-0.015444304122021	0.903636337623261	1	pseudobulk	DESeq2	LRT	0.044006313263006
217	KC_AB1	alpha-Catr	0.0071584483675418	0.976896410689978	1	pseudobulk	DESeq2	LRT	0.010151486077441
218	KC_AB1	alpha-Est10	-0.82542030717083	1	1	pseudobulk	DESeq2	LRT	0
219	KC_AB1	alpha-Est5	-0.850996402080018	0.900449987182352	1	pseudobulk	DESeq2	LRT	0.045540403770502
220	KC_AB1	alpha-Est8	-0.773639052665765	0.0228392211353321	1	pseudobulk	DESeq2	LRT	1.64131871051354
221	KC_AB1	alpha-Est9	-0.0847487246072534	0.781771062194653	1	pseudobulk	DESeq2	LRT	0.10692040932175
222	KC_AB1	alpha-Man-Ia	0.116614333369887	0.465833009558052	1	pseudobulk	DESeq2	LRT	0.331769740015657
223	KC_AB1	alpha-Man-Ib	-0.0036046651968645	0.912915507157129	1	pseudobulk	DESeq2	LRT	0.039569415755370
224	KC_AB1	alpha-Man-IIa	-0.551886730090779	0.388722724716804	1	pseudobulk	DESeq2	LRT	0.410360069786465
225	KC_AB1	alpha-Man-IIb	0.159644393447164	0.669271654210498	1	pseudobulk	DESeq2	LRT	0.174397568363682
226	KC_AB1	alpha-PheRS	-0.661265133714219	0.259707081420711	1	pseudobulk	DESeq2	LRT	0.585516208322143
227	KC_AB1	alpha-Spec	0.079402970691228	0.656954406333229	1	pseudobulk	DESeq2	LRT	0.182464770112952
228	KC_AB1	alpha4GT1	-0.0675306044591041	0.880486552298151	1	pseudobulk	DESeq2	LRT	0.055277272640834
229	KC_AB1	alpha4GT2	1.70421517321918	0.264978264845543	1	pseudobulk	DESeq2	LRT	0.576789748118945
230	KC_AB1	alphaCOP	0.107469990061703	0.822687125418228	1	pseudobulk	DESeq2	LRT	0.084765299100236
231	KC_AB1	alphaSnap	-0.0725785072625341	0.645822450977401	1	pseudobulk	DESeq2	LRT	0.189886861508205
232	KC_AB1	alphaTub67C	-0.109530186160918	0.880083557309963	1	pseudobulk	DESeq2	LRT	0.055476092899915
233	KC_AB1	alphaTub84B	-0.0724954450849467	0.540863234905596	1	pseudobulk	DESeq2	LRT	0.266912538654602
234	KC_AB1	alphaTub84D	-0.0206244256862604	0.920245494050234	1	pseudobulk	DESeq2	LRT	0.036096300383779
235	KC_AB1	Alr	0.191690687055442	0.776702269783121	1	pseudobulk	DESeq2	LRT	0.109745425690265
236	KC_AB1	alrm	-1.51194245640947	0.550007888855835	1	pseudobulk	DESeq2	LRT	0.259631081302143
237	KC_AB1	Als2	0.214081205568031	0.628810293315982	1	pseudobulk	DESeq2	LRT	0.201480357722874
238	KC_AB1	alt	-0.0077496264325989	0.969161989400724	1	pseudobulk	DESeq2	LRT	0.013603627259700
239	KC_AB1	aly	-0.82542030717083	1	1	pseudobulk	DESeq2	LRT	0
240	KC_AB1	Amacr	-0.0697530907920435	0.889218057175777	1	pseudobulk	DESeq2	LRT	0.050991726751079
241	KC_AB1	amn	0.851915531595632	0.369253904500011	1	pseudobulk	DESeq2	LRT	0.432674903751032
242	KC_AB1	Amnionless	0.599234701197507	0.624825804227426	1	pseudobulk	DESeq2	LRT	0.204241043147769
243	KC_AB1	amon	-0.0054347723035112	0.962563594822796	1	pseudobulk	DESeq2	LRT	0.016570567826492
244	KC_AB1	AMPdeam	0.406782821089584	0.167158778304562	1	pseudobulk	DESeq2	LRT	0.776870811598323
245	KC_AB1	Amph	0.464065357743168	0.0998666184775919	1	pseudobulk	DESeq2	LRT	1.00057965525419
246	KC_AB1	AMPKalpha	0.051434815595636	0.880884902339513	1	pseudobulk	DESeq2	LRT	0.055080833408980
247	KC_AB1	Amun	-0.167123978943051	0.453580350917301	1	pseudobulk	DESeq2	LRT	0.343345767327282
248	KC_AB1	amx	-0.0495441853986298	0.885019164791493	1	pseudobulk	DESeq2	LRT	0.053047324699831
249	KC_AB1	Amyrel	2.76821230112469	0.228830322536972	1	pseudobulk	DESeq2	LRT	0.64048642726567
250	KC_AB1	ana1	1.24522514393301	0.608213953617254	1	pseudobulk	DESeq2	LRT	0.215943620513061
251	KC_AB1	ana2	0.899120070663023	0.559470205872958	1	pseudobulk	DESeq2	LRT	0.252223036516329
252	KC_AB1	ana3	1.7107723845566	0.486724792253923	1	pseudobulk	DESeq2	LRT	0.312716531576823
253	KC_AB1	anchor	0.13900371371318	0.714275941634575	1	pseudobulk	DESeq2	LRT	0.146133977610735
254	KC_AB1	angel	-0.541247460454687	0.566278788050941	1	pseudobulk	DESeq2	LRT	0.246969706084866
255	KC_AB1	Ank	-0.051046681615708	0.739485480184118	1	pseudobulk	DESeq2	LRT	0.131070348966376
256	KC_AB1	Ank2	-0.164971305144867	0.208426699444912	1	pseudobulk	DESeq2	LRT	0.681046648717633
257	KC_AB1	Ankle2	0.208908950035932	0.651039639797115	1	pseudobulk	DESeq2	LRT	0.186392567774288
258	KC_AB1	anne	0.0591808956456692	0.668230142964381	1	pseudobulk	DESeq2	LRT	0.175073937815161

259	KC_AB1	Ant2	1.17276926104076	0.551679293648606	1	pseudobulk	DESeq2	LRT	0.258313316246919
260	KC_AB1	antr	1.08899820779554	0.878177167143508	1	pseudobulk	DESeq2	LRT	0.056417858865076
261	KC_AB1	AnxB10	0.0690140035665839	0.893394571959283	1	pseudobulk	DESeq2	LRT	0.048956690520205
262	KC_AB1	AnxB11	0.132004363191668	0.596122235504051	1	pseudobulk	DESeq2	LRT	0.224664678578827
263	KC_AB1	AnxB9	1.95275942187961	0.438420007953907	1	pseudobulk	DESeq2	LRT	0.358109634392079
264	KC_AB1	aop	-0.249317656010936	0.556455698596881	1	pseudobulk	DESeq2	LRT	0.254569405670281
265	KC_AB1	Aos1	-0.0334269215583582	0.9400180893206	1	pseudobulk	DESeq2	LRT	0.026863788935910
266	KC_AB1	ap	4.83542310224335	0.216563047187184	1	pseudobulk	DESeq2	LRT	0.664415646380195
267	KC_AB1	AP-1-2beta	-0.181579527878868	0.525033831204147	1	pseudobulk	DESeq2	LRT	0.279812711390432
268	KC_AB1	AP-1gamma	-0.0457384907664749	0.761645133924043	1	pseudobulk	DESeq2	LRT	0.118247328235226
269	KC_AB1	AP-1mu	0.164163126294581	0.56261340188132	1	pseudobulk	DESeq2	LRT	0.249789926821047
270	KC_AB1	AP-1sigma	0.139235250750246	0.488536837890887	1	pseudobulk	DESeq2	LRT	0.311102682938979
271	KC_AB1	AP-2alpha	-0.0030838887720150	0.94029986790523	1	pseudobulk	DESeq2	LRT	0.026733624901823
272	KC_AB1	AP-2mu	0.0941520746279515	0.700242860749564	1	pseudobulk	DESeq2	LRT	0.154751310284294
273	KC_AB1	AP-2sigma	-0.226194624301784	0.214099473229133	1	pseudobulk	DESeq2	LRT	0.669384401243451
274	KC_AB1	Apc	-0.137190125830363	0.524086285849228	1	pseudobulk	DESeq2	LRT	0.280597204651406
275	KC_AB1	APC10	-0.50582904479135	0.334552071770423	1	pseudobulk	DESeq2	LRT	0.475536276334015
276	KC_AB1	APC4	0.446873906534943	0.708834526491679	1	pseudobulk	DESeq2	LRT	0.149455136635081
277	KC_AB1	ApepP	-0.429299012303637	0.651491821409439	1	pseudobulk	DESeq2	LRT	0.186091031891604
278	KC_AB1	Apf	-0.191219351686791	0.724454984142616	1	pseudobulk	DESeq2	LRT	0.139988595349254
279	KC_AB1	aph-1	-0.0225723001320822	0.943645381048924	1	pseudobulk	DESeq2	LRT	0.025191181536744
280	KC_AB1	aPKC	-0.182825755320203	0.26176939285372	1	pseudobulk	DESeq2	LRT	0.582081134300975
281	KC_AB1	Apl	-0.763644203070602	0.167792368539527	1	pseudobulk	DESeq2	LRT	0.77522779545333
282	KC_AB1	Aplip1	-0.0503542062287586	0.752872730617073	1	pseudobulk	DESeq2	LRT	0.123278432912471
283	KC_AB1	apolpp	-1.18041319423287	0.348001047601127	1	pseudobulk	DESeq2	LRT	0.458419448677833
284	KC_AB1	Apoltp	1.23131300041807	0.0576458156482986	1	pseudobulk	DESeq2	LRT	1.23923221147088
285	KC_AB1	app	-2.93037476368149	0.182596501207181	1	pseudobulk	DESeq2	LRT	0.738507548384262
286	KC_AB1	APP-BP1	0.198524088107476	0.828366844902042	1	pseudobulk	DESeq2	LRT	0.081777291917944
287	KC_AB1	Appl	-0.09883007143012	0.268174191375158	1	pseudobulk	DESeq2	LRT	0.57158302023044
288	KC_AB1	Aprt	-1.07670795837504	0.210279181063899	1	pseudobulk	DESeq2	LRT	0.67720372301809
289	KC_AB1	Aps	-0.154842685630796	0.465902166302661	1	pseudobulk	DESeq2	LRT	0.331705270210495
290	KC_AB1	apt	1.08899820779554	0.878177167143508	1	pseudobulk	DESeq2	LRT	0.056417858865076
291	KC_AB1	aqz	0.205134007834448	0.0118404572136685	1	pseudobulk	DESeq2	LRT	1.92663152721281
292	KC_AB1	aralar1	-0.0416313209346183	0.912410038300293	1	pseudobulk	DESeq2	LRT	0.039809945277849
293	KC_AB1	Arc1	-1.6747012584303	0.563129890257477	1	pseudobulk	DESeq2	LRT	0.249391420218277
294	KC_AB1	Arc2	0.201015108276127	0.907885876043949	1	pseudobulk	DESeq2	LRT	0.041968740156634
295	KC_AB1	Arc42	0.403977127571024	0.643205912415886	1	pseudobulk	DESeq2	LRT	0.191649972161047
296	KC_AB1	Archease	0.0559386659533778	0.900298261716641	1	pseudobulk	DESeq2	LRT	0.045613588384419
297	KC_AB1	Arf102F	0.264832141206005	0.233003238752525	1	pseudobulk	DESeq2	LRT	0.632638042224737
298	KC_AB1	Arf51F	0.172889069293381	0.539430898614436	1	pseudobulk	DESeq2	LRT	0.268064180772602
299	KC_AB1	Arf79F	0.0230563418288344	0.866383192560601	1	pseudobulk	DESeq2	LRT	0.062289981416193
300	KC_AB1	ArfGAP1	0.0522608746137199	0.872164171802255	1	pseudobulk	DESeq2	LRT	0.059401757960886
301	KC_AB1	ArfGAP3	0.0966540297068007	0.841529916129967	1	pseudobulk	DESeq2	LRT	0.074930440378325
302	KC_AB1	Arfip	0.192023303188866	0.572683943939886	1	pseudobulk	DESeq2	LRT	0.242084992818324
303	KC_AB1	Arfrp1	0.331178239446449	0.685348907294701	1	pseudobulk	DESeq2	LRT	0.164088275317679
304	KC_AB1	arg	-0.494100298096412	0.416789103124433	1	pseudobulk	DESeq2	LRT	0.380083644110626
305	KC_AB1	Argk	-0.0596572821599572	0.609620320816324	1	pseudobulk	DESeq2	LRT	0.214940564845445
306	KC_AB1	Argl	-0.838580640014249	0.695856596814275	1	pseudobulk	DESeq2	LRT	0.157480251235852
307	KC_AB1	ArgRS	-0.0548104412124916	0.897427441910973	1	pseudobulk	DESeq2	LRT	0.047000654561924
308	KC_AB1	ArgRS-m	-0.624827064197955	0.59694226978911	1	pseudobulk	DESeq2	LRT	0.224067667403864
309	KC_AB1	ari-1	0.0415912221380329	0.797339088973459	1	pseudobulk	DESeq2	LRT	0.098356944411159
310	KC_AB1	ari-2	-0.28156068742721	0.420352720797474	1	pseudobulk	DESeq2	LRT	0.376386137200059

311	KC_AB1	Arl1	0.0639293242359764	0.865464911983831	1	pseudobulk	DESeq2	LRT	0.062750534768023
312	KC_AB1	Arl2	0.146289634723291	0.747365838640987	1	pseudobulk	DESeq2	LRT	0.126466757207254
313	KC_AB1	Arl4	0.0890945362494907	0.566145681903293	1	pseudobulk	DESeq2	LRT	0.247071800780328
314	KC_AB1	Arl5	-0.0954782099865063	0.797194751712972	1	pseudobulk	DESeq2	LRT	0.098435569115552
315	KC_AB1	Arl6	0.135795303631797	0.946582799906172	1	pseudobulk	DESeq2	LRT	0.023841391241048
316	KC_AB1	Arl6IP1	-0.153226196437507	0.357945425721119	1	pseudobulk	DESeq2	LRT	0.446183183174267
317	KC_AB1	Arl8	0.122842007739564	0.569369689463246	1	pseudobulk	DESeq2	LRT	0.244605656348242
318	KC_AB1	arm	0.0608687328623142	0.692533494590369	1	pseudobulk	DESeq2	LRT	0.159559216972551
319	KC_AB1	armi	-0.881996409411637	0.593501682860195	1	pseudobulk	DESeq2	LRT	0.226578045275759
320	KC_AB1	Arp1	-0.151284280448632	0.572166410802781	1	pseudobulk	DESeq2	LRT	0.242477641167562
321	KC_AB1	Arp10	0.241880113285969	0.696429515134845	1	pseudobulk	DESeq2	LRT	0.157122831482666
322	KC_AB1	Arp2	0.0027975567909347	0.981303456446886	1	pseudobulk	DESeq2	LRT	0.008196671436446
323	KC_AB1	Arp3	0.25193820334389	0.545643082640062	1	pseudobulk	DESeq2	LRT	0.263091346170207
324	KC_AB1	Arp5	-0.0648255795604926	0.871620123887632	1	pseudobulk	DESeq2	LRT	0.059672751357790
325	KC_AB1	Arp6	0.280238310536849	0.686761943828693	1	pseudobulk	DESeq2	LRT	0.16319377880396
326	KC_AB1	Arp8	0.251661510438103	0.777322598302266	1	pseudobulk	DESeq2	LRT	0.109398706313151
327	KC_AB1	Arpc1	-0.145632958885776	0.553814352867366	1	pseudobulk	DESeq2	LRT	0.256635793100516
328	KC_AB1	Arpc2	0.424375815855147	0.333972073933457	1	pseudobulk	DESeq2	LRT	0.476289846492783
329	KC_AB1	Arpc3A	0.210808140120148	0.672663748333608	1	pseudobulk	DESeq2	LRT	0.172201976989276
330	KC_AB1	Arpc4	-0.0747551419595637	0.83649289206134	1	pseudobulk	DESeq2	LRT	0.077537745020359
331	KC_AB1	Arpc5	-0.146850735704637	0.692489465521464	1	pseudobulk	DESeq2	LRT	0.15958682890712
332	KC_AB1	arr	0.110464321670125	0.670220738618664	1	pseudobulk	DESeq2	LRT	0.173782137932708
333	KC_AB1	Ars2	-0.041739217534801	0.834650272121948	1	pseudobulk	DESeq2	LRT	0.078495460688736
334	KC_AB1	Art1	-0.378788436319351	0.551380472488875	1	pseudobulk	DESeq2	LRT	0.258548618789729
335	KC_AB1	Art2	1.92519139383419	0.160399182455744	1	pseudobulk	DESeq2	LRT	0.794797849617091
336	KC_AB1	Art3	-2.30942458854814	0.0685709058975807	1	pseudobulk	DESeq2	LRT	1.16386011298582
337	KC_AB1	Art4	-0.521740197285787	0.491580878845676	1	pseudobulk	DESeq2	LRT	0.308405018326906
338	KC_AB1	Art7	1.8767568197016	0.171163386301947	1	pseudobulk	DESeq2	LRT	0.766589130007093
339	KC_AB1	Art8	-0.516149264712251	0.740635150913599	1	pseudobulk	DESeq2	LRT	0.130395679964754
340	KC_AB1	Arts	0.326164302114813	0.710453929358608	1	pseudobulk	DESeq2	LRT	0.148464079417095
341	KC_AB1	aru	1.08899820779554	0.670846322504933	1	pseudobulk	DESeq2	LRT	0.173376956629912
342	KC_AB1	Arv1	-0.237368157934929	0.673603371262308	1	pseudobulk	DESeq2	LRT	0.171595747938108
343	KC_AB1	arx	2.90826578475952	0.111231024705509	1	pseudobulk	DESeq2	LRT	0.953774061862259
344	KC_AB1	Asap	0.123530025459388	0.700532098714061	1	pseudobulk	DESeq2	LRT	0.1545719603425
345	KC_AB1	Asator	0.0729559258046623	0.556642662211694	1	pseudobulk	DESeq2	LRT	0.254423511524979
346	KC_AB1	Asciz	-0.565664665276523	0.448370628909746	1	pseudobulk	DESeq2	LRT	0.348362844159591
347	KC_AB1	asf1	0.183560990462633	0.642380688726447	1	pseudobulk	DESeq2	LRT	0.192207523310949
348	KC_AB1	ash1	-0.178231947830941	0.59598041800861	1	pseudobulk	DESeq2	LRT	0.224768009539154
349	KC_AB1	ash2	0.596816415398369	0.438529226558413	1	pseudobulk	DESeq2	LRT	0.358001457007033
350	KC_AB1	Ask1	-0.726974773234988	0.322746351820786	1	pseudobulk	DESeq2	LRT	0.49113865810277
351	KC_AB1	asl	0.247727543350143	0.822525487543146	1	pseudobulk	DESeq2	LRT	0.084850635714954
352	KC_AB1	AsnRS	-0.321166375617008	0.541815656648533	1	pseudobulk	DESeq2	LRT	0.266148449472989
353	KC_AB1	AsnRS-m	-1.09227137052841	0.322096465223483	1	pseudobulk	DESeq2	LRT	0.492014041223027
354	KC_AB1	AsnS	0.923124750607267	0.707531785811194	1	pseudobulk	DESeq2	LRT	0.150254044719123
355	KC_AB1	Asph	-0.355480763104499	0.839612936202973	1	pseudobulk	DESeq2	LRT	0.075920878714830
356	KC_AB1	ASPP	-0.850996402080018	0.900449987182352	1	pseudobulk	DESeq2	LRT	0.045540403770502
357	KC_AB1	AspRS	0.356237001599714	0.665448704871293	1	pseudobulk	DESeq2	LRT	0.176885415846595
358	KC_AB1	AspRS-m	-0.0196032287870942	0.924599110554636	1	pseudobulk	DESeq2	LRT	0.034046528680112
359	KC_AB1	asrij	0.52111726247134	0.31952833482184	1	pseudobulk	DESeq2	LRT	0.49549062385569
360	KC_AB1	asRNA:CR17025	0.135879393043758	1	1	pseudobulk	DESeq2	LRT	0
361	KC_AB1	asRNA:CR30267	0.69794357772624	0.730620326456649	1	pseudobulk	DESeq2	LRT	0.136308249527385
362	KC_AB1	asRNA:CR31144	1.96835645445386	0.334600076334956	1	pseudobulk	DESeq2	LRT	0.475473964294586

363	KC_AB1	asRNA:CR31429	0.187499809431982	0.910303214736121	1	pseudobulk	DESeq2	LRT	0.040813923554985
364	KC_AB1	asRNA:CR31912	-0.632979844271142	0.739322933378285	1	pseudobulk	DESeq2	LRT	0.131165822024903
365	KC_AB1	asRNA:CR32252	1.68130633771206	0.562684503730219	1	pseudobulk	DESeq2	LRT	0.249735045103917
366	KC_AB1	asRNA:CR42254	0.125249587973413	0.857341357004883	1	pseudobulk	DESeq2	LRT	0.066846225980729
367	KC_AB1	asRNA:CR42547	-0.0831002292589573	0.84159073957966	1	pseudobulk	DESeq2	LRT	0.074899051911320
368	KC_AB1	asRNA:CR43152	-2.61889767292382	0.248831098743653	1	pseudobulk	DESeq2	LRT	0.604095342756375
369	KC_AB1	asRNA:CR43159	1.08899820779554	0.670846322504933	1	pseudobulk	DESeq2	LRT	0.173376956629912
370	KC_AB1	asRNA:CR43425	0.531225647872925	0.192913281503381	1	pseudobulk	DESeq2	LRT	0.71463787144856
371	KC_AB1	asRNA:CR43426	0.73362978992059	0.719815137332351	1	pseudobulk	DESeq2	LRT	0.142779024603383
372	KC_AB1	asRNA:CR43430	1.08899820779554	0.878177167143508	1	pseudobulk	DESeq2	LRT	0.056417858865076
373	KC_AB1	asRNA:CR43460	0.111831901354254	1	1	pseudobulk	DESeq2	LRT	0
374	KC_AB1	asRNA:CR43464	1.08899820779554	0.697631521651407	1	pseudobulk	DESeq2	LRT	0.156373904552
375	KC_AB1	asRNA:CR43466	0.283804810706531	0.899195232875688	1	pseudobulk	DESeq2	LRT	0.046146004200805
376	KC_AB1	asRNA:CR43468	0.111831901354254	1	1	pseudobulk	DESeq2	LRT	0
377	KC_AB1	asRNA:CR43480	0.111831901354254	1	1	pseudobulk	DESeq2	LRT	0
378	KC_AB1	asRNA:CR43481	1.08899820779554	0.670846322504933	1	pseudobulk	DESeq2	LRT	0.173376956629912
379	KC_AB1	asRNA:CR43609	2.47206067484574	0.237892268034413	1	pseudobulk	DESeq2	LRT	0.623619673156337
380	KC_AB1	asRNA:CR43629	-0.357151916635175	0.646402517018283	1	pseudobulk	DESeq2	LRT	0.189496961136591
381	KC_AB1	asRNA:CR43683	1.08899820779554	0.878177167143508	1	pseudobulk	DESeq2	LRT	0.056417858865076
382	KC_AB1	asRNA:CR43730	-2.85058847969495	0.116276754490938	1	pseudobulk	DESeq2	LRT	0.934507098731223
383	KC_AB1	asRNA:CR43767	0.591698504190091	0.564333272933449	1	pseudobulk	DESeq2	LRT	0.24846434309272
384	KC_AB1	asRNA:CR43786	0.135879393043758	1	1	pseudobulk	DESeq2	LRT	0
385	KC_AB1	asRNA:CR43806	-1.56855358782635	0.573026303465967	1	pseudobulk	DESeq2	LRT	0.241825442277049
386	KC_AB1	asRNA:CR43809	-1.96719661029409	0.414177861019091	1	pseudobulk	DESeq2	LRT	0.382813119093482
387	KC_AB1	asRNA:CR43833	1.08899820779554	0.697631521651407	1	pseudobulk	DESeq2	LRT	0.156373904552
388	KC_AB1	asRNA:CR43842	0.512065610279438	0.799951782310952	1	pseudobulk	DESeq2	LRT	0.096936189642279
389	KC_AB1	asRNA:CR43865	1.68130633771206	0.562684503730219	1	pseudobulk	DESeq2	LRT	0.249735045103917
390	KC_AB1	asRNA:CR43872	1.88952324269138	0.456767015650974	1	pseudobulk	DESeq2	LRT	0.340305265178099
391	KC_AB1	asRNA:CR43889	2.10449501374856	0.335478261052675	1	pseudobulk	DESeq2	LRT	0.4743356168087
392	KC_AB1	asRNA:CR43906	-0.543761513828981	0.37083850612558	1	pseudobulk	DESeq2	LRT	0.43081517709062
393	KC_AB1	asRNA:CR43933	2.60936870382413	0.0889952143735695	1	pseudobulk	DESeq2	LRT	1.0506333464678
394	KC_AB1	asRNA:CR43936	-0.850996402080018	0.900449987182352	1	pseudobulk	DESeq2	LRT	0.045540403770502
395	KC_AB1	asRNA:CR43944	1.18021523819004	0.147480074380486	1	pseudobulk	DESeq2	LRT	0.831266652032653
396	KC_AB1	asRNA:CR43957	1.1500340395912	0.47418850444273	1	pseudobulk	DESeq2	LRT	0.324048978653072
397	KC_AB1	asRNA:CR43959	-0.666202553059316	0.402807892683871	1	pseudobulk	DESeq2	LRT	0.394902028397788
398	KC_AB1	asRNA:CR43971	1.08899820779554	0.670846322504933	1	pseudobulk	DESeq2	LRT	0.173376956629912
399	KC_AB1	asRNA:CR43979	0.135879393043758	1	1	pseudobulk	DESeq2	LRT	0
400	KC_AB1	asRNA:CR43981	-0.850932661185203	0.660736244265418	1	pseudobulk	DESeq2	LRT	0.179971869591347
401	KC_AB1	asRNA:CR44018	0.279145366656992	0.515323798990001	1	pseudobulk	DESeq2	LRT	0.287919800229858
402	KC_AB1	asRNA:CR44021	-1.56855358782635	0.573026303465967	1	pseudobulk	DESeq2	LRT	0.241825442277049
403	KC_AB1	asRNA:CR44027	-0.121651740976222	0.825377092114092	1	pseudobulk	DESeq2	LRT	0.083347588895120
404	KC_AB1	asRNA:CR44029	0.160010779988566	0.721335503713913	1	pseudobulk	DESeq2	LRT	0.141862691562083
405	KC_AB1	asRNA:CR44030	-0.483524472897784	0.632784673062549	1	pseudobulk	DESeq2	LRT	0.198744048607998
406	KC_AB1	asRNA:CR44031	0.0570710079969697	0.929693487103464	1	pseudobulk	DESeq2	LRT	0.031660211447136
407	KC_AB1	asRNA:CR44046	-0.0737946396162282	0.93108081082744	1	pseudobulk	DESeq2	LRT	0.031012623880773
408	KC_AB1	asRNA:CR44049	1.08899820779554	0.697631521651407	1	pseudobulk	DESeq2	LRT	0.156373904552
409	KC_AB1	asRNA:CR44065	-1.99275908811736	0.412167844949191	1	pseudobulk	DESeq2	LRT	0.384925892483647
410	KC_AB1	asRNA:CR44071	-0.850996402080018	0.900449987182352	1	pseudobulk	DESeq2	LRT	0.045540403770502
411	KC_AB1	asRNA:CR44093	-0.683793083446628	0.598065526113343	1	pseudobulk	DESeq2	LRT	0.223251230609531
412	KC_AB1	asRNA:CR44095	-0.82542030717083	1	1	pseudobulk	DESeq2	LRT	0
413	KC_AB1	asRNA:CR44106	1.19732100989811	0.526547493354687	1	pseudobulk	DESeq2	LRT	0.278562450363344
414	KC_AB1	asRNA:CR44107	-1.05813155791189	0.687053260159203	1	pseudobulk	DESeq2	LRT	0.163009595259685

415	KC_AB1	asRNA:CR44108	1.08899820779554	0.878177167143508	1	pseudobulk	DESeq2	LRT	0.056417858865076
416	KC_AB1	asRNA:CR44116	0.923124750607267	0.707531785811194	1	pseudobulk	DESeq2	LRT	0.150254044719123
417	KC_AB1	asRNA:CR44119	0.280931390658159	0.820746644191521	1	pseudobulk	DESeq2	LRT	0.085790884306309
418	KC_AB1	asRNA:CR44137	1.08899820779554	0.697631521651407	1	pseudobulk	DESeq2	LRT	0.156373904552
419	KC_AB1	asRNA:CR44165	-0.850996402080018	0.900449987182352	1	pseudobulk	DESeq2	LRT	0.045540403770502
420	KC_AB1	asRNA:CR44179	-0.141082556924054	0.874144325212205	1	pseudobulk	DESeq2	LRT	0.058416857447258
421	KC_AB1	asRNA:CR44205	0.135795303631797	0.946582799906172	1	pseudobulk	DESeq2	LRT	0.023841391241048
422	KC_AB1	asRNA:CR44228	-0.327396427897997	0.834560042472953	1	pseudobulk	DESeq2	LRT	0.078542412517823
423	KC_AB1	asRNA:CR44340	0.44736324331837	0.68256613061919	1	pseudobulk	DESeq2	LRT	0.165855265500871
424	KC_AB1	asRNA:CR44351	1.08899820779554	0.670846322504933	1	pseudobulk	DESeq2	LRT	0.173376956629912
425	KC_AB1	asRNA:CR44368	0.978387385707312	0.806388900818397	1	pseudobulk	DESeq2	LRT	0.093455458510773
426	KC_AB1	asRNA:CR44381	0.135795303631797	0.946582799906172	1	pseudobulk	DESeq2	LRT	0.023841391241048
427	KC_AB1	asRNA:CR44390	1.68130633771206	0.562684503730219	1	pseudobulk	DESeq2	LRT	0.249735045103917
428	KC_AB1	asRNA:CR44429	1.95275942187961	0.438420007953907	1	pseudobulk	DESeq2	LRT	0.358109634392079
429	KC_AB1	asRNA:CR44431	-2.36817011682091	0.282654768599882	1	pseudobulk	DESeq2	LRT	0.548743683265438
430	KC_AB1	asRNA:CR44599	1.08899820779554	0.697631521651407	1	pseudobulk	DESeq2	LRT	0.156373904552
431	KC_AB1	asRNA:CR44690	1.61328694041235	0.301544710925827	1	pseudobulk	DESeq2	LRT	0.520648284622551
432	KC_AB1	asRNA:CR44724	-0.850932661185203	0.660736244265418	1	pseudobulk	DESeq2	LRT	0.179971869591347
433	KC_AB1	asRNA:CR44840	-0.414396371597331	0.768056844382232	1	pseudobulk	DESeq2	LRT	0.114606636364386
434	KC_AB1	asRNA:CR44843	0.187499809431982	0.910303214736121	1	pseudobulk	DESeq2	LRT	0.040813923554985
435	KC_AB1	asRNA:CR44847	-0.82542030717083	1	1	pseudobulk	DESeq2	LRT	0
436	KC_AB1	asRNA:CR44892	-0.311560601561585	0.740302651392201	1	pseudobulk	DESeq2	LRT	0.130590695190349
437	KC_AB1	asRNA:CR44894	0.241875799164083	0.57731195407542	1	pseudobulk	DESeq2	LRT	0.238589449715571
438	KC_AB1	asRNA:CR44960	1.45568099092791	0.336613567628375	1	pseudobulk	DESeq2	LRT	0.472868383228332
439	KC_AB1	asRNA:CR44961	-1.77113182578892	0.387859174780729	1	pseudobulk	DESeq2	LRT	0.411325930893727
440	KC_AB1	asRNA:CR44968	-1.6747012584303	0.563129890257477	1	pseudobulk	DESeq2	LRT	0.249391420218277
441	KC_AB1	asRNA:CR44981	-0.417549989552149	0.734060089331172	1	pseudobulk	DESeq2	LRT	0.134268387772204
442	KC_AB1	asRNA:CR44987	-0.0358971148200924	0.937753068006931	1	pseudobulk	DESeq2	LRT	0.027911506312082
443	KC_AB1	asRNA:CR44989	-1.67405691780143	0.195343215405437	1	pseudobulk	DESeq2	LRT	0.709201667946484
444	KC_AB1	asRNA:CR45000	0.457654306495041	0.635689879476558	1	pseudobulk	DESeq2	LRT	0.196754702715323
445	KC_AB1	asRNA:CR45007	1.08899820779554	0.878177167143508	1	pseudobulk	DESeq2	LRT	0.056417858865076
446	KC_AB1	asRNA:CR45009	0.573163093277814	0.324704922004223	1	pseudobulk	DESeq2	LRT	0.488511128118463
447	KC_AB1	asRNA:CR45016	1.08899820779554	0.670846322504933	1	pseudobulk	DESeq2	LRT	0.173376956629912
448	KC_AB1	asRNA:CR45024	-0.850996402080018	0.900449987182352	1	pseudobulk	DESeq2	LRT	0.045540403770502
449	KC_AB1	asRNA:CR45041	1.37689666173222	0.399537346082775	1	pseudobulk	DESeq2	LRT	0.398442619504896
450	KC_AB1	asRNA:CR45053	1.7107723845566	0.486724792253923	1	pseudobulk	DESeq2	LRT	0.312716531576823
451	KC_AB1	asRNA:CR45056	1.08899820779554	0.878177167143508	1	pseudobulk	DESeq2	LRT	0.056417858865076
452	KC_AB1	asRNA:CR45121	-1.82709236256241	0.44317674354685	1	pseudobulk	DESeq2	LRT	0.353423038023035
453	KC_AB1	asRNA:CR45124	1.49217975990061	0.505914797175642	1	pseudobulk	DESeq2	LRT	0.295922618006439
454	KC_AB1	asRNA:CR45126	-0.378204873658276	0.468821337104715	1	pseudobulk	DESeq2	LRT	0.328992630829635
455	KC_AB1	asRNA:CR45129	-0.555783156553894	0.525478864433749	1	pseudobulk	DESeq2	LRT	0.279444747274921
456	KC_AB1	asRNA:CR45131	1.16523413356038	0.63328025468345	1	pseudobulk	DESeq2	LRT	0.198404052815419
457	KC_AB1	asRNA:CR45133	0.942080999092271	0.681532122211136	1	pseudobulk	DESeq2	LRT	0.166513670026992
458	KC_AB1	asRNA:CR45134	0.152188543173232	0.825197657326593	1	pseudobulk	DESeq2	LRT	0.083442013626149
459	KC_AB1	asRNA:CR45135	2.9997434502155	0.180805914486867	1	pseudobulk	DESeq2	LRT	0.742787367074168
460	KC_AB1	asRNA:CR45137	0.292732419254208	0.805926411622281	1	pseudobulk	DESeq2	LRT	0.093704611402608
461	KC_AB1	asRNA:CR45139	-0.82806355307977	0.480449663845762	1	pseudobulk	DESeq2	LRT	0.318352106141422
462	KC_AB1	asRNA:CR45140	-0.850996402080018	0.900449987182352	1	pseudobulk	DESeq2	LRT	0.045540403770502
463	KC_AB1	asRNA:CR45141	-0.715773599702744	0.835451286644154	1	pseudobulk	DESeq2	LRT	0.078078867803391
464	KC_AB1	asRNA:CR45145	-0.566370708252565	0.625342472630152	1	pseudobulk	DESeq2	LRT	0.203882073474315
465	KC_AB1	asRNA:CR45161	1.08899820779554	0.878177167143508	1	pseudobulk	DESeq2	LRT	0.056417858865076
466	KC_AB1	asRNA:CR45162	-0.178723235721682	0.886688907600286	1	pseudobulk	DESeq2	LRT	0.052228724486788

467	KC_AB1	asRNA:CR45164	0.177391395189428	0.881966904771762	1	pseudobulk	DESeq2	LRT	0.054547711177327
468	KC_AB1	asRNA:CR45167	-1.56855358782635	0.573026303465967	1	pseudobulk	DESeq2	LRT	0.241825442277049
469	KC_AB1	asRNA:CR45171	-1.57108292043403	0.471241990605733	1	pseudobulk	DESeq2	LRT	0.326756018144865
470	KC_AB1	asRNA:CR45175	1.08899820779554	0.878177167143508	1	pseudobulk	DESeq2	LRT	0.056417858865076
471	KC_AB1	asRNA:CR45182	0.0844348191643991	0.889241029460093	1	pseudobulk	DESeq2	LRT	0.050980507222453
472	KC_AB1	asRNA:CR45185	-0.82542030717083	1	1	pseudobulk	DESeq2	LRT	0
473	KC_AB1	asRNA:CR45190	-1.61862498639867	0.500791630206422	1	pseudobulk	DESeq2	LRT	0.300342938155337
474	KC_AB1	asRNA:CR45198	0.754591343752014	0.421692418596878	1	pseudobulk	DESeq2	LRT	0.375004206884445
475	KC_AB1	asRNA:CR45208	-1.6747012584303	0.563129890257477	1	pseudobulk	DESeq2	LRT	0.249391420218277
476	KC_AB1	asRNA:CR45275	0.13617601020169	1	1	pseudobulk	DESeq2	LRT	0
477	KC_AB1	asRNA:CR45281	0.543281933770898	0.747876331659472	1	pseudobulk	DESeq2	LRT	0.126170210849752
478	KC_AB1	asRNA:CR45350	0.11269840515639	0.977007463492936	1	pseudobulk	DESeq2	LRT	0.010102118633921
479	KC_AB1	asRNA:CR45367	0.144068105809641	0.913017658949245	1	pseudobulk	DESeq2	LRT	0.039520822564225
480	KC_AB1	asRNA:CR45445	1.08899820779554	0.878177167143508	1	pseudobulk	DESeq2	LRT	0.056417858865076
481	KC_AB1	asRNA:CR45467	-1.6747012584303	0.563129890257477	1	pseudobulk	DESeq2	LRT	0.249391420218277
482	KC_AB1	asRNA:CR45468	1.08899820779554	0.878177167143508	1	pseudobulk	DESeq2	LRT	0.056417858865076
483	KC_AB1	asRNA:CR45469	-1.25404308034563	0.29239446240069	1	pseudobulk	DESeq2	LRT	0.534030856651238
484	KC_AB1	asRNA:CR45479	-0.850996402080018	0.900449987182352	1	pseudobulk	DESeq2	LRT	0.045540403770502
485	KC_AB1	asRNA:CR45501	-0.850996402080018	0.900449987182352	1	pseudobulk	DESeq2	LRT	0.045540403770502
486	KC_AB1	asRNA:CR45520	0.0668408038645169	0.947668556444641	1	pseudobulk	DESeq2	LRT	0.023343528987016
487	KC_AB1	asRNA:CR45528	1.22236326706149	0.63482863141329	1	pseudobulk	DESeq2	LRT	0.197343494355134
488	KC_AB1	asRNA:CR45549	-0.850932661185203	0.660736244265418	1	pseudobulk	DESeq2	LRT	0.179971869591347
489	KC_AB1	asRNA:CR45604	1.08899820779554	0.670846322504933	1	pseudobulk	DESeq2	LRT	0.173376956629912
490	KC_AB1	asRNA:CR45612	1.08899820779554	0.697631521651407	1	pseudobulk	DESeq2	LRT	0.156373904552
491	KC_AB1	asRNA:CR45620	0.0592301179651143	0.933622839719758	1	pseudobulk	DESeq2	LRT	0.029828532452265
492	KC_AB1	asRNA:CR45623	0.113316967542732	1	1	pseudobulk	DESeq2	LRT	0
493	KC_AB1	asRNA:CR45683	2.09447112639525	0.299159443021327	1	pseudobulk	DESeq2	LRT	0.524097284022574
494	KC_AB1	asRNA:CR45713	1.08899820779554	0.670846322504933	1	pseudobulk	DESeq2	LRT	0.173376956629912
495	KC_AB1	asRNA:CR45789	-0.281809071603848	0.841702290754529	1	pseudobulk	DESeq2	LRT	0.074841490858202
496	KC_AB1	asRNA:CR45797	-0.850996402080018	0.900449987182352	1	pseudobulk	DESeq2	LRT	0.045540403770502
497	KC_AB1	asRNA:CR45798	-1.56855358782635	0.573026303465967	1	pseudobulk	DESeq2	LRT	0.241825442277049
498	KC_AB1	asRNA:CR45804	0.679575154642495	0.647419021413336	1	pseudobulk	DESeq2	LRT	0.188814545011459
499	KC_AB1	asRNA:CR45812	-0.82542030717083	1	1	pseudobulk	DESeq2	LRT	0
500	KC_AB1	asRNA:CR45813	-2.36817011682091	0.282654768599882	1	pseudobulk	DESeq2	LRT	0.548743683265438
501	KC_AB1	asRNA:CR45815	-1.21975155535148	0.660614825582151	1	pseudobulk	DESeq2	LRT	0.180051684056245
502	KC_AB1	asRNA:CR45822	0.678429120484887	0.353795226219586	1	pseudobulk	DESeq2	LRT	0.451248031348508
503	KC_AB1	asRNA:CR45828	1.08899820779554	0.878177167143508	1	pseudobulk	DESeq2	LRT	0.056417858865076
504	KC_AB1	asRNA:CR45832	0.135795303631797	0.946582799906172	1	pseudobulk	DESeq2	LRT	0.023841391241048
505	KC_AB1	asRNA:CR45835	-0.429556661844806	0.537879743900667	1	pseudobulk	DESeq2	LRT	0.26931481057558
506	KC_AB1	asRNA:CR45865	1.68130633771206	0.562684503730219	1	pseudobulk	DESeq2	LRT	0.249735045103917
507	KC_AB1	asRNA:CR45873	1.20497046556671	0.583313363570901	1	pseudobulk	DESeq2	LRT	0.234098073872369
508	KC_AB1	asRNA:CR45874	0.198576390707481	0.677105302004414	1	pseudobulk	DESeq2	LRT	0.169343785490354
509	KC_AB1	asRNA:CR45875	1.08899820779554	0.878177167143508	1	pseudobulk	DESeq2	LRT	0.056417858865076
510	KC_AB1	asRNA:CR45882	0.977851105145882	0.69132261808108	1	pseudobulk	DESeq2	LRT	0.160319234022942
511	KC_AB1	asRNA:CR45889	-1.49710585464092	0.405043932251866	1	pseudobulk	DESeq2	LRT	0.392497869378382
512	KC_AB1	asRNA:CR45891	2.15213953086308	0.324747696360003	1	pseudobulk	DESeq2	LRT	0.488453920968368
513	KC_AB1	asRNA:CR45894	-0.850932661185203	0.660736244265418	1	pseudobulk	DESeq2	LRT	0.179971869591347
514	KC_AB1	asRNA:CR45896	1.45141434898336	0.518035873737501	1	pseudobulk	DESeq2	LRT	0.285640164527385
515	KC_AB1	asRNA:CR45907	0.348283376139838	0.784223599014706	1	pseudobulk	DESeq2	LRT	0.105560092962033
516	KC_AB1	asRNA:CR45908	2.211521544984	0.400335997833192	1	pseudobulk	DESeq2	LRT	0.39757535679119
517	KC_AB1	asRNA:CR45912	0.112935832476632	0.940353958317081	1	pseudobulk	DESeq2	LRT	0.026708642986417
518	KC_AB1	asRNA:CR45925	-1.97437890458968	0.359999599262835	1	pseudobulk	DESeq2	LRT	0.443697982671702

519	KC_AB1	asRNA:CR45928	-0.395139673960286	0.815006432716876	1	pseudobulk	DESeq2	LRT	0.088838963428835
520	KC_AB1	asRNA:CR45943	-1.44925888589622	0.6315588144614	1	pseudobulk	DESeq2	LRT	0.199586199175701
521	KC_AB1	asRNA:CR45944	2.10549911548503	0.428045198278114	1	pseudobulk	DESeq2	LRT	0.368510370411162
522	KC_AB1	asRNA:CR45951	1.01257433700954	0.323675694737486	1	pseudobulk	DESeq2	LRT	0.48988991115566
523	KC_AB1	asRNA:CR45952	0.111831901354254	1	1	pseudobulk	DESeq2	LRT	0
524	KC_AB1	asRNA:CR45960	-0.82542030717083	1	1	pseudobulk	DESeq2	LRT	0
525	KC_AB1	asRNA:CR46020	-0.82542030717083	1	1	pseudobulk	DESeq2	LRT	0
526	KC_AB1	asRNA:CR46029	-0.230442022118086	0.875301546346612	1	pseudobulk	DESeq2	LRT	0.057842304287899
527	KC_AB1	asRNA:CR46033	3.80066119564989	0.0571915232053293	1	pseudobulk	DESeq2	LRT	1.24266833655597
528	KC_AB1	asRNA:CR46050	-0.501300761870089	0.863550576027942	1	pseudobulk	DESeq2	LRT	0.063712221794699
529	KC_AB1	asRNA:CR46058	-0.850932661185203	0.660736244265418	1	pseudobulk	DESeq2	LRT	0.179971869591347
530	KC_AB1	asRNA:CR46066	-0.618422766585323	0.856874262644257	1	pseudobulk	DESeq2	LRT	0.067082901584478
531	KC_AB1	asRNA:CR46067	0.997694962638592	0.665140342223729	1	pseudobulk	DESeq2	LRT	0.177086710452097
532	KC_AB1	asRNA:CR46068	1.08899820779554	0.697631521651407	1	pseudobulk	DESeq2	LRT	0.156373904552
533	KC_AB1	asRNA:CR46091	-0.850996402080018	0.900449987182352	1	pseudobulk	DESeq2	LRT	0.045540403770502
534	KC_AB1	asRNA:CR46093	-1.44925888589622	0.6315588144614	1	pseudobulk	DESeq2	LRT	0.199586199175701
535	KC_AB1	asRNA:CR46096	-0.850932661185203	0.660736244265418	1	pseudobulk	DESeq2	LRT	0.179971869591347
536	KC_AB1	asRNA:CR46104	0.135879393043758	1	1	pseudobulk	DESeq2	LRT	0
537	KC_AB1	asRNA:CR46105	1.08899820779554	0.670846322504933	1	pseudobulk	DESeq2	LRT	0.173376956629912
538	KC_AB1	asRNA:CR46107	-0.850932661185203	0.660736244265418	1	pseudobulk	DESeq2	LRT	0.179971869591347
539	KC_AB1	asRNA:CR46133	-0.850996402080018	0.900449987182352	1	pseudobulk	DESeq2	LRT	0.045540403770502
540	KC_AB1	asRNA:CR46134	-0.82542030717083	1	1	pseudobulk	DESeq2	LRT	0
541	KC_AB1	asRNA:CR46135	-0.331373312393185	0.757147527276348	1	pseudobulk	DESeq2	LRT	0.120819491649572
542	KC_AB1	asRNA:CR46137	0.111831901354254	1	1	pseudobulk	DESeq2	LRT	0
543	KC_AB1	asRNA:CR46139	-2.9044344670839	0.215443081422891	1	pseudobulk	DESeq2	LRT	0.666667447958097
544	KC_AB1	asRNA:CR46140	1.08899820779554	0.697631521651407	1	pseudobulk	DESeq2	LRT	0.156373904552
545	KC_AB1	asRNA:CR46246	2.59276232077997	0.188638260008516	1	pseudobulk	DESeq2	LRT	0.724370218144794
546	KC_AB1	asRNA:CR46251	1.08899820779554	0.878177167143508	1	pseudobulk	DESeq2	LRT	0.056417858865076
547	KC_AB1	asRNA:CR46256	1.08899820779554	0.878177167143508	1	pseudobulk	DESeq2	LRT	0.056417858865076
548	KC_AB1	asRNA:CR46262	0.915117603444762	0.658527786599951	1	pseudobulk	DESeq2	LRT	0.181425895246723
549	KC_AB1	asRNA:CR46354	-0.666761472045792	0.682228153659215	1	pseudobulk	DESeq2	LRT	0.166070362432943
550	KC_AB1	ast	-0.888140693355049	0.398987636652794	1	pseudobulk	DESeq2	LRT	0.399040561497857
551	KC_AB1	AstA-R1	-1.1105307848878	0.369906482067883	1	pseudobulk	DESeq2	LRT	0.4319080582449
552	KC_AB1	AstA-R2	-1.50603936527667	0.486905287062083	1	pseudobulk	DESeq2	LRT	0.312555509640957
553	KC_AB1	AstC	-1.44925888589622	0.6315588144614	1	pseudobulk	DESeq2	LRT	0.199586199175701
554	KC_AB1	AstC-R1	2.10549911548503	0.428045198278114	1	pseudobulk	DESeq2	LRT	0.368510370411162
555	KC_AB1	asun	-0.621664724376261	0.617026479816111	1	pseudobulk	DESeq2	LRT	0.209696197731342
556	KC_AB1	Asx	-0.0594550522858865	0.853079999782982	1	pseudobulk	DESeq2	LRT	0.069010239834619
557	KC_AB1	Atac1	0.595167565405605	0.530646801499774	1	pseudobulk	DESeq2	LRT	0.275194449158176
558	KC_AB1	Atac2	0.837577873744335	0.418281716093556	1	pseudobulk	DESeq2	LRT	0.378531118860018
559	KC_AB1	Atac3	0.514347498064074	0.367858515082517	1	pseudobulk	DESeq2	LRT	0.434319186582067
560	KC_AB1	ATbp	1.22292870131877	0.179856182021965	1	pseudobulk	DESeq2	LRT	0.745074630006245
561	KC_AB1	Ate1	-0.252445076630189	0.438480953402186	1	pseudobulk	DESeq2	LRT	0.358049266635413
562	KC_AB1	Atet	0.0911385546544128	0.635026098704271	1	pseudobulk	DESeq2	LRT	0.197208425431914
563	KC_AB1	Atf-2	0.186368681361993	0.706718658819673	1	pseudobulk	DESeq2	LRT	0.150753442270323
564	KC_AB1	Atf3	1.32160363646547	0.183955016608971	1	pseudobulk	DESeq2	LRT	0.735288364093325
565	KC_AB1	Atf6	-0.0427579509427786	0.804772004871408	1	pseudobulk	DESeq2	LRT	0.094327139571117
566	KC_AB1	Atg1	0.0836211118954707	0.629933027290213	1	pseudobulk	DESeq2	LRT	0.200705621061352
567	KC_AB1	Atg10	-0.125138906702836	0.94828766407765	1	pseudobulk	DESeq2	LRT	0.023059898979777
568	KC_AB1	Atg101	-0.336061789822809	0.450676958495023	1	pseudobulk	DESeq2	LRT	0.346134645288835
569	KC_AB1	Atg12	-0.0062634718856996	0.978124646610622	1	pseudobulk	DESeq2	LRT	0.009605797680892
570	KC_AB1	Atg13	0.0817783015743754	0.80988888557996	1	pseudobulk	DESeq2	LRT	0.091574560985115

571	KC_AB1	Atg14	0.22446387309004	0.580125196388359	1	pseudobulk	DESeq2	LRT	0.236478271552244
572	KC_AB1	Atg16	0.673853400000724	0.23084007372727	1	pseudobulk	DESeq2	LRT	0.636688795655433
573	KC_AB1	Atg17	0.251074031397752	0.200939761256697	1	pseudobulk	DESeq2	LRT	0.696934118076095
574	KC_AB1	Atg18a	0.0412890419757624	0.887805861096605	1	pseudobulk	DESeq2	LRT	0.051681992185724
575	KC_AB1	Atg18b	-0.0493279774519398	0.842813226550537	1	pseudobulk	DESeq2	LRT	0.074268657481545
576	KC_AB1	Atg2	0.439301930170957	0.584290351342528	1	pseudobulk	DESeq2	LRT	0.233371285336654
577	KC_AB1	Atg3	0.331101240419636	0.379862347248152	1	pseudobulk	DESeq2	LRT	0.420373752491003
578	KC_AB1	Atg4a	0.157069165232733	0.741705797458592	1	pseudobulk	DESeq2	LRT	0.129768326361759
579	KC_AB1	Atg4b	-0.660899174557829	0.299297400554009	1	pseudobulk	DESeq2	LRT	0.523897055060751
580	KC_AB1	Atg5	-0.138968846859525	0.757102530008726	1	pseudobulk	DESeq2	LRT	0.120845302531917
581	KC_AB1	Atg6	0.209207105840122	0.669607696816388	1	pseudobulk	DESeq2	LRT	0.174179562981233
582	KC_AB1	Atg7	0.746585616302679	0.340909477323947	1	pseudobulk	DESeq2	LRT	0.467360925165122
583	KC_AB1	Atg8a	-0.0723109000113294	0.505309933484322	1	pseudobulk	DESeq2	LRT	0.296442164229564
584	KC_AB1	Atg9	0.495831018054014	0.56849546722712	1	pseudobulk	DESeq2	LRT	0.245272993713445
585	KC_AB1	atilla	-0.82542030717083	1	1	pseudobulk	DESeq2	LRT	0
586	KC_AB1	atl	0.228817104000497	0.548402236601916	1	pseudobulk	DESeq2	LRT	0.260900782653697
587	KC_AB1	atms	-0.0196181588780602	0.971265864077133	1	pseudobulk	DESeq2	LRT	0.012661874626145
588	KC_AB1	Atox1	-0.243571818757438	0.205951154878823	1	pseudobulk	DESeq2	LRT	0.686235768378145
589	KC_AB1	ATP6AP2	-0.282909138008826	0.101466697811548	1	pseudobulk	DESeq2	LRT	0.993676473315793
590	KC_AB1	ATP7	-0.037760130559083	0.96034182167917	1	pseudobulk	DESeq2	LRT	0.017574157745603
591	KC_AB1	ATP8A	0.155853101091816	0.431701435277425	1	pseudobulk	DESeq2	LRT	0.364816507442807
592	KC_AB1	Atpalpha	-0.167548937226376	0.0805548505100823	1	pseudobulk	DESeq2	LRT	1.09390830395562
593	KC_AB1	ATPCL	0.0066887338009635	0.969846053355118	1	pseudobulk	DESeq2	LRT	0.013297197160972
594	KC_AB1	ATPsynB	-0.172549397023032	0.241174925151337	1	pseudobulk	DESeq2	LRT	0.617667847587013
595	KC_AB1	ATPsynbeta	-0.142105422448722	0.274626557192247	1	pseudobulk	DESeq2	LRT	0.561257467520381
596	KC_AB1	ATPsynC	-0.2136653626979	0.0858264430342409	1	pseudobulk	DESeq2	LRT	1.06637888587437
597	KC_AB1	ATPsynCF6	-0.24346327769842	0.127326063369758	1	pseudobulk	DESeq2	LRT	0.895082688105331
598	KC_AB1	ATPsynCF6L	1.18616812058418	0.386649243155644	1	pseudobulk	DESeq2	LRT	0.412682835592357
599	KC_AB1	ATPsynD	-0.176646810431236	0.269320509864807	1	pseudobulk	DESeq2	LRT	0.569730572017612
600	KC_AB1	ATPsyndelta	-0.214126460224219	0.227693676015471	1	pseudobulk	DESeq2	LRT	0.642649031351149
601	KC_AB1	ATPsynE	-0.102555091713748	0.494874447723957	1	pseudobulk	DESeq2	LRT	0.30550496990874
602	KC_AB1	ATPsynF	-0.129851613287633	0.301020993983031	1	pseudobulk	DESeq2	LRT	0.521403214528966
603	KC_AB1	ATPsynG	-0.0485617502350876	0.730128103354521	1	pseudobulk	DESeq2	LRT	0.136600934812335
604	KC_AB1	ATPsyngamma	-0.17850018436889	0.320806375490846	1	pseudobulk	DESeq2	LRT	0.493757009424718
605	KC_AB1	ATPsynO	-0.130178919556909	0.459119637877361	1	pseudobulk	DESeq2	LRT	0.338074130803191
606	KC_AB1	AttA	-1.05091146457495	0.579718062776486	1	pseudobulk	DESeq2	LRT	0.236783167730066
607	KC_AB1	AttC	1.08899820779554	0.878177167143508	1	pseudobulk	DESeq2	LRT	0.056417858865076
608	KC_AB1	Atu	-0.176420132114883	0.808854583517888	1	pseudobulk	DESeq2	LRT	0.092129549156970
609	KC_AB1	Atx2	-0.102836250654868	0.558036605623549	1	pseudobulk	DESeq2	LRT	0.2533373116379
610	KC_AB1	Atxn7	-0.137115946222835	0.660395809074723	1	pseudobulk	DESeq2	LRT	0.180195691468446
611	KC_AB1	aub	0.827801041443763	0.687474828940444	1	pseudobulk	DESeq2	LRT	0.162743198373905
612	KC_AB1	aurA	0.468423502875156	0.727644518582349	1	pseudobulk	DESeq2	LRT	0.138080737888935
613	KC_AB1	aux	-0.455580359150725	0.194260405609221	1	pseudobulk	DESeq2	LRT	0.711615708806945
614	KC_AB1	ave	-0.0520020156356166	0.881724340963778	1	pseudobulk	DESeq2	LRT	0.054667169868564
615	KC_AB1	Aven	0.20439153829736	0.874025851137484	1	pseudobulk	DESeq2	LRT	0.058475722010531
616	KC_AB1	awd	-0.144789536331373	0.440519256976296	1	pseudobulk	DESeq2	LRT	0.356035101972148
617	KC_AB1	axed	-0.0144009168374601	0.93070562394823	1	pseudobulk	DESeq2	LRT	0.031187661780379
618	KC_AB1	Axn	-0.198431314033155	0.63585796638958	1	pseudobulk	DESeq2	LRT	0.196639883254194
619	KC_AB1	axo	0.44144756552762	0.600411062080775	1	pseudobulk	DESeq2	LRT	0.221551314835859
620	KC_AB1	Axud1	0.0990323123423947	0.722242568224917	1	pseudobulk	DESeq2	LRT	0.141316918290293
621	KC_AB1	az2	-0.0755156661328086	0.863288219962815	1	pseudobulk	DESeq2	LRT	0.063844185232768
622	KC_AB1	azot	-0.373753020939138	0.810781873518248	1	pseudobulk	DESeq2	LRT	0.091095969301919

623	KC_AB1	B4	-0.060196410907728	0.698385147053859	1	pseudobulk	DESeq2	LRT	0.155905005587055
624	KC_AB1	B52	-0.0022945268805798	0.949664148797189	1	pseudobulk	DESeq2	LRT	0.022429956935015
625	KC_AB1	babo	0.464344397663173	0.0166358807694838	1	pseudobulk	DESeq2	LRT	1.77895420091475
626	KC_AB1	babos	0.111831901354254	1	1	pseudobulk	DESeq2	LRT	0
627	KC_AB1	Bacc	0.111321854339542	0.197149369007694	1	pseudobulk	DESeq2	LRT	0.70520460858272
628	KC_AB1	baf	-0.367375976908591	0.515172357954529	1	pseudobulk	DESeq2	LRT	0.288047447491945
629	KC_AB1	bai	-0.0653541518635198	0.822569067429305	1	pseudobulk	DESeq2	LRT	0.084827626090336
630	KC_AB1	Baldspot	-0.131593788695605	0.426856161884015	1	pseudobulk	DESeq2	LRT	0.369718444937141
631	KC_AB1	ball	0.65673938062526	0.490908252893747	1	pseudobulk	DESeq2	LRT	0.30899966670623
632	KC_AB1	Bap111	-0.815593131511812	0.0907236896192212	1	pseudobulk	DESeq2	LRT	1.04227929587749
633	KC_AB1	Bap170	0.366601699281423	0.606589365208477	1	pseudobulk	DESeq2	LRT	0.217105208057273
634	KC_AB1	Bap55	0.36319371244263	0.361022982886448	1	pseudobulk	DESeq2	LRT	0.442465149831052
635	KC_AB1	Bap60	0.146461799567551	0.633869507525803	1	pseudobulk	DESeq2	LRT	0.198000139585968
636	KC_AB1	barc	-0.0949550484478551	0.733741357259831	1	pseudobulk	DESeq2	LRT	0.134457001269331
637	KC_AB1	barr	-0.958636528207196	0.711263785178276	1	pseudobulk	DESeq2	LRT	0.147969303347184
638	KC_AB1	baz	0.501972100566564	0.738623645956202	1	pseudobulk	DESeq2	LRT	0.131576793159562
639	KC_AB1	bbc	-0.17303247970903	0.513611788415579	1	pseudobulk	DESeq2	LRT	0.289365016899711
640	KC_AB1	bbg	-0.82542030717083	1	1	pseudobulk	DESeq2	LRT	0
641	KC_AB1	BBS5	1.82617801372066	0.317334072118348	1	pseudobulk	DESeq2	LRT	0.498483295253628
642	KC_AB1	bbx	-0.908263419633038	0.397762502719816	1	pseudobulk	DESeq2	LRT	0.400376160450994
643	KC_AB1	bc10	0.478395387131148	0.122513865053375	1	pseudobulk	DESeq2	LRT	0.911814758846976
644	KC_AB1	BCAS2	-0.0102990793641038	0.916205632032958	1	pseudobulk	DESeq2	LRT	0.038007042888119
645	KC_AB1	bcd	-1.05972628603973	0.705203047462737	1	pseudobulk	DESeq2	LRT	0.151685819609541
646	KC_AB1	bchs	-0.0252696007183625	0.915921891148435	1	pseudobulk	DESeq2	LRT	0.038141560927791
647	KC_AB1	BCL7-like	0.201202924245765	0.717681371533033	1	pseudobulk	DESeq2	LRT	0.144068326368222
648	KC_AB1	bcn92	-0.0197618807997685	0.921347702514691	1	pseudobulk	DESeq2	LRT	0.035576442782143
649	KC_AB1	Bdbt	0.495074130904316	0.352955387625031	1	pseudobulk	DESeq2	LRT	0.452280184506466
650	KC_AB1	bdg	-0.691340072146256	0.261629206282472	1	pseudobulk	DESeq2	LRT	0.582313776329006
651	KC_AB1	bdl	-1.61862498639867	0.500791630206422	1	pseudobulk	DESeq2	LRT	0.300342938155337
652	KC_AB1	Bdp1	0.601616658140292	0.509755076954359	1	pseudobulk	DESeq2	LRT	0.292638440130925
653	KC_AB1	be	0.548456830129877	0.533024896141326	1	pseudobulk	DESeq2	LRT	0.273252505787144
654	KC_AB1	BEAF-32	-0.216869626509681	0.441917712578928	1	pseudobulk	DESeq2	LRT	0.354658591057926
655	KC_AB1	beag	0.0177912300607144	0.917185722777659	1	pseudobulk	DESeq2	LRT	0.037542714263881
656	KC_AB1	beat-Ia	-0.26739497124034	0.857779682292576	1	pseudobulk	DESeq2	LRT	0.066624244845279
657	KC_AB1	beat-Ib	0.0072311651964269	0.922042133982102	1	pseudobulk	DESeq2	LRT	0.035249232809893
658	KC_AB1	beat-Ic	0.101384238996347	0.883924705801437	1	pseudobulk	DESeq2	LRT	0.053584727348499
659	KC_AB1	beat-IIa	0.135879393043758	1	1	pseudobulk	DESeq2	LRT	0
660	KC_AB1	beat-IIIa	1.08899820779554	0.670846322504933	1	pseudobulk	DESeq2	LRT	0.173376956629912
661	KC_AB1	beat-IIIb	0.278048986111134	0.148513028363262	1	pseudobulk	DESeq2	LRT	0.828235446022969
662	KC_AB1	beat-IIIc	-0.878671969620744	0.691590630900759	1	pseudobulk	DESeq2	LRT	0.160150898821571
663	KC_AB1	beat-IV	-0.388174913877192	0.284455342023798	1	pseudobulk	DESeq2	LRT	0.545985905841815
664	KC_AB1	beat-Va	-0.850932661185203	0.660736244265418	1	pseudobulk	DESeq2	LRT	0.179971869591347
665	KC_AB1	beat-Vb	-1.56855358782635	0.573026303465967	1	pseudobulk	DESeq2	LRT	0.241825442277049
666	KC_AB1	beat-VI	-0.0948009083275992	0.923956092054964	1	pseudobulk	DESeq2	LRT	0.034348666692586
667	KC_AB1	beat-VII	0.0269798683764043	0.847461709992494	1	pseudobulk	DESeq2	LRT	0.071879914971057
668	KC_AB1	beg	-0.0511354598331084	0.930404068352593	1	pseudobulk	DESeq2	LRT	0.031328399247773
669	KC_AB1	bel	-0.0405428482377764	0.761186314368098	1	pseudobulk	DESeq2	LRT	0.118509028645457
670	KC_AB1	Bem46	-0.307087385511731	0.636320626704249	1	pseudobulk	DESeq2	LRT	0.196323998626666
671	KC_AB1	ben	-0.128691287669099	0.400021904516978	1	pseudobulk	DESeq2	LRT	0.397916226796064
672	KC_AB1	Best1	-1.13282616228887	0.138257918778905	1	pseudobulk	DESeq2	LRT	0.859309984916626
673	KC_AB1	Bet1	0.304969481825524	0.431046809911588	1	pseudobulk	DESeq2	LRT	0.365475564682835
674	KC_AB1	Bet3	-0.121286213221885	0.696562139113554	1	pseudobulk	DESeq2	LRT	0.157040134846086

675	KC_AB1	Bet5	-0.081972796640287	0.817206073812849	1	pseudobulk	DESeq2	LRT	0.087668414171746
676	KC_AB1	beta-PheRS	-0.933411816761683	0.203243866294877	1	pseudobulk	DESeq2	LRT	0.69198255212742
677	KC_AB1	beta-Spec	-0.1786009601521	0.274406393801896	1	pseudobulk	DESeq2	LRT	0.561605773575595
678	KC_AB1	beta'COP	0.0265195724112254	0.903343543891384	1	pseudobulk	DESeq2	LRT	0.044147054955267
679	KC_AB1	beta3GalTII	1.59422578437749	0.0860755211378618	1	pseudobulk	DESeq2	LRT	1.06512033921158
680	KC_AB1	beta4GalNAcTA	-0.639325549289818	0.100550382332865	1	pseudobulk	DESeq2	LRT	0.997616273702366
681	KC_AB1	beta4GalNAcTB	-0.77864421012471	0.320966791741088	1	pseudobulk	DESeq2	LRT	0.493539898777854
682	KC_AB1	beta4GalT7	1.5234920499224	0.0915245028237606	1	pseudobulk	DESeq2	LRT	1.03846262159949
683	KC_AB1	betaCOP	0.0985767035736617	0.832390362107048	1	pseudobulk	DESeq2	LRT	0.079672956941859
684	KC_AB1	betaggt-I	-0.11221479125194	0.884176064159914	1	pseudobulk	DESeq2	LRT	0.053461246209124
685	KC_AB1	betaggt-II	-0.204292065177116	0.683605704435624	1	pseudobulk	DESeq2	LRT	0.165194321910082
686	KC_AB1	betaGlu	-0.0957801435018513	0.921063173017913	1	pseudobulk	DESeq2	LRT	0.035710581798594
687	KC_AB1	betaNACtes4	1.08899820779554	0.697631521651407	1	pseudobulk	DESeq2	LRT	0.156373904552
688	KC_AB1	betaTub56D	-0.102668708138811	0.422958368396287	1	pseudobulk	DESeq2	LRT	0.373702377933605
689	KC_AB1	betaTub85D	-0.626150683486409	0.646678972988005	1	pseudobulk	DESeq2	LRT	0.189311260073051
690	KC_AB1	betaTub97EF	3.08854013156813	0.16347020152329	1	pseudobulk	DESeq2	LRT	0.786561401985031
691	KC_AB1	Bgb	0.0148282296869897	0.942246501642155	1	pseudobulk	DESeq2	LRT	0.025835466317018
692	KC_AB1	BHD	0.524495947855497	0.601469717208259	1	pseudobulk	DESeq2	LRT	0.220786233628249
693	KC_AB1	BI-1	-0.0909332535522912	0.618088394834606	1	pseudobulk	DESeq2	LRT	0.208949410601036
694	KC_AB1	bib	0.291098811057067	0.881956311467151	1	pseudobulk	DESeq2	LRT	0.054552927519734
695	KC_AB1	bic	-0.024918777440103	0.838457277556887	1	pseudobulk	DESeq2	LRT	0.076519061375116
696	KC_AB1	BicD	0.152103415963236	0.379684520750631	1	pseudobulk	DESeq2	LRT	0.420577108126513
697	KC_AB1	bif	0.130830453134809	0.596418866525973	1	pseudobulk	DESeq2	LRT	0.224448626958603
698	KC_AB1	bigmax	1.40634173620353	0.0114242441328789	1	pseudobulk	DESeq2	LRT	1.942172524718
699	KC_AB1	Bili	0.236848296432806	0.231926911022855	1	pseudobulk	DESeq2	LRT	0.634648856232004
700	KC_AB1	Bin1	0.489238744068994	0.265948076606754	1	pseudobulk	DESeq2	LRT	0.575203146242419
701	KC_AB1	bin3	0.164114331611367	0.473048652277016	1	pseudobulk	DESeq2	LRT	0.325094190490857
702	KC_AB1	bip1	0.33801878461149	0.868910254227304	1	pseudobulk	DESeq2	LRT	0.06102507752903
703	KC_AB1	bip2	0.0837463266920414	0.527854409459526	1	pseudobulk	DESeq2	LRT	0.277485846192216
704	KC_AB1	Bka	0.43522959578278	0.477584335249261	1	pseudobulk	DESeq2	LRT	0.320949926490027
705	KC_AB1	Blimp-1	0.303209152736054	0.063442663379152	1	pseudobulk	DESeq2	LRT	1.19761859324107
706	KC_AB1	Blm	1.07815650932759	0.224191426134697	1	pseudobulk	DESeq2	LRT	0.649381000365445
707	KC_AB1	Blos1	-0.0292351705879892	0.92739122126595	1	pseudobulk	DESeq2	LRT	0.032737019475151
708	KC_AB1	Blos2	-1.00739107213036	0.114418319605575	1	pseudobulk	DESeq2	LRT	0.94150443475061
709	KC_AB1	Blos3	0.262763562767491	0.498181405107719	1	pseudobulk	DESeq2	LRT	0.302612486775068
710	KC_AB1	Blos4	0.0886331009813008	0.869974297466277	1	pseudobulk	DESeq2	LRT	0.060493577994550
711	KC_AB1	blot	-2.93502495981086	0.156327481571882	1	pseudobulk	DESeq2	LRT	0.805964668520183
712	KC_AB1	blp	-0.391711427664327	0.478288853731668	1	pseudobulk	DESeq2	LRT	0.320309740012911
713	KC_AB1	blw	-0.187856644406873	0.236378352420802	1	pseudobulk	DESeq2	LRT	0.626392298749629
714	KC_AB1	bma	-0.0701702332930687	0.653918705275361	1	pseudobulk	DESeq2	LRT	0.18447623951186
715	KC_AB1	Bmcp	-0.503344530902084	0.235888948187628	1	pseudobulk	DESeq2	LRT	0.62729240609857
716	KC_AB1	bmm	0.174489483572011	0.743634603556574	1	pseudobulk	DESeq2	LRT	0.128640409400386
717	KC_AB1	bnb	-0.630058486312384	0.702450306767029	1	pseudobulk	DESeq2	LRT	0.153384393501482
718	KC_AB1	boca	-0.0480607404088211	0.867331084606614	1	pseudobulk	DESeq2	LRT	0.061815088486644
719	KC_AB1	bocks	0.810599480615165	0.143502891190578	1	pseudobulk	DESeq2	LRT	0.843139348996434
720	KC_AB1	BOD1	-0.17424648296855	0.711766172817499	1	pseudobulk	DESeq2	LRT	0.147662655989088
721	KC_AB1	boi	-0.195616682121027	0.891793983773985	1	pseudobulk	DESeq2	LRT	0.049735461816612
722	KC_AB1	bol	-0.0425227163033792	0.667706730076504	1	pseudobulk	DESeq2	LRT	0.175414246321021
723	KC_AB1	Bom068	-0.850996402080018	0.900449987182352	1	pseudobulk	DESeq2	LRT	0.045540403770502
724	KC_AB1	bon	0.0614213155834061	0.816109548154461	1	pseudobulk	DESeq2	LRT	0.088251541043054
725	KC_AB1	bonsai	-0.33355294600261	0.433050139468813	1	pseudobulk	DESeq2	LRT	0.363461817194008
726	KC_AB1	bor	-0.451854721260523	0.478471836305203	1	pseudobulk	DESeq2	LRT	0.320143620472744

727	KC_AB1	BORCS5	-0.261827677127137	0.444551270036656	1	pseudobulk	DESeq2	LRT	0.352078144682579
728	KC_AB1	BORCS6	0.242518719534738	0.565404484745351	1	pseudobulk	DESeq2	LRT	0.247640751042053
729	KC_AB1	botv	-0.0788202119879667	0.901678578009967	1	pseudobulk	DESeq2	LRT	0.044948248127537
730	KC_AB1	br	-0.0871316363975462	0.866742658485517	1	pseudobulk	DESeq2	LRT	0.062109828232548
731	KC_AB1	Br140	-0.0811981582354551	0.896754467044009	1	pseudobulk	DESeq2	LRT	0.047326451275657
732	KC_AB1	brat	0.301033826577662	0.0100827847547978	1	pseudobulk	DESeq2	LRT	1.99641950394062
733	KC_AB1	Brca2	-0.0562133848527164	0.93940028370641	1	pseudobulk	DESeq2	LRT	0.027149312992338
734	KC_AB1	Brd8	-0.92648325905096	0.351436459877456	1	pseudobulk	DESeq2	LRT	0.454153184496406
735	KC_AB1	Bre1	-0.276358440870664	0.498585266123871	1	pseudobulk	DESeq2	LRT	0.302260559638195
736	KC_AB1	Brf	-0.169398266361645	0.754344418903116	1	pseudobulk	DESeq2	LRT	0.122430318485115
737	KC_AB1	brk	-0.431834396981437	0.797253406317302	1	pseudobulk	DESeq2	LRT	0.098403616529471
738	KC_AB1	brm	-0.206425755628355	0.53407362083252	1	pseudobulk	DESeq2	LRT	0.272398872339413
739	KC_AB1	Brms1	-0.0656354990281899	0.848023090659767	1	pseudobulk	DESeq2	LRT	0.071592322260276
740	KC_AB1	brn	-0.948576374010963	0.512682565522919	1	pseudobulk	DESeq2	LRT	0.290151451090774
741	KC_AB1	brp	0.0136878226589573	0.872120842665447	1	pseudobulk	DESeq2	LRT	0.059423334257041
742	KC_AB1	bru1	-0.500630347651429	0.00625843541353561	1	pseudobulk	DESeq2	LRT	2.20353422528734
743	KC_AB1	bru2	-0.413658186085007	0.00958057874783684	1	pseudobulk	DESeq2	LRT	2.01860825507594
744	KC_AB1	bru3	0.0747854497881386	0.441269371088022	1	pseudobulk	DESeq2	LRT	0.355296216285187
745	KC_AB1	Bruce	-0.0158946185247055	0.950596916062808	1	pseudobulk	DESeq2	LRT	0.022003598982010
746	KC_AB1	brun	-0.313924160914515	0.594387838483708	1	pseudobulk	DESeq2	LRT	0.225930085070917
747	KC_AB1	brv3	-0.82542030717083	1	1	pseudobulk	DESeq2	LRT	0
748	KC_AB1	BRWD3	0.333300885817104	0.390024076390406	1	pseudobulk	DESeq2	LRT	0.40890858292028
749	KC_AB1	brwl	0.416224402270783	0.401906910848015	1	pseudobulk	DESeq2	LRT	0.395874525986732
750	KC_AB1	bs	0.0233247885345317	0.966628425799289	1	pseudobulk	DESeq2	LRT	0.014740437640905
751	KC_AB1	bsf	-1.15840648792497	0.106497295601675	1	pseudobulk	DESeq2	LRT	0.972661420583761
752	KC_AB1	Bsg	-0.0658334234542105	0.540284526931908	1	pseudobulk	DESeq2	LRT	0.267377469929458
753	KC_AB1	Bsg25D	0.0158282922792324	0.94995017351687	1	pseudobulk	DESeq2	LRT	0.022299173589232
754	KC_AB1	bsk	-0.0833026501137544	0.57830391568267	1	pseudobulk	DESeq2	LRT	0.237843867088383
755	KC_AB1	bt	-0.399013626028224	0.274990017795365	1	pseudobulk	DESeq2	LRT	0.560683070879097
756	KC_AB1	BTBD9	0.243857553977914	0.711453180745034	1	pseudobulk	DESeq2	LRT	0.147853674656452
757	KC_AB1	BtbVII	0.111233998578926	0.505504294894409	1	pseudobulk	DESeq2	LRT	0.296275150179769
758	KC_AB1	Btk29A	0.483880417039301	0.833651335462188	1	pseudobulk	DESeq2	LRT	0.079015549776920
759	KC_AB1	btl	0.566461463755043	0.809160703341751	1	pseudobulk	DESeq2	LRT	0.091965216776465
760	KC_AB1	btsz	0.00985048121658859	0.948305432871511	1	pseudobulk	DESeq2	LRT	0.023051761346948
761	KC_AB1	btz	-0.107298524656632	0.86976435093536	1	pseudobulk	DESeq2	LRT	0.060598396750400
762	KC_AB1	Bub1	-0.26131339456595	0.672253761574831	1	pseudobulk	DESeq2	LRT	0.172466759016714
763	KC_AB1	Bub3	-0.0098987034840128	0.932650378832302	1	pseudobulk	DESeq2	LRT	0.030281129029565
764	KC_AB1	BubR1	0.135795303631797	0.946582799906172	1	pseudobulk	DESeq2	LRT	0.023841391241048
765	KC_AB1	bug	-0.463304309750748	0.531046928472858	1	pseudobulk	DESeq2	LRT	0.274867098737254
766	KC_AB1	Bug22	-0.0643804730340686	0.877604839657865	1	pseudobulk	DESeq2	LRT	0.056700990457377
767	KC_AB1	BuGZ	0.0894555155353901	0.623138883844129	1	pseudobulk	DESeq2	LRT	0.205415147939503
768	KC_AB1	bun	0.0212102935127071	0.825591528081795	1	pseudobulk	DESeq2	LRT	0.083234772265077
769	KC_AB1	bur	0.148208136659421	0.70194036977327	1	pseudobulk	DESeq2	LRT	0.153699779862186
770	KC_AB1	bves	0.0070657299186691	0.945199668913956	1	pseudobulk	DESeq2	LRT	0.024476439165004
771	KC_AB1	bw	-0.82542030717083	1	1	pseudobulk	DESeq2	LRT	0
772	KC_AB1	bwa	0.601971760440312	0.263054828045795	1	pseudobulk	DESeq2	LRT	0.579953722855968
773	KC_AB1	Bx	0.0081825789465917	0.945078064732968	1	pseudobulk	DESeq2	LRT	0.024532316694440
774	KC_AB1	Bx42	0.0244783297513248	0.942897432115489	1	pseudobulk	DESeq2	LRT	0.025535547017654
775	KC_AB1	by	0.725476977798061	0.78671877098961	1	pseudobulk	DESeq2	LRT	0.104180487507299
776	KC_AB1	bys	-0.239224817469574	0.765947210749463	1	pseudobulk	DESeq2	LRT	0.115801161007382
777	KC_AB1	c(3)G	-1.39634342310828	0.327112434511188	1	pseudobulk	DESeq2	LRT	0.485302946743397
778	KC_AB1	c11.1	-0.260941999807494	0.434330448788856	1	pseudobulk	DESeq2	LRT	0.362179723303109

779	KC_AB1	c12.1	-0.071209616109269	0.806577835545253	1	pseudobulk	DESeq2	LRT	0.093353716413011
780	KC_AB1	c12.2	-0.187671401028414	0.708848998692869	1	pseudobulk	DESeq2	LRT	0.14944626978009
781	KC_AB1	C1GalTA	0.0411555306512201	0.883797722417641	1	pseudobulk	DESeq2	LRT	0.053647121966671
782	KC_AB1	C3G	0.0626648370553717	0.779232716553328	1	pseudobulk	DESeq2	LRT	0.10833282163214
783	KC_AB1	C901	-0.686589209834713	0.609389421662804	1	pseudobulk	DESeq2	LRT	0.2151050889275
784	KC_AB1	ca	0.164181190039018	0.690258818165555	1	pseudobulk	DESeq2	LRT	0.160988036472577
785	KC_AB1	Ca-alpha1D	-0.060918707693917	0.627962104904933	1	pseudobulk	DESeq2	LRT	0.20206656347179
786	KC_AB1	Ca-alpha1T	0.0108134327173715	0.936091349375333	1	pseudobulk	DESeq2	LRT	0.02868176814883
787	KC_AB1	Ca-beta	-0.078726575125815	0.511271645092458	1	pseudobulk	DESeq2	LRT	0.291348292392472
788	KC_AB1	Ca-Ma2d	0.239778522286642	0.409636012205633	1	pseudobulk	DESeq2	LRT	0.387601870355726
789	KC_AB1	CaBP1	0.643337339777019	0.30761475971011	1	pseudobulk	DESeq2	LRT	0.511992830421738
790	KC_AB1	cac	-0.041351583922274	0.642990496690461	1	pseudobulk	DESeq2	LRT	0.191795445839996
791	KC_AB1	cact	-0.068884993667352	0.669766067112139	1	pseudobulk	DESeq2	LRT	0.17407685924353
792	KC_AB1	cactin	0.244984561057858	0.678201715740608	1	pseudobulk	DESeq2	LRT	0.168641115861772
793	KC_AB1	Cad86C	-1.54496158472927	0.407729625456069	1	pseudobulk	DESeq2	LRT	0.38962773175447
794	KC_AB1	Cad96Ca	0.377223875544296	0.761509813638716	1	pseudobulk	DESeq2	LRT	0.118324495503672
795	KC_AB1	Cad96Cb	-0.82542030717083	1	1	pseudobulk	DESeq2	LRT	0
796	KC_AB1	CadN	0.351595789275973	0.0518724220854371	1	pseudobulk	DESeq2	LRT	1.28506347296687
797	KC_AB1	CadN2	-1.64900969927877	0.352626394698632	1	pseudobulk	DESeq2	LRT	0.45268518312308
798	KC_AB1	Cadps	-0.030554494673631	0.77114569371203	1	pseudobulk	DESeq2	LRT	0.11286356228940
799	KC_AB1	Caf1-105	0.142878862734744	0.887315161582805	1	pseudobulk	DESeq2	LRT	0.05192209761382
800	KC_AB1	Caf1-55	0.126866076075563	0.739102461202007	1	pseudobulk	DESeq2	LRT	0.13129535152383
801	KC_AB1	cag	-0.220734076027523	0.659656594295758	1	pseudobulk	DESeq2	LRT	0.180682091754047
802	KC_AB1	CAH1	0.185511947497157	0.891811752338048	1	pseudobulk	DESeq2	LRT	0.049726808795187
803	KC_AB1	CAH13	1.08899820779554	0.878177167143508	1	pseudobulk	DESeq2	LRT	0.056417858865076
804	KC_AB1	CAH2	-3.33587100902895	0.056714912771367	1	pseudobulk	DESeq2	LRT	1.24630273153458
805	KC_AB1	CAH3	0.141954365836233	0.61800826289354	1	pseudobulk	DESeq2	LRT	0.209005718268661
806	KC_AB1	CAH7	0.0103321335792304	0.935527119640646	1	pseudobulk	DESeq2	LRT	0.028943618383565
807	KC_AB1	CAH8	0.230451300633456	0.558154854613733	1	pseudobulk	DESeq2	LRT	0.253245293581949
808	KC_AB1	CAHbeta	-0.526258673073626	0.42270480527338	1	pseudobulk	DESeq2	LRT	0.37396281511905
809	KC_AB1	cal1	0.28018762613334	0.546543682086882	1	pseudobulk	DESeq2	LRT	0.262375121671911
810	KC_AB1	CalpA	0.239634834197333	0.58490285523335	1	pseudobulk	DESeq2	LRT	0.232916258600668
811	KC_AB1	CalpB	-0.644131577611039	0.323289181851953	1	pseudobulk	DESeq2	LRT	0.490408827836971
812	KC_AB1	Calr	0.0436452699344905	0.881778029820635	1	pseudobulk	DESeq2	LRT	0.05464072615670
813	KC_AB1	Cals	-0.078591603536759	0.470106725356336	1	pseudobulk	DESeq2	LRT	0.327803535741649
814	KC_AB1	Calx	-0.042294031762874	0.834721083670855	1	pseudobulk	DESeq2	LRT	0.078458616801536
815	KC_AB1	calypso	0.340806188898605	0.550368222795409	1	pseudobulk	DESeq2	LRT	0.259346649378302
816	KC_AB1	Cam	-0.166910514749463	0.108273459000316	1	pseudobulk	DESeq2	LRT	0.965477988641037
817	KC_AB1	CaMKI	0.0248726169610505	0.835915674483621	1	pseudobulk	DESeq2	LRT	0.07783753111788
818	KC_AB1	CaMKII	-0.054743439827356	0.576094194834697	1	pseudobulk	DESeq2	LRT	0.23950650103447
819	KC_AB1	Camp	-0.748726340555386	0.227052780197465	1	pseudobulk	DESeq2	LRT	0.64387317591661
820	KC_AB1	Camta	-0.087677838647774	0.740159076665524	1	pseudobulk	DESeq2	LRT	0.13067493068330
821	KC_AB1	can	-0.163850383885756	0.940952236747614	1	pseudobulk	DESeq2	LRT	0.02643242103970
822	KC_AB1	CanA-14F	-0.176279680429579	0.0938570719200228	1	pseudobulk	DESeq2	LRT	1.02753299869387
823	KC_AB1	CanB	-0.1334667688146	0.268825814960839	1	pseudobulk	DESeq2	LRT	0.57052902893397
824	KC_AB1	CanB2	-0.023917815040873	0.896274469887958	1	pseudobulk	DESeq2	LRT	0.04755897415009
825	KC_AB1	Cand1	0.440170248203601	0.365945072377427	1	pseudobulk	DESeq2	LRT	0.436584096445955
826	KC_AB1	CAP	0.0652595410402082	0.911822108274839	1	pseudobulk	DESeq2	LRT	0.04008988198998
827	KC_AB1	Cap-D3	1.25340713202048	0.214959457103388	1	pseudobulk	DESeq2	LRT	0.66764344341756
828	KC_AB1	Cap-G	-0.073767183430237	0.868949319809816	1	pseudobulk	DESeq2	LRT	0.061005552399182
829	KC_AB1	Cap-H2	0.328050151409727	0.520901249441624	1	pseudobulk	DESeq2	LRT	0.28324460086073
830	KC_AB1	Caper	-0.039034211024915	0.735036850754942	1	pseudobulk	DESeq2	LRT	0.13369088720377

831	KC_AB1	Capr	0.0225684386476712	0.937333335301838	1	pseudobulk	DESeq2	LRT	0.028105937459809
832	KC_AB1	caps	0.0974727334547671	0.530583895154785	1	pseudobulk	DESeq2	LRT	0.275245936320437
833	KC_AB1	capt	-0.155082688801424	0.584636358002799	1	pseudobulk	DESeq2	LRT	0.233114179760325
834	KC_AB1	capu	0.276305174425169	0.227063865403562	1	pseudobulk	DESeq2	LRT	0.643851973241546
835	KC_AB1	car	0.0802250658358642	0.862612411377856	1	pseudobulk	DESeq2	LRT	0.064184297460484
836	KC_AB1	CARPA	1.08899820779554	0.697631521651407	1	pseudobulk	DESeq2	LRT	0.156373904552
837	KC_AB1	CARPB	0.316714010331109	0.585833140622574	1	pseudobulk	DESeq2	LRT	0.23222606388965
838	KC_AB1	CarT	1.88952324269138	0.456767015650974	1	pseudobulk	DESeq2	LRT	0.340305265178099
839	KC_AB1	CASK	0.309580987766918	0.0127790846093412	1	pseudobulk	DESeq2	LRT	1.89350025442098
840	KC_AB1	casp	0.0204399929829134	0.932988961964863	1	pseudobulk	DESeq2	LRT	0.030123494287913
841	KC_AB1	cass	0.0618014079129096	0.664226375625566	1	pseudobulk	DESeq2	LRT	0.177683883086912
842	KC_AB1	Cat	-0.29855577167089	0.49590131038909	1	pseudobulk	DESeq2	LRT	0.304604744110586
843	KC_AB1	cathD	-0.10289139390838	0.745687417180498	1	pseudobulk	DESeq2	LRT	0.127443185185038
844	KC_AB1	Catsup	0.676110053694814	0.278623343527593	1	pseudobulk	DESeq2	LRT	0.554982500472996
845	KC_AB1	cav	-0.583328991211611	0.657212714871809	1	pseudobulk	DESeq2	LRT	0.182294043007444
846	KC_AB1	caz	-0.0597290389858311	0.770135459476595	1	pseudobulk	DESeq2	LRT	0.1134328798546
847	KC_AB1	cbc	-0.0590385286305175	0.929422303522397	1	pseudobulk	DESeq2	LRT	0.031786909885468
848	KC_AB1	Cbl	-0.108301413643235	0.627582487467999	1	pseudobulk	DESeq2	LRT	0.202329183794673
849	KC_AB1	Cbp20	-0.511493415516751	0.250613330312347	1	pseudobulk	DESeq2	LRT	0.600995832275933
850	KC_AB1	Cbp53E	0.116076970522083	0.805806756236904	1	pseudobulk	DESeq2	LRT	0.093769095617155
851	KC_AB1	Cbp80	-0.120076696097251	0.839674995276201	1	pseudobulk	DESeq2	LRT	0.075888779498773
852	KC_AB1	cbs	0.0139683962816698	0.956699039704775	1	pseudobulk	DESeq2	LRT	0.019224661964133
853	KC_AB1	Cbs	-0.574533264645872	0.179760699843093	1	pseudobulk	DESeq2	LRT	0.745305249793412
854	KC_AB1	cbt	0.0189497284060716	0.928572564371152	1	pseudobulk	DESeq2	LRT	0.032184152156207
855	KC_AB1	CCAP-R	-0.974475768564286	0.0663556503071537	1	pseudobulk	DESeq2	LRT	1.17812209024132
856	KC_AB1	CCDC53	0.543810228872929	0.546042515509156	1	pseudobulk	DESeq2	LRT	0.262773541302261
857	KC_AB1	Ccdc58	-0.304261625417379	0.51980740583079	1	pseudobulk	DESeq2	LRT	0.284157537284921
858	KC_AB1	Cchl	0.0923288919067655	0.719783805593828	1	pseudobulk	DESeq2	LRT	0.142797928758902
859	KC_AB1	CCKLR-17D1	-1.1344768037299	0.067590459771323	1	pseudobulk	DESeq2	LRT	1.17011459934036
860	KC_AB1	CCKLR-17D3	-1.43119657064981	0.111741477569813	1	pseudobulk	DESeq2	LRT	0.951785590214321
861	KC_AB1	Ccm3	0.476235236299131	0.125449546419291	1	pseudobulk	DESeq2	LRT	0.90153090460011
862	KC_AB1	Ccn	-0.279039075074501	0.126707288157668	1	pseudobulk	DESeq2	LRT	0.897198403935612
863	KC_AB1	Ccs	0.0204743247230122	0.932409457877532	1	pseudobulk	DESeq2	LRT	0.030393329871333
864	KC_AB1	CCT1	-0.0717711531719012	0.886944012541097	1	pseudobulk	DESeq2	LRT	0.052103793710407
865	KC_AB1	CCT2	0.0692693444869477	0.867363486462716	1	pseudobulk	DESeq2	LRT	0.061798864365644
866	KC_AB1	CCT3	-0.0360565909381788	0.926932778540997	1	pseudobulk	DESeq2	LRT	0.032951759887067
867	KC_AB1	CCT4	-0.104851972559955	0.743764500698177	1	pseudobulk	DESeq2	LRT	0.128564554020622
868	KC_AB1	CCT5	-0.644808043669049	0.315563302130431	1	pseudobulk	DESeq2	LRT	0.500913508025791
869	KC_AB1	CCT6	0.306313391344229	0.535158653563099	1	pseudobulk	DESeq2	LRT	0.271517447600167
870	KC_AB1	CCT7	1.30860281146335	0.0741162798010298	1	pseudobulk	DESeq2	LRT	1.13008638782092
871	KC_AB1	CCT8	0.0763530664658321	0.842907217644814	1	pseudobulk	DESeq2	LRT	0.074220227371113
872	KC_AB1	Ccz1	0.0767106553746161	0.893760854259683	1	pseudobulk	DESeq2	LRT	0.048778670878738
873	KC_AB1	CD98hc	0.282631135182986	0.297262912945016	1	pseudobulk	DESeq2	LRT	0.526859270775009
874	KC_AB1	Cda4	1.08899820779554	0.878177167143508	1	pseudobulk	DESeq2	LRT	0.056417858865076
875	KC_AB1	Cda5	-0.0683537656973059	0.81827505288843	1	pseudobulk	DESeq2	LRT	0.087100689149027
876	KC_AB1	CDase	1.08899820779554	0.878177167143508	1	pseudobulk	DESeq2	LRT	0.056417858865076
877	KC_AB1	cdc14	0.0562635252360774	0.771123322147158	1	pseudobulk	DESeq2	LRT	0.112876161708928
878	KC_AB1	Cdc16	-0.332919312684286	0.741207733345361	1	pseudobulk	DESeq2	LRT	0.130060058144699
879	KC_AB1	Cdc23	0.840786134165709	0.369321419683423	1	pseudobulk	DESeq2	LRT	0.432595503666241
880	KC_AB1	Cdc27	0.415221902800606	0.188479043847346	1	pseudobulk	DESeq2	LRT	0.724736930059774
881	KC_AB1	Cdc2rk	-0.589213112115915	0.318257316562083	1	pseudobulk	DESeq2	LRT	0.49722160336633
882	KC_AB1	Cdc37	0.227973006604503	0.334842388989488	1	pseudobulk	DESeq2	LRT	0.475159568184971

883	KC_AB1	Cdc42	0.128384245883576	0.591597803754086	1	pseudobulk	DESeq2	LRT	0.227973446945686
884	KC_AB1	CDC45L	0.428468128039178	0.604762991330262	1	pseudobulk	DESeq2	LRT	0.218414793487051
885	KC_AB1	Cdc5	0.0591256321573904	0.893638760900192	1	pseudobulk	DESeq2	LRT	0.048838002291744
886	KC_AB1	CDC50	-0.114819975037035	0.740512849493203	1	pseudobulk	DESeq2	LRT	0.130467401132541
887	KC_AB1	Cdc7	0.240235654060465	0.427736693273509	1	pseudobulk	DESeq2	LRT	0.368823492313521
888	KC_AB1	Cdep	0.0122045572641611	0.926515923287369	1	pseudobulk	DESeq2	LRT	0.033147112409397
889	KC_AB1	CdGAPr	0.0305013099957583	0.921668410820227	1	pseudobulk	DESeq2	LRT	0.035425297224822
890	KC_AB1	cdi	0.0454087699399182	0.83528368036677	1	pseudobulk	DESeq2	LRT	0.078166003684403
891	KC_AB1	Cdk1	-0.82542030717083	1	1	pseudobulk	DESeq2	LRT	0
892	KC_AB1	Cdk12	-0.125352096412428	0.660291543291176	1	pseudobulk	DESeq2	LRT	0.180264264934314
893	KC_AB1	Cdk2	0.245639415735161	0.611710192293605	1	pseudobulk	DESeq2	LRT	0.213454283242604
894	KC_AB1	CDK2AP1	0.301208926225364	0.483453047423775	1	pseudobulk	DESeq2	LRT	0.315645697868311
895	KC_AB1	Cdk5	0.401997079891715	0.339390701184429	1	pseudobulk	DESeq2	LRT	0.469300060897582
896	KC_AB1	Cdk5alpha	0.0351476074864126	0.816449125730671	1	pseudobulk	DESeq2	LRT	0.088070871677828
897	KC_AB1	Cdk7	-0.393054262680478	0.591459544726212	1	pseudobulk	DESeq2	LRT	0.228074955352239
898	KC_AB1	Cdk8	0.858214112467428	0.437951465857434	1	pseudobulk	DESeq2	LRT	0.358574015701846
899	KC_AB1	Cdk9	-0.561659524097708	0.359084751058312	1	pseudobulk	DESeq2	LRT	0.444803037290648
900	KC_AB1	cdm	0.0263450577494789	0.922613651273909	1	pseudobulk	DESeq2	LRT	0.034980123726160
901	KC_AB1	Cds	0.0005226532730468	0.922752466341251	1	pseudobulk	DESeq2	LRT	0.034914785344481
902	KC_AB1	Ced-12	-0.053841261906467	0.880422338815905	1	pseudobulk	DESeq2	LRT	0.055308946694109
903	KC_AB1	Cen	0.217762046879265	0.498054020129583	1	pseudobulk	DESeq2	LRT	0.302723550067904
904	KC_AB1	CenB1A	-0.079254226606763	0.867464902593678	1	pseudobulk	DESeq2	LRT	0.061748087624404
905	KC_AB1	CenG1A	-0.106421389576571	0.683937298513396	1	pseudobulk	DESeq2	LRT	0.164983711376124
906	KC_AB1	Cenp-C	2.61681794392005	0.166588350430006	1	pseudobulk	DESeq2	LRT	0.77835537220211
907	KC_AB1	Cep135	1.08899820779554	0.697631521651407	1	pseudobulk	DESeq2	LRT	0.156373904552
908	KC_AB1	Cep97	0.367545806972701	0.267026850435352	1	pseudobulk	DESeq2	LRT	0.573445066689303
909	KC_AB1	cer	-0.213184072149392	0.56301525318795	1	pseudobulk	DESeq2	LRT	0.249479839099588
910	KC_AB1	Cerk	-0.242021864043685	0.535942659507089	1	pseudobulk	DESeq2	LRT	0.270881672978463
911	KC_AB1	cert	0.321512581359496	0.52130018452912	1	pseudobulk	DESeq2	LRT	0.282912121341992
912	KC_AB1	cerv	0.447624632979645	0.495332134260774	1	pseudobulk	DESeq2	LRT	0.305103496609829
913	KC_AB1	Cf2	0.138562336501292	0.457933981338364	1	pseudobulk	DESeq2	LRT	0.339197128125142
914	KC_AB1	Cfp1	0.382462306900993	0.484712533726549	1	pseudobulk	DESeq2	LRT	0.314515750118585
915	KC_AB1	cg	0.0861944960323697	0.556095483037115	1	pseudobulk	DESeq2	LRT	0.25485063252609
916	KC_AB1	CG10011	0.069716660167162	0.794996439697522	1	pseudobulk	DESeq2	LRT	0.099634816278350
917	KC_AB1	CG10019	-0.922562085100224	0.132374871757359	1	pseudobulk	DESeq2	LRT	0.878194447621744
918	KC_AB1	CG10038	0.0507036338625758	0.934509163797719	1	pseudobulk	DESeq2	LRT	0.029416435572180
919	KC_AB1	CG10050	-0.749770609511111	0.497948350142809	1	pseudobulk	DESeq2	LRT	0.302815702242839
920	KC_AB1	CG10051	-0.82542030717083	1	1	pseudobulk	DESeq2	LRT	0
921	KC_AB1	CG10053	-0.670647458239279	0.436953707253281	1	pseudobulk	DESeq2	LRT	0.359564571609447
922	KC_AB1	CG10055	-0.276548221884401	0.677700055077746	1	pseudobulk	DESeq2	LRT	0.16896247906418
923	KC_AB1	CG10068	0.505534121983005	0.403469698261171	1	pseudobulk	DESeq2	LRT	0.394189076486212
924	KC_AB1	CG10075	-0.26938259263869	0.56156556618579	1	pseudobulk	DESeq2	LRT	0.250599529927355
925	KC_AB1	CG10077	0.22444739965968	0.0269691627935626	1	pseudobulk	DESeq2	LRT	1.56913253518521
926	KC_AB1	CG10082	0.259221963374251	0.0790915709753786	1	pseudobulk	DESeq2	LRT	1.10186979809447
927	KC_AB1	CG10089	0.0537899706159946	0.766007635899491	1	pseudobulk	DESeq2	LRT	0.115766901108278
928	KC_AB1	CG10098	0.0821264632194572	0.810347694818203	1	pseudobulk	DESeq2	LRT	0.091328598971725
929	KC_AB1	CG10103	0.154880068429047	0.611262821752381	1	pseudobulk	DESeq2	LRT	0.213772018080338
930	KC_AB1	CG10116	0.111831901354254	1	1	pseudobulk	DESeq2	LRT	0
931	KC_AB1	CG10126	-0.82542030717083	1	1	pseudobulk	DESeq2	LRT	0
932	KC_AB1	CG10132	-0.106282442760649	0.622741388999295	1	pseudobulk	DESeq2	LRT	0.205692268987675
933	KC_AB1	CG10137	-0.199923486545557	0.423771009226446	1	pseudobulk	DESeq2	LRT	0.372868757308186
934	KC_AB1	CG10139	-0.102808891139903	0.701021772595193	1	pseudobulk	DESeq2	LRT	0.154268493344172

935	KC_AB1	CG10147	-0.488472280315003	0.49071640183229	1	pseudobulk	DESeq2	LRT	0.309169425805082
936	KC_AB1	CG10151	-0.318581692672487	0.490788893837414	1	pseudobulk	DESeq2	LRT	0.309105273572784
937	KC_AB1	CG10165	2.32496151311036	0.230919791244108	1	pseudobulk	DESeq2	LRT	0.636538843783717
938	KC_AB1	CG10166	0.0104102181374167	0.934041213290632	1	pseudobulk	DESeq2	LRT	0.029633960697295
939	KC_AB1	CG10171	-0.399111036651782	0.699960994570579	1	pseudobulk	DESeq2	LRT	0.154926160435373
940	KC_AB1	CG10188	0.207963833832621	0.376273317272176	1	pseudobulk	DESeq2	LRT	0.424496577779852
941	KC_AB1	CG10189	0.0746274789864981	0.914026427309486	1	pseudobulk	DESeq2	LRT	0.039041247298161
942	KC_AB1	CG10194	-0.112264680374638	0.843201559580776	1	pseudobulk	DESeq2	LRT	0.074068598862199
943	KC_AB1	CG10208	-0.82542030717083	1	1	pseudobulk	DESeq2	LRT	0
944	KC_AB1	CG10209	0.0130665026785485	0.946802013914389	1	pseudobulk	DESeq2	LRT	0.023740826967103
945	KC_AB1	CG10214	-0.41116108619504	0.452989350632607	1	pseudobulk	DESeq2	LRT	0.3439120077352
946	KC_AB1	CG10222	0.242959972460426	0.837318382551301	1	pseudobulk	DESeq2	LRT	0.077109374153036
947	KC_AB1	CG10232	-0.850996402080018	0.900449987182352	1	pseudobulk	DESeq2	LRT	0.045540403770502
948	KC_AB1	CG1024	0.127312776303862	0.833890491718247	1	pseudobulk	DESeq2	LRT	0.078890978097411
949	KC_AB1	CG10252	-0.607123990271071	0.71186498650553	1	pseudobulk	DESeq2	LRT	0.14760236756503
950	KC_AB1	CG10254	0.0789608792230578	0.826202854283796	1	pseudobulk	DESeq2	LRT	0.082913308999276
951	KC_AB1	CG10257	-0.635509335194001	0.417117889018271	1	pseudobulk	DESeq2	LRT	0.379741184080513
952	KC_AB1	CG10265	0.159027727191272	0.734481007541753	1	pseudobulk	DESeq2	LRT	0.134019429862094
953	KC_AB1	CG10268	-1.16636088527961	0.320511344559026	1	pseudobulk	DESeq2	LRT	0.494156593938644
954	KC_AB1	CG10274	0.128612119252374	0.907146396205412	1	pseudobulk	DESeq2	LRT	0.042322620400016
955	KC_AB1	CG10283	0.192749565896034	0.672351786786289	1	pseudobulk	DESeq2	LRT	0.172403436641538
956	KC_AB1	CG10286	0.012944782836182	0.984762874812275	1	pseudobulk	DESeq2	LRT	0.006668332505746
957	KC_AB1	CG10311	0.13617601020169	1	1	pseudobulk	DESeq2	LRT	0
958	KC_AB1	CG10321	0.130128822030788	0.729621589321475	1	pseudobulk	DESeq2	LRT	0.13690232382374
959	KC_AB1	CG10324	-0.620860802130259	0.38592384883933	1	pseudobulk	DESeq2	LRT	0.413498382612105
960	KC_AB1	CG10333	0.0141008561111252	0.932985054927029	1	pseudobulk	DESeq2	LRT	0.030125312968084
961	KC_AB1	CG10336	-1.83138402360123	0.394392020662989	1	pseudobulk	DESeq2	LRT	0.404071880285311
962	KC_AB1	CG10338	-0.102485378251422	0.906031887443468	1	pseudobulk	DESeq2	LRT	0.042856517227115
963	KC_AB1	CG10340	-0.305538489567258	0.486321608105833	1	pseudobulk	DESeq2	LRT	0.313076433552026
964	KC_AB1	CG10341	0.380421625327504	0.62174898400976	1	pseudobulk	DESeq2	LRT	0.206384915731232
965	KC_AB1	CG10343	0.558320197782173	0.336789457157331	1	pseudobulk	DESeq2	LRT	0.472641512095368
966	KC_AB1	CG10347	0.351901280527438	0.536147635850469	1	pseudobulk	DESeq2	LRT	0.270715604697982
967	KC_AB1	CG10353	-0.188368402058756	0.571519669416435	1	pseudobulk	DESeq2	LRT	0.242968818336028
968	KC_AB1	CG10359	1.58039152950664	0.424549083540724	1	pseudobulk	DESeq2	LRT	0.372072092278466
969	KC_AB1	CG10362	0.563131013098422	0.157495843563987	1	pseudobulk	DESeq2	LRT	0.802730903087358
970	KC_AB1	CG10365	0.900870256283409	0.1688171708196	1	pseudobulk	DESeq2	LRT	0.772583382278229
971	KC_AB1	CG10366	0.0268384984133428	0.895913542004855	1	pseudobulk	DESeq2	LRT	0.047733898866781
972	KC_AB1	CG10375	0.0848466725473934	0.761503285507635	1	pseudobulk	DESeq2	LRT	0.118328218559379
973	KC_AB1	CG10376	0.293860297074131	0.681142241069475	1	pseudobulk	DESeq2	LRT	0.16676218608865
974	KC_AB1	CG10383	-0.386490492650734	0.615484047261952	1	pseudobulk	DESeq2	LRT	0.210783199070424
975	KC_AB1	CG10384	-0.211031752329223	0.853705430000631	1	pseudobulk	DESeq2	LRT	0.068691956235804
976	KC_AB1	CG10395	-0.0438904306535686	0.880617189785549	1	pseudobulk	DESeq2	LRT	0.055212841297404
977	KC_AB1	CG10413	0.153932800191453	0.818135632618689	1	pseudobulk	DESeq2	LRT	0.087174691909454
978	KC_AB1	CG10417	0.0484180330752874	0.802738893921951	1	pseudobulk	DESeq2	LRT	0.095425694284385
979	KC_AB1	CG10418	0.301366643868574	0.525560583991114	1	pseudobulk	DESeq2	LRT	0.279377213457894
980	KC_AB1	CG10425	0.593998096904338	0.567764180234634	1	pseudobulk	DESeq2	LRT	0.245832010220076
981	KC_AB1	CG10428	0.116983257022744	0.809769644983182	1	pseudobulk	DESeq2	LRT	0.091638507221085
982	KC_AB1	CG10431	0.688452679199384	0.626302567188469	1	pseudobulk	DESeq2	LRT	0.203215808139821
983	KC_AB1	CG10433	2.43184435054116	0.269625356413713	1	pseudobulk	DESeq2	LRT	0.569239267801659
984	KC_AB1	CG10435	0.0984239071905789	0.827695586573003	1	pseudobulk	DESeq2	LRT	0.082129360534028
985	KC_AB1	CG10444	-0.530381563074275	0.446672957668856	1	pseudobulk	DESeq2	LRT	0.350010339649714
986	KC_AB1	CG10463	1.60844721198074	0.100207779495502	1	pseudobulk	DESeq2	LRT	0.999098561294931

987	KC_AB1	CG10465	-0.125096824020588	0.584120620058059	1	pseudobulk	DESeq2	LRT	0.23349746244826
988	KC_AB1	CG10466	-0.284979784755746	0.678383222533506	1	pseudobulk	DESeq2	LRT	0.168524901392881
989	KC_AB1	CG10467	-0.58257482632813	0.711976821591527	1	pseudobulk	DESeq2	LRT	0.147534144591727
990	KC_AB1	CG10469	0.113316967542732	1	1	pseudobulk	DESeq2	LRT	0
991	KC_AB1	CG10470	0.220050507164433	0.570645043831856	1	pseudobulk	DESeq2	LRT	0.24363395031735
992	KC_AB1	CG10472	0.167904355330047	0.911111240962872	1	pseudobulk	DESeq2	LRT	0.040428595159865
993	KC_AB1	CG10479	-0.82542030717083	1	1	pseudobulk	DESeq2	LRT	0
994	KC_AB1	CG10481	1.08899820779554	0.878177167143508	1	pseudobulk	DESeq2	LRT	0.056417858865076
995	KC_AB1	CG10483	-0.490490102668732	0.204405073468989	1	pseudobulk	DESeq2	LRT	0.689508328927535
996	KC_AB1	CG10492	-0.402717652146666	0.402708763473146	1	pseudobulk	DESeq2	LRT	0.395008919469752
997	KC_AB1	CG10494	-0.419737235666155	0.46078521429	1	pseudobulk	DESeq2	LRT	0.336501465034687
998	KC_AB1	CG10495	-0.377450194575298	0.618467572028928	1	pseudobulk	DESeq2	LRT	0.208683066702771
999	KC_AB1	CG10508	-0.0119353760226405	0.883140001505855	1	pseudobulk	DESeq2	LRT	0.053970443577779
100	KC_AB1	CG10510	1.68130633771206	0.562684503730219	1	pseudobulk	DESeq2	LRT	0.249735045103917
100	KC_AB1	CG10512	-0.528824970372891	0.684584982541699	1	pseudobulk	DESeq2	LRT	0.164572632032508
100	KC_AB1	CG10513	-0.615767568564188	0.806245159731688	1	pseudobulk	DESeq2	LRT	0.093532879622572
100	KC_AB1	CG10514	1.88952324269138	0.456767015650974	1	pseudobulk	DESeq2	LRT	0.340305265178099
100	KC_AB1	CG10516	0.0868735403015698	0.90415638835952	1	pseudobulk	DESeq2	LRT	0.043756444826153
100	KC_AB1	CG10527	-0.82542030717083	1	1	pseudobulk	DESeq2	LRT	0
100	KC_AB1	CG10543	-0.0249018599474502	0.878884246171651	1	pseudobulk	DESeq2	LRT	0.056068320101727
100	KC_AB1	CG10550	1.08899820779554	0.878177167143508	1	pseudobulk	DESeq2	LRT	0.056417858865076
100	KC_AB1	CG10555	0.00859932139476203	0.968620608995084	1	pseudobulk	DESeq2	LRT	0.013846294862445
100	KC_AB1	CG10561	-0.805629746289662	0.102822143028039	1	pseudobulk	DESeq2	LRT	0.987913348771329
101	KC_AB1	CG10562	0.135879393043758	1	1	pseudobulk	DESeq2	LRT	0
101	KC_AB1	CG10565	0.0316868317384434	0.919834607664043	1	pseudobulk	DESeq2	LRT	0.036290254650238
101	KC_AB1	CG10576	0.144414686160724	0.72358512750736	1	pseudobulk	DESeq2	LRT	0.140510368163612
101	KC_AB1	CG10581	-1.98353794242956	0.116338971190123	1	pseudobulk	DESeq2	LRT	0.93427478107769
101	KC_AB1	CG10584	-0.958636528207196	0.711263785178276	1	pseudobulk	DESeq2	LRT	0.147969303347184
101	KC_AB1	CG10600	0.268110800240763	0.3589391262527	1	pseudobulk	DESeq2	LRT	0.444979198703579
101	KC_AB1	CG10602	0.0659886068016529	0.892397571675811	1	pseudobulk	DESeq2	LRT	0.049441620155603
101	KC_AB1	CG10616	-0.232066422476891	0.540538872647746	1	pseudobulk	DESeq2	LRT	0.26717306846486
101	KC_AB1	CG10623	-0.21802689201555	0.826023240657456	1	pseudobulk	DESeq2	LRT	0.083007733372962
101	KC_AB1	CG10628	-0.307895992956037	0.643967855267718	1	pseudobulk	DESeq2	LRT	0.191135810634399
102	KC_AB1	CG10631	0.125755242705021	0.765093762739079	1	pseudobulk	DESeq2	LRT	0.116285338513156
102	KC_AB1	CG10638	-0.0097044093930436	0.902619786153797	1	pseudobulk	DESeq2	LRT	0.044495150626167
102	KC_AB1	CG10646	-0.521647427615084	0.437638152844117	1	pseudobulk	DESeq2	LRT	0.35888482367785
102	KC_AB1	CG10654	0.289903967762345	0.549040108388991	1	pseudobulk	DESeq2	LRT	0.260395928380943
102	KC_AB1	CG10663	1.08899820779554	0.697631521651407	1	pseudobulk	DESeq2	LRT	0.156373904552
102	KC_AB1	CG10669	-0.0968658668972928	0.904511982865074	1	pseudobulk	DESeq2	LRT	0.043585675285419
102	KC_AB1	CG10672	0.309932216225948	0.610854357069919	1	pseudobulk	DESeq2	LRT	0.21406232406298
102	KC_AB1	CG10674	-0.685609852012165	0.0812178358897248	1	pseudobulk	DESeq2	LRT	1.09034858679467
102	KC_AB1	CG10681	0.525058222464293	0.422723678545972	1	pseudobulk	DESeq2	LRT	0.373943424813896
102	KC_AB1	CG10694	-0.850996402080018	0.900449987182352	1	pseudobulk	DESeq2	LRT	0.045540403770502
103	KC_AB1	CG10710	-0.850932661185203	0.660736244265418	1	pseudobulk	DESeq2	LRT	0.179971869591347
103	KC_AB1	CG10713	0.116455602118501	0.796050280980261	1	pseudobulk	DESeq2	LRT	0.099059500022859
103	KC_AB1	CG10731	-0.646932192652102	0.354039444238386	1	pseudobulk	DESeq2	LRT	0.450948349666948
103	KC_AB1	CG10734	0.942080999092271	0.681532122211136	1	pseudobulk	DESeq2	LRT	0.166513670026992
103	KC_AB1	CG10737	-0.139970183731061	0.653625634667152	1	pseudobulk	DESeq2	LRT	0.18467092346436
103	KC_AB1	CG10738	-0.296194208739866	0.370550852454687	1	pseudobulk	DESeq2	LRT	0.431152183298738
103	KC_AB1	CG1074	0.156662429221675	0.744783371954151	1	pseudobulk	DESeq2	LRT	0.127970027984246
103	KC_AB1	CG10747	-0.352343735349504	0.595846324247885	1	pseudobulk	DESeq2	LRT	0.224865735456053
103	KC_AB1	CG10764	-0.82542030717083	1	1	pseudobulk	DESeq2	LRT	0

103	KC_AB1	CG10778	0.26786245540663	0.742932944664876	1	pseudobulk	DESeq2	LRT	0.129050382842678
104	KC_AB1	CG10795	-0.76764681216928	0.209216259526916	1	pseudobulk	DESeq2	LRT	0.679404566705258
104	KC_AB1	CG10801	-0.615767568564188	0.806245159731688	1	pseudobulk	DESeq2	LRT	0.093532879622572
104	KC_AB1	CG10802	0.831563112044077	0.18850905021342	1	pseudobulk	DESeq2	LRT	0.724667794723985
104	KC_AB1	CG10803	0.083564332752511	0.733152382515049	1	pseudobulk	DESeq2	LRT	0.134805749768897
104	KC_AB1	CG10804	0.0512099284522249	0.796526067108211	1	pseudobulk	DESeq2	LRT	0.098800006911172
104	KC_AB1	CG10809	0.082343297528283	0.77125955493135	1	pseudobulk	DESeq2	LRT	0.112799442565502
104	KC_AB1	CG10863	1.45124784972704	0.133495151980669	1	pseudobulk	DESeq2	LRT	0.874534505881987
104	KC_AB1	CG10864	0.69794357772624	0.730620326456649	1	pseudobulk	DESeq2	LRT	0.136308249527385
104	KC_AB1	CG10866	-0.0905114841896409	0.922873817891714	1	pseudobulk	DESeq2	LRT	0.034857674856553
104	KC_AB1	CG10869	1.88952324269138	0.456767015650974	1	pseudobulk	DESeq2	LRT	0.340305265178099
105	KC_AB1	CG10874	0.201467601930148	0.688264624597426	1	pseudobulk	DESeq2	LRT	0.162244551726157
105	KC_AB1	CG10889	0.172145074441099	0.828864459100325	1	pseudobulk	DESeq2	LRT	0.081516482088436
105	KC_AB1	CG10898	1.08271001751184	0.0983806028333887	1	pseudobulk	DESeq2	LRT	1.00709052060153
105	KC_AB1	CG1090	0.0187424066653603	0.893674357463075	1	pseudobulk	DESeq2	LRT	0.048820703262628
105	KC_AB1	CG10903	0.0866441290660682	0.881480773354412	1	pseudobulk	DESeq2	LRT	0.054787155987629
105	KC_AB1	CG10904	-0.615950818602513	0.318004618722709	1	pseudobulk	DESeq2	LRT	0.497566572244438
105	KC_AB1	CG10907	-0.443897081769887	0.610890872567766	1	pseudobulk	DESeq2	LRT	0.214036363692784
105	KC_AB1	CG10914	-0.560040837453559	0.535192419440682	1	pseudobulk	DESeq2	LRT	0.271490046619899
105	KC_AB1	CG10916	-0.0984152583576259	0.862015588165572	1	pseudobulk	DESeq2	LRT	0.064484880587027
105	KC_AB1	CG1092	-0.82542030717083	1	1	pseudobulk	DESeq2	LRT	0
106	KC_AB1	CG10927	-0.724155123897022	0.752037323693379	1	pseudobulk	DESeq2	LRT	0.123760604791558
106	KC_AB1	CG10932	-0.687419326030303	0.338234439586676	1	pseudobulk	DESeq2	LRT	0.470782173908716
106	KC_AB1	CG10939	-0.82542030717083	1	1	pseudobulk	DESeq2	LRT	0
106	KC_AB1	CG10947	-0.0356810755195623	0.88067588361014	1	pseudobulk	DESeq2	LRT	0.055183896195040
106	KC_AB1	CG10948	-0.0972395234639001	0.659612010154636	1	pseudobulk	DESeq2	LRT	0.180711445361662
106	KC_AB1	CG10949	-1.17653357633994	0.103584901718242	1	pseudobulk	DESeq2	LRT	0.984703541678662
106	KC_AB1	CG10958	1.0605775435428	0.518305079793661	1	pseudobulk	DESeq2	LRT	0.285414534715272
106	KC_AB1	CG10959	-0.743202087471744	0.454837265992047	1	pseudobulk	DESeq2	LRT	0.34214395965784
106	KC_AB1	CG10960	0.234149902279437	0.31982683514644	1	pseudobulk	DESeq2	LRT	0.49508509947948
106	KC_AB1	CG10973	0.189693497187473	0.716684738036614	1	pseudobulk	DESeq2	LRT	0.144671843831081
107	KC_AB1	CG10979	-0.108515410938272	0.808191376872174	1	pseudobulk	DESeq2	LRT	0.092485787643445
107	KC_AB1	CG10984	-0.228924792024433	0.483010384610288	1	pseudobulk	DESeq2	LRT	0.316043531918338
107	KC_AB1	CG10996	0.112935832476632	0.940353958317081	1	pseudobulk	DESeq2	LRT	0.026708642986417
107	KC_AB1	CG11000	0.23960697106659	0.170729816151417	1	pseudobulk	DESeq2	LRT	0.767690627337146
107	KC_AB1	CG11007	-0.166534675713275	0.743294244328389	1	pseudobulk	DESeq2	LRT	0.128839230127359
107	KC_AB1	CG11029	-0.850996402080018	0.900449987182352	1	pseudobulk	DESeq2	LRT	0.045540403770502
107	KC_AB1	CG1103	-0.695480275097161	0.355047809194845	1	pseudobulk	DESeq2	LRT	0.449713162799845
107	KC_AB1	CG11030	0.0778732939092436	0.788308007122968	1	pseudobulk	DESeq2	LRT	0.103304062132207
107	KC_AB1	CG11035	-0.550829987409505	0.333434516128117	1	pseudobulk	DESeq2	LRT	0.476989445335599
107	KC_AB1	CG11041	-0.850996402080018	0.900449987182352	1	pseudobulk	DESeq2	LRT	0.045540403770502
108	KC_AB1	CG1105	-0.013049626625153	0.977558624141911	1	pseudobulk	DESeq2	LRT	0.009857188532708
108	KC_AB1	CG11050	-0.0491174181055718	0.83100452799392	1	pseudobulk	DESeq2	LRT	0.080396609817072
108	KC_AB1	CG11052	-0.850932661185203	0.660736244265418	1	pseudobulk	DESeq2	LRT	0.179971869591347
108	KC_AB1	CG11070	-0.160056398709045	0.768153960321906	1	pseudobulk	DESeq2	LRT	0.114551726040058
108	KC_AB1	CG11076	-0.0776425982438045	0.775773400799071	1	pseudobulk	DESeq2	LRT	0.110265115280208
108	KC_AB1	CG11077	-0.178348883461618	0.494466018830236	1	pseudobulk	DESeq2	LRT	0.305863549044772
108	KC_AB1	CG11089	-1.62324810528694	0.0549445494332604	1	pseudobulk	DESeq2	LRT	1.26007538364934
108	KC_AB1	CG11095	0.282748172511425	0.642686064443116	1	pseudobulk	DESeq2	LRT	0.192001116926494
108	KC_AB1	CG11099	0.343708056828528	0.183311327885113	1	pseudobulk	DESeq2	LRT	0.736810696598142
108	KC_AB1	CG11103	0.257686357341321	0.76064026486463	1	pseudobulk	DESeq2	LRT	0.118820688725016
109	KC_AB1	CG11109	-0.396715217309285	0.581305805417608	1	pseudobulk	DESeq2	LRT	0.235595339785324

109	KC_AB1	CG11110	0.0526431744404081	0.928180105246779	1	pseudobulk	DESeq2	LRT	0.032367744551054
109	KC_AB1	CG11122	-0.0220116586045396	0.906772613457937	1	pseudobulk	DESeq2	LRT	0.042501605003068
109	KC_AB1	CG11123	0.113833774319439	0.933048003186676	1	pseudobulk	DESeq2	LRT	0.030096012220488
109	KC_AB1	CG11127	0.234230123608677	0.81728204351743	1	pseudobulk	DESeq2	LRT	0.087628042849171
109	KC_AB1	CG1113	0.909917542530533	0.594514857344515	1	pseudobulk	DESeq2	LRT	0.225837287585051
109	KC_AB1	CG11131	-0.82542030717083	1	1	pseudobulk	DESeq2	LRT	0
109	KC_AB1	CG11134	-0.525895859226578	0.597988433554184	1	pseudobulk	DESeq2	LRT	0.223307216165725
109	KC_AB1	CG11137	-0.0107483936355488	0.958427347113419	1	pseudobulk	DESeq2	LRT	0.018440802914893
109	KC_AB1	CG11141	-0.0920171765496628	0.844210444919735	1	pseudobulk	DESeq2	LRT	0.073549278877958
110	KC_AB1	CG11151	0.0176632146017285	0.948416177835793	1	pseudobulk	DESeq2	LRT	0.023001046548667
110	KC_AB1	CG11155	0.0629649147608204	0.568513075410178	1	pseudobulk	DESeq2	LRT	0.245259542386648
110	KC_AB1	CG11158	1.08899820779554	0.670846322504933	1	pseudobulk	DESeq2	LRT	0.173376956629912
110	KC_AB1	CG1116	0.028285291942947	0.950889827890603	1	pseudobulk	DESeq2	LRT	0.021869798426307
110	KC_AB1	CG11168	0.381277038507367	0.758554273137856	1	pseudobulk	DESeq2	LRT	0.120013340808624
110	KC_AB1	CG11170	-1.41839063522747	0.214149525157554	1	pseudobulk	DESeq2	LRT	0.66928288424515
110	KC_AB1	CG11178	-0.113331988024292	0.831772685515089	1	pseudobulk	DESeq2	LRT	0.079995345482081
110	KC_AB1	CG11180	1.68067122661377	0.0500308476755125	1	pseudobulk	DESeq2	LRT	1.30076213877802
110	KC_AB1	CG11191	0.0924393244969255	0.521390962899241	1	pseudobulk	DESeq2	LRT	0.282836500588106
110	KC_AB1	CG11200	-0.681253717429508	0.434605933065962	1	pseudobulk	DESeq2	LRT	0.361904349160869
111	KC_AB1	CG11241	0.493639873756039	0.738069084669706	1	pseudobulk	DESeq2	LRT	0.131902985485061
111	KC_AB1	CG11247	0.153014227167899	0.562482787163679	1	pseudobulk	DESeq2	LRT	0.249890763090753
111	KC_AB1	CG11248	0.393927653353106	0.415543739946209	1	pseudobulk	DESeq2	LRT	0.381383255832972
111	KC_AB1	CG11257	1.31308042838553	0.0767624392525765	1	pseudobulk	DESeq2	LRT	1.11485123329688
111	KC_AB1	CG11267	-0.108024075044227	0.582987242710932	1	pseudobulk	DESeq2	LRT	0.234340948638696
111	KC_AB1	CG11269	1.08899820779554	0.878177167143508	1	pseudobulk	DESeq2	LRT	0.056417858865076
111	KC_AB1	CG11279	0.313834267126442	0.420084390967183	1	pseudobulk	DESeq2	LRT	0.376663455197929
111	KC_AB1	CG11291	-0.850996402080018	0.900449987182352	1	pseudobulk	DESeq2	LRT	0.045540403770502
111	KC_AB1	CG11307	-0.425428284956404	0.49890295081252	1	pseudobulk	DESeq2	LRT	0.301983927374098
111	KC_AB1	CG11309	0.556638328291118	0.385251495748958	1	pseudobulk	DESeq2	LRT	0.414255666446641
112	KC_AB1	CG11317	-0.0712755652712802	0.773378112982301	1	pseudobulk	DESeq2	LRT	0.111608122857103
112	KC_AB1	CG11319	-0.16128097989016	0.115268773457841	1	pseudobulk	DESeq2	LRT	0.938288328019295
112	KC_AB1	CG11334	-0.0256705530325198	0.938495798005158	1	pseudobulk	DESeq2	LRT	0.027567667536250
112	KC_AB1	CG11342	1.72480714872207	0.350671956418813	1	pseudobulk	DESeq2	LRT	0.455098963686617
112	KC_AB1	CG11356	-0.82542030717083	1	1	pseudobulk	DESeq2	LRT	0
112	KC_AB1	CG11357	0.264105205361004	0.246902360632753	1	pseudobulk	DESeq2	LRT	0.607474757759937
112	KC_AB1	CG11360	0.424078220497772	0.0260471877325609	1	pseudobulk	DESeq2	LRT	1.58423915981788
112	KC_AB1	CG11362	-0.82542030717083	1	1	pseudobulk	DESeq2	LRT	0
112	KC_AB1	CG11367	-0.634099472222903	0.339480037752986	1	pseudobulk	DESeq2	LRT	0.469185758198246
112	KC_AB1	CG11374	0.866208808329645	0.505790600936228	1	pseudobulk	DESeq2	LRT	0.296029245374094
113	KC_AB1	CG11377	-0.199339143174085	0.732106641914896	1	pseudobulk	DESeq2	LRT	0.135425653064745
113	KC_AB1	CG11378	1.08899820779554	0.878177167143508	1	pseudobulk	DESeq2	LRT	0.056417858865076
113	KC_AB1	CG11396	0.71179405452973	0.228079288384874	1	pseudobulk	DESeq2	LRT	0.641914150710017
113	KC_AB1	CG11398	0.657315010810756	0.484873671095331	1	pseudobulk	DESeq2	LRT	0.314371397671352
113	KC_AB1	CG11399	0.0776004580299185	0.823393812007889	1	pseudobulk	DESeq2	LRT	0.084392401148256
113	KC_AB1	CG11403	-1.73774824727488	0.415725963735328	1	pseudobulk	DESeq2	LRT	0.381192851233832
113	KC_AB1	CG11406	-0.947474892419104	0.118599759770075	1	pseudobulk	DESeq2	LRT	0.925916190656716
113	KC_AB1	CG11407	-1.44925888589622	0.6315588144614	1	pseudobulk	DESeq2	LRT	0.199586199175701
113	KC_AB1	CG11409	-0.82542030717083	1	1	pseudobulk	DESeq2	LRT	0
113	KC_AB1	CG11413	-0.368328291494384	0.589120294723786	1	pseudobulk	DESeq2	LRT	0.229796015908151
114	KC_AB1	CG11414	0.0427543975728549	0.917999250991376	1	pseudobulk	DESeq2	LRT	0.037157673145647
114	KC_AB1	CG11417	-1.04847436584437	0.223855248529567	1	pseudobulk	DESeq2	LRT	0.650032718655084
114	KC_AB1	CG1142	-0.270560254092017	0.54419830469335	1	pseudobulk	DESeq2	LRT	0.264242815484358

114	KC_AB1	CG1143	-0.850932661185203	0.660736244265418	1	pseudobulk	DESeq2	LRT	0.179971869591347
114	KC_AB1	CG11436	-0.186682613695878	0.78975687888017	1	pseudobulk	DESeq2	LRT	0.102506582647645
114	KC_AB1	CG11444	0.0414369165878944	0.88582475552252	1	pseudobulk	DESeq2	LRT	0.052652186958691
114	KC_AB1	CG11447	-0.640416972753449	0.251611556703142	1	pseudobulk	DESeq2	LRT	0.599269415304915
114	KC_AB1	CG11454	-0.310591975967665	0.4072038562258	1	pseudobulk	DESeq2	LRT	0.390188117877447
114	KC_AB1	CG11456	0.103282336245085	0.786663702081082	1	pseudobulk	DESeq2	LRT	0.1042108884092
114	KC_AB1	CG11458	1.08899820779554	0.878177167143508	1	pseudobulk	DESeq2	LRT	0.056417858865076
115	KC_AB1	CG1146	-0.0192163804976479	0.954541837386204	1	pseudobulk	DESeq2	LRT	0.020205031809055
115	KC_AB1	CG11474	0.597237554262819	0.201213872288651	1	pseudobulk	DESeq2	LRT	0.696342081018054
115	KC_AB1	CG11475	0.135879393043758	1	1	pseudobulk	DESeq2	LRT	0
115	KC_AB1	CG11486	-0.0074598820748494	0.95571085076488	1	pseudobulk	DESeq2	LRT	0.019673483154621
115	KC_AB1	CG11504	0.208753567468616	0.585556049603349	1	pseudobulk	DESeq2	LRT	0.232431527801871
115	KC_AB1	CG11523	-0.201583713956566	0.53988269806592	1	pseudobulk	DESeq2	LRT	0.267700590392993
115	KC_AB1	CG11537	0.0169084872683127	0.900986076665363	1	pseudobulk	DESeq2	LRT	0.045281920312972
115	KC_AB1	CG11539	-1.66533205080857	0.399465001813759	1	pseudobulk	DESeq2	LRT	0.39852126437246
115	KC_AB1	CG11555	0.357898789956006	0.449111203430509	1	pseudobulk	DESeq2	LRT	0.347646110986361
115	KC_AB1	CG11560	-0.687857952304781	0.322712013777456	1	pseudobulk	DESeq2	LRT	0.491184866575837
116	KC_AB1	CG11562	-0.0523779500656122	0.92352161469142	1	pseudobulk	DESeq2	LRT	0.034552935598553
116	KC_AB1	CG11563	0.434827950554309	0.371926526115909	1	pseudobulk	DESeq2	LRT	0.429542846284904
116	KC_AB1	CG11583	0.177598613645268	0.715588367930995	1	pseudobulk	DESeq2	LRT	0.145336727613385
116	KC_AB1	CG11586	-0.0485374762969458	0.877985961104133	1	pseudobulk	DESeq2	LRT	0.056512428357852
116	KC_AB1	CG11590	-0.815555156919355	0.154311299906517	1	pseudobulk	DESeq2	LRT	0.811602270259864
116	KC_AB1	CG11593	-0.403996052667601	0.474697305442151	1	pseudobulk	DESeq2	LRT	0.323583233491091
116	KC_AB1	CG11594	-0.82542030717083	1	1	pseudobulk	DESeq2	LRT	0
116	KC_AB1	CG11596	-0.491683518505957	0.501802535092062	1	pseudobulk	DESeq2	LRT	0.299467148972559
116	KC_AB1	CG11597	-0.959478430682113	0.47449512329348	1	pseudobulk	DESeq2	LRT	0.323768246751157
116	KC_AB1	CG11601	2.48891624893288	0.00684644747071185	1	pseudobulk	DESeq2	LRT	2.16453471961383
117	KC_AB1	CG1161	0.197229614906346	0.42936139651113	1	pseudobulk	DESeq2	LRT	0.367177005162582
117	KC_AB1	CG11617	1.08899820779554	0.878177167143508	1	pseudobulk	DESeq2	LRT	0.056417858865076
117	KC_AB1	CG11619	0.11269840515639	0.977007463492936	1	pseudobulk	DESeq2	LRT	0.010102118633921
117	KC_AB1	CG11626	-2.78647806120231	0.114453813979484	1	pseudobulk	DESeq2	LRT	0.941369730620319
117	KC_AB1	CG11638	0.267840415677195	0.710994554540159	1	pseudobulk	DESeq2	LRT	0.148133725489948
117	KC_AB1	CG11655	0.111831901354254	1	1	pseudobulk	DESeq2	LRT	0
117	KC_AB1	CG11658	0.0574142282825484	0.816789995831891	1	pseudobulk	DESeq2	LRT	0.087889590193444
117	KC_AB1	CG11679	-1.69852946273637	0.349615407583913	1	pseudobulk	DESeq2	LRT	0.456409436217198
117	KC_AB1	CG11686	1.08899820779554	0.878177167143508	1	pseudobulk	DESeq2	LRT	0.056417858865076
117	KC_AB1	CG11695	-0.484846223879029	0.523178283061179	1	pseudobulk	DESeq2	LRT	0.281350291714448
118	KC_AB1	CG11696	-0.553028899822201	0.400908584858744	1	pseudobulk	DESeq2	LRT	0.396954643882026
118	KC_AB1	CG11699	-0.215336189817111	0.491750708435153	1	pseudobulk	DESeq2	LRT	0.308255005746692
118	KC_AB1	CG11700	-1.39069491230407	0.559139881025611	1	pseudobulk	DESeq2	LRT	0.252479530292469
118	KC_AB1	CG11710	-0.123491076880669	0.802860635617003	1	pseudobulk	DESeq2	LRT	0.095359835089103
118	KC_AB1	CG1172	0.110626479508681	0.794324933684008	1	pseudobulk	DESeq2	LRT	0.1000018048289
118	KC_AB1	CG11722	-0.153428126558658	0.746161746724405	1	pseudobulk	DESeq2	LRT	0.127167019584054
118	KC_AB1	CG11723	0.223082771114435	0.762959179273147	1	pseudobulk	DESeq2	LRT	0.117498697550583
118	KC_AB1	CG11737	0.254916550399883	0.701794441353504	1	pseudobulk	DESeq2	LRT	0.153790075988182
118	KC_AB1	CG11752	0.0177292995108666	0.924290794223163	1	pseudobulk	DESeq2	LRT	0.034191372439964
118	KC_AB1	CG11753	-0.347170971133763	0.239635234670362	1	pseudobulk	DESeq2	LRT	0.620449325273224
119	KC_AB1	CG11755	-0.0039811712825206	0.942525515757881	1	pseudobulk	DESeq2	LRT	0.025706883877763
119	KC_AB1	CG11760	-0.0147445298560239	0.927515535122681	1	pseudobulk	DESeq2	LRT	0.032678807576470
119	KC_AB1	CG11768	0.130750088802639	0.512733165352835	1	pseudobulk	DESeq2	LRT	0.290108589983276
119	KC_AB1	CG11771	-0.395864328768069	0.453063687676762	1	pseudobulk	DESeq2	LRT	0.343840744427679
119	KC_AB1	CG11777	-0.578078733784584	0.44618593661184	1	pseudobulk	DESeq2	LRT	0.350484122416429

119	KC_AB1	CG11779	0.0704099999056436	0.874008751748177	1	pseudobulk	DESeq2	LRT	0.058484218604829
119	KC_AB1	CG11781	-0.003972163265331	0.972927348221443	1	pseudobulk	DESeq2	LRT	0.011919588759968
119	KC_AB1	CG11788	0.342090151582592	0.677159167156448	1	pseudobulk	DESeq2	LRT	0.169309237824311
119	KC_AB1	CG11790	0.785384074860177	0.207317972335311	1	pseudobulk	DESeq2	LRT	0.683363047410608
119	KC_AB1	CG11791	-0.31131387894845	0.871589143496327	1	pseudobulk	DESeq2	LRT	0.059688187958827
120	KC_AB1	CG11807	-0.611265225866246	0.157782284838725	1	pseudobulk	DESeq2	LRT	0.801941759229734
120	KC_AB1	CG11808	-0.517560015322184	0.546073219696731	1	pseudobulk	DESeq2	LRT	0.262749121437573
120	KC_AB1	CG11811	-0.186531352772125	0.64066432283351	1	pseudobulk	DESeq2	LRT	0.193369460225328
120	KC_AB1	CG11835	-4.33519370782265	0.248955327594649	1	pseudobulk	DESeq2	LRT	0.603878575473275
120	KC_AB1	CG11836	-0.31131387894845	0.871589143496327	1	pseudobulk	DESeq2	LRT	0.059688187958827
120	KC_AB1	CG11837	0.322535552474986	0.696258295609057	1	pseudobulk	DESeq2	LRT	0.157229617361087
120	KC_AB1	CG11839	1.15876370642731	0.228771937851198	1	pseudobulk	DESeq2	LRT	0.640597249039039
120	KC_AB1	CG11852	-0.850996402080018	0.900449987182352	1	pseudobulk	DESeq2	LRT	0.045540403770502
120	KC_AB1	CG11857	-0.16427021275943	0.596073748001958	1	pseudobulk	DESeq2	LRT	0.224700004741476
120	KC_AB1	CG11858	0.0018247195796905	0.994385197020298	1	pseudobulk	DESeq2	LRT	0.002445349471250
121	KC_AB1	CG11873	0.0667411262989883	0.696913559780216	1	pseudobulk	DESeq2	LRT	0.156821085371861
121	KC_AB1	CG11882	-0.921304049283659	0.202405042661568	1	pseudobulk	DESeq2	LRT	0.693778671808463
121	KC_AB1	CG11883	0.151518698885765	0.521225754207073	1	pseudobulk	DESeq2	LRT	0.282974133570187
121	KC_AB1	CG11885	0.180817638377923	0.742696549336356	1	pseudobulk	DESeq2	LRT	0.129188593867708
121	KC_AB1	CG11893	-2.36817011682091	0.282654768599882	1	pseudobulk	DESeq2	LRT	0.548743683265438
121	KC_AB1	CG11898	-1.24030067431808	0.294858320455586	1	pseudobulk	DESeq2	LRT	0.530386612580211
121	KC_AB1	CG11899	2.5678226185994	0.252470751860913	1	pseudobulk	DESeq2	LRT	0.597788926619178
121	KC_AB1	CG11902	-0.843903138012026	0.178625653920608	1	pseudobulk	DESeq2	LRT	0.748056168313825
121	KC_AB1	CG11906	0.228984357089728	0.815406753529544	1	pseudobulk	DESeq2	LRT	0.088625695865873
121	KC_AB1	CG11911	0.33801878461149	0.868910254227304	1	pseudobulk	DESeq2	LRT	0.06102507752903
122	KC_AB1	CG11920	0.12534258062751	0.911832473333177	1	pseudobulk	DESeq2	LRT	0.040084945213374
122	KC_AB1	CG11927	0.488809415970779	0.329885099230171	1	pseudobulk	DESeq2	LRT	0.481637300908927
122	KC_AB1	CG11961	0.997694962638592	0.665140342223729	1	pseudobulk	DESeq2	LRT	0.177086710452097
122	KC_AB1	CG11964	-0.277169669244456	0.618757681632397	1	pseudobulk	DESeq2	LRT	0.208479396430329
122	KC_AB1	CG11970	-0.033095050482386	0.955773491986826	1	pseudobulk	DESeq2	LRT	0.019645018639969
122	KC_AB1	CG11975	0.0659488585973238	0.85154130295897	1	pseudobulk	DESeq2	LRT	0.069794282273149
122	KC_AB1	CG11980	-0.484085803729789	0.381219684879242	1	pseudobulk	DESeq2	LRT	0.41882468197467
122	KC_AB1	CG11983	-0.894033925257157	0.583084274108837	1	pseudobulk	DESeq2	LRT	0.234268671423842
122	KC_AB1	CG11986	0.814871552093028	0.638192090213231	1	pseudobulk	DESeq2	LRT	0.1950485827781
122	KC_AB1	CG11999	-0.428503413835609	0.456882219442276	1	pseudobulk	DESeq2	LRT	0.340195743122588
123	KC_AB1	CG12003	1.08899820779554	0.670846322504933	1	pseudobulk	DESeq2	LRT	0.173376956629912
123	KC_AB1	CG12004	0.012948000926065	0.948265377947085	1	pseudobulk	DESeq2	LRT	0.023070105647677
123	KC_AB1	CG12007	-0.253809537467211	0.665250447586954	1	pseudobulk	DESeq2	LRT	0.177014824578935
123	KC_AB1	CG12010	0.221595025659774	0.862485078279408	1	pseudobulk	DESeq2	LRT	0.064248409851177
123	KC_AB1	CG12012	-0.283735577303343	0.478494817263142	1	pseudobulk	DESeq2	LRT	0.320122761849955
123	KC_AB1	CG12017	-2.45509113940241	0.186119518080447	1	pseudobulk	DESeq2	LRT	0.730208080656681
123	KC_AB1	CG12024	0.30900553861261	0.367930176176526	1	pseudobulk	DESeq2	LRT	0.434234591593958
123	KC_AB1	CG12025	-0.014094909055472	0.943990390140561	1	pseudobulk	DESeq2	LRT	0.025032426814398
123	KC_AB1	CG12038	0.134332788216641	0.576242604018847	1	pseudobulk	DESeq2	LRT	0.239394635666358
123	KC_AB1	CG12054	-0.165320005819604	0.242817652865886	1	pseudobulk	DESeq2	LRT	0.614719743199917
124	KC_AB1	CG12056	0.179764031830323	0.753091478879328	1	pseudobulk	DESeq2	LRT	0.123152266349823
124	KC_AB1	CG12065	-1.44925888589622	0.6315588144614	1	pseudobulk	DESeq2	LRT	0.199586199175701
124	KC_AB1	CG12071	-0.031015439322205	0.87286083418567	1	pseudobulk	DESeq2	LRT	0.059054993136425
124	KC_AB1	CG12081	0.461561064622796	0.353194386464094	1	pseudobulk	DESeq2	LRT	0.451986207549286
124	KC_AB1	CG12084	-0.10651498691197	0.737362303470998	1	pseudobulk	DESeq2	LRT	0.132319068808837
124	KC_AB1	CG12091	0.120176717358904	0.624265661265706	1	pseudobulk	DESeq2	LRT	0.204630553476482
124	KC_AB1	CG12093	-0.390083701908718	0.444917752120836	1	pseudobulk	DESeq2	LRT	0.351720265651568

124	KC_AB1	CG12096	0.139997663784917	0.817685568920626	1	pseudobulk	DESeq2	LRT	0.087413666909072
124	KC_AB1	CG12099	0.243048004365195	0.357526665287382	1	pseudobulk	DESeq2	LRT	0.446691561820097
124	KC_AB1	CG12104	-0.427757117395555	0.479529017042128	1	pseudobulk	DESeq2	LRT	0.319185107867691
125	KC_AB1	CG12106	0.385131047556683	0.497782318366251	1	pseudobulk	DESeq2	LRT	0.302960533947493
125	KC_AB1	CG12107	-0.134865306857035	0.727088886341438	1	pseudobulk	DESeq2	LRT	0.138412493559236
125	KC_AB1	CG12112	-1.11207965551997	0.461175844672008	1	pseudobulk	DESeq2	LRT	0.336133448125789
125	KC_AB1	CG12118	-0.178885167415196	0.734494034329105	1	pseudobulk	DESeq2	LRT	0.134011727264429
125	KC_AB1	CG12121	-0.23025218770534	0.535324911426426	1	pseudobulk	DESeq2	LRT	0.271382546186343
125	KC_AB1	CG12123	0.105704511131182	0.791089556718337	1	pseudobulk	DESeq2	LRT	0.10177434863004
125	KC_AB1	CG12128	0.327902282776446	0.623167522221479	1	pseudobulk	DESeq2	LRT	0.205395188980461
125	KC_AB1	CG12129	-0.431027090809741	0.435610581810924	1	pseudobulk	DESeq2	LRT	0.360901578947773
125	KC_AB1	CG12134	-0.29363273043693	0.780147224455856	1	pseudobulk	DESeq2	LRT	0.107823432264212
125	KC_AB1	CG12147	-0.82542030717083	1	1	pseudobulk	DESeq2	LRT	0
126	KC_AB1	CG12155	0.370058859042306	0.444659375534278	1	pseudobulk	DESeq2	LRT	0.351972546258751
126	KC_AB1	CG12158	0.145581871004682	0.920463899771264	1	pseudobulk	DESeq2	LRT	0.035993239683134
126	KC_AB1	CG12159	0.272252251457833	0.373200977470773	1	pseudobulk	DESeq2	LRT	0.428057227439989
126	KC_AB1	CG12163	-0.188856111747572	0.601401757511233	1	pseudobulk	DESeq2	LRT	0.220835307069696
126	KC_AB1	CG12170	0.342805608574608	0.675093124819581	1	pseudobulk	DESeq2	LRT	0.170636314864271
126	KC_AB1	CG12171	-0.421212447673593	0.83467750543181	1	pseudobulk	DESeq2	LRT	0.078481290582233
126	KC_AB1	CG12173	0.217811412739406	0.672363590655495	1	pseudobulk	DESeq2	LRT	0.172395812194986
126	KC_AB1	CG12177	-0.82542030717083	1	1	pseudobulk	DESeq2	LRT	0
126	KC_AB1	CG12179	-0.0409744222480786	0.90815137510853	1	pseudobulk	DESeq2	LRT	0.041841755118803
126	KC_AB1	CG1218	0.522701323966415	0.45922706063017	1	pseudobulk	DESeq2	LRT	0.337972528414759
127	KC_AB1	CG12182	0.588767048594354	0.579421979644761	1	pseudobulk	DESeq2	LRT	0.237005034404296
127	KC_AB1	CG12184	-0.850996402080018	0.900449987182352	1	pseudobulk	DESeq2	LRT	0.045540403770502
127	KC_AB1	CG12194	0.178125175930247	0.344812599302774	1	pseudobulk	DESeq2	LRT	0.462416873625455
127	KC_AB1	CG12204	-0.0195137790680613	0.918683573130569	1	pseudobulk	DESeq2	LRT	0.036834049122223
127	KC_AB1	CG12206	-0.262039810552863	0.76849159375171	1	pseudobulk	DESeq2	LRT	0.114360878726648
127	KC_AB1	CG12213	-0.177200024192702	0.593658856391078	1	pseudobulk	DESeq2	LRT	0.226463048869955
127	KC_AB1	CG12219	-0.109705442831419	0.833448007363681	1	pseudobulk	DESeq2	LRT	0.079121487400872
127	KC_AB1	CG12236	0.0131179356977599	0.969377764141317	1	pseudobulk	DESeq2	LRT	0.013506946467480
127	KC_AB1	CG12237	0.230684705437874	0.498163989856257	1	pseudobulk	DESeq2	LRT	0.302627668955163
127	KC_AB1	CG12239	-2.36817011682091	0.282654768599882	1	pseudobulk	DESeq2	LRT	0.548743683265438
128	KC_AB1	CG12241	-0.407522549805591	0.400640068450773	1	pseudobulk	DESeq2	LRT	0.397245618604928
128	KC_AB1	CG12253	0.196814287785866	0.693954223723429	1	pseudobulk	DESeq2	LRT	0.158669176576847
128	KC_AB1	CG12259	-0.115606175436573	0.836624429222431	1	pseudobulk	DESeq2	LRT	0.077469458280848
128	KC_AB1	CG12267	-0.207464747306644	0.850590514648359	1	pseudobulk	DESeq2	LRT	0.070279464625576
128	KC_AB1	CG1227	-2.83262282567345	0.0386987730674153	1	pseudobulk	DESeq2	LRT	1.41230280393453
128	KC_AB1	CG12279	-0.166723359550029	0.613041604440289	1	pseudobulk	DESeq2	LRT	0.212510050823006
128	KC_AB1	CG12288	-0.044907667890203	0.94749610301104	1	pseudobulk	DESeq2	LRT	0.023422567580767
128	KC_AB1	CG12299	-0.0099717712207056	0.916294122281696	1	pseudobulk	DESeq2	LRT	0.037965099278938
128	KC_AB1	CG12301	0.655524627786934	0.342576687048153	1	pseudobulk	DESeq2	LRT	0.46524219487558
128	KC_AB1	CG1231	-0.105548151517707	0.902457912113297	1	pseudobulk	DESeq2	LRT	0.044573043121109
129	KC_AB1	CG12316	-0.429476147071309	0.406437510542802	1	pseudobulk	DESeq2	LRT	0.391006217382627
129	KC_AB1	CG12320	-0.0288304943606188	0.96969981221402	1	pseudobulk	DESeq2	LRT	0.013362688492581
129	KC_AB1	CG12321	-0.198241035603336	0.695801517387233	1	pseudobulk	DESeq2	LRT	0.157514628488424
129	KC_AB1	CG12325	0.721721663397875	0.365907883737384	1	pseudobulk	DESeq2	LRT	0.436628233238844
129	KC_AB1	CG1233	0.0592427306280681	0.936839769440886	1	pseudobulk	DESeq2	LRT	0.028334681468866
129	KC_AB1	CG12333	0.071864371020429	0.842333157997159	1	pseudobulk	DESeq2	LRT	0.074516103200468
129	KC_AB1	CG12338	-0.927632369368702	0.44566997527006	1	pseudobulk	DESeq2	LRT	0.350986623292691
129	KC_AB1	CG1234	-0.0993825521363124	0.839192755086928	1	pseudobulk	DESeq2	LRT	0.076138274146422
129	KC_AB1	CG12341	-0.285333583182984	0.662063448306785	1	pseudobulk	DESeq2	LRT	0.179100388310884

129	KC_AB1	CG12343	0.0767925559425212	0.793508275401626	1	pseudobulk	DESeq2	LRT	0.100448539681003
130	KC_AB1	CG12347	-0.619148458022618	0.879843167001942	1	pseudobulk	DESeq2	LRT	0.055594734413205
130	KC_AB1	CG12355	-0.545650564597275	0.283936031158966	1	pseudobulk	DESeq2	LRT	0.546779492501939
130	KC_AB1	CG1236	-0.230607253606254	0.807424751547873	1	pseudobulk	DESeq2	LRT	0.092897941455434
130	KC_AB1	CG12362	1.08899820779554	0.878177167143508	1	pseudobulk	DESeq2	LRT	0.056417858865076
130	KC_AB1	CG12375	-0.0498576768931343	0.892130816093304	1	pseudobulk	DESeq2	LRT	0.049571458917660
130	KC_AB1	CG12379	-0.642125190206587	0.469930851893846	1	pseudobulk	DESeq2	LRT	0.327966041745691
130	KC_AB1	CG12384	0.134757588736202	0.507566079813566	1	pseudobulk	DESeq2	LRT	0.294507409118751
130	KC_AB1	CG1239	0.139239088096501	0.847647710534031	1	pseudobulk	DESeq2	LRT	0.071784606658606
130	KC_AB1	CG12391	-0.0063610259257708	0.936647781760692	1	pseudobulk	DESeq2	LRT	0.028423691070600
130	KC_AB1	CG12402	0.0492600517277352	0.887214804180533	1	pseudobulk	DESeq2	LRT	0.051971220104755
131	KC_AB1	CG12420	0.113316967542732	1	1	pseudobulk	DESeq2	LRT	0
131	KC_AB1	CG12470	1.88952324269138	0.456767015650974	1	pseudobulk	DESeq2	LRT	0.340305265178099
131	KC_AB1	CG12483	1.08899820779554	0.697631521651407	1	pseudobulk	DESeq2	LRT	0.156373904552
131	KC_AB1	CG12499	1.44874597285247	0.219782082011849	1	pseudobulk	DESeq2	LRT	0.658007716828713
131	KC_AB1	CG12502	-0.0867294161059657	0.87315087963799	1	pseudobulk	DESeq2	LRT	0.058910704138127
131	KC_AB1	CG12531	-0.238882379214309	0.0994722387137451	1	pseudobulk	DESeq2	LRT	1.00229810775361
131	KC_AB1	CG12535	-0.0044446694628382	0.953887397252032	1	pseudobulk	DESeq2	LRT	0.020502889067778
131	KC_AB1	CG12539	-0.507979848677457	0.940470850565906	1	pseudobulk	DESeq2	LRT	0.026654660650085
131	KC_AB1	CG12547	-0.0795932767431324	0.753335537260007	1	pseudobulk	DESeq2	LRT	0.123011545016784
131	KC_AB1	CG12560	0.852677560647305	0.301855501564006	1	pseudobulk	DESeq2	LRT	0.520200904365366
132	KC_AB1	CG12567	0.0029466513091699	0.87341043285108	1	pseudobulk	DESeq2	LRT	0.058781624754278
132	KC_AB1	CG12576	4.88016105397078E-0	0.944086100578743	1	pseudobulk	DESeq2	LRT	0.024988396272836
132	KC_AB1	CG12594	1.95275942187961	0.438420007953907	1	pseudobulk	DESeq2	LRT	0.358109634392079
132	KC_AB1	CG12605	0.16265830446458	0.26781972684771	1	pseudobulk	DESeq2	LRT	0.572157437235968
132	KC_AB1	CG12607	0.10745091433163	0.925708810521252	1	pseudobulk	DESeq2	LRT	0.033525602812194
132	KC_AB1	CG12608	0.230057833930904	0.7352832870381	1	pseudobulk	DESeq2	LRT	0.133545305413571
132	KC_AB1	CG12617	3.17000212185395	0.408004972110056	1	pseudobulk	DESeq2	LRT	0.389334544393445
132	KC_AB1	CG12640	-0.618422766585323	0.856874262644257	1	pseudobulk	DESeq2	LRT	0.067082901584478
132	KC_AB1	CG12645	1.08899820779554	0.697631521651407	1	pseudobulk	DESeq2	LRT	0.156373904552
132	KC_AB1	CG1265	0.28762251055877	0.290616778909053	1	pseudobulk	DESeq2	LRT	0.536679315099075
133	KC_AB1	CG12655	-0.244584508994539	0.90053809697704	1	pseudobulk	DESeq2	LRT	0.045497909766236
133	KC_AB1	CG12659	0.0113531386789511	0.974161932116289	1	pseudobulk	DESeq2	LRT	0.011368845606845
133	KC_AB1	CG12672	-0.222918852911873	0.751397899911976	1	pseudobulk	DESeq2	LRT	0.124130023084073
133	KC_AB1	CG12674	-0.82542030717083	1	1	pseudobulk	DESeq2	LRT	0
133	KC_AB1	CG12693	1.40154326353935	0.370592109227183	1	pseudobulk	DESeq2	LRT	0.431103832064006
133	KC_AB1	CG12698	-1.56855358782635	0.573026303465967	1	pseudobulk	DESeq2	LRT	0.241825442277049
133	KC_AB1	CG1271	-0.886674045312369	0.361785885246312	1	pseudobulk	DESeq2	LRT	0.44154838075762
133	KC_AB1	CG12713	-0.781691516493505	0.474616553742394	1	pseudobulk	DESeq2	LRT	0.323657118470972
133	KC_AB1	CG12715	-1.05813155791189	0.687053260159203	1	pseudobulk	DESeq2	LRT	0.163009595259685
133	KC_AB1	CG12717	-0.030846788948437	0.892569938386582	1	pseudobulk	DESeq2	LRT	0.049357744218183
134	KC_AB1	CG12728	0.365472548277422	0.483448104832074	1	pseudobulk	DESeq2	LRT	0.31565013790915
134	KC_AB1	CG1273	1.08899820779554	0.878177167143508	1	pseudobulk	DESeq2	LRT	0.056417858865076
134	KC_AB1	CG12730	0.138886974760333	0.73786589602318	1	pseudobulk	DESeq2	LRT	0.132022562173307
134	KC_AB1	CG12744	1.08899820779554	0.670846322504933	1	pseudobulk	DESeq2	LRT	0.173376956629912
134	KC_AB1	CG12746	0.190361318154592	0.798747050813758	1	pseudobulk	DESeq2	LRT	0.09759073236115
134	KC_AB1	CG1275	-0.520244089045079	0.029083827323908	1	pseudobulk	DESeq2	LRT	1.53634844250763
134	KC_AB1	CG12768	0.448405137888618	0.729228923208129	1	pseudobulk	DESeq2	LRT	0.137136114371723
134	KC_AB1	CG12769	0.241461252806008	0.352831494001388	1	pseudobulk	DESeq2	LRT	0.452432656361765
134	KC_AB1	CG12773	-0.574774846840595	0.388084048894718	1	pseudobulk	DESeq2	LRT	0.411074207348543
134	KC_AB1	CG12795	-0.468227939665563	0.312456169444113	1	pseudobulk	DESeq2	LRT	0.505210895771472
135	KC_AB1	CG12814	-3.10449612243424	0.145535010276424	1	pseudobulk	DESeq2	LRT	0.837032519111992

135	KC_AB1	CG12817	-0.326567269668703	0.438688239613075	1	pseudobulk	DESeq2	LRT	0.357844008037741
135	KC_AB1	CG12818	0.0290889410298097	0.95723046184461	1	pseudobulk	DESeq2	LRT	0.018983489330934
135	KC_AB1	CG12822	0.0606449446111502	0.821681083557831	1	pseudobulk	DESeq2	LRT	0.085296711080429
135	KC_AB1	CG12825	-1.62582030451705	0.456245907193616	1	pseudobulk	DESeq2	LRT	0.340801018406593
135	KC_AB1	CG12836	2.81802837506375	0.354271613205394	1	pseudobulk	DESeq2	LRT	0.450663645076239
135	KC_AB1	CG12848	-0.130371517940179	0.738311636168902	1	pseudobulk	DESeq2	LRT	0.131760286820497
135	KC_AB1	CG12851	-0.131341645326867	0.654466564871788	1	pseudobulk	DESeq2	LRT	0.184112535610691
135	KC_AB1	CG12857	1.08899820779554	0.670846322504933	1	pseudobulk	DESeq2	LRT	0.173376956629912
135	KC_AB1	CG12863	0.571983172542909	0.650023990775532	1	pseudobulk	DESeq2	LRT	0.187070614327672
136	KC_AB1	CG12866	-0.850996402080018	0.900449987182352	1	pseudobulk	DESeq2	LRT	0.045540403770502
136	KC_AB1	CG12868	-0.113110200004956	0.794263862051838	1	pseudobulk	DESeq2	LRT	0.100035196821764
136	KC_AB1	CG12877	0.459815438165891	0.387225673281685	1	pseudobulk	DESeq2	LRT	0.41203585643869
136	KC_AB1	CG12883	-0.127188597785637	0.887882607949037	1	pseudobulk	DESeq2	LRT	0.051644450989600
136	KC_AB1	CG12885	0.798344842149955	0.62645177897689	1	pseudobulk	DESeq2	LRT	0.203112353129905
136	KC_AB1	CG12895	0.0517997641310587	0.879251193624545	1	pseudobulk	DESeq2	LRT	0.055887033435584
136	KC_AB1	CG12909	-0.486968979396144	0.703463937728129	1	pseudobulk	DESeq2	LRT	0.15275816126706
136	KC_AB1	CG12910	0.126155685988783	0.783901151888258	1	pseudobulk	DESeq2	LRT	0.105738697386988
136	KC_AB1	CG12913	0.312111829085283	0.483307863247588	1	pseudobulk	DESeq2	LRT	0.31577613898639
136	KC_AB1	CG12923	-2.20800634893943	0.293235984441374	1	pseudobulk	DESeq2	LRT	0.532782736331732
137	KC_AB1	CG12926	1.08899820779554	0.878177167143508	1	pseudobulk	DESeq2	LRT	0.056417858865076
137	KC_AB1	CG12929	-0.003416028077954	0.90025556888022	1	pseudobulk	DESeq2	LRT	0.045634183451295
137	KC_AB1	CG12935	-0.246957516679892	0.72926681844111	1	pseudobulk	DESeq2	LRT	0.13711354633795
137	KC_AB1	CG12942	0.360265750402903	0.715990187487174	1	pseudobulk	DESeq2	LRT	0.145092929576747
137	KC_AB1	CG12945	0.243114092015119	0.391610151272748	1	pseudobulk	DESeq2	LRT	0.407146058991989
137	KC_AB1	CG12947	-0.592452038069654	0.78094862978074	1	pseudobulk	DESeq2	LRT	0.107377532750746
137	KC_AB1	CG12948	0.470842688065501	0.553818797032582	1	pseudobulk	DESeq2	LRT	0.256632308054171
137	KC_AB1	CG12970	-0.850996402080018	0.900449987182352	1	pseudobulk	DESeq2	LRT	0.045540403770502
137	KC_AB1	CG12975	0.0428758006422085	0.89492343772549	1	pseudobulk	DESeq2	LRT	0.048214117751823
137	KC_AB1	CG12990	-0.850996402080018	0.900449987182352	1	pseudobulk	DESeq2	LRT	0.045540403770502
138	KC_AB1	CG12991	-0.020694523070353	0.934109608510002	1	pseudobulk	DESeq2	LRT	0.029602160624167
138	KC_AB1	CG12992	-0.190833323967761	0.857013817137965	1	pseudobulk	DESeq2	LRT	0.067012176141678
138	KC_AB1	CG12994	-1.08057839120766	0.439965504157848	1	pseudobulk	DESeq2	LRT	0.356581373380159
138	KC_AB1	CG13001	0.32879000790662	0.43943180037469	1	pseudobulk	DESeq2	LRT	0.357108517676051
138	KC_AB1	CG13005	0.0359537155990283	0.939798084432024	1	pseudobulk	DESeq2	LRT	0.026965444524228
138	KC_AB1	CG13014	0.521919449909147	0.563573785907153	1	pseudobulk	DESeq2	LRT	0.249049215883193
138	KC_AB1	CG13016	-0.072841738692699	0.908884047165953	1	pseudobulk	DESeq2	LRT	0.041491519295189
138	KC_AB1	CG13018	0.131469150981278	0.786070696632889	1	pseudobulk	DESeq2	LRT	0.104538393175807
138	KC_AB1	CG13025	-0.627379389712276	0.443687609360203	1	pseudobulk	DESeq2	LRT	0.352922699436768
138	KC_AB1	CG13029	-0.82542030717083	1	1	pseudobulk	DESeq2	LRT	0
139	KC_AB1	CG13054	-0.404955690647059	0.769052205834085	1	pseudobulk	DESeq2	LRT	0.114044177836931
139	KC_AB1	CG13055	-0.188831243696652	0.664385761717456	1	pseudobulk	DESeq2	LRT	0.17757968337567
139	KC_AB1	CG1307	-0.420261886763685	0.283250608268639	1	pseudobulk	DESeq2	LRT	0.547829148834628
139	KC_AB1	CG13085	0.463657374761214	0.815431268768723	1	pseudobulk	DESeq2	LRT	0.088612638979839
139	KC_AB1	CG13088	0.536822480739811	0.77353335804325	1	pseudobulk	DESeq2	LRT	0.111520952938458
139	KC_AB1	CG1309	0.124205650159507	0.800073683056815	1	pseudobulk	DESeq2	LRT	0.096870014668797
139	KC_AB1	CG13096	-0.390485781927721	0.572219397186235	1	pseudobulk	DESeq2	LRT	0.242437424496216
139	KC_AB1	CG13097	-0.557278449388791	0.455691229624378	1	pseudobulk	DESeq2	LRT	0.341329329904254
139	KC_AB1	CG13108	0.0367344660891334	0.907902450129777	1	pseudobulk	DESeq2	LRT	0.041960811882270
139	KC_AB1	CG13116	1.08899820779554	0.670846322504933	1	pseudobulk	DESeq2	LRT	0.173376956629912
140	KC_AB1	CG13123	-0.629234269374762	0.491122255925444	1	pseudobulk	DESeq2	LRT	0.308810384730948
140	KC_AB1	CG13124	0.276837720658869	0.889347567833129	1	pseudobulk	DESeq2	LRT	0.050928478296095
140	KC_AB1	CG13126	1.10797237014336	0.31163120974516	1	pseudobulk	DESeq2	LRT	0.506359054396915

140	KC_AB1	CG13135	0.111831901354254	1	1	pseudobulk	DESeq2	LRT	0
140	KC_AB1	CG13137	-0.850996402080018	0.900449987182352	1	pseudobulk	DESeq2	LRT	0.045540403770502
140	KC_AB1	CG1314	0.10745091433163	0.925708810521252	1	pseudobulk	DESeq2	LRT	0.033525602812194
140	KC_AB1	CG13144	1.08899820779554	0.878177167143508	1	pseudobulk	DESeq2	LRT	0.056417858865076
140	KC_AB1	CG13148	0.0770121649025143	0.866083609193095	1	pseudobulk	DESeq2	LRT	0.062440180432466
140	KC_AB1	CG13151	0.807599266171632	0.453609367775709	1	pseudobulk	DESeq2	LRT	0.343317985130827
140	KC_AB1	CG13154	1.08899820779554	0.878177167143508	1	pseudobulk	DESeq2	LRT	0.056417858865076
141	KC_AB1	CG1316	0.203663828142228	0.245570643410227	1	pseudobulk	DESeq2	LRT	0.609823551891519
141	KC_AB1	CG13168	0.942080999092271	0.681532122211136	1	pseudobulk	DESeq2	LRT	0.166513670026992
141	KC_AB1	CG1317	-0.111306099501747	0.728063912836422	1	pseudobulk	DESeq2	LRT	0.137830494624015
141	KC_AB1	CG13185	1.16216269187709	0.152637589248418	1	pseudobulk	DESeq2	LRT	0.816338501814358
141	KC_AB1	CG13192	0.949280821888685	0.159737735271579	1	pseudobulk	DESeq2	LRT	0.796592477196829
141	KC_AB1	CG13197	-0.531427725733317	0.217874480612461	1	pseudobulk	DESeq2	LRT	0.661793635188484
141	KC_AB1	CG13204	0.169617025214784	0.500028094049544	1	pseudobulk	DESeq2	LRT	0.301005594168129
141	KC_AB1	CG13220	0.0770198494876843	0.846136870556983	1	pseudobulk	DESeq2	LRT	0.072559380081882
141	KC_AB1	CG13229	1.88952324269138	0.456767015650974	1	pseudobulk	DESeq2	LRT	0.340305265178099
141	KC_AB1	CG13248	-0.231574253785655	0.392435894282826	1	pseudobulk	DESeq2	LRT	0.406231276582535
142	KC_AB1	CG13255	1.08899820779554	0.878177167143508	1	pseudobulk	DESeq2	LRT	0.056417858865076
142	KC_AB1	CG13258	-0.850996402080018	0.900449987182352	1	pseudobulk	DESeq2	LRT	0.045540403770502
142	KC_AB1	CG13284	0.895342957975216	0.386484597100939	1	pseudobulk	DESeq2	LRT	0.41286780970779
142	KC_AB1	CG13293	0.267025552086647	0.156462565331699	1	pseudobulk	DESeq2	LRT	0.805589553418462
142	KC_AB1	CG13295	-0.412281843870963	0.72949951413208	1	pseudobulk	DESeq2	LRT	0.136974993023205
142	KC_AB1	CG13300	-0.496737806053583	0.737014967771613	1	pseudobulk	DESeq2	LRT	0.132523692121124
142	KC_AB1	CG13314	1.08899820779554	0.878177167143508	1	pseudobulk	DESeq2	LRT	0.056417858865076
142	KC_AB1	CG13319	-0.678566913506527	0.494294698909323	1	pseudobulk	DESeq2	LRT	0.306014047129299
142	KC_AB1	CG13325	-0.82542030717083	1	1	pseudobulk	DESeq2	LRT	0
142	KC_AB1	CG13334	-0.82542030717083	1	1	pseudobulk	DESeq2	LRT	0
143	KC_AB1	CG13339	0.0377110548191889	0.923144415033908	1	pseudobulk	DESeq2	LRT	0.034730353420156
143	KC_AB1	CG13344	-0.20861381460189	0.561636843018249	1	pseudobulk	DESeq2	LRT	0.250544410503087
143	KC_AB1	CG13364	-0.0536520029762257	0.770185422618791	1	pseudobulk	DESeq2	LRT	0.113404705573235
143	KC_AB1	CG13365	-0.794396620861095	0.0435717621597043	1	pseudobulk	DESeq2	LRT	1.36079487566764
143	KC_AB1	CG13366	0.390642824366647	0.248204950927255	1	pseudobulk	DESeq2	LRT	0.605189559908432
143	KC_AB1	CG13367	0.0573048332618858	0.906054772061058	1	pseudobulk	DESeq2	LRT	0.042845547924861
143	KC_AB1	CG13369	0.119242627734003	0.81877228526117	1	pseudobulk	DESeq2	LRT	0.086836866256050
143	KC_AB1	CG13375	-0.165630912773981	0.583086569545507	1	pseudobulk	DESeq2	LRT	0.234266961733559
143	KC_AB1	CG13376	0.99339361936951	0.567742685631369	1	pseudobulk	DESeq2	LRT	0.245848452194792
143	KC_AB1	CG13377	-1.68123190487689	0.343686575071797	1	pseudobulk	DESeq2	LRT	0.463837431744382
144	KC_AB1	CG13384	-0.131354725184235	0.685679266720558	1	pseudobulk	DESeq2	LRT	0.163878982348811
144	KC_AB1	CG13390	-0.0838997233913553	0.92569871520742	1	pseudobulk	DESeq2	LRT	0.033530339034802
144	KC_AB1	CG13392	-0.151541014261095	0.859552482810675	1	pseudobulk	DESeq2	LRT	0.065727600885938
144	KC_AB1	CG13398	-0.280436554417737	0.664928578378854	1	pseudobulk	DESeq2	LRT	0.177225000834871
144	KC_AB1	CG13404	-0.0785051350603707	0.785886061667577	1	pseudobulk	DESeq2	LRT	0.104640413726369
144	KC_AB1	CG13405	1.08899820779554	0.697631521651407	1	pseudobulk	DESeq2	LRT	0.156373904552
144	KC_AB1	CG13408	0.187499809431982	0.910303214736121	1	pseudobulk	DESeq2	LRT	0.040813923554985
144	KC_AB1	CG13409	-0.0063110116708083	0.894049771054867	1	pseudobulk	DESeq2	LRT	0.048638303695331
144	KC_AB1	CG13436	-1.46189868005265	0.463922482191506	1	pseudobulk	DESeq2	LRT	0.33355458058509
144	KC_AB1	CG1344	0.222500655741248	0.576535767195457	1	pseudobulk	DESeq2	LRT	0.239173744720062
145	KC_AB1	CG13445	-0.82542030717083	1	1	pseudobulk	DESeq2	LRT	0
145	KC_AB1	CG13482	-0.151123574592873	0.90568935937435	1	pseudobulk	DESeq2	LRT	0.043020734600344
145	KC_AB1	CG13484	-0.160115728216072	0.730696925339682	1	pseudobulk	DESeq2	LRT	0.136262720095345
145	KC_AB1	CG13502	1.88952324269138	0.456767015650974	1	pseudobulk	DESeq2	LRT	0.340305265178099
145	KC_AB1	CG13506	0.0007996769086529	0.981507755816923	1	pseudobulk	DESeq2	LRT	0.008106264280802

145	KC_AB1	CG13526	1.51625991547077	0.440450719950563	1	pseudobulk	DESeq2	LRT	0.356102675789467
145	KC_AB1	CG13531	-0.223549813235181	0.798265244649707	1	pseudobulk	DESeq2	LRT	0.097852778890581
145	KC_AB1	CG13532	-0.159778718764433	0.64545648704114	1	pseudobulk	DESeq2	LRT	0.190133030043398
145	KC_AB1	CG1354	-0.350723575708459	0.329526197415242	1	pseudobulk	DESeq2	LRT	0.482110053177361
145	KC_AB1	CG13551	-0.0179729409461815	0.900938332546777	1	pseudobulk	DESeq2	LRT	0.045304934604964
146	KC_AB1	CG13578	-0.49078105603539	0.243718143411325	1	pseudobulk	DESeq2	LRT	0.613112138890288
146	KC_AB1	CG13579	-1.99275908811736	0.412167844949191	1	pseudobulk	DESeq2	LRT	0.384925892483647
146	KC_AB1	CG1358	0.400453905463738	0.232415886560448	1	pseudobulk	DESeq2	LRT	0.633734189492846
146	KC_AB1	CG13585	-0.0647794299779333	0.76425212591222	1	pseudobulk	DESeq2	LRT	0.116763344527733
146	KC_AB1	CG13594	1.08899820779554	0.670846322504933	1	pseudobulk	DESeq2	LRT	0.173376956629912
146	KC_AB1	CG1360	0.330646958073183	0.742477235301799	1	pseudobulk	DESeq2	LRT	0.129316857477919
146	KC_AB1	CG13601	-0.581323368870613	0.162029583881311	1	pseudobulk	DESeq2	LRT	0.790405683337554
146	KC_AB1	CG13603	-0.308383285043586	0.352788712687989	1	pseudobulk	DESeq2	LRT	0.452485318369413
146	KC_AB1	CG13604	-0.529908456833418	0.579492041970734	1	pseudobulk	DESeq2	LRT	0.236952523724419
146	KC_AB1	CG13605	0.555965447805693	0.30525039531715	1	pseudobulk	DESeq2	LRT	0.515343764945912
147	KC_AB1	CG13609	0.750520698123525	0.478174377497079	1	pseudobulk	DESeq2	LRT	0.320413698838729
147	KC_AB1	CG13623	0.303945251481849	0.45268043573993	1	pseudobulk	DESeq2	LRT	0.344208274742201
147	KC_AB1	CG13625	0.0910997858981867	0.862037306006235	1	pseudobulk	DESeq2	LRT	0.064473938998876
147	KC_AB1	CG13630	0.733246346972593	0.214506276422333	1	pseudobulk	DESeq2	LRT	0.668559995900205
147	KC_AB1	CG13631	1.14247969565044	0.639889789651426	1	pseudobulk	DESeq2	LRT	0.19389481955965
147	KC_AB1	CG13654	0.478460027824876	0.278118432471812	1	pseudobulk	DESeq2	LRT	0.555770227045521
147	KC_AB1	CG13658	2.1559833397943	0.381743505411375	1	pseudobulk	DESeq2	LRT	0.418228342845202
147	KC_AB1	CG13659	-0.850996402080018	0.900449987182352	1	pseudobulk	DESeq2	LRT	0.045540403770502
147	KC_AB1	CG13663	0.934974959654698	0.103485999254297	1	pseudobulk	DESeq2	LRT	0.985118402457289
147	KC_AB1	CG13667	3.05562915577017	0.145714142161275	1	pseudobulk	DESeq2	LRT	0.836498296105453
148	KC_AB1	CG13671	-0.867276514825298	0.386085810784715	1	pseudobulk	DESeq2	LRT	0.413316159036754
148	KC_AB1	CG13679	-0.968336042648622	0.663512636338631	1	pseudobulk	DESeq2	LRT	0.178150801752249
148	KC_AB1	CG13685	-0.403387061979666	0.845209033867155	1	pseudobulk	DESeq2	LRT	0.073035869715218
148	KC_AB1	CG13689	-0.207562985823249	0.763798934132014	1	pseudobulk	DESeq2	LRT	0.117020952012593
148	KC_AB1	CG13693	-2.08238890537319	0.386092553331648	1	pseudobulk	DESeq2	LRT	0.413308574646766
148	KC_AB1	CG13698	1.08899820779554	0.878177167143508	1	pseudobulk	DESeq2	LRT	0.056417858865076
148	KC_AB1	CG1371	0.139935205535189	0.778282572686131	1	pseudobulk	DESeq2	LRT	0.108862694170251
148	KC_AB1	CG13737	-1.33515844615533	0.121008710632539	1	pseudobulk	DESeq2	LRT	0.917183366514243
148	KC_AB1	CG13738	-1.44925888589622	0.6315588144614	1	pseudobulk	DESeq2	LRT	0.199586199175701
148	KC_AB1	CG13739	0.252889332931783	0.0066050838921193	1	pseudobulk	DESeq2	LRT	2.18012166197782
149	KC_AB1	CG13741	0.364225774475942	0.834910345031795	1	pseudobulk	DESeq2	LRT	0.07836015774808
149	KC_AB1	CG13743	-1.38499545253713	0.290465311097172	1	pseudobulk	DESeq2	LRT	0.536905725919532
149	KC_AB1	CG13751	0.213246802423401	0.537517287503963	1	pseudobulk	DESeq2	LRT	0.269607563511255
149	KC_AB1	CG13760	-0.255908974790237	0.595366117072759	1	pseudobulk	DESeq2	LRT	0.225215885152935
149	KC_AB1	CG13766	0.523516370771059	0.261487471055853	1	pseudobulk	DESeq2	LRT	0.582549115139581
149	KC_AB1	CG13773	-0.923790808404168	0.254869274734593	1	pseudobulk	DESeq2	LRT	0.593682516894434
149	KC_AB1	CG13775	-0.511556918198203	0.803093880303542	1	pseudobulk	DESeq2	LRT	0.095233683470231
149	KC_AB1	CG13784	-0.189362354964081	0.24427585165758	1	pseudobulk	DESeq2	LRT	0.612119463891169
149	KC_AB1	CG13796	-0.850932661185203	0.660736244265418	1	pseudobulk	DESeq2	LRT	0.179971869591347
149	KC_AB1	CG13807	-0.757394755692634	0.197161501177167	1	pseudobulk	DESeq2	LRT	0.705177883809675
150	KC_AB1	CG13827	0.794337344688767	0.490125219161175	1	pseudobulk	DESeq2	LRT	0.309692950495425
150	KC_AB1	CG13841	-1.56855358782635	0.573026303465967	1	pseudobulk	DESeq2	LRT	0.241825442277049
150	KC_AB1	CG13843	-1.44925888589622	0.6315588144614	1	pseudobulk	DESeq2	LRT	0.199586199175701
150	KC_AB1	CG13850	0.164590926730249	0.799528112240817	1	pseudobulk	DESeq2	LRT	0.097166261403152
150	KC_AB1	CG13868	0.0669324963939754	0.637949552232993	1	pseudobulk	DESeq2	LRT	0.195213663054825
150	KC_AB1	CG13869	-0.82542030717083	1	1	pseudobulk	DESeq2	LRT	0
150	KC_AB1	CG13870	1.45141434898336	0.518035873737501	1	pseudobulk	DESeq2	LRT	0.285640164527385

150	KC_AB1	CG13871	1.08899820779554	0.878177167143508	1	pseudobulk	DESeq2	LRT	0.056417858865076
150	KC_AB1	CG13875	0.114184054362142	1	1	pseudobulk	DESeq2	LRT	0
150	KC_AB1	CG13876	0.120782867578953	0.880791857892742	1	pseudobulk	DESeq2	LRT	0.055126708669134
151	KC_AB1	CG13887	-0.146129806983753	0.613066810157504	1	pseudobulk	DESeq2	LRT	0.212492194809997
151	KC_AB1	CG13893	-0.158008609400026	0.766319068485738	1	pseudobulk	DESeq2	LRT	0.115590367663854
151	KC_AB1	CG13895	-0.930489713393763	0.369567582125547	1	pseudobulk	DESeq2	LRT	0.432306131395278
151	KC_AB1	CG13896	0.31699699808461	0.772447690352406	1	pseudobulk	DESeq2	LRT	0.112130921042933
151	KC_AB1	CG13898	0.11269840515639	0.977007463492936	1	pseudobulk	DESeq2	LRT	0.010102118633921
151	KC_AB1	CG13901	-0.171337215423465	0.744829350283916	1	pseudobulk	DESeq2	LRT	0.127943218150237
151	KC_AB1	CG13907	-0.0521759615739863	0.893268142752859	1	pseudobulk	DESeq2	LRT	0.049018154283164
151	KC_AB1	CG13912	-0.82542030717083	1	1	pseudobulk	DESeq2	LRT	0
151	KC_AB1	CG13917	0.023204691487984	0.839555779291847	1	pseudobulk	DESeq2	LRT	0.075950444452787
151	KC_AB1	CG13919	0.853839657374096	0.162811839883802	1	pseudobulk	DESeq2	LRT	0.788314015852034
152	KC_AB1	CG13920	-0.0422204948251791	0.833188130853201	1	pseudobulk	DESeq2	LRT	0.079256925404170
152	KC_AB1	CG13921	0.130633887668985	0.520311513199403	1	pseudobulk	DESeq2	LRT	0.283736564141154
152	KC_AB1	CG13924	1.11665486949011	0.786340792605537	1	pseudobulk	DESeq2	LRT	0.104389194073077
152	KC_AB1	CG13926	-0.351941712777927	0.393038968473773	1	pseudobulk	DESeq2	LRT	0.405564388672584
152	KC_AB1	CG13928	0.116808402868854	0.362042948555313	1	pseudobulk	DESeq2	LRT	0.441239906775709
152	KC_AB1	CG13933	0.493733755794413	0.27802577687002	1	pseudobulk	DESeq2	LRT	0.555914937055162
152	KC_AB1	CG13937	0.394145169184451	0.405017667730459	1	pseudobulk	DESeq2	LRT	0.392526031524869
152	KC_AB1	CG13954	0.182792491011096	0.260463280644195	1	pseudobulk	DESeq2	LRT	0.584253493622169
152	KC_AB1	CG13962	-1.59780289500814	0.36188740655407	1	pseudobulk	DESeq2	LRT	0.441426529793811
152	KC_AB1	CG13982	-1.12995011128703	0.207608750935881	1	pseudobulk	DESeq2	LRT	0.682754344450429
153	KC_AB1	CG13994	-0.0590842379069055	0.904149612641278	1	pseudobulk	DESeq2	LRT	0.043759699426914
153	KC_AB1	CG13995	-1.05181095576566	0.374374888858446	1	pseudobulk	DESeq2	LRT	0.426693289196618
153	KC_AB1	CG13999	0.130222828994215	0.635821786525893	1	pseudobulk	DESeq2	LRT	0.196664595002767
153	KC_AB1	CG14005	0.0536667412918433	0.906481240153232	1	pseudobulk	DESeq2	LRT	0.042641179303979
153	KC_AB1	CG14006	-0.82542030717083	1	1	pseudobulk	DESeq2	LRT	0
153	KC_AB1	CG14015	0.014913416456804	0.912555705482225	1	pseudobulk	DESeq2	LRT	0.039740615260388
153	KC_AB1	CG14024	-0.311544740500836	0.3419178775285	1	pseudobulk	DESeq2	LRT	0.466078191075307
153	KC_AB1	CG14043	-0.361113598539116	0.491981183516234	1	pseudobulk	DESeq2	LRT	0.308051507093085
153	KC_AB1	CG14044	0.464646188450318	0.753023724259594	1	pseudobulk	DESeq2	LRT	0.123191340993559
153	KC_AB1	CG14050	-1.96719661029409	0.414177861019091	1	pseudobulk	DESeq2	LRT	0.382813119093482
154	KC_AB1	CG14052	0.11400025262841	1	1	pseudobulk	DESeq2	LRT	0
154	KC_AB1	CG14057	0.141860390436725	0.850553504488659	1	pseudobulk	DESeq2	LRT	0.070298361683030
154	KC_AB1	CG1407	-0.299050308005242	0.53434438103287	1	pseudobulk	DESeq2	LRT	0.272178753129061
154	KC_AB1	CG14073	0.0526887074873319	0.783086821980155	1	pseudobulk	DESeq2	LRT	0.106190084408806
154	KC_AB1	CG14074	0.193677146927987	0.703801329729324	1	pseudobulk	DESeq2	LRT	0.152549916964364
154	KC_AB1	CG14082	-0.637928827034845	0.363820123803194	1	pseudobulk	DESeq2	LRT	0.439113282691525
154	KC_AB1	CG14100	0.185587087530302	0.774201999281851	1	pseudobulk	DESeq2	LRT	0.111145711503302
154	KC_AB1	CG14102	-0.0093570960596061	0.989975720097911	1	pseudobulk	DESeq2	LRT	0.004375456671949
154	KC_AB1	CG14104	0.226330843613473	0.760618143858307	1	pseudobulk	DESeq2	LRT	0.118833319098562
154	KC_AB1	CG14105	-0.968336042648622	0.663512636338631	1	pseudobulk	DESeq2	LRT	0.178150801752249
155	KC_AB1	CG14117	1.08899820779554	0.697631521651407	1	pseudobulk	DESeq2	LRT	0.156373904552
155	KC_AB1	CG14118	-1.44925888589622	0.6315588144614	1	pseudobulk	DESeq2	LRT	0.199586199175701
155	KC_AB1	CG14130	-0.0940980598833644	0.850709749723541	1	pseudobulk	DESeq2	LRT	0.070218589850710
155	KC_AB1	CG14135	-0.673572337088939	0.250785410890732	1	pseudobulk	DESeq2	LRT	0.600697731613014
155	KC_AB1	CG14137	0.111831901354254	1	1	pseudobulk	DESeq2	LRT	0
155	KC_AB1	CG14141	2.92221825271647	0.170141571531611	1	pseudobulk	DESeq2	LRT	0.769189560103148
155	KC_AB1	CG14142	0.0454176375469165	0.956078680358933	1	pseudobulk	DESeq2	LRT	0.019506366050621
155	KC_AB1	CG14153	-1.89854133735591	0.478664405825708	1	pseudobulk	DESeq2	LRT	0.319968866080244
155	KC_AB1	CG1416	-0.108449730411094	0.699802998659744	1	pseudobulk	DESeq2	LRT	0.155024200894853

155	KC_AB1	CG14174	0.347991103818142	0.783608895530203	1	pseudobulk	DESeq2	LRT	0.105900642537239
156	KC_AB1	CG1418	0.086086711254976	0.877461546816262	1	pseudobulk	DESeq2	LRT	0.056771906624448
156	KC_AB1	CG14182	0.0602420513380249	0.901756985186099	1	pseudobulk	DESeq2	LRT	0.044910484866512
156	KC_AB1	CG14183	0.276789356640319	0.790297254115524	1	pseudobulk	DESeq2	LRT	0.102209527012499
156	KC_AB1	CG14184	-0.376772682444039	0.299928516965336	1	pseudobulk	DESeq2	LRT	0.522982239902691
156	KC_AB1	CG14185	1.08899820779554	0.697631521651407	1	pseudobulk	DESeq2	LRT	0.156373904552
156	KC_AB1	CG14186	0.187624234579633	0.215370190951457	1	pseudobulk	DESeq2	LRT	0.666814406893019
156	KC_AB1	CG14190	0.135879393043758	1	1	pseudobulk	DESeq2	LRT	0
156	KC_AB1	CG14194	-0.383945098404902	0.440298131620473	1	pseudobulk	DESeq2	LRT	0.356253157439953
156	KC_AB1	CG14200	0.185596434139494	0.518394851019902	1	pseudobulk	DESeq2	LRT	0.285339320765452
156	KC_AB1	CG14207	0.0677152006877068	0.799514914030623	1	pseudobulk	DESeq2	LRT	0.097173430578420
157	KC_AB1	CG14210	-0.170849920173898	0.794150567526228	1	pseudobulk	DESeq2	LRT	0.100097149403495
157	KC_AB1	CG14220	-0.0248909746522462	0.860404622359697	1	pseudobulk	DESeq2	LRT	0.065297265114052
157	KC_AB1	CG14221	1.88952324269138	0.456767015650974	1	pseudobulk	DESeq2	LRT	0.340305265178099
157	KC_AB1	CG14223	1.68130633771206	0.562684503730219	1	pseudobulk	DESeq2	LRT	0.249735045103917
157	KC_AB1	CG14229	-0.0910275417567472	0.824479522335757	1	pseudobulk	DESeq2	LRT	0.083820126492506
157	KC_AB1	CG14230	0.759548810733774	0.434856053306119	1	pseudobulk	DESeq2	LRT	0.36165448005834
157	KC_AB1	CG14231	0.149868556216594	0.862393946096732	1	pseudobulk	DESeq2	LRT	0.064294300842414
157	KC_AB1	CG14232	0.114366130528371	0.782070189490742	1	pseudobulk	DESeq2	LRT	0.106754267988003
157	KC_AB1	CG14234	0.0153515069662479	0.897298549001846	1	pseudobulk	DESeq2	LRT	0.047063034534766
157	KC_AB1	CG14252	0.0779809861740743	0.918150202820761	1	pseudobulk	DESeq2	LRT	0.037086265508816
158	KC_AB1	CG14265	-1.6747012584303	0.563129890257477	1	pseudobulk	DESeq2	LRT	0.249391420218277
158	KC_AB1	CG14270	0.488869583879698	0.34819102949321	1	pseudobulk	DESeq2	LRT	0.458182421879752
158	KC_AB1	CG14285	-0.432156810228358	0.828919178818359	1	pseudobulk	DESeq2	LRT	0.081487811915733
158	KC_AB1	CG14286	-0.892019746462564	0.296022273493757	1	pseudobulk	DESeq2	LRT	0.528675610253556
158	KC_AB1	CG14304	1.06659676108873	0.534361150970224	1	pseudobulk	DESeq2	LRT	0.272165123385343
158	KC_AB1	CG14312	0.210262188972811	0.418316491631784	1	pseudobulk	DESeq2	LRT	0.378495013536754
158	KC_AB1	CG14314	-0.679348556987842	0.365710494583557	1	pseudobulk	DESeq2	LRT	0.436862576784371
158	KC_AB1	CG14321	-0.236121767552285	0.476443575092456	1	pseudobulk	DESeq2	LRT	0.321988525197719
158	KC_AB1	CG14322	-0.527941630652961	0.401084336205315	1	pseudobulk	DESeq2	LRT	0.39676429845822
158	KC_AB1	CG1434	0.709819741553208	0.559147532612497	1	pseudobulk	DESeq2	LRT	0.252473587201414
159	KC_AB1	CG14340	0.720904127654186	0.269328220879032	1	pseudobulk	DESeq2	LRT	0.569718137751134
159	KC_AB1	CG14341	-0.128443828702411	0.748695533211308	1	pseudobulk	DESeq2	LRT	0.125694757906377
159	KC_AB1	CG14352	0.231093597087479	0.817307640567312	1	pseudobulk	DESeq2	LRT	0.087614441077881
159	KC_AB1	CG14356	0.38066359041065	0.867123912282251	1	pseudobulk	DESeq2	LRT	0.061918837269822
159	KC_AB1	CG14367	1.36007871193763	0.401932820325473	1	pseudobulk	DESeq2	LRT	0.395846529502714
159	KC_AB1	CG1440	0.221727440948079	0.585921507774936	1	pseudobulk	DESeq2	LRT	0.232160559789832
159	KC_AB1	CG14406	0.135795303631797	0.946582799906172	1	pseudobulk	DESeq2	LRT	0.023841391241048
159	KC_AB1	CG14407	0.0306504533797709	0.881728541762359	1	pseudobulk	DESeq2	LRT	0.054665100764636
159	KC_AB1	CG14408	0.0125314547101417	0.920371977352365	1	pseudobulk	DESeq2	LRT	0.036036612804900
159	KC_AB1	CG1441	-0.82542030717083	1	1	pseudobulk	DESeq2	LRT	0
160	KC_AB1	CG14410	0.190148400713034	0.870453682911839	1	pseudobulk	DESeq2	LRT	0.060254332864818
160	KC_AB1	CG14411	0.162269886154905	0.648280955249551	1	pseudobulk	DESeq2	LRT	0.188236736613228
160	KC_AB1	CG14414	-0.0870150779931858	0.729851396799925	1	pseudobulk	DESeq2	LRT	0.136765556481604
160	KC_AB1	CG14431	-0.136630555792466	0.509792843550956	1	pseudobulk	DESeq2	LRT	0.292606265430393
160	KC_AB1	CG14434	0.568992074144604	0.330008423837266	1	pseudobulk	DESeq2	LRT	0.481474974124088
160	KC_AB1	CG14435	0.312896101074318	0.260556534942411	1	pseudobulk	DESeq2	LRT	0.584098029947469
160	KC_AB1	CG14440	0.148504752089555	0.492401300273813	1	pseudobulk	DESeq2	LRT	0.307680808906854
160	KC_AB1	CG14441	-0.31721443384254	0.744116124373329	1	pseudobulk	DESeq2	LRT	0.128359284562717
160	KC_AB1	CG14442	0.0168825324262982	0.932158101981274	1	pseudobulk	DESeq2	LRT	0.030510421342568
160	KC_AB1	CG14445	1.08899820779554	0.878177167143508	1	pseudobulk	DESeq2	LRT	0.056417858865076
161	KC_AB1	CG14448	-0.82542030717083	1	1	pseudobulk	DESeq2	LRT	0

161	KC_AB1	CG14450	-0.0456233442193694	0.900933099784554	1	pseudobulk	DESeq2	LRT	0.045307457048816
161	KC_AB1	CG14459	0.13617601020169	1	1	pseudobulk	DESeq2	LRT	0
161	KC_AB1	CG14464	0.354951412744939	0.043390756979202	1	pseudobulk	DESeq2	LRT	1.36260277326275
161	KC_AB1	CG14478	0.278157612971424	0.0467596083573132	1	pseudobulk	DESeq2	LRT	1.33012913499886
161	KC_AB1	CG14483	-0.433803736085733	0.218239548564821	1	pseudobulk	DESeq2	LRT	0.661066545374101
161	KC_AB1	CG14490	2.21745641838459	0.396737788462723	1	pseudobulk	DESeq2	LRT	0.401496431894786
161	KC_AB1	CG14492	1.95275942187961	0.438420007953907	1	pseudobulk	DESeq2	LRT	0.358109634392079
161	KC_AB1	CG14502	1.43130777531804	0.34310153378153	1	pseudobulk	DESeq2	LRT	0.464577340494203
161	KC_AB1	CG14505	-1.6747012584303	0.563129890257477	1	pseudobulk	DESeq2	LRT	0.249391420218277
162	KC_AB1	CG14511	1.08899820779554	0.878177167143508	1	pseudobulk	DESeq2	LRT	0.056417858865076
162	KC_AB1	CG14512	0.751906519349475	0.438433134258436	1	pseudobulk	DESeq2	LRT	0.358096631800536
162	KC_AB1	CG14535	-0.204354223752996	0.556636513917469	1	pseudobulk	DESeq2	LRT	0.254428308471356
162	KC_AB1	CG14543	0.500384241538014	0.41517002900902	1	pseudobulk	DESeq2	LRT	0.381774005607112
162	KC_AB1	CG14544	-1.44925888589622	0.6315588144614	1	pseudobulk	DESeq2	LRT	0.199586199175701
162	KC_AB1	CG14561	1.16949295019	0.219233441835646	1	pseudobulk	DESeq2	LRT	0.65909319792072
162	KC_AB1	CG14562	0.387366591493712	0.24590989354004	1	pseudobulk	DESeq2	LRT	0.60922399820959
162	KC_AB1	CG14563	-0.801412448245884	0.53756988979645	1	pseudobulk	DESeq2	LRT	0.269565064845354
162	KC_AB1	CG14565	1.08899820779554	0.670846322504933	1	pseudobulk	DESeq2	LRT	0.173376956629912
162	KC_AB1	CG14589	0.239786206930574	0.818873364334042	1	pseudobulk	DESeq2	LRT	0.086783255044551
163	KC_AB1	CG14591	-0.21355302808282	0.592376120450273	1	pseudobulk	DESeq2	LRT	0.227402456845131
163	KC_AB1	CG14598	-1.6747012584303	0.563129890257477	1	pseudobulk	DESeq2	LRT	0.249391420218277
163	KC_AB1	CG14609	0.855700630127339	0.665189798240232	1	pseudobulk	DESeq2	LRT	0.177054420008663
163	KC_AB1	CG1461	-0.650903169014973	0.653865808133273	1	pseudobulk	DESeq2	LRT	0.184511372114662
163	KC_AB1	CG14613	-0.0508663245947184	0.928744327474957	1	pseudobulk	DESeq2	LRT	0.032103825779253
163	KC_AB1	CG14615	-1.56855358782635	0.573026303465967	1	pseudobulk	DESeq2	LRT	0.241825442277049
163	KC_AB1	CG14618	0.99119462055958	0.209360416990467	1	pseudobulk	DESeq2	LRT	0.6791054253678
163	KC_AB1	CG14621	0.123542734715083	0.759031922294707	1	pseudobulk	DESeq2	LRT	0.119739958775261
163	KC_AB1	CG14627	1.08899820779554	0.670846322504933	1	pseudobulk	DESeq2	LRT	0.173376956629912
163	KC_AB1	CG1463	0.235996667509943	0.623931804396164	1	pseudobulk	DESeq2	LRT	0.204862876011392
164	KC_AB1	CG14630	-0.82542030717083	1	1	pseudobulk	DESeq2	LRT	0
164	KC_AB1	CG14641	-0.0537896476239481	0.839280893301973	1	pseudobulk	DESeq2	LRT	0.076092663730661
164	KC_AB1	CG14644	-0.82542030717083	1	1	pseudobulk	DESeq2	LRT	0
164	KC_AB1	CG14646	-0.185634602413243	0.770890763368723	1	pseudobulk	DESeq2	LRT	0.113007157911761
164	KC_AB1	CG14647	-0.281067941331221	0.485360984622126	1	pseudobulk	DESeq2	LRT	0.313935137036477
164	KC_AB1	CG14650	0.315571773910049	0.466129038596609	1	pseudobulk	DESeq2	LRT	0.331493840836057
164	KC_AB1	CG14657	0.0358242509160539	0.920453858799572	1	pseudobulk	DESeq2	LRT	0.035997977253380
164	KC_AB1	CG14662	1.08899820779554	0.670846322504933	1	pseudobulk	DESeq2	LRT	0.173376956629912
164	KC_AB1	CG14667	-0.342524271872513	0.427128233814501	1	pseudobulk	DESeq2	LRT	0.369441720110122
164	KC_AB1	CG14669	-0.201369000069698	0.456776306479378	1	pseudobulk	DESeq2	LRT	0.340296431540086
165	KC_AB1	CG14671	-0.061411552324865	0.887446164944616	1	pseudobulk	DESeq2	LRT	0.051857983035267
165	KC_AB1	CG14683	0.148917847108677	0.875718341296999	1	pseudobulk	DESeq2	LRT	0.057635554208434
165	KC_AB1	CG14688	-0.850996402080018	0.900449987182352	1	pseudobulk	DESeq2	LRT	0.045540403770502
165	KC_AB1	CG14692	0.69226361051485	0.664001405019094	1	pseudobulk	DESeq2	LRT	0.17783100166904
165	KC_AB1	CG14693	-1.14160466569893	0.58139554263009	1	pseudobulk	DESeq2	LRT	0.235528302146906
165	KC_AB1	CG14694	-0.402295890857678	0.825105804395784	1	pseudobulk	DESeq2	LRT	0.083490357730083
165	KC_AB1	CG14701	-0.420489888679539	0.484906411706138	1	pseudobulk	DESeq2	LRT	0.314342073359861
165	KC_AB1	CG14710	-0.190407756918373	0.746137019526576	1	pseudobulk	DESeq2	LRT	0.127181411991008
165	KC_AB1	CG14711	0.280078811536822	0.675503731504586	1	pseudobulk	DESeq2	LRT	0.170372247578479
165	KC_AB1	CG14712	-0.887520048063626	0.313523279684521	1	pseudobulk	DESeq2	LRT	0.503730206465907
166	KC_AB1	CG14715	0.170160295294885	0.600068790706404	1	pseudobulk	DESeq2	LRT	0.221798960096853
166	KC_AB1	CG14721	-0.132390840017128	0.765036992975128	1	pseudobulk	DESeq2	LRT	0.116317564249607
166	KC_AB1	CG14722	-0.318383614654571	0.555033243929389	1	pseudobulk	DESeq2	LRT	0.255681003863437

166	KC_AB1	CG14742	-0.82542030717083	1	1	pseudobulk	DESeq2	LRT	0
166	KC_AB1	CG14757	-0.363042995565852	0.7045109490756	1	pseudobulk	DESeq2	LRT	0.152112252964612
166	KC_AB1	CG14760	-1.56855358782635	0.573026303465967	1	pseudobulk	DESeq2	LRT	0.241825442277049
166	KC_AB1	CG14762	-0.212537325215478	0.0987071970305861	1	pseudobulk	DESeq2	LRT	1.00565118049432
166	KC_AB1	CG14763	-0.82542030717083	1	1	pseudobulk	DESeq2	LRT	0
166	KC_AB1	CG14764	0.0441567051085203	0.832973857044192	1	pseudobulk	DESeq2	LRT	0.079368628748813
166	KC_AB1	CG14767	-0.115442614112755	0.559093009163421	1	pseudobulk	DESeq2	LRT	0.252515938077101
167	KC_AB1	CG14777	0.100476640731786	0.751902962036127	1	pseudobulk	DESeq2	LRT	0.123838204317328
167	KC_AB1	CG14778	1.21433710563586	0.747381629023987	1	pseudobulk	DESeq2	LRT	0.126457581508545
167	KC_AB1	CG14780	-0.850996402080018	0.900449987182352	1	pseudobulk	DESeq2	LRT	0.045540403770502
167	KC_AB1	CG14785	-1.82709236256241	0.44317674354685	1	pseudobulk	DESeq2	LRT	0.353423038023035
167	KC_AB1	CG14795	-1.89854133735591	0.478664405825708	1	pseudobulk	DESeq2	LRT	0.319968866080244
167	KC_AB1	CG14797	0.625337119397526	0.691061835327573	1	pseudobulk	DESeq2	LRT	0.160483090773339
167	KC_AB1	CG14803	0.272529800757858	0.80155057910477	1	pseudobulk	DESeq2	LRT	0.096069067778913
167	KC_AB1	CG14805	0.109378248113199	0.832950266917616	1	pseudobulk	DESeq2	LRT	0.079380928302739
167	KC_AB1	CG14806	-0.256923590923042	0.398422297451726	1	pseudobulk	DESeq2	LRT	0.39965636455238
167	KC_AB1	CG14811	2.28292029397527	0.0293929480265167	1	pseudobulk	DESeq2	LRT	1.53175685327952
168	KC_AB1	CG14812	0.358737616369789	0.32425290506171	1	pseudobulk	DESeq2	LRT	0.489116124275128
168	KC_AB1	CG14817	0.0177036126870553	0.903904314023102	1	pseudobulk	DESeq2	LRT	0.043877540864468
168	KC_AB1	CG14818	-0.119420884061004	0.533903106466807	1	pseudobulk	DESeq2	LRT	0.272537552231681
168	KC_AB1	CG14820	1.08899820779554	0.878177167143508	1	pseudobulk	DESeq2	LRT	0.056417858865076
168	KC_AB1	CG14830	-0.246438421307241	0.838795417218959	1	pseudobulk	DESeq2	LRT	0.076343950979649
168	KC_AB1	CG14834	-1.97437890458968	0.359999599262835	1	pseudobulk	DESeq2	LRT	0.443697982671702
168	KC_AB1	CG14850	1.08899820779554	0.878177167143508	1	pseudobulk	DESeq2	LRT	0.056417858865076
168	KC_AB1	CG14853	0.302895477652562	0.199981744773003	1	pseudobulk	DESeq2	LRT	0.699009646867008
168	KC_AB1	CG14857	1.08899820779554	0.670846322504933	1	pseudobulk	DESeq2	LRT	0.173376956629912
168	KC_AB1	CG1486	-0.0861685681794891	0.841227409531087	1	pseudobulk	DESeq2	LRT	0.075086585231828
169	KC_AB1	CG14861	-0.82542030717083	1	1	pseudobulk	DESeq2	LRT	0
169	KC_AB1	CG14864	-0.607123990271071	0.71186498650553	1	pseudobulk	DESeq2	LRT	0.14760236756503
169	KC_AB1	CG14868	-0.170064145528805	0.609248596847133	1	pseudobulk	DESeq2	LRT	0.215205462360307
169	KC_AB1	CG14881	-0.265215522270536	0.689539427070732	1	pseudobulk	DESeq2	LRT	0.161440896319587
169	KC_AB1	CG14882	-0.714138042173079	0.36979713772907	1	pseudobulk	DESeq2	LRT	0.432036454651377
169	KC_AB1	CG14883	-0.0511630060966455	0.895830982181121	1	pseudobulk	DESeq2	LRT	0.047773921621606
169	KC_AB1	CG14894	-0.0249693731374661	0.944339000498101	1	pseudobulk	DESeq2	LRT	0.024872073904483
169	KC_AB1	CG14903	-0.184812163522803	0.526141285868358	1	pseudobulk	DESeq2	LRT	0.278897618139535
169	KC_AB1	CG14906	0.555174800129279	0.574700594854019	1	pseudobulk	DESeq2	LRT	0.240558353341882
169	KC_AB1	CG1492	-0.167743888127816	0.730488742409742	1	pseudobulk	DESeq2	LRT	0.136386472607168
170	KC_AB1	CG14937	-1.96719661029409	0.414177861019091	1	pseudobulk	DESeq2	LRT	0.382813119093482
170	KC_AB1	CG1494	0.13617601020169	1	1	pseudobulk	DESeq2	LRT	0
170	KC_AB1	CG14965	-0.0795254062657544	0.935263211254364	1	pseudobulk	DESeq2	LRT	0.029066148369698
170	KC_AB1	CG14966	-1.24907299141191	0.229216919671792	1	pseudobulk	DESeq2	LRT	0.63975332802713
170	KC_AB1	CG14969	-0.0028233167433636	0.936847991331448	1	pseudobulk	DESeq2	LRT	0.028330870031574
170	KC_AB1	CG14971	-0.185795084241427	0.754494539410542	1	pseudobulk	DESeq2	LRT	0.122343899046047
170	KC_AB1	CG14977	-0.121141742152277	0.743130940464239	1	pseudobulk	DESeq2	LRT	0.128934656346028
170	KC_AB1	CG14982	-0.168269192204985	0.477632665043977	1	pseudobulk	DESeq2	LRT	0.32090597969434
170	KC_AB1	CG14984	1.57630725943786	0.50105907583397	1	pseudobulk	DESeq2	LRT	0.300111066954744
170	KC_AB1	CG14985	-2.91519351765031	0.119638594470422	1	pseudobulk	DESeq2	LRT	0.922128697758118
171	KC_AB1	CG14989	1.5940412424434	0.475208010505809	1	pseudobulk	DESeq2	LRT	0.323116247132191
171	KC_AB1	CG14995	0.159322415749422	0.603101754683899	1	pseudobulk	DESeq2	LRT	0.219609407976691
171	KC_AB1	CG14997	-0.120658858679741	0.89254044765331	1	pseudobulk	DESeq2	LRT	0.049372093653186
171	KC_AB1	CG15011	0.0850244059591282	0.926570760641095	1	pseudobulk	DESeq2	LRT	0.033121408743898
171	KC_AB1	CG15012	0.0505518318614697	0.834018113909996	1	pseudobulk	DESeq2	LRT	0.078824516885369

171	KC_AB1	CG15014	-0.46762220433626	0.56692366250813	1	pseudobulk	DESeq2	LRT	0.246475415857428
171	KC_AB1	CG15019	-0.318685958469846	0.414535608131093	1	pseudobulk	DESeq2	LRT	0.382438158115788
171	KC_AB1	CG15027	-0.0939602153182453	0.874831340724279	1	pseudobulk	DESeq2	LRT	0.058075666809752
171	KC_AB1	CG1503	1.08899820779554	0.878177167143508	1	pseudobulk	DESeq2	LRT	0.056417858865076
171	KC_AB1	CG1504	-2.86378688787447	0.227637018922626	1	pseudobulk	DESeq2	LRT	0.642757110438212
172	KC_AB1	CG15040	-0.850932661185203	0.660736244265418	1	pseudobulk	DESeq2	LRT	0.179971869591347
172	KC_AB1	CG15047	0.144938062156184	0.939011437924561	1	pseudobulk	DESeq2	LRT	0.027329117640952
172	KC_AB1	CG15073	0.332102555746816	0.64551172712271	1	pseudobulk	DESeq2	LRT	0.190095863423249
172	KC_AB1	CG15084	0.91813490979844	0.333786399998414	1	pseudobulk	DESeq2	LRT	0.47653136246195
172	KC_AB1	CG15093	-0.82542030717083	1	1	pseudobulk	DESeq2	LRT	0
172	KC_AB1	CG15097	-0.488070142378525	0.0462876741478528	1	pseudobulk	DESeq2	LRT	1.33453464099577
172	KC_AB1	CG15098	0.810936078232138	0.0459390032162628	1	pseudobulk	DESeq2	LRT	1.3378184323533
172	KC_AB1	CG15099	0.0435563698013079	0.884544886692693	1	pseudobulk	DESeq2	LRT	0.053280123694538
172	KC_AB1	CG15107	-0.802577998512225	0.304282452719214	1	pseudobulk	DESeq2	LRT	0.516723091700155
172	KC_AB1	CG15109	-1.46848161684268	0.554701175609767	1	pseudobulk	DESeq2	LRT	0.25594091369518
173	KC_AB1	CG15111	-0.373073038582727	0.356234748606819	1	pseudobulk	DESeq2	LRT	0.448263719931322
173	KC_AB1	CG15117	0.135795303631797	0.946582799906172	1	pseudobulk	DESeq2	LRT	0.023841391241048
173	KC_AB1	CG15118	0.463785229481641	0.342756880243962	1	pseudobulk	DESeq2	LRT	0.465013818749984
173	KC_AB1	CG1513	-0.0373887863771138	0.849543337487883	1	pseudobulk	DESeq2	LRT	0.070814461701612
173	KC_AB1	CG15136	1.08899820779554	0.878177167143508	1	pseudobulk	DESeq2	LRT	0.056417858865076
173	KC_AB1	CG15141	-0.140983751175753	0.749206876082195	1	pseudobulk	DESeq2	LRT	0.125398245404047
173	KC_AB1	CG15143	-1.44925888589622	0.6315588144614	1	pseudobulk	DESeq2	LRT	0.199586199175701
173	KC_AB1	CG15160	-0.222141120022418	0.594055469641237	1	pseudobulk	DESeq2	LRT	0.226173001088901
173	KC_AB1	CG15168	0.121853469744959	0.666019749980432	1	pseudobulk	DESeq2	LRT	0.176512892180535
173	KC_AB1	CG15201	0.323111129108613	0.799141134060853	1	pseudobulk	DESeq2	LRT	0.097376514389311
174	KC_AB1	CG15202	1.08899820779554	0.878177167143508	1	pseudobulk	DESeq2	LRT	0.056417858865076
174	KC_AB1	CG15203	2.21745641838459	0.396737788462723	1	pseudobulk	DESeq2	LRT	0.401496431894786
174	KC_AB1	CG15209	2.10358249103202	0.214483331288803	1	pseudobulk	DESeq2	LRT	0.668606453645144
174	KC_AB1	CG15221	1.08899820779554	0.697631521651407	1	pseudobulk	DESeq2	LRT	0.156373904552
174	KC_AB1	CG1523	-0.468682301907437	0.509072709833443	1	pseudobulk	DESeq2	LRT	0.293220183823825
174	KC_AB1	CG15236	-0.835003521415096	0.452804965316428	1	pseudobulk	DESeq2	LRT	0.344088819457368
174	KC_AB1	CG15237	0.453382178893613	0.155869891045712	1	pseudobulk	DESeq2	LRT	0.807237768169489
174	KC_AB1	CG15252	-0.850932661185203	0.660736244265418	1	pseudobulk	DESeq2	LRT	0.179971869591347
174	KC_AB1	CG15270	0.586401590480775	0.142565257875529	1	pseudobulk	DESeq2	LRT	0.845986296021701
174	KC_AB1	CG15286	2.1559833397943	0.381743505411375	1	pseudobulk	DESeq2	LRT	0.418228342845202
175	KC_AB1	CG1529	-0.850996402080018	0.900449987182352	1	pseudobulk	DESeq2	LRT	0.045540403770502
175	KC_AB1	CG15293	-0.82542030717083	1	1	pseudobulk	DESeq2	LRT	0
175	KC_AB1	CG15309	-0.0014195661843778	0.957762830488501	1	pseudobulk	DESeq2	LRT	0.018742021362078
175	KC_AB1	CG15312	0.133290652974635	0.588413918409196	1	pseudobulk	DESeq2	LRT	0.230317062972347
175	KC_AB1	CG15317	-0.346839282934268	0.753311392035512	1	pseudobulk	DESeq2	LRT	0.123025464851033
175	KC_AB1	CG1532	0.659076708792435	0.234729553537076	1	pseudobulk	DESeq2	LRT	0.629432227273798
175	KC_AB1	CG15332	0.977851105145882	0.69132261808108	1	pseudobulk	DESeq2	LRT	0.160319234022942
175	KC_AB1	CG15343	-0.848585259864657	0.446231127262499	1	pseudobulk	DESeq2	LRT	0.350440138384689
175	KC_AB1	CG15356	-0.470886148321673	0.265504761529144	1	pseudobulk	DESeq2	LRT	0.57592768592982
175	KC_AB1	CG15362	-1.56855358782635	0.573026303465967	1	pseudobulk	DESeq2	LRT	0.241825442277049
176	KC_AB1	CG15365	-2.85374641634447	0.199169359211768	1	pseudobulk	DESeq2	LRT	0.70077747388823
176	KC_AB1	CG15373	1.82443202324224	0.401185591318225	1	pseudobulk	DESeq2	LRT	0.396654673167464
176	KC_AB1	CG15382	-1.16016107924111	0.376093226299368	1	pseudobulk	DESeq2	LRT	0.424704488452895
176	KC_AB1	CG15385	0.10941237501304	0.771701656943041	1	pseudobulk	DESeq2	LRT	0.112550567262749
176	KC_AB1	CG15387	0.161296968137452	0.793919588720373	1	pseudobulk	DESeq2	LRT	0.100223482388385
176	KC_AB1	CG15390	-0.438378734488673	0.684622147446866	1	pseudobulk	DESeq2	LRT	0.164549055594897
176	KC_AB1	CG15403	0.111831901354254	1	1	pseudobulk	DESeq2	LRT	0

176	KC_AB1	CG1542	-0.0334557813116779	0.957979366902044	1	pseudobulk	DESeq2	LRT	0.018643844717996
176	KC_AB1	CG15431	-0.0505377258154598	0.73779391034777	1	pseudobulk	DESeq2	LRT	0.132064933702973
176	KC_AB1	CG15432	-0.180418560616665	0.641856997314983	1	pseudobulk	DESeq2	LRT	0.192561719898229
177	KC_AB1	CG15435	-2.04938849026729	0.199165941686355	1	pseudobulk	DESeq2	LRT	0.700784925964033
177	KC_AB1	CG15436	-1.99275908811736	0.412167844949191	1	pseudobulk	DESeq2	LRT	0.384925892483647
177	KC_AB1	CG15439	-0.521040908981741	0.510950075013876	1	pseudobulk	DESeq2	LRT	0.291621532752236
177	KC_AB1	CG15443	0.014152927066728	0.96965324813749	1	pseudobulk	DESeq2	LRT	0.013383543407459
177	KC_AB1	CG15445	-0.371063367169376	0.637490173625511	1	pseudobulk	DESeq2	LRT	0.195526505126333
177	KC_AB1	CG15446	1.47845164298158	0.630642748871417	1	pseudobulk	DESeq2	LRT	0.200216593420489
177	KC_AB1	CG15449	0.174747669734623	0.676715490282364	1	pseudobulk	DESeq2	LRT	0.169593882232125
177	KC_AB1	CG1545	0.135879393043758	1	1	pseudobulk	DESeq2	LRT	0
177	KC_AB1	CG15450	-0.725033943492997	0.739093404909074	1	pseudobulk	DESeq2	LRT	0.131300673008131
177	KC_AB1	CG15456	0.0678025696879385	0.837957344520372	1	pseudobulk	DESeq2	LRT	0.076778088184661
178	KC_AB1	CG15458	1.54813506565411	0.388185325694878	1	pseudobulk	DESeq2	LRT	0.410960885975464
178	KC_AB1	CG15459	0.482947637418516	0.566299936282866	1	pseudobulk	DESeq2	LRT	0.246953487238032
178	KC_AB1	CG15473	1.08899820779554	0.697631521651407	1	pseudobulk	DESeq2	LRT	0.156373904552
178	KC_AB1	CG15497	-0.82542030717083	1	1	pseudobulk	DESeq2	LRT	0
178	KC_AB1	CG15514	0.0198990491509941	0.960747245820114	1	pseudobulk	DESeq2	LRT	0.017390851856422
178	KC_AB1	CG1552	2.03222352020069	0.190946264929109	1	pseudobulk	DESeq2	LRT	0.719088832375986
178	KC_AB1	CG15522	0.31888482699571	0.644909936533575	1	pseudobulk	DESeq2	LRT	0.190500931562494
178	KC_AB1	CG15525	0.253791939726509	0.657413092313008	1	pseudobulk	DESeq2	LRT	0.182161651239066
178	KC_AB1	CG15535	0.347073713191228	0.360510915354203	1	pseudobulk	DESeq2	LRT	0.443081581412423
178	KC_AB1	CG15536	-0.274532591140384	0.647142114398612	1	pseudobulk	DESeq2	LRT	0.189000336453828
179	KC_AB1	CG15539	0.404995576066175	0.716223806030939	1	pseudobulk	DESeq2	LRT	0.144951247892604
179	KC_AB1	CG15547	0.516794653828635	0.814433332789471	1	pseudobulk	DESeq2	LRT	0.089144460019238
179	KC_AB1	CG15554	0.406024713202589	0.845216792824485	1	pseudobulk	DESeq2	LRT	0.073031882941825
179	KC_AB1	CG15561	-0.273042831922132	0.701932812372323	1	pseudobulk	DESeq2	LRT	0.153704455694121
179	KC_AB1	CG15579	-1.35710895788342	0.248306867582695	1	pseudobulk	DESeq2	LRT	0.605011268717594
179	KC_AB1	CG15601	0.00483592309430141	0.934178329051428	1	pseudobulk	DESeq2	LRT	0.029570211638844
179	KC_AB1	CG15602	-0.0357961242747206	0.923201291649659	1	pseudobulk	DESeq2	LRT	0.034703596567105
179	KC_AB1	CG15614	-2.80036860845584	0.209900133609211	1	pseudobulk	DESeq2	LRT	0.677987284972985
179	KC_AB1	CG15628	-0.0395717703219309	0.7852204121962	1	pseudobulk	DESeq2	LRT	0.105008419223651
179	KC_AB1	CG15629	-0.850996402080018	0.900449987182352	1	pseudobulk	DESeq2	LRT	0.045540403770502
180	KC_AB1	CG15631	-2.29603295607844	0.327786153610032	1	pseudobulk	DESeq2	LRT	0.484409395908184
180	KC_AB1	CG15638	-0.569658384202923	0.762736196533549	1	pseudobulk	DESeq2	LRT	0.117625643172353
180	KC_AB1	CG15642	-0.140251455316915	0.808111375381913	1	pseudobulk	DESeq2	LRT	0.092528779843692
180	KC_AB1	CG15643	0.658353976562747	0.322515521338131	1	pseudobulk	DESeq2	LRT	0.491449379728573
180	KC_AB1	CG15651	-0.38325052685516	0.345766147323716	1	pseudobulk	DESeq2	LRT	0.461217529136691
180	KC_AB1	CG15653	-0.345852786206648	0.674083778228034	1	pseudobulk	DESeq2	LRT	0.171286123997271
180	KC_AB1	CG15674	-0.507979848677457	0.940470850565906	1	pseudobulk	DESeq2	LRT	0.026654660650085
180	KC_AB1	CG15705	1.08899820779554	0.697631521651407	1	pseudobulk	DESeq2	LRT	0.156373904552
180	KC_AB1	CG15706	0.190073829964272	0.885829419857183	1	pseudobulk	DESeq2	LRT	0.052649900175148
180	KC_AB1	CG15715	-0.0515580870708307	0.83063184640991	1	pseudobulk	DESeq2	LRT	0.080591422064385
181	KC_AB1	CG15717	1.08899820779554	0.670846322504933	1	pseudobulk	DESeq2	LRT	0.173376956629912
181	KC_AB1	CG15719	-1.54496158472927	0.407729625456069	1	pseudobulk	DESeq2	LRT	0.38962773175447
181	KC_AB1	CG1572	-0.690431739854444	0.64757522093734	1	pseudobulk	DESeq2	LRT	0.188709777608699
181	KC_AB1	CG15725	0.73362978992059	0.719815137332351	1	pseudobulk	DESeq2	LRT	0.142779024603383
181	KC_AB1	CG15743	-0.299754606876129	0.526198076239971	1	pseudobulk	DESeq2	LRT	0.278850744011419
181	KC_AB1	CG15744	0.618012917963853	0.496098505354593	1	pseudobulk	DESeq2	LRT	0.30443208140411
181	KC_AB1	CG15747	-0.0319290306231174	0.934680501636161	1	pseudobulk	DESeq2	LRT	0.029336817030133
181	KC_AB1	CG1575	-0.905537697035476	0.41108078880191	1	pseudobulk	DESeq2	LRT	0.386072818798035
181	KC_AB1	CG15760	0.00064389663028362	0.93032954286971	1	pseudobulk	DESeq2	LRT	0.031363187683688

181	KC_AB1	CG15765	-0.191344118402801	0.197112928672699	1	pseudobulk	DESeq2	LRT	0.705284889332593
182	KC_AB1	CG15767	1.88952324269138	0.456767015650974	1	pseudobulk	DESeq2	LRT	0.340305265178099
182	KC_AB1	CG15771	0.579503379671003	0.0673249676014546	1	pseudobulk	DESeq2	LRT	1.17182384691033
182	KC_AB1	CG15772	0.108964295252264	0.636772489256066	1	pseudobulk	DESeq2	LRT	0.196015707868333
182	KC_AB1	CG1578	-0.143260914321733	0.503121820968882	1	pseudobulk	DESeq2	LRT	0.298326846417317
182	KC_AB1	CG15784	1.08899820779554	0.878177167143508	1	pseudobulk	DESeq2	LRT	0.056417858865076
182	KC_AB1	CG15803	0.12941689520101	0.568625748911755	1	pseudobulk	DESeq2	LRT	0.245173478171611
182	KC_AB1	CG15812	0.119410992241382	0.828944194312895	1	pseudobulk	DESeq2	LRT	0.081474705779948
182	KC_AB1	CG15814	-0.16823881113974	0.618943538479866	1	pseudobulk	DESeq2	LRT	0.208348966561512
182	KC_AB1	CG1582	-0.536952453248902	0.563583402653173	1	pseudobulk	DESeq2	LRT	0.249041805204766
182	KC_AB1	CG15864	-0.968336042648622	0.663512636338631	1	pseudobulk	DESeq2	LRT	0.178150801752249
183	KC_AB1	CG15877	1.11392209758678	0.100631363704007	1	pseudobulk	DESeq2	LRT	0.997266641936898
183	KC_AB1	CG15880	-0.82542030717083	1	1	pseudobulk	DESeq2	LRT	0
183	KC_AB1	CG15890	1.08899820779554	0.878177167143508	1	pseudobulk	DESeq2	LRT	0.056417858865076
183	KC_AB1	CG15891	0.235287092505409	0.374716262250519	1	pseudobulk	DESeq2	LRT	0.426297458621155
183	KC_AB1	CG15894	0.124073977879645	0.351835559831565	1	pseudobulk	DESeq2	LRT	0.453660268749593
183	KC_AB1	CG15908	-0.820863598292701	0.15649538712044	1	pseudobulk	DESeq2	LRT	0.805498459252764
183	KC_AB1	CG15912	0.574096803000732	0.42140860573656	1	pseudobulk	DESeq2	LRT	0.375296599753693
183	KC_AB1	CG15914	-0.82542030717083	1	1	pseudobulk	DESeq2	LRT	0
183	KC_AB1	CG15916	0.0881465411764719	0.696114174201762	1	pseudobulk	DESeq2	LRT	0.157319523091764
183	KC_AB1	CG15922	-0.277782807549626	0.441814351595679	1	pseudobulk	DESeq2	LRT	0.354760180910085
184	KC_AB1	CG15923	0.827712394573779	0.700305254150331	1	pseudobulk	DESeq2	LRT	0.154712615277118
184	KC_AB1	CG1598	0.449738785105129	0.191065350410178	1	pseudobulk	DESeq2	LRT	0.718818064855306
184	KC_AB1	CG1602	0.171854383373116	0.891287590957884	1	pseudobulk	DESeq2	LRT	0.049982139943667
184	KC_AB1	CG1603	-0.334589304045476	0.714242862405651	1	pseudobulk	DESeq2	LRT	0.146154090928878
184	KC_AB1	CG1607	-0.264939249899814	0.284450127822445	1	pseudobulk	DESeq2	LRT	0.545993866738643
184	KC_AB1	CG1620	0.332834129692308	0.426313599317071	1	pseudobulk	DESeq2	LRT	0.370270813226293
184	KC_AB1	CG1622	-0.25013355994309	0.53193160288137	1	pseudobulk	DESeq2	LRT	0.274144206804306
184	KC_AB1	CG1628	-0.308759453806314	0.859503253373532	1	pseudobulk	DESeq2	LRT	0.065752475091531
184	KC_AB1	CG1635	-0.321721040513722	0.533651684730554	1	pseudobulk	DESeq2	LRT	0.27274211517422
184	KC_AB1	CG1636	-0.25169022652546	0.269727478889846	1	pseudobulk	DESeq2	LRT	0.569074806894106
185	KC_AB1	CG1637	-0.108901433411903	0.749570501271074	1	pseudobulk	DESeq2	LRT	0.125187513085164
185	KC_AB1	CG1638	-1.44925888589622	0.6315588144614	1	pseudobulk	DESeq2	LRT	0.199586199175701
185	KC_AB1	CG1640	0.101767806888636	0.64948964772879	1	pseudobulk	DESeq2	LRT	0.187427766792789
185	KC_AB1	CG1646	0.204940687784397	0.344767474155077	1	pseudobulk	DESeq2	LRT	0.462473712862615
185	KC_AB1	CG1647	0.258072211484263	0.5745505005093	1	pseudobulk	DESeq2	LRT	0.2406717926874
185	KC_AB1	CG1648	0.793002777853068	0.349127450418911	1	pseudobulk	DESeq2	LRT	0.457016003099956
185	KC_AB1	CG1662	-0.137173789219705	0.840686950424381	1	pseudobulk	DESeq2	LRT	0.075365693873033
185	KC_AB1	CG1663	-2.13460583577721	0.0960049900510338	1	pseudobulk	DESeq2	LRT	1.01770619305099
185	KC_AB1	CG1671	0.753861682997673	0.542939116169123	1	pseudobulk	DESeq2	LRT	0.265248868374825
185	KC_AB1	CG16710	-0.506516985469986	0.909346984303859	1	pseudobulk	DESeq2	LRT	0.041270369127443
186	KC_AB1	CG16711	-0.0027784722751885	0.979880764243842	1	pseudobulk	DESeq2	LRT	0.008826767757937
186	KC_AB1	CG16717	0.0113795180840289	0.950920265674192	1	pseudobulk	DESeq2	LRT	0.021855896973686
186	KC_AB1	CG16721	0.38145004070377	0.397789352159688	1	pseudobulk	DESeq2	LRT	0.400346846048641
186	KC_AB1	CG16734	0.257512489794058	0.861192158530684	1	pseudobulk	DESeq2	LRT	0.064899933240512
186	KC_AB1	CG16736	-0.82542030717083	1	1	pseudobulk	DESeq2	LRT	0
186	KC_AB1	CG16739	2.3024431441448	0.1594987164786	1	pseudobulk	DESeq2	LRT	0.797242807456358
186	KC_AB1	CG1674	1.68130633771206	0.562684503730219	1	pseudobulk	DESeq2	LRT	0.249735045103917
186	KC_AB1	CG16743	-1.56855358782635	0.573026303465967	1	pseudobulk	DESeq2	LRT	0.241825442277049
186	KC_AB1	CG16749	0.11269840515639	0.977007463492936	1	pseudobulk	DESeq2	LRT	0.010102118633921
186	KC_AB1	CG16753	0.243523282327987	0.712563737513668	1	pseudobulk	DESeq2	LRT	0.147176282740591
187	KC_AB1	CG16758	-0.618422766585323	0.856874262644257	1	pseudobulk	DESeq2	LRT	0.067082901584478

187	KC_AB1	CG1677	0.00084276905244309	0.977830602864463	1	pseudobulk	DESeq2	LRT	0.009736374878290
187	KC_AB1	CG16779	0.128584793014787	0.265669598153788	1	pseudobulk	DESeq2	LRT	0.575658141146675
187	KC_AB1	CG16787	-0.0269736685077846	0.937044829256206	1	pseudobulk	DESeq2	LRT	0.028239631489084
187	KC_AB1	CG16789	1.68130633771206	0.562684503730219	1	pseudobulk	DESeq2	LRT	0.249735045103917
187	KC_AB1	CG16790	-0.253243846242334	0.814818575790549	1	pseudobulk	DESeq2	LRT	0.088939078749715
187	KC_AB1	CG16791	-0.06637111517118861	0.565441204192665	1	pseudobulk	DESeq2	LRT	0.247612547276484
187	KC_AB1	CG16798	1.08899820779554	0.670846322504933	1	pseudobulk	DESeq2	LRT	0.173376956629912
187	KC_AB1	CG16812	0.282204124864987	0.421601432926893	1	pseudobulk	DESeq2	LRT	0.375097921725336
187	KC_AB1	CG16825	0.135795303631797	0.946582799906172	1	pseudobulk	DESeq2	LRT	0.023841391241048
188	KC_AB1	CG16852	-0.850996402080018	0.900449987182352	1	pseudobulk	DESeq2	LRT	0.045540403770502
188	KC_AB1	CG16854	-0.0301644501249645	0.914851892701034	1	pseudobulk	DESeq2	LRT	0.038649209090946
188	KC_AB1	CG16863	0.192832440797711	0.855561485913267	1	pseudobulk	DESeq2	LRT	0.067748773927319
188	KC_AB1	CG16865	-0.776475249935848	0.342496044011646	1	pseudobulk	DESeq2	LRT	0.465344440445526
188	KC_AB1	CG16868	-0.190813465599305	0.77169344205359	1	pseudobulk	DESeq2	LRT	0.112555190422639
188	KC_AB1	CG1688	-0.5953266343461	0.0187211182171951	1	pseudobulk	DESeq2	LRT	1.72766821430232
188	KC_AB1	CG16890	-0.0790642205359274	0.940424185082035	1	pseudobulk	DESeq2	LRT	0.026676210563036
188	KC_AB1	CG16896	-0.233943581150613	0.766390812359858	1	pseudobulk	DESeq2	LRT	0.115549710303902
188	KC_AB1	CG16903	-0.478817540036218	0.265585196454845	1	pseudobulk	DESeq2	LRT	0.575796135919785
188	KC_AB1	CG16908	-1.72932662710037	0.304509913622206	1	pseudobulk	DESeq2	LRT	0.51639856391342
189	KC_AB1	CG16935	0.0928371151922746	0.809556194646344	1	pseudobulk	DESeq2	LRT	0.091752999686675
189	KC_AB1	CG1695	0.0141375701298817	0.951106124035122	1	pseudobulk	DESeq2	LRT	0.021771021956955
189	KC_AB1	CG16952	0.0487557672618135	0.850945261275691	1	pseudobulk	DESeq2	LRT	0.070098375861958
189	KC_AB1	CG16953	0.224386170147209	0.483063084370788	1	pseudobulk	DESeq2	LRT	0.315996149983235
189	KC_AB1	CG16972	-0.146385233670282	0.774511889552757	1	pseudobulk	DESeq2	LRT	0.110971910987382
189	KC_AB1	CG16974	0.220617767775846	0.488818283189391	1	pseudobulk	DESeq2	LRT	0.310852558617041
189	KC_AB1	CG16979	-0.957178964189317	0.289999847517623	1	pseudobulk	DESeq2	LRT	0.537602230453706
189	KC_AB1	CG16984	-0.507979848677457	0.940470850565906	1	pseudobulk	DESeq2	LRT	0.026654660650085
189	KC_AB1	CG16985	-0.321104735766149	0.839796900271028	1	pseudobulk	DESeq2	LRT	0.075825732696021
189	KC_AB1	CG16986	0.248705738013303	0.644012875854959	1	pseudobulk	DESeq2	LRT	0.191105449632644
190	KC_AB1	CG16989	-0.309915650027848	0.62132184185835	1	pseudobulk	DESeq2	LRT	0.206683378992997
190	KC_AB1	CG17002	0.0526456520862024	0.843971659080549	1	pseudobulk	DESeq2	LRT	0.073672136918878
190	KC_AB1	CG17005	1.08899820779554	0.697631521651407	1	pseudobulk	DESeq2	LRT	0.156373904552
190	KC_AB1	CG1701	-5.63584805078408	0.172705286548062	1	pseudobulk	DESeq2	LRT	0.762694368376305
190	KC_AB1	CG17010	0.89025409057689	0.522065044454364	1	pseudobulk	DESeq2	LRT	0.282275384569024
190	KC_AB1	CG17019	-0.239974346200453	0.521617228808142	1	pseudobulk	DESeq2	LRT	0.282648072466446
190	KC_AB1	CG17026	-0.427574686712982	0.578818845152916	1	pseudobulk	DESeq2	LRT	0.237457337586366
190	KC_AB1	CG17027	-0.850996402080018	0.900449987182352	1	pseudobulk	DESeq2	LRT	0.045540403770502
190	KC_AB1	CG17029	0.0557659075901032	0.945317281545337	1	pseudobulk	DESeq2	LRT	0.024422402604407
190	KC_AB1	CG1703	-0.328594242868038	0.429955998379242	1	pseudobulk	DESeq2	LRT	0.366575987766684
191	KC_AB1	CG17030	1.19732100989811	0.526547493354687	1	pseudobulk	DESeq2	LRT	0.278562450363344
191	KC_AB1	CG17048	-0.829971373743754	0.758323735176306	1	pseudobulk	DESeq2	LRT	0.120145350587648
191	KC_AB1	CG17059	-0.118456534806685	0.578516169940864	1	pseudobulk	DESeq2	LRT	0.237684497701088
191	KC_AB1	CG17065	0.126627134181511	0.647934717606975	1	pseudobulk	DESeq2	LRT	0.188468749085108
191	KC_AB1	CG17068	0.162403929580034	0.88965126967691	1	pseudobulk	DESeq2	LRT	0.050780197101753
191	KC_AB1	CG17075	0.580347103675937	0.0737980038241201	1	pseudobulk	DESeq2	LRT	1.13195538533
191	KC_AB1	CG17078	-0.424788487260331	0.299144388366731	1	pseudobulk	DESeq2	LRT	0.524119139652026
191	KC_AB1	CG17109	-0.850996402080018	0.900449987182352	1	pseudobulk	DESeq2	LRT	0.045540403770502
191	KC_AB1	CG17111	-2.78897630828524	0.16221016923357	1	pseudobulk	DESeq2	LRT	0.789921922605846
191	KC_AB1	CG17118	0.563960670394673	0.765273331028603	1	pseudobulk	DESeq2	LRT	0.116183421113154
192	KC_AB1	CG17119	1.08899820779554	0.670846322504933	1	pseudobulk	DESeq2	LRT	0.173376956629912
192	KC_AB1	CG17122	-0.82542030717083	1	1	pseudobulk	DESeq2	LRT	0
192	KC_AB1	CG17124	0.0730320806511384	0.65624246714559	1	pseudobulk	DESeq2	LRT	0.182935668722565

192	KC_AB1	CG17141	-2.00055697119191	0.0456709508158236	1	pseudobulk	DESeq2	LRT	1.3403599467953
192	KC_AB1	CG17162	-0.95415957444639	0.173945920552851	1	pseudobulk	DESeq2	LRT	0.75958575203948
192	KC_AB1	CG17163	-0.307552455416794	0.339191112455078	1	pseudobulk	DESeq2	LRT	0.469555535726304
192	KC_AB1	CG17167	-0.11903230687324	0.903517683195478	1	pseudobulk	DESeq2	LRT	0.044063343225831
192	KC_AB1	CG17168	-0.344203959875678	0.254953812764853	1	pseudobulk	DESeq2	LRT	0.593538488892214
192	KC_AB1	CG17169	2.6288931366292	0.260231277769534	1	pseudobulk	DESeq2	LRT	0.584640505828425
192	KC_AB1	CG17186	0.536915069438059	0.629029752894302	1	pseudobulk	DESeq2	LRT	0.201328812086974
193	KC_AB1	CG17187	0.556025179573894	0.514096752206085	1	pseudobulk	DESeq2	LRT	0.288955139769284
193	KC_AB1	CG17202	-0.121355739663939	0.613316406485331	1	pseudobulk	DESeq2	LRT	0.212315417587504
193	KC_AB1	CG17211	-1.43837617865047	0.407477429236323	1	pseudobulk	DESeq2	LRT	0.389896442456755
193	KC_AB1	CG17221	-1.60976773952503	0.1478038408066	1	pseudobulk	DESeq2	LRT	0.830314280288692
193	KC_AB1	CG17230	-0.331856340816813	0.454934910089328	1	pseudobulk	DESeq2	LRT	0.342050735675139
193	KC_AB1	CG17233	-0.314545232337558	0.297975629259009	1	pseudobulk	DESeq2	LRT	0.525819254417517
193	KC_AB1	CG17249	-0.416984259995727	0.561025666453164	1	pseudobulk	DESeq2	LRT	0.251017269681455
193	KC_AB1	CG17260	-0.405266634264114	0.726055463073829	1	pseudobulk	DESeq2	LRT	0.139030202458473
193	KC_AB1	CG17261	-0.850996402080018	0.900449987182352	1	pseudobulk	DESeq2	LRT	0.045540403770502
193	KC_AB1	CG17264	1.08899820779554	0.697631521651407	1	pseudobulk	DESeq2	LRT	0.156373904552
194	KC_AB1	CG17266	2.2333809898129	0.108785787476695	1	pseudobulk	DESeq2	LRT	0.963427840149156
194	KC_AB1	CG17267	-0.850996402080018	0.900449987182352	1	pseudobulk	DESeq2	LRT	0.045540403770502
194	KC_AB1	CG17270	0.32742517513573	0.286197632551882	1	pseudobulk	DESeq2	LRT	0.543333963077662
194	KC_AB1	CG17271	0.102842731205616	0.767132659686567	1	pseudobulk	DESeq2	LRT	0.115129527331279
194	KC_AB1	CG17272	0.0028715053558968(	0.962123698750999	1	pseudobulk	DESeq2	LRT	0.016769087804835
194	KC_AB1	CG17294	-0.185932909899075	0.607594187534183	1	pseudobulk	DESeq2	LRT	0.216386389407515
194	KC_AB1	CG17298	1.08899820779554	0.697631521651407	1	pseudobulk	DESeq2	LRT	0.156373904552
194	KC_AB1	CG17321	0.216852906572002	0.222355233137615	1	pseudobulk	DESeq2	LRT	0.652952644960067
194	KC_AB1	CG17328	-0.882971817583479	0.304014974984969	1	pseudobulk	DESeq2	LRT	0.517105023650581
194	KC_AB1	CG17329	-1.89854133735591	0.478664405825708	1	pseudobulk	DESeq2	LRT	0.319968866080244
195	KC_AB1	CG17333	0.0834341681082088	0.940394100468447	1	pseudobulk	DESeq2	LRT	0.026690104070743
195	KC_AB1	CG17343	-0.118964292426325	0.817833075143221	1	pseudobulk	DESeq2	LRT	0.087335329509586
195	KC_AB1	CG17350	-0.850996402080018	0.900449987182352	1	pseudobulk	DESeq2	LRT	0.045540403770502
195	KC_AB1	CG17359	0.497278507558497	0.556827179494322	1	pseudobulk	DESeq2	LRT	0.254279574373935
195	KC_AB1	CG17361	-0.169241340473846	0.80765418323678	1	pseudobulk	DESeq2	LRT	0.092774553161537
195	KC_AB1	CG17364	0.063388303581331	0.805935501148609	1	pseudobulk	DESeq2	LRT	0.093699713301727
195	KC_AB1	CG1737	0.0908681340288523	0.735462082137307	1	pseudobulk	DESeq2	LRT	0.133439713069734
195	KC_AB1	CG17378	0.368971276310164	0.146515457380454	1	pseudobulk	DESeq2	LRT	0.834116554825847
195	KC_AB1	CG1738	-0.419073161146367	0.527209283523355	1	pseudobulk	DESeq2	LRT	0.278016950941611
195	KC_AB1	CG17385	-0.0757794251451269	0.785025892692459	1	pseudobulk	DESeq2	LRT	0.105116018582129
196	KC_AB1	CG17454	0.304522679129475	0.119293068066275	1	pseudobulk	DESeq2	LRT	0.923384791769992
196	KC_AB1	CG17486	0.144739306238433	0.594431368156899	1	pseudobulk	DESeq2	LRT	0.225898280913028
196	KC_AB1	CG17490	2.49031626112382	0.279391560018803	1	pseudobulk	DESeq2	LRT	0.553786717379403
196	KC_AB1	CG17493	0.0279515040550151	0.867885393211453	1	pseudobulk	DESeq2	LRT	0.061537620885778
196	KC_AB1	CG1750	-0.156865042523534	0.825209364785528	1	pseudobulk	DESeq2	LRT	0.083435852134172
196	KC_AB1	CG17508	0.101513809157226	0.688422050783753	1	pseudobulk	DESeq2	LRT	0.16214522727485
196	KC_AB1	CG17528	0.46739221277608	0.055723276210151	1	pseudobulk	DESeq2	LRT	1.25396335746412
196	KC_AB1	CG17544	-0.27558185548321	0.725672794862815	1	pseudobulk	DESeq2	LRT	0.139259158112376
196	KC_AB1	CG17556	0.519698674073341	0.245007755745483	1	pseudobulk	DESeq2	LRT	0.610820167781776
196	KC_AB1	CG17565	0.191563810809451	0.65234163441683	1	pseudobulk	DESeq2	LRT	0.185524902605117
197	KC_AB1	CG17568	-0.393607501450433	0.436976100998731	1	pseudobulk	DESeq2	LRT	0.359542314718818
197	KC_AB1	CG17574	-0.0623396271700933	0.883862230090781	1	pseudobulk	DESeq2	LRT	0.053615424324307
197	KC_AB1	CG17593	-0.157246676283727	0.649953011077822	1	pseudobulk	DESeq2	LRT	0.18711803992217
197	KC_AB1	CG17598	-0.126534922160693	0.736192811901695	1	pseudobulk	DESeq2	LRT	0.133008427267876
197	KC_AB1	CG17600	-0.178307127052485	0.683566670125212	1	pseudobulk	DESeq2	LRT	0.165219121102834

197	KC_AB1	CG17612	0.119781993212677	0.861475792688879	1	pseudobulk	DESeq2	LRT	0.064756921639456
197	KC_AB1	CG17637	0.7010250583415	0.742783423431957	1	pseudobulk	DESeq2	LRT	0.129137796898277
197	KC_AB1	CG1764	0.295433629888971	0.399551187978782	1	pseudobulk	DESeq2	LRT	0.398427573715101
197	KC_AB1	CG17646	1.14832302679308	0.116777765553835	1	pseudobulk	DESeq2	LRT	0.93263983887171
197	KC_AB1	CG17650	-1.35597590423535	0.632167787041343	1	pseudobulk	DESeq2	LRT	0.199167637991254
198	KC_AB1	CG17652	0.579466310317673	0.374276152667735	1	pseudobulk	DESeq2	LRT	0.426807843456642
198	KC_AB1	CG17660	-0.260804559762163	0.608435846745307	1	pseudobulk	DESeq2	LRT	0.215785206872381
198	KC_AB1	CG17666	2.21745641838459	0.396737788462723	1	pseudobulk	DESeq2	LRT	0.401496431894786
198	KC_AB1	CG17683	0.11798037934744	0.665645804435742	1	pseudobulk	DESeq2	LRT	0.176756801006323
198	KC_AB1	CG17684	0.181060272638258	0.080483580867666	1	pseudobulk	DESeq2	LRT	1.09429270927036
198	KC_AB1	CG17687	-1.6747012584303	0.563129890257477	1	pseudobulk	DESeq2	LRT	0.249391420218277
198	KC_AB1	CG17691	0.426105738702196	0.231599341636052	1	pseudobulk	DESeq2	LRT	0.635262679505207
198	KC_AB1	CG17698	-0.0788748855141956	0.601401692783719	1	pseudobulk	DESeq2	LRT	0.220835353811834
198	KC_AB1	CG17707	-1.56855358782635	0.573026303465967	1	pseudobulk	DESeq2	LRT	0.241825442277049
198	KC_AB1	CG17712	0.0429962552980561	0.926039327575683	1	pseudobulk	DESeq2	LRT	0.033370569056268
199	KC_AB1	CG17715	0.161006364463455	0.390881558939035	1	pseudobulk	DESeq2	LRT	0.407954818285931
199	KC_AB1	CG17716	1.61144947759219	0.550510687016443	1	pseudobulk	DESeq2	LRT	0.259234245689108
199	KC_AB1	CG17721	0.513596032659297	0.582589967817752	1	pseudobulk	DESeq2	LRT	0.23463699818227
199	KC_AB1	CG17724	0.410467282860249	0.0899534575469866	1	pseudobulk	DESeq2	LRT	1.04598213899193
199	KC_AB1	CG17726	0.181418649088132	0.850157753319435	1	pseudobulk	DESeq2	LRT	0.070500480121788
199	KC_AB1	CG17734	-0.140251208448119	0.532285131704453	1	pseudobulk	DESeq2	LRT	0.273855664785154
199	KC_AB1	CG17739	-0.850932661185203	0.660736244265418	1	pseudobulk	DESeq2	LRT	0.179971869591347
199	KC_AB1	CG1774	-0.82542030717083	1	1	pseudobulk	DESeq2	LRT	0
199	KC_AB1	CG17746	-0.243254891979682	0.626906629649949	1	pseudobulk	DESeq2	LRT	0.202797137394785
199	KC_AB1	CG17754	0.449756352711084	0.276758656645504	1	pseudobulk	DESeq2	LRT	0.557898786078591
200	KC_AB1	CG17760	-0.0648610729589713	0.959494932948847	1	pseudobulk	DESeq2	LRT	0.017957314412788
200	KC_AB1	CG17765	0.0428149567739444	0.84852074414416	1	pseudobulk	DESeq2	LRT	0.071337535836328
200	KC_AB1	CG17776	0.0468354053930309	0.8558061763346	1	pseudobulk	DESeq2	LRT	0.067624583546925
200	KC_AB1	CG17777	-0.850996402080018	0.900449987182352	1	pseudobulk	DESeq2	LRT	0.045540403770502
200	KC_AB1	CG17778	-0.0631610755807914	0.656003544307258	1	pseudobulk	DESeq2	LRT	0.183093814177806
200	KC_AB1	CG17782	1.01357809590017	0.532795976371359	1	pseudobulk	DESeq2	LRT	0.273439063579336
200	KC_AB1	CG17786	-0.464779313391341	0.563135837738842	1	pseudobulk	DESeq2	LRT	0.249386833452874
200	KC_AB1	CG17802	0.300055831253664	0.55004001937042	1	pseudobulk	DESeq2	LRT	0.259605711306737
200	KC_AB1	CG17803	1.22373295626637	0.130634037801071	1	pseudobulk	DESeq2	LRT	0.883943649223498
200	KC_AB1	CG17806	-0.768458419276056	0.286381142395997	1	pseudobulk	DESeq2	LRT	0.543055582813182
201	KC_AB1	CG17807	-0.375356410119531	0.67524342528718	1	pseudobulk	DESeq2	LRT	0.170539635760132
201	KC_AB1	CG17829	0.278796197519469	0.640396407216445	1	pseudobulk	DESeq2	LRT	0.193551113246257
201	KC_AB1	CG17839	-0.873956330993575	0.0321581518227785	1	pseudobulk	DESeq2	LRT	1.49270891876169
201	KC_AB1	CG17841	-0.13194891091925	0.3289078022643	1	pseudobulk	DESeq2	LRT	0.482925824175779
201	KC_AB1	CG1785	-1.4569786900155	0.212145104813022	1	pseudobulk	DESeq2	LRT	0.673366985001397
201	KC_AB1	CG17883	0.265734700446118	0.224470463032855	1	pseudobulk	DESeq2	LRT	0.64884079758171
201	KC_AB1	CG1789	0.155373643871863	0.716821181898001	1	pseudobulk	DESeq2	LRT	0.144589169856115
201	KC_AB1	CG17896	-1.52983429187015	0.378746459096567	1	pseudobulk	DESeq2	LRT	0.421651418692966
201	KC_AB1	CG17904	-0.323376468711586	0.444866164967967	1	pseudobulk	DESeq2	LRT	0.351770623981097
201	KC_AB1	CG1792	-0.312088281227961	0.655173503993472	1	pseudobulk	DESeq2	LRT	0.183643674285038
202	KC_AB1	CG17931	-0.0018000533400119	0.986949781428883	1	pseudobulk	DESeq2	LRT	0.005704944800658
202	KC_AB1	CG17977	0.0086555632162797	0.96005702142989	1	pseudobulk	DESeq2	LRT	0.017702971796973
202	KC_AB1	CG17982	-0.884310964316533	0.344818065263218	1	pseudobulk	DESeq2	LRT	0.462409989255078
202	KC_AB1	CG17985	0.0008007235144437	0.985185864613691	1	pseudobulk	DESeq2	LRT	0.006481828018799
202	KC_AB1	CG17996	0.0794699095923335	0.8644269899813	1	pseudobulk	DESeq2	LRT	0.063271681611850
202	KC_AB1	CG18004	0.0355387896634716	0.908038143593668	1	pseudobulk	DESeq2	LRT	0.041895907861829
202	KC_AB1	CG18011	0.952970829717089	0.598065999552866	1	pseudobulk	DESeq2	LRT	0.223250886814275

202	KC_AB1	CG18048	-0.270593264525873	0.690001256410402	1	pseudobulk	DESeq2	LRT	0.161150118463313
202	KC_AB1	CG18063	-0.850996402080018	0.900449987182352	1	pseudobulk	DESeq2	LRT	0.045540403770502
202	KC_AB1	CG18081	0.681558931704701	0.147561634438877	1	pseudobulk	DESeq2	LRT	0.831026543036899
203	KC_AB1	CG18094	1.08899820779554	0.697631521651407	1	pseudobulk	DESeq2	LRT	0.156373904552
203	KC_AB1	CG18095	-2.3118469151042	0.353435572380865	1	pseudobulk	DESeq2	LRT	0.451689742007392
203	KC_AB1	CG1812	0.476075136473836	0.489242074971681	1	pseudobulk	DESeq2	LRT	0.31047620056816
203	KC_AB1	CG18135	0.437752370856254	0.129222329155113	1	pseudobulk	DESeq2	LRT	0.888662435327293
203	KC_AB1	CG1814	-0.298482752070152	0.23489552984641	1	pseudobulk	DESeq2	LRT	0.629125247937208
203	KC_AB1	CG1815	-0.0010161800314362	0.984535448098599	1	pseudobulk	DESeq2	LRT	0.006768642515740
203	KC_AB1	CG18171	1.19415263249269	0.561944806229977	1	pseudobulk	DESeq2	LRT	0.25030633839338
203	KC_AB1	CG18178	-0.423716092793461	0.434948407148528	1	pseudobulk	DESeq2	LRT	0.361562255274231
203	KC_AB1	CG18213	-0.82542030717083	1	1	pseudobulk	DESeq2	LRT	0
203	KC_AB1	CG18223	-0.82542030717083	1	1	pseudobulk	DESeq2	LRT	0
204	KC_AB1	CG1824	-0.0001780031647499	0.990405714571854	1	pseudobulk	DESeq2	LRT	0.004186862467876
204	KC_AB1	CG18259	0.302177234878734	0.561164725671071	1	pseudobulk	DESeq2	LRT	0.250909636160927
204	KC_AB1	CG18262	-0.482345907168606	0.604667391579494	1	pseudobulk	DESeq2	LRT	0.218483451336073
204	KC_AB1	CG18265	0.204947590873202	0.497193759121592	1	pseudobulk	DESeq2	LRT	0.303474331349913
204	KC_AB1	CG18268	1.08899820779554	0.878177167143508	1	pseudobulk	DESeq2	LRT	0.056417858865076
204	KC_AB1	CG1827	0.775178373764451	0.706702588695047	1	pseudobulk	DESeq2	LRT	0.150763317834966
204	KC_AB1	CG18273	-1.47921378495508	0.357598966472388	1	pseudobulk	DESeq2	LRT	0.446603745066241
204	KC_AB1	CG18281	-0.44493998250221	0.735125998188153	1	pseudobulk	DESeq2	LRT	0.133638217884329
204	KC_AB1	CG18324	-0.373532854927737	0.417576930042581	1	pseudobulk	DESeq2	LRT	0.37926350289843
204	KC_AB1	CG18327	-0.258437620760278	0.794381732213576	1	pseudobulk	DESeq2	LRT	0.099970751534404
205	KC_AB1	CG18335	1.08899820779554	0.878177167143508	1	pseudobulk	DESeq2	LRT	0.056417858865076
205	KC_AB1	CG18336	0.144938062156184	0.939011437924561	1	pseudobulk	DESeq2	LRT	0.027329117640952
205	KC_AB1	CG18368	1.08899820779554	0.697631521651407	1	pseudobulk	DESeq2	LRT	0.156373904552
205	KC_AB1	CG18371	-0.850932661185203	0.660736244265418	1	pseudobulk	DESeq2	LRT	0.179971869591347
205	KC_AB1	CG1840	-0.174491952638705	0.65620378159373	1	pseudobulk	DESeq2	LRT	0.182961271175577
205	KC_AB1	CG18420	-1.44925888589622	0.6315588144614	1	pseudobulk	DESeq2	LRT	0.199586199175701
205	KC_AB1	CG18428	0.18993975290825	0.468821715395045	1	pseudobulk	DESeq2	LRT	0.328992280399048
205	KC_AB1	CG18467	-0.0354660486137945	0.874562896541526	1	pseudobulk	DESeq2	LRT	0.058208951607084
205	KC_AB1	CG1847	0.0232983472864382	0.934567138461998	1	pseudobulk	DESeq2	LRT	0.029389493839769
205	KC_AB1	CG18476	-0.199748733895487	0.815076496869362	1	pseudobulk	DESeq2	LRT	0.088801629776332
206	KC_AB1	CG18507	4.69658047115784	0.284325907870278	1	pseudobulk	DESeq2	LRT	0.546183565450404
206	KC_AB1	CG18508	0.0685932066686079	0.820864588713679	1	pseudobulk	DESeq2	LRT	0.085728478964282
206	KC_AB1	CG18528	0.29608556765303	0.750005976503145	1	pseudobulk	DESeq2	LRT	0.124935275872306
206	KC_AB1	CG18537	-0.82542030717083	1	1	pseudobulk	DESeq2	LRT	0
206	KC_AB1	CG18549	0.707696058377932	0.127320340344434	1	pseudobulk	DESeq2	LRT	0.895102209121784
206	KC_AB1	CG18563	-1.56855358782635	0.573026303465967	1	pseudobulk	DESeq2	LRT	0.241825442277049
206	KC_AB1	CG18586	1.08899820779554	0.878177167143508	1	pseudobulk	DESeq2	LRT	0.056417858865076
206	KC_AB1	CG18596	-0.467203033794754	0.633487949416234	1	pseudobulk	DESeq2	LRT	0.198261642109364
206	KC_AB1	CG18598	0.516794653828635	0.814433332789471	1	pseudobulk	DESeq2	LRT	0.089144460019238
206	KC_AB1	CG18599	-0.850996402080018	0.900449987182352	1	pseudobulk	DESeq2	LRT	0.045540403770502
207	KC_AB1	CG18600	-0.507823378540224	0.589378096187075	1	pseudobulk	DESeq2	LRT	0.229606008428154
207	KC_AB1	CG18609	1.08899820779554	0.878177167143508	1	pseudobulk	DESeq2	LRT	0.056417858865076
207	KC_AB1	CG18628	-1.89189631351533	0.272977974932945	1	pseudobulk	DESeq2	LRT	0.563872392340062
207	KC_AB1	CG18659	0.0232512149179744	0.951329286778658	1	pseudobulk	DESeq2	LRT	0.021669133243175
207	KC_AB1	CG18661	0.108586480966263	0.883615201232661	1	pseudobulk	DESeq2	LRT	0.053736821371590
207	KC_AB1	CG18675	-0.597401359775062	0.539270675645017	1	pseudobulk	DESeq2	LRT	0.268193195049887
207	KC_AB1	CG18731	-0.0891134531654782	0.828579233858634	1	pseudobulk	DESeq2	LRT	0.081665955340909
207	KC_AB1	CG18744	1.08899820779554	0.878177167143508	1	pseudobulk	DESeq2	LRT	0.056417858865076
207	KC_AB1	CG18754	0.114184054362142	1	1	pseudobulk	DESeq2	LRT	0

207	KC_AB1	CG18764	1.09853581628742	0.304985007817006	1	pseudobulk	DESeq2	LRT	0.515721508792149
208	KC_AB1	CG18766	0.0766347370777282	0.799190312038274	1	pseudobulk	DESeq2	LRT	0.097349789363931
208	KC_AB1	CG18787	-0.300969580318417	0.701814504233814	1	pseudobulk	DESeq2	LRT	0.153777660566868
208	KC_AB1	CG18789	0.855700630127339	0.665189798240232	1	pseudobulk	DESeq2	LRT	0.177054420008663
208	KC_AB1	CG18809	-0.01998546766337	0.962454123871189	1	pseudobulk	DESeq2	LRT	0.016619962312372
208	KC_AB1	CG18815	-0.122457217540435	0.504701545629328	1	pseudobulk	DESeq2	LRT	0.29696536525391
208	KC_AB1	CG1882	-0.021923588528152	0.92682647022996	1	pseudobulk	DESeq2	LRT	0.033001571223620
208	KC_AB1	CG18858	-2.36817011682091	0.282654768599882	1	pseudobulk	DESeq2	LRT	0.548743683265438
208	KC_AB1	CG18870	0.480745583931813	0.422910185396402	1	pseudobulk	DESeq2	LRT	0.373751855150603
208	KC_AB1	CG1888	0.480680882359805	0.280756184544598	1	pseudobulk	DESeq2	LRT	0.551670668249019
208	KC_AB1	CG1890	-1.46149620197804	0.0366997937479596	1	pseudobulk	DESeq2	LRT	1.43533637646657
209	KC_AB1	CG1896	-0.215211939022511	0.558729717819353	1	pseudobulk	DESeq2	LRT	0.252798228690449
209	KC_AB1	CG1902	-0.0339311853275407	0.909023515540132	1	pseudobulk	DESeq2	LRT	0.041424881863597
209	KC_AB1	CG1907	-0.017380429509735	0.907727699913991	1	pseudobulk	DESeq2	LRT	0.042044411567657
209	KC_AB1	CG1909	-0.0360146645511054	0.794081411965756	1	pseudobulk	DESeq2	LRT	0.100134969921798
209	KC_AB1	CG1910	0.0592989230468782	0.788569431922753	1	pseudobulk	DESeq2	LRT	0.103160061912268
209	KC_AB1	CG1924	-1.82709236256241	0.44317674354685	1	pseudobulk	DESeq2	LRT	0.353423038023035
209	KC_AB1	CG1927	0.0123663407585138	0.965480793631254	1	pseudobulk	DESeq2	LRT	0.015256361245466
209	KC_AB1	CG1943	0.114490104241442	0.535359804503985	1	pseudobulk	DESeq2	LRT	0.271354239307912
209	KC_AB1	CG1951	-0.0550485888193539	0.878656683570442	1	pseudobulk	DESeq2	LRT	0.056180783124206
209	KC_AB1	CG1958	1.08899820779554	0.878177167143508	1	pseudobulk	DESeq2	LRT	0.056417858865076
210	KC_AB1	CG1965	0.0580493289952988	0.808567321231678	1	pseudobulk	DESeq2	LRT	0.092283814938465
210	KC_AB1	CG1983	0.114532354749379	0.763970935588483	1	pseudobulk	DESeq2	LRT	0.116923163353162
210	KC_AB1	CG1998	0.136457692251067	0.522638649486258	1	pseudobulk	DESeq2	LRT	0.281798477046592
210	KC_AB1	CG2003	0.710071387175138	0.500724783936384	1	pseudobulk	DESeq2	LRT	0.300400912175226
210	KC_AB1	CG2004	0.785251537395176	0.611382519317276	1	pseudobulk	DESeq2	LRT	0.213686982804202
210	KC_AB1	CG2006	0.526202898899209	0.574346097827385	1	pseudobulk	DESeq2	LRT	0.240826325232639
210	KC_AB1	CG2016	1.08899820779554	0.670846322504933	1	pseudobulk	DESeq2	LRT	0.173376956629912
210	KC_AB1	CG2017	-0.251785095546436	0.350820650484001	1	pseudobulk	DESeq2	LRT	0.454914850518966
210	KC_AB1	CG2021	-0.21761043760162	0.60654017842114	1	pseudobulk	DESeq2	LRT	0.217140425319019
210	KC_AB1	CG2023	-0.635741385864236	0.371650381818889	1	pseudobulk	DESeq2	LRT	0.429865416683845
211	KC_AB1	CG2034	0.507080721395074	0.500304884249198	1	pseudobulk	DESeq2	LRT	0.300765257276254
211	KC_AB1	CG2059	-0.400378201545332	0.331084362001821	1	pseudobulk	DESeq2	LRT	0.480061331651148
211	KC_AB1	CG2061	0.0344932887999985	0.916880368723216	1	pseudobulk	DESeq2	LRT	0.037687325826912
211	KC_AB1	CG2063	0.0348612913289427	0.933486190000249	1	pseudobulk	DESeq2	LRT	0.029892102622295
211	KC_AB1	CG2064	1.13983444729496	0.164840245743595	1	pseudobulk	DESeq2	LRT	0.782936746701393
211	KC_AB1	CG2065	1.08899820779554	0.670846322504933	1	pseudobulk	DESeq2	LRT	0.173376956629912
211	KC_AB1	CG2076	0.228959814185785	0.551262511239806	1	pseudobulk	DESeq2	LRT	0.258641540833402
211	KC_AB1	CG2082	-0.178088900908889	0.917437930365065	1	pseudobulk	DESeq2	LRT	0.037423308451134
211	KC_AB1	CG2091	0.446875258640178	0.358073737932018	1	pseudobulk	DESeq2	LRT	0.446027530117534
211	KC_AB1	CG2100	-0.20282299578342	0.816182764359485	1	pseudobulk	DESeq2	LRT	0.088212580626951
212	KC_AB1	CG2104	0.33801878461149	0.868910254227304	1	pseudobulk	DESeq2	LRT	0.06102507752903
212	KC_AB1	CG2111	2.00676409265136	0.376151646279052	1	pseudobulk	DESeq2	LRT	0.424637033091521
212	KC_AB1	CG2116	-0.109857571641844	0.881546658694922	1	pseudobulk	DESeq2	LRT	0.054754696322719
212	KC_AB1	CG2120	-0.82542030717083	1	1	pseudobulk	DESeq2	LRT	0
212	KC_AB1	CG2124	0.282405443373498	0.836932826257467	1	pseudobulk	DESeq2	LRT	0.077309397868658
212	KC_AB1	CG2126	-0.111564266896394	0.856341051151203	1	pseudobulk	DESeq2	LRT	0.067353236334271
212	KC_AB1	CG2129	-1.38775589774078	0.46068469705374	1	pseudobulk	DESeq2	LRT	0.336596213828282
212	KC_AB1	CG2145	1.08899820779554	0.697631521651407	1	pseudobulk	DESeq2	LRT	0.156373904552
212	KC_AB1	CG2147	0.345619481458254	0.560467328795313	1	pseudobulk	DESeq2	LRT	0.251449698568492
212	KC_AB1	CG2162	-0.162176304468477	0.6257596563592	1	pseudobulk	DESeq2	LRT	0.203592439888084
213	KC_AB1	CG2182	0.482732107138837	0.0810676771807937	1	pseudobulk	DESeq2	LRT	1.09115227057488

213	KC_AB1	CG2186	-0.040963938816569	0.846035128160983	1	pseudobulk	DESeq2	LRT	0.072611604278057
213	KC_AB1	CG2199	-0.039674094777178	0.929844510168763	1	pseudobulk	DESeq2	LRT	0.031589668673458
213	KC_AB1	CG2200	0.985058142162852	0.145244101737292	1	pseudobulk	DESeq2	LRT	0.837901494978651
213	KC_AB1	CG2201	0.680357868705269	0.0874553395239384	1	pseudobulk	DESeq2	LRT	1.05821366983321
213	KC_AB1	CG2202	-2.13918050309547	0.0308869377042297	1	pseudobulk	DESeq2	LRT	1.51022514783586
213	KC_AB1	CG2209	0.274006407164787	0.740167401959087	1	pseudobulk	DESeq2	LRT	0.130670045775675
213	KC_AB1	CG2211	0.568202825405389	0.451056039626525	1	pseudobulk	DESeq2	LRT	0.345769497615087
213	KC_AB1	CG2218	0.0577031938779181	0.888654280478158	1	pseudobulk	DESeq2	LRT	0.051267162857286
213	KC_AB1	CG2224	-0.270727812607625	0.684720296504287	1	pseudobulk	DESeq2	LRT	0.164486798568828
214	KC_AB1	CG2225	0.0428804606566615	0.784085066279188	1	pseudobulk	DESeq2	LRT	0.10563681765799
214	KC_AB1	CG2233	1.26418346555976	0.618943317621274	1	pseudobulk	DESeq2	LRT	0.20834912153152
214	KC_AB1	CG2246	0.419347766424955	0.0673097007289721	1	pseudobulk	DESeq2	LRT	1.17192234039029
214	KC_AB1	CG2247	0.356772350396067	0.48957519175867	1	pseudobulk	DESeq2	LRT	0.31018059730712
214	KC_AB1	CG2256	-1.71421461715908	0.404851648933651	1	pseudobulk	DESeq2	LRT	0.392704087531862
214	KC_AB1	CG2258	0.25073953097216	0.320447316562385	1	pseudobulk	DESeq2	LRT	0.494243360862653
214	KC_AB1	CG2260	-0.748310946516826	0.461601463968214	1	pseudobulk	DESeq2	LRT	0.335732822485972
214	KC_AB1	CG2269	-0.261497850374882	0.0574884590146368	1	pseudobulk	DESeq2	LRT	1.24041933251613
214	KC_AB1	CG2277	-0.285565750273493	0.595680708489277	1	pseudobulk	DESeq2	LRT	0.224986464585258
214	KC_AB1	CG2278	0.261722858802386	0.517291644233059	1	pseudobulk	DESeq2	LRT	0.286264536655256
215	KC_AB1	CG2310	1.08899820779554	0.878177167143508	1	pseudobulk	DESeq2	LRT	0.056417858865076
215	KC_AB1	CG2321	-0.132896148240469	0.84372802488718	1	pseudobulk	DESeq2	LRT	0.073797525324793
215	KC_AB1	CG2444	1.08899820779554	0.878177167143508	1	pseudobulk	DESeq2	LRT	0.056417858865076
215	KC_AB1	CG2519	0.0782342910860811	0.768877110061097	1	pseudobulk	DESeq2	LRT	0.11414306811434
215	KC_AB1	CG2528	1.64750305707911	0.566713307590673	1	pseudobulk	DESeq2	LRT	0.246636589113683
215	KC_AB1	CG2540	-1.78686736724522	0.0230252077854248	1	pseudobulk	DESeq2	LRT	1.6377964419278
215	KC_AB1	CG2556	-1.56855358782635	0.573026303465967	1	pseudobulk	DESeq2	LRT	0.241825442277049
215	KC_AB1	CG2608	-0.318629649579752	0.532274984973025	1	pseudobulk	DESeq2	LRT	0.273863943640242
215	KC_AB1	CG2611	-0.501773093294086	0.524135449136518	1	pseudobulk	DESeq2	LRT	0.280556466430311
215	KC_AB1	CG2614	-1.34212606730294	0.118743321784637	1	pseudobulk	DESeq2	LRT	0.925390806070569
216	KC_AB1	CG2617	-0.175589756662561	0.82340389022612	1	pseudobulk	DESeq2	LRT	0.084387085480692
216	KC_AB1	CG2652	-0.850932661185203	0.660736244265418	1	pseudobulk	DESeq2	LRT	0.179971869591347
216	KC_AB1	CG2656	-0.076782048958807	0.837555941996006	1	pseudobulk	DESeq2	LRT	0.076986175946294
216	KC_AB1	CG2662	0.176084376930194	0.817098897439922	1	pseudobulk	DESeq2	LRT	0.087725375520133
216	KC_AB1	CG2680	-0.205589562320426	0.849343440552622	1	pseudobulk	DESeq2	LRT	0.070916662904724
216	KC_AB1	CG2681	1.08899820779554	0.670846322504933	1	pseudobulk	DESeq2	LRT	0.173376956629912
216	KC_AB1	CG2685	-0.747847731942873	0.290369800857001	1	pseudobulk	DESeq2	LRT	0.537048553272051
216	KC_AB1	CG2691	-0.175278905154576	0.840398383180457	1	pseudobulk	DESeq2	LRT	0.075514791792597
216	KC_AB1	CG2694	-0.766318063248534	0.71163005426468	1	pseudobulk	DESeq2	LRT	0.147745718646455
216	KC_AB1	CG2698	0.369121166686253	0.278040673651726	1	pseudobulk	DESeq2	LRT	0.555891667929366
217	KC_AB1	CG2701	-0.037595400094251	0.936419302794421	1	pseudobulk	DESeq2	LRT	0.028529642593205
217	KC_AB1	CG2712	-1.09427928545388	0.380609951533811	1	pseudobulk	DESeq2	LRT	0.419519860716052
217	KC_AB1	CG2747	0.132277445002687	0.644949203266892	1	pseudobulk	DESeq2	LRT	0.190474489410664
217	KC_AB1	CG2765	0.253746432961893	0.408363514727006	1	pseudobulk	DESeq2	LRT	0.388953066929307
217	KC_AB1	CG2767	0.599083466581894	0.373631411973079	1	pseudobulk	DESeq2	LRT	0.427556618862982
217	KC_AB1	CG2772	0.621369219015482	0.0519956770216096	1	pseudobulk	DESeq2	LRT	1.28403276259029
217	KC_AB1	CG2790	0.493480776676849	0.218432130992758	1	pseudobulk	DESeq2	LRT	0.660683477292966
217	KC_AB1	CG2794	-0.939884034469772	0.408292675495491	1	pseudobulk	DESeq2	LRT	0.389028410967092
217	KC_AB1	CG2811	-0.044535213809897	0.896284806200424	1	pseudobulk	DESeq2	LRT	0.047553965664709
217	KC_AB1	CG2812	-0.506516985469986	0.909346984303859	1	pseudobulk	DESeq2	LRT	0.041270369127443
218	KC_AB1	CG2818	0.117484801011668	0.755788823681344	1	pseudobulk	DESeq2	LRT	0.121599534553707
218	KC_AB1	CG2839	2.21745641838459	0.396737788462723	1	pseudobulk	DESeq2	LRT	0.401496431894786
218	KC_AB1	CG2841	2.95289133500521	0.186463231545554	1	pseudobulk	DESeq2	LRT	0.729406793405742

218	KC_AB1	CG2846	0.674401442359234	0.143927551415657	1	pseudobulk	DESeq2	LRT	0.84185606303024
218	KC_AB1	CG2852	-0.299621599762953	0.121523509142231	1	pseudobulk	DESeq2	LRT	0.915339698171743
218	KC_AB1	CG2865	-0.362184972768211	0.313469427920019	1	pseudobulk	DESeq2	LRT	0.503804808686756
218	KC_AB1	CG2875	-0.0116975879036681	0.98082202144956	1	pseudobulk	DESeq2	LRT	0.00840979192163
218	KC_AB1	CG2889	-0.477668667336464	0.364039879064358	1	pseudobulk	DESeq2	LRT	0.438851038579151
218	KC_AB1	CG2906	-0.0252398775879618	0.969620142579904	1	pseudobulk	DESeq2	LRT	0.013398371188885
218	KC_AB1	CG2909	-0.294712082623673	0.66897276000666	1	pseudobulk	DESeq2	LRT	0.174591565968572
219	KC_AB1	CG2911	-0.958830208790218	0.37543958723207	1	pseudobulk	DESeq2	LRT	0.425459936269812
219	KC_AB1	CG2915	-0.525334123198883	0.46636280026813	1	pseudobulk	DESeq2	LRT	0.331276098649117
219	KC_AB1	CG2918	0.0236685983174909	0.948433429700176	1	pseudobulk	DESeq2	LRT	0.022993146724164
219	KC_AB1	CG2921	0.287056918986646	0.545685577579377	1	pseudobulk	DESeq2	LRT	0.263057524429176
219	KC_AB1	CG2924	-0.0302735234919527	0.848182498018895	1	pseudobulk	DESeq2	LRT	0.071510693314929
219	KC_AB1	CG2926	0.0698549484166717	0.720267631015359	1	pseudobulk	DESeq2	LRT	0.142506102073633
219	KC_AB1	CG2931	0.046421151078367	0.835938789247997	1	pseudobulk	DESeq2	LRT	0.077825522160359
219	KC_AB1	CG2938	-0.225353704003527	0.596035641112522	1	pseudobulk	DESeq2	LRT	0.224727769998526
219	KC_AB1	CG2962	0.11269840515639	0.977007463492936	1	pseudobulk	DESeq2	LRT	0.010102118633921
219	KC_AB1	CG2972	0.233855587190062	0.755468149486525	1	pseudobulk	DESeq2	LRT	0.121783840780724
220	KC_AB1	CG2991	0.198254880647978	0.456638298769745	1	pseudobulk	DESeq2	LRT	0.34042766654961
220	KC_AB1	CG2993	0.207533082088679	0.200513347387487	1	pseudobulk	DESeq2	LRT	0.697856712800419
220	KC_AB1	CG30001	-0.642749171356925	0.752779105808464	1	pseudobulk	DESeq2	LRT	0.123332443722695
220	KC_AB1	CG30008	-1.68203279576935	0.480780548244404	1	pseudobulk	DESeq2	LRT	0.318053111639696
220	KC_AB1	CG30010	-1.28321267334249	0.224061348637002	1	pseudobulk	DESeq2	LRT	0.649633054314616
220	KC_AB1	CG30015	-1.6747012584303	0.563129890257477	1	pseudobulk	DESeq2	LRT	0.249391420218277
220	KC_AB1	CG30020	-0.58090955073695	0.297619258411676	1	pseudobulk	DESeq2	LRT	0.52633896979514
220	KC_AB1	CG30022	-0.306749672961153	0.568279309623546	1	pseudobulk	DESeq2	LRT	0.245438155815027
220	KC_AB1	CG30054	0.51897310101609	0.696247050690259	1	pseudobulk	DESeq2	LRT	0.157236631490256
220	KC_AB1	CG30060	0.717462828913306	0.406865841979617	1	pseudobulk	DESeq2	LRT	0.390548769381075
221	KC_AB1	CG30069	1.10989969347445	0.155224944484984	1	pseudobulk	DESeq2	LRT	0.809038486804056
221	KC_AB1	CG30076	2.54858448870456	0.280000067828146	1	pseudobulk	DESeq2	LRT	0.552841863452831
221	KC_AB1	CG3008	-0.54217674874545	0.295872426400548	1	pseudobulk	DESeq2	LRT	0.528895506688215
221	KC_AB1	CG30089	0.10540316571799	0.743015074250489	1	pseudobulk	DESeq2	LRT	0.129002375206589
221	KC_AB1	CG3009	0.537812637845797	0.509681430599975	1	pseudobulk	DESeq2	LRT	0.292701188924283
221	KC_AB1	CG30094	-0.0864893765051196	0.818496429519212	1	pseudobulk	DESeq2	LRT	0.086983210746219
221	KC_AB1	CG30096	0.0300432230512911	0.960462359540107	1	pseudobulk	DESeq2	LRT	0.017519650442416
221	KC_AB1	CG30099	1.33514302811076	0.295266536115487	1	pseudobulk	DESeq2	LRT	0.529785770792834
221	KC_AB1	CG30105	0.11400025262841	1	1	pseudobulk	DESeq2	LRT	0
221	KC_AB1	CG30109	0.0957673732658755	0.868106041958853	1	pseudobulk	DESeq2	LRT	0.061427221108442
222	KC_AB1	CG30110	1.03379335559591	0.428550197932582	1	pseudobulk	DESeq2	LRT	0.36799829997586
222	KC_AB1	CG30116	-0.0062833925563918	0.935574004590387	1	pseudobulk	DESeq2	LRT	0.028921853792975
222	KC_AB1	CG30122	-0.293442029245595	0.264959214913676	1	pseudobulk	DESeq2	LRT	0.576820971727806
222	KC_AB1	CG30156	0.101253751624946	0.87814625096636	1	pseudobulk	DESeq2	LRT	0.056433148446709
222	KC_AB1	CG30157	0.547962416103743	0.787939377744845	1	pseudobulk	DESeq2	LRT	0.103507194850131
222	KC_AB1	CG30158	-0.317749138671637	0.0810512226726244	1	pseudobulk	DESeq2	LRT	1.09124042935379
222	KC_AB1	CG30172	0.211199926385634	0.181689294441161	1	pseudobulk	DESeq2	LRT	0.740670661586311
222	KC_AB1	CG30183	0.678286941471034	0.584317781956446	1	pseudobulk	DESeq2	LRT	0.233350897040659
222	KC_AB1	CG30187	0.934204698285863	0.661333165276948	1	pseudobulk	DESeq2	LRT	0.179579697263353
222	KC_AB1	CG30197	-1.30223380965193	0.57947592928145	1	pseudobulk	DESeq2	LRT	0.236964599385212
223	KC_AB1	CG3021	-0.467090395719644	0.507043700120032	1	pseudobulk	DESeq2	LRT	0.294954608905099
223	KC_AB1	CG30270	-0.82542030717083	1	1	pseudobulk	DESeq2	LRT	0
223	KC_AB1	CG30291	0.426144589549679	0.500750456575067	1	pseudobulk	DESeq2	LRT	0.300378646052476
223	KC_AB1	CG3032	-2.21154086892511	0.254486012135975	1	pseudobulk	DESeq2	LRT	0.594336083736296
223	KC_AB1	CG30324	-0.268359571488918	0.874433266607883	1	pseudobulk	DESeq2	LRT	0.058273328609344

223	KC_AB1	CG30325	0.187499809431982	0.910303214736121	1	pseudobulk	DESeq2	LRT	0.040813923554985
223	KC_AB1	CG30334	1.47845164298158	0.630642748871417	1	pseudobulk	DESeq2	LRT	0.200216593420489
223	KC_AB1	CG30338	-1.12344067462071	0.383671542928858	1	pseudobulk	DESeq2	LRT	0.416040411401849
223	KC_AB1	CG30344	-0.606506423917245	0.499693238389451	1	pseudobulk	DESeq2	LRT	0.301296527183424
223	KC_AB1	CG30345	0.927282623097541	0.829267166618274	1	pseudobulk	DESeq2	LRT	0.081305529407006
224	KC_AB1	CG30349	1.09039751614319	0.234870266415054	1	pseudobulk	DESeq2	LRT	0.62917195959195
224	KC_AB1	CG30356	-0.619148458022618	0.879843167001942	1	pseudobulk	DESeq2	LRT	0.055594734413205
224	KC_AB1	CG30357	-0.850996402080018	0.900449987182352	1	pseudobulk	DESeq2	LRT	0.045540403770502
224	KC_AB1	CG3036	-1.56855358782635	0.573026303465967	1	pseudobulk	DESeq2	LRT	0.241825442277049
224	KC_AB1	CG30373	-0.0108221162192246	0.979523187627473	1	pseudobulk	DESeq2	LRT	0.008985278771372
224	KC_AB1	CG3038	1.08899820779554	0.670846322504933	1	pseudobulk	DESeq2	LRT	0.173376956629912
224	KC_AB1	CG30380	1.08899820779554	0.670846322504933	1	pseudobulk	DESeq2	LRT	0.173376956629912
224	KC_AB1	CG30389	-0.0588286352920682	0.729039962670451	1	pseudobulk	DESeq2	LRT	0.137248664972048
224	KC_AB1	CG30392	0.188859650768739	0.594988125112687	1	pseudobulk	DESeq2	LRT	0.225491701917664
224	KC_AB1	CG30394	-0.549948123910182	0.447749997565959	1	pseudobulk	DESeq2	LRT	0.348964407839826
225	KC_AB1	CG3040	-0.26471641741178	0.561727883636635	1	pseudobulk	DESeq2	LRT	0.250474017624724
225	KC_AB1	CG30403	1.41080707540048	0.24136758538779	1	pseudobulk	DESeq2	LRT	0.617321054174237
225	KC_AB1	CG30414	-0.850996402080018	0.900449987182352	1	pseudobulk	DESeq2	LRT	0.045540403770502
225	KC_AB1	CG30423	-0.0273350966992319	0.886104154287483	1	pseudobulk	DESeq2	LRT	0.052515227352458
225	KC_AB1	CG30424	3.031977956515	0.0787074090405708	1	pseudobulk	DESeq2	LRT	1.10398438385461
225	KC_AB1	CG30428	0.111492020545228	0.66564616967509	1	pseudobulk	DESeq2	LRT	0.176756562709335
225	KC_AB1	CG3045	0.287423898507149	0.772486683967159	1	pseudobulk	DESeq2	LRT	0.112108998154813
225	KC_AB1	CG30460	0.291609378257244	0.72773143204764	1	pseudobulk	DESeq2	LRT	0.138028866702789
225	KC_AB1	CG30466	1.38619582403046	0.295430699868849	1	pseudobulk	DESeq2	LRT	0.52954437669208
225	KC_AB1	CG30467	0.148348334078817	0.81504303251263	1	pseudobulk	DESeq2	LRT	0.088819460843575
226	KC_AB1	CG30472	-0.850996402080018	0.900449987182352	1	pseudobulk	DESeq2	LRT	0.045540403770502
226	KC_AB1	CG30479	1.08899820779554	0.878177167143508	1	pseudobulk	DESeq2	LRT	0.056417858865076
226	KC_AB1	CG30480	-1.20120449962996	0.506796796872571	1	pseudobulk	DESeq2	LRT	0.295166138668161
226	KC_AB1	CG30484	1.95275942187961	0.438420007953907	1	pseudobulk	DESeq2	LRT	0.358109634392079
226	KC_AB1	CG30485	1.21433710563586	0.747381629023987	1	pseudobulk	DESeq2	LRT	0.126457581508545
226	KC_AB1	CG30491	-0.207105268539856	0.754126692618441	1	pseudobulk	DESeq2	LRT	0.122555686911377
226	KC_AB1	CG30495	-0.434529171007376	0.698996519694173	1	pseudobulk	DESeq2	LRT	0.1555249866025
226	KC_AB1	CG30496	-2.5499728768689	0.191972425411594	1	pseudobulk	DESeq2	LRT	0.716761148127771
226	KC_AB1	CG3061	-0.0252677275927594	0.877524632314753	1	pseudobulk	DESeq2	LRT	0.056740683946777
226	KC_AB1	CG3065	0.142951975453306	0.737656904957212	1	pseudobulk	DESeq2	LRT	0.132145587947309
227	KC_AB1	CG3071	0.364029586221256	0.732417067503567	1	pseudobulk	DESeq2	LRT	0.135241543894845
227	KC_AB1	CG3078	-0.247369753845092	0.445512685905953	1	pseudobulk	DESeq2	LRT	0.351139924981736
227	KC_AB1	CG3088	-0.82542030717083	1	1	pseudobulk	DESeq2	LRT	0
227	KC_AB1	CG3097	-2.74419724648772	0.136655816030679	1	pseudobulk	DESeq2	LRT	0.86437188013466
227	KC_AB1	CG31019	1.08899820779554	0.670846322504933	1	pseudobulk	DESeq2	LRT	0.173376956629912
227	KC_AB1	CG31030	-0.274631946359594	0.0937137811556376	1	pseudobulk	DESeq2	LRT	1.02819653889071
227	KC_AB1	CG31038	-0.294838592183076	0.6330161693825	1	pseudobulk	DESeq2	LRT	0.198585196485159
227	KC_AB1	CG3104	-0.0340299175644155	0.88095998529456	1	pseudobulk	DESeq2	LRT	0.055043817536232
227	KC_AB1	CG31041	2.10549911548503	0.428045198278114	1	pseudobulk	DESeq2	LRT	0.368510370411162
227	KC_AB1	CG31051	-0.316219729955055	0.394214113145735	1	pseudobulk	DESeq2	LRT	0.404267831726799
228	KC_AB1	CG31064	-0.0874688834899939	0.724734069769767	1	pseudobulk	DESeq2	LRT	0.139821322005557
228	KC_AB1	CG31068	0.111831901354254	1	1	pseudobulk	DESeq2	LRT	0
228	KC_AB1	CG3107	-0.197624310014795	0.341997313847505	1	pseudobulk	DESeq2	LRT	0.465977305013419
228	KC_AB1	CG31098	-1.43572388380703	0.181685478061569	1	pseudobulk	DESeq2	LRT	0.740679784028059
228	KC_AB1	CG31109	0.0218227778854752	0.925543595699756	1	pseudobulk	DESeq2	LRT	0.033603119940872
228	KC_AB1	CG31111	-0.162216324222471	0.837324171292453	1	pseudobulk	DESeq2	LRT	0.077106371699368
228	KC_AB1	CG31115	-0.480508829474867	0.790489736644791	1	pseudobulk	DESeq2	LRT	0.102103764374996

228	KC_AB1	CG31122	-0.19983111476302	0.512589112094253	1	pseudobulk	DESeq2	LRT	0.290230622903247
228	KC_AB1	CG31125	0.0070023473387183	0.962674710533732	1	pseudobulk	DESeq2	LRT	0.016520436951751
228	KC_AB1	CG31126	-0.137336054786522	0.740327578003974	1	pseudobulk	DESeq2	LRT	0.130576072376724
229	KC_AB1	CG31140	-0.0409533407012793	0.696834382411368	1	pseudobulk	DESeq2	LRT	0.156870429006214
229	KC_AB1	CG31142	-0.608329710967242	0.531204609185541	1	pseudobulk	DESeq2	LRT	0.274738165313303
229	KC_AB1	CG31145	-0.82542030717083	1	1	pseudobulk	DESeq2	LRT	0
229	KC_AB1	CG31156	-0.450072239530249	0.526535536794201	1	pseudobulk	DESeq2	LRT	0.278572312203471
229	KC_AB1	CG31157	-2.21082868077468	0.352001909964874	1	pseudobulk	DESeq2	LRT	0.453454980030519
229	KC_AB1	CG31161	-1.0035696844245	0.534278373660666	1	pseudobulk	DESeq2	LRT	0.272232404686496
229	KC_AB1	CG31174	-0.755103345909353	0.212757097762968	1	pseudobulk	DESeq2	LRT	0.672115942557721
229	KC_AB1	CG31191	-0.17080925089351	0.313831435075726	1	pseudobulk	DESeq2	LRT	0.503303557255872
229	KC_AB1	CG31195	-0.836532679853019	0.420560802566966	1	pseudobulk	DESeq2	LRT	0.376171207213542
229	KC_AB1	CG31199	0.736294445752933	1	1	pseudobulk	DESeq2	LRT	0
230	KC_AB1	CG31200	0.112935832476632	0.940353958317081	1	pseudobulk	DESeq2	LRT	0.026708642986417
230	KC_AB1	CG31211	0.263082821860281	0.176016751640371	1	pseudobulk	DESeq2	LRT	0.754445998101904
230	KC_AB1	CG31213	-0.506516985469986	0.909346984303859	1	pseudobulk	DESeq2	LRT	0.041270369127443
230	KC_AB1	CG31223	0.293516037499815	0.71537117704429	1	pseudobulk	DESeq2	LRT	0.145468561954435
230	KC_AB1	CG31224	-0.111493860675535	0.686818822222018	1	pseudobulk	DESeq2	LRT	0.163157811537986
230	KC_AB1	CG31229	-0.0437824411214937	0.896755192271779	1	pseudobulk	DESeq2	LRT	0.047326100050987
230	KC_AB1	CG31248	-1.01987673318539	0.607334833550393	1	pseudobulk	DESeq2	LRT	0.216571809302465
230	KC_AB1	CG31249	-0.700422729390092	0.155414887585839	1	pseudobulk	DESeq2	LRT	0.808507381373643
230	KC_AB1	CG31251	-0.0729244462101597	0.903939708190853	1	pseudobulk	DESeq2	LRT	0.043860535534849
230	KC_AB1	CG31278	-0.0137804575495152	0.960802758115147	1	pseudobulk	DESeq2	LRT	0.017365758901914
231	KC_AB1	CG31300	1.15613149945626	0.580035201991247	1	pseudobulk	DESeq2	LRT	0.236545648563562
231	KC_AB1	CG31301	-0.445574916983117	0.290312527139522	1	pseudobulk	DESeq2	LRT	0.537134223726758
231	KC_AB1	CG31324	-1.91084301577124	0.0501074757293486	1	pseudobulk	DESeq2	LRT	1.30009747521458
231	KC_AB1	CG31327	-0.850932661185203	0.660736244265418	1	pseudobulk	DESeq2	LRT	0.179971869591347
231	KC_AB1	CG31342	-0.0006792062379514	0.958848629150758	1	pseudobulk	DESeq2	LRT	0.018249948317693
231	KC_AB1	CG31344	0.632983974886073	0.0378826694430165	1	pseudobulk	DESeq2	LRT	1.42155942556305
231	KC_AB1	CG31345	-1.99275908811736	0.412167844949191	1	pseudobulk	DESeq2	LRT	0.384925892483647
231	KC_AB1	CG31357	0.537076358263542	0.220345696985745	1	pseudobulk	DESeq2	LRT	0.656895426177859
231	KC_AB1	CG31360	0.554719043670635	0.163646279299374	1	pseudobulk	DESeq2	LRT	0.786093864466091
231	KC_AB1	CG31365	0.447644157639616	0.5622671180356	1	pseudobulk	DESeq2	LRT	0.250057313739846
232	KC_AB1	CG31368	0.0663259011920649	0.911515303034965	1	pseudobulk	DESeq2	LRT	0.040236035766160
232	KC_AB1	CG31371	0.371786291484431	0.863065234100387	1	pseudobulk	DESeq2	LRT	0.063956377240616
232	KC_AB1	CG31381	1.71170801572932	0.339711972006452	1	pseudobulk	DESeq2	LRT	0.468889147611218
232	KC_AB1	CG31388	0.342420010567347	0.389724327863825	1	pseudobulk	DESeq2	LRT	0.409242483282896
232	KC_AB1	CG31415	-0.477394605854819	0.802183186155542	1	pseudobulk	DESeq2	LRT	0.095726445117267
232	KC_AB1	CG31431	0.135879393043758	1	1	pseudobulk	DESeq2	LRT	0
232	KC_AB1	CG31436	1.08899820779554	0.670846322504933	1	pseudobulk	DESeq2	LRT	0.173376956629912
232	KC_AB1	CG31441	-0.654047020686207	0.572337805207487	1	pseudobulk	DESeq2	LRT	0.242347566251789
232	KC_AB1	CG31446	1.08899820779554	0.697631521651407	1	pseudobulk	DESeq2	LRT	0.156373904552
232	KC_AB1	CG31457	-0.600173329577453	0.546644111748994	1	pseudobulk	DESeq2	LRT	0.262295325595805
233	KC_AB1	CG31460	-0.45540412110841	0.425777954137489	1	pseudobulk	DESeq2	LRT	0.370816829145475
233	KC_AB1	CG31467	1.10931513936091	0.376827418739629	1	pseudobulk	DESeq2	LRT	0.423857504567873
233	KC_AB1	CG31469	1.08899820779554	0.878177167143508	1	pseudobulk	DESeq2	LRT	0.056417858865076
233	KC_AB1	CG31473	0.660802221865477	0.771695266873055	1	pseudobulk	DESeq2	LRT	0.112554163449953
233	KC_AB1	CG31475	-0.0145700953214856	0.953620672776855	1	pseudobulk	DESeq2	LRT	0.020624342780434
233	KC_AB1	CG3149	-0.469544637999656	0.614329604440644	1	pseudobulk	DESeq2	LRT	0.211598555597876
233	KC_AB1	CG31510	-0.0404193760709339	0.896994321374926	1	pseudobulk	DESeq2	LRT	0.047210306346477
233	KC_AB1	CG31522	-0.0139257056504531	0.935968556317856	1	pseudobulk	DESeq2	LRT	0.028738741055725
233	KC_AB1	CG31523	0.230195396187684	0.342817764461689	1	pseudobulk	DESeq2	LRT	0.464936681475239

233	KC_AB1	CG31538	-3.70956928380912	0.0444574819898486	1	pseudobulk	DESeq2	LRT	1.35205513877788
234	KC_AB1	CG31549	2.62490424814164	0.295548209233383	1	pseudobulk	DESeq2	LRT	0.529371667756191
234	KC_AB1	CG31550	0.159064253714999	0.569837668090507	1	pseudobulk	DESeq2	LRT	0.24424884589481
234	KC_AB1	CG3156	0.0102217477597997	0.929273470138147	1	pseudobulk	DESeq2	LRT	0.031856461361892
234	KC_AB1	CG31612	1.10055402149342	0.505288769654201	1	pseudobulk	DESeq2	LRT	0.296460354109702
234	KC_AB1	CG31626	0.11400025262841	1	1	pseudobulk	DESeq2	LRT	0
234	KC_AB1	CG31627	1.08899820779554	0.878177167143508	1	pseudobulk	DESeq2	LRT	0.056417858865076
234	KC_AB1	CG3163	-0.059660223387752	0.885458359796503	1	pseudobulk	DESeq2	LRT	0.052831857439254
234	KC_AB1	CG31633	-0.237251080488376	0.803460908951146	1	pseudobulk	DESeq2	LRT	0.095035248259437
234	KC_AB1	CG31635	0.0739758126946991	0.654818539950938	1	pseudobulk	DESeq2	LRT	0.18387903285753
234	KC_AB1	CG31636	1.08899820779554	0.697631521651407	1	pseudobulk	DESeq2	LRT	0.156373904552
235	KC_AB1	CG31637	-0.119133417698733	0.601636391413225	1	pseudobulk	DESeq2	LRT	0.220665902283513
235	KC_AB1	CG31638	0.0094750478518480	0.926224223319849	1	pseudobulk	DESeq2	LRT	0.033283865194232
235	KC_AB1	CG3164	0.811685789421454	0.135445346320496	1	pseudobulk	DESeq2	LRT	0.868235912019142
235	KC_AB1	CG31642	0.787932527644325	0.691824901730782	1	pseudobulk	DESeq2	LRT	0.160003809928432
235	KC_AB1	CG31643	-0.59183424176151	0.689190912044155	1	pseudobulk	DESeq2	LRT	0.161660457972554
235	KC_AB1	CG31648	-0.10668404684648	0.874152439520469	1	pseudobulk	DESeq2	LRT	0.058412826095860
235	KC_AB1	CG3165	-0.0968235460384704	0.811006996523319	1	pseudobulk	DESeq2	LRT	0.090975399132416
235	KC_AB1	CG31650	-0.399403903198487	0.0612507623922311	1	pseudobulk	DESeq2	LRT	1.21288850123807
235	KC_AB1	CG31663	2.94535899049764	0.222980779577595	1	pseudobulk	DESeq2	LRT	0.651732570508521
235	KC_AB1	CG31673	1.08899820779554	0.878177167143508	1	pseudobulk	DESeq2	LRT	0.056417858865076
236	KC_AB1	CG31676	-1.42283760776358	0.45392880280356	1	pseudobulk	DESeq2	LRT	0.343012259421103
236	KC_AB1	CG31678	-0.102119880293881	0.812198274293882	1	pseudobulk	DESeq2	LRT	0.090337937605697
236	KC_AB1	CG3168	-0.82542030717083	1	1	pseudobulk	DESeq2	LRT	0
236	KC_AB1	CG31683	1.08899820779554	0.878177167143508	1	pseudobulk	DESeq2	LRT	0.056417858865076
236	KC_AB1	CG31687	-0.185600573382316	0.598040590694554	1	pseudobulk	DESeq2	LRT	0.223269338224957
236	KC_AB1	CG31688	0.0716388377477541	0.701171040280148	1	pseudobulk	DESeq2	LRT	0.154176029409752
236	KC_AB1	CG31690	-0.234085015791891	0.580308893003131	1	pseudobulk	DESeq2	LRT	0.236340773994156
236	KC_AB1	CG31694	0.291516714656487	0.306699096655182	1	pseudobulk	DESeq2	LRT	0.513287503177109
236	KC_AB1	CG31697	1.08899820779554	0.878177167143508	1	pseudobulk	DESeq2	LRT	0.056417858865076
236	KC_AB1	CG31705	0.744782276103072	0.764014638758689	1	pseudobulk	DESeq2	LRT	0.116898320126909
237	KC_AB1	CG31706	1.73977881795246	0.325147220140179	1	pseudobulk	DESeq2	LRT	0.487919954659133
237	KC_AB1	CG31712	0.0545227524314774	0.675900572960475	1	pseudobulk	DESeq2	LRT	0.170117185405202
237	KC_AB1	CG31715	0.308605616242384	0.186975883814092	1	pseudobulk	DESeq2	LRT	0.728214405238228
237	KC_AB1	CG31717	0.173154411139993	0.66635150775094	1	pseudobulk	DESeq2	LRT	0.176296615266421
237	KC_AB1	CG31729	0.0278106345469944	0.915169188826766	1	pseudobulk	DESeq2	LRT	0.038498609778942
237	KC_AB1	CG31751	0.2420824057708	0.777443882086622	1	pseudobulk	DESeq2	LRT	0.109330949670515
237	KC_AB1	CG3176	2.11724188091509	0.0326620635962842	1	pseudobulk	DESeq2	LRT	1.48595637991266
237	KC_AB1	CG31760	-0.252707367206656	0.155060840645373	1	pseudobulk	DESeq2	LRT	0.809497865880138
237	KC_AB1	CG31788	1.08899820779554	0.697631521651407	1	pseudobulk	DESeq2	LRT	0.156373904552
237	KC_AB1	CG31793	-0.658567387591498	0.64254072798774	1	pseudobulk	DESeq2	LRT	0.192099339000963
238	KC_AB1	CG31800	-0.54145237322562	0.232141841536857	1	pseudobulk	DESeq2	LRT	0.634246574706333
238	KC_AB1	CG31802	-0.82542030717083	1	1	pseudobulk	DESeq2	LRT	0
238	KC_AB1	CG31806	0.531012068059879	0.823796455286506	1	pseudobulk	DESeq2	LRT	0.084180081101162
238	KC_AB1	CG31808	-0.0502262191378738	0.774603187943947	1	pseudobulk	DESeq2	LRT	0.11092071997114
238	KC_AB1	CG31815	-0.927992165273896	0.328745495419183	1	pseudobulk	DESeq2	LRT	0.483140189309776
238	KC_AB1	CG31817	-1.76646499465866	0.241954616875565	1	pseudobulk	DESeq2	LRT	0.616266086452852
238	KC_AB1	CG31832	1.08899820779554	0.878177167143508	1	pseudobulk	DESeq2	LRT	0.056417858865076
238	KC_AB1	CG3184	-0.210204357367727	0.726844012770334	1	pseudobulk	DESeq2	LRT	0.138558782623559
238	KC_AB1	CG31849	-0.947902801659235	0.425108145215459	1	pseudobulk	DESeq2	LRT	0.371500573724389
238	KC_AB1	CG31855	0.0019216886692629	0.995057257135718	1	pseudobulk	DESeq2	LRT	0.002151928558143
239	KC_AB1	CG31861	0.980603726925276	0.631823227524267	1	pseudobulk	DESeq2	LRT	0.199404412302045

239	KC_AB1	CG31875	1.95275942187961	0.438420007953907	1	pseudobulk	DESeq2	LRT	0.358109634392079
239	KC_AB1	CG31882	-0.850996402080018	0.900449987182352	1	pseudobulk	DESeq2	LRT	0.045540403770502
239	KC_AB1	CG31883	0.135879393043758	1	1	pseudobulk	DESeq2	LRT	0
239	KC_AB1	CG31886	0.762312975937231	0.503652169931833	1	pseudobulk	DESeq2	LRT	0.297869290597481
239	KC_AB1	CG31897	-1.19370766665661	0.521181031066061	1	pseudobulk	DESeq2	LRT	0.28301139927805
239	KC_AB1	CG31898	-2.94050702023793	0.101592819155394	1	pseudobulk	DESeq2	LRT	0.993136988030454
239	KC_AB1	CG31908	0.170449389000653	0.691580780661627	1	pseudobulk	DESeq2	LRT	0.160157084467782
239	KC_AB1	CG31909	-1.6747012584303	0.563129890257477	1	pseudobulk	DESeq2	LRT	0.249391420218277
239	KC_AB1	CG3191	-1.27122344771782	0.188918005548793	1	pseudobulk	DESeq2	LRT	0.723726648018722
240	KC_AB1	CG31913	0.113316967542732	1	1	pseudobulk	DESeq2	LRT	0
240	KC_AB1	CG31915	0.0019588373899799	0.996347353549511	1	pseudobulk	DESeq2	LRT	0.001589228412719
240	KC_AB1	CG31921	-0.828237688596619	0.337708054082214	1	pseudobulk	DESeq2	LRT	0.471458581645454
240	KC_AB1	CG31922	0.417637570555346	0.423036716136862	1	pseudobulk	DESeq2	LRT	0.373621937770013
240	KC_AB1	CG31935	0.260150758382017	0.678690963827105	1	pseudobulk	DESeq2	LRT	0.168327933018862
240	KC_AB1	CG31937	0.554034171112158	0.557626663573255	1	pseudobulk	DESeq2	LRT	0.253656468113282
240	KC_AB1	CG31957	-0.805973110107074	0.197023112783725	1	pseudobulk	DESeq2	LRT	0.705482823758988
240	KC_AB1	CG31974	0.11400025262841	1	1	pseudobulk	DESeq2	LRT	0
240	KC_AB1	CG3198	0.15108959159357	0.379941400359712	1	pseudobulk	DESeq2	LRT	0.420283380917134
240	KC_AB1	CG31997	0.400664460818111	0.0163223261384098	1	pseudobulk	DESeq2	LRT	1.78721794870183
241	KC_AB1	CG31998	0.065146994371164	0.70934938709406	1	pseudobulk	DESeq2	LRT	0.149139802176348
241	KC_AB1	CG31999	0.577838190757919	0.12248933685524	1	pseudobulk	DESeq2	LRT	0.911901716576946
241	KC_AB1	CG32006	-0.850996402080018	0.900449987182352	1	pseudobulk	DESeq2	LRT	0.045540403770502
241	KC_AB1	CG32017	-0.132869425839212	0.412817771027849	1	pseudobulk	DESeq2	LRT	0.38424161541861
241	KC_AB1	CG32022	0.0863208007227067	0.880290416532762	1	pseudobulk	DESeq2	LRT	0.055374026156438
241	KC_AB1	CG32036	1.333128595275	0.431201289874457	1	pseudobulk	DESeq2	LRT	0.365319948692094
241	KC_AB1	CG32039	0.0302793998646364	0.919863455011898	1	pseudobulk	DESeq2	LRT	0.036276634758751
241	KC_AB1	CG32052	0.0139229403912084	0.897767767452613	1	pseudobulk	DESeq2	LRT	0.046835991125007
241	KC_AB1	CG32055	-1.88067393919781	0.247113996760149	1	pseudobulk	DESeq2	LRT	0.607102655069219
241	KC_AB1	CG32066	-0.198199854547597	0.0757038193672831	1	pseudobulk	DESeq2	LRT	1.12088220916186
242	KC_AB1	CG32069	0.308539912340666	0.476474010885488	1	pseudobulk	DESeq2	LRT	0.321960782825943
242	KC_AB1	CG32075	1.00001625110887	0.381651261161436	1	pseudobulk	DESeq2	LRT	0.418333298159765
242	KC_AB1	CG32085	0.0434466204770194	0.809324927069896	1	pseudobulk	DESeq2	LRT	0.091877083205385
242	KC_AB1	CG32095	-0.655296950508904	0.412575208202369	1	pseudobulk	DESeq2	LRT	0.384496872495673
242	KC_AB1	CG32099	-0.0804571988685912	0.912207727319845	1	pseudobulk	DESeq2	LRT	0.039906253167044
242	KC_AB1	CG32100	-0.249232251828838	0.564361747590325	1	pseudobulk	DESeq2	LRT	0.248442430377358
242	KC_AB1	CG32104	-0.439188399305189	0.585956435945334	1	pseudobulk	DESeq2	LRT	0.232134671237612
242	KC_AB1	CG32107	1.08899820779554	0.878177167143508	1	pseudobulk	DESeq2	LRT	0.056417858865076
242	KC_AB1	CG32109	0.428434779228948	0.295789625517828	1	pseudobulk	DESeq2	LRT	0.529017062453302
242	KC_AB1	CG32110	1.22236326706149	0.63482863141329	1	pseudobulk	DESeq2	LRT	0.197343494355134
243	KC_AB1	CG32112	-0.448932251208446	0.408529342239523	1	pseudobulk	DESeq2	LRT	0.388776745214632
243	KC_AB1	CG32119	-0.850932661185203	0.660736244265418	1	pseudobulk	DESeq2	LRT	0.179971869591347
243	KC_AB1	CG32137	0.73362978992059	0.719815137332351	1	pseudobulk	DESeq2	LRT	0.142779024603383
243	KC_AB1	CG32147	0.232033719135798	0.675906791635959	1	pseudobulk	DESeq2	LRT	0.170113189663621
243	KC_AB1	CG32161	-0.850996402080018	0.900449987182352	1	pseudobulk	DESeq2	LRT	0.045540403770502
243	KC_AB1	CG32163	1.11432527768905	0.127811924178956	1	pseudobulk	DESeq2	LRT	0.893428626901047
243	KC_AB1	CG32176	0.257239331960004	0.605476816119287	1	pseudobulk	DESeq2	LRT	0.217902481462672
243	KC_AB1	CG32187	0.178262308018008	0.799338146048985	1	pseudobulk	DESeq2	LRT	0.097269461115850
243	KC_AB1	CG32192	0.744782276103072	0.764014638758689	1	pseudobulk	DESeq2	LRT	0.116898320126909
243	KC_AB1	CG32195	1.08899820779554	0.670846322504933	1	pseudobulk	DESeq2	LRT	0.173376956629912
244	KC_AB1	CG32196	0.135445777568018	0.887160706670073	1	pseudobulk	DESeq2	LRT	0.051997701816367
244	KC_AB1	CG32202	0.0419693607446588	0.930593573553135	1	pseudobulk	DESeq2	LRT	0.031239950921651
244	KC_AB1	CG32204	-0.100431509948458	0.476979579678965	1	pseudobulk	DESeq2	LRT	0.321500213460049

244	KC_AB1	CG32206	-0.180899160241185	0.412877149831072	1	pseudobulk	DESeq2	LRT	0.384179151944572
244	KC_AB1	CG3222	-0.170583349778313	0.904650643099319	1	pseudobulk	DESeq2	LRT	0.043519103741385
244	KC_AB1	CG32221	0.603644967504369	0.580249500745036	1	pseudobulk	DESeq2	LRT	0.236385224545064
244	KC_AB1	CG32225	-0.164313350322543	0.667409842826624	1	pseudobulk	DESeq2	LRT	0.175607392746003
244	KC_AB1	CG32228	1.08899820779554	0.878177167143508	1	pseudobulk	DESeq2	LRT	0.056417858865076
244	KC_AB1	CG3223	-0.0531182159995662	0.914193455528483	1	pseudobulk	DESeq2	LRT	0.038961892036012
244	KC_AB1	CG32232	-2.3118469151042	0.353435572380865	1	pseudobulk	DESeq2	LRT	0.451689742007392
245	KC_AB1	CG3224	0.496998615557292	0.0923015640378161	1	pseudobulk	DESeq2	LRT	1.03479093984908
245	KC_AB1	CG32243	-0.374116541113743	0.228421632874204	1	pseudobulk	DESeq2	LRT	0.641262768232678
245	KC_AB1	CG3225	2.10549911548503	0.428045198278114	1	pseudobulk	DESeq2	LRT	0.368510370411162
245	KC_AB1	CG3226	0.103200969009646	0.754913856399157	1	pseudobulk	DESeq2	LRT	0.122102603105844
245	KC_AB1	CG32260	1.08899820779554	0.670846322504933	1	pseudobulk	DESeq2	LRT	0.173376956629912
245	KC_AB1	CG32262	-0.378659499645014	0.545808442927307	1	pseudobulk	DESeq2	LRT	0.262959750658829
245	KC_AB1	CG32264	-0.314850015302791	0.00749759070801882	1	pseudobulk	DESeq2	LRT	2.12507827131652
245	KC_AB1	CG32267	-0.104131917577403	0.582061450708926	1	pseudobulk	DESeq2	LRT	0.235031162609598
245	KC_AB1	CG32276	0.0077397493757939	0.913313514151804	1	pseudobulk	DESeq2	LRT	0.039380116119600
245	KC_AB1	CG32278	0.171700185961843	0.408868711491425	1	pseudobulk	DESeq2	LRT	0.388416122379223
246	KC_AB1	CG32280	-0.0658158312196102	0.897186418638294	1	pseudobulk	DESeq2	LRT	0.047117309269953
246	KC_AB1	CG32281	-0.249727220890869	0.804291821537306	1	pseudobulk	DESeq2	LRT	0.094586347409949
246	KC_AB1	CG32295	-1.44925888589622	0.6315588144614	1	pseudobulk	DESeq2	LRT	0.199586199175701
246	KC_AB1	CG32313	-0.850996402080018	0.900449987182352	1	pseudobulk	DESeq2	LRT	0.045540403770502
246	KC_AB1	CG32333	0.0695310412069151	0.937985723801927	1	pseudobulk	DESeq2	LRT	0.027803771558279
246	KC_AB1	CG32344	0.496445925906063	0.505506338637567	1	pseudobulk	DESeq2	LRT	0.296273394339926
246	KC_AB1	CG32365	-0.38808986118587	0.784905011711296	1	pseudobulk	DESeq2	LRT	0.105182897886234
246	KC_AB1	CG32368	1.51489279024391	0.450802353566048	1	pseudobulk	DESeq2	LRT	0.346013825238888
246	KC_AB1	CG32369	-0.162110975642915	0.423152154434672	1	pseudobulk	DESeq2	LRT	0.373503443618663
246	KC_AB1	CG32373	-0.281751114627366	0.405602274183527	1	pseudobulk	DESeq2	LRT	0.391899618609169
247	KC_AB1	CG32392	-0.409547340692355	0.440541906125193	1	pseudobulk	DESeq2	LRT	0.356012773441963
247	KC_AB1	CG32396	0.918586694154703	0.520164224249543	1	pseudobulk	DESeq2	LRT	0.283859520931582
247	KC_AB1	CG32407	-1.6747012584303	0.563129890257477	1	pseudobulk	DESeq2	LRT	0.249391420218277
247	KC_AB1	CG32409	-0.6403004729707	0.493905924326903	1	pseudobulk	DESeq2	LRT	0.306355764510547
247	KC_AB1	CG32425	-0.299759628158253	0.251297781582992	1	pseudobulk	DESeq2	LRT	0.599811345273392
247	KC_AB1	CG32432	0.0007401160898945	0.96756499891413	1	pseudobulk	DESeq2	LRT	0.014319850369469
247	KC_AB1	CG32436	0.11269840515639	0.977007463492936	1	pseudobulk	DESeq2	LRT	0.010102118633921
247	KC_AB1	CG32437	-3.73119162468188	0.296754785036902	1	pseudobulk	DESeq2	LRT	0.527602269514508
247	KC_AB1	CG32441	-0.390732792367655	0.0998614081991316	1	pseudobulk	DESeq2	LRT	1.00060231401894
247	KC_AB1	CG32448	0.0885236273165393	0.779057457364721	1	pseudobulk	DESeq2	LRT	0.108430510881694
248	KC_AB1	CG32452	0.0931551874657317	0.911711086291797	1	pseudobulk	DESeq2	LRT	0.040142764202172
248	KC_AB1	CG32457	2.32496151311036	0.230919791244108	1	pseudobulk	DESeq2	LRT	0.636538843783717
248	KC_AB1	CG32459	0.104666760163359	0.896374213271674	1	pseudobulk	DESeq2	LRT	0.047510645660964
248	KC_AB1	CG3246	1.08899820779554	0.878177167143508	1	pseudobulk	DESeq2	LRT	0.056417858865076
248	KC_AB1	CG32461	1.08899820779554	0.878177167143508	1	pseudobulk	DESeq2	LRT	0.056417858865076
248	KC_AB1	CG32473	1.01508984635183	0.199480914132997	1	pseudobulk	DESeq2	LRT	0.700098650269161
248	KC_AB1	CG32485	0.0126131047579628	0.88155515242142	1	pseudobulk	DESeq2	LRT	0.054750511903503
248	KC_AB1	CG32486	0.255285319744696	0.0439907048859085	1	pseudobulk	DESeq2	LRT	1.35663907904137
248	KC_AB1	CG32500	-1.74282330322402	0.460467112295906	1	pseudobulk	DESeq2	LRT	0.336801382749348
248	KC_AB1	CG32506	-0.0056129347069837	0.968771182247935	1	pseudobulk	DESeq2	LRT	0.013778788503367
249	KC_AB1	CG3251	-0.850996402080018	0.900449987182352	1	pseudobulk	DESeq2	LRT	0.045540403770502
249	KC_AB1	CG32512	-1.44925888589622	0.6315588144614	1	pseudobulk	DESeq2	LRT	0.199586199175701
249	KC_AB1	CG32521	0.831741038490028	0.235480709140402	1	pseudobulk	DESeq2	LRT	0.628044664998055
249	KC_AB1	CG3253	-0.220713214442016	0.645119983216991	1	pseudobulk	DESeq2	LRT	0.19035950520492
249	KC_AB1	CG32533	0.930878251424284	0.437844423701863	1	pseudobulk	DESeq2	LRT	0.358680177007879

249	KC_AB1	CG32536	0.723571820138588	0.690288484863566	1	pseudobulk	DESeq2	LRT	0.160969371290708
249	KC_AB1	CG32537	-0.331257163442156	0.678404046817702	1	pseudobulk	DESeq2	LRT	0.168511570088967
249	KC_AB1	CG32544	0.0116948897109434	0.92558265484376	1	pseudobulk	DESeq2	LRT	0.033584792535374
249	KC_AB1	CG32547	-0.160659079412443	0.499867377659301	1	pseudobulk	DESeq2	LRT	0.301145205245507
249	KC_AB1	CG32549	0.289623893277539	0.229245259206893	1	pseudobulk	DESeq2	LRT	0.639699636777135
250	KC_AB1	CG32554	-0.591551605715815	0.738495477481815	1	pseudobulk	DESeq2	LRT	0.131652159946759
250	KC_AB1	CG3257	0.0305189616404	0.9092867431353	1	pseudobulk	DESeq2	LRT	0.041299140624093
250	KC_AB1	CG32576	-0.739915691869114	0.239953280366697	1	pseudobulk	DESeq2	LRT	0.619873308513736
250	KC_AB1	CG32579	1.08899820779554	0.878177167143508	1	pseudobulk	DESeq2	LRT	0.056417858865076
250	KC_AB1	CG32581	-0.0394874885209461	0.873187610484991	1	pseudobulk	DESeq2	LRT	0.058892435051967
250	KC_AB1	CG32590	-0.181295373235731	0.686856709161302	1	pseudobulk	DESeq2	LRT	0.1631338552422
250	KC_AB1	CG3262	0.37828862534731	0.0565805752701463	1	pseudobulk	DESeq2	LRT	1.24733264126609
250	KC_AB1	CG32638	0.26400162516801	0.636016797547394	1	pseudobulk	DESeq2	LRT	0.196531414248114
250	KC_AB1	CG32641	-1.04865228821592	0.338874000885872	1	pseudobulk	DESeq2	LRT	0.469961749855256
250	KC_AB1	CG32647	0.0654989308616322	0.755660135244495	1	pseudobulk	DESeq2	LRT	0.121673488328743
251	KC_AB1	CG32650	0.654214717103117	0.730426816812749	1	pseudobulk	DESeq2	LRT	0.136423290539469
251	KC_AB1	CG32651	-0.82542030717083	1	1	pseudobulk	DESeq2	LRT	0
251	KC_AB1	CG32655	1.16523413356038	0.63328025468345	1	pseudobulk	DESeq2	LRT	0.198404052815419
251	KC_AB1	CG32679	-1.82709236256241	0.44317674354685	1	pseudobulk	DESeq2	LRT	0.353423038023035
251	KC_AB1	CG32681	1.08899820779554	0.697631521651407	1	pseudobulk	DESeq2	LRT	0.156373904552
251	KC_AB1	CG32683	-0.525620989024958	0.0200206079944836	1	pseudobulk	DESeq2	LRT	1.69852273781375
251	KC_AB1	CG32686	0.187499809431982	0.910303214736121	1	pseudobulk	DESeq2	LRT	0.040813923554985
251	KC_AB1	CG3270	1.88952324269138	0.456767015650974	1	pseudobulk	DESeq2	LRT	0.340305265178099
251	KC_AB1	CG32700	-0.143647823873685	0.214749913525546	1	pseudobulk	DESeq2	LRT	0.668067002376282
251	KC_AB1	CG32706	-0.460292796507708	0.702103663156099	1	pseudobulk	DESeq2	LRT	0.153598761071108
252	KC_AB1	CG32708	-0.406458043016611	0.592278814916724	1	pseudobulk	DESeq2	LRT	0.227473801259631
252	KC_AB1	CG32755	1.2263877406838	0.532581859110394	1	pseudobulk	DESeq2	LRT	0.273613630654175
252	KC_AB1	CG32756	0.0372601088274243	0.921893391309301	1	pseudobulk	DESeq2	LRT	0.035319298298924
252	KC_AB1	CG32762	2.10449501374856	0.335478261052675	1	pseudobulk	DESeq2	LRT	0.4743356168087
252	KC_AB1	CG32767	0.209342783172876	0.0437256022005325	1	pseudobulk	DESeq2	LRT	1.35926420058989
252	KC_AB1	CG3277	-0.850996402080018	0.900449987182352	1	pseudobulk	DESeq2	LRT	0.045540403770502
252	KC_AB1	CG32772	-0.0546697142845275	0.821040843563034	1	pseudobulk	DESeq2	LRT	0.085635237895372
252	KC_AB1	CG32795	-0.301085374213514	0.181306474925994	1	pseudobulk	DESeq2	LRT	0.741586685841518
252	KC_AB1	CG32797	0.135795303631797	0.946582799906172	1	pseudobulk	DESeq2	LRT	0.023841391241048
252	KC_AB1	CG32803	0.835579594462006	0.119272197359932	1	pseudobulk	DESeq2	LRT	0.92346077963524
253	KC_AB1	CG32808	0.121699299087022	0.880567987584901	1	pseudobulk	DESeq2	LRT	0.055237107052668
253	KC_AB1	CG32809	-0.0064539763040906	0.957170372033342	1	pseudobulk	DESeq2	LRT	0.019010752872612
253	KC_AB1	CG3281	0.358274904840108	0.685130963871937	1	pseudobulk	DESeq2	LRT	0.16422640449223
253	KC_AB1	CG32813	0.230676958798888	0.548268761086784	1	pseudobulk	DESeq2	LRT	0.261006498369895
253	KC_AB1	CG32815	-0.0566634012135214	0.792968448843226	1	pseudobulk	DESeq2	LRT	0.100744092336725
253	KC_AB1	CG32816	2.93932813264263	0.198229201291412	1	pseudobulk	DESeq2	LRT	0.702832368894213
253	KC_AB1	CG32817	1.08899820779554	0.697631521651407	1	pseudobulk	DESeq2	LRT	0.156373904552
253	KC_AB1	CG32829	0.551224032451582	0.80069218923956	1	pseudobulk	DESeq2	LRT	0.096534408019249
253	KC_AB1	CG32846	-1.56855358782635	0.573026303465967	1	pseudobulk	DESeq2	LRT	0.241825442277049
253	KC_AB1	CG32850	-0.341669716332684	0.0158229363486411	1	pseudobulk	DESeq2	LRT	1.80071291896237
254	KC_AB1	CG32856	0.0226123321350875	0.951348592816342	1	pseudobulk	DESeq2	LRT	0.021660319869431
254	KC_AB1	CG3288	1.08899820779554	0.878177167143508	1	pseudobulk	DESeq2	LRT	0.056417858865076
254	KC_AB1	CG3294	-0.714096431213227	0.357270821770137	1	pseudobulk	DESeq2	LRT	0.447002451084614
254	KC_AB1	CG32944	0.11269840515639	0.977007463492936	1	pseudobulk	DESeq2	LRT	0.010102118633921
254	KC_AB1	CG3295	-0.230356166863351	0.667503095344393	1	pseudobulk	DESeq2	LRT	0.175546716048971
254	KC_AB1	CG32982	-0.150687712076891	0.560237042695708	1	pseudobulk	DESeq2	LRT	0.251628179149579
254	KC_AB1	CG33051	0.939792195455099	0.467039387753225	1	pseudobulk	DESeq2	LRT	0.330646491676826

254	KC_AB1	CG33054	0.428684270947735	0.779961843253277	1	pseudobulk	DESeq2	LRT	0.107926643040143
254	KC_AB1	CG33056	-0.640538970262739	0.605131334623748	1	pseudobulk	DESeq2	LRT	0.218150358054439
254	KC_AB1	CG3308	0.342216697710184	0.533550379040738	1	pseudobulk	DESeq2	LRT	0.272824567230298
255	KC_AB1	CG33080	-0.850932661185203	0.660736244265418	1	pseudobulk	DESeq2	LRT	0.179971869591347
255	KC_AB1	CG33082	-2.5192264985262	0.273630365215179	1	pseudobulk	DESeq2	LRT	0.562835709899105
255	KC_AB1	CG3309	-0.239440928072281	0.745940894004354	1	pseudobulk	DESeq2	LRT	0.127295583284316
255	KC_AB1	CG33090	-0.300865314873563	0.058146783447885	1	pseudobulk	DESeq2	LRT	1.2354743044862
255	KC_AB1	CG33096	0.166778955791672	0.810283320845341	1	pseudobulk	DESeq2	LRT	0.091363100670060
255	KC_AB1	CG33098	-0.477417351510566	0.688152521832718	1	pseudobulk	DESeq2	LRT	0.162315294251201
255	KC_AB1	CG33107	-0.0300685328806457	0.924151263348106	1	pseudobulk	DESeq2	LRT	0.034256938454284
255	KC_AB1	CG33108	-0.50991679990519	0.56083973349628	1	pseudobulk	DESeq2	LRT	0.251161225735224
255	KC_AB1	CG33111	-0.131566469124447	0.861523161052892	1	pseudobulk	DESeq2	LRT	0.064733042554598
255	KC_AB1	CG33116	-0.0180654466517743	0.915237643038174	1	pseudobulk	DESeq2	LRT	0.038466125977228
256	KC_AB1	CG33124	1.08899820779554	0.697631521651407	1	pseudobulk	DESeq2	LRT	0.156373904552
256	KC_AB1	CG33129	-0.136788104323386	0.506687528765536	1	pseudobulk	DESeq2	LRT	0.295259784982991
256	KC_AB1	CG33143	-1.33877264045386	0.46237644618641	1	pseudobulk	DESeq2	LRT	0.335004297320679
256	KC_AB1	CG33144	-0.233477397399837	0.359638974410823	1	pseudobulk	DESeq2	LRT	0.444133249490646
256	KC_AB1	CG33155	0.162489296131754	0.882547255390542	1	pseudobulk	DESeq2	LRT	0.054262031315885
256	KC_AB1	CG33156	-0.318170049098121	0.487890496789605	1	pseudobulk	DESeq2	LRT	0.311677641063855
256	KC_AB1	CG33158	-0.0273991744511173	0.9454525171351	1	pseudobulk	DESeq2	LRT	0.024360277568845
256	KC_AB1	CG33169	-0.117855851303519	0.712477204903152	1	pseudobulk	DESeq2	LRT	0.147229025976493
256	KC_AB1	CG33170	-0.22547873331079	0.353611633275003	1	pseudobulk	DESeq2	LRT	0.451473455808192
256	KC_AB1	CG33172	-0.506516985469986	0.909346984303859	1	pseudobulk	DESeq2	LRT	0.041270369127443
257	KC_AB1	CG33177	1.08899820779554	0.670846322504933	1	pseudobulk	DESeq2	LRT	0.173376956629912
257	KC_AB1	CG33181	-0.163408469945735	0.18418591147535	1	pseudobulk	DESeq2	LRT	0.734743592393864
257	KC_AB1	CG33199	-0.112442693381111	0.614583169506702	1	pseudobulk	DESeq2	LRT	0.211419337165307
257	KC_AB1	CG33203	0.217243340947265	0.569375642258573	1	pseudobulk	DESeq2	LRT	0.244601115796302
257	KC_AB1	CG33213	-0.156445356455009	0.859049973270071	1	pseudobulk	DESeq2	LRT	0.06598157134436
257	KC_AB1	CG33217	-0.437556342336324	0.316424626085343	1	pseudobulk	DESeq2	LRT	0.499729724425971
257	KC_AB1	CG33223	-1.81798203917607	0.270649263745959	1	pseudobulk	DESeq2	LRT	0.567593149993582
257	KC_AB1	CG33228	-0.273550363903798	0.694470946915818	1	pseudobulk	DESeq2	LRT	0.15834591818852
257	KC_AB1	CG33229	-0.0580978841992558	0.735218527493156	1	pseudobulk	DESeq2	LRT	0.133583557271216
257	KC_AB1	CG33230	-0.559436985386915	0.569034796023783	1	pseudobulk	DESeq2	LRT	0.244861176031859
258	KC_AB1	CG33233	0.644002677963084	0.764564505832492	1	pseudobulk	DESeq2	LRT	0.116585867550177
258	KC_AB1	CG33234	0.347477059951964	0.84999925484814	1	pseudobulk	DESeq2	LRT	0.070581455009804
258	KC_AB1	CG33258	-1.6747012584303	0.563129890257477	1	pseudobulk	DESeq2	LRT	0.249391420218277
258	KC_AB1	CG33267	0.170497817732956	0.843934850760444	1	pseudobulk	DESeq2	LRT	0.073691078311922
258	KC_AB1	CG33268	-0.850932661185203	0.660736244265418	1	pseudobulk	DESeq2	LRT	0.179971869591347
258	KC_AB1	CG3328	-0.82542030717083	1	1	pseudobulk	DESeq2	LRT	0
258	KC_AB1	CG33288	-1.44925888589622	0.6315588144614	1	pseudobulk	DESeq2	LRT	0.199586199175701
258	KC_AB1	CG33298	-0.110649781627976	0.663624039241148	1	pseudobulk	DESeq2	LRT	0.178077890398302
258	KC_AB1	CG33303	0.288124126944399	0.217230096288158	1	pseudobulk	DESeq2	LRT	0.663080005296866
258	KC_AB1	CG33306	1.08899820779554	0.697631521651407	1	pseudobulk	DESeq2	LRT	0.156373904552
259	KC_AB1	CG33307	1.08899820779554	0.878177167143508	1	pseudobulk	DESeq2	LRT	0.056417858865076
259	KC_AB1	CG33310	0.273862584018816	0.628611325165001	1	pseudobulk	DESeq2	LRT	0.201617798927156
259	KC_AB1	CG33331	0.178262157811492	0.782679616428052	1	pseudobulk	DESeq2	LRT	0.106415976500918
259	KC_AB1	CG33332	0.158703764914761	0.907760319240135	1	pseudobulk	DESeq2	LRT	0.042028805413059
259	KC_AB1	CG3335	-0.262686087438639	0.706979091072385	1	pseudobulk	DESeq2	LRT	0.15059343028537
259	KC_AB1	CG3337	-0.215875889381987	0.685854234082862	1	pseudobulk	DESeq2	LRT	0.163768175926176
259	KC_AB1	CG3342	0.493756927287365	0.464108124854336	1	pseudobulk	DESeq2	LRT	0.333380828589119
259	KC_AB1	CG3345	-0.375792913009251	0.840089697917738	1	pseudobulk	DESeq2	LRT	0.075674341044102
259	KC_AB1	CG33459	1.08899820779554	0.697631521651407	1	pseudobulk	DESeq2	LRT	0.156373904552

259	KC_AB1	CG33468	2.58753908880453	0.243878358825908	1	pseudobulk	DESeq2	LRT	0.61282673621552
260	KC_AB1	CG33475	-0.490258259926738	0.745068981940488	1	pseudobulk	DESeq2	LRT	0.127803516393701
260	KC_AB1	CG33476	1.28116770184993	0.592386603154796	1	pseudobulk	DESeq2	LRT	0.227394771625737
260	KC_AB1	CG33477	-0.82542030717083	1	1	pseudobulk	DESeq2	LRT	0
260	KC_AB1	CG3348	-2.44844178584521	0.29018040253795	1	pseudobulk	DESeq2	LRT	0.537331921178161
260	KC_AB1	CG33493	-0.7522811187128	0.543765010446936	1	pseudobulk	DESeq2	LRT	0.264588741321357
260	KC_AB1	CG33506	0.544815929843905	0.384341094423119	1	pseudobulk	DESeq2	LRT	0.415283177526172
260	KC_AB1	CG33521	-0.0111477008976106	0.984080346426928	1	pseudobulk	DESeq2	LRT	0.006969441624212
260	KC_AB1	CG33523	-0.195055569200992	0.694257673621421	1	pseudobulk	DESeq2	LRT	0.15847931129967
260	KC_AB1	CG3356	0.190915639957462	0.67253180623023	1	pseudobulk	DESeq2	LRT	0.172287171632414
260	KC_AB1	CG3358	-0.851687725049452	0.218594766853155	1	pseudobulk	DESeq2	LRT	0.660360239248173
261	KC_AB1	CG33635	0.525117034071923	0.15306910902206	1	pseudobulk	DESeq2	LRT	0.815112445715148
261	KC_AB1	CG33639	0.135795303631797	0.946582799906172	1	pseudobulk	DESeq2	LRT	0.023841391241048
261	KC_AB1	CG33640	-0.850996402080018	0.900449987182352	1	pseudobulk	DESeq2	LRT	0.045540403770502
261	KC_AB1	CG33641	1.08899820779554	0.878177167143508	1	pseudobulk	DESeq2	LRT	0.056417858865076
261	KC_AB1	CG33642	-1.99275908811736	0.412167844949191	1	pseudobulk	DESeq2	LRT	0.384925892483647
261	KC_AB1	CG33643	0.827712394573779	0.700305254150331	1	pseudobulk	DESeq2	LRT	0.154712615277118
261	KC_AB1	CG33644	1.08899820779554	0.670846322504933	1	pseudobulk	DESeq2	LRT	0.173376956629912
261	KC_AB1	CG33654	2.10549911548503	0.428045198278114	1	pseudobulk	DESeq2	LRT	0.368510370411162
261	KC_AB1	CG3368	0.179239081518682	0.793982678783897	1	pseudobulk	DESeq2	LRT	0.100188971868404
261	KC_AB1	CG33695	0.280407023872497	0.598135718921425	1	pseudobulk	DESeq2	LRT	0.2232002620131
262	KC_AB1	CG33700	0.0352149254077214	0.969579999079126	1	pseudobulk	DESeq2	LRT	0.013416351902170
262	KC_AB1	CG33703	-0.82542030717083	1	1	pseudobulk	DESeq2	LRT	0
262	KC_AB1	CG33774	-0.135123488583508	0.65938132763956	1	pseudobulk	DESeq2	LRT	0.180863355369878
262	KC_AB1	CG33777	1.08899820779554	0.878177167143508	1	pseudobulk	DESeq2	LRT	0.056417858865076
262	KC_AB1	CG3386	-0.170339685507484	0.852728251707622	1	pseudobulk	DESeq2	LRT	0.069189348184201
262	KC_AB1	CG33914	1.22236326706149	0.63482863141329	1	pseudobulk	DESeq2	LRT	0.197343494355134
262	KC_AB1	CG33927	1.08899820779554	0.878177167143508	1	pseudobulk	DESeq2	LRT	0.056417858865076
262	KC_AB1	CG33941	0.434642928972476	0.434690605652872	1	pseudobulk	DESeq2	LRT	0.361819745494015
262	KC_AB1	CG33947	-0.133274057621861	0.797134051514553	1	pseudobulk	DESeq2	LRT	0.098468638531576
262	KC_AB1	CG33969	0.585090268623989	0.367019246564017	1	pseudobulk	DESeq2	LRT	0.435311160659685
263	KC_AB1	CG33970	1.04931673169927	0.0240943787132685	1	pseudobulk	DESeq2	LRT	1.61808426773911
263	KC_AB1	CG33977	-0.0270688153765474	0.892173431843955	1	pseudobulk	DESeq2	LRT	0.049550713817295
263	KC_AB1	CG33993	-0.442950628405716	0.329527908758341	1	pseudobulk	DESeq2	LRT	0.482107797742314
263	KC_AB1	CG33998	-0.671685282238562	0.694797615546385	1	pseudobulk	DESeq2	LRT	0.158141680663661
263	KC_AB1	CG34001	-1.84294760878525	0.275315031123039	1	pseudobulk	DESeq2	LRT	0.560170077180626
263	KC_AB1	CG34008	0.114184054362142	1	1	pseudobulk	DESeq2	LRT	0
263	KC_AB1	CG34012	-0.82542030717083	1	1	pseudobulk	DESeq2	LRT	0
263	KC_AB1	CG34015	-0.0585917324986122	0.899555611964473	1	pseudobulk	DESeq2	LRT	0.045971982710062
263	KC_AB1	CG3402	0.174979539179547	0.723726173237367	1	pseudobulk	DESeq2	LRT	0.140425721021839
263	KC_AB1	CG34025	2.21745641838459	0.396737788462723	1	pseudobulk	DESeq2	LRT	0.401496431894786
264	KC_AB1	CG34026	1.28116770184993	0.592386603154796	1	pseudobulk	DESeq2	LRT	0.227394771625737
264	KC_AB1	CG34027	-0.82542030717083	1	1	pseudobulk	DESeq2	LRT	0
264	KC_AB1	CG34028	-0.0894765763163675	0.862792041873547	1	pseudobulk	DESeq2	LRT	0.064093869348750
264	KC_AB1	CG34031	2.05296423313396	0.327185303142031	1	pseudobulk	DESeq2	LRT	0.485206212664739
264	KC_AB1	CG34039	0.517949537442392	0.417858962988358	1	pseudobulk	DESeq2	LRT	0.378970277870058
264	KC_AB1	CG34041	0.629233713384603	0.462557674497588	1	pseudobulk	DESeq2	LRT	0.334834109077525
264	KC_AB1	CG34045	1.53996927327048	0.26335671139004	1	pseudobulk	DESeq2	LRT	0.579455609592354
264	KC_AB1	CG34051	-0.850996402080018	0.900449987182352	1	pseudobulk	DESeq2	LRT	0.045540403770502
264	KC_AB1	CG34056	-1.82709236256241	0.44317674354685	1	pseudobulk	DESeq2	LRT	0.353423038023035
264	KC_AB1	CG34057	1.17276926104076	0.551679293648606	1	pseudobulk	DESeq2	LRT	0.258313316246919
265	KC_AB1	CG3407	-0.519166192858502	0.634093466777106	1	pseudobulk	DESeq2	LRT	0.197846721434185

265	KC_AB1	CG3408	-0.605787710153141	0.677829307687401	1	pseudobulk	DESeq2	LRT	0.168879657257649
265	KC_AB1	CG34109	0.214927510352243	0.877287275313415	1	pseudobulk	DESeq2	LRT	0.056858169856353
265	KC_AB1	CG34112	0.111831901354254	1	1	pseudobulk	DESeq2	LRT	0
265	KC_AB1	CG34116	0.292421637398504	0.727588978327112	1	pseudobulk	DESeq2	LRT	0.138113888344083
265	KC_AB1	CG34117	-0.0684880411555716	0.863043965339223	1	pseudobulk	DESeq2	LRT	0.063967079814490
265	KC_AB1	CG34124	-2.29603295607844	0.327786153610032	1	pseudobulk	DESeq2	LRT	0.484409395908184
265	KC_AB1	CG34125	-0.0530278871955756	0.859858237689688	1	pseudobulk	DESeq2	LRT	0.065573143714552
265	KC_AB1	CG34126	-0.0969808175228931	0.734552936538839	1	pseudobulk	DESeq2	LRT	0.133976900733787
265	KC_AB1	CG34132	0.566789311842158	0.203081679392164	1	pseudobulk	DESeq2	LRT	0.692329253836793
266	KC_AB1	CG34133	0.400063442783037	0.362776152125084	1	pseudobulk	DESeq2	LRT	0.440361269960601
266	KC_AB1	CG34134	-0.318064573992823	0.552003801006803	1	pseudobulk	DESeq2	LRT	0.25805793178059
266	KC_AB1	CG34136	-1.93786892659159	0.381996981812635	1	pseudobulk	DESeq2	LRT	0.417940068468648
266	KC_AB1	CG34141	0.942080999092271	0.681532122211136	1	pseudobulk	DESeq2	LRT	0.166513670026992
266	KC_AB1	CG34148	0.173892472655344	0.809635394084933	1	pseudobulk	DESeq2	LRT	0.091710514437349
266	KC_AB1	CG34149	0.261203690816479	0.817506071416267	1	pseudobulk	DESeq2	LRT	0.087509013257534
266	KC_AB1	CG34150	0.0258291234745721	0.906182763662404	1	pseudobulk	DESeq2	LRT	0.042784202714654
266	KC_AB1	CG34155	-1.96719661029409	0.414177861019091	1	pseudobulk	DESeq2	LRT	0.382813119093482
266	KC_AB1	CG34159	-0.0284861768827285	0.898241604521043	1	pseudobulk	DESeq2	LRT	0.046606833268565
266	KC_AB1	CG34163	-1.68458827954395	0.0377814171830661	1	pseudobulk	DESeq2	LRT	1.42272175569841
267	KC_AB1	CG34169	-0.82542030717083	1	1	pseudobulk	DESeq2	LRT	0
267	KC_AB1	CG34172	0.135879393043758	1	1	pseudobulk	DESeq2	LRT	0
267	KC_AB1	CG34174	-0.698839062177131	0.572174590769518	1	pseudobulk	DESeq2	LRT	0.242471432328743
267	KC_AB1	CG34183	-0.186180861043162	0.693488819404314	1	pseudobulk	DESeq2	LRT	0.15896053633569
267	KC_AB1	CG34188	-0.82542030717083	1	1	pseudobulk	DESeq2	LRT	0
267	KC_AB1	CG34189	-2.3118469151042	0.353435572380865	1	pseudobulk	DESeq2	LRT	0.451689742007392
267	KC_AB1	CG34190	-0.850996402080018	0.900449987182352	1	pseudobulk	DESeq2	LRT	0.045540403770502
267	KC_AB1	CG34195	-0.295274707384035	0.658945252737494	1	pseudobulk	DESeq2	LRT	0.181150666470769
267	KC_AB1	CG3420	-0.369590994146826	0.405972210877002	1	pseudobulk	DESeq2	LRT	0.391503693210541
267	KC_AB1	CG34200	-0.115607704866971	0.608310504435411	1	pseudobulk	DESeq2	LRT	0.215874683982671
268	KC_AB1	CG34205	0.135879393043758	1	1	pseudobulk	DESeq2	LRT	0
268	KC_AB1	CG34213	0.374541802991104	0.651569507511955	1	pseudobulk	DESeq2	LRT	0.186039248226577
268	KC_AB1	CG34214	-0.850996402080018	0.900449987182352	1	pseudobulk	DESeq2	LRT	0.045540403770502
268	KC_AB1	CG34219	0.00635561734005138	0.974045615871089	1	pseudobulk	DESeq2	LRT	0.011420704048270
268	KC_AB1	CG34222	0.13617601020169	1	1	pseudobulk	DESeq2	LRT	0
268	KC_AB1	CG34228	-0.803299594702468	0.0637770206315119	1	pseudobulk	DESeq2	LRT	1.19533577285594
268	KC_AB1	CG34229	-0.300812676181874	0.600554713323314	1	pseudobulk	DESeq2	LRT	0.2214474202145
268	KC_AB1	CG34231	0.235643658590502	0.827523995392858	1	pseudobulk	DESeq2	LRT	0.082219404300739
268	KC_AB1	CG34232	0.647509711057945	0.6306647836225	1	pseudobulk	DESeq2	LRT	0.200201419372036
268	KC_AB1	CG34234	-0.138601142423443	0.924727839446494	1	pseudobulk	DESeq2	LRT	0.033986067497209
269	KC_AB1	CG34242	0.239553724568706	0.655234802028811	1	pseudobulk	DESeq2	LRT	0.183603043592457
269	KC_AB1	CG34250	-0.0728131772723116	0.78078859437745	1	pseudobulk	DESeq2	LRT	0.107466539392486
269	KC_AB1	CG34261	-0.124183063686109	0.929571469269125	1	pseudobulk	DESeq2	LRT	0.031717214266494
269	KC_AB1	CG34269	-0.82542030717083	1	1	pseudobulk	DESeq2	LRT	0
269	KC_AB1	CG34279	1.06659676108873	0.534361150970224	1	pseudobulk	DESeq2	LRT	0.272165123385343
269	KC_AB1	CG34286	-0.793942924944103	0.552493454240934	1	pseudobulk	DESeq2	LRT	0.257672862989245
269	KC_AB1	CG34287	0.113316967542732	1	1	pseudobulk	DESeq2	LRT	0
269	KC_AB1	CG34293	-0.542166737287123	0.319479356643156	1	pseudobulk	DESeq2	LRT	0.495557198806389
269	KC_AB1	CG3430	-3.53280310927706	0.0623676923058829	1	pseudobulk	DESeq2	LRT	1.20504032517065
269	KC_AB1	CG34309	-0.82542030717083	1	1	pseudobulk	DESeq2	LRT	0
270	KC_AB1	CG34310	-0.615767568564188	0.806245159731688	1	pseudobulk	DESeq2	LRT	0.093532879622572
270	KC_AB1	CG34316	-0.850996402080018	0.900449987182352	1	pseudobulk	DESeq2	LRT	0.045540403770502
270	KC_AB1	CG34330	1.1064467668528	0.476287176463694	1	pseudobulk	DESeq2	LRT	0.322131111257731

270 KC_AB1	CG34331	1.08899820779554	0.878177167143508	1	pseudobulk	DESeq2	LRT	0.056417858865076
270 KC_AB1	CG34334	-0.850932661185203	0.660736244265418	1	pseudobulk	DESeq2	LRT	0.179971869591347
270 KC_AB1	CG3434	-0.809959036894988	0.457589125899528	1	pseudobulk	DESeq2	LRT	0.339524304621378
270 KC_AB1	CG34348	0.197665778928297	0.759283603136983	1	pseudobulk	DESeq2	LRT	0.119595978686575
270 KC_AB1	CG34351	-0.996825493437144	0.25712857372773	1	pseudobulk	DESeq2	LRT	0.589849659169082
270 KC_AB1	CG34353	-0.034932605242637	0.906260043535316	1	pseudobulk	DESeq2	LRT	0.042747167369588
270 KC_AB1	CG34354	-0.249185484133503	0.0400696653795468	1	pseudobulk	DESeq2	LRT	1.39718428433104
271 KC_AB1	CG34355	-0.0437579119154087	0.798241645403853	1	pseudobulk	DESeq2	LRT	0.097865618199094
271 KC_AB1	CG34357	-0.369110044010574	0.0344420961394165	1	pseudobulk	DESeq2	LRT	1.46291042533454
271 KC_AB1	CG3436	-0.0473008329776479	0.898412738607629	1	pseudobulk	DESeq2	LRT	0.046524098835001
271 KC_AB1	CG3437	-0.0636622117498004	0.908672697615708	1	pseudobulk	DESeq2	LRT	0.041592520756740
271 KC_AB1	CG34370	-0.142629824788092	0.553932408645035	1	pseudobulk	DESeq2	LRT	0.256543225061007
271 KC_AB1	CG34376	1.24208856332173	0.085905847588112	1	pseudobulk	DESeq2	LRT	1.06597727285312
271 KC_AB1	CG34377	0.880402921372305	0.19191154555973	1	pseudobulk	DESeq2	LRT	0.71689889695786
271 KC_AB1	CG34384	0.227373250687939	0.139412744101882	1	pseudobulk	DESeq2	LRT	0.855697524371829
271 KC_AB1	CG34401	0.0682503813206218	0.624314171926631	1	pseudobulk	DESeq2	LRT	0.204596806475604
271 KC_AB1	CG34402	-0.487687788131568	0.0125025881144965	1	pseudobulk	DESeq2	LRT	1.90300007599207
272 KC_AB1	CG34404	-0.0891967055363513	0.789530031838186	1	pseudobulk	DESeq2	LRT	0.102631345815856
272 KC_AB1	CG34408	0.339228960982206	0.38123112560761	1	pseudobulk	DESeq2	LRT	0.418811648621879
272 KC_AB1	CG34409	-0.993760379257954	0.248444705332768	1	pseudobulk	DESeq2	LRT	0.604770254178116
272 KC_AB1	CG34417	0.466903895658733	0.815224421820197	1	pseudobulk	DESeq2	LRT	0.088722818571206
272 KC_AB1	CG34425	0.725476977798061	0.78671877098961	1	pseudobulk	DESeq2	LRT	0.104180487507299
272 KC_AB1	CG34430	-1.56855358782635	0.573026303465967	1	pseudobulk	DESeq2	LRT	0.241825442277049
272 KC_AB1	CG34431	-0.0232052177874785	0.959153840461279	1	pseudobulk	DESeq2	LRT	0.018111729946036
272 KC_AB1	CG34457	1.08899820779554	0.670846322504933	1	pseudobulk	DESeq2	LRT	0.173376956629912
272 KC_AB1	CG3448	0.44529037224658	0.751625109208805	1	pseudobulk	DESeq2	LRT	0.123998720044794
272 KC_AB1	CG3491	-0.82542030717083	1	1	pseudobulk	DESeq2	LRT	0
273 KC_AB1	CG3500	-0.474435968299951	0.361709726241313	1	pseudobulk	DESeq2	LRT	0.441639813062711
273 KC_AB1	CG3511	-0.608312399285793	0.189566293979826	1	pseudobulk	DESeq2	LRT	0.722238880288477
273 KC_AB1	CG3520	-2.53053775813952	0.189674204430594	1	pseudobulk	DESeq2	LRT	0.721991728864674
273 KC_AB1	CG3527	0.568895608823718	0.349952497626022	1	pseudobulk	DESeq2	LRT	0.455990902561115
273 KC_AB1	CG3528	1.68130633771206	0.562684503730219	1	pseudobulk	DESeq2	LRT	0.249735045103917
273 KC_AB1	CG3529	0.063270897652329	0.841154511588383	1	pseudobulk	DESeq2	LRT	0.075124221364124
273 KC_AB1	CG3530	-0.0894925760057459	0.669787358900692	1	pseudobulk	DESeq2	LRT	0.174063053289853
273 KC_AB1	CG3534	0.27381181072236	0.776605311282546	1	pseudobulk	DESeq2	LRT	0.109799643595774
273 KC_AB1	CG3542	-0.170428183786993	0.69909677970858	1	pseudobulk	DESeq2	LRT	0.1554626983831
273 KC_AB1	CG3548	-0.0815646219034289	0.852665114568576	1	pseudobulk	DESeq2	LRT	0.069221505118367
274 KC_AB1	CG3552	0.243698951050303	0.69327206966879	1	pseudobulk	DESeq2	LRT	0.159096296170857
274 KC_AB1	CG3556	-0.0405744107301826	0.858334373199925	1	pseudobulk	DESeq2	LRT	0.066343495197152
274 KC_AB1	CG3558	-0.111386162899733	0.586724915156251	1	pseudobulk	DESeq2	LRT	0.231565469163715
274 KC_AB1	CG3566	0.166189379714393	0.507298228474692	1	pseudobulk	DESeq2	LRT	0.294736654270432
274 KC_AB1	CG3568	0.512549080974617	0.385619592916888	1	pseudobulk	DESeq2	LRT	0.413840908172952
274 KC_AB1	CG3581	0.11400025262841	1	1	pseudobulk	DESeq2	LRT	0
274 KC_AB1	CG3587	0.27041639638331	0.779957778594288	1	pseudobulk	DESeq2	LRT	0.107928906309282
274 KC_AB1	CG3589	0.299502618295837	0.750419902536844	1	pseudobulk	DESeq2	LRT	0.124695656175761
274 KC_AB1	CG3594	-0.0316375546253087	0.924010415512307	1	pseudobulk	DESeq2	LRT	0.034323133353457
274 KC_AB1	CG3609	0.485835982700236	0.655479511044526	1	pseudobulk	DESeq2	LRT	0.183440878923357
275 KC_AB1	CG3610	1.08899820779554	0.697631521651407	1	pseudobulk	DESeq2	LRT	0.156373904552
275 KC_AB1	CG3618	-0.26277588610494	0.36563319273069	1	pseudobulk	DESeq2	LRT	0.436954385242824
275 KC_AB1	CG3625	-0.241152019768597	0.0961565464899768	1	pseudobulk	DESeq2	LRT	1.0170211429628
275 KC_AB1	CG3626	0.659307109461888	0.700956938990666	1	pseudobulk	DESeq2	LRT	0.154308660682627
275 KC_AB1	CG3631	-0.538982688712863	0.533008366375858	1	pseudobulk	DESeq2	LRT	0.273265974008414

275	KC_AB1	CG3632	-0.383733517897204	0.315249399346889	1	pseudobulk	DESeq2	LRT	0.50134573222698
275	KC_AB1	CG3634	-0.188693138858753	0.651945605924683	1	pseudobulk	DESeq2	LRT	0.185788637446312
275	KC_AB1	CG3638	0.0780318268578321	0.652874695785029	1	pseudobulk	DESeq2	LRT	0.185170163519742
275	KC_AB1	CG3645	0.134489152907369	0.792655751877802	1	pseudobulk	DESeq2	LRT	0.100915384586541
275	KC_AB1	CG3651	-0.263350956087072	0.453302413537535	1	pseudobulk	DESeq2	LRT	0.34361196860237
276	KC_AB1	CG3652	0.276640560362164	0.394709002261544	1	pseudobulk	DESeq2	LRT	0.403722968390578
276	KC_AB1	CG3655	0.144938062156184	0.939011437924561	1	pseudobulk	DESeq2	LRT	0.027329117640952
276	KC_AB1	CG3662	0.0790479448991948	0.524649342331684	1	pseudobulk	DESeq2	LRT	0.280130867206095
276	KC_AB1	CG3678	-0.236626638434349	0.660847024887989	1	pseudobulk	DESeq2	LRT	0.17989906084093
276	KC_AB1	CG3700	-1.50365500551025	0.175363419454376	1	pseudobulk	DESeq2	LRT	0.756060994698416
276	KC_AB1	CG3702	-0.340063191806152	0.445633124252779	1	pseudobulk	DESeq2	LRT	0.351022535191741
276	KC_AB1	CG3703	0.317753760376659	0.293407189046365	1	pseudobulk	DESeq2	LRT	0.532529249303518
276	KC_AB1	CG3704	0.597572014237675	0.350102174209429	1	pseudobulk	DESeq2	LRT	0.455805192164915
276	KC_AB1	CG3709	0.459271309445925	0.781975522426583	1	pseudobulk	DESeq2	LRT	0.106806841110794
276	KC_AB1	CG3713	0.259460037534651	0.872806002160514	1	pseudobulk	DESeq2	LRT	0.059082275829169
277	KC_AB1	CG3719	0.0629730224709802	0.828903393634726	1	pseudobulk	DESeq2	LRT	0.081496082303966
277	KC_AB1	CG3726	-1.60313212205239	0.304649542944806	1	pseudobulk	DESeq2	LRT	0.516199469096044
277	KC_AB1	CG3732	0.0287151459443338	0.918816166148976	1	pseudobulk	DESeq2	LRT	0.036771372197594
277	KC_AB1	CG3735	0.278843606289763	0.757994479041634	1	pseudobulk	DESeq2	LRT	0.120333957600776
277	KC_AB1	CG3740	0.256923703811155	0.741383480121621	1	pseudobulk	DESeq2	LRT	0.12995709536601
277	KC_AB1	CG3744	-0.258528990959362	0.415326694420484	1	pseudobulk	DESeq2	LRT	0.381610154450108
277	KC_AB1	CG3756	0.0332272727470557	0.918096636473053	1	pseudobulk	DESeq2	LRT	0.03711160368093
277	KC_AB1	CG3760	0.224174566788547	0.586960421170379	1	pseudobulk	DESeq2	LRT	0.231391182307437
277	KC_AB1	CG3764	-0.127214359823667	0.756291144336588	1	pseudobulk	DESeq2	LRT	0.121310984872
277	KC_AB1	CG3776	0.516248680365708	0.228050277650653	1	pseudobulk	DESeq2	LRT	0.641969394670255
278	KC_AB1	CG3777	-0.053026907507618	0.933252559863258	1	pseudobulk	DESeq2	LRT	0.030000810152836
278	KC_AB1	CG3781	-0.489239358440261	0.413167520945315	1	pseudobulk	DESeq2	LRT	0.383873825653452
278	KC_AB1	CG3792	-0.078362337692900	0.865670165298579	1	pseudobulk	DESeq2	LRT	0.062647549872650
278	KC_AB1	CG3793	0.116009565560165	0.731585727354068	1	pseudobulk	DESeq2	LRT	0.135734775847116
278	KC_AB1	CG3803	0.658466140618604	0.314572120378935	1	pseudobulk	DESeq2	LRT	0.50227977027636
278	KC_AB1	CG3808	0.321859439895203	0.850325745950471	1	pseudobulk	DESeq2	LRT	0.070414671264569
278	KC_AB1	CG3815	-0.24990373856777	0.70746715826115	1	pseudobulk	DESeq2	LRT	0.150293715969055
278	KC_AB1	CG3817	-0.024972466150221	0.944433181334258	1	pseudobulk	DESeq2	LRT	0.024828762998200
278	KC_AB1	CG3819	-0.724900536262134	0.610770426695333	1	pseudobulk	DESeq2	LRT	0.214121999503572
278	KC_AB1	CG3823	-2.0299211165401	0.127389564810846	1	pseudobulk	DESeq2	LRT	0.89486614602485
279	KC_AB1	CG3842	-0.619148458022618	0.879843167001942	1	pseudobulk	DESeq2	LRT	0.055594734413205
279	KC_AB1	CG3847	-0.045514228679823	0.90599828087959	1	pseudobulk	DESeq2	LRT	0.042872626390754
279	KC_AB1	CG3857	-0.717361243679298	0.545261464444652	1	pseudobulk	DESeq2	LRT	0.263395194355026
279	KC_AB1	CG3860	-0.032712688377416	0.916383344264474	1	pseudobulk	DESeq2	LRT	0.037922812935026
279	KC_AB1	CG3862	0.127793895628567	0.870909249590572	1	pseudobulk	DESeq2	LRT	0.060027096950313
279	KC_AB1	CG3894	-0.036038121685132	0.894566873062148	1	pseudobulk	DESeq2	LRT	0.048387188337170
279	KC_AB1	CG3902	-1.56855358782635	0.573026303465967	1	pseudobulk	DESeq2	LRT	0.241825442277049
279	KC_AB1	CG3907	0.078144336426544	0.796371950176892	1	pseudobulk	DESeq2	LRT	0.098884045101090
279	KC_AB1	CG3909	-0.515800573927313	0.560070071698549	1	pseudobulk	DESeq2	LRT	0.251757633979079
279	KC_AB1	CG3918	-0.809817814051329	0.0762145543228286	1	pseudobulk	DESeq2	LRT	1.11796208563518
280	KC_AB1	CG3919	-0.556516675261754	0.598265183321835	1	pseudobulk	DESeq2	LRT	0.223106270651483
280	KC_AB1	CG3955	-0.264986551376847	0.207363310203684	1	pseudobulk	DESeq2	LRT	0.683268082979064
280	KC_AB1	CG3961	0.215233454401562	0.779120954459783	1	pseudobulk	DESeq2	LRT	0.108395115142915
280	KC_AB1	CG3982	1.68130633771206	0.562684503730219	1	pseudobulk	DESeq2	LRT	0.249735045103917
280	KC_AB1	CG3995	-0.258865269914175	0.627235786813246	1	pseudobulk	DESeq2	LRT	0.20256917103131
280	KC_AB1	CG4000	0.113924349464167	0.898882770622153	1	pseudobulk	DESeq2	LRT	0.046296943852606
280	KC_AB1	CG40006	-0.615767568564188	0.806245159731688	1	pseudobulk	DESeq2	LRT	0.093532879622572

280	KC_AB1	CG4004	0.680348179820086	0.105893118337923	1	pseudobulk	DESeq2	LRT	0.975132262413536
280	KC_AB1	CG40045	0.112485105203425	0.505964796688768	1	pseudobulk	DESeq2	LRT	0.295879698843333
280	KC_AB1	CG40191	0.189376522216255	0.530372918682223	1	pseudobulk	DESeq2	LRT	0.27541865950362
281	KC_AB1	CG4022	-0.0272362121984771	0.94744966877969	1	pseudobulk	DESeq2	LRT	0.023443851704832
281	KC_AB1	CG40228	0.0160375167200247	0.896216117768279	1	pseudobulk	DESeq2	LRT	0.047587249895325
281	KC_AB1	CG4025	0.0454580225440951	0.918655469572613	1	pseudobulk	DESeq2	LRT	0.036847334879353
281	KC_AB1	CG4036	0.324810950813173	0.604131987612201	1	pseudobulk	DESeq2	LRT	0.218868168614709
281	KC_AB1	CG4038	-0.0318091191330758	0.942505584554903	1	pseudobulk	DESeq2	LRT	0.025716067823291
281	KC_AB1	CG4041	0.0547354259712371	0.925881557419771	1	pseudobulk	DESeq2	LRT	0.033444566502749
281	KC_AB1	CG4042	-0.495524277663059	0.469352070993224	1	pseudobulk	DESeq2	LRT	0.328501261490584
281	KC_AB1	CG40439	0.786789470620264	0.250238757601831	1	pseudobulk	DESeq2	LRT	0.601645424822326
281	KC_AB1	CG4045	0.306323064298755	0.605503104498506	1	pseudobulk	DESeq2	LRT	0.217883625827099
281	KC_AB1	CG40470	-1.44925888589622	0.6315588144614	1	pseudobulk	DESeq2	LRT	0.199586199175701
282	KC_AB1	CG40472	-0.128189962428028	0.732933425254425	1	pseudobulk	DESeq2	LRT	0.134935471958778
282	KC_AB1	CG4049	-0.194843194191925	0.574734710841884	1	pseudobulk	DESeq2	LRT	0.240532573056197
282	KC_AB1	CG40498	0.0253567224310979	0.835080554738336	1	pseudobulk	DESeq2	LRT	0.078271628958392
282	KC_AB1	CG4069	0.210060093080186	0.815042533370768	1	pseudobulk	DESeq2	LRT	0.088819726810664
282	KC_AB1	CG4074	-0.280334561958352	0.638808772220338	1	pseudobulk	DESeq2	LRT	0.194629128693966
282	KC_AB1	CG4078	2.4580402721534	0.29687568141055	1	pseudobulk	DESeq2	LRT	0.527425376205788
282	KC_AB1	CG4080	-0.0860011670452981	0.740956529742268	1	pseudobulk	DESeq2	LRT	0.130207270356488
282	KC_AB1	CG4098	0.111831901354254	1	1	pseudobulk	DESeq2	LRT	0
282	KC_AB1	CG4101	0.588972050361215	0.112647356644853	1	pseudobulk	DESeq2	LRT	0.948278994868009
282	KC_AB1	CG41099	-0.235509911433623	0.283294756934075	1	pseudobulk	DESeq2	LRT	0.547761463092237
283	KC_AB1	CG41128	-0.0751992507634414	0.553648796943566	1	pseudobulk	DESeq2	LRT	0.256765639451973
283	KC_AB1	CG4119	-0.166330647775578	0.684104875353671	1	pseudobulk	DESeq2	LRT	0.164877314513834
283	KC_AB1	CG4133	0.0758224006227219	0.763130248372961	1	pseudobulk	DESeq2	LRT	0.117401331871956
283	KC_AB1	CG41378	-0.0996845382202089	0.849658116336395	1	pseudobulk	DESeq2	LRT	0.070755789646557
283	KC_AB1	CG41520	0.262615403777483	0.80378180509137	1	pseudobulk	DESeq2	LRT	0.094861828994758
283	KC_AB1	CG4168	-0.850932661185203	0.660736244265418	1	pseudobulk	DESeq2	LRT	0.179971869591347
283	KC_AB1	CG4186	-0.21552606419077	0.723280643338258	1	pseudobulk	DESeq2	LRT	0.140693157471486
283	KC_AB1	CG4199	0.152601625895913	0.745353112736418	1	pseudobulk	DESeq2	LRT	0.127637930498609
283	KC_AB1	CG4210	-0.82542030717083	1	1	pseudobulk	DESeq2	LRT	0
283	KC_AB1	CG42231	-0.943478159386651	0.508247536021103	1	pseudobulk	DESeq2	LRT	0.293924718140062
284	KC_AB1	CG42232	-0.189080743853226	0.556999695059995	1	pseudobulk	DESeq2	LRT	0.254145042588924
284	KC_AB1	CG42233	0.572067121824985	0.444489991870101	1	pseudobulk	DESeq2	LRT	0.352138013151633
284	KC_AB1	CG42237	-1.05972628603973	0.705203047462737	1	pseudobulk	DESeq2	LRT	0.151685819609541
284	KC_AB1	CG42238	-4.12764356808243E-	0.949436201390609	1	pseudobulk	DESeq2	LRT	0.022534212933185
284	KC_AB1	CG42239	-0.520369519514284	0.508459753811796	1	pseudobulk	DESeq2	LRT	0.293743417154577
284	KC_AB1	CG42240	0.102095453359741	0.882641597985816	1	pseudobulk	DESeq2	LRT	0.054215608556647
284	KC_AB1	CG42258	0.0624597037685184	0.629088809638236	1	pseudobulk	DESeq2	LRT	0.201288040066288
284	KC_AB1	CG42259	-0.201132718258865	0.7507554846948	1	pseudobulk	DESeq2	LRT	0.124501486349779
284	KC_AB1	CG42260	0.0574199187125822	0.604308087932691	1	pseudobulk	DESeq2	LRT	0.218741593206397
284	KC_AB1	CG42264	1.08899820779554	0.878177167143508	1	pseudobulk	DESeq2	LRT	0.056417858865076
285	KC_AB1	CG42266	1.08899820779554	0.697631521651407	1	pseudobulk	DESeq2	LRT	0.156373904552
285	KC_AB1	CG42268	0.0789633805267177	0.628471278366376	1	pseudobulk	DESeq2	LRT	0.201714565125227
285	KC_AB1	CG42271	-0.0685883197796185	0.873038417594681	1	pseudobulk	DESeq2	LRT	0.058966644974527
285	KC_AB1	CG4230	0.0210544472395801	0.894616228625529	1	pseudobulk	DESeq2	LRT	0.048363227850608
285	KC_AB1	CG42302	-0.850932661185203	0.660736244265418	1	pseudobulk	DESeq2	LRT	0.179971869591347
285	KC_AB1	CG42307	0.501150806363932	0.482108604590279	1	pseudobulk	DESeq2	LRT	0.316855117235663
285	KC_AB1	CG42322	0.117184810394406	0.397671833209087	1	pseudobulk	DESeq2	LRT	0.40047516866972
285	KC_AB1	CG42323	-0.501300761870089	0.863550576027942	1	pseudobulk	DESeq2	LRT	0.063712221794699
285	KC_AB1	CG42324	-0.0342830419357734	0.824064110242742	1	pseudobulk	DESeq2	LRT	0.084038999902909

285	KC_AB1	CG42327	-2.41936212248622	0.195645360860811	1	pseudobulk	DESeq2	LRT	0.70853044562395
286	KC_AB1	CG42336	-0.030880907210455	0.872721528796905	1	pseudobulk	DESeq2	LRT	0.05912431047492
286	KC_AB1	CG42337	0.0219573962242765	0.878427165560578	1	pseudobulk	DESeq2	LRT	0.05629424203033
286	KC_AB1	CG42339	-0.261161783498692	0.123199507850738	1	pseudobulk	DESeq2	LRT	0.90939102705905
286	KC_AB1	CG42340	-0.052088529156668	0.882536665390116	1	pseudobulk	DESeq2	LRT	0.05426724260209
286	KC_AB1	CG42346	0.257033524339741	0.263589276423632	1	pseudobulk	DESeq2	LRT	0.57907226207793
286	KC_AB1	CG42354	-0.638308674827923	0.720264967776621	1	pseudobulk	DESeq2	LRT	0.14250770791009
286	KC_AB1	CG42359	0.497844253238369	0.4464616973584	1	pseudobulk	DESeq2	LRT	0.35021579397459
286	KC_AB1	CG42360	0.0992455619449859	0.816930196986316	1	pseudobulk	DESeq2	LRT	0.08781505039386
286	KC_AB1	CG42361	-0.279574515589409	0.171761359612472	1	pseudobulk	DESeq2	LRT	0.76507453081064
286	KC_AB1	CG42364	0.13617601020169	1	1	pseudobulk	DESeq2	LRT	0
287	KC_AB1	CG42365	-0.856221751335553	0.199798965427083	1	pseudobulk	DESeq2	LRT	0.6994067649112
287	KC_AB1	CG42376	0.024031174523507	0.933817959013998	1	pseudobulk	DESeq2	LRT	0.02973777805387
287	KC_AB1	CG4238	-0.507721933756028	0.0757196589567083	1	pseudobulk	DESeq2	LRT	1.12079135077735
287	KC_AB1	CG42382	0.0015635485148084	0.93455004008483	1	pseudobulk	DESeq2	LRT	0.02939743954904
287	KC_AB1	CG4239	-0.773447448734387	0.142491319981179	1	pseudobulk	DESeq2	LRT	0.84621159038593
287	KC_AB1	CG42392	-0.007664897216812	0.960786470028926	1	pseudobulk	DESeq2	LRT	0.01737312137653
287	KC_AB1	CG42393	1.62909730677576	0.443426561013432	1	pseudobulk	DESeq2	LRT	0.353178296435601
287	KC_AB1	CG42394	0.895112034594156	0.481556457136687	1	pseudobulk	DESeq2	LRT	0.31735278935859
287	KC_AB1	CG42399	-0.519101403976899	0.436725333608676	1	pseudobulk	DESeq2	LRT	0.35979161474836
287	KC_AB1	CG42402	-0.479952722772758	0.0217389117860183	1	pseudobulk	DESeq2	LRT	1.66276219976932
288	KC_AB1	CG42404	-0.006826801325832	0.972032096692179	1	pseudobulk	DESeq2	LRT	0.01231939434722
288	KC_AB1	CG42450	0.0682202425686106	0.613890019277543	1	pseudobulk	DESeq2	LRT	0.21190942739169
288	KC_AB1	CG42486	-0.82542030717083	1	1	pseudobulk	DESeq2	LRT	0
288	KC_AB1	CG42489	-0.850996402080018	0.900449987182352	1	pseudobulk	DESeq2	LRT	0.04554040377050
288	KC_AB1	CG42495	-0.26549468753927	0.723680578637889	1	pseudobulk	DESeq2	LRT	0.14045308234641
288	KC_AB1	CG4250	2.33333760328394	0.29311636197001	1	pseudobulk	DESeq2	LRT	0.53295993824885
288	KC_AB1	CG42514	-0.848066911524363	0.506550956494707	1	pseudobulk	DESeq2	LRT	0.29537686025169
288	KC_AB1	CG42518	-0.134157149022937	0.901001015508819	1	pseudobulk	DESeq2	LRT	0.04527471953189
288	KC_AB1	CG42526	0.557334180568749	0.718407986413044	1	pseudobulk	DESeq2	LRT	0.14362884833986
288	KC_AB1	CG42537	1.08899820779554	0.878177167143508	1	pseudobulk	DESeq2	LRT	0.05641785886507
289	KC_AB1	CG42540	0.245296359243317	0.0116479280422845	1	pseudobulk	DESeq2	LRT	1.93375132097535
289	KC_AB1	CG42541	0.17556488165223	0.123875829433174	1	pseudobulk	DESeq2	LRT	0.90701342459914
289	KC_AB1	CG42542	-0.056088087169748	0.860399519536737	1	pseudobulk	DESeq2	LRT	0.06529984080270
289	KC_AB1	CG42553	0.834289268636494	0.444231517589005	1	pseudobulk	DESeq2	LRT	0.35239063212386
289	KC_AB1	CG42554	0.74243078832247	0.122639203923343	1	pseudobulk	DESeq2	LRT	0.91137067724061
289	KC_AB1	CG42556	0.997694962638592	0.665140342223729	1	pseudobulk	DESeq2	LRT	0.17708671045209
289	KC_AB1	CG42561	-2.80036860845584	0.209900133609211	1	pseudobulk	DESeq2	LRT	0.67798728497298
289	KC_AB1	CG42566	1.08899820779554	0.878177167143508	1	pseudobulk	DESeq2	LRT	0.05641785886507
289	KC_AB1	CG42578	-0.968336042648622	0.663512636338631	1	pseudobulk	DESeq2	LRT	0.17815080175224
289	KC_AB1	CG42581	1.08899820779554	0.878177167143508	1	pseudobulk	DESeq2	LRT	0.05641785886507
290	KC_AB1	CG42588	-0.041327083456052	0.899789670687516	1	pseudobulk	DESeq2	LRT	0.04585899671075
290	KC_AB1	CG42590	1.08899820779554	0.697631521651407	1	pseudobulk	DESeq2	LRT	0.156373904552
290	KC_AB1	CG42594	-0.230950335906351	0.721917221447364	1	pseudobulk	DESeq2	LRT	0.14151259790081
290	KC_AB1	CG42613	0.100753015073644	0.446291542746079	1	pseudobulk	DESeq2	LRT	0.35038134299032
290	KC_AB1	CG42633	1.68130633771206	0.562684503730219	1	pseudobulk	DESeq2	LRT	0.24973504510391
290	KC_AB1	CG4266	-0.074658748130777	0.816389376142526	1	pseudobulk	DESeq2	LRT	0.08810265549038
290	KC_AB1	CG42663	0.157912427555467	0.929571689575968	1	pseudobulk	DESeq2	LRT	0.03171711133945
290	KC_AB1	CG42668	-0.057322059160844	0.716750781526541	1	pseudobulk	DESeq2	LRT	0.14463182483773
290	KC_AB1	CG42671	0.154654826947504	0.577807741666516	1	pseudobulk	DESeq2	LRT	0.23821664362625
290	KC_AB1	CG42672	0.112180502085377	0.765723955692605	1	pseudobulk	DESeq2	LRT	0.11592776578007
291	KC_AB1	CG42673	-0.581171820322958	0.0353684845485066	1	pseudobulk	DESeq2	LRT	1.45138354822373

291	KC_AB1	CG42674	0.460426908444563	0.017225212482314	1	pseudobulk	DESeq2	LRT	1.76383541212993
291	KC_AB1	CG42675	0.111831901354254	1	1	pseudobulk	DESeq2	LRT	0
291	KC_AB1	CG42676	0.660689231435588	0.251768705991387	1	pseudobulk	DESeq2	LRT	0.598998252226046
291	KC_AB1	CG4269	-0.850932661185203	0.660736244265418	1	pseudobulk	DESeq2	LRT	0.179971869591347
291	KC_AB1	CG42692	-0.268537367244746	0.801444457189982	1	pseudobulk	DESeq2	LRT	0.096126570342583
291	KC_AB1	CG42697	0.923124750607267	0.707531785811194	1	pseudobulk	DESeq2	LRT	0.150254044719123
291	KC_AB1	CG42699	-0.0388876809460714	0.906963368211505	1	pseudobulk	DESeq2	LRT	0.042410253518602
291	KC_AB1	CG42700	-0.0985050356285426	0.71447739204288	1	pseudobulk	DESeq2	LRT	0.146011508883318
291	KC_AB1	CG42709	-0.39350685308655	0.431738906688769	1	pseudobulk	DESeq2	LRT	0.364778812592578
292	KC_AB1	CG42724	-0.104108274404454	0.551608996599617	1	pseudobulk	DESeq2	LRT	0.258368659208104
292	KC_AB1	CG42733	1.08899820779554	0.670846322504933	1	pseudobulk	DESeq2	LRT	0.173376956629912
292	KC_AB1	CG42747	0.997694962638592	0.665140342223729	1	pseudobulk	DESeq2	LRT	0.177086710452097
292	KC_AB1	CG42748	0.021141181156699	0.859101837949558	1	pseudobulk	DESeq2	LRT	0.065955351840394
292	KC_AB1	CG42750	-0.174266448309725	0.27547968533401	1	pseudobulk	DESeq2	LRT	0.559910421758233
292	KC_AB1	CG42766	-0.728397227558699	0.719090563612935	1	pseudobulk	DESeq2	LRT	0.143216410311794
292	KC_AB1	CG4278	-0.3378917154399	0.558444153139052	1	pseudobulk	DESeq2	LRT	0.253020251726982
292	KC_AB1	CG42784	0.0994453243744977	0.485100049900321	1	pseudobulk	DESeq2	LRT	0.31416868069978
292	KC_AB1	CG42788	-0.0802319443342495	0.677275343299691	1	pseudobulk	DESeq2	LRT	0.169234734911585
292	KC_AB1	CG42789	2.12777997112157	0.4429581475599	1	pseudobulk	DESeq2	LRT	0.353637305701188
293	KC_AB1	CG42795	-0.0156378602169765	0.896583220917667	1	pseudobulk	DESeq2	LRT	0.047409392986460
293	KC_AB1	CG42797	-0.490834699444855	0.228457449226327	1	pseudobulk	DESeq2	LRT	0.641194676500182
293	KC_AB1	CG42806	-0.275585751481609	0.629670158537909	1	pseudobulk	DESeq2	LRT	0.200886888386701
293	KC_AB1	CG42809	0.369324777633601	0.49188415084872	1	pseudobulk	DESeq2	LRT	0.308137170751983
293	KC_AB1	CG4281	0.717099496189458	0.137050648375585	1	pseudobulk	DESeq2	LRT	0.86311890550739
293	KC_AB1	CG42810	-1.61862498639867	0.500791630206422	1	pseudobulk	DESeq2	LRT	0.300342938155337
293	KC_AB1	CG42812	-0.850932661185203	0.660736244265418	1	pseudobulk	DESeq2	LRT	0.179971869591347
293	KC_AB1	CG42813	-0.0492597862461553	0.882938448135305	1	pseudobulk	DESeq2	LRT	0.054069571130167
293	KC_AB1	CG4282	-0.315510271461001	0.66787854421623	1	pseudobulk	DESeq2	LRT	0.175302508142045
293	KC_AB1	CG42846	1.08899820779554	0.878177167143508	1	pseudobulk	DESeq2	LRT	0.056417858865076
294	KC_AB1	CG42847	-2.9044344670839	0.215443081422891	1	pseudobulk	DESeq2	LRT	0.666667447958097
294	KC_AB1	CG42855	0.798453933822545	0.303775243001253	1	pseudobulk	DESeq2	LRT	0.517447623054067
294	KC_AB1	CG4287	-0.714484371634344	0.117996433346047	1	pseudobulk	DESeq2	LRT	0.928131119825577
294	KC_AB1	CG42878	-0.850932661185203	0.660736244265418	1	pseudobulk	DESeq2	LRT	0.179971869591347
294	KC_AB1	CG4291	0.225025231349584	0.699374028101374	1	pseudobulk	DESeq2	LRT	0.15529049965287
294	KC_AB1	CG4293	-0.556157122397543	0.371586457330308	1	pseudobulk	DESeq2	LRT	0.429940122487582
294	KC_AB1	CG4294	0.0748616838525204	0.744524965591283	1	pseudobulk	DESeq2	LRT	0.128120734799993
294	KC_AB1	CG4297	0.128207293017683	0.515923908847955	1	pseudobulk	DESeq2	LRT	0.28741434566781
294	KC_AB1	CG43049	-2.3118469151042	0.353435572380865	1	pseudobulk	DESeq2	LRT	0.451689742007392
294	KC_AB1	CG43051	0.644002677963084	0.764564505832492	1	pseudobulk	DESeq2	LRT	0.116585867550177
295	KC_AB1	CG43055	1.12049781000607	0.666276071830617	1	pseudobulk	DESeq2	LRT	0.176345783397819
295	KC_AB1	CG43056	0.113316967542732	1	1	pseudobulk	DESeq2	LRT	0
295	KC_AB1	CG43060	-0.850932661185203	0.660736244265418	1	pseudobulk	DESeq2	LRT	0.179971869591347
295	KC_AB1	CG43066	0.205044760056541	0.237849156214401	1	pseudobulk	DESeq2	LRT	0.62369838492972
295	KC_AB1	CG43074	-0.850996402080018	0.900449987182352	1	pseudobulk	DESeq2	LRT	0.045540403770502
295	KC_AB1	CG43078	0.124834323577413	0.621878822257498	1	pseudobulk	DESeq2	LRT	0.206294232595243
295	KC_AB1	CG43085	2.1559833397943	0.381743505411375	1	pseudobulk	DESeq2	LRT	0.418228342845202
295	KC_AB1	CG43093	-1.82709236256241	0.44317674354685	1	pseudobulk	DESeq2	LRT	0.353423038023035
295	KC_AB1	CG43094	0.942080999092271	0.681532122211136	1	pseudobulk	DESeq2	LRT	0.166513670026992
295	KC_AB1	CG43102	0.222681893347647	0.17598387568291	1	pseudobulk	DESeq2	LRT	0.754527122087747
296	KC_AB1	CG43110	1.88952324269138	0.456767015650974	1	pseudobulk	DESeq2	LRT	0.340305265178099
296	KC_AB1	CG43117	0.687160826507298	0.578246224844598	1	pseudobulk	DESeq2	LRT	0.237887193895884
296	KC_AB1	CG43125	1.08899820779554	0.697631521651407	1	pseudobulk	DESeq2	LRT	0.156373904552

296	KC_AB1	CG4313	0.280578546680952	0.729637870261796	1	pseudobulk	DESeq2	LRT	0.136892632987069
296	KC_AB1	CG43155	-1.21673908022264	0.60839381642346	1	pseudobulk	DESeq2	LRT	0.215815208667062
296	KC_AB1	CG43163	0.499478800638951	0.731945909372716	1	pseudobulk	DESeq2	LRT	0.135521012013917
296	KC_AB1	CG43175	1.08899820779554	0.670846322504933	1	pseudobulk	DESeq2	LRT	0.173376956629912
296	KC_AB1	CG43191	-0.82542030717083	1	1	pseudobulk	DESeq2	LRT	0
296	KC_AB1	CG43192	1.08899820779554	0.670846322504933	1	pseudobulk	DESeq2	LRT	0.173376956629912
296	KC_AB1	CG43219	0.152838635984156	0.870417721069575	1	pseudobulk	DESeq2	LRT	0.060272275637180
297	KC_AB1	CG43222	-0.467179387388911	0.0141238069271879	1	pseudobulk	DESeq2	LRT	1.85004822788535
297	KC_AB1	CG43229	-0.850996402080018	0.900449987182352	1	pseudobulk	DESeq2	LRT	0.045540403770502
297	KC_AB1	CG43232	-1.08519402035964	0.583003469479504	1	pseudobulk	DESeq2	LRT	0.234328860727572
297	KC_AB1	CG43245	1.30411745457488	0.514755047492433	1	pseudobulk	DESeq2	LRT	0.288399386162357
297	KC_AB1	CG43273	0.927282623097541	0.829267166618274	1	pseudobulk	DESeq2	LRT	0.081305529407006
297	KC_AB1	CG43290	-0.850996402080018	0.900449987182352	1	pseudobulk	DESeq2	LRT	0.045540403770502
297	KC_AB1	CG4332	1.12336271955431	0.421459062985956	1	pseudobulk	DESeq2	LRT	0.375244602730141
297	KC_AB1	CG43322	-0.488305690378364	0.739203336023166	1	pseudobulk	DESeq2	LRT	0.131236081813405
297	KC_AB1	CG43324	-0.0420552736036137	0.756674576439704	1	pseudobulk	DESeq2	LRT	0.121090857676986
297	KC_AB1	CG43335	1.08899820779554	0.697631521651407	1	pseudobulk	DESeq2	LRT	0.156373904552
298	KC_AB1	CG43340	0.725476977798061	0.78671877098961	1	pseudobulk	DESeq2	LRT	0.104180487507299
298	KC_AB1	CG43342	2.24119773531942	0.25573199814213	1	pseudobulk	DESeq2	LRT	0.59221492802715
298	KC_AB1	CG43347	0.0385355112717266	0.817245311998561	1	pseudobulk	DESeq2	LRT	0.087647562003629
298	KC_AB1	CG4335	1.88952324269138	0.456767015650974	1	pseudobulk	DESeq2	LRT	0.340305265178099
298	KC_AB1	CG43366	0.843059322176753	0.676170389573906	1	pseudobulk	DESeq2	LRT	0.169943851503148
298	KC_AB1	CG43367	-0.26431206658144	0.525636470089068	1	pseudobulk	DESeq2	LRT	0.279314509869593
298	KC_AB1	CG43373	-0.107558986171674	0.563495438302657	1	pseudobulk	DESeq2	LRT	0.249109595372982
298	KC_AB1	CG4338	-0.621883534239884	0.327102384841944	1	pseudobulk	DESeq2	LRT	0.48531628950412
298	KC_AB1	CG43394	-0.507131305163188	0.571923767425607	1	pseudobulk	DESeq2	LRT	0.242661855110417
298	KC_AB1	CG43396	-0.82542030717083	1	1	pseudobulk	DESeq2	LRT	0
299	KC_AB1	CG4341	-0.072190411957551	0.812330697122441	1	pseudobulk	DESeq2	LRT	0.090267134923983
299	KC_AB1	CG43428	-0.474018358882842	0.779166127793705	1	pseudobulk	DESeq2	LRT	0.108369935533997
299	KC_AB1	CG43445	-0.506516985469986	0.909346984303859	1	pseudobulk	DESeq2	LRT	0.041270369127443
299	KC_AB1	CG4360	0.57011378001029	0.176860263802898	1	pseudobulk	DESeq2	LRT	0.752369731531349
299	KC_AB1	CG4364	-0.0797077799192999	0.816676345883271	1	pseudobulk	DESeq2	LRT	0.087950023083150
299	KC_AB1	CG43647	-0.503543407493149	0.8143511552897	1	pseudobulk	DESeq2	LRT	0.089188283170770
299	KC_AB1	CG43658	-1.50962343931436	0.188264913491653	1	pseudobulk	DESeq2	LRT	0.725230610927576
299	KC_AB1	CG43675	0.113316967542732	1	1	pseudobulk	DESeq2	LRT	0
299	KC_AB1	CG43679	1.08899820779554	0.697631521651407	1	pseudobulk	DESeq2	LRT	0.156373904552
299	KC_AB1	CG43693	1.27633831633667	0.74136702739884	1	pseudobulk	DESeq2	LRT	0.129966733301293
300	KC_AB1	CG43707	-0.159088789719034	0.594908750066777	1	pseudobulk	DESeq2	LRT	0.225549643316133
300	KC_AB1	CG43736	0.338302963789846	0.0209540947444809	1	pseudobulk	DESeq2	LRT	1.67873109674705
300	KC_AB1	CG43737	-0.242088812044567	0.0396957851527855	1	pseudobulk	DESeq2	LRT	1.40125560361631
300	KC_AB1	CG43740	-0.23694049579015	0.484664573931725	1	pseudobulk	DESeq2	LRT	0.314558723434332
300	KC_AB1	CG4375	-0.838580640014249	0.695856596814275	1	pseudobulk	DESeq2	LRT	0.157480251235852
300	KC_AB1	CG43755	-0.770011685721021	0.708224801478314	1	pseudobulk	DESeq2	LRT	0.149828868658713
300	KC_AB1	CG43759	-0.0688950441092799	0.565463965348514	1	pseudobulk	DESeq2	LRT	0.247595065626113
300	KC_AB1	CG43778	0.096881309056414	0.599164355237276	1	pseudobulk	DESeq2	LRT	0.222454031063294
300	KC_AB1	CG43779	-0.615767568564188	0.806245159731688	1	pseudobulk	DESeq2	LRT	0.093532879622572
300	KC_AB1	CG43783	-0.143814440807975	0.749535243347329	1	pseudobulk	DESeq2	LRT	0.125207941693063
301	KC_AB1	CG43788	1.08899820779554	0.878177167143508	1	pseudobulk	DESeq2	LRT	0.056417858865076
301	KC_AB1	CG43789	-0.879108772441003	0.578499634006938	1	pseudobulk	DESeq2	LRT	0.237696911472504
301	KC_AB1	CG43795	-0.82542030717083	1	1	pseudobulk	DESeq2	LRT	0
301	KC_AB1	CG43800	-0.233866811812165	0.773341945225661	1	pseudobulk	DESeq2	LRT	0.111628433522954
301	KC_AB1	CG43843	-0.508514723156182	0.765250990125593	1	pseudobulk	DESeq2	LRT	0.116196099815171

301	KC_AB1	CG43845	-0.253763730474027	0.881712611470969	1	pseudobulk	DESeq2	LRT	0.054672947284077
301	KC_AB1	CG43850	0.31221119133396	0.39427089961385	1	pseudobulk	DESeq2	LRT	0.404205276194626
301	KC_AB1	CG43861	-0.184392709008341	0.412200022781717	1	pseudobulk	DESeq2	LRT	0.384891988553958
301	KC_AB1	CG43867	-0.12902630132868	0.411794044568561	1	pseudobulk	DESeq2	LRT	0.385319938508939
301	KC_AB1	CG43897	0.114184054362142	1	1	pseudobulk	DESeq2	LRT	0
302	KC_AB1	CG4390	-0.0688919401015694	0.875925137510392	1	pseudobulk	DESeq2	LRT	0.057533009991122
302	KC_AB1	CG43901	-0.208736329078087	0.346113111108185	1	pseudobulk	DESeq2	LRT	0.460781948888229
302	KC_AB1	CG43902	0.688171909625677	0.466961895553728	1	pseudobulk	DESeq2	LRT	0.330718556748493
302	KC_AB1	CG43919	1.08899820779554	0.670846322504933	1	pseudobulk	DESeq2	LRT	0.173376956629912
302	KC_AB1	CG43925	2.6288931366292	0.260231277769534	1	pseudobulk	DESeq2	LRT	0.584640505828425
302	KC_AB1	CG43980	0.111831901354254	1	1	pseudobulk	DESeq2	LRT	0
302	KC_AB1	CG44002	-0.749759853648093	0.569678458666663	1	pseudobulk	DESeq2	LRT	0.244370202272432
302	KC_AB1	CG44004	1.08899820779554	0.878177167143508	1	pseudobulk	DESeq2	LRT	0.056417858865076
302	KC_AB1	CG44006	1.08899820779554	0.670846322504933	1	pseudobulk	DESeq2	LRT	0.173376956629912
302	KC_AB1	CG44008	-2.36817011682091	0.282654768599882	1	pseudobulk	DESeq2	LRT	0.548743683265438
303	KC_AB1	CG4404	0.364582778501962	0.188909574904556	1	pseudobulk	DESeq2	LRT	0.723746029252179
303	KC_AB1	CG4407	0.100067473796172	0.836800127558493	1	pseudobulk	DESeq2	LRT	0.077378262274667
303	KC_AB1	CG44085	1.75953787297189	0.424665700986932	1	pseudobulk	DESeq2	LRT	0.371952814294562
303	KC_AB1	CG4409	1.68960204341841	0.469390402876188	1	pseudobulk	DESeq2	LRT	0.328465794201684
303	KC_AB1	CG44098	0.444111670180509	0.655872950668826	1	pseudobulk	DESeq2	LRT	0.183180279781767
303	KC_AB1	CG44153	-0.748349447066606	0.398835305001567	1	pseudobulk	DESeq2	LRT	0.399206404801922
303	KC_AB1	CG44158	0.527560905317856	0.444746072715054	1	pseudobulk	DESeq2	LRT	0.351887878220342
303	KC_AB1	CG44195	0.13617601020169	1	1	pseudobulk	DESeq2	LRT	0
303	KC_AB1	CG4424	0.098072450303724	0.895000708134486	1	pseudobulk	DESeq2	LRT	0.048176621065341
303	KC_AB1	CG44242	-0.34707347683768	0.077053571160286	1	pseudobulk	DESeq2	LRT	1.1132072284671
304	KC_AB1	CG44243	0.0898283107600077	0.892055311690173	1	pseudobulk	DESeq2	LRT	0.049608216456740
304	KC_AB1	CG44245	0.771231299457179	0.6353142701336	1	pseudobulk	DESeq2	LRT	0.197011389634554
304	KC_AB1	CG44247	0.0148952598383585	0.920743689365117	1	pseudobulk	DESeq2	LRT	0.035861249050078
304	KC_AB1	CG44249	-1.29467356980949	0.510838645677309	1	pseudobulk	DESeq2	LRT	0.291716255164423
304	KC_AB1	CG44296	-1.44925888589622	0.6315588144614	1	pseudobulk	DESeq2	LRT	0.199586199175701
304	KC_AB1	CG44303	0.135879393043758	1	1	pseudobulk	DESeq2	LRT	0
304	KC_AB1	CG44325	0.13617601020169	1	1	pseudobulk	DESeq2	LRT	0
304	KC_AB1	CG4438	0.119923880065143	0.930603642043932	1	pseudobulk	DESeq2	LRT	0.031235252128898
304	KC_AB1	CG44405	1.08899820779554	0.878177167143508	1	pseudobulk	DESeq2	LRT	0.056417858865076
304	KC_AB1	CG44422	-2.31863758588079	0.151572621408696	1	pseudobulk	DESeq2	LRT	0.819379238314392
305	KC_AB1	CG44437	-1.56855358782635	0.573026303465967	1	pseudobulk	DESeq2	LRT	0.241825442277049
305	KC_AB1	CG44438	-0.82542030717083	1	1	pseudobulk	DESeq2	LRT	0
305	KC_AB1	CG4452	0.133233350529554	0.725453110701745	1	pseudobulk	DESeq2	LRT	0.139390652725321
305	KC_AB1	CG44521	-0.850932661185203	0.660736244265418	1	pseudobulk	DESeq2	LRT	0.179971869591347
305	KC_AB1	CG4455	-1.48328515816818	0.259020404595336	1	pseudobulk	DESeq2	LRT	0.586666022582417
305	KC_AB1	CG4459	-0.850996402080018	0.900449987182352	1	pseudobulk	DESeq2	LRT	0.045540403770502
305	KC_AB1	CG4467	0.0296848721170938	0.790375969276418	1	pseudobulk	DESeq2	LRT	0.102166272581994
305	KC_AB1	CG4476	0.483880417039301	0.833651335462188	1	pseudobulk	DESeq2	LRT	0.079015549776920
305	KC_AB1	CG4477	1.08899820779554	0.878177167143508	1	pseudobulk	DESeq2	LRT	0.056417858865076
305	KC_AB1	CG44774	-0.0060625943413273	0.946151817671183	1	pseudobulk	DESeq2	LRT	0.024039171963132
306	KC_AB1	CG44812	-0.82542030717083	1	1	pseudobulk	DESeq2	LRT	0
306	KC_AB1	CG44837	0.0501883098190489	0.925163053272541	1	pseudobulk	DESeq2	LRT	0.033781719265287
306	KC_AB1	CG44838	0.238029870083122	0.186893289984458	1	pseudobulk	DESeq2	LRT	0.728406290782942
306	KC_AB1	CG4496	1.11782151172364	0.570340019529147	1	pseudobulk	DESeq2	LRT	0.24386615383854
306	KC_AB1	CG4497	-0.179229633186134	0.715206546438205	1	pseudobulk	DESeq2	LRT	0.145568519003837
306	KC_AB1	CG45002	-0.927497090455248	0.077254345236062	1	pseudobulk	DESeq2	LRT	1.11207708395527
306	KC_AB1	CG4502	-0.0327069491748776	0.793919324912774	1	pseudobulk	DESeq2	LRT	0.100223626697967

306	KC_AB1	CG45049	0.124043145480366	0.45862324886153	1	pseudobulk	DESeq2	LRT	0.338543933477972
306	KC_AB1	CG45050	0.0938727387621952	0.447397232353377	1	pseudobulk	DESeq2	LRT	0.349306706822701
306	KC_AB1	CG45066	0.135879393043758	1	1	pseudobulk	DESeq2	LRT	0
307	KC_AB1	CG45067	-1.68203279576935	0.480780548244404	1	pseudobulk	DESeq2	LRT	0.318053111639696
307	KC_AB1	CG45071	0.597659737120955	0.167402316625726	1	pseudobulk	DESeq2	LRT	0.776238536242354
307	KC_AB1	CG45076	1.08899820779554	0.878177167143508	1	pseudobulk	DESeq2	LRT	0.056417858865076
307	KC_AB1	CG45081	-0.865921657347254	0.62304665676012	1	pseudobulk	DESeq2	LRT	0.205479430042665
307	KC_AB1	CG4511	0.0502592825827393	0.888181765408233	1	pseudobulk	DESeq2	LRT	0.051498147244165
307	KC_AB1	CG4520	1.08899820779554	0.878177167143508	1	pseudobulk	DESeq2	LRT	0.056417858865076
307	KC_AB1	CG45218	0.541364420187768	0.788064344760044	1	pseudobulk	DESeq2	LRT	0.103438321300584
307	KC_AB1	CG45263	-0.112551510047266	0.214913542337995	1	pseudobulk	DESeq2	LRT	0.667736217466404
307	KC_AB1	CG4537	-0.0771935336506159	0.925084780429285	1	pseudobulk	DESeq2	LRT	0.033818464033540
307	KC_AB1	CG4538	-0.236823883773755	0.550162287274638	1	pseudobulk	DESeq2	LRT	0.259509183102763
308	KC_AB1	CG4552	-0.340095422470643	0.492972525304502	1	pseudobulk	DESeq2	LRT	0.307177284457344
308	KC_AB1	CG4553	-0.0931070296089717	0.895504481267481	1	pseudobulk	DESeq2	LRT	0.047932236520846
308	KC_AB1	CG4554	0.27363688613553	0.77986603453499	1	pseudobulk	DESeq2	LRT	0.107979994051899
308	KC_AB1	CG4557	0.882463351422246	0.131829827484981	1	pseudobulk	DESeq2	LRT	0.879986316247579
308	KC_AB1	CG4558	0.499477936435072	0.272995039057438	1	pseudobulk	DESeq2	LRT	0.563845245008899
308	KC_AB1	CG4562	-0.356731018621518	0.261336758260966	1	pseudobulk	DESeq2	LRT	0.582799500386914
308	KC_AB1	CG4565	-0.359494365428323	0.831434659614021	1	pseudobulk	DESeq2	LRT	0.080171875228688
308	KC_AB1	CG4570	1.08899820779554	0.878177167143508	1	pseudobulk	DESeq2	LRT	0.056417858865076
308	KC_AB1	CG4572	0.462389625211321	0.52310420430053	1	pseudobulk	DESeq2	LRT	0.281411789440767
308	KC_AB1	CG4576	-0.82542030717083	1	1	pseudobulk	DESeq2	LRT	0
309	KC_AB1	CG4577	-0.0960069366483648	0.501777276262583	1	pseudobulk	DESeq2	LRT	0.299489010253825
309	KC_AB1	CG4587	-0.041872322478846	0.762383880250738	1	pseudobulk	DESeq2	LRT	0.117826294940775
309	KC_AB1	CG4593	0.180114908484585	0.769220535155871	1	pseudobulk	DESeq2	LRT	0.113949130328206
309	KC_AB1	CG4596	-0.0407441049823675	0.861811910997574	1	pseudobulk	DESeq2	LRT	0.064587507874973
309	KC_AB1	CG4598	-1.48096148824369	0.0312831875920573	1	pseudobulk	DESeq2	LRT	1.50468900103708
309	KC_AB1	CG4603	0.162848522664742	0.745152296459223	1	pseudobulk	DESeq2	LRT	0.127754955772119
309	KC_AB1	CG4607	-2.61627160405009	0.247973659858837	1	pseudobulk	DESeq2	LRT	0.605594448147543
309	KC_AB1	CG4610	-0.884652269578608	0.434027087984385	1	pseudobulk	DESeq2	LRT	0.362483164968984
309	KC_AB1	CG4611	0.197171892658449	0.875596500104751	1	pseudobulk	DESeq2	LRT	0.057695983043155
309	KC_AB1	CG4612	-0.138454997535203	0.314378435495489	1	pseudobulk	DESeq2	LRT	0.502547251648248
310	KC_AB1	CG4615	0.242557145302639	0.447623884669741	1	pseudobulk	DESeq2	LRT	0.349086748096501
310	KC_AB1	CG4617	-0.569172725191739	0.270166086197327	1	pseudobulk	DESeq2	LRT	0.568369168643957
310	KC_AB1	CG4619	0.22080650506924	0.84164029116836	1	pseudobulk	DESeq2	LRT	0.074873482062434
310	KC_AB1	CG4622	-0.156480100966311	0.69719910360198	1	pseudobulk	DESeq2	LRT	0.15664317993979
310	KC_AB1	CG4627	-0.107511126558159	0.843893348313601	1	pseudobulk	DESeq2	LRT	0.073712436272007
310	KC_AB1	CG46280	-0.187333120263489	0.558214742203193	1	pseudobulk	DESeq2	LRT	0.253198698171726
310	KC_AB1	CG46281	0.46201371474032	0.737401434049852	1	pseudobulk	DESeq2	LRT	0.132296022141712
310	KC_AB1	CG4630	1.08899820779554	0.670846322504933	1	pseudobulk	DESeq2	LRT	0.173376956629912
310	KC_AB1	CG46301	-0.0947718669524915	0.686630691694272	1	pseudobulk	DESeq2	LRT	0.163276787954317
310	KC_AB1	CG46304	0.898121657436304	0.639192109007287	1	pseudobulk	DESeq2	LRT	0.194368595139224
311	KC_AB1	CG4631	2.01869853195404	0.144151928592452	1	pseudobulk	DESeq2	LRT	0.841179542859841
311	KC_AB1	CG46338	0.0582448574761012	0.80215510093429	1	pseudobulk	DESeq2	LRT	0.095741650459821
311	KC_AB1	CG46385	-0.30214799036372	0.0401036090231851	1	pseudobulk	DESeq2	LRT	1.39681654238416
311	KC_AB1	CG46387	1.99053915838216	0.26047207498336	1	pseudobulk	DESeq2	LRT	0.584238830255859
311	KC_AB1	CG46394	-0.82542030717083	1	1	pseudobulk	DESeq2	LRT	0
311	KC_AB1	CG46448	-0.619148458022618	0.879843167001942	1	pseudobulk	DESeq2	LRT	0.055594734413205
311	KC_AB1	CG4645	0.062177215094455	0.866326188953254	1	pseudobulk	DESeq2	LRT	0.062318556724196
311	KC_AB1	CG4646	-0.415004688580611	0.306163354955233	1	pseudobulk	DESeq2	LRT	0.514046791734166
311	KC_AB1	CG4658	-0.135837927645292	0.5804929725236	1	pseudobulk	DESeq2	LRT	0.236203033483885

311	KC_AB1	CG4660	0.0398007639040127	0.822184292169294	1	pseudobulk	DESeq2	LRT	0.085030824672384
312	KC_AB1	CG4661	-1.97413723473692	0.323179316657538	1	pseudobulk	DESeq2	LRT	0.490556441675099
312	KC_AB1	CG4666	-0.82542030717083	1	1	pseudobulk	DESeq2	LRT	0
312	KC_AB1	CG4676	-0.028468772539064	0.957000748787466	1	pseudobulk	DESeq2	LRT	0.01908772241737
312	KC_AB1	CG4678	-1.13107982665014	0.0617432206942355	1	pseudobulk	DESeq2	LRT	1.20941072028228
312	KC_AB1	CG4679	-0.428133557380708	0.506337464911591	1	pseudobulk	DESeq2	LRT	0.295559937115629
312	KC_AB1	CG4686	-0.423334476324192	0.193321713214802	1	pseudobulk	DESeq2	LRT	0.713719364803393
312	KC_AB1	CG4707	0.173272561769786	0.840621592400628	1	pseudobulk	DESeq2	LRT	0.075399458798810
312	KC_AB1	CG4709	0.114751567613342	0.893385844822197	1	pseudobulk	DESeq2	LRT	0.048960932952507
312	KC_AB1	CG4730	-0.383564052525988	0.766039529900579	1	pseudobulk	DESeq2	LRT	0.11574881891213
312	KC_AB1	CG4743	0.218194244756784	0.827522790080981	1	pseudobulk	DESeq2	LRT	0.082220036863276
313	KC_AB1	CG4751	-0.347956506612587	0.553142098434387	1	pseudobulk	DESeq2	LRT	0.257163287070539
313	KC_AB1	CG4763	2.61681794392005	0.166588350430006	1	pseudobulk	DESeq2	LRT	0.77835537220211
313	KC_AB1	CG4766	-0.850996402080018	0.900449987182352	1	pseudobulk	DESeq2	LRT	0.045540403770502
313	KC_AB1	CG4768	0.132777749987086	0.624341439006498	1	pseudobulk	DESeq2	LRT	0.204577838968115
313	KC_AB1	CG4783	-0.82542030717083	1	1	pseudobulk	DESeq2	LRT	0
313	KC_AB1	CG4788	-0.474407066236339	0.447538308248538	1	pseudobulk	DESeq2	LRT	0.349169784149446
313	KC_AB1	CG4789	0.071459386675607	0.824490417745102	1	pseudobulk	DESeq2	LRT	0.083814387374961
313	KC_AB1	CG4797	-1.89854133735591	0.478664405825708	1	pseudobulk	DESeq2	LRT	0.319968866080244
313	KC_AB1	CG4806	0.0273248552053605	0.938515112926886	1	pseudobulk	DESeq2	LRT	0.027558729533944
313	KC_AB1	CG4813	-0.367875801423868	0.695071359548112	1	pseudobulk	DESeq2	LRT	0.157970606248631
314	KC_AB1	CG4820	0.273134718264308	0.714201300580716	1	pseudobulk	DESeq2	LRT	0.146179363280014
314	KC_AB1	CG4825	0.335535498001009	0.585288817814578	1	pseudobulk	DESeq2	LRT	0.232629773182617
314	KC_AB1	CG4829	-0.663028950496329	0.570989846037436	1	pseudobulk	DESeq2	LRT	0.243371614782385
314	KC_AB1	CG4839	1.08899820779554	0.878177167143508	1	pseudobulk	DESeq2	LRT	0.056417858865076
314	KC_AB1	CG4849	-0.129188575837186	0.840917528662711	1	pseudobulk	DESeq2	LRT	0.075246594693141
314	KC_AB1	CG4853	-0.317905731628546	0.463827212246691	1	pseudobulk	DESeq2	LRT	0.333643775357599
314	KC_AB1	CG4854	-0.059836179688834	0.917375142652432	1	pseudobulk	DESeq2	LRT	0.037453031759235
314	KC_AB1	CG4858	0.213456058181878	0.636999290597851	1	pseudobulk	DESeq2	LRT	0.195861051321809
314	KC_AB1	CG4866	-1.46433560842486	0.0559351276738584	1	pseudobulk	DESeq2	LRT	1.25231536628974
314	KC_AB1	CG4880	-0.849009005497671	0.665790727018774	1	pseudobulk	DESeq2	LRT	0.176662257891611
315	KC_AB1	CG4882	0.274954501696718	0.606424787969809	1	pseudobulk	DESeq2	LRT	0.217223054970851
315	KC_AB1	CG4887	-0.342659684882897	0.189911510559209	1	pseudobulk	DESeq2	LRT	0.721448711825115
315	KC_AB1	CG4896	-0.455285651456036	0.108922711677207	1	pseudobulk	DESeq2	LRT	0.962881555251755
315	KC_AB1	CG4901	0.789778488839138	0.279943759584225	1	pseudobulk	DESeq2	LRT	0.552929209213266
315	KC_AB1	CG4908	-0.434174029389439	0.466486713194795	1	pseudobulk	DESeq2	LRT	0.331160721624577
315	KC_AB1	CG4911	0.0943012743522961	0.716636136387061	1	pseudobulk	DESeq2	LRT	0.144701296312514
315	KC_AB1	CG4928	-0.715916138796926	0.857202297388202	1	pseudobulk	DESeq2	LRT	0.066916673675843
315	KC_AB1	CG4935	-0.281169309644767	0.733372306830971	1	pseudobulk	DESeq2	LRT	0.134675493635807
315	KC_AB1	CG4936	-0.064306760763040	0.908964718444057	1	pseudobulk	DESeq2	LRT	0.041452973634389
315	KC_AB1	CG4942	-0.855135436518178	0.619979188836491	1	pseudobulk	DESeq2	LRT	0.207622888445563
316	KC_AB1	CG4949	-0.781534866287576	0.398527063864241	1	pseudobulk	DESeq2	LRT	0.399542180446551
316	KC_AB1	CG4951	-0.418193008993117	0.516362965104409	1	pseudobulk	DESeq2	LRT	0.287044914019046
316	KC_AB1	CG4953	1.13686662187473	0.094998336326169	1	pseudobulk	DESeq2	LRT	1.02228400029737
316	KC_AB1	CG4957	-0.517313715108522	0.383189151014107	1	pseudobulk	DESeq2	LRT	0.416586795317413
316	KC_AB1	CG4968	0.380516145897027	0.273092025604097	1	pseudobulk	DESeq2	LRT	0.56369098124694
316	KC_AB1	CG4972	0.135344417338744	0.838649465164031	1	pseudobulk	DESeq2	LRT	0.076419525650272
316	KC_AB1	CG4975	1.76548660144211	0.307591712555849	1	pseudobulk	DESeq2	LRT	0.512025369910105
316	KC_AB1	CG4984	0.0057685083369781	0.967171184455792	1	pseudobulk	DESeq2	LRT	0.014496651165342
316	KC_AB1	CG5003	-0.247434825933218	0.660785279477905	1	pseudobulk	DESeq2	LRT	0.179939640495068
316	KC_AB1	CG5004	-0.059718879899879	0.882163798945413	1	pseudobulk	DESeq2	LRT	0.054450768186300
317	KC_AB1	CG5009	-0.092117754084607	0.909455286744236	1	pseudobulk	DESeq2	LRT	0.041218648109676

317	KC_AB1	CG5013	-0.330631857108351	0.658289794769749	1	pseudobulk	DESeq2	LRT	0.181582877567102
317	KC_AB1	CG5021	-0.0398726662510093	0.889062082952007	1	pseudobulk	DESeq2	LRT	0.051067911310921
317	KC_AB1	CG5022	-0.25091679084765	0.60111337236382	1	pseudobulk	DESeq2	LRT	0.221043610611953
317	KC_AB1	CG5027	-0.148329696504473	0.716699497800162	1	pseudobulk	DESeq2	LRT	0.144662899845647
317	KC_AB1	CG5028	-0.0535864652267948	0.826616445424867	1	pseudobulk	DESeq2	LRT	0.082695958737076
317	KC_AB1	CG5033	-0.503663865323928	0.544416143680229	1	pseudobulk	DESeq2	LRT	0.264069005057077
317	KC_AB1	CG5037	-0.13591123923621	0.723965892654438	1	pseudobulk	DESeq2	LRT	0.140281893721208
317	KC_AB1	CG5038	2.01248326573443	0.142425746697586	1	pseudobulk	DESeq2	LRT	0.846411494983158
317	KC_AB1	CG5039	-0.184246036503057	0.832839981959561	1	pseudobulk	DESeq2	LRT	0.079438433920655
318	KC_AB1	CG5044	0.0156690934823544	0.97392966562011	1	pseudobulk	DESeq2	LRT	0.011472405478688
318	KC_AB1	CG5048	-1.44925888589622	0.6315588144614	1	pseudobulk	DESeq2	LRT	0.199586199175701
318	KC_AB1	CG5050	1.11665486949011	0.786340792605537	1	pseudobulk	DESeq2	LRT	0.104389194073077
318	KC_AB1	CG5056	0.610335125035977	0.103788122163906	1	pseudobulk	DESeq2	LRT	0.983852345657481
318	KC_AB1	CG5059	0.580300829534189	0.00809253847492371	1	pseudobulk	DESeq2	LRT	2.09191522712374
318	KC_AB1	CG5065	-0.382174282755504	0.0555264170227676	1	pseudobulk	DESeq2	LRT	1.25550034953201
318	KC_AB1	CG5068	-0.387882770547906	0.316446098512766	1	pseudobulk	DESeq2	LRT	0.499700254405051
318	KC_AB1	CG5071	-0.639599218891621	0.788513993518674	1	pseudobulk	DESeq2	LRT	0.103190594974246
318	KC_AB1	CG5078	1.64954027140197	0.477780694739395	1	pseudobulk	DESeq2	LRT	0.320771402383411
318	KC_AB1	CG5079	1.81406626695653	0.511455069952588	1	pseudobulk	DESeq2	LRT	0.291192511959086
319	KC_AB1	CG5087	-0.386196520398104	0.436827059811141	1	pseudobulk	DESeq2	LRT	0.359690466542654
319	KC_AB1	CG5096	0.113316967542732	1	1	pseudobulk	DESeq2	LRT	0
319	KC_AB1	CG5098	-0.0936416479678823	0.753351828055925	1	pseudobulk	DESeq2	LRT	0.123002153549203
319	KC_AB1	CG5104	1.16523413356038	0.63328025468345	1	pseudobulk	DESeq2	LRT	0.198404052815419
319	KC_AB1	CG5110	0.0544451541961319	0.874264065671346	1	pseudobulk	DESeq2	LRT	0.058357371779015
319	KC_AB1	CG5112	2.39533548415136	0.318016613010836	1	pseudobulk	DESeq2	LRT	0.497550192120982
319	KC_AB1	CG5114	-0.227756719684028	0.773034134624255	1	pseudobulk	DESeq2	LRT	0.111801328653925
319	KC_AB1	CG5116	-0.813279733705338	0.630745084827421	1	pseudobulk	DESeq2	LRT	0.200146125099957
319	KC_AB1	CG5126	-1.12239841934071	0.150901770865808	1	pseudobulk	DESeq2	LRT	0.821305663652269
319	KC_AB1	CG5131	-0.833123035198192	0.320433835129751	1	pseudobulk	DESeq2	LRT	0.494261632305918
320	KC_AB1	CG5149	-0.119140362755402	0.75190254176274	1	pseudobulk	DESeq2	LRT	0.123838447064694
320	KC_AB1	CG5151	0.0400025173255896	0.861331827436935	1	pseudobulk	DESeq2	LRT	0.064829504688103
320	KC_AB1	CG5160	-0.941710893225668	0.522642215230855	1	pseudobulk	DESeq2	LRT	0.281795514047355
320	KC_AB1	CG5174	-0.0094696388353027	0.966423751245597	1	pseudobulk	DESeq2	LRT	0.014832405183884
320	KC_AB1	CG5177	1.88952324269138	0.456767015650974	1	pseudobulk	DESeq2	LRT	0.340305265178099
320	KC_AB1	CG5180	0.479047657608386	0.413355374934558	1	pseudobulk	DESeq2	LRT	0.383676410789947
320	KC_AB1	CG5181	0.0055945472141042	0.976126110532644	1	pseudobulk	DESeq2	LRT	0.010494070068615
320	KC_AB1	CG5188	1.20432066570211	0.370428364806413	1	pseudobulk	DESeq2	LRT	0.431295765491036
320	KC_AB1	CG5191	0.264386358344114	0.720198569173	1	pseudobulk	DESeq2	LRT	0.142547745781851
320	KC_AB1	CG5196	0.697631420705367	0.013042416281178	1	pseudobulk	DESeq2	LRT	1.884641942318
321	KC_AB1	CG5199	0.973349700839609	0.338287269215553	1	pseudobulk	DESeq2	LRT	0.470714345733951
321	KC_AB1	CG5214	-0.439844529322735	0.0842426833043104	1	pseudobulk	DESeq2	LRT	1.07446780842162
321	KC_AB1	CG5217	-4.79126156432836	0.259788766451793	1	pseudobulk	DESeq2	LRT	0.585379632221715
321	KC_AB1	CG5220	0.487284681080717	0.377423109782196	1	pseudobulk	DESeq2	LRT	0.423171511315731
321	KC_AB1	CG5245	-3.32605984644888	0.138359851832382	1	pseudobulk	DESeq2	LRT	0.858989911737277
321	KC_AB1	CG5262	-0.578133650055872	0.363812264853192	1	pseudobulk	DESeq2	LRT	0.439122664073313
321	KC_AB1	CG5270	-0.209577017939083	0.748098197263264	1	pseudobulk	DESeq2	LRT	0.126041391800638
321	KC_AB1	CG5273	-0.16282586298818	0.622993284687661	1	pseudobulk	DESeq2	LRT	0.205516634623031
321	KC_AB1	CG5274	0.586163237450093	0.337974928994789	1	pseudobulk	DESeq2	LRT	0.471115514524276
321	KC_AB1	CG5276	0.219133489987847	0.743358843055941	1	pseudobulk	DESeq2	LRT	0.128801487803787
322	KC_AB1	CG5280	1.08899820779554	0.697631521651407	1	pseudobulk	DESeq2	LRT	0.156373904552
322	KC_AB1	CG5281	-0.0036499643822076	0.985976003285434	1	pseudobulk	DESeq2	LRT	0.006133654802744
322	KC_AB1	CG5282	-0.0439548243486292	0.846239119544026	1	pseudobulk	DESeq2	LRT	0.072506902179611

322	KC_AB1	CG5285	-0.236516790769319	0.698748713425743	1	pseudobulk	DESeq2	LRT	0.155678978751481
322	KC_AB1	CG5287	-0.731694784563039	0.343243672802876	1	pseudobulk	DESeq2	LRT	0.464397459603583
322	KC_AB1	CG5290	-0.234117749818084	0.825338347354061	1	pseudobulk	DESeq2	LRT	0.083367975977091
322	KC_AB1	CG5292	-0.256940293376103	0.559798324417691	1	pseudobulk	DESeq2	LRT	0.251968405793472
322	KC_AB1	CG5316	0.134630784906488	0.676737368616691	1	pseudobulk	DESeq2	LRT	0.169579841639858
322	KC_AB1	CG5323	0.267731154980086	0.628003087391824	1	pseudobulk	DESeq2	LRT	0.202038221176917
322	KC_AB1	CG5339	-0.347279618538929	0.477337563915139	1	pseudobulk	DESeq2	LRT	0.321174387634296
323	KC_AB1	CG5346	-0.850932661185203	0.660736244265418	1	pseudobulk	DESeq2	LRT	0.179971869591347
323	KC_AB1	CG5347	1.08899820779554	0.697631521651407	1	pseudobulk	DESeq2	LRT	0.156373904552
323	KC_AB1	CG5348	0.916062021341639	0.213362287312319	1	pseudobulk	DESeq2	LRT	0.670882341516746
323	KC_AB1	CG5355	0.202282292280883	0.717321421406448	1	pseudobulk	DESeq2	LRT	0.144286199612489
323	KC_AB1	CG5359	0.141594469684296	0.806443633420017	1	pseudobulk	DESeq2	LRT	0.093425982335946
323	KC_AB1	CG5377	-0.82542030717083	1	1	pseudobulk	DESeq2	LRT	0
323	KC_AB1	CG5380	0.852118321250365	0.247547029858721	1	pseudobulk	DESeq2	LRT	0.606342280092773
323	KC_AB1	CG5382	-0.133115284114939	0.788843778015152	1	pseudobulk	DESeq2	LRT	0.103008995603819
323	KC_AB1	CG5388	-0.850932661185203	0.660736244265418	1	pseudobulk	DESeq2	LRT	0.179971869591347
323	KC_AB1	CG5404	-0.850996402080018	0.900449987182352	1	pseudobulk	DESeq2	LRT	0.045540403770502
324	KC_AB1	CG5412	-0.0591589842363915	0.872667650710761	1	pseudobulk	DESeq2	LRT	0.059151122782221
324	KC_AB1	CG5435	0.0944367054142478	0.950578142812657	1	pseudobulk	DESeq2	LRT	0.022012175908065
324	KC_AB1	CG5439	-0.211681628675964	0.701698491301832	1	pseudobulk	DESeq2	LRT	0.153849457232042
324	KC_AB1	CG5445	-0.0872452586574361	0.78878599298658	1	pseudobulk	DESeq2	LRT	0.103040810063102
324	KC_AB1	CG5446	-0.272557955963919	0.396035726555491	1	pseudobulk	DESeq2	LRT	0.402265634412769
324	KC_AB1	CG5447	0.074559252069368	0.814654718089566	1	pseudobulk	DESeq2	LRT	0.089026422918977
324	KC_AB1	CG5455	0.487001616206472	0.0893203258924203	1	pseudobulk	DESeq2	LRT	1.04904970103923
324	KC_AB1	CG5458	2.76048105199981	0.230323384636431	1	pseudobulk	DESeq2	LRT	0.637661966061005
324	KC_AB1	CG5466	0.0182918567041018	0.944133800743355	1	pseudobulk	DESeq2	LRT	0.024966453999834
324	KC_AB1	CG5498	0.840653493177473	0.186184134910443	1	pseudobulk	DESeq2	LRT	0.730057328802588
325	KC_AB1	CG5500	0.316271173200566	0.38269247901299	1	pseudobulk	DESeq2	LRT	0.417150072803599
325	KC_AB1	CG5504	0.0827012737544132	0.869265491462108	1	pseudobulk	DESeq2	LRT	0.060847560872992
325	KC_AB1	CG5510	0.18207710133127	0.739267540815893	1	pseudobulk	DESeq2	LRT	0.131198362048143
325	KC_AB1	CG5515	-0.365614304896171	0.328323788006749	1	pseudobulk	DESeq2	LRT	0.483697650241108
325	KC_AB1	CG5516	-0.349586178960103	0.415745382340263	1	pseudobulk	DESeq2	LRT	0.38117256576516
325	KC_AB1	CG5521	0.55418796175958	0.089060151765812	1	pseudobulk	DESeq2	LRT	1.0503165691307
325	KC_AB1	CG5522	0.188222699068774	0.570482258309825	1	pseudobulk	DESeq2	LRT	0.243757857359621
325	KC_AB1	CG5532	-0.06572137935621	0.801268052513701	1	pseudobulk	DESeq2	LRT	0.096222172738317
325	KC_AB1	CG5535	-0.824109655547633	0.0369041605204378	1	pseudobulk	DESeq2	LRT	1.43292466936368
325	KC_AB1	CG5541	-1.83278265827949	0.273892500836766	1	pseudobulk	DESeq2	LRT	0.56241985854486
326	KC_AB1	CG5543	-0.176546515060318	0.842854672041523	1	pseudobulk	DESeq2	LRT	0.074247301497898
326	KC_AB1	CG5554	-0.218547763928007	0.49310869097638	1	pseudobulk	DESeq2	LRT	0.307057343017793
326	KC_AB1	CG5555	0.0306435619334986	0.910910367916701	1	pseudobulk	DESeq2	LRT	0.040524354788009
326	KC_AB1	CG5567	-0.426852471906816	0.47095444491414	1	pseudobulk	DESeq2	LRT	0.327021099833402
326	KC_AB1	CG5569	-0.31697414949251	0.469864904110688	1	pseudobulk	DESeq2	LRT	0.328026992772939
326	KC_AB1	CG5577	-0.619148458022618	0.879843167001942	1	pseudobulk	DESeq2	LRT	0.055594734413205
326	KC_AB1	CG5589	-0.241721409035627	0.768719366369335	1	pseudobulk	DESeq2	LRT	0.114232177599407
326	KC_AB1	CG5590	-1.08115230084383	0.238621856655557	1	pseudobulk	DESeq2	LRT	0.622289779483086
326	KC_AB1	CG5599	0.274288971571316	0.837427149387951	1	pseudobulk	DESeq2	LRT	0.077052963389566
326	KC_AB1	CG5608	-0.0422630199682275	0.935492999505322	1	pseudobulk	DESeq2	LRT	0.028959458070528
327	KC_AB1	CG5611	-0.011078325827749	0.930996105027249	1	pseudobulk	DESeq2	LRT	0.031052135956048
327	KC_AB1	CG5612	-0.82542030717083	1	1	pseudobulk	DESeq2	LRT	0
327	KC_AB1	CG5613	-0.501300761870089	0.863550576027942	1	pseudobulk	DESeq2	LRT	0.063712221794699
327	KC_AB1	CG5618	0.197860874846153	0.843747532269876	1	pseudobulk	DESeq2	LRT	0.073787484351310
327	KC_AB1	CG5641	-0.330001766987916	0.305005957865713	1	pseudobulk	DESeq2	LRT	0.515691677233767

327	KC_AB1	CG5644	0.469662304766959	0.261438009052561	1	pseudobulk	DESeq2	LRT	0.58263127244869
327	KC_AB1	CG5645	-0.404229663494436	0.71341228716479	1	pseudobulk	DESeq2	LRT	0.14665941503983
327	KC_AB1	CG5646	-0.137049559302796	0.77247762998628	1	pseudobulk	DESeq2	LRT	0.11211408836162
327	KC_AB1	CG5662	1.08899820779554	0.697631521651407	1	pseudobulk	DESeq2	LRT	0.156373904552
327	KC_AB1	CG5664	-0.246808116989894	0.774555509625904	1	pseudobulk	DESeq2	LRT	0.11094745245564
328	KC_AB1	CG5674	-0.021973703758314	0.945174571373313	1	pseudobulk	DESeq2	LRT	0.02448797098042
328	KC_AB1	CG5676	-0.011383095206947	0.878210448262889	1	pseudobulk	DESeq2	LRT	0.05640140030390
328	KC_AB1	CG5697	-0.82542030717083	1	1	pseudobulk	DESeq2	LRT	0
328	KC_AB1	CG5708	-0.146786986575151	0.529042628620766	1	pseudobulk	DESeq2	LRT	0.27650933244709
328	KC_AB1	CG5715	0.353415723811213	0.861040573565567	1	pseudobulk	DESeq2	LRT	0.06497638343546
328	KC_AB1	CG5721	0.0147330871352509	0.979837979409749	1	pseudobulk	DESeq2	LRT	0.00884573090498
328	KC_AB1	CG5726	0.0954967654256133	0.778219735863819	1	pseudobulk	DESeq2	LRT	0.10889775956662
328	KC_AB1	CG5727	-0.165177793303255	0.670475238965223	1	pseudobulk	DESeq2	LRT	0.17361725626031
328	KC_AB1	CG5728	0.336187545148936	0.6673294132285	1	pseudobulk	DESeq2	LRT	0.17565973275253
328	KC_AB1	CG5742	0.308514372780838	0.3839450670402	1	pseudobulk	DESeq2	LRT	0.41573090789265
329	KC_AB1	CG5745	0.488432887201875	0.560136590504157	1	pseudobulk	DESeq2	LRT	0.25170605644213
329	KC_AB1	CG5746	0.0770344518301061	0.657772714842915	1	pseudobulk	DESeq2	LRT	0.18192414550664
329	KC_AB1	CG5757	-1.96719661029409	0.414177861019091	1	pseudobulk	DESeq2	LRT	0.38281311909348
329	KC_AB1	CG5758	-0.056685461089993	0.849557213210655	1	pseudobulk	DESeq2	LRT	0.07080736836057
329	KC_AB1	CG5768	0.135795303631797	0.946582799906172	1	pseudobulk	DESeq2	LRT	0.02384139124104
329	KC_AB1	CG5776	-1.15984078380938	0.518928978299158	1	pseudobulk	DESeq2	LRT	0.28489207653185
329	KC_AB1	CG5787	0.561853532693213	0.315938617709809	1	pseudobulk	DESeq2	LRT	0.50039728630368
329	KC_AB1	CG5789	0.118654937915836	0.926407789219637	1	pseudobulk	DESeq2	LRT	0.03319780206127
329	KC_AB1	CG5793	-1.44925888589622	0.6315588144614	1	pseudobulk	DESeq2	LRT	0.19958619917570
329	KC_AB1	CG5800	-1.82907532466841	0.269385984986087	1	pseudobulk	DESeq2	LRT	0.56962500253112
330	KC_AB1	CG5805	-0.434633745571146	0.348620556652871	1	pseudobulk	DESeq2	LRT	0.45764700799570
330	KC_AB1	CG5808	-0.132710183833492	0.871233865801606	1	pseudobulk	DESeq2	LRT	0.05986525142846
330	KC_AB1	CG5810	1.08899820779554	0.878177167143508	1	pseudobulk	DESeq2	LRT	0.05641785886507
330	KC_AB1	CG5815	0.129006141195618	0.843829247347944	1	pseudobulk	DESeq2	LRT	0.07374542593008
330	KC_AB1	CG5823	0.375851961695868	0.127265565156804	1	pseudobulk	DESeq2	LRT	0.89528908955456
330	KC_AB1	CG5828	-0.101236754671027	0.860887320244465	1	pseudobulk	DESeq2	LRT	0.06505368873042
330	KC_AB1	CG5830	-0.093878365857998	0.402375113097133	1	pseudobulk	DESeq2	LRT	0.39536888823747
330	KC_AB1	CG5846	0.32509672124178	0.638538666869803	1	pseudobulk	DESeq2	LRT	0.19481279879252
330	KC_AB1	CG5850	-0.049724681896669	0.924443796388646	1	pseudobulk	DESeq2	LRT	0.03411948759864
330	KC_AB1	CG5854	-0.857205359615719	0.419148400151699	1	pseudobulk	DESeq2	LRT	0.37763218716150
331	KC_AB1	CG5861	0.183973697494048	0.538979183438811	1	pseudobulk	DESeq2	LRT	0.26842800789718
331	KC_AB1	CG5862	-0.124957946526256	0.837200730417279	1	pseudobulk	DESeq2	LRT	0.07717040143218
331	KC_AB1	CG5877	1.19912948549208	0.102844073570756	1	pseudobulk	DESeq2	LRT	0.98782072963455
331	KC_AB1	CG5880	-0.529837152230451	0.52844389884854	1	pseudobulk	DESeq2	LRT	0.27700111188306
331	KC_AB1	CG5882	1.08899820779554	0.878177167143508	1	pseudobulk	DESeq2	LRT	0.05641785886507
331	KC_AB1	CG5885	-0.034186110572365	0.925044833189289	1	pseudobulk	DESeq2	LRT	0.03383721825029
331	KC_AB1	CG5886	0.692365276558378	0.206412047475629	1	pseudobulk	DESeq2	LRT	0.68526495821007
331	KC_AB1	CG5888	0.27472260691171	0.653006913252556	1	pseudobulk	DESeq2	LRT	0.18508222091245
331	KC_AB1	CG5890	0.104974646913572	0.378988963713712	1	pseudobulk	DESeq2	LRT	0.42137343664939
331	KC_AB1	CG5892	1.08899820779554	0.878177167143508	1	pseudobulk	DESeq2	LRT	0.05641785886507
332	KC_AB1	CG5902	0.0311145907288135	0.92431037263408	1	pseudobulk	DESeq2	LRT	0.03418217327253
332	KC_AB1	CG5903	-0.215482712952143	0.229588326456546	1	pseudobulk	DESeq2	LRT	0.63905019765207
332	KC_AB1	CG5910	0.735557303417222	0.613788434479203	1	pseudobulk	DESeq2	LRT	0.21198129917103
332	KC_AB1	CG5913	-0.330903000160343	0.690434782568161	1	pseudobulk	DESeq2	LRT	0.16087733795201
332	KC_AB1	CG5916	-0.056024249924674	0.862131504543688	1	pseudobulk	DESeq2	LRT	0.06442648435892
332	KC_AB1	CG5934	0.134528407109786	0.583600909749888	1	pseudobulk	DESeq2	LRT	0.23388403977541
332	KC_AB1	CG5937	-0.106268759127205	0.70401801358	1	pseudobulk	DESeq2	LRT	0.15241622850233

332	KC_AB1	CG5938	-0.23411849944469	0.338826774417095	1	pseudobulk	DESeq2	LRT	0.470022278618304
332	KC_AB1	CG5946	0.0281058494530885	0.902391569951167	1	pseudobulk	DESeq2	LRT	0.044604970475971
332	KC_AB1	CG5953	-1.44971445679075	0.160397240784715	1	pseudobulk	DESeq2	LRT	0.794803106889009
333	KC_AB1	CG5955	2.21745641838459	0.396737788462723	1	pseudobulk	DESeq2	LRT	0.401496431894786
333	KC_AB1	CG5961	-0.233995920787222	0.630026569914898	1	pseudobulk	DESeq2	LRT	0.200641134793875
333	KC_AB1	CG5968	0.137402834129399	0.922366574821677	1	pseudobulk	DESeq2	LRT	0.035096443607464
333	KC_AB1	CG5969	-0.0545757115654102	0.89173265758675	1	pseudobulk	DESeq2	LRT	0.049765328064870
333	KC_AB1	CG5973	-0.604990252529976	0.725821804414012	1	pseudobulk	DESeq2	LRT	0.139169989298251
333	KC_AB1	CG5986	0.0941938205906775	0.75032160531004	1	pseudobulk	DESeq2	LRT	0.124752547976281
333	KC_AB1	CG5989	-0.356955828046959	0.477924780503733	1	pseudobulk	DESeq2	LRT	0.320640450632129
333	KC_AB1	CG6000	-1.96719661029409	0.414177861019091	1	pseudobulk	DESeq2	LRT	0.382813119093482
333	KC_AB1	CG6005	-0.0623318854445272	0.884997673981697	1	pseudobulk	DESeq2	LRT	0.053057870746515
333	KC_AB1	CG6006	0.19117323012314	0.534123632851644	1	pseudobulk	DESeq2	LRT	0.272358205797957
334	KC_AB1	CG6013	-0.154975282623786	0.641320545025771	1	pseudobulk	DESeq2	LRT	0.192924847018046
334	KC_AB1	CG6024	0.0578763710075579	0.675719680453044	1	pseudobulk	DESeq2	LRT	0.170233431988612
334	KC_AB1	CG6028	-0.981872959661444	0.236599484257979	1	pseudobulk	DESeq2	LRT	0.625986206386693
334	KC_AB1	CG6034	0.112935832476632	0.940353958317081	1	pseudobulk	DESeq2	LRT	0.026708642986417
334	KC_AB1	CG6040	-1.99275908811736	0.412167844949191	1	pseudobulk	DESeq2	LRT	0.384925892483647
334	KC_AB1	CG6041	-0.0717108677088818	0.855276015161199	1	pseudobulk	DESeq2	LRT	0.067893706889614
334	KC_AB1	CG6051	0.398462367736685	0.163518149207254	1	pseudobulk	DESeq2	LRT	0.786434037113051
334	KC_AB1	CG6052	-0.82542030717083	1	1	pseudobulk	DESeq2	LRT	0
334	KC_AB1	CG6055	-0.82542030717083	1	1	pseudobulk	DESeq2	LRT	0
334	KC_AB1	CG6066	-0.249556810766918	0.742076603360312	1	pseudobulk	DESeq2	LRT	0.129551260894157
335	KC_AB1	CG6071	-0.850996402080018	0.900449987182352	1	pseudobulk	DESeq2	LRT	0.045540403770502
335	KC_AB1	CG6073	-0.922668544634759	0.670519447274774	1	pseudobulk	DESeq2	LRT	0.173588621657807
335	KC_AB1	CG6083	-1.5279157440969	0.390878977997195	1	pseudobulk	DESeq2	LRT	0.407957685887317
335	KC_AB1	CG6084	-0.294307808381028	0.794477120220143	1	pseudobulk	DESeq2	LRT	0.099918605322828
335	KC_AB1	CG6094	0.0340798671430597	0.88841923238922	1	pseudobulk	DESeq2	LRT	0.051382048468786
335	KC_AB1	CG6115	-0.173301922270072	0.474474627969697	1	pseudobulk	DESeq2	LRT	0.323787006055156
335	KC_AB1	CG6123	2.1559833397943	0.381743505411375	1	pseudobulk	DESeq2	LRT	0.418228342845202
335	KC_AB1	CG6126	1.35022418894863	0.496574784606603	1	pseudobulk	DESeq2	LRT	0.304015337105745
335	KC_AB1	CG6136	0.188909446416406	0.790679398980205	1	pseudobulk	DESeq2	LRT	0.101999576525342
335	KC_AB1	CG6144	-0.243702892051867	0.514811884043427	1	pseudobulk	DESeq2	LRT	0.288351436291909
336	KC_AB1	CG6145	0.271344462812217	0.639047462589281	1	pseudobulk	DESeq2	LRT	0.194466885229487
336	KC_AB1	CG6153	0.717143939099732	0.431824474214	1	pseudobulk	DESeq2	LRT	0.36469274709934
336	KC_AB1	CG6154	-0.246211179888675	0.149104215962392	1	pseudobulk	DESeq2	LRT	0.826510076578414
336	KC_AB1	CG6163	-2.05361031456501	0.181943759894861	1	pseudobulk	DESeq2	LRT	0.740062834772447
336	KC_AB1	CG6171	-0.488314663222733	0.266087080147385	1	pseudobulk	DESeq2	LRT	0.574976212096177
336	KC_AB1	CG6178	0.0133462885669943	0.959624628927126	1	pseudobulk	DESeq2	LRT	0.017898614320417
336	KC_AB1	CG6179	-0.471925401904919	0.370442757995591	1	pseudobulk	DESeq2	LRT	0.431278891078078
336	KC_AB1	CG6180	-0.223855082492204	0.481121046859463	1	pseudobulk	DESeq2	LRT	0.317745644271838
336	KC_AB1	CG6184	-0.250282616616282	0.458039458802819	1	pseudobulk	DESeq2	LRT	0.339097107144242
336	KC_AB1	CG6191	0.227620100580544	0.341668400069129	1	pseudobulk	DESeq2	LRT	0.466395185995554
337	KC_AB1	CG6195	0.179914487730899	0.717850047448043	1	pseudobulk	DESeq2	LRT	0.143966266575608
337	KC_AB1	CG6201	0.589917931288472	0.407288545910459	1	pseudobulk	DESeq2	LRT	0.390097803314026
337	KC_AB1	CG6204	-0.718411511674438	0.420452374347377	1	pseudobulk	DESeq2	LRT	0.37628319066161
337	KC_AB1	CG6218	0.491691163964068	0.575653926108586	1	pseudobulk	DESeq2	LRT	0.239838528991175
337	KC_AB1	CG6220	-0.82542030717083	1	1	pseudobulk	DESeq2	LRT	0
337	KC_AB1	CG6227	0.345854726124468	0.513247623622105	1	pseudobulk	DESeq2	LRT	0.289673052771196
337	KC_AB1	CG6230	-0.243999359078634	0.763113152590736	1	pseudobulk	DESeq2	LRT	0.117411061124682
337	KC_AB1	CG6231	-1.39069491230407	0.559139881025611	1	pseudobulk	DESeq2	LRT	0.252479530292469
337	KC_AB1	CG6236	-0.0213424083672042	0.967605356400436	1	pseudobulk	DESeq2	LRT	0.014301736167212

337	KC_AB1	CG6244	1.08899820779554	0.697631521651407	1	pseudobulk	DESeq2	LRT	0.156373904552
338	KC_AB1	CG6254	0.190288485111253	0.745811390686262	1	pseudobulk	DESeq2	LRT	0.12737098799749
338	KC_AB1	CG6276	-0.0774391469849596	0.839284377543811	1	pseudobulk	DESeq2	LRT	0.0760908607778299
338	KC_AB1	CG6282	-0.0712359681599465	0.651456266436991	1	pseudobulk	DESeq2	LRT	0.186114734030601
338	KC_AB1	CG6287	0.276278978962869	0.878844651226061	1	pseudobulk	DESeq2	LRT	0.0560878861069399
338	KC_AB1	CG6293	-0.0767277494956071	0.941932501921592	1	pseudobulk	DESeq2	LRT	0.0259802172636799
338	KC_AB1	CG6294	-0.850996402080018	0.900449987182352	1	pseudobulk	DESeq2	LRT	0.045540403770502
338	KC_AB1	CG6310	-0.831765148915113	0.743013616167264	1	pseudobulk	DESeq2	LRT	0.129003227461331
338	KC_AB1	CG6321	-0.0637308313890977	0.937495912990482	1	pseudobulk	DESeq2	LRT	0.028030616901097
338	KC_AB1	CG6325	-1.20397689624494	0.205572123173361	1	pseudobulk	DESeq2	LRT	0.68703577864962
338	KC_AB1	CG6329	-0.0504480619555459	0.638299956648707	1	pseudobulk	DESeq2	LRT	0.194975185066087
339	KC_AB1	CG6330	0.417438172513336	0.18985433810055	1	pseudobulk	DESeq2	LRT	0.721579474944484
339	KC_AB1	CG6340	-0.0310366448313156	0.871003961876882	1	pseudobulk	DESeq2	LRT	0.0599798695418722
339	KC_AB1	CG6345	-2.98353809966973	0.1675887052005	1	pseudobulk	DESeq2	LRT	0.77575525440736
339	KC_AB1	CG6356	-0.82542030717083	1	1	pseudobulk	DESeq2	LRT	0
339	KC_AB1	CG6357	0.0861653185070086	0.957012149157673	1	pseudobulk	DESeq2	LRT	0.0190825488703399
339	KC_AB1	CG6362	0.11269840515639	0.977007463492936	1	pseudobulk	DESeq2	LRT	0.0101021186339211
339	KC_AB1	CG6379	-0.175882212473076	0.687813447656755	1	pseudobulk	DESeq2	LRT	0.162529337403172
339	KC_AB1	CG6388	1.04583354539212	0.287012130023154	1	pseudobulk	DESeq2	LRT	0.542099748245803
339	KC_AB1	CG6418	-0.0580260417330769	0.867802143397844	1	pseudobulk	DESeq2	LRT	0.0615792815348700
339	KC_AB1	CG6420	-0.119073888595598	0.766474743091647	1	pseudobulk	DESeq2	LRT	0.115502151464074
340	KC_AB1	CG6424	0.736294445752933	1	1	pseudobulk	DESeq2	LRT	0
340	KC_AB1	CG6425	0.52349811891942	0.759092093118787	1	pseudobulk	DESeq2	LRT	0.119705532263832
340	KC_AB1	CG6428	0.072986995709084	0.893310751599889	1	pseudobulk	DESeq2	LRT	0.0489974389515248
340	KC_AB1	CG6435	1.08899820779554	0.697631521651407	1	pseudobulk	DESeq2	LRT	0.156373904552
340	KC_AB1	CG6443	0.154610848932236	0.662584057397446	1	pseudobulk	DESeq2	LRT	0.178759017997695
340	KC_AB1	CG6454	0.0549812905804791	0.8435139770896	1	pseudobulk	DESeq2	LRT	0.0739077167232322
340	KC_AB1	CG6472	-0.326645811571136	0.719958470938346	1	pseudobulk	DESeq2	LRT	0.142692554072178
340	KC_AB1	CG6479	1.51230729949974	0.302811773873315	1	pseudobulk	DESeq2	LRT	0.518827242683186
340	KC_AB1	CG6481	1.38912802193265	0.323527728077971	1	pseudobulk	DESeq2	LRT	0.490088492011654
340	KC_AB1	CG6495	0.180635554320584	0.461551997541129	1	pseudobulk	DESeq2	LRT	0.335779365118889
341	KC_AB1	CG6511	-1.40964828681911	0.20398117835851	1	pseudobulk	DESeq2	LRT	0.69040990371208
341	KC_AB1	CG6512	-0.304533691746804	0.419002801950224	1	pseudobulk	DESeq2	LRT	0.377783072815442
341	KC_AB1	CG6523	-0.0567728452211537	0.850840147171778	1	pseudobulk	DESeq2	LRT	0.0701520259580644
341	KC_AB1	CG6550	-0.0685113148484196	0.899287463015351	1	pseudobulk	DESeq2	LRT	0.0461014610524644
341	KC_AB1	CG6565	0.146429329815074	0.838075857568174	1	pseudobulk	DESeq2	LRT	0.0767166698753866
341	KC_AB1	CG6567	0.0061739818666775	0.966593454137931	1	pseudobulk	DESeq2	LRT	0.0147561502703866
341	KC_AB1	CG6574	-1.10851121899538	0.21921387953598	1	pseudobulk	DESeq2	LRT	0.659131951943791
341	KC_AB1	CG6583	-0.132398780866897	0.708352132583155	1	pseudobulk	DESeq2	LRT	0.14975079425975
341	KC_AB1	CG6607	0.387080101856573	0.364713278729267	1	pseudobulk	DESeq2	LRT	0.438048424309296
341	KC_AB1	CG6610	-0.291701474201308	0.70703987048638	1	pseudobulk	DESeq2	LRT	0.150556095334644
342	KC_AB1	CG6617	-0.434868097630132	0.14386968924853	1	pseudobulk	DESeq2	LRT	0.842030694449047
342	KC_AB1	CG6621	0.498103327883461	0.236816557497626	1	pseudobulk	DESeq2	LRT	0.625587936330594
342	KC_AB1	CG6628	-2.08238890537319	0.386092553331648	1	pseudobulk	DESeq2	LRT	0.413308574646766
342	KC_AB1	CG6638	0.942897398615768	0.492870613443234	1	pseudobulk	DESeq2	LRT	0.307267075129728
342	KC_AB1	CG6650	0.424443009691292	0.563875382574644	1	pseudobulk	DESeq2	LRT	0.248816865236729
342	KC_AB1	CG6654	-0.294143130428285	0.770453757956963	1	pseudobulk	DESeq2	LRT	0.113253422184737
342	KC_AB1	CG6656	0.287129740171306	0.880420973702385	1	pseudobulk	DESeq2	LRT	0.0553096200774420
342	KC_AB1	CG6659	-1.29641750343237	0.0588152470165665	1	pseudobulk	DESeq2	LRT	1.23051007465862
342	KC_AB1	CG6660	-0.82542030717083	1	1	pseudobulk	DESeq2	LRT	0
342	KC_AB1	CG6664	-0.323575156404542	0.444201397188162	1	pseudobulk	DESeq2	LRT	0.352420079758381
343	KC_AB1	CG6665	0.0381037437399238	0.960495669163191	1	pseudobulk	DESeq2	LRT	0.0175045890144436

343	KC_AB1	CG6674	0.331874760241207	0.526972068117418	1	pseudobulk	DESeq2	LRT	0.278212403732347
343	KC_AB1	CG6678	-0.82542030717083	1	1	pseudobulk	DESeq2	LRT	0
343	KC_AB1	CG6683	-2.37432853298142	0.308079992266679	1	pseudobulk	DESeq2	LRT	0.51133650528684
343	KC_AB1	CG6685	-0.088053937724544	0.889390145878347	1	pseudobulk	DESeq2	LRT	0.050907686686461
343	KC_AB1	CG6686	-0.238948653385346	0.418237959278338	1	pseudobulk	DESeq2	LRT	0.378576553161139
343	KC_AB1	CG6689	0.257922841621176	0.611335388228925	1	pseudobulk	DESeq2	LRT	0.213720463579254
343	KC_AB1	CG6693	0.293583913925918	0.421537062734161	1	pseudobulk	DESeq2	LRT	0.375164234957862
343	KC_AB1	CG6695	-0.241253807777243	0.696452339826708	1	pseudobulk	DESeq2	LRT	0.157108598204096
343	KC_AB1	CG6700	0.144720017471384	0.228169095465075	1	pseudobulk	DESeq2	LRT	0.641743179277562
344	KC_AB1	CG6707	-0.0995067505367702	0.659333956404885	1	pseudobulk	DESeq2	LRT	0.180894557049388
344	KC_AB1	CG6712	0.303691884738476	0.683626590555227	1	pseudobulk	DESeq2	LRT	0.165181053167782
344	KC_AB1	CG6723	0.10745091433163	0.925708810521252	1	pseudobulk	DESeq2	LRT	0.033525602812194
344	KC_AB1	CG6724	-0.652455998549525	0.397841558869431	1	pseudobulk	DESeq2	LRT	0.400289852068951
344	KC_AB1	CG6729	0.111782576465535	0.890459378415786	1	pseudobulk	DESeq2	LRT	0.050385887691204
344	KC_AB1	CG6738	1.08899820779554	0.697631521651407	1	pseudobulk	DESeq2	LRT	0.156373904552
344	KC_AB1	CG6749	-1.6747012584303	0.563129890257477	1	pseudobulk	DESeq2	LRT	0.249391420218277
344	KC_AB1	CG6752	-0.616127752675454	0.558532155402043	1	pseudobulk	DESeq2	LRT	0.252951818946449
344	KC_AB1	CG6758	0.398428113822955	0.4770849746825	1	pseudobulk	DESeq2	LRT	0.321404260899119
344	KC_AB1	CG6762	-0.426692637215461	0.410726296446538	1	pseudobulk	DESeq2	LRT	0.386447490874066
345	KC_AB1	CG6763	0.516125129258093	0.806329321809124	1	pseudobulk	DESeq2	LRT	0.093487546986755
345	KC_AB1	CG6765	0.539902575700017	0.0108471565529135	1	pseudobulk	DESeq2	LRT	1.96468409178974
345	KC_AB1	CG6766	-0.138430357692672	0.753750913558264	1	pseudobulk	DESeq2	LRT	0.122772148479557
345	KC_AB1	CG6767	-0.171279711341798	0.506514672884113	1	pseudobulk	DESeq2	LRT	0.295407969335675
345	KC_AB1	CG6769	0.042246774127582	0.938022185470491	1	pseudobulk	DESeq2	LRT	0.027786889858173
345	KC_AB1	CG6785	0.135879393043758	1	1	pseudobulk	DESeq2	LRT	0
345	KC_AB1	CG6791	0.147874406984493	0.63738039821713	1	pseudobulk	DESeq2	LRT	0.195601296803668
345	KC_AB1	CG6805	0.642398899642368	0.357908688917058	1	pseudobulk	DESeq2	LRT	0.446227758153929
345	KC_AB1	CG6808	0.294150951295732	0.739617563644643	1	pseudobulk	DESeq2	LRT	0.130992784230845
345	KC_AB1	CG6813	0.0341451290823185	0.954196224752166	1	pseudobulk	DESeq2	LRT	0.020362306046371
346	KC_AB1	CG6833	-0.308701671579639	0.590752441282229	1	pseudobulk	DESeq2	LRT	0.228594474971581
346	KC_AB1	CG6836	-1.39069491230407	0.559139881025611	1	pseudobulk	DESeq2	LRT	0.252479530292469
346	KC_AB1	CG6841	-0.102141215462085	0.831294683480644	1	pseudobulk	DESeq2	LRT	0.080244997006108
346	KC_AB1	CG6843	0.11827961702306	0.746706689445024	1	pseudobulk	DESeq2	LRT	0.126849957984658
346	KC_AB1	CG6847	-1.99275908811736	0.412167844949191	1	pseudobulk	DESeq2	LRT	0.384925892483647
346	KC_AB1	CG6867	-0.121047714335275	0.708062678876583	1	pseudobulk	DESeq2	LRT	0.149928296144456
346	KC_AB1	CG6878	-0.363185133464438	0.361152761252338	1	pseudobulk	DESeq2	LRT	0.442309060321704
346	KC_AB1	CG6891	-0.82542030717083	1	1	pseudobulk	DESeq2	LRT	0
346	KC_AB1	CG6903	0.366755188489567	0.462234781883184	1	pseudobulk	DESeq2	LRT	0.335137378174096
346	KC_AB1	CG6907	-0.0096328751136128	0.90669477937305	1	pseudobulk	DESeq2	LRT	0.042538884872256
347	KC_AB1	CG6908	1.08899820779554	0.878177167143508	1	pseudobulk	DESeq2	LRT	0.056417858865076
347	KC_AB1	CG6912	0.11400025262841	1	1	pseudobulk	DESeq2	LRT	0
347	KC_AB1	CG6923	-0.0014767325799659	0.968180509836639	1	pseudobulk	DESeq2	LRT	0.014043664264429
347	KC_AB1	CG6928	-1.9020722008881	0.367842149552769	1	pseudobulk	DESeq2	LRT	0.434338508188274
347	KC_AB1	CG6937	0.522253215375054	0.474535214215671	1	pseudobulk	DESeq2	LRT	0.323731554001506
347	KC_AB1	CG6938	-1.54170648922113	0.692203976511459	1	pseudobulk	DESeq2	LRT	0.15976591013865
347	KC_AB1	CG6951	-1.19366644108336	0.524852736756964	1	pseudobulk	DESeq2	LRT	0.279962533899444
347	KC_AB1	CG6959	-0.351536747486572	0.87091076975573	1	pseudobulk	DESeq2	LRT	0.060026338893416
347	KC_AB1	CG6961	1.06981800776423	0.192155571607761	1	pseudobulk	DESeq2	LRT	0.716347018519823
347	KC_AB1	CG6962	0.477028581170134	0.782340637227571	1	pseudobulk	DESeq2	LRT	0.106604110549928
348	KC_AB1	CG6966	0.12448015082156	0.57939268961149	1	pseudobulk	DESeq2	LRT	0.237026988734293
348	KC_AB1	CG6983	0.485701650082394	0.102228714736955	1	pseudobulk	DESeq2	LRT	0.990427099306768
348	KC_AB1	CG6984	-0.602018630239726	0.499735005447667	1	pseudobulk	DESeq2	LRT	0.301260228023318

348	KC_AB1	CG6999	0.496095474257362	0.726634238725904	1	pseudobulk	DESeq2	LRT	0.138684142202185
348	KC_AB1	CG7006	0.197765967798806	0.848631048896877	1	pseudobulk	DESeq2	LRT	0.071281082729780
348	KC_AB1	CG7009	0.0839100084531289	0.920934348812531	1	pseudobulk	DESeq2	LRT	0.035771328505320
348	KC_AB1	CG7011	0.316270145028854	0.648834030734402	1	pseudobulk	DESeq2	LRT	0.187866379862595
348	KC_AB1	CG7028	-0.467413115371049	0.13148558729356	1	pseudobulk	DESeq2	LRT	0.881121849470203
348	KC_AB1	CG7029	-0.0438437793025208	0.829819365139024	1	pseudobulk	DESeq2	LRT	0.081016434444517
348	KC_AB1	CG7044	0.36423824162021	0.763606576317955	1	pseudobulk	DESeq2	LRT	0.117130340039633
349	KC_AB1	CG7049	-0.240894629071929	0.707334886338007	1	pseudobulk	DESeq2	LRT	0.150374921628488
349	KC_AB1	CG7058	-0.406482323992274	0.337539212677579	1	pseudobulk	DESeq2	LRT	0.471675766950713
349	KC_AB1	CG7059	-0.0541867847124427	0.913137847975466	1	pseudobulk	DESeq2	LRT	0.039463656095378
349	KC_AB1	CG7065	0.233558694703028	0.446451581452076	1	pseudobulk	DESeq2	LRT	0.350225634308252
349	KC_AB1	CG7069	0.958055496267105	0.419419659793679	1	pseudobulk	DESeq2	LRT	0.377351216360423
349	KC_AB1	CG7083	0.0988673147518985	0.799311461121741	1	pseudobulk	DESeq2	LRT	0.097283959748448
349	KC_AB1	CG7099	-0.685949155549487	0.604994916420792	1	pseudobulk	DESeq2	LRT	0.218248274570132
349	KC_AB1	CG7101	-1.25902596951628	0.169423055964555	1	pseudobulk	DESeq2	LRT	0.771027488947255
349	KC_AB1	CG7102	-1.32489642030663	0.55306199336977	1	pseudobulk	DESeq2	LRT	0.257226185387141
349	KC_AB1	CG7110	0.515669274249832	0.754717696421651	1	pseudobulk	DESeq2	LRT	0.122215466666653
350	KC_AB1	CG7115	-0.0220208955633577	0.928249912521767	1	pseudobulk	DESeq2	LRT	0.032335083029695
350	KC_AB1	CG7120	-0.0345766092268363	0.894309222987367	1	pseudobulk	DESeq2	LRT	0.048512290357104
350	KC_AB1	CG7130	0.11400025262841	1	1	pseudobulk	DESeq2	LRT	0
350	KC_AB1	CG7131	0.13617601020169	1	1	pseudobulk	DESeq2	LRT	0
350	KC_AB1	CG7133	0.0094441323320162	0.914749164493296	1	pseudobulk	DESeq2	LRT	0.038697978513797
350	KC_AB1	CG7137	0.204805144277426	0.870306052814937	1	pseudobulk	DESeq2	LRT	0.060327996030717
350	KC_AB1	CG7139	0.0302397090374683	0.937223880730187	1	pseudobulk	DESeq2	LRT	0.028156653987567
350	KC_AB1	CG7142	1.08899820779554	0.878177167143508	1	pseudobulk	DESeq2	LRT	0.056417858865076
350	KC_AB1	CG7148	-0.276130377953361	0.582123393347831	1	pseudobulk	DESeq2	LRT	0.234984947704064
350	KC_AB1	CG7149	0.875047377070068	0.274524073528457	1	pseudobulk	DESeq2	LRT	0.561419565450151
351	KC_AB1	CG7154	-0.377014939723003	0.226057489264714	1	pseudobulk	DESeq2	LRT	0.645781100253398
351	KC_AB1	CG7156	0.48378127104965	0.604988918101455	1	pseudobulk	DESeq2	LRT	0.218252580473922
351	KC_AB1	CG7166	-0.231369834928755	0.23213542987509	1	pseudobulk	DESeq2	LRT	0.634258569906171
351	KC_AB1	CG7168	0.126943174328827	0.827034377086608	1	pseudobulk	DESeq2	LRT	0.082476437885515
351	KC_AB1	CG7182	-0.326272862803443	0.662871766027572	1	pseudobulk	DESeq2	LRT	0.178570478673768
351	KC_AB1	CG7183	0.304868626124092	0.701244871116808	1	pseudobulk	DESeq2	LRT	0.154130302140509
351	KC_AB1	CG7191	-0.82542030717083	1	1	pseudobulk	DESeq2	LRT	0
351	KC_AB1	CG7206	0.253711018048184	0.521730459169889	1	pseudobulk	DESeq2	LRT	0.282553807971397
351	KC_AB1	CG7218	-0.684504226389428	0.320263694277682	1	pseudobulk	DESeq2	LRT	0.494492291022537
351	KC_AB1	CG7220	-0.349825148793852	0.178129223409357	1	pseudobulk	DESeq2	LRT	0.749264825487363
352	KC_AB1	CG7222	0.36525222685311	0.356275136838897	1	pseudobulk	DESeq2	LRT	0.448214484442235
352	KC_AB1	CG7231	-0.0975690973099205	0.769702088817852	1	pseudobulk	DESeq2	LRT	0.113677334850374
352	KC_AB1	CG7239	0.446703550772879	0.466958857942199	1	pseudobulk	DESeq2	LRT	0.330721381865979
352	KC_AB1	CG7246	-1.54488962734574	0.280472598224451	1	pseudobulk	DESeq2	LRT	0.552109562290973
352	KC_AB1	CG7265	0.12585845034288	0.8385828944648	1	pseudobulk	DESeq2	LRT	0.076454000643772
352	KC_AB1	CG7272	-0.541648180583293	0.446371665493261	1	pseudobulk	DESeq2	LRT	0.350303381074234
352	KC_AB1	CG7275	0.571168642239416	0.513796545874221	1	pseudobulk	DESeq2	LRT	0.28920881971313
352	KC_AB1	CG7289	-0.640944942037029	0.275934320382306	1	pseudobulk	DESeq2	LRT	0.559194279130559
352	KC_AB1	CG7324	0.0991273940285338	0.753260779752282	1	pseudobulk	DESeq2	LRT	0.123054644515629
352	KC_AB1	CG7326	0.0835811668013768	0.868082106777802	1	pseudobulk	DESeq2	LRT	0.06143919552154
353	KC_AB1	CG7332	0.114768783274897	0.661274868576247	1	pseudobulk	DESeq2	LRT	0.179617982129006
353	KC_AB1	CG7339	0.241892136634314	0.629817868423567	1	pseudobulk	DESeq2	LRT	0.200785022251914
353	KC_AB1	CG7341	-0.850996402080018	0.900449987182352	1	pseudobulk	DESeq2	LRT	0.045540403770502
353	KC_AB1	CG7352	-1.68017023177981	0.493199771721807	1	pseudobulk	DESeq2	LRT	0.306977133090058
353	KC_AB1	CG7362	1.88952324269138	0.456767015650974	1	pseudobulk	DESeq2	LRT	0.340305265178099

353	KC_AB1	CG7367	3.30486943280718	0.102310375740275	1	pseudobulk	DESeq2	LRT	0.990080320361677
353	KC_AB1	CG7368	0.341093174589245	0.332321113895866	1	pseudobulk	DESeq2	LRT	0.478442065109598
353	KC_AB1	CG7369	0.178535249169241	0.71102540944618	1	pseudobulk	DESeq2	LRT	0.148114878897402
353	KC_AB1	CG7376	0.80993006670105	0.458429792263454	1	pseudobulk	DESeq2	LRT	0.338727166356575
353	KC_AB1	CG7378	0.0505012837277194	0.663342925892407	1	pseudobulk	DESeq2	LRT	0.17826189796018
354	KC_AB1	CG7379	-0.0709562766252385	0.850906518677761	1	pseudobulk	DESeq2	LRT	0.070118149260202
354	KC_AB1	CG7381	-0.506516985469986	0.909346984303859	1	pseudobulk	DESeq2	LRT	0.041270369127443
354	KC_AB1	CG7382	0.207007751767383	0.559873018281768	1	pseudobulk	DESeq2	LRT	0.251910461766843
354	KC_AB1	CG7386	-1.62522384115856	0.257590201287305	1	pseudobulk	DESeq2	LRT	0.58907066152881
354	KC_AB1	CG7394	-0.468342315575428	0.198680932830504	1	pseudobulk	DESeq2	LRT	0.701843809608013
354	KC_AB1	CG7407	0.160194827260051	0.520541476328982	1	pseudobulk	DESeq2	LRT	0.283544660537386
354	KC_AB1	CG7414	-0.177795302191957	0.595833793980142	1	pseudobulk	DESeq2	LRT	0.224874868487736
354	KC_AB1	CG7420	-1.15792644572218	0.167739309543716	1	pseudobulk	DESeq2	LRT	0.775365148977863
354	KC_AB1	CG7430	-0.366721505502797	0.130540974134333	1	pseudobulk	DESeq2	LRT	0.884253150797489
354	KC_AB1	CG7453	-0.0325608976684843	0.931083466638066	1	pseudobulk	DESeq2	LRT	0.031011385102956
355	KC_AB1	CG7460	1.08899820779554	0.878177167143508	1	pseudobulk	DESeq2	LRT	0.056417858865076
355	KC_AB1	CG7461	-1.07070997412897	0.196137284561118	1	pseudobulk	DESeq2	LRT	0.707439841620294
355	KC_AB1	CG7483	0.216540504500092	0.636707447372783	1	pseudobulk	DESeq2	LRT	0.196060070296377
355	KC_AB1	CG7484	-0.0054539882726576	0.984810839517043	1	pseudobulk	DESeq2	LRT	0.006647179901546
355	KC_AB1	CG7488	-0.128755595767543	0.797188052987303	1	pseudobulk	DESeq2	LRT	0.098439218451941
355	KC_AB1	CG7492	0.151180677905396	0.631377479634498	1	pseudobulk	DESeq2	LRT	0.199710912853833
355	KC_AB1	CG7497	-0.850932661185203	0.660736244265418	1	pseudobulk	DESeq2	LRT	0.179971869591347
355	KC_AB1	CG7504	0.133503378651854	0.860079064904595	1	pseudobulk	DESeq2	LRT	0.065461623322186
355	KC_AB1	CG7506	-0.292243055997576	0.480592101705336	1	pseudobulk	DESeq2	LRT	0.318223370889538
355	KC_AB1	CG7518	0.112999811771546	0.738255698079169	1	pseudobulk	DESeq2	LRT	0.131793192334722
356	KC_AB1	CG7519	-0.377911765908786	0.412028586934516	1	pseudobulk	DESeq2	LRT	0.385072651158085
356	KC_AB1	CG7523	0.708198751949222	0.199860542155823	1	pseudobulk	DESeq2	LRT	0.699272938825695
356	KC_AB1	CG7536	0.274325729173996	0.780102498959642	1	pseudobulk	DESeq2	LRT	0.10784833088899
356	KC_AB1	CG7544	0.338644998367473	0.733629543199566	1	pseudobulk	DESeq2	LRT	0.134523187983884
356	KC_AB1	CG7546	-0.148386547862988	0.613454842206626	1	pseudobulk	DESeq2	LRT	0.212217401155874
356	KC_AB1	CG7550	0.139575741484968	0.805559885875668	1	pseudobulk	DESeq2	LRT	0.093902168294307
356	KC_AB1	CG7556	-0.165824981942232	0.739078030923117	1	pseudobulk	DESeq2	LRT	0.131309706922742
356	KC_AB1	CG7560	-1.14396378458764	0.660650724079103	1	pseudobulk	DESeq2	LRT	0.180028084683443
356	KC_AB1	CG7564	0.221665421998069	0.382912997476886	1	pseudobulk	DESeq2	LRT	0.41689989184848
356	KC_AB1	CG7565	0.402439503335367	0.403699941716678	1	pseudobulk	DESeq2	LRT	0.393941313290025
357	KC_AB1	CG7568	-0.134157149022937	0.901001015508819	1	pseudobulk	DESeq2	LRT	0.045274719531894
357	KC_AB1	CG7582	-0.0612922082498501	0.804242812632065	1	pseudobulk	DESeq2	LRT	0.094612811617389
357	KC_AB1	CG7600	-0.210907671127429	0.681680850653154	1	pseudobulk	DESeq2	LRT	0.1664189057558
357	KC_AB1	CG7601	0.242181119447351	0.762860050244079	1	pseudobulk	DESeq2	LRT	0.117555127813813
357	KC_AB1	CG7607	2.09856955000568	0.2176189688001	1	pseudobulk	DESeq2	LRT	0.662303251960228
357	KC_AB1	CG7611	0.383386916109913	0.369602746886576	1	pseudobulk	DESeq2	LRT	0.432264809764568
357	KC_AB1	CG7627	-0.324254218813778	0.848824744254821	1	pseudobulk	DESeq2	LRT	0.071181968719472
357	KC_AB1	CG7630	-0.178388847050512	0.220719591437336	1	pseudobulk	DESeq2	LRT	0.656159116428267
357	KC_AB1	CG7632	0.176670654922672	0.852459103069639	1	pseudobulk	DESeq2	LRT	0.069326447217842
357	KC_AB1	CG7637	-0.0260066984795697	0.909746945470063	1	pseudobulk	DESeq2	LRT	0.041079393909051
358	KC_AB1	CG7638	-0.442505243006212	0.302914342744595	1	pseudobulk	DESeq2	LRT	0.518680162694341
358	KC_AB1	CG7639	-0.133372220098241	0.739982575234363	1	pseudobulk	DESeq2	LRT	0.130778506713161
358	KC_AB1	CG7646	-0.0286241132006325	0.77573939929009	1	pseudobulk	DESeq2	LRT	0.110284150466677
358	KC_AB1	CG7650	-0.404181387511008	0.515293250114399	1	pseudobulk	DESeq2	LRT	0.287945546375078
358	KC_AB1	CG7655	-0.0216567095211703	0.964019940113036	1	pseudobulk	DESeq2	LRT	0.015913982910968
358	KC_AB1	CG7656	-0.122130286053634	0.698231464847353	1	pseudobulk	DESeq2	LRT	0.15600058419317
358	KC_AB1	CG7668	-0.811524680672023	0.219130700923127	1	pseudobulk	DESeq2	LRT	0.659296772108648

358	KC_AB1	CG7685	-0.050418530987818	0.940342948485165	1	pseudobulk	DESeq2	LRT	0.026713727813462
358	KC_AB1	CG7692	-0.0339488951817989	0.949174788806071	1	pseudobulk	DESeq2	LRT	0.022653805671528
358	KC_AB1	CG7694	-0.533030384270574	0.168375948065634	1	pseudobulk	DESeq2	LRT	0.773719945902875
359	KC_AB1	CG7702	-0.960531374449184	0.550146433218986	1	pseudobulk	DESeq2	LRT	0.259521698370084
359	KC_AB1	CG7706	0.0597307419866766	0.902005108506197	1	pseudobulk	DESeq2	LRT	0.044791002824167
359	KC_AB1	CG7718	0.758209772456866	0.451556566206946	1	pseudobulk	DESeq2	LRT	0.34528783814612
359	KC_AB1	CG7720	-2.28196308857196	0.366390875301127	1	pseudobulk	DESeq2	LRT	0.43605535065843
359	KC_AB1	CG7728	-0.279892448441299	0.786138877074399	1	pseudobulk	DESeq2	LRT	0.104500725946152
359	KC_AB1	CG7737	-0.238351196043149	0.763516173796328	1	pseudobulk	DESeq2	LRT	0.117181758717451
359	KC_AB1	CG7739	0.267692951235952	0.356130803277701	1	pseudobulk	DESeq2	LRT	0.448390460712249
359	KC_AB1	CG7741	0.0911418218195351	0.896458072466248	1	pseudobulk	DESeq2	LRT	0.047470017671635
359	KC_AB1	CG7744	0.497662278741489	0.736955839944538	1	pseudobulk	DESeq2	LRT	0.132558535265785
359	KC_AB1	CG7745	-0.303578796321484	0.569548171837662	1	pseudobulk	DESeq2	LRT	0.244469537821008
360	KC_AB1	CG7747	-0.0167026998578006	0.938274080462091	1	pseudobulk	DESeq2	LRT	0.027670280762355
360	KC_AB1	CG7766	0.119710440205053	0.650448473246846	1	pseudobulk	DESeq2	LRT	0.186787101363572
360	KC_AB1	CG7772	-0.0445492113559113	0.913955118427902	1	pseudobulk	DESeq2	LRT	0.039075130631251
360	KC_AB1	CG7781	-0.0928065080966115	0.5074874611512	1	pseudobulk	DESeq2	LRT	0.294574683700693
360	KC_AB1	CG7785	-0.0867744623502877	0.81332679297113	1	pseudobulk	DESeq2	LRT	0.089734920743189
360	KC_AB1	CG7789	0.109546589835848	0.77964319831894	1	pseudobulk	DESeq2	LRT	0.108104105583423
360	KC_AB1	CG7791	0.0345522358282003	0.966340797905383	1	pseudobulk	DESeq2	LRT	0.014869684611374
360	KC_AB1	CG7806	0.566068921671376	0.612603968331796	1	pseudobulk	DESeq2	LRT	0.212820194243421
360	KC_AB1	CG7810	-0.717342679598583	0.256592345908905	1	pseudobulk	DESeq2	LRT	0.590756302673113
360	KC_AB1	CG7824	1.08899820779554	0.670846322504933	1	pseudobulk	DESeq2	LRT	0.173376956629912
361	KC_AB1	CG7834	-0.279730265347498	0.514769555178599	1	pseudobulk	DESeq2	LRT	0.288387146322666
361	KC_AB1	CG7839	0.200373485143099	0.714660772814756	1	pseudobulk	DESeq2	LRT	0.145900055337806
361	KC_AB1	CG7840	-0.341328756571168	0.754955953786175	1	pseudobulk	DESeq2	LRT	0.122078385570312
361	KC_AB1	CG7841	-0.237038349407331	0.706548850532096	1	pseudobulk	DESeq2	LRT	0.150857805813874
361	KC_AB1	CG7849	0.027482788443832	0.968730195084734	1	pseudobulk	DESeq2	LRT	0.013797163198754
361	KC_AB1	CG7857	-0.0122150499373421	0.97466956687799	1	pseudobulk	DESeq2	LRT	0.011142594152136
361	KC_AB1	CG7872	-0.0451424420755726	0.908956563936606	1	pseudobulk	DESeq2	LRT	0.041456869796036
361	KC_AB1	CG7878	0.0914296902673949	0.82415068554179	1	pseudobulk	DESeq2	LRT	0.083993375783072
361	KC_AB1	CG7879	-0.0058356029296933	0.97854781197956	1	pseudobulk	DESeq2	LRT	0.009417949799944
361	KC_AB1	CG7882	1.08899820779554	0.878177167143508	1	pseudobulk	DESeq2	LRT	0.056417858865076
362	KC_AB1	CG7888	1.45928532158392	0.459969586225171	1	pseudobulk	DESeq2	LRT	0.337270883473328
362	KC_AB1	CG7889	0.0687369761409892	0.909866798027106	1	pseudobulk	DESeq2	LRT	0.041022182531717
362	KC_AB1	CG7896	-0.82542030717083	1	1	pseudobulk	DESeq2	LRT	0
362	KC_AB1	CG7900	-0.850996402080018	0.900449987182352	1	pseudobulk	DESeq2	LRT	0.045540403770502
362	KC_AB1	CG7903	-0.0622019613137649	0.83386175704123	1	pseudobulk	DESeq2	LRT	0.078905943523695
362	KC_AB1	CG7910	0.114184054362142	1	1	pseudobulk	DESeq2	LRT	0
362	KC_AB1	CG7914	1.67772976848679	0.360833731704885	1	pseudobulk	DESeq2	LRT	0.442692870219512
362	KC_AB1	CG7920	-0.108671704655002	0.641594451542517	1	pseudobulk	DESeq2	LRT	0.192739400451585
362	KC_AB1	CG7943	-0.074348254854058	0.860963119721785	1	pseudobulk	DESeq2	LRT	0.065015451617617
362	KC_AB1	CG7945	0.0180258381178364	0.936661013147021	1	pseudobulk	DESeq2	LRT	0.02841755613109
363	KC_AB1	CG7946	0.345472912876777	0.493359947812444	1	pseudobulk	DESeq2	LRT	0.306836110521367
363	KC_AB1	CG7956	-0.0774572622970683	0.859135271998451	1	pseudobulk	DESeq2	LRT	0.065938450537472
363	KC_AB1	CG7968	1.08899820779554	0.670846322504933	1	pseudobulk	DESeq2	LRT	0.173376956629912
363	KC_AB1	CG7970	-0.247377072787572	0.641961382585597	1	pseudobulk	DESeq2	LRT	0.192491096283815
363	KC_AB1	CG7971	-0.0594354392123823	0.58263529502127	1	pseudobulk	DESeq2	LRT	0.234603210115694
363	KC_AB1	CG7974	-0.10051176127827	0.856680344734248	1	pseudobulk	DESeq2	LRT	0.067181197230565
363	KC_AB1	CG7985	0.133909954200343	0.404613923809536	1	pseudobulk	DESeq2	LRT	0.392959176113903
363	KC_AB1	CG7987	-0.396978708260993	0.415187900891038	1	pseudobulk	DESeq2	LRT	0.381755310874483
363	KC_AB1	CG7988	-1.41102679647305	0.315111544708667	1	pseudobulk	DESeq2	LRT	0.501535685333068

363	KC_AB1	CG7990	-0.0959615867985893	0.564717615191319	1	pseudobulk	DESeq2	LRT	0.24816866513886
364	KC_AB1	CG7991	0.0258369990569015	0.989350770674444	1	pseudobulk	DESeq2	LRT	0.004649703590146
364	KC_AB1	CG8001	0.717394534850857	0.203695673718962	1	pseudobulk	DESeq2	LRT	0.69101819485813
364	KC_AB1	CG8003	-1.70149557824952	0.0201579566018651	1	pseudobulk	DESeq2	LRT	1.69555349412692
364	KC_AB1	CG8004	-0.271205900346634	0.46292251125457	1	pseudobulk	DESeq2	LRT	0.334491699588916
364	KC_AB1	CG8005	-0.0156092991584433	0.963970074226862	1	pseudobulk	DESeq2	LRT	0.015936448254714
364	KC_AB1	CG8009	0.0813037106417649	0.806248076619798	1	pseudobulk	DESeq2	LRT	0.093531308405549
364	KC_AB1	CG8010	-0.527168882854495	0.49633110940052	1	pseudobulk	DESeq2	LRT	0.304228502925672
364	KC_AB1	CG8012	0.353823986020041	0.67042482752342	1	pseudobulk	DESeq2	LRT	0.173649911059213
364	KC_AB1	CG8027	-0.0748369075705925	0.920044846955346	1	pseudobulk	DESeq2	LRT	0.036191002751686
364	KC_AB1	CG8031	0.182727362607257	0.78698044907331	1	pseudobulk	DESeq2	LRT	0.104036056668948
365	KC_AB1	CG8032	0.198633449275435	0.686738526655613	1	pseudobulk	DESeq2	LRT	0.163208587607116
365	KC_AB1	CG8034	-1.8174850951113	0.277997151620396	1	pseudobulk	DESeq2	LRT	0.555959653872866
365	KC_AB1	CG8036	0.0045305775751199	0.933769105195056	1	pseudobulk	DESeq2	LRT	0.029760499290702
365	KC_AB1	CG8038	0.0160456965095137	0.961672135659152	1	pseudobulk	DESeq2	LRT	0.016972967404857
365	KC_AB1	CG8042	0.407355108341343	0.343219824641139	1	pseudobulk	DESeq2	LRT	0.46442763491542
365	KC_AB1	CG8043	0.206101404561438	0.7897047300788	1	pseudobulk	DESeq2	LRT	0.102535260694079
365	KC_AB1	CG8060	0.439157310820296	0.614326866589834	1	pseudobulk	DESeq2	LRT	0.211600491099828
365	KC_AB1	CG8064	0.254133249914099	0.76784420200844	1	pseudobulk	DESeq2	LRT	0.11472689073932
365	KC_AB1	CG8066	0.421802616977574	0.713045811886423	1	pseudobulk	DESeq2	LRT	0.146882566627336
365	KC_AB1	CG8067	-0.214563711320888	0.802013824326144	1	pseudobulk	DESeq2	LRT	0.095818145709831
366	KC_AB1	CG8072	-0.82542030717083	1	1	pseudobulk	DESeq2	LRT	0
366	KC_AB1	CG8078	0.0527958350437824	0.933709385811351	1	pseudobulk	DESeq2	LRT	0.029788275566508
366	KC_AB1	CG8079	-0.424201462687928	0.304359048123251	1	pseudobulk	DESeq2	LRT	0.516613782816545
366	KC_AB1	CG8080	-0.299196843983369	0.65713216584716	1	pseudobulk	DESeq2	LRT	0.182347274084743
366	KC_AB1	CG8097	-2.85072110802759	0.101728196504568	1	pseudobulk	DESeq2	LRT	0.992558654854038
366	KC_AB1	CG8108	-0.0601592054790931	0.665332382956163	1	pseudobulk	DESeq2	LRT	0.176961338124806
366	KC_AB1	CG8111	-0.570200320873278	0.291932650055314	1	pseudobulk	DESeq2	LRT	0.53471733034296
366	KC_AB1	CG8112	-0.615767568564188	0.806245159731688	1	pseudobulk	DESeq2	LRT	0.093532879622572
366	KC_AB1	CG8128	-1.69192034359908	0.384518758327288	1	pseudobulk	DESeq2	LRT	0.4150824687642
366	KC_AB1	CG8132	-0.268630174263106	0.562229569392393	1	pseudobulk	DESeq2	LRT	0.250086317237564
367	KC_AB1	CG8134	-0.399623342041262	0.403790569656035	1	pseudobulk	DESeq2	LRT	0.393843828022818
367	KC_AB1	CG8142	-0.785842599200624	0.560512963680741	1	pseudobulk	DESeq2	LRT	0.251414338483988
367	KC_AB1	CG8149	-0.346912113404367	0.414552457306342	1	pseudobulk	DESeq2	LRT	0.382420506181955
367	KC_AB1	CG8152	-0.678189315631742	0.389320453673637	1	pseudobulk	DESeq2	LRT	0.409692779214664
367	KC_AB1	CG8155	0.447015278609781	0.413986452769025	1	pseudobulk	DESeq2	LRT	0.383013870434467
367	KC_AB1	CG8157	-0.82542030717083	1	1	pseudobulk	DESeq2	LRT	0
367	KC_AB1	CG8159	0.288082718844297	0.755435172106355	1	pseudobulk	DESeq2	LRT	0.12180279883586
367	KC_AB1	CG8160	0.111831901354254	1	1	pseudobulk	DESeq2	LRT	0
367	KC_AB1	CG8173	0.943812156732743	0.302814314631795	1	pseudobulk	DESeq2	LRT	0.518823598727257
367	KC_AB1	CG8176	0.0766090261921324	0.771960102028866	1	pseudobulk	DESeq2	LRT	0.112405145151921
368	KC_AB1	CG8177	-0.216333060939887	0.224644587267399	1	pseudobulk	DESeq2	LRT	0.64850404111823
368	KC_AB1	CG8187	-0.0425910276684876	0.865792406524376	1	pseudobulk	DESeq2	LRT	0.062586227507643
368	KC_AB1	CG8188	0.273297598533382	0.158705413967039	1	pseudobulk	DESeq2	LRT	0.79940825777001
368	KC_AB1	CG8195	-0.0958121644465079	0.775595022011179	1	pseudobulk	DESeq2	LRT	0.110364987009544
368	KC_AB1	CG8199	-1.82709236256241	0.44317674354685	1	pseudobulk	DESeq2	LRT	0.353423038023035
368	KC_AB1	CG8202	0.696475039884811	0.416614185277626	1	pseudobulk	DESeq2	LRT	0.380265946863272
368	KC_AB1	CG8204	0.461946457303192	0.555159637835936	1	pseudobulk	DESeq2	LRT	0.255582116224544
368	KC_AB1	CG8206	-0.0961155331354651	0.811480999112518	1	pseudobulk	DESeq2	LRT	0.090721644756041
368	KC_AB1	CG8207	-0.213861841652772	0.796705870544468	1	pseudobulk	DESeq2	LRT	0.098701982714343
368	KC_AB1	CG8209	0.0614841350688746	0.889036160585514	1	pseudobulk	DESeq2	LRT	0.051080574211599
369	KC_AB1	CG8223	-0.113463268758101	0.748981347143871	1	pseudobulk	DESeq2	LRT	0.125528997965902

369	KC_AB1	CG8229	-0.106228963429619	0.818862818276264	1	pseudobulk	DESeq2	LRT	0.086788848246616
369	KC_AB1	CG8230	0.153879250266448	0.793529881445878	1	pseudobulk	DESeq2	LRT	0.100436714652405
369	KC_AB1	CG8236	-0.850932661185203	0.660736244265418	1	pseudobulk	DESeq2	LRT	0.179971869591347
369	KC_AB1	CG8237	-0.0430947006296673	0.927665352665772	1	pseudobulk	DESeq2	LRT	0.032608663545959
369	KC_AB1	CG8243	-0.0235545630257133	0.913674971451901	1	pseudobulk	DESeq2	LRT	0.039208271672675
369	KC_AB1	CG8245	0.0486151824777265	0.794565649468996	1	pseudobulk	DESeq2	LRT	0.099870214222177
369	KC_AB1	CG8248	0.0751060346637999	0.798266522990829	1	pseudobulk	DESeq2	LRT	0.097852083412411
369	KC_AB1	CG8260	1.08899820779554	0.670846322504933	1	pseudobulk	DESeq2	LRT	0.173376956629912
369	KC_AB1	CG8270	0.0218158896114089	0.95797918795867	1	pseudobulk	DESeq2	LRT	0.018643925840962
370	KC_AB1	CG8272	0.732256821579389	0.32708276106401	1	pseudobulk	DESeq2	LRT	0.485342344814385
370	KC_AB1	CG8281	0.369901884105538	0.510232010141925	1	pseudobulk	DESeq2	LRT	0.292232298780094
370	KC_AB1	CG8289	0.113050807005526	0.697110210970337	1	pseudobulk	DESeq2	LRT	0.156698555858251
370	KC_AB1	CG8292	-0.829971373743754	0.758323735176306	1	pseudobulk	DESeq2	LRT	0.120145350587648
370	KC_AB1	CG8298	0.531496480256323	0.485418587032733	1	pseudobulk	DESeq2	LRT	0.313883598232343
370	KC_AB1	CG8300	0.45995048711731	0.290614055907727	1	pseudobulk	DESeq2	LRT	0.536683384341032
370	KC_AB1	CG8301	0.134238266241356	0.692744964966069	1	pseudobulk	DESeq2	LRT	0.159426622089071
370	KC_AB1	CG8311	0.321697194801566	0.510933557728894	1	pseudobulk	DESeq2	LRT	0.291635572248504
370	KC_AB1	CG8312	0.221821332470352	0.892939800691619	1	pseudobulk	DESeq2	LRT	0.049177818948751
370	KC_AB1	CG8314	-0.0335959768183255	0.887421825501222	1	pseudobulk	DESeq2	LRT	0.051869894327844
371	KC_AB1	CG8319	0.268929223302319	0.747656838715326	1	pseudobulk	DESeq2	LRT	0.126297689901414
371	KC_AB1	CG8320	0.079902254708948	0.890585159497966	1	pseudobulk	DESeq2	LRT	0.050324546125795
371	KC_AB1	CG8321	0.558961564253855	0.0363727586305229	1	pseudobulk	DESeq2	LRT	1.43922375936376
371	KC_AB1	CG8323	-0.619374744783652	0.464801342645446	1	pseudobulk	DESeq2	LRT	0.332732626093917
371	KC_AB1	CG8326	-0.7370304645148	0.390911373555048	1	pseudobulk	DESeq2	LRT	0.407921693598606
371	KC_AB1	CG8336	0.315778921094173	0.636372511706064	1	pseudobulk	DESeq2	LRT	0.196288588095542
371	KC_AB1	CG8353	0.238609769685464	0.751302214835772	1	pseudobulk	DESeq2	LRT	0.124185330860048
371	KC_AB1	CG8360	-0.0986081301059784	0.7950998227834	1	pseudobulk	DESeq2	LRT	0.099578343340401
371	KC_AB1	CG8366	-0.205740432328245	0.699275921912619	1	pseudobulk	DESeq2	LRT	0.15535142551425
371	KC_AB1	CG8369	1.51840051802115	0.0529895902354082	1	pseudobulk	DESeq2	LRT	1.27580943883987
372	KC_AB1	CG8370	-0.0834959616178025	0.921022996344024	1	pseudobulk	DESeq2	LRT	0.035729526089110
372	KC_AB1	CG8372	0.0074838531566420	0.973982339769124	1	pseudobulk	DESeq2	LRT	0.011448917670000
372	KC_AB1	CG8379	-0.188688453515843	0.732142256973961	1	pseudobulk	DESeq2	LRT	0.135404526296421
372	KC_AB1	CG8388	0.0084005277976167	0.984906044779292	1	pseudobulk	DESeq2	LRT	0.006605197096398
372	KC_AB1	CG8389	0.349039179220809	0.488301709919502	1	pseudobulk	DESeq2	LRT	0.311311754914517
372	KC_AB1	CG8397	-0.446816622596661	0.0851240853686497	1	pseudobulk	DESeq2	LRT	1.06994754141082
372	KC_AB1	CG8399	-0.850996402080018	0.900449987182352	1	pseudobulk	DESeq2	LRT	0.045540403770502
372	KC_AB1	CG8401	2.0723532127365	0.0244233825482848	1	pseudobulk	DESeq2	LRT	1.61219418804375
372	KC_AB1	CG8405	-0.0797537408916375	0.800612239325976	1	pseudobulk	DESeq2	LRT	0.096577774921516
372	KC_AB1	CG8407	0.111831901354254	1	1	pseudobulk	DESeq2	LRT	0
373	KC_AB1	CG8412	0.186592789723651	0.654014806683722	1	pseudobulk	DESeq2	LRT	0.184412419277807
373	KC_AB1	CG8414	-0.408594189768397	0.504406721201437	1	pseudobulk	DESeq2	LRT	0.297219135102033
373	KC_AB1	CG8435	-0.209692014171095	0.728824746304745	1	pseudobulk	DESeq2	LRT	0.137376889875422
373	KC_AB1	CG8441	-0.183114746402597	0.725034892638024	1	pseudobulk	DESeq2	LRT	0.139641092304168
373	KC_AB1	CG8460	0.196054986836826	0.834406267612619	1	pseudobulk	DESeq2	LRT	0.078622442381918
373	KC_AB1	CG8461	0.0580551166197663	0.921036416782252	1	pseudobulk	DESeq2	LRT	0.035723197930283
373	KC_AB1	CG8475	0.544320353825758	0.25947064285239	1	pseudobulk	DESeq2	LRT	0.585911772183337
373	KC_AB1	CG8485	-0.0833112422968963	0.723855046845936	1	pseudobulk	DESeq2	LRT	0.140348393274786
373	KC_AB1	CG8490	-0.229251657840112	0.653961272854725	1	pseudobulk	DESeq2	LRT	0.184447969540392
373	KC_AB1	CG8500	0.0024416431431578	0.951252341498422	1	pseudobulk	DESeq2	LRT	0.021704261208395
374	KC_AB1	CG8507	0.132519117832247	0.759925863637556	1	pseudobulk	DESeq2	LRT	0.11922877427657
374	KC_AB1	CG8516	-0.11704997612427	0.847567966059165	1	pseudobulk	DESeq2	LRT	0.071825465867143
374	KC_AB1	CG8519	-0.855747055024491	0.32189373598003	1	pseudobulk	DESeq2	LRT	0.492287474559422

374	KC_AB1	CG8520	0.0321090625739217	0.915314153835226	1	pseudobulk	DESeq2	LRT	0.038429821932686
374	KC_AB1	CG8526	0.111831901354254	1	1	pseudobulk	DESeq2	LRT	0
374	KC_AB1	CG8531	0.0799258339442901	0.883986642288162	1	pseudobulk	DESeq2	LRT	0.053554297457935
374	KC_AB1	CG8545	0.0325552229100177	0.96399251610871	1	pseudobulk	DESeq2	LRT	0.015926337700184
374	KC_AB1	CG8547	-1.56855358782635	0.573026303465967	1	pseudobulk	DESeq2	LRT	0.241825442277049
374	KC_AB1	CG8549	-0.114561925084936	0.803632928543115	1	pseudobulk	DESeq2	LRT	0.094942276513652
374	KC_AB1	CG8563	-3.32605984644888	0.138359851832382	1	pseudobulk	DESeq2	LRT	0.858989911737277
375	KC_AB1	CG8565	1.08899820779554	0.878177167143508	1	pseudobulk	DESeq2	LRT	0.056417858865076
375	KC_AB1	CG8569	-0.70541605034385	0.356157143101257	1	pseudobulk	DESeq2	LRT	0.448358341005358
375	KC_AB1	CG8578	0.0007400440185940	0.960056046036614	1	pseudobulk	DESeq2	LRT	0.017703413029236
375	KC_AB1	CG8607	0.453321124912592	0.828810605733731	1	pseudobulk	DESeq2	LRT	0.081544700187927
375	KC_AB1	CG8611	0.224902217319205	0.747379592670212	1	pseudobulk	DESeq2	LRT	0.126458764810861
375	KC_AB1	CG8613	0.374861741065635	0.175958808685057	1	pseudobulk	DESeq2	LRT	0.754588987041035
375	KC_AB1	CG8617	0.0877895391467177	0.767755498327835	1	pseudobulk	DESeq2	LRT	0.114777064647332
375	KC_AB1	CG8635	-0.0645545105994671	0.850370967085589	1	pseudobulk	DESeq2	LRT	0.070391575683444
375	KC_AB1	CG8641	-0.725094826118835	0.437323415349774	1	pseudobulk	DESeq2	LRT	0.359197268859228
375	KC_AB1	CG8642	-1.56855358782635	0.573026303465967	1	pseudobulk	DESeq2	LRT	0.241825442277049
376	KC_AB1	CG8646	1.25086272048698	0.523255167846221	1	pseudobulk	DESeq2	LRT	0.281286473727902
376	KC_AB1	CG8664	1.08899820779554	0.670846322504933	1	pseudobulk	DESeq2	LRT	0.173376956629912
376	KC_AB1	CG8668	-0.0758619413717964	0.786248572811272	1	pseudobulk	DESeq2	LRT	0.104440129871955
376	KC_AB1	CG8671	0.172038771606325	0.361375597615814	1	pseudobulk	DESeq2	LRT	0.442041177117105
376	KC_AB1	CG8673	1.72858030091037	0.219513596899958	1	pseudobulk	DESeq2	LRT	0.658538573937868
376	KC_AB1	CG8675	-0.16616723205357	0.765687035379655	1	pseudobulk	DESeq2	LRT	0.115948706322208
376	KC_AB1	CG8677	-0.234442860681463	0.159613666952475	1	pseudobulk	DESeq2	LRT	0.796929924840405
376	KC_AB1	CG8679	0.873441510982583	0.545835130567871	1	pseudobulk	DESeq2	LRT	0.262938516081324
376	KC_AB1	CG8712	-0.067874316626605	0.961378182062558	1	pseudobulk	DESeq2	LRT	0.017105738164680
376	KC_AB1	CG8726	-0.160740114279248	0.342609444878901	1	pseudobulk	DESeq2	LRT	0.465200668801187
377	KC_AB1	CG8728	-0.192083291085939	0.667257903391073	1	pseudobulk	DESeq2	LRT	0.175706273473308
377	KC_AB1	CG8738	1.08899820779554	0.697631521651407	1	pseudobulk	DESeq2	LRT	0.156373904552
377	KC_AB1	CG8740	0.111831901354254	1	1	pseudobulk	DESeq2	LRT	0
377	KC_AB1	CG8745	-2.37432853298142	0.308079992266679	1	pseudobulk	DESeq2	LRT	0.51133650528684
377	KC_AB1	CG8765	0.181268720660238	0.737082512372641	1	pseudobulk	DESeq2	LRT	0.132483892517741
377	KC_AB1	CG8768	-0.669460791739276	0.260075540249882	1	pseudobulk	DESeq2	LRT	0.584900490687696
377	KC_AB1	CG8777	0.172087035070151	0.85341226281322	1	pseudobulk	DESeq2	LRT	0.068841120987075
377	KC_AB1	CG8778	-0.0624385716568171	0.917136094523103	1	pseudobulk	DESeq2	LRT	0.037566214259249
377	KC_AB1	CG8813	1.08899820779554	0.697631521651407	1	pseudobulk	DESeq2	LRT	0.156373904552
377	KC_AB1	CG8814	-0.32025281209626	0.442025951810315	1	pseudobulk	DESeq2	LRT	0.354552232014821
378	KC_AB1	CG8818	-0.0007038970842782	0.915883387730163	1	pseudobulk	DESeq2	LRT	0.038159818132621
378	KC_AB1	CG8833	0.56062895094895	0.450167735879857	1	pseudobulk	DESeq2	LRT	0.346625634683042
378	KC_AB1	CG8837	1.08899820779554	0.670846322504933	1	pseudobulk	DESeq2	LRT	0.173376956629912
378	KC_AB1	CG8839	-0.386179485376053	0.603976574476659	1	pseudobulk	DESeq2	LRT	0.218979905373593
378	KC_AB1	CG8841	0.284223654249568	0.714728317774782	1	pseudobulk	DESeq2	LRT	0.145859010665457
378	KC_AB1	CG8850	-0.487823502542862	0.767206051182811	1	pseudobulk	DESeq2	LRT	0.115087980413853
378	KC_AB1	CG8858	0.108479391135036	0.87669789488607	1	pseudobulk	DESeq2	LRT	0.057150036265853
378	KC_AB1	CG8860	-0.0154850981659683	0.894899502217938	1	pseudobulk	DESeq2	LRT	0.048225733491705
378	KC_AB1	CG8861	-2.91460844145047	0.506163602165981	1	pseudobulk	DESeq2	LRT	0.295709087836007
378	KC_AB1	CG8878	-0.285046293925874	0.310057502618875	1	pseudobulk	DESeq2	LRT	0.50855775534572
379	KC_AB1	CG8888	-2.73875389726269	0.196003139872929	1	pseudobulk	DESeq2	LRT	0.707736971405949
379	KC_AB1	CG8891	-0.841999180331673	0.139470109450018	1	pseudobulk	DESeq2	LRT	0.855518858281876
379	KC_AB1	CG8892	-0.157971904361469	0.765068939215059	1	pseudobulk	DESeq2	LRT	0.116299429458506
379	KC_AB1	CG8908	-0.850996402080018	0.900449987182352	1	pseudobulk	DESeq2	LRT	0.045540403770502
379	KC_AB1	CG8910	0.125535432457787	0.476525837957622	1	pseudobulk	DESeq2	LRT	0.321913546278129

379	KC_AB1	CG8915	-1.56311639744371	0.0936112821073507	1	pseudobulk	DESeq2	LRT	1.02867180658568
379	KC_AB1	CG8924	0.0980655046116724	0.768915370256325	1	pseudobulk	DESeq2	LRT	0.11412145766705
379	KC_AB1	CG8929	-0.00501433161516	0.976093913982523	1	pseudobulk	DESeq2	LRT	0.01050839507694
379	KC_AB1	CG8939	-0.900969518836191	0.355233415009875	1	pseudobulk	DESeq2	LRT	0.44948618908756
379	KC_AB1	CG8944	-0.18174098600413	0.775550290202266	1	pseudobulk	DESeq2	LRT	0.11039003531111
380	KC_AB1	CG8950	0.0528686460929599	0.934961413081487	1	pseudobulk	DESeq2	LRT	0.02920631258373
380	KC_AB1	CG8960	1.08899820779554	0.670846322504933	1	pseudobulk	DESeq2	LRT	0.17337695662991
380	KC_AB1	CG8963	0.339484966224844	0.18716051268245	1	pseudobulk	DESeq2	LRT	0.72778577383007
380	KC_AB1	CG8974	0.0334963209316565	0.873235574854089	1	pseudobulk	DESeq2	LRT	0.05886857982488
380	KC_AB1	CG8993	-0.199483288017564	0.376958048824201	1	pseudobulk	DESeq2	LRT	0.42370697917859
380	KC_AB1	CG8997	1.08899820779554	0.697631521651407	1	pseudobulk	DESeq2	LRT	0.156373904552
380	KC_AB1	CG9003	-0.05198829554621	0.809007086168019	1	pseudobulk	DESeq2	LRT	0.09204767434546
380	KC_AB1	CG9004	0.795110255027384	0.252996061844818	1	pseudobulk	DESeq2	LRT	0.59688623903120
380	KC_AB1	CG9005	0.259156513932242	0.169593461609234	1	pseudobulk	DESeq2	LRT	0.77059089525010
380	KC_AB1	CG9008	-1.32252668385619	0.602163303828335	1	pseudobulk	DESeq2	LRT	0.22028571416775
381	KC_AB1	CG9018	-0.109905341136855	0.776610751522226	1	pseudobulk	DESeq2	LRT	0.10979660130677
381	KC_AB1	CG9034	-0.347041704724237	0.0885909611464398	1	pseudobulk	DESeq2	LRT	1.05261058651561
381	KC_AB1	CG9044	0.363923952371055	0.417361247812351	1	pseudobulk	DESeq2	LRT	0.37948787784498
381	KC_AB1	CG9062	-0.390168031410268	0.62141562008607	1	pseudobulk	DESeq2	LRT	0.20661783438981
381	KC_AB1	CG9065	-0.19415399556155	0.353709563395532	1	pseudobulk	DESeq2	LRT	0.45135319782234
381	KC_AB1	CG9067	-0.320861357101273	0.580953900346385	1	pseudobulk	DESeq2	LRT	0.23585832822843
381	KC_AB1	CG9072	1.95275942187961	0.438420007953907	1	pseudobulk	DESeq2	LRT	0.35810963439207
381	KC_AB1	CG9084	-0.850932661185203	0.660736244265418	1	pseudobulk	DESeq2	LRT	0.17997186959134
381	KC_AB1	CG9098	0.453985126424884	0.600033846686338	1	pseudobulk	DESeq2	LRT	0.22182425125882
381	KC_AB1	CG9106	-0.850996402080018	0.900449987182352	1	pseudobulk	DESeq2	LRT	0.04554040377050
382	KC_AB1	CG9107	0.102727688241089	0.86251117089859	1	pseudobulk	DESeq2	LRT	0.06423527141040
382	KC_AB1	CG9114	-0.850996402080018	0.900449987182352	1	pseudobulk	DESeq2	LRT	0.04554040377050
382	KC_AB1	CG9117	-1.51071978438463	0.535151592350864	1	pseudobulk	DESeq2	LRT	0.27152317798632
382	KC_AB1	CG9119	1.08899820779554	0.697631521651407	1	pseudobulk	DESeq2	LRT	0.156373904552
382	KC_AB1	CG9121	-0.16031924711287	0.720929211738525	1	pseudobulk	DESeq2	LRT	0.14210737669613
382	KC_AB1	CG9123	-0.243088600213091	0.423192208461999	1	pseudobulk	DESeq2	LRT	0.37346233684505
382	KC_AB1	CG9125	-0.187136768113457	0.774462248590566	1	pseudobulk	DESeq2	LRT	0.11099974721192
382	KC_AB1	CG9132	-0.148655310772826	0.492740691899338	1	pseudobulk	DESeq2	LRT	0.30738157099562
382	KC_AB1	CG9135	0.634369481750878	0.311322049622869	1	pseudobulk	DESeq2	LRT	0.50679011898848
382	KC_AB1	CG9143	-0.09518528140777	0.876659918352226	1	pseudobulk	DESeq2	LRT	0.05716884931013
383	KC_AB1	CG9147	0.352445646473567	0.613763820531044	1	pseudobulk	DESeq2	LRT	0.21199871545913
383	KC_AB1	CG9149	0.113180122560035	0.802080755605238	1	pseudobulk	DESeq2	LRT	0.09578190360097
383	KC_AB1	CG9154	0.187189953130343	0.738031032151957	1	pseudobulk	DESeq2	LRT	0.13192537691813
383	KC_AB1	CG9164	0.53117416822345	0.334670666198139	1	pseudobulk	DESeq2	LRT	0.47538235177898
383	KC_AB1	CG9170	0.00891416370035	0.956452586796849	1	pseudobulk	DESeq2	LRT	0.01933655391986
383	KC_AB1	CG9171	-0.25685696528594	0.147922375035321	1	pseudobulk	DESeq2	LRT	0.82996612877929
383	KC_AB1	CG9175	0.155270589479632	0.680528571608701	1	pseudobulk	DESeq2	LRT	0.16715363646871
383	KC_AB1	CG9205	0.309928576625406	0.344060979740395	1	pseudobulk	DESeq2	LRT	0.46336457830711
383	KC_AB1	CG9213	-0.537439770158979	0.511704673368906	1	pseudobulk	DESeq2	LRT	0.29098061662362
383	KC_AB1	CG9215	0.0198585490809503	0.962451101034869	1	pseudobulk	DESeq2	LRT	0.01662132632875
384	KC_AB1	CG9231	0.115621346078609	0.671822908831435	1	pseudobulk	DESeq2	LRT	0.17274519101319
384	KC_AB1	CG9240	-0.411327907061519	0.211025691819116	1	pseudobulk	DESeq2	LRT	0.67566466728037
384	KC_AB1	CG9246	-0.58668767181798	0.394505922370159	1	pseudobulk	DESeq2	LRT	0.40394647272484
384	KC_AB1	CG9248	-2.14757529320053	0.345787765116326	1	pseudobulk	DESeq2	LRT	0.46119037727513
384	KC_AB1	CG9253	0.61249587134349	0.254122731166915	1	pseudobulk	DESeq2	LRT	0.59495648576103
384	KC_AB1	CG9254	-2.19369050038995	0.291918361597822	1	pseudobulk	DESeq2	LRT	0.53473858712978
384	KC_AB1	CG9257	0.12686955160695	0.789435594499633	1	pseudobulk	DESeq2	LRT	0.10268329579422

384	KC_AB1	CG9263	0.169014373152776	0.908833701841318	1	pseudobulk	DESeq2	LRT	0.041515576601881
384	KC_AB1	CG9265	-0.343072688687792	0.193564243568772	1	pseudobulk	DESeq2	LRT	0.713174865286811
384	KC_AB1	CG9270	-0.831765148915113	0.743013616167264	1	pseudobulk	DESeq2	LRT	0.129003227461331
385	KC_AB1	CG9272	-1.68833667049648	0.359710228646132	1	pseudobulk	DESeq2	LRT	0.444047212497273
385	KC_AB1	CG9281	0.277002498777788	0.222876155307185	1	pseudobulk	DESeq2	LRT	0.651936392589511
385	KC_AB1	CG9286	0.52023770067697	0.494545911560791	1	pseudobulk	DESeq2	LRT	0.305793384123534
385	KC_AB1	CG9289	-1.96719661029409	0.414177861019091	1	pseudobulk	DESeq2	LRT	0.382813119093482
385	KC_AB1	CG9300	-0.658279118297612	0.590368289804804	1	pseudobulk	DESeq2	LRT	0.228876977629189
385	KC_AB1	CG9302	0.624433683068624	0.516567648174085	1	pseudobulk	DESeq2	LRT	0.286872796496881
385	KC_AB1	CG9304	-0.270018239924189	0.698419429392654	1	pseudobulk	DESeq2	LRT	0.155883687457347
385	KC_AB1	CG9313	1.08899820779554	0.878177167143508	1	pseudobulk	DESeq2	LRT	0.056417858865076
385	KC_AB1	CG9328	-0.107180874268051	0.481597349289231	1	pseudobulk	DESeq2	LRT	0.317315912099543
385	KC_AB1	CG9330	0.0020618095786104	0.947970935517237	1	pseudobulk	DESeq2	LRT	0.023204977786388
386	KC_AB1	CG9331	-1.13912913574204	0.60803432663814	1	pseudobulk	DESeq2	LRT	0.216071901897157
386	KC_AB1	CG9336	-1.6747012584303	0.563129890257477	1	pseudobulk	DESeq2	LRT	0.249391420218277
386	KC_AB1	CG9344	0.0502713155617649	0.862698980482557	1	pseudobulk	DESeq2	LRT	0.064140715200860
386	KC_AB1	CG9346	-0.125448217979846	0.756663837357323	1	pseudobulk	DESeq2	LRT	0.121097021432794
386	KC_AB1	CG9356	-0.046875735642054	0.896803300488559	1	pseudobulk	DESeq2	LRT	0.047302802084212
386	KC_AB1	CG9368	-0.043295919738701	0.823024151135216	1	pseudobulk	DESeq2	LRT	0.084587420497496
386	KC_AB1	CG9380	0.563960670394673	0.765273331028603	1	pseudobulk	DESeq2	LRT	0.116183421113154
386	KC_AB1	CG9384	-0.329948133661594	0.405539172857295	1	pseudobulk	DESeq2	LRT	0.391967188964348
386	KC_AB1	CG9386	0.567900338556815	0.481436021479035	1	pseudobulk	DESeq2	LRT	0.317461418538941
386	KC_AB1	CG9391	0.0772534817162194	0.783919320826814	1	pseudobulk	DESeq2	LRT	0.105728631604719
387	KC_AB1	CG9393	0.591877559573987	0.385423018875024	1	pseudobulk	DESeq2	LRT	0.41406235124811
387	KC_AB1	CG9394	-0.615767568564188	0.806245159731688	1	pseudobulk	DESeq2	LRT	0.093532879622572
387	KC_AB1	CG9395	-0.152561913927531	0.685922841924559	1	pseudobulk	DESeq2	LRT	0.163724734455093
387	KC_AB1	CG9399	-0.113256769658792	0.860907196883577	1	pseudobulk	DESeq2	LRT	0.065043661616733
387	KC_AB1	CG9410	-0.031914989813455	0.894512151165647	1	pseudobulk	DESeq2	LRT	0.048413755545634
387	KC_AB1	CG9422	-0.251197427764041	0.440646085652683	1	pseudobulk	DESeq2	LRT	0.355910083449477
387	KC_AB1	CG9425	-0.0276602281405471	0.841119641231062	1	pseudobulk	DESeq2	LRT	0.075142225568425
387	KC_AB1	CG9426	-0.0917808286622726	0.79637620241802	1	pseudobulk	DESeq2	LRT	0.098881726184751
387	KC_AB1	CG9467	-0.348389618667354	0.487112049929	1	pseudobulk	DESeq2	LRT	0.312371126940357
387	KC_AB1	CG9471	0.553639541626629	0.355482265678612	1	pseudobulk	DESeq2	LRT	0.449182060503928
388	KC_AB1	CG9485	0.295343402821542	0.825937076880018	1	pseudobulk	DESeq2	LRT	0.083053037673214
388	KC_AB1	CG9503	1.08899820779554	0.878177167143508	1	pseudobulk	DESeq2	LRT	0.056417858865076
388	KC_AB1	CG9512	1.95275942187961	0.438420007953907	1	pseudobulk	DESeq2	LRT	0.358109634392079
388	KC_AB1	CG9514	0.255834644427643	0.855198708192687	1	pseudobulk	DESeq2	LRT	0.067932963815662
388	KC_AB1	CG9515	-0.386373117119278	0.71989443396505	1	pseudobulk	DESeq2	LRT	0.142731184274005
388	KC_AB1	CG9518	0.318491578108543	0.766087968953381	1	pseudobulk	DESeq2	LRT	0.115721357994728
388	KC_AB1	CG9519	-0.850932661185203	0.660736244265418	1	pseudobulk	DESeq2	LRT	0.179971869591347
388	KC_AB1	CG9521	-0.82542030717083	1	1	pseudobulk	DESeq2	LRT	0
388	KC_AB1	CG9522	0.114184054362142	1	1	pseudobulk	DESeq2	LRT	0
388	KC_AB1	CG9527	0.658788807506573	0.291333015441373	1	pseudobulk	DESeq2	LRT	0.535610295971299
389	KC_AB1	CG9536	-0.11563366326619	0.840851618855332	1	pseudobulk	DESeq2	LRT	0.075280635352889
389	KC_AB1	CG9541	-1.44925888589622	0.6315588144614	1	pseudobulk	DESeq2	LRT	0.199586199175701
389	KC_AB1	CG9570	-0.82542030717083	1	1	pseudobulk	DESeq2	LRT	0
389	KC_AB1	CG9577	0.375122969690604	0.601412446894703	1	pseudobulk	DESeq2	LRT	0.220827587938614
389	KC_AB1	CG9578	0.396545224556217	0.446332642409173	1	pseudobulk	DESeq2	LRT	0.350341349996188
389	KC_AB1	CG9581	0.212006323953766	0.797975570250382	1	pseudobulk	DESeq2	LRT	0.098010404223027
389	KC_AB1	CG9586	0.234754538050988	0.585809480202205	1	pseudobulk	DESeq2	LRT	0.232243604374819
389	KC_AB1	CG9588	0.582851212142754	0.492066717108829	1	pseudobulk	DESeq2	LRT	0.307976009208578
389	KC_AB1	CG9590	0.0049564513471157	0.965585430233515	1	pseudobulk	DESeq2	LRT	0.015209295952262

389	KC_AB1	CG9593	1.08899820779554	0.878177167143508	1	pseudobulk	DESeq2	LRT	0.056417858865076
390	KC_AB1	CG9596	0.486634837095397	0.598756445022252	1	pseudobulk	DESeq2	LRT	0.222749798800703
390	KC_AB1	CG9601	-0.631157498022954	0.186311382748251	1	pseudobulk	DESeq2	LRT	0.729760610945196
390	KC_AB1	CG9609	-0.0151399139644782	0.904133827997806	1	pseudobulk	DESeq2	LRT	0.043767281405939
390	KC_AB1	CG9628	2.2820936629399	0.0669261143739883	1	pseudobulk	DESeq2	LRT	1.17440438872609
390	KC_AB1	CG9630	-1.08602501521527	0.456034772456624	1	pseudobulk	DESeq2	LRT	0.341002041304252
390	KC_AB1	CG9636	0.341835099843731	0.260160421387766	1	pseudobulk	DESeq2	LRT	0.584758772648782
390	KC_AB1	CG9641	0.0929332382910059	0.812393885678373	1	pseudobulk	DESeq2	LRT	0.090233353886305
390	KC_AB1	CG9643	-0.583598008741896	0.348615368435709	1	pseudobulk	DESeq2	LRT	0.457653471271627
390	KC_AB1	CG9646	0.0330005440850007	0.858034201931073	1	pseudobulk	DESeq2	LRT	0.066495400480092
390	KC_AB1	CG9650	-0.92179759686814	0.460131053978356	1	pseudobulk	DESeq2	LRT	0.337118455463023
391	KC_AB1	CG9657	-0.499771239170307	0.801310913577201	1	pseudobulk	DESeq2	LRT	0.096198942278209
391	KC_AB1	CG9662	-0.329144132848785	0.391532530068244	1	pseudobulk	DESeq2	LRT	0.407232149206987
391	KC_AB1	CG9663	-0.939384626430541	0.63182432676416	1	pseudobulk	DESeq2	LRT	0.199403656721395
391	KC_AB1	CG9667	0.0120816367087953	0.959653438923711	1	pseudobulk	DESeq2	LRT	0.017885576061135
391	KC_AB1	CG9673	-0.850932661185203	0.660736244265418	1	pseudobulk	DESeq2	LRT	0.179971869591347
391	KC_AB1	CG9674	0.0297837164538354	0.859693236498527	1	pseudobulk	DESeq2	LRT	0.065656489991909
391	KC_AB1	CG9684	0.0672222416463946	0.896960085338298	1	pseudobulk	DESeq2	LRT	0.047226882600295
391	KC_AB1	CG9686	-0.80254487358381	0.696363446032399	1	pseudobulk	DESeq2	LRT	0.157164034227709
391	KC_AB1	CG9705	0.689932149233537	0.0249618041325482	1	pseudobulk	DESeq2	LRT	1.60272402890569
391	KC_AB1	CG9706	-1.24474852523676	0.483713709830666	1	pseudobulk	DESeq2	LRT	0.315411603288768
392	KC_AB1	CG9723	-0.644328402956081	0.621730681653805	1	pseudobulk	DESeq2	LRT	0.206397700197946
392	KC_AB1	CG9727	-0.0043902917572400	0.983941779800992	1	pseudobulk	DESeq2	LRT	0.007030598173539
392	KC_AB1	CG9743	-0.349507503089684	0.681579911517858	1	pseudobulk	DESeq2	LRT	0.166483218190755
392	KC_AB1	CG9752	-0.31131387894845	0.871589143496327	1	pseudobulk	DESeq2	LRT	0.059688187958827
392	KC_AB1	CG9760	-0.848649487787247	0.320425387545232	1	pseudobulk	DESeq2	LRT	0.494273081745086
392	KC_AB1	CG9766	0.964220711946188	0.590461768762227	1	pseudobulk	DESeq2	LRT	0.228808216853371
392	KC_AB1	CG9773	-0.338054337988144	0.546484820978642	1	pseudobulk	DESeq2	LRT	0.262421896398207
392	KC_AB1	CG9775	0.247730743420574	0.186402757392553	1	pseudobulk	DESeq2	LRT	0.72954766756405
392	KC_AB1	CG9776	0.214908440797482	0.313431347304282	1	pseudobulk	DESeq2	LRT	0.50385757046966
392	KC_AB1	CG9777	-0.715773599702744	0.835451286644154	1	pseudobulk	DESeq2	LRT	0.078078867803391
393	KC_AB1	CG9782	1.34806076738172	0.218704682117102	1	pseudobulk	DESeq2	LRT	0.660141919313279
393	KC_AB1	CG9784	0.158815871484031	0.628032558067454	1	pseudobulk	DESeq2	LRT	0.202017841258795
393	KC_AB1	CG9795	0.174419409068447	0.571062407794858	1	pseudobulk	DESeq2	LRT	0.243316427866405
393	KC_AB1	CG9799	-0.331831924919237	0.703618574327027	1	pseudobulk	DESeq2	LRT	0.152662704431663
393	KC_AB1	CG9801	0.316381950315664	0.686502181267579	1	pseudobulk	DESeq2	LRT	0.163358078513216
393	KC_AB1	CG9804	-0.0911619361492252	0.876328841646126	1	pseudobulk	DESeq2	LRT	0.057332894615545
393	KC_AB1	CG9813	-0.125306842075997	0.314339006972804	1	pseudobulk	DESeq2	LRT	0.502601723144337
393	KC_AB1	CG9837	0.779013907895398	0.0193806457227255	1	pseudobulk	DESeq2	LRT	1.71263175725715
393	KC_AB1	CG9839	0.804302228135395	0.205235513170277	1	pseudobulk	DESeq2	LRT	0.687747488397329
393	KC_AB1	CG9849	-0.40197442144651	0.464011787509211	1	pseudobulk	DESeq2	LRT	0.333470986718434
394	KC_AB1	CG9853	-0.194787989325727	0.626355143900017	1	pseudobulk	DESeq2	LRT	0.203179351611412
394	KC_AB1	CG9855	0.302759744700739	0.467673522286857	1	pseudobulk	DESeq2	LRT	0.330057217327376
394	KC_AB1	CG9864	1.68130633771206	0.562684503730219	1	pseudobulk	DESeq2	LRT	0.249735045103917
394	KC_AB1	CG9886	-1.10523278471011	0.266550159032961	1	pseudobulk	DESeq2	LRT	0.574221054023966
394	KC_AB1	CG9890	-0.518005925534838	0.552050597045544	1	pseudobulk	DESeq2	LRT	0.258021116092073
394	KC_AB1	CG9902	0.665367330006672	0.303946311111172	1	pseudobulk	DESeq2	LRT	0.517203123126009
394	KC_AB1	CG9911	0.175516520161909	0.610871871094998	1	pseudobulk	DESeq2	LRT	0.214049872428117
394	KC_AB1	CG9915	0.565077300543277	0.213418340882148	1	pseudobulk	DESeq2	LRT	0.670768260630223
394	KC_AB1	CG9917	-0.0648361725357566	0.865101971017213	1	pseudobulk	DESeq2	LRT	0.062932698500518
394	KC_AB1	CG9919	-0.0298749294522619	0.902861900864848	1	pseudobulk	DESeq2	LRT	0.044378673029763
395	KC_AB1	CG9921	-0.0821115142631213	0.826956330281499	1	pseudobulk	DESeq2	LRT	0.082517423964413

395	KC_AB1	CG9922	-0.498554958342956	0.218167149300798	1	pseudobulk	DESeq2	LRT	0.66121064307609
395	KC_AB1	CG9932	0.0588879637962869	0.81871104535525	1	pseudobulk	DESeq2	LRT	0.086869350437999
395	KC_AB1	CG9934	0.349030309186986	0.445732911227915	1	pseudobulk	DESeq2	LRT	0.350925298072256
395	KC_AB1	CG9941	-0.221351797953939	0.574401034316363	1	pseudobulk	DESeq2	LRT	0.240784786736709
395	KC_AB1	CG9945	-0.199790975099843	0.699556284092322	1	pseudobulk	DESeq2	LRT	0.155177337802104
395	KC_AB1	CG9948	0.343356807682103	0.617425161520811	1	pseudobulk	DESeq2	LRT	0.209415675976404
395	KC_AB1	CG9951	-0.241292353335714	0.778344852232401	1	pseudobulk	DESeq2	LRT	0.108827942549071
395	KC_AB1	CG9953	-0.400655859513707	0.321979256291417	1	pseudobulk	DESeq2	LRT	0.492172107087917
395	KC_AB1	CG9967	-0.656514556277586	0.57641843084227	1	pseudobulk	DESeq2	LRT	0.23926214117324
396	KC_AB1	CG9986	0.312103150031121	0.624828170361157	1	pseudobulk	DESeq2	LRT	0.20423939853439
396	KC_AB1	CG9992	0.261821564462822	0.538816924456965	1	pseudobulk	DESeq2	LRT	0.268558771369929
396	KC_AB1	Charon	-0.458655352097447	0.579715384187894	1	pseudobulk	DESeq2	LRT	0.236785174393316
396	KC_AB1	chas	1.08899820779554	0.878177167143508	1	pseudobulk	DESeq2	LRT	0.056417858865076
396	KC_AB1	ChAT	-0.112912089518187	0.662259636839398	1	pseudobulk	DESeq2	LRT	0.17897171336939
396	KC_AB1	chb	0.0529331547144491	0.847679948642848	1	pseudobulk	DESeq2	LRT	0.071768089694830
396	KC_AB1	Chc	0.0504000895023485	0.819246365251346	1	pseudobulk	DESeq2	LRT	0.086585476774741
396	KC_AB1	Chchd2	-0.151533391184091	0.322410727260058	1	pseudobulk	DESeq2	LRT	0.491590516766623
396	KC_AB1	Chchd3	-0.108908116627415	0.687957021745534	1	pseudobulk	DESeq2	LRT	0.162438692290323
396	KC_AB1	Chd1	0.151873641866106	0.550379639138236	1	pseudobulk	DESeq2	LRT	0.259337640858068
397	KC_AB1	Chd64	-0.624147277501105	0.358932486960704	1	pseudobulk	DESeq2	LRT	0.44498723191683
397	KC_AB1	CheB42c	1.07906814060865	0.366769679379559	1	pseudobulk	DESeq2	LRT	0.435606574371779
397	KC_AB1	cher	0.134463298809937	0.934388988157475	1	pseudobulk	DESeq2	LRT	0.029472288393731
397	KC_AB1	CHES-1-like	0.0291060605538077	0.854648698374351	1	pseudobulk	DESeq2	LRT	0.068212364464418
397	KC_AB1	Chi	0.260157267755052	0.441736876099514	1	pseudobulk	DESeq2	LRT	0.354836344418207
397	KC_AB1	chic	-0.106336023738102	0.562556888633966	1	pseudobulk	DESeq2	LRT	0.249833552913396
397	KC_AB1	chico	-0.0580066747120817	0.851309834780515	1	pseudobulk	DESeq2	LRT	0.069912349379581
397	KC_AB1	chif	-0.179222243141339	0.579271536462042	1	pseudobulk	DESeq2	LRT	0.23711781080902
397	KC_AB1	chinmo	0.189673828193004	0.191356711172875	1	pseudobulk	DESeq2	LRT	0.71815630179989
397	KC_AB1	CHKov1	1.88952324269138	0.456767015650974	1	pseudobulk	DESeq2	LRT	0.340305265178099
398	KC_AB1	chm	-0.423515996947993	0.382402151956783	1	pseudobulk	DESeq2	LRT	0.417479672409728
398	KC_AB1	Chmp1	0.142653899394991	0.596162514044238	1	pseudobulk	DESeq2	LRT	0.224635335340552
398	KC_AB1	CHMP2B	0.372850483064625	0.459108686550279	1	pseudobulk	DESeq2	LRT	0.338084490102279
398	KC_AB1	chn	-0.0618237567897515	0.590405896192733	1	pseudobulk	DESeq2	LRT	0.228849314004829
398	KC_AB1	CHOp24	0.0352492834154751	0.875202677911601	1	pseudobulk	DESeq2	LRT	0.057891362171005
398	KC_AB1	CHORD	-0.524971713923285	0.413745208199921	1	pseudobulk	DESeq2	LRT	0.383267022979085
398	KC_AB1	Chrac-14	-0.640927738516237	0.387770228306866	1	pseudobulk	DESeq2	LRT	0.411425537646124
398	KC_AB1	Chrac-16	0.243529985474202	0.767421801713736	1	pseudobulk	DESeq2	LRT	0.114965867074793
398	KC_AB1	chrb	-0.195955180114943	0.149814780715025	1	pseudobulk	DESeq2	LRT	0.824445337061748
398	KC_AB1	Chro	0.235920126346047	0.348943146275831	1	pseudobulk	DESeq2	LRT	0.457245327396297
399	KC_AB1	ChT	-0.218140687710788	0.303713295964936	1	pseudobulk	DESeq2	LRT	0.517536195115629
399	KC_AB1	Cht10	-0.615767568564188	0.806245159731688	1	pseudobulk	DESeq2	LRT	0.093532879622572
399	KC_AB1	Cht11	-1.40820837414628	0.431115263155535	1	pseudobulk	DESeq2	LRT	0.365406601161693
399	KC_AB1	Cht2	0.901916968543292	0.24196028030668	1	pseudobulk	DESeq2	LRT	0.616255921041965
399	KC_AB1	ci	0.516794653828635	0.814433332789471	1	pseudobulk	DESeq2	LRT	0.089144460019238
399	KC_AB1	CIA30	0.0829579511098341	0.895443845930169	1	pseudobulk	DESeq2	LRT	0.047961643949316
399	KC_AB1	Ciao1	-0.768761011907497	0.300228526309807	1	pseudobulk	DESeq2	LRT	0.522548045502622
399	KC_AB1	CIAPIN1	0.104581744700303	0.843031956005925	1	pseudobulk	DESeq2	LRT	0.074155962677751
399	KC_AB1	cib	0.321885299861383	0.434924378467024	1	pseudobulk	DESeq2	LRT	0.361586248492457
399	KC_AB1	cic	0.0775307253854685	0.595564668318531	1	pseudobulk	DESeq2	LRT	0.22507107453544
400	KC_AB1	cin	-0.155164408698302	0.716555683575948	1	pseudobulk	DESeq2	LRT	0.144750054910588
400	KC_AB1	cindr	0.299891184717552	0.299461117306175	1	pseudobulk	DESeq2	LRT	0.523659559370213
400	KC_AB1	Cip4	-0.180826042979757	0.196811951732189	1	pseudobulk	DESeq2	LRT	0.705948531851157

400	KC_AB1	Cirl	0.105616872385302	0.444670003630045	1	pseudobulk	DESeq2	LRT	0.351962166025166
400	KC_AB1	Cisd2	-0.138759449080487	0.532640757748564	1	pseudobulk	DESeq2	LRT	0.273565604348369
400	KC_AB1	ck	1.68130633771206	0.562684503730219	1	pseudobulk	DESeq2	LRT	0.249735045103917
400	KC_AB1	Cka	-0.0528718603966435	0.750049112085994	1	pseudobulk	DESeq2	LRT	0.124910298728796
400	KC_AB1	CkIalpha	0.0697427668397929	0.615436624717137	1	pseudobulk	DESeq2	LRT	0.2108166623959
400	KC_AB1	CkIIalpha	0.118058876497528	0.425430202744411	1	pseudobulk	DESeq2	LRT	0.371171681288911
400	KC_AB1	CkIIalpha-i1	0.0684664864229973	0.910801617969796	1	pseudobulk	DESeq2	LRT	0.040576206565096
401	KC_AB1	CkIIalpha-i3	0.355162186449227	0.523745592228652	1	pseudobulk	DESeq2	LRT	0.280879618973293
401	KC_AB1	CkIIbeta	-0.0604563813467113	0.702153350989166	1	pseudobulk	DESeq2	LRT	0.153568027164835
401	KC_AB1	ckn	0.17684598352521	0.320111239161464	1	pseudobulk	DESeq2	LRT	0.494699077433139
401	KC_AB1	Cks85A	0.0686146312323497	0.6940081531029	1	pseudobulk	DESeq2	LRT	0.158635427489236
401	KC_AB1	cl	0.177106462796453	0.55312129991191	1	pseudobulk	DESeq2	LRT	0.257179617148092
401	KC_AB1	Clamp	0.0311364836410876	0.909141779769142	1	pseudobulk	DESeq2	LRT	0.041368383697814
401	KC_AB1	Clbn	-0.0662302330017301	0.866337661261756	1	pseudobulk	DESeq2	LRT	0.062312805625648
401	KC_AB1	Clc	-0.113898928412392	0.597288715792781	1	pseudobulk	DESeq2	LRT	0.223815690033139
401	KC_AB1	ClC-b	-0.070249185858778	0.899019527145975	1	pseudobulk	DESeq2	LRT	0.046230875074732
401	KC_AB1	ClC-c	-0.646153837828165	0.0635519466319835	1	pseudobulk	DESeq2	LRT	1.19687114222121
402	KC_AB1	Clic	0.110153252347962	0.73353357173432	1	pseudobulk	DESeq2	LRT	0.134580004949103
402	KC_AB1	CLIP-190	-0.228616309832929	0.196626684194441	1	pseudobulk	DESeq2	LRT	0.70635754442857
402	KC_AB1	Cln7	1.08899820779554	0.878177167143508	1	pseudobulk	DESeq2	LRT	0.056417858865076
402	KC_AB1	clos	0.13617601020169	1	1	pseudobulk	DESeq2	LRT	0
402	KC_AB1	Clp	0.168461698104607	0.868425702327305	1	pseudobulk	DESeq2	LRT	0.061267331447770
402	KC_AB1	ClpP	0.268161048232788	0.477184594111671	1	pseudobulk	DESeq2	LRT	0.321313585957885
402	KC_AB1	CLS	1.00120301880542	0.163382218143414	1	pseudobulk	DESeq2	LRT	0.786795212077926
402	KC_AB1	clt	-0.517398835373713	0.623256966245996	1	pseudobulk	DESeq2	LRT	0.205332858615945
402	KC_AB1	clu	0.235541739972698	0.463283490683408	1	pseudobulk	DESeq2	LRT	0.33415317580126
402	KC_AB1	cm	-0.129942024251579	0.800716126478422	1	pseudobulk	DESeq2	LRT	0.096521424683707
403	KC_AB1	cmb	0.298969253653896	0.652058494211932	1	pseudobulk	DESeq2	LRT	0.185713443265034
403	KC_AB1	cmpy	0.0269760440842243	0.904029973778841	1	pseudobulk	DESeq2	LRT	0.043817169932879
403	KC_AB1	cN-IIIB	-0.575018240714526	0.437359129812272	1	pseudobulk	DESeq2	LRT	0.359161803205818
403	KC_AB1	CNBP	0.095417879511146	0.587410094915745	1	pseudobulk	DESeq2	LRT	0.23105859419638
403	KC_AB1	cnc	-0.51700269844474	0.106030791013149	1	pseudobulk	DESeq2	LRT	0.974567998647797
403	KC_AB1	Cndp2	0.849749029388997	0.171383007171296	1	pseudobulk	DESeq2	LRT	0.766032241091867
403	KC_AB1	CngA	-0.605472210897017	0.351236522074101	1	pseudobulk	DESeq2	LRT	0.454400331858819
403	KC_AB1	CngB	0.515006330272197	0.0615919007606143	1	pseudobulk	DESeq2	LRT	1.21047639313156
403	KC_AB1	CngI	0.275930161225222	0.047890866285092	1	pseudobulk	DESeq2	LRT	1.31974730704943
403	KC_AB1	cni	-0.0788594370010276	0.794698359717295	1	pseudobulk	DESeq2	LRT	0.099797683379813
404	KC_AB1	cnir	-0.212610699981896	0.671718051065693	1	pseudobulk	DESeq2	LRT	0.172812980757602
404	KC_AB1	cnk	-0.0564542416998747	0.930709776667574	1	pseudobulk	DESeq2	LRT	0.031185724004324
404	KC_AB1	CNMaR	-0.798280436496897	0.438403256385613	1	pseudobulk	DESeq2	LRT	0.358126228645379
404	KC_AB1	cnn	0.181152121878012	0.631951722953066	1	pseudobulk	DESeq2	LRT	0.199316097755458
404	KC_AB1	cno	0.868398007004907	0.247300727860991	1	pseudobulk	DESeq2	LRT	0.606774605411664
404	KC_AB1	Cnot4	-0.0101260998919419	0.95294247504657	1	pseudobulk	DESeq2	LRT	0.02093331502157
404	KC_AB1	CNPYb	0.735213250088382	0.129320171972864	1	pseudobulk	DESeq2	LRT	0.888333726525559
404	KC_AB1	Cnx14D	1.08063427044639	0.313273672635323	1	pseudobulk	DESeq2	LRT	0.504076101478447
404	KC_AB1	Cnx99A	-0.0840248052107035	0.772000485063075	1	pseudobulk	DESeq2	LRT	0.112382426788421
404	KC_AB1	Coa7	-0.185570946387777	0.536369141466883	1	pseudobulk	DESeq2	LRT	0.270536216048179
405	KC_AB1	Cog1	-0.720132508025407	0.344423226566516	1	pseudobulk	DESeq2	LRT	0.462907569095433
405	KC_AB1	Cog2	-1.17614061360565	0.153976041820568	1	pseudobulk	DESeq2	LRT	0.812546848738646
405	KC_AB1	Cog3	-1.13345971167842	0.165733914892073	1	pseudobulk	DESeq2	LRT	0.780588610812152
405	KC_AB1	Cog4	0.433063139930504	0.568243506758705	1	pseudobulk	DESeq2	LRT	0.245465518198637
405	KC_AB1	Cog6	0.0757226832884406	0.891750029112379	1	pseudobulk	DESeq2	LRT	0.049756867811480

405	KC_AB1	Cog7	-0.324810959131156	0.616222493963676	1	pseudobulk	DESeq2	LRT	0.2102624526825
405	KC_AB1	Cog8	-0.046844936705674	0.950112388513085	1	pseudobulk	DESeq2	LRT	0.022225019106830
405	KC_AB1	coil	-0.0962700020974192	0.862894527795023	1	pseudobulk	DESeq2	LRT	0.064042285160748
405	KC_AB1	Col4a1	0.135879393043758	1	1	pseudobulk	DESeq2	LRT	0
405	KC_AB1	cold	0.747021965388735	0.238588276229098	1	pseudobulk	DESeq2	LRT	0.622350900540375
406	KC_AB1	colt	0.441099646530591	0.567494378829347	1	pseudobulk	DESeq2	LRT	0.246038435906623
406	KC_AB1	comm	-0.0631004285208702	0.75160562815576	1	pseudobulk	DESeq2	LRT	0.124009976485511
406	KC_AB1	comt	-0.385948543395699	0.131847198795013	1	pseudobulk	DESeq2	LRT	0.879929092725244
406	KC_AB1	Con	-0.0526230720408826	0.671812014015312	1	pseudobulk	DESeq2	LRT	0.172752233936001
406	KC_AB1	Cont	0.702533893509828	0.36145981926045	1	pseudobulk	DESeq2	LRT	0.441939972901502
406	KC_AB1	conu	0.252739732555104	0.180622509856578	1	pseudobulk	DESeq2	LRT	0.743228127237795
406	KC_AB1	conv	1.08899820779554	0.878177167143508	1	pseudobulk	DESeq2	LRT	0.056417858865076
406	KC_AB1	Coop	0.359026773898891	0.856962521129231	1	pseudobulk	DESeq2	LRT	0.067038171335481
406	KC_AB1	Coprox	0.26199099195593	0.760889709436616	1	pseudobulk	DESeq2	LRT	0.118678289424372
406	KC_AB1	Coq2	-0.964274563059771	0.239812229493625	1	pseudobulk	DESeq2	LRT	0.620128673338212
407	KC_AB1	Coq3	0.0653431858793438	0.920678670519443	1	pseudobulk	DESeq2	LRT	0.035891918087798
407	KC_AB1	Coq5	-0.793390925087398	0.534098652079951	1	pseudobulk	DESeq2	LRT	0.272378518070889
407	KC_AB1	Coq6	-0.0059132529675041	0.979589465894526	1	pseudobulk	DESeq2	LRT	0.008955893747892
407	KC_AB1	Coq7	0.6097559295279	0.059952192243513	1	pseudobulk	DESeq2	LRT	1.22219493163347
407	KC_AB1	Coq8	0.495050785916752	0.506812054043508	1	pseudobulk	DESeq2	LRT	0.295153064383851
407	KC_AB1	Coq9	-0.207000632206819	0.754917450286709	1	pseudobulk	DESeq2	LRT	0.12210053558277
407	KC_AB1	cora	0.159756109048611	0.26066535298564	1	pseudobulk	DESeq2	LRT	0.583916690377941
407	KC_AB1	CoRest	-0.210539524682674	0.349104896296669	1	pseudobulk	DESeq2	LRT	0.457044060037975
407	KC_AB1	Corin	0.408745392863502	0.1552232541028	1	pseudobulk	DESeq2	LRT	0.809043216247861
407	KC_AB1	corn	-0.340606696585298	0.0234536628558452	1	pseudobulk	DESeq2	LRT	1.62978932208078
408	KC_AB1	coro	0.183464468640937	0.393950747198697	1	pseudobulk	DESeq2	LRT	0.404558071466051
408	KC_AB1	Corp	-0.82542030717083	1	1	pseudobulk	DESeq2	LRT	0
408	KC_AB1	cort	0.0944367054142478	0.950578142812657	1	pseudobulk	DESeq2	LRT	0.022012175908065
408	KC_AB1	Cortactin	-0.340944359188037	0.45514610649505	1	pseudobulk	DESeq2	LRT	0.341849168048571
408	KC_AB1	corto	-0.0142457224330932	0.913693970435868	1	pseudobulk	DESeq2	LRT	0.039199241034461
408	KC_AB1	cos	-0.113451539206826	0.774482026428603	1	pseudobulk	DESeq2	LRT	0.11098865655352
408	KC_AB1	Cow	0.0652917103942904	0.549214626768288	1	pseudobulk	DESeq2	LRT	0.260257905083923
408	KC_AB1	COX4	-0.202056503326702	0.134576794204256	1	pseudobulk	DESeq2	LRT	0.871029821374332
408	KC_AB1	COX5A	-0.365740887080797	0.0171685236845126	1	pseudobulk	DESeq2	LRT	1.7652670480677
408	KC_AB1	COX5B	-0.13826534530804	0.405986279408479	1	pseudobulk	DESeq2	LRT	0.391488643461908
409	KC_AB1	COX6B	-0.244175629736034	0.0411622894693324	1	pseudobulk	DESeq2	LRT	1.38550047753824
409	KC_AB1	COX7A	-0.243184017501757	0.0497347604558663	1	pseudobulk	DESeq2	LRT	1.30333996946742
409	KC_AB1	COX7C	0.0356332522054939	0.818322204861145	1	pseudobulk	DESeq2	LRT	0.087075664248771
409	KC_AB1	COX8	-0.300200400841079	0.0297781506647924	1	pseudobulk	DESeq2	LRT	1.52610227706789
409	KC_AB1	Cp1	-0.0818316691723947	0.714397141984441	1	pseudobulk	DESeq2	LRT	0.146060291553538
409	KC_AB1	Cp110	0.0897048283590542	0.648827446763952	1	pseudobulk	DESeq2	LRT	0.18787078683943
409	KC_AB1	Cp18	-1.56855358782635	0.573026303465967	1	pseudobulk	DESeq2	LRT	0.241825442277049
409	KC_AB1	Cp190	0.015606141226848	0.930829975681517	1	pseudobulk	DESeq2	LRT	0.031129639498482
409	KC_AB1	Cp7Fa	-1.16459802684386	0.334081247553207	1	pseudobulk	DESeq2	LRT	0.476147901233253
409	KC_AB1	Cp7Fb	-0.708924106411694	0.611629417233884	1	pseudobulk	DESeq2	LRT	0.213511634713103
410	KC_AB1	Cp7Fc	-0.850996402080018	0.900449987182352	1	pseudobulk	DESeq2	LRT	0.045540403770502
410	KC_AB1	cpa	0.372874662329378	0.499913934943466	1	pseudobulk	DESeq2	LRT	0.301104757256802
410	KC_AB1	cpb	0.277454319750704	0.485879604599058	1	pseudobulk	DESeq2	LRT	0.313471330605623
410	KC_AB1	Cpes	-0.122712777982161	0.839658328266332	1	pseudobulk	DESeq2	LRT	0.075897400051126
410	KC_AB1	cpo	-0.221629831531767	0.0638869243517933	1	pseudobulk	DESeq2	LRT	1.19458801919603
410	KC_AB1	Cpr	0.0421092338418537	0.961177567741255	1	pseudobulk	DESeq2	LRT	0.017196373448528
410	KC_AB1	Cpr11B	-2.37432853298142	0.308079992266679	1	pseudobulk	DESeq2	LRT	0.51133650528684

410	KC_AB1	Cpr64Ab	-0.850996402080018	0.900449987182352	1	pseudobulk	DESeq2	LRT	0.045540403770502
410	KC_AB1	Cpr64Ad	2.1559833397943	0.381743505411375	1	pseudobulk	DESeq2	LRT	0.418228342845202
410	KC_AB1	Cpr67B	0.0321463419253685	0.952650405727522	1	pseudobulk	DESeq2	LRT	0.021066443242050
411	KC_AB1	Cpr73D	-0.850996402080018	0.900449987182352	1	pseudobulk	DESeq2	LRT	0.045540403770502
411	KC_AB1	Cpr78Ca	0.11269840515639	0.977007463492936	1	pseudobulk	DESeq2	LRT	0.010102118633921
411	KC_AB1	Cpr92F	1.68130633771206	0.562684503730219	1	pseudobulk	DESeq2	LRT	0.249735045103917
411	KC_AB1	Cpsf100	-1.43226923910682	0.137840112290261	1	pseudobulk	DESeq2	LRT	0.860624381765622
411	KC_AB1	Cpsf160	-0.0095292751023242	0.980885950318599	1	pseudobulk	DESeq2	LRT	0.008381486021406
411	KC_AB1	Cpsf5	0.0788295417018622	0.735745388244336	1	pseudobulk	DESeq2	LRT	0.133272451438768
411	KC_AB1	Cpsf6	0.0665998401068398	0.73785751363686	1	pseudobulk	DESeq2	LRT	0.132027495921868
411	KC_AB1	Cpsf73	-0.239077822893309	0.7381976572358	1	pseudobulk	DESeq2	LRT	0.131827337426976
411	KC_AB1	cpx	-0.0332107591584647	0.738014569119163	1	pseudobulk	DESeq2	LRT	0.131935064700529
411	KC_AB1	CR12842	0.308775298587704	0.771224784003174	1	pseudobulk	DESeq2	LRT	0.112819022436569
412	KC_AB1	CR14578	-0.800430865882526	0.644018727773926	1	pseudobulk	DESeq2	LRT	0.191101503369045
412	KC_AB1	CR14798	-0.758869005107591	0.608496930574265	1	pseudobulk	DESeq2	LRT	0.215741608129103
412	KC_AB1	CR15345	-0.0945354857349814	0.883246920262812	1	pseudobulk	DESeq2	LRT	0.053917868204125
412	KC_AB1	CR17024	1.08899820779554	0.697631521651407	1	pseudobulk	DESeq2	LRT	0.156373904552
412	KC_AB1	CR18166	0.396826813486293	0.380599521589517	1	pseudobulk	DESeq2	LRT	0.419531761953264
412	KC_AB1	CR18217	0.466903895658733	0.815224421820197	1	pseudobulk	DESeq2	LRT	0.088722818571206
412	KC_AB1	CR18228	1.48399207075518	0.347434732735957	1	pseudobulk	DESeq2	LRT	0.459126767692134
412	KC_AB1	CR18275	0.480627294213205	0.355542223973873	1	pseudobulk	DESeq2	LRT	0.449108815328221
412	KC_AB1	CR31032	0.300912782029917	0.643778016859347	1	pseudobulk	DESeq2	LRT	0.191263857286445
412	KC_AB1	CR32009	-1.57536227797437	0.533448959696272	1	pseudobulk	DESeq2	LRT	0.272907127472266
413	KC_AB1	CR32010	-0.850932661185203	0.660736244265418	1	pseudobulk	DESeq2	LRT	0.179971869591347
413	KC_AB1	CR32733	0.635343606245865	0.736278607579394	1	pseudobulk	DESeq2	LRT	0.132957817675126
413	KC_AB1	CR32745	0.177900222546369	0.683789095141517	1	pseudobulk	DESeq2	LRT	0.165077829479214
413	KC_AB1	CR40100	-0.850932661185203	0.660736244265418	1	pseudobulk	DESeq2	LRT	0.179971869591347
413	KC_AB1	CR40190	2.48532174800475	0.247437616432353	1	pseudobulk	DESeq2	LRT	0.606534276545381
413	KC_AB1	CR40461	1.08899820779554	0.878177167143508	1	pseudobulk	DESeq2	LRT	0.056417858865076
413	KC_AB1	CR41243	1.08899820779554	0.878177167143508	1	pseudobulk	DESeq2	LRT	0.056417858865076
413	KC_AB1	CR41320	0.316765464003935	0.711106712065522	1	pseudobulk	DESeq2	LRT	0.14806522207797
413	KC_AB1	CR41440	-0.82542030717083	1	1	pseudobulk	DESeq2	LRT	0
413	KC_AB1	CR41597	-0.160371953541682	0.588315968806439	1	pseudobulk	DESeq2	LRT	0.230389363289594
414	KC_AB1	CR42786	-1.82709236256241	0.44317674354685	1	pseudobulk	DESeq2	LRT	0.353423038023035
414	KC_AB1	CR43087	0.114184054362142	1	1	pseudobulk	DESeq2	LRT	0
414	KC_AB1	CR43170	-0.451135134101306	0.69269955518547	1	pseudobulk	DESeq2	LRT	0.159455091243713
414	KC_AB1	CR43177	-0.82542030717083	1	1	pseudobulk	DESeq2	LRT	0
414	KC_AB1	CR43186	0.135795303631797	0.946582799906172	1	pseudobulk	DESeq2	LRT	0.023841391241048
414	KC_AB1	CR43212	-1.89854133735591	0.478664405825708	1	pseudobulk	DESeq2	LRT	0.319968866080244
414	KC_AB1	CR43281	0.097251922312974	0.791910490788407	1	pseudobulk	DESeq2	LRT	0.101323903705341
414	KC_AB1	CR43361	2.13275063396931	0.192303080670683	1	pseudobulk	DESeq2	LRT	0.716013758363904
414	KC_AB1	CR43670	-0.291094383292077	0.642793336148477	1	pseudobulk	DESeq2	LRT	0.191928634225037
414	KC_AB1	CR43671	0.813435280898331	0.560391650297353	1	pseudobulk	DESeq2	LRT	0.251508344222808
415	KC_AB1	CR43972	2.10549911548503	0.428045198278114	1	pseudobulk	DESeq2	LRT	0.368510370411162
415	KC_AB1	CR43975	-0.850996402080018	0.900449987182352	1	pseudobulk	DESeq2	LRT	0.045540403770502
415	KC_AB1	CR44003	0.207860326655067	0.760854966797915	1	pseudobulk	DESeq2	LRT	0.118698119999781
415	KC_AB1	CR44383	-1.3553650301445	0.299408724046632	1	pseudobulk	DESeq2	LRT	0.52373554951697
415	KC_AB1	CR45033	1.08899820779554	0.670846322504933	1	pseudobulk	DESeq2	LRT	0.173376956629912
415	KC_AB1	CR45470	-0.0356194766761602	0.964042745765415	1	pseudobulk	DESeq2	LRT	0.015903709003323
415	KC_AB1	CR45496	-4.12518017936767	0.334353457753199	1	pseudobulk	DESeq2	LRT	0.475794181136523
415	KC_AB1	CR4575	1.68130633771206	0.562684503730219	1	pseudobulk	DESeq2	LRT	0.249735045103917
415	KC_AB1	CR46405	-0.501300761870089	0.863550576027942	1	pseudobulk	DESeq2	LRT	0.063712221794699

415	KC_AB1	CR46425	-0.82542030717083	1	1	pseudobulk	DESeq2	LRT	0
416	KC_AB1	CR6900	1.40558781813143	0.23390193481869	1	pseudobulk	DESeq2	LRT	0.630966185725668
416	KC_AB1	CR7448	0.130024002626362	0.938395636387274	1	pseudobulk	DESeq2	LRT	0.027614020390965
416	KC_AB1	Crag	-0.228501030280211	0.566309587675484	1	pseudobulk	DESeq2	LRT	0.246946085664113
416	KC_AB1	Cralbp	1.08899820779554	0.670846322504933	1	pseudobulk	DESeq2	LRT	0.173376956629912
416	KC_AB1	CRAT	-0.520384665049269	0.550075979184662	1	pseudobulk	DESeq2	LRT	0.259577319484563
416	KC_AB1	crb	-0.0745829031945398	0.609701106081113	1	pseudobulk	DESeq2	LRT	0.214883017107835
416	KC_AB1	crc	0.0708564837214167	0.798470789561214	1	pseudobulk	DESeq2	LRT	0.097740967020076
416	KC_AB1	CrebA	-0.281184891236575	0.254479972554762	1	pseudobulk	DESeq2	LRT	0.594346390738621
416	KC_AB1	CrebB	-0.0796873790771986	0.771559740164735	1	pseudobulk	DESeq2	LRT	0.112630441837461
416	KC_AB1	CREG	0.111831901354254	1	1	pseudobulk	DESeq2	LRT	0
417	KC_AB1	Crg-1	0.459548134205734	0.541002132004906	1	pseudobulk	DESeq2	LRT	0.266801023403335
417	KC_AB1	CRIF	-0.143140473287409	0.767973992036479	1	pseudobulk	DESeq2	LRT	0.114653487398565
417	KC_AB1	crim	-0.0356846875382009	0.890723301272221	1	pseudobulk	DESeq2	LRT	0.050257186416097
417	KC_AB1	Crk	-0.202691596362953	0.314951655901218	1	pseudobulk	DESeq2	LRT	0.501756103945729
417	KC_AB1	crm	-0.339930636586344	0.608008932302976	1	pseudobulk	DESeq2	LRT	0.216090040428913
417	KC_AB1	CRMP	-0.313806581806288	0.120779950536921	1	pseudobulk	DESeq2	LRT	0.918005152584522
417	KC_AB1	crn	0.406733992221389	0.442399491729435	1	pseudobulk	DESeq2	LRT	0.354185380661831
417	KC_AB1	crok	-2.5192264985262	0.273630365215179	1	pseudobulk	DESeq2	LRT	0.562835709899105
417	KC_AB1	crol	0.136077706546394	0.384156375746939	1	pseudobulk	DESeq2	LRT	0.415491954539573
417	KC_AB1	CROT	-0.547592924797915	0.565130188608145	1	pseudobulk	DESeq2	LRT	0.24785149256806
418	KC_AB1	crp	0.493815188530485	0.0095338322498735	1	pseudobulk	DESeq2	LRT	2.02073249386012
418	KC_AB1	crq	-0.16883670209607	0.627939280329759	1	pseudobulk	DESeq2	LRT	0.202082349085486
418	KC_AB1	Crtc	0.0655678249290526	0.67677442272577	1	pseudobulk	DESeq2	LRT	0.16955606291076
418	KC_AB1	cry	0.0944367054142478	0.950578142812657	1	pseudobulk	DESeq2	LRT	0.022012175908065
418	KC_AB1	CrzR	1.08899820779554	0.878177167143508	1	pseudobulk	DESeq2	LRT	0.056417858865076
418	KC_AB1	Csas	-0.501300761870089	0.863550576027942	1	pseudobulk	DESeq2	LRT	0.063712221794699
418	KC_AB1	Cse1	0.523527233923966	0.386539939181892	1	pseudobulk	DESeq2	LRT	0.41280562601429
418	KC_AB1	Csk	0.184277734354092	0.330981715228948	1	pseudobulk	DESeq2	LRT	0.480195997748489
418	KC_AB1	Csl4	-0.605301229642098	0.420590096952302	1	pseudobulk	DESeq2	LRT	0.376140957255028
418	KC_AB1	CSN1b	0.655969749620006	0.302412726380854	1	pseudobulk	DESeq2	LRT	0.519399936448998
419	KC_AB1	CSN3	0.601833771486702	0.364460591889712	1	pseudobulk	DESeq2	LRT	0.438349423862031
419	KC_AB1	CSN4	0.108706683831175	0.779054689284668	1	pseudobulk	DESeq2	LRT	0.108432053982291
419	KC_AB1	CSN5	0.0992182812225618	0.817060652749633	1	pseudobulk	DESeq2	LRT	0.087745703349146
419	KC_AB1	CSN6	0.0779429362275726	0.856621005569887	1	pseudobulk	DESeq2	LRT	0.067211280288222
419	KC_AB1	CSN7	0.462351823880714	0.140202678168429	1	pseudobulk	DESeq2	LRT	0.853243690344671
419	KC_AB1	CSN8	-0.346705278287983	0.303176766700229	1	pseudobulk	DESeq2	LRT	0.518304082988841
419	KC_AB1	Csp	-0.179661494713633	0.314507030608919	1	pseudobulk	DESeq2	LRT	0.502369641727361
419	KC_AB1	CstF50	-0.600536652396408	0.536603623994011	1	pseudobulk	DESeq2	LRT	0.270346398612856
419	KC_AB1	CstF64	0.259955894440214	0.732131852303899	1	pseudobulk	DESeq2	LRT	0.135410698215689
419	KC_AB1	csul	-0.0153177233082921	0.911811070668782	1	pseudobulk	DESeq2	LRT	0.040095139156237
420	KC_AB1	csw	0.128108773250463	0.35151307279337	1	pseudobulk	DESeq2	LRT	0.454058518904612
420	KC_AB1	cta	0.0880519244395072	0.704787872946386	1	pseudobulk	DESeq2	LRT	0.151941577294331
420	KC_AB1	CtBP	0.0930221662005521	0.434635268823624	1	pseudobulk	DESeq2	LRT	0.361875035415382
420	KC_AB1	CTCF	-0.413595828137551	0.433666009122398	1	pseudobulk	DESeq2	LRT	0.362844616654765
420	KC_AB1	CtIP	0.278444001901118	0.649618201025321	1	pseudobulk	DESeq2	LRT	0.187341815518536
420	KC_AB1	Ctl1	-0.21095101596279	0.501083928824945	1	pseudobulk	DESeq2	LRT	0.300089526083249
420	KC_AB1	Ctl2	-0.115183344596763	0.807046136317397	1	pseudobulk	DESeq2	LRT	0.093101637303542
420	KC_AB1	ctp	-0.0677143780798602	0.424650100674365	1	pseudobulk	DESeq2	LRT	0.371968768618387
420	KC_AB1	CTPsyn	0.149466292659367	0.621882202444131	1	pseudobulk	DESeq2	LRT	0.206291872018937
420	KC_AB1	Ctr1A	-0.193614344269628	0.541890350892826	1	pseudobulk	DESeq2	LRT	0.266088582132673
421	KC_AB1	Ctr9	-0.0344683488552427	0.897362234771141	1	pseudobulk	DESeq2	LRT	0.047032211575417

421	KC_AB1	ctrip	0.235048600517878	0.196983880265584	1	pseudobulk	DESeq2	LRT	0.70556931190004З
421	KC_AB1	CtsB1	0.0921791104060169	0.719483097212592	1	pseudobulk	DESeq2	LRT	0.142979404470292
421	KC_AB1	cu	-1.08285034588325	0.454581667037628	1	pseudobulk	DESeq2	LRT	0.342388083052187
421	KC_AB1	Cubn	-0.299982604576125	0.809694522443502	1	pseudobulk	DESeq2	LRT	0.091678798701600
421	KC_AB1	cue	0.0239884622199893	0.927087350845416	1	pseudobulk	DESeq2	LRT	0.032879344385255
421	KC_AB1	Cul1	0.158638773252278	0.467960760983204	1	pseudobulk	DESeq2	LRT	0.32979056146202
421	KC_AB1	Cul2	0.0614272084298812	0.808435664946133	1	pseudobulk	DESeq2	LRT	0.092354535399633
421	KC_AB1	Cul3	-0.468411798067345	0.137331813584665	1	pseudobulk	DESeq2	LRT	0.862228844672783
421	KC_AB1	Cul4	-0.436319655770779	0.414758164830496	1	pseudobulk	DESeq2	LRT	0.382205055801667
422	KC_AB1	Cul5	0.244387481453559	0.741692861819248	1	pseudobulk	DESeq2	LRT	0.129775900693517
422	KC_AB1	Cul6	-0.850996402080018	0.900449987182352	1	pseudobulk	DESeq2	LRT	0.045540403770502
422	KC_AB1	cv	1.08899820779554	0.878177167143508	1	pseudobulk	DESeq2	LRT	0.056417858865076
422	KC_AB1	cv-c	0.217519828884633	0.133415692601986	1	pseudobulk	DESeq2	LRT	0.874793084890049
422	KC_AB1	Cwc25	-0.333334172811957	0.451608129047507	1	pseudobulk	DESeq2	LRT	0.345238249278732
422	KC_AB1	cwo	-0.0588707474348825	0.828328172038012	1	pseudobulk	DESeq2	LRT	0.081797567721612
422	KC_AB1	cyc	0.180926351961663	0.680180169716703	1	pseudobulk	DESeq2	LRT	0.16737603383839
422	KC_AB1	CycB3	1.08899820779554	0.697631521651407	1	pseudobulk	DESeq2	LRT	0.156373904552
422	KC_AB1	CycC	-0.136813696488638	0.786751027995841	1	pseudobulk	DESeq2	LRT	0.104162680950326
422	KC_AB1	CycD	-2.08238890537319	0.386092553331648	1	pseudobulk	DESeq2	LRT	0.413308574646766
423	KC_AB1	CycE	1.08899820779554	0.697631521651407	1	pseudobulk	DESeq2	LRT	0.156373904552
423	KC_AB1	CycG	0.0150903233179591	0.889403614008009	1	pseudobulk	DESeq2	LRT	0.050901110168682
423	KC_AB1	CycH	0.191204813900271	0.850712101304745	1	pseudobulk	DESeq2	LRT	0.070217389350388
423	KC_AB1	CycJ	-0.639599218891621	0.788513993518674	1	pseudobulk	DESeq2	LRT	0.103190594974246
423	KC_AB1	CycK	-0.0356625272650233	0.891838229345656	1	pseudobulk	DESeq2	LRT	0.049713915213576
423	KC_AB1	CycT	0.0788802819112443	0.618972468687089	1	pseudobulk	DESeq2	LRT	0.208328667560147
423	KC_AB1	CycY	-0.0610543238200584	0.864849107542567	1	pseudobulk	DESeq2	LRT	0.063059658405751
423	KC_AB1	CYLD	-0.257747043921286	0.48721262284239	1	pseudobulk	DESeq2	LRT	0.312281468405189
423	KC_AB1	Cyp1	-0.248786841789988	0.082886087896937	1	pseudobulk	DESeq2	LRT	1.08151835794939
423	KC_AB1	Cyp12b2	-0.346019579042185	0.609801811930518	1	pseudobulk	DESeq2	LRT	0.21481128953073
424	KC_AB1	Cyp28a5	-0.82542030717083	1	1	pseudobulk	DESeq2	LRT	0
424	KC_AB1	Cyp28d1	0.113316967542732	1	1	pseudobulk	DESeq2	LRT	0
424	KC_AB1	Cyp28d2	1.08899820779554	0.697631521651407	1	pseudobulk	DESeq2	LRT	0.156373904552
424	KC_AB1	Cyp301a1	1.08899820779554	0.697631521651407	1	pseudobulk	DESeq2	LRT	0.156373904552
424	KC_AB1	Cyp303a1	1.64750305707911	0.566713307590673	1	pseudobulk	DESeq2	LRT	0.246636589113683
424	KC_AB1	Cyp313a1	-0.850932661185203	0.660736244265418	1	pseudobulk	DESeq2	LRT	0.179971869591347
424	KC_AB1	cyp33	0.0759662662814021	0.889213899451606	1	pseudobulk	DESeq2	LRT	0.050993757390101
424	KC_AB1	Cyp49a1	0.0953083840434401	0.855978634098311	1	pseudobulk	DESeq2	LRT	0.067537075520288
424	KC_AB1	Cyp4ac3	-0.82542030717083	1	1	pseudobulk	DESeq2	LRT	0
424	KC_AB1	Cyp4ae1	1.19871948081564	0.398432485715629	1	pseudobulk	DESeq2	LRT	0.399645259124171
425	KC_AB1	Cyp4c3	1.68130633771206	0.562684503730219	1	pseudobulk	DESeq2	LRT	0.249735045103917
425	KC_AB1	Cyp4d2	3.05562915577017	0.145714142161275	1	pseudobulk	DESeq2	LRT	0.836498296105453
425	KC_AB1	Cyp4s3	0.737878214276948	0.722402866174353	1	pseudobulk	DESeq2	LRT	0.141220539603138
425	KC_AB1	Cyp6a14	-0.850996402080018	0.900449987182352	1	pseudobulk	DESeq2	LRT	0.045540403770502
425	KC_AB1	Cyp6g1	2.10549911548503	0.428045198278114	1	pseudobulk	DESeq2	LRT	0.368510370411162
425	KC_AB1	Cyp6t2Psi	1.88952324269138	0.456767015650974	1	pseudobulk	DESeq2	LRT	0.340305265178099
425	KC_AB1	Cyp6u1	0.439150795340864	0.530878636035314	1	pseudobulk	DESeq2	LRT	0.275004751469284
425	KC_AB1	Cyp6w1	-0.82542030717083	1	1	pseudobulk	DESeq2	LRT	0
425	KC_AB1	Cyp9c1	1.08899820779554	0.878177167143508	1	pseudobulk	DESeq2	LRT	0.056417858865076
425	KC_AB1	Cyp9f2	0.744782276103072	0.764014638758689	1	pseudobulk	DESeq2	LRT	0.116898320126909
426	KC_AB1	Cyp9f3	0.855700630127339	0.665189798240232	1	pseudobulk	DESeq2	LRT	0.177054420008663
426	KC_AB1	cype	-0.272924465752043	0.0475204564440827	1	pseudobulk	DESeq2	LRT	1.32311939651604
426	KC_AB1	Cypl	0.964369054566296	0.0588421557554768	1	pseudobulk	DESeq2	LRT	1.23031142473517

426	KC_AB1	cyr	0.135879393043758	1	1	pseudobulk	DESeq2	LRT	0
426	KC_AB1	Cys	1.73414032718858	0.323835454499663	1	pseudobulk	DESeq2	LRT	0.489675605086543
426	KC_AB1	CysRS	0.21494148630938	0.762353163362323	1	pseudobulk	DESeq2	LRT	0.117843793269681
426	KC_AB1	CysRS-m	0.445878056009699	0.637550768924702	1	pseudobulk	DESeq2	LRT	0.195485226131738
426	KC_AB1	Cyt-b5	0.106171817331888	0.598094281387476	1	pseudobulk	DESeq2	LRT	0.223230350026843
426	KC_AB1	Cyt-c-d	0.477028581170134	0.782340637227571	1	pseudobulk	DESeq2	LRT	0.106604110549928
426	KC_AB1	Cyt-c-p	-0.0279257256423059	0.811421273226215	1	pseudobulk	DESeq2	LRT	0.090753610479797
427	KC_AB1	Cyt-c1	-0.284369804358461	0.181178856176733	1	pseudobulk	DESeq2	LRT	0.741892486469163
427	KC_AB1	D1	0.146664053168492	0.42591172816684	1	pseudobulk	DESeq2	LRT	0.370680400762099
427	KC_AB1	D12	0.742048708036775	0.312554142239708	1	pseudobulk	DESeq2	LRT	0.505074741074321
427	KC_AB1	D19A	0.653106716130813	0.21935540482615	1	pseudobulk	DESeq2	LRT	0.658851660291385
427	KC_AB1	D19B	-0.555663573423599	0.307440437757847	1	pseudobulk	DESeq2	LRT	0.512239010160668
427	KC_AB1	d4	0.240305236189038	0.150412509129771	1	pseudobulk	DESeq2	LRT	0.822716043929734
427	KC_AB1	da	-0.0159407195667004	0.909991037894045	1	pseudobulk	DESeq2	LRT	0.040962884835311
427	KC_AB1	DAAM	0.267348085790026	0.382005733361215	1	pseudobulk	DESeq2	LRT	0.417930118898624
427	KC_AB1	Dab	-0.0880284816863597	0.648206533020232	1	pseudobulk	DESeq2	LRT	0.188286596197454
427	KC_AB1	dac	-0.121368555958583	0.346130287546928	1	pseudobulk	DESeq2	LRT	0.460760396837084
428	KC_AB1	Dad	-0.80300747829697	0.164669422530593	1	pseudobulk	DESeq2	LRT	0.783387037497549
428	KC_AB1	Dad1	-0.183971073022971	0.503696884129152	1	pseudobulk	DESeq2	LRT	0.297830735681302
428	KC_AB1	daed	-0.206605606369991	0.857091545596683	1	pseudobulk	DESeq2	LRT	0.066972788783742
428	KC_AB1	Dak1	-0.0185183820230893	0.93923026532881	1	pseudobulk	DESeq2	LRT	0.027227921363157
428	KC_AB1	dally	1.2148106084576	0.534316412108626	1	pseudobulk	DESeq2	LRT	0.272201485785755
428	KC_AB1	Damm	-0.82542030717083	1	1	pseudobulk	DESeq2	LRT	0
428	KC_AB1	danr	-0.82542030717083	1	1	pseudobulk	DESeq2	LRT	0
428	KC_AB1	dao	0.151630718379552	0.567762563054697	1	pseudobulk	DESeq2	LRT	0.245833247235984
428	KC_AB1	Dap160	-0.163281263527683	0.398233255355515	1	pseudobulk	DESeq2	LRT	0.399862476065652
428	KC_AB1	dare	0.147107679195442	0.832626182022754	1	pseudobulk	DESeq2	LRT	0.079549936796312
429	KC_AB1	Dark	-0.16993497413683	0.662975414566	1	pseudobulk	DESeq2	LRT	0.17850257644635
429	KC_AB1	DAT	-2.58068486840805	0.292900969777782	1	pseudobulk	DESeq2	LRT	0.533279190392686
429	KC_AB1	dati	0.111676028498986	0.349505505050441	1	pseudobulk	DESeq2	LRT	0.456545979305424
429	KC_AB1	Daxx	0.107759419994101	0.781740471807457	1	pseudobulk	DESeq2	LRT	0.106937403422621
429	KC_AB1	dbe	-1.23553904470811	0.181436830668285	1	pseudobulk	DESeq2	LRT	0.741274548960561
429	KC_AB1	dbo	-0.314912717551322	0.187606958271936	1	pseudobulk	DESeq2	LRT	0.726751057838279
429	KC_AB1	Dbp21E2	1.59790324933398	0.200980011174992	1	pseudobulk	DESeq2	LRT	0.696847133962996
429	KC_AB1	Dbp45A	0.483653974922569	0.449225621171413	1	pseudobulk	DESeq2	LRT	0.347535482116622
429	KC_AB1	Dbp73D	-0.833649591159108	0.406185864202564	1	pseudobulk	DESeq2	LRT	0.391275194682189
429	KC_AB1	Dbp80	-0.01201645973916	0.929188414489084	1	pseudobulk	DESeq2	LRT	0.031896213803817
430	KC_AB1	dbr	0.129212200009391	0.744113943157868	1	pseudobulk	DESeq2	LRT	0.128360557604915
430	KC_AB1	DCAF12	-0.0293036484063858	0.955363452624055	1	pseudobulk	DESeq2	LRT	0.019831376646232
430	KC_AB1	Dci	-0.365386792940488	0.669495773595578	1	pseudobulk	DESeq2	LRT	0.174252160264986
430	KC_AB1	dco	0.298050567276195	0.148313125680534	1	pseudobulk	DESeq2	LRT	0.828820412300162
430	KC_AB1	Dcp-1	-0.813516557182265	0.0895107363985048	1	pseudobulk	DESeq2	LRT	1.04812486994692
430	KC_AB1	DCP1	-0.110811241169596	0.825900838028831	1	pseudobulk	DESeq2	LRT	0.083072093214877
430	KC_AB1	DCP2	0.257119925940189	0.141059156423249	1	pseudobulk	DESeq2	LRT	0.850598717697029
430	KC_AB1	Dcr-1	1.34862113172824	0.250209365521738	1	pseudobulk	DESeq2	LRT	0.601696438374465
430	KC_AB1	Dcr-2	-0.28447712267207	0.678705491838483	1	pseudobulk	DESeq2	LRT	0.168318636641008
430	KC_AB1	DCTN1-p150	0.366044624084447	0.348145825036098	1	pseudobulk	DESeq2	LRT	0.458238808515564
431	KC_AB1	DCTN2-p50	0.396708920342762	0.399120858881505	1	pseudobulk	DESeq2	LRT	0.39889557449507
431	KC_AB1	DCTN3-p24	0.188362436782774	0.695387320149746	1	pseudobulk	DESeq2	LRT	0.157773232604734
431	KC_AB1	DCTN4-p62	0.0623877072013537	0.90757369743522	1	pseudobulk	DESeq2	LRT	0.042118098975146
431	KC_AB1	DCTN5-p25	0.53863328046871	0.248288720366779	1	pseudobulk	DESeq2	LRT	0.605043009779855
431	KC_AB1	DCTN6-p27	0.0624480167555798	0.862238252952647	1	pseudobulk	DESeq2	LRT	0.064372713704667

431	KC_AB1	Dd	-0.107770664822099	0.567709028725102	1	pseudobulk	DESeq2	LRT	0.24587419879148
431	KC_AB1	Ddr	1.08899820779554	0.697631521651407	1	pseudobulk	DESeq2	LRT	0.156373904552
431	KC_AB1	Ddx1	-0.39713964594821	0.63530501247791	1	pseudobulk	DESeq2	LRT	0.197017718121515
431	KC_AB1	Deaf1	0.0307875536973438	0.871446032891199	1	pseudobulk	DESeq2	LRT	0.059759502809027
431	KC_AB1	DEF8	-0.387000364612723	0.541947930325979	1	pseudobulk	DESeq2	LRT	0.266042437923622
432	KC_AB1	defl	0.592431324960374	0.589478902081111	1	pseudobulk	DESeq2	LRT	0.229531734035765
432	KC_AB1	Dek	0.185219720642484	0.271253930922443	1	pseudobulk	DESeq2	LRT	0.566623959445512
432	KC_AB1	del	0.346160424670311	0.811793675977412	1	pseudobulk	DESeq2	LRT	0.090554336239737
432	KC_AB1	deltaCOP	0.0521582693122121	0.865934744068821	1	pseudobulk	DESeq2	LRT	0.062514834724735
432	KC_AB1	Den1	0.0088981018673143	0.941234142130821	1	pseudobulk	DESeq2	LRT	0.026302327702897
432	KC_AB1	DENR	0.211548815875632	0.519691279138909	1	pseudobulk	DESeq2	LRT	0.28425457094629
432	KC_AB1	Der-1	-0.119870283499725	0.75045970101643	1	pseudobulk	DESeq2	LRT	0.12467262400178
432	KC_AB1	Der-2	-0.226973073999699	0.558463911282828	1	pseudobulk	DESeq2	LRT	0.253004886357012
432	KC_AB1	Desat1	-0.0645050233023349	0.649170702117943	1	pseudobulk	DESeq2	LRT	0.187641088654227
432	KC_AB1	Det	-1.05220943051072	0.633614666162374	1	pseudobulk	DESeq2	LRT	0.198174778767564
433	KC_AB1	Df31	0.159151247859733	0.291555154152801	1	pseudobulk	DESeq2	LRT	0.535279276656469
433	KC_AB1	Dg	-0.205606506204636	0.249323628892567	1	pseudobulk	DESeq2	LRT	0.603236560600239
433	KC_AB1	Dgk	-0.568542198693811	0.0655685183599121	1	pseudobulk	DESeq2	LRT	1.18330462989738
433	KC_AB1	Dgkepsilon	-0.16485982283664	0.654899185009035	1	pseudobulk	DESeq2	LRT	0.18382555002974
433	KC_AB1	Dgp-1	0.587337503083031	0.156329917134211	1	pseudobulk	DESeq2	LRT	0.805957902319963
433	KC_AB1	dgrn	0.0368218213519368	0.932393488427645	1	pseudobulk	DESeq2	LRT	0.030400768130672
433	KC_AB1	dgt1	0.130958349917052	0.800145443376028	1	pseudobulk	DESeq2	LRT	0.096831063614957
433	KC_AB1	dgt2	-1.93786892659159	0.381996981812635	1	pseudobulk	DESeq2	LRT	0.417940068468648
433	KC_AB1	dgt6	0.149541386031591	0.937254640168289	1	pseudobulk	DESeq2	LRT	0.028142400792253
433	KC_AB1	Dh31	-0.850996402080018	0.900449987182352	1	pseudobulk	DESeq2	LRT	0.045540403770502
434	KC_AB1	Dh44-R1	-0.111342073423271	0.678121085916677	1	pseudobulk	DESeq2	LRT	0.168692751191664
434	KC_AB1	Dh44-R2	0.367996908786689	0.139723941256345	1	pseudobulk	DESeq2	LRT	0.854729172520925
434	KC_AB1	Dhap-at	-0.221884762693925	0.63962355040686	1	pseudobulk	DESeq2	LRT	0.194075554269997
434	KC_AB1	Dhc64C	-0.0602940989157771	0.780106481242023	1	pseudobulk	DESeq2	LRT	0.107846113899746
434	KC_AB1	Dhc98D	1.12049781000607	0.666276071830617	1	pseudobulk	DESeq2	LRT	0.176345783397819
434	KC_AB1	Dhit	0.101625911248748	0.406504125469695	1	pseudobulk	DESeq2	LRT	0.390935042543143
434	KC_AB1	Dhod	0.392426733116047	0.569955699770715	1	pseudobulk	DESeq2	LRT	0.244158898876285
434	KC_AB1	DhpD	-0.371468295277386	0.72031458147586	1	pseudobulk	DESeq2	LRT	0.142477793622023
434	KC_AB1	Dhpr	-0.0481124695994266	0.828746349592568	1	pseudobulk	DESeq2	LRT	0.081578371534737
434	KC_AB1	Dhx15	-0.034418063400634	0.897977635453434	1	pseudobulk	DESeq2	LRT	0.046734479502087
435	KC_AB1	dia	-0.740743158931843	0.611087382978319	1	pseudobulk	DESeq2	LRT	0.21389668299505
435	KC_AB1	Diap1	0.265250218727041	0.137032383332829	1	pseudobulk	DESeq2	LRT	0.863176788749871
435	KC_AB1	Diap2	-0.139607065312555	0.614264550576699	1	pseudobulk	DESeq2	LRT	0.211644547244587
435	KC_AB1	Dic1	-0.324610117778243	0.357224949234607	1	pseudobulk	DESeq2	LRT	0.447058216814483
435	KC_AB1	Dic3	-0.850932661185203	0.660736244265418	1	pseudobulk	DESeq2	LRT	0.179971869591347
435	KC_AB1	didum	0.179515283008461	0.568830278519519	1	pseudobulk	DESeq2	LRT	0.245017294409157
435	KC_AB1	Diedel3	-0.82542030717083	1	1	pseudobulk	DESeq2	LRT	0
435	KC_AB1	Dif	-0.132668069080724	0.412667482666032	1	pseudobulk	DESeq2	LRT	0.384399751270609
435	KC_AB1	dikar	-0.113210609485873	0.505017387479977	1	pseudobulk	DESeq2	LRT	0.296693669095965
435	KC_AB1	Dim1	-0.269161152454363	0.579658437373641	1	pseudobulk	DESeq2	LRT	0.236827838263678
436	KC_AB1	dind	-0.408014664568635	0.416056952086135	1	pseudobulk	DESeq2	LRT	0.380847216767956
436	KC_AB1	DIP-alpha	-0.621619104877798	0.428154474957921	1	pseudobulk	DESeq2	LRT	0.36839951248682
436	KC_AB1	Dip-B	-0.0053201376421719	0.95993054468122	1	pseudobulk	DESeq2	LRT	0.017760188994714
436	KC_AB1	DIP-beta	0.135795303631797	0.946582799906172	1	pseudobulk	DESeq2	LRT	0.023841391241048
436	KC_AB1	Dip-C	0.0306429186582863	0.951972334823463	1	pseudobulk	DESeq2	LRT	0.021375672422343
436	KC_AB1	DIP-epsilon	1.08899820779554	0.697631521651407	1	pseudobulk	DESeq2	LRT	0.156373904552
436	KC_AB1	DIP-eta	-1.96719661029409	0.414177861019091	1	pseudobulk	DESeq2	LRT	0.382813119093482

436	KC_AB1	DIP-gamma	-0.619148458022618	0.879843167001942	1	pseudobulk	DESeq2	LRT	0.055594734413205
436	KC_AB1	DIP-kappa	-0.128727592553353	0.334791312468568	1	pseudobulk	DESeq2	LRT	0.475225820081149
436	KC_AB1	DIP-lambda	1.15933124763035	0.353550244517355	1	pseudobulk	DESeq2	LRT	0.451548858059363
437	KC_AB1	DIP1	0.0649945893645308	0.695839109881878	1	pseudobulk	DESeq2	LRT	0.157491165228342
437	KC_AB1	DIP2	-0.195234791562287	0.449253386095708	1	pseudobulk	DESeq2	LRT	0.347508640858634
437	KC_AB1	Dis3	1.49051704380498	0.393073691324644	1	pseudobulk	DESeq2	LRT	0.405526022816619
437	KC_AB1	Dis3l2	0.016863087150943	0.96664279451535	1	pseudobulk	DESeq2	LRT	0.014733981998228
437	KC_AB1	disp	-0.015733869941088	0.945606489828796	1	pseudobulk	DESeq2	LRT	0.024289555829187
437	KC_AB1	DJ-1alpha	0.536560205000711	0.407507871420948	1	pseudobulk	DESeq2	LRT	0.38986399801194
437	KC_AB1	dj-1beta	0.154299992694777	0.589026270930464	1	pseudobulk	DESeq2	LRT	0.229865334982508
437	KC_AB1	dl	0.260349418001312	0.79621259179839	1	pseudobulk	DESeq2	LRT	0.098970958496060
437	KC_AB1	Dl	0.736294445752933	1	1	pseudobulk	DESeq2	LRT	0
437	KC_AB1	Dlc90F	-0.156902818635532	0.585983667027439	1	pseudobulk	DESeq2	LRT	0.232114488791995
438	KC_AB1	dlg1	-0.0047989873531261	0.959862073930195	1	pseudobulk	DESeq2	LRT	0.017791167829685
438	KC_AB1	Dlg5	-0.255838405745431	0.456793607274818	1	pseudobulk	DESeq2	LRT	0.340279982574594
438	KC_AB1	Dlic	0.238011589086026	0.269783006744251	1	pseudobulk	DESeq2	LRT	0.568985409406987
438	KC_AB1	Dlip1	-0.44165459062224	0.584501490411904	1	pseudobulk	DESeq2	LRT	0.233214377099506
438	KC_AB1	Dlip2	0.654380240152332	0.203937630391558	1	pseudobulk	DESeq2	LRT	0.690502631193194
438	KC_AB1	Dlip3	0.0035594742677969	0.9642227076709	1	pseudobulk	DESeq2	LRT	0.015822644995675
438	KC_AB1	dlp	-0.648626015842063	0.613344961193751	1	pseudobulk	DESeq2	LRT	0.212295198230104
438	KC_AB1	dlt	-2.69356630720211	0.0540397730192333	1	pseudobulk	DESeq2	LRT	1.26728648379326
438	KC_AB1	DMAP1	-0.0078863983534290	0.930083898434818	1	pseudobulk	DESeq2	LRT	0.031477874044080
438	KC_AB1	dmpd	-0.0845613026262344	0.796225229592523	1	pseudobulk	DESeq2	LRT	0.098964065260818
439	KC_AB1	dmrt11E	1.23644181501379	0.546580817719643	1	pseudobulk	DESeq2	LRT	0.262345613961858
439	KC_AB1	dmt	1.61194964757856	0.0808027286082652	1	pseudobulk	DESeq2	LRT	1.09257397338996
439	KC_AB1	Dmtn	0.162927818402678	0.531581803185252	1	pseudobulk	DESeq2	LRT	0.274429894003748
439	KC_AB1	DnaJ-1	0.0377985239339507	0.839959052885403	1	pseudobulk	DESeq2	LRT	0.075741884818316
439	KC_AB1	DnaJ-H	0.332995404262064	0.371014234637774	1	pseudobulk	DESeq2	LRT	0.43060942756543
439	KC_AB1	DNAlig1	-0.19846797131013	0.888540109380929	1	pseudobulk	DESeq2	LRT	0.051322963030717
439	KC_AB1	DNAlig3	0.542090619416477	0.336157839613572	1	pseudobulk	DESeq2	LRT	0.473456756010586
439	KC_AB1	DNAlig4	-1.63514875833007	0.232685153787067	1	pseudobulk	DESeq2	LRT	0.63323132549729
439	KC_AB1	DNApol-alpha50	0.11269840515639	0.977007463492936	1	pseudobulk	DESeq2	LRT	0.010102118633921
439	KC_AB1	DNApol-delta	-0.185753414263509	0.860244816470241	1	pseudobulk	DESeq2	LRT	0.065377935603462
440	KC_AB1	DNApol-eta	-0.947393391213101	0.184217387633811	1	pseudobulk	DESeq2	LRT	0.734669380669933
440	KC_AB1	DNApol-iota	-0.0595062240934329	0.925712543262756	1	pseudobulk	DESeq2	LRT	0.033523851607332
440	KC_AB1	DNApol-theta	1.19732100989811	0.526547493354687	1	pseudobulk	DESeq2	LRT	0.278562450363344
440	KC_AB1	DNApol-zeta	0.73362978992059	0.719815137332351	1	pseudobulk	DESeq2	LRT	0.142779024603383
440	KC_AB1	DNaseII	0.942080999092271	0.681532122211136	1	pseudobulk	DESeq2	LRT	0.166513670026992
440	KC_AB1	dnc	-0.138972483107295	0.131564214154758	1	pseudobulk	DESeq2	LRT	0.880862224009348
440	KC_AB1	dnd	1.08899820779554	0.878177167143508	1	pseudobulk	DESeq2	LRT	0.056417858865076
440	KC_AB1	dnk	-0.736482445015055	0.217067606129679	1	pseudobulk	DESeq2	LRT	0.66340498321911
440	KC_AB1	dnr1	0.605738917599904	0.528483106143347	1	pseudobulk	DESeq2	LRT	0.276968891092581
440	KC_AB1	dnt	-0.159942953909973	0.80908007968533	1	pseudobulk	DESeq2	LRT	0.092008491435578
441	KC_AB1	Dnz1	0.255880142360593	0.492749991163578	1	pseudobulk	DESeq2	LRT	0.307373374836666
441	KC_AB1	Doa	0.0189351562469576	0.941153656348669	1	pseudobulk	DESeq2	LRT	0.026339466204443
441	KC_AB1	dob	-0.850996402080018	0.900449987182352	1	pseudobulk	DESeq2	LRT	0.045540403770502
441	KC_AB1	dock	0.0179295623901697	0.902072776496017	1	pseudobulk	DESeq2	LRT	0.044758423482844
441	KC_AB1	dod	0.130418062980052	0.634719702078502	1	pseudobulk	DESeq2	LRT	0.197418020716807
441	KC_AB1	dom	0.031109512848456	0.904834619088323	1	pseudobulk	DESeq2	LRT	0.043430791602268
441	KC_AB1	dome	-0.111582095299442	0.837982165489758	1	pseudobulk	DESeq2	LRT	0.076765224223908
441	KC_AB1	dop	-0.132654872622474	0.725340673049881	1	pseudobulk	DESeq2	LRT	0.139457969048849
441	KC_AB1	Dop1R1	-0.0016610693576693	0.987218206527711	1	pseudobulk	DESeq2	LRT	0.005586843868362

441	KC_AB1	Dop1R2	-0.0558325452846426	0.597562596419635	1	pseudobulk	DESeq2	LRT	0.223616594386995
442	KC_AB1	Dop2R	-0.0977367207436773	0.259876828675243	1	pseudobulk	DESeq2	LRT	0.585232441649804
442	KC_AB1	dor	0.0412323277192985	0.899180236003417	1	pseudobulk	DESeq2	LRT	0.046153247470062
442	KC_AB1	DOR	0.155182816333986	0.223219001494805	1	pseudobulk	DESeq2	LRT	0.651268838887255
442	KC_AB1	dos	-0.0244827270463679	0.913864927525742	1	pseudobulk	DESeq2	LRT	0.039117989786061
442	KC_AB1	Dp	0.0331888274634676	0.880811772697719	1	pseudobulk	DESeq2	LRT	0.055116889332072
442	KC_AB1	Dp1	-0.842810318710423	0.0482697746025235	1	pseudobulk	DESeq2	LRT	1.31632472913053
442	KC_AB1	Dpck	-0.342949082951138	0.533427495957209	1	pseudobulk	DESeq2	LRT	0.272924602004369
442	KC_AB1	DPCoAC	0.248401051981236	0.760710206859612	1	pseudobulk	DESeq2	LRT	0.118780756542314
442	KC_AB1	Dph1	0.329911458952913	0.732406415997904	1	pseudobulk	DESeq2	LRT	0.135247859864223
442	KC_AB1	Dph5	0.723588012520705	0.164765270578964	1	pseudobulk	DESeq2	LRT	0.783134324103641
443	KC_AB1	Dpit47	-0.412098667230818	0.586681417547169	1	pseudobulk	DESeq2	LRT	0.231597667339372
443	KC_AB1	dpn	1.08899820779554	0.670846322504933	1	pseudobulk	DESeq2	LRT	0.173376956629912
443	KC_AB1	DppIII	-0.245019340758242	0.599110091712496	1	pseudobulk	DESeq2	LRT	0.222493364872768
443	KC_AB1	dpr1	-1.08859929459317	0.107610336140477	1	pseudobulk	DESeq2	LRT	0.968146012004366
443	KC_AB1	dpr12	-0.194972975606708	0.129959001257765	1	pseudobulk	DESeq2	LRT	0.886193634891752
443	KC_AB1	dpr13	1.68130633771206	0.562684503730219	1	pseudobulk	DESeq2	LRT	0.249735045103917
443	KC_AB1	dpr14	0.0809936549584166	0.642896576750759	1	pseudobulk	DESeq2	LRT	0.19185888673223
443	KC_AB1	dpr17	-1.62855372038815	0.482600569056906	1	pseudobulk	DESeq2	LRT	0.316412170329992
443	KC_AB1	dpr19	-0.040207155478036	0.84264701143414	1	pseudobulk	DESeq2	LRT	0.074354315163011
443	KC_AB1	dpr2	-0.109631437126432	0.90678780613409	1	pseudobulk	DESeq2	LRT	0.042494328603178
444	KC_AB1	dpr21	0.0218262015075618	0.836728144224555	1	pseudobulk	DESeq2	LRT	0.077415622820365
444	KC_AB1	dpr3	0.167227797442402	0.780900746279989	1	pseudobulk	DESeq2	LRT	0.107404162130602
444	KC_AB1	dpr4	-0.187974783025214	0.316945807132718	1	pseudobulk	DESeq2	LRT	0.499014989123646
444	KC_AB1	dpr5	-0.0688235261207868	0.821333818393061	1	pseudobulk	DESeq2	LRT	0.085480294743466
444	KC_AB1	dpr6	-0.256743745409416	0.101703621592945	1	pseudobulk	DESeq2	LRT	0.992663581887211
444	KC_AB1	dpr7	-0.0596544515506464	0.714173524071298	1	pseudobulk	DESeq2	LRT	0.146196254063246
444	KC_AB1	dpr8	0.0284492029783492	0.788175368200053	1	pseudobulk	DESeq2	LRT	0.103377141688065
444	KC_AB1	dpr9	-0.0840670783390289	0.457361080753015	1	pseudobulk	DESeq2	LRT	0.339740794546372
444	KC_AB1	dpy	-0.962792406507331	0.306392055929998	1	pseudobulk	DESeq2	LRT	0.513722499191168
444	KC_AB1	Dpy-30L1	0.0304484385543054	0.891117027807598	1	pseudobulk	DESeq2	LRT	0.050065257586465
445	KC_AB1	Drak	0.296559331472044	0.115515704972795	1	pseudobulk	DESeq2	LRT	0.93735896711941
445	KC_AB1	Drat	0.224603468996514	0.177937569974973	1	pseudobulk	DESeq2	LRT	0.749732344720133
445	KC_AB1	drd	0.11269840515639	0.977007463492936	1	pseudobulk	DESeq2	LRT	0.010102118633921
445	KC_AB1	dre4	-0.201002053904257	0.644849720031226	1	pseudobulk	DESeq2	LRT	0.190541484381115
445	KC_AB1	Dredd	-0.496684465173279	0.74350888412972	1	pseudobulk	DESeq2	LRT	0.1287138377601
445	KC_AB1	Dref	-0.0720266075310172	0.85978471327615	1	pseudobulk	DESeq2	LRT	0.065610280780809
445	KC_AB1	Drep1	0.154252567622912	0.85839293712848	1	pseudobulk	DESeq2	LRT	0.066313864409703
445	KC_AB1	Drep2	0.215739645738954	0.217939511449176	1	pseudobulk	DESeq2	LRT	0.661664026987448
445	KC_AB1	Drep3	-0.850932661185203	0.660736244265418	1	pseudobulk	DESeq2	LRT	0.179971869591347
445	KC_AB1	Drep4	-0.772055205420475	0.0934886481516146	1	pseudobulk	DESeq2	LRT	1.0292411200838
446	KC_AB1	Drice	-0.155341455533121	0.876609366075157	1	pseudobulk	DESeq2	LRT	0.057193893466422
446	KC_AB1	Drip	-0.850996402080018	0.900449987182352	1	pseudobulk	DESeq2	LRT	0.045540403770502
446	KC_AB1	drk	0.200592174004274	0.468499831757861	1	pseudobulk	DESeq2	LRT	0.329290560734891
446	KC_AB1	drl	0.0962880133290425	0.785556250459198	1	pseudobulk	DESeq2	LRT	0.104822711465034
446	KC_AB1	Drl-2	0.254565529359293	0.645102524300205	1	pseudobulk	DESeq2	LRT	0.190371258698874
446	KC_AB1	Droj2	-0.201915148585502	0.266309834144791	1	pseudobulk	DESeq2	LRT	0.574612795874393
446	KC_AB1	Dronc	0.366812360270598	0.598247853242269	1	pseudobulk	DESeq2	LRT	0.223118851137775
446	KC_AB1	drongo	0.215805293069213	0.548659690753462	1	pseudobulk	DESeq2	LRT	0.260696945629324
446	KC_AB1	drosha	-0.810770636228522	0.271127828439016	1	pseudobulk	DESeq2	LRT	0.566825904340407
446	KC_AB1	Drp1	-0.284598488964357	0.41793330838612	1	pseudobulk	DESeq2	LRT	0.378893015140564
447	KC_AB1	drpr	0.166552391896383	0.6148196339817	1	pseudobulk	DESeq2	LRT	0.211252271956683

447	KC_AB1	Drsl2	1.08899820779554	0.670846322504933	1	pseudobulk	DESeq2	LRT	0.173376956629912
447	KC_AB1	Drsl5	1.08899820779554	0.878177167143508	1	pseudobulk	DESeq2	LRT	0.056417858865076
447	KC_AB1	ds	1.19732100989811	0.526547493354687	1	pseudobulk	DESeq2	LRT	0.278562450363344
447	KC_AB1	Dscam1	-0.200724231090348	0.238914142690869	1	pseudobulk	DESeq2	LRT	0.621758141121586
447	KC_AB1	Dscam2	0.319082349992349	0.0339305516877715	1	pseudobulk	DESeq2	LRT	1.46940907896357
447	KC_AB1	Dscam3	-0.986531296966105	0.481872440684143	1	pseudobulk	DESeq2	LRT	0.317067911219143
447	KC_AB1	Dscam4	0.0050798673312084	0.948443642249937	1	pseudobulk	DESeq2	LRT	0.022988470349429
447	KC_AB1	dsd	-0.0608046597882485	0.740431621243378	1	pseudobulk	DESeq2	LRT	0.130515042325585
447	KC_AB1	dsh	-0.349657890738843	0.13006401770052	1	pseudobulk	DESeq2	LRT	0.885842834687523
448	KC_AB1	Dsor1	-0.131098688062402	0.637159211361049	1	pseudobulk	DESeq2	LRT	0.195752033950333
448	KC_AB1	Dsp1	0.314874214856528	0.00663203578899667	1	pseudobulk	DESeq2	LRT	2.17835313883266
448	KC_AB1	dsx	-0.850996402080018	0.900449987182352	1	pseudobulk	DESeq2	LRT	0.045540403770502
448	KC_AB1	Dtd	0.127820420239955	0.886868246690138	1	pseudobulk	DESeq2	LRT	0.052140894244398
448	KC_AB1	dtn	-0.119632578725961	0.44950139672112	1	pseudobulk	DESeq2	LRT	0.34726895445949
448	KC_AB1	dtr	1.09905394375445	0.509517834685999	1	pseudobulk	DESeq2	LRT	0.292840609753421
448	KC_AB1	Duba	0.138388228518932	0.657474687777468	1	pseudobulk	DESeq2	LRT	0.182120962488665
448	KC_AB1	DUBAI	-0.0082308672637344	0.975925767263368	1	pseudobulk	DESeq2	LRT	0.010583215216476
448	KC_AB1	dunk	1.18623224275774	0.259299761858322	1	pseudobulk	DESeq2	LRT	0.586197882087818
448	KC_AB1	dup	1.35623815927509	0.242578426496802	1	pseudobulk	DESeq2	LRT	0.615147825357349
449	KC_AB1	dUTPase	-1.47113868087471	0.131260804635073	1	pseudobulk	DESeq2	LRT	0.881864937824783
449	KC_AB1	dve	-0.82542030717083	1	1	pseudobulk	DESeq2	LRT	0
449	KC_AB1	dwg	-0.305214941364309	0.714418725972503	1	pseudobulk	DESeq2	LRT	0.146047170468975
449	KC_AB1	dx	1.08899820779554	0.670846322504933	1	pseudobulk	DESeq2	LRT	0.173376956629912
449	KC_AB1	dy	1.08899820779554	0.697631521651407	1	pseudobulk	DESeq2	LRT	0.156373904552
449	KC_AB1	Dyb	-0.146066581059398	0.262850201084807	1	pseudobulk	DESeq2	LRT	0.580291686402875
449	KC_AB1	Dyrk2	-0.0205983449725331	0.905816708440734	1	pseudobulk	DESeq2	LRT	0.042959672705074
449	KC_AB1	Dyrk3	-0.0419795732077366	0.774065162324644	1	pseudobulk	DESeq2	LRT	0.111222478016296
449	KC_AB1	Dys	-0.027845022532336	0.877959369790077	1	pseudobulk	DESeq2	LRT	0.056525581911961
449	KC_AB1	Dysb	1.92195783642536	0.38798652042754	1	pseudobulk	DESeq2	LRT	0.411183362564631
450	KC_AB1	dysc	-0.0474693956276998	0.597729208896608	1	pseudobulk	DESeq2	LRT	0.223495521223918
450	KC_AB1	dyw	1.8368666240224	0.42868209358391	1	pseudobulk	DESeq2	LRT	0.367864656960828
450	KC_AB1	e	1.08899820779554	0.697631521651407	1	pseudobulk	DESeq2	LRT	0.156373904552
450	KC_AB1	E(bx)	-0.135098923673176	0.413915004510949	1	pseudobulk	DESeq2	LRT	0.383088830042349
450	KC_AB1	E(Pc)	-0.0752853975114918	0.625958426826768	1	pseudobulk	DESeq2	LRT	0.203454509599922
450	KC_AB1	e(r)	0.187483705022074	0.390467274872632	1	pseudobulk	DESeq2	LRT	0.408415358554384
450	KC_AB1	E(spl)mbeta-HLH	1.08899820779554	0.878177167143508	1	pseudobulk	DESeq2	LRT	0.056417858865076
450	KC_AB1	E(var)3-9	0.336058558673573	0.568914041069763	1	pseudobulk	DESeq2	LRT	0.244953347505093
450	KC_AB1	e(y)1	0.702823485133916	0.394650337264679	1	pseudobulk	DESeq2	LRT	0.403787521714244
450	KC_AB1	e(y)2	-0.466816446350881	0.468189718393265	1	pseudobulk	DESeq2	LRT	0.329578127790396
451	KC_AB1	e(y)2b	-2.85374641634447	0.199169359211768	1	pseudobulk	DESeq2	LRT	0.70077747388823
451	KC_AB1	e(y)3	-0.0042171096874277	0.968172545253885	1	pseudobulk	DESeq2	LRT	0.014047236933505
451	KC_AB1	E(z)	-0.250107861921189	0.580844387163971	1	pseudobulk	DESeq2	LRT	0.235940202976352
451	KC_AB1	E23	-0.850996402080018	0.900449987182352	1	pseudobulk	DESeq2	LRT	0.045540403770502
451	KC_AB1	E2f1	-0.218007064223827	0.652429941357494	1	pseudobulk	DESeq2	LRT	0.185466116503681
451	KC_AB1	E2f2	0.476859382461276	0.293682684143169	1	pseudobulk	DESeq2	LRT	0.532121659206739
451	KC_AB1	Eaat1	-1.99275908811736	0.412167844949191	1	pseudobulk	DESeq2	LRT	0.384925892483647
451	KC_AB1	Eaat2	-0.0532878565861561	0.848527216151846	1	pseudobulk	DESeq2	LRT	0.071334223310942
451	KC_AB1	Eaf	-0.473080051240519	0.18179491558489	1	pseudobulk	DESeq2	LRT	0.740418267232336
451	KC_AB1	Eaf6	0.149266455773156	0.810408674451972	1	pseudobulk	DESeq2	LRT	0.091295919021834
452	KC_AB1	eag	0.218279905739446	0.0883611781494732	1	pseudobulk	DESeq2	LRT	1.05373850216679
452	KC_AB1	ear	-0.0122059057138425	0.965204391668839	1	pseudobulk	DESeq2	LRT	0.015380710723877
452	KC_AB1	eas	-0.155764859153949	0.59209110286836	1	pseudobulk	DESeq2	LRT	0.22761146484978

452	KC_AB1	east	0.124981837786208	0.482248136193561	1	pseudobulk	DESeq2	LRT	0.316729442154613
452	KC_AB1	Eato	-0.436675893145052	0.486902193313103	1	pseudobulk	DESeq2	LRT	0.31255826911475
452	KC_AB1	Eb1	-0.0621469969852972	0.712717766912794	1	pseudobulk	DESeq2	LRT	0.14708241480202
452	KC_AB1	ebd1	0.527139374995416	0.47218886647202	1	pseudobulk	DESeq2	LRT	0.325884257169976
452	KC_AB1	ebi	-0.0980158464800926	0.846721370799116	1	pseudobulk	DESeq2	LRT	0.072259478727220
452	KC_AB1	ebo	0.0995617361100388	0.833370487613079	1	pseudobulk	DESeq2	LRT	0.079161883400857
452	KC_AB1	eca	-0.0057740583116355	0.97520964546305	1	pseudobulk	DESeq2	LRT	0.010902011911145
453	KC_AB1	ecd	-0.383908070933002	0.467953241400657	1	pseudobulk	DESeq2	LRT	0.329797540122875
453	KC_AB1	Echs1	0.190208264363704	0.754037737851378	1	pseudobulk	DESeq2	LRT	0.122606918148156
453	KC_AB1	eco	-1.44925888589622	0.6315588144614	1	pseudobulk	DESeq2	LRT	0.199586199175701
453	KC_AB1	EcR	0.0489764921912086	0.703836995834155	1	pseudobulk	DESeq2	LRT	0.152527909049062
453	KC_AB1	ECSIT	0.552756661509169	0.443674723431176	1	pseudobulk	DESeq2	LRT	0.352935312746267
453	KC_AB1	ect	0.11400025262841	1	1	pseudobulk	DESeq2	LRT	0
453	KC_AB1	Ect4	1.36906298087032	0.324269065477555	1	pseudobulk	DESeq2	LRT	0.489094480044873
453	KC_AB1	ed	0.235976571228065	0.638016360724034	1	pseudobulk	DESeq2	LRT	0.19516818447466
453	KC_AB1	Edc3	0.100169103571076	0.634486473045303	1	pseudobulk	DESeq2	LRT	0.197577632425705
453	KC_AB1	Edem1	0.121313097613512	0.862465359365562	1	pseudobulk	DESeq2	LRT	0.064258339197882
454	KC_AB1	Edem2	-0.0413889927695513	0.912939230122019	1	pseudobulk	DESeq2	LRT	0.039558130352938
454	KC_AB1	Edg78E	-0.85447878276006	0.594429112578802	1	pseudobulk	DESeq2	LRT	0.225899928852609
454	KC_AB1	EDTP	0.0727879514004697	0.815863128729977	1	pseudobulk	DESeq2	LRT	0.088382693480367
454	KC_AB1	eEF1alpha1	0.023421733311508	0.918285282209842	1	pseudobulk	DESeq2	LRT	0.037022376269166
454	KC_AB1	eEF1alpha2	0.0299809533813931	0.765975623256811	1	pseudobulk	DESeq2	LRT	0.11578505132498
454	KC_AB1	eEF1beta	-0.0514898302433267	0.829022051756582	1	pseudobulk	DESeq2	LRT	0.081433917183349
454	KC_AB1	eEF1delta	0.191816442974585	0.561570900282142	1	pseudobulk	DESeq2	LRT	0.250595404749907
454	KC_AB1	eEF1gamma	-0.0507884481417281	0.849109251816836	1	pseudobulk	DESeq2	LRT	0.071036427060391
454	KC_AB1	eEF2	-0.0783687387634323	0.564621731700259	1	pseudobulk	DESeq2	LRT	0.248242410326765
454	KC_AB1	eEF5	0.01883008537789	0.889853376903743	1	pseudobulk	DESeq2	LRT	0.050681547116306
455	KC_AB1	eEFSec	0.813606094179434	0.688917500160909	1	pseudobulk	DESeq2	LRT	0.161832782983688
455	KC_AB1	Efa6	-0.113713105836596	0.712174616322672	1	pseudobulk	DESeq2	LRT	0.147413509723058
455	KC_AB1	eff	-0.0281445194343286	0.813425010195703	1	pseudobulk	DESeq2	LRT	0.089682478571459
455	KC_AB1	Efhc1.2	-2.67994893257349	0.234161893695881	1	pseudobulk	DESeq2	LRT	0.630483778367458
455	KC_AB1	Efr	-0.633064287432129	0.365235736181292	1	pseudobulk	DESeq2	LRT	0.437426735882967
455	KC_AB1	egg	-0.484836401742086	0.359639134701597	1	pseudobulk	DESeq2	LRT	0.444133055926021
455	KC_AB1	egh	0.263122550977582	0.0768066676277601	1	pseudobulk	DESeq2	LRT	1.11460107699647
455	KC_AB1	egl	0.0129286744273172	0.872647872754508	1	pseudobulk	DESeq2	LRT	0.059160965652635
455	KC_AB1	Eglp4	0.114184054362142	1	1	pseudobulk	DESeq2	LRT	0
455	KC_AB1	Egm	-0.577747700115152	0.468887439577849	1	pseudobulk	DESeq2	LRT	0.328931400861262
456	KC_AB1	Ehbp1	-0.238841673395245	0.283476484369273	1	pseudobulk	DESeq2	LRT	0.547482961928909
456	KC_AB1	eIF1	-0.0991145986600039	0.529830414796175	1	pseudobulk	DESeq2	LRT	0.275863114745827
456	KC_AB1	eIF1A	-0.0497842540306164	0.692777218935144	1	pseudobulk	DESeq2	LRT	0.159406401956974
456	KC_AB1	eIF2alpha	-0.294631183606281	0.329356346306687	1	pseudobulk	DESeq2	LRT	0.482333963801577
456	KC_AB1	eIF2Balpha	-0.0924273544060345	0.881957565864057	1	pseudobulk	DESeq2	LRT	0.054552309827837
456	KC_AB1	eIF2Bbeta	0.121407311982845	0.792442291906319	1	pseudobulk	DESeq2	LRT	0.101032354623374
456	KC_AB1	eIF2Bdelta	-0.0980679814115575	0.818141523331659	1	pseudobulk	DESeq2	LRT	0.087171564928029
456	KC_AB1	eIF2Bepsilon	0.244019896183859	0.687851551098709	1	pseudobulk	DESeq2	LRT	0.162505279053791
456	KC_AB1	eIF2beta	0.382363567986069	0.0457728565226225	1	pseudobulk	DESeq2	LRT	1.33939198393928
456	KC_AB1	eIF2Bgamma	-0.264195054648905	0.720393102895348	1	pseudobulk	DESeq2	LRT	0.142430453804795
457	KC_AB1	eIF2D	-0.642977864883503	0.235859172525852	1	pseudobulk	DESeq2	LRT	0.627347229446258
457	KC_AB1	eIF2gamma	-0.170107822107599	0.709365469933045	1	pseudobulk	DESeq2	LRT	0.149129955675918
457	KC_AB1	eIF3a	0.139599001139962	0.586134085306122	1	pseudobulk	DESeq2	LRT	0.232003022467665
457	KC_AB1	eIF3b	-0.0671914384832269	0.777570034645678	1	pseudobulk	DESeq2	LRT	0.10926048424277
457	KC_AB1	eIF3c	0.040046984693156	0.85772918879564	1	pseudobulk	DESeq2	LRT	0.066649810492263

457	KC_AB1	eIF3d1	-0.318907016448341	0.237866702526313	1	pseudobulk	DESeq2	LRT	0.623666347879432
457	KC_AB1	eIF3e	-0.0097928908916463	0.946363315729942	1	pseudobulk	DESeq2	LRT	0.023942102789297
457	KC_AB1	eIF3f1	-0.264736160576886	0.158277600209208	1	pseudobulk	DESeq2	LRT	0.800580543090111
457	KC_AB1	eIF3g1	0.3199707426737	0.154017316512235	1	pseudobulk	DESeq2	LRT	0.812430447715991
457	KC_AB1	eIF3g2	-1.62582030451705	0.456245907193616	1	pseudobulk	DESeq2	LRT	0.340801018406593
458	KC_AB1	eIF3h	-0.126632046125971	0.525173237217799	1	pseudobulk	DESeq2	LRT	0.27969741362745
458	KC_AB1	eIF3i	0.319144314563702	0.0822429385150686	1	pseudobulk	DESeq2	LRT	1.08490138086242
458	KC_AB1	eIF3j	0.146419154919715	0.573434353575303	1	pseudobulk	DESeq2	LRT	0.241516292691811
458	KC_AB1	eIF3k	0.140240125516925	0.572169938608285	1	pseudobulk	DESeq2	LRT	0.24247496344704
458	KC_AB1	eIF3l	0.066455949155837	0.700304259906547	1	pseudobulk	DESeq2	LRT	0.154713231858092
458	KC_AB1	eIF3m	-0.0757661691808702	0.649361819471814	1	pseudobulk	DESeq2	LRT	0.187513250172863
458	KC_AB1	eIF4A	0.0533344492997461	0.615060789426248	1	pseudobulk	DESeq2	LRT	0.211081958683625
458	KC_AB1	eIF4B	-0.0396618470467844	0.810752411414398	1	pseudobulk	DESeq2	LRT	0.091111750934472
458	KC_AB1	eIF4E1	-0.0151183088356928	0.932869893594288	1	pseudobulk	DESeq2	LRT	0.030178922635268
458	KC_AB1	eIF4E4	1.08899820779554	0.878177167143508	1	pseudobulk	DESeq2	LRT	0.056417858865076
459	KC_AB1	eIF4EHP	-0.0763424652176534	0.570161702009639	1	pseudobulk	DESeq2	LRT	0.24400195778152
459	KC_AB1	eIF4G1	-0.0744674545583762	0.695672392848403	1	pseudobulk	DESeq2	LRT	0.15759523089777
459	KC_AB1	eIF4G2	0.0891269781192273	0.58522544580802	1	pseudobulk	DESeq2	LRT	0.232676798859513
459	KC_AB1	eIF4H1	0.121918314357744	0.549739302295083	1	pseudobulk	DESeq2	LRT	0.259843213080257
459	KC_AB1	eIF5	-0.178022310186869	0.402213914211656	1	pseudobulk	DESeq2	LRT	0.39554290947071
459	KC_AB1	eIF5B	-0.158993774393024	0.483344544653645	1	pseudobulk	DESeq2	LRT	0.315743178778322
459	KC_AB1	eIF6	0.175779768150836	0.807893650666852	1	pseudobulk	DESeq2	LRT	0.092645805027863
459	KC_AB1	Eip55E	-0.129298758402396	0.849918456963134	1	pseudobulk	DESeq2	LRT	0.070622739450092
459	KC_AB1	Eip63E	0.0206161074569853	0.907442576578313	1	pseudobulk	DESeq2	LRT	0.042180847795122
459	KC_AB1	Eip63F-1	1.08899820779554	0.670846322504933	1	pseudobulk	DESeq2	LRT	0.173376956629912
460	KC_AB1	Eip71CD	-0.45327030367319	0.298735421510722	1	pseudobulk	DESeq2	LRT	0.524713279389187
460	KC_AB1	Eip74EF	0.929418481906961	0.0204393863793505	1	pseudobulk	DESeq2	LRT	1.68953214650515
460	KC_AB1	Eip75B	0.191781949118477	0.0978012022160243	1	pseudobulk	DESeq2	LRT	1.009655806638
460	KC_AB1	Eip78C	0.0179547500634338	0.963598924873844	1	pseudobulk	DESeq2	LRT	0.016103693233280
460	KC_AB1	Eip93F	0.131774791943373	0.142743555829334	1	pseudobulk	DESeq2	LRT	0.845443488903009
460	KC_AB1	Ekar	-2.08835743421929	0.343040066823548	1	pseudobulk	DESeq2	LRT	0.464655151723601
460	KC_AB1	elav	0.144089637797134	0.22753735644058	1	pseudobulk	DESeq2	LRT	0.642947291911685
460	KC_AB1	elB	0.745814467397354	0.0190139416621134	1	pseudobulk	DESeq2	LRT	1.7209278429106
460	KC_AB1	elgi	-0.0743211176103357	0.861176417444005	1	pseudobulk	DESeq2	LRT	0.064907871456733
460	KC_AB1	Elk	-0.0568537632167924	0.692333712173446	1	pseudobulk	DESeq2	LRT	0.159684520541628
461	KC_AB1	elm	-0.0231492401812055	0.906046674939081	1	pseudobulk	DESeq2	LRT	0.042849429093213
461	KC_AB1	EloA	0.129755812002073	0.690885683001546	1	pseudobulk	DESeq2	LRT	0.160593806965622
461	KC_AB1	EloB	0.0055110471899918	0.945690333647769	1	pseudobulk	DESeq2	LRT	0.024251050072192
461	KC_AB1	EloC	-0.219985364291681	0.257217009335557	1	pseudobulk	DESeq2	LRT	0.589700315622151
461	KC_AB1	Elp1	-0.209524966952971	0.801594048968119	1	pseudobulk	DESeq2	LRT	0.096045515665827
461	KC_AB1	Elp2	-1.7605211492876	0.272108755066944	1	pseudobulk	DESeq2	LRT	0.565257484621959
461	KC_AB1	Elp3	0.116639664292639	0.863431848697515	1	pseudobulk	DESeq2	LRT	0.063771935922415
461	KC_AB1	Elys	0.0393887460206718	0.938102180680226	1	pseudobulk	DESeq2	LRT	0.027749854490140
461	KC_AB1	ema	0.0362471196717134	0.917831782393386	1	pseudobulk	DESeq2	LRT	0.037236907766784
461	KC_AB1	emb	0.171914383783133	0.418694514696585	1	pseudobulk	DESeq2	LRT	0.378102728749585
462	KC_AB1	emc	-0.0370129361021679	0.786272757472698	1	pseudobulk	DESeq2	LRT	0.104426771369527
462	KC_AB1	EMC1	-0.695413001040916	0.0843373774269953	1	pseudobulk	DESeq2	LRT	1.07397990802465
462	KC_AB1	EMC3	-0.0410955492572728	0.910048691834817	1	pseudobulk	DESeq2	LRT	0.040935370283912
462	KC_AB1	EMC8-9	-0.0713648040381237	0.772739139630914	1	pseudobulk	DESeq2	LRT	0.111967089961208
462	KC_AB1	emp	1.08899820779554	0.697631521651407	1	pseudobulk	DESeq2	LRT	0.156373904552
462	KC_AB1	EMRE	-0.324923404661937	0.134530497462491	1	pseudobulk	DESeq2	LRT	0.871179251882534
462	KC_AB1	ena	-0.34350292499211	0.207196759774524	1	pseudobulk	DESeq2	LRT	0.683617040543829

462	KC_AB1	enc	0.11284832957596	0.406409625873626	1	pseudobulk	DESeq2	LRT	0.391036014271649
462	KC_AB1	EndoA	0.0056146855022759	0.939172837484964	1	pseudobulk	DESeq2	LRT	0.027254476468033
462	KC_AB1	EndoB	-0.325144267665378	0.334572247602626	1	pseudobulk	DESeq2	LRT	0.475510086125881
463	KC_AB1	EndoG	0.0857759977112317	0.860589759206246	1	pseudobulk	DESeq2	LRT	0.065203826213816
463	KC_AB1	EndoGI	-0.229506974219352	0.818380920637066	1	pseudobulk	DESeq2	LRT	0.087044504123155
463	KC_AB1	endos	-0.0789915759802612	0.681330915581146	1	pseudobulk	DESeq2	LRT	0.166641904379732
463	KC_AB1	Eno	-0.219023782842431	0.125692974493274	1	pseudobulk	DESeq2	LRT	0.900688996173011
463	KC_AB1	enok	-0.215743782677182	0.596971100292827	1	pseudobulk	DESeq2	LRT	0.224046692802204
463	KC_AB1	ens	0.0466499550822719	0.795313788864134	1	pseudobulk	DESeq2	LRT	0.099461487840602
463	KC_AB1	Ent1	0.77107370657056	0.186714690302832	1	pseudobulk	DESeq2	LRT	0.728821511368159
463	KC_AB1	Ent2	0.199936714808815	0.734274702178494	1	pseudobulk	DESeq2	LRT	0.134141434196575
463	KC_AB1	Ent3	-0.112128246639497	0.661366234526027	1	pseudobulk	DESeq2	LRT	0.179557981380816
463	KC_AB1	Eogt	0.304341842582023	0.740303618144667	1	pseudobulk	DESeq2	LRT	0.130590128050698
464	KC_AB1	Epac	-1.20678039963028	0.296907302374798	1	pseudobulk	DESeq2	LRT	0.527379120888026
464	KC_AB1	Epg5	0.463475865186169	0.426778306090545	1	pseudobulk	DESeq2	LRT	0.369797664656706
464	KC_AB1	Eph	0.0771588940042488	0.62439451840595	1	pseudobulk	DESeq2	LRT	0.204540918288188
464	KC_AB1	Ephrin	-0.0447601013789414	0.818802381015836	1	pseudobulk	DESeq2	LRT	0.086820903111955
464	KC_AB1	Eps-15	-0.182518734686672	0.462717904662819	1	pseudobulk	DESeq2	LRT	0.334683695343807
464	KC_AB1	epsilonCOP	-0.705439412382079	0.148085897822786	1	pseudobulk	DESeq2	LRT	0.829486297247559
464	KC_AB1	Ercc1	-0.515331394350481	0.609642989592515	1	pseudobulk	DESeq2	LRT	0.214924415873638
464	KC_AB1	eRF1	0.136723978679376	0.551627939402136	1	pseudobulk	DESeq2	LRT	0.258353745358979
464	KC_AB1	eRF3	0.0454070316908802	0.859098472966582	1	pseudobulk	DESeq2	LRT	0.065957052915093
464	KC_AB1	ergic53	-0.341446287164748	0.495326785335259	1	pseudobulk	DESeq2	LRT	0.305108186435539
465	KC_AB1	Ero1L	-0.125963132881474	0.778535161479086	1	pseudobulk	DESeq2	LRT	0.108721768334778
465	KC_AB1	ERp60	-0.116374324741925	0.732520538173968	1	pseudobulk	DESeq2	LRT	0.135180194194906
465	KC_AB1	ERR	-0.128400777239193	0.79313661832612	1	pseudobulk	DESeq2	LRT	0.100651998716661
465	KC_AB1	Es2	-0.0861438676277046	0.879065884441816	1	pseudobulk	DESeq2	LRT	0.055978574098877
465	KC_AB1	esc	-1.56855358782635	0.573026303465967	1	pseudobulk	DESeq2	LRT	0.241825442277049
465	KC_AB1	escl	-1.15876668317227	0.390364597149859	1	pseudobulk	DESeq2	LRT	0.408529576147875
465	KC_AB1	esn	0.0140980285640273	0.976044014724272	1	pseudobulk	DESeq2	LRT	0.010530597373407
465	KC_AB1	Est-P	-0.82542030717083	1	1	pseudobulk	DESeq2	LRT	0
465	KC_AB1	Est-Q	2.58753908880453	0.243878358825908	1	pseudobulk	DESeq2	LRT	0.61282673621552
465	KC_AB1	Esyt2	-0.0040675420247396	0.967410416613104	1	pseudobulk	DESeq2	LRT	0.014389240646927
466	KC_AB1	Etf-QO	1.34875264083576	0.0438427786422915	1	pseudobulk	DESeq2	LRT	1.35810192918671
466	KC_AB1	ETHR	0.171167499316231	0.866200993927579	1	pseudobulk	DESeq2	LRT	0.062381322272055
466	KC_AB1	Etl1	-0.181544536942645	0.775303954409496	1	pseudobulk	DESeq2	LRT	0.110528000921704
466	KC_AB1	Ets21C	-0.850932661185203	0.660736244265418	1	pseudobulk	DESeq2	LRT	0.179971869591347
466	KC_AB1	Ets97D	0.424313969863552	0.5082411516337	1	pseudobulk	DESeq2	LRT	0.293930173595205
466	KC_AB1	euc	0.0152798113134633	0.972346859796394	1	pseudobulk	DESeq2	LRT	0.012178784020206
466	KC_AB1	Evi5	0.0207090327255255	0.905258796433595	1	pseudobulk	DESeq2	LRT	0.043227246434717
466	KC_AB1	ewg	0.199630982626123	0.400040084765242	1	pseudobulk	DESeq2	LRT	0.397896489371695
466	KC_AB1	ex	0.112935832476632	0.940353958317081	1	pseudobulk	DESeq2	LRT	0.026708642986417
466	KC_AB1	exd	-0.0272335778864833	0.897801979197421	1	pseudobulk	DESeq2	LRT	0.046819441534532
467	KC_AB1	Exd2	-0.779291886055509	0.663007185306945	1	pseudobulk	DESeq2	LRT	0.17848176492517
467	KC_AB1	Exn	-0.58661592452754	0.0898970090708152	1	pseudobulk	DESeq2	LRT	1.04625475727288
467	KC_AB1	Exo70	0.606718758354611	0.324262515585731	1	pseudobulk	DESeq2	LRT	0.489103252422223
467	KC_AB1	Exo84	-0.199188977841833	0.681864720414815	1	pseudobulk	DESeq2	LRT	0.166301779309781
467	KC_AB1	exp	4.03660590748071	0.271189362610558	1	pseudobulk	DESeq2	LRT	0.566727349650625
467	KC_AB1	ey	-0.118515832200882	0.292514661476172	1	pseudobulk	DESeq2	LRT	0.533852361244634
467	KC_AB1	f-cup	0.173502847209402	0.691006416534438	1	pseudobulk	DESeq2	LRT	0.160517919843513
467	KC_AB1	fabp	0.212143825747567	0.600332505528861	1	pseudobulk	DESeq2	LRT	0.221608140752704
467	KC_AB1	Fadd	0.411745953290541	0.770578549020229	1	pseudobulk	DESeq2	LRT	0.113183084827057

467	KC_AB1	faf	-0.0560952467208057	0.790538097529677	1	pseudobulk	DESeq2	LRT	0.102077195752992
468	KC_AB1	Faf2	-0.0158866583830012	0.91490008349737	1	pseudobulk	DESeq2	LRT	0.038626332769779
468	KC_AB1	Fak	-0.0384508085849001	0.860215095156911	1	pseudobulk	DESeq2	LRT	0.065392940663781
468	KC_AB1	fal	-0.322100137516813	0.671867470766145	1	pseudobulk	DESeq2	LRT	0.172716385266497
468	KC_AB1	FAM21	0.222862832021144	0.684296647986759	1	pseudobulk	DESeq2	LRT	0.164755587381183
468	KC_AB1	Fancm	-1.4722520190256	0.507123370735961	1	pseudobulk	DESeq2	LRT	0.29488637456791
468	KC_AB1	fand	0.237672466775067	0.746007149559641	1	pseudobulk	DESeq2	LRT	0.127257010329715
468	KC_AB1	FarO	-1.82709236256241	0.44317674354685	1	pseudobulk	DESeq2	LRT	0.353423038023035
468	KC_AB1	Fas1	-0.0558053811574431	0.646137423921999	1	pseudobulk	DESeq2	LRT	0.189675104135309
468	KC_AB1	Fas2	0.041127327199131	0.694431061649369	1	pseudobulk	DESeq2	LRT	0.158370861562532
468	KC_AB1	Fas3	-2.39936861215526	0.0721530173083759	1	pseudobulk	DESeq2	LRT	1.14174550278285
469	KC_AB1	FASN1	-0.287052365453035	0.335183284501286	1	pseudobulk	DESeq2	LRT	0.474717647648755
469	KC_AB1	FASN3	-0.859869546740353	0.687511302578539	1	pseudobulk	DESeq2	LRT	0.162720157705494
469	KC_AB1	Fatp1	-0.110483235616471	0.785361671604952	1	pseudobulk	DESeq2	LRT	0.104930297637347
469	KC_AB1	fax	0.258608083053479	0.0166677181798388	1	pseudobulk	DESeq2	LRT	1.77812385126585
469	KC_AB1	fbl	-0.197310291354174	0.715694626225781	1	pseudobulk	DESeq2	LRT	0.145272243662475
469	KC_AB1	Fbl6	-0.154978197096825	0.642188060892183	1	pseudobulk	DESeq2	LRT	0.192337772791382
469	KC_AB1	fbp	-0.793241477410878	0.662253224746005	1	pseudobulk	DESeq2	LRT	0.178975918291606
469	KC_AB1	Fbp2	-0.850932661185203	0.660736244265418	1	pseudobulk	DESeq2	LRT	0.179971869591347
469	KC_AB1	Fbw5	-0.694402247656641	0.55036203008819	1	pseudobulk	DESeq2	LRT	0.259351536058878
469	KC_AB1	Fbxl4	-0.106728750004132	0.849344690685754	1	pseudobulk	DESeq2	LRT	0.070916023675061
470	KC_AB1	FBXO11	-0.0196509445868938	0.946364557481428	1	pseudobulk	DESeq2	LRT	0.02394153293895
470	KC_AB1	Fcp1	-0.358525027046215	0.364084364626718	1	pseudobulk	DESeq2	LRT	0.438797971168063
470	KC_AB1	Fcp3C	-0.850996402080018	0.900449987182352	1	pseudobulk	DESeq2	LRT	0.045540403770502
470	KC_AB1	Fdh	0.832682857980659	0.578316565835221	1	pseudobulk	DESeq2	LRT	0.237834367184938
470	KC_AB1	fdl	0.13956282485092	0.549590042906317	1	pseudobulk	DESeq2	LRT	0.259961144125389
470	KC_AB1	Fdx1	0.289256336427593	0.570840590803935	1	pseudobulk	DESeq2	LRT	0.243485153049683
470	KC_AB1	Fdx2	0.318423659722267	0.563961309418583	1	pseudobulk	DESeq2	LRT	0.248750689779754
470	KC_AB1	FeCH	-0.401065230722041	0.546787759077397	1	pseudobulk	DESeq2	LRT	0.262181216534073
470	KC_AB1	Fem-1	-0.240960845867818	0.509637127787765	1	pseudobulk	DESeq2	LRT	0.292738940551052
470	KC_AB1	Femcoat	1.08899820779554	0.878177167143508	1	pseudobulk	DESeq2	LRT	0.056417858865076
471	KC_AB1	Fen1	0.135795303631797	0.946582799906172	1	pseudobulk	DESeq2	LRT	0.023841391241048
471	KC_AB1	fend	-0.183537870348565	0.167941292092997	1	pseudobulk	DESeq2	LRT	0.774842509797925
471	KC_AB1	feo	1.08899820779554	0.697631521651407	1	pseudobulk	DESeq2	LRT	0.156373904552
471	KC_AB1	FER	0.108644062800065	0.949090645333146	1	pseudobulk	DESeq2	LRT	0.022692307184905
471	KC_AB1	Fer1HCH	0.0746636338272442	0.590138590091188	1	pseudobulk	DESeq2	LRT	0.229045985232746
471	KC_AB1	Fer2LCH	0.0804568152668629	0.537701426822205	1	pseudobulk	DESeq2	LRT	0.269458811095527
471	KC_AB1	fest	0.703864109154222	0.125726491209714	1	pseudobulk	DESeq2	LRT	0.90057320462086
471	KC_AB1	Fgop2	-0.44851858851249	0.436417563899169	1	pseudobulk	DESeq2	LRT	0.360097779277571
471	KC_AB1	fh	-0.6290304967593	0.417585322742254	1	pseudobulk	DESeq2	LRT	0.379254774287567
471	KC_AB1	Fhos	-2.50879299177917	0.0947294479397957	1	pseudobulk	DESeq2	LRT	1.02351499365015
472	KC_AB1	Fib	-0.0520416769699924	0.925574254973088	1	pseudobulk	DESeq2	LRT	0.033588733873315
472	KC_AB1	Fibp	-0.20534921082355	0.485898608747449	1	pseudobulk	DESeq2	LRT	0.313454344431892
472	KC_AB1	Fic	-0.0354652657180172	0.950845622636839	1	pseudobulk	DESeq2	LRT	0.021889988508770
472	KC_AB1	fid	0.55501556616015	0.116546315494197	1	pseudobulk	DESeq2	LRT	0.933501451751576
472	KC_AB1	fidipidine	-0.131223907515208	0.899077233042613	1	pseudobulk	DESeq2	LRT	0.046202999653290
472	KC_AB1	Fie	-0.0828584518940655	0.815360749240576	1	pseudobulk	DESeq2	LRT	0.088650198940424
472	KC_AB1	Fife	-0.116978260049562	0.318596815805963	1	pseudobulk	DESeq2	LRT	0.496758569039628
472	KC_AB1	FIG4	-0.922175227142389	0.380947491115179	1	pseudobulk	DESeq2	LRT	0.419134882304702
472	KC_AB1	Fili	-2.67928706258793	0.23532931449478	1	pseudobulk	DESeq2	LRT	0.628323970272578
472	KC_AB1	Fim	0.042660493415371	0.793343746230116	1	pseudobulk	DESeq2	LRT	0.100538597368274
473	KC_AB1	Fip1	-0.164993770708885	0.68506163437053	1	pseudobulk	DESeq2	LRT	0.164270353671285

473	KC_AB1	fipi	-0.466760828749815	0.0750709627597648	1	pseudobulk	DESeq2	LRT	1.12452801441772
473	KC_AB1	FipoQ	1.08899820779554	0.878177167143508	1	pseudobulk	DESeq2	LRT	0.056417858865076
473	KC_AB1	Fis1	-0.058118273614734	0.736863564938596	1	pseudobulk	DESeq2	LRT	0.13261291713349
473	KC_AB1	Fit1	-0.123569175622761	0.731088354790971	1	pseudobulk	DESeq2	LRT	0.13603013374066
473	KC_AB1	Fitm	-0.494565952842654	0.533695121072597	1	pseudobulk	DESeq2	LRT	0.27270676740614
473	KC_AB1	Fkbp14	1.88952324269138	0.456767015650974	1	pseudobulk	DESeq2	LRT	0.34030526517809
473	KC_AB1	Fkbp39	0.0547565513860214	0.887691213444455	1	pseudobulk	DESeq2	LRT	0.05173807883714
473	KC_AB1	Fkbp59	-0.170810116717686	0.675200766317956	1	pseudobulk	DESeq2	LRT	0.170567073480341
473	KC_AB1	fl(2)d	-0.025869880927352	0.882177371145669	1	pseudobulk	DESeq2	LRT	0.05444408656283
474	KC_AB1	Flacc	-0.284550026526242	0.362857695831665	1	pseudobulk	DESeq2	LRT	0.44026366155368
474	KC_AB1	FLASH	-0.199263722327497	0.77901981216052	1	pseudobulk	DESeq2	LRT	0.10845149713799
474	KC_AB1	flfl	0.21741085390428	0.3126394787544	1	pseudobulk	DESeq2	LRT	0.50495618203462
474	KC_AB1	fliI	-0.198758404722764	0.716449933744351	1	pseudobulk	DESeq2	LRT	0.14481415314977
474	KC_AB1	fln	1.08899820779554	0.878177167143508	1	pseudobulk	DESeq2	LRT	0.056417858865076
474	KC_AB1	Flo1	0.0102137817093204	0.949626798487666	1	pseudobulk	DESeq2	LRT	0.02244703808137
474	KC_AB1	Flo2	0.0162139705877306	0.914452860205414	1	pseudobulk	DESeq2	LRT	0.03883867737218
474	KC_AB1	flr	0.003116963697728	0.996557262408974	1	pseudobulk	DESeq2	LRT	0.00149774158592
474	KC_AB1	flw	-0.093659552247954	0.522781010421009	1	pseudobulk	DESeq2	LRT	0.28168019618550
474	KC_AB1	Fmo-1	-0.850996402080018	0.900449987182352	1	pseudobulk	DESeq2	LRT	0.04554040377050
475	KC_AB1	Fmr1	-0.199530743586488	0.416443548736395	1	pseudobulk	DESeq2	LRT	0.38044386132532
475	KC_AB1	FMRFaR	-0.596280969352969	0.27215342626403	1	pseudobulk	DESeq2	LRT	0.56518619378050
475	KC_AB1	fmt	-0.026479457007633	0.892503201312508	1	pseudobulk	DESeq2	LRT	0.04939021744561
475	KC_AB1	fne	0.121941111804319	0.242499963230366	1	pseudobulk	DESeq2	LRT	0.61528832291264
475	KC_AB1	fng	-0.82542030717083	1	1	pseudobulk	DESeq2	LRT	0
475	KC_AB1	Fnta	-0.58258998936233	0.254675762285504	1	pseudobulk	DESeq2	LRT	0.59401238525445
475	KC_AB1	fog	0.490585233042531	0.459263694868924	1	pseudobulk	DESeq2	LRT	0.33793788452171
475	KC_AB1	foi	0.296688327200697	0.409347944885425	1	pseudobulk	DESeq2	LRT	0.38790738562659
475	KC_AB1	fok	0.238639070927176	0.112773857260205	1	pseudobulk	DESeq2	LRT	0.94779156492279
475	KC_AB1	fon	1.08899820779554	0.878177167143508	1	pseudobulk	DESeq2	LRT	0.056417858865076
476	KC_AB1	for	-1.6747012584303	0.563129890257477	1	pseudobulk	DESeq2	LRT	0.24939142021827
476	KC_AB1	form3	1.08899820779554	0.878177167143508	1	pseudobulk	DESeq2	LRT	0.056417858865076
476	KC_AB1	FoxK	0.236011933912219	0.185682663333545	1	pseudobulk	DESeq2	LRT	0.73122864321798
476	KC_AB1	FoxP	0.298211351362847	0.051119517756768	1	pseudobulk	DESeq2	LRT	1.29141325180902
476	KC_AB1	Fpgs	1.06888150341759	0.59077249842156	1	pseudobulk	DESeq2	LRT	0.22857973012000
476	KC_AB1	Fpps	-0.447010249045803	0.634144199081616	1	pseudobulk	DESeq2	LRT	0.19781197595940
476	KC_AB1	fra	2.40036907428623	0.0238621479905755	1	pseudobulk	DESeq2	LRT	1.62229046517253
476	KC_AB1	fray	-0.129033962891919	0.846647827751419	1	pseudobulk	DESeq2	LRT	0.07229720155794
476	KC_AB1	fred	1.08899820779554	0.670846322504933	1	pseudobulk	DESeq2	LRT	0.17337695662991
476	KC_AB1	FRG1	0.254249504050895	0.629689155531881	1	pseudobulk	DESeq2	LRT	0.20087378602653
477	KC_AB1	frj	-0.3780996248877	0.21658303949483	1	pseudobulk	DESeq2	LRT	0.66437555575412
477	KC_AB1	Frl	-0.074675200476333	0.699576716435832	1	pseudobulk	DESeq2	LRT	0.15516465329814
477	KC_AB1	Frq1	-0.691870405962795	0.137286379514775	1	pseudobulk	DESeq2	LRT	0.862372547941681
477	KC_AB1	Frq2	0.209277078048543	0.584025399856948	1	pseudobulk	DESeq2	LRT	0.23356826456939
477	KC_AB1	frtz	-0.089882241693436	0.914553023616489	1	pseudobulk	DESeq2	LRT	0.03879111009299
477	KC_AB1	fru	-1.87842684310973	0.010843209884666	1	pseudobulk	DESeq2	LRT	1.96484213580118
477	KC_AB1	fry	-0.044982478658941	0.885095815152608	1	pseudobulk	DESeq2	LRT	0.05300971264720
477	KC_AB1	fs(1)h	0.102452069926061	0.268274058883115	1	pseudobulk	DESeq2	LRT	0.57142131998029
477	KC_AB1	fs(1)K10	0.150697060117856	0.81861089933515	1	pseudobulk	DESeq2	LRT	0.08692247727003
477	KC_AB1	Fs(2)Ket	-0.114731582091279	0.301949184802519	1	pseudobulk	DESeq2	LRT	0.52006613855523
478	KC_AB1	fs(2)ltoPP43	-0.076282691304055	0.866307868195677	1	pseudobulk	DESeq2	LRT	0.06232774112627
478	KC_AB1	fsd	0.0130375186846266	0.989110907826917	1	pseudobulk	DESeq2	LRT	0.00475500874994
478	KC_AB1	Fsn	-0.473265664486729	0.223610707966865	1	pseudobulk	DESeq2	LRT	0.65050740338143

478	KC_AB1	fu	0.154403438237345	0.845135388153622	1	pseudobulk	DESeq2	LRT	0.073073712804007
478	KC_AB1	FucT6	-0.463889206845042	0.227124495016834	1	pseudobulk	DESeq2	LRT	0.643736025282791
478	KC_AB1	FucTA	-0.441038926746026	0.228165135018314	1	pseudobulk	DESeq2	LRT	0.641750717612666
478	KC_AB1	FucTB	-0.529088509345319	0.616503938929442	1	pseudobulk	DESeq2	LRT	0.210064144291516
478	KC_AB1	FucTC	-0.850996402080018	0.900449987182352	1	pseudobulk	DESeq2	LRT	0.045540403770502
478	KC_AB1	Fum1	-0.0115802377235919	0.956886602385792	1	pseudobulk	DESeq2	LRT	0.019139526043539
478	KC_AB1	Fum2	0.111831901354254	1	1	pseudobulk	DESeq2	LRT	0
479	KC_AB1	Fur1	-0.0158359375397757	0.897971986799358	1	pseudobulk	DESeq2	LRT	0.046737211404213
479	KC_AB1	Fur2	0.182438519828479	0.531190891750663	1	pseudobulk	DESeq2	LRT	0.274749380357547
479	KC_AB1	fuss	0.0537593469077913	0.948198438824073	1	pseudobulk	DESeq2	LRT	0.023100764067313
479	KC_AB1	futsch	-0.000749569982815З	0.985250379932563	1	pseudobulk	DESeq2	LRT	0.006453388989551
479	KC_AB1	fw	-0.82542030717083	1	1	pseudobulk	DESeq2	LRT	0
479	KC_AB1	fwd	-0.0131338865389472	0.920831575372964	1	pseudobulk	DESeq2	LRT	0.035819797137680
479	KC_AB1	fwe	-0.120371269124477	0.414935314582599	1	pseudobulk	DESeq2	LRT	0.382019601382927
479	KC_AB1	fws	-0.497898963493722	0.414039833874236	1	pseudobulk	DESeq2	LRT	0.382957874339084
479	KC_AB1	fy	0.0858551531072512	0.91027743668332	1	pseudobulk	DESeq2	LRT	0.040826222121465
479	KC_AB1	fz	-0.0162095138229792	0.895458509198492	1	pseudobulk	DESeq2	LRT	0.047954532253359
480	KC_AB1	fz2	-0.0498902117141806	0.855412102263428	1	pseudobulk	DESeq2	LRT	0.067824609691734
480	KC_AB1	fz3	-0.333529190359999	0.755109376309679	1	pseudobulk	DESeq2	LRT	0.121990136996877
480	KC_AB1	fz4	-0.598355880690337	0.513862871253366	1	pseudobulk	DESeq2	LRT	0.289152760778169
480	KC_AB1	fzo	-0.82542030717083	1	1	pseudobulk	DESeq2	LRT	0
480	KC_AB1	fzr	-0.0979960822012483	0.68936207164662	1	pseudobulk	DESeq2	LRT	0.16155261493404
480	KC_AB1	fzy	2.21745641838459	0.396737788462723	1	pseudobulk	DESeq2	LRT	0.401496431894786
480	KC_AB1	g	-0.043162400053331	0.773837121298414	1	pseudobulk	DESeq2	LRT	0.11135044082261
480	KC_AB1	G6P	-0.372127483827144	0.645694186477619	1	pseudobulk	DESeq2	LRT	0.189973123756862
480	KC_AB1	G9a	0.0983381621323472	0.657898336145973	1	pseudobulk	DESeq2	LRT	0.181841211951403
480	KC_AB1	GAA1	-0.199861479487712	0.694746321378613	1	pseudobulk	DESeq2	LRT	0.158173744096037
481	KC_AB1	GABA-B-R1	0.300869262889533	0.0470917385738043	1	pseudobulk	DESeq2	LRT	1.3270552755995
481	KC_AB1	GABA-B-R2	-0.0933105782681657	0.650888475681302	1	pseudobulk	DESeq2	LRT	0.186493417799529
481	KC_AB1	Gabat	0.0917438603529261	0.687794996358863	1	pseudobulk	DESeq2	LRT	0.162540987952272
481	KC_AB1	GABPI	-0.185377298905914	0.616767163502249	1	pseudobulk	DESeq2	LRT	0.209878756048846
481	KC_AB1	Gad1	0.113316967542732	1	1	pseudobulk	DESeq2	LRT	0
481	KC_AB1	Gadd45	-3.69724907782354	0.0688641167018797	1	pseudobulk	DESeq2	LRT	1.16200701869975
481	KC_AB1	Gale	0.499456078163389	0.403266456029326	1	pseudobulk	DESeq2	LRT	0.394407901392417
481	KC_AB1	galectin	0.166391193971918	0.526950166254458	1	pseudobulk	DESeq2	LRT	0.278230454130977
481	KC_AB1	Galk	-0.215076314536203	0.806786855787681	1	pseudobulk	DESeq2	LRT	0.093241185949331
481	KC_AB1	galla-1	-0.591009200873434	0.140031067880552	1	pseudobulk	DESeq2	LRT	0.853775599234518
482	KC_AB1	galla-2	-0.512817798959131	0.193154178884894	1	pseudobulk	DESeq2	LRT	0.714095891469368
482	KC_AB1	Galphaf	1.16523413356038	0.63328025468345	1	pseudobulk	DESeq2	LRT	0.198404052815419
482	KC_AB1	Galphao	-0.0801264087760482	0.435522012310523	1	pseudobulk	DESeq2	LRT	0.360989889833351
482	KC_AB1	Galphaq	-0.0351501596150187	0.823078428883828	1	pseudobulk	DESeq2	LRT	0.084558780087102
482	KC_AB1	Galphas	-0.0019826583258303	0.95461491149679	1	pseudobulk	DESeq2	LRT	0.020171786049605
482	KC_AB1	Galt	-0.0953763887219622	0.874839192818673	1	pseudobulk	DESeq2	LRT	0.058071768794438
482	KC_AB1	GalT1	-0.553243531197342	0.464623950157357	1	pseudobulk	DESeq2	LRT	0.332898407206698
482	KC_AB1	gammaCOP	0.16129521034688	0.750573524714328	1	pseudobulk	DESeq2	LRT	0.124606758698928
482	KC_AB1	gammaSnap1	-0.375598640507856	0.0514037126604811	1	pseudobulk	DESeq2	LRT	1.28900551272202
482	KC_AB1	gammaSnap2	-0.610874715247911	0.128213860343673	1	pseudobulk	DESeq2	LRT	0.89206502360503
483	KC_AB1	gammaTub23C	-0.451539532394989	0.383076383177411	1	pseudobulk	DESeq2	LRT	0.416714621631873
483	KC_AB1	GAPcenA	0.196485196343349	0.501450991891016	1	pseudobulk	DESeq2	LRT	0.299771505298268
483	KC_AB1	Gapdh1	-0.189094575162225	0.455604461047473	1	pseudobulk	DESeq2	LRT	0.341412032181137
483	KC_AB1	Gapdh2	-0.240872560099229	0.16833248567504	1	pseudobulk	DESeq2	LRT	0.773832063537118
483	KC_AB1	GAPsec	-0.294176420370501	0.647608240914748	1	pseudobulk	DESeq2	LRT	0.188687633419237

483	KC_AB1	Gapvd1	-0.273532134879358	0.418784311609322	1	pseudobulk	DESeq2	LRT	0.37800959611717
483	KC_AB1	Gart	1.6730745812192	0.494924933360665	1	pseudobulk	DESeq2	LRT	0.305460666721901
483	KC_AB1	garz	0.589248822952551	0.16795641003965	1	pseudobulk	DESeq2	LRT	0.774803416700402
483	KC_AB1	Gas41	0.167433904944627	0.720969775220808	1	pseudobulk	DESeq2	LRT	0.142082941563459
483	KC_AB1	Gas8	1.16523413356038	0.63328025468345	1	pseudobulk	DESeq2	LRT	0.198404052815419
484	KC_AB1	Gasz	0.0333887759675067	0.920108665439976	1	pseudobulk	DESeq2	LRT	0.036160879160863
484	KC_AB1	Gat	0.38066359041065	0.867123912282251	1	pseudobulk	DESeq2	LRT	0.061918837269822
484	KC_AB1	GatA	-0.340222559590003	0.593918660057151	1	pseudobulk	DESeq2	LRT	0.226273029610091
484	KC_AB1	GATAd	0.110101885291719	0.667763368053947	1	pseudobulk	DESeq2	LRT	0.17537740901389
484	KC_AB1	GatB	0.309280274911316	0.707000167080448	1	pseudobulk	DESeq2	LRT	0.150580483569285
484	KC_AB1	gb	0.0305058368326915	0.968220022579583	1	pseudobulk	DESeq2	LRT	0.014025940486790
484	KC_AB1	Gba1a	-0.82542030717083	1	1	pseudobulk	DESeq2	LRT	0
484	KC_AB1	gbb	0.143496810234727	0.913040183696488	1	pseudobulk	DESeq2	LRT	0.039510108365359
484	KC_AB1	Gbeta13F	-0.0921387153468904	0.327601464884903	1	pseudobulk	DESeq2	LRT	0.484654164939678
484	KC_AB1	Gbeta5	-0.0579439820802854	0.785679908148536	1	pseudobulk	DESeq2	LRT	0.104754352737492
485	KC_AB1	Gbs-76A	-1.16285098436786	0.216037021940614	1	pseudobulk	DESeq2	LRT	0.665471818076978
485	KC_AB1	GC	0.534670251215794	0.0160775694060256	1	pseudobulk	DESeq2	LRT	1.7937796069146
485	KC_AB1	GC1	-0.0122082359840589	0.942365600852716	1	pseudobulk	DESeq2	LRT	0.025780575307938
485	KC_AB1	GCC185	-0.272909704912698	0.603099589660277	1	pseudobulk	DESeq2	LRT	0.21961096701626
485	KC_AB1	GCC88	0.119292594692616	0.838659006247659	1	pseudobulk	DESeq2	LRT	0.076414584829854
485	KC_AB1	gce	0.0276499702843025	0.863059872877981	1	pseudobulk	DESeq2	LRT	0.063959075016458
485	KC_AB1	GckIII	0.107790306138873	0.754314911007135	1	pseudobulk	DESeq2	LRT	0.122447307232866
485	KC_AB1	gcl	0.29718256538102	0.32681581443283	1	pseudobulk	DESeq2	LRT	0.485696936366251
485	KC_AB1	Gclc	-0.011216660341684	0.929556337792599	1	pseudobulk	DESeq2	LRT	0.031724283728564
485	KC_AB1	Gclm	-0.0987373822881026	0.838673353681407	1	pseudobulk	DESeq2	LRT	0.076407155161823
486	KC_AB1	Gcn2	0.194466456460639	0.767724272910055	1	pseudobulk	DESeq2	LRT	0.114794728216586
486	KC_AB1	Gcn5	0.092471536194386	0.871271736340447	1	pseudobulk	DESeq2	LRT	0.059846374053235
486	KC_AB1	GCS1	0.626199440526468	0.337827677045516	1	pseudobulk	DESeq2	LRT	0.471304773072435
486	KC_AB1	GCS2alpha	-0.351050376509561	0.129319347739418	1	pseudobulk	DESeq2	LRT	0.888336494548308
486	KC_AB1	GCS2beta	0.218634197631235	0.676800221777459	1	pseudobulk	DESeq2	LRT	0.169539507657108
486	KC_AB1	Gdap2	0.089742926077654	0.384291356617993	1	pseudobulk	DESeq2	LRT	0.415339383465198
486	KC_AB1	Gdh	-0.0685744725849838	0.680071837147453	1	pseudobulk	DESeq2	LRT	0.167445209604201
486	KC_AB1	Gdi	-0.161200287194181	0.34007985271173	1	pseudobulk	DESeq2	LRT	0.468419096133432
486	KC_AB1	gdl	0.205471478125546	0.823125531305072	1	pseudobulk	DESeq2	LRT	0.084533927368812
486	KC_AB1	Gdn1	-0.0754309069223191	0.891170201461728	1	pseudobulk	DESeq2	LRT	0.050039343666195
487	KC_AB1	Ge-1	-0.0973017533426053	0.823879169116384	1	pseudobulk	DESeq2	LRT	0.084136477664429
487	KC_AB1	GEFmeso	-0.321104735766149	0.839796900271028	1	pseudobulk	DESeq2	LRT	0.075825732696021
487	KC_AB1	gek	-0.440450617574679	0.396582978035215	1	pseudobulk	DESeq2	LRT	0.401665930332493
487	KC_AB1	Gel	-0.151845111369959	0.841599838082183	1	pseudobulk	DESeq2	LRT	0.074894356745494
487	KC_AB1	Gem2	-0.13809686869765	0.825640913784817	1	pseudobulk	DESeq2	LRT	0.083208794168027
487	KC_AB1	Gem3	1.17886577340032	0.284993933096111	1	pseudobulk	DESeq2	LRT	0.545164385082458
487	KC_AB1	geminin	0.184798011909705	0.718670258125973	1	pseudobulk	DESeq2	LRT	0.14347032787046
487	KC_AB1	Gen	1.08899820779554	0.697631521651407	1	pseudobulk	DESeq2	LRT	0.156373904552
487	KC_AB1	Gfat1	-1.6747012584303	0.563129890257477	1	pseudobulk	DESeq2	LRT	0.249391420218277
487	KC_AB1	Gfat2	-0.710142337869308	0.100881063821615	1	pseudobulk	DESeq2	LRT	0.996190346642932
488	KC_AB1	Gfrl	-0.219691509018388	0.0578017142575001	1	pseudobulk	DESeq2	LRT	1.23805928127613
488	KC_AB1	gfzf	-0.539240951583327	0.411625843091533	1	pseudobulk	DESeq2	LRT	0.385497366757714
488	KC_AB1	Gga	-0.136679435998588	0.837375428003283	1	pseudobulk	DESeq2	LRT	0.077079787222140
488	KC_AB1	Ggamma1	-0.0567345051837142	0.596160076196767	1	pseudobulk	DESeq2	LRT	0.224637111275542
488	KC_AB1	Ggamma30A	0.100183291133467	0.404094388915019	1	pseudobulk	DESeq2	LRT	0.393517179946274
488	KC_AB1	ghi	0.338512620376488	0.18928659342295	1	pseudobulk	DESeq2	LRT	0.722880144665999
488	KC_AB1	gig	0.0142040444968942	0.975846106146208	1	pseudobulk	DESeq2	LRT	0.010618666473967

488	KC_AB1	GIIIspla2	0.0713989800250509	0.87775213757372	1	pseudobulk	DESeq2	LRT	0.056628104231918
488	KC_AB1	GILT1	-0.508390840396301	0.492954449223608	1	pseudobulk	DESeq2	LRT	0.307193209251757
488	KC_AB1	GILT2	1.08899820779554	0.697631521651407	1	pseudobulk	DESeq2	LRT	0.156373904552
489	KC_AB1	GILT3	1.68130633771206	0.562684503730219	1	pseudobulk	DESeq2	LRT	0.249735045103917
489	KC_AB1	Gip	-0.604990252529976	0.725821804414012	1	pseudobulk	DESeq2	LRT	0.139169989298251
489	KC_AB1	Girdin	0.119412363534099	0.772625661506121	1	pseudobulk	DESeq2	LRT	0.112030871566001
489	KC_AB1	gish	-0.129342748073984	0.245873666881848	1	pseudobulk	DESeq2	LRT	0.609287981795237
489	KC_AB1	Git	-0.136723705334682	0.825521148163243	1	pseudobulk	DESeq2	LRT	0.083271796522383
489	KC_AB1	Gk1	0.343919594674957	0.521980895949537	1	pseudobulk	DESeq2	LRT	0.282345391510236
489	KC_AB1	Gk2	-1.00026176406151	0.52880523164933	1	pseudobulk	DESeq2	LRT	0.276704256878702
489	KC_AB1	gkt	-0.168161925770529	0.807215623832633	1	pseudobulk	DESeq2	LRT	0.093010440826358
489	KC_AB1	GLaz	1.95275942187961	0.438420007953907	1	pseudobulk	DESeq2	LRT	0.358109634392079
489	KC_AB1	GlcAT-I	-0.0288765661149711	0.884018320976381	1	pseudobulk	DESeq2	LRT	0.053538734289534
490	KC_AB1	GlcAT-P	-0.0285806886135793	0.799813646899172	1	pseudobulk	DESeq2	LRT	0.097011189946880
490	KC_AB1	GlcAT-S	0.352446286459907	0.371603729527488	1	pseudobulk	DESeq2	LRT	0.429919935945673
490	KC_AB1	GlcT	0.167925169575073	0.829633015194693	1	pseudobulk	DESeq2	LRT	0.081113973552990
490	KC_AB1	Gld	-2.00068388878968	0.376599576446518	1	pseudobulk	DESeq2	LRT	0.424120172760787
490	KC_AB1	Gle1	0.059529106985223	0.903429319143805	1	pseudobulk	DESeq2	LRT	0.044105819313974
490	KC_AB1	glec	0.213908818914446	0.594879116876252	1	pseudobulk	DESeq2	LRT	0.225571276636637
490	KC_AB1	Glg1	0.167189048840367	0.766678739649359	1	pseudobulk	DESeq2	LRT	0.115386579760574
490	KC_AB1	GlnRS	0.21846530208189	0.699015033844418	1	pseudobulk	DESeq2	LRT	0.155513483702873
490	KC_AB1	glo	-0.070371390406442	0.631744104192656	1	pseudobulk	DESeq2	LRT	0.199458802480535
490	KC_AB1	Glo1	-0.475323430405453	0.378631973523469	1	pseudobulk	DESeq2	LRT	0.421782714887553
491	KC_AB1	Glos	-1.43550446490422	0.510445781841267	1	pseudobulk	DESeq2	LRT	0.292050380702718
491	KC_AB1	GLS	-0.320177634238918	0.670360159669456	1	pseudobulk	DESeq2	LRT	0.173691804267182
491	KC_AB1	glu	0.12291274656792	0.824899256153312	1	pseudobulk	DESeq2	LRT	0.083599088017724
491	KC_AB1	GluClalpha	0.0483815878148645	0.598585888630234	1	pseudobulk	DESeq2	LRT	0.222873525655298
491	KC_AB1	GluProRS	-0.0627515759493779	0.907092382474248	1	pseudobulk	DESeq2	LRT	0.042348480133226
491	KC_AB1	GluRIA	0.0207901746200949	0.861158146593498	1	pseudobulk	DESeq2	LRT	0.064917085612590
491	KC_AB1	GluRIB	-0.0413084006201056	0.702672517860814	1	pseudobulk	DESeq2	LRT	0.153247031769167
491	KC_AB1	GluRIIE	-0.341884002218887	0.868674590665903	1	pseudobulk	DESeq2	LRT	0.061142881715951
491	KC_AB1	GluRS-m	-1.14396378458764	0.660650724079103	1	pseudobulk	DESeq2	LRT	0.180028084683443
491	KC_AB1	Glut1	0.251558456392053	0.131126682710752	1	pseudobulk	DESeq2	LRT	0.882308925587287
492	KC_AB1	Glut4EF	0.113273649230891	0.262907341219095	1	pseudobulk	DESeq2	LRT	0.580197286824677
492	KC_AB1	GlyP	-0.240692786429492	0.335412504137703	1	pseudobulk	DESeq2	LRT	0.474420750982228
492	KC_AB1	GlyRS	0.0395794414233026	0.88695529914848	1	pseudobulk	DESeq2	LRT	0.052098267228409
492	KC_AB1	GlyS	-0.25749604989208	0.812492169663235	1	pseudobulk	DESeq2	LRT	0.090180815811777
492	KC_AB1	GlyT	-0.0589118697098261	0.735271594899031	1	pseudobulk	DESeq2	LRT	0.133552211421014
492	KC_AB1	GM130	0.435470526191113	0.251689381052731	1	pseudobulk	DESeq2	LRT	0.599135107246582
492	KC_AB1	Gmap	-0.231880944105656	0.643113126026169	1	pseudobulk	DESeq2	LRT	0.191712626317466
492	KC_AB1	Gmd	-0.244409107164394	0.868337109258456	1	pseudobulk	DESeq2	LRT	0.061311638569862
492	KC_AB1	Gmer	-0.0272643116440244	0.931474532752522	1	pseudobulk	DESeq2	LRT	0.030829014558663
492	KC_AB1	GMF	0.0716822230307898	0.898538819511799	1	pseudobulk	DESeq2	LRT	0.046463155354586
493	KC_AB1	GNBP3	1.95275942187961	0.438420007953907	1	pseudobulk	DESeq2	LRT	0.358109634392079
493	KC_AB1	Gnf1	-0.0791774481333089	0.882019712200886	1	pseudobulk	DESeq2	LRT	0.054521708740734
493	KC_AB1	Gnpnat	0.236288153201182	0.633943713103649	1	pseudobulk	DESeq2	LRT	0.197949300751828
493	KC_AB1	gny	-0.115523498187253	0.895290982602627	1	pseudobulk	DESeq2	LRT	0.048035789711983
493	KC_AB1	goddard	1.08899820779554	0.697631521651407	1	pseudobulk	DESeq2	LRT	0.156373904552
493	KC_AB1	goe	1.88952324269138	0.456767015650974	1	pseudobulk	DESeq2	LRT	0.340305265178099
493	KC_AB1	gogo	0.0983754006856757	0.877440472558388	1	pseudobulk	DESeq2	LRT	0.056782337330897
493	KC_AB1	gol	0.300724885210681	0.135345754205198	1	pseudobulk	DESeq2	LRT	0.868555363492851
493	KC_AB1	Golgin104	-0.0967844416439141	0.895888787627084	1	pseudobulk	DESeq2	LRT	0.047745898728029

493	KC_AB1	Golgin245	-0.222698827404035	0.591077340592295	1	pseudobulk	DESeq2	LRT	0.228355689347366
494	KC_AB1	Golgin84	-0.740932258441461	0.202279205476381	1	pseudobulk	DESeq2	LRT	0.69404876088239
494	KC_AB1	gom	-0.0931702797748925	0.86827117369496	1	pseudobulk	DESeq2	LRT	0.061344617165764
494	KC_AB1	Gorab	0.105651927454987	0.885476686448829	1	pseudobulk	DESeq2	LRT	0.052822868782110
494	KC_AB1	Gos28	-0.116295131970085	0.766833070693423	1	pseudobulk	DESeq2	LRT	0.115299165864446
494	KC_AB1	Got1	-0.200260360508639	0.664373249575525	1	pseudobulk	DESeq2	LRT	0.177587862366972
494	KC_AB1	Got2	-0.105669428533626	0.7698532279737	1	pseudobulk	DESeq2	LRT	0.113592064901474
494	KC_AB1	Gp150	-0.850932661185203	0.660736244265418	1	pseudobulk	DESeq2	LRT	0.179971869591347
494	KC_AB1	Gp210	-0.818681297419949	0.242899518163084	1	pseudobulk	DESeq2	LRT	0.614573346698869
494	KC_AB1	Gp93	-0.0264980267735826	0.950880320447746	1	pseudobulk	DESeq2	LRT	0.021874140728108
494	KC_AB1	Gpat4	-0.277021407240124	0.359071094364592	1	pseudobulk	DESeq2	LRT	0.444819554671276
495	KC_AB1	Gpb5	-0.0344538432976624	0.9365429590594	1	pseudobulk	DESeq2	LRT	0.028472296820997
495	KC_AB1	Gpdh1	0.0418742186660656	0.795225054638621	1	pseudobulk	DESeq2	LRT	0.099509945361389
495	KC_AB1	Gpdh3	0.952906404360553	0.288528360189174	1	pseudobulk	DESeq2	LRT	0.539811492496723
495	KC_AB1	GPHR	0.110267675658335	0.835889330255739	1	pseudobulk	DESeq2	LRT	0.077851218303610
495	KC_AB1	Gpo1	-0.360949012000493	0.35954636143693	1	pseudobulk	DESeq2	LRT	0.444245101892891
495	KC_AB1	Gpo2	0.11269840515639	0.977007463492936	1	pseudobulk	DESeq2	LRT	0.010102118633921
495	KC_AB1	gpp	0.189910839076412	0.179716265710879	1	pseudobulk	DESeq2	LRT	0.745412614105321
495	KC_AB1	Gprk1	-0.0078603471915758	0.924319514357283	1	pseudobulk	DESeq2	LRT	0.034177877983387
495	KC_AB1	Gprk2	-0.0380538400201499	0.761463024488842	1	pseudobulk	DESeq2	LRT	0.118351180509603
495	KC_AB1	gprs	-0.0022888746197161	0.937748769587758	1	pseudobulk	DESeq2	LRT	0.027913497010988
496	KC_AB1	Gr32a	-0.507979848677457	0.940470850565906	1	pseudobulk	DESeq2	LRT	0.026654660650085
496	KC_AB1	Gr39a	1.05570414071799	0.599063570399837	1	pseudobulk	DESeq2	LRT	0.222527089448809
496	KC_AB1	Gr47a	1.08899820779554	0.878177167143508	1	pseudobulk	DESeq2	LRT	0.056417858865076
496	KC_AB1	Gr47b	1.22236326706149	0.63482863141329	1	pseudobulk	DESeq2	LRT	0.197343494355134
496	KC_AB1	Gr77a	-1.6747012584303	0.563129890257477	1	pseudobulk	DESeq2	LRT	0.249391420218277
496	KC_AB1	Graf	0.0537157292379492	0.777797152052812	1	pseudobulk	DESeq2	LRT	0.109133651378856
496	KC_AB1	GramD1B	0.180245479660764	0.372152697122702	1	pseudobulk	DESeq2	LRT	0.429278829174429
496	KC_AB1	Grasp65	0.391533722736947	0.480200625486796	1	pseudobulk	DESeq2	LRT	0.318577278587015
496	KC_AB1	grass	-0.184873771334748	0.924239397505881	1	pseudobulk	DESeq2	LRT	0.034215522773899
496	KC_AB1	grau	0.2941248525306	0.652274723828009	1	pseudobulk	DESeq2	LRT	0.185569450413134
497	KC_AB1	Grd	1.08899820779554	0.878177167143508	1	pseudobulk	DESeq2	LRT	0.056417858865076
497	KC_AB1	Grik	-0.126189338111231	0.851407664187163	1	pseudobulk	DESeq2	LRT	0.069862444716538
497	KC_AB1	grim	0.13617601020169	1	1	pseudobulk	DESeq2	LRT	0
497	KC_AB1	Grip128	-0.0657886766579838	0.935493793453531	1	pseudobulk	DESeq2	LRT	0.028959089487140
497	KC_AB1	Grip163	-0.349786693589143	0.53255574031748	1	pseudobulk	DESeq2	LRT	0.273634929775801
497	KC_AB1	Grip71	-1.09167609484485	0.494655414747475	1	pseudobulk	DESeq2	LRT	0.305697232554269
497	KC_AB1	Grip75	0.472813443388081	0.446053215320107	1	pseudobulk	DESeq2	LRT	0.350613325724603
497	KC_AB1	Grip84	0.220442429204711	0.723001107932941	1	pseudobulk	DESeq2	LRT	0.140861037188465
497	KC_AB1	Grip91	0.304327663434786	0.620918315526359	1	pseudobulk	DESeq2	LRT	0.206965529371743
497	KC_AB1	grk	-0.850996402080018	0.900449987182352	1	pseudobulk	DESeq2	LRT	0.045540403770502
498	KC_AB1	grn	1.08899820779554	0.878177167143508	1	pseudobulk	DESeq2	LRT	0.056417858865076
498	KC_AB1	gro	0.0438480886645977	0.79216363094769	1	pseudobulk	DESeq2	LRT	0.101185100383259
498	KC_AB1	grp	0.125875407068619	0.433960272811672	1	pseudobulk	DESeq2	LRT	0.3625500264515
498	KC_AB1	grsm	-0.0045117226758625	0.950875767291732	1	pseudobulk	DESeq2	LRT	0.021876220290829
498	KC_AB1	Grx1	0.186453028615897	0.498602626307175	1	pseudobulk	DESeq2	LRT	0.3022454382516
498	KC_AB1	gry	-0.336804343278315	0.652252203858874	1	pseudobulk	DESeq2	LRT	0.185584444811673
498	KC_AB1	Gs1	-0.305656492333264	0.155329871694042	1	pseudobulk	DESeq2	LRT	0.808745016490569
498	KC_AB1	Gs1l	-0.466764999413278	0.0991126224597518	1	pseudobulk	DESeq2	LRT	1.00387103254265
498	KC_AB1	Gs2	-0.505821227970159	0.693836129734331	1	pseudobulk	DESeq2	LRT	0.15874308913548
498	KC_AB1	GstD1	-1.70563479171624	0.0062115595689754	1	pseudobulk	DESeq2	LRT	2.20679934552956
499	KC_AB1	GstD10	-0.82542030717083	1	1	pseudobulk	DESeq2	LRT	0

499	KC_AB1	GstD11	0.363534555998765	0.677053138089835	1	pseudobulk	DESeq2	LRT	0.16937724464786
499	KC_AB1	GstD3	-1.44925888589622	0.6315588144614	1	pseudobulk	DESeq2	LRT	0.199586199175701
499	KC_AB1	GstD6	1.08899820779554	0.670846322504933	1	pseudobulk	DESeq2	LRT	0.173376956629912
499	KC_AB1	GstD9	-0.80354010716979	0.584973613134267	1	pseudobulk	DESeq2	LRT	0.232863723539425
499	KC_AB1	GstE12	-0.195096576551817	0.890148018003158	1	pseudobulk	DESeq2	LRT	0.050537770819105
499	KC_AB1	GstE13	-0.0272643116440244	0.931474532752522	1	pseudobulk	DESeq2	LRT	0.030829014558663
499	KC_AB1	GstE2	0.11269840515639	0.977007463492936	1	pseudobulk	DESeq2	LRT	0.010102118633921
499	KC_AB1	GstE7	1.08899820779554	0.670846322504933	1	pseudobulk	DESeq2	LRT	0.173376956629912
499	KC_AB1	GstE8	-0.82542030717083	1	1	pseudobulk	DESeq2	LRT	0
500	KC_AB1	GstO1	-1.66609159573062	0.36570524490273	1	pseudobulk	DESeq2	LRT	0.436868811015581
500	KC_AB1	GstO2	-0.277674889972017	0.553568649958269	1	pseudobulk	DESeq2	LRT	0.256828513088247
500	KC_AB1	GstO3	2.09447112639525	0.299159443021327	1	pseudobulk	DESeq2	LRT	0.524097284022574
500	KC_AB1	GstS1	1.08899820779554	0.670846322504933	1	pseudobulk	DESeq2	LRT	0.173376956629912
500	KC_AB1	GstT1	-0.226518520109668	0.832853755254687	1	pseudobulk	DESeq2	LRT	0.079431251728711
500	KC_AB1	GstT2	-0.501566158316869	0.648487297427995	1	pseudobulk	DESeq2	LRT	0.188098526458787
500	KC_AB1	GstZ1	-0.850932661185203	0.660736244265418	1	pseudobulk	DESeq2	LRT	0.179971869591347
500	KC_AB1	GstZ2	0.66817593025734	0.240134571737395	1	pseudobulk	DESeq2	LRT	0.619545310855356
500	KC_AB1	gt	2.07187506576596	0.225471795980193	1	pseudobulk	DESeq2	LRT	0.646907775809237
500	KC_AB1	Gtp-bp	0.974247229230793	0.169424566376695	1	pseudobulk	DESeq2	LRT	0.771023617214865
501	KC_AB1	Gug	0.058379903189496	0.640959596600908	1	pseudobulk	DESeq2	LRT	0.193169345717233
501	KC_AB1	gukh	-1.05900802849316	0.403117893422581	1	pseudobulk	DESeq2	LRT	0.394567924143603
501	KC_AB1	gus	0.130051182998491	0.403474304731959	1	pseudobulk	DESeq2	LRT	0.394184118112801
501	KC_AB1	gw	-0.0226698905891462	0.838495422801681	1	pseudobulk	DESeq2	LRT	0.076499303788245
501	KC_AB1	GXIVsPLA2	-0.0463566576533241	0.899867884284889	1	pseudobulk	DESeq2	LRT	0.045821247602677
501	KC_AB1	Gyc88E	-0.258773678193516	0.190687527177237	1	pseudobulk	DESeq2	LRT	0.719677713116837
501	KC_AB1	Gyc89Da	0.510800944861603	0.614152859222605	1	pseudobulk	DESeq2	LRT	0.211723521929423
501	KC_AB1	Gyc89Db	0.24999439403008	0.521955436211502	1	pseudobulk	DESeq2	LRT	0.28236657483991
501	KC_AB1	Gycalpha99B	-0.0788857551998532	0.659711557672401	1	pseudobulk	DESeq2	LRT	0.180645907325673
501	KC_AB1	Gycbeta100B	-0.125935575946571	0.522138864951997	1	pseudobulk	DESeq2	LRT	0.282213979254338
502	KC_AB1	Gyf	0.199080280411903	0.245011697742056	1	pseudobulk	DESeq2	LRT	0.610813180355677
502	KC_AB1	Gyg	-0.682027003474804	0.636673046431254	1	pseudobulk	DESeq2	LRT	0.196083535614331
502	KC_AB1	gzl	-0.0915586066016233	0.648947537286647	1	pseudobulk	DESeq2	LRT	0.187790411347338
502	KC_AB1	H	0.112940642096725	0.698207122643267	1	pseudobulk	DESeq2	LRT	0.15601572511667
502	KC_AB1	h-cup	-0.850996402080018	0.900449987182352	1	pseudobulk	DESeq2	LRT	0.045540403770502
502	KC_AB1	Hacd1	-0.271152400266514	0.539457609333568	1	pseudobulk	DESeq2	LRT	0.268042676571114
502	KC_AB1	Hacd2	0.217856390011377	0.551480445965049	1	pseudobulk	DESeq2	LRT	0.258469881882053
502	KC_AB1	Hacl	-0.305801367363977	0.570169709068486	1	pseudobulk	DESeq2	LRT	0.243995858815132
502	KC_AB1	Had1	-2.61627160405009	0.247973659858837	1	pseudobulk	DESeq2	LRT	0.605594448147543
502	KC_AB1	haf	-0.306243588936002	0.518124391712995	1	pseudobulk	DESeq2	LRT	0.285565961974591
503	KC_AB1	Hakai	0.198900563003713	0.730122841172455	1	pseudobulk	DESeq2	LRT	0.136604064872471
503	KC_AB1	hang	0.23841819059563	0.239861317913204	1	pseudobulk	DESeq2	LRT	0.620039784426417
503	KC_AB1	Hasp	-0.129313495066021	0.427195580153696	1	pseudobulk	DESeq2	LRT	0.369373249249141
503	KC_AB1	Haspin	0.0839863479524601	0.659845552184139	1	pseudobulk	DESeq2	LRT	0.180557706403078
503	KC_AB1	Hat1	0.0782383382437653	0.847012264608959	1	pseudobulk	DESeq2	LRT	0.072110301105620
503	KC_AB1	hay	0.80172944818131	0.324338857394924	1	pseudobulk	DESeq2	LRT	0.489001017588918
503	KC_AB1	hb	0.112935832476632	0.940353958317081	1	pseudobulk	DESeq2	LRT	0.026708642986417
503	KC_AB1	hbs	-0.82542030717083	1	1	pseudobulk	DESeq2	LRT	0
503	KC_AB1	HBS1	-0.713132403830086	0.260333847627952	1	pseudobulk	DESeq2	LRT	0.584469362884254
503	KC_AB1	Hcf	-0.0344454671601328	0.851815787360022	1	pseudobulk	DESeq2	LRT	0.069654315072549
504	KC_AB1	Hcs	0.101693849959272	0.817703667031704	1	pseudobulk	DESeq2	LRT	0.087404054628885
504	KC_AB1	hd	0.912619318367561	0.590161437887788	1	pseudobulk	DESeq2	LRT	0.229029171419727
504	KC_AB1	HDAC1	0.0037931597499528	0.885582411467101	1	pseudobulk	DESeq2	LRT	0.052771017556882

504	KC_AB1	HDAC11	0.276709375859245	0.64039979870887	1	pseudobulk	DESeq2	LRT	0.193548813260609
504	KC_AB1	HDAC4	0.112091065767045	0.336489629527819	1	pseudobulk	DESeq2	LRT	0.473028316011313
504	KC_AB1	HDAC6	-0.452101967886937	0.178586772442396	1	pseudobulk	DESeq2	LRT	0.748150711561799
504	KC_AB1	hdc	-0.215969733682228	0.580794133634915	1	pseudobulk	DESeq2	LRT	0.235977778917556
504	KC_AB1	Hdc	-1.44925888589622	0.6315588144614	1	pseudobulk	DESeq2	LRT	0.199586199175701
504	KC_AB1	hdly	0.696014114897656	0.471953418377794	1	pseudobulk	DESeq2	LRT	0.326100863950241
504	KC_AB1	hdm	0.11269840515639	0.977007463492936	1	pseudobulk	DESeq2	LRT	0.010102118633921
505	KC_AB1	HEATR2	1.13227237519976	0.38441967997557	1	pseudobulk	DESeq2	LRT	0.415194387168905
505	KC_AB1	hec	1.06141506222607	0.363464582821421	1	pseudobulk	DESeq2	LRT	0.439537901818996
505	KC_AB1	heix	-0.314769313173822	0.528748254677689	1	pseudobulk	DESeq2	LRT	0.276751053158461
505	KC_AB1	Hel25E	-0.166099801414772	0.539471210253637	1	pseudobulk	DESeq2	LRT	0.268031727184226
505	KC_AB1	Hel89B	-0.118791552760285	0.740010028978719	1	pseudobulk	DESeq2	LRT	0.130762394457399
505	KC_AB1	Hem	-0.0782897403877813	0.875645828397666	1	pseudobulk	DESeq2	LRT	0.057671516976898
505	KC_AB1	HemK1	-0.586751199540085	0.213751242137688	1	pseudobulk	DESeq2	LRT	0.670091352847854
505	KC_AB1	Hen1	0.0514533628350485	0.925342191998732	1	pseudobulk	DESeq2	LRT	0.033697635244751
505	KC_AB1	hep	0.0864694711999909	0.692359898190775	1	pseudobulk	DESeq2	LRT	0.159668094607767
505	KC_AB1	heph	0.105504830929424	0.332064588492737	1	pseudobulk	DESeq2	LRT	0.478777435278411
506	KC_AB1	her	0.098301499397949	0.841754157008022	1	pseudobulk	DESeq2	LRT	0.074814730168638
506	KC_AB1	HERC2	-0.062323080582645	0.770451499123484	1	pseudobulk	DESeq2	LRT	0.113254695460833
506	KC_AB1	Herc4	-0.365969788984854	0.324377128908276	1	pseudobulk	DESeq2	LRT	0.488949774495074
506	KC_AB1	Herp	-0.211716253681953	0.608387562994503	1	pseudobulk	DESeq2	LRT	0.215819672623751
506	KC_AB1	Hers	0.0849866729150779	0.594266220709468	1	pseudobulk	DESeq2	LRT	0.22601895521447
506	KC_AB1	Hex-A	-0.0393414823463556	0.797233357731895	1	pseudobulk	DESeq2	LRT	0.098414537899544
506	KC_AB1	Hexim	0.269125266142	0.609982282247633	1	pseudobulk	DESeq2	LRT	0.21468277947093
506	KC_AB1	Hexo1	0.0969469790037064	0.876371614152049	1	pseudobulk	DESeq2	LRT	0.057311697766715
506	KC_AB1	Hey	0.333889580062801	0.524550630353941	1	pseudobulk	DESeq2	LRT	0.280212586741883
506	KC_AB1	Hf	-0.324254218813778	0.848824744254821	1	pseudobulk	DESeq2	LRT	0.071181968719472
507	KC_AB1	hfp	0.108898576469044	0.501314495084159	1	pseudobulk	DESeq2	LRT	0.299889737948177
507	KC_AB1	hfw	-0.642749171356925	0.752779105808464	1	pseudobulk	DESeq2	LRT	0.123332443722695
507	KC_AB1	hgo	-0.850996402080018	0.900449987182352	1	pseudobulk	DESeq2	LRT	0.045540403770502
507	KC_AB1	HGTX	-0.850996402080018	0.900449987182352	1	pseudobulk	DESeq2	LRT	0.045540403770502
507	KC_AB1	hig	-1.92255931202893	0.364128068789703	1	pseudobulk	DESeq2	LRT	0.438745842212356
507	KC_AB1	Him	-1.96719661029409	0.414177861019091	1	pseudobulk	DESeq2	LRT	0.382813119093482
507	KC_AB1	HINT1	-0.288758137912558	0.262031941355008	1	pseudobulk	DESeq2	LRT	0.581645765517264
507	KC_AB1	HIP	0.222231096365427	0.680968373349805	1	pseudobulk	DESeq2	LRT	0.166873057835547
507	KC_AB1	HIP-R	-0.117922707383009	0.739118364592001	1	pseudobulk	DESeq2	LRT	0.131286006838114
507	KC_AB1	Hip1	-1.36816707453198	0.0251327295998159	1	pseudobulk	DESeq2	LRT	1.59976034126173
508	KC_AB1	Hip14	-0.217474577683889	0.568061292310282	1	pseudobulk	DESeq2	LRT	0.245604802535404
508	KC_AB1	HipHop	-0.586257496419322	0.662751549717692	1	pseudobulk	DESeq2	LRT	0.178649248079131
508	KC_AB1	Hipk	-0.0729098023214732	0.564261646403565	1	pseudobulk	DESeq2	LRT	0.248519468286349
508	KC_AB1	HIPP1	0.222264607091991	0.396082102680171	1	pseudobulk	DESeq2	LRT	0.402214781132309
508	KC_AB1	Hira	-0.718053131205487	0.514443331198002	1	pseudobulk	DESeq2	LRT	0.288662458226078
508	KC_AB1	His-Psi:CR31614	1.08899820779554	0.670846322504933	1	pseudobulk	DESeq2	LRT	0.173376956629912
508	KC_AB1	His-Psi:CR33867	-0.82542030717083	1	1	pseudobulk	DESeq2	LRT	0
508	KC_AB1	His1:CG33834	1.08899820779554	0.878177167143508	1	pseudobulk	DESeq2	LRT	0.056417858865076
508	KC_AB1	His2Av	0.13421583936626	0.507810814378796	1	pseudobulk	DESeq2	LRT	0.29429805459458
508	KC_AB1	His2B:CG33868	2.88885723090896	0.199893040000958	1	pseudobulk	DESeq2	LRT	0.69920232715152
509	KC_AB1	His3.3A	0.30269130901797	0.046309420459239	1	pseudobulk	DESeq2	LRT	1.33433065396463
509	KC_AB1	His3.3B	0.138974822101207	0.127701040731085	1	pseudobulk	DESeq2	LRT	0.893805563332311
509	KC_AB1	His4r	0.0803254387668205	0.419533150238223	1	pseudobulk	DESeq2	LRT	0.377233716845492
509	KC_AB1	HisCl1	-0.598574955868036	0.607168240584035	1	pseudobulk	DESeq2	LRT	0.216690953350772
509	KC_AB1	HisRS	-0.412727146527764	0.443584127648118	1	pseudobulk	DESeq2	LRT	0.353024002194121

509	KC_AB1	hiw	-0.132270612979751	0.527080677596776	1	pseudobulk	DESeq2	LRT	0.278122904420426
509	KC_AB1	Hk	0.024716093348627	0.84670754366678	1	pseudobulk	DESeq2	LRT	0.072266570902419
509	KC_AB1	hkb	0.13617601020169	1	1	pseudobulk	DESeq2	LRT	0
509	KC_AB1	Hlc	-0.177847356604499	0.583564850714067	1	pseudobulk	DESeq2	LRT	0.233910874421902
509	KC_AB1	hll	-1.28957086838014	0.627702624447357	1	pseudobulk	DESeq2	LRT	0.20224605554457
510	KC_AB1	Hmg-2	0.742229550903523	0.281871952002799	1	pseudobulk	DESeq2	LRT	0.549948136957201
510	KC_AB1	Hmgcr	-0.24355791131332	0.585700518012092	1	pseudobulk	DESeq2	LRT	0.232324391868073
510	KC_AB1	HmgD	0.644386370460379	0.0255269622639367	1	pseudobulk	DESeq2	LRT	1.59300086363304
510	KC_AB1	Hmgs	-0.0701116095883042	0.878682127214559	1	pseudobulk	DESeq2	LRT	0.056168207251902
510	KC_AB1	HmgZ	0.305651382690579	0.0111855113263755	1	pseudobulk	DESeq2	LRT	1.9513441580897
510	KC_AB1	Hmr	-0.0739601829833246	0.936062733447563	1	pseudobulk	DESeq2	LRT	0.028695044555553
510	KC_AB1	Hmt-1	0.44954571566491	0.424620632841595	1	pseudobulk	DESeq2	LRT	0.371998906751413
510	KC_AB1	Hmt4-20	0.227035472385045	0.331990134165051	1	pseudobulk	DESeq2	LRT	0.478874822143378
510	KC_AB1	Hmu	-0.0529650039437085	0.916152591039103	1	pseudobulk	DESeq2	LRT	0.03803218580031
510	KC_AB1	hmw	-0.82542030717083	1	1	pseudobulk	DESeq2	LRT	0
511	KC_AB1	Hnf4	1.16523413356038	0.63328025468345	1	pseudobulk	DESeq2	LRT	0.198404052815419
511	KC_AB1	hng1	-0.661022915638341	0.322169212613136	1	pseudobulk	DESeq2	LRT	0.491915964323635
511	KC_AB1	hng2	0.732546427073525	0.503864755165286	1	pseudobulk	DESeq2	LRT	0.297686019045623
511	KC_AB1	hng3	0.0257892550108557	0.965744460316161	1	pseudobulk	DESeq2	LRT	0.015137774367219
511	KC_AB1	HnRNP-K	0.162167180406954	0.340619705132778	1	pseudobulk	DESeq2	LRT	0.467730231350261
511	KC_AB1	Ho	0.166027278527003	0.674462988095384	1	pseudobulk	DESeq2	LRT	0.17104187772962
511	KC_AB1	hob	-0.203270429043305	0.786714354642529	1	pseudobulk	DESeq2	LRT	0.104182925482115
511	KC_AB1	hoe1	-0.0755419409356789	0.792152017030523	1	pseudobulk	DESeq2	LRT	0.101191467624718
511	KC_AB1	hoe2	-0.481293450654593	0.0917212593032923	1	pseudobulk	DESeq2	LRT	1.03752999117807
511	KC_AB1	hog	0.959088647258781	0.657893326835362	1	pseudobulk	DESeq2	LRT	0.181844518730155
512	KC_AB1	hoip	-0.101287236163978	0.725218346665555	1	pseudobulk	DESeq2	LRT	0.139531217600763
512	KC_AB1	holn1	-0.112330463830378	0.872545899692595	1	pseudobulk	DESeq2	LRT	0.059211717997551
512	KC_AB1	homer	-0.222677997119639	0.585884220639272	1	pseudobulk	DESeq2	LRT	0.232188198496279
512	KC_AB1	hook	-0.0295617227870592	0.883154353882656	1	pseudobulk	DESeq2	LRT	0.053963385685047
512	KC_AB1	hop	-0.587614566332161	0.283638416899525	1	pseudobulk	DESeq2	LRT	0.547234947263511
512	KC_AB1	how	-0.850996402080018	0.900449987182352	1	pseudobulk	DESeq2	LRT	0.045540403770502
512	KC_AB1	HP1b	-0.0567398533330693	0.871067264986706	1	pseudobulk	DESeq2	LRT	0.059948306892915
512	KC_AB1	HP1c	0.105489511445384	0.755252871141869	1	pseudobulk	DESeq2	LRT	0.121907615053424
512	KC_AB1	HP4	0.62726727096513	0.153221070132726	1	pseudobulk	DESeq2	LRT	0.814681508768861
512	KC_AB1	HP5	-0.0898201234450806	0.779460100280367	1	pseudobulk	DESeq2	LRT	0.108206110971054
513	KC_AB1	HP6	-0.850996402080018	0.900449987182352	1	pseudobulk	DESeq2	LRT	0.045540403770502
513	KC_AB1	Hpd	-1.82709236256241	0.44317674354685	1	pseudobulk	DESeq2	LRT	0.353423038023035
513	KC_AB1	Hph	0.586487423434186	0.247732128527632	1	pseudobulk	DESeq2	LRT	0.606017665850897
513	KC_AB1	hpo	0.0375487885585092	0.950728986254804	1	pseudobulk	DESeq2	LRT	0.021943264921837
513	KC_AB1	hppy	-0.114007282790333	0.53188190451083	1	pseudobulk	DESeq2	LRT	0.274184784834104
513	KC_AB1	Hpr1	-0.294263605459984	0.628959915077736	1	pseudobulk	DESeq2	LRT	0.20137703216249
513	KC_AB1	hpRNA:CR18854	-0.233276868857139	0.197720439991207	1	pseudobulk	DESeq2	LRT	0.703948431766082
513	KC_AB1	hpRNA:CR32207	-0.507979848677457	0.940470850565906	1	pseudobulk	DESeq2	LRT	0.026654660650085
513	KC_AB1	hpRNA:CR33940	1.08899820779554	0.670846322504933	1	pseudobulk	DESeq2	LRT	0.173376956629912
513	KC_AB1	hpRNA:CR46342	0.300043336959098	0.369806286923994	1	pseudobulk	DESeq2	LRT	0.432025709852915
514	KC_AB1	HPS1	0.0319551019300294	0.945082619880149	1	pseudobulk	DESeq2	LRT	0.024530223459403
514	KC_AB1	HPS4	-0.20267195214807	0.575049453719757	1	pseudobulk	DESeq2	LRT	0.240294804781583
514	KC_AB1	Hr3	0.026658058606086	0.88009442990282	1	pseudobulk	DESeq2	LRT	0.055470727638997
514	KC_AB1	Hr38	0.0424801761366885	0.750378777169406	1	pseudobulk	DESeq2	LRT	0.124719457529521
514	KC_AB1	Hr39	0.0078515101607897	0.966533672007004	1	pseudobulk	DESeq2	LRT	0.014783011462529
514	KC_AB1	Hr4	0.4650126833937	0.616348793099436	1	pseudobulk	DESeq2	LRT	0.210173450093364
514	KC_AB1	Hr51	-0.132942789768953	0.42567934039835	1	pseudobulk	DESeq2	LRT	0.370917427033857

514	KC_AB1	Hr78	0.398818160434418	0.22399753448184	1	pseudobulk	DESeq2	LRT	0.649756761874876
514	KC_AB1	Hr96	-0.385600942640958	0.540564129019345	1	pseudobulk	DESeq2	LRT	0.267152776776087
514	KC_AB1	Hrb27C	-0.0378151002511566	0.697175733549097	1	pseudobulk	DESeq2	LRT	0.15665773769677
515	KC_AB1	Hrb87F	0.401053750721529	0.152876337663802	1	pseudobulk	DESeq2	LRT	0.815659729874112
515	KC_AB1	Hrb98DE	-9.03772468368807E-	0.955905026366363	1	pseudobulk	DESeq2	LRT	0.019585254767795
515	KC_AB1	Hrd3	0.579700875086754	0.228977674956984	1	pseudobulk	DESeq2	LRT	0.640206858776407
515	KC_AB1	hrg	0.0178293470973243	0.904630905735473	1	pseudobulk	DESeq2	LRT	0.043528579135871
515	KC_AB1	hrm	-0.0626108404167456	0.745758664944421	1	pseudobulk	DESeq2	LRT	0.127401691883853
515	KC_AB1	Hrs	-0.218529932561529	0.606300900646581	1	pseudobulk	DESeq2	LRT	0.217311786629705
515	KC_AB1	Hs2st	-0.715158184318518	0.283291996438414	1	pseudobulk	DESeq2	LRT	0.547765694987972
515	KC_AB1	Hs3st-A	0.105748360087276	0.344517256410095	1	pseudobulk	DESeq2	LRT	0.462789019979905
515	KC_AB1	Hs3st-B	-1.05645262975921	0.337441742298117	1	pseudobulk	DESeq2	LRT	0.471801195225189
515	KC_AB1	Hs6st	-0.345938575044808	0.226520671916073	1	pseudobulk	DESeq2	LRT	0.644892158819259
516	KC_AB1	Hsc20	-0.0747571062058667	0.92141072735109	1	pseudobulk	DESeq2	LRT	0.035546735862084
516	KC_AB1	Hsc70-2	0.544688880513973	0.885243342345618	1	pseudobulk	DESeq2	LRT	0.052937330758423
516	KC_AB1	Hsc70-3	-0.310920476729558	0.139074605195069	1	pseudobulk	DESeq2	LRT	0.856752164260379
516	KC_AB1	Hsc70-4	0.0576097569686192	0.69549391236913	1	pseudobulk	DESeq2	LRT	0.157706667017823
516	KC_AB1	Hsc70-5	-0.179440015110014	0.480043728118834	1	pseudobulk	DESeq2	LRT	0.318719200091644
516	KC_AB1	Hsc70Cb	0.13230511805367	0.574400313111795	1	pseudobulk	DESeq2	LRT	0.240785332027094
516	KC_AB1	Hsepi	0.0089528719119064	0.945551260842278	1	pseudobulk	DESeq2	LRT	0.024314921924728
516	KC_AB1	Hsf	-0.0026501870134324	0.963551895643538	1	pseudobulk	DESeq2	LRT	0.016124889846432
516	KC_AB1	Hsl	0.0363956059026609	0.928202309493863	1	pseudobulk	DESeq2	LRT	0.032357355331777
516	KC_AB1	Hsp23	-1.86202252563672	0.02940891286027	1	pseudobulk	DESeq2	LRT	1.53152102947159
517	KC_AB1	Hsp26	-0.800722984095163	0.0538349776001916	1	pseudobulk	DESeq2	LRT	1.26893546331351
517	KC_AB1	Hsp60A	-0.166152502822718	0.431840679176449	1	pseudobulk	DESeq2	LRT	0.36467644975136
517	KC_AB1	Hsp60D	-1.04171374991564	0.34682943513389	1	pseudobulk	DESeq2	LRT	0.459884051409946
517	KC_AB1	Hsp67Bc	0.0848284990483924	0.956053969845324	1	pseudobulk	DESeq2	LRT	0.019517590836417
517	KC_AB1	Hsp68	-0.989414155271523	0.588375161649144	1	pseudobulk	DESeq2	LRT	0.230345669368036
517	KC_AB1	Hsp70Aa	-0.594653421178588	0.71980809855257	1	pseudobulk	DESeq2	LRT	0.14278327141344
517	KC_AB1	Hsp70Ab	-0.114152308184255	0.758403263478593	1	pseudobulk	DESeq2	LRT	0.120099806857101
517	KC_AB1	Hsp70Ba	-2.55314344521075	0.0970980464148837	1	pseudobulk	DESeq2	LRT	1.01278950788572
517	KC_AB1	Hsp70Bb	0.308339154067054	0.23778588833422	1	pseudobulk	DESeq2	LRT	0.623813922637554
517	KC_AB1	Hsp70Bbb	0.13617601020169	1	1	pseudobulk	DESeq2	LRT	0
518	KC_AB1	Hsp70Bc	0.463557965862676	0.15954486834808	1	pseudobulk	DESeq2	LRT	0.797117160031756
518	KC_AB1	Hsp83	-0.179760668721927	0.236921797759733	1	pseudobulk	DESeq2	LRT	0.62539498059732
518	KC_AB1	HSPBAP1	-0.530932167980835	0.624762071119862	1	pseudobulk	DESeq2	LRT	0.204285344052891
518	KC_AB1	HSPC300	0.191241361200593	0.640555550452425	1	pseudobulk	DESeq2	LRT	0.193443201268512
518	KC_AB1	hth	0.199554258591388	0.312681642092864	1	pseudobulk	DESeq2	LRT	0.50489761594865
518	KC_AB1	htk	-0.103935525698007	0.665408684355985	1	pseudobulk	DESeq2	LRT	0.176911535382929
518	KC_AB1	htl	1.88952324269138	0.456767015650974	1	pseudobulk	DESeq2	LRT	0.340305265178099
518	KC_AB1	HtrA2	0.599493575966779	0.461912549009701	1	pseudobulk	DESeq2	LRT	0.335440238897349
518	KC_AB1	hts	0.0343772179816471	0.840126964152214	1	pseudobulk	DESeq2	LRT	0.075655076242887
518	KC_AB1	htt	-0.274163666794979	0.406066934571811	1	pseudobulk	DESeq2	LRT	0.391402373025724
519	KC_AB1	Hug	0.13962132150037	0.82754014533641	1	pseudobulk	DESeq2	LRT	0.082210928700071
519	KC_AB1	Hus1-like	-0.850932661185203	0.660736244265418	1	pseudobulk	DESeq2	LRT	0.179971869591347
519	KC_AB1	HUWE1	-0.0795753245785761	0.713661724518986	1	pseudobulk	DESeq2	LRT	0.146507594928584
519	KC_AB1	hwt	0.135534958746191	0.38163992136994	1	pseudobulk	DESeq2	LRT	0.418346202301685
519	KC_AB1	Hyccin	-0.338117853065538	0.497748762773452	1	pseudobulk	DESeq2	LRT	0.302989810800966
519	KC_AB1	hyd	-0.312050495900565	0.319161814356469	1	pseudobulk	DESeq2	LRT	0.49598907473455
519	KC_AB1	Hydr1	-0.761800975484312	0.546422009523928	1	pseudobulk	DESeq2	LRT	0.262471815874325
519	KC_AB1	Hydr2	0.127229680693662	0.729662413642493	1	pseudobulk	DESeq2	LRT	0.136878024540922
519	KC_AB1	hyx	0.0040864199724009	0.9790441936796	1	pseudobulk	DESeq2	LRT	0.009197703867960

519	KC_AB1	I-2	0.344410986188465	0.220399629017347	1	pseudobulk	DESeq2	LRT	0.656789140835928
520	KC_AB1	I-3	-1.9753321838664	0.109100319015489	1	pseudobulk	DESeq2	LRT	0.962173979508228
520	KC_AB1	IA-2	-0.246285043636394	0.0537599809009216	1	pseudobulk	DESeq2	LRT	1.26954089424415
520	KC_AB1	Ibf1	0.0967778319494028	0.83625309983401	1	pseudobulk	DESeq2	LRT	0.077662259372628
520	KC_AB1	Ibf2	-0.0474320463430006	0.901998075521101	1	pseudobulk	DESeq2	LRT	0.044794389056127
520	KC_AB1	ICA69	-0.0402089390102794	0.854000126839846	1	pseudobulk	DESeq2	LRT	0.068542064807668
520	KC_AB1	Ice1	-0.173651826606853	0.883722078181816	1	pseudobulk	DESeq2	LRT	0.05368429481669
520	KC_AB1	Ice2	-2.32215638867048	0.0647794107387688	1	pseudobulk	DESeq2	LRT	1.18856300683626
520	KC_AB1	icln	-0.214958243272145	0.790824765156472	1	pseudobulk	DESeq2	LRT	0.101919738950687
520	KC_AB1	ics	-0.34448417034444	0.251289729154606	1	pseudobulk	DESeq2	LRT	0.599825261756152
520	KC_AB1	ida	0.249465038656821	0.845193977687552	1	pseudobulk	DESeq2	LRT	0.073043606113787
521	KC_AB1	Ide	-0.500806822148603	0.163439228043065	1	pseudobulk	DESeq2	LRT	0.786643697632517
521	KC_AB1	Idgf3	1.08899820779554	0.878177167143508	1	pseudobulk	DESeq2	LRT	0.056417858865076
521	KC_AB1	Idgf6	1.36311663992408	0.315113569293236	1	pseudobulk	DESeq2	LRT	0.501532895009779
521	KC_AB1	Idh	0.0698684603386959	0.892668498236023	1	pseudobulk	DESeq2	LRT	0.049309790961168
521	KC_AB1	Idh3a	-0.239946205635533	0.277214588408625	1	pseudobulk	DESeq2	LRT	0.557183918725975
521	KC_AB1	Idh3b	-0.0879541776441888	0.693926270930257	1	pseudobulk	DESeq2	LRT	0.158686670509324
521	KC_AB1	Idi	0.116621155478255	0.819035812972122	1	pseudobulk	DESeq2	LRT	0.086697107963480
521	KC_AB1	Ids	0.505522014943202	0.691765552187514	1	pseudobulk	DESeq2	LRT	0.160041068322472
521	KC_AB1	ifc	0.0564313369488275	0.896909671287013	1	pseudobulk	DESeq2	LRT	0.047251293006021
521	KC_AB1	IFT43	0.419169214450219	0.605983963879936	1	pseudobulk	DESeq2	LRT	0.217538868392465
522	KC_AB1	IFT46	1.08899820779554	0.878177167143508	1	pseudobulk	DESeq2	LRT	0.056417858865076
522	KC_AB1	Ih	0.132840836077608	0.275381831052053	1	pseudobulk	DESeq2	LRT	0.560064716708479
522	KC_AB1	ihog	1.77020821532336	0.521955920340528	1	pseudobulk	DESeq2	LRT	0.282366172019185
522	KC_AB1	IKKepsilon	-0.345485414797687	0.615070957692263	1	pseudobulk	DESeq2	LRT	0.211074778929372
522	KC_AB1	IleRS	-0.118124351379145	0.861606801928211	1	pseudobulk	DESeq2	LRT	0.064690881172205
522	KC_AB1	IleRS-m	-0.99085997564486	0.467891906677227	1	pseudobulk	DESeq2	LRT	0.329854466916859
522	KC_AB1	Ilk	0.82498316435056	0.153679364698073	1	pseudobulk	DESeq2	LRT	0.813384443491831
522	KC_AB1	Ilp1	2.1559833397943	0.381743505411375	1	pseudobulk	DESeq2	LRT	0.418228342845202
522	KC_AB1	Ilp2	-1.44146469927985	0.560160415033477	1	pseudobulk	DESeq2	LRT	0.251687584801904
522	KC_AB1	Ilp7	3.98171353883062	0.339838837136866	1	pseudobulk	DESeq2	LRT	0.468726991004283
523	KC_AB1	imd	-0.0671256740475228	0.92219458446296	1	pseudobulk	DESeq2	LRT	0.035177432479611
523	KC_AB1	Iml1	0.0116403623922466	0.976613390446674	1	pseudobulk	DESeq2	LRT	0.010277325354749
523	KC_AB1	Imp	-0.135131741221972	0.851837929140855	1	pseudobulk	DESeq2	LRT	0.069643026331014
523	KC_AB1	Impbeta11	0.864442718142122	0.363200819342342	1	pseudobulk	DESeq2	LRT	0.439853180427779
523	KC_AB1	ImpE3	-1.6747012584303	0.563129890257477	1	pseudobulk	DESeq2	LRT	0.249391420218277
523	KC_AB1	inaC	3.1582405794361	0.148073259549147	1	pseudobulk	DESeq2	LRT	0.82952336334779
523	KC_AB1	inaE	0.286937136851585	0.127603682531373	1	pseudobulk	DESeq2	LRT	0.894136792072714
523	KC_AB1	inc	-0.149916171787834	0.653991949704439	1	pseudobulk	DESeq2	LRT	0.184427597578573
523	KC_AB1	ind	1.08899820779554	0.878177167143508	1	pseudobulk	DESeq2	LRT	0.056417858865076
523	KC_AB1	ine	-0.82542030717083	1	1	pseudobulk	DESeq2	LRT	0
524	KC_AB1	Ing3	-0.221461862630503	0.682109883562349	1	pseudobulk	DESeq2	LRT	0.166145657629837
524	KC_AB1	Ing5	0.0582092161613541	0.88743985425063	1	pseudobulk	DESeq2	LRT	0.051861071345779
524	KC_AB1	Ino80	1.07522700623795	0.166108739521606	1	pseudobulk	DESeq2	LRT	0.779607517300811
524	KC_AB1	Inos	0.0782036689968812	0.777947050473019	1	pseudobulk	DESeq2	LRT	0.109049961454975
524	KC_AB1	INPP5E	-0.259633648195884	0.635962279280164	1	pseudobulk	DESeq2	LRT	0.196568642820607
524	KC_AB1	InR	0.80223858131072	0.00923769271886145	1	pseudobulk	DESeq2	LRT	2.03443648815165
524	KC_AB1	insv	-2.71583325990106	0.126602926665699	1	pseudobulk	DESeq2	LRT	0.897556254665645
524	KC_AB1	IntS1	0.999905664250816	0.204739120437598	1	pseudobulk	DESeq2	LRT	0.688799166780988
524	KC_AB1	IntS10	-1.28655869378407	0.380016565662533	1	pseudobulk	DESeq2	LRT	0.420197471227884
524	KC_AB1	IntS11	0.0692566232176769	0.897196188869338	1	pseudobulk	DESeq2	LRT	0.047112579891267
525	KC_AB1	IntS12	-1.28203610931285	0.358627095264387	1	pseudobulk	DESeq2	LRT	0.445356901390482

525	KC_AB1	IntS14	-0.60200974879641	0.545010720120225	1	pseudobulk	DESeq2	LRT	0.26359495525864
525	KC_AB1	IntS2	0.210430132016578	0.746270377981052	1	pseudobulk	DESeq2	LRT	0.127103796668081
525	KC_AB1	IntS3	0.282600822244574	0.360755141579608	1	pseudobulk	DESeq2	LRT	0.44278747050823
525	KC_AB1	IntS4	-0.136267569207005	0.862333733813864	1	pseudobulk	DESeq2	LRT	0.064324624310296
525	KC_AB1	IntS6	0.0411169306760164	0.879785958194819	1	pseudobulk	DESeq2	LRT	0.055622973851735
525	KC_AB1	IntS8	0.605526915836591	0.509362451662401	1	pseudobulk	DESeq2	LRT	0.292973072793795
525	KC_AB1	IntS9	-0.848817632166672	0.568488405682699	1	pseudobulk	DESeq2	LRT	0.24527838831903
525	KC_AB1	Invadolysin	-0.127380917110697	0.906924314031547	1	pseudobulk	DESeq2	LRT	0.042428954803164
525	KC_AB1	Inx2	0.0478629758791133	0.925977551895549	1	pseudobulk	DESeq2	LRT	0.033399541618349
526	KC_AB1	Inx3	0.536822480739811	0.77353335804325	1	pseudobulk	DESeq2	LRT	0.11152095293845
526	KC_AB1	Inx7	0.652334304641712	0.421918994633695	1	pseudobulk	DESeq2	LRT	0.374770922404544
526	KC_AB1	Ip259	0.208930844202072	0.150118708375423	1	pseudobulk	DESeq2	LRT	0.823565180921889
526	KC_AB1	IP3K1	1.64954027140197	0.477780694739395	1	pseudobulk	DESeq2	LRT	0.320771402383411
526	KC_AB1	IP3K2	-0.82542030717083	1	1	pseudobulk	DESeq2	LRT	0
526	KC_AB1	IPIP	0.260319727857318	0.479186788115384	1	pseudobulk	DESeq2	LRT	0.319495164591374
526	KC_AB1	Ipk2	0.219538566562472	0.76767670261153	1	pseudobulk	DESeq2	LRT	0.114821639125837
526	KC_AB1	iPLA2-VIA	0.118333413168765	0.773909825637318	1	pseudobulk	DESeq2	LRT	0.111309639458689
526	KC_AB1	Ipod	-1.56855358782635	0.573026303465967	1	pseudobulk	DESeq2	LRT	0.241825442277049
526	KC_AB1	Ipp	-0.0471035473588642	0.841698446051721	1	pseudobulk	DESeq2	LRT	0.074843474620211
527	KC_AB1	Ir25a	-0.850996402080018	0.900449987182352	1	pseudobulk	DESeq2	LRT	0.045540403770502
527	KC_AB1	Ir40a	-1.56855358782635	0.573026303465967	1	pseudobulk	DESeq2	LRT	0.241825442277049
527	KC_AB1	Ir41a	1.01524755243299	0.465895281012719	1	pseudobulk	DESeq2	LRT	0.331711688436766
527	KC_AB1	Ir47a	0.584955017462298	0.728403848706781	1	pseudobulk	DESeq2	LRT	0.13762776816583
527	KC_AB1	Ir47b	-0.673472237912633	0.63622717472733	1	pseudobulk	DESeq2	LRT	0.196387785115356
527	KC_AB1	Ir51a	-0.850996402080018	0.900449987182352	1	pseudobulk	DESeq2	LRT	0.045540403770502
527	KC_AB1	Ir52c	-0.850932661185203	0.660736244265418	1	pseudobulk	DESeq2	LRT	0.179971869591347
527	KC_AB1	Ir56a	0.380370988489192	0.535643314500968	1	pseudobulk	DESeq2	LRT	0.271124311256452
527	KC_AB1	Ir62a	-1.44925888589622	0.6315588144614	1	pseudobulk	DESeq2	LRT	0.199586199175701
527	KC_AB1	Ir68a	-0.70924130443717	0.299301275942684	1	pseudobulk	DESeq2	LRT	0.523891431727514
528	KC_AB1	Ir76a	0.535124599493902	0.435215208248936	1	pseudobulk	DESeq2	LRT	0.361295936969826
528	KC_AB1	Ir93a	0.13617601020169	1	1	pseudobulk	DESeq2	LRT	0
528	KC_AB1	Irbp	1.74286091722126	0.11535610249394	1	pseudobulk	DESeq2	LRT	0.937959425769319
528	KC_AB1	Irbp18	0.119436752192568	0.749698900276025	1	pseudobulk	DESeq2	LRT	0.125113126214584
528	KC_AB1	Ire1	0.182767412681594	0.631076691585448	1	pseudobulk	DESeq2	LRT	0.199917859918328
528	KC_AB1	Iris	0.144938062156184	0.939011437924561	1	pseudobulk	DESeq2	LRT	0.027329117640952
528	KC_AB1	Irk1	0.243199347770039	0.883207012028292	1	pseudobulk	DESeq2	LRT	0.053937491615436
528	KC_AB1	Irk2	3.51727516456755	0.0735103265174962	1	pseudobulk	DESeq2	LRT	1.13365164820085
528	KC_AB1	Irp-1A	0.0304803239879316	0.92601391195577	1	pseudobulk	DESeq2	LRT	0.033382488652581
528	KC_AB1	Irp-1B	-1.2594239325953	0.143657922801468	1	pseudobulk	DESeq2	LRT	0.842670417468237
529	KC_AB1	Iru	-0.156961709484937	0.647761836013499	1	pseudobulk	DESeq2	LRT	0.188584642767629
529	KC_AB1	IscU	0.0046108669575315	0.925558229897189	1	pseudobulk	DESeq2	LRT	0.033596253164442
529	KC_AB1	isopeptidase-T-3	-0.155349025343589	0.784011579263746	1	pseudobulk	DESeq2	LRT	0.105677523064014
529	KC_AB1	isoQC	0.265483599873216	0.686282491633104	1	pseudobulk	DESeq2	LRT	0.163497080643239
529	KC_AB1	Iswi	0.0845456075900922	0.819262145370415	1	pseudobulk	DESeq2	LRT	0.086577111583360
529	KC_AB1	Itgbn	-0.95606620823724	0.726305878557626	1	pseudobulk	DESeq2	LRT	0.138880440741803
529	KC_AB1	ITP	1.09246423455511	0.165966390181021	1	pseudobulk	DESeq2	LRT	0.779979851940629
529	KC_AB1	Itpr	-0.184884053148648	0.570821257420153	1	pseudobulk	DESeq2	LRT	0.243499862101942
529	KC_AB1	ix	0.119652691726643	0.778398149983318	1	pseudobulk	DESeq2	LRT	0.108798204924945
529	KC_AB1	Jafrac1	-0.0859936922298125	0.758864620379368	1	pseudobulk	DESeq2	LRT	0.119835694284098
530	KC_AB1	Jafrac2	0.173292854796881	0.81045753235882	1	pseudobulk	DESeq2	LRT	0.091269737071395
530	KC_AB1	jagn	-0.16326977551429	0.654979444006751	1	pseudobulk	DESeq2	LRT	0.183772329770851
530	KC_AB1	janA	-0.325499211474307	0.524320257982001	1	pseudobulk	DESeq2	LRT	0.28040336227515

530	KC_AB1	janB	0.112935832476632	0.940353958317081	1	pseudobulk	DESeq2	LRT	0.026708642986417
530	KC_AB1	jar	-0.173319064136142	0.447511185431607	1	pseudobulk	DESeq2	LRT	0.349196105129042
530	KC_AB1	Jarid2	0.0388221240692127	0.842340730898606	1	pseudobulk	DESeq2	LRT	0.07451219874236
530	KC_AB1	jbug	-1.56855358782635	0.573026303465967	1	pseudobulk	DESeq2	LRT	0.24182544227704
530	KC_AB1	jdp	0.044073031336653	0.624093687864567	1	pseudobulk	DESeq2	LRT	0.20475020988739
530	KC_AB1	jeb	-0.621493805425444	0.182657348382865	1	pseudobulk	DESeq2	LRT	0.738362851248761
530	KC_AB1	jef	0.132153959319097	0.425874822718884	1	pseudobulk	DESeq2	LRT	0.37071803421003
531	KC_AB1	jet	-0.211303269271931	0.73689031258131	1	pseudobulk	DESeq2	LRT	0.132597152827372
531	KC_AB1	JHDM2	-0.438110285474311	0.744845939421166	1	pseudobulk	DESeq2	LRT	0.12793354547751
531	KC_AB1	JhI-21	-0.332339938382811	0.855195084351371	1	pseudobulk	DESeq2	LRT	0.06793480411032
531	KC_AB1	jigr1	-0.025823946579780	0.887227550195079	1	pseudobulk	DESeq2	LRT	0.05196498093472
531	KC_AB1	JIL-1	-0.108384969675237	0.547995594111	1	pseudobulk	DESeq2	LRT	0.26122293323273
531	KC_AB1	jim	0.289092264610334	0.0280836282180056	1	pseudobulk	DESeq2	LRT	1.55154678495859
531	KC_AB1	jing	0.443768773848714	0.0616414458615468	1	pseudobulk	DESeq2	LRT	1.21012718302424
531	KC_AB1	JMJD4	0.713239800520783	0.447192372527529	1	pseudobulk	DESeq2	LRT	0.34950561253991
531	KC_AB1	JMJD5	0.0921829404143341	0.871011556576532	1	pseudobulk	DESeq2	LRT	0.05997608273731
531	KC_AB1	JMJD7	-0.482552245863007	0.479762004966911	1	pseudobulk	DESeq2	LRT	0.31897414921228
532	KC_AB1	jnj	-0.200275544380475	0.754594340390151	1	pseudobulk	DESeq2	LRT	0.12228645641907
532	KC_AB1	Jon74E	-0.850996402080018	0.900449987182352	1	pseudobulk	DESeq2	LRT	0.045540403770502
532	KC_AB1	jp	-0.218017234507426	0.317264767385933	1	pseudobulk	DESeq2	LRT	0.49857815411911
532	KC_AB1	Jra	0.107095544473642	0.796414417965743	1	pseudobulk	DESeq2	LRT	0.09886088628113
532	KC_AB1	JTBR	-0.085438065512506	0.865213179020753	1	pseudobulk	DESeq2	LRT	0.06287687396202
532	KC_AB1	jub	-0.251391836970304	0.679180456747136	1	pseudobulk	DESeq2	LRT	0.16801481930266
532	KC_AB1	jumu	0.809125913417745	0.114852474807712	1	pseudobulk	DESeq2	LRT	0.93985964231699
532	KC_AB1	Jupiter	0.0956844012115723	0.44334069197614	1	pseudobulk	DESeq2	LRT	0.35326240520045
532	KC_AB1	jus	-0.028316453676119	0.762913756120304	1	pseudobulk	DESeq2	LRT	0.11752455425749
532	KC_AB1	jv	-0.340144935324874	0.767471064046867	1	pseudobulk	DESeq2	LRT	0.11493798974220
533	KC_AB1	jvl	-0.009915144261284	0.937941764540843	1	pseudobulk	DESeq2	LRT	0.02782412550547
533	KC_AB1	Jwa	0.0450805196392052	0.861167242901565	1	pseudobulk	DESeq2	LRT	0.06491249823737
533	KC_AB1	KaiR1D	-0.144435067076584	0.714570062049278	1	pseudobulk	DESeq2	LRT	0.14595518315059
533	KC_AB1	Kank	-0.231763284581386	0.759342880441134	1	pseudobulk	DESeq2	LRT	0.119562074620781
533	KC_AB1	Kap-alpha1	0.0852005552389855	0.811355602256949	1	pseudobulk	DESeq2	LRT	0.09078876077065
533	KC_AB1	Kap-alpha3	-0.200238406164073	0.314969855602116	1	pseudobulk	DESeq2	LRT	0.50173100866153
533	KC_AB1	Kap3	0.388363861586972	0.202678974932993	1	pseudobulk	DESeq2	LRT	0.693191300858921
533	KC_AB1	kappaB-Ras	-0.82542030717083	1	1	pseudobulk	DESeq2	LRT	0
533	KC_AB1	kar	1.08899820779554	0.878177167143508	1	pseudobulk	DESeq2	LRT	0.05641785886507
533	KC_AB1	Karybeta3	0.023337512860204	0.935357759466747	1	pseudobulk	DESeq2	LRT	0.02902224661986
534	KC_AB1	kat-60L1	-0.270326388725376	0.393798252279795	1	pseudobulk	DESeq2	LRT	0.40472621564057
534	KC_AB1	Kat60	0.16203607950335	0.726341331940554	1	pseudobulk	DESeq2	LRT	0.13885924191463
534	KC_AB1	kat80	-0.014040836917035	0.964138570840696	1	pseudobulk	DESeq2	LRT	0.01586054262566
534	KC_AB1	kay	0.447922141092432	0.256958205698808	1	pseudobulk	DESeq2	LRT	0.59013750900503
534	KC_AB1	Kaz1-ORFB	0.520713377223975	0.636334399925995	1	pseudobulk	DESeq2	LRT	0.19631459838217
534	KC_AB1	kcc	-0.076636089630865	0.663922205906315	1	pseudobulk	DESeq2	LRT	0.17788280545867
534	KC_AB1	Kcmf1	-0.014022123601630	0.951128973285087	1	pseudobulk	DESeq2	LRT	0.02176058864806
534	KC_AB1	KCNQ	-0.031632442583200	0.810766787915431	1	pseudobulk	DESeq2	LRT	0.09110404996482
534	KC_AB1	KdelR	0.101958274857625	0.75210654360292	1	pseudobulk	DESeq2	LRT	0.12372063278432
534	KC_AB1	Kdm2	0.263548867720943	0.372599654484927	1	pseudobulk	DESeq2	LRT	0.428757552164841
535	KC_AB1	Kdm4A	-0.885285832782994	0.107589298547612	1	pseudobulk	DESeq2	LRT	0.96823092395634
535	KC_AB1	Kdm4B	0.121701434474941	0.491408638140943	1	pseudobulk	DESeq2	LRT	0.30855721361911
535	KC_AB1	kdn	0.002582943311865	0.91065772513103	1	pseudobulk	DESeq2	LRT	0.04064482392537
535	KC_AB1	Keap1	-0.181597449854503	0.765823269972783	1	pseudobulk	DESeq2	LRT	0.11587144150461
535	KC_AB1	Kebab	0.135879393043758	1	1	pseudobulk	DESeq2	LRT	0

535	KC_AB1	kek2	0.445982509103692	0.804553940113815	1	pseudobulk	DESeq2	LRT	0.094444833965836
535	KC_AB1	kek3	-0.297569392805985	0.0775471535010887	1	pseudobulk	DESeq2	LRT	1.11043413906841
535	KC_AB1	kek4	0.067425284061244	0.825687480004527	1	pseudobulk	DESeq2	LRT	0.083184300611997
535	KC_AB1	kek5	0.0680305129570023	0.940651258616759	1	pseudobulk	DESeq2	LRT	0.026571359070847
535	KC_AB1	kek6	-0.212307795687745	0.413426098398036	1	pseudobulk	DESeq2	LRT	0.383602111084414
536	KC_AB1	kel	0.106630276406237	0.856525520973975	1	pseudobulk	DESeq2	LRT	0.067259692297951
536	KC_AB1	ken	1.08899820779554	0.697631521651407	1	pseudobulk	DESeq2	LRT	0.156373904552
536	KC_AB1	kermit	-0.82542030717083	1	1	pseudobulk	DESeq2	LRT	0
536	KC_AB1	key	0.101521407762362	0.760214076235518	1	pseudobulk	DESeq2	LRT	0.119064093198037
536	KC_AB1	KFase	0.866143204781726	0.641643690690731	1	pseudobulk	DESeq2	LRT	0.192706071812621
536	KC_AB1	Khc	0.327315180264462	0.275393753988348	1	pseudobulk	DESeq2	LRT	0.560045913894475
536	KC_AB1	Khc-73	-0.0281387991172921	0.861821170976087	1	pseudobulk	DESeq2	LRT	0.064582841501819
536	KC_AB1	kibra	-1.97114790339378	0.230503052311109	1	pseudobulk	DESeq2	LRT	0.637323319328151
536	KC_AB1	Kif19A	-1.21673908022264	0.60839381642346	1	pseudobulk	DESeq2	LRT	0.215815208667062
536	KC_AB1	Kif3C	0.261698975662625	0.629729993255914	1	pseudobulk	DESeq2	LRT	0.200845621299606
537	KC_AB1	kin17	0.164695692211349	0.75347370972535	1	pseudobulk	DESeq2	LRT	0.122931896528381
537	KC_AB1	kirre	0.0071622266448084	0.959560694354655	1	pseudobulk	DESeq2	LRT	0.017927549964828
537	KC_AB1	kis	0.0037464049956684	0.943224743875179	1	pseudobulk	DESeq2	LRT	0.025384814796452
537	KC_AB1	kl-2	1.08899820779554	0.670846322504933	1	pseudobulk	DESeq2	LRT	0.173376956629912
537	KC_AB1	kl-3	2.44062030581626	0.306343935355429	1	pseudobulk	DESeq2	LRT	0.51379071290912
537	KC_AB1	kl-5	0.114184054362142	1	1	pseudobulk	DESeq2	LRT	0
537	KC_AB1	klar	-0.193116329521852	0.452671996086779	1	pseudobulk	DESeq2	LRT	0.344216371688019
537	KC_AB1	Klc	0.0541392813169906	0.835677032135306	1	pseudobulk	DESeq2	LRT	0.077961533879219
537	KC_AB1	klg	-0.209323322194236	0.213695828211603	1	pseudobulk	DESeq2	LRT	0.670203956087523
537	KC_AB1	KLHL18	-0.203852425481516	0.598425181974217	1	pseudobulk	DESeq2	LRT	0.222990139471238
538	KC_AB1	Klp10A	0.189662575522874	0.497752235672785	1	pseudobulk	DESeq2	LRT	0.302986780646261
538	KC_AB1	Klp31E	0.29471015933484	0.675085752604738	1	pseudobulk	DESeq2	LRT	0.170641057513233
538	KC_AB1	Klp54D	1.33048802031544	0.119177819612816	1	pseudobulk	DESeq2	LRT	0.923804564361682
538	KC_AB1	Klp64D	0.124316394895084	0.680916809311802	1	pseudobulk	DESeq2	LRT	0.166905944568533
538	KC_AB1	Klp68D	0.24358065173108	0.449331302820752	1	pseudobulk	DESeq2	LRT	0.347433325077636
538	KC_AB1	Klp98A	-0.0788988621894777	0.756310407218123	1	pseudobulk	DESeq2	LRT	0.121299923448523
538	KC_AB1	klu	1.68130633771206	0.562684503730219	1	pseudobulk	DESeq2	LRT	0.249735045103917
538	KC_AB1	Kmn1	-0.0769326150246702	0.939750576698345	1	pseudobulk	DESeq2	LRT	0.026987399099829
538	KC_AB1	Kmn2	3.39385523352305	0.108103110209592	1	pseudobulk	DESeq2	LRT	0.966161810880806
538	KC_AB1	kmr	0.15064113144186	0.428986919636436	1	pseudobulk	DESeq2	LRT	0.367555949811366
539	KC_AB1	knk	-0.501300761870089	0.863550576027942	1	pseudobulk	DESeq2	LRT	0.063712221794699
539	KC_AB1	knon	-0.850932661185203	0.660736244265418	1	pseudobulk	DESeq2	LRT	0.179971869591347
539	KC_AB1	ko	-0.883805746545996	0.461618696969106	1	pseudobulk	DESeq2	LRT	0.335716609241262
539	KC_AB1	koi	0.201310983678913	0.490466202474617	1	pseudobulk	DESeq2	LRT	0.309390914042422
539	KC_AB1	koko	-0.223357041804379	0.772877784501388	1	pseudobulk	DESeq2	LRT	0.111889175826843
539	KC_AB1	Kr-h1	0.318976418305078	0.276904907472089	1	pseudobulk	DESeq2	LRT	0.557669347363265
539	KC_AB1	Kr-h2	0.227448635586	0.462456518487737	1	pseudobulk	DESeq2	LRT	0.334929094640769
539	KC_AB1	kra	-0.100487353898817	0.478847426928705	1	pseudobulk	DESeq2	LRT	0.319802841905184
539	KC_AB1	kraken	-0.195555038542612	0.616234892578986	1	pseudobulk	DESeq2	LRT	0.210253714611275
539	KC_AB1	kri	0.114735954015547	0.728750659444475	1	pseudobulk	DESeq2	LRT	0.137421039240865
540	KC_AB1	Krn	-0.116300217629876	0.89825874951453	1	pseudobulk	DESeq2	LRT	0.046598543845134
540	KC_AB1	KrT95D	0.143310116024882	0.309739540320029	1	pseudobulk	DESeq2	LRT	0.509003350514427
540	KC_AB1	krz	0.061063590116055	0.816560054778771	1	pseudobulk	DESeq2	LRT	0.088011869102555
540	KC_AB1	ksh	0.0376602595176255	0.902870591672803	1	pseudobulk	DESeq2	LRT	0.044374492598881
540	KC_AB1	ksr	0.469836272615898	0.348950574925825	1	pseudobulk	DESeq2	LRT	0.457236081800291
540	KC_AB1	kst	1.08899820779554	0.697631521651407	1	pseudobulk	DESeq2	LRT	0.156373904552
540	KC_AB1	Ktl	-0.301276775471147	0.0871434937862807	1	pseudobulk	DESeq2	LRT	1.05976503217069

540	KC_AB1	kto	0.587985825558095	0.344952502144852	1	pseudobulk	DESeq2	LRT	0.462240700510803
540	KC_AB1	ktub	2.45879058325456	0.260906079711047	1	pseudobulk	DESeq2	LRT	0.583515800722718
540	KC_AB1	Ku80	-1.37548852721203	0.287844783245504	1	pseudobulk	DESeq2	LRT	0.540841637072913
541	KC_AB1	Kua	-0.748375356063598	0.707269124804655	1	pseudobulk	DESeq2	LRT	0.150415300237416
541	KC_AB1	kud	0.0349712731365002	0.893271400671582	1	pseudobulk	DESeq2	LRT	0.04901657033152
541	KC_AB1	kug	-0.0313310943743668	0.853647860570109	1	pseudobulk	DESeq2	LRT	0.068721243772426
541	KC_AB1	kuk	0.273222542632613	0.420153810156233	1	pseudobulk	DESeq2	LRT	0.376591693712268
541	KC_AB1	Kul	0.280974593982104	0.343804210970722	1	pseudobulk	DESeq2	LRT	0.463688808305894
541	KC_AB1	kune	-0.0454962168859866	0.861169070948662	1	pseudobulk	DESeq2	LRT	0.064911576337561
541	KC_AB1	kuz	-0.0784228880269966	0.588312392097801	1	pseudobulk	DESeq2	LRT	0.230392003621711
541	KC_AB1	Kyat	-0.62655242716653	0.381643643137745	1	pseudobulk	DESeq2	LRT	0.418341967065148
541	KC_AB1	kz	0.45719775015443	0.565733625614739	1	pseudobulk	DESeq2	LRT	0.247388007250522
541	KC_AB1	l(1)10Bb	-0.0241957382572256	0.94069676598431	1	pseudobulk	DESeq2	LRT	0.026550349030856
542	KC_AB1	l(1)G0004	0.228969356132523	0.787739773965144	1	pseudobulk	DESeq2	LRT	0.103617225906213
542	KC_AB1	l(1)G0007	-0.09291660923663	0.821353455956527	1	pseudobulk	DESeq2	LRT	0.085469911165795
542	KC_AB1	l(1)G0020	2.39533548415136	0.318016613010836	1	pseudobulk	DESeq2	LRT	0.497550192120982
542	KC_AB1	l(1)G0193	0.303194733833815	0.644676119056155	1	pseudobulk	DESeq2	LRT	0.190658417200445
542	KC_AB1	l(1)G0196	-0.0721269595528085	0.729580433459982	1	pseudobulk	DESeq2	LRT	0.136926821820961
542	KC_AB1	l(1)G0289	-0.136220405010669	0.369308984885367	1	pseudobulk	DESeq2	LRT	0.432610126308761
542	KC_AB1	l(1)G0320	-0.0401719636667819	0.841077552472204	1	pseudobulk	DESeq2	LRT	0.075163957759864
542	KC_AB1	l(2)05287	-0.0288437734468504	0.945090772141806	1	pseudobulk	DESeq2	LRT	0.024526477261020
542	KC_AB1	l(2)05714	0.449233456864479	0.428253894001842	1	pseudobulk	DESeq2	LRT	0.368298679417154
542	KC_AB1	l(2)09851	0.08187353385771	0.899400269704615	1	pseudobulk	DESeq2	LRT	0.046046986535467
543	KC_AB1	l(2)10685	0.530798652010862	0.664357930525457	1	pseudobulk	DESeq2	LRT	0.177597876399926
543	KC_AB1	l(2)34Fd	0.47555388028613	0.666418096141823	1	pseudobulk	DESeq2	LRT	0.176253218462264
543	KC_AB1	l(2)35Be	0.3038397736737	0.591378731790513	1	pseudobulk	DESeq2	LRT	0.228134298396494
543	KC_AB1	l(2)37Bb	-0.230954088533027	0.68615105101226	1	pseudobulk	DESeq2	LRT	0.163580267092318
543	KC_AB1	l(2)37Cb	-1.04400437590145	0.337577868189795	1	pseudobulk	DESeq2	LRT	0.471626033723264
543	KC_AB1	l(2)37Cc	-0.249044729976202	0.518449092756961	1	pseudobulk	DESeq2	LRT	0.285293881165295
543	KC_AB1	l(2)37Cd	1.51196084809339	0.227472651995571	1	pseudobulk	DESeq2	LRT	0.643070809122694
543	KC_AB1	l(2)37Cg	-0.539869261833284	0.44683632330142	1	pseudobulk	DESeq2	LRT	0.349851530345643
543	KC_AB1	l(2)41Ab	0.118570175053961	0.850668473294418	1	pseudobulk	DESeq2	LRT	0.070239662326144
543	KC_AB1	l(2)dtl	0.141165457253645	0.878486655741235	1	pseudobulk	DESeq2	LRT	0.056264831074733
544	KC_AB1	l(2)efl	0.0277788983132372	0.883091685165871	1	pseudobulk	DESeq2	LRT	0.053994204356169
544	KC_AB1	l(2)gd1	0.866214172712939	0.124901474196646	1	pseudobulk	DESeq2	LRT	0.903432435671577
544	KC_AB1	l(2)gl	-0.850996402080018	0.900449987182352	1	pseudobulk	DESeq2	LRT	0.045540403770502
544	KC_AB1	l(2)k01209	0.627395499462269	0.0628729666996911	1	pseudobulk	DESeq2	LRT	1.20153604671348
544	KC_AB1	l(2)k05819	-0.26566169184421	0.413110544608724	1	pseudobulk	DESeq2	LRT	0.383933719555157
544	KC_AB1	l(2)k09022	-0.770549948825493	0.502528028352981	1	pseudobulk	DESeq2	LRT	0.298839710584946
544	KC_AB1	l(2)k09848	-0.394034902489078	0.597672006376531	1	pseudobulk	DESeq2	LRT	0.223537085074653
544	KC_AB1	l(2)k09913	0.0593727050399333	0.690132231533702	1	pseudobulk	DESeq2	LRT	0.161067689083861
544	KC_AB1	l(2)k10201	0.945517553524618	0.501050392334321	1	pseudobulk	DESeq2	LRT	0.300118593469762
544	KC_AB1	l(2)k14505	-0.0642691965307514	0.841971941535729	1	pseudobulk	DESeq2	LRT	0.074702380993512
545	KC_AB1	l(2)SH0834	-0.109084470477222	0.702721074035307	1	pseudobulk	DESeq2	LRT	0.15321702212806
545	KC_AB1	l(3)02640	-0.0203101197082053	0.947166358490081	1	pseudobulk	DESeq2	LRT	0.023573735643941
545	KC_AB1	l(3)04053	-0.194843886972909	0.665912182693073	1	pseudobulk	DESeq2	LRT	0.176583039728302
545	KC_AB1	l(3)05822	0.283688866034606	0.426623438460437	1	pseudobulk	DESeq2	LRT	0.369955288327735
545	KC_AB1	l(3)07882	0.652650010846077	0.347514765013116	1	pseudobulk	DESeq2	LRT	0.459026738620638
545	KC_AB1	l(3)72Ab	-0.668739704377722	0.227039092786027	1	pseudobulk	DESeq2	LRT	0.643899357258384
545	KC_AB1	l(3)72Dn	0.211000513416313	0.831698987340343	1	pseudobulk	DESeq2	LRT	0.080033827304359
545	KC_AB1	l(3)72Dp	3.21696230837417	0.13645887550041	1	pseudobulk	DESeq2	LRT	0.864998211877592
545	KC_AB1	l(3)72Dr	-1.05220943051072	0.633614666162374	1	pseudobulk	DESeq2	LRT	0.198174778767564

545	KC_AB1	l(3)73Ah	0.0916401279068575	0.804273019944548	1	pseudobulk	DESeq2	LRT	0.094596499848664
546	KC_AB1	l(3)76BDm	-0.0283320530682301	0.897409642539931	1	pseudobulk	DESeq2	LRT	0.047009268344971
546	KC_AB1	l(3)80Fg	0.0280960745709895	0.752451538517587	1	pseudobulk	DESeq2	LRT	0.123521465477816
546	KC_AB1	l(3)80Fj	0.23577021120855	0.0320237447497559	1	pseudobulk	DESeq2	LRT	1.49452788450095
546	KC_AB1	l(3)87Df	0.231860345720309	0.488148611444218	1	pseudobulk	DESeq2	LRT	0.311447941717048
546	KC_AB1	l(3)L1231	-0.0140070192741862	0.91487625673986	1	pseudobulk	DESeq2	LRT	0.038637643255199
546	KC_AB1	l(3)mbt	-0.29855561578169	0.5456450979358	1	pseudobulk	DESeq2	LRT	0.26308974213594
546	KC_AB1	l(3)neo43	-0.106201597495185	0.746384896226087	1	pseudobulk	DESeq2	LRT	0.12703715751455
546	KC_AB1	l(3)psg2	0.242338870481142	0.465046143634756	1	pseudobulk	DESeq2	LRT	0.332503952633276
546	KC_AB1	L2HGDH	-0.226155096262135	0.644547026565969	1	pseudobulk	DESeq2	LRT	0.190745390749626
546	KC_AB1	La	-0.0298291193240204	0.931843681987455	1	pseudobulk	DESeq2	LRT	0.030656935010940
547	KC_AB1	Lac	0.631006901357798	0.693991797348321	1	pseudobulk	DESeq2	LRT	0.15864566266822
547	KC_AB1	lace	0.198654772233569	0.680470813027745	1	pseudobulk	DESeq2	LRT	0.167190497959303
547	KC_AB1	Lam	0.0414854419135317	0.842636135590224	1	pseudobulk	DESeq2	LRT	0.074359920534437
547	KC_AB1	LamC	-0.655131685049161	0.54180643419788	1	pseudobulk	DESeq2	LRT	0.266155841827689
547	KC_AB1	Lamp1	0.0533956220198142	0.750054529541964	1	pseudobulk	DESeq2	LRT	0.124907161917221
547	KC_AB1	lap	-0.174284590889354	0.239475189563263	1	pseudobulk	DESeq2	LRT	0.620739474290814
547	KC_AB1	Lap1	0.220215647715418	0.711374615907491	1	pseudobulk	DESeq2	LRT	0.147901635872183
547	KC_AB1	Lar	-0.174277220600962	0.261638684748675	1	pseudobulk	DESeq2	LRT	0.582298042722416
547	KC_AB1	lark	-0.106148880206293	0.608930787818634	1	pseudobulk	DESeq2	LRT	0.215432067263074
547	KC_AB1	larp	-0.0909139646241083	0.551603811252776	1	pseudobulk	DESeq2	LRT	0.258372741770424
548	KC_AB1	Larp4B	-0.147828335072497	0.232893470878616	1	pseudobulk	DESeq2	LRT	0.632842686652677
548	KC_AB1	Larp7	0.13825483194754	0.843768659117252	1	pseudobulk	DESeq2	LRT	0.073776610057739
548	KC_AB1	Las	0.0753137226606306	0.896229665662303	1	pseudobulk	DESeq2	LRT	0.047580684814617
548	KC_AB1	lawc	0.254486471720533	0.229266860655879	1	pseudobulk	DESeq2	LRT	0.639658715764598
548	KC_AB1	lbk	0.23742387177324	0.285206007956587	1	pseudobulk	DESeq2	LRT	0.544841330170415
548	KC_AB1	lbm	-0.242967177946648	0.311904295598026	1	pseudobulk	DESeq2	LRT	0.505978644025566
548	KC_AB1	LBR	-0.154078403257169	0.839330793119325	1	pseudobulk	DESeq2	LRT	0.076066843327400
548	KC_AB1	Lcch3	-0.0877296754236227	0.723036837513528	1	pseudobulk	DESeq2	LRT	0.14083957556674
548	KC_AB1	ldbr	-0.219499078010674	0.908159027051922	1	pseudobulk	DESeq2	LRT	0.041838095835911
548	KC_AB1	lds	0.11269840515639	0.977007463492936	1	pseudobulk	DESeq2	LRT	0.010102118633921
549	KC_AB1	Leash	0.12801225956701	0.84615081203551	1	pseudobulk	DESeq2	LRT	0.072552224434227
549	KC_AB1	lectin-30A	0.13617601020169	1	1	pseudobulk	DESeq2	LRT	0
549	KC_AB1	lectin-46Cb	1.08899820779554	0.670846322504933	1	pseudobulk	DESeq2	LRT	0.173376956629912
549	KC_AB1	Lerp	-0.100147358740443	0.692939128526052	1	pseudobulk	DESeq2	LRT	0.159304914459409
549	KC_AB1	Letm1	-0.0516999511782113	0.904088941690822	1	pseudobulk	DESeq2	LRT	0.043788842770796
549	KC_AB1	LeuRS	-0.234386331629434	0.790619289554759	1	pseudobulk	DESeq2	LRT	0.102032593933102
549	KC_AB1	LeuRS-m	0.0546477660375442	0.949667858192561	1	pseudobulk	DESeq2	LRT	0.022428260580786
549	KC_AB1	levy	-0.146016351906673	0.290049889738186	1	pseudobulk	DESeq2	LRT	0.537527295292424
549	KC_AB1	Lgr3	1.28389016842472	0.514221177656897	1	pseudobulk	DESeq2	LRT	0.288850041364617
549	KC_AB1	Lgr4	-0.82542030717083	1	1	pseudobulk	DESeq2	LRT	0
550	KC_AB1	lgs	-0.0377261529324657	0.822086156761797	1	pseudobulk	DESeq2	LRT	0.085082664886424
550	KC_AB1	Lhr	0.389733297448039	0.639979746458748	1	pseudobulk	DESeq2	LRT	0.193833769985468
550	KC_AB1	lic	-0.182597982917163	0.649613768284831	1	pseudobulk	DESeq2	LRT	0.187344778984307
550	KC_AB1	lid	0.204855877504308	0.286904818569866	1	pseudobulk	DESeq2	LRT	0.542262157711722
550	KC_AB1	lig	0.0055306395553818	0.973142300480132	1	pseudobulk	DESeq2	LRT	0.011823649151369
550	KC_AB1	lili	0.561225490065003	0.326266114226278	1	pseudobulk	DESeq2	LRT	0.486428029395495
550	KC_AB1	lilli	0.216596234903968	0.127314464112703	1	pseudobulk	DESeq2	LRT	0.895122253632362
550	KC_AB1	LIMK1	-0.0310422320192224	0.901402604726597	1	pseudobulk	DESeq2	LRT	0.045081191309690
550	KC_AB1	lin	0.572610764121255	0.400682920614666	1	pseudobulk	DESeq2	LRT	0.39719916927404
550	KC_AB1	lin-28	0.997694962638592	0.665140342223729	1	pseudobulk	DESeq2	LRT	0.177086710452097
551	KC_AB1	lin-52	-0.0173671298618207	0.90191053400728	1	pseudobulk	DESeq2	LRT	0.044836540633229

551	KC_AB1	link	0.754666262784078	0.69777646569361	1	pseudobulk	DESeq2	LRT	0.156283682339557
551	KC_AB1	Lint-1	0.197515557386956	0.580342796020254	1	pseudobulk	DESeq2	LRT	0.236315402225175
551	KC_AB1	Liprin-alpha	0.292393125746063	0.290280405526893	1	pseudobulk	DESeq2	LRT	0.53718227887295
551	KC_AB1	Liprin-beta	-0.409137166291148	0.51532201325867	1	pseudobulk	DESeq2	LRT	0.287921305176085
551	KC_AB1	Liprin-gamma	0.0802493173872728	0.580005384314674	1	pseudobulk	DESeq2	LRT	0.236567974769308
551	KC_AB1	Lis-1	-0.0080560068974662	0.937823010684675	1	pseudobulk	DESeq2	LRT	0.027879115496958
551	KC_AB1	List	1.08899820779554	0.697631521651407	1	pseudobulk	DESeq2	LRT	0.156373904552
551	KC_AB1	Lk6	0.204934384675666	0.0910096275102938	1	pseudobulk	DESeq2	LRT	1.04091266313568
551	KC_AB1	Lkb1	-0.0337569072780117	0.940093450499307	1	pseudobulk	DESeq2	LRT	0.026828972975913
552	KC_AB1	LKRSDH	0.888933280325755	0.641693803207784	1	pseudobulk	DESeq2	LRT	0.192672154640023
552	KC_AB1	lms	-0.850996402080018	0.900449987182352	1	pseudobulk	DESeq2	LRT	0.045540403770502
552	KC_AB1	lncRNA:alphagamr	-0.128942263488751	0.559879214351858	1	pseudobulk	DESeq2	LRT	0.251905655491005
552	KC_AB1	lncRNA:cherub	-0.493917181159795	0.297012444803641	1	pseudobulk	DESeq2	LRT	0.527225353388782
552	KC_AB1	lncRNA:CR30055	0.369319631999528	0.135543179173923	1	pseudobulk	DESeq2	LRT	0.867922332171723
552	KC_AB1	lncRNA:CR30121	-0.838580640014249	0.695856596814275	1	pseudobulk	DESeq2	LRT	0.157480251235852
552	KC_AB1	lncRNA:CR31044	-1.02514982632322	0.127993923575442	1	pseudobulk	DESeq2	LRT	0.89281064769819
552	KC_AB1	lncRNA:CR31386	-1.96719661029409	0.414177861019091	1	pseudobulk	DESeq2	LRT	0.382813119093482
552	KC_AB1	lncRNA:CR31451	-0.143574523280661	0.231517545882463	1	pseudobulk	DESeq2	LRT	0.635416089781331
552	KC_AB1	lncRNA:CR32218	0.147132311389587	0.721135107345394	1	pseudobulk	DESeq2	LRT	0.141983360970436
553	KC_AB1	lncRNA:CR32553	-0.903100498097436	0.648988249826069	1	pseudobulk	DESeq2	LRT	0.187763166191766
553	KC_AB1	lncRNA:CR32582	0.320855530390182	0.798208865580763	1	pseudobulk	DESeq2	LRT	0.097883452884466
553	KC_AB1	lncRNA:CR32773	-0.660121547679352	0.74106204897254	1	pseudobulk	DESeq2	LRT	0.130145427110827
553	KC_AB1	lncRNA:CR33938	-0.95606620823724	0.726305878557626	1	pseudobulk	DESeq2	LRT	0.138880440741803
553	KC_AB1	lncRNA:CR34335	0.0013686123165688	0.9906611762937	1	pseudobulk	DESeq2	LRT	0.004074856540906
553	KC_AB1	lncRNA:CR40172	2.69141570238421	0.27890455639465	1	pseudobulk	DESeq2	LRT	0.554544390714624
553	KC_AB1	lncRNA:CR40341	-0.807185913698031	0.687648214393182	1	pseudobulk	DESeq2	LRT	0.162633680398578
553	KC_AB1	lncRNA:CR40469	0.381431038398219	0.47366113744122	1	pseudobulk	DESeq2	LRT	0.324532246446203
553	KC_AB1	lncRNA:CR41257	1.08899820779554	0.670846322504933	1	pseudobulk	DESeq2	LRT	0.173376956629912
553	KC_AB1	lncRNA:CR42491	-0.909323037328223	0.594008737361652	1	pseudobulk	DESeq2	LRT	0.226207166870584
554	KC_AB1	lncRNA:CR42549	-0.113051734244233	0.826427897983678	1	pseudobulk	DESeq2	LRT	0.082795030627031
554	KC_AB1	lncRNA:CR42736	-0.850996402080018	0.900449987182352	1	pseudobulk	DESeq2	LRT	0.045540403770502
554	KC_AB1	lncRNA:CR42861	0.16182425216112	0.507776112066767	1	pseudobulk	DESeq2	LRT	0.294327734028683
554	KC_AB1	lncRNA:CR42862	0.19794709516016	0.0313030557931693	1	pseudobulk	DESeq2	LRT	1.50441326471129
554	KC_AB1	lncRNA:CR43146	0.929128749160313	0.514398349143244	1	pseudobulk	DESeq2	LRT	0.288700433861303
554	KC_AB1	lncRNA:CR43148	0.390961762688467	0.634343880033737	1	pseudobulk	DESeq2	LRT	0.197675245724743
554	KC_AB1	lncRNA:CR43166	1.08899820779554	0.697631521651407	1	pseudobulk	DESeq2	LRT	0.156373904552
554	KC_AB1	lncRNA:CR43174	-0.82542030717083	1	1	pseudobulk	DESeq2	LRT	0
554	KC_AB1	lncRNA:CR43241	-0.28666927188319	0.576885008135814	1	pseudobulk	DESeq2	LRT	0.238910747172019
554	KC_AB1	lncRNA:CR43256	0.445781060933151	0.692254001333419	1	pseudobulk	DESeq2	LRT	0.159734525286972
555	KC_AB1	lncRNA:CR43285	-0.850932661185203	0.660736244265418	1	pseudobulk	DESeq2	LRT	0.179971869591347
555	KC_AB1	lncRNA:CR43287	-1.6747012584303	0.563129890257477	1	pseudobulk	DESeq2	LRT	0.249391420218277
555	KC_AB1	lncRNA:CR43301	1.44429060067088	0.481823685817173	1	pseudobulk	DESeq2	LRT	0.317111854468736
555	KC_AB1	lncRNA:CR43309	-0.850996402080018	0.900449987182352	1	pseudobulk	DESeq2	LRT	0.045540403770502
555	KC_AB1	lncRNA:CR43334	0.30418546481037	0.661076579488793	1	pseudobulk	DESeq2	LRT	0.179748228679232
555	KC_AB1	lncRNA:CR43363	-0.424182005897328	0.832276680909294	1	pseudobulk	DESeq2	LRT	0.079732273448622
555	KC_AB1	lncRNA:CR43404	-0.850932661185203	0.660736244265418	1	pseudobulk	DESeq2	LRT	0.179971869591347
555	KC_AB1	lncRNA:CR43408	-0.82542030717083	1	1	pseudobulk	DESeq2	LRT	0
555	KC_AB1	lncRNA:CR43410	-0.850996402080018	0.900449987182352	1	pseudobulk	DESeq2	LRT	0.045540403770502
555	KC_AB1	lncRNA:CR43414	1.08899820779554	0.697631521651407	1	pseudobulk	DESeq2	LRT	0.156373904552
556	KC_AB1	lncRNA:CR43423	1.08899820779554	0.670846322504933	1	pseudobulk	DESeq2	LRT	0.173376956629912
556	KC_AB1	lncRNA:CR43424	1.24522514393301	0.608213953617254	1	pseudobulk	DESeq2	LRT	0.215943620513061
556	KC_AB1	lncRNA:CR43459	0.309492641245689	0.40217887881224	1	pseudobulk	DESeq2	LRT	0.395580740940061

556	KC_AB1	lncRNA:CR43461	-1.96719661029409	0.414177861019091	1	pseudobulk	DESeq2	LRT	0.382813119093482
556	KC_AB1	lncRNA:CR43492	-0.0322470405402642	0.970888926409382	1	pseudobulk	DESeq2	LRT	0.012830452282245
556	KC_AB1	lncRNA:CR43493	-0.770011685721021	0.708224801478314	1	pseudobulk	DESeq2	LRT	0.149828868658713
556	KC_AB1	lncRNA:CR43496	1.16580575930825	0.214668347187734	1	pseudobulk	DESeq2	LRT	0.6682319874926
556	KC_AB1	lncRNA:CR43604	0.687133299156391	0.686602411048791	1	pseudobulk	DESeq2	LRT	0.163294675854418
556	KC_AB1	lncRNA:CR43605	-0.850996402080018	0.900449987182352	1	pseudobulk	DESeq2	LRT	0.045540403770502
556	KC_AB1	lncRNA:CR43610	-0.82542030717083	1	1	pseudobulk	DESeq2	LRT	0
557	KC_AB1	lncRNA:CR43635	2.31142484995088	0.24780120124803	1	pseudobulk	DESeq2	LRT	0.605896592656783
557	KC_AB1	lncRNA:CR43651	-0.107598480545954	0.733201002336443	1	pseudobulk	DESeq2	LRT	0.134776949998843
557	KC_AB1	lncRNA:CR43654	-0.82542030717083	1	1	pseudobulk	DESeq2	LRT	0
557	KC_AB1	lncRNA:CR43656	-0.82542030717083	1	1	pseudobulk	DESeq2	LRT	0
557	KC_AB1	lncRNA:CR43682	1.08899820779554	0.670846322504933	1	pseudobulk	DESeq2	LRT	0.173376956629912
557	KC_AB1	lncRNA:CR43685	0.379040235013039	0.312336973477213	1	pseudobulk	DESeq2	LRT	0.505376602299799
557	KC_AB1	lncRNA:CR43686	1.08899820779554	0.878177167143508	1	pseudobulk	DESeq2	LRT	0.056417858865076
557	KC_AB1	lncRNA:CR43700	-2.78758686077382	0.170950619440775	1	pseudobulk	DESeq2	LRT	0.767129321195417
557	KC_AB1	lncRNA:CR43785	-1.44925888589622	0.6315588144614	1	pseudobulk	DESeq2	LRT	0.199586199175701
557	KC_AB1	lncRNA:CR43821	1.08899820779554	0.670846322504933	1	pseudobulk	DESeq2	LRT	0.173376956629912
558	KC_AB1	lncRNA:CR43827	1.08899820779554	0.697631521651407	1	pseudobulk	DESeq2	LRT	0.156373904552
558	KC_AB1	lncRNA:CR43828	1.08899820779554	0.670846322504933	1	pseudobulk	DESeq2	LRT	0.173376956629912
558	KC_AB1	lncRNA:CR43836	0.386708584704644	0.808722705392886	1	pseudobulk	DESeq2	LRT	0.092200363630555
558	KC_AB1	lncRNA:CR43837	1.16832976536133	0.106189169139911	1	pseudobulk	DESeq2	LRT	0.973919777253098
558	KC_AB1	lncRNA:CR43838	1.08899820779554	0.878177167143508	1	pseudobulk	DESeq2	LRT	0.056417858865076
558	KC_AB1	lncRNA:CR43849	2.44062030581626	0.306343935355429	1	pseudobulk	DESeq2	LRT	0.51379071290912
558	KC_AB1	lncRNA:CR43855	-0.415345049822811	0.856507449263177	1	pseudobulk	DESeq2	LRT	0.067268855512453
558	KC_AB1	lncRNA:CR43856	0.359285705684311	0.671164369477936	1	pseudobulk	DESeq2	LRT	0.173171107229796
558	KC_AB1	lncRNA:CR43870	1.08899820779554	0.670846322504933	1	pseudobulk	DESeq2	LRT	0.173376956629912
558	KC_AB1	lncRNA:CR43878	-0.243923859827955	0.653749657505189	1	pseudobulk	DESeq2	LRT	0.18458852564841
559	KC_AB1	lncRNA:CR43883	-0.82542030717083	1	1	pseudobulk	DESeq2	LRT	0
559	KC_AB1	lncRNA:CR43884	1.08899820779554	0.670846322504933	1	pseudobulk	DESeq2	LRT	0.173376956629912
559	KC_AB1	lncRNA:CR43887	1.08899820779554	0.670846322504933	1	pseudobulk	DESeq2	LRT	0.173376956629912
559	KC_AB1	lncRNA:CR43893	0.114184054362142	1	1	pseudobulk	DESeq2	LRT	0
559	KC_AB1	lncRNA:CR43900	0.143359888036467	0.92098577622951	1	pseudobulk	DESeq2	LRT	0.035747077026535
559	KC_AB1	lncRNA:CR43907	-0.506516985469986	0.909346984303859	1	pseudobulk	DESeq2	LRT	0.041270369127443
559	KC_AB1	lncRNA:CR43909	1.08899820779554	0.670846322504933	1	pseudobulk	DESeq2	LRT	0.173376956629912
559	KC_AB1	lncRNA:CR43910	1.15872431447787	0.736254552082691	1	pseudobulk	DESeq2	LRT	0.132972007059467
559	KC_AB1	lncRNA:CR43943	1.52216698204519	0.281949074406922	1	pseudobulk	DESeq2	LRT	0.549829326791628
559	KC_AB1	lncRNA:CR43951	-0.850996402080018	0.900449987182352	1	pseudobulk	DESeq2	LRT	0.045540403770502
560	KC_AB1	lncRNA:CR43956	0.144068105809641	0.913017658949245	1	pseudobulk	DESeq2	LRT	0.039520822564225
560	KC_AB1	lncRNA:CR43958	1.55603224697666	0.223978132864999	1	pseudobulk	DESeq2	LRT	0.649794380057083
560	KC_AB1	lncRNA:CR43960	1.08899820779554	0.878177167143508	1	pseudobulk	DESeq2	LRT	0.056417858865076
560	KC_AB1	lncRNA:CR43963	0.453321124912592	0.828810605733731	1	pseudobulk	DESeq2	LRT	0.081544700187927
560	KC_AB1	lncRNA:CR43966	1.08899820779554	0.670846322504933	1	pseudobulk	DESeq2	LRT	0.173376956629912
560	KC_AB1	lncRNA:CR43973	-0.298682689277034	0.88695443716659	1	pseudobulk	DESeq2	LRT	0.052098689294956
560	KC_AB1	lncRNA:CR43974	2.69141570238421	0.27890455639465	1	pseudobulk	DESeq2	LRT	0.554544390714624
560	KC_AB1	lncRNA:CR43995	-0.160907475787787	0.78757287770005	1	pseudobulk	DESeq2	LRT	0.103709248435424
560	KC_AB1	lncRNA:CR44017	3.16357068718239	0.145854314781226	1	pseudobulk	DESeq2	LRT	0.836080718698852
560	KC_AB1	lncRNA:CR44022	1.08899820779554	0.670846322504933	1	pseudobulk	DESeq2	LRT	0.173376956629912
561	KC_AB1	lncRNA:CR44023	0.259008000028412	0.670099176559435	1	pseudobulk	DESeq2	LRT	0.173860915739527
561	KC_AB1	lncRNA:CR44024	0.317296716683713	0.0345949151945844	1	pseudobulk	DESeq2	LRT	1.46098772968165
561	KC_AB1	lncRNA:CR44042	2.10549911548503	0.428045198278114	1	pseudobulk	DESeq2	LRT	0.368510370411162
561	KC_AB1	lncRNA:CR44043	1.14720029796356	0.236087113141951	1	pseudobulk	DESeq2	LRT	0.626927718317653
561	KC_AB1	lncRNA:CR44048	-0.850932661185203	0.660736244265418	1	pseudobulk	DESeq2	LRT	0.179971869591347

561	KC_AB1	lncRNA:CR44050	0.79472212735697	0.43670383769134	1	pseudobulk	DESeq2	LRT	0.359812991543627
561	KC_AB1	lncRNA:CR44051	0.725476977798061	0.78671877098961	1	pseudobulk	DESeq2	LRT	0.104180487507299
561	KC_AB1	lncRNA:CR44056	1.08899820779554	0.697631521651407	1	pseudobulk	DESeq2	LRT	0.156373904552
561	KC_AB1	lncRNA:CR44058	-0.850932661185203	0.660736244265418	1	pseudobulk	DESeq2	LRT	0.179971869591347
561	KC_AB1	lncRNA:CR44070	-2.70176410047587	0.132235476106443	1	pseudobulk	DESeq2	LRT	0.87865201678397
562	KC_AB1	lncRNA:CR44074	-0.202179091938154	0.899499805559398	1	pseudobulk	DESeq2	LRT	0.045998926197795
562	KC_AB1	lncRNA:CR44092	-0.39945653822425	0.596566248469236	1	pseudobulk	DESeq2	LRT	0.224341321068173
562	KC_AB1	lncRNA:CR44097	1.95275942187961	0.438420007953907	1	pseudobulk	DESeq2	LRT	0.358109634392079
562	KC_AB1	lncRNA:CR44109	-0.82542030717083	1	1	pseudobulk	DESeq2	LRT	0
562	KC_AB1	lncRNA:CR44125	-0.850996402080018	0.900449987182352	1	pseudobulk	DESeq2	LRT	0.045540403770502
562	KC_AB1	lncRNA:CR44126	1.08899820779554	0.670846322504933	1	pseudobulk	DESeq2	LRT	0.173376956629912
562	KC_AB1	lncRNA:CR44127	-1.29467356980949	0.510838645677309	1	pseudobulk	DESeq2	LRT	0.291716255164423
562	KC_AB1	lncRNA:CR44129	1.08899820779554	0.878177167143508	1	pseudobulk	DESeq2	LRT	0.056417858865076
562	KC_AB1	lncRNA:CR44151	1.08899820779554	0.697631521651407	1	pseudobulk	DESeq2	LRT	0.156373904552
562	KC_AB1	lncRNA:CR44156	1.08899820779554	0.878177167143508	1	pseudobulk	DESeq2	LRT	0.056417858865076
563	KC_AB1	lncRNA:CR44157	0.135879393043758	1	1	pseudobulk	DESeq2	LRT	0
563	KC_AB1	lncRNA:CR44161	2.39533548415136	0.318016613010836	1	pseudobulk	DESeq2	LRT	0.497550192120982
563	KC_AB1	lncRNA:CR44171	1.68130633771206	0.562684503730219	1	pseudobulk	DESeq2	LRT	0.249735045103917
563	KC_AB1	lncRNA:CR44178	1.11131485757568	0.399727047951532	1	pseudobulk	DESeq2	LRT	0.398236463752257
563	KC_AB1	lncRNA:CR44186	-0.850996402080018	0.900449987182352	1	pseudobulk	DESeq2	LRT	0.045540403770502
563	KC_AB1	lncRNA:CR44187	0.521490819370241	0.379638141043885	1	pseudobulk	DESeq2	LRT	0.420630161859137
563	KC_AB1	lncRNA:CR44188	0.12227511087111	0.915966715186365	1	pseudobulk	DESeq2	LRT	0.038120307635133
563	KC_AB1	lncRNA:CR44206	-0.555543108545147	0.270758892132851	1	pseudobulk	DESeq2	LRT	0.567417271578671
563	KC_AB1	lncRNA:CR44208	0.11400025262841	1	1	pseudobulk	DESeq2	LRT	0
563	KC_AB1	lncRNA:CR44218	0.463322901273212	0.832129216819238	1	pseudobulk	DESeq2	LRT	0.079809229245096
564	KC_AB1	lncRNA:CR44221	1.47494494152369	0.532234504947988	1	pseudobulk	DESeq2	LRT	0.273896973409681
564	KC_AB1	lncRNA:CR44222	-0.262860257075161	0.88861278106432	1	pseudobulk	DESeq2	LRT	0.051287444520999
564	KC_AB1	lncRNA:CR44260	0.13617601020169	1	1	pseudobulk	DESeq2	LRT	0
564	KC_AB1	lncRNA:CR44268	0.21107388748849	0.860664550365775	1	pseudobulk	DESeq2	LRT	0.065166084681122
564	KC_AB1	lncRNA:CR44272	2.02886365389065	0.256330895777222	1	pseudobulk	DESeq2	LRT	0.591199044783779
564	KC_AB1	lncRNA:CR44294	0.301490118816988	0.720611033207977	1	pseudobulk	DESeq2	LRT	0.142299092720445
564	KC_AB1	lncRNA:CR44295	1.08899820779554	0.697631521651407	1	pseudobulk	DESeq2	LRT	0.156373904552
564	KC_AB1	lncRNA:CR44299	-1.71331293139832	0.446929317282518	1	pseudobulk	DESeq2	LRT	0.349761155931696
564	KC_AB1	lncRNA:CR44300	1.08899820779554	0.878177167143508	1	pseudobulk	DESeq2	LRT	0.056417858865076
564	KC_AB1	lncRNA:CR44315	1.08899820779554	0.697631521651407	1	pseudobulk	DESeq2	LRT	0.156373904552
565	KC_AB1	lncRNA:CR44317	-0.586626921271722	0.649881383010807	1	pseudobulk	DESeq2	LRT	0.187165903979971
565	KC_AB1	lncRNA:CR44322	1.08899820779554	0.670846322504933	1	pseudobulk	DESeq2	LRT	0.173376956629912
565	KC_AB1	lncRNA:CR44331	-1.14160466569893	0.58139554263009	1	pseudobulk	DESeq2	LRT	0.235528302146906
565	KC_AB1	lncRNA:CR44332	-0.65542576849484	0.727232890812946	1	pseudobulk	DESeq2	LRT	0.138326487355475
565	KC_AB1	lncRNA:CR44347	0.254279415389557	0.614828543202174	1	pseudobulk	DESeq2	LRT	0.211245978733366
565	KC_AB1	lncRNA:CR44348	-1.13912913574204	0.60803432663814	1	pseudobulk	DESeq2	LRT	0.216071901897157
565	KC_AB1	lncRNA:CR44357	-0.727587819808342	0.1687182781196	1	pseudobulk	DESeq2	LRT	0.772837865505247
565	KC_AB1	lncRNA:CR44360	-0.431834396981437	0.797253406317302	1	pseudobulk	DESeq2	LRT	0.098403616529471
565	KC_AB1	lncRNA:CR44363	-1.99275908811736	0.412167844949191	1	pseudobulk	DESeq2	LRT	0.384925892483647
565	KC_AB1	lncRNA:CR44365	-0.82542030717083	1	1	pseudobulk	DESeq2	LRT	0
566	KC_AB1	lncRNA:CR44366	-0.82542030717083	1	1	pseudobulk	DESeq2	LRT	0
566	KC_AB1	lncRNA:CR44384	-0.587144259037263	0.571286071031511	1	pseudobulk	DESeq2	LRT	0.243146364680741
566	KC_AB1	lncRNA:CR44392	-1.30223380965193	0.57947592928145	1	pseudobulk	DESeq2	LRT	0.236964599385212
566	KC_AB1	lncRNA:CR44408	-0.82542030717083	1	1	pseudobulk	DESeq2	LRT	0
566	KC_AB1	lncRNA:CR44409	-1.44925888589622	0.6315588144614	1	pseudobulk	DESeq2	LRT	0.199586199175701
566	KC_AB1	lncRNA:CR44410	1.73751002447134	0.534537772665177	1	pseudobulk	DESeq2	LRT	0.27202160031407
566	KC_AB1	lncRNA:CR44411	2.10549911548503	0.428045198278114	1	pseudobulk	DESeq2	LRT	0.368510370411162

566	KC_AB1	lncRNA:CR44417	0.0689807433050679	0.747307129988703	1	pseudobulk	DESeq2	LRT	0.126500874160293
566	KC_AB1	lncRNA:CR44440	0.11269840515639	0.977007463492936	1	pseudobulk	DESeq2	LRT	0.010102118633921
566	KC_AB1	lncRNA:CR44441	-1.35952627225718	0.34441887195214	1	pseudobulk	DESeq2	LRT	0.462913060005464
567	KC_AB1	lncRNA:CR44444	1.88952324269138	0.456767015650974	1	pseudobulk	DESeq2	LRT	0.340305265178099
567	KC_AB1	lncRNA:CR44445	0.606047205968107	0.776409367274311	1	pseudobulk	DESeq2	LRT	0.109909233547711
567	KC_AB1	lncRNA:CR44457	1.08899820779554	0.670846322504933	1	pseudobulk	DESeq2	LRT	0.173376956629912
567	KC_AB1	lncRNA:CR44458	-0.829971373743754	0.758323735176306	1	pseudobulk	DESeq2	LRT	0.120145350587648
567	KC_AB1	lncRNA:CR44464	-0.958636528207196	0.711263785178276	1	pseudobulk	DESeq2	LRT	0.147969303347184
567	KC_AB1	lncRNA:CR44472	-1.56855358782635	0.573026303465967	1	pseudobulk	DESeq2	LRT	0.241825442277049
567	KC_AB1	lncRNA:CR44473	0.112935832476632	0.940353958317081	1	pseudobulk	DESeq2	LRT	0.026708642986417
567	KC_AB1	lncRNA:CR44474	1.72714441644398	0.349445883472448	1	pseudobulk	DESeq2	LRT	0.45662007121743
567	KC_AB1	lncRNA:CR44506	0.105420075099043	0.884711836471071	1	pseudobulk	DESeq2	LRT	0.053198162307284
567	KC_AB1	lncRNA:CR44517	1.08899820779554	0.878177167143508	1	pseudobulk	DESeq2	LRT	0.056417858865076
568	KC_AB1	lncRNA:CR44539	-0.506516985469986	0.909346984303859	1	pseudobulk	DESeq2	LRT	0.041270369127443
568	KC_AB1	lncRNA:CR44554	-0.850996402080018	0.900449987182352	1	pseudobulk	DESeq2	LRT	0.045540403770502
568	KC_AB1	lncRNA:CR44565	0.135795303631797	0.946582799906172	1	pseudobulk	DESeq2	LRT	0.023841391241048
568	KC_AB1	lncRNA:CR44566	-0.967393394131364	0.739767738650682	1	pseudobulk	DESeq2	LRT	0.130904612235943
568	KC_AB1	lncRNA:CR44589	2.18861624283415	0.137663822629939	1	pseudobulk	DESeq2	LRT	0.861180175185893
568	KC_AB1	lncRNA:CR44595	0.213948755818873	0.783605706825496	1	pseudobulk	DESeq2	LRT	0.105902409795988
568	KC_AB1	lncRNA:CR44596	0.238327176373016	0.793347878040036	1	pseudobulk	DESeq2	LRT	0.100536335527068
568	KC_AB1	lncRNA:CR44597	0.256634444027936	0.500799365440156	1	pseudobulk	DESeq2	LRT	0.300336230089187
568	KC_AB1	lncRNA:CR44598	0.561063793334978	0.0961184020226896	1	pseudobulk	DESeq2	LRT	1.01719345799399
568	KC_AB1	lncRNA:CR44603	2.04303378166351	0.338946812768934	1	pseudobulk	DESeq2	LRT	0.469868445552658
569	KC_AB1	lncRNA:CR44606	1.08899820779554	0.697631521651407	1	pseudobulk	DESeq2	LRT	0.156373904552
569	KC_AB1	lncRNA:CR44619	-0.82542030717083	1	1	pseudobulk	DESeq2	LRT	0
569	KC_AB1	lncRNA:CR44628	-2.08336392144876	0.0921903770056227	1	pseudobulk	DESeq2	LRT	1.03531440900566
569	KC_AB1	lncRNA:CR44642	0.736294445752933	1	1	pseudobulk	DESeq2	LRT	0
569	KC_AB1	lncRNA:CR44657	1.08899820779554	0.697631521651407	1	pseudobulk	DESeq2	LRT	0.156373904552
569	KC_AB1	lncRNA:CR44661	1.18653848769441	0.60665041788826	1	pseudobulk	DESeq2	LRT	0.21706149890377
569	KC_AB1	lncRNA:CR44662	1.63393762750153	0.222972253302954	1	pseudobulk	DESeq2	LRT	0.651749177252977
569	KC_AB1	lncRNA:CR44663	0.184932491516167	0.658123802097881	1	pseudobulk	DESeq2	LRT	0.181692401962752
569	KC_AB1	lncRNA:CR44664	1.08899820779554	0.670846322504933	1	pseudobulk	DESeq2	LRT	0.173376956629912
569	KC_AB1	lncRNA:CR44671	-0.850996402080018	0.900449987182352	1	pseudobulk	DESeq2	LRT	0.045540403770502
570	KC_AB1	lncRNA:CR44702	-1.6747012584303	0.563129890257477	1	pseudobulk	DESeq2	LRT	0.249391420218277
570	KC_AB1	lncRNA:CR44708	0.389105908944397	0.611108348813431	1	pseudobulk	DESeq2	LRT	0.213881783014201
570	KC_AB1	lncRNA:CR44716	1.08899820779554	0.670846322504933	1	pseudobulk	DESeq2	LRT	0.173376956629912
570	KC_AB1	lncRNA:CR44717	-1.6747012584303	0.563129890257477	1	pseudobulk	DESeq2	LRT	0.249391420218277
570	KC_AB1	lncRNA:CR44718	0.302057587989584	0.811905961739269	1	pseudobulk	DESeq2	LRT	0.090494269604609
570	KC_AB1	lncRNA:CR44727	-1.9020722008881	0.367842149552769	1	pseudobulk	DESeq2	LRT	0.434338508188274
570	KC_AB1	lncRNA:CR44728	1.08899820779554	0.697631521651407	1	pseudobulk	DESeq2	LRT	0.156373904552
570	KC_AB1	lncRNA:CR44730	1.08899820779554	0.697631521651407	1	pseudobulk	DESeq2	LRT	0.156373904552
570	KC_AB1	lncRNA:CR44732	1.08899820779554	0.878177167143508	1	pseudobulk	DESeq2	LRT	0.056417858865076
570	KC_AB1	lncRNA:CR44740	-0.850996402080018	0.900449987182352	1	pseudobulk	DESeq2	LRT	0.045540403770502
571	KC_AB1	lncRNA:CR44749	2.95121152231888	0.455808202241228	1	pseudobulk	DESeq2	LRT	0.341217863982204
571	KC_AB1	lncRNA:CR44753	1.08899820779554	0.697631521651407	1	pseudobulk	DESeq2	LRT	0.156373904552
571	KC_AB1	lncRNA:CR44760	-0.850932661185203	0.660736244265418	1	pseudobulk	DESeq2	LRT	0.179971869591347
571	KC_AB1	lncRNA:CR44761	-0.82542030717083	1	1	pseudobulk	DESeq2	LRT	0
571	KC_AB1	lncRNA:CR44763	-0.850996402080018	0.900449987182352	1	pseudobulk	DESeq2	LRT	0.045540403770502
571	KC_AB1	lncRNA:CR44775	1.68130633771206	0.562684503730219	1	pseudobulk	DESeq2	LRT	0.249735045103917
571	KC_AB1	lncRNA:CR44779	-1.1745213467153	0.516550129166316	1	pseudobulk	DESeq2	LRT	0.286887525521141
571	KC_AB1	lncRNA:CR44809	2.1559833397943	0.381743505411375	1	pseudobulk	DESeq2	LRT	0.418228342845202
571	KC_AB1	lncRNA:CR44811	2.56158413313683	0.171827136117536	1	pseudobulk	DESeq2	LRT	0.764908248330923

571	KC_AB1	lncRNA:CR44813	-2.78645437352956	0.139521418140034	1	pseudobulk	DESeq2	LRT	0.855359118081941
572	KC_AB1	lncRNA:CR44814	-0.968336042648622	0.663512636338631	1	pseudobulk	DESeq2	LRT	0.178150801752249
572	KC_AB1	lncRNA:CR44816	-0.533608045955084	0.595417112858135	1	pseudobulk	DESeq2	LRT	0.225178687470525
572	KC_AB1	lncRNA:CR44832	2.66517577143659	0.03913270133756	1	pseudobulk	DESeq2	LRT	1.40746017163669
572	KC_AB1	lncRNA:CR44833	-0.584377212850125	0.703543417272399	1	pseudobulk	DESeq2	LRT	0.152709096097053
572	KC_AB1	lncRNA:CR44834	1.08899820779554	0.697631521651407	1	pseudobulk	DESeq2	LRT	0.156373904552
572	KC_AB1	lncRNA:CR44851	0.997694962638592	0.665140342223729	1	pseudobulk	DESeq2	LRT	0.177086710452097
572	KC_AB1	lncRNA:CR44886	0.0641182900671293	0.851673783569944	1	pseudobulk	DESeq2	LRT	0.069726721107356
572	KC_AB1	lncRNA:CR44900	0.730615352574358	0.672979690651201	1	pseudobulk	DESeq2	LRT	0.171998041825359
572	KC_AB1	lncRNA:CR44901	-1.43275332122645	0.424117212325715	1	pseudobulk	DESeq2	LRT	0.372514101823738
572	KC_AB1	lncRNA:CR44914	-2.9044344670839	0.215443081422891	1	pseudobulk	DESeq2	LRT	0.666667447958097
573	KC_AB1	lncRNA:CR44915	1.08899820779554	0.697631521651407	1	pseudobulk	DESeq2	LRT	0.156373904552
573	KC_AB1	lncRNA:CR44922	0.0773305557341167	0.784514503645939	1	pseudobulk	DESeq2	LRT	0.105399023020099
573	KC_AB1	lncRNA:CR44938	1.07720815880413	0.530784089859304	1	pseudobulk	DESeq2	LRT	0.275082103497632
573	KC_AB1	lncRNA:CR44947	-0.831765148915113	0.743013616167264	1	pseudobulk	DESeq2	LRT	0.129003227461331
573	KC_AB1	lncRNA:CR44951	-0.0932139484671917	0.957501957161841	1	pseudobulk	DESeq2	LRT	0.018860329647883
573	KC_AB1	lncRNA:CR44953	-0.850932661185203	0.660736244265418	1	pseudobulk	DESeq2	LRT	0.179971869591347
573	KC_AB1	lncRNA:CR44957	1.08899820779554	0.878177167143508	1	pseudobulk	DESeq2	LRT	0.056417858865076
573	KC_AB1	lncRNA:CR44973	-1.56855358782635	0.573026303465967	1	pseudobulk	DESeq2	LRT	0.241825442277049
573	KC_AB1	lncRNA:CR44977	-0.82542030717083	1	1	pseudobulk	DESeq2	LRT	0
573	KC_AB1	lncRNA:CR44978	1.0175508810467	0.282552285130324	1	pseudobulk	DESeq2	LRT	0.548901176012598
574	KC_AB1	lncRNA:CR44979	-0.615767568564188	0.806245159731688	1	pseudobulk	DESeq2	LRT	0.093532879622572
574	KC_AB1	lncRNA:CR44985	-0.0812488279201191	0.961823864172591	1	pseudobulk	DESeq2	LRT	0.016904451685275
574	KC_AB1	lncRNA:CR44986	1.15581674750619	0.119744043427476	1	pseudobulk	DESeq2	LRT	0.921746081010842
574	KC_AB1	lncRNA:CR44993	1.49369967524616	0.423637886253356	1	pseudobulk	DESeq2	LRT	0.373005207547052
574	KC_AB1	lncRNA:CR44994	-0.82542030717083	1	1	pseudobulk	DESeq2	LRT	0
574	KC_AB1	lncRNA:CR44997	-0.0545939390934685	0.812141982232815	1	pseudobulk	DESeq2	LRT	0.090368038850073
574	KC_AB1	lncRNA:CR44999	0.061238185207589	0.815579456499929	1	pseudobulk	DESeq2	LRT	0.088533722130147
574	KC_AB1	lncRNA:CR45004	-0.850932661185203	0.660736244265418	1	pseudobulk	DESeq2	LRT	0.179971869591347
574	KC_AB1	lncRNA:CR45013	0.114184054362142	1	1	pseudobulk	DESeq2	LRT	0
574	KC_AB1	lncRNA:CR45014	-0.82542030717083	1	1	pseudobulk	DESeq2	LRT	0
575	KC_AB1	lncRNA:CR45018	-0.82542030717083	1	1	pseudobulk	DESeq2	LRT	0
575	KC_AB1	lncRNA:CR45025	-3.36909185124163	0.0827546078154964	1	pseudobulk	DESeq2	LRT	1.08220781515792
575	KC_AB1	lncRNA:CR45029	0.736294445752933	1	1	pseudobulk	DESeq2	LRT	0
575	KC_AB1	lncRNA:CR45030	-2.92324379669808	0.159135916396977	1	pseudobulk	DESeq2	LRT	0.79823179060706
575	KC_AB1	lncRNA:CR45035	0.342690176339656	0.752014146758075	1	pseudobulk	DESeq2	LRT	0.12377398945998
575	KC_AB1	lncRNA:CR45048	1.08899820779554	0.878177167143508	1	pseudobulk	DESeq2	LRT	0.056417858865076
575	KC_AB1	lncRNA:CR45054	0.190135656172753	0.225478808522616	1	pseudobulk	DESeq2	LRT	0.646894426749048
575	KC_AB1	lncRNA:CR45102	-0.0946213550723651	0.692921832083458	1	pseudobulk	DESeq2	LRT	0.159315755012378
575	KC_AB1	lncRNA:CR45120	0.744782276103072	0.764014638758689	1	pseudobulk	DESeq2	LRT	0.116898320126909
575	KC_AB1	lncRNA:CR45128	-0.26568241954666	0.813741906030431	1	pseudobulk	DESeq2	LRT	0.089513318158071
576	KC_AB1	lncRNA:CR45138	-0.82542030717083	1	1	pseudobulk	DESeq2	LRT	0
576	KC_AB1	lncRNA:CR45155	0.323976697797843	0.880042989168869	1	pseudobulk	DESeq2	LRT	0.055496112505729
576	KC_AB1	lncRNA:CR45156	-0.640805784516564	0.709439957027445	1	pseudobulk	DESeq2	LRT	0.14908435487191
576	KC_AB1	lncRNA:CR45165	-0.82542030717083	1	1	pseudobulk	DESeq2	LRT	0
576	KC_AB1	lncRNA:CR45169	1.08899820779554	0.878177167143508	1	pseudobulk	DESeq2	LRT	0.056417858865076
576	KC_AB1	lncRNA:CR45170	1.21109074570152	0.211224222949671	1	pseudobulk	DESeq2	LRT	0.675256278893198
576	KC_AB1	lncRNA:CR45177	2.21745641838459	0.396737788462723	1	pseudobulk	DESeq2	LRT	0.401496431894786
576	KC_AB1	lncRNA:CR45181	0.11400025262841	1	1	pseudobulk	DESeq2	LRT	0
576	KC_AB1	lncRNA:CR45187	0.628159706651807	0.742228541592568	1	pseudobulk	DESeq2	LRT	0.129462349343438
576	KC_AB1	lncRNA:CR45188	0.0902306960627484	0.951865156056514	1	pseudobulk	DESeq2	LRT	0.02142457065797
577	KC_AB1	lncRNA:CR45196	0.855700630127339	0.665189798240232	1	pseudobulk	DESeq2	LRT	0.177054420008663

577	KC_AB1	lncRNA:CR45199	-2.61627160405009	0.247973659858837	1	pseudobulk	DESeq2	LRT	0.605594448147543
577	KC_AB1	lncRNA:CR45200	1.5940412424434	0.475208010505809	1	pseudobulk	DESeq2	LRT	0.323116247132191
577	KC_AB1	lncRNA:CR45203	0.144938062156184	0.939011437924561	1	pseudobulk	DESeq2	LRT	0.027329117640952
577	KC_AB1	lncRNA:CR45211	-0.82542030717083	1	1	pseudobulk	DESeq2	LRT	0
577	KC_AB1	lncRNA:CR45217	-0.618422766585323	0.856874262644257	1	pseudobulk	DESeq2	LRT	0.067082901584478
577	KC_AB1	lncRNA:CR45223	0.11269840515639	0.977007463492936	1	pseudobulk	DESeq2	LRT	0.010102118633921
577	KC_AB1	lncRNA:CR45233	1.82443202324224	0.401185591318225	1	pseudobulk	DESeq2	LRT	0.396654673167464
577	KC_AB1	lncRNA:CR45240	0.979190749421256	0.391873289012283	1	pseudobulk	DESeq2	LRT	0.406854338027176
577	KC_AB1	lncRNA:CR45242	0.11400025262841	1	1	pseudobulk	DESeq2	LRT	0
578	KC_AB1	lncRNA:CR45245	1.08899820779554	0.878177167143508	1	pseudobulk	DESeq2	LRT	0.056417858865076
578	KC_AB1	lncRNA:CR45247	1.88952324269138	0.456767015650974	1	pseudobulk	DESeq2	LRT	0.340305265178099
578	KC_AB1	lncRNA:CR45251	-0.82542030717083	1	1	pseudobulk	DESeq2	LRT	0
578	KC_AB1	lncRNA:CR45262	-0.850932661185203	0.660736244265418	1	pseudobulk	DESeq2	LRT	0.179971869591347
578	KC_AB1	lncRNA:CR45282	-0.850996402080018	0.900449987182352	1	pseudobulk	DESeq2	LRT	0.045540403770502
578	KC_AB1	lncRNA:CR45284	0.113316967542732	1	1	pseudobulk	DESeq2	LRT	0
578	KC_AB1	lncRNA:CR45290	-1.05972628603973	0.705203047462737	1	pseudobulk	DESeq2	LRT	0.151685819609541
578	KC_AB1	lncRNA:CR45306	-1.09135202595011	0.51817666449975	1	pseudobulk	DESeq2	LRT	0.285522148870814
578	KC_AB1	lncRNA:CR45308	1.08899820779554	0.697631521651407	1	pseudobulk	DESeq2	LRT	0.156373904552
578	KC_AB1	lncRNA:CR45312	0.457036265917753	0.245177586348392	1	pseudobulk	DESeq2	LRT	0.610519234684359
579	KC_AB1	lncRNA:CR45339	2.10549911548503	0.428045198278114	1	pseudobulk	DESeq2	LRT	0.368510370411162
579	KC_AB1	lncRNA:CR45358	0.876133856950809	0.645550575991126	1	pseudobulk	DESeq2	LRT	0.190069727040604
579	KC_AB1	lncRNA:CR45387	1.61229351901473	0.468469427117158	1	pseudobulk	DESeq2	LRT	0.329318746435887
579	KC_AB1	lncRNA:CR45388	0.290391508206503	0.459201132920382	1	pseudobulk	DESeq2	LRT	0.337997049136948
579	KC_AB1	lncRNA:CR45402	1.08899820779554	0.878177167143508	1	pseudobulk	DESeq2	LRT	0.056417858865076
579	KC_AB1	lncRNA:CR45405	1.08899820779554	0.697631521651407	1	pseudobulk	DESeq2	LRT	0.156373904552
579	KC_AB1	lncRNA:CR45409	0.247439089076456	0.615014282413129	1	pseudobulk	DESeq2	LRT	0.211114798531597
579	KC_AB1	lncRNA:CR45417	-0.850932661185203	0.660736244265418	1	pseudobulk	DESeq2	LRT	0.179971869591347
579	KC_AB1	lncRNA:CR45418	1.40898611248717	0.402331566972175	1	pseudobulk	DESeq2	LRT	0.395415891306459
579	KC_AB1	lncRNA:CR45444	0.0397164163085418	0.97506527848086	1	pseudobulk	DESeq2	LRT	0.010966308265549
580	KC_AB1	lncRNA:CR45456	1.08899820779554	0.878177167143508	1	pseudobulk	DESeq2	LRT	0.056417858865076
580	KC_AB1	lncRNA:CR45464	1.08899820779554	0.670846322504933	1	pseudobulk	DESeq2	LRT	0.173376956629912
580	KC_AB1	lncRNA:CR45472	1.88952324269138	0.456767015650974	1	pseudobulk	DESeq2	LRT	0.340305265178099
580	KC_AB1	lncRNA:CR45473	0.767659719836499	0.254941065470334	1	pseudobulk	DESeq2	LRT	0.593560203484401
580	KC_AB1	lncRNA:CR45475	1.08899820779554	0.670846322504933	1	pseudobulk	DESeq2	LRT	0.173376956629912
580	KC_AB1	lncRNA:CR45480	-0.850996402080018	0.900449987182352	1	pseudobulk	DESeq2	LRT	0.045540403770502
580	KC_AB1	lncRNA:CR45483	1.08899820779554	0.878177167143508	1	pseudobulk	DESeq2	LRT	0.056417858865076
580	KC_AB1	lncRNA:CR45504	-1.44925888589622	0.6315588144614	1	pseudobulk	DESeq2	LRT	0.199586199175701
580	KC_AB1	lncRNA:CR45522	0.105822668164031	0.886297613045522	1	pseudobulk	DESeq2	LRT	0.052420420325096
580	KC_AB1	lncRNA:CR45527	1.59417244189775	0.390930118679682	1	pseudobulk	DESeq2	LRT	0.407900868650651
581	KC_AB1	lncRNA:CR45530	1.30038315571453	0.411731545928205	1	pseudobulk	DESeq2	LRT	0.385385857079044
581	KC_AB1	lncRNA:CR45531	1.43521109676237	0.425120197375181	1	pseudobulk	DESeq2	LRT	0.371488261299125
581	KC_AB1	lncRNA:CR45539	-0.62904432427458	0.666034141052074	1	pseudobulk	DESeq2	LRT	0.176503508231364
581	KC_AB1	lncRNA:CR45540	-1.44925888589622	0.6315588144614	1	pseudobulk	DESeq2	LRT	0.199586199175701
581	KC_AB1	lncRNA:CR45556	-0.171943842351679	0.864333395045414	1	pseudobulk	DESeq2	LRT	0.063318706942348
581	KC_AB1	lncRNA:CR45602	-0.850996402080018	0.900449987182352	1	pseudobulk	DESeq2	LRT	0.045540403770502
581	KC_AB1	lncRNA:CR45605	2.76821230112469	0.228830322536972	1	pseudobulk	DESeq2	LRT	0.64048642726567
581	KC_AB1	lncRNA:CR45639	-0.82542030717083	1	1	pseudobulk	DESeq2	LRT	0
581	KC_AB1	lncRNA:CR45641	0.771231299457179	0.6353142701336	1	pseudobulk	DESeq2	LRT	0.197011389634554
581	KC_AB1	lncRNA:CR45643	0.197626311084782	0.908444903824526	1	pseudobulk	DESeq2	LRT	0.041701407035715
582	KC_AB1	lncRNA:CR45660	-0.0963965775035873	0.752922029161534	1	pseudobulk	DESeq2	LRT	0.123249995988124
582	KC_AB1	lncRNA:CR45668	-0.733504476742314	0.185874000021893	1	pseudobulk	DESeq2	LRT	0.730781354911911
582	KC_AB1	lncRNA:CR45669	-1.05220943051072	0.633614666162374	1	pseudobulk	DESeq2	LRT	0.198174778767564

582	KC_AB1	lncRNA:CR45670	-0.850932661185203	0.660736244265418	1	pseudobulk	DESeq2	LRT	0.179971869591347
582	KC_AB1	lncRNA:CR45674	-2.9852027467753	0.168870698096331	1	pseudobulk	DESeq2	LRT	0.772445701281961
582	KC_AB1	lncRNA:CR45684	0.684356557028805	0.0690473745928766	1	pseudobulk	DESeq2	LRT	1.16085283006874
582	KC_AB1	lncRNA:CR45689	1.08899820779554	0.697631521651407	1	pseudobulk	DESeq2	LRT	0.156373904552
582	KC_AB1	lncRNA:CR45736	1.12269801611019	0.394819679254069	1	pseudobulk	DESeq2	LRT	0.403601208640572
582	KC_AB1	lncRNA:CR45740	-1.82709236256241	0.44317674354685	1	pseudobulk	DESeq2	LRT	0.353423038023035
582	KC_AB1	lncRNA:CR45745	1.08899820779554	0.878177167143508	1	pseudobulk	DESeq2	LRT	0.056417858865076
583	KC_AB1	lncRNA:CR45758	-0.0469797759508188	0.846864334357822	1	pseudobulk	DESeq2	LRT	0.072186157035123
583	KC_AB1	lncRNA:CR45793	1.08899820779554	0.670846322504933	1	pseudobulk	DESeq2	LRT	0.173376956629912
583	KC_AB1	lncRNA:CR45805	2.88885723090896	0.199893040000958	1	pseudobulk	DESeq2	LRT	0.69920232715152
583	KC_AB1	lncRNA:CR45808	0.321859439895203	0.850325745950471	1	pseudobulk	DESeq2	LRT	0.070414671264569
583	KC_AB1	lncRNA:CR45810	0.13617601020169	1	1	pseudobulk	DESeq2	LRT	0
583	KC_AB1	lncRNA:CR45819	2.05296423313396	0.327185303142031	1	pseudobulk	DESeq2	LRT	0.485206212664739
583	KC_AB1	lncRNA:CR45820	1.88952324269138	0.456767015650974	1	pseudobulk	DESeq2	LRT	0.340305265178099
583	KC_AB1	lncRNA:CR45821	1.24175920901764	0.364175148264511	1	pseudobulk	DESeq2	LRT	0.438689694290255
583	KC_AB1	lncRNA:CR45825	0.885068635902682	0.414470127451357	1	pseudobulk	DESeq2	LRT	0.382506765356291
583	KC_AB1	lncRNA:CR45878	0.135879393043758	1	1	pseudobulk	DESeq2	LRT	0
584	KC_AB1	lncRNA:CR45880	0.111831901354254	1	1	pseudobulk	DESeq2	LRT	0
584	KC_AB1	lncRNA:CR45883	2.28149241197936	0.150170313915864	1	pseudobulk	DESeq2	LRT	0.823415911384836
584	KC_AB1	lncRNA:CR45906	1.08899820779554	0.670846322504933	1	pseudobulk	DESeq2	LRT	0.173376956629912
584	KC_AB1	lncRNA:CR45910	1.58523147777669	0.407958777449897	1	pseudobulk	DESeq2	LRT	0.389383718357442
584	KC_AB1	lncRNA:CR45911	-0.0526041507728341	0.931602179361519	1	pseudobulk	DESeq2	LRT	0.030769504160999
584	KC_AB1	lncRNA:CR45916	-0.850996402080018	0.900449987182352	1	pseudobulk	DESeq2	LRT	0.045540403770502
584	KC_AB1	lncRNA:CR45918	0.468936568037825	0.65388413257048	1	pseudobulk	DESeq2	LRT	0.184499201283349
584	KC_AB1	lncRNA:CR45919	0.177227737800529	0.781874633229645	1	pseudobulk	DESeq2	LRT	0.106862876686091
584	KC_AB1	lncRNA:CR45921	2.10549911548503	0.428045198278114	1	pseudobulk	DESeq2	LRT	0.368510370411162
584	KC_AB1	lncRNA:CR45922	2.05198382624807	0.391545551880393	1	pseudobulk	DESeq2	LRT	0.407217705433648
585	KC_AB1	lncRNA:CR45938	0.341798989761231	0.804586799396378	1	pseudobulk	DESeq2	LRT	0.094427097039850
585	KC_AB1	lncRNA:CR45939	-0.715916138796926	0.857202297388202	1	pseudobulk	DESeq2	LRT	0.066916673675843
585	KC_AB1	lncRNA:CR45941	1.88952324269138	0.456767015650974	1	pseudobulk	DESeq2	LRT	0.340305265178099
585	KC_AB1	lncRNA:CR45950	1.08899820779554	0.697631521651407	1	pseudobulk	DESeq2	LRT	0.156373904552
585	KC_AB1	lncRNA:CR45953	2.10549911548503	0.428045198278114	1	pseudobulk	DESeq2	LRT	0.368510370411162
585	KC_AB1	lncRNA:CR45954	1.88952324269138	0.456767015650974	1	pseudobulk	DESeq2	LRT	0.340305265178099
585	KC_AB1	lncRNA:CR45957	1.08899820779554	0.670846322504933	1	pseudobulk	DESeq2	LRT	0.173376956629912
585	KC_AB1	lncRNA:CR45959	1.95275942187961	0.438420007953907	1	pseudobulk	DESeq2	LRT	0.358109634392079
585	KC_AB1	lncRNA:CR45961	-0.375236740187668	0.741529462296401	1	pseudobulk	DESeq2	LRT	0.12987158899358
585	KC_AB1	lncRNA:CR45962	-0.615767568564188	0.806245159731688	1	pseudobulk	DESeq2	LRT	0.093532879622572
586	KC_AB1	lncRNA:CR45972	-0.850996402080018	0.900449987182352	1	pseudobulk	DESeq2	LRT	0.045540403770502
586	KC_AB1	lncRNA:CR45973	1.5940412424434	0.475208010505809	1	pseudobulk	DESeq2	LRT	0.323116247132191
586	KC_AB1	lncRNA:CR45979	0.146842246349189	0.733081031521563	1	pseudobulk	DESeq2	LRT	0.134848017722248
586	KC_AB1	lncRNA:CR45981	-1.99275908811736	0.412167844949191	1	pseudobulk	DESeq2	LRT	0.384925892483647
586	KC_AB1	lncRNA:CR45982	1.26269203670432	0.541057763329599	1	pseudobulk	DESeq2	LRT	0.266756367137151
586	KC_AB1	lncRNA:CR45993	0.0651876034097768	0.940772142186263	1	pseudobulk	DESeq2	LRT	0.026515551252960
586	KC_AB1	lncRNA:CR45994	-0.0199252112394237	0.936867535051078	1	pseudobulk	DESeq2	LRT	0.028321810246914
586	KC_AB1	lncRNA:CR46003	0.431185682717556	0.17741266367151	1	pseudobulk	DESeq2	LRT	0.751015383566486
586	KC_AB1	lncRNA:CR46004	-0.140609995909315	0.496320552578152	1	pseudobulk	DESeq2	LRT	0.304237740344751
586	KC_AB1	lncRNA:CR46006	0.0747679750599549	0.623173880052024	1	pseudobulk	DESeq2	LRT	0.205390758138771
587	KC_AB1	lncRNA:CR46008	0.113316967542732	1	1	pseudobulk	DESeq2	LRT	0
587	KC_AB1	lncRNA:CR46009	-0.421212447673593	0.83467750543181	1	pseudobulk	DESeq2	LRT	0.078481290582233
587	KC_AB1	lncRNA:CR46010	-0.82542030717083	1	1	pseudobulk	DESeq2	LRT	0
587	KC_AB1	lncRNA:CR46016	1.08899820779554	0.697631521651407	1	pseudobulk	DESeq2	LRT	0.156373904552
587	KC_AB1	lncRNA:CR46021	-0.850996402080018	0.900449987182352	1	pseudobulk	DESeq2	LRT	0.045540403770502

587	KC_AB1	lncRNA:CR46024	1.68130633771206	0.562684503730219	1	pseudobulk	DESeq2	LRT	0.249735045103917
587	KC_AB1	lncRNA:CR46035	0.135795303631797	0.946582799906172	1	pseudobulk	DESeq2	LRT	0.023841391241048
587	KC_AB1	lncRNA:CR46036	-1.44925888589622	0.6315588144614	1	pseudobulk	DESeq2	LRT	0.199586199175701
587	KC_AB1	lncRNA:CR46061	-0.507979848677457	0.940470850565906	1	pseudobulk	DESeq2	LRT	0.026654660650085
587	KC_AB1	lncRNA:CR46064	0.215299415203599	0.457953005755887	1	pseudobulk	DESeq2	LRT	0.339179086162173
588	KC_AB1	lncRNA:CR46073	1.08899820779554	0.878177167143508	1	pseudobulk	DESeq2	LRT	0.056417858865076
588	KC_AB1	lncRNA:CR46078	1.08899820779554	0.697631521651407	1	pseudobulk	DESeq2	LRT	0.156373904552
588	KC_AB1	lncRNA:CR46079	0.275953357280421	0.861551631718739	1	pseudobulk	DESeq2	LRT	0.064718690707393
588	KC_AB1	lncRNA:CR46080	-0.325915115793007	0.853378889642572	1	pseudobulk	DESeq2	LRT	0.068858104651230
588	KC_AB1	lncRNA:CR46084	0.183015778123224	0.916988170923185	1	pseudobulk	DESeq2	LRT	0.037636266659228
588	KC_AB1	lncRNA:CR46094	-0.82542030717083	1	1	pseudobulk	DESeq2	LRT	0
588	KC_AB1	lncRNA:CR46095	0.744782276103072	0.764014638758689	1	pseudobulk	DESeq2	LRT	0.116898320126909
588	KC_AB1	lncRNA:CR46110	1.08899820779554	0.670846322504933	1	pseudobulk	DESeq2	LRT	0.173376956629912
588	KC_AB1	lncRNA:CR46112	1.08899820779554	0.878177167143508	1	pseudobulk	DESeq2	LRT	0.056417858865076
588	KC_AB1	lncRNA:CR46125	0.11400025262841	1	1	pseudobulk	DESeq2	LRT	0
589	KC_AB1	lncRNA:CR46127	-0.850932661185203	0.660736244265418	1	pseudobulk	DESeq2	LRT	0.179971869591347
589	KC_AB1	lncRNA:CR46194	1.68130633771206	0.562684503730219	1	pseudobulk	DESeq2	LRT	0.249735045103917
589	KC_AB1	lncRNA:CR46197	-0.82542030717083	1	1	pseudobulk	DESeq2	LRT	0
589	KC_AB1	lncRNA:CR46208	-0.296961497530524	0.543630673321522	1	pseudobulk	DESeq2	LRT	0.264696047012284
589	KC_AB1	lncRNA:CR46233	1.08899820779554	0.878177167143508	1	pseudobulk	DESeq2	LRT	0.056417858865076
589	KC_AB1	lncRNA:CR46250	-0.968336042648622	0.663512636338631	1	pseudobulk	DESeq2	LRT	0.178150801752249
589	KC_AB1	lncRNA:CR46253	-0.82542030717083	1	1	pseudobulk	DESeq2	LRT	0
589	KC_AB1	lncRNA:CR46254	0.11269840515639	0.977007463492936	1	pseudobulk	DESeq2	LRT	0.010102118633921
589	KC_AB1	lncRNA:CR46268	-0.041784840022778	0.934876191145394	1	pseudobulk	DESeq2	LRT	0.029245900424432
589	KC_AB1	lncRNA:CR46271	-0.28125582460092	0.805629977391475	1	pseudobulk	DESeq2	LRT	0.093864382110001
590	KC_AB1	lncRNA:CR46322	-0.495003095270453	0.617857124246702	1	pseudobulk	DESeq2	LRT	0.209111941294784
590	KC_AB1	lncRNA:CR46348	-1.89854133735591	0.478664405825708	1	pseudobulk	DESeq2	LRT	0.319968866080244
590	KC_AB1	lncRNA:CR46352	-0.850932661185203	0.660736244265418	1	pseudobulk	DESeq2	LRT	0.179971869591347
590	KC_AB1	lncRNA:CR46391	0.113316967542732	1	1	pseudobulk	DESeq2	LRT	0
590	KC_AB1	lncRNA:CR46414	1.08899820779554	0.697631521651407	1	pseudobulk	DESeq2	LRT	0.156373904552
590	KC_AB1	lncRNA:flam	-1.374446199942	0.245504955615302	1	pseudobulk	DESeq2	LRT	0.609939737045459
590	KC_AB1	lncRNA:Hsromega	0.219473595707866	0.0666949469463039	1	pseudobulk	DESeq2	LRT	1.17590706857921
590	KC_AB1	lncRNA:noe	0.0466478831453893	0.70751415539932	1	pseudobulk	DESeq2	LRT	0.150264866686619
590	KC_AB1	lncRNA:roX1	-0.038882247552100	0.938950130681099	1	pseudobulk	DESeq2	LRT	0.027357473277181
590	KC_AB1	lncRNA:roX2	2.32496151311036	0.230919791244108	1	pseudobulk	DESeq2	LRT	0.636538843783717
591	KC_AB1	lncRNA:sphinx	1.08899820779554	0.878177167143508	1	pseudobulk	DESeq2	LRT	0.056417858865076
591	KC_AB1	Lnk	0.143641908979851	0.572833063062864	1	pseudobulk	DESeq2	LRT	0.241971923152443
591	KC_AB1	Lnpk	-0.721924241063815	0.131172458015809	1	pseudobulk	DESeq2	LRT	0.882157343218085
591	KC_AB1	loh	-0.821518422757893	0.520730071190508	1	pseudobulk	DESeq2	LRT	0.283387341903708
591	KC_AB1	loj	-0.153165631923376	0.759369097274266	1	pseudobulk	DESeq2	LRT	0.11954708056512
591	KC_AB1	lola	0.0208706220019465	0.82222850948149	1	pseudobulk	DESeq2	LRT	0.085007468815902
591	KC_AB1	lolal	0.189172044636244	0.168838023643554	1	pseudobulk	DESeq2	LRT	0.772529740180992
591	KC_AB1	Lon	-0.495914755971158	0.433346004929408	1	pseudobulk	DESeq2	LRT	0.363165202896385
591	KC_AB1	loqs	-0.193434444318319	0.319682884899939	1	pseudobulk	DESeq2	LRT	0.495280614252188
591	KC_AB1	lost	0.192543781969163	0.500839511234297	1	pseudobulk	DESeq2	LRT	0.300301416949876
592	KC_AB1	lovit	0.835894319682986	0.0236748180222034	1	pseudobulk	DESeq2	LRT	1.62571335054039
592	KC_AB1	Loxl1	0.814871552093028	0.638192090213231	1	pseudobulk	DESeq2	LRT	0.1950485827781
592	KC_AB1	LPCAT	-0.123877651097636	0.674884788700303	1	pseudobulk	DESeq2	LRT	0.170770360357602
592	KC_AB1	Lpin	0.058881137137271	0.828377799093823	1	pseudobulk	DESeq2	LRT	0.081771548914565
592	KC_AB1	LpR2	1.35698301658672	0.435008042059517	1	pseudobulk	DESeq2	LRT	0.361502714103324
592	KC_AB1	Lpt	-0.219136063543965	0.765365510390145	1	pseudobulk	DESeq2	LRT	0.116131112250961
592	KC_AB1	lqf	-0.012196408819631	0.928021520644912	1	pseudobulk	DESeq2	LRT	0.032441952456825

592	KC_AB1	IqfR	-0.101816098967493	0.785025522312047	1	pseudobulk	DESeq2	LRT	0.10511622348518
592	KC_AB1	Lrch	0.118813721267158	0.660238608970974	1	pseudobulk	DESeq2	LRT	0.18029908289457
592	KC_AB1	LRP1	0.412914426829281	0.218037901922731	1	pseudobulk	DESeq2	LRT	0.661468005637941
593	KC_AB1	Lrp4	-0.185970470763561	0.319445169744317	1	pseudobulk	DESeq2	LRT	0.49560367434745
593	KC_AB1	Lrpprc2	-0.402127804860487	0.712204231251592	1	pseudobulk	DESeq2	LRT	0.14739545048237
593	KC_AB1	LRR	-0.156231806079305	0.491467613023219	1	pseudobulk	DESeq2	LRT	0.3085050962423
593	KC_AB1	Lrr47	-3.43655902851875	0.0341344410774438	1	pseudobulk	DESeq2	LRT	1.46680720395832
593	KC_AB1	Lrrk	0.18325328014109	0.748465692512875	1	pseudobulk	DESeq2	LRT	0.12582810165869
593	KC_AB1	Lsd-2	1.35094210236122	0.293897630439703	1	pseudobulk	DESeq2	LRT	0.53180391542827
593	KC_AB1	LSm1	-0.386149420746292	0.495747409015388	1	pseudobulk	DESeq2	LRT	0.30473954692247
593	KC_AB1	Lsm10	-0.793645619651263	0.70312343095238	1	pseudobulk	DESeq2	LRT	0.15296842935269
593	KC_AB1	Lsm11	0.329610044997077	0.742116263938903	1	pseudobulk	DESeq2	LRT	0.12952805047344
593	KC_AB1	Lsm12a	-0.0263455205637764	0.895223819228888	1	pseudobulk	DESeq2	LRT	0.04806837104846
594	KC_AB1	LSm3	-0.312873212710452	0.56623225522918	1	pseudobulk	DESeq2	LRT	0.24700539482776
594	KC_AB1	LSm7	-0.0013547345769845	0.992660981163737	1	pseudobulk	DESeq2	LRT	0.00319904873428
594	KC_AB1	lsn	0.0155712347260046	0.966502817382179	1	pseudobulk	DESeq2	LRT	0.01479687565327
594	KC_AB1	Lst8	-0.850023564159619	0.215127007866785	1	pseudobulk	DESeq2	LRT	0.66730506320060
594	KC_AB1	lt	-0.323908258290684	0.280896047758275	1	pseudobulk	DESeq2	LRT	0.55145437133187
594	KC_AB1	Ltn1	-0.345317365248042	0.538223290878068	1	pseudobulk	DESeq2	LRT	0.26903751266486
594	KC_AB1	LTV1	0.476242435778933	0.170564852462378	1	pseudobulk	DESeq2	LRT	0.76811045707391
594	KC_AB1	luna	0.409343829968013	0.691309218639887	1	pseudobulk	DESeq2	LRT	0.16032765174227
594	KC_AB1	lute	0.0082336602600787	0.966264276876215	1	pseudobulk	DESeq2	LRT	0.014904076180731
594	KC_AB1	lva	0.0087179111789682	0.929841301420524	1	pseudobulk	DESeq2	LRT	0.0315911673585
595	KC_AB1	lwr	-0.191099641346923	0.284673607990039	1	pseudobulk	DESeq2	LRT	0.54565279430577
595	KC_AB1	LysRS	0.624650246188784	0.110096738101178	1	pseudobulk	DESeq2	LRT	0.95822554792783
595	KC_AB1	Lztr1	0.411993684362428	0.261786223610322	1	pseudobulk	DESeq2	LRT	0.58205321174798
595	KC_AB1	m-cup	-0.346329189342787	0.393703327434926	1	pseudobulk	DESeq2	LRT	0.40483091469790
595	KC_AB1	M1BP	0.218710647285566	0.560229859439288	1	pseudobulk	DESeq2	LRT	0.25163374762932
595	KC_AB1	M6	0.297162131174203	0.482999574144373	1	pseudobulk	DESeq2	LRT	0.31605325216118
595	KC_AB1	mAChR-A	0.0057596773947638	0.960167408899097	1	pseudobulk	DESeq2	LRT	0.01765303943693
595	KC_AB1	mAChR-B	-0.144000575465009	0.312239575324555	1	pseudobulk	DESeq2	LRT	0.50551205240810
595	KC_AB1	mAcon1	-0.162500938738022	0.583976049955434	1	pseudobulk	DESeq2	LRT	0.23360496382164
595	KC_AB1	Mad	-0.0690528404519598	0.760214899733515	1	pseudobulk	DESeq2	LRT	0.11906362275102
596	KC_AB1	Mad1	-0.175877244375568	0.761676590069469	1	pseudobulk	DESeq2	LRT	0.11822939212866
596	KC_AB1	mad2	-0.82542030717083	1	1	pseudobulk	DESeq2	LRT	0
596	KC_AB1	Madm	-0.253554732063157	0.587185766147487	1	pseudobulk	DESeq2	LRT	0.23122448060991
596	KC_AB1	mael	0.14264558573029	0.85685669814237	1	pseudobulk	DESeq2	LRT	0.067091803992641
596	KC_AB1	maf-S	0.245925388170489	0.278350357345529	1	pseudobulk	DESeq2	LRT	0.55540821681045
596	KC_AB1	Maf1	0.0711347251497878	0.611440851557181	1	pseudobulk	DESeq2	LRT	0.21364554857824
596	KC_AB1	MAGE	-2.19286887839518	0.260100170239712	1	pseudobulk	DESeq2	LRT	0.58485936355136
596	KC_AB1	Magi	-0.189995031195264	0.462164350683301	1	pseudobulk	DESeq2	LRT	0.335203557123091
596	KC_AB1	mago	-0.0638175945028732	0.829241959737635	1	pseudobulk	DESeq2	LRT	0.08131873067191
596	KC_AB1	MagR	-0.533466767178862	0.0738167019474097	1	pseudobulk	DESeq2	LRT	1.13184536252749
597	KC_AB1	magu	-0.82542030717083	1	1	pseudobulk	DESeq2	LRT	0
597	KC_AB1	mahe	-0.0348161993187527	0.817369133253018	1	pseudobulk	DESeq2	LRT	0.08758176680915
597	KC_AB1	mahj	-0.0206708371664587	0.922444207042833	1	pseudobulk	DESeq2	LRT	0.03505989216748
597	KC_AB1	mal	0.413603855636154	0.696514493206204	1	pseudobulk	DESeq2	LRT	0.15706984226371
597	KC_AB1	Mal-A5	1.08899820779554	0.697631521651407	1	pseudobulk	DESeq2	LRT	0.156373904552
597	KC_AB1	Mal-B1	-0.850996402080018	0.900449987182352	1	pseudobulk	DESeq2	LRT	0.04554040377050
597	KC_AB1	Mal-B2	-0.82542030717083	1	1	pseudobulk	DESeq2	LRT	0
597	KC_AB1	mam	0.0310000223773914	0.874613750158986	1	pseudobulk	DESeq2	LRT	0.05818369921705
597	KC_AB1	mamo	-1.79757124301353	0.0156475366070967	1	pseudobulk	DESeq2	LRT	1.80555402375029

597	KC_AB1	MAN1	-0.0160692929771382	0.968852940679225	1	pseudobulk	DESeq2	LRT	0.013742138221035
598	KC_AB1	Manf	0.445269440578954	0.174052601775469	1	pseudobulk	DESeq2	LRT	0.759319480404491
598	KC_AB1	Map205	-0.216006334590408	0.465740025406908	1	pseudobulk	DESeq2	LRT	0.33185643746688
598	KC_AB1	Map60	0.311024880569528	0.457988362872928	1	pseudobulk	DESeq2	LRT	0.33914555693997
598	KC_AB1	MAPk-Ak2	0.175434454342047	0.643540513980939	1	pseudobulk	DESeq2	LRT	0.191424106965492
598	KC_AB1	Mapmodulin	0.0648157651553467	0.68811400929504	1	pseudobulk	DESeq2	LRT	0.162339600273652
598	KC_AB1	Marc	1.08899820779554	0.878177167143508	1	pseudobulk	DESeq2	LRT	0.056417858865076
598	KC_AB1	Marcal1	-0.63446524291639	0.576255926974597	1	pseudobulk	DESeq2	LRT	0.239384594723121
598	KC_AB1	Marf	-0.0224823266493041	0.908202071397107	1	pseudobulk	DESeq2	LRT	0.04181751190949
598	KC_AB1	Marf1	-0.752923220468911	0.277922683101896	1	pseudobulk	DESeq2	LRT	0.556076006141065
598	KC_AB1	mars	0.997694962638592	0.665140342223729	1	pseudobulk	DESeq2	LRT	0.177086710452097
599	KC_AB1	mask	0.109618697677272	0.481382551145317	1	pseudobulk	DESeq2	LRT	0.31750965581138
599	KC_AB1	Mat1	-0.743382709642376	0.284771140022235	1	pseudobulk	DESeq2	LRT	0.54550402614536
599	KC_AB1	Mat89Ba	0.501196062229388	0.53513682039447	1	pseudobulk	DESeq2	LRT	0.271535166117885
599	KC_AB1	mats	0.393537556198287	0.278041721915011	1	pseudobulk	DESeq2	LRT	0.555890030564241
599	KC_AB1	Mau2	0.458501696593855	0.595553564511476	1	pseudobulk	DESeq2	LRT	0.225079171669712
599	KC_AB1	mav	1.08899820779554	0.878177167143508	1	pseudobulk	DESeq2	LRT	0.056417858865076
599	KC_AB1	Max	0.193669077796987	0.604721999777824	1	pseudobulk	DESeq2	LRT	0.21844423147952
599	KC_AB1	mbc	0.330591175838013	0.359695933987623	1	pseudobulk	DESeq2	LRT	0.444064471430082
599	KC_AB1	MBD-like	0.245026413275843	0.479016042940076	1	pseudobulk	DESeq2	LRT	0.319649941191546
599	KC_AB1	MBD-R2	-0.0297133774037243	0.941238719194982	1	pseudobulk	DESeq2	LRT	0.026300215806625
600	KC_AB1	mbf1	-0.113689223144232	0.534226365503581	1	pseudobulk	DESeq2	LRT	0.272274682188552
600	KC_AB1	mbl	-0.491384657632753	0.479500276770343	1	pseudobulk	DESeq2	LRT	0.319211137815949
600	KC_AB1	mbm	-0.308735235662593	0.719999067239547	1	pseudobulk	DESeq2	LRT	0.14266806619787
600	KC_AB1	mbo	-0.418707504793284	0.706987117782198	1	pseudobulk	DESeq2	LRT	0.150588499537014
600	KC_AB1	Mbs	0.394722724393815	0.0995976017746305	1	pseudobulk	DESeq2	LRT	1.0017511188914
600	KC_AB1	mbt	0.0109040813697128	0.948306606130879	1	pseudobulk	DESeq2	LRT	0.023051224030872
600	KC_AB1	Mcad	1.06929790548588	0.394737401809759	1	pseudobulk	DESeq2	LRT	0.403691721767578
600	KC_AB1	Mccc1	1.08899820779554	0.878177167143508	1	pseudobulk	DESeq2	LRT	0.056417858865076
600	KC_AB1	Mccc2	-1.13912913574204	0.60803432663814	1	pseudobulk	DESeq2	LRT	0.216071901897157
600	KC_AB1	Mcm3	1.08899820779554	0.697631521651407	1	pseudobulk	DESeq2	LRT	0.156373904552
601	KC_AB1	Mcm6	1.08899820779554	0.697631521651407	1	pseudobulk	DESeq2	LRT	0.156373904552
601	KC_AB1	Mco1	-6.47841500423359	0.137668044013494	1	pseudobulk	DESeq2	LRT	0.861166857994488
601	KC_AB1	MCPH1	-0.903187606287057	0.642949210949681	1	pseudobulk	DESeq2	LRT	0.191823332325345
601	KC_AB1	Mcr	0.770814719110316	0.210152805196314	1	pseudobulk	DESeq2	LRT	0.677464808498664
601	KC_AB1	Mct1	-0.949827474458335	0.0612579486377312	1	pseudobulk	DESeq2	LRT	1.21283755062821
601	KC_AB1	Mctp	-0.473354381714837	0.280008055144447	1	pseudobulk	DESeq2	LRT	0.552829474891835
601	KC_AB1	MCTS1	0.464359484136126	0.114280131838984	1	pseudobulk	DESeq2	LRT	0.942029267266279
601	KC_AB1	MCU	-0.25337006272819	0.380243209644304	1	pseudobulk	DESeq2	LRT	0.419938532805116
601	KC_AB1	Mdh1	-0.106919015944311	0.733599011992451	1	pseudobulk	DESeq2	LRT	0.134541262243318
601	KC_AB1	Mdh2	0.0222036202633543	0.896791421251853	1	pseudobulk	DESeq2	LRT	0.047308554874280
602	KC_AB1	mdlc	0.08074235219556	0.933707552515847	1	pseudobulk	DESeq2	LRT	0.029789128284618
602	KC_AB1	mdy	-0.0767032436280026	0.812586679305516	1	pseudobulk	DESeq2	LRT	0.090130301319879
602	KC_AB1	me31B	0.0084263050110878	0.957843834319067	1	pseudobulk	DESeq2	LRT	0.018705291988251
602	KC_AB1	Med	0.04511828255273	0.882918659631404	1	pseudobulk	DESeq2	LRT	0.054079304690161
602	KC_AB1	MED1	0.0601548738736636	0.872952058352177	1	pseudobulk	DESeq2	LRT	0.059009606655049
602	KC_AB1	MED10	-0.0459074158354713	0.894301599998312	1	pseudobulk	DESeq2	LRT	0.048515992249142
602	KC_AB1	MED11	0.206066165194143	0.62499358860249	1	pseudobulk	DESeq2	LRT	0.204124437774071
602	KC_AB1	MED14	-0.237306006704913	0.603968440326677	1	pseudobulk	DESeq2	LRT	0.218985754342559
602	KC_AB1	MED15	0.132144897399339	0.817090359736862	1	pseudobulk	DESeq2	LRT	0.087729913400077
602	KC_AB1	MED16	1.01359866022623	0.467019177582225	1	pseudobulk	DESeq2	LRT	0.330665285285858
603	KC_AB1	MED17	-0.394327114038087	0.607084870635532	1	pseudobulk	DESeq2	LRT	0.21675059018977

603	KC_AB1	MED18	0.152996737634398	0.757123994839421	1	pseudobulk	DESeq2	LRT	0.120832989899198
603	KC_AB1	MED19	0.0935606439173648	0.747846878169531	1	pseudobulk	DESeq2	LRT	0.126187314934348
603	KC_AB1	MED20	-0.802667744324883	0.308129556871063	1	pseudobulk	DESeq2	LRT	0.511266640630383
603	KC_AB1	MED21	0.255501583126163	0.153207477847182	1	pseudobulk	DESeq2	LRT	0.81472003686718
603	KC_AB1	MED22	-0.0155414702877616	0.894456900251761	1	pseudobulk	DESeq2	LRT	0.048440581238353
603	KC_AB1	MED23	1.14247969565044	0.639889789651426	1	pseudobulk	DESeq2	LRT	0.19389481955965
603	KC_AB1	MED24	0.0353266585731492	0.878984215734835	1	pseudobulk	DESeq2	LRT	0.056018923652975
603	KC_AB1	MED25	-1.37165876747677	0.179813253801168	1	pseudobulk	DESeq2	LRT	0.745178300141329
603	KC_AB1	MED26	0.236693309448068	0.103506992479531	1	pseudobulk	DESeq2	LRT	0.98503031018101
604	KC_AB1	MED27	-0.225863906145359	0.745888959219701	1	pseudobulk	DESeq2	LRT	0.127325821304228
604	KC_AB1	MED28	0.488808913613103	0.249448393009916	1	pseudobulk	DESeq2	LRT	0.603019289516638
604	KC_AB1	MED30	0.358733946315895	0.618152993816424	1	pseudobulk	DESeq2	LRT	0.208904023055168
604	KC_AB1	MED31	0.0962182473533777	0.724452832489935	1	pseudobulk	DESeq2	LRT	0.139989885218597
604	KC_AB1	MED4	-0.965636114565803	0.0954151029688868	1	pseudobulk	DESeq2	LRT	1.020382876691
604	KC_AB1	MED6	0.0598680944016526	0.862241228988313	1	pseudobulk	DESeq2	LRT	0.064371214729606
604	KC_AB1	MED7	-0.0666367149227681	0.866611440843879	1	pseudobulk	DESeq2	LRT	0.062175581788411
604	KC_AB1	MED8	-0.613827482881009	0.245987069384759	1	pseudobulk	DESeq2	LRT	0.609087721524435
604	KC_AB1	Mef2	-0.0890992755039803	0.438458611652386	1	pseudobulk	DESeq2	LRT	0.358071395637326
604	KC_AB1	mEFG1	0.24177522269703	0.780063963473515	1	pseudobulk	DESeq2	LRT	0.107869784688169
605	KC_AB1	mEFTs	-0.282843425409655	0.401609775629468	1	pseudobulk	DESeq2	LRT	0.396195724522737
605	KC_AB1	mEFTu1	-0.0539512802875024	0.903469147404446	1	pseudobulk	DESeq2	LRT	0.044086673585376
605	KC_AB1	mEFTu2	0.297228800722039	0.661796613604636	1	pseudobulk	DESeq2	LRT	0.179275459443592
605	KC_AB1	Megf8	0.141517599586823	0.760716746612451	1	pseudobulk	DESeq2	LRT	0.118777022970269
605	KC_AB1	mei-218	1.12049781000607	0.666276071830617	1	pseudobulk	DESeq2	LRT	0.176345783397819
605	KC_AB1	mei-41	-1.80108203135475	0.436835374552077	1	pseudobulk	DESeq2	LRT	0.359682200086492
605	KC_AB1	mei-9	-0.235337114522827	0.771960288084647	1	pseudobulk	DESeq2	LRT	0.112405040479427
605	KC_AB1	mei-P26	0.116005044861887	0.160238599061364	1	pseudobulk	DESeq2	LRT	0.795232860660179
605	KC_AB1	mei-S332	-0.107209672908198	0.929892633731216	1	pseudobulk	DESeq2	LRT	0.031567192596453
605	KC_AB1	mei-W68	-0.619148458022618	0.879843167001942	1	pseudobulk	DESeq2	LRT	0.055594734413205
606	KC_AB1	Meics	-0.240085568330753	0.735808611765189	1	pseudobulk	DESeq2	LRT	0.133235133574456
606	KC_AB1	meigo	0.373078776450635	0.615600610586355	1	pseudobulk	DESeq2	LRT	0.210700958083325
606	KC_AB1	Mekk1	0.294730018487159	0.570983110300413	1	pseudobulk	DESeq2	LRT	0.243376738009523
606	KC_AB1	Meltrin	0.23872988729142	0.839227320749543	1	pseudobulk	DESeq2	LRT	0.076120386280619
606	KC_AB1	Membrin	-0.0062164060204785	0.978104921804916	1	pseudobulk	DESeq2	LRT	0.009614555726893
606	KC_AB1	Men	0.397298978242687	0.263380203478617	1	pseudobulk	DESeq2	LRT	0.579416871147578
606	KC_AB1	Men-b	-0.314400523630583	0.159705157242236	1	pseudobulk	DESeq2	LRT	0.796681059279921
606	KC_AB1	meng	-0.0024069549052315	0.971626902137731	1	pseudobulk	DESeq2	LRT	0.012500469074582
606	KC_AB1	MEP-1	-0.438252367549852	0.473384758527236	1	pseudobulk	DESeq2	LRT	0.324785729072919
606	KC_AB1	Mer	0.438913542691387	0.468519246539585	1	pseudobulk	DESeq2	LRT	0.329272563806619
607	KC_AB1	Mes4	0.715702913673658	0.0216380891357912	1	pseudobulk	DESeq2	LRT	1.66478109450927
607	KC_AB1	Mesh1	0.342722149474347	0.823151944836974	1	pseudobulk	DESeq2	LRT	0.084519991381325
607	KC_AB1	MESK2	-0.124280246873217	0.332024503755327	1	pseudobulk	DESeq2	LRT	0.478829863726611
607	KC_AB1	meso18E	-1.96719661029409	0.414177861019091	1	pseudobulk	DESeq2	LRT	0.382813119093482
607	KC_AB1	MESR3	-0.703552208446388	0.15954442424089	1	pseudobulk	DESeq2	LRT	0.79711836893037
607	KC_AB1	MESR4	0.0646417456395078	0.794862710665689	1	pseudobulk	DESeq2	LRT	0.099707876562613
607	KC_AB1	MESR6	-0.71341472681977	0.376580831166378	1	pseudobulk	DESeq2	LRT	0.424141790348408
607	KC_AB1	Met	0.204206206751388	0.363679583184801	1	pseudobulk	DESeq2	LRT	0.439281079366148
607	KC_AB1	metl	0.0703823255594509	0.874608740551728	1	pseudobulk	DESeq2	LRT	0.058186186773443
607	KC_AB1	metro	0.221555246154469	0.171641099576539	1	pseudobulk	DESeq2	LRT	0.765378711929543
608	KC_AB1	MetRS	-0.346540370340117	0.56083853196769	1	pseudobulk	DESeq2	LRT	0.251162156157525
608	KC_AB1	MetRS-m	-0.349909276226678	0.59497877757815	1	pseudobulk	DESeq2	LRT	0.225498524935447
608	KC_AB1	Mettl14	0.80508456342636	0.460543987523963	1	pseudobulk	DESeq2	LRT	0.336728883107542

608	KC_AB1	Mettl3	0.0931551874657317	0.911711086291797	1	pseudobulk	DESeq2	LRT	0.040142764202172
608	KC_AB1	mew	-1.49770207624262	0.0602421443380111	1	pseudobulk	DESeq2	LRT	1.220099577684
608	KC_AB1	Mf	-0.443008213084181	0.715315546778846	1	pseudobulk	DESeq2	LRT	0.145502335829482
608	KC_AB1	Mfap1	-0.266245485900464	0.595783330165306	1	pseudobulk	DESeq2	LRT	0.224911652377272
608	KC_AB1	Mfe2	0.233714000098109	0.765334043349352	1	pseudobulk	DESeq2	LRT	0.116148968089682
608	KC_AB1	mfrn	0.0845141712605447	0.827242334389643	1	pseudobulk	DESeq2	LRT	0.082367248523144
608	KC_AB1	MFS10	0.304791024818544	0.587305435068353	1	pseudobulk	DESeq2	LRT	0.231135980070933
609	KC_AB1	MFS12	1.08899820779554	0.697631521651407	1	pseudobulk	DESeq2	LRT	0.156373904552
609	KC_AB1	MFS15	-0.433804123620093	0.597276502026931	1	pseudobulk	DESeq2	LRT	0.22382457087285
609	KC_AB1	MFS16	-0.393029759610734	0.525371697145571	1	pseudobulk	DESeq2	LRT	0.279533327257193
609	KC_AB1	MFS18	0.0098931531182496	0.915608281687559	1	pseudobulk	DESeq2	LRT	0.038290287781088
609	KC_AB1	MFS3	-0.850932661185203	0.660736244265418	1	pseudobulk	DESeq2	LRT	0.179971869591347
609	KC_AB1	MFS9	-0.174680016714631	0.858282157269743	1	pseudobulk	DESeq2	LRT	0.066369915879873
609	KC_AB1	Mgat1	0.0176505435581006	0.948990122816151	1	pseudobulk	DESeq2	LRT	0.022738307729467
609	KC_AB1	Mgat2	-0.075111615608464	0.804771759493556	1	pseudobulk	DESeq2	LRT	0.094327271989072
609	KC_AB1	mge	-0.018968458132504	0.897414230493368	1	pseudobulk	DESeq2	LRT	0.047007048045931
609	KC_AB1	mGluR	-0.026830346773355	0.941945620418109	1	pseudobulk	DESeq2	LRT	0.025974168793153
610	KC_AB1	mgr	-0.57298703196981	0.107971340967156	1	pseudobulk	DESeq2	LRT	0.966691504804885
610	KC_AB1	Mgstl	-0.814230936473363	0.0924423424329258	1	pseudobulk	DESeq2	LRT	1.03412905829456
610	KC_AB1	mh	-0.519299816988039	0.707538521336163	1	pseudobulk	DESeq2	LRT	0.150249910364367
610	KC_AB1	Mhcl	0.0932275256279298	0.735437513696121	1	pseudobulk	DESeq2	LRT	0.133454221114291
610	KC_AB1	mi	0.035165024264767	0.954417234882809	1	pseudobulk	DESeq2	LRT	0.020261726769695
610	KC_AB1	Mi-2	-0.244062670247417	0.181846666632054	1	pseudobulk	DESeq2	LRT	0.740294655437277
610	KC_AB1	mia	2.54858448870456	0.280000067828146	1	pseudobulk	DESeq2	LRT	0.552841863452831
610	KC_AB1	mib1	0.0808085127888817	0.876965084250537	1	pseudobulk	DESeq2	LRT	0.057017697419804
610	KC_AB1	mib2	0.231154416142753	0.307458170236089	1	pseudobulk	DESeq2	LRT	0.512213961746659
610	KC_AB1	Mical	-0.127060349255455	0.645278878241924	1	pseudobulk	DESeq2	LRT	0.190252550316877
611	KC_AB1	MICAL-like	-0.261767143122516	0.603808116571625	1	pseudobulk	DESeq2	LRT	0.219101053355689
611	KC_AB1	Mics1	2.10549911548503	0.428045198278114	1	pseudobulk	DESeq2	LRT	0.368510370411162
611	KC_AB1	MICU1	0.307928533981552	0.287238269604709	1	pseudobulk	DESeq2	LRT	0.541757698235739
611	KC_AB1	MICU3	-0.023910584487833	0.908050443976075	1	pseudobulk	DESeq2	LRT	0.041890024901888
611	KC_AB1	Mid1	-0.248410769665789	0.0870064682468937	1	pseudobulk	DESeq2	LRT	1.06044845980077
611	KC_AB1	mIF2	-1.35824333625894	0.239879450280158	1	pseudobulk	DESeq2	LRT	0.620006955084249
611	KC_AB1	mIF3	-0.674680071043128	0.381648911211674	1	pseudobulk	DESeq2	LRT	0.418335972259028
611	KC_AB1	Miga	-0.324576072361739	0.420933080152535	1	pseudobulk	DESeq2	LRT	0.375786942716754
611	KC_AB1	milt	-0.242246230618893	0.192590380324116	1	pseudobulk	DESeq2	LRT	0.715365409197414
611	KC_AB1	mim	0.0536411500169236	0.80374404441513	1	pseudobulk	DESeq2	LRT	0.094882232092266
612	KC_AB1	mino	0.796076280502459	0.536793549180797	1	pseudobulk	DESeq2	LRT	0.270192711862941
612	KC_AB1	mio	-0.201808325384113	0.81369045114699	1	pseudobulk	DESeq2	LRT	0.089540780524607
612	KC_AB1	mip120	-0.349780353236279	0.470617563387809	1	pseudobulk	DESeq2	LRT	0.327331869042541
612	KC_AB1	mip130	-0.172947582724529	0.764895495145834	1	pseudobulk	DESeq2	LRT	0.116397896848746
612	KC_AB1	mip40	0.398449476282533	0.61620059346371	1	pseudobulk	DESeq2	LRT	0.210277887749214
612	KC_AB1	miple1	0.10320923719841	0.311287047592138	1	pseudobulk	DESeq2	LRT	0.506838949591463
612	KC_AB1	miple2	0.345857426764128	0.688554559439479	1	pseudobulk	DESeq2	LRT	0.16206164157305
612	KC_AB1	Mipp1	-0.82542030717083	1	1	pseudobulk	DESeq2	LRT	0
612	KC_AB1	Mipp2	0.175081024848555	0.879203234151306	1	pseudobulk	DESeq2	LRT	0.055910723028284
612	KC_AB1	mira	-1.13912913574204	0.60803432663814	1	pseudobulk	DESeq2	LRT	0.216071901897157
613	KC_AB1	Miro	-0.165617605098977	0.651403121145959	1	pseudobulk	DESeq2	LRT	0.186150164878883
613	KC_AB1	mirr	1.88952324269138	0.456767015650974	1	pseudobulk	DESeq2	LRT	0.340305265178099
613	KC_AB1	Mis12	-1.07863060369249	0.0604098887441414	1	pseudobulk	DESeq2	LRT	1.21889196410251
613	KC_AB1	Mitf	-0.029074502434802	0.869209043677781	1	pseudobulk	DESeq2	LRT	0.060875763714923
613	KC_AB1	Mitofilin	-0.179774783132246	0.668731192569969	1	pseudobulk	DESeq2	LRT	0.174748418919034

613	KC_AB1	mkg-p	0.13617601020169	1	1	pseudobulk	DESeq2	LRT	0
613	KC_AB1	Mkk4	0.0018771114775660	0.953666569331923	1	pseudobulk	DESeq2	LRT	0.020603441240101
613	KC_AB1	Mkp	1.08899820779554	0.697631521651407	1	pseudobulk	DESeq2	LRT	0.156373904552
613	KC_AB1	MKP-4	0.182365741207012	0.70593413131066	1	pseudobulk	DESeq2	LRT	0.15123581983043
613	KC_AB1	Mkp3	0.0010081732507209	0.950581615599667	1	pseudobulk	DESeq2	LRT	0.02201058928471
614	KC_AB1	Mkrn1	0.062832154865956	0.83322490050879	1	pseudobulk	DESeq2	LRT	0.07923775985730
614	KC_AB1	Mlc-c	-0.0225306645518488	0.949089223725262	1	pseudobulk	DESeq2	LRT	0.02269295769908
614	KC_AB1	mld	0.301334042893548	0.23073979200394	1	pseudobulk	DESeq2	LRT	0.63687750319229
614	KC_AB1	mldr	0.045707994267386	0.959806512825956	1	pseudobulk	DESeq2	LRT	0.01781630746175
614	KC_AB1	mle	-0.0653158366337325	0.791151057415506	1	pseudobulk	DESeq2	LRT	0.101740587123492
614	KC_AB1	Mlf	-0.0161613545084107	0.902418367664425	1	pseudobulk	DESeq2	LRT	0.044592073717422
614	KC_AB1	Mlh1	1.26269203670432	0.541057763329599	1	pseudobulk	DESeq2	LRT	0.266756367137151
614	KC_AB1	Mlp84B	0.685669198120902	0.393447347493426	1	pseudobulk	DESeq2	LRT	0.40511337823006
614	KC_AB1	mlt	0.195932161810328	0.592196474520408	1	pseudobulk	DESeq2	LRT	0.22753418239233
614	KC_AB1	mmd	0.107423887373079	0.25552590815069	1	pseudobulk	DESeq2	LRT	0.59256505953535
615	KC_AB1	MME1	-0.0492885114339589	0.941715337395017	1	pseudobulk	DESeq2	LRT	0.026080356317281
615	KC_AB1	Mmp1	-0.501300761870089	0.863550576027942	1	pseudobulk	DESeq2	LRT	0.06371222179469
615	KC_AB1	Mmp2	-0.159593584323763	0.417742655006095	1	pseudobulk	DESeq2	LRT	0.379091177385177
615	KC_AB1	Mms19	0.788550667893827	0.396884951385389	1	pseudobulk	DESeq2	LRT	0.40133536784563
615	KC_AB1	mmy	-0.475605336105722	0.222882366172955	1	pseudobulk	DESeq2	LRT	0.65192429032067
615	KC_AB1	mnb	0.0412144039493232	0.817274911080193	1	pseudobulk	DESeq2	LRT	0.08763183296260
615	KC_AB1	mnd	1.88952324269138	0.456767015650974	1	pseudobulk	DESeq2	LRT	0.34030526517809
615	KC_AB1	Mnn1	0.251787793779538	0.0492622937922318	1	pseudobulk	DESeq2	LRT	1.30748537004227
615	KC_AB1	Mnt	0.136997467579241	0.359694399948439	1	pseudobulk	DESeq2	LRT	0.44406632362275
615	KC_AB1	Mo25	-0.237298935488082	0.350618084470122	1	pseudobulk	DESeq2	LRT	0.45516568726381
616	KC_AB1	Mob2	0.215010481825654	0.175194142663016	1	pseudobulk	DESeq2	LRT	0.756480417869161
616	KC_AB1	Mob3	0.0741048600919213	0.844646371795521	1	pseudobulk	DESeq2	LRT	0.07332507912461
616	KC_AB1	Mob4	0.0955349163351219	0.665382884085834	1	pseudobulk	DESeq2	LRT	0.17692837485685
616	KC_AB1	Moca-cyp	-0.311216784561539	0.174695654262335	1	pseudobulk	DESeq2	LRT	0.75771789841353
616	KC_AB1	Mocs1	-0.82542030717083	1	1	pseudobulk	DESeq2	LRT	0
616	KC_AB1	mod	0.414377588299958	0.244682792398373	1	pseudobulk	DESeq2	LRT	0.61139657183826
616	KC_AB1	Moe	0.105212305520031	0.552043043824804	1	pseudobulk	DESeq2	LRT	0.25802705820054
616	KC_AB1	mof	-0.151609705827895	0.867821962214939	1	pseudobulk	DESeq2	LRT	0.06156936325489
616	KC_AB1	Mon1	0.15446167280808	0.86812296984117	1	pseudobulk	DESeq2	LRT	0.06141875254141
616	KC_AB1	mon2	0.0517540528017069	0.89518830454702	1	pseudobulk	DESeq2	LRT	0.04808560041164
617	KC_AB1	Mondo	0.179883540361746	0.599708839251663	1	pseudobulk	DESeq2	LRT	0.222059549945061
617	KC_AB1	moody	1.08899820779554	0.697631521651407	1	pseudobulk	DESeq2	LRT	0.156373904552
617	KC_AB1	moon	-0.850996402080018	0.900449987182352	1	pseudobulk	DESeq2	LRT	0.04554040377050
617	KC_AB1	mop	0.46999814583401	0.284708392582828	1	pseudobulk	DESeq2	LRT	0.54559973060966
617	KC_AB1	mor	-0.0491130546511169	0.901676965430582	1	pseudobulk	DESeq2	LRT	0.04494902482887
617	KC_AB1	morgue	-0.132139898234117	0.782937159713785	1	pseudobulk	DESeq2	LRT	0.10627309398898
617	KC_AB1	Mos	1.08899820779554	0.697631521651407	1	pseudobulk	DESeq2	LRT	0.156373904552
617	KC_AB1	Mp	0.294344816136114	0.131351394821524	1	pseudobulk	DESeq2	LRT	0.88156531107680
617	KC_AB1	MP1	-0.322246135217121	0.797158409460509	1	pseudobulk	DESeq2	LRT	0.09845536804102
617	KC_AB1	Mpc1	-0.210957632635715	0.701756214683957	1	pseudobulk	DESeq2	LRT	0.15381373260731
618	KC_AB1	Mpcp2	-0.169640841678638	0.188162015833501	1	pseudobulk	DESeq2	LRT	0.72546804285801
618	KC_AB1	Mpi	-0.43990276256609	0.484033294000084	1	pseudobulk	DESeq2	LRT	0.31512476458861
618	KC_AB1	Mpp6	-0.751250153435237	0.324325285781272	1	pseudobulk	DESeq2	LRT	0.489019190558651
618	KC_AB1	Mppe	-0.149444072285606	0.856580694292009	1	pseudobulk	DESeq2	LRT	0.06723171800497
618	KC_AB1	Mps1	0.483335712218914	0.723265436195069	1	pseudobulk	DESeq2	LRT	0.14070228870963
618	KC_AB1	mr	0.0732184278935561	0.89298869037277	1	pseudobulk	DESeq2	LRT	0.04915404137998
618	KC_AB1	mre11	-0.519051611439418	0.553041286196302	1	pseudobulk	DESeq2	LRT	0.25724244609505

618	KC_AB1	mRF1	0.505164367823093	0.686648276393157	1	pseudobulk	DESeq2	LRT	0.16326566576011
618	KC_AB1	MRG15	0.2648447148174	0.547443438323807	1	pseudobulk	DESeq2	LRT	0.261660745333156
618	KC_AB1	MrgBP	0.039572015883399	0.890547927457494	1	pseudobulk	DESeq2	LRT	0.050342702736154
619	KC_AB1	mri	0.27808286854311	0.391400939530574	1	pseudobulk	DESeq2	LRT	0.407378136183244
619	KC_AB1	mrj	0.220544471162681	0.321943874587365	1	pseudobulk	DESeq2	LRT	0.492219833526395
619	KC_AB1	mrn	0.310345458164229	0.734964733605155	1	pseudobulk	DESeq2	LRT	0.133733499511903
619	KC_AB1	mRNA-cap	0.381284699465238	0.317391824529949	1	pseudobulk	DESeq2	LRT	0.498404264119476
619	KC_AB1	MRP	0.0200699256160541	0.956921773412658	1	pseudobulk	DESeq2	LRT	0.019123563543755
619	KC_AB1	mRpL1	0.159377910091369	0.705061289769628	1	pseudobulk	DESeq2	LRT	0.151773128892583
619	KC_AB1	mRpL10	0.385472988382957	0.453173297837931	1	pseudobulk	DESeq2	LRT	0.343735687827364
619	KC_AB1	mRpL11	0.393938823806942	0.54312312078118	1	pseudobulk	DESeq2	LRT	0.265101708873819
619	KC_AB1	mRpL12	-0.0912772516203034	0.718132176728736	1	pseudobulk	DESeq2	LRT	0.143795613778795
619	KC_AB1	mRpL13	-0.431761021690535	0.338765071145626	1	pseudobulk	DESeq2	LRT	0.470101374583121
620	KC_AB1	mRpL14	-0.82706092757276	0.0658825811952437	1	pseudobulk	DESeq2	LRT	1.18122939406678
620	KC_AB1	mRpL15	0.114417494802682	0.807124696784758	1	pseudobulk	DESeq2	LRT	0.093059363738938
620	KC_AB1	mRpL16	-0.0685730886265325	0.869144939803975	1	pseudobulk	DESeq2	LRT	0.060907793967610
620	KC_AB1	mRpL17	0.0693960052663718	0.825616427294583	1	pseudobulk	DESeq2	LRT	0.083221674471207
620	KC_AB1	mRpL18	-0.0343885914064745	0.933682571916636	1	pseudobulk	DESeq2	LRT	0.029800747641764
620	KC_AB1	mRpL19	0.020200701103007	0.953362663527087	1	pseudobulk	DESeq2	LRT	0.020741860319265
620	KC_AB1	mRpL2	-0.127717649611847	0.723993798838322	1	pseudobulk	DESeq2	LRT	0.140265153612013
620	KC_AB1	mRpL20	0.0422261506926964	0.919191834531681	1	pseudobulk	DESeq2	LRT	0.036593842276237
620	KC_AB1	mRpL21	-0.449359878414337	0.281415316030651	1	pseudobulk	DESeq2	LRT	0.55065226977766
620	KC_AB1	mRpL22	-0.302854976234651	0.385405290540202	1	pseudobulk	DESeq2	LRT	0.414082327987141
621	KC_AB1	mRpL23	-0.131638113283275	0.667161570590566	1	pseudobulk	DESeq2	LRT	0.175768977599821
621	KC_AB1	mRpL24	0.0594032076647024	0.827137768931944	1	pseudobulk	DESeq2	LRT	0.0824221478794
621	KC_AB1	mRpL27	-0.0208153917253116	0.93228145776839	1	pseudobulk	DESeq2	LRT	0.030452953416130
621	KC_AB1	mRpL28	0.0298354407691946	0.926401297616501	1	pseudobulk	DESeq2	LRT	0.033200845297023
621	KC_AB1	mRpL3	0.654942791551844	0.405148349709325	1	pseudobulk	DESeq2	LRT	0.392385925764873
621	KC_AB1	mRpL30	0.0395806368317412	0.918726301555428	1	pseudobulk	DESeq2	LRT	0.036813850341977
621	KC_AB1	mRpL32	-0.297428548948597	0.562022718691515	1	pseudobulk	DESeq2	LRT	0.250246128553823
621	KC_AB1	mRpL33	0.0281373114654854	0.905128017357844	1	pseudobulk	DESeq2	LRT	0.043289991733803
621	KC_AB1	mRpL34	0.223716237944396	0.478128343826665	1	pseudobulk	DESeq2	LRT	0.320455510220444
621	KC_AB1	mRpL35	-0.414210222443494	0.311460145796127	1	pseudobulk	DESeq2	LRT	0.506597517441936
622	KC_AB1	mRpL36	-0.115559352147982	0.719218861442297	1	pseudobulk	DESeq2	LRT	0.14313893179638
622	KC_AB1	mRpL37	0.314194251033287	0.549913465863716	1	pseudobulk	DESeq2	LRT	0.259705645514151
622	KC_AB1	mRpL38	0.135730306665714	0.77548351459023	1	pseudobulk	DESeq2	LRT	0.110427430085829
622	KC_AB1	mRpL39	0.102863843910741	0.803855322114274	1	pseudobulk	DESeq2	LRT	0.094822108541859
622	KC_AB1	mRpL4	0.0552006709826733	0.854484390836996	1	pseudobulk	DESeq2	LRT	0.068295866278851
622	KC_AB1	mRpL40	-0.0301403934390724	0.93843776247339	1	pseudobulk	DESeq2	LRT	0.027594524652282
622	KC_AB1	mRpL41	-0.178898976952251	0.64472320905012	1	pseudobulk	DESeq2	LRT	0.190626695569923
622	KC_AB1	mRpL42	-0.247581044354794	0.489897935477842	1	pseudobulk	DESeq2	LRT	0.309894390738546
622	KC_AB1	mRpL43	-0.127236674577216	0.758264992612407	1	pseudobulk	DESeq2	LRT	0.120178993949094
622	KC_AB1	mRpL44	-0.188877390149664	0.743939081350158	1	pseudobulk	DESeq2	LRT	0.12846262590125
623	KC_AB1	mRpL45	-0.647883346208882	0.4808918159322	1	pseudobulk	DESeq2	LRT	0.317952613913361
623	KC_AB1	mRpL46	-0.331576381605988	0.61751843566134	1	pseudobulk	DESeq2	LRT	0.209350072260241
623	KC_AB1	mRpL47	-0.198650535860598	0.581578789465757	1	pseudobulk	DESeq2	LRT	0.235391440845639
623	KC_AB1	mRpL48	-0.222172052031785	0.524520126041702	1	pseudobulk	DESeq2	LRT	0.280237843102147
623	KC_AB1	mRpL49	-0.133524856670533	0.7343137283015	1	pseudobulk	DESeq2	LRT	0.134118352399764
623	KC_AB1	mRpL50	-0.0655936164851384	0.858041294842516	1	pseudobulk	DESeq2	LRT	0.066491810413914
623	KC_AB1	mRpL51	-0.14636918967185	0.812975726448855	1	pseudobulk	DESeq2	LRT	0.089922421228434
623	KC_AB1	mRpL52	-0.528090603828888	0.182825739639124	1	pseudobulk	DESeq2	LRT	0.73796266092004
623	KC_AB1	mRpL54	-0.435387140382876	0.271629324571432	1	pseudobulk	DESeq2	LRT	0.566023346276357

623	KC_AB1	mRpL55	-0.134410950595746	0.599029547810352	1	pseudobulk	DESeq2	LRT	0.222551755015538
624	KC_AB1	mRpL9	0.465929233216025	0.382409531049421	1	pseudobulk	DESeq2	LRT	0.417471292048169
624	KC_AB1	mRpS10	-0.153318733867167	0.687419690257735	1	pseudobulk	DESeq2	LRT	0.162778032210709
624	KC_AB1	mRpS11	0.153970665128687	0.64001858260362	1	pseudobulk	DESeq2	LRT	0.19380741632072
624	KC_AB1	mRpS14	-0.0930313120697473	0.814780675474695	1	pseudobulk	DESeq2	LRT	0.088959279910250
624	KC_AB1	mRpS16	-0.421586407719108	0.319769492497827	1	pseudobulk	DESeq2	LRT	0.495162972334155
624	KC_AB1	mRpS17	0.140203590354633	0.724712011649314	1	pseudobulk	DESeq2	LRT	0.139834540462066
624	KC_AB1	mRpS18A	-0.316744315633543	0.383398054054388	1	pseudobulk	DESeq2	LRT	0.416350095723961
624	KC_AB1	mRpS18B	-1.21595917607942	0.0609684567210428	1	pseudobulk	DESeq2	LRT	1.21489479802413
624	KC_AB1	mRpS18C	0.170598734716555	0.592966996273566	1	pseudobulk	DESeq2	LRT	0.226969478195859
624	KC_AB1	mRpS2	-0.167986403279989	0.705495652100086	1	pseudobulk	DESeq2	LRT	0.151505658415316
625	KC_AB1	mRpS21	0.180974437262779	0.626181304021236	1	pseudobulk	DESeq2	LRT	0.203299903313839
625	KC_AB1	mRpS22	0.180652499546493	0.754950951255094	1	pseudobulk	DESeq2	LRT	0.122081263326068
625	KC_AB1	mRpS23	0.0222829984644659	0.961129153031219	1	pseudobulk	DESeq2	LRT	0.017218249501074
625	KC_AB1	mRpS24	0.0166173042184903	0.941168542877029	1	pseudobulk	DESeq2	LRT	0.026332596884063
625	KC_AB1	mRpS25	-0.0149299977977688	0.917922350235564	1	pseudobulk	DESeq2	LRT	0.037194055498685
625	KC_AB1	mRpS26	-0.58385922707346	0.169953169524508	1	pseudobulk	DESeq2	LRT	0.769670731674222
625	KC_AB1	mRpS28	-0.0745606351520415	0.820336498441505	1	pseudobulk	DESeq2	LRT	0.086007965373184
625	KC_AB1	mRpS29	-0.23464790343586	0.664303595456203	1	pseudobulk	DESeq2	LRT	0.177633396990088
625	KC_AB1	mRpS30	-0.459140227625316	0.532892259224591	1	pseudobulk	DESeq2	LRT	0.273360588260612
625	KC_AB1	mRpS31	0.50175397936632	0.317169223510111	1	pseudobulk	DESeq2	LRT	0.498708961039797
626	KC_AB1	mRpS33	0.0813431485930426	0.789825838625593	1	pseudobulk	DESeq2	LRT	0.102468662712718
626	KC_AB1	mRpS34	0.0873210931065155	0.828996154448649	1	pseudobulk	DESeq2	LRT	0.081447484052386
626	KC_AB1	mRpS35	-0.713287065253692	0.195588215116891	1	pseudobulk	DESeq2	LRT	0.708657316541735
626	KC_AB1	mRpS5	0.167143788607922	0.34939736290242	1	pseudobulk	DESeq2	LRT	0.456680377204402
626	KC_AB1	mRpS6	-0.189771754991562	0.604294932342898	1	pseudobulk	DESeq2	LRT	0.218751047758401
626	KC_AB1	mRpS7	0.0673508390217663	0.817106899746299	1	pseudobulk	DESeq2	LRT	0.087721122252267
626	KC_AB1	mRpS9	0.138946035860502	0.692941932474751	1	pseudobulk	DESeq2	LRT	0.159303157108824
626	KC_AB1	mRRF1	-0.611121352574148	0.398974912692802	1	pseudobulk	DESeq2	LRT	0.399054411635605
626	KC_AB1	mRRF2	-0.12479488481263	0.896695597429279	1	pseudobulk	DESeq2	LRT	0.047354962522604
626	KC_AB1	mrt	-0.318915996668363	0.508807039446104	1	pseudobulk	DESeq2	LRT	0.293446888767956
627	KC_AB1	Mrtf	-0.185771012455049	0.456527554333696	1	pseudobulk	DESeq2	LRT	0.340533004911859
627	KC_AB1	mrva	0.336893868449255	0.173931155684598	1	pseudobulk	DESeq2	LRT	0.759622617364907
627	KC_AB1	Ms	-1.44925888589622	0.6315588144614	1	pseudobulk	DESeq2	LRT	0.199586199175701
627	KC_AB1	ms(3)76Ba	0.112935832476632	0.940353958317081	1	pseudobulk	DESeq2	LRT	0.026708642986417
627	KC_AB1	MSBP	-0.0287087210985648	0.894970801590915	1	pseudobulk	DESeq2	LRT	0.048191133303973
627	KC_AB1	msd1	0.111831901354254	1	1	pseudobulk	DESeq2	LRT	0
627	KC_AB1	msd5	0.161900740917243	0.826632327468123	1	pseudobulk	DESeq2	LRT	0.082687614579971
627	KC_AB1	Msh6	1.11663863281567	0.31912412005698	1	pseudobulk	DESeq2	LRT	0.496040369696271
627	KC_AB1	msi	0.797255582562714	0.00908642282880753	1	pseudobulk	DESeq2	LRT	2.04160705753402
627	KC_AB1	msk	0.203640855971945	0.646424738693008	1	pseudobulk	DESeq2	LRT	0.18948203145334
628	KC_AB1	msl-1	-0.0554180274463323	0.884841262585577	1	pseudobulk	DESeq2	LRT	0.053134633219093
628	KC_AB1	msl-2	0.15081795038288	0.813189460362797	1	pseudobulk	DESeq2	LRT	0.089808258828811
628	KC_AB1	msl-3	-0.276857100477155	0.682762376834394	1	pseudobulk	DESeq2	LRT	0.165730418405862
628	KC_AB1	msn	-0.157824514640865	0.374871537426481	1	pseudobulk	DESeq2	LRT	0.426117532658939
628	KC_AB1	Msp300	-0.0599758174838716	0.73837766980205	1	pseudobulk	DESeq2	LRT	0.13172144582341
628	KC_AB1	mspo	0.0638339520770737	0.619337354840507	1	pseudobulk	DESeq2	LRT	0.208072725073819
628	KC_AB1	msps	0.0799818015798365	0.713308618101953	1	pseudobulk	DESeq2	LRT	0.146722528858172
628	KC_AB1	Msr-110	0.536822480739811	0.77353335804325	1	pseudobulk	DESeq2	LRT	0.111520952938458
628	KC_AB1	MsR1	1.88952324269138	0.456767015650974	1	pseudobulk	DESeq2	LRT	0.340305265178099
628	KC_AB1	mst	0.521792914923747	0.761731134322654	1	pseudobulk	DESeq2	LRT	0.118198293076445
629	KC_AB1	Mst36Fa	-0.850996402080018	0.900449987182352	1	pseudobulk	DESeq2	LRT	0.045540403770502

629	KC_AB1	Mst77F	0.0541157279124217	0.902999106619151	1	pseudobulk	DESeq2	LRT	0.044312679354893
629	KC_AB1	Mst85C	-0.244809044538984	0.561031954128225	1	pseudobulk	DESeq2	LRT	0.251012402368881
629	KC_AB1	Mst89B	1.08899820779554	0.697631521651407	1	pseudobulk	DESeq2	LRT	0.156373904552
629	KC_AB1	mt:ATPase6	0.31807913839353	0.0175563288133087	1	pseudobulk	DESeq2	LRT	1.7555662938291
629	KC_AB1	mt:ATPase8	0.928444401186144	0.252080001230013	1	pseudobulk	DESeq2	LRT	0.598461607714937
629	KC_AB1	mt:CoI	0.274776897173907	0.0308432797473534	1	pseudobulk	DESeq2	LRT	1.5108394470788
629	KC_AB1	mt:CoIII	0.26614748469586	0.0282837508436441	1	pseudobulk	DESeq2	LRT	1.54846299718491
629	KC_AB1	mt:Cyt-b	0.214622775256494	0.0943138250981607	1	pseudobulk	DESeq2	LRT	1.02542464105103
629	KC_AB1	mt:lrRNA	0.0497039213665415	0.60576183878155	1	pseudobulk	DESeq2	LRT	0.217698089419499
630	KC_AB1	mt:ND1	-0.314035571380363	0.110304043918746	1	pseudobulk	DESeq2	LRT	0.957408565353077
630	KC_AB1	mt:ND2	0.070397934806023	0.760229532781415	1	pseudobulk	DESeq2	LRT	0.119055263284776
630	KC_AB1	mt:ND3	0.269000210167969	0.223114011480637	1	pseudobulk	DESeq2	LRT	0.651473155320607
630	KC_AB1	mt:ND4	0.175938416167789	0.333030213504472	1	pseudobulk	DESeq2	LRT	0.477516364202271
630	KC_AB1	mt:ND4L	1.67535466974767	0.127819942771655	1	pseudobulk	DESeq2	LRT	0.893401381232464
630	KC_AB1	mt:ND5	0.150056819821728	0.431352764372106	1	pseudobulk	DESeq2	LRT	0.365167414392753
630	KC_AB1	mt:ND6	0.113432811556453	0.65866158009843	1	pseudobulk	DESeq2	LRT	0.181337668329967
630	KC_AB1	mt:srRNA	0.166764423299	0.338814090221138	1	pseudobulk	DESeq2	LRT	0.470038537012978
630	KC_AB1	mt:tRNA:Ala-TGC	-0.850996402080018	0.900449987182352	1	pseudobulk	DESeq2	LRT	0.045540403770502
630	KC_AB1	mt:tRNA:Arg-TCG	0.959088647258781	0.657893326835362	1	pseudobulk	DESeq2	LRT	0.181844518730155
631	KC_AB1	mt:tRNA:Asn-GTT	-0.850932661185203	0.660736244265418	1	pseudobulk	DESeq2	LRT	0.179971869591347
631	KC_AB1	mt:tRNA:Asp-GTC	0.11269840515639	0.977007463492936	1	pseudobulk	DESeq2	LRT	0.010102118633921
631	KC_AB1	mt:tRNA:Ser-GCT	0.221643939736625	0.905680069226275	1	pseudobulk	DESeq2	LRT	0.043025189417767
631	KC_AB1	mt:tRNA:Ser-TGA	0.111831901354254	1	1	pseudobulk	DESeq2	LRT	0
631	KC_AB1	mt:tRNA:Tyr-GTA	-0.82542030717083	1	1	pseudobulk	DESeq2	LRT	0
631	KC_AB1	mt:tRNA:Val-TAC	1.08899820779554	0.878177167143508	1	pseudobulk	DESeq2	LRT	0.056417858865076
631	KC_AB1	MTA1-like	-0.335018056938674	0.244780396524477	1	pseudobulk	DESeq2	LRT	0.611223366028722
631	KC_AB1	Mtap	0.247740265607575	0.672503274269134	1	pseudobulk	DESeq2	LRT	0.172305596836996
631	KC_AB1	Mtch	-0.0838973904580941	0.742797409565367	1	pseudobulk	DESeq2	LRT	0.129129619489915
631	KC_AB1	mtd	-0.0581888286549749	0.532910172123673	1	pseudobulk	DESeq2	LRT	0.273345989920436
632	KC_AB1	mtDNA-helicase	-0.588354800448092	0.805189412611406	1	pseudobulk	DESeq2	LRT	0.094101944258636
632	KC_AB1	mTerf3	0.926905908134647	0.0244758642297798	1	pseudobulk	DESeq2	LRT	1.61126196454636
632	KC_AB1	mTerf5	0.202667801369938	0.775354559126942	1	pseudobulk	DESeq2	LRT	0.110499655093966
632	KC_AB1	MTF-1	0.0137261221825561	0.926334827056697	1	pseudobulk	DESeq2	LRT	0.033232007637909
632	KC_AB1	mtg	1.06911625683957	0.319540492961539	1	pseudobulk	DESeq2	LRT	0.495474099147388
632	KC_AB1	mtgo	-0.193842025027444	0.192090408659786	1	pseudobulk	DESeq2	LRT	0.716494319522071
632	KC_AB1	Mthfs	2.21745641838459	0.396737788462723	1	pseudobulk	DESeq2	LRT	0.401496431894786
632	KC_AB1	mthl1	0.315546443544659	0.834249450196689	1	pseudobulk	DESeq2	LRT	0.078704070890278
632	KC_AB1	mthl14	0.942080999092271	0.681532122211136	1	pseudobulk	DESeq2	LRT	0.166513670026992
632	KC_AB1	mthl15	1.08899820779554	0.878177167143508	1	pseudobulk	DESeq2	LRT	0.056417858865076
633	KC_AB1	mthl8	0.324770180282829	0.930964781781981	1	pseudobulk	DESeq2	LRT	0.031066747984338
633	KC_AB1	Mtl	-0.0235534743349404	0.951146488231315	1	pseudobulk	DESeq2	LRT	0.021752591231653
633	KC_AB1	mtm	0.219473313686384	0.679541398638327	1	pseudobulk	DESeq2	LRT	0.167784080281725
633	KC_AB1	MtnA	0.463458124577148	0.436081700771197	1	pseudobulk	DESeq2	LRT	0.360432137176228
633	KC_AB1	MtnE	-2.36817011682091	0.282654768599882	1	pseudobulk	DESeq2	LRT	0.548743683265438
633	KC_AB1	Mtor	0.182003581673025	0.654370812480401	1	pseudobulk	DESeq2	LRT	0.184176080151827
633	KC_AB1	Mtp	0.980907182251946	0.486536175321043	1	pseudobulk	DESeq2	LRT	0.312884863189614
633	KC_AB1	Mtpalpha	-0.189126327826457	0.72935583465494	1	pseudobulk	DESeq2	LRT	0.137060538453516
633	KC_AB1	MTPAP	-0.184026661869972	0.847604474133986	1	pseudobulk	DESeq2	LRT	0.071806759504219
633	KC_AB1	Mtpbeta	-0.349714191223589	0.577068577279445	1	pseudobulk	DESeq2	LRT	0.238772573387896
634	KC_AB1	Mtr3	0.444367718283026	0.693866588655881	1	pseudobulk	DESeq2	LRT	0.15872402432948
634	KC_AB1	Mtr4	-0.0551180188564645	0.943261254673264	1	pseudobulk	DESeq2	LRT	0.025368004241640
634	KC_AB1	mtRNApol	-0.260286541074514	0.695300383050012	1	pseudobulk	DESeq2	LRT	0.157827531355848

634	KC_AB1	mts	-0.078619271676631	0.498130988617174	1	pseudobulk	DESeq2	LRT	0.302656440064813
634	KC_AB1	mtSSB	-0.805506012216404	0.169330424523258	1	pseudobulk	DESeq2	LRT	0.771265002815754
634	KC_AB1	mtt	-1.58257984633167	0.12271512106868	1	pseudobulk	DESeq2	LRT	0.911101919815165
634	KC_AB1	mTTF	-0.271326640120362	0.734690438004819	1	pseudobulk	DESeq2	LRT	0.133895612461009
634	KC_AB1	mtTFB2	-1.04463445516174	0.338278236500797	1	pseudobulk	DESeq2	LRT	0.470725942122303
634	KC_AB1	mu2	0.138589196718824	0.898064130577098	1	pseudobulk	DESeq2	LRT	0.046692649344617
634	KC_AB1	mub	-0.0505350749781754	0.532350044905148	1	pseudobulk	DESeq2	LRT	0.273802704962825
635	KC_AB1	muc	-0.0133410289422027	0.88329217660299	1	pseudobulk	DESeq2	LRT	0.053895616130735
635	KC_AB1	Muc11A	-1.6747012584303	0.563129890257477	1	pseudobulk	DESeq2	LRT	0.249391420218277
635	KC_AB1	Muc12Ea	0.10745091433163	0.925708810521252	1	pseudobulk	DESeq2	LRT	0.033525602812194
635	KC_AB1	Muc14A	0.595035684970554	0.793463972381063	1	pseudobulk	DESeq2	LRT	0.100472787814383
635	KC_AB1	Muc4B	1.08899820779554	0.697631521651407	1	pseudobulk	DESeq2	LRT	0.156373904552
635	KC_AB1	Muc91C	2.1559833397943	0.381743505411375	1	pseudobulk	DESeq2	LRT	0.418228342845202
635	KC_AB1	Muc96D	-1.44925888589622	0.6315588144614	1	pseudobulk	DESeq2	LRT	0.199586199175701
635	KC_AB1	mud	-0.82542030717083	1	1	pseudobulk	DESeq2	LRT	0
635	KC_AB1	Mul1	0.0417590149732853	0.885765855381629	1	pseudobulk	DESeq2	LRT	0.052681064969247
635	KC_AB1	Mulk	0.209994542506968	0.77224291635531	1	pseudobulk	DESeq2	LRT	0.112246066713025
636	KC_AB1	Mur2B	-0.55840360662797	0.535677291173661	1	pseudobulk	DESeq2	LRT	0.271096764168511
636	KC_AB1	mura	-0.716485177537603	0.19671375941717	1	pseudobulk	DESeq2	LRT	0.706165261687378
636	KC_AB1	mus101	0.768123422515191	0.670507976041533	1	pseudobulk	DESeq2	LRT	0.17359605162264
636	KC_AB1	mus201	0.136871973658506	0.84527506195815	1	pseudobulk	DESeq2	LRT	0.073001943769797
636	KC_AB1	mus304	0.13617601020169	1	1	pseudobulk	DESeq2	LRT	0
636	KC_AB1	mus81	-0.477767482101864	0.68047224371164	1	pseudobulk	DESeq2	LRT	0.167189584859937
636	KC_AB1	muskelin	2.17001672698427	0.124223132757636	1	pseudobulk	DESeq2	LRT	0.905797522569147
636	KC_AB1	mute	0.488125151061213	0.426747433143993	1	pseudobulk	DESeq2	LRT	0.369829082456351
636	KC_AB1	mv	-0.358576708110258	0.671797552141066	1	pseudobulk	DESeq2	LRT	0.172761582949794
636	KC_AB1	Mvb12	0.193930943254559	0.697626242695311	1	pseudobulk	DESeq2	LRT	0.156377190857314
637	KC_AB1	Mvd	-0.253828890366754	0.783749859546582	1	pseudobulk	DESeq2	LRT	0.105822523989808
637	KC_AB1	Mvl	-0.416078896762688	0.420408424306593	1	pseudobulk	DESeq2	LRT	0.376328589995816
637	KC_AB1	mwh	-0.619148458022618	0.879843167001942	1	pseudobulk	DESeq2	LRT	0.055594734413205
637	KC_AB1	mxc	-0.592947640509791	0.317527237421873	1	pseudobulk	DESeq2	LRT	0.498219015081741
637	KC_AB1	mxt	0.124295780545347	0.676064402730016	1	pseudobulk	DESeq2	LRT	0.170011930658946
637	KC_AB1	Myb	0.936217080330666	0.552814743184166	1	pseudobulk	DESeq2	LRT	0.257420383148062
637	KC_AB1	Mybbp1A	-0.86836635807659	0.364794730500346	1	pseudobulk	DESeq2	LRT	0.437951443729452
637	KC_AB1	Myc	0.133606561112346	0.587690926642476	1	pseudobulk	DESeq2	LRT	0.230851014304358
637	KC_AB1	Myd88	-2.44844178584521	0.29018040253795	1	pseudobulk	DESeq2	LRT	0.537331921178161
637	KC_AB1	myo	-0.375885983273109	0.570752466867312	1	pseudobulk	DESeq2	LRT	0.243552202755737
638	KC_AB1	Myo10A	1.08899820779554	0.878177167143508	1	pseudobulk	DESeq2	LRT	0.056417858865076
638	KC_AB1	Myo28B1	0.360799461596268	0.644946251949054	1	pseudobulk	DESeq2	LRT	0.190476476767152
638	KC_AB1	Myo31DF	0.612384615961542	0.732285882307898	1	pseudobulk	DESeq2	LRT	0.135319338517633
638	KC_AB1	Myo81F	-0.421463521078145	0.507750783445565	1	pseudobulk	DESeq2	LRT	0.294349397818137
638	KC_AB1	Myo95E	0.0163883418865989	0.956698667413489	1	pseudobulk	DESeq2	LRT	0.019224830966166
638	KC_AB1	MYPT-75D	0.0942344616969833	0.618427124507613	1	pseudobulk	DESeq2	LRT	0.208711470309593
638	KC_AB1	mys	0.153793280123646	0.525695924739784	1	pseudobulk	DESeq2	LRT	0.279265389673385
638	KC_AB1	Mys45A	-0.438815875229451	0.431926655822305	1	pseudobulk	DESeq2	LRT	0.364589993175663
638	KC_AB1	Myt1	-0.569562797622717	0.608006514556093	1	pseudobulk	DESeq2	LRT	0.2160917674039
638	KC_AB1	N	0.812016835280145	0.706157120454203	1	pseudobulk	DESeq2	LRT	0.151098657369794
639	KC_AB1	na	0.19858441872733	0.360355071618954	1	pseudobulk	DESeq2	LRT	0.443269361323073
639	KC_AB1	Naa15-16	-0.469540672339328	0.428702916874756	1	pseudobulk	DESeq2	LRT	0.36784356155981
639	KC_AB1	Naa20A	0.36054937860327	0.274891669457483	1	pseudobulk	DESeq2	LRT	0.560838421173141
639	KC_AB1	Naa30A	-0.122236411863759	0.689208312654167	1	pseudobulk	DESeq2	LRT	0.161649493095652
639	KC_AB1	Naa35	0.0271685951463898	0.906192050074728	1	pseudobulk	DESeq2	LRT	0.042779752158843

639	KC_AB1	Naa40	0.472208495374525	0.771131949183679	1	pseudobulk	DESeq2	LRT	0.11287130301345
639	KC_AB1	Naa60	-0.0433732705174969	0.89320580709225	1	pseudobulk	DESeq2	LRT	0.0490484620673224
639	KC_AB1	Naa80	0.145370627392804	0.587147966413776	1	pseudobulk	DESeq2	LRT	0.23125243895824
639	KC_AB1	Naam	1.29691405984246	0.374549054930397	1	pseudobulk	DESeq2	LRT	0.426491294412037
639	KC_AB1	NAAT1	1.08899820779554	0.878177167143508	1	pseudobulk	DESeq2	LRT	0.056417858865076
640	KC_AB1	nab	2.211521544984	0.400335997833192	1	pseudobulk	DESeq2	LRT	0.39757535679119
640	KC_AB1	Nab2	0.216377209045675	0.550041969816403	1	pseudobulk	DESeq2	LRT	0.259604171298018
640	KC_AB1	nac	0.866208808329645	0.505790600936228	1	pseudobulk	DESeq2	LRT	0.296029245374094
640	KC_AB1	Nacalpha	-0.0662544737095088	0.553514116940757	1	pseudobulk	DESeq2	LRT	0.256871298305964
640	KC_AB1	nAChRalpha1	-0.12850889131647	0.314581815040327	1	pseudobulk	DESeq2	LRT	0.502266386149805
640	KC_AB1	nAChRalpha2	-0.155601344973889	0.562718631536751	1	pseudobulk	DESeq2	LRT	0.24970870517719
640	KC_AB1	nAChRalpha3	-1.51197249083442	0.0840485402129615	1	pseudobulk	DESeq2	LRT	1.07546982515088
640	KC_AB1	nAChRalpha4	-0.109381313533191	0.440309653503913	1	pseudobulk	DESeq2	LRT	0.356241792810923
640	KC_AB1	nAChRalpha5	-0.167828003187469	0.0623789249698379	1	pseudobulk	DESeq2	LRT	1.20496211408818
640	KC_AB1	nAChRalpha6	-0.105874498967533	0.292274434685961	1	pseudobulk	DESeq2	LRT	0.534209170822471
641	KC_AB1	nAChRalpha7	-0.524811161454388	0.248465301301732	1	pseudobulk	DESeq2	LRT	0.604734252827543
641	KC_AB1	nAChRbeta1	-0.171903074437242	0.0701065145330013	1	pseudobulk	DESeq2	LRT	1.15424162405502
641	KC_AB1	nAChRbeta2	-0.0734669307005464	0.759778791960308	1	pseudobulk	DESeq2	LRT	0.119312833265055
641	KC_AB1	NaCP60E	-0.163486246469021	0.215109737191775	1	pseudobulk	DESeq2	LRT	0.667339930324092
641	KC_AB1	Nadsyn	0.0035159173977702	0.921544347077053	1	pseudobulk	DESeq2	LRT	0.035483760577916
641	KC_AB1	Nak	-0.222198718499093	0.457967821302293	1	pseudobulk	DESeq2	LRT	0.339165036235888
641	KC_AB1	Nap1	0.0880963179720337	0.668443224611131	1	pseudobulk	DESeq2	LRT	0.174935474393346
641	KC_AB1	NaPi-III	-0.0694240576453229	0.657709964987985	1	pseudobulk	DESeq2	LRT	0.181965578079151
641	KC_AB1	Naprt	-0.693143660785917	0.152116429812764	1	pseudobulk	DESeq2	LRT	0.817823876074097
641	KC_AB1	NAT1	-0.096677950819636	0.495447879794464	1	pseudobulk	DESeq2	LRT	0.305002025756356
642	KC_AB1	Naus	-0.238966092654882	0.519229459377547	1	pseudobulk	DESeq2	LRT	0.284640675063319
642	KC_AB1	Naxd	0.0450485402107229	0.925229032824247	1	pseudobulk	DESeq2	LRT	0.033750747931750
642	KC_AB1	Naxe	-0.80707464408581	0.363356011399411	1	pseudobulk	DESeq2	LRT	0.439667650398486
642	KC_AB1	naz	0.980603726925276	0.631823227524267	1	pseudobulk	DESeq2	LRT	0.199404412302045
642	KC_AB1	Nbr	-0.307179567911828	0.546979037646316	1	pseudobulk	DESeq2	LRT	0.262029317193036
642	KC_AB1	nbs	0.0807302232471743	0.886977255052146	1	pseudobulk	DESeq2	LRT	0.052087516731965
642	KC_AB1	NC2alpha	0.410040074983546	0.53158558049823	1	pseudobulk	DESeq2	LRT	0.274426808005799
642	KC_AB1	NC2beta	-0.023444376206375	0.938582308496411	1	pseudobulk	DESeq2	LRT	0.027527636139715
642	KC_AB1	Nc73EF	-0.204436960425215	0.232421136270414	1	pseudobulk	DESeq2	LRT	0.633724379946885
642	KC_AB1	Nca	0.0412469063618382	0.782588370993772	1	pseudobulk	DESeq2	LRT	0.10646660986079
643	KC_AB1	Ncc69	0.0034655162769823	0.948583204657749	1	pseudobulk	DESeq2	LRT	0.022924569109653
643	KC_AB1	ncd	-1.79679308474825	0.39394949584736	1	pseudobulk	DESeq2	LRT	0.404559450968051
643	KC_AB1	Nckx30C	-0.155155729687491	0.169919616909204	1	pseudobulk	DESeq2	LRT	0.769756479732372
643	KC_AB1	nclb	-0.106656796079056	0.864111788683265	1	pseudobulk	DESeq2	LRT	0.063430069942656
643	KC_AB1	ncm	-0.269163172361147	0.465297016985196	1	pseudobulk	DESeq2	LRT	0.332269731715651
643	KC_AB1	Ncoa6	0.126666184189246	0.454332202345934	1	pseudobulk	DESeq2	LRT	0.34262647999034
643	KC_AB1	Nct	-0.0816326005917564	0.90125880933013	1	pseudobulk	DESeq2	LRT	0.045150477251969
643	KC_AB1	ND-13A	-0.0264107672311395	0.888533178282161	1	pseudobulk	DESeq2	LRT	0.051326350778391
643	KC_AB1	ND-13B	-0.0344957184309434	0.851091598093123	1	pseudobulk	DESeq2	LRT	0.070023696783045
643	KC_AB1	ND-15	-0.176121914924349	0.35864284456879	1	pseudobulk	DESeq2	LRT	0.445337829530314
644	KC_AB1	ND-18	-0.0307184914035067	0.888128397513484	1	pseudobulk	DESeq2	LRT	0.051524243342348
644	KC_AB1	ND-19	-0.107358526931804	0.65198307774245	1	pseudobulk	DESeq2	LRT	0.185763676259567
644	KC_AB1	ND-20	0.0056159534589238	0.92562430630897	1	pseudobulk	DESeq2	LRT	0.033565249608098
644	KC_AB1	ND-23	-0.148254615154014	0.522523101800643	1	pseudobulk	DESeq2	LRT	0.281894503757102
644	KC_AB1	ND-24	-0.117223317763846	0.697815144287247	1	pseudobulk	DESeq2	LRT	0.156259609538218
644	KC_AB1	ND-30	-0.144987736557496	0.525789928440753	1	pseudobulk	DESeq2	LRT	0.279187737104698
644	KC_AB1	ND-39	-0.155245399559069	0.502302179816232	1	pseudobulk	DESeq2	LRT	0.299034937149814

644	KC_AB1	ND-42	0.0731249616087216	0.776993843090277	1	pseudobulk	DESeq2	LRT	0.1095824225408
644	KC_AB1	ND-49	0.0196842283270525	0.90019518962206	1	pseudobulk	DESeq2	LRT	0.045663312133128
644	KC_AB1	ND-51	0.0666304420791558	0.735155153502234	1	pseudobulk	DESeq2	LRT	0.13362099397853
645	KC_AB1	ND-51L1	-0.881996409411637	0.593501682860195	1	pseudobulk	DESeq2	LRT	0.226578045275759
645	KC_AB1	ND-75	-0.438713799190231	0.0656203431932235	1	pseudobulk	DESeq2	LRT	1.18296150259164
645	KC_AB1	ND-ACP	-0.217179257046126	0.268014647842848	1	pseudobulk	DESeq2	LRT	0.571841469764196
645	KC_AB1	ND-AGGG	-0.0867427626047315	0.623080427356073	1	pseudobulk	DESeq2	LRT	0.205455890896997
645	KC_AB1	ND-ASHI	-0.330093312871177	0.175300827996717	1	pseudobulk	DESeq2	LRT	0.756216032602405
645	KC_AB1	ND-B12	-0.137855205302209	0.396846687754118	1	pseudobulk	DESeq2	LRT	0.401377240143812
645	KC_AB1	ND-B14	-0.144205937299313	0.461073887929927	1	pseudobulk	DESeq2	LRT	0.336229472551916
645	KC_AB1	ND-B14.5A	-0.225384695021643	0.369349081487223	1	pseudobulk	DESeq2	LRT	0.432562976663372
645	KC_AB1	ND-B14.5B	-0.07916719276241	0.737053099082027	1	pseudobulk	DESeq2	LRT	0.132501223392473
645	KC_AB1	ND-B14.7	0.031257931471449	0.885545592919713	1	pseudobulk	DESeq2	LRT	0.052789073950237
646	KC_AB1	ND-B15	-0.315092856150817	0.141353641840086	1	pseudobulk	DESeq2	LRT	0.849692997852363
646	KC_AB1	ND-B16.6	-0.0698146994991848	0.683095602432769	1	pseudobulk	DESeq2	LRT	0.16551851051548
646	KC_AB1	ND-B17	-0.144375082896988	0.549768478568317	1	pseudobulk	DESeq2	LRT	0.259820164413042
646	KC_AB1	ND-B17.2	-0.318638042822392	0.1263045233062	1	pseudobulk	DESeq2	LRT	0.898581095907485
646	KC_AB1	ND-B18	-0.039181652475813	0.851601050394073	1	pseudobulk	DESeq2	LRT	0.069763811559609
646	KC_AB1	ND-B22	-0.0921506696345083	0.617897222675473	1	pseudobulk	DESeq2	LRT	0.209083756847935
646	KC_AB1	ND-MLRQ	-0.229203834532249	0.104633514079308	1	pseudobulk	DESeq2	LRT	0.980329188810207
646	KC_AB1	ND-MNLL	-0.0625204430439131	0.917183067799505	1	pseudobulk	DESeq2	LRT	0.037543971418241
646	KC_AB1	ND-MWFE	0.130312314776402	0.52068200579309	1	pseudobulk	DESeq2	LRT	0.283427430812498
646	KC_AB1	ND-PDSW	-0.157995602646097	0.449723390188148	1	pseudobulk	DESeq2	LRT	0.347054524116574
647	KC_AB1	ND-SGDH	-0.245687829782771	0.152061317448724	1	pseudobulk	DESeq2	LRT	0.817981251135649
647	KC_AB1	Ndae1	-0.14873912714612	0.287224306286122	1	pseudobulk	DESeq2	LRT	0.5417788108111
647	KC_AB1	Ndc1	-0.588354800448092	0.805189412611406	1	pseudobulk	DESeq2	LRT	0.094101944258636
647	KC_AB1	Ndf	0.0823684248894893	0.731146537704207	1	pseudobulk	DESeq2	LRT	0.135995572237176
647	KC_AB1	Ndfip	-0.142417840901041	0.560469953447978	1	pseudobulk	DESeq2	LRT	0.251447664784466
647	KC_AB1	neb	3.09851320464003	0.0747016608924983	1	pseudobulk	DESeq2	LRT	1.12666974211461
647	KC_AB1	Neb-cGP	-0.237458256599925	0.11053229064686	1	pseudobulk	DESeq2	LRT	0.956510829660946
647	KC_AB1	Nedd4	0.0104793523501541	0.960427553912759	1	pseudobulk	DESeq2	LRT	0.017535388868436
647	KC_AB1	Nedd8	0.317578215768422	0.344331807465894	1	pseudobulk	DESeq2	LRT	0.463022857722629
647	KC_AB1	nej	0.0140711145875595	0.914210222487452	1	pseudobulk	DESeq2	LRT	0.038953926838997
648	KC_AB1	Nelf-A	0.0733008850752063	0.810323850305305	1	pseudobulk	DESeq2	LRT	0.091341378292011
648	KC_AB1	NELF-B	-0.743028717059259	0.41644682542764	1	pseudobulk	DESeq2	LRT	0.380440444191523
648	KC_AB1	Nelf-E	0.313247055777373	0.303168612167705	1	pseudobulk	DESeq2	LRT	0.518315764346039
648	KC_AB1	nemy	1.31111728351591	0.118063238291209	1	pseudobulk	DESeq2	LRT	0.927885308928519
648	KC_AB1	Neos	-0.248532707783446	0.616995754383619	1	pseudobulk	DESeq2	LRT	0.209717824385089
648	KC_AB1	Nep1	-0.306126823063993	0.00986218379340483	1	pseudobulk	DESeq2	LRT	2.00602690814054
648	KC_AB1	Nep3	0.111167023925759	0.697513947352408	1	pseudobulk	DESeq2	LRT	0.15644710390006
648	KC_AB1	Nep4	1.08899820779554	0.697631521651407	1	pseudobulk	DESeq2	LRT	0.156373904552
648	KC_AB1	Nep6	-0.82542030717083	1	1	pseudobulk	DESeq2	LRT	0
648	KC_AB1	Nepl11	-2.16549748155799	0.305318288651619	1	pseudobulk	DESeq2	LRT	0.515247180560194
649	KC_AB1	Nepl16	-0.147741717272769	0.639201350197288	1	pseudobulk	DESeq2	LRT	0.194362316324521
649	KC_AB1	Nepl18	1.08899820779554	0.670846322504933	1	pseudobulk	DESeq2	LRT	0.173376956629912
649	KC_AB1	Nepl3	-0.299183599703628	0.804367158009037	1	pseudobulk	DESeq2	LRT	0.094545669784176
649	KC_AB1	nero	-0.307592780568204	0.532626275804658	1	pseudobulk	DESeq2	LRT	0.273577412520644
649	KC_AB1	nes	0.0791673311295827	0.893969543217643	1	pseudobulk	DESeq2	LRT	0.048677276995840
649	KC_AB1	nesd	-0.82542030717083	1	1	pseudobulk	DESeq2	LRT	0
649	KC_AB1	net	0.683310856523788	0.613126138402442	1	pseudobulk	DESeq2	LRT	0.212450168912771
649	KC_AB1	NetA	1.08899820779554	0.878177167143508	1	pseudobulk	DESeq2	LRT	0.056417858865076
649	KC_AB1	NetB	0.202749589337417	0.175723925354404	1	pseudobulk	DESeq2	LRT	0.755169103945778

649	KC_AB1	Neto	0.135795303631797	0.946582799906172	1	pseudobulk	DESeq2	LRT	0.023841391241048
650	KC_AB1	neur	-0.177656878617664	0.503762436210472	1	pseudobulk	DESeq2	LRT	0.297774219439584
650	KC_AB1	Neurl4	0.568511532392743	0.34956421942117	1	pseudobulk	DESeq2	LRT	0.45647302713375
650	KC_AB1	Nf-YA	-0.132890921854095	0.802330316924733	1	pseudobulk	DESeq2	LRT	0.095646797197527
650	KC_AB1	Nf-YB	-0.117444184852618	0.791868936982564	1	pseudobulk	DESeq2	LRT	0.101346692975447
650	KC_AB1	Nf-YC	-0.183966365887422	0.729105228721104	1	pseudobulk	DESeq2	LRT	0.137209787244589
650	KC_AB1	Nf1	0.109993143388867	0.666888756203227	1	pseudobulk	DESeq2	LRT	0.175946604758673
650	KC_AB1	NFAT	-0.143818972327742	0.337823876222338	1	pseudobulk	DESeq2	LRT	0.471309659249415
650	KC_AB1	Nfl	-0.031024019299705	0.839627943714574	1	pseudobulk	DESeq2	LRT	0.075913116064988
650	KC_AB1	Nfs1	0.0128220111079982	0.95430131487433	1	pseudobulk	DESeq2	LRT	0.020314477784341
650	KC_AB1	ng1	1.08899820779554	0.697631521651407	1	pseudobulk	DESeq2	LRT	0.156373904552
651	KC_AB1	ng4	-0.82542030717083	1	1	pseudobulk	DESeq2	LRT	0
651	KC_AB1	Nha1	-1.43574395249627	0.405285633839357	1	pseudobulk	DESeq2	LRT	0.392238790422806
651	KC_AB1	Nhe1	0.273830364302417	0.706826193277652	1	pseudobulk	DESeq2	LRT	0.150687364958754
651	KC_AB1	Nhe2	0.0535793746489299	0.877310590691601	1	pseudobulk	DESeq2	LRT	0.056846627906728
651	KC_AB1	Nhe3	-0.159287410071209	0.408146020995616	1	pseudobulk	DESeq2	LRT	0.389184433068936
651	KC_AB1	NHP2	-0.19439319906058	0.710690024625352	1	pseudobulk	DESeq2	LRT	0.148319780351253
651	KC_AB1	NimC3	1.08899820779554	0.670846322504933	1	pseudobulk	DESeq2	LRT	0.173376956629912
651	KC_AB1	NimC4	1.14247969565044	0.639889789651426	1	pseudobulk	DESeq2	LRT	0.19389481955965
651	KC_AB1	ninaA	-2.28196308857196	0.366390875301127	1	pseudobulk	DESeq2	LRT	0.43605535065843
651	KC_AB1	ninaC	2.1559833397943	0.381743505411375	1	pseudobulk	DESeq2	LRT	0.418228342845202
652	KC_AB1	NiPp1	0.290783095787708	0.491123654621986	1	pseudobulk	DESeq2	LRT	0.308809147879395
652	KC_AB1	Nipped-A	0.0919087078490103	0.622293459227837	1	pseudobulk	DESeq2	LRT	0.206004763753094
652	KC_AB1	Nipped-B	0.125841378884652	0.309192013036702	1	pseudobulk	DESeq2	LRT	0.509771733184788
652	KC_AB1	Nipsnap	-0.0073531398725641	0.981436968234811	1	pseudobulk	DESeq2	LRT	0.008137587278271
652	KC_AB1	NitFhit	0.914263901111014	0.461934526337336	1	pseudobulk	DESeq2	LRT	0.335419576100712
652	KC_AB1	nito	-0.0901602108585189	0.835898612715641	1	pseudobulk	DESeq2	LRT	0.077846395537504
652	KC_AB1	NK7.1	0.0807130425141876	0.669897195214801	1	pseudobulk	DESeq2	LRT	0.173991840547883
652	KC_AB1	NKAIN	0.409317116044912	0.267344516767642	1	pseudobulk	DESeq2	LRT	0.572928718846141
652	KC_AB1	nkd	-0.295144814140714	0.831020139355425	1	pseudobulk	DESeq2	LRT	0.080388451179851
652	KC_AB1	NLaz	2.52826608569039	0.205364170950721	1	pseudobulk	DESeq2	LRT	0.687475323716788
653	KC_AB1	Nle	-0.469247128246128	0.459777185643653	1	pseudobulk	DESeq2	LRT	0.337452582424916
653	KC_AB1	Nlg1	-0.850932661185203	0.660736244265418	1	pseudobulk	DESeq2	LRT	0.179971869591347
653	KC_AB1	Nlg2	-0.127261883986761	0.396579690098792	1	pseudobulk	DESeq2	LRT	0.401669530937269
653	KC_AB1	Nlg3	-0.316580095668862	0.0186227177216962	1	pseudobulk	DESeq2	LRT	1.72995693960557
653	KC_AB1	Nlg4	0.186213622009187	0.899256886708457	1	pseudobulk	DESeq2	LRT	0.046116227573361
653	KC_AB1	Nlp	-0.0551594698092061	0.844476567017202	1	pseudobulk	DESeq2	LRT	0.073412396958950
653	KC_AB1	nmd	-0.646759806012607	0.246425132932414	1	pseudobulk	DESeq2	LRT	0.60831500049614
653	KC_AB1	Nmd3	-0.120446528875518	0.854325517970121	1	pseudobulk	DESeq2	LRT	0.068376621439798
653	KC_AB1	Nmda1	0.0369840842868145	0.885426054727382	1	pseudobulk	DESeq2	LRT	0.052847702531281
653	KC_AB1	Nmdar1	-0.285299087060809	0.398457638437698	1	pseudobulk	DESeq2	LRT	0.399617843328512
654	KC_AB1	Nmdar2	0.368370111000333	0.227695618599768	1	pseudobulk	DESeq2	LRT	0.642645326153637
654	KC_AB1	Nmdmc	-0.403387061979666	0.845209033867155	1	pseudobulk	DESeq2	LRT	0.073035869715218
654	KC_AB1	nmdyn-D6	0.696845475033456	0.548868510847791	1	pseudobulk	DESeq2	LRT	0.260531684425758
654	KC_AB1	Nmnat	-0.219054817878006	0.651876763050061	1	pseudobulk	DESeq2	LRT	0.185834499645498
654	KC_AB1	nmo	-0.21966872262372	0.110837334075134	1	pseudobulk	DESeq2	LRT	0.955313928670952
654	KC_AB1	Nmt	0.287916603460249	0.807404808072382	1	pseudobulk	DESeq2	LRT	0.092908668706870
654	KC_AB1	Nna1	-0.0318520669281351	0.950764179688867	1	pseudobulk	DESeq2	LRT	0.021927188803881
654	KC_AB1	Nnf1a	-1.27385069327018	0.464072392776944	1	pseudobulk	DESeq2	LRT	0.333414266576129
654	KC_AB1	Nnp-1	-0.0585355471564364	0.889460780938818	1	pseudobulk	DESeq2	LRT	0.050873196537179
654	KC_AB1	NO66	-0.139046432129716	0.880171822233335	1	pseudobulk	DESeq2	LRT	0.055432539027399
655	KC_AB1	Noa36	-0.269563506962137	0.577374984718232	1	pseudobulk	DESeq2	LRT	0.238542036240158

655	KC_AB1	noc	0.449914761437036	0.418038319286124	1	pseudobulk	DESeq2	LRT	0.378783907000665
655	KC_AB1	nocte	-0.0469567354425158	0.744720730017119	1	pseudobulk	DESeq2	LRT	0.128006556984157
655	KC_AB1	nod	-0.82542030717083	1	1	pseudobulk	DESeq2	LRT	0
655	KC_AB1	noi	-0.235329219631788	0.753469473971299	1	pseudobulk	DESeq2	LRT	0.122934337980229
655	KC_AB1	nolo	0.114184054362142	1	1	pseudobulk	DESeq2	LRT	0
655	KC_AB1	nom	0.900455242893563	0.357287268677295	1	pseudobulk	DESeq2	LRT	0.446982458865312
655	KC_AB1	Non1	-0.0308271951590525	0.871058107457232	1	pseudobulk	DESeq2	LRT	0.059952872654462
655	KC_AB1	Non2	-0.26144983250306	0.368601652046144	1	pseudobulk	DESeq2	LRT	0.433442722635892
655	KC_AB1	Non3	-0.108940838595488	0.837343783517125	1	pseudobulk	DESeq2	LRT	0.077096199556372
656	KC_AB1	nonA	0.00533557735832929	0.940448731960594	1	pseudobulk	DESeq2	LRT	0.026664874790364
656	KC_AB1	nonA-l	-0.450953054375648	0.251608660616643	1	pseudobulk	DESeq2	LRT	0.599274414127864
656	KC_AB1	nonC	0.441369610553055	0.330517468708962	1	pseudobulk	DESeq2	LRT	0.480805581977374
656	KC_AB1	Nop17l	0.289799283926974	0.329371918299127	1	pseudobulk	DESeq2	LRT	0.482313430811712
656	KC_AB1	nop5	-0.595182407217755	0.523513877382618	1	pseudobulk	DESeq2	LRT	0.281071801490749
656	KC_AB1	Nop56	0.191157006363368	0.642203944083614	1	pseudobulk	DESeq2	LRT	0.192327031549819
656	KC_AB1	Nop60B	0.118427015387164	0.842463951287389	1	pseudobulk	DESeq2	LRT	0.074448673355037
656	KC_AB1	Nopp140	-0.0581731421805663	0.938390057044834	1	pseudobulk	DESeq2	LRT	0.027616602547940
656	KC_AB1	nord	-0.653651259784102	0.690025728911805	1	pseudobulk	DESeq2	LRT	0.161134715471297
656	KC_AB1	norpA	-0.288270401973909	0.0488885953485059	1	pseudobulk	DESeq2	LRT	1.31079244056714
657	KC_AB1	Nos	0.516794653828635	0.814433332789471	1	pseudobulk	DESeq2	LRT	0.089144460019238
657	KC_AB1	Nost	-1.29467356980949	0.510838645677309	1	pseudobulk	DESeq2	LRT	0.291716255164423
657	KC_AB1	not	0.0124903659126146	0.924227681694935	1	pseudobulk	DESeq2	LRT	0.034221027997229
657	KC_AB1	Not1	0.0805591443649374	0.613848685116541	1	pseudobulk	DESeq2	LRT	0.211938670092895
657	KC_AB1	Not10	0.302702465955641	0.603994168643233	1	pseudobulk	DESeq2	LRT	0.218967254323059
657	KC_AB1	Not11	-0.252956956150428	0.540046267269211	1	pseudobulk	DESeq2	LRT	0.267569031364164
657	KC_AB1	Not3	-0.252900725113265	0.200544099095769	1	pseudobulk	DESeq2	LRT	0.69779011238016
657	KC_AB1	NP15.6	-0.324838064559617	0.221649165939616	1	pseudobulk	DESeq2	LRT	0.654333898601072
657	KC_AB1	Npc1a	-0.254903601210621	0.44239311907564	1	pseudobulk	DESeq2	LRT	0.354191636610049
657	KC_AB1	Npc2a	2.211521544984	0.400335997833192	1	pseudobulk	DESeq2	LRT	0.39757535679119
658	KC_AB1	Npc2f	-0.82542030717083	1	1	pseudobulk	DESeq2	LRT	0
658	KC_AB1	NPFR	-0.850996402080018	0.900449987182352	1	pseudobulk	DESeq2	LRT	0.045540403770502
658	KC_AB1	Nph	-0.438508917321181	0.275529300530609	1	pseudobulk	DESeq2	LRT	0.559832210307195
658	KC_AB1	Npl4	0.184535167309605	0.768647130719884	1	pseudobulk	DESeq2	LRT	0.114272989655654
658	KC_AB1	Nplp1	0.287412002255847	0.393203461445744	1	pseudobulk	DESeq2	LRT	0.405382667645169
658	KC_AB1	Nplp2	-0.82542030717083	1	1	pseudobulk	DESeq2	LRT	0
658	KC_AB1	Nprl2	-0.806560543810071	0.481512238716629	1	pseudobulk	DESeq2	LRT	0.317392669828028
658	KC_AB1	Nprl3	0.0187992543719286	0.950337297856203	1	pseudobulk	DESeq2	LRT	0.022122225659784
658	KC_AB1	Nrd1	-0.201086991738211	0.771962678445264	1	pseudobulk	DESeq2	LRT	0.112403695696755
658	KC_AB1	Nrg	-0.0115096419692161	0.913815266123926	1	pseudobulk	DESeq2	LRT	0.039141590931221
659	KC_AB1	Nrk	1.0523846466244	0.377962974356669	1	pseudobulk	DESeq2	LRT	0.422550742015076
659	KC_AB1	nrm	0.169017230295646	0.424679821618663	1	pseudobulk	DESeq2	LRT	0.371938373734674
659	KC_AB1	Nrt	-0.271162227360012	0.379950411885738	1	pseudobulk	DESeq2	LRT	0.420273080356018
659	KC_AB1	nrv1	-0.850932661185203	0.660736244265418	1	pseudobulk	DESeq2	LRT	0.179971869591347
659	KC_AB1	nrv2	0.126612969266757	0.759225592387706	1	pseudobulk	DESeq2	LRT	0.119629160900273
659	KC_AB1	nrv3	-0.0311430538943049	0.760501266989595	1	pseudobulk	DESeq2	LRT	0.118900058078923
659	KC_AB1	Nrx-1	0.0339807795412817	0.762727195584446	1	pseudobulk	DESeq2	LRT	0.117630768254407
659	KC_AB1	Nrx-IV	-0.476231344756525	0.151567080263426	1	pseudobulk	DESeq2	LRT	0.819395115408693
659	KC_AB1	Ns1	-0.0617914390685591	0.865115813472998	1	pseudobulk	DESeq2	LRT	0.062925749430618
659	KC_AB1	Ns2	-0.754881827702311	0.0950856369961278	1	pseudobulk	DESeq2	LRT	1.02188507974418
660	KC_AB1	Ns3	0.0186011381204473	0.962446489044026	1	pseudobulk	DESeq2	LRT	0.016623407439134
660	KC_AB1	Ns4	-0.258844137675745	0.678670062874172	1	pseudobulk	DESeq2	LRT	0.16834130774872
660	KC_AB1	NSD	0.898065008034705	0.387907191927096	1	pseudobulk	DESeq2	LRT	0.411272168362569

660	KC_AB1	Nse1	1.16523413356038	0.63328025468345	1	pseudobulk	DESeq2	LRT	0.198404052815419
660	KC_AB1	Nse4	1.01984525082149	0.169033641279446	1	pseudobulk	DESeq2	LRT	0.772026852971775
660	KC_AB1	Nsf2	-0.271985118920297	0.510227365124882	1	pseudobulk	DESeq2	LRT	0.292236252499995
660	KC_AB1	nsl1	-0.140708290721882	0.498465190553235	1	pseudobulk	DESeq2	LRT	0.30236516449072
660	KC_AB1	nSMase	0.176541915985959	0.680027355755662	1	pseudobulk	DESeq2	LRT	0.167473616389691
660	KC_AB1	nst	0.0949593409102393	0.839430008150696	1	pseudobulk	DESeq2	LRT	0.076015509580908
660	KC_AB1	Nsun2	-0.542382569585348	0.650036853097831	1	pseudobulk	DESeq2	LRT	0.187062020828952
661	KC_AB1	Nsun5	-0.129504412738401	0.855252512870536	1	pseudobulk	DESeq2	LRT	0.067905641113429
661	KC_AB1	nSyb	-0.138414932151831	0.161197778819095	1	pseudobulk	DESeq2	LRT	0.792640946732271
661	KC_AB1	NT5E-2	-2.4495425631334	0.286967014245434	1	pseudobulk	DESeq2	LRT	0.542168020877472
661	KC_AB1	Ntan1	0.174889084844064	0.736723398152758	1	pseudobulk	DESeq2	LRT	0.132695536840838
661	KC_AB1	ntc	-0.452554351003196	0.559736997925834	1	pseudobulk	DESeq2	LRT	0.252015985814402
661	KC_AB1	Ntf-2	0.0416305299419616	0.815208599139191	1	pseudobulk	DESeq2	LRT	0.088731247869389
661	KC_AB1	Ntl	1.08899820779554	0.878177167143508	1	pseudobulk	DESeq2	LRT	0.056417858865076
661	KC_AB1	Ntmt	-1.11658980988338	0.210590970253876	1	pseudobulk	DESeq2	LRT	0.676560254484759
661	KC_AB1	NTPase	0.0686548075979158	0.878073617589487	1	pseudobulk	DESeq2	LRT	0.056469071368845
661	KC_AB1	Nuak1	0.0826065158911681	0.783524855461517	1	pseudobulk	DESeq2	LRT	0.1059472220179
662	KC_AB1	nub	1.08899820779554	0.670846322504933	1	pseudobulk	DESeq2	LRT	0.173376956629912
662	KC_AB1	NUCB1	0.0290178111510475	0.884970526995783	1	pseudobulk	DESeq2	LRT	0.053071192778150
662	KC_AB1	nudC	0.073603893054531	0.866935578950505	1	pseudobulk	DESeq2	LRT	0.062013173280173
662	KC_AB1	nudE	0.306982721178277	0.209284248360039	1	pseudobulk	DESeq2	LRT	0.67926345731705
662	KC_AB1	nuf	0.219617996189768	0.853437053806947	1	pseudobulk	DESeq2	LRT	0.068828505237492
662	KC_AB1	Nuf2	1.08899820779554	0.697631521651407	1	pseudobulk	DESeq2	LRT	0.156373904552
662	KC_AB1	Nufip	0.617131146985272	0.456285836299341	1	pseudobulk	DESeq2	LRT	0.340763012078773
662	KC_AB1	nullo	0.113316967542732	1	1	pseudobulk	DESeq2	LRT	0
662	KC_AB1	Nulp1	0.130776269610063	0.69682976283621	1	pseudobulk	DESeq2	LRT	0.156873308115942
662	KC_AB1	numb	-0.428754475734283	0.18412732263004	1	pseudobulk	DESeq2	LRT	0.734881761810966
663	KC_AB1	Nup107	-0.387517173264366	0.654966381572483	1	pseudobulk	DESeq2	LRT	0.183780991110904
663	KC_AB1	Nup133	0.790591501136915	0.474020952478569	1	pseudobulk	DESeq2	LRT	0.324202461396137
663	KC_AB1	Nup153	-0.000886478354837	0.996871275234414	1	pseudobulk	DESeq2	LRT	0.001360917981884
663	KC_AB1	Nup154	0.31366347509552	0.750049768443724	1	pseudobulk	DESeq2	LRT	0.124909918683795
663	KC_AB1	Nup160	0.305092652085552	0.764713074778322	1	pseudobulk	DESeq2	LRT	0.116501484342868
663	KC_AB1	Nup188	0.35733923886768	0.760031110146721	1	pseudobulk	DESeq2	LRT	0.119168630497461
663	KC_AB1	Nup205	-0.394108448797443	0.714180061985086	1	pseudobulk	DESeq2	LRT	0.146192278325152
663	KC_AB1	Nup214	-0.869142680302344	0.230745570653452	1	pseudobulk	DESeq2	LRT	0.636866626853413
663	KC_AB1	Nup35	-0.592379974011507	0.490984912148191	1	pseudobulk	DESeq2	LRT	0.308931853440092
663	KC_AB1	Nup358	0.165934449844383	0.635638686143614	1	pseudobulk	DESeq2	LRT	0.196789678696546
664	KC_AB1	Nup37	-0.473256489116353	0.569014000311889	1	pseudobulk	DESeq2	LRT	0.244877047869609
664	KC_AB1	Nup43	-0.614659769023728	0.492583801116972	1	pseudobulk	DESeq2	LRT	0.307519874269524
664	KC_AB1	Nup44A	0.454797985153297	0.359628621544199	1	pseudobulk	DESeq2	LRT	0.444145751632747
664	KC_AB1	Nup50	-0.163894978954912	0.810140117028308	1	pseudobulk	DESeq2	LRT	0.091439861628009
664	KC_AB1	Nup54	0.0650751315917617	0.933695596339895	1	pseudobulk	DESeq2	LRT	0.029794689484667
664	KC_AB1	Nup58	0.277960093465119	0.620858482061844	1	pseudobulk	DESeq2	LRT	0.207007381246481
664	KC_AB1	Nup62	-0.00610510579893	0.975251766033524	1	pseudobulk	DESeq2	LRT	0.010883254573802
664	KC_AB1	Nup75	0.636512371815627	0.525461087814547	1	pseudobulk	DESeq2	LRT	0.279459439432376
664	KC_AB1	Nup93-1	-0.32828333001852	0.742241276010325	1	pseudobulk	DESeq2	LRT	0.129454898215369
664	KC_AB1	Nup93-2	-0.136457699207945	0.891605616428791	1	pseudobulk	DESeq2	LRT	0.049827204462381
665	KC_AB1	Nup98-96	-0.096623522895977	0.830282403833903	1	pseudobulk	DESeq2	LRT	0.080774165977526
665	KC_AB1	Nurf-38	0.055394724144724	0.843793196921138	1	pseudobulk	DESeq2	LRT	0.073763980437333
665	KC_AB1	nvd	0.111831901354254	1	1	pseudobulk	DESeq2	LRT	0
665	KC_AB1	nvy	0.284786010699894	0.756228396117172	1	pseudobulk	DESeq2	LRT	0.121347019058307
665	KC_AB1	nwk	-0.248998937194742	0.265963725476273	1	pseudobulk	DESeq2	LRT	0.575177592315413

665	KC_AB1	nxf2	-0.850996402080018	0.900449987182352	1	pseudobulk	DESeq2	LRT	0.045540403770502
665	KC_AB1	Nxf3	0.559821911656834	0.460999370651864	1	pseudobulk	DESeq2	LRT	0.336299667501045
665	KC_AB1	Nxt1	-0.0283554366763615	0.932071416951515	1	pseudobulk	DESeq2	LRT	0.030550809963961
665	KC_AB1	O-fut1	0.0187836262802805	0.973616435527368	1	pseudobulk	DESeq2	LRT	0.011612103431857
665	KC_AB1	O-fut2	0.642571165315524	0.70675985264817	1	pseudobulk	DESeq2	LRT	0.150728128475646
666	KC_AB1	oaf	0.0324327495762743	0.926425809637409	1	pseudobulk	DESeq2	LRT	0.033189354278375
666	KC_AB1	Oamb	-0.0341756981895417	0.791063311681197	1	pseudobulk	DESeq2	LRT	0.101788756940433
666	KC_AB1	Oat	-0.82542030717083	1	1	pseudobulk	DESeq2	LRT	0
666	KC_AB1	Oatp30B	0.0038884489628632	0.917963162027849	1	pseudobulk	DESeq2	LRT	0.037174746738884
666	KC_AB1	Oatp33Ea	-0.82542030717083	1	1	pseudobulk	DESeq2	LRT	0
666	KC_AB1	Oatp58Dc	-0.850996402080018	0.900449987182352	1	pseudobulk	DESeq2	LRT	0.045540403770502
666	KC_AB1	Oatp74D	4.24128603939507	0.252482137728416	1	pseudobulk	DESeq2	LRT	0.597769341348825
666	KC_AB1	obe	0.457635537693964	0.685681782808	1	pseudobulk	DESeq2	LRT	0.163877388716131
666	KC_AB1	Obp18a	-1.34702019872736	0.563386203015265	1	pseudobulk	DESeq2	LRT	0.249193792827183
666	KC_AB1	Obp19a	0.927689595940524	0.592543308205765	1	pseudobulk	DESeq2	LRT	0.227279902149211
667	KC_AB1	Obp44a	0.005404587971036	0.944590233826062	1	pseudobulk	DESeq2	LRT	0.024756548932277
667	KC_AB1	Obp49a	-1.96719661029409	0.414177861019091	1	pseudobulk	DESeq2	LRT	0.382813119093482
667	KC_AB1	Obp50e	1.08899820779554	0.670846322504933	1	pseudobulk	DESeq2	LRT	0.173376956629912
667	KC_AB1	Obp56a	-1.67764803720201	0.365676506919753	1	pseudobulk	DESeq2	LRT	0.436902940243999
667	KC_AB1	Obp56h	1.08899820779554	0.670846322504933	1	pseudobulk	DESeq2	LRT	0.173376956629912
667	KC_AB1	Obp57e	-0.850932661185203	0.660736244265418	1	pseudobulk	DESeq2	LRT	0.179971869591347
667	KC_AB1	Obp73a	-1.21673908022264	0.60839381642346	1	pseudobulk	DESeq2	LRT	0.215815208667062
667	KC_AB1	Obp83b	0.135795303631797	0.946582799906172	1	pseudobulk	DESeq2	LRT	0.023841391241048
667	KC_AB1	Obp85a	-0.482474259035348	0.726718158336436	1	pseudobulk	DESeq2	LRT	0.13863398805665
667	KC_AB1	Obp99c	1.08899820779554	0.878177167143508	1	pseudobulk	DESeq2	LRT	0.056417858865076
668	KC_AB1	obst-E	0.407864582631758	0.803728246440601	1	pseudobulk	DESeq2	LRT	0.094890768442414
668	KC_AB1	ocm	0.420339959754924	0.455530080494419	1	pseudobulk	DESeq2	LRT	0.341482939522153
668	KC_AB1	Ocrl	0.253390966759445	0.477163071554308	1	pseudobulk	DESeq2	LRT	0.321333174475229
668	KC_AB1	Oct-TyrR	0.744782276103072	0.764014638758689	1	pseudobulk	DESeq2	LRT	0.116898320126909
668	KC_AB1	Octbeta1R	-0.10661972850161	0.293389052547436	1	pseudobulk	DESeq2	LRT	0.532556095356202
668	KC_AB1	Octbeta2R	-0.409189427687465	0.91475832753349	1	pseudobulk	DESeq2	LRT	0.038693628208798
668	KC_AB1	Octbeta3R	-0.246961299591322	0.107917220721239	1	pseudobulk	DESeq2	LRT	0.966909247926334
668	KC_AB1	Oda	0.0727453332209923	0.530389910642979	1	pseudobulk	DESeq2	LRT	0.275404745903728
668	KC_AB1	Odc1	-0.850996402080018	0.900449987182352	1	pseudobulk	DESeq2	LRT	0.045540403770502
668	KC_AB1	Odj	-0.0170681828297335	0.919473207934267	1	pseudobulk	DESeq2	LRT	0.036460920929250
669	KC_AB1	Oga	-0.750161310531045	0.28190424884491	1	pseudobulk	DESeq2	LRT	0.549898378417177
669	KC_AB1	Ogg1	0.358057346461737	0.654060764919117	1	pseudobulk	DESeq2	LRT	0.184381902068448
669	KC_AB1	ogre	2.80766675271277	0.116606272431683	1	pseudobulk	DESeq2	LRT	0.933278087577001
669	KC_AB1	ohgt	0.120223493113469	0.740772163902059	1	pseudobulk	DESeq2	LRT	0.130315345563254
669	KC_AB1	okr	-0.236281432489516	0.877584852693914	1	pseudobulk	DESeq2	LRT	0.056710881386194
669	KC_AB1	olf186-F	0.0278233233206666	0.931791883282658	1	pseudobulk	DESeq2	LRT	0.030681076953805
669	KC_AB1	olf413	-0.190926066946819	0.270255773507889	1	pseudobulk	DESeq2	LRT	0.568225019388216
669	KC_AB1	Oli	0.0199791187559067	0.923646126271025	1	pseudobulk	DESeq2	LRT	0.034494386836751
669	KC_AB1	omd	0.746211905143301	0.250336742882271	1	pseudobulk	DESeq2	LRT	0.601475402649427
669	KC_AB1	ome	-0.328243714498621	0.132431546267704	1	pseudobulk	DESeq2	LRT	0.878008550107124
670	KC_AB1	onecut	0.163481105401002	0.306034090147031	1	pseudobulk	DESeq2	LRT	0.514230193328504
670	KC_AB1	Opa1	-0.147776419792392	0.490402342233286	1	pseudobulk	DESeq2	LRT	0.309447464230746
670	KC_AB1	Opbp	0.386929021915723	0.623563805727987	1	pseudobulk	DESeq2	LRT	0.205119101034191
670	KC_AB1	opm	-0.265999038397916	0.630937554983098	1	pseudobulk	DESeq2	LRT	0.200013621529641
670	KC_AB1	or	0.395859937046226	0.419284713405737	1	pseudobulk	DESeq2	LRT	0.377490971139376
670	KC_AB1	Or22a	1.95275942187961	0.438420007953907	1	pseudobulk	DESeq2	LRT	0.358109634392079
670	KC_AB1	Or22c	0.13617601020169	1	1	pseudobulk	DESeq2	LRT	0

670	KC_AB1	Or43b	-1.03965604878779	0.184498606725885	1	pseudobulk	DESeq2	LRT	0.734006909144728
670	KC_AB1	Or45a	1.27267724533804	0.244605088322962	1	pseudobulk	DESeq2	LRT	0.611534512926881
670	KC_AB1	Or45b	-0.850996402080018	0.900449987182352	1	pseudobulk	DESeq2	LRT	0.045540403770502
671	KC_AB1	Or63a	-0.0614188704808313	0.94675570134426	1	pseudobulk	DESeq2	LRT	0.023762070886411
671	KC_AB1	Or67d	-1.82709236256241	0.44317674354685	1	pseudobulk	DESeq2	LRT	0.353423038023035
671	KC_AB1	Or92a	-0.82542030717083	1	1	pseudobulk	DESeq2	LRT	0
671	KC_AB1	orb	0.180204740373118	0.632587709805452	1	pseudobulk	DESeq2	LRT	0.198879250004115
671	KC_AB1	orb2	-0.273934989666895	0.0335872092800367	1	pseudobulk	DESeq2	LRT	1.47382607965434
671	KC_AB1	Orc4	0.216915664064821	0.79136872148065	1	pseudobulk	DESeq2	LRT	0.101621119039419
671	KC_AB1	Orc5	0.13617601020169	1	1	pseudobulk	DESeq2	LRT	0
671	KC_AB1	Orc6	1.24522514393301	0.608213953617254	1	pseudobulk	DESeq2	LRT	0.215943620513061
671	KC_AB1	Orcokinin	1.08899820779554	0.878177167143508	1	pseudobulk	DESeq2	LRT	0.056417858865076
671	KC_AB1	Orct2	1.5940412424434	0.475208010505809	1	pseudobulk	DESeq2	LRT	0.323116247132191
672	KC_AB1	ORMDL	-0.0408675586784568	0.888404951443992	1	pseudobulk	DESeq2	LRT	0.051389029616122
672	KC_AB1	ORY	0.112935832476632	0.940353958317081	1	pseudobulk	DESeq2	LRT	0.026708642986417
672	KC_AB1	osa	-0.0108737140929868	0.931038148456887	1	pseudobulk	DESeq2	LRT	0.031032523825432
672	KC_AB1	Osbp	-0.145818246520799	0.796997057194579	1	pseudobulk	DESeq2	LRT	0.098543282175424
672	KC_AB1	Oscillin	0.394124561183153	0.647213124130435	1	pseudobulk	DESeq2	LRT	0.188952684721436
672	KC_AB1	Oseg1	-1.38516836286199	0.297324227641843	1	pseudobulk	DESeq2	LRT	0.526769700608525
672	KC_AB1	Oseg2	0.135795303631797	0.946582799906172	1	pseudobulk	DESeq2	LRT	0.023841391241048
672	KC_AB1	Oseg4	1.08899820779554	0.878177167143508	1	pseudobulk	DESeq2	LRT	0.056417858865076
672	KC_AB1	Oseg6	-0.850996402080018	0.900449987182352	1	pseudobulk	DESeq2	LRT	0.045540403770502
672	KC_AB1	Osi24	0.978387385707312	0.806388900818397	1	pseudobulk	DESeq2	LRT	0.093455458510773
673	KC_AB1	osp	-1.52790282045146	0.136462314584812	1	pseudobulk	DESeq2	LRT	0.864987266773781
673	KC_AB1	Ost48	-0.362429989421101	0.359652324553406	1	pseudobulk	DESeq2	LRT	0.444117128363461
673	KC_AB1	OstDelta	-0.0983334946998241	0.856559325292293	1	pseudobulk	DESeq2	LRT	0.067242552424296
673	KC_AB1	Ostgamma	0.124313145606135	0.780748933017845	1	pseudobulk	DESeq2	LRT	0.107488600610129
673	KC_AB1	Ote	-0.82542030717083	1	1	pseudobulk	DESeq2	LRT	0
673	KC_AB1	otk	0.769657469907831	0.0363727612235646	1	pseudobulk	DESeq2	LRT	1.43922372840258
673	KC_AB1	otk2	0.427452000631992	0.531793964085595	1	pseudobulk	DESeq2	LRT	0.274256596262059
673	KC_AB1	OtopLa	0.264825368743979	0.0647440881817314	1	pseudobulk	DESeq2	LRT	1.18879988110412
673	KC_AB1	OtopLc	0.171041377399065	0.412587240366635	1	pseudobulk	DESeq2	LRT	0.384484207104621
673	KC_AB1	otu	0.11269840515639	0.977007463492936	1	pseudobulk	DESeq2	LRT	0.010102118633921
674	KC_AB1	ova	-0.0986446467151657	0.715639821291446	1	pseudobulk	DESeq2	LRT	0.145305501411027
674	KC_AB1	ovo	-0.850996402080018	0.900449987182352	1	pseudobulk	DESeq2	LRT	0.045540403770502
674	KC_AB1	ox	-0.218487797881511	0.161140828002286	1	pseudobulk	DESeq2	LRT	0.792794409117563
674	KC_AB1	OXA1L	-0.218794363765948	0.751472380381966	1	pseudobulk	DESeq2	LRT	0.124086976844385
674	KC_AB1	oxt	0.405474399143697	0.347020619898587	1	pseudobulk	DESeq2	LRT	0.459644718747706
674	KC_AB1	oys	-0.281236379811977	0.466116369075064	1	pseudobulk	DESeq2	LRT	0.331505645245604
674	KC_AB1	p	-0.523628459131239	0.212951527154958	1	pseudobulk	DESeq2	LRT	0.671719241091086
674	KC_AB1	p115	-0.300725003228842	0.723865619050353	1	pseudobulk	DESeq2	LRT	0.140342050269332
674	KC_AB1	p120ctn	0.112643718944641	0.426389583019514	1	pseudobulk	DESeq2	LRT	0.370193413954292
674	KC_AB1	p130CAS	-0.0618707363823037	0.849252098493723	1	pseudobulk	DESeq2	LRT	0.070963371319143
675	KC_AB1	p23	-0.0619308428380816	0.713885126495643	1	pseudobulk	DESeq2	LRT	0.146371666294885
675	KC_AB1	p24-1	0.338079780712138	0.389499031248401	1	pseudobulk	DESeq2	LRT	0.409493618155722
675	KC_AB1	P32	0.228534015529155	0.591205696948394	1	pseudobulk	DESeq2	LRT	0.228261389665107
675	KC_AB1	p38b	0.170594586435928	0.707563567930775	1	pseudobulk	DESeq2	LRT	0.150234536776958
675	KC_AB1	p47	-0.080113458977017	0.847403377450252	1	pseudobulk	DESeq2	LRT	0.071909809387325
675	KC_AB1	p53	-0.958139826622859	0.622272246409331	1	pseudobulk	DESeq2	LRT	0.206019568290904
675	KC_AB1	P58IPK	0.0194998283309525	0.96863255799164	1	pseudobulk	DESeq2	LRT	0.013840937397188
675	KC_AB1	P5CDh1	-0.0502711352865911	0.815122042692432	1	pseudobulk	DESeq2	LRT	0.088777362425874
675	KC_AB1	P5cr	-0.268249732753818	0.530222933956742	1	pseudobulk	DESeq2	LRT	0.275541491473816

675	KC_AB1	P5cr-2	-0.507979848677457	0.940470850565906	1	pseudobulk	DESeq2	LRT	0.02665466065008
676	KC_AB1	Pa1	0.263700691762933	0.402081450949632	1	pseudobulk	DESeq2	LRT	0.39568596155521
676	KC_AB1	pAbp	0.110755375226505	0.257281864106107	1	pseudobulk	DESeq2	LRT	0.58959082628960
676	KC_AB1	Pabp2	-0.020251532161060	0.921893198929084	1	pseudobulk	DESeq2	LRT	0.03531938892727
676	KC_AB1	pad	-0.062829800316453	0.906297263140118	1	pseudobulk	DESeq2	LRT	0.04272933149876
676	KC_AB1	Paf-AHalpha	0.261695678530752	0.377895793080538	1	pseudobulk	DESeq2	LRT	0.42262794283312
676	KC_AB1	Paics	-0.463187216132628	0.565042504441201	1	pseudobulk	DESeq2	LRT	0.24791888182664
676	KC_AB1	Paip2	0.21634039528769	0.241108664110811	1	pseudobulk	DESeq2	LRT	0.61778718320235
676	KC_AB1	Pak	0.295390448846287	0.160854319377242	1	pseudobulk	DESeq2	LRT	0.79356727261395
676	KC_AB1	Pak3	-0.161615921398727	0.701103174921794	1	pseudobulk	DESeq2	LRT	0.15421806619592
676	KC_AB1	Pal1	0.0245403320193047	0.910449831075669	1	pseudobulk	DESeq2	LRT	0.04074398033285
677	KC_AB1	Pal2	-0.257612379168568	0.083356208606447	1	pseudobulk	DESeq2	LRT	1.0790620471485
677	KC_AB1	pall	-0.003257672722098	0.987133511149769	1	pseudobulk	DESeq2	LRT	0.00562410443847
677	KC_AB1	pan	-0.079162178570994	0.362510815282152	1	pseudobulk	DESeq2	LRT	0.44067903199701
677	KC_AB1	PAN2	-0.063280649383837	0.89394205844457	1	pseudobulk	DESeq2	LRT	0.04869062942922
677	KC_AB1	Panx	0.239841919022938	0.654667990540801	1	pseudobulk	DESeq2	LRT	0.18397889303454
677	KC_AB1	papi	0.281608936249673	0.674993996058367	1	pseudobulk	DESeq2	LRT	0.17070009011759
677	KC_AB1	PAPLA1	0.0255666514743313	0.920731187393394	1	pseudobulk	DESeq2	LRT	0.03586714599431
677	KC_AB1	Papss	0.434547295825344	0.457873941107187	1	pseudobulk	DESeq2	LRT	0.33925407268849
677	KC_AB1	Papst2	0.0371413869521646	0.930292055110539	1	pseudobulk	DESeq2	LRT	0.03138068799322
677	KC_AB1	par-1	-0.156194668486149	0.287248808522686	1	pseudobulk	DESeq2	LRT	0.54174176404297
678	KC_AB1	par-6	-0.479212420620296	0.156951190685693	1	pseudobulk	DESeq2	LRT	0.80423538524418
678	KC_AB1	para	-0.092140326575992	0.328957582082934	1	pseudobulk	DESeq2	LRT	0.48286009917763
678	KC_AB1	Parg	0.567957041591724	0.497882460583563	1	pseudobulk	DESeq2	LRT	0.30287317279251
678	KC_AB1	park	0.54652160362316	0.499744345739745	1	pseudobulk	DESeq2	LRT	0.30125211092254
678	KC_AB1	Parp	0.0781141776819386	0.672924597370132	1	pseudobulk	DESeq2	LRT	0.17203359666857
678	KC_AB1	parvin	0.404916484472813	0.345163356588428	1	pseudobulk	DESeq2	LRT	0.46197531630604
678	KC_AB1	pasha	-0.20574232303729	0.726555076511838	1	pseudobulk	DESeq2	LRT	0.13873145842233
678	KC_AB1	pasi1	-0.199580401210225	0.783181048759029	1	pseudobulk	DESeq2	LRT	0.10613783003913
678	KC_AB1	pasi2	0.0056609992388701	0.952056770817825	1	pseudobulk	DESeq2	LRT	0.02133715401278
678	KC_AB1	Pask	0.525848652898736	0.422814617267989	1	pseudobulk	DESeq2	LRT	0.37385000696278
679	KC_AB1	Past1	-0.423667361816967	0.135477736474021	1	pseudobulk	DESeq2	LRT	0.86813206804345
679	KC_AB1	Pat1	-0.076475343650815	0.700862659578524	1	pseudobulk	DESeq2	LRT	0.15436707765518
679	KC_AB1	path	-0.052569790830679	0.844178622266698	1	pseudobulk	DESeq2	LRT	0.07356564998919
679	KC_AB1	Patj	0.0061371778344735	0.970114084517091	1	pseudobulk	DESeq2	LRT	0.01317771901019
679	KC_AB1	Patr-1	-0.160595481384516	0.699449287426549	1	pseudobulk	DESeq2	LRT	0.15524376793299
679	KC_AB1	Patronin	-0.228563201345762	0.297880679381713	1	pseudobulk	DESeq2	LRT	0.52595766432446
679	KC_AB1	Patsas	-0.003054409623963	0.992812471069598	1	pseudobulk	DESeq2	LRT	0.00313277614803
679	KC_AB1	Pax	0.0554129038673219	0.885105730234987	1	pseudobulk	DESeq2	LRT	0.05300484759037
679	KC_AB1	pb	-0.341884002218887	0.868674590665903	1	pseudobulk	DESeq2	LRT	0.06114288171595
679	KC_AB1	Pbgs	-0.466654968177324	0.569377515777192	1	pseudobulk	DESeq2	LRT	0.24459968676176
680	KC_AB1	Pbp45	-0.415345049822811	0.856507449263177	1	pseudobulk	DESeq2	LRT	0.06726885551245
680	KC_AB1	Pbp95	0.233007490930005	0.850718356135831	1	pseudobulk	DESeq2	LRT	0.07021419622707
680	KC_AB1	Pc	0.302774622958171	0.332495194383272	1	pseudobulk	DESeq2	LRT	0.47821462725684
680	KC_AB1	PCB	-0.074901950652467	0.876537464547888	1	pseudobulk	DESeq2	LRT	0.05722951676505
680	KC_AB1	Pcd	0.271220089569312	0.119309245400168	1	pseudobulk	DESeq2	LRT	0.92332590108489
680	KC_AB1	Pcf11	-0.091260221672288	0.600703269997204	1	pseudobulk	DESeq2	LRT	0.22134000391434
680	KC_AB1	pch2	0.686073439784065	0.672590900444057	1	pseudobulk	DESeq2	LRT	0.17224901259924
680	KC_AB1	PCID2	-0.061694921041847	0.911244030979792	1	pseudobulk	DESeq2	LRT	0.04036530347070
680	KC_AB1	pck	0.2375575823199	0.58529848871679	1	pseudobulk	DESeq2	LRT	0.23262259726426
680	KC_AB1	Pcl	-0.005177921691511	0.959982227103929	1	pseudobulk	DESeq2	LRT	0.01773680731682
681	KC_AB1	pcm	-0.080894772833840	0.835690231908208	1	pseudobulk	DESeq2	LRT	0.07795467411995

681	KC_AB1	Pcmt	0.21789375158326	0.446914329308435	1	pseudobulk	DESeq2	LRT	0.349775720435152
681	KC_AB1	PCNA2	-0.134729850363246	0.824983694893144	1	pseudobulk	DESeq2	LRT	0.083554634829360
681	KC_AB1	pcs	0.137411111327077	0.578130251950548	1	pseudobulk	DESeq2	LRT	0.237974304606135
681	KC_AB1	pcx	-0.25532685659325	0.554673109483902	1	pseudobulk	DESeq2	LRT	0.255962888178919
681	KC_AB1	Pcyt1	0.101092971547631	0.588314126962309	1	pseudobulk	DESeq2	LRT	0.230390722939913
681	KC_AB1	Pcyt2	0.243798755538464	0.628645721439963	1	pseudobulk	DESeq2	LRT	0.201594035906765
681	KC_AB1	PDCD-5	0.130389475758749	0.713536990729976	1	pseudobulk	DESeq2	LRT	0.146583507547946
681	KC_AB1	Pdcd4	0.0116119105166919	0.880425977641021	1	pseudobulk	DESeq2	LRT	0.055307151739196
681	KC_AB1	Pde11	1.08899820779554	0.670846322504933	1	pseudobulk	DESeq2	LRT	0.173376956629912
682	KC_AB1	Pde1c	-0.0599085601381346	0.618272259565849	1	pseudobulk	DESeq2	LRT	0.208820238844397
682	KC_AB1	Pde6	-0.192943487010506	0.0704085656982092	1	pseudobulk	DESeq2	LRT	1.15237450265942
682	KC_AB1	Pde8	-0.264185528784258	0.0813026378107492	1	pseudobulk	DESeq2	LRT	1.08989536377778
682	KC_AB1	Pde9	-0.0620189150604322	0.529666871798896	1	pseudobulk	DESeq2	LRT	0.275997189316352
682	KC_AB1	pdgy	-0.0340633972589304	0.955003023976034	1	pseudobulk	DESeq2	LRT	0.019995253239263
682	KC_AB1	Pdha	-0.0275545939041179	0.910673237004499	1	pseudobulk	DESeq2	LRT	0.040637426345045
682	KC_AB1	Pdhb	-0.159086149587398	0.537396895227204	1	pseudobulk	DESeq2	LRT	0.269704847001451
682	KC_AB1	Pdi	-0.0386784858630034	0.877887340654774	1	pseudobulk	DESeq2	LRT	0.056561213560020
682	KC_AB1	Pdk	0.342885114418783	0.264765556177354	1	pseudobulk	DESeq2	LRT	0.577138513700142
682	KC_AB1	Pdk1	0.390446171860546	0.032225017794357	1	pseudobulk	DESeq2	LRT	1.49180683416109
683	KC_AB1	pdm3	0.167904355330047	0.911111240962872	1	pseudobulk	DESeq2	LRT	0.040428595159865
683	KC_AB1	Pdp	-0.251819802014802	0.631677531559455	1	pseudobulk	DESeq2	LRT	0.199504570459258
683	KC_AB1	Pdp1	0.155881638207903	0.162635978208582	1	pseudobulk	DESeq2	LRT	0.788783373815519
683	KC_AB1	Pdrg1	-0.424907520353804	0.707197970629552	1	pseudobulk	DESeq2	LRT	0.150458994241663
683	KC_AB1	pds5	0.174288323125217	0.66180507288821	1	pseudobulk	DESeq2	LRT	0.179269908196243
683	KC_AB1	Pdss2	0.0311482327378141	0.924325146810995	1	pseudobulk	DESeq2	LRT	0.034175231565048
683	KC_AB1	Pdxk	1.09352205905303	0.319471701273473	1	pseudobulk	DESeq2	LRT	0.495567605502706
683	KC_AB1	PDZ-GEF	0.0820076911682744	0.608348134839601	1	pseudobulk	DESeq2	LRT	0.215847819131069
683	KC_AB1	pea	-0.0672950647995185	0.903657666110641	1	pseudobulk	DESeq2	LRT	0.043996062751071
683	KC_AB1	Pect	0.0865981960718719	0.665120470107079	1	pseudobulk	DESeq2	LRT	0.177099685878458
684	KC_AB1	PEK	0.661058977184856	0.212032614474624	1	pseudobulk	DESeq2	LRT	0.673597331538507
684	KC_AB1	pelo	-0.0664256584802323	0.882844406456423	1	pseudobulk	DESeq2	LRT	0.054115830252165
684	KC_AB1	pen-2	0.595517180403666	0.244826549797048	1	pseudobulk	DESeq2	LRT	0.611141487649303
684	KC_AB1	peng	-0.155615223073084	0.797324085273705	1	pseudobulk	DESeq2	LRT	0.098365116699975
684	KC_AB1	Pep	0.0339653990760966	0.799136655721408	1	pseudobulk	DESeq2	LRT	0.097378948156609
684	KC_AB1	per	0.277850890450932	0.502521426977012	1	pseudobulk	DESeq2	LRT	0.298845415659739
684	KC_AB1	pes	-0.0784704507985625	0.920322868469869	1	pseudobulk	DESeq2	LRT	0.036059786354534
684	KC_AB1	Pex1	0.786350086625093	0.470934995660907	1	pseudobulk	DESeq2	LRT	0.327039035491227
684	KC_AB1	Pex10	0.21883025292131	0.762836408074607	1	pseudobulk	DESeq2	LRT	0.117568587455391
684	KC_AB1	Pex11	0.0353000291904139	0.940836703533245	1	pseudobulk	DESeq2	LRT	0.026485748420332
685	KC_AB1	Pex12	0.948865750546245	0.472138971450056	1	pseudobulk	DESeq2	LRT	0.325930150411419
685	KC_AB1	Pex13	-0.154472147100044	0.652045063391973	1	pseudobulk	DESeq2	LRT	0.185722388767808
685	KC_AB1	Pex14	0.170576660658578	0.729434635968453	1	pseudobulk	DESeq2	LRT	0.137013618794596
685	KC_AB1	Pex16	-0.0593018973807343	0.880112622234802	1	pseudobulk	DESeq2	LRT	0.055461750479880
685	KC_AB1	Pex19	-0.184571084055531	0.514305424230124	1	pseudobulk	DESeq2	LRT	0.288778895276832
685	KC_AB1	Pex2	-0.819817140550221	0.267408342776136	1	pseudobulk	DESeq2	LRT	0.572825047466259
685	KC_AB1	Pex23	0.248600368177778	0.520486619854801	1	pseudobulk	DESeq2	LRT	0.283590430414573
685	KC_AB1	Pex3	-0.0892694473819914	0.821295332756033	1	pseudobulk	DESeq2	LRT	0.085500645167401
685	KC_AB1	Pex5	0.21759994181503	0.712334237455204	1	pseudobulk	DESeq2	LRT	0.147316181332069
685	KC_AB1	Pex6	0.747219044831433	0.270693818695695	1	pseudobulk	DESeq2	LRT	0.567521661248336
686	KC_AB1	Pez	-0.850996402080018	0.900449987182352	1	pseudobulk	DESeq2	LRT	0.045540403770502
686	KC_AB1	Pfas	-0.850996402080018	0.900449987182352	1	pseudobulk	DESeq2	LRT	0.045540403770502
686	KC_AB1	Pfdn1	0.0784666775201901	0.790784274617422	1	pseudobulk	DESeq2	LRT	0.101941975568258

686	KC_AB1	Pfdn2	0.20693240831586	0.504501755453345	1	pseudobulk	DESeq2	LRT	0.2971373182628
686	KC_AB1	Pfdn4	0.334885576879746	0.176144867436674	1	pseudobulk	DESeq2	LRT	0.75413000690457
686	KC_AB1	Pfdn5	-0.074903866840498	0.821909493891086	1	pseudobulk	DESeq2	LRT	0.085176002980319
686	KC_AB1	Pfdn6	0.0691392374616514	0.771580118731475	1	pseudobulk	DESeq2	LRT	0.112618971329414
686	KC_AB1	Pfk	0.180896261108623	0.511801687193934	1	pseudobulk	DESeq2	LRT	0.290898286761049
686	KC_AB1	Pfrx	0.19099026414719	0.293311828754753	1	pseudobulk	DESeq2	LRT	0.532670422326884
686	KC_AB1	Pgam5	0.0628565774398771	0.89355310824865	1	pseudobulk	DESeq2	LRT	0.048879630137747
687	KC_AB1	Pgant1	-0.355255261868806	0.47460556907539	1	pseudobulk	DESeq2	LRT	0.323667170028244
687	KC_AB1	Pgant2	-0.148819295504554	0.415844107203193	1	pseudobulk	DESeq2	LRT	0.381069448388287
687	KC_AB1	Pgant3	-0.0043381184169996	0.898008617230786	1	pseudobulk	DESeq2	LRT	0.046719495851823
687	KC_AB1	Pgant35A	0.205183639303411	0.837838845468354	1	pseudobulk	DESeq2	LRT	0.076839507926268
687	KC_AB1	Pgant5	-0.356663157943399	0.23214275577714	1	pseudobulk	DESeq2	LRT	0.634244864335098
687	KC_AB1	Pgant6	-0.157159382167793	0.844382617960968	1	pseudobulk	DESeq2	LRT	0.073460715441755
687	KC_AB1	Pgant7	-0.461724404487113	0.535410482093872	1	pseudobulk	DESeq2	LRT	0.271313130588449
687	KC_AB1	Pgant9	0.0158525143804795	0.895398223812829	1	pseudobulk	DESeq2	LRT	0.047983771454754
687	KC_AB1	PGAP1	1.36085120387006	0.145923527810408	1	pseudobulk	DESeq2	LRT	0.835874679494873
687	KC_AB1	PGAP2	-0.2793062493413	0.39636795911337	1	pseudobulk	DESeq2	LRT	0.401901459491174
688	KC_AB1	PGAP3	-0.179368303033705	0.744926658380818	1	pseudobulk	DESeq2	LRT	0.127886483531489
688	KC_AB1	PGAP5	0.466596487029106	0.413318526060231	1	pseudobulk	DESeq2	LRT	0.383715128022404
688	KC_AB1	Pgd	0.111831901354254	1	1	pseudobulk	DESeq2	LRT	0
688	KC_AB1	Pgi	0.15233877515456	0.571771793720975	1	pseudobulk	DESeq2	LRT	0.242777272786147
688	KC_AB1	Pgk	0.0512881602906523	0.807745400122106	1	pseudobulk	DESeq2	LRT	0.092725506485527
688	KC_AB1	Pglym78	-0.130679562078961	0.440002746031347	1	pseudobulk	DESeq2	LRT	0.356544613098949
688	KC_AB1	Pgm1	0.103638824591021	0.887139941226545	1	pseudobulk	DESeq2	LRT	0.052007867306133
688	KC_AB1	Pgm2a	-0.0768676739092982	0.875827365157285	1	pseudobulk	DESeq2	LRT	0.057581489434898
688	KC_AB1	PGRP-LA	-0.850996402080018	0.900449987182352	1	pseudobulk	DESeq2	LRT	0.045540403770502
688	KC_AB1	PGRP-LB	0.0057685083369781	0.967171184455792	1	pseudobulk	DESeq2	LRT	0.014496651165342
689	KC_AB1	PGRP-LE	-0.0823076204126743	0.852659204878163	1	pseudobulk	DESeq2	LRT	0.069224515156876
689	KC_AB1	ph-d	0.0790825018243635	0.857860682788758	1	pseudobulk	DESeq2	LRT	0.066583236170891
689	KC_AB1	ph-p	0.15979600050968	0.700217207819483	1	pseudobulk	DESeq2	LRT	0.154767220664361
689	KC_AB1	PH4alphaEFB	-0.191422293987726	0.597618588016063	1	pseudobulk	DESeq2	LRT	0.22357590291411
689	KC_AB1	PH4alphaNE1	3.02740643558141	0.150851323028781	1	pseudobulk	DESeq2	LRT	0.821450876529235
689	KC_AB1	PH4alphaSG1	-0.850996402080018	0.900449987182352	1	pseudobulk	DESeq2	LRT	0.045540403770502
689	KC_AB1	Phax	-0.422492682422994	0.606742513546298	1	pseudobulk	DESeq2	LRT	0.216995573620816
689	KC_AB1	Phb2	-0.0246093776727456	0.912656618169309	1	pseudobulk	DESeq2	LRT	0.039692592548036
689	KC_AB1	pHCl-1	0.0887160845649268	0.494561978612805	1	pseudobulk	DESeq2	LRT	0.305779274778942
689	KC_AB1	PheRS-m	0.316947868530784	0.460629277649836	1	pseudobulk	DESeq2	LRT	0.336648461687309
690	KC_AB1	Phf5a	-0.402093316084037	0.332847124414375	1	pseudobulk	DESeq2	LRT	0.477755190721816
690	KC_AB1	PHGPx	-0.38604300977207	0.176168827783262	1	pseudobulk	DESeq2	LRT	0.754070935421032
690	KC_AB1	PhKgamma	0.0580125428938366	0.823561734626894	1	pseudobulk	DESeq2	LRT	0.084303840327993
690	KC_AB1	Phm	-0.0771803166145639	0.712122759055498	1	pseudobulk	DESeq2	LRT	0.147445134193312
690	KC_AB1	pho	0.136213480502259	0.467811732880973	1	pseudobulk	DESeq2	LRT	0.329928890133612
690	KC_AB1	phol	0.0590323180718271	0.887248735896521	1	pseudobulk	DESeq2	LRT	0.051954610738956
690	KC_AB1	phr	1.08899820779554	0.878177167143508	1	pseudobulk	DESeq2	LRT	0.056417858865076
690	KC_AB1	phr6-4	-0.82542030717083	1	1	pseudobulk	DESeq2	LRT	0
690	KC_AB1	phtf	0.0279447834795363	0.936864828870098	1	pseudobulk	DESeq2	LRT	0.028323064726464
690	KC_AB1	phu	-0.850932661185203	0.660736244265418	1	pseudobulk	DESeq2	LRT	0.179971869591347
691	KC_AB1	phyl	0.0076625002490807	0.883064986381493	1	pseudobulk	DESeq2	LRT	0.054007334714214
691	KC_AB1	PI31	-0.0924313430055701	0.68886899383398	1	pseudobulk	DESeq2	LRT	0.161863362511392
691	KC_AB1	Pi3K21B	-0.0165908815989582	0.881775511243625	1	pseudobulk	DESeq2	LRT	0.054641966611356
691	KC_AB1	Pi3K59F	0.969444724173131	0.219890787546392	1	pseudobulk	DESeq2	LRT	0.6577929652803
691	KC_AB1	Pi3K68D	-0.278091752744488	0.348665961216755	1	pseudobulk	DESeq2	LRT	0.45759044888816

691	KC_AB1	Pi3K92E	0.323531428056113	0.55694353509221	1	pseudobulk	DESeq2	LRT	0.25418883290691
691	KC_AB1	Pi4KIIalpha	-0.0095123221798969	0.914223452066376	1	pseudobulk	DESeq2	LRT	0.038947642188693
691	KC_AB1	PI4KIIIalpha	-0.166298942584749	0.466105290403222	1	pseudobulk	DESeq2	LRT	0.331515967696276
691	KC_AB1	pic	-0.58710139363702	0.331508605800461	1	pseudobulk	DESeq2	LRT	0.479505193041588
691	KC_AB1	PICK1	0.0781863603130219	0.789514113497793	1	pseudobulk	DESeq2	LRT	0.1026401020592
692	KC_AB1	pico	-0.0099814788627528	0.907233884342238	1	pseudobulk	DESeq2	LRT	0.042280737653061
692	KC_AB1	Picot	-0.258422843011608	0.634068882425499	1	pseudobulk	DESeq2	LRT	0.197863559732928
692	KC_AB1	pie	-2.86378688787447	0.227637018922626	1	pseudobulk	DESeq2	LRT	0.642757110438212
692	KC_AB1	Piezo	-1.62938097278138	0.0817796320490776	1	pseudobulk	DESeq2	LRT	1.08735484779751
692	KC_AB1	PIG-A	0.322356392469037	0.652579826548624	1	pseudobulk	DESeq2	LRT	0.185366355853127
692	KC_AB1	PIG-B	0.0492135142045082	0.893014721639279	1	pseudobulk	DESeq2	LRT	0.049141381566096
692	KC_AB1	PIG-C	-0.104298688036742	0.927187989122899	1	pseudobulk	DESeq2	LRT	0.032832202898034
692	KC_AB1	PIG-F	-0.212385587658239	0.620094535634043	1	pseudobulk	DESeq2	LRT	0.207542095704232
692	KC_AB1	PIG-G	-1.00052378071217	0.395811546068601	1	pseudobulk	DESeq2	LRT	0.402511541305105
692	KC_AB1	PIG-H	0.429545341111071	0.37319822271823	1	pseudobulk	DESeq2	LRT	0.428060433161077
693	KC_AB1	PIG-K	-0.244476506360501	0.502625114621651	1	pseudobulk	DESeq2	LRT	0.298755814849898
693	KC_AB1	PIG-L	0.0239734336095057	0.945262686782764	1	pseudobulk	DESeq2	LRT	0.024447485070665
693	KC_AB1	PIG-N	-2.74134617855854	0.217733240445677	1	pseudobulk	DESeq2	LRT	0.662075263930316
693	KC_AB1	PIG-O	-0.296193732484131	0.632169298929601	1	pseudobulk	DESeq2	LRT	0.199166599336713
693	KC_AB1	PIG-Q	-0.777813849967516	0.504162336272277	1	pseudobulk	DESeq2	LRT	0.297429601658557
693	KC_AB1	PIG-S	-0.124737552061458	0.865320083703482	1	pseudobulk	DESeq2	LRT	0.062823216381606
693	KC_AB1	PIG-T	-0.388697122413406	0.180055602021228	1	pseudobulk	DESeq2	LRT	0.744593361995785
693	KC_AB1	PIG-U	-0.548488781452926	0.471402491795226	1	pseudobulk	DESeq2	LRT	0.326608126160323
693	KC_AB1	PIG-V	0.107158642746409	0.891717050276338	1	pseudobulk	DESeq2	LRT	0.049772929253465
693	KC_AB1	PIG-Wa	-1.11449270027482	0.554063550316889	1	pseudobulk	DESeq2	LRT	0.25644041944292
694	KC_AB1	PIG-X	-0.491498385165196	0.616446400515875	1	pseudobulk	DESeq2	LRT	0.210104678958206
694	KC_AB1	Pig1	0.11400025262841	1	1	pseudobulk	DESeq2	LRT	0
694	KC_AB1	pigeon	-0.114785590908323	0.822734631706	1	pseudobulk	DESeq2	LRT	0.084740221373445
694	KC_AB1	pigs	-0.101963332553568	0.672553073323378	1	pseudobulk	DESeq2	LRT	0.172273438400644
694	KC_AB1	Pih1D1	0.637179384254528	0.0188914563912281	1	pseudobulk	DESeq2	LRT	1.72373455990281
694	KC_AB1	Pink1	0.241496319062776	0.331210156014945	1	pseudobulk	DESeq2	LRT	0.479896354756649
694	KC_AB1	Pino	-0.0295539691189212	0.92090780822623	1	pseudobulk	DESeq2	LRT	0.035783844702904
694	KC_AB1	pins	-0.266869292492284	0.527413105091819	1	pseudobulk	DESeq2	LRT	0.277849083116056
694	KC_AB1	pinta	-0.850996402080018	0.900449987182352	1	pseudobulk	DESeq2	LRT	0.045540403770502
694	KC_AB1	pip	1.68130633771206	0.562684503730219	1	pseudobulk	DESeq2	LRT	0.249735045103917
695	KC_AB1	PIP4K	-0.0301784559266655	0.845485958450064	1	pseudobulk	DESeq2	LRT	0.072893600624835
695	KC_AB1	PIP5K59B	-0.0022612668282281	0.986038530427239	1	pseudobulk	DESeq2	LRT	0.006106114242367
695	KC_AB1	PIP82	-0.82542030717083	1	1	pseudobulk	DESeq2	LRT	0
695	KC_AB1	Pis	0.0305079184711588	0.879863117160881	1	pseudobulk	DESeq2	LRT	0.055584887038084
695	KC_AB1	Pisd	0.176397791521923	0.703741735609049	1	pseudobulk	DESeq2	LRT	0.152586692247824
695	KC_AB1	pit	-1.10586206060768	0.155998279008043	1	pseudobulk	DESeq2	LRT	0.806880192808571
695	KC_AB1	pita	0.478360215712489	0.456019429865677	1	pseudobulk	DESeq2	LRT	0.341016652722236
695	KC_AB1	Pits	0.0746067658975252	0.55859130708034	1	pseudobulk	DESeq2	LRT	0.252905827167802
695	KC_AB1	Pitslre	0.0024679023954764	0.980761821695532	1	pseudobulk	DESeq2	LRT	0.008436448361624
695	KC_AB1	piwi	1.47845164298158	0.630642748871417	1	pseudobulk	DESeq2	LRT	0.200216593420489
696	KC_AB1	pix	0.162817293643216	0.590412980249595	1	pseudobulk	DESeq2	LRT	0.228844103100996
696	KC_AB1	pk	1.26269203670432	0.541057763329599	1	pseudobulk	DESeq2	LRT	0.266756367137151
696	KC_AB1	Pka-C1	0.0834942642288186	0.383431247980377	1	pseudobulk	DESeq2	LRT	0.416312496902788
696	KC_AB1	Pka-C3	0.0261578395138203	0.965447038386915	1	pseudobulk	DESeq2	LRT	0.015271545361734
696	KC_AB1	Pka-R1	0.0040552922702162	0.956224336079999	1	pseudobulk	DESeq2	LRT	0.019440207627510
696	KC_AB1	Pka-R2	-0.0004551728809109	0.985378514672397	1	pseudobulk	DESeq2	LRT	0.006396911374024
696	KC_AB1	pkaap	0.0604878390468458	0.910238483268236	1	pseudobulk	DESeq2	LRT	0.040844807241254

696	KC_AB1	Pkc53E	-0.222435440820759	0.0167336606927484	1	pseudobulk	DESeq2	LRT	1.77640904141945
696	KC_AB1	Pkc98E	-0.0647408170209221	0.582283435015891	1	pseudobulk	DESeq2	LRT	0.234865564663605
696	KC_AB1	PKD	-0.349808318050635	0.498178322085227	1	pseudobulk	DESeq2	LRT	0.302615174438206
697	KC_AB1	Pkd2	-0.838580640014249	0.695856596814275	1	pseudobulk	DESeq2	LRT	0.157480251235852
697	KC_AB1	Pkn	-0.159870711697365	0.530801149080549	1	pseudobulk	DESeq2	LRT	0.275068145644072
697	KC_AB1	Plap	-0.256302770391341	0.730852572737514	1	pseudobulk	DESeq2	LRT	0.136170219901071
697	KC_AB1	PLCXD	0.69794357772624	0.730620326456649	1	pseudobulk	DESeq2	LRT	0.136308249527385
697	KC_AB1	Pld	-0.82542030717083	1	1	pseudobulk	DESeq2	LRT	0
697	KC_AB1	Pldn	0.0166321249955104	0.959679168289788	1	pseudobulk	DESeq2	LRT	0.017873932303644
697	KC_AB1	Plekhm1	-0.225202296332851	0.749906969775556	1	pseudobulk	DESeq2	LRT	0.124992609967104
697	KC_AB1	PlexA	0.0485989746760586	0.718671177864991	1	pseudobulk	DESeq2	LRT	0.14346977206991
697	KC_AB1	PlexB	0.10870108249124	0.552652326760154	1	pseudobulk	DESeq2	LRT	0.257547997184055
697	KC_AB1	plh	-1.14754125710473	0.554391346921886	1	pseudobulk	DESeq2	LRT	0.256183556919705
698	KC_AB1	Pli	-0.196632701666054	0.284393750002535	1	pseudobulk	DESeq2	LRT	0.5460799521379
698	KC_AB1	pll	-1.16607616091846	0.360459325970383	1	pseudobulk	DESeq2	LRT	0.443143733727377
698	KC_AB1	Plod	-1.09109960610624	0.255262902383649	1	pseudobulk	DESeq2	LRT	0.593012297067083
698	KC_AB1	Plp	0.909481402033944	0.0761623836691601	1	pseudobulk	DESeq2	LRT	1.11825947221073
698	KC_AB1	plum	1.2571587669669	0.440643871012018	1	pseudobulk	DESeq2	LRT	0.355912266173138
698	KC_AB1	plx	0.261215601449643	0.123388258495142	1	pseudobulk	DESeq2	LRT	0.908726165370705
698	KC_AB1	Plzf	0.605806905875835	0.540441179767758	1	pseudobulk	DESeq2	LRT	0.267251566636441
698	KC_AB1	PMCA	-0.0888143457069657	0.40077898266248	1	pseudobulk	DESeq2	LRT	0.397095061474741
698	KC_AB1	Pmm2	0.883768621942469	0.119315528269816	1	pseudobulk	DESeq2	LRT	0.923303031576611
698	KC_AB1	PMP34	0.061266562213143	0.853913790258817	1	pseudobulk	DESeq2	LRT	0.068585972759215
699	KC_AB1	Pmp70	0.26671655029777	0.580788235266316	1	pseudobulk	DESeq2	LRT	0.23598218950264
699	KC_AB1	Pms2	0.835069068173119	0.558091057294559	1	pseudobulk	DESeq2	LRT	0.253294936449013
699	KC_AB1	pn	-0.762062822991129	0.35132138315087	1	pseudobulk	DESeq2	LRT	0.454295416100917
699	KC_AB1	Pngl	-0.242347117447662	0.66257500576607	1	pseudobulk	DESeq2	LRT	0.178764950981392
699	KC_AB1	Pnn	-0.146730560999975	0.559794038676958	1	pseudobulk	DESeq2	LRT	0.251971730706382
699	KC_AB1	PNPase	-3.63633757073294	0.0553234594523172	1	pseudobulk	DESeq2	LRT	1.25709067067993
699	KC_AB1	pns	0.17172822782491	0.659839188929532	1	pseudobulk	DESeq2	LRT	0.180561894564503
699	KC_AB1	pnut	-0.266747705626029	0.322162241343043	1	pseudobulk	DESeq2	LRT	0.491925361922075
699	KC_AB1	PNUTS	0.26312512470185	0.050465927992035	1	pseudobulk	DESeq2	LRT	1.2970017363191
699	KC_AB1	pod1	0.407063612298332	0.172840025803569	1	pseudobulk	DESeq2	LRT	0.762355677524271
700	KC_AB1	poe	0.0042219666118630	0.979571499853966	1	pseudobulk	DESeq2	LRT	0.008963858945661
700	KC_AB1	Pof	0.28850538696817	0.625691862762616	1	pseudobulk	DESeq2	LRT	0.203639493065109
700	KC_AB1	Pol31	-0.0870883367120457	0.892183774249022	1	pseudobulk	DESeq2	LRT	0.049545679343889
700	KC_AB1	Pol32	-0.0081321240646828	0.960791071464209	1	pseudobulk	DESeq2	LRT	0.017371041441789
700	KC_AB1	POLDIP2	-0.147214678746212	0.71966828729515	1	pseudobulk	DESeq2	LRT	0.14286763439208
700	KC_AB1	poly	0.0382107781260341	0.902627605686657	1	pseudobulk	DESeq2	LRT	0.044491388283136
700	KC_AB1	polybromo	-0.473715942906544	0.477887161697146	1	pseudobulk	DESeq2	LRT	0.320674636520385
700	KC_AB1	polyph	-1.6747012584303	0.563129890257477	1	pseudobulk	DESeq2	LRT	0.249391420218277
700	KC_AB1	Pomp	0.0509495831270108	0.841104717478447	1	pseudobulk	DESeq2	LRT	0.075149931203380
700	KC_AB1	pont	-0.456091513185118	0.46373971832156	1	pseudobulk	DESeq2	LRT	0.333725706111585
701	KC_AB1	Pop2	0.094064795109052	0.73526747049034	1	pseudobulk	DESeq2	LRT	0.133554647545259
701	KC_AB1	por	-0.104861456891555	0.938553150635704	1	pseudobulk	DESeq2	LRT	0.027541128078135
701	KC_AB1	Porin2	-0.82542030717083	1	1	pseudobulk	DESeq2	LRT	0
701	KC_AB1	POSH	-0.849060238057152	0.297576059502812	1	pseudobulk	DESeq2	LRT	0.526402011446215
701	KC_AB1	Poxn	1.08899820779554	0.697631521651407	1	pseudobulk	DESeq2	LRT	0.156373904552
701	KC_AB1	Pp1-87B	0.0542849363125559	0.709254769486822	1	pseudobulk	DESeq2	LRT	0.149197735045901
701	KC_AB1	Pp1alpha-96A	0.0831327560760795	0.604612305090364	1	pseudobulk	DESeq2	LRT	0.21852301829207
701	KC_AB1	Pp2A-29B	-0.112632040367811	0.589208971850149	1	pseudobulk	DESeq2	LRT	0.229730648802681
701	KC_AB1	Pp2B-14D	-0.110124362964033	0.530911326956399	1	pseudobulk	DESeq2	LRT	0.274978008917301

701	KC_AB1	Pp2C1	0.0497780610125125	0.830496289025045	1	pseudobulk	DESeq2	LRT	0.080662303802046
702	KC_AB1	Pp4-19C	-0.011738083972894	0.892217659699599	1	pseudobulk	DESeq2	LRT	0.049529185001389
702	KC_AB1	Ppa	0.443498606559838	0.272956185360081	1	pseudobulk	DESeq2	LRT	0.563907059858421
702	KC_AB1	ppan	-0.384504950336115	0.665450302110857	1	pseudobulk	DESeq2	LRT	0.176884373434924
702	KC_AB1	Ppat-Dpck	0.08453651763296	0.934184361576495	1	pseudobulk	DESeq2	LRT	0.029567407159433
702	KC_AB1	Ppcs	0.0574232553140011	0.676145594239292	1	pseudobulk	DESeq2	LRT	0.169959777480701
702	KC_AB1	PpD3	-0.279440652355296	0.588214770380879	1	pseudobulk	DESeq2	LRT	0.230464074330186
702	KC_AB1	ppk19	-0.850932661185203	0.660736244265418	1	pseudobulk	DESeq2	LRT	0.179971869591347
702	KC_AB1	ppk21	0.190780379489138	0.895949733933807	1	pseudobulk	DESeq2	LRT	0.047716355167721
702	KC_AB1	ppk25	1.16523413356038	0.63328025468345	1	pseudobulk	DESeq2	LRT	0.198404052815419
702	KC_AB1	ppk28	1.88952324269138	0.456767015650974	1	pseudobulk	DESeq2	LRT	0.340305265178099
703	KC_AB1	ppk29	1.68130633771206	0.562684503730219	1	pseudobulk	DESeq2	LRT	0.249735045103917
703	KC_AB1	ppk8	-0.737210588914118	0.478639259771479	1	pseudobulk	DESeq2	LRT	0.319991681813617
703	KC_AB1	ppk9	1.08295541925661	0.422468547586575	1	pseudobulk	DESeq2	LRT	0.374205618354652
703	KC_AB1	ppl	-0.1811712400088	0.753461134209175	1	pseudobulk	DESeq2	LRT	0.122939144986805
703	KC_AB1	Ppm1	-0.82542030717083	1	1	pseudobulk	DESeq2	LRT	0
703	KC_AB1	Ppn	1.08899820779554	0.697631521651407	1	pseudobulk	DESeq2	LRT	0.156373904552
703	KC_AB1	PPO2	-1.44925888589622	0.6315588144614	1	pseudobulk	DESeq2	LRT	0.199586199175701
703	KC_AB1	Ppox	0.0155492772904725	0.966303583360214	1	pseudobulk	DESeq2	LRT	0.014886409955955
703	KC_AB1	PPP1R15	0.108622971419288	0.629148673893838	1	pseudobulk	DESeq2	LRT	0.201246714456089
703	KC_AB1	PPP4R2r	-0.227633465649088	0.50222220609671	1	pseudobulk	DESeq2	LRT	0.29910408857241
704	KC_AB1	Ppr-Y	-1.76926247187208	0.440254843124717	1	pseudobulk	DESeq2	LRT	0.356295857778015
704	KC_AB1	pps	-0.014320153458995	0.935022994559049	1	pseudobulk	DESeq2	LRT	0.029177708606292
704	KC_AB1	Ppt1	1.08899820779554	0.878177167143508	1	pseudobulk	DESeq2	LRT	0.056417858865076
704	KC_AB1	Ppt2	-0.299910622559163	0.643136167683321	1	pseudobulk	DESeq2	LRT	0.191697066558398
704	KC_AB1	PpV	0.27764748499545	0.537451778109792	1	pseudobulk	DESeq2	LRT	0.269660495952469
704	KC_AB1	PQBP1	0.438114055879914	0.52874754544257	1	pseudobulk	DESeq2	LRT	0.276751635698644
704	KC_AB1	pr	1.01749950384476	0.0290075402813636	1	pseudobulk	DESeq2	LRT	1.53748909599985
704	KC_AB1	PR-Set7	-0.044062178198966	0.878407891793413	1	pseudobulk	DESeq2	LRT	0.056303771087362
704	KC_AB1	prage	0.505541508408209	0.154378965101078	1	pseudobulk	DESeq2	LRT	0.811411874744252
704	KC_AB1	PRAS40	-0.043753530610430	0.865228324105157	1	pseudobulk	DESeq2	LRT	0.062869271940725
705	KC_AB1	PrBP	-0.556183384298781	0.0633261162251178	1	pseudobulk	DESeq2	LRT	1.19841714632799
705	KC_AB1	prd1	-0.061658344606401	0.80353791739291	1	pseudobulk	DESeq2	LRT	0.094993624904404
705	KC_AB1	pre-lola-G	-0.792908472039481	0.38770001607294	1	pseudobulk	DESeq2	LRT	0.411504180988175
705	KC_AB1	pre-mod(mdg4)-AE	1.18653848769441	0.60665041788826	1	pseudobulk	DESeq2	LRT	0.21706149890377
705	KC_AB1	pre-mod(mdg4)-B	-0.72174557449871	0.536974984608643	1	pseudobulk	DESeq2	LRT	0.270045945770612
705	KC_AB1	pre-mod(mdg4)-I	-0.850996402080018	0.900449987182352	1	pseudobulk	DESeq2	LRT	0.045540403770502
705	KC_AB1	pre-mod(mdg4)-O	-0.850932661185203	0.660736244265418	1	pseudobulk	DESeq2	LRT	0.179971869591347
705	KC_AB1	pre-rRNA:CR4584	0.261926662915498	0.67970729515757	1	pseudobulk	DESeq2	LRT	0.167678068860032
705	KC_AB1	pre-rRNA:CR4584	-0.82542030717083	1	1	pseudobulk	DESeq2	LRT	0
705	KC_AB1	pre-rRNA:CR4585	0.311999813123359	0.759772010525728	1	pseudobulk	DESeq2	LRT	0.119316709594313
706	KC_AB1	prel	0.0502399551430666	0.841596177596761	1	pseudobulk	DESeq2	LRT	0.074896245686063
706	KC_AB1	Prestin	-0.152848251445298	0.798802928262248	1	pseudobulk	DESeq2	LRT	0.097560351756011
706	KC_AB1	prg	0.0424419997045465	0.90289583245283	1	pseudobulk	DESeq2	LRT	0.044362351569633
706	KC_AB1	prim	-0.058269842259058	0.960292065035846	1	pseudobulk	DESeq2	LRT	0.017596659728711
706	KC_AB1	PRL-1	-0.092696682024519	0.758247940331137	1	pseudobulk	DESeq2	LRT	0.120188760712698
706	KC_AB1	Proc	0.112935832476632	0.940353958317081	1	pseudobulk	DESeq2	LRT	0.026708642986417
706	KC_AB1	Proc-R	0.114184054362142	1	1	pseudobulk	DESeq2	LRT	0
706	KC_AB1	prod	0.0677282535693338	0.855038340507055	1	pseudobulk	DESeq2	LRT	0.068014410772039
706	KC_AB1	prominin-like	-0.095831778852922	0.568434477040854	1	pseudobulk	DESeq2	LRT	0.245319588847129
706	KC_AB1	ProRS-m	0.0755814161199455	0.908358166452599	1	pseudobulk	DESeq2	LRT	0.041742874999577
707	KC_AB1	pros	0.0821518149389779	0.301791219066995	1	pseudobulk	DESeq2	LRT	0.520293400631432

707	KC_AB1	Prosalpha1	-0.902823120079472	0.462427669230504	1	pseudobulk	DESeq2	LRT	0.334956187920974
707	KC_AB1	Prosalpha2	-0.246765412520347	0.487478891989834	1	pseudobulk	DESeq2	LRT	0.312044184664533
707	KC_AB1	Prosalpha3	-0.0107098577670643	0.870961015347149	1	pseudobulk	DESeq2	LRT	0.060001283796610
707	KC_AB1	Prosalpha4	-0.11262108584069	0.780738689227468	1	pseudobulk	DESeq2	LRT	0.107494298793798
707	KC_AB1	Prosalpha5	-0.0454730479992775	0.901810546845317	1	pseudobulk	DESeq2	LRT	0.044884689843444
707	KC_AB1	Prosalpha6	-0.471241919660015	0.244160226719658	1	pseudobulk	DESeq2	LRT	0.612325080452561
707	KC_AB1	Prosalpha7	-0.0476526370158873	0.889100388858744	1	pseudobulk	DESeq2	LRT	0.051049199810497
707	KC_AB1	Prosap	0.145313328613362	0.289694910054694	1	pseudobulk	DESeq2	LRT	0.538059135291851
707	KC_AB1	Prosbeta1	-0.304599716271992	0.364340920463834	1	pseudobulk	DESeq2	LRT	0.438492048814055
708	KC_AB1	Prosbeta2	-0.0427684465692741	0.868902317086419	1	pseudobulk	DESeq2	LRT	0.061029044650162
708	KC_AB1	Prosbeta3	0.0922687906296932	0.800385572578867	1	pseudobulk	DESeq2	LRT	0.096700748378976
708	KC_AB1	Prosbeta4	-0.199710847137079	0.513995827683908	1	pseudobulk	DESeq2	LRT	0.289040406337817
708	KC_AB1	Prosbeta5	0.0703292817424646	0.811292393810152	1	pseudobulk	DESeq2	LRT	0.090822595687085
708	KC_AB1	Prosbeta6	0.46512162942785	0.232084230029996	1	pseudobulk	DESeq2	LRT	0.634354368564476
708	KC_AB1	Prosbeta7	0.174534314144401	0.683905943899512	1	pseudobulk	DESeq2	LRT	0.165003621750898
708	KC_AB1	Prp18	0.0216859425000566	0.890279810280617	1	pseudobulk	DESeq2	LRT	0.050473475419928
708	KC_AB1	Prp19	-0.0521868792470518	0.928821892500104	1	pseudobulk	DESeq2	LRT	0.032067556749307
708	KC_AB1	Prp3	-0.185573288955087	0.597822553671654	1	pseudobulk	DESeq2	LRT	0.223427704635681
708	KC_AB1	Prp31	0.086388402050825	0.840964750157696	1	pseudobulk	DESeq2	LRT	0.075222207689409
709	KC_AB1	Prp38	0.505296550263704	0.4286762753614	1	pseudobulk	DESeq2	LRT	0.367870551396625
709	KC_AB1	Prp8	-0.0137128611219996	0.887970628984765	1	pseudobulk	DESeq2	LRT	0.051601398952028
709	KC_AB1	Prpk	-0.0095632888526133	0.968133331723692	1	pseudobulk	DESeq2	LRT	0.014064827356572
709	KC_AB1	prt	-0.323700950159246	0.0302837131281052	1	pseudobulk	DESeq2	LRT	1.51879087645791
709	KC_AB1	prtp	0.131984152179434	0.639327999238287	1	pseudobulk	DESeq2	LRT	0.19427627531209
709	KC_AB1	Prx2540-1	1.08899820779554	0.670846322504933	1	pseudobulk	DESeq2	LRT	0.173376956629912
709	KC_AB1	Prx2540-2	1.08899820779554	0.878177167143508	1	pseudobulk	DESeq2	LRT	0.056417858865076
709	KC_AB1	Prx3	-0.182071229623962	0.592828844028535	1	pseudobulk	DESeq2	LRT	0.22707067395977
709	KC_AB1	Prx6005	-0.4305141744936	0.607220240348577	1	pseudobulk	DESeq2	LRT	0.216653760621807
709	KC_AB1	PRY	1.32487122469648	0.272983231810605	1	pseudobulk	DESeq2	LRT	0.563864028987751
710	KC_AB1	ps	0.0215065550697039	0.829173446654503	1	pseudobulk	DESeq2	LRT	0.081354614147005
710	KC_AB1	Psa	-0.218750296400862	0.574049519480212	1	pseudobulk	DESeq2	LRT	0.241050642254668
710	KC_AB1	Psc	-0.014224979351137	0.942817658854331	1	pseudobulk	DESeq2	LRT	0.025572291791359
710	KC_AB1	Psf3	0.475169443676198	0.498768898219657	1	pseudobulk	DESeq2	LRT	0.302100635692112
710	KC_AB1	PsGEF	0.0777849095876583	0.747112206131759	1	pseudobulk	DESeq2	LRT	0.126614168138624
710	KC_AB1	Psi	0.119024716548062	0.46293669584634	1	pseudobulk	DESeq2	LRT	0.334478392403791
710	KC_AB1	psidin	0.194200710595799	0.740906763189322	1	pseudobulk	DESeq2	LRT	0.130236440846694
710	KC_AB1	PSMG1	-0.0447878726074209	0.933751287621586	1	pseudobulk	DESeq2	LRT	0.029768786294013
710	KC_AB1	Psn	-0.409281219426847	0.406591565219724	1	pseudobulk	DESeq2	LRT	0.390841635084419
710	KC_AB1	psq	0.0285745114444096	0.823316524616654	1	pseudobulk	DESeq2	LRT	0.084433167865768
711	KC_AB1	PSR	0.171552703439885	0.790231054026541	1	pseudobulk	DESeq2	LRT	0.102245907674858
711	KC_AB1	Pstk	0.135795303631797	0.946582799906172	1	pseudobulk	DESeq2	LRT	0.023841391241048
711	KC_AB1	ptc	-1.6747012584303	0.563129890257477	1	pseudobulk	DESeq2	LRT	0.249391420218277
711	KC_AB1	Pten	-0.0632944786530346	0.849829236758242	1	pseudobulk	DESeq2	LRT	0.070668331914006
711	KC_AB1	Ptip	-0.138855540549914	0.783691352807804	1	pseudobulk	DESeq2	LRT	0.105854945178707
711	KC_AB1	PTP-ER	-0.0626648190592542	0.82697238751378	1	pseudobulk	DESeq2	LRT	0.082508991234151
711	KC_AB1	Ptp10D	2.3117138799137	0.300080866425249	1	pseudobulk	DESeq2	LRT	0.522761694914513
711	KC_AB1	Ptp36E	0.111862091912243	0.618053833840101	1	pseudobulk	DESeq2	LRT	0.20897369526482
711	KC_AB1	Ptp4E	-0.129991372824188	0.691860503536952	1	pseudobulk	DESeq2	LRT	0.159981461397603
711	KC_AB1	Ptp61F	0.166033708079861	0.693625832778282	1	pseudobulk	DESeq2	LRT	0.158874740756555
712	KC_AB1	Ptp69D	0.227460294975183	0.70954445157995	1	pseudobulk	DESeq2	LRT	0.149020391649553
712	KC_AB1	Ptp99A	-0.0680043043323574	0.500790396493255	1	pseudobulk	DESeq2	LRT	0.300344008052372
712	KC_AB1	Ptpa	-0.703534317420522	0.155521767913657	1	pseudobulk	DESeq2	LRT	0.808208815491477

712	KC_AB1	Ptpmeg	0.180278170441404	0.469131659033103	1	pseudobulk	DESeq2	LRT	0.328705257994142
712	KC_AB1	Ptpmeg2	0.0499876884300911	0.702466201299925	1	pseudobulk	DESeq2	LRT	0.153374566714068
712	KC_AB1	PTPMT1	-0.214285884903357	0.69398964833161	1	pseudobulk	DESeq2	LRT	0.158647007507666
712	KC_AB1	Ptth	-0.850996402080018	0.900449987182352	1	pseudobulk	DESeq2	LRT	0.045540403770502
712	KC_AB1	Pu	0.394872873693106	0.466235280547644	1	pseudobulk	DESeq2	LRT	0.331394866020453
712	KC_AB1	puc	0.298364162870095	0.25395722098083	1	pseudobulk	DESeq2	LRT	0.595239433996859
712	KC_AB1	puf	-0.568614445861657	0.0833026451976368	1	pseudobulk	DESeq2	LRT	1.07934120775891
713	KC_AB1	pug	-2.29603295607844	0.327786153610032	1	pseudobulk	DESeq2	LRT	0.484409395908184
713	KC_AB1	pum	-0.0522560086745872	0.544281196446133	1	pseudobulk	DESeq2	LRT	0.264176669211762
713	KC_AB1	Pur-alpha	0.0409015406985336	0.74008565632381	1	pseudobulk	DESeq2	LRT	0.130718012815038
713	KC_AB1	Pus1	-1.50598856144355	0.172554928901449	1	pseudobulk	DESeq2	LRT	0.76307263090087
713	KC_AB1	Pus7	-0.425924340433473	0.603711044171552	1	pseudobulk	DESeq2	LRT	0.219170879175698
713	KC_AB1	put	0.181908159050327	0.497477005955326	1	pseudobulk	DESeq2	LRT	0.303226988119266
713	KC_AB1	Pvf3	0.365314514790064	0.134350488049277	1	pseudobulk	DESeq2	LRT	0.87176075158494
713	KC_AB1	Pvr	-0.850996402080018	0.900449987182352	1	pseudobulk	DESeq2	LRT	0.045540403770502
713	KC_AB1	PVRAP	-0.191249494566098	0.272499845553386	1	pseudobulk	DESeq2	LRT	0.564633739535343
713	KC_AB1	px	0.0715054571492019	0.738744799365058	1	pseudobulk	DESeq2	LRT	0.131505563457568
714	KC_AB1	pxb	0.0894143247818974	0.708200914850398	1	pseudobulk	DESeq2	LRT	0.149843516558107
714	KC_AB1	Pxn	-0.445904093077	0.0115729274574595	1	pseudobulk	DESeq2	LRT	1.93655676915501
714	KC_AB1	pyd	0.293981955186928	0.341114273018362	1	pseudobulk	DESeq2	LRT	0.467100108316352
714	KC_AB1	pyd3	-0.829971373743754	0.758323735176306	1	pseudobulk	DESeq2	LRT	0.120145350587648
714	KC_AB1	pygo	0.0649577460339435	0.854600355943682	1	pseudobulk	DESeq2	LRT	0.068236930633794
714	KC_AB1	PyK	-0.111344539884611	0.505764087393116	1	pseudobulk	DESeq2	LRT	0.296052011687383
714	KC_AB1	Pym	-0.137177665561497	0.799742277574619	1	pseudobulk	DESeq2	LRT	0.097049944833004
714	KC_AB1	pyr	-0.82542030717083	1	1	pseudobulk	DESeq2	LRT	0
714	KC_AB1	Pyroxd1	-0.0882307884299879	0.892267654195366	1	pseudobulk	DESeq2	LRT	0.049504850440074
714	KC_AB1	pzg	-0.306482949334866	0.483153962699966	1	pseudobulk	DESeq2	LRT	0.315914454142487
715	KC_AB1	Pzl	0.4869605596855	0.366192250035119	1	pseudobulk	DESeq2	LRT	0.436290851193794
715	KC_AB1	QIL1	-0.0531770753131499	0.856530980222654	1	pseudobulk	DESeq2	LRT	0.067256924237966
715	KC_AB1	qin	-0.82542030717083	1	1	pseudobulk	DESeq2	LRT	0
715	KC_AB1	qjt	0.744782276103072	0.764014638758689	1	pseudobulk	DESeq2	LRT	0.116898320126909
715	KC_AB1	qkr54B	-0.317075035021442	0.229420571222475	1	pseudobulk	DESeq2	LRT	0.639367643244436
715	KC_AB1	qkr58E-1	0.0741357366162539	0.658346292729847	1	pseudobulk	DESeq2	LRT	0.181545605685286
715	KC_AB1	qkr58E-2	0.165288874948552	0.600751193236422	1	pseudobulk	DESeq2	LRT	0.221305357909865
715	KC_AB1	qkr58E-3	-0.0532735392640495	0.710323794311983	1	pseudobulk	DESeq2	LRT	0.148543637157716
715	KC_AB1	qless	0.416561079669791	0.380018956968726	1	pseudobulk	DESeq2	LRT	0.42019473837908
715	KC_AB1	qm	-0.0240711387432121	0.856522414816463	1	pseudobulk	DESeq2	LRT	0.067261267253373
716	KC_AB1	Qsox1	0.0294841764352009	0.911665989859092	1	pseudobulk	DESeq2	LRT	0.040164246464001
716	KC_AB1	qtc	-0.238183348064573	0.283092833741516	1	pseudobulk	DESeq2	LRT	0.548071124297586
716	KC_AB1	Qtzl	-0.481220615277407	0.230594770394009	1	pseudobulk	DESeq2	LRT	0.637150546194737
716	KC_AB1	qvr	-0.127440094976049	0.34031649279251	1	pseudobulk	DESeq2	LRT	0.468117003136893
716	KC_AB1	r	-1.89854133735591	0.478664405825708	1	pseudobulk	DESeq2	LRT	0.319968866080244
716	KC_AB1	r-l	-0.178088900908889	0.917437930365065	1	pseudobulk	DESeq2	LRT	0.037423308451134
716	KC_AB1	r2d2	-0.112729300063133	0.734524213477301	1	pseudobulk	DESeq2	LRT	0.133993883187201
716	KC_AB1	Rab1	0.15582411104571	0.337837898187743	1	pseudobulk	DESeq2	LRT	0.471291633478438
716	KC_AB1	Rab10	-0.0815770730434004	0.824783325998193	1	pseudobulk	DESeq2	LRT	0.083660127428919
716	KC_AB1	Rab11	0.104213737936608	0.604189011132312	1	pseudobulk	DESeq2	LRT	0.218827177851217
717	KC_AB1	Rab14	-0.155069359301334	0.414304366590787	1	pseudobulk	DESeq2	LRT	0.382680489399534
717	KC_AB1	Rab18	0.0156748602386971	0.92056031212696	1	pseudobulk	DESeq2	LRT	0.035947752661471
717	KC_AB1	Rab19	0.130593888788449	0.841600277141888	1	pseudobulk	DESeq2	LRT	0.074894130175634
717	KC_AB1	Rab2	0.0347515353702541	0.80147657576744	1	pseudobulk	DESeq2	LRT	0.096109165965609
717	KC_AB1	Rab21	-0.420499032587288	0.137931768850694	1	pseudobulk	DESeq2	LRT	0.86033569432057

717	KC_AB1	Rab23	1.88952324269138	0.456767015650974	1	pseudobulk	DESeq2	LRT	0.340305265178099
717	KC_AB1	Rab26	-0.0844126988079306	0.50139701023821	1	pseudobulk	DESeq2	LRT	0.299818260008673
717	KC_AB1	Rab27	-0.115706006821185	0.405788966066445	1	pseudobulk	DESeq2	LRT	0.391699766177996
717	KC_AB1	Rab3	-0.198640795365485	0.261523474891005	1	pseudobulk	DESeq2	LRT	0.582489321871084
717	KC_AB1	Rab3-GAP	-0.112299344867994	0.909659143485592	1	pseudobulk	DESeq2	LRT	0.041121310793486
718	KC_AB1	Rab3-GEF	0.105225305537131	0.444802037939628	1	pseudobulk	DESeq2	LRT	0.351833231624049
718	KC_AB1	Rab30	-0.504416492549612	0.203618037551087	1	pseudobulk	DESeq2	LRT	0.691183752553732
718	KC_AB1	Rab35	0.174740121247177	0.426953394602179	1	pseudobulk	DESeq2	LRT	0.369619529124309
718	KC_AB1	Rab39	0.035712894574679	0.879904642016333	1	pseudobulk	DESeq2	LRT	0.055564391134015
718	KC_AB1	Rab4	0.0680355837775628	0.755086761883634	1	pseudobulk	DESeq2	LRT	0.122003143678851
718	KC_AB1	Rab40	0.106112291834229	0.786955911309853	1	pseudobulk	DESeq2	LRT	0.104049598023651
718	KC_AB1	Rab5	0.0336393011691939	0.828675629414692	1	pseudobulk	DESeq2	LRT	0.081615433168308
718	KC_AB1	Rab6	-0.0777049951884854	0.828870627388217	1	pseudobulk	DESeq2	LRT	0.081513250144543
718	KC_AB1	Rab7	0.360424344041405	0.128398597709176	1	pseudobulk	DESeq2	LRT	0.891439719339537
718	KC_AB1	Rab8	-0.0730632473008791	0.756017308501005	1	pseudobulk	DESeq2	LRT	0.12146826150881
719	KC_AB1	Rab9	-0.226199873039523	0.793771887052297	1	pseudobulk	DESeq2	LRT	0.10030428652518
719	KC_AB1	Rab9E	-0.850996402080018	0.900449987182352	1	pseudobulk	DESeq2	LRT	0.045540403770502
719	KC_AB1	Rabex-5	-0.162132040387422	0.768924570288556	1	pseudobulk	DESeq2	LRT	0.114116261387354
719	KC_AB1	RabX1	0.539779608124939	0.214960673540768	1	pseudobulk	DESeq2	LRT	0.667640985788785
719	KC_AB1	RabX4	0.274376363356738	0.293363285120711	1	pseudobulk	DESeq2	LRT	0.532594239733406
719	KC_AB1	RabX6	0.213507090520478	0.75784381539736	1	pseudobulk	DESeq2	LRT	0.120420289223071
719	KC_AB1	Rac1	-0.196336262236192	0.369611970705941	1	pseudobulk	DESeq2	LRT	0.432253971631189
719	KC_AB1	Rack1	0.0195996718116677	0.869946555427744	1	pseudobulk	DESeq2	LRT	0.060507427146634
719	KC_AB1	rad	-0.123288916674919	0.196382992345061	1	pseudobulk	DESeq2	LRT	0.706896126785962
719	KC_AB1	Rad17	-0.150440508625614	0.833909678547905	1	pseudobulk	DESeq2	LRT	0.078880985612227
720	KC_AB1	Rad23	0.215752592633935	0.282680496238822	1	pseudobulk	DESeq2	LRT	0.548704154963113
720	KC_AB1	rad50	0.335163510657121	0.637108327245488	1	pseudobulk	DESeq2	LRT	0.195786718488622
720	KC_AB1	Rad60	-0.396737485351554	0.413686207838695	1	pseudobulk	DESeq2	LRT	0.383328958097471
720	KC_AB1	Rad9	-0.397608166404753	0.717745549598631	1	pseudobulk	DESeq2	LRT	0.144029491678198
720	KC_AB1	Rae1	-0.0125932514794445	0.912331858037907	1	pseudobulk	DESeq2	LRT	0.039847159589240
720	KC_AB1	Raf	0.0633506310139968	0.888748628043209	1	pseudobulk	DESeq2	LRT	0.051221056679869
720	KC_AB1	RAF2	0.58108729139818	0.488780283642541	1	pseudobulk	DESeq2	LRT	0.310886320928239
720	KC_AB1	RagA-B	-0.0912021038361115	0.875646718881972	1	pseudobulk	DESeq2	LRT	0.057671075323194
720	KC_AB1	RagC-D	0.314800937025184	0.410759293168439	1	pseudobulk	DESeq2	LRT	0.386412602144595
720	KC_AB1	Rala	0.0566406185913485	0.825744643969219	1	pseudobulk	DESeq2	LRT	0.083154234593444
721	KC_AB1	Ran	0.230485301441817	0.284929563222115	1	pseudobulk	DESeq2	LRT	0.545262487657195
721	KC_AB1	Ranbp16	0.133236503471455	0.650608388186107	1	pseudobulk	DESeq2	LRT	0.186680341731301
721	KC_AB1	Ranbp21	-0.462816772960833	0.651806573112779	1	pseudobulk	DESeq2	LRT	0.185881264226482
721	KC_AB1	RanBP3	-0.443259328158087	0.315315273238619	1	pseudobulk	DESeq2	LRT	0.501254992390047
721	KC_AB1	Ranbp9	-1.20466899848857	0.532614628324918	1	pseudobulk	DESeq2	LRT	0.273586909783019
721	KC_AB1	RanBPM	0.266153442190522	0.354755238650681	1	pseudobulk	DESeq2	LRT	0.450071182620919
721	KC_AB1	RanGAP	-0.801678920860384	0.517514225241959	1	pseudobulk	DESeq2	LRT	0.286077707978915
721	KC_AB1	ranshi	0.86849124484675	0.457945211310116	1	pseudobulk	DESeq2	LRT	0.33918647799835
721	KC_AB1	Rap1	-0.0117465653978891	0.90311198734644	1	pseudobulk	DESeq2	LRT	0.044258393129420
721	KC_AB1	Rap2l	0.0371136838436228	0.867152474286362	1	pseudobulk	DESeq2	LRT	0.061904532374853
722	KC_AB1	RapGAP1	-1.6408406578498	0.0566337043752037	1	pseudobulk	DESeq2	LRT	1.24692503051379
722	KC_AB1	raptor	-0.0601880774061604	0.820634800434408	1	pseudobulk	DESeq2	LRT	0.085850069971876
722	KC_AB1	ras	0.0175160808209792	0.955376747297002	1	pseudobulk	DESeq2	LRT	0.019825333121215
722	KC_AB1	Ras64B	-0.0394032167339565	0.940803995199197	1	pseudobulk	DESeq2	LRT	0.026500846997867
722	KC_AB1	Ras85D	0.116986590621052	0.669866072141146	1	pseudobulk	DESeq2	LRT	0.174012018111308
722	KC_AB1	RasGAP1	0.317185721671621	0.270985804637872	1	pseudobulk	DESeq2	LRT	0.567053458678773
722	KC_AB1	raskol	-0.071231449321191	0.687157022021624	1	pseudobulk	DESeq2	LRT	0.162944011112991

722	KC_AB1	rasp	-0.82542030717083	1	1	pseudobulk	DESeq2	LRT	0
722	KC_AB1	Rassf	0.466006115697011	0.432128395696675	1	pseudobulk	DESeq2	LRT	0.364387194718188
722	KC_AB1	RASSF8	-0.29315904145134	0.277217224953891	1	pseudobulk	DESeq2	LRT	0.557179788237671
723	KC_AB1	Rat1	0.215468726195686	0.741804828761695	1	pseudobulk	DESeq2	LRT	0.129710343968135
723	KC_AB1	Ravus	0.16707968532909	0.729877003780093	1	pseudobulk	DESeq2	LRT	0.136750319441369
723	KC_AB1	raw	0.114483799709242	0.612598937131475	1	pseudobulk	DESeq2	LRT	0.212823761036361
723	KC_AB1	rb	-0.0518929911921982	0.874009496145723	1	pseudobulk	DESeq2	LRT	0.058483848714244
723	KC_AB1	Rb97D	0.392368595874457	0.104212754436654	1	pseudobulk	DESeq2	LRT	0.982079125158577
723	KC_AB1	Rbbp5	0.234297025092859	0.71702906565708	1	pseudobulk	DESeq2	LRT	0.144463239313185
723	KC_AB1	Rbcn-3A	-0.147833025184653	0.557631937807243	1	pseudobulk	DESeq2	LRT	0.253652360418951
723	KC_AB1	Rbcn-3B	-0.144391549538834	0.602609365118505	1	pseudobulk	DESeq2	LRT	0.219964123263915
723	KC_AB1	Rbf	-0.0670940237130399	0.86792938900674	1	pseudobulk	DESeq2	LRT	0.061515605713054
723	KC_AB1	Rbf2	1.14247969565044	0.639889789651426	1	pseudobulk	DESeq2	LRT	0.19389481955965
724	KC_AB1	Rbfox1	-0.0075335303743343	0.931778398929197	1	pseudobulk	DESeq2	LRT	0.030687361857955
724	KC_AB1	Rbm13	0.62173128738282	0.545612915078967	1	pseudobulk	DESeq2	LRT	0.263115358144202
724	KC_AB1	Rbp	-0.107904855044541	0.291640065760987	1	pseudobulk	DESeq2	LRT	0.535152812509006
724	KC_AB1	Rbp1	0.0802853261391895	0.658647649644251	1	pseudobulk	DESeq2	LRT	0.181346853598306
724	KC_AB1	Rbp1-like	0.0319625871805089	0.727913850320129	1	pseudobulk	DESeq2	LRT	0.137920017039324
724	KC_AB1	Rbp6	-1.97198433270073	0.0121131046312134	1	pseudobulk	DESeq2	LRT	1.91674453139044
724	KC_AB1	Rbp9	0.158456907419646	0.291190533791439	1	pseudobulk	DESeq2	LRT	0.535822747452975
724	KC_AB1	Rbpn-5	-0.734853183996426	0.143034845127472	1	pseudobulk	DESeq2	LRT	0.844558149925636
724	KC_AB1	Rbsn-5	-0.134407919448014	0.801306092252628	1	pseudobulk	DESeq2	LRT	0.096201555347520
724	KC_AB1	Rca1	-0.0607616187773451	0.896788989260852	1	pseudobulk	DESeq2	LRT	0.047309732630533
725	KC_AB1	Rcc1	0.0076337369815265	0.979918560746302	1	pseudobulk	DESeq2	LRT	0.008810016234200
725	KC_AB1	Rcd-1	0.0562522258219988	0.857499411307834	1	pseudobulk	DESeq2	LRT	0.066766169437783
725	KC_AB1	Rcd1	0.152817206288062	0.622925496647407	1	pseudobulk	DESeq2	LRT	0.205563892875
725	KC_AB1	Rcd4	0.46373008223136	0.421085792056764	1	pseudobulk	DESeq2	LRT	0.37562941195291
725	KC_AB1	Rcd5	-0.0689356531613576	0.881361645237435	1	pseudobulk	DESeq2	LRT	0.054845852877805
725	KC_AB1	Rcd6	1.18653848769441	0.60665041788826	1	pseudobulk	DESeq2	LRT	0.21706149890377
725	KC_AB1	Rchy1	0.10745091433163	0.925708810521252	1	pseudobulk	DESeq2	LRT	0.033525602812194
725	KC_AB1	Rcp	0.454262001165142	0.503332651755765	1	pseudobulk	DESeq2	LRT	0.298144895517044
725	KC_AB1	rdgA	0.104214459115473	0.813140265113645	1	pseudobulk	DESeq2	LRT	0.089834532990703
725	KC_AB1	rdgB	0.0669693277787105	0.587188849114197	1	pseudobulk	DESeq2	LRT	0.231222200391296
726	KC_AB1	rdgBbeta	0.16091002455396	0.552052985250895	1	pseudobulk	DESeq2	LRT	0.258019237311092
726	KC_AB1	rdgC	0.227908561303259	0.472538892883011	1	pseudobulk	DESeq2	LRT	0.325562440552936
726	KC_AB1	Rdh	0.167260225563399	0.276111177325087	1	pseudobulk	DESeq2	LRT	0.558916012222188
726	KC_AB1	Rdl	-0.033156691733891	0.711084715150895	1	pseudobulk	DESeq2	LRT	0.148078656470077
726	KC_AB1	rdo	-0.0718201978037517	0.925299011125846	1	pseudobulk	DESeq2	LRT	0.033717901966134
726	KC_AB1	rdog	0.901182577436173	0.268781610155472	1	pseudobulk	DESeq2	LRT	0.570600448717968
726	KC_AB1	rdx	0.167073933978565	0.262976226394526	1	pseudobulk	DESeq2	LRT	0.58008351087278
726	KC_AB1	rec	-0.489627937984416	0.466030101715388	1	pseudobulk	DESeq2	LRT	0.331586030548894
726	KC_AB1	RecQ5	-0.96946195626779	0.214732357126772	1	pseudobulk	DESeq2	LRT	0.668102508601496
726	KC_AB1	red	-0.187798628961165	0.654641952943284	1	pseudobulk	DESeq2	LRT	0.183996166232506
727	KC_AB1	ReepA	0.25510654408537	0.380410285644533	1	pseudobulk	DESeq2	LRT	0.419747748994684
727	KC_AB1	ReepB	0.0511071761617111	0.830400871800329	1	pseudobulk	DESeq2	LRT	0.080712203549238
727	KC_AB1	ref(2)P	0.110987374068014	0.666410024105722	1	pseudobulk	DESeq2	LRT	0.176258478916607
727	KC_AB1	Ref1	0.0877678080041712	0.670649146157245	1	pseudobulk	DESeq2	LRT	0.173504624001707
727	KC_AB1	REG	-0.297577557176033	0.317064447842708	1	pseudobulk	DESeq2	LRT	0.4988524523007
727	KC_AB1	regucalcin	-0.187346449698364	0.84843975992388	1	pseudobulk	DESeq2	LRT	0.071378987596615
727	KC_AB1	Rel	-0.97612569349808	0.313560994886921	1	pseudobulk	DESeq2	LRT	0.503677966264804
727	KC_AB1	rempA	0.977851105145882	0.69132261808108	1	pseudobulk	DESeq2	LRT	0.160319234022942
727	KC_AB1	Rep	-0.0158030550355817	0.952301890032615	1	pseudobulk	DESeq2	LRT	0.021225353725195

727	KC_AB1	Reph	0.276956528081696	0.0494065814163747	1	pseudobulk	DESeq2	LRT	1.30621519515668
728	KC_AB1	repo	1.08899820779554	0.878177167143508	1	pseudobulk	DESeq2	LRT	0.056417858865076
728	KC_AB1	Reps	-0.182833401569039	0.65435646174843	1	pseudobulk	DESeq2	LRT	0.184185604586489
728	KC_AB1	rept	-0.187480682796385	0.813400448266099	1	pseudobulk	DESeq2	LRT	0.089695592591082
728	KC_AB1	REPTOR	0.287606940041099	0.0627707552020895	1	pseudobulk	DESeq2	LRT	1.20224264627159
728	KC_AB1	resilin	1.08899820779554	0.878177167143508	1	pseudobulk	DESeq2	LRT	0.056417858865076
728	KC_AB1	retm	0.257676223138683	0.398206308453577	1	pseudobulk	DESeq2	LRT	0.399891864085415
728	KC_AB1	retn	-0.0165310379755968	0.953142724558705	1	pseudobulk	DESeq2	LRT	0.020842062795843
728	KC_AB1	Rev1	-0.14291477696336	0.902281322892435	1	pseudobulk	DESeq2	LRT	0.044658032379325
728	KC_AB1	Rev7	-0.626597539839171	0.461017096344384	1	pseudobulk	DESeq2	LRT	0.33628296894798
728	KC_AB1	Rexo5	0.168445469974069	0.806315362912112	1	pseudobulk	DESeq2	LRT	0.093495065409137
729	KC_AB1	RfC3	-0.71118587074844	0.543722008398127	1	pseudobulk	DESeq2	LRT	0.264623087575048
729	KC_AB1	RfC38	-0.189068948006717	0.823169855474434	1	pseudobulk	DESeq2	LRT	0.084510541842340
729	KC_AB1	RfC4	1.01359952645655	0.208028477678906	1	pseudobulk	DESeq2	LRT	0.68187720901685
729	KC_AB1	RFeSP	-0.175567259867549	0.303415833018906	1	pseudobulk	DESeq2	LRT	0.517961760354394
729	KC_AB1	Rfx	0.213786490084723	0.714987238608989	1	pseudobulk	DESeq2	LRT	0.145701709599139
729	KC_AB1	rg	-0.179595514800411	0.768646254244273	1	pseudobulk	DESeq2	LRT	0.114273484874752
729	KC_AB1	Rga	0.119026743874313	0.59979332436307	1	pseudobulk	DESeq2	LRT	0.221998372201726
729	KC_AB1	Rgk1	-0.0569761902001803	0.546720548588539	1	pseudobulk	DESeq2	LRT	0.26223460276675
729	KC_AB1	Rgk3	-0.238312116747867	0.148106672219832	1	pseudobulk	DESeq2	LRT	0.829425376029885
729	KC_AB1	Rgl	-0.102727842536694	0.59254997234535	1	pseudobulk	DESeq2	LRT	0.227275017809563
730	KC_AB1	rgn	-0.233197772959657	0.077756344259249	1	pseudobulk	DESeq2	LRT	1.10926416610335
730	KC_AB1	rgr	-2.39599323879458	0.0215225125178428	1	pseudobulk	DESeq2	LRT	1.66710703091731
730	KC_AB1	Rh2	-0.850996402080018	0.900449987182352	1	pseudobulk	DESeq2	LRT	0.045540403770502
730	KC_AB1	Rh4	1.08899820779554	0.670846322504933	1	pseudobulk	DESeq2	LRT	0.173376956629912
730	KC_AB1	Rh5	3.29233314315581	0.0541994257579063	1	pseudobulk	DESeq2	LRT	1.26600531478069
730	KC_AB1	Rh50	-0.615767568564188	0.806245159731688	1	pseudobulk	DESeq2	LRT	0.093532879622572
730	KC_AB1	Rh7	-0.590750588069125	0.661175010642884	1	pseudobulk	DESeq2	LRT	0.179683569112978
730	KC_AB1	rha	1.08899820779554	0.697631521651407	1	pseudobulk	DESeq2	LRT	0.156373904552
730	KC_AB1	Rhau	-0.242626492111986	0.782264677124058	1	pseudobulk	DESeq2	LRT	0.106646279720036
730	KC_AB1	rhea	0.0177737037994807	0.914237980316713	1	pseudobulk	DESeq2	LRT	0.038940740715310
731	KC_AB1	Rheb	0.18228002655051	0.552758588848618	1	pseudobulk	DESeq2	LRT	0.257464500561856
731	KC_AB1	rho	-1.32489642030663	0.55306199336977	1	pseudobulk	DESeq2	LRT	0.257226185387141
731	KC_AB1	rho-4	-0.321453434871889	0.651887543111545	1	pseudobulk	DESeq2	LRT	0.185827317793387
731	KC_AB1	rho-5	0.318608511898098	0.287863563394462	1	pseudobulk	DESeq2	LRT	0.540813302882129
731	KC_AB1	rho-7	0.0886364251946818	0.849670624202155	1	pseudobulk	DESeq2	LRT	0.070749396419690
731	KC_AB1	Rho1	-0.276988869407716	0.12670968937706	1	pseudobulk	DESeq2	LRT	0.897190173734604
731	KC_AB1	RhoBTB	0.183988023854874	0.477864813396163	1	pseudobulk	DESeq2	LRT	0.320694946693006
731	KC_AB1	RhoGAP100F	0.00705402308996200	0.956933962647477	1	pseudobulk	DESeq2	LRT	0.019118031551640
731	KC_AB1	RhoGAP102A	-0.452753913190324	0.117001355282005	1	pseudobulk	DESeq2	LRT	0.931809107586427
731	KC_AB1	RhoGAP15B	-1.99275908811736	0.412167844949191	1	pseudobulk	DESeq2	LRT	0.384925892483647
732	KC_AB1	RhoGAP16F	0.488542553981275	0.482079022236527	1	pseudobulk	DESeq2	LRT	0.316881766515623
732	KC_AB1	RhoGAP18B	-0.82542030717083	1	1	pseudobulk	DESeq2	LRT	0
732	KC_AB1	RhoGAP19D	0.149157192535313	0.341117367246879	1	pseudobulk	DESeq2	LRT	0.467096168873233
732	KC_AB1	RhoGAP1A	-0.0711581920361165	0.79904668918514	1	pseudobulk	DESeq2	LRT	0.097427843635871
732	KC_AB1	RhoGAP54D	0.187499809431982	0.910303214736121	1	pseudobulk	DESeq2	LRT	0.040813923554985
732	KC_AB1	RhoGAP5A	0.373879951327731	0.557409707264725	1	pseudobulk	DESeq2	LRT	0.253825472338188
732	KC_AB1	RhoGAP68F	0.304692639157106	0.325803930885001	1	pseudobulk	DESeq2	LRT	0.487043680152229
732	KC_AB1	RhoGAP71E	0.0927262981166356	0.477538071338767	1	pseudobulk	DESeq2	LRT	0.320991998921615
732	KC_AB1	RhoGAP92B	-0.0730276023066689	0.840635361849055	1	pseudobulk	DESeq2	LRT	0.075392345078976
732	KC_AB1	RhoGAP93B	0.0487365638616188	0.768838184539	1	pseudobulk	DESeq2	LRT	0.114165055460781
733	KC_AB1	RhoGAPp190	0.229824547005552	0.109632487781669	1	pseudobulk	DESeq2	LRT	0.960060730764265

733	KC_AB1	RhoGDI	-0.082072871969409	0.59449328664018	1	pseudobulk	DESeq2	LRT	0.225853045320457
733	KC_AB1	RhoGEF2	0.718303495083666	0.399137072525786	1	pseudobulk	DESeq2	LRT	0.398877932337149
733	KC_AB1	RhoGEF3	0.0771568184561576	0.76392195655925	1	pseudobulk	DESeq2	LRT	0.116951007349612
733	KC_AB1	RhoGEF64C	1.64954027140197	0.477780694739395	1	pseudobulk	DESeq2	LRT	0.320771402383411
733	KC_AB1	RhoU	0.67282618419264	0.257863094290357	1	pseudobulk	DESeq2	LRT	0.588610810219428
733	KC_AB1	Rhp	0.457146793672889	0.54406427248271	1	pseudobulk	DESeq2	LRT	0.264349792331661
733	KC_AB1	Ric	-0.0098302352833904	0.92859653453535	1	pseudobulk	DESeq2	LRT	0.03217294142688
733	KC_AB1	RIC-3	-0.115894309139931	0.611728845231172	1	pseudobulk	DESeq2	LRT	0.213441040461216
733	KC_AB1	ric8a	0.775404039936966	0.24150016608399	1	pseudobulk	DESeq2	LRT	0.617082566240272
734	KC_AB1	Rich	0.0729385684645381	0.91215710531057	1	pseudobulk	DESeq2	LRT	0.039930354551705
734	KC_AB1	rictor	0.230638948068413	0.661078316925788	1	pseudobulk	DESeq2	LRT	0.179747087270911
734	KC_AB1	Rif1	0.177626491261002	0.919633239009805	1	pseudobulk	DESeq2	LRT	0.036385340080376
734	KC_AB1	Rift	0.181292912762134	0.759663307463333	1	pseudobulk	DESeq2	LRT	0.11937884996934
734	KC_AB1	rig	-0.240142025331779	0.727955556821833	1	pseudobulk	DESeq2	LRT	0.137895134445507
734	KC_AB1	Rilpl	-0.164999366855705	0.578710715422891	1	pseudobulk	DESeq2	LRT	0.237538476153908
734	KC_AB1	Rim	-0.0755026078632202	0.482332370542543	1	pseudobulk	DESeq2	LRT	0.316653590501409
734	KC_AB1	Rim2	0.0171151776976247	0.922697353977853	1	pseudobulk	DESeq2	LRT	0.034940724814687
734	KC_AB1	rin	-0.0323002449348677	0.812675814344514	1	pseudobulk	DESeq2	LRT	0.090082664884815
734	KC_AB1	ringer	0.119453573175033	0.4031912509279	1	pseudobulk	DESeq2	LRT	0.394488900458762
735	KC_AB1	Rint1	0.391478831819632	0.705243260250529	1	pseudobulk	DESeq2	LRT	0.151661055544792
735	KC_AB1	RIOK1	-0.207977835800412	0.830901204447662	1	pseudobulk	DESeq2	LRT	0.080450611491382
735	KC_AB1	RIOK2	0.56767555914453	0.327816410880503	1	pseudobulk	DESeq2	LRT	0.484369308921574
735	KC_AB1	Rip11	0.248173763337996	0.479331030662914	1	pseudobulk	DESeq2	LRT	0.319364454985995
735	KC_AB1	Ripalpha	-0.0878430463488033	0.811209865533351	1	pseudobulk	DESeq2	LRT	0.090866776303833
735	KC_AB1	rk	-0.23756292170449	0.552485772557363	1	pseudobulk	DESeq2	LRT	0.25767890131595
735	KC_AB1	rl	0.0685013398916369	0.60843093485587	1	pseudobulk	DESeq2	LRT	0.215788712936496
735	KC_AB1	Rlb1	0.0995266514739726	0.859083221593785	1	pseudobulk	DESeq2	LRT	0.065964762911152
735	KC_AB1	Rlip	0.15308437149815	0.505384668176029	1	pseudobulk	DESeq2	LRT	0.296377937381571
735	KC_AB1	RluA-2	0.106734888899055	0.886030384983745	1	pseudobulk	DESeq2	LRT	0.052551384428409
736	KC_AB1	Rm62	0.100583202265219	0.416415930903684	1	pseudobulk	DESeq2	LRT	0.380472663956854
736	KC_AB1	Rme-8	0.244676781580641	0.623011765719903	1	pseudobulk	DESeq2	LRT	0.205503751512613
736	KC_AB1	rn	-1.87372289452956	0.439914075902828	1	pseudobulk	DESeq2	LRT	0.356632141707781
736	KC_AB1	RN-tre	-0.0583680959816622	0.84766953238885	1	pseudobulk	DESeq2	LRT	0.071773426319147
736	KC_AB1	RNASEK	-0.213917395101524	0.0645113238548343	1	pseudobulk	DESeq2	LRT	1.19036404573655
736	KC_AB1	RNaseMRP:RNA	-0.368712653985904	0.547078033792567	1	pseudobulk	DESeq2	LRT	0.261950722609104
736	KC_AB1	RNaseP:RNA	-1.29589268592755	0.501430226769231	1	pseudobulk	DESeq2	LRT	0.299789489836497
736	KC_AB1	RNaseX25	0.329549415682736	0.441240774694655	1	pseudobulk	DESeq2	LRT	0.355324361583924
736	KC_AB1	RNaseZ	0.49045854069518	0.557917985869783	1	pseudobulk	DESeq2	LRT	0.25342963780438
736	KC_AB1	Rnb	0.21503919871424	0.790456484637505	1	pseudobulk	DESeq2	LRT	0.102122033387673
737	KC_AB1	Rnf146	0.0556747848994497	0.877930511682016	1	pseudobulk	DESeq2	LRT	0.056539857200204
737	KC_AB1	rngo	0.514443894734283	0.574404199835361	1	pseudobulk	DESeq2	LRT	0.240782393350272
737	KC_AB1	rnh1	-0.183681197108315	0.782488756454591	1	pseudobulk	DESeq2	LRT	0.10652189409437
737	KC_AB1	Rnmt	-0.38288650920409	0.621449729956132	1	pseudobulk	DESeq2	LRT	0.206593996363681
737	KC_AB1	rno	0.113954799290476	0.588782039329052	1	pseudobulk	DESeq2	LRT	0.230045446529347
737	KC_AB1	Rnp4F	-0.355593950139605	0.38616797203001	1	pseudobulk	DESeq2	LRT	0.413223748545833
737	KC_AB1	RnpS1	-0.213846499513948	0.485085844114891	1	pseudobulk	DESeq2	LRT	0.314181398869076
737	KC_AB1	ro	-0.82542030717083	1	1	pseudobulk	DESeq2	LRT	0
737	KC_AB1	robl	-0.337716021424622	0.127548416420229	1	pseudobulk	DESeq2	LRT	0.894324929012055
737	KC_AB1	robo1	-0.0427127642879712	0.863197089993088	1	pseudobulk	DESeq2	LRT	0.063890032414666
738	KC_AB1	robo2	-1.6747012584303	0.563129890257477	1	pseudobulk	DESeq2	LRT	0.249391420218277
738	KC_AB1	robo3	-0.0991508572497728	0.903937877844827	1	pseudobulk	DESeq2	LRT	0.043861414918703
738	KC_AB1	Roc1a	-0.154863157539465	0.541677185225543	1	pseudobulk	DESeq2	LRT	0.266259455975125

738	KC_AB1	Roc1b	-2.71522716927994	0.148736388787011	1	pseudobulk	DESeq2	LRT	0.827582767079494
738	KC_AB1	Roc2	0.0154342200626255	0.932134561435214	1	pseudobulk	DESeq2	LRT	0.030521389072534
738	KC_AB1	rod	1.88952324269138	0.456767015650974	1	pseudobulk	DESeq2	LRT	0.340305265178099
738	KC_AB1	Roe1	-0.036289877638435	0.909899703732253	1	pseudobulk	DESeq2	LRT	0.041006476378036
738	KC_AB1	rogdi	-0.073068350059176	0.832980841350055	1	pseudobulk	DESeq2	LRT	0.079364987298659
738	KC_AB1	roh	-0.281025301368259	0.0788782960091105	1	pseudobulk	DESeq2	LRT	1.10304247993594
738	KC_AB1	Rok	0.301172445729699	0.312495613998619	1	pseudobulk	DESeq2	LRT	0.505156073774513
739	KC_AB1	Rop	-0.430523837391837	0.0145312817456254	1	pseudobulk	DESeq2	LRT	1.83769607665164
739	KC_AB1	roq	0.109768926291163	0.704713419626196	1	pseudobulk	DESeq2	LRT	0.151987458296807
739	KC_AB1	Ror	-0.346502470683412	0.520052978131549	1	pseudobulk	DESeq2	LRT	0.283952412253224
739	KC_AB1	row	0.0965171804053238	0.776229186439028	1	pseudobulk	DESeq2	LRT	0.110010031692484
739	KC_AB1	Rox8	0.15358085291675	0.497100910267539	1	pseudobulk	DESeq2	LRT	0.303555441600877
739	KC_AB1	RpA-70	-0.087711224462254	0.877995426071064	1	pseudobulk	DESeq2	LRT	0.056507746550926
739	KC_AB1	RPA2	-0.751981682263106	0.306608910480826	1	pseudobulk	DESeq2	LRT	0.513415228097355
739	KC_AB1	RPA3	0.0907437941086417	0.837702717406013	1	pseudobulk	DESeq2	LRT	0.076910075756690
739	KC_AB1	Rpb10	0.110398350616497	0.696927241102933	1	pseudobulk	DESeq2	LRT	0.156812559688071
739	KC_AB1	Rpb11	0.0693589004061557	0.808425786092999	1	pseudobulk	DESeq2	LRT	0.092359842386699
740	KC_AB1	Rpb12	0.168969802796325	0.460641313648046	1	pseudobulk	DESeq2	LRT	0.33663711395157
740	KC_AB1	Rpb4	-0.970244467477917	0.553481626179277	1	pseudobulk	DESeq2	LRT	0.25689679173449
740	KC_AB1	Rpb5	-0.199648778050184	0.719108384675878	1	pseudobulk	DESeq2	LRT	0.143205647420739
740	KC_AB1	Rpb7	0.355985890718613	0.316204715276066	1	pseudobulk	DESeq2	LRT	0.500031658113147
740	KC_AB1	Rpb8	0.357869802384399	0.487340686199438	1	pseudobulk	DESeq2	LRT	0.312167329529246
740	KC_AB1	Rpe	0.568115879134733	0.588604355853384	1	pseudobulk	DESeq2	LRT	0.23017652831169
740	KC_AB1	Rph	0.212637120809213	0.564383154794963	1	pseudobulk	DESeq2	LRT	0.248425957158601
740	KC_AB1	Rpi	1.27763142692614	0.178853650888728	1	pseudobulk	DESeq2	LRT	0.74750219029474
740	KC_AB1	RpI1	0.169779487434368	0.849664728402501	1	pseudobulk	DESeq2	LRT	0.070752409966430
740	KC_AB1	RpI12	0.866143204781726	0.641643690690731	1	pseudobulk	DESeq2	LRT	0.192706071812621
741	KC_AB1	RpI135	1.39856744191113	0.333620653613134	1	pseudobulk	DESeq2	LRT	0.476747071126138
741	KC_AB1	RpII140	-0.095800871564363	0.851800225830998	1	pseudobulk	DESeq2	LRT	0.069662249121223
741	KC_AB1	RpII15	-0.245983650477658	0.643823837408291	1	pseudobulk	DESeq2	LRT	0.191232947713728
741	KC_AB1	RpII18	0.255410031094647	0.319758717389316	1	pseudobulk	DESeq2	LRT	0.495177606779036
741	KC_AB1	RpII215	0.0527767406934849	0.825257405109924	1	pseudobulk	DESeq2	LRT	0.083410570016192
741	KC_AB1	RpII33	-0.197095813962714	0.630034399612246	1	pseudobulk	DESeq2	LRT	0.200635737603682
741	KC_AB1	RpIII128	0.647835165111655	0.688037038550395	1	pseudobulk	DESeq2	LRT	0.162388182105937
741	KC_AB1	RpIIIC160	-1.29094316301828	0.345115806504968	1	pseudobulk	DESeq2	LRT	0.462035149312743
741	KC_AB1	RpIIIC53	-0.141942714014203	0.846321467463001	1	pseudobulk	DESeq2	LRT	0.072464642839402
741	KC_AB1	RpL10	-0.055901748655480	0.635815733763183	1	pseudobulk	DESeq2	LRT	0.196668729327845
742	KC_AB1	RpL10Ab	0.0493656707141915	0.694571369506254	1	pseudobulk	DESeq2	LRT	0.158283122438606
742	KC_AB1	RpL11	0.0428688646902258	0.794568618752875	1	pseudobulk	DESeq2	LRT	0.099868591271077
742	KC_AB1	RpL12	-0.073483889990324	0.562157085273664	1	pseudobulk	DESeq2	LRT	0.250142311236964
742	KC_AB1	RpL13	-0.038059834479046	0.744624541560303	1	pseudobulk	DESeq2	LRT	0.128062654273179
742	KC_AB1	RpL13A	0.0665056292968778	0.582831581980055	1	pseudobulk	DESeq2	LRT	0.234456923085907
742	KC_AB1	RpL14	0.004677655724780	0.958649189261799	1	pseudobulk	DESeq2	LRT	0.018340290682338
742	KC_AB1	RpL15	0.115289485449354	0.266248129549793	1	pseudobulk	DESeq2	LRT	0.574713434554359
742	KC_AB1	RpL17	-0.053721363770701	0.66446070886742	1	pseudobulk	DESeq2	LRT	0.177530694821433
742	KC_AB1	RpL18	0.04411597376261	0.720425420636241	1	pseudobulk	DESeq2	LRT	0.142410971244971
742	KC_AB1	RpL18A	0.0685100757140553	0.540157248271099	1	pseudobulk	DESeq2	LRT	0.26747979181589
743	KC_AB1	RpL19	-0.105852352712712	0.3244884523093	1	pseudobulk	DESeq2	LRT	0.488800753987142
743	KC_AB1	RpL21	0.0856444833414127	0.408314935973386	1	pseudobulk	DESeq2	LRT	0.389004733493159
743	KC_AB1	RpL22	-0.152183088034198	0.429798679001646	1	pseudobulk	DESeq2	LRT	0.366734923658855
743	KC_AB1	RpL23	-0.105228690012771	0.32670304653825	1	pseudobulk	DESeq2	LRT	0.485846815666645
743	KC_AB1	RpL23A	0.008971609046120	0.941264610667712	1	pseudobulk	DESeq2	LRT	0.026288269454598

743	KC_AB1	RpL24	0.0380697485152097	0.723917481210976	1	pseudobulk	DESeq2	LRT	0.140310935870796
743	KC_AB1	RpL24-like	0.226963557880069	0.166847408333567	1	pseudobulk	DESeq2	LRT	0.77768053491972
743	KC_AB1	RpL26	-0.0788759913090916	0.445297765155331	1	pseudobulk	DESeq2	LRT	0.351349484485088
743	KC_AB1	RpL27	-0.0369449635029228	0.762651205464706	1	pseudobulk	DESeq2	LRT	0.117674038946698
743	KC_AB1	RpL27A	-0.0748464872535807	0.499001452111968	1	pseudobulk	DESeq2	LRT	0.301898190562388
744	KC_AB1	RpL28	0.0650305771848119	0.566227412543532	1	pseudobulk	DESeq2	LRT	0.247009109135156
744	KC_AB1	RpL29	-0.0816890018288255	0.435808202604432	1	pseudobulk	DESeq2	LRT	0.36070459989904
744	KC_AB1	RpL3	-0.0215305244076682	0.883014460955496	1	pseudobulk	DESeq2	LRT	0.054032184008219
744	KC_AB1	RpL30	-0.0486166814434135	0.803576830021852	1	pseudobulk	DESeq2	LRT	0.094972593997860
744	KC_AB1	RpL31	-0.031163030288467	0.78163717795782	1	pseudobulk	DESeq2	LRT	0.106994791922429
744	KC_AB1	RpL32	0.123235823036451	0.289372407911457	1	pseudobulk	DESeq2	LRT	0.538542881866197
744	KC_AB1	RpL34a	0.412460213072092	0.0731551881406515	1	pseudobulk	DESeq2	LRT	1.13575486843194
744	KC_AB1	RpL34b	0.0195550941136943	0.876972522876876	1	pseudobulk	DESeq2	LRT	0.057014013646380
744	KC_AB1	RpL35	-0.0468168171604793	0.73147582223072	1	pseudobulk	DESeq2	LRT	0.135800024214427
744	KC_AB1	RpL35A	-0.0527620461402032	0.693675760717721	1	pseudobulk	DESeq2	LRT	0.158843480893953
745	KC_AB1	RpL36	0.0325402345106777	0.741781161817059	1	pseudobulk	DESeq2	LRT	0.129724200156486
745	KC_AB1	RpL36A	-0.0795610340873707	0.458557623916097	1	pseudobulk	DESeq2	LRT	0.338606081637776
745	KC_AB1	RpL37a	-0.0456238778705288	0.674001543784469	1	pseudobulk	DESeq2	LRT	0.171339108722383
745	KC_AB1	RpL37A	-0.0872073168382847	0.437817623763624	1	pseudobulk	DESeq2	LRT	0.358706760471967
745	KC_AB1	RpL38	0.0208071845844553	0.82386431914067	1	pseudobulk	DESeq2	LRT	0.084144305657748
745	KC_AB1	RpL39	-0.0955531449945108	0.396050396982382	1	pseudobulk	DESeq2	LRT	0.402249547057475
745	KC_AB1	RpL4	-0.0285180901319155	0.816215689857334	1	pseudobulk	DESeq2	LRT	0.08819506117636
745	KC_AB1	RpL40	0.0073180468675741	0.924319219542034	1	pseudobulk	DESeq2	LRT	0.034178016503297
745	KC_AB1	RpL41	0.0458490441593262	0.622084549729677	1	pseudobulk	DESeq2	LRT	0.206150584785718
745	KC_AB1	RpL5	0.128705815763712	0.306508541239074	1	pseudobulk	DESeq2	LRT	0.513557418824802
746	KC_AB1	RpL6	-0.0154921336902509	0.881759189937567	1	pseudobulk	DESeq2	LRT	0.054650005299967
746	KC_AB1	RpL7	0.0631784515836287	0.584009649774961	1	pseudobulk	DESeq2	LRT	0.233579976844118
746	KC_AB1	RpL7-like	1.18653848769441	0.60665041788826	1	pseudobulk	DESeq2	LRT	0.21706149890377
746	KC_AB1	RpL7A	-0.11048214995575	0.344006629519818	1	pseudobulk	DESeq2	LRT	0.463433187846692
746	KC_AB1	RpL8	0.0752163632324861	0.496008968224018	1	pseudobulk	DESeq2	LRT	0.304510471060221
746	KC_AB1	RpL9	0.0547905245965366	0.637012086729361	1	pseudobulk	DESeq2	LRT	0.195852327240684
746	KC_AB1	RpLP0	-0.0897734500202504	0.484492869905241	1	pseudobulk	DESeq2	LRT	0.314712609910624
746	KC_AB1	RpLP0-like	0.14730468512827	0.828632236091265	1	pseudobulk	DESeq2	LRT	0.081638175449706
746	KC_AB1	RpLP1	0.0079796164813910	0.946589695927787	1	pseudobulk	DESeq2	LRT	0.023838227341149
746	KC_AB1	RpLP2	-0.226450551715375	0.0800853691628247	1	pseudobulk	DESeq2	LRT	1.09644681815086
747	KC_AB1	Rpn1	0.288247987045007	0.530743836501107	1	pseudobulk	DESeq2	LRT	0.275115040570538
747	KC_AB1	Rpn10	0.0681284388529326	0.843385870678994	1	pseudobulk	DESeq2	LRT	0.073973679035580
747	KC_AB1	Rpn11	-0.316971825500134	0.398830692019227	1	pseudobulk	DESeq2	LRT	0.399211427938881
747	KC_AB1	Rpn12	-0.129519169073633	0.717901037215318	1	pseudobulk	DESeq2	LRT	0.14393541920268
747	KC_AB1	Rpn13	0.0592935420246001	0.846388104471404	1	pseudobulk	DESeq2	LRT	0.072430449040851
747	KC_AB1	Rpn13R	0.125541479044925	0.922109998361781	1	pseudobulk	DESeq2	LRT	0.035217268933259
747	KC_AB1	Rpn2	-0.370207211942817	0.273892054834771	1	pseudobulk	DESeq2	LRT	0.56242056574335
747	KC_AB1	Rpn3	-0.0135623321293649	0.938909624231521	1	pseudobulk	DESeq2	LRT	0.027376209210458
747	KC_AB1	Rpn5	-0.173364007335965	0.553512067904058	1	pseudobulk	DESeq2	LRT	0.256872906010179
747	KC_AB1	Rpn6	-0.286830309430851	0.388619430155438	1	pseudobulk	DESeq2	LRT	0.410475489381284
748	KC_AB1	Rpn7	-0.3152131811916	0.507604379361734	1	pseudobulk	DESeq2	LRT	0.294474639681291
748	KC_AB1	Rpn8	-0.0394321071809557	0.89957973938225	1	pseudobulk	DESeq2	LRT	0.045960334443137
748	KC_AB1	Rpn9	-0.216802741832704	0.528246244677596	1	pseudobulk	DESeq2	LRT	0.277163581680467
748	KC_AB1	Rpp30	-0.57077478707295	0.502686950061781	1	pseudobulk	DESeq2	LRT	0.298702389070159
748	KC_AB1	RpS10b	-0.134418251500534	0.23564922958224	1	pseudobulk	DESeq2	LRT	0.627733975758912
748	KC_AB1	RpS11	0.0235799774543535	0.819915517422195	1	pseudobulk	DESeq2	LRT	0.086230894210849
748	KC_AB1	RpS12	0.0062338683077410	0.953621008050099	1	pseudobulk	DESeq2	LRT	0.020624190091531

748	KC_AB1	RpS13	-0.0425348115913935	0.739724149385179	1	pseudobulk	DESeq2	LRT	0.13093020288315
748	KC_AB1	RpS14a	0.0624717645687708	0.601958842496178	1	pseudobulk	DESeq2	LRT	0.220433201579131
748	KC_AB1	RpS14b	-0.003213295548972	0.986803769317672	1	pseudobulk	DESeq2	LRT	0.00576920029424
749	KC_AB1	RpS15	-0.0957638441702067	0.482575695042349	1	pseudobulk	DESeq2	LRT	0.3164345551475
749	KC_AB1	RpS15Aa	-0.0255236614821696	0.829609924705837	1	pseudobulk	DESeq2	LRT	0.08112606107996
749	KC_AB1	RpS15Ab	0.0117819169675853	0.969266147781151	1	pseudobulk	DESeq2	LRT	0.01355695500083
749	KC_AB1	RpS16	-0.0535688233560487	0.676844482950111	1	pseudobulk	DESeq2	LRT	0.169511106728881
749	KC_AB1	RpS17	-0.041616824445929	0.70818648176679	1	pseudobulk	DESeq2	LRT	0.149852367538542
749	KC_AB1	RpS18	0.0239036506735035	0.863847908342704	1	pseudobulk	DESeq2	LRT	0.06356271398160
749	KC_AB1	RpS19a	-0.168929139195772	0.232178065956022	1	pseudobulk	DESeq2	LRT	0.634178810799891
749	KC_AB1	RpS2	-0.083429378128712	0.468156664907047	1	pseudobulk	DESeq2	LRT	0.329608789406512
749	KC_AB1	RpS20	-0.166770926968554	0.147813479317952	1	pseudobulk	DESeq2	LRT	0.830285960213986
749	KC_AB1	RpS21	-0.0603824639342667	0.620708001542201	1	pseudobulk	DESeq2	LRT	0.20711265609160
750	KC_AB1	RpS23	-0.0637572820956321	0.569817262427571	1	pseudobulk	DESeq2	LRT	0.244264398087981
750	KC_AB1	RpS24	-0.103760863655486	0.3751522364705	1	pseudobulk	DESeq2	LRT	0.425792460159032
750	KC_AB1	RpS25	-0.0433271716907354	0.650680733655827	1	pseudobulk	DESeq2	LRT	0.186632052326902
750	KC_AB1	RpS26	0.0746377796115359	0.442460171739271	1	pseudobulk	DESeq2	LRT	0.354125816429077
750	KC_AB1	RpS27	-0.056770063677533	0.588477672100653	1	pseudobulk	DESeq2	LRT	0.230270010420266
750	KC_AB1	RpS27A	-0.002921386927037	0.975678496495899	1	pseudobulk	DESeq2	LRT	0.010693266554666
750	KC_AB1	RpS28b	-0.0577167146442208	0.563112511720812	1	pseudobulk	DESeq2	LRT	0.249404823021376
750	KC_AB1	RpS29	0.056057392856647	0.569499038861729	1	pseudobulk	DESeq2	LRT	0.24450700453905
750	KC_AB1	RpS3	-0.029183867671578	0.828976573721671	1	pseudobulk	DESeq2	LRT	0.081457742124228
750	KC_AB1	RpS30	-0.0612773613236191	0.557030949501411	1	pseudobulk	DESeq2	LRT	0.254120674082525
751	KC_AB1	RpS3A	0.0477219903330015	0.6671481603534	1	pseudobulk	DESeq2	LRT	0.175777707195158
751	KC_AB1	RpS4	-0.139358613104186	0.200605883681589	1	pseudobulk	DESeq2	LRT	0.697656333464281
751	KC_AB1	RpS5a	0.0975704951093746	0.520229012661367	1	pseudobulk	DESeq2	LRT	0.283805431287811
751	KC_AB1	RpS5b	1.08899820779554	0.878177167143508	1	pseudobulk	DESeq2	LRT	0.056417858865076
751	KC_AB1	RpS6	0.0247948660882807	0.816756979807	1	pseudobulk	DESeq2	LRT	0.087907145461386
751	KC_AB1	RpS7	0.0055096956858959	0.962538763303419	1	pseudobulk	DESeq2	LRT	0.016581771585915
751	KC_AB1	RpS8	0.001766043034412	0.98562814311273	1	pseudobulk	DESeq2	LRT	0.00628690438412
751	KC_AB1	RpS9	-0.243426866136855	0.0741099443804122	1	pseudobulk	DESeq2	LRT	1.13012351267156
751	KC_AB1	Rpt1	-0.220978852740334	0.460713475592678	1	pseudobulk	DESeq2	LRT	0.33656908470857
751	KC_AB1	Rpt2	-0.017065410665319	0.942746078978524	1	pseudobulk	DESeq2	LRT	0.025605265214075
752	KC_AB1	Rpt3	0.116910345507163	0.649540311444883	1	pseudobulk	DESeq2	LRT	0.187393890788332
752	KC_AB1	Rpt4	-0.0625634519992798	0.837987946004199	1	pseudobulk	DESeq2	LRT	0.076762228412101
752	KC_AB1	Rpt5	-0.278834978475369	0.475456968741461	1	pseudobulk	DESeq2	LRT	0.322888782793438
752	KC_AB1	Rpt6	-0.115569254129776	0.706170226806718	1	pseudobulk	DESeq2	LRT	0.15109059689205
752	KC_AB1	Rrp1	-0.723760200430699	0.377695481519986	1	pseudobulk	DESeq2	LRT	0.422858210731582
752	KC_AB1	Rrp4	0.339232933052958	0.750941585809324	1	pseudobulk	DESeq2	LRT	0.124393844550225
752	KC_AB1	Rrp40	0.972176376307916	0.358777492102507	1	pseudobulk	DESeq2	LRT	0.445174810227424
752	KC_AB1	Rrp42	0.224541392050063	0.793578833082096	1	pseudobulk	DESeq2	LRT	0.100409924521016
752	KC_AB1	Rrp45	-0.30053136469145	0.862895403201731	1	pseudobulk	DESeq2	LRT	0.06404184456911
752	KC_AB1	Rrp46	-0.555489529439484	0.650299082300517	1	pseudobulk	DESeq2	LRT	0.186886858866318
753	KC_AB1	Rrp47	-0.199596099975597	0.569579182189901	1	pseudobulk	DESeq2	LRT	0.244445892308827
753	KC_AB1	Rrp6	-0.091811126376614	0.889225085940972	1	pseudobulk	DESeq2	LRT	0.05098829391269
753	KC_AB1	Rs1	-0.129420329738187	0.81637037146345	1	pseudobulk	DESeq2	LRT	0.088112765523125
753	KC_AB1	Rsf1	-0.0158536368175687	0.89799072452745	1	pseudobulk	DESeq2	LRT	0.046728149198341
753	KC_AB1	RSG7	-0.177741751229119	0.201866752087739	1	pseudobulk	DESeq2	LRT	0.69493520445341
753	KC_AB1	rswl	0.518295969372636	0.410588920497047	1	pseudobulk	DESeq2	LRT	0.386592773991037
753	KC_AB1	rt	-0.0720846258511338	0.920805862288647	1	pseudobulk	DESeq2	LRT	0.035831924443961
753	KC_AB1	Rtc1	-0.3454905219473	0.735065759445203	1	pseudobulk	DESeq2	LRT	0.133673806920302
753	KC_AB1	Rtca	0.0861287474598586	0.80243196332415	1	pseudobulk	DESeq2	LRT	0.095591780362978

753	KC_AB1	RtcB	0.0568462092248729	0.92080646763979	1	pseudobulk	DESeq2	LRT	0.035831638932557
754	KC_AB1	rtet	-0.457656316822457	0.641533640029913	1	pseudobulk	DESeq2	LRT	0.192780565639306
754	KC_AB1	Rtf1	0.238499444149534	0.569313872492281	1	pseudobulk	DESeq2	LRT	0.244648233589744
754	KC_AB1	RtGEF	-0.131239747087604	0.800339291749513	1	pseudobulk	DESeq2	LRT	0.096725861387789
754	KC_AB1	Rtnl1	0.128468889962451	0.250257568703113	1	pseudobulk	DESeq2	LRT	0.601612778998333
754	KC_AB1	Rubicon	-0.198023482197332	0.608802039726892	1	pseudobulk	DESeq2	LRT	0.215523901177284
754	KC_AB1	rudhira	0.231417457118241	0.0994584117278033	1	pseudobulk	DESeq2	LRT	1.00235848038791
754	KC_AB1	rumi	-0.0568293849921701	0.945629034116223	1	pseudobulk	DESeq2	LRT	0.024279201899701
754	KC_AB1	rump	-0.0107816986984314	0.95592806635127	1	pseudobulk	DESeq2	LRT	0.019574787182160
754	KC_AB1	rush	-0.0225678472435924	0.962120061628636	1	pseudobulk	DESeq2	LRT	0.016770729574142
754	KC_AB1	rut	0.0459748342767551	0.653940879655333	1	pseudobulk	DESeq2	LRT	0.184461512837875
755	KC_AB1	rux	-0.0447253773463071	0.911258231095255	1	pseudobulk	DESeq2	LRT	0.040358535817338
755	KC_AB1	RYa-R	-0.700952324425246	0.670670247568059	1	pseudobulk	DESeq2	LRT	0.173490959506404
755	KC_AB1	RYBP	0.0336732113870079	0.934896152619683	1	pseudobulk	DESeq2	LRT	0.029236627468648
755	KC_AB1	RyR	-0.303977781771641	0.243522809110295	1	pseudobulk	DESeq2	LRT	0.613460355160574
755	KC_AB1	S	-0.278233161220048	0.289946599739484	1	pseudobulk	DESeq2	LRT	0.537681979942281
755	KC_AB1	s-cup	1.46212838815867	0.153730288311474	1	pseudobulk	DESeq2	LRT	0.813240558325672
755	KC_AB1	S-Lap1	-0.93665198022647	0.628709176344405	1	pseudobulk	DESeq2	LRT	0.201550200844889
755	KC_AB1	S-Lap4	2.211521544984	0.400335997833192	1	pseudobulk	DESeq2	LRT	0.39757535679119
755	KC_AB1	S1P	0.137007703896631	0.846095133200617	1	pseudobulk	DESeq2	LRT	0.072580803035145
755	KC_AB1	S2P	0.0978610256502183	0.886084814164352	1	pseudobulk	DESeq2	LRT	0.052524706374058
756	KC_AB1	S6k	0.178316693907936	0.474411252058968	1	pseudobulk	DESeq2	LRT	0.323845018949932
756	KC_AB1	S6kII	-0.208778472890997	0.475832620292838	1	pseudobulk	DESeq2	LRT	0.32254578859642
756	KC_AB1	S6KL	0.372696400073351	0.435015605780387	1	pseudobulk	DESeq2	LRT	0.36149516285516
756	KC_AB1	SA	-0.257607992728661	0.612288779511845	1	pseudobulk	DESeq2	LRT	0.21304369914787
756	KC_AB1	Sac1	0.101966379586425	0.801333724378037	1	pseudobulk	DESeq2	LRT	0.096186579456537
756	KC_AB1	Saf-B	0.023271017383489	0.885607883320671	1	pseudobulk	DESeq2	LRT	0.052758526199536
756	KC_AB1	Saf6	-0.0353701835946941	0.946230277347016	1	pseudobulk	DESeq2	LRT	0.024003159569678
756	KC_AB1	sage	0.967400803665067	0.478945344451838	1	pseudobulk	DESeq2	LRT	0.319714043907544
756	KC_AB1	SAK	-0.129140245876792	0.848841265046931	1	pseudobulk	DESeq2	LRT	0.071173516068820
756	KC_AB1	sals	0.628583476920153	0.324677835650749	1	pseudobulk	DESeq2	LRT	0.488547357764898
757	KC_AB1	Sam-S	0.215158821156337	0.248860553700604	1	pseudobulk	DESeq2	LRT	0.604043936930215
757	KC_AB1	SamDC	-0.0573903265996569	0.878501247653815	1	pseudobulk	DESeq2	LRT	0.056257617380089
757	KC_AB1	Samtor	0.38968830294911	0.612807230124622	1	pseudobulk	DESeq2	LRT	0.212676119379527
757	KC_AB1	san	-0.041137452675467	0.876888838025455	1	pseudobulk	DESeq2	LRT	0.057055458050172
757	KC_AB1	sano	-1.6747012584303	0.563129890257477	1	pseudobulk	DESeq2	LRT	0.249391420218277
757	KC_AB1	Sap-r	0.0699515255422277	0.80240630743471	1	pseudobulk	DESeq2	LRT	0.095605666137534
757	KC_AB1	Sap130	-0.598001423123526	0.135105893519185	1	pseudobulk	DESeq2	LRT	0.869325705996213
757	KC_AB1	Sap30	0.139183241488352	0.736918732673879	1	pseudobulk	DESeq2	LRT	0.13258040345347
757	KC_AB1	Sap47	-0.212113809845421	0.0638103593121186	1	pseudobulk	DESeq2	LRT	1.19510880988582
757	KC_AB1	Sar1	-0.127280728244527	0.559799561622904	1	pseudobulk	DESeq2	LRT	0.251967445964226
758	KC_AB1	Sara	0.264068305367054	0.52033382478467	1	pseudobulk	DESeq2	LRT	0.283717941469219
758	KC_AB1	Sas-4	0.607099950721679	0.732113787889941	1	pseudobulk	DESeq2	LRT	0.135421414006927
758	KC_AB1	Sas-6	-0.754795611867936	0.522993788646795	1	pseudobulk	DESeq2	LRT	0.281503469015022
758	KC_AB1	Sas10	-0.161834913572507	0.831084901942464	1	pseudobulk	DESeq2	LRT	0.080354607308977
758	KC_AB1	sau	0.29461620210849	0.206540063679649	1	pseudobulk	DESeq2	LRT	0.684995693415055
758	KC_AB1	sav	0.135879393043758	1	1	pseudobulk	DESeq2	LRT	0
758	KC_AB1	sax	0.187805362884331	0.659244227532063	1	pseudobulk	DESeq2	LRT	0.180953664283026
758	KC_AB1	sba	0.19740998770241	0.399759286075075	1	pseudobulk	DESeq2	LRT	0.398201439165661
758	KC_AB1	Sbat	-0.337893945591258	0.712077759119855	1	pseudobulk	DESeq2	LRT	0.147472578676716
758	KC_AB1	sbb	-0.0291433652773774	0.794018978446583	1	pseudobulk	DESeq2	LRT	0.100169117048819
759	KC_AB1	Sbf	0.0634094038592915	0.826905092262147	1	pseudobulk	DESeq2	LRT	0.082544333581835

759	KC_AB1	sbm	0.153419894024899	0.486251700055876	1	pseudobulk	DESeq2	LRT	0.313138867262956
759	KC_AB1	Sbp2	0.240342042809881	0.730121851370263	1	pseudobulk	DESeq2	LRT	0.136604653630823
759	KC_AB1	sbr	0.106852811616401	0.432479644626366	1	pseudobulk	DESeq2	LRT	0.36403432851011
759	KC_AB1	Sc2	0.279871330402705	0.483993020999496	1	pseudobulk	DESeq2	LRT	0.315160900676374
759	KC_AB1	SC35	0.112862096295744	0.594108246330704	1	pseudobulk	DESeq2	LRT	0.226134419494941
759	KC_AB1	sca	0.325612409011196	0.186955003289461	1	pseudobulk	DESeq2	LRT	0.728262907766094
759	KC_AB1	scaf	2.72432631526949	0.238668299696773	1	pseudobulk	DESeq2	LRT	0.622205260846706
759	KC_AB1	scaf6	-0.116928289585031	0.568032512618095	1	pseudobulk	DESeq2	LRT	0.245626805756363
759	KC_AB1	SCaMC	-0.0258346999035619	0.917944794221318	1	pseudobulk	DESeq2	LRT	0.037183436757351
760	KC_AB1	Scamp	-0.0177498793600612	0.933430514682117	1	pseudobulk	DESeq2	LRT	0.029918005742011
760	KC_AB1	SCAP	0.226062543255051	0.331446795657141	1	pseudobulk	DESeq2	LRT	0.479586175274144
760	KC_AB1	SCAR	0.0207127842380559	0.929505651149368	1	pseudobulk	DESeq2	LRT	0.031747965488073
760	KC_AB1	scaRNA:MeU2-G2	0.135879393043758	1	1	pseudobulk	DESeq2	LRT	0
760	KC_AB1	scaRNA:PsiU5-44	1.08899820779554	0.670846322504933	1	pseudobulk	DESeq2	LRT	0.173376956629912
760	KC_AB1	scat	-0.0917776179271833	0.856599225582756	1	pseudobulk	DESeq2	LRT	0.067222322567616
760	KC_AB1	scb	0.658946517350553	0.588194537575052	1	pseudobulk	DESeq2	LRT	0.230479013001388
760	KC_AB1	Sccpdh1	-0.569965335579363	0.27293827000589	1	pseudobulk	DESeq2	LRT	0.563935565513289
760	KC_AB1	Sccpdh2	1.08899820779554	0.878177167143508	1	pseudobulk	DESeq2	LRT	0.056417858865076
760	KC_AB1	SCCRO	-0.220183029131792	0.567900745468548	1	pseudobulk	DESeq2	LRT	0.245727561229088
761	KC_AB1	SCCRO3	-0.307762984145709	0.536840005257939	1	pseudobulk	DESeq2	LRT	0.270155128056282
761	KC_AB1	SCCRO4	0.0155803378077848	0.925215991544928	1	pseudobulk	DESeq2	LRT	0.033756869438275
761	KC_AB1	Sce	0.0720907358275386	0.84351539014877	1	pseudobulk	DESeq2	LRT	0.073906989191380
761	KC_AB1	sced	-0.387410505007673	0.41337442404184	1	pseudobulk	DESeq2	LRT	0.383656397184077
761	KC_AB1	scf	-0.147629796127866	0.682182664910596	1	pseudobulk	DESeq2	LRT	0.166099320738245
761	KC_AB1	Scgalpha	-0.0993628673532521	0.767621027876136	1	pseudobulk	DESeq2	LRT	0.114853136899714
761	KC_AB1	Scgbeta	-0.0777040275378453	0.722888989036026	1	pseudobulk	DESeq2	LRT	0.140928390327472
761	KC_AB1	Scgdelta	0.0979848735870313	0.764776406182425	1	pseudobulk	DESeq2	LRT	0.116465518774367
761	KC_AB1	Schip1	0.21659598206337	0.294749412980158	1	pseudobulk	DESeq2	LRT	0.53054705116835
761	KC_AB1	schlank	0.238799018243253	0.368083192079952	1	pseudobulk	DESeq2	LRT	0.434054013451428
762	KC_AB1	Scm	0.101458421186695	0.713620229389083	1	pseudobulk	DESeq2	LRT	0.14653284727049
762	KC_AB1	scny	0.0532316556033829	0.857997689956449	1	pseudobulk	DESeq2	LRT	0.066513881429287
762	KC_AB1	SCOT	0.41353982688909	0.656703051072151	1	pseudobulk	DESeq2	LRT	0.182630965955864
762	KC_AB1	Scox	-0.304076446526097	0.493239256426551	1	pseudobulk	DESeq2	LRT	0.306942365630722
762	KC_AB1	Scp1	0.343592353814171	0.83258514384092	1	pseudobulk	DESeq2	LRT	0.079571342675127
762	KC_AB1	ScpX	0.323230655485803	0.685890807078194	1	pseudobulk	DESeq2	LRT	0.163745017905426
762	KC_AB1	scramb1	0.187085738729133	0.604584540916727	1	pseudobulk	DESeq2	LRT	0.218542961823096
762	KC_AB1	scramb2	-0.118860927346435	0.752870614039577	1	pseudobulk	DESeq2	LRT	0.123279653861486
762	KC_AB1	scrib	-0.0329756800350411	0.759196999727022	1	pseudobulk	DESeq2	LRT	0.119645516867082
762	KC_AB1	scro	0.0651113812657852	0.90969123134843	1	pseudobulk	DESeq2	LRT	0.041105991499346
763	KC_AB1	scrt	0.221876850301105	0.0889592939522391	1	pseudobulk	DESeq2	LRT	1.05080867263548
763	KC_AB1	Scsalpha1	-0.217743843214087	0.25181739435756	1	pseudobulk	DESeq2	LRT	0.59891427417815
763	KC_AB1	ScsbetaA	-0.414065460300338	0.0352839603300274	1	pseudobulk	DESeq2	LRT	1.45242267487387
763	KC_AB1	ScsbetaG	-0.0798718159591312	0.902456012140349	1	pseudobulk	DESeq2	LRT	0.044573957455866
763	KC_AB1	scu	-0.952667754204591	0.0469953684076414	1	pseudobulk	DESeq2	LRT	1.32794494151365
763	KC_AB1	scyl	-0.147384045338831	0.248706219696349	1	pseudobulk	DESeq2	LRT	0.604313353668434
763	KC_AB1	sd	-0.137169757021114	0.383619342200058	1	pseudobulk	DESeq2	LRT	0.416099503693817
763	KC_AB1	Sdc	0.0526986128525093	0.748674060420917	1	pseudobulk	DESeq2	LRT	0.125707213768201
763	KC_AB1	SdhA	-0.337388957368421	0.222606659950816	1	pseudobulk	DESeq2	LRT	0.652461846575338
763	KC_AB1	Sdhaf3	-0.252625718351666	0.629924873791891	1	pseudobulk	DESeq2	LRT	0.200711242360907
764	KC_AB1	SdhB	-0.248241751472516	0.271896755744359	1	pseudobulk	DESeq2	LRT	0.56559597435707
764	KC_AB1	SdhC	-0.0469286226729443	0.797650074703547	1	pseudobulk	DESeq2	LRT	0.098187589795699
764	KC_AB1	SdhD	-0.167832305077091	0.43522016838417	1	pseudobulk	DESeq2	LRT	0.361290987356288

764	KC_AB1	Sdic2	1.08899820779554	0.878177167143508	1	pseudobulk	DESeq2	LRT	0.056417858865076
764	KC_AB1	SdicB	1.08899820779554	0.670846322504933	1	pseudobulk	DESeq2	LRT	0.173376956629912
764	KC_AB1	sdk	1.17647985872266	0.412434272979288	1	pseudobulk	DESeq2	LRT	0.384645252342649
764	KC_AB1	sds22	-0.312353824928558	0.574535334163263	1	pseudobulk	DESeq2	LRT	0.240683256862132
764	KC_AB1	sdt	-0.198455100948075	0.0615089599928551	1	pseudobulk	DESeq2	LRT	1.21106161606165
764	KC_AB1	sea	-0.506516985469986	0.909346984303859	1	pseudobulk	DESeq2	LRT	0.041270369127443
764	KC_AB1	Sec10	-0.492402324060679	0.445894043645536	1	pseudobulk	DESeq2	LRT	0.350768329016515
765	KC_AB1	Sec13	-0.246068855753594	0.445268246071214	1	pseudobulk	DESeq2	LRT	0.351378275108547
765	KC_AB1	Sec15	-0.0052745595360268	0.992729610552855	1	pseudobulk	DESeq2	LRT	0.003169024047830
765	KC_AB1	Sec16	0.0086281390826067	0.969286822591727	1	pseudobulk	DESeq2	LRT	0.013547691435395
765	KC_AB1	Sec22	0.19146092700796	0.549377517779097	1	pseudobulk	DESeq2	LRT	0.260129117220264
765	KC_AB1	Sec23	0.252974445631753	0.49391137112549	1	pseudobulk	DESeq2	LRT	0.306350975133845
765	KC_AB1	Sec24AB	-0.0256549306170426	0.898358297625624	1	pseudobulk	DESeq2	LRT	0.046550416509991
765	KC_AB1	Sec24CD	-0.162355539274655	0.670358351925269	1	pseudobulk	DESeq2	LRT	0.173692975420288
765	KC_AB1	Sec3	1.1741071963587	0.239648112282826	1	pseudobulk	DESeq2	LRT	0.620425987612652
765	KC_AB1	Sec31	-0.177584861535995	0.741525626379227	1	pseudobulk	DESeq2	LRT	0.129873835596116
765	KC_AB1	Sec5	-0.805753461849651	0.292196838385028	1	pseudobulk	DESeq2	LRT	0.534324487509747
766	KC_AB1	Sec6	-0.122773103882128	0.835202390563047	1	pseudobulk	DESeq2	LRT	0.078208271278050
766	KC_AB1	Sec61alpha	0.0672015488883581	0.871791506630207	1	pseudobulk	DESeq2	LRT	0.059587366382342
766	KC_AB1	Sec61beta	-0.194700344814505	0.503739198016501	1	pseudobulk	DESeq2	LRT	0.297794253589544
766	KC_AB1	Sec61gamma	-0.0919171837742022	0.692492556728775	1	pseudobulk	DESeq2	LRT	0.15958489026206
766	KC_AB1	Sec63	0.10041557030247	0.789615242494157	1	pseudobulk	DESeq2	LRT	0.102584476767992
766	KC_AB1	Sec71	-0.383168841632361	0.54374230104038	1	pseudobulk	DESeq2	LRT	0.2646068792591
766	KC_AB1	Sec8	0.102998979105698	0.810143448758046	1	pseudobulk	DESeq2	LRT	0.091438075580343
766	KC_AB1	Secp43	-0.791711874523882	0.136272210754794	1	pseudobulk	DESeq2	LRT	0.865592698430322
766	KC_AB1	SecS	0.0269163539351012	0.902495998864156	1	pseudobulk	DESeq2	LRT	0.044554714824044
766	KC_AB1	sei	-0.136209090513586	0.707197769244464	1	pseudobulk	DESeq2	LRT	0.150459117913462
767	KC_AB1	sel	0.377927059189578	0.679517552552556	1	pseudobulk	DESeq2	LRT	0.167799320567597
767	KC_AB1	SelG	-0.089209440761328	0.778809409686699	1	pseudobulk	DESeq2	LRT	0.108568809912249
767	KC_AB1	SelR	-0.0302635952408242	0.847350987919907	1	pseudobulk	DESeq2	LRT	0.071936659867147
767	KC_AB1	SelT	0.062255853137447	0.828275674920595	1	pseudobulk	DESeq2	LRT	0.081825092960624
767	KC_AB1	Sem1	-0.243025255327779	0.412942412640299	1	pseudobulk	DESeq2	LRT	0.384110509158017
767	KC_AB1	Sema1a	0.139435945027482	0.233024258752715	1	pseudobulk	DESeq2	LRT	0.632598864836503
767	KC_AB1	Sema1b	0.298430345856745	0.369265569768351	1	pseudobulk	DESeq2	LRT	0.43266118397289
767	KC_AB1	Sema2a	0.0265994091261491	0.862885544752134	1	pseudobulk	DESeq2	LRT	0.064046806346291
767	KC_AB1	Sema2b	-0.0343756945651841	0.868363183004704	1	pseudobulk	DESeq2	LRT	0.061298598111276
767	KC_AB1	senju	-1.11754068086064	0.23828155494504	1	pseudobulk	DESeq2	LRT	0.622909574504671
768	KC_AB1	Sep1	-0.0407689579846593	0.90110112570362	1	pseudobulk	DESeq2	LRT	0.045226467766098
768	KC_AB1	Sep2	-0.234209337685397	0.499180173208263	1	pseudobulk	DESeq2	LRT	0.30174267259896
768	KC_AB1	Sep4	0.172679153474518	0.380168979464708	1	pseudobulk	DESeq2	LRT	0.42002332302272
768	KC_AB1	Sep5	1.08899820779554	0.670846322504933	1	pseudobulk	DESeq2	LRT	0.173376956629912
768	KC_AB1	SERCA	-0.325713665781651	0.145047468571761	1	pseudobulk	DESeq2	LRT	0.838489846281579
768	KC_AB1	SerRS	-0.0604007436695719	0.889078158029344	1	pseudobulk	DESeq2	LRT	0.051060058929852
768	KC_AB1	SerRS-m	0.0520629659617215	0.860152934309452	1	pseudobulk	DESeq2	LRT	0.065424324777635
768	KC_AB1	sesB	-0.314293537800429	0.0112977357541802	1	pseudobulk	DESeq2	LRT	1.94700858731287
768	KC_AB1	Sesn	0.172786110957146	0.862430806832974	1	pseudobulk	DESeq2	LRT	0.064275738476210
768	KC_AB1	Set	0.146533286908083	0.721778264729843	1	pseudobulk	DESeq2	LRT	0.141596200206712
769	KC_AB1	Set1	0.0896615997940694	0.60951137463302	1	pseudobulk	DESeq2	LRT	0.215018185214984
769	KC_AB1	Set2	0.0422961560109266	0.91327009367837	1	pseudobulk	DESeq2	LRT	0.039400763706644
769	KC_AB1	Setd3	0.174602069457071	0.732948691056726	1	pseudobulk	DESeq2	LRT	0.134926426410534
769	KC_AB1	SF1	-0.149362313497109	0.632068920743957	1	pseudobulk	DESeq2	LRT	0.199235563700457
769	KC_AB1	SF2	0.0940525059624692	0.581196503510627	1	pseudobulk	DESeq2	LRT	0.235677007090135

769	KC_AB1	Sf3a1	-0.0312114344586351	0.929077902483817	1	pseudobulk	DESeq2	LRT	0.03194786921369
769	KC_AB1	Sf3a2	0.659510439238563	0.151186798290714	1	pseudobulk	DESeq2	LRT	0.820486129997578
769	KC_AB1	Sf3b1	0.366839412512699	0.505702360744776	1	pseudobulk	DESeq2	LRT	0.29610501896774
769	KC_AB1	Sf3b2	-0.149678727083014	0.660840252758357	1	pseudobulk	DESeq2	LRT	0.179903511361991
769	KC_AB1	Sf3b3	0.23575773176021	0.780266338450702	1	pseudobulk	DESeq2	LRT	0.107757128622653
770	KC_AB1	Sf3b5	0.179091517303995	0.526717188941236	1	pseudobulk	DESeq2	LRT	0.278422508601852
770	KC_AB1	Sf3b6	-0.213586956438574	0.444414584261595	1	pseudobulk	DESeq2	LRT	0.352211697345023
770	KC_AB1	sff	-0.160195611569696	0.373150669789116	1	pseudobulk	DESeq2	LRT	0.428115774503304
770	KC_AB1	sfl	-0.112597449259043	0.511207596883667	1	pseudobulk	DESeq2	LRT	0.291402700897893
770	KC_AB1	Sfmbt	-0.119429928361953	0.765601152990238	1	pseudobulk	DESeq2	LRT	0.115997421186123
770	KC_AB1	Sfp33A2	1.08899820779554	0.878177167143508	1	pseudobulk	DESeq2	LRT	0.056417858865076
770	KC_AB1	Sfxn1-3	0.226611152940897	0.600401106649063	1	pseudobulk	DESeq2	LRT	0.22155851594386
770	KC_AB1	Sfxn2	-0.450485974313866	0.334667207831598	1	pseudobulk	DESeq2	LRT	0.475386839645463
770	KC_AB1	Sgf11	-0.171432468271426	0.78327472074252	1	pseudobulk	DESeq2	LRT	0.106085889567682
770	KC_AB1	Sgf29	0.370278055774584	0.425272626284774	1	pseudobulk	DESeq2	LRT	0.371332570815419
771	KC_AB1	sgg	-0.0646764214828549	0.574331653903503	1	pseudobulk	DESeq2	LRT	0.240837247210417
771	KC_AB1	sgl	-0.502723858286887	0.483876725486512	1	pseudobulk	DESeq2	LRT	0.315265266992163
771	KC_AB1	sgll	0.791675943694005	0.234544956641756	1	pseudobulk	DESeq2	LRT	0.629773901135941
771	KC_AB1	Sgp	-0.82542030717083	1	1	pseudobulk	DESeq2	LRT	0
771	KC_AB1	Sgs4	-0.648495897878299	0.705704185599103	1	pseudobulk	DESeq2	LRT	0.151377306716067
771	KC_AB1	Sgt	-0.110036484169333	0.721870741961908	1	pseudobulk	DESeq2	LRT	0.141540560156456
771	KC_AB1	Sgt1	-0.248765220892199	0.55414421256397	1	pseudobulk	DESeq2	LRT	0.256377198140849
771	KC_AB1	Sh	0.0136099240517119	0.8732641251558	1	pseudobulk	DESeq2	LRT	0.058854380866259
771	KC_AB1	Sh3beta	-0.135383001129224	0.499648130449469	1	pseudobulk	DESeq2	LRT	0.301335733264647
771	KC_AB1	SH3PX1	-0.952366702551854	0.146645590955145	1	pseudobulk	DESeq2	LRT	0.833730989980642
772	KC_AB1	Shab	0.181158778120276	0.118170412309648	1	pseudobulk	DESeq2	LRT	0.927491249167038
772	KC_AB1	shakB	-0.0774305695060016	0.559730688328383	1	pseudobulk	DESeq2	LRT	0.252020881397168
772	KC_AB1	Shal	-0.0439884291084234	0.775610113258983	1	pseudobulk	DESeq2	LRT	0.110356536746587
772	KC_AB1	shams	0.482001858313954	0.603967004496573	1	pseudobulk	DESeq2	LRT	0.218986786803513
772	KC_AB1	Shark	-0.507979848677457	0.940470850565906	1	pseudobulk	DESeq2	LRT	0.026654660650085
772	KC_AB1	Shaw	-0.179430241110856	0.341806954766263	1	pseudobulk	DESeq2	LRT	0.46621910490995
772	KC_AB1	Shawl	-0.130789875839492	0.343801200802724	1	pseudobulk	DESeq2	LRT	0.463692610775534
772	KC_AB1	Shc	0.692945929469811	0.197749929102654	1	pseudobulk	DESeq2	LRT	0.703883663533606
772	KC_AB1	shd	1.08899820779554	0.878177167143508	1	pseudobulk	DESeq2	LRT	0.056417858865076
772	KC_AB1	shep	-0.0452198888289683	0.6979836928295	1	pseudobulk	DESeq2	LRT	0.156154723790726
773	KC_AB1	shg	-1.44925888589622	0.6315588144614	1	pseudobulk	DESeq2	LRT	0.199586199175701
773	KC_AB1	shi	-0.131102002230103	0.276097394352464	1	pseudobulk	DESeq2	LRT	0.558937691961557
773	KC_AB1	Shmt	-0.0435568762008058	0.947766969857575	1	pseudobulk	DESeq2	LRT	0.023298430747981
773	KC_AB1	shn	0.453660437895368	0.341499213772932	1	pseudobulk	DESeq2	LRT	0.466610291849125
773	KC_AB1	shop	-0.669162705170721	0.415798617963254	1	pseudobulk	DESeq2	LRT	0.381116958515685
773	KC_AB1	shot	-0.141960390242041	0.213155374030452	1	pseudobulk	DESeq2	LRT	0.671303713533623
773	KC_AB1	shrb	0.245234123292373	0.354842013178994	1	pseudobulk	DESeq2	LRT	0.449964965470537
773	KC_AB1	Shrm	-1.8548675720385	0.420450816907966	1	pseudobulk	DESeq2	LRT	0.376284799377927
773	KC_AB1	shtd	-0.221169148171213	0.856718464103484	1	pseudobulk	DESeq2	LRT	0.067161873029299
773	KC_AB1	shv	0.134970056819326	0.877998962757863	1	pseudobulk	DESeq2	LRT	0.056505997156315
774	KC_AB1	SiaT	2.1559833397943	0.381743505411375	1	pseudobulk	DESeq2	LRT	0.418228342845202
774	KC_AB1	sicily	-0.0431810402952193	0.919249399595415	1	pseudobulk	DESeq2	LRT	0.036566645116955
774	KC_AB1	sick	0.096774380264216	0.641435596144639	1	pseudobulk	DESeq2	LRT	0.192846942785758
774	KC_AB1	side	0.114184054362142	1	1	pseudobulk	DESeq2	LRT	0
774	KC_AB1	side-III	-0.0554834091507234	0.659509402349102	1	pseudobulk	DESeq2	LRT	0.180779008519096
774	KC_AB1	side-IV	0.350214453675714	0.220292546456165	1	pseudobulk	DESeq2	LRT	0.657000196847051
774	KC_AB1	side-V	-0.501705230630191	0.543934172568283	1	pseudobulk	DESeq2	LRT	0.264453655853632

774	KC_AB1	side-VI	-0.101049010760793	0.379020105361549	1	pseudobulk	DESeq2	LRT	0.421337751995027
774	KC_AB1	side-VII	-0.0885281576720787	0.671933529645879	1	pseudobulk	DESeq2	LRT	0.172673686969705
774	KC_AB1	side-VIII	-0.0950938548426246	0.914974665004444	1	pseudobulk	DESeq2	LRT	0.038590931071364
775	KC_AB1	SIDL	0.430464892180326	0.411421666633146	1	pseudobulk	DESeq2	LRT	0.3857128408585
775	KC_AB1	Sidpn	1.16540490369572	0.0102782769529763	1	pseudobulk	DESeq2	LRT	1.98807968422539
775	KC_AB1	SIFaR	0.375295384970325	0.664457135512723	1	pseudobulk	DESeq2	LRT	0.177533030388276
775	KC_AB1	sigmar	0.0586577509955765	0.881956819926509	1	pseudobulk	DESeq2	LRT	0.054552677143353
775	KC_AB1	Sik2	-0.244027238969942	0.601400420032077	1	pseudobulk	DESeq2	LRT	0.220836272914002
775	KC_AB1	Sik3	0.246267841546265	0.375658883169245	1	pseudobulk	DESeq2	LRT	0.425206336983226
775	KC_AB1	Sil1	-0.0744742119405852	0.914746643295147	1	pseudobulk	DESeq2	LRT	0.038699175501994
775	KC_AB1	sima	-0.056346782256538	0.779135315073241	1	pseudobulk	DESeq2	LRT	0.108387110381049
775	KC_AB1	simj	0.162788687168272	0.398947823830129	1	pseudobulk	DESeq2	LRT	0.399083899562308
775	KC_AB1	Sin	0.510894557102471	0.503286127997177	1	pseudobulk	DESeq2	LRT	0.298185039833945
776	KC_AB1	Sin1	0.120321752452016	0.818285062863081	1	pseudobulk	DESeq2	LRT	0.087095376448671
776	KC_AB1	Sin3A	0.0767023926547077	0.634709185940399	1	pseudobulk	DESeq2	LRT	0.197425216237237
776	KC_AB1	sina	0.185099460180235	0.32394795919974	1	pseudobulk	DESeq2	LRT	0.489524751668904
776	KC_AB1	sing	1.2881130239535	0.320380504184431	1	pseudobulk	DESeq2	LRT	0.494333919500196
776	KC_AB1	sinu	-0.285025360300616	0.662022220181068	1	pseudobulk	DESeq2	LRT	0.179127433611547
776	KC_AB1	sip1	-1.18687113923728	0.258279311965252	1	pseudobulk	DESeq2	LRT	0.587910379189275
776	KC_AB1	sip3	0.0020710887989069	0.942420524897953	1	pseudobulk	DESeq2	LRT	0.025755263990701
776	KC_AB1	Sirt1	-0.150189354087999	0.512282607683307	1	pseudobulk	DESeq2	LRT	0.290490388444149
776	KC_AB1	Sirt2	0.272590588906162	0.560956466635801	1	pseudobulk	DESeq2	LRT	0.251070841120752
776	KC_AB1	Sirt4	0.0274719805666437	0.951599332896878	1	pseudobulk	DESeq2	LRT	0.021545871089695
777	KC_AB1	Sirt6	0.344362044565422	0.798093938526408	1	pseudobulk	DESeq2	LRT	0.097945987618459
777	KC_AB1	Sirt7	-0.0818172547609453	0.910526443025864	1	pseudobulk	DESeq2	LRT	0.040707437135992
777	KC_AB1	Sirup	-0.0901486532045033	0.841599364905654	1	pseudobulk	DESeq2	LRT	0.074894600920937
777	KC_AB1	sisRNA:1	-0.272598564737279	0.856109435916694	1	pseudobulk	DESeq2	LRT	0.067470716190938
777	KC_AB1	sisRNA:CR46364	-1.14524220236926	0.677974618202316	1	pseudobulk	DESeq2	LRT	0.168786564806998
777	KC_AB1	siz	-0.0559724018799717	0.741568486860619	1	pseudobulk	DESeq2	LRT	0.129848733924685
777	KC_AB1	SK	0.0564947783435072	0.68879751239985	1	pseudobulk	DESeq2	LRT	0.161908430011093
777	KC_AB1	Sk1	2.07920493043213	0.319384403960823	1	pseudobulk	DESeq2	LRT	0.49568629495748
777	KC_AB1	Sk2	-0.277441450265721	0.697154685955416	1	pseudobulk	DESeq2	LRT	0.156670849156817
777	KC_AB1	Skadu	0.111831901354254	1	1	pseudobulk	DESeq2	LRT	0
778	KC_AB1	skd	-0.0775422886753844	0.582194760189171	1	pseudobulk	DESeq2	LRT	0.234931707573212
778	KC_AB1	Ski6	0.791030394629192	0.386111482781617	1	pseudobulk	DESeq2	LRT	0.413287282461581
778	KC_AB1	SKIP	0.426476101831308	0.60835840850201	1	pseudobulk	DESeq2	LRT	0.215840484914092
778	KC_AB1	skl	1.08899820779554	0.697631521651407	1	pseudobulk	DESeq2	LRT	0.156373904552
778	KC_AB1	SkpA	-0.0035859205114092	0.919150734728651	1	pseudobulk	DESeq2	LRT	0.036613261309409
778	KC_AB1	sky	-0.210380456907114	0.244112195795017	1	pseudobulk	DESeq2	LRT	0.612410522777944
778	KC_AB1	sl	0.33938404237119	0.57946642280711	1	pseudobulk	DESeq2	LRT	0.236971724173328
778	KC_AB1	Slbp	-0.292164327482108	0.515717786996439	1	pseudobulk	DESeq2	LRT	0.287587889610954
778	KC_AB1	SLC22A	0.080312904132091	0.944500614111958	1	pseudobulk	DESeq2	LRT	0.024797755364860
778	KC_AB1	Slc25A46a	-0.593678663641617	0.318253106627242	1	pseudobulk	DESeq2	LRT	0.497227348288656
779	KC_AB1	SLC5A11	0.864968630796036	0.425603978835783	1	pseudobulk	DESeq2	LRT	0.370994320613507
779	KC_AB1	Sld5	0.135879393043758	1	1	pseudobulk	DESeq2	LRT	0
779	KC_AB1	sle	0.468163154351214	0.477133817459825	1	pseudobulk	DESeq2	LRT	0.321359801182195
779	KC_AB1	slgA	0.0122898619672385	0.946915890118852	1	pseudobulk	DESeq2	LRT	0.023688595526325
779	KC_AB1	Slh	0.113661492666109	0.866268929335861	1	pseudobulk	DESeq2	LRT	0.062347262260321
779	KC_AB1	slif	-0.56323908337126	0.709837113177923	1	pseudobulk	DESeq2	LRT	0.148841297708894
779	KC_AB1	Slik	-0.159629012367964	0.510376450109915	1	pseudobulk	DESeq2	LRT	0.292109373125592
779	KC_AB1	slim	0.638207843060246	0.0329386697576689	1	pseudobulk	DESeq2	LRT	1.48229394398137
779	KC_AB1	Slimp	-1.47195632525567	0.432779672012829	1	pseudobulk	DESeq2	LRT	0.363733146561353

779	KC_AB1	Slip1	-0.0380457157799129	0.823389755767327	1	pseudobulk	DESeq2	LRT	0.084394540595026
780	KC_AB1	SLIRP2	0.384473243407308	0.473817412886912	1	pseudobulk	DESeq2	LRT	0.324388982908864
780	KC_AB1	sll	-0.242870460675401	0.754916613775988	1	pseudobulk	DESeq2	LRT	0.122101016817097
780	KC_AB1	Slmap	0.0192812863284806	0.899048714844033	1	pseudobulk	DESeq2	LRT	0.046216775436118
780	KC_AB1	slmb	-0.110143636379398	0.698719010093759	1	pseudobulk	DESeq2	LRT	0.155697440706572
780	KC_AB1	slmo	-0.25167278340423	0.655116940491368	1	pseudobulk	DESeq2	LRT	0.183681170119583
780	KC_AB1	Sln	0.487122753778877	0.222269586286652	1	pseudobulk	DESeq2	LRT	0.653119958855292
780	KC_AB1	slo	-0.201133830430341	0.0237599934822326	1	pseudobulk	DESeq2	LRT	1.62415368282514
780	KC_AB1	SLO2	-0.05370552740279	0.633663825076634	1	pseudobulk	DESeq2	LRT	0.198141085391786
780	KC_AB1	Slob	-0.0627895731853634	0.56722051377707	1	pseudobulk	DESeq2	LRT	0.246248071091768
780	KC_AB1	slpr	0.186285630151933	0.474361934679381	1	pseudobulk	DESeq2	LRT	0.323890168341031
781	KC_AB1	sls	-1.65563143601174	0.492109880929607	1	pseudobulk	DESeq2	LRT	0.307937914807165
781	KC_AB1	Slu7	-0.496173576395552	0.377967807555725	1	pseudobulk	DESeq2	LRT	0.422545188512745
781	KC_AB1	slv	0.0987990546084043	0.869727668315945	1	pseudobulk	DESeq2	LRT	0.060616713660187
781	KC_AB1	slx1	-0.517891365451089	0.801051704895929	1	pseudobulk	DESeq2	LRT	0.096339450924157
781	KC_AB1	sm	0.0420155659852223	0.772921357532399	1	pseudobulk	DESeq2	LRT	0.111864692015954
781	KC_AB1	smal	-1.24441419239342	0.552647239366035	1	pseudobulk	DESeq2	LRT	0.257551995063473
781	KC_AB1	smash	0.287782185309122	0.187865500937898	1	pseudobulk	DESeq2	LRT	0.726152965125188
781	KC_AB1	SmB	-0.0344761570311654	0.900571985777191	1	pseudobulk	DESeq2	LRT	0.045481566824129
781	KC_AB1	SMC1	0.162413229352204	0.630856266619099	1	pseudobulk	DESeq2	LRT	0.200069578513061
781	KC_AB1	SMC2	0.194676637300056	0.832908505934096	1	pseudobulk	DESeq2	LRT	0.079402702735243
782	KC_AB1	SMC3	0.12165774541669	0.772397774418625	1	pseudobulk	DESeq2	LRT	0.112158986263827
782	KC_AB1	SMC5	-0.209590086371302	0.709904257708934	1	pseudobulk	DESeq2	LRT	0.1488002191013
782	KC_AB1	SmD1	-0.0966931288430937	0.855159183354655	1	pseudobulk	DESeq2	LRT	0.067953036128180
782	KC_AB1	SmD2	-0.142880484106401	0.680275725736921	1	pseudobulk	DESeq2	LRT	0.167315025682963
782	KC_AB1	SmD3	0.553526323969858	0.286637352068449	1	pseudobulk	DESeq2	LRT	0.542667216799009
782	KC_AB1	SmE	0.335952317437059	0.290270214071142	1	pseudobulk	DESeq2	LRT	0.537197526787073
782	KC_AB1	SmF	0.139629338145497	0.744943877392617	1	pseudobulk	DESeq2	LRT	0.127876444911852
782	KC_AB1	smg	0.421730901584582	0.223008537949409	1	pseudobulk	DESeq2	LRT	0.651678509537787
782	KC_AB1	Smg5	-0.194422153994944	0.306269981270632	1	pseudobulk	DESeq2	LRT	0.513895568023332
782	KC_AB1	Smg6	-0.0087291852227148	0.91257111971382	1	pseudobulk	DESeq2	LRT	0.039733279533760
783	KC_AB1	smid	0.555499807293009	0.374102395673831	1	pseudobulk	DESeq2	LRT	0.427009510661474
783	KC_AB1	Smn	0.208238823550214	0.682356992560383	1	pseudobulk	DESeq2	LRT	0.16598835359069
783	KC_AB1	smo	-0.339760360582209	0.626250935646395	1	pseudobulk	DESeq2	LRT	0.203251612269188
783	KC_AB1	smog	0.228599542147095	0.118667893118404	1	pseudobulk	DESeq2	LRT	0.925666768220359
783	KC_AB1	Smox	-0.189038691531964	0.186180184386428	1	pseudobulk	DESeq2	LRT	0.730066543921713
783	KC_AB1	smp-30	0.0687885917589341	0.911153227577622	1	pseudobulk	DESeq2	LRT	0.040408582087758
783	KC_AB1	Smr	0.154102278090626	0.221652730092828	1	pseudobulk	DESeq2	LRT	0.654326915134111
783	KC_AB1	Sms	0.0791137237851508	0.758121634616625	1	pseudobulk	DESeq2	LRT	0.120261109664931
783	KC_AB1	SMSr	-0.289819438363933	0.461709400109796	1	pseudobulk	DESeq2	LRT	0.335631283399355
783	KC_AB1	smt3	0.113934238817471	0.439761546671792	1	pseudobulk	DESeq2	LRT	0.356782748595893
784	KC_AB1	Smu1	-0.684404907446999	0.336593909913671	1	pseudobulk	DESeq2	LRT	0.472893746098799
784	KC_AB1	Smurf	-0.199549939449596	0.444018979189826	1	pseudobulk	DESeq2	LRT	0.352598465963647
784	KC_AB1	Smyd3	-0.104843923544561	0.860334037817026	1	pseudobulk	DESeq2	LRT	0.065332894554032
784	KC_AB1	Smyd4-1	-0.91458584899595	0.460350861531989	1	pseudobulk	DESeq2	LRT	0.336911039743432
784	KC_AB1	Smyd4-4	-0.10536830751914	0.853078906370887	1	pseudobulk	DESeq2	LRT	0.06901079648012
784	KC_AB1	Smyd5	0.48676912366156	0.551372025036729	1	pseudobulk	DESeq2	LRT	0.258555272471061
784	KC_AB1	SmydA-1	0.44870192113835	0.419840362942143	1	pseudobulk	DESeq2	LRT	0.3769158112029
784	KC_AB1	SmydA-3	0.982290489828387	0.384874185633079	1	pseudobulk	DESeq2	LRT	0.414681217024593
784	KC_AB1	SmydA-4	0.759963669898173	0.468244572505265	1	pseudobulk	DESeq2	LRT	0.329527247897573
784	KC_AB1	SmydA-7	-0.478575756560405	0.486967042480992	1	pseudobulk	DESeq2	LRT	0.312500430475276
785	KC_AB1	sn	0.676653144948615	0.231409274224299	1	pseudobulk	DESeq2	LRT	0.635619239750191

785	KC_AB1	snama	-0.0938643765213554	0.786447882686322	1	pseudobulk	DESeq2	LRT	0.10433005245799
785	KC_AB1	Snap24	0.0493033540532595	0.769490552506309	1	pseudobulk	DESeq2	LRT	0.113796707891717
785	KC_AB1	Snap25	-0.0444567865114765	0.631249818681928	1	pseudobulk	DESeq2	LRT	0.199798733618549
785	KC_AB1	Snap29	-0.0146034798155919	0.942197666107408	1	pseudobulk	DESeq2	LRT	0.025857975875676
785	KC_AB1	snf	-0.0661370968077161	0.8122619967029	1	pseudobulk	DESeq2	LRT	0.090303865623613
785	KC_AB1	SNF4Agamma	-0.11438723621832	0.671076640338428	1	pseudobulk	DESeq2	LRT	0.1732278783774
785	KC_AB1	sni	-0.495485915607652	0.447243205492019	1	pseudobulk	DESeq2	LRT	0.349456248501758
785	KC_AB1	Snm1	-0.1590670292588	0.889946817399492	1	pseudobulk	DESeq2	LRT	0.050635945714691
785	KC_AB1	Snmp1	-2.3118469151042	0.353435572380865	1	pseudobulk	DESeq2	LRT	0.451689742007392
786	KC_AB1	snmRNA:CR43603	1.08899820779554	0.697631521651407	1	pseudobulk	DESeq2	LRT	0.156373904552
786	KC_AB1	sno	0.12233371739612	0.481631886773637	1	pseudobulk	DESeq2	LRT	0.317284768030527
786	KC_AB1	Snoo	0.107558954399332	0.478599675077375	1	pseudobulk	DESeq2	LRT	0.32002760056619
786	KC_AB1	snoRNA:2R:94452	1.08899820779554	0.670846322504933	1	pseudobulk	DESeq2	LRT	0.173376956629912
786	KC_AB1	snoRNA:Dek-a	2.72432631526949	0.238668299696773	1	pseudobulk	DESeq2	LRT	0.622205260846706
786	KC_AB1	snoRNA:Irbp-a	-0.850996402080018	0.900449987182352	1	pseudobulk	DESeq2	LRT	0.045540403770502
786	KC_AB1	snoRNA:lola-a	1.08899820779554	0.697631521651407	1	pseudobulk	DESeq2	LRT	0.156373904552
786	KC_AB1	snoRNA:lola-c	2.58753908880453	0.243878358825908	1	pseudobulk	DESeq2	LRT	0.61282673621552
786	KC_AB1	snoRNA:lola-d	1.08899820779554	0.878177167143508	1	pseudobulk	DESeq2	LRT	0.056417858865076
786	KC_AB1	snoRNA:Me28S-A	-0.850996402080018	0.900449987182352	1	pseudobulk	DESeq2	LRT	0.045540403770502
787	KC_AB1	snoRNA:Me28S-C	1.08899820779554	0.878177167143508	1	pseudobulk	DESeq2	LRT	0.056417858865076
787	KC_AB1	snoRNA:nop5-x16	-0.850996402080018	0.900449987182352	1	pseudobulk	DESeq2	LRT	0.045540403770502
787	KC_AB1	snoRNA:Or-CD2	1.08899820779554	0.670846322504933	1	pseudobulk	DESeq2	LRT	0.173376956629912
787	KC_AB1	snoRNA:Psi18S-1	-1.96719661029409	0.414177861019091	1	pseudobulk	DESeq2	LRT	0.382813119093482
787	KC_AB1	snoRNA:Psi18S-1	-0.850996402080018	0.900449987182352	1	pseudobulk	DESeq2	LRT	0.045540403770502
787	KC_AB1	snoRNA:Psi18S-1	-0.639599218891621	0.788513993518674	1	pseudobulk	DESeq2	LRT	0.103190594974246
787	KC_AB1	snoRNA:Psi18S-9	-1.6747012584303	0.563129890257477	1	pseudobulk	DESeq2	LRT	0.249391420218277
787	KC_AB1	snoRNA:Psi28S-1	1.08899820779554	0.670846322504933	1	pseudobulk	DESeq2	LRT	0.173376956629912
787	KC_AB1	snoRNA:Psi28S-1	0.135879393043758	1	1	pseudobulk	DESeq2	LRT	0
787	KC_AB1	snoRNA:Psi28S-1	1.45928532158392	0.459969586225171	1	pseudobulk	DESeq2	LRT	0.337270883473328
788	KC_AB1	snoRNA:Psi28S-1	-0.850996402080018	0.900449987182352	1	pseudobulk	DESeq2	LRT	0.045540403770502
788	KC_AB1	snoRNA:Psi28S-1	-0.850996402080018	0.900449987182352	1	pseudobulk	DESeq2	LRT	0.045540403770502
788	KC_AB1	snoRNA:Psi28S-1	-0.82542030717083	1	1	pseudobulk	DESeq2	LRT	0
788	KC_AB1	snoRNA:Psi28S-2	-0.850996402080018	0.900449987182352	1	pseudobulk	DESeq2	LRT	0.045540403770502
788	KC_AB1	snoRNA:Psi28S-2	0.395588220531637	0.81564783073045	1	pseudobulk	DESeq2	LRT	0.088497314510842
788	KC_AB1	snoRNA:Psi28S-3	-1.56855358782635	0.573026303465967	1	pseudobulk	DESeq2	LRT	0.241825442277049
788	KC_AB1	snoRNA:Psi28S-3	1.08899820779554	0.670846322504933	1	pseudobulk	DESeq2	LRT	0.173376956629912
788	KC_AB1	snoRNA:Psi28S-3	1.08899820779554	0.697631521651407	1	pseudobulk	DESeq2	LRT	0.156373904552
788	KC_AB1	snoRNA:Psi28S-3	1.08899820779554	0.878177167143508	1	pseudobulk	DESeq2	LRT	0.056417858865076
788	KC_AB1	snoRNA:Psi28S-3	-0.979852967857776	0.553157418973078	1	pseudobulk	DESeq2	LRT	0.257151258455668
789	KC_AB1	snoRNA:Psi28S-3	1.08899820779554	0.670846322504933	1	pseudobulk	DESeq2	LRT	0.173376956629912
789	KC_AB1	snoRNA:Psi28S-3	-0.82542030717083	1	1	pseudobulk	DESeq2	LRT	0
789	KC_AB1	snoRNA:Tudor-SN	-0.452707468590943	0.834941154904573	1	pseudobulk	DESeq2	LRT	0.078344131702964
789	KC_AB1	snoRNA:U29:54Eb	1.08899820779554	0.697631521651407	1	pseudobulk	DESeq2	LRT	0.156373904552
789	KC_AB1	Snp	0.0388496548232144	0.881002793991202	1	pseudobulk	DESeq2	LRT	0.055022714274629
789	KC_AB1	sNPF	-0.135104099887282	0.270234103486317	1	pseudobulk	DESeq2	LRT	0.568259843984187
789	KC_AB1	sNPF-R	-0.850932661185203	0.660736244265418	1	pseudobulk	DESeq2	LRT	0.179971869591347
789	KC_AB1	Snr1	-0.111841601903739	0.750353053689456	1	pseudobulk	DESeq2	LRT	0.124734345686311
789	KC_AB1	snRNA:7SK	0.188318388286188	0.247422295706912	1	pseudobulk	DESeq2	LRT	0.606561167818473
789	KC_AB1	snRNA:LU	1.68130633771206	0.562684503730219	1	pseudobulk	DESeq2	LRT	0.249735045103917
790	KC_AB1	snRNA:U1:82Eb	0.135795303631797	0.946582799906172	1	pseudobulk	DESeq2	LRT	0.023841391241048
790	KC_AB1	snRNA:U11	0.135879393043758	1	1	pseudobulk	DESeq2	LRT	0
790	KC_AB1	snRNA:U12	2.11901358407042	0.0276809657806436	1	pseudobulk	DESeq2	LRT	1.55781876154594

790	KC_AB1	snRNA:U2:14B	-0.5932280843359	0.73100346344671	1	pseudobulk	DESeq2	LRT	0.136080565378348
790	KC_AB1	snRNA:U2:34ABa	1.08899820779554	0.878177167143508	1	pseudobulk	DESeq2	LRT	0.056417858865076
790	KC_AB1	snRNA:U4:39B	1.08899820779554	0.670846322504933	1	pseudobulk	DESeq2	LRT	0.173376956629912
790	KC_AB1	snRNA:U5:14B	0.13617601020169	1	1	pseudobulk	DESeq2	LRT	0
790	KC_AB1	snRNA:U7	-0.850996402080018	0.900449987182352	1	pseudobulk	DESeq2	LRT	0.045540403770502
790	KC_AB1	snRNP-U1-70K	0.218969316946024	0.362660801916946	1	pseudobulk	DESeq2	LRT	0.440499382470652
790	KC_AB1	snRNP-U1-C	-0.0887252862437992	0.825238596203199	1	pseudobulk	DESeq2	LRT	0.083420468379344
791	KC_AB1	SNRPG	0.172183273485683	0.488688890755854	1	pseudobulk	DESeq2	LRT	0.310967533569064
791	KC_AB1	sns	1.34653517418009	0.0574859616999662	1	pseudobulk	DESeq2	LRT	1.24043819879916
791	KC_AB1	Snx1	-0.111712015303463	0.760243559351122	1	pseudobulk	DESeq2	LRT	0.119047250434229
791	KC_AB1	Snx16	-0.353177829971102	0.563364280981703	1	pseudobulk	DESeq2	LRT	0.249210692075676
791	KC_AB1	Snx17	-0.0375820669502969	0.911286310012017	1	pseudobulk	DESeq2	LRT	0.040345153956611
791	KC_AB1	Snx21	0.013855824916307	0.974309364121796	1	pseudobulk	DESeq2	LRT	0.011303123410482
791	KC_AB1	Snx27	-0.167996368709679	0.462034656438069	1	pseudobulk	DESeq2	LRT	0.335325447527591
791	KC_AB1	Snx3	0.186267165555536	0.515727528292178	1	pseudobulk	DESeq2	LRT	0.287579686382099
791	KC_AB1	Snx6	0.364803738196823	0.283997617058771	1	pseudobulk	DESeq2	LRT	0.54668530397609
791	KC_AB1	snz	0.0282681589448642	0.940254289260439	1	pseudobulk	DESeq2	LRT	0.026754676732586
792	KC_AB1	Socs16D	-0.0260972097225239	0.917095967069109	1	pseudobulk	DESeq2	LRT	0.037585216360685
792	KC_AB1	Socs36E	0.145109278201976	0.562398419477051	1	pseudobulk	DESeq2	LRT	0.249955908495629
792	KC_AB1	Socs44A	-0.0596272245077593	0.877398477229709	1	pseudobulk	DESeq2	LRT	0.056803123670882
792	KC_AB1	Sod1	-0.172794761640205	0.2274924016318	1	pseudobulk	DESeq2	LRT	0.643033104433198
792	KC_AB1	Sod2	-0.156090574777979	0.529845236390088	1	pseudobulk	DESeq2	LRT	0.275850965865285
792	KC_AB1	Sod3	0.13617601020169	1	1	pseudobulk	DESeq2	LRT	0
792	KC_AB1	sofe	0.527002072710335	0.727035853176201	1	pseudobulk	DESeq2	LRT	0.138444171737049
792	KC_AB1	sog	0.13617601020169	1	1	pseudobulk	DESeq2	LRT	0
792	KC_AB1	sol	-0.199635781752065	0.410500496106253	1	pseudobulk	DESeq2	LRT	0.386686313681987
792	KC_AB1	Son	-0.233786067586079	0.560529235812611	1	pseudobulk	DESeq2	LRT	0.251401730756851
793	KC_AB1	Sos	-0.0147931591466488	0.968907951764636	1	pseudobulk	DESeq2	LRT	0.013717479852774
793	KC_AB1	soti	3.06362957045066	0.168201180880496	1	pseudobulk	DESeq2	LRT	0.774170959500753
793	KC_AB1	sotv	-0.213326333606789	0.569899109041641	1	pseudobulk	DESeq2	LRT	0.244202021984937
793	KC_AB1	sov	-0.0329468100522	0.883381986936063	1	pseudobulk	DESeq2	LRT	0.053851460696858
793	KC_AB1	sowah	-0.239034198410364	0.437322404992735	1	pseudobulk	DESeq2	LRT	0.359198272219622
793	KC_AB1	Sox100B	-0.850996402080018	0.900449987182352	1	pseudobulk	DESeq2	LRT	0.045540403770502
793	KC_AB1	Sox102F	3.4671146592944	0.486909188710335	1	pseudobulk	DESeq2	LRT	0.312552029585263
793	KC_AB1	SoxN	0.135879393043758	1	1	pseudobulk	DESeq2	LRT	0
793	KC_AB1	SP1173	0.0545806793303371	0.66946345714881	1	pseudobulk	DESeq2	LRT	0.174273124091496
793	KC_AB1	SP2353	0.60214667105663	0.118133434286	1	pseudobulk	DESeq2	LRT	0.927627170371742
794	KC_AB1	spab	-0.0873641852338057	0.664896249496142	1	pseudobulk	DESeq2	LRT	0.177246116781988
794	KC_AB1	spag	-0.241796975539621	0.737640473110407	1	pseudobulk	DESeq2	LRT	0.132155262282126
794	KC_AB1	spag4	-0.82542030717083	1	1	pseudobulk	DESeq2	LRT	0
794	KC_AB1	SPARC	0.660755449641801	0.674466818467947	1	pseudobulk	DESeq2	LRT	0.171039411315638
794	KC_AB1	spartin	-0.542956546487509	0.251641609834909	1	pseudobulk	DESeq2	LRT	0.599217545152182
794	KC_AB1	spas	0.100573217241955	0.819782739006812	1	pseudobulk	DESeq2	LRT	0.086301230241294
794	KC_AB1	Spase12	-0.0708675038001294	0.783786495352566	1	pseudobulk	DESeq2	LRT	0.105802223690766
794	KC_AB1	Spase22-23	-0.147595867064572	0.679184559906047	1	pseudobulk	DESeq2	LRT	0.168012195590698
794	KC_AB1	Spase25	0.491704190377784	0.142821398329991	1	pseudobulk	DESeq2	LRT	0.845206719167524
794	KC_AB1	spdo	0.923124750607267	0.707531785811194	1	pseudobulk	DESeq2	LRT	0.150254044719123
795	KC_AB1	SpdS	0.312572690449045	0.414149256256422	1	pseudobulk	DESeq2	LRT	0.382843114225427
795	KC_AB1	SPE	1.08899820779554	0.670846322504933	1	pseudobulk	DESeq2	LRT	0.173376956629912
795	KC_AB1	Spec2	-0.115897328164819	0.794655817276995	1	pseudobulk	DESeq2	LRT	0.099820933008298
795	KC_AB1	spel1	0.409405379492751	0.636745379393594	1	pseudobulk	DESeq2	LRT	0.196034197854172
795	KC_AB1	spen	0.200804896214049	0.0262980918034447	1	pseudobulk	DESeq2	LRT	1.58007576289046

795	KC_AB1	Spf45	0.237686717447007	0.33836025017332	1	pseudobulk	DESeq2	LRT	0.470620662608077
795	KC_AB1	spg	0.402774078992747	0.319244590720808	1	pseudobulk	DESeq2	LRT	0.495876452686337
795	KC_AB1	Spg7	0.21585697916594	0.727389064680624	1	pseudobulk	DESeq2	LRT	0.138233232267506
795	KC_AB1	spi	0.270681469723024	0.327853314741763	1	pseudobulk	DESeq2	LRT	0.484320421066311
795	KC_AB1	spict	0.750302365325013	0.333041358720004	1	pseudobulk	DESeq2	LRT	0.477501830311897
796	KC_AB1	spidey	0.132325767877583	0.55625140065558	1	pseudobulk	DESeq2	LRT	0.254728882444767
796	KC_AB1	spin	0.169040596516124	0.725184052288565	1	pseudobulk	DESeq2	LRT	0.139551755154618
796	KC_AB1	Spindly	1.08899820779554	0.697631521651407	1	pseudobulk	DESeq2	LRT	0.156373904552
796	KC_AB1	spir	-0.10952655317129	0.440529367618326	1	pseudobulk	DESeq2	LRT	0.356025134313339
796	KC_AB1	sPLA2	1.87556107715387	0.0921356173133526	1	pseudobulk	DESeq2	LRT	1.03557245001579
796	KC_AB1	Sply	-0.0621847629227115	0.893195179218936	1	pseudobulk	DESeq2	LRT	0.049053629582087
796	KC_AB1	Spn	-0.0430726958962832	0.738761682808956	1	pseudobulk	DESeq2	LRT	0.131495638104808
796	KC_AB1	spn-A	-1.1207746378843	0.567941967719394	1	pseudobulk	DESeq2	LRT	0.245696038209441
796	KC_AB1	spn-B	1.08899820779554	0.697631521651407	1	pseudobulk	DESeq2	LRT	0.156373904552
796	KC_AB1	spn-F	0.0312459224584414	0.953230047571985	1	pseudobulk	DESeq2	LRT	0.020802276345479
797	KC_AB1	Spn42Da	0.602404053732299	0.220897941287157	1	pseudobulk	DESeq2	LRT	0.655808331624236
797	KC_AB1	Spn43Ab	1.08899820779554	0.878177167143508	1	pseudobulk	DESeq2	LRT	0.056417858865076
797	KC_AB1	Spn55B	-0.82542030717083	1	1	pseudobulk	DESeq2	LRT	0
797	KC_AB1	SPoCk	0.0194057451019529	0.891714847140752	1	pseudobulk	DESeq2	LRT	0.049774002251688
797	KC_AB1	spok	1.08899820779554	0.878177167143508	1	pseudobulk	DESeq2	LRT	0.056417858865076
797	KC_AB1	spoon	-0.182853453175606	0.602453736222753	1	pseudobulk	DESeq2	LRT	0.22007629792257
797	KC_AB1	Spp	0.682287361192608	0.181566476467056	1	pseudobulk	DESeq2	LRT	0.740964334390634
797	KC_AB1	SppL	0.199706654529662	0.552352244374154	1	pseudobulk	DESeq2	LRT	0.257783876982138
797	KC_AB1	Spps	0.339317102100664	0.532105315685308	1	pseudobulk	DESeq2	LRT	0.274002402489926
797	KC_AB1	SPR	-0.913086822549633	0.0708241986655284	1	pseudobulk	DESeq2	LRT	1.14981833056832
798	KC_AB1	Spred	-0.0773595528486969	0.499678026056413	1	pseudobulk	DESeq2	LRT	0.301309748760892
798	KC_AB1	spri	-0.66908007593739	0.687716665757612	1	pseudobulk	DESeq2	LRT	0.162590451070413
798	KC_AB1	sprt	-1.14661508406441	0.331123574823976	1	pseudobulk	DESeq2	LRT	0.480009897920579
798	KC_AB1	Sps1	-0.409968712739343	0.437047149478961	1	pseudobulk	DESeq2	LRT	0.359471707986187
798	KC_AB1	Sps2	-0.069774856485395	0.8928609946544	1	pseudobulk	DESeq2	LRT	0.049216149122213
798	KC_AB1	Spt	0.620441612395759	0.524836900501793	1	pseudobulk	DESeq2	LRT	0.279975637959904
798	KC_AB1	Spt-I	0.0631331559250343	0.885187318849633	1	pseudobulk	DESeq2	LRT	0.052964816381785
798	KC_AB1	Spt20	-0.392009687628989	0.332314909764727	1	pseudobulk	DESeq2	LRT	0.478450173065902
798	KC_AB1	Spt3	-0.112652576426859	0.873615702073739	1	pseudobulk	DESeq2	LRT	0.058679568706766
798	KC_AB1	spt4	0.290995072447172	0.486494776749158	1	pseudobulk	DESeq2	LRT	0.31292181817301
799	KC_AB1	Spt5	-0.151286751986786	0.635981423205107	1	pseudobulk	DESeq2	LRT	0.19655556975621
799	KC_AB1	Spt6	0.190808606740642	0.618007349820804	1	pseudobulk	DESeq2	LRT	0.209006359915008
799	KC_AB1	Spt7	0.90657404534109	0.376223927138186	1	pseudobulk	DESeq2	LRT	0.424553587590432
799	KC_AB1	Sptr	0.0176758029832879	0.963528878846911	1	pseudobulk	DESeq2	LRT	0.016135264157565
799	KC_AB1	Spx	0.294536573648704	0.664058079359017	1	pseudobulk	DESeq2	LRT	0.177793935026471
799	KC_AB1	spz	-0.82542030717083	1	1	pseudobulk	DESeq2	LRT	0
799	KC_AB1	spz4	-1.56855358782635	0.573026303465967	1	pseudobulk	DESeq2	LRT	0.241825442277049
799	KC_AB1	spz5	-0.82542030717083	1	1	pseudobulk	DESeq2	LRT	0
799	KC_AB1	sqd	-0.0663725179320987	0.537090716578125	1	pseudobulk	DESeq2	LRT	0.269952354185172
799	KC_AB1	sqh	-0.143404648206434	0.575156486263081	1	pseudobulk	DESeq2	LRT	0.240213978137471
800	KC_AB1	squ	0.341666058290915	0.766344489654726	1	pseudobulk	DESeq2	LRT	0.115575961014257
800	KC_AB1	sqz	0.0675176462883088	0.882475808518626	1	pseudobulk	DESeq2	LRT	0.054297191176256
800	KC_AB1	sr	0.119783155070496	0.543782093786958	1	pseudobulk	DESeq2	LRT	0.264575097406025
800	KC_AB1	Sr-CII	1.01200857549066	0.622374419095099	1	pseudobulk	DESeq2	LRT	0.20594826607324
800	KC_AB1	sra	-0.478849399129196	0.0569295531583164	1	pseudobulk	DESeq2	LRT	1.24466222496483
800	KC_AB1	Sra-1	-0.547804971614643	0.444492766582571	1	pseudobulk	DESeq2	LRT	0.352135302092722
800	KC_AB1	Sras	0.846548738459834	0.205364977471802	1	pseudobulk	DESeq2	LRT	0.687473618127319

800	KC_AB1	Src42A	0.197614977176521	0.306400734227449	1	pseudobulk	DESeq2	LRT	0.51371019833921
800	KC_AB1	Src64B	0.0167962405997685	0.909847017466893	1	pseudobulk	DESeq2	LRT	0.04103162422312
800	KC_AB1	SREBP	-0.157298907862038	0.744503730141912	1	pseudobulk	DESeq2	LRT	0.12813312198703
801	KC_AB1	srl	-1.0955807077656	0.18193421542816	1	pseudobulk	DESeq2	LRT	0.74008561773543
801	KC_AB1	Srlp	-0.744870027570323	0.0710853491242504	1	pseudobulk	DESeq2	LRT	1.14821989927224
801	KC_AB1	SRm160	-0.163504175520448	0.589779194521884	1	pseudobulk	DESeq2	LRT	0.22931055200075
801	KC_AB1	Srp14	-0.161935168441204	0.609409599418293	1	pseudobulk	DESeq2	LRT	0.21509070905401
801	KC_AB1	Srp19	-0.061198877643373	0.890734384753148	1	pseudobulk	DESeq2	LRT	0.05025178242065
801	KC_AB1	Srp54	-0.029436834220719	0.889607370503842	1	pseudobulk	DESeq2	LRT	0.05080162756520
801	KC_AB1	Srp54k	-0.057422711217110	0.900583262617201	1	pseudobulk	DESeq2	LRT	0.04547612868169
801	KC_AB1	Srp68	-0.277488045249581	0.670605794500686	1	pseudobulk	DESeq2	LRT	0.17353269828447
801	KC_AB1	Srp72	-0.439577916889435	0.487085986809528	1	pseudobulk	DESeq2	LRT	0.31239436465700
801	KC_AB1	Srp9	-0.343955159942588	0.283737205374413	1	pseudobulk	DESeq2	LRT	0.54708371309599
802	KC_AB1	SRPK	-0.111808313325048	0.584656107576062	1	pseudobulk	DESeq2	LRT	0.23309950912668
802	KC_AB1	Srpk79D	-0.004256218130039	0.988754610620444	1	pseudobulk	DESeq2	LRT	0.00491147834903
802	KC_AB1	SrpRbeta	-0.072451594707246	0.864684662925738	1	pseudobulk	DESeq2	LRT	0.06314224411885
802	KC_AB1	Sry-delta	-0.781374335910712	0.276674304779205	1	pseudobulk	DESeq2	LRT	0.55803117266525
802	KC_AB1	Ssadh	-0.145635945727881	0.738949343751589	1	pseudobulk	DESeq2	LRT	0.13138533221843
802	KC_AB1	Ssb-c31a	-0.214790028177336	0.452600670804013	1	pseudobulk	DESeq2	LRT	0.34428480670858
802	KC_AB1	Ssdp	-0.024106771817713	0.872457851431623	1	pseudobulk	DESeq2	LRT	0.05925554469292
802	KC_AB1	Sse	-0.82542030717083	1	1	pseudobulk	DESeq2	LRT	0
802	KC_AB1	ssh	-0.351227567501985	0.312645137825429	1	pseudobulk	DESeq2	LRT	0.50494832096382
802	KC_AB1	Ssl1	-0.076936221691709	0.887549322700504	1	pseudobulk	DESeq2	LRT	0.05180750308249
803	KC_AB1	ssp	0.244431799206067	0.75545701105306	1	pseudobulk	DESeq2	LRT	0.12179024395715
803	KC_AB1	ssp2	-0.850996402080018	0.900449987182352	1	pseudobulk	DESeq2	LRT	0.04554040377050
803	KC_AB1	ssp3	-0.220321788882681	0.501270195808495	1	pseudobulk	DESeq2	LRT	0.29992811661316
803	KC_AB1	ssp6	-0.521146219260969	0.809140205763683	1	pseudobulk	DESeq2	LRT	0.09197621842022
803	KC_AB1	ssp7	1.68130633771206	0.562684503730219	1	pseudobulk	DESeq2	LRT	0.24973504510391
803	KC_AB1	SsRbeta	0.185625692251052	0.566825908835735	1	pseudobulk	DESeq2	LRT	0.24655030696510
803	KC_AB1	Ssrp	-0.959170384959842	0.185382065254536	1	pseudobulk	DESeq2	LRT	0.73193228387948
803	KC_AB1	sstn	-0.668744571658464	0.488936893082427	1	pseudobulk	DESeq2	LRT	0.31074719149949
803	KC_AB1	Ssu72	0.347387613060561	0.520911623988653	1	pseudobulk	DESeq2	LRT	0.2832359513064
803	KC_AB1	ssx	0.047885469889764	0.800194676169795	1	pseudobulk	DESeq2	LRT	0.09680434238188
804	KC_AB1	St1	1.28116770184993	0.592386603154796	1	pseudobulk	DESeq2	LRT	0.22739477162573
804	KC_AB1	St3	0.023809720710605	0.915281212048531	1	pseudobulk	DESeq2	LRT	0.03844545229694
804	KC_AB1	sta	-0.029854667844350	0.810287785439189	1	pseudobulk	DESeq2	LRT	0.09136070775010
804	KC_AB1	stac	0.845726476905101	0.694547825938244	1	pseudobulk	DESeq2	LRT	0.15829784376951
804	KC_AB1	Stacl	-0.153582350338909	0.106982467814034	1	pseudobulk	DESeq2	LRT	0.97068738825390
804	KC_AB1	stai	-0.098849952514054	0.279349614882118	1	pseudobulk	DESeq2	LRT	0.55385192303188
804	KC_AB1	Stam	0.429506642118219	0.294260341895133	1	pseudobulk	DESeq2	LRT	0.53126826476639
804	KC_AB1	stan	-0.356005114886445	0.771522655313492	1	pseudobulk	DESeq2	LRT	0.11265131660903
804	KC_AB1	Start1	-0.454102488618258	0.324442232992139	1	pseudobulk	DESeq2	LRT	0.48886261820435
804	KC_AB1	stas	-0.206371574036546	0.406148717497442	1	pseudobulk	DESeq2	LRT	0.39131491380637
805	KC_AB1	Stat92E	0.100643281631452	0.535846115800539	1	pseudobulk	DESeq2	LRT	0.27095991301996
805	KC_AB1	stau	-0.019155806424426	0.935293071782709	1	pseudobulk	DESeq2	LRT	0.02905228269475
805	KC_AB1	stc	-0.234606002673196	0.6965136825143	1	pseudobulk	DESeq2	LRT	0.15707034775101
805	KC_AB1	stck	-0.22762631000426	0.337651265279489	1	pseudobulk	DESeq2	LRT	0.47153161851044
805	KC_AB1	ste14	-0.279425250017497	0.753740152950567	1	pseudobulk	DESeq2	LRT	0.12277834854620
805	KC_AB1	ste24a	-0.070046105482492	0.899110827334332	1	pseudobulk	DESeq2	LRT	0.04618677241228
805	KC_AB1	step	0.0080430727257275	0.959544980733871	1	pseudobulk	DESeq2	LRT	0.01793466196380
805	KC_AB1	stet	-0.184873771334748	0.924239397505881	1	pseudobulk	DESeq2	LRT	0.03421552277389
805	KC_AB1	Stim	-0.223299203940767	0.517017921751681	1	pseudobulk	DESeq2	LRT	0.28649440239356

805	KC_AB1	Stip1	0.122023354420789	0.828084669374329	1	pseudobulk	DESeq2	LRT	0.081925255531419
806	KC_AB1	stj	-0.0288963803486985	0.864195333703299	1	pseudobulk	DESeq2	LRT	0.063388083028699
806	KC_AB1	Stlk	-0.0468417784098422	0.8036140608541	1	pseudobulk	DESeq2	LRT	0.094952472996557
806	KC_AB1	stmA	-0.236482383194377	0.173717177607858	1	pseudobulk	DESeq2	LRT	0.760157235257831
806	KC_AB1	Stoml2	0.254180397437906	0.438381290870386	1	pseudobulk	DESeq2	LRT	0.358147988841968
806	KC_AB1	stops	0.257621583013889	0.721627298543436	1	pseudobulk	DESeq2	LRT	0.141687046166597
806	KC_AB1	strat	-0.0824538216543003	0.863845418399147	1	pseudobulk	DESeq2	LRT	0.063563965787967
806	KC_AB1	Strica	-4.62089750393184	0.228870625533508	1	pseudobulk	DESeq2	LRT	0.640409943399431
806	KC_AB1	Strip	0.235451108916843	0.586983610197897	1	pseudobulk	DESeq2	LRT	0.23137402498745€
806	KC_AB1	Strump	-0.213861346693293	0.81549536127676	1	pseudobulk	DESeq2	LRT	0.088578504982547
806	KC_AB1	Stt3A	-0.148349456150524	0.752965773268817	1	pseudobulk	DESeq2	LRT	0.123224764593882
807	KC_AB1	Stt3B	0.391111283890832	0.531510730259737	1	pseudobulk	DESeq2	LRT	0.274487963416219
807	KC_AB1	STUB1	0.113821971318901	0.669621195712889	1	pseudobulk	DESeq2	LRT	0.174170807948698
807	KC_AB1	sturkopf	-0.302605211258134	0.551005854527528	1	pseudobulk	DESeq2	LRT	0.258843786673638
807	KC_AB1	stv	0.0703294490405957	0.716989585060523	1	pseudobulk	DESeq2	LRT	0.144487152816991
807	KC_AB1	stwl	0.147862137390477	0.809952341387125	1	pseudobulk	DESeq2	LRT	0.091540534802238
807	KC_AB1	stx	0.127086001558693	0.498079610958434	1	pseudobulk	DESeq2	LRT	0.302701235881507
807	KC_AB1	sty	-0.0724884985537775	0.9381996526153	1	pseudobulk	DESeq2	LRT	0.027704732193898
807	KC_AB1	Su(dx)	-0.0171596205425132	0.95659023740436	1	pseudobulk	DESeq2	LRT	0.019274055685516
807	KC_AB1	su(f)	0.259359840833056	0.496339680718565	1	pseudobulk	DESeq2	LRT	0.304221003004919
807	KC_AB1	Su(fu)	-0.224137185852432	0.733102385368044	1	pseudobulk	DESeq2	LRT	0.134835367384524
808	KC_AB1	Su(H)	0.107099001883801	0.861851951941338	1	pseudobulk	DESeq2	LRT	0.064567330436316
808	KC_AB1	su(Hw)	0.0888096147340924	0.84361694223565	1	pseudobulk	DESeq2	LRT	0.073854706972416
808	KC_AB1	Su(P)	-0.211455496728838	0.537738321013548	1	pseudobulk	DESeq2	LRT	0.26942901315705
808	KC_AB1	su(sable)	0.0048512792569036	0.986098588994435	1	pseudobulk	DESeq2	LRT	0.006079662628962
808	KC_AB1	Su(Tpl)	-0.0193166609291539	0.890683518012285	1	pseudobulk	DESeq2	LRT	0.050276584175304
808	KC_AB1	Su(var)2-10	0.0160560888239176	0.936784952457502	1	pseudobulk	DESeq2	LRT	0.028360093936076
808	KC_AB1	Su(var)2-HP2	0.459537362588494	0.130508230529307	1	pseudobulk	DESeq2	LRT	0.884362098588179
808	KC_AB1	Su(var)205	0.0561613658236464	0.812248552569318	1	pseudobulk	DESeq2	LRT	0.090311053897067
808	KC_AB1	Su(var)3-3	0.255946781986999	0.502629322785859	1	pseudobulk	DESeq2	LRT	0.298752178790357
808	KC_AB1	Su(var)3-7	-0.099044637688189	0.870420102242587	1	pseudobulk	DESeq2	LRT	0.060271087553735
809	KC_AB1	Su(var)3-9	0.113316967542732	1	1	pseudobulk	DESeq2	LRT	0
809	KC_AB1	su(w[a])	0.0749385039479302	0.759125038020118	1	pseudobulk	DESeq2	LRT	0.119686684118118
809	KC_AB1	Su(z)12	0.459142976489874	0.235119306089629	1	pseudobulk	DESeq2	LRT	0.62871170867196
809	KC_AB1	Su(z)2	0.326511375069439	0.234874755930738	1	pseudobulk	DESeq2	LRT	0.62916365818674
809	KC_AB1	subdued	-0.805996893655701	0.325168728892027	1	pseudobulk	DESeq2	LRT	0.487891226677733
809	KC_AB1	sud1	0.459782251261447	0.617743923547103	1	pseudobulk	DESeq2	LRT	0.209191517852944
809	KC_AB1	sug	-1.05813155791189	0.687053260159203	1	pseudobulk	DESeq2	LRT	0.163009595259685
809	KC_AB1	Sugb	0.0095452833462145	0.95700771492713	1	pseudobulk	DESeq2	LRT	0.019084561139796
809	KC_AB1	Sulf1	-0.615767568564188	0.806245159731688	1	pseudobulk	DESeq2	LRT	0.093532879622572
809	KC_AB1	sun	-0.0213388930018065	0.880973037582224	1	pseudobulk	DESeq2	LRT	0.055037383084484
810	KC_AB1	Sur-8	-0.0186577873423037	0.923821145053348	1	pseudobulk	DESeq2	LRT	0.034412101545098
810	KC_AB1	Surf1	-0.518961076304718	0.440867017033778	1	pseudobulk	DESeq2	LRT	0.355692391207795
810	KC_AB1	Surf4	0.343710342369062	0.277123691158067	1	pseudobulk	DESeq2	LRT	0.557326345024668
810	KC_AB1	Surf6	-1.21274856028049	0.0292211258484684	1	pseudobulk	DESeq2	LRT	1.53430305532993
810	KC_AB1	sut2	0.749696100593861	0.527164011121058	1	pseudobulk	DESeq2	LRT	0.278054246185358
810	KC_AB1	Suv3	-0.521364568625632	0.393843089606626	1	pseudobulk	DESeq2	LRT	0.404676770283631
810	KC_AB1	sv	0.917527351802732	0.557334218557009	1	pseudobulk	DESeq2	LRT	0.253884291818605
810	KC_AB1	Svil	-0.117389274563072	0.709375808506216	1	pseudobulk	DESeq2	LRT	0.149123626142342
810	KC_AB1	svr	0.0174374421688503	0.893276824713051	1	pseudobulk	DESeq2	LRT	0.049013933256017
810	KC_AB1	sw	0.119866075110287	0.672378812880529	1	pseudobulk	DESeq2	LRT	0.172385979935191
811	KC_AB1	swi2	-0.850932661185203	0.660736244265418	1	pseudobulk	DESeq2	LRT	0.179971869591347

811	KC_AB1	SWIP	0.130979350961249	0.882599141012361	1	pseudobulk	DESeq2	LRT	0.05423649956475
811	KC_AB1	Swip-1	0.0326973708666334	0.903899191119081	1	pseudobulk	DESeq2	LRT	0.04388000224806
811	KC_AB1	swm	-4.34453488478134E-	0.993736179661515	1	pseudobulk	DESeq2	LRT	0.00272889822325
811	KC_AB1	sws	-0.205388844776332	0.349983446445255	1	pseudobulk	DESeq2	LRT	0.45595249647113
811	KC_AB1	sxc	0.0636023471339153	0.709336302815584	1	pseudobulk	DESeq2	LRT	0.14914781301339
811	KC_AB1	sxe2	1.08899820779554	0.670846322504933	1	pseudobulk	DESeq2	LRT	0.17337695662991
811	KC_AB1	Sxl	-0.021877033385076	0.828968011145934	1	pseudobulk	DESeq2	LRT	0.08146222801528
811	KC_AB1	Syb	0.593091571723423	0.731093409620853	1	pseudobulk	DESeq2	LRT	0.13602713098851
811	KC_AB1	syd	0.201417884175528	0.327088057652875	1	pseudobulk	DESeq2	LRT	0.48533531215792
812	KC_AB1	Sym	0.612807172673998	0.515241198574365	1	pseudobulk	DESeq2	LRT	0.28798941816734
812	KC_AB1	Syn	-0.101169413161456	0.547226072493796	1	pseudobulk	DESeq2	LRT	0.26183321890775
812	KC_AB1	Syn1	0.0405825075237046	0.87739782274744	1	pseudobulk	DESeq2	LRT	0.05680344762647
812	KC_AB1	Syn2	0.347235886838676	0.258657878274268	1	pseudobulk	DESeq2	LRT	0.58727428919019
812	KC_AB1	Synd	-0.310779203144538	0.363668098963064	1	pseudobulk	DESeq2	LRT	0.43929479367156
812	KC_AB1	Syngr	-0.011815684618399	0.943413023958687	1	pseudobulk	DESeq2	LRT	0.02529813254829
812	KC_AB1	Synj	-0.1002132815172	0.693987086516757	1	pseudobulk	DESeq2	LRT	0.15864861067870
812	KC_AB1	Syp	0.15069342840272	0.238004248936562	1	pseudobulk	DESeq2	LRT	0.62341528969461
812	KC_AB1	Syt1	-0.119123066394582	0.236040911560265	1	pseudobulk	DESeq2	LRT	0.62701271683922
812	KC_AB1	Syt12	-0.1388762264071	0.470009084059759	1	pseudobulk	DESeq2	LRT	0.32789374819428
813	KC_AB1	Syt14	-0.207256440784536	0.433560011851053	1	pseudobulk	DESeq2	LRT	0.36295078051322
813	KC_AB1	Syt4	-0.142449961937576	0.322340164922104	1	pseudobulk	DESeq2	LRT	0.49168557622135
813	KC_AB1	Syt7	-0.048559136493155	0.645656341810309	1	pseudobulk	DESeq2	LRT	0.18999857885960
813	KC_AB1	Sytalpha	-0.074139212510261	0.563032442500329	1	pseudobulk	DESeq2	LRT	0.24946657993917
813	KC_AB1	Sytbeta	0.145486671621255	0.576833378054755	1	pseudobulk	DESeq2	LRT	0.23894961741901
813	KC_AB1	Syx13	-0.260793960629256	0.498161839064572	1	pseudobulk	DESeq2	LRT	0.30262954399831
813	KC_AB1	Syx16	0.0312394677775194	0.904591576700837	1	pseudobulk	DESeq2	LRT	0.04354746059781
813	KC_AB1	Syx17	0.46823556372091	0.211367649703903	1	pseudobulk	DESeq2	LRT	0.67496148169361
813	KC_AB1	Syx18	0.352680762097287	0.513163931164249	1	pseudobulk	DESeq2	LRT	0.28974387655047
813	KC_AB1	Syx1A	-0.020846337084016	0.85469751637148	1	pseudobulk	DESeq2	LRT	0.06818755803656
814	KC_AB1	Syx4	1.95275942187961	0.438420007953907	1	pseudobulk	DESeq2	LRT	0.35810963439207
814	KC_AB1	Syx5	0.644012853977174	0.127820275007281	1	pseudobulk	DESeq2	LRT	0.89340025239522
814	KC_AB1	Syx6	0.133367521395071	0.557408150749077	1	pseudobulk	DESeq2	LRT	0.25382668506751
814	KC_AB1	Syx7	-0.043153221515975	0.868380785429736	1	pseudobulk	DESeq2	LRT	0.06128979469960
814	KC_AB1	Syx8	0.37318170233634	0.360802055502206	1	pseudobulk	DESeq2	LRT	0.44273099693186
814	KC_AB1	t-cup	-0.618422766585323	0.856874262644257	1	pseudobulk	DESeq2	LRT	0.06708290158447
814	KC_AB1	T48	1.08899820779554	0.878177167143508	1	pseudobulk	DESeq2	LRT	0.05641785886507
814	KC_AB1	Tab2	-0.82542030717083	1	1	pseudobulk	DESeq2	LRT	0
814	KC_AB1	tacc	0.0678082101639706	0.672316143595856	1	pseudobulk	DESeq2	LRT	0.17242646037933
814	KC_AB1	Tace	-0.151206457610721	0.669274255587409	1	pseudobulk	DESeq2	LRT	0.17439588031752
815	KC_AB1	tadr	-1.00997296364438	0.40814960779223	1	pseudobulk	DESeq2	LRT	0.38918061649581
815	KC_AB1	Taf1	-0.321842472055349	0.475481640776936	1	pseudobulk	DESeq2	LRT	0.32286624731373
815	KC_AB1	Taf10	-0.332741368345954	0.282277690269786	1	pseudobulk	DESeq2	LRT	0.54932344482721
815	KC_AB1	Taf10b	-0.127191365281555	0.567204483079848	1	pseudobulk	DESeq2	LRT	0.24626034522776
815	KC_AB1	Taf11	0.0914731617608992	0.834853546992627	1	pseudobulk	DESeq2	LRT	0.07838970333248
815	KC_AB1	Taf12	0.0122802774127737	0.95095986253145	1	pseudobulk	DESeq2	LRT	0.02183781308253
815	KC_AB1	Taf13	-0.033603126299365	0.930985609063732	1	pseudobulk	DESeq2	LRT	0.03105703217925
815	KC_AB1	TAF1B	-1.11297944558263	0.34987513549639	1	pseudobulk	DESeq2	LRT	0.45608692033621
815	KC_AB1	TAF1C-like	-0.60932435831973	0.610475001893519	1	pseudobulk	DESeq2	LRT	0.21433211511300
815	KC_AB1	Taf2	-0.483658855636439	0.576385291056934	1	pseudobulk	DESeq2	LRT	0.23928711060321
816	KC_AB1	Taf4	-0.203721110308034	0.350166279296736	1	pseudobulk	DESeq2	LRT	0.45572567841381
816	KC_AB1	Taf5	0.453157149311311	0.473729368707802	1	pseudobulk	DESeq2	LRT	0.32446969048342
816	KC_AB1	Taf6	-0.326420423758454	0.397474088196118	1	pseudobulk	DESeq2	LRT	0.40069117825340

816	KC_AB1	Taf7	0.439323872113259	0.508652188388234	1	pseudobulk	DESeq2	LRT	0.29357908268517
816	KC_AB1	Taf8	-0.313684076735059	0.616834129218932	1	pseudobulk	DESeq2	LRT	0.20983160492889
816	KC_AB1	tai	0.199189111673981	0.0655311020315293	1	pseudobulk	DESeq2	LRT	1.18355252848537
816	KC_AB1	Tailor	-0.005052662839437	0.972930231000541	1	pseudobulk	DESeq2	LRT	0.01191830194939
816	KC_AB1	Tak1	-0.562951673031012	0.279891165090526	1	pseudobulk	DESeq2	LRT	0.55301081004755
816	KC_AB1	Takl1	1.08899820779554	0.697631521651407	1	pseudobulk	DESeq2	LRT	0.156373904552
816	KC_AB1	Takl2	-0.850932661185203	0.660736244265418	1	pseudobulk	DESeq2	LRT	0.17997186959134
817	KC_AB1	Taldo	0.51611007135081	0.60756022019249	1	pseudobulk	DESeq2	LRT	0.21641066916813
817	KC_AB1	tam	0.545617592249095	0.683893247237766	1	pseudobulk	DESeq2	LRT	0.16501168446971
817	KC_AB1	tamo	-0.391772430941648	0.313001968107533	1	pseudobulk	DESeq2	LRT	0.50445293166903
817	KC_AB1	Tango1	-0.162607054226272	0.647165305618244	1	pseudobulk	DESeq2	LRT	0.18898477319805
817	KC_AB1	Tango10	-0.281422061276186	0.445484779433682	1	pseudobulk	DESeq2	LRT	0.35116712960889
817	KC_AB1	Tango11	-0.090325940651831	0.723835464373734	1	pseudobulk	DESeq2	LRT	0.14036014241530
817	KC_AB1	Tango14	-0.479404408091322	0.29293380065435	1	pseudobulk	DESeq2	LRT	0.53323051363512
817	KC_AB1	Tango2	0.210594227283806	0.850260588385007	1	pseudobulk	DESeq2	LRT	0.07044795104646
817	KC_AB1	Tango4	-0.260367293507668	0.687807429531328	1	pseudobulk	DESeq2	LRT	0.16253313734356
817	KC_AB1	Tango5	-0.018103110562075	0.917296981450144	1	pseudobulk	DESeq2	LRT	0.03749003562322
818	KC_AB1	Tango6	0.698494621547974	0.53656027769592	1	pseudobulk	DESeq2	LRT	0.27038148189892
818	KC_AB1	Tango8	-0.850996402080018	0.900449987182352	1	pseudobulk	DESeq2	LRT	0.04554040377050
818	KC_AB1	Tango9	-0.157442315103515	0.698028606108359	1	pseudobulk	DESeq2	LRT	0.15612677906675
818	KC_AB1	tank	0.52969618186738	0.420616619755137	1	pseudobulk	DESeq2	LRT	0.37611357110441
818	KC_AB1	tant	-0.104430779511593	0.838239045927819	1	pseudobulk	DESeq2	LRT	0.07663211320386
818	KC_AB1	Tao	-0.138096096548874	0.571571591339659	1	pseudobulk	DESeq2	LRT	0.24292936495984
818	KC_AB1	Tap42	0.0591238093970666	0.920159188695747	1	pseudobulk	DESeq2	LRT	0.03613703266357
818	KC_AB1	tapas	0.031059548452237	0.892512005943998	1	pseudobulk	DESeq2	LRT	0.04938593310914
818	KC_AB1	Tapdelta	0.304239834497932	0.355589627093024	1	pseudobulk	DESeq2	LRT	0.44905091632377
818	KC_AB1	tara	0.377021679083149	0.0873067674240976	1	pseudobulk	DESeq2	LRT	1.05895209144871
819	KC_AB1	Task6	-0.126624851947523	0.279671123940426	1	pseudobulk	DESeq2	LRT	0.55335237224386
819	KC_AB1	Task7	0.0986363763056394	0.699692105918948	1	pseudobulk	DESeq2	LRT	0.15509302586659
819	KC_AB1	Tasp1	-0.29700081948127	0.75763136435278	1	pseudobulk	DESeq2	LRT	0.12054205475004
819	KC_AB1	Tat	-0.357094613298592	0.14159322474709	1	pseudobulk	DESeq2	LRT	0.84895752719250
819	KC_AB1	tau	0.107846452446949	0.548897905724621	1	pseudobulk	DESeq2	LRT	0.26050842623054
819	KC_AB1	tay	-0.014033143357983	0.895073868761359	1	pseudobulk	DESeq2	LRT	0.04814112169903
819	KC_AB1	Taz	-0.297308113888848	0.614346593103604	1	pseudobulk	DESeq2	LRT	0.21158654578993
819	KC_AB1	Tbc1d15-17	0.0060889336613257	0.950431734334652	1	pseudobulk	DESeq2	LRT	0.02207907129350
819	KC_AB1	TBC1D16	-0.13395078373577	0.60831691478559	1	pseudobulk	DESeq2	LRT	0.21587010743003
819	KC_AB1	TBC1D5	0.0117584791335123	0.986480747657878	1	pseudobulk	DESeq2	LRT	0.00591138610113
820	KC_AB1	TBC1d7	0.810260665324239	0.29812585217671	1	pseudobulk	DESeq2	LRT	0.52560036220662
820	KC_AB1	TBCB	-0.328439298636364	0.408580153964557	1	pseudobulk	DESeq2	LRT	0.38872273225326
820	KC_AB1	TBCC	0.374278879233346	0.340498514079348	1	pseudobulk	DESeq2	LRT	0.46788477898922
820	KC_AB1	TBCD	0.471332296867904	0.554545163205786	1	pseudobulk	DESeq2	LRT	0.25606307831134
820	KC_AB1	Tbce	0.156518353522245	0.686402648365425	1	pseudobulk	DESeq2	LRT	0.16342104950627
820	KC_AB1	Tbh	1.26418346555976	0.618943317621274	1	pseudobulk	DESeq2	LRT	0.20834912153152
820	KC_AB1	Tbp	-0.19627651596518	0.787878071236955	1	pseudobulk	DESeq2	LRT	0.10354098693450
820	KC_AB1	TBPH	-0.036389623096359	0.845232238068619	1	pseudobulk	DESeq2	LRT	0.07302394684416
820	KC_AB1	tbrd-1	-0.850996402080018	0.900449987182352	1	pseudobulk	DESeq2	LRT	0.04554040377050
820	KC_AB1	Tcs3	-0.882919306583677	0.210117244560621	1	pseudobulk	DESeq2	LRT	0.67753830309053
821	KC_AB1	Tctp	-0.007849200529934	0.960024376213295	1	pseudobulk	DESeq2	LRT	0.01771773954322
821	KC_AB1	Tdrd3	-0.116800985567204	0.812368748317675	1	pseudobulk	DESeq2	LRT	0.09024679217792
821	KC_AB1	tea	0.278490252439381	0.623331673196384	1	pseudobulk	DESeq2	LRT	0.20528080485045
821	KC_AB1	tef	-0.023361126597737	0.958623852244691	1	pseudobulk	DESeq2	LRT	0.01835176920050
821	KC_AB1	tefu	0.563960670394673	0.765273331028603	1	pseudobulk	DESeq2	LRT	0.11618342111315

821	KC_AB1	Teh1	0.72364088990596	0.14631256385617	1	pseudobulk	DESeq2	LRT	0.834718379417217
821	KC_AB1	Teh2	0.0477230378230292	0.780464415937924	1	pseudobulk	DESeq2	LRT	0.107646893132118
821	KC_AB1	Teh3	-1.36912927419679	0.343010395498608	1	pseudobulk	DESeq2	LRT	0.464692717743618
821	KC_AB1	Teh4	-0.0475369231121604	0.826589304092418	1	pseudobulk	DESeq2	LRT	0.082710218705527
821	KC_AB1	Tehao	-2.39796000128417	0.112520153479531	1	pseudobulk	DESeq2	LRT	0.948769684120879
822	KC_AB1	Tektin-C	1.08899820779554	0.670846322504933	1	pseudobulk	DESeq2	LRT	0.173376956629912
822	KC_AB1	temp	-0.255697173870781	0.572189327711947	1	pseudobulk	DESeq2	LRT	0.242460246773739
822	KC_AB1	Ten-a	-0.073557464492928	0.434885172164803	1	pseudobulk	DESeq2	LRT	0.361625399777263
822	KC_AB1	Ten-m	-0.430050147969919	0.438651600409646	1	pseudobulk	DESeq2	LRT	0.357880281789722
822	KC_AB1	Tengl4	-0.82542030717083	1	1	pseudobulk	DESeq2	LRT	0
822	KC_AB1	teq	0.994343727405496	0.644884606210224	1	pseudobulk	DESeq2	LRT	0.190517989813701
822	KC_AB1	TER94	-0.0011155118901226	0.90919091561359	1	pseudobulk	DESeq2	LRT	0.041344912276806
822	KC_AB1	Tes	-0.05172926823694	0.950494605063742	1	pseudobulk	DESeq2	LRT	0.022050343814525
822	KC_AB1	tex	-1.56642216281807	0.260568321891143	1	pseudobulk	DESeq2	LRT	0.584078383957734
822	KC_AB1	TFAM	0.250766187153878	0.57718205404028	1	pseudobulk	DESeq2	LRT	0.238687180614652
823	KC_AB1	Tfb1	-0.0961051822673182	0.842877848135533	1	pseudobulk	DESeq2	LRT	0.074235359805345
823	KC_AB1	Tfb4	-0.30066348659609	0.742129082294611	1	pseudobulk	DESeq2	LRT	0.129520549097522
823	KC_AB1	TfIIA-L	-0.44069739019941	0.255044981278836	1	pseudobulk	DESeq2	LRT	0.593383218003614
823	KC_AB1	TfIIA-S	-0.143087357250002	0.480505064486425	1	pseudobulk	DESeq2	LRT	0.318302030540874
823	KC_AB1	TfIIB	-0.0284774548373322	0.890181141062474	1	pseudobulk	DESeq2	LRT	0.050521610704203
823	KC_AB1	TfIIEalpha	0.0428679633974233	0.900429909753464	1	pseudobulk	DESeq2	LRT	0.045550087388596
823	KC_AB1	TfIIEbeta	0.0361589768945943	0.949488105984022	1	pseudobulk	DESeq2	LRT	0.022510471198538
823	KC_AB1	TfIIFalpha	0.0853629937755748	0.75841158485903	1	pseudobulk	DESeq2	LRT	0.120095041701209
823	KC_AB1	TfIIFbeta	-0.143713905514278	0.771561484664812	1	pseudobulk	DESeq2	LRT	0.112629459896775
823	KC_AB1	TfIIS	0.194955579883575	0.489287185176444	1	pseudobulk	DESeq2	LRT	0.31043615861169
824	KC_AB1	Tg	-3.19465691336346	0.109017056567507	1	pseudobulk	DESeq2	LRT	0.962505547989357
824	KC_AB1	Tgi	0.119608457226894	0.759987737881765	1	pseudobulk	DESeq2	LRT	0.119193414841902
824	KC_AB1	tgo	-0.498370226527706	0.505667923246829	1	pseudobulk	DESeq2	LRT	0.296134594713777
824	KC_AB1	Tgt	0.685369048915063	0.587557205420701	1	pseudobulk	DESeq2	LRT	0.230949843457788
824	KC_AB1	TH1	0.232355458847061	0.666414544358242	1	pseudobulk	DESeq2	LRT	0.176255533111475
824	KC_AB1	THADA	0.785279928379204	0.598955895140116	1	pseudobulk	DESeq2	LRT	0.222605156245894
824	KC_AB1	Thd1	0.0662622429226096	0.635684336736968	1	pseudobulk	DESeq2	LRT	0.196758489454409
824	KC_AB1	Theg	-1.35597590423535	0.632167787041343	1	pseudobulk	DESeq2	LRT	0.199167637991254
824	KC_AB1	THG	0.305425265002219	0.80584075578903	1	pseudobulk	DESeq2	LRT	0.093750771737067
824	KC_AB1	tho2	-0.105391159427467	0.740958542224442	1	pseudobulk	DESeq2	LRT	0.130206090788348
825	KC_AB1	thoc5	-0.0956577930398655	0.883185814213391	1	pseudobulk	DESeq2	LRT	0.053947915224390
825	KC_AB1	thoc6	-0.0642550994447604	0.925613762028336	1	pseudobulk	DESeq2	LRT	0.033570196916576
825	KC_AB1	thoc7	0.166232413301354	0.632769710828301	1	pseudobulk	DESeq2	LRT	0.19875431765033
825	KC_AB1	ThrRS	-0.297767910044358	0.36767227400583	1	pseudobulk	DESeq2	LRT	0.43453911884007
825	KC_AB1	ths	-0.291184974677637	0.599671423422295	1	pseudobulk	DESeq2	LRT	0.222086646419367
825	KC_AB1	Tif-IA	0.141654726574792	0.60851523589426	1	pseudobulk	DESeq2	LRT	0.215728543511209
825	KC_AB1	tim	-0.484513118378328	0.785166097958822	1	pseudobulk	DESeq2	LRT	0.105038460711449
825	KC_AB1	Tim10	0.111831901354254	1	1	pseudobulk	DESeq2	LRT	0
825	KC_AB1	Tim17b	-0.173973233514422	0.271811432338566	1	pseudobulk	DESeq2	LRT	0.565732280873798
825	KC_AB1	Tim17b1	0.207536541292615	0.907538084137969	1	pseudobulk	DESeq2	LRT	0.042135141075404
826	KC_AB1	Tim23	-0.0630366159885346	0.785410953697636	1	pseudobulk	DESeq2	LRT	0.104903046155406
826	KC_AB1	Tim8	-0.221250881723429	0.383081565722859	1	pseudobulk	DESeq2	LRT	0.416708746209205
826	KC_AB1	Tim9a	-0.0593583223780005	0.861974536406746	1	pseudobulk	DESeq2	LRT	0.064505563480833
826	KC_AB1	timeout	0.402598552024792	0.512358057977321	1	pseudobulk	DESeq2	LRT	0.290426429150895
826	KC_AB1	Timp	-0.194511947676764	0.833300043150978	1	pseudobulk	DESeq2	LRT	0.079198595685168
826	KC_AB1	tin	-0.850932661185203	0.660736244265418	1	pseudobulk	DESeq2	LRT	0.179971869591347
826	KC_AB1	Tina-1	-0.0200247934123267	0.876055990568993	1	pseudobulk	DESeq2	LRT	0.057468136277033

826	KC_AB1	tinc	0.0952094734138765	0.62141729446586	1	pseudobulk	DESeq2	LRT	0.206616664202105
826	KC_AB1	Tip60	0.347486804966068	0.545774147608019	1	pseudobulk	DESeq2	LRT	0.262987039968966
826	KC_AB1	tipE	-0.086285482084607	0.720481418053272	1	pseudobulk	DESeq2	LRT	0.14237721560085
827	KC_AB1	Tis11	-0.506516985469986	0.909346984303859	1	pseudobulk	DESeq2	LRT	0.041270369127443
827	KC_AB1	tko	-0.306746353402062	0.38305913622398	1	pseudobulk	DESeq2	LRT	0.416734174978549
827	KC_AB1	TkR86C	-4.11107823992848	0.0266466534757747	1	pseudobulk	DESeq2	LRT	1.57435732578431
827	KC_AB1	tkv	0.254226963760035	0.424603525999946	1	pseudobulk	DESeq2	LRT	0.372016403679296
827	KC_AB1	Tlk	0.291478833477074	0.0217944003604533	1	pseudobulk	DESeq2	LRT	1.66165507542945
827	KC_AB1	Tm1	-0.0258796616651482	0.868072219134027	1	pseudobulk	DESeq2	LRT	0.061444142258205
827	KC_AB1	Tm2	-0.753287160385906	0.653144847306474	1	pseudobulk	DESeq2	LRT	0.18499049497292
827	KC_AB1	TM4SF	-0.0592723065876891	0.640784147202361	1	pseudobulk	DESeq2	LRT	0.193288241099381
827	KC_AB1	TM9SF2	-0.157332540546914	0.633010536101992	1	pseudobulk	DESeq2	LRT	0.198589061336713
827	KC_AB1	TM9SF3	-0.232353884252103	0.63874082341501	1	pseudobulk	DESeq2	LRT	0.194675326182243
828	KC_AB1	TM9SF4	-0.357242979609321	0.408637314905505	1	pseudobulk	DESeq2	LRT	0.388661978091194
828	KC_AB1	Tmc	-0.157023397055232	0.606355789871889	1	pseudobulk	DESeq2	LRT	0.217272471153413
828	KC_AB1	Tmem18	0.116839268577407	0.870227126849105	1	pseudobulk	DESeq2	LRT	0.060367382940273
828	KC_AB1	TMEM216	0.531815801656096	0.488578462033969	1	pseudobulk	DESeq2	LRT	0.311065681910202
828	KC_AB1	Tmem63	0.220345680403698	0.668227677471361	1	pseudobulk	DESeq2	LRT	0.175075540185243
828	KC_AB1	tmod	0.0971418364640656	0.659775837400147	1	pseudobulk	DESeq2	LRT	0.180603593422282
828	KC_AB1	TMS1	-0.185500498794973	0.303532759711543	1	pseudobulk	DESeq2	LRT	0.517794429484412
828	KC_AB1	Tmtc3	-0.55329981685545	0.122848110346688	1	pseudobulk	DESeq2	LRT	0.910631519463249
828	KC_AB1	tna	0.19550949801762	0.0965374544280712	1	pseudobulk	DESeq2	LRT	1.01530415716864
828	KC_AB1	tnc	-2.94844098471155	0.105635897115187	1	pseudobulk	DESeq2	LRT	0.976188475080009
829	KC_AB1	Tnks	-0.0948655749114719	0.7665010742357	1	pseudobulk	DESeq2	LRT	0.115487232154277
829	KC_AB1	Tnpo	-0.0557049302059287	0.770190641314459	1	pseudobulk	DESeq2	LRT	0.113401762849333
829	KC_AB1	Tnpo-SR	-0.263172918454491	0.740104199542352	1	pseudobulk	DESeq2	LRT	0.130707131484568
829	KC_AB1	Tob	-0.04074227121403	0.68646642744496	1	pseudobulk	DESeq2	LRT	0.163380697658002
829	KC_AB1	toc	0.0088585054453997	0.930285263945863	1	pseudobulk	DESeq2	LRT	0.031383858369770
829	KC_AB1	tok	-0.235187948618382	0.509371712080336	1	pseudobulk	DESeq2	LRT	0.29296517721406
829	KC_AB1	Toll-7	0.333501690638235	0.707277494842632	1	pseudobulk	DESeq2	LRT	0.150410160694814
829	KC_AB1	Tom20	-0.204688629040518	0.292472487334326	1	pseudobulk	DESeq2	LRT	0.533914981413818
829	KC_AB1	Tom40	-0.384517004238952	0.188036720956982	1	pseudobulk	DESeq2	LRT	0.725757330780451
829	KC_AB1	Tom7	-0.214561681720342	0.232703769580934	1	pseudobulk	DESeq2	LRT	0.633196581496515
830	KC_AB1	Tom70	0.0858912850144616	0.856351873543452	1	pseudobulk	DESeq2	LRT	0.067347747778597
830	KC_AB1	tomboy20	-0.850996402080018	0.900449987182352	1	pseudobulk	DESeq2	LRT	0.045540403770502
830	KC_AB1	tomboy40	-0.82542030717083	1	1	pseudobulk	DESeq2	LRT	0
830	KC_AB1	Tomosyn	0.0243585298580779	0.806628355185164	1	pseudobulk	DESeq2	LRT	0.093326515425346
830	KC_AB1	Top1	0.132558124645863	0.384737473968011	1	pseudobulk	DESeq2	LRT	0.41483551074308
830	KC_AB1	Top2	-0.291859130846149	0.501354819634743	1	pseudobulk	DESeq2	LRT	0.299854805733625
830	KC_AB1	Top3alpha	0.55308882610582	0.569246198174086	1	pseudobulk	DESeq2	LRT	0.244699861226705
830	KC_AB1	Top3beta	-0.449058662878231	0.358481457097245	1	pseudobulk	DESeq2	LRT	0.445533303841702
830	KC_AB1	Topors	-0.196630993036183	0.778816440747614	1	pseudobulk	DESeq2	LRT	0.108564889136337
830	KC_AB1	Tor	0.500892654438922	0.414488941542945	1	pseudobulk	DESeq2	LRT	0.382487051822859
831	KC_AB1	TORIP	-0.760864811482459	0.17369343822339	1	pseudobulk	DESeq2	LRT	0.760216587984687
831	KC_AB1	Torsin	0.038889561385712	0.920856245593304	1	pseudobulk	DESeq2	LRT	0.035808162005559
831	KC_AB1	tos	-2.19286887839518	0.260100170239712	1	pseudobulk	DESeq2	LRT	0.584859363551365
831	KC_AB1	TotA	1.20203426906033	0.130216262380925	1	pseudobulk	DESeq2	LRT	0.885334774437096
831	KC_AB1	TotB	-0.850996402080018	0.900449987182352	1	pseudobulk	DESeq2	LRT	0.045540403770502
831	KC_AB1	tou	0.118712894393981	0.484890925884042	1	pseudobulk	DESeq2	LRT	0.314355943076396
831	KC_AB1	tow	0.0931888722005012	0.493456329947506	1	pseudobulk	DESeq2	LRT	0.306751275622536
831	KC_AB1	toy	-0.0698917751902107	0.505304379135088	1	pseudobulk	DESeq2	LRT	0.296446938005658
831	KC_AB1	Tpc1	0.852536599157774	0.245970510941064	1	pseudobulk	DESeq2	LRT	0.609116956730948

831	KC_AB1	Tpc2	2.1559833397943	0.381743505411375	1	pseudobulk	DESeq2	LRT	0.418228342845202
832	KC_AB1	Tpi	-0.302867313955091	0.192078773028303	1	pseudobulk	DESeq2	LRT	0.71652062715313
832	KC_AB1	TpnC41C	1.08899820779554	0.697631521651407	1	pseudobulk	DESeq2	LRT	0.156373904552
832	KC_AB1	TppII	0.0461121122651123	0.930544323617837	1	pseudobulk	DESeq2	LRT	0.031262935758155
832	KC_AB1	Tpr2	0.0377549256547886	0.846633262416592	1	pseudobulk	DESeq2	LRT	0.072304673022170
832	KC_AB1	Tps1	-0.850996402080018	0.900449987182352	1	pseudobulk	DESeq2	LRT	0.045540403770502
832	KC_AB1	Tpst	0.454499020334851	0.250146698916958	1	pseudobulk	DESeq2	LRT	0.601805223948308
832	KC_AB1	tra	-0.091414094356558	0.794717670335842	1	pseudobulk	DESeq2	LRT	0.099787130453557
832	KC_AB1	tra2	0.105918304422389	0.778834482793618	1	pseudobulk	DESeq2	LRT	0.108554828395636
832	KC_AB1	Traf4	-0.82542030717083	1	1	pseudobulk	DESeq2	LRT	0
832	KC_AB1	Traf6	-0.378538122158266	0.570100833879601	1	pseudobulk	DESeq2	LRT	0.244048323758113
833	KC_AB1	tral	0.0718770003185582	0.60983888033897	1	pseudobulk	DESeq2	LRT	0.214784890599715
833	KC_AB1	TRAM	0.146971658717208	0.811144394918724	1	pseudobulk	DESeq2	LRT	0.090901828483702
833	KC_AB1	Trap1	0.43185362316706	0.354107542465494	1	pseudobulk	DESeq2	LRT	0.450864822701764
833	KC_AB1	Trax	0.798908623573605	0.282380980272246	1	pseudobulk	DESeq2	LRT	0.549164558473582
833	KC_AB1	trbd	-0.318758608603918	0.365610159861182	1	pseudobulk	DESeq2	LRT	0.436981744255656
833	KC_AB1	trc	-0.225338386122809	0.506911782548795	1	pseudobulk	DESeq2	LRT	0.29506761401145
833	KC_AB1	Trc8	0.345271177099722	0.394388154067658	1	pseudobulk	DESeq2	LRT	0.404076138102811
833	KC_AB1	Tre1	1.08899820779554	0.697631521651407	1	pseudobulk	DESeq2	LRT	0.156373904552
833	KC_AB1	Treh	-0.222222114620994	0.721517587250217	1	pseudobulk	DESeq2	LRT	0.141753078357555
833	KC_AB1	trem	-0.0535750765453332	0.948213133907973	1	pseudobulk	DESeq2	LRT	0.023094033467331
834	KC_AB1	Tret1-1	1.68130633771206	0.562684503730219	1	pseudobulk	DESeq2	LRT	0.249735045103917
834	KC_AB1	Trf	0.0173435162062991	0.974871175277721	1	pseudobulk	DESeq2	LRT	0.011052770519393
834	KC_AB1	Trf2	-0.0190053390557017	0.905650154263682	1	pseudobulk	DESeq2	LRT	0.043039534569207
834	KC_AB1	Trf4-1	-0.255552362880179	0.196542973455251	1	pseudobulk	DESeq2	LRT	0.706542477888965
834	KC_AB1	Trim9	-0.82542030717083	1	1	pseudobulk	DESeq2	LRT	0
834	KC_AB1	trio	-0.194483712133787	0.388470195136627	1	pseudobulk	DESeq2	LRT	0.410642296256229
834	KC_AB1	TrissinR	0.420479375459253	0.85373567265173	1	pseudobulk	DESeq2	LRT	0.068676571556975
834	KC_AB1	Trl	0.647622069737913	0.221601871383977	1	pseudobulk	DESeq2	LRT	0.654426576397113
834	KC_AB1	trn	-0.365515258416992	0.0774682349135935	1	pseudobulk	DESeq2	LRT	1.11087633916713
834	KC_AB1	tRNA:Ala-CGC-1-1	-0.850996402080018	0.900449987182352	1	pseudobulk	DESeq2	LRT	0.045540403770502
835	KC_AB1	tRNA:Arg-TCG-2-3	1.08899820779554	0.697631521651407	1	pseudobulk	DESeq2	LRT	0.156373904552
835	KC_AB1	tRNA:Arg-TCG-2-4	1.14496938012276	0.577963365460694	1	pseudobulk	DESeq2	LRT	0.238099688711815
835	KC_AB1	tRNA:Glu-TTC-1-2	1.08899820779554	0.697631521651407	1	pseudobulk	DESeq2	LRT	0.156373904552
835	KC_AB1	tRNA:Gly-GCC-1-1	-0.615767568564188	0.806245159731688	1	pseudobulk	DESeq2	LRT	0.093532879622572
835	KC_AB1	tRNA:Ile-TAT-1-2	1.08899820779554	0.878177167143508	1	pseudobulk	DESeq2	LRT	0.056417858865076
835	KC_AB1	tRNA:Lys-CTT-2-1	0.221643939736625	0.905680069226275	1	pseudobulk	DESeq2	LRT	0.043025189417767
835	KC_AB1	tRNA:Lys-TTT-2-1	-0.850996402080018	0.900449987182352	1	pseudobulk	DESeq2	LRT	0.045540403770502
835	KC_AB1	tRNA:Pro-CGG-1-1	1.08899820779554	0.697631521651407	1	pseudobulk	DESeq2	LRT	0.156373904552
835	KC_AB1	tRNA:SeC-TCA-1-	1.08899820779554	0.878177167143508	1	pseudobulk	DESeq2	LRT	0.056417858865076
835	KC_AB1	trol	0.13617601020169	1	1	pseudobulk	DESeq2	LRT	0
836	KC_AB1	trp	-1.82709236256241	0.44317674354685	1	pseudobulk	DESeq2	LRT	0.353423038023035
836	KC_AB1	Trp1	0.125432523487978	0.642318924767454	1	pseudobulk	DESeq2	LRT	0.192249282094575
836	KC_AB1	trpl	1.08899820779554	0.878177167143508	1	pseudobulk	DESeq2	LRT	0.056417858865076
836	KC_AB1	Trpm	-0.0976951697747186	0.673887225330972	1	pseudobulk	DESeq2	LRT	0.171412776334711
836	KC_AB1	TrpRS	0.461638588190256	0.632775069532958	1	pseudobulk	DESeq2	LRT	0.19875063977832
836	KC_AB1	TrpRS-m	-0.420160340936842	0.71326005597168	1	pseudobulk	DESeq2	LRT	0.146752096681437
836	KC_AB1	trr	-0.0642666608278363	0.827056253678792	1	pseudobulk	DESeq2	LRT	0.082464950143438
836	KC_AB1	Trs20	0.0031638623592869	0.961517809117401	1	pseudobulk	DESeq2	LRT	0.017042667400762
836	KC_AB1	Trs23	-0.0153136593936421	0.914596570922734	1	pseudobulk	DESeq2	LRT	0.038770431243251
836	KC_AB1	Trs31	0.283276129637638	0.43070890880731	1	pseudobulk	DESeq2	LRT	0.365816145172991
837	KC_AB1	Trs33	0.0213390878515267	0.958260624523936	1	pseudobulk	DESeq2	LRT	0.018516356893293

837	KC_AB1	trsn	0.23244659343832	0.572147625454628	1	pseudobulk	DESeq2	LRT	0.242491900143307
837	KC_AB1	trus	-0.384950200502729	0.468883778518847	1	pseudobulk	DESeq2	LRT	0.328934791832731
837	KC_AB1	trv	0.0321127453171675	0.774942482303003	1	pseudobulk	DESeq2	LRT	0.110730530455512
837	KC_AB1	trx	-0.050728898816441	0.773828860414952	1	pseudobulk	DESeq2	LRT	0.111355077037588
837	KC_AB1	Trx-2	0.160078236748864	0.454585676486014	1	pseudobulk	DESeq2	LRT	0.342384252555366
837	KC_AB1	Trxr-1	-0.109717856510758	0.71113227957442	1	pseudobulk	DESeq2	LRT	0.148049607503912
837	KC_AB1	Try29F	1.98602877977907	0.170620575097334	1	pseudobulk	DESeq2	LRT	0.767968598535196
837	KC_AB1	Ts	2.1559833397943	0.381743505411375	1	pseudobulk	DESeq2	LRT	0.418228342845202
837	KC_AB1	Tsc1	-0.0697642200232762	0.895840879988348	1	pseudobulk	DESeq2	LRT	0.047769123239544
838	KC_AB1	Tsen15	0.420564027906161	0.737188344536495	1	pseudobulk	DESeq2	LRT	0.132421539898298
838	KC_AB1	Tsen2	1.30676635984905	0.145529847386115	1	pseudobulk	DESeq2	LRT	0.837047926088912
838	KC_AB1	Tsen34	-0.267822596722221	0.556620623854313	1	pseudobulk	DESeq2	LRT	0.254440706266101
838	KC_AB1	Tsen54	0.870419417370055	0.364447551334806	1	pseudobulk	DESeq2	LRT	0.438364963381214
838	KC_AB1	Tsf1	1.18643961142846	0.570345860202777	1	pseudobulk	DESeq2	LRT	0.243861706387452
838	KC_AB1	Tsf2	0.536327490124491	0.400993864282542	1	pseudobulk	DESeq2	LRT	0.396862272588349
838	KC_AB1	TSG101	0.0454156014856919	0.88764451079383	1	pseudobulk	DESeq2	LRT	0.051760928265775
838	KC_AB1	tsl	1.08899820779554	0.878177167143508	1	pseudobulk	DESeq2	LRT	0.056417858865076
838	KC_AB1	Tsp26A	0.426690387772466	0.318805711965611	1	pseudobulk	DESeq2	LRT	0.496473906055966
838	KC_AB1	Tsp2A	-0.850996402080018	0.900449987182352	1	pseudobulk	DESeq2	LRT	0.045540403770502
839	KC_AB1	Tsp39D	0.0678061562919375	0.710618525759444	1	pseudobulk	DESeq2	LRT	0.148363474682007
839	KC_AB1	Tsp3A	0.485904776271933	0.37697255666082	1	pseudobulk	DESeq2	LRT	0.423690264978794
839	KC_AB1	Tsp42Ea	-0.82542030717083	1	1	pseudobulk	DESeq2	LRT	0
839	KC_AB1	Tsp42Ee	0.215666095345363	0.417989947092251	1	pseudobulk	DESeq2	LRT	0.378834163140906
839	KC_AB1	Tsp42Ej	-0.145493894802758	0.333724528031024	1	pseudobulk	DESeq2	LRT	0.476611872467016
839	KC_AB1	Tsp42Ek	-0.457920057450178	0.41595264434427	1	pseudobulk	DESeq2	LRT	0.380956110408471
839	KC_AB1	Tsp42El	-2.3118469151042	0.353435572380865	1	pseudobulk	DESeq2	LRT	0.451689742007392
839	KC_AB1	Tsp42En	0.363358099020769	0.0374795970252913	1	pseudobulk	DESeq2	LRT	1.42620508722485
839	KC_AB1	Tsp42Eo	0.554711015364136	0.504895018354167	1	pseudobulk	DESeq2	LRT	0.296798914335152
839	KC_AB1	Tsp42Ep	-0.398697488139235	0.814270144992142	1	pseudobulk	DESeq2	LRT	0.089231488212028
840	KC_AB1	Tsp5D	1.45141434898336	0.518035873737501	1	pseudobulk	DESeq2	LRT	0.285640164527385
840	KC_AB1	Tsp66E	-0.0386299975656576	0.794976100565634	1	pseudobulk	DESeq2	LRT	0.099645927379356
840	KC_AB1	Tsp86D	0.196260675968405	0.681653181100685	1	pseudobulk	DESeq2	LRT	0.16643653420734
840	KC_AB1	Tsp96F	0.0996366899299103	0.644219620231994	1	pseudobulk	DESeq2	LRT	0.190966052523575
840	KC_AB1	Tsp97E	-0.0763060638419747	0.688827845267015	1	pseudobulk	DESeq2	LRT	0.161889305222545
840	KC_AB1	Tspo	-0.296244282378343	0.495116508491539	1	pseudobulk	DESeq2	LRT	0.305292592902503
840	KC_AB1	tsr	-0.10538256313429	0.602811704661052	1	pseudobulk	DESeq2	LRT	0.219818323675991
840	KC_AB1	Tsr1	0.0348791764039771	0.91789478865031	1	pseudobulk	DESeq2	LRT	0.037207095843647
840	KC_AB1	tst	0.177179136308329	0.819007821401076	1	pseudobulk	DESeq2	LRT	0.086711950773026
840	KC_AB1	tsu	-0.0826118488169163	0.826976718112993	1	pseudobulk	DESeq2	LRT	0.082506716973915
841	KC_AB1	Ttc19	-0.529802326220956	0.289381018279405	1	pseudobulk	DESeq2	LRT	0.538529959488142
841	KC_AB1	Ttc30	-0.850932661185203	0.660736244265418	1	pseudobulk	DESeq2	LRT	0.179971869591347
841	KC_AB1	Ttc7	-0.29679743245523	0.45454274534005	1	pseudobulk	DESeq2	LRT	0.342425269334427
841	KC_AB1	Ttd14	0.196708411102255	0.378130576031162	1	pseudobulk	DESeq2	LRT	0.422358203714638
841	KC_AB1	tth	0.133321349068886	0.591577961035873	1	pseudobulk	DESeq2	LRT	0.227988013814419
841	KC_AB1	TTLL12	0.604429684343425	0.549210875999564	1	pseudobulk	DESeq2	LRT	0.260260871035015
841	KC_AB1	TTLL15	0.0526546887140693	0.939627958166124	1	pseudobulk	DESeq2	LRT	0.027044069484226
841	KC_AB1	TTLL3A	0.311608533279536	0.620607396350791	1	pseudobulk	DESeq2	LRT	0.207183052833076
841	KC_AB1	TTLL4A	0.106937740008268	0.728171745102357	1	pseudobulk	DESeq2	LRT	0.137766176794826
841	KC_AB1	TTLL5	-0.176331472447189	0.76803492736579	1	pseudobulk	DESeq2	LRT	0.114619029425109
842	KC_AB1	ttm2	-1.99275908811736	0.412167844949191	1	pseudobulk	DESeq2	LRT	0.384925892483647
842	KC_AB1	ttm50	-0.305014198227007	0.44602977256346	1	pseudobulk	DESeq2	LRT	0.350636151089492
842	KC_AB1	ttv	0.184213518227994	0.297537615908	1	pseudobulk	DESeq2	LRT	0.526458121200719

842	KC_AB1	tty	-0.0291416295557878	0.841574365411658	1	pseudobulk	DESeq2	LRT	0.074907501719111
842	KC_AB1	tub	0.215033071406597	0.654656370810706	1	pseudobulk	DESeq2	LRT	0.183986601414238
842	KC_AB1	tud	0.286562262390888	0.347006052972935	1	pseudobulk	DESeq2	LRT	0.459662949561238
842	KC_AB1	Tudor-SN	-0.403308416156352	0.309331424105012	1	pseudobulk	DESeq2	LRT	0.50957595900689
842	KC_AB1	tum	-0.82542030717083	1	1	pseudobulk	DESeq2	LRT	0
842	KC_AB1	tun	0.0626835665797841	0.787141700694354	1	pseudobulk	DESeq2	LRT	0.103947079216375
842	KC_AB1	Tusp	0.0665335168684978	0.796637744388279	1	pseudobulk	DESeq2	LRT	0.098739120734601
843	KC_AB1	tut	-0.605926254839629	0.624893224278942	1	pseudobulk	DESeq2	LRT	0.204194184364789
843	KC_AB1	tutl	0.077139320371914	0.456848320537546	1	pseudobulk	DESeq2	LRT	0.340227967299474
843	KC_AB1	tw	-0.268359571488918	0.874433266607883	1	pseudobulk	DESeq2	LRT	0.058273328609344
843	KC_AB1	TwdlG	0.111831901354254	1	1	pseudobulk	DESeq2	LRT	0
843	KC_AB1	TwdlM	0.11269840515639	0.977007463492936	1	pseudobulk	DESeq2	LRT	0.010102118633921
843	KC_AB1	TwdlP	1.08899820779554	0.670846322504933	1	pseudobulk	DESeq2	LRT	0.173376956629912
843	KC_AB1	twe	2.10549911548503	0.428045198278114	1	pseudobulk	DESeq2	LRT	0.368510370411162
843	KC_AB1	tweek	-0.0576720738711345	0.844307683894971	1	pseudobulk	DESeq2	LRT	0.073499258271490
843	KC_AB1	twf	-0.817769359748822	0.108760830892762	1	pseudobulk	DESeq2	LRT	0.963527483221692
843	KC_AB1	twin	0.0143298525611142	0.952048656614004	1	pseudobulk	DESeq2	LRT	0.021340855440127
844	KC_AB1	twit	-0.850996402080018	0.900449987182352	1	pseudobulk	DESeq2	LRT	0.045540403770502
844	KC_AB1	twr	0.136227800259203	0.684612034157614	1	pseudobulk	DESeq2	LRT	0.164555471072651
844	KC_AB1	tws	-0.0130470580848292	0.947688138294619	1	pseudobulk	DESeq2	LRT	0.023334555172837
844	KC_AB1	twz	0.11400025262841	1	1	pseudobulk	DESeq2	LRT	0
844	KC_AB1	tx	-2.35428651150093	0.278147550140927	1	pseudobulk	DESeq2	LRT	0.555724760871319
844	KC_AB1	Txl	-0.0304932295376835	0.907660909458558	1	pseudobulk	DESeq2	LRT	0.042076368060170
844	KC_AB1	tyf	-0.159780250599204	0.3779984910758	1	pseudobulk	DESeq2	LRT	0.422509933810293
844	KC_AB1	tyn	0.350425646620167	0.378568275457635	1	pseudobulk	DESeq2	LRT	0.421855783322493
844	KC_AB1	TyrR	-0.337480708121392	0.721226999379419	1	pseudobulk	DESeq2	LRT	0.141928023682835
844	KC_AB1	TyrRII	-0.850932661185203	0.660736244265418	1	pseudobulk	DESeq2	LRT	0.179971869591347
845	KC_AB1	TyrRS	0.529935337130453	0.302172093702008	1	pseudobulk	DESeq2	LRT	0.519745646253928
845	KC_AB1	TyrRS-m	0.235085710500472	0.794027017792505	1	pseudobulk	DESeq2	LRT	0.100164719892086
845	KC_AB1	tzn	0.112685816026386	0.710577863364711	1	pseudobulk	DESeq2	LRT	0.148388326213848
845	KC_AB1	U2A	0.819405208090544	0.25378328512312	1	pseudobulk	DESeq2	LRT	0.595536985148502
845	KC_AB1	U2af38	-0.0694128300992271	0.832438633652369	1	pseudobulk	DESeq2	LRT	0.079647772294277
845	KC_AB1	U2af50	-0.172011888689614	0.527454861174992	1	pseudobulk	DESeq2	LRT	0.27781470073438
845	KC_AB1	U3-55K	-0.887898655721216	0.447333160339661	1	pseudobulk	DESeq2	LRT	0.349368906837982
845	KC_AB1	U4-U6-60K	0.0728348347268231	0.876004470347578	1	pseudobulk	DESeq2	LRT	0.057493677573549
845	KC_AB1	Uba1	0.111137785187766	0.716126296581037	1	pseudobulk	DESeq2	LRT	0.145010378430612
845	KC_AB1	Uba2	0.0138268276493558	0.96247587502454	1	pseudobulk	DESeq2	LRT	0.016610147507801
846	KC_AB1	Uba3	0.0769487923349573	0.906663049322528	1	pseudobulk	DESeq2	LRT	0.042554083401345
846	KC_AB1	Uba4	-2.2791923705273	0.129137467853913	1	pseudobulk	DESeq2	LRT	0.888947733555504
846	KC_AB1	Uba5	0.632421285426692	0.561677846570323	1	pseudobulk	DESeq2	LRT	0.250512705021841
846	KC_AB1	Ubc10	0.0332218049361133	0.915899962526649	1	pseudobulk	DESeq2	LRT	0.038151958750504
846	KC_AB1	Ubc2	0.126178516337047	0.712048436529433	1	pseudobulk	DESeq2	LRT	0.147490462821051
846	KC_AB1	Ubc4	0.232770626525018	0.410450318498455	1	pseudobulk	DESeq2	LRT	0.386739402997014
846	KC_AB1	Ubc6	-0.0405572563833498	0.826787260285371	1	pseudobulk	DESeq2	LRT	0.082606223905406
846	KC_AB1	Ubc7	-0.308058139404152	0.471085301195269	1	pseudobulk	DESeq2	LRT	0.32690044640786
846	KC_AB1	UbcE2H	0.0429619024822927	0.807375197177658	1	pseudobulk	DESeq2	LRT	0.092924596385120
846	KC_AB1	UbcE2M	0.364070937847139	0.235331663650502	1	pseudobulk	DESeq2	LRT	0.628319634984909
847	KC_AB1	Ube3a	0.109657450101004	0.810531785182809	1	pseudobulk	DESeq2	LRT	0.091229949527581
847	KC_AB1	Ubi-p5E	-0.19278532281824	0.126682985665137	1	pseudobulk	DESeq2	LRT	0.897281709725338
847	KC_AB1	Ubi-p63E	0.188953443238652	0.290958055097607	1	pseudobulk	DESeq2	LRT	0.536169614975066
847	KC_AB1	ubl	0.287772256756105	0.423000320937038	1	pseudobulk	DESeq2	LRT	0.373659303118736
847	KC_AB1	UBL3	0.0899745134030338	0.539491379647488	1	pseudobulk	DESeq2	LRT	0.268015490372059

847	KC_AB1	Ublcp1	-0.348313469360245	0.409495325702762	1	pseudobulk	DESeq2	LRT	0.387751051249309
847	KC_AB1	Ubqn	-0.2874668397173	0.295868285281871	1	pseudobulk	DESeq2	LRT	0.528901585245782
847	KC_AB1	Ubr1	0.0231786152614098	0.890360848256488	1	pseudobulk	DESeq2	LRT	0.050433945438849
847	KC_AB1	Ubr3	0.0264087453208142	0.876201848815903	1	pseudobulk	DESeq2	LRT	0.057395834780649
847	KC_AB1	Uch	-0.066089831385549	0.871213538627799	1	pseudobulk	DESeq2	LRT	0.059875384278842
848	KC_AB1	Uch-L5	-0.278575942706675	0.511246619465362	1	pseudobulk	DESeq2	LRT	0.291369550676324
848	KC_AB1	Uch-L5R	1.08899820779554	0.670846322504933	1	pseudobulk	DESeq2	LRT	0.173376956629912
848	KC_AB1	Uck	-0.378650905482488	0.193558817799838	1	pseudobulk	DESeq2	LRT	0.713187039097898
848	KC_AB1	Ucp4A	0.169494181633156	0.535993778014952	1	pseudobulk	DESeq2	LRT	0.27084025170547
848	KC_AB1	udd	0.446822249911907	0.365225990216549	1	pseudobulk	DESeq2	LRT	0.437438324768616
848	KC_AB1	udt	-0.142641681263047	0.883675708323065	1	pseudobulk	DESeq2	LRT	0.053707083318452
848	KC_AB1	Uev1A	-0.198328621090004	0.389556839861859	1	pseudobulk	DESeq2	LRT	0.409429165880068
848	KC_AB1	uex	-0.0650222261511548	0.628103671819109	1	pseudobulk	DESeq2	LRT	0.201968667754793
848	KC_AB1	Ufc1	0.0271596063592093	0.959136437008525	1	pseudobulk	DESeq2	LRT	0.018119610112646
848	KC_AB1	Ufd1-like	0.283431390340334	0.409815208051849	1	pseudobulk	DESeq2	LRT	0.387411929169458
849	KC_AB1	Ufd4	-0.108719264360012	0.647026823678627	1	pseudobulk	DESeq2	LRT	0.189077714487195
849	KC_AB1	Ufl1	0.0049221639500470	0.921097417849374	1	pseudobulk	DESeq2	LRT	0.035694435170105
849	KC_AB1	Ufm1	0.102158656828266	0.776214244093214	1	pseudobulk	DESeq2	LRT	0.110018391904787
849	KC_AB1	Ugalt	0.302621334849538	0.769742350050785	1	pseudobulk	DESeq2	LRT	0.113654618562783
849	KC_AB1	Uggt	0.184174057190373	0.823078223551051	1	pseudobulk	DESeq2	LRT	0.084558888430245
849	KC_AB1	UGP	-0.600663614734299	0.39074242326817	1	pseudobulk	DESeq2	LRT	0.408109434459709
849	KC_AB1	Ugt316A1	-0.0693782677072405	0.848865814824489	1	pseudobulk	DESeq2	LRT	0.071160955794962
849	KC_AB1	Ugt35B1	0.0103321335792304	0.935527119640646	1	pseudobulk	DESeq2	LRT	0.028943618383565
849	KC_AB1	Ugt35E2	1.08899820779554	0.878177167143508	1	pseudobulk	DESeq2	LRT	0.056417858865076
849	KC_AB1	Ugt37A1	0.942080999092271	0.681532122211136	1	pseudobulk	DESeq2	LRT	0.166513670026992
850	KC_AB1	Ugt50B3	-1.82709236256241	0.44317674354685	1	pseudobulk	DESeq2	LRT	0.353423038023035
850	KC_AB1	Uhg1	-0.478225532101114	0.17300353335607	1	pseudobulk	DESeq2	LRT	0.761945026921069
850	KC_AB1	Uhg2	0.61490868247212	0.118724411103791	1	pseudobulk	DESeq2	LRT	0.925459975925842
850	KC_AB1	Uhg3	0.256690970910017	0.510196806479984	1	pseudobulk	DESeq2	LRT	0.2922622641356
850	KC_AB1	Uhg4	0.576654152207748	0.259846394489094	1	pseudobulk	DESeq2	LRT	0.585283304872888
850	KC_AB1	Uhg5	0.42476883636016	0.151937369122598	1	pseudobulk	DESeq2	LRT	0.818335397909558
850	KC_AB1	Uhg8	1.17905339705432	0.209007138975183	1	pseudobulk	DESeq2	LRT	0.679838879608668
850	KC_AB1	UK114	1.08899820779554	0.878177167143508	1	pseudobulk	DESeq2	LRT	0.056417858865076
850	KC_AB1	Ulp1	0.25099953922491	0.533412480001707	1	pseudobulk	DESeq2	LRT	0.272936827542898
850	KC_AB1	unc-104	-0.0162154036272263	0.883578796513522	1	pseudobulk	DESeq2	LRT	0.053754714561199
851	KC_AB1	Unc-115a	0.232154677617677	0.471571159796253	1	pseudobulk	DESeq2	LRT	0.326452763212955
851	KC_AB1	Unc-115b	-0.204045654034498	0.815178770227615	1	pseudobulk	DESeq2	LRT	0.088747139223730
851	KC_AB1	unc-119	0.0776306428126407	0.689111339307011	1	pseudobulk	DESeq2	LRT	0.161710603723469
851	KC_AB1	unc-13	0.07347585076726	0.39131292322721	1	pseudobulk	DESeq2	LRT	0.407475809156341
851	KC_AB1	unc-13-4A	-0.046322464459936	0.724375964105225	1	pseudobulk	DESeq2	LRT	0.140035968669481
851	KC_AB1	unc-45	0.0342079836063834	0.945266477094065	1	pseudobulk	DESeq2	LRT	0.024445743641531
851	KC_AB1	unc-5	0.0353456191255707	0.869265432651818	1	pseudobulk	DESeq2	LRT	0.060847590255253
851	KC_AB1	Unc-76	-0.100977925257449	0.463129882104098	1	pseudobulk	DESeq2	LRT	0.334297196518012
851	KC_AB1	unc79	0.0942291074056907	0.510241902996344	1	pseudobulk	DESeq2	LRT	0.292223878354977
851	KC_AB1	unc80	-0.228769364345705	0.220927736325732	1	pseudobulk	DESeq2	LRT	0.655749757297228
852	KC_AB1	und	-0.28504255204232	0.353454909727229	1	pseudobulk	DESeq2	LRT	0.451665981317341
852	KC_AB1	unk	0.615626465941949	0.00919923573115003	1	pseudobulk	DESeq2	LRT	2.03624825216875
852	KC_AB1	Unr	-0.0332046548045358	0.739317815814922	1	pseudobulk	DESeq2	LRT	0.131168828204099
852	KC_AB1	Updo	-0.252814373339908	0.29225832431507	1	pseudobulk	DESeq2	LRT	0.534233110097296
852	KC_AB1	Upf1	0.0569463144704202	0.874277401529086	1	pseudobulk	DESeq2	LRT	0.058350747184132
852	KC_AB1	Upf2	0.16440616234442	0.74695369987475	1	pseudobulk	DESeq2	LRT	0.126706317210158
852	KC_AB1	Upf3	-0.0691640739977503	0.917958035034402	1	pseudobulk	DESeq2	LRT	0.037177172360383

852	KC_AB1	upSET	-0.0102894183837923	0.932346904735609	1	pseudobulk	DESeq2	LRT	0.030422466636777
852	KC_AB1	UQCR-11	-0.252453190112431	0.099102637137517	1	pseudobulk	DESeq2	LRT	1.00391478871318
852	KC_AB1	UQCR-11L	-1.4722520190256	0.507123370735961	1	pseudobulk	DESeq2	LRT	0.29488637456791
853	KC_AB1	UQCR-14	-0.163728506374796	0.280051600925177	1	pseudobulk	DESeq2	LRT	0.552761940327921
853	KC_AB1	UQCR-6.4	-0.155883403298972	0.298144232293837	1	pseudobulk	DESeq2	LRT	0.525573587818119
853	KC_AB1	UQCR-C1	-0.254635800806594	0.1756506722665	1	pseudobulk	DESeq2	LRT	0.755350183696763
853	KC_AB1	UQCR-C2	-0.304284896450739	0.0590275588404014	1	pseudobulk	DESeq2	LRT	1.22894517720681
853	KC_AB1	UQCR-Q	-0.219512595779711	0.146408568620976	1	pseudobulk	DESeq2	LRT	0.834433505272778
853	KC_AB1	uri	-0.503813407307424	0.680507416109859	1	pseudobulk	DESeq2	LRT	0.16716713753186
853	KC_AB1	Urm1	0.236981975882868	0.688735774246056	1	pseudobulk	DESeq2	LRT	0.161947358347408
853	KC_AB1	Uros1	0.427608546274419	0.671939886558027	1	pseudobulk	DESeq2	LRT	0.172669578291262
853	KC_AB1	Uros2	-0.850996402080018	0.900449987182352	1	pseudobulk	DESeq2	LRT	0.045540403770502
853	KC_AB1	Use1	-0.0400674819915342	0.931202424083407	1	pseudobulk	DESeq2	LRT	0.030955902147926
854	KC_AB1	Usf	0.0202439947403994	0.922281001876386	1	pseudobulk	DESeq2	LRT	0.035136737329080
854	KC_AB1	ush	0.677508217693963	0.507936620018214	1	pseudobulk	DESeq2	LRT	0.294190475301893
854	KC_AB1	usp	0.109193260100675	0.693735065623326	1	pseudobulk	DESeq2	LRT	0.158806353039995
854	KC_AB1	Usp1	-0.123451043495816	0.664633150554625	1	pseudobulk	DESeq2	LRT	0.177418000788439
854	KC_AB1	Usp10	-0.1278534254031	0.688897774983549	1	pseudobulk	DESeq2	LRT	0.161845217938994
854	KC_AB1	Usp12-46	-0.42404939049565	0.570258366532353	1	pseudobulk	DESeq2	LRT	0.243928334263188
854	KC_AB1	Usp14	-0.251017564811242	0.557662883986159	1	pseudobulk	DESeq2	LRT	0.253628259608152
854	KC_AB1	Usp15-31	-0.0644409791281379	0.885019955483873	1	pseudobulk	DESeq2	LRT	0.053046936693336
854	KC_AB1	Usp16-45	0.849709115971462	0.168663510879115	1	pseudobulk	DESeq2	LRT	0.772978863693747
854	KC_AB1	Usp2	0.0414660372294022	0.785248689781835	1	pseudobulk	DESeq2	LRT	0.104992779566189
855	KC_AB1	Usp20-33	-0.932015751213514	0.143190817264118	1	pseudobulk	DESeq2	LRT	0.844084832163595
855	KC_AB1	Usp30	-0.256238754318429	0.480924430416126	1	pseudobulk	DESeq2	LRT	0.317923160698241
855	KC_AB1	Usp32	-0.0459719598883069	0.909679384905876	1	pseudobulk	DESeq2	LRT	0.041111647130569
855	KC_AB1	Usp39	0.147807605250189	0.827092124982091	1	pseudobulk	DESeq2	LRT	0.082446114216402
855	KC_AB1	Usp47	0.118355720158997	0.771873733120367	1	pseudobulk	DESeq2	LRT	0.112453737869031
855	KC_AB1	Usp5	-0.0996381569570955	0.873954523288003	1	pseudobulk	DESeq2	LRT	0.058511165533703
855	KC_AB1	Usp7	0.0006825217190826	0.994365004916854	1	pseudobulk	DESeq2	LRT	0.002454168395915
855	KC_AB1	Usp8	0.148954708389017	0.750834165263138	1	pseudobulk	DESeq2	LRT	0.124455973866799
855	KC_AB1	Utx	-0.0423669954154539	0.877957857183442	1	pseudobulk	DESeq2	LRT	0.056526330143945
855	KC_AB1	Uvrag	-0.253665452832902	0.730158921523772	1	pseudobulk	DESeq2	LRT	0.136582603949126
856	KC_AB1	Uxs	0.203840911741394	0.78194540915823	1	pseudobulk	DESeq2	LRT	0.106823565775085
856	KC_AB1	Uxt	-0.0799596107650319	0.852597065077199	1	pseudobulk	DESeq2	LRT	0.069256166672432
856	KC_AB1	uzip	0.196270572711181	0.493791778952713	1	pseudobulk	DESeq2	LRT	0.306456144831776
856	KC_AB1	v	1.08899820779554	0.697631521651407	1	pseudobulk	DESeq2	LRT	0.156373904552
856	KC_AB1	VAChT	-0.0444329472516738	0.710775783153597	1	pseudobulk	DESeq2	LRT	0.148267377464832
856	KC_AB1	ValRS	0.0146635163324668	0.893276728080452	1	pseudobulk	DESeq2	LRT	0.049013980236982
856	KC_AB1	ValRS-m	1.95275942187961	0.438420007953907	1	pseudobulk	DESeq2	LRT	0.358109634392079
856	KC_AB1	Vamp7	0.0202084684039922	0.948688228255208	1	pseudobulk	DESeq2	LRT	0.022876488304659
856	KC_AB1	Vang	-0.142234607790406	0.770672741130126	1	pseudobulk	DESeq2	LRT	0.113130001836789
856	KC_AB1	vanin-like	-0.850932661185203	0.660736244265418	1	pseudobulk	DESeq2	LRT	0.179971869591347
857	KC_AB1	vap	-0.116990759130773	0.718066302918324	1	pseudobulk	DESeq2	LRT	0.143835453164213
857	KC_AB1	Vap33	-0.102472933016225	0.414315818347629	1	pseudobulk	DESeq2	LRT	0.382668485263272
857	KC_AB1	vari	0.291948671932598	0.851644899441512	1	pseudobulk	DESeq2	LRT	0.069741450256656
857	KC_AB1	Vav	-0.0778351376423706	0.802573323404785	1	pseudobulk	DESeq2	LRT	0.095515279801083
857	KC_AB1	veli	-0.0937911644263323	0.664136357121197	1	pseudobulk	DESeq2	LRT	0.177742744328368
857	KC_AB1	velo	0.121712202455497	0.704569308539235	1	pseudobulk	DESeq2	LRT	0.152076278870303
857	KC_AB1	ver	0.045707994267386	0.959806512825956	1	pseudobulk	DESeq2	LRT	0.017816307461757
857	KC_AB1	vers	0.281887045390791	0.530986312094427	1	pseudobulk	DESeq2	LRT	0.274916674132239
857	KC_AB1	VGAT	-0.110446767852955	0.700673487420932	1	pseudobulk	DESeq2	LRT	0.154484315337351

857	KC_AB1	VGlut	0.135879393043758	1	1	pseudobulk	DESeq2	LRT	0
858	KC_AB1	Vha100-1	0.0566707565518816	0.7254892315424	1	pseudobulk	DESeq2	LRT	0.139369029423932
858	KC_AB1	Vha13	-0.192257539775365	0.127106050952539	1	pseudobulk	DESeq2	LRT	0.895833774129345
858	KC_AB1	Vha14-1	-0.305150038328148	0.0562910852118189	1	pseudobulk	DESeq2	LRT	1.24956037868104
858	KC_AB1	Vha14-2	-0.82542030717083	1	1	pseudobulk	DESeq2	LRT	0
858	KC_AB1	Vha16-1	-0.12171644230238	0.267962781774076	1	pseudobulk	DESeq2	LRT	0.57192552236153
858	KC_AB1	Vha26	-0.0934219861807486	0.575876891225274	1	pseudobulk	DESeq2	LRT	0.239670348479482
858	KC_AB1	Vha36-1	-0.263305854015481	0.126713283885577	1	pseudobulk	DESeq2	LRT	0.897177853815902
858	KC_AB1	Vha44	-0.117690475329565	0.388859312260738	1	pseudobulk	DESeq2	LRT	0.410207496258743
858	KC_AB1	Vha55	-0.152649763179921	0.280902875694025	1	pseudobulk	DESeq2	LRT	0.551443814761918
858	KC_AB1	Vha68-1	-0.189907530597017	0.259872091037306	1	pseudobulk	DESeq2	LRT	0.585240359049669
859	KC_AB1	Vha68-2	1.08899820779554	0.697631521651407	1	pseudobulk	DESeq2	LRT	0.156373904552
859	KC_AB1	VhaAC39-1	-0.223688006941671	0.269019256419269	1	pseudobulk	DESeq2	LRT	0.570216632052195
859	KC_AB1	VhaAC45	-0.0694064804084521	0.793960670356554	1	pseudobulk	DESeq2	LRT	0.100201010255738
859	KC_AB1	VhaM9.7-a	-0.0458781080337923	0.705351915524863	1	pseudobulk	DESeq2	LRT	0.151594149905276
859	KC_AB1	VhaM9.7-b	-0.0189157057638401	0.928273794544845	1	pseudobulk	DESeq2	LRT	0.032323909640634
859	KC_AB1	VhaM9.7-c	0.789622060587802	0.115545941707809	1	pseudobulk	DESeq2	LRT	0.937245303537154
859	KC_AB1	VhaPPA1-1	-0.271776750483625	0.133392848257297	1	pseudobulk	DESeq2	LRT	0.874867454120009
859	KC_AB1	VhaSFD	-0.380572566917839	0.0707582902581968	1	pseudobulk	DESeq2	LRT	1.15022266954979
859	KC_AB1	Vhl	-0.57190169671657	0.227121188099619	1	pseudobulk	DESeq2	LRT	0.643742348626973
859	KC_AB1	viaf	0.138137807011495	0.570136036841026	1	pseudobulk	DESeq2	LRT	0.244021507484598
860	KC_AB1	vib	0.0963589936828126	0.605546202523175	1	pseudobulk	DESeq2	LRT	0.217852715055434
860	KC_AB1	vig	-0.150236083948672	0.302098142309957	1	pseudobulk	DESeq2	LRT	0.5198519453226
860	KC_AB1	vig2	0.244052587467729	0.384981956963613	1	pseudobulk	DESeq2	LRT	0.41455962419218
860	KC_AB1	vih	0.0411531172971812	0.865787513059374	1	pseudobulk	DESeq2	LRT	0.062588682150162
860	KC_AB1	vimar	0.237376491664839	0.520180389121872	1	pseudobulk	DESeq2	LRT	0.283846024798159
860	KC_AB1	Vinc	-0.735093855283643	0.12307218629843	1	pseudobulk	DESeq2	LRT	0.909840084371246
860	KC_AB1	vir	1.00099447279522	0.377920138838586	1	pseudobulk	DESeq2	LRT	0.422599964517413
860	KC_AB1	vir-1	-0.0349398172948144	0.932844196917205	1	pseudobulk	DESeq2	LRT	0.030190885802741
860	KC_AB1	vis	0.888933280325755	0.641693803207784	1	pseudobulk	DESeq2	LRT	0.192672154640023
860	KC_AB1	vito	0.417326242006611	0.442520415159102	1	pseudobulk	DESeq2	LRT	0.354066688838638
861	KC_AB1	vkg	1.08899820779554	0.878177167143508	1	pseudobulk	DESeq2	LRT	0.056417858865076
861	KC_AB1	Vkor	1.70481383703091	0.360957792074198	1	pseudobulk	DESeq2	LRT	0.442543578540209
861	KC_AB1	vlc	-0.014792743215932	0.939564236453216	1	pseudobulk	DESeq2	LRT	0.027073522552819
861	KC_AB1	Vlet	0.146309767202408	0.7941034319507	1	pseudobulk	DESeq2	LRT	0.100122927044056
861	KC_AB1	vls	0.179931560694602	0.805659472802244	1	pseudobulk	DESeq2	LRT	0.093848482180738
861	KC_AB1	Vm26Ab	1.08899820779554	0.878177167143508	1	pseudobulk	DESeq2	LRT	0.056417858865076
861	KC_AB1	Vm32E	-0.82542030717083	1	1	pseudobulk	DESeq2	LRT	0
861	KC_AB1	Vmat	1.08899820779554	0.670846322504933	1	pseudobulk	DESeq2	LRT	0.173376956629912
861	KC_AB1	vnd	0.0863138234318787	0.952426238244534	1	pseudobulk	DESeq2	LRT	0.021168648790201
861	KC_AB1	Vps11	0.238945731972195	0.25504332480121	1	pseudobulk	DESeq2	LRT	0.593386038688125
862	KC_AB1	Vps13	-0.334382446333264	0.519184692310428	1	pseudobulk	DESeq2	LRT	0.284678120797666
862	KC_AB1	Vps13B	0.676632248672807	0.47386546134081	1	pseudobulk	DESeq2	LRT	0.324344944593986
862	KC_AB1	Vps13D	0.320234917007127	0.676454312789812	1	pseudobulk	DESeq2	LRT	0.169761529995124
862	KC_AB1	Vps15	0.94546562029473	0.254474189668782	1	pseudobulk	DESeq2	LRT	0.594356259900354
862	KC_AB1	Vps16A	0.221817731933454	0.664466872343907	1	pseudobulk	DESeq2	LRT	0.177526666364404
862	KC_AB1	Vps16B	0.917856700303682	0.233940725619807	1	pseudobulk	DESeq2	LRT	0.630894167364211
862	KC_AB1	Vps2	0.199780174568556	0.54164871965503	1	pseudobulk	DESeq2	LRT	0.266282279098148
862	KC_AB1	Vps20	-0.120862091182075	0.778242149851789	1	pseudobulk	DESeq2	LRT	0.108885251362755
862	KC_AB1	Vps24	-0.0590870600109075	0.772783804987892	1	pseudobulk	DESeq2	LRT	0.111941987884088
862	KC_AB1	Vps25	0.184835244636676	0.647791284751922	1	pseudobulk	DESeq2	LRT	0.188564899193409
863	KC_AB1	Vps26	-0.241139627917052	0.529731069235284	1	pseudobulk	DESeq2	LRT	0.275944554529499

863	KC_AB1	Vps28	0.515082102531332	0.15037148957702	1	pseudobulk	DESeq2	LRT	0.822834498140043
863	KC_AB1	Vps29	0.192422166358866	0.609668574143688	1	pseudobulk	DESeq2	LRT	0.214906190459021
863	KC_AB1	Vps33B	0.824328516523741	0.47207552208246	1	pseudobulk	DESeq2	LRT	0.325988517891388
863	KC_AB1	Vps35	-0.0328057058745915	0.918401231792513	1	pseudobulk	DESeq2	LRT	0.036967542454450
863	KC_AB1	Vps36	-0.0845298331857917	0.932599110683416	1	pseudobulk	DESeq2	LRT	0.030305003019923
863	KC_AB1	Vps37B	-0.0628107220624339	0.855087066844024	1	pseudobulk	DESeq2	LRT	0.067989662201923
863	KC_AB1	Vps39	-0.038082016289828	0.926827382349152	1	pseudobulk	DESeq2	LRT	0.033001143820919
863	KC_AB1	Vps4	0.252723690967218	0.379587879531239	1	pseudobulk	DESeq2	LRT	0.420687663306756
863	KC_AB1	Vps45	-0.187257769112508	0.767508892806112	1	pseudobulk	DESeq2	LRT	0.114916583832211
864	KC_AB1	Vps50	-0.620683465246408	0.32526360105903	1	pseudobulk	DESeq2	LRT	0.487764534147209
864	KC_AB1	Vps51	0.202140680618703	0.755411290242526	1	pseudobulk	DESeq2	LRT	0.121816528572054
864	KC_AB1	Vps52	0.129592867800577	0.843590022436482	1	pseudobulk	DESeq2	LRT	0.073868565521460
864	KC_AB1	Vps53	-0.140771187751742	0.80660480890337	1	pseudobulk	DESeq2	LRT	0.093339193097093
864	KC_AB1	Vps60	0.121969026332621	0.694006705137904	1	pseudobulk	DESeq2	LRT	0.158636333593668
864	KC_AB1	Vps8	0.227646953197448	0.792700979645318	1	pseudobulk	DESeq2	LRT	0.100890605091206
864	KC_AB1	vri	1.08899820779554	0.878177167143508	1	pseudobulk	DESeq2	LRT	0.056417858865076
864	KC_AB1	Vrp1	-0.104183223273267	0.614263237541867	1	pseudobulk	DESeq2	LRT	0.211645475581394
864	KC_AB1	vrs	-0.850996402080018	0.900449987182352	1	pseudobulk	DESeq2	LRT	0.045540403770502
864	KC_AB1	vsg	0.131619821524551	0.464130618762635	1	pseudobulk	DESeq2	LRT	0.333359780167429
865	KC_AB1	Vsp37A	-0.222324295982303	0.478479878988153	1	pseudobulk	DESeq2	LRT	0.3201363204329
865	KC_AB1	Vsx1	0.178413682955283	0.506608505455188	1	pseudobulk	DESeq2	LRT	0.295327523109929
865	KC_AB1	Vsx2	1.07044812341491	0.419126285850109	1	pseudobulk	DESeq2	LRT	0.377655101176007
865	KC_AB1	Vta1	-0.371684854408224	0.201791088665875	1	pseudobulk	DESeq2	LRT	0.695098016635733
865	KC_AB1	vtd	-0.0777532319483349	0.671131236328147	1	pseudobulk	DESeq2	LRT	0.173192547431287
865	KC_AB1	Vti1a	0.117923138990961	0.739733626405697	1	pseudobulk	DESeq2	LRT	0.130924638928874
865	KC_AB1	Vti1b	0.157871657047039	0.613882238636988	1	pseudobulk	DESeq2	LRT	0.211914931815231
865	KC_AB1	w	0.707687178002089	0.0842008144979342	1	pseudobulk	DESeq2	LRT	1.07468370742853
865	KC_AB1	wac	0.0631930092326502	0.921137961850191	1	pseudobulk	DESeq2	LRT	0.035675319224308
865	KC_AB1	wake	-0.267670187521156	0.507010434538539	1	pseudobulk	DESeq2	LRT	0.294983102568288
866	KC_AB1	wal	-0.0503495163035739	0.932405794662567	1	pseudobulk	DESeq2	LRT	0.030395036114398
866	KC_AB1	wap	0.140409965873626	0.638222808367847	1	pseudobulk	DESeq2	LRT	0.195027679347623
866	KC_AB1	wapl	0.131344240870424	0.667833645192385	1	pseudobulk	DESeq2	LRT	0.175331705147899
866	KC_AB1	wash	0.265790035067218	0.698379553249564	1	pseudobulk	DESeq2	LRT	0.155908484137648
866	KC_AB1	WASp	-0.0400946975611868	0.890659556684878	1	pseudobulk	DESeq2	LRT	0.050288267800340
866	KC_AB1	wat	1.08899820779554	0.670846322504933	1	pseudobulk	DESeq2	LRT	0.173376956629912
866	KC_AB1	wb	1.24522514393301	0.608213953617254	1	pseudobulk	DESeq2	LRT	0.215943620513061
866	KC_AB1	Wbp2	0.193629810635618	0.540009653747523	1	pseudobulk	DESeq2	LRT	0.267598476229247
866	KC_AB1	wcd	0.43284312989469	0.655492091180867	1	pseudobulk	DESeq2	LRT	0.183432543908647
866	KC_AB1	wcy	0.0228565484003059	0.898299382529068	1	pseudobulk	DESeq2	LRT	0.046578898843189
867	KC_AB1	wda	-1.0059448407689	0.458654373932873	1	pseudobulk	DESeq2	LRT	0.338514460510568
867	KC_AB1	wdb	-0.016185866412258	0.872738847521821	1	pseudobulk	DESeq2	LRT	0.059115692202330
867	KC_AB1	wde	0.037726243588465	0.881046939775315	1	pseudobulk	DESeq2	LRT	0.055000952947379
867	KC_AB1	Wdfy2	0.472907179633561	0.212822438867055	1	pseudobulk	DESeq2	LRT	0.671982584260496
867	KC_AB1	wdn	-0.235196168092599	0.708921580189782	1	pseudobulk	DESeq2	LRT	0.149401803144807
867	KC_AB1	Wdr24	-0.0725227054705634	0.903076824097955	1	pseudobulk	DESeq2	LRT	0.044275302994754
867	KC_AB1	Wdr33	-0.301625125563896	0.454737314955491	1	pseudobulk	DESeq2	LRT	0.342239406880577
867	KC_AB1	Wdr59	0.30980846318213	0.7827431339552	1	pseudobulk	DESeq2	LRT	0.10638073322679
867	KC_AB1	Wdr62	-0.136447555395442	0.439700477932811	1	pseudobulk	DESeq2	LRT	0.356843062323279
867	KC_AB1	WDR79	1.02634755917486	0.32555570683181	1	pseudobulk	DESeq2	LRT	0.487374687281173
868	KC_AB1	Wdr81	-0.320468262590604	0.627116466905366	1	pseudobulk	DESeq2	LRT	0.202651795314203
868	KC_AB1	Wdr82	-0.397069922484895	0.369701821384307	1	pseudobulk	DESeq2	LRT	0.432148409813138
868	KC_AB1	Wdr92	-0.615665282962158	0.557958180526244	1	pseudobulk	DESeq2	LRT	0.253398350609553

868	KC_AB1	wds	0.365003263211	0.288331697108649	1	pseudobulk	DESeq2	LRT	0.540107611782135
868	KC_AB1	WDY	-0.420535986292194	0.660762552102851	1	pseudobulk	DESeq2	LRT	0.179954578090726
868	KC_AB1	wech	0.170924220927808	0.482076198713744	1	pseudobulk	DESeq2	LRT	0.316884310173195
868	KC_AB1	Wee1	-0.292443837221726	0.48523665769466	1	pseudobulk	DESeq2	LRT	0.314046397349694
868	KC_AB1	wek	0.265899907894825	0.766451799611131	1	pseudobulk	DESeq2	LRT	0.115515151730828
868	KC_AB1	wfs1	-0.0120378239381555	0.936534180045521	1	pseudobulk	DESeq2	LRT	0.028476367851714
868	KC_AB1	wge	0.169480889428322	0.392114444886941	1	pseudobulk	DESeq2	LRT	0.40658715867113
869	KC_AB1	whd	0.681462689492728	0.396267226731612	1	pseudobulk	DESeq2	LRT	0.402011844491899
869	KC_AB1	wisp	1.84493075739606	0.499944822388828	1	pseudobulk	DESeq2	LRT	0.301077924972768
869	KC_AB1	wit	-0.246462395230337	0.435719947720855	1	pseudobulk	DESeq2	LRT	0.360792557147593
869	KC_AB1	wkd	0.153745974902144	0.687869454738234	1	pseudobulk	DESeq2	LRT	0.162493975233002
869	KC_AB1	wls	-0.485934551651567	0.530860409687751	1	pseudobulk	DESeq2	LRT	0.275019662105196
869	KC_AB1	wmd	0.223744781240674	0.372670925039177	1	pseudobulk	DESeq2	LRT	0.428674488618855
869	KC_AB1	wnd	0.0976212264497257	0.681265772237584	1	pseudobulk	DESeq2	LRT	0.166683430085637
869	KC_AB1	Wnk	0.169266813958081	0.494461510626525	1	pseudobulk	DESeq2	LRT	0.305867508663525
869	KC_AB1	Wnt5	0.193725894320849	0.64631703297376	1	pseudobulk	DESeq2	LRT	0.18955439857363
869	KC_AB1	Wnt6	0.766816096708399	0.564856087275611	1	pseudobulk	DESeq2	LRT	0.248062186628824
870	KC_AB1	woc	-0.151093790164174	0.623128814401606	1	pseudobulk	DESeq2	LRT	0.205422165859215
870	KC_AB1	wol	0.440017961726838	0.424616761283005	1	pseudobulk	DESeq2	LRT	0.372002866531195
870	KC_AB1	wrd	-0.0450984800415186	0.75455659245684	1	pseudobulk	DESeq2	LRT	0.12230818217067
870	KC_AB1	wry	-0.194027804890169	0.10233157349558	1	pseudobulk	DESeq2	LRT	0.989990347917365
870	KC_AB1	wtrw	-0.391363811231866	0.79478993219354	1	pseudobulk	DESeq2	LRT	0.099747642846172
870	KC_AB1	wts	-0.0126844455260928	0.947956749930147	1	pseudobulk	DESeq2	LRT	0.023211476686365
870	KC_AB1	wuho	0.284947554794106	0.554828481337089	1	pseudobulk	DESeq2	LRT	0.255841253132189
870	KC_AB1	wun	0.75255978646787	0.10035688646233	1	pseudobulk	DESeq2	LRT	0.998452820984416
870	KC_AB1	wun2	2.39533548415136	0.318016613010836	1	pseudobulk	DESeq2	LRT	0.497550192120982
870	KC_AB1	wupA	-0.06757997812928	0.968127782473684	1	pseudobulk	DESeq2	LRT	0.014067316699192
871	KC_AB1	wus	-1.56855358782635	0.573026303465967	1	pseudobulk	DESeq2	LRT	0.241825442277049
871	KC_AB1	Wwox	0.441905274244545	0.54842732197684	1	pseudobulk	DESeq2	LRT	0.260880917324899
871	KC_AB1	X11L	0.187114482766272	0.34601629230932	1	pseudobulk	DESeq2	LRT	0.460903451804182
871	KC_AB1	X11Lbeta	0.0355837587166222	0.83001336846049	1	pseudobulk	DESeq2	LRT	0.080914912681918
871	KC_AB1	x16	-0.0218386542266539	0.898699350263113	1	pseudobulk	DESeq2	LRT	0.046385572293165
871	KC_AB1	Xbp1	0.110760145343948	0.646315092384954	1	pseudobulk	DESeq2	LRT	0.189555702559496
871	KC_AB1	Xe7	0.0481304426241689	0.711178983985253	1	pseudobulk	DESeq2	LRT	0.148021085662832
871	KC_AB1	xit	0.213440871668443	0.867376773950383	1	pseudobulk	DESeq2	LRT	0.061792211285982
871	KC_AB1	xmas	0.899486492465562	0.537355403971286	1	pseudobulk	DESeq2	LRT	0.269738379236767
871	KC_AB1	XNP	0.0587497713577722	0.80127637604103	1	pseudobulk	DESeq2	LRT	0.096217661335170
872	KC_AB1	Xpac	0.622865547838829	0.399809354649092	1	pseudobulk	DESeq2	LRT	0.398147048574701
872	KC_AB1	Xpc	0.0425924784946144	0.854793737652724	1	pseudobulk	DESeq2	LRT	0.068138668202900
872	KC_AB1	Xpd	0.527012038974436	0.711489211530043	1	pseudobulk	DESeq2	LRT	0.147831680834079
872	KC_AB1	XRCC1	0.109431965263518	0.904877179085277	1	pseudobulk	DESeq2	LRT	0.043410364513436
872	KC_AB1	Xrp1	0.135846532232642	0.451662784306999	1	pseudobulk	DESeq2	LRT	0.345185692560072
872	KC_AB1	Xxylt	0.137127095558546	0.651918772625264	1	pseudobulk	DESeq2	LRT	0.18580651285431
872	KC_AB1	yata	-0.211631233681095	0.64846457052465	1	pseudobulk	DESeq2	LRT	0.188113747021644
872	KC_AB1	yellow-b	-0.82542030717083	1	1	pseudobulk	DESeq2	LRT	0
872	KC_AB1	yellow-h	1.68197474101089	0.597975566962289	1	pseudobulk	DESeq2	LRT	0.223316560744423
872	KC_AB1	yem	0.407273874847539	0.624383673915501	1	pseudobulk	DESeq2	LRT	0.204548461184741
873	KC_AB1	Yeti	0.0784765190812261	0.644877021491187	1	pseudobulk	DESeq2	LRT	0.19052309773683
873	KC_AB1	Yif1	-0.274802125607015	0.613857132737325	1	pseudobulk	DESeq2	LRT	0.211932693489969
873	KC_AB1	Yip1d1	-0.0967081849885588	0.82402549396274	1	pseudobulk	DESeq2	LRT	0.084059351754261
873	KC_AB1	yip2	-3.15129201279641	0.119387362832502	1	pseudobulk	DESeq2	LRT	0.923041640899841
873	KC_AB1	Yippee	0.332097233852178	0.165634765446096	1	pseudobulk	DESeq2	LRT	0.780848502957135

873	KC_AB1	yki	-0.0039638616425114	0.921424886440364	1	pseudobulk	DESeq2	LRT	0.035540062218169
873	KC_AB1	Ykt6	0.238799935699066	0.409022040369551	1	pseudobulk	DESeq2	LRT	0.388253289173651
873	KC_AB1	YL-1	-0.084181137490451	0.818482738726325	1	pseudobulk	DESeq2	LRT	0.086990475146303
873	KC_AB1	YME1L	0.0845876357376402	0.816974187706681	1	pseudobulk	DESeq2	LRT	0.087791664781714
873	KC_AB1	ymp	-1.89854133735591	0.478664405825708	1	pseudobulk	DESeq2	LRT	0.319968866080244
874	KC_AB1	Yp2	-0.82542030717083	1	1	pseudobulk	DESeq2	LRT	0
874	KC_AB1	Yp3	-0.850996402080018	0.900449987182352	1	pseudobulk	DESeq2	LRT	0.045540403770502
874	KC_AB1	yps	-0.15368832508035	0.408833301570826	1	pseudobulk	DESeq2	LRT	0.388453735917859
874	KC_AB1	yrt	0.407337939724291	0.147657438698883	1	pseudobulk	DESeq2	LRT	0.830744669223788
874	KC_AB1	Ythdc1	0.0644156521039395	0.677417118898656	1	pseudobulk	DESeq2	LRT	0.169143832566969
874	KC_AB1	Ythdf	0.0336954435044488	0.87156412847951	1	pseudobulk	DESeq2	LRT	0.059700652592825
874	KC_AB1	ytr	0.0443232645359455	0.859779120613954	1	pseudobulk	DESeq2	LRT	0.065613105755704
874	KC_AB1	yuri	1.95275942187961	0.438420007953907	1	pseudobulk	DESeq2	LRT	0.358109634392079
874	KC_AB1	z	-0.169265106712961	0.610901081253293	1	pseudobulk	DESeq2	LRT	0.214029106195666
874	KC_AB1	ZAP3	0.0562620101208476	0.815969491218005	1	pseudobulk	DESeq2	LRT	0.088326079044385
875	KC_AB1	Zasp52	1.0977666759669	0.581338566910467	1	pseudobulk	DESeq2	LRT	0.235570864312153
875	KC_AB1	Zasp66	1.08899820779554	0.878177167143508	1	pseudobulk	DESeq2	LRT	0.056417858865076
875	KC_AB1	Zasp67	1.08899820779554	0.878177167143508	1	pseudobulk	DESeq2	LRT	0.056417858865076
875	KC_AB1	ZC3H3	-0.0189218043472822	0.959860382891502	1	pseudobulk	DESeq2	LRT	0.017791932949424
875	KC_AB1	Zcchc7	-0.118806265663991	0.756893049078178	1	pseudobulk	DESeq2	LRT	0.120965483084671
875	KC_AB1	zda	-0.214059305844667	0.54558123788414	1	pseudobulk	DESeq2	LRT	0.263140573145367
875	KC_AB1	Zdhhc8	0.175112501677509	0.224457018112753	1	pseudobulk	DESeq2	LRT	0.648866810935121
875	KC_AB1	zetaCOP	-0.124507877799362	0.661865234733639	1	pseudobulk	DESeq2	LRT	0.179230430151961
875	KC_AB1	zf30C	0.233261811165212	0.560753478332495	1	pseudobulk	DESeq2	LRT	0.251228023824489
875	KC_AB1	zfh2	0.604353817145893	0.277686345300951	1	pseudobulk	DESeq2	LRT	0.556445475331661
876	KC_AB1	Zfrp8	-0.346263013974794	0.63252247491187	1	pseudobulk	DESeq2	LRT	0.198924038441798
876	KC_AB1	Zif	0.10371453131301	0.757274609381498	1	pseudobulk	DESeq2	LRT	0.120746604366946
876	KC_AB1	zip	-0.0694331202294252	0.749364615784341	1	pseudobulk	DESeq2	LRT	0.125306817690596
876	KC_AB1	Zip102B	-0.0053837474406846	0.979224554186887	1	pseudobulk	DESeq2	LRT	0.009117705069659
876	KC_AB1	Zip48C	-0.412282079464988	0.550088489924472	1	pseudobulk	DESeq2	LRT	0.259567442151825
876	KC_AB1	Zip71B	-0.615767568564188	0.806245159731688	1	pseudobulk	DESeq2	LRT	0.093532879622572
876	KC_AB1	Zip88E	-0.521470089167407	0.430962904366388	1	pseudobulk	DESeq2	LRT	0.36556011063352
876	KC_AB1	Zip99C	0.223214977346341	0.634913030947762	1	pseudobulk	DESeq2	LRT	0.197285759379214
876	KC_AB1	ZIPIC	-0.177257833906122	0.846978812001453	1	pseudobulk	DESeq2	LRT	0.072127453832158
876	KC_AB1	Zir	0.139262595384496	0.697820877846803	1	pseudobulk	DESeq2	LRT	0.156256041196275
877	KC_AB1	Ziz	0.163083881258918	0.513565830375142	1	pseudobulk	DESeq2	LRT	0.2894038793574
877	KC_AB1	zld	-0.506516985469986	0.909346984303859	1	pseudobulk	DESeq2	LRT	0.041270369127443
877	KC_AB1	Zn72D	-0.0937847667076226	0.734600387143126	1	pseudobulk	DESeq2	LRT	0.133948847112316
877	KC_AB1	ZnT33D	-0.866024557185641	0.590851077432179	1	pseudobulk	DESeq2	LRT	0.228521968187266
877	KC_AB1	ZnT41F	-0.82542030717083	1	1	pseudobulk	DESeq2	LRT	0
877	KC_AB1	ZnT49B	0.067579974019105	0.852355043941944	1	pseudobulk	DESeq2	LRT	0.069379464498448
877	KC_AB1	ZnT63C	0.0003925726473956	0.957885014214094	1	pseudobulk	DESeq2	LRT	0.018686621077498
877	KC_AB1	ZnT77C	-1.96719661029409	0.414177861019091	1	pseudobulk	DESeq2	LRT	0.382813119093482
877	KC_AB1	ZnT86D	0.579443644152958	0.477676399715181	1	pseudobulk	DESeq2	LRT	0.320866215125813
877	KC_AB1	zormin	1.08899820779554	0.670846322504933	1	pseudobulk	DESeq2	LRT	0.173376956629912
878	KC_AB1	Zpr1	0.188752366802649	0.825374845598901	1	pseudobulk	DESeq2	LRT	0.083348770961461
878	KC_AB1	Zw10	0.152802925834735	0.816042471625971	1	pseudobulk	DESeq2	LRT	0.088287237431444
878	KC_AB1	zye	1.08899820779554	0.697631521651407	1	pseudobulk	DESeq2	LRT	0.156373904552
878	KC_AB1	Zyx	-0.175172788665522	0.332378800625274	1	pseudobulk	DESeq2	LRT	0.478366683639625
878	KC_AB2	128up	-0.684201050410529	0.696816590394116	1	pseudobulk	DESeq2	LRT	0.156881517829837
878	KC_AB2	14-3-3epsilon	0.228215190671912	0.335330812796071	1	pseudobulk	DESeq2	LRT	0.474526538392016
878	KC_AB2	14-3-3zeta	-0.0641136238880567	0.691581062484634	1	pseudobulk	DESeq2	LRT	0.160156907490406

878	KC_AB2	140up	-1.16016699006818	0.583985689825587	1	pseudobulk	DESeq2	LRT	0.233597794849804
878	KC_AB2	18SrRNA-Psi:CR4	-0.870211945215634	0.632219765227438	1	pseudobulk	DESeq2	LRT	0.199131930839008
878	KC_AB2	18w	-1.02762996864212	0.611704153801032	1	pseudobulk	DESeq2	LRT	0.213458570398483
879	KC_AB2	26-29-p	-0.26851753505932	0.793207005351784	1	pseudobulk	DESeq2	LRT	0.100613458899174
879	KC_AB2	28SrRNA-Psi:CR4	1.4129164084859	0.215635697726238	1	pseudobulk	DESeq2	LRT	0.666279341619851
879	KC_AB2	28SrRNA-Psi:CR4	1.50730388760116	0.557960431011111	1	pseudobulk	DESeq2	LRT	0.253396598917002
879	KC_AB2	28SrRNA-Psi:CR4	-0.729379579623002	0.772694994316916	1	pseudobulk	DESeq2	LRT	0.1119919011982
879	KC_AB2	28SrRNA-Psi:CR4	-0.721175887303532	0.733409564198845	1	pseudobulk	DESeq2	LRT	0.134653430819787
879	KC_AB2	28SrRNA-Psi:CR4	-0.729379579623002	0.772694994316916	1	pseudobulk	DESeq2	LRT	0.1119919011982
879	KC_AB2	28SrRNA-Psi:CR4	1.10706867860301	1	1	pseudobulk	DESeq2	LRT	0
879	KC_AB2	28SrRNA-Psi:CR4	-0.729379579623002	1	1	pseudobulk	DESeq2	LRT	0
879	KC_AB2	28SrRNA-Psi:CR4	0.794883107974144	0.504175680337019	1	pseudobulk	DESeq2	LRT	0.297418106993894
879	KC_AB2	28SrRNA-Psi:CR4	0.221486168185573	0.903697778547821	1	pseudobulk	DESeq2	LRT	0.0439767852922
880	KC_AB2	2mit	0.0267984807869226	0.876097652101551	1	pseudobulk	DESeq2	LRT	0.057447483551998
880	KC_AB2	312	-1.8486129258364	0.285061695422884	1	pseudobulk	DESeq2	LRT	0.545061136194929
880	KC_AB2	4E-T	0.305081443917741	0.703874628148628	1	pseudobulk	DESeq2	LRT	0.152504689085198
880	KC_AB2	5-HT1A	0.251293315964024	0.320791948343773	1	pseudobulk	DESeq2	LRT	0.493776540742465
880	KC_AB2	5-HT1B	0.0379838032898312	0.908484549079869	1	pseudobulk	DESeq2	LRT	0.041682454493993
880	KC_AB2	5-HT2A	-0.729379579623002	0.772694994316916	1	pseudobulk	DESeq2	LRT	0.1119919011982
880	KC_AB2	5-HT2B	-1.6052707068109	0.52265609184397	1	pseudobulk	DESeq2	LRT	0.281783983297782
880	KC_AB2	5.8SrRNA-Psi:CR4	1.10706867860301	1	1	pseudobulk	DESeq2	LRT	0
880	KC_AB2	5PtaseI	-0.325969057328661	0.70307964064599	1	pseudobulk	DESeq2	LRT	0.152995477918892
880	KC_AB2	7B2	-0.402731805376778	0.0831672470272829	1	pseudobulk	DESeq2	LRT	1.08004767414061
881	KC_AB2	a	0.453095733081312	0.319300563041563	1	pseudobulk	DESeq2	LRT	0.49580031564274
881	KC_AB2	A16	-0.570791843289369	0.809824469773434	1	pseudobulk	DESeq2	LRT	0.091609104664395
881	KC_AB2	a6	-0.160057240907588	0.913227577530507	1	pseudobulk	DESeq2	LRT	0.039420982214122
881	KC_AB2	AANAT1	-0.0281211457303824	0.862289544226825	1	pseudobulk	DESeq2	LRT	0.064346879946129
881	KC_AB2	AANATL7	-0.690174510442813	0.72579365052962	1	pseudobulk	DESeq2	LRT	0.139186835463116
881	KC_AB2	Aatf	-0.432812902426798	0.764335622495468	1	pseudobulk	DESeq2	LRT	0.116715899288902
881	KC_AB2	ab	-1.48503611119644	0.147032490177941	1	pseudobulk	DESeq2	LRT	0.832586687388666
881	KC_AB2	ABCA	-0.258774545877841	0.792035014850529	1	pseudobulk	DESeq2	LRT	0.101255618384965
881	KC_AB2	ABCB7	-0.253213284608431	0.865729347344558	1	pseudobulk	DESeq2	LRT	0.062617860091847
881	KC_AB2	ABCD	-0.400378961105968	0.48690126606586	1	pseudobulk	DESeq2	LRT	0.312559096177659
882	KC_AB2	Abi	0.965935795500056	0.498470258902688	1	pseudobulk	DESeq2	LRT	0.30236074864574
882	KC_AB2	Abl	-0.0487066086835069	0.896294978905547	1	pseudobulk	DESeq2	LRT	0.047549036512057
882	KC_AB2	abo	-1.52244591594958	0.547176617578731	1	pseudobulk	DESeq2	LRT	0.261872469536296
882	KC_AB2	Abp1	0.548070338633157	0.632219552033216	1	pseudobulk	DESeq2	LRT	0.199132077289807
882	KC_AB2	abs	-1.33570647694682	0.512066034235951	1	pseudobulk	DESeq2	LRT	0.290674030322833
882	KC_AB2	Ac13E	0.147593560254927	1	1	pseudobulk	DESeq2	LRT	0
882	KC_AB2	Ac3	-0.623390033075642	0.419388404422683	1	pseudobulk	DESeq2	LRT	0.377383581416838
882	KC_AB2	Ac76E	-0.116102703374448	0.905622904757188	1	pseudobulk	DESeq2	LRT	0.043052601964260
882	KC_AB2	Ac78C	-0.975875124114442	0.112297125101284	1	pseudobulk	DESeq2	LRT	0.949631361891973
882	KC_AB2	Acbp1	0.0916577336608694	0.852323457610566	1	pseudobulk	DESeq2	LRT	0.069395558758345
883	KC_AB2	ACC	-0.437181720415318	0.694616746833156	1	pseudobulk	DESeq2	LRT	0.158254750294543
883	KC_AB2	AcCoAS	-0.324939216649003	0.50400961112566	1	pseudobulk	DESeq2	LRT	0.297561181770664
883	KC_AB2	Ace	-0.692222106591127	0.578029247879483	1	pseudobulk	DESeq2	LRT	0.238050186023588
883	KC_AB2	Acer	0.894072396527432	0.660151861263169	1	pseudobulk	DESeq2	LRT	0.180356147909384
883	KC_AB2	Acf	0.357125501160608	0.826882908673327	1	pseudobulk	DESeq2	LRT	0.082555984664330
883	KC_AB2	achi	-0.729379579623002	1	1	pseudobulk	DESeq2	LRT	0
883	KC_AB2	Ack	-0.467031669438227	0.808037298456944	1	pseudobulk	DESeq2	LRT	0.092568592022230
883	KC_AB2	Ack-like	0.505711789439636	0.488104448993685	1	pseudobulk	DESeq2	LRT	0.311487233800964
883	KC_AB2	Acn	0.0738672794471182	0.87218662897867	1	pseudobulk	DESeq2	LRT	0.059390575545294

883	KC_AB2	Acph-1	-0.17796351416645	0.874755835152541	1	pseudobulk	DESeq2	LRT	0.058113151827511
884	KC_AB2	Acsl	-0.356746469736066	0.685944655994639	1	pseudobulk	DESeq2	LRT	0.163710923018816
884	KC_AB2	Act42A	-0.697429596943154	0.630579475679167	1	pseudobulk	DESeq2	LRT	0.200260168926431
884	KC_AB2	Act5C	-0.988345222691588	0.0215717190087828	1	pseudobulk	DESeq2	LRT	1.6661152454268
884	KC_AB2	Actbeta	0.222062561396013	0.681310005308673	1	pseudobulk	DESeq2	LRT	0.166655233226403
884	KC_AB2	Actn	-1.07983333094255	0.445116055530038	1	pseudobulk	DESeq2	LRT	0.351526740243783
884	KC_AB2	Acyp2	1.67332285989993	0.370463604811074	1	pseudobulk	DESeq2	LRT	0.431254451668764
884	KC_AB2	Ada1-2	0.843244047257989	0.721056826075985	1	pseudobulk	DESeq2	LRT	0.142030507432132
884	KC_AB2	Ada2b	0.575318463019561	0.628988507523228	1	pseudobulk	DESeq2	LRT	0.201357289633972
884	KC_AB2	Ada3	1.53722766603166	0.41188885531382	1	pseudobulk	DESeq2	LRT	0.385219958811221
884	KC_AB2	Adar	1.09606898238413	0.181047694302251	1	pseudobulk	DESeq2	LRT	0.742207001684779
885	KC_AB2	Adck5	-0.729379579623002	0.823527397767669	1	pseudobulk	DESeq2	LRT	0.084321947797948
885	KC_AB2	ADD1	2.57479073852543	0.111765811659112	1	pseudobulk	DESeq2	LRT	0.951691023627406
885	KC_AB2	AdenoK	-0.0458625429834585	0.968970809044799	1	pseudobulk	DESeq2	LRT	0.013689306191466
885	KC_AB2	Adf1	1.2223135447749	0.197765467297782	1	pseudobulk	DESeq2	LRT	0.703849540197485
885	KC_AB2	Adgf-B	1.10706867860301	1	1	pseudobulk	DESeq2	LRT	0
885	KC_AB2	Adi1	1.96523470879118	0.284326997065582	1	pseudobulk	DESeq2	LRT	0.546181901758876
885	KC_AB2	AdipoR	0.579955401664068	0.370596088583129	1	pseudobulk	DESeq2	LRT	0.431099168707698
885	KC_AB2	Adk1	-0.0036763039867276	0.981558414511276	1	pseudobulk	DESeq2	LRT	0.008083849558613
885	KC_AB2	Adk2	0.134037283499509	0.819726370840876	1	pseudobulk	DESeq2	LRT	0.086331093306139
885	KC_AB2	Adk3	1.09863729809763	0.612627088385388	1	pseudobulk	DESeq2	LRT	0.212803804009689
886	KC_AB2	AdoR	1.38446765109058	0.642794900879723	1	pseudobulk	DESeq2	LRT	0.191927577037166
886	KC_AB2	adp	-1.90302989316	0.39316374119602	1	pseudobulk	DESeq2	LRT	0.405426541004158
886	KC_AB2	ADPS	-1.31655680132672	0.188449837175558	1	pseudobulk	DESeq2	LRT	0.724804233453364
886	KC_AB2	AdSL	-0.968109268377054	0.551802346970015	1	pseudobulk	DESeq2	LRT	0.25821645667677
886	KC_AB2	AdSS	0.374140908250023	0.727706333901042	1	pseudobulk	DESeq2	LRT	0.138043844995684
886	KC_AB2	Aduk	0.0028788792586350	0.88552575685708	1	pseudobulk	DESeq2	LRT	0.052798802177782
886	KC_AB2	Ady43A	-1.56907089061617	0.306963150636533	1	pseudobulk	DESeq2	LRT	0.512913756235338
886	KC_AB2	Aef1	-0.311173834756602	0.678206478079871	1	pseudobulk	DESeq2	LRT	0.168638066252264
886	KC_AB2	aft	-0.721175887303532	0.733409564198845	1	pseudobulk	DESeq2	LRT	0.134653430819787
886	KC_AB2	Afti	-0.529161330453081	0.620555937870655	1	pseudobulk	DESeq2	LRT	0.207219064425372
887	KC_AB2	Ag5r	-1.78975280219494	0.468855431150075	1	pseudobulk	DESeq2	LRT	0.32896104882553
887	KC_AB2	AGBE	-1.6052707068109	0.52265609184397	1	pseudobulk	DESeq2	LRT	0.281783983297782
887	KC_AB2	ago	0.29662113530374	0.83167926927431	1	pseudobulk	DESeq2	LRT	0.080044123756460
887	KC_AB2	AGO1	0.324115688216457	0.169536799790642	1	pseudobulk	DESeq2	LRT	0.77073601892781
887	KC_AB2	AGO2	0.157266117658411	0.860172516348403	1	pseudobulk	DESeq2	LRT	0.065414437844371
887	KC_AB2	AGO3	-1.6052707068109	0.52265609184397	1	pseudobulk	DESeq2	LRT	0.281783983297782
887	KC_AB2	Agpat1	2.31644847791259	0.145006038491478	1	pseudobulk	DESeq2	LRT	0.838613912048315
887	KC_AB2	Agpat2	-1.78975280219494	0.468855431150075	1	pseudobulk	DESeq2	LRT	0.32896104882553
887	KC_AB2	Agpat3	-1.36585445481012	0.208436248944377	1	pseudobulk	DESeq2	LRT	0.681026751075305
887	KC_AB2	agt	-0.242904908965546	0.933507439612501	1	pseudobulk	DESeq2	LRT	0.029882216579625
888	KC_AB2	Ahcy	-0.0185848432475528	0.967527788606754	1	pseudobulk	DESeq2	LRT	0.014336552649866
888	KC_AB2	AhcyL1	-0.55637332818372	0.614561776908255	1	pseudobulk	DESeq2	LRT	0.211434454483223
888	KC_AB2	AIF	-1.09051709252325	0.659544943795745	1	pseudobulk	DESeq2	LRT	0.180755604700739
888	KC_AB2	AIMP1	2.03913993462321	0.360583814303595	1	pseudobulk	DESeq2	LRT	0.442993771558146
888	KC_AB2	AIMP2	-1.38863402716319	0.294812368866123	1	pseudobulk	DESeq2	LRT	0.530454299586333
888	KC_AB2	AIMP3	-0.330573516906517	0.673548833947633	1	pseudobulk	DESeq2	LRT	0.171630911381024
888	KC_AB2	Ak6	1.30866632083937	0.511483045170782	1	pseudobulk	DESeq2	LRT	0.291168757867386
888	KC_AB2	Akap200	-0.224094162086639	0.853658100123691	1	pseudobulk	DESeq2	LRT	0.068716034417195
888	KC_AB2	akirin	0.0249201593711778	0.932122233653962	1	pseudobulk	DESeq2	LRT	0.030527132795595
888	KC_AB2	Akt1	-1.58518843043162	0.17267589985301	1	pseudobulk	DESeq2	LRT	0.762768272109548
889	KC_AB2	Aladin	-0.277947413912466	0.859179291176031	1	pseudobulk	DESeq2	LRT	0.065916199331162

889	KC_AB2	AlaRS	2.24081982404879	0.300870360367611	1	pseudobulk	DESeq2	LRT	0.521620593791382
889	KC_AB2	Alas	-0.0671752922339567	0.900053395585923	1	pseudobulk	DESeq2	LRT	0.045731725315723
889	KC_AB2	alc	-0.338505851126486	0.616150753611948	1	pseudobulk	DESeq2	LRT	0.210313015998414
889	KC_AB2	Ald1	-0.277524717551312	0.489189568600639	1	pseudobulk	DESeq2	LRT	0.310522812362413
889	KC_AB2	Aldh-III	0.018466438710614	0.895151406593711	1	pseudobulk	DESeq2	LRT	0.048103501570200
889	KC_AB2	Alg-2	0.654926045180157	0.325079047875191	1	pseudobulk	DESeq2	LRT	0.488011020923502
889	KC_AB2	Alg1	-0.893328173497901	0.679679924321693	1	pseudobulk	DESeq2	LRT	0.167695557626761
889	KC_AB2	Alg10	-0.0519406878405818	0.974292486183317	1	pseudobulk	DESeq2	LRT	0.011310646748866
889	KC_AB2	Alg11	0.155610210398682	0.861545540228706	1	pseudobulk	DESeq2	LRT	0.064721761341541
890	KC_AB2	Alg14	2.40959160372577	0.287675536018374	1	pseudobulk	DESeq2	LRT	0.541097069023718
890	KC_AB2	Alg2	0.0055004516115709	0.960229690405946	1	pseudobulk	DESeq2	LRT	0.017624869726863
890	KC_AB2	Alg3	2.32149674095703	0.249331547667048	1	pseudobulk	DESeq2	LRT	0.603222767180568
890	KC_AB2	Alg9	0.393976029558784	0.939642609940555	1	pseudobulk	DESeq2	LRT	0.027037297511213
890	KC_AB2	Alh	1.29184776913119	0.185501354411923	1	pseudobulk	DESeq2	LRT	0.731652915097576
890	KC_AB2	alien	-1.35635530942244	0.492839867522929	1	pseudobulk	DESeq2	LRT	0.307294167838575
890	KC_AB2	ALiX	0.562862214454635	0.699709144744904	1	pseudobulk	DESeq2	LRT	0.155082450103441
890	KC_AB2	Alk	0.336944903294454	0.263869532624386	1	pseudobulk	DESeq2	LRT	0.578610752170915
890	KC_AB2	Alp6	1.38446765109058	0.642794900879723	1	pseudobulk	DESeq2	LRT	0.191927577037166
890	KC_AB2	alph	-0.640637165121884	0.248400713636977	1	pseudobulk	DESeq2	LRT	0.604847160797567
891	KC_AB2	alpha-Cat	0.206239695026546	0.622226139397704	1	pseudobulk	DESeq2	LRT	0.206051748357933
891	KC_AB2	alpha-Catr	0.499171738808674	0.579817941546379	1	pseudobulk	DESeq2	LRT	0.236708350219321
891	KC_AB2	alpha-Est8	0.281700311435579	0.637897969335276	1	pseudobulk	DESeq2	LRT	0.195248780367
891	KC_AB2	alpha-Est9	-0.806502802298727	0.436009138126119	1	pseudobulk	DESeq2	LRT	0.360504408446644
891	KC_AB2	alpha-Man-Ia	-0.390540075460282	0.228741014528169	1	pseudobulk	DESeq2	LRT	0.640655957010056
891	KC_AB2	alpha-Man-Ib	-0.321993762241313	0.748208118404204	1	pseudobulk	DESeq2	LRT	0.125977583816216
891	KC_AB2	alpha-Man-IIa	0.33333575867548	0.858979456944999	1	pseudobulk	DESeq2	LRT	0.066017222480879
891	KC_AB2	alpha-Man-IIb	0.20227273596828	0.891300700872092	1	pseudobulk	DESeq2	LRT	0.049975751970152
891	KC_AB2	alpha-PheRS	0.63497111999374	0.776284876380326	1	pseudobulk	DESeq2	LRT	0.109978874700361
891	KC_AB2	alpha-Spec	-0.181932262379234	0.71643383849697	1	pseudobulk	DESeq2	LRT	0.144823909805909
892	KC_AB2	alpha4GT1	-0.405618042774821	0.716267919007105	1	pseudobulk	DESeq2	LRT	0.14492450006321
892	KC_AB2	alpha4GT2	-0.729379579623002	0.823527397767669	1	pseudobulk	DESeq2	LRT	0.084321947797948
892	KC_AB2	alphaCOP	-0.604368479427411	0.663725688979734	1	pseudobulk	DESeq2	LRT	0.178011373006988
892	KC_AB2	alphaSnap	-0.0164337907425746	0.91113787566131	1	pseudobulk	DESeq2	LRT	0.040415899527359
892	KC_AB2	alphaTub67C	-2.1751167112989	0.359363815234217	1	pseudobulk	DESeq2	LRT	0.444465654648455
892	KC_AB2	alphaTub84B	-0.111399062369993	0.71134156399512	1	pseudobulk	DESeq2	LRT	0.147921814546418
892	KC_AB2	alphaTub84D	-0.660252985788033	0.29735880814349	1	pseudobulk	DESeq2	LRT	0.52671919262306
892	KC_AB2	Alr	1.47789775877809	0.581821529618366	1	pseudobulk	DESeq2	LRT	0.235210212237369
892	KC_AB2	Als2	0.944738735067321	0.534624117944108	1	pseudobulk	DESeq2	LRT	0.271951453260403
892	KC_AB2	alt	-1.10430938728734	0.0788348154203215	1	pseudobulk	DESeq2	LRT	1.10328194487437
893	KC_AB2	Amacr	-0.768120147155614	0.627624899526867	1	pseudobulk	DESeq2	LRT	0.202299835141485
893	KC_AB2	amn	3.30354169368959	0.127303602791195	1	pseudobulk	DESeq2	LRT	0.895159305300041
893	KC_AB2	Amnionless	0.388447834546628	0.84487503289525	1	pseudobulk	DESeq2	LRT	0.073207523639173
893	KC_AB2	amon	0.0516321565026895	0.842061649364145	1	pseudobulk	DESeq2	LRT	0.074656111589966
893	KC_AB2	AMPdeam	0.687930224833601	0.521160131561907	1	pseudobulk	DESeq2	LRT	0.283028814956631
893	KC_AB2	Amph	-1.24428225442477	0.287852768228258	1	pseudobulk	DESeq2	LRT	0.540829589656907
893	KC_AB2	AMPKalpha	0.699271245104251	0.489846579437251	1	pseudobulk	DESeq2	LRT	0.30993992025103
893	KC_AB2	Amun	0.270168281473351	0.69906953680607	1	pseudobulk	DESeq2	LRT	0.155479622610344
893	KC_AB2	amx	-0.115183097143228	0.910399790291748	1	pseudobulk	DESeq2	LRT	0.040767850987610
893	KC_AB2	ana	1.10706867860301	1	1	pseudobulk	DESeq2	LRT	0
894	KC_AB2	anchor	-0.121327009109861	0.897563217938633	1	pseudobulk	DESeq2	LRT	0.046934953072753
894	KC_AB2	angel	-1.63739047546051	0.431761148313956	1	pseudobulk	DESeq2	LRT	0.364756439889685
894	KC_AB2	Ank	-0.174944391390928	0.739656559915246	1	pseudobulk	DESeq2	LRT	0.130969886696343

894	KC_AB2	Ank2	0.0202041565182291	0.919840647364968	1	pseudobulk	DESeq2	LRT	0.036287403050450
894	KC_AB2	Ankle2	-0.034050306591806	0.980236861315582	1	pseudobulk	DESeq2	LRT	0.008668970090120
894	KC_AB2	anne	0.02354311517221	0.893291041480737	1	pseudobulk	DESeq2	LRT	0.049007021384519
894	KC_AB2	Ant2	0.146930209264112	1	1	pseudobulk	DESeq2	LRT	0
894	KC_AB2	AnxB10	-1.02187704029498	0.501916101653985	1	pseudobulk	DESeq2	LRT	0.299368871767022
894	KC_AB2	AnxB11	0.318485131944161	0.656459990215636	1	pseudobulk	DESeq2	LRT	0.182791738055958
894	KC_AB2	aop	-0.823923029966701	0.567603460454628	1	pseudobulk	DESeq2	LRT	0.245954965484175
895	KC_AB2	aos	1.10706867860301	1	1	pseudobulk	DESeq2	LRT	0
895	KC_AB2	Aos1	0.0801046780703637	0.951818018243433	1	pseudobulk	DESeq2	LRT	0.021446078115083
895	KC_AB2	AP-1-2beta	-0.648700943760337	0.433203767448627	1	pseudobulk	DESeq2	LRT	0.363307775096037
895	KC_AB2	AP-1gamma	0.382020215773745	0.408609528353561	1	pseudobulk	DESeq2	LRT	0.388691510285254
895	KC_AB2	AP-1mu	0.787057912345252	0.36846955424679	1	pseudobulk	DESeq2	LRT	0.433598391031171
895	KC_AB2	AP-1sigma	-0.307793827259098	0.567857885084686	1	pseudobulk	DESeq2	LRT	0.245760339369991
895	KC_AB2	AP-2alpha	-0.0106604598593416	0.913081941771375	1	pseudobulk	DESeq2	LRT	0.039490246274813
895	KC_AB2	AP-2mu	0.148124715766616	0.799218854488135	1	pseudobulk	DESeq2	LRT	0.097334279156988
895	KC_AB2	AP-2sigma	-0.363949906533385	0.445993029729985	1	pseudobulk	DESeq2	LRT	0.350671928672297
895	KC_AB2	Apc	-0.335916186123079	0.631086713188092	1	pseudobulk	DESeq2	LRT	0.199910963304606
896	KC_AB2	APC10	0.27555101894953	0.799629953874363	1	pseudobulk	DESeq2	LRT	0.097110945721139
896	KC_AB2	APC4	-0.242904908965546	0.933507439612501	1	pseudobulk	DESeq2	LRT	0.029882216579625
896	KC_AB2	ApepP	-1.33407500886303	0.63128114564055	1	pseudobulk	DESeq2	LRT	0.19977718147345
896	KC_AB2	Apf	-1.28620650693415	0.420524804750522	1	pseudobulk	DESeq2	LRT	0.376208382152492
896	KC_AB2	aph-1	-0.127755677887302	0.880037531788115	1	pseudobulk	DESeq2	LRT	0.055498805689726
896	KC_AB2	aPKC	0.122073037647583	0.794135700682113	1	pseudobulk	DESeq2	LRT	0.100105279661235
896	KC_AB2	Apl	-0.194402616845475	0.899217068637067	1	pseudobulk	DESeq2	LRT	0.046135458064451
896	KC_AB2	Aplip1	-0.48021161732827	0.322498022115643	1	pseudobulk	DESeq2	LRT	0.491472944553973
896	KC_AB2	apolpp	0.145321756433347	1	1	pseudobulk	DESeq2	LRT	0
896	KC_AB2	Apoltp	-0.0915019784319721	0.807728103502277	1	pseudobulk	DESeq2	LRT	0.092734806330471
897	KC_AB2	app	-0.496925528804606	0.620349788332291	1	pseudobulk	DESeq2	LRT	0.207363361620383
897	KC_AB2	APP-BP1	2.33862076287154	0.37727071943742	1	pseudobulk	DESeq2	LRT	0.423346899755834
897	KC_AB2	Appl	0.0567840774146717	0.765064184041849	1	pseudobulk	DESeq2	LRT	0.116302128760226
897	KC_AB2	Aprt	-1.33407500886303	0.63128114564055	1	pseudobulk	DESeq2	LRT	0.19977718147345
897	KC_AB2	Aps	0.480906445592567	0.485950226046979	1	pseudobulk	DESeq2	LRT	0.31340821151991
897	KC_AB2	aqz	0.207638661791701	0.338100814523093	1	pseudobulk	DESeq2	LRT	0.470953782968456
897	KC_AB2	aralar1	-1.68583823399618	0.353560440138572	1	pseudobulk	DESeq2	LRT	0.45153633413028
897	KC_AB2	Arc2	-0.729379579623002	1	1	pseudobulk	DESeq2	LRT	0
897	KC_AB2	Arc42	0.690387450349416	0.75906361064041	1	pseudobulk	DESeq2	LRT	0.119721828066906
897	KC_AB2	Archease	2.077280204983	0.235792119260643	1	pseudobulk	DESeq2	LRT	0.62747071416379
898	KC_AB2	Arf102F	-0.927563906400923	0.238551303349087	1	pseudobulk	DESeq2	LRT	0.622418206286904
898	KC_AB2	Arf51F	-0.186345169992104	0.823221613575138	1	pseudobulk	DESeq2	LRT	0.084483235751462
898	KC_AB2	Arf79F	-0.569400901978117	0.170102208259308	1	pseudobulk	DESeq2	LRT	0.769290048359172
898	KC_AB2	ArfGAP1	-0.0285360800339529	0.901333120098553	1	pseudobulk	DESeq2	LRT	0.045114670194183
898	KC_AB2	ArfGAP3	-0.775829472274612	0.655781491761667	1	pseudobulk	DESeq2	LRT	0.183240844664528
898	KC_AB2	Arfip	-1.11940611455449	0.233928384258721	1	pseudobulk	DESeq2	LRT	0.630917078836274
898	KC_AB2	Arfrp1	-0.729379579623002	0.823527397767669	1	pseudobulk	DESeq2	LRT	0.084321947797948
898	KC_AB2	arg	0.518865066730979	0.817181531186031	1	pseudobulk	DESeq2	LRT	0.087681457255733
898	KC_AB2	Argk	-0.479892903372382	0.136403852995655	1	pseudobulk	DESeq2	LRT	0.865173362002719
898	KC_AB2	ArgRS	-1.33407500886303	0.63128114564055	1	pseudobulk	DESeq2	LRT	0.19977718147345
899	KC_AB2	ArgRS-m	-0.729379579623002	0.772694994316916	1	pseudobulk	DESeq2	LRT	0.1119919011982
899	KC_AB2	ari-1	0.666614941071611	0.214206605916395	1	pseudobulk	DESeq2	LRT	0.669167140092395
899	KC_AB2	ari-2	-0.139544798598615	0.902874491355144	1	pseudobulk	DESeq2	LRT	0.044372616796435
899	KC_AB2	Arl1	0.514566852681691	0.642493561314044	1	pseudobulk	DESeq2	LRT	0.192131220214244
899	KC_AB2	Arl2	-0.0715864007502557	0.909238575309591	1	pseudobulk	DESeq2	LRT	0.041322147200579

899	KC_AB2	Arl4	-0.441267466610877	0.304657730904419	1	pseudobulk	DESeq2	LRT	0.516187796871436
899	KC_AB2	Arl5	4.08180847551845	0.00800771480909098	1	pseudobulk	DESeq2	LRT	2.09649140244227
899	KC_AB2	Arl6IP1	-0.204814560346598	0.603684209878644	1	pseudobulk	DESeq2	LRT	0.219190183517501
899	KC_AB2	Arl8	-0.124720222179393	0.853441579055248	1	pseudobulk	DESeq2	LRT	0.068826202448848
899	KC_AB2	arm	0.11744546872275	0.744207784603973	1	pseudobulk	DESeq2	LRT	0.128305791459248
900	KC_AB2	armi	1.10706867860301	1	1	pseudobulk	DESeq2	LRT	0
900	KC_AB2	Arp1	-0.229592082349186	0.752200416150154	1	pseudobulk	DESeq2	LRT	0.12366643064286
900	KC_AB2	Arp10	-2.22728608909242	0.317088151657018	1	pseudobulk	DESeq2	LRT	0.498819985554471
900	KC_AB2	Arp2	0.0201310491006901	0.931569831431552	1	pseudobulk	DESeq2	LRT	0.030784584386985
900	KC_AB2	Arp3	-1.27490506506191	0.382243885886127	1	pseudobulk	DESeq2	LRT	0.41765945253998
900	KC_AB2	Arp5	2.9023801951582	0.206074905726345	1	pseudobulk	DESeq2	LRT	0.685974890162107
900	KC_AB2	Arp6	0.306101013060283	0.832815272420834	1	pseudobulk	DESeq2	LRT	0.079451319199637
900	KC_AB2	Arp8	0.690387450349416	0.75906361064041	1	pseudobulk	DESeq2	LRT	0.119721828066906
900	KC_AB2	Arpc1	-0.206100103091349	0.769749553016928	1	pseudobulk	DESeq2	LRT	0.113650554613576
900	KC_AB2	Arpc2	0.356944388250469	0.746766974321085	1	pseudobulk	DESeq2	LRT	0.126814896919083
901	KC_AB2	Arpc3A	-0.522455994596459	0.70483042036727	1	pseudobulk	DESeq2	LRT	0.151915360111704
901	KC_AB2	Arpc4	0.0443288857893974	0.926889643930769	1	pseudobulk	DESeq2	LRT	0.032971970154218
901	KC_AB2	Arpc5	-0.111902223823945	0.901074571754341	1	pseudobulk	DESeq2	LRT	0.045239265889678
901	KC_AB2	arr	0.873834738456049	0.278107766298986	1	pseudobulk	DESeq2	LRT	0.555786883075072
901	KC_AB2	Ars2	-1.04675655940712	0.138197659570467	1	pseudobulk	DESeq2	LRT	0.859499311840533
901	KC_AB2	Art1	0.53853850907868	0.816883535008605	1	pseudobulk	DESeq2	LRT	0.087839857431584
901	KC_AB2	Art3	-0.729379579623002	1	1	pseudobulk	DESeq2	LRT	0
901	KC_AB2	Art4	-0.721175887303532	0.733409564198845	1	pseudobulk	DESeq2	LRT	0.134653430819787
901	KC_AB2	Art7	1.38446765109058	0.642794900879723	1	pseudobulk	DESeq2	LRT	0.191927577037166
901	KC_AB2	Art8	1.02605719768525	0.762384804222891	1	pseudobulk	DESeq2	LRT	0.117825768597322
902	KC_AB2	Arts	-0.721175887303532	0.733409564198845	1	pseudobulk	DESeq2	LRT	0.134653430819787
902	KC_AB2	Arv1	-2.98267449058754	0.168067967023006	1	pseudobulk	DESeq2	LRT	0.774515053192418
902	KC_AB2	Asap	0.366280452532096	0.676805255769052	1	pseudobulk	DESeq2	LRT	0.169536277417858
902	KC_AB2	Asator	0.230485182885415	0.538179313496555	1	pseudobulk	DESeq2	LRT	0.269072999636103
902	KC_AB2	Asciz	-1.00833870937463	0.651152668420623	1	pseudobulk	DESeq2	LRT	0.186317175365195
902	KC_AB2	asf1	0.538543435593964	0.689840506042511	1	pseudobulk	DESeq2	LRT	0.161251308324288
902	KC_AB2	ash1	-0.723012353830105	0.537485449939725	1	pseudobulk	DESeq2	LRT	0.269633287870808
902	KC_AB2	ash2	2.47215486522296	0.30601789350912	1	pseudobulk	DESeq2	LRT	0.514253178665238
902	KC_AB2	Ask1	0.90329921765475	0.676169871361722	1	pseudobulk	DESeq2	LRT	0.169944184343482
902	KC_AB2	asl	-1.52244591594958	0.547176617578731	1	pseudobulk	DESeq2	LRT	0.261872469536296
903	KC_AB2	AsnRS	0.383351023029283	0.794098563169369	1	pseudobulk	DESeq2	LRT	0.100125589784529
903	KC_AB2	AsnRS-m	-1.59499420489131	0.469581734450344	1	pseudobulk	DESeq2	LRT	0.328288804396094
903	KC_AB2	AsnS	1.10706867860301	1	1	pseudobulk	DESeq2	LRT	0
903	KC_AB2	Asph	-0.729379579623002	1	1	pseudobulk	DESeq2	LRT	0
903	KC_AB2	AspRS	-0.893328173497901	0.679679924321693	1	pseudobulk	DESeq2	LRT	0.167695557626761
903	KC_AB2	AspRS-m	-0.439853737731089	0.751903088223596	1	pseudobulk	DESeq2	LRT	0.123838131432236
903	KC_AB2	asrij	1.02719911966085	0.343926411613571	1	pseudobulk	DESeq2	LRT	0.463534471524341
903	KC_AB2	asRNA:CR30267	1.61205113368698	0.631647504209	1	pseudobulk	DESeq2	LRT	0.199525215522471
903	KC_AB2	asRNA:CR31144	-0.729379579623002	0.823527397767669	1	pseudobulk	DESeq2	LRT	0.084321947797948
903	KC_AB2	asRNA:CR31845	-0.729379579623002	1	1	pseudobulk	DESeq2	LRT	0
904	KC_AB2	asRNA:CR31912	1.10706867860301	1	1	pseudobulk	DESeq2	LRT	0
904	KC_AB2	asRNA:CR42254	1.35534537220437	0.537584927319277	1	pseudobulk	DESeq2	LRT	0.269552916430853
904	KC_AB2	asRNA:CR42547	0.843244047257989	0.721056826075985	1	pseudobulk	DESeq2	LRT	0.142030507432132
904	KC_AB2	asRNA:CR43425	0.527269226237322	0.644408055545173	1	pseudobulk	DESeq2	LRT	0.190839039238309
904	KC_AB2	asRNA:CR43426	-0.729379579623002	1	1	pseudobulk	DESeq2	LRT	0
904	KC_AB2	asRNA:CR43629	0.393976029558784	0.939642609940555	1	pseudobulk	DESeq2	LRT	0.027037297511213
904	KC_AB2	asRNA:CR43767	-0.729379579623002	0.772694994316916	1	pseudobulk	DESeq2	LRT	0.1119919011982

904	KC_AB2	asRNA:CR43786	-0.729379579623002	1	1	pseudobulk	DESeq2	LRT	0
904	KC_AB2	asRNA:CR43806	1.10706867860301	1	1	pseudobulk	DESeq2	LRT	0
904	KC_AB2	asRNA:CR43809	-0.729379579623002	0.823527397767669	1	pseudobulk	DESeq2	LRT	0.084321947797948
905	KC_AB2	asRNA:CR43820	-0.729379579623002	1	1	pseudobulk	DESeq2	LRT	0
905	KC_AB2	asRNA:CR43833	-0.729379579623002	0.772694994316916	1	pseudobulk	DESeq2	LRT	0.1119919011982
905	KC_AB2	asRNA:CR43889	-0.729379579623002	0.823527397767669	1	pseudobulk	DESeq2	LRT	0.084321947797948
905	KC_AB2	asRNA:CR43906	-0.263487579709627	0.844957435629643	1	pseudobulk	DESeq2	LRT	0.073165167898547
905	KC_AB2	asRNA:CR43933	1.10706867860301	1	1	pseudobulk	DESeq2	LRT	0
905	KC_AB2	asRNA:CR43944	1.94871771291584	0.412369540323867	1	pseudobulk	DESeq2	LRT	0.38471342136576
905	KC_AB2	asRNA:CR43957	-0.729379579623002	1	1	pseudobulk	DESeq2	LRT	0
905	KC_AB2	asRNA:CR43959	-0.161754810636277	0.996285598531333	1	pseudobulk	DESeq2	LRT	0.001616147433209
905	KC_AB2	asRNA:CR43971	1.38069928703004	0.566368801250645	1	pseudobulk	DESeq2	LRT	0.246900678019328
905	KC_AB2	asRNA:CR44018	1.05959893905903	0.541908746999156	1	pseudobulk	DESeq2	LRT	0.266073838943674
906	KC_AB2	asRNA:CR44027	2.47215486522296	0.30601789350912	1	pseudobulk	DESeq2	LRT	0.514253178665238
906	KC_AB2	asRNA:CR44029	1.37200440170938	0.48893549025908	1	pseudobulk	DESeq2	LRT	0.310748437548461
906	KC_AB2	asRNA:CR44030	-0.828938418224188	0.492788665102001	1	pseudobulk	DESeq2	LRT	0.307339290170474
906	KC_AB2	asRNA:CR44031	-0.734540122400504	0.833184184675853	1	pseudobulk	DESeq2	LRT	0.079258982331102
906	KC_AB2	asRNA:CR44053	-0.734071467759236	0.785001998672014	1	pseudobulk	DESeq2	LRT	0.105129237508095
906	KC_AB2	asRNA:CR44069	1.10706867860301	1	1	pseudobulk	DESeq2	LRT	0
906	KC_AB2	asRNA:CR44093	1.10706867860301	1	1	pseudobulk	DESeq2	LRT	0
906	KC_AB2	asRNA:CR44106	0.147593560254927	1	1	pseudobulk	DESeq2	LRT	0
906	KC_AB2	asRNA:CR44114	-0.729379579623002	1	1	pseudobulk	DESeq2	LRT	0
906	KC_AB2	asRNA:CR44119	-0.729379579623002	0.772694994316916	1	pseudobulk	DESeq2	LRT	0.1119919011982
907	KC_AB2	asRNA:CR44179	1.61205113368698	0.631647504209	1	pseudobulk	DESeq2	LRT	0.199525215522471
907	KC_AB2	asRNA:CR44228	2.48384162006255	0.303740962766822	1	pseudobulk	DESeq2	LRT	0.517496634805608
907	KC_AB2	asRNA:CR44291	1.38446765109058	0.642794900879723	1	pseudobulk	DESeq2	LRT	0.191927577037166
907	KC_AB2	asRNA:CR44340	-0.729379579623002	0.823527397767669	1	pseudobulk	DESeq2	LRT	0.084321947797948
907	KC_AB2	asRNA:CR44351	-0.729379579623002	0.823527397767669	1	pseudobulk	DESeq2	LRT	0.084321947797948
907	KC_AB2	asRNA:CR44381	-0.729379579623002	0.772694994316916	1	pseudobulk	DESeq2	LRT	0.1119919011982
907	KC_AB2	asRNA:CR44390	1.10706867860301	1	1	pseudobulk	DESeq2	LRT	0
907	KC_AB2	asRNA:CR44599	1.10706867860301	1	1	pseudobulk	DESeq2	LRT	0
907	KC_AB2	asRNA:CR44690	1.10706867860301	1	1	pseudobulk	DESeq2	LRT	0
907	KC_AB2	asRNA:CR44892	-0.729379579623002	0.823527397767669	1	pseudobulk	DESeq2	LRT	0.084321947797948
908	KC_AB2	asRNA:CR44894	0.0506112603868946	1	1	pseudobulk	DESeq2	LRT	0
908	KC_AB2	asRNA:CR44960	-0.729379579623002	0.772694994316916	1	pseudobulk	DESeq2	LRT	0.1119919011982
908	KC_AB2	asRNA:CR44961	-1.71999636108657	0.519869997311849	1	pseudobulk	DESeq2	LRT	0.284105245805297
908	KC_AB2	asRNA:CR44984	1.38069928703004	0.566368801250645	1	pseudobulk	DESeq2	LRT	0.246900678019328
908	KC_AB2	asRNA:CR44987	-0.729379579623002	1	1	pseudobulk	DESeq2	LRT	0
908	KC_AB2	asRNA:CR45000	0.38605638660934	0.863777890371283	1	pseudobulk	DESeq2	LRT	0.063597916533759
908	KC_AB2	asRNA:CR45007	-0.729379579623002	1	1	pseudobulk	DESeq2	LRT	0
908	KC_AB2	asRNA:CR45009	2.16548735034375	0.319952228384207	1	pseudobulk	DESeq2	LRT	0.494914860736039
908	KC_AB2	asRNA:CR45121	1.38446765109058	0.642794900879723	1	pseudobulk	DESeq2	LRT	0.191927577037166
908	KC_AB2	asRNA:CR45126	-0.785201786757791	0.678486044454677	1	pseudobulk	DESeq2	LRT	0.168459080765713
909	KC_AB2	asRNA:CR45129	1.40009820523851	0.478755394339011	1	pseudobulk	DESeq2	LRT	0.319886319616599
909	KC_AB2	asRNA:CR45134	1.78258764191389	0.445483464074049	1	pseudobulk	DESeq2	LRT	0.351168411929411
909	KC_AB2	asRNA:CR45140	1.10706867860301	1	1	pseudobulk	DESeq2	LRT	0
909	KC_AB2	asRNA:CR45145	-0.0425218595985072	0.974719112664924	1	pseudobulk	DESeq2	LRT	0.011120518039663
909	KC_AB2	asRNA:CR45162	1.10706867860301	1	1	pseudobulk	DESeq2	LRT	0
909	KC_AB2	asRNA:CR45164	0.147593560254927	1	1	pseudobulk	DESeq2	LRT	0
909	KC_AB2	asRNA:CR45167	1.10706867860301	1	1	pseudobulk	DESeq2	LRT	0
909	KC_AB2	asRNA:CR45182	1.80483863294315	0.422281715013702	1	pseudobulk	DESeq2	LRT	0.374397723312422
909	KC_AB2	asRNA:CR45198	1.4492427647578	0.495795124936081	1	pseudobulk	DESeq2	LRT	0.304697747886407

909	KC_AB2	asRNA:CR45281	-0.729379579623002	0.823527397767669	1	pseudobulk	DESeq2	LRT	0.084321947797948
910	KC_AB2	asRNA:CR45367	-0.729379579623002	0.772694994316916	1	pseudobulk	DESeq2	LRT	0.1119919011982
910	KC_AB2	asRNA:CR45384	0.145321756433347	1	1	pseudobulk	DESeq2	LRT	0
910	KC_AB2	asRNA:CR45520	0.146930209264112	1	1	pseudobulk	DESeq2	LRT	0
910	KC_AB2	asRNA:CR45528	1.38069928703004	0.566368801250645	1	pseudobulk	DESeq2	LRT	0.246900678019328
910	KC_AB2	asRNA:CR45620	-0.729379579623002	1	1	pseudobulk	DESeq2	LRT	0
910	KC_AB2	asRNA:CR45683	-1.33407500886303	0.63128114564055	1	pseudobulk	DESeq2	LRT	0.19977718147345
910	KC_AB2	asRNA:CR45789	-0.729379579623002	1	1	pseudobulk	DESeq2	LRT	0
910	KC_AB2	asRNA:CR45798	1.10706867860301	1	1	pseudobulk	DESeq2	LRT	0
910	KC_AB2	asRNA:CR45815	1.10706867860301	1	1	pseudobulk	DESeq2	LRT	0
910	KC_AB2	asRNA:CR45822	0.0667588332006575	0.944703280098873	1	pseudobulk	DESeq2	LRT	0.024704576729781
911	KC_AB2	asRNA:CR45835	0.0107214417965268	0.937943862816314	1	pseudobulk	DESeq2	LRT	0.027823153943621
911	KC_AB2	asRNA:CR45873	1.10706867860301	1	1	pseudobulk	DESeq2	LRT	0
911	KC_AB2	asRNA:CR45874	-0.170595056242129	0.879706463696137	1	pseudobulk	DESeq2	LRT	0.055662217012676
911	KC_AB2	asRNA:CR45882	-0.729379579623002	1	1	pseudobulk	DESeq2	LRT	0
911	KC_AB2	asRNA:CR45907	-1.78975280219494	0.468855431150075	1	pseudobulk	DESeq2	LRT	0.32896104882553
911	KC_AB2	asRNA:CR45912	1.10706867860301	1	1	pseudobulk	DESeq2	LRT	0
911	KC_AB2	asRNA:CR45928	1.10706867860301	1	1	pseudobulk	DESeq2	LRT	0
911	KC_AB2	asRNA:CR45968	1.38446765109058	0.642794900879723	1	pseudobulk	DESeq2	LRT	0.191927577037166
911	KC_AB2	asRNA:CR46029	1.37221885443763	0.558734269457382	1	pseudobulk	DESeq2	LRT	0.252794690766539
911	KC_AB2	asRNA:CR46067	1.38069928703004	0.566368801250645	1	pseudobulk	DESeq2	LRT	0.246900678019328
912	KC_AB2	asRNA:CR46068	1.10706867860301	1	1	pseudobulk	DESeq2	LRT	0
912	KC_AB2	asRNA:CR46093	-0.729379579623002	0.772694994316916	1	pseudobulk	DESeq2	LRT	0.1119919011982
912	KC_AB2	asRNA:CR46135	2.48384162006255	0.303740962766822	1	pseudobulk	DESeq2	LRT	0.517496634805608
912	KC_AB2	asRNA:CR46140	-0.729379579623002	0.772694994316916	1	pseudobulk	DESeq2	LRT	0.1119919011982
912	KC_AB2	asRNA:CR46251	-0.729379579623002	0.772694994316916	1	pseudobulk	DESeq2	LRT	0.1119919011982
912	KC_AB2	ast	0.661678460170035	0.878003361430606	1	pseudobulk	DESeq2	LRT	0.056503821396894
912	KC_AB2	AstA-R1	0.38605638660934	0.863777890371283	1	pseudobulk	DESeq2	LRT	0.063597916533759
912	KC_AB2	asun	-2.28054226263626	0.331630256012328	1	pseudobulk	DESeq2	LRT	0.479345853816062
912	KC_AB2	Asx	-0.57771599022483	0.58090999938353	1	pseudobulk	DESeq2	LRT	0.235891147814159
912	KC_AB2	Atac1	1.38069928703004	0.566368801250645	1	pseudobulk	DESeq2	LRT	0.246900678019328
913	KC_AB2	Atac2	-0.112490013242319	0.888963332509736	1	pseudobulk	DESeq2	LRT	0.051116152209881
913	KC_AB2	Atac3	0.676006581667494	0.697279153755715	1	pseudobulk	DESeq2	LRT	0.156593318509685
913	KC_AB2	ATbp	0.729840118778791	0.723180990776418	1	pseudobulk	DESeq2	LRT	0.140752998060082
913	KC_AB2	Ate1	-0.883247510422591	0.469952300411005	1	pseudobulk	DESeq2	LRT	0.327946220191009
913	KC_AB2	Atet	0.0514943654098845	0.863947105931143	1	pseudobulk	DESeq2	LRT	0.063512845837564
913	KC_AB2	Atf-2	0.0051010923713501	0.987795542617535	1	pseudobulk	DESeq2	LRT	0.005332937904148
913	KC_AB2	Atf3	1.99339641594643	0.465071165814147	1	pseudobulk	DESeq2	LRT	0.332480585700252
913	KC_AB2	Atf6	-0.256253593555991	0.587292561523669	1	pseudobulk	DESeq2	LRT	0.231145499769477
913	KC_AB2	Atg1	0.205185042834918	0.648699792569179	1	pseudobulk	DESeq2	LRT	0.187956240941479
913	KC_AB2	Atg101	-0.108012843038738	0.912388349052183	1	pseudobulk	DESeq2	LRT	0.039820269180848
914	KC_AB2	Atg12	-0.789919846287602	0.346369629852588	1	pseudobulk	DESeq2	LRT	0.460460194500127
914	KC_AB2	Atg13	0.0450543274824054	0.930987684656819	1	pseudobulk	DESeq2	LRT	0.031056063939131
914	KC_AB2	Atg14	-0.819116208432312	0.521368633634294	1	pseudobulk	DESeq2	LRT	0.28285510022811
914	KC_AB2	Atg16	-0.866792081985791	0.699019818888033	1	pseudobulk	DESeq2	LRT	0.15551051078981
914	KC_AB2	Atg17	-0.286707077531016	0.639033408900671	1	pseudobulk	DESeq2	LRT	0.194476436173485
914	KC_AB2	Atg18a	-1.24816655109457	0.274432555898961	1	pseudobulk	DESeq2	LRT	0.561564369610351
914	KC_AB2	Atg18b	0.0785694505616203	0.859201097368544	1	pseudobulk	DESeq2	LRT	0.065905176964818
914	KC_AB2	Atg2	-2.30556784634639	0.359761931587247	1	pseudobulk	DESeq2	LRT	0.443984793676075
914	KC_AB2	Atg3	-0.428220081896663	0.7061939742646	1	pseudobulk	DESeq2	LRT	0.151075992457995
914	KC_AB2	Atg4a	-0.277947413912466	0.859179291176031	1	pseudobulk	DESeq2	LRT	0.065916199331162
915	KC_AB2	Atg4b	0.268781370907002	0.88698811189061	1	pseudobulk	DESeq2	LRT	0.052082200884065

915	KC_AB2	Atg5	0.138981759612247	0.880186741433333	1	pseudobulk	DESeq2	LRT	0.055425177656406
915	KC_AB2	Atg6	1.00268106024788	0.627374080284178	1	pseudobulk	DESeq2	LRT	0.202473427975038
915	KC_AB2	Atg7	-0.647621034217714	0.754702560287896	1	pseudobulk	DESeq2	LRT	0.122224176685402
915	KC_AB2	Atg8a	-0.223145654610394	0.429957912591501	1	pseudobulk	DESeq2	LRT	0.366574054243314
915	KC_AB2	Atg9	1.10706867860301	1	1	pseudobulk	DESeq2	LRT	0
915	KC_AB2	atl	-1.22570198695868	0.411906850415908	1	pseudobulk	DESeq2	LRT	0.38520098523929
915	KC_AB2	atms	-0.279636156373759	0.830588778741131	1	pseudobulk	DESeq2	LRT	0.080613940507294
915	KC_AB2	Atox1	-0.0531267490890858	0.885783440998267	1	pseudobulk	DESeq2	LRT	0.05267244275785
915	KC_AB2	ATP6AP2	-0.417249138006627	0.351560813872572	1	pseudobulk	DESeq2	LRT	0.453999538795459
916	KC_AB2	ATP7	-0.856567423351035	0.665132979544327	1	pseudobulk	DESeq2	LRT	0.177091517841652
916	KC_AB2	ATP8A	0.430165408717545	0.527629793738406	1	pseudobulk	DESeq2	LRT	0.277670689071736
916	KC_AB2	ATP8B	0.942758880874816	0.658080412798793	1	pseudobulk	DESeq2	LRT	0.181721035411818
916	KC_AB2	Atpalpha	-0.275369409701744	0.250954917094723	1	pseudobulk	DESeq2	LRT	0.600404290533093
916	KC_AB2	ATPCL	-0.175945585588723	0.75957894092253	1	pseudobulk	DESeq2	LRT	0.119427084440445
916	KC_AB2	ATPsynB	-0.395202138099589	0.259463338494967	1	pseudobulk	DESeq2	LRT	0.585923998178306
916	KC_AB2	ATPsynbeta	-0.345623248406645	0.272264229670075	1	pseudobulk	DESeq2	LRT	0.565009412928671
916	KC_AB2	ATPsynC	-0.402305694036779	0.137427675868135	1	pseudobulk	DESeq2	LRT	0.861925798088253
916	KC_AB2	ATPsynCF6	-0.314277152966753	0.443335623422107	1	pseudobulk	DESeq2	LRT	0.353267370360813
916	KC_AB2	ATPsynCF6L	1.38069928703004	0.566368801250645	1	pseudobulk	DESeq2	LRT	0.246900678019328
917	KC_AB2	ATPsynD	-0.706579910808681	0.0429984601995296	1	pseudobulk	DESeq2	LRT	1.36654709648602
917	KC_AB2	ATPsyndelta	-0.437126980086031	0.380502014419853	1	pseudobulk	DESeq2	LRT	0.419643039684182
917	KC_AB2	ATPsynE	-0.115539055636979	0.726346710277002	1	pseudobulk	DESeq2	LRT	0.138856026108115
917	KC_AB2	ATPsynF	-0.152225733019058	0.62352508464015	1	pseudobulk	DESeq2	LRT	0.205146070009565
917	KC_AB2	ATPsynG	-0.379461885723885	0.29207438282269	1	pseudobulk	DESeq2	LRT	0.534506532333623
917	KC_AB2	ATPsyngamma	-0.384761970419791	0.371028589756387	1	pseudobulk	DESeq2	LRT	0.430592624360557
917	KC_AB2	ATPsynO	-0.361733836977747	0.381407892083058	1	pseudobulk	DESeq2	LRT	0.418610324785919
917	KC_AB2	AttA	1.61205113368698	0.631647504209	1	pseudobulk	DESeq2	LRT	0.199525215522471
917	KC_AB2	Atu	-0.620563475958957	0.7478554969218	1	pseudobulk	DESeq2	LRT	0.126182309825559
917	KC_AB2	Atx2	-0.0533499425863027	0.864965550701628	1	pseudobulk	DESeq2	LRT	0.063001188995435
918	KC_AB2	Atxn7	-0.570319610955485	0.538579259104945	1	pseudobulk	DESeq2	LRT	0.268750375450528
918	KC_AB2	aux	0.938659765582738	0.485707879095811	1	pseudobulk	DESeq2	LRT	0.313624851396063
918	KC_AB2	ave	-1.16818510865211	0.423906256750554	1	pseudobulk	DESeq2	LRT	0.372730173306308
918	KC_AB2	Aven	2.01814786626755	0.401718761445944	1	pseudobulk	DESeq2	LRT	0.396077884966921
918	KC_AB2	awd	-0.0211141783251746	0.912259247926815	1	pseudobulk	DESeq2	LRT	0.039881725328918
918	KC_AB2	axed	-0.358159633680718	0.658019562408671	1	pseudobulk	DESeq2	LRT	0.181761194957701
918	KC_AB2	Axn	1.11298662269478	0.485488564462183	1	pseudobulk	DESeq2	LRT	0.313820995312026
918	KC_AB2	axo	-1.6052707068109	0.52265609184397	1	pseudobulk	DESeq2	LRT	0.281783983297782
918	KC_AB2	Axud1	-0.0419941545665593	0.95735715272114	1	pseudobulk	DESeq2	LRT	0.018926013613191
918	KC_AB2	az2	3.08473756969698	0.0966943291744697	1	pseudobulk	DESeq2	LRT	1.01459899520813
919	KC_AB2	B4	0.145590264264213	0.777476746267592	1	pseudobulk	DESeq2	LRT	0.109312591521309
919	KC_AB2	B52	0.0028130458454547	0.924272532497557	1	pseudobulk	DESeq2	LRT	0.034199953121563
919	KC_AB2	bab1	3.78823588484119	0.0697241250253772	1	pseudobulk	DESeq2	LRT	1.15661692703014
919	KC_AB2	babo	-0.200290711200514	0.755575706906583	1	pseudobulk	DESeq2	LRT	0.121722013860347
919	KC_AB2	Bacc	0.268697285335036	0.205317479221548	1	pseudobulk	DESeq2	LRT	0.687574076414451
919	KC_AB2	baf	-2.20860336617865	0.299155245452459	1	pseudobulk	DESeq2	LRT	0.524103377742256
919	KC_AB2	bai	-1.80534772157314	0.100486171877555	1	pseudobulk	DESeq2	LRT	0.997893698347647
919	KC_AB2	Baldspot	-0.868620444126706	0.122721070226195	1	pseudobulk	DESeq2	LRT	0.911080865982252
919	KC_AB2	ball	1.10706867860301	1	1	pseudobulk	DESeq2	LRT	0
919	KC_AB2	Bap111	-0.983836207333212	0.536211115297032	1	pseudobulk	DESeq2	LRT	0.270664187626299
920	KC_AB2	Bap170	2.05028198082901	0.383573006114165	1	pseudobulk	DESeq2	LRT	0.416151963827146
920	KC_AB2	Bap55	0.595077121471768	0.560175581349743	1	pseudobulk	DESeq2	LRT	0.251675826458895
920	KC_AB2	Bap60	-0.799094300231758	0.372855536350548	1	pseudobulk	DESeq2	LRT	0.428459403897878

920	KC_AB2	barc	0.269264829240557	0.703402621731331	1	pseudobulk	DESeq2	LRT	0.152796017308061
920	KC_AB2	baz	1.10706867860301	1	1	pseudobulk	DESeq2	LRT	0
920	KC_AB2	bbc	-0.132608015844891	0.851146278074278	1	pseudobulk	DESeq2	LRT	0.069995795613148
920	KC_AB2	BBS5	1.38069928703004	0.566368801250645	1	pseudobulk	DESeq2	LRT	0.246900678019328
920	KC_AB2	bbx	1.99339641594643	0.465071165814147	1	pseudobulk	DESeq2	LRT	0.332480585700252
920	KC_AB2	bc10	-1.51990061850752	0.120572761861299	1	pseudobulk	DESeq2	LRT	0.918750790947105
920	KC_AB2	BCAS2	-0.438383410849624	0.799654701834727	1	pseudobulk	DESeq2	LRT	0.097097504833569
921	KC_AB2	bchs	0.0998928774202958	0.893251547819267	1	pseudobulk	DESeq2	LRT	0.049026222582802
921	KC_AB2	BCL7-like	1.09863729809763	0.612627088385388	1	pseudobulk	DESeq2	LRT	0.212803804009689
921	KC_AB2	bcn92	-0.192722226338661	0.79977903891912	1	pseudobulk	DESeq2	LRT	0.097029982299336
921	KC_AB2	Bdbt	-1.30838602435545	0.360947148589123	1	pseudobulk	DESeq2	LRT	0.442556384677351
921	KC_AB2	bdg	0.308729460136586	0.863002669209548	1	pseudobulk	DESeq2	LRT	0.063987861038921
921	KC_AB2	Bdp1	-0.729379579623002	1	1	pseudobulk	DESeq2	LRT	0
921	KC_AB2	be	0.433531960331872	0.825287783380275	1	pseudobulk	DESeq2	LRT	0.083394583643596
921	KC_AB2	BEAF-32	0.302620256419864	0.677759040820548	1	pseudobulk	DESeq2	LRT	0.168924680531264
921	KC_AB2	beag	0.627456353463085	0.584935119130598	1	pseudobulk	DESeq2	LRT	0.232892303090845
921	KC_AB2	beat-Ia	-1.5569401799281	0.491369326863142	1	pseudobulk	DESeq2	LRT	0.308591957318393
922	KC_AB2	beat-Ib	-0.161063677344028	0.844305071941177	1	pseudobulk	DESeq2	LRT	0.073500601808800
922	KC_AB2	beat-Ic	1.10706867860301	1	1	pseudobulk	DESeq2	LRT	0
922	KC_AB2	beat-IIIb	0.0254804284054044	0.972149791157622	1	pseudobulk	DESeq2	LRT	0.012266812788603
922	KC_AB2	beat-IV	-0.195959163613398	0.663774939623096	1	pseudobulk	DESeq2	LRT	0.177979148114297
922	KC_AB2	beat-VII	-0.239209471500153	0.520775931754591	1	pseudobulk	DESeq2	LRT	0.283349095384005
922	KC_AB2	beg	-2.35262986616105	0.205208696428774	1	pseudobulk	DESeq2	LRT	0.687804238438389
922	KC_AB2	bel	-0.363168596541015	0.367108604882342	1	pseudobulk	DESeq2	LRT	0.435205435683644
922	KC_AB2	Bem46	-1.12801111853905	0.441951678096085	1	pseudobulk	DESeq2	LRT	0.354625212740181
922	KC_AB2	ben	-0.175683727974925	0.721706552520334	1	pseudobulk	DESeq2	LRT	0.141639351636542
922	KC_AB2	Best1	-0.71425875868894	0.728191074001245	1	pseudobulk	DESeq2	LRT	0.137754648851352
923	KC_AB2	Bet1	-0.351397904745341	0.745625416911225	1	pseudobulk	DESeq2	LRT	0.1274792961528
923	KC_AB2	Bet3	-0.623783123878172	0.474016232604155	1	pseudobulk	DESeq2	LRT	0.324206785731607
923	KC_AB2	Bet5	-0.513766872743829	0.602466001496648	1	pseudobulk	DESeq2	LRT	0.220067456270127
923	KC_AB2	beta-PheRS	0.221486168185573	0.903697778547821	1	pseudobulk	DESeq2	LRT	0.0439767852922
923	KC_AB2	beta-Spec	0.0259473801864009	0.864557650024349	1	pseudobulk	DESeq2	LRT	0.063206042005232
923	KC_AB2	beta'COP	-0.104028296136761	0.940902960108381	1	pseudobulk	DESeq2	LRT	0.026455165162150
923	KC_AB2	beta3GalTII	-1.15253484707392	0.661144484795202	1	pseudobulk	DESeq2	LRT	0.179703620556297
923	KC_AB2	beta4GalNAcTA	-0.114126996294144	0.901360482269285	1	pseudobulk	DESeq2	LRT	0.045101486323394
923	KC_AB2	beta4GalNAcTB	-0.0763111680965758	0.917521349699159	1	pseudobulk	DESeq2	LRT	0.037383821409759
923	KC_AB2	beta4GalT7	-1.37870954886799	0.465061382101824	1	pseudobulk	DESeq2	LRT	0.332489722058901
924	KC_AB2	betaCOP	0.620674600121139	0.690148278062962	1	pseudobulk	DESeq2	LRT	0.161057591253654
924	KC_AB2	betaggt-I	1.9465755490013	0.430520135983084	1	pseudobulk	DESeq2	LRT	0.36600653122117
924	KC_AB2	betaggt-II	1.02928733579242	0.697125366781187	1	pseudobulk	DESeq2	LRT	0.156689114003336
924	KC_AB2	betaGlu	-0.470100723599697	0.833985291815347	1	pseudobulk	DESeq2	LRT	0.078841608522644
924	KC_AB2	betaNACtes4	1.10706867860301	1	1	pseudobulk	DESeq2	LRT	0
924	KC_AB2	betaTub56D	0.0907646923980882	0.786845224901189	1	pseudobulk	DESeq2	LRT	0.104110686423548
924	KC_AB2	betaTub85D	-0.729379579623002	1	1	pseudobulk	DESeq2	LRT	0
924	KC_AB2	Bgb	-0.634860880175475	0.404458844688731	1	pseudobulk	DESeq2	LRT	0.393125663010684
924	KC_AB2	BHD	-0.470100723599697	0.833985291815347	1	pseudobulk	DESeq2	LRT	0.078841608522644
924	KC_AB2	BI-1	0.117098078656894	0.795183749356625	1	pseudobulk	DESeq2	LRT	0.099532503908605
925	KC_AB2	bic	0.036945393611266	0.864932472634233	1	pseudobulk	DESeq2	LRT	0.063017797630105
925	KC_AB2	BicD	-0.0864399708627018	0.861110108714669	1	pseudobulk	DESeq2	LRT	0.064941312483914
925	KC_AB2	bif	0.411421068197501	0.583960054084829	1	pseudobulk	DESeq2	LRT	0.23361685988054
925	KC_AB2	bigmax	0.71416779160008	0.619010762631397	1	pseudobulk	DESeq2	LRT	0.208301799912367
925	KC_AB2	Bili	0.271768007922536	0.725804366028675	1	pseudobulk	DESeq2	LRT	0.139180423657564

925	KC_AB2	Bin1	0.78993828277269	0.564598236040941	1	pseudobulk	DESeq2	LRT	0.24826048304153
925	KC_AB2	bin3	0.410390563703573	0.530033719073646	1	pseudobulk	DESeq2	LRT	0.275696501075047
925	KC_AB2	bip2	-0.0577885634181563	0.862624626722566	1	pseudobulk	DESeq2	LRT	0.064178147515074
925	KC_AB2	Bka	0.844198923204955	0.7046337711535	1	pseudobulk	DESeq2	LRT	0.152036546119094
925	KC_AB2	Blimp-1	-0.378238257903549	0.404264598635768	1	pseudobulk	DESeq2	LRT	0.393334288075908
926	KC_AB2	Blm	-1.00833870937463	0.651152668420623	1	pseudobulk	DESeq2	LRT	0.186317175365195
926	KC_AB2	Blos1	-0.149699401554828	0.892826970081888	1	pseudobulk	DESeq2	LRT	0.049232699252342
926	KC_AB2	Blos2	-0.778523756993934	0.689563908531076	1	pseudobulk	DESeq2	LRT	0.161425477368935
926	KC_AB2	Blos3	-1.16512759261893	0.243826847467485	1	pseudobulk	DESeq2	LRT	0.612918476465625
926	KC_AB2	Blos4	-0.508638613984787	0.885316207746818	1	pseudobulk	DESeq2	LRT	0.052901584949501
926	KC_AB2	blp	1.09368415924037	0.473559655321076	1	pseudobulk	DESeq2	LRT	0.324625304211185
926	KC_AB2	blw	-0.596401015583752	0.122195464185974	1	pseudobulk	DESeq2	LRT	0.912944914515415
926	KC_AB2	bma	-0.104980015894298	0.698610038611554	1	pseudobulk	DESeq2	LRT	0.155765178099631
926	KC_AB2	Bmcp	0.331127769465248	0.775291049590764	1	pseudobulk	DESeq2	LRT	0.110535229748769
926	KC_AB2	bmm	-0.83320718254958	0.715059964627045	1	pseudobulk	DESeq2	LRT	0.14565753691968
927	KC_AB2	bnb	-0.729379579623002	0.772694994316916	1	pseudobulk	DESeq2	LRT	0.1119919011982
927	KC_AB2	boca	-0.285010290259498	0.717815288490895	1	pseudobulk	DESeq2	LRT	0.143987296021554
927	KC_AB2	bocks	-0.668125427806539	0.661700106990187	1	pseudobulk	DESeq2	LRT	0.179338795135392
927	KC_AB2	BOD1	0.473703858752358	0.73942922935742	1	pseudobulk	DESeq2	LRT	0.131103385927185
927	KC_AB2	boi	-0.883890872531852	0.676366690187261	1	pseudobulk	DESeq2	LRT	0.169817788750343
927	KC_AB2	boil	1.38446765109058	0.642794900879723	1	pseudobulk	DESeq2	LRT	0.191927577037166
927	KC_AB2	bol	-0.035208910810846	0.862633645371847	1	pseudobulk	DESeq2	LRT	0.064173607035911
927	KC_AB2	bon	0.757215755801676	0.322416696266552	1	pseudobulk	DESeq2	LRT	0.491582476455407
927	KC_AB2	bonsai	-0.291205585676428	0.810781907345635	1	pseudobulk	DESeq2	LRT	0.091095951182314
927	KC_AB2	bor	1.95432904991983	0.509212778150152	1	pseudobulk	DESeq2	LRT	0.293100706726022
928	KC_AB2	BORCS5	-0.126920620643582	0.907566607745001	1	pseudobulk	DESeq2	LRT	0.042121491565054
928	KC_AB2	BORCS6	0.306101013060283	0.832815272420834	1	pseudobulk	DESeq2	LRT	0.079451319199637
928	KC_AB2	botv	-0.311977968454154	0.857344273924811	1	pseudobulk	DESeq2	LRT	0.066844748389494
928	KC_AB2	br	1.18967990203526	0.257321958021629	1	pseudobulk	DESeq2	LRT	0.589523152613059
928	KC_AB2	Br140	-1.80075127281421	0.363208620892189	1	pseudobulk	DESeq2	LRT	0.439843851886983
928	KC_AB2	brat	0.471151302227765	0.175585895367892	1	pseudobulk	DESeq2	LRT	0.755510373452225
928	KC_AB2	Brca2	2.33862076287154	0.37727071943742	1	pseudobulk	DESeq2	LRT	0.423346899755834
928	KC_AB2	Brd8	0.378320304847799	0.842666545020638	1	pseudobulk	DESeq2	LRT	0.074344247803384
928	KC_AB2	Bre1	0.821921508763912	0.532341342109372	1	pseudobulk	DESeq2	LRT	0.27380980481585
928	KC_AB2	Brf	1.6004373554983	0.287436645179574	1	pseudobulk	DESeq2	LRT	0.541457864647345
929	KC_AB2	brk	-0.729379579623002	1	1	pseudobulk	DESeq2	LRT	0
929	KC_AB2	brm	-0.120985560753425	0.896679574676134	1	pseudobulk	DESeq2	LRT	0.047362722854502
929	KC_AB2	Brms1	-0.258788999780871	0.816707712242975	1	pseudobulk	DESeq2	LRT	0.087933343311029
929	KC_AB2	brn	1.95432904991983	0.509212778150152	1	pseudobulk	DESeq2	LRT	0.293100706726022
929	KC_AB2	brp	0.227147607870691	0.285406448205866	1	pseudobulk	DESeq2	LRT	0.544536219067936
929	KC_AB2	bru1	-0.147887837367307	0.746586332453823	1	pseudobulk	DESeq2	LRT	0.126919964843973
929	KC_AB2	bru2	-1.6910968260663	0.0386699895406851	1	pseudobulk	DESeq2	LRT	1.41262594539327
929	KC_AB2	bru3	-0.0682380952190239	0.678478711315265	1	pseudobulk	DESeq2	LRT	0.168463774685681
929	KC_AB2	Bruce	0.593754446749882	0.462818930178195	1	pseudobulk	DESeq2	LRT	0.334588885883217
929	KC_AB2	brun	-1.17849149785232	0.564162873029578	1	pseudobulk	DESeq2	LRT	0.248595497707284
930	KC_AB2	BRWD3	0.701389122208031	0.513448522747379	1	pseudobulk	DESeq2	LRT	0.289503091321651
930	KC_AB2	brwl	-0.0670487401443239	0.958587665045141	1	pseudobulk	DESeq2	LRT	0.018368163741159
930	KC_AB2	bs	-0.311977968454154	0.857344273924811	1	pseudobulk	DESeq2	LRT	0.066844748389494
930	KC_AB2	bsf	-0.277947413912466	0.859179291176031	1	pseudobulk	DESeq2	LRT	0.065916199331162
930	KC_AB2	Bsg	-0.049925248690517	0.831831575397888	1	pseudobulk	DESeq2	LRT	0.079964598324538
930	KC_AB2	Bsg25D	-0.583286998860159	0.651683942320859	1	pseudobulk	DESeq2	LRT	0.185962980013086
930	KC_AB2	bsk	-0.340535500267084	0.40820166729198	1	pseudobulk	DESeq2	LRT	0.389125225748739

930	KC_AB2	bt	0.263892964475114	0.844937848772347	1	pseudobulk	DESeq2	LRT	0.073175235343697
930	KC_AB2	BTBD9	-1.29052728408004	0.545673810438722	1	pseudobulk	DESeq2	LRT	0.263066889637944
930	KC_AB2	BtbVII	-0.0339921273556848	0.91180564581114	1	pseudobulk	DESeq2	LRT	0.040097723016889
931	KC_AB2	btl	-0.729379579623002	0.823527397767669	1	pseudobulk	DESeq2	LRT	0.084321947797948
931	KC_AB2	btsz	0.278980881113711	0.570879532765074	1	pseudobulk	DESeq2	LRT	0.243455527087077
931	KC_AB2	btz	0.424912966262768	0.824492810492961	1	pseudobulk	DESeq2	LRT	0.083813127013820
931	KC_AB2	Bub1	0.474396347993209	0.808657101852946	1	pseudobulk	DESeq2	LRT	0.092235595003270
931	KC_AB2	Bub3	-0.769275671960077	0.399427151010061	1	pseudobulk	DESeq2	LRT	0.398562417349456
931	KC_AB2	bug	0.661678460170035	0.878003361430606	1	pseudobulk	DESeq2	LRT	0.056503821396894
931	KC_AB2	Bug22	-0.0763111680965758	0.917521349699159	1	pseudobulk	DESeq2	LRT	0.037383821409759
931	KC_AB2	BuGZ	0.131993221811584	0.785650599679431	1	pseudobulk	DESeq2	LRT	0.104770553665742
931	KC_AB2	bun	0.295899567905621	0.193959593964676	1	pseudobulk	DESeq2	LRT	0.712288733708325
931	KC_AB2	bur	-0.173287638493015	0.86407190217763	1	pseudobulk	DESeq2	LRT	0.063450116989037
932	KC_AB2	bves	0.630481613849642	0.427937018000429	1	pseudobulk	DESeq2	LRT	0.368620143948522
932	KC_AB2	bw	-0.729379579623002	0.823527397767669	1	pseudobulk	DESeq2	LRT	0.084321947797948
932	KC_AB2	bwa	0.0988919064041738	0.880137787395857	1	pseudobulk	DESeq2	LRT	0.055449332825524
932	KC_AB2	Bx	0.0803920773530619	0.762178369366642	1	pseudobulk	DESeq2	LRT	0.117943380672758
932	KC_AB2	Bx42	0.524673000927914	0.675924867018301	1	pseudobulk	DESeq2	LRT	0.170101575734092
932	KC_AB2	bys	0.661678460170035	0.878003361430606	1	pseudobulk	DESeq2	LRT	0.056503821396894
932	KC_AB2	c(3)G	-1.33407500886303	0.63128114564055	1	pseudobulk	DESeq2	LRT	0.19977718147345
932	KC_AB2	c11.1	-0.699596040121543	0.525721109903176	1	pseudobulk	DESeq2	LRT	0.279244583890087
932	KC_AB2	c12.1	0.025536500158434	0.914461738131088	1	pseudobulk	DESeq2	LRT	0.038834461063632
932	KC_AB2	c12.2	-1.6052707068109	0.52265609184397	1	pseudobulk	DESeq2	LRT	0.281783983297782
933	KC_AB2	C1GalTA	-0.394896974274258	0.613608552853086	1	pseudobulk	DESeq2	LRT	0.212108595553514
933	KC_AB2	C3G	0.28375398924093	0.705083260262156	1	pseudobulk	DESeq2	LRT	0.151759596005235
933	KC_AB2	ca	-0.128826574422099	0.928562034864685	1	pseudobulk	DESeq2	LRT	0.032189076846704
933	KC_AB2	Ca-alpha1D	0.0612703764438255	0.836194983935115	1	pseudobulk	DESeq2	LRT	0.077692441970768
933	KC_AB2	Ca-alpha1T	0.0249545670706415	0.90925400174798	1	pseudobulk	DESeq2	LRT	0.041314778881178
933	KC_AB2	Ca-beta	0.0850244569043412	0.782478564345671	1	pseudobulk	DESeq2	LRT	0.106527550923882
933	KC_AB2	Ca-Ma2d	-0.030226653481207	0.912749346870321	1	pseudobulk	DESeq2	LRT	0.039648469143194
933	KC_AB2	CaBP1	0.684456561102218	0.674249847892975	1	pseudobulk	DESeq2	LRT	0.171179142849486
933	KC_AB2	cac	0.0321987606826334	0.857471434786589	1	pseudobulk	DESeq2	LRT	0.066780338831774
933	KC_AB2	cact	0.207177750088952	0.635113515947481	1	pseudobulk	DESeq2	LRT	0.197148644875554
934	KC_AB2	cactin	-1.63739047546051	0.431761148313956	1	pseudobulk	DESeq2	LRT	0.364756439889685
934	KC_AB2	Cad86C	1.61205113368698	0.631647504209	1	pseudobulk	DESeq2	LRT	0.199525215522471
934	KC_AB2	Cad87A	0.146930209264112	1	1	pseudobulk	DESeq2	LRT	0
934	KC_AB2	Cad96Ca	-1.71999636108657	0.519869997311849	1	pseudobulk	DESeq2	LRT	0.284105245805297
934	KC_AB2	CadN	0.456020290879447	0.616176622469586	1	pseudobulk	DESeq2	LRT	0.210294782690699
934	KC_AB2	CadN2	-0.729379579623002	1	1	pseudobulk	DESeq2	LRT	0
934	KC_AB2	Cadps	0.0199131360872874	0.930353600054377	1	pseudobulk	DESeq2	LRT	0.031351957504503
934	KC_AB2	Caf1-105	3.765322106338	0.0505474862851322	1	pseudobulk	DESeq2	LRT	1.29630043690069
934	KC_AB2	Caf1-55	-0.188611049317074	0.875715896912589	1	pseudobulk	DESeq2	LRT	0.057636766452249
934	KC_AB2	cag	-3.23029384923107	0.0446430462792732	1	pseudobulk	DESeq2	LRT	1.35024617838186
935	KC_AB2	CAH2	-1.6052707068109	0.52265609184397	1	pseudobulk	DESeq2	LRT	0.281783983297782
935	KC_AB2	CAH3	0.0945694987408347	0.862373284974318	1	pseudobulk	DESeq2	LRT	0.064304705737977
935	KC_AB2	CAH8	-0.768120147155614	0.627624899526867	1	pseudobulk	DESeq2	LRT	0.202299835141485
935	KC_AB2	CAHbeta	0.820459634962717	0.607908177995275	1	pseudobulk	DESeq2	LRT	0.216162014150793
935	KC_AB2	cal1	-2.39970914040097	0.301766661516198	1	pseudobulk	DESeq2	LRT	0.520328741761584
935	KC_AB2	CalpA	0.92969828370937	0.5180568042086	1	pseudobulk	DESeq2	LRT	0.285622617857484
935	KC_AB2	CalpB	1.37221885443763	0.558734269457382	1	pseudobulk	DESeq2	LRT	0.252794690766539
935	KC_AB2	Calr	0.191081573822002	0.800530915136881	1	pseudobulk	DESeq2	LRT	0.096621891709576
935	KC_AB2	Cals	-0.222676833180088	0.411241299695743	1	pseudobulk	DESeq2	LRT	0.385903276966829

935	KC_AB2	Calx	0.702486200935543	0.343458188147613	1	pseudobulk	DESeq2	LRT	0.464126125451659
936	KC_AB2	calypso	0.186034431065808	0.881762404370314	1	pseudobulk	DESeq2	LRT	0.054648422092356
936	KC_AB2	Cam	-0.297166759254396	0.0930549615845015	1	pseudobulk	DESeq2	LRT	1.03126046582903
936	KC_AB2	CaMKI	-0.095412668212313	0.795853649594019	1	pseudobulk	DESeq2	LRT	0.099166787811787
936	KC_AB2	CaMKII	-0.0867832280223965	0.693572388294503	1	pseudobulk	DESeq2	LRT	0.158908204819661
936	KC_AB2	Camp	-1.57685088282527	0.45256857946707	1	pseudobulk	DESeq2	LRT	0.344315601149633
936	KC_AB2	Camta	-0.0571584938388483	0.942010288964085	1	pseudobulk	DESeq2	LRT	0.025944353665802
936	KC_AB2	CanA-14F	-0.0337111163171444	0.871004324723628	1	pseudobulk	DESeq2	LRT	0.059979688621562
936	KC_AB2	CanB	-0.21424292275432	0.427946387557493	1	pseudobulk	DESeq2	LRT	0.368610635300722
936	KC_AB2	CanB2	-0.221750404620148	0.753780750541704	1	pseudobulk	DESeq2	LRT	0.122754957414752
936	KC_AB2	Cand1	0.301183407903318	0.898796313604012	1	pseudobulk	DESeq2	LRT	0.046338717499814
937	KC_AB2	CAP	1.38446765109058	0.642794900879723	1	pseudobulk	DESeq2	LRT	0.191927577037166
937	KC_AB2	Cap-D3	-0.729379579623002	0.772694994316916	1	pseudobulk	DESeq2	LRT	0.1119919011982
937	KC_AB2	Cap-G	1.02605719768525	0.762384804222891	1	pseudobulk	DESeq2	LRT	0.117825768597322
937	KC_AB2	Cap-H2	0.53463990667909	0.768615909510603	1	pseudobulk	DESeq2	LRT	0.114290630356765
937	KC_AB2	Caper	-0.0662746609210819	0.830534489546326	1	pseudobulk	DESeq2	LRT	0.080642327922231
937	KC_AB2	Capr	-0.493306740395542	0.675710301944197	1	pseudobulk	DESeq2	LRT	0.170239459729145
937	KC_AB2	caps	0.338168824735213	0.490424878369542	1	pseudobulk	DESeq2	LRT	0.30942750695506
937	KC_AB2	capt	-0.398355642893704	0.677765458236509	1	pseudobulk	DESeq2	LRT	0.168920568398519
937	KC_AB2	capu	1.2899029710342	0.138946542015242	1	pseudobulk	DESeq2	LRT	0.857152257108174
937	KC_AB2	car	-1.25712302539349	0.399136678917837	1	pseudobulk	DESeq2	LRT	0.398878360615694
938	KC_AB2	CARPB	3.76115270095386	0.0661348612476696	1	pseudobulk	DESeq2	LRT	1.179569553276
938	KC_AB2	CASK	0.317460234057963	0.282121560370631	1	pseudobulk	DESeq2	LRT	0.549563722805315
938	KC_AB2	casp	-1.01038402177403	0.46495178240523	1	pseudobulk	DESeq2	LRT	0.332592083069445
938	KC_AB2	cass	0.141529619787914	0.685042905705166	1	pseudobulk	DESeq2	LRT	0.164282226861664
938	KC_AB2	Cat	0.20387726057939	0.834555812037157	1	pseudobulk	DESeq2	LRT	0.078544613988641
938	KC_AB2	cathD	-0.288363903425341	0.759712866479579	1	pseudobulk	DESeq2	LRT	0.119350518332105
938	KC_AB2	Catsup	0.0838459745380907	0.944863813211315	1	pseudobulk	DESeq2	LRT	0.024630783483369
938	KC_AB2	cav	1.04237305304479	0.767386654171747	1	pseudobulk	DESeq2	LRT	0.114985758004482
938	KC_AB2	caz	-0.134208412703314	0.794703428198397	1	pseudobulk	DESeq2	LRT	0.099794913515841
938	KC_AB2	cbc	1.55243756765955	0.43008786988567	1	pseudobulk	DESeq2	LRT	0.366442806029975
939	KC_AB2	Cbl	-0.418511032633534	0.508190636312045	1	pseudobulk	DESeq2	LRT	0.293973341323181
939	KC_AB2	Cbp20	-0.626366290949993	0.518488749714651	1	pseudobulk	DESeq2	LRT	0.285260662591921
939	KC_AB2	Cbp53E	0.155760047114596	0.59203388614174	1	pseudobulk	DESeq2	LRT	0.227653434927084
939	KC_AB2	Cbp80	0.469663871581374	0.73730566635323	1	pseudobulk	DESeq2	LRT	0.132352428437219
939	KC_AB2	cbs	-0.433082522164763	0.658456996235855	1	pseudobulk	DESeq2	LRT	0.181472583507557
939	KC_AB2	Cbs	-3.13971392010881	0.0533458498351595	1	pseudobulk	DESeq2	LRT	1.27289936187771
939	KC_AB2	cbt	-1.16417338217288	0.258010203673724	1	pseudobulk	DESeq2	LRT	0.588363118410542
939	KC_AB2	CCAP-R	0.403294498144138	0.89479244414645	1	pseudobulk	DESeq2	LRT	0.048277691852293
939	KC_AB2	CCDC53	-0.729379579623002	0.823527397767669	1	pseudobulk	DESeq2	LRT	0.084321947797948
939	KC_AB2	Ccdc58	-1.98576727392759	0.252151004940763	1	pseudobulk	DESeq2	LRT	0.598339296632913
940	KC_AB2	Cchl	-0.397183620822373	0.580608641002525	1	pseudobulk	DESeq2	LRT	0.236116504997991
940	KC_AB2	CCKLR-17D1	0.667244748290888	0.924085151242949	1	pseudobulk	DESeq2	LRT	0.034288008205147
940	KC_AB2	CCKLR-17D3	-0.729379579623002	0.772694994316916	1	pseudobulk	DESeq2	LRT	0.1119919011982
940	KC_AB2	Ccm3	0.175167544593768	0.813292002905299	1	pseudobulk	DESeq2	LRT	0.089753498093526
940	KC_AB2	Ccn	0.0177136835362786	0.939578222505736	1	pseudobulk	DESeq2	LRT	0.027067057832271
940	KC_AB2	Ccs	-0.785201786757791	0.678486044454677	1	pseudobulk	DESeq2	LRT	0.168459080765713
940	KC_AB2	CCT1	1.18387387643018	0.485014283325132	1	pseudobulk	DESeq2	LRT	0.314245471546298
940	KC_AB2	CCT2	-1.8300051836134	0.374346570306812	1	pseudobulk	DESeq2	LRT	0.426726141442446
940	KC_AB2	CCT3	-0.214740596650046	0.837929925080374	1	pseudobulk	DESeq2	LRT	0.076792299297431
940	KC_AB2	CCT4	-0.391125650931222	0.710921410171032	1	pseudobulk	DESeq2	LRT	0.148178406324414
941	KC_AB2	CCT5	-0.568755105949716	0.726721465982017	1	pseudobulk	DESeq2	LRT	0.138632011377055

941	KC_AB2	CCT6	-0.0806397617506279	0.958764917307812	1	pseudobulk	DESeq2	LRT	0.018287865854886
941	KC_AB2	CCT7	0.474396347993209	0.808657101852946	1	pseudobulk	DESeq2	LRT	0.092235595003270
941	KC_AB2	CCT8	-0.399268350175093	0.713025128755375	1	pseudobulk	DESeq2	LRT	0.146895164275079
941	KC_AB2	Ccz1	1.08937410746753	0.523121629807279	1	pseudobulk	DESeq2	LRT	0.281397322580594
941	KC_AB2	CD98hc	-0.188341082563116	0.756143799583506	1	pseudobulk	DESeq2	LRT	0.121395604727764
941	KC_AB2	Cda5	1.27732525054232	0.432088672740696	1	pseudobulk	DESeq2	LRT	0.364427118624413
941	KC_AB2	cdc14	-0.0219111732104455	0.965268850268027	1	pseudobulk	DESeq2	LRT	0.015351708494433
941	KC_AB2	Cdc16	-1.71999636108657	0.519869997311849	1	pseudobulk	DESeq2	LRT	0.284105245805297
941	KC_AB2	Cdc23	1.99339641594643	0.465071165814147	1	pseudobulk	DESeq2	LRT	0.332480585700252
942	KC_AB2	Cdc27	-0.562407428229251	0.586429319938405	1	pseudobulk	DESeq2	LRT	0.231784324231299
942	KC_AB2	Cdc2rk	-0.982583391612964	0.651268810528278	1	pseudobulk	DESeq2	LRT	0.186239719829609
942	KC_AB2	Cdc37	0.0760051983333371	0.891954080444417	1	pseudobulk	DESeq2	LRT	0.049657503385014
942	KC_AB2	Cdc42	-0.551410201516943	0.408385658477713	1	pseudobulk	DESeq2	LRT	0.388929517695201
942	KC_AB2	CDC45L	-0.729379579623002	1	1	pseudobulk	DESeq2	LRT	0
942	KC_AB2	Cdc5	-1.08790260676404	0.510159451910456	1	pseudobulk	DESeq2	LRT	0.292294062604603
942	KC_AB2	CDC50	0.622571702847818	0.50528833884978	1	pseudobulk	DESeq2	LRT	0.296460724385223
942	KC_AB2	Cdc7	-0.026770492726296	0.949053031791482	1	pseudobulk	DESeq2	LRT	0.022709519110207
942	KC_AB2	Cdep	0.267177859555622	0.506748191700382	1	pseudobulk	DESeq2	LRT	0.295207792385219
942	KC_AB2	CdGAPr	0.432716590030631	0.706597993315722	1	pseudobulk	DESeq2	LRT	0.150827600262547
943	KC_AB2	cdi	-0.480932018521462	0.500855901280077	1	pseudobulk	DESeq2	LRT	0.300287204832396
943	KC_AB2	Cdk12	-0.653557004300122	0.454072422009255	1	pseudobulk	DESeq2	LRT	0.342874874079884
943	KC_AB2	Cdk2	1.72664113248049	0.368460738421108	1	pseudobulk	DESeq2	LRT	0.433608781876778
943	KC_AB2	CDK2AP1	-0.488494306467694	0.706865156065109	1	pseudobulk	DESeq2	LRT	0.15066342575363
943	KC_AB2	Cdk5	1.19570443259055	0.250240436843326	1	pseudobulk	DESeq2	LRT	0.601642510474145
943	KC_AB2	Cdk5alpha	0.19249921734705	0.622577915903806	1	pseudobulk	DESeq2	LRT	0.205806288682766
943	KC_AB2	Cdk7	1.00268106024788	0.627374080284178	1	pseudobulk	DESeq2	LRT	0.202473427975038
943	KC_AB2	Cdk8	1.02928733579242	0.697125366781187	1	pseudobulk	DESeq2	LRT	0.156689114003336
943	KC_AB2	Cdk9	-0.708434668538857	0.655580595423002	1	pseudobulk	DESeq2	LRT	0.183373909624774
943	KC_AB2	cdm	-0.661624135834514	0.385237308084789	1	pseudobulk	DESeq2	LRT	0.414271660512304
944	KC_AB2	Cds	-0.159169499220701	0.740193935080368	1	pseudobulk	DESeq2	LRT	0.130654477700675
944	KC_AB2	Ced-12	0.403294498144138	0.89479244414645	1	pseudobulk	DESeq2	LRT	0.048277691852293
944	KC_AB2	ced-6	1.10706867860301	1	1	pseudobulk	DESeq2	LRT	0
944	KC_AB2	Cen	0.804058711536506	0.430966430986292	1	pseudobulk	DESeq2	LRT	0.365556556765468
944	KC_AB2	CenB1A	0.267668900308628	0.828553981517339	1	pseudobulk	DESeq2	LRT	0.081679191395052
944	KC_AB2	CenG1A	0.472584332987542	0.458666983549643	1	pseudobulk	DESeq2	LRT	0.338502520775517
944	KC_AB2	Cep97	0.12634902710124	0.89239230450279	1	pseudobulk	DESeq2	LRT	0.049444183487240
944	KC_AB2	cer	-0.635687562968924	0.708468237112161	1	pseudobulk	DESeq2	LRT	0.149679615785099
944	KC_AB2	Cerk	-1.14407523891035	0.306397147028046	1	pseudobulk	DESeq2	LRT	0.513715282889777
944	KC_AB2	cert	-0.47913552965934	0.763084002517908	1	pseudobulk	DESeq2	LRT	0.117427651007234
945	KC_AB2	cerv	1.10706867860301	1	1	pseudobulk	DESeq2	LRT	0
945	KC_AB2	Cf2	0.0280360664777274	0.960060188205896	1	pseudobulk	DESeq2	LRT	0.017701539266356
945	KC_AB2	Cfp1	-2.56293201114635	0.174375435858672	1	pseudobulk	DESeq2	LRT	0.758514693843806
945	KC_AB2	cg	-0.271024915578933	0.491383341707193	1	pseudobulk	DESeq2	LRT	0.308579570540659
945	KC_AB2	CG10011	-0.286085234530416	0.738519015996094	1	pseudobulk	DESeq2	LRT	0.131638317635922
945	KC_AB2	CG10019	-0.452024649891782	0.594013119268369	1	pseudobulk	DESeq2	LRT	0.226203963162004
945	KC_AB2	CG10038	-1.52244591594958	0.547176617578731	1	pseudobulk	DESeq2	LRT	0.261872469536296
945	KC_AB2	CG10053	-1.6052707068109	0.52265609184397	1	pseudobulk	DESeq2	LRT	0.281783983297782
945	KC_AB2	CG10055	-1.45675668689178	0.495625979996641	1	pseudobulk	DESeq2	LRT	0.304845936611032
945	KC_AB2	CG10068	0.205564527517923	0.908209004553827	1	pseudobulk	DESeq2	LRT	0.041814196545762
946	KC_AB2	CG10075	0.577507210111775	0.718433673375617	1	pseudobulk	DESeq2	LRT	0.143613320245971
946	KC_AB2	CG10077	0.146939796943528	0.481124550854632	1	pseudobulk	DESeq2	LRT	0.317742481325143
946	KC_AB2	CG10082	0.292971261480724	0.534577305138642	1	pseudobulk	DESeq2	LRT	0.271989482658176

946	KC_AB2	CG10089	0.265036417995678	0.57759096254904	1	pseudobulk	DESeq2	LRT	0.238379610704672
946	KC_AB2	CG10098	-0.145312529473359	0.869670053843339	1	pseudobulk	DESeq2	LRT	0.060645484133117
946	KC_AB2	CG10103	-0.79746146370714	0.574085868110089	1	pseudobulk	DESeq2	LRT	0.241023143739151
946	KC_AB2	CG10132	0.012726764589972	0.92839075101398	1	pseudobulk	DESeq2	LRT	0.032269194803366
946	KC_AB2	CG10137	-0.391377465778084	0.599641168840839	1	pseudobulk	DESeq2	LRT	0.222108557967477
946	KC_AB2	CG10139	-0.953491205581094	0.227401239801382	1	pseudobulk	DESeq2	LRT	0.643207171849555
946	KC_AB2	CG10147	1.02928733579242	0.697125366781187	1	pseudobulk	DESeq2	LRT	0.156689114003336
947	KC_AB2	CG10151	-0.344614925271821	0.832814556155584	1	pseudobulk	DESeq2	LRT	0.079451692716058
947	KC_AB2	CG10165	-0.729379579623002	1	1	pseudobulk	DESeq2	LRT	0
947	KC_AB2	CG10166	-1.6478169722696	0.335693539726267	1	pseudobulk	DESeq2	LRT	0.474057016476833
947	KC_AB2	CG10171	-0.875392059107729	0.660698616093063	1	pseudobulk	DESeq2	LRT	0.179996602868957
947	KC_AB2	CG10188	0.547022121728146	0.498662161044738	1	pseudobulk	DESeq2	LRT	0.302193585206423
947	KC_AB2	CG10189	0.843700179273233	0.692173184066514	1	pseudobulk	DESeq2	LRT	0.159785230002206
947	KC_AB2	CG10194	0.977308131755069	0.560554164401135	1	pseudobulk	DESeq2	LRT	0.251382416674709
947	KC_AB2	CG10209	0.0055004516115709	0.960229690405946	1	pseudobulk	DESeq2	LRT	0.017624869726863
947	KC_AB2	CG10214	0.622780400536336	0.65892468760795	1	pseudobulk	DESeq2	LRT	0.181164220649384
947	KC_AB2	CG10222	1.10706867860301	1	1	pseudobulk	DESeq2	LRT	0
948	KC_AB2	CG1024	-0.729379579623002	1	1	pseudobulk	DESeq2	LRT	0
948	KC_AB2	CG10252	0.709584622582653	0.756945022027757	1	pseudobulk	DESeq2	LRT	0.120935662768153
948	KC_AB2	CG10254	0.678369113582151	0.542277605975902	1	pseudobulk	DESeq2	LRT	0.265778329921738
948	KC_AB2	CG10257	-0.922865356673073	0.518786479010401	1	pseudobulk	DESeq2	LRT	0.28501135133833
948	KC_AB2	CG10265	-0.0108012841198609	0.920410649261457	1	pseudobulk	DESeq2	LRT	0.036018365135150
948	KC_AB2	CG10268	1.35983586014236	0.633699807227832	1	pseudobulk	DESeq2	LRT	0.198116424987592
948	KC_AB2	CG10274	1.38446765109058	0.642794900879723	1	pseudobulk	DESeq2	LRT	0.191927577037166
948	KC_AB2	CG10283	-1.94796815983293	0.315261868985908	1	pseudobulk	DESeq2	LRT	0.501328554118708
948	KC_AB2	CG10286	1.09863729809763	0.612627088385388	1	pseudobulk	DESeq2	LRT	0.212803804009689
948	KC_AB2	CG10321	0.424582837269606	0.741689755946897	1	pseudobulk	DESeq2	LRT	0.129777719325078
949	KC_AB2	CG10324	-1.89982409916076	0.314815504664147	1	pseudobulk	DESeq2	LRT	0.50194388678227
949	KC_AB2	CG10333	-0.0019687088198954	0.883863315423397	1	pseudobulk	DESeq2	LRT	0.053614891035678
949	KC_AB2	CG10340	0.389447494329595	0.671807089389233	1	pseudobulk	DESeq2	LRT	0.172755417483967
949	KC_AB2	CG10341	-0.647621034217714	0.754702560287896	1	pseudobulk	DESeq2	LRT	0.122224176685402
949	KC_AB2	CG10343	-0.326002591878444	0.83169727708792	1	pseudobulk	DESeq2	LRT	0.080034720360579
949	KC_AB2	CG10347	-1.16016699006818	0.583985689825587	1	pseudobulk	DESeq2	LRT	0.233597794849804
949	KC_AB2	CG10353	-0.699682262218102	0.528364141410614	1	pseudobulk	DESeq2	LRT	0.27706666440335
949	KC_AB2	CG10362	0.264088868452584	0.855966783964928	1	pseudobulk	DESeq2	LRT	0.067543087917043
949	KC_AB2	CG10365	2.76364737573217	0.211914962801091	1	pseudobulk	DESeq2	LRT	0.673838377727484
949	KC_AB2	CG10366	-0.0965849293754046	0.935156467321203	1	pseudobulk	DESeq2	LRT	0.029115718315662
950	KC_AB2	CG10375	-0.98920822088019	0.24193211329295	1	pseudobulk	DESeq2	LRT	0.616306480954913
950	KC_AB2	CG10376	-0.729379579623002	1	1	pseudobulk	DESeq2	LRT	0
950	KC_AB2	CG10383	0.0772645829333075	0.941528584941732	1	pseudobulk	DESeq2	LRT	0.026166490204928
950	KC_AB2	CG10395	-1.40140710510656	0.166571089268784	1	pseudobulk	DESeq2	LRT	0.778400374241274
950	KC_AB2	CG10413	-0.232770980261393	0.885244835973594	1	pseudobulk	DESeq2	LRT	0.052936597995114
950	KC_AB2	CG10417	-0.417342386643296	0.470633749837769	1	pseudobulk	DESeq2	LRT	0.32731693214779
950	KC_AB2	CG10418	-1.31781572888606	0.358648281388581	1	pseudobulk	DESeq2	LRT	0.445331245925001
950	KC_AB2	CG10425	-1.09586535083235	0.704995102869895	1	pseudobulk	DESeq2	LRT	0.151813899751819
950	KC_AB2	CG10428	1.89949765046429	0.307764128341067	1	pseudobulk	DESeq2	LRT	0.511782001045796
950	KC_AB2	CG10431	-0.729379579623002	1	1	pseudobulk	DESeq2	LRT	0
951	KC_AB2	CG10433	1.10706867860301	1	1	pseudobulk	DESeq2	LRT	0
951	KC_AB2	CG10435	0.922242888484304	0.482869908113842	1	pseudobulk	DESeq2	LRT	0.316169858478954
951	KC_AB2	CG10444	0.481451988221553	0.763332199187401	1	pseudobulk	DESeq2	LRT	0.117286417646612
951	KC_AB2	CG10463	1.38446765109058	0.642794900879723	1	pseudobulk	DESeq2	LRT	0.191927577037166
951	KC_AB2	CG10465	-0.0649651885433712	0.871373744811558	1	pseudobulk	DESeq2	LRT	0.059795529843508

951	KC_AB2	CG10466	-2.69831193693389	0.203295284067868	1	pseudobulk	DESeq2	LRT	0.691872695769522
951	KC_AB2	CG10467	3.02241878477171	0.183885600586836	1	pseudobulk	DESeq2	LRT	0.735452277453175
951	KC_AB2	CG10469	-1.33407500886303	0.63128114564055	1	pseudobulk	DESeq2	LRT	0.19977718147345
951	KC_AB2	CG10470	-0.569610615423936	0.608729393475991	1	pseudobulk	DESeq2	LRT	0.21557572713442
951	KC_AB2	CG10479	-0.502610851884822	0.843360912667957	1	pseudobulk	DESeq2	LRT	0.073986531144244
952	KC_AB2	CG10483	-1.08870813339097	0.298181914082684	1	pseudobulk	DESeq2	LRT	0.525518701769317
952	KC_AB2	CG10492	0.83268418032005	0.569764643143929	1	pseudobulk	DESeq2	LRT	0.244304504489166
952	KC_AB2	CG10494	-0.039982284792722	0.927109137386119	1	pseudobulk	DESeq2	LRT	0.032869138590489
952	KC_AB2	CG10508	-0.0361169366213687	0.943649203107967	1	pseudobulk	DESeq2	LRT	0.025189422511772
952	KC_AB2	CG10512	0.145321756433347	1	1	pseudobulk	DESeq2	LRT	0
952	KC_AB2	CG10516	-1.52244591594958	0.547176617578731	1	pseudobulk	DESeq2	LRT	0.261872469536296
952	KC_AB2	CG10543	-0.0381224633827157	0.913309947645079	1	pseudobulk	DESeq2	LRT	0.039381812051162
952	KC_AB2	CG10555	0.336386480546444	0.709556977875827	1	pseudobulk	DESeq2	LRT	0.149012724683591
952	KC_AB2	CG10561	-2.20709059110951	0.184116801012711	1	pseudobulk	DESeq2	LRT	0.734906579479761
952	KC_AB2	CG10565	-0.940829471809576	0.405867451496222	1	pseudobulk	DESeq2	LRT	0.391615775493192
953	KC_AB2	CG10576	0.389904703913888	0.7708853144989	1	pseudobulk	DESeq2	LRT	0.113010227636516
953	KC_AB2	CG10581	-0.508638613984787	0.885316207746818	1	pseudobulk	DESeq2	LRT	0.052901584949501
953	KC_AB2	CG10600	0.0050365616915060	0.990083146329284	1	pseudobulk	DESeq2	LRT	0.004328332194658
953	KC_AB2	CG10602	0.145321756433347	1	1	pseudobulk	DESeq2	LRT	0
953	KC_AB2	CG10616	0.208364413394472	0.82606573199822	1	pseudobulk	DESeq2	LRT	0.082985393468596
953	KC_AB2	CG10623	3.24003735334713	0.16854266152517	1	pseudobulk	DESeq2	LRT	0.77329015224124
953	KC_AB2	CG10628	0.575162595042011	0.797021781108777	1	pseudobulk	DESeq2	LRT	0.098529809988961
953	KC_AB2	CG10631	0.785434690746334	0.501562267224235	1	pseudobulk	DESeq2	LRT	0.299675143135658
953	KC_AB2	CG10638	-0.630483529706862	0.743785125112415	1	pseudobulk	DESeq2	LRT	0.128552511304008
953	KC_AB2	CG10646	-1.72205629554805	0.342494289164649	1	pseudobulk	DESeq2	LRT	0.465346665645885
954	KC_AB2	CG10654	-0.553497952975019	0.749989464590024	1	pseudobulk	DESeq2	LRT	0.124944837278371
954	KC_AB2	CG10669	-1.38764087950137	0.516647656322572	1	pseudobulk	DESeq2	LRT	0.286805536367625
954	KC_AB2	CG10672	-0.930140310998572	0.5815426884533	1	pseudobulk	DESeq2	LRT	0.235418400145771
954	KC_AB2	CG10674	0.624085895386376	0.691638938857648	1	pseudobulk	DESeq2	LRT	0.160120564190825
954	KC_AB2	CG10681	0.537493212081734	0.748601384394283	1	pseudobulk	DESeq2	LRT	0.125749374076737
954	KC_AB2	CG10710	-0.729379579623002	0.772694994316916	1	pseudobulk	DESeq2	LRT	0.1119919011982
954	KC_AB2	CG10713	1.37646356319375	0.523574002340058	1	pseudobulk	DESeq2	LRT	0.281021926140695
954	KC_AB2	CG10731	-0.357615107700012	0.853922387948944	1	pseudobulk	DESeq2	LRT	0.068581600057164
954	KC_AB2	CG10734	1.10706867860301	1	1	pseudobulk	DESeq2	LRT	0
954	KC_AB2	CG10737	-0.621462716639656	0.546069440774998	1	pseudobulk	DESeq2	LRT	0.262752126841382
955	KC_AB2	CG10738	-1.13465563304706	0.53966505508667	1	pseudobulk	DESeq2	LRT	0.267875702880455
955	KC_AB2	CG1074	0.628255288245608	0.600696982120429	1	pseudobulk	DESeq2	LRT	0.221344549926687
955	KC_AB2	CG10747	0.0055004516115709	0.960229690405946	1	pseudobulk	DESeq2	LRT	0.017624869726863
955	KC_AB2	CG10778	-0.987864420262975	0.622985879197186	1	pseudobulk	DESeq2	LRT	0.205521797090819
955	KC_AB2	CG10795	-1.78975280219494	0.468855431150075	1	pseudobulk	DESeq2	LRT	0.32896104882553
955	KC_AB2	CG10802	-0.33572805049699	0.856817945219033	1	pseudobulk	DESeq2	LRT	0.067111446205159
955	KC_AB2	CG10803	0.878680207199768	0.391696883914978	1	pseudobulk	DESeq2	LRT	0.407049883402129
955	KC_AB2	CG10804	0.72113915842785	0.151978998219004	1	pseudobulk	DESeq2	LRT	0.818216422501111
955	KC_AB2	CG10809	-0.481611563683199	0.573368001367387	1	pseudobulk	DESeq2	LRT	0.241566547903911
955	KC_AB2	CG10863	0.301183407903318	0.898796313604012	1	pseudobulk	DESeq2	LRT	0.046338717499814
956	KC_AB2	CG10866	0.396659246788372	0.915482501218565	1	pseudobulk	DESeq2	LRT	0.038349952505755
956	KC_AB2	CG10874	-1.82903348227644	0.286298658634215	1	pseudobulk	DESeq2	LRT	0.543180686729094
956	KC_AB2	CG10889	1.02648689492346	0.73959154128541	1	pseudobulk	DESeq2	LRT	0.131008064514051
956	KC_AB2	CG10898	0.182464987382747	0.906839011984365	1	pseudobulk	DESeq2	LRT	0.042469804905139
956	KC_AB2	CG1090	-0.0902129600053301	0.87072277839171	1	pseudobulk	DESeq2	LRT	0.060120094106667
956	KC_AB2	CG10903	0.834440811967584	0.650917543165916	1	pseudobulk	DESeq2	LRT	0.186474023438173
956	KC_AB2	CG10904	1.68881465104187	0.456529340335333	1	pseudobulk	DESeq2	LRT	0.340531305892531

956	KC_AB2	CG10907	1.99339641594643	0.465071165814147	1	pseudobulk	DESeq2	LRT	0.332480585700252
956	KC_AB2	CG10914	-2.35203494925393	0.313819523334105	1	pseudobulk	DESeq2	LRT	0.503320041588723
956	KC_AB2	CG10916	1.9465755490013	0.430520135983084	1	pseudobulk	DESeq2	LRT	0.36600653122117
957	KC_AB2	CG10927	-0.432213394558105	0.865061123889812	1	pseudobulk	DESeq2	LRT	0.062953204870221
957	KC_AB2	CG10932	-1.98028140890842	0.328533066956313	1	pseudobulk	DESeq2	LRT	0.483420912028321
957	KC_AB2	CG10947	-0.622805369041268	0.594867574809253	1	pseudobulk	DESeq2	LRT	0.225579703062266
957	KC_AB2	CG10948	0.720817030181438	0.385263758181268	1	pseudobulk	DESeq2	LRT	0.414241843211708
957	KC_AB2	CG10949	1.16419655301094	0.553019346454958	1	pseudobulk	DESeq2	LRT	0.257259675364985
957	KC_AB2	CG10958	0.661678460170035	0.878003361430606	1	pseudobulk	DESeq2	LRT	0.056503821396894
957	KC_AB2	CG10959	-0.470100723599697	0.833985291815347	1	pseudobulk	DESeq2	LRT	0.078841608522644
957	KC_AB2	CG10960	0.218673142619457	0.771701978041569	1	pseudobulk	DESeq2	LRT	0.112550386556529
957	KC_AB2	CG10973	1.53722766603166	0.41188885531382	1	pseudobulk	DESeq2	LRT	0.385219958811221
957	KC_AB2	CG10979	0.942758880874816	0.658080412798793	1	pseudobulk	DESeq2	LRT	0.181721035411818
958	KC_AB2	CG10984	-0.380385000817002	0.735159553671553	1	pseudobulk	DESeq2	LRT	0.13361839457686
958	KC_AB2	CG11000	0.354424624842075	0.622533442482962	1	pseudobulk	DESeq2	LRT	0.20583731331545
958	KC_AB2	CG11007	-1.34289675692482	0.601340585891312	1	pseudobulk	DESeq2	LRT	0.220879483608669
958	KC_AB2	CG11029	1.38069928703004	0.566368801250645	1	pseudobulk	DESeq2	LRT	0.246900678019328
958	KC_AB2	CG1103	0.603292063141174	0.663469122189784	1	pseudobulk	DESeq2	LRT	0.178179284366444
958	KC_AB2	CG11030	-1.2806546998074	0.0597913875331606	1	pseudobulk	DESeq2	LRT	1.22336136812186
958	KC_AB2	CG11035	0.686075985438457	0.543873057328898	1	pseudobulk	DESeq2	LRT	0.264502454961642
958	KC_AB2	CG1105	0.423375734291782	0.738774256868618	1	pseudobulk	DESeq2	LRT	0.131488246278214
958	KC_AB2	CG11050	0.543563629762462	0.441885117602841	1	pseudobulk	DESeq2	LRT	0.354690624940994
958	KC_AB2	CG11070	-0.67912253320804	0.649181460226402	1	pseudobulk	DESeq2	LRT	0.1876338915512
959	KC_AB2	CG11076	-0.0724833384914041	0.907089458428917	1	pseudobulk	DESeq2	LRT	0.042349880099562
959	KC_AB2	CG11077	-0.376721639421346	0.597351348883651	1	pseudobulk	DESeq2	LRT	0.223770151286234
959	KC_AB2	CG11089	-2.2834221630407	0.364642497726807	1	pseudobulk	DESeq2	LRT	0.438132717324805
959	KC_AB2	CG11095	-0.647621034217714	0.754702560287896	1	pseudobulk	DESeq2	LRT	0.122224176685402
959	KC_AB2	CG11099	0.0540256890355292	0.924643383739934	1	pseudobulk	DESeq2	LRT	0.034025733570618
959	KC_AB2	CG11103	-0.0575053354053096	0.968247930601512	1	pseudobulk	DESeq2	LRT	0.014013422541522
959	KC_AB2	CG11109	0.834440811967584	0.650917543165916	1	pseudobulk	DESeq2	LRT	0.186474023438173
959	KC_AB2	CG11110	0.690387450349416	0.75906361064041	1	pseudobulk	DESeq2	LRT	0.119721828066906
959	KC_AB2	CG11122	0.656169451367115	0.22717433316926	1	pseudobulk	DESeq2	LRT	0.643640738069484
959	KC_AB2	CG11123	-0.729379579623002	1	1	pseudobulk	DESeq2	LRT	0
960	KC_AB2	CG11127	1.61871478396052	0.412432734304544	1	pseudobulk	DESeq2	LRT	0.384646872574624
960	KC_AB2	CG1113	0.403294498144138	0.89479244414645	1	pseudobulk	DESeq2	LRT	0.048277691852293
960	KC_AB2	CG11134	-0.729379579623002	0.772694994316916	1	pseudobulk	DESeq2	LRT	0.1119919011982
960	KC_AB2	CG11137	-0.236518757561611	0.707983087272977	1	pseudobulk	DESeq2	LRT	0.149977116874852
960	KC_AB2	CG11141	1.28688440374599	0.433125359669712	1	pseudobulk	DESeq2	LRT	0.363386387403411
960	KC_AB2	CG11151	0.578732977123705	0.422596853062007	1	pseudobulk	DESeq2	LRT	0.374073741318002
960	KC_AB2	CG11155	0.216387872472257	0.459643362831968	1	pseudobulk	DESeq2	LRT	0.337579006636718
960	KC_AB2	CG1116	-1.00833870937463	0.651152668420623	1	pseudobulk	DESeq2	LRT	0.186317175365195
960	KC_AB2	CG11168	-0.729379579623002	0.823527397767669	1	pseudobulk	DESeq2	LRT	0.084321947797948
960	KC_AB2	CG11170	1.10706867860301	1	1	pseudobulk	DESeq2	LRT	0
961	KC_AB2	CG11178	0.570932193837037	0.728731777446908	1	pseudobulk	DESeq2	LRT	0.137432291997184
961	KC_AB2	CG11180	-0.169524318341919	0.912374154556569	1	pseudobulk	DESeq2	LRT	0.039827025776412
961	KC_AB2	CG11191	-0.0922481708196421	0.764790861725068	1	pseudobulk	DESeq2	LRT	0.116457309965857
961	KC_AB2	CG11200	2.48384162006255	0.303740962766822	1	pseudobulk	DESeq2	LRT	0.517496634805608
961	KC_AB2	CG11241	-0.729379579623002	0.823527397767669	1	pseudobulk	DESeq2	LRT	0.084321947797948
961	KC_AB2	CG11247	-0.601385939995913	0.543350716645895	1	pseudobulk	DESeq2	LRT	0.264919755795538
961	KC_AB2	CG11248	1.12784076416542	0.561851145028072	1	pseudobulk	DESeq2	LRT	0.250378729725996
961	KC_AB2	CG11257	-1.02019568450201	0.730699957834935	1	pseudobulk	DESeq2	LRT	0.136260917715759
961	KC_AB2	CG11267	0.0377791746775589	0.861713901508845	1	pseudobulk	DESeq2	LRT	0.064636900787880

961	KC_AB2	CG11269	1.10706867860301	1	1	pseudobulk	DESeq2	LRT	0
962	KC_AB2	CG11279	2.64310175501159	0.0375743033314134	1	pseudobulk	DESeq2	LRT	1.42510906296002
962	KC_AB2	CG1129	0.204804967402211	0.778065642455356	1	pseudobulk	DESeq2	LRT	0.108983761681849
962	KC_AB2	CG11307	0.253580511664603	0.902661095927063	1	pseudobulk	DESeq2	LRT	0.044475274930634
962	KC_AB2	CG11309	0.364136077420657	0.814057422322293	1	pseudobulk	DESeq2	LRT	0.089344959584829
962	KC_AB2	CG11317	0.387635511634525	0.668810988401111	1	pseudobulk	DESeq2	LRT	0.174696600158208
962	KC_AB2	CG11319	-0.163611994126011	0.55766217794374	1	pseudobulk	DESeq2	LRT	0.253628809457406
962	KC_AB2	CG11334	-0.400818874228283	0.829617251912903	1	pseudobulk	DESeq2	LRT	0.081122225359766
962	KC_AB2	CG11357	-0.0911771412264657	0.908165533255061	1	pseudobulk	DESeq2	LRT	0.041834984488762
962	KC_AB2	CG11360	-0.0470538204089722	0.919780066132101	1	pseudobulk	DESeq2	LRT	0.036316006876181
962	KC_AB2	CG11367	0.0585026687147193	0.972772634716089	1	pseudobulk	DESeq2	LRT	0.011988655134416
963	KC_AB2	CG11374	1.10706867860301	1	1	pseudobulk	DESeq2	LRT	0
963	KC_AB2	CG11377	1.32439637941804	0.44655862524333	1	pseudobulk	DESeq2	LRT	0.350121517854179
963	KC_AB2	CG11396	0.0512684897050687	0.898258174589374	1	pseudobulk	DESeq2	LRT	0.046598821812817
963	KC_AB2	CG11398	-2.35203494925393	0.313819523334105	1	pseudobulk	DESeq2	LRT	0.503320041588723
963	KC_AB2	CG11399	-0.601000921402953	0.610058071910912	1	pseudobulk	DESeq2	LRT	0.214628822185866
963	KC_AB2	CG11403	-0.729379579623002	1	1	pseudobulk	DESeq2	LRT	0
963	KC_AB2	CG11406	-0.112490013242319	0.888963332509736	1	pseudobulk	DESeq2	LRT	0.051116152209881
963	KC_AB2	CG11413	-0.18317077492817	0.899664519927029	1	pseudobulk	DESeq2	LRT	0.045919406456170
963	KC_AB2	CG11414	1.26966455883425	0.41821041121263	1	pseudobulk	DESeq2	LRT	0.378605159763413
963	KC_AB2	CG11417	1.78258764191389	0.445483464074049	1	pseudobulk	DESeq2	LRT	0.351168411929411
964	KC_AB2	CG1142	0.16772780138044	0.858500526890365	1	pseudobulk	DESeq2	LRT	0.066259433961995
964	KC_AB2	CG11436	-1.78975280219494	0.468855431150075	1	pseudobulk	DESeq2	LRT	0.32896104882553
964	KC_AB2	CG11444	-1.01774524073266	0.349234047442098	1	pseudobulk	DESeq2	LRT	0.456883422768828
964	KC_AB2	CG11447	-1.13951370469285	0.507398845204455	1	pseudobulk	DESeq2	LRT	0.29465052552955
964	KC_AB2	CG11454	-0.727278929324385	0.511273827124764	1	pseudobulk	DESeq2	LRT	0.29134643889135
964	KC_AB2	CG11456	1.68283803538613	0.257745602445591	1	pseudobulk	DESeq2	LRT	0.588808735743402
964	KC_AB2	CG11474	1.47962494894969	0.449379558710309	1	pseudobulk	DESeq2	LRT	0.347386686570256
964	KC_AB2	CG11486	-0.19448866535615	0.533017458861469	1	pseudobulk	DESeq2	LRT	0.273258565526867
964	KC_AB2	CG11504	0.417848929975184	0.736971060561246	1	pseudobulk	DESeq2	LRT	0.132549565716691
964	KC_AB2	CG11523	-0.419133701212731	0.647124942068475	1	pseudobulk	DESeq2	LRT	0.189011860888732
965	KC_AB2	CG11537	0.236864796976001	0.558848419465363	1	pseudobulk	DESeq2	LRT	0.252705973002316
965	KC_AB2	CG11555	-1.42503705481346	0.420454133680305	1	pseudobulk	DESeq2	LRT	0.376281373411833
965	KC_AB2	CG11560	1.10706867860301	1	1	pseudobulk	DESeq2	LRT	0
965	KC_AB2	CG11562	-0.0606401002171201	0.879673416741016	1	pseudobulk	DESeq2	LRT	0.055678531977299
965	KC_AB2	CG11563	0.314354712497931	0.853507994456407	1	pseudobulk	DESeq2	LRT	0.068792406673391
965	KC_AB2	CG11583	1.2856232851715	0.552348637441447	1	pseudobulk	DESeq2	LRT	0.257786712991409
965	KC_AB2	CG11586	0.736806881147161	0.490864033358947	1	pseudobulk	DESeq2	LRT	0.309038788405299
965	KC_AB2	CG11590	-0.527510649809022	0.761880031680828	1	pseudobulk	DESeq2	LRT	0.118113408819
965	KC_AB2	CG11593	2.27929825416414	0.184122049210094	1	pseudobulk	DESeq2	LRT	0.734894200214716
965	KC_AB2	CG11596	1.67841060813711	0.505968139121766	1	pseudobulk	DESeq2	LRT	0.295876829878097
966	KC_AB2	CG11601	1.48091416250208	0.564234146509008	1	pseudobulk	DESeq2	LRT	0.248540634607989
966	KC_AB2	CG1161	-1.28333109944422	0.175169646655631	1	pseudobulk	DESeq2	LRT	0.756541146067552
966	KC_AB2	CG11626	-1.33407500886303	0.63128114564055	1	pseudobulk	DESeq2	LRT	0.19977718147345
966	KC_AB2	CG11638	-0.0297174987241083	0.948723593811725	1	pseudobulk	DESeq2	LRT	0.022860298813408
966	KC_AB2	CG11658	1.00278481426558	0.286763030001609	1	pseudobulk	DESeq2	LRT	0.542476839410193
966	KC_AB2	CG11695	-1.89324719569543	0.358469656860786	1	pseudobulk	DESeq2	LRT	0.445547599872436
966	KC_AB2	CG11696	0.601001254660651	0.777533255868503	1	pseudobulk	DESeq2	LRT	0.109281026698154
966	KC_AB2	CG11699	0.424066975332598	0.64763254307623	1	pseudobulk	DESeq2	LRT	0.188671336380475
966	KC_AB2	CG11700	1.10706867860301	1	1	pseudobulk	DESeq2	LRT	0
966	KC_AB2	CG11710	-0.517253550924715	0.704875831988562	1	pseudobulk	DESeq2	LRT	0.151887379790959
967	KC_AB2	CG1172	1.14459500861008	0.327342125398332	1	pseudobulk	DESeq2	LRT	0.48499810208332

967	KC_AB2	CG11722	-0.748174191105629	0.624208266171741	1	pseudobulk	DESeq2	LRT	0.204670484422683
967	KC_AB2	CG11723	-1.05725921281211	0.56361554652405	1	pseudobulk	DESeq2	LRT	0.249017036008941
967	KC_AB2	CG11737	0.364136077420657	0.814057422322293	1	pseudobulk	DESeq2	LRT	0.089344959584829
967	KC_AB2	CG11752	-0.273055896405795	0.647460068414101	1	pseudobulk	DESeq2	LRT	0.188787011188298
967	KC_AB2	CG11753	0.264889475379475	0.738013671850131	1	pseudobulk	DESeq2	LRT	0.131935592710737
967	KC_AB2	CG11755	0.661678460170035	0.878003361430606	1	pseudobulk	DESeq2	LRT	0.056503821396894
967	KC_AB2	CG11768	0.0984233562523555	0.825220820162237	1	pseudobulk	DESeq2	LRT	0.083429823394588
967	KC_AB2	CG11771	1.3074674942285	0.349092484575371	1	pseudobulk	DESeq2	LRT	0.457059500777854
967	KC_AB2	CG11777	0.643658223820713	0.775115890013421	1	pseudobulk	DESeq2	LRT	0.110633359905791
968	KC_AB2	CG11779	-0.469251302609692	0.79168155293543	1	pseudobulk	DESeq2	LRT	0.10144947448897
968	KC_AB2	CG11781	0.0206495023669072	0.983343449308345	1	pseudobulk	DESeq2	LRT	0.007294770989436
968	KC_AB2	CG11788	0.147593560254927	1	1	pseudobulk	DESeq2	LRT	0
968	KC_AB2	CG11790	-0.804360183303268	0.597663709269567	1	pseudobulk	DESeq2	LRT	0.223543114155392
968	KC_AB2	CG11791	-1.71999636108657	0.519869997311849	1	pseudobulk	DESeq2	LRT	0.284105245805297
968	KC_AB2	CG11807	-0.885316208992894	0.530593159252183	1	pseudobulk	DESeq2	LRT	0.27523835352101
968	KC_AB2	CG11808	0.358105185835313	0.820492657446879	1	pseudobulk	DESeq2	LRT	0.085925301075936
968	KC_AB2	CG11811	-1.08078402834235	0.306489657218196	1	pseudobulk	DESeq2	LRT	0.513584176574128
968	KC_AB2	CG11835	-0.669196903545793	0.258148862514555	1	pseudobulk	DESeq2	LRT	0.588129784251237
968	KC_AB2	CG11837	-0.961033333862793	0.70039622209097	1	pseudobulk	DESeq2	LRT	0.154656205149228
969	KC_AB2	CG11839	-1.78975280219494	0.468855431150075	1	pseudobulk	DESeq2	LRT	0.32896104882553
969	KC_AB2	CG11857	-0.0760584236168179	0.94975982560029	1	pseudobulk	DESeq2	LRT	0.022386204820449
969	KC_AB2	CG11858	-0.786800864663039	0.527583660804651	1	pseudobulk	DESeq2	LRT	0.277708662959371
969	KC_AB2	CG11873	-0.191891163156336	0.724660861766014	1	pseudobulk	DESeq2	LRT	0.139865193874288
969	KC_AB2	CG11882	0.146930209264112	1	1	pseudobulk	DESeq2	LRT	0
969	KC_AB2	CG11883	-0.687337820235226	0.319252674272885	1	pseudobulk	DESeq2	LRT	0.495865456109676
969	KC_AB2	CG11885	0.470168023740528	0.84771616298758	1	pseudobulk	DESeq2	LRT	0.071749536284240
969	KC_AB2	CG11898	0.146930209264112	1	1	pseudobulk	DESeq2	LRT	0
969	KC_AB2	CG11899	-0.729379579623002	0.772694994316916	1	pseudobulk	DESeq2	LRT	0.1119919011982
969	KC_AB2	CG11902	0.548404510208272	0.790217697249523	1	pseudobulk	DESeq2	LRT	0.102253248342565
970	KC_AB2	CG11906	-0.729379579623002	0.823527397767669	1	pseudobulk	DESeq2	LRT	0.084321947797948
970	KC_AB2	CG11920	0.39439088612385	0.813541888331052	1	pseudobulk	DESeq2	LRT	0.089620080830767
970	KC_AB2	CG11927	0.536217279712505	0.686415050289259	1	pseudobulk	DESeq2	LRT	0.163413202743968
970	KC_AB2	CG11961	1.10706867860301	1	1	pseudobulk	DESeq2	LRT	0
970	KC_AB2	CG11964	-0.358730459332058	0.77871178211666	1	pseudobulk	DESeq2	LRT	0.108623254262963
970	KC_AB2	CG11970	3.12100799138407	0.161346113416639	1	pseudobulk	DESeq2	LRT	0.792241491629998
970	KC_AB2	CG11975	0.193283627299478	0.848534008274878	1	pseudobulk	DESeq2	LRT	0.071330746970182
970	KC_AB2	CG11980	-1.33570647694682	0.512066034235951	1	pseudobulk	DESeq2	LRT	0.290674030322833
970	KC_AB2	CG11983	-0.729379579623002	0.823527397767669	1	pseudobulk	DESeq2	LRT	0.084321947797948
970	KC_AB2	CG11986	-1.6052707068109	0.52265609184397	1	pseudobulk	DESeq2	LRT	0.281783983297782
971	KC_AB2	CG11999	-1.14525798232617	0.550703157242018	1	pseudobulk	DESeq2	LRT	0.259082433653354
971	KC_AB2	CG12004	0.174178742066711	0.745488099927324	1	pseudobulk	DESeq2	LRT	0.127559284708835
971	KC_AB2	CG12007	0.947760643287124	0.515683007341942	1	pseudobulk	DESeq2	LRT	0.287617179121141
971	KC_AB2	CG12010	-0.729379579623002	1	1	pseudobulk	DESeq2	LRT	0
971	KC_AB2	CG12012	-0.899222402532833	0.440644997113638	1	pseudobulk	DESeq2	LRT	0.355911156299322
971	KC_AB2	CG12017	-0.729379579623002	0.823527397767669	1	pseudobulk	DESeq2	LRT	0.084321947797948
971	KC_AB2	CG12024	-0.894195498205969	0.368184778844705	1	pseudobulk	DESeq2	LRT	0.433934169662512
971	KC_AB2	CG12025	-0.765813783510337	0.305247529890512	1	pseudobulk	DESeq2	LRT	0.515347841745932
971	KC_AB2	CG12038	-0.447110298481768	0.495385188273142	1	pseudobulk	DESeq2	LRT	0.305056982706595
971	KC_AB2	CG12054	0.0344376520025225	0.878596283302282	1	pseudobulk	DESeq2	LRT	0.05621063824885
972	KC_AB2	CG12056	-1.66891092733648	0.324235974754614	1	pseudobulk	DESeq2	LRT	0.489138800805688
972	KC_AB2	CG12071	0.230852771450545	0.637367044869255	1	pseudobulk	DESeq2	LRT	0.195610395524091
972	KC_AB2	CG12081	0.225684255865163	0.866276002928094	1	pseudobulk	DESeq2	LRT	0.062343716006464

972	KC_AB2	CG12084	-0.786568866141875	0.459686184715675	1	pseudobulk	DESeq2	LRT	0.337538548222515
972	KC_AB2	CG12091	0.89571037465875	0.25913571070536	1	pseudobulk	DESeq2	LRT	0.586472734086301
972	KC_AB2	CG12093	0.25288967549489	0.842779437310897	1	pseudobulk	DESeq2	LRT	0.074286069138157
972	KC_AB2	CG12096	-0.294038124956324	0.857416604202101	1	pseudobulk	DESeq2	LRT	0.066808110464381
972	KC_AB2	CG12099	0.487758944196455	0.598537399426908	1	pseudobulk	DESeq2	LRT	0.222908707651435
972	KC_AB2	CG12104	-0.169524318341919	0.912374154556569	1	pseudobulk	DESeq2	LRT	0.039827025776412
972	KC_AB2	CG12106	-0.519497269246583	0.732385281090826	1	pseudobulk	DESeq2	LRT	0.13526039239501
973	KC_AB2	CG12107	-0.556889561404948	0.682529320025758	1	pseudobulk	DESeq2	LRT	0.165878687507882
973	KC_AB2	CG12112	1.63577765519498	0.536426415953348	1	pseudobulk	DESeq2	LRT	0.270489843757993
973	KC_AB2	CG12118	-0.169524318341919	0.912374154556569	1	pseudobulk	DESeq2	LRT	0.039827025776412
973	KC_AB2	CG12121	-1.00035474028366	0.276204120664905	1	pseudobulk	DESeq2	LRT	0.558769846508001
973	KC_AB2	CG12123	0.301206009968849	0.824594764252616	1	pseudobulk	DESeq2	LRT	0.083759427069727
973	KC_AB2	CG12128	0.0487673033804221	0.942805703571486	1	pseudobulk	DESeq2	LRT	0.025577798843798
973	KC_AB2	CG12129	-2.28945135223953	0.244879642118917	1	pseudobulk	DESeq2	LRT	0.611047318112997
973	KC_AB2	CG12134	-0.893328173497901	0.679679924321693	1	pseudobulk	DESeq2	LRT	0.167695557626761
973	KC_AB2	CG12155	-1.8317720234636	0.266960063916526	1	pseudobulk	DESeq2	LRT	0.573553702372925
973	KC_AB2	CG12159	0.459847948764022	0.664509522594691	1	pseudobulk	DESeq2	LRT	0.177498791123513
974	KC_AB2	CG12163	-0.15348383106247	0.8628021409862	1	pseudobulk	DESeq2	LRT	0.064088785895247
974	KC_AB2	CG12170	-1.38764087950137	0.516647656322572	1	pseudobulk	DESeq2	LRT	0.286805536367625
974	KC_AB2	CG12171	1.99339641594643	0.465071165814147	1	pseudobulk	DESeq2	LRT	0.332480585700252
974	KC_AB2	CG12173	-1.09893957618674	0.427209306250733	1	pseudobulk	DESeq2	LRT	0.369359295283529
974	KC_AB2	CG12179	-0.729379579623002	1	1	pseudobulk	DESeq2	LRT	0
974	KC_AB2	CG1218	0.467937907784063	0.752586835116196	1	pseudobulk	DESeq2	LRT	0.123443382995158
974	KC_AB2	CG12182	1.10706867860301	1	1	pseudobulk	DESeq2	LRT	0
974	KC_AB2	CG12194	3.30110175219668	0.111422080617826	1	pseudobulk	DESeq2	LRT	0.953028736090146
974	KC_AB2	CG12204	2.33862076287154	0.37727071943742	1	pseudobulk	DESeq2	LRT	0.423346899755834
974	KC_AB2	CG12206	0.145321756433347	1	1	pseudobulk	DESeq2	LRT	0
975	KC_AB2	CG12213	-1.14740055622928	0.26650384929159	1	pseudobulk	DESeq2	LRT	0.57429651378929
975	KC_AB2	CG12219	1.48493682276439	0.491226398984852	1	pseudobulk	DESeq2	LRT	0.308718301831515
975	KC_AB2	CG12236	0.28855822520198	0.78828760371794	1	pseudobulk	DESeq2	LRT	0.103315302917295
975	KC_AB2	CG12237	-1.11714674443802	0.261009838229326	1	pseudobulk	DESeq2	LRT	0.583343122515428
975	KC_AB2	CG12239	1.38069928703004	0.566368801250645	1	pseudobulk	DESeq2	LRT	0.246900678019328
975	KC_AB2	CG12241	-1.08622409048678	0.462964493164969	1	pseudobulk	DESeq2	LRT	0.334452315707402
975	KC_AB2	CG12253	-0.954521953068045	0.535586403508224	1	pseudobulk	DESeq2	LRT	0.271170456597113
975	KC_AB2	CG12259	0.630933534679724	0.705154990606571	1	pseudobulk	DESeq2	LRT	0.15171541610475
975	KC_AB2	CG12267	-1.78975280219494	0.468855431150075	1	pseudobulk	DESeq2	LRT	0.32896104882553
975	KC_AB2	CG1227	1.61205113368698	0.631647504209	1	pseudobulk	DESeq2	LRT	0.199525215522471
976	KC_AB2	CG12279	0.163586665441299	0.856869720176133	1	pseudobulk	DESeq2	LRT	0.067085203875672
976	KC_AB2	CG12288	-2.80050663093489	0.203902275365684	1	pseudobulk	DESeq2	LRT	0.690577927859686
976	KC_AB2	CG12299	-0.597425699804926	0.627388675275849	1	pseudobulk	DESeq2	LRT	0.202463324831127
976	KC_AB2	CG12301	-0.242904908965546	0.933507439612501	1	pseudobulk	DESeq2	LRT	0.029882216579625
976	KC_AB2	CG1231	-1.17211486980499	0.550804693380036	1	pseudobulk	DESeq2	LRT	0.259002367797164
976	KC_AB2	CG12310	-0.729379579623002	0.823527397767669	1	pseudobulk	DESeq2	LRT	0.084321947797948
976	KC_AB2	CG12316	1.61194132148607	0.388116896275451	1	pseudobulk	DESeq2	LRT	0.411037450278626
976	KC_AB2	CG12320	1.04324661842443	0.68096673636569	1	pseudobulk	DESeq2	LRT	0.166874101840012
976	KC_AB2	CG12321	0.50708159733276	0.79362281859345	1	pseudobulk	DESeq2	LRT	0.100385853647791
976	KC_AB2	CG12325	2.65300248092945	0.16643904427923	1	pseudobulk	DESeq2	LRT	0.778744786663328
977	KC_AB2	CG1233	-1.11167283223406	0.564212144344824	1	pseudobulk	DESeq2	LRT	0.248557570138858
977	KC_AB2	CG12333	-0.250360529998988	0.826638335586768	1	pseudobulk	DESeq2	LRT	0.082684458057772
977	KC_AB2	CG12338	-2.22728608909242	0.317088151657018	1	pseudobulk	DESeq2	LRT	0.498819985554471
977	KC_AB2	CG1234	0.404449229411186	0.828138828172865	1	pseudobulk	DESeq2	LRT	0.081896852520118
977	KC_AB2	CG12341	0.77520441832633	0.572652341710047	1	pseudobulk	DESeq2	LRT	0.242108959009233

977	KC_AB2	CG12343	-0.0031962033005064	0.952829756142698	1	pseudobulk	DESeq2	LRT	0.020984688629825
977	KC_AB2	CG12355	-1.9966181735319	0.275830635927913	1	pseudobulk	DESeq2	LRT	0.559357499307618
977	KC_AB2	CG1236	-0.83320718254958	0.715059964627045	1	pseudobulk	DESeq2	LRT	0.14565753691968
977	KC_AB2	CG12375	0.282812098998667	0.789542978965984	1	pseudobulk	DESeq2	LRT	0.102624224085422
977	KC_AB2	CG12379	1.61205113368698	0.631647504209	1	pseudobulk	DESeq2	LRT	0.199525215522471
978	KC_AB2	CG12384	0.0197753459112026	0.931178952878641	1	pseudobulk	DESeq2	LRT	0.030966848793807
978	KC_AB2	CG1239	0.473249351445965	0.789307127259556	1	pseudobulk	DESeq2	LRT	0.10275397559889
978	KC_AB2	CG12391	0.0547092441385226	0.898896908613856	1	pseudobulk	DESeq2	LRT	0.046290113147093
978	KC_AB2	CG12402	0.527597997907203	0.635469448756762	1	pseudobulk	DESeq2	LRT	0.196905324032545
978	KC_AB2	CG12470	1.61205113368698	0.631647504209	1	pseudobulk	DESeq2	LRT	0.199525215522471
978	KC_AB2	CG12499	-1.86716056129969	0.477680380390446	1	pseudobulk	DESeq2	LRT	0.320862595985116
978	KC_AB2	CG12502	-0.888013106938667	0.5634094233233	1	pseudobulk	DESeq2	LRT	0.249175893482504
978	KC_AB2	CG12531	-0.182101621791779	0.677370574542599	1	pseudobulk	DESeq2	LRT	0.169173673341439
978	KC_AB2	CG12535	-0.18317077492817	0.899664519927029	1	pseudobulk	DESeq2	LRT	0.045919406456170
978	KC_AB2	CG12541	3.83612728639432	0.0815829828358478	1	pseudobulk	DESeq2	LRT	1.08840042006002
979	KC_AB2	CG12547	-0.774308968267904	0.421092625984752	1	pseudobulk	DESeq2	LRT	0.375622363714315
979	KC_AB2	CG12560	2.08285472251636	0.233200441895513	1	pseudobulk	DESeq2	LRT	0.63227063096019
979	KC_AB2	CG12567	-0.145078495308522	0.845942206244238	1	pseudobulk	DESeq2	LRT	0.072659306425019
979	KC_AB2	CG12576	-0.788064501027982	0.298953378516213	1	pseudobulk	DESeq2	LRT	0.524396534190118
979	KC_AB2	CG12590	1.10706867860301	1	1	pseudobulk	DESeq2	LRT	0
979	KC_AB2	CG12605	0.445413357829625	0.248052167712454	1	pseudobulk	DESeq2	LRT	0.605456973338766
979	KC_AB2	CG12608	0.79613745118511	0.665952465403801	1	pseudobulk	DESeq2	LRT	0.17655676895219
979	KC_AB2	CG12637	-1.6052707068109	0.52265609184397	1	pseudobulk	DESeq2	LRT	0.281783983297782
979	KC_AB2	CG1265	0.669705838925604	0.417815285773633	1	pseudobulk	DESeq2	LRT	0.379015675399981
979	KC_AB2	CG12655	-0.729379579623002	1	1	pseudobulk	DESeq2	LRT	0
980	KC_AB2	CG12659	1.10706867860301	1	1	pseudobulk	DESeq2	LRT	0
980	KC_AB2	CG12672	-1.34387033725355	0.647060155693926	1	pseudobulk	DESeq2	LRT	0.189055342095618
980	KC_AB2	CG1271	-1.33407500886303	0.63128114564055	1	pseudobulk	DESeq2	LRT	0.19977718147345
980	KC_AB2	CG12713	0.631690707653592	0.771422696955948	1	pseudobulk	DESeq2	LRT	0.11270758738116
980	KC_AB2	CG12717	1.69347526891562	0.497235149290649	1	pseudobulk	DESeq2	LRT	0.303438178897214
980	KC_AB2	CG12728	-1.53390420416511	0.550292219668796	1	pseudobulk	DESeq2	LRT	0.259406627437427
980	KC_AB2	CG12730	-0.0285360800339529	0.901333120098553	1	pseudobulk	DESeq2	LRT	0.045114670194183
980	KC_AB2	CG12746	2.47215486522296	0.30601789350912	1	pseudobulk	DESeq2	LRT	0.514253178665238
980	KC_AB2	CG1275	-0.551359387489622	0.451892826423795	1	pseudobulk	DESeq2	LRT	0.344964552825225
980	KC_AB2	CG12768	1.61205113368698	0.631647504209	1	pseudobulk	DESeq2	LRT	0.199525215522471
981	KC_AB2	CG12769	0.0433310387475314	0.951385000919325	1	pseudobulk	DESeq2	LRT	0.021643699741131
981	KC_AB2	CG12773	0.124462516295993	0.952307241396434	1	pseudobulk	DESeq2	LRT	0.021222913258289
981	KC_AB2	CG12795	-1.58268409616911	0.264051534605388	1	pseudobulk	DESeq2	LRT	0.578311304150813
981	KC_AB2	CG12814	1.38446765109058	0.642794900879723	1	pseudobulk	DESeq2	LRT	0.191927577037166
981	KC_AB2	CG12817	0.429863682023003	0.658370823546547	1	pseudobulk	DESeq2	LRT	0.181529423624312
981	KC_AB2	CG12818	0.145321756433347	1	1	pseudobulk	DESeq2	LRT	0
981	KC_AB2	CG12822	-0.495762144334328	0.511157991256763	1	pseudobulk	DESeq2	LRT	0.291444845215547
981	KC_AB2	CG12825	-1.33407500886303	0.63128114564055	1	pseudobulk	DESeq2	LRT	0.19977718147345
981	KC_AB2	CG12831	-1.33407500886303	0.63128114564055	1	pseudobulk	DESeq2	LRT	0.19977718147345
981	KC_AB2	CG12848	-0.501825532277803	0.678685849318107	1	pseudobulk	DESeq2	LRT	0.168331205806513
982	KC_AB2	CG12851	0.0496544419969787	0.863113189836534	1	pseudobulk	DESeq2	LRT	0.063932246581467
982	KC_AB2	CG12868	-1.45678625006774	0.434437731616474	1	pseudobulk	DESeq2	LRT	0.362072462606123
982	KC_AB2	CG12877	-0.242059415553701	0.873035398122288	1	pseudobulk	DESeq2	LRT	0.058968147018927
982	KC_AB2	CG12883	1.38446765109058	0.642794900879723	1	pseudobulk	DESeq2	LRT	0.191927577037166
982	KC_AB2	CG12895	-0.87789204122309	0.321476337246175	1	pseudobulk	DESeq2	LRT	0.492850988467958
982	KC_AB2	CG12909	1.02754127378565	0.694973473680879	1	pseudobulk	DESeq2	LRT	0.15803177160251
982	KC_AB2	CG12910	1.02648689492346	0.73959154128541	1	pseudobulk	DESeq2	LRT	0.131008064514051

982	KC_AB2	CG12913	0.528241556797397	0.677537941848443	1	pseudobulk	DESeq2	LRT	0.169066379458687
982	KC_AB2	CG12923	1.38446765109058	0.642794900879723	1	pseudobulk	DESeq2	LRT	0.191927577037166
982	KC_AB2	CG12929	-0.153926777936872	0.909116280188619	1	pseudobulk	DESeq2	LRT	0.041380564946842
983	KC_AB2	CG12935	-0.427478535071269	0.813013586949621	1	pseudobulk	DESeq2	LRT	0.089902196487186
983	KC_AB2	CG12942	-2.28054226263626	0.331630256012328	1	pseudobulk	DESeq2	LRT	0.479345853816062
983	KC_AB2	CG12945	-0.589959803728696	0.55024070437241	1	pseudobulk	DESeq2	LRT	0.259447285574012
983	KC_AB2	CG12948	-0.883890872531852	0.676366690187261	1	pseudobulk	DESeq2	LRT	0.169817788750343
983	KC_AB2	CG12975	0.0598926141106683	0.911434085100927	1	pseudobulk	DESeq2	LRT	0.040274734043619
983	KC_AB2	CG12991	0.326245098062304	0.71035307826645	1	pseudobulk	DESeq2	LRT	0.148525733213363
983	KC_AB2	CG12992	-0.729379579623002	1	1	pseudobulk	DESeq2	LRT	0
983	KC_AB2	CG12994	1.10706867860301	1	1	pseudobulk	DESeq2	LRT	0
983	KC_AB2	CG13001	1.13884246904056	0.429577063587174	1	pseudobulk	DESeq2	LRT	0.36695891497947
983	KC_AB2	CG13012	1.10706867860301	1	1	pseudobulk	DESeq2	LRT	0
984	KC_AB2	CG13014	0.388447834546628	0.84487503289525	1	pseudobulk	DESeq2	LRT	0.073207523639173
984	KC_AB2	CG13016	0.0455261422590676	0.956410711868198	1	pseudobulk	DESeq2	LRT	0.019355568399845
984	KC_AB2	CG13018	-1.1197120523618	0.494064443571732	1	pseudobulk	DESeq2	LRT	0.306216399938223
984	KC_AB2	CG13025	0.0506112603868946	1	1	pseudobulk	DESeq2	LRT	0
984	KC_AB2	CG13054	-0.18317077492817	0.899664519927029	1	pseudobulk	DESeq2	LRT	0.045919406456170
984	KC_AB2	CG13055	0.179215130238249	0.689587347184975	1	pseudobulk	DESeq2	LRT	0.161410715713155
984	KC_AB2	CG1307	-0.36499178955896	0.750029705458507	1	pseudobulk	DESeq2	LRT	0.124921535726655
984	KC_AB2	CG13085	-0.729379579623002	1	1	pseudobulk	DESeq2	LRT	0
984	KC_AB2	CG13088	-1.78975280219494	0.468855431150075	1	pseudobulk	DESeq2	LRT	0.32896104882553
984	KC_AB2	CG1309	0.15704602031289	0.920277811941327	1	pseudobulk	DESeq2	LRT	0.036081048762977
985	KC_AB2	CG13096	0.966681968407098	0.635049159877072	1	pseudobulk	DESeq2	LRT	0.197192654177373
985	KC_AB2	CG13097	0.972230061963122	0.501663282959177	1	pseudobulk	DESeq2	LRT	0.299587684086372
985	KC_AB2	CG13108	-0.376222978888125	0.742994696822562	1	pseudobulk	DESeq2	LRT	0.129014286036083
985	KC_AB2	CG13123	1.70114581505585	0.381085348812214	1	pseudobulk	DESeq2	LRT	0.418977747770267
985	KC_AB2	CG13124	-0.729379579623002	0.823527397767669	1	pseudobulk	DESeq2	LRT	0.084321947797948
985	KC_AB2	CG13126	-0.729379579623002	0.772694994316916	1	pseudobulk	DESeq2	LRT	0.1119919011982
985	KC_AB2	CG1314	1.38069928703004	0.566368801250645	1	pseudobulk	DESeq2	LRT	0.246900678019328
985	KC_AB2	CG13148	1.40779529144543	0.48386893053043	1	pseudobulk	DESeq2	LRT	0.315272263265186
985	KC_AB2	CG13151	1.38446765109058	0.642794900879723	1	pseudobulk	DESeq2	LRT	0.191927577037166
985	KC_AB2	CG1316	-0.0482133447130334	0.911911448806423	1	pseudobulk	DESeq2	LRT	0.040047331805218
986	KC_AB2	CG1317	1.30263537950461	0.482611790937213	1	pseudobulk	DESeq2	LRT	0.316402071825463
986	KC_AB2	CG13185	0.145321756433347	1	1	pseudobulk	DESeq2	LRT	0
986	KC_AB2	CG13192	-0.465155737991212	0.842472988529882	1	pseudobulk	DESeq2	LRT	0.074444014635232
986	KC_AB2	CG13197	-0.799132886553859	0.550015802756192	1	pseudobulk	DESeq2	LRT	0.25962483241263
986	KC_AB2	CG13204	0.336430634648092	0.656897086095268	1	pseudobulk	DESeq2	LRT	0.182502664598298
986	KC_AB2	CG13220	-1.17390124951674	0.298822684071622	1	pseudobulk	DESeq2	LRT	0.524586437669225
986	KC_AB2	CG13248	-0.747273174212981	0.337961934901655	1	pseudobulk	DESeq2	LRT	0.471132212127994
986	KC_AB2	CG13255	0.403294498144138	0.89479244414645	1	pseudobulk	DESeq2	LRT	0.048277691852293
986	KC_AB2	CG13284	1.10706867860301	1	1	pseudobulk	DESeq2	LRT	0
986	KC_AB2	CG13293	0.387743015274735	0.468779757025113	1	pseudobulk	DESeq2	LRT	0.329031150407424
987	KC_AB2	CG13295	2.64741945039871	0.292550810913063	1	pseudobulk	DESeq2	LRT	0.533798693743382
987	KC_AB2	CG13300	0.14630031414499	0.901902924779258	1	pseudobulk	DESeq2	LRT	0.044840204699183
987	KC_AB2	CG13314	1.10706867860301	1	1	pseudobulk	DESeq2	LRT	0
987	KC_AB2	CG13318	1.99339641594643	0.465071165814147	1	pseudobulk	DESeq2	LRT	0.332480585700252
987	KC_AB2	CG13319	-1.6052707068109	0.52265609184397	1	pseudobulk	DESeq2	LRT	0.281783983297782
987	KC_AB2	CG13325	1.28515114261845	0.257758620424008	1	pseudobulk	DESeq2	LRT	0.588786801350075
987	KC_AB2	CG13339	0.157877382047148	0.846704117606095	1	pseudobulk	DESeq2	LRT	0.072268328206073
987	KC_AB2	CG13344	-0.593911142405807	0.458537305341535	1	pseudobulk	DESeq2	LRT	0.338625325544884
987	KC_AB2	CG13364	-0.283033709488692	0.607916947282255	1	pseudobulk	DESeq2	LRT	0.216155749346972

987	KC_AB2	CG13365	0.0509686445515292	0.950939781967902	1	pseudobulk	DESeq2	LRT	0.021846983785067
988	KC_AB2	CG13366	-0.639508585699781	0.584069300051646	1	pseudobulk	DESeq2	LRT	0.233535620619678
988	KC_AB2	CG13367	-0.242904908965546	0.933507439612501	1	pseudobulk	DESeq2	LRT	0.029882216579625
988	KC_AB2	CG13369	2.09318022853789	0.126737321736537	1	pseudobulk	DESeq2	LRT	0.897095474797436
988	KC_AB2	CG13375	-0.0092134682466518	0.882543912889025	1	pseudobulk	DESeq2	LRT	0.054263676137400
988	KC_AB2	CG13377	-1.52244591594958	0.547176617578731	1	pseudobulk	DESeq2	LRT	0.261872469536296
988	KC_AB2	CG13384	0.253314003454277	0.772800656463697	1	pseudobulk	DESeq2	LRT	0.111932517676362
988	KC_AB2	CG13390	-0.303672526813818	0.918092162121602	1	pseudobulk	DESeq2	LRT	0.037113720223793
988	KC_AB2	CG13392	0.412266503805257	0.859735858836361	1	pseudobulk	DESeq2	LRT	0.065634958838131
988	KC_AB2	CG13398	0.293338761753077	0.863749499656955	1	pseudobulk	DESeq2	LRT	0.063612191190869
988	KC_AB2	CG13404	0.451319178886345	0.609439298035871	1	pseudobulk	DESeq2	LRT	0.215069544911737
989	KC_AB2	CG13409	-0.921493429177312	0.431787783488166	1	pseudobulk	DESeq2	LRT	0.364729649261473
989	KC_AB2	CG1344	0.940879444410985	0.473520111468633	1	pseudobulk	DESeq2	LRT	0.324661570801436
989	KC_AB2	CG13445	2.01814786626755	0.401718761445944	1	pseudobulk	DESeq2	LRT	0.396077884966921
989	KC_AB2	CG13482	0.403294498144138	0.89479244414645	1	pseudobulk	DESeq2	LRT	0.048277691852293
989	KC_AB2	CG13484	0.397754829178693	0.823169963702779	1	pseudobulk	DESeq2	LRT	0.084510484742375
989	KC_AB2	CG13506	0.267151962897292	0.793742219437549	1	pseudobulk	DESeq2	LRT	0.100320518798425
989	KC_AB2	CG13526	1.10706867860301	1	1	pseudobulk	DESeq2	LRT	0
989	KC_AB2	CG13527	1.10706867860301	1	1	pseudobulk	DESeq2	LRT	0
989	KC_AB2	CG13531	-2.28054226263626	0.331630256012328	1	pseudobulk	DESeq2	LRT	0.479345853816062
989	KC_AB2	CG13532	0.45276247211033	0.67268444178824	1	pseudobulk	DESeq2	LRT	0.172188616800107
990	KC_AB2	CG1354	-1.18823353600293	0.276137435171391	1	pseudobulk	DESeq2	LRT	0.55887471329447
990	KC_AB2	CG13551	-0.36644993762952	0.498960182311113	1	pseudobulk	DESeq2	LRT	0.301934110273472
990	KC_AB2	CG13578	-0.672424505335587	0.790771522020703	1	pseudobulk	DESeq2	LRT	0.101948979282445
990	KC_AB2	CG13579	1.10706867860301	1	1	pseudobulk	DESeq2	LRT	0
990	KC_AB2	CG1358	1.06892952192513	0.275293482897876	1	pseudobulk	DESeq2	LRT	0.560204069663669
990	KC_AB2	CG13585	-1.10390460329745	0.163495476479084	1	pseudobulk	DESeq2	LRT	0.786494258706137
990	KC_AB2	CG13601	-2.23048278007313	0.125361622761933	1	pseudobulk	DESeq2	LRT	0.901835394714389
990	KC_AB2	CG13603	-0.114237405518136	0.870573213480357	1	pseudobulk	DESeq2	LRT	0.060194699706347
990	KC_AB2	CG13604	2.48384162006255	0.303740962766822	1	pseudobulk	DESeq2	LRT	0.517496634805608
990	KC_AB2	CG13605	1.69347526891562	0.497235149290649	1	pseudobulk	DESeq2	LRT	0.303438178897214
991	KC_AB2	CG13609	1.02648689492346	0.73959154128541	1	pseudobulk	DESeq2	LRT	0.131008064514051
991	KC_AB2	CG13623	0.693822832067212	0.67608449237572	1	pseudobulk	DESeq2	LRT	0.169999025538356
991	KC_AB2	CG13625	0.277796426002785	0.850612929600354	1	pseudobulk	DESeq2	LRT	0.070268020150787
991	KC_AB2	CG13630	-1.58259623014096	0.413154296397809	1	pseudobulk	DESeq2	LRT	0.383887726649135
991	KC_AB2	CG13631	1.10706867860301	1	1	pseudobulk	DESeq2	LRT	0
991	KC_AB2	CG13654	-1.02031505357459	0.442939041875032	1	pseudobulk	DESeq2	LRT	0.35365603810854
991	KC_AB2	CG13658	1.10706867860301	1	1	pseudobulk	DESeq2	LRT	0
991	KC_AB2	CG13663	1.66610102613172	0.254141991773108	1	pseudobulk	DESeq2	LRT	0.594923570728323
991	KC_AB2	CG13671	-1.78975280219494	0.468855431150075	1	pseudobulk	DESeq2	LRT	0.32896104882553
991	KC_AB2	CG13679	-0.729379579623002	1	1	pseudobulk	DESeq2	LRT	0
992	KC_AB2	CG13685	1.99339641594643	0.465071165814147	1	pseudobulk	DESeq2	LRT	0.332480585700252
992	KC_AB2	CG13689	-1.50128259374009	0.482047325167343	1	pseudobulk	DESeq2	LRT	0.316910322653065
992	KC_AB2	CG1371	0.0492326208273354	0.937631563904513	1	pseudobulk	DESeq2	LRT	0.027967781233381
992	KC_AB2	CG13737	-0.115183097143228	0.910399790291748	1	pseudobulk	DESeq2	LRT	0.040767850987610
992	KC_AB2	CG13739	0.187274644654336	0.433670547174665	1	pseudobulk	DESeq2	LRT	0.362840072049688
992	KC_AB2	CG13743	1.10706867860301	1	1	pseudobulk	DESeq2	LRT	0
992	KC_AB2	CG13751	-0.356435333713412	0.722229730296956	1	pseudobulk	DESeq2	LRT	0.141324637983193
992	KC_AB2	CG13760	-0.572541005784988	0.666802454731832	1	pseudobulk	DESeq2	LRT	0.176002810052001
992	KC_AB2	CG13766	-1.08912079905666	0.411013228084275	1	pseudobulk	DESeq2	LRT	0.386144200528897
992	KC_AB2	CG13773	0.403294498144138	0.89479244414645	1	pseudobulk	DESeq2	LRT	0.048277691852293
993	KC_AB2	CG13784	-0.284427682619786	0.509840309034312	1	pseudobulk	DESeq2	LRT	0.292565831285103

993	KC_AB2	CG13796	-0.729379579623002	0.823527397767669	1	pseudobulk	DESeq2	LRT	0.084321947797948
993	KC_AB2	CG13807	-2.78159444941183	0.131398655790444	1	pseudobulk	DESeq2	LRT	0.881409077590385
993	KC_AB2	CG13827	-0.672424505335587	0.790771522020703	1	pseudobulk	DESeq2	LRT	0.101948979282445
993	KC_AB2	CG13850	-0.137776128546849	0.933125921491185	1	pseudobulk	DESeq2	LRT	0.030059746051232
993	KC_AB2	CG13868	0.710332255153969	0.0638129928607421	1	pseudobulk	DESeq2	LRT	1.1950908862752
993	KC_AB2	CG13876	0.720557559963523	0.758319263499194	1	pseudobulk	DESeq2	LRT	0.120147911539297
993	KC_AB2	CG13887	-1.03963563652602	0.258763231542074	1	pseudobulk	DESeq2	LRT	0.58709743384888
993	KC_AB2	CG13893	-1.38624700933171	0.495854866941722	1	pseudobulk	DESeq2	LRT	0.304645419698777
993	KC_AB2	CG13895	-0.432213394558105	0.865061123889812	1	pseudobulk	DESeq2	LRT	0.062953204870221
994	KC_AB2	CG13896	0.388447834546628	0.84487503289525	1	pseudobulk	DESeq2	LRT	0.073207523639173
994	KC_AB2	CG13901	-0.176134029389346	0.905414860151646	1	pseudobulk	DESeq2	LRT	0.043152381909576
994	KC_AB2	CG13907	0.500961740139739	0.69305969424905	1	pseudobulk	DESeq2	LRT	0.159229357355787
994	KC_AB2	CG13917	0.0391578108957072	0.895161194063282	1	pseudobulk	DESeq2	LRT	0.048098753076529
994	KC_AB2	CG13919	1.6633702262396	0.376942695982191	1	pseudobulk	DESeq2	LRT	0.423724667593454
994	KC_AB2	CG13920	0.0482378918551103	0.905250337046487	1	pseudobulk	DESeq2	LRT	0.043231304812633
994	KC_AB2	CG13921	-0.660543756312884	0.280360873045493	1	pseudobulk	DESeq2	LRT	0.552282596290684
994	KC_AB2	CG13926	-0.786788685384813	0.571390337399051	1	pseudobulk	DESeq2	LRT	0.243067108107554
994	KC_AB2	CG13928	0.536870973619152	0.144292482631624	1	pseudobulk	DESeq2	LRT	0.84075629424709
994	KC_AB2	CG13933	0.140480931853221	0.905093203795789	1	pseudobulk	DESeq2	LRT	0.043306696142878
995	KC_AB2	CG13937	-1.22541146518754	0.451155261903437	1	pseudobulk	DESeq2	LRT	0.345673973013598
995	KC_AB2	CG13954	0.425113031076896	0.411678916349361	1	pseudobulk	DESeq2	LRT	0.385441374313169
995	KC_AB2	CG13982	0.145321756433347	1	1	pseudobulk	DESeq2	LRT	0
995	KC_AB2	CG13994	0.28281488118536	0.84926261051605	1	pseudobulk	DESeq2	LRT	0.070957995665619
995	KC_AB2	CG13995	0.107051933064737	0.915300717187672	1	pseudobulk	DESeq2	LRT	0.038436197344555
995	KC_AB2	CG13999	-0.122950787951818	0.8306704623542	1	pseudobulk	DESeq2	LRT	0.080571232251329
995	KC_AB2	CG14005	-0.690174510442813	0.72579365052962	1	pseudobulk	DESeq2	LRT	0.139186835463116
995	KC_AB2	CG14015	-0.533556358262089	0.731803920350555	1	pseudobulk	DESeq2	LRT	0.135605268281971
995	KC_AB2	CG14024	-0.592553806576769	0.39542108472416	1	pseudobulk	DESeq2	LRT	0.402940176865503
995	KC_AB2	CG14043	1.07688429474991	0.512450546968336	1	pseudobulk	DESeq2	LRT	0.290348038984478
996	KC_AB2	CG14057	1.61205113368698	0.631647504209	1	pseudobulk	DESeq2	LRT	0.199525215522471
996	KC_AB2	CG1407	-0.442404358903885	0.770176697825333	1	pseudobulk	DESeq2	LRT	0.113409625363984
996	KC_AB2	CG14073	0.362394264807245	0.437911708627851	1	pseudobulk	DESeq2	LRT	0.358613442740083
996	KC_AB2	CG14074	-1.27615292748028	0.43417368673181	1	pseudobulk	DESeq2	LRT	0.362336500673076
996	KC_AB2	CG14082	-2.706489000177	0.06421351377199	1	pseudobulk	DESeq2	LRT	1.19237356478163
996	KC_AB2	CG14086	2.7605424445074	0.237810191029118	1	pseudobulk	DESeq2	LRT	0.623769538224996
996	KC_AB2	CG14100	-1.5033370644665	0.499350065149774	1	pseudobulk	DESeq2	LRT	0.30159488912562
996	KC_AB2	CG14102	-1.29989014053669	0.524724068065777	1	pseudobulk	DESeq2	LRT	0.280069015107107
996	KC_AB2	CG14104	-0.502610851884822	0.843360912667957	1	pseudobulk	DESeq2	LRT	0.073986531144244
996	KC_AB2	CG14130	-2.69831193693389	0.203295284067868	1	pseudobulk	DESeq2	LRT	0.691872695769522
997	KC_AB2	CG14135	-1.19881828456548	0.535965909404053	1	pseudobulk	DESeq2	LRT	0.270862833121686
997	KC_AB2	CG14141	1.10706867860301	1	1	pseudobulk	DESeq2	LRT	0
997	KC_AB2	CG14142	-0.729379579623002	0.772694994316916	1	pseudobulk	DESeq2	LRT	0.1119919011982
997	KC_AB2	CG1416	0.243435487534524	0.77318551779708	1	pseudobulk	DESeq2	LRT	0.111716289149068
997	KC_AB2	CG14174	-0.729379579623002	1	1	pseudobulk	DESeq2	LRT	0
997	KC_AB2	CG1418	0.538910257240981	0.732798465306709	1	pseudobulk	DESeq2	LRT	0.135015448878314
997	KC_AB2	CG14182	-0.753475542599712	0.653209841727513	1	pseudobulk	DESeq2	LRT	0.18494728049423
997	KC_AB2	CG14183	-2.28054226263626	0.331630256012328	1	pseudobulk	DESeq2	LRT	0.479345853816062
997	KC_AB2	CG14184	-0.225690218523003	0.785504954858395	1	pseudobulk	DESeq2	LRT	0.104851071144857
997	KC_AB2	CG14186	-0.187518751222279	0.608015399166879	1	pseudobulk	DESeq2	LRT	0.216085421239584
998	KC_AB2	CG14194	0.294165444687412	0.794584536694726	1	pseudobulk	DESeq2	LRT	0.099859890946271
998	KC_AB2	CG14200	0.58086962615023	0.522398809192065	1	pseudobulk	DESeq2	LRT	0.281997821706
998	KC_AB2	CG14207	-0.381517504837496	0.616829916736895	1	pseudobulk	DESeq2	LRT	0.209834570821776

998	KC_AB2	CG14210	1.67808859760333	0.523397782439413	1	pseudobulk	DESeq2	LRT	0.28116812173504
998	KC_AB2	CG14220	0.145321756433347	1	1	pseudobulk	DESeq2	LRT	0
998	KC_AB2	CG14229	0.771447503907791	0.452424052923823	1	pseudobulk	DESeq2	LRT	0.34445431405956
998	KC_AB2	CG14230	1.38446765109058	0.642794900879723	1	pseudobulk	DESeq2	LRT	0.19192757703716
998	KC_AB2	CG14231	1.61205113368698	0.631647504209	1	pseudobulk	DESeq2	LRT	0.199525215522471
998	KC_AB2	CG14232	0.581429473954394	0.651864853931615	1	pseudobulk	DESeq2	LRT	0.18584243383177
998	KC_AB2	CG14234	0.82716146708627	0.311327915795729	1	pseudobulk	DESeq2	LRT	0.50678193575024
999	KC_AB2	CG14252	-0.115183097143228	0.910399790291748	1	pseudobulk	DESeq2	LRT	0.04076785098761
999	KC_AB2	CG14270	-1.00986291845828	0.52844279420318	1	pseudobulk	DESeq2	LRT	0.27700201972188
999	KC_AB2	CG14304	1.99339641594643	0.465071165814147	1	pseudobulk	DESeq2	LRT	0.332480585700252
999	KC_AB2	CG14312	0.313371088736037	0.623204382926995	1	pseudobulk	DESeq2	LRT	0.20536950098004
999	KC_AB2	CG14314	-0.163037718133371	0.861101562546744	1	pseudobulk	DESeq2	LRT	0.06494562270156
999	KC_AB2	CG14321	0.0820304947243216	0.901292650940666	1	pseudobulk	DESeq2	LRT	0.04513417011728
999	KC_AB2	CG14322	0.145321756433347	1	1	pseudobulk	DESeq2	LRT	0
999	KC_AB2	CG1434	1.10706867860301	1	1	pseudobulk	DESeq2	LRT	0
999	KC_AB2	CG14341	-0.276223388832025	0.775422855394474	1	pseudobulk	DESeq2	LRT	0.11046140241906
999	KC_AB2	CG14352	-1.38764087950137	0.516647656322572	1	pseudobulk	DESeq2	LRT	0.28680553636762
100	KC_AB2	CG14367	1.99339641594643	0.465071165814147	1	pseudobulk	DESeq2	LRT	0.332480585700252
100	KC_AB2	CG1440	0.582580151882302	0.684404612100314	1	pseudobulk	DESeq2	LRT	0.16468707247552
100	KC_AB2	CG14407	-1.19070629571991	0.0850557026526872	1	pseudobulk	DESeq2	LRT	1.07029656332633
100	KC_AB2	CG14408	0.325523811952154	0.473695913582803	1	pseudobulk	DESeq2	LRT	0.32450036177002
100	KC_AB2	CG14410	-0.729379579623002	0.823527397767669	1	pseudobulk	DESeq2	LRT	0.08432194779794
100	KC_AB2	CG14411	-0.796856898494309	0.43690249346359	1	pseudobulk	DESeq2	LRT	0.35961547670405
100	KC_AB2	CG14414	-0.798758533794184	0.314202631768941	1	pseudobulk	DESeq2	LRT	0.502790181619591
100	KC_AB2	CG14431	-1.66666907493776	0.0705869228962428	1	pseudobulk	DESeq2	LRT	1.15127574994041
100	KC_AB2	CG14434	0.967710497961774	0.46704736379021	1	pseudobulk	DESeq2	LRT	0.33063907491635
100	KC_AB2	CG14435	0.0816514008233141	0.913095616862885	1	pseudobulk	DESeq2	LRT	0.03948374196009
100	KC_AB2	CG14440	-0.608825130214916	0.402007319578046	1	pseudobulk	DESeq2	LRT	0.39576603939459
100	KC_AB2	CG14442	-0.52945469738486	0.408280201113604	1	pseudobulk	DESeq2	LRT	0.38904167997314
100	KC_AB2	CG14450	-0.881925904751173	0.620461367982025	1	pseudobulk	DESeq2	LRT	0.20728525396305
100	KC_AB2	CG14464	0.357659774159018	0.453139484152	1	pseudobulk	DESeq2	LRT	0.34376809407278
100	KC_AB2	CG14478	0.105596153208191	0.793465621316337	1	pseudobulk	DESeq2	LRT	0.10047188528727
100	KC_AB2	CG14483	0.530451694308181	0.616543626868794	1	pseudobulk	DESeq2	LRT	0.210036187133101
100	KC_AB2	CG14502	-0.68407403394891	0.724748624101311	1	pseudobulk	DESeq2	LRT	0.13981260045834
100	KC_AB2	CG14511	-0.729379579623002	1	1	pseudobulk	DESeq2	LRT	0
100	KC_AB2	CG14512	0.532937724813817	0.83094555443934	1	pseudobulk	DESeq2	LRT	0.08042743130757
100	KC_AB2	CG14535	-0.523932678074134	0.727325231237361	1	pseudobulk	DESeq2	LRT	0.13827134629754
100	KC_AB2	CG14543	-0.346264706712265	0.818049737766862	1	pseudobulk	DESeq2	LRT	0.08722029023942
100	KC_AB2	CG14544	1.38446765109058	0.642794900879723	1	pseudobulk	DESeq2	LRT	0.19192757703716
100	KC_AB2	CG14561	-2.82805375419762	0.203719330373857	1	pseudobulk	DESeq2	LRT	0.69096776002097
100	KC_AB2	CG14562	-0.14366556261111	0.880823595816413	1	pseudobulk	DESeq2	LRT	0.05511105984510
100	KC_AB2	CG14563	1.25392847690597	0.375812985539373	1	pseudobulk	DESeq2	LRT	0.42502821771430
100	KC_AB2	CG14591	0.646072526572719	0.866480302386618	1	pseudobulk	DESeq2	LRT	0.06224130561253
100	KC_AB2	CG14613	-0.53822110192213	0.740417532237098	1	pseudobulk	DESeq2	LRT	0.13052330620265
100	KC_AB2	CG14618	-0.470100723599697	0.833985291815347	1	pseudobulk	DESeq2	LRT	0.07884160852264
100	KC_AB2	CG14621	0.165517037562896	0.885235583659123	1	pseudobulk	DESeq2	LRT	0.052941137134971
100	KC_AB2	CG1463	-0.816874200745502	0.668198591964671	1	pseudobulk	DESeq2	LRT	0.17509444384697
100	KC_AB2	CG14641	-0.169185231818752	0.829740477781052	1	pseudobulk	DESeq2	LRT	0.08105772291559
100	KC_AB2	CG14646	-0.19215578423827	0.90837035950385	1	pseudobulk	DESeq2	LRT	0.04173704542757
100	KC_AB2	CG14647	-1.03568753816011	0.557792821075362	1	pseudobulk	DESeq2	LRT	0.25352707950600
100	KC_AB2	CG14650	0.0656041073316128	0.955453378559894	1	pseudobulk	DESeq2	LRT	0.01979049953325
100	KC_AB2	CG14657	-0.0827565535052576	0.921775711104865	1	pseudobulk	DESeq2	LRT	0.03537473976986

10(	KC_AB2	CG14667	0.729840118778791	0.723180990776418	1	pseudobulk	DESeq2	LRT	0.140752998060082
10(	KC_AB2	CG14669	-0.329190731009387	0.758648315540928	1	pseudobulk	DESeq2	LRT	0.119959502122774
10(	KC_AB2	CG14671	-2.02328388573499	0.328513671102738	1	pseudobulk	DESeq2	LRT	0.483446552554919
10(	KC_AB2	CG14683	-2.24039602753331	0.186451734675393	1	pseudobulk	DESeq2	LRT	0.729433571774669
10(	KC_AB2	CG14688	1.10706867860301	1	1	pseudobulk	DESeq2	LRT	0
10(	KC_AB2	CG14701	-0.729379579623002	0.823527397767669	1	pseudobulk	DESeq2	LRT	0.084321947797948
10(	KC_AB2	CG14710	0.843244047257989	0.721056826075985	1	pseudobulk	DESeq2	LRT	0.142030507432132
10(	KC_AB2	CG14711	-0.051768802237555	0.953597974616862	1	pseudobulk	DESeq2	LRT	0.020634680017486
10(	KC_AB2	CG14712	0.38605638660934	0.863777890371283	1	pseudobulk	DESeq2	LRT	0.063597916533759
10(	KC_AB2	CG14715	-0.121671596731652	0.812403437130018	1	pseudobulk	DESeq2	LRT	0.090228247843002
10(	KC_AB2	CG14721	-0.808528166986178	0.528167573134558	1	pseudobulk	DESeq2	LRT	0.277228265834557
10(	KC_AB2	CG14722	-0.47813475921538	0.796890486478401	1	pseudobulk	DESeq2	LRT	0.098601357883741
10(	KC_AB2	CG14757	-1.3058424967125	0.239425935089313	1	pseudobulk	DESeq2	LRT	0.620828807747011
10(	KC_AB2	CG14762	-0.172036390110867	0.533563146375011	1	pseudobulk	DESeq2	LRT	0.272814175116074
10(	KC_AB2	CG14764	0.0578795279300234	0.930587394654395	1	pseudobulk	DESeq2	LRT	0.031242834533372
10(	KC_AB2	CG14767	-0.551185442599474	0.277077435883316	1	pseudobulk	DESeq2	LRT	0.557398840043714
10(	KC_AB2	CG14777	-0.0772664897366678	0.936338135993574	1	pseudobulk	DESeq2	LRT	0.028567287931585
10(	KC_AB2	CG14780	1.10706867860301	1	1	pseudobulk	DESeq2	LRT	0
10(	KC_AB2	CG14785	1.38069928703004	0.566368801250645	1	pseudobulk	DESeq2	LRT	0.246900678019328
10(	KC_AB2	CG14797	0.737139165475597	0.741715516711887	1	pseudobulk	DESeq2	LRT	0.129762635438979
10(	KC_AB2	CG14805	2.17096815696177	0.180850076469475	1	pseudobulk	DESeq2	LRT	0.742681303268057
10(	KC_AB2	CG14806	-0.542076197751696	0.534749196610173	1	pseudobulk	DESeq2	LRT	0.27184985922587
10(	KC_AB2	CG14811	-0.795404628052927	0.767318925015269	1	pseudobulk	DESeq2	LRT	0.115024090306574
10(	KC_AB2	CG14812	-0.171828911750878	0.848677637080618	1	pseudobulk	DESeq2	LRT	0.071257241467089
10(	KC_AB2	CG14817	-0.376183671907339	0.636956362294356	1	pseudobulk	DESeq2	LRT	0.195890320041312
10(	KC_AB2	CG14818	-0.394522600788446	0.437892604443711	1	pseudobulk	DESeq2	LRT	0.358632389533632
10(	KC_AB2	CG14834	0.38605638660934	0.863777890371283	1	pseudobulk	DESeq2	LRT	0.063597916533759
10(	KC_AB2	CG14853	-0.0327993000332368	0.945649517664533	1	pseudobulk	DESeq2	LRT	0.024269794621230
10(	KC_AB2	CG1486	-0.31282836821297	0.770955795006068	1	pseudobulk	DESeq2	LRT	0.112970522773055
10(	KC_AB2	CG14868	-0.0407677829300683	0.966280556614589	1	pseudobulk	DESeq2	LRT	0.014896759195975
10(	KC_AB2	CG14879	1.10706867860301	1	1	pseudobulk	DESeq2	LRT	0
10(	KC_AB2	CG14881	0.700413062980526	0.686018079735201	1	pseudobulk	DESeq2	LRT	0.163664438483097
10(	KC_AB2	CG14882	-0.0966389603663488	0.917681995065894	1	pseudobulk	DESeq2	LRT	0.037307789076129
10(	KC_AB2	CG14883	-0.912725601732069	0.520455874213664	1	pseudobulk	DESeq2	LRT	0.28361608536164
10(	KC_AB2	CG14894	0.205617634492819	0.81599478672436	1	pseudobulk	DESeq2	LRT	0.088312615883486
10(	KC_AB2	CG14903	-0.155094299622426	0.844020579850802	1	pseudobulk	DESeq2	LRT	0.073646963792975
10(	KC_AB2	CG14906	-0.729379579623002	0.823527397767669	1	pseudobulk	DESeq2	LRT	0.084321947797948
10(	KC_AB2	CG1492	-0.242904908965546	0.933507439612501	1	pseudobulk	DESeq2	LRT	0.029882216579625
10(	KC_AB2	CG14937	-0.502610851884822	0.843360912667957	1	pseudobulk	DESeq2	LRT	0.073986531144244
10(	KC_AB2	CG14966	-2.4120103603151	0.297158744475551	1	pseudobulk	DESeq2	LRT	0.527011485255674
10(	KC_AB2	CG14969	0.651827642906759	0.682475183689276	1	pseudobulk	DESeq2	LRT	0.16591313591029
10(	KC_AB2	CG14971	0.802422437972137	0.703555366906532	1	pseudobulk	DESeq2	LRT	0.152701719699286
10(	KC_AB2	CG14977	-0.429791665455161	0.703176157050731	1	pseudobulk	DESeq2	LRT	0.152935863527077
10(	KC_AB2	CG14982	-1.03170130301494	0.164452126899944	1	pseudobulk	DESeq2	LRT	0.783960505307285
10(	KC_AB2	CG14995	0.662293293205564	0.591698536638416	1	pseudobulk	DESeq2	LRT	0.227899504798781
10(	KC_AB2	CG15011	1.38069928703004	0.566368801250645	1	pseudobulk	DESeq2	LRT	0.246900678019328
10(	KC_AB2	CG15012	-0.254612306810776	0.706119645223089	1	pseudobulk	DESeq2	LRT	0.151121705665256
10(	KC_AB2	CG15014	0.182390943420167	0.910204700850011	1	pseudobulk	DESeq2	LRT	0.040860925863301
10(	KC_AB2	CG15019	0.127989839589053	0.925831082834506	1	pseudobulk	DESeq2	LRT	0.033468242783167
10(	KC_AB2	CG15027	1.71107910732564	0.330424909656551	1	pseudobulk	DESeq2	LRT	0.480927220072587
10(	KC_AB2	CG15040	1.38446765109058	0.642794900879723	1	pseudobulk	DESeq2	LRT	0.191927577037166
10(	KC_AB2	CG15047	2.33862076287154	0.37727071943742	1	pseudobulk	DESeq2	LRT	0.423346899755834

100	KC_AB2	CG15073	1.02928733579242	0.697125366781187	1	pseudobulk	DESeq2	LRT	0.156689114003336
100	KC_AB2	CG15084	2.7605424445074	0.237810191029118	1	pseudobulk	DESeq2	LRT	0.623769538224996
100	KC_AB2	CG15097	-0.118744032512272	0.867556744732751	1	pseudobulk	DESeq2	LRT	0.061702109484242
100	KC_AB2	CG15098	1.23680723027495	0.298053451423188	1	pseudobulk	DESeq2	LRT	0.525705844727419
100	KC_AB2	CG15099	-0.619271096989902	0.620925632671823	1	pseudobulk	DESeq2	LRT	0.206960411505038
100	KC_AB2	CG15107	0.146930209264112	1	1	pseudobulk	DESeq2	LRT	0
100	KC_AB2	CG15111	-0.653079383968656	0.629688236051715	1	pseudobulk	DESeq2	LRT	0.20087442018936
100	KC_AB2	CG15117	-0.729379579623002	0.772694994316916	1	pseudobulk	DESeq2	LRT	0.1119919011982
100	KC_AB2	CG15118	0.233975265394779	0.832979109160646	1	pseudobulk	DESeq2	LRT	0.079365890417971
100	KC_AB2	CG1513	-0.0212676530706411	0.960564198119556	1	pseudobulk	DESeq2	LRT	0.017473604298009
100	KC_AB2	CG15141	-0.0204111639550705	0.923091270985538	1	pseudobulk	DESeq2	LRT	0.034755355826008
100	KC_AB2	CG15160	-0.735336443051548	0.509759314812578	1	pseudobulk	DESeq2	LRT	0.29263482963076
100	KC_AB2	CG15168	0.638025866424136	0.516648513993177	1	pseudobulk	DESeq2	LRT	0.286804815409546
101	KC_AB2	CG15201	3.47260925290815	0.101409732776213	1	pseudobulk	DESeq2	LRT	0.993920361687507
101	KC_AB2	CG1523	-0.075926582162442	0.914355817015571	1	pseudobulk	DESeq2	LRT	0.038884767837982
101	KC_AB2	CG15236	-2.03522979197945	0.396502959232805	1	pseudobulk	DESeq2	LRT	0.401753567050837
101	KC_AB2	CG15237	0.458071527844498	0.641887090650337	1	pseudobulk	DESeq2	LRT	0.192541358566697
101	KC_AB2	CG15270	-0.143246576575619	0.838400415012357	1	pseudobulk	DESeq2	LRT	0.076548515382376
101	KC_AB2	CG15309	0.164207125045112	0.849250193734677	1	pseudobulk	DESeq2	LRT	0.070964345384761
101	KC_AB2	CG15312	0.661678460170035	0.878003361430606	1	pseudobulk	DESeq2	LRT	0.056503821396894
101	KC_AB2	CG15317	1.37221885443763	0.558734269457382	1	pseudobulk	DESeq2	LRT	0.252794690766539
101	KC_AB2	CG1532	0.236952465879932	0.881832493036225	1	pseudobulk	DESeq2	LRT	0.054613902690072
101	KC_AB2	CG15332	1.10706867860301	1	1	pseudobulk	DESeq2	LRT	0
101	KC_AB2	CG15356	-0.349531820170131	0.784598116065649	1	pseudobulk	DESeq2	LRT	0.105352739009536
101	KC_AB2	CG15365	-2.30556784634639	0.359761931587247	1	pseudobulk	DESeq2	LRT	0.443984793676075
101	KC_AB2	CG15385	-1.85990224091138	0.176997186577609	1	pseudobulk	DESeq2	LRT	0.752033636821981
101	KC_AB2	CG15387	-0.938147182841625	0.609043095688634	1	pseudobulk	DESeq2	LRT	0.21535197574549
101	KC_AB2	CG15390	-0.620563475958957	0.7478554969218	1	pseudobulk	DESeq2	LRT	0.126182309825559
101	KC_AB2	CG15399	1.38069928703004	0.566368801250645	1	pseudobulk	DESeq2	LRT	0.246900678019328
101	KC_AB2	CG1542	-0.678142266099058	0.711549586694344	1	pseudobulk	DESeq2	LRT	0.147794829272831
101	KC_AB2	CG15431	-0.352330182346172	0.532930526306023	1	pseudobulk	DESeq2	LRT	0.273329402621616
101	KC_AB2	CG15432	0.00660049267025280	0.909395525502645	1	pseudobulk	DESeq2	LRT	0.041247186984100
101	KC_AB2	CG15435	1.10706867860301	1	1	pseudobulk	DESeq2	LRT	0
101	KC_AB2	CG15439	1.38069928703004	0.566368801250645	1	pseudobulk	DESeq2	LRT	0.246900678019328
101	KC_AB2	CG15443	0.676006581667494	0.697279153755715	1	pseudobulk	DESeq2	LRT	0.156593318509685
101	KC_AB2	CG15445	1.38069928703004	0.566368801250645	1	pseudobulk	DESeq2	LRT	0.246900678019328
101	KC_AB2	CG15449	1.56534206539159	0.237770553199951	1	pseudobulk	DESeq2	LRT	0.623841931780582
101	KC_AB2	CG15456	-0.338305114541556	0.779824974316307	1	pseudobulk	DESeq2	LRT	0.10800286040979
101	KC_AB2	CG15459	-0.670541955584215	0.735328220685952	1	pseudobulk	DESeq2	LRT	0.133518766201526
101	KC_AB2	CG15514	-0.929774044730819	0.553641242794361	1	pseudobulk	DESeq2	LRT	0.256771565135755
101	KC_AB2	CG1552	1.95432904991983	0.509212778150152	1	pseudobulk	DESeq2	LRT	0.293100706726022
101	KC_AB2	CG15525	-1.46127416469959	0.407826953327994	1	pseudobulk	DESeq2	LRT	0.389524075039504
101	KC_AB2	CG15535	0.222467809157701	0.815668692004814	1	pseudobulk	DESeq2	LRT	0.088486206995879
101	KC_AB2	CG15536	-0.672007877941243	0.713146579991023	1	pseudobulk	DESeq2	LRT	0.146821196180962
101	KC_AB2	CG15544	-0.729379579623002	1	1	pseudobulk	DESeq2	LRT	0
101	KC_AB2	CG15547	0.403294498144138	0.89479244414645	1	pseudobulk	DESeq2	LRT	0.048277691852293
101	KC_AB2	CG15561	0.182390943420167	0.910204700850011	1	pseudobulk	DESeq2	LRT	0.040860925863301
101	KC_AB2	CG15577	1.10706867860301	1	1	pseudobulk	DESeq2	LRT	0
101	KC_AB2	CG15579	-1.33407500886303	0.63128114564055	1	pseudobulk	DESeq2	LRT	0.19977718147345
101	KC_AB2	CG15601	-1.30325400719404	0.631049090267552	1	pseudobulk	DESeq2	LRT	0.199936855014109
101	KC_AB2	CG15602	-0.787646498937377	0.588254633591863	1	pseudobulk	DESeq2	LRT	0.230434643267195
101	KC_AB2	CG15614	0.146930209264112	1	1	pseudobulk	DESeq2	LRT	0

101	KC_AB2	CG15628	-0.128898944915801	0.758952834030879	1	pseudobulk	DESeq2	LRT	0.119785212980156
101	KC_AB2	CG15631	1.10706867860301	1	1	pseudobulk	DESeq2	LRT	0
101	KC_AB2	CG15642	-1.86026162883275	0.308151899369447	1	pseudobulk	DESeq2	LRT	0.511235151045011
101	KC_AB2	CG15643	-0.427692090780194	0.83957454067488	1	pseudobulk	DESeq2	LRT	0.075940739470360
101	KC_AB2	CG15651	-0.398122703741194	0.67413105678199	1	pseudobulk	DESeq2	LRT	0.171255664734295
101	KC_AB2	CG15653	0.306101013060283	0.832815272420834	1	pseudobulk	DESeq2	LRT	0.079451319199637
101	KC_AB2	CG15715	0.462593909757001	0.619102741323812	1	pseudobulk	DESeq2	LRT	0.208237272971155
101	KC_AB2	CG15719	-0.729379579623002	1	1	pseudobulk	DESeq2	LRT	0
101	KC_AB2	CG1572	1.38446765109058	0.642794900879723	1	pseudobulk	DESeq2	LRT	0.191927577037166
101	KC_AB2	CG15743	0.26403132383208	0.833218175111959	1	pseudobulk	DESeq2	LRT	0.079241265290852
101	KC_AB2	CG15747	-0.755185876061967	0.58336111309029	1	pseudobulk	DESeq2	LRT	0.234062524362697
101	KC_AB2	CG1575	0.375534485637874	0.901061951722844	1	pseudobulk	DESeq2	LRT	0.045245348458862
101	KC_AB2	CG15760	0.582819499765789	0.617924078267374	1	pseudobulk	DESeq2	LRT	0.209064881569829
101	KC_AB2	CG15765	-0.312978081339991	0.258592592212467	1	pseudobulk	DESeq2	LRT	0.587383920319232
101	KC_AB2	CG15771	-0.0364622849090942	0.960330548112701	1	pseudobulk	DESeq2	LRT	0.017579256009925
101	KC_AB2	CG15772	-0.318631595810384	0.581462502467981	1	pseudobulk	DESeq2	LRT	0.235478286948129
101	KC_AB2	CG1578	-0.101247750011288	0.852636138643853	1	pseudobulk	DESeq2	LRT	0.069236263899776
101	KC_AB2	CG15803	1.21437748278777	0.134049295311702	1	pseudobulk	DESeq2	LRT	0.872735464732528
101	KC_AB2	CG15812	0.474396347993209	0.808657101852946	1	pseudobulk	DESeq2	LRT	0.092235595003270
101	KC_AB2	CG15814	-0.161915895016358	0.880887200173539	1	pseudobulk	DESeq2	LRT	0.055079700530742
101	KC_AB2	CG1582	2.31968409897139	0.349905511974677	1	pseudobulk	DESeq2	LRT	0.456049216130035
101	KC_AB2	CG15864	-0.672424505335587	0.790771522020703	1	pseudobulk	DESeq2	LRT	0.101948979282445
101	KC_AB2	CG15877	1.94531887231797	0.378646728337548	1	pseudobulk	DESeq2	LRT	0.421765791304938
101	KC_AB2	CG15891	-0.110417121215155	0.891175612672276	1	pseudobulk	DESeq2	LRT	0.050036706625883
101	KC_AB2	CG15894	0.552334373584163	0.356585070133707	1	pseudobulk	DESeq2	LRT	0.447836844262972
101	KC_AB2	CG15908	1.49784664065061	0.474481812020792	1	pseudobulk	DESeq2	LRT	0.323780430424038
101	KC_AB2	CG15912	0.221486168185573	0.903697778547821	1	pseudobulk	DESeq2	LRT	0.0439767852922
101	KC_AB2	CG15916	0.253628094118105	0.706028580934363	1	pseudobulk	DESeq2	LRT	0.151177717799697
101	KC_AB2	CG15922	-0.522083809197933	0.634863608535027	1	pseudobulk	DESeq2	LRT	0.197319566712687
101	KC_AB2	CG1598	0.204873679635555	0.820203647107746	1	pseudobulk	DESeq2	LRT	0.086078303915582
101	KC_AB2	CG1602	-0.729379579623002	1	1	pseudobulk	DESeq2	LRT	0
101	KC_AB2	CG1603	1.04560394656545	0.638145957880541	1	pseudobulk	DESeq2	LRT	0.195079977309609
101	KC_AB2	CG1607	0.267615943746048	0.707093563155896	1	pseudobulk	DESeq2	LRT	0.150523116225961
101	KC_AB2	CG1620	-0.808528166986178	0.528167573134558	1	pseudobulk	DESeq2	LRT	0.277228265834557
101	KC_AB2	CG1622	0.190487921357337	0.881060239292312	1	pseudobulk	DESeq2	LRT	0.054994397265735
101	KC_AB2	CG1628	-0.729379579623002	0.823527397767669	1	pseudobulk	DESeq2	LRT	0.084321947797948
101	KC_AB2	CG1635	-1.47881549346798	0.326068240365326	1	pseudobulk	DESeq2	LRT	0.486691500199862
101	KC_AB2	CG1636	-0.98705699396297	0.16369117877772	1	pseudobulk	DESeq2	LRT	0.785974723834139
101	KC_AB2	CG1637	0.288900475524326	0.691223296803423	1	pseudobulk	DESeq2	LRT	0.160381632938496
101	KC_AB2	CG1638	-0.729379579623002	0.823527397767669	1	pseudobulk	DESeq2	LRT	0.084321947797948
101	KC_AB2	CG1640	0.559429816251837	0.376057187646141	1	pseudobulk	DESeq2	LRT	0.424746106161145
101	KC_AB2	CG1646	0.27451707947738	0.671056271138019	1	pseudobulk	DESeq2	LRT	0.173241060725085
101	KC_AB2	CG1647	-2.14688448203575	0.212129471698107	1	pseudobulk	DESeq2	LRT	0.67339898963199
101	KC_AB2	CG1648	1.25324204241104	0.113621549773836	1	pseudobulk	DESeq2	LRT	0.944539291333757
101	KC_AB2	CG1662	1.54283623725204	0.409626837565336	1	pseudobulk	DESeq2	LRT	0.387611597382053
101	KC_AB2	CG1663	0.145321756433347	1	1	pseudobulk	DESeq2	LRT	0
101	KC_AB2	CG1671	-1.33407500886303	0.63128114564055	1	pseudobulk	DESeq2	LRT	0.19977718147345
101	KC_AB2	CG16711	-0.247908842622736	0.5629254012423	1	pseudobulk	DESeq2	LRT	0.249549153950444
101	KC_AB2	CG16717	0.0554216016470706	0.871820024577925	1	pseudobulk	DESeq2	LRT	0.059573160021454
101	KC_AB2	CG16721	0.890849638667045	0.607482269011261	1	pseudobulk	DESeq2	LRT	0.216466393586352
101	KC_AB2	CG16739	1.99339641594643	0.465071165814147	1	pseudobulk	DESeq2	LRT	0.332480585700252
101	KC_AB2	CG16753	0.342015047887936	0.779731611827791	1	pseudobulk	DESeq2	LRT	0.108054858284042

101	KC_AB2	CG1677	0.512460788506192	0.317662586860216	1	pseudobulk	DESeq2	LRT	0.498033931779574
101	KC_AB2	CG16779	0.106368637161582	0.731131718790435	1	pseudobulk	DESeq2	LRT	0.136004374628172
101	KC_AB2	CG16787	1.79603169052098	0.328707702555358	1	pseudobulk	DESeq2	LRT	0.483190119045559
101	KC_AB2	CG16790	1.10706867860301	1	1	pseudobulk	DESeq2	LRT	0
101	KC_AB2	CG16791	-0.10667353619615	0.735991309172815	1	pseudobulk	DESeq2	LRT	0.133127313924151
101	KC_AB2	CG16812	0.0849376620542084	0.928048005568608	1	pseudobulk	DESeq2	LRT	0.032429558248469
101	KC_AB2	CG16854	0.0554216016470706	0.871820024577925	1	pseudobulk	DESeq2	LRT	0.059573160021454
101	KC_AB2	CG16863	1.10706867860301	1	1	pseudobulk	DESeq2	LRT	0
101	KC_AB2	CG16865	-2.45801888341184	0.286003272809243	1	pseudobulk	DESeq2	LRT	0.543628997098713
102	KC_AB2	CG16868	0.0121062175346913	0.937598285304008	1	pseudobulk	DESeq2	LRT	0.027983195570532
102	KC_AB2	CG1688	0.19398841824435	0.776232213387626	1	pseudobulk	DESeq2	LRT	0.11000833814046
102	KC_AB2	CG16890	-0.83320718254958	0.715059964627045	1	pseudobulk	DESeq2	LRT	0.14565753691968
102	KC_AB2	CG16896	0.54125854781789	0.798649923599016	1	pseudobulk	DESeq2	LRT	0.097643545549271
102	KC_AB2	CG16903	-0.212831808289283	0.884127614550083	1	pseudobulk	DESeq2	LRT	0.053485044608074
102	KC_AB2	CG16908	-0.729379579623002	0.823527397767669	1	pseudobulk	DESeq2	LRT	0.084321947797948
102	KC_AB2	CG16935	-2.57262117149999	0.0719319012424261	1	pseudobulk	DESeq2	LRT	1.1430784606476
102	KC_AB2	CG1695	-1.18264855164563	0.17105223372715	1	pseudobulk	DESeq2	LRT	0.766871250101887
102	KC_AB2	CG16952	-0.221860555467093	0.774761902810381	1	pseudobulk	DESeq2	LRT	0.110831742890992
102	KC_AB2	CG16953	0.209706538694255	0.800353615780569	1	pseudobulk	DESeq2	LRT	0.09671808869433
102	KC_AB2	CG16972	1.99339641594643	0.465071165814147	1	pseudobulk	DESeq2	LRT	0.332480585700252
102	KC_AB2	CG16974	-0.04183345482911	0.954369294831676	1	pseudobulk	DESeq2	LRT	0.020283541780802
102	KC_AB2	CG16979	-0.795404628052927	0.767318925015269	1	pseudobulk	DESeq2	LRT	0.115024090306574
102	KC_AB2	CG16985	-0.729379579623002	0.772694994316916	1	pseudobulk	DESeq2	LRT	0.1119919011982
102	KC_AB2	CG16986	-1.27937117069897	0.417728943904111	1	pseudobulk	DESeq2	LRT	0.379105431983276
102	KC_AB2	CG16989	-0.986923023579537	0.558458157332019	1	pseudobulk	DESeq2	LRT	0.253009360991646
102	KC_AB2	CG17002	0.328398766233159	0.820002707666631	1	pseudobulk	DESeq2	LRT	0.086184713564167
102	KC_AB2	CG1701	0.388447834546628	0.84487503289525	1	pseudobulk	DESeq2	LRT	0.073207523639173
102	KC_AB2	CG17019	1.00863904899666	0.497041012870148	1	pseudobulk	DESeq2	LRT	0.303607774388769
102	KC_AB2	CG17026	-0.432213394558105	0.865061123889812	1	pseudobulk	DESeq2	LRT	0.062953204870221
102	KC_AB2	CG17029	2.64389091645132	0.267289646604	1	pseudobulk	DESeq2	LRT	0.573017863203188
102	KC_AB2	CG1703	-0.0584082789679553	0.960381816589906	1	pseudobulk	DESeq2	LRT	0.017556071261224
102	KC_AB2	CG17030	1.95432904991983	0.509212778150152	1	pseudobulk	DESeq2	LRT	0.293100706726022
102	KC_AB2	CG17059	-0.282215522146706	0.621534649056557	1	pseudobulk	DESeq2	LRT	0.206534655478304
102	KC_AB2	CG17065	0.688697455388298	0.427321017389214	1	pseudobulk	DESeq2	LRT	0.369245746284871
102	KC_AB2	CG17068	1.2301645998707	0.540755082270647	1	pseudobulk	DESeq2	LRT	0.266999390165847
102	KC_AB2	CG17075	2.86712222569521	0.016024006136294	1	pseudobulk	DESeq2	LRT	1.79522889740406
102	KC_AB2	CG17078	-0.131343354896782	0.907625001180452	1	pseudobulk	DESeq2	LRT	0.042093549669906
102	KC_AB2	CG17118	0.145321756433347	1	1	pseudobulk	DESeq2	LRT	0
102	KC_AB2	CG17124	0.0191234867358223	0.894391277937526	1	pseudobulk	DESeq2	LRT	0.048472444656749
102	KC_AB2	CG17141	-0.348901974470907	0.89890135131336	1	pseudobulk	DESeq2	LRT	0.046287966699494
102	KC_AB2	CG17162	2.25461030872415	0.305019014551908	1	pseudobulk	DESeq2	LRT	0.515673086366102
102	KC_AB2	CG17163	0.0583948511573435	0.932500490660047	1	pseudobulk	DESeq2	LRT	0.030350931003569
102	KC_AB2	CG17167	-0.729379579623002	0.772694994316916	1	pseudobulk	DESeq2	LRT	0.1119919011982
102	KC_AB2	CG17168	0.255412710100475	0.782590922157591	1	pseudobulk	DESeq2	LRT	0.106465194104303
102	KC_AB2	CG17169	-0.729379579623002	1	1	pseudobulk	DESeq2	LRT	0
102	KC_AB2	CG17186	0.147593560254927	1	1	pseudobulk	DESeq2	LRT	0
102	KC_AB2	CG17187	-1.53462310097022	0.464407033597658	1	pseudobulk	DESeq2	LRT	0.33310121135052
102	KC_AB2	CG17202	-0.378935607292562	0.548125629458388	1	pseudobulk	DESeq2	LRT	0.261119890553013
102	KC_AB2	CG17221	-0.18317077492817	0.899664519927029	1	pseudobulk	DESeq2	LRT	0.045919406456170
102	KC_AB2	CG17230	0.521384630086089	0.694934012485361	1	pseudobulk	DESeq2	LRT	0.158056431919312
102	KC_AB2	CG17233	-0.703285503731457	0.534075156318327	1	pseudobulk	DESeq2	LRT	0.272397623724937
102	KC_AB2	CG17249	0.667244748290888	0.924085151242949	1	pseudobulk	DESeq2	LRT	0.034288008205147

102	KC_AB2	CG17260	-0.721175887303532	0.733409564198845	1	pseudobulk	DESeq2	LRT	0.134653430819787
102	KC_AB2	CG17266	-0.729379579623002	0.823527397767669	1	pseudobulk	DESeq2	LRT	0.084321947797948
102	KC_AB2	CG17270	-0.333020558715455	0.68448341801557	1	pseudobulk	DESeq2	LRT	0.164637068423441
102	KC_AB2	CG17271	0.5818308306584	0.605909308283728	1	pseudobulk	DESeq2	LRT	0.217592375601824
102	KC_AB2	CG17272	-0.327149428496313	0.532929248242393	1	pseudobulk	DESeq2	LRT	0.273330444139451
102	KC_AB2	CG17294	0.132850428664825	0.883788646423664	1	pseudobulk	DESeq2	LRT	0.053651581894820
102	KC_AB2	CG17321	0.180424900477599	0.726802793508488	1	pseudobulk	DESeq2	LRT	0.138583412124887
102	KC_AB2	CG17343	1.65789039649356	0.27077765798482	1	pseudobulk	DESeq2	LRT	0.567387172387916
102	KC_AB2	CG17359	2.88790980540653	0.211468500133428	1	pseudobulk	DESeq2	LRT	0.674754314989032
102	KC_AB2	CG17361	0.381651071578796	0.797969014042512	1	pseudobulk	DESeq2	LRT	0.098013972423237
102	KC_AB2	CG17364	0.404554993103252	0.681237080452057	1	pseudobulk	DESeq2	LRT	0.166701720959829
102	KC_AB2	CG1737	0.266214941495982	0.753507424082627	1	pseudobulk	DESeq2	LRT	0.122912464355011
102	KC_AB2	CG17378	-0.441116621084317	0.610060093063105	1	pseudobulk	DESeq2	LRT	0.214627383349416
102	KC_AB2	CG1738	2.47215486522296	0.30601789350912	1	pseudobulk	DESeq2	LRT	0.514253178665238
102	KC_AB2	CG17385	0.340914340711767	0.644275675526407	1	pseudobulk	DESeq2	LRT	0.190928265028129
102	KC_AB2	CG17454	-0.0358892258255115	0.949305788664965	1	pseudobulk	DESeq2	LRT	0.022593870883031
102	KC_AB2	CG17486	0.553893021573489	0.525455979831208	1	pseudobulk	DESeq2	LRT	0.279463661209805
102	KC_AB2	CG17493	-0.0339138208576165	0.926194661671238	1	pseudobulk	DESeq2	LRT	0.033297726487672
102	KC_AB2	CG1750	0.661678460170035	0.878003361430606	1	pseudobulk	DESeq2	LRT	0.056503821396894
102	KC_AB2	CG17508	-0.271984304724183	0.661638588185287	1	pseudobulk	DESeq2	LRT	0.179379173728745
102	KC_AB2	CG17528	0.74627710730258	0.363751261057987	1	pseudobulk	DESeq2	LRT	0.439195492385209
102	KC_AB2	CG17544	-1.38764087950137	0.516647656322572	1	pseudobulk	DESeq2	LRT	0.286805536367625
102	KC_AB2	CG17556	-0.266776735269636	0.826613001919923	1	pseudobulk	DESeq2	LRT	0.082697767917523
102	KC_AB2	CG17565	0.198188248334986	0.877322320096044	1	pseudobulk	DESeq2	LRT	0.056840821546213
102	KC_AB2	CG17568	2.32149674095703	0.249331547667048	1	pseudobulk	DESeq2	LRT	0.603222767180568
102	KC_AB2	CG17574	-0.193641391041381	0.868231175467069	1	pseudobulk	DESeq2	LRT	0.061364624060278
102	KC_AB2	CG17593	0.757023142398258	0.505730857689513	1	pseudobulk	DESeq2	LRT	0.296080546633575
102	KC_AB2	CG17598	-2.25108031028047	0.14961857016504	1	pseudobulk	DESeq2	LRT	0.825014499922838
102	KC_AB2	CG17600	0.527588587463093	0.723013253311418	1	pseudobulk	DESeq2	LRT	0.140853741727238
102	KC_AB2	CG1764	-0.118879957862716	0.891678855299997	1	pseudobulk	DESeq2	LRT	0.049791531816600
102	KC_AB2	CG17646	2.25688970431185	0.32072335275684	1	pseudobulk	DESeq2	LRT	0.493869416734511
102	KC_AB2	CG17650	1.10706867860301	1	1	pseudobulk	DESeq2	LRT	0
102	KC_AB2	CG17652	0.126408876402088	0.941796133627145	1	pseudobulk	DESeq2	LRT	0.026043096806699
102	KC_AB2	CG17660	0.222105744543024	0.91314726950204	1	pseudobulk	DESeq2	LRT	0.039459175177297
102	KC_AB2	CG17672	1.61205113368698	0.631647504209	1	pseudobulk	DESeq2	LRT	0.199525215522471
102	KC_AB2	CG17683	-0.294102969523811	0.775810645263781	1	pseudobulk	DESeq2	LRT	0.110244265535653
102	KC_AB2	CG17684	0.148864317153272	0.598922704326169	1	pseudobulk	DESeq2	LRT	0.222629223104351
102	KC_AB2	CG17691	-0.859381927637536	0.821881224561477	1	pseudobulk	DESeq2	LRT	0.085190940665171
102	KC_AB2	CG17698	0.369840242225304	0.390905967771315	1	pseudobulk	DESeq2	LRT	0.407927699354482
102	KC_AB2	CG17712	-0.0906239011354687	0.938107922169492	1	pseudobulk	DESeq2	LRT	0.027747196475345
102	KC_AB2	CG17715	0.522757123216195	0.41326631617077	1	pseudobulk	DESeq2	LRT	0.383769991034076
102	KC_AB2	CG17716	-1.17211486980499	0.550804693380036	1	pseudobulk	DESeq2	LRT	0.259002367797164
102	KC_AB2	CG17721	1.38069928703004	0.566368801250645	1	pseudobulk	DESeq2	LRT	0.246900678019328
102	KC_AB2	CG17724	-0.139400834849924	0.815879434087187	1	pseudobulk	DESeq2	LRT	0.088374014014839
102	KC_AB2	CG17726	-0.432213394558105	0.865061123889812	1	pseudobulk	DESeq2	LRT	0.062953204870221
102	KC_AB2	CG17734	-0.529055466750102	0.440692028051109	1	pseudobulk	DESeq2	LRT	0.355864805638771
102	KC_AB2	CG17746	-0.753475542599712	0.653209841727513	1	pseudobulk	DESeq2	LRT	0.18494728049423
102	KC_AB2	CG17754	1.50542607031961	0.439834541648453	1	pseudobulk	DESeq2	LRT	0.356710667066508
102	KC_AB2	CG17760	-0.729379579623002	0.823527397767669	1	pseudobulk	DESeq2	LRT	0.084321947797948
102	KC_AB2	CG17765	0.141673869940369	0.84640058578329	1	pseudobulk	DESeq2	LRT	0.072424044738990
102	KC_AB2	CG17776	0.453288392826674	0.557533950608194	1	pseudobulk	DESeq2	LRT	0.253728681440596
102	KC_AB2	CG17778	-0.163141064113542	0.668907894795217	1	pseudobulk	DESeq2	LRT	0.17463367825014

102	KC_AB2	CG17786	-0.50491169497659	0.835055051214058	1	pseudobulk	DESeq2	LRT	0.078284892599768
102	KC_AB2	CG17802	-0.903092823655932	0.666117165665049	1	pseudobulk	DESeq2	LRT	0.176449374543784
102	KC_AB2	CG17803	0.412266503805257	0.859735858836361	1	pseudobulk	DESeq2	LRT	0.065634958838131
102	KC_AB2	CG17806	-0.470100723599697	0.833985291815347	1	pseudobulk	DESeq2	LRT	0.078841608522644
102	KC_AB2	CG17807	-0.332717163850492	0.865408112046236	1	pseudobulk	DESeq2	LRT	0.062779038186976
103	KC_AB2	CG17816	-0.267479626433177	0.292818777533214	1	pseudobulk	DESeq2	LRT	0.53340107680225
103	KC_AB2	CG17829	-0.893328173497901	0.679679924321693	1	pseudobulk	DESeq2	LRT	0.167695557626761
103	KC_AB2	CG17839	0.31085907316198	0.515073405311878	1	pseudobulk	DESeq2	LRT	0.288130873385723
103	KC_AB2	CG17841	-0.190179187946428	0.51355697728433	1	pseudobulk	DESeq2	LRT	0.289411365995723
103	KC_AB2	CG17883	0.242928481943873	0.673028342412925	1	pseudobulk	DESeq2	LRT	0.171966646482773
103	KC_AB2	CG1789	-0.835873429549362	0.623589417931435	1	pseudobulk	DESeq2	LRT	0.205101263228191
103	KC_AB2	CG17896	1.38446765109058	0.642794900879723	1	pseudobulk	DESeq2	LRT	0.191927577037166
103	KC_AB2	CG17904	-0.182070071591186	0.875731836715799	1	pseudobulk	DESeq2	LRT	0.057628861484897
103	KC_AB2	CG1792	3.765322106338	0.0505474862851322	1	pseudobulk	DESeq2	LRT	1.29630043690069
103	KC_AB2	CG17931	-0.0893243891172594	0.905349515306559	1	pseudobulk	DESeq2	LRT	0.043183726579307
103	KC_AB2	CG17977	-0.31789625112827	0.814908077985632	1	pseudobulk	DESeq2	LRT	0.088891377119025
103	KC_AB2	CG17982	-0.470100723599697	0.833985291815347	1	pseudobulk	DESeq2	LRT	0.078841608522644
103	KC_AB2	CG17985	1.02648689492346	0.73959154128541	1	pseudobulk	DESeq2	LRT	0.131008064514051
103	KC_AB2	CG17996	0.196114241773109	0.865433511748925	1	pseudobulk	DESeq2	LRT	0.062766291843730
103	KC_AB2	CG18004	0.804768576027188	0.673413155402318	1	pseudobulk	DESeq2	LRT	0.171718403744147
103	KC_AB2	CG18011	-1.33407500886303	0.63128114564055	1	pseudobulk	DESeq2	LRT	0.19977718147345
103	KC_AB2	CG18048	-1.00833870937463	0.651152668420623	1	pseudobulk	DESeq2	LRT	0.186317175365195
103	KC_AB2	CG18081	-0.889337947640851	0.66589289368567	1	pseudobulk	DESeq2	LRT	0.176595619811718
103	KC_AB2	CG1812	-0.856567423351035	0.665132979544327	1	pseudobulk	DESeq2	LRT	0.177091517841652
103	KC_AB2	CG18135	-0.0814427507605245	0.928732040439955	1	pseudobulk	DESeq2	LRT	0.032109571415205
103	KC_AB2	CG1814	0.114987842088876	0.847221010641888	1	pseudobulk	DESeq2	LRT	0.072003282488995
103	KC_AB2	CG1815	0.695699391471633	0.614717492322291	1	pseudobulk	DESeq2	LRT	0.21132442847511
103	KC_AB2	CG18178	-1.44959158709828	0.438539757302766	1	pseudobulk	DESeq2	LRT	0.357991028080125
103	KC_AB2	CG1824	0.652135568475544	0.695836151616136	1	pseudobulk	DESeq2	LRT	0.157493011576443
103	KC_AB2	CG18259	-0.203959824699505	0.914908889316588	1	pseudobulk	DESeq2	LRT	0.038622152750168
103	KC_AB2	CG18262	0.147593560254927	1	1	pseudobulk	DESeq2	LRT	0
103	KC_AB2	CG18265	-0.0064039149458334	0.950497845146721	1	pseudobulk	DESeq2	LRT	0.022048863377127
103	KC_AB2	CG1827	1.38446765109058	0.642794900879723	1	pseudobulk	DESeq2	LRT	0.191927577037166
103	KC_AB2	CG18273	1.10706867860301	1	1	pseudobulk	DESeq2	LRT	0
103	KC_AB2	CG18281	-1.78975280219494	0.468855431150075	1	pseudobulk	DESeq2	LRT	0.32896104882553
103	KC_AB2	CG18324	-0.452523470392016	0.67491985790363	1	pseudobulk	DESeq2	LRT	0.170747793593543
103	KC_AB2	CG18327	0.147593560254927	1	1	pseudobulk	DESeq2	LRT	0
103	KC_AB2	CG18335	-1.71999636108657	0.519869997311849	1	pseudobulk	DESeq2	LRT	0.284105245805297
103	KC_AB2	CG18336	-1.52244591594958	0.547176617578731	1	pseudobulk	DESeq2	LRT	0.261872469536296
103	KC_AB2	CG18368	1.10706867860301	1	1	pseudobulk	DESeq2	LRT	0
103	KC_AB2	CG1840	1.35202980410346	0.354653603812669	1	pseudobulk	DESeq2	LRT	0.45019562270956
103	KC_AB2	CG18420	-0.432213394558105	0.865061123889812	1	pseudobulk	DESeq2	LRT	0.062953204870221
103	KC_AB2	CG18428	-0.911927747394975	0.262377024388649	1	pseudobulk	DESeq2	LRT	0.58107419756656
103	KC_AB2	CG18467	-0.352979017506003	0.549200116034461	1	pseudobulk	DESeq2	LRT	0.260269379677961
103	KC_AB2	CG1847	0.183688935492983	0.839364004698953	1	pseudobulk	DESeq2	LRT	0.076049659017556
103	KC_AB2	CG18476	-0.386855787837361	0.81807320393191	1	pseudobulk	DESeq2	LRT	0.087207832464117
103	KC_AB2	CG18507	0.974788993263208	0.52914604093027	1	pseudobulk	DESeq2	LRT	0.276424448915803
103	KC_AB2	CG18508	-0.309763830670367	0.741290112908638	1	pseudobulk	DESeq2	LRT	0.13001179232107
103	KC_AB2	CG18528	-1.71999636108657	0.519869997311849	1	pseudobulk	DESeq2	LRT	0.284105245805297
103	KC_AB2	CG18538	-0.729379579623002	1	1	pseudobulk	DESeq2	LRT	0
103	KC_AB2	CG18549	-0.755295215690878	0.556519839351879	1	pseudobulk	DESeq2	LRT	0.25451934890795
103	KC_AB2	CG18598	-1.33407500886303	0.63128114564055	1	pseudobulk	DESeq2	LRT	0.19977718147345

103	KC_AB2	CG18600	1.61205113368698	0.631647504209	1	pseudobulk	DESeq2	LRT	0.199525215522471
103	KC_AB2	CG18628	2.50905915816151	0.218124909391685	1	pseudobulk	DESeq2	LRT	0.661294736101097
103	KC_AB2	CG18659	-0.501233018705808	0.638205326234444	1	pseudobulk	DESeq2	LRT	0.195039575660252
103	KC_AB2	CG18661	-0.0045035166158998	1	1	pseudobulk	DESeq2	LRT	0
103	KC_AB2	CG18675	-0.729379579623002	1	1	pseudobulk	DESeq2	LRT	0
103	KC_AB2	CG18731	-1.28181745855224	0.349762948376081	1	pseudobulk	DESeq2	LRT	0.456226198768402
103	KC_AB2	CG18764	-1.50128259374009	0.482047325167343	1	pseudobulk	DESeq2	LRT	0.316910322653065
103	KC_AB2	CG18766	-0.355028021774633	0.686001507831792	1	pseudobulk	DESeq2	LRT	0.163674929712625
103	KC_AB2	CG18787	0.372319056733928	0.867976741609407	1	pseudobulk	DESeq2	LRT	0.061491912065472
103	KC_AB2	CG18809	0.278431659889372	0.836181048314113	1	pseudobulk	DESeq2	LRT	0.077699679772777
103	KC_AB2	CG18815	0.0928992400303663	0.821874369233325	1	pseudobulk	DESeq2	LRT	0.085194563139460
103	KC_AB2	CG1882	0.542242771487179	0.637304146316505	1	pseudobulk	DESeq2	LRT	0.195653255980325
103	KC_AB2	CG18870	0.2214068670955	0.897346822679319	1	pseudobulk	DESeq2	LRT	0.047039670596594
103	KC_AB2	CG1888	-0.874142951047079	0.445073426843211	1	pseudobulk	DESeq2	LRT	0.351568334542166
103	KC_AB2	CG1890	-1.52100148215139	0.440665145004373	1	pseudobulk	DESeq2	LRT	0.355891299231187
103	KC_AB2	CG1896	1.51550881085529	0.13030530078883	1	pseudobulk	DESeq2	LRT	0.88503791693121
103	KC_AB2	CG1902	-0.0524336825523165	0.886594274654936	1	pseudobulk	DESeq2	LRT	0.052275077563863
103	KC_AB2	CG1907	1.90941952383975	0.0643692138306113	1	pseudobulk	DESeq2	LRT	1.19132179506728
103	KC_AB2	CG1909	0.0585519927571097	0.860876052734527	1	pseudobulk	DESeq2	LRT	0.065059372923113
103	KC_AB2	CG1910	0.311173067627833	0.627602330416152	1	pseudobulk	DESeq2	LRT	0.202315452457637
103	KC_AB2	CG1924	1.10706867860301	1	1	pseudobulk	DESeq2	LRT	0
103	KC_AB2	CG1927	-0.806621335522765	0.423905085382075	1	pseudobulk	DESeq2	LRT	0.372731373381942
103	KC_AB2	CG1943	-0.27686476960372	0.554037759334674	1	pseudobulk	DESeq2	LRT	0.256460635791666
103	KC_AB2	CG1951	0.506475365647763	0.690261265132365	1	pseudobulk	DESeq2	LRT	0.160986496901808
103	KC_AB2	CG1965	-0.599891491835932	0.362332080112168	1	pseudobulk	DESeq2	LRT	0.440893212752902
103	KC_AB2	CG1983	-1.89786841617053	0.124323350015068	1	pseudobulk	DESeq2	LRT	0.905447295893093
103	KC_AB2	CG1998	0.243240057341611	0.690869338325517	1	pseudobulk	DESeq2	LRT	0.160604081439025
103	KC_AB2	CG2003	-0.729379579623002	1	1	pseudobulk	DESeq2	LRT	0
103	KC_AB2	CG2004	1.61205113368698	0.631647504209	1	pseudobulk	DESeq2	LRT	0.199525215522471
103	KC_AB2	CG2006	-1.52244591594958	0.547176617578731	1	pseudobulk	DESeq2	LRT	0.261872469536296
103	KC_AB2	CG2016	-0.729379579623002	1	1	pseudobulk	DESeq2	LRT	0
103	KC_AB2	CG2017	0.615039434824284	0.468358708100355	1	pseudobulk	DESeq2	LRT	0.329421400607887
103	KC_AB2	CG2021	-0.50538289494301	0.757010667786811	1	pseudobulk	DESeq2	LRT	0.12089800038328
103	KC_AB2	CG2023	-0.470100723599697	0.833985291815347	1	pseudobulk	DESeq2	LRT	0.078841608522644
103	KC_AB2	CG2034	1.94826447887871	0.376549774012609	1	pseudobulk	DESeq2	LRT	0.424177608705598
103	KC_AB2	CG2059	-0.99905311401359	0.49283820666151	1	pseudobulk	DESeq2	LRT	0.307295631405571
103	KC_AB2	CG2061	0.678304986774265	0.489722229203251	1	pseudobulk	DESeq2	LRT	0.310050182277063
103	KC_AB2	CG2063	-0.604591387832362	0.63716123588174	1	pseudobulk	DESeq2	LRT	0.195750654017733
103	KC_AB2	CG2064	0.301183407903318	0.898796313604012	1	pseudobulk	DESeq2	LRT	0.046338717499814
103	KC_AB2	CG2076	-0.106134428961607	0.915782084982458	1	pseudobulk	DESeq2	LRT	0.038207856625490
103	KC_AB2	CG2082	1.99339641594643	0.465071165814147	1	pseudobulk	DESeq2	LRT	0.332480585700252
103	KC_AB2	CG2091	-1.29698133384253	0.323811873749427	1	pseudobulk	DESeq2	LRT	0.489707230291386
103	KC_AB2	CG2100	-0.647621034217714	0.754702560287896	1	pseudobulk	DESeq2	LRT	0.122224176685402
103	KC_AB2	CG2116	-2.39970914040097	0.301766661516198	1	pseudobulk	DESeq2	LRT	0.520328741761584
103	KC_AB2	CG2124	-0.729379579623002	0.823527397767669	1	pseudobulk	DESeq2	LRT	0.084321947797948
103	KC_AB2	CG2126	0.734253571864123	0.751957650179593	1	pseudobulk	DESeq2	LRT	0.123806617933922
103	KC_AB2	CG2129	-0.729379579623002	0.823527397767669	1	pseudobulk	DESeq2	LRT	0.084321947797948
103	KC_AB2	CG2145	-0.729379579623002	1	1	pseudobulk	DESeq2	LRT	0
103	KC_AB2	CG2147	1.53600323097801	0.43223306245546	1	pseudobulk	DESeq2	LRT	0.364282016044215
103	KC_AB2	CG2162	-0.60049492049297	0.573260028874051	1	pseudobulk	DESeq2	LRT	0.241648338784571
103	KC_AB2	CG2182	0.585637995622367	0.415798066637734	1	pseudobulk	DESeq2	LRT	0.381117534366059
103	KC_AB2	CG2186	0.0701951551233316	0.897623985873752	1	pseudobulk	DESeq2	LRT	0.046905550925887

103	KC_AB2	CG2199	-0.134510699892525	0.879995414101603	1	pseudobulk	DESeq2	LRT	0.055519591072056
104	KC_AB2	CG2200	-0.179181196994628	0.898891728962341	1	pseudobulk	DESeq2	LRT	0.046292615659374
104	KC_AB2	CG2201	0.476481310221852	0.754952114852412	1	pseudobulk	DESeq2	LRT	0.122080593953437
104	KC_AB2	CG2202	-0.856567423351035	0.665132979544327	1	pseudobulk	DESeq2	LRT	0.177091517841652
104	KC_AB2	CG2209	-0.729379579623002	0.823527397767669	1	pseudobulk	DESeq2	LRT	0.084321947797948
104	KC_AB2	CG2211	-0.750218004183046	0.684438648478708	1	pseudobulk	DESeq2	LRT	0.164665474953366
104	KC_AB2	CG2218	0.80457744263352	0.646920291795168	1	pseudobulk	DESeq2	LRT	0.18914922623398
104	KC_AB2	CG2224	0.518865066730979	0.817181531186031	1	pseudobulk	DESeq2	LRT	0.087681457255733
104	KC_AB2	CG2225	0.189617988877605	0.699828784293375	1	pseudobulk	DESeq2	LRT	0.155008198745522
104	KC_AB2	CG2246	-0.432244409795114	0.559753660565097	1	pseudobulk	DESeq2	LRT	0.252003057627406
104	KC_AB2	CG2247	-0.440473110398812	0.725342262186638	1	pseudobulk	DESeq2	LRT	0.13945701756137
104	KC_AB2	CG2256	-0.721175887303532	0.733409564198845	1	pseudobulk	DESeq2	LRT	0.134653430819787
104	KC_AB2	CG2258	0.713422668222254	0.441016232838011	1	pseudobulk	DESeq2	LRT	0.355545424812824
104	KC_AB2	CG2260	-0.108150992064891	0.905978176473916	1	pseudobulk	DESeq2	LRT	0.042882263637873
104	KC_AB2	CG2269	-0.439548999267755	0.0752365433275202	1	pseudobulk	DESeq2	LRT	1.1235711659163
104	KC_AB2	CG2277	0.606304442210622	0.645408910759428	1	pseudobulk	DESeq2	LRT	0.190165042856914
104	KC_AB2	CG2278	-0.752669219984623	0.498477286815098	1	pseudobulk	DESeq2	LRT	0.30235462558823
104	KC_AB2	CG2321	0.680883174950485	0.711135058021138	1	pseudobulk	DESeq2	LRT	0.148047910686379
104	KC_AB2	CG2519	-1.29154688524372	0.147461354817115	1	pseudobulk	DESeq2	LRT	0.831321780287159
104	KC_AB2	CG2528	-0.729379579623002	1	1	pseudobulk	DESeq2	LRT	0
104	KC_AB2	CG2540	1.37686412499759	0.622972562188606	1	pseudobulk	DESeq2	LRT	0.205531080712408
104	KC_AB2	CG2608	0.0455261422590676	0.956410711868198	1	pseudobulk	DESeq2	LRT	0.019355568399845
104	KC_AB2	CG2611	1.64462134930074	0.494740966888383	1	pseudobulk	DESeq2	LRT	0.305622126512861
104	KC_AB2	CG2614	-0.33572805049699	0.856817945219033	1	pseudobulk	DESeq2	LRT	0.067111446205159
104	KC_AB2	CG2617	2.76364737573217	0.211914962801091	1	pseudobulk	DESeq2	LRT	0.673838377727484
104	KC_AB2	CG2656	0.942758880874816	0.658080412798793	1	pseudobulk	DESeq2	LRT	0.181721035411818
104	KC_AB2	CG2662	-1.29052728408004	0.545673810438722	1	pseudobulk	DESeq2	LRT	0.263066889637944
104	KC_AB2	CG2680	0.295920449873446	0.879415268446587	1	pseudobulk	DESeq2	LRT	0.055805998410949
104	KC_AB2	CG2681	-0.729379579623002	0.772694994316916	1	pseudobulk	DESeq2	LRT	0.1119919011982
104	KC_AB2	CG2685	-0.311256113568336	0.867408333719348	1	pseudobulk	DESeq2	LRT	0.061776409636023
104	KC_AB2	CG2691	1.10706867860301	1	1	pseudobulk	DESeq2	LRT	0
104	KC_AB2	CG2698	0.613729681066337	0.552560233052794	1	pseudobulk	DESeq2	LRT	0.257620373829908
104	KC_AB2	CG2701	-0.602160707538023	0.619312190664346	1	pseudobulk	DESeq2	LRT	0.208090371167324
104	KC_AB2	CG2712	1.38446765109058	0.642794900879723	1	pseudobulk	DESeq2	LRT	0.191927577037166
104	KC_AB2	CG2747	0.249994841105475	0.803015352520257	1	pseudobulk	DESeq2	LRT	0.095276151544365
104	KC_AB2	CG2765	0.837185936403776	0.522115338193839	1	pseudobulk	DESeq2	LRT	0.28223354832328
104	KC_AB2	CG2767	-1.38764087950137	0.516647656322572	1	pseudobulk	DESeq2	LRT	0.286805536367625
104	KC_AB2	CG2772	1.98205447381612	0.114044537223298	1	pseudobulk	DESeq2	LRT	0.942925512759953
104	KC_AB2	CG2790	-0.456878578495147	0.669922796362626	1	pseudobulk	DESeq2	LRT	0.173975243635907
104	KC_AB2	CG2794	1.3632625091553	0.58937724908886	1	pseudobulk	DESeq2	LRT	0.229606632629065
104	KC_AB2	CG2811	-0.640794273038907	0.729795302113197	1	pseudobulk	DESeq2	LRT	0.136798936631664
104	KC_AB2	CG2818	0.530422715334198	0.67667720807568	1	pseudobulk	DESeq2	LRT	0.169618451229937
104	KC_AB2	CG2846	0.0278727729680457	0.950451746862329	1	pseudobulk	DESeq2	LRT	0.022069926776835
104	KC_AB2	CG2852	-0.866215214377299	0.0795190991996539	1	pseudobulk	DESeq2	LRT	1.09952854831441
104	KC_AB2	CG2865	-0.763156271648455	0.0835104108065714	1	pseudobulk	DESeq2	LRT	1.07825937991751
104	KC_AB2	CG2889	-0.609958788825387	0.734505775184742	1	pseudobulk	DESeq2	LRT	0.134004785141247
104	KC_AB2	CG2909	2.04956921837453	0.224095519142189	1	pseudobulk	DESeq2	LRT	0.649566827227889
104	KC_AB2	CG2915	-1.33570647694682	0.512066034235951	1	pseudobulk	DESeq2	LRT	0.290674030322833
104	KC_AB2	CG2918	0.276236285558167	0.78437518673804	1	pseudobulk	DESeq2	LRT	0.10547615344535
104	KC_AB2	CG2921	0.274361831777722	0.820483725155243	1	pseudobulk	DESeq2	LRT	0.085930029047659
104	KC_AB2	CG2924	-0.0176432721232035	0.95349602384147	1	pseudobulk	DESeq2	LRT	0.020681113658677
104	KC_AB2	CG2926	-0.172955009513632	0.800577167062841	1	pseudobulk	DESeq2	LRT	0.096596800391319

104 KC_AB2	CG2931	-0.7607288587042	0.468806187024391	1	pseudobulk	DESeq2	LRT	0.32900666539265
104 KC_AB2	CG2938	0.238696574483959	0.843239767129946	1	pseudobulk	DESeq2	LRT	0.074048920350398
104 KC_AB2	CG2972	2.7605424445074	0.237810191029118	1	pseudobulk	DESeq2	LRT	0.623769538224996
104 KC_AB2	CG2991	-0.262303950187049	0.754303892163585	1	pseudobulk	DESeq2	LRT	0.122453651344368
104 KC_AB2	CG2993	0.158798270412244	0.803023054736408	1	pseudobulk	DESeq2	LRT	0.095271985977766
104 KC_AB2	CG30010	-2.87023745984587	0.189640517887019	1	pseudobulk	DESeq2	LRT	0.722068867341863
104 KC_AB2	CG30020	0.965778378667528	0.555908821592816	1	pseudobulk	DESeq2	LRT	0.254996434184712
104 KC_AB2	CG30022	0.607253890471962	0.694507960473751	1	pseudobulk	DESeq2	LRT	0.158322771999785
104 KC_AB2	CG30054	1.38069928703004	0.566368801250645	1	pseudobulk	DESeq2	LRT	0.246900678019328
104 KC_AB2	CG30060	-1.71999636108657	0.519869997311849	1	pseudobulk	DESeq2	LRT	0.284105245805297
104 KC_AB2	CG30069	2.16659757126187	0.312659446690657	1	pseudobulk	DESeq2	LRT	0.504928445014192
104 KC_AB2	CG3008	0.736983593872263	0.738143027923711	1	pseudobulk	DESeq2	LRT	0.13185947798781
104 KC_AB2	CG30089	-0.0889148712101084	0.896579061669394	1	pseudobulk	DESeq2	LRT	0.047411407682610
104 KC_AB2	CG3009	1.38069928703004	0.566368801250645	1	pseudobulk	DESeq2	LRT	0.246900678019328
104 KC_AB2	CG30094	-0.0987329796554643	0.9174582054765	1	pseudobulk	DESeq2	LRT	0.037413710775446
104 KC_AB2	CG30096	3.10652105827792	0.167663820143121	1	pseudobulk	DESeq2	LRT	0.775560642864307
104 KC_AB2	CG30109	-0.0244723505894313	0.895278953000377	1	pseudobulk	DESeq2	LRT	0.048041625161103
104 KC_AB2	CG30110	2.31968409897139	0.349905511974677	1	pseudobulk	DESeq2	LRT	0.456049216130035
104 KC_AB2	CG30116	0.353016298338766	0.405719324014549	1	pseudobulk	DESeq2	LRT	0.391774306781686
104 KC_AB2	CG30122	0.464816201230116	0.598463068014726	1	pseudobulk	DESeq2	LRT	0.222962645344965
104 KC_AB2	CG30156	-0.18317077492817	0.899664519927029	1	pseudobulk	DESeq2	LRT	0.045919406456170
104 KC_AB2	CG30158	0.0901224268536526	0.80713319704562	1	pseudobulk	DESeq2	LRT	0.093054789976094
104 KC_AB2	CG30172	0.217636867436851	0.565383113683408	1	pseudobulk	DESeq2	LRT	0.247657166740789
104 KC_AB2	CG30183	1.08937410746753	0.523121629807279	1	pseudobulk	DESeq2	LRT	0.281397322580594
104 KC_AB2	CG30187	-0.729379579623002	1	1	pseudobulk	DESeq2	LRT	0
104 KC_AB2	CG30197	1.10706867860301	1	1	pseudobulk	DESeq2	LRT	0
104 KC_AB2	CG3021	0.375532389921205	0.823694144494803	1	pseudobulk	DESeq2	LRT	0.084234021332856
104 KC_AB2	CG30291	-2.67387083344122	0.142993910035898	1	pseudobulk	DESeq2	LRT	0.844682458298044
104 KC_AB2	CG30344	-0.539059754135504	0.759052767771577	1	pseudobulk	DESeq2	LRT	0.119728031804866
104 KC_AB2	CG30349	1.80622761856892	0.461120290665938	1	pseudobulk	DESeq2	LRT	0.336185767109657
104 KC_AB2	CG30373	-0.930063893886844	0.450975843805415	1	pseudobulk	DESeq2	LRT	0.345846720168549
104 KC_AB2	CG3038	1.38446765109058	0.642794900879723	1	pseudobulk	DESeq2	LRT	0.191927577037166
104 KC_AB2	CG30389	0.119608051094263	0.817300798813315	1	pseudobulk	DESeq2	LRT	0.087618076610326
104 KC_AB2	CG30392	1.47899978336446	0.199197197302617	1	pseudobulk	DESeq2	LRT	0.700716776377033
104 KC_AB2	CG30394	-0.427478535071269	0.813013586949621	1	pseudobulk	DESeq2	LRT	0.089902196487186
104 KC_AB2	CG3040	-1.00341811101709	0.47611956805908	1	pseudobulk	DESeq2	LRT	0.322283969070666
104 KC_AB2	CG30403	-0.729379579623002	1	1	pseudobulk	DESeq2	LRT	0
104 KC_AB2	CG30419	-0.751114358558354	0.264254744068566	1	pseudobulk	DESeq2	LRT	0.577977207220893
104 KC_AB2	CG30423	-1.12585891060072	0.132069268982488	1	pseudobulk	DESeq2	LRT	0.879198226018901
104 KC_AB2	CG30424	0.50708159733276	0.79362281859345	1	pseudobulk	DESeq2	LRT	0.100385853647791
104 KC_AB2	CG30428	0.325800290023004	0.713124051474392	1	pseudobulk	DESeq2	LRT	0.146834915891995
104 KC_AB2	CG3045	1.10706867860301	1	1	pseudobulk	DESeq2	LRT	0
104 KC_AB2	CG30466	1.10706867860301	1	1	pseudobulk	DESeq2	LRT	0
104 KC_AB2	CG30467	-0.163182249325424	0.877914621987962	1	pseudobulk	DESeq2	LRT	0.056547717581868
104 KC_AB2	CG30491	-2.22728608909242	0.317088151657018	1	pseudobulk	DESeq2	LRT	0.498819985554471
104 KC_AB2	CG30495	-1.33570647694682	0.512066034235951	1	pseudobulk	DESeq2	LRT	0.290674030322833
104 KC_AB2	CG3061	0.139928555037267	0.818475757789018	1	pseudobulk	DESeq2	LRT	0.086994179311751
104 KC_AB2	CG3065	-0.89709111669284	0.544023224615042	1	pseudobulk	DESeq2	LRT	0.264382559664211
104 KC_AB2	CG3071	0.403294498144138	0.89479244414645	1	pseudobulk	DESeq2	LRT	0.048277691852293
105 KC_AB2	CG3078	-0.120784139988103	0.887912802185582	1	pseudobulk	DESeq2	LRT	0.051629682182064
105 KC_AB2	CG3097	1.10706867860301	1	1	pseudobulk	DESeq2	LRT	0
105 KC_AB2	CG31030	-0.303185611829616	0.443246818369665	1	pseudobulk	DESeq2	LRT	0.353354373086226

105	KC_AB2	CG31038	1.16404872429817	0.502029431853011	1	pseudobulk	DESeq2	LRT	0.299270821267993
105	KC_AB2	CG3104	-0.536567692923415	0.475526404163048	1	pseudobulk	DESeq2	LRT	0.322825363344989
105	KC_AB2	CG31051	0.48541240285688	0.679129059461705	1	pseudobulk	DESeq2	LRT	0.168047685975821
105	KC_AB2	CG31064	-0.240967367421568	0.735080302853697	1	pseudobulk	DESeq2	LRT	0.133665214410728
105	KC_AB2	CG3107	0.258782195698882	0.681741171580941	1	pseudobulk	DESeq2	LRT	0.166380477381209
105	KC_AB2	CG31098	-0.778523756993934	0.689563908531076	1	pseudobulk	DESeq2	LRT	0.161425477368935
105	KC_AB2	CG31109	-0.333187175027435	0.774400213956779	1	pseudobulk	DESeq2	LRT	0.111034535709689
105	KC_AB2	CG31111	-2.98842125158759	0.170667429115194	1	pseudobulk	DESeq2	LRT	0.767849353549797
105	KC_AB2	CG31115	-0.729379579623002	0.772694994316916	1	pseudobulk	DESeq2	LRT	0.1119919011982
105	KC_AB2	CG31122	0.0399976032068958	0.941783024269033	1	pseudobulk	DESeq2	LRT	0.026049142023185
105	KC_AB2	CG31125	1.13118962049082	0.587800658503729	1	pseudobulk	DESeq2	LRT	0.230769931729933
105	KC_AB2	CG31126	-0.766169857266325	0.481669201304215	1	pseudobulk	DESeq2	LRT	0.317251122278574
105	KC_AB2	CG31140	-0.0066767925513210	0.959064688226311	1	pseudobulk	DESeq2	LRT	0.018152098989650
105	KC_AB2	CG31142	1.10706867860301	1	1	pseudobulk	DESeq2	LRT	0
105	KC_AB2	CG31156	-1.12396603762283	0.539446936247551	1	pseudobulk	DESeq2	LRT	0.268051269105794
105	KC_AB2	CG31174	0.225287527156343	0.907574049327722	1	pseudobulk	DESeq2	LRT	0.042117930586680
105	KC_AB2	CG31191	-0.200564079358281	0.749170362137902	1	pseudobulk	DESeq2	LRT	0.125419412042351
105	KC_AB2	CG31195	-0.347135499964956	0.854806062097812	1	pseudobulk	DESeq2	LRT	0.068132406575477
105	KC_AB2	CG31211	0.242724340301368	0.706776522594159	1	pseudobulk	DESeq2	LRT	0.150717885137471
105	KC_AB2	CG31221	-0.497969227378184	0.0593514083502455	1	pseudobulk	DESeq2	LRT	1.22656897120841
105	KC_AB2	CG31223	-1.6052707068109	0.52265609184397	1	pseudobulk	DESeq2	LRT	0.281783983297782
105	KC_AB2	CG31224	0.0249255357090242	0.93323750749261	1	pseudobulk	DESeq2	LRT	0.030007814917154
105	KC_AB2	CG31229	-1.03881944901687	0.459057664109497	1	pseudobulk	DESeq2	LRT	0.338132757531142
105	KC_AB2	CG31248	1.10706867860301	1	1	pseudobulk	DESeq2	LRT	0
105	KC_AB2	CG31249	0.86041887782826	0.524076728930733	1	pseudobulk	DESeq2	LRT	0.280605124253429
105	KC_AB2	CG31251	2.7605424445074	0.237810191029118	1	pseudobulk	DESeq2	LRT	0.623769538224996
105	KC_AB2	CG31278	-1.38890204751614	0.560346778081821	1	pseudobulk	DESeq2	LRT	0.251543120857772
105	KC_AB2	CG31301	-0.0701990329703557	0.934773550025546	1	pseudobulk	DESeq2	LRT	0.029293584727037
105	KC_AB2	CG31324	-0.729379579623002	1	1	pseudobulk	DESeq2	LRT	0
105	KC_AB2	CG31342	-0.0172834102449847	0.935729041235848	1	pseudobulk	DESeq2	LRT	0.028849891574006
105	KC_AB2	CG31344	0.992608104940911	0.224959022966636	1	pseudobulk	DESeq2	LRT	0.647896582867241
105	KC_AB2	CG31345	0.861234190921189	0.667616957316091	1	pseudobulk	DESeq2	LRT	0.175472640873442
105	KC_AB2	CG31357	0.633537578391533	0.649154746686913	1	pseudobulk	DESeq2	LRT	0.187651762951122
105	KC_AB2	CG31360	-0.357096327924547	0.780220733179465	1	pseudobulk	DESeq2	LRT	0.107782513155482
105	KC_AB2	CG31365	-1.50703928374247	0.391926990106867	1	pseudobulk	DESeq2	LRT	0.406794827741947
105	KC_AB2	CG31368	-0.647621034217714	0.754702560287896	1	pseudobulk	DESeq2	LRT	0.122224176685402
105	KC_AB2	CG31381	-0.729379579623002	0.772694994316916	1	pseudobulk	DESeq2	LRT	0.1119919011982
105	KC_AB2	CG31388	0.753086547482185	0.539972375107059	1	pseudobulk	DESeq2	LRT	0.267628458038982
105	KC_AB2	CG31415	-0.729379579623002	0.823527397767669	1	pseudobulk	DESeq2	LRT	0.084321947797948
105	KC_AB2	CG31457	-2.69831193693389	0.203295284067868	1	pseudobulk	DESeq2	LRT	0.691872695769522
105	KC_AB2	CG31460	-0.999629084374803	0.500534763907515	1	pseudobulk	DESeq2	LRT	0.300565753851207
105	KC_AB2	CG31467	-0.729379579623002	1	1	pseudobulk	DESeq2	LRT	0
105	KC_AB2	CG31473	1.10706867860301	1	1	pseudobulk	DESeq2	LRT	0
105	KC_AB2	CG31475	-1.12188775721553	0.217890778220192	1	pseudobulk	DESeq2	LRT	0.661761149987034
105	KC_AB2	CG3149	1.10706867860301	1	1	pseudobulk	DESeq2	LRT	0
105	KC_AB2	CG31510	-0.97307188652369	0.298486406728938	1	pseudobulk	DESeq2	LRT	0.525075442146122
105	KC_AB2	CG31522	-0.418445353267511	0.641584384783271	1	pseudobulk	DESeq2	LRT	0.192746214681788
105	KC_AB2	CG31523	0.0918238584729809	0.875914931872603	1	pseudobulk	DESeq2	LRT	0.057538070101635
105	KC_AB2	CG31550	-0.561189391511934	0.517319961827728	1	pseudobulk	DESeq2	LRT	0.286240763144431
105	KC_AB2	CG3156	1.37686412499759	0.622972562188606	1	pseudobulk	DESeq2	LRT	0.205531080712408
105	KC_AB2	CG31612	1.61205113368698	0.631647504209	1	pseudobulk	DESeq2	LRT	0.199525215522471
105	KC_AB2	CG3163	0.285678246402494	0.846020939984343	1	pseudobulk	DESeq2	LRT	0.072618887543594

105	KC_AB2	CG31633	-0.576411864802883	0.767001992742844	1	pseudobulk	DESeq2	LRT	0.11520350771191
105	KC_AB2	CG31635	0.198325551802138	0.721860087843024	1	pseudobulk	DESeq2	LRT	0.141546969973224
105	KC_AB2	CG31637	-0.432828084162617	0.256790870054547	1	pseudobulk	DESeq2	LRT	0.590420421239543
105	KC_AB2	CG31638	-0.0747046318403578	0.921734221155546	1	pseudobulk	DESeq2	LRT	0.035394288192940
105	KC_AB2	CG3164	0.364136077420657	0.814057422322293	1	pseudobulk	DESeq2	LRT	0.089344959584829
105	KC_AB2	CG31648	-1.34134752679715	0.479477589337917	1	pseudobulk	DESeq2	LRT	0.319231686833943
105	KC_AB2	CG3165	1.23182740906698	0.233042868084089	1	pseudobulk	DESeq2	LRT	0.632564183437766
105	KC_AB2	CG31650	-0.298634668114667	0.57833512985126	1	pseudobulk	DESeq2	LRT	0.237820426515057
105	KC_AB2	CG31663	1.10706867860301	1	1	pseudobulk	DESeq2	LRT	0
105	KC_AB2	CG31676	1.99339641594643	0.465071165814147	1	pseudobulk	DESeq2	LRT	0.332480585700252
105	KC_AB2	CG31678	-0.542879284844619	0.678261132100168	1	pseudobulk	DESeq2	LRT	0.168603069560468
105	KC_AB2	CG31687	-0.917278061484809	0.455093486912102	1	pseudobulk	DESeq2	LRT	0.341899379871272
105	KC_AB2	CG31688	-0.439796050806081	0.429696931252093	1	pseudobulk	DESeq2	LRT	0.366837747887056
105	KC_AB2	CG31690	-1.21363965631423	0.568763379520138	1	pseudobulk	DESeq2	LRT	0.24506837392442
105	KC_AB2	CG31694	-0.690400461061978	0.487086062028863	1	pseudobulk	DESeq2	LRT	0.312394297590124
105	KC_AB2	CG31705	1.10706867860301	1	1	pseudobulk	DESeq2	LRT	0
105	KC_AB2	CG31706	1.61205113368698	0.631647504209	1	pseudobulk	DESeq2	LRT	0.199525215522471
105	KC_AB2	CG31712	-0.230824116147087	0.559283785828597	1	pseudobulk	DESeq2	LRT	0.252367771101291
105	KC_AB2	CG31715	-0.318296202750505	0.607335220152564	1	pseudobulk	DESeq2	LRT	0.216571532850124
105	KC_AB2	CG31717	0.391019586407292	0.697267075629371	1	pseudobulk	DESeq2	LRT	0.156600841334691
105	KC_AB2	CG31729	0.342388702966436	0.685807028421124	1	pseudobulk	DESeq2	LRT	0.163798068376996
105	KC_AB2	CG31751	-0.470100723599697	0.833985291815347	1	pseudobulk	DESeq2	LRT	0.078841608522644
105	KC_AB2	CG3176	-0.503853048997234	0.794184118624158	1	pseudobulk	DESeq2	LRT	0.10007880181363
105	KC_AB2	CG31760	-0.702606989796916	0.156938897555588	1	pseudobulk	DESeq2	LRT	0.804269402492726
105	KC_AB2	CG31793	-2.45801888341184	0.286003272809243	1	pseudobulk	DESeq2	LRT	0.543628997098713
105	KC_AB2	CG31800	-1.10471018259914	0.549639707377377	1	pseudobulk	DESeq2	LRT	0.259921900271704
105	KC_AB2	CG31808	0.0751911610961867	0.901400604531353	1	pseudobulk	DESeq2	LRT	0.045082155001958
105	KC_AB2	CG31815	-1.16016699006818	0.583985689825587	1	pseudobulk	DESeq2	LRT	0.233597794849804
105	KC_AB2	CG31817	-2.03522979197945	0.396502959232805	1	pseudobulk	DESeq2	LRT	0.401753567050837
105	KC_AB2	CG3184	-0.671423393675388	0.671717273984619	1	pseudobulk	DESeq2	LRT	0.17281348317408
105	KC_AB2	CG31855	0.678170015295371	0.464885361189203	1	pseudobulk	DESeq2	LRT	0.332654129133855
105	KC_AB2	CG31883	-0.729379579623002	0.772694994316916	1	pseudobulk	DESeq2	LRT	0.1119919011982
105	KC_AB2	CG31886	1.63577765519498	0.536426415953348	1	pseudobulk	DESeq2	LRT	0.270489843757993
105	KC_AB2	CG31897	-0.729379579623002	1	1	pseudobulk	DESeq2	LRT	0
105	KC_AB2	CG31898	-1.33407500886303	0.63128114564055	1	pseudobulk	DESeq2	LRT	0.19977718147345
105	KC_AB2	CG31908	0.559810551039353	0.658696503992676	1	pseudobulk	DESeq2	LRT	0.181314641554264
105	KC_AB2	CG3191	-0.502610851884822	0.843360912667957	1	pseudobulk	DESeq2	LRT	0.073986531144244
105	KC_AB2	CG31915	-1.16016699006818	0.583985689825587	1	pseudobulk	DESeq2	LRT	0.233597794849804
105	KC_AB2	CG31921	2.28200477535996	0.396216942607302	1	pseudobulk	DESeq2	LRT	0.402066957561886
105	KC_AB2	CG31922	0.0584696104942761	0.935210792156753	1	pseudobulk	DESeq2	LRT	0.029090490140611
105	KC_AB2	CG31935	-0.160057240907588	0.913227577530507	1	pseudobulk	DESeq2	LRT	0.039420982214122
105	KC_AB2	CG31937	1.38069928703004	0.566368801250645	1	pseudobulk	DESeq2	LRT	0.246900678019328
105	KC_AB2	CG31957	-2.09428695548101	0.380540277844868	1	pseudobulk	DESeq2	LRT	0.419599369064229
105	KC_AB2	CG3198	0.136356160670367	0.758201697705447	1	pseudobulk	DESeq2	LRT	0.120215247470542
105	KC_AB2	CG31997	0.0091340201361105	0.923668486099611	1	pseudobulk	DESeq2	LRT	0.034483873467662
106	KC_AB2	CG31998	-0.379409681089995	0.504469999577406	1	pseudobulk	DESeq2	LRT	0.297164655800494
106	KC_AB2	CG31999	0.280700541159967	0.828339256746653	1	pseudobulk	DESeq2	LRT	0.081791756020708
106	KC_AB2	CG32017	0.580605884776133	0.237154590205426	1	pseudobulk	DESeq2	LRT	0.624968465017613
106	KC_AB2	CG32022	0.66954517615027	1	1	pseudobulk	DESeq2	LRT	0
106	KC_AB2	CG32039	-0.354086205994976	0.723125429351045	1	pseudobulk	DESeq2	LRT	0.14078636584456
106	KC_AB2	CG32052	1.21934034355239	0.58319143853755	1	pseudobulk	DESeq2	LRT	0.23418886024425
106	KC_AB2	CG32055	1.04560394656545	0.638145957880541	1	pseudobulk	DESeq2	LRT	0.195079977309609

106	KC_AB2	CG32066	0.285424000539115	0.331660213198904	1	pseudobulk	DESeq2	LRT	0.479306624424307
106	KC_AB2	CG32069	1.17869847424234	0.313328194538307	1	pseudobulk	DESeq2	LRT	0.504000523785787
106	KC_AB2	CG32075	-0.729379579623002	1	1	pseudobulk	DESeq2	LRT	0
106	KC_AB2	CG32085	0.113831369930349	0.85129975364687	1	pseudobulk	DESeq2	LRT	0.069917492285792
106	KC_AB2	CG32095	1.10450497242239	0.58963576011552	1	pseudobulk	DESeq2	LRT	0.229416185342543
106	KC_AB2	CG32099	1.94531887231797	0.378646728337548	1	pseudobulk	DESeq2	LRT	0.421765791304938
106	KC_AB2	CG32100	-0.0670422462985607	0.94702436040848	1	pseudobulk	DESeq2	LRT	0.023638849449832
106	KC_AB2	CG32104	1.04324661842443	0.68096673636569	1	pseudobulk	DESeq2	LRT	0.166874101840012
106	KC_AB2	CG32109	-0.692964876485196	0.546562277070797	1	pseudobulk	DESeq2	LRT	0.262360345979146
106	KC_AB2	CG32112	-1.57664552787045	0.348372935477857	1	pseudobulk	DESeq2	LRT	0.457955591980057
106	KC_AB2	CG32137	4.06839000002171	0.0277361388700711	1	pseudobulk	DESeq2	LRT	1.55695399690853
106	KC_AB2	CG32147	0.927871940478661	0.61140509600946	1	pseudobulk	DESeq2	LRT	0.213670945787285
106	KC_AB2	CG32163	0.39439088612385	0.813541888331052	1	pseudobulk	DESeq2	LRT	0.089620080830767
106	KC_AB2	CG32176	-2.23097939393208	0.209019602347337	1	pseudobulk	DESeq2	LRT	0.679812982826386
106	KC_AB2	CG32187	-2.03522979197945	0.396502959232805	1	pseudobulk	DESeq2	LRT	0.401753567050837
106	KC_AB2	CG32196	2.64741945039871	0.292550810913063	1	pseudobulk	DESeq2	LRT	0.533798693743382
106	KC_AB2	CG32202	-0.853831505315869	0.473570928624891	1	pseudobulk	DESeq2	LRT	0.324614965755805
106	KC_AB2	CG32204	-0.0535180059844141	0.851904534684529	1	pseudobulk	DESeq2	LRT	0.069609070001685
106	KC_AB2	CG32206	-0.234496663747115	0.601786453601895	1	pseudobulk	DESeq2	LRT	0.220557592588231
106	KC_AB2	CG32221	-0.729379579623002	1	1	pseudobulk	DESeq2	LRT	0
106	KC_AB2	CG32225	-0.349333337077732	0.756614839902855	1	pseudobulk	DESeq2	LRT	0.121125144903283
106	KC_AB2	CG32228	1.10706867860301	1	1	pseudobulk	DESeq2	LRT	0
106	KC_AB2	CG3223	0.416805714779571	0.785623510133657	1	pseudobulk	DESeq2	LRT	0.104785528570725
106	KC_AB2	CG3224	-0.0683329778422532	0.902491001061132	1	pseudobulk	DESeq2	LRT	0.044557119847765
106	KC_AB2	CG32243	0.103346149534817	0.914109934808685	1	pseudobulk	DESeq2	LRT	0.039001570995149
106	KC_AB2	CG3225	-0.729379579623002	0.823527397767669	1	pseudobulk	DESeq2	LRT	0.084321947797948
106	KC_AB2	CG3226	-0.994533171768777	0.314415052719991	1	pseudobulk	DESeq2	LRT	0.502496670149362
106	KC_AB2	CG32262	-3.76368007159067	0.0447259338696091	1	pseudobulk	DESeq2	LRT	1.34944058267375
106	KC_AB2	CG32264	0.0938655890822122	0.700138583416505	1	pseudobulk	DESeq2	LRT	0.15481598847695
106	KC_AB2	CG32267	-0.156252504983917	0.752185732620213	1	pseudobulk	DESeq2	LRT	0.123674908487506
106	KC_AB2	CG32276	-0.145196171458335	0.809445988285384	1	pseudobulk	DESeq2	LRT	0.091812125010920
106	KC_AB2	CG32278	0.197512108646567	0.713454681833901	1	pseudobulk	DESeq2	LRT	0.146633607769417
106	KC_AB2	CG32280	1.23218135953961	0.352670198403399	1	pseudobulk	DESeq2	LRT	0.452631237853877
106	KC_AB2	CG32281	1.10706867860301	1	1	pseudobulk	DESeq2	LRT	0
106	KC_AB2	CG32333	-0.575223092470286	0.681939408985712	1	pseudobulk	DESeq2	LRT	0.166254211138465
106	KC_AB2	CG32344	2.31968409897139	0.349905511974677	1	pseudobulk	DESeq2	LRT	0.456049216130035
106	KC_AB2	CG32368	1.95432904991983	0.509212778150152	1	pseudobulk	DESeq2	LRT	0.293100706726022
106	KC_AB2	CG32369	0.0463748149395136	0.893583340875403	1	pseudobulk	DESeq2	LRT	0.048864936393483
106	KC_AB2	CG32373	-0.214492652571554	0.764845647093485	1	pseudobulk	DESeq2	LRT	0.116426200636202
106	KC_AB2	CG32392	-0.169524318341919	0.912374154556569	1	pseudobulk	DESeq2	LRT	0.039827025776412
106	KC_AB2	CG32409	1.38069928703004	0.566368801250645	1	pseudobulk	DESeq2	LRT	0.246900678019328
106	KC_AB2	CG32425	-0.402793473853903	0.478832569401754	1	pseudobulk	DESeq2	LRT	0.319816317266463
106	KC_AB2	CG32428	-0.397976480196999	0.570648919357431	1	pseudobulk	DESeq2	LRT	0.243631000824185
106	KC_AB2	CG32432	0.505005889265475	0.188092254807277	1	pseudobulk	DESeq2	LRT	0.725629087297652
106	KC_AB2	CG32436	-0.729379579623002	0.772694994316916	1	pseudobulk	DESeq2	LRT	0.1119919011982
106	KC_AB2	CG32441	-0.444876835432003	0.419362056349307	1	pseudobulk	DESeq2	LRT	0.377410866821532
106	KC_AB2	CG32448	0.718447742075755	0.536000121793235	1	pseudobulk	DESeq2	LRT	0.270835111624163
106	KC_AB2	CG32452	1.50776466886541	0.515809803599324	1	pseudobulk	DESeq2	LRT	0.287510407819198
106	KC_AB2	CG32459	0.145321756433347	1	1	pseudobulk	DESeq2	LRT	0
106	KC_AB2	CG32473	-0.647621034217714	0.754702560287896	1	pseudobulk	DESeq2	LRT	0.122224176685402
106	KC_AB2	CG32485	-0.74383858954646	0.185261228288564	1	pseudobulk	DESeq2	LRT	0.732215460887161
106	KC_AB2	CG32486	0.555613003790288	0.0642182114469751	1	pseudobulk	DESeq2	LRT	1.19234179421527

106	KC_AB2	CG32506	-0.17796351416645	0.874755835152541	1	pseudobulk	DESeq2	LRT	0.058113151827511
106	KC_AB2	CG32521	2.76364737573217	0.211914962801091	1	pseudobulk	DESeq2	LRT	0.673838377727484
106	KC_AB2	CG3253	-1.19592003493553	0.344808344470233	1	pseudobulk	DESeq2	LRT	0.462422232657285
106	KC_AB2	CG32533	0.146930209264112	1	1	pseudobulk	DESeq2	LRT	0
106	KC_AB2	CG32537	-1.21363965631423	0.568763379520138	1	pseudobulk	DESeq2	LRT	0.24506837392442
106	KC_AB2	CG32544	0.125964133022283	0.842596304185635	1	pseudobulk	DESeq2	LRT	0.074380450116962
106	KC_AB2	CG32547	-0.740042394976751	0.530099752596237	1	pseudobulk	DESeq2	LRT	0.275642398463847
106	KC_AB2	CG32549	0.0929143300674959	0.876810962001585	1	pseudobulk	DESeq2	LRT	0.057094029221203
106	KC_AB2	CG32554	1.10706867860301	1	1	pseudobulk	DESeq2	LRT	0
106	KC_AB2	CG3257	-0.1757578306877	0.823972088891451	1	pseudobulk	DESeq2	LRT	0.084087499280709
106	KC_AB2	CG32576	-1.62998836015156	0.458034020100829	1	pseudobulk	DESeq2	LRT	0.339102263932028
106	KC_AB2	CG32581	0.132623924928099	0.828844912508682	1	pseudobulk	DESeq2	LRT	0.081526723904084
106	KC_AB2	CG32590	0.67264277115268	0.624430052120725	1	pseudobulk	DESeq2	LRT	0.204516203693974
106	KC_AB2	CG3262	0.395369052993315	0.539478399883629	1	pseudobulk	DESeq2	LRT	0.268025939302006
106	KC_AB2	CG32638	2.3587276565843	0.260468459248785	1	pseudobulk	DESeq2	LRT	0.584244858942325
106	KC_AB2	CG32641	-1.33407500886303	0.63128114564055	1	pseudobulk	DESeq2	LRT	0.19977718147345
106	KC_AB2	CG32647	0.223823829014903	0.666526228981987	1	pseudobulk	DESeq2	LRT	0.176182755660459
106	KC_AB2	CG32650	0.38605638660934	0.863777890371283	1	pseudobulk	DESeq2	LRT	0.063597916533759
106	KC_AB2	CG32655	-0.729379579623002	1	1	pseudobulk	DESeq2	LRT	0
106	KC_AB2	CG32683	-0.315361128834952	0.384176561897365	1	pseudobulk	DESeq2	LRT	0.415469134396603
106	KC_AB2	CG32686	1.38446765109058	0.642794900879723	1	pseudobulk	DESeq2	LRT	0.191927577037166
106	KC_AB2	CG32700	0.119712824844784	0.572336520231151	1	pseudobulk	DESeq2	LRT	0.242348541303155
106	KC_AB2	CG32708	-1.29052728408004	0.545673810438722	1	pseudobulk	DESeq2	LRT	0.263066889637944
106	KC_AB2	CG32719	1.10706867860301	1	1	pseudobulk	DESeq2	LRT	0
106	KC_AB2	CG32755	1.61205113368698	0.631647504209	1	pseudobulk	DESeq2	LRT	0.199525215522471
106	KC_AB2	CG32756	2.04395492862141	0.135963186173148	1	pseudobulk	DESeq2	LRT	0.866578666675008
106	KC_AB2	CG32767	0.0562829180465596	0.839330560662441	1	pseudobulk	DESeq2	LRT	0.076066963607458
106	KC_AB2	CG32772	1.65434015532876	0.116765809237326	1	pseudobulk	DESeq2	LRT	0.932684306481604
106	KC_AB2	CG32795	-0.676432335835217	0.322653434211714	1	pseudobulk	DESeq2	LRT	0.491263708051789
106	KC_AB2	CG32803	-2.3709810723925	0.217929575422311	1	pseudobulk	DESeq2	LRT	0.661683827252117
106	KC_AB2	CG32808	-0.729379579623002	0.823527397767669	1	pseudobulk	DESeq2	LRT	0.084321947797948
106	KC_AB2	CG32809	0.038670622367303	0.898639033056113	1	pseudobulk	DESeq2	LRT	0.046414721428817
106	KC_AB2	CG3281	-1.71999636108657	0.519869997311849	1	pseudobulk	DESeq2	LRT	0.284105245805297
106	KC_AB2	CG32813	0.093766168218877	0.945400178701012	1	pseudobulk	DESeq2	LRT	0.024384319942079
106	KC_AB2	CG32815	0.0912414061567595	0.883587296345962	1	pseudobulk	DESeq2	LRT	0.053750536764510
106	KC_AB2	CG32817	-0.729379579623002	1	1	pseudobulk	DESeq2	LRT	0
106	KC_AB2	CG32850	-0.321192411255074	0.457411728633987	1	pseudobulk	DESeq2	LRT	0.339692703708903
106	KC_AB2	CG32856	-1.45675668689178	0.495625979996641	1	pseudobulk	DESeq2	LRT	0.304845936611032
106	KC_AB2	CG3294	-1.2593686706548	0.647053428594028	1	pseudobulk	DESeq2	LRT	0.189059857220721
106	KC_AB2	CG3295	2.80011757288138	0.131360758885593	1	pseudobulk	DESeq2	LRT	0.881534351250262
106	KC_AB2	CG32971	2.64741945039871	0.292550810913063	1	pseudobulk	DESeq2	LRT	0.533798693743382
107	KC_AB2	CG32982	-0.142061573604699	0.879203316178379	1	pseudobulk	DESeq2	LRT	0.055910682509890
107	KC_AB2	CG33051	1.90334599570774	0.391638683118061	1	pseudobulk	DESeq2	LRT	0.407114418413825
107	KC_AB2	CG33056	0.403294498144138	0.89479244414645	1	pseudobulk	DESeq2	LRT	0.048277691852293
107	KC_AB2	CG3308	1.48454497256533	0.330319414077846	1	pseudobulk	DESeq2	LRT	0.481065900486297
107	KC_AB2	CG3309	1.37646356319375	0.523574002340058	1	pseudobulk	DESeq2	LRT	0.281021926140695
107	KC_AB2	CG33090	-0.220361266599945	0.663504885933307	1	pseudobulk	DESeq2	LRT	0.178155874719574
107	KC_AB2	CG33096	2.47215486522296	0.30601789350912	1	pseudobulk	DESeq2	LRT	0.514253178665238
107	KC_AB2	CG33098	0.145321756433347	1	1	pseudobulk	DESeq2	LRT	0
107	KC_AB2	CG33107	-0.336234585808749	0.828307287695249	1	pseudobulk	DESeq2	LRT	0.081808517571523
107	KC_AB2	CG33108	1.78124453243794	0.460163425723109	1	pseudobulk	DESeq2	LRT	0.33708790248149
107	KC_AB2	CG33111	-1.45220519246244	0.536855403325269	1	pseudobulk	DESeq2	LRT	0.270142671458964

107	KC_AB2	CG33116	-0.79811112711199	0.289168941646461	1	pseudobulk	DESeq2	LRT	0.538848354516862
107	KC_AB2	CG33129	0.00403703794362762	0.977437643356336	1	pseudobulk	DESeq2	LRT	0.009910939313241
107	KC_AB2	CG33144	-1.25224039389589	0.078709417463057	1	pseudobulk	DESeq2	LRT	1.10397330185248
107	KC_AB2	CG33155	2.01814786626755	0.401718761445944	1	pseudobulk	DESeq2	LRT	0.396077884966921
107	KC_AB2	CG33156	-0.73888450387487	0.561853769296618	1	pseudobulk	DESeq2	LRT	0.250376701247996
107	KC_AB2	CG33158	-0.369854891353399	0.811074780250121	1	pseudobulk	DESeq2	LRT	0.090939102443862
107	KC_AB2	CG33169	-1.02144198660749	0.315759912157156	1	pseudobulk	DESeq2	LRT	0.500643007431098
107	KC_AB2	CG33170	0.0206504911472485	0.915724666093963	1	pseudobulk	DESeq2	LRT	0.038235087435776
107	KC_AB2	CG33172	2.88969196614093	0.207685089444696	1	pseudobulk	DESeq2	LRT	0.682594682108805
107	KC_AB2	CG33181	0.0735465927232841	0.817049474542697	1	pseudobulk	DESeq2	LRT	0.087751644972674
107	KC_AB2	CG33199	-0.367827343043921	0.574539144782976	1	pseudobulk	DESeq2	LRT	0.240680376402867
107	KC_AB2	CG33203	0.967583857015351	0.443160972387322	1	pseudobulk	DESeq2	LRT	0.353438493367953
107	KC_AB2	CG33213	0.211319900981398	0.958606495011728	1	pseudobulk	DESeq2	LRT	0.018359632784041
107	KC_AB2	CG33217	-0.0693351641514065	0.938604324684617	1	pseudobulk	DESeq2	LRT	0.027517449076924
107	KC_AB2	CG33223	1.02648689492346	0.73959154128541	1	pseudobulk	DESeq2	LRT	0.131008064514051
107	KC_AB2	CG33228	-1.16016699006818	0.583985689825587	1	pseudobulk	DESeq2	LRT	0.233597794849804
107	KC_AB2	CG33229	0.0478512211110975	0.917024987659571	1	pseudobulk	DESeq2	LRT	0.037618830246211
107	KC_AB2	CG33230	-2.4120103603151	0.297158744475551	1	pseudobulk	DESeq2	LRT	0.527011485255674
107	KC_AB2	CG33267	1.10706867860301	1	1	pseudobulk	DESeq2	LRT	0
107	KC_AB2	CG33288	-0.729379579623002	0.772694994316916	1	pseudobulk	DESeq2	LRT	0.1119919011982
107	KC_AB2	CG33298	-0.125118956043906	0.907940475564685	1	pseudobulk	DESeq2	LRT	0.041942622823821
107	KC_AB2	CG33303	0.166349051115832	0.7838596893127	1	pseudobulk	DESeq2	LRT	0.105761668961862
107	KC_AB2	CG33307	-0.729379579623002	0.823527397767669	1	pseudobulk	DESeq2	LRT	0.084321947797948
107	KC_AB2	CG33310	1.44502939524275	0.501852597187124	1	pseudobulk	DESeq2	LRT	0.299423823947943
107	KC_AB2	CG33331	0.643689786032629	0.725039051770887	1	pseudobulk	DESeq2	LRT	0.139638600998867
107	KC_AB2	CG33332	-2.75585076225026	0.218954080020334	1	pseudobulk	DESeq2	LRT	0.659646957693561
107	KC_AB2	CG3335	1.03254930292854	0.645433035572793	1	pseudobulk	DESeq2	LRT	0.190148809617279
107	KC_AB2	CG3337	-0.584018577036285	0.753106093268505	1	pseudobulk	DESeq2	LRT	0.123143838572892
107	KC_AB2	CG3342	-0.727850568319075	0.713539842709958	1	pseudobulk	DESeq2	LRT	0.146581771693029
107	KC_AB2	CG3345	1.38069928703004	0.566368801250645	1	pseudobulk	DESeq2	LRT	0.246900678019328
107	KC_AB2	CG33468	-0.729379579623002	0.823527397767669	1	pseudobulk	DESeq2	LRT	0.084321947797948
107	KC_AB2	CG33493	-0.729379579623002	1	1	pseudobulk	DESeq2	LRT	0
107	KC_AB2	CG33506	0.448149827291886	0.779466629056405	1	pseudobulk	DESeq2	LRT	0.108202473325546
107	KC_AB2	CG33521	-0.83320718254958	0.715059964627045	1	pseudobulk	DESeq2	LRT	0.14565753691968
107	KC_AB2	CG33523	-1.73305743602016	0.350811935727641	1	pseudobulk	DESeq2	LRT	0.45492563898767
107	KC_AB2	CG33543	0.615918587839124	0.252683664642055	1	pseudobulk	DESeq2	LRT	0.597422833209196
107	KC_AB2	CG3356	-0.0458625429834585	0.968970809044799	1	pseudobulk	DESeq2	LRT	0.013689306191466
107	KC_AB2	CG3358	2.16811029366837	0.310827889047297	1	pseudobulk	DESeq2	LRT	0.507480021029577
107	KC_AB2	CG33635	-0.0178968628095796	0.924910508657509	1	pseudobulk	DESeq2	LRT	0.033900286154389
107	KC_AB2	CG33639	-2.5216736243906	0.267985186584202	1	pseudobulk	DESeq2	LRT	0.571889211801602
107	KC_AB2	CG33641	-0.729379579623002	1	1	pseudobulk	DESeq2	LRT	0
107	KC_AB2	CG33644	1.38069928703004	0.566368801250645	1	pseudobulk	DESeq2	LRT	0.246900678019328
107	KC_AB2	CG33667	-0.729379579623002	0.823527397767669	1	pseudobulk	DESeq2	LRT	0.084321947797948
107	KC_AB2	CG3368	0.211319900981398	0.958606495011728	1	pseudobulk	DESeq2	LRT	0.018359632784041
107	KC_AB2	CG33695	-1.43319088453329	0.468553403065376	1	pseudobulk	DESeq2	LRT	0.329240903527803
107	KC_AB2	CG33700	-1.6052707068109	0.52265609184397	1	pseudobulk	DESeq2	LRT	0.281783983297782
107	KC_AB2	CG33774	0.303640673227841	0.767279806188404	1	pseudobulk	DESeq2	LRT	0.11504623171762
107	KC_AB2	CG3386	-1.98028140890842	0.328533066956313	1	pseudobulk	DESeq2	LRT	0.483420912028321
107	KC_AB2	CG33941	0.128145084209758	0.885567431998245	1	pseudobulk	DESeq2	LRT	0.052778363632868
107	KC_AB2	CG33947	-1.0653296100089	0.511938287305544	1	pseudobulk	DESeq2	LRT	0.290782388824992
107	KC_AB2	CG33969	0.45823239341792	0.802310608403512	1	pseudobulk	DESeq2	LRT	0.095657465381141
107	KC_AB2	CG33970	-0.744467116302025	0.71305844217897	1	pseudobulk	DESeq2	LRT	0.146874873969043

107	KC_AB2	CG33977	-0.129002215624387	0.824454332251108	1	pseudobulk	DESeq2	LRT	0.083833395569045
107	KC_AB2	CG33978	1.95432904991983	0.509212778150152	1	pseudobulk	DESeq2	LRT	0.293100706726022
107	KC_AB2	CG33993	-0.319628607137448	0.839117678448347	1	pseudobulk	DESeq2	LRT	0.076177129139189
107	KC_AB2	CG34001	-1.78975280219494	0.468855431150075	1	pseudobulk	DESeq2	LRT	0.32896104882553
107	KC_AB2	CG34015	-0.263915929773877	0.823781554475256	1	pseudobulk	DESeq2	LRT	0.084187936680763
107	KC_AB2	CG3402	0.124462516295993	0.952307241396434	1	pseudobulk	DESeq2	LRT	0.021222913258289
107	KC_AB2	CG34026	1.99339641594643	0.465071165814147	1	pseudobulk	DESeq2	LRT	0.332480585700252
107	KC_AB2	CG34027	0.38605638660934	0.863777890371283	1	pseudobulk	DESeq2	LRT	0.063597916533759
107	KC_AB2	CG34028	-1.06378174651514	0.614782299510286	1	pseudobulk	DESeq2	LRT	0.211278644971369
107	KC_AB2	CG34039	0.619440369709763	0.722026032044035	1	pseudobulk	DESeq2	LRT	0.141447144024172
107	KC_AB2	CG34045	-1.41713457097052	0.533151600466288	1	pseudobulk	DESeq2	LRT	0.273149282747435
107	KC_AB2	CG3407	0.393976029558784	0.939642609940555	1	pseudobulk	DESeq2	LRT	0.027037297511213
107	KC_AB2	CG3408	1.3632625091553	0.58937724908886	1	pseudobulk	DESeq2	LRT	0.229606632629065
107	KC_AB2	CG34109	2.33331203280489	0.344281080698468	1	pseudobulk	DESeq2	LRT	0.463086842453662
107	KC_AB2	CG34116	-0.303672526813818	0.918092162121602	1	pseudobulk	DESeq2	LRT	0.037113720223793
107	KC_AB2	CG34117	-1.38723819085141	0.247964309103696	1	pseudobulk	DESeq2	LRT	0.605610825120527
107	KC_AB2	CG34125	-0.141655807052538	0.855869022350367	1	pseudobulk	DESeq2	LRT	0.067592692358819
107	KC_AB2	CG34126	-1.23570555233712	0.238637961027305	1	pseudobulk	DESeq2	LRT	0.62226047033311
107	KC_AB2	CG34132	-3.12088662121595	0.0438633960219208	1	pseudobulk	DESeq2	LRT	1.35789774711165
107	KC_AB2	CG34133	-0.103248527019908	0.937029228976571	1	pseudobulk	DESeq2	LRT	0.028246861849413
107	KC_AB2	CG34134	-1.75538554779205	0.282373786199827	1	pseudobulk	DESeq2	LRT	0.549175622907703
107	KC_AB2	CG34148	1.61663811589523	0.397581307715032	1	pseudobulk	DESeq2	LRT	0.400574042149588
107	KC_AB2	CG34149	-0.844143140939175	0.641163764521006	1	pseudobulk	DESeq2	LRT	0.193031029850728
107	KC_AB2	CG34150	2.27430245073838	0.146283804514179	1	pseudobulk	DESeq2	LRT	0.834803753160161
107	KC_AB2	CG34159	0.256190534677083	0.760123544902556	1	pseudobulk	DESeq2	LRT	0.119115814944371
107	KC_AB2	CG34163	-0.729379579623002	1	1	pseudobulk	DESeq2	LRT	0
107	KC_AB2	CG34183	1.13746102707583	0.612851413153431	1	pseudobulk	DESeq2	LRT	0.212644808140106
107	KC_AB2	CG34184	-0.729379579623002	0.772694994316916	1	pseudobulk	DESeq2	LRT	0.1119919011982
107	KC_AB2	CG34195	-2.36590551523243	0.207929435083995	1	pseudobulk	DESeq2	LRT	0.682084026350702
107	KC_AB2	CG3420	0.867600116512273	0.489656364952738	1	pseudobulk	DESeq2	LRT	0.31010859580737
107	KC_AB2	CG34200	-0.732588500198081	0.212052534468936	1	pseudobulk	DESeq2	LRT	0.673556532450696
107	KC_AB2	CG34213	1.99339641594643	0.465071165814147	1	pseudobulk	DESeq2	LRT	0.332480585700252
107	KC_AB2	CG34219	-0.491409334419632	0.418672401193793	1	pseudobulk	DESeq2	LRT	0.378125666776054
107	KC_AB2	CG34228	-0.576016220255167	0.653661754415435	1	pseudobulk	DESeq2	LRT	0.18464692475145
107	KC_AB2	CG34229	-1.46690171509567	0.421286230321457	1	pseudobulk	DESeq2	LRT	0.375422735492514
107	KC_AB2	CG34231	0.145321756433347	1	1	pseudobulk	DESeq2	LRT	0
107	KC_AB2	CG34234	-0.729379579623002	1	1	pseudobulk	DESeq2	LRT	0
108	KC_AB2	CG34242	0.857710569376359	0.523398214296606	1	pseudobulk	DESeq2	LRT	0.281167763397424
108	KC_AB2	CG34250	-0.273766758400573	0.741062663772384	1	pseudobulk	DESeq2	LRT	0.13014506681162
108	KC_AB2	CG34283	-0.729379579623002	0.823527397767669	1	pseudobulk	DESeq2	LRT	0.084321947797948
108	KC_AB2	CG34293	-0.3910841108731	0.76197506003524	1	pseudobulk	DESeq2	LRT	0.118059243183142
108	KC_AB2	CG34309	-0.729379579623002	1	1	pseudobulk	DESeq2	LRT	0
108	KC_AB2	CG34310	-0.729379579623002	1	1	pseudobulk	DESeq2	LRT	0
108	KC_AB2	CG34330	-0.729379579623002	0.823527397767669	1	pseudobulk	DESeq2	LRT	0.084321947797948
108	KC_AB2	CG3434	1.94629698203144	0.451780032596131	1	pseudobulk	DESeq2	LRT	0.345072967583315
108	KC_AB2	CG34348	0.844198923204955	0.7046337711535	1	pseudobulk	DESeq2	LRT	0.152036546119094
108	KC_AB2	CG34351	-2.66356999955445	0.123361559834606	1	pseudobulk	DESeq2	LRT	0.908820147859273
108	KC_AB2	CG34353	-0.544138476417757	0.569383190132492	1	pseudobulk	DESeq2	LRT	0.244595358651066
108	KC_AB2	CG34354	0.24961368471395	0.504267081178244	1	pseudobulk	DESeq2	LRT	0.297339381888992
108	KC_AB2	CG34355	-0.0839582259507212	0.861460209334815	1	pseudobulk	DESeq2	LRT	0.064764777723108
108	KC_AB2	CG34357	0.131915986642598	0.759305917397308	1	pseudobulk	DESeq2	LRT	0.119583215579762
108	KC_AB2	CG3436	-0.563646813160038	0.657289234484393	1	pseudobulk	DESeq2	LRT	0.18224348082219

108	KC_AB2	CG3437	0.144723777698294	0.930326572940145	1	pseudobulk	DESeq2	LRT	0.031364574102179
108	KC_AB2	CG34370	-0.64573669162536	0.50150508839869	1	pseudobulk	DESeq2	LRT	0.299724656158495
108	KC_AB2	CG34376	-0.68407403394891	0.724748624101311	1	pseudobulk	DESeq2	LRT	0.13981260045834
108	KC_AB2	CG34384	0.0720009343162316	0.923895249289585	1	pseudobulk	DESeq2	LRT	0.034377266046534
108	KC_AB2	CG34401	-0.176016006899583	0.669927233105467	1	pseudobulk	DESeq2	LRT	0.173972367414104
108	KC_AB2	CG34402	-0.376998738495709	0.389792481196387	1	pseudobulk	DESeq2	LRT	0.409166542351318
108	KC_AB2	CG34404	0.0785333430304297	0.911513756353191	1	pseudobulk	DESeq2	LRT	0.040236772688434
108	KC_AB2	CG34408	-0.708181405046695	0.684469381783492	1	pseudobulk	DESeq2	LRT	0.164645974293883
108	KC_AB2	CG34409	-0.824996233971657	0.664071509040402	1	pseudobulk	DESeq2	LRT	0.177785152093585
108	KC_AB2	CG34417	-0.729379579623002	0.823527397767669	1	pseudobulk	DESeq2	LRT	0.084321947797948
108	KC_AB2	CG34431	1.10706867860301	1	1	pseudobulk	DESeq2	LRT	0
108	KC_AB2	CG3500	-1.22554233339765	0.393675348198849	1	pseudobulk	DESeq2	LRT	0.404861779714239
108	KC_AB2	CG3511	1.1674499696881	0.576329272969097	1	pseudobulk	DESeq2	LRT	0.23932932113341
108	KC_AB2	CG3527	0.682569013985824	0.677625635090906	1	pseudobulk	DESeq2	LRT	0.169010172677449
108	KC_AB2	CG3529	0.504530475698193	0.640821539378281	1	pseudobulk	DESeq2	LRT	0.193262899114958
108	KC_AB2	CG3530	-0.617998105698206	0.411539836072349	1	pseudobulk	DESeq2	LRT	0.385588119749288
108	KC_AB2	CG3534	1.38446765109058	0.642794900879723	1	pseudobulk	DESeq2	LRT	0.191927577037166
108	KC_AB2	CG3542	-0.0806397617506279	0.958764917307812	1	pseudobulk	DESeq2	LRT	0.018287865854886
108	KC_AB2	CG3548	-0.977411425594654	0.386632815549623	1	pseudobulk	DESeq2	LRT	0.412701287898515
108	KC_AB2	CG3552	0.145321756433347	1	1	pseudobulk	DESeq2	LRT	0
108	KC_AB2	CG3556	-0.226728112741044	0.700709462474748	1	pseudobulk	DESeq2	LRT	0.154462017695778
108	KC_AB2	CG3558	-0.217994587646663	0.7109852586096	1	pseudobulk	DESeq2	LRT	0.148139403729974
108	KC_AB2	CG3566	-0.350310084284624	0.60375608444505	1	pseudobulk	DESeq2	LRT	0.219138479548762
108	KC_AB2	CG3568	0.726677935264657	0.544465798031125	1	pseudobulk	DESeq2	LRT	0.264029396335901
108	KC_AB2	CG3587	0.403294498144138	0.89479244414645	1	pseudobulk	DESeq2	LRT	0.048277691852293
108	KC_AB2	CG3589	1.02605719768525	0.762384804222891	1	pseudobulk	DESeq2	LRT	0.117825768597322
108	KC_AB2	CG3594	-2.45416506545088	0.321711556460218	1	pseudobulk	DESeq2	LRT	0.492533338175562
108	KC_AB2	CG3609	-0.729379579623002	1	1	pseudobulk	DESeq2	LRT	0
108	KC_AB2	CG3618	0.217439372228412	0.806502475198953	1	pseudobulk	DESeq2	LRT	0.093394295400131
108	KC_AB2	CG3625	-0.283194401287457	0.404175005318622	1	pseudobulk	DESeq2	LRT	0.393430547297305
108	KC_AB2	CG3626	-0.729379579623002	0.823527397767669	1	pseudobulk	DESeq2	LRT	0.084321947797948
108	KC_AB2	CG3631	-0.729379579623002	0.823527397767669	1	pseudobulk	DESeq2	LRT	0.084321947797948
108	KC_AB2	CG3632	1.96283802215484	0.156411141865356	1	pseudobulk	DESeq2	LRT	0.805732313435432
108	KC_AB2	CG3634	-0.402474667185194	0.816087557225511	1	pseudobulk	DESeq2	LRT	0.088263243721688
108	KC_AB2	CG3638	0.347913339909292	0.550335988217476	1	pseudobulk	DESeq2	LRT	0.259372086365253
108	KC_AB2	CG3645	-1.69521547874114	0.344174360916368	1	pseudobulk	DESeq2	LRT	0.463221485336979
108	KC_AB2	CG3651	0.341223134051336	0.741452937077436	1	pseudobulk	DESeq2	LRT	0.129916410132882
108	KC_AB2	CG3652	-0.0476644833628728	0.932920115308014	1	pseudobulk	DESeq2	LRT	0.030155542712507
108	KC_AB2	CG3662	-0.278414680082812	0.365094444501752	1	pseudobulk	DESeq2	LRT	0.437594775487934
108	KC_AB2	CG3678	-0.474969207769783	0.756786055709287	1	pseudobulk	DESeq2	LRT	0.121026878699687
108	KC_AB2	CG3700	0.145321756433347	1	1	pseudobulk	DESeq2	LRT	0
108	KC_AB2	CG3702	-2.01117131132678	0.147773620298675	1	pseudobulk	DESeq2	LRT	0.830403086788544
108	KC_AB2	CG3703	-0.785818497659063	0.404025678229205	1	pseudobulk	DESeq2	LRT	0.393591032020939
108	KC_AB2	CG3704	0.360638004629589	0.793307033433948	1	pseudobulk	DESeq2	LRT	0.100558695256226
108	KC_AB2	CG3709	1.38069928703004	0.566368801250645	1	pseudobulk	DESeq2	LRT	0.246900678019328
108	KC_AB2	CG3719	-0.351540609288244	0.720529592614175	1	pseudobulk	DESeq2	LRT	0.14234817772977
108	KC_AB2	CG3726	0.182789314645945	0.626185773324088	1	pseudobulk	DESeq2	LRT	0.203296803593954
108	KC_AB2	CG3732	-0.0536744891561543	0.927907977153501	1	pseudobulk	DESeq2	LRT	0.032495091664856
108	KC_AB2	CG3735	0.942310192709897	0.647802620308151	1	pseudobulk	DESeq2	LRT	0.188557299636942
108	KC_AB2	CG3740	1.05564509663658	0.613237310736703	1	pseudobulk	DESeq2	LRT	0.212371429561112
108	KC_AB2	CG3744	0.387556506521735	0.682352458465683	1	pseudobulk	DESeq2	LRT	0.165991239380493
108	KC_AB2	CG3756	1.3723515946239	0.644983770400341	1	pseudobulk	DESeq2	LRT	0.190451213293808

108	KC_AB2	CG3760	-1.34884876838071	0.310063606987421	1	pseudobulk	DESeq2	LRT	0.50854920510115
108	KC_AB2	CG3764	0.602291076327263	0.742123520577962	1	pseudobulk	DESeq2	LRT	0.129523803830369
108	KC_AB2	CG3776	-0.144019713618319	0.898086069474563	1	pseudobulk	DESeq2	LRT	0.046682040051354
108	KC_AB2	CG3777	1.32787468450772	0.343278553083851	1	pseudobulk	DESeq2	LRT	0.464353329019034
108	KC_AB2	CG3781	0.51139704840244	0.757128120108083	1	pseudobulk	DESeq2	LRT	0.120830623607088
108	KC_AB2	CG3788	1.10706867860301	1	1	pseudobulk	DESeq2	LRT	0
108	KC_AB2	CG3792	0.554899539621602	0.733466939489885	1	pseudobulk	DESeq2	LRT	0.134619456900038
108	KC_AB2	CG3793	0.231714543773121	0.817097395566714	1	pseudobulk	DESeq2	LRT	0.087726173778274
108	KC_AB2	CG3803	-0.138068503460402	0.92071191727055	1	pseudobulk	DESeq2	LRT	0.035876235504598
108	KC_AB2	CG3808	1.38069928703004	0.566368801250645	1	pseudobulk	DESeq2	LRT	0.246900678019328
108	KC_AB2	CG3815	2.62919123543814	0.269826951733818	1	pseudobulk	DESeq2	LRT	0.568914672882509
108	KC_AB2	CG3817	-0.366480273760048	0.825711009569154	1	pseudobulk	DESeq2	LRT	0.083171924725566
108	KC_AB2	CG3819	-1.86716056129969	0.477680380390446	1	pseudobulk	DESeq2	LRT	0.320862595985116
108	KC_AB2	CG3847	0.209618550936829	0.87158278053514	1	pseudobulk	DESeq2	LRT	0.059691358499717
108	KC_AB2	CG3857	1.04560394656545	0.638145957880541	1	pseudobulk	DESeq2	LRT	0.195079977309609
108	KC_AB2	CG3860	-0.730096365151101	0.429202019977372	1	pseudobulk	DESeq2	LRT	0.367338242756968
108	KC_AB2	CG3862	-0.801863169396014	0.647277270195681	1	pseudobulk	DESeq2	LRT	0.188909643409864
108	KC_AB2	CG3894	0.545769785153131	0.732095334837864	1	pseudobulk	DESeq2	LRT	0.135432360610889
108	KC_AB2	CG3907	0.0259417477594563	0.918192152696699	1	pseudobulk	DESeq2	LRT	0.037066423239661
108	KC_AB2	CG3909	-1.16016699006818	0.583985689825587	1	pseudobulk	DESeq2	LRT	0.233597794849804
108	KC_AB2	CG3918	-0.0667564969473319	0.915510114323073	1	pseudobulk	DESeq2	LRT	0.038336853360717
108	KC_AB2	CG3919	-0.729379579623002	1	1	pseudobulk	DESeq2	LRT	0
108	KC_AB2	CG3955	-0.384672389544246	0.476411110885896	1	pseudobulk	DESeq2	LRT	0.322018118431569
108	KC_AB2	CG3961	2.14464629004459	0.0675116553791575	1	pseudobulk	DESeq2	LRT	1.17062124302259
108	KC_AB2	CG3987	1.10706867860301	1	1	pseudobulk	DESeq2	LRT	0
108	KC_AB2	CG3995	-0.298130645044972	0.86764568568639	1	pseudobulk	DESeq2	LRT	0.061657588378534
108	KC_AB2	CG3999	-0.729379579623002	0.772694994316916	1	pseudobulk	DESeq2	LRT	0.1119919011982
108	KC_AB2	CG4004	-0.568684676206718	0.584485434229854	1	pseudobulk	DESeq2	LRT	0.233226307277882
108	KC_AB2	CG40045	-0.453660767215254	0.311293233154747	1	pseudobulk	DESeq2	LRT	0.506830319842684
108	KC_AB2	CG40191	-0.63550311037393	0.47258445970463	1	pseudobulk	DESeq2	LRT	0.325520563650523
108	KC_AB2	CG40198	-0.729379579623002	0.823527397767669	1	pseudobulk	DESeq2	LRT	0.084321947797948
108	KC_AB2	CG4022	-0.0011353152861548	0.951846469955695	1	pseudobulk	DESeq2	LRT	0.021433096393027
108	KC_AB2	CG40228	-0.637580185114783	0.211452769558674	1	pseudobulk	DESeq2	LRT	0.674786622192784
109	KC_AB2	CG4025	1.20557793383574	0.389187330544644	1	pseudobulk	DESeq2	LRT	0.409841306029864
109	KC_AB2	CG4036	-0.969404222682795	0.749350307836562	1	pseudobulk	DESeq2	LRT	0.125315109945074
109	KC_AB2	CG4038	-2.39970914040097	0.301766661516198	1	pseudobulk	DESeq2	LRT	0.520328741761584
109	KC_AB2	CG4041	-0.311256113568336	0.867408333719348	1	pseudobulk	DESeq2	LRT	0.061776409636023
109	KC_AB2	CG4042	-1.90302989316	0.39316374119602	1	pseudobulk	DESeq2	LRT	0.405426541004158
109	KC_AB2	CG40439	1.0379688136061	0.529179100938792	1	pseudobulk	DESeq2	LRT	0.276397315894561
109	KC_AB2	CG4045	2.64741945039871	0.292550810913063	1	pseudobulk	DESeq2	LRT	0.533798693743382
109	KC_AB2	CG40472	0.269129963583811	0.787533228059098	1	pseudobulk	DESeq2	LRT	0.103731113146907
109	KC_AB2	CG4049	0.874350016157299	0.438408508472906	1	pseudobulk	DESeq2	LRT	0.358121025812522
109	KC_AB2	CG40498	-0.193653492285031	0.534955095035334	1	pseudobulk	DESeq2	LRT	0.271682671804669
109	KC_AB2	CG4069	1.10706867860301	1	1	pseudobulk	DESeq2	LRT	0
109	KC_AB2	CG4074	-0.429347771485688	0.829237007768346	1	pseudobulk	DESeq2	LRT	0.081321324148204
109	KC_AB2	CG4080	0.572303969192865	0.456320386428264	1	pseudobulk	DESeq2	LRT	0.340730128388143
109	KC_AB2	CG4101	-0.145079396343263	0.887451496929993	1	pseudobulk	DESeq2	LRT	0.051855373699668
109	KC_AB2	CG41099	-0.078032860513564	0.900807232382812	1	pseudobulk	DESeq2	LRT	0.045368135624015
109	KC_AB2	CG41128	-0.397445383103172	0.308621983455802	1	pseudobulk	DESeq2	LRT	0.510573141936162
109	KC_AB2	CG4119	-0.376788556687894	0.730719278952839	1	pseudobulk	DESeq2	LRT	0.136249434282834
109	KC_AB2	CG4133	-0.327743901526407	0.679756688679922	1	pseudobulk	DESeq2	LRT	0.167646510342169
109	KC_AB2	CG41378	-0.264428973154515	0.624384172316112	1	pseudobulk	DESeq2	LRT	0.204548114518808

109	KC_AB2	CG4168	-1.24366137053543	0.459915587343369	1	pseudobulk	DESeq2	LRT	0.33732187117722
109	KC_AB2	CG4186	-0.701783287992825	0.611696426517263	1	pseudobulk	DESeq2	LRT	0.213464056609222
109	KC_AB2	CG4199	-0.459534289464394	0.777543471499966	1	pseudobulk	DESeq2	LRT	0.109275320751565
109	KC_AB2	CG42232	0.513490750057229	0.626815496166353	1	pseudobulk	DESeq2	LRT	0.202860275419228
109	KC_AB2	CG42238	-0.100836909777471	0.780817752272242	1	pseudobulk	DESeq2	LRT	0.107450321332357
109	KC_AB2	CG42239	0.146930209264112	1	1	pseudobulk	DESeq2	LRT	0
109	KC_AB2	CG42240	1.53600323097801	0.43223306245546	1	pseudobulk	DESeq2	LRT	0.364282016044215
109	KC_AB2	CG42258	0.187466990252239	0.594188489838085	1	pseudobulk	DESeq2	LRT	0.22607576526918
109	KC_AB2	CG42259	0.162385149200511	0.91636577592647	1	pseudobulk	DESeq2	LRT	0.037931139041603
109	KC_AB2	CG42260	0.136466092464798	0.596483993601903	1	pseudobulk	DESeq2	LRT	0.224401205947769
109	KC_AB2	CG42268	-0.519453428168175	0.213727933236769	1	pseudobulk	DESeq2	LRT	0.670138713878012
109	KC_AB2	CG42271	-0.153570174523651	0.914413875965522	1	pseudobulk	DESeq2	LRT	0.03885719226989
109	KC_AB2	CG42288	1.10706867860301	1	1	pseudobulk	DESeq2	LRT	0
109	KC_AB2	CG4230	-0.306243895792852	0.509472167486368	1	pseudobulk	DESeq2	LRT	0.292879536558934
109	KC_AB2	CG42302	-0.729379579623002	0.823527397767669	1	pseudobulk	DESeq2	LRT	0.084321947797948
109	KC_AB2	CG42307	-0.155918236559262	0.912058701751563	1	pseudobulk	DESeq2	LRT	0.039977208791815
109	KC_AB2	CG42322	0.0781215665892838	0.830182464884812	1	pseudobulk	DESeq2	LRT	0.080826444029279
109	KC_AB2	CG42324	0.376472019028585	0.425970010601607	1	pseudobulk	DESeq2	LRT	0.370620975283893
109	KC_AB2	CG42336	0.992979485516005	0.275069776739582	1	pseudobulk	DESeq2	LRT	0.560557125045779
109	KC_AB2	CG42337	-0.379833149324498	0.461275639826894	1	pseudobulk	DESeq2	LRT	0.336039480072216
109	KC_AB2	CG42339	-0.123895043894826	0.803996278578545	1	pseudobulk	DESeq2	LRT	0.094745961446254
109	KC_AB2	CG42340	-0.39841325970222	0.485798363932005	1	pseudobulk	DESeq2	LRT	0.313543952142042
109	KC_AB2	CG42342	0.395435693465223	0.691893220408741	1	pseudobulk	DESeq2	LRT	0.159960924857553
109	KC_AB2	CG42346	0.235578350921222	0.853934808110951	1	pseudobulk	DESeq2	LRT	0.068575283360620
109	KC_AB2	CG42354	-2.28054226263626	0.331630256012328	1	pseudobulk	DESeq2	LRT	0.479345853816062
109	KC_AB2	CG42359	-0.630017695052784	0.718014678875224	1	pseudobulk	DESeq2	LRT	0.14386667708173
109	KC_AB2	CG42360	0.0886882400035385	0.942568237390891	1	pseudobulk	DESeq2	LRT	0.025687199159752
109	KC_AB2	CG42361	-0.337732735745181	0.452516919372062	1	pseudobulk	DESeq2	LRT	0.344365178110594
109	KC_AB2	CG42364	-1.78975280219494	0.468855431150075	1	pseudobulk	DESeq2	LRT	0.32896104882553
109	KC_AB2	CG42365	0.579478098174944	0.746740024728732	1	pseudobulk	DESeq2	LRT	0.126830570175729
109	KC_AB2	CG42376	-1.39580133385922	0.128905024499755	1	pseudobulk	DESeq2	LRT	0.88973015425257
109	KC_AB2	CG4238	-0.89573354749665	0.254743986196031	1	pseudobulk	DESeq2	LRT	0.593896059697226
109	KC_AB2	CG42382	-0.0254956210965036	0.974202778523158	1	pseudobulk	DESeq2	LRT	0.011350636111452
109	KC_AB2	CG4239	-2.5216736243906	0.267985186584202	1	pseudobulk	DESeq2	LRT	0.571889211801602
109	KC_AB2	CG42391	-2.6040519176076	0.285294059094629	1	pseudobulk	DESeq2	LRT	0.544707271908587
109	KC_AB2	CG42392	-0.159705334856502	0.691574447348583	1	pseudobulk	DESeq2	LRT	0.160161061639471
109	KC_AB2	CG42393	1.10706867860301	1	1	pseudobulk	DESeq2	LRT	0
109	KC_AB2	CG42394	0.149388946327995	0.909949433493333	1	pseudobulk	DESeq2	LRT	0.040982741047027
109	KC_AB2	CG42399	0.266062070939207	0.86119631447911	1	pseudobulk	DESeq2	LRT	0.064897837423574
109	KC_AB2	CG42402	-0.220892022110242	0.53421594931387	1	pseudobulk	DESeq2	LRT	0.272283150018123
109	KC_AB2	CG42404	-0.300471953365536	0.631067528142876	1	pseudobulk	DESeq2	LRT	0.199924166063825
109	KC_AB2	CG42450	0.16790678934606	0.627821245692179	1	pseudobulk	DESeq2	LRT	0.202163991709001
109	KC_AB2	CG42486	-0.729379579623002	1	1	pseudobulk	DESeq2	LRT	0
109	KC_AB2	CG42495	0.268781370907002	0.88698811189061	1	pseudobulk	DESeq2	LRT	0.052082200884065
109	KC_AB2	CG42514	-0.600496552110112	0.825488100373549	1	pseudobulk	DESeq2	LRT	0.083289182824389
109	KC_AB2	CG42518	-0.729379579623002	0.772694994316916	1	pseudobulk	DESeq2	LRT	0.1119919011982
109	KC_AB2	CG42540	0.454598705983135	0.0457870642726099	1	pseudobulk	DESeq2	LRT	1.33925720122178
109	KC_AB2	CG42541	0.341984306306011	0.257539316238915	1	pseudobulk	DESeq2	LRT	0.589156461682159
109	KC_AB2	CG42542	0.521524772036218	0.562428928119087	1	pseudobulk	DESeq2	LRT	0.249932349795855
109	KC_AB2	CG42544	1.61205113368698	0.631647504209	1	pseudobulk	DESeq2	LRT	0.199525215522471
109	KC_AB2	CG42553	0.667244748290888	0.924085151242949	1	pseudobulk	DESeq2	LRT	0.034288008205147
109	KC_AB2	CG42554	0.113573166238829	0.943397466455626	1	pseudobulk	DESeq2	LRT	0.025305294409819

109	KC_AB2	CG42556	-0.729379579623002	0.823527397767669	1	pseudobulk	DESeq2	LRT	0.084321947797948
109	KC_AB2	CG42561	-0.729379579623002	1	1	pseudobulk	DESeq2	LRT	0
109	KC_AB2	CG42588	-0.9470931325005	0.428616290669404	1	pseudobulk	DESeq2	LRT	0.367931326496765
109	KC_AB2	CG42594	0.472914638015187	0.469105168539134	1	pseudobulk	DESeq2	LRT	0.32872978202692
109	KC_AB2	CG42598	1.10706867860301	1	1	pseudobulk	DESeq2	LRT	0
109	KC_AB2	CG42613	0.465422543489337	0.267264560610105	1	pseudobulk	DESeq2	LRT	0.573058625051407
109	KC_AB2	CG4266	-0.199576421327841	0.856559352895608	1	pseudobulk	DESeq2	LRT	0.067242538428807
109	KC_AB2	CG42663	-1.71999636108657	0.519869997311849	1	pseudobulk	DESeq2	LRT	0.284105245805297
109	KC_AB2	CG42668	0.0493273442488156	0.885363803319363	1	pseudobulk	DESeq2	LRT	0.052878237428328
109	KC_AB2	CG42671	0.645122580715638	0.471405610253867	1	pseudobulk	DESeq2	LRT	0.326605253190995
109	KC_AB2	CG42672	-0.282586778090566	0.8022517451286	1	pseudobulk	DESeq2	LRT	0.095689329515852
109	KC_AB2	CG42673	0.248703504226533	0.669033021150762	1	pseudobulk	DESeq2	LRT	0.174552446433003
109	KC_AB2	CG42674	0.0580318492183272	0.895608106802326	1	pseudobulk	DESeq2	LRT	0.047881983959039
109	KC_AB2	CG42676	-2.918325684408	0.155417960611578	1	pseudobulk	DESeq2	LRT	0.808498794133725
109	KC_AB2	CG42692	1.10706867860301	1	1	pseudobulk	DESeq2	LRT	0
109	KC_AB2	CG42699	0.10732471338735	0.921578622592797	1	pseudobulk	DESeq2	LRT	0.035467607920064
109	KC_AB2	CG42700	-0.217547432396009	0.682938080724805	1	pseudobulk	DESeq2	LRT	0.165618670284152
109	KC_AB2	CG42709	-0.0636000315128116	0.950802782015263	1	pseudobulk	DESeq2	LRT	0.021909556211726
109	KC_AB2	CG42716	1.10706867860301	1	1	pseudobulk	DESeq2	LRT	0
109	KC_AB2	CG42717	1.10706867860301	1	1	pseudobulk	DESeq2	LRT	0
109	KC_AB2	CG42724	0.948660480412611	0.0718169744288129	1	pseudobulk	DESeq2	LRT	1.14377289517972
109	KC_AB2	CG42747	-0.193943514347788	0.799891202364794	1	pseudobulk	DESeq2	LRT	0.096969079790369
109	KC_AB2	CG42748	0.290899400908915	0.486338739353517	1	pseudobulk	DESeq2	LRT	0.313061135290188
109	KC_AB2	CG42750	0.0157015976017106	0.941237318599253	1	pseudobulk	DESeq2	LRT	0.026300862052298
109	KC_AB2	CG42762	1.10706867860301	1	1	pseudobulk	DESeq2	LRT	0
109	KC_AB2	CG4278	-0.470100723599697	0.833985291815347	1	pseudobulk	DESeq2	LRT	0.078841608522644
109	KC_AB2	CG42784	0.965099267198389	0.0608724205487987	1	pseudobulk	DESeq2	LRT	1.21557942849037
109	KC_AB2	CG42788	-0.211231661978362	0.719548953186882	1	pseudobulk	DESeq2	LRT	0.142939654297602
109	KC_AB2	CG42795	-0.118480554610146	0.725236006123728	1	pseudobulk	DESeq2	LRT	0.139520642424477
110	KC_AB2	CG42797	-1.36024886179603	0.301308885444416	1	pseudobulk	DESeq2	LRT	0.520988061016388
110	KC_AB2	CG42806	2.48384162006255	0.303740962766822	1	pseudobulk	DESeq2	LRT	0.517496634805608
110	KC_AB2	CG42809	1.01752317615527	0.366691963298366	1	pseudobulk	DESeq2	LRT	0.435698608265249
110	KC_AB2	CG4281	0.517191850030707	0.657846744198505	1	pseudobulk	DESeq2	LRT	0.181875270369621
110	KC_AB2	CG42813	0.887845942488872	0.420864547070188	1	pseudobulk	DESeq2	LRT	0.375857656956363
110	KC_AB2	CG4282	1.10706867860301	1	1	pseudobulk	DESeq2	LRT	0
110	KC_AB2	CG42847	-1.86716056129969	0.477680380390446	1	pseudobulk	DESeq2	LRT	0.320862595985116
110	KC_AB2	CG42855	-0.893328173497901	0.679679924321693	1	pseudobulk	DESeq2	LRT	0.167695557626761
110	KC_AB2	CG4287	-0.0827062312514116	0.906918445385998	1	pseudobulk	DESeq2	LRT	0.042431765102309
110	KC_AB2	CG4291	1.11918848303371	0.620073586240309	1	pseudobulk	DESeq2	LRT	0.207556768240863
110	KC_AB2	CG4293	-0.397682675562939	0.799957429487472	1	pseudobulk	DESeq2	LRT	0.096933123796314
110	KC_AB2	CG4294	-0.531169300375544	0.527562153148251	1	pseudobulk	DESeq2	LRT	0.277726367918005
110	KC_AB2	CG4297	0.260151194116645	0.680772671860937	1	pseudobulk	DESeq2	LRT	0.166997886381473
110	KC_AB2	CG43049	-0.729379579623002	0.823527397767669	1	pseudobulk	DESeq2	LRT	0.084321947797948
110	KC_AB2	CG43056	-0.729379579623002	1	1	pseudobulk	DESeq2	LRT	0
110	KC_AB2	CG43066	0.182479223831216	0.750537150491097	1	pseudobulk	DESeq2	LRT	0.124627805947072
110	KC_AB2	CG43078	0.251576092899875	0.740145723342178	1	pseudobulk	DESeq2	LRT	0.130682765927295
110	KC_AB2	CG43085	-0.729379579623002	1	1	pseudobulk	DESeq2	LRT	0
110	KC_AB2	CG43102	-0.163373014409188	0.729264391518103	1	pseudobulk	DESeq2	LRT	0.137114991626556
110	KC_AB2	CG43117	-1.00833870937463	0.651152668420623	1	pseudobulk	DESeq2	LRT	0.186317175365195
110	KC_AB2	CG4313	0.96999401900141	0.689523601131123	1	pseudobulk	DESeq2	LRT	0.161450864128313
110	KC_AB2	CG43147	1.10706867860301	1	1	pseudobulk	DESeq2	LRT	0
110	KC_AB2	CG43163	-0.729379579623002	1	1	pseudobulk	DESeq2	LRT	0

110	KC_AB2	CG43219	3.33831449681749	0.155514673862792	1	pseudobulk	DESeq2	LRT	0.808228626077838
110	KC_AB2	CG43222	-0.220262658076413	0.61359778813985	1	pseudobulk	DESeq2	LRT	0.212116214574703
110	KC_AB2	CG43232	-2.30556784634639	0.359761931587247	1	pseudobulk	DESeq2	LRT	0.443984793676075
110	KC_AB2	CG4332	-1.71506977335689	0.409450762061488	1	pseudobulk	DESeq2	LRT	0.387798316249551
110	KC_AB2	CG43324	-0.305257971989512	0.28823514580969	1	pseudobulk	DESeq2	LRT	0.54025306481075
110	KC_AB2	CG43347	-0.465078404319926	0.382567513333871	1	pseudobulk	DESeq2	LRT	0.417291911932506
110	KC_AB2	CG43366	2.47215486522296	0.30601789350912	1	pseudobulk	DESeq2	LRT	0.514253178665238
110	KC_AB2	CG43367	0.139763241092823	0.887655599010552	1	pseudobulk	DESeq2	LRT	0.051755503209684
110	KC_AB2	CG43373	0.0592336724753468	0.888903113721817	1	pseudobulk	DESeq2	LRT	0.051145572515738
110	KC_AB2	CG4338	-0.277301926447655	0.873415243299635	1	pseudobulk	DESeq2	LRT	0.058779232814096
110	KC_AB2	CG43394	-0.729379579623002	1	1	pseudobulk	DESeq2	LRT	0
110	KC_AB2	CG4341	-0.780460565334942	0.450366582777553	1	pseudobulk	DESeq2	LRT	0.34643384163301
110	KC_AB2	CG4360	-1.26897809221856	0.309255329263239	1	pseudobulk	DESeq2	LRT	0.509682807625519
110	KC_AB2	CG4364	0.534077007852162	0.594167823155427	1	pseudobulk	DESeq2	LRT	0.226090870883784
110	KC_AB2	CG43658	-0.218231475329656	0.798114464382208	1	pseudobulk	DESeq2	LRT	0.097934818317641
110	KC_AB2	CG43679	1.10706867860301	1	1	pseudobulk	DESeq2	LRT	0
110	KC_AB2	CG43707	0.191381324977272	0.797315750498135	1	pseudobulk	DESeq2	LRT	0.098369656592879
110	KC_AB2	CG43736	0.481116286946053	0.281274941488725	1	pseudobulk	DESeq2	LRT	0.550868956984906
110	KC_AB2	CG43737	0.007156627804229	0.947469750009134	1	pseudobulk	DESeq2	LRT	0.023434646915381
110	KC_AB2	CG43740	-0.295133262884712	0.85360339346359	1	pseudobulk	DESeq2	LRT	0.068743867061114
110	KC_AB2	CG43755	-0.729379579623002	0.823527397767669	1	pseudobulk	DESeq2	LRT	0.084321947797948
110	KC_AB2	CG43759	0.146216892469931	0.651662188166111	1	pseudobulk	DESeq2	LRT	0.185977477634471
110	KC_AB2	CG43778	0.106667775807755	0.877299547527769	1	pseudobulk	DESeq2	LRT	0.056852094631228
110	KC_AB2	CG43783	-1.52634500267137	0.318476446710359	1	pseudobulk	DESeq2	LRT	0.496922680885815
110	KC_AB2	CG43789	-0.729379579623002	0.772694994316916	1	pseudobulk	DESeq2	LRT	0.1119919011982
110	KC_AB2	CG43795	-0.27773517532214	0.545123779002416	1	pseudobulk	DESeq2	LRT	0.263504873073586
110	KC_AB2	CG43800	0.643658223820713	0.775115890013421	1	pseudobulk	DESeq2	LRT	0.110633359905791
110	KC_AB2	CG43850	1.61205113368698	0.631647504209	1	pseudobulk	DESeq2	LRT	0.199525215522471
110	KC_AB2	CG43861	-0.531376324838281	0.58613895232681	1	pseudobulk	DESeq2	LRT	0.231999416276683
110	KC_AB2	CG43867	-0.123304940079545	0.793119260333479	1	pseudobulk	DESeq2	LRT	0.100661503463685
110	KC_AB2	CG4390	0.496777630414709	0.68742316384948	1	pseudobulk	DESeq2	LRT	0.162775837688299
110	KC_AB2	CG43901	-0.0315600948017758	0.927827312581264	1	pseudobulk	DESeq2	LRT	0.032532847242199
110	KC_AB2	CG43902	-2.2834221630407	0.364642497726807	1	pseudobulk	DESeq2	LRT	0.438132717324805
110	KC_AB2	CG4393	-0.538984994779925	0.77085744091785	1	pseudobulk	DESeq2	LRT	0.113025931089507
110	KC_AB2	CG44002	1.10706867860301	1	1	pseudobulk	DESeq2	LRT	0
110	KC_AB2	CG4404	0.171162206976819	0.855946625368347	1	pseudobulk	DESeq2	LRT	0.067553315966208
110	KC_AB2	CG4407	0.156924061709912	0.875701538314711	1	pseudobulk	DESeq2	LRT	0.057643887381504
110	KC_AB2	CG44085	0.39439088612385	0.813541888331052	1	pseudobulk	DESeq2	LRT	0.089620080830767
110	KC_AB2	CG4409	1.10706867860301	1	1	pseudobulk	DESeq2	LRT	0
110	KC_AB2	CG44098	0.676006581667494	0.697279153755715	1	pseudobulk	DESeq2	LRT	0.156593318509685
110	KC_AB2	CG44102	0.147593560254927	1	1	pseudobulk	DESeq2	LRT	0
110	KC_AB2	CG44153	0.575388667929316	0.340216905901632	1	pseudobulk	DESeq2	LRT	0.468244109426077
110	KC_AB2	CG44195	1.10706867860301	1	1	pseudobulk	DESeq2	LRT	0
110	KC_AB2	CG4424	-0.23099159550585	0.863223945713159	1	pseudobulk	DESeq2	LRT	0.063876520889037
110	KC_AB2	CG44242	-0.611692498930628	0.219451692849266	1	pseudobulk	DESeq2	LRT	0.658661064660614
110	KC_AB2	CG44243	0.388447834546628	0.84487503289525	1	pseudobulk	DESeq2	LRT	0.073207523639173
110	KC_AB2	CG44247	-0.0202507384918799	0.882200986854632	1	pseudobulk	DESeq2	LRT	0.054432460743418
110	KC_AB2	CG44325	-0.729379579623002	0.772694994316916	1	pseudobulk	DESeq2	LRT	0.1119919011982
110	KC_AB2	CG4438	2.01814786626755	0.401718761445944	1	pseudobulk	DESeq2	LRT	0.396077884966921
110	KC_AB2	CG44385	1.10706867860301	1	1	pseudobulk	DESeq2	LRT	0
110	KC_AB2	CG44405	-0.729379579623002	0.823527397767669	1	pseudobulk	DESeq2	LRT	0.084321947797948
110	KC_AB2	CG44422	1.68881465104187	0.456529340335333	1	pseudobulk	DESeq2	LRT	0.340531305892531

110	KC_AB2	CG4452	-0.827401499718414	0.476602238187202	1	pseudobulk	DESeq2	LRT	0.321843922480396
110	KC_AB2	CG4467	-0.0500926068661328	0.841095523604819	1	pseudobulk	DESeq2	LRT	0.075154678377333
110	KC_AB2	CG44774	-0.0509232332375836	0.887713992970931	1	pseudobulk	DESeq2	LRT	0.051726934313503
110	KC_AB2	CG44837	-0.0946875354404391	0.888233358073739	1	pseudobulk	DESeq2	LRT	0.051472920697007
110	KC_AB2	CG44838	-0.130490258873453	0.848388643792905	1	pseudobulk	DESeq2	LRT	0.071405153417009
110	KC_AB2	CG4496	1.38069928703004	0.566368801250645	1	pseudobulk	DESeq2	LRT	0.246900678019328
110	KC_AB2	CG4497	-1.1840994928738	0.429054743421061	1	pseudobulk	DESeq2	LRT	0.367487292316668
110	KC_AB2	CG45002	1.38069928703004	0.566368801250645	1	pseudobulk	DESeq2	LRT	0.246900678019328
110	KC_AB2	CG4502	-0.272018091386982	0.445706088443643	1	pseudobulk	DESeq2	LRT	0.350951433314727
110	KC_AB2	CG45049	-0.189644145035221	0.677241985763995	1	pseudobulk	DESeq2	LRT	0.169256125547755
110	KC_AB2	CG45050	0.268679682604158	0.436236543151108	1	pseudobulk	DESeq2	LRT	0.360277956767106
110	KC_AB2	CG45067	1.10706867860301	1	1	pseudobulk	DESeq2	LRT	0
110	KC_AB2	CG45071	-1.33570647694682	0.512066034235951	1	pseudobulk	DESeq2	LRT	0.290674030322833
110	KC_AB2	CG45081	1.10706867860301	1	1	pseudobulk	DESeq2	LRT	0
110	KC_AB2	CG4511	-0.923735108754637	0.52412296241937	1	pseudobulk	DESeq2	LRT	0.280566812948488
110	KC_AB2	CG45218	-1.33407500886303	0.63128114564055	1	pseudobulk	DESeq2	LRT	0.19977718147345
110	KC_AB2	CG45263	-0.228049420403089	0.33092780794708	1	pseudobulk	DESeq2	LRT	0.48026673743203
110	KC_AB2	CG4537	-0.600496552110112	0.825488100373549	1	pseudobulk	DESeq2	LRT	0.083289182824389
110	KC_AB2	CG4538	0.716361034148989	0.519446534222574	1	pseudobulk	DESeq2	LRT	0.284459147023182
110	KC_AB2	CG4552	-0.89141608166444	0.554365338995139	1	pseudobulk	DESeq2	LRT	0.256203931270937
110	KC_AB2	CG4553	2.92170020483689	0.236715975474367	1	pseudobulk	DESeq2	LRT	0.625772431440413
110	KC_AB2	CG4557	-0.335599982168614	0.806744406445812	1	pseudobulk	DESeq2	LRT	0.093264037090019
110	KC_AB2	CG4558	2.55650109639203	0.116313891638074	1	pseudobulk	DESeq2	LRT	0.93436841337581
110	KC_AB2	CG4562	0.130422798158781	0.886161961682786	1	pseudobulk	DESeq2	LRT	0.052486895904290
110	KC_AB2	CG4572	0.396659246788372	0.915482501218565	1	pseudobulk	DESeq2	LRT	0.038349952505755
111	KC_AB2	CG4577	-0.25411527711704	0.41435263368759	1	pseudobulk	DESeq2	LRT	0.382629896370535
111	KC_AB2	CG4582	-0.729379579623002	0.772694994316916	1	pseudobulk	DESeq2	LRT	0.1119919011982
111	KC_AB2	CG4587	-0.125585734367398	0.776899731872394	1	pseudobulk	DESeq2	LRT	0.109635028437781
111	KC_AB2	CG4593	0.341310869435805	0.849261178469218	1	pseudobulk	DESeq2	LRT	0.070958727983931
111	KC_AB2	CG4596	0.743285948069022	0.322322852799386	1	pseudobulk	DESeq2	LRT	0.491708901770249
111	KC_AB2	CG4598	-0.721175887303532	0.733409564198845	1	pseudobulk	DESeq2	LRT	0.134653430819787
111	KC_AB2	CG4603	-0.741654928529208	0.613330133745365	1	pseudobulk	DESeq2	LRT	0.212305697308534
111	KC_AB2	CG4607	-0.729379579623002	1	1	pseudobulk	DESeq2	LRT	0
111	KC_AB2	CG4610	2.9023801951582	0.206074905726345	1	pseudobulk	DESeq2	LRT	0.685974890162107
111	KC_AB2	CG4611	-1.86716056129969	0.477680380390446	1	pseudobulk	DESeq2	LRT	0.320862595985116
111	KC_AB2	CG4612	0.104884281133479	0.795355080391687	1	pseudobulk	DESeq2	LRT	0.099438940492230
111	KC_AB2	CG4615	-1.21429618231524	0.229601951211058	1	pseudobulk	DESeq2	LRT	0.639024425522346
111	KC_AB2	CG4617	-1.6755901375031	0.304466255318887	1	pseudobulk	DESeq2	LRT	0.516460834199912
111	KC_AB2	CG4619	0.150205259410532	1	1	pseudobulk	DESeq2	LRT	0
111	KC_AB2	CG4622	-0.539059754135504	0.759052767771577	1	pseudobulk	DESeq2	LRT	0.119728031804866
111	KC_AB2	CG4627	0.123942181828955	0.937551441668936	1	pseudobulk	DESeq2	LRT	0.028004894032192
111	KC_AB2	CG46280	-0.266411652085228	0.769134575759716	1	pseudobulk	DESeq2	LRT	0.113997664884618
111	KC_AB2	CG46281	-1.6052707068109	0.52265609184397	1	pseudobulk	DESeq2	LRT	0.281783983297782
111	KC_AB2	CG4629	-0.171465679035952	0.829007505887957	1	pseudobulk	DESeq2	LRT	0.081441537301398
111	KC_AB2	CG46298	1.38446765109058	0.642794900879723	1	pseudobulk	DESeq2	LRT	0.191927577037166
111	KC_AB2	CG46301	-0.0813003529621807	0.827551040168707	1	pseudobulk	DESeq2	LRT	0.082205211111108
111	KC_AB2	CG46304	1.38069928703004	0.566368801250645	1	pseudobulk	DESeq2	LRT	0.246900678019328
111	KC_AB2	CG4631	1.02648689492346	0.73959154128541	1	pseudobulk	DESeq2	LRT	0.131008064514051
111	KC_AB2	CG46338	0.142045623816765	0.835198214593416	1	pseudobulk	DESeq2	LRT	0.078210442733882
111	KC_AB2	CG46339	-0.574236439274169	0.043963453710065	1	pseudobulk	DESeq2	LRT	1.3569081973153
111	KC_AB2	CG46385	-0.491715752422702	0.146151606863514	1	pseudobulk	DESeq2	LRT	0.835196405430588
111	KC_AB2	CG4645	-0.803974026335663	0.54420823957816	1	pseudobulk	DESeq2	LRT	0.26423488707622

111	KC_AB2	CG4646	0.892343896250246	0.400616898896202	1	pseudobulk	DESeq2	LRT	0.39727073516581
111	KC_AB2	CG4650	-0.729379579623002	0.823527397767669	1	pseudobulk	DESeq2	LRT	0.084321947797948
111	KC_AB2	CG4658	-0.145691096518754	0.850623338281473	1	pseudobulk	DESeq2	LRT	0.070262705859222
111	KC_AB2	CG4660	-0.146391334358304	0.752412039016021	1	pseudobulk	DESeq2	LRT	0.123544264109968
111	KC_AB2	CG4661	1.10706867860301	1	1	pseudobulk	DESeq2	LRT	0
111	KC_AB2	CG4676	0.745657604431715	0.622136033480232	1	pseudobulk	DESeq2	LRT	0.206114644038413
111	KC_AB2	CG4679	1.50776466886541	0.515809803599324	1	pseudobulk	DESeq2	LRT	0.287510407819198
111	KC_AB2	CG4686	-0.554311180566228	0.530719273145405	1	pseudobulk	DESeq2	LRT	0.275135140618751
111	KC_AB2	CG4707	-1.52244591594958	0.547176617578731	1	pseudobulk	DESeq2	LRT	0.261872469536296
111	KC_AB2	CG4709	3.66164120763063	0.105565969618823	1	pseudobulk	DESeq2	LRT	0.976476058967247
111	KC_AB2	CG4743	-0.729379579623002	1	1	pseudobulk	DESeq2	LRT	0
111	KC_AB2	CG4751	-0.38544845948724	0.816777438605556	1	pseudobulk	DESeq2	LRT	0.087896267033335
111	KC_AB2	CG4768	-0.267384009454791	0.69844131539626	1	pseudobulk	DESeq2	LRT	0.155870078412017
111	KC_AB2	CG4788	-0.0782888181561201	0.946466373177535	1	pseudobulk	DESeq2	LRT	0.023894811391539
111	KC_AB2	CG4789	-1.44877365249764	0.201138455055199	1	pseudobulk	DESeq2	LRT	0.696504890011498
111	KC_AB2	CG4806	0.801055576594028	0.541976011462716	1	pseudobulk	DESeq2	LRT	0.266019935454098
111	KC_AB2	CG4813	1.38069928703004	0.566368801250645	1	pseudobulk	DESeq2	LRT	0.246900678019328
111	KC_AB2	CG4820	-0.349350206779871	0.84336127815354	1	pseudobulk	DESeq2	LRT	0.073986342934979
111	KC_AB2	CG4825	0.410383189737304	0.807369555435922	1	pseudobulk	DESeq2	LRT	0.092927631140056
111	KC_AB2	CG4829	-0.766139465682707	0.397812484566673	1	pseudobulk	DESeq2	LRT	0.400321591514914
111	KC_AB2	CG4849	0.562131270932052	0.685681719658593	1	pseudobulk	DESeq2	LRT	0.16387742871346
111	KC_AB2	CG4853	0.146041763837985	0.835183108761391	1	pseudobulk	DESeq2	LRT	0.078218297682212
111	KC_AB2	CG4854	-1.33407500886303	0.63128114564055	1	pseudobulk	DESeq2	LRT	0.19977718147345
111	KC_AB2	CG4858	-0.67912253320804	0.649181460226402	1	pseudobulk	DESeq2	LRT	0.1876338915512
111	KC_AB2	CG4866	0.375534485637874	0.901061951722844	1	pseudobulk	DESeq2	LRT	0.045245348458862
111	KC_AB2	CG4882	3.12312407990434	0.163309574433488	1	pseudobulk	DESeq2	LRT	0.786988352913433
111	KC_AB2	CG4887	-0.275235095034659	0.708940901378419	1	pseudobulk	DESeq2	LRT	0.149389966897331
111	KC_AB2	CG4896	0.0597794378911414	0.92774094967281	1	pseudobulk	DESeq2	LRT	0.032573273601469
111	KC_AB2	CG4901	-1.86716056129969	0.477680380390446	1	pseudobulk	DESeq2	LRT	0.320862595985116
111	KC_AB2	CG4908	0.361456813127167	0.738630583973854	1	pseudobulk	DESeq2	LRT	0.131572713776718
111	KC_AB2	CG4911	1.11078664374735	0.226731925471306	1	pseudobulk	DESeq2	LRT	0.644487323808969
111	KC_AB2	CG4928	1.38446765109058	0.642794900879723	1	pseudobulk	DESeq2	LRT	0.191927577037166
111	KC_AB2	CG4935	-0.441081437403916	0.911612819631012	1	pseudobulk	DESeq2	LRT	0.040189576146487
111	KC_AB2	CG4936	0.250084140754706	0.843601155788686	1	pseudobulk	DESeq2	LRT	0.073862833920335
111	KC_AB2	CG4949	-1.15253484707392	0.661144484795202	1	pseudobulk	DESeq2	LRT	0.179703620556297
111	KC_AB2	CG4951	1.29323210685144	0.489272320226568	1	pseudobulk	DESeq2	LRT	0.31044935303813
111	KC_AB2	CG4953	-0.961033333862793	0.70039622209097	1	pseudobulk	DESeq2	LRT	0.154656205149228
111	KC_AB2	CG4957	-0.502135367801406	0.803973561278985	1	pseudobulk	DESeq2	LRT	0.094758232818016
111	KC_AB2	CG4968	-0.845156976316614	0.36006786782364	1	pseudobulk	DESeq2	LRT	0.44361563300118
111	KC_AB2	CG4972	-2.39970914040097	0.301766661516198	1	pseudobulk	DESeq2	LRT	0.520328741761584
111	KC_AB2	CG4984	-0.729379579623002	0.823527397767669	1	pseudobulk	DESeq2	LRT	0.084321947797948
111	KC_AB2	CG5003	-0.579667190256024	0.691760758742976	1	pseudobulk	DESeq2	LRT	0.160044077685569
111	KC_AB2	CG5004	0.0583291182537053	0.88046533188367	1	pseudobulk	DESeq2	LRT	0.055287739603612
111	KC_AB2	CG5009	-0.214327473302368	0.89086923253313	1	pseudobulk	DESeq2	LRT	0.050186039798350
111	KC_AB2	CG5013	-2.60549329027675	0.248372566101959	1	pseudobulk	DESeq2	LRT	0.604896375679482
111	KC_AB2	CG5021	-0.821571766574591	0.330345324970217	1	pseudobulk	DESeq2	LRT	0.481031834924996
111	KC_AB2	CG5022	-0.358730459332058	0.77871178211666	1	pseudobulk	DESeq2	LRT	0.108623254262963
111	KC_AB2	CG5026	1.99339641594643	0.465071165814147	1	pseudobulk	DESeq2	LRT	0.332480585700252
111	KC_AB2	CG5027	-0.0658781080812471	0.911488112206675	1	pseudobulk	DESeq2	LRT	0.040248991119492
111	KC_AB2	CG5028	-0.36159094629469	0.57489874625261	1	pseudobulk	DESeq2	LRT	0.240408638468597
111	KC_AB2	CG5033	-0.485288229662457	0.799307674653955	1	pseudobulk	DESeq2	LRT	0.097286017076586
111	KC_AB2	CG5037	-0.338680379490913	0.745218533239756	1	pseudobulk	DESeq2	LRT	0.127716352938877

111	KC_AB2	CG5044	2.62919123543814	0.269826951733818	1	pseudobulk	DESeq2	LRT	0.568914672882509
111	KC_AB2	CG5056	0.0216501615815407	0.877174999413108	1	pseudobulk	DESeq2	LRT	0.056913754755182
111	KC_AB2	CG5059	0.615225907057051	0.335886768289175	1	pseudobulk	DESeq2	LRT	0.473807104143397
111	KC_AB2	CG5065	-0.307791671306085	0.591090152191431	1	pseudobulk	DESeq2	LRT	0.228346276118118
111	KC_AB2	CG5068	-0.28498767899769	0.790392145538176	1	pseudobulk	DESeq2	LRT	0.102157384167363
111	KC_AB2	CG5071	0.145321756433347	1	1	pseudobulk	DESeq2	LRT	0
111	KC_AB2	CG5079	1.61205113368698	0.631647504209	1	pseudobulk	DESeq2	LRT	0.199525215522471
111	KC_AB2	CG5087	-1.02685802591585	0.433847382537978	1	pseudobulk	DESeq2	LRT	0.362663018363706
111	KC_AB2	CG5098	0.265863885034613	0.795830488794415	1	pseudobulk	DESeq2	LRT	0.099179426760963
111	KC_AB2	CG5110	0.0997078945960814	0.881121326475729	1	pseudobulk	DESeq2	LRT	0.054964287057799
111	KC_AB2	CG5112	-0.729379579623002	1	1	pseudobulk	DESeq2	LRT	0
111	KC_AB2	CG5114	-0.470100723599697	0.833985291815347	1	pseudobulk	DESeq2	LRT	0.078841608522644
111	KC_AB2	CG5116	-0.729379579623002	1	1	pseudobulk	DESeq2	LRT	0
111	KC_AB2	CG5126	-1.52244591594958	0.547176617578731	1	pseudobulk	DESeq2	LRT	0.261872469536296
111	KC_AB2	CG5131	1.38446765109058	0.642794900879723	1	pseudobulk	DESeq2	LRT	0.191927577037166
111	KC_AB2	CG5149	0.0872694456224651	0.920505680068557	1	pseudobulk	DESeq2	LRT	0.035973527295957
111	KC_AB2	CG5151	-0.369854891353399	0.811074780250121	1	pseudobulk	DESeq2	LRT	0.090939102443862
111	KC_AB2	CG5160	-0.729379579623002	1	1	pseudobulk	DESeq2	LRT	0
111	KC_AB2	CG5174	-0.501768903536672	0.380765566300135	1	pseudobulk	DESeq2	LRT	0.419342332981904
111	KC_AB2	CG5180	0.587965191329595	0.710213770999975	1	pseudobulk	DESeq2	LRT	0.148610911010302
111	KC_AB2	CG5181	-0.718345386525883	0.591117622871783	1	pseudobulk	DESeq2	LRT	0.228326092923346
112	KC_AB2	CG5188	1.99339641594643	0.465071165814147	1	pseudobulk	DESeq2	LRT	0.332480585700252
112	KC_AB2	CG5191	1.04237305304479	0.767386654171747	1	pseudobulk	DESeq2	LRT	0.114985758004482
112	KC_AB2	CG5196	-0.245857142950352	0.760371231523198	1	pseudobulk	DESeq2	LRT	0.118974322931106
112	KC_AB2	CG5199	-0.729379579623002	0.772694994316916	1	pseudobulk	DESeq2	LRT	0.1119919011982
112	KC_AB2	CG5214	-0.83397994933874	0.276018793263317	1	pseudobulk	DESeq2	LRT	0.559061347164155
112	KC_AB2	CG5217	-2.87093509453135	0.225888123098267	1	pseudobulk	DESeq2	LRT	0.646106603114541
112	KC_AB2	CG5220	-1.12682145651608	0.374401531768056	1	pseudobulk	DESeq2	LRT	0.426662383125563
112	KC_AB2	CG5262	-0.169524318341919	0.912374154556569	1	pseudobulk	DESeq2	LRT	0.039827025776412
112	KC_AB2	CG5270	-0.678142266099058	0.711549586694344	1	pseudobulk	DESeq2	LRT	0.147794829272831
112	KC_AB2	CG5273	0.656870482313845	0.483344725465054	1	pseudobulk	DESeq2	LRT	0.315743016315782
112	KC_AB2	CG5274	2.13440729018205	0.325457877627974	1	pseudobulk	DESeq2	LRT	0.487505212003632
112	KC_AB2	CG5276	1.02928733579242	0.697125366781187	1	pseudobulk	DESeq2	LRT	0.156689114003336
112	KC_AB2	CG5281	0.0345815909508346	0.969810009212676	1	pseudobulk	DESeq2	LRT	0.01331333793198
112	KC_AB2	CG5282	-0.0272750763970026	0.965475286126351	1	pseudobulk	DESeq2	LRT	0.015258838649290
112	KC_AB2	CG5285	-0.814568847540344	0.67522668438328	1	pseudobulk	DESeq2	LRT	0.17055040309535
112	KC_AB2	CG5287	2.20535415462806	0.17435449065714	1	pseudobulk	DESeq2	LRT	0.758566862497926
112	KC_AB2	CG5290	-0.729379579623002	1	1	pseudobulk	DESeq2	LRT	0
112	KC_AB2	CG5292	-0.272194856628724	0.809818174303051	1	pseudobulk	DESeq2	LRT	0.091612480826487
112	KC_AB2	CG5316	-0.296919122797428	0.729009914355394	1	pseudobulk	DESeq2	LRT	0.137266565343808
112	KC_AB2	CG5323	-0.811860000959043	0.627237119867208	1	pseudobulk	DESeq2	LRT	0.202568248033329
112	KC_AB2	CG5335	-0.729379579623002	0.772694994316916	1	pseudobulk	DESeq2	LRT	0.1119919011982
112	KC_AB2	CG5339	-1.50128259374009	0.482047325167343	1	pseudobulk	DESeq2	LRT	0.316910322653065
112	KC_AB2	CG5348	-2.13593891537277	0.345336770345161	1	pseudobulk	DESeq2	LRT	0.46175717691322
112	KC_AB2	CG5355	-1.57685088282527	0.45256857946707	1	pseudobulk	DESeq2	LRT	0.344315601149633
112	KC_AB2	CG5359	-0.824996233971657	0.664071509040402	1	pseudobulk	DESeq2	LRT	0.177785152093585
112	KC_AB2	CG5380	-0.734490307341015	0.691285120313097	1	pseudobulk	DESeq2	LRT	0.160342791064134
112	KC_AB2	CG5382	-1.13684113428818	0.451950671894501	1	pseudobulk	DESeq2	LRT	0.344908963624635
112	KC_AB2	CG5412	-0.372854217900195	0.814483485662669	1	pseudobulk	DESeq2	LRT	0.089117716951892
112	KC_AB2	CG5439	-1.21363965631423	0.568763379520138	1	pseudobulk	DESeq2	LRT	0.24506837392442
112	KC_AB2	CG5445	0.0996908277343758	0.904431071871425	1	pseudobulk	DESeq2	LRT	0.043624525815289
112	KC_AB2	CG5446	-0.221364733460059	0.814597515359962	1	pseudobulk	DESeq2	LRT	0.089056918908876

112	KC_AB2	CG5447	-0.0953518600803178	0.916314497664432	1	pseudobulk	DESeq2	LRT	0.037955442098248
112	KC_AB2	CG5455	-2.34208170650179	0.0474758880410467	1	pseudobulk	DESeq2	LRT	1.32352690299496
112	KC_AB2	CG5466	-1.2469628188333	0.229942745568503	1	pseudobulk	DESeq2	LRT	0.638380287369639
112	KC_AB2	CG5498	1.10706867860301	1	1	pseudobulk	DESeq2	LRT	0
112	KC_AB2	CG5500	0.209080069562842	0.842803510501132	1	pseudobulk	DESeq2	LRT	0.074273664107963
112	KC_AB2	CG5504	-0.191152107167456	0.880350455807559	1	pseudobulk	DESeq2	LRT	0.055344406571704
112	KC_AB2	CG5510	-0.554575191474136	0.674484959286385	1	pseudobulk	DESeq2	LRT	0.171027730457248
112	KC_AB2	CG5515	0.467791553006124	0.636546433849689	1	pseudobulk	DESeq2	LRT	0.196169910581956
112	KC_AB2	CG5516	0.0889254883999854	0.939560617253976	1	pseudobulk	DESeq2	LRT	0.027075195457369
112	KC_AB2	CG5521	0.356857420492616	0.662776501315291	1	pseudobulk	DESeq2	LRT	0.178632897853808
112	KC_AB2	CG5522	0.501217635448163	0.655253528701462	1	pseudobulk	DESeq2	LRT	0.183590631592431
112	KC_AB2	CG5532	0.0619598637376955	0.885363955868922	1	pseudobulk	DESeq2	LRT	0.052878162598721
112	KC_AB2	CG5535	-0.389511547063832	0.74958911411283	1	pseudobulk	DESeq2	LRT	0.125176729104079
112	KC_AB2	CG5541	-0.729379579623002	0.823527397767669	1	pseudobulk	DESeq2	LRT	0.084321947797948
112	KC_AB2	CG5554	0.505759695055143	0.716765854757615	1	pseudobulk	DESeq2	LRT	0.144622691743753
112	KC_AB2	CG5555	-0.0664815203066391	0.924072821440784	1	pseudobulk	DESeq2	LRT	0.034293802910061
112	KC_AB2	CG5567	-0.350224135645729	0.825372845318855	1	pseudobulk	DESeq2	LRT	0.083349823467051
112	KC_AB2	CG5569	0.38245451307505	0.830649933977651	1	pseudobulk	DESeq2	LRT	0.080581965112585
112	KC_AB2	CG5577	1.38446765109058	0.642794900879723	1	pseudobulk	DESeq2	LRT	0.191927577037166
112	KC_AB2	CG5589	0.643658223820713	0.775115890013421	1	pseudobulk	DESeq2	LRT	0.110633359905791
112	KC_AB2	CG5590	-1.16016699006818	0.583985689825587	1	pseudobulk	DESeq2	LRT	0.233597794849804
112	KC_AB2	CG5599	1.02605719768525	0.762384804222891	1	pseudobulk	DESeq2	LRT	0.117825768597322
112	KC_AB2	CG5608	0.39439088612385	0.813541888331052	1	pseudobulk	DESeq2	LRT	0.089620080830767
112	KC_AB2	CG5611	2.16548735034375	0.319952228384207	1	pseudobulk	DESeq2	LRT	0.494914860736039
112	KC_AB2	CG5613	-0.729379579623002	1	1	pseudobulk	DESeq2	LRT	0
112	KC_AB2	CG5618	0.720557559963523	0.758319263499194	1	pseudobulk	DESeq2	LRT	0.120147911539297
112	KC_AB2	CG5641	0.100798925087121	0.884275961173455	1	pseudobulk	DESeq2	LRT	0.053412181014068
112	KC_AB2	CG5644	0.688404500789555	0.496861283464158	1	pseudobulk	DESeq2	LRT	0.303764843127609
112	KC_AB2	CG5645	-1.52244591594958	0.547176617578731	1	pseudobulk	DESeq2	LRT	0.261872469536296
112	KC_AB2	CG5646	1.07598520382199	0.506093322421112	1	pseudobulk	DESeq2	LRT	0.29576939289235
112	KC_AB2	CG5664	-0.795404628052927	0.767318925015269	1	pseudobulk	DESeq2	LRT	0.115024090306574
112	KC_AB2	CG5674	1.52502387524934	0.222942995874938	1	pseudobulk	DESeq2	LRT	0.651806167183613
112	KC_AB2	CG5676	-0.440850141497748	0.469563090018325	1	pseudobulk	DESeq2	LRT	0.328306048113429
112	KC_AB2	CG5708	-0.387400744692138	0.523683073938622	1	pseudobulk	DESeq2	LRT	0.280931462787172
112	KC_AB2	CG5721	-3.22751840048416	0.100841934252849	1	pseudobulk	DESeq2	LRT	0.996358832697273
112	KC_AB2	CG5726	1.65756225530857	0.29153466566651	1	pseudobulk	DESeq2	LRT	0.535309796951078
112	KC_AB2	CG5727	-0.346214264300902	0.777111323786513	1	pseudobulk	DESeq2	LRT	0.109516762610842
112	KC_AB2	CG5728	-1.63222955096776	0.49068522332581	1	pseudobulk	DESeq2	LRT	0.309197020324952
112	KC_AB2	CG5742	-0.0136973295613676	0.980232707007433	1	pseudobulk	DESeq2	LRT	0.008670810662536
112	KC_AB2	CG5745	-0.311256113568336	0.867408333719348	1	pseudobulk	DESeq2	LRT	0.061776409636023
112	KC_AB2	CG5746	-0.387480974776974	0.42121579503976	1	pseudobulk	DESeq2	LRT	0.375495351707841
112	KC_AB2	CG5758	-1.36067772985929	0.291773947712763	1	pseudobulk	DESeq2	LRT	0.534953488555312
112	KC_AB2	CG5776	-0.729379579623002	1	1	pseudobulk	DESeq2	LRT	0
112	KC_AB2	CG5787	-1.41813487571249	0.473691038140433	1	pseudobulk	DESeq2	LRT	0.324504831702221
112	KC_AB2	CG5805	-0.812894984236214	0.48936078133982	1	pseudobulk	DESeq2	LRT	0.310370839091705
112	KC_AB2	CG5808	3.4911662448989	0.102118164682477	1	pseudobulk	DESeq2	LRT	0.990896999150157
112	KC_AB2	CG5815	2.58961269685837	0.206717236663161	1	pseudobulk	DESeq2	LRT	0.684623309170508
112	KC_AB2	CG5823	-0.291488209687637	0.701455773870106	1	pseudobulk	DESeq2	LRT	0.153999705632988
112	KC_AB2	CG5828	-1.5033370644665	0.499350065149774	1	pseudobulk	DESeq2	LRT	0.30159488912562
112	KC_AB2	CG5830	0.110461825633594	0.581059748539845	1	pseudobulk	DESeq2	LRT	0.235779208182799
112	KC_AB2	CG5846	1.61205113368698	0.631647504209	1	pseudobulk	DESeq2	LRT	0.199525215522471
112	KC_AB2	CG5850	-0.256235494911363	1	1	pseudobulk	DESeq2	LRT	0

112	KC_AB2	CG5854	1.38446765109058	0.642794900879723	1	pseudobulk	DESeq2	LRT	0.191927577037166
112	KC_AB2	CG5861	-1.36966261209577	0.144975089882603	1	pseudobulk	DESeq2	LRT	0.838706613322935
112	KC_AB2	CG5862	-2.81379877664096	0.111128458567084	1	pseudobulk	DESeq2	LRT	0.954174709592246
112	KC_AB2	CG5877	-2.62316164937934	0.247937455125287	1	pseudobulk	DESeq2	LRT	0.605657860785625
112	KC_AB2	CG5880	-0.672424505335587	0.790771522020703	1	pseudobulk	DESeq2	LRT	0.101948979282445
112	KC_AB2	CG5885	0.693105884974498	0.47074624791638	1	pseudobulk	DESeq2	LRT	0.327213132842271
112	KC_AB2	CG5886	0.475798194954997	0.693681155260972	1	pseudobulk	DESeq2	LRT	0.158840103507299
112	KC_AB2	CG5888	0.562421548979232	0.776682669513024	1	pseudobulk	DESeq2	LRT	0.1097563853551
112	KC_AB2	CG5890	-0.165143913118108	0.539084249328691	1	pseudobulk	DESeq2	LRT	0.268343356966698
112	KC_AB2	CG5895	1.10706867860301	1	1	pseudobulk	DESeq2	LRT	0
112	KC_AB2	CG5902	-0.0686652919111462	0.961242182172663	1	pseudobulk	DESeq2	LRT	0.017167179313352
112	KC_AB2	CG5903	-0.499854042753533	0.277487200863426	1	pseudobulk	DESeq2	LRT	0.556757043974029
112	KC_AB2	CG5913	-0.962513467986815	0.647790837194539	1	pseudobulk	DESeq2	LRT	0.188565199246487
112	KC_AB2	CG5916	-0.735954819961447	0.401624573544266	1	pseudobulk	DESeq2	LRT	0.396179722585699
112	KC_AB2	CG5934	-0.0502545703899777	0.940417421816198	1	pseudobulk	DESeq2	LRT	0.026679333897847
112	KC_AB2	CG5937	0.727534378883475	0.410348526396414	1	pseudobulk	DESeq2	LRT	0.3868471218339
112	KC_AB2	CG5938	-0.483531169028956	0.483080503864056	1	pseudobulk	DESeq2	LRT	0.315980489392207
113	KC_AB2	CG5946	0.599442403941293	0.366313273703726	1	pseudobulk	DESeq2	LRT	0.436147343984003
113	KC_AB2	CG5953	-1.50762400794157	0.534858155142685	1	pseudobulk	DESeq2	LRT	0.271761377991121
113	KC_AB2	CG5961	0.978708996173918	0.428398782315165	1	pseudobulk	DESeq2	LRT	0.368151772281256
113	KC_AB2	CG5968	1.10706867860301	1	1	pseudobulk	DESeq2	LRT	0
113	KC_AB2	CG5969	-1.68061190037671	0.257410652872157	1	pseudobulk	DESeq2	LRT	0.589373483896823
113	KC_AB2	CG5973	-0.729379579623002	0.823527397767669	1	pseudobulk	DESeq2	LRT	0.084321947797948
113	KC_AB2	CG5986	-0.808932771582168	0.364995190712156	1	pseudobulk	DESeq2	LRT	0.437712857902245
113	KC_AB2	CG5989	-1.22853269946298	0.392792625633018	1	pseudobulk	DESeq2	LRT	0.405836674339648
113	KC_AB2	CG6005	0.603822174712652	0.611286408498732	1	pseudobulk	DESeq2	LRT	0.213755260319531
113	KC_AB2	CG6006	1.95432904991983	0.509212778150152	1	pseudobulk	DESeq2	LRT	0.293100706726022
113	KC_AB2	CG6013	-0.919814048681515	0.268794447733302	1	pseudobulk	DESeq2	LRT	0.57057970639088
113	KC_AB2	CG6024	-0.160687731027628	0.703213404975037	1	pseudobulk	DESeq2	LRT	0.152912859135649
113	KC_AB2	CG6028	2.16446168587495	0.353340127823748	1	pseudobulk	DESeq2	LRT	0.451807038186996
113	KC_AB2	CG6040	0.145321756433347	1	1	pseudobulk	DESeq2	LRT	0
113	KC_AB2	CG6041	1.61152931644013	0.194014297518101	1	pseudobulk	DESeq2	LRT	0.712166264376897
113	KC_AB2	CG6044	-0.877061295727796	0.000522372200537176	1	pseudobulk	DESeq2	LRT	3.28201994328457
113	KC_AB2	CG6051	0.981107816003812	0.263639328544919	1	pseudobulk	DESeq2	LRT	0.578989803122018
113	KC_AB2	CG6066	2.16987893176541	0.343129001386579	1	pseudobulk	DESeq2	LRT	0.464542573666734
113	KC_AB2	CG6083	-1.8431539046271	0.376153284809138	1	pseudobulk	DESeq2	LRT	0.424635141293275
113	KC_AB2	CG6084	-1.34487879216434	0.263753185805343	1	pseudobulk	DESeq2	LRT	0.578802285933777
113	KC_AB2	CG6094	-0.349350206779871	0.84336127815354	1	pseudobulk	DESeq2	LRT	0.073986342934979
113	KC_AB2	CG6115	-0.0025221520131404	0.920931850039021	1	pseudobulk	DESeq2	LRT	0.035772506879241
113	KC_AB2	CG6136	1.43061234575529	0.44489683943264	1	pseudobulk	DESeq2	LRT	0.351740679488727
113	KC_AB2	CG6144	-0.808673106901415	0.446552832240018	1	pseudobulk	DESeq2	LRT	0.350127151796843
113	KC_AB2	CG6145	-0.650816137511009	0.638665625326302	1	pseudobulk	DESeq2	LRT	0.194726458091035
113	KC_AB2	CG6153	-0.256235494911363	1	1	pseudobulk	DESeq2	LRT	0
113	KC_AB2	CG6154	-2.45398554909617	0.206959437181751	1	pseudobulk	DESeq2	LRT	0.684114765337089
113	KC_AB2	CG6171	-0.9877321374250620	0.473411331929981	1	pseudobulk	DESeq2	LRT	0.324761350682901
113	KC_AB2	CG6175	1.10706867860301	1	1	pseudobulk	DESeq2	LRT	0
113	KC_AB2	CG6178	-1.57890769361772	0.356175270517934	1	pseudobulk	DESeq2	LRT	0.448336237176064
113	KC_AB2	CG6179	0.667244748290888	0.924085151242949	1	pseudobulk	DESeq2	LRT	0.034288008205147
113	KC_AB2	CG6180	0.330135187775193	0.666985457539036	1	pseudobulk	DESeq2	LRT	0.175883635018085
113	KC_AB2	CG6184	-0.558656529654356	0.602141887989759	1	pseudobulk	DESeq2	LRT	0.220301160054034
113	KC_AB2	CG6191	0.047919343606564	0.905180980522816	1	pseudobulk	DESeq2	LRT	0.043264579928056
113	KC_AB2	CG6195	0.214138303528418	0.89870431627244	1	pseudobulk	DESeq2	LRT	0.046383172486725

113	KC_AB2	CG6201	-2.46448661783174	0.198851523452658	1	pseudobulk	DESeq2	LRT	0.701471077423547
113	KC_AB2	CG6218	-2.28054226263626	0.331630256012328	1	pseudobulk	DESeq2	LRT	0.479345853816062
113	KC_AB2	CG6227	-0.290826466720231	0.817647422079908	1	pseudobulk	DESeq2	LRT	0.087433928180042
113	KC_AB2	CG6230	-1.57685088282527	0.45256857946707	1	pseudobulk	DESeq2	LRT	0.344315601149633
113	KC_AB2	CG6236	-0.783393898289894	0.657611202088378	1	pseudobulk	DESeq2	LRT	0.182030797408803
113	KC_AB2	CG6254	1.17552337668403	0.55284650689925	1	pseudobulk	DESeq2	LRT	0.257395430105368
113	KC_AB2	CG6276	0.447439455579362	0.682948265042077	1	pseudobulk	DESeq2	LRT	0.165612193913982
113	KC_AB2	CG6282	0.202140881803361	0.669410739760647	1	pseudobulk	DESeq2	LRT	0.174307324273774
113	KC_AB2	CG6287	1.10706867860301	1	1	pseudobulk	DESeq2	LRT	0
113	KC_AB2	CG6293	-0.729379579623002	1	1	pseudobulk	DESeq2	LRT	0
113	KC_AB2	CG6321	-0.968936593053995	0.673838136795855	1	pseudobulk	DESeq2	LRT	0.171444413165975
113	KC_AB2	CG6325	-3.06712017766516	0.131619576286216	1	pseudobulk	DESeq2	LRT	0.880679511627911
113	KC_AB2	CG6329	-0.104282829421692	0.64969029341667	1	pseudobulk	DESeq2	LRT	0.18729362168633
113	KC_AB2	CG6330	-0.0038122329896682	0.984200155045491	1	pseudobulk	DESeq2	LRT	0.006916570885563
113	KC_AB2	CG6337	-0.729379579623002	0.823527397767669	1	pseudobulk	DESeq2	LRT	0.084321947797948
113	KC_AB2	CG6340	0.0943193565377805	0.877800527222208	1	pseudobulk	DESeq2	LRT	0.056604162646175
113	KC_AB2	CG6356	1.10706867860301	1	1	pseudobulk	DESeq2	LRT	0
113	KC_AB2	CG6379	-0.225432490582687	0.85645651204832	1	pseudobulk	DESeq2	LRT	0.067294684136805
113	KC_AB2	CG6388	1.01111288088582	0.663328951158366	1	pseudobulk	DESeq2	LRT	0.178271047396428
113	KC_AB2	CG6403	1.38446765109058	0.642794900879723	1	pseudobulk	DESeq2	LRT	0.191927577037166
113	KC_AB2	CG6418	-0.0648343390289289	0.947738168880787	1	pseudobulk	DESeq2	LRT	0.023311628396500
113	KC_AB2	CG6420	-0.578154003412626	0.617750029404837	1	pseudobulk	DESeq2	LRT	0.209187225253511
113	KC_AB2	CG6425	0.388447834546628	0.84487503289525	1	pseudobulk	DESeq2	LRT	0.073207523639173
113	KC_AB2	CG6428	0.640234219807562	0.725658688827097	1	pseudobulk	DESeq2	LRT	0.139267600254702
113	KC_AB2	CG6443	-0.66832934274197	0.519763194615216	1	pseudobulk	DESeq2	LRT	0.284194476934745
113	KC_AB2	CG6454	0.226738553137184	0.835950721106365	1	pseudobulk	DESeq2	LRT	0.077819323257602
113	KC_AB2	CG6472	-0.969404222682795	0.749350307836562	1	pseudobulk	DESeq2	LRT	0.125315109945074
113	KC_AB2	CG6479	0.39439088612385	0.813541888331052	1	pseudobulk	DESeq2	LRT	0.089620080830767
113	KC_AB2	CG6495	0.246883658031511	0.758964256465575	1	pseudobulk	DESeq2	LRT	0.119778676786065
113	KC_AB2	CG6511	1.10706867860301	1	1	pseudobulk	DESeq2	LRT	0
113	KC_AB2	CG6512	-0.0008200417956720	0.934746201574799	1	pseudobulk	DESeq2	LRT	0.029306290964816
113	KC_AB2	CG6523	-0.987706070754354	0.312486839679359	1	pseudobulk	DESeq2	LRT	0.505168268159859
113	KC_AB2	CG6550	1.37686412499759	0.622972562188606	1	pseudobulk	DESeq2	LRT	0.205531080712408
113	KC_AB2	CG6565	0.532937724813817	0.83094555443934	1	pseudobulk	DESeq2	LRT	0.080427431307578
113	KC_AB2	CG6567	2.04030215638816	0.327981738462365	1	pseudobulk	DESeq2	LRT	0.484150336488967
113	KC_AB2	CG6574	1.10706867860301	1	1	pseudobulk	DESeq2	LRT	0
113	KC_AB2	CG6583	-0.24599992179689	0.804336411164268	1	pseudobulk	DESeq2	LRT	0.094562270959529
113	KC_AB2	CG6607	-0.711058602249914	0.578675547304245	1	pseudobulk	DESeq2	LRT	0.237564868932815
113	KC_AB2	CG6610	-1.93857404258473	0.269912219391932	1	pseudobulk	DESeq2	LRT	0.568777453737807
113	KC_AB2	CG6617	-0.274798096832635	0.716694647461139	1	pseudobulk	DESeq2	LRT	0.14466583898905
113	KC_AB2	CG6621	0.264088868452584	0.855966783964928	1	pseudobulk	DESeq2	LRT	0.067543087917043
113	KC_AB2	CG6638	1.38446765109058	0.642794900879723	1	pseudobulk	DESeq2	LRT	0.191927577037166
113	KC_AB2	CG6650	-1.35408099671499	0.617991261467677	1	pseudobulk	DESeq2	LRT	0.209017665887235
113	KC_AB2	CG6654	0.0107214417965268	0.937943862816314	1	pseudobulk	DESeq2	LRT	0.027823153943621
113	KC_AB2	CG6659	0.145321756433347	1	1	pseudobulk	DESeq2	LRT	0
113	KC_AB2	CG6664	0.148116938421691	0.900263843490553	1	pseudobulk	DESeq2	LRT	0.045630191694714
113	KC_AB2	CG6665	-1.01397824610998	0.639310995576363	1	pseudobulk	DESeq2	LRT	0.194287826025898
113	KC_AB2	CG6674	-1.37138537813394	0.262136289356352	1	pseudobulk	DESeq2	LRT	0.581472852474753
113	KC_AB2	CG6685	1.37200440170938	0.48893549025908	1	pseudobulk	DESeq2	LRT	0.310748437548461
113	KC_AB2	CG6686	-0.144252726819504	0.839516507844971	1	pseudobulk	DESeq2	LRT	0.075970759686156
113	KC_AB2	CG6689	0.760706891684122	0.701177403860344	1	pseudobulk	DESeq2	LRT	0.154172087924629
113	KC_AB2	CG6693	-1.10908348749736	0.263973805334839	1	pseudobulk	DESeq2	LRT	0.578439166929252

113	KC_AB2	CG6695	-2.39970914040097	0.301766661516198	1	pseudobulk	DESeq2	LRT	0.520328741761584
113	KC_AB2	CG6700	0.279017131088034	0.416788194591608	1	pseudobulk	DESeq2	LRT	0.380084590803377
113	KC_AB2	CG6707	0.1741568802092	0.767874140151892	1	pseudobulk	DESeq2	LRT	0.114709957985254
113	KC_AB2	CG6712	-2.45416506545088	0.321711556460218	1	pseudobulk	DESeq2	LRT	0.492533338175562
113	KC_AB2	CG6724	0.216598803308022	0.899737058780134	1	pseudobulk	DESeq2	LRT	0.045884391233312
113	KC_AB2	CG6729	1.10706867860301	1	1	pseudobulk	DESeq2	LRT	0
113	KC_AB2	CG6752	1.10706867860301	1	1	pseudobulk	DESeq2	LRT	0
113	KC_AB2	CG6758	-0.136415338406465	0.925618576865565	1	pseudobulk	DESeq2	LRT	0.033567937819005
113	KC_AB2	CG6762	-0.859988838384602	0.577476476840146	1	pseudobulk	DESeq2	LRT	0.238465701802207
113	KC_AB2	CG6765	0.332685795479674	0.590621756583076	1	pseudobulk	DESeq2	LRT	0.228690559081164
113	KC_AB2	CG6766	-2.53890838511639	0.162059043960256	1	pseudobulk	DESeq2	LRT	0.790326727467717
113	KC_AB2	CG6767	-0.0355618483022867	0.898835064600692	1	pseudobulk	DESeq2	LRT	0.046319993589859
113	KC_AB2	CG6769	-0.768549197643566	0.635329691590297	1	pseudobulk	DESeq2	LRT	0.197000847808915
114	KC_AB2	CG6770	0.864610055004827	0.0120492209992095	1	pseudobulk	DESeq2	LRT	1.91904102999208
114	KC_AB2	CG6791	-0.354944735900431	0.786229574057537	1	pseudobulk	DESeq2	LRT	0.104450624204009
114	KC_AB2	CG6805	-1.00833870937463	0.651152668420623	1	pseudobulk	DESeq2	LRT	0.186317175365195
114	KC_AB2	CG6808	-1.42073461079967	0.447791631833179	1	pseudobulk	DESeq2	LRT	0.348924026618158
114	KC_AB2	CG6813	-0.176134029389346	0.905414860151646	1	pseudobulk	DESeq2	LRT	0.043152381909576
114	KC_AB2	CG6833	0.38245451307505	0.830649933977651	1	pseudobulk	DESeq2	LRT	0.080581965112585
114	KC_AB2	CG6836	-0.729379579623002	0.772694994316916	1	pseudobulk	DESeq2	LRT	0.1119919011982
114	KC_AB2	CG6841	0.293315361421469	0.82161693884506	1	pseudobulk	DESeq2	LRT	0.085330615697162
114	KC_AB2	CG6843	0.636992642553719	0.511827813196138	1	pseudobulk	DESeq2	LRT	0.290876117844289
114	KC_AB2	CG6867	-0.765170686641463	0.518852172795412	1	pseudobulk	DESeq2	LRT	0.284956360232681
114	KC_AB2	CG6878	-0.912425681204123	0.331569153222189	1	pseudobulk	DESeq2	LRT	0.479425879836266
114	KC_AB2	CG6903	0.105513328384322	0.921783749038961	1	pseudobulk	DESeq2	LRT	0.035370952714989
114	KC_AB2	CG6907	1.95432904991983	0.509212778150152	1	pseudobulk	DESeq2	LRT	0.293100706726022
114	KC_AB2	CG6908	1.10706867860301	1	1	pseudobulk	DESeq2	LRT	0
114	KC_AB2	CG6923	-0.190367160542965	0.873455141329984	1	pseudobulk	DESeq2	LRT	0.058759394485420
114	KC_AB2	CG6928	-0.734071467759236	0.785001998672014	1	pseudobulk	DESeq2	LRT	0.105129237508095
114	KC_AB2	CG6937	1.94871771291584	0.412369540323867	1	pseudobulk	DESeq2	LRT	0.38471342136576
114	KC_AB2	CG6938	0.396659246788372	0.915482501218565	1	pseudobulk	DESeq2	LRT	0.038349952505755
114	KC_AB2	CG6951	-0.427692090780194	0.83957454067488	1	pseudobulk	DESeq2	LRT	0.075940739470360
114	KC_AB2	CG6961	-0.108150992064891	0.905978176473916	1	pseudobulk	DESeq2	LRT	0.042882263637873
114	KC_AB2	CG6962	-0.729379579623002	0.823527397767669	1	pseudobulk	DESeq2	LRT	0.084321947797948
114	KC_AB2	CG6966	0.46894999588844	0.459792108204932	1	pseudobulk	DESeq2	LRT	0.337438487160831
114	KC_AB2	CG6983	-0.11954425546865	0.880897511518124	1	pseudobulk	DESeq2	LRT	0.055074616867540
114	KC_AB2	CG6984	-0.647621034217714	0.754702560287896	1	pseudobulk	DESeq2	LRT	0.122224176685402
114	KC_AB2	CG7006	2.48384162006255	0.303740962766822	1	pseudobulk	DESeq2	LRT	0.517496634805608
114	KC_AB2	CG7009	-1.93335857128809	0.384223899985767	1	pseudobulk	DESeq2	LRT	0.415415624094039
114	KC_AB2	CG7011	-0.720747651968478	0.756336938577071	1	pseudobulk	DESeq2	LRT	0.121284688671136
114	KC_AB2	CG7028	0.441670335719955	0.588734550688692	1	pseudobulk	DESeq2	LRT	0.230080476276948
114	KC_AB2	CG7029	-0.122534394370892	0.842601952155963	1	pseudobulk	DESeq2	LRT	0.074377539026426
114	KC_AB2	CG7044	0.147593560254927	1	1	pseudobulk	DESeq2	LRT	0
114	KC_AB2	CG7049	-1.67513874790658	0.39900884344985	1	pseudobulk	DESeq2	LRT	0.39901747870195
114	KC_AB2	CG7058	-0.653079383968656	0.629688236051715	1	pseudobulk	DESeq2	LRT	0.20087442018936
114	KC_AB2	CG7059	-0.311256113568336	0.867408333719348	1	pseudobulk	DESeq2	LRT	0.061776409636023
114	KC_AB2	CG7065	0.585108878598068	0.609550393086064	1	pseudobulk	DESeq2	LRT	0.214990384328591
114	KC_AB2	CG7069	-0.729379579623002	0.772694994316916	1	pseudobulk	DESeq2	LRT	0.1119919011982
114	KC_AB2	CG7083	1.78997141568059	0.233488522617501	1	pseudobulk	DESeq2	LRT	0.631734462791209
114	KC_AB2	CG7099	1.38446765109058	0.642794900879723	1	pseudobulk	DESeq2	LRT	0.191927577037166
114	KC_AB2	CG7101	0.403294498144138	0.89479244414645	1	pseudobulk	DESeq2	LRT	0.048277691852293
114	KC_AB2	CG7110	1.38446765109058	0.642794900879723	1	pseudobulk	DESeq2	LRT	0.191927577037166

114	KC_AB2	CG7115	0.18500285695668	0.818771014523935	1	pseudobulk	DESeq2	LRT	0.086837540283013
114	KC_AB2	CG7120	-0.47035829084506	0.578505235294341	1	pseudobulk	DESeq2	LRT	0.237692706463043
114	KC_AB2	CG7133	-0.29071286829462	0.858030201396282	1	pseudobulk	DESeq2	LRT	0.066497425357691
114	KC_AB2	CG7137	1.10706867860301	1	1	pseudobulk	DESeq2	LRT	0
114	KC_AB2	CG7139	1.18022061141176	0.413794048199544	1	pseudobulk	DESeq2	LRT	0.383215760293495
114	KC_AB2	CG7148	-1.68583823399618	0.353560440138572	1	pseudobulk	DESeq2	LRT	0.45153633413028
114	KC_AB2	CG7149	1.37554157525252	0.602797270183386	1	pseudobulk	DESeq2	LRT	0.219828723090944
114	KC_AB2	CG7154	0.389896191428959	0.637284857256948	1	pseudobulk	DESeq2	LRT	0.19566640081698
114	KC_AB2	CG7156	-1.74578352204418	0.47644265335571	1	pseudobulk	DESeq2	LRT	0.321989365392845
114	KC_AB2	CG7166	0.91799008206237	0.0966394432977496	1	pseudobulk	DESeq2	LRT	1.01484558051735
114	KC_AB2	CG7168	-0.924513449326059	0.611719569564352	1	pseudobulk	DESeq2	LRT	0.213447625734232
114	KC_AB2	CG7182	-1.63344800533453	0.320520193665452	1	pseudobulk	DESeq2	LRT	0.494144603520508
114	KC_AB2	CG7183	-1.92832080064225	0.352157092425653	1	pseudobulk	DESeq2	LRT	0.453263560515136
114	KC_AB2	CG7194	1.10706867860301	1	1	pseudobulk	DESeq2	LRT	0
114	KC_AB2	CG7206	0.135763089443803	0.914099572816242	1	pseudobulk	DESeq2	LRT	0.039006494015318
114	KC_AB2	CG7218	1.69157571537722	0.366387042685409	1	pseudobulk	DESeq2	LRT	0.436059893600615
114	KC_AB2	CG7220	0.612429674225294	0.451440731219198	1	pseudobulk	DESeq2	LRT	0.345399259290812
114	KC_AB2	CG7222	-0.13348406150289	0.877543828722951	1	pseudobulk	DESeq2	LRT	0.056731183583300
114	KC_AB2	CG7231	-1.09783264521657	0.224255365248406	1	pseudobulk	DESeq2	LRT	0.649257157782631
114	KC_AB2	CG7239	-0.81845626141508	0.531690049999436	1	pseudobulk	DESeq2	LRT	0.274341466958293
114	KC_AB2	CG7246	-0.441081437403916	0.911612819631012	1	pseudobulk	DESeq2	LRT	0.040189576146487
114	KC_AB2	CG7265	-3.09998517663387	0.147473502521391	1	pseudobulk	DESeq2	LRT	0.831286005058787
114	KC_AB2	CG7272	-0.893328173497901	0.679679924321693	1	pseudobulk	DESeq2	LRT	0.167695557626761
114	KC_AB2	CG7275	3.20939298820062	0.0799645076148474	1	pseudobulk	DESeq2	LRT	1.09710273259947
114	KC_AB2	CG7289	-1.80075127281421	0.363208620892189	1	pseudobulk	DESeq2	LRT	0.439843851886983
114	KC_AB2	CG7324	0.612209495157057	0.551965686393035	1	pseudobulk	DESeq2	LRT	0.258087919867461
114	KC_AB2	CG7326	0.769674397209766	0.611362411965087	1	pseudobulk	DESeq2	LRT	0.213701266260839
114	KC_AB2	CG7332	-0.131316354303648	0.840920753803362	1	pseudobulk	DESeq2	LRT	0.075244929062381
114	KC_AB2	CG7339	-0.232710601599884	0.833905611801002	1	pseudobulk	DESeq2	LRT	0.078883103551485
114	KC_AB2	CG7362	1.10706867860301	1	1	pseudobulk	DESeq2	LRT	0
114	KC_AB2	CG7366	-0.729379579623002	0.823527397767669	1	pseudobulk	DESeq2	LRT	0.084321947797948
114	KC_AB2	CG7368	0.44569415845966	0.617120967219518	1	pseudobulk	DESeq2	LRT	0.209629697803659
114	KC_AB2	CG7369	1.35716268081763	0.496675741906945	1	pseudobulk	DESeq2	LRT	0.303927050822623
114	KC_AB2	CG7376	-0.729379579623002	0.772694994316916	1	pseudobulk	DESeq2	LRT	0.1119919011982
114	KC_AB2	CG7378	0.375933254170021	0.220739595020075	1	pseudobulk	DESeq2	LRT	0.656119758562923
114	KC_AB2	CG7379	0.580248830891951	0.581975145144243	1	pseudobulk	DESeq2	LRT	0.235095562699255
114	KC_AB2	CG7381	-1.32478208616813	0.398829425117082	1	pseudobulk	DESeq2	LRT	0.399212807495409
114	KC_AB2	CG7382	0.0968460507593213	0.93608858286138	1	pseudobulk	DESeq2	LRT	0.028683051659804
114	KC_AB2	CG7394	0.763913018493835	0.476327968189163	1	pseudobulk	DESeq2	LRT	0.322093917599014
114	KC_AB2	CG7407	-0.445432562367104	0.552201732981448	1	pseudobulk	DESeq2	LRT	0.257902234742936
114	KC_AB2	CG7414	1.43171555405068	0.185081995866009	1	pseudobulk	DESeq2	LRT	0.732635825851844
114	KC_AB2	CG7420	-0.729379579623002	1	1	pseudobulk	DESeq2	LRT	0
114	KC_AB2	CG7430	-0.325656095026817	0.598811765165157	1	pseudobulk	DESeq2	LRT	0.222709675436348
114	KC_AB2	CG7453	0.650722636393607	0.598112548047932	1	pseudobulk	DESeq2	LRT	0.22321708625064
114	KC_AB2	CG7461	1.38446765109058	0.642794900879723	1	pseudobulk	DESeq2	LRT	0.191927577037166
114	KC_AB2	CG7483	-0.959667740872212	0.472845956573839	1	pseudobulk	DESeq2	LRT	0.325280320374501
114	KC_AB2	CG7484	0.575318463019561	0.628988507523228	1	pseudobulk	DESeq2	LRT	0.201357289633972
114	KC_AB2	CG7488	-1.21055130536385	0.537502581213114	1	pseudobulk	DESeq2	LRT	0.269619445823668
114	KC_AB2	CG7492	-0.311893501023333	0.665909363762579	1	pseudobulk	DESeq2	LRT	0.176584878181752
114	KC_AB2	CG7504	-1.766017528816	0.373098694305789	1	pseudobulk	DESeq2	LRT	0.428176270811176
114	KC_AB2	CG7506	-2.05519017029786	0.138369876440415	1	pseudobulk	DESeq2	LRT	0.858958446870952
114	KC_AB2	CG7518	0.723059834220254	0.578502261217918	1	pseudobulk	DESeq2	LRT	0.237694939162457

114	KC_AB2	CG7519	-0.833099610885199	0.538745139248118	1	pseudobulk	DESeq2	LRT	0.268616635174123
114	KC_AB2	CG7523	0.308729460136586	0.863002669209548	1	pseudobulk	DESeq2	LRT	0.063987861038921
114	KC_AB2	CG7530	-0.729379579623002	0.823527397767669	1	pseudobulk	DESeq2	LRT	0.084321947797948
114	KC_AB2	CG7544	1.38069928703004	0.566368801250645	1	pseudobulk	DESeq2	LRT	0.246900678019328
114	KC_AB2	CG7546	-1.3221892601294	0.185786082376971	1	pseudobulk	DESeq2	LRT	0.730986823029538
114	KC_AB2	CG7550	-0.107809350480725	0.891562794400617	1	pseudobulk	DESeq2	LRT	0.049848063255561
114	KC_AB2	CG7556	-0.744467116302025	0.71305844217897	1	pseudobulk	DESeq2	LRT	0.146874873969043
114	KC_AB2	CG7560	0.145321756433347	1	1	pseudobulk	DESeq2	LRT	0
114	KC_AB2	CG7564	-0.454590868708328	0.52971121042417	1	pseudobulk	DESeq2	LRT	0.275960835873467
115	KC_AB2	CG7565	-2.14235399364046	0.232855022260984	1	pseudobulk	DESeq2	LRT	0.632914390685039
115	KC_AB2	CG7568	-0.836295728681047	0.685981024666688	1	pseudobulk	DESeq2	LRT	0.163687897407719
115	KC_AB2	CG7582	-0.420395877977812	0.603682860181324	1	pseudobulk	DESeq2	LRT	0.219191154499918
115	KC_AB2	CG7600	0.409929667627161	0.75560513891205	1	pseudobulk	DESeq2	LRT	0.121705097079536
115	KC_AB2	CG7601	-0.903092823655932	0.666117165665049	1	pseudobulk	DESeq2	LRT	0.176449374543784
115	KC_AB2	CG7611	1.11394331170633	0.315125346250431	1	pseudobulk	DESeq2	LRT	0.501516664125275
115	KC_AB2	CG7627	1.99339641594643	0.465071165814147	1	pseudobulk	DESeq2	LRT	0.332480585700252
115	KC_AB2	CG7630	0.0541925562699935	0.853781172061484	1	pseudobulk	DESeq2	LRT	0.068653426669530
115	KC_AB2	CG7632	-0.968936593053995	0.673838136795855	1	pseudobulk	DESeq2	LRT	0.171444413165975
115	KC_AB2	CG7637	0.675944063194313	0.47129880889775	1	pseudobulk	DESeq2	LRT	0.326703657821074
115	KC_AB2	CG7638	0.0501888431609012	0.952925113905255	1	pseudobulk	DESeq2	LRT	0.020941227268902
115	KC_AB2	CG7639	-0.716128054415663	0.5271174110119	1	pseudobulk	DESeq2	LRT	0.278092638534596
115	KC_AB2	CG7646	-0.132185085624619	0.616009956524925	1	pseudobulk	DESeq2	LRT	0.210412268308158
115	KC_AB2	CG7650	-0.347135499964956	0.854806062097812	1	pseudobulk	DESeq2	LRT	0.068132406575477
115	KC_AB2	CG7655	0.412266503805257	0.859735858836361	1	pseudobulk	DESeq2	LRT	0.065634958838131
115	KC_AB2	CG7656	-0.202607093281416	0.8141335826766	1	pseudobulk	DESeq2	LRT	0.089304330418426
115	KC_AB2	CG7668	-2.22838112499084	0.263713055867331	1	pseudobulk	DESeq2	LRT	0.578868368686737
115	KC_AB2	CG7685	0.0163607969601969	0.982626433786628	1	pseudobulk	DESeq2	LRT	0.007611557020600
115	KC_AB2	CG7692	0.337983997995624	0.80435534907223	1	pseudobulk	DESeq2	LRT	0.094552045720449
115	KC_AB2	CG7694	-1.16124795352912	0.265785991380811	1	pseudobulk	DESeq2	LRT	0.575467912882901
115	KC_AB2	CG7702	0.146930209264112	1	1	pseudobulk	DESeq2	LRT	0
115	KC_AB2	CG7706	-0.571551052146302	0.612223284266052	1	pseudobulk	DESeq2	LRT	0.213090157201991
115	KC_AB2	CG7718	-2.09428695548101	0.380540277844868	1	pseudobulk	DESeq2	LRT	0.419599369064229
115	KC_AB2	CG7720	1.61205113368698	0.631647504209	1	pseudobulk	DESeq2	LRT	0.199525215522471
115	KC_AB2	CG7728	1.61205113368698	0.631647504209	1	pseudobulk	DESeq2	LRT	0.199525215522471
115	KC_AB2	CG7737	0.146930209264112	1	1	pseudobulk	DESeq2	LRT	0
115	KC_AB2	CG7739	-0.530842470134094	0.449042136852088	1	pseudobulk	DESeq2	LRT	0.347712904110889
115	KC_AB2	CG7741	1.04560394656545	0.638145957880541	1	pseudobulk	DESeq2	LRT	0.195079977309609
115	KC_AB2	CG7744	-0.729379579623002	0.823527397767669	1	pseudobulk	DESeq2	LRT	0.084321947797948
115	KC_AB2	CG7745	0.133204037681734	0.900596101178174	1	pseudobulk	DESeq2	LRT	0.045469937497969
115	KC_AB2	CG7747	0.858103339274647	0.6980413252262	1	pseudobulk	DESeq2	LRT	0.156118865648421
115	KC_AB2	CG7766	-0.0417533431049832	0.948148803895907	1	pseudobulk	DESeq2	LRT	0.023123498485284
115	KC_AB2	CG7772	-0.646397844902821	0.631150469804279	1	pseudobulk	DESeq2	LRT	0.199867090176811
115	KC_AB2	CG7781	-0.281844864898082	0.350461686169333	1	pseudobulk	DESeq2	LRT	0.455359453859242
115	KC_AB2	CG7785	-0.0994571885158658	0.921199016059611	1	pseudobulk	DESeq2	LRT	0.035646534581134
115	KC_AB2	CG7789	0.496201244031516	0.745052592578305	1	pseudobulk	DESeq2	LRT	0.127813069721
115	KC_AB2	CG7791	-1.72205629554805	0.342494289164649	1	pseudobulk	DESeq2	LRT	0.465346665645885
115	KC_AB2	CG7806	1.99339641594643	0.465071165814147	1	pseudobulk	DESeq2	LRT	0.332480585700252
115	KC_AB2	CG7810	-0.833859461282172	0.651573152703153	1	pseudobulk	DESeq2	LRT	0.1860368185825
115	KC_AB2	CG7834	-1.42073461079967	0.447791631833179	1	pseudobulk	DESeq2	LRT	0.348924026618158
115	KC_AB2	CG7839	1.83731691536722	0.353433188403798	1	pseudobulk	DESeq2	LRT	0.451692671400067
115	KC_AB2	CG7840	-2.09428695548101	0.380540277844868	1	pseudobulk	DESeq2	LRT	0.419599369064229
115	KC_AB2	CG7841	-0.575782176207854	0.674281855263163	1	pseudobulk	DESeq2	LRT	0.171158526909611

115	KC_AB2	CG7849	-0.0045035166158998	1	1	pseudobulk	DESeq2	LRT	0
115	KC_AB2	CG7857	1.78258764191389	0.445483464074049	1	pseudobulk	DESeq2	LRT	0.351168411929411
115	KC_AB2	CG7872	0.130057097686319	0.936253762795319	1	pseudobulk	DESeq2	LRT	0.028606423863414
115	KC_AB2	CG7878	0.338167141160421	0.762269568945396	1	pseudobulk	DESeq2	LRT	0.117891417633736
115	KC_AB2	CG7879	0.643880740690417	0.287834941542074	1	pseudobulk	DESeq2	LRT	0.54085648629309
115	KC_AB2	CG7881	-0.729379579623002	1	1	pseudobulk	DESeq2	LRT	0
115	KC_AB2	CG7889	0.661678460170035	0.878003361430606	1	pseudobulk	DESeq2	LRT	0.056503821396894
115	KC_AB2	CG7903	0.552953166943826	0.592599142532749	1	pseudobulk	DESeq2	LRT	0.227238981262253
115	KC_AB2	CG7914	-0.729379579623002	0.823527397767669	1	pseudobulk	DESeq2	LRT	0.084321947797948
115	KC_AB2	CG7920	0.596863712993731	0.360590749887559	1	pseudobulk	DESeq2	LRT	0.442985418280053
115	KC_AB2	CG7943	-0.0117223952846826	0.962293604444399	1	pseudobulk	DESeq2	LRT	0.016692400586156
115	KC_AB2	CG7945	-0.428159808498798	0.657660758324992	1	pseudobulk	DESeq2	LRT	0.181998071097039
115	KC_AB2	CG7946	-0.707193359789462	0.620370926614278	1	pseudobulk	DESeq2	LRT	0.207348563383981
115	KC_AB2	CG7956	-1.4891308841529	0.252198134116299	1	pseudobulk	DESeq2	LRT	0.598258130871553
115	KC_AB2	CG7970	0.400955890185621	0.808852698639772	1	pseudobulk	DESeq2	LRT	0.092130561196878
115	KC_AB2	CG7971	-0.209201616283592	0.402772858922313	1	pseudobulk	DESeq2	LRT	0.39493980231258
115	KC_AB2	CG7974	-2.38810703322659	0.197475441859044	1	pseudobulk	DESeq2	LRT	0.704486905750214
115	KC_AB2	CG7985	3.2288849799448	0.0063641954578118	1	pseudobulk	DESeq2	LRT	2.19625649072477
115	KC_AB2	CG7987	-0.298142386667798	0.845357701573608	1	pseudobulk	DESeq2	LRT	0.072959486377769
115	KC_AB2	CG7988	-0.729379579623002	0.772694994316916	1	pseudobulk	DESeq2	LRT	0.1119919011982
115	KC_AB2	CG7990	-0.320269782945464	0.55240216743905	1	pseudobulk	DESeq2	LRT	0.257744626065577
115	KC_AB2	CG7991	0.843244047257989	0.721056826075985	1	pseudobulk	DESeq2	LRT	0.142030507432132
115	KC_AB2	CG8001	2.1640607613294	0.366992469753458	1	pseudobulk	DESeq2	LRT	0.435342846859824
115	KC_AB2	CG8003	-0.194402616845475	0.899217068637067	1	pseudobulk	DESeq2	LRT	0.046135458064451
115	KC_AB2	CG8004	-0.585025207488445	0.600444985153176	1	pseudobulk	DESeq2	LRT	0.221526778001173
115	KC_AB2	CG8005	-1.52244591594958	0.547176617578731	1	pseudobulk	DESeq2	LRT	0.261872469536296
115	KC_AB2	CG8009	0.1859244655857	0.823635494930322	1	pseudobulk	DESeq2	LRT	0.084264945540885
115	KC_AB2	CG8010	0.942758880874816	0.658080412798793	1	pseudobulk	DESeq2	LRT	0.181721035411818
115	KC_AB2	CG8012	1.10706867860301	1	1	pseudobulk	DESeq2	LRT	0
115	KC_AB2	CG8031	1.10706867860301	1	1	pseudobulk	DESeq2	LRT	0
115	KC_AB2	CG8032	0.782644711136243	0.552419959403454	1	pseudobulk	DESeq2	LRT	0.257730638380545
115	KC_AB2	CG8036	0.680883174950485	0.711135058021138	1	pseudobulk	DESeq2	LRT	0.148047910686379
115	KC_AB2	CG8038	-1.86979501903407	0.475964703567026	1	pseudobulk	DESeq2	LRT	0.322425252351861
115	KC_AB2	CG8042	1.06336461269421	0.53538060482756	1	pseudobulk	DESeq2	LRT	0.271337366001044
115	KC_AB2	CG8043	1.29237120107421	0.456093415264977	1	pseudobulk	DESeq2	LRT	0.340946197732156
115	KC_AB2	CG8060	-1.6052707068109	0.52265609184397	1	pseudobulk	DESeq2	LRT	0.281783983297782
115	KC_AB2	CG8064	0.518865066730979	0.817181531186031	1	pseudobulk	DESeq2	LRT	0.087681457255733
115	KC_AB2	CG8078	0.229116498387851	0.859640013003888	1	pseudobulk	DESeq2	LRT	0.065683377938202
115	KC_AB2	CG8079	1.85214185136044	0.211569004698825	1	pseudobulk	DESeq2	LRT	0.674547957025585
115	KC_AB2	CG8080	1.10706867860301	1	1	pseudobulk	DESeq2	LRT	0
115	KC_AB2	CG8108	-0.135153354308486	0.734675745591722	1	pseudobulk	DESeq2	LRT	0.133904297612478
115	KC_AB2	CG8111	-1.17730169668773	0.519431033867205	1	pseudobulk	DESeq2	LRT	0.284472106623065
115	KC_AB2	CG8128	-0.729379579623002	1	1	pseudobulk	DESeq2	LRT	0
115	KC_AB2	CG8132	-1.00938763126439	0.547247229125383	1	pseudobulk	DESeq2	LRT	0.261816428715906
115	KC_AB2	CG8134	-0.13751229470955	0.892153252778423	1	pseudobulk	DESeq2	LRT	0.049560536745894
115	KC_AB2	CG8136	-0.729379579623002	1	1	pseudobulk	DESeq2	LRT	0
115	KC_AB2	CG8142	0.145321756433347	1	1	pseudobulk	DESeq2	LRT	0
115	KC_AB2	CG8149	1.35397486222867	0.277845170754137	1	pseudobulk	DESeq2	LRT	0.556197147315012
115	KC_AB2	CG8152	-1.25711388229196	0.605528431072361	1	pseudobulk	DESeq2	LRT	0.217865460831475
115	KC_AB2	CG8155	0.455421473386798	0.766210125336229	1	pseudobulk	DESeq2	LRT	0.115652113187454
115	KC_AB2	CG8159	-0.729379579623002	0.772694994316916	1	pseudobulk	DESeq2	LRT	0.1119919011982
115	KC_AB2	CG8160	0.38605638660934	0.863777890371283	1	pseudobulk	DESeq2	LRT	0.063597916533759

115	KC_AB2	CG8173	1.39644850851527	0.256800278460569	1	pseudobulk	DESeq2	LRT	0.590404509677082
115	KC_AB2	CG8176	0.277905059966436	0.691816456014588	1	pseudobulk	DESeq2	LRT	0.160009111776225
115	KC_AB2	CG8177	-0.0166933022997337	0.889354164366255	1	pseudobulk	DESeq2	LRT	0.050925257027587
115	KC_AB2	CG8187	0.822517763603507	0.560995324378554	1	pseudobulk	DESeq2	LRT	0.251040758360693
115	KC_AB2	CG8188	-0.0277731936239633	0.953965658314694	1	pseudobulk	DESeq2	LRT	0.020467259124914
116	KC_AB2	CG8195	0.344986417489335	0.711581133913478	1	pseudobulk	DESeq2	LRT	0.147775574845963
116	KC_AB2	CG8202	0.183896532101256	0.900821579250278	1	pseudobulk	DESeq2	LRT	0.045361218810371
116	KC_AB2	CG8204	1.37221885443763	0.558734269457382	1	pseudobulk	DESeq2	LRT	0.252794690766539
116	KC_AB2	CG8206	-1.52314349080222	0.21067370786178	1	pseudobulk	DESeq2	LRT	0.676389661084043
116	KC_AB2	CG8207	-1.09586535083235	0.704995102869895	1	pseudobulk	DESeq2	LRT	0.151813899751819
116	KC_AB2	CG8209	0.446246512543113	0.726398363719835	1	pseudobulk	DESeq2	LRT	0.138825142774756
116	KC_AB2	CG8223	-0.724195215807031	0.503875408202964	1	pseudobulk	DESeq2	LRT	0.297676837005158
116	KC_AB2	CG8229	-0.0153776093856792	0.985351349916652	1	pseudobulk	DESeq2	LRT	0.006408884099190
116	KC_AB2	CG8230	-0.257188986071904	0.88741862317942	1	pseudobulk	DESeq2	LRT	0.051871461511743
116	KC_AB2	CG8237	0.361186577594284	0.788380600099349	1	pseudobulk	DESeq2	LRT	0.10326407106619
116	KC_AB2	CG8243	0.333756393617997	0.7653111741346	1	pseudobulk	DESeq2	LRT	0.116161945589182
116	KC_AB2	CG8245	-0.313224210620533	0.54849970053943	1	pseudobulk	DESeq2	LRT	0.260823605197946
116	KC_AB2	CG8248	-0.838920879114343	0.421718064336983	1	pseudobulk	DESeq2	LRT	0.374977795537561
116	KC_AB2	CG8270	0.396659246788372	0.915482501218565	1	pseudobulk	DESeq2	LRT	0.038349952505755
116	KC_AB2	CG8272	-0.171666651011574	0.92393267296366	1	pseudobulk	DESeq2	LRT	0.034359674695143
116	KC_AB2	CG8281	-0.0694577164119598	0.943774232507176	1	pseudobulk	DESeq2	LRT	0.025131884200859
116	KC_AB2	CG8289	-0.262078597898261	0.784972151365143	1	pseudobulk	DESeq2	LRT	0.105145750545173
116	KC_AB2	CG8292	-0.729379579623002	0.823527397767669	1	pseudobulk	DESeq2	LRT	0.084321947797948
116	KC_AB2	CG8298	0.707492165822316	0.787718970936811	1	pseudobulk	DESeq2	LRT	0.103628695124867
116	KC_AB2	CG8300	0.433944133623326	0.512784486510482	1	pseudobulk	DESeq2	LRT	0.29006512218816
116	KC_AB2	CG8301	0.0514390141153702	0.957383303111777	1	pseudobulk	DESeq2	LRT	0.018914150939773
116	KC_AB2	CG8311	-0.154668464776196	0.907130501727862	1	pseudobulk	DESeq2	LRT	0.042330229915300
116	KC_AB2	CG8312	-0.729379579623002	1	1	pseudobulk	DESeq2	LRT	0
116	KC_AB2	CG8314	-0.170106272378047	0.887522063629523	1	pseudobulk	DESeq2	LRT	0.051820841660533
116	KC_AB2	CG8319	1.61205113368698	0.631647504209	1	pseudobulk	DESeq2	LRT	0.199525215522471
116	KC_AB2	CG8320	0.402930403301789	0.839430910337632	1	pseudobulk	DESeq2	LRT	0.076015042818232
116	KC_AB2	CG8321	0.576577225376985	0.494997464357038	1	pseudobulk	DESeq2	LRT	0.305397025750405
116	KC_AB2	CG8323	1.61205113368698	0.631647504209	1	pseudobulk	DESeq2	LRT	0.199525215522471
116	KC_AB2	CG8326	0.396659246788372	0.915482501218565	1	pseudobulk	DESeq2	LRT	0.038349952505755
116	KC_AB2	CG8336	0.843244047257989	0.721056826075985	1	pseudobulk	DESeq2	LRT	0.142030507432132
116	KC_AB2	CG8353	-1.00833870937463	0.651152668420623	1	pseudobulk	DESeq2	LRT	0.186317175365195
116	KC_AB2	CG8360	0.186148960011836	0.867144366210932	1	pseudobulk	DESeq2	LRT	0.061908593147307
116	KC_AB2	CG8366	-1.28272554373657	0.555763353522301	1	pseudobulk	DESeq2	LRT	0.255110093556768
116	KC_AB2	CG8369	2.50876149578457	0.243275226083277	1	pseudobulk	DESeq2	LRT	0.613902115167576
116	KC_AB2	CG8370	1.38446765109058	0.642794900879723	1	pseudobulk	DESeq2	LRT	0.191927577037166
116	KC_AB2	CG8372	1.08079701715022	0.206560135566065	1	pseudobulk	DESeq2	LRT	0.684953490048731
116	KC_AB2	CG8379	-0.647621034217714	0.754702560287896	1	pseudobulk	DESeq2	LRT	0.122224176685402
116	KC_AB2	CG8388	-0.729379579623002	0.823527397767669	1	pseudobulk	DESeq2	LRT	0.084321947797948
116	KC_AB2	CG8389	0.280700541159967	0.828339256746653	1	pseudobulk	DESeq2	LRT	0.081791756020708
116	KC_AB2	CG8397	-0.150807281805405	0.82916465808905	1	pseudobulk	DESeq2	LRT	0.081359217340370
116	KC_AB2	CG8405	-0.0174951021334739	0.938547406537411	1	pseudobulk	DESeq2	LRT	0.027543786039300
116	KC_AB2	CG8412	-1.00035612719924	0.389970004974585	1	pseudobulk	DESeq2	LRT	0.408968795986298
116	KC_AB2	CG8414	0.38605638660934	0.863777890371283	1	pseudobulk	DESeq2	LRT	0.063597916533759
116	KC_AB2	CG8435	1.11533217645914	0.572587072943417	1	pseudobulk	DESeq2	LRT	0.242158461086888
116	KC_AB2	CG8441	-0.107809350480725	0.891562794400617	1	pseudobulk	DESeq2	LRT	0.049848063255561
116	KC_AB2	CG8460	0.190977459119008	0.884361551215505	1	pseudobulk	DESeq2	LRT	0.053370147208116
116	KC_AB2	CG8461	1.99339641594643	0.465071165814147	1	pseudobulk	DESeq2	LRT	0.332480585700252

116	KC_AB2	CG8475	0.0872694456224651	0.920505680068557	1	pseudobulk	DESeq2	LRT	0.035973527295957
116	KC_AB2	CG8485	0.341022524114114	0.647785258855402	1	pseudobulk	DESeq2	LRT	0.188568939114663
116	KC_AB2	CG8490	-1.30549715978175	0.485559362626414	1	pseudobulk	DESeq2	LRT	0.313757667331498
116	KC_AB2	CG8500	0.288335368363471	0.529221395061017	1	pseudobulk	DESeq2	LRT	0.276362606719767
116	KC_AB2	CG8507	-0.652437582918878	0.569730988459822	1	pseudobulk	DESeq2	LRT	0.244330158020775
116	KC_AB2	CG8516	0.372319056733928	0.867976741609407	1	pseudobulk	DESeq2	LRT	0.061491912065472
116	KC_AB2	CG8519	0.0487673033804221	0.942805703571486	1	pseudobulk	DESeq2	LRT	0.025577798843798
116	KC_AB2	CG8520	0.661362391586437	0.730605074655461	1	pseudobulk	DESeq2	LRT	0.136317315579878
116	KC_AB2	CG8531	-1.63222955096776	0.49068522332581	1	pseudobulk	DESeq2	LRT	0.309197020324952
116	KC_AB2	CG8545	1.38446765109058	0.642794900879723	1	pseudobulk	DESeq2	LRT	0.191927577037166
116	KC_AB2	CG8549	-0.266769469189161	0.856762563156764	1	pseudobulk	DESeq2	LRT	0.067139518564678
116	KC_AB2	CG8569	0.33333575867548	0.858979456944999	1	pseudobulk	DESeq2	LRT	0.066017222480879
116	KC_AB2	CG8578	-0.480935836028446	0.514095970916012	1	pseudobulk	DESeq2	LRT	0.288955799781672
116	KC_AB2	CG8607	-0.729379579623002	1	1	pseudobulk	DESeq2	LRT	0
116	KC_AB2	CG8611	-0.647621034217714	0.754702560287896	1	pseudobulk	DESeq2	LRT	0.122224176685402
116	KC_AB2	CG8613	0.284365508337905	0.687922336038235	1	pseudobulk	DESeq2	LRT	0.162460589284705
116	KC_AB2	CG8617	-0.190828951056817	0.81064334588716	1	pseudobulk	DESeq2	LRT	0.091170177825629
116	KC_AB2	CG8635	0.390313129008281	0.733084903002083	1	pseudobulk	DESeq2	LRT	0.134845724171994
116	KC_AB2	CG8641	1.38446765109058	0.642794900879723	1	pseudobulk	DESeq2	LRT	0.191927577037166
116	KC_AB2	CG8646	-0.729379579623002	1	1	pseudobulk	DESeq2	LRT	0
116	KC_AB2	CG8661	1.38069928703004	0.566368801250645	1	pseudobulk	DESeq2	LRT	0.246900678019328
116	KC_AB2	CG8668	0.137466182332914	0.858456262267112	1	pseudobulk	DESeq2	LRT	0.066281826932800
116	KC_AB2	CG8671	-0.0438654346665025	0.920415877996398	1	pseudobulk	DESeq2	LRT	0.036015897970864
116	KC_AB2	CG8673	2.47215486522296	0.30601789350912	1	pseudobulk	DESeq2	LRT	0.514253178665238
116	KC_AB2	CG8675	1.53600323097801	0.43223306245546	1	pseudobulk	DESeq2	LRT	0.364282016044215
116	KC_AB2	CG8677	-0.0744653787749284	0.875834851098471	1	pseudobulk	DESeq2	LRT	0.057577777414488
116	KC_AB2	CG8679	-0.729379579623002	1	1	pseudobulk	DESeq2	LRT	0
116	KC_AB2	CG8712	-2.6040519176076	0.285294059094629	1	pseudobulk	DESeq2	LRT	0.544707271908587
116	KC_AB2	CG8726	-0.125547090964385	0.763230036021972	1	pseudobulk	DESeq2	LRT	0.117344546818214
116	KC_AB2	CG8728	-0.691104742118331	0.662234072315643	1	pseudobulk	DESeq2	LRT	0.178988478313647
116	KC_AB2	CG8745	-3.31404967532551	0.0923604341214165	1	pseudobulk	DESeq2	LRT	1.03451403443727
116	KC_AB2	CG8757	1.38446765109058	0.642794900879723	1	pseudobulk	DESeq2	LRT	0.191927577037166
116	KC_AB2	CG8765	-0.278644223999356	0.847511366025468	1	pseudobulk	DESeq2	LRT	0.071854468736630
116	KC_AB2	CG8768	0.796171800233555	0.593036468603691	1	pseudobulk	DESeq2	LRT	0.22691859900276
116	KC_AB2	CG8777	2.88969196614093	0.207685089444696	1	pseudobulk	DESeq2	LRT	0.682594682108805
116	KC_AB2	CG8778	-0.586200653393176	0.673535756140177	1	pseudobulk	DESeq2	LRT	0.171639343842735
116	KC_AB2	CG8800	-0.729379579623002	1	1	pseudobulk	DESeq2	LRT	0
116	KC_AB2	CG8814	-1.20066215866911	0.392344575344341	1	pseudobulk	DESeq2	LRT	0.406332347678953
116	KC_AB2	CG8818	-0.469960375967025	0.443560785613442	1	pseudobulk	DESeq2	LRT	0.353046855995584
116	KC_AB2	CG8833	-1.04646554409772	0.565967303815696	1	pseudobulk	DESeq2	LRT	0.247208657473528
116	KC_AB2	CG8839	0.354023967257127	0.816950640622699	1	pseudobulk	DESeq2	LRT	0.087804182332658
116	KC_AB2	CG8841	-0.473795964521473	0.80663503472328	1	pseudobulk	DESeq2	LRT	0.093322919129089
116	KC_AB2	CG8850	0.0455261422590676	0.956410711868198	1	pseudobulk	DESeq2	LRT	0.019355568399845
116	KC_AB2	CG8858	-0.419730715833961	0.871309690495475	1	pseudobulk	DESeq2	LRT	0.059827455820959
116	KC_AB2	CG8860	-0.1815129227217	0.806738336994528	1	pseudobulk	DESeq2	LRT	0.093267304468256
116	KC_AB2	CG8878	-0.352820919850586	0.642652043867619	1	pseudobulk	DESeq2	LRT	0.192024106905734
116	KC_AB2	CG8891	0.0163607969601969	0.982626433786628	1	pseudobulk	DESeq2	LRT	0.007611557020600
116	KC_AB2	CG8892	-0.436587925451555	0.824276803233537	1	pseudobulk	DESeq2	LRT	0.083926921883127
116	KC_AB2	CG8910	0.119684083947501	0.721942033533776	1	pseudobulk	DESeq2	LRT	0.141497671581401
116	KC_AB2	CG8915	0.619440369709763	0.722026032044035	1	pseudobulk	DESeq2	LRT	0.141447144024172
116	KC_AB2	CG8924	1.74534392953525	0.130615066762946	1	pseudobulk	DESeq2	LRT	0.884006723255478
116	KC_AB2	CG8929	0.492773613169869	0.363633380544309	1	pseudobulk	DESeq2	LRT	0.439336256581026

116	KC_AB2	CG8944	-1.29052728408004	0.545673810438722	1	pseudobulk	DESeq2	LRT	0.263066889637944
117	KC_AB2	CG8950	1.61205113368698	0.631647504209	1	pseudobulk	DESeq2	LRT	0.199525215522471
117	KC_AB2	CG8963	-0.398668729164087	0.6115061575316	1	pseudobulk	DESeq2	LRT	0.213599165498453
117	KC_AB2	CG8974	0.287310000458216	0.628570218663818	1	pseudobulk	DESeq2	LRT	0.201646199481917
117	KC_AB2	CG8993	-0.529663390627992	0.362470520385568	1	pseudobulk	DESeq2	LRT	0.440727308691574
117	KC_AB2	CG9003	-0.439307021122851	0.433357680314349	1	pseudobulk	DESeq2	LRT	0.363153502114814
117	KC_AB2	CG9004	-0.801863169396014	0.647277270195681	1	pseudobulk	DESeq2	LRT	0.188909643409864
117	KC_AB2	CG9005	0.321833443942497	0.511870589889931	1	pseudobulk	DESeq2	LRT	0.29083982261901
117	KC_AB2	CG9008	-0.0640829416272726	0.874619979600749	1	pseudobulk	DESeq2	LRT	0.058180605962980
117	KC_AB2	CG9018	-0.0577279350192099	0.925958576931456	1	pseudobulk	DESeq2	LRT	0.033408441193309
117	KC_AB2	CG9034	-0.125011341672721	0.780714706535116	1	pseudobulk	DESeq2	LRT	0.107507639635067
117	KC_AB2	CG9044	-0.408063964725295	0.773965109315406	1	pseudobulk	DESeq2	LRT	0.111278617060431
117	KC_AB2	CG9062	-0.473795964521473	0.80663503472328	1	pseudobulk	DESeq2	LRT	0.093322919129089
117	KC_AB2	CG9065	0.256747570112935	0.665679929878954	1	pseudobulk	DESeq2	LRT	0.176734536745517
117	KC_AB2	CG9067	-0.946620268344687	0.577248833899267	1	pseudobulk	DESeq2	LRT	0.238636935728456
117	KC_AB2	CG9098	-0.729379579623002	0.772694994316916	1	pseudobulk	DESeq2	LRT	0.1119919011982
117	KC_AB2	CG9101	1.10706867860301	1	1	pseudobulk	DESeq2	LRT	0
117	KC_AB2	CG9107	0.667244748290888	0.924085151242949	1	pseudobulk	DESeq2	LRT	0.034288008205147
117	KC_AB2	CG9121	-0.510341532713416	0.689890944421392	1	pseudobulk	DESeq2	LRT	0.161219555609473
117	KC_AB2	CG9123	1.3364440484199	0.139765960462128	1	pseudobulk	DESeq2	LRT	0.854598586698463
117	KC_AB2	CG9125	-1.12267264858809	0.544098381430871	1	pseudobulk	DESeq2	LRT	0.264322566019636
117	KC_AB2	CG9132	-0.0472387940847226	0.925597066683792	1	pseudobulk	DESeq2	LRT	0.033578030379839
117	KC_AB2	CG9135	0.552348055732357	0.708916361110231	1	pseudobulk	DESeq2	LRT	0.149405000431864
117	KC_AB2	CG9143	-0.522739678289228	0.786544031773407	1	pseudobulk	DESeq2	LRT	0.104276959981635
117	KC_AB2	CG9147	-0.818820863403386	0.701088644508524	1	pseudobulk	DESeq2	LRT	0.154227067073285
117	KC_AB2	CG9149	-0.323295323850604	0.852742509148022	1	pseudobulk	DESeq2	LRT	0.069182086930788
117	KC_AB2	CG9154	-0.387139839985679	0.842914941402511	1	pseudobulk	DESeq2	LRT	0.074216247846548
117	KC_AB2	CG9164	-0.824996233971657	0.664071509040402	1	pseudobulk	DESeq2	LRT	0.177785152093585
117	KC_AB2	CG9170	-0.569834115115807	0.400578209740408	1	pseudobulk	DESeq2	LRT	0.397312678724133
117	KC_AB2	CG9171	0.101903940892188	0.788188476145911	1	pseudobulk	DESeq2	LRT	0.103369919106076
117	KC_AB2	CG9175	-0.0391265659417848	0.900374520306502	1	pseudobulk	DESeq2	LRT	0.045576803594728
117	KC_AB2	CG9205	-0.0995437773680735	0.909128144869492	1	pseudobulk	DESeq2	LRT	0.041374897100031
117	KC_AB2	CG9213	-1.6052707068109	0.52265609184397	1	pseudobulk	DESeq2	LRT	0.281783983297782
117	KC_AB2	CG9231	-0.114247426711895	0.813686076902626	1	pseudobulk	DESeq2	LRT	0.089543115215020
117	KC_AB2	CG9240	-0.831572874905813	0.312245307248688	1	pseudobulk	DESeq2	LRT	0.505504079939622
117	KC_AB2	CG9246	0.488398599996631	0.774777494238074	1	pseudobulk	DESeq2	LRT	0.110823003169981
117	KC_AB2	CG9253	-1.49317447962368	0.332650442874122	1	pseudobulk	DESeq2	LRT	0.478011894019647
117	KC_AB2	CG9257	-0.152109526738888	0.884694909799742	1	pseudobulk	DESeq2	LRT	0.053206471487715
117	KC_AB2	CG9265	0.330228806794547	0.694907402032754	1	pseudobulk	DESeq2	LRT	0.158073062266643
117	KC_AB2	CG9272	-0.729379579623002	1	1	pseudobulk	DESeq2	LRT	0
117	KC_AB2	CG9281	-0.209415425169906	0.775306308548465	1	pseudobulk	DESeq2	LRT	0.110526682228557
117	KC_AB2	CG9286	2.3587276565843	0.260468459248785	1	pseudobulk	DESeq2	LRT	0.584244858942325
117	KC_AB2	CG9289	-0.729379579623002	1	1	pseudobulk	DESeq2	LRT	0
117	KC_AB2	CG9300	1.02754127378565	0.694973473680879	1	pseudobulk	DESeq2	LRT	0.15803177160251
117	KC_AB2	CG9302	1.10706867860301	1	1	pseudobulk	DESeq2	LRT	0
117	KC_AB2	CG9304	0.0667588332006575	0.944703280098873	1	pseudobulk	DESeq2	LRT	0.024704576729781
117	KC_AB2	CG9313	-0.729379579623002	1	1	pseudobulk	DESeq2	LRT	0
117	KC_AB2	CG9328	-0.0696866286902897	0.845137354279901	1	pseudobulk	DESeq2	LRT	0.073072702460798
117	KC_AB2	CG9330	0.254967458656047	0.806924692422748	1	pseudobulk	DESeq2	LRT	0.093166994635244
117	KC_AB2	CG9344	-0.242300885458739	0.819460504246819	1	pseudobulk	DESeq2	LRT	0.086471973394296
117	KC_AB2	CG9346	-0.564573822202069	0.622626391690327	1	pseudobulk	DESeq2	LRT	0.205772474526368
117	KC_AB2	CG9360	1.61205113368698	0.631647504209	1	pseudobulk	DESeq2	LRT	0.199525215522471

117	KC_AB2	CG9368	-0.174536921754589	0.71099295748699	1	pseudobulk	DESeq2	LRT	0.148134701013815
117	KC_AB2	CG9380	2.01814786626755	0.401718761445944	1	pseudobulk	DESeq2	LRT	0.396077884966921
117	KC_AB2	CG9384	0.858534494841453	0.474471762002089	1	pseudobulk	DESeq2	LRT	0.323789629330677
117	KC_AB2	CG9386	-1.53018569546598	0.511598982887392	1	pseudobulk	DESeq2	LRT	0.291070327615651
117	KC_AB2	CG9391	-0.853700897868846	0.246147036342936	1	pseudobulk	DESeq2	LRT	0.608805388847787
117	KC_AB2	CG9393	3.02241878477171	0.183885600586836	1	pseudobulk	DESeq2	LRT	0.735452277453175
117	KC_AB2	CG9394	1.61205113368698	0.631647504209	1	pseudobulk	DESeq2	LRT	0.199525215522471
117	KC_AB2	CG9395	-0.85557082864671	0.515319465225203	1	pseudobulk	DESeq2	LRT	0.287923452570494
117	KC_AB2	CG9399	-1.30993282377647	0.512878470536922	1	pseudobulk	DESeq2	LRT	0.289985531238904
117	KC_AB2	CG9410	-0.467001929615833	0.606133430228883	1	pseudobulk	DESeq2	LRT	0.2174317625771
117	KC_AB2	CG9422	-0.771016880199008	0.446001839587279	1	pseudobulk	DESeq2	LRT	0.350663349985265
117	KC_AB2	CG9425	-0.043128531613491	0.850763556302567	1	pseudobulk	DESeq2	LRT	0.070191122008240
117	KC_AB2	CG9426	-0.957583700163573	0.336908673027671	1	pseudobulk	DESeq2	LRT	0.47248780884798
117	KC_AB2	CG9467	-0.701070017390609	0.705653816598702	1	pseudobulk	DESeq2	LRT	0.151408305199667
117	KC_AB2	CG9471	-1.37141410468602	0.460853212743517	1	pseudobulk	DESeq2	LRT	0.336437380568744
117	KC_AB2	CG9485	-1.00833870937463	0.651152668420623	1	pseudobulk	DESeq2	LRT	0.186317175365195
117	KC_AB2	CG9503	0.39439088612385	0.813541888331052	1	pseudobulk	DESeq2	LRT	0.089620080830767
117	KC_AB2	CG9515	0.393976029558784	0.939642609940555	1	pseudobulk	DESeq2	LRT	0.027037297511213
117	KC_AB2	CG9527	-0.39935006418526	0.791560209514437	1	pseudobulk	DESeq2	LRT	0.101516045216834
117	KC_AB2	CG9536	-0.744467116302025	0.71305844217897	1	pseudobulk	DESeq2	LRT	0.146874873969043
117	KC_AB2	CG9577	-2.4120103603151	0.297158744475551	1	pseudobulk	DESeq2	LRT	0.527011485255674
117	KC_AB2	CG9578	-1.15551468259112	0.482538481234403	1	pseudobulk	DESeq2	LRT	0.316468047042753
117	KC_AB2	CG9581	-0.674317410868381	0.830763653594129	1	pseudobulk	DESeq2	LRT	0.080522512364194
117	KC_AB2	CG9586	0.325459253007928	0.741438134874976	1	pseudobulk	DESeq2	LRT	0.129925080378418
117	KC_AB2	CG9588	-0.12147046555065	0.915022431235692	1	pseudobulk	DESeq2	LRT	0.038568259329753
117	KC_AB2	CG9590	1.31626977258328	0.323671959874643	1	pseudobulk	DESeq2	LRT	0.489894922467007
117	KC_AB2	CG9601	0.33016386306824	0.770937898811582	1	pseudobulk	DESeq2	LRT	0.11298060416653
117	KC_AB2	CG9609	-0.0275528699222174	0.972799550665241	1	pseudobulk	DESeq2	LRT	0.011976638671311
117	KC_AB2	CG9636	-0.0688871286040953	0.868011010656569	1	pseudobulk	DESeq2	LRT	0.061474765794636
117	KC_AB2	CG9641	0.320660690297792	0.773236190339645	1	pseudobulk	DESeq2	LRT	0.111687827565126
117	KC_AB2	CG9643	1.2303076704182	0.460609241134141	1	pseudobulk	DESeq2	LRT	0.336667353099309
117	KC_AB2	CG9646	0.401404311214918	0.424324044053245	1	pseudobulk	DESeq2	LRT	0.372302358514339
117	KC_AB2	CG9650	-0.729379579623002	0.823527397767669	1	pseudobulk	DESeq2	LRT	0.084321947797948
117	KC_AB2	CG9662	-1.8459701174818	0.0876304087406796	1	pseudobulk	DESeq2	LRT	1.05734516259176
117	KC_AB2	CG9663	-0.729379579623002	0.823527397767669	1	pseudobulk	DESeq2	LRT	0.084321947797948
117	KC_AB2	CG9667	-1.01004712586775	0.602923322647095	1	pseudobulk	DESeq2	LRT	0.219737916166115
117	KC_AB2	CG9674	0.781243774812381	0.121399559008045	1	pseudobulk	DESeq2	LRT	0.915782890861425
117	KC_AB2	CG9684	-0.729379579623002	0.823527397767669	1	pseudobulk	DESeq2	LRT	0.084321947797948
117	KC_AB2	CG9686	1.10706867860301	1	1	pseudobulk	DESeq2	LRT	0
117	KC_AB2	CG9705	0.588956606622794	0.45976281034619	1	pseudobulk	DESeq2	LRT	0.337466161197761
117	KC_AB2	CG9706	-0.729379579623002	1	1	pseudobulk	DESeq2	LRT	0
117	KC_AB2	CG9722	1.10706867860301	1	1	pseudobulk	DESeq2	LRT	0
117	KC_AB2	CG9723	0.145321756433347	1	1	pseudobulk	DESeq2	LRT	0
117	KC_AB2	CG9727	1.10932077638921	0.486267207234822	1	pseudobulk	DESeq2	LRT	0.31312501728596
117	KC_AB2	CG9760	0.942758880874816	0.658080412798793	1	pseudobulk	DESeq2	LRT	0.181721035411818
117	KC_AB2	CG9766	0.145321756433347	1	1	pseudobulk	DESeq2	LRT	0
117	KC_AB2	CG9773	0.067455944572346	0.958004189544848	1	pseudobulk	DESeq2	LRT	0.018632591660254
117	KC_AB2	CG9775	-0.16114449363871	0.74513636164056	1	pseudobulk	DESeq2	LRT	0.12776424310552
117	KC_AB2	CG9776	-0.668865739479816	0.333184158931138	1	pseudobulk	DESeq2	LRT	0.477315655081388
118	KC_AB2	CG9777	-0.729379579623002	0.823527397767669	1	pseudobulk	DESeq2	LRT	0.084321947797948
118	KC_AB2	CG9782	0.393976029558784	0.939642609940555	1	pseudobulk	DESeq2	LRT	0.027037297511213
118	KC_AB2	CG9784	0.180719366556618	0.853788719187443	1	pseudobulk	DESeq2	LRT	0.068649587675677

118	KC_AB2	CG9795	1.04842560579296	0.303339220869606	1	pseudobulk	DESeq2	LRT	0.518071433058894
118	KC_AB2	CG9799	0.150205259410532	1	1	pseudobulk	DESeq2	LRT	0
118	KC_AB2	CG9801	2.05028198082901	0.383573006114165	1	pseudobulk	DESeq2	LRT	0.416151963827146
118	KC_AB2	CG9804	-1.19881828456548	0.535965909404053	1	pseudobulk	DESeq2	LRT	0.270862833121686
118	KC_AB2	CG9813	-0.521522290665005	0.0523063706171781	1	pseudobulk	DESeq2	LRT	1.28144541332404
118	KC_AB2	CG9837	0.154131207583266	0.842859891141018	1	pseudobulk	DESeq2	LRT	0.074244612280921
118	KC_AB2	CG9839	1.02973255321436	0.490557632099677	1	pseudobulk	DESeq2	LRT	0.309309963141207
118	KC_AB2	CG9849	-0.358886256476938	0.787970713011382	1	pseudobulk	DESeq2	LRT	0.103489923898663
118	KC_AB2	CG9853	0.455792306014321	0.662454294529058	1	pseudobulk	DESeq2	LRT	0.178884080136153
118	KC_AB2	CG9855	-0.678142266099058	0.711549586694344	1	pseudobulk	DESeq2	LRT	0.147794829272831
118	KC_AB2	CG9886	3.12312407990434	0.163309574433488	1	pseudobulk	DESeq2	LRT	0.786988352913433
118	KC_AB2	CG9890	-0.647621034217714	0.754702560287896	1	pseudobulk	DESeq2	LRT	0.122224176685402
118	KC_AB2	CG9902	2.62919123543814	0.269826951733818	1	pseudobulk	DESeq2	LRT	0.568914672882509
118	KC_AB2	CG9911	-1.33211241356416	0.123483104327243	1	pseudobulk	DESeq2	LRT	0.908392461023073
118	KC_AB2	CG9915	0.441207660578267	0.716583370378644	1	pseudobulk	DESeq2	LRT	0.144733274646872
118	KC_AB2	CG9917	-0.0269285582245244	0.924578300622982	1	pseudobulk	DESeq2	LRT	0.034056303446375
118	KC_AB2	CG9919	-0.515744254806809	0.533971006894739	1	pseudobulk	DESeq2	LRT	0.272482323285047
118	KC_AB2	CG9921	-1.03816115823942	0.364985178590705	1	pseudobulk	DESeq2	LRT	0.437724771124249
118	KC_AB2	CG9922	-0.758589464399842	0.511783937802472	1	pseudobulk	DESeq2	LRT	0.290913348447305
118	KC_AB2	CG9932	0.869551209232091	0.263334926255143	1	pseudobulk	DESeq2	LRT	0.579491536359374
118	KC_AB2	CG9934	-0.438513833729198	0.779049377836182	1	pseudobulk	DESeq2	LRT	0.108435014930558
118	KC_AB2	CG9941	-0.942285745979106	0.330245988398668	1	pseudobulk	DESeq2	LRT	0.481162449192325
118	KC_AB2	CG9945	1.56595988235652	0.313751675894324	1	pseudobulk	DESeq2	LRT	0.503413945734927
118	KC_AB2	CG9948	-1.33407500886303	0.63128114564055	1	pseudobulk	DESeq2	LRT	0.19977718147345
118	KC_AB2	CG9951	-0.647621034217714	0.754702560287896	1	pseudobulk	DESeq2	LRT	0.122224176685402
118	KC_AB2	CG9953	0.638010440290858	0.498577306525888	1	pseudobulk	DESeq2	LRT	0.30226749292987
118	KC_AB2	CG9967	-1.00833870937463	0.651152668420623	1	pseudobulk	DESeq2	LRT	0.186317175365195
118	KC_AB2	CG9986	0.374382595316825	0.84032162522171	1	pseudobulk	DESeq2	LRT	0.075554459979782
118	KC_AB2	CG9992	-0.107621645975096	0.923882022158427	1	pseudobulk	DESeq2	LRT	0.034383483754872
118	KC_AB2	Charon	-1.52244591594958	0.547176617578731	1	pseudobulk	DESeq2	LRT	0.261872469536296
118	KC_AB2	ChAT	-0.757285597585281	0.174135623690676	1	pseudobulk	DESeq2	LRT	0.759112374260758
118	KC_AB2	chb	-0.241403335464168	0.789874468740111	1	pseudobulk	DESeq2	LRT	0.102441923729004
118	KC_AB2	Chc	-0.0132549456310426	0.9111006566152	1	pseudobulk	DESeq2	LRT	0.040433640373097
118	KC_AB2	Chchd2	-0.449207520295014	0.212167741395663	1	pseudobulk	DESeq2	LRT	0.673320646819429
118	KC_AB2	Chchd3	-0.118156509450221	0.851495727979487	1	pseudobulk	DESeq2	LRT	0.06981752658522
118	KC_AB2	Chd1	-0.212827578510989	0.779840500180169	1	pseudobulk	DESeq2	LRT	0.107994213944106
118	KC_AB2	Chd64	-1.78975280219494	0.468855431150075	1	pseudobulk	DESeq2	LRT	0.32896104882553
118	KC_AB2	CheB42c	-0.729379579623002	1	1	pseudobulk	DESeq2	LRT	0
118	KC_AB2	CHES-1-like	-0.0881869316017131	0.862697823697655	1	pseudobulk	DESeq2	LRT	0.064141297542612
118	KC_AB2	Chi	0.308481105017443	0.774233516032795	1	pseudobulk	DESeq2	LRT	0.111128032302729
118	KC_AB2	chic	0.727067349328716	0.164555628717276	1	pseudobulk	DESeq2	LRT	0.783687257829986
118	KC_AB2	chico	0.835765752514657	0.44638161654242	1	pseudobulk	DESeq2	LRT	0.350293699371963
118	KC_AB2	chif	1.86147218450859	0.0696909595468318	1	pseudobulk	DESeq2	LRT	1.15682355581264
118	KC_AB2	chinmo	0.102948038974107	0.692501166028748	1	pseudobulk	DESeq2	LRT	0.159579491000807
118	KC_AB2	chm	0.127692145716859	0.903043895149941	1	pseudobulk	DESeq2	LRT	0.044291138990949
118	KC_AB2	Chmp1	0.106051949797129	0.883924111417854	1	pseudobulk	DESeq2	LRT	0.053585019384229
118	KC_AB2	CHMP2B	-2.4523940842299	0.179170102332144	1	pseudobulk	DESeq2	LRT	0.746734458264563
118	KC_AB2	chn	0.0349825288158536	0.902463619743517	1	pseudobulk	DESeq2	LRT	0.044570296417426
118	KC_AB2	CHOp24	-0.576800116907905	0.504890931047319	1	pseudobulk	DESeq2	LRT	0.296802430119485
118	KC_AB2	CHORD	-0.734071467759236	0.785001998672014	1	pseudobulk	DESeq2	LRT	0.105129237508095
118	KC_AB2	Chrac-14	-2.12405037015269	0.265879278548733	1	pseudobulk	DESeq2	LRT	0.575315508334519
118	KC_AB2	Chrac-16	0.375534485637874	0.901061951722844	1	pseudobulk	DESeq2	LRT	0.045245348458862

118	KC_AB2	chrb	0.208663169091197	0.450845234378002	1	pseudobulk	DESeq2	LRT	0.345972516638334
118	KC_AB2	Chro	0.135040154315426	0.89128724570206	1	pseudobulk	DESeq2	LRT	0.049982308175257
118	KC_AB2	ChT	0.0009195712368456	0.899543913622572	1	pseudobulk	DESeq2	LRT	0.045977630563627
118	KC_AB2	Cht11	-2.28054226263626	0.331630256012328	1	pseudobulk	DESeq2	LRT	0.479345853816062
118	KC_AB2	Cht7	-0.729379579623002	1	1	pseudobulk	DESeq2	LRT	0
118	KC_AB2	CIA30	0.575162595042011	0.797021781108777	1	pseudobulk	DESeq2	LRT	0.098529809988961
118	KC_AB2	Ciao1	-1.86499814887644	0.364960432713785	1	pseudobulk	DESeq2	LRT	0.437754217147716
118	KC_AB2	CIAPIN1	0.165517037562896	0.885235583659123	1	pseudobulk	DESeq2	LRT	0.052941137134971
118	KC_AB2	cib	1.27175296938886	0.322589238332178	1	pseudobulk	DESeq2	LRT	0.491350124891598
118	KC_AB2	cic	-0.284449209232483	0.418863794507804	1	pseudobulk	DESeq2	LRT	0.37792717729698
118	KC_AB2	cin	0.144723777698294	0.930326572940145	1	pseudobulk	DESeq2	LRT	0.031364574102179
118	KC_AB2	cindr	0.294378766974587	0.73952483063423	1	pseudobulk	DESeq2	LRT	0.131047239346081
118	KC_AB2	Cip4	0.113546748547892	0.759612662137169	1	pseudobulk	DESeq2	LRT	0.119407804532086
118	KC_AB2	Cirl	0.374970142670125	0.351731442966648	1	pseudobulk	DESeq2	LRT	0.453788806273543
118	KC_AB2	Cisd2	-0.289712652912831	0.60238104110721	1	pseudobulk	DESeq2	LRT	0.220128705253723
118	KC_AB2	Cka	0.312063514761474	0.566657273724245	1	pseudobulk	DESeq2	LRT	0.246679532171162
118	KC_AB2	CkIalpha	-0.0024284555704577	0.92797982238534	1	pseudobulk	DESeq2	LRT	0.032461466800506
118	KC_AB2	CkIIalpha	-0.393574277703502	0.362285098230579	1	pseudobulk	DESeq2	LRT	0.440949529308905
118	KC_AB2	CkIIalpha-i1	0.518865066730979	0.817181531186031	1	pseudobulk	DESeq2	LRT	0.087681457255733
118	KC_AB2	CkIIalpha-i3	-1.96604153319074	0.257523789779084	1	pseudobulk	DESeq2	LRT	0.589182645098332
118	KC_AB2	CkIIbeta	0.300062675173604	0.559677422722277	1	pseudobulk	DESeq2	LRT	0.252062212094339
118	KC_AB2	ckn	0.0194084526447339	0.932467338393049	1	pseudobulk	DESeq2	LRT	0.030366371319909
118	KC_AB2	Cks85A	-0.641216805015087	0.440690125042413	1	pseudobulk	DESeq2	LRT	0.355866681025453
118	KC_AB2	cl	-0.868594177140863	0.412335210381286	1	pseudobulk	DESeq2	LRT	0.384749578074262
118	KC_AB2	Clamp	0.197062745472506	0.823143563884196	1	pseudobulk	DESeq2	LRT	0.084524413189591
118	KC_AB2	Clbn	-0.0620089354703304	0.935501241565665	1	pseudobulk	DESeq2	LRT	0.028955631782614
118	KC_AB2	Clc	-0.553496595029512	0.34491996364947	1	pseudobulk	DESeq2	LRT	0.46228166834096
118	KC_AB2	ClC-b	1.89827754571339	0.428537461411561	1	pseudobulk	DESeq2	LRT	0.368011207408929
118	KC_AB2	ClC-c	0.0705112577709372	0.921396628927158	1	pseudobulk	DESeq2	LRT	0.035553381014326
118	KC_AB2	Clic	0.0594326503261174	0.918987790249335	1	pseudobulk	DESeq2	LRT	0.036690258649323
118	KC_AB2	CLIP-190	0.117784774626674	0.764275363555799	1	pseudobulk	DESeq2	LRT	0.116750139687847
118	KC_AB2	Clp	1.10706867860301	1	1	pseudobulk	DESeq2	LRT	0
118	KC_AB2	ClpP	-0.350540376023258	0.708429429301522	1	pseudobulk	DESeq2	LRT	0.149703405813722
118	KC_AB2	CLS	0.734253571864123	0.751957650179593	1	pseudobulk	DESeq2	LRT	0.123806617933922
118	KC_AB2	clt	1.35842679064456	0.654374436372797	1	pseudobulk	DESeq2	LRT	0.184173675044382
118	KC_AB2	clu	0.870773507162111	0.504432967702403	1	pseudobulk	DESeq2	LRT	0.297196537437276
118	KC_AB2	cm	-1.80660582003933	0.419701418835015	1	pseudobulk	DESeq2	LRT	0.377059562617968
118	KC_AB2	cmb	2.01814786626755	0.401718761445944	1	pseudobulk	DESeq2	LRT	0.396077884966921
118	KC_AB2	cmpy	-0.0236245860493506	0.947173046586851	1	pseudobulk	DESeq2	LRT	0.023570669030309
118	KC_AB2	cN-IIIB	2.74393498453441	0.166840603680413	1	pseudobulk	DESeq2	LRT	0.777698247412889
118	KC_AB2	CNBP	0.166007382992958	0.735002086235087	1	pseudobulk	DESeq2	LRT	0.133711428209546
118	KC_AB2	cnc	-0.481627348141	0.663491708215301	1	pseudobulk	DESeq2	LRT	0.178164500228094
118	KC_AB2	Cndp2	-1.53671722310391	0.188287976688622	1	pseudobulk	DESeq2	LRT	0.725177411393314
118	KC_AB2	CngA	-1.30129323996907	0.145788025966898	1	pseudobulk	DESeq2	LRT	0.836278144536927
118	KC_AB2	CngB	-0.78608453235502	0.281178024602003	1	pseudobulk	DESeq2	LRT	0.55101862450223
119	KC_AB2	Cngl	0.0497985503615559	0.834910504327144	1	pseudobulk	DESeq2	LRT	0.078360074887585
119	KC_AB2	cni	0.311679158495458	0.707607036510279	1	pseudobulk	DESeq2	LRT	0.150207857076063
119	KC_AB2	cnir	-0.326002591878444	0.83169727708792	1	pseudobulk	DESeq2	LRT	0.080034720360579
119	KC_AB2	cnk	-0.903412938451153	0.664037271512513	1	pseudobulk	DESeq2	LRT	0.177807543586898
119	KC_AB2	CNMaR	-0.700378979054034	0.550623629222948	1	pseudobulk	DESeq2	LRT	0.259145155418271
119	KC_AB2	cnn	0.245942347661955	0.793121243355125	1	pseudobulk	DESeq2	LRT	0.100660417606457
119	KC_AB2	cno	0.0528268105208937	0.956985759332489	1	pseudobulk	DESeq2	LRT	0.019094524803443

119	KC_AB2	Cnot4	-0.812296299587881	0.291277849272684	1	pseudobulk	DESeq2	LRT	0.535692540790858
119	KC_AB2	CNPYb	0.151408359271651	0.912261442481077	1	pseudobulk	DESeq2	LRT	0.039880680580222
119	KC_AB2	Cnx99A	-0.0266926878078313	0.955225907246321	1	pseudobulk	DESeq2	LRT	0.019893907297684
119	KC_AB2	Coa7	-0.910968734894303	0.287390833800024	1	pseudobulk	DESeq2	LRT	0.541527087605941
119	KC_AB2	Cog1	-0.987864420262975	0.622985879197186	1	pseudobulk	DESeq2	LRT	0.205521797090819
119	KC_AB2	Cog2	2.7605424445074	0.237810191029118	1	pseudobulk	DESeq2	LRT	0.623769538224996
119	KC_AB2	Cog3	-0.0690899661576322	0.963748143254324	1	pseudobulk	DESeq2	LRT	0.016036445646748
119	KC_AB2	Cog4	1.61205113368698	0.631647504209	1	pseudobulk	DESeq2	LRT	0.199525215522471
119	KC_AB2	Cog6	-0.311977968454154	0.857344273924811	1	pseudobulk	DESeq2	LRT	0.066844748389494
119	KC_AB2	Cog7	-2.03522979197945	0.396502959232805	1	pseudobulk	DESeq2	LRT	0.401753567050837
119	KC_AB2	Cog8	0.146930209264112	1	1	pseudobulk	DESeq2	LRT	0
119	KC_AB2	coil	0.879713421944237	0.683210282761593	1	pseudobulk	DESeq2	LRT	0.16544560585387
119	KC_AB2	cold	1.13465310574527	0.53172465627308	1	pseudobulk	DESeq2	LRT	0.274313200819788
119	KC_AB2	colt	0.0455261422590676	0.956410711868198	1	pseudobulk	DESeq2	LRT	0.019355568399845
119	KC_AB2	comm	-0.0936307667982717	0.841515397861468	1	pseudobulk	DESeq2	LRT	0.074937932991677
119	KC_AB2	comt	-0.175121550576811	0.782090056663848	1	pseudobulk	DESeq2	LRT	0.106743235609865
119	KC_AB2	Con	0.0796942984609813	0.80917018122081	1	pseudobulk	DESeq2	LRT	0.091960129818521
119	KC_AB2	Cont	0.760706891684122	0.701177403860344	1	pseudobulk	DESeq2	LRT	0.154172087924629
119	KC_AB2	conu	-0.161831395282586	0.772177454479476	1	pseudobulk	DESeq2	LRT	0.112282882766483
119	KC_AB2	Coop	1.38069928703004	0.566368801250645	1	pseudobulk	DESeq2	LRT	0.246900678019328
119	KC_AB2	Coprox	1.99339641594643	0.465071165814147	1	pseudobulk	DESeq2	LRT	0.332480585700252
119	KC_AB2	Coq2	0.145321756433347	1	1	pseudobulk	DESeq2	LRT	0
119	KC_AB2	Coq3	-0.467031669438227	0.808037298456944	1	pseudobulk	DESeq2	LRT	0.092568592022230
119	KC_AB2	Coq5	0.145321756433347	1	1	pseudobulk	DESeq2	LRT	0
119	KC_AB2	Coq6	-1.02685802591585	0.433847382537978	1	pseudobulk	DESeq2	LRT	0.362663018363706
119	KC_AB2	Coq7	-0.0260532125295181	0.958630028259348	1	pseudobulk	DESeq2	LRT	0.018348971230854
119	KC_AB2	Coq8	1.61205113368698	0.631647504209	1	pseudobulk	DESeq2	LRT	0.199525215522471
119	KC_AB2	Coq9	-0.467031669438227	0.808037298456944	1	pseudobulk	DESeq2	LRT	0.092568592022230
119	KC_AB2	cora	0.379731819995679	0.311073995007713	1	pseudobulk	DESeq2	LRT	0.507136293284439
119	KC_AB2	CoRest	0.246459534080667	0.735329960147012	1	pseudobulk	DESeq2	LRT	0.133517738854247
119	KC_AB2	Corin	1.45252577379083	0.301981278966806	1	pseudobulk	DESeq2	LRT	0.520019979868763
119	KC_AB2	corn	0.0361966433957074	0.871380081500106	1	pseudobulk	DESeq2	LRT	0.059792371636275
119	KC_AB2	coro	-0.0659313318619964	0.941614676914042	1	pseudobulk	DESeq2	LRT	0.026126780779423
119	KC_AB2	Cortactin	0.412080430143068	0.771777791047019	1	pseudobulk	DESeq2	LRT	0.112507722997719
119	KC_AB2	corto	0.368624269323261	0.451081956731913	1	pseudobulk	DESeq2	LRT	0.345744544324336
119	KC_AB2	cos	-0.733603641829958	0.372584801867924	1	pseudobulk	DESeq2	LRT	0.428774864414317
119	KC_AB2	Cow	0.436762583081758	0.475467715346967	1	pseudobulk	DESeq2	LRT	0.322878966681713
119	KC_AB2	COX4	-0.446175461253275	0.233550656036272	1	pseudobulk	DESeq2	LRT	0.631618908455271
119	KC_AB2	COX5A	-0.435353544618862	0.23560362217732	1	pseudobulk	DESeq2	LRT	0.627818036976914
119	KC_AB2	COX5B	-0.387900270529858	0.339392699799282	1	pseudobulk	DESeq2	LRT	0.469297503417829
119	KC_AB2	COX6B	-0.396743767061539	0.173322495059936	1	pseudobulk	DESeq2	LRT	0.761145067716577
119	KC_AB2	COX7A	-0.394000421623629	0.182522613525959	1	pseudobulk	DESeq2	LRT	0.738683321228677
119	KC_AB2	COX7C	0.0537757240167285	0.833725602204801	1	pseudobulk	DESeq2	LRT	0.078976861900414
119	KC_AB2	COX8	-0.324555551374915	0.292809105410229	1	pseudobulk	DESeq2	LRT	0.533415422258686
119	KC_AB2	Cp1	-0.169208556217154	0.781248981506762	1	pseudobulk	DESeq2	LRT	0.107210535825508
119	KC_AB2	Cp110	-0.578994096539406	0.307074299023712	1	pseudobulk	DESeq2	LRT	0.512756530868129
119	KC_AB2	Cp190	-0.355611311718458	0.674078494769824	1	pseudobulk	DESeq2	LRT	0.171289528004032
119	KC_AB2	Cp7Fa	-1.80075127281421	0.363208620892189	1	pseudobulk	DESeq2	LRT	0.439843851886983
119	KC_AB2	Cp7Fb	1.50730388760116	0.557960431011111	1	pseudobulk	DESeq2	LRT	0.253396598917002
119	KC_AB2	Cp7Fc	0.667244748290888	0.924085151242949	1	pseudobulk	DESeq2	LRT	0.034288008205147
119	KC_AB2	cpa	-0.051768802237555	0.953597974616862	1	pseudobulk	DESeq2	LRT	0.020634680017486
119	KC_AB2	cpb	-1.63535597681683	0.27453563416456	1	pseudobulk	DESeq2	LRT	0.561401277018503

119	KC_AB2	Cpes	0.252150834562007	0.850246474602041	1	pseudobulk	DESeq2	LRT	0.070455160117444
119	KC_AB2	cpo	0.0019159491631379	0.9269649332328	1	pseudobulk	DESeq2	LRT	0.032936694756850
119	KC_AB2	Cpr	-0.729379579623002	1	1	pseudobulk	DESeq2	LRT	0
119	KC_AB2	Cpr11B	0.393976029558784	0.939642609940555	1	pseudobulk	DESeq2	LRT	0.027037297511213
119	KC_AB2	Cpsf100	-0.470100723599697	0.833985291815347	1	pseudobulk	DESeq2	LRT	0.078841608522644
119	KC_AB2	Cpsf160	1.10706867860301	1	1	pseudobulk	DESeq2	LRT	0
119	KC_AB2	Cpsf5	0.278887848500386	0.652800315342287	1	pseudobulk	DESeq2	LRT	0.185219644463475
119	KC_AB2	Cpsf6	-0.589105112514072	0.358398329113974	1	pseudobulk	DESeq2	LRT	0.445634023726075
119	KC_AB2	Cpsf73	-1.86499814887644	0.364960432713785	1	pseudobulk	DESeq2	LRT	0.437754217147716
119	KC_AB2	cpx	0.0103491576755273	0.935877646136312	1	pseudobulk	DESeq2	LRT	0.028780925921311
119	KC_AB2	CR12842	1.26443978446157	0.293173504364488	1	pseudobulk	DESeq2	LRT	0.532875281739818
119	KC_AB2	CR15345	2.88969196614093	0.207685089444696	1	pseudobulk	DESeq2	LRT	0.682594682108805
119	KC_AB2	CR18166	1.68450022891931	0.207640602448559	1	pseudobulk	DESeq2	LRT	0.68268771972911
119	KC_AB2	CR18228	1.10706867860301	1	1	pseudobulk	DESeq2	LRT	0
119	KC_AB2	CR18275	0.208973860059943	0.870115574843371	1	pseudobulk	DESeq2	LRT	0.060423057515809
119	KC_AB2	CR31032	-0.206397962031176	0.915265628332718	1	pseudobulk	DESeq2	LRT	0.038452846723171
119	KC_AB2	CR32745	-0.602102016830105	0.757200167957577	1	pseudobulk	DESeq2	LRT	0.120789298375646
119	KC_AB2	CR33222	1.10706867860301	1	1	pseudobulk	DESeq2	LRT	0
119	KC_AB2	CR33294	1.38446765109058	0.642794900879723	1	pseudobulk	DESeq2	LRT	0.191927577037166
119	KC_AB2	CR41440	-0.729379579623002	1	1	pseudobulk	DESeq2	LRT	0
119	KC_AB2	CR41597	-0.93143827784496	0.338085721026006	1	pseudobulk	DESeq2	LRT	0.470973171176159
119	KC_AB2	CR43281	-1.02095645511662	0.300814660610402	1	pseudobulk	DESeq2	LRT	0.521701001634107
119	KC_AB2	CR43670	1.22041736249262	0.453005178211119	1	pseudobulk	DESeq2	LRT	0.343896833625884
119	KC_AB2	CR43671	-1.33407500886303	0.63128114564055	1	pseudobulk	DESeq2	LRT	0.19977718147345
119	KC_AB2	CR43972	1.10706867860301	1	1	pseudobulk	DESeq2	LRT	0
119	KC_AB2	CR44003	-2.70996484816113	0.227318796562982	1	pseudobulk	DESeq2	LRT	0.643364651797815
119	KC_AB2	CR44383	-0.729379579623002	1	1	pseudobulk	DESeq2	LRT	0
119	KC_AB2	CR45470	0.146930209264112	1	1	pseudobulk	DESeq2	LRT	0
119	KC_AB2	CR45496	-1.86716056129969	0.477680380390446	1	pseudobulk	DESeq2	LRT	0.320862595985116
119	KC_AB2	CR7448	0.145321756433347	1	1	pseudobulk	DESeq2	LRT	0
119	KC_AB2	Crag	0.439029583588558	0.751679973895002	1	pseudobulk	DESeq2	LRT	0.123967019985046
119	KC_AB2	CRAT	-0.83320718254958	0.715059964627045	1	pseudobulk	DESeq2	LRT	0.14565753691968
119	KC_AB2	crb	-0.0552676714803418	0.879729353021634	1	pseudobulk	DESeq2	LRT	0.055650917131584
119	KC_AB2	crc	0.31694554775778	0.653468733343219	1	pseudobulk	DESeq2	LRT	0.184775187366313
119	KC_AB2	CrebA	-0.784039942166074	0.233038179664711	1	pseudobulk	DESeq2	LRT	0.632572920779372
119	KC_AB2	CrebB	-0.412378257096071	0.625035279347774	1	pseudobulk	DESeq2	LRT	0.204095468746085
119	KC_AB2	CREG	1.10706867860301	1	1	pseudobulk	DESeq2	LRT	0
119	KC_AB2	Crg-1	2.64741945039871	0.292550810913063	1	pseudobulk	DESeq2	LRT	0.533798693743382
119	KC_AB2	CRIF	-1.16621284326036	0.430986520377392	1	pseudobulk	DESeq2	LRT	0.365536312709948
119	KC_AB2	crim	1.69530465237941	0.515035175910199	1	pseudobulk	DESeq2	LRT	0.288163108469491
119	KC_AB2	Crk	-0.402568802424824	0.404425650157557	1	pseudobulk	DESeq2	LRT	0.393161307659132
120	KC_AB2	crm	1.32278695949127	0.504433031325968	1	pseudobulk	DESeq2	LRT	0.297196482660203
120	KC_AB2	CRMP	0.210574228037583	0.688748893212239	1	pseudobulk	DESeq2	LRT	0.161939086030835
120	KC_AB2	crn	-0.39935006418526	0.791560209514437	1	pseudobulk	DESeq2	LRT	0.101516045216834
120	KC_AB2	crok	1.10706867860301	1	1	pseudobulk	DESeq2	LRT	0
120	KC_AB2	crol	0.437933347488002	0.312869953437518	1	pseudobulk	DESeq2	LRT	0.50463614245576
120	KC_AB2	CROT	-1.78975280219494	0.468855431150075	1	pseudobulk	DESeq2	LRT	0.32896104882553
120	KC_AB2	crp	0.842697335419995	0.103365274660178	1	pseudobulk	DESeq2	LRT	0.98562533702933
120	KC_AB2	crq	-0.632623756314477	0.513540999749075	1	pseudobulk	DESeq2	LRT	0.289424877764905
120	KC_AB2	Crtc	-0.211382899825553	0.637926760349711	1	pseudobulk	DESeq2	LRT	0.195229179275394
120	KC_AB2	Cse1	-2.476920539158	0.196065088561075	1	pseudobulk	DESeq2	LRT	0.70759973012142
120	KC_AB2	Csk	0.303167280502324	0.578820855255708	1	pseudobulk	DESeq2	LRT	0.237455829385584

120	KC_AB2	CSN1b	1.05478158788551	0.603428439295934	1	pseudobulk	DESeq2	LRT	0.21937422558589
120	KC_AB2	CSN3	2.47215486522296	0.30601789350912	1	pseudobulk	DESeq2	LRT	0.514253178665238
120	KC_AB2	CSN4	-0.465993033452039	0.714246829467516	1	pseudobulk	DESeq2	LRT	0.146151678768557
120	KC_AB2	CSN5	-1.3285271727402	0.24187313145089	1	pseudobulk	DESeq2	LRT	0.616412372691079
120	KC_AB2	CSN6	0.423170152652793	0.774866969542059	1	pseudobulk	DESeq2	LRT	0.110772851497467
120	KC_AB2	CSN7	0.159661089654143	0.865333109999793	1	pseudobulk	DESeq2	LRT	0.062816678678237
120	KC_AB2	CSN8	-1.03535473005918	0.401659676088632	1	pseudobulk	DESeq2	LRT	0.396141766304754
120	KC_AB2	Csp	-0.400870255289175	0.430214072905869	1	pseudobulk	DESeq2	LRT	0.366315387334424
120	KC_AB2	CstF50	1.04232157414887	0.700586543363791	1	pseudobulk	DESeq2	LRT	0.154538208723974
120	KC_AB2	CstF64	-0.50491169497659	0.835055051214058	1	pseudobulk	DESeq2	LRT	0.078284892599768
120	KC_AB2	csul	-1.71999636108657	0.519869997311849	1	pseudobulk	DESeq2	LRT	0.284105245805297
120	KC_AB2	csw	0.341306095336718	0.334631397760781	1	pseudobulk	DESeq2	LRT	0.4754333125233
120	KC_AB2	cta	0.163070410808799	0.809942535727886	1	pseudobulk	DESeq2	LRT	0.091545792604777
120	KC_AB2	CtBP	0.0245281491908081	0.88579604570462	1	pseudobulk	DESeq2	LRT	0.052666262787427
120	KC_AB2	CTCF	-0.0636000315128116	0.950802782015263	1	pseudobulk	DESeq2	LRT	0.021909556211726
120	KC_AB2	CtIP	-0.277947413912466	0.859179291176031	1	pseudobulk	DESeq2	LRT	0.065916199331162
120	KC_AB2	Ctl1	-0.449745927741117	0.631443976760157	1	pseudobulk	DESeq2	LRT	0.199665175058333
120	KC_AB2	Ctl2	0.181696615742416	0.829585765294942	1	pseudobulk	DESeq2	LRT	0.081138708531860
120	KC_AB2	ctp	-0.332848501636547	0.0667186464207609	1	pseudobulk	DESeq2	LRT	1.17575277319102
120	KC_AB2	CTPsyn	0.2894174731325	0.782831492052293	1	pseudobulk	DESeq2	LRT	0.106331711693862
120	KC_AB2	Ctr1A	-1.59648763579568	0.158895630536394	1	pseudobulk	DESeq2	LRT	0.79888804527232
120	KC_AB2	Ctr9	0.19495538195298	0.855422196204219	1	pseudobulk	DESeq2	LRT	0.067819485007490
120	KC_AB2	ctrip	0.67301700384705	0.257700725807209	1	pseudobulk	DESeq2	LRT	0.588884358268568
120	KC_AB2	CtsB1	0.215484316849599	0.744517458002914	1	pseudobulk	DESeq2	LRT	0.128125114130157
120	KC_AB2	cu	1.10706867860301	1	1	pseudobulk	DESeq2	LRT	0
120	KC_AB2	Cubn	-1.52244591594958	0.547176617578731	1	pseudobulk	DESeq2	LRT	0.261872469536296
120	KC_AB2	cue	-0.633849510305765	0.516397031341264	1	pseudobulk	DESeq2	LRT	0.287016263066759
120	KC_AB2	Cul1	-0.101398072959197	0.876782683146789	1	pseudobulk	DESeq2	LRT	0.057108036286782
120	KC_AB2	Cul2	-0.191345722804506	0.832822885958063	1	pseudobulk	DESeq2	LRT	0.079447348928916
120	KC_AB2	Cul3	-0.557560529027423	0.542080799287326	1	pseudobulk	DESeq2	LRT	0.265935975326383
120	KC_AB2	Cul4	1.15632841424846	0.438791956847973	1	pseudobulk	DESeq2	LRT	0.357741341736699
120	KC_AB2	Cul5	-0.729379579623002	0.772694994316916	1	pseudobulk	DESeq2	LRT	0.1119919011982
120	KC_AB2	cv-c	1.03728849428735	0.032215286157269	1	pseudobulk	DESeq2	LRT	1.4919380066125
120	KC_AB2	Cwc25	-1.51663092729204	0.281614279390474	1	pseudobulk	DESeq2	LRT	0.550345327856412
120	KC_AB2	cwo	0.583650793046094	0.482327373873702	1	pseudobulk	DESeq2	LRT	0.31665808955036
120	KC_AB2	cyc	0.351785237379586	0.789012940114753	1	pseudobulk	DESeq2	LRT	0.102915874136145
120	KC_AB2	CycC	1.82149197575781	0.300294174408485	1	pseudobulk	DESeq2	LRT	0.522453092865252
120	KC_AB2	CycD	1.10706867860301	1	1	pseudobulk	DESeq2	LRT	0
120	KC_AB2	CycG	-0.160106867190832	0.537924865508508	1	pseudobulk	DESeq2	LRT	0.269278380046951
120	KC_AB2	CycH	1.10706867860301	1	1	pseudobulk	DESeq2	LRT	0
120	KC_AB2	CycJ	1.38069928703004	0.566368801250645	1	pseudobulk	DESeq2	LRT	0.246900678019328
120	KC_AB2	CycK	-0.175153270001846	0.835089254200842	1	pseudobulk	DESeq2	LRT	0.078267104713562
120	KC_AB2	CycT	-0.249256041619449	0.610366498876006	1	pseudobulk	DESeq2	LRT	0.21440931147612
120	KC_AB2	CycY	0.272906773409039	0.741912053628353	1	pseudobulk	DESeq2	LRT	0.129647573006173
120	KC_AB2	CYLD	0.481239150645989	0.636212830924614	1	pseudobulk	DESeq2	LRT	0.196397576436505
120	KC_AB2	Cyp1	-0.303741207171881	0.378746070957365	1	pseudobulk	DESeq2	LRT	0.421651863757985
120	KC_AB2	Cyp12a4	1.38069928703004	0.566368801250645	1	pseudobulk	DESeq2	LRT	0.246900678019328
120	KC_AB2	Cyp12b2	-1.78975280219494	0.468855431150075	1	pseudobulk	DESeq2	LRT	0.32896104882553
120	KC_AB2	cyp33	-1.5028496659223	0.439431583548731	1	pseudobulk	DESeq2	LRT	0.357108731967189
120	KC_AB2	Cyp49a1	-0.104826770756343	0.940331830280396	1	pseudobulk	DESeq2	LRT	0.026718862752298
120	KC_AB2	Cyp4ae1	1.38446765109058	0.642794900879723	1	pseudobulk	DESeq2	LRT	0.191927577037166
120	KC_AB2	Cyp6a14	1.10706867860301	1	1	pseudobulk	DESeq2	LRT	0

120	KC_AB2	Cyp6d4	1.10706867860301	1	1	pseudobulk	DESeq2	LRT	0
120	KC_AB2	Cyp6u1	-0.729379579623002	1	1	pseudobulk	DESeq2	LRT	0
120	KC_AB2	Cyp9f2	1.10706867860301	1	1	pseudobulk	DESeq2	LRT	0
120	KC_AB2	Cyp9f3	1.10706867860301	1	1	pseudobulk	DESeq2	LRT	0
120	KC_AB2	cype	-0.203496373774568	0.492447270972752	1	pseudobulk	DESeq2	LRT	0.307640264966444
120	KC_AB2	Cypl	0.102887055144363	0.881341887563236	1	pseudobulk	DESeq2	LRT	0.054855588660052
120	KC_AB2	Cys	1.95432904991983	0.509212778150152	1	pseudobulk	DESeq2	LRT	0.293100706726022
120	KC_AB2	CysRS	2.57605741848544	0.2267635073907	1	pseudobulk	DESeq2	LRT	0.644426834320657
120	KC_AB2	CysRS-m	-2.03522979197945	0.396502959232805	1	pseudobulk	DESeq2	LRT	0.401753567050837
120	KC_AB2	Cyt-b5	0.265053321604339	0.660911213462947	1	pseudobulk	DESeq2	LRT	0.179856879535581
120	KC_AB2	Cyt-c-p	-0.590407556953897	0.073127490335423	1	pseudobulk	DESeq2	LRT	1.13591933088561
120	KC_AB2	Cyt-c1	-0.663932066061388	0.216658995517289	1	pseudobulk	DESeq2	LRT	0.66422327467753
120	KC_AB2	D1	0.185670755129398	0.708104832249227	1	pseudobulk	DESeq2	LRT	0.149902441890642
120	KC_AB2	D12	0.942758880874816	0.658080412798793	1	pseudobulk	DESeq2	LRT	0.181721035411818
120	KC_AB2	D19A	-1.686554135871	0.462587037417107	1	pseudobulk	DESeq2	LRT	0.334806541165695
120	KC_AB2	D19B	2.02556328998816	0.265962460108277	1	pseudobulk	DESeq2	LRT	0.575179658550741
120	KC_AB2	d4	0.281268921094517	0.582293677131629	1	pseudobulk	DESeq2	LRT	0.234857925677227
120	KC_AB2	da	-0.354305617187611	0.473577603458108	1	pseudobulk	DESeq2	LRT	0.324608844554884
120	KC_AB2	DAAM	-0.0905202933917032	0.931974600370995	1	pseudobulk	DESeq2	LRT	0.030595923556999
120	KC_AB2	Dab	0.300693013501162	0.589185540874275	1	pseudobulk	DESeq2	LRT	0.229747919663024
120	KC_AB2	dac	0.38413583883507	0.179072485644348	1	pseudobulk	DESeq2	LRT	0.746971138052636
120	KC_AB2	Dad	-0.961033333862793	0.70039622209097	1	pseudobulk	DESeq2	LRT	0.154656205149228
120	KC_AB2	Dad1	-0.270812396734498	0.721058066623697	1	pseudobulk	DESeq2	LRT	0.142029760247524
120	KC_AB2	daed	1.10706867860301	1	1	pseudobulk	DESeq2	LRT	0
120	KC_AB2	Dak1	0.366078382323169	0.620729867924999	1	pseudobulk	DESeq2	LRT	0.207097356978175
120	KC_AB2	dally	1.92129177323338	0.410879922887257	1	pseudobulk	DESeq2	LRT	0.386285079454371
120	KC_AB2	dao	-0.507378677169564	0.554050821212029	1	pseudobulk	DESeq2	LRT	0.256450397077255
120	KC_AB2	Dap160	-0.348018207565133	0.552471592713311	1	pseudobulk	DESeq2	LRT	0.257690047860097
120	KC_AB2	dare	-1.53678895984791	0.404729832843378	1	pseudobulk	DESeq2	LRT	0.392834782359752
120	KC_AB2	Dark	1.08849128365667	0.368158226181737	1	pseudobulk	DESeq2	LRT	0.433965491138869
120	KC_AB2	DAT	-0.729379579623002	0.823527397767669	1	pseudobulk	DESeq2	LRT	0.084321947797948
120	KC_AB2	dati	0.209798476679985	0.511693422038473	1	pseudobulk	DESeq2	LRT	0.290990165968582
120	KC_AB2	Daxx	0.470444693724321	0.595012712116287	1	pseudobulk	DESeq2	LRT	0.225473755711817
120	KC_AB2	dbe	1.04237305304479	0.767386654171747	1	pseudobulk	DESeq2	LRT	0.114985758004482
120	KC_AB2	dbo	-0.279452907893302	0.689623749440598	1	pseudobulk	DESeq2	LRT	0.161387790580392
120	KC_AB2	Dbp21E2	-0.729379579623002	0.772694994316916	1	pseudobulk	DESeq2	LRT	0.1119919011982
120	KC_AB2	Dbp45A	-1.52244591594958	0.547176617578731	1	pseudobulk	DESeq2	LRT	0.261872469536296
121	KC_AB2	Dbp73D	1.10706867860301	1	1	pseudobulk	DESeq2	LRT	0
121	KC_AB2	Dbp80	-0.147377458268778	0.70983330358629	1	pseudobulk	DESeq2	LRT	0.14884362850991
121	KC_AB2	dbr	-0.750218004183046	0.684438648478708	1	pseudobulk	DESeq2	LRT	0.164665474953366
121	KC_AB2	DCAF12	0.375534485637874	0.901061951722844	1	pseudobulk	DESeq2	LRT	0.045245348458862
121	KC_AB2	Dci	2.88790980540653	0.211468500133428	1	pseudobulk	DESeq2	LRT	0.674754314989032
121	KC_AB2	dco	0.0296940344886825	0.915006795136265	1	pseudobulk	DESeq2	LRT	0.038575680710354
121	KC_AB2	Dcp-1	-1.50967690248522	0.449786644124088	1	pseudobulk	DESeq2	LRT	0.346993444563629
121	KC_AB2	DCP1	0.911597717409207	0.507825313121622	1	pseudobulk	DESeq2	LRT	0.294285655027779
121	KC_AB2	DCP2	0.148699560839076	0.803557830292256	1	pseudobulk	DESeq2	LRT	0.094982862555841
121	KC_AB2	Dcr-1	-2.35203494925393	0.313819523334105	1	pseudobulk	DESeq2	LRT	0.503320041588723
121	KC_AB2	Dcr-2	-0.486384853384213	0.819854886224541	1	pseudobulk	DESeq2	LRT	0.086263010651694
121	KC_AB2	DCTN1-p150	-0.100047767754773	0.903337212004383	1	pseudobulk	DESeq2	LRT	0.044150099105237
121	KC_AB2	DCTN2-p50	-0.159195421264669	0.911769936225633	1	pseudobulk	DESeq2	LRT	0.040114731882429
121	KC_AB2	DCTN3-p24	-0.01495700991388	0.988442406777497	1	pseudobulk	DESeq2	LRT	0.005048630495385
121	KC_AB2	DCTN4-p62	0.881552417140076	0.514041096709213	1	pseudobulk	DESeq2	LRT	0.289002158513372

121	KC_AB2	DCTN5-p25	0.961298419535005	0.451595697094379	1	pseudobulk	DESeq2	LRT	0.345250204783098
121	KC_AB2	DCTN6-p27	-1.01651277871371	0.392005670878965	1	pseudobulk	DESeq2	LRT	0.406707650291716
121	KC_AB2	Dd	-0.306123122769319	0.574860669229072	1	pseudobulk	DESeq2	LRT	0.240437403862457
121	KC_AB2	Ddc	-1.14086593652881	0.409283734327952	1	pseudobulk	DESeq2	LRT	0.387975514655935
121	KC_AB2	Ddr	0.667244748290888	0.924085151242949	1	pseudobulk	DESeq2	LRT	0.034288008205147
121	KC_AB2	Ddx1	-0.516135479975958	0.798595883210028	1	pseudobulk	DESeq2	LRT	0.097672932939297
121	KC_AB2	Deaf1	-0.313411133373326	0.58704972586973	1	pseudobulk	DESeq2	LRT	0.231325110411366
121	KC_AB2	DEF8	-0.729379579623002	0.823527397767669	1	pseudobulk	DESeq2	LRT	0.084321947797948
121	KC_AB2	defl	1.10706867860301	1	1	pseudobulk	DESeq2	LRT	0
121	KC_AB2	Dek	-0.572006334881562	0.281427801548361	1	pseudobulk	DESeq2	LRT	0.550633001916772
121	KC_AB2	deltaCOP	0.60010426771657	0.586261343427883	1	pseudobulk	DESeq2	LRT	0.231908741137457
121	KC_AB2	Den1	-0.189435222083962	0.818541353940311	1	pseudobulk	DESeq2	LRT	0.086959374487352
121	KC_AB2	DENR	-0.50735696915275	0.637772631951847	1	pseudobulk	DESeq2	LRT	0.195334121103268
121	KC_AB2	Der-1	-1.38539385387624	0.380717857380081	1	pseudobulk	DESeq2	LRT	0.419396752343114
121	KC_AB2	Der-2	-1.06947806997529	0.376432044709299	1	pseudobulk	DESeq2	LRT	0.424313413278646
121	KC_AB2	Desat1	0.20393834335244	0.582957313441402	1	pseudobulk	DESeq2	LRT	0.234363244924637
121	KC_AB2	Df31	-0.0767062837526093	0.80797910187552	1	pseudobulk	DESeq2	LRT	0.092599871969864
121	KC_AB2	Dg	0.0458367783444841	0.852540341533698	1	pseudobulk	DESeq2	LRT	0.069285061378337
121	KC_AB2	Dgk	-0.0816624197001391	0.883720664992052	1	pseudobulk	DESeq2	LRT	0.053684989312190
121	KC_AB2	Dgkepsilon	-0.667939119979437	0.589601326933866	1	pseudobulk	DESeq2	LRT	0.229441547741597
121	KC_AB2	Dgp-1	-0.378264198690272	0.782647677137681	1	pseudobulk	DESeq2	LRT	0.106433699385612
121	KC_AB2	dgrn	-0.994575578221731	0.616586547219332	1	pseudobulk	DESeq2	LRT	0.210005955010331
121	KC_AB2	dgt1	-0.486384853384213	0.819854886224541	1	pseudobulk	DESeq2	LRT	0.086263010651694
121	KC_AB2	Dh44	1.10706867860301	1	1	pseudobulk	DESeq2	LRT	0
121	KC_AB2	Dh44-R1	-0.632123393991588	0.485503818641624	1	pseudobulk	DESeq2	LRT	0.313807349878605
121	KC_AB2	Dh44-R2	1.61205113368698	0.631647504209	1	pseudobulk	DESeq2	LRT	0.199525215522471
121	KC_AB2	Dhap-at	-0.844053880567611	0.477334380694763	1	pseudobulk	DESeq2	LRT	0.321177283822985
121	KC_AB2	Dhc64C	0.107120869194979	0.858092754992346	1	pseudobulk	DESeq2	LRT	0.066465764827186
121	KC_AB2	Dhit	0.0724149837212194	0.842575124912376	1	pseudobulk	DESeq2	LRT	0.074391366563026
121	KC_AB2	Dhod	0.229116498387851	0.859640013003888	1	pseudobulk	DESeq2	LRT	0.065683377938202
121	KC_AB2	DhpD	-0.729379579623002	1	1	pseudobulk	DESeq2	LRT	0
121	KC_AB2	Dhpr	-0.46386223939949	0.461442094335877	1	pseudobulk	DESeq2	LRT	0.335882790170534
121	KC_AB2	Dhx15	2.58564464312165	0.314515351959278	1	pseudobulk	DESeq2	LRT	0.502358151145649
121	KC_AB2	dia	-0.729379579623002	0.772694994316916	1	pseudobulk	DESeq2	LRT	0.1119919011982
121	KC_AB2	Diap1	0.0478359630300141	0.9111255648547	1	pseudobulk	DESeq2	LRT	0.040421767521261
121	KC_AB2	Diap2	0.891178438100988	0.412043021329387	1	pseudobulk	DESeq2	LRT	0.385057436999558
121	KC_AB2	Dic1	-1.03699647685996	0.172507455126082	1	pseudobulk	DESeq2	LRT	0.763192131604289
121	KC_AB2	didum	-0.0667348347358648	0.946291061943005	1	pseudobulk	DESeq2	LRT	0.023975261955967
121	KC_AB2	Dif	0.308003446669754	0.580703026588621	1	pseudobulk	DESeq2	LRT	0.236045910444527
121	KC_AB2	dikar	-0.0750489657970134	0.851444449170621	1	pseudobulk	DESeq2	LRT	0.069843681471967
121	KC_AB2	Dim1	-0.723947534526386	0.604706513785067	1	pseudobulk	DESeq2	LRT	0.218455353230217
121	KC_AB2	dind	0.271600213663012	0.867668243784365	1	pseudobulk	DESeq2	LRT	0.061646297214086
121	KC_AB2	DIP-alpha	-2.94953869446039	0.149849840797516	1	pseudobulk	DESeq2	LRT	0.824343714118383
121	KC_AB2	Dip-B	2.48384162006255	0.303740962766822	1	pseudobulk	DESeq2	LRT	0.517496634805608
121	KC_AB2	Dip-C	-0.289322388441402	0.778900486699501	1	pseudobulk	DESeq2	LRT	0.108518024789519
121	KC_AB2	DIP-gamma	-0.729379579623002	0.823527397767669	1	pseudobulk	DESeq2	LRT	0.084321947797948
121	KC_AB2	DIP-kappa	-0.285651358278501	0.234783342075835	1	pseudobulk	DESeq2	LRT	0.629332719610033
121	KC_AB2	DIP-lambda	-2.09428695548101	0.380540277844868	1	pseudobulk	DESeq2	LRT	0.419599369064229
121	KC_AB2	DIP1	0.0062669502161422	0.980517743407454	1	pseudobulk	DESeq2	LRT	0.008544542950583
121	KC_AB2	DIP2	0.519158430980687	0.547326802095258	1	pseudobulk	DESeq2	LRT	0.261753284331332
121	KC_AB2	Dis3	-1.33407500886303	0.63128114564055	1	pseudobulk	DESeq2	LRT	0.19977718147345
121	KC_AB2	Dis3l2	0.89698058322345	0.673674818076485	1	pseudobulk	DESeq2	LRT	0.171549686245101

121	KC_AB2	disp	-0.277947413912466	0.859179291176031	1	pseudobulk	DESeq2	LRT	0.065916199331162
121	KC_AB2	DJ-1alpha	1.37221885443763	0.558734269457382	1	pseudobulk	DESeq2	LRT	0.252794690766539
121	KC_AB2	dj-1beta	0.655457402474653	0.45345395602842	1	pseudobulk	DESeq2	LRT	0.343466804872868
121	KC_AB2	dl	1.13954879384451	0.318722457381926	1	pseudobulk	DESeq2	LRT	0.496587334789728
121	KC_AB2	Dlc90F	0.347335651640711	0.645290145871692	1	pseudobulk	DESeq2	LRT	0.190244966887644
121	KC_AB2	dlg1	-0.110414991637605	0.679000647588149	1	pseudobulk	DESeq2	LRT	0.168129811516515
121	KC_AB2	Dlg5	-0.815032814367807	0.48757459761281	1	pseudobulk	DESeq2	LRT	0.31195892898467
121	KC_AB2	Dlic	-0.272803254577437	0.663984778280361	1	pseudobulk	DESeq2	LRT	0.177841876632914
121	KC_AB2	Dlip1	-2.39970914040097	0.301766661516198	1	pseudobulk	DESeq2	LRT	0.520328741761584
121	KC_AB2	Dlip2	2.36270654664069	0.296460111017658	1	pseudobulk	DESeq2	LRT	0.528033733093372
121	KC_AB2	Dlip3	0.622674322077138	0.61296354301558	1	pseudobulk	DESeq2	LRT	0.212565355070492
121	KC_AB2	dlp	1.66565955991481	0.485757178015219	1	pseudobulk	DESeq2	LRT	0.313580773127835
121	KC_AB2	dlt	-0.729379579623002	0.823527397767669	1	pseudobulk	DESeq2	LRT	0.084321947797948
121	KC_AB2	DMAP1	-1.47477682440444	0.369703721748464	1	pseudobulk	DESeq2	LRT	0.432146177431532
121	KC_AB2	dmpd	0.914032205632245	0.414871179294766	1	pseudobulk	DESeq2	LRT	0.382086734146679
121	KC_AB2	dmt	1.61205113368698	0.631647504209	1	pseudobulk	DESeq2	LRT	0.199525215522471
121	KC_AB2	Dmtn	0.0898881043229587	0.914158537321752	1	pseudobulk	DESeq2	LRT	0.038978480509744
121	KC_AB2	DnaJ-1	-0.847744224818049	0.26054992727288	1	pseudobulk	DESeq2	LRT	0.58410904372152
121	KC_AB2	DnaJ-H	-0.858958047691042	0.496002230748259	1	pseudobulk	DESeq2	LRT	0.304516370285043
121	KC_AB2	DNAlig1	-0.729379579623002	1	1	pseudobulk	DESeq2	LRT	0
121	KC_AB2	DNAlig3	-0.656452603741136	0.790618269930056	1	pseudobulk	DESeq2	LRT	0.102033154022734
121	KC_AB2	DNAlig4	-0.729379579623002	0.823527397767669	1	pseudobulk	DESeq2	LRT	0.084321947797948
121	KC_AB2	DNApol-delta	-0.729379579623002	0.823527397767669	1	pseudobulk	DESeq2	LRT	0.084321947797948
121	KC_AB2	DNApol-eta	2.68075286224744	0.203957449403284	1	pseudobulk	DESeq2	LRT	0.690460427754798
121	KC_AB2	DNApol-iota	-0.400818874228283	0.829617251912903	1	pseudobulk	DESeq2	LRT	0.081122225359766
121	KC_AB2	DNApol-theta	-0.729379579623002	1	1	pseudobulk	DESeq2	LRT	0
121	KC_AB2	DNApol-zeta	-0.729379579623002	1	1	pseudobulk	DESeq2	LRT	0
121	KC_AB2	dnc	-0.0137472257206178	0.936978088958648	1	pseudobulk	DESeq2	LRT	0.028270564881286
121	KC_AB2	dnk	0.901518178659393	0.609547797017874	1	pseudobulk	DESeq2	LRT	0.214992233987788
121	KC_AB2	dnt	-0.527510649809022	0.761880031680828	1	pseudobulk	DESeq2	LRT	0.118113408819
121	KC_AB2	Dnz1	-0.372243158427753	0.715722407075097	1	pseudobulk	DESeq2	LRT	0.145255386143972
121	KC_AB2	Doa	-0.554541541479981	0.544922240094403	1	pseudobulk	DESeq2	LRT	0.26366546672762
121	KC_AB2	dock	0.0275462389542446	0.938166162911958	1	pseudobulk	DESeq2	LRT	0.027720234920757
122	KC_AB2	dod	-0.398937652120596	0.604123400609423	1	pseudobulk	DESeq2	LRT	0.2188743416274
122	KC_AB2	dom	-0.471128456904913	0.595747852867042	1	pseudobulk	DESeq2	LRT	0.224937514218058
122	KC_AB2	dome	-1.29052728408004	0.545673810438722	1	pseudobulk	DESeq2	LRT	0.263066889637944
122	KC_AB2	dop	1.14524880071631	0.31396345800733	1	pseudobulk	DESeq2	LRT	0.503120896223877
122	KC_AB2	Dop1R1	0.278967552534032	0.317023112468226	1	pseudobulk	DESeq2	LRT	0.498909074528273
122	KC_AB2	Dop1R2	-0.286682395085276	0.37693409933253	1	pseudobulk	DESeq2	LRT	0.423734572335273
122	KC_AB2	Dop2R	0.107295260953733	0.569660155980787	1	pseudobulk	DESeq2	LRT	0.244384155552657
122	KC_AB2	DopEcR	-0.319219145175015	0.377456804293315	1	pseudobulk	DESeq2	LRT	0.423132741333947
122	KC_AB2	dor	-1.63598319081091	0.397013186805603	1	pseudobulk	DESeq2	LRT	0.401195067892312
122	KC_AB2	DOR	0.0841869154842343	0.790050845510552	1	pseudobulk	DESeq2	LRT	0.102344957805659
122	KC_AB2	dos	0.209013533085127	0.828091995610101	1	pseudobulk	DESeq2	LRT	0.081921413255609
122	KC_AB2	Dp	1.17476689057623	0.112784268646812	1	pseudobulk	DESeq2	LRT	0.947751472305981
122	KC_AB2	Dp1	0.749863021798967	0.538479168069221	1	pseudobulk	DESeq2	LRT	0.268831093420011
122	KC_AB2	Dpck	1.03858245968159	0.742043499884082	1	pseudobulk	DESeq2	LRT	0.129570634874779
122	KC_AB2	DPCoAC	-1.67513874790658	0.39900884344985	1	pseudobulk	DESeq2	LRT	0.39901747870195
122	KC_AB2	Dph1	-0.729379579623002	0.772694994316916	1	pseudobulk	DESeq2	LRT	0.1119919011982
122	KC_AB2	Dph5	0.0544232158395821	0.92882168767184	1	pseudobulk	DESeq2	LRT	0.032067652522022
122	KC_AB2	DppIII	-0.690458796728516	0.723861755771348	1	pseudobulk	DESeq2	LRT	0.140344368110085
122	KC_AB2	dpr1	-1.6052707068109	0.52265609184397	1	pseudobulk	DESeq2	LRT	0.281783983297782

122	KC_AB2	dpr10	0.0389945323765715	0.882247394318705	1	pseudobulk	DESeq2	LRT	0.054409615636941
122	KC_AB2	dpr12	-0.170446693127997	0.590969888256224	1	pseudobulk	DESeq2	LRT	0.228434647202202
122	KC_AB2	dpr14	0.0072810407890707	0.882235089944832	1	pseudobulk	DESeq2	LRT	0.054415672621608
122	KC_AB2	dpr16	-0.871839595111151	0.46653718248204	1	pseudobulk	DESeq2	LRT	0.331113737762579
122	KC_AB2	dpr17	-0.588625341219296	0.205015339859846	1	pseudobulk	DESeq2	LRT	0.688213642518674
122	KC_AB2	dpr19	-0.517172999008093	0.308687549041238	1	pseudobulk	DESeq2	LRT	0.51048088749794
122	KC_AB2	dpr2	-1.12170703143393	0.555119388710213	1	pseudobulk	DESeq2	LRT	0.255613603756584
122	KC_AB2	dpr20	3.09862063360264	0.123011857074261	1	pseudobulk	DESeq2	LRT	0.910053025034281
122	KC_AB2	dpr21	-0.0726623161264175	0.783627793355109	1	pseudobulk	DESeq2	LRT	0.105890169044454
122	KC_AB2	dpr3	-0.0372098554831334	0.938217941140456	1	pseudobulk	DESeq2	LRT	0.027696266481804
122	KC_AB2	dpr4	-0.196790561964548	0.682697899484169	1	pseudobulk	DESeq2	LRT	0.165771433377761
122	KC_AB2	dpr5	-0.0116608928244716	0.965978862134819	1	pseudobulk	DESeq2	LRT	0.015032376854327
122	KC_AB2	dpr6	0.124760549995029	0.850354187262515	1	pseudobulk	DESeq2	LRT	0.070400145421535
122	KC_AB2	dpr7	-0.4050855261448	0.382582051845763	1	pseudobulk	DESeq2	LRT	0.41727540798144
122	KC_AB2	dpr8	0.151877909258461	0.598118562309967	1	pseudobulk	DESeq2	LRT	0.22321271926705
122	KC_AB2	dpr9	-0.310353938079699	0.306516809620954	1	pseudobulk	DESeq2	LRT	0.513545703444287
122	KC_AB2	dpy	-1.16016699006818	0.583985689825587	1	pseudobulk	DESeq2	LRT	0.233597794849804
122	KC_AB2	Dpy-30L1	-0.264661158980782	0.779498056899491	1	pseudobulk	DESeq2	LRT	0.108184963065108
122	KC_AB2	Drak	0.283929910092396	0.621481904995014	1	pseudobulk	DESeq2	LRT	0.206571511712954
122	KC_AB2	Drat	-0.236214440583594	0.579087187463282	1	pseudobulk	DESeq2	LRT	0.237256043900252
122	KC_AB2	dre4	-1.2241657876264	0.517395327738637	1	pseudobulk	DESeq2	LRT	0.286177497433333
122	KC_AB2	Dref	0.600815629217726	0.635492320765682	1	pseudobulk	DESeq2	LRT	0.196889693055276
122	KC_AB2	Drep1	2.62919123543814	0.269826951733818	1	pseudobulk	DESeq2	LRT	0.568914672882509
122	KC_AB2	Drep2	-0.0365556007069147	0.913340750994835	1	pseudobulk	DESeq2	LRT	0.039367164779153
122	KC_AB2	Drep4	-0.317241443206136	0.855681736781888	1	pseudobulk	DESeq2	LRT	0.067687737263355
122	KC_AB2	Drice	0.147593560254927	1	1	pseudobulk	DESeq2	LRT	0
122	KC_AB2	drk	0.333224709610205	0.698264291007882	1	pseudobulk	DESeq2	LRT	0.155980167059322
122	KC_AB2	drl	2.01814786626755	0.401718761445944	1	pseudobulk	DESeq2	LRT	0.396077884966921
122	KC_AB2	Drl-2	-0.0544073435710988	0.918236529058472	1	pseudobulk	DESeq2	LRT	0.037045434230678
122	KC_AB2	Droj2	-1.17314467920338	0.0193096463598856	1	pseudobulk	DESeq2	LRT	1.71422567989003
122	KC_AB2	Dronc	-1.52244591594958	0.547176617578731	1	pseudobulk	DESeq2	LRT	0.261872469536296
122	KC_AB2	drongo	0.268770435406075	0.825028394016754	1	pseudobulk	DESeq2	LRT	0.083531104598443
122	KC_AB2	drosha	2.74363758354933	0.142615141839741	1	pseudobulk	DESeq2	LRT	0.845834361802974
122	KC_AB2	Drp1	-0.436015688431305	0.615559155514748	1	pseudobulk	DESeq2	LRT	0.210730204829926
122	KC_AB2	drpr	0.159809030812634	0.875550259399342	1	pseudobulk	DESeq2	LRT	0.057718918965680
122	KC_AB2	Dscam1	0.514778569371473	0.254092336295208	1	pseudobulk	DESeq2	LRT	0.595008433552126
122	KC_AB2	Dscam2	0.511752278908108	0.306004666100572	1	pseudobulk	DESeq2	LRT	0.514271951144955
122	KC_AB2	Dscam3	-0.560125320539469	0.178891866602925	1	pseudobulk	DESeq2	LRT	0.747409404372628
122	KC_AB2	Dscam4	0.720687702883897	0.167265320928639	1	pseudobulk	DESeq2	LRT	0.776594091855959
122	KC_AB2	dsd	0.487027485112868	0.406868038975419	1	pseudobulk	DESeq2	LRT	0.390546424282326
122	KC_AB2	dsh	-0.136761416842417	0.843027447715452	1	pseudobulk	DESeq2	LRT	0.074158285164932
122	KC_AB2	Dsor1	-0.726899502363916	0.438105174079685	1	pseudobulk	DESeq2	LRT	0.358421617716627
122	KC_AB2	Dsp1	0.227945351456956	0.470104646138322	1	pseudobulk	DESeq2	LRT	0.327805456571236
122	KC_AB2	dsx-c73A	1.38446765109058	0.642794900879723	1	pseudobulk	DESeq2	LRT	0.191927577037166
122	KC_AB2	Dtd	-2.62316164937934	0.247937455125287	1	pseudobulk	DESeq2	LRT	0.605657860785625
122	KC_AB2	dtn	0.339510752485451	0.387534687802756	1	pseudobulk	DESeq2	LRT	0.411689418197431
122	KC_AB2	dtr	1.10706867860301	1	1	pseudobulk	DESeq2	LRT	0
122	KC_AB2	Duba	0.962943929100069	0.287734870892736	1	pseudobulk	DESeq2	LRT	0.541007502313413
122	KC_AB2	DUBAI	-1.58613696021611	0.394222143993741	1	pseudobulk	DESeq2	LRT	0.404258984459966
122	KC_AB2	dunk	1.38446765109058	0.642794900879723	1	pseudobulk	DESeq2	LRT	0.191927577037166
122	KC_AB2	dup	0.145321756433347	1	1	pseudobulk	DESeq2	LRT	0
122	KC_AB2	dwg	1.10706867860301	1	1	pseudobulk	DESeq2	LRT	0

122	KC_AB2	Dyb	-0.653373143389891	0.0843230319139154	1	pseudobulk	DESeq2	LRT	1.07405378638425
122	KC_AB2	Dyrk2	-0.60052337749037	0.20924465734264	1	pseudobulk	DESeq2	LRT	0.679345622062215
122	KC_AB2	Dyrk3	0.49570336460572	0.274017536398825	1	pseudobulk	DESeq2	LRT	0.562221642590063
122	KC_AB2	Dys	0.967622088124255	0.106271258997117	1	pseudobulk	DESeq2	LRT	0.973584174297843
122	KC_AB2	Dysb	0.147593560254927	1	1	pseudobulk	DESeq2	LRT	0
122	KC_AB2	dysc	-0.0535479200644484	0.816868128532539	1	pseudobulk	DESeq2	LRT	0.087848048330716
122	KC_AB2	E(bx)	0.0116786817324332	0.892227475542794	1	pseudobulk	DESeq2	LRT	0.049524407083088
122	KC_AB2	E(Pc)	0.0916546909030843	0.815166612096324	1	pseudobulk	DESeq2	LRT	0.088753616635678
122	KC_AB2	e(r)	0.270470180237476	0.688403363229519	1	pseudobulk	DESeq2	LRT	0.162157016571593
122	KC_AB2	E(spl)m4-BFM	1.10706867860301	1	1	pseudobulk	DESeq2	LRT	0
122	KC_AB2	E(var)3-9	0.471164545572349	0.766071580717929	1	pseudobulk	DESeq2	LRT	0.115730648567928
122	KC_AB2	e(y)1	-0.883890872531852	0.676366690187261	1	pseudobulk	DESeq2	LRT	0.169817788750343
122	KC_AB2	e(y)2	0.2272557679205	0.88342832984611	1	pseudobulk	DESeq2	LRT	0.053828677866215
122	KC_AB2	e(y)2b	1.61205113368698	0.631647504209	1	pseudobulk	DESeq2	LRT	0.199525215522471
122	KC_AB2	e(y)3	0.897377785913111	0.314209519169617	1	pseudobulk	DESeq2	LRT	0.502780661879707
122	KC_AB2	E(z)	0.0230985328136399	0.91416542764115	1	pseudobulk	DESeq2	LRT	0.038975207098958
122	KC_AB2	E2f1	0.169025192386537	0.820273278611217	1	pseudobulk	DESeq2	LRT	0.086041435883769
122	KC_AB2	E2f2	-1.19287235924974	0.576214109151084	1	pseudobulk	DESeq2	LRT	0.239416111811524
122	KC_AB2	Eaat1	1.10706867860301	1	1	pseudobulk	DESeq2	LRT	0
122	KC_AB2	Eaat2	-0.327862718828606	0.719156627659881	1	pseudobulk	DESeq2	LRT	0.143176512787592
122	KC_AB2	Eaf	-0.576040043132414	0.541559564903338	1	pseudobulk	DESeq2	LRT	0.266353769350457
122	KC_AB2	Eaf6	-1.34387033725355	0.647060155693926	1	pseudobulk	DESeq2	LRT	0.189055342095618
122	KC_AB2	eag	0.58126704729314	0.29019518721786	1	pseudobulk	DESeq2	LRT	0.537309794455341
122	KC_AB2	ear	0.0108473848464778	0.981595008998383	1	pseudobulk	DESeq2	LRT	0.008067658481920
122	KC_AB2	eas	-0.435946902912835	0.631900532742801	1	pseudobulk	DESeq2	LRT	0.199351278490797
122	KC_AB2	east	1.10374826535824	0.0405042321027108	1	pseudobulk	DESeq2	LRT	1.39249959696237
122	KC_AB2	Eato	1.21989951758771	0.461754102446995	1	pseudobulk	DESeq2	LRT	0.335589237388148
122	KC_AB2	Eb1	-0.316510228398873	0.506304931555648	1	pseudobulk	DESeq2	LRT	0.2955878424394
122	KC_AB2	ebd1	-0.19215578423827	0.90837035950385	1	pseudobulk	DESeq2	LRT	0.041737045427574
123	KC_AB2	ebd2	0.360893881225285	0.708778966251189	1	pseudobulk	DESeq2	LRT	0.149489179067653
123	KC_AB2	ebi	-0.848248965067998	0.702637594633186	1	pseudobulk	DESeq2	LRT	0.153268616990703
123	KC_AB2	ebo	0.678936903976771	0.651686408107832	1	pseudobulk	DESeq2	LRT	0.185961336769206
123	KC_AB2	eca	-1.24816655109457	0.274432555898961	1	pseudobulk	DESeq2	LRT	0.561564369610351
123	KC_AB2	ecd	-0.481711097359631	0.768222482590664	1	pseudobulk	DESeq2	LRT	0.114512987040425
123	KC_AB2	Echs1	1.04560394656545	0.638145957880541	1	pseudobulk	DESeq2	LRT	0.195079977309609
123	KC_AB2	EcR	0.744810596211478	0.0470668980109686	1	pseudobulk	DESeq2	LRT	1.32728442334438
123	KC_AB2	ECSIT	0.160151903119214	0.951581073906036	1	pseudobulk	DESeq2	LRT	0.021554204276544
123	KC_AB2	ect	1.10706867860301	1	1	pseudobulk	DESeq2	LRT	0
123	KC_AB2	Ect4	1.61205113368698	0.631647504209	1	pseudobulk	DESeq2	LRT	0.199525215522471
123	KC_AB2	ed	0.149876377686925	0.831615184792173	1	pseudobulk	DESeq2	LRT	0.080077589312620
123	KC_AB2	Edc3	1.27202903106577	0.0768504505708237	1	pseudobulk	DESeq2	LRT	1.11435358190726
123	KC_AB2	Edem1	1.94531887231797	0.378646728337548	1	pseudobulk	DESeq2	LRT	0.421765791304938
123	KC_AB2	Edem2	0.0911864476975538	0.953272652059487	1	pseudobulk	DESeq2	LRT	0.020782866046366
123	KC_AB2	Edg78E	0.39439088612385	0.813541888331052	1	pseudobulk	DESeq2	LRT	0.089620080830767
123	KC_AB2	EDTP	0.393976029558784	0.939642609940555	1	pseudobulk	DESeq2	LRT	0.027037297511213
123	KC_AB2	eEF1alpha1	-0.572255005363621	0.426864841480039	1	pseudobulk	DESeq2	LRT	0.369709614181792
123	KC_AB2	eEF1alpha2	0.18407054542725	0.385297410356841	1	pseudobulk	DESeq2	LRT	0.414203909937203
123	KC_AB2	eEF1beta	-0.54168279096228	0.336188380788833	1	pseudobulk	DESeq2	LRT	0.473417300552986
123	KC_AB2	eEF1delta	-0.596694475951283	0.456249311514836	1	pseudobulk	DESeq2	LRT	0.340797777890062
123	KC_AB2	eEF1gamma	-1.3421561153701	0.116486142745293	1	pseudobulk	DESeq2	LRT	0.933725735472292
123	KC_AB2	eEF2	0.0737062019883706	0.836540683061191	1	pseudobulk	DESeq2	LRT	0.077512933363457
123	KC_AB2	eEF5	0.0272022741183221	0.943402501569525	1	pseudobulk	DESeq2	LRT	0.025302976493538

123	KC_AB2	eEFSec	1.38069928703004	0.566368801250645	1	pseudobulk	DESeq2	LRT	0.246900678019328
123	KC_AB2	Efa6	-0.342664726954474	0.67720277228765	1	pseudobulk	DESeq2	LRT	0.16928127267361
123	KC_AB2	eff	-0.19946544581082	0.499885255142287	1	pseudobulk	DESeq2	LRT	0.301129673218969
123	KC_AB2	Efr	0.389544867459584	0.848941394447694	1	pseudobulk	DESeq2	LRT	0.071122289669451
123	KC_AB2	egg	-0.387887463341148	0.79673485976169	1	pseudobulk	DESeq2	LRT	0.098686180611590
123	KC_AB2	egh	0.0992966646716195	0.781472886356895	1	pseudobulk	DESeq2	LRT	0.107086085475706
123	KC_AB2	egl	1.51551107307959	0.248956584174168	1	pseudobulk	DESeq2	LRT	0.603876383416655
123	KC_AB2	Egm	-0.670541955584215	0.735328220685952	1	pseudobulk	DESeq2	LRT	0.133518766201526
123	KC_AB2	Ehbp1	0.354438083177995	0.585475521631713	1	pseudobulk	DESeq2	LRT	0.23249125779487
123	KC_AB2	eIF1	0.14363516614848	0.742255662698292	1	pseudobulk	DESeq2	LRT	0.129446480469217
123	KC_AB2	eIF1A	-0.145221865471197	0.638374113472633	1	pseudobulk	DESeq2	LRT	0.194924732252458
123	KC_AB2	eIF2alpha	0.206576924451102	0.786880319552223	1	pseudobulk	DESeq2	LRT	0.104091316574283
123	KC_AB2	eIF2Balpha	2.58564464312165	0.314515351959278	1	pseudobulk	DESeq2	LRT	0.502358151145649
123	KC_AB2	eIF2Bbeta	0.0442718227672004	0.970023086008469	1	pseudobulk	DESeq2	LRT	0.013217929644293
123	KC_AB2	eIF2Bdelta	-3.59418805397463	0.0571820857937072	1	pseudobulk	DESeq2	LRT	1.24274000721209
123	KC_AB2	eIF2Bepsilon	2.99957963596671	0.187362043973732	1	pseudobulk	DESeq2	LRT	0.727318384431879
123	KC_AB2	eIF2beta	0.447656433218065	0.452964862251276	1	pseudobulk	DESeq2	LRT	0.34393548611575
123	KC_AB2	eIF2Bgamma	0.413763223382476	0.860970715086041	1	pseudobulk	DESeq2	LRT	0.065011620315023
123	KC_AB2	eIF2D	0.815920546158336	0.664120266731846	1	pseudobulk	DESeq2	LRT	0.177753266342234
123	KC_AB2	eIF2gamma	0.76365578227251	0.558011856360394	1	pseudobulk	DESeq2	LRT	0.253356573289678
123	KC_AB2	eIF3a	0.77579954982982	0.218798163232715	1	pseudobulk	DESeq2	LRT	0.659956328139543
123	KC_AB2	eIF3b	0.616991536856857	0.374746446980946	1	pseudobulk	DESeq2	LRT	0.426262476061762
123	KC_AB2	eIF3c	0.565120249141373	0.356620291534667	1	pseudobulk	DESeq2	LRT	0.447793949296343
123	KC_AB2	eIF3d1	0.430446287270684	0.509786714532672	1	pseudobulk	DESeq2	LRT	0.292611486796003
123	KC_AB2	eIF3e	-0.196239543989667	0.737377268043126	1	pseudobulk	DESeq2	LRT	0.132310255007099
123	KC_AB2	eIF3f1	-0.405294245007591	0.466507897404191	1	pseudobulk	DESeq2	LRT	0.331140999784512
123	KC_AB2	eIF3g1	-0.369480140494548	0.596309284213226	1	pseudobulk	DESeq2	LRT	0.224528428871292
123	KC_AB2	eIF3h	-0.176825178154556	0.709391897332269	1	pseudobulk	DESeq2	LRT	0.149113776343328
123	KC_AB2	eIF3i	-0.0986428794218158	0.840484618600683	1	pseudobulk	DESeq2	LRT	0.075470230015077
123	KC_AB2	eIF3j	0.388738671587703	0.609073998303737	1	pseudobulk	DESeq2	LRT	0.215329940367636
123	KC_AB2	eIF3k	-0.0632094957268718	0.925455257918623	1	pseudobulk	DESeq2	LRT	0.033644572814648
123	KC_AB2	eIF3l	0.732268518124069	0.116547093560702	1	pseudobulk	DESeq2	LRT	0.933498552398986
123	KC_AB2	eIF3m	-0.0731181929795848	0.860159141686937	1	pseudobulk	DESeq2	LRT	0.065421190660486
123	KC_AB2	eIF4A	0.0258445430117552	0.904153376861659	1	pseudobulk	DESeq2	LRT	0.043757891344807
123	KC_AB2	eIF4B	-0.171891705520485	0.684199406778611	1	pseudobulk	DESeq2	LRT	0.164817306697123
123	KC_AB2	eIF4E1	-0.149478732131545	0.790690078931852	1	pseudobulk	DESeq2	LRT	0.10199371041482
123	KC_AB2	eIF4EHP	0.210491871105194	0.534504979241389	1	pseudobulk	DESeq2	LRT	0.272048244717154
123	KC_AB2	eIF4G1	0.2506894265202	0.658290984979169	1	pseudobulk	DESeq2	LRT	0.181582092349223
123	KC_AB2	eIF4G2	-0.0460231297359605	0.873907537299256	1	pseudobulk	DESeq2	LRT	0.058534514922770
123	KC_AB2	eIF4H1	0.184908988156387	0.739348011599636	1	pseudobulk	DESeq2	LRT	0.131151090778324
123	KC_AB2	eIF5	0.567068459483073	0.348027861086272	1	pseudobulk	DESeq2	LRT	0.458385987571551
123	KC_AB2	eIF5B	0.178655701183327	0.715153999814359	1	pseudobulk	DESeq2	LRT	0.145600428033836
123	KC_AB2	eIF6	2.80624269445693	0.17636147375696	1	pseudobulk	DESeq2	LRT	0.753596280667665
123	KC_AB2	Eip55E	0.372319056733928	0.867976741609407	1	pseudobulk	DESeq2	LRT	0.061491912065472
123	KC_AB2	Eip63E	0.130374134347897	0.78845094104021	1	pseudobulk	DESeq2	LRT	0.103225324146572
123	KC_AB2	Eip63F-1	1.61205113368698	0.631647504209	1	pseudobulk	DESeq2	LRT	0.199525215522471
123	KC_AB2	Eip71CD	-0.0905869313547174	0.889185368337937	1	pseudobulk	DESeq2	LRT	0.051007692287010
123	KC_AB2	Eip74EF	2.48384162006255	0.303740962766822	1	pseudobulk	DESeq2	LRT	0.517496634805608
123	KC_AB2	Eip75B	0.232223590233942	0.397418949063478	1	pseudobulk	DESeq2	LRT	0.400751429431763
123	KC_AB2	Eip78C	-0.0099592595164595	0.895773935588191	1	pseudobulk	DESeq2	LRT	0.047801578411653
123	KC_AB2	Eip93F	0.301670894487436	0.203957308722469	1	pseudobulk	DESeq2	LRT	0.690460727311999
123	KC_AB2	Ekar	-0.729379579623002	0.823527397767669	1	pseudobulk	DESeq2	LRT	0.084321947797948

123	KC_AB2	elav	0.254971225314636	0.439962795178625	1	pseudobulk	DESeq2	LRT	0.356584047449691
123	KC_AB2	elB	-0.014924844531416	0.940535633602451	1	pseudobulk	DESeq2	LRT	0.026624745904399
123	KC_AB2	elgi	-0.50579352507058	0.696908682991729	1	pseudobulk	DESeq2	LRT	0.156824124442791
123	KC_AB2	Elk	0.388565581682978	0.280616310766964	1	pseudobulk	DESeq2	LRT	0.551887089294062
123	KC_AB2	elm	-0.0282705387044268	0.922421236780221	1	pseudobulk	DESeq2	LRT	0.035070706894740
123	KC_AB2	EloA	-0.538831791326431	0.615684432154401	1	pseudobulk	DESeq2	LRT	0.2106418275924
123	KC_AB2	EloB	0.126780315579586	0.855341681616255	1	pseudobulk	DESeq2	LRT	0.067860363870721
123	KC_AB2	EloC	-0.40095902330897	0.427724640593534	1	pseudobulk	DESeq2	LRT	0.368835729949982
123	KC_AB2	Elp1	0.661678460170035	0.878003361430606	1	pseudobulk	DESeq2	LRT	0.056503821396894
123	KC_AB2	Elp2	1.10706867860301	1	1	pseudobulk	DESeq2	LRT	0
123	KC_AB2	Elp3	-1.43319088453329	0.468553403065376	1	pseudobulk	DESeq2	LRT	0.329240903527803
123	KC_AB2	Elys	1.80622761856892	0.461120290665938	1	pseudobulk	DESeq2	LRT	0.336185767109657
123	KC_AB2	ema	0.0411618608380074	0.938717111374813	1	pseudobulk	DESeq2	LRT	0.027465265538930
123	KC_AB2	emb	-0.0707800741490709	0.884307781001995	1	pseudobulk	DESeq2	LRT	0.053396553621797
123	KC_AB2	emc	0.127874669507859	0.737223013149819	1	pseudobulk	DESeq2	LRT	0.13240111630703
123	KC_AB2	EMC1	-0.118670605758625	0.892512479157994	1	pseudobulk	DESeq2	LRT	0.049385702844266
123	KC_AB2	EMC3	-0.91856277527982	0.367930653875992	1	pseudobulk	DESeq2	LRT	0.434234027731245
123	KC_AB2	EMC8-9	-0.548716701421621	0.490343440575068	1	pseudobulk	DESeq2	LRT	0.30949962997159
123	KC_AB2	EMRE	-0.33773854720952	0.584025928025896	1	pseudobulk	DESeq2	LRT	0.233567871811205
123	KC_AB2	ena	-0.891735881455027	0.284211087882371	1	pseudobulk	DESeq2	LRT	0.546358983015877
123	KC_AB2	enc	0.0098648689326537	0.882872304586082	1	pseudobulk	DESeq2	LRT	0.054102106642152
123	KC_AB2	EndoA	-0.156208224362588	0.660421386138206	1	pseudobulk	DESeq2	LRT	0.180178871612412
123	KC_AB2	EndoB	-0.412045365433139	0.692414824498271	1	pseudobulk	DESeq2	LRT	0.159633642516928
123	KC_AB2	EndoG	0.366693140476542	0.851488552788718	1	pseudobulk	DESeq2	LRT	0.069821186214721
123	KC_AB2	EndoGI	0.146930209264112	1	1	pseudobulk	DESeq2	LRT	0
124	KC_AB2	endos	0.170344221377888	0.737135221924285	1	pseudobulk	DESeq2	LRT	0.132452836769462
124	KC_AB2	Eno	-0.197123644603375	0.527906605837251	1	pseudobulk	DESeq2	LRT	0.277442903521457
124	KC_AB2	enok	-0.322652041277399	0.793525763927723	1	pseudobulk	DESeq2	LRT	0.100438968152992
124	KC_AB2	ens	-0.39694098088706	0.404408375351215	1	pseudobulk	DESeq2	LRT	0.393179858691444
124	KC_AB2	Ent1	-0.963919078111595	0.599234761105423	1	pseudobulk	DESeq2	LRT	0.222403001519551
124	KC_AB2	Ent2	-2.20804467954271	0.294131125906175	1	pseudobulk	DESeq2	LRT	0.531459014615604
124	KC_AB2	Ent3	0.100836860144623	0.871065706537589	1	pseudobulk	DESeq2	LRT	0.059949083901172
124	KC_AB2	Eogt	2.01814786626755	0.401718761445944	1	pseudobulk	DESeq2	LRT	0.396077884966921
124	KC_AB2	Epac	-0.0080725147954082	0.964579212139485	1	pseudobulk	DESeq2	LRT	0.015662101889975
124	KC_AB2	Epg5	-1.47740131595573	0.429760234207816	1	pseudobulk	DESeq2	LRT	0.366773772332455
124	KC_AB2	Eph	0.0077417930570338	0.898563912712821	1	pseudobulk	DESeq2	LRT	0.046451027123359
124	KC_AB2	Ephrin	-0.870237418080371	0.176077659646114	1	pseudobulk	DESeq2	LRT	0.754295742885041
124	KC_AB2	Eps-15	-0.187539165942485	0.808583080762492	1	pseudobulk	DESeq2	LRT	0.09227535032409
124	KC_AB2	epsilonCOP	0.248560193114587	0.813133922820121	1	pseudobulk	DESeq2	LRT	0.089837920393763
124	KC_AB2	Ercc1	1.99339641594643	0.465071165814147	1	pseudobulk	DESeq2	LRT	0.332480585700252
124	KC_AB2	eRF1	-0.39061139134142	0.624210675948647	1	pseudobulk	DESeq2	LRT	0.204668807817535
124	KC_AB2	eRF3	-0.816874200745502	0.668198591964671	1	pseudobulk	DESeq2	LRT	0.175094443846972
124	KC_AB2	ergic53	-0.292821059595112	0.80179510686463	1	pseudobulk	DESeq2	LRT	0.095936598457378
124	KC_AB2	Ero1L	2.05082272821134	0.146542805116936	1	pseudobulk	DESeq2	LRT	0.834035499468594
124	KC_AB2	ERp60	-1.07195248400437	0.258299654304656	1	pseudobulk	DESeq2	LRT	0.587876175064317
124	KC_AB2	ERR	0.841909814302928	0.523195817243198	1	pseudobulk	DESeq2	LRT	0.28133573669346
124	KC_AB2	Es2	1.50730388760116	0.557960431011111	1	pseudobulk	DESeq2	LRT	0.253396598917002
124	KC_AB2	escl	-2.22728608909242	0.317088151657018	1	pseudobulk	DESeq2	LRT	0.498819985554471
124	KC_AB2	Esyt2	0.335161373128942	0.55051525966274	1	pseudobulk	DESeq2	LRT	0.259230638371659
124	KC_AB2	Etf-QO	0.645208851633052	0.724910106700029	1	pseudobulk	DESeq2	LRT	0.139715845269899
124	KC_AB2	ETHR	1.61205113368698	0.631647504209	1	pseudobulk	DESeq2	LRT	0.199525215522471
124	KC_AB2	Etl1	0.387107135046641	0.828037304932132	1	pseudobulk	DESeq2	LRT	0.081950096836841

124	KC_AB2	Ets97D	2.7605424445074	0.237810191029118	1	pseudobulk	DESeq2	LRT	0.623769538224996
124	KC_AB2	Evi5	-0.253767561818461	0.590659604495385	1	pseudobulk	DESeq2	LRT	0.228662729742592
124	KC_AB2	ewg	-0.389354226247045	0.633280380185034	1	pseudobulk	DESeq2	LRT	0.198403966748248
124	KC_AB2	exd	-0.271916029268566	0.646839773366735	1	pseudobulk	DESeq2	LRT	0.189203283721372
124	KC_AB2	Exd2	0.145321756433347	1	1	pseudobulk	DESeq2	LRT	0
124	KC_AB2	Exn	0.903225270002794	0.602964880476285	1	pseudobulk	DESeq2	LRT	0.219707982485932
124	KC_AB2	Exo70	-0.400818874228283	0.829617251912903	1	pseudobulk	DESeq2	LRT	0.081122225359766
124	KC_AB2	Exo84	-0.752030063326928	0.545573718389609	1	pseudobulk	DESeq2	LRT	0.263146558867125
124	KC_AB2	exp	1.10706867860301	1	1	pseudobulk	DESeq2	LRT	0
124	KC_AB2	ey	0.226596047447842	0.536056086228542	1	pseudobulk	DESeq2	LRT	0.270789768767554
124	KC_AB2	eys	0.119590642108532	0.7863580634614	1	pseudobulk	DESeq2	LRT	0.104379655517911
124	KC_AB2	f-cup	-0.560823129224834	0.720653166429987	1	pseudobulk	DESeq2	LRT	0.14227370080988
124	KC_AB2	fabp	-0.930034222747166	0.386767127894455	1	pseudobulk	DESeq2	LRT	0.412550444570189
124	KC_AB2	Fadd	-1.6052707068109	0.52265609184397	1	pseudobulk	DESeq2	LRT	0.281783983297782
124	KC_AB2	faf	-0.389331167754659	0.527303218392855	1	pseudobulk	DESeq2	LRT	0.277939577946002
124	KC_AB2	Faf2	1.18477765922077	0.365941768066623	1	pseudobulk	DESeq2	LRT	0.436588017937282
124	KC_AB2	Fak	0.859063849362067	0.231724738247124	1	pseudobulk	DESeq2	LRT	0.63502759974168
124	KC_AB2	fal	0.566540747623509	0.699295514819186	1	pseudobulk	DESeq2	LRT	0.155339257253147
124	KC_AB2	FAM21	1.20032875138118	0.560039268060527	1	pseudobulk	DESeq2	LRT	0.251781520665037
124	KC_AB2	Fancm	-0.729379579623002	1	1	pseudobulk	DESeq2	LRT	0
124	KC_AB2	fand	-1.65769740794391	0.396624755891695	1	pseudobulk	DESeq2	LRT	0.401620182184119
124	KC_AB2	Fas1	0.0294257414941364	0.883405539631259	1	pseudobulk	DESeq2	LRT	0.053839881709114
124	KC_AB2	Fas2	-0.150717631916464	0.546257081199117	1	pseudobulk	DESeq2	LRT	0.262602920171144
124	KC_AB2	Fas3	-1.61440587605172	0.00762315458264323	1	pseudobulk	DESeq2	LRT	2.11786527349925
124	KC_AB2	FASN1	-0.146905408291932	0.845847091379072	1	pseudobulk	DESeq2	LRT	0.072708139765188
124	KC_AB2	Fatp1	0.408731147072076	0.798102826073857	1	pseudobulk	DESeq2	LRT	0.097941151356539
124	KC_AB2	fax	0.14469186182668	0.590024762949441	1	pseudobulk	DESeq2	LRT	0.229129760922912
124	KC_AB2	fbl	0.991856157082641	0.508367795450838	1	pseudobulk	DESeq2	LRT	0.293821969331623
124	KC_AB2	Fbl6	-0.673119739151887	0.619324335128745	1	pseudobulk	DESeq2	LRT	0.208081854909707
124	KC_AB2	fbp	2.01814786626755	0.401718761445944	1	pseudobulk	DESeq2	LRT	0.396077884966921
124	KC_AB2	Fbp2	-2.87093509453135	0.225888123098267	1	pseudobulk	DESeq2	LRT	0.646106603114541
124	KC_AB2	Fbw5	-0.115183097143228	0.910399790291748	1	pseudobulk	DESeq2	LRT	0.040767850987610
124	KC_AB2	Fbxl4	-0.729379579623002	1	1	pseudobulk	DESeq2	LRT	0
124	KC_AB2	FBXO11	0.11195081305639	0.86354192553839	1	pseudobulk	DESeq2	LRT	0.063716572296915
124	KC_AB2	Fcp1	-0.004950350487396	0.928699845863342	1	pseudobulk	DESeq2	LRT	0.032124626531977
124	KC_AB2	Fdh	1.10706867860301	1	1	pseudobulk	DESeq2	LRT	0
124	KC_AB2	fdl	-0.277620368033949	0.764077184255034	1	pseudobulk	DESeq2	LRT	0.116862768383832
124	KC_AB2	Fdx1	-0.651886994612862	0.678865794276369	1	pseudobulk	DESeq2	LRT	0.168216073386695
124	KC_AB2	Fdx2	-0.825534617607436	0.564953635876577	1	pseudobulk	DESeq2	LRT	0.247987192019813
124	KC_AB2	FeCH	-1.29230303935953	0.506845634274986	1	pseudobulk	DESeq2	LRT	0.295124289957523
124	KC_AB2	Fem-1	-0.420970781641909	0.700289153759858	1	pseudobulk	DESeq2	LRT	0.154722600053153
124	KC_AB2	Fen1	0.145321756433347	1	1	pseudobulk	DESeq2	LRT	0
124	KC_AB2	fend	-0.372537952675953	0.229903454344521	1	pseudobulk	DESeq2	LRT	0.638454503321875
124	KC_AB2	FER	-1.71999636108657	0.519869997311849	1	pseudobulk	DESeq2	LRT	0.284105245805297
124	KC_AB2	Fer1HCH	-0.301815372086192	0.387059917331289	1	pseudobulk	DESeq2	LRT	0.412221800479422
124	KC_AB2	Fer2LCH	-0.151861991751384	0.597433703228652	1	pseudobulk	DESeq2	LRT	0.22371028103997
124	KC_AB2	fest	0.0658198284109504	0.910613435229985	1	pseudobulk	DESeq2	LRT	0.040665946381000
124	KC_AB2	Fgop2	-1.12956848412701	0.556577485235076	1	pseudobulk	DESeq2	LRT	0.254474365799203
124	KC_AB2	fh	-1.33570647694682	0.512066034235951	1	pseudobulk	DESeq2	LRT	0.290674030322833
124	KC_AB2	Fhos	-2.09428695548101	0.380540277844868	1	pseudobulk	DESeq2	LRT	0.419599369064229
124	KC_AB2	Fib	-0.721175887303532	0.733409564198845	1	pseudobulk	DESeq2	LRT	0.134653430819787
124	KC_AB2	Fibp	0.310156371104469	0.755200363896789	1	pseudobulk	DESeq2	LRT	0.121937809443132

124	KC_AB2	Fic	-0.83320718254958	0.715059964627045	1	pseudobulk	DESeq2	LRT	0.14565753691968
124	KC_AB2	fid	-0.309032874152101	0.76069556653909	1	pseudobulk	DESeq2	LRT	0.118789114878436
124	KC_AB2	fidipidine	-2.1751167112989	0.359363815234217	1	pseudobulk	DESeq2	LRT	0.444465654648455
124	KC_AB2	Fie	-1.06962301654648	0.311894251896467	1	pseudobulk	DESeq2	LRT	0.505992629066675
124	KC_AB2	Fife	-0.0038648667488533	0.86553611622212	1	pseudobulk	DESeq2	LRT	0.062714805606353
124	KC_AB2	FIG4	-0.729379579623002	1	1	pseudobulk	DESeq2	LRT	0
124	KC_AB2	Fim	0.288805962500535	0.613771425992312	1	pseudobulk	DESeq2	LRT	0.211993333927529
124	KC_AB2	Fip1	0.618516526982311	0.619312448809666	1	pseudobulk	DESeq2	LRT	0.208090190142203
124	KC_AB2	fipi	0.0180405264935355	0.977618213954976	1	pseudobulk	DESeq2	LRT	0.009830715707850
124	KC_AB2	Fis1	-0.433059148809479	0.3037502849579	1	pseudobulk	DESeq2	LRT	0.517483305967836
124	KC_AB2	Fit1	1.57464926876229	0.187112843307371	1	pseudobulk	DESeq2	LRT	0.727896401777976
124	KC_AB2	Fitm	0.682569013985824	0.677625635090906	1	pseudobulk	DESeq2	LRT	0.169010172677449
124	KC_AB2	Fkbp12	-0.435391305550133	0.19149755230427	1	pseudobulk	DESeq2	LRT	0.717836772753022
124	KC_AB2	Fkbp39	-0.454690977487992	0.662838060740794	1	pseudobulk	DESeq2	LRT	0.178592561967964
124	KC_AB2	Fkbp59	-1.46573123613579	0.239147792076225	1	pseudobulk	DESeq2	LRT	0.621333624382393
124	KC_AB2	fl(2)d	0.141231912918731	0.798284955767852	1	pseudobulk	DESeq2	LRT	0.097842055231731
124	KC_AB2	Flacc	0.463392806003924	0.58200357429978	1	pseudobulk	DESeq2	LRT	0.235074348178455
124	KC_AB2	FLASH	-1.16016699006818	0.583985689825587	1	pseudobulk	DESeq2	LRT	0.233597794849804
124	KC_AB2	flfl	0.729254298605143	0.275306246379827	1	pseudobulk	DESeq2	LRT	0.560183934856159
124	KC_AB2	fliI	0.33333575867548	0.858979456944999	1	pseudobulk	DESeq2	LRT	0.066017222480879
124	KC_AB2	Flo1	-0.183449961594842	0.716462334619109	1	pseudobulk	DESeq2	LRT	0.144806636106901
125	KC_AB2	Flo2	0.290498250421158	0.629092634829907	1	pseudobulk	DESeq2	LRT	0.201285399334869
125	KC_AB2	flr	-2.22728608909242	0.317088151657018	1	pseudobulk	DESeq2	LRT	0.498819985554471
125	KC_AB2	flw	0.41694168649296	0.386475697789537	1	pseudobulk	DESeq2	LRT	0.412877810019217
125	KC_AB2	Fmr1	-0.0810076306205449	0.879985456120576	1	pseudobulk	DESeq2	LRT	0.055524505552985
125	KC_AB2	FMRFaR	-1.6052707068109	0.52265609184397	1	pseudobulk	DESeq2	LRT	0.281783983297782
125	KC_AB2	fmt	0.0200546275023179	0.860461154598076	1	pseudobulk	DESeq2	LRT	0.065268731059258
125	KC_AB2	fne	0.242131443414289	0.313948215366847	1	pseudobulk	DESeq2	LRT	0.503141981338021
125	KC_AB2	fng	1.38069928703004	0.566368801250645	1	pseudobulk	DESeq2	LRT	0.246900678019328
125	KC_AB2	Fnta	0.511332240287867	0.722710953390183	1	pseudobulk	DESeq2	LRT	0.141035363075178
125	KC_AB2	fog	1.02648689492346	0.73959154128541	1	pseudobulk	DESeq2	LRT	0.131008064514051
125	KC_AB2	foi	-0.660557272500536	0.48841843015468	1	pseudobulk	DESeq2	LRT	0.31120795659558
125	KC_AB2	fok	0.336788739264714	0.262718599376252	1	pseudobulk	DESeq2	LRT	0.580509179895558
125	KC_AB2	form3	1.10706867860301	1	1	pseudobulk	DESeq2	LRT	0
125	KC_AB2	FoxK	0.92729226394184	0.092662939699248	1	pseudobulk	DESeq2	LRT	1.03309392607426
125	KC_AB2	FoxP	0.229414664770658	0.547205704812665	1	pseudobulk	DESeq2	LRT	0.261849383591036
125	KC_AB2	Fpgs	-1.00833870937463	0.651152668420623	1	pseudobulk	DESeq2	LRT	0.186317175365195
125	KC_AB2	Fpps	-0.485288229662457	0.799307674653955	1	pseudobulk	DESeq2	LRT	0.097286017076586
125	KC_AB2	fra	0.396659246788372	0.915482501218565	1	pseudobulk	DESeq2	LRT	0.038349952505755
125	KC_AB2	fray	-1.74251194973247	0.331715316505189	1	pseudobulk	DESeq2	LRT	0.479234475058412
125	KC_AB2	FRG1	0.595145833393607	0.623509213728424	1	pseudobulk	DESeq2	LRT	0.205157124475981
125	KC_AB2	frj	-0.494271296748179	0.607149461352772	1	pseudobulk	DESeq2	LRT	0.216704385941764
125	KC_AB2	Frl	-0.0104182715191306	0.985954201145499	1	pseudobulk	DESeq2	LRT	0.006143258133578
125	KC_AB2	Frq1	-0.19196739904406	0.514380971996265	1	pseudobulk	DESeq2	LRT	0.288715105227435
125	KC_AB2	Frq2	-0.506702152863133	0.323284864373601	1	pseudobulk	DESeq2	LRT	0.490414627813121
125	KC_AB2	frtz	-0.729379579623002	0.772694994316916	1	pseudobulk	DESeq2	LRT	0.1119919011982
125	KC_AB2	fru	-1.32050922299706	0.178841219504391	1	pseudobulk	DESeq2	LRT	0.747532377373555
125	KC_AB2	fry	-0.484430753425092	0.664882709893157	1	pseudobulk	DESeq2	LRT	0.177254960620014
125	KC_AB2	fs(1)h	0.320211354926077	0.218744930538886	1	pseudobulk	DESeq2	LRT	0.660062003057647
125	KC_AB2	fs(1)K10	3.30110175219668	0.111422080617826	1	pseudobulk	DESeq2	LRT	0.953028736090146
125	KC_AB2	Fs(2)Ket	-0.0527577863816251	0.8404712826069	1	pseudobulk	DESeq2	LRT	0.075477121032868
125	KC_AB2	fs(2)ltoPP43	-0.928591663175024	0.513299001024196	1	pseudobulk	DESeq2	LRT	0.289629580956657

125	KC_AB2	fsd	1.61205113368698	0.631647504209	1	pseudobulk	DESeq2	LRT	0.199525215522471
125	KC_AB2	Fsn	0.049633710086246	0.957435610366892	1	pseudobulk	DESeq2	LRT	0.018890423628427
125	KC_AB2	ftz-f1	0.477698868114667	0.149194482803644	1	pseudobulk	DESeq2	LRT	0.826247236730882
125	KC_AB2	fu	-2.09428695548101	0.380540277844868	1	pseudobulk	DESeq2	LRT	0.419599369064229
125	KC_AB2	FucT6	-0.552880000366928	0.628936107633007	1	pseudobulk	DESeq2	LRT	0.201393471425653
125	KC_AB2	FucTA	0.31548080200679	0.724774292886508	1	pseudobulk	DESeq2	LRT	0.139797219105429
125	KC_AB2	FucTB	-1.8030374808264	0.318160221392724	1	pseudobulk	DESeq2	LRT	0.497354119816881
125	KC_AB2	Fum1	-0.693727421447918	0.355484059374595	1	pseudobulk	DESeq2	LRT	0.449179869142001
125	KC_AB2	Fur1	-0.0396220553976863	0.869117136167679	1	pseudobulk	DESeq2	LRT	0.060921687115240
125	KC_AB2	Fur2	0.49100344064803	0.648581846405458	1	pseudobulk	DESeq2	LRT	0.188035211253966
125	KC_AB2	fuss	0.388447834546628	0.84487503289525	1	pseudobulk	DESeq2	LRT	0.073207523639173
125	KC_AB2	futsch	0.168769068036258	0.472510844280536	1	pseudobulk	DESeq2	LRT	0.325588219837992
125	KC_AB2	fwd	0.0276416871352975	0.965973465841234	1	pseudobulk	DESeq2	LRT	0.015034802980990
125	KC_AB2	fwe	-0.132993715920554	0.696041849310548	1	pseudobulk	DESeq2	LRT	0.157364647777009
125	KC_AB2	fws	-1.2593686706548	0.647053428594028	1	pseudobulk	DESeq2	LRT	0.189059857220721
125	KC_AB2	fz	0.0831917458222305	0.813924861708462	1	pseudobulk	DESeq2	LRT	0.089415685591858
125	KC_AB2	fz2	-0.249060247835705	0.799789355903826	1	pseudobulk	DESeq2	LRT	0.097024380026195
125	KC_AB2	fz3	0.416402335790242	0.850714640962553	1	pseudobulk	DESeq2	LRT	0.070216092839237
125	KC_AB2	fz4	-0.729379579623002	0.823527397767669	1	pseudobulk	DESeq2	LRT	0.084321947797948
125	KC_AB2	fzr	-0.24277571490725	0.768508435907067	1	pseudobulk	DESeq2	LRT	0.114351360893213
125	KC_AB2	g	0.342584553675287	0.341036502334006	1	pseudobulk	DESeq2	LRT	0.46719913445672
125	KC_AB2	G6P	2.51016341631255	0.22350281941927	1	pseudobulk	DESeq2	LRT	0.650716994006281
125	KC_AB2	G9a	0.322760696438755	0.585380433182344	1	pseudobulk	DESeq2	LRT	0.232561798306322
125	KC_AB2	GAA1	0.860167988964514	0.585942207833506	1	pseudobulk	DESeq2	LRT	0.23214521684322
125	KC_AB2	GABA-B-R1	0.387590871394639	0.3766869573536	1	pseudobulk	DESeq2	LRT	0.424019416822012
125	KC_AB2	GABA-B-R2	-0.389003768516787	0.51431630249459	1	pseudobulk	DESeq2	LRT	0.28876970945058
125	KC_AB2	Gabat	-0.569789903850799	0.320102584526786	1	pseudobulk	DESeq2	LRT	0.494710819322939
125	KC_AB2	GABPI	-0.107042985749518	0.909870962194343	1	pseudobulk	DESeq2	LRT	0.041020194910322
125	KC_AB2	Gadd45	-1.78975280219494	0.468855431150075	1	pseudobulk	DESeq2	LRT	0.32896104882553
125	KC_AB2	Gale	-0.620563475958957	0.7478554969218	1	pseudobulk	DESeq2	LRT	0.126182309825559
125	KC_AB2	galectin	0.265282637653864	0.750918571017528	1	pseudobulk	DESeq2	LRT	0.124407154973349
125	KC_AB2	Galk	1.04560394656545	0.638145957880541	1	pseudobulk	DESeq2	LRT	0.195079977309609
125	KC_AB2	galla-1	-1.58188057031999	0.196603775503197	1	pseudobulk	DESeq2	LRT	0.706408146399809
125	KC_AB2	galla-2	0.519649940040782	0.704166561838508	1	pseudobulk	DESeq2	LRT	0.152324601750658
125	KC_AB2	Galphao	-0.0180478713197666	0.937325470855657	1	pseudobulk	DESeq2	LRT	0.028109581307498
125	KC_AB2	Galphaq	-0.240324001067433	0.594551005553188	1	pseudobulk	DESeq2	LRT	0.225810882037549
125	KC_AB2	Galphas	-0.196305891128101	0.59264001127109	1	pseudobulk	DESeq2	LRT	0.227209031074573
125	KC_AB2	Galt	-2.02037282360048	0.249801402390061	1	pseudobulk	DESeq2	LRT	0.602405127817146
125	KC_AB2	GalT1	-0.670541955584215	0.735328220685952	1	pseudobulk	DESeq2	LRT	0.133518766201526
125	KC_AB2	gammaCOP	0.521397928253146	0.65181493807211	1	pseudobulk	DESeq2	LRT	0.185875690744229
125	KC_AB2	gammaSnap1	-0.723587586189127	0.1649175585163	1	pseudobulk	DESeq2	LRT	0.782733103234899
125	KC_AB2	gammaSnap2	0.263892964475114	0.844937848772347	1	pseudobulk	DESeq2	LRT	0.073175235343697
125	KC_AB2	gammaTub23C	0.466361351705936	0.831209435945037	1	pseudobulk	DESeq2	LRT	0.080289535282928
125	KC_AB2	GAPcenA	0.0080762034593200	0.959736774927901	1	pseudobulk	DESeq2	LRT	0.017847863701735
125	KC_AB2	Gapdh1	0.512523355481183	0.415883100915522	1	pseudobulk	DESeq2	LRT	0.381028726493078
125	KC_AB2	Gapdh2	-0.294819531571622	0.544964360488855	1	pseudobulk	DESeq2	LRT	0.26363189873094
125	KC_AB2	GAPsec	1.68881465104187	0.456529340335333	1	pseudobulk	DESeq2	LRT	0.340531305892531
125	KC_AB2	Gapvd1	0.099497411576047	0.919126721662107	1	pseudobulk	DESeq2	LRT	0.036624607520785
125	KC_AB2	Gart	-0.729379579623002	0.823527397767669	1	pseudobulk	DESeq2	LRT	0.084321947797948
125	KC_AB2	garz	-0.195607417027587	0.880277718811865	1	pseudobulk	DESeq2	LRT	0.055380290668453
125	KC_AB2	gas	1.38446765109058	0.642794900879723	1	pseudobulk	DESeq2	LRT	0.191927577037166
125	KC_AB2	Gas41	-0.651886994612862	0.678865794276369	1	pseudobulk	DESeq2	LRT	0.168216073386695

125	KC_AB2	Gas8	1.10706867860301	1	1	pseudobulk	DESeq2	LRT	0
125	KC_AB2	Gasz	-0.325143278256431	0.788706970234127	1	pseudobulk	DESeq2	LRT	0.103084321059555
125	KC_AB2	GatA	1.08974813354727	0.517763588289733	1	pseudobulk	DESeq2	LRT	0.285868494574904
125	KC_AB2	GATAd	0.259354316724594	0.77002602869003	1	pseudobulk	DESeq2	LRT	0.113494594404926
125	KC_AB2	GatB	-0.855653026423282	0.660415060584474	1	pseudobulk	DESeq2	LRT	0.180183031329669
125	KC_AB2	gb	-0.956935179051669	0.636976450719219	1	pseudobulk	DESeq2	LRT	0.195876623413077
125	KC_AB2	Gba1a	1.10706867860301	1	1	pseudobulk	DESeq2	LRT	0
125	KC_AB2	gbb	1.10706867860301	1	1	pseudobulk	DESeq2	LRT	0
125	KC_AB2	Gbeta13F	0.129636392007023	0.561850375713063	1	pseudobulk	DESeq2	LRT	0.250379324384377
125	KC_AB2	Gbeta5	-0.26620115593501	0.677977603252528	1	pseudobulk	DESeq2	LRT	0.168784652658746
125	KC_AB2	Gbs-76A	-0.643725547303771	0.468753619515246	1	pseudobulk	DESeq2	LRT	0.329055365814775
125	KC_AB2	GC	0.709738435463797	0.186836445197978	1	pseudobulk	DESeq2	LRT	0.728538404312904
125	KC_AB2	GC1	-0.478273111093814	0.76088385487653	1	pseudobulk	DESeq2	LRT	0.118681631055741
125	KC_AB2	GC2	-0.729379579623002	0.823527397767669	1	pseudobulk	DESeq2	LRT	0.084321947797948
125	KC_AB2	GCC185	-1.39744542985847	0.428051868432218	1	pseudobulk	DESeq2	LRT	0.368503602928283
125	KC_AB2	GCC88	1.67841060813711	0.505968139121766	1	pseudobulk	DESeq2	LRT	0.295876829878097
125	KC_AB2	gce	-0.139731873580778	0.763620265580395	1	pseudobulk	DESeq2	LRT	0.117122554462564
126	KC_AB2	GckIII	-0.0555725555448067	0.873300202230004	1	pseudobulk	DESeq2	LRT	0.058836439272003
126	KC_AB2	gcl	0.095990250642614	0.909131218441482	1	pseudobulk	DESeq2	LRT	0.041373428843761
126	KC_AB2	Gclc	-0.0155451176553194	0.930726093304866	1	pseudobulk	DESeq2	LRT	0.031178110284557
126	KC_AB2	Gclm	-1.1704891662601	0.466091984999642	1	pseudobulk	DESeq2	LRT	0.331528365208064
126	KC_AB2	Gcn2	-0.883890872531852	0.676366690187261	1	pseudobulk	DESeq2	LRT	0.169817788750343
126	KC_AB2	Gcn5	0.145321756433347	1	1	pseudobulk	DESeq2	LRT	0
126	KC_AB2	GCS1	0.0487673033804221	0.942805703571486	1	pseudobulk	DESeq2	LRT	0.025577798843798
126	KC_AB2	GCS2alpha	-0.781188486572787	0.235042135487812	1	pseudobulk	DESeq2	LRT	0.628854275730301
126	KC_AB2	GCS2beta	0.247259187844349	0.882369575364555	1	pseudobulk	DESeq2	LRT	0.054349475045523
126	KC_AB2	Gdap2	0.152239445542163	0.563685480572815	1	pseudobulk	DESeq2	LRT	0.248963151605779
126	KC_AB2	Gdh	-0.593230714281811	0.174478065673681	1	pseudobulk	DESeq2	LRT	0.75825916214682
126	KC_AB2	Gdi	-0.0040192351271649	0.953958236568865	1	pseudobulk	DESeq2	LRT	0.020470637900418
126	KC_AB2	gdl	0.147593560254927	1	1	pseudobulk	DESeq2	LRT	0
126	KC_AB2	Gdn1	1.10706867860301	1	1	pseudobulk	DESeq2	LRT	0
126	KC_AB2	Ge-1	0.0910705918438462	0.924377900027839	1	pseudobulk	DESeq2	LRT	0.034150446155597
126	KC_AB2	gek	0.33412391470694	0.812574763640844	1	pseudobulk	DESeq2	LRT	0.090136669803991
126	KC_AB2	Gel	0.147593560254927	1	1	pseudobulk	DESeq2	LRT	0
126	KC_AB2	Gem2	0.66954517615027	1	1	pseudobulk	DESeq2	LRT	0
126	KC_AB2	Gem3	-0.729379579623002	0.772694994316916	1	pseudobulk	DESeq2	LRT	0.1119919011982
126	KC_AB2	geminin	0.0927653025881295	0.948322606574911	1	pseudobulk	DESeq2	LRT	0.023043896394553
126	KC_AB2	Gfat2	0.334359046631833	0.809614125523131	1	pseudobulk	DESeq2	LRT	0.091721923202851
126	KC_AB2	Gfrl	-0.0414679268301646	0.838200729995134	1	pseudobulk	DESeq2	LRT	0.076651965271775
126	KC_AB2	gfzf	-1.97344956369647	0.36861920379507	1	pseudobulk	DESeq2	LRT	0.433422043275976
126	KC_AB2	Gga	0.306602564063627	0.858036922935618	1	pseudobulk	DESeq2	LRT	0.066494023244333
126	KC_AB2	Ggamma1	-0.0216109046692449	0.906873645027798	1	pseudobulk	DESeq2	LRT	0.042453219102974
126	KC_AB2	Ggamma30A	-0.364763415727336	0.0779781739743615	1	pseudobulk	DESeq2	LRT	1.10802693896265
126	KC_AB2	ghi	0.27318984422794	0.685901829210882	1	pseudobulk	DESeq2	LRT	0.163738038933126
126	KC_AB2	gig	-1.86604523843964	0.375091528268488	1	pseudobulk	DESeq2	LRT	0.425862744614398
126	KC_AB2	GIIIspla2	0.274300365849694	0.875467438164865	1	pseudobulk	DESeq2	LRT	0.057760002279909
126	KC_AB2	GILT1	0.728917240532532	0.670274586078713	1	pseudobulk	DESeq2	LRT	0.173747246867673
126	KC_AB2	Gip	-0.729379579623002	0.823527397767669	1	pseudobulk	DESeq2	LRT	0.084321947797948
126	KC_AB2	Girdin	1.13407472886881	0.477543805429445	1	pseudobulk	DESeq2	LRT	0.320986784114381
126	KC_AB2	gish	-0.050465954754367	0.844689469270505	1	pseudobulk	DESeq2	LRT	0.073302920125652
126	KC_AB2	Git	0.932634638337851	0.56938095269347	1	pseudobulk	DESeq2	LRT	0.244597065251027
126	KC_AB2	Gk1	-0.830495090747097	0.644120225631779	1	pseudobulk	DESeq2	LRT	0.191033063610174

126	KC_AB2	Gk2	-0.729379579623002	0.823527397767669	1	pseudobulk	DESeq2	LRT	0.084321947797948
126	KC_AB2	gkt	-1.15253484707392	0.661144484795202	1	pseudobulk	DESeq2	LRT	0.179703620556297
126	KC_AB2	GLaz	1.10706867860301	1	1	pseudobulk	DESeq2	LRT	0
126	KC_AB2	GlcAT-I	0.063618914990959	0.944678467344679	1	pseudobulk	DESeq2	LRT	0.024715983680485
126	KC_AB2	GlcAT-P	0.151400345999726	0.591416026110411	1	pseudobulk	DESeq2	LRT	0.228106911198106
126	KC_AB2	GlcAT-S	0.962469923008377	0.349450625685333	1	pseudobulk	DESeq2	LRT	0.456614177592663
126	KC_AB2	GlcT	-2.22728608909242	0.317088151657018	1	pseudobulk	DESeq2	LRT	0.498819985554471
126	KC_AB2	Gle1	-0.824996233971657	0.664071509040402	1	pseudobulk	DESeq2	LRT	0.177785152093585
126	KC_AB2	glec	0.221230803247962	0.734495574288555	1	pseudobulk	DESeq2	LRT	0.134010816712186
126	KC_AB2	Glg1	-0.160504810314691	0.909647360934701	1	pseudobulk	DESeq2	LRT	0.041126936120304
126	KC_AB2	Gli	1.61205113368698	0.631647504209	1	pseudobulk	DESeq2	LRT	0.199525215522471
126	KC_AB2	GlnRS	-0.687449350922837	0.61172968486158	1	pseudobulk	DESeq2	LRT	0.213440444369327
126	KC_AB2	glo	-0.356935989507589	0.415223975660703	1	pseudobulk	DESeq2	LRT	0.381717577614992
126	KC_AB2	Glo1	-0.176210328030041	0.876386810395005	1	pseudobulk	DESeq2	LRT	0.057304167185923
126	KC_AB2	GLS	0.900647852796099	0.491264769589157	1	pseudobulk	DESeq2	LRT	0.3086843796096
126	KC_AB2	glu	0.393976029558784	0.939642609940555	1	pseudobulk	DESeq2	LRT	0.027037297511213
126	KC_AB2	GluClalpha	0.135968254198018	0.69533708579854	1	pseudobulk	DESeq2	LRT	0.157804606903548
126	KC_AB2	GluProRS	0.72249280293873	0.661418891608578	1	pseudobulk	DESeq2	LRT	0.179523404819672
126	KC_AB2	GluRIA	-0.522444735484822	0.165259149168704	1	pseudobulk	DESeq2	LRT	0.781834487524679
126	KC_AB2	GluRIB	-0.20867122870815	0.524208934030944	1	pseudobulk	DESeq2	LRT	0.280495581696788
126	KC_AB2	GluRIIE	1.10706867860301	1	1	pseudobulk	DESeq2	LRT	0
126	KC_AB2	Glut1	0.0495393067983027	0.901061993525322	1	pseudobulk	DESeq2	LRT	0.045245328310874
126	KC_AB2	Glut4EF	0.320627132876447	0.195302715097567	1	pseudobulk	DESeq2	LRT	0.709291719110021
126	KC_AB2	GlyP	-0.4251685866153	0.607856786357751	1	pseudobulk	DESeq2	LRT	0.216198730301135
126	KC_AB2	GlyRS	0.972382740300136	0.462680858767213	1	pseudobulk	DESeq2	LRT	0.334718467009132
126	KC_AB2	GlyS	1.02754127378565	0.694973473680879	1	pseudobulk	DESeq2	LRT	0.15803177160251
126	KC_AB2	GlyT	-0.226128275035522	0.656636041547369	1	pseudobulk	DESeq2	LRT	0.182675283325288
126	KC_AB2	GM130	0.184408229501965	0.858202132185184	1	pseudobulk	DESeq2	LRT	0.066410410806482
126	KC_AB2	Gmap	1.82407290294359	0.316656092844161	1	pseudobulk	DESeq2	LRT	0.499412151207984
126	KC_AB2	Gmer	1.61205113368698	0.631647504209	1	pseudobulk	DESeq2	LRT	0.199525215522471
126	KC_AB2	GMF	0.545769785153131	0.732095334837864	1	pseudobulk	DESeq2	LRT	0.135432360610889
126	KC_AB2	Gnf1	0.828164348785031	0.592051051829226	1	pseudobulk	DESeq2	LRT	0.227640842986904
126	KC_AB2	Gnpnat	-1.07194103900864	0.379986138763665	1	pseudobulk	DESeq2	LRT	0.420232245404896
126	KC_AB2	gny	0.162385149200511	0.91636577592647	1	pseudobulk	DESeq2	LRT	0.037931139041603
126	KC_AB2	gogo	-1.14860503318904	0.622840789129387	1	pseudobulk	DESeq2	LRT	0.205622953724741
126	KC_AB2	gol	0.283461237795263	0.660335110056182	1	pseudobulk	DESeq2	LRT	0.180235610650236
126	KC_AB2	Golgin104	-0.843122195863938	0.503674147087682	1	pseudobulk	DESeq2	LRT	0.297850340318205
126	KC_AB2	Golgin245	-1.66296247985993	0.208675770920662	1	pseudobulk	DESeq2	LRT	0.680527973389336
126	KC_AB2	Golgin84	-3.32552274381165	0.0889569567695445	1	pseudobulk	DESeq2	LRT	1.05082008278541
126	KC_AB2	gom	2.05028198082901	0.383573006114165	1	pseudobulk	DESeq2	LRT	0.416151963827146
126	KC_AB2	Gorab	1.92865759370794	0.259657072286117	1	pseudobulk	DESeq2	LRT	0.585599844018257
126	KC_AB2	Gos28	-0.393459153237157	0.716294278670823	1	pseudobulk	DESeq2	LRT	0.144908517711424
126	KC_AB2	Got1	-0.53065443859286	0.665651110659245	1	pseudobulk	DESeq2	LRT	0.176753339023408
126	KC_AB2	Got2	-1.24292003858607	0.43948094240754	1	pseudobulk	DESeq2	LRT	0.357059952869376
126	KC_AB2	Gp210	0.433531960331872	0.825287783380275	1	pseudobulk	DESeq2	LRT	0.083394583643596
126	KC_AB2	Gp93	-0.155733442257507	0.86477703810067	1	pseudobulk	DESeq2	LRT	0.063095850459192
126	KC_AB2	Gpat4	-1.51679754016797	0.127869452985746	1	pseudobulk	DESeq2	LRT	0.89323319269275
126	KC_AB2	Gpb5	0.229580178396302	0.822625397812194	1	pseudobulk	DESeq2	LRT	0.084797886172073
126	KC_AB2	Gpdh1	0.182730989605097	0.669214737367772	1	pseudobulk	DESeq2	LRT	0.174434503622634
126	KC_AB2	Gpdh3	-0.729379579623002	1	1	pseudobulk	DESeq2	LRT	0
126	KC_AB2	GPHR	0.600457737989162	0.742742932528136	1	pseudobulk	DESeq2	LRT	0.129161471976179
126	KC_AB2	Gpo1	-1.5533547320928	0.197748167359864	1	pseudobulk	DESeq2	LRT	0.703887532655501

126	KC_AB2	gpp	0.281829188030053	0.438449507548684	1	pseudobulk	DESeq2	LRT	0.358080413371143
126	KC_AB2	Gprk1	-0.201196796759233	0.524499664982335	1	pseudobulk	DESeq2	LRT	0.280254784870556
126	KC_AB2	Gprk2	0.254820291682645	0.501268104229295	1	pseudobulk	DESeq2	LRT	0.299929928736061
126	KC_AB2	gprs	0.222270425802941	0.611029104788338	1	pseudobulk	DESeq2	LRT	0.213938102772291
126	KC_AB2	Graf	0.452323095869811	0.411982102812471	1	pseudobulk	DESeq2	LRT	0.385121650031456
126	KC_AB2	GramD1B	-0.0538025965136587	0.918759681182479	1	pseudobulk	DESeq2	LRT	0.036798071622603
126	KC_AB2	Grasp65	0.441408821018942	0.732820590051061	1	pseudobulk	DESeq2	LRT	0.135002336802088
126	KC_AB2	grau	-1.57685088282527	0.45256857946707	1	pseudobulk	DESeq2	LRT	0.344315601149633
126	KC_AB2	Grik	-1.6052707068109	0.52265609184397	1	pseudobulk	DESeq2	LRT	0.281783983297782
126	KC_AB2	grim	-0.729379579623002	1	1	pseudobulk	DESeq2	LRT	0
126	KC_AB2	Grip163	1.70788892736497	0.247428705247058	1	pseudobulk	DESeq2	LRT	0.606549917450538
126	KC_AB2	Grip71	-1.20549938956585	0.694957040684221	1	pseudobulk	DESeq2	LRT	0.158042040834959
126	KC_AB2	Grip75	0.132435945738664	0.922629103672155	1	pseudobulk	DESeq2	LRT	0.034972850004291
127	KC_AB2	Grip84	-0.961033333862793	0.70039622209097	1	pseudobulk	DESeq2	LRT	0.154656205149228
127	KC_AB2	Grip91	-2.09428695548101	0.380540277844868	1	pseudobulk	DESeq2	LRT	0.419599369064229
127	KC_AB2	gro	-0.587813204656848	0.228875931210122	1	pseudobulk	DESeq2	LRT	0.640399875705672
127	KC_AB2	grp	-0.241073070480004	0.5987229027128	1	pseudobulk	DESeq2	LRT	0.222774128639772
127	KC_AB2	grsm	-0.975501265622955	0.530259126554907	1	pseudobulk	DESeq2	LRT	0.275511847887638
127	KC_AB2	Grx1	-0.157804587450962	0.82301749218889	1	pseudobulk	DESeq2	LRT	0.084590934313390
127	KC_AB2	gry	-0.729379579623002	0.823527397767669	1	pseudobulk	DESeq2	LRT	0.084321947797948
127	KC_AB2	Gs1	0.173057636006003	0.796241560031282	1	pseudobulk	DESeq2	LRT	0.098955158049080
127	KC_AB2	Gs1l	-0.814220232235486	0.198077217032879	1	pseudobulk	DESeq2	LRT	0.703165474415603
127	KC_AB2	Gs2	0.667244748290888	0.924085151242949	1	pseudobulk	DESeq2	LRT	0.034288008205147
127	KC_AB2	GstD1	0.0855715838743956	0.87625568920544	1	pseudobulk	DESeq2	LRT	0.057369149302023
127	KC_AB2	GstD11	1.48091416250208	0.564234146509008	1	pseudobulk	DESeq2	LRT	0.248540634607989
127	KC_AB2	GstD9	0.403294498144138	0.89479244414645	1	pseudobulk	DESeq2	LRT	0.048277691852293
127	KC_AB2	GstE2	0.146930209264112	1	1	pseudobulk	DESeq2	LRT	0
127	KC_AB2	GstE4	0.805610891257279	0.699374928055192	1	pseudobulk	DESeq2	LRT	0.155289940803513
127	KC_AB2	GstO2	0.428112055785329	0.72572539368729	1	pseudobulk	DESeq2	LRT	0.139227680356079
127	KC_AB2	GstT1	-0.438383410849624	0.799654701834727	1	pseudobulk	DESeq2	LRT	0.097097504833569
127	KC_AB2	GstT2	1.95432904991983	0.509212778150152	1	pseudobulk	DESeq2	LRT	0.293100706726022
127	KC_AB2	GstZ2	-0.893328173497901	0.679679924321693	1	pseudobulk	DESeq2	LRT	0.167695557626761
127	KC_AB2	gt	1.10706867860301	1	1	pseudobulk	DESeq2	LRT	0
127	KC_AB2	Gtp-bp	-1.00833870937463	0.651152668420623	1	pseudobulk	DESeq2	LRT	0.186317175365195
127	KC_AB2	Gug	0.155401391386086	0.618628646646599	1	pseudobulk	DESeq2	LRT	0.208569973126106
127	KC_AB2	gukh	0.700471302628362	0.628618961300458	1	pseudobulk	DESeq2	LRT	0.201612523312135
127	KC_AB2	gus	0.195027560152255	0.648504499805012	1	pseudobulk	DESeq2	LRT	0.188087006112351
127	KC_AB2	gw	-0.419802133444392	0.218954493954594	1	pseudobulk	DESeq2	LRT	0.659646136657501
127	KC_AB2	GXIVsPLA2	2.50905915816151	0.218124909391685	1	pseudobulk	DESeq2	LRT	0.661294736101097
127	KC_AB2	Gyc88E	0.252821962904788	0.669723186610351	1	pseudobulk	DESeq2	LRT	0.174104665013928
127	KC_AB2	Gyc89Db	0.651441472997861	0.624442687835305	1	pseudobulk	DESeq2	LRT	0.20450741557499
127	KC_AB2	Gycalpha99B	-0.047729999885191	0.917254719416048	1	pseudobulk	DESeq2	LRT	0.037510045054625
127	KC_AB2	Gycbeta100B	-0.456284439567993	0.473557110473336	1	pseudobulk	DESeq2	LRT	0.324627638059281
127	KC_AB2	Gyf	-0.0611249302395204	0.859010724716532	1	pseudobulk	DESeq2	LRT	0.066001413984666
127	KC_AB2	Gyg	1.99339641594643	0.465071165814147	1	pseudobulk	DESeq2	LRT	0.332480585700252
127	KC_AB2	gzl	-0.0035832889055728	0.987199371068783	1	pseudobulk	DESeq2	LRT	0.005595129993819
127	KC_AB2	H	-0.676671275010194	0.477505815098287	1	pseudobulk	DESeq2	LRT	0.321021335180297
127	KC_AB2	Hacd1	-1.37085429034816	0.434930498232122	1	pseudobulk	DESeq2	LRT	0.361580137633545
127	KC_AB2	Hacd2	0.139350852739509	0.868572032189223	1	pseudobulk	DESeq2	LRT	0.061194158926636
127	KC_AB2	Hacl	1.0568863089215	0.41271654323331	1	pseudobulk	DESeq2	LRT	0.38434812261533
127	KC_AB2	Hakai	1.55400543303111	0.396514916577079	1	pseudobulk	DESeq2	LRT	0.401740470224661
127	KC_AB2	hang	0.382018439519746	0.548171491467453	1	pseudobulk	DESeq2	LRT	0.261083554386298

127	KC_AB2	Hasp	-0.204144800494331	0.61817562685125	1	pseudobulk	DESeq2	LRT	0.208888122101376
127	KC_AB2	Haspin	0.100028468141298	0.858276477970992	1	pseudobulk	DESeq2	LRT	0.066372789639101
127	KC_AB2	Hat1	0.0034771034327136	0.969363964762302	1	pseudobulk	DESeq2	LRT	0.013513128821809
127	KC_AB2	hay	-0.0725725628699797	0.965504001499157	1	pseudobulk	DESeq2	LRT	0.015245921961850
127	KC_AB2	HBS1	-0.856567423351035	0.665132979544327	1	pseudobulk	DESeq2	LRT	0.177091517841652
127	KC_AB2	Hcf	-0.0360436916212101	0.952478881816111	1	pseudobulk	DESeq2	LRT	0.021144644641744
127	KC_AB2	Hcs	0.508355021850741	0.726459199370163	1	pseudobulk	DESeq2	LRT	0.138788772262013
127	KC_AB2	HDAC1	-0.560521727984731	0.587309052030063	1	pseudobulk	DESeq2	LRT	0.231133305446145
127	KC_AB2	HDAC11	0.350408177089567	0.81405906359628	1	pseudobulk	DESeq2	LRT	0.089344083976429
127	KC_AB2	HDAC4	-0.168808740513537	0.591387230501247	1	pseudobulk	DESeq2	LRT	0.228128057190215
127	KC_AB2	HDAC6	-1.880710221184	0.0806050595146293	1	pseudobulk	DESeq2	LRT	1.09363769702467
127	KC_AB2	hdc	0.150154720939107	0.821861108172254	1	pseudobulk	DESeq2	LRT	0.085201570599878
127	KC_AB2	Hdc	1.61205113368698	0.631647504209	1	pseudobulk	DESeq2	LRT	0.199525215522471
127	KC_AB2	heix	0.245201072196277	0.850737093521509	1	pseudobulk	DESeq2	LRT	0.070204630836217
127	KC_AB2	Hel25E	-1.13826098328519	0.147199968547802	1	pseudobulk	DESeq2	LRT	0.832092282794155
127	KC_AB2	Hel89B	-0.162126549030456	0.852837710412813	1	pseudobulk	DESeq2	LRT	0.069133604445428
127	KC_AB2	Hem	-0.470100723599697	0.833985291815347	1	pseudobulk	DESeq2	LRT	0.078841608522644
127	KC_AB2	HemK1	-1.08527194611241	0.395540690490958	1	pseudobulk	DESeq2	LRT	0.40280883265508
127	KC_AB2	Hen1	1.94826447887871	0.376549774012609	1	pseudobulk	DESeq2	LRT	0.424177608705598
127	KC_AB2	hep	-0.250659358857274	0.728326689001297	1	pseudobulk	DESeq2	LRT	0.137673775349013
127	KC_AB2	heph	0.657845987961826	0.293625198023363	1	pseudobulk	DESeq2	LRT	0.532206677322299
127	KC_AB2	her	-0.488817088764194	0.713979219849849	1	pseudobulk	DESeq2	LRT	0.146314428049978
127	KC_AB2	HERC2	-0.217613288021394	0.760198012097906	1	pseudobulk	DESeq2	LRT	0.119073270402527
127	KC_AB2	Herc4	-1.28828568961021	0.287385175785937	1	pseudobulk	DESeq2	LRT	0.541535637873369
127	KC_AB2	Herp	0.174504709489041	0.89177129624092	1	pseudobulk	DESeq2	LRT	0.049746510552007
127	KC_AB2	Hers	0.19209466030517	0.700855759277435	1	pseudobulk	DESeq2	LRT	0.154371353496392
127	KC_AB2	Hex-A	0.0961573949725711	0.852069966314294	1	pseudobulk	DESeq2	LRT	0.069524742396873
127	KC_AB2	Hexim	-2.0645275095224	0.171080473028167	1	pseudobulk	DESeq2	LRT	0.766799557613036
127	KC_AB2	Hexo1	-0.299056004457792	0.875974510877379	1	pseudobulk	DESeq2	LRT	0.057508530756982
127	KC_AB2	Hey	0.924052757865169	0.615501166509999	1	pseudobulk	DESeq2	LRT	0.210771119648383
127	KC_AB2	hfp	0.0036567966498928	0.957163909582974	1	pseudobulk	DESeq2	LRT	0.019013685073703
127	KC_AB2	hfw	1.78258764191389	0.445483464074049	1	pseudobulk	DESeq2	LRT	0.351168411929411
127	KC_AB2	hig	-1.86716056129969	0.477680380390446	1	pseudobulk	DESeq2	LRT	0.320862595985116
127	KC_AB2	Him	-1.71999636108657	0.519869997311849	1	pseudobulk	DESeq2	LRT	0.284105245805297
127	KC_AB2	HINT1	-0.144158261746568	0.81995806118584	1	pseudobulk	DESeq2	LRT	0.086208360130130
127	KC_AB2	HIP	1.11579041078589	0.42485837578952	1	pseudobulk	DESeq2	LRT	0.371755815510683
127	KC_AB2	HIP-R	-0.285737639721385	0.752660749633098	1	pseudobulk	DESeq2	LRT	0.123400731318219
127	KC_AB2	Hip1	0.378595056850824	0.784696103698346	1	pseudobulk	DESeq2	LRT	0.10529850381553
127	KC_AB2	Hip14	0.787418100663283	0.5219888481459	1	pseudobulk	DESeq2	LRT	0.282338775236117
127	KC_AB2	HipHop	0.145321756433347	1	1	pseudobulk	DESeq2	LRT	0
127	KC_AB2	Hipk	0.158588366609398	0.681357933156449	1	pseudobulk	DESeq2	LRT	0.166624683157322
127	KC_AB2	HIPP1	-0.69036162796314	0.457950093654536	1	pseudobulk	DESeq2	LRT	0.339181847828878
127	KC_AB2	Hira	0.147593560254927	1	1	pseudobulk	DESeq2	LRT	0
127	KC_AB2	His2Av	-0.296276965775732	0.597080115475651	1	pseudobulk	DESeq2	LRT	0.223967391861255
127	KC_AB2	His3.3A	0.317579304185047	0.479129561517697	1	pseudobulk	DESeq2	LRT	0.319547033049285
127	KC_AB2	His3.3B	-0.145105826768631	0.462285314222642	1	pseudobulk	DESeq2	LRT	0.335089902913584
127	KC_AB2	His4r	0.138308425950175	0.581523703333859	1	pseudobulk	DESeq2	LRT	0.23543257841224
127	KC_AB2	HisCl1	1.10706867860301	1	1	pseudobulk	DESeq2	LRT	0
127	KC_AB2	HisRS	-2.5586123064881	0.152808704629224	1	pseudobulk	DESeq2	LRT	0.815851905807685
127	KC_AB2	hiw	0.0563815288419409	0.907074560527461	1	pseudobulk	DESeq2	LRT	0.042357012945685
127	KC_AB2	Hk	0.344094430127422	0.424595108444348	1	pseudobulk	DESeq2	LRT	0.37202501343866
127	KC_AB2	Hlc	0.490250913498221	0.547470455067905	1	pseudobulk	DESeq2	LRT	0.261639313103729

127	KC_AB2	Hmg-2	0.268781370907002	0.88698811189061	1	pseudobulk	DESeq2	LRT	0.052082200884065
127	KC_AB2	Hmgcr	0.0135536646801672	0.975358044495833	1	pseudobulk	DESeq2	LRT	0.010835929732593
127	KC_AB2	HmgD	1.11063693283264	0.242658358269988	1	pseudobulk	DESeq2	LRT	0.615004744991525
127	KC_AB2	Hmgs	1.88501939819828	0.320834444570065	1	pseudobulk	DESeq2	LRT	0.493719012321529
127	KC_AB2	HmgZ	0.332900338557374	0.349886667303583	1	pseudobulk	DESeq2	LRT	0.456072606321906
127	KC_AB2	Hmr	-0.866792081985791	0.699019818888033	1	pseudobulk	DESeq2	LRT	0.15551051078981
127	KC_AB2	Hmt-1	0.182057766966765	0.894170520212044	1	pseudobulk	DESeq2	LRT	0.048579652428491
127	KC_AB2	Hmt4-20	0.708649617190342	0.328734878595578	1	pseudobulk	DESeq2	LRT	0.483154215060473
127	KC_AB2	Hmu	-0.142302079545238	0.890329550010427	1	pseudobulk	DESeq2	LRT	0.050449212164129
128	KC_AB2	Hnf4	-1.86716056129969	0.477680380390446	1	pseudobulk	DESeq2	LRT	0.320862595985116
128	KC_AB2	hng1	-0.427478535071269	0.813013586949621	1	pseudobulk	DESeq2	LRT	0.089902196487186
128	KC_AB2	hng2	1.10706867860301	1	1	pseudobulk	DESeq2	LRT	0
128	KC_AB2	hng3	2.47215486522296	0.30601789350912	1	pseudobulk	DESeq2	LRT	0.514253178665238
128	KC_AB2	HnRNP-K	0.108529879915392	0.814455090450691	1	pseudobulk	DESeq2	LRT	0.089132857957276
128	KC_AB2	Ho	-0.206604242905277	0.847449761374964	1	pseudobulk	DESeq2	LRT	0.071886038262742
128	KC_AB2	hob	1.61205113368698	0.631647504209	1	pseudobulk	DESeq2	LRT	0.199525215522471
128	KC_AB2	hoe1	-0.344645837554029	0.633806691164981	1	pseudobulk	DESeq2	LRT	0.198043180228253
128	KC_AB2	hoe2	0.135854638855446	0.857789087976816	1	pseudobulk	DESeq2	LRT	0.066619482766563
128	KC_AB2	hoip	0.766618888456018	0.172080416577298	1	pseudobulk	DESeq2	LRT	0.764268551266557
128	KC_AB2	holn1	-0.785201786757791	0.678486044454677	1	pseudobulk	DESeq2	LRT	0.168459080765713
128	KC_AB2	homer	-0.935325715625522	0.400691511185762	1	pseudobulk	DESeq2	LRT	0.397189858176815
128	KC_AB2	hook	-1.39645530548754	0.147749971682457	1	pseudobulk	DESeq2	LRT	0.830472593682942
128	KC_AB2	hop	2.25444644428762	0.265526921939049	1	pseudobulk	DESeq2	LRT	0.575891438963608
128	KC_AB2	HP1b	-0.0987433913440183	0.903435462012518	1	pseudobulk	DESeq2	LRT	0.044102866338176
128	KC_AB2	HP1c	0.984049965705698	0.410428704689561	1	pseudobulk	DESeq2	LRT	0.386762273012382
128	KC_AB2	HP4	0.64386856569417	0.676859099940543	1	pseudobulk	DESeq2	LRT	0.16950172789829
128	KC_AB2	HP5	-1.35379244188507	0.183514743662842	1	pseudobulk	DESeq2	LRT	0.736329038582492
128	KC_AB2	Hph	0.322283893990939	0.824209177228156	1	pseudobulk	DESeq2	LRT	0.083962554095150
128	KC_AB2	hpo	-1.24134078108148	0.492384277960687	1	pseudobulk	DESeq2	LRT	0.307695822726763
128	KC_AB2	hppy	0.317980397682664	0.509523090399865	1	pseudobulk	DESeq2	LRT	0.292836129997065
128	KC_AB2	Hpr1	0.0558380315193336	0.977897506507802	1	pseudobulk	DESeq2	LRT	0.009706661256041
128	KC_AB2	hpRNA:CR18854	-0.202197325021276	0.677626969339637	1	pseudobulk	DESeq2	LRT	0.169009317549985
128	KC_AB2	hpRNA:CR33940	1.38446765109058	0.642794900879723	1	pseudobulk	DESeq2	LRT	0.191927577037166
128	KC_AB2	hpRNA:CR46342	0.0782484979608839	0.885735454520585	1	pseudobulk	DESeq2	LRT	0.052695970886758
128	KC_AB2	HPS1	0.23858349385628	0.829218459981331	1	pseudobulk	DESeq2	LRT	0.081331038248714
128	KC_AB2	HPS4	-0.204535454614829	0.785682078889027	1	pseudobulk	DESeq2	LRT	0.104753152834956
128	KC_AB2	Hr3	0.559587288322522	0.305366734434916	1	pseudobulk	DESeq2	LRT	0.515178275197836
128	KC_AB2	Hr38	0.301283839655864	0.303253524327326	1	pseudobulk	DESeq2	LRT	0.518194143183514
128	KC_AB2	Hr39	-0.723502980439558	0.277918719483211	1	pseudobulk	DESeq2	LRT	0.55608219991402
128	KC_AB2	Hr51	-0.133597397402702	0.781517477637793	1	pseudobulk	DESeq2	LRT	0.107061305096139
128	KC_AB2	Hr78	-1.14694249494482	0.188938006860349	1	pseudobulk	DESeq2	LRT	0.72368067040311
128	KC_AB2	Hr96	1.76518413240156	0.320576124727132	1	pseudobulk	DESeq2	LRT	0.494068825354827
128	KC_AB2	Hrb27C	0.0982458514977432	0.660791213370229	1	pseudobulk	DESeq2	LRT	0.179935740521504
128	KC_AB2	Hrb87F	0.552959144325488	0.540566723301173	1	pseudobulk	DESeq2	LRT	0.267150692509822
128	KC_AB2	Hrb98DE	0.220859765629301	0.601099306958542	1	pseudobulk	DESeq2	LRT	0.221053772753816
128	KC_AB2	Hrd3	1.49049211286363	0.478258655503098	1	pseudobulk	DESeq2	LRT	0.320337161388031
128	KC_AB2	hrg	0.472086899439847	0.401680374622815	1	pseudobulk	DESeq2	LRT	0.396119386593514
128	KC_AB2	hrm	0.239395155123982	0.699034026835158	1	pseudobulk	DESeq2	LRT	0.155501683614728
128	KC_AB2	Hrs	0.90296778402159	0.480007273426019	1	pseudobulk	DESeq2	LRT	0.318752181822638
128	KC_AB2	Hs2st	-0.191760953214709	0.878004114747556	1	pseudobulk	DESeq2	LRT	0.056503448777302
128	KC_AB2	Hs3st-A	0.516738589475573	0.0458396043121337	1	pseudobulk	DESeq2	LRT	1.33875913985826
128	KC_AB2	Hs3st-B	1.10706867860301	1	1	pseudobulk	DESeq2	LRT	0

128	KC_AB2	Hs6st	0.139321135726814	0.874857203244782	1	pseudobulk	DESeq2	LRT	0.058062828010547
128	KC_AB2	Hsc20	-2.39970914040097	0.301766661516198	1	pseudobulk	DESeq2	LRT	0.520328741761584
128	KC_AB2	Hsc70-2	-0.222368670234767	0.919128948430502	1	pseudobulk	DESeq2	LRT	0.036623555356777
128	KC_AB2	Hsc70-3	-0.0710454017109047	0.871353979848996	1	pseudobulk	DESeq2	LRT	0.059805380853571
128	KC_AB2	Hsc70-4	-0.0850947072343014	0.834335149087128	1	pseudobulk	DESeq2	LRT	0.078659459960425
128	KC_AB2	Hsc70-5	-0.447134615711444	0.502889058936199	1	pseudobulk	DESeq2	LRT	0.298527812967905
128	KC_AB2	Hsc70Cb	-0.221272389663479	0.742096521539939	1	pseudobulk	DESeq2	LRT	0.129539604092997
128	KC_AB2	Hsepi	0.600457737989162	0.742742932528136	1	pseudobulk	DESeq2	LRT	0.129161471976179
128	KC_AB2	Hsf	0.46951690653708	0.572627343122544	1	pseudobulk	DESeq2	LRT	0.24212791813119
128	KC_AB2	Hsl	0.970782606828758	0.624232946975692	1	pseudobulk	DESeq2	LRT	0.204653313030305
128	KC_AB2	Hsp23	-5.60499172463478	0.0231729256485406	1	pseudobulk	DESeq2	LRT	1.63501913182048
128	KC_AB2	Hsp27	-2.49053021141053	0.0016034985768915	1	pseudobulk	DESeq2	LRT	2.79493142117267
128	KC_AB2	Hsp60A	-0.469601590028815	0.378318139874626	1	pseudobulk	DESeq2	LRT	0.422142834338823
128	KC_AB2	Hsp60D	0.858103339274647	0.6980413252262	1	pseudobulk	DESeq2	LRT	0.156118865648421
128	KC_AB2	Hsp68	-4.34919443251642	0.0929057715256098	1	pseudobulk	DESeq2	LRT	1.03195730577098
128	KC_AB2	Hsp70Aa	-0.945898486863123	0.650373526616336	1	pseudobulk	DESeq2	LRT	0.18683714496386
128	KC_AB2	Hsp70Ab	-0.232606117344034	0.792761593070193	1	pseudobulk	DESeq2	LRT	0.100857398282773
128	KC_AB2	Hsp70Ba	-0.83320718254958	0.715059964627045	1	pseudobulk	DESeq2	LRT	0.14565753691968
128	KC_AB2	Hsp70Bb	0.932229473263668	0.273704007676963	1	pseudobulk	DESeq2	LRT	0.56271884344027
128	KC_AB2	Hsp70Bc	0.907292367512464	0.220542387567597	1	pseudobulk	DESeq2	LRT	0.656507928112021
128	KC_AB2	Hsp83	0.149430885678337	0.687613471693769	1	pseudobulk	DESeq2	LRT	0.162655623222693
128	KC_AB2	HSPBAP1	1.61205113368698	0.631647504209	1	pseudobulk	DESeq2	LRT	0.199525215522471
128	KC_AB2	HSPC300	1.25903626699711	0.368374708827082	1	pseudobulk	DESeq2	LRT	0.433710194419498
128	KC_AB2	hth	0.497544024660572	0.404187668690759	1	pseudobulk	DESeq2	LRT	0.393416940452706
128	KC_AB2	htk	-0.213833503327162	0.811519053521517	1	pseudobulk	DESeq2	LRT	0.090701278989797
128	KC_AB2	htl	-0.729379579623002	1	1	pseudobulk	DESeq2	LRT	0
128	KC_AB2	hts	-0.52822662795562	0.329304040777965	1	pseudobulk	DESeq2	LRT	0.482402940175411
128	KC_AB2	htt	-0.531905051878191	0.661731322275534	1	pseudobulk	DESeq2	LRT	0.179318308050259
128	KC_AB2	Hug	-2.39970914040097	0.301766661516198	1	pseudobulk	DESeq2	LRT	0.520328741761584
128	KC_AB2	Hus1-like	-0.729379579623002	0.823527397767669	1	pseudobulk	DESeq2	LRT	0.084321947797948
128	KC_AB2	HUWE1	0.569155703922799	0.391502089387044	1	pseudobulk	DESeq2	LRT	0.407265915836325
128	KC_AB2	hwt	0.410015911767962	0.313329544342512	1	pseudobulk	DESeq2	LRT	0.503998652868118
128	KC_AB2	Hyccin	0.492486263870049	0.738213908214142	1	pseudobulk	DESeq2	LRT	0.131817776799865
128	KC_AB2	hyd	0.85582993482162	0.378016905374744	1	pseudobulk	DESeq2	LRT	0.422488777552209
128	KC_AB2	Hydr1	1.10706867860301	1	1	pseudobulk	DESeq2	LRT	0
128	KC_AB2	Hydr2	-1.29785233166196	0.191141972974605	1	pseudobulk	DESeq2	LRT	0.718643935500216
128	KC_AB2	hyx	-0.744467116302025	0.71305844217897	1	pseudobulk	DESeq2	LRT	0.146874873969043
128	KC_AB2	I-2	-0.573701807745216	0.480710218439882	1	pseudobulk	DESeq2	LRT	0.318116645990998
128	KC_AB2	I-3	0.38605638660934	0.863777890371283	1	pseudobulk	DESeq2	LRT	0.063597916533759
128	KC_AB2	IA-2	-0.271830397032305	0.191843778365712	1	pseudobulk	DESeq2	LRT	0.717052280725522
128	KC_AB2	Ibf1	0.89698058322345	0.673674818076485	1	pseudobulk	DESeq2	LRT	0.171549686245101
128	KC_AB2	Ibf2	-1.76087072987779	0.317269399629035	1	pseudobulk	DESeq2	LRT	0.498571813222998
128	KC_AB2	ICA69	-0.547775357009626	0.458744947597096	1	pseudobulk	DESeq2	LRT	0.338428705830393
128	KC_AB2	Ice1	-0.299056004457792	0.875974510877379	1	pseudobulk	DESeq2	LRT	0.057508530756982
128	KC_AB2	icln	-0.68003817249802	0.743702263962739	1	pseudobulk	DESeq2	LRT	0.128600896440406
128	KC_AB2	ics	-0.0956476251326481	0.893150855228327	1	pseudobulk	DESeq2	LRT	0.049075181581722
128	KC_AB2	ida	0.145321756433347	1	1	pseudobulk	DESeq2	LRT	0
128	KC_AB2	Ide	-1.70729982174102	0.162076263952897	1	pseudobulk	DESeq2	LRT	0.790280582863246
128	KC_AB2	Idgf6	-0.152789803467358	0.917227842241703	1	pseudobulk	DESeq2	LRT	0.037522770832185
128	KC_AB2	Idh	-0.555954927549371	0.694568097535988	1	pseudobulk	DESeq2	LRT	0.158285168307528
128	KC_AB2	Idh3a	0.104173647951404	0.848840254155878	1	pseudobulk	DESeq2	LRT	0.071174033273491
128	KC_AB2	Idh3b	-0.229882420592419	0.68692659336163	1	pseudobulk	DESeq2	LRT	0.163089670220378

128	KC_AB2	Idi	0.0237795764023947	0.988035687382292	1	pseudobulk	DESeq2	LRT	0.00522736861753
128	KC_AB2	Ids	1.10706867860301	1	1	pseudobulk	DESeq2	LRT	0
128	KC_AB2	ifc	-0.597488066650183	0.701294907970441	1	pseudobulk	DESeq2	LRT	0.15409931445693
128	KC_AB2	igl	-0.359196856199215	0.233085622006352	1	pseudobulk	DESeq2	LRT	0.63248451530777
128	KC_AB2	Ih	-0.020282424270249	0.869720437113682	1	pseudobulk	DESeq2	LRT	0.06062032454254
129	KC_AB2	IKKepsilon	1.10706867860301	1	1	pseudobulk	DESeq2	LRT	0
129	KC_AB2	IleRS	-1.20441416492585	0.509438913975176	1	pseudobulk	DESeq2	LRT	0.29290788410894
129	KC_AB2	IleRS-m	-0.824996233971657	0.664071509040402	1	pseudobulk	DESeq2	LRT	0.17778515209358
129	KC_AB2	Ilk	-0.259755476903462	0.89089279884898	1	pseudobulk	DESeq2	LRT	0.05017455148425
129	KC_AB2	Ilp2	-2.87093509453135	0.225888123098267	1	pseudobulk	DESeq2	LRT	0.64610660311454
129	KC_AB2	Ilp3	-4.0723755557937	0.269776801772911	1	pseudobulk	DESeq2	LRT	0.56899539823406
129	KC_AB2	Ilp5	-0.729379579623002	0.772694994316916	1	pseudobulk	DESeq2	LRT	0.1119919011982
129	KC_AB2	Ilp7	1.38069928703004	0.566368801250645	1	pseudobulk	DESeq2	LRT	0.24690067801932
129	KC_AB2	imd	1.34950042090258	0.513284828877346	1	pseudobulk	DESeq2	LRT	0.28964157196020
129	KC_AB2	Iml1	-0.134051763797116	0.916486491927382	1	pseudobulk	DESeq2	LRT	0.03787393171063
129	KC_AB2	Imp	2.64741945039871	0.292550810913063	1	pseudobulk	DESeq2	LRT	0.53379869374338
129	KC_AB2	Impbeta11	2.33862076287154	0.37727071943742	1	pseudobulk	DESeq2	LRT	0.42334689975583
129	KC_AB2	inaE	0.178997585464988	0.785036339663297	1	pseudobulk	DESeq2	LRT	0.10511023911467
129	KC_AB2	inc	-0.647086810898795	0.610213520970147	1	pseudobulk	DESeq2	LRT	0.21451817359025
129	KC_AB2	Ing3	-0.800746076174387	0.620012708340552	1	pseudobulk	DESeq2	LRT	0.2075994087185
129	KC_AB2	Ing5	0.101364409018496	0.892031415147758	1	pseudobulk	DESeq2	LRT	0.04961985057334
129	KC_AB2	Ino80	0.759515370974497	0.597995982708436	1	pseudobulk	DESeq2	LRT	0.22330173355911
129	KC_AB2	Inos	-0.089224327918158	0.90144233223	1	pseudobulk	DESeq2	LRT	0.04506205107731
129	KC_AB2	INPP5E	1.62307726862426	0.360835495440644	1	pseudobulk	DESeq2	LRT	0.44269074741676
129	KC_AB2	InR	0.566266233312595	0.587417962004939	1	pseudobulk	DESeq2	LRT	0.23105277779898
129	KC_AB2	insv	-0.729379579623002	1	1	pseudobulk	DESeq2	LRT	0
129	KC_AB2	IntS1	-3.07118289746962	0.157022295854486	1	pseudobulk	DESeq2	LRT	0.80403867702331
129	KC_AB2	IntS10	-1.33407500886303	0.63128114564055	1	pseudobulk	DESeq2	LRT	0.19977718147345
129	KC_AB2	IntS11	0.0487673033804221	0.942805703571486	1	pseudobulk	DESeq2	LRT	0.02557779884379
129	KC_AB2	IntS12	-0.206397962031176	0.915265628332718	1	pseudobulk	DESeq2	LRT	0.03845284672317
129	KC_AB2	IntS14	1.04560394656545	0.638145957880541	1	pseudobulk	DESeq2	LRT	0.19507997730960
129	KC_AB2	IntS2	0.211319900981398	0.958606495011728	1	pseudobulk	DESeq2	LRT	0.01835963278404
129	KC_AB2	IntS3	0.244718703508961	0.796507500846594	1	pseudobulk	DESeq2	LRT	0.09881013001859
129	KC_AB2	IntS4	0.375534485637874	0.901061951722844	1	pseudobulk	DESeq2	LRT	0.04524534845886
129	KC_AB2	IntS6	0.53725623715534	0.574398478391904	1	pseudobulk	DESeq2	LRT	0.24078671923037
129	KC_AB2	IntS8	0.39439088612385	0.813541888331052	1	pseudobulk	DESeq2	LRT	0.08962008083076
129	KC_AB2	IntS9	-1.91525348325759	0.346063376217872	1	pseudobulk	DESeq2	LRT	0.46084435952728
129	KC_AB2	Invadolysin	-1.74576972023816	0.482521062123617	1	pseudobulk	DESeq2	LRT	0.31648372488096
129	KC_AB2	Inx3	0.147593560254927	1	1	pseudobulk	DESeq2	LRT	0
129	KC_AB2	Inx7	2.64389091645132	0.267289646604	1	pseudobulk	DESeq2	LRT	0.57301786320318
129	KC_AB2	Ip259	0.036448237901419	0.887610337261109	1	pseudobulk	DESeq2	LRT	0.05177764854309
129	KC_AB2	IPIP	-0.175714474517952	0.876848828393286	1	pseudobulk	DESeq2	LRT	0.05707527396997
129	KC_AB2	Ipk2	-0.647621034217714	0.754702560287896	1	pseudobulk	DESeq2	LRT	0.12222417668540
129	KC_AB2	iPLA2-VIA	-0.312643756799871	0.807450541450584	1	pseudobulk	DESeq2	LRT	0.09288406990450
129	KC_AB2	Ipp	0.137115091363509	0.805524789620138	1	pseudobulk	DESeq2	LRT	0.09392108984491
129	KC_AB2	Ir41a	0.66954517615027	1	1	pseudobulk	DESeq2	LRT	0
129	KC_AB2	Ir47a	1.99339641594643	0.465071165814147	1	pseudobulk	DESeq2	LRT	0.33248058570025
129	KC_AB2	Ir56a	1.90703728954173	0.262339779882953	1	pseudobulk	DESeq2	LRT	0.58113585018831
129	KC_AB2	Ir68a	1.10706867860301	1	1	pseudobulk	DESeq2	LRT	0
129	KC_AB2	Ir76a	0.661678460170035	0.878003361430606	1	pseudobulk	DESeq2	LRT	0.05650382139689
129	KC_AB2	Irbp	1.10706867860301	1	1	pseudobulk	DESeq2	LRT	0
129	KC_AB2	Irbp18	-0.461840586776517	0.786016377315427	1	pseudobulk	DESeq2	LRT	0.10456840497379

129	KC_AB2	Ire1	0.0648047793803259	0.938605025639256	1	pseudobulk	DESeq2	LRT	0.027517124743646
129	KC_AB2	Irp-1A	-0.121694666120293	0.872468543141359	1	pseudobulk	DESeq2	LRT	0.059250222576698
129	KC_AB2	Irp-1B	-0.469722102860755	0.72345646312798	1	pseudobulk	DESeq2	LRT	0.140587599157512
129	KC_AB2	IRSp53	0.0384695982190361	0.977248984064524	1	pseudobulk	DESeq2	LRT	0.009994772382438
129	KC_AB2	Iru	-0.0347618596612806	0.963572981456878	1	pseudobulk	DESeq2	LRT	0.016115386100734
129	KC_AB2	IscU	-0.909509917284779	0.0884443130514862	1	pseudobulk	DESeq2	LRT	1.05333008688949
129	KC_AB2	isopeptidase-T-3	3.82540775711006	0.0471362960284793	1	pseudobulk	DESeq2	LRT	1.32664454734401
129	KC_AB2	isoQC	-0.317241443206136	0.855681736781888	1	pseudobulk	DESeq2	LRT	0.067687737263355
129	KC_AB2	Iswi	0.547704228278546	0.671693609012576	1	pseudobulk	DESeq2	LRT	0.172828783879031
129	KC_AB2	ITP	-0.729379579623002	0.772694994316916	1	pseudobulk	DESeq2	LRT	0.1119919011982
129	KC_AB2	Itpr	0.986695214759282	0.345262232120143	1	pseudobulk	DESeq2	LRT	0.461850926078296
129	KC_AB2	ix	-0.867983160258898	0.607581956589965	1	pseudobulk	DESeq2	LRT	0.216395131895767
129	KC_AB2	Jafrac1	-0.676000635713435	0.295365639796327	1	pseudobulk	DESeq2	LRT	0.529640028031486
129	KC_AB2	jagn	0.167026756429215	0.870486799671492	1	pseudobulk	DESeq2	LRT	0.060237810271287
129	KC_AB2	janA	-0.78106359304094	0.494441571145442	1	pseudobulk	DESeq2	LRT	0.30588502222328
129	KC_AB2	jar	0.59758936393988	0.425914527980502	1	pseudobulk	DESeq2	LRT	0.370677545852158
129	KC_AB2	Jarid2	-0.0494467812774498	0.924786601725698	1	pseudobulk	DESeq2	LRT	0.033958470920461
129	KC_AB2	jdp	0.223068952536941	0.231206103076863	1	pseudobulk	DESeq2	LRT	0.63600070616145
129	KC_AB2	jeb	-0.52238592696395	0.120537095215164	1	pseudobulk	DESeq2	LRT	0.918879278665566
129	KC_AB2	jef	-0.508944913428487	0.325279090350976	1	pseudobulk	DESeq2	LRT	0.487743853216932
129	KC_AB2	jet	2.32149674095703	0.249331547667048	1	pseudobulk	DESeq2	LRT	0.603222767180568
129	KC_AB2	JHDM2	-0.0045035166158998	1	1	pseudobulk	DESeq2	LRT	0
129	KC_AB2	Jheh1	1.99339641594643	0.465071165814147	1	pseudobulk	DESeq2	LRT	0.332480585700252
129	KC_AB2	Jheh2	-0.729379579623002	0.823527397767669	1	pseudobulk	DESeq2	LRT	0.084321947797948
129	KC_AB2	Jhl-21	1.10706867860301	1	1	pseudobulk	DESeq2	LRT	0
129	KC_AB2	jigr1	1.01608407489484	0.230003414102789	1	pseudobulk	DESeq2	LRT	0.638265717395463
129	KC_AB2	JIL-1	-0.223435025573048	0.691434383095632	1	pseudobulk	DESeq2	LRT	0.160249028008213
129	KC_AB2	jim	0.289617373562272	0.136118494155563	1	pseudobulk	DESeq2	LRT	0.866082864039547
129	KC_AB2	jing	1.05388012269339	0.317738304584715	1	pseudobulk	DESeq2	LRT	0.497930426132884
129	KC_AB2	JMJD4	-2.1751167112989	0.359363815234217	1	pseudobulk	DESeq2	LRT	0.444465654648455
129	KC_AB2	JMJD5	0.616166021714033	0.74314492175284	1	pseudobulk	DESeq2	LRT	0.128926485593015
129	KC_AB2	JMJD7	1.17971048284228	0.415139856423882	1	pseudobulk	DESeq2	LRT	0.381805569213581
129	KC_AB2	jnj	0.0597970208643884	0.934860154330338	1	pseudobulk	DESeq2	LRT	0.029253350352122
129	KC_AB2	jp	0.602475421119997	0.435127437841367	1	pseudobulk	DESeq2	LRT	0.361383530526184
129	KC_AB2	Jra	-0.316478514343741	0.781568927978056	1	pseudobulk	DESeq2	LRT	0.107032714739526
129	KC_AB2	JTBR	-0.754342923618676	0.670634839187245	1	pseudobulk	DESeq2	LRT	0.173513888912333
129	KC_AB2	jub	0.0163607969601969	0.982626433786628	1	pseudobulk	DESeq2	LRT	0.007611557020600
129	KC_AB2	jumu	0.0843130080157558	0.943549788858392	1	pseudobulk	DESeq2	LRT	0.025235178216641
129	KC_AB2	Jupiter	-0.217118872992529	0.491307930328388	1	pseudobulk	DESeq2	LRT	0.308646225749032
129	KC_AB2	jus	0.0634645145513867	0.786329619577678	1	pseudobulk	DESeq2	LRT	0.104395364958353
129	KC_AB2	jv	-0.115183097143228	0.910399790291748	1	pseudobulk	DESeq2	LRT	0.040767850987610
129	KC_AB2	jvl	-0.125336120073078	0.782813907097712	1	pseudobulk	DESeq2	LRT	0.106341467477315
129	KC_AB2	Jwa	-0.817407947341251	0.329621372889922	1	pseudobulk	DESeq2	LRT	0.481984636091369
129	KC_AB2	KaiR1D	0.151408359271651	0.912261442481077	1	pseudobulk	DESeq2	LRT	0.039880680580222
129	KC_AB2	Kank	0.366498153868735	0.660212985014005	1	pseudobulk	DESeq2	LRT	0.180315938254007
129	KC_AB2	Kap-alpha1	-0.961238693752317	0.377344738245963	1	pseudobulk	DESeq2	LRT	0.423261701500529
129	KC_AB2	Kap-alpha3	0.443532530652069	0.461400694641908	1	pseudobulk	DESeq2	LRT	0.335921755981115
129	KC_AB2	Kap3	0.772584056371047	0.348817731191368	1	pseudobulk	DESeq2	LRT	0.457401447011124
129	KC_AB2	Karybeta3	0.306148766833519	0.771530422731855	1	pseudobulk	DESeq2	LRT	0.112646944307156
129	KC_AB2	kat-60L1	-0.686418115209095	0.637324867484452	1	pseudobulk	DESeq2	LRT	0.195639135653859
129	KC_AB2	Kat60	-0.743747489175216	0.65419637449897	1	pseudobulk	DESeq2	LRT	0.18429186702769
129	KC_AB2	kat80	0.393554365786369	0.91735792252201	1	pseudobulk	DESeq2	LRT	0.037461184016107

129	KC_AB2	kay	-0.347567282060173	0.815850230698437	1	pseudobulk	DESeq2	LRT	0.088389559323430
130	KC_AB2	Kaz1-ORFB	1.38069928703004	0.566368801250645	1	pseudobulk	DESeq2	LRT	0.246900678019328
130	KC_AB2	kcc	0.112599499217975	0.809662408215618	1	pseudobulk	DESeq2	LRT	0.091696024097309
130	KC_AB2	Kcmf1	0.477927883274424	0.491181367356097	1	pseudobulk	DESeq2	LRT	0.30875811623153
130	KC_AB2	KCNQ	0.117619114433816	0.760268090146431	1	pseudobulk	DESeq2	LRT	0.119033237270905
130	KC_AB2	KdelR	-0.888586824318219	0.225062810777564	1	pseudobulk	DESeq2	LRT	0.647696261589497
130	KC_AB2	Kdm2	0.262244976831293	0.774120762709563	1	pseudobulk	DESeq2	LRT	0.111191284165503
130	KC_AB2	Kdm4A	-1.9798493942058	0.248312565686225	1	pseudobulk	DESeq2	LRT	0.605001302716449
130	KC_AB2	Kdm4B	0.380540980271964	0.423133841511805	1	pseudobulk	DESeq2	LRT	0.37352223915915
130	KC_AB2	kdn	-0.573056360133939	0.441878255151126	1	pseudobulk	DESeq2	LRT	0.354697369562924
130	KC_AB2	Keap1	0.170298757134349	0.84973283257208	1	pseudobulk	DESeq2	LRT	0.070717600848496
130	KC_AB2	kek1	-0.315389522117815	0.314771694680081	1	pseudobulk	DESeq2	LRT	0.502004327764497
130	KC_AB2	kek2	-0.940330394521192	0.145086598636672	1	pseudobulk	DESeq2	LRT	0.838372700635539
130	KC_AB2	kek3	0.0065992585447106	0.968886179763182	1	pseudobulk	DESeq2	LRT	0.013727238846196
130	KC_AB2	kek4	0.08604158138726	0.926363792213924	1	pseudobulk	DESeq2	LRT	0.033218428086631
130	KC_AB2	kek6	-0.401904003113228	0.659674097244534	1	pseudobulk	DESeq2	LRT	0.180670568586853
130	KC_AB2	kel	-1.5033370644665	0.499350065149774	1	pseudobulk	DESeq2	LRT	0.30159488912562
130	KC_AB2	key	0.113489910552412	0.912190300059717	1	pseudobulk	DESeq2	LRT	0.039914550219326
130	KC_AB2	KFase	0.396659246788372	0.915482501218565	1	pseudobulk	DESeq2	LRT	0.038349952505755
130	KC_AB2	Khc	-0.584209491231891	0.569193985475375	1	pseudobulk	DESeq2	LRT	0.244739697640207
130	KC_AB2	Khc-73	-0.28780240577249	0.56037656137108	1	pseudobulk	DESeq2	LRT	0.251520038054504
130	KC_AB2	Kif3C	-1.89324719569543	0.358469656860786	1	pseudobulk	DESeq2	LRT	0.445547599872436
130	KC_AB2	kin17	0.915096279045815	0.463608034060672	1	pseudobulk	DESeq2	LRT	0.333849046569562
130	KC_AB2	kirre	-0.305056235408384	0.774912926363009	1	pseudobulk	DESeq2	LRT	0.110747094557655
130	KC_AB2	kis	0.377640310935502	0.249998508603105	1	pseudobulk	DESeq2	LRT	0.602062582157458
130	KC_AB2	kl-3	-0.729379579623002	0.823527397767669	1	pseudobulk	DESeq2	LRT	0.084321947797948
130	KC_AB2	kl-5	-0.729379579623002	1	1	pseudobulk	DESeq2	LRT	0
130	KC_AB2	klar	0.506607835909096	0.501434218155139	1	pseudobulk	DESeq2	LRT	0.299786032865052
130	KC_AB2	Klc	0.475448119865524	0.586629245801991	1	pseudobulk	DESeq2	LRT	0.231636289507847
130	KC_AB2	klg	-1.56677493270283	0.0623753509512718	1	pseudobulk	DESeq2	LRT	1.20498699782912
130	KC_AB2	KLHL18	-0.799132886553859	0.550015802756192	1	pseudobulk	DESeq2	LRT	0.25962483241263
130	KC_AB2	Klp10A	-0.688264808523459	0.384100058577326	1	pseudobulk	DESeq2	LRT	0.415555626602573
130	KC_AB2	Klp31E	1.05127534969664	0.531418750285872	1	pseudobulk	DESeq2	LRT	0.27456312624861
130	KC_AB2	Klp64D	-0.284107593338793	0.716034081836453	1	pseudobulk	DESeq2	LRT	0.145066305623378
130	KC_AB2	Klp68D	0.0881090349774954	0.90461435641552	1	pseudobulk	DESeq2	LRT	0.043536524192835
130	KC_AB2	Klp98A	0.139813752237469	0.861060300082039	1	pseudobulk	DESeq2	LRT	0.064966433824053
130	KC_AB2	kmr	-0.0662608016766596	0.912844426642062	1	pseudobulk	DESeq2	LRT	0.039603231674877
130	KC_AB2	knk	-0.729379579623002	1	1	pseudobulk	DESeq2	LRT	0
130	KC_AB2	ko	-1.26196526765428	0.507633790087936	1	pseudobulk	DESeq2	LRT	0.294449477278052
130	KC_AB2	koi	-1.27901364030294	0.305569793679598	1	pseudobulk	DESeq2	LRT	0.514889579044785
130	KC_AB2	koko	1.50713387114482	0.576249954186023	1	pseudobulk	DESeq2	LRT	0.239389096130292
130	KC_AB2	Kr-h1	0.330433790849642	0.665436196312775	1	pseudobulk	DESeq2	LRT	0.1768935794345
130	KC_AB2	Kr-h2	-0.301680293590872	0.729763093667701	1	pseudobulk	DESeq2	LRT	0.136818104004744
130	KC_AB2	kra	-0.169335189559172	0.63518969628384	1	pseudobulk	DESeq2	LRT	0.197096555422531
130	KC_AB2	kraken	0.544355192570787	0.705704397239955	1	pseudobulk	DESeq2	LRT	0.151377176471069
130	KC_AB2	kri	0.0172371992010125	0.941184547254938	1	pseudobulk	DESeq2	LRT	0.026325211858322
130	KC_AB2	Krn	1.2308286027964	0.515118054682673	1	pseudobulk	DESeq2	LRT	0.28809322800402
130	KC_AB2	KrT95D	0.673549871243026	0.25667098875996	1	pseudobulk	DESeq2	LRT	0.590623216383036
130	KC_AB2	krz	0.932750721665479	0.24848293192217	1	pseudobulk	DESeq2	LRT	0.604703437218753
130	KC_AB2	ksh	-0.0863964714761508	0.923516373579793	1	pseudobulk	DESeq2	LRT	0.034555400286202
130	KC_AB2	ksr	-1.635281488391	0.185700662878052	1	pseudobulk	DESeq2	LRT	0.731186545997611
130	KC_AB2	Ktl	-0.408212552549047	0.35790895081769	1	pseudobulk	DESeq2	LRT	0.446227440357906

130	KC_AB2	kto	0.0333202646883626	0.981578461551168	1	pseudobulk	DESeq2	LRT	0.00807497975548
130	KC_AB2	ktub	1.39739628414676	0.420366776722904	1	pseudobulk	DESeq2	LRT	0.37637161532713
130	KC_AB2	Ku80	1.99339641594643	0.465071165814147	1	pseudobulk	DESeq2	LRT	0.33248058570025
130	KC_AB2	Kua	-1.69195327381593	0.501212413776909	1	pseudobulk	DESeq2	LRT	0.29997818115744
130	KC_AB2	kud	0.784726727348189	0.38706565119638	1	pseudobulk	DESeq2	LRT	0.41221536693408
130	KC_AB2	kug	1.95432904991983	0.509212778150152	1	pseudobulk	DESeq2	LRT	0.29310070672602
130	KC_AB2	kuk	0.820010573653782	0.333540740669702	1	pseudobulk	DESeq2	LRT	0.47685111117050
130	KC_AB2	Kul	0.0308094967350868	0.970707816873857	1	pseudobulk	DESeq2	LRT	0.01291147309400
130	KC_AB2	kune	0.423170152652793	0.774866969542059	1	pseudobulk	DESeq2	LRT	0.11077285149746
130	KC_AB2	kuz	0.37705588680115	0.311293872220906	1	pseudobulk	DESeq2	LRT	0.50682942826332
130	KC_AB2	Kyat	0.403294498144138	0.89479244414645	1	pseudobulk	DESeq2	LRT	0.04827769185229
130	KC_AB2	l(1)10Bb	-0.0424203624657978	0.96253603572174	1	pseudobulk	DESeq2	LRT	0.01658300226398
130	KC_AB2	l(1)G0004	-0.720747651968478	0.756336938577071	1	pseudobulk	DESeq2	LRT	0.12128468867113
130	KC_AB2	l(1)G0007	-0.845593431183803	0.515636981606144	1	pseudobulk	DESeq2	LRT	0.28765594249877
130	KC_AB2	l(1)G0193	0.755826059109958	0.586489867039965	1	pseudobulk	DESeq2	LRT	0.23173948691869
130	KC_AB2	l(1)G0196	-0.352072054923239	0.575385805851068	1	pseudobulk	DESeq2	LRT	0.24004085589128
130	KC_AB2	l(1)G0289	0.325122024251575	0.422772919625478	1	pseudobulk	DESeq2	LRT	0.37389283885250
130	KC_AB2	l(1)G0320	0.254979446110438	0.638248007099534	1	pseudobulk	DESeq2	LRT	0.19501053258642
130	KC_AB2	l(1)G0469	0.661032491972075	0.225730623813858	1	pseudobulk	DESeq2	LRT	0.64640951823205
130	KC_AB2	l(2)05287	0.38245451307505	0.830649933977651	1	pseudobulk	DESeq2	LRT	0.08058196511258
130	KC_AB2	l(2)05714	-2.0846073628073	0.240650955303512	1	pseudobulk	DESeq2	LRT	0.61861240997989
130	KC_AB2	l(2)09851	-1.54352099991151	0.432364700375742	1	pseudobulk	DESeq2	LRT	0.36414977044912
130	KC_AB2	l(2)10685	-0.729379579623002	1	1	pseudobulk	DESeq2	LRT	0
130	KC_AB2	l(2)34Fd	0.205564527517923	0.908209004553827	1	pseudobulk	DESeq2	LRT	0.04181419654576
130	KC_AB2	l(2)35Be	0.478177610186217	0.726346233096467	1	pseudobulk	DESeq2	LRT	0.13885631142219
130	KC_AB2	l(2)37Bb	-0.348005420194213	0.842767341459593	1	pseudobulk	DESeq2	LRT	0.07429230232200
130	KC_AB2	l(2)37Cb	-0.945898486863123	0.650373526616336	1	pseudobulk	DESeq2	LRT	0.18683714496386
130	KC_AB2	l(2)37Cc	-0.954316587621425	0.346188814339318	1	pseudobulk	DESeq2	LRT	0.46068696866510
130	KC_AB2	l(2)37Cd	-0.729379579623002	1	1	pseudobulk	DESeq2	LRT	0
130	KC_AB2	l(2)37Cg	0.195816521025938	0.911784622069932	1	pseudobulk	DESeq2	LRT	0.04010773677376
130	KC_AB2	l(2)41Ab	3.39861498430041	0.0517608448503605	1	pseudobulk	DESeq2	LRT	1.28599864364432
130	KC_AB2	l(2)dtl	1.38069928703004	0.566368801250645	1	pseudobulk	DESeq2	LRT	0.24690067801932
130	KC_AB2	l(2)efl	1.1674499696881	0.576329272969097	1	pseudobulk	DESeq2	LRT	0.23932932113341
130	KC_AB2	l(2)gd1	-0.37266060448874	0.830246527888934	1	pseudobulk	DESeq2	LRT	0.08079293195580
130	KC_AB2	l(2)k01209	0.127747059643972	0.860467324311413	1	pseudobulk	DESeq2	LRT	0.06526561707458
130	KC_AB2	l(2)k05819	-0.152830551181244	0.887268376737041	1	pseudobulk	DESeq2	LRT	0.05194499695885
130	KC_AB2	l(2)k09022	-2.5216736243906	0.267985186584202	1	pseudobulk	DESeq2	LRT	0.57188921180160
130	KC_AB2	l(2)k09848	-0.620563475958957	0.7478554969218	1	pseudobulk	DESeq2	LRT	0.12618230982555
130	KC_AB2	l(2)k09913	-0.314081555736992	0.44629998121813	1	pseudobulk	DESeq2	LRT	0.35037313143610
130	KC_AB2	l(2)k10201	1.50776466886541	0.515809803599324	1	pseudobulk	DESeq2	LRT	0.28751040781919
130	KC_AB2	l(2)k14505	-1.63837116939414	0.174896747756094	1	pseudobulk	DESeq2	LRT	0.75721826624867
130	KC_AB2	l(2)SH0834	0.209377103689203	0.784777134227168	1	pseudobulk	DESeq2	LRT	0.10525365932794
130	KC_AB2	l(3)02640	-0.816544044741322	0.435586616421664	1	pseudobulk	DESeq2	LRT	0.36092547258823
130	KC_AB2	l(3)04053	-0.219456293688254	0.891746992784615	1	pseudobulk	DESeq2	LRT	0.04975834654721
130	KC_AB2	l(3)05822	-0.416297631704092	0.707325261298839	1	pseudobulk	DESeq2	LRT	0.15038083131839
130	KC_AB2	l(3)07882	-0.427478535071269	0.813013586949621	1	pseudobulk	DESeq2	LRT	0.08990219648718
130	KC_AB2	l(3)72Ab	-0.247174672626038	0.830711838660583	1	pseudobulk	DESeq2	LRT	0.08054960026266
130	KC_AB2	l(3)72Dn	-1.19881828456548	0.535965909404053	1	pseudobulk	DESeq2	LRT	0.27086283312168
130	KC_AB2	l(3)72Dp	0.403294498144138	0.89479244414645	1	pseudobulk	DESeq2	LRT	0.04827769185229
131	KC_AB2	l(3)72Dr	-0.729379579623002	0.772694994316916	1	pseudobulk	DESeq2	LRT	0.1119919011982
131	KC_AB2	l(3)73Ah	-0.224963958910798	0.871574355471199	1	pseudobulk	DESeq2	LRT	0.05969555658226
131	KC_AB2	l(3)76BDm	-0.508782221997665	0.782587401008762	1	pseudobulk	DESeq2	LRT	0.10646714815066

131	KC_AB2	l(3)80Fg	0.0717565656483602	0.745217207318486	1	pseudobulk	DESeq2	LRT	0.12771712565289
131	KC_AB2	l(3)80Fj	0.396915944425138	0.202068660039581	1	pseudobulk	DESeq2	LRT	0.694501038425834
131	KC_AB2	l(3)87Df	-0.319114037816349	0.734898164701034	1	pseudobulk	DESeq2	LRT	0.133772837204934
131	KC_AB2	l(3)L1231	-0.0821949392611723	0.820256567770863	1	pseudobulk	DESeq2	LRT	0.086050283544505
131	KC_AB2	l(3)mbt	1.04324661842443	0.68096673636569	1	pseudobulk	DESeq2	LRT	0.166874101840012
131	KC_AB2	l(3)neo38	0.449031824352272	0.297706226554728	1	pseudobulk	DESeq2	LRT	0.526212081946892
131	KC_AB2	l(3)neo43	0.0614919633754454	0.877995864787549	1	pseudobulk	DESeq2	LRT	0.056507529542847
131	KC_AB2	l(3)psg2	-0.14015098440472	0.886030882518793	1	pseudobulk	DESeq2	LRT	0.052551140557918
131	KC_AB2	L2HGDH	-0.959227828465055	0.533870741157631	1	pseudobulk	DESeq2	LRT	0.272563880044853
131	KC_AB2	La	-0.719007585913654	0.58089524401527	1	pseudobulk	DESeq2	LRT	0.235902179224201
131	KC_AB2	lace	-0.59027993502747	0.690627657677954	1	pseudobulk	DESeq2	LRT	0.160756033376661
131	KC_AB2	Lam	0.218045969826031	0.8401307331839	1	pseudobulk	DESeq2	LRT	0.075653127887387
131	KC_AB2	LamC	1.61205113368698	0.631647504209	1	pseudobulk	DESeq2	LRT	0.199525215522471
131	KC_AB2	Lamp1	-0.0045522874943932	0.97324077440155	1	pseudobulk	DESeq2	LRT	0.011779704378861
131	KC_AB2	lap	-0.341438347908855	0.42494615507663	1	pseudobulk	DESeq2	LRT	0.371666095913367
131	KC_AB2	Lap1	-0.263403332390688	0.833333003477391	1	pseudobulk	DESeq2	LRT	0.079181417953197
131	KC_AB2	Lar	-0.215962097038762	0.486419004269235	1	pseudobulk	DESeq2	LRT	0.312989465626709
131	KC_AB2	lark	-0.276155430598845	0.707996310912984	1	pseudobulk	DESeq2	LRT	0.149969005240045
131	KC_AB2	larp	-0.116003820712829	0.795560255757462	1	pseudobulk	DESeq2	LRT	0.099326921294668
131	KC_AB2	Larp4B	0.0311512831268662	0.892238420431477	1	pseudobulk	DESeq2	LRT	0.049519079657362
131	KC_AB2	Larp7	-1.57685088282527	0.45256857946707	1	pseudobulk	DESeq2	LRT	0.344315601149633
131	KC_AB2	Las	0.474396347993209	0.808657101852946	1	pseudobulk	DESeq2	LRT	0.092235595003270
131	KC_AB2	lawc	0.776612938753513	0.209307890937514	1	pseudobulk	DESeq2	LRT	0.679214398388407
131	KC_AB2	lbk	0.12117711987238	0.780487359414607	1	pseudobulk	DESeq2	LRT	0.107634126273491
131	KC_AB2	lbm	-0.819230343735736	0.460894533184783	1	pseudobulk	DESeq2	LRT	0.336398443148561
131	KC_AB2	LBR	2.16811029366837	0.310827889047297	1	pseudobulk	DESeq2	LRT	0.507480021029577
131	KC_AB2	Lcch3	-0.23989524546028	0.779628197894937	1	pseudobulk	DESeq2	LRT	0.108112461539143
131	KC_AB2	Ldh	1.10706867860301	1	1	pseudobulk	DESeq2	LRT	0
131	KC_AB2	Leash	0.145321756433347	1	1	pseudobulk	DESeq2	LRT	0
131	KC_AB2	Lerp	-0.226523240170566	0.758982164640799	1	pseudobulk	DESeq2	LRT	0.119768429491993
131	KC_AB2	Letm1	2.38648468442401	0.0683881928061303	1	pseudobulk	DESeq2	LRT	1.16501887257484
131	KC_AB2	LeuRS	1.61205113368698	0.631647504209	1	pseudobulk	DESeq2	LRT	0.199525215522471
131	KC_AB2	LeuRS-m	1.10706867860301	1	1	pseudobulk	DESeq2	LRT	0
131	KC_AB2	levy	-0.446185285055066	0.183168781312128	1	pseudobulk	DESeq2	LRT	0.737148544091797
131	KC_AB2	Lgr1	-1.87429678068092	0.0032132120097353	1	pseudobulk	DESeq2	LRT	2.49306061848194
131	KC_AB2	lgs	-0.453159580826885	0.443496966545615	1	pseudobulk	DESeq2	LRT	0.353109346332832
131	KC_AB2	Lhr	1.50776466886541	0.515809803599324	1	pseudobulk	DESeq2	LRT	0.287510407819198
131	KC_AB2	lic	-1.44016316499563	0.46417400647559	1	pseudobulk	DESeq2	LRT	0.333319183484357
131	KC_AB2	lid	0.617539408722623	0.246912989064738	1	pseudobulk	DESeq2	LRT	0.607456063041898
131	KC_AB2	lig	-0.536756561725755	0.458187036496848	1	pseudobulk	DESeq2	LRT	0.338957202483001
131	KC_AB2	lili	0.330550203569517	0.80327521305228	1	pseudobulk	DESeq2	LRT	0.095135634008465
131	KC_AB2	lilli	0.479636278990933	0.197971431429073	1	pseudobulk	DESeq2	LRT	0.703397476747985
131	KC_AB2	LIMK1	-0.835755539813264	0.398119122931769	1	pseudobulk	DESeq2	LRT	0.3999869613652
131	KC_AB2	lin	1.68881465104187	0.456529340335333	1	pseudobulk	DESeq2	LRT	0.340531305892531
131	KC_AB2	lin-52	-0.338631457797787	0.765516668966024	1	pseudobulk	DESeq2	LRT	0.116045348190872
131	KC_AB2	link	-0.729379579623002	0.823527397767669	1	pseudobulk	DESeq2	LRT	0.084321947797948
131	KC_AB2	Lint-1	1.35476372790059	0.34371679329243	1	pseudobulk	DESeq2	LRT	0.463799248613236
131	KC_AB2	Liprin-alpha	-0.35682771191779	0.713993055083679	1	pseudobulk	DESeq2	LRT	0.146306012528453
131	KC_AB2	Liprin-beta	3.5498422368588	0.0335595002218962	1	pseudobulk	DESeq2	LRT	1.47418451543836
131	KC_AB2	Liprin-gamma	0.180643917766967	0.630707830102831	1	pseudobulk	DESeq2	LRT	0.200171777300094
131	KC_AB2	Lis-1	-0.134924989642181	0.620502365631952	1	pseudobulk	DESeq2	LRT	0.207256558437702
131	KC_AB2	List	1.10706867860301	1	1	pseudobulk	DESeq2	LRT	0

131	KC_AB2	Lk6	0.0382929735518944	0.891143306493985	1	pseudobulk	DESeq2	LRT	0.050052450603926
131	KC_AB2	Lkb1	-2.0236867780796	0.291626479478556	1	pseudobulk	DESeq2	LRT	0.535173044931177
131	KC_AB2	LKRSDH	1.10706867860301	1	1	pseudobulk	DESeq2	LRT	0
131	KC_AB2	Lmpt	-0.761610561539828	0.197545735009504	1	pseudobulk	DESeq2	LRT	0.704332342250987
131	KC_AB2	lncRNA:alphagamr	0.121401859498698	0.797102245300379	1	pseudobulk	DESeq2	LRT	0.098485967535232
131	KC_AB2	lncRNA:cherub	2.23683931246812	0.0955950084148564	1	pseudobulk	DESeq2	LRT	1.0195647842352
131	KC_AB2	lncRNA:CR30055	0.586539677293533	0.482876335456888	1	pseudobulk	DESeq2	LRT	0.316164077747966
131	KC_AB2	lncRNA:CR30121	-0.729379579623002	0.823527397767669	1	pseudobulk	DESeq2	LRT	0.084321947797948
131	KC_AB2	lncRNA:CR31451	-0.0410759697305986	0.868724655239055	1	pseudobulk	DESeq2	LRT	0.061117852618101
131	KC_AB2	lncRNA:CR32218	1.56714941850574	0.301876446160354	1	pseudobulk	DESeq2	LRT	0.520170771381159
131	KC_AB2	lncRNA:CR32582	-0.427692090780194	0.83957454067488	1	pseudobulk	DESeq2	LRT	0.075940739470360
131	KC_AB2	lncRNA:CR32773	0.39439088612385	0.813541888331052	1	pseudobulk	DESeq2	LRT	0.089620080830767
131	KC_AB2	lncRNA:CR33963	-0.729379579623002	1	1	pseudobulk	DESeq2	LRT	0
131	KC_AB2	lncRNA:CR34335	0.254596929989986	0.241192772754054	1	pseudobulk	DESeq2	LRT	0.61763570979913
131	KC_AB2	lncRNA:CR40465	1.38446765109058	0.642794900879723	1	pseudobulk	DESeq2	LRT	0.191927577037166
131	KC_AB2	lncRNA:CR40469	0.66954517615027	1	1	pseudobulk	DESeq2	LRT	0
131	KC_AB2	lncRNA:CR42549	-0.778523756993934	0.689563908531076	1	pseudobulk	DESeq2	LRT	0.161425477368935
131	KC_AB2	lncRNA:CR42861	0.047624341136464	0.930860339293037	1	pseudobulk	DESeq2	LRT	0.031115473072596
131	KC_AB2	lncRNA:CR42862	0.219452699623042	0.351172511248181	1	pseudobulk	DESeq2	LRT	0.454479486730802
131	KC_AB2	lncRNA:CR43146	-1.86716056129969	0.477680380390446	1	pseudobulk	DESeq2	LRT	0.320862595985116
131	KC_AB2	lncRNA:CR43148	3.5554947126272	0.0906963498444766	1	pseudobulk	DESeq2	LRT	1.04241019115712
131	KC_AB2	lncRNA:CR43174	1.10706867860301	1	1	pseudobulk	DESeq2	LRT	0
131	KC_AB2	lncRNA:CR43241	-1.53462310097022	0.464407033597658	1	pseudobulk	DESeq2	LRT	0.33310121135052
131	KC_AB2	lncRNA:CR43242	2.93902422553353	0.147531775884011	1	pseudobulk	DESeq2	LRT	0.831114429822618
131	KC_AB2	lncRNA:CR43264	0.840382761514741	0.456314517756448	1	pseudobulk	DESeq2	LRT	0.34073571382384
131	KC_AB2	lncRNA:CR43285	1.38446765109058	0.642794900879723	1	pseudobulk	DESeq2	LRT	0.191927577037166
131	KC_AB2	lncRNA:CR43309	-0.729379579623002	1	1	pseudobulk	DESeq2	LRT	0
131	KC_AB2	lncRNA:CR43334	0.0795822821047814	0.956193137189769	1	pseudobulk	DESeq2	LRT	0.019454377656867
131	KC_AB2	lncRNA:CR43363	-0.729379579623002	1	1	pseudobulk	DESeq2	LRT	0
131	KC_AB2	lncRNA:CR43399	-0.729379579623002	0.823527397767669	1	pseudobulk	DESeq2	LRT	0.084321947797948
131	KC_AB2	lncRNA:CR43410	-0.729379579623002	1	1	pseudobulk	DESeq2	LRT	0
131	KC_AB2	lncRNA:CR43424	1.38446765109058	0.642794900879723	1	pseudobulk	DESeq2	LRT	0.191927577037166
131	KC_AB2	lncRNA:CR43459	0.107149955670703	0.925145349866943	1	pseudobulk	DESeq2	LRT	0.033790029762445
131	KC_AB2	lncRNA:CR43492	1.02648689492346	0.73959154128541	1	pseudobulk	DESeq2	LRT	0.131008064514051
131	KC_AB2	lncRNA:CR43493	1.38446765109058	0.642794900879723	1	pseudobulk	DESeq2	LRT	0.191927577037166
131	KC_AB2	lncRNA:CR43496	0.145321756433347	1	1	pseudobulk	DESeq2	LRT	0
131	KC_AB2	lncRNA:CR43604	-0.729379579623002	0.772694994316916	1	pseudobulk	DESeq2	LRT	0.1119919011982
131	KC_AB2	lncRNA:CR43605	-0.729379579623002	0.823527397767669	1	pseudobulk	DESeq2	LRT	0.084321947797948
131	KC_AB2	lncRNA:CR43611	-0.729379579623002	0.772694994316916	1	pseudobulk	DESeq2	LRT	0.1119919011982
131	KC_AB2	lncRNA:CR43651	-0.0994073884600092	0.905106171670297	1	pseudobulk	DESeq2	LRT	0.043300473760507
131	KC_AB2	lncRNA:CR43684	0.147593560254927	1	1	pseudobulk	DESeq2	LRT	0
131	KC_AB2	lncRNA:CR43685	0.325306630788908	0.792541889867698	1	pseudobulk	DESeq2	LRT	0.100977773832487
131	KC_AB2	lncRNA:CR43792	1.38069928703004	0.566368801250645	1	pseudobulk	DESeq2	LRT	0.246900678019328
131	KC_AB2	lncRNA:CR43836	-1.37442195900011	0.0920239925955804	1	pseudobulk	DESeq2	LRT	1.03609892816084
131	KC_AB2	lncRNA:CR43837	-0.729379579623002	1	1	pseudobulk	DESeq2	LRT	0
132	KC_AB2	lncRNA:CR43849	2.01814786626755	0.401718761445944	1	pseudobulk	DESeq2	LRT	0.396077884966921
132	KC_AB2	lncRNA:CR43855	-0.729379579623002	0.823527397767669	1	pseudobulk	DESeq2	LRT	0.084321947797948
132	KC_AB2	lncRNA:CR43870	1.38069928703004	0.566368801250645	1	pseudobulk	DESeq2	LRT	0.246900678019328
132	KC_AB2	lncRNA:CR43878	2.12184972583613	0.126686206542117	1	pseudobulk	DESeq2	LRT	0.897270668058536
132	KC_AB2	lncRNA:CR43900	0.396659246788372	0.915482501218565	1	pseudobulk	DESeq2	LRT	0.038349952505755
132	KC_AB2	lncRNA:CR43943	-0.729379579623002	1	1	pseudobulk	DESeq2	LRT	0
132	KC_AB2	lncRNA:CR43956	2.01814786626755	0.401718761445944	1	pseudobulk	DESeq2	LRT	0.396077884966921

132	KC_AB2	lncRNA:CR43958	-0.729379579623002	1	1	pseudobulk	DESeq2	LRT	0
132	KC_AB2	lncRNA:CR43973	1.10706867860301	1	1	pseudobulk	DESeq2	LRT	0
132	KC_AB2	lncRNA:CR43974	1.38446765109058	0.642794900879723	1	pseudobulk	DESeq2	LRT	0.191927577037166
132	KC_AB2	lncRNA:CR43995	-1.53674535567238	0.381797606203516	1	pseudobulk	DESeq2	LRT	0.418166798875466
132	KC_AB2	lncRNA:CR44017	1.10706867860301	1	1	pseudobulk	DESeq2	LRT	0
132	KC_AB2	lncRNA:CR44023	1.50776466886541	0.515809803599324	1	pseudobulk	DESeq2	LRT	0.287510407819198
132	KC_AB2	lncRNA:CR44024	0.336833380727331	0.414491170299054	1	pseudobulk	DESeq2	LRT	0.382484716576293
132	KC_AB2	lncRNA:CR44092	0.80215911270901	0.68127594793042	1	pseudobulk	DESeq2	LRT	0.166676943315826
132	KC_AB2	lncRNA:CR44125	-0.729379579623002	0.823527397767669	1	pseudobulk	DESeq2	LRT	0.084321947797948
132	KC_AB2	lncRNA:CR44129	1.10706867860301	1	1	pseudobulk	DESeq2	LRT	0
132	KC_AB2	lncRNA:CR44171	1.38069928703004	0.566368801250645	1	pseudobulk	DESeq2	LRT	0.246900678019328
132	KC_AB2	lncRNA:CR44178	1.61205113368698	0.631647504209	1	pseudobulk	DESeq2	LRT	0.199525215522471
132	KC_AB2	lncRNA:CR44187	-0.274459162011074	0.891545093103103	1	pseudobulk	DESeq2	LRT	0.049856685925997
132	KC_AB2	lncRNA:CR44188	-2.39970914040097	0.301766661516198	1	pseudobulk	DESeq2	LRT	0.520328741761584
132	KC_AB2	lncRNA:CR44206	-0.222764485729612	0.908104953152855	1	pseudobulk	DESeq2	LRT	0.041863955508560
132	KC_AB2	lncRNA:CR44260	1.61205113368698	0.631647504209	1	pseudobulk	DESeq2	LRT	0.199525215522471
132	KC_AB2	lncRNA:CR44272	-0.729379579623002	0.772694994316916	1	pseudobulk	DESeq2	LRT	0.1119919011982
132	KC_AB2	lncRNA:CR44294	0.388447834546628	0.84487503289525	1	pseudobulk	DESeq2	LRT	0.073207523639173
132	KC_AB2	lncRNA:CR44299	1.38069928703004	0.566368801250645	1	pseudobulk	DESeq2	LRT	0.246900678019328
132	KC_AB2	lncRNA:CR44300	1.99339641594643	0.465071165814147	1	pseudobulk	DESeq2	LRT	0.332480585700252
132	KC_AB2	lncRNA:CR44317	1.10706867860301	1	1	pseudobulk	DESeq2	LRT	0
132	KC_AB2	lncRNA:CR44330	-0.729379579623002	1	1	pseudobulk	DESeq2	LRT	0
132	KC_AB2	lncRNA:CR44332	1.10706867860301	1	1	pseudobulk	DESeq2	LRT	0
132	KC_AB2	lncRNA:CR44347	0.910547638448857	0.404031966444549	1	pseudobulk	DESeq2	LRT	0.393584272757556
132	KC_AB2	lncRNA:CR44357	-1.23843665632218	0.484129370698825	1	pseudobulk	DESeq2	LRT	0.315038569196538
132	KC_AB2	lncRNA:CR44366	0.39439088612385	0.813541888331052	1	pseudobulk	DESeq2	LRT	0.089620080830767
132	KC_AB2	lncRNA:CR44384	1.64462134930074	0.494740966888383	1	pseudobulk	DESeq2	LRT	0.305622126512861
132	KC_AB2	lncRNA:CR44392	-0.729379579623002	0.772694994316916	1	pseudobulk	DESeq2	LRT	0.1119919011982
132	KC_AB2	lncRNA:CR44417	1.88121523815628	0.0239038446073908	1	pseudobulk	DESeq2	LRT	1.62153224308977
132	KC_AB2	lncRNA:CR44444	1.38446765109058	0.642794900879723	1	pseudobulk	DESeq2	LRT	0.191927577037166
132	KC_AB2	lncRNA:CR44506	1.04232157414887	0.700586543363791	1	pseudobulk	DESeq2	LRT	0.154538208723974
132	KC_AB2	lncRNA:CR44589	0.221486168185573	0.903697778547821	1	pseudobulk	DESeq2	LRT	0.0439767852922
132	KC_AB2	lncRNA:CR44595	0.575162595042011	0.797021781108777	1	pseudobulk	DESeq2	LRT	0.098529809988961
132	KC_AB2	lncRNA:CR44596	1.38446765109058	0.642794900879723	1	pseudobulk	DESeq2	LRT	0.191927577037166
132	KC_AB2	lncRNA:CR44597	-0.88830345940619	0.681892771765791	1	pseudobulk	DESeq2	LRT	0.166283913161275
132	KC_AB2	lncRNA:CR44598	-1.50967690248522	0.449786644124088	1	pseudobulk	DESeq2	LRT	0.346993444563629
132	KC_AB2	lncRNA:CR44662	-0.729379579623002	0.772694994316916	1	pseudobulk	DESeq2	LRT	0.1119919011982
132	KC_AB2	lncRNA:CR44663	4.04707686358712	0.252920616416493	1	pseudobulk	DESeq2	LRT	0.597015768397822
132	KC_AB2	lncRNA:CR44708	-2.12405037015269	0.265879278548733	1	pseudobulk	DESeq2	LRT	0.575315508334519
132	KC_AB2	lncRNA:CR44727	-0.729379579623002	1	1	pseudobulk	DESeq2	LRT	0
132	KC_AB2	lncRNA:CR44728	-1.86716056129969	0.477680380390446	1	pseudobulk	DESeq2	LRT	0.320862595985116
132	KC_AB2	lncRNA:CR44761	1.38069928703004	0.566368801250645	1	pseudobulk	DESeq2	LRT	0.246900678019328
132	KC_AB2	lncRNA:CR44779	0.393976029558784	0.939642609940555	1	pseudobulk	DESeq2	LRT	0.027037297511213
132	KC_AB2	lncRNA:CR44811	0.145321756433347	1	1	pseudobulk	DESeq2	LRT	0
132	KC_AB2	lncRNA:CR44816	-1.28292259562998	0.530493603495545	1	pseudobulk	DESeq2	LRT	0.275319848300503
132	KC_AB2	lncRNA:CR44832	2.63571935317989	0.482917648654329	1	pseudobulk	DESeq2	LRT	0.316126922632104
132	KC_AB2	lncRNA:CR44886	-0.169643420358751	0.832294701541504	1	pseudobulk	DESeq2	LRT	0.079722870113671
132	KC_AB2	lncRNA:CR44901	0.146930209264112	1	1	pseudobulk	DESeq2	LRT	0
132	KC_AB2	lncRNA:CR44914	-0.729379579623002	0.772694994316916	1	pseudobulk	DESeq2	LRT	0.1119919011982
132	KC_AB2	lncRNA:CR44922	0.427594942590856	0.64087504254188	1	pseudobulk	DESeq2	LRT	0.193226640722673
132	KC_AB2	lncRNA:CR44951	1.38446765109058	0.642794900879723	1	pseudobulk	DESeq2	LRT	0.191927577037166
132	KC_AB2	lncRNA:CR44978	-0.83320718254958	0.715059964627045	1	pseudobulk	DESeq2	LRT	0.14565753691968

132	KC_AB2	lncRNA:CR44985	2.01814786626755	0.401718761445944	1	pseudobulk	DESeq2	LRT	0.396077884966921
132	KC_AB2	lncRNA:CR44986	-0.778523756993934	0.689563908531076	1	pseudobulk	DESeq2	LRT	0.161425477368935
132	KC_AB2	lncRNA:CR44994	-0.729379579623002	1	1	pseudobulk	DESeq2	LRT	0
132	KC_AB2	lncRNA:CR44997	0.0504936981435261	0.9594252810246	1	pseudobulk	DESeq2	LRT	0.0179888419835
132	KC_AB2	lncRNA:CR44999	0.90292058629339	0.553675136302447	1	pseudobulk	DESeq2	LRT	0.256744978762038
132	KC_AB2	lncRNA:CR45020	1.99339641594643	0.465071165814147	1	pseudobulk	DESeq2	LRT	0.332480585700252
132	KC_AB2	lncRNA:CR45029	-0.729379579623002	1	1	pseudobulk	DESeq2	LRT	0
132	KC_AB2	lncRNA:CR45035	-0.672424505335587	0.790771522020703	1	pseudobulk	DESeq2	LRT	0.101948979282445
132	KC_AB2	lncRNA:CR45054	0.0321990279533526	0.924134268818545	1	pseudobulk	DESeq2	LRT	0.034264924915556
132	KC_AB2	lncRNA:CR45102	-0.670217415789661	0.486031649777666	1	pseudobulk	DESeq2	LRT	0.313335449099342
132	KC_AB2	lncRNA:CR45156	1.38446765109058	0.642794900879723	1	pseudobulk	DESeq2	LRT	0.191927577037166
132	KC_AB2	lncRNA:CR45169	0.396659246788372	0.915482501218565	1	pseudobulk	DESeq2	LRT	0.038349952505755
132	KC_AB2	lncRNA:CR45178	-0.729379579623002	0.772694994316916	1	pseudobulk	DESeq2	LRT	0.1119919011982
132	KC_AB2	lncRNA:CR45188	-1.6052707068109	0.52265609184397	1	pseudobulk	DESeq2	LRT	0.281783983297782
132	KC_AB2	lncRNA:CR45196	1.10706867860301	1	1	pseudobulk	DESeq2	LRT	0
132	KC_AB2	lncRNA:CR45199	1.10706867860301	1	1	pseudobulk	DESeq2	LRT	0
132	KC_AB2	lncRNA:CR45200	-0.729379579623002	1	1	pseudobulk	DESeq2	LRT	0
132	KC_AB2	lncRNA:CR45240	-1.09051709252325	0.659544943795745	1	pseudobulk	DESeq2	LRT	0.180755604700739
132	KC_AB2	lncRNA:CR45308	1.10706867860301	1	1	pseudobulk	DESeq2	LRT	0
132	KC_AB2	lncRNA:CR45312	0.709584622582653	0.756945022027757	1	pseudobulk	DESeq2	LRT	0.120935662768153
132	KC_AB2	lncRNA:CR45358	2.33862076287154	0.37727071943742	1	pseudobulk	DESeq2	LRT	0.423346899755834
132	KC_AB2	lncRNA:CR45387	-0.729379579623002	1	1	pseudobulk	DESeq2	LRT	0
132	KC_AB2	lncRNA:CR45388	0.663884212739971	0.687096402397815	1	pseudobulk	DESeq2	LRT	0.16298232539719
132	KC_AB2	lncRNA:CR45409	-0.2987810728647	0.821683175992749	1	pseudobulk	DESeq2	LRT	0.085295605138233
132	KC_AB2	lncRNA:CR45418	0.388447834546628	0.84487503289525	1	pseudobulk	DESeq2	LRT	0.073207523639173
132	KC_AB2	lncRNA:CR45464	-0.729379579623002	1	1	pseudobulk	DESeq2	LRT	0
132	KC_AB2	lncRNA:CR45473	0.854931530788726	0.576724343514581	1	pseudobulk	DESeq2	LRT	0.239031716647153
132	KC_AB2	lncRNA:CR45522	-1.52244591594958	0.547176617578731	1	pseudobulk	DESeq2	LRT	0.261872469536296
132	KC_AB2	lncRNA:CR45527	0.39439088612385	0.813541888331052	1	pseudobulk	DESeq2	LRT	0.089620080830767
132	KC_AB2	lncRNA:CR45530	-0.734071467759236	0.785001998672014	1	pseudobulk	DESeq2	LRT	0.105129237508095
132	KC_AB2	lncRNA:CR45531	1.38069928703004	0.566368801250645	1	pseudobulk	DESeq2	LRT	0.246900678019328
132	KC_AB2	lncRNA:CR45556	-0.729379579623002	1	1	pseudobulk	DESeq2	LRT	0
132	KC_AB2	lncRNA:CR45562	1.38446765109058	0.642794900879723	1	pseudobulk	DESeq2	LRT	0.191927577037166
132	KC_AB2	lncRNA:CR45622	-0.729379579623002	1	1	pseudobulk	DESeq2	LRT	0
132	KC_AB2	lncRNA:CR45643	1.38446765109058	0.642794900879723	1	pseudobulk	DESeq2	LRT	0.191927577037166
132	KC_AB2	lncRNA:CR45660	1.03155030078665	0.428701675083744	1	pseudobulk	DESeq2	LRT	0.367844819549303
132	KC_AB2	lncRNA:CR45668	0.91162481720165	0.679563587018482	1	pseudobulk	DESeq2	LRT	0.167769899933384
132	KC_AB2	lncRNA:CR45674	-0.729379579623002	0.772694994316916	1	pseudobulk	DESeq2	LRT	0.1119919011982
132	KC_AB2	lncRNA:CR45684	0.822509830775414	0.32586820500732	1	pseudobulk	DESeq2	LRT	0.486958011622902
132	KC_AB2	lncRNA:CR45758	-2.20080260477502	0.0131070421286647	1	pseudobulk	DESeq2	LRT	1.88249530465887
132	KC_AB2	lncRNA:CR45819	1.38446765109058	0.642794900879723	1	pseudobulk	DESeq2	LRT	0.191927577037166
133	KC_AB2	lncRNA:CR45821	-0.647621034217714	0.754702560287896	1	pseudobulk	DESeq2	LRT	0.122224176685402
133	KC_AB2	lncRNA:CR45825	-0.50491169497659	0.835055051214058	1	pseudobulk	DESeq2	LRT	0.078284892599768
133	KC_AB2	lncRNA:CR45910	-0.729379579623002	1	1	pseudobulk	DESeq2	LRT	0
133	KC_AB2	lncRNA:CR45911	-2.1751167112989	0.359363815234217	1	pseudobulk	DESeq2	LRT	0.444465654648455
133	KC_AB2	lncRNA:CR45916	1.10706867860301	1	1	pseudobulk	DESeq2	LRT	0
133	KC_AB2	lncRNA:CR45918	-0.729379579623002	1	1	pseudobulk	DESeq2	LRT	0
133	KC_AB2	lncRNA:CR45919	0.28281488118536	0.84926261051605	1	pseudobulk	DESeq2	LRT	0.070957995665619
133	KC_AB2	lncRNA:CR45938	1.10706867860301	1	1	pseudobulk	DESeq2	LRT	0
133	KC_AB2	lncRNA:CR45939	1.10706867860301	1	1	pseudobulk	DESeq2	LRT	0
133	KC_AB2	lncRNA:CR45961	1.10706867860301	1	1	pseudobulk	DESeq2	LRT	0
133	KC_AB2	lncRNA:CR45962	1.10706867860301	1	1	pseudobulk	DESeq2	LRT	0

133	KC_AB2	lncRNA:CR45979	-0.367664335173966	0.849318376240959	1	pseudobulk	DESeq2	LRT	0.070929479219474
133	KC_AB2	lncRNA:CR45993	1.10706867860301	1	1	pseudobulk	DESeq2	LRT	0
133	KC_AB2	lncRNA:CR45994	1.37686412499759	0.622972562188606	1	pseudobulk	DESeq2	LRT	0.205531080712408
133	KC_AB2	lncRNA:CR46003	2.7605424445074	0.237810191029118	1	pseudobulk	DESeq2	LRT	0.623769538224996
133	KC_AB2	lncRNA:CR46004	0.41283485533865	0.796318858951703	1	pseudobulk	DESeq2	LRT	0.098912998901807
133	KC_AB2	lncRNA:CR46006	0.161907671210261	0.606376958536672	1	pseudobulk	DESeq2	LRT	0.217257309636059
133	KC_AB2	lncRNA:CR46008	-0.729379579623002	0.772694994316916	1	pseudobulk	DESeq2	LRT	0.1119919011982
133	KC_AB2	lncRNA:CR46016	-0.729379579623002	1	1	pseudobulk	DESeq2	LRT	0
133	KC_AB2	lncRNA:CR46019	1.10706867860301	1	1	pseudobulk	DESeq2	LRT	0
133	KC_AB2	lncRNA:CR46024	-0.729379579623002	1	1	pseudobulk	DESeq2	LRT	0
133	KC_AB2	lncRNA:CR46064	-0.225323113990486	0.794168295191615	1	pseudobulk	DESeq2	LRT	0.100087454842238
133	KC_AB2	lncRNA:CR46084	0.393976029558784	0.939642609940555	1	pseudobulk	DESeq2	LRT	0.027037297511213
133	KC_AB2	lncRNA:CR46204	1.10706867860301	1	1	pseudobulk	DESeq2	LRT	0
133	KC_AB2	lncRNA:CR46208	-0.714420715613035	0.400829846084618	1	pseudobulk	DESeq2	LRT	0.397039948050829
133	KC_AB2	lncRNA:CR46268	1.31526223725409	0.425509885688155	1	pseudobulk	DESeq2	LRT	0.371090345685285
133	KC_AB2	lncRNA:CR46322	1.38069928703004	0.566368801250645	1	pseudobulk	DESeq2	LRT	0.246900678019328
133	KC_AB2	lncRNA:CR46348	1.95432904991983	0.509212778150152	1	pseudobulk	DESeq2	LRT	0.293100706726022
133	KC_AB2	lncRNA:CR46391	1.10706867860301	1	1	pseudobulk	DESeq2	LRT	0
133	KC_AB2	lncRNA:CR46414	1.38069928703004	0.566368801250645	1	pseudobulk	DESeq2	LRT	0.246900678019328
133	KC_AB2	lncRNA:flam	0.145321756433347	1	1	pseudobulk	DESeq2	LRT	0
133	KC_AB2	lncRNA:Hsromega	0.497911818782636	0.0123400095298695	1	pseudobulk	DESeq2	LRT	1.90868450490828
133	KC_AB2	lncRNA:let7C	0.469503957303859	0.556443859935749	1	pseudobulk	DESeq2	LRT	0.254578645435296
133	KC_AB2	lncRNA:noe	0.0316054479357948	0.884236294382764	1	pseudobulk	DESeq2	LRT	0.053431663003249
133	KC_AB2	lncRNA:roX1	3.13194326631394	0.164331439568934	1	pseudobulk	DESeq2	LRT	0.784279340246697
133	KC_AB2	Lnk	-0.395972366006324	0.652107803285732	1	pseudobulk	DESeq2	LRT	0.185680602884616
133	KC_AB2	Lnpk	-1.20836194102535	0.409316996491066	1	pseudobulk	DESeq2	LRT	0.387940221323396
133	KC_AB2	loh	-0.729379579623002	1	1	pseudobulk	DESeq2	LRT	0
133	KC_AB2	loj	-0.473795964521473	0.80663503472328	1	pseudobulk	DESeq2	LRT	0.093322919129089
133	KC_AB2	lola	-0.0109863027130108	0.957298785746701	1	pseudobulk	DESeq2	LRT	0.018952491952639
133	KC_AB2	lolal	0.118436075056897	0.732256946284385	1	pseudobulk	DESeq2	LRT	0.135336499853029
133	KC_AB2	Lon	-1.18297446092115	0.509878010701894	1	pseudobulk	DESeq2	LRT	0.292533717267183
133	KC_AB2	loqs	-0.101890349325339	0.81890671934705	1	pseudobulk	DESeq2	LRT	0.086765565370291
133	KC_AB2	lost	-0.620837149959983	0.474217643578602	1	pseudobulk	DESeq2	LRT	0.324022291846059
133	KC_AB2	lovit	0.735561494123178	0.478698592208263	1	pseudobulk	DESeq2	LRT	0.319937849721064
133	KC_AB2	LPCAT	-1.49551927482761	0.116608667807463	1	pseudobulk	DESeq2	LRT	0.933269166206165
133	KC_AB2	Lpin	-0.309716536511236	0.692057401973206	1	pseudobulk	DESeq2	LRT	0.159857881950301
133	KC_AB2	LpR1	2.01814786626755	0.401718761445944	1	pseudobulk	DESeq2	LRT	0.396077884966921
133	KC_AB2	LpR2	-0.729379579623002	0.823527397767669	1	pseudobulk	DESeq2	LRT	0.084321947797948
133	KC_AB2	Lpt	1.80483863294315	0.422281715013702	1	pseudobulk	DESeq2	LRT	0.374397723312422
133	KC_AB2	lqf	-0.453170146874691	0.313017680416878	1	pseudobulk	DESeq2	LRT	0.504431131173589
133	KC_AB2	lqfR	0.869058021798837	0.381505118278302	1	pseudobulk	DESeq2	LRT	0.418499631168831
133	KC_AB2	Lrch	-0.678034526981037	0.480338462552408	1	pseudobulk	DESeq2	LRT	0.318452636335277
133	KC_AB2	LRP1	-1.04494347046261	0.346652165983512	1	pseudobulk	DESeq2	LRT	0.460106081858551
133	KC_AB2	Lrp4	0.342029236486381	0.508439397084394	1	pseudobulk	DESeq2	LRT	0.293760804944455
133	KC_AB2	Lrpprc2	-0.729379579623002	0.823527397767669	1	pseudobulk	DESeq2	LRT	0.084321947797948
133	KC_AB2	LRR	-0.481724437512295	0.609935799471602	1	pseudobulk	DESeq2	LRT	0.214715875485238
133	KC_AB2	Lrr47	0.403294498144138	0.89479244414645	1	pseudobulk	DESeq2	LRT	0.048277691852293
133	KC_AB2	Lrrk	0.194591674821004	0.857623365754983	1	pseudobulk	DESeq2	LRT	0.066703395235005
133	KC_AB2	Lrt	1.61205113368698	0.631647504209	1	pseudobulk	DESeq2	LRT	0.199525215522471
133	KC_AB2	LSm1	-1.15253484707392	0.661144484795202	1	pseudobulk	DESeq2	LRT	0.179703620556297
133	KC_AB2	Lsm10	-0.729379579623002	1	1	pseudobulk	DESeq2	LRT	0
133	KC_AB2	Lsm11	-1.15253484707392	0.661144484795202	1	pseudobulk	DESeq2	LRT	0.179703620556297

133	KC_AB2	Lsm12a	-0.314237600383047	0.683924723525899	1	pseudobulk	DESeq2	LRT	0.164991696461743
133	KC_AB2	LSm3	0.533662367061994	0.766762619563229	1	pseudobulk	DESeq2	LRT	0.115339067563836
133	KC_AB2	LSm7	0.843700179273233	0.692173184066514	1	pseudobulk	DESeq2	LRT	0.159785230002206
133	KC_AB2	lsn	-1.0574711251031	0.488047020435937	1	pseudobulk	DESeq2	LRT	0.311538334283918
133	KC_AB2	Lst8	2.35046557194152	0.149362374283698	1	pseudobulk	DESeq2	LRT	0.825758791402624
133	KC_AB2	lt	-0.171662026399962	0.829124289104619	1	pseudobulk	DESeq2	LRT	0.081380362059704
133	KC_AB2	Ltn1	-1.10406512773501	0.518005793689334	1	pseudobulk	DESeq2	LRT	0.285665382816086
133	KC_AB2	LTV1	-0.885513408133007	0.462207838926153	1	pseudobulk	DESeq2	LRT	0.335162693271658
133	KC_AB2	luna	0.147593560254927	1	1	pseudobulk	DESeq2	LRT	0
133	KC_AB2	lute	0.0205558847982094	0.969219929444718	1	pseudobulk	DESeq2	LRT	0.013577664325202
133	KC_AB2	lva	-0.135770176049446	0.92698095239034	1	pseudobulk	DESeq2	LRT	0.032929189649200
133	KC_AB2	lwr	-0.485817204168525	0.357749743310252	1	pseudobulk	DESeq2	LRT	0.446420669179593
133	KC_AB2	LysRS	1.12866861538443	0.0254808202270766	1	pseudobulk	DESeq2	LRT	1.59378659618128
133	KC_AB2	Lztr1	-0.310814887396079	0.73999912290202	1	pseudobulk	DESeq2	LRT	0.130768795024481
133	KC_AB2	m-cup	0.360971210600822	0.775409366427584	1	pseudobulk	DESeq2	LRT	0.110468957309662
133	KC_AB2	M1BP	-0.737994360320996	0.470928832151873	1	pseudobulk	DESeq2	LRT	0.327044719493273
133	KC_AB2	M6	0.41206906355439	0.776546966506845	1	pseudobulk	DESeq2	LRT	0.109832272481568
133	KC_AB2	mAChR-A	0.180123306792636	0.684037822182896	1	pseudobulk	DESeq2	LRT	0.16491988437462
133	KC_AB2	mAChR-B	0.0378032758846298	0.933871460492323	1	pseudobulk	DESeq2	LRT	0.029712896618477
133	KC_AB2	mAcon1	0.367577715381962	0.587889817029313	1	pseudobulk	DESeq2	LRT	0.230704062257387
133	KC_AB2	Mad	0.0337799730354194	0.960348920965181	1	pseudobulk	DESeq2	LRT	0.017570947254010
133	KC_AB2	Mad1	-1.00833870937463	0.651152668420623	1	pseudobulk	DESeq2	LRT	0.186317175365195
133	KC_AB2	Madm	-0.173497269645805	0.874321488012558	1	pseudobulk	DESeq2	LRT	0.058328847917677
133	KC_AB2	mael	-0.729379579623002	1	1	pseudobulk	DESeq2	LRT	0
133	KC_AB2	maf-S	0.419885457820552	0.522358426047886	1	pseudobulk	DESeq2	LRT	0.282031395393927
133	KC_AB2	Maf1	0.469589689235337	0.269568796612768	1	pseudobulk	DESeq2	LRT	0.569330380101005
133	KC_AB2	Magi	-0.589888465795039	0.650780283169212	1	pseudobulk	DESeq2	LRT	0.186565613449571
133	KC_AB2	mago	-0.653920496003178	0.399981691226625	1	pseudobulk	DESeq2	LRT	0.397959887625108
133	KC_AB2	MagR	0.0570650273289936	0.860164706939821	1	pseudobulk	DESeq2	LRT	0.065418380772546
133	KC_AB2	mahe	-0.126454813645789	0.767062836733517	1	pseudobulk	DESeq2	LRT	0.115169057786571
133	KC_AB2	mahj	1.50713387114482	0.576249954186023	1	pseudobulk	DESeq2	LRT	0.239389096130292
133	KC_AB2	mal	-1.6052707068109	0.52265609184397	1	pseudobulk	DESeq2	LRT	0.281783983297782
133	KC_AB2	Mal-B1	-0.729379579623002	0.823527397767669	1	pseudobulk	DESeq2	LRT	0.084321947797948
133	KC_AB2	mam	-0.275665807911593	0.552790945946624	1	pseudobulk	DESeq2	LRT	0.257439078798875
133	KC_AB2	mamo	-0.670541955584215	0.735328220685952	1	pseudobulk	DESeq2	LRT	0.133518766201526
133	KC_AB2	MAN1	-0.855866430859675	0.527268919460851	1	pseudobulk	DESeq2	LRT	0.277967827956362
133	KC_AB2	Manf	-1.03271861730972	0.255914936861687	1	pseudobulk	DESeq2	LRT	0.591904365119878
134	KC_AB2	Map205	0.136986354115095	0.864512166376611	1	pseudobulk	DESeq2	LRT	0.063228890472132
134	KC_AB2	Map60	0.830820049222858	0.520542380425724	1	pseudobulk	DESeq2	LRT	0.283543906238443
134	KC_AB2	MAPk-Ak2	0.694807427514077	0.581095020105131	1	pseudobulk	DESeq2	LRT	0.235752846382134
134	KC_AB2	Mapmodulin	-0.0334240365352886	0.917353531529745	1	pseudobulk	DESeq2	LRT	0.037463262799347
134	KC_AB2	Marcal1	1.3723515946239	0.644983770400341	1	pseudobulk	DESeq2	LRT	0.190451213293808
134	KC_AB2	Marf	0.194478021486559	0.707058902596913	1	pseudobulk	DESeq2	LRT	0.150544405146177
134	KC_AB2	Marf1	0.39439088612385	0.813541888331052	1	pseudobulk	DESeq2	LRT	0.089620080830767
134	KC_AB2	mask	0.0366582064563066	0.9156262551612	1	pseudobulk	DESeq2	LRT	0.038281762624680
134	KC_AB2	Mat1	0.261445104008245	0.836157004452423	1	pseudobulk	DESeq2	LRT	0.077712167817810
134	KC_AB2	mats	-0.453025957378819	0.668782051745079	1	pseudobulk	DESeq2	LRT	0.174715390671712
134	KC_AB2	Max	-0.248126166705663	0.840836052091855	1	pseudobulk	DESeq2	LRT	0.075288675561063
134	KC_AB2	mbc	1.11078347876117	0.302118938197332	1	pseudobulk	DESeq2	LRT	0.51982205030828
134	KC_AB2	MBD-like	-0.075767891212254	0.898660218237683	1	pseudobulk	DESeq2	LRT	0.046404483169999
134	KC_AB2	MBD-R2	-1.00336248278043	0.50562925824916	1	pseudobulk	DESeq2	LRT	0.296167803537941
134	KC_AB2	mbf1	-0.116338119284076	0.785326870167174	1	pseudobulk	DESeq2	LRT	0.104949542792564

134	KC_AB2	mbl	1.93194446698954	0.467042697610198	1	pseudobulk	DESeq2	LRT	0.33064341389032
134	KC_AB2	mbm	0.145321756433347	1	1	pseudobulk	DESeq2	LRT	0
134	KC_AB2	mbo	0.661678460170035	0.878003361430606	1	pseudobulk	DESeq2	LRT	0.056503821396894
134	KC_AB2	Mbs	0.0811255827347406	0.871840843127377	1	pseudobulk	DESeq2	LRT	0.059562789448465
134	KC_AB2	mbt	-0.0481725671816659	0.89993479032926	1	pseudobulk	DESeq2	LRT	0.045788958589776
134	KC_AB2	Mcad	2.47215486522296	0.30601789350912	1	pseudobulk	DESeq2	LRT	0.514253178665238
134	KC_AB2	Mco1	-1.86716056129969	0.477680380390446	1	pseudobulk	DESeq2	LRT	0.320862595985116
134	KC_AB2	MCPH1	1.38446765109058	0.642794900879723	1	pseudobulk	DESeq2	LRT	0.191927577037166
134	KC_AB2	Mcr	-1.04864158320383	0.625596054654345	1	pseudobulk	DESeq2	LRT	0.203705998834478
134	KC_AB2	Mctp	-0.228425745978469	0.878696405310875	1	pseudobulk	DESeq2	LRT	0.056161150265228
134	KC_AB2	MCTS1	-0.632973207650494	0.5054703379965	1	pseudobulk	DESeq2	LRT	0.296304324588127
134	KC_AB2	MCU	0.352359980143466	0.642285913223837	1	pseudobulk	DESeq2	LRT	0.192271602937236
134	KC_AB2	Mdh1	-0.418216082708585	0.655774931305154	1	pseudobulk	DESeq2	LRT	0.183245189380489
134	KC_AB2	Mdh2	-0.542946124146913	0.265307729114566	1	pseudobulk	DESeq2	LRT	0.57625009768857
134	KC_AB2	mdlc	-0.729379579623002	0.823527397767669	1	pseudobulk	DESeq2	LRT	0.084321947797948
134	KC_AB2	mdy	1.3900428777958	0.249389652844457	1	pseudobulk	DESeq2	LRT	0.603121569324903
134	KC_AB2	me31B	-0.588775944121327	0.473235025221472	1	pseudobulk	DESeq2	LRT	0.324923119712981
134	KC_AB2	Med	-0.928962135469931	0.334352341066149	1	pseudobulk	DESeq2	LRT	0.475795631613024
134	KC_AB2	MED1	-0.764922546284053	0.683967866249129	1	pseudobulk	DESeq2	LRT	0.164964301552553
134	KC_AB2	MED10	0.122231257978813	0.833279967205424	1	pseudobulk	DESeq2	LRT	0.079209058876037
134	KC_AB2	MED11	-0.207888845720414	0.870166725915329	1	pseudobulk	DESeq2	LRT	0.060397527602309
134	KC_AB2	MED14	-1.09161297849732	0.469600442072018	1	pseudobulk	DESeq2	LRT	0.328271502924523
134	KC_AB2	MED15	-0.508638613984787	0.885316207746818	1	pseudobulk	DESeq2	LRT	0.052901584949501
134	KC_AB2	MED16	-1.6052707068109	0.52265609184397	1	pseudobulk	DESeq2	LRT	0.281783983297782
134	KC_AB2	MED17	0.501866148889605	0.79402377265782	1	pseudobulk	DESeq2	LRT	0.10016649482787
134	KC_AB2	MED18	0.186034431065808	0.881762404370314	1	pseudobulk	DESeq2	LRT	0.054648422092356
134	KC_AB2	MED19	-0.336760822156157	0.693388461473015	1	pseudobulk	DESeq2	LRT	0.159023389619762
134	KC_AB2	MED20	-0.470100723599697	0.833985291815347	1	pseudobulk	DESeq2	LRT	0.078841608522644
134	KC_AB2	MED21	-0.352892251958853	0.540980804609595	1	pseudobulk	DESeq2	LRT	0.266818144505313
134	KC_AB2	MED22	-0.369459886786198	0.756924274417054	1	pseudobulk	DESeq2	LRT	0.12094756679826
134	KC_AB2	MED23	1.10706867860301	1	1	pseudobulk	DESeq2	LRT	0
134	KC_AB2	MED24	-1.79272292774872	0.331210168534235	1	pseudobulk	DESeq2	LRT	0.479896338340912
134	KC_AB2	MED25	1.48850410624721	0.522705582363401	1	pseudobulk	DESeq2	LRT	0.281742861722271
134	KC_AB2	MED26	-0.404185098232504	0.368142701336454	1	pseudobulk	DESeq2	LRT	0.433983805266661
134	KC_AB2	MED27	1.02754127378565	0.694973473680879	1	pseudobulk	DESeq2	LRT	0.15803177160251
134	KC_AB2	MED28	-0.175340134630287	0.924111562598935	1	pseudobulk	DESeq2	LRT	0.034275595775150
134	KC_AB2	MED30	2.25461030872415	0.305019014551908	1	pseudobulk	DESeq2	LRT	0.515673086366102
134	KC_AB2	MED31	-0.131097241164822	0.851095767066932	1	pseudobulk	DESeq2	LRT	0.070021569446939
134	KC_AB2	MED4	-0.200092076072252	0.905136363317163	1	pseudobulk	DESeq2	LRT	0.043285987231348
134	KC_AB2	MED6	-0.627634629474375	0.577077738217013	1	pseudobulk	DESeq2	LRT	0.238765679037359
134	KC_AB2	MED7	-0.20696697698772	0.862472435853242	1	pseudobulk	DESeq2	LRT	0.064254775846718
134	KC_AB2	MED8	2.13281973364574	0.231889130679496	1	pseudobulk	DESeq2	LRT	0.634719607535133
134	KC_AB2	Mef2	0.0026123823153675	0.92844504630676	1	pseudobulk	DESeq2	LRT	0.032243796600587
134	KC_AB2	mEFG1	-0.18317077492817	0.899664519927029	1	pseudobulk	DESeq2	LRT	0.045919406456170
134	KC_AB2	mEFTs	-0.447552921940633	0.681773686282523	1	pseudobulk	DESeq2	LRT	0.166359764800925
134	KC_AB2	mEFTu1	-0.163492821395991	0.868777629688441	1	pseudobulk	DESeq2	LRT	0.061091370338041
134	KC_AB2	mEFTu2	0.145321756433347	1	1	pseudobulk	DESeq2	LRT	0
134	KC_AB2	Megf8	1.24311771188368	0.402016204915003	1	pseudobulk	DESeq2	LRT	0.395756440539107
134	KC_AB2	mei-P26	-0.210039530834762	0.417589703090308	1	pseudobulk	DESeq2	LRT	0.379250218689207
134	KC_AB2	mei-S332	-0.604527079544814	0.878338577287641	1	pseudobulk	DESeq2	LRT	0.056338042290314
134	KC_AB2	Meics	3.05945358553285	0.105141712594729	1	pseudobulk	DESeq2	LRT	0.97822495327933
134	KC_AB2	meigo	1.10706867860301	1	1	pseudobulk	DESeq2	LRT	0

134	KC_AB2	Mekk1	-0.454921796474203	0.696977490792764	1	pseudobulk	DESeq2	LRT	0.156781247414684
134	KC_AB2	Meltrin	-0.729379579623002	0.823527397767669	1	pseudobulk	DESeq2	LRT	0.084321947797948
134	KC_AB2	Membrin	-0.274459162011074	0.891545093103103	1	pseudobulk	DESeq2	LRT	0.049856685925997
134	KC_AB2	Men	-1.18843965564845	0.276033300363731	1	pseudobulk	DESeq2	LRT	0.559038521949897
134	KC_AB2	Men-b	0.243117486723429	0.696664704169186	1	pseudobulk	DESeq2	LRT	0.156976192010083
134	KC_AB2	meng	-0.473811103729645	0.54159149374706	1	pseudobulk	DESeq2	LRT	0.266328165312063
134	KC_AB2	MEP-1	1.02928733579242	0.697125366781187	1	pseudobulk	DESeq2	LRT	0.156689114003336
134	KC_AB2	Mer	-0.470100723599697	0.833985291815347	1	pseudobulk	DESeq2	LRT	0.078841608522644
134	KC_AB2	Mes4	0.539525380290298	0.55225702660436	1	pseudobulk	DESeq2	LRT	0.257858749705352
134	KC_AB2	MESK2	-0.246713401309427	0.505921836967552	1	pseudobulk	DESeq2	LRT	0.295916574851493
134	KC_AB2	MESR3	1.02754127378565	0.694973473680879	1	pseudobulk	DESeq2	LRT	0.15803177160251
134	KC_AB2	MESR4	-0.928947707392267	0.294050050546599	1	pseudobulk	DESeq2	LRT	0.531578741607609
134	KC_AB2	MESR6	0.145321756433347	1	1	pseudobulk	DESeq2	LRT	0
134	KC_AB2	Met	-0.0544331846145436	0.915338847279362	1	pseudobulk	DESeq2	LRT	0.038418105647271
134	KC_AB2	metl	0.0411618608380074	0.938717111374813	1	pseudobulk	DESeq2	LRT	0.027465265538930
134	KC_AB2	metro	0.486254613687282	0.34092428731716	1	pseudobulk	DESeq2	LRT	0.467342058694542
134	KC_AB2	MetRS	0.813801838352253	0.552517771286784	1	pseudobulk	DESeq2	LRT	0.257653748687803
134	KC_AB2	MetRS-m	0.668261508471375	0.750218387944288	1	pseudobulk	DESeq2	LRT	0.124812295444066
134	KC_AB2	Mettl14	0.412266503805257	0.859735858836361	1	pseudobulk	DESeq2	LRT	0.065634958838131
134	KC_AB2	Mettl3	0.145321756433347	1	1	pseudobulk	DESeq2	LRT	0
134	KC_AB2	mew	-2.97225596971586	0.00111407819345882	1	pseudobulk	DESeq2	LRT	2.95308432640114
134	KC_AB2	Mf	-2.66109654779851	0.211158123098611	1	pseudobulk	DESeq2	LRT	0.675392206926521
134	KC_AB2	Mfap1	2.08461878438749	0.242596306397263	1	pseudobulk	DESeq2	LRT	0.615115815685021
134	KC_AB2	Mfe2	1.16419655301094	0.553019346454958	1	pseudobulk	DESeq2	LRT	0.257259675364985
134	KC_AB2	mfrn	-1.88071858220318	0.237582109301097	1	pseudobulk	DESeq2	LRT	0.624186266234406
134	KC_AB2	MFS10	0.946740860353036	0.645788323904481	1	pseudobulk	DESeq2	LRT	0.18990981145527
134	KC_AB2	MFS15	-0.115183097143228	0.910399790291748	1	pseudobulk	DESeq2	LRT	0.040767850987610
134	KC_AB2	MFS16	2.9023801951582	0.206074905726345	1	pseudobulk	DESeq2	LRT	0.685974890162107
134	KC_AB2	MFS17	0.0242856514582813	0.928027089551915	1	pseudobulk	DESeq2	LRT	0.032439346333722
134	KC_AB2	MFS18	-1.01779627149618	0.493130631495869	1	pseudobulk	DESeq2	LRT	0.30703801982447
134	KC_AB2	MFS9	-2.03522979197945	0.396502959232805	1	pseudobulk	DESeq2	LRT	0.401753567050837
134	KC_AB2	Mgat1	-0.505908253351192	0.703182667959701	1	pseudobulk	DESeq2	LRT	0.152931842288987
134	KC_AB2	Mgat2	-0.848506167881681	0.319104923551602	1	pseudobulk	DESeq2	LRT	0.496066494913991
135	KC_AB2	mge	-0.343763373515647	0.528255561086735	1	pseudobulk	DESeq2	LRT	0.27715592231811
135	KC_AB2	mgl	-0.281407505330603	0.474632497368106	1	pseudobulk	DESeq2	LRT	0.323642529615369
135	KC_AB2	mGluR	0.667244748290888	0.924085151242949	1	pseudobulk	DESeq2	LRT	0.034288008205147
135	KC_AB2	mgr	-0.116338218778214	0.890618547425787	1	pseudobulk	DESeq2	LRT	0.050308264784407
135	KC_AB2	Mgstl	-1.52117895717465	0.398474933007116	1	pseudobulk	DESeq2	LRT	0.399598993713538
135	KC_AB2	Mhcl	-0.562813035852612	0.532020502866472	1	pseudobulk	DESeq2	LRT	0.274071630655714
135	KC_AB2	mi	-0.647621034217714	0.754702560287896	1	pseudobulk	DESeq2	LRT	0.122224176685402
135	KC_AB2	Mi-2	0.0413451762935405	0.905760481130965	1	pseudobulk	DESeq2	LRT	0.042986631766507
135	KC_AB2	mib1	-1.53280421938336	0.363696097106752	1	pseudobulk	DESeq2	LRT	0.439261359418469
135	KC_AB2	mib2	-0.808098980311534	0.376184924592683	1	pseudobulk	DESeq2	LRT	0.424598612553304
135	KC_AB2	Mical	0.489409874104102	0.637996082230714	1	pseudobulk	DESeq2	LRT	0.195181988160979
135	KC_AB2	MICAL-like	-0.721175887303532	0.733409564198845	1	pseudobulk	DESeq2	LRT	0.134653430819787
135	KC_AB2	MICU1	-0.310347802308359	0.764457800045755	1	pseudobulk	DESeq2	LRT	0.116646483715506
135	KC_AB2	MICU3	0.097379223228496	0.881800314701328	1	pseudobulk	DESeq2	LRT	0.054629750516471
135	KC_AB2	Mid1	-0.0698331981056856	0.830923349639446	1	pseudobulk	DESeq2	LRT	0.080439036822360
135	KC_AB2	mIF2	0.145321756433347	1	1	pseudobulk	DESeq2	LRT	0
135	KC_AB2	mIF3	-0.729379579623002	0.823527397767669	1	pseudobulk	DESeq2	LRT	0.084321947797948
135	KC_AB2	Miga	-0.486847299090882	0.726255334680593	1	pseudobulk	DESeq2	LRT	0.138910664496504
135	KC_AB2	milt	-0.129590974019882	0.803180698339619	1	pseudobulk	DESeq2	LRT	0.095186736834162

135	KC_AB2	mim	-0.104896416982516	0.855245817879244	1	pseudobulk	DESeq2	LRT	0.067909040821833
135	KC_AB2	mino	1.03858245968159	0.742043499884082	1	pseudobulk	DESeq2	LRT	0.129570634874779
135	KC_AB2	mip120	-0.961033333862793	0.70039622209097	1	pseudobulk	DESeq2	LRT	0.154656205149228
135	KC_AB2	mip130	1.72408848693846	0.341137389564752	1	pseudobulk	DESeq2	LRT	0.467070678154164
135	KC_AB2	mip40	0.270409256503617	0.846783059700464	1	pseudobulk	DESeq2	LRT	0.072227838834251
135	KC_AB2	miple1	-0.300751728158664	0.311276138178395	1	pseudobulk	DESeq2	LRT	0.50685417020938
135	KC_AB2	miple2	-2.03522979197945	0.396502959232805	1	pseudobulk	DESeq2	LRT	0.401753567050837
135	KC_AB2	Mipp2	1.38069928703004	0.566368801250645	1	pseudobulk	DESeq2	LRT	0.246900678019328
135	KC_AB2	Miro	-0.533556358262089	0.731803920350555	1	pseudobulk	DESeq2	LRT	0.135605268281971
135	KC_AB2	Mis12	-0.0222819650520445	0.929953624955713	1	pseudobulk	DESeq2	LRT	0.031538708357899
135	KC_AB2	Mitf	-1.788706033112	0.212352638851972	1	pseudobulk	DESeq2	LRT	0.672942337773234
135	KC_AB2	Mitofilin	-0.216344828250288	0.847241872251284	1	pseudobulk	DESeq2	LRT	0.071992588738266
135	KC_AB2	Mkk4	-0.0041247518113727	0.977085094716419	1	pseudobulk	DESeq2	LRT	0.010067611760787
135	KC_AB2	MKP-4	-0.324987845889662	0.80717194386703	1	pseudobulk	DESeq2	LRT	0.093033941958819
135	KC_AB2	Mkp3	-0.138624228264565	0.594674583073546	1	pseudobulk	DESeq2	LRT	0.225720623240758
135	KC_AB2	Mkrn1	0.462288877555678	0.662113907332246	1	pseudobulk	DESeq2	LRT	0.179067289910225
135	KC_AB2	Mlc-c	-0.353837457826184	0.739240413926395	1	pseudobulk	DESeq2	LRT	0.131214298463478
135	KC_AB2	mld	0.552627499291508	0.40096519697652	1	pseudobulk	DESeq2	LRT	0.396893321686546
135	KC_AB2	mldr	-2.66109654779851	0.211158123098611	1	pseudobulk	DESeq2	LRT	0.675392206926521
135	KC_AB2	mle	0.166181523782748	0.81801167117338	1	pseudobulk	DESeq2	LRT	0.087240499886089
135	KC_AB2	Mlf	-0.255777365646709	0.542024596398531	1	pseudobulk	DESeq2	LRT	0.265981005273821
135	KC_AB2	Mlh1	0.38605638660934	0.863777890371283	1	pseudobulk	DESeq2	LRT	0.063597916533759
135	KC_AB2	Mlp84B	0.667244748290888	0.924085151242949	1	pseudobulk	DESeq2	LRT	0.034288008205147
135	KC_AB2	mlt	-0.535729505451777	0.566316413022002	1	pseudobulk	DESeq2	LRT	0.246940851437938
135	KC_AB2	mmd	-0.297670767252687	0.221695511959977	1	pseudobulk	DESeq2	LRT	0.654243098724689
135	KC_AB2	MME1	1.99339641594643	0.465071165814147	1	pseudobulk	DESeq2	LRT	0.332480585700252
135	KC_AB2	Mmp2	-0.333794761478093	0.562588036398064	1	pseudobulk	DESeq2	LRT	0.249809507473961
135	KC_AB2	Mms19	0.147593560254927	1	1	pseudobulk	DESeq2	LRT	0
135	KC_AB2	mmy	-0.233979871128455	0.812287589363261	1	pseudobulk	DESeq2	LRT	0.090290182137103
135	KC_AB2	mnb	-0.257360847777672	0.662948215343337	1	pseudobulk	DESeq2	LRT	0.178520394172877
135	KC_AB2	Mnn1	0.0075910152915640	0.925337860935222	1	pseudobulk	DESeq2	LRT	0.033699667964540
135	KC_AB2	Mnt	0.312033396729955	0.491305107134141	1	pseudobulk	DESeq2	LRT	0.308648721335059
135	KC_AB2	Mo25	-0.595461111041501	0.478134168375407	1	pseudobulk	DESeq2	LRT	0.320450219687048
135	KC_AB2	Mob2	0.562847274084703	0.23031434936249	1	pseudobulk	DESeq2	LRT	0.637679003178576
135	KC_AB2	Mob3	-0.66822880316352	0.575843906315352	1	pseudobulk	DESeq2	LRT	0.239695224585566
135	KC_AB2	Mob4	-0.284560232949202	0.653694959416638	1	pseudobulk	DESeq2	LRT	0.184624863827075
135	KC_AB2	Moca-cyp	-0.807321804773894	0.312862707090004	1	pseudobulk	DESeq2	LRT	0.504646201220271
135	KC_AB2	mod	0.899154075804704	0.432831605004956	1	pseudobulk	DESeq2	LRT	0.363681034920631
135	KC_AB2	Moe	0.0895514225067091	0.858853730039745	1	pseudobulk	DESeq2	LRT	0.066080793847050
135	KC_AB2	mof	0.145321756433347	1	1	pseudobulk	DESeq2	LRT	0
135	KC_AB2	Mon1	-0.721175887303532	0.733409564198845	1	pseudobulk	DESeq2	LRT	0.134653430819787
135	KC_AB2	mon2	0.743088595948166	0.567883920501187	1	pseudobulk	DESeq2	LRT	0.245740428087926
135	KC_AB2	Mondo	0.420569983525916	0.769225719824499	1	pseudobulk	DESeq2	LRT	0.113946203124258
135	KC_AB2	mop	1.13139976753416	0.365349113095474	1	pseudobulk	DESeq2	LRT	0.437291942590583
135	KC_AB2	mor	-0.760094433048362	0.619952293639431	1	pseudobulk	DESeq2	LRT	0.207641728898992
135	KC_AB2	morgue	0.801624979004844	0.485267064865267	1	pseudobulk	DESeq2	LRT	0.314019183303829
135	KC_AB2	Mp	-0.361010532303942	0.468095760695944	1	pseudobulk	DESeq2	LRT	0.329665292037898
135	KC_AB2	Mpc1	-1.04495448968502	0.532458724665933	1	pseudobulk	DESeq2	LRT	0.273714052386107
135	KC_AB2	Mpcp2	-0.414242067358835	0.257945536119761	1	pseudobulk	DESeq2	LRT	0.588471983415136
135	KC_AB2	Mpi	-2.35203494925393	0.313819523334105	1	pseudobulk	DESeq2	LRT	0.503320041588723
135	KC_AB2	Mpp6	1.3663630016865	0.328028700282877	1	pseudobulk	DESeq2	LRT	0.484088156809075
135	KC_AB2	Mppe	2.04030215638816	0.327981738462365	1	pseudobulk	DESeq2	LRT	0.484150336488967

135	KC_AB2	mr	0.424912966262768	0.824492810492961	1	pseudobulk	DESeq2	LRT	0.083813127013820
135	KC_AB2	mre11	-0.347135499964956	0.854806062097812	1	pseudobulk	DESeq2	LRT	0.068132406575477
135	KC_AB2	MRG15	0.149370476025787	0.870020283749287	1	pseudobulk	DESeq2	LRT	0.060470622073682
135	KC_AB2	MrgBP	0.540824915900089	0.515181833656672	1	pseudobulk	DESeq2	LRT	0.288039459471541
135	KC_AB2	mri	-1.71626504223523	0.174185223614675	1	pseudobulk	DESeq2	LRT	0.75898868959699
135	KC_AB2	mrj	-0.37721788577986	0.504779471698108	1	pseudobulk	DESeq2	LRT	0.296898315232852
135	KC_AB2	mrn	0.254640737012646	0.905542112804522	1	pseudobulk	DESeq2	LRT	0.043091347742705
135	KC_AB2	mRNA-cap	0.462129756918411	0.674072216828538	1	pseudobulk	DESeq2	LRT	0.171293572767329
135	KC_AB2	MRP	-0.0807305778852393	0.95673095170253	1	pseudobulk	DESeq2	LRT	0.019210175722572
135	KC_AB2	mRpL1	-0.284621405584683	0.816681899180356	1	pseudobulk	DESeq2	LRT	0.087947069944991
135	KC_AB2	mRpL10	0.963204843529826	0.506537038452862	1	pseudobulk	DESeq2	LRT	0.295388793131766
135	KC_AB2	mRpL11	-0.988283900777906	0.610993900099061	1	pseudobulk	DESeq2	LRT	0.213963125545567
135	KC_AB2	mRpL12	0.250944335294567	0.703990158911109	1	pseudobulk	DESeq2	LRT	0.15243341182447
135	KC_AB2	mRpL13	0.436682894037495	0.74882619483016	1	pseudobulk	DESeq2	LRT	0.125618971867156
135	KC_AB2	mRpL14	-0.940841838016682	0.504359124167161	1	pseudobulk	DESeq2	LRT	0.297260118110035
135	KC_AB2	mRpL15	-3.32552274381165	0.0889569567695445	1	pseudobulk	DESeq2	LRT	1.05082008278541
135	KC_AB2	mRpL16	0.35883964106547	0.781966783180373	1	pseudobulk	DESeq2	LRT	0.106811694750716
135	KC_AB2	mRpL17	-0.935541872328128	0.454774492070336	1	pseudobulk	DESeq2	LRT	0.342203902523812
135	KC_AB2	mRpL18	0.543136893302163	0.652116044205316	1	pseudobulk	DESeq2	LRT	0.18567511458457
135	KC_AB2	mRpL19	0.297016636476532	0.792491344360866	1	pseudobulk	DESeq2	LRT	0.101005472475311
135	KC_AB2	mRpL2	-0.573390824704325	0.663664088032278	1	pseudobulk	DESeq2	LRT	0.178051682118965
135	KC_AB2	mRpL20	-0.589000134376956	0.687747681153777	1	pseudobulk	DESeq2	LRT	0.162570865225908
135	KC_AB2	mRpL21	-1.41306189037252	0.397276709719391	1	pseudobulk	DESeq2	LRT	0.400906894639666
135	KC_AB2	mRpL22	-1.32672795781899	0.308847330776785	1	pseudobulk	DESeq2	LRT	0.510256147714413
135	KC_AB2	mRpL23	-0.401555812789024	0.655272294516081	1	pseudobulk	DESeq2	LRT	0.183578194005024
135	KC_AB2	mRpL24	-0.154508653834565	0.826643964329561	1	pseudobulk	DESeq2	LRT	0.082681500871410
135	KC_AB2	mRpL27	-0.888754216803971	0.383422604156994	1	pseudobulk	DESeq2	LRT	0.41632228746402
135	KC_AB2	mRpL28	-0.61795023560206	0.577043668334815	1	pseudobulk	DESeq2	LRT	0.238791319947853
135	KC_AB2	mRpL3	-1.67513874790658	0.39900884344985	1	pseudobulk	DESeq2	LRT	0.39901747870195
136	KC_AB2	mRpL30	-0.314299857094133	0.774627995892214	1	pseudobulk	DESeq2	LRT	0.11090681119477
136	KC_AB2	mRpL32	-0.254781446179545	0.844251824042837	1	pseudobulk	DESeq2	LRT	0.073527992379393
136	KC_AB2	mRpL33	-0.315271510000839	0.650961419481219	1	pseudobulk	DESeq2	LRT	0.186444749992367
136	KC_AB2	mRpL34	-1.24038923850004	0.193431463545738	1	pseudobulk	DESeq2	LRT	0.713472882202829
136	KC_AB2	mRpL35	0.295577280669977	0.791165311545057	1	pseudobulk	DESeq2	LRT	0.101732762531736
136	KC_AB2	mRpL36	-2.73137323424294	0.0967634661473128	1	pseudobulk	DESeq2	LRT	1.01428858324432
136	KC_AB2	mRpL37	-1.66167910657999	0.360792435054962	1	pseudobulk	DESeq2	LRT	0.442742577139878
136	KC_AB2	mRpL38	-0.361559777502133	0.770101633798288	1	pseudobulk	DESeq2	LRT	0.113451955236568
136	KC_AB2	mRpL39	0.0565652011607359	0.947429291070731	1	pseudobulk	DESeq2	LRT	0.023453192593345
136	KC_AB2	mRpL4	-0.86575475118938	0.463725808430078	1	pseudobulk	DESeq2	LRT	0.33373873298739
136	KC_AB2	mRpL40	-1.13639901356451	0.298184263475006	1	pseudobulk	DESeq2	LRT	0.525515279951784
136	KC_AB2	mRpL41	-0.941343812223838	0.415569196471841	1	pseudobulk	DESeq2	LRT	0.381356651437517
136	KC_AB2	mRpL42	-0.353244825923096	0.706921274996446	1	pseudobulk	DESeq2	LRT	0.150628947926305
136	KC_AB2	mRpL43	-0.610963696765406	0.652845824221811	1	pseudobulk	DESeq2	LRT	0.185189369407066
136	KC_AB2	mRpL44	-0.302260333582416	0.830396080330023	1	pseudobulk	DESeq2	LRT	0.080714709465583
136	KC_AB2	mRpL45	0.39439088612385	0.813541888331052	1	pseudobulk	DESeq2	LRT	0.089620080830767
136	KC_AB2	mRpL46	-1.52185899549078	0.327020515096113	1	pseudobulk	DESeq2	LRT	0.485425001732474
136	KC_AB2	mRpL47	0.0734233346443849	0.944933283407473	1	pseudobulk	DESeq2	LRT	0.024598853576274
136	KC_AB2	mRpL48	0.0929257771226804	0.890328394128088	1	pseudobulk	DESeq2	LRT	0.050449775993160
136	KC_AB2	mRpL49	0.15745826620835	0.899236654973073	1	pseudobulk	DESeq2	LRT	0.046125998563173
136	KC_AB2	mRpL50	-0.245011366979911	0.795168127757445	1	pseudobulk	DESeq2	LRT	0.099541035824617
136	KC_AB2	mRpL51	-0.210215900124525	0.911549335431309	1	pseudobulk	DESeq2	LRT	0.040219821221017
136	KC_AB2	mRpL52	-1.07217020556534	0.491983432847904	1	pseudobulk	DESeq2	LRT	0.308049521508815

136	KC_AB2	mRpL54	1.69936944919821	0.204554778472558	1	pseudobulk	DESeq2	LRT	0.689190370773165
136	KC_AB2	mRpL55	0.0827518718797437	0.909618169020174	1	pseudobulk	DESeq2	LRT	0.041140873489190
136	KC_AB2	mRpL9	-0.987864420262975	0.622985879197186	1	pseudobulk	DESeq2	LRT	0.205521797090819
136	KC_AB2	mRpS10	-1.13460058177666	0.379768440910495	1	pseudobulk	DESeq2	LRT	0.420481128350707
136	KC_AB2	mRpS11	0.0214314340670984	0.951691364385052	1	pseudobulk	DESeq2	LRT	0.021503871443963
136	KC_AB2	mRpS14	-0.672007877941243	0.713146579991023	1	pseudobulk	DESeq2	LRT	0.146821196180962
136	KC_AB2	mRpS16	-1.99428646066943	0.202207762330168	1	pseudobulk	DESeq2	LRT	0.694202176774433
136	KC_AB2	mRpS17	0.771862725762229	0.647957631206405	1	pseudobulk	DESeq2	LRT	0.18845339094243
136	KC_AB2	mRpS18A	-1.27528656247235	0.312621155754402	1	pseudobulk	DESeq2	LRT	0.504981635669007
136	KC_AB2	mRpS18B	-0.277947413912466	0.859179291176031	1	pseudobulk	DESeq2	LRT	0.065916199331162
136	KC_AB2	mRpS18C	-0.479345893861304	0.671106496804665	1	pseudobulk	DESeq2	LRT	0.173208556873235
136	KC_AB2	mRpS2	-0.274509907139123	0.839542725793588	1	pseudobulk	DESeq2	LRT	0.075957196959858
136	KC_AB2	mRpS21	-1.16034079612193	0.349070929890048	1	pseudobulk	DESeq2	LRT	0.45708631708104
136	KC_AB2	mRpS22	2.48384162006255	0.303740962766822	1	pseudobulk	DESeq2	LRT	0.517496634805608
136	KC_AB2	mRpS23	-0.792211336445826	0.634123659645977	1	pseudobulk	DESeq2	LRT	0.197826042646503
136	KC_AB2	mRpS24	-1.86796888043117	0.151273025181132	1	pseudobulk	DESeq2	LRT	0.820238507928075
136	KC_AB2	mRpS25	0.324471565839802	0.778942657264009	1	pseudobulk	DESeq2	LRT	0.108494512225318
136	KC_AB2	mRpS26	1.26983941004435	0.422661171138466	1	pseudobulk	DESeq2	LRT	0.374007647924158
136	KC_AB2	mRpS28	-1.12300291927447	0.196648343912809	1	pseudobulk	DESeq2	LRT	0.706309706678883
136	KC_AB2	mRpS29	0.481785761401232	0.709666562394285	1	pseudobulk	DESeq2	LRT	0.14894565709288
136	KC_AB2	mRpS30	0.17924906163653	0.890812680764919	1	pseudobulk	DESeq2	LRT	0.050213609389421
136	KC_AB2	mRpS31	-1.73214592314926	0.248010263183689	1	pseudobulk	DESeq2	LRT	0.605530346787427
136	KC_AB2	mRpS33	-0.0362289366020788	0.912947087150058	1	pseudobulk	DESeq2	LRT	0.039554392700829
136	KC_AB2	mRpS34	-0.537501088179251	0.645752769154598	1	pseudobulk	DESeq2	LRT	0.189933722783785
136	KC_AB2	mRpS35	-1.601549006404	0.385495268880093	1	pseudobulk	DESeq2	LRT	0.413980947604923
136	KC_AB2	mRpS5	-0.647191651632794	0.222543922723158	1	pseudobulk	DESeq2	LRT	0.65258426102574
136	KC_AB2	mRpS6	-0.450487832778861	0.74388900762558	1	pseudobulk	DESeq2	LRT	0.128491858770883
136	KC_AB2	mRpS7	-0.854028658029915	0.390615370328482	1	pseudobulk	DESeq2	LRT	0.408250671653616
136	KC_AB2	mRpS9	-0.0778424710895956	0.928402234704575	1	pseudobulk	DESeq2	LRT	0.032263822849147
136	KC_AB2	mRRF1	0.0215953682293528	0.916154148963656	1	pseudobulk	DESeq2	LRT	0.038031447279819
136	KC_AB2	mRRF2	1.38069928703004	0.566368801250645	1	pseudobulk	DESeq2	LRT	0.246900678019328
136	KC_AB2	mrt	0.19023961044933	0.891037993113229	1	pseudobulk	DESeq2	LRT	0.050103777615601
136	KC_AB2	Mrtf	0.723203551901333	0.330679339725049	1	pseudobulk	DESeq2	LRT	0.48059293820906
136	KC_AB2	mrva	-0.266854441072505	0.671783408059587	1	pseudobulk	DESeq2	LRT	0.172770726718157
136	KC_AB2	MSBP	-0.548230282667098	0.440177432943351	1	pseudobulk	DESeq2	LRT	0.356372226660917
136	KC_AB2	msd5	-0.441081437403916	0.911612819631012	1	pseudobulk	DESeq2	LRT	0.040189576146487
136	KC_AB2	Msh6	1.10706867860301	1	1	pseudobulk	DESeq2	LRT	0
136	KC_AB2	msi	-0.0504040786984956	0.943885987474892	1	pseudobulk	DESeq2	LRT	0.025080461211332
136	KC_AB2	msk	-0.235449563676684	0.855153811360479	1	pseudobulk	DESeq2	LRT	0.067955764315868
136	KC_AB2	msl-1	-0.206387957994511	0.869577730195087	1	pseudobulk	DESeq2	LRT	0.060691591020693
136	KC_AB2	msl-2	1.10706867860301	1	1	pseudobulk	DESeq2	LRT	0
136	KC_AB2	msl-3	2.31968409897139	0.349905511974677	1	pseudobulk	DESeq2	LRT	0.456049216130035
136	KC_AB2	msn	-0.310858972324567	0.531768687055416	1	pseudobulk	DESeq2	LRT	0.274277239474222
136	KC_AB2	Msp300	0.391668066009903	0.123815943098903	1	pseudobulk	DESeq2	LRT	0.90722343000026
136	KC_AB2	mspo	-0.12128094372848	0.745401611747618	1	pseudobulk	DESeq2	LRT	0.127609672526182
136	KC_AB2	msps	0.124431757711564	0.835976421300163	1	pseudobulk	DESeq2	LRT	0.077805971656246
136	KC_AB2	mst	1.99339641594643	0.465071165814147	1	pseudobulk	DESeq2	LRT	0.332480585700252
136	KC_AB2	Mst36Fa	-0.729379579623002	1	1	pseudobulk	DESeq2	LRT	0
136	KC_AB2	Mst77F	-0.332187485045999	0.832221724989265	1	pseudobulk	DESeq2	LRT	0.079760951220545
136	KC_AB2	Mst85C	0.693704603845161	0.570152465653813	1	pseudobulk	DESeq2	LRT	0.244008993208215
136	KC_AB2	mt:ATPase6	-0.116551308412384	0.760044881725379	1	pseudobulk	DESeq2	LRT	0.119160761258489
136	KC_AB2	mt:ATPase8	-0.294729952624233	0.892189018547858	1	pseudobulk	DESeq2	LRT	0.049543126547685

136	KC_AB2	mt:CoI	-0.306662997225779	0.431015480753416	1	pseudobulk	DESeq2	LRT	0.365507131034061
136	KC_AB2	mt:CoII	-0.0035343130951791	0.922204658827423	1	pseudobulk	DESeq2	LRT	0.035172688126228
136	KC_AB2	mt:CoIII	-0.119545751504413	0.741929880820259	1	pseudobulk	DESeq2	LRT	0.129637137594027
136	KC_AB2	mt:Cyt-b	-0.259018861695681	0.510468899395431	1	pseudobulk	DESeq2	LRT	0.292030712406445
136	KC_AB2	mt:lrRNA	0.0551853345442726	0.784870787937544	1	pseudobulk	DESeq2	LRT	0.105201834598211
136	KC_AB2	mt:ND1	0.45353128529926	0.367155833088284	1	pseudobulk	DESeq2	LRT	0.435149567666079
136	KC_AB2	mt:ND2	-0.411748657818794	0.60500267668877	1	pseudobulk	DESeq2	LRT	0.218242703911843
136	KC_AB2	mt:ND3	-0.552793984865028	0.411557974833341	1	pseudobulk	DESeq2	LRT	0.385568978491216
136	KC_AB2	mt:ND4	0.642261221220078	0.233786922575704	1	pseudobulk	DESeq2	LRT	0.631179785783474
136	KC_AB2	mt:ND4L	0.779558650624278	0.72455574449411	1	pseudobulk	DESeq2	LRT	0.139928195983184
136	KC_AB2	mt:ND5	0.357719479730279	0.517565057116387	1	pseudobulk	DESeq2	LRT	0.286035052304199
136	KC_AB2	mt:ND6	-0.747307913363504	0.31779184319158	1	pseudobulk	DESeq2	LRT	0.497857254083796
136	KC_AB2	mt:srRNA	0.196619974017622	0.642519888199875	1	pseudobulk	DESeq2	LRT	0.192113424881054
136	KC_AB2	mt:tRNA:Ala-TGC	1.10706867860301	1	1	pseudobulk	DESeq2	LRT	0
136	KC_AB2	mt:tRNA:Arg-TCG	-0.729379579623002	1	1	pseudobulk	DESeq2	LRT	0
136	KC_AB2	Mt2	1.10706867860301	1	1	pseudobulk	DESeq2	LRT	0
136	KC_AB2	MTA1-like	-0.24280347280765	0.783767287194657	1	pseudobulk	DESeq2	LRT	0.105812867021898
136	KC_AB2	Mtap	-1.93335857128809	0.384223899985767	1	pseudobulk	DESeq2	LRT	0.415415624094039
136	KC_AB2	Mtch	-0.283868670977199	0.711780585684753	1	pseudobulk	DESeq2	LRT	0.147653861857557
136	KC_AB2	mtd	0.161744228181596	0.501506539871312	1	pseudobulk	DESeq2	LRT	0.299723399210853
136	KC_AB2	mTerf3	-0.127583525754786	0.90344164270244	1	pseudobulk	DESeq2	LRT	0.044099895201378
136	KC_AB2	mTerf5	-1.00833870937463	0.651152668420623	1	pseudobulk	DESeq2	LRT	0.186317175365195
136	KC_AB2	MTF-1	0.261197697050516	0.848771410582132	1	pseudobulk	DESeq2	LRT	0.071209257329534
136	KC_AB2	mtg	-0.604527079544814	0.878338577287641	1	pseudobulk	DESeq2	LRT	0.056338042290314
136	KC_AB2	mtgo	-0.253204826493869	0.427857687569437	1	pseudobulk	DESeq2	LRT	0.368700660381498
137	KC_AB2	mthl1	-0.729379579623002	0.823527397767669	1	pseudobulk	DESeq2	LRT	0.084321947797948
137	KC_AB2	mthl13	-0.729379579623002	1	1	pseudobulk	DESeq2	LRT	0
137	KC_AB2	mthl15	-0.729379579623002	1	1	pseudobulk	DESeq2	LRT	0
137	KC_AB2	mthl8	-1.13293713463756	0.772968209818576	1	pseudobulk	DESeq2	LRT	0.111838367122136
137	KC_AB2	Mtl	-3.03373899387034	0.157377531834931	1	pseudobulk	DESeq2	LRT	0.803057270049482
137	KC_AB2	mtm	-0.632028176875517	0.611021437383352	1	pseudobulk	DESeq2	LRT	0.213943552484167
137	KC_AB2	MtnA	1.04237305304479	0.767386654171747	1	pseudobulk	DESeq2	LRT	0.114985758004482
137	KC_AB2	MtnE	1.38446765109058	0.642794900879723	1	pseudobulk	DESeq2	LRT	0.191927577037166
137	KC_AB2	Mtor	-0.193641391041381	0.868231175467069	1	pseudobulk	DESeq2	LRT	0.061364624060278
137	KC_AB2	Mtpalpha	-0.419730715833961	0.871309690495475	1	pseudobulk	DESeq2	LRT	0.059827455820959
137	KC_AB2	MTPAP	1.95432904991983	0.509212778150152	1	pseudobulk	DESeq2	LRT	0.293100706726022
137	KC_AB2	Mtpbeta	-2.20640656535147	0.271085832356937	1	pseudobulk	DESeq2	LRT	0.566893179173424
137	KC_AB2	Mtr3	-0.0379277777728407	0.95506882437494	1	pseudobulk	DESeq2	LRT	0.019965331066243
137	KC_AB2	Mtr4	-0.600496552110112	0.825488100373549	1	pseudobulk	DESeq2	LRT	0.083289182824389
137	KC_AB2	mtRNApol	-0.436587925451555	0.824276803233537	1	pseudobulk	DESeq2	LRT	0.083926921883127
137	KC_AB2	mts	0.127560172481593	0.672528612789871	1	pseudobulk	DESeq2	LRT	0.172289233834984
137	KC_AB2	mtSSB	-0.481711097359631	0.768222482590664	1	pseudobulk	DESeq2	LRT	0.114512987040425
137	KC_AB2	mtt	0.634281715201455	0.447521868653158	1	pseudobulk	DESeq2	LRT	0.349185737547314
137	KC_AB2	mTTF	2.7605424445074	0.237810191029118	1	pseudobulk	DESeq2	LRT	0.623769538224996
137	KC_AB2	mtTFB2	-0.721175887303532	0.733409564198845	1	pseudobulk	DESeq2	LRT	0.134653430819787
137	KC_AB2	mu2	-0.729379579623002	0.772694994316916	1	pseudobulk	DESeq2	LRT	0.1119919011982
137	KC_AB2	mub	0.132321238902684	0.47779479829639	1	pseudobulk	DESeq2	LRT	0.320758582680472
137	KC_AB2	muc	-0.0391454942885205	0.936969883674715	1	pseudobulk	DESeq2	LRT	0.028274368092033
137	KC_AB2	Muc14A	-0.729379579623002	0.823527397767669	1	pseudobulk	DESeq2	LRT	0.084321947797948
137	KC_AB2	Muc91C	-1.86979501903407	0.475964703567026	1	pseudobulk	DESeq2	LRT	0.322425252351861
137	KC_AB2	Mul1	1.04324661842443	0.68096673636569	1	pseudobulk	DESeq2	LRT	0.166874101840012
137	KC_AB2	Mulk	-1.47740131595573	0.429760234207816	1	pseudobulk	DESeq2	LRT	0.366773772332455

137	KC_AB2	Mur2B	1.04237305304479	0.767386654171747	1	pseudobulk	DESeq2	LRT	0.114985758004482
137	KC_AB2	mura	-0.682977036507264	0.103501316421307	1	pseudobulk	DESeq2	LRT	0.985054126430505
137	KC_AB2	mus201	-0.729379579623002	0.772694994316916	1	pseudobulk	DESeq2	LRT	0.1119919011982
137	KC_AB2	mus301	1.10706867860301	1	1	pseudobulk	DESeq2	LRT	0
137	KC_AB2	muskelin	1.10706867860301	1	1	pseudobulk	DESeq2	LRT	0
137	KC_AB2	mv	1.50776466886541	0.515809803599324	1	pseudobulk	DESeq2	LRT	0.287510407819198
137	KC_AB2	Mvb12	-0.373388555047944	0.786758140677013	1	pseudobulk	DESeq2	LRT	0.104158754696515
137	KC_AB2	Mvd	0.393976029558784	0.939642609940555	1	pseudobulk	DESeq2	LRT	0.027037297511213
137	KC_AB2	Mvl	1.00543216571798	0.570813853402089	1	pseudobulk	DESeq2	LRT	0.24350549529272
137	KC_AB2	mxc	-0.0966758989148081	0.92865541834516	1	pseudobulk	DESeq2	LRT	0.032145402983705
137	KC_AB2	mxt	-0.748810456096544	0.482560431002365	1	pseudobulk	DESeq2	LRT	0.316448292252884
137	KC_AB2	Myb	-0.729379579623002	0.823527397767669	1	pseudobulk	DESeq2	LRT	0.084321947797948
137	KC_AB2	Myc	-0.232410822263004	0.783427318228766	1	pseudobulk	DESeq2	LRT	0.106001288610014
137	KC_AB2	myo	1.02928733579242	0.697125366781187	1	pseudobulk	DESeq2	LRT	0.156689114003336
137	KC_AB2	Myo28B1	-0.68327639629059	0.749130702734851	1	pseudobulk	DESeq2	LRT	0.125442403229247
137	KC_AB2	Myo31DF	1.38069928703004	0.566368801250645	1	pseudobulk	DESeq2	LRT	0.246900678019328
137	KC_AB2	Myo81F	0.245560770723763	0.689469983939792	1	pseudobulk	DESeq2	LRT	0.161484636077065
137	KC_AB2	Myo95E	0.603825202674264	0.687306921305535	1	pseudobulk	DESeq2	LRT	0.162849282644604
137	KC_AB2	MYPT-75D	-1.12874692488189	0.0244803052554559	1	pseudobulk	DESeq2	LRT	1.61118317108802
137	KC_AB2	mys	0.381205157794408	0.531090213131011	1	pseudobulk	DESeq2	LRT	0.274831701635579
137	KC_AB2	Mys45A	-0.404770109376765	0.762063667943323	1	pseudobulk	DESeq2	LRT	0.118008743251782
137	KC_AB2	N	-0.729379579623002	0.772694994316916	1	pseudobulk	DESeq2	LRT	0.1119919011982
137	KC_AB2	na	0.109122677434755	0.861861325153054	1	pseudobulk	DESeq2	LRT	0.064562607221405
137	KC_AB2	Naa15-16	-0.519696389650941	0.752299890889585	1	pseudobulk	DESeq2	LRT	0.123609001169055
137	KC_AB2	Naa20A	-1.12878389929345	0.301153242334547	1	pseudobulk	DESeq2	LRT	0.521212456682602
137	KC_AB2	Naa30A	-0.522683132766544	0.547946795171782	1	pseudobulk	DESeq2	LRT	0.261261608824164
137	KC_AB2	Naa35	-0.934096005146402	0.570093089366374	1	pseudobulk	DESeq2	LRT	0.244054223455157
137	KC_AB2	Naa60	1.20182371803081	0.208617543914672	1	pseudobulk	DESeq2	LRT	0.680649171916435
137	KC_AB2	Naa80	-0.682895545282	0.462977174249106	1	pseudobulk	DESeq2	LRT	0.334440420087928
137	KC_AB2	Nab2	-0.876884788957675	0.525571321913295	1	pseudobulk	DESeq2	LRT	0.27936834031776
137	KC_AB2	nac	2.33862076287154	0.37727071943742	1	pseudobulk	DESeq2	LRT	0.423346899755834
137	KC_AB2	Nacalpha	-0.205569887546608	0.476462947450276	1	pseudobulk	DESeq2	LRT	0.321970866995356
137	KC_AB2	nAChRalpha1	0.152559611393321	0.68314699431446	1	pseudobulk	DESeq2	LRT	0.165485838117918
137	KC_AB2	nAChRalpha2	-0.506675896572835	0.385906855289	1	pseudobulk	DESeq2	LRT	0.413517506508311
137	KC_AB2	nAChRalpha3	-2.25772321511932	0.261792990170142	1	pseudobulk	DESeq2	LRT	0.582041986399411
137	KC_AB2	nAChRalpha4	0.184349882639644	0.614256144978216	1	pseudobulk	DESeq2	LRT	0.211650490172616
137	KC_AB2	nAChRalpha5	0.00498662872092335	0.973357981154939	1	pseudobulk	DESeq2	LRT	0.011727405725988
137	KC_AB2	nAChRalpha6	-0.190580184319983	0.402335711276842	1	pseudobulk	DESeq2	LRT	0.395411417783807
137	KC_AB2	nAChRalpha7	0.423674287923816	0.653954985091294	1	pseudobulk	DESeq2	LRT	0.184452145252629
137	KC_AB2	nAChRbeta1	-0.0499237361183198	0.810364825462547	1	pseudobulk	DESeq2	LRT	0.091319418140187
137	KC_AB2	nAChRbeta2	-0.104492454779226	0.858974142015019	1	pseudobulk	DESeq2	LRT	0.066019909683563
137	KC_AB2	NaCP60E	0.158626591416508	0.688243073253759	1	pseudobulk	DESeq2	LRT	0.162258150821386
137	KC_AB2	Nadsyn	-0.361765815314725	0.538268407803624	1	pseudobulk	DESeq2	LRT	0.269001109166641
137	KC_AB2	Nak	0.0804775657697612	0.898464767899594	1	pseudobulk	DESeq2	LRT	0.046498948501356
137	KC_AB2	Nap1	0.798429870664168	0.23454370284239	1	pseudobulk	DESeq2	LRT	0.629776222736069
137	KC_AB2	NaPi-III	0.270090408745135	0.512195571188295	1	pseudobulk	DESeq2	LRT	0.29056418107415
137	KC_AB2	NaPi-T	-0.729379579623002	0.772694994316916	1	pseudobulk	DESeq2	LRT	0.1119919011982
137	KC_AB2	Naprt	-0.297160422424282	0.799290755397741	1	pseudobulk	DESeq2	LRT	0.097295210053976
137	KC_AB2	NAT1	-0.0840008964412313	0.805085650461572	1	pseudobulk	DESeq2	LRT	0.094157913987285
137	KC_AB2	Naus	-0.103469730314101	0.889169561914455	1	pseudobulk	DESeq2	LRT	0.051015412503464
137	KC_AB2	Naxd	-0.572908453396411	0.75445548997041	1	pseudobulk	DESeq2	LRT	0.122366376869758
137	KC_AB2	Naxe	0.147593560254927	1	1	pseudobulk	DESeq2	LRT	0

137	KC_AB2	naz	-0.729379579623002	1	1	pseudobulk	DESeq2	LRT	0
137	KC_AB2	Nbr	-0.733536332022347	0.565590488666871	1	pseudobulk	DESeq2	LRT	0.247497902524851
137	KC_AB2	nbs	-0.620563475958957	0.7478554969218	1	pseudobulk	DESeq2	LRT	0.126182309825559
137	KC_AB2	NC2alpha	1.80622761856892	0.461120290665938	1	pseudobulk	DESeq2	LRT	0.336185767109657
137	KC_AB2	NC2beta	0.296840527920674	0.813513722081578	1	pseudobulk	DESeq2	LRT	0.089635117129029
137	KC_AB2	Nc73EF	-0.340587212043991	0.519885978760672	1	pseudobulk	DESeq2	LRT	0.284091895259214
137	KC_AB2	Nca	-0.220306049873137	0.60883705388437	1	pseudobulk	DESeq2	LRT	0.215498924227274
137	KC_AB2	Ncc69	-1.33407500886303	0.63128114564055	1	pseudobulk	DESeq2	LRT	0.19977718147345
137	KC_AB2	Nckx30C	-0.118622806883869	0.65241323083356	1	pseudobulk	DESeq2	LRT	0.185477240121429
137	KC_AB2	nclb	-1.80075127281421	0.363208620892189	1	pseudobulk	DESeq2	LRT	0.439843851886983
137	KC_AB2	ncm	0.400129610077907	0.727706928203675	1	pseudobulk	DESeq2	LRT	0.138043490316555
137	KC_AB2	Ncoa6	0.311428812319014	0.500905832746756	1	pseudobulk	DESeq2	LRT	0.300243911183351
137	KC_AB2	Nct	-2.32772888956263	0.206870311121279	1	pseudobulk	DESeq2	LRT	0.684301832395529
137	KC_AB2	ND-13A	-0.57183711565786	0.411451040845394	1	pseudobulk	DESeq2	LRT	0.385681834706081
137	KC_AB2	ND-13B	-0.102791362837493	0.834629798652989	1	pseudobulk	DESeq2	LRT	0.078506113801716
137	KC_AB2	ND-15	-0.339794431194682	0.49430842491731	1	pseudobulk	DESeq2	LRT	0.306001987427313
137	KC_AB2	ND-18	-0.388183408878604	0.481911747776993	1	pseudobulk	DESeq2	LRT	0.317032486580021
137	KC_AB2	ND-19	-0.0327287911135084	0.86772855963943	1	pseudobulk	DESeq2	LRT	0.061616108333958
137	KC_AB2	ND-20	0.256494890776465	0.655852667413678	1	pseudobulk	DESeq2	LRT	0.18319371080067
137	KC_AB2	ND-23	0.400837916834807	0.548362442120411	1	pseudobulk	DESeq2	LRT	0.260932298117443
137	KC_AB2	ND-24	-0.124141985549922	0.86107961909564	1	pseudobulk	DESeq2	LRT	0.064956689968853
138	KC_AB2	ND-30	-0.90601176949555	0.152422276057009	1	pseudobulk	DESeq2	LRT	0.816951557526187
138	KC_AB2	ND-39	-0.0124070012153162	0.921962973435295	1	pseudobulk	DESeq2	LRT	0.035286520112998
138	KC_AB2	ND-42	-0.535731437911001	0.457157098688497	1	pseudobulk	DESeq2	LRT	0.339934532163597
138	KC_AB2	ND-49	-0.151892107179489	0.748904235456498	1	pseudobulk	DESeq2	LRT	0.125573713237528
138	KC_AB2	ND-51	-0.714475806177475	0.201624152025838	1	pseudobulk	DESeq2	LRT	0.695457446118909
138	KC_AB2	ND-75	-0.222838816886613	0.79011275429499	1	pseudobulk	DESeq2	LRT	0.102310927603369
138	KC_AB2	ND-ACP	-1.05848446916042	0.054127804874813	1	pseudobulk	DESeq2	LRT	1.26657958514035
138	KC_AB2	ND-AGGG	-0.157818048557144	0.738785978809776	1	pseudobulk	DESeq2	LRT	0.131481355494015
138	KC_AB2	ND-ASHI	-0.384720347812184	0.535989521796224	1	pseudobulk	DESeq2	LRT	0.270843700364246
138	KC_AB2	ND-B12	-0.30039652339858	0.519511296038936	1	pseudobulk	DESeq2	LRT	0.284405004884394
138	KC_AB2	ND-B14	-0.700331367891566	0.160912767751714	1	pseudobulk	DESeq2	LRT	0.793409495092237
138	KC_AB2	ND-B14.5A	-0.0340632594591692	0.952995500026925	1	pseudobulk	DESeq2	LRT	0.020909150062711
138	KC_AB2	ND-B14.5B	-0.217329447566895	0.684985308472938	1	pseudobulk	DESeq2	LRT	0.164318743131745
138	KC_AB2	ND-B14.7	-0.554473589587056	0.385560299790311	1	pseudobulk	DESeq2	LRT	0.413907690717417
138	KC_AB2	ND-B15	-0.23810440008368	0.681394999364698	1	pseudobulk	DESeq2	LRT	0.16660105796551
138	KC_AB2	ND-B16.6	-0.0541849019827449	0.885593215147875	1	pseudobulk	DESeq2	LRT	0.052765719404769
138	KC_AB2	ND-B17	-0.736540107514346	0.313859408200242	1	pseudobulk	DESeq2	LRT	0.503264848475085
138	KC_AB2	ND-B17.2	-0.0969454949481857	0.856640942278683	1	pseudobulk	DESeq2	LRT	0.067201172782329
138	KC_AB2	ND-B18	-0.292431566719455	0.602255982375007	1	pseudobulk	DESeq2	LRT	0.220218877341159
138	KC_AB2	ND-B22	-0.318534737191287	0.515176603273071	1	pseudobulk	DESeq2	LRT	0.28804386866868
138	KC_AB2	ND-MLRQ	-0.555344735734412	0.0958460262495935	1	pseudobulk	DESeq2	LRT	1.0184258881448
138	KC_AB2	ND-MNLL	0.999852773071968	0.507455995214527	1	pseudobulk	DESeq2	LRT	0.294601612260276
138	KC_AB2	ND-MWFE	-0.0161645149255619	0.938748462769226	1	pseudobulk	DESeq2	LRT	0.027450761158363
138	KC_AB2	ND-PDSW	-0.742419302493301	0.114769058949828	1	pseudobulk	DESeq2	LRT	0.940175179346838
138	KC_AB2	ND-SGDH	-0.235460393741438	0.657174001475184	1	pseudobulk	DESeq2	LRT	0.18231962606352
138	KC_AB2	Ndae1	0.448633924071326	0.286607074417708	1	pseudobulk	DESeq2	LRT	0.542713093971283
138	KC_AB2	Ndc1	2.01814786626755	0.401718761445944	1	pseudobulk	DESeq2	LRT	0.396077884966921
138	KC_AB2	Ndf	-0.217550632758632	0.722016063267497	1	pseudobulk	DESeq2	LRT	0.141453140226992
138	KC_AB2	Ndfip	-0.0288924121407775	0.928367357576671	1	pseudobulk	DESeq2	LRT	0.032280138222102
138	KC_AB2	Neb-cGP	-0.423662163551322	0.237232124317726	1	pseudobulk	DESeq2	LRT	0.624826502199516
138	KC_AB2	Nedd4	0.651016662353975	0.354849381198696	1	pseudobulk	DESeq2	LRT	0.449955947775452

138	KC_AB2	Nedd8	0.111621847628581	0.893258433147478	1	pseudobulk	DESeq2	LRT	0.049022874983203
138	KC_AB2	nej	0.36648574889496	0.347500123919234	1	pseudobulk	DESeq2	LRT	0.459045036203566
138	KC_AB2	Nelf-A	0.654894427236772	0.555075637945224	1	pseudobulk	DESeq2	LRT	0.255647833266475
138	KC_AB2	NELF-B	1.95432904991983	0.509212778150152	1	pseudobulk	DESeq2	LRT	0.293100706726022
138	KC_AB2	Nelf-E	-0.168363321945366	0.840460274001163	1	pseudobulk	DESeq2	LRT	0.075482809517573
138	KC_AB2	nemy	1.10706867860301	1	1	pseudobulk	DESeq2	LRT	0
138	KC_AB2	Neos	0.474396347993209	0.808657101852946	1	pseudobulk	DESeq2	LRT	0.092235595003270
138	KC_AB2	Nep1	-0.508474977956062	0.0930434431029735	1	pseudobulk	DESeq2	LRT	1.03131422677322
138	KC_AB2	Nep2	-0.729379579623002	1	1	pseudobulk	DESeq2	LRT	0
138	KC_AB2	Nep3	-0.359400552486599	0.708591529849598	1	pseudobulk	DESeq2	LRT	0.149604043311854
138	KC_AB2	Nepl16	0.12197258710267	0.894807401685852	1	pseudobulk	DESeq2	LRT	0.048270432154724
138	KC_AB2	nero	1.04237305304479	0.767386654171747	1	pseudobulk	DESeq2	LRT	0.114985758004482
138	KC_AB2	nes	-1.12415610630345	0.500458372619195	1	pseudobulk	DESeq2	LRT	0.300632040649461
138	KC_AB2	nesd	1.38446765109058	0.642794900879723	1	pseudobulk	DESeq2	LRT	0.191927577037166
138	KC_AB2	net	1.04232157414887	0.700586543363791	1	pseudobulk	DESeq2	LRT	0.154538208723974
138	KC_AB2	NetB	-0.0494087753565114	0.890571167989638	1	pseudobulk	DESeq2	LRT	0.050331369148308
138	KC_AB2	neur	0.190233709954232	0.746338426387101	1	pseudobulk	DESeq2	LRT	0.127064197481498
138	KC_AB2	Neurl4	0.848906987400183	0.548350603200166	1	pseudobulk	DESeq2	LRT	0.260941674458421
138	KC_AB2	Nf-YA	0.0500234823321031	0.961664685731833	1	pseudobulk	DESeq2	LRT	0.016976331830983
138	KC_AB2	Nf-YB	1.02361249949071	0.526357773685645	1	pseudobulk	DESeq2	LRT	0.278718958662514
138	KC_AB2	Nf-YC	-1.38624700933171	0.495854866941722	1	pseudobulk	DESeq2	LRT	0.304645419698777
138	KC_AB2	Nf1	0.699877525562416	0.348110879862994	1	pseudobulk	DESeq2	LRT	0.458282403056927
138	KC_AB2	NFAT	-0.142374834926019	0.727593517656032	1	pseudobulk	DESeq2	LRT	0.138111178848186
138	KC_AB2	NfI	-0.0415835601498217	0.855559921730807	1	pseudobulk	DESeq2	LRT	0.067749567928034
138	KC_AB2	Nfs1	-0.387032506944607	0.752348692357982	1	pseudobulk	DESeq2	LRT	0.12358082952994
138	KC_AB2	Nhe1	1.37686412499759	0.622972562188606	1	pseudobulk	DESeq2	LRT	0.205531080712408
138	KC_AB2	Nhe2	0.363958766697548	0.747125119588835	1	pseudobulk	DESeq2	LRT	0.126606661642763
138	KC_AB2	Nhe3	-0.271592779564863	0.653184018556876	1	pseudobulk	DESeq2	LRT	0.184964449681648
138	KC_AB2	NHP2	0.360170294777902	0.785552702640174	1	pseudobulk	DESeq2	LRT	0.104824672879896
138	KC_AB2	NimC4	1.38446765109058	0.642794900879723	1	pseudobulk	DESeq2	LRT	0.191927577037166
138	KC_AB2	NiPp1	0.244210846527922	0.848335459592238	1	pseudobulk	DESeq2	LRT	0.071432379534485
138	KC_AB2	Nipped-A	-0.185730591686316	0.728080312932539	1	pseudobulk	DESeq2	LRT	0.137820711978696
138	KC_AB2	Nipped-B	0.0958363974847487	0.782990077736501	1	pseudobulk	DESeq2	LRT	0.106243741405133
138	KC_AB2	Nipsnap	-0.156211983176898	0.890699935291607	1	pseudobulk	DESeq2	LRT	0.050268579235319
138	KC_AB2	nito	0.584907330311258	0.56188964389012	1	pseudobulk	DESeq2	LRT	0.250348972252532
138	KC_AB2	NK7.1	-0.399922199780013	0.694232773877396	1	pseudobulk	DESeq2	LRT	0.158494887671053
138	KC_AB2	NKAIN	0.761320848740786	0.481252353894019	1	pseudobulk	DESeq2	LRT	0.317627133263841
138	KC_AB2	nkd	1.49831363725381	0.0973089604375589	1	pseudobulk	DESeq2	LRT	1.01184716703469
138	KC_AB2	Nle	-0.768511978427448	0.660852672472004	1	pseudobulk	DESeq2	LRT	0.179895349385784
138	KC_AB2	Nlg2	-0.280346448672608	0.524157447577346	1	pseudobulk	DESeq2	LRT	0.280538239078979
138	KC_AB2	Nlg3	-0.410961610765897	0.152271346780895	1	pseudobulk	DESeq2	LRT	0.817381811080721
138	KC_AB2	Nlp	0.0159871443730439	0.941411902244503	1	pseudobulk	DESeq2	LRT	0.026220315221651
138	KC_AB2	nmd	-1.74285916595985	0.366221301489537	1	pseudobulk	DESeq2	LRT	0.436256398291263
138	KC_AB2	Nmd3	-0.83320718254958	0.715059964627045	1	pseudobulk	DESeq2	LRT	0.14565753691968
138	KC_AB2	Nmda1	0.530053167453316	0.47659196062515	1	pseudobulk	DESeq2	LRT	0.321853287809082
138	KC_AB2	Nmdar1	-2.07543831712051	0.148895553628423	1	pseudobulk	DESeq2	LRT	0.827118271109293
138	KC_AB2	Nmdar2	-0.0598914836386775	0.954123872813099	1	pseudobulk	DESeq2	LRT	0.020395237678697
138	KC_AB2	nmdyn-D6	-0.729379579623002	0.772694994316916	1	pseudobulk	DESeq2	LRT	0.1119919011982
138	KC_AB2	Nmnat	-1.54352099991151	0.432364700375742	1	pseudobulk	DESeq2	LRT	0.364149770449125
138	KC_AB2	nmo	-0.832429630185681	0.0351337511686543	1	pseudobulk	DESeq2	LRT	1.45427547887648
138	KC_AB2	Nmt	0.0455261422590676	0.956410711868198	1	pseudobulk	DESeq2	LRT	0.019355568399845
138	KC_AB2	Nna1	-0.729379579623002	0.772694994316916	1	pseudobulk	DESeq2	LRT	0.1119919011982

138	KC_AB2	Nnf1a	1.38446765109058	0.642794900879723	1	pseudobulk	DESeq2	LRT	0.191927577037166
138	KC_AB2	Nnp-1	0.111430571057559	0.929560676204963	1	pseudobulk	DESeq2	LRT	0.031722256800151
138	KC_AB2	NO66	1.04324661842443	0.68096673636569	1	pseudobulk	DESeq2	LRT	0.166874101840012
138	KC_AB2	Noa36	-0.963919078111595	0.599234761105423	1	pseudobulk	DESeq2	LRT	0.222403001519551
138	KC_AB2	noc	-1.17300420086321	0.534249222133528	1	pseudobulk	DESeq2	LRT	0.272256101495877
138	KC_AB2	nocte	-0.299352953028699	0.461195923037346	1	pseudobulk	DESeq2	LRT	0.336114540514826
138	KC_AB2	noi	-1.12288499100873	0.591469288777357	1	pseudobulk	DESeq2	LRT	0.228067800589375
138	KC_AB2	nom	3.56851677823906	0.093980116830568	1	pseudobulk	DESeq2	LRT	1.02696401942331
138	KC_AB2	Non1	0.250495727513639	0.594102988213689	1	pseudobulk	DESeq2	LRT	0.22613826320747
138	KC_AB2	Non2	0.240512272478465	0.782518300054642	1	pseudobulk	DESeq2	LRT	0.106505497206668
138	KC_AB2	Non3	-2.12405037015269	0.265879278548733	1	pseudobulk	DESeq2	LRT	0.575315508334519
138	KC_AB2	nonA	-0.0349767299154801	0.920923994698877	1	pseudobulk	DESeq2	LRT	0.035776211328628
138	KC_AB2	nonA-l	0.502525628695585	0.61006384698678	1	pseudobulk	DESeq2	LRT	0.214624710984283
138	KC_AB2	nonC	0.31401700421368	0.839826222640754	1	pseudobulk	DESeq2	LRT	0.075810569123675
138	KC_AB2	Nop17l	-0.175465652222972	0.843179015037507	1	pseudobulk	DESeq2	LRT	0.074080210678519
138	KC_AB2	nop5	-1.86716056129969	0.477680380390446	1	pseudobulk	DESeq2	LRT	0.320862595985116
138	KC_AB2	Nop56	0.260374079648564	0.844792087460066	1	pseudobulk	DESeq2	LRT	0.073250162505012
139	KC_AB2	Nop60B	0.575162595042011	0.797021781108777	1	pseudobulk	DESeq2	LRT	0.098529809988961
139	KC_AB2	Nopp140	-1.86908183888077	0.404263068385338	1	pseudobulk	DESeq2	LRT	0.393335932000648
139	KC_AB2	nord	1.10706867860301	1	1	pseudobulk	DESeq2	LRT	0
139	KC_AB2	norpA	-0.287342386079209	0.852663842823148	1	pseudobulk	DESeq2	LRT	0.069222152866732
139	KC_AB2	Nost	1.61205113368698	0.631647504209	1	pseudobulk	DESeq2	LRT	0.199525215522471
139	KC_AB2	not	-1.31474375032394	0.452590555181994	1	pseudobulk	DESeq2	LRT	0.344294513295876
139	KC_AB2	Not1	0.036116155192175	0.913636473076894	1	pseudobulk	DESeq2	LRT	0.039226571379471
139	KC_AB2	Not10	-1.80075127281421	0.363208620892189	1	pseudobulk	DESeq2	LRT	0.439843851886983
139	KC_AB2	Not11	-0.388343937341001	0.807863855425201	1	pseudobulk	DESeq2	LRT	0.092661822170273
139	KC_AB2	Not3	0.0696638482426827	0.907498224447431	1	pseudobulk	DESeq2	LRT	0.042154216003626
139	KC_AB2	NP15.6	-0.377076724540637	0.570199400965647	1	pseudobulk	DESeq2	LRT	0.243973243283049
139	KC_AB2	Npc1a	-0.423552079205093	0.637195353750274	1	pseudobulk	DESeq2	LRT	0.195727399612862
139	KC_AB2	Npc2a	-0.729379579623002	0.823527397767669	1	pseudobulk	DESeq2	LRT	0.084321947797948
139	KC_AB2	Nph	0.668176891401745	0.45931186149022	1	pseudobulk	DESeq2	LRT	0.337892339007869
139	KC_AB2	Npl4	0.757737109895719	0.539963588931202	1	pseudobulk	DESeq2	LRT	0.267635524731487
139	KC_AB2	Nplp1	2.09752700723056	0.207662333127874	1	pseudobulk	DESeq2	LRT	0.682642270909394
139	KC_AB2	Nplp2	-0.729379579623002	0.772694994316916	1	pseudobulk	DESeq2	LRT	0.1119919011982
139	KC_AB2	Nprl2	1.99339641594643	0.465071165814147	1	pseudobulk	DESeq2	LRT	0.332480585700252
139	KC_AB2	Nprl3	-0.637636877597799	0.73776154810985	1	pseudobulk	DESeq2	LRT	0.132083983805458
139	KC_AB2	Nrd1	-0.893328173497901	0.679679924321693	1	pseudobulk	DESeq2	LRT	0.167695557626761
139	KC_AB2	Nrg	0.0186444714291041	0.948601785060369	1	pseudobulk	DESeq2	LRT	0.022916062436478
139	KC_AB2	Nrk	0.66954517615027	1	1	pseudobulk	DESeq2	LRT	0
139	KC_AB2	nrm	0.157430198868658	0.750266889465721	1	pseudobulk	DESeq2	LRT	0.124784219269715
139	KC_AB2	Nrt	-0.43851858340217	0.515316290480045	1	pseudobulk	DESeq2	LRT	0.287926128150681
139	KC_AB2	nrv2	0.14037760707246	0.916534277079674	1	pseudobulk	DESeq2	LRT	0.037851288401501
139	KC_AB2	nrv3	-0.147193183770509	0.511766815740442	1	pseudobulk	DESeq2	LRT	0.290927878292618
139	KC_AB2	Nrx-1	-0.0657641144361083	0.799753023172844	1	pseudobulk	DESeq2	LRT	0.097044109549823
139	KC_AB2	Nrx-IV	0.573318695449307	0.631377801070543	1	pseudobulk	DESeq2	LRT	0.199710691753351
139	KC_AB2	Ns1	0.724530792294316	0.639652227032513	1	pseudobulk	DESeq2	LRT	0.19405608372189
139	KC_AB2	Ns2	-1.24292003858607	0.43948094240754	1	pseudobulk	DESeq2	LRT	0.357059952869376
139	KC_AB2	Ns3	-0.0166754065092529	0.974446601891568	1	pseudobulk	DESeq2	LRT	0.011241954534277
139	KC_AB2	Ns4	0.301183407903318	0.898796313604012	1	pseudobulk	DESeq2	LRT	0.046338717499814
139	KC_AB2	NSD	-0.729379579623002	0.772694994316916	1	pseudobulk	DESeq2	LRT	0.1119919011982
139	KC_AB2	Nse4	2.47215486522296	0.30601789350912	1	pseudobulk	DESeq2	LRT	0.514253178665238
139	KC_AB2	Nsf2	-1.1223589129859	0.424392318855662	1	pseudobulk	DESeq2	LRT	0.372232485064733

139	KC_AB2	nsl1	0.553831050514354	0.408642436478483	1	pseudobulk	DESeq2	LRT	0.388656534983422
139	KC_AB2	nSMase	0.0933678712029918	0.906838782482696	1	pseudobulk	DESeq2	LRT	0.042469914815850
139	KC_AB2	nst	-0.661305861948952	0.585566046575873	1	pseudobulk	DESeq2	LRT	0.232424113323027
139	KC_AB2	Nsun2	1.17332755916016	0.471181529420146	1	pseudobulk	DESeq2	LRT	0.326811742474208
139	KC_AB2	Nsun5	-0.721175887303532	0.733409564198845	1	pseudobulk	DESeq2	LRT	0.134653430819787
139	KC_AB2	nSyb	-0.282402669168473	0.201614866308032	1	pseudobulk	DESeq2	LRT	0.695477447834125
139	KC_AB2	NT5E-2	1.10706867860301	1	1	pseudobulk	DESeq2	LRT	0
139	KC_AB2	Ntan1	0.0900166539187119	0.881474581787942	1	pseudobulk	DESeq2	LRT	0.054790206505209
139	KC_AB2	ntc	-0.721175887303532	0.733409564198845	1	pseudobulk	DESeq2	LRT	0.134653430819787
139	KC_AB2	Ntf-2	0.175457889140397	0.734032399637289	1	pseudobulk	DESeq2	LRT	0.134284770229509
139	KC_AB2	Ntl	-0.729379579623002	1	1	pseudobulk	DESeq2	LRT	0
139	KC_AB2	Ntmt	-0.729379579623002	0.823527397767669	1	pseudobulk	DESeq2	LRT	0.084321947797948
139	KC_AB2	NTPase	-1.34610742914652	0.346700295514816	1	pseudobulk	DESeq2	LRT	0.460045788166507
139	KC_AB2	Nuak1	-0.489436946725218	0.602114178648578	1	pseudobulk	DESeq2	LRT	0.22032114585989
139	KC_AB2	NUCB1	-0.667627347909082	0.390009247685932	1	pseudobulk	DESeq2	LRT	0.408925095098232
139	KC_AB2	nudC	-0.254351467699333	0.836935923244272	1	pseudobulk	DESeq2	LRT	0.077307790808032
139	KC_AB2	nudE	-0.436146009779873	0.556069558849726	1	pseudobulk	DESeq2	LRT	0.254870879038269
139	KC_AB2	nuf	0.0667588332006575	0.944703280098873	1	pseudobulk	DESeq2	LRT	0.024704576729781
139	KC_AB2	Nufip	0.39439088612385	0.813541888331052	1	pseudobulk	DESeq2	LRT	0.089620080830767
139	KC_AB2	Nulp1	0.27612057840723	0.77653142540786	1	pseudobulk	DESeq2	LRT	0.109840964140106
139	KC_AB2	numb	0.583678195130401	0.376568373706478	1	pseudobulk	DESeq2	LRT	0.424156157238948
139	KC_AB2	Nup107	1.50713387114482	0.576249954186023	1	pseudobulk	DESeq2	LRT	0.239389096130292
139	KC_AB2	Nup133	-0.729379579623002	0.823527397767669	1	pseudobulk	DESeq2	LRT	0.084321947797948
139	KC_AB2	Nup153	-0.506980025547369	0.491797611471011	1	pseudobulk	DESeq2	LRT	0.308213584843903
139	KC_AB2	Nup154	-0.729379579623002	1	1	pseudobulk	DESeq2	LRT	0
139	KC_AB2	Nup160	1.03254930292854	0.645433035572793	1	pseudobulk	DESeq2	LRT	0.190148809617279
139	KC_AB2	Nup188	0.147593560254927	1	1	pseudobulk	DESeq2	LRT	0
139	KC_AB2	Nup205	1.38069928703004	0.566368801250645	1	pseudobulk	DESeq2	LRT	0.246900678019328
139	KC_AB2	Nup214	-0.721175887303532	0.733409564198845	1	pseudobulk	DESeq2	LRT	0.134653430819787
139	KC_AB2	Nup35	3.39822824606857	0.116524932483093	1	pseudobulk	DESeq2	LRT	0.933581140034328
139	KC_AB2	Nup358	-0.417754783660064	0.710858919026289	1	pseudobulk	DESeq2	LRT	0.148216583191529
139	KC_AB2	Nup37	-2.35203494925393	0.313819523334105	1	pseudobulk	DESeq2	LRT	0.503320041588723
139	KC_AB2	Nup43	1.67808859760333	0.523397782439413	1	pseudobulk	DESeq2	LRT	0.281168121735049
139	KC_AB2	Nup44A	-1.26150651003313	0.399493018430534	1	pseudobulk	DESeq2	LRT	0.398490806046081
139	KC_AB2	Nup50	-0.818820863403386	0.701088644508524	1	pseudobulk	DESeq2	LRT	0.154227067073285
139	KC_AB2	Nup54	1.38069928703004	0.566368801250645	1	pseudobulk	DESeq2	LRT	0.246900678019328
139	KC_AB2	Nup58	-1.766017528816	0.373098694305789	1	pseudobulk	DESeq2	LRT	0.428176270811176
139	KC_AB2	Nup62	-0.519696389650941	0.752299890889585	1	pseudobulk	DESeq2	LRT	0.123609001169055
139	KC_AB2	Nup75	1.38069928703004	0.566368801250645	1	pseudobulk	DESeq2	LRT	0.246900678019328
139	KC_AB2	Nup93-1	0.145321756433347	1	1	pseudobulk	DESeq2	LRT	0
139	KC_AB2	Nup93-2	1.48850410624721	0.522705582363401	1	pseudobulk	DESeq2	LRT	0.281742861722271
139	KC_AB2	Nup98-96	0.376597696097159	0.825096774316101	1	pseudobulk	DESeq2	LRT	0.083495110738990
139	KC_AB2	Nurf-38	-0.0303082318718535	0.932069719741568	1	pseudobulk	DESeq2	LRT	0.030551600772018
139	KC_AB2	nvd	-0.729379579623002	0.823527397767669	1	pseudobulk	DESeq2	LRT	0.084321947797948
139	KC_AB2	nwk	-0.27325217479115	0.669285240100749	1	pseudobulk	DESeq2	LRT	0.174388752485638
139	KC_AB2	Nxf3	3.47212988600903	0.084654023808664	1	pseudobulk	DESeq2	LRT	1.07235239400671
139	KC_AB2	Nxt1	0.800656956056621	0.463613374476614	1	pseudobulk	DESeq2	LRT	0.333844043852513
139	KC_AB2	O-fut2	2.76364737573217	0.211914962801091	1	pseudobulk	DESeq2	LRT	0.673838377727484
139	KC_AB2	oaf	0.357125501160608	0.826882908673327	1	pseudobulk	DESeq2	LRT	0.082555984664330
139	KC_AB2	Oamb	0.138651484432347	0.707272381172104	1	pseudobulk	DESeq2	LRT	0.150413300688575
139	KC_AB2	Oatp30B	0.147650649941619	0.881769324210645	1	pseudobulk	DESeq2	LRT	0.054645013876419
139	KC_AB2	obe	-2.09428695548101	0.380540277844868	1	pseudobulk	DESeq2	LRT	0.419599369064229

139	KC_AB2	Obp19a	1.10706867860301	1	1	pseudobulk	DESeq2	LRT	0
139	KC_AB2	Obp44a	2.01814786626755	0.401718761445944	1	pseudobulk	DESeq2	LRT	0.396077884966921
139	KC_AB2	Obp85a	0.145321756433347	1	1	pseudobulk	DESeq2	LRT	0
139	KC_AB2	ocm	-0.603647963308095	0.706080294682827	1	pseudobulk	DESeq2	LRT	0.15114590864391
139	KC_AB2	Ocrl	-0.305804501037192	0.742218187417572	1	pseudobulk	DESeq2	LRT	0.129468407844531
139	KC_AB2	Octalpha2R	0.140173331667209	0.780211439782588	1	pseudobulk	DESeq2	LRT	0.107787686172357
139	KC_AB2	Octbeta1R	-0.076599130563077	0.794371191873622	1	pseudobulk	DESeq2	LRT	0.099976514055951
139	KC_AB2	Octbeta2R	-1.86979501903407	0.475964703567026	1	pseudobulk	DESeq2	LRT	0.322425252351861
139	KC_AB2	Octbeta3R	-0.395517961695295	0.327857258219222	1	pseudobulk	DESeq2	LRT	0.484315197327148
139	KC_AB2	Oda	-0.259680847919756	0.369319966628464	1	pseudobulk	DESeq2	LRT	0.432597212354009
139	KC_AB2	Odj	-1.41813487571249	0.473691038140433	1	pseudobulk	DESeq2	LRT	0.324504831702221
139	KC_AB2	Oga	-2.03522979197945	0.396502959232805	1	pseudobulk	DESeq2	LRT	0.401753567050837
139	KC_AB2	Ogg1	-0.670541955584215	0.735328220685952	1	pseudobulk	DESeq2	LRT	0.133518766201526
140	KC_AB2	ohgt	1.19367667085681	0.270987626609024	1	pseudobulk	DESeq2	LRT	0.567050538712694
140	KC_AB2	olf186-F	0.987997075282065	0.492921267772312	1	pseudobulk	DESeq2	LRT	0.307222443202763
140	KC_AB2	olf413	-0.0107084033230793	0.85881446966383	1	pseudobulk	DESeq2	LRT	0.066100646999858
140	KC_AB2	Oli	-0.443110654163094	0.637901461332241	1	pseudobulk	DESeq2	LRT	0.195246402947828
140	KC_AB2	omd	0.162385149200511	0.91636577592647	1	pseudobulk	DESeq2	LRT	0.037931139041603
140	KC_AB2	ome	-0.196044154232892	0.721343899745596	1	pseudobulk	DESeq2	LRT	0.14185763659253
140	KC_AB2	onecut	-0.248215167764652	0.602549103382631	1	pseudobulk	DESeq2	LRT	0.220007555459872
140	KC_AB2	Opa1	-0.0512292176914448	0.936740022660255	1	pseudobulk	DESeq2	LRT	0.028380923936509
140	KC_AB2	Opbp	-0.968936593053995	0.673838136795855	1	pseudobulk	DESeq2	LRT	0.171444413165975
140	KC_AB2	opm	0.96901565995684	0.569525848090615	1	pseudobulk	DESeq2	LRT	0.244486560560513
140	KC_AB2	or	0.528236031995133	0.729334342456385	1	pseudobulk	DESeq2	LRT	0.137073336158448
140	KC_AB2	Or13a	1.10706867860301	1	1	pseudobulk	DESeq2	LRT	0
140	KC_AB2	Or2a	-0.729379579623002	1	1	pseudobulk	DESeq2	LRT	0
140	KC_AB2	Or43b	-0.347135499964956	0.854806062097812	1	pseudobulk	DESeq2	LRT	0.068132406575477
140	KC_AB2	Or45a	1.10706867860301	1	1	pseudobulk	DESeq2	LRT	0
140	KC_AB2	Or92a	-0.729379579623002	1	1	pseudobulk	DESeq2	LRT	0
140	KC_AB2	orb	0.788339339749612	0.656388976933639	1	pseudobulk	DESeq2	LRT	0.182838720880814
140	KC_AB2	orb2	-0.0535021577601418	0.897394651515619	1	pseudobulk	DESeq2	LRT	0.047016523196275
140	KC_AB2	Orc4	1.04232157414887	0.700586543363791	1	pseudobulk	DESeq2	LRT	0.154538208723974
140	KC_AB2	Orc5	-0.729379579623002	1	1	pseudobulk	DESeq2	LRT	0
140	KC_AB2	Orc6	-0.729379579623002	0.772694994316916	1	pseudobulk	DESeq2	LRT	0.1119919011982
140	KC_AB2	Orct2	1.10706867860301	1	1	pseudobulk	DESeq2	LRT	0
140	KC_AB2	ORMDL	0.360971210600822	0.775409366427584	1	pseudobulk	DESeq2	LRT	0.110468957309662
140	KC_AB2	osa	-0.611792609016439	0.159650542016156	1	pseudobulk	DESeq2	LRT	0.796829602685287
140	KC_AB2	Osbp	1.98471335244306	0.308905241676658	1	pseudobulk	DESeq2	LRT	0.510174722284711
140	KC_AB2	Oscillin	1.38446765109058	0.642794900879723	1	pseudobulk	DESeq2	LRT	0.191927577037166
140	KC_AB2	Oseg1	-1.78975280219494	0.468855431150075	1	pseudobulk	DESeq2	LRT	0.32896104882553
140	KC_AB2	Oseg4	1.10706867860301	1	1	pseudobulk	DESeq2	LRT	0
140	KC_AB2	Osi24	-0.729379579623002	0.823527397767669	1	pseudobulk	DESeq2	LRT	0.084321947797948
140	KC_AB2	osp	0.720557559963523	0.758319263499194	1	pseudobulk	DESeq2	LRT	0.120147911539297
140	KC_AB2	Ost48	-0.359273515233454	0.736485774691001	1	pseudobulk	DESeq2	LRT	0.132835637189124
140	KC_AB2	OstDelta	-2.15713211248321	0.284597948138266	1	pseudobulk	DESeq2	LRT	0.545768235367128
140	KC_AB2	Ostgamma	-0.0965483561300648	0.922118774049871	1	pseudobulk	DESeq2	LRT	0.035213135787774
140	KC_AB2	otk	3.39822824606857	0.116524932483093	1	pseudobulk	DESeq2	LRT	0.933581140034328
140	KC_AB2	otk2	1.50776466886541	0.515809803599324	1	pseudobulk	DESeq2	LRT	0.287510407819198
140	KC_AB2	OtopLa	0.382873548253534	0.223256420820137	1	pseudobulk	DESeq2	LRT	0.651196042017666
140	KC_AB2	OtopLc	0.23070880126401	0.690910270049976	1	pseudobulk	DESeq2	LRT	0.160578351688832
140	KC_AB2	ova	-0.123719186762323	0.87741977924758	1	pseudobulk	DESeq2	LRT	0.056792579731325
140	KC_AB2	ox	-0.542876065371212	0.242368753616951	1	pseudobulk	DESeq2	LRT	0.615523370505901

140	KC_AB2	OXA1L	-1.98028140890842	0.328533066956313	1	pseudobulk	DESeq2	LRT	0.483420912028321
140	KC_AB2	oxt	0.463809157915508	0.662956932770052	1	pseudobulk	DESeq2	LRT	0.178514683462236
140	KC_AB2	oys	-0.196868539515769	0.886177906859106	1	pseudobulk	DESeq2	LRT	0.052479081486512
140	KC_AB2	p	-0.240478378824993	0.802782712975552	1	pseudobulk	DESeq2	LRT	0.095401988127950
140	KC_AB2	p115	-0.348901974470907	0.89890135131336	1	pseudobulk	DESeq2	LRT	0.046287966699494
140	KC_AB2	p120ctn	-0.109142229015271	0.712445788699202	1	pseudobulk	DESeq2	LRT	0.147248176322275
140	KC_AB2	p130CAS	0.233974419361612	0.807781752923074	1	pseudobulk	DESeq2	LRT	0.092705961384307
140	KC_AB2	p23	-0.700683416375765	0.272505905405849	1	pseudobulk	DESeq2	LRT	0.564624081800608
140	KC_AB2	p24-1	-0.236901216944185	0.790845192994392	1	pseudobulk	DESeq2	LRT	0.101908520810973
140	KC_AB2	P32	-2.10789216924743	0.165298993325177	1	pseudobulk	DESeq2	LRT	0.781729791284282
140	KC_AB2	p38b	-0.656296399573135	0.564627428144581	1	pseudobulk	DESeq2	LRT	0.248238028770553
140	KC_AB2	p47	0.285438622063816	0.787056625172708	1	pseudobulk	DESeq2	LRT	0.103994020988609
140	KC_AB2	P58IPK	-0.297948803095252	0.868972202454058	1	pseudobulk	DESeq2	LRT	0.060994115972393
140	KC_AB2	P5CDh1	-0.223997592721118	0.691558414125205	1	pseudobulk	DESeq2	LRT	0.160171130289612
140	KC_AB2	P5cr	0.21401591726502	0.859119634462673	1	pseudobulk	DESeq2	LRT	0.065946355412905
140	KC_AB2	Pa1	-0.442646935322895	0.605808594648106	1	pseudobulk	DESeq2	LRT	0.217664569593839
140	KC_AB2	pAbp	0.0418695216204296	0.855135030536041	1	pseudobulk	DESeq2	LRT	0.067965302363734
140	KC_AB2	Pabp2	0.0122286048397962	0.929406395365524	1	pseudobulk	DESeq2	LRT	0.031794343410409
140	KC_AB2	pad	2.01814786626755	0.401718761445944	1	pseudobulk	DESeq2	LRT	0.396077884966921
140	KC_AB2	Paf-AHalpha	0.212373013699175	0.806896960551672	1	pseudobulk	DESeq2	LRT	0.093181920446359
140	KC_AB2	Paics	1.13746102707583	0.612851413153431	1	pseudobulk	DESeq2	LRT	0.212644808140106
140	KC_AB2	Paip2	-0.0437478805789688	0.903893946388926	1	pseudobulk	DESeq2	LRT	0.043882522179486
140	KC_AB2	Pak	-0.525965202461392	0.423015035307439	1	pseudobulk	DESeq2	LRT	0.373644196135575
140	KC_AB2	Pak3	0.742538672709105	0.528298426387999	1	pseudobulk	DESeq2	LRT	0.277120682919016
140	KC_AB2	Pal1	0.634972336942021	0.633428957531517	1	pseudobulk	DESeq2	LRT	0.198302086514026
140	KC_AB2	Pal2	-0.415580435468158	0.319587070889542	1	pseudobulk	DESeq2	LRT	0.49541079867878
140	KC_AB2	pall	0.713594606398094	0.595066221598323	1	pseudobulk	DESeq2	LRT	0.22543470137326
140	KC_AB2	pan	0.196903397708988	0.35463022768308	1	pseudobulk	DESeq2	LRT	0.45022424911733
140	KC_AB2	PAN2	-3.12892256584057	0.142517299650159	1	pseudobulk	DESeq2	LRT	0.846132415047834
140	KC_AB2	Panx	-1.13684113428818	0.451950671894501	1	pseudobulk	DESeq2	LRT	0.344908963624635
140	KC_AB2	papi	0.681680287540556	0.717771302843503	1	pseudobulk	DESeq2	LRT	0.144013909148294
140	KC_AB2	PAPLA1	1.2400276916476	0.290449393224262	1	pseudobulk	DESeq2	LRT	0.536929526468282
140	KC_AB2	Papss	2.03913993462321	0.360583814303595	1	pseudobulk	DESeq2	LRT	0.442993771558146
140	KC_AB2	Papst2	-1.18702567043751	0.403921765998825	1	pseudobulk	DESeq2	LRT	0.393702743516105
140	KC_AB2	par-1	0.0792480890682327	0.841375710895724	1	pseudobulk	DESeq2	LRT	0.075010029490660
140	KC_AB2	par-6	-0.220831446630607	0.842589214517586	1	pseudobulk	DESeq2	LRT	0.074384104318447
140	KC_AB2	para	-0.0733396920784242	0.699085040877319	1	pseudobulk	DESeq2	LRT	0.15546999086762
140	KC_AB2	Parg	-0.729379579623002	0.823527397767669	1	pseudobulk	DESeq2	LRT	0.084321947797948
140	KC_AB2	Parp	0.0074992779505245:	0.922145234770369	1	pseudobulk	DESeq2	LRT	0.035200673640440
140	KC_AB2	parvin	-0.586200653393176	0.673535756140177	1	pseudobulk	DESeq2	LRT	0.171639343842735
140	KC_AB2	pasha	0.280403434799392	0.873908442859524	1	pseudobulk	DESeq2	LRT	0.058534064898475
140	KC_AB2	pasi1	0.0843130080157558	0.943549788858392	1	pseudobulk	DESeq2	LRT	0.025235178216641
140	KC_AB2	pasi2	3.63594195979476	0.0797044662899187	1	pseudobulk	DESeq2	LRT	1.09851734195747
140	KC_AB2	Pask	-0.794131483073487	0.650767771801022	1	pseudobulk	DESeq2	LRT	0.18657396291949
140	KC_AB2	Past1	-0.0032219210735671	0.887704234753852	1	pseudobulk	DESeq2	LRT	0.051731708332114
140	KC_AB2	Pat1	-0.270012346626457	0.693824720610677	1	pseudobulk	DESeq2	LRT	0.15875023053382
140	KC_AB2	path	0.473052454753245	0.672732522212462	1	pseudobulk	DESeq2	LRT	0.172157576515207
140	KC_AB2	Patj	-1.11167283223406	0.564212144344824	1	pseudobulk	DESeq2	LRT	0.248557570138858
140	KC_AB2	Patr-1	0.80209323348837	0.653341122229668	1	pseudobulk	DESeq2	LRT	0.184860005830735
140	KC_AB2	Patronin	-0.101749408729067	0.884131393146983	1	pseudobulk	DESeq2	LRT	0.053483188518241
140	KC_AB2	Patsas	-1.11167283223406	0.564212144344824	1	pseudobulk	DESeq2	LRT	0.248557570138858
140	KC_AB2	Pax	-1.13248612328926	0.530057852990008	1	pseudobulk	DESeq2	LRT	0.275676726883872

140	KC_AB2	Pbgs	0.147593560254927	1	1	pseudobulk	DESeq2	LRT	0
140	KC_AB2	Pbp45	-0.729379579623002	0.823527397767669	1	pseudobulk	DESeq2	LRT	0.084321947797948
140	KC_AB2	Pc	-0.0586660628284225	0.935275195916326	1	pseudobulk	DESeq2	LRT	0.029060583263375
140	KC_AB2	PCB	-0.994598991352086	0.355825625633681	1	pseudobulk	DESeq2	LRT	0.448762778369716
140	KC_AB2	Pcd	-0.585785646779873	0.157147910015069	1	pseudobulk	DESeq2	LRT	0.80369139049723€
140	KC_AB2	Pcf11	0.608108892067477	0.19615469884606	1	pseudobulk	DESeq2	LRT	0.707401283973533
140	KC_AB2	PCID2	0.341310869435805	0.849261178469218	1	pseudobulk	DESeq2	LRT	0.070958727983931
140	KC_AB2	pck	-0.132280652090949	0.911172139618949	1	pseudobulk	DESeq2	LRT	0.040399567895959
140	KC_AB2	Pcl	0.122976979799163	0.872489640843667	1	pseudobulk	DESeq2	LRT	0.059239720759774
141	KC_AB2	pcm	2.50658737188506	0.249588261752715	1	pseudobulk	DESeq2	LRT	0.60277584357254
141	KC_AB2	Pcmt	0.314295002907474	0.689872586872963	1	pseudobulk	DESeq2	LRT	0.161231112056362
141	KC_AB2	PCNA2	-2.5216736243906	0.267985186584202	1	pseudobulk	DESeq2	LRT	0.571889211801602
141	KC_AB2	pcs	-0.36395412346892	0.570983310873186	1	pseudobulk	DESeq2	LRT	0.243376585452238
141	KC_AB2	pcx	-0.919269376899882	0.505523091509982	1	pseudobulk	DESeq2	LRT	0.296259001722204
141	KC_AB2	Pcyt1	0.175545782450655	0.798261452293563	1	pseudobulk	DESeq2	LRT	0.097854842118649
141	KC_AB2	Pcyt2	-0.689996600097885	0.601950657474518	1	pseudobulk	DESeq2	LRT	0.220439106856465
141	KC_AB2	PDCD-5	-0.615825575154615	0.575530010712768	1	pseudobulk	DESeq2	LRT	0.239932025384085
141	KC_AB2	Pdcd4	-0.241740397925419	0.612049209549126	1	pseudobulk	DESeq2	LRT	0.213213658611835
141	KC_AB2	Pde11	3.5554947126272	0.0906963498444766	1	pseudobulk	DESeq2	LRT	1.04241019115712
141	KC_AB2	Pde1c	-0.15731263974586	0.506427539441149	1	pseudobulk	DESeq2	LRT	0.295482685490459
141	KC_AB2	Pde6	-1.2855154833102	0.0224220225605878	1	pseudobulk	DESeq2	LRT	1.64932521478813
141	KC_AB2	Pde8	-0.0755144250092419	0.865342983454271	1	pseudobulk	DESeq2	LRT	0.062811723404615
141	KC_AB2	Pde9	0.0114636452239663	0.937796437881914	1	pseudobulk	DESeq2	LRT	0.027891421214536
141	KC_AB2	Pdha	-0.398989359385897	0.552516679828063	1	pseudobulk	DESeq2	LRT	0.257654606605852
141	KC_AB2	Pdhb	-0.186650811543081	0.763033785075795	1	pseudobulk	DESeq2	LRT	0.11745623223234
141	KC_AB2	Pdi	0.0641939325912252	0.924232181496402	1	pseudobulk	DESeq2	LRT	0.034218913546182
141	KC_AB2	Pdk	0.305372297257597	0.674142110298506	1	pseudobulk	DESeq2	LRT	0.171248543801615
141	KC_AB2	Pdk1	1.09970369755254	0.0575131823142015	1	pseudobulk	DESeq2	LRT	1.24023260139444
141	KC_AB2	pdm3	-2.06066018765151	0.373409266927379	1	pseudobulk	DESeq2	LRT	0.427814908332614
141	KC_AB2	Pdp	0.344007688005072	0.827505590432938	1	pseudobulk	DESeq2	LRT	0.082229063551065
141	KC_AB2	Pdp1	-0.25113143219006	0.381874294885525	1	pseudobulk	DESeq2	LRT	0.418079574318727
141	KC_AB2	pds5	-0.0082824264629634	0.942089653069169	1	pseudobulk	DESeq2	LRT	0.025907766017609
141	KC_AB2	Pdss2	-0.376821744321153	0.759011700224948	1	pseudobulk	DESeq2	LRT	0.119751529369385
141	KC_AB2	Pdxk	-0.470100723599697	0.833985291815347	1	pseudobulk	DESeq2	LRT	0.078841608522644
141	KC_AB2	PDZ-GEF	0.196029351107096	0.667649772495239	1	pseudobulk	DESeq2	LRT	0.175451294649633
141	KC_AB2	pea	3.64432457146037	0.0814062568706535	1	pseudobulk	DESeq2	LRT	1.08934221403009
141	KC_AB2	Pect	-0.616792320092902	0.282829925586982	1	pseudobulk	DESeq2	LRT	0.548474640738986
141	KC_AB2	PEK	-1.15253484707392	0.661144484795202	1	pseudobulk	DESeq2	LRT	0.179703620556297
141	KC_AB2	pelo	-0.982687249906883	0.564766895025148	1	pseudobulk	DESeq2	LRT	0.248130768275784
141	KC_AB2	pen-2	0.280127094018748	0.822047833946926	1	pseudobulk	DESeq2	LRT	0.085102910665814
141	KC_AB2	peng	-2.47900728534227	0.218260881703082	1	pseudobulk	DESeq2	LRT	0.661024094720707
141	KC_AB2	Pep	-0.219125031448885	0.500489481588755	1	pseudobulk	DESeq2	LRT	0.300605045329446
141	KC_AB2	per	0.436595940151346	0.695711487605498	1	pseudobulk	DESeq2	LRT	0.157570825501789
141	KC_AB2	pes	1.68881465104187	0.456529340335333	1	pseudobulk	DESeq2	LRT	0.340531305892531
141	KC_AB2	Pex1	1.10706867860301	1	1	pseudobulk	DESeq2	LRT	0
141	KC_AB2	Pex10	-0.115183097143228	0.910399790291748	1	pseudobulk	DESeq2	LRT	0.040767850987610
141	KC_AB2	Pex11	-1.19881828456548	0.535965909404053	1	pseudobulk	DESeq2	LRT	0.270862833121686
141	KC_AB2	Pex12	0.145321756433347	1	1	pseudobulk	DESeq2	LRT	0
141	KC_AB2	Pex13	-0.542769384404681	0.626131825778583	1	pseudobulk	DESeq2	LRT	0.203334220813605
141	KC_AB2	Pex14	-1.23843665632218	0.484129370698825	1	pseudobulk	DESeq2	LRT	0.315038569196538
141	KC_AB2	Pex16	0.403412342391299	0.806687565169252	1	pseudobulk	DESeq2	LRT	0.093294637515942
141	KC_AB2	Pex19	-0.461170341800682	0.577613648655123	1	pseudobulk	DESeq2	LRT	0.238362553205778

141	KC_AB2	Pex2	-2.0963685236798	0.327463702938206	1	pseudobulk	DESeq2	LRT	0.484836831511152
141	KC_AB2	Pex23	-0.62878936280848	0.58768758705628	1	pseudobulk	DESeq2	LRT	0.230853482213785
141	KC_AB2	Pex3	-1.57222074873527	0.359423533061397	1	pseudobulk	DESeq2	LRT	0.444393491099843
141	KC_AB2	Pex5	-1.23187249882758	0.525233583714338	1	pseudobulk	DESeq2	LRT	0.279647512674874
141	KC_AB2	Pex6	1.05564509663658	0.613237310736703	1	pseudobulk	DESeq2	LRT	0.212371429561112
141	KC_AB2	Pfdn1	-0.852737710458097	0.434345850931902	1	pseudobulk	DESeq2	LRT	0.362164322708578
141	KC_AB2	Pfdn2	0.0219558727795613	0.907766412152221	1	pseudobulk	DESeq2	LRT	0.042025890426403
141	KC_AB2	Pfdn4	-0.254504145550625	0.746131947549871	1	pseudobulk	DESeq2	LRT	0.127184364181992
141	KC_AB2	Pfdn5	-0.851740869740002	0.396749369666639	1	pseudobulk	DESeq2	LRT	0.401483754555516
141	KC_AB2	Pfdn6	0.0712015630802045	0.884094123440317	1	pseudobulk	DESeq2	LRT	0.053501496169384
141	KC_AB2	Pfk	0.0009829673605504	0.889089625693938	1	pseudobulk	DESeq2	LRT	0.051054457272348
141	KC_AB2	Pfrx	-0.343621124839582	0.583447022094425	1	pseudobulk	DESeq2	LRT	0.233998572449124
141	KC_AB2	Pgam5	2.08439339128058	0.210251523153406	1	pseudobulk	DESeq2	LRT	0.677260849300689
141	KC_AB2	Pgant1	-0.0686652919111462	0.961242182172663	1	pseudobulk	DESeq2	LRT	0.017167179313352
141	KC_AB2	Pgant2	-0.22281297568827	0.624365877345349	1	pseudobulk	DESeq2	LRT	0.204560839891434
141	KC_AB2	Pgant3	-1.21104514705662	0.64032599509333	1	pseudobulk	DESeq2	LRT	0.193598866914817
141	KC_AB2	Pgant35A	0.145321756433347	1	1	pseudobulk	DESeq2	LRT	0
141	KC_AB2	Pgant5	0.41112287599156	0.552131728471811	1	pseudobulk	DESeq2	LRT	0.257957295235705
141	KC_AB2	Pgant6	-1.01397824610998	0.639310995576363	1	pseudobulk	DESeq2	LRT	0.194287826025898
141	KC_AB2	Pgant7	-2.1751167112989	0.359363815234217	1	pseudobulk	DESeq2	LRT	0.444465654648455
141	KC_AB2	Pgant9	-0.0410946076568079	0.882606522125415	1	pseudobulk	DESeq2	LRT	0.054232867606464
141	KC_AB2	PGAP2	-1.41091034403069	0.269007832981323	1	pseudobulk	DESeq2	LRT	0.570235074008604
141	KC_AB2	PGAP3	-0.146783720166155	0.920449649997984	1	pseudobulk	DESeq2	LRT	0.035999963081857
141	KC_AB2	PGAP5	-1.12956848412701	0.556577485235076	1	pseudobulk	DESeq2	LRT	0.254474365799203
141	KC_AB2	Pgi	-0.435000035956653	0.602513647857459	1	pseudobulk	DESeq2	LRT	0.220033111206117
141	KC_AB2	Pgk	0.0674706466072958	0.897951992193336	1	pseudobulk	DESeq2	LRT	0.046746881687772
141	KC_AB2	Pglym78	-0.0892278042210459	0.823042310090662	1	pseudobulk	DESeq2	LRT	0.084577838461371
141	KC_AB2	Pgm1	0.474396347993209	0.808657101852946	1	pseudobulk	DESeq2	LRT	0.092235595003270
141	KC_AB2	Pgm2a	-0.388225598920598	0.784399171918626	1	pseudobulk	DESeq2	LRT	0.105462873483806
141	KC_AB2	PGRP-LE	-0.198420145317587	0.888264607462347	1	pseudobulk	DESeq2	LRT	0.051457641831171
141	KC_AB2	ph-d	0.745855653721021	0.621012836666784	1	pseudobulk	DESeq2	LRT	0.206899422631903
141	KC_AB2	ph-p	0.600428627163984	0.736132202233469	1	pseudobulk	DESeq2	LRT	0.133044183563834
141	KC_AB2	PH4alphaEFB	-0.765882155791966	0.591796441596247	1	pseudobulk	DESeq2	LRT	0.227827650530169
141	KC_AB2	PH4alphaSG1	-0.729379579623002	1	1	pseudobulk	DESeq2	LRT	0
141	KC_AB2	Phax	-1.63598319081091	0.397013186805603	1	pseudobulk	DESeq2	LRT	0.401195067892312
141	KC_AB2	Phb2	-0.530574379816464	0.479679401358711	1	pseudobulk	DESeq2	LRT	0.319048930832253
141	KC_AB2	pHCl-1	0.29945177837049	0.288368476032427	1	pseudobulk	DESeq2	LRT	0.540052217716337
141	KC_AB2	PheRS-m	-1.50961686094135	0.269044884929906	1	pseudobulk	DESeq2	LRT	0.570175260327516
141	KC_AB2	Phf5a	0.0295996189736942	0.969990760988217	1	pseudobulk	DESeq2	LRT	0.013232402301739
141	KC_AB2	PHGPx	-0.564214967713104	0.494594385614565	1	pseudobulk	DESeq2	LRT	0.305750817838152
141	KC_AB2	PhKgamma	-0.339403236464946	0.678018208623544	1	pseudobulk	DESeq2	LRT	0.168758642713259
141	KC_AB2	Phm	-0.585686095126761	0.294543675952426	1	pseudobulk	DESeq2	LRT	0.530850297417714
141	KC_AB2	pho	0.657502475201304	0.423268452210662	1	pseudobulk	DESeq2	LRT	0.373384099920527
141	KC_AB2	phol	-1.27799160288928	0.564705366038248	1	pseudobulk	DESeq2	LRT	0.248178085417489
141	KC_AB2	phr	1.38069928703004	0.566368801250645	1	pseudobulk	DESeq2	LRT	0.246900678019328
141	KC_AB2	phtf	-0.405496671036891	0.65146993267518	1	pseudobulk	DESeq2	LRT	0.186105623504098
141	KC_AB2	phyl	0.265733933647134	0.56881890348242	1	pseudobulk	DESeq2	LRT	0.245025979188124
141	KC_AB2	PI31	0.433809307969685	0.530635584941734	1	pseudobulk	DESeq2	LRT	0.275203629164035
141	KC_AB2	Pi3K21B	-0.545430917770912	0.626537856427188	1	pseudobulk	DESeq2	LRT	0.203052683104418
141	KC_AB2	Pi3K59F	1.61205113368698	0.631647504209	1	pseudobulk	DESeq2	LRT	0.199525215522471
141	KC_AB2	Pi3K68D	-0.209065158074281	0.765024788343755	1	pseudobulk	DESeq2	LRT	0.116324492602597
141	KC_AB2	Pi3K92E	0.729262424309725	0.695351091939929	1	pseudobulk	DESeq2	LRT	0.157795859018845

141	KC_AB2	Pi4KIIalpha	1.35087689131302	0.401056815464057	1	pseudobulk	DESeq2	LRT	0.396794098964144
141	KC_AB2	PI4KIIIalpha	0.0685918628264749	0.90073008545964	1	pseudobulk	DESeq2	LRT	0.045405331060067
141	KC_AB2	pic	1.50713387114482	0.576249954186023	1	pseudobulk	DESeq2	LRT	0.239389096130292
141	KC_AB2	PICK1	-0.732989004083733	0.474187699224746	1	pseudobulk	DESeq2	LRT	0.32404971612777
141	KC_AB2	pico	0.761680655092855	0.415338573982033	1	pseudobulk	DESeq2	LRT	0.381597732531858
142	KC_AB2	Picot	0.387107135046641	0.828037304932132	1	pseudobulk	DESeq2	LRT	0.081950096836841
142	KC_AB2	Piezo	1.03858245968159	0.742043499884082	1	pseudobulk	DESeq2	LRT	0.129570634874779
142	KC_AB2	Pif1A	-0.729379579623002	1	1	pseudobulk	DESeq2	LRT	0
142	KC_AB2	PIG-A	1.94622644967243	0.415302697062391	1	pseudobulk	DESeq2	LRT	0.381635248481959
142	KC_AB2	PIG-B	-0.983286961722016	0.354059673176353	1	pseudobulk	DESeq2	LRT	0.450923535863568
142	KC_AB2	PIG-C	-1.71999636108657	0.519869997311849	1	pseudobulk	DESeq2	LRT	0.284105245805297
142	KC_AB2	PIG-F	-0.412238379136225	0.684968424498327	1	pseudobulk	DESeq2	LRT	0.164329448043642
142	KC_AB2	PIG-G	2.33862076287154	0.37727071943742	1	pseudobulk	DESeq2	LRT	0.423346899755834
142	KC_AB2	PIG-H	-0.566467411810672	0.829044799078116	1	pseudobulk	DESeq2	LRT	0.081422000852936
142	KC_AB2	PIG-K	1.0206123313166	0.272979749591742	1	pseudobulk	DESeq2	LRT	0.563869568954703
142	KC_AB2	PIG-L	0.0142358340639796	0.913972295601378	1	pseudobulk	DESeq2	LRT	0.039066968434439
142	KC_AB2	PIG-O	1.2887322017375	0.528378805215652	1	pseudobulk	DESeq2	LRT	0.277054611501303
142	KC_AB2	PIG-S	0.38605638660934	0.863777890371283	1	pseudobulk	DESeq2	LRT	0.063597916533759
142	KC_AB2	PIG-T	-0.472824668242616	0.566071725306496	1	pseudobulk	DESeq2	LRT	0.247128537131016
142	KC_AB2	PIG-U	-1.33407500886303	0.63128114564055	1	pseudobulk	DESeq2	LRT	0.19977718147345
142	KC_AB2	PIG-V	-1.57685088282527	0.45256857946707	1	pseudobulk	DESeq2	LRT	0.344315601149633
142	KC_AB2	PIG-X	0.393976029558784	0.939642609940555	1	pseudobulk	DESeq2	LRT	0.027037297511213
142	KC_AB2	Pig1	1.10706867860301	1	1	pseudobulk	DESeq2	LRT	0
142	KC_AB2	pigeon	-1.17849149785232	0.564162873029578	1	pseudobulk	DESeq2	LRT	0.248595497707284
142	KC_AB2	pigs	0.0585137093973692	0.86437966276614	1	pseudobulk	DESeq2	LRT	0.063295459804004
142	KC_AB2	Pih1D1	1.36574740718695	0.173307579309706	1	pseudobulk	DESeq2	LRT	0.761182443746535
142	KC_AB2	Pink1	0.371001983481998	0.668361705919041	1	pseudobulk	DESeq2	LRT	0.174988441161169
142	KC_AB2	Pino	-0.13966292405656	0.889350719029382	1	pseudobulk	DESeq2	LRT	0.050926939477337
142	KC_AB2	pins	-0.374381411314494	0.77074989813858	1	pseudobulk	DESeq2	LRT	0.113086523997309
142	KC_AB2	PIP4K	0.0700847720563224	0.830757759431985	1	pseudobulk	DESeq2	LRT	0.080525593639072
142	KC_AB2	PIP5K59B	-0.331277009221012	0.510648395805664	1	pseudobulk	DESeq2	LRT	0.291878028083781
142	KC_AB2	pirk	1.10706867860301	1	1	pseudobulk	DESeq2	LRT	0
142	KC_AB2	Pis	-0.570220752173281	0.304556751547	1	pseudobulk	DESeq2	LRT	0.516331768426209
142	KC_AB2	Pisd	2.10297294330985	0.195322193481196	1	pseudobulk	DESeq2	LRT	0.709248407204674
142	KC_AB2	pit	-0.620563475958957	0.7478554969218	1	pseudobulk	DESeq2	LRT	0.126182309825559
142	KC_AB2	pita	-1.25711388229196	0.605528431072361	1	pseudobulk	DESeq2	LRT	0.217865460831475
142	KC_AB2	Pits	0.238912223531004	0.48146510819	1	pseudobulk	DESeq2	LRT	0.317435180750484
142	KC_AB2	Pitslre	0.29916616945067	0.328407256949201	1	pseudobulk	DESeq2	LRT	0.483587254664134
142	KC_AB2	pix	-0.13810141878515	0.880501752744263	1	pseudobulk	DESeq2	LRT	0.055269775180629
142	KC_AB2	Pka-C1	0.012983772668631	0.931939648055018	1	pseudobulk	DESeq2	LRT	0.030612211428582
142	KC_AB2	Pka-C3	1.99339641594643	0.465071165814147	1	pseudobulk	DESeq2	LRT	0.332480585700252
142	KC_AB2	Pka-R1	0.0126041334044241	0.947436419892665	1	pseudobulk	DESeq2	LRT	0.023449924807126
142	KC_AB2	Pka-R2	0.105306840276194	0.557574313400077	1	pseudobulk	DESeq2	LRT	0.253697241734941
142	KC_AB2	pkaap	1.50776466886541	0.515809803599324	1	pseudobulk	DESeq2	LRT	0.287510407819198
142	KC_AB2	Pkc53E	0.107978238329381	0.6082451662972	1	pseudobulk	DESeq2	LRT	0.215921333706301
142	KC_AB2	Pkc98E	-0.19323114525202	0.534348809962509	1	pseudobulk	DESeq2	LRT	0.272175153480969
142	KC_AB2	PKD	1.95432904991983	0.509212778150152	1	pseudobulk	DESeq2	LRT	0.293100706726022
142	KC_AB2	Pkn	-0.174229460071671	0.841757910290364	1	pseudobulk	DESeq2	LRT	0.074812793705168
142	KC_AB2	Plap	-0.969404222682795	0.749350307836562	1	pseudobulk	DESeq2	LRT	0.125315109945074
142	KC_AB2	Plc21C	-0.396134880894242	0.198144666803842	1	pseudobulk	DESeq2	LRT	0.703017612498769
142	KC_AB2	PLCXD	1.10706867860301	1	1	pseudobulk	DESeq2	LRT	0
142	KC_AB2	Pldn	0.311721346440043	0.760289290404259	1	pseudobulk	DESeq2	LRT	0.119021127034083

142	KC_AB2	Plekhm1	-0.348901974470907	0.89890135131336	1	pseudobulk	DESeq2	LRT	0.046287966699494
142	KC_AB2	PlexA	-0.0565485699459047	0.868752990113444	1	pseudobulk	DESeq2	LRT	0.061103687624811
142	KC_AB2	PlexB	-0.0622582132140668	0.851409999881799	1	pseudobulk	DESeq2	LRT	0.069861253303842
142	KC_AB2	Pli	-0.104136151773731	0.829981641028072	1	pseudobulk	DESeq2	LRT	0.080931513995472
142	KC_AB2	pll	-0.729379579623002	0.772694994316916	1	pseudobulk	DESeq2	LRT	0.1119919011982
142	KC_AB2	Plod	0.403294498144138	0.89479244414645	1	pseudobulk	DESeq2	LRT	0.048277691852293
142	KC_AB2	Plp	0.269661021625649	0.847353854477266	1	pseudobulk	DESeq2	LRT	0.071935190667306
142	KC_AB2	plum	-3.60578477977519	0.070843435062868	1	pseudobulk	DESeq2	LRT	1.14970038886141
142	KC_AB2	plx	0.686240786452798	0.172068216089056	1	pseudobulk	DESeq2	LRT	0.764299343803381
142	KC_AB2	Plzf	1.67919315647972	0.465129744086312	1	pseudobulk	DESeq2	LRT	0.332425887365465
142	KC_AB2	PMCA	-0.21740472087111	0.487444389812053	1	pseudobulk	DESeq2	LRT	0.312074923709768
142	KC_AB2	Pmm2	-1.44016316499563	0.46417400647559	1	pseudobulk	DESeq2	LRT	0.333319183484357
142	KC_AB2	PMP34	0.818098881605262	0.406501272059711	1	pseudobulk	DESeq2	LRT	0.390938091035107
142	KC_AB2	Pmp70	0.271600213663012	0.867668243784365	1	pseudobulk	DESeq2	LRT	0.061646297214086
142	KC_AB2	Pms2	1.38446765109058	0.642794900879723	1	pseudobulk	DESeq2	LRT	0.191927577037166
142	KC_AB2	pn	-0.61781286788334	0.716977077809616	1	pseudobulk	DESeq2	LRT	0.144494728767247
142	KC_AB2	Pngl	-0.544536893163093	0.646400464146631	1	pseudobulk	DESeq2	LRT	0.189498340389027
142	KC_AB2	Pnn	-0.172562019235754	0.815825788320107	1	pseudobulk	DESeq2	LRT	0.088402570717740
142	KC_AB2	PNPase	-0.729379579623002	0.772694994316916	1	pseudobulk	DESeq2	LRT	0.1119919011982
142	KC_AB2	pns	-0.37266060448874	0.830246527888934	1	pseudobulk	DESeq2	LRT	0.080792931955809
142	KC_AB2	pnut	-0.363599667583481	0.602870043631382	1	pseudobulk	DESeq2	LRT	0.219776295515658
142	KC_AB2	PNUTS	-0.0483855007400297	0.876834411015804	1	pseudobulk	DESeq2	LRT	0.057082414811997
142	KC_AB2	pod1	-0.601458551717588	0.423003978518127	1	pseudobulk	DESeq2	LRT	0.373655547896014
142	KC_AB2	poe	-0.341282597129365	0.664750265921303	1	pseudobulk	DESeq2	LRT	0.17734148026472
142	KC_AB2	Pof	-1.3559506281947	0.361966187719165	1	pseudobulk	DESeq2	LRT	0.441331996242596
142	KC_AB2	Pol31	1.10706867860301	1	1	pseudobulk	DESeq2	LRT	0
142	KC_AB2	Pol32	0.147593560254927	1	1	pseudobulk	DESeq2	LRT	0
142	KC_AB2	POLDIP2	-0.255227196297625	0.853214308021342	1	pseudobulk	DESeq2	LRT	0.068941870237829
142	KC_AB2	poly	0.0028904956327288	0.996275594940229	1	pseudobulk	DESeq2	LRT	0.001620508156915
142	KC_AB2	polybromo	0.685291418384569	0.66285075245084	1	pseudobulk	DESeq2	LRT	0.178584246382007
142	KC_AB2	Pomp	0.0978621844868149	0.8890343822971	1	pseudobulk	DESeq2	LRT	0.051081442906993
142	KC_AB2	pont	0.412266503805257	0.859735858836361	1	pseudobulk	DESeq2	LRT	0.065634958838131
142	KC_AB2	Pop2	-0.512966689837212	0.537400548756694	1	pseudobulk	DESeq2	LRT	0.269701894430796
142	KC_AB2	por	-1.6052707068109	0.52265609184397	1	pseudobulk	DESeq2	LRT	0.281783983297782
142	KC_AB2	porin	-0.287996317403467	0.342361107168389	1	pseudobulk	DESeq2	LRT	0.46551557781947
142	KC_AB2	POSH	1.90334599570774	0.391638683118061	1	pseudobulk	DESeq2	LRT	0.407114418413825
142	KC_AB2	Pp1-87B	-0.203257195198732	0.582712143044915	1	pseudobulk	DESeq2	LRT	0.234545931622478
142	KC_AB2	Pp1alpha-96A	-0.286037097527535	0.550948438446489	1	pseudobulk	DESeq2	LRT	0.258889043518408
142	KC_AB2	Pp2A-29B	-0.666859210567136	0.204083618094705	1	pseudobulk	DESeq2	LRT	0.690191854943951
142	KC_AB2	Pp2B-14D	0.0928133458883462	0.842158532763022	1	pseudobulk	DESeq2	LRT	0.074606146714811
142	KC_AB2	Pp2C1	0.442453368682651	0.562707494580148	1	pseudobulk	DESeq2	LRT	0.249717300532649
142	KC_AB2	Pp4-19C	-0.163182249325424	0.877914621987962	1	pseudobulk	DESeq2	LRT	0.056547717581868
142	KC_AB2	Ppa	3.3202388026444	0.127266776114093	1	pseudobulk	DESeq2	LRT	0.895284957175427
142	KC_AB2	ppan	1.10706867860301	1	1	pseudobulk	DESeq2	LRT	0
142	KC_AB2	Ppat-Dpck	-2.56512514058146	0.231651930984972	1	pseudobulk	DESeq2	LRT	0.635164075289781
142	KC_AB2	Ppcs	0.384750476829812	0.271075373534147	1	pseudobulk	DESeq2	LRT	0.566909935108657
142	KC_AB2	PpD3	-1.46127416469959	0.407826953327994	1	pseudobulk	DESeq2	LRT	0.389524075039504
142	KC_AB2	ppk25	1.10706867860301	1	1	pseudobulk	DESeq2	LRT	0
142	KC_AB2	ppk8	-1.21363965631423	0.568763379520138	1	pseudobulk	DESeq2	LRT	0.24506837392442
142	KC_AB2	ppk9	1.38446765109058	0.642794900879723	1	pseudobulk	DESeq2	LRT	0.191927577037166
142	KC_AB2	ppl	-0.0617078421419387	0.896967203653297	1	pseudobulk	DESeq2	LRT	0.047223436033711
142	KC_AB2	PPP1R15	0.0062120454690689	0.928104367647592	1	pseudobulk	DESeq2	LRT	0.032403183538879

142	KC_AB2	PPP4R2r	-0.384177594190889	0.670410241250976	1	pseudobulk	DESeq2	LRT	0.17365936000305€
143	KC_AB2	pps	0.7259166145049	0.582756182098947	1	pseudobulk	DESeq2	LRT	0.234513110622112
143	KC_AB2	Ppt2	0.580533807341923	0.749481737832834	1	pseudobulk	DESeq2	LRT	0.125238944877148
143	KC_AB2	PpV	0.365419243159056	0.768983683682382	1	pseudobulk	DESeq2	LRT	0.114082874973448
143	KC_AB2	PQBP1	1.16252919734411	0.573641555183235	1	pseudobulk	DESeq2	LRT	0.241359395462503
143	KC_AB2	pr	1.6946105618747	0.281789774742277	1	pseudobulk	DESeq2	LRT	0.550074770110889
143	KC_AB2	PR-Set7	0.0003455780081085	0.958237606765067	1	pseudobulk	DESeq2	LRT	0.018526788925527
143	KC_AB2	prage	0.601576977934365	0.524594714179432	1	pseudobulk	DESeq2	LRT	0.280176089678319
143	KC_AB2	PRAS40	0.309376533786041	0.742310381552654	1	pseudobulk	DESeq2	LRT	0.129414465596442
143	KC_AB2	PrBP	-0.625613208876848	0.405403279575277	1	pseudobulk	DESeq2	LRT	0.392112742333477
143	KC_AB2	prd1	-0.392508880632505	0.602127641682546	1	pseudobulk	DESeq2	LRT	0.22031143531627
143	KC_AB2	pre-lola-G	-0.729379579623002	0.772694994316916	1	pseudobulk	DESeq2	LRT	0.1119919011982
143	KC_AB2	pre-mod(mdg4)-B	1.38069928703004	0.566368801250645	1	pseudobulk	DESeq2	LRT	0.246900678019328
143	KC_AB2	pre-mod(mdg4)-L	-0.729379579623002	0.823527397767669	1	pseudobulk	DESeq2	LRT	0.084321947797948
143	KC_AB2	pre-rRNA:CR4584	0.54063808950709	0.517535681282476	1	pseudobulk	DESeq2	LRT	0.286059702586243
143	KC_AB2	prel	-0.156036135369665	0.804413191468722	1	pseudobulk	DESeq2	LRT	0.094520816077409
143	KC_AB2	Prestin	0.0843130080157558	0.943549788858392	1	pseudobulk	DESeq2	LRT	0.025235178216641
143	KC_AB2	prg	-1.29052728408004	0.545673810438722	1	pseudobulk	DESeq2	LRT	0.263066889637944
143	KC_AB2	prim	-0.256235494911363	1	1	pseudobulk	DESeq2	LRT	0
143	KC_AB2	PRL-1	0.220065946405105	0.809745005317391	1	pseudobulk	DESeq2	LRT	0.09165172213197
143	KC_AB2	prod	-0.302042743829094	0.763632414925526	1	pseudobulk	DESeq2	LRT	0.117115644808888
143	KC_AB2	prominin-like	0.0980126563859072	0.852245560973318	1	pseudobulk	DESeq2	LRT	0.069435252169841
143	KC_AB2	ProRS-m	-0.205504300584806	0.898556422545151	1	pseudobulk	DESeq2	LRT	0.046454647291050
143	KC_AB2	pros	0.142415773886613	0.423583926293548	1	pseudobulk	DESeq2	LRT	0.373060528390952
143	KC_AB2	Prosalpha1	2.48384162006255	0.303740962766822	1	pseudobulk	DESeq2	LRT	0.517496634805608
143	KC_AB2	Prosalpha2	-0.374493058573818	0.676354844184069	1	pseudobulk	DESeq2	LRT	0.169825395125622
143	KC_AB2	Prosalpha3	-0.393128637059214	0.677620491824853	1	pseudobulk	DESeq2	LRT	0.169013469041145
143	KC_AB2	Prosalpha4	0.04829761439066	0.959285729622318	1	pseudobulk	DESeq2	LRT	0.018052016066229
143	KC_AB2	Prosalpha5	-1.59202358644187	0.163812823651184	1	pseudobulk	DESeq2	LRT	0.785652103656461
143	KC_AB2	Prosalpha6	-1.88305091590504	0.0860008741074053	1	pseudobulk	DESeq2	LRT	1.06549713459255
143	KC_AB2	Prosalpha7	-0.662704651821465	0.524940176720919	1	pseudobulk	DESeq2	LRT	0.279890186880439
143	KC_AB2	Prosap	0.038955014798686	0.866237197209234	1	pseudobulk	DESeq2	LRT	0.062363171107376
143	KC_AB2	Prosbeta1	-0.0098425593495380	0.94398449122389	1	pseudobulk	DESeq2	LRT	0.025035140692623
143	KC_AB2	Prosbeta2	0.174809841078227	0.817308850551359	1	pseudobulk	DESeq2	LRT	0.087613798126586
143	KC_AB2	Prosbeta3	-0.28614777227181	0.826735031058453	1	pseudobulk	DESeq2	LRT	0.082633659719450
143	KC_AB2	Prosbeta4	-0.16151282414178	0.836209593932172	1	pseudobulk	DESeq2	LRT	0.077684854044752
143	KC_AB2	Prosbeta5	0.514255222498719	0.472917599919221	1	pseudobulk	DESeq2	LRT	0.325214523147677
143	KC_AB2	Prosbeta6	0.690023378982225	0.57613733738886	1	pseudobulk	DESeq2	LRT	0.239473978797935
143	KC_AB2	Prosbeta7	-0.21850755887893	0.817018425588811	1	pseudobulk	DESeq2	LRT	0.087768149047372
143	KC_AB2	Prp18	1.10706867860301	1	1	pseudobulk	DESeq2	LRT	0
143	KC_AB2	Prp19	-0.268366549469347	0.872907090216809	1	pseudobulk	DESeq2	LRT	0.059031978921532
143	KC_AB2	Prp3	1.0983018024877	0.311924477578316	1	pseudobulk	DESeq2	LRT	0.50595054361387
143	KC_AB2	Prp31	-0.219456293688254	0.891746992784615	1	pseudobulk	DESeq2	LRT	0.049758346547219
143	KC_AB2	Prp38	-1.29052728408004	0.545673810438722	1	pseudobulk	DESeq2	LRT	0.263066889637944
143	KC_AB2	Prp8	0.604656088523711	0.6532381005693	1	pseudobulk	DESeq2	LRT	0.184928492667089
143	KC_AB2	Prpk	0.413763223382476	0.860970715086041	1	pseudobulk	DESeq2	LRT	0.065011620315023
143	KC_AB2	prt	-0.357652446064921	0.140844841580188	1	pseudobulk	DESeq2	LRT	0.851259054352926
143	KC_AB2	prtp	-0.144848023095751	0.86044312663913	1	pseudobulk	DESeq2	LRT	0.065277830278983
143	KC_AB2	Prx3	0.959370489633142	0.387750681288793	1	pseudobulk	DESeq2	LRT	0.411447430446135
143	KC_AB2	Prx6005	1.38069928703004	0.566368801250645	1	pseudobulk	DESeq2	LRT	0.246900678019328
143	KC_AB2	PRY	0.38605638660934	0.863777890371283	1	pseudobulk	DESeq2	LRT	0.063597916533759
143	KC_AB2	ps	0.0130979243736079	0.924829090204447	1	pseudobulk	DESeq2	LRT	0.033938518114104

143	KC_AB2	Psa	0.624630843502781	0.541761876173304	1	pseudobulk	DESeq2	LRT	0.266191559565137
143	KC_AB2	Psc	2.57879055756044	0.202758645671951	1	pseudobulk	DESeq2	LRT	0.693020618312527
143	KC_AB2	Psf3	-0.721175887303532	0.733409564198845	1	pseudobulk	DESeq2	LRT	0.134653430819787
143	KC_AB2	PsGEF	0.334942191333399	0.661049386882984	1	pseudobulk	DESeq2	LRT	0.179766093238213
143	KC_AB2	Psi	0.0787222339161133	0.838312734538041	1	pseudobulk	DESeq2	LRT	0.076593936563943
143	KC_AB2	psidin	0.379872020897556	0.78438553194688	1	pseudobulk	DESeq2	LRT	0.105470425526413
143	KC_AB2	PSMG1	-0.470273284461303	0.785773350633378	1	pseudobulk	DESeq2	LRT	0.104702704296327
143	KC_AB2	Psn	-1.12801111853905	0.441951678096085	1	pseudobulk	DESeq2	LRT	0.354625212740181
143	KC_AB2	psq	-0.0529929856512181	0.861197559469978	1	pseudobulk	DESeq2	LRT	0.064897209584991
143	KC_AB2	PSR	0.166718223031801	0.877957544909355	1	pseudobulk	DESeq2	LRT	0.056526484614841
143	KC_AB2	Pstk	-1.86716056129969	0.477680380390446	1	pseudobulk	DESeq2	LRT	0.320862595985116
143	KC_AB2	Pten	-0.268648041990477	0.824329397351738	1	pseudobulk	DESeq2	LRT	0.083899212008792
143	KC_AB2	Ptip	-0.733805188447709	0.62451251615905	1	pseudobulk	DESeq2	LRT	0.204458853295707
143	KC_AB2	PTP-ER	-0.312118636625111	0.725110852558068	1	pseudobulk	DESeq2	LRT	0.139595594843984
143	KC_AB2	Ptp10D	-2.2834221630407	0.364642497726807	1	pseudobulk	DESeq2	LRT	0.438132717324805
143	KC_AB2	Ptp36E	0.387762601660152	0.608594708464169	1	pseudobulk	DESeq2	LRT	0.21567182801269
143	KC_AB2	Ptp4E	-0.056169603621905	0.931682099800997	1	pseudobulk	DESeq2	LRT	0.030732248433286
143	KC_AB2	Ptp61F	-1.00506307555096	0.516197764794289	1	pseudobulk	DESeq2	LRT	0.287183880341565
143	KC_AB2	Ptp69D	-0.45893662982697	0.760413513779769	1	pseudobulk	DESeq2	LRT	0.118950173621649
143	KC_AB2	Ptp99A	-0.137191755313198	0.64227479380623	1	pseudobulk	DESeq2	LRT	0.192279121619198
143	KC_AB2	Ptpa	0.68360439929388	0.576514705122231	1	pseudobulk	DESeq2	LRT	0.239189610707544
143	KC_AB2	Ptpmeg	0.214003701106126	0.819498404132776	1	pseudobulk	DESeq2	LRT	0.086451887825031
143	KC_AB2	Ptpmeg2	-0.121796803488822	0.710543358767837	1	pseudobulk	DESeq2	LRT	0.148409415415518
143	KC_AB2	PTPMT1	1.36091968839315	0.394623794738619	1	pseudobulk	DESeq2	LRT	0.403816731521433
143	KC_AB2	Pu	-0.796599527000822	0.622738419829118	1	pseudobulk	DESeq2	LRT	0.205694339666323
143	KC_AB2	puc	-0.430914305356977	0.624683680864335	1	pseudobulk	DESeq2	LRT	0.204339839344745
143	KC_AB2	puf	0.131750854631975	0.869830750717548	1	pseudobulk	DESeq2	LRT	0.060565242990665
143	KC_AB2	pum	0.0058077285768494	0.936746262203601	1	pseudobulk	DESeq2	LRT	0.028378031148591
143	KC_AB2	Pur-alpha	-0.0969499993697781	0.76607503557659	1	pseudobulk	DESeq2	LRT	0.115728689974623
143	KC_AB2	Pus1	1.38069928703004	0.566368801250645	1	pseudobulk	DESeq2	LRT	0.246900678019328
143	KC_AB2	Pus7	0.186034431065808	0.881762404370314	1	pseudobulk	DESeq2	LRT	0.054648422092356
143	KC_AB2	put	-0.0885436376543557	0.895208882374419	1	pseudobulk	DESeq2	LRT	0.048075617334198
143	KC_AB2	Pvf3	1.13637463199394	0.573539159699493	1	pseudobulk	DESeq2	LRT	0.241436924300117
143	KC_AB2	PVRAP	-0.0006156522015205	0.951734481941945	1	pseudobulk	DESeq2	LRT	0.021484195639863
143	KC_AB2	px	0.0517729768980139	0.924530272536715	1	pseudobulk	DESeq2	LRT	0.034078863866190
143	KC_AB2	pxb	0.390738358981617	0.490539430955671	1	pseudobulk	DESeq2	LRT	0.309326077054304
143	KC_AB2	Pxn	-1.21119125881877	0.0492132315422816	1	pseudobulk	DESeq2	LRT	1.30791811647573
143	KC_AB2	pyd	0.706303649518545	0.126885445750601	1	pseudobulk	DESeq2	LRT	0.896588190298521
143	KC_AB2	pygo	-0.942189863667974	0.619669494410584	1	pseudobulk	DESeq2	LRT	0.207839883122948
143	KC_AB2	PyK	-0.194837838968572	0.591861074274335	1	pseudobulk	DESeq2	LRT	0.227780221919957
143	KC_AB2	Pym	-0.893328173497901	0.679679924321693	1	pseudobulk	DESeq2	LRT	0.167695557626761
143	KC_AB2	Pyroxd1	0.496222601129902	0.801826919465134	1	pseudobulk	DESeq2	LRT	0.095919367418357
143	KC_AB2	pzg	0.196910705509526	0.822882862222784	1	pseudobulk	DESeq2	LRT	0.084661982419496
143	KC_AB2	Pzl	-1.73187581389555	0.432606897969795	1	pseudobulk	DESeq2	LRT	0.363906559963191
143	KC_AB2	QIL1	-0.123966695114135	0.876229447604667	1	pseudobulk	DESeq2	LRT	0.057382155497823
143	KC_AB2	qjt	0.145321756433347	1	1	pseudobulk	DESeq2	LRT	0
143	KC_AB2	qkr54B	-0.103091431839625	0.855375815306465	1	pseudobulk	DESeq2	LRT	0.067843033045138
143	KC_AB2	qkr58E-1	0.0834900603418807	0.835562871600049	1	pseudobulk	DESeq2	LRT	0.078020866223402
143	KC_AB2	qkr58E-2	-0.357103987382082	0.738781488963514	1	pseudobulk	DESeq2	LRT	0.13148399485296
144	KC_AB2	qkr58E-3	0.0481784357373069	0.904825058691043	1	pseudobulk	DESeq2	LRT	0.043435380341062
144	KC_AB2	qless	0.117121282388885	0.869614642512364	1	pseudobulk	DESeq2	LRT	0.060673156239168
144	KC_AB2	qm	-0.21556360037832	0.46354307629233	1	pseudobulk	DESeq2	LRT	0.333909901375384

144	KC_AB2	Qsox1	0.341310869435805	0.849261178469218	1	pseudobulk	DESeq2	LRT	0.070958727983931
144	KC_AB2	Qsox3	0.146930209264112	1	1	pseudobulk	DESeq2	LRT	0
144	KC_AB2	qtc	0.26268165729588	0.642357285658843	1	pseudobulk	DESeq2	LRT	0.192223345717267
144	KC_AB2	Qtzl	-0.179281539279264	0.908092942104804	1	pseudobulk	DESeq2	LRT	0.041869699741504
144	KC_AB2	qvr	0.168456928709982	0.631154854115668	1	pseudobulk	DESeq2	LRT	0.199864073343709
144	KC_AB2	r-cup	-0.729379579623002	1	1	pseudobulk	DESeq2	LRT	0
144	KC_AB2	r2d2	-0.943904419644962	0.459819021289797	1	pseudobulk	DESeq2	LRT	0.337413067276543
144	KC_AB2	Rab1	0.0191597181510885	0.945687204790289	1	pseudobulk	DESeq2	LRT	0.024252486956696
144	KC_AB2	Rab10	0.814839142543963	0.448119932338242	1	pseudobulk	DESeq2	LRT	0.348605738273998
144	KC_AB2	Rab11	-0.0410276537148984	0.905357881151471	1	pseudobulk	DESeq2	LRT	0.043179713517573
144	KC_AB2	Rab14	-0.340955130998537	0.545845728356582	1	pseudobulk	DESeq2	LRT	0.262930084017833
144	KC_AB2	Rab18	-0.130210318837865	0.891057362272103	1	pseudobulk	DESeq2	LRT	0.050094337134436
144	KC_AB2	Rab19	3.30354169368959	0.127303602791195	1	pseudobulk	DESeq2	LRT	0.895159305300041
144	KC_AB2	Rab2	-0.362923802560945	0.301062315890725	1	pseudobulk	DESeq2	LRT	0.521343601926433
144	KC_AB2	Rab21	-0.675354717599273	0.506229988583371	1	pseudobulk	DESeq2	LRT	0.29565213122338
144	KC_AB2	Rab23	-0.354609979998418	0.657522563966947	1	pseudobulk	DESeq2	LRT	0.182089339053882
144	KC_AB2	Rab26	-0.232327201619081	0.445312462054072	1	pseudobulk	DESeq2	LRT	0.35133515098244
144	KC_AB2	Rab27	-0.119684068584212	0.705843915388853	1	pseudobulk	DESeq2	LRT	0.151291324698449
144	KC_AB2	Rab3	-0.20055145534127	0.561793078291541	1	pseudobulk	DESeq2	LRT	0.250423615932425
144	KC_AB2	Rab3-GEF	0.25701006983465	0.499477296753056	1	pseudobulk	DESeq2	LRT	0.30148424741588
144	KC_AB2	Rab30	-1.85417217891375	0.121076613745571	1	pseudobulk	DESeq2	LRT	0.916939733835201
144	KC_AB2	Rab35	-0.256300180959698	0.688671917636653	1	pseudobulk	DESeq2	LRT	0.161987626124741
144	KC_AB2	Rab39	-0.178991796419641	0.842400544292633	1	pseudobulk	DESeq2	LRT	0.074481361215719
144	KC_AB2	Rab4	-0.901056239806365	0.151041228636366	1	pseudobulk	DESeq2	LRT	0.820904490287638
144	KC_AB2	Rab40	0.0457556725838857	0.939492207930456	1	pseudobulk	DESeq2	LRT	0.027106817550511
144	KC_AB2	Rab5	0.222886173753448	0.589966175236144	1	pseudobulk	DESeq2	LRT	0.229172887221241
144	KC_AB2	Rab6	0.215321475382467	0.829410502862347	1	pseudobulk	DESeq2	LRT	0.081230469446704
144	KC_AB2	Rab7	0.707215133082514	0.278695833890678	1	pseudobulk	DESeq2	LRT	0.554869523327645
144	KC_AB2	Rab8	-0.0241998012523816	0.901173311573943	1	pseudobulk	DESeq2	LRT	0.045191678474832
144	KC_AB2	Rab9	0.147593560254927	1	1	pseudobulk	DESeq2	LRT	0
144	KC_AB2	Rabex-5	0.243248195589571	0.846044164126029	1	pseudobulk	DESeq2	LRT	0.072606965878692
144	KC_AB2	RabX1	-0.639847617121441	0.663966055550718	1	pseudobulk	DESeq2	LRT	0.177854122836558
144	KC_AB2	RabX4	0.068475085944989	0.900666569385409	1	pseudobulk	DESeq2	LRT	0.045435956941921
144	KC_AB2	RabX6	-1.29052728408004	0.545673810438722	1	pseudobulk	DESeq2	LRT	0.263066889637944
144	KC_AB2	Rac1	-0.188521608675845	0.689848832344989	1	pseudobulk	DESeq2	LRT	0.161246066467124
144	KC_AB2	Rack1	0.0371738626154745	0.903682679190339	1	pseudobulk	DESeq2	LRT	0.043984041725239
144	KC_AB2	rad	-0.0629755967153482	0.777748341349807	1	pseudobulk	DESeq2	LRT	0.10916090640813
144	KC_AB2	Rad17	-2.28054226263626	0.331630256012328	1	pseudobulk	DESeq2	LRT	0.479345853816062
144	KC_AB2	Rad23	0.320647726633985	0.568517847175243	1	pseudobulk	DESeq2	LRT	0.245255897188975
144	KC_AB2	rad50	2.0338726988452	0.404823271651394	1	pseudobulk	DESeq2	LRT	0.392734529618649
144	KC_AB2	Rad51D	1.10706867860301	1	1	pseudobulk	DESeq2	LRT	0
144	KC_AB2	Rad60	0.8525897920314	0.590610951174645	1	pseudobulk	DESeq2	LRT	0.228698504559181
144	KC_AB2	Rad9	0.0055775572564737	0.991207809741364	1	pseudobulk	DESeq2	LRT	0.003835284805704
144	KC_AB2	Rae1	2.74658080486175	0.146111265263646	1	pseudobulk	DESeq2	LRT	0.835316298417319
144	KC_AB2	Raf	-2.90714682159232	0.085574939407195	1	pseudobulk	DESeq2	LRT	1.06765339969964
144	KC_AB2	RAF2	1.80677871671018	0.477532853980226	1	pseudobulk	DESeq2	LRT	0.320996743846761
144	KC_AB2	RagA-B	0.0176675625996484	0.927240462356208	1	pseudobulk	DESeq2	LRT	0.032807625152085
144	KC_AB2	RagC-D	-0.799969675193766	0.523893239377363	1	pseudobulk	DESeq2	LRT	0.280757205904125
144	KC_AB2	Rala	-0.661736378943764	0.32231591403562	1	pseudobulk	DESeq2	LRT	0.491718251089495
144	KC_AB2	Ran	-0.302340678152595	0.557266587176283	1	pseudobulk	DESeq2	LRT	0.253936995773846
144	KC_AB2	Ranbp16	-0.106161604904106	0.905212229147653	1	pseudobulk	DESeq2	LRT	0.043249587488554
144	KC_AB2	Ranbp21	-0.903412938451153	0.664037271512513	1	pseudobulk	DESeq2	LRT	0.177807543586898

144	KC_AB2	RanBP3	0.0656041073316128	0.955453378559894	1	pseudobulk	DESeq2	LRT	0.019790499533255
144	KC_AB2	RanBPM	-0.48984358977588	0.51729426637049	1	pseudobulk	DESeq2	LRT	0.286262335233789
144	KC_AB2	RanGAP	-0.729379579623002	1	1	pseudobulk	DESeq2	LRT	0
144	KC_AB2	Rap1	-0.594174877604395	0.0533884964764153	1	pseudobulk	DESeq2	LRT	1.27255230954461
144	KC_AB2	Rap2l	-0.0647208685421904	0.918256340597439	1	pseudobulk	DESeq2	LRT	0.037036064151225
144	KC_AB2	RapGAP1	-3.59165791647264	0.0622234296730551	1	pseudobulk	DESeq2	LRT	1.20604605483078
144	KC_AB2	raptor	-0.0218745540528002	0.965786728409952	1	pseudobulk	DESeq2	LRT	0.015118766856490
144	KC_AB2	ras	-0.0731913250317784	0.875099421633111	1	pseudobulk	DESeq2	LRT	0.057942603190522
144	KC_AB2	Ras64B	-0.0606401002171201	0.879673416741016	1	pseudobulk	DESeq2	LRT	0.055678531977299
144	KC_AB2	Ras85D	0.901467745674981	0.275163278757221	1	pseudobulk	DESeq2	LRT	0.560409524278095
144	KC_AB2	RasGAP1	0.587376610701748	0.401762351078626	1	pseudobulk	DESeq2	LRT	0.396030763169867
144	KC_AB2	raskol	0.102711044302216	0.825287521035022	1	pseudobulk	DESeq2	LRT	0.083394721698606
144	KC_AB2	rasp	-0.729379579623002	1	1	pseudobulk	DESeq2	LRT	0
144	KC_AB2	Rassf	2.33534228727018	0.263553805171469	1	pseudobulk	DESeq2	LRT	0.5791307090903
144	KC_AB2	RASSF8	0.480279898597536	0.534247201315909	1	pseudobulk	DESeq2	LRT	0.272257744234068
144	KC_AB2	Rat1	1.95291936742543	0.413271664647853	1	pseudobulk	DESeq2	LRT	0.383764370447636
144	KC_AB2	Ravus	1.78195549754972	0.30997562247767	1	pseudobulk	DESeq2	LRT	0.50867245919708
144	KC_AB2	raw	0.384142986188599	0.559184225000366	1	pseudobulk	DESeq2	LRT	0.252445088857382
144	KC_AB2	rb	-0.964702223922912	0.473656302383358	1	pseudobulk	DESeq2	LRT	0.324536679678108
144	KC_AB2	Rb97D	-0.145058702829173	0.821472513881293	1	pseudobulk	DESeq2	LRT	0.085406963293296
144	KC_AB2	Rbbp5	1.04324661842443	0.68096673636569	1	pseudobulk	DESeq2	LRT	0.166874101840012
144	KC_AB2	Rbcn-3A	-0.251266446470382	0.723021959623263	1	pseudobulk	DESeq2	LRT	0.140848512112968
144	KC_AB2	Rbcn-3B	0.206756442353511	0.810799592790208	1	pseudobulk	DESeq2	LRT	0.091086478095716
144	KC_AB2	Rbf	2.12661202377811	0.1978348019443	1	pseudobulk	DESeq2	LRT	0.703697307466434
144	KC_AB2	Rbf2	1.10706867860301	1	1	pseudobulk	DESeq2	LRT	0
144	KC_AB2	Rbfox1	0.0526936983105982	0.80685711566836	1	pseudobulk	DESeq2	LRT	0.093203366604994
144	KC_AB2	Rbm13	0.0843130080157558	0.943549788858392	1	pseudobulk	DESeq2	LRT	0.025235178216641
144	KC_AB2	Rbp	-0.0563731781542051	0.818028830660268	1	pseudobulk	DESeq2	LRT	0.08723138975653
144	KC_AB2	Rbp1	-0.0134387990705387	0.971534205251974	1	pseudobulk	DESeq2	LRT	0.012541904389288
144	KC_AB2	Rbp1-like	0.0347818274454296	0.867675827393535	1	pseudobulk	DESeq2	LRT	0.061642501402442
144	KC_AB2	Rbp4	-0.729379579623002	0.823527397767669	1	pseudobulk	DESeq2	LRT	0.084321947797948
144	KC_AB2	Rbp6	0.221486168185573	0.903697778547821	1	pseudobulk	DESeq2	LRT	0.0439767852922
144	KC_AB2	Rbp9	0.216849162247345	0.680161795423008	1	pseudobulk	DESeq2	LRT	0.167387765968348
144	KC_AB2	Rbpn-5	2.18060033169204	0.183615079904277	1	pseudobulk	DESeq2	LRT	0.73609165401563
144	KC_AB2	Rbsn-5	-2.10393889394842	0.297207953190465	1	pseudobulk	DESeq2	LRT	0.526939573173515
144	KC_AB2	Rca1	0.145321756433347	1	1	pseudobulk	DESeq2	LRT	0
144	KC_AB2	Rcc1	0.60571064033538	0.556302167413051	1	pseudobulk	DESeq2	LRT	0.254689247997884
144	KC_AB2	Rcd-1	-0.565080227446421	0.49307715716292	1	pseudobulk	DESeq2	LRT	0.307085116608764
144	KC_AB2	Rcd1	0.00106937074358749	0.972584366501137	1	pseudobulk	DESeq2	LRT	0.012072715640612
144	KC_AB2	Rcd4	-1.03058036490783	0.572631373472145	1	pseudobulk	DESeq2	LRT	0.242124861426942
144	KC_AB2	Rcd5	0.776537766225463	0.630520992324023	1	pseudobulk	DESeq2	LRT	0.200300449616328
144	KC_AB2	Rcd6	1.10706867860301	1	1	pseudobulk	DESeq2	LRT	0
144	KC_AB2	Rcp	-1.25063166152606	0.382198759069416	1	pseudobulk	DESeq2	LRT	0.417710727354737
144	KC_AB2	rdgB	0.238677142062383	0.406415546312472	1	pseudobulk	DESeq2	LRT	0.391029687661674
144	KC_AB2	rdgBbeta	-0.0999020451991876	0.892875288730514	1	pseudobulk	DESeq2	LRT	0.049209196428875
145	KC_AB2	rdgC	0.0150951065873752	0.97134111987654	1	pseudobulk	DESeq2	LRT	0.012628225845203
145	KC_AB2	Rdh	0.00391527881230625	0.900108194969906	1	pseudobulk	DESeq2	LRT	0.045705284278199
145	KC_AB2	Rdl	-0.0027135055729927	0.96502913899368	1	pseudobulk	DESeq2	LRT	0.015459572963871
145	KC_AB2	rdog	3.12100799138407	0.161346113416639	1	pseudobulk	DESeq2	LRT	0.792241491629998
145	KC_AB2	rdx	0.288983513356623	0.4724388847819	1	pseudobulk	DESeq2	LRT	0.325654364337064
145	KC_AB2	rec	0.147593560254927	1	1	pseudobulk	DESeq2	LRT	0
145	KC_AB2	RecQ5	1.10706867860301	1	1	pseudobulk	DESeq2	LRT	0

145	KC_AB2	red	-0.180056185154597	0.880456541348976	1	pseudobulk	DESeq2	LRT	0.055292075605985
145	KC_AB2	ReepA	-0.254059869358903	0.768708315748681	1	pseudobulk	DESeq2	LRT	0.114238420785512
145	KC_AB2	ReepB	-0.707027948868801	0.191662500496571	1	pseudobulk	DESeq2	LRT	0.717462850191848
145	KC_AB2	ref(2)P	0.290736957255233	0.674030195608909	1	pseudobulk	DESeq2	LRT	0.171320647244231
145	KC_AB2	Ref1	0.375092386921007	0.52979389644683	1	pseudobulk	DESeq2	LRT	0.27589304935085
145	KC_AB2	REG	0.474334941409859	0.552159134631023	1	pseudobulk	DESeq2	LRT	0.257935738698173
145	KC_AB2	regucalcin	-0.399761693026551	0.829842811978585	1	pseudobulk	DESeq2	LRT	0.081004163473736
145	KC_AB2	Rel	0.39439088612385	0.813541888331052	1	pseudobulk	DESeq2	LRT	0.089620080830767
145	KC_AB2	Rep	1.08937410746753	0.523121629807279	1	pseudobulk	DESeq2	LRT	0.281397322580594
145	KC_AB2	Reph	-0.0779602723245465	0.848715170208259	1	pseudobulk	DESeq2	LRT	0.071238035034546
145	KC_AB2	Reps	-0.0276480481565866	0.893477009000603	1	pseudobulk	DESeq2	LRT	0.048916618303984
145	KC_AB2	rept	0.124462516295993	0.952307241396434	1	pseudobulk	DESeq2	LRT	0.021222913258289
145	KC_AB2	REPTOR	1.00684811929375	0.0582040177461529	1	pseudobulk	DESeq2	LRT	1.23504703554326
145	KC_AB2	REPTOR-BP	0.617996643256693	0.233475834018304	1	pseudobulk	DESeq2	LRT	0.631758064543837
145	KC_AB2	retm	-0.529092620537046	0.52592636518506	1	pseudobulk	DESeq2	LRT	0.279075057048541
145	KC_AB2	retn	-0.432213394558105	0.865061123889812	1	pseudobulk	DESeq2	LRT	0.062953204870221
145	KC_AB2	Rev7	0.182464987382747	0.906839011984365	1	pseudobulk	DESeq2	LRT	0.042469804905139
145	KC_AB2	Rexo5	0.89698058322345	0.673674818076485	1	pseudobulk	DESeq2	LRT	0.171549686245101
145	KC_AB2	RfC3	-0.721175887303532	0.733409564198845	1	pseudobulk	DESeq2	LRT	0.134653430819787
145	KC_AB2	RfC38	0.211319900981398	0.958606495011728	1	pseudobulk	DESeq2	LRT	0.018359632784041
145	KC_AB2	RfC4	2.7605424445074	0.237810191029118	1	pseudobulk	DESeq2	LRT	0.623769538224996
145	KC_AB2	RFeSP	-0.372512417937399	0.403654911375292	1	pseudobulk	DESeq2	LRT	0.393989758973426
145	KC_AB2	Rfx	-2.39970914040097	0.301766661516198	1	pseudobulk	DESeq2	LRT	0.520328741761584
145	KC_AB2	rg	-0.647621034217714	0.754702560287896	1	pseudobulk	DESeq2	LRT	0.122224176685402
145	KC_AB2	Rga	-0.445633133302264	0.502909949902793	1	pseudobulk	DESeq2	LRT	0.298509771925043
145	KC_AB2	Rgk1	0.189232650866854	0.390831303520835	1	pseudobulk	DESeq2	LRT	0.40801065886855
145	KC_AB2	Rgk2	-0.770608174088596	0.198805776506925	1	pseudobulk	DESeq2	LRT	0.701571000881183
145	KC_AB2	Rgk3	-0.167078689913558	0.640402864657788	1	pseudobulk	DESeq2	LRT	0.193546734057099
145	KC_AB2	Rgl	-0.23856270666887	0.680117827091023	1	pseudobulk	DESeq2	LRT	0.167415841378213
145	KC_AB2	rgn	-0.119323050823371	0.749068617493001	1	pseudobulk	DESeq2	LRT	0.125478397475933
145	KC_AB2	Rh7	0.151938707176109	0.9107789483196	1	pseudobulk	DESeq2	LRT	0.040587016192920
145	KC_AB2	Rhau	1.38446765109058	0.642794900879723	1	pseudobulk	DESeq2	LRT	0.191927577037166
145	KC_AB2	rhea	0.101252900269648	0.861988654312084	1	pseudobulk	DESeq2	LRT	0.064498450419002
145	KC_AB2	Rheb	-0.207695974288867	0.787591333992828	1	pseudobulk	DESeq2	LRT	0.103699071126861
145	KC_AB2	rho	-0.624774120894754	0.63338309101159	1	pseudobulk	DESeq2	LRT	0.198333534867457
145	KC_AB2	rho-4	3.00763170468485	0.160686826767478	1	pseudobulk	DESeq2	LRT	0.794019725580746
145	KC_AB2	rho-5	-0.269808471055824	0.747972499255155	1	pseudobulk	DESeq2	LRT	0.126114369570424
145	KC_AB2	rho-7	-2.49384569936698	0.114134063717321	1	pseudobulk	DESeq2	LRT	0.942584719502625
145	KC_AB2	Rho1	-0.335346239006879	0.49831147286182	1	pseudobulk	DESeq2	LRT	0.302499113745617
145	KC_AB2	RhoBTB	0.509472873750921	0.515763607774057	1	pseudobulk	DESeq2	LRT	0.287549304890008
145	KC_AB2	RhoGAP100F	0.126331842900651	0.749827765648699	1	pseudobulk	DESeq2	LRT	0.125038481966275
145	KC_AB2	RhoGAP102A	1.30850668796832	0.376247906633674	1	pseudobulk	DESeq2	LRT	0.424525907716965
145	KC_AB2	RhoGAP16F	1.61205113368698	0.631647504209	1	pseudobulk	DESeq2	LRT	0.199525215522471
145	KC_AB2	RhoGAP18B	1.10706867860301	1	1	pseudobulk	DESeq2	LRT	0
145	KC_AB2	RhoGAP19D	-0.192241129680586	0.678952627847535	1	pseudobulk	DESeq2	LRT	0.168160526429549
145	KC_AB2	RhoGAP1A	-0.972294677528956	0.300169942018619	1	pseudobulk	DESeq2	LRT	0.522632798665103
145	KC_AB2	RhoGAP5A	2.49625423211926	0.231240584281302	1	pseudobulk	DESeq2	LRT	0.635935941952931
145	KC_AB2	RhoGAP68F	-0.356573595115632	0.653958936817695	1	pseudobulk	DESeq2	LRT	0.184449520901071
145	KC_AB2	RhoGAP71E	-0.0210964030907362	0.954195295371841	1	pseudobulk	DESeq2	LRT	0.020362729046308
145	KC_AB2	RhoGAP92B	-0.329360369939155	0.761951159801211	1	pseudobulk	DESeq2	LRT	0.118072865548347
145	KC_AB2	RhoGAP93B	0.343340320619964	0.514531763834231	1	pseudobulk	DESeq2	LRT	0.288587809566039
145	KC_AB2	RhoGAPp190	0.158315332788309	0.635088530281373	1	pseudobulk	DESeq2	LRT	0.197165730561963

145	KC_AB2	RhoGDI	-0.0439957799126478	0.905793821572444	1	pseudobulk	DESeq2	LRT	0.042970645969406
145	KC_AB2	RhoGEF2	1.99608423660526	0.125202400317658	1	pseudobulk	DESeq2	LRT	0.902387344969669
145	KC_AB2	RhoGEF3	0.295136689952485	0.697061323711954	1	pseudobulk	DESeq2	LRT	0.156729013324971
145	KC_AB2	RhoU	-0.729379579623002	1	1	pseudobulk	DESeq2	LRT	0
145	KC_AB2	Rhp	0.145321756433347	1	1	pseudobulk	DESeq2	LRT	0
145	KC_AB2	Ric	-0.199668837436588	0.402616770619225	1	pseudobulk	DESeq2	LRT	0.395108138946386
145	KC_AB2	RIC-3	-0.202342450403091	0.741354527535842	1	pseudobulk	DESeq2	LRT	0.129974055811048
145	KC_AB2	ric8a	-0.875592753870058	0.690854542500206	1	pseudobulk	DESeq2	LRT	0.160613382494579
145	KC_AB2	Rich	-0.701506377582755	0.690302159658499	1	pseudobulk	DESeq2	LRT	0.160960767888962
145	KC_AB2	rictor	-0.0373209911830363	0.875406061977526	1	pseudobulk	DESeq2	LRT	0.057790450327115
145	KC_AB2	Rift	1.49784664065061	0.474481812020792	1	pseudobulk	DESeq2	LRT	0.323780430424038
145	KC_AB2	rig	1.0198925688614	0.53375600619583	1	pseudobulk	DESeq2	LRT	0.272657224954217
145	KC_AB2	Rilpl	-0.516343045266129	0.560526178093923	1	pseudobulk	DESeq2	LRT	0.251404099864277
145	KC_AB2	Rim	-0.0596961965985989	0.783334492334882	1	pseudobulk	DESeq2	LRT	0.106052749876516
145	KC_AB2	Rim2	0.866324335112618	0.361481249080321	1	pseudobulk	DESeq2	LRT	0.441914225705973
145	KC_AB2	rin	0.198107123288645	0.655803723620621	1	pseudobulk	DESeq2	LRT	0.183226121757386
145	KC_AB2	ringer	-0.0802481527551558	0.846662815474288	1	pseudobulk	DESeq2	LRT	0.072289513558861
145	KC_AB2	Rint1	-2.09428695548101	0.380540277844868	1	pseudobulk	DESeq2	LRT	0.419599369064229
145	KC_AB2	RIOK2	0.906901817385668	0.481777754093939	1	pseudobulk	DESeq2	LRT	0.31715325725859
145	KC_AB2	Rip11	0.313876611022331	0.777611895409328	1	pseudobulk	DESeq2	LRT	0.109237104471806
145	KC_AB2	Ripalpha	-0.237162796700251	0.833382985353084	1	pseudobulk	DESeq2	LRT	0.079155370500649
145	KC_AB2	rk	-0.384684712653267	0.638491825525478	1	pseudobulk	DESeq2	LRT	0.194844658544502
145	KC_AB2	rl	0.0861490009920496	0.795722225802786	1	pseudobulk	DESeq2	LRT	0.099238511225461
145	KC_AB2	Rlb1	0.0385345362073844	0.875592295603491	1	pseudobulk	DESeq2	LRT	0.057698068474150
145	KC_AB2	Rlip	-0.115708024352806	0.827647784231104	1	pseudobulk	DESeq2	LRT	0.082154443297738
145	KC_AB2	RluA-1	-0.729379579623002	0.772694994316916	1	pseudobulk	DESeq2	LRT	0.1119919011982
145	KC_AB2	RluA-2	-0.564200282882025	0.722085743523957	1	pseudobulk	DESeq2	LRT	0.141411229399432
145	KC_AB2	Rm62	-0.0282951212717888	0.896044806611268	1	pseudobulk	DESeq2	LRT	0.047670272952283
145	KC_AB2	Rme-8	0.387107135046641	0.828037304932132	1	pseudobulk	DESeq2	LRT	0.081950096836841
145	KC_AB2	rn	0.0079921442148758	0.937700252291879	1	pseudobulk	DESeq2	LRT	0.027935967143352
145	KC_AB2	RN-tre	-0.708933340516178	0.470553454748599	1	pseudobulk	DESeq2	LRT	0.327391033695421
145	KC_AB2	RNASEK	-0.533051906965894	0.0657160711815027	1	pseudobulk	DESeq2	LRT	1.18232840864533
145	KC_AB2	RNaseMRP:RNA	-0.322868029972598	0.843777553813732	1	pseudobulk	DESeq2	LRT	0.073772031910402
145	KC_AB2	RNaseX25	0.663907848612739	0.642317268420375	1	pseudobulk	DESeq2	LRT	0.192250402010704
145	KC_AB2	RNaseZ	-1.52244591594958	0.547176617578731	1	pseudobulk	DESeq2	LRT	0.261872469536296
145	KC_AB2	Rnb	-1.86499814887644	0.364960432713785	1	pseudobulk	DESeq2	LRT	0.437754217147716
145	KC_AB2	Rnf146	-2.49384569936698	0.114134063717321	1	pseudobulk	DESeq2	LRT	0.942584719502625
145	KC_AB2	rngo	1.15557239320604	0.572679486029256	1	pseudobulk	DESeq2	LRT	0.242088373484996
145	KC_AB2	rnh1	1.14512083299725	0.513915745794639	1	pseudobulk	DESeq2	LRT	0.289108075821415
145	KC_AB2	Rnmt	-1.29052728408004	0.545673810438722	1	pseudobulk	DESeq2	LRT	0.263066889637944
145	KC_AB2	rno	-0.672518038256325	0.319135154399332	1	pseudobulk	DESeq2	LRT	0.496025353372381
146	KC_AB2	Rnp4F	-1.60535096369336	0.34475699460483	1	pseudobulk	DESeq2	LRT	0.462486913875927
146	KC_AB2	RnpS1	-0.55731411122295	0.602927349890484	1	pseudobulk	DESeq2	LRT	0.219735015293526
146	KC_AB2	robl	0.174607033633289	0.770496366142111	1	pseudobulk	DESeq2	LRT	0.113229405184564
146	KC_AB2	robo1	0.178717961077203	0.794616484008433	1	pseudobulk	DESeq2	LRT	0.099842429917898
146	KC_AB2	Roc1a	-0.484226677433187	0.478339725516175	1	pseudobulk	DESeq2	LRT	0.320263550016509
146	KC_AB2	Roc1b	0.145321756433347	1	1	pseudobulk	DESeq2	LRT	0
146	KC_AB2	Roc2	-0.260569849119732	0.698048584424991	1	pseudobulk	DESeq2	LRT	0.156114349277439
146	KC_AB2	Roe1	0.216577798381059	0.780667247647333	1	pseudobulk	DESeq2	LRT	0.107534040776738
146	KC_AB2	rogdi	-0.572833391689806	0.506254866630519	1	pseudobulk	DESeq2	LRT	0.295630788882221
146	KC_AB2	roh	-0.672306625966779	0.118493434522141	1	pseudobulk	DESeq2	LRT	0.926305712352591
146	KC_AB2	Rok	-0.600393395884272	0.506608104071975	1	pseudobulk	DESeq2	LRT	0.295327867199263

146	KC_AB2	Rop	-0.453695540744355	0.343620222982413	1	pseudobulk	DESeq2	LRT	0.463921284680083
146	KC_AB2	roq	-0.0404208484117342	0.939438536262686	1	pseudobulk	DESeq2	LRT	0.027131628799383
146	KC_AB2	Ror	-0.647621034217714	0.754702560287896	1	pseudobulk	DESeq2	LRT	0.122224176685402
146	KC_AB2	row	-0.0736460134636664	0.935339549141061	1	pseudobulk	DESeq2	LRT	0.029030701909687
146	KC_AB2	Rox8	-0.828794901665361	0.256561136975808	1	pseudobulk	DESeq2	LRT	0.590809128456818
146	KC_AB2	RpA-70	1.13966997725421	0.492673952049737	1	pseudobulk	DESeq2	LRT	0.307440398512806
146	KC_AB2	RPA2	-2.29555162987475	0.241638147791594	1	pseudobulk	DESeq2	LRT	0.616834501890482
146	KC_AB2	RPA3	-0.0482867344442866	0.887419910385345	1	pseudobulk	DESeq2	LRT	0.051870831565503
146	KC_AB2	Rpb10	-0.0356785368171669	0.960697060298336	1	pseudobulk	DESeq2	LRT	0.017413538223251
146	KC_AB2	Rpb11	-0.0479891280163599	0.944111366897865	1	pseudobulk	DESeq2	LRT	0.024976773523455
146	KC_AB2	Rpb12	-1.13053699949495	0.130332813110465	1	pseudobulk	DESeq2	LRT	0.884946230804912
146	KC_AB2	Rpb4	1.10706867860301	1	1	pseudobulk	DESeq2	LRT	0
146	KC_AB2	Rpb5	0.681680287540556	0.717771302843503	1	pseudobulk	DESeq2	LRT	0.144013909148294
146	KC_AB2	Rpb7	0.138265758373108	0.883045927063996	1	pseudobulk	DESeq2	LRT	0.054016708255088
146	KC_AB2	Rpb8	-0.592789099888785	0.75266656177414	1	pseudobulk	DESeq2	LRT	0.123397377654516
146	KC_AB2	Rpe	-0.729379579623002	1	1	pseudobulk	DESeq2	LRT	0
146	KC_AB2	Rph	-0.132229573483494	0.90918403499286	1	pseudobulk	DESeq2	LRT	0.041348198964861
146	KC_AB2	Rpi	0.145321756433347	1	1	pseudobulk	DESeq2	LRT	0
146	KC_AB2	RpI135	1.38446765109058	0.642794900879723	1	pseudobulk	DESeq2	LRT	0.191927577037166
146	KC_AB2	RpII140	-1.07510159026337	0.484763747919268	1	pseudobulk	DESeq2	LRT	0.314469865465738
146	KC_AB2	RpII15	-1.28292259562998	0.530493603495545	1	pseudobulk	DESeq2	LRT	0.275319848300503
146	KC_AB2	RpII18	-0.300425209500835	0.731540728462537	1	pseudobulk	DESeq2	LRT	0.13576148956036
146	KC_AB2	RpII215	-0.420246074132895	0.611541516514853	1	pseudobulk	DESeq2	LRT	0.21357405411213
146	KC_AB2	RpII33	-0.163968651110663	0.879651377806168	1	pseudobulk	DESeq2	LRT	0.055689412728621
146	KC_AB2	RpIII128	1.10706867860301	1	1	pseudobulk	DESeq2	LRT	0
146	KC_AB2	RpIIIC160	0.150205259410532	1	1	pseudobulk	DESeq2	LRT	0
146	KC_AB2	RpIIIC53	-0.429347771485688	0.829237007768346	1	pseudobulk	DESeq2	LRT	0.081321324148204
146	KC_AB2	RpL10	0.0232734093240238	0.881824890746402	1	pseudobulk	DESeq2	LRT	0.054617646764800
146	KC_AB2	RpL10Ab	-0.0563562713471604	0.839659357809118	1	pseudobulk	DESeq2	LRT	0.075896867543483
146	KC_AB2	RpL11	0.0606897435510109	0.877440114344022	1	pseudobulk	DESeq2	LRT	0.056782514631306
146	KC_AB2	RpL12	-0.202527486636282	0.512451646219305	1	pseudobulk	DESeq2	LRT	0.290347107386061
146	KC_AB2	RpL13	-0.167472971523256	0.522765182157817	1	pseudobulk	DESeq2	LRT	0.281693345537325
146	KC_AB2	RpL13A	-0.451079483483875	0.197138458152429	1	pseudobulk	DESeq2	LRT	0.705228644446417
146	KC_AB2	RpL14	0.0062109489572953	0.906107265739016	1	pseudobulk	DESeq2	LRT	0.042820387134376
146	KC_AB2	RpL15	0.0012810303917376	0.922052912616453	1	pseudobulk	DESeq2	LRT	0.035244155955064
146	KC_AB2	RpL17	-0.0922543765874554	0.770332388358283	1	pseudobulk	DESeq2	LRT	0.113321841993952
146	KC_AB2	RpL18	-0.328640964952491	0.299389538284124	1	pseudobulk	DESeq2	LRT	0.523763379493492
146	KC_AB2	RpL18A	0.137530112170522	0.674103119424683	1	pseudobulk	DESeq2	LRT	0.171273663150792
146	KC_AB2	RpL19	-0.125927399692585	0.667759176922898	1	pseudobulk	DESeq2	LRT	0.175380134816003
146	KC_AB2	RpL21	0.0905841033288593	0.715490314056695	1	pseudobulk	DESeq2	LRT	0.145396241121818
146	KC_AB2	RpL22	-0.549795039817593	0.341004458315196	1	pseudobulk	DESeq2	LRT	0.467239942975153
146	KC_AB2	RpL23	0.0610579888392445	0.830468948075026	1	pseudobulk	DESeq2	LRT	0.080676601541046
146	KC_AB2	RpL23A	-0.331029235691636	0.320905844188727	1	pseudobulk	DESeq2	LRT	0.493622373662352
146	KC_AB2	RpL24	0.200297124948144	0.473194387632507	1	pseudobulk	DESeq2	LRT	0.324960415004984
146	KC_AB2	RpL24-like	0.326975986833435	0.408601776023828	1	pseudobulk	DESeq2	LRT	0.388699750000017
146	KC_AB2	RpL26	-0.107401966234037	0.687375710746848	1	pseudobulk	DESeq2	LRT	0.162805818249109
146	KC_AB2	RpL27	0.290770268165451	0.381388820660205	1	pseudobulk	DESeq2	LRT	0.418632041223786
146	KC_AB2	RpL27A	-0.416884761320411	0.172394069995074	1	pseudobulk	DESeq2	LRT	0.763477677100112
146	KC_AB2	RpL28	0.0074587795607191	0.893406971693923	1	pseudobulk	DESeq2	LRT	0.048950662837555
146	KC_AB2	RpL29	0.291704166026523	0.249327291398505	1	pseudobulk	DESeq2	LRT	0.603230180962484
146	KC_AB2	RpL3	-0.622509832340309	0.0782061338847743	1	pseudobulk	DESeq2	LRT	1.10675918290129
146	KC_AB2	RpL30	-0.590674124225715	0.287808575884369	1	pseudobulk	DESeq2	LRT	0.540896269455114

146	KC_AB2	RpL31	-0.255062509073387	0.325713469223864	1	pseudobulk	DESeq2	LRT	0.487164281691041
146	KC_AB2	RpL32	-0.16103164268703	0.597664899963705	1	pseudobulk	DESeq2	LRT	0.22354224893408
146	KC_AB2	RpL34a	-0.826096708057625	0.208062776298225	1	pseudobulk	DESeq2	LRT	0.681805610774886
146	KC_AB2	RpL34b	-0.271998616040422	0.395302362905337	1	pseudobulk	DESeq2	LRT	0.403070589669977
146	KC_AB2	RpL35	-0.281174599092281	0.458672973844017	1	pseudobulk	DESeq2	LRT	0.338496848828557
146	KC_AB2	RpL35A	-0.14098162535505	0.68253004658844	1	pseudobulk	DESeq2	LRT	0.165878225195148
146	KC_AB2	RpL36	-0.300363658850264	0.177617441430323	1	pseudobulk	DESeq2	LRT	0.750514390218296
146	KC_AB2	RpL36A	-0.265330511822177	0.368384119624566	1	pseudobulk	DESeq2	LRT	0.43369909972403
146	KC_AB2	RpL37a	-0.14273215480722	0.618913170206456	1	pseudobulk	DESeq2	LRT	0.208370275608799
146	KC_AB2	RpL37A	-0.231479191720133	0.406080763813242	1	pseudobulk	DESeq2	LRT	0.39138758270309
146	KC_AB2	RpL38	0.0202323840504866	0.898408584925407	1	pseudobulk	DESeq2	LRT	0.046526106737785
146	KC_AB2	RpL39	-0.0223376741760062	0.91457166802788	1	pseudobulk	DESeq2	LRT	0.038782256497581
146	KC_AB2	RpL4	-0.23387772197411	0.528518183386422	1	pseudobulk	DESeq2	LRT	0.27694006642933
146	KC_AB2	RpL40	0.195266003477303	0.50274434417383	1	pseudobulk	DESeq2	LRT	0.298652806475453
146	KC_AB2	RpL41	0.0098205973008957	0.931917049366073	1	pseudobulk	DESeq2	LRT	0.030622742802503
146	KC_AB2	RpL5	0.355274348404906	0.207543205627892	1	pseudobulk	DESeq2	LRT	0.682891479614363
146	KC_AB2	RpL6	-0.0876171117745507	0.762678977601878	1	pseudobulk	DESeq2	LRT	0.117658224290881
146	KC_AB2	RpL7	-0.348462189190718	0.259800105755452	1	pseudobulk	DESeq2	LRT	0.585360676477001
146	KC_AB2	RpL7A	-0.416257004742079	0.164426639132522	1	pseudobulk	DESeq2	LRT	0.784027820062223
146	KC_AB2	RpL8	0.0596479485100968	0.810194194742936	1	pseudobulk	DESeq2	LRT	0.091410872976845
146	KC_AB2	RpL9	-0.177146159593967	0.515802012398278	1	pseudobulk	DESeq2	LRT	0.287516967797611
146	KC_AB2	RpLP0	0.0164165235877725	0.958620399431342	1	pseudobulk	DESeq2	LRT	0.018353333464196
146	KC_AB2	RpLP0-like	-1.0778469209274	0.476351080090846	1	pseudobulk	DESeq2	LRT	0.322072845714654
146	KC_AB2	RpLP1	0.115102255871289	0.682547486752693	1	pseudobulk	DESeq2	LRT	0.16586712814474
146	KC_AB2	RpLP2	-0.195066995967357	0.539034659216756	1	pseudobulk	DESeq2	LRT	0.268383309353796
146	KC_AB2	Rpn1	-0.740724624894638	0.588359936328425	1	pseudobulk	DESeq2	LRT	0.230356907704999
146	KC_AB2	Rpn10	-0.493960362839363	0.588449532398991	1	pseudobulk	DESeq2	LRT	0.230290777919072
146	KC_AB2	Rpn11	-0.22705512327485	0.826400773491547	1	pseudobulk	DESeq2	LRT	0.082809284998898
146	KC_AB2	Rpn12	0.0279468929992046	0.9818784703387	1	pseudobulk	DESeq2	LRT	0.007942262648380
146	KC_AB2	Rpn13	-0.521016983093415	0.648284115485053	1	pseudobulk	DESeq2	LRT	0.188234619522661
146	KC_AB2	Rpn13R	2.47215486522296	0.30601789350912	1	pseudobulk	DESeq2	LRT	0.514253178665238
146	KC_AB2	Rpn2	-0.0085837851043342	0.923679802016673	1	pseudobulk	DESeq2	LRT	0.034478552932958
146	KC_AB2	Rpn3	-0.375224690839849	0.774399475306582	1	pseudobulk	DESeq2	LRT	0.111034949955258
146	KC_AB2	Rpn5	-0.178912131565591	0.829105675051237	1	pseudobulk	DESeq2	LRT	0.081390112191887
146	KC_AB2	Rpn6	-0.52418076991543	0.584363146885199	1	pseudobulk	DESeq2	LRT	0.233317180843615
146	KC_AB2	Rpn7	-0.530195555423761	0.686703437006413	1	pseudobulk	DESeq2	LRT	0.16323077892124
146	KC_AB2	Rpn8	-0.257919425352015	0.77129973288702	1	pseudobulk	DESeq2	LRT	0.112776819040321
147	KC_AB2	Rpn9	-0.708608700488007	0.445949060377918	1	pseudobulk	DESeq2	LRT	0.350714746804109
147	KC_AB2	Rpp30	0.489566863927151	0.82181613769085	1	pseudobulk	DESeq2	LRT	0.085225334914893
147	KC_AB2	RpS10a	0.403294498144138	0.89479244414645	1	pseudobulk	DESeq2	LRT	0.048277691852293
147	KC_AB2	RpS10b	-0.161212753291223	0.580242412464889	1	pseudobulk	DESeq2	LRT	0.236390529883117
147	KC_AB2	RpS11	-0.0893720063928067	0.718829419131596	1	pseudobulk	DESeq2	LRT	0.143374157070769
147	KC_AB2	RpS12	-0.0580821055204725	0.849230960565221	1	pseudobulk	DESeq2	LRT	0.070974181065709
147	KC_AB2	RpS13	0.0889582088357652	0.759361620069461	1	pseudobulk	DESeq2	LRT	0.119551356910821
147	KC_AB2	RpS14a	0.111635273577549	0.684033289995987	1	pseudobulk	DESeq2	LRT	0.164922761862125
147	KC_AB2	RpS14b	0.0945422877850871	0.869098449014384	1	pseudobulk	DESeq2	LRT	0.060931025114080
147	KC_AB2	RpS15	-0.0343278787766548	0.888865984422314	1	pseudobulk	DESeq2	LRT	0.051163713285437
147	KC_AB2	RpS15Aa	-0.537365734716242	0.0861404649823098	1	pseudobulk	DESeq2	LRT	1.06479278826524
147	KC_AB2	RpS15Ab	1.26482114150091	0.415166155087822	1	pseudobulk	DESeq2	LRT	0.381778057996088
147	KC_AB2	RpS16	-0.0031286880013959	0.932374881747254	1	pseudobulk	DESeq2	LRT	0.030409434921411
147	KC_AB2	RpS17	-0.0327779483865545	0.865585194391235	1	pseudobulk	DESeq2	LRT	0.062690180667703
147	KC_AB2	RpS18	-0.166564491830884	0.496981217546866	1	pseudobulk	DESeq2	LRT	0.303660024284559

147	KC_AB2	RpS19a	0.180697106708811	0.652478072504959	1	pseudobulk	DESeq2	LRT	0.185434078855756
147	KC_AB2	RpS2	-0.24750498637296	0.420544627795576	1	pseudobulk	DESeq2	LRT	0.376187910503541
147	KC_AB2	RpS20	-0.191945482183116	0.455898768922289	1	pseudobulk	DESeq2	LRT	0.341131580535433
147	KC_AB2	RpS21	-0.309138721082284	0.313338118532237	1	pseudobulk	DESeq2	LRT	0.503986768663762
147	KC_AB2	RpS23	-0.132278426836589	0.632750516702721	1	pseudobulk	DESeq2	LRT	0.198767491525616
147	KC_AB2	RpS24	-0.251965470498899	0.39385190538006	1	pseudobulk	DESeq2	LRT	0.404667049156086
147	KC_AB2	RpS25	-0.33486337070247	0.166316019050622	1	pseudobulk	DESeq2	LRT	0.779065918848779
147	KC_AB2	RpS26	0.252332313631008	0.305582811725962	1	pseudobulk	DESeq2	LRT	0.51487107739511
147	KC_AB2	RpS27	0.187281692730429	0.478195345830719	1	pseudobulk	DESeq2	LRT	0.32039465509158
147	KC_AB2	RpS27A	-0.160673877618168	0.532080440812157	1	pseudobulk	DESeq2	LRT	0.274022705374237
147	KC_AB2	RpS28b	-0.113254644638761	0.678652323081227	1	pseudobulk	DESeq2	LRT	0.168352659943698
147	KC_AB2	RpS29	0.0141418452960778	0.939615252599441	1	pseudobulk	DESeq2	LRT	0.027049942015752
147	KC_AB2	RpS3	-0.059905019050898	0.827713619735723	1	pseudobulk	DESeq2	LRT	0.082119898579810
147	KC_AB2	RpS30	0.15724389990573	0.544595454125693	1	pseudobulk	DESeq2	LRT	0.263925988145461
147	KC_AB2	RpS3A	0.21562532193472	0.458415457023587	1	pseudobulk	DESeq2	LRT	0.33874074709024
147	KC_AB2	RpS4	-0.122208072241759	0.667792147902749	1	pseudobulk	DESeq2	LRT	0.175358691814377
147	KC_AB2	RpS5a	-0.279620130398432	0.538103572430401	1	pseudobulk	DESeq2	LRT	0.269134124694521
147	KC_AB2	RpS5b	-0.729379579623002	0.823527397767669	1	pseudobulk	DESeq2	LRT	0.084321947797948
147	KC_AB2	RpS6	-0.0871952669129463	0.72223494379417	1	pseudobulk	DESeq2	LRT	0.141321502990538
147	KC_AB2	RpS7	-0.261314510067553	0.465206409188055	1	pseudobulk	DESeq2	LRT	0.332354310590555
147	KC_AB2	RpS8	-0.0654982580241576	0.781809298101746	1	pseudobulk	DESeq2	LRT	0.106899168784473
147	KC_AB2	RpS9	-0.19993391155734	0.498579026169781	1	pseudobulk	DESeq2	LRT	0.302265995006568
147	KC_AB2	Rpt1	0.0364685997625096	0.946812085786652	1	pseudobulk	DESeq2	LRT	0.023736207062185
147	KC_AB2	Rpt2	-0.389627831942898	0.672163796192326	1	pseudobulk	DESeq2	LRT	0.172524883025906
147	KC_AB2	Rpt3	-0.33444714501023	0.643282605108515	1	pseudobulk	DESeq2	LRT	0.191598192121355
147	KC_AB2	Rpt4	0.177700856956277	0.83576471558096	1	pseudobulk	DESeq2	LRT	0.077915967903990
147	KC_AB2	Rpt5	-0.299783143069153	0.809143806406698	1	pseudobulk	DESeq2	LRT	0.091974285830665
147	KC_AB2	Rpt6	-0.759588017500183	0.439170012075831	1	pseudobulk	DESeq2	LRT	0.357367322553016
147	KC_AB2	Rrp1	0.182464987382747	0.906839011984365	1	pseudobulk	DESeq2	LRT	0.042469804905139
147	KC_AB2	Rrp4	1.10706867860301	1	1	pseudobulk	DESeq2	LRT	0
147	KC_AB2	Rrp42	-1.86604523843964	0.375091528268488	1	pseudobulk	DESeq2	LRT	0.425862744614398
147	KC_AB2	Rrp45	-0.729379579623002	1	1	pseudobulk	DESeq2	LRT	0
147	KC_AB2	Rrp46	1.10706867860301	1	1	pseudobulk	DESeq2	LRT	0
147	KC_AB2	Rrp47	-0.268505655393316	0.813712442516395	1	pseudobulk	DESeq2	LRT	0.089529043135642
147	KC_AB2	Rrp6	-0.179517854375827	0.901955620076629	1	pseudobulk	DESeq2	LRT	0.044814831005728
147	KC_AB2	Rs1	-0.347135499964956	0.854806062097812	1	pseudobulk	DESeq2	LRT	0.068132406575477
147	KC_AB2	Rsf1	0.262805619057193	0.529505247253481	1	pseudobulk	DESeq2	LRT	0.276129731794569
147	KC_AB2	RSG7	0.0469365598245502	0.886872546664094	1	pseudobulk	DESeq2	LRT	0.052138788576006
147	KC_AB2	rswl	0.796171800233555	0.593036468603691	1	pseudobulk	DESeq2	LRT	0.22691859900276
147	KC_AB2	rt	-0.18317077492817	0.899664519927029	1	pseudobulk	DESeq2	LRT	0.045919406456170
147	KC_AB2	Rtc1	0.396659246788372	0.915482501218565	1	pseudobulk	DESeq2	LRT	0.038349952505755
147	KC_AB2	Rtca	0.553657416284704	0.503087303325656	1	pseudobulk	DESeq2	LRT	0.298356643050294
147	KC_AB2	RtcB	-2.95242274408022	0.0840075705146754	1	pseudobulk	DESeq2	LRT	1.07568157483575
147	KC_AB2	rtet	0.150205259410532	1	1	pseudobulk	DESeq2	LRT	0
147	KC_AB2	Rtf1	-0.444686072737572	0.742009345714434	1	pseudobulk	DESeq2	LRT	0.12959062468506
147	KC_AB2	RtGEF	-2.4120103603151	0.297158744475551	1	pseudobulk	DESeq2	LRT	0.527011485255674
147	KC_AB2	Rtnl1	-0.184051213217122	0.483661171295387	1	pseudobulk	DESeq2	LRT	0.315458776719517
147	KC_AB2	Rubicon	-3.06511173096008	0.0634176626219939	1	pseudobulk	DESeq2	LRT	1.19778976875921
147	KC_AB2	rudhira	0.676526282205688	0.0518152721981394	1	pseudobulk	DESeq2	LRT	1.28554221604973
147	KC_AB2	rump	1.00519042355029	0.173371264334147	1	pseudobulk	DESeq2	LRT	0.761022883649272
147	KC_AB2	rush	-1.40133744391925	0.428660057955876	1	pseudobulk	DESeq2	LRT	0.367886981657322
147	KC_AB2	rut	0.197485331233807	0.367480745519868	1	pseudobulk	DESeq2	LRT	0.434765411228873

147	KC_AB2	rux	0.052584888526993	0.943723369149211	1	pseudobulk	DESeq2	LRT	0.025155290509488
147	KC_AB2	RYa-R	1.99339641594643	0.465071165814147	1	pseudobulk	DESeq2	LRT	0.332480585700252
147	KC_AB2	RYBP	3.29581267878089	0.13415686913437	1	pseudobulk	DESeq2	LRT	0.872387085565452
147	KC_AB2	RyR	-0.501697391067604	0.730274567944797	1	pseudobulk	DESeq2	LRT	0.136513823545266
147	KC_AB2	S	-0.630581853637629	0.417834610969137	1	pseudobulk	DESeq2	LRT	0.378995588457343
147	KC_AB2	s-cup	0.145321756433347	1	1	pseudobulk	DESeq2	LRT	0
147	KC_AB2	S-Lap1	1.99339641594643	0.465071165814147	1	pseudobulk	DESeq2	LRT	0.332480585700252
147	KC_AB2	S1P	-0.976691273560528	0.623102301228213	1	pseudobulk	DESeq2	LRT	0.2054406448151
147	KC_AB2	S2P	-0.729379579623002	0.823527397767669	1	pseudobulk	DESeq2	LRT	0.084321947797948
147	KC_AB2	S6k	0.312216098760424	0.740816967150122	1	pseudobulk	DESeq2	LRT	0.130289079437236
147	KC_AB2	S6kII	-1.10301481191544	0.281381396853759	1	pseudobulk	DESeq2	LRT	0.5507046187379
147	KC_AB2	S6KL	-0.578591498905967	0.625340878731614	1	pseudobulk	DESeq2	LRT	0.203883180423311
147	KC_AB2	SA	-0.647621034217714	0.754702560287896	1	pseudobulk	DESeq2	LRT	0.122224176685402
147	KC_AB2	Sac1	-0.0514351391734618	0.956344182265455	1	pseudobulk	DESeq2	LRT	0.019385779734768
147	KC_AB2	Saf-B	-0.227967134328458	0.684570630658684	1	pseudobulk	DESeq2	LRT	0.164581736831902
147	KC_AB2	Saf6	-0.795404628052927	0.767318925015269	1	pseudobulk	DESeq2	LRT	0.115024090306574
147	KC_AB2	sage	0.403294498144138	0.89479244414645	1	pseudobulk	DESeq2	LRT	0.048277691852293
147	KC_AB2	SAK	0.132730453525954	0.936793672000375	1	pseudobulk	DESeq2	LRT	0.028356051565710
147	KC_AB2	sals	2.63815056834323	0.043990636414995	1	pseudobulk	DESeq2	LRT	1.35663975501515
147	KC_AB2	Sam-S	-0.215127182948168	0.652603045939696	1	pseudobulk	DESeq2	LRT	0.185350903530499
147	KC_AB2	SamDC	0.177427281908243	0.847684073738986	1	pseudobulk	DESeq2	LRT	0.071765976276748
147	KC_AB2	Samtor	-0.672424505335587	0.790771522020703	1	pseudobulk	DESeq2	LRT	0.101948979282445
147	KC_AB2	san	-0.679476194260674	0.491462099560796	1	pseudobulk	DESeq2	LRT	0.308509968343074
147	KC_AB2	sano	-0.18914985872157	0.817315884017605	1	pseudobulk	DESeq2	LRT	0.087610060760431
147	KC_AB2	Sap-r	-0.276465024963307	0.692879589318616	1	pseudobulk	DESeq2	LRT	0.15934223182124
147	KC_AB2	Sap130	-1.97832784139986	0.174264025088346	1	pseudobulk	DESeq2	LRT	0.758792259006927
147	KC_AB2	Sap30	-0.774070552872373	0.608275281417677	1	pseudobulk	DESeq2	LRT	0.215899831674547
147	KC_AB2	Sap47	-0.0492718918928257	0.82857706419619	1	pseudobulk	DESeq2	LRT	0.081667092557028
147	KC_AB2	Sar1	0.447579986135381	0.551615679396693	1	pseudobulk	DESeq2	LRT	0.258363397719748
147	KC_AB2	Sara	0.547478514889769	0.645512209756485	1	pseudobulk	DESeq2	LRT	0.190095538711709
147	KC_AB2	Sas-6	1.02605719768525	0.762384804222891	1	pseudobulk	DESeq2	LRT	0.117825768597322
147	KC_AB2	Sas10	-1.5569401799281	0.491369326863142	1	pseudobulk	DESeq2	LRT	0.308591957318393
147	KC_AB2	sau	0.528739319059408	0.457375884363731	1	pseudobulk	DESeq2	LRT	0.33972673777007
148	KC_AB2	sax	0.406466497954158	0.722818652232339	1	pseudobulk	DESeq2	LRT	0.140970649059276
148	KC_AB2	sba	0.609532394629758	0.386797606530505	1	pseudobulk	DESeq2	LRT	0.412516221956695
148	KC_AB2	Sbat	1.02648689492346	0.73959154128541	1	pseudobulk	DESeq2	LRT	0.131008064514051
148	KC_AB2	sbb	0.218799303365631	0.436711482015347	1	pseudobulk	DESeq2	LRT	0.359805389460954
148	KC_AB2	Sbf	-0.733064587541404	0.476763892728295	1	pseudobulk	DESeq2	LRT	0.321696642912626
148	KC_AB2	sbm	0.848405451787743	0.288719562216178	1	pseudobulk	DESeq2	LRT	0.539523789466291
148	KC_AB2	Sbp2	-0.674317410868381	0.830763653594129	1	pseudobulk	DESeq2	LRT	0.080522512364194
148	KC_AB2	sbr	0.462179181635442	0.25047805051556	1	pseudobulk	DESeq2	LRT	0.601230325515819
148	KC_AB2	Sc2	-1.12489422583229	0.522558265669225	1	pseudobulk	DESeq2	LRT	0.281865278331085
148	KC_AB2	SC35	-0.151380333461024	0.788262874746985	1	pseudobulk	DESeq2	LRT	0.10332892716311
148	KC_AB2	sca	0.426505816547008	0.62787414900636	1	pseudobulk	DESeq2	LRT	0.202127397456073
148	KC_AB2	scaf6	0.0493067858544995	0.904804129748864	1	pseudobulk	DESeq2	LRT	0.043445425851113
148	KC_AB2	SCaMC	0.0116969443348265	0.936483121123282	1	pseudobulk	DESeq2	LRT	0.028500045805112
148	KC_AB2	Scamp	-0.250235673515224	0.684935743293306	1	pseudobulk	DESeq2	LRT	0.164350169590942
148	KC_AB2	SCAP	0.621967675302016	0.285967236998562	1	pseudobulk	DESeq2	LRT	0.543683720737368
148	KC_AB2	SCAR	-0.572541478005558	0.476312106555406	1	pseudobulk	DESeq2	LRT	0.322108379766202
148	KC_AB2	scat	0.396659246788372	0.915482501218565	1	pseudobulk	DESeq2	LRT	0.038349952505755
148	KC_AB2	Sccpdh1	-0.804525100198913	0.541211287470496	1	pseudobulk	DESeq2	LRT	0.266633154360017
148	KC_AB2	SCCRO	-0.775775885996245	0.523664124435433	1	pseudobulk	DESeq2	LRT	0.280947178043171

148	KC_AB2	SCCRO3	0.0527381103135567	0.952938732623559	1	pseudobulk	DESeq2	LRT	0.020935020598660
148	KC_AB2	SCCRO4	-0.0962486177519835	0.909817317165856	1	pseudobulk	DESeq2	LRT	0.041045801207977
148	KC_AB2	Sce	-0.644078006814249	0.663164551483348	1	pseudobulk	DESeq2	LRT	0.178378696430382
148	KC_AB2	sced	-0.65339711380866	0.644103135128829	1	pseudobulk	DESeq2	LRT	0.191044586939669
148	KC_AB2	scf	-1.3686873869832	0.184172262963321	1	pseudobulk	DESeq2	LRT	0.734775775599638
148	KC_AB2	Scgalpha	-0.714920904179902	0.469869917475766	1	pseudobulk	DESeq2	LRT	0.328022358961913
148	KC_AB2	Scgbeta	-1.00675813830613	0.0957946434832311	1	pseudobulk	DESeq2	LRT	1.01865877454059
148	KC_AB2	Scgdelta	-0.721175887303532	0.733409564198845	1	pseudobulk	DESeq2	LRT	0.134653430819787
148	KC_AB2	Schip1	0.166897025599263	0.767971822108649	1	pseudobulk	DESeq2	LRT	0.114654714509149
148	KC_AB2	schlank	0.367021524382852	0.619743770522502	1	pseudobulk	DESeq2	LRT	0.207787829935372
148	KC_AB2	Scm	-0.110607701667472	0.914986308981522	1	pseudobulk	DESeq2	LRT	0.038585404270449
148	KC_AB2	scny	-0.183947198811148	0.836693750568343	1	pseudobulk	DESeq2	LRT	0.077433474833840
148	KC_AB2	SCOT	-0.427692090780194	0.83957454067488	1	pseudobulk	DESeq2	LRT	0.075940739470360
148	KC_AB2	Scox	0.440138724847644	0.687054625436004	1	pseudobulk	DESeq2	LRT	0.163008732252948
148	KC_AB2	Scp1	-0.729379579623002	0.823527397767669	1	pseudobulk	DESeq2	LRT	0.084321947797948
148	KC_AB2	ScpX	1.15196837059098	0.569232633023539	1	pseudobulk	DESeq2	LRT	0.244710210597932
148	KC_AB2	scramb1	0.224780078996072	0.827961573603656	1	pseudobulk	DESeq2	LRT	0.081989818721335
148	KC_AB2	scramb2	-1.40066889480218	0.329351545815205	1	pseudobulk	DESeq2	LRT	0.482340293851622
148	KC_AB2	scrib	0.372661179250235	0.194710712766728	1	pseudobulk	DESeq2	LRT	0.710610153423543
148	KC_AB2	scrt	0.0374096170453201	0.909822364608823	1	pseudobulk	DESeq2	LRT	0.041043391855564
148	KC_AB2	Scsalpha1	-0.335876468427705	0.422596434427128	1	pseudobulk	DESeq2	LRT	0.374074171541054
148	KC_AB2	ScsbetaA	-0.37668620856008	0.536869373496549	1	pseudobulk	DESeq2	LRT	0.270131370297941
148	KC_AB2	ScsbetaG	-0.470100723599697	0.833985291815347	1	pseudobulk	DESeq2	LRT	0.078841608522644
148	KC_AB2	scu	0.633515361335381	0.692346491558974	1	pseudobulk	DESeq2	LRT	0.159676504226234
148	KC_AB2	scyl	-0.388719120723441	0.266031855990159	1	pseudobulk	DESeq2	LRT	0.575066355653221
148	KC_AB2	sd	0.165356554371255	0.683774681013038	1	pseudobulk	DESeq2	LRT	0.165086984410881
148	KC_AB2	Sdc	-0.10020007903136	0.733687783497626	1	pseudobulk	DESeq2	LRT	0.134488712223767
148	KC_AB2	SdhA	-0.291778686614376	0.712839664857508	1	pseudobulk	DESeq2	LRT	0.14700814265599
148	KC_AB2	Sdhaf3	-0.0537336713563124	0.963611243864229	1	pseudobulk	DESeq2	LRT	0.016098141094096
148	KC_AB2	SdhB	-0.454823904656678	0.40524255136184	1	pseudobulk	DESeq2	LRT	0.392284959038806
148	KC_AB2	SdhC	-0.247689994655694	0.580292576687676	1	pseudobulk	DESeq2	LRT	0.236352985051876
148	KC_AB2	SdhD	-1.07895371457401	0.0617372917224977	1	pseudobulk	DESeq2	LRT	1.20945242596783
148	KC_AB2	sdk	-0.729379579623002	0.823527397767669	1	pseudobulk	DESeq2	LRT	0.084321947797948
148	KC_AB2	sds22	-0.893365233820797	0.449159514221872	1	pseudobulk	DESeq2	LRT	0.347599396538894
148	KC_AB2	sdt	0.15141810212627	0.521453079593413	1	pseudobulk	DESeq2	LRT	0.282784763345632
148	KC_AB2	Sec10	-0.203536419534241	0.877659317741844	1	pseudobulk	DESeq2	LRT	0.056674032086279
148	KC_AB2	Sec13	-1.73049674299479	0.207614707294417	1	pseudobulk	DESeq2	LRT	0.682741884587704
148	KC_AB2	Sec15	-1.57003801835154	0.383914332729826	1	pseudobulk	DESeq2	LRT	0.415765674000646
148	KC_AB2	Sec16	0.400126095774684	0.654013819468991	1	pseudobulk	DESeq2	LRT	0.18441307483213
148	KC_AB2	Sec22	-0.907840262567122	0.383622373875533	1	pseudobulk	DESeq2	LRT	0.416096071555281
148	KC_AB2	Sec23	-0.0846732002757032	0.931341319543643	1	pseudobulk	DESeq2	LRT	0.030891128869704
148	KC_AB2	Sec24AB	0.217539487810903	0.869333958258249	1	pseudobulk	DESeq2	LRT	0.060813355464981
148	KC_AB2	Sec24CD	0.443385409515957	0.721577249134799	1	pseudobulk	DESeq2	LRT	0.141717168275119
148	KC_AB2	Sec3	-0.115183097143228	0.910399790291748	1	pseudobulk	DESeq2	LRT	0.040767850987610
148	KC_AB2	Sec31	1.29323210685144	0.489272320226568	1	pseudobulk	DESeq2	LRT	0.31044935303813
148	KC_AB2	Sec5	-0.938142572757362	0.608739806489362	1	pseudobulk	DESeq2	LRT	0.215568298093509
148	KC_AB2	Sec6	-0.824996233971657	0.664071509040402	1	pseudobulk	DESeq2	LRT	0.177785152093585
148	KC_AB2	Sec61alpha	1.61780397615607	0.377839752653399	1	pseudobulk	DESeq2	LRT	0.422692351735028
148	KC_AB2	Sec61beta	-3.06376964427821	0.028550907426714	1	pseudobulk	DESeq2	LRT	1.54438008412034
148	KC_AB2	Sec61gamma	-0.158257557663935	0.803269018123999	1	pseudobulk	DESeq2	LRT	0.095138983338183
148	KC_AB2	Sec63	2.08710145465109	0.0335911227120397	1	pseudobulk	DESeq2	LRT	1.47377548054371
148	KC_AB2	Sec71	-0.0274154595993649	0.957527839269979	1	pseudobulk	DESeq2	LRT	0.018848590450127

148	KC_AB2	Sec8	0.227800203919916	0.882554963386119	1	pseudobulk	DESeq2	LRT	0.054258238289158
148	KC_AB2	SecCl	1.38069928703004	0.566368801250645	1	pseudobulk	DESeq2	LRT	0.246900678019328
148	KC_AB2	Secp43	-0.62294681620203	0.567715547295039	1	pseudobulk	DESeq2	LRT	0.245869212147693
148	KC_AB2	SecS	0.431362405382703	0.673764609945635	1	pseudobulk	DESeq2	LRT	0.171491804439748
148	KC_AB2	sei	0.257943341580225	0.828385502068825	1	pseudobulk	DESeq2	LRT	0.081767510486704
148	KC_AB2	sel	-0.729379579623002	0.823527397767669	1	pseudobulk	DESeq2	LRT	0.084321947797948
148	KC_AB2	SelG	0.0308590232760449	0.972599184638301	1	pseudobulk	DESeq2	LRT	0.012066098850953
148	KC_AB2	SelR	-0.145965288504499	0.718977086196132	1	pseudobulk	DESeq2	LRT	0.143284950364199
148	KC_AB2	SelT	-0.308196400293963	0.717903255490098	1	pseudobulk	DESeq2	LRT	0.143934077258654
148	KC_AB2	Sem1	0.0323546715886188	0.909908739839718	1	pseudobulk	DESeq2	LRT	0.041002163471780
148	KC_AB2	Sema1a	0.687685035473887	0.0614844353289862	1	pseudobulk	DESeq2	LRT	1.21123481115561
148	KC_AB2	Sema1b	0.0788000589042686	0.930221750095779	1	pseudobulk	DESeq2	LRT	0.031413510195233
148	KC_AB2	Sema2a	0.533971347890876	0.489265090077467	1	pseudobulk	DESeq2	LRT	0.310455770807803
148	KC_AB2	Sema2b	-1.11167283223406	0.564212144344824	1	pseudobulk	DESeq2	LRT	0.248557570138858
148	KC_AB2	senju	-0.729379579623002	0.823527397767669	1	pseudobulk	DESeq2	LRT	0.084321947797948
148	KC_AB2	Sep1	-1.29120778207021	0.303544303859722	1	pseudobulk	DESeq2	LRT	0.517777912438542
148	KC_AB2	Sep2	-0.0971691262923304	0.914937315513765	1	pseudobulk	DESeq2	LRT	0.038608659440682
148	KC_AB2	Sep4	0.0704420273975093	0.890377682819643	1	pseudobulk	DESeq2	LRT	0.050425734061653
148	KC_AB2	SERCA	-0.114021044965891	0.866480410974772	1	pseudobulk	DESeq2	LRT	0.062241251186333
148	KC_AB2	SerRS	0.629049291096712	0.778060243600628	1	pseudobulk	DESeq2	LRT	0.108986775182027
148	KC_AB2	SerRS-m	-0.547422375819482	0.70386793097862	1	pseudobulk	DESeq2	LRT	0.152508821295253
148	KC_AB2	sesB	-0.283504352027589	0.286295228133397	1	pseudobulk	DESeq2	LRT	0.543185890583379
148	KC_AB2	Set	-0.675019960887318	0.552766045872075	1	pseudobulk	DESeq2	LRT	0.257458641725179
148	KC_AB2	Set1	-0.129276957307298	0.82819113012513	1	pseudobulk	DESeq2	LRT	0.081869425075296
148	KC_AB2	Set2	-0.056601183218691	0.971866364361422	1	pseudobulk	DESeq2	LRT	0.012393448253165
148	KC_AB2	Setd3	1.64541678555507	0.422372189619268	1	pseudobulk	DESeq2	LRT	0.374304684916733
148	KC_AB2	SF1	0.683695285108428	0.480280978265584	1	pseudobulk	DESeq2	LRT	0.318504613439909
148	KC_AB2	SF2	-0.208692152063859	0.686412062845715	1	pseudobulk	DESeq2	LRT	0.163415092902217
148	KC_AB2	Sf3a1	0.358043878278745	0.770536363569391	1	pseudobulk	DESeq2	LRT	0.113206861001527
149	KC_AB2	Sf3a2	-0.401554048784752	0.777386001083676	1	pseudobulk	DESeq2	LRT	0.109363284269265
149	KC_AB2	Sf3b1	1.77162356870521	0.315794714675844	1	pseudobulk	DESeq2	LRT	0.500595142872134
149	KC_AB2	Sf3b2	-1.18244204779995	0.281851268386738	1	pseudobulk	DESeq2	LRT	0.549980006427892
149	KC_AB2	Sf3b3	-0.242904908965546	0.933507439612501	1	pseudobulk	DESeq2	LRT	0.029882216579625
149	KC_AB2	Sf3b5	-0.74682456857537	0.339361794381046	1	pseudobulk	DESeq2	LRT	0.469337052482256
149	KC_AB2	Sf3b6	-0.39962620011379	0.652562978363803	1	pseudobulk	DESeq2	LRT	0.185377568532184
149	KC_AB2	sff	0.0056035251610318	0.976525763601171	1	pseudobulk	DESeq2	LRT	0.010316294268372
149	KC_AB2	sfl	0.271876375558956	0.6819719322375	1	pseudobulk	DESeq2	LRT	0.166233499134157
149	KC_AB2	Sfmbt	-0.349207592987077	0.800445356263882	1	pseudobulk	DESeq2	LRT	0.096668310569269
149	KC_AB2	Sfxn1-3	-0.750122436773216	0.494019035790617	1	pseudobulk	DESeq2	LRT	0.306256316299947
149	KC_AB2	Sfxn2	-0.310888275536833	0.773491483805488	1	pseudobulk	DESeq2	LRT	0.111544463550449
149	KC_AB2	Sgf11	-1.76515524916613	0.251970844229659	1	pseudobulk	DESeq2	LRT	0.598649708911802
149	KC_AB2	Sgf29	-0.393263742573289	0.769952807609556	1	pseudobulk	DESeq2	LRT	0.113535893039015
149	KC_AB2	sgg	-0.170007145317338	0.604991854143695	1	pseudobulk	DESeq2	LRT	0.218250472825647
149	KC_AB2	sgl	0.834440811967584	0.650917543165916	1	pseudobulk	DESeq2	LRT	0.186474023438173
149	KC_AB2	sgll	1.09928730399921	0.571654169938736	1	pseudobulk	DESeq2	LRT	0.242866624193882
149	KC_AB2	Sgs4	-0.347135499964956	0.854806062097812	1	pseudobulk	DESeq2	LRT	0.068132406575477
149	KC_AB2	Sgs7	-0.729379579623002	1	1	pseudobulk	DESeq2	LRT	0
149	KC_AB2	Sgt	-0.647244049280499	0.466178029446247	1	pseudobulk	DESeq2	LRT	0.331448198243802
149	KC_AB2	Sgt1	-0.522442169664214	0.690814651870884	1	pseudobulk	DESeq2	LRT	0.16063845981449
149	KC_AB2	Sh	0.226465556766406	0.276785494974821	1	pseudobulk	DESeq2	LRT	0.557856672948858
149	KC_AB2	Sh3beta	0.197048519922587	0.730064508990902	1	pseudobulk	DESeq2	LRT	0.136638763631608
149	KC_AB2	SH3PX1	-0.115183097143228	0.910399790291748	1	pseudobulk	DESeq2	LRT	0.040767850987610

149	KC_AB2	Shab	0.385962640775938	0.251165542226074	1	pseudobulk	DESeq2	LRT	0.600039942353435
149	KC_AB2	shakB	-0.0439362625924703	0.862338087111756	1	pseudobulk	DESeq2	LRT	0.064322431877823
149	KC_AB2	Shal	-0.22694878875416	0.559857515442846	1	pseudobulk	DESeq2	LRT	0.251922487512672
149	KC_AB2	Shark	1.38446765109058	0.642794900879723	1	pseudobulk	DESeq2	LRT	0.191927577037166
149	KC_AB2	Shaw	1.05920687175603	0.104987227958005	1	pseudobulk	DESeq2	LRT	0.978863531070463
149	KC_AB2	Shawl	0.352376793038294	0.39831580266619	1	pseudobulk	DESeq2	LRT	0.399772463174947
149	KC_AB2	Shc	1.72505947263211	0.330914788486869	1	pseudobulk	DESeq2	LRT	0.480283823910774
149	KC_AB2	shep	-0.168195579313674	0.517674033206744	1	pseudobulk	DESeq2	LRT	0.285943618904284
149	KC_AB2	shg	-2.73972386905082	0.222008923900166	1	pseudobulk	DESeq2	LRT	0.653629568239874
149	KC_AB2	shi	-0.0406624773502597	0.847747561837354	1	pseudobulk	DESeq2	LRT	0.071733450599838
149	KC_AB2	shn	1.48240474096234	0.460720464508086	1	pseudobulk	DESeq2	LRT	0.336562496613076
149	KC_AB2	shop	0.779558650624278	0.72455574449411	1	pseudobulk	DESeq2	LRT	0.139928195983184
149	KC_AB2	shot	-0.093900720889009	0.767041825132054	1	pseudobulk	DESeq2	LRT	0.115180954266386
149	KC_AB2	shrb	0.290235216909206	0.758428946245643	1	pseudobulk	DESeq2	LRT	0.120085100043863
149	KC_AB2	shtd	1.61205113368698	0.631647504209	1	pseudobulk	DESeq2	LRT	0.199525215522471
149	KC_AB2	shv	1.90334599570774	0.391638683118061	1	pseudobulk	DESeq2	LRT	0.407114418413825
149	KC_AB2	sicily	-0.908980957545978	0.520016879952817	1	pseudobulk	DESeq2	LRT	0.283982558766393
149	KC_AB2	sick	-0.170952924697387	0.741670949252607	1	pseudobulk	DESeq2	LRT	0.129788731674974
149	KC_AB2	side	-0.729379579623002	1	1	pseudobulk	DESeq2	LRT	0
149	KC_AB2	side-II	-0.610586574809789	0.0675673970991044	1	pseudobulk	DESeq2	LRT	1.17026281109233
149	KC_AB2	side-III	0.131329986081263	0.753046090800848	1	pseudobulk	DESeq2	LRT	0.123178441637435
149	KC_AB2	side-IV	0.955056545516501	0.484550136565945	1	pseudobulk	DESeq2	LRT	0.314661279691834
149	KC_AB2	side-V	0.601247184230854	0.485910678622647	1	pseudobulk	DESeq2	LRT	0.31344355655352
149	KC_AB2	side-VI	-0.0522309580464838	0.833764615294992	1	pseudobulk	DESeq2	LRT	0.078956540138262
149	KC_AB2	side-VII	-0.227004681161408	0.723716213119353	1	pseudobulk	DESeq2	LRT	0.140431697942694
149	KC_AB2	SIDL	1.596304964814	0.385040369086843	1	pseudobulk	DESeq2	LRT	0.414493735030307
149	KC_AB2	Sidpn	2.9023801951582	0.206074905726345	1	pseudobulk	DESeq2	LRT	0.685974890162107
149	KC_AB2	sif	-0.349596923140661	0.341097857335321	1	pseudobulk	DESeq2	LRT	0.467121008679291
149	KC_AB2	SIFaR	1.95472979411273	0.428935700137652	1	pseudobulk	DESeq2	LRT	0.367607806109828
149	KC_AB2	sigmar	-1.38953729358954	0.220153014933235	1	pseudobulk	DESeq2	LRT	0.657275362623709
149	KC_AB2	Sik2	0.694456221555189	0.531894999250534	1	pseudobulk	DESeq2	LRT	0.27417409279299
149	KC_AB2	Sik3	0.23522877521955	0.750701514228899	1	pseudobulk	DESeq2	LRT	0.124532708123768
149	KC_AB2	Sil1	-1.5033370644665	0.499350065149774	1	pseudobulk	DESeq2	LRT	0.30159488912562
149	KC_AB2	sima	-0.0878283092306746	0.880565959145356	1	pseudobulk	DESeq2	LRT	0.055238107476404
149	KC_AB2	simj	0.0714217113794404	0.894939169801683	1	pseudobulk	DESeq2	LRT	0.048206483250884
149	KC_AB2	Sin	0.720557559963523	0.758319263499194	1	pseudobulk	DESeq2	LRT	0.120147911539297
149	KC_AB2	Sin1	-1.68863556777156	0.392835953858398	1	pseudobulk	DESeq2	LRT	0.405788770763573
149	KC_AB2	Sin3A	0.0527938539557334	0.904703710488417	1	pseudobulk	DESeq2	LRT	0.043493628494687
149	KC_AB2	sina	-0.0923194805640291	0.840705623135023	1	pseudobulk	DESeq2	LRT	0.075356047755497
149	KC_AB2	sinu	0.280403434799392	0.873908442859524	1	pseudobulk	DESeq2	LRT	0.058534064898475
149	KC_AB2	sip3	-2.37214467400967	0.202483703497177	1	pseudobulk	DESeq2	LRT	0.69360992438043
149	KC_AB2	Sirt1	0.259499037414192	0.701913518822916	1	pseudobulk	DESeq2	LRT	0.153716393014977
149	KC_AB2	Sirt2	-1.52931829402953	0.39682439841363	1	pseudobulk	DESeq2	LRT	0.401401633466815
149	KC_AB2	Sirt4	-0.304943709632738	0.805572874475077	1	pseudobulk	DESeq2	LRT	0.093895165920337
149	KC_AB2	Sirt6	-0.600496552110112	0.825488100373549	1	pseudobulk	DESeq2	LRT	0.083289182824389
149	KC_AB2	Sirt7	1.78603717283625	0.408528783698185	1	pseudobulk	DESeq2	LRT	0.388777338982477
149	KC_AB2	Sirup	-1.11749855077849	0.339939400948675	1	pseudobulk	DESeq2	LRT	0.468598495249581
149	KC_AB2	siz	-0.529904406563754	0.282164541781947	1	pseudobulk	DESeq2	LRT	0.549497562785695
149	KC_AB2	SK	-0.157051091837997	0.592563857012545	1	pseudobulk	DESeq2	LRT	0.227264841513925
149	KC_AB2	Sk2	-2.88484645392124	0.187038310290045	1	pseudobulk	DESeq2	LRT	0.728069429594469
149	KC_AB2	skd	-0.0420539431924798	0.90865156373056	1	pseudobulk	DESeq2	LRT	0.041602621683577
149	KC_AB2	Ski6	-2.4120103603151	0.297158744475551	1	pseudobulk	DESeq2	LRT	0.527011485255674

149	KC_AB2	SKIP	-2.99942866014429	0.083191834560958	1	pseudobulk	DESeq2	LRT	1.07991929845863
149	KC_AB2	skl	-0.729379579623002	1	1	pseudobulk	DESeq2	LRT	0
149	KC_AB2	SkpA	-0.184454902261421	0.673515711600935	1	pseudobulk	DESeq2	LRT	0.17165226871308
149	KC_AB2	sky	-0.912315008811473	0.106282243490264	1	pseudobulk	DESeq2	LRT	0.97353928673110
149	KC_AB2	sl	-0.161754810636277	0.996285598531333	1	pseudobulk	DESeq2	LRT	0.00161614743320
149	KC_AB2	Slbp	-1.64836926504166	0.36756202899651	1	pseudobulk	DESeq2	LRT	0.434669359769711
149	KC_AB2	SLC22A	-1.6052707068109	0.52265609184397	1	pseudobulk	DESeq2	LRT	0.28178398329778
149	KC_AB2	Slc25A46a	0.402559906720127	0.794596007534613	1	pseudobulk	DESeq2	LRT	0.09985362139749
149	KC_AB2	sle	-0.942189863667974	0.619669494410584	1	pseudobulk	DESeq2	LRT	0.20783988312294
149	KC_AB2	slgA	-0.459924118970496	0.463506244446535	1	pseudobulk	DESeq2	LRT	0.33394441058009
149	KC_AB2	Slh	1.10706867860301	1	1	pseudobulk	DESeq2	LRT	0
149	KC_AB2	Slik	-0.364682881972356	0.633156959246756	1	pseudobulk	DESeq2	LRT	0.19848861528307
149	KC_AB2	slim	0.616664203771071	0.578497773251455	1	pseudobulk	DESeq2	LRT	0.23769830839151
149	KC_AB2	Slimp	-0.729379579623002	1	1	pseudobulk	DESeq2	LRT	0
149	KC_AB2	Slip1	-0.606602984306574	0.334736880406674	1	pseudobulk	DESeq2	LRT	0.47529643561137
149	KC_AB2	SLIRP2	0.661362391586437	0.730605074655461	1	pseudobulk	DESeq2	LRT	0.13631731557987
149	KC_AB2	sll	-1.45220519246244	0.536855403325269	1	pseudobulk	DESeq2	LRT	0.27014267145896
149	KC_AB2	Slmap	-0.6585664754327	0.480944932424233	1	pseudobulk	DESeq2	LRT	0.31790464693881
149	KC_AB2	slmb	-0.656029581992703	0.416120723548333	1	pseudobulk	DESeq2	LRT	0.38078065503480
149	KC_AB2	slmo	1.27972286293695	0.574832240983415	1	pseudobulk	DESeq2	LRT	0.24045888130241
149	KC_AB2	Sln	0.0928190508532855	0.911926292736257	1	pseudobulk	DESeq2	LRT	0.04004026249562
149	KC_AB2	slo	-0.202044287737776	0.302523170381056	1	pseudobulk	DESeq2	LRT	0.51924135693458
149	KC_AB2	SLO2	-0.038385148678157	0.869741305619801	1	pseudobulk	DESeq2	LRT	0.06060990398907
149	KC_AB2	Slob	0.428806412284471	0.196344189925599	1	pseudobulk	DESeq2	LRT	0.70698194552972
149	KC_AB2	slpr	-0.129906540021745	0.818869218501379	1	pseudobulk	DESeq2	LRT	0.08678545381774
150	KC_AB2	sls	1.38069928703004	0.566368801250645	1	pseudobulk	DESeq2	LRT	0.24690067801932
150	KC_AB2	Slu7	2.0316093444095	0.258997628658257	1	pseudobulk	DESeq2	LRT	0.58670421223283
150	KC_AB2	slv	1.08110106417042	0.536647447380605	1	pseudobulk	DESeq2	LRT	0.27031093206547
150	KC_AB2	slx1	1.38446765109058	0.642794900879723	1	pseudobulk	DESeq2	LRT	0.19192757703716
150	KC_AB2	sm	-0.140793227290371	0.6907073654888	1	pseudobulk	DESeq2	LRT	0.16070591278770
150	KC_AB2	smal	0.38519248585154	0.808654012475167	1	pseudobulk	DESeq2	LRT	0.09223725417658
150	KC_AB2	smash	0.397015013648192	0.637894351451632	1	pseudobulk	DESeq2	LRT	0.19525124350598
150	KC_AB2	SmB	-0.637619981713867	0.477207785920384	1	pseudobulk	DESeq2	LRT	0.32129247917882
150	KC_AB2	SMC1	-0.363206148306063	0.752932472152863	1	pseudobulk	DESeq2	LRT	0.1232439723869
150	KC_AB2	SMC2	1.02928733579242	0.697125366781187	1	pseudobulk	DESeq2	LRT	0.15668911400333
150	KC_AB2	SMC3	0.995343390570734	0.444513011097334	1	pseudobulk	DESeq2	LRT	0.35211552251136
150	KC_AB2	SMC5	2.13773457369525	0.349253628402833	1	pseudobulk	DESeq2	LRT	0.45685907329640
150	KC_AB2	SmD1	0.114597490839075	0.912621611939741	1	pseudobulk	DESeq2	LRT	0.03970925084383
150	KC_AB2	SmD2	-0.729498312556753	0.454024163218034	1	pseudobulk	DESeq2	LRT	0.34292103332569
150	KC_AB2	SmD3	0.584603311544642	0.64971890497703	1	pseudobulk	DESeq2	LRT	0.18727449631335
150	KC_AB2	SmE	0.532070401313025	0.582511536851187	1	pseudobulk	DESeq2	LRT	0.23469546885783
150	KC_AB2	SmF	-0.263223434935314	0.867338064879219	1	pseudobulk	DESeq2	LRT	0.06181159330270
150	KC_AB2	smg	0.0401171459707668	0.950605979684428	1	pseudobulk	DESeq2	LRT	0.021999458150051
150	KC_AB2	Smg5	-0.202755806396855	0.714228927975495	1	pseudobulk	DESeq2	LRT	0.146162563824411
150	KC_AB2	Smg6	1.96986501883708	0.167971668059916	1	pseudobulk	DESeq2	LRT	0.77476396495794
150	KC_AB2	smid	-0.344614925271821	0.832814556155584	1	pseudobulk	DESeq2	LRT	0.07945169271605
150	KC_AB2	Smn	0.236702594812255	0.863907252404348	1	pseudobulk	DESeq2	LRT	0.06353288012655
150	KC_AB2	smo	0.53853850907868	0.816883535008605	1	pseudobulk	DESeq2	LRT	0.08783985743158
150	KC_AB2	smog	0.26190986175353	0.530870938757394	1	pseudobulk	DESeq2	LRT	0.27501104840672
150	KC_AB2	Smox	-0.012543975673641	0.953178455810505	1	pseudobulk	DESeq2	LRT	0.02082578234357
150	KC_AB2	smp-30	-1.16016699006818	0.583985689825587	1	pseudobulk	DESeq2	LRT	0.23359779484980
150	KC_AB2	Smr	0.39687085427125	0.201528618153331	1	pseudobulk	DESeq2	LRT	0.69566327297914

150	KC_AB2	Sms	0.761203963154521	0.290253628524965	1	pseudobulk	DESeq2	LRT	0.537222342343843
150	KC_AB2	SMSr	-0.663730855950887	0.58493717739693	1	pseudobulk	DESeq2	LRT	0.23289077490061
150	KC_AB2	smt3	0.0792437639782282	0.816490958552773	1	pseudobulk	DESeq2	LRT	0.088048620079033
150	KC_AB2	Smu1	-0.529476089419283	0.791653821988583	1	pseudobulk	DESeq2	LRT	0.1014646871816
150	KC_AB2	Smurf	0.511149437490523	0.543601905841285	1	pseudobulk	DESeq2	LRT	0.264719029321989
150	KC_AB2	Smyd3	3.13194326631394	0.164331439568934	1	pseudobulk	DESeq2	LRT	0.784279340246697
150	KC_AB2	Smyd4-1	-0.729379579623002	1	1	pseudobulk	DESeq2	LRT	0
150	KC_AB2	Smyd4-4	-0.197453836303281	0.886689244650687	1	pseudobulk	DESeq2	LRT	0.052228559401717
150	KC_AB2	Smyd5	1.95432904991983	0.509212778150152	1	pseudobulk	DESeq2	LRT	0.293100706726022
150	KC_AB2	SmydA-1	0.802422437972137	0.703555366906532	1	pseudobulk	DESeq2	LRT	0.152701719699286
150	KC_AB2	SmydA-3	1.10706867860301	1	1	pseudobulk	DESeq2	LRT	0
150	KC_AB2	SmydA-7	-0.807405154693935	0.828356177178021	1	pseudobulk	DESeq2	LRT	0.081782884806574
150	KC_AB2	sn	0.100464947902714	0.888809964858645	1	pseudobulk	DESeq2	LRT	0.051191084964058
150	KC_AB2	snama	1.45575281328437	0.365475312980312	1	pseudobulk	DESeq2	LRT	0.437141953312197
150	KC_AB2	Snap24	-0.261296211478543	0.598173453068397	1	pseudobulk	DESeq2	LRT	0.223172864861597
150	KC_AB2	Snap25	-0.194149769587501	0.481351586989249	1	pseudobulk	DESeq2	LRT	0.317537592001848
150	KC_AB2	Snap29	0.683135944059007	0.645602449622687	1	pseudobulk	DESeq2	LRT	0.190034830430352
150	KC_AB2	snf	0.383858264910519	0.637889935023819	1	pseudobulk	DESeq2	LRT	0.195254250331278
150	KC_AB2	SNF4Agamma	0.415954851811401	0.679691719411509	1	pseudobulk	DESeq2	LRT	0.167688020994017
150	KC_AB2	sni	2.7605424445074	0.237810191029118	1	pseudobulk	DESeq2	LRT	0.623769538224996
150	KC_AB2	Snm1	1.10706867860301	1	1	pseudobulk	DESeq2	LRT	0
150	KC_AB2	sno	0.389941987259549	0.447055983531362	1	pseudobulk	DESeq2	LRT	0.349638088017192
150	KC_AB2	Snoo	0.421427166336771	0.303903568372545	1	pseudobulk	DESeq2	LRT	0.517264200494565
150	KC_AB2	snoRNA:Me18S-U	-0.729379579623002	1	1	pseudobulk	DESeq2	LRT	0
150	KC_AB2	snoRNA:Me28S-C	-0.729379579623002	0.823527397767669	1	pseudobulk	DESeq2	LRT	0.084321947797948
150	KC_AB2	snoRNA:Psi18S-13	1.38069928703004	0.566368801250645	1	pseudobulk	DESeq2	LRT	0.246900678019328
150	KC_AB2	snoRNA:Psi18S-52	1.10706867860301	1	1	pseudobulk	DESeq2	LRT	0
150	KC_AB2	snoRNA:Psi18S-53	-0.729379579623002	0.823527397767669	1	pseudobulk	DESeq2	LRT	0.084321947797948
150	KC_AB2	snoRNA:Psi28S-11	-0.18317077492817	0.899664519927029	1	pseudobulk	DESeq2	LRT	0.045919406456170
150	KC_AB2	snoRNA:Psi28S-35	-0.729379579623002	0.823527397767669	1	pseudobulk	DESeq2	LRT	0.084321947797948
150	KC_AB2	Snp	-2.12853982341084	0.1620721594835	1	pseudobulk	DESeq2	LRT	0.790291581210186
150	KC_AB2	sNPF	-0.230902933164099	0.25371824980411	1	pseudobulk	DESeq2	LRT	0.595648293108717
150	KC_AB2	Snr1	1.17415993305184	0.420066457017294	1	pseudobulk	DESeq2	LRT	0.376681996190884
150	KC_AB2	snRNA:7SK	0.151610056372889	0.75197119728364	1	pseudobulk	DESeq2	LRT	0.123798793850298
150	KC_AB2	snRNA:U12	-0.729379579623002	0.823527397767669	1	pseudobulk	DESeq2	LRT	0.084321947797948
150	KC_AB2	snRNA:U4:39B	-0.729379579623002	1	1	pseudobulk	DESeq2	LRT	0
150	KC_AB2	snRNP-U1-70K	0.225775423852334	0.76601497590602	1	pseudobulk	DESeq2	LRT	0.115762739649683
150	KC_AB2	snRNP-U1-C	0.00711441129717403	0.986910345585398	1	pseudobulk	DESeq2	LRT	0.005722298380056
150	KC_AB2	SNRPG	-1.00290711568156	0.230880557180907	1	pseudobulk	DESeq2	LRT	0.636612638172352
150	KC_AB2	Snx1	-0.0479878212768124	0.863664202501683	1	pseudobulk	DESeq2	LRT	0.063655080840107
150	KC_AB2	Snx16	-0.488143361449198	0.801070470903415	1	pseudobulk	DESeq2	LRT	0.096329276951632
150	KC_AB2	Snx17	0.72453489533559	0.471801417454087	1	pseudobulk	DESeq2	LRT	0.326240758678139
150	KC_AB2	Snx21	-1.86979501903407	0.475964703567026	1	pseudobulk	DESeq2	LRT	0.322425252351861
150	KC_AB2	Snx27	-0.426705161765772	0.561282429091767	1	pseudobulk	DESeq2	LRT	0.250818553126719
150	KC_AB2	Snx3	0.473671220272108	0.540417289430088	1	pseudobulk	DESeq2	LRT	0.267270765157096
150	KC_AB2	Snx6	-0.204976680830207	0.850600069849676	1	pseudobulk	DESeq2	LRT	0.070274585958549
150	KC_AB2	snz	-0.720959562417885	0.557245770987604	1	pseudobulk	DESeq2	LRT	0.253953218753901
150	KC_AB2	Socs16D	-1.04560353577914	0.174986534073347	1	pseudobulk	DESeq2	LRT	0.756995370757442
150	KC_AB2	Socs36E	-0.0454647697120307	0.915618492408933	1	pseudobulk	DESeq2	LRT	0.038285444623472
150	KC_AB2	Socs44A	0.424122354430647	0.723430760770878	1	pseudobulk	DESeq2	LRT	0.140603028683294
150	KC_AB2	Sod1	-0.352735575475806	0.328557036306005	1	pseudobulk	DESeq2	LRT	0.483389227617739
150	KC_AB2	Sod2	-0.633687649715934	0.365928288888272	1	pseudobulk	DESeq2	LRT	0.436604015128926

150	KC_AB2	Sod3	1.95432904991983	0.509212778150152	1	pseudobulk	DESeq2	LRT	0.293100706726022
150	KC_AB2	Sodh-2	-0.729379579623002	1	1	pseudobulk	DESeq2	LRT	0
150	KC_AB2	sol	0.131260298514448	0.855352305708999	1	pseudobulk	DESeq2	LRT	0.067854969586427
150	KC_AB2	Son	-0.502461501812022	0.668658319992718	1	pseudobulk	DESeq2	LRT	0.17479574717743
150	KC_AB2	Sos	-0.0700115001409927	0.951922212247406	1	pseudobulk	DESeq2	LRT	0.021398539191134
150	KC_AB2	soti	1.10706867860301	1	1	pseudobulk	DESeq2	LRT	0
150	KC_AB2	sotv	-1.3680787780191	0.194105547132077	1	pseudobulk	DESeq2	LRT	0.711962053202942
150	KC_AB2	sov	-0.550774089176127	0.450139273089396	1	pseudobulk	DESeq2	LRT	0.346653094722156
150	KC_AB2	sowah	0.193486814807763	0.850869521670009	1	pseudobulk	DESeq2	LRT	0.070137032586721
150	KC_AB2	SP1173	0.099845136781298	0.807198785944274	1	pseudobulk	DESeq2	LRT	0.093019499965024
150	KC_AB2	SP2353	0.701074858445968	0.690777205145172	1	pseudobulk	DESeq2	LRT	0.160662002087504
150	KC_AB2	spab	-0.0904281365187314	0.794947301726047	1	pseudobulk	DESeq2	LRT	0.099661660435554
150	KC_AB2	spag	2.42873859307181	0.25128535823465	1	pseudobulk	DESeq2	LRT	0.599832815916576
150	KC_AB2	SPARC	1.10706867860301	1	1	pseudobulk	DESeq2	LRT	0
150	KC_AB2	spartin	-0.33572805049699	0.856817945219033	1	pseudobulk	DESeq2	LRT	0.067111446205159
150	KC_AB2	spas	0.651848917923957	0.592908153591024	1	pseudobulk	DESeq2	LRT	0.227012577256249
150	KC_AB2	Spase12	-0.536577991285068	0.489226735019616	1	pseudobulk	DESeq2	LRT	0.310489817878118
150	KC_AB2	Spase22-23	-0.223234901647449	0.807561466260654	1	pseudobulk	DESeq2	LRT	0.092824412102870
150	KC_AB2	Spase25	-0.478999687767479	0.614715554073137	1	pseudobulk	DESeq2	LRT	0.211325797839493
150	KC_AB2	SpdS	-0.21471049466397	0.881001728025935	1	pseudobulk	DESeq2	LRT	0.055023239747552
150	KC_AB2	Spec2	1.90088310496714	0.267187927990492	1	pseudobulk	DESeq2	LRT	0.573183167923859
151	KC_AB2	spel1	-0.729379579623002	0.772694994316916	1	pseudobulk	DESeq2	LRT	0.1119919011982
151	KC_AB2	spen	0.141920324557495	0.530507164549064	1	pseudobulk	DESeq2	LRT	0.275308746535283
151	KC_AB2	Spf45	0.467959076186781	0.586384546142824	1	pseudobulk	DESeq2	LRT	0.231817483819408
151	KC_AB2	spg	0.677317578043817	0.586008187777756	1	pseudobulk	DESeq2	LRT	0.232096315924134
151	KC_AB2	Spg7	0.400955890185621	0.808852698639772	1	pseudobulk	DESeq2	LRT	0.092130561196878
151	KC_AB2	spi	0.24779467828891	0.745831392102741	1	pseudobulk	DESeq2	LRT	0.127359341100649
151	KC_AB2	spict	1.67481482242989	0.527609545588562	1	pseudobulk	DESeq2	LRT	0.277687355735723
151	KC_AB2	spidey	-0.183573101451929	0.808193803144143	1	pseudobulk	DESeq2	LRT	0.092484483849596
151	KC_AB2	spin	-0.223427513467405	0.891901096070693	1	pseudobulk	DESeq2	LRT	0.049683302364040
151	KC_AB2	spir	-0.139486331501247	0.71629956661497	1	pseudobulk	DESeq2	LRT	0.144905311603885
151	KC_AB2	sPLA2	2.62919123543814	0.269826951733818	1	pseudobulk	DESeq2	LRT	0.568914672882509
151	KC_AB2	Sply	1.12078839091449	0.409004081784114	1	pseudobulk	DESeq2	LRT	0.388272357793461
151	KC_AB2	Spn	0.521536146776724	0.119651300633948	1	pseudobulk	DESeq2	LRT	0.92208257615563
151	KC_AB2	spn-F	-0.348901974470907	0.89890135131336	1	pseudobulk	DESeq2	LRT	0.046287966699494
151	KC_AB2	Spn42Da	0.220455551236389	0.8740400417874	1	pseudobulk	DESeq2	LRT	0.05846867087936
151	KC_AB2	SPoCk	0.00905884485327122	0.952339068601783	1	pseudobulk	DESeq2	LRT	0.021208398878809
151	KC_AB2	spoon	-0.180924183580589	0.858216874297901	1	pseudobulk	DESeq2	LRT	0.066402950602207
151	KC_AB2	Spp	-0.903092823655932	0.666117165665049	1	pseudobulk	DESeq2	LRT	0.176449374543784
151	KC_AB2	SppL	-0.440929747516667	0.62358966583229	1	pseudobulk	DESeq2	LRT	0.205101090579411
151	KC_AB2	Spps	0.803430897925083	0.714923169369323	1	pseudobulk	DESeq2	LRT	0.145740628005649
151	KC_AB2	SPR	-3.25318851699	0.100220177608641	1	pseudobulk	DESeq2	LRT	0.99904483194293
151	KC_AB2	Spred	-0.407374136883131	0.200257725517342	1	pseudobulk	DESeq2	LRT	0.69841072076266
151	KC_AB2	spri	-1.27114501616082	0.190653221416634	1	pseudobulk	DESeq2	LRT	0.719755852170283
151	KC_AB2	sprt	0.145321756433347	1	1	pseudobulk	DESeq2	LRT	0
151	KC_AB2	Sps1	-0.0682925271728545	0.957247720575207	1	pseudobulk	DESeq2	LRT	0.018975659133096
151	KC_AB2	Sps2	0.220179503755738	0.872977028443869	1	pseudobulk	DESeq2	LRT	0.058997184188276
151	KC_AB2	Spt	-0.299056004457792	0.875974510877379	1	pseudobulk	DESeq2	LRT	0.057508530756982
151	KC_AB2	Spt-I	-0.930985816944667	0.391230385694323	1	pseudobulk	DESeq2	LRT	0.407567422220582
151	KC_AB2	Spt20	-0.454116902864077	0.714315920223388	1	pseudobulk	DESeq2	LRT	0.146109670485679
151	KC_AB2	Spt3	1.74085835635066	0.363742845362722	1	pseudobulk	DESeq2	LRT	0.43920554027421
151	KC_AB2	spt4	1.21717517252866	0.354588234312383	1	pseudobulk	DESeq2	LRT	0.450275678938094

151	KC_AB2	Spt5	0.39368862399003	0.659154652376919	1	pseudobulk	DESeq2	LRT	0.181012678282883
151	KC_AB2	Spt6	-1.87763741045983	0.11469925590327	1	pseudobulk	DESeq2	LRT	0.940439399519412
151	KC_AB2	Spt7	0.145321756433347	1	1	pseudobulk	DESeq2	LRT	0
151	KC_AB2	Sptr	-0.684795146295678	0.506015468353304	1	pseudobulk	DESeq2	LRT	0.295836207038057
151	KC_AB2	Spx	1.38707906116813	0.39532490201099	1	pseudobulk	DESeq2	LRT	0.403045828041712
151	KC_AB2	spz	2.64741945039871	0.292550810913063	1	pseudobulk	DESeq2	LRT	0.533798693743382
151	KC_AB2	spz4	-0.729379579623002	1	1	pseudobulk	DESeq2	LRT	0
151	KC_AB2	sqd	0.104830649109261	0.742057252934877	1	pseudobulk	DESeq2	LRT	0.129562585725747
151	KC_AB2	sqh	0.181821994122438	0.790738017595967	1	pseudobulk	DESeq2	LRT	0.101967380419359
151	KC_AB2	sr	0.228304505884471	0.54755746749794	1	pseudobulk	DESeq2	LRT	0.261570293825732
151	KC_AB2	sra	-0.920556947193668	0.0236690564362697	1	pseudobulk	DESeq2	LRT	1.62581905481656
151	KC_AB2	Sra-1	-1.93335857128809	0.384223899985767	1	pseudobulk	DESeq2	LRT	0.415415624094039
151	KC_AB2	Sras	-1.52244591594958	0.547176617578731	1	pseudobulk	DESeq2	LRT	0.261872469536296
151	KC_AB2	Src42A	0.207082459205547	0.734554363997003	1	pseudobulk	DESeq2	LRT	0.133976056769287
151	KC_AB2	Src64B	-0.364163620200527	0.57729638994343	1	pseudobulk	DESeq2	LRT	0.238601158303464
151	KC_AB2	SREBP	0.865671279435945	0.549091830856377	1	pseudobulk	DESeq2	LRT	0.260355017482371
151	KC_AB2	srl	0.274300365849694	0.875467438164865	1	pseudobulk	DESeq2	LRT	0.057760002279909
151	KC_AB2	Srlp	-0.922612823666893	0.495676196729969	1	pseudobulk	DESeq2	LRT	0.304801936202853
151	KC_AB2	SRm160	-0.30004153765668	0.729834080119332	1	pseudobulk	DESeq2	LRT	0.136775860809414
151	KC_AB2	Srp14	-0.851356265083539	0.37160584318954	1	pseudobulk	DESeq2	LRT	0.429917465709013
151	KC_AB2	Srp19	-0.173201479640044	0.882419055451432	1	pseudobulk	DESeq2	LRT	0.054325122068260
151	KC_AB2	Srp54	0.938714245845031	0.321759492807827	1	pseudobulk	DESeq2	LRT	0.492468631328809
151	KC_AB2	Srp54k	-0.0782888181561201	0.946466373177535	1	pseudobulk	DESeq2	LRT	0.023894811391539
151	KC_AB2	Srp68	-2.28054226263626	0.331630256012328	1	pseudobulk	DESeq2	LRT	0.479345853816062
151	KC_AB2	Srp72	0.596011866041249	0.776574358233591	1	pseudobulk	DESeq2	LRT	0.109816953554982
151	KC_AB2	Srp9	-0.540068832200325	0.537106759455549	1	pseudobulk	DESeq2	LRT	0.269939382020751
151	KC_AB2	SRPK	-0.476783129300954	0.366086794697425	1	pseudobulk	DESeq2	LRT	0.436415936492612
151	KC_AB2	Srpk79D	-0.52418076991543	0.584363146885199	1	pseudobulk	DESeq2	LRT	0.233317180843615
151	KC_AB2	SrpRbeta	1.04903678246879	0.41016282609429	1	pseudobulk	DESeq2	LRT	0.387043703191689
151	KC_AB2	Sry-delta	-3.43739847134205	0.0790331247108727	1	pseudobulk	DESeq2	LRT	1.10219084714008
151	KC_AB2	Ssadh	-0.294635686636112	0.788728604918834	1	pseudobulk	DESeq2	LRT	0.103072408276131
151	KC_AB2	Ssb-c31a	-0.58976325824356	0.498185259687206	1	pseudobulk	DESeq2	LRT	0.302609126520935
151	KC_AB2	Ssdp	0.110273006672835	0.768233796067488	1	pseudobulk	DESeq2	LRT	0.114506591309386
151	KC_AB2	ssh	-1.49953119799486	0.369727503720536	1	pseudobulk	DESeq2	LRT	0.432118241421021
151	KC_AB2	Ssk	1.10706867860301	1	1	pseudobulk	DESeq2	LRT	0
151	KC_AB2	Ssl1	0.0772645829333075	0.941528584941732	1	pseudobulk	DESeq2	LRT	0.026166490204928
151	KC_AB2	ssp	-0.115183097143228	0.910399790291748	1	pseudobulk	DESeq2	LRT	0.040767850987610
151	KC_AB2	ssp3	-0.397627732647636	0.721528111041953	1	pseudobulk	DESeq2	LRT	0.141746743943219
151	KC_AB2	ssp6	-0.393911587834317	0.804923707221212	1	pseudobulk	DESeq2	LRT	0.094245281250800
151	KC_AB2	SsRbeta	0.0782281624331927	0.89221316770358	1	pseudobulk	DESeq2	LRT	0.049531371523897
151	KC_AB2	Ssrp	0.124462516295993	0.952307241396434	1	pseudobulk	DESeq2	LRT	0.021222913258289
151	KC_AB2	sstn	-0.427692090780194	0.83957454067488	1	pseudobulk	DESeq2	LRT	0.075940739470360
151	KC_AB2	Ssu72	1.49872525918912	0.359519724086426	1	pseudobulk	DESeq2	LRT	0.444277278224314
151	KC_AB2	ssx	-0.204874672320071	0.649212481590785	1	pseudobulk	DESeq2	LRT	0.187613139132475
151	KC_AB2	St3	-0.16847100897959	0.816369949151496	1	pseudobulk	DESeq2	LRT	0.088112990185613
151	KC_AB2	sta	-0.173919575965081	0.597681926668114	1	pseudobulk	DESeq2	LRT	0.223529876619023
151	KC_AB2	Stacl	0.0881597634405027	0.704126680707873	1	pseudobulk	DESeq2	LRT	0.152349199120649
151	KC_AB2	stai	-0.0701839363631934	0.757559671347604	1	pseudobulk	DESeq2	LRT	0.120583153035118
151	KC_AB2	Stam	0.976556236804431	0.517356115262484	1	pseudobulk	DESeq2	LRT	0.286210413090785
151	KC_AB2	stan	-0.972519827806649	0.400854945389737	1	pseudobulk	DESeq2	LRT	0.397012754096716
151	KC_AB2	Start1	-0.179936670791578	0.904057061354521	1	pseudobulk	DESeq2	LRT	0.043804157301940
151	KC_AB2	stas	-0.32846740170202	0.611030210676815	1	pseudobulk	DESeq2	LRT	0.213937316752731

151	KC_AB2	Stat92E	0.349860189004484	0.432227721767297	1	pseudobulk	DESeq2	LRT	0.364287382236507
151	KC_AB2	stau	0.644861466948746	0.429447673229058	1	pseudobulk	DESeq2	LRT	0.367089745947562
151	KC_AB2	stc	2.97581486371455	0.10006305883964	1	pseudobulk	DESeq2	LRT	0.999726225249612
151	KC_AB2	stck	0.0924730787225688	0.89741923891541	1	pseudobulk	DESeq2	LRT	0.047004624277829
151	KC_AB2	ste14	-2.72113288127959	0.259052821623388	1	pseudobulk	DESeq2	LRT	0.586611672981979
151	KC_AB2	ste24a	-1.09986023604032	0.583781937504295	1	pseudobulk	DESeq2	LRT	0.233749346421605
151	KC_AB2	step	0.369237648837091	0.613026174566505	1	pseudobulk	DESeq2	LRT	0.21252098188107
151	KC_AB2	Stim	0.272959970364326	0.824922747145961	1	pseudobulk	DESeq2	LRT	0.083586720612680
151	KC_AB2	Stip1	-0.589518398213179	0.670314665270482	1	pseudobulk	DESeq2	LRT	0.173721278925871
151	KC_AB2	stj	0.183897228912932	0.638953144834823	1	pseudobulk	DESeq2	LRT	0.194530987985218
151	KC_AB2	Stlk	0.0903898091163122	0.830985689506532	1	pseudobulk	DESeq2	LRT	0.080406455183230
151	KC_AB2	stmA	0.443571605958416	0.35174158513091	1	pseudobulk	DESeq2	LRT	0.453776283587391
151	KC_AB2	Stoml2	0.0256806332848898	0.870617830861328	1	pseudobulk	DESeq2	LRT	0.060172442433178
151	KC_AB2	stops	1.10706867860301	1	1	pseudobulk	DESeq2	LRT	0
151	KC_AB2	strat	2.41500687297367	0.248312426104075	1	pseudobulk	DESeq2	LRT	0.605001546843339
151	KC_AB2	Strica	-3.04352251165992	0.191291787639632	1	pseudobulk	DESeq2	LRT	0.718303674297579
151	KC_AB2	Strip	0.491569970061496	0.707883458417109	1	pseudobulk	DESeq2	LRT	0.15003823600016
152	KC_AB2	Strn-Mlck	-0.729379579623002	1	1	pseudobulk	DESeq2	LRT	0
152	KC_AB2	Strump	-1.57685088282527	0.45256857946707	1	pseudobulk	DESeq2	LRT	0.344315601149633
152	KC_AB2	Stt3A	0.140563494705281	0.91703866696139	1	pseudobulk	DESeq2	LRT	0.037612351904762
152	KC_AB2	Stt3B	-1.33211738749109	0.350789079498422	1	pseudobulk	DESeq2	LRT	0.454953935224462
152	KC_AB2	STUB1	-0.214751250105487	0.810889218109186	1	pseudobulk	DESeq2	LRT	0.091038474087488
152	KC_AB2	stumps	1.10706867860301	1	1	pseudobulk	DESeq2	LRT	0
152	KC_AB2	sturkopf	-1.04379861518972	0.402268500721624	1	pseudobulk	DESeq2	LRT	0.395483973141806
152	KC_AB2	stv	-0.301617309523196	0.52457512991227	1	pseudobulk	DESeq2	LRT	0.280192303143106
152	KC_AB2	stw	-1.21363965631423	0.568763379520138	1	pseudobulk	DESeq2	LRT	0.24506837392442
152	KC_AB2	stwl	0.0168318308959245	0.949877659726892	1	pseudobulk	DESeq2	LRT	0.022332326423730
152	KC_AB2	stx	0.517610901469609	0.386511240790806	1	pseudobulk	DESeq2	LRT	0.412837871105474
152	KC_AB2	sty	0.208703363949235	0.489914620119031	1	pseudobulk	DESeq2	LRT	0.309879600057287
152	KC_AB2	Su(dx)	-0.89359437936909	0.465525694018826	1	pseudobulk	DESeq2	LRT	0.332056343763966
152	KC_AB2	su(f)	-0.230864488500055	0.844923566944523	1	pseudobulk	DESeq2	LRT	0.073182576204856
152	KC_AB2	Su(fu)	0.39439088612385	0.813541888331052	1	pseudobulk	DESeq2	LRT	0.089620080830767
152	KC_AB2	Su(H)	-0.172789369632759	0.89047881884407	1	pseudobulk	DESeq2	LRT	0.050376406317013
152	KC_AB2	su(Hw)	2.49920391921416	0.199705285650508	1	pseudobulk	DESeq2	LRT	0.699610440394844
152	KC_AB2	Su(P)	-0.362831301919876	0.710063144600187	1	pseudobulk	DESeq2	LRT	0.148703028559751
152	KC_AB2	su(sable)	-0.0856769585824242	0.91992181366535	1	pseudobulk	DESeq2	LRT	0.036249082802981
152	KC_AB2	Su(Tpl)	-0.670709751321305	0.115662794183831	1	pseudobulk	DESeq2	LRT	0.936806320211904
152	KC_AB2	Su(var)2-10	0.373020218137956	0.690071175367533	1	pseudobulk	DESeq2	LRT	0.16110611292188
152	KC_AB2	Su(var)2-HP2	-0.600821395463247	0.475857403423227	1	pseudobulk	DESeq2	LRT	0.322523169515613
152	KC_AB2	Su(var)205	0.632808651594702	0.395044207023657	1	pseudobulk	DESeq2	LRT	0.403354302368019
152	KC_AB2	Su(var)3-3	1.05034007824951	0.343881108696138	1	pseudobulk	DESeq2	LRT	0.463591681736508
152	KC_AB2	Su(var)3-7	-0.881925904751173	0.620461367982025	1	pseudobulk	DESeq2	LRT	0.207285253963056
152	KC_AB2	su(w[a])	-0.486700914972471	0.556797522030278	1	pseudobulk	DESeq2	LRT	0.25430270617582
152	KC_AB2	Su(z)12	-0.518381487905311	0.639372249428466	1	pseudobulk	DESeq2	LRT	0.194246217269252
152	KC_AB2	Su(z)2	0.313449497085399	0.660119773629033	1	pseudobulk	DESeq2	LRT	0.18037725793262
152	KC_AB2	subdued	-0.889337947640851	0.66589289368567	1	pseudobulk	DESeq2	LRT	0.176595619811718
152	KC_AB2	sud1	1.04560394656545	0.638145957880541	1	pseudobulk	DESeq2	LRT	0.195079977309609
152	KC_AB2	sug	-0.729379579623002	0.772694994316916	1	pseudobulk	DESeq2	LRT	0.1119919011982
152	KC_AB2	Sugb	-0.729379579623002	0.772694994316916	1	pseudobulk	DESeq2	LRT	0.1119919011982
152	KC_AB2	Sulf1	1.38446765109058	0.642794900879723	1	pseudobulk	DESeq2	LRT	0.191927577037166
152	KC_AB2	sun	-0.640635151723327	0.0825756176824688	1	pseudobulk	DESeq2	LRT	1.08314816901306
152	KC_AB2	Sur-8	-0.328361558848144	0.549476283411391	1	pseudobulk	DESeq2	LRT	0.260051047928105

152	KC_AB2	Surf1	1.39378040467077	0.297543710219509	1	pseudobulk	DESeq2	LRT	0.526449225859047
152	KC_AB2	Surf4	-1.13847914738189	0.264240922594139	1	pseudobulk	DESeq2	LRT	0.577999922979812
152	KC_AB2	Surf6	-3.56501124222263	0.0574567840040706	1	pseudobulk	DESeq2	LRT	1.2406586861821
152	KC_AB2	sut2	2.01814786626755	0.401718761445944	1	pseudobulk	DESeq2	LRT	0.396077884966921
152	KC_AB2	Suv3	-0.349350206779871	0.84336127815354	1	pseudobulk	DESeq2	LRT	0.073986342934979
152	KC_AB2	sv	1.10706867860301	1	1	pseudobulk	DESeq2	LRT	0
152	KC_AB2	Svil	-0.0571849230644027	0.879622653373041	1	pseudobulk	DESeq2	LRT	0.055703594558834
152	KC_AB2	svr	0.15577004122971	0.6591637770109	1	pseudobulk	DESeq2	LRT	0.181006666414886
152	KC_AB2	sw	-0.251350300086539	0.780580476159574	1	pseudobulk	DESeq2	LRT	0.107582315471118
152	KC_AB2	SWIP	0.0029723196026293	0.946828684594097	1	pseudobulk	DESeq2	LRT	0.023728593400088
152	KC_AB2	Swip-1	-1.36937908893147	0.144948612790543	1	pseudobulk	DESeq2	LRT	0.838785936640791
152	KC_AB2	swm	0.211571788448636	0.69622768606992	1	pseudobulk	DESeq2	LRT	0.157248710628981
152	KC_AB2	sws	0.0324428536610063	0.95810242393985	1	pseudobulk	DESeq2	LRT	0.018588061094554
152	KC_AB2	sxc	-0.0667869776034637	0.877173365293161	1	pseudobulk	DESeq2	LRT	0.056914563818297
152	KC_AB2	Sxl	0.0839289172050128	0.765611234447219	1	pseudobulk	DESeq2	LRT	0.115991702422126
152	KC_AB2	syd	-0.711160555661046	0.25023706375797	1	pseudobulk	DESeq2	LRT	0.601648364532941
152	KC_AB2	Sym	1.99339641594643	0.465071165814147	1	pseudobulk	DESeq2	LRT	0.332480585700252
152	KC_AB2	Syn	0.15854232133547	0.689565138358264	1	pseudobulk	DESeq2	LRT	0.161424702811742
152	KC_AB2	Syn1	-0.743639432774865	0.302421543488514	1	pseudobulk	DESeq2	LRT	0.519387274397942
152	KC_AB2	Syn2	1.12851214227813	0.246454104192567	1	pseudobulk	DESeq2	LRT	0.608263945156476
152	KC_AB2	Synd	0.282204736926559	0.759657626904715	1	pseudobulk	DESeq2	LRT	0.119382097519224
152	KC_AB2	Syngr	-0.373597449830092	0.46887103302251	1	pseudobulk	DESeq2	LRT	0.328946597261315
152	KC_AB2	Synj	-0.0435824775048043	0.942900842123065	1	pseudobulk	DESeq2	LRT	0.025533976385742
152	KC_AB2	Syp	0.129898747925391	0.70294861187096	1	pseudobulk	DESeq2	LRT	0.153076422343769
152	KC_AB2	Syt1	-0.227467069697371	0.398142896729233	1	pseudobulk	DESeq2	LRT	0.399961028120111
152	KC_AB2	Syt12	0.400163797152349	0.52029374251976	1	pseudobulk	DESeq2	LRT	0.283751397254993
152	KC_AB2	Syt14	-0.0471372646074281	0.94518738079851	1	pseudobulk	DESeq2	LRT	0.024482085268781
152	KC_AB2	Syt4	-0.106859155731238	0.735556015932484	1	pseudobulk	DESeq2	LRT	0.1333842481791
152	KC_AB2	Syt7	0.0283814377046762	0.904030151686862	1	pseudobulk	DESeq2	LRT	0.043817084466173
152	KC_AB2	Sytalpha	0.0395491304350905	0.900564200659079	1	pseudobulk	DESeq2	LRT	0.045485321158602
152	KC_AB2	Sytbeta	0.0034776592469391	0.924646736874941	1	pseudobulk	DESeq2	LRT	0.034024158644094
152	KC_AB2	Syx13	-0.526780667149081	0.558600965785661	1	pseudobulk	DESeq2	LRT	0.252898317766735
152	KC_AB2	Syx16	-0.5283343718985	0.632653655702511	1	pseudobulk	DESeq2	LRT	0.198833978105798
152	KC_AB2	Syx17	0.756548449222262	0.507889995217987	1	pseudobulk	DESeq2	LRT	0.29423034213186
152	KC_AB2	Syx18	-0.051768802237555	0.953597974616862	1	pseudobulk	DESeq2	LRT	0.020634680017486
152	KC_AB2	Syx1A	-0.150292194759585	0.615394882781559	1	pseudobulk	DESeq2	LRT	0.210846119380356
152	KC_AB2	Syx5	-0.219849246145077	0.852148744942043	1	pseudobulk	DESeq2	LRT	0.069484591302273
152	KC_AB2	Syx6	0.178150688316904	0.749209549480317	1	pseudobulk	DESeq2	LRT	0.125396695711942
152	KC_AB2	Syx7	-0.548382630928614	0.418658008745383	1	pseudobulk	DESeq2	LRT	0.378140596511919
152	KC_AB2	Syx8	0.331108688848472	0.790189933097074	1	pseudobulk	DESeq2	LRT	0.102268507466822
152	KC_AB2	tacc	-0.0432503819686289	0.918851702672799	1	pseudobulk	DESeq2	LRT	0.036754575564789
152	KC_AB2	Tace	0.364101718164429	0.755047287036943	1	pseudobulk	DESeq2	LRT	0.122025848561385
152	KC_AB2	Taf1	-0.709993947624295	0.68530477226094	1	pseudobulk	DESeq2	LRT	0.164116243872995
152	KC_AB2	Taf10	-0.391037036686065	0.661790865198892	1	pseudobulk	DESeq2	LRT	0.17927923176833
152	KC_AB2	Taf10b	0.315157223853895	0.691264073139233	1	pseudobulk	DESeq2	LRT	0.160356013988154
152	KC_AB2	Taf11	-0.438383410849624	0.799654701834727	1	pseudobulk	DESeq2	LRT	0.097097504833569
152	KC_AB2	Taf12	-0.679941334196101	0.346363000133918	1	pseudobulk	DESeq2	LRT	0.460468507231794
152	KC_AB2	Taf13	-1.22001029899593	0.333114244017732	1	pseudobulk	DESeq2	LRT	0.477406796411074
152	KC_AB2	TAF1C-like	1.10706867860301	1	1	pseudobulk	DESeq2	LRT	0
152	KC_AB2	Taf2	1.10706867860301	1	1	pseudobulk	DESeq2	LRT	0
152	KC_AB2	Taf4	0.115848904357874	0.850091365550188	1	pseudobulk	DESeq2	LRT	0.070534394965848
152	KC_AB2	Taf5	-2.39436951131995	0.167953878436944	1	pseudobulk	DESeq2	LRT	0.77480996285944

152	KC_AB2	Taf6	-1.21165387060851	0.422183932935778	1	pseudobulk	DESeq2	LRT	0.374498298669547
152	KC_AB2	Taf7	1.46149998396542	0.402709938159564	1	pseudobulk	DESeq2	LRT	0.39500765265082
152	KC_AB2	Taf8	1.33500753071363	0.412776777727525	1	pseudobulk	DESeq2	LRT	0.384284743523482
152	KC_AB2	tai	0.0739091843930038	0.804954772149175	1	pseudobulk	DESeq2	LRT	0.094228520573557
152	KC_AB2	Tailor	-0.100331036370678	0.93575632258968	1	pseudobulk	DESeq2	LRT	0.028837229822375
152	KC_AB2	Tak1	1.10546419043115	0.471164865757229	1	pseudobulk	DESeq2	LRT	0.326827101872602
152	KC_AB2	Taldo	1.10706867860301	1	1	pseudobulk	DESeq2	LRT	0
152	KC_AB2	tamo	-1.38131409835323	0.278970865197347	1	pseudobulk	DESeq2	LRT	0.554441150650144
152	KC_AB2	Tango1	0.323561093332177	0.742357677510918	1	pseudobulk	DESeq2	LRT	0.129386795608431
152	KC_AB2	Tango10	-0.471778911842301	0.635534526627818	1	pseudobulk	DESeq2	LRT	0.196860850591289
152	KC_AB2	Tango11	-1.16941991930243	0.200685597501499	1	pseudobulk	DESeq2	LRT	0.697483794179512
152	KC_AB2	Tango14	-0.49383505563944	0.707393381349206	1	pseudobulk	DESeq2	LRT	0.1503390079323
152	KC_AB2	Tango2	-0.115183097143228	0.910399790291748	1	pseudobulk	DESeq2	LRT	0.040767850987610
153	KC_AB2	Tango4	1.2615777461901	0.501768267196502	1	pseudobulk	DESeq2	LRT	0.29949680778272
153	KC_AB2	Tango5	0.128994276241785	0.87822236138799	1	pseudobulk	DESeq2	LRT	0.056395509040132
153	KC_AB2	Tango6	-1.6052707068109	0.52265609184397	1	pseudobulk	DESeq2	LRT	0.281783983297782
153	KC_AB2	Tango9	-0.550436667985956	0.633311407697041	1	pseudobulk	DESeq2	LRT	0.19838268905265
153	KC_AB2	tank	2.16548735034375	0.319952228384207	1	pseudobulk	DESeq2	LRT	0.494914860736039
153	KC_AB2	tant	-0.165517720345711	0.921104572835441	1	pseudobulk	DESeq2	LRT	0.035691061630197
153	KC_AB2	Tao	-0.177649511238846	0.786724080458831	1	pseudobulk	DESeq2	LRT	0.104177556516596
153	KC_AB2	Tap42	0.619440369709763	0.722026032044035	1	pseudobulk	DESeq2	LRT	0.141447144024172
153	KC_AB2	tapas	0.186419645963252	0.783991721140987	1	pseudobulk	DESeq2	LRT	0.105688523389247
153	KC_AB2	Tapdelta	0.110550242897393	0.899199104933026	1	pseudobulk	DESeq2	LRT	0.046144134073547
153	KC_AB2	tara	0.538253057091917	0.618236036738803	1	pseudobulk	DESeq2	LRT	0.208845683677977
153	KC_AB2	Task6	0.181329750854214	0.554663804334399	1	pseudobulk	DESeq2	LRT	0.255970173927788
153	KC_AB2	Task7	-0.0837236257234964	0.888093876292369	1	pseudobulk	DESeq2	LRT	0.051541124535723
153	KC_AB2	Tasp1	-0.729379579623002	1	1	pseudobulk	DESeq2	LRT	0
153	KC_AB2	Tat	-0.432917134717355	0.482363021174241	1	pseudobulk	DESeq2	LRT	0.316625993395976
153	KC_AB2	tau	0.343140790165539	0.415832523599952	1	pseudobulk	DESeq2	LRT	0.381081546106997
153	KC_AB2	tay	-0.160432977035988	0.704552927414299	1	pseudobulk	DESeq2	LRT	0.152086376265669
153	KC_AB2	Taz	0.268781370907002	0.88698811189061	1	pseudobulk	DESeq2	LRT	0.052082200884065
153	KC_AB2	Tbc1d15-17	0.375534485637874	0.901061951722844	1	pseudobulk	DESeq2	LRT	0.045245348458862
153	KC_AB2	TBC1D16	-0.208858989507537	0.785564525711797	1	pseudobulk	DESeq2	LRT	0.104818136518799
153	KC_AB2	TBC1D5	-0.169524318341919	0.912374154556569	1	pseudobulk	DESeq2	LRT	0.039827025776412
153	KC_AB2	TBC1d7	0.375534485637874	0.901061951722844	1	pseudobulk	DESeq2	LRT	0.045245348458862
153	KC_AB2	TBCB	-0.196275339414538	0.851401749050717	1	pseudobulk	DESeq2	LRT	0.069865461978597
153	KC_AB2	TBCC	0.0275411683831844	0.908791032127495	1	pseudobulk	DESeq2	LRT	0.041535967192871
153	KC_AB2	TBCD	-1.78975280219494	0.468855431150075	1	pseudobulk	DESeq2	LRT	0.32896104882553
153	KC_AB2	Tbce	0.599472024155989	0.724516344410118	1	pseudobulk	DESeq2	LRT	0.139951812805562
153	KC_AB2	Tbh	1.10706867860301	1	1	pseudobulk	DESeq2	LRT	0
153	KC_AB2	Tbp	-0.542302065913131	0.799614055888811	1	pseudobulk	DESeq2	LRT	0.097119580310179
153	KC_AB2	TBPH	-0.396415017978575	0.454830065546574	1	pseudobulk	DESeq2	LRT	0.342150834948681
153	KC_AB2	Tcs3	0.661678460170035	0.878003361430606	1	pseudobulk	DESeq2	LRT	0.056503821396894
153	KC_AB2	Tctp	0.064114390937833	0.858885094398361	1	pseudobulk	DESeq2	LRT	0.066064934197512
153	KC_AB2	Tdrd3	0.376739013127128	0.752336912062366	1	pseudobulk	DESeq2	LRT	0.123587629777601
153	KC_AB2	tea	1.26851375151404	0.439871453988053	1	pseudobulk	DESeq2	LRT	0.356674221195976
153	KC_AB2	tef	-2.56512514058146	0.231651930984972	1	pseudobulk	DESeq2	LRT	0.635164075289781
153	KC_AB2	tefu	1.38069928703004	0.566368801250645	1	pseudobulk	DESeq2	LRT	0.246900678019328
153	KC_AB2	Teh1	1.47222230513241	0.175018091592002	1	pseudobulk	DESeq2	LRT	0.756917056042455
153	KC_AB2	Teh2	0.0484967070859195	0.894289161859622	1	pseudobulk	DESeq2	LRT	0.048522032552059
153	KC_AB2	Teh3	0.147593560254927	1	1	pseudobulk	DESeq2	LRT	0
153	KC_AB2	Teh4	0.23671535392724	0.858022725612787	1	pseudobulk	DESeq2	LRT	0.066501209263734

153	KC_AB2	Tehao	1.10706867860301	1	1	pseudobulk	DESeq2	LRT	0
153	KC_AB2	temp	0.616408419475858	0.607735621567611	1	pseudobulk	DESeq2	LRT	0.216285307347174
153	KC_AB2	Ten-a	0.0273220763696315	0.879589284069676	1	pseudobulk	DESeq2	LRT	0.055720070236391
153	KC_AB2	Ten-m	0.0808420423671674	0.892581851575715	1	pseudobulk	DESeq2	LRT	0.049351947700115
153	KC_AB2	TER94	0.542751363852359	0.551072130777103	1	pseudobulk	DESeq2	LRT	0.258791551877389
153	KC_AB2	Tes	-0.441081437403916	0.911612819631012	1	pseudobulk	DESeq2	LRT	0.040189576146487
153	KC_AB2	Tet	0.838196546160542	0.211527643334164	1	pseudobulk	DESeq2	LRT	0.67463286912582
153	KC_AB2	tex	-2.2834221630407	0.364642497726807	1	pseudobulk	DESeq2	LRT	0.438132717324805
153	KC_AB2	TFAM	-0.248548513307421	0.862005195210534	1	pseudobulk	DESeq2	LRT	0.064490116722307
153	KC_AB2	Tfb1	0.66954517615027	1	1	pseudobulk	DESeq2	LRT	0
153	KC_AB2	Tfb4	1.02605719768525	0.762384804222891	1	pseudobulk	DESeq2	LRT	0.117825768597322
153	KC_AB2	TfIIA-L	0.607253890471962	0.694507960473751	1	pseudobulk	DESeq2	LRT	0.158322771999785
153	KC_AB2	TfIIA-S	0.0962709916994106	0.856433943331786	1	pseudobulk	DESeq2	LRT	0.067306128498614
153	KC_AB2	TfIIB	0.0245556823770887	0.967728367379246	1	pseudobulk	DESeq2	LRT	0.014246528128696
153	KC_AB2	TfIIEalpha	-1.01811685651142	0.497628311669954	1	pseudobulk	DESeq2	LRT	0.303094919208998
153	KC_AB2	TfIIEbeta	0.130057097686319	0.936253762795319	1	pseudobulk	DESeq2	LRT	0.028606423863414
153	KC_AB2	TfIIFalpha	-0.223881477339706	0.771643861463335	1	pseudobulk	DESeq2	LRT	0.112583094338025
153	KC_AB2	TfIIFbeta	0.0872694456224651	0.920505680068557	1	pseudobulk	DESeq2	LRT	0.035973527295957
153	KC_AB2	TfIIS	-0.51528440963685	0.573045310981758	1	pseudobulk	DESeq2	LRT	0.241811036791179
153	KC_AB2	Tg	1.10706867860301	1	1	pseudobulk	DESeq2	LRT	0
153	KC_AB2	Tgi	0.944548510431109	0.554160497248544	1	pseudobulk	DESeq2	LRT	0.256364435678345
153	KC_AB2	tgo	-2.1751167112989	0.359363815234217	1	pseudobulk	DESeq2	LRT	0.444465654648455
153	KC_AB2	Tgt	-0.432213394558105	0.865061123889812	1	pseudobulk	DESeq2	LRT	0.062953204870221
153	KC_AB2	TH1	0.607253890471962	0.694507960473751	1	pseudobulk	DESeq2	LRT	0.158322771999785
153	KC_AB2	THADA	-0.647621034217714	0.754702560287896	1	pseudobulk	DESeq2	LRT	0.122224176685402
153	KC_AB2	Thd1	0.284385344877852	0.466699534755802	1	pseudobulk	DESeq2	LRT	0.330962632054824
153	KC_AB2	THG	0.145321756433347	1	1	pseudobulk	DESeq2	LRT	0
153	KC_AB2	tho2	-0.399348248026132	0.733189879720452	1	pseudobulk	DESeq2	LRT	0.134783538270509
153	KC_AB2	thoc5	0.66954517615027	1	1	pseudobulk	DESeq2	LRT	0
153	KC_AB2	thoc6	0.0163626323653186	0.9421667738388	1	pseudobulk	DESeq2	LRT	0.025872215522219
153	KC_AB2	thoc7	-0.838168720083395	0.349135796410212	1	pseudobulk	DESeq2	LRT	0.457005621290561
153	KC_AB2	ThrRS	0.22492643493772	0.770857320056374	1	pseudobulk	DESeq2	LRT	0.113025999181834
153	KC_AB2	ths	0.789463356632272	0.477812140456472	1	pseudobulk	DESeq2	LRT	0.320742819704873
153	KC_AB2	Tif-IA	0.0972752079902775	0.906915888718177	1	pseudobulk	DESeq2	LRT	0.042432989411173
153	KC_AB2	Tim10	-0.729379579623002	1	1	pseudobulk	DESeq2	LRT	0
153	KC_AB2	Tim17b	-0.142517431926735	0.730617006436061	1	pseudobulk	DESeq2	LRT	0.136310223014632
153	KC_AB2	Tim17b1	-1.86979501903407	0.475964703567026	1	pseudobulk	DESeq2	LRT	0.322425252351861
153	KC_AB2	Tim23	0.0357061758237396	0.902468415161973	1	pseudobulk	DESeq2	LRT	0.044567988714162
153	KC_AB2	Tim8	-1.01697588889375	0.168729429654416	1	pseudobulk	DESeq2	LRT	0.772809161502076
153	KC_AB2	Tim9a	-0.525192580227396	0.60505692677503	1	pseudobulk	DESeq2	LRT	0.21820376283315
153	KC_AB2	timeout	-0.115183097143228	0.910399790291748	1	pseudobulk	DESeq2	LRT	0.040767850987610
153	KC_AB2	Timp	-0.893328173497901	0.679679924321693	1	pseudobulk	DESeq2	LRT	0.167695557626761
153	KC_AB2	Tina-1	-0.396060592881156	0.613255274618893	1	pseudobulk	DESeq2	LRT	0.212358707731396
153	KC_AB2	tinc	0.111648676946371	0.799730961802312	1	pseudobulk	DESeq2	LRT	0.097056089827932
153	KC_AB2	Tip60	3.70038173442613	0.0606577068174169	1	pseudobulk	DESeq2	LRT	1.21711401234661
153	KC_AB2	tipE	-0.244189013639684	0.749631122708641	1	pseudobulk	DESeq2	LRT	0.125152390983622
153	KC_AB2	Tis11	-0.729379579623002	1	1	pseudobulk	DESeq2	LRT	0
153	KC_AB2	tko	1.09650608298016	0.232193964616781	1	pseudobulk	DESeq2	LRT	0.63414907300267
153	KC_AB2	TkR86C	-0.211471510045543	0.900971302272423	1	pseudobulk	DESeq2	LRT	0.045289041943487
153	KC_AB2	tkv	-0.444840533150009	0.637927776856058	1	pseudobulk	DESeq2	LRT	0.195228487248075
153	KC_AB2	Tl	-0.36933621622144	0.377900650889683	1	pseudobulk	DESeq2	LRT	0.422622360060878
153	KC_AB2	Tlk	0.372706367170863	0.212842214182763	1	pseudobulk	DESeq2	LRT	0.671942231788501

153	KC_AB2	Tm1	-0.293755669771262	0.518593495878665	1	pseudobulk	DESeq2	LRT	0.285172934388339
153	KC_AB2	Tm2	-1.33407500886303	0.63128114564055	1	pseudobulk	DESeq2	LRT	0.19977718147345
153	KC_AB2	TM4SF	-0.18607436690008	0.511495045699678	1	pseudobulk	DESeq2	LRT	0.291158568473252
153	KC_AB2	TM9SF2	1.36649682481774	0.213295082818375	1	pseudobulk	DESeq2	LRT	0.671019156409274
153	KC_AB2	TM9SF3	-0.968109268377054	0.551802346970015	1	pseudobulk	DESeq2	LRT	0.25821645667677
153	KC_AB2	TM9SF4	-0.229919238066944	0.817175278810368	1	pseudobulk	DESeq2	LRT	0.087684780119177
153	KC_AB2	Tmc	-0.726593383514666	0.644495492459859	1	pseudobulk	DESeq2	LRT	0.190780115714233
153	KC_AB2	Tmem18	-1.26422566708958	0.436438769436775	1	pseudobulk	DESeq2	LRT	0.360076677413225
153	KC_AB2	TMEM216	-1.05611573998168	0.533854163205506	1	pseudobulk	DESeq2	LRT	0.272577366127457
154	KC_AB2	Tmem63	-1.47740131595573	0.429760234207816	1	pseudobulk	DESeq2	LRT	0.366773772332455
154	KC_AB2	tmod	0.0933522625953401	0.87111265099596	1	pseudobulk	DESeq2	LRT	0.059925679042372
154	KC_AB2	TMS1	-0.352822571140286	0.491356070814374	1	pseudobulk	DESeq2	LRT	0.308603673773155
154	KC_AB2	Tmtc3	-0.991110864030464	0.293262338371235	1	pseudobulk	DESeq2	LRT	0.532743706837947
154	KC_AB2	tna	-0.117631545837455	0.655469041870521	1	pseudobulk	DESeq2	LRT	0.183447815434314
154	KC_AB2	tnc	-0.729379579623002	1	1	pseudobulk	DESeq2	LRT	0
154	KC_AB2	Tnks	0.215865261740404	0.845523150758998	1	pseudobulk	DESeq2	LRT	0.072874496748393
154	KC_AB2	Tnpo	-0.160558334306782	0.77945442356185	1	pseudobulk	DESeq2	LRT	0.108209273899476
154	KC_AB2	Tnpo-SR	-0.647621034217714	0.754702560287896	1	pseudobulk	DESeq2	LRT	0.122224176685402
154	KC_AB2	Tob	-0.0748628693701214	0.760703894752784	1	pseudobulk	DESeq2	LRT	0.118784360180747
154	KC_AB2	toc	0.728985451732277	0.119279779506155	1	pseudobulk	DESeq2	LRT	0.923433172366504
154	KC_AB2	tok	0.728917240532532	0.670274586078713	1	pseudobulk	DESeq2	LRT	0.173747246867673
154	KC_AB2	Toll-6	-1.5675434670589	0.217205539182513	1	pseudobulk	DESeq2	LRT	0.663129103550385
154	KC_AB2	Toll-7	-1.78975280219494	0.468855431150075	1	pseudobulk	DESeq2	LRT	0.32896104882553
154	KC_AB2	Tom20	-0.436747962680157	0.393241085446424	1	pseudobulk	DESeq2	LRT	0.405341113803999
154	KC_AB2	Tom40	-0.479125739384219	0.542484471415467	1	pseudobulk	DESeq2	LRT	0.265612688954266
154	KC_AB2	Tom7	-0.298364534577745	0.449215785501073	1	pseudobulk	DESeq2	LRT	0.347544990977908
154	KC_AB2	Tom70	-0.618087445124117	0.582281891333539	1	pseudobulk	DESeq2	LRT	0.234866716016324
154	KC_AB2	Tomosyn	0.260627229935975	0.284128115241044	1	pseudobulk	DESeq2	LRT	0.546485789543865
154	KC_AB2	Top1	-0.120422168713666	0.779827343470939	1	pseudobulk	DESeq2	LRT	0.10800154099934
154	KC_AB2	Top2	0.564369349228282	0.635586319302459	1	pseudobulk	DESeq2	LRT	0.196825459349167
154	KC_AB2	Top3alpha	1.03858245968159	0.742043499884082	1	pseudobulk	DESeq2	LRT	0.129570634874779
154	KC_AB2	Top3beta	0.843700179273233	0.692173184066514	1	pseudobulk	DESeq2	LRT	0.159785230002206
154	KC_AB2	Topors	-1.82538827363707	0.311491423661201	1	pseudobulk	DESeq2	LRT	0.506553906333513
154	KC_AB2	Tor	1.69530465237941	0.515035175910199	1	pseudobulk	DESeq2	LRT	0.288163108469491
154	KC_AB2	TORIP	-0.0366670563693456	0.971208108011187	1	pseudobulk	DESeq2	LRT	0.012687700599728
154	KC_AB2	Torsin	-0.809871825471265	0.516805364896433	1	pseudobulk	DESeq2	LRT	0.286672986629026
154	KC_AB2	TotA	-1.21363965631423	0.568763379520138	1	pseudobulk	DESeq2	LRT	0.24506837392442
154	KC_AB2	tou	0.486125940346971	0.340506586245305	1	pseudobulk	DESeq2	LRT	0.467874483333196
154	KC_AB2	tow	-0.170965399977415	0.594374548448869	1	pseudobulk	DESeq2	LRT	0.2259397956554
154	KC_AB2	toy	0.196549315551696	0.477489820383888	1	pseudobulk	DESeq2	LRT	0.321035882715272
154	KC_AB2	Tpc1	-0.786027860261208	0.690326116112976	1	pseudobulk	DESeq2	LRT	0.160945696263719
154	KC_AB2	Tpi	0.0225741129802735	0.913195876009412	1	pseudobulk	DESeq2	LRT	0.039436058450308
154	KC_AB2	TppII	0.476565864525631	0.76932609253243	1	pseudobulk	DESeq2	LRT	0.113889537742045
154	KC_AB2	Tpr2	0.148589968585047	0.797604559333495	1	pseudobulk	DESeq2	LRT	0.098212372139080
154	KC_AB2	Tpst	1.19463076268389	0.490028525107175	1	pseudobulk	DESeq2	LRT	0.30977863846931
154	KC_AB2	tra	0.727014701651327	0.502479273826426	1	pseudobulk	DESeq2	LRT	0.298881847237728
154	KC_AB2	tra2	0.151900854520341	0.903357565928682	1	pseudobulk	DESeq2	LRT	0.044140313725751
154	KC_AB2	Traf6	1.02928733579242	0.697125366781187	1	pseudobulk	DESeq2	LRT	0.156689114003336
154	KC_AB2	tral	-0.100516588558066	0.789244917988376	1	pseudobulk	DESeq2	LRT	0.102788205883906
154	KC_AB2	TRAM	2.05113488428426	0.398118996741336	1	pseudobulk	DESeq2	LRT	0.399987099022033
154	KC_AB2	Trap1	0.624363112469519	0.679622483225035	1	pseudobulk	DESeq2	LRT	0.167732262264653
154	KC_AB2	Trax	-0.400818874228283	0.829617251912903	1	pseudobulk	DESeq2	LRT	0.081122225359766

154	KC_AB2	trbd	-0.79593009731915	0.528231024715658	1	pseudobulk	DESeq2	LRT	0.27717609486115
154	KC_AB2	trc	-0.329101097438098	0.712969325893958	1	pseudobulk	DESeq2	LRT	0.14692915441399
154	KC_AB2	Trc8	0.144131965390257	0.847536727168985	1	pseudobulk	DESeq2	LRT	0.07184147299364
154	KC_AB2	Treh	0.147593560254927	1	1	pseudobulk	DESeq2	LRT	0
154	KC_AB2	trem	0.267620161836785	0.85360591461508	1	pseudobulk	DESeq2	LRT	0.06874258435702
154	KC_AB2	Trf	1.35931792282326	0.578661801806309	1	pseudobulk	DESeq2	LRT	0.23757518501552
154	KC_AB2	Trf2	0.422841624443723	0.327035002234885	1	pseudobulk	DESeq2	LRT	0.48540576274048
154	KC_AB2	Trf4-1	0.175529939072201	0.730207230192709	1	pseudobulk	DESeq2	LRT	0.13655387117093
154	KC_AB2	trio	0.432956603170403	0.497977606338649	1	pseudobulk	DESeq2	LRT	0.30279018668249
154	KC_AB2	TrissinR	1.10706867860301	1	1	pseudobulk	DESeq2	LRT	0
154	KC_AB2	trk	-0.729379579623002	0.823527397767669	1	pseudobulk	DESeq2	LRT	0.08432194779794
154	KC_AB2	Trl	-0.951016782992348	0.621785407713543	1	pseudobulk	DESeq2	LRT	0.20635947435434
154	KC_AB2	trn	-0.432044363385868	0.639088967096516	1	pseudobulk	DESeq2	LRT	0.19443867982347
154	KC_AB2	tRNA:Arg-TCG-2-4	-0.729379579623002	0.823527397767669	1	pseudobulk	DESeq2	LRT	0.08432194779794
154	KC_AB2	tRNA:Cys-GCA-4-	1.10706867860301	1	1	pseudobulk	DESeq2	LRT	0
154	KC_AB2	Trp1	0.251464937537886	0.762018052844234	1	pseudobulk	DESeq2	LRT	0.11803473973945
154	KC_AB2	Trpm	0.295713194376029	0.446011918210789	1	pseudobulk	DESeq2	LRT	0.3506535360322
154	KC_AB2	TrpRS	-1.33407500886303	0.63128114564055	1	pseudobulk	DESeq2	LRT	0.19977718147345
154	KC_AB2	TrpRS-m	1.38446765109058	0.642794900879723	1	pseudobulk	DESeq2	LRT	0.19192757703716
154	KC_AB2	trr	0.156832622553597	0.847788856986229	1	pseudobulk	DESeq2	LRT	0.07171229593151
154	KC_AB2	Trs20	-0.019408203493835	0.883057474557444	1	pseudobulk	DESeq2	LRT	0.05401102907154
154	KC_AB2	Trs23	2.18581715476656	0.115531069557504	1	pseudobulk	DESeq2	LRT	0.93730120605021
154	KC_AB2	Trs31	1.04040805529344	0.303552433487547	1	pseudobulk	DESeq2	LRT	0.51776628117028
154	KC_AB2	Trs33	0.592767088540896	0.778649537027528	1	pseudobulk	DESeq2	LRT	0.10865797028989
154	KC_AB2	trsn	-0.923010883589963	0.420474008061671	1	pseudobulk	DESeq2	LRT	0.37626084529835
154	KC_AB2	trus	-1.15450353316387	0.53254210359827	1	pseudobulk	DESeq2	LRT	0.27364605054172
154	KC_AB2	trv	0.0348693043140682	0.861536405131097	1	pseudobulk	DESeq2	LRT	0.06472636625600
154	KC_AB2	trx	0.231014796452106	0.634138980206141	1	pseudobulk	DESeq2	LRT	0.19781555012779
154	KC_AB2	Trx-2	0.0369673981017689	0.938382516314629	1	pseudobulk	DESeq2	LRT	0.02762009247268
154	KC_AB2	Trxr-1	0.11548876190738	0.887703864608974	1	pseudobulk	DESeq2	LRT	0.05173188941935
154	KC_AB2	Try29F	-0.620563475958957	0.7478554969218	1	pseudobulk	DESeq2	LRT	0.12618230982555
154	KC_AB2	Tsc1	0.556882826741579	0.730652485787502	1	pseudobulk	DESeq2	LRT	0.13628913383616
154	KC_AB2	Tsen15	1.10706867860301	1	1	pseudobulk	DESeq2	LRT	0
154	KC_AB2	Tsen2	-2.62316164937934	0.247937455125287	1	pseudobulk	DESeq2	LRT	0.60565786078562
154	KC_AB2	Tsen34	0.806303238957506	0.585021205705234	1	pseudobulk	DESeq2	LRT	0.23282839143259
154	KC_AB2	Tsen54	2.92170020483689	0.236715975474367	1	pseudobulk	DESeq2	LRT	0.62577243144041
154	KC_AB2	Tsf1	1.38069928703004	0.566368801250645	1	pseudobulk	DESeq2	LRT	0.24690067801932
154	KC_AB2	Tsf2	1.61205113368698	0.631647504209	1	pseudobulk	DESeq2	LRT	0.19952521552247
154	KC_AB2	TSG101	-0.863830094023461	0.458556795820242	1	pseudobulk	DESeq2	LRT	0.33860686591823
154	KC_AB2	Tsp26A	-0.53634997502674	0.706795726247064	1	pseudobulk	DESeq2	LRT	0.15070608518809
154	KC_AB2	Tsp39D	-0.396698317889348	0.683074318749864	1	pseudobulk	DESeq2	LRT	0.16553204234032
154	KC_AB2	Tsp3A	-1.23858725735555	0.43823303111687	1	pseudobulk	DESeq2	LRT	0.35829489128661
154	KC_AB2	Tsp42Ee	-0.2080479074253	0.760413694546885	1	pseudobulk	DESeq2	LRT	0.11895007038025
154	KC_AB2	Tsp42Ej	-0.256444942432941	0.503750851997521	1	pseudobulk	DESeq2	LRT	0.29778420632476
154	KC_AB2	Tsp42Ek	-0.598942063397819	0.649678852722839	1	pseudobulk	DESeq2	LRT	0.18730126944403
154	KC_AB2	Tsp42En	0.0207715601562503	0.918964277309547	1	pseudobulk	DESeq2	LRT	0.03670137051691
154	KC_AB2	Tsp42Eo	-0.729379579623002	0.823527397767669	1	pseudobulk	DESeq2	LRT	0.08432194779794
154	KC_AB2	Tsp42Ep	1.38069928703004	0.566368801250645	1	pseudobulk	DESeq2	LRT	0.24690067801932
154	KC_AB2	Tsp66E	-0.342512968968251	0.396421510405158	1	pseudobulk	DESeq2	LRT	0.40184278809996
154	KC_AB2	Tsp86D	-1.03520656023961	0.395743791122147	1	pseudobulk	DESeq2	LRT	0.40258589011701
154	KC_AB2	Tsp96F	-0.257809926306611	0.62729867440583	1	pseudobulk	DESeq2	LRT	0.20252563020352
154	KC_AB2	Tsp97E	-0.274803492783973	0.585900943690743	1	pseudobulk	DESeq2	LRT	0.23217580248855

154	KC_AB2	Tspo	-1.54234522913661	0.310415432478807	1	pseudobulk	DESeq2	LRT	0.508056695681303
154	KC_AB2	tsr	-0.277616162625835	0.549600668568652	1	pseudobulk	DESeq2	LRT	0.259952747645195
154	KC_AB2	Tsr1	0.145321756433347	1	1	pseudobulk	DESeq2	LRT	0
154	KC_AB2	tst	-0.108150992064891	0.905978176473916	1	pseudobulk	DESeq2	LRT	0.042882263637873
154	KC_AB2	tsu	-0.0215474289875123	0.944379404353694	1	pseudobulk	DESeq2	LRT	0.024853492869333
155	KC_AB2	Ttc19	-0.0831039926956772	0.951661252363334	1	pseudobulk	DESeq2	LRT	0.021517612970107
155	KC_AB2	Ttc7	-0.470922007332909	0.67120080748279	1	pseudobulk	DESeq2	LRT	0.173147529704521
155	KC_AB2	Ttd14	-0.876611697597589	0.0870383930921476	1	pseudobulk	DESeq2	LRT	1.06028913554175
155	KC_AB2	tth	-0.689334691999768	0.479326780778342	1	pseudobulk	DESeq2	LRT	0.31936830558085
155	KC_AB2	TTLL12	-0.348901974470907	0.89890135131336	1	pseudobulk	DESeq2	LRT	0.046287966699494
155	KC_AB2	TTLL3A	-2.45416506545088	0.321711556460218	1	pseudobulk	DESeq2	LRT	0.492533338175562
155	KC_AB2	TTLL4A	1.33889695233627	0.201353772350065	1	pseudobulk	DESeq2	LRT	0.696040229500948
155	KC_AB2	TTLL5	1.78160130202253	0.317924271926985	1	pseudobulk	DESeq2	LRT	0.497676314609412
155	KC_AB2	ttm50	-0.038762175951681	0.95554308772421	1	pseudobulk	DESeq2	LRT	0.019749724790073
155	KC_AB2	ttv	-0.349855669694757	0.617290481199669	1	pseudobulk	DESeq2	LRT	0.209510419929588
155	KC_AB2	tty	0.141212298206977	0.754140684502029	1	pseudobulk	DESeq2	LRT	0.122547629191736
155	KC_AB2	tub	-0.0166754065092529	0.974446601891568	1	pseudobulk	DESeq2	LRT	0.011241954534277
155	KC_AB2	tud	1.46056508509605	0.122801743396624	1	pseudobulk	DESeq2	LRT	0.91079546754209
155	KC_AB2	Tudor-SN	-0.155754406913422	0.890387319974852	1	pseudobulk	DESeq2	LRT	0.050421033426452
155	KC_AB2	tun	0.29862061453089	0.590246463946193	1	pseudobulk	DESeq2	LRT	0.228966606016717
155	KC_AB2	Tusp	0.40851943051918	0.683035256734964	1	pseudobulk	DESeq2	LRT	0.165556878441077
155	KC_AB2	tut	-0.350224135645729	0.825372845318855	1	pseudobulk	DESeq2	LRT	0.083349823467051
155	KC_AB2	tutl	0.0573550147422581	0.826256910697392	1	pseudobulk	DESeq2	LRT	0.082884895112727
155	KC_AB2	tweek	0.612914564319176	0.504959751543676	1	pseudobulk	DESeq2	LRT	0.296743236493394
155	KC_AB2	twf	-0.360422521010947	0.827739787150686	1	pseudobulk	DESeq2	LRT	0.082106168970897
155	KC_AB2	twin	-0.57653944514359	0.482856584421709	1	pseudobulk	DESeq2	LRT	0.316181842008488
155	KC_AB2	twr	-0.217727152868307	0.810128016780351	1	pseudobulk	DESeq2	LRT	0.091446348296243
155	KC_AB2	tws	0.254434026261761	0.681595990548263	1	pseudobulk	DESeq2	LRT	0.166472972947762
155	KC_AB2	Txl	-0.0066762825747908	0.909419075863724	1	pseudobulk	DESeq2	LRT	0.041235940327232
155	KC_AB2	tyf	0.253417234285009	0.628974485201803	1	pseudobulk	DESeq2	LRT	0.201366971662543
155	KC_AB2	tyn	0.449794795206973	0.700364059541001	1	pseudobulk	DESeq2	LRT	0.154676148630152
155	KC_AB2	TyrR	0.62494562711567	0.750264017873708	1	pseudobulk	DESeq2	LRT	0.124785881503475
155	KC_AB2	TyrRII	-0.729379579623002	1	1	pseudobulk	DESeq2	LRT	0
155	KC_AB2	TyrRS	0.0139998711376467	0.909104143159845	1	pseudobulk	DESeq2	LRT	0.041386362972819
155	KC_AB2	TyrRS-m	0.575162595042011	0.797021781108777	1	pseudobulk	DESeq2	LRT	0.098529809988961
155	KC_AB2	tzn	-0.284415224551566	0.801688245587064	1	pseudobulk	DESeq2	LRT	0.095994484014062
155	KC_AB2	U2A	-0.720747651968478	0.756336938577071	1	pseudobulk	DESeq2	LRT	0.121284688671136
155	KC_AB2	U2af38	-0.572523996236299	0.60524800437431	1	pseudobulk	DESeq2	LRT	0.218066633841048
155	KC_AB2	U2af50	0.0134344950093324	0.892810029334968	1	pseudobulk	DESeq2	LRT	0.049240939754649
155	KC_AB2	U4-U6-60K	-0.778523756993934	0.689563908531076	1	pseudobulk	DESeq2	LRT	0.161425477368935
155	KC_AB2	Uba1	-1.34488796852161	0.21467006999824	1	pseudobulk	DESeq2	LRT	0.668228502097089
155	KC_AB2	Uba2	-1.07207849860966	0.386862540072729	1	pseudobulk	DESeq2	LRT	0.412443321008472
155	KC_AB2	Uba3	-1.21363965631423	0.568763379520138	1	pseudobulk	DESeq2	LRT	0.24506837392442
155	KC_AB2	Uba4	0.403294498144138	0.89479244414645	1	pseudobulk	DESeq2	LRT	0.048277691852293
155	KC_AB2	Uba5	1.3632625091553	0.58937724908886	1	pseudobulk	DESeq2	LRT	0.229606632629065
155	KC_AB2	Ubc10	-0.872523231487524	0.422080521568191	1	pseudobulk	DESeq2	LRT	0.37460468947232
155	KC_AB2	Ubc2	0.903124268828636	0.369199231998477	1	pseudobulk	DESeq2	LRT	0.432739211056663
155	KC_AB2	Ubc4	0.247429252989441	0.772036852687499	1	pseudobulk	DESeq2	LRT	0.112361968398946
155	KC_AB2	Ubc6	0.412416320875213	0.508867699152598	1	pseudobulk	DESeq2	LRT	0.293395115495345
155	KC_AB2	Ubc7	0.666189970424501	0.620040042418525	1	pseudobulk	DESeq2	LRT	0.207580262695516
155	KC_AB2	UbcE2H	-0.0007119504685601	0.94922123341181	1	pseudobulk	DESeq2	LRT	0.022632555483733
155	KC_AB2	UbcE2M	-0.376903260282124	0.66416139641658	1	pseudobulk	DESeq2	LRT	0.177726370849172

155	KC_AB2	Ube3a	-0.172425315789086	0.893480880137585	1	pseudobulk	DESeq2	LRT	0.048914736655356
155	KC_AB2	Ubi-p5E	-0.393540493375354	0.229262808502589	1	pseudobulk	DESeq2	LRT	0.639666391725095
155	KC_AB2	Ubi-p63E	0.0857568558843193	0.825722911600437	1	pseudobulk	DESeq2	LRT	0.083165664727580
155	KC_AB2	ubl	-0.144172803762084	0.885836553842226	1	pseudobulk	DESeq2	LRT	0.052646402619306
155	KC_AB2	UBL3	0.0359721724549303	0.914559901389109	1	pseudobulk	DESeq2	LRT	0.038787844052215
155	KC_AB2	Ublcp1	-0.609958788825387	0.734505775184742	1	pseudobulk	DESeq2	LRT	0.134004785141247
155	KC_AB2	Ubqn	-0.441447804138103	0.599549949645843	1	pseudobulk	DESeq2	LRT	0.222174629159126
155	KC_AB2	Ubr1	-0.187791705442536	0.728786585180242	1	pseudobulk	DESeq2	LRT	0.13739963004688
155	KC_AB2	Ubr3	-0.513398489757113	0.30329894645557	1	pseudobulk	DESeq2	LRT	0.518129098259488
155	KC_AB2	Uch	0.682573942368388	0.673741806646811	1	pseudobulk	DESeq2	LRT	0.1715065032148
155	KC_AB2	Uch-L5	0.861353293395997	0.448389548593514	1	pseudobulk	DESeq2	LRT	0.348344518826983
155	KC_AB2	Uck	-1.37686213004096	0.17380380575138	1	pseudobulk	DESeq2	LRT	0.759940718113313
155	KC_AB2	Ucp4A	-0.395348084444288	0.588199414146608	1	pseudobulk	DESeq2	LRT	0.230475412391041
155	KC_AB2	udd	2.66940639653205	0.184529810964056	1	pseudobulk	DESeq2	LRT	0.733933463156114
155	KC_AB2	udt	-0.729379579623002	1	1	pseudobulk	DESeq2	LRT	0
155	KC_AB2	Uev1A	-0.672471967896213	0.265450800136004	1	pseudobulk	DESeq2	LRT	0.576015961247188
155	KC_AB2	uex	0.0641793024601905	0.864964532888075	1	pseudobulk	DESeq2	LRT	0.063001700034365
155	KC_AB2	Ufc1	-0.617335520876419	0.647455594555672	1	pseudobulk	DESeq2	LRT	0.188790012112735
155	KC_AB2	Ufd1-like	-0.891374397608492	0.307992286916517	1	pseudobulk	DESeq2	LRT	0.511460159446111
155	KC_AB2	Ufd4	-0.0515566780343379	0.862322673117401	1	pseudobulk	DESeq2	LRT	0.064330194810507
155	KC_AB2	Ufl1	-0.961033333862793	0.70039622209097	1	pseudobulk	DESeq2	LRT	0.154656205149228
155	KC_AB2	Ufm1	-0.663921406845277	0.583358486752655	1	pseudobulk	DESeq2	LRT	0.23406447959503
155	KC_AB2	Ugalt	-1.33407500886303	0.63128114564055	1	pseudobulk	DESeq2	LRT	0.19977718147345
155	KC_AB2	Uggt	-0.101465738941791	0.948482558802264	1	pseudobulk	DESeq2	LRT	0.022970650737972
155	KC_AB2	UGP	-1.15826224286613	0.568080627318958	1	pseudobulk	DESeq2	LRT	0.24559002077719
155	KC_AB2	Ugt316A1	0.194078352352745	0.850240669783232	1	pseudobulk	DESeq2	LRT	0.070458125151064
155	KC_AB2	Uhg1	1.78871362473311	0.162665531848195	1	pseudobulk	DESeq2	LRT	0.788704462514818
155	KC_AB2	Uhg2	-0.0093009102043394	0.948321402376152	1	pseudobulk	DESeq2	LRT	0.023044447870606
155	KC_AB2	Uhg3	-1.3291832230123	0.422800327239599	1	pseudobulk	DESeq2	LRT	0.37386468522862
155	KC_AB2	Uhg4	-0.0352584701112013	0.886404676829863	1	pseudobulk	DESeq2	LRT	0.052367961194115
155	KC_AB2	Uhg5	0.638615986054243	0.422071870582155	1	pseudobulk	DESeq2	LRT	0.374613590887594
155	KC_AB2	uif	1.38069928703004	0.566368801250645	1	pseudobulk	DESeq2	LRT	0.246900678019328
155	KC_AB2	Ulp1	-0.0219888814108787	0.967194870285222	1	pseudobulk	DESeq2	LRT	0.014486015510321
155	KC_AB2	unc-104	0.147750483098444	0.60834224591885	1	pseudobulk	DESeq2	LRT	0.215852023201105
155	KC_AB2	Unc-115a	1.347023790459	0.334339314710653	1	pseudobulk	DESeq2	LRT	0.475812552040438
155	KC_AB2	Unc-115b	1.37221885443763	0.558734269457382	1	pseudobulk	DESeq2	LRT	0.252794690766539
155	KC_AB2	unc-119	-0.151823180305342	0.773947650027931	1	pseudobulk	DESeq2	LRT	0.111288414088283
155	KC_AB2	unc-13	0.345950075222173	0.0994031133158013	1	pseudobulk	DESeq2	LRT	1.00260001324153
155	KC_AB2	unc-13-4A	0.243902187331757	0.490557433989077	1	pseudobulk	DESeq2	LRT	0.309310138530096
155	KC_AB2	unc-45	1.48091416250208	0.564234146509008	1	pseudobulk	DESeq2	LRT	0.248540634607989
155	KC_AB2	unc-5	1.03842924766642	0.292401734148785	1	pseudobulk	DESeq2	LRT	0.534020056033552
155	KC_AB2	Unc-76	-0.0060286521838968	0.968792668894875	1	pseudobulk	DESeq2	LRT	0.013769156271433
155	KC_AB2	unc79	0.011327521124853	0.854124275762822	1	pseudobulk	DESeq2	LRT	0.068478934520207
155	KC_AB2	unc80	-0.130268928537195	0.778841095496122	1	pseudobulk	DESeq2	LRT	0.108551141029347
155	KC_AB2	und	-0.861411092459907	0.287033663810911	1	pseudobulk	DESeq2	LRT	0.542067165461287
155	KC_AB2	unk	0.175920357109554	0.751922333691868	1	pseudobulk	DESeq2	LRT	0.123827015513392
155	KC_AB2	Unr	0.0518335280297921	0.818825161527421	1	pseudobulk	DESeq2	LRT	0.086808820449420
155	KC_AB2	Updo	0.205457650794445	0.755746524556302	1	pseudobulk	DESeq2	LRT	0.121623841331079
155	KC_AB2	Upf1	-1.00027641441447	0.535914832041766	1	pseudobulk	DESeq2	LRT	0.270904223205024
155	KC_AB2	Upf2	0.810457230400821	0.627759605077236	1	pseudobulk	DESeq2	LRT	0.202206633612843
155	KC_AB2	Upf3	-2.31224825938678	0.239115849320636	1	pseudobulk	DESeq2	LRT	0.621391636580306
155	KC_AB2	upSET	0.451150393220808	0.334004218773463	1	pseudobulk	DESeq2	LRT	0.476248047624171

155	KC_AB2	UQCR-11	-0.612742828808203	0.0862426342737591	1	pseudobulk	DESeq2	LRT	1.06427798646758
156	KC_AB2	UQCR-14	-0.321756305070647	0.386249683989884	1	pseudobulk	DESeq2	LRT	0.41313186288492
156	KC_AB2	UQCR-6.4	-0.675317809999111	0.0985478282656094	1	pseudobulk	DESeq2	LRT	1.00635294198479
156	KC_AB2	UQCR-C1	-0.550223613312408	0.264224084661443	1	pseudobulk	DESeq2	LRT	0.57802759792955
156	KC_AB2	UQCR-C2	-0.344660111680932	0.436404993996131	1	pseudobulk	DESeq2	LRT	0.36011028821075
156	KC_AB2	UQCR-Q	-0.237224108450945	0.542046747859531	1	pseudobulk	DESeq2	LRT	0.26596325688976
156	KC_AB2	uri	0.378320304847799	0.842666545020638	1	pseudobulk	DESeq2	LRT	0.07434424780338
156	KC_AB2	Urm1	-3.16336897464401	0.112753768916637	1	pseudobulk	DESeq2	LRT	0.94786893244475
156	KC_AB2	Uros1	-0.893328173497901	0.679679924321693	1	pseudobulk	DESeq2	LRT	0.16769555762676
156	KC_AB2	Uros2	-0.729379579623002	1	1	pseudobulk	DESeq2	LRT	0
156	KC_AB2	Use1	0.775582208069803	0.605942378009312	1	pseudobulk	DESeq2	LRT	0.21756867303243
156	KC_AB2	Usf	-0.548413455643832	0.529667626353542	1	pseudobulk	DESeq2	LRT	0.27599657062807
156	KC_AB2	ush	-1.02019568450201	0.730699957834935	1	pseudobulk	DESeq2	LRT	0.13626091771575
156	KC_AB2	usp	-0.551936519290039	0.511280228478554	1	pseudobulk	DESeq2	LRT	0.29134100138393
156	KC_AB2	Usp1	0.223967543274013	0.764009020675413	1	pseudobulk	DESeq2	LRT	0.11690151366720
156	KC_AB2	Usp10	-1.02031505357459	0.442939041875032	1	pseudobulk	DESeq2	LRT	0.35365603810854
156	KC_AB2	Usp12-46	1.04232157414887	0.700586543363791	1	pseudobulk	DESeq2	LRT	0.15453820872397
156	KC_AB2	Usp14	-2.01037164510964	0.252449294890841	1	pseudobulk	DESeq2	LRT	0.59782583798273
156	KC_AB2	Usp15-31	0.965778378667528	0.555908821592816	1	pseudobulk	DESeq2	LRT	0.25499643418471
156	KC_AB2	Usp16-45	0.542750838737471	0.754609686636466	1	pseudobulk	DESeq2	LRT	0.12227762422677
156	KC_AB2	Usp2	-0.0079797108006423	0.948056889884857	1	pseudobulk	DESeq2	LRT	0.02316560124657
156	KC_AB2	Usp20-33	-3.02385141335684	0.137379093426569	1	pseudobulk	DESeq2	LRT	0.86207935388721
156	KC_AB2	Usp30	-1.16259488550784	0.320619974917718	1	pseudobulk	DESeq2	LRT	0.49400942419551
156	KC_AB2	Usp32	-0.10908367772688	0.908177621734931	1	pseudobulk	DESeq2	LRT	0.04182920368672
156	KC_AB2	Usp39	-2.09428695548101	0.380540277844868	1	pseudobulk	DESeq2	LRT	0.41959936906422
156	KC_AB2	Usp47	-0.520778442345317	0.677867998619702	1	pseudobulk	DESeq2	LRT	0.16885486815712
156	KC_AB2	Usp5	-0.477131411897435	0.824127670300531	1	pseudobulk	DESeq2	LRT	0.08400550406497
156	KC_AB2	Usp7	-0.0807994098669526	0.855953427739257	1	pseudobulk	DESeq2	LRT	0.06754986455893
156	KC_AB2	Usp8	-0.609408981115751	0.659293530721663	1	pseudobulk	DESeq2	LRT	0.18092118572401
156	KC_AB2	Utx	0.0571231278369023	0.902708970599834	1	pseudobulk	DESeq2	LRT	0.04445224175134
156	KC_AB2	Uvrag	-1.17970554645024	0.419712160339806	1	pseudobulk	DESeq2	LRT	0.37704844777214
156	KC_AB2	Uxs	-1.65749370833214	0.513027701278311	1	pseudobulk	DESeq2	LRT	0.28985918423027
156	KC_AB2	Uxt	-0.778523756993934	0.689563908531076	1	pseudobulk	DESeq2	LRT	0.16142547736893
156	KC_AB2	uzip	0.770018348823893	0.354858333725405	1	pseudobulk	DESeq2	LRT	0.44994499105942
156	KC_AB2	VAChT	0.393812193832272	0.27065307118276	1	pseudobulk	DESeq2	LRT	0.56758704047331
156	KC_AB2	ValRS	-1.36089652170387	0.447957456601475	1	pseudobulk	DESeq2	LRT	0.34876322984239
156	KC_AB2	Vamp7	-0.0359880206543801	0.971985853620892	1	pseudobulk	DESeq2	LRT	0.01234005579295
156	KC_AB2	Vang	0.843700179273233	0.692173184066514	1	pseudobulk	DESeq2	LRT	0.15978523000220
156	KC_AB2	vap	-0.819459759816161	0.442094423588542	1	pseudobulk	DESeq2	LRT	0.35448496308552
156	KC_AB2	Vap33	-0.268501254720794	0.480934990042871	1	pseudobulk	DESeq2	LRT	0.31791362502696
156	KC_AB2	vari	1.04232157414887	0.700586543363791	1	pseudobulk	DESeq2	LRT	0.15453820872397
156	KC_AB2	Vav	-1.81504288794368	0.13458632530444	1	pseudobulk	DESeq2	LRT	0.87099906452900
156	KC_AB2	veli	-0.231755999349377	0.674877179129637	1	pseudobulk	DESeq2	LRT	0.17077525721294
156	KC_AB2	velo	0.498967092651496	0.639910848178298	1	pseudobulk	DESeq2	LRT	0.19388052733045
156	KC_AB2	VepD	-1.06260274209206	0.0403599469330376	1	pseudobulk	DESeq2	LRT	1.39404941346347
156	KC_AB2	ver	1.38069928703004	0.566368801250645	1	pseudobulk	DESeq2	LRT	0.24690067801932
156	KC_AB2	vers	-1.15826224286613	0.568080627318958	1	pseudobulk	DESeq2	LRT	0.24559002077719
156	KC_AB2	VGAT	-0.117799200223991	0.866077291415121	1	pseudobulk	DESeq2	LRT	0.06244334847085
156	KC_AB2	Vha100-1	-0.41004227514046	0.33820279067541	1	pseudobulk	DESeq2	LRT	0.47082281314888
156	KC_AB2	Vha13	-0.330908421088112	0.229176966789397	1	pseudobulk	DESeq2	LRT	0.63982903286053
156	KC_AB2	Vha14-1	-0.320677462529699	0.41576295623807	1	pseudobulk	DESeq2	LRT	0.38115420816971
156	KC_AB2	Vha14-2	1.38446765109058	0.642794900879723	1	pseudobulk	DESeq2	LRT	0.19192757703716

156	KC_AB2	Vha16-1	-0.297861140517848	0.176811470080352	1	pseudobulk	DESeq2	LRT	0.752489564939325
156	KC_AB2	Vha26	-0.583890217911229	0.185605349558314	1	pseudobulk	DESeq2	LRT	0.731409510605438
156	KC_AB2	Vha36-1	-0.227480888049089	0.555059392796067	1	pseudobulk	DESeq2	LRT	0.255660543753834
156	KC_AB2	Vha44	0.207107992818018	0.609441318605926	1	pseudobulk	DESeq2	LRT	0.215068105029254
156	KC_AB2	Vha55	-0.131625556308997	0.710339462061834	1	pseudobulk	DESeq2	LRT	0.148534057945648
156	KC_AB2	Vha68-1	-0.439427486358343	0.366250553532109	1	pseudobulk	DESeq2	LRT	0.436221710267162
156	KC_AB2	VhaAC39-1	-0.585265138103948	0.246033108478363	1	pseudobulk	DESeq2	LRT	0.609006446303369
156	KC_AB2	VhaAC45	0.0695131409074483	0.89481916518139	1	pseudobulk	DESeq2	LRT	0.048264722783513
156	KC_AB2	VhaM9.7-a	-0.261797858960396	0.411988096648215	1	pseudobulk	DESeq2	LRT	0.385115331623846
156	KC_AB2	VhaM9.7-b	-0.327833865612648	0.6104062896662	1	pseudobulk	DESeq2	LRT	0.214381000031468
156	KC_AB2	VhaM9.7-c	-0.750406468722284	0.464641528652681	1	pseudobulk	DESeq2	LRT	0.332881976501599
156	KC_AB2	VhaPPA1-1	-0.4487309966363	0.362295121188209	1	pseudobulk	DESeq2	LRT	0.440937514310221
156	KC_AB2	VhaSFD	-0.165295951959725	0.799920816738503	1	pseudobulk	DESeq2	LRT	0.096953001202466
156	KC_AB2	Vhl	1.07261497208913	0.300174707293958	1	pseudobulk	DESeq2	LRT	0.522625904182787
156	KC_AB2	viaf	-1.05302035339529	0.254122361287582	1	pseudobulk	DESeq2	LRT	0.594957117883437
156	KC_AB2	vib	-0.0403030709770147	0.875931281122251	1	pseudobulk	DESeq2	LRT	0.057529963923304
156	KC_AB2	vig	0.131830002762617	0.694618641654953	1	pseudobulk	DESeq2	LRT	0.158253565598764
156	KC_AB2	vig2	-0.460075129685562	0.530409856869178	1	pseudobulk	DESeq2	LRT	0.27538841381875
156	KC_AB2	vih	-0.282940340680471	0.770508175194701	1	pseudobulk	DESeq2	LRT	0.113222748998629
156	KC_AB2	vimar	-0.122661754480986	0.862458846847928	1	pseudobulk	DESeq2	LRT	0.064261618588897
156	KC_AB2	Vinc	0.146930209264112	1	1	pseudobulk	DESeq2	LRT	0
156	KC_AB2	vir	0.661678460170035	0.878003361430606	1	pseudobulk	DESeq2	LRT	0.056503821396894
156	KC_AB2	vir-1	-0.729379579623002	1	1	pseudobulk	DESeq2	LRT	0
156	KC_AB2	vito	-0.142583703271328	0.882509341137768	1	pseudobulk	DESeq2	LRT	0.054280689018760
156	KC_AB2	vlc	-0.0290084406213922	0.937293366425203	1	pseudobulk	DESeq2	LRT	0.028124456626912
156	KC_AB2	Vlet	-0.603647963308095	0.706080294682827	1	pseudobulk	DESeq2	LRT	0.15114590864391
156	KC_AB2	vls	-0.23099159550585	0.863223945713159	1	pseudobulk	DESeq2	LRT	0.063876520889037
156	KC_AB2	Vps11	1.0339497049177	0.145669909909336	1	pseudobulk	DESeq2	LRT	0.83663014836676
156	KC_AB2	Vps13	1.20107281905329	0.762593168320301	1	pseudobulk	DESeq2	LRT	0.117707089665659
156	KC_AB2	Vps13D	1.80677871671018	0.477532853980226	1	pseudobulk	DESeq2	LRT	0.320996743846761
156	KC_AB2	Vps15	0.396659246788372	0.915482501218565	1	pseudobulk	DESeq2	LRT	0.038349952505755
156	KC_AB2	Vps16A	-0.0405873896132414	0.965036510824772	1	pseudobulk	DESeq2	LRT	0.015456255412921
156	KC_AB2	Vps16B	-0.18317077492817	0.899664519927029	1	pseudobulk	DESeq2	LRT	0.045919406456170
156	KC_AB2	Vps2	0.150969331366648	0.88153209624233	1	pseudobulk	DESeq2	LRT	0.054761870582844
156	KC_AB2	Vps20	-0.551341853851541	0.691236584581417	1	pseudobulk	DESeq2	LRT	0.160373284329271
156	KC_AB2	Vps24	-0.264323156136889	0.65821605300626	1	pseudobulk	DESeq2	LRT	0.181631530052857
156	KC_AB2	Vps25	-1.24519816906373	0.308187439449887	1	pseudobulk	DESeq2	LRT	0.511185065451358
156	KC_AB2	Vps26	0.696022867081849	0.668116349046407	1	pseudobulk	DESeq2	LRT	0.175147900774716
156	KC_AB2	Vps28	0.792724262197627	0.492489880995935	1	pseudobulk	DESeq2	LRT	0.307602688359821
156	KC_AB2	Vps29	-1.32060483020588	0.399828599226881	1	pseudobulk	DESeq2	LRT	0.398126144579584
156	KC_AB2	Vps33B	1.47789775877809	0.581821529618366	1	pseudobulk	DESeq2	LRT	0.235210212237369
156	KC_AB2	Vps35	1.30771590840378	0.516263367871464	1	pseudobulk	DESeq2	LRT	0.287128689780778
156	KC_AB2	Vps36	1.38446765109058	0.642794900879723	1	pseudobulk	DESeq2	LRT	0.191927577037166
156	KC_AB2	Vps37B	1.59958358224302	0.170159738483136	1	pseudobulk	DESeq2	LRT	0.769143190567822
156	KC_AB2	Vps39	0.661678460170035	0.878003361430606	1	pseudobulk	DESeq2	LRT	0.056503821396894
156	KC_AB2	Vps4	0.232525737904775	0.775664949054798	1	pseudobulk	DESeq2	LRT	0.110325833122037
156	KC_AB2	Vps45	0.162385149200511	0.91636577592647	1	pseudobulk	DESeq2	LRT	0.037931139041603
156	KC_AB2	Vps50	0.0300355375033142	0.954625634966385	1	pseudobulk	DESeq2	LRT	0.020166907519573
156	KC_AB2	Vps51	1.78124453243794	0.460163425723109	1	pseudobulk	DESeq2	LRT	0.33708790248149
157	KC_AB2	Vps52	1.69530465237941	0.515035175910199	1	pseudobulk	DESeq2	LRT	0.288163108469491
157	KC_AB2	Vps53	-0.721175887303532	0.733409564198845	1	pseudobulk	DESeq2	LRT	0.134653430819787
157	KC_AB2	Vps60	-0.123860999432975	0.884055868062701	1	pseudobulk	DESeq2	LRT	0.053520288805172

157	KC_AB2	Vps8	-0.473795964521473	0.80663503472328	1	pseudobulk	DESeq2	LRT	0.093322919129089
157	KC_AB2	Vrp1	-0.323554775507352	0.613388812860322	1	pseudobulk	DESeq2	LRT	0.212264149053461
157	KC_AB2	vsg	-0.0620768936684052	0.846426934683736	1	pseudobulk	DESeq2	LRT	0.072410525131197
157	KC_AB2	Vsp37A	-0.753061126838944	0.443174556851735	1	pseudobulk	DESeq2	LRT	0.353425180897133
157	KC_AB2	Vsx1	0.151904459063767	0.855326048080542	1	pseudobulk	DESeq2	LRT	0.067868301774939
157	KC_AB2	Vta1	-1.12133702346516	0.26586200983361	1	pseudobulk	DESeq2	LRT	0.575343716441935
157	KC_AB2	vtd	-0.171542418384427	0.762728393032488	1	pseudobulk	DESeq2	LRT	0.117630086431769
157	KC_AB2	Vti1a	-0.314736662348435	0.769892145176576	1	pseudobulk	DESeq2	LRT	0.113570111237241
157	KC_AB2	Vti1b	-0.0522186559507013	0.944312552869609	1	pseudobulk	DESeq2	LRT	0.024884237142422
157	KC_AB2	w	1.73298758435161	0.156596538916436	1	pseudobulk	DESeq2	LRT	0.805217840912202
157	KC_AB2	wac	-1.5033370644665	0.499350065149774	1	pseudobulk	DESeq2	LRT	0.30159488912562
157	KC_AB2	wake	0.425449780259029	0.569268858627088	1	pseudobulk	DESeq2	LRT	0.244682573252095
157	KC_AB2	wal	-0.536576864477712	0.776032296287497	1	pseudobulk	DESeq2	LRT	0.110120204247509
157	KC_AB2	wap	0.154786780479112	0.880996790325504	1	pseudobulk	DESeq2	LRT	0.055025673820031
157	KC_AB2	wapl	0.0769493221325992	0.907902216383054	1	pseudobulk	DESeq2	LRT	0.041960923694861
157	KC_AB2	wash	2.01814786626755	0.401718761445944	1	pseudobulk	DESeq2	LRT	0.396077884966921
157	KC_AB2	WASp	-0.561428883744135	0.571324528312714	1	pseudobulk	DESeq2	LRT	0.243117130250338
157	KC_AB2	wb	2.64741945039871	0.292550810913063	1	pseudobulk	DESeq2	LRT	0.533798693743382
157	KC_AB2	Wbp2	0.370493087615216	0.670839362752115	1	pseudobulk	DESeq2	LRT	0.173381462278705
157	KC_AB2	wcd	-0.674317410868381	0.830763653594129	1	pseudobulk	DESeq2	LRT	0.080522512364194
157	KC_AB2	wcy	-0.522464037651805	0.421057923630879	1	pseudobulk	DESeq2	LRT	0.375658155511427
157	KC_AB2	wda	1.99339641594643	0.465071165814147	1	pseudobulk	DESeq2	LRT	0.332480585700252
157	KC_AB2	wdb	0.153376830884139	0.554848633145464	1	pseudobulk	DESeq2	LRT	0.255825479500266
157	KC_AB2	wde	-0.507566860442248	0.55322503522084	1	pseudobulk	DESeq2	LRT	0.257098174889103
157	KC_AB2	Wdfy2	-0.0260572929384691	0.917216315950487	1	pseudobulk	DESeq2	LRT	0.037528228403242
157	KC_AB2	wdn	-1.53390420416511	0.550292219668796	1	pseudobulk	DESeq2	LRT	0.259406627437427
157	KC_AB2	Wdr24	-0.729379579623002	0.823527397767669	1	pseudobulk	DESeq2	LRT	0.084321947797948
157	KC_AB2	Wdr33	-0.59147693594622	0.573569318791414	1	pseudobulk	DESeq2	LRT	0.241414087877147
157	KC_AB2	Wdr59	-0.729379579623002	1	1	pseudobulk	DESeq2	LRT	0
157	KC_AB2	Wdr62	-1.73929447458106	0.0476361909189905	1	pseudobulk	DESeq2	LRT	1.322062972819
157	KC_AB2	Wdr81	-1.52244591594958	0.547176617578731	1	pseudobulk	DESeq2	LRT	0.261872469536296
157	KC_AB2	Wdr82	-0.704841387243703	0.560397835348987	1	pseudobulk	DESeq2	LRT	0.251503550934096
157	KC_AB2	Wdr92	-0.729379579623002	1	1	pseudobulk	DESeq2	LRT	0
157	KC_AB2	wds	0.751062180338627	0.48460188981128	1	pseudobulk	DESeq2	LRT	0.314614896568693
157	KC_AB2	WDY	0.39439088612385	0.813541888331052	1	pseudobulk	DESeq2	LRT	0.089620080830767
157	KC_AB2	wech	0.111832800843381	0.87428592306827	1	pseudobulk	DESeq2	LRT	0.058346514157632
157	KC_AB2	Wee1	-1.59499420489131	0.469581734450344	1	pseudobulk	DESeq2	LRT	0.328288804396094
157	KC_AB2	wek	-1.52244591594958	0.547176617578731	1	pseudobulk	DESeq2	LRT	0.261872469536296
157	KC_AB2	wfs1	-1.6052707068109	0.52265609184397	1	pseudobulk	DESeq2	LRT	0.281783983297782
157	KC_AB2	wge	-0.702801286543829	0.206474698662951	1	pseudobulk	DESeq2	LRT	0.685133159040436
157	KC_AB2	whd	1.38069928703004	0.566368801250645	1	pseudobulk	DESeq2	LRT	0.246900678019328
157	KC_AB2	wit	0.891696636492894	0.341186757695715	1	pseudobulk	DESeq2	LRT	0.467007833223399
157	KC_AB2	wkd	0.222243581431297	0.817792858193197	1	pseudobulk	DESeq2	LRT	0.087356686470395
157	KC_AB2	wls	0.146930209264112	1	1	pseudobulk	DESeq2	LRT	0
157	KC_AB2	wmd	-1.04886595502375	0.238497298706795	1	pseudobulk	DESeq2	LRT	0.622516535547878
157	KC_AB2	wnd	-0.0148447282247095	0.963133280584825	1	pseudobulk	DESeq2	LRT	0.016313610053703
157	KC_AB2	Wnk	0.148001780807642	0.802532613858123	1	pseudobulk	DESeq2	LRT	0.095537309414294
157	KC_AB2	Wnt5	0.112474594344709	0.849633684704559	1	pseudobulk	DESeq2	LRT	0.070768277817082
157	KC_AB2	Wnt6	1.10706867860301	1	1	pseudobulk	DESeq2	LRT	0
157	KC_AB2	woc	0.561641945052231	0.500513366117973	1	pseudobulk	DESeq2	LRT	0.30058432027503
157	KC_AB2	wol	-1.20441416492585	0.509438913975176	1	pseudobulk	DESeq2	LRT	0.292907884108946
157	KC_AB2	wrapper	1.10706867860301	1	1	pseudobulk	DESeq2	LRT	0

157	KC_AB2	wrd	0.0050386622446235	0.953158863198747	1	pseudobulk	DESeq2	LRT	0.020834709371432
157	KC_AB2	wry	0.0964236717031351	0.872813173028305	1	pseudobulk	DESeq2	LRT	0.059078707733268
157	KC_AB2	Wsck	-0.729379579623002	0.823527397767669	1	pseudobulk	DESeq2	LRT	0.084321947797948
157	KC_AB2	wts	-0.343817928764839	0.565789621966543	1	pseudobulk	DESeq2	LRT	0.247345022881147
157	KC_AB2	wuho	0.52303597090985	0.755188486132328	1	pseudobulk	DESeq2	LRT	0.121944640065011
157	KC_AB2	wun	0.64899489158548	0.565672786484226	1	pseudobulk	DESeq2	LRT	0.247434713895294
157	KC_AB2	Wwox	-0.969404222682795	0.749350307836562	1	pseudobulk	DESeq2	LRT	0.125315109945074
157	KC_AB2	X11L	0.755072002102048	0.186088970977645	1	pseudobulk	DESeq2	LRT	0.730279365641333
157	KC_AB2	X11Lbeta	-0.0050020318103685	0.889874287989458	1	pseudobulk	DESeq2	LRT	0.050671341544615
157	KC_AB2	x16	-0.122036144854655	0.848747311788291	1	pseudobulk	DESeq2	LRT	0.071221588237050
157	KC_AB2	Xbp1	-0.740121068665944	0.283540811373721	1	pseudobulk	DESeq2	LRT	0.547384422207463
157	KC_AB2	Xe7	0.201082572881073	0.546410996694012	1	pseudobulk	DESeq2	LRT	0.262480568924857
157	KC_AB2	xit	-1.52244591594958	0.547176617578731	1	pseudobulk	DESeq2	LRT	0.261872469536296
157	KC_AB2	xmas	-0.729379579623002	0.772694994316916	1	pseudobulk	DESeq2	LRT	0.1119919011982
157	KC_AB2	XNP	0.110542714830025	0.86197053359096	1	pseudobulk	DESeq2	LRT	0.064507580251365
157	KC_AB2	Xpac	-0.721175887303532	0.733409564198845	1	pseudobulk	DESeq2	LRT	0.134653430819787
157	KC_AB2	Xpc	-0.296077157330819	0.700796347694507	1	pseudobulk	DESeq2	LRT	0.154408170225043
157	KC_AB2	XRCC1	-0.729379579623002	1	1	pseudobulk	DESeq2	LRT	0
157	KC_AB2	Xrp1	-0.0643753997862357	0.844734976048684	1	pseudobulk	DESeq2	LRT	0.073279523586420
157	KC_AB2	Xxylt	-0.65467796138591	0.436494103990213	1	pseudobulk	DESeq2	LRT	0.360021618216852
157	KC_AB2	yata	-0.828100747512996	0.609865574570427	1	pseudobulk	DESeq2	LRT	0.214765880818492
157	KC_AB2	yellow-c	-0.729379579623002	1	1	pseudobulk	DESeq2	LRT	0
157	KC_AB2	yem	1.80677871671018	0.477532853980226	1	pseudobulk	DESeq2	LRT	0.320996743846761
157	KC_AB2	Yeti	-0.204600047926828	0.666490548001936	1	pseudobulk	DESeq2	LRT	0.176206005259439
157	KC_AB2	Yif1	-0.159640693216623	0.913321571945367	1	pseudobulk	DESeq2	LRT	0.039376284532977
157	KC_AB2	Yip1d1	-0.956455048496356	0.376829684534279	1	pseudobulk	DESeq2	LRT	0.423854893242088
157	KC_AB2	Yippee	0.348188413790247	0.589393678287955	1	pseudobulk	DESeq2	LRT	0.229594526611952
157	KC_AB2	yki	-0.594761941930707	0.535609718995649	1	pseudobulk	DESeq2	LRT	0.271151551025443
157	KC_AB2	Ykt6	-0.179879875271918	0.812370139018769	1	pseudobulk	DESeq2	LRT	0.090246048706074
157	KC_AB2	YL-1	-0.558216076994733	0.655324374018266	1	pseudobulk	DESeq2	LRT	0.183543678671704
157	KC_AB2	YME1L	0.53549124953756	0.663063572337917	1	pseudobulk	DESeq2	LRT	0.178444830883339
157	KC_AB2	yps	-1.03856402411711	0.0568432678545003	1	pseudobulk	DESeq2	LRT	1.24532096293348
157	KC_AB2	yrt	-0.190097883870666	0.791386890849091	1	pseudobulk	DESeq2	LRT	0.101611148003597
157	KC_AB2	Ythdc1	0.355248249827363	0.445627382265239	1	pseudobulk	DESeq2	LRT	0.351028131116806
157	KC_AB2	Ythdf	-1.36888258668893	0.181063305274107	1	pseudobulk	DESeq2	LRT	0.742169555935017
157	KC_AB2	ytr	0.808585271248031	0.333057791947472	1	pseudobulk	DESeq2	LRT	0.477480401490066
157	KC_AB2	z	-0.723166044958219	0.530194246656589	1	pseudobulk	DESeq2	LRT	0.275564989275224
157	KC_AB2	ZAP3	0.358894934632907	0.651664136166696	1	pseudobulk	DESeq2	LRT	0.185976179408709
157	KC_AB2	Zasp52	-0.734540122400504	0.833184184675853	1	pseudobulk	DESeq2	LRT	0.079258982331102
157	KC_AB2	ZC3H3	0.572616942423986	0.599463869834473	1	pseudobulk	DESeq2	LRT	0.22223698705115
157	KC_AB2	Zcchc7	1.06983801284922	0.358616223682248	1	pseudobulk	DESeq2	LRT	0.445370066987172
157	KC_AB2	zda	0.643222179038489	0.475489245194661	1	pseudobulk	DESeq2	LRT	0.322859301661235
157	KC_AB2	Zdhhc8	0.160480674249781	0.642928788223294	1	pseudobulk	DESeq2	LRT	0.191837127533571
157	KC_AB2	zetaCOP	-0.497838698697654	0.611532027425925	1	pseudobulk	DESeq2	LRT	0.213580792969299
157	KC_AB2	zf30C	-0.750660512063168	0.681375449302725	1	pseudobulk	DESeq2	LRT	0.166613518587978
158	KC_AB2	zfh2	-0.470100723599697	0.833985291815347	1	pseudobulk	DESeq2	LRT	0.078841608522644
158	KC_AB2	Zfrp8	-1.78975280219494	0.468855431150075	1	pseudobulk	DESeq2	LRT	0.32896104882553
158	KC_AB2	Zif	-0.676838372398416	0.534310209879328	1	pseudobulk	DESeq2	LRT	0.272206527011389
158	KC_AB2	zip	-0.596512535003252	0.391354521712288	1	pseudobulk	DESeq2	LRT	0.407429643974286
158	KC_AB2	Zip102B	0.147792430327622	0.827133626950398	1	pseudobulk	DESeq2	LRT	0.082424322661281
158	KC_AB2	Zip71B	-0.729379579623002	0.823527397767669	1	pseudobulk	DESeq2	LRT	0.084321947797948
158	KC_AB2	Zip88E	-0.347135499964956	0.854806062097812	1	pseudobulk	DESeq2	LRT	0.068132406575477

158	KC_AB2	Zip99C	0.600457737989162	0.742742932528136	1	pseudobulk	DESeq2	LRT	0.12916147197617
158	KC_AB2	ZIPIC	0.145321756433347	1	1	pseudobulk	DESeq2	LRT	0
158	KC_AB2	Zir	0.0161039229410589	0.953129939856944	1	pseudobulk	DESeq2	LRT	0.020847888117196
158	KC_AB2	Ziz	0.372080743447726	0.648593568940423	1	pseudobulk	DESeq2	LRT	0.188027361841791
158	KC_AB2	Zn72D	-0.293589575580907	0.726714492578552	1	pseudobulk	DESeq2	LRT	0.13863617875798
158	KC_AB2	ZnT33D	-3.15861521307311	0.1419450179671	1	pseudobulk	DESeq2	LRT	0.847879845881112
158	KC_AB2	ZnT49B	-0.652729121726214	0.538577590539707	1	pseudobulk	DESeq2	LRT	0.26875172093456
158	KC_AB2	ZnT63C	0.318781417357197	0.67284044960858	1	pseudobulk	DESeq2	LRT	0.172087907647502
158	KC_AB2	ZnT86D	-1.6052707068109	0.52265609184397	1	pseudobulk	DESeq2	LRT	0.281783983297782
158	KC_AB2	Zpr1	-0.729379579623002	0.823527397767669	1	pseudobulk	DESeq2	LRT	0.084321947797948
158	KC_AB2	Zw10	-1.02138183124508	0.522464423422091	1	pseudobulk	DESeq2	LRT	0.281943276963334
158	KC_AB2	Zyx	-0.616558594155099	0.263449061433367	1	pseudobulk	DESeq2	LRT	0.579303344310915
158	KC_ABp1	128up	0.277282302601699	0.84216594397728	1	pseudobulk	DESeq2	LRT	0.074602324827163
158	KC_ABp1	14-3-3epsilon	0.104032500460128	0.591210009201659	1	pseudobulk	DESeq2	LRT	0.22825822193350
158	KC_ABp1	14-3-3zeta	0.0789461577200761	0.463706543279817	1	pseudobulk	DESeq2	LRT	0.333756775809461
158	KC_ABp1	140up	0.0298093333474787	0.982026648769609	1	pseudobulk	DESeq2	LRT	0.007876726819406
158	KC_ABp1	18SrRNA-Psi:CR4	-2.02056439956913	0.551734546998444	1	pseudobulk	DESeq2	LRT	0.258269821731848
158	KC_ABp1	18w	1.08728530506484	0.256972261642425	1	pseudobulk	DESeq2	LRT	0.59011375318926
158	KC_ABp1	26-29-p	0.17346351651014	0.855568448108301	1	pseudobulk	DESeq2	LRT	0.067745239838148
158	KC_ABp1	28SrRNA-Psi:CR4	-0.494292870072392	0.816493640274756	1	pseudobulk	DESeq2	LRT	0.088047193663794
158	KC_ABp1	28SrRNA-Psi:CR4	0.95393172603581	0.606576326267775	1	pseudobulk	DESeq2	LRT	0.21711454353393
158	KC_ABp1	28SrRNA-Psi:CR4	1.26892581352586	0.510463056733843	1	pseudobulk	DESeq2	LRT	0.292035683228784
158	KC_ABp1	28SrRNA-Psi:CR4	0.400640538490757	1	1	pseudobulk	DESeq2	LRT	0
158	KC_ABp1	28SrRNA-Psi:CR4	-1.51023398327283	0.845597316437363	1	pseudobulk	DESeq2	LRT	0.072836403962567
158	KC_ABp1	28SrRNA-Psi:CR4	0.400640538490757	1	1	pseudobulk	DESeq2	LRT	0
158	KC_ABp1	28SrRNA-Psi:CR4	-0.376805201627888	0.720786072498754	1	pseudobulk	DESeq2	LRT	0.142193613670746
158	KC_ABp1	28SrRNA-Psi:CR4	1.09722696841801	0.609256248199107	1	pseudobulk	DESeq2	LRT	0.215200008233546
158	KC_ABp1	2mit	0.0602828268097268	0.920047337746251	1	pseudobulk	DESeq2	LRT	0.036189827009780
158	KC_ABp1	312	0.552426144712001	0.574221677033504	1	pseudobulk	DESeq2	LRT	0.24092041678841
158	KC_ABp1	4E-T	0.205008661154925	0.754843020145014	1	pseudobulk	DESeq2	LRT	0.122143356414928
158	KC_ABp1	5-HT1A	0.0433551841014044	0.887841838285215	1	pseudobulk	DESeq2	LRT	0.051664393318003
158	KC_ABp1	5-HT1B	-0.188914166240936	0.64967108136302	1	pseudobulk	DESeq2	LRT	0.187306464439777
158	KC_ABp1	5-HT2A	0.400640538490757	1	1	pseudobulk	DESeq2	LRT	0
158	KC_ABp1	5-HT2B	0.295850504875218	1	1	pseudobulk	DESeq2	LRT	0
158	KC_ABp1	5.8SrRNA-Psi:CR4	0.295850504875218	1	1	pseudobulk	DESeq2	LRT	0
158	KC_ABp1	5.8SrRNA-Psi:CR4	1.70517510654842	0.489009985553122	1	pseudobulk	DESeq2	LRT	0.310682272519852
158	KC_ABp1	5PtaseI	-0.276358377002383	0.64717693937373	1	pseudobulk	DESeq2	LRT	0.188976966181273
158	KC_ABp1	7B2	-0.0058375714682157	0.965992199445521	1	pseudobulk	DESeq2	LRT	0.015026380573579
158	KC_ABp1	a	-0.856224073285002	0.272798158311267	1	pseudobulk	DESeq2	LRT	0.56415856597286
158	KC_ABp1	A16	0.520795866549737	0.754252938159858	1	pseudobulk	DESeq2	LRT	0.122482989374216
158	KC_ABp1	a6	1.35317872288937	0.342346541980747	1	pseudobulk	DESeq2	LRT	0.46553405455367
158	KC_ABp1	AANAT1	-0.204158342948521	0.618768258585852	1	pseudobulk	DESeq2	LRT	0.208471972727322
158	KC_ABp1	AANATL2	0.295850504875218	1	1	pseudobulk	DESeq2	LRT	0
158	KC_ABp1	Aasdh	-0.424145909178609	0.848850391788508	1	pseudobulk	DESeq2	LRT	0.071168846559503
158	KC_ABp1	Aatf	0.552926642163555	0.668161626402058	1	pseudobulk	DESeq2	LRT	0.175118470212987
158	KC_ABp1	ab	2.899005774642	0.424623374325033	1	pseudobulk	DESeq2	LRT	0.371996102819634
158	KC_ABp1	ABCA	0.230938390589738	0.81517367990452	1	pseudobulk	DESeq2	LRT	0.088749851151732
158	KC_ABp1	ABCB7	-0.0696888680386012	0.917130952938375	1	pseudobulk	DESeq2	LRT	0.037568648977660
158	KC_ABp1	ABCD	0.0450908853469764	0.882827030883596	1	pseudobulk	DESeq2	LRT	0.054124377839490
158	KC_ABp1	Abi	0.583042795119855	0.513907719240122	1	pseudobulk	DESeq2	LRT	0.289114858870166
158	KC_ABp1	Abl	-0.0333880955362762	0.895372942025896	1	pseudobulk	DESeq2	LRT	0.047996034038346
158	KC_ABp1	abo	1.513267077797	0.518264337008286	1	pseudobulk	DESeq2	LRT	0.285448674960176

158	KC_ABp1	Abp1	0.256004724799166	0.734645188787414	1	pseudobulk	DESeq2	LRT	0.133922361265131
158	KC_ABp1	abs	-1.04646382435696	0.518862574767641	1	pseudobulk	DESeq2	LRT	0.284947653564204
158	KC_ABp1	Ac3	-0.126471524074165	0.822307995577393	1	pseudobulk	DESeq2	LRT	0.084965486928897
158	KC_ABp1	Ac76E	2.36777936152995	0.109467096125376	1	pseudobulk	DESeq2	LRT	0.960716402455131
158	KC_ABp1	Ac78C	0.687353794178817	0.540711126375215	1	pseudobulk	DESeq2	LRT	0.267034693724462
158	KC_ABp1	Acbp1	0.88649152374539	0.244637075729983	1	pseudobulk	DESeq2	LRT	0.611477723241117
158	KC_ABp1	Acbp2	0.400640538490757	1	1	pseudobulk	DESeq2	LRT	0
158	KC_ABp1	ACC	-0.143534887790493	0.779089970803629	1	pseudobulk	DESeq2	LRT	0.108412386271849
158	KC_ABp1	AcCoAS	0.537876735024818	0.184228399526804	1	pseudobulk	DESeq2	LRT	0.734643420789104
158	KC_ABp1	Ace	0.350282104067057	0.319771681225935	1	pseudobulk	DESeq2	LRT	0.495159999726359
158	KC_ABp1	Acer	0.556897978800036	0.75025082400973	1	pseudobulk	DESeq2	LRT	0.124793518911864
158	KC_ABp1	Acf	1.11835354977117	0.254816418472305	1	pseudobulk	DESeq2	LRT	0.59377259273307
158	KC_ABp1	achi	0.681929511787139	0.697230806723761	1	pseudobulk	DESeq2	LRT	0.15662343209768
158	KC_ABp1	Ack	2.13370700280468	0.252757669629696	1	pseudobulk	DESeq2	LRT	0.597295657389508
158	KC_ABp1	Ack-like	-0.179527658219132	0.772271081146557	1	pseudobulk	DESeq2	LRT	0.112230227667936
158	KC_ABp1	Acn	0.222153360479004	0.585208562708284	1	pseudobulk	DESeq2	LRT	0.232689327950432
158	KC_ABp1	Acp36DE	0.295850504875218	1	1	pseudobulk	DESeq2	LRT	0
158	KC_ABp1	Acp53C14b	-1.75348438488736	0.474001036022703	1	pseudobulk	DESeq2	LRT	0.324220709088676
158	KC_ABp1	Acph-1	2.38016512652036	0.267714971078639	1	pseudobulk	DESeq2	LRT	0.572327341644445
158	KC_ABp1	Acsl	0.449925228201482	0.518309071310083	1	pseudobulk	DESeq2	LRT	0.285411190185286
158	KC_ABp1	Act42A	1.17479427547106	0.524941894231034	1	pseudobulk	DESeq2	LRT	0.279888765949106
158	KC_ABp1	Act5C	0.412000511018821	0.213922547217213	1	pseudobulk	DESeq2	LRT	0.669743438822771
158	KC_ABp1	Actbeta	0.199935580341207	0.675011478450824	1	pseudobulk	DESeq2	LRT	0.170688842005318
158	KC_ABp1	Actn	0.959253924802099	0.527386370400359	1	pseudobulk	DESeq2	LRT	0.277871098161093
158	KC_ABp1	ACXB	-1.76541829495743	0.569721440048943	1	pseudobulk	DESeq2	LRT	0.244337436643475
158	KC_ABp1	ACXC	0.26083682599798	0.874826757055689	1	pseudobulk	DESeq2	LRT	0.058077942296578
158	KC_ABp1	Acyp2	-0.297398843922861	0.751317901652296	1	pseudobulk	DESeq2	LRT	0.124176263101962
158	KC_ABp1	Ada1-1	-1.51023398327283	0.845597316437363	1	pseudobulk	DESeq2	LRT	0.072836403962567
158	KC_ABp1	Ada1-2	1.41919504844869	0.32728061787373	1	pseudobulk	DESeq2	LRT	0.485079713603492
158	KC_ABp1	Ada2a	-2.02056439956913	0.551734546998444	1	pseudobulk	DESeq2	LRT	0.258269821731848
158	KC_ABp1	Ada2b	1.23831260238656	0.341430267307204	1	pseudobulk	DESeq2	LRT	0.466697981927073
158	KC_ABp1	Ada3	-1.45346649494553	0.305499121845656	1	pseudobulk	DESeq2	LRT	0.514990033795025
158	KC_ABp1	Adar	-0.302078920744646	0.495427151236096	1	pseudobulk	DESeq2	LRT	0.305020196157742
158	KC_ABp1	Adat1	-0.584613676872836	1	1	pseudobulk	DESeq2	LRT	0
158	KC_ABp1	Adck1	-2.3250534469118	0.266822310336738	1	pseudobulk	DESeq2	LRT	0.573777859721606
158	KC_ABp1	Adck5	-2.02056439956913	0.551734546998444	1	pseudobulk	DESeq2	LRT	0.258269821731848
158	KC_ABp1	ADD1	0.879063852013378	0.363491709848968	1	pseudobulk	DESeq2	LRT	0.439505489640992
158	KC_ABp1	AdenoK	0.176146554950191	0.891554164080225	1	pseudobulk	DESeq2	LRT	0.049852267242861
158	KC_ABp1	Adf1	0.704360865925517	0.350238931396257	1	pseudobulk	DESeq2	LRT	0.455635580837293
158	KC_ABp1	Adi1	-0.490084820079513	0.746297752331536	1	pseudobulk	DESeq2	LRT	0.127087866367654
158	KC_ABp1	AdipoR	0.118000557366101	0.83805857557995	1	pseudobulk	DESeq2	LRT	0.076725625567691
159	KC_ABp1	Adk1	-0.250559355658094	0.748894708824858	1	pseudobulk	DESeq2	LRT	0.125579237828887
159	KC_ABp1	Adk2	-0.195946954757814	0.629414158974991	1	pseudobulk	DESeq2	LRT	0.201063491649136
159	KC_ABp1	Adk3	2.4905005807226	0.24044178561213	1	pseudobulk	DESeq2	LRT	0.618990055455479
159	KC_ABp1	adp	-0.489049563805578	0.723593219264098	1	pseudobulk	DESeq2	LRT	0.140505511533041
159	KC_ABp1	ADPS	0.263794543718869	0.802545697738879	1	pseudobulk	DESeq2	LRT	0.095530229065412
159	KC_ABp1	AdSL	0.168166623510894	0.891099449136215	1	pseudobulk	DESeq2	LRT	0.050073824806082
159	KC_ABp1	AdSS	0.77148647025502	0.267587309504367	1	pseudobulk	DESeq2	LRT	0.572534487103448
159	KC_ABp1	Aduk	0.442082711764194	0.621848618402979	1	pseudobulk	DESeq2	LRT	0.206315326231877
159	KC_ABp1	Ady43A	-1.22385493799666	0.188627972918954	1	pseudobulk	DESeq2	LRT	0.724393902354209
159	KC_ABp1	Aef1	-0.0166842050612612	0.924300362445913	1	pseudobulk	DESeq2	LRT	0.034186876663462
159	KC_ABp1	Afti	0.116482670827954	0.88449007324537	1	pseudobulk	DESeq2	LRT	0.053307036874044

159	KC_ABp1	Ag5r	1.10067831374717	0.673764308950964	1	pseudobulk	DESeq2	LRT	0.171491998454619
159	KC_ABp1	ago	2.16402579552821	0.0793445251240166	1	pseudobulk	DESeq2	LRT	1.10048303476243
159	KC_ABp1	AGO1	-0.061736345092635	0.745090666400579	1	pseudobulk	DESeq2	LRT	0.127790876887093
159	KC_ABp1	AGO2	0.697019843712919	0.352936623070822	1	pseudobulk	DESeq2	LRT	0.452303273993885
159	KC_ABp1	AGO3	-0.486167498126476	1	1	pseudobulk	DESeq2	LRT	0
159	KC_ABp1	Agpat1	0.698637644803432	0.487118697600964	1	pseudobulk	DESeq2	LRT	0.312365200116021
159	KC_ABp1	Agpat3	0.632416744040433	0.358121642319785	1	pseudobulk	DESeq2	LRT	0.445969432531677
159	KC_ABp1	agt	1.22695834703904	0.565162108618297	1	pseudobulk	DESeq2	LRT	0.247826963188697
159	KC_ABp1	Ahcy	0.310645321882168	0.851835655473561	1	pseudobulk	DESeq2	LRT	0.069644185521563
159	KC_ABp1	AhcyL1	0.643729162717006	0.34904015463423	1	pseudobulk	DESeq2	LRT	0.457124607616991
159	KC_ABp1	AIF	1.3610129170307	0.437165011316831	1	pseudobulk	DESeq2	LRT	0.359354604270592
159	KC_ABp1	AIMP1	-0.0337974374901381	0.843703883925745	1	pseudobulk	DESeq2	LRT	0.073809951647046
159	KC_ABp1	AIMP2	-0.992191975287121	0.438501445665082	1	pseudobulk	DESeq2	LRT	0.358028970500146
159	KC_ABp1	AIMP3	0.112481306663173	0.828711239050545	1	pseudobulk	DESeq2	LRT	0.081596771178582
159	KC_ABp1	Ak6	1.52058571881041	0.434338725773257	1	pseudobulk	DESeq2	LRT	0.362171447082983
159	KC_ABp1	Akap200	-0.163972597134025	0.801292495769687	1	pseudobulk	DESeq2	LRT	0.096208924476082
159	KC_ABp1	akirin	0.378699192788453	0.413228556024386	1	pseudobulk	DESeq2	LRT	0.383809674337079
159	KC_ABp1	Akt1	0.353795216598118	0.582971719134343	1	pseudobulk	DESeq2	LRT	0.234352513031395
159	KC_ABp1	Aladin	-2.02056439956913	0.551734546998444	1	pseudobulk	DESeq2	LRT	0.258269821731848
159	KC_ABp1	AlaRS	1.10625008795045	0.461042912157403	1	pseudobulk	DESeq2	LRT	0.336258650215924
159	KC_ABp1	AlaRS-m	0.0511840554088066	0.918039339430788	1	pseudobulk	DESeq2	LRT	0.037138708197831
159	KC_ABp1	Alas	0.106708821471325	0.850122619937685	1	pseudobulk	DESeq2	LRT	0.070518428025048
159	KC_ABp1	alc	0.230215066644773	0.550638978734422	1	pseudobulk	DESeq2	LRT	0.259133048935974
159	KC_ABp1	Ald1	0.304717543547641	0.332909996259681	1	pseudobulk	DESeq2	LRT	0.477673164124678
159	KC_ABp1	Aldh	-3.02990869154163	0.208852371682276	1	pseudobulk	DESeq2	LRT	0.680160588616829
159	KC_ABp1	Aldh-III	-0.117921736493977	0.831858287647939	1	pseudobulk	DESeq2	LRT	0.079950652236321
159	KC_ABp1	Alg-2	0.0109961275558603	0.861535584336645	1	pseudobulk	DESeq2	LRT	0.064726780012946
159	KC_ABp1	Alg1	0.17340092779666	0.852683850928201	1	pseudobulk	DESeq2	LRT	0.069211962089039
159	KC_ABp1	Alg10	0.400640538490757	0.854745824291873	1	pseudobulk	DESeq2	LRT	0.068163012194342
159	KC_ABp1	Alg11	0.346626284139021	0.681424989214594	1	pseudobulk	DESeq2	LRT	0.16658194403053
159	KC_ABp1	Alg14	-0.712425324840931	0.73711055633684	1	pseudobulk	DESeq2	LRT	0.132467369120416
159	KC_ABp1	Alg2	0.282505275384056	0.805898883419826	1	pseudobulk	DESeq2	LRT	0.093719445946353
159	KC_ABp1	Alg3	0.390229982907281	0.811617608001738	1	pseudobulk	DESeq2	LRT	0.090648539540317
159	KC_ABp1	Alg9	2.41120908737014	0.259039902194963	1	pseudobulk	DESeq2	LRT	0.586633332566133
159	KC_ABp1	Alh	0.951988984751734	0.192399182661688	1	pseudobulk	DESeq2	LRT	0.715796777237254
159	KC_ABp1	alien	-2.12850741312116	0.149048043267186	1	pseudobulk	DESeq2	LRT	0.826673721099594
159	KC_ABp1	ALiX	0.601243684215429	0.639606154736513	1	pseudobulk	DESeq2	LRT	0.194087365821293
159	KC_ABp1	Alk	0.268878964169793	0.32609038273372	1	pseudobulk	DESeq2	LRT	0.486662009495997
159	KC_ABp1	AlkB	-0.491049632507942	1	1	pseudobulk	DESeq2	LRT	0
159	KC_ABp1	alph	0.140969200172928	0.702067924002945	1	pseudobulk	DESeq2	LRT	0.153620868507475
159	KC_ABp1	alpha-Cat	0.534618679731713	0.142595070752774	1	pseudobulk	DESeq2	LRT	0.845895486979739
159	KC_ABp1	alpha-Catr	-0.452362176342501	0.556487839877808	1	pseudobulk	DESeq2	LRT	0.254544321233436
159	KC_ABp1	alpha-Est8	-2.49285270663435	0.286521467899626	1	pseudobulk	DESeq2	LRT	0.542842832541076
159	KC_ABp1	alpha-Est9	-0.54908135541674	0.561425237202675	1	pseudobulk	DESeq2	LRT	0.250708068845307
159	KC_ABp1	alpha-Man-Ia	-0.331002655735878	0.538776562401722	1	pseudobulk	DESeq2	LRT	0.26859130500732
159	KC_ABp1	alpha-Man-Ib	0.503149738844203	0.570897328291665	1	pseudobulk	DESeq2	LRT	0.243441989417388
159	KC_ABp1	alpha-Man-IIa	1.87090255016732	0.304302950848324	1	pseudobulk	DESeq2	LRT	0.516693836235555
159	KC_ABp1	alpha-Man-IIb	0.595136751250954	0.449126630199127	1	pseudobulk	DESeq2	LRT	0.347631193421693
159	KC_ABp1	alpha-PheRS	-1.15716734203882	0.462840537597807	1	pseudobulk	DESeq2	LRT	0.334568610645002
159	KC_ABp1	alpha-Spec	-0.0207562884961364	0.957714496915406	1	pseudobulk	DESeq2	LRT	0.018763938618722
159	KC_ABp1	alpha4GT1	-0.0449326693479714	0.957530249396123	1	pseudobulk	DESeq2	LRT	0.018847497319332
159	KC_ABp1	alpha4GT2	-1.75283054089783	0.460938311409796	1	pseudobulk	DESeq2	LRT	0.33635719349755

159	KC_ABp1	alphaCOP	-0.171961245091816	0.912349345029262	1	pseudobulk	DESeq2	LRT	0.039838835391196
159	KC_ABp1	alphaSnap	-0.0396559579110561	0.855230233777561	1	pseudobulk	DESeq2	LRT	0.067916954512714
159	KC_ABp1	alphaTub67C	-0.396771373099634	0.882552835457681	1	pseudobulk	DESeq2	LRT	0.054259285417928
159	KC_ABp1	alphaTub84B	0.0180973604152746	0.870727391272466	1	pseudobulk	DESeq2	LRT	0.060117793324603
159	KC_ABp1	alphaTub84D	-0.0004882579510724	0.922697620926775	1	pseudobulk	DESeq2	LRT	0.034940599167392
159	KC_ABp1	Alr	-0.549751953743039	0.688453576388781	1	pseudobulk	DESeq2	LRT	0.162125339645155
159	KC_ABp1	Als2	0.0544256311668363	0.811581205473894	1	pseudobulk	DESeq2	LRT	0.090668018875616
159	KC_ABp1	alt	0.341033597722911	0.474088765203769	1	pseudobulk	DESeq2	LRT	0.324140336323779
159	KC_ABp1	Amacr	1.74233076384957	0.250563393645961	1	pseudobulk	DESeq2	LRT	0.601082377471234
159	KC_ABp1	amn	0.400640538490757	1	1	pseudobulk	DESeq2	LRT	0
159	KC_ABp1	amon	-0.216017956243059	0.423926361834973	1	pseudobulk	DESeq2	LRT	0.372709576016156
159	KC_ABp1	AMPdeam	0.0989311495547667	0.904791831573332	1	pseudobulk	DESeq2	LRT	0.043451328859171
159	KC_ABp1	Amph	-0.206118487920803	0.823504870081317	1	pseudobulk	DESeq2	LRT	0.084333828135771
159	KC_ABp1	AMPKalpha	0.961206997714992	0.281501522105059	1	pseudobulk	DESeq2	LRT	0.550519252535402
159	KC_ABp1	Amun	0.552844611129506	0.248204165842545	1	pseudobulk	DESeq2	LRT	0.605190933605835
159	KC_ABp1	amx	-1.39634963098245	0.413707427525001	1	pseudobulk	DESeq2	LRT	0.383306681898384
159	KC_ABp1	anchor	-0.652173959732705	0.53771330317156	1	pseudobulk	DESeq2	LRT	0.269449218827825
159	KC_ABp1	angel	0.94181418288183	0.680159143588432	1	pseudobulk	DESeq2	LRT	0.167389459211597
159	KC_ABp1	Ank	0.0139444120631743	0.970722303559873	1	pseudobulk	DESeq2	LRT	0.012904991801961
159	KC_ABp1	Ank2	0.163292457073504	0.36412845395037	1	pseudobulk	DESeq2	LRT	0.438745382832598
159	KC_ABp1	Ankle2	0.636947695904133	0.608068418228737	1	pseudobulk	DESeq2	LRT	0.21604755232679
159	KC_ABp1	anne	0.224780479897953	0.470191667872315	1	pseudobulk	DESeq2	LRT	0.327725071147145
159	KC_ABp1	Ant2	-1.88188546139885	0.432736380884179	1	pseudobulk	DESeq2	LRT	0.363776591389998
159	KC_ABp1	AnxB10	-0.0178761201817129	0.883221225208233	1	pseudobulk	DESeq2	LRT	0.053930502703587
159	KC_ABp1	AnxB11	-0.312241530995323	0.806743512153785	1	pseudobulk	DESeq2	LRT	0.093264518514252
159	KC_ABp1	aop	1.49013548325744	0.173029043166081	1	pseudobulk	DESeq2	LRT	0.761880993818442
159	KC_ABp1	aos	0.400640538490757	0.854745824291873	1	pseudobulk	DESeq2	LRT	0.068163012194342
159	KC_ABp1	Aos1	0.434270212980653	0.702471848628168	1	pseudobulk	DESeq2	LRT	0.153371075309599
159	KC_ABp1	ap	1.70517510654842	0.489009985553122	1	pseudobulk	DESeq2	LRT	0.310682272519852
159	KC_ABp1	AP-1-2beta	-0.554590056238662	0.392029506712844	1	pseudobulk	DESeq2	LRT	0.406681243897145
159	KC_ABp1	AP-1gamma	0.0760179313253362	0.78780257113515	1	pseudobulk	DESeq2	LRT	0.10358260612654
159	KC_ABp1	AP-1mu	0.833748024908182	0.424123935725137	1	pseudobulk	DESeq2	LRT	0.372507217141769
159	KC_ABp1	AP-1sigma	-0.394045537162599	0.409095502679045	1	pseudobulk	DESeq2	LRT	0.388175294816977
159	KC_ABp1	AP-2alpha	0.0349326754679166	0.799272580758802	1	pseudobulk	DESeq2	LRT	0.097305085352907
159	KC_ABp1	AP-2mu	-0.514788588107424	0.306400978292357	1	pseudobulk	DESeq2	LRT	0.513709852400093
159	KC_ABp1	AP-2sigma	0.151768526759864	0.727328947064655	1	pseudobulk	DESeq2	LRT	0.13826912753887
159	KC_ABp1	Apc	0.199632574521987	0.718932836127104	1	pseudobulk	DESeq2	LRT	0.143311680217733
160	KC_ABp1	APC10	0.321242995198308	0.848133343462872	1	pseudobulk	DESeq2	LRT	0.071535862628244
160	KC_ABp1	APC4	0.400640538490757	1	1	pseudobulk	DESeq2	LRT	0
160	KC_ABp1	ApepP	0.0293119929085274	0.983753047576729	1	pseudobulk	DESeq2	LRT	0.007113909226316
160	KC_ABp1	Apf	0.404617347656451	0.778279448786586	1	pseudobulk	DESeq2	LRT	0.108864437361098
160	KC_ABp1	aph-1	0.247026724636619	0.777792469637108	1	pseudobulk	DESeq2	LRT	0.109136265882416
160	KC_ABp1	aPKC	-0.193414072044621	0.621175031349788	1	pseudobulk	DESeq2	LRT	0.20678600942278
160	KC_ABp1	Apl	-1.06576904479449	0.38285388916824	1	pseudobulk	DESeq2	LRT	0.416966936827253
160	KC_ABp1	Aplip1	-0.164747574275083	0.681875396445762	1	pseudobulk	DESeq2	LRT	0.166294979566528
160	KC_ABp1	apolpp	1.12865749489541	0.576186843684875	1	pseudobulk	DESeq2	LRT	0.239436662369893
160	KC_ABp1	Apoltp	-0.0136309118093082	0.868458639018127	1	pseudobulk	DESeq2	LRT	0.061250860318705
160	KC_ABp1	app	-0.0733036136209092	0.806763829681236	1	pseudobulk	DESeq2	LRT	0.093253581111206
160	KC_ABp1	APP-BP1	0.400640538490757	0.854745824291873	1	pseudobulk	DESeq2	LRT	0.068163012194342
160	KC_ABp1	Appl	0.286855211501264	0.0330748159358078	1	pseudobulk	DESeq2	LRT	1.48050256401984
160	KC_ABp1	Aprt	0.819316850599535	0.717765361506106	1	pseudobulk	DESeq2	LRT	0.144017504026936
160	KC_ABp1	Aps	0.613284097049777	0.299203181741632	1	pseudobulk	DESeq2	LRT	0.524033792473731

160	KC_ABp1	aqz	0.380940332546477	0.00307132119362628	1	pseudobulk	DESeq2	LRT	2.51267476339239
160	KC_ABp1	aralar1	-0.555220556745873	0.516447620293847	1	pseudobulk	DESeq2	LRT	0.286973719392965
160	KC_ABp1	Arc42	0.400640538490757	0.854745824291873	1	pseudobulk	DESeq2	LRT	0.068163012194342
160	KC_ABp1	Archease	1.63485744373813	0.176164528278611	1	pseudobulk	DESeq2	LRT	0.754081534764595
160	KC_ABp1	Arf102F	0.015406171879071	0.886732730156321	1	pseudobulk	DESeq2	LRT	0.052207261013165
160	KC_ABp1	Arf51F	0.427089730287784	0.536362253272214	1	pseudobulk	DESeq2	LRT	0.270541793408829
160	KC_ABp1	Arf79F	-0.0505361436053392	0.86936639828222	1	pseudobulk	DESeq2	LRT	0.060797149655449
160	KC_ABp1	ArfGAP1	-0.424598274202498	0.705291321416626	1	pseudobulk	DESeq2	LRT	0.151631460099903
160	KC_ABp1	ArfGAP3	0.896428307036196	0.450282782771196	1	pseudobulk	DESeq2	LRT	0.346514658612051
160	KC_ABp1	Arfip	0.18921358776129	0.830734417549977	1	pseudobulk	DESeq2	LRT	0.080537796225030
160	KC_ABp1	Arfrp1	-0.368074841405707	0.837314367022967	1	pseudobulk	DESeq2	LRT	0.077111456904371
160	KC_ABp1	arg	-0.553969417757635	0.70443643139102	1	pseudobulk	DESeq2	LRT	0.152158191684107
160	KC_ABp1	Argk	-0.176996415605483	0.321182188626705	1	pseudobulk	DESeq2	LRT	0.493248546816827
160	KC_ABp1	Argl	-1.76541829495743	0.569721440048943	1	pseudobulk	DESeq2	LRT	0.244337436643475
160	KC_ABp1	ArgRS	-0.526968065172491	0.756230440008884	1	pseudobulk	DESeq2	LRT	0.121345845273116
160	KC_ABp1	ArgRS-m	0.797330470441812	0.681869674788157	1	pseudobulk	DESeq2	LRT	0.166298623773026
160	KC_ABp1	ari-1	0.334137591664494	0.486073729362462	1	pseudobulk	DESeq2	LRT	0.313297850435903
160	KC_ABp1	ari-2	-0.244289104852165	0.805406952020662	1	pseudobulk	DESeq2	LRT	0.093984626018404
160	KC_ABp1	Arl1	0.661630740340469	0.451822029667914	1	pseudobulk	DESeq2	LRT	0.345032597829181
160	KC_ABp1	Arl2	-0.180213397845471	0.886533749698872	1	pseudobulk	DESeq2	LRT	0.052304726471426
160	KC_ABp1	Arl4	-0.0687220009053363	0.84364644589205	1	pseudobulk	DESeq2	LRT	0.0738395187389
160	KC_ABp1	Arl5	-0.422122734005519	0.577256285526574	1	pseudobulk	DESeq2	LRT	0.238631329515775
160	KC_ABp1	Arl6IP1	0.0368475554548096	0.91332307791946	1	pseudobulk	DESeq2	LRT	0.039375568426275
160	KC_ABp1	Arl8	0.360869360843664	0.483304902541928	1	pseudobulk	DESeq2	LRT	0.315778799448109
160	KC_ABp1	arm	-0.396072782390263	0.241157460683032	1	pseudobulk	DESeq2	LRT	0.617699297775474
160	KC_ABp1	armi	0.842405823124912	0.788653372765757	1	pseudobulk	DESeq2	LRT	0.103113835031894
160	KC_ABp1	Arp1	-0.167870230539697	0.73425626554817	1	pseudobulk	DESeq2	LRT	0.134152338872051
160	KC_ABp1	Arp10	0.556384509334529	0.759891754990402	1	pseudobulk	DESeq2	LRT	0.119248267664546
160	KC_ABp1	Arp2	0.600712608208013	0.405753490356055	1	pseudobulk	DESeq2	LRT	0.391737735615459
160	KC_ABp1	Arp3	0.404640832633775	0.69770752012192	1	pseudobulk	DESeq2	LRT	0.156326596026371
160	KC_ABp1	Arp5	-0.0921379055312957	0.960162781311171	1	pseudobulk	DESeq2	LRT	0.017655132551874
160	KC_ABp1	Arp6	-0.406087015976841	0.699836183773297	1	pseudobulk	DESeq2	LRT	0.155003606856217
160	KC_ABp1	Arp8	1.10112221034529	0.542062627808734	1	pseudobulk	DESeq2	LRT	0.265950533866672
160	KC_ABp1	Arpc1	0.111032943574107	0.841955369340952	1	pseudobulk	DESeq2	LRT	0.074710929121093
160	KC_ABp1	Arpc2	0.408358020554887	0.644713358338084	1	pseudobulk	DESeq2	LRT	0.190633331196659
160	KC_ABp1	Arpc3A	1.14036890754671	0.465092516812651	1	pseudobulk	DESeq2	LRT	0.332460648089253
160	KC_ABp1	Arpc4	0.286540483013863	0.684437835943718	1	pseudobulk	DESeq2	LRT	0.164665990528681
160	KC_ABp1	Arpc5	-0.102077525893489	0.878817507130196	1	pseudobulk	DESeq2	LRT	0.056101299982860
160	KC_ABp1	arr	0.111781491714045	0.839119402603051	1	pseudobulk	DESeq2	LRT	0.076176236785084
160	KC_ABp1	Ars2	0.0156150062717828	0.974428053542309	1	pseudobulk	DESeq2	LRT	0.011250221300649
160	KC_ABp1	Art1	0.306062464724015	0.802729431752884	1	pseudobulk	DESeq2	LRT	0.095430813498195
160	KC_ABp1	Art3	-1.51023398327283	0.845597316437363	1	pseudobulk	DESeq2	LRT	0.072836403962567
160	KC_ABp1	Art4	-0.0588597866621421	0.956570013315577	1	pseudobulk	DESeq2	LRT	0.019283237572038
160	KC_ABp1	Art7	-2.02056439956913	0.551734546998444	1	pseudobulk	DESeq2	LRT	0.258269821731848
160	KC_ABp1	Arts	0.82060646502571	0.593653231055307	1	pseudobulk	DESeq2	LRT	0.226467164135537
160	KC_ABp1	aru	0.400640538490757	0.854745824291873	1	pseudobulk	DESeq2	LRT	0.068163012194342
160	KC_ABp1	Arv1	-0.314309832215102	0.767606447226456	1	pseudobulk	DESeq2	LRT	0.11486138622537
160	KC_ABp1	Asap	0.505621073102498	0.526134950512292	1	pseudobulk	DESeq2	LRT	0.27890284758421
160	KC_ABp1	Asator	0.250341748892195	0.422022712138098	1	pseudobulk	DESeq2	LRT	0.374664175837324
160	KC_ABp1	Asciz	1.92075549174513	0.318594324504082	1	pseudobulk	DESeq2	LRT	0.496761965065062
160	KC_ABp1	asf1	0.758967101654168	0.504817836628077	1	pseudobulk	DESeq2	LRT	0.296865308652397
160	KC_ABp1	ash1	-0.30040205814783	0.702021603325847	1	pseudobulk	DESeq2	LRT	0.153649523111156

160	KC_ABp1	ash2	-0.811113612176247	0.72211469299442	1	pseudobulk	DESeq2	LRT	0.141393818249345
160	KC_ABp1	Ask1	3.50427182182601	0.0561226829004679	1	pseudobulk	DESeq2	LRT	1.25086157604321
160	KC_ABp1	asl	0.400640538490757	0.854745824291873	1	pseudobulk	DESeq2	LRT	0.068163012194342
160	KC_ABp1	AsnRS	1.27054412955232	0.355012072397935	1	pseudobulk	DESeq2	LRT	0.449756878250088
160	KC_ABp1	AsnRS-m	-1.75348438488736	0.474001036022703	1	pseudobulk	DESeq2	LRT	0.324220709088676
160	KC_ABp1	AsnS	-2.02056439956913	0.551734546998444	1	pseudobulk	DESeq2	LRT	0.258269821731848
160	KC_ABp1	Asph	0.400640538490757	1	1	pseudobulk	DESeq2	LRT	0
160	KC_ABp1	AspRS	-0.879785807477258	0.688402928617533	1	pseudobulk	DESeq2	LRT	0.16215729075626
160	KC_ABp1	AspRS-m	2.95160621540517	0.125757797906737	1	pseudobulk	DESeq2	LRT	0.900465075990783
160	KC_ABp1	asrij	1.8248252244751	0.11592369672104	1	pseudobulk	DESeq2	LRT	0.935827777985899
160	KC_ABp1	asRNA:ASTR	0.295850504875218	1	1	pseudobulk	DESeq2	LRT	0
160	KC_ABp1	asRNA:CR17025	0.400640538490757	0.854745824291873	1	pseudobulk	DESeq2	LRT	0.068163012194342
160	KC_ABp1	asRNA:CR30267	0.400640538490757	1	1	pseudobulk	DESeq2	LRT	0
160	KC_ABp1	asRNA:CR32252	0.295850504875218	1	1	pseudobulk	DESeq2	LRT	0
160	KC_ABp1	asRNA:CR42254	0.781489261617598	0.516976259513429	1	pseudobulk	DESeq2	LRT	0.286529400037677
160	KC_ABp1	asRNA:CR42547	-0.106417528703525	0.935972058462851	1	pseudobulk	DESeq2	LRT	0.028737116044510
160	KC_ABp1	asRNA:CR43425	1.62719822818589	0.116799443280119	1	pseudobulk	DESeq2	LRT	0.932559227265988
160	KC_ABp1	asRNA:CR43426	0.634020948563258	0.724023316408978	1	pseudobulk	DESeq2	LRT	0.140247447580887
160	KC_ABp1	asRNA:CR43629	0.996463889987528	0.561881797344915	1	pseudobulk	DESeq2	LRT	0.25035503702898
160	KC_ABp1	asRNA:CR43637	1.10067831374717	0.673764308950964	1	pseudobulk	DESeq2	LRT	0.171491998454619
160	KC_ABp1	asRNA:CR43683	-2.84770290365933	0.223489999778285	1	pseudobulk	DESeq2	LRT	0.650741904918056
160	KC_ABp1	asRNA:CR43730	0.842405823124912	0.788653372765757	1	pseudobulk	DESeq2	LRT	0.103113835031894
160	KC_ABp1	asRNA:CR43767	1.90090186132448	0.432852416067745	1	pseudobulk	DESeq2	LRT	0.363660154022951
160	KC_ABp1	asRNA:CR43786	-0.817002774795447	0.779755878997874	1	pseudobulk	DESeq2	LRT	0.108041342179397
160	KC_ABp1	asRNA:CR43806	-1.76541829495743	0.569721440048943	1	pseudobulk	DESeq2	LRT	0.244337436643475
160	KC_ABp1	asRNA:CR43809	1.10067831374717	0.673764308950964	1	pseudobulk	DESeq2	LRT	0.171491998454619
160	KC_ABp1	asRNA:CR43889	-1.75283054089783	0.460938311409796	1	pseudobulk	DESeq2	LRT	0.33635719349755
160	KC_ABp1	asRNA:CR43906	-0.711951099345206	0.678136671327284	1	pseudobulk	DESeq2	LRT	0.168682769817859
160	KC_ABp1	asRNA:CR43933	0.842405823124912	0.788653372765757	1	pseudobulk	DESeq2	LRT	0.103113835031894
160	KC_ABp1	asRNA:CR43936	0.400640538490757	1	1	pseudobulk	DESeq2	LRT	0
160	KC_ABp1	asRNA:CR43944	2.00922679421407	0.369170119796718	1	pseudobulk	DESeq2	LRT	0.432773457517613
160	KC_ABp1	asRNA:CR43959	0.127581302198757	0.891926597962237	1	pseudobulk	DESeq2	LRT	0.049670884874600
160	KC_ABp1	asRNA:CR44018	-0.274923196050815	0.74510142487397	1	pseudobulk	DESeq2	LRT	0.127784606090657
161	KC_ABp1	asRNA:CR44027	0.451114445327168	0.685178155856128	1	pseudobulk	DESeq2	LRT	0.164196491214945
161	KC_ABp1	asRNA:CR44029	0.390699849529891	0.662597276128123	1	pseudobulk	DESeq2	LRT	0.178750353792057
161	KC_ABp1	asRNA:CR44030	0.833519884218563	0.484299543706894	1	pseudobulk	DESeq2	LRT	0.314885940133042
161	KC_ABp1	asRNA:CR44031	-1.51023398327283	0.845597316437363	1	pseudobulk	DESeq2	LRT	0.072836403962567
161	KC_ABp1	asRNA:CR44046	1.97255813851701	0.407506488865353	1	pseudobulk	DESeq2	LRT	0.389865471449207
161	KC_ABp1	asRNA:CR44053	-1.75283054089783	0.460938311409796	1	pseudobulk	DESeq2	LRT	0.33635719349755
161	KC_ABp1	asRNA:CR44093	-1.70112733289603	0.411943372629136	1	pseudobulk	DESeq2	LRT	0.385162479707002
161	KC_ABp1	asRNA:CR44106	0.842405823124912	0.788653372765757	1	pseudobulk	DESeq2	LRT	0.103113835031894
161	KC_ABp1	asRNA:CR44119	-0.0295035337095442	1	1	pseudobulk	DESeq2	LRT	0
161	KC_ABp1	asRNA:CR44179	0.400640538490757	0.854745824291873	1	pseudobulk	DESeq2	LRT	0.068163012194342
161	KC_ABp1	asRNA:CR44840	0.400640538490757	1	1	pseudobulk	DESeq2	LRT	0
161	KC_ABp1	asRNA:CR44892	2.24629070315823	0.194509500856497	1	pseudobulk	DESeq2	LRT	0.711059180617057
161	KC_ABp1	asRNA:CR44894	0.22497700646999	0.923707879872993	1	pseudobulk	DESeq2	LRT	0.034465351526254
161	KC_ABp1	asRNA:CR44960	-2.42721364822641	0.302598871124693	1	pseudobulk	DESeq2	LRT	0.519132696488745
161	KC_ABp1	asRNA:CR44961	0.842405823124912	0.788653372765757	1	pseudobulk	DESeq2	LRT	0.103113835031894
161	KC_ABp1	asRNA:CR44981	1.10067831374717	0.673764308950964	1	pseudobulk	DESeq2	LRT	0.171491998454619
161	KC_ABp1	asRNA:CR44987	0.326162780164558	0.850310530222794	1	pseudobulk	DESeq2	LRT	0.070422442598934
161	KC_ABp1	asRNA:CR45000	-2.42721364822641	0.302598871124693	1	pseudobulk	DESeq2	LRT	0.519132696488745
161	KC_ABp1	asRNA:CR45007	-1.76541829495743	0.569721440048943	1	pseudobulk	DESeq2	LRT	0.244337436643475

161	KC_ABp1	asRNA:CR45009	-0.618022582288293	0.64698934980052	1	pseudobulk	DESeq2	LRT	0.189102868266283
161	KC_ABp1	asRNA:CR45121	0.295850504875218	1	1	pseudobulk	DESeq2	LRT	0
161	KC_ABp1	asRNA:CR45126	0.410426615507629	0.81538411933238	1	pseudobulk	DESeq2	LRT	0.0886377512521211
161	KC_ABp1	asRNA:CR45129	1.71856490340698	0.298956778810029	1	pseudobulk	DESeq2	LRT	0.52439159455555
161	KC_ABp1	asRNA:CR45134	-0.528897106516391	0.714320187392245	1	pseudobulk	DESeq2	LRT	0.146107076112097
161	KC_ABp1	asRNA:CR45135	0.295850504875218	1	1	pseudobulk	DESeq2	LRT	0
161	KC_ABp1	asRNA:CR45139	-2.39593946583853	0.39950212853761	1	pseudobulk	DESeq2	LRT	0.398480902433404
161	KC_ABp1	asRNA:CR45145	-2.58737442373349	0.202895337944512	1	pseudobulk	DESeq2	LRT	0.692727931913101
161	KC_ABp1	asRNA:CR45162	0.295850504875218	1	1	pseudobulk	DESeq2	LRT	0
161	KC_ABp1	asRNA:CR45164	1.32823068455131	0.607220246153186	1	pseudobulk	DESeq2	LRT	0.21665375647025
161	KC_ABp1	asRNA:CR45182	-0.584613676872836	1	1	pseudobulk	DESeq2	LRT	0
161	KC_ABp1	asRNA:CR45190	-1.51023398327283	0.845597316437363	1	pseudobulk	DESeq2	LRT	0.072836403962567
161	KC_ABp1	asRNA:CR45275	0.400640538490757	1	1	pseudobulk	DESeq2	LRT	0
161	KC_ABp1	asRNA:CR45305	0.400640538490757	1	1	pseudobulk	DESeq2	LRT	0
161	KC_ABp1	asRNA:CR45469	1.36466217916099	0.492093788464712	1	pseudobulk	DESeq2	LRT	0.307952116885296
161	KC_ABp1	asRNA:CR45479	-1.51023398327283	0.845597316437363	1	pseudobulk	DESeq2	LRT	0.072836403962567
161	KC_ABp1	asRNA:CR45520	0.400640538490757	0.854745824291873	1	pseudobulk	DESeq2	LRT	0.068163012194342
161	KC_ABp1	asRNA:CR45620	1.50346570824895	0.512804951187805	1	pseudobulk	DESeq2	LRT	0.290047790308346
161	KC_ABp1	asRNA:CR45789	-1.51023398327283	0.845597316437363	1	pseudobulk	DESeq2	LRT	0.072836403962567
161	KC_ABp1	asRNA:CR45798	0.400640538490757	0.854745824291873	1	pseudobulk	DESeq2	LRT	0.068163012194342
161	KC_ABp1	asRNA:CR45801	-1.51023398327283	0.845597316437363	1	pseudobulk	DESeq2	LRT	0.072836403962567
161	KC_ABp1	asRNA:CR45804	0.400640538490757	0.854745824291873	1	pseudobulk	DESeq2	LRT	0.068163012194342
161	KC_ABp1	asRNA:CR45807	0.400640538490757	1	1	pseudobulk	DESeq2	LRT	0
161	KC_ABp1	asRNA:CR45815	0.295850504875218	1	1	pseudobulk	DESeq2	LRT	0
161	KC_ABp1	asRNA:CR45822	0.617506036250969	0.733737500923007	1	pseudobulk	DESeq2	LRT	0.134459283804161
161	KC_ABp1	asRNA:CR45831	0.400640538490757	1	1	pseudobulk	DESeq2	LRT	0
161	KC_ABp1	asRNA:CR45835	-1.64398569370659	0.293788333017896	1	pseudobulk	DESeq2	LRT	0.531965454994216
161	KC_ABp1	asRNA:CR45873	0.295850504875218	1	1	pseudobulk	DESeq2	LRT	0
161	KC_ABp1	asRNA:CR45874	-0.0735621202531379	0.923401042942602	1	pseudobulk	DESeq2	LRT	0.034609639267267
161	KC_ABp1	asRNA:CR45882	0.295850504875218	1	1	pseudobulk	DESeq2	LRT	0
161	KC_ABp1	asRNA:CR45889	1.22889843787771	0.595717321482481	1	pseudobulk	DESeq2	LRT	0.224959771875484
161	KC_ABp1	asRNA:CR45891	0.400640538490757	1	1	pseudobulk	DESeq2	LRT	0
161	KC_ABp1	asRNA:CR45896	0.400640538490757	0.854745824291873	1	pseudobulk	DESeq2	LRT	0.068163012194342
161	KC_ABp1	asRNA:CR45907	-2.72346463950539	0.26106630045163	1	pseudobulk	DESeq2	LRT	0.583249185137864
161	KC_ABp1	asRNA:CR45908	0.842405823124912	0.788653372765757	1	pseudobulk	DESeq2	LRT	0.103113835031894
161	KC_ABp1	asRNA:CR45912	0.295850504875218	1	1	pseudobulk	DESeq2	LRT	0
161	KC_ABp1	asRNA:CR45925	-1.51023398327283	0.845597316437363	1	pseudobulk	DESeq2	LRT	0.072836403962567
161	KC_ABp1	asRNA:CR45927	0.400640538490757	1	1	pseudobulk	DESeq2	LRT	0
161	KC_ABp1	asRNA:CR45928	0.295850504875218	1	1	pseudobulk	DESeq2	LRT	0
161	KC_ABp1	asRNA:CR45943	0.400640538490757	1	1	pseudobulk	DESeq2	LRT	0
161	KC_ABp1	asRNA:CR45951	0.987429041292792	0.60343242224665	1	pseudobulk	DESeq2	LRT	0.219371359019291
161	KC_ABp1	asRNA:CR46020	-1.76541829495743	0.569721440048943	1	pseudobulk	DESeq2	LRT	0.244337436643475
161	KC_ABp1	asRNA:CR46050	1.09609931916753	0.587351075528798	1	pseudobulk	DESeq2	LRT	0.231102231651792
161	KC_ABp1	asRNA:CR46104	-1.51023398327283	0.845597316437363	1	pseudobulk	DESeq2	LRT	0.072836403962567
161	KC_ABp1	asRNA:CR46135	-0.228299552556886	1	1	pseudobulk	DESeq2	LRT	0
161	KC_ABp1	asRNA:CR46144	-1.51023398327283	0.845597316437363	1	pseudobulk	DESeq2	LRT	0.072836403962567
161	KC_ABp1	asRNA:CR46354	-0.631785388714633	0.805865402687387	1	pseudobulk	DESeq2	LRT	0.093737488903968
161	KC_ABp1	ast	0.812167726963992	0.743112627884761	1	pseudobulk	DESeq2	LRT	0.128945358565215
161	KC_ABp1	AstA-R1	-3.51510680322635	0.102063541149827	1	pseudobulk	DESeq2	LRT	0.991129367660165
161	KC_ABp1	AstA-R2	0.400640538490757	0.854745824291873	1	pseudobulk	DESeq2	LRT	0.068163012194342
161	KC_ABp1	asun	0.0932981124673794	0.873204907300119	1	pseudobulk	DESeq2	LRT	0.058883832276487
161	KC_ABp1	Asx	-0.107578321688304	0.884390952792554	1	pseudobulk	DESeq2	LRT	0.053355708848149

161	KC_ABp1	Atac1	2.13079680834456	0.26184590397909	1	pseudobulk	DESeq2	LRT	0.58195421532061
161	KC_ABp1	Atac2	0.295850504875218	1	1	pseudobulk	DESeq2	LRT	0
161	KC_ABp1	Atac3	0.39325077980097	0.789715249808113	1	pseudobulk	DESeq2	LRT	0.102529475455891
161	KC_ABp1	ATbp	1.85895029951982	0.405908856945625	1	pseudobulk	DESeq2	LRT	0.39157147225836
161	KC_ABp1	Ate1	0.190670779677526	0.809281421591846	1	pseudobulk	DESeq2	LRT	0.091900429448985
161	KC_ABp1	Atet	0.113476498351303	0.789126090480029	1	pseudobulk	DESeq2	LRT	0.102853597519958
161	KC_ABp1	Atf-2	-1.01257861607368	0.443747517001122	1	pseudobulk	DESeq2	LRT	0.352864064034266
161	KC_ABp1	Atf3	-0.631785388714633	0.805865402687387	1	pseudobulk	DESeq2	LRT	0.093737488903968
161	KC_ABp1	Atf6	-0.136837359000031	0.717138941850657	1	pseudobulk	DESeq2	LRT	0.144396693937181
161	KC_ABp1	Atg1	0.178443212590382	0.654118550953835	1	pseudobulk	DESeq2	LRT	0.184343534001133
161	KC_ABp1	Atg101	-0.444102780180545	0.529508263001047	1	pseudobulk	DESeq2	LRT	0.276127258318047
161	KC_ABp1	Atg12	-0.0813508279148313	0.883709404282288	1	pseudobulk	DESeq2	LRT	0.053690523295342
161	KC_ABp1	Atg13	-0.117921736493977	0.831858287647939	1	pseudobulk	DESeq2	LRT	0.079950652236321
161	KC_ABp1	Atg14	-0.262950920141043	0.748885369795802	1	pseudobulk	DESeq2	LRT	0.1255846536958
161	KC_ABp1	Atg16	1.47863010120697	0.437708761008955	1	pseudobulk	DESeq2	LRT	0.358814760629823
161	KC_ABp1	Atg17	0.309897386262735	0.478639165367458	1	pseudobulk	DESeq2	LRT	0.31999176747134
161	KC_ABp1	Atg18a	0.288471167864867	0.706739163990865	1	pseudobulk	DESeq2	LRT	0.150740841565079
161	KC_ABp1	Atg18b	0.379070282762858	0.592779251392202	1	pseudobulk	DESeq2	LRT	0.227107006046904
161	KC_ABp1	Atg2	1.36845791403606	0.578882070925646	1	pseudobulk	DESeq2	LRT	0.23740990114772
161	KC_ABp1	Atg3	-1.25424272363114	0.283196080106916	1	pseudobulk	DESeq2	LRT	0.547912762280707
161	KC_ABp1	Atg4a	-0.366170411527131	0.701123549748475	1	pseudobulk	DESeq2	LRT	0.154205445305673
161	KC_ABp1	Atg4b	0.979736530429057	0.52342802920959	1	pseudobulk	DESeq2	LRT	0.28114302490377
161	KC_ABp1	Atg5	-0.0456192811864003	0.904615861949542	1	pseudobulk	DESeq2	LRT	0.043535801404645
161	KC_ABp1	Atg6	-0.0171931660809865	0.959395034076865	1	pseudobulk	DESeq2	LRT	0.018002533815292
161	KC_ABp1	Atg7	-1.8308760579733	0.440222376269376	1	pseudobulk	DESeq2	LRT	0.356327886263966
161	KC_ABp1	Atg8a	0.269966302904065	0.28317905137394	1	pseudobulk	DESeq2	LRT	0.547938877427212
161	KC_ABp1	Atg9	-0.236391460934091	0.959743486014838	1	pseudobulk	DESeq2	LRT	0.017844826850475
161	KC_ABp1	atl	-0.642039245632535	0.542991196148209	1	pseudobulk	DESeq2	LRT	0.265207211838866
161	KC_ABp1	atms	0.096031298130996	0.841318046576117	1	pseudobulk	DESeq2	LRT	0.075039795210901
162	KC_ABp1	Atox1	-0.082869426600752	0.797536173262607	1	pseudobulk	DESeq2	LRT	0.098249609848126
162	KC_ABp1	ATP6AP2	-0.205158672774569	0.612296305353872	1	pseudobulk	DESeq2	LRT	0.213038361124886
162	KC_ABp1	ATP7	1.22889843787771	0.595717321482481	1	pseudobulk	DESeq2	LRT	0.224959771875484
162	KC_ABp1	ATP8A	-0.422913553081034	0.397056250474608	1	pseudobulk	DESeq2	LRT	0.401147962908894
162	KC_ABp1	ATP8B	-0.213724454107	0.608324808598421	1	pseudobulk	DESeq2	LRT	0.215864471852548
162	KC_ABp1	Atpalpha	0.12302089020916	0.328003462225464	1	pseudobulk	DESeq2	LRT	0.484121572088692
162	KC_ABp1	ATPCL	-0.0304052559091305	0.943069143437414	1	pseudobulk	DESeq2	LRT	0.025456464725565
162	KC_ABp1	ATPsynB	-0.122202531070333	0.631736702660924	1	pseudobulk	DESeq2	LRT	0.199463890716734
162	KC_ABp1	ATPsynbeta	0.0669425940449993	0.708377447240753	1	pseudobulk	DESeq2	LRT	0.149735273985077
162	KC_ABp1	ATPsynC	-0.0942318162088241	0.59084132196749	1	pseudobulk	DESeq2	LRT	0.228529138825725
162	KC_ABp1	ATPsynCF6	-0.39775503033145	0.137892114139931	1	pseudobulk	DESeq2	LRT	0.860460569817192
162	KC_ABp1	ATPsynD	0.0269485252364248	0.870727376439874	1	pseudobulk	DESeq2	LRT	0.060117800722686
162	KC_ABp1	ATPsyndelta	0.010445046410994	0.96612180418023	1	pseudobulk	DESeq2	LRT	0.014968116287837
162	KC_ABp1	ATPsynE	-0.034600602376097	0.891046591658144	1	pseudobulk	DESeq2	LRT	0.050099586680288
162	KC_ABp1	ATPsynF	0.0835444298907439	0.667806442220264	1	pseudobulk	DESeq2	LRT	0.175349395693124
162	KC_ABp1	ATPsynG	0.298262615731932	0.220687368932818	1	pseudobulk	DESeq2	LRT	0.65622252302212
162	KC_ABp1	ATPsyngamma	0.0295452017402625	0.834967310772752	1	pseudobulk	DESeq2	LRT	0.078330526946232
162	KC_ABp1	ATPsynO	0.198893574939108	0.518004048774512	1	pseudobulk	DESeq2	LRT	0.28566684574983
162	KC_ABp1	Atu	-1.27835327547055	0.417276398747615	1	pseudobulk	DESeq2	LRT	0.379576178392867
162	KC_ABp1	Atx2	0.296044021165887	0.469877037822177	1	pseudobulk	DESeq2	LRT	0.3280157777708
162	KC_ABp1	Atxn7	-0.368202019604363	0.565522743305486	1	pseudobulk	DESeq2	LRT	0.247549924613936
162	KC_ABp1	aux	-0.96676215701227	0.40682878285777	1	pseudobulk	DESeq2	LRT	0.390588328625151
162	KC_ABp1	ave	-0.478825560086462	0.600956496295873	1	pseudobulk	DESeq2	LRT	0.221156965771774

162	KC_ABp1	Aven	0.295850504875218	1	1	pseudobulk	DESeq2	LRT	0
162	KC_ABp1	awd	-0.088790902258521	0.828819776717309	1	pseudobulk	DESeq2	LRT	0.08153989464429
162	KC_ABp1	axed	-0.588372277913786	0.319488424525677	1	pseudobulk	DESeq2	LRT	0.49554487226518
162	KC_ABp1	Axn	-0.439103000892621	0.706681438691043	1	pseudobulk	DESeq2	LRT	0.15077631547729
162	KC_ABp1	axo	0.909904497842359	0.691488213862076	1	pseudobulk	DESeq2	LRT	0.16021521786600
162	KC_ABp1	Axud1	0.916926479851	0.261071058800229	1	pseudobulk	DESeq2	LRT	0.58324126950224
162	KC_ABp1	az2	-1.40645798779945	0.604944642110073	1	pseudobulk	DESeq2	LRT	0.21828436539067
162	KC_ABp1	B4	0.306822307873207	0.271577580089982	1	pseudobulk	DESeq2	LRT	0.56610608580944
162	KC_ABp1	B52	-0.255359752652892	0.305598979027044	1	pseudobulk	DESeq2	LRT	0.51484810102456
162	KC_ABp1	bab1	4.24053299354692	0.256852968979401	1	pseudobulk	DESeq2	LRT	0.59031540987377
162	KC_ABp1	bab2	0.400640538490757	0.854745824291873	1	pseudobulk	DESeq2	LRT	0.06816301219434
162	KC_ABp1	babo	0.728359020849251	0.19842685846881	1	pseudobulk	DESeq2	LRT	0.70239954339491
162	KC_ABp1	Bacc	0.160105521487021	0.310335453425322	1	pseudobulk	DESeq2	LRT	0.50816860679597
162	KC_ABp1	baf	0.299737760991461	0.823610352704386	1	pseudobulk	DESeq2	LRT	0.08427820297905
162	KC_ABp1	bai	-0.531766824460465	0.507896546560456	1	pseudobulk	DESeq2	LRT	0.29422474014410
162	KC_ABp1	Baldspot	-0.044213454883454	0.884584565719766	1	pseudobulk	DESeq2	LRT	0.05326064249435
162	KC_ABp1	ball	0.123827804220246	0.928992020461119	1	pseudobulk	DESeq2	LRT	0.03198801634498
162	KC_ABp1	Bap111	1.37638104031529	0.335543152542639	1	pseudobulk	DESeq2	LRT	0.47425161944131
162	KC_ABp1	Bap170	-0.266573545997244	0.81013116921501	1	pseudobulk	DESeq2	LRT	0.09144465833825
162	KC_ABp1	Bap55	-0.670739886398215	0.412737749554516	1	pseudobulk	DESeq2	LRT	0.38432580814349
162	KC_ABp1	Bap60	-0.601671364277596	0.443293177437811	1	pseudobulk	DESeq2	LRT	0.35330895271580
162	KC_ABp1	barc	0.121704627154862	0.788234991964969	1	pseudobulk	DESeq2	LRT	0.10334428949074
162	KC_ABp1	barr	-1.51023398327283	0.845597316437363	1	pseudobulk	DESeq2	LRT	0.07283640396256
162	KC_ABp1	bbc	0.243060694687971	0.670834809698647	1	pseudobulk	DESeq2	LRT	0.17338440988854
162	KC_ABp1	bbx	1.09588221270465	0.548255846318272	1	pseudobulk	DESeq2	LRT	0.26101672853283
162	KC_ABp1	bc10	0.608737977773068	0.405645805003296	1	pseudobulk	DESeq2	LRT	0.39185301093065
162	KC_ABp1	BCAS2	0.584353171048288	0.713448643412246	1	pseudobulk	DESeq2	LRT	0.14663728349594
162	KC_ABp1	bchs	0.223840522312681	0.637995322758873	1	pseudobulk	DESeq2	LRT	0.19518250514632
162	KC_ABp1	BCL7-like	0.0702047328047288	0.928330951319301	1	pseudobulk	DESeq2	LRT	0.03229716956920
162	KC_ABp1	bcn92	0.171571904275633	0.807827117604443	1	pseudobulk	DESeq2	LRT	0.09268157227484
162	KC_ABp1	Bdbt	-0.048015612495413	0.942998325920543	1	pseudobulk	DESeq2	LRT	0.02548907825325
162	KC_ABp1	bdg	-0.250931678713561	0.885855279102727	1	pseudobulk	DESeq2	LRT	0.05263722238021
162	KC_ABp1	Bdp1	0.218060112955718	0.926586010097	1	pseudobulk	DESeq2	LRT	0.03311426120553
162	KC_ABp1	be	-3.04213321156523	0.18856594146886	1	pseudobulk	DESeq2	LRT	0.72453674621088
162	KC_ABp1	BEAF-32	-1.06842750185563	0.184383561682498	1	pseudobulk	DESeq2	LRT	0.73427780014143
162	KC_ABp1	beag	-0.406368957050228	0.673485976788797	1	pseudobulk	DESeq2	LRT	0.17167144265316
162	KC_ABp1	beat-Ib	-1.51023398327283	0.845597316437363	1	pseudobulk	DESeq2	LRT	0.07283640396256
162	KC_ABp1	beat-IIa	-1.76541829495743	0.569721440048943	1	pseudobulk	DESeq2	LRT	0.24433743664347
162	KC_ABp1	beat-IIIa	0.295850504875218	1	1	pseudobulk	DESeq2	LRT	0
162	KC_ABp1	beat-IIIb	-0.27056510585383	0.656415453385646	1	pseudobulk	DESeq2	LRT	0.18282120330280
162	KC_ABp1	beat-IV	0.230012984081907	0.575768833446723	1	pseudobulk	DESeq2	LRT	0.23975184732254
162	KC_ABp1	beat-Va	-0.242577553663919	0.813043356284901	1	pseudobulk	DESeq2	LRT	0.08988629463565
162	KC_ABp1	beat-Vb	-0.383194597103193	0.780291871649904	1	pseudobulk	DESeq2	LRT	0.10774291713416
162	KC_ABp1	beat-Vc	1.10206463661056	0.649272665626896	1	pseudobulk	DESeq2	LRT	0.18757288053597
162	KC_ABp1	beat-VI	0.889498583380155	0.142507852613165	1	pseudobulk	DESeq2	LRT	0.84616120405824
162	KC_ABp1	beat-VII	-1.39584306873666E-	0.889814602727557	1	pseudobulk	DESeq2	LRT	0.05070047133245
162	KC_ABp1	beg	-2.06436254104119	0.378741194036943	1	pseudobulk	DESeq2	LRT	0.42165745598303
162	KC_ABp1	bel	-0.046269103858489	0.854632966059859	1	pseudobulk	DESeq2	LRT	0.06822035900097
162	KC_ABp1	Bem46	0.244631509358063	0.823564009087109	1	pseudobulk	DESeq2	LRT	0.08430264092287
162	KC_ABp1	ben	0.0511699527942324	0.846426664898371	1	pseudobulk	DESeq2	LRT	0.07241066355580
162	KC_ABp1	Best1	2.28889991689751	0.266180374047543	1	pseudobulk	DESeq2	LRT	0.57482396898557
162	KC_ABp1	Bet1	0.19608513271143	0.810709149924911	1	pseudobulk	DESeq2	LRT	0.09113492536748

162	KC_ABp1	Bet3	-0.331841192077238	0.693931020386353	1	pseudobulk	DESeq2	LRT	0.15868369806743
162	KC_ABp1	Bet5	0.649758331626784	0.466386270331685	1	pseudobulk	DESeq2	LRT	0.33125424299808
162	KC_ABp1	beta-PheRS	1.02429215250316	0.565975685830988	1	pseudobulk	DESeq2	LRT	0.247202225589551
162	KC_ABp1	beta-Spec	0.448334691635723	0.286400256017223	1	pseudobulk	DESeq2	LRT	0.54302659814205
162	KC_ABp1	beta'COP	0.749741776304036	0.493733953931954	1	pseudobulk	DESeq2	LRT	0.30650700545570
162	KC_ABp1	beta3GalTII	1.9488906802722	0.307136832681869	1	pseudobulk	DESeq2	LRT	0.51266809866179
162	KC_ABp1	beta4GalNAcTA	-0.334034751232535	0.685973630820565	1	pseudobulk	DESeq2	LRT	0.16369257847579
162	KC_ABp1	beta4GalNAcTB	1.1147167439515	0.514800206730425	1	pseudobulk	DESeq2	LRT	0.28836128736620
162	KC_ABp1	beta4GalT7	1.07001666442999	0.542127893829044	1	pseudobulk	DESeq2	LRT	0.26589824661282
162	KC_ABp1	betaCOP	-0.150141519572539	0.859803059950626	1	pseudobulk	DESeq2	LRT	0.06560101360688
162	KC_ABp1	betaggt-I	0.490733042290333	0.75024415919128	1	pseudobulk	DESeq2	LRT	0.12479737696391
162	KC_ABp1	betaggt-II	0.929098767145583	0.535819020299262	1	pseudobulk	DESeq2	LRT	0.27098187403426
162	KC_ABp1	betaGlu	0.0669834804425677	0.909713634927058	1	pseudobulk	DESeq2	LRT	0.04109529596834
162	KC_ABp1	betaTub56D	0.451991350835631	0.189834556199642	1	pseudobulk	DESeq2	LRT	0.72162472868006
162	KC_ABp1	betaTub60D	-1.51023398327283	0.845597316437363	1	pseudobulk	DESeq2	LRT	0.07283640396256
162	KC_ABp1	betaTub85D	-0.817002774795447	0.779755878997874	1	pseudobulk	DESeq2	LRT	0.10804134217939
162	KC_ABp1	Bgb	0.0725888930471052	0.907027450828171	1	pseudobulk	DESeq2	LRT	0.04237956898974
162	KC_ABp1	BHD	-0.402496272095792	0.836484707942874	1	pseudobulk	DESeq2	LRT	0.07754199411190
162	KC_ABp1	bi	0.400640538490757	0.854745824291873	1	pseudobulk	DESeq2	LRT	0.06816301219434
162	KC_ABp1	BI-1	-0.0155040809672467	0.929999003637205	1	pseudobulk	DESeq2	LRT	0.03151751673111
162	KC_ABp1	bic	-0.032242903826952	0.843277246264554	1	pseudobulk	DESeq2	LRT	0.07402961786934
162	KC_ABp1	BicD	0.336451563217625	0.397111509740977	1	pseudobulk	DESeq2	LRT	0.40108752531447
162	KC_ABp1	bif	-0.0535666811197735	0.899097243672137	1	pseudobulk	DESeq2	LRT	0.04619333373255
162	KC_ABp1	bigmax	-0.639686532542405	0.571205363404213	1	pseudobulk	DESeq2	LRT	0.24320772335037
162	KC_ABp1	Bili	0.474862264826134	0.365655563227605	1	pseudobulk	DESeq2	LRT	0.43692781466235
163	KC_ABp1	Bin1	2.45617742720074	0.0808932413561233	1	pseudobulk	DESeq2	LRT	1.09208776224876
163	KC_ABp1	bin3	-0.0747822992290861	0.82300770574486	1	pseudobulk	DESeq2	LRT	0.08459609850971
163	KC_ABp1	bip2	0.112180908084644	0.688347128329351	1	pseudobulk	DESeq2	LRT	0.16219249505080
163	KC_ABp1	Bka	1.69871589336531	0.249079946105177	1	pseudobulk	DESeq2	LRT	0.60366123692135
163	KC_ABp1	Blimp-1	0.452764874402639	0.238110335270407	1	pseudobulk	DESeq2	LRT	0.62322175346747
163	KC_ABp1	Blm	-1.14005166467136	0.534316479201624	1	pseudobulk	DESeq2	LRT	0.27220143125230
163	KC_ABp1	Blos1	0.361916938502762	0.700682419472695	1	pseudobulk	DESeq2	LRT	0.15447877906955
163	KC_ABp1	Blos2	0.549175156636922	0.648686355785232	1	pseudobulk	DESeq2	LRT	0.18796523675375
163	KC_ABp1	Blos3	-0.490179114703493	0.638537562914439	1	pseudobulk	DESeq2	LRT	0.19481354963529
163	KC_ABp1	Blos4	0.235385875695668	0.862911742158911	1	pseudobulk	DESeq2	LRT	0.06403362126448
163	KC_ABp1	blot	0.295850504875218	1	1	pseudobulk	DESeq2	LRT	0
163	KC_ABp1	blp	0.792564266541876	0.531817956055111	1	pseudobulk	DESeq2	LRT	0.27423700343919
163	KC_ABp1	blw	0.0112519309561668	0.807415907839353	1	pseudobulk	DESeq2	LRT	0.09290269830099
163	KC_ABp1	bma	-0.731373686978631	0.304531533785641	1	pseudobulk	DESeq2	LRT	0.51636773015745
163	KC_ABp1	Bmcp	0.421986854838949	0.710407755644667	1	pseudobulk	DESeq2	LRT	0.1484923059354
163	KC_ABp1	bmm	0.0989870647562853	0.939817982781136	1	pseudobulk	DESeq2	LRT	0.02695624930253
163	KC_ABp1	bnb	0.218060112955718	0.926586010097	1	pseudobulk	DESeq2	LRT	0.03311426120553
163	KC_ABp1	bnk	0.400640538490757	1	1	pseudobulk	DESeq2	LRT	0
163	KC_ABp1	boca	0.0073004532693866	0.848504057799546	1	pseudobulk	DESeq2	LRT	0.07134607641555
163	KC_ABp1	bocks	1.24171981453361	0.571708867051231	1	pseudobulk	DESeq2	LRT	0.24282507194252
163	KC_ABp1	BOD1	1.13380813456518	0.490370446289327	1	pseudobulk	DESeq2	LRT	0.30947571181800
163	KC_ABp1	boil	-1.75283054089783	0.460938311409796	1	pseudobulk	DESeq2	LRT	0.33635719349755
163	KC_ABp1	bol	1.64705394628082	0.213720640814142	1	pseudobulk	DESeq2	LRT	0.67015353231044
163	KC_ABp1	bon	-0.0932356759391142	0.883194563204215	1	pseudobulk	DESeq2	LRT	0.05394361304974
163	KC_ABp1	bonsai	-0.447040740130697	0.63188534019039	1	pseudobulk	DESeq2	LRT	0.19936172019903
163	KC_ABp1	bor	-0.379578787476505	0.768058509511155	1	pseudobulk	DESeq2	LRT	0.11460569482532
163	KC_ABp1	BORCS5	-0.62866722592986	0.529323601820347	1	pseudobulk	DESeq2	LRT	0.27627874097601

163	KC_ABp1	BORCS6	-0.861789891602172	0.433047625944541	1	pseudobulk	DESeq2	LRT	0.36346433794805
163	KC_ABp1	botv	-1.6421260587743	0.379038182010144	1	pseudobulk	DESeq2	LRT	0.421317039633277
163	KC_ABp1	Br140	0.181645597533981	0.841812723184187	1	pseudobulk	DESeq2	LRT	0.074784514597823
163	KC_ABp1	brat	0.272204316480648	0.290951020238593	1	pseudobulk	DESeq2	LRT	0.536180115586177
163	KC_ABp1	Brca2	-0.933645186511125	0.70752579026696	1	pseudobulk	DESeq2	LRT	0.150257724896976
163	KC_ABp1	Brd8	-0.007498960327057	0.985670023864724	1	pseudobulk	DESeq2	LRT	0.006268450981396
163	KC_ABp1	Bre1	0.324780568751038	0.80512983328771	1	pseudobulk	DESeq2	LRT	0.094134080708093
163	KC_ABp1	Brf	-2.0637278012646	0.236391392354458	1	pseudobulk	DESeq2	LRT	0.626368341331477
163	KC_ABp1	brk	-0.712425324840931	0.73711055633684	1	pseudobulk	DESeq2	LRT	0.132467369120416
163	KC_ABp1	brm	0.540544852678889	0.546021767389937	1	pseudobulk	DESeq2	LRT	0.26279004361596
163	KC_ABp1	Brms1	0.412936111319124	0.676728063976448	1	pseudobulk	DESeq2	LRT	0.169585812910075
163	KC_ABp1	brn	0.295850504875218	1	1	pseudobulk	DESeq2	LRT	0
163	KC_ABp1	brp	0.232935314368534	0.244071790020187	1	pseudobulk	DESeq2	LRT	0.612482413727239
163	KC_ABp1	bru1	1.00292348061784	0.459678207034196	1	pseudobulk	DESeq2	LRT	0.337546085305908
163	KC_ABp1	bru2	0.862397351438396	0.699757606492085	1	pseudobulk	DESeq2	LRT	0.155052371976363
163	KC_ABp1	bru3	0.164719265092298	0.280728656665051	1	pseudobulk	DESeq2	LRT	0.551713252501765
163	KC_ABp1	Bruce	-0.107298536399931	0.852560784272467	1	pseudobulk	DESeq2	LRT	0.069274647721917
163	KC_ABp1	brun	-0.535692113031391	0.765311938306389	1	pseudobulk	DESeq2	LRT	0.116161511941489
163	KC_ABp1	BRWD3	0.285967958421747	0.733260776026703	1	pseudobulk	DESeq2	LRT	0.134741545893206
163	KC_ABp1	brwl	0.233747908948658	0.824590863225438	1	pseudobulk	DESeq2	LRT	0.083761481652970
163	KC_ABp1	bs	-0.504969249990942	0.640788604816223	1	pseudobulk	DESeq2	LRT	0.193285219941043
163	KC_ABp1	bsf	-0.228299552556886	1	1	pseudobulk	DESeq2	LRT	0
163	KC_ABp1	Bsg	0.0931809647946499	0.576643122326852	1	pseudobulk	DESeq2	LRT	0.23909288347342
163	KC_ABp1	Bsg25D	1.26676181016425	0.148385692012079	1	pseudobulk	DESeq2	LRT	0.828607973583786
163	KC_ABp1	bsk	0.204365498221067	0.453483236547055	1	pseudobulk	DESeq2	LRT	0.343438762428961
163	KC_ABp1	bt	-1.53478401302219	0.442568884116552	1	pseudobulk	DESeq2	LRT	0.354019123466868
163	KC_ABp1	BTBD9	1.89663391286907	0.313455232577263	1	pseudobulk	DESeq2	LRT	0.503824475991033
163	KC_ABp1	BtbVII	-0.17640842443654	0.626822138485017	1	pseudobulk	DESeq2	LRT	0.20285567325627
163	KC_ABp1	Btk29A	0.400640538490757	1	1	pseudobulk	DESeq2	LRT	0
163	KC_ABp1	btsz	-0.058960869742446	0.853896258415606	1	pseudobulk	DESeq2	LRT	0.068594889421545
163	KC_ABp1	btz	0.303332864886297	0.812126155584219	1	pseudobulk	DESeq2	LRT	0.090376502263216
163	KC_ABp1	Bub1	-1.1758348612624	0.459548338890082	1	pseudobulk	DESeq2	LRT	0.337668799383278
163	KC_ABp1	Bub3	-0.20337137851442	0.816755989889491	1	pseudobulk	DESeq2	LRT	0.087907671830897
163	KC_ABp1	BubR1	-1.75283054089783	0.460938311409796	1	pseudobulk	DESeq2	LRT	0.33635719349755
163	KC_ABp1	bug	1.04609816125407	0.479027193019007	1	pseudobulk	DESeq2	LRT	0.319639832216145
163	KC_ABp1	Bug22	0.411805226164904	0.746173459870851	1	pseudobulk	DESeq2	LRT	0.12716020214137
163	KC_ABp1	BuGZ	-0.073010313384997	0.814434673830639	1	pseudobulk	DESeq2	LRT	0.089143744913070
163	KC_ABp1	bun	0.284614105593799	0.0708711707341829	1	pseudobulk	DESeq2	LRT	1.1495303929871
163	KC_ABp1	bur	0.606024447358256	0.498114091451421	1	pseudobulk	DESeq2	LRT	0.302671172074022
163	KC_ABp1	bves	0.156381719717277	0.658275751783407	1	pseudobulk	DESeq2	LRT	0.181592142265687
163	KC_ABp1	bw	-1.76541829495743	0.569721440048943	1	pseudobulk	DESeq2	LRT	0.244337436643475
163	KC_ABp1	bwa	0.285007958132404	0.80454047826751	1	pseudobulk	DESeq2	LRT	0.094452100668771
163	KC_ABp1	Bx	-0.029157261676375	0.957830268970142	1	pseudobulk	DESeq2	LRT	0.018711442675545
163	KC_ABp1	Bx42	-0.434009899168532	0.624688966214846	1	pseudobulk	DESeq2	LRT	0.204336164862888
163	KC_ABp1	bys	-1.00403795712626	0.642593163840201	1	pseudobulk	DESeq2	LRT	0.192063898956101
163	KC_ABp1	c11.1	-0.358295106185512	0.727905351798787	1	pseudobulk	DESeq2	LRT	0.137925087532855
163	KC_ABp1	c12.1	0.470898980616441	0.622793879782861	1	pseudobulk	DESeq2	LRT	0.205655663909941
163	KC_ABp1	c12.2	0.492593511947829	0.72119948944652	1	pseudobulk	DESeq2	LRT	0.141944589396443
163	KC_ABp1	C1GalTA	0.73157618794998	0.395688601367502	1	pseudobulk	DESeq2	LRT	0.402646460308895
163	KC_ABp1	C3G	0.232096584810011	0.467501548284321	1	pseudobulk	DESeq2	LRT	0.330216946480825
163	KC_ABp1	C901	-1.75283054089783	0.460938311409796	1	pseudobulk	DESeq2	LRT	0.33635719349755
163	KC_ABp1	ca	0.628796095634193	0.728315685809712	1	pseudobulk	DESeq2	LRT	0.137680336500153

163	KC_ABp1	Ca-alpha1D	0.130320372768981	0.591199518938277	1	pseudobulk	DESeq2	LRT	0.228265928000472
163	KC_ABp1	Ca-alpha1T	-2.69620756325413	0.299115455459286	1	pseudobulk	DESeq2	LRT	0.524161146155349
163	KC_ABp1	Ca-beta	-0.239627231869525	0.401781763398694	1	pseudobulk	DESeq2	LRT	0.396009779471931
163	KC_ABp1	Ca-Ma2d	0.190548848915632	0.524755144568404	1	pseudobulk	DESeq2	LRT	0.280043295010035
163	KC_ABp1	CaBP1	0.278658212726369	0.836088971607298	1	pseudobulk	DESeq2	LRT	0.077747505070421
163	KC_ABp1	cac	0.0927877163885741	0.518452246266723	1	pseudobulk	DESeq2	LRT	0.285291239540993
163	KC_ABp1	cact	0.528085346183954	0.247835131948173	1	pseudobulk	DESeq2	LRT	0.605837130043269
163	KC_ABp1	cactin	1.40068053309973	0.418995264369971	1	pseudobulk	DESeq2	LRT	0.377790885553066
163	KC_ABp1	Cad87A	0.0575903271729706	0.951950021006582	1	pseudobulk	DESeq2	LRT	0.021385852215140
163	KC_ABp1	Cad96Ca	-2.39593946583853	0.39950212853761	1	pseudobulk	DESeq2	LRT	0.398480902433404
163	KC_ABp1	CadN	-0.245497213831353	0.47633780682885	1	pseudobulk	DESeq2	LRT	0.322084947261174
163	KC_ABp1	CadN2	-0.584613676872836	1	1	pseudobulk	DESeq2	LRT	0
163	KC_ABp1	Cadps	0.644415145379798	0.0127719063722827	1	pseudobulk	DESeq2	LRT	1.89374427382739
163	KC_ABp1	Caf1-105	-0.584613676872836	1	1	pseudobulk	DESeq2	LRT	0
163	KC_ABp1	Caf1-55	0.468754037031879	0.681977381546342	1	pseudobulk	DESeq2	LRT	0.166230028910247
163	KC_ABp1	cag	0.665144245585208	0.60632104772978	1	pseudobulk	DESeq2	LRT	0.217297355475672
163	KC_ABp1	CAH2	1.90090186132448	0.432852416067745	1	pseudobulk	DESeq2	LRT	0.363660154022951
163	KC_ABp1	CAH3	-0.112873062590949	0.837534051567152	1	pseudobulk	DESeq2	LRT	0.076997526849822
163	KC_ABp1	CAH8	-0.537006304934948	0.55663478724508	1	pseudobulk	DESeq2	LRT	0.254429655643945
163	KC_ABp1	CAHbeta	-0.229954536708027	0.904966571493722	1	pseudobulk	DESeq2	LRT	0.043367462876507
163	KC_ABp1	cal1	-1.06152100873948	0.374761681370356	1	pseudobulk	DESeq2	LRT	0.426244821253211
164	KC_ABp1	CalpA	-1.22002885007556	0.438177974255428	1	pseudobulk	DESeq2	LRT	0.358349456761029
164	KC_ABp1	CalpB	-1.33858350751393	0.51093307655033	1	pseudobulk	DESeq2	LRT	0.291635981251378
164	KC_ABp1	Calr	0.44133585875096	0.419513470854973	1	pseudobulk	DESeq2	LRT	0.377254089128364
164	KC_ABp1	Cals	0.0615103985098513	0.774233005292564	1	pseudobulk	DESeq2	LRT	0.111128318794767
164	KC_ABp1	Calx	0.622825729602387	0.115050800303089	1	pseudobulk	DESeq2	LRT	0.939110355995714
164	KC_ABp1	calypso	-0.541006216775032	0.656311501653012	1	pseudobulk	DESeq2	LRT	0.182889984801942
164	KC_ABp1	Cam	-0.0711422199154318	0.592992648900231	1	pseudobulk	DESeq2	LRT	0.22695069038254
164	KC_ABp1	CaMKI	0.237858817072226	0.359179730676027	1	pseudobulk	DESeq2	LRT	0.444688179532382
164	KC_ABp1	CaMKII	0.156193367570373	0.363824874275115	1	pseudobulk	DESeq2	LRT	0.439107612058876
164	KC_ABp1	Camp	-0.635135934241451	0.55402937655866	1	pseudobulk	DESeq2	LRT	0.256467206861681
164	KC_ABp1	Camta	-0.162696949966189	0.828094379723547	1	pseudobulk	DESeq2	LRT	0.081920162904405
164	KC_ABp1	CanA-14F	0.0129093849235122	0.865113538550313	1	pseudobulk	DESeq2	LRT	0.062926891459988
164	KC_ABp1	CanB	0.00196113568326462	0.888042667247618	1	pseudobulk	DESeq2	LRT	0.051566167436446
164	KC_ABp1	CanB2	-0.0967643214302726	0.80828423706812	1	pseudobulk	DESeq2	LRT	0.092435890607716
164	KC_ABp1	Cand1	0.696668493052567	0.500539449816502	1	pseudobulk	DESeq2	LRT	0.300561688089873
164	KC_ABp1	CAP	2.31802751202782	0.074063739803817	1	pseudobulk	DESeq2	LRT	1.13039436229529
164	KC_ABp1	Cap-D3	-1.51023398327283	0.845597316437363	1	pseudobulk	DESeq2	LRT	0.072836403962567
164	KC_ABp1	Cap-G	-0.584613676872836	1	1	pseudobulk	DESeq2	LRT	0
164	KC_ABp1	Cap-H2	-0.153283834849882	0.84704382086286	1	pseudobulk	DESeq2	LRT	0.072094121349758
164	KC_ABp1	Caper	0.108528496521918	0.65677938723158	1	pseudobulk	DESeq2	LRT	0.182580485839982
164	KC_ABp1	Capr	0.77965102505799	0.304448619273477	1	pseudobulk	DESeq2	LRT	0.516485991204696
164	KC_ABp1	caps	-0.0094429562339404	0.877973862845009	1	pseudobulk	DESeq2	LRT	0.056518412785407
164	KC_ABp1	capt	-0.232933808692448	0.83503278609745	1	pseudobulk	DESeq2	LRT	0.078296472370684
164	KC_ABp1	capu	-0.774314447235651	0.131262309279843	1	pseudobulk	DESeq2	LRT	0.881859959528503
164	KC_ABp1	car	-1.16285325720926	0.29935498249522	1	pseudobulk	DESeq2	LRT	0.523813509015968
164	KC_ABp1	CARPB	3.23578104050438	0.0832629434372393	1	pseudobulk	DESeq2	LRT	1.07954824038708
164	KC_ABp1	CASK	0.176351289996947	0.630127728298652	1	pseudobulk	DESeq2	LRT	0.200571409161038
164	KC_ABp1	casp	-0.0599080746989411	0.826803839079738	1	pseudobulk	DESeq2	LRT	0.082597515490232
164	KC_ABp1	cass	0.0207952118625891	0.937119902280264	1	pseudobulk	DESeq2	LRT	0.028204838602892
164	KC_ABp1	Cat	-0.848730778766596	0.629998703588267	1	pseudobulk	DESeq2	LRT	0.20066034423696
164	KC_ABp1	cathD	-0.463352230960558	0.643122945377748	1	pseudobulk	DESeq2	LRT	0.191705995357098

164	KC_ABp1	Catsup	1.25924989875084	0.448807470923981	1	pseudobulk	DESeq2	LRT	0.347939922353378
164	KC_ABp1	cav	0.295850504875218	1	1	pseudobulk	DESeq2	LRT	0
164	KC_ABp1	caz	0.252650992606719	0.682302352921373	1	pseudobulk	DESeq2	LRT	0.166023131051903
164	KC_ABp1	cbc	0.17340092779666	0.852683850928201	1	pseudobulk	DESeq2	LRT	0.069211962089039
164	KC_ABp1	Cbl	-0.569099717712958	0.32366002665073	1	pseudobulk	DESeq2	LRT	0.489910934446657
164	KC_ABp1	Cbp20	1.37964735060349	0.158089645415119	1	pseudobulk	DESeq2	LRT	0.801096574636696
164	KC_ABp1	Cbp53E	-0.0555236254559382	0.853844734831368	1	pseudobulk	DESeq2	LRT	0.068621095278777
164	KC_ABp1	Cbp80	-1.37724725087845	0.274345111714025	1	pseudobulk	DESeq2	LRT	0.561702773665599
164	KC_ABp1	cbs	1.52946610165515	0.19934628001955	1	pseudobulk	DESeq2	LRT	0.700391864250676
164	KC_ABp1	Cbs	-1.02243221465913	0.7970385187166	1	pseudobulk	DESeq2	LRT	0.098520689818640
164	KC_ABp1	cbt	-0.0057532554341146	0.886114734822979	1	pseudobulk	DESeq2	LRT	0.052510041685823
164	KC_ABp1	CCDC53	3.10364094562471	0.118080838846288	1	pseudobulk	DESeq2	LRT	0.927820570282042
164	KC_ABp1	Ccdc58	0.0635740798955028	0.942660466186692	1	pseudobulk	DESeq2	LRT	0.025644706217602
164	KC_ABp1	Cchl	-0.166324074149823	0.711551804249838	1	pseudobulk	DESeq2	LRT	0.147793475789347
164	KC_ABp1	CCKLR-17D1	-1.76541829495743	0.569721440048943	1	pseudobulk	DESeq2	LRT	0.244337436643475
164	KC_ABp1	CCKLR-17D3	0.295850504875218	1	1	pseudobulk	DESeq2	LRT	0
164	KC_ABp1	Ccm3	0.103718684829724	0.877410529578352	1	pseudobulk	DESeq2	LRT	0.056797158043296
164	KC_ABp1	Ccn	-0.0151655886111964	0.942875421570138	1	pseudobulk	DESeq2	LRT	0.025545685098039
164	KC_ABp1	Ccs	0.556897978800036	0.75025082400973	1	pseudobulk	DESeq2	LRT	0.124793518911864
164	KC_ABp1	CCT1	0.810246036941512	0.630663030155404	1	pseudobulk	DESeq2	LRT	0.200202626863182
164	KC_ABp1	CCT2	-0.439458602012499	0.689665650785132	1	pseudobulk	DESeq2	LRT	0.161361403771767
164	KC_ABp1	CCT3	-0.705766765127154	0.509810897426989	1	pseudobulk	DESeq2	LRT	0.292590885536374
164	KC_ABp1	CCT4	-0.607903498690944	0.508318859020396	1	pseudobulk	DESeq2	LRT	0.293863777339288
164	KC_ABp1	CCT5	0.982859018468178	0.58619943926276	1	pseudobulk	DESeq2	LRT	0.231954601327822
164	KC_ABp1	CCT6	0.226099455287585	0.852384718723171	1	pseudobulk	DESeq2	LRT	0.069364344778685
164	KC_ABp1	CCT7	2.61634889846779	0.191622466808554	1	pseudobulk	DESeq2	LRT	0.717553573342204
164	KC_ABp1	CCT8	0.300952478262104	0.837313893571583	1	pseudobulk	DESeq2	LRT	0.077111702472093
164	KC_ABp1	Ccz1	-2.28138995062764	0.0684941468210259	1	pseudobulk	DESeq2	LRT	1.1643465396308
164	KC_ABp1	CD98hc	-0.849412627809915	0.220899899787815	1	pseudobulk	DESeq2	LRT	0.65580448114807
164	KC_ABp1	Cda5	-0.673833847002232	0.232894252183622	1	pseudobulk	DESeq2	LRT	0.632841229695201
164	KC_ABp1	CDase	0.400640538490757	1	1	pseudobulk	DESeq2	LRT	0
164	KC_ABp1	cdc14	-0.0432664777029516	0.926497043016145	1	pseudobulk	DESeq2	LRT	0.033155962425839
164	KC_ABp1	Cdc16	-1.96852075540546	0.461031367303002	1	pseudobulk	DESeq2	LRT	0.336269525406075
164	KC_ABp1	Cdc23	-0.817002774795447	0.779755878997874	1	pseudobulk	DESeq2	LRT	0.108041342179397
164	KC_ABp1	Cdc27	0.369643495115873	0.638599397610507	1	pseudobulk	DESeq2	LRT	0.194771495465468
164	KC_ABp1	Cdc2rk	0.0113700659643092	0.955200909578721	1	pseudobulk	DESeq2	LRT	0.019905272662747
164	KC_ABp1	Cdc37	0.0443678204463207	0.884420152423464	1	pseudobulk	DESeq2	LRT	0.053341370133809
164	KC_ABp1	Cdc42	0.073229633944647	0.882817429227421	1	pseudobulk	DESeq2	LRT	0.054129101266417
164	KC_ABp1	CDC45L	1.63259667120755	0.407355166729606	1	pseudobulk	DESeq2	LRT	0.390026770901327
164	KC_ABp1	Cdc5	1.77590247163198	0.132253873745902	1	pseudobulk	DESeq2	LRT	0.878591598522787
164	KC_ABp1	CDC50	-0.276716167316332	0.75539733584882	1	pseudobulk	DESeq2	LRT	0.121824551184687
164	KC_ABp1	Cdc7	0.645232045118366	0.571197090055471	1	pseudobulk	DESeq2	LRT	0.243214013725003
164	KC_ABp1	Cdep	0.436094253266776	0.319699708118084	1	pseudobulk	DESeq2	LRT	0.495257760233672
164	KC_ABp1	CdGAPr	0.325735103965678	0.736778165476689	1	pseudobulk	DESeq2	LRT	0.132663252997821
164	KC_ABp1	cdi	-0.133386980654573	0.794299619041012	1	pseudobulk	DESeq2	LRT	0.100015645745241
164	KC_ABp1	Cdk12	0.338472807106553	0.621729587451645	1	pseudobulk	DESeq2	LRT	0.206398464526284
164	KC_ABp1	Cdk2	-0.684690434515359	0.683859295026911	1	pseudobulk	DESeq2	LRT	0.165033245764419
164	KC_ABp1	CDK2AP1	-0.159644752733724	0.834598811494909	1	pseudobulk	DESeq2	LRT	0.078522238078144
164	KC_ABp1	Cdk5	0.558899401303168	0.602157986961864	1	pseudobulk	DESeq2	LRT	0.220289548835109
164	KC_ABp1	Cdk5alpha	-0.23380385338649	0.444142465473101	1	pseudobulk	DESeq2	LRT	0.352477700956148
164	KC_ABp1	Cdk7	0.445590887953102	0.752434656866821	1	pseudobulk	DESeq2	LRT	0.123531209214978
164	KC_ABp1	Cdk8	0.842405823124912	0.788653372765757	1	pseudobulk	DESeq2	LRT	0.103113835031894

164	KC_ABp1	Cdk9	0.476031969324445	0.682254498057982	1	pseudobulk	DESeq2	LRT	0.166053592374341
164	KC_ABp1	cdm	0.0279079790528367	0.962321900596444	1	pseudobulk	DESeq2	LRT	0.016679630385915
164	KC_ABp1	Cds	-0.14717583931951	0.725420390829137	1	pseudobulk	DESeq2	LRT	0.139410241007979
164	KC_ABp1	Ced-12	0.789663895977649	0.6834062990289	1	pseudobulk	DESeq2	LRT	0.165321022580152
164	KC_ABp1	Cen	0.607171037007349	0.510817408400339	1	pseudobulk	DESeq2	LRT	0.291734310618924
164	KC_ABp1	CenB1A	0.274929312521294	0.87073888194867	1	pseudobulk	DESeq2	LRT	0.060112062134346
164	KC_ABp1	CenG1A	0.851532471358299	0.214405815522761	1	pseudobulk	DESeq2	LRT	0.668763439057666
164	KC_ABp1	Cep97	-0.253857212703778	0.76625327100956	1	pseudobulk	DESeq2	LRT	0.115627658537646
164	KC_ABp1	cer	-1.45675306120982	0.226449623615784	1	pseudobulk	DESeq2	LRT	0.645028396825687
164	KC_ABp1	Cerk	0.577667376709909	0.557255327074835	1	pseudobulk	DESeq2	LRT	0.253945771195598
164	KC_ABp1	cert	-0.843026869841655	0.506541670320037	1	pseudobulk	DESeq2	LRT	0.295384821881876
164	KC_ABp1	cerv	2.3203768617626	0.177607275973132	1	pseudobulk	DESeq2	LRT	0.750539246607782
164	KC_ABp1	Cf2	-0.159426310874122	0.752760326691637	1	pseudobulk	DESeq2	LRT	0.123343277934856
164	KC_ABp1	Cfp1	0.402050576021211	0.706435300103106	1	pseudobulk	DESeq2	LRT	0.150927607481235
164	KC_ABp1	cg	0.0616588266175579	0.816790655546587	1	pseudobulk	DESeq2	LRT	0.087889239417433
164	KC_ABp1	CG10011	-0.416790741682605	0.363633770504882	1	pseudobulk	DESeq2	LRT	0.439335790843712
164	KC_ABp1	CG10019	0.450415085511891	0.54997330447467	1	pseudobulk	DESeq2	LRT	0.259658390507057
165	KC_ABp1	CG10035	0.295850504875218	1	1	pseudobulk	DESeq2	LRT	0
165	KC_ABp1	CG10038	1.66859086492535	0.429630482476201	1	pseudobulk	DESeq2	LRT	0.366904912826277
165	KC_ABp1	CG10050	0.400640538490757	0.854745824291873	1	pseudobulk	DESeq2	LRT	0.068163012194342
165	KC_ABp1	CG10053	-0.566571997783829	0.701642563990323	1	pseudobulk	DESeq2	LRT	0.153884073083544
165	KC_ABp1	CG10055	1.54606463491882	0.332691807948304	1	pseudobulk	DESeq2	LRT	0.477957892869227
165	KC_ABp1	CG10068	0.840049292044347	0.594670035065474	1	pseudobulk	DESeq2	LRT	0.225723944691545
165	KC_ABp1	CG10075	0.550703727339748	0.651190091177581	1	pseudobulk	DESeq2	LRT	0.186292216502822
165	KC_ABp1	CG10077	0.164397376406393	0.234102599692922	1	pseudobulk	DESeq2	LRT	0.630593763466888
165	KC_ABp1	CG10082	-0.308795241867197	0.365797919643863	1	pseudobulk	DESeq2	LRT	0.436758768760531
165	KC_ABp1	CG10089	0.323079236933894	0.630795676264925	1	pseudobulk	DESeq2	LRT	0.200111292167358
165	KC_ABp1	CG10098	0.534680134640384	0.543422332832811	1	pseudobulk	DESeq2	LRT	0.26486251750673
165	KC_ABp1	CG10103	0.0452544085559351	0.940992449248347	1	pseudobulk	DESeq2	LRT	0.026413861443023
165	KC_ABp1	CG10132	0.321900605030753	0.623276179865102	1	pseudobulk	DESeq2	LRT	0.205319470494206
165	KC_ABp1	CG10137	-0.0631337742782595	0.919456050194719	1	pseudobulk	DESeq2	LRT	0.036469025114417
165	KC_ABp1	CG10139	-0.369319850778204	0.669136844687214	1	pseudobulk	DESeq2	LRT	0.174485055901779
165	KC_ABp1	CG10147	2.03224944717325	0.256153892434314	1	pseudobulk	DESeq2	LRT	0.591499040334586
165	KC_ABp1	CG10151	-0.107987646739473	0.901491296218769	1	pseudobulk	DESeq2	LRT	0.045038461978082
165	KC_ABp1	CG10166	0.886457746341452	0.577497022571705	1	pseudobulk	DESeq2	LRT	0.238450250542333
165	KC_ABp1	CG10171	-0.546601890199522	0.855848288403055	1	pseudobulk	DESeq2	LRT	0.067603213534073
165	KC_ABp1	CG10188	0.39101160819926	0.610036267436481	1	pseudobulk	DESeq2	LRT	0.21464434485919
165	KC_ABp1	CG10189	-0.646873269590272	0.731466100082903	1	pseudobulk	DESeq2	LRT	0.1358057965217
165	KC_ABp1	CG10194	-0.302259490936107	0.796283118893455	1	pseudobulk	DESeq2	LRT	0.098932491167042
165	KC_ABp1	CG10209	0.891294121207791	0.564657059619344	1	pseudobulk	DESeq2	LRT	0.248215237726981
165	KC_ABp1	CG10214	-1.06261012059527	0.265173781323842	1	pseudobulk	DESeq2	LRT	0.576469418386713
165	KC_ABp1	CG1024	1.77817710329883	0.361290029179325	1	pseudobulk	DESeq2	LRT	0.442144023851717
165	KC_ABp1	CG10252	-1.51023398327283	0.845597316437363	1	pseudobulk	DESeq2	LRT	0.072836403962567
165	KC_ABp1	CG10254	-0.324766095495528	0.822288094211073	1	pseudobulk	DESeq2	LRT	0.084975997781537
165	KC_ABp1	CG10257	0.400640538490757	0.854745824291873	1	pseudobulk	DESeq2	LRT	0.068163012194342
165	KC_ABp1	CG10265	-0.276888095586284	0.809546985335006	1	pseudobulk	DESeq2	LRT	0.091757940141546
165	KC_ABp1	CG10268	-0.944929376095243	0.624417382220801	1	pseudobulk	DESeq2	LRT	0.204525015767366
165	KC_ABp1	CG10274	-1.80293735913889	0.383363463040706	1	pseudobulk	DESeq2	LRT	0.416389280492733
165	KC_ABp1	CG10283	0.263085179468712	0.621580966327818	1	pseudobulk	DESeq2	LRT	0.206502292703537
165	KC_ABp1	CG10286	-0.188880073581604	0.940427510379179	1	pseudobulk	DESeq2	LRT	0.026674674920217
165	KC_ABp1	CG10321	0.234722248855123	0.791818100608817	1	pseudobulk	DESeq2	LRT	0.101374574692076
165	KC_ABp1	CG10324	-1.4456730064023	0.567254902835535	1	pseudobulk	DESeq2	LRT	0.246221741780238

165	KC_ABp1	CG10333	0.212829610708963	0.792346147943498	1	pseudobulk	DESeq2	LRT	0.101085049092267
165	KC_ABp1	CG10336	-1.51023398327283	0.845597316437363	1	pseudobulk	DESeq2	LRT	0.072836403962567
165	KC_ABp1	CG10337	0.842405823124912	0.788653372765757	1	pseudobulk	DESeq2	LRT	0.103113835031894
165	KC_ABp1	CG10338	2.68982024117334	0.193606417131331	1	pseudobulk	DESeq2	LRT	0.713080251993847
165	KC_ABp1	CG10340	-0.564747831511404	0.438344583331897	1	pseudobulk	DESeq2	LRT	0.358184355697079
165	KC_ABp1	CG10341	-2.89521061460743	0.148373880464856	1	pseudobulk	DESeq2	LRT	0.828642544935547
165	KC_ABp1	CG10343	0.0686270586096658	0.956014749395227	1	pseudobulk	DESeq2	LRT	0.019535407377094
165	KC_ABp1	CG10347	0.121060331686199	0.877392969562322	1	pseudobulk	DESeq2	LRT	0.056805849863284
165	KC_ABp1	CG10353	-0.615397331554466	0.414673663613605	1	pseudobulk	DESeq2	LRT	0.382293546286547
165	KC_ABp1	CG10362	-0.677496876706122	0.462353241889292	1	pseudobulk	DESeq2	LRT	0.335026092875265
165	KC_ABp1	CG10365	-0.0279705882949645	0.9247180762944	1	pseudobulk	DESeq2	LRT	0.033990652744114
165	KC_ABp1	CG10366	0.0996549363696755	0.890437482556358	1	pseudobulk	DESeq2	LRT	0.050396566862058
165	KC_ABp1	CG10375	0.482852108171106	0.485321396683963	1	pseudobulk	DESeq2	LRT	0.313970561235855
165	KC_ABp1	CG10376	2.16232673790001	0.328986484433671	1	pseudobulk	DESeq2	LRT	0.482821943558981
165	KC_ABp1	CG10383	-0.276751475697207	0.812938522597624	1	pseudobulk	DESeq2	LRT	0.089942296110898
165	KC_ABp1	CG10384	-0.103035271576912	0.857270887913423	1	pseudobulk	DESeq2	LRT	0.066881924241573
165	KC_ABp1	CG10395	0.531031868882429	0.512019159701364	1	pseudobulk	DESeq2	LRT	0.290713787467742
165	KC_ABp1	CG10413	-1.30646356168432	0.441869046051455	1	pseudobulk	DESeq2	LRT	0.354706420704954
165	KC_ABp1	CG10417	-0.0754366473407044	0.885664221791245	1	pseudobulk	DESeq2	LRT	0.052730899177322
165	KC_ABp1	CG10418	0.788809061759142	0.479800182253664	1	pseudobulk	DESeq2	LRT	0.318939591399982
165	KC_ABp1	CG10425	-1.33940988009516	0.439102009387311	1	pseudobulk	DESeq2	LRT	0.357434575503618
165	KC_ABp1	CG10428	0.0557621478166742	0.955863250806697	1	pseudobulk	DESeq2	LRT	0.019604234991770
165	KC_ABp1	CG10431	-0.549208653246393	0.87387374417952	1	pseudobulk	DESeq2	LRT	0.058551308975419
165	KC_ABp1	CG10435	-1.00400536597084	0.412278208539291	1	pseudobulk	DESeq2	LRT	0.384809619749326
165	KC_ABp1	CG10444	-0.804052214782513	0.679213304465596	1	pseudobulk	DESeq2	LRT	0.167993815697846
165	KC_ABp1	CG10463	-1.36597518135172	0.630522975737565	1	pseudobulk	DESeq2	LRT	0.200299083469582
165	KC_ABp1	CG10465	0.0837655781592529	0.855453821259677	1	pseudobulk	DESeq2	LRT	0.067803429388104
165	KC_ABp1	CG10466	-2.06956992793787	0.419099033258735	1	pseudobulk	DESeq2	LRT	0.377683340957807
165	KC_ABp1	CG10467	0.400640538490757	1	1	pseudobulk	DESeq2	LRT	0
165	KC_ABp1	CG10469	0.295850504875218	1	1	pseudobulk	DESeq2	LRT	0
165	KC_ABp1	CG10470	-0.649241973074111	0.47493824930882	1	pseudobulk	DESeq2	LRT	0.323362852959918
165	KC_ABp1	CG10479	-0.625837088675441	0.694294927973356	1	pseudobulk	DESeq2	LRT	0.158456007379525
165	KC_ABp1	CG10483	0.349587069953557	0.698809793389155	1	pseudobulk	DESeq2	LRT	0.155641017276591
165	KC_ABp1	CG10492	0.0961382038388089	0.921817770216268	1	pseudobulk	DESeq2	LRT	0.035354924078193
165	KC_ABp1	CG10494	-0.354299178291619	0.793606145996128	1	pseudobulk	DESeq2	LRT	0.100394977494668
165	KC_ABp1	CG10495	-2.84770290365933	0.223489999778285	1	pseudobulk	DESeq2	LRT	0.650741904918056
165	KC_ABp1	CG10508	0.0390567203704284	0.912746461310267	1	pseudobulk	DESeq2	LRT	0.039649842121213
165	KC_ABp1	CG10512	2.00082579141071	0.368240584073267	1	pseudobulk	DESeq2	LRT	0.433868349270948
165	KC_ABp1	CG10513	0.400640538490757	1	1	pseudobulk	DESeq2	LRT	0
165	KC_ABp1	CG10516	2.59639044293808	0.191741755583031	1	pseudobulk	DESeq2	LRT	0.717283300556408
165	KC_ABp1	CG10543	0.0754627180288981	0.778215585218551	1	pseudobulk	DESeq2	LRT	0.108900075888176
165	KC_ABp1	CG10555	0.109624178651511	0.869755757246701	1	pseudobulk	DESeq2	LRT	0.060602687809234
165	KC_ABp1	CG10561	-0.569854406557957	0.547775979994595	1	pseudobulk	DESeq2	LRT	0.261397015500305
165	KC_ABp1	CG10565	0.673562732322876	0.469901473244953	1	pseudobulk	DESeq2	LRT	0.327993193365746
165	KC_ABp1	CG10576	-0.418814989698953	0.689083120781021	1	pseudobulk	DESeq2	LRT	0.161728388078988
165	KC_ABp1	CG10581	-1.76541829495743	0.569721440048943	1	pseudobulk	DESeq2	LRT	0.244337436643475
165	KC_ABp1	CG10584	0.295850504875218	1	1	pseudobulk	DESeq2	LRT	0
165	KC_ABp1	CG10600	0.149880005690654	0.824367365548634	1	pseudobulk	DESeq2	LRT	0.083879209084332
165	KC_ABp1	CG10602	-1.77175424419709	0.182839970784163	1	pseudobulk	DESeq2	LRT	0.737928856778231
165	KC_ABp1	CG10616	-0.398059353943635	0.685924445087449	1	pseudobulk	DESeq2	LRT	0.163723719408006
165	KC_ABp1	CG10623	0.400640538490757	1	1	pseudobulk	DESeq2	LRT	0
165	KC_ABp1	CG10628	1.47863010120697	0.437708761008955	1	pseudobulk	DESeq2	LRT	0.358814760629823

165	KC_ABp1	CG10631	1.22388802561056	0.169447104375493	1	pseudobulk	DESeq2	LRT	0.770965848275349
165	KC_ABp1	CG10638	-0.321121593834955	0.826391977881708	1	pseudobulk	DESeq2	LRT	0.082813907338650
165	KC_ABp1	CG10646	-0.545703191618431	0.739011330612462	1	pseudobulk	DESeq2	LRT	0.131348902897806
165	KC_ABp1	CG10654	-2.04185063924413	0.0742493627149664	1	pseudobulk	DESeq2	LRT	1.1293072695604
165	KC_ABp1	CG10669	0.147714867069266	0.97877687198138	1	pseudobulk	DESeq2	LRT	0.009316301365134
165	KC_ABp1	CG10672	0.726567150156693	0.499991831114897	1	pseudobulk	DESeq2	LRT	0.301037091125391
165	KC_ABp1	CG10674	0.451842529049418	0.640536820833255	1	pseudobulk	DESeq2	LRT	0.193455900072184
165	KC_ABp1	CG10681	-1.71345797286108	0.355382733520169	1	pseudobulk	DESeq2	LRT	0.449303676471839
165	KC_ABp1	CG10713	0.411140290556064	0.758851472491603	1	pseudobulk	DESeq2	LRT	0.119843218820646
165	KC_ABp1	CG10731	-0.655217611760584	0.604308906887488	1	pseudobulk	DESeq2	LRT	0.218741004653447
165	KC_ABp1	CG10737	0.664040107753029	0.567040758073436	1	pseudobulk	DESeq2	LRT	0.24638572352295
165	KC_ABp1	CG10738	-0.829256503624173	0.599947273168892	1	pseudobulk	DESeq2	LRT	0.221886916246383
165	KC_ABp1	CG1074	0.365616955112549	0.763670253405766	1	pseudobulk	DESeq2	LRT	0.117094125769452
166	KC_ABp1	CG10747	-1.31418300799745	0.335052541367161	1	pseudobulk	DESeq2	LRT	0.474887083600178
166	KC_ABp1	CG10778	0.572943232322451	0.745322802236824	1	pseudobulk	DESeq2	LRT	0.127655591859588
166	KC_ABp1	CG10795	-1.58305763264611	0.196993879986059	1	pseudobulk	DESeq2	LRT	0.70554726586668
166	KC_ABp1	CG10802	-0.691771035910761	0.630251402294302	1	pseudobulk	DESeq2	LRT	0.200486179355603
166	KC_ABp1	CG10803	0.901957564387197	0.267032875080624	1	pseudobulk	DESeq2	LRT	0.573435268271365
166	KC_ABp1	CG10804	0.264197294578655	0.503755695326317	1	pseudobulk	DESeq2	LRT	0.297780030806546
166	KC_ABp1	CG10809	0.178705480628618	0.767260626189416	1	pseudobulk	DESeq2	LRT	0.115057088085491
166	KC_ABp1	CG10863	-0.174880928544256	0.821775712075991	1	pseudobulk	DESeq2	LRT	0.085246698639065
166	KC_ABp1	CG10866	0.194179896432272	0.882522135034132	1	pseudobulk	DESeq2	LRT	0.054274393019320
166	KC_ABp1	CG10874	-0.970687841044315	0.4025893916358	1	pseudobulk	DESeq2	LRT	0.395137673100454
166	KC_ABp1	CG10889	-1.2710602462342	0.568055123161665	1	pseudobulk	DESeq2	LRT	0.245609519001368
166	KC_ABp1	CG10898	-0.543035387118704	0.705856057513031	1	pseudobulk	DESeq2	LRT	0.151283853907728
166	KC_ABp1	CG1090	-0.329407247014655	0.51320471817083	1	pseudobulk	DESeq2	LRT	0.289709359572758
166	KC_ABp1	CG10903	0.662046796791161	0.706352715680322	1	pseudobulk	DESeq2	LRT	0.150978380787275
166	KC_ABp1	CG10904	-0.239190065655376	0.862286773611066	1	pseudobulk	DESeq2	LRT	0.064348275376558
166	KC_ABp1	CG10907	0.634020948563258	0.724023316408978	1	pseudobulk	DESeq2	LRT	0.140247447580887
166	KC_ABp1	CG10914	1.29969445817716	0.460551035978938	1	pseudobulk	DESeq2	LRT	0.336722236442381
166	KC_ABp1	CG10916	2.73255099795875	0.0947547175613903	1	pseudobulk	DESeq2	LRT	1.02339915856436
166	KC_ABp1	CG10919	-0.584613676872836	1	1	pseudobulk	DESeq2	LRT	0
166	KC_ABp1	CG1092	1.10067831374717	0.673764308950964	1	pseudobulk	DESeq2	LRT	0.171491998454619
166	KC_ABp1	CG10927	1.10067831374717	0.673764308950964	1	pseudobulk	DESeq2	LRT	0.171491998454619
166	KC_ABp1	CG10932	0.459542441629015	0.770191330441885	1	pseudobulk	DESeq2	LRT	0.113401374264885
166	KC_ABp1	CG10947	-0.329913584231585	0.60897418878584	1	pseudobulk	DESeq2	LRT	0.215401114436808
166	KC_ABp1	CG10948	0.489108773871175	0.537090739875513	1	pseudobulk	DESeq2	LRT	0.269952335346777
166	KC_ABp1	CG10949	-1.4524490200424	0.29173522517058	1	pseudobulk	DESeq2	LRT	0.535011129419248
166	KC_ABp1	CG10958	1.90090186132448	0.432852416067745	1	pseudobulk	DESeq2	LRT	0.363660154022951
166	KC_ABp1	CG10959	-2.02056439956913	0.551734546998444	1	pseudobulk	DESeq2	LRT	0.258269821731848
166	KC_ABp1	CG10960	-0.156877423144472	0.641175255914636	1	pseudobulk	DESeq2	LRT	0.193023246185467
166	KC_ABp1	CG10962	0.400640538490757	0.854745824291873	1	pseudobulk	DESeq2	LRT	0.068163012194342
166	KC_ABp1	CG10973	-0.0931628780578538	0.936992984333493	1	pseudobulk	DESeq2	LRT	0.028263660848248
166	KC_ABp1	CG10979	-0.80736476286774	0.497252834216039	1	pseudobulk	DESeq2	LRT	0.303422732827201
166	KC_ABp1	CG10984	-0.0227156122084031	0.923960542355598	1	pseudobulk	DESeq2	LRT	0.034346574887174
166	KC_ABp1	CG11000	0.24869058573774	0.533583022541046	1	pseudobulk	DESeq2	LRT	0.272797997183785
166	KC_ABp1	CG11007	0.0695216339027072	0.951132135107648	1	pseudobulk	DESeq2	LRT	0.021759144932389
166	KC_ABp1	CG1103	1.65402532028382	0.202527561662378	1	pseudobulk	DESeq2	LRT	0.69351586596356
166	KC_ABp1	CG11030	-0.373719194702894	0.679607932183691	1	pseudobulk	DESeq2	LRT	0.167741560816085
166	KC_ABp1	CG11035	-0.351989205469897	0.747613209782501	1	pseudobulk	DESeq2	LRT	0.1263230335573
166	KC_ABp1	CG1105	-0.905390095058659	0.428570226424807	1	pseudobulk	DESeq2	LRT	0.36797800349614
166	KC_ABp1	CG11050	-0.183213332085375	0.769955920180773	1	pseudobulk	DESeq2	LRT	0.113534137386255

166	KC_ABp1	CG11052	-0.718744503482587	0.834003203510492	1	pseudobulk	DESeq2	LRT	0.078832281179698
166	KC_ABp1	CG11070	0.224708527921892	0.856901838828719	1	pseudobulk	DESeq2	LRT	0.067068925214129
166	KC_ABp1	CG11076	0.724993901516732	0.439426621985999	1	pseudobulk	DESeq2	LRT	0.357113635555283
166	KC_ABp1	CG11077	-0.0162396099699782	0.888750020065218	1	pseudobulk	DESeq2	LRT	0.051220376457155
166	KC_ABp1	CG11095	-0.433603363252163	0.886641747525372	1	pseudobulk	DESeq2	LRT	0.052251823800215
166	KC_ABp1	CG11099	0.15860642035466	0.782745156126071	1	pseudobulk	DESeq2	LRT	0.106379611253976
166	KC_ABp1	CG11103	0.535802688654979	0.688764229120499	1	pseudobulk	DESeq2	LRT	0.161929415995429
166	KC_ABp1	CG11109	-0.200568682130691	0.870409320508799	1	pseudobulk	DESeq2	LRT	0.060276467112959
166	KC_ABp1	CG11110	0.181726175135085	0.842428369023126	1	pseudobulk	DESeq2	LRT	0.074467016581888
166	KC_ABp1	CG11122	-0.28620674007034	0.575522774988955	1	pseudobulk	DESeq2	LRT	0.239937485489761
166	KC_ABp1	CG11123	-2.02056439956913	0.551734546998444	1	pseudobulk	DESeq2	LRT	0.258269821731848
166	KC_ABp1	CG11127	-3.22584568007573	0.398257103620311	1	pseudobulk	DESeq2	LRT	0.39983646904701
166	KC_ABp1	CG11134	-0.811113612176247	0.72211469299442	1	pseudobulk	DESeq2	LRT	0.141393818249345
166	KC_ABp1	CG11137	-0.581683391683447	0.358567145626505	1	pseudobulk	DESeq2	LRT	0.445429505980703
166	KC_ABp1	CG11141	0.139979168119371	0.902350647464814	1	pseudobulk	DESeq2	LRT	0.044624665709828
166	KC_ABp1	CG11151	0.164229069554989	0.765825343971797	1	pseudobulk	DESeq2	LRT	0.115870265351909
166	KC_ABp1	CG11155	0.182680811560538	0.452568631295417	1	pseudobulk	DESeq2	LRT	0.344315551414046
166	KC_ABp1	CG11158	0.295850504875218	1	1	pseudobulk	DESeq2	LRT	0
166	KC_ABp1	CG1116	0.535264099255538	0.728413001870977	1	pseudobulk	DESeq2	LRT	0.137622310830956
166	KC_ABp1	CG11168	0.673196662949902	0.7313687318949	1	pseudobulk	DESeq2	LRT	0.135863610932324
166	KC_ABp1	CG11170	-2.02056439956913	0.551734546998444	1	pseudobulk	DESeq2	LRT	0.258269821731848
166	KC_ABp1	CG11178	-0.483612558857755	0.710780042274975	1	pseudobulk	DESeq2	LRT	0.148264775086678
166	KC_ABp1	CG11180	-3.0120367293273	0.19415545747128	1	pseudobulk	DESeq2	LRT	0.711850397462252
166	KC_ABp1	CG11191	0.0649564526351041	0.809017055341552	1	pseudobulk	DESeq2	LRT	0.092042322686063
166	KC_ABp1	CG11200	2.03672877216785	0.36268011289938	1	pseudobulk	DESeq2	LRT	0.440476257749733
166	KC_ABp1	CG11241	0.987429041292792	0.60343242224665	1	pseudobulk	DESeq2	LRT	0.219371359019291
166	KC_ABp1	CG11247	0.66531774311483	0.255308692442254	1	pseudobulk	DESeq2	LRT	0.592934398609616
166	KC_ABp1	CG11248	-0.110143220099627	0.867646644810605	1	pseudobulk	DESeq2	LRT	0.061657108295326
166	KC_ABp1	CG11257	0.657117986908535	0.730938424265734	1	pseudobulk	DESeq2	LRT	0.136119207348541
166	KC_ABp1	CG11267	0.499044009371562	0.230107723439778	1	pseudobulk	DESeq2	LRT	0.638068804230163
166	KC_ABp1	CG11279	0.621789280654577	0.658249637856965	1	pseudobulk	DESeq2	LRT	0.181609371153554
166	KC_ABp1	CG1129	0.218322878143344	0.736830327443345	1	pseudobulk	DESeq2	LRT	0.132632507172994
166	KC_ABp1	CG11307	0.521188273538617	0.818300014350056	1	pseudobulk	DESeq2	LRT	0.087087441207930
166	KC_ABp1	CG11309	0.751311405186704	0.628648169823892	1	pseudobulk	DESeq2	LRT	0.20159234446511
166	KC_ABp1	CG11317	-0.213102198538866	0.692817323896673	1	pseudobulk	DESeq2	LRT	0.159381261321736
166	KC_ABp1	CG11319	-0.112371999836281	0.737380698157942	1	pseudobulk	DESeq2	LRT	0.132308234770598
166	KC_ABp1	CG11334	0.337103503630046	0.823991888025558	1	pseudobulk	DESeq2	LRT	0.084077063792057
166	KC_ABp1	CG11357	-0.0372714547516171	0.966275790068697	1	pseudobulk	DESeq2	LRT	0.014898901523760
166	KC_ABp1	CG11360	0.300427432234341	0.487104104146002	1	pseudobulk	DESeq2	LRT	0.312378211219746
166	KC_ABp1	CG11362	-1.51023398327283	0.845597316437363	1	pseudobulk	DESeq2	LRT	0.072836403962567
166	KC_ABp1	CG11367	0.034597143421512	0.946694489432362	1	pseudobulk	DESeq2	LRT	0.023790150838767
166	KC_ABp1	CG11377	-0.775458923455661	0.500737558826611	1	pseudobulk	DESeq2	LRT	0.30038983224921
166	KC_ABp1	CG11396	-0.609539944343713	0.594711895569607	1	pseudobulk	DESeq2	LRT	0.225693374551721
166	KC_ABp1	CG11398	1.00818416031385	0.633942837086	1	pseudobulk	DESeq2	LRT	0.197949900883805
166	KC_ABp1	CG11399	-1.08122693913234	0.307979078026861	1	pseudobulk	DESeq2	LRT	0.511478785467439
166	KC_ABp1	CG11413	-1.51023398327283	0.845597316437363	1	pseudobulk	DESeq2	LRT	0.072836403962567
166	KC_ABp1	CG11414	0.157337135978556	0.83541032713115	1	pseudobulk	DESeq2	LRT	0.078100160399182
166	KC_ABp1	CG11417	-1.49678648501033	0.560524490544228	1	pseudobulk	DESeq2	LRT	0.251405407376134
166	KC_ABp1	CG1142	-2.78540052590447	0.157470524062141	1	pseudobulk	DESeq2	LRT	0.802800727177103
166	KC_ABp1	CG11436	-0.0155184523084071	0.978623921589579	1	pseudobulk	DESeq2	LRT	0.009384172504778
166	KC_ABp1	CG11444	-0.588434309476029	0.484960253596024	1	pseudobulk	DESeq2	LRT	0.314293853874579
166	KC_ABp1	CG11447	-1.17165849560558	0.525950705487356	1	pseudobulk	DESeq2	LRT	0.279054958009838

166	KC_ABp1	CG11454	-0.0961723866170551	0.900772260952288	1	pseudobulk	DESeq2	LRT	0.045384996272528
166	KC_ABp1	CG11456	0.575655515216934	0.601444454133934	1	pseudobulk	DESeq2	LRT	0.220804475351621
166	KC_ABp1	CG11474	0.729332194125817	0.6233887678096	1	pseudobulk	DESeq2	LRT	0.20524102708686
166	KC_ABp1	CG11486	0.22186302932646	0.436214969590405	1	pseudobulk	DESeq2	LRT	0.360299434816564
166	KC_ABp1	CG11504	-0.322062985342857	0.721348172235127	1	pseudobulk	DESeq2	LRT	0.141855064292226
166	KC_ABp1	CG11523	-0.011310430919638	0.919372246429333	1	pseudobulk	DESeq2	LRT	0.036508610662411
166	KC_ABp1	CG11537	-0.0130878823554842	0.955214820735543	1	pseudobulk	DESeq2	LRT	0.019898947820934
166	KC_ABp1	CG11539	0.400640538490757	1	1	pseudobulk	DESeq2	LRT	0
166	KC_ABp1	CG11555	-0.29595577233763	0.805324368639219	1	pseudobulk	DESeq2	LRT	0.094029159214511
167	KC_ABp1	CG11560	0.446647467407347	0.793927489874372	1	pseudobulk	DESeq2	LRT	0.100219160274967
167	KC_ABp1	CG11562	0.805668087064073	0.461486719383217	1	pseudobulk	DESeq2	LRT	0.335840792539641
167	KC_ABp1	CG11563	-0.628754386092485	0.669277134727908	1	pseudobulk	DESeq2	LRT	0.174394012036876
167	KC_ABp1	CG11583	-0.156820399322952	0.840097095934469	1	pseudobulk	DESeq2	LRT	0.075670516567139
167	KC_ABp1	CG11586	1.14565310501574	0.192099211652833	1	pseudobulk	DESeq2	LRT	0.716474417415907
167	KC_ABp1	CG11590	-0.631273094547312	0.634624642523633	1	pseudobulk	DESeq2	LRT	0.197483068220086
167	KC_ABp1	CG11593	-0.652499302457841	0.541997092929105	1	pseudobulk	DESeq2	LRT	0.266003042849499
167	KC_ABp1	CG11596	-1.97531498634805	0.418961214978195	1	pseudobulk	DESeq2	LRT	0.377826179660417
167	KC_ABp1	CG11597	-0.39141522202559	0.871679849683438	1	pseudobulk	DESeq2	LRT	0.059642993331117
167	KC_ABp1	CG11601	0.302222827399987	0.832506282698367	1	pseudobulk	DESeq2	LRT	0.079612480307004
167	KC_ABp1	CG1161	0.0861182872204801	0.884614400760409	1	pseudobulk	DESeq2	LRT	0.053245994968818
167	KC_ABp1	CG11626	-1.76541829495743	0.569721440048943	1	pseudobulk	DESeq2	LRT	0.244337436643475
167	KC_ABp1	CG11638	-0.0951301161305075	0.846531300813643	1	pseudobulk	DESeq2	LRT	0.072356979056140
167	KC_ABp1	CG11655	-1.75283054089783	0.460938311409796	1	pseudobulk	DESeq2	LRT	0.33635719349755
167	KC_ABp1	CG11658	0.0222095420757178	0.973065954299922	1	pseudobulk	DESeq2	LRT	0.011857722303320
167	KC_ABp1	CG11679	-2.02056439956913	0.551734546998444	1	pseudobulk	DESeq2	LRT	0.258269821731848
167	KC_ABp1	CG11695	-1.76541829495743	0.569721440048943	1	pseudobulk	DESeq2	LRT	0.244337436643475
167	KC_ABp1	CG11696	-1.88936695114569	0.190397372869943	1	pseudobulk	DESeq2	LRT	0.720339048367292
167	KC_ABp1	CG11699	0.661244572504176	0.333525659315207	1	pseudobulk	DESeq2	LRT	0.476870748642992
167	KC_ABp1	CG11700	0.400640538490757	1	1	pseudobulk	DESeq2	LRT	0
167	KC_ABp1	CG11710	-0.825200906688751	0.463585327982631	1	pseudobulk	DESeq2	LRT	0.333870317481983
167	KC_ABp1	CG1172	-0.048119529888121	0.951013388899815	1	pseudobulk	DESeq2	LRT	0.021813368778278
167	KC_ABp1	CG11722	-0.606621978858135	0.628483522444824	1	pseudobulk	DESeq2	LRT	0.201706104143651
167	KC_ABp1	CG11723	0.43143638126085	0.854037960782142	1	pseudobulk	DESeq2	LRT	0.068522825105123
167	KC_ABp1	CG11737	2.84956721136844	0.163313208999115	1	pseudobulk	DESeq2	LRT	0.786978687501978
167	KC_ABp1	CG11752	-0.363438348986845	0.353611861035784	1	pseudobulk	DESeq2	LRT	0.451473176079785
167	KC_ABp1	CG11753	0.383569549757025	0.66803993822949	1	pseudobulk	DESeq2	LRT	0.175197572802602
167	KC_ABp1	CG11755	1.52373887470721	0.368632299955946	1	pseudobulk	DESeq2	LRT	0.433406614103232
167	KC_ABp1	CG11768	0.816898736439022	0.800081777561681	1	pseudobulk	DESeq2	LRT	0.096865620847217
167	KC_ABp1	CG11771	-0.441494293995906	0.733232799376769	1	pseudobulk	DESeq2	LRT	0.134758116173354
167	KC_ABp1	CG11777	-0.459363159795541	0.763063091478384	1	pseudobulk	DESeq2	LRT	0.117439552283478
167	KC_ABp1	CG11779	-0.268059280981327	0.757884166810113	1	pseudobulk	DESeq2	LRT	0.120397165818204
167	KC_ABp1	CG11781	-0.36788109669089	0.646352265289464	1	pseudobulk	DESeq2	LRT	0.189530724768862
167	KC_ABp1	CG11790	-0.249558935242125	0.848973270986605	1	pseudobulk	DESeq2	LRT	0.071105982837035
167	KC_ABp1	CG11791	1.32823068455131	0.607220246153186	1	pseudobulk	DESeq2	LRT	0.21665375647025
167	KC_ABp1	CG11807	-1.99700631327622	0.139775891597147	1	pseudobulk	DESeq2	LRT	0.854567728799313
167	KC_ABp1	CG11808	-2.39593946583853	0.39950212853761	1	pseudobulk	DESeq2	LRT	0.398480902433404
167	KC_ABp1	CG11811	0.501419958101442	0.611747752249394	1	pseudobulk	DESeq2	LRT	0.213427617705603
167	KC_ABp1	CG11836	-2.37872251454671	0.371481306607683	1	pseudobulk	DESeq2	LRT	0.430063035581449
167	KC_ABp1	CG11837	-2.02056439956913	0.551734546998444	1	pseudobulk	DESeq2	LRT	0.258269821731848
167	KC_ABp1	CG11839	-0.108795934239537	0.945904061246687	1	pseudobulk	DESeq2	LRT	0.024152909881582
167	KC_ABp1	CG11857	-0.175558972550309	0.78316972645206	1	pseudobulk	DESeq2	LRT	0.106144108601403
167	KC_ABp1	CG11858	-0.070821316878686	0.90130902118514	1	pseudobulk	DESeq2	LRT	0.045126282066418

167	KC_ABp1	CG11873	0.303790250561759	0.450548228475332	1	pseudobulk	DESeq2	LRT	0.34625871358696
167	KC_ABp1	CG11882	0.295850504875218	1	1	pseudobulk	DESeq2	LRT	0
167	KC_ABp1	CG11883	0.411748705480489	0.753402991330149	1	pseudobulk	DESeq2	LRT	0.122972659795615
167	KC_ABp1	CG11885	1.62040905205773	0.278322410173747	1	pseudobulk	DESeq2	LRT	0.555451823407397
167	KC_ABp1	CG11898	-2.88847209274501	0.216066922480215	1	pseudobulk	DESeq2	LRT	0.665411713837973
167	KC_ABp1	CG11902	-0.870081633678332	0.532046908423681	1	pseudobulk	DESeq2	LRT	0.274050076029253
167	KC_ABp1	CG11906	0.600326153075906	0.678593606950301	1	pseudobulk	DESeq2	LRT	0.168390236172312
167	KC_ABp1	CG11920	0.532099693644146	0.768186641922076	1	pseudobulk	DESeq2	LRT	0.114533249097387
167	KC_ABp1	CG11927	-0.855010247463926	0.481874476662216	1	pseudobulk	DESeq2	LRT	0.317066076268439
167	KC_ABp1	CG11961	0.400640538490757	0.854745824291873	1	pseudobulk	DESeq2	LRT	0.068163012194342
167	KC_ABp1	CG11964	1.60875763568915	0.360260962940573	1	pseudobulk	DESeq2	LRT	0.443382794491341
167	KC_ABp1	CG11970	-0.213620807987907	0.875502133948888	1	pseudobulk	DESeq2	LRT	0.057742791029881
167	KC_ABp1	CG11975	0.656637426427931	0.484374094840533	1	pseudobulk	DESeq2	LRT	0.314819091723558
167	KC_ABp1	CG11980	-0.281916011274866	0.852090910950044	1	pseudobulk	DESeq2	LRT	0.069514067184393
167	KC_ABp1	CG11983	0.218060112955718	0.926586010097	1	pseudobulk	DESeq2	LRT	0.033114261205539
167	KC_ABp1	CG11999	0.395664647573929	0.731941672282492	1	pseudobulk	DESeq2	LRT	0.135523526066125
167	KC_ABp1	CG12004	-0.215547267454988	0.633600604731381	1	pseudobulk	DESeq2	LRT	0.198184416911971
167	KC_ABp1	CG12007	0.938362670607093	0.486849430808091	1	pseudobulk	DESeq2	LRT	0.312605333405547
167	KC_ABp1	CG12010	-0.0295035337095442	1	1	pseudobulk	DESeq2	LRT	0
167	KC_ABp1	CG12012	-0.189479868268179	0.832442763025174	1	pseudobulk	DESeq2	LRT	0.079645617950037
167	KC_ABp1	CG12024	-0.34965937360877	0.669812992508423	1	pseudobulk	DESeq2	LRT	0.174046432609844
167	KC_ABp1	CG12025	0.446204867903921	0.362860311835012	1	pseudobulk	DESeq2	LRT	0.440260530541899
167	KC_ABp1	CG12038	0.256304281872776	0.635070789487052	1	pseudobulk	DESeq2	LRT	0.197177862471652
167	KC_ABp1	CG12054	0.157389872122243	0.658193016752585	1	pseudobulk	DESeq2	LRT	0.181646729762561
167	KC_ABp1	CG12056	1.11325719108385	0.45482052765407	1	pseudobulk	DESeq2	LRT	0.342159942300701
167	KC_ABp1	CG12071	-0.311075249654432	0.462424471706155	1	pseudobulk	DESeq2	LRT	0.33495919092463
167	KC_ABp1	CG12081	-0.902721280952588	0.432903130659879	1	pseudobulk	DESeq2	LRT	0.363609273453663
167	KC_ABp1	CG12084	0.287849394409509	0.701446979072437	1	pseudobulk	DESeq2	LRT	0.154005150817395
167	KC_ABp1	CG12091	-0.160556690086154	0.75274273763502	1	pseudobulk	DESeq2	LRT	0.123353425812215
167	KC_ABp1	CG12093	-0.257157366768849	0.824762697732492	1	pseudobulk	DESeq2	LRT	0.083670989498649
167	KC_ABp1	CG12096	-0.220080077043622	0.857921325919194	1	pseudobulk	DESeq2	LRT	0.066552536493670
167	KC_ABp1	CG12099	0.839886857207403	0.239667777045311	1	pseudobulk	DESeq2	LRT	0.620390352249711
167	KC_ABp1	CG12104	0.690976509258701	0.671305966660401	1	pseudobulk	DESeq2	LRT	0.173079492728424
167	KC_ABp1	CG12106	1.58001492720401	0.249997470846518	1	pseudobulk	DESeq2	LRT	0.602064384939792
167	KC_ABp1	CG12107	0.148079614483731	0.884006925048349	1	pseudobulk	DESeq2	LRT	0.053544332839297
167	KC_ABp1	CG12112	-0.584613676872836	1	1	pseudobulk	DESeq2	LRT	0
167	KC_ABp1	CG12118	-1.3755832762945	0.385446791446311	1	pseudobulk	DESeq2	LRT	0.414035565151687
167	KC_ABp1	CG12121	0.425021166432048	0.557388311427335	1	pseudobulk	DESeq2	LRT	0.253842142791418
167	KC_ABp1	CG12123	-0.45608386975785	0.621059833702662	1	pseudobulk	DESeq2	LRT	0.206866557319197
167	KC_ABp1	CG12128	-0.546601890199522	0.855848288403055	1	pseudobulk	DESeq2	LRT	0.067603213534073
167	KC_ABp1	CG12129	1.17722560577903	0.390839972843	1	pseudobulk	DESeq2	LRT	0.40800102556388
167	KC_ABp1	CG12134	0.400640538490757	0.854745824291873	1	pseudobulk	DESeq2	LRT	0.068163012194342
167	KC_ABp1	CG12155	-0.94891080681207	0.471407382527055	1	pseudobulk	DESeq2	LRT	0.326603620442037
167	KC_ABp1	CG12159	0.0702198305320965	0.919943413994396	1	pseudobulk	DESeq2	LRT	0.036238885421595
167	KC_ABp1	CG12163	0.568006638346436	0.416048288710224	1	pseudobulk	DESeq2	LRT	0.380856259990301
167	KC_ABp1	CG12170	1.49700930425751	0.231750011218547	1	pseudobulk	DESeq2	LRT	0.634980236160151
167	KC_ABp1	CG12171	-0.584613676872836	1	1	pseudobulk	DESeq2	LRT	0
167	KC_ABp1	CG12173	-0.216236854253498	0.812362824168897	1	pseudobulk	DESeq2	LRT	0.090249959255078
167	KC_ABp1	CG12179	0.0305710196345365	0.911897373843046	1	pseudobulk	DESeq2	LRT	0.040054035006619
167	KC_ABp1	CG1218	-0.939073844242465	0.616219751380642	1	pseudobulk	DESeq2	LRT	0.210264385574174
167	KC_ABp1	CG12182	0.0198862537038369	1	1	pseudobulk	DESeq2	LRT	0
167	KC_ABp1	CG12194	0.119309234506315	0.693168112626484	1	pseudobulk	DESeq2	LRT	0.1591614240725

167	KC_ABp1	CG12204	0.122720335974157	0.930351673940671	1	pseudobulk	DESeq2	LRT	0.031352856626535
167	KC_ABp1	CG12206	1.70517510654842	0.489009985553122	1	pseudobulk	DESeq2	LRT	0.310682272519852
167	KC_ABp1	CG12213	-0.986136626673404	0.352871411322983	1	pseudobulk	DESeq2	LRT	0.452383525566387
167	KC_ABp1	CG12219	0.0972295387953128	0.927894828933504	1	pseudobulk	DESeq2	LRT	0.03250124555079
167	KC_ABp1	CG12236	0.202296264614772	0.783887555049203	1	pseudobulk	DESeq2	LRT	0.105746230330868
168	KC_ABp1	CG12237	1.20791601282125	0.267915472790444	1	pseudobulk	DESeq2	LRT	0.572002204072028
168	KC_ABp1	CG12239	0.113065313169369	0.971312496720761	1	pseudobulk	DESeq2	LRT	0.012641023678542
168	KC_ABp1	CG12241	0.380091888738479	0.791855393767422	1	pseudobulk	DESeq2	LRT	0.101354120712066
168	KC_ABp1	CG12253	-0.235310012799337	0.854209627867877	1	pseudobulk	DESeq2	LRT	0.068435537912323
168	KC_ABp1	CG12259	-0.426943879249595	0.773227455507295	1	pseudobulk	DESeq2	LRT	0.111692733583447
168	KC_ABp1	CG12267	1.10067831374717	0.673764308950964	1	pseudobulk	DESeq2	LRT	0.171491998454619
168	KC_ABp1	CG1227	0.326162780164558	0.850310530222794	1	pseudobulk	DESeq2	LRT	0.070422442598934
168	KC_ABp1	CG12279	-0.583287015395893	0.580185164791549	1	pseudobulk	DESeq2	LRT	0.236433380206809
168	KC_ABp1	CG12288	-0.309353506685618	0.843790025162851	1	pseudobulk	DESeq2	LRT	0.073765612922301
168	KC_ABp1	CG12299	-0.0299296987826945	0.878754245904724	1	pseudobulk	DESeq2	LRT	0.056132563572645
168	KC_ABp1	CG12301	-1.18884049019016	0.532343884935051	1	pseudobulk	DESeq2	LRT	0.273807730333867
168	KC_ABp1	CG1231	2.96414098907676	0.144181322379525	1	pseudobulk	DESeq2	LRT	0.841090995600518
168	KC_ABp1	CG12316	0.455696216519348	0.72327899478506	1	pseudobulk	DESeq2	LRT	0.140694147347794
168	KC_ABp1	CG12320	-0.718744503482587	0.834003203510492	1	pseudobulk	DESeq2	LRT	0.078832281179698
168	KC_ABp1	CG12321	-0.267053214659678	0.827410769712075	1	pseudobulk	DESeq2	LRT	0.082278830556670
168	KC_ABp1	CG12325	0.295850504875218	1	1	pseudobulk	DESeq2	LRT	0
168	KC_ABp1	CG1233	0.684496455792122	0.675584031836112	1	pseudobulk	DESeq2	LRT	0.170320624002478
168	KC_ABp1	CG12333	0.153839767805239	0.877647998098596	1	pseudobulk	DESeq2	LRT	0.056679633451414
168	KC_ABp1	CG12338	0.326162780164558	0.850310530222794	1	pseudobulk	DESeq2	LRT	0.070422442598934
168	KC_ABp1	CG1234	1.09599059461232	0.469063405901426	1	pseudobulk	DESeq2	LRT	0.328768447323568
168	KC_ABp1	CG12341	1.97162525132653	0.215782565172766	1	pseudobulk	DESeq2	LRT	0.665983648416538
168	KC_ABp1	CG12343	-0.207730585708928	0.821075860956304	1	pseudobulk	DESeq2	LRT	0.085616715630020
168	KC_ABp1	CG12355	-0.155181309333815	0.770137978289617	1	pseudobulk	DESeq2	LRT	0.113431459448886
168	KC_ABp1	CG1236	2.23406790830633	0.338204825165653	1	pseudobulk	DESeq2	LRT	0.470820200618027
168	KC_ABp1	CG12375	-0.326874379011231	0.758261542280598	1	pseudobulk	DESeq2	LRT	0.120180970122977
168	KC_ABp1	CG12379	1.3210887790502	0.567025539115222	1	pseudobulk	DESeq2	LRT	0.24639737982486
168	KC_ABp1	CG12384	0.283218748659879	0.444965856373948	1	pseudobulk	DESeq2	LRT	0.351673312518589
168	KC_ABp1	CG1239	0.48909724424222	0.701600527195429	1	pseudobulk	DESeq2	LRT	0.153910093305101
168	KC_ABp1	CG12391	0.934568630864741	0.338114329362933	1	pseudobulk	DESeq2	LRT	0.470936423344318
168	KC_ABp1	CG12402	0.312898692994941	0.696565439594468	1	pseudobulk	DESeq2	LRT	0.157038077058026
168	KC_ABp1	CG12470	-2.39593946583853	0.39950212853761	1	pseudobulk	DESeq2	LRT	0.398480902433404
168	KC_ABp1	CG12499	1.26676202814031	0.62240272781904	1	pseudobulk	DESeq2	LRT	0.20592851262158
168	KC_ABp1	CG12502	1.57769114991325	0.266780937212512	1	pseudobulk	DESeq2	LRT	0.573845206086789
168	KC_ABp1	CG12531	0.066632788303685	0.866670949363532	1	pseudobulk	DESeq2	LRT	0.062145760656128
168	KC_ABp1	CG12535	0.18797367449671	0.901146219696155	1	pseudobulk	DESeq2	LRT	0.045204734820085
168	KC_ABp1	CG12541	0.960565319834105	0.613128326141245	1	pseudobulk	DESeq2	LRT	0.212448619278634
168	KC_ABp1	CG12547	-0.122321300229352	0.857878841954432	1	pseudobulk	DESeq2	LRT	0.06657404313741
168	KC_ABp1	CG12560	-0.584613676872836	1	1	pseudobulk	DESeq2	LRT	0
168	KC_ABp1	CG12567	0.867943164154383	0.150356237387908	1	pseudobulk	DESeq2	LRT	0.82287855088929
168	KC_ABp1	CG12576	-0.0117147401056151	0.985134327334872	1	pseudobulk	DESeq2	LRT	0.006504547529972
168	KC_ABp1	CG12605	0.325994222316936	0.190671023961862	1	pseudobulk	DESeq2	LRT	0.71971530113134
168	KC_ABp1	CG12608	-2.06956992793787	0.419099033258735	1	pseudobulk	DESeq2	LRT	0.377683340957807
168	KC_ABp1	CG1265	0.498515581594688	0.49353899106355	1	pseudobulk	DESeq2	LRT	0.306678531069678
168	KC_ABp1	CG12655	-0.0295035337095442	1	1	pseudobulk	DESeq2	LRT	0
168	KC_ABp1	CG12659	0.235385875695668	0.862911742158911	1	pseudobulk	DESeq2	LRT	0.064033621264485
168	KC_ABp1	CG12662	0.400640538490757	0.854745824291873	1	pseudobulk	DESeq2	LRT	0.068163012194342
168	KC_ABp1	CG12672	0.532099693644146	0.768186641922076	1	pseudobulk	DESeq2	LRT	0.114533249097387

168	KC_ABp1	CG1271	-0.410989085971238	0.782745749437301	1	pseudobulk	DESeq2	LRT	0.106379282064209
168	KC_ABp1	CG12713	1.10067831374717	0.673764308950964	1	pseudobulk	DESeq2	LRT	0.171491998454619
168	KC_ABp1	CG12715	0.400640538490757	0.854745824291873	1	pseudobulk	DESeq2	LRT	0.068163012194342
168	KC_ABp1	CG12717	0.975676391298004	0.570122095752154	1	pseudobulk	DESeq2	LRT	0.244032127076418
168	KC_ABp1	CG12728	0.0458249728657489	0.966397123770882	1	pseudobulk	DESeq2	LRT	0.014844371285333
168	KC_ABp1	CG1273	-2.02056439956913	0.551734546998444	1	pseudobulk	DESeq2	LRT	0.258269821731848
168	KC_ABp1	CG12730	-1.76541829495743	0.569721440048943	1	pseudobulk	DESeq2	LRT	0.244337436643475
168	KC_ABp1	CG12746	-1.10907402927	0.368200809675418	1	pseudobulk	DESeq2	LRT	0.433915260815221
168	KC_ABp1	CG1275	-0.647646364975956	0.405626643652655	1	pseudobulk	DESeq2	LRT	0.391873526033473
168	KC_ABp1	CG12768	-3.02990869154163	0.208852371682276	1	pseudobulk	DESeq2	LRT	0.680160588616829
168	KC_ABp1	CG12769	0.49064821217195	0.398538295752004	1	pseudobulk	DESeq2	LRT	0.399529940680217
168	KC_ABp1	CG12773	2.06342303012375	0.192331260936542	1	pseudobulk	DESeq2	LRT	0.715950121123757
168	KC_ABp1	CG12795	-0.027933457568979	0.897509010933349	1	pseudobulk	DESeq2	LRT	0.046961182438796
168	KC_ABp1	CG12814	0.400640538490757	1	1	pseudobulk	DESeq2	LRT	0
168	KC_ABp1	CG12817	-0.619321288427446	0.363398796653965	1	pseudobulk	DESeq2	LRT	0.439616515133125
168	KC_ABp1	CG12818	-0.309353506685618	0.843790025162851	1	pseudobulk	DESeq2	LRT	0.073765612922301
168	KC_ABp1	CG12822	0.579794891859041	0.378779822336259	1	pseudobulk	DESeq2	LRT	0.421613163991416
168	KC_ABp1	CG12848	0.321436872123383	0.670167765503942	1	pseudobulk	DESeq2	LRT	0.173816465191341
168	KC_ABp1	CG12851	-0.343111751514144	0.67385644107872	1	pseudobulk	DESeq2	LRT	0.17143261605827
168	KC_ABp1	CG12866	-2.37872251454671	0.371481306607683	1	pseudobulk	DESeq2	LRT	0.430063035581449
168	KC_ABp1	CG12868	1.3164974534447	0.447201844524519	1	pseudobulk	DESeq2	LRT	0.349496413831006
168	KC_ABp1	CG12877	1.92267819634924	0.232957574724318	1	pseudobulk	DESeq2	LRT	0.632723163698309
168	KC_ABp1	CG12883	-1.19019811203007	0.584022864016683	1	pseudobulk	DESeq2	LRT	0.233570150281596
168	KC_ABp1	CG12895	0.0010945926804490	0.937567728167683	1	pseudobulk	DESeq2	LRT	0.027997349832709
168	KC_ABp1	CG12910	-1.51023398327283	0.845597316437363	1	pseudobulk	DESeq2	LRT	0.072836403962567
168	KC_ABp1	CG12913	0.493031600119714	0.654027119881634	1	pseudobulk	DESeq2	LRT	0.184404242851961
168	KC_ABp1	CG12923	1.513267077797	0.518264337008286	1	pseudobulk	DESeq2	LRT	0.285448674960176
168	KC_ABp1	CG12926	1.10067831374717	0.673764308950964	1	pseudobulk	DESeq2	LRT	0.171491998454619
168	KC_ABp1	CG12929	1.36453060896236	0.406007287559892	1	pseudobulk	DESeq2	LRT	0.39146617105669
168	KC_ABp1	CG12935	0.490733042290333	0.75024415919128	1	pseudobulk	DESeq2	LRT	0.124797376963917
168	KC_ABp1	CG12942	-0.811113612176247	0.72211469299442	1	pseudobulk	DESeq2	LRT	0.141393818249345
168	KC_ABp1	CG12945	1.91759982628891	0.0397502893270316	1	pseudobulk	DESeq2	LRT	1.4006597059339
168	KC_ABp1	CG12948	-2.6710190790924	0.180527645036365	1	pseudobulk	DESeq2	LRT	0.743456283135472
168	KC_ABp1	CG12964	-1.76541829495743	0.569721440048943	1	pseudobulk	DESeq2	LRT	0.244337436643475
168	KC_ABp1	CG12975	-0.0484632737075044	0.944857207121586	1	pseudobulk	DESeq2	LRT	0.024633819898041
168	KC_ABp1	CG12991	0.529633508896451	0.440736798009544	1	pseudobulk	DESeq2	LRT	0.35582068784878
168	KC_ABp1	CG12992	0.780319451757274	0.71518159883725	1	pseudobulk	DESeq2	LRT	0.145583668186203
168	KC_ABp1	CG12994	0.45714217746972	0.838963214018292	1	pseudobulk	DESeq2	LRT	0.076257081244136
168	KC_ABp1	CG13001	0.616935332995323	0.664955483628531	1	pseudobulk	DESeq2	LRT	0.177207428173006
168	KC_ABp1	CG13005	-1.51023398327283	0.845597316437363	1	pseudobulk	DESeq2	LRT	0.072836403962567
168	KC_ABp1	CG13008	-1.75283054089783	0.460938311409796	1	pseudobulk	DESeq2	LRT	0.33635719349755
168	KC_ABp1	CG13014	-1.26377724241541	0.545809526720244	1	pseudobulk	DESeq2	LRT	0.262958888296161
168	KC_ABp1	CG13016	-1.22746033122518	0.43704788328047	1	pseudobulk	DESeq2	LRT	0.359470978807015
168	KC_ABp1	CG13018	1.23743683658153	0.372937396351109	1	pseudobulk	DESeq2	LRT	0.428364065513412
168	KC_ABp1	CG13025	-0.712425324840931	0.73711055633684	1	pseudobulk	DESeq2	LRT	0.132467369120416
168	KC_ABp1	CG13055	1.72551572988559	0.155211195455306	1	pseudobulk	DESeq2	LRT	0.809076956086182
168	KC_ABp1	CG1307	-0.342280041402962	0.707568457255495	1	pseudobulk	DESeq2	LRT	0.150231535775343
168	KC_ABp1	CG1309	1.29368375666945	0.433897888326472	1	pseudobulk	DESeq2	LRT	0.362612463469296
168	KC_ABp1	CG13096	-3.49327352740568	0.104510949263223	1	pseudobulk	DESeq2	LRT	0.980838207586452
168	KC_ABp1	CG13097	1.06398294192051	0.576197676029729	1	pseudobulk	DESeq2	LRT	0.239428497686117
168	KC_ABp1	CG13108	0.0986271205058234	0.850214727374907	1	pseudobulk	DESeq2	LRT	0.070471376477507
168	KC_ABp1	CG13123	2.32638803143179	0.197774045360251	1	pseudobulk	DESeq2	LRT	0.703830703115085

168	KC_ABp1	CG13126	0.797330470441812	0.681869674788157	1	pseudobulk	DESeq2	LRT	0.166298623773026
169	KC_ABp1	CG13148	-0.0940619464014284	0.934106572356045	1	pseudobulk	DESeq2	LRT	0.0296035722219500
169	KC_ABp1	CG13151	-2.42721364822641	0.302598871124693	1	pseudobulk	DESeq2	LRT	0.519132696488745
169	KC_ABp1	CG1316	-0.0668526731871496	0.869758195477703	1	pseudobulk	DESeq2	LRT	0.060601470330906
169	KC_ABp1	CG13168	-0.584613676872836	1	1	pseudobulk	DESeq2	LRT	0
169	KC_ABp1	CG1317	-0.153227426813503	0.836482485148656	1	pseudobulk	DESeq2	LRT	0.077543148165932
169	KC_ABp1	CG13185	-0.817002774795447	0.779755878997874	1	pseudobulk	DESeq2	LRT	0.108041342179397
169	KC_ABp1	CG13192	-1.47801162537062	0.198149080997387	1	pseudobulk	DESeq2	LRT	0.703007937554819
169	KC_ABp1	CG13197	0.774965262941668	0.436530618534572	1	pseudobulk	DESeq2	LRT	0.35998528919953
169	KC_ABp1	CG13204	-0.243855256276095	0.758384820568564	1	pseudobulk	DESeq2	LRT	0.120110368192782
169	KC_ABp1	CG13220	-0.320905021209963	0.713011818585135	1	pseudobulk	DESeq2	LRT	0.146903271405121
169	KC_ABp1	CG13229	0.295850504875218	1	1	pseudobulk	DESeq2	LRT	0
169	KC_ABp1	CG13248	-0.416083971779807	0.474836368646663	1	pseudobulk	DESeq2	LRT	0.323456024979302
169	KC_ABp1	CG13252	0.838553191314359	0.489412790120734	1	pseudobulk	DESeq2	LRT	0.310324685155306
169	KC_ABp1	CG13255	0.295850504875218	1	1	pseudobulk	DESeq2	LRT	0
169	KC_ABp1	CG13284	-1.4568477020856	0.451682792630576	1	pseudobulk	DESeq2	LRT	0.345166454065426
169	KC_ABp1	CG13288	-1.48452149696191	0.498183500182326	1	pseudobulk	DESeq2	LRT	0.302610660377256
169	KC_ABp1	CG13293	0.168157000351714	0.706133468881039	1	pseudobulk	DESeq2	LRT	0.15111320359391
169	KC_ABp1	CG13295	0.400640538490757	0.854745824291873	1	pseudobulk	DESeq2	LRT	0.068163012194342
169	KC_ABp1	CG13300	-0.160145820752557	0.770519786386916	1	pseudobulk	DESeq2	LRT	0.113216204436385
169	KC_ABp1	CG13319	1.58473780507819	0.427908000542383	1	pseudobulk	DESeq2	LRT	0.368649593490531
169	KC_ABp1	CG13325	-0.508108381139994	0.623548536950574	1	pseudobulk	DESeq2	LRT	0.205129735434236
169	KC_ABp1	CG13339	-0.351606645780379	0.765335340020421	1	pseudobulk	DESeq2	LRT	0.116148232284685
169	KC_ABp1	CG13344	-0.594141995572261	0.438520859041833	1	pseudobulk	DESeq2	LRT	0.358009743800085
169	KC_ABp1	CG13360	0.295850504875218	1	1	pseudobulk	DESeq2	LRT	0
169	KC_ABp1	CG13364	0.169992392778133	0.6738437830586	1	pseudobulk	DESeq2	LRT	0.171440774116259
169	KC_ABp1	CG13365	-0.0072294574235169	0.836457985533963	1	pseudobulk	DESeq2	LRT	0.077555868340567
169	KC_ABp1	CG13366	-0.230584060414668	0.739904077908413	1	pseudobulk	DESeq2	LRT	0.13082457910048
169	KC_ABp1	CG13367	-0.208641691520638	0.84392371477426	1	pseudobulk	DESeq2	LRT	0.073696809002217
169	KC_ABp1	CG13369	-0.103374803876383	0.92123933443892	1	pseudobulk	DESeq2	LRT	0.035627527106988
169	KC_ABp1	CG13373	0.400640538490757	1	1	pseudobulk	DESeq2	LRT	0
169	KC_ABp1	CG13375	1.35316755063699	0.0709498197511128	1	pseudobulk	DESeq2	LRT	1.14904870353556
169	KC_ABp1	CG13377	-2.02056439956913	0.551734546998444	1	pseudobulk	DESeq2	LRT	0.258269821731848
169	KC_ABp1	CG13384	-0.155081825301571	0.836549077156136	1	pseudobulk	DESeq2	LRT	0.077508575546917
169	KC_ABp1	CG13390	0.278609059428086	0.922782656077873	1	pseudobulk	DESeq2	LRT	0.034900576743553
169	KC_ABp1	CG13392	0.218060112955718	0.926586010097	1	pseudobulk	DESeq2	LRT	0.033114261205539
169	KC_ABp1	CG13398	0.947032427647804	0.513315117532653	1	pseudobulk	DESeq2	LRT	0.28961594523791
169	KC_ABp1	CG13404	-0.253760336757324	0.751224196412894	1	pseudobulk	DESeq2	LRT	0.124230432191139
169	KC_ABp1	CG13409	-0.775452048432378	0.413338977037587	1	pseudobulk	DESeq2	LRT	0.383693639687552
169	KC_ABp1	CG13437	0.295850504875218	1	1	pseudobulk	DESeq2	LRT	0
169	KC_ABp1	CG1344	-0.439463927624798	0.679050080657179	1	pseudobulk	DESeq2	LRT	0.168098194863814
169	KC_ABp1	CG13458	-1.76541829495743	0.569721440048943	1	pseudobulk	DESeq2	LRT	0.244337436643475
169	KC_ABp1	CG13481	0.400640538490757	1	1	pseudobulk	DESeq2	LRT	0
169	KC_ABp1	CG13482	-0.491049632507942	1	1	pseudobulk	DESeq2	LRT	0
169	KC_ABp1	CG13484	0.322500017868688	0.81651006821752	1	pseudobulk	DESeq2	LRT	0.088038455698466
169	KC_ABp1	CG13502	1.90090186132448	0.432852416067745	1	pseudobulk	DESeq2	LRT	0.363660154022951
169	KC_ABp1	CG13506	-0.0957721943995552	0.837186474577068	1	pseudobulk	DESeq2	LRT	0.077177796654414
169	KC_ABp1	CG13532	-0.542896132173624	0.550758576738308	1	pseudobulk	DESeq2	LRT	0.259038731033995
169	KC_ABp1	CG1354	-1.87615518447871	0.154415309894939	1	pseudobulk	DESeq2	LRT	0.811309642644948
169	KC_ABp1	CG13551	-0.215382012569626	0.582915111992061	1	pseudobulk	DESeq2	LRT	0.23439468551142
169	KC_ABp1	CG1358	0.528285299027531	0.213188957951478	1	pseudobulk	DESeq2	LRT	0.671235293195796
169	KC_ABp1	CG13585	0.180741534613178	0.677650751834878	1	pseudobulk	DESeq2	LRT	0.168994075498428

169	KC_ABp1	CG13594	-0.653355646545221	0.659842136899533	1	pseudobulk	DESeq2	LRT	0.180559954267131
169	KC_ABp1	CG1360	0.411191767407229	0.81245359084367	1	pseudobulk	DESeq2	LRT	0.090201437507425
169	KC_ABp1	CG13601	-0.230615259042772	0.778608540692876	1	pseudobulk	DESeq2	LRT	0.108680836739814
169	KC_ABp1	CG13603	-0.76393785778064	0.291344039664359	1	pseudobulk	DESeq2	LRT	0.535593862308197
169	KC_ABp1	CG13604	0.0644720208414366	0.889299344095513	1	pseudobulk	DESeq2	LRT	0.050952027998970
169	KC_ABp1	CG13605	-1.07855442624534	0.40206216051417	1	pseudobulk	DESeq2	LRT	0.395706797956964
169	KC_ABp1	CG13609	-2.39593946583853	0.39950212853761	1	pseudobulk	DESeq2	LRT	0.398480902433404
169	KC_ABp1	CG13623	0.434819275545601	0.576349801281744	1	pseudobulk	DESeq2	LRT	0.239313852243404
169	KC_ABp1	CG13625	1.47333772349469	0.294894659250755	1	pseudobulk	DESeq2	LRT	0.530333092754847
169	KC_ABp1	CG13630	0.385265140850509	0.742462559489322	1	pseudobulk	DESeq2	LRT	0.129325441831842
169	KC_ABp1	CG13654	1.4794834203706	0.293079360399315	1	pseudobulk	DESeq2	LRT	0.533014764912994
169	KC_ABp1	CG13663	-0.131598492016444	0.920855488127423	1	pseudobulk	DESeq2	LRT	0.035808519241978
169	KC_ABp1	CG13667	0.295850504875218	1	1	pseudobulk	DESeq2	LRT	0
169	KC_ABp1	CG13671	-1.5186153921777	0.380926128425436	1	pseudobulk	DESeq2	LRT	0.419159237258171
169	KC_ABp1	CG13679	0.295850504875218	1	1	pseudobulk	DESeq2	LRT	0
169	KC_ABp1	CG1368	1.26833180271543	0.163578134772811	1	pseudobulk	DESeq2	LRT	0.786274748232787
169	KC_ABp1	CG13685	0.363195683479391	0.539359284639898	1	pseudobulk	DESeq2	LRT	0.26812184083384
169	KC_ABp1	CG13689	-0.400911419260453	0.818851024272015	1	pseudobulk	DESeq2	LRT	0.086795103394288
169	KC_ABp1	CG1371	-0.193279640708224	0.84405561786581	1	pseudobulk	DESeq2	LRT	0.073628935203273
169	KC_ABp1	CG13737	1.98207544136045	0.211491571386406	1	pseudobulk	DESeq2	LRT	0.674706935964183
169	KC_ABp1	CG13739	0.113242670112756	0.393900722415259	1	pseudobulk	DESeq2	LRT	0.404613222692577
169	KC_ABp1	CG13743	-0.464762780901259	0.746462735270588	1	pseudobulk	DESeq2	LRT	0.126991868140309
169	KC_ABp1	CG13751	-0.102824631708183	0.877768707997316	1	pseudobulk	DESeq2	LRT	0.056619905589847
169	KC_ABp1	CG13760	0.0474289293875647	0.891725485843275	1	pseudobulk	DESeq2	LRT	0.049768820884288
169	KC_ABp1	CG13766	0.423384779095433	0.713564139655184	1	pseudobulk	DESeq2	LRT	0.146566983661646
169	KC_ABp1	CG13773	-1.88217380369668	0.210082468402463	1	pseudobulk	DESeq2	LRT	0.677610188402011
169	KC_ABp1	CG13775	0.400640538490757	1	1	pseudobulk	DESeq2	LRT	0
169	KC_ABp1	CG13784	0.173153736515366	0.585848560041703	1	pseudobulk	DESeq2	LRT	0.232214633193688
169	KC_ABp1	CG13807	-1.7011596512709	0.331217949079445	1	pseudobulk	DESeq2	LRT	0.479886136334804
169	KC_ABp1	CG13827	-0.449741320065298	0.823189377956997	1	pseudobulk	DESeq2	LRT	0.084500242138268
169	KC_ABp1	CG13850	-1.4137380240586	0.479069955692572	1	pseudobulk	DESeq2	LRT	0.319601064554227
169	KC_ABp1	CG13868	0.451450590663263	0.221938091474252	1	pseudobulk	DESeq2	LRT	0.653768152939895
169	KC_ABp1	CG13870	0.400640538490757	1	1	pseudobulk	DESeq2	LRT	0
169	KC_ABp1	CG13875	-1.51023398327283	0.845597316437363	1	pseudobulk	DESeq2	LRT	0.072836403962567
169	KC_ABp1	CG13876	0.666554460641469	0.754480641201238	1	pseudobulk	DESeq2	LRT	0.122351899065778
169	KC_ABp1	CG13887	-0.307124834715952	0.64561932572115	1	pseudobulk	DESeq2	LRT	0.190023478087046
169	KC_ABp1	CG13893	1.313583495879	0.367913546469432	1	pseudobulk	DESeq2	LRT	0.434254221278375
169	KC_ABp1	CG13894	3.09265241527641	0.0125729629247814	1	pseudobulk	DESeq2	LRT	1.90056236509444
169	KC_ABp1	CG13895	2.42000968389812	0.264039447107927	1	pseudobulk	DESeq2	LRT	0.578331185321406
169	KC_ABp1	CG13896	1.09609931916753	0.587351075528798	1	pseudobulk	DESeq2	LRT	0.231102231651792
169	KC_ABp1	CG13898	-0.486167498126476	1	1	pseudobulk	DESeq2	LRT	0
169	KC_ABp1	CG13901	1.85895029951982	0.405908856945625	1	pseudobulk	DESeq2	LRT	0.39157147225836
169	KC_ABp1	CG13907	0.0185330440656323	0.948765772939746	1	pseudobulk	DESeq2	LRT	0.022840991023994
169	KC_ABp1	CG13917	0.0107992127512392	0.877994295157149	1	pseudobulk	DESeq2	LRT	0.056508305950183
169	KC_ABp1	CG13919	1.18999254199982	0.484028196080295	1	pseudobulk	DESeq2	LRT	0.315129338674986
169	KC_ABp1	CG13920	0.194739215439539	0.67380214789751	1	pseudobulk	DESeq2	LRT	0.171467608940311
169	KC_ABp1	CG13921	-0.021856014182217	0.96456023598293	1	pseudobulk	DESeq2	LRT	0.015670645844729
[truncated: 572,549 more chars]
